# Structural analysis of hubs in human NR-RTK network

**DOI:** 10.1186/1745-6150-6-49

**Published:** 2011-10-05

**Authors:** Mouna Choura, Ahmed Rebaï

**Affiliations:** 1 Molecular and Cellular Diagnosis Processes, Centre of Biotechnology of Sfax, University of Sfax, Route Sidi Mansour, Po Box 1177, 3018 Sfax, Tunisia

## Abstract

**Background:**

Currently a huge amount of protein-protein interaction data is available therefore extracting meaningful ones are a challenging task. In a protein-protein interaction network, hubs are considered as key proteins maintaining function and stability of the network. Therefore, studying protein-protein complexes from a structural perspective provides valuable information for predicted interactions.

**Results:**

In this study, we have predicted by comparative modelling and docking methods protein-protein complexes of hubs of human NR-RTK network inferred from our earlier study. We found that some interactions are mutually excluded while others could occur simultaneously. This study revealed by structural analysis the key role played by Estrogen receptor (ESR1) in mediating the signal transduction between human Receptor Tyrosine kinases (RTKs) and nuclear receptors (NRs).

**Conclusions:**

Although the methods require human intervention and judgment, they can identify the interactions that could occur together or ones that are mutually exclusive. This adds a fourth dimension to interaction network, that of time, and can assist in obtaining concrete predictions consistent with experiments.

**Open peer review:**

This article was reviewed by Dr. Anthony Almudevar, Prof. James Faeder and Prof. Eugene Koonin. For the full reviews, please go to the Reviewers' comments.

## Background

Protein-protein interactions are the major mechanism that controls biological processes and their studies have recently become very attractive not only for understanding cellular functions but also for therapeutic reasons. With the tremendous increase in human protein interaction data, network approaches are used to understand molecular mechanisms of disease [1] particularly to analyze and identify cancer related subnetworks [2].

Protein-protein interactions are usually shown as an interaction network where the proteins are represented as nodes and the connections between the interacting proteins are shown as edges. Many biological networks are known as scale-free networks and are characterized by a power-law degree distribution [3]. This means that most of the proteins share a few interactions whereas, a small number of proteins have a large number of interactions in the network. Such proteins called hubs are central to the normal function and stability of the protein-protein interaction network in any organism. The deletion of a hub has been shown to be lethal to the organism [4]. Moreover, several well-known and extensively studied proteins involved in diseases are hubs (eg. p53, p21, p27, BRCA1, kalirin, ubiquitin, calmodulin). This makes hubs important and attractive targets for in depth studies in biological networks.

It is clear that hubs in protein-protein network are able to recognize and bind to many other proteins. Interactions in proteins are mediated by the recognition of distinct binding regions by the protein on the surface of its interaction partner. Such molecular recognition must be specific enough and of sufficient affinity for the interaction to take place. In order to recognize and bind several other proteins, it is imperative for hubs to have some structural characteristics [5] and specificities such as interfaces [6]. Since a single protein cannot interact with a large number of partners at the same time, this presents a challenge.

Currently, the number of protein-protein interactions derived from high throughput experimental methods and prediction approaches has dramatically increased in protein interaction databases. Therefore, in extracting meaningful information from this interaction data set there is a strong need to avoid the huge amount of false positives.

The prediction of the structure of a protein-protein complex by docking methods is one of the major challenges in current computational structural biology [7-9]. Accurate predictions, properly integrated with experimental data could give new insights into validation of protein-protein interaction. Moreover, looking at 3D structure of each protein, especially the binding sites, in an interacting cluster can reveal information that can aid in figuring out which interactions can occur simultaneously and which are excluded.

In this study, we predict protein-protein interaction complexes of previously identified protein hubs of NR-RTK network and their interactions through docking of their molecular structures.

## Results

**Figure 1:**
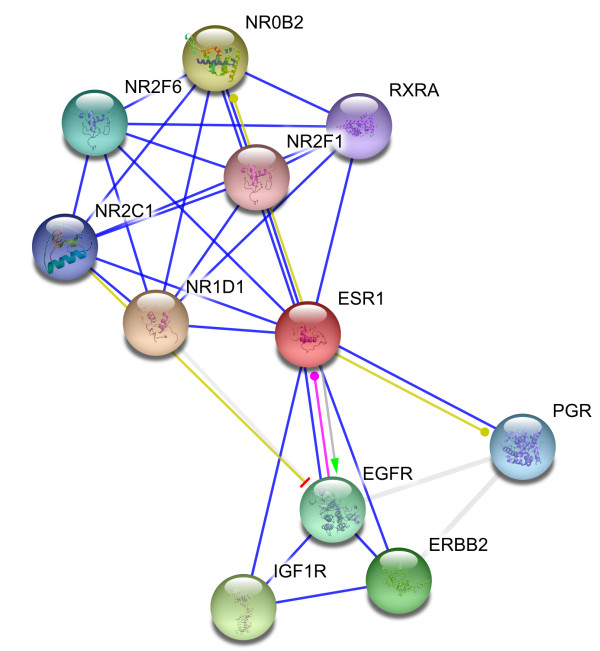
**Hubs of NR-RTK network previously inferred in **[10].

**Figure 2:**
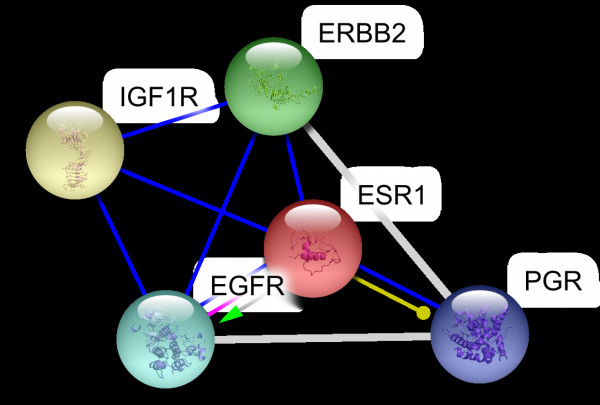
**Hubs protein cluster selected in this study**.

**Figure 3:**
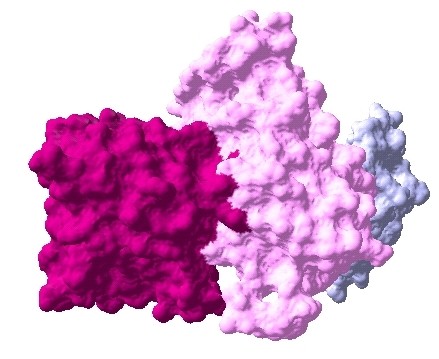
**Docked complex of Erbb2-ESR1-PGR interactions, Erbb2: prune, ESR1: pink, PGR: azure**.

**Figure 4:**
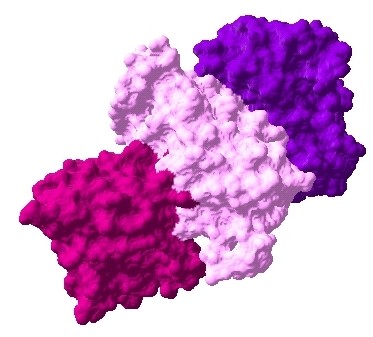
**Docked complex of Erbb2-ESR1-IGF1R interactions, Erbb2: prune, ESR1: pink, IGF1R: purple**.

**Figure 5:**
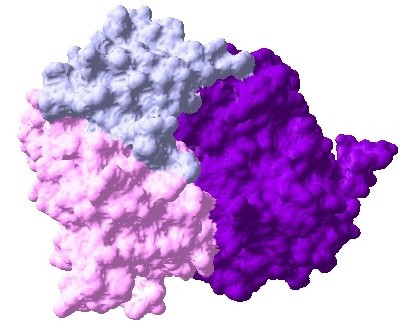
**Docked complex of ESR1-IGF1R-PGR interactions, ESR1: pink, IGF1R: purple, PGR: azure**.

**Figure 6:**
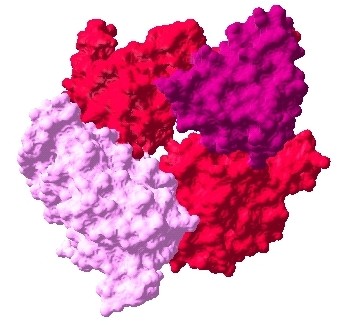
**Docked complex of ESR1-EGFR-Erbb2 interactions, ESR1: pink, EGFR: red, Erbb2: prune**.

**Figure 7:**
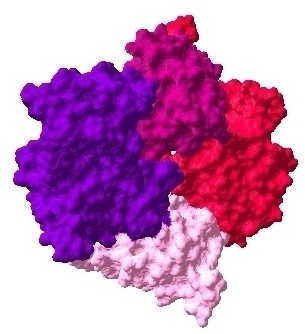
**Docked complex of ESR1-IGF1R-EGFR-Erbb2 interactions, ESR1: pink, IGF1R: purple, EGFR: red, Erbb2: prune**.

**Figure 8:**
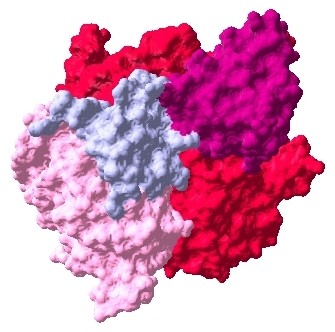
**Docked complex of ESR1-PGR-EGFR-Erbb2 interactions, ESR1: pink, PGR: azure, EGFR: red, Erbb2: prune**.

The interaction data are extracted from previously identified hub proteins of NR-RTK network [10]. The proteins and their interactions are shown in Figure 1. We have noticed that this subnetwork can be divided into two protein clusters linked by estrogen receptor protein (ESR1). In order to understand the signal transmission between transmembrane receptors (RTK) and nuclear receptors (NR), we have removed the cluster composed only of nuclear receptors. Therefore, we have considered the cluster protein shown in Figure 2 for further structural analysis. It is worthy to note that studied interactions have been reported in interaction databases and tested by *in vivo *or *in vitro *assays but no experimental complex structure are available in PDB (Table 1). Consequently, we have predicted its structures by comparative modelling by I-TASSER server (Table 2) and additional files 1, 2, 3, 4, 5. Then, we have performed docking for ESR1-EGFR (additional file 6), ESR1-Erbb2 (additional file 7), ESR1-PGR (additional file 8) and ESR1-IGF1-R (additional file 9) interactions. It is evident from Figure 3, Figure 4 and Figure 5 that ESR1 has at least two binding sites. {IGF1-R and PGR}, {Erbb2, IGF1R} and {EGFR and PGR or IGF1R} bind to ESR1 at overlapping sites but Erbb2 and PGR bind to ESR1 at different sites. Thus, the following sets of interactions {ESR1-IGF1R, ESR1-PGR} (additional file 10), {ESR1-Erbb2, ESR1-PGR} (additional file 11), {ESR1-Erbb2, ESR1-IGF1R} (additional file 12), {ESR1-IGF1R, ESR1-EGFR} (additional file 13) and {ESR1-EGFR, ESR1-PGR} (additional file 14) could occur simultaneously. The docked complexes involving ESR1-EGFR and Erbb2 shown in Figure 6, Figure 7 and Figure 8 revealed that ESR1-EGFR and ESR1-Erbb2 interactions are mutually exclusive because of shared binding sites of the interacting proteins, and hence EGFR and Erbb2 cannot bind to ESR1 at the same time (see additional files 15, 16, 17). This is consistent with previous findings proving that any member of EGFR family can have a homodimer or a herterodimer preferentially with Erbb2 [11].

**Table 1:** List of studied protein interactions, their detection method and interaction databases taken from.

Node 1	Node 2	Detection method	Interaction database
ESR1	PGR	In vivo, in vitro and yeast 2-hybrid assays	HPRD [22]
ESR1	IGF1R	Affinity capture-western assay	BIOGRID [23]
ESR1	EGFR	Anti-bait coimmunoprecipitation assay	HPRD, MINT [24]
ESR1	Erbb2	in vitro and in vivo assays	HPRD

**Table 2:** List of homology modelling details performed by I-TASSER.

Protein	PDB homolog	Identity (%)*	C-score
EGFR	1yy9A	50	-2.51
ESR1	3dzyA	25	-2.29
Erbb2	1n8yC	41	-2.72
IGF1-R	2dtgE	33	-2.03
PGR	3hq5B	27	-2.71

* The percentage sequence identity of the whole template chains with query sequence.

## Discussion

One of our goals in this paper is to test the validity of a reported interaction by using structural information about the interacting proteins in a cluster. Although the interaction data have been obtained from high-throughput screening methods such as the yeast two-hybrid method and affinity purification techniques, these interactions require more validation. Therefore prediction of interaction complex structure could constitute complementary validation. At this end, we prioritize for structural analysis hubs proteins that seem to play 'switch' role in the signal transmission from RTK network to NR network.

It is evident from our models that ESR1 is a flexible protein. This can be explained by the fact that ESR1 is a regulatory protein. In fact, in breast cancer cells estrogens activate the Src/Erk pathway through an interaction of the estrogen receptor alpha (ESR1) with the SH2 domain of c-Src [12]. Moreover, it has been proved that IGF1R serves as an anchor for ESR1 in the plasma membrane of breast cancer cells [13]. Furthermore, in confirmation of a direct interaction between ESR and EGFR, activation of affinity-purified EGFR tyrosine kinase in vitro stimulated the phosphorylation of recombinant ESR [14].

These theoretical predictions might be useful for crystallographers to select targets for the X-ray crystallographic determination of such protein complexes considered as potential cancer therapeutic targets. More interestingly, including microarray data can help in determining if two proteins bind to ESR1 at the same time by looking at the correlation of their expression patterns. If their expression is correlated, most likely these two interactions can occur simultaneously.

## Conclusions

This work has taken the approach of predicting protein-protein complex interactions of previously predicted hub proteins of NR-RTK network through docking of their molecular structures. Since studied complexes are not available in PDB, we have relied upon comparative modelling and docking methods. This methodology has the advantage that it can also identify interactions that could occur together or ones that are mutually exclusive. In addition indirect interactions through another intermediate protein can be identified. We believe that the correctness of our results depends on experimental validation which is an important task.

## Methods

### 1. Interaction complex within protein subnetwork

We previously determined potential protein hubs of NR-RTK network [10]. Out of these, we selected for structural analysis the top five hub proteins involved in the signal transmission between transmembrane receptors (RTK) and nuclear receptors (NR) based on statistical criteria given in [10].

### 2. Comparative modelling

To predict an interaction complex or predict a new interaction, we require the protein structures of both interacting proteins. We used comparative modelling approaches because the studied protein structures are not available in PDB. To predict the structure of the protein, we have relied upon Zhang's I-TASSER server [15,16] (http://zhanglab.ccmb.med.umich.edu/I-TASSER/), which gave the best protein models at the Critical Assessment of Structure Prediction (CASP 7 and CASP 8), a community-wide, worldwide experiment designed to obtain an objective assessment of the state-of-the-art in structure prediction [17,18]. The I-TASSER algorithm consists of three consecutive steps: threading, fragment assembly and iteration. During the threading, I-TASSER generates the template alignments by a simple sequence Profile-Profile Alignment approach constrained with the secondary structure matches. Fragment assembly is performed on the basis of threaded alignments and the target sequences are divided into aligned and unaligned regions. The fragments in the aligned regions are used directly from the template structures and the unaligned regions are modelled with ab initio simulations. Clusters of decoys are generated with the use of a knowledge-based force field. The cluster centroids are generated by averaging the coordinates of all clustered decoys and ranked based on the structure density. In the iteration phase, the steric clashes of the cluster centroids are removed and the topology is refined. The conformations with the lowest energy are selected.

The I-TASSER server returns the best five models with a c-score attached for each model. Also it returns the top ten templates used in the threading. The c-score is a confidence score that I-TASSER uses to estimate the quality of the predicted model. The calculation of c-score is based on the significance of the threading template alignments and the convergence parameters of the structure assembly simulations. C-score is typically in the range of [5,2], where a C-score a higher value a model with high confidence and vice-versa [16]. When selecting one of these models, we select the model that comes from the largest cluster and has the best c-score.

### 3. Docking

After having both structures of an interacting pair we used docking to predict the protein complex formed in a protein-protein interaction. We used the Cluspro server [19,20] for docking the interacting proteins to predict the protein complex. Cluspro is the first fully automated web-based program for docking proteins and was one of the top performers at CAPRI (Critical Assessment of Predicted Interactions) rounds 1-12, the community-wide experiment devoted to protein docking [21]. The Cluspro server is based on a Fast Fourier Transform correlation approach, which makes it feasible to generate and evaluate billions of docked conformations by simple scoring functions. It is an implementation of a multistage protocol: rigid body docking, an energy based filtering, ranking the retained structures based on clustering properties, and finally, the refinement of a limited number of structures by energy minimization. The server (http://cluspro.bu.edu/) returns the top models based on energy and cluster size. We select one of the returned models after considering the energy and the size of the cluster preferring lower energies and larger cluster sizes.

## List of abbreviations

EGFR: Epidermal growth factor receptor; ESR1: Estrogen Receptor1; Erbb2: Receptor tyrosine-kinase Erbb2; IGF1-R: Insulin-like growth factor 1; NR: Nuclear Receptor; PGR: Progesterone receptor; RTK: Receptor Tyrosine Kinase

## Authors' contributions

MC did the comparative modelling and docking. She also wrote the manuscript. AR supervised the work and corrected the manuscript. All authors read and approved the final manuscript.

## Reviewers' comments

### Reviewer's report 1

Anthony Almudevar, Department of Biostatistics and Computational Biology University of Rochester Medical Center, Rochester, NY

The authors propose the use of structural analysis for the validation of PPI networks compiled using high-throughput data. The object is to reduce false positives, as well as to introduce additional structure, for example, determination of interactions which are mutually exclusive due to shared binding sites. Published software applications (I-TASSER, Cluspro) are used to predict structure, then to predict binding sites of protein complexes. The method is demonstrated using a PPI network compiled by the authors in an earlier paper (Choura and Rebai (2010) Biology Direct).

The paper is interesting, but application of the method is limited to a small subset of a PPI network. Additionally, interactions among the exemplary proteins are already described in literature cited by the authors, so that validity is more likely to occur than for a randomly selected interaction. Thus, it is difficult to assess the value of the method with respect to the reduction of false positives. Would it be possible to systematically apply the method over a larger portion of the network?

A report on the resulting concordance would be interesting.

**Author's response**: We agree with the reviewer. We think that this approach could not be systematically applied because of the lengthy computational time and it requires human judgement. Nonetheless, many such cases can be investigated and the results can provide new information.

Minor Points

Page 4: "Results of the docking of these interactions are shown in Figure 2". This needs to be clearly annotated.

Page 6: Is it possible to give more interpretation of the c-score?

Page 6 "select the model "repeated.

Table 2: Interpret "Identity %".

**Author's response**: We have corrected these points accordingly.

**Quality of written English**: Needs some language corrections before being published

**Author's response**: We have corrected the manuscript. (This response is also for the reviewer 2).

### Second report

The paper is an interesting contribution to PPI network reconstruction, and I think would be of interest to readers of Biology Direct. The only concern I have at this point is in the reference on page 3 to the motivation of false positive control. It is difficult to evaluate the methodology from this point of view given the limited application demonstrated. The authors, in their earlier response, point out that this is difficult due to the need for human judgement, and because of the lengthy computation time. Some comment on the required computational burden should therefore be provided. More generally, it would be good for the authors to predict, for example, with how much certainty their validation method will detect a false positive selected from a high-throughput screening.

**Author's response**: At least homology modelling takes one day for one protein, similarly for docking of a pair wise interaction. Regarding the false positives estimation, at this time it is difficult to give a certainty value for false positive detection because studied complexes are not validated experimentally.

minor corrections

page 2 - commas around 'that of time'

page 3 - 'target' -> 'targets'

page 3 - 'Therefore, extract' -> 'Therefore, in extracting'

page 3 - 'set is a strong need' -> 'set there is a strong need'

page 4 - 'only by nuclear' -> 'only of nuclear'

**Quality of written English**: Needs some language corrections before being published

**Author's response**: done

### Third report

The comments in my second review still apply.

**Author's response**: We thank very much the reviewer. We agree with his comments that we will consider carefully in our upcoming work.

**Quality of written English**: Acceptable

### Reviewer's report 2

Prof James Faeder, Department of Computational Biology, University of Pittsburgh School of Medicine, Pittsburgh, USA.

The goal of this study is to use structure prediction methods to determine which of a possible set of complexes that can form between a set of proteins based on their known interactions can actually form. In particular a hub protein, ESR1, that has been shown to form a hub linking growth factor and estrogen signalling networks has been studied in detail. Its potential interactions with four different receptor proteins have been studied. It is found that because some of the interaction pairs have overlapping sites, a number of the interactions are mutually exclusive, with the result that ESR1 is at most able to form ternary complexes of the involved proteins. This is an interesting and to my knowledge novel finding that could be of interest both to experimentalists and modelers studying these networks. However, not enough detail about either the methods or the results is provided to give sufficient confidence that obtained results are valid. Specifically:

-No evidence is provide that the combination of structure prediction methods for proteins with unknown or partially known structures and docking can be used to accurately predict the structure of complexes, particularly complexes involving potentially three or more proteins. No evidence has been provided that the predictions from this approach have been validated. For example, can docking of KNOWN structures be used to accurately predict ternary complexes and to rule out possible complexes?

**Author's response**: The following articles illustrate some examples of docking application to accurate prediction of protein complexes:

* Prediction of multimolecular assemblies by multiple docking (PMID: 15890207).

* Pushing structure by high throughput experiments (PMID: 19714207).

-How are the interfaces between the involved proteins determined and how reliable are those predictions likely to be?

**Author's response**: the interfaces between proteins are studied in our paper currently under preparation.

-Structures of both individual proteins and complexes should be provided as supplemental material. It is also not clear what portions of the receptor protein were used in study - presumably the cytosolic portions. Large portions of these regions are known to be disordered how is that handled? How many docked structures were obtained and how prevalent were the final structures reported in the docking runs?

**Author's response**: All pdb files obtained in this work are deposited as supplemental files.

Portions of receptors used correspond to extracellular domain for EGFR and IGF1R (receptor tyrosine kinases) and ligand binding domain for the other receptors (nuclear receptors).

As Cluspro server implements rigid body docking, when a partner protein in a complex is structurally flexible Cluspro is not so able to account for his flexibility.

-Figures 6, 7, 8 show complexes containing four or more proteins - presumably to argue that these complexes are not likely to form. It is not clear, however, how this assessment is made.

**Author's response**: Figures 6, 7, 8 correspond to interactions involving EGFR protein showing that in tetramer case Erbb2 cannot bind directly ESR1 because of shared binding sites. This binding occurs in heterodimer state with EGFR which is consistent with previously validated findings.

A few minor comments

- The abstract should give more concrete details about the methods being employed.

**Author's response**: done.

-The work of Gerstein and co-workers (http://www.sciencemag.org/content/314/5807/1938.abstract) and determining interaction interfaces involving hub proteins in yeast signaling networks may be relevant to the current study.

**Author's response**: We thank very much the reviewer for this relevant reference.

**Quality of written English**: Needs some language corrections before being published

### Second report

It is difficult to tell from the response letter or the revised manuscript what changes were made in response to my critique or that of Reviewer 1. My impression is that only a few revisions were made. In the future, it would be very helpful to the Reviewers to highlight any changes. The main new item in the revision is the set of pdb files of the docked structures, which are now provided as Supplemental information. While these are helpful, they are not really a substitute for further clarification of the methodology employed or validation of the procedures. I will leave it to readers to judge whether the obtained structures are meaningful.

**Quality of written English**: Acceptable

**Author's response**: We thank the reviewer for his comments.

### Third report

I have no additional comments.

**Quality of written English**: Acceptable

### Reviewer's report 3

Prof Eugene Koonin, National Center for Biotechnology Information, NIH, Bethesda

Maryland, USA.

This reviewer provided no comments for publication

**Quality of written English**: acceptable.

## Supplementary Material

Additional file 1 **EGFR**. EGFR structure. Click here for file

Additional file 2 **ESR1**. ESR1 structure. Click here for file

Additional file 3 **PGR**. PGR structure. Click here for file

Additional file 4 **IGF1R**. IGF1R structure. Click here for file

Additional file 5 **Erbb2**. Erbb2 structure. Click here for file

Additional file 6 **ESR1-EGFR**. ESR1-EGFR complex structure. Click here for file

Additional file 7 **ESR1-Erbb2**. ESR1-Erbb2 complex structure. Click here for file

Additional file 8 **ESR1-PGR**. ESR1-PGR complex structure. Click here for file

Additional file 9 **ESR1-IGF1-R**. ESR1-IGF1-R complex structure. Click here for file

Additional file 10 **ESR1-IGF1R-PGR**. ESR1-IGF1-R-PGR complex structure. Click here for file

Additional file 11 **ESR1-Erbb2-PGR**. ESR1-Erbb2-PGR complex structure. Click here for file

Additional file 12 **ESR1-Erbb2-IGF1R**. ESR1-Erbb2-IGF1R complex structure. Click here for file

Additional file 13 **ESR1-IGF1R-EGFR**. ESR1-IGF1R-EGFR complex structure. Click here for file

Additional file 14 **ESR1-EGFR-PGR**. ESR1-EGFR-PGR complex structure. Click here for file

Additional file 15 **ESR1-EGFR-Erbb2**. ESR1-EGFR-Erbb2 complex structure. Click here for file

Additional file 16 **ESR1-EGFR-Erbb2-IGF1R**. ESR1-EGFR-Erbb2-IGF1R complex structure. Click here for file

Additional file 17 **ESR1-EGFR-Erbb2-PGR**. ESR1-EGFR-Erbb2-PGR complex structure. Click here for file
