# Supplementary material for: Structural analysis of hubs in human NR-RTK network
Source: Biol Direct. 2011 Oct 5;6:49. doi: 10.1186/1745-6150-6-49 (PMC3220635; doi:10.1186/1745-6150-6-49)
Supplement: Additional file 1 — EGFR. EGFR structure. [file 1745-6150-6-49-S1.PDF]

```
HEADER EGFR
REMARK PARENT 1yy9_A 1n8y_C 1yy9 1mox
ATOM      1  N   MET A   1     126.154  38.467 113.977  1.00  0.00
ATOM      2  CA  MET A   1     126.316  37.771 112.691  1.00  0.00
ATOM      3  HA  MET A   1     126.856  36.841 112.874  1.00  0.00
ATOM      4  CB  MET A   1     124.942  37.415 112.093  1.00  0.00
ATOM      5  HB1 MET A   1     125.082  37.003 111.092  1.00  0.00
ATOM      6  HB2 MET A   1     124.337  38.319 112.011  1.00  0.00
ATOM      7  CG  MET A   1     124.186  36.376 112.930  1.00  0.00
ATOM      8  HG1 MET A   1     123.199  36.239 112.487  1.00  0.00
ATOM      9  HG2 MET A   1     124.050  36.760 113.941  1.00  0.00
ATOM     10  SD  MET A   1     124.995  34.753 113.022  1.00  0.00
ATOM     11  CE  MET A   1     123.757  33.834 113.974  1.00  0.00
ATOM     12  HE1 MET A   1     124.101  32.811 114.123  1.00  0.00
ATOM     13  HE2 MET A   1     123.612  34.309 114.943  1.00  0.00
ATOM     14  HE3 MET A   1     122.813  33.819 113.430  1.00  0.00
ATOM     15  C   MET A   1     127.184  38.602 111.745  1.00  0.00
ATOM     16  O   MET A   1     126.783  39.006 110.654  1.00  0.00
ATOM     17  N   ARG A   2     128.361  38.968 112.254  1.00  0.00
ATOM     18  H   ARG A   2     128.608  38.548 113.141  1.00  0.00
ATOM     19  CA  ARG A   2     129.397  39.768 111.590  1.00  0.00
ATOM     20  HA  ARG A   2     129.062  40.057 110.594  1.00  0.00
ATOM     21  CB  ARG A   2     129.681  41.031 112.430  1.00  0.00
ATOM     22  HB1 ARG A   2     130.722  41.312 112.296  1.00  0.00
ATOM     23  HB2 ARG A   2     129.555  40.802 113.489  1.00  0.00
ATOM     24  CG  ARG A   2     128.837  42.270 112.081  1.00  0.00
ATOM     25  HG1 ARG A   2     129.143  42.626 111.096  1.00  0.00
ATOM     26  HG2 ARG A   2     129.081  43.054 112.797  1.00  0.00
ATOM     27  CD  ARG A   2     127.308  42.083 112.059  1.00  0.00
ATOM     28  HD1 ARG A   2     127.050  41.479 111.187  1.00  0.00
ATOM     29  HD2 ARG A   2     126.832  43.056 111.921  1.00  0.00
ATOM     30  NE  ARG A   2     126.775  41.422 113.268  1.00  0.00
ATOM     31  HE  ARG A   2     126.529  40.446 113.162  1.00  0.00
ATOM     32  CZ  ARG A   2     126.571  41.926 114.466  1.00  0.00
ATOM     33  NH1 ARG A   2     126.806  43.177 114.743  1.00  0.00
ATOM     34 1HH1 ARG A   2     127.141  43.766 114.004  1.00  0.00
ATOM     35 2HH1 ARG A   2     126.634  43.536 115.662  1.00  0.00
ATOM     36  NH2 ARG A   2     126.125  41.153 115.415  1.00  0.00
ATOM     37 1HH2 ARG A   2     125.962  40.172 115.199  1.00  0.00
ATOM     38 2HH2 ARG A   2     125.964  41.500 116.340  1.00  0.00
ATOM     39  C   ARG A   2     130.635  38.862 111.456  1.00  0.00
ATOM     40  O   ARG A   2     130.862  38.041 112.344  1.00  0.00
ATOM     41  N   PRO A   3     131.439  38.959 110.384  1.00  0.00
ATOM     42  CD  PRO A   3     131.152  39.666 109.144  1.00  0.00
ATOM     43  HD1 PRO A   3     131.292  40.736 109.286  1.00  0.00
ATOM     44  HD2 PRO A   3     130.140  39.462 108.793  1.00  0.00
ATOM     45  CG  PRO A   3     132.162  39.135 108.128  1.00  0.00
ATOM     46  HG1 PRO A   3     132.434  39.895 107.394  1.00  0.00
ATOM     47  HG2 PRO A   3     131.750  38.255 107.632  1.00  0.00
ATOM     48  CB  PRO A   3     133.352  38.724 108.992  1.00  0.00
ATOM     49  HB1 PRO A   3     133.971  39.602 109.186  1.00  0.00
ATOM     50  HB2 PRO A   3     133.944  37.940 108.518  1.00  0.00
ATOM     51  CA  PRO A   3     132.709  38.236 110.298  1.00  0.00
ATOM     52  HA  PRO A   3     132.492  37.170 110.215  1.00  0.00
ATOM     53  C   PRO A   3     133.646  38.479 111.488  1.00  0.00
ATOM     54  O   PRO A   3     133.638  39.565 112.063  1.00  0.00
ATOM     55  N   SER A   4     134.499  37.502 111.818  1.00  0.00
ATOM     56  H   SER A   4     134.456  36.637 111.296  1.00  0.00
ATOM     57  CA  SER A   4     135.241  37.405 113.091  1.00  0.00
ATOM     58  HA  SER A   4     134.520  37.120 113.857  1.00  0.00
ATOM     59  CB  SER A   4     136.275  36.274 113.010  1.00  0.00
```

ATOM	60	HB1	SER	A	4	136.625	36.035	114.016	1.00	0.00
ATOM	61	HB2	SER	A	4	137.125	36.598	112.407	1.00	0.00
ATOM	62	OG	SER	A	4	135.705	35.118	112.414	1.00	0.00
ATOM	63	HG	SER	A	4	136.289	34.366	112.547	1.00	0.00
ATOM	64	C	SER	A	4	135.923	38.691	113.588	1.00	0.00
ATOM	65	O	SER	A	4	135.845	38.971	114.782	1.00	0.00
ATOM	66	N	GLY	A	5	136.527	39.494	112.704	1.00	0.00
ATOM	67	H	GLY	A	5	136.533	39.212	111.738	1.00	0.00
ATOM	68	CA	GLY	A	5	137.133	40.779	113.073	1.00	0.00
ATOM	69	HA1	GLY	A	5	137.719	41.156	112.235	1.00	0.00
ATOM	70	HA2	GLY	A	5	137.810	40.633	113.916	1.00	0.00
ATOM	71	C	GLY	A	5	136.110	41.855	113.462	1.00	0.00
ATOM	72	O	GLY	A	5	136.130	42.369	114.581	1.00	0.00
ATOM	73	N	THR	A	6	135.159	42.167	112.575	1.00	0.00
ATOM	74	H	THR	A	6	135.128	41.653	111.708	1.00	0.00
ATOM	75	CA	THR	A	6	134.081	43.134	112.876	1.00	0.00
ATOM	76	HA	THR	A	6	134.567	44.030	113.263	1.00	0.00
ATOM	77	CB	THR	A	6	133.312	43.589	111.614	1.00	0.00
ATOM	78	HB	THR	A	6	133.916	44.342	111.107	1.00	0.00
ATOM	79	CG2	THR	A	6	133.016	42.483	110.604	1.00	0.00
ATOM	80	1HG2	THR	A	6	132.370	42.874	109.817	1.00	0.00
ATOM	81	2HG2	THR	A	6	133.941	42.135	110.148	1.00	0.00
ATOM	82	3HG2	THR	A	6	132.523	41.652	111.096	1.00	0.00
ATOM	83	OG1	THR	A	6	132.071	44.171	111.941	1.00	0.00
ATOM	84	HG1	THR	A	6	132.232	44.871	112.583	1.00	0.00
ATOM	85	C	THR	A	6	133.147	42.662	113.998	1.00	0.00
ATOM	86	O	THR	A	6	132.713	43.490	114.804	1.00	0.00
ATOM	87	N	ALA	A	7	132.898	41.355	114.130	1.00	0.00
ATOM	88	H	ALA	A	7	133.270	40.714	113.438	1.00	0.00
ATOM	89	CA	ALA	A	7	132.234	40.792	115.306	1.00	0.00
ATOM	90	HA	ALA	A	7	131.290	41.316	115.462	1.00	0.00
ATOM	91	CB	ALA	A	7	131.929	39.312	115.095	1.00	0.00
ATOM	92	HB1	ALA	A	7	131.629	38.855	116.038	1.00	0.00
ATOM	93	HB2	ALA	A	7	131.100	39.218	114.407	1.00	0.00
ATOM	94	HB3	ALA	A	7	132.800	38.783	114.709	1.00	0.00
ATOM	95	C	ALA	A	7	133.063	40.974	116.580	1.00	0.00
ATOM	96	O	ALA	A	7	132.501	41.373	117.593	1.00	0.00
ATOM	97	N	GLY	A	8	134.377	40.740	116.539	1.00	0.00
ATOM	98	H	GLY	A	8	134.798	40.377	115.690	1.00	0.00
ATOM	99	CA	GLY	A	8	135.262	40.973	117.678	1.00	0.00
ATOM	100	HA1	GLY	A	8	136.285	40.736	117.387	1.00	0.00
ATOM	101	HA2	GLY	A	8	134.972	40.326	118.506	1.00	0.00
ATOM	102	C	GLY	A	8	135.225	42.427	118.156	1.00	0.00
ATOM	103	O	GLY	A	8	135.020	42.677	119.340	1.00	0.00
ATOM	104	N	ALA	A	9	135.320	43.389	117.235	1.00	0.00
ATOM	105	H	ALA	A	9	135.508	43.115	116.276	1.00	0.00
ATOM	106	CA	ALA	A	9	135.166	44.812	117.543	1.00	0.00
ATOM	107	HA	ALA	A	9	135.928	45.092	118.272	1.00	0.00
ATOM	108	CB	ALA	A	9	135.421	45.609	116.257	1.00	0.00
ATOM	109	HB1	ALA	A	9	135.354	46.676	116.470	1.00	0.00
ATOM	110	HB2	ALA	A	9	136.416	45.384	115.873	1.00	0.00
ATOM	111	HB3	ALA	A	9	134.680	45.351	115.500	1.00	0.00
ATOM	112	C	ALA	A	9	133.793	45.154	118.166	1.00	0.00
ATOM	113	O	ALA	A	9	133.725	45.856	119.178	1.00	0.00
ATOM	114	N	ALA	A	10	132.701	44.629	117.595	1.00	0.00
ATOM	115	H	ALA	A	10	132.807	44.072	116.756	1.00	0.00
ATOM	116	CA	ALA	A	10	131.354	44.837	118.126	1.00	0.00
ATOM	117	HA	ALA	A	10	131.170	45.911	118.185	1.00	0.00
ATOM	118	CB	ALA	A	10	130.345	44.233	117.140	1.00	0.00
ATOM	119	HB1	ALA	A	10	129.332	44.434	117.489	1.00	0.00
ATOM	120	HB2	ALA	A	10	130.479	44.680	116.155	1.00	0.00

ATOM	121	HB3	ALA	A	10	130.491	43.155	117.071	1.00	0.00
ATOM	122	C	ALA	A	10	131.160	44.268	119.547	1.00	0.00
ATOM	123	O	ALA	A	10	130.474	44.877	120.368	1.00	0.00
ATOM	124	N	LEU	A	11	131.774	43.120	119.848	1.00	0.00
ATOM	125	H	LEU	A	11	132.296	42.659	119.108	1.00	0.00
ATOM	126	CA	LEU	A	11	131.725	42.455	121.150	1.00	0.00
ATOM	127	HA	LEU	A	11	130.696	42.463	121.508	1.00	0.00
ATOM	128	CB	LEU	A	11	132.185	40.994	120.988	1.00	0.00
ATOM	129	HB1	LEU	A	11	132.358	40.566	121.977	1.00	0.00
ATOM	130	HB2	LEU	A	11	133.136	40.989	120.454	1.00	0.00
ATOM	131	CG	LEU	A	11	131.171	40.110	120.237	1.00	0.00
ATOM	132	HG	LEU	A	11	130.812	40.619	119.344	1.00	0.00
ATOM	133	CD1	LEU	A	11	131.830	38.800	119.806	1.00	0.00
ATOM	134	1HD1	LEU	A	11	131.116	38.193	119.252	1.00	0.00
ATOM	135	2HD1	LEU	A	11	132.679	39.017	119.159	1.00	0.00
ATOM	136	3HD1	LEU	A	11	132.173	38.247	120.679	1.00	0.00
ATOM	137	CD2	LEU	A	11	129.952	39.767	121.099	1.00	0.00
ATOM	138	1HD2	LEU	A	11	129.291	39.100	120.550	1.00	0.00
ATOM	139	2HD2	LEU	A	11	130.274	39.279	122.017	1.00	0.00
ATOM	140	3HD2	LEU	A	11	129.404	40.674	121.342	1.00	0.00
ATOM	141	C	LEU	A	11	132.559	43.191	122.204	1.00	0.00
ATOM	142	O	LEU	A	11	132.084	43.375	123.325	1.00	0.00
ATOM	143	N	LEU	A	12	133.751	43.675	121.841	1.00	0.00
ATOM	144	H	LEU	A	12	134.090	43.457	120.908	1.00	0.00
ATOM	145	CA	LEU	A	12	134.567	44.540	122.702	1.00	0.00
ATOM	146	HA	LEU	A	12	134.745	44.024	123.645	1.00	0.00
ATOM	147	CB	LEU	A	12	135.918	44.841	122.021	1.00	0.00
ATOM	148	HB1	LEU	A	12	136.387	45.683	122.532	1.00	0.00
ATOM	149	HB2	LEU	A	12	135.723	45.149	120.992	1.00	0.00
ATOM	150	CG	LEU	A	12	136.914	43.666	122.019	1.00	0.00
ATOM	151	HG	LEU	A	12	136.435	42.776	121.616	1.00	0.00
ATOM	152	CD1	LEU	A	12	138.113	44.012	121.133	1.00	0.00
ATOM	153	1HD1	LEU	A	12	138.804	43.170	121.111	1.00	0.00
ATOM	154	2HD1	LEU	A	12	137.774	44.208	120.117	1.00	0.00
ATOM	155	3HD1	LEU	A	12	138.628	44.892	121.517	1.00	0.00
ATOM	156	CD2	LEU	A	12	137.432	43.349	123.427	1.00	0.00
ATOM	157	1HD2	LEU	A	12	138.181	42.559	123.371	1.00	0.00
ATOM	158	2HD2	LEU	A	12	137.878	44.239	123.872	1.00	0.00
ATOM	159	3HD2	LEU	A	12	136.618	42.999	124.058	1.00	0.00
ATOM	160	C	LEU	A	12	133.832	45.838	123.064	1.00	0.00
ATOM	161	O	LEU	A	12	133.771	46.198	124.244	1.00	0.00
ATOM	162	N	ALA	A	13	133.236	46.510	122.072	1.00	0.00
ATOM	163	H	ALA	A	13	133.340	46.171	121.121	1.00	0.00
ATOM	164	CA	ALA	A	13	132.440	47.716	122.300	1.00	0.00
ATOM	165	HA	ALA	A	13	133.066	48.456	122.802	1.00	0.00
ATOM	166	CB	ALA	A	13	132.015	48.293	120.944	1.00	0.00
ATOM	167	HB1	ALA	A	13	131.449	49.211	121.101	1.00	0.00
ATOM	168	HB2	ALA	A	13	132.899	48.520	120.347	1.00	0.00
ATOM	169	HB3	ALA	A	13	131.396	47.574	120.407	1.00	0.00
ATOM	170	C	ALA	A	13	131.225	47.458	123.217	1.00	0.00
ATOM	171	O	ALA	A	13	130.996	48.203	124.167	1.00	0.00
ATOM	172	N	LEU	A	14	130.481	46.372	122.962	1.00	0.00
ATOM	173	H	LEU	A	14	130.707	45.812	122.147	1.00	0.00
ATOM	174	CA	LEU	A	14	129.333	45.969	123.777	1.00	0.00
ATOM	175	HA	LEU	A	14	128.615	46.791	123.771	1.00	0.00
ATOM	176	CB	LEU	A	14	128.658	44.739	123.144	1.00	0.00
ATOM	177	HB1	LEU	A	14	129.414	43.971	122.980	1.00	0.00
ATOM	178	HB2	LEU	A	14	128.259	45.028	122.171	1.00	0.00
ATOM	179	CG	LEU	A	14	127.525	44.115	123.987	1.00	0.00
ATOM	180	HG	LEU	A	14	127.939	43.715	124.913	1.00	0.00
ATOM	181	CD1	LEU	A	14	126.401	45.096	124.340	1.00	0.00

```
ATOM    182 1HD1 LEU A  14      125.620  44.574 124.891  1.00  0.00
ATOM    183 2HD1 LEU A  14      126.781  45.900 124.969  1.00  0.00
ATOM    184 3HD1 LEU A  14      125.979  45.524 123.431  1.00  0.00
ATOM    185  CD2 LEU A  14      126.922  42.952 123.197  1.00  0.00
ATOM    186 1HD2 LEU A  14      126.165  42.453 123.802  1.00  0.00
ATOM    187 2HD2 LEU A  14      126.464  43.319 122.279  1.00  0.00
ATOM    188 3HD2 LEU A  14      127.702  42.232 122.952  1.00  0.00
ATOM    189  C   LEU A  14      129.717  45.721 125.237  1.00  0.00
ATOM    190  O   LEU A  14      129.110  46.310 126.124  1.00  0.00
ATOM    191  N   LEU A  15      130.732  44.898 125.506  1.00  0.00
ATOM    192  H   LEU A  15      131.227  44.471 124.731  1.00  0.00
ATOM    193  CA  LEU A  15      131.159  44.598 126.876  1.00  0.00
ATOM    194  HA  LEU A  15      130.296  44.250 127.442  1.00  0.00
ATOM    195  CB  LEU A  15      132.227  43.491 126.850  1.00  0.00
ATOM    196  HB1 LEU A  15      132.659  43.396 127.848  1.00  0.00
ATOM    197  HB2 LEU A  15      133.024  43.793 126.168  1.00  0.00
ATOM    198  CG  LEU A  15      131.697  42.106 126.419  1.00  0.00
ATOM    199  HG  LEU A  15      131.187  42.189 125.461  1.00  0.00
ATOM    200  CD1 LEU A  15      132.867  41.136 126.262  1.00  0.00
ATOM    201 1HD1 LEU A  15      132.500  40.167 125.927  1.00  0.00
ATOM    202 2HD1 LEU A  15      133.560  41.520 125.514  1.00  0.00
ATOM    203 3HD1 LEU A  15      133.389  41.017 127.211  1.00  0.00
ATOM    204  CD2 LEU A  15      130.720  41.505 127.434  1.00  0.00
ATOM    205 1HD2 LEU A  15      130.434  40.503 127.118  1.00  0.00
ATOM    206 2HD2 LEU A  15      131.184  41.453 128.419  1.00  0.00
ATOM    207 3HD2 LEU A  15      129.817  42.110 127.491  1.00  0.00
ATOM    208  C   LEU A  15      131.664  45.854 127.605  1.00  0.00
ATOM    209  O   LEU A  15      131.312  46.071 128.768  1.00  0.00
ATOM    210  N   ALA A  16      132.426  46.711 126.915  1.00  0.00
ATOM    211  H   ALA A  16      132.678  46.487 125.958  1.00  0.00
ATOM    212  CA  ALA A  16      132.896  47.975 127.478  1.00  0.00
ATOM    213  HA  ALA A  16      133.427  47.758 128.406  1.00  0.00
ATOM    214  CB  ALA A  16      133.889  48.608 126.496  1.00  0.00
ATOM    215  HB1 ALA A  16      134.283  49.534 126.916  1.00  0.00
ATOM    216  HB2 ALA A  16      134.717  47.924 126.311  1.00  0.00
ATOM    217  HB3 ALA A  16      133.390  48.831 125.552  1.00  0.00
ATOM    218  C   ALA A  16      131.763  48.962 127.824  1.00  0.00
ATOM    219  O   ALA A  16      131.876  49.680 128.820  1.00  0.00
ATOM    220  N   ALA A  17      130.684  49.003 127.035  1.00  0.00
ATOM    221  H   ALA A  17      130.696  48.463 126.176  1.00  0.00
ATOM    222  CA  ALA A  17      129.519  49.855 127.284  1.00  0.00
ATOM    223  HA  ALA A  17      129.854  50.822 127.659  1.00  0.00
ATOM    224  CB  ALA A  17      128.813  50.096 125.945  1.00  0.00
ATOM    225  HB1 ALA A  17      127.965  50.765 126.098  1.00  0.00
ATOM    226  HB2 ALA A  17      129.506  50.560 125.242  1.00  0.00
ATOM    227  HB3 ALA A  17      128.458  49.154 125.527  1.00  0.00
ATOM    228  C   ALA A  17      128.542  49.260 128.316  1.00  0.00
ATOM    229  O   ALA A  17      128.010  49.972 129.171  1.00  0.00
ATOM    230  N   LEU A  18      128.320  47.944 128.250  1.00  0.00
ATOM    231  H   LEU A  18      128.782  47.431 127.507  1.00  0.00
ATOM    232  CA  LEU A  18      127.367  47.212 129.084  1.00  0.00
ATOM    233  HA  LEU A  18      126.412  47.735 129.032  1.00  0.00
ATOM    234  CB  LEU A  18      127.195  45.797 128.503  1.00  0.00
ATOM    235  HB1 LEU A  18      128.162  45.293 128.530  1.00  0.00
ATOM    236  HB2 LEU A  18      126.893  45.888 127.460  1.00  0.00
ATOM    237  CG  LEU A  18      126.163  44.909 129.222  1.00  0.00
ATOM    238  HG  LEU A  18      126.438  44.791 130.269  1.00  0.00
ATOM    239  CD1 LEU A  18      124.743  45.477 129.135  1.00  0.00
ATOM    240 1HD1 LEU A  18      124.040  44.777 129.583  1.00  0.00
ATOM    241 2HD1 LEU A  18      124.687  46.415 129.687  1.00  0.00
ATOM    242 3HD1 LEU A  18      124.472  45.647 128.094  1.00  0.00
```

ATOM	243	CD2	LEU	A	18	126.166	43.524	128.571	1.00	0.00
ATOM	244	1HD2	LEU	A	18	125.457	42.876	129.085	1.00	0.00
ATOM	245	2HD2	LEU	A	18	125.888	43.606	127.521	1.00	0.00
ATOM	246	3HD2	LEU	A	18	127.161	43.087	128.648	1.00	0.00
ATOM	247	C	LEU	A	18	127.776	47.167	130.558	1.00	0.00
ATOM	248	O	LEU	A	18	126.919	47.268	131.430	1.00	0.00
ATOM	249	N	CYS	A	19	129.074	47.017	130.834	1.00	0.00
ATOM	250	H	CYS	A	19	129.722	46.912	130.062	1.00	0.00
ATOM	251	CA	CYS	A	19	129.588	46.931	132.200	1.00	0.00
ATOM	252	HA	CYS	A	19	129.098	46.076	132.669	1.00	0.00
ATOM	253	CB	CYS	A	19	131.095	46.608	132.138	1.00	0.00
ATOM	254	HB1	CYS	A	19	131.633	47.389	131.603	1.00	0.00
ATOM	255	HB2	CYS	A	19	131.235	45.676	131.589	1.00	0.00
ATOM	256	SG	CYS	A	19	131.800	46.431	133.804	1.00	0.00
ATOM	257	HG	CYS	A	19	130.946	45.511	134.269	1.00	0.00
ATOM	258	C	CYS	A	19	129.231	48.142	133.092	1.00	0.00
ATOM	259	O	CYS	A	19	128.718	47.913	134.189	1.00	0.00
ATOM	260	N	PRO	A	20	129.459	49.419	132.703	1.00	0.00
ATOM	261	CD	PRO	A	20	130.245	49.839	131.557	1.00	0.00
ATOM	262	HD1	PRO	A	20	129.673	50.570	130.988	1.00	0.00
ATOM	263	HD2	PRO	A	20	130.551	49.031	130.902	1.00	0.00
ATOM	264	CG	PRO	A	20	131.461	50.505	132.184	1.00	0.00
ATOM	265	HG1	PRO	A	20	132.010	51.120	131.475	1.00	0.00
ATOM	266	HG2	PRO	A	20	132.115	49.748	132.622	1.00	0.00
ATOM	267	CB	PRO	A	20	130.791	51.324	133.287	1.00	0.00
ATOM	268	HB1	PRO	A	20	130.448	52.271	132.875	1.00	0.00
ATOM	269	HB2	PRO	A	20	131.477	51.505	134.114	1.00	0.00
ATOM	270	CA	PRO	A	20	129.593	50.460	133.723	1.00	0.00
ATOM	271	HA	PRO	A	20	129.878	49.986	134.664	1.00	0.00
ATOM	272	C	PRO	A	20	128.365	51.324	134.036	1.00	0.00
ATOM	273	O	PRO	A	20	128.443	52.081	135.001	1.00	0.00
ATOM	274	N	ALA	A	21	127.285	51.288	133.238	1.00	0.00
ATOM	275	H	ALA	A	21	127.274	50.548	132.545	1.00	0.00
ATOM	276	CA	ALA	A	21	126.169	52.269	133.183	1.00	0.00
ATOM	277	HA	ALA	A	21	125.557	52.000	132.320	1.00	0.00
ATOM	278	CB	ALA	A	21	125.270	52.099	134.414	1.00	0.00
ATOM	279	HB1	ALA	A	21	124.391	52.737	134.316	1.00	0.00
ATOM	280	HB2	ALA	A	21	124.949	51.060	134.494	1.00	0.00
ATOM	281	HB3	ALA	A	21	125.811	52.374	135.320	1.00	0.00
ATOM	282	C	ALA	A	21	126.599	53.744	132.944	1.00	0.00
ATOM	283	O	ALA	A	21	126.138	54.374	131.996	1.00	0.00
ATOM	284	N	SER	A	22	127.613	54.241	133.653	1.00	0.00
ATOM	285	H	SER	A	22	127.923	53.679	134.439	1.00	0.00
ATOM	286	CA	SER	A	22	128.504	55.337	133.247	1.00	0.00
ATOM	287	HA	SER	A	22	127.888	56.181	132.930	1.00	0.00
ATOM	288	CB	SER	A	22	129.339	55.782	134.459	1.00	0.00
ATOM	289	HB1	SER	A	22	130.042	54.994	134.726	1.00	0.00
ATOM	290	HB2	SER	A	22	128.677	55.963	135.305	1.00	0.00
ATOM	291	OG	SER	A	22	130.048	56.974	134.177	1.00	0.00
ATOM	292	HG	SER	A	22	130.467	56.868	133.308	1.00	0.00
ATOM	293	C	SER	A	22	129.421	54.967	132.054	1.00	0.00
ATOM	294	O	SER	A	22	130.471	55.581	131.863	1.00	0.00
ATOM	295	N	ARG	A	23	129.051	53.939	131.273	1.00	0.00
ATOM	296	H	ARG	A	23	128.256	53.401	131.574	1.00	0.00
ATOM	297	CA	ARG	A	23	129.438	53.801	129.861	1.00	0.00
ATOM	298	HA	ARG	A	23	129.690	54.811	129.537	1.00	0.00
ATOM	299	CB	ARG	A	23	130.730	52.977	129.683	1.00	0.00
ATOM	300	HB1	ARG	A	23	130.496	51.932	129.496	1.00	0.00
ATOM	301	HB2	ARG	A	23	131.331	53.064	130.586	1.00	0.00
ATOM	302	CG	ARG	A	23	131.561	53.553	128.525	1.00	0.00
ATOM	303	HG1	ARG	A	23	131.707	54.617	128.722	1.00	0.00

ATOM	304	HG2	ARG	A	23	131.006	53.449	127.593	1.00	0.00
ATOM	305	CD	ARG	A	23	132.948	52.926	128.343	1.00	0.00
ATOM	306	HD1	ARG	A	23	132.837	51.880	128.059	1.00	0.00
ATOM	307	HD2	ARG	A	23	133.493	52.993	129.284	1.00	0.00
ATOM	308	NE	ARG	A	23	133.657	53.656	127.277	1.00	0.00
ATOM	309	HE	ARG	A	23	133.066	54.245	126.689	1.00	0.00
ATOM	310	CZ	ARG	A	23	134.933	53.955	127.153	1.00	0.00
ATOM	311	NH1	ARG	A	23	135.825	53.553	128.004	1.00	0.00
ATOM	312	1HH1	ARG	A	23	135.566	52.945	128.748	1.00	0.00
ATOM	313	2HH1	ARG	A	23	136.713	54.055	127.977	1.00	0.00
ATOM	314	NH2	ARG	A	23	135.337	54.703	126.171	1.00	0.00
ATOM	315	1HH2	ARG	A	23	134.649	55.187	125.585	1.00	0.00
ATOM	316	2HH2	ARG	A	23	136.336	54.864	126.030	1.00	0.00
ATOM	317	C	ARG	A	23	128.269	53.436	128.921	1.00	0.00
ATOM	318	O	ARG	A	23	128.476	53.096	127.765	1.00	0.00
ATOM	319	N	ALA	A	24	127.032	53.571	129.415	1.00	0.00
ATOM	320	H	ALA	A	24	126.942	53.866	130.373	1.00	0.00
ATOM	321	CA	ALA	A	24	125.836	53.739	128.588	1.00	0.00
ATOM	322	HA	ALA	A	24	125.962	53.175	127.662	1.00	0.00
ATOM	323	CB	ALA	A	24	124.616	53.170	129.324	1.00	0.00
ATOM	324	HB1	ALA	A	24	123.751	53.195	128.661	1.00	0.00
ATOM	325	HB2	ALA	A	24	124.807	52.134	129.608	1.00	0.00
ATOM	326	HB3	ALA	A	24	124.393	53.757	130.214	1.00	0.00
ATOM	327	C	ALA	A	24	125.638	55.216	128.198	1.00	0.00
ATOM	328	O	ALA	A	24	125.407	55.524	127.033	1.00	0.00
ATOM	329	N	LEU	A	25	125.828	56.144	129.145	1.00	0.00
ATOM	330	H	LEU	A	25	125.958	55.831	130.097	1.00	0.00
ATOM	331	CA	LEU	A	25	126.362	57.470	128.818	1.00	0.00
ATOM	332	HA	LEU	A	25	125.874	57.853	127.920	1.00	0.00
ATOM	333	CB	LEU	A	25	126.084	58.436	129.992	1.00	0.00
ATOM	334	HB1	LEU	A	25	126.499	58.013	130.908	1.00	0.00
ATOM	335	HB2	LEU	A	25	125.001	58.470	130.121	1.00	0.00
ATOM	336	CG	LEU	A	25	126.569	59.896	129.854	1.00	0.00
ATOM	337	HG	LEU	A	25	125.963	60.495	130.534	1.00	0.00
ATOM	338	CD1	LEU	A	25	128.022	60.097	130.283	1.00	0.00
ATOM	339	1HD1	LEU	A	25	128.264	61.160	130.271	1.00	0.00
ATOM	340	2HD1	LEU	A	25	128.172	59.708	131.289	1.00	0.00
ATOM	341	3HD1	LEU	A	25	128.697	59.589	129.597	1.00	0.00
ATOM	342	CD2	LEU	A	25	126.381	60.478	128.451	1.00	0.00
ATOM	343	1HD2	LEU	A	25	126.595	61.546	128.473	1.00	0.00
ATOM	344	2HD2	LEU	A	25	127.072	60.011	127.749	1.00	0.00
ATOM	345	3HD2	LEU	A	25	125.355	60.329	128.120	1.00	0.00
ATOM	346	C	LEU	A	25	127.851	57.258	128.528	1.00	0.00
ATOM	347	O	LEU	A	25	128.596	56.923	129.446	1.00	0.00
ATOM	348	N	GLU	A	26	128.261	57.321	127.259	1.00	0.00
ATOM	349	H	GLU	A	26	127.593	57.525	126.534	1.00	0.00
ATOM	350	CA	GLU	A	26	129.683	57.219	126.903	1.00	0.00
ATOM	351	HA	GLU	A	26	130.089	56.347	127.415	1.00	0.00
ATOM	352	CB	GLU	A	26	129.848	56.995	125.380	1.00	0.00
ATOM	353	HB1	GLU	A	26	129.180	57.687	124.865	1.00	0.00
ATOM	354	HB2	GLU	A	26	129.506	55.985	125.150	1.00	0.00
ATOM	355	CG	GLU	A	26	131.260	57.187	124.773	1.00	0.00
ATOM	356	HG1	GLU	A	26	131.539	58.239	124.861	1.00	0.00
ATOM	357	HG2	GLU	A	26	131.189	56.969	123.706	1.00	0.00
ATOM	358	CD	GLU	A	26	132.385	56.328	125.377	1.00	0.00
ATOM	359	OE1	GLU	A	26	132.225	55.795	126.497	1.00	0.00
ATOM	360	OE2	GLU	A	26	133.479	56.227	124.777	1.00	0.00
ATOM	361	C	GLU	A	26	130.485	58.440	127.389	1.00	0.00
ATOM	362	O	GLU	A	26	130.021	59.575	127.331	1.00	0.00
ATOM	363	N	GLU	A	27	131.726	58.201	127.817	1.00	0.00
ATOM	364	H	GLU	A	27	132.045	57.237	127.738	1.00	0.00

ATOM	365	CA	GLU	A	27	132.725	59.229	128.122	1.00	0.00
ATOM	366	HA	GLU	A	27	132.721	59.952	127.305	1.00	0.00
ATOM	367	CB	GLU	A	27	132.389	60.023	129.412	1.00	0.00
ATOM	368	HB1	GLU	A	27	131.594	60.736	129.196	1.00	0.00
ATOM	369	HB2	GLU	A	27	133.268	60.613	129.676	1.00	0.00
ATOM	370	CG	GLU	A	27	131.961	59.201	130.646	1.00	0.00
ATOM	371	HG1	GLU	A	27	132.397	58.204	130.593	1.00	0.00
ATOM	372	HG2	GLU	A	27	130.878	59.073	130.630	1.00	0.00
ATOM	373	CD	GLU	A	27	132.382	59.865	131.969	1.00	0.00
ATOM	374	OE1	GLU	A	27	131.938	60.990	132.290	1.00	0.00
ATOM	375	OE2	GLU	A	27	133.163	59.252	132.739	1.00	0.00
ATOM	376	C	GLU	A	27	134.151	58.647	128.208	1.00	0.00
ATOM	377	O	GLU	A	27	134.595	58.202	129.268	1.00	0.00
ATOM	378	N	LYS	A	28	134.887	58.642	127.089	1.00	0.00
ATOM	379	H	LYS	A	28	134.430	58.922	126.235	1.00	0.00
ATOM	380	CA	LYS	A	28	136.305	58.230	127.009	1.00	0.00
ATOM	381	HA	LYS	A	28	136.325	57.164	127.240	1.00	0.00
ATOM	382	CB	LYS	A	28	136.843	58.371	125.572	1.00	0.00
ATOM	383	HB1	LYS	A	28	136.454	57.528	124.999	1.00	0.00
ATOM	384	HB2	LYS	A	28	137.931	58.288	125.595	1.00	0.00
ATOM	385	CG	LYS	A	28	136.442	59.677	124.858	1.00	0.00
ATOM	386	HG1	LYS	A	28	136.700	60.528	125.488	1.00	0.00
ATOM	387	HG2	LYS	A	28	135.362	59.679	124.716	1.00	0.00
ATOM	388	CD	LYS	A	28	137.088	59.883	123.479	1.00	0.00
ATOM	389	HD1	LYS	A	28	138.118	60.221	123.608	1.00	0.00
ATOM	390	HD2	LYS	A	28	136.550	60.687	122.975	1.00	0.00
ATOM	391	CE	LYS	A	28	137.051	58.645	122.572	1.00	0.00
ATOM	392	HE1	LYS	A	28	136.900	58.977	121.541	1.00	0.00
ATOM	393	HE2	LYS	A	28	136.217	57.996	122.853	1.00	0.00
ATOM	394	NZ	LYS	A	28	138.335	57.909	122.637	1.00	0.00
ATOM	395	HZ1	LYS	A	28	139.057	58.513	122.237	1.00	0.00
ATOM	396	HZ2	LYS	A	28	138.587	57.674	123.583	1.00	0.00
ATOM	397	HZ3	LYS	A	28	138.305	57.028	122.128	1.00	0.00
ATOM	398	C	LYS	A	28	137.214	58.899	128.054	1.00	0.00
ATOM	399	O	LYS	A	28	137.210	60.122	128.212	1.00	0.00
ATOM	400	N	LYS	A	29	137.982	58.089	128.791	1.00	0.00
ATOM	401	H	LYS	A	29	137.945	57.092	128.580	1.00	0.00
ATOM	402	CA	LYS	A	29	138.758	58.531	129.960	1.00	0.00
ATOM	403	HA	LYS	A	29	138.144	59.269	130.469	1.00	0.00
ATOM	404	CB	LYS	A	29	138.975	57.346	130.929	1.00	0.00
ATOM	405	HB1	LYS	A	29	140.044	57.170	131.055	1.00	0.00
ATOM	406	HB2	LYS	A	29	138.548	56.433	130.513	1.00	0.00
ATOM	407	CG	LYS	A	29	138.356	57.601	132.320	1.00	0.00
ATOM	408	HG1	LYS	A	29	138.876	58.450	132.767	1.00	0.00
ATOM	409	HG2	LYS	A	29	138.556	56.732	132.949	1.00	0.00
ATOM	410	CD	LYS	A	29	136.839	57.898	132.366	1.00	0.00
ATOM	411	HD1	LYS	A	29	136.599	58.752	131.735	1.00	0.00
ATOM	412	HD2	LYS	A	29	136.578	58.176	133.387	1.00	0.00
ATOM	413	CE	LYS	A	29	135.960	56.713	131.954	1.00	0.00
ATOM	414	HE1	LYS	A	29	136.039	55.939	132.722	1.00	0.00
ATOM	415	HE2	LYS	A	29	136.341	56.304	131.013	1.00	0.00
ATOM	416	NZ	LYS	A	29	134.547	57.133	131.776	1.00	0.00
ATOM	417	HZ1	LYS	A	29	133.894	56.368	131.719	1.00	0.00
ATOM	418	HZ2	LYS	A	29	134.207	57.793	132.479	1.00	0.00
ATOM	419	HZ3	LYS	A	29	134.456	57.652	130.905	1.00	0.00
ATOM	420	C	LYS	A	29	140.039	59.292	129.588	1.00	0.00
ATOM	421	O	LYS	A	29	140.984	58.748	129.022	1.00	0.00
ATOM	422	N	VAL	A	30	140.011	60.594	129.868	1.00	0.00
ATOM	423	H	VAL	A	30	139.166	60.949	130.278	1.00	0.00
ATOM	424	CA	VAL	A	30	141.114	61.538	129.662	1.00	0.00
ATOM	425	HA	VAL	A	30	141.579	61.322	128.705	1.00	0.00

ATOM	426	CB	VAL	A	30	140.601	63.001	129.643	1.00	0.00
ATOM	427	HB	VAL	A	30	141.462	63.667	129.589	1.00	0.00
ATOM	428	CG1	VAL	A	30	139.724	63.288	128.420	1.00	0.00
ATOM	429	1HG1	VAL	A	30	139.295	64.286	128.483	1.00	0.00
ATOM	430	2HG1	VAL	A	30	140.335	63.249	127.521	1.00	0.00
ATOM	431	3HG1	VAL	A	30	138.915	62.561	128.340	1.00	0.00
ATOM	432	CG2	VAL	A	30	139.788	63.400	130.889	1.00	0.00
ATOM	433	1HG2	VAL	A	30	139.527	64.455	130.833	1.00	0.00
ATOM	434	2HG2	VAL	A	30	138.875	62.810	130.952	1.00	0.00
ATOM	435	3HG2	VAL	A	30	140.381	63.244	131.788	1.00	0.00
ATOM	436	C	VAL	A	30	142.191	61.414	130.736	1.00	0.00
ATOM	437	O	VAL	A	30	141.903	61.236	131.917	1.00	0.00
ATOM	438	N	CYS	A	31	143.431	61.648	130.317	1.00	0.00
ATOM	439	H	CYS	A	31	143.575	61.762	129.319	1.00	0.00
ATOM	440	CA	CYS	A	31	144.601	61.822	131.167	1.00	0.00
ATOM	441	HA	CYS	A	31	144.278	62.008	132.192	1.00	0.00
ATOM	442	CB	CYS	A	31	145.455	60.556	131.153	1.00	0.00
ATOM	443	HB1	CYS	A	31	146.404	60.745	131.657	1.00	0.00
ATOM	444	HB2	CYS	A	31	145.676	60.296	130.119	1.00	0.00
ATOM	445	SG	CYS	A	31	144.661	59.145	131.962	1.00	0.00
ATOM	446	C	CYS	A	31	145.387	63.050	130.689	1.00	0.00
ATOM	447	O	CYS	A	31	145.384	63.401	129.505	1.00	0.00
ATOM	448	N	GLN	A	32	146.085	63.705	131.617	1.00	0.00
ATOM	449	H	GLN	A	32	146.116	63.321	132.550	1.00	0.00
ATOM	450	CA	GLN	A	32	147.173	64.583	131.217	1.00	0.00
ATOM	451	HA	GLN	A	32	146.844	65.200	130.380	1.00	0.00
ATOM	452	CB	GLN	A	32	147.562	65.504	132.381	1.00	0.00
ATOM	453	HB1	GLN	A	32	148.176	64.947	133.091	1.00	0.00
ATOM	454	HB2	GLN	A	32	146.663	65.848	132.896	1.00	0.00
ATOM	455	CG	GLN	A	32	148.326	66.734	131.875	1.00	0.00
ATOM	456	HG1	GLN	A	32	147.634	67.423	131.389	1.00	0.00
ATOM	457	HG2	GLN	A	32	149.074	66.439	131.139	1.00	0.00
ATOM	458	CD	GLN	A	32	149.031	67.447	133.013	1.00	0.00
ATOM	459	OE1	GLN	A	32	148.447	68.237	133.752	1.00	0.00
ATOM	460	NE2	GLN	A	32	150.296	67.153	133.222	1.00	0.00
ATOM	461	1HE2	GLN	A	32	150.768	66.502	132.605	1.00	0.00
ATOM	462	2HE2	GLN	A	32	150.782	67.647	133.947	1.00	0.00
ATOM	463	C	GLN	A	32	148.348	63.711	130.774	1.00	0.00
ATOM	464	O	GLN	A	32	148.622	62.694	131.404	1.00	0.00
ATOM	465	N	GLY	A	33	149.045	64.120	129.716	1.00	0.00
ATOM	466	H	GLY	A	33	148.744	64.952	129.232	1.00	0.00
ATOM	467	CA	GLY	A	33	150.311	63.485	129.349	1.00	0.00
ATOM	468	HA1	GLY	A	33	150.568	63.767	128.332	1.00	0.00
ATOM	469	HA2	GLY	A	33	150.197	62.399	129.381	1.00	0.00
ATOM	470	C	GLY	A	33	151.469	63.882	130.280	1.00	0.00
ATOM	471	O	GLY	A	33	151.377	64.828	131.074	1.00	0.00
ATOM	472	N	THR	A	34	152.581	63.167	130.119	1.00	0.00
ATOM	473	H	THR	A	34	152.561	62.493	129.363	1.00	0.00
ATOM	474	CA	THR	A	34	153.913	63.592	130.584	1.00	0.00
ATOM	475	HA	THR	A	34	153.802	64.194	131.485	1.00	0.00
ATOM	476	CB	THR	A	34	154.764	62.363	130.975	1.00	0.00
ATOM	477	HB	THR	A	34	154.190	61.753	131.671	1.00	0.00
ATOM	478	CG2	THR	A	34	155.155	61.496	129.788	1.00	0.00
ATOM	479	1HG2	THR	A	34	155.650	60.596	130.150	1.00	0.00
ATOM	480	2HG2	THR	A	34	154.271	61.204	129.224	1.00	0.00
ATOM	481	3HG2	THR	A	34	155.837	62.035	129.130	1.00	0.00
ATOM	482	OG1	THR	A	34	155.955	62.763	131.623	1.00	0.00
ATOM	483	HG1	THR	A	34	156.642	62.102	131.406	1.00	0.00
ATOM	484	C	THR	A	34	154.602	64.474	129.527	1.00	0.00
ATOM	485	O	THR	A	34	153.996	64.819	128.509	1.00	0.00
ATOM	486	N	SER	A	35	155.852	64.875	129.774	1.00	0.00

ATOM	487	H	SER	A	35	156.241	64.521	130.641	1.00	0.00
ATOM	488	CA	SER	A	35	156.774	65.540	128.830	1.00	0.00
ATOM	489	HA	SER	A	35	156.753	64.990	127.888	1.00	0.00
ATOM	490	CB	SER	A	35	156.340	66.988	128.543	1.00	0.00
ATOM	491	HB1	SER	A	35	157.138	67.527	128.030	1.00	0.00
ATOM	492	HB2	SER	A	35	156.111	67.499	129.478	1.00	0.00
ATOM	493	OG	SER	A	35	155.210	66.981	127.696	1.00	0.00
ATOM	494	HG	SER	A	35	154.658	66.214	127.948	1.00	0.00
ATOM	495	C	SER	A	35	158.233	65.486	129.330	1.00	0.00
ATOM	496	O	SER	A	35	159.009	66.427	129.165	1.00	0.00
ATOM	497	N	ASN	A	36	158.621	64.410	130.022	1.00	0.00
ATOM	498	H	ASN	A	36	158.001	63.611	130.057	1.00	0.00
ATOM	499	CA	ASN	A	36	159.939	64.261	130.655	1.00	0.00
ATOM	500	HA	ASN	A	36	160.156	65.191	131.182	1.00	0.00
ATOM	501	CB	ASN	A	36	159.863	63.148	131.721	1.00	0.00
ATOM	502	HB1	ASN	A	36	159.134	63.425	132.483	1.00	0.00
ATOM	503	HB2	ASN	A	36	160.830	63.037	132.214	1.00	0.00
ATOM	504	CG	ASN	A	36	159.436	61.803	131.155	1.00	0.00
ATOM	505	OD1	ASN	A	36	158.282	61.611	130.811	1.00	0.00
ATOM	506	ND2	ASN	A	36	160.327	60.849	131.058	1.00	0.00
ATOM	507	1HD2	ASN	A	36	161.268	60.992	131.407	1.00	0.00
ATOM	508	2HD2	ASN	A	36	160.101	60.115	130.410	1.00	0.00
ATOM	509	C	ASN	A	36	161.133	64.068	129.685	1.00	0.00
ATOM	510	O	ASN	A	36	162.284	64.097	130.130	1.00	0.00
ATOM	511	N	LYS	A	37	160.885	63.935	128.377	1.00	0.00
ATOM	512	H	LYS	A	37	159.925	63.682	128.148	1.00	0.00
ATOM	513	CA	LYS	A	37	161.832	63.861	127.246	1.00	0.00
ATOM	514	HA	LYS	A	37	161.335	63.266	126.479	1.00	0.00
ATOM	515	CB	LYS	A	37	162.076	65.262	126.628	1.00	0.00
ATOM	516	HB1	LYS	A	37	161.162	65.578	126.126	1.00	0.00
ATOM	517	HB2	LYS	A	37	162.838	65.167	125.852	1.00	0.00
ATOM	518	CG	LYS	A	37	162.468	66.383	127.620	1.00	0.00
ATOM	519	HG1	LYS	A	37	162.734	65.970	128.585	1.00	0.00
ATOM	520	HG2	LYS	A	37	161.589	67.010	127.779	1.00	0.00
ATOM	521	CD	LYS	A	37	163.626	67.285	127.162	1.00	0.00
ATOM	522	HD1	LYS	A	37	163.668	68.156	127.818	1.00	0.00
ATOM	523	HD2	LYS	A	37	163.419	67.646	126.151	1.00	0.00
ATOM	524	CE	LYS	A	37	165.010	66.604	127.173	1.00	0.00
ATOM	525	HE1	LYS	A	37	165.754	67.338	126.848	1.00	0.00
ATOM	526	HE2	LYS	A	37	164.997	65.791	126.439	1.00	0.00
ATOM	527	NZ	LYS	A	37	165.377	66.057	128.506	1.00	0.00
ATOM	528	HZ1	LYS	A	37	166.322	65.702	128.522	1.00	0.00
ATOM	529	HZ2	LYS	A	37	165.251	66.720	129.256	1.00	0.00
ATOM	530	HZ3	LYS	A	37	164.791	65.243	128.702	1.00	0.00
ATOM	531	C	LYS	A	37	163.137	63.115	127.522	1.00	0.00
ATOM	532	O	LYS	A	37	164.163	63.742	127.781	1.00	0.00
ATOM	533	N	LEU	A	38	163.107	61.786	127.474	1.00	0.00
ATOM	534	H	LEU	A	38	162.172	61.382	127.398	1.00	0.00
ATOM	535	CA	LEU	A	38	164.210	60.855	127.774	1.00	0.00
ATOM	536	HA	LEU	A	38	163.714	59.889	127.866	1.00	0.00
ATOM	537	CB	LEU	A	38	165.158	60.668	126.568	1.00	0.00
ATOM	538	HB1	LEU	A	38	164.538	60.398	125.712	1.00	0.00
ATOM	539	HB2	LEU	A	38	165.787	59.802	126.784	1.00	0.00
ATOM	540	CG	LEU	A	38	166.072	61.834	126.140	1.00	0.00
ATOM	541	HG	LEU	A	38	165.472	62.697	125.857	1.00	0.00
ATOM	542	CD1	LEU	A	38	167.084	62.246	127.209	1.00	0.00
ATOM	543	1HD1	LEU	A	38	167.794	62.960	126.794	1.00	0.00
ATOM	544	2HD1	LEU	A	38	166.577	62.721	128.045	1.00	0.00
ATOM	545	3HD1	LEU	A	38	167.624	61.371	127.574	1.00	0.00
ATOM	546	CD2	LEU	A	38	166.865	61.395	124.913	1.00	0.00
ATOM	547	1HD2	LEU	A	38	167.523	62.198	124.582	1.00	0.00

ATOM	548	2HD2	LEU	A	38	167.457	60.510	125.143	1.00	0.00
ATOM	549	3HD2	LEU	A	38	166.172	61.171	124.104	1.00	0.00
ATOM	550	C	LEU	A	38	164.884	61.008	129.152	1.00	0.00
ATOM	551	O	LEU	A	38	165.828	60.286	129.462	1.00	0.00
ATOM	552	N	THR	A	39	164.387	61.906	130.008	1.00	0.00
ATOM	553	H	THR	A	39	163.590	62.457	129.715	1.00	0.00
ATOM	554	CA	THR	A	39	164.843	62.030	131.395	1.00	0.00
ATOM	555	HA	THR	A	39	165.908	61.811	131.428	1.00	0.00
ATOM	556	CB	THR	A	39	164.638	63.446	131.974	1.00	0.00
ATOM	557	HB	THR	A	39	163.641	63.525	132.412	1.00	0.00
ATOM	558	CG2	THR	A	39	165.667	63.757	133.061	1.00	0.00
ATOM	559	1HG2	THR	A	39	165.487	64.758	133.452	1.00	0.00
ATOM	560	2HG2	THR	A	39	165.567	63.041	133.876	1.00	0.00
ATOM	561	3HG2	THR	A	39	166.673	63.701	132.648	1.00	0.00
ATOM	562	OG1	THR	A	39	164.750	64.446	130.979	1.00	0.00
ATOM	563	HG1	THR	A	39	163.847	64.451	130.604	1.00	0.00
ATOM	564	C	THR	A	39	164.102	60.999	132.235	1.00	0.00
ATOM	565	O	THR	A	39	162.882	61.086	132.398	1.00	0.00
ATOM	566	N	GLN	A	40	164.824	59.980	132.708	1.00	0.00
ATOM	567	H	GLN	A	40	165.805	59.926	132.482	1.00	0.00
ATOM	568	CA	GLN	A	40	164.253	58.929	133.548	1.00	0.00
ATOM	569	HA	GLN	A	40	163.279	58.668	133.137	1.00	0.00
ATOM	570	CB	GLN	A	40	165.121	57.660	133.499	1.00	0.00
ATOM	571	HB1	GLN	A	40	165.280	57.369	132.460	1.00	0.00
ATOM	572	HB2	GLN	A	40	164.563	56.856	133.982	1.00	0.00
ATOM	573	CG	GLN	A	40	166.487	57.799	134.198	1.00	0.00
ATOM	574	HG1	GLN	A	40	166.375	58.276	135.170	1.00	0.00
ATOM	575	HG2	GLN	A	40	167.148	58.414	133.587	1.00	0.00
ATOM	576	CD	GLN	A	40	167.121	56.435	134.421	1.00	0.00
ATOM	577	OE1	GLN	A	40	168.007	56.014	133.702	1.00	0.00
ATOM	578	NE2	GLN	A	40	166.678	55.691	135.409	1.00	0.00
ATOM	579	1HE2	GLN	A	40	165.964	56.051	136.038	1.00	0.00
ATOM	580	2HE2	GLN	A	40	167.063	54.766	135.470	1.00	0.00
ATOM	581	C	GLN	A	40	164.018	59.372	134.998	1.00	0.00
ATOM	582	O	GLN	A	40	164.630	60.324	135.485	1.00	0.00
ATOM	583	N	LEU	A	41	163.192	58.599	135.701	1.00	0.00
ATOM	584	H	LEU	A	41	162.685	57.882	135.198	1.00	0.00
ATOM	585	CA	LEU	A	41	163.045	58.639	137.157	1.00	0.00
ATOM	586	HA	LEU	A	41	163.408	59.597	137.531	1.00	0.00
ATOM	587	CB	LEU	A	41	161.536	58.541	137.491	1.00	0.00
ATOM	588	HB1	LEU	A	41	161.417	58.176	138.509	1.00	0.00
ATOM	589	HB2	LEU	A	41	161.066	57.817	136.830	1.00	0.00
ATOM	590	CG	LEU	A	41	160.782	59.886	137.394	1.00	0.00
ATOM	591	HG	LEU	A	41	161.276	60.614	138.038	1.00	0.00
ATOM	592	CD1	LEU	A	41	160.685	60.464	135.981	1.00	0.00
ATOM	593	1HD1	LEU	A	41	160.033	61.337	135.975	1.00	0.00
ATOM	594	2HD1	LEU	A	41	161.667	60.785	135.637	1.00	0.00
ATOM	595	3HD1	LEU	A	41	160.286	59.713	135.297	1.00	0.00
ATOM	596	CD2	LEU	A	41	159.351	59.708	137.902	1.00	0.00
ATOM	597	1HD2	LEU	A	41	158.842	60.671	137.924	1.00	0.00
ATOM	598	2HD2	LEU	A	41	158.805	59.027	137.249	1.00	0.00
ATOM	599	3HD2	LEU	A	41	159.358	59.298	138.912	1.00	0.00
ATOM	600	C	LEU	A	41	163.931	57.555	137.826	1.00	0.00
ATOM	601	O	LEU	A	41	164.768	56.921	137.182	1.00	0.00
ATOM	602	N	GLY	A	42	163.785	57.387	139.148	1.00	0.00
ATOM	603	H	GLY	A	42	163.048	57.886	139.620	1.00	0.00
ATOM	604	CA	GLY	A	42	164.647	56.534	139.970	1.00	0.00
ATOM	605	HA1	GLY	A	42	164.532	56.818	141.016	1.00	0.00
ATOM	606	HA2	GLY	A	42	165.685	56.705	139.684	1.00	0.00
ATOM	607	C	GLY	A	42	164.318	55.028	139.859	1.00	0.00
ATOM	608	O	GLY	A	42	163.178	54.559	139.996	1.00	0.00

ATOM	609	N	THR	A	43	165.404	54.293	139.600	1.00	0.00
ATOM	610	H	THR	A	43	166.275	54.795	139.629	1.00	0.00
ATOM	611	CA	THR	A	43	165.460	53.006	138.888	1.00	0.00
ATOM	612	HA	THR	A	43	166.447	53.009	138.427	1.00	0.00
ATOM	613	CB	THR	A	43	165.505	51.793	139.831	1.00	0.00
ATOM	614	HB	THR	A	43	166.171	52.045	140.656	1.00	0.00
ATOM	615	CG2	THR	A	43	164.173	51.383	140.442	1.00	0.00
ATOM	616	1HG2	THR	A	43	164.291	50.442	140.982	1.00	0.00
ATOM	617	2HG2	THR	A	43	163.843	52.154	141.138	1.00	0.00
ATOM	618	3HG2	THR	A	43	163.429	51.269	139.666	1.00	0.00
ATOM	619	OG1	THR	A	43	166.109	50.703	139.164	1.00	0.00
ATOM	620	HG1	THR	A	43	165.440	50.057	138.838	1.00	0.00
ATOM	621	C	THR	A	43	164.501	52.868	137.689	1.00	0.00
ATOM	622	O	THR	A	43	163.497	53.572	137.539	1.00	0.00
ATOM	623	N	PHE	A	44	164.802	51.946	136.772	1.00	0.00
ATOM	624	H	PHE	A	44	165.565	51.301	136.932	1.00	0.00
ATOM	625	CA	PHE	A	44	163.890	51.696	135.653	1.00	0.00
ATOM	626	HA	PHE	A	44	163.704	52.642	135.150	1.00	0.00
ATOM	627	CB	PHE	A	44	164.536	50.749	134.635	1.00	0.00
ATOM	628	HB1	PHE	A	44	163.825	50.564	133.830	1.00	0.00
ATOM	629	HB2	PHE	A	44	164.734	49.795	135.123	1.00	0.00
ATOM	630	CG	PHE	A	44	165.820	51.266	134.003	1.00	0.00
ATOM	631	CD1	PHE	A	44	165.869	52.547	133.419	1.00	0.00
ATOM	632	HD1	PHE	A	44	165.001	53.184	133.423	1.00	0.00
ATOM	633	CE1	PHE	A	44	167.052	53.006	132.815	1.00	0.00
ATOM	634	HE1	PHE	A	44	167.092	53.989	132.366	1.00	0.00
ATOM	635	CZ	PHE	A	44	168.191	52.185	132.785	1.00	0.00
ATOM	636	HZ	PHE	A	44	169.100	52.541	132.315	1.00	0.00
ATOM	637	CE2	PHE	A	44	168.148	50.903	133.362	1.00	0.00
ATOM	638	HE2	PHE	A	44	169.017	50.266	133.329	1.00	0.00
ATOM	639	CD2	PHE	A	44	166.967	50.448	133.974	1.00	0.00
ATOM	640	HD2	PHE	A	44	166.940	49.460	134.412	1.00	0.00
ATOM	641	C	PHE	A	44	162.516	51.186	136.112	1.00	0.00
ATOM	642	O	PHE	A	44	161.528	51.308	135.400	1.00	0.00
ATOM	643	N	GLU	A	45	162.452	50.646	137.322	1.00	0.00
ATOM	644	H	GLU	A	45	163.344	50.409	137.745	1.00	0.00
ATOM	645	CA	GLU	A	45	161.277	50.041	137.918	1.00	0.00
ATOM	646	HA	GLU	A	45	160.696	49.568	137.124	1.00	0.00
ATOM	647	CB	GLU	A	45	161.722	48.908	138.863	1.00	0.00
ATOM	648	HB1	GLU	A	45	160.833	48.510	139.352	1.00	0.00
ATOM	649	HB2	GLU	A	45	162.373	49.300	139.642	1.00	0.00
ATOM	650	CG	GLU	A	45	162.429	47.732	138.142	1.00	0.00
ATOM	651	HG1	GLU	A	45	161.718	47.297	137.437	1.00	0.00
ATOM	652	HG2	GLU	A	45	162.655	46.965	138.886	1.00	0.00
ATOM	653	CD	GLU	A	45	163.735	48.085	137.393	1.00	0.00
ATOM	654	OE1	GLU	A	45	164.516	48.922	137.918	1.00	0.00
ATOM	655	OE2	GLU	A	45	163.923	47.563	136.262	1.00	0.00
ATOM	656	C	GLU	A	45	160.344	51.079	138.570	1.00	0.00
ATOM	657	O	GLU	A	45	159.141	50.913	138.358	1.00	0.00
ATOM	658	N	ASP	A	46	160.809	52.187	139.214	1.00	0.00
ATOM	659	H	ASP	A	46	161.801	52.358	139.295	1.00	0.00
ATOM	660	CA	ASP	A	46	159.842	53.237	139.617	1.00	0.00
ATOM	661	HA	ASP	A	46	158.871	52.752	139.668	1.00	0.00
ATOM	662	CB	ASP	A	46	160.013	53.787	141.039	1.00	0.00
ATOM	663	HB1	ASP	A	46	160.799	54.541	141.056	1.00	0.00
ATOM	664	HB2	ASP	A	46	160.285	52.975	141.715	1.00	0.00
ATOM	665	CG	ASP	A	46	158.682	54.402	141.527	1.00	0.00
ATOM	666	OD1	ASP	A	46	157.658	53.682	141.605	1.00	0.00
ATOM	667	OD2	ASP	A	46	158.625	55.624	141.778	1.00	0.00
ATOM	668	C	ASP	A	46	159.646	54.318	138.545	1.00	0.00
ATOM	669	O	ASP	A	46	158.603	54.981	138.505	1.00	0.00

ATOM	670	N	HIS	A	47	160.549	54.397	137.566	1.00	0.00
ATOM	671	H	HIS	A	47	161.437	53.919	137.695	1.00	0.00
ATOM	672	CA	HIS	A	47	160.234	54.974	136.257	1.00	0.00
ATOM	673	HA	HIS	A	47	159.928	56.010	136.391	1.00	0.00
ATOM	674	CB	HIS	A	47	161.526	54.956	135.434	1.00	0.00
ATOM	675	HB1	HIS	A	47	161.840	53.927	135.280	1.00	0.00
ATOM	676	HB2	HIS	A	47	162.310	55.470	135.991	1.00	0.00
ATOM	677	CG	HIS	A	47	161.406	55.629	134.101	1.00	0.00
ATOM	678	ND1	HIS	A	47	161.210	56.993	133.905	1.00	0.00
ATOM	679	CE1	HIS	A	47	161.117	57.157	132.576	1.00	0.00
ATOM	680	HE1	HIS	A	47	160.940	58.107	132.080	1.00	0.00
ATOM	681	NE2	HIS	A	47	161.275	55.977	131.951	1.00	0.00
ATOM	682	HE2	HIS	A	47	161.208	55.811	130.949	1.00	0.00
ATOM	683	CD2	HIS	A	47	161.461	54.999	132.895	1.00	0.00
ATOM	684	HD2	HIS	A	47	161.588	53.936	132.724	1.00	0.00
ATOM	685	C	HIS	A	47	159.049	54.269	135.560	1.00	0.00
ATOM	686	O	HIS	A	47	158.049	54.913	135.243	1.00	0.00
ATOM	687	N	PHE	A	48	159.096	52.941	135.394	1.00	0.00
ATOM	688	H	PHE	A	48	159.952	52.455	135.646	1.00	0.00
ATOM	689	CA	PHE	A	48	158.015	52.139	134.805	1.00	0.00
ATOM	690	HA	PHE	A	48	157.780	52.550	133.824	1.00	0.00
ATOM	691	CB	PHE	A	48	158.475	50.685	134.602	1.00	0.00
ATOM	692	HB1	PHE	A	48	158.918	50.325	135.531	1.00	0.00
ATOM	693	HB2	PHE	A	48	159.250	50.662	133.835	1.00	0.00
ATOM	694	CG	PHE	A	48	157.348	49.747	134.200	1.00	0.00
ATOM	695	CD1	PHE	A	48	156.640	49.966	133.002	1.00	0.00
ATOM	696	HD1	PHE	A	48	156.923	50.772	132.343	1.00	0.00
ATOM	697	CE1	PHE	A	48	155.523	49.174	132.681	1.00	0.00
ATOM	698	HE1	PHE	A	48	154.970	49.354	131.769	1.00	0.00
ATOM	699	CZ	PHE	A	48	155.095	48.171	133.565	1.00	0.00
ATOM	700	HZ	PHE	A	48	154.220	47.580	133.325	1.00	0.00
ATOM	701	CE2	PHE	A	48	155.798	47.946	134.763	1.00	0.00
ATOM	702	HE2	PHE	A	48	155.464	47.181	135.447	1.00	0.00
ATOM	703	CD2	PHE	A	48	156.931	48.724	135.072	1.00	0.00
ATOM	704	HD2	PHE	A	48	157.457	48.563	136.002	1.00	0.00
ATOM	705	C	PHE	A	48	156.712	52.173	135.616	1.00	0.00
ATOM	706	O	PHE	A	48	155.636	52.294	135.035	1.00	0.00
ATOM	707	N	LEU	A	49	156.778	52.106	136.950	1.00	0.00
ATOM	708	H	LEU	A	49	157.672	51.921	137.396	1.00	0.00
ATOM	709	CA	LEU	A	49	155.581	52.225	137.787	1.00	0.00
ATOM	710	HA	LEU	A	49	154.816	51.578	137.361	1.00	0.00
ATOM	711	CB	LEU	A	49	155.875	51.747	139.224	1.00	0.00
ATOM	712	HB1	LEU	A	49	155.347	52.388	139.926	1.00	0.00
ATOM	713	HB2	LEU	A	49	156.935	51.866	139.437	1.00	0.00
ATOM	714	CG	LEU	A	49	155.473	50.291	139.529	1.00	0.00
ATOM	715	HG	LEU	A	49	155.876	50.035	140.508	1.00	0.00
ATOM	716	CD1	LEU	A	49	153.950	50.145	139.604	1.00	0.00
ATOM	717	1HD1	LEU	A	49	153.697	49.121	139.877	1.00	0.00
ATOM	718	2HD1	LEU	A	49	153.554	50.817	140.363	1.00	0.00
ATOM	719	3HD1	LEU	A	49	153.489	50.376	138.645	1.00	0.00
ATOM	720	CD2	LEU	A	49	156.010	49.275	138.519	1.00	0.00
ATOM	721	1HD2	LEU	A	49	155.767	48.266	138.847	1.00	0.00
ATOM	722	2HD2	LEU	A	49	155.577	49.442	137.534	1.00	0.00
ATOM	723	3HD2	LEU	A	49	157.093	49.366	138.457	1.00	0.00
ATOM	724	C	LEU	A	49	154.968	53.636	137.756	1.00	0.00
ATOM	725	O	LEU	A	49	153.746	53.742	137.828	1.00	0.00
ATOM	726	N	SER	A	50	155.769	54.698	137.585	1.00	0.00
ATOM	727	H	SER	A	50	156.772	54.551	137.591	1.00	0.00
ATOM	728	CA	SER	A	50	155.278	56.068	137.342	1.00	0.00
ATOM	729	HA	SER	A	50	154.599	56.356	138.133	1.00	0.00
ATOM	730	CB	SER	A	50	156.427	57.085	137.349	1.00	0.00

ATOM	731	HB1	SER	A	50	156.027	58.070	137.109	1.00	0.00
ATOM	732	HB2	SER	A	50	157.176	56.823	136.603	1.00	0.00
ATOM	733	OG	SER	A	50	157.019	57.134	138.625	1.00	0.00
ATOM	734	HG	SER	A	50	157.685	56.411	138.666	1.00	0.00
ATOM	735	C	SER	A	50	154.533	56.193	136.012	1.00	0.00
ATOM	736	O	SER	A	50	153.386	56.646	135.975	1.00	0.00
ATOM	737	N	LEU	A	51	155.169	55.716	134.934	1.00	0.00
ATOM	738	H	LEU	A	51	156.117	55.374	135.055	1.00	0.00
ATOM	739	CA	LEU	A	51	154.555	55.540	133.619	1.00	0.00
ATOM	740	HA	LEU	A	51	154.366	56.525	133.186	1.00	0.00
ATOM	741	CB	LEU	A	51	155.589	54.805	132.724	1.00	0.00
ATOM	742	HB1	LEU	A	51	156.045	54.005	133.297	1.00	0.00
ATOM	743	HB2	LEU	A	51	156.393	55.515	132.527	1.00	0.00
ATOM	744	CG	LEU	A	51	155.184	54.201	131.361	1.00	0.00
ATOM	745	HG	LEU	A	51	156.107	53.925	130.849	1.00	0.00
ATOM	746	CD1	LEU	A	51	154.345	52.922	131.469	1.00	0.00
ATOM	747	1HD1	LEU	A	51	154.366	52.402	130.513	1.00	0.00
ATOM	748	2HD1	LEU	A	51	154.757	52.266	132.237	1.00	0.00
ATOM	749	3HD1	LEU	A	51	153.305	53.150	131.698	1.00	0.00
ATOM	750	CD2	LEU	A	51	154.448	55.194	130.480	1.00	0.00
ATOM	751	1HD2	LEU	A	51	154.267	54.757	129.498	1.00	0.00
ATOM	752	2HD2	LEU	A	51	153.498	55.452	130.936	1.00	0.00
ATOM	753	3HD2	LEU	A	51	155.048	56.097	130.355	1.00	0.00
ATOM	754	C	LEU	A	51	153.196	54.848	133.766	1.00	0.00
ATOM	755	O	LEU	A	51	152.175	55.409	133.372	1.00	0.00
ATOM	756	N	GLN	A	52	153.136	53.680	134.402	1.00	0.00
ATOM	757	H	GLN	A	52	153.995	53.238	134.717	1.00	0.00
ATOM	758	CA	GLN	A	52	151.898	52.919	134.418	1.00	0.00
ATOM	759	HA	GLN	A	52	151.545	52.956	133.390	1.00	0.00
ATOM	760	CB	GLN	A	52	152.160	51.448	134.789	1.00	0.00
ATOM	761	HB1	GLN	A	52	152.374	51.368	135.857	1.00	0.00
ATOM	762	HB2	GLN	A	52	153.031	51.094	134.234	1.00	0.00
ATOM	763	CG	GLN	A	52	150.960	50.558	134.422	1.00	0.00
ATOM	764	HG1	GLN	A	52	150.116	50.783	135.075	1.00	0.00
ATOM	765	HG2	GLN	A	52	151.231	49.513	134.574	1.00	0.00
ATOM	766	CD	GLN	A	52	150.551	50.750	132.965	1.00	0.00
ATOM	767	OE1	GLN	A	52	151.174	50.253	132.051	1.00	0.00
ATOM	768	NE2	GLN	A	52	149.571	51.585	132.680	1.00	0.00
ATOM	769	1HE2	GLN	A	52	149.146	52.147	133.404	1.00	0.00
ATOM	770	2HE2	GLN	A	52	149.413	51.736	131.698	1.00	0.00
ATOM	771	C	GLN	A	52	150.807	53.566	135.274	1.00	0.00
ATOM	772	O	GLN	A	52	149.641	53.551	134.868	1.00	0.00
ATOM	773	N	ARG	A	53	151.168	54.198	136.400	1.00	0.00
ATOM	774	H	ARG	A	53	152.152	54.172	136.661	1.00	0.00
ATOM	775	CA	ARG	A	53	150.232	54.941	137.266	1.00	0.00
ATOM	776	HA	ARG	A	53	149.288	54.398	137.250	1.00	0.00
ATOM	777	CB	ARG	A	53	150.704	54.915	138.738	1.00	0.00
ATOM	778	HB1	ARG	A	53	150.993	53.887	138.969	1.00	0.00
ATOM	779	HB2	ARG	A	53	149.856	55.161	139.380	1.00	0.00
ATOM	780	CG	ARG	A	53	151.862	55.864	139.091	1.00	0.00
ATOM	781	HG1	ARG	A	53	151.476	56.870	139.258	1.00	0.00
ATOM	782	HG2	ARG	A	53	152.539	55.902	138.249	1.00	0.00
ATOM	783	CD	ARG	A	53	152.620	55.376	140.342	1.00	0.00
ATOM	784	HD1	ARG	A	53	152.849	54.317	140.213	1.00	0.00
ATOM	785	HD2	ARG	A	53	151.975	55.486	141.216	1.00	0.00
ATOM	786	NE	ARG	A	53	153.884	56.112	140.566	1.00	0.00
ATOM	787	HE	ARG	A	53	153.949	57.050	140.210	1.00	0.00
ATOM	788	CZ	ARG	A	53	154.984	55.661	141.157	1.00	0.00
ATOM	789	NH1	ARG	A	53	155.066	54.487	141.703	1.00	0.00
ATOM	790	1HH1	ARG	A	53	154.301	53.853	141.685	1.00	0.00
ATOM	791	2HH1	ARG	A	53	156.018	54.148	141.891	1.00	0.00

ATOM	792	NH2	ARG	A	53	156.054	56.389	141.202	1.00	0.00
ATOM	793	1HH2	ARG	A	53	156.198	57.138	140.547	1.00	0.00
ATOM	794	2HH2	ARG	A	53	156.919	55.983	141.582	1.00	0.00
ATOM	795	C	ARG	A	53	149.858	56.340	136.755	1.00	0.00
ATOM	796	O	ARG	A	53	149.052	56.994	137.411	1.00	0.00
ATOM	797	N	MET	A	54	150.334	56.759	135.573	1.00	0.00
ATOM	798	H	MET	A	54	151.126	56.271	135.174	1.00	0.00
ATOM	799	CA	MET	A	54	149.775	57.922	134.844	1.00	0.00
ATOM	800	HA	MET	A	54	148.865	58.226	135.363	1.00	0.00
ATOM	801	CB	MET	A	54	150.754	59.108	134.925	1.00	0.00
ATOM	802	HB1	MET	A	54	151.472	59.050	134.107	1.00	0.00
ATOM	803	HB2	MET	A	54	151.307	59.057	135.865	1.00	0.00
ATOM	804	CG	MET	A	54	150.021	60.461	134.897	1.00	0.00
ATOM	805	HG1	MET	A	54	149.327	60.483	135.738	1.00	0.00
ATOM	806	HG2	MET	A	54	149.435	60.541	133.982	1.00	0.00
ATOM	807	SD	MET	A	54	151.083	61.928	135.019	1.00	0.00
ATOM	808	CE	MET	A	54	151.732	61.965	133.327	1.00	0.00
ATOM	809	HE1	MET	A	54	152.373	62.837	133.206	1.00	0.00
ATOM	810	HE2	MET	A	54	152.304	61.059	133.131	1.00	0.00
ATOM	811	HE3	MET	A	54	150.903	62.027	132.620	1.00	0.00
ATOM	812	C	MET	A	54	149.310	57.622	133.391	1.00	0.00
ATOM	813	O	MET	A	54	148.888	58.531	132.686	1.00	0.00
ATOM	814	N	PHE	A	55	149.383	56.359	132.939	1.00	0.00
ATOM	815	H	PHE	A	55	149.882	55.690	133.507	1.00	0.00
ATOM	816	CA	PHE	A	55	148.977	55.928	131.585	1.00	0.00
ATOM	817	HA	PHE	A	55	148.526	56.769	131.059	1.00	0.00
ATOM	818	CB	PHE	A	55	150.196	55.490	130.743	1.00	0.00
ATOM	819	HB1	PHE	A	55	149.806	54.969	129.868	1.00	0.00
ATOM	820	HB2	PHE	A	55	150.759	54.745	131.305	1.00	0.00
ATOM	821	CG	PHE	A	55	151.170	56.553	130.229	1.00	0.00
ATOM	822	CD1	PHE	A	55	151.613	56.495	128.890	1.00	0.00
ATOM	823	HD1	PHE	A	55	151.186	55.771	128.212	1.00	0.00
ATOM	824	CE1	PHE	A	55	152.659	57.326	128.441	1.00	0.00
ATOM	825	HE1	PHE	A	55	153.030	57.227	127.435	1.00	0.00
ATOM	826	CZ	PHE	A	55	153.257	58.243	129.320	1.00	0.00
ATOM	827	HZ	PHE	A	55	154.096	58.844	129.007	1.00	0.00
ATOM	828	CE2	PHE	A	55	152.801	58.336	130.642	1.00	0.00
ATOM	829	HE2	PHE	A	55	153.278	59.011	131.337	1.00	0.00
ATOM	830	CD2	PHE	A	55	151.769	57.496	131.091	1.00	0.00
ATOM	831	HD2	PHE	A	55	151.500	57.539	132.132	1.00	0.00
ATOM	832	C	PHE	A	55	147.904	54.809	131.603	1.00	0.00
ATOM	833	O	PHE	A	55	147.325	54.475	130.569	1.00	0.00
ATOM	834	N	ASN	A	56	147.608	54.209	132.765	1.00	0.00
ATOM	835	H	ASN	A	56	148.095	54.506	133.598	1.00	0.00
ATOM	836	CA	ASN	A	56	146.543	53.205	132.910	1.00	0.00
ATOM	837	HA	ASN	A	56	146.761	52.374	132.235	1.00	0.00
ATOM	838	CB	ASN	A	56	146.543	52.683	134.359	1.00	0.00
ATOM	839	HB1	ASN	A	56	146.347	53.513	135.038	1.00	0.00
ATOM	840	HB2	ASN	A	56	147.507	52.247	134.604	1.00	0.00
ATOM	841	CG	ASN	A	56	145.501	51.601	134.577	1.00	0.00
ATOM	842	OD1	ASN	A	56	145.190	50.830	133.686	1.00	0.00
ATOM	843	ND2	ASN	A	56	144.906	51.523	135.743	1.00	0.00
ATOM	844	1HD2	ASN	A	56	145.141	52.152	136.487	1.00	0.00
ATOM	845	2HD2	ASN	A	56	144.237	50.780	135.846	1.00	0.00
ATOM	846	C	ASN	A	56	145.157	53.765	132.527	1.00	0.00
ATOM	847	O	ASN	A	56	144.801	54.859	132.956	1.00	0.00
ATOM	848	N	ASN	A	57	144.358	53.013	131.764	1.00	0.00
ATOM	849	H	ASN	A	57	144.688	52.082	131.554	1.00	0.00
ATOM	850	CA	ASN	A	57	143.074	53.389	131.141	1.00	0.00
ATOM	851	HA	ASN	A	57	142.961	52.692	130.310	1.00	0.00
ATOM	852	CB	ASN	A	57	141.880	53.070	132.083	1.00	0.00

ATOM	853	HB1	ASN	A	57	142.166	52.272	132.765	1.00	0.00
ATOM	854	HB2	ASN	A	57	141.068	52.696	131.460	1.00	0.00
ATOM	855	CG	ASN	A	57	141.289	54.216	132.901	1.00	0.00
ATOM	856	OD1	ASN	A	57	140.083	54.349	133.053	1.00	0.00
ATOM	857	ND2	ASN	A	57	142.101	55.034	133.533	1.00	0.00
ATOM	858	1HD2	ASN	A	57	143.100	55.006	133.360	1.00	0.00
ATOM	859	2HD2	ASN	A	57	141.682	55.795	134.032	1.00	0.00
ATOM	860	C	ASN	A	57	143.009	54.779	130.442	1.00	0.00
ATOM	861	O	ASN	A	57	141.920	55.275	130.163	1.00	0.00
ATOM	862	N	CYS	A	58	144.157	55.397	130.154	1.00	0.00
ATOM	863	H	CYS	A	58	145.024	54.946	130.413	1.00	0.00
ATOM	864	CA	CYS	A	58	144.259	56.718	129.543	1.00	0.00
ATOM	865	HA	CYS	A	58	143.525	57.376	130.012	1.00	0.00
ATOM	866	CB	CYS	A	58	145.653	57.285	129.829	1.00	0.00
ATOM	867	HB1	CYS	A	58	145.768	58.212	129.267	1.00	0.00
ATOM	868	HB2	CYS	A	58	146.403	56.584	129.463	1.00	0.00
ATOM	869	SG	CYS	A	58	145.960	57.643	131.573	1.00	0.00
ATOM	870	C	CYS	A	58	143.967	56.712	128.047	1.00	0.00
ATOM	871	O	CYS	A	58	144.871	56.768	127.214	1.00	0.00
ATOM	872	N	GLU	A	59	142.680	56.625	127.709	1.00	0.00
ATOM	873	H	GLU	A	59	141.996	56.574	128.458	1.00	0.00
ATOM	874	CA	GLU	A	59	142.193	56.592	126.332	1.00	0.00
ATOM	875	HA	GLU	A	59	142.559	55.682	125.869	1.00	0.00
ATOM	876	CB	GLU	A	59	140.664	56.570	126.294	1.00	0.00
ATOM	877	HB1	GLU	A	59	140.338	56.583	125.254	1.00	0.00
ATOM	878	HB2	GLU	A	59	140.262	57.455	126.787	1.00	0.00
ATOM	879	CG	GLU	A	59	140.122	55.314	126.982	1.00	0.00
ATOM	880	HG1	GLU	A	59	140.386	55.332	128.039	1.00	0.00
ATOM	881	HG2	GLU	A	59	140.567	54.425	126.531	1.00	0.00
ATOM	882	CD	GLU	A	59	138.607	55.252	126.848	1.00	0.00
ATOM	883	OE1	GLU	A	59	138.098	55.082	125.721	1.00	0.00
ATOM	884	OE2	GLU	A	59	137.898	55.383	127.868	1.00	0.00
ATOM	885	C	GLU	A	59	142.751	57.733	125.499	1.00	0.00
ATOM	886	O	GLU	A	59	143.236	57.471	124.396	1.00	0.00
ATOM	887	N	VAL	A	60	142.722	58.950	126.055	1.00	0.00
ATOM	888	H	VAL	A	60	142.388	59.019	127.011	1.00	0.00
ATOM	889	CA	VAL	A	60	143.013	60.199	125.349	1.00	0.00
ATOM	890	HA	VAL	A	60	143.592	59.925	124.478	1.00	0.00
ATOM	891	CB	VAL	A	60	141.704	60.820	124.808	1.00	0.00
ATOM	892	HB	VAL	A	60	141.405	60.217	123.952	1.00	0.00
ATOM	893	CG1	VAL	A	60	140.523	60.778	125.786	1.00	0.00
ATOM	894	1HG1	VAL	A	60	139.667	61.301	125.360	1.00	0.00
ATOM	895	2HG1	VAL	A	60	140.229	59.749	125.985	1.00	0.00
ATOM	896	3HG1	VAL	A	60	140.808	61.249	126.716	1.00	0.00
ATOM	897	CG2	VAL	A	60	141.891	62.257	124.312	1.00	0.00
ATOM	898	1HG2	VAL	A	60	141.003	62.571	123.765	1.00	0.00
ATOM	899	2HG2	VAL	A	60	142.056	62.931	125.153	1.00	0.00
ATOM	900	3HG2	VAL	A	60	142.747	62.307	123.640	1.00	0.00
ATOM	901	C	VAL	A	60	143.892	61.147	126.178	1.00	0.00
ATOM	902	O	VAL	A	60	143.606	61.438	127.340	1.00	0.00
ATOM	903	N	VAL	A	61	144.987	61.614	125.566	1.00	0.00
ATOM	904	H	VAL	A	61	145.126	61.325	124.603	1.00	0.00
ATOM	905	CA	VAL	A	61	146.145	62.189	126.269	1.00	0.00
ATOM	906	HA	VAL	A	61	145.934	62.168	127.339	1.00	0.00
ATOM	907	CB	VAL	A	61	147.393	61.308	126.083	1.00	0.00
ATOM	908	HB	VAL	A	61	147.668	61.265	125.031	1.00	0.00
ATOM	909	CG1	VAL	A	61	148.581	61.853	126.890	1.00	0.00
ATOM	910	1HG1	VAL	A	61	149.435	61.183	126.798	1.00	0.00
ATOM	911	2HG1	VAL	A	61	148.872	62.836	126.520	1.00	0.00
ATOM	912	3HG1	VAL	A	61	148.305	61.920	127.942	1.00	0.00
ATOM	913	CG2	VAL	A	61	147.090	59.886	126.574	1.00	0.00

```
ATOM    914 1HG2 VAL A  61     147.995  59.293 126.585  1.00  0.00
ATOM    915 2HG2 VAL A  61     146.684  59.911 127.584  1.00  0.00
ATOM    916 3HG2 VAL A  61     146.376  59.396 125.912  1.00  0.00
ATOM    917  C   VAL A  61     146.385  63.651 125.892  1.00  0.00
ATOM    918  O   VAL A  61     146.902  63.993 124.821  1.00  0.00
ATOM    919  N   LEU A  62     145.992  64.506 126.835  1.00  0.00
ATOM    920  H   LEU A  62     145.577  64.088 127.665  1.00  0.00
ATOM    921  CA  LEU A  62     146.058  65.967 126.809  1.00  0.00
ATOM    922  HA  LEU A  62     146.092  66.302 125.772  1.00  0.00
ATOM    923  CB  LEU A  62     144.778  66.531 127.459  1.00  0.00
ATOM    924  HB1 LEU A  62     144.825  67.621 127.418  1.00  0.00
ATOM    925  HB2 LEU A  62     144.759  66.236 128.510  1.00  0.00
ATOM    926  CG  LEU A  62     143.464  66.060 126.796  1.00  0.00
ATOM    927  HG  LEU A  62     143.344  64.986 126.943  1.00  0.00
ATOM    928  CD1 LEU A  62     142.273  66.752 127.458  1.00  0.00
ATOM    929 1HD1 LEU A  62     141.344  66.422 126.994  1.00  0.00
ATOM    930 2HD1 LEU A  62     142.245  66.488 128.514  1.00  0.00
ATOM    931 3HD1 LEU A  62     142.370  67.832 127.363  1.00  0.00
ATOM    932  CD2 LEU A  62     143.433  66.348 125.294  1.00  0.00
ATOM    933 1HD2 LEU A  62     142.448  66.115 124.889  1.00  0.00
ATOM    934 2HD2 LEU A  62     143.675  67.393 125.097  1.00  0.00
ATOM    935 3HD2 LEU A  62     144.154  65.712 124.783  1.00  0.00
ATOM    936  C   LEU A  62     147.360  66.442 127.477  1.00  0.00
ATOM    937  O   LEU A  62     147.407  66.914 128.613  1.00  0.00
ATOM    938  N   GLY A  63     148.440  66.200 126.743  1.00  0.00
ATOM    939  H   GLY A  63     148.264  65.736 125.855  1.00  0.00
ATOM    940  CA  GLY A  63     149.862  66.405 127.040  1.00  0.00
ATOM    941  HA1 GLY A  63     150.106  66.082 128.051  1.00  0.00
ATOM    942  HA2 GLY A  63     150.132  67.453 126.913  1.00  0.00
ATOM    943  C   GLY A  63     150.622  65.548 126.011  1.00  0.00
ATOM    944  O   GLY A  63     150.055  65.261 124.950  1.00  0.00
ATOM    945  N   ASN A  64     151.840  65.079 126.306  1.00  0.00
ATOM    946  H   ASN A  64     152.289  65.305 127.183  1.00  0.00
ATOM    947  CA  ASN A  64     152.495  64.073 125.459  1.00  0.00
ATOM    948  HA  ASN A  64     152.006  64.047 124.483  1.00  0.00
ATOM    949  CB  ASN A  64     153.977  64.437 125.196  1.00  0.00
ATOM    950  HB1 ASN A  64     154.455  63.585 124.716  1.00  0.00
ATOM    951  HB2 ASN A  64     154.502  64.608 126.129  1.00  0.00
ATOM    952  CG  ASN A  64     154.190  65.628 124.285  1.00  0.00
ATOM    953  OD1 ASN A  64     153.264  66.196 123.739  1.00  0.00
ATOM    954  ND2 ASN A  64     155.412  66.033 124.048  1.00  0.00
ATOM    955 1HD2 ASN A  64     156.192  65.529 124.433  1.00  0.00
ATOM    956 2HD2 ASN A  64     155.548  66.814 123.422  1.00  0.00
ATOM    957  C   ASN A  64     152.397  62.656 126.044  1.00  0.00
ATOM    958  O   ASN A  64     152.368  62.435 127.260  1.00  0.00
ATOM    959  N   LEU A  65     152.415  61.678 125.139  1.00  0.00
ATOM    960  H   LEU A  65     152.492  61.946 124.161  1.00  0.00
ATOM    961  CA  LEU A  65     152.779  60.308 125.473  1.00  0.00
ATOM    962  HA  LEU A  65     152.446  60.066 126.481  1.00  0.00
ATOM    963  CB  LEU A  65     152.132  59.355 124.459  1.00  0.00
ATOM    964  HB1 LEU A  65     152.663  58.400 124.461  1.00  0.00
ATOM    965  HB2 LEU A  65     152.234  59.785 123.462  1.00  0.00
ATOM    966  CG  LEU A  65     150.654  59.096 124.764  1.00  0.00
ATOM    967  HG  LEU A  65     150.136  60.038 124.935  1.00  0.00
ATOM    968  CD1 LEU A  65     149.998  58.411 123.574  1.00  0.00
ATOM    969 1HD1 LEU A  65     148.925  58.386 123.752  1.00  0.00
ATOM    970 2HD1 LEU A  65     150.187  58.975 122.666  1.00  0.00
ATOM    971 3HD1 LEU A  65     150.391  57.401 123.458  1.00  0.00
ATOM    972  CD2 LEU A  65     150.501  58.186 125.984  1.00  0.00
ATOM    973 1HD2 LEU A  65     149.473  57.844 126.074  1.00  0.00
ATOM    974 2HD2 LEU A  65     151.136  57.308 125.880  1.00  0.00
```

ATOM	975	3HD2	LEU	A	65	150.759	58.726	126.894	1.00	0.00
ATOM	976	C	LEU	A	65	154.298	60.150	125.425	1.00	0.00
ATOM	977	O	LEU	A	65	154.931	60.547	124.453	1.00	0.00
ATOM	978	N	GLU	A	66	154.854	59.481	126.432	1.00	0.00
ATOM	979	H	GLU	A	66	154.278	59.175	127.199	1.00	0.00
ATOM	980	CA	GLU	A	66	156.270	59.127	126.487	1.00	0.00
ATOM	981	HA	GLU	A	66	156.667	59.117	125.476	1.00	0.00
ATOM	982	CB	GLU	A	66	157.069	60.163	127.283	1.00	0.00
ATOM	983	HB1	GLU	A	66	156.933	60.014	128.351	1.00	0.00
ATOM	984	HB2	GLU	A	66	156.693	61.147	127.026	1.00	0.00
ATOM	985	CG	GLU	A	66	158.565	60.064	126.958	1.00	0.00
ATOM	986	HG1	GLU	A	66	158.702	60.043	125.876	1.00	0.00
ATOM	987	HG2	GLU	A	66	158.958	59.129	127.368	1.00	0.00
ATOM	988	CD	GLU	A	66	159.351	61.252	127.517	1.00	0.00
ATOM	989	OE1	GLU	A	66	158.854	62.401	127.403	1.00	0.00
ATOM	990	OE2	GLU	A	66	160.488	61.035	127.994	1.00	0.00
ATOM	991	C	GLU	A	66	156.436	57.728	127.091	1.00	0.00
ATOM	992	O	GLU	A	66	156.363	57.533	128.307	1.00	0.00
ATOM	993	N	ILE	A	67	156.650	56.744	126.221	1.00	0.00
ATOM	994	H	ILE	A	67	156.731	56.985	125.235	1.00	0.00
ATOM	995	CA	ILE	A	67	157.223	55.469	126.647	1.00	0.00
ATOM	996	HA	ILE	A	67	156.968	55.285	127.690	1.00	0.00
ATOM	997	CB	ILE	A	67	156.735	54.249	125.825	1.00	0.00
ATOM	998	HB	ILE	A	67	157.274	54.231	124.880	1.00	0.00
ATOM	999	CG2	ILE	A	67	157.086	52.960	126.598	1.00	0.00
ATOM	1000	1HG2	ILE	A	67	156.876	52.083	125.988	1.00	0.00
ATOM	1001	2HG2	ILE	A	67	158.147	52.938	126.842	1.00	0.00
ATOM	1002	3HG2	ILE	A	67	156.508	52.908	127.520	1.00	0.00
ATOM	1003	CG1	ILE	A	67	155.220	54.304	125.525	1.00	0.00
ATOM	1004	1HG1	ILE	A	67	154.989	55.227	124.995	1.00	0.00
ATOM	1005	2HG1	ILE	A	67	154.672	54.311	126.466	1.00	0.00
ATOM	1006	CD	ILE	A	67	154.722	53.150	124.645	1.00	0.00
ATOM	1007	HD1	ILE	A	67	154.749	52.207	125.190	1.00	0.00
ATOM	1008	HD2	ILE	A	67	153.695	53.343	124.336	1.00	0.00
ATOM	1009	HD3	ILE	A	67	155.343	53.070	123.755	1.00	0.00
ATOM	1010	C	ILE	A	67	158.736	55.617	126.523	1.00	0.00
ATOM	1011	O	ILE	A	67	159.258	55.930	125.451	1.00	0.00
ATOM	1012	N	THR	A	68	159.472	55.398	127.606	1.00	0.00
ATOM	1013	H	THR	A	68	159.029	55.159	128.479	1.00	0.00
ATOM	1014	CA	THR	A	68	160.928	55.379	127.495	1.00	0.00
ATOM	1015	HA	THR	A	68	161.137	54.911	126.540	1.00	0.00
ATOM	1016	CB	THR	A	68	161.534	56.797	127.471	1.00	0.00
ATOM	1017	HB	THR	A	68	161.043	57.376	126.687	1.00	0.00
ATOM	1018	CG2	THR	A	68	161.402	57.567	128.785	1.00	0.00
ATOM	1019	1HG2	THR	A	68	161.721	58.597	128.625	1.00	0.00
ATOM	1020	2HG2	THR	A	68	160.364	57.573	129.113	1.00	0.00
ATOM	1021	3HG2	THR	A	68	162.037	57.115	129.545	1.00	0.00
ATOM	1022	OG1	THR	A	68	162.907	56.722	127.167	1.00	0.00
ATOM	1023	HG1	THR	A	68	162.995	56.172	126.368	1.00	0.00
ATOM	1024	C	THR	A	68	161.584	54.497	128.528	1.00	0.00
ATOM	1025	O	THR	A	68	161.063	54.374	129.636	1.00	0.00
ATOM	1026	N	TYR	A	69	162.700	53.864	128.159	1.00	0.00
ATOM	1027	H	TYR	A	69	163.071	54.069	127.238	1.00	0.00
ATOM	1028	CA	TYR	A	69	163.495	53.001	129.070	1.00	0.00
ATOM	1029	HA	TYR	A	69	164.283	52.578	128.451	1.00	0.00
ATOM	1030	CB	TYR	A	69	164.225	53.851	130.125	1.00	0.00
ATOM	1031	HB1	TYR	A	69	164.577	53.209	130.931	1.00	0.00
ATOM	1032	HB2	TYR	A	69	163.537	54.563	130.580	1.00	0.00
ATOM	1033	CG	TYR	A	69	165.446	54.586	129.607	1.00	0.00
ATOM	1034	CD1	TYR	A	69	166.647	53.882	129.382	1.00	0.00
ATOM	1035	HD1	TYR	A	69	166.689	52.813	129.550	1.00	0.00

ATOM	1036	CE1	TYR	A	69	167.798	54.572	128.952	1.00	0.00
ATOM	1037	HE1	TYR	A	69	168.722	54.041	128.776	1.00	0.00
ATOM	1038	CZ	TYR	A	69	167.749	55.968	128.755	1.00	0.00
ATOM	1039	OH	TYR	A	69	168.846	56.639	128.323	1.00	0.00
ATOM	1040	HH	TYR	A	69	168.653	57.573	128.266	1.00	0.00
ATOM	1041	CE2	TYR	A	69	166.545	56.669	128.974	1.00	0.00
ATOM	1042	HE2	TYR	A	69	166.504	57.736	128.824	1.00	0.00
ATOM	1043	CD2	TYR	A	69	165.396	55.975	129.388	1.00	0.00
ATOM	1044	HD2	TYR	A	69	164.474	56.511	129.546	1.00	0.00
ATOM	1045	C	TYR	A	69	162.836	51.745	129.696	1.00	0.00
ATOM	1046	O	TYR	A	69	163.521	51.007	130.404	1.00	0.00
ATOM	1047	N	VAL	A	70	161.561	51.429	129.450	1.00	0.00
ATOM	1048	H	VAL	A	70	161.003	52.079	128.919	1.00	0.00
ATOM	1049	CA	VAL	A	70	160.919	50.214	129.997	1.00	0.00
ATOM	1050	HA	VAL	A	70	161.031	50.259	131.081	1.00	0.00
ATOM	1051	CB	VAL	A	70	159.406	50.177	129.705	1.00	0.00
ATOM	1052	HB	VAL	A	70	159.239	50.056	128.635	1.00	0.00
ATOM	1053	CG1	VAL	A	70	158.769	49.016	130.468	1.00	0.00
ATOM	1054	1HG1	VAL	A	70	157.688	49.030	130.358	1.00	0.00
ATOM	1055	2HG1	VAL	A	70	159.136	48.063	130.089	1.00	0.00
ATOM	1056	3HG1	VAL	A	70	159.001	49.100	131.527	1.00	0.00
ATOM	1057	CG2	VAL	A	70	158.709	51.464	130.166	1.00	0.00
ATOM	1058	1HG2	VAL	A	70	157.632	51.378	130.022	1.00	0.00
ATOM	1059	2HG2	VAL	A	70	158.915	51.652	131.220	1.00	0.00
ATOM	1060	3HG2	VAL	A	70	159.054	52.320	129.587	1.00	0.00
ATOM	1061	C	VAL	A	70	161.619	48.937	129.516	1.00	0.00
ATOM	1062	O	VAL	A	70	161.732	48.716	128.314	1.00	0.00
ATOM	1063	N	GLN	A	71	162.117	48.120	130.452	1.00	0.00
ATOM	1064	H	GLN	A	71	162.004	48.363	131.421	1.00	0.00
ATOM	1065	CA	GLN	A	71	162.937	46.938	130.162	1.00	0.00
ATOM	1066	HA	GLN	A	71	163.672	47.209	129.404	1.00	0.00
ATOM	1067	CB	GLN	A	71	163.688	46.480	131.430	1.00	0.00
ATOM	1068	HB1	GLN	A	71	164.300	45.621	131.153	1.00	0.00
ATOM	1069	HB2	GLN	A	71	162.972	46.135	132.176	1.00	0.00
ATOM	1070	CG	GLN	A	71	164.610	47.524	132.089	1.00	0.00
ATOM	1071	HG1	GLN	A	71	164.028	48.148	132.768	1.00	0.00
ATOM	1072	HG2	GLN	A	71	165.052	48.159	131.322	1.00	0.00
ATOM	1073	CD	GLN	A	71	165.753	46.841	132.846	1.00	0.00
ATOM	1074	OE1	GLN	A	71	166.619	46.221	132.262	1.00	0.00
ATOM	1075	NE2	GLN	A	71	165.817	46.909	134.162	1.00	0.00
ATOM	1076	1HE2	GLN	A	71	165.105	47.356	134.746	1.00	0.00
ATOM	1077	2HE2	GLN	A	71	166.598	46.457	134.599	1.00	0.00
ATOM	1078	C	GLN	A	71	162.134	45.732	129.653	1.00	0.00
ATOM	1079	O	GLN	A	71	160.962	45.562	130.006	1.00	0.00
ATOM	1080	N	ARG	A	72	162.852	44.858	128.937	1.00	0.00
ATOM	1081	H	ARG	A	72	163.691	45.281	128.562	1.00	0.00
ATOM	1082	CA	ARG	A	72	162.856	43.382	128.755	1.00	0.00
ATOM	1083	HA	ARG	A	72	162.921	43.196	127.679	1.00	0.00
ATOM	1084	CB	ARG	A	72	164.195	42.942	129.403	1.00	0.00
ATOM	1085	HB1	ARG	A	72	164.112	43.061	130.484	1.00	0.00
ATOM	1086	HB2	ARG	A	72	164.972	43.633	129.072	1.00	0.00
ATOM	1087	CG	ARG	A	72	164.752	41.531	129.132	1.00	0.00
ATOM	1088	HG1	ARG	A	72	164.138	40.782	129.634	1.00	0.00
ATOM	1089	HG2	ARG	A	72	165.750	41.481	129.570	1.00	0.00
ATOM	1090	CD	ARG	A	72	164.831	41.204	127.639	1.00	0.00
ATOM	1091	HD1	ARG	A	72	164.793	42.130	127.061	1.00	0.00
ATOM	1092	HD2	ARG	A	72	163.950	40.611	127.387	1.00	0.00
ATOM	1093	NE	ARG	A	72	166.061	40.472	127.257	1.00	0.00
ATOM	1094	HE	ARG	A	72	166.848	40.527	127.877	1.00	0.00
ATOM	1095	CZ	ARG	A	72	166.267	39.938	126.063	1.00	0.00
ATOM	1096	NH1	ARG	A	72	165.331	39.901	125.168	1.00	0.00

ATOM	1097	1HH1	ARG	A	72	164.466	40.412	125.394	1.00	0.00
ATOM	1098	2HH1	ARG	A	72	165.611	39.931	124.206	1.00	0.00
ATOM	1099	NH2	ARG	A	72	167.438	39.475	125.723	1.00	0.00
ATOM	1100	1HH2	ARG	A	72	168.261	39.719	126.273	1.00	0.00
ATOM	1101	2HH2	ARG	A	72	167.556	39.116	124.798	1.00	0.00
ATOM	1102	C	ARG	A	72	161.708	42.465	129.216	1.00	0.00
ATOM	1103	O	ARG	A	72	161.698	41.302	128.812	1.00	0.00
ATOM	1104	N	ASN	A	73	160.787	42.878	130.081	1.00	0.00
ATOM	1105	H	ASN	A	73	160.842	43.835	130.401	1.00	0.00
ATOM	1106	CA	ASN	A	73	159.776	41.977	130.655	1.00	0.00
ATOM	1107	HA	ASN	A	73	159.448	41.292	129.871	1.00	0.00
ATOM	1108	CB	ASN	A	73	160.449	41.177	131.799	1.00	0.00
ATOM	1109	HB1	ASN	A	73	159.983	41.423	132.755	1.00	0.00
ATOM	1110	HB2	ASN	A	73	161.510	41.418	131.875	1.00	0.00
ATOM	1111	CG	ASN	A	73	160.350	39.674	131.606	1.00	0.00
ATOM	1112	OD1	ASN	A	73	159.982	38.933	132.497	1.00	0.00
ATOM	1113	ND2	ASN	A	73	160.699	39.168	130.446	1.00	0.00
ATOM	1114	1HD2	ASN	A	73	161.039	39.772	129.701	1.00	0.00
ATOM	1115	2HD2	ASN	A	73	160.620	38.174	130.349	1.00	0.00
ATOM	1116	C	ASN	A	73	158.474	42.654	131.132	1.00	0.00
ATOM	1117	O	ASN	A	73	157.582	41.955	131.606	1.00	0.00
ATOM	1118	N	TYR	A	74	158.348	43.985	131.074	1.00	0.00
ATOM	1119	H	TYR	A	74	159.057	44.535	130.608	1.00	0.00
ATOM	1120	CA	TYR	A	74	157.307	44.709	131.824	1.00	0.00
ATOM	1121	HA	TYR	A	74	156.934	44.069	132.623	1.00	0.00
ATOM	1122	CB	TYR	A	74	157.945	45.946	132.486	1.00	0.00
ATOM	1123	HB1	TYR	A	74	157.195	46.402	133.123	1.00	0.00
ATOM	1124	HB2	TYR	A	74	158.173	46.655	131.694	1.00	0.00
ATOM	1125	CG	TYR	A	74	159.215	45.764	133.326	1.00	0.00
ATOM	1126	CD1	TYR	A	74	159.601	44.508	133.842	1.00	0.00
ATOM	1127	HD1	TYR	A	74	158.985	43.636	133.685	1.00	0.00
ATOM	1128	CE1	TYR	A	74	160.803	44.370	134.560	1.00	0.00
ATOM	1129	HE1	TYR	A	74	161.117	43.412	134.937	1.00	0.00
ATOM	1130	CZ	TYR	A	74	161.613	45.491	134.818	1.00	0.00
ATOM	1131	OH	TYR	A	74	162.766	45.328	135.517	1.00	0.00
ATOM	1132	HH	TYR	A	74	163.215	46.170	135.744	1.00	0.00
ATOM	1133	CE2	TYR	A	74	161.211	46.763	134.354	1.00	0.00
ATOM	1134	HE2	TYR	A	74	161.820	47.630	134.576	1.00	0.00
ATOM	1135	CD2	TYR	A	74	160.020	46.892	133.608	1.00	0.00
ATOM	1136	HD2	TYR	A	74	159.714	47.866	133.259	1.00	0.00
ATOM	1137	C	TYR	A	74	156.092	45.087	130.942	1.00	0.00
ATOM	1138	O	TYR	A	74	156.273	45.397	129.767	1.00	0.00
ATOM	1139	N	ASP	A	75	154.851	45.032	131.467	1.00	0.00
ATOM	1140	H	ASP	A	75	154.760	44.821	132.446	1.00	0.00
ATOM	1141	CA	ASP	A	75	153.649	44.829	130.618	1.00	0.00
ATOM	1142	HA	ASP	A	75	154.045	44.437	129.680	1.00	0.00
ATOM	1143	CB	ASP	A	75	152.755	43.691	131.132	1.00	0.00
ATOM	1144	HB1	ASP	A	75	152.219	44.024	132.021	1.00	0.00
ATOM	1145	HB2	ASP	A	75	153.378	42.836	131.400	1.00	0.00
ATOM	1146	CG	ASP	A	75	151.754	43.245	130.049	1.00	0.00
ATOM	1147	OD1	ASP	A	75	150.717	42.643	130.411	1.00	0.00
ATOM	1148	OD2	ASP	A	75	151.964	43.572	128.856	1.00	0.00
ATOM	1149	C	ASP	A	75	152.844	46.101	130.202	1.00	0.00
ATOM	1150	O	ASP	A	75	151.843	46.551	130.784	1.00	0.00
ATOM	1151	N	LEU	A	76	153.251	46.664	129.069	1.00	0.00
ATOM	1152	H	LEU	A	76	154.072	46.276	128.631	1.00	0.00
ATOM	1153	CA	LEU	A	76	152.565	47.774	128.404	1.00	0.00
ATOM	1154	HA	LEU	A	76	152.401	48.559	129.144	1.00	0.00
ATOM	1155	CB	LEU	A	76	153.465	48.340	127.281	1.00	0.00
ATOM	1156	HB1	LEU	A	76	152.871	49.043	126.698	1.00	0.00
ATOM	1157	HB2	LEU	A	76	153.735	47.527	126.610	1.00	0.00

ATOM	1158	CG	LEU	A	76	154.751	49.091	127.676	1.00	0.00
ATOM	1159	HG	LEU	A	76	155.209	49.451	126.755	1.00	0.00
ATOM	1160	CD1	LEU	A	76	154.504	50.306	128.568	1.00	0.00
ATOM	1161	1HD1	LEU	A	76	153.833	50.991	128.056	1.00	0.00
ATOM	1162	2HD1	LEU	A	76	154.057	50.003	129.514	1.00	0.00
ATOM	1163	3HD1	LEU	A	76	155.444	50.818	128.763	1.00	0.00
ATOM	1164	CD2	LEU	A	76	155.786	48.228	128.383	1.00	0.00
ATOM	1165	1HD2	LEU	A	76	156.722	48.772	128.419	1.00	0.00
ATOM	1166	2HD2	LEU	A	76	155.474	48.001	129.402	1.00	0.00
ATOM	1167	3HD2	LEU	A	76	155.940	47.302	127.831	1.00	0.00
ATOM	1168	C	LEU	A	76	151.148	47.418	127.852	1.00	0.00
ATOM	1169	O	LEU	A	76	150.501	48.237	127.181	1.00	0.00
ATOM	1170	N	SER	A	77	150.618	46.223	128.139	1.00	0.00
ATOM	1171	H	SER	A	77	151.168	45.557	128.677	1.00	0.00
ATOM	1172	CA	SER	A	77	149.235	45.840	127.833	1.00	0.00
ATOM	1173	HA	SER	A	77	149.174	45.708	126.755	1.00	0.00
ATOM	1174	CB	SER	A	77	148.886	44.485	128.462	1.00	0.00
ATOM	1175	HB1	SER	A	77	149.573	43.725	128.089	1.00	0.00
ATOM	1176	HB2	SER	A	77	147.876	44.203	128.166	1.00	0.00
ATOM	1177	OG	SER	A	77	148.953	44.522	129.873	1.00	0.00
ATOM	1178	HG	SER	A	77	149.540	43.776	130.159	1.00	0.00
ATOM	1179	C	SER	A	77	148.180	46.892	128.186	1.00	0.00
ATOM	1180	O	SER	A	77	147.259	47.114	127.400	1.00	0.00
ATOM	1181	N	PHE	A	78	148.367	47.632	129.281	1.00	0.00
ATOM	1182	H	PHE	A	78	149.108	47.353	129.914	1.00	0.00
ATOM	1183	CA	PHE	A	78	147.542	48.794	129.618	1.00	0.00
ATOM	1184	HA	PHE	A	78	146.505	48.469	129.697	1.00	0.00
ATOM	1185	CB	PHE	A	78	147.982	49.337	130.974	1.00	0.00
ATOM	1186	HB1	PHE	A	78	147.381	50.213	131.219	1.00	0.00
ATOM	1187	HB2	PHE	A	78	149.017	49.656	130.877	1.00	0.00
ATOM	1188	CG	PHE	A	78	147.871	48.343	132.115	1.00	0.00
ATOM	1189	CD1	PHE	A	78	146.623	48.116	132.726	1.00	0.00
ATOM	1190	HD1	PHE	A	78	145.753	48.654	132.387	1.00	0.00
ATOM	1191	CE1	PHE	A	78	146.506	47.176	133.765	1.00	0.00
ATOM	1192	HE1	PHE	A	78	145.541	47.002	134.221	1.00	0.00
ATOM	1193	CZ	PHE	A	78	147.634	46.453	134.190	1.00	0.00
ATOM	1194	HZ	PHE	A	78	147.534	45.717	134.975	1.00	0.00
ATOM	1195	CE2	PHE	A	78	148.880	46.673	133.578	1.00	0.00
ATOM	1196	HE2	PHE	A	78	149.743	46.098	133.885	1.00	0.00
ATOM	1197	CD2	PHE	A	78	148.999	47.619	132.545	1.00	0.00
ATOM	1198	HD2	PHE	A	78	149.959	47.769	132.069	1.00	0.00
ATOM	1199	C	PHE	A	78	147.590	49.899	128.571	1.00	0.00
ATOM	1200	O	PHE	A	78	146.557	50.512	128.308	1.00	0.00
ATOM	1201	N	LEU	A	79	148.746	50.153	127.946	1.00	0.00
ATOM	1202	H	LEU	A	79	149.538	49.541	128.101	1.00	0.00
ATOM	1203	CA	LEU	A	79	148.838	51.176	126.881	1.00	0.00
ATOM	1204	HA	LEU	A	79	148.369	52.079	127.278	1.00	0.00
ATOM	1205	CB	LEU	A	79	150.300	51.547	126.570	1.00	0.00
ATOM	1206	HB1	LEU	A	79	150.334	51.940	125.555	1.00	0.00
ATOM	1207	HB2	LEU	A	79	150.937	50.666	126.592	1.00	0.00
ATOM	1208	CG	LEU	A	79	150.859	52.642	127.502	1.00	0.00
ATOM	1209	HG	LEU	A	79	150.144	53.459	127.569	1.00	0.00
ATOM	1210	CD1	LEU	A	79	151.122	52.157	128.930	1.00	0.00
ATOM	1211	1HD1	LEU	A	79	151.647	52.928	129.493	1.00	0.00
ATOM	1212	2HD1	LEU	A	79	150.176	51.948	129.426	1.00	0.00
ATOM	1213	3HD1	LEU	A	79	151.722	51.249	128.923	1.00	0.00
ATOM	1214	CD2	LEU	A	79	152.141	53.201	126.901	1.00	0.00
ATOM	1215	1HD2	LEU	A	79	152.622	53.879	127.602	1.00	0.00
ATOM	1216	2HD2	LEU	A	79	152.820	52.388	126.657	1.00	0.00
ATOM	1217	3HD2	LEU	A	79	151.905	53.747	125.988	1.00	0.00
ATOM	1218	C	LEU	A	79	148.024	50.866	125.627	1.00	0.00

ATOM	1219	O	LEU	A	79	147.686	51.809	124.904	1.00	0.00
ATOM	1220	N	LYS	A	80	147.559	49.625	125.420	1.00	0.00
ATOM	1221	H	LYS	A	80	147.836	48.886	126.052	1.00	0.00
ATOM	1222	CA	LYS	A	80	146.480	49.367	124.416	1.00	0.00
ATOM	1223	HA	LYS	A	80	146.885	49.580	123.425	1.00	0.00
ATOM	1224	CB	LYS	A	80	146.034	47.900	124.454	1.00	0.00
ATOM	1225	HB1	LYS	A	80	145.227	47.764	123.731	1.00	0.00
ATOM	1226	HB2	LYS	A	80	145.642	47.661	125.443	1.00	0.00
ATOM	1227	CG	LYS	A	80	147.160	46.923	124.094	1.00	0.00
ATOM	1228	HG1	LYS	A	80	147.968	47.007	124.821	1.00	0.00
ATOM	1229	HG2	LYS	A	80	147.546	47.182	123.111	1.00	0.00
ATOM	1230	CD	LYS	A	80	146.633	45.480	124.095	1.00	0.00
ATOM	1231	HD1	LYS	A	80	145.744	45.413	123.465	1.00	0.00
ATOM	1232	HD2	LYS	A	80	146.350	45.218	125.117	1.00	0.00
ATOM	1233	CE	LYS	A	80	147.683	44.484	123.598	1.00	0.00
ATOM	1234	HE1	LYS	A	80	147.331	43.472	123.819	1.00	0.00
ATOM	1235	HE2	LYS	A	80	148.619	44.649	124.141	1.00	0.00
ATOM	1236	NZ	LYS	A	80	147.893	44.617	122.137	1.00	0.00
ATOM	1237	HZ1	LYS	A	80	148.548	43.960	121.747	1.00	0.00
ATOM	1238	HZ2	LYS	A	80	147.008	44.542	121.622	1.00	0.00
ATOM	1239	HZ3	LYS	A	80	148.151	45.566	121.860	1.00	0.00
ATOM	1240	C	LYS	A	80	145.239	50.274	124.558	1.00	0.00
ATOM	1241	O	LYS	A	80	144.502	50.440	123.596	1.00	0.00
ATOM	1242	N	THR	A	81	144.970	50.865	125.724	1.00	0.00
ATOM	1243	H	THR	A	81	145.596	50.689	126.504	1.00	0.00
ATOM	1244	CA	THR	A	81	143.797	51.735	125.958	1.00	0.00
ATOM	1245	HA	THR	A	81	142.897	51.164	125.725	1.00	0.00
ATOM	1246	CB	THR	A	81	143.749	52.083	127.455	1.00	0.00
ATOM	1247	HB	THR	A	81	143.890	51.158	128.016	1.00	0.00
ATOM	1248	CG2	THR	A	81	144.826	53.076	127.888	1.00	0.00
ATOM	1249	1HG2	THR	A	81	144.940	53.047	128.968	1.00	0.00
ATOM	1250	2HG2	THR	A	81	145.790	52.811	127.458	1.00	0.00
ATOM	1251	3HG2	THR	A	81	144.572	54.083	127.568	1.00	0.00
ATOM	1252	OG1	THR	A	81	142.490	52.602	127.804	1.00	0.00
ATOM	1253	HG1	THR	A	81	142.334	53.394	127.278	1.00	0.00
ATOM	1254	C	THR	A	81	143.752	53.028	125.114	1.00	0.00
ATOM	1255	O	THR	A	81	142.668	53.563	124.879	1.00	0.00
ATOM	1256	N	ILE	A	82	144.922	53.535	124.706	1.00	0.00
ATOM	1257	H	ILE	A	82	145.742	52.964	124.870	1.00	0.00
ATOM	1258	CA	ILE	A	82	145.188	54.799	124.040	1.00	0.00
ATOM	1259	HA	ILE	A	82	144.705	55.591	124.606	1.00	0.00
ATOM	1260	CB	ILE	A	82	146.710	55.064	124.051	1.00	0.00
ATOM	1261	HB	ILE	A	82	147.207	54.201	123.608	1.00	0.00
ATOM	1262	CG2	ILE	A	82	147.066	56.307	123.221	1.00	0.00
ATOM	1263	1HG2	ILE	A	82	148.143	56.410	123.189	1.00	0.00
ATOM	1264	2HG2	ILE	A	82	146.736	56.203	122.190	1.00	0.00
ATOM	1265	3HG2	ILE	A	82	146.613	57.199	123.659	1.00	0.00
ATOM	1266	CG1	ILE	A	82	147.235	55.264	125.494	1.00	0.00
ATOM	1267	1HG1	ILE	A	82	146.766	54.555	126.173	1.00	0.00
ATOM	1268	2HG1	ILE	A	82	146.977	56.266	125.837	1.00	0.00
ATOM	1269	CD	ILE	A	82	148.748	55.079	125.640	1.00	0.00
ATOM	1270	HD1	ILE	A	82	149.290	55.744	124.974	1.00	0.00
ATOM	1271	HD2	ILE	A	82	149.035	55.305	126.667	1.00	0.00
ATOM	1272	HD3	ILE	A	82	149.017	54.050	125.410	1.00	0.00
ATOM	1273	C	ILE	A	82	144.640	54.792	122.610	1.00	0.00
ATOM	1274	O	ILE	A	82	144.919	53.904	121.807	1.00	0.00
ATOM	1275	N	GLN	A	83	143.921	55.859	122.294	1.00	0.00
ATOM	1276	H	GLN	A	83	143.765	56.521	123.044	1.00	0.00
ATOM	1277	CA	GLN	A	83	143.188	56.089	121.057	1.00	0.00
ATOM	1278	HA	GLN	A	83	143.400	55.300	120.335	1.00	0.00
ATOM	1279	CB	GLN	A	83	141.687	56.079	121.367	1.00	0.00

ATOM	1280	HB1	GLN	A	83	141.126	56.405	120.491	1.00	0.00
ATOM	1281	HB2	GLN	A	83	141.487	56.779	122.180	1.00	0.00
ATOM	1282	CG	GLN	A	83	141.210	54.676	121.764	1.00	0.00
ATOM	1283	HG1	GLN	A	83	141.981	54.179	122.342	1.00	0.00
ATOM	1284	HG2	GLN	A	83	141.024	54.090	120.866	1.00	0.00
ATOM	1285	CD	GLN	A	83	139.951	54.721	122.605	1.00	0.00
ATOM	1286	OE1	GLN	A	83	138.945	55.300	122.219	1.00	0.00
ATOM	1287	NE2	GLN	A	83	139.978	54.174	123.801	1.00	0.00
ATOM	1288	1HE2	GLN	A	83	140.849	53.829	124.190	1.00	0.00
ATOM	1289	2HE2	GLN	A	83	139.157	54.321	124.393	1.00	0.00
ATOM	1290	C	GLN	A	83	143.589	57.429	120.432	1.00	0.00
ATOM	1291	O	GLN	A	83	143.724	57.526	119.217	1.00	0.00
ATOM	1292	N	GLU	A	84	143.808	58.459	121.253	1.00	0.00
ATOM	1293	H	GLU	A	84	143.698	58.325	122.254	1.00	0.00
ATOM	1294	CA	GLU	A	84	144.025	59.831	120.785	1.00	0.00
ATOM	1295	HA	GLU	A	84	144.374	59.824	119.754	1.00	0.00
ATOM	1296	CB	GLU	A	84	142.676	60.604	120.863	1.00	0.00
ATOM	1297	HB1	GLU	A	84	142.870	61.661	120.680	1.00	0.00
ATOM	1298	HB2	GLU	A	84	142.316	60.518	121.885	1.00	0.00
ATOM	1299	CG	GLU	A	84	141.550	60.151	119.894	1.00	0.00
ATOM	1300	HG1	GLU	A	84	141.617	59.079	119.723	1.00	0.00
ATOM	1301	HG2	GLU	A	84	141.694	60.641	118.931	1.00	0.00
ATOM	1302	CD	GLU	A	84	140.118	60.431	120.409	1.00	0.00
ATOM	1303	OE1	GLU	A	84	139.325	61.120	119.708	1.00	0.00
ATOM	1304	OE2	GLU	A	84	139.794	59.893	121.494	1.00	0.00
ATOM	1305	C	GLU	A	84	145.105	60.526	121.637	1.00	0.00
ATOM	1306	O	GLU	A	84	145.065	60.504	122.868	1.00	0.00
ATOM	1307	N	VAL	A	85	146.061	61.199	121.002	1.00	0.00
ATOM	1308	H	VAL	A	85	146.098	61.163	119.985	1.00	0.00
ATOM	1309	CA	VAL	A	85	147.066	62.035	121.670	1.00	0.00
ATOM	1310	HA	VAL	A	85	146.701	62.267	122.672	1.00	0.00
ATOM	1311	CB	VAL	A	85	148.399	61.290	121.862	1.00	0.00
ATOM	1312	HB	VAL	A	85	148.182	60.402	122.454	1.00	0.00
ATOM	1313	CG1	VAL	A	85	148.999	60.839	120.539	1.00	0.00
ATOM	1314	1HG1	VAL	A	85	149.868	60.229	120.738	1.00	0.00
ATOM	1315	2HG1	VAL	A	85	148.284	60.233	119.988	1.00	0.00
ATOM	1316	3HG1	VAL	A	85	149.287	61.700	119.941	1.00	0.00
ATOM	1317	CG2	VAL	A	85	149.458	62.103	122.618	1.00	0.00
ATOM	1318	1HG2	VAL	A	85	150.326	61.479	122.823	1.00	0.00
ATOM	1319	2HG2	VAL	A	85	149.793	62.957	122.031	1.00	0.00
ATOM	1320	3HG2	VAL	A	85	149.062	62.456	123.568	1.00	0.00
ATOM	1321	C	VAL	A	85	147.194	63.367	120.936	1.00	0.00
ATOM	1322	O	VAL	A	85	146.972	63.461	119.718	1.00	0.00
ATOM	1323	N	ALA	A	86	147.474	64.402	121.739	1.00	0.00
ATOM	1324	H	ALA	A	86	147.621	64.199	122.719	1.00	0.00
ATOM	1325	CA	ALA	A	86	147.179	65.788	121.410	1.00	0.00
ATOM	1326	HA	ALA	A	86	146.663	65.809	120.455	1.00	0.00
ATOM	1327	CB	ALA	A	86	146.191	66.327	122.452	1.00	0.00
ATOM	1328	HB1	ALA	A	86	145.889	67.340	122.184	1.00	0.00
ATOM	1329	HB2	ALA	A	86	145.304	65.695	122.482	1.00	0.00
ATOM	1330	HB3	ALA	A	86	146.657	66.346	123.437	1.00	0.00
ATOM	1331	C	ALA	A	86	148.408	66.694	121.290	1.00	0.00
ATOM	1332	O	ALA	A	86	148.487	67.495	120.360	1.00	0.00
ATOM	1333	N	GLY	A	87	149.399	66.524	122.165	1.00	0.00
ATOM	1334	H	GLY	A	87	149.280	65.872	122.930	1.00	0.00
ATOM	1335	CA	GLY	A	87	150.766	66.912	121.841	1.00	0.00
ATOM	1336	HA1	GLY	A	87	151.350	66.943	122.750	1.00	0.00
ATOM	1337	HA2	GLY	A	87	150.793	67.901	121.381	1.00	0.00
ATOM	1338	C	GLY	A	87	151.334	65.888	120.839	1.00	0.00
ATOM	1339	O	GLY	A	87	150.785	65.676	119.753	1.00	0.00
ATOM	1340	N	TYR	A	88	152.419	65.234	121.238	1.00	0.00

ATOM	1341	H	TYR	A	88	152.798	65.511	122.134	1.00	0.00
ATOM	1342	CA	TYR	A	88	153.184	64.238	120.483	1.00	0.00
ATOM	1343	HA	TYR	A	88	152.733	64.076	119.503	1.00	0.00
ATOM	1344	CB	TYR	A	88	154.621	64.772	120.279	1.00	0.00
ATOM	1345	HB1	TYR	A	88	155.273	63.939	120.025	1.00	0.00
ATOM	1346	HB2	TYR	A	88	154.984	65.175	121.225	1.00	0.00
ATOM	1347	CG	TYR	A	88	154.891	65.800	119.196	1.00	0.00
ATOM	1348	CD1	TYR	A	88	153.866	66.469	118.506	1.00	0.00
ATOM	1349	HD1	TYR	A	88	152.848	66.267	118.736	1.00	0.00
ATOM	1350	CE1	TYR	A	88	154.182	67.413	117.515	1.00	0.00
ATOM	1351	HE1	TYR	A	88	153.431	67.916	116.960	1.00	0.00
ATOM	1352	CZ	TYR	A	88	155.508	67.709	117.208	1.00	0.00
ATOM	1353	OH	TYR	A	88	155.767	68.690	116.312	1.00	0.00
ATOM	1354	HH	TYR	A	88	154.956	69.067	115.956	1.00	0.00
ATOM	1355	CE2	TYR	A	88	156.543	66.999	117.844	1.00	0.00
ATOM	1356	HE2	TYR	A	88	157.567	67.183	117.582	1.00	0.00
ATOM	1357	CD2	TYR	A	88	156.230	66.069	118.853	1.00	0.00
ATOM	1358	HD2	TYR	A	88	157.020	65.534	119.353	1.00	0.00
ATOM	1359	C	TYR	A	88	153.244	62.861	121.174	1.00	0.00
ATOM	1360	O	TYR	A	88	152.795	62.672	122.314	1.00	0.00
ATOM	1361	N	VAL	A	89	153.898	61.919	120.489	1.00	0.00
ATOM	1362	H	VAL	A	89	154.208	62.165	119.554	1.00	0.00
ATOM	1363	CA	VAL	A	89	154.370	60.631	121.017	1.00	0.00
ATOM	1364	HA	VAL	A	89	154.054	60.506	122.053	1.00	0.00
ATOM	1365	CB	VAL	A	89	153.801	59.468	120.170	1.00	0.00
ATOM	1366	HB	VAL	A	89	154.162	59.571	119.146	1.00	0.00
ATOM	1367	CG1	VAL	A	89	154.238	58.100	120.701	1.00	0.00
ATOM	1368	1HG1	VAL	A	89	153.748	57.303	120.143	1.00	0.00
ATOM	1369	2HG1	VAL	A	89	155.312	57.981	120.564	1.00	0.00
ATOM	1370	3HG1	VAL	A	89	153.986	58.008	121.758	1.00	0.00
ATOM	1371	CG2	VAL	A	89	152.277	59.467	120.130	1.00	0.00
ATOM	1372	1HG2	VAL	A	89	151.913	58.624	119.544	1.00	0.00
ATOM	1373	2HG2	VAL	A	89	151.892	59.395	121.144	1.00	0.00
ATOM	1374	3HG2	VAL	A	89	151.918	60.384	119.664	1.00	0.00
ATOM	1375	C	VAL	A	89	155.902	60.562	120.943	1.00	0.00
ATOM	1376	O	VAL	A	89	156.493	60.859	119.903	1.00	0.00
ATOM	1377	N	LEU	A	90	156.541	60.048	121.989	1.00	0.00
ATOM	1378	H	LEU	A	90	156.027	59.917	122.855	1.00	0.00
ATOM	1379	CA	LEU	A	90	157.941	59.615	121.961	1.00	0.00
ATOM	1380	HA	LEU	A	90	158.285	59.575	120.928	1.00	0.00
ATOM	1381	CB	LEU	A	90	158.793	60.662	122.706	1.00	0.00
ATOM	1382	HB1	LEU	A	90	158.373	60.800	123.702	1.00	0.00
ATOM	1383	HB2	LEU	A	90	158.704	61.605	122.171	1.00	0.00
ATOM	1384	CG	LEU	A	90	160.289	60.334	122.848	1.00	0.00
ATOM	1385	HG	LEU	A	90	160.408	59.404	123.403	1.00	0.00
ATOM	1386	CD1	LEU	A	90	160.988	60.197	121.493	1.00	0.00
ATOM	1387	1HD1	LEU	A	90	160.821	61.100	120.915	1.00	0.00
ATOM	1388	2HD1	LEU	A	90	162.057	60.061	121.642	1.00	0.00
ATOM	1389	3HD1	LEU	A	90	160.594	59.343	120.947	1.00	0.00
ATOM	1390	CD2	LEU	A	90	161.005	61.447	123.619	1.00	0.00
ATOM	1391	1HD2	LEU	A	90	162.047	61.174	123.782	1.00	0.00
ATOM	1392	2HD2	LEU	A	90	160.957	62.378	123.054	1.00	0.00
ATOM	1393	3HD2	LEU	A	90	160.516	61.588	124.583	1.00	0.00
ATOM	1394	C	LEU	A	90	158.065	58.200	122.546	1.00	0.00
ATOM	1395	O	LEU	A	90	157.553	57.944	123.641	1.00	0.00
ATOM	1396	N	ILE	A	91	158.746	57.288	121.840	1.00	0.00
ATOM	1397	H	ILE	A	91	159.070	57.565	120.920	1.00	0.00
ATOM	1398	CA	ILE	A	91	158.887	55.864	122.209	1.00	0.00
ATOM	1399	HA	ILE	A	91	158.657	55.778	123.263	1.00	0.00
ATOM	1400	CB	ILE	A	91	157.832	54.968	121.510	1.00	0.00
ATOM	1401	HB	ILE	A	91	158.079	53.932	121.755	1.00	0.00

ATOM	1402	CG2	ILE	A	91	156.436	55.258	122.078	1.00	0.00
ATOM	1403	1HG2	ILE	A	91	155.741	54.461	121.819	1.00	0.00
ATOM	1404	2HG2	ILE	A	91	156.474	55.348	123.157	1.00	0.00
ATOM	1405	3HG2	ILE	A	91	156.061	56.197	121.687	1.00	0.00
ATOM	1406	CG1	ILE	A	91	157.786	55.114	119.976	1.00	0.00
ATOM	1407	1HG1	ILE	A	91	158.782	54.969	119.581	1.00	0.00
ATOM	1408	2HG1	ILE	A	91	157.459	56.118	119.707	1.00	0.00
ATOM	1409	CD	ILE	A	91	156.865	54.106	119.282	1.00	0.00
ATOM	1410	HD1	ILE	A	91	156.965	54.211	118.202	1.00	0.00
ATOM	1411	HD2	ILE	A	91	157.139	53.091	119.569	1.00	0.00
ATOM	1412	HD3	ILE	A	91	155.822	54.289	119.536	1.00	0.00
ATOM	1413	C	ILE	A	91	160.329	55.347	122.072	1.00	0.00
ATOM	1414	O	ILE	A	91	160.778	54.877	121.022	1.00	0.00
ATOM	1415	N	ALA	A	92	161.114	55.544	123.121	1.00	0.00
ATOM	1416	H	ALA	A	92	160.648	55.789	123.990	1.00	0.00
ATOM	1417	CA	ALA	A	92	162.556	55.784	123.042	1.00	0.00
ATOM	1418	HA	ALA	A	92	162.934	55.567	122.051	1.00	0.00
ATOM	1419	CB	ALA	A	92	162.714	57.277	123.381	1.00	0.00
ATOM	1420	HB1	ALA	A	92	163.738	57.512	123.661	1.00	0.00
ATOM	1421	HB2	ALA	A	92	162.406	57.880	122.530	1.00	0.00
ATOM	1422	HB3	ALA	A	92	162.092	57.536	124.240	1.00	0.00
ATOM	1423	C	ALA	A	92	163.335	54.937	124.035	1.00	0.00
ATOM	1424	O	ALA	A	92	162.927	54.929	125.175	1.00	0.00
ATOM	1425	N	LEU	A	93	164.474	54.307	123.708	1.00	0.00
ATOM	1426	H	LEU	A	93	164.732	54.261	122.731	1.00	0.00
ATOM	1427	CA	LEU	A	93	165.312	53.579	124.720	1.00	0.00
ATOM	1428	HA	LEU	A	93	166.126	53.112	124.174	1.00	0.00
ATOM	1429	CB	LEU	A	93	165.986	54.593	125.684	1.00	0.00
ATOM	1430	HB1	LEU	A	93	166.291	54.070	126.587	1.00	0.00
ATOM	1431	HB2	LEU	A	93	165.261	55.335	126.012	1.00	0.00
ATOM	1432	CG	LEU	A	93	167.240	55.353	125.188	1.00	0.00
ATOM	1433	HG	LEU	A	93	167.399	56.182	125.876	1.00	0.00
ATOM	1434	CD1	LEU	A	93	168.493	54.486	125.225	1.00	0.00
ATOM	1435	1HD1	LEU	A	93	169.354	55.049	124.870	1.00	0.00
ATOM	1436	2HD1	LEU	A	93	168.690	54.175	126.250	1.00	0.00
ATOM	1437	3HD1	LEU	A	93	168.374	53.595	124.613	1.00	0.00
ATOM	1438	CD2	LEU	A	93	167.089	55.966	123.796	1.00	0.00
ATOM	1439	1HD2	LEU	A	93	167.960	56.585	123.577	1.00	0.00
ATOM	1440	2HD2	LEU	A	93	167.011	55.187	123.046	1.00	0.00
ATOM	1441	3HD2	LEU	A	93	166.199	56.589	123.779	1.00	0.00
ATOM	1442	C	LEU	A	93	164.649	52.384	125.487	1.00	0.00
ATOM	1443	O	LEU	A	93	165.284	51.777	126.344	1.00	0.00
ATOM	1444	N	ASN	A	94	163.405	51.986	125.191	1.00	0.00
ATOM	1445	H	ASN	A	94	162.939	52.466	124.426	1.00	0.00
ATOM	1446	CA	ASN	A	94	162.673	50.893	125.852	1.00	0.00
ATOM	1447	HA	ASN	A	94	163.041	50.818	126.877	1.00	0.00
ATOM	1448	CB	ASN	A	94	161.174	51.275	125.968	1.00	0.00
ATOM	1449	HB1	ASN	A	94	161.095	52.087	126.687	1.00	0.00
ATOM	1450	HB2	ASN	A	94	160.636	50.431	126.387	1.00	0.00
ATOM	1451	CG	ASN	A	94	160.417	51.733	124.720	1.00	0.00
ATOM	1452	OD1	ASN	A	94	159.842	52.805	124.709	1.00	0.00
ATOM	1453	ND2	ASN	A	94	160.333	50.947	123.674	1.00	0.00
ATOM	1454	1HD2	ASN	A	94	160.957	50.152	123.608	1.00	0.00
ATOM	1455	2HD2	ASN	A	94	159.834	51.317	122.888	1.00	0.00
ATOM	1456	C	ASN	A	94	162.897	49.501	125.237	1.00	0.00
ATOM	1457	O	ASN	A	94	162.393	49.207	124.151	1.00	0.00
ATOM	1458	N	THR	A	95	163.593	48.600	125.930	1.00	0.00
ATOM	1459	H	THR	A	95	163.945	48.860	126.842	1.00	0.00
ATOM	1460	CA	THR	A	95	163.848	47.212	125.481	1.00	0.00
ATOM	1461	HA	THR	A	95	163.947	47.240	124.402	1.00	0.00
ATOM	1462	CB	THR	A	95	165.186	46.701	126.032	1.00	0.00

ATOM	1463	HB	THR	A	95	165.332	45.670	125.719	1.00	0.00
ATOM	1464	CG2	THR	A	95	166.342	47.548	125.495	1.00	0.00
ATOM	1465	1HG2	THR	A	95	166.633	48.310	126.219	1.00	0.00
ATOM	1466	2HG2	THR	A	95	167.178	46.889	125.275	1.00	0.00
ATOM	1467	3HG2	THR	A	95	166.066	48.062	124.579	1.00	0.00
ATOM	1468	OG1	THR	A	95	165.245	46.796	127.438	1.00	0.00
ATOM	1469	HG1	THR	A	95	166.152	46.591	127.689	1.00	0.00
ATOM	1470	C	THR	A	95	162.690	46.242	125.743	1.00	0.00
ATOM	1471	O	THR	A	95	162.898	45.078	126.055	1.00	0.00
ATOM	1472	N	VAL	A	96	161.447	46.707	125.636	1.00	0.00
ATOM	1473	H	VAL	A	96	161.358	47.674	125.367	1.00	0.00
ATOM	1474	CA	VAL	A	96	160.206	45.956	125.922	1.00	0.00
ATOM	1475	HA	VAL	A	96	160.213	45.668	126.975	1.00	0.00
ATOM	1476	CB	VAL	A	96	158.972	46.851	125.663	1.00	0.00
ATOM	1477	HB	VAL	A	96	158.068	46.244	125.706	1.00	0.00
ATOM	1478	CG1	VAL	A	96	158.838	47.918	126.739	1.00	0.00
ATOM	1479	1HG1	VAL	A	96	159.746	48.508	126.794	1.00	0.00
ATOM	1480	2HG1	VAL	A	96	157.998	48.572	126.507	1.00	0.00
ATOM	1481	3HG1	VAL	A	96	158.670	47.436	127.702	1.00	0.00
ATOM	1482	CG2	VAL	A	96	159.004	47.560	124.301	1.00	0.00
ATOM	1483	1HG2	VAL	A	96	158.046	48.040	124.121	1.00	0.00
ATOM	1484	2HG2	VAL	A	96	159.780	48.322	124.280	1.00	0.00
ATOM	1485	3HG2	VAL	A	96	159.191	46.852	123.498	1.00	0.00
ATOM	1486	C	VAL	A	96	159.991	44.662	125.125	1.00	0.00
ATOM	1487	O	VAL	A	96	159.086	43.904	125.463	1.00	0.00
ATOM	1488	N	GLU	A	97	160.685	44.466	124.001	1.00	0.00
ATOM	1489	H	GLU	A	97	161.556	44.977	123.892	1.00	0.00
ATOM	1490	CA	GLU	A	97	160.319	43.550	122.906	1.00	0.00
ATOM	1491	HA	GLU	A	97	160.923	43.848	122.054	1.00	0.00
ATOM	1492	CB	GLU	A	97	160.787	42.118	123.254	1.00	0.00
ATOM	1493	HB1	GLU	A	97	160.282	41.424	122.580	1.00	0.00
ATOM	1494	HB2	GLU	A	97	160.486	41.847	124.266	1.00	0.00
ATOM	1495	CG	GLU	A	97	162.299	41.889	123.057	1.00	0.00
ATOM	1496	HG1	GLU	A	97	162.648	42.468	122.201	1.00	0.00
ATOM	1497	HG2	GLU	A	97	162.438	40.839	122.791	1.00	0.00
ATOM	1498	CD	GLU	A	97	163.195	42.161	124.280	1.00	0.00
ATOM	1499	OE1	GLU	A	97	164.205	42.887	124.124	1.00	0.00
ATOM	1500	OE2	GLU	A	97	163.050	41.450	125.295	1.00	0.00
ATOM	1501	C	GLU	A	97	158.866	43.594	122.368	1.00	0.00
ATOM	1502	O	GLU	A	97	158.553	42.913	121.398	1.00	0.00
ATOM	1503	N	ARG	A	98	157.982	44.413	122.944	1.00	0.00
ATOM	1504	H	ARG	A	98	158.325	44.884	123.766	1.00	0.00
ATOM	1505	CA	ARG	A	98	156.574	44.676	122.587	1.00	0.00
ATOM	1506	HA	ARG	A	98	156.210	43.959	121.858	1.00	0.00
ATOM	1507	CB	ARG	A	98	155.738	44.503	123.884	1.00	0.00
ATOM	1508	HB1	ARG	A	98	154.816	45.075	123.810	1.00	0.00
ATOM	1509	HB2	ARG	A	98	156.288	44.879	124.749	1.00	0.00
ATOM	1510	CG	ARG	A	98	155.311	43.044	124.125	1.00	0.00
ATOM	1511	HG1	ARG	A	98	154.746	42.735	123.245	1.00	0.00
ATOM	1512	HG2	ARG	A	98	154.613	43.031	124.963	1.00	0.00
ATOM	1513	CD	ARG	A	98	156.420	42.010	124.418	1.00	0.00
ATOM	1514	HD1	ARG	A	98	156.604	41.982	125.495	1.00	0.00
ATOM	1515	HD2	ARG	A	98	157.358	42.276	123.945	1.00	0.00
ATOM	1516	NE	ARG	A	98	156.016	40.674	123.937	1.00	0.00
ATOM	1517	HE	ARG	A	98	155.547	40.086	124.599	1.00	0.00
ATOM	1518	CZ	ARG	A	98	155.922	40.343	122.658	1.00	0.00
ATOM	1519	NH1	ARG	A	98	156.619	40.907	121.721	1.00	0.00
ATOM	1520	1HH1	ARG	A	98	157.336	41.591	121.917	1.00	0.00
ATOM	1521	2HH1	ARG	A	98	156.281	40.777	120.768	1.00	0.00
ATOM	1522	NH2	ARG	A	98	155.055	39.471	122.241	1.00	0.00
ATOM	1523	1HH2	ARG	A	98	154.427	39.031	122.879	1.00	0.00

```
ATOM   1524 2HH2 ARG A  98      154.825  39.541 121.248  1.00  0.00
ATOM   1525  C   ARG A  98      156.515  46.062 121.904  1.00  0.00
ATOM   1526  O   ARG A  98      157.521  46.480 121.312  1.00  0.00
ATOM   1527  N   ILE A  99      155.345  46.723 122.014  1.00  0.00
ATOM   1528  H   ILE A  99      154.554  46.127 122.201  1.00  0.00
ATOM   1529  CA  ILE A  99      155.175  48.103 122.511  1.00  0.00
ATOM   1530  HA  ILE A  99      155.928  48.213 123.286  1.00  0.00
ATOM   1531  CB  ILE A  99      155.485  49.192 121.448  1.00  0.00
ATOM   1532  HB  ILE A  99      156.357  48.862 120.882  1.00  0.00
ATOM   1533  CG2 ILE A  99      154.335  49.404 120.461  1.00  0.00
ATOM   1534 1HG2 ILE A  99      154.642  50.088 119.671  1.00  0.00
ATOM   1535 2HG2 ILE A  99      154.069  48.458 120.009  1.00  0.00
ATOM   1536 3HG2 ILE A  99      153.472  49.834 120.962  1.00  0.00
ATOM   1537  CG1 ILE A  99      155.826  50.576 122.045  1.00  0.00
ATOM   1538 1HG1 ILE A  99      154.954  50.977 122.561  1.00  0.00
ATOM   1539 2HG1 ILE A  99      156.069  51.252 121.224  1.00  0.00
ATOM   1540  CD  ILE A  99      157.013  50.589 123.015  1.00  0.00
ATOM   1541  HD1 ILE A  99      157.315  51.618 123.203  1.00  0.00
ATOM   1542  HD2 ILE A  99      156.731  50.135 123.964  1.00  0.00
ATOM   1543  HD3 ILE A  99      157.851  50.047 122.582  1.00  0.00
ATOM   1544  C   ILE A  99      153.833  48.294 123.247  1.00  0.00
ATOM   1545  O   ILE A  99      153.812  49.089 124.177  1.00  0.00
ATOM   1546  N   PRO A 100      152.806  47.439 123.011  1.00  0.00
ATOM   1547  CD  PRO A 100      152.157  46.743 124.114  1.00  0.00
ATOM   1548  HD1 PRO A 100      151.834  47.455 124.875  1.00  0.00
ATOM   1549  HD2 PRO A 100      152.836  46.015 124.557  1.00  0.00
ATOM   1550  CG  PRO A 100      150.937  46.027 123.530  1.00  0.00
ATOM   1551  HG1 PRO A 100      150.035  46.519 123.889  1.00  0.00
ATOM   1552  HG2 PRO A 100      150.931  44.971 123.800  1.00  0.00
ATOM   1553  CB  PRO A 100      151.058  46.209 122.016  1.00  0.00
ATOM   1554  HB1 PRO A 100      150.088  46.285 121.528  1.00  0.00
ATOM   1555  HB2 PRO A 100      151.631  45.380 121.593  1.00  0.00
ATOM   1556  CA  PRO A 100      151.863  47.498 121.874  1.00  0.00
ATOM   1557  HA  PRO A 100      152.389  47.486 120.924  1.00  0.00
ATOM   1558  C   PRO A 100      150.965  48.745 121.957  1.00  0.00
ATOM   1559  O   PRO A 100      150.541  49.117 123.045  1.00  0.00
ATOM   1560  N   LEU A 101      150.640  49.389 120.834  1.00  0.00
ATOM   1561  H   LEU A 101      150.939  49.035 119.931  1.00  0.00
ATOM   1562  CA  LEU A 101      149.923  50.676 120.840  1.00  0.00
ATOM   1563  HA  LEU A 101      149.389  50.758 121.785  1.00  0.00
ATOM   1564  CB  LEU A 101      150.982  51.801 120.759  1.00  0.00
ATOM   1565  HB1 LEU A 101      151.314  51.901 119.728  1.00  0.00
ATOM   1566  HB2 LEU A 101      151.846  51.484 121.345  1.00  0.00
ATOM   1567  CG  LEU A 101      150.565  53.180 121.298  1.00  0.00
ATOM   1568  HG  LEU A 101      149.794  53.620 120.666  1.00  0.00
ATOM   1569  CD1 LEU A 101      150.060  53.103 122.738  1.00  0.00
ATOM   1570 1HD1 LEU A 101      149.977  54.103 123.159  1.00  0.00
ATOM   1571 2HD1 LEU A 101      149.070  52.649 122.759  1.00  0.00
ATOM   1572 3HD1 LEU A 101      150.752  52.512 123.338  1.00  0.00
ATOM   1573  CD2 LEU A 101      151.790  54.102 121.305  1.00  0.00
ATOM   1574 1HD2 LEU A 101      152.540  53.724 121.999  1.00  0.00
ATOM   1575 2HD2 LEU A 101      152.226  54.153 120.308  1.00  0.00
ATOM   1576 3HD2 LEU A 101      151.491  55.102 121.615  1.00  0.00
ATOM   1577  C   LEU A 101      148.831  50.693 119.750  1.00  0.00
ATOM   1578  O   LEU A 101      148.515  51.740 119.183  1.00  0.00
ATOM   1579  N   GLU A 102      148.261  49.520 119.432  1.00  0.00
ATOM   1580  H   GLU A 102      148.454  48.710 120.014  1.00  0.00
ATOM   1581  CA  GLU A 102      147.572  49.246 118.164  1.00  0.00
ATOM   1582  HA  GLU A 102      148.225  49.626 117.398  1.00  0.00
ATOM   1583  CB  GLU A 102      147.473  47.737 117.862  1.00  0.00
ATOM   1584  HB1 GLU A 102      148.432  47.261 118.065  1.00  0.00
```

ATOM	1585	HB2	GLU	A	102	147.307	47.642	116.787	1.00	0.00
ATOM	1586	CG	GLU	A	102	146.341	46.971	118.562	1.00	0.00
ATOM	1587	HG1	GLU	A	102	146.047	46.150	117.904	1.00	0.00
ATOM	1588	HG2	GLU	A	102	145.463	47.602	118.697	1.00	0.00
ATOM	1589	CD	GLU	A	102	146.744	46.369	119.912	1.00	0.00
ATOM	1590	OE1	GLU	A	102	147.698	46.852	120.566	1.00	0.00
ATOM	1591	OE2	GLU	A	102	146.125	45.365	120.326	1.00	0.00
ATOM	1592	C	GLU	A	102	146.240	49.964	117.903	1.00	0.00
ATOM	1593	O	GLU	A	102	145.628	49.772	116.858	1.00	0.00
ATOM	1594	N	ASN	A	103	145.816	50.788	118.852	1.00	0.00
ATOM	1595	H	ASN	A	103	146.450	50.936	119.625	1.00	0.00
ATOM	1596	CA	ASN	A	103	144.508	51.424	118.947	1.00	0.00
ATOM	1597	HA	ASN	A	103	143.818	50.955	118.245	1.00	0.00
ATOM	1598	CB	ASN	A	103	144.051	51.093	120.384	1.00	0.00
ATOM	1599	HB1	ASN	A	103	144.760	51.530	121.084	1.00	0.00
ATOM	1600	HB2	ASN	A	103	144.085	50.013	120.527	1.00	0.00
ATOM	1601	CG	ASN	A	103	142.657	51.539	120.772	1.00	0.00
ATOM	1602	OD1	ASN	A	103	141.774	51.732	119.953	1.00	0.00
ATOM	1603	ND2	ASN	A	103	142.408	51.635	122.061	1.00	0.00
ATOM	1604	1HD2	ASN	A	103	143.150	51.379	122.708	1.00	0.00
ATOM	1605	2HD2	ASN	A	103	141.482	51.872	122.355	1.00	0.00
ATOM	1606	C	ASN	A	103	144.570	52.929	118.624	1.00	0.00
ATOM	1607	O	ASN	A	103	143.603	53.477	118.097	1.00	0.00
ATOM	1608	N	LEU	A	104	145.746	53.547	118.801	1.00	0.00
ATOM	1609	H	LEU	A	104	146.518	52.985	119.130	1.00	0.00
ATOM	1610	CA	LEU	A	104	146.018	54.966	118.569	1.00	0.00
ATOM	1611	HA	LEU	A	104	145.367	55.527	119.236	1.00	0.00
ATOM	1612	CB	LEU	A	104	147.468	55.245	119.003	1.00	0.00
ATOM	1613	HB1	LEU	A	104	148.167	54.846	118.265	1.00	0.00
ATOM	1614	HB2	LEU	A	104	147.639	54.704	119.936	1.00	0.00
ATOM	1615	CG	LEU	A	104	147.768	56.730	119.268	1.00	0.00
ATOM	1616	HG	LEU	A	104	146.953	57.166	119.844	1.00	0.00
ATOM	1617	CD1	LEU	A	104	149.054	56.855	120.086	1.00	0.00
ATOM	1618	1HD1	LEU	A	104	149.218	57.898	120.349	1.00	0.00
ATOM	1619	2HD1	LEU	A	104	148.973	56.274	121.002	1.00	0.00
ATOM	1620	3HD1	LEU	A	104	149.899	56.483	119.509	1.00	0.00
ATOM	1621	CD2	LEU	A	104	147.969	57.538	117.987	1.00	0.00
ATOM	1622	1HD2	LEU	A	104	148.214	58.565	118.245	1.00	0.00
ATOM	1623	2HD2	LEU	A	104	148.777	57.112	117.395	1.00	0.00
ATOM	1624	3HD2	LEU	A	104	147.061	57.556	117.394	1.00	0.00
ATOM	1625	C	LEU	A	104	145.681	55.388	117.122	1.00	0.00
ATOM	1626	O	LEU	A	104	146.405	55.059	116.188	1.00	0.00
ATOM	1627	N	GLN	A	105	144.567	56.108	116.955	1.00	0.00
ATOM	1628	H	GLN	A	105	144.034	56.310	117.794	1.00	0.00
ATOM	1629	CA	GLN	A	105	144.080	56.670	115.687	1.00	0.00
ATOM	1630	HA	GLN	A	105	144.333	55.985	114.878	1.00	0.00
ATOM	1631	CB	GLN	A	105	142.544	56.837	115.736	1.00	0.00
ATOM	1632	HB1	GLN	A	105	142.245	57.727	115.178	1.00	0.00
ATOM	1633	HB2	GLN	A	105	142.231	56.993	116.772	1.00	0.00
ATOM	1634	CG	GLN	A	105	141.753	55.643	115.163	1.00	0.00
ATOM	1635	HG1	GLN	A	105	140.811	55.566	115.708	1.00	0.00
ATOM	1636	HG2	GLN	A	105	142.299	54.716	115.325	1.00	0.00
ATOM	1637	CD	GLN	A	105	141.403	55.806	113.679	1.00	0.00
ATOM	1638	OE1	GLN	A	105	140.414	56.425	113.318	1.00	0.00
ATOM	1639	NE2	GLN	A	105	142.177	55.285	112.754	1.00	0.00
ATOM	1640	1HE2	GLN	A	105	143.077	54.875	112.990	1.00	0.00
ATOM	1641	2HE2	GLN	A	105	141.923	55.483	111.803	1.00	0.00
ATOM	1642	C	GLN	A	105	144.719	58.021	115.328	1.00	0.00
ATOM	1643	O	GLN	A	105	144.975	58.264	114.142	1.00	0.00
ATOM	1644	N	ILE	A	106	144.982	58.896	116.309	1.00	0.00
ATOM	1645	H	ILE	A	106	144.816	58.604	117.265	1.00	0.00

ATOM	1646	CA	ILE	A	106	145.308	60.312	116.051	1.00	0.00
ATOM	1647	HA	ILE	A	106	145.722	60.374	115.042	1.00	0.00
ATOM	1648	CB	ILE	A	106	143.992	61.146	116.077	1.00	0.00
ATOM	1649	HB	ILE	A	106	143.167	60.436	116.030	1.00	0.00
ATOM	1650	CG2	ILE	A	106	143.754	61.957	117.363	1.00	0.00
ATOM	1651	1HG2	ILE	A	106	142.766	62.416	117.339	1.00	0.00
ATOM	1652	2HG2	ILE	A	106	143.799	61.287	118.215	1.00	0.00
ATOM	1653	3HG2	ILE	A	106	144.512	62.730	117.495	1.00	0.00
ATOM	1654	CG1	ILE	A	106	143.778	62.043	114.837	1.00	0.00
ATOM	1655	1HG1	ILE	A	106	142.769	62.451	114.888	1.00	0.00
ATOM	1656	2HG1	ILE	A	106	143.819	61.407	113.954	1.00	0.00
ATOM	1657	CD	ILE	A	106	144.746	63.218	114.654	1.00	0.00
ATOM	1658	HD1	ILE	A	106	144.423	63.819	113.804	1.00	0.00
ATOM	1659	HD2	ILE	A	106	144.766	63.850	115.540	1.00	0.00
ATOM	1660	HD3	ILE	A	106	145.746	62.849	114.449	1.00	0.00
ATOM	1661	C	ILE	A	106	146.379	60.867	117.001	1.00	0.00
ATOM	1662	O	ILE	A	106	146.418	60.563	118.192	1.00	0.00
ATOM	1663	N	ILE	A	107	147.205	61.738	116.442	1.00	0.00
ATOM	1664	H	ILE	A	107	147.091	61.862	115.440	1.00	0.00
ATOM	1665	CA	ILE	A	107	148.273	62.542	116.995	1.00	0.00
ATOM	1666	HA	ILE	A	107	148.243	62.534	118.076	1.00	0.00
ATOM	1667	CB	ILE	A	107	149.663	62.040	116.533	1.00	0.00
ATOM	1668	HB	ILE	A	107	149.733	62.138	115.449	1.00	0.00
ATOM	1669	CG2	ILE	A	107	150.743	62.928	117.167	1.00	0.00
ATOM	1670	1HG2	ILE	A	107	151.726	62.647	116.798	1.00	0.00
ATOM	1671	2HG2	ILE	A	107	150.590	63.969	116.895	1.00	0.00
ATOM	1672	3HG2	ILE	A	107	150.735	62.830	118.253	1.00	0.00
ATOM	1673	CG1	ILE	A	107	149.872	60.549	116.889	1.00	0.00
ATOM	1674	1HG1	ILE	A	107	149.122	59.957	116.363	1.00	0.00
ATOM	1675	2HG1	ILE	A	107	149.724	60.413	117.957	1.00	0.00
ATOM	1676	CD	ILE	A	107	151.239	59.964	116.523	1.00	0.00
ATOM	1677	HD1	ILE	A	107	151.229	58.891	116.709	1.00	0.00
ATOM	1678	HD2	ILE	A	107	151.449	60.142	115.471	1.00	0.00
ATOM	1679	HD3	ILE	A	107	152.022	60.410	117.132	1.00	0.00
ATOM	1680	C	ILE	A	107	147.922	63.923	116.429	1.00	0.00
ATOM	1681	O	ILE	A	107	147.951	64.101	115.219	1.00	0.00
ATOM	1682	N	ARG	A	108	147.445	64.869	117.249	1.00	0.00
ATOM	1683	H	ARG	A	108	147.314	64.640	118.231	1.00	0.00
ATOM	1684	CA	ARG	A	108	147.162	66.234	116.752	1.00	0.00
ATOM	1685	HA	ARG	A	108	146.643	66.155	115.798	1.00	0.00
ATOM	1686	CB	ARG	A	108	146.291	67.038	117.736	1.00	0.00
ATOM	1687	HB1	ARG	A	108	146.327	68.094	117.464	1.00	0.00
ATOM	1688	HB2	ARG	A	108	146.722	66.963	118.726	1.00	0.00
ATOM	1689	CG	ARG	A	108	144.814	66.621	117.802	1.00	0.00
ATOM	1690	HG1	ARG	A	108	144.335	67.199	118.594	1.00	0.00
ATOM	1691	HG2	ARG	A	108	144.732	65.561	118.046	1.00	0.00
ATOM	1692	CD	ARG	A	108	144.112	66.911	116.469	1.00	0.00
ATOM	1693	HD1	ARG	A	108	144.399	66.142	115.752	1.00	0.00
ATOM	1694	HD2	ARG	A	108	144.456	67.874	116.088	1.00	0.00
ATOM	1695	NE	ARG	A	108	142.643	66.938	116.585	1.00	0.00
ATOM	1696	HE	ARG	A	108	142.158	66.069	116.445	1.00	0.00
ATOM	1697	CZ	ARG	A	108	141.900	67.982	116.893	1.00	0.00
ATOM	1698	NH1	ARG	A	108	142.395	69.151	117.189	1.00	0.00
ATOM	1699	1HH1	ARG	A	108	143.393	69.294	117.147	1.00	0.00
ATOM	1700	2HH1	ARG	A	108	141.763	69.907	117.426	1.00	0.00
ATOM	1701	NH2	ARG	A	108	140.609	67.873	116.910	1.00	0.00
ATOM	1702	1HH2	ARG	A	108	140.120	67.087	116.504	1.00	0.00
ATOM	1703	2HH2	ARG	A	108	140.077	68.678	117.206	1.00	0.00
ATOM	1704	C	ARG	A	108	148.426	67.022	116.422	1.00	0.00
ATOM	1705	O	ARG	A	108	148.354	68.027	115.723	1.00	0.00
ATOM	1706	N	GLY	A	109	149.565	66.574	116.949	1.00	0.00

ATOM	1707	H	GLY	A	109	149.519	65.787	117.575	1.00	0.00
ATOM	1708	CA	GLY	A	109	150.873	67.141	116.656	1.00	0.00
ATOM	1709	HA1	GLY	A	109	150.986	67.174	115.579	1.00	0.00
ATOM	1710	HA2	GLY	A	109	151.637	66.492	117.060	1.00	0.00
ATOM	1711	C	GLY	A	109	151.039	68.573	117.191	1.00	0.00
ATOM	1712	O	GLY	A	109	151.694	69.404	116.559	1.00	0.00
ATOM	1713	N	ASN	A	110	150.434	68.898	118.337	1.00	0.00
ATOM	1714	H	ASN	A	110	149.873	68.197	118.803	1.00	0.00
ATOM	1715	CA	ASN	A	110	150.304	70.291	118.765	1.00	0.00
ATOM	1716	HA	ASN	A	110	150.311	70.911	117.863	1.00	0.00
ATOM	1717	CB	ASN	A	110	148.921	70.480	119.421	1.00	0.00
ATOM	1718	HB1	ASN	A	110	149.004	70.371	120.502	1.00	0.00
ATOM	1719	HB2	ASN	A	110	148.236	69.716	119.053	1.00	0.00
ATOM	1720	CG	ASN	A	110	148.271	71.814	119.096	1.00	0.00
ATOM	1721	OD1	ASN	A	110	147.151	71.883	118.620	1.00	0.00
ATOM	1722	ND2	ASN	A	110	148.935	72.922	119.333	1.00	0.00
ATOM	1723	1HD2	ASN	A	110	149.887	72.877	119.686	1.00	0.00
ATOM	1724	2HD2	ASN	A	110	148.487	73.778	119.068	1.00	0.00
ATOM	1725	C	ASN	A	110	151.477	70.808	119.638	1.00	0.00
ATOM	1726	O	ASN	A	110	151.480	71.981	120.024	1.00	0.00
ATOM	1727	N	MET	A	111	152.483	69.979	119.944	1.00	0.00
ATOM	1728	H	MET	A	111	152.464	69.034	119.591	1.00	0.00
ATOM	1729	CA	MET	A	111	153.665	70.392	120.715	1.00	0.00
ATOM	1730	HA	MET	A	111	153.928	71.414	120.444	1.00	0.00
ATOM	1731	CB	MET	A	111	153.324	70.356	122.219	1.00	0.00
ATOM	1732	HB1	MET	A	111	153.082	69.334	122.510	1.00	0.00
ATOM	1733	HB2	MET	A	111	152.447	70.979	122.401	1.00	0.00
ATOM	1734	CG	MET	A	111	154.474	70.876	123.093	1.00	0.00
ATOM	1735	HG1	MET	A	111	154.709	71.892	122.778	1.00	0.00
ATOM	1736	HG2	MET	A	111	155.361	70.261	122.940	1.00	0.00
ATOM	1737	SD	MET	A	111	154.125	70.919	124.872	1.00	0.00
ATOM	1738	CE	MET	A	111	154.248	69.155	125.277	1.00	0.00
ATOM	1739	HE1	MET	A	111	154.047	69.010	126.338	1.00	0.00
ATOM	1740	HE2	MET	A	111	153.522	68.591	124.694	1.00	0.00
ATOM	1741	HE3	MET	A	111	155.250	68.788	125.054	1.00	0.00
ATOM	1742	C	MET	A	111	154.882	69.493	120.471	1.00	0.00
ATOM	1743	O	MET	A	111	154.787	68.289	120.630	1.00	0.00
ATOM	1744	N	TYR	A	112	156.013	70.112	120.135	1.00	0.00
ATOM	1745	H	TYR	A	112	155.975	71.117	120.077	1.00	0.00
ATOM	1746	CA	TYR	A	112	157.298	69.513	119.740	1.00	0.00
ATOM	1747	HA	TYR	A	112	157.175	69.101	118.750	1.00	0.00
ATOM	1748	CB	TYR	A	112	158.324	70.660	119.601	1.00	0.00
ATOM	1749	HB1	TYR	A	112	159.080	70.369	118.873	1.00	0.00
ATOM	1750	HB2	TYR	A	112	158.833	70.787	120.558	1.00	0.00
ATOM	1751	CG	TYR	A	112	157.739	72.007	119.193	1.00	0.00
ATOM	1752	CD1	TYR	A	112	157.271	72.216	117.881	1.00	0.00
ATOM	1753	HD1	TYR	A	112	157.380	71.438	117.138	1.00	0.00
ATOM	1754	CE1	TYR	A	112	156.655	73.436	117.535	1.00	0.00
ATOM	1755	HE1	TYR	A	112	156.293	73.602	116.534	1.00	0.00
ATOM	1756	CZ	TYR	A	112	156.493	74.450	118.505	1.00	0.00
ATOM	1757	OH	TYR	A	112	155.879	75.617	118.174	1.00	0.00
ATOM	1758	HH	TYR	A	112	155.806	76.215	118.919	1.00	0.00
ATOM	1759	CE2	TYR	A	112	156.956	74.238	119.821	1.00	0.00
ATOM	1760	HE2	TYR	A	112	156.836	75.007	120.566	1.00	0.00
ATOM	1761	CD2	TYR	A	112	157.577	73.020	120.161	1.00	0.00
ATOM	1762	HD2	TYR	A	112	157.927	72.862	121.171	1.00	0.00
ATOM	1763	C	TYR	A	112	157.854	68.417	120.700	1.00	0.00
ATOM	1764	O	TYR	A	112	157.547	68.397	121.892	1.00	0.00
ATOM	1765	N	TYR	A	113	158.802	67.612	120.191	1.00	0.00
ATOM	1766	H	TYR	A	113	158.973	67.670	119.205	1.00	0.00
ATOM	1767	CA	TYR	A	113	159.790	66.893	121.008	1.00	0.00

ATOM	1768	HA	TYR	A	113	159.656	67.193	122.049	1.00	0.00
ATOM	1769	CB	TYR	A	113	159.631	65.368	120.969	1.00	0.00
ATOM	1770	HB1	TYR	A	113	160.630	64.938	120.973	1.00	0.00
ATOM	1771	HB2	TYR	A	113	159.144	65.064	120.045	1.00	0.00
ATOM	1772	CG	TYR	A	113	158.931	64.772	122.174	1.00	0.00
ATOM	1773	CD1	TYR	A	113	157.683	64.140	122.031	1.00	0.00
ATOM	1774	HD1	TYR	A	113	157.221	64.069	121.062	1.00	0.00
ATOM	1775	CE1	TYR	A	113	157.053	63.559	123.148	1.00	0.00
ATOM	1776	HE1	TYR	A	113	156.107	63.053	123.053	1.00	0.00
ATOM	1777	CZ	TYR	A	113	157.686	63.592	124.407	1.00	0.00
ATOM	1778	OH	TYR	A	113	157.080	63.039	125.480	1.00	0.00
ATOM	1779	HH	TYR	A	113	157.698	62.913	126.226	1.00	0.00
ATOM	1780	CE2	TYR	A	113	158.944	64.205	124.551	1.00	0.00
ATOM	1781	HE2	TYR	A	113	159.416	64.214	125.516	1.00	0.00
ATOM	1782	CD2	TYR	A	113	159.565	64.797	123.433	1.00	0.00
ATOM	1783	HD2	TYR	A	113	160.539	65.261	123.536	1.00	0.00
ATOM	1784	C	TYR	A	113	161.231	67.313	120.667	1.00	0.00
ATOM	1785	O	TYR	A	113	161.981	67.647	121.579	1.00	0.00
ATOM	1786	N	GLU	A	114	161.591	67.433	119.382	1.00	0.00
ATOM	1787	H	GLU	A	114	160.996	67.034	118.676	1.00	0.00
ATOM	1788	CA	GLU	A	114	162.699	68.306	118.958	1.00	0.00
ATOM	1789	HA	GLU	A	114	162.877	69.048	119.738	1.00	0.00
ATOM	1790	CB	GLU	A	114	164.036	67.581	118.720	1.00	0.00
ATOM	1791	HB1	GLU	A	114	164.756	68.327	118.379	1.00	0.00
ATOM	1792	HB2	GLU	A	114	163.894	66.887	117.899	1.00	0.00
ATOM	1793	CG	GLU	A	114	164.695	66.828	119.881	1.00	0.00
ATOM	1794	HG1	GLU	A	114	163.947	66.228	120.399	1.00	0.00
ATOM	1795	HG2	GLU	A	114	165.124	67.542	120.587	1.00	0.00
ATOM	1796	CD	GLU	A	114	165.787	65.906	119.318	1.00	0.00
ATOM	1797	OE1	GLU	A	114	165.785	64.700	119.647	1.00	0.00
ATOM	1798	OE2	GLU	A	114	166.526	66.307	118.393	1.00	0.00
ATOM	1799	C	GLU	A	114	162.292	69.099	117.708	1.00	0.00
ATOM	1800	O	GLU	A	114	161.505	70.036	117.817	1.00	0.00
ATOM	1801	N	ASN	A	115	162.769	68.721	116.516	1.00	0.00
ATOM	1802	H	ASN	A	115	163.422	67.949	116.520	1.00	0.00
ATOM	1803	CA	ASN	A	115	162.514	69.377	115.228	1.00	0.00
ATOM	1804	HA	ASN	A	115	162.634	70.453	115.360	1.00	0.00
ATOM	1805	CB	ASN	A	115	163.537	68.875	114.180	1.00	0.00
ATOM	1806	HB1	ASN	A	115	163.525	69.539	113.316	1.00	0.00
ATOM	1807	HB2	ASN	A	115	163.236	67.885	113.831	1.00	0.00
ATOM	1808	CG	ASN	A	115	164.965	68.741	114.661	1.00	0.00
ATOM	1809	OD1	ASN	A	115	165.502	67.649	114.734	1.00	0.00
ATOM	1810	ND2	ASN	A	115	165.616	69.812	115.043	1.00	0.00
ATOM	1811	1HD2	ASN	A	115	165.198	70.721	114.980	1.00	0.00
ATOM	1812	2HD2	ASN	A	115	166.551	69.661	115.381	1.00	0.00
ATOM	1813	C	ASN	A	115	161.070	69.132	114.717	1.00	0.00
ATOM	1814	O	ASN	A	115	160.873	68.685	113.590	1.00	0.00
ATOM	1815	N	SER	A	116	160.060	69.340	115.562	1.00	0.00
ATOM	1816	H	SER	A	116	160.287	69.764	116.454	1.00	0.00
ATOM	1817	CA	SER	A	116	158.650	69.054	115.269	1.00	0.00
ATOM	1818	HA	SER	A	116	158.116	69.250	116.194	1.00	0.00
ATOM	1819	CB	SER	A	116	158.089	70.032	114.223	1.00	0.00
ATOM	1820	HB1	SER	A	116	157.030	69.826	114.062	1.00	0.00
ATOM	1821	HB2	SER	A	116	158.622	69.899	113.282	1.00	0.00
ATOM	1822	OG	SER	A	116	158.240	71.363	114.672	1.00	0.00
ATOM	1823	HG	SER	A	116	158.082	71.955	113.932	1.00	0.00
ATOM	1824	C	SER	A	116	158.347	67.584	114.899	1.00	0.00
ATOM	1825	O	SER	A	116	157.415	67.289	114.147	1.00	0.00
ATOM	1826	N	TYR	A	117	159.103	66.619	115.439	1.00	0.00
ATOM	1827	H	TYR	A	117	159.907	66.868	115.988	1.00	0.00
ATOM	1828	CA	TYR	A	117	158.806	65.197	115.229	1.00	0.00

ATOM	1829	HA	TYR	A	117	158.540	65.127	114.186	1.00	0.00
ATOM	1830	CB	TYR	A	117	160.061	64.305	115.385	1.00	0.00
ATOM	1831	HB1	TYR	A	117	160.717	64.547	114.549	1.00	0.00
ATOM	1832	HB2	TYR	A	117	159.745	63.277	115.213	1.00	0.00
ATOM	1833	CG	TYR	A	117	160.907	64.318	116.660	1.00	0.00
ATOM	1834	CD1	TYR	A	117	160.458	63.705	117.848	1.00	0.00
ATOM	1835	HD1	TYR	A	117	159.474	63.270	117.919	1.00	0.00
ATOM	1836	CE1	TYR	A	117	161.324	63.479	118.929	1.00	0.00
ATOM	1837	HE1	TYR	A	117	160.970	62.976	119.815	1.00	0.00
ATOM	1838	CZ	TYR	A	117	162.675	63.836	118.847	1.00	0.00
ATOM	1839	OH	TYR	A	117	163.512	63.519	119.874	1.00	0.00
ATOM	1840	HH	TYR	A	117	164.412	63.910	119.773	1.00	0.00
ATOM	1841	CE2	TYR	A	117	163.127	64.473	117.677	1.00	0.00
ATOM	1842	HE2	TYR	A	117	164.161	64.752	117.609	1.00	0.00
ATOM	1843	CD2	TYR	A	117	162.253	64.729	116.599	1.00	0.00
ATOM	1844	HD2	TYR	A	117	162.639	65.172	115.689	1.00	0.00
ATOM	1845	C	TYR	A	117	157.553	64.664	115.966	1.00	0.00
ATOM	1846	O	TYR	A	117	157.623	64.180	117.087	1.00	0.00
ATOM	1847	N	ALA	A	118	156.369	64.734	115.347	1.00	0.00
ATOM	1848	H	ALA	A	118	156.317	65.261	114.489	1.00	0.00
ATOM	1849	CA	ALA	A	118	155.096	64.340	115.989	1.00	0.00
ATOM	1850	HA	ALA	A	118	154.954	64.974	116.849	1.00	0.00
ATOM	1851	CB	ALA	A	118	153.946	64.623	115.020	1.00	0.00
ATOM	1852	HB1	ALA	A	118	153.010	64.243	115.430	1.00	0.00
ATOM	1853	HB2	ALA	A	118	153.835	65.697	114.891	1.00	0.00
ATOM	1854	HB3	ALA	A	118	154.134	64.150	114.057	1.00	0.00
ATOM	1855	C	ALA	A	118	155.017	62.902	116.529	1.00	0.00
ATOM	1856	O	ALA	A	118	154.269	62.632	117.474	1.00	0.00
ATOM	1857	N	LEU	A	119	155.826	62.011	115.959	1.00	0.00
ATOM	1858	H	LEU	A	119	156.434	62.356	115.235	1.00	0.00
ATOM	1859	CA	LEU	A	119	156.232	60.757	116.573	1.00	0.00
ATOM	1860	HA	LEU	A	119	156.047	60.821	117.640	1.00	0.00
ATOM	1861	CB	LEU	A	119	155.385	59.592	116.005	1.00	0.00
ATOM	1862	HB1	LEU	A	119	155.544	59.509	114.929	1.00	0.00
ATOM	1863	HB2	LEU	A	119	154.338	59.861	116.156	1.00	0.00
ATOM	1864	CG	LEU	A	119	155.628	58.212	116.670	1.00	0.00
ATOM	1865	HG	LEU	A	119	155.964	58.361	117.698	1.00	0.00
ATOM	1866	CD1	LEU	A	119	154.340	57.386	116.710	1.00	0.00
ATOM	1867	1HD1	LEU	A	119	154.516	56.441	117.227	1.00	0.00
ATOM	1868	2HD1	LEU	A	119	153.568	57.934	117.248	1.00	0.00
ATOM	1869	3HD1	LEU	A	119	153.991	57.182	115.698	1.00	0.00
ATOM	1870	CD2	LEU	A	119	156.665	57.377	115.912	1.00	0.00
ATOM	1871	1HD2	LEU	A	119	156.800	56.422	116.420	1.00	0.00
ATOM	1872	2HD2	LEU	A	119	156.334	57.194	114.891	1.00	0.00
ATOM	1873	3HD2	LEU	A	119	157.624	57.886	115.883	1.00	0.00
ATOM	1874	C	LEU	A	119	157.735	60.614	116.349	1.00	0.00
ATOM	1875	O	LEU	A	119	158.237	60.946	115.265	1.00	0.00
ATOM	1876	N	ALA	A	120	158.419	60.077	117.354	1.00	0.00
ATOM	1877	H	ALA	A	120	157.932	59.904	118.225	1.00	0.00
ATOM	1878	CA	ALA	A	120	159.766	59.546	117.229	1.00	0.00
ATOM	1879	HA	ALA	A	120	159.927	59.291	116.182	1.00	0.00
ATOM	1880	CB	ALA	A	120	160.792	60.596	117.617	1.00	0.00
ATOM	1881	HB1	ALA	A	120	160.731	61.400	116.891	1.00	0.00
ATOM	1882	HB2	ALA	A	120	160.572	60.951	118.620	1.00	0.00
ATOM	1883	HB3	ALA	A	120	161.799	60.180	117.592	1.00	0.00
ATOM	1884	C	ALA	A	120	159.963	58.258	118.022	1.00	0.00
ATOM	1885	O	ALA	A	120	159.245	57.979	118.980	1.00	0.00
ATOM	1886	N	VAL	A	121	160.987	57.510	117.621	1.00	0.00
ATOM	1887	H	VAL	A	121	161.553	57.853	116.851	1.00	0.00
ATOM	1888	CA	VAL	A	121	161.307	56.190	118.156	1.00	0.00
ATOM	1889	HA	VAL	A	121	160.734	56.032	119.059	1.00	0.00

ATOM	1890	CB	VAL	A	121	160.943	55.070	117.149	1.00	0.00
ATOM	1891	HB	VAL	A	121	161.607	55.112	116.286	1.00	0.00
ATOM	1892	CG1	VAL	A	121	161.082	53.689	117.793	1.00	0.00
ATOM	1893	1HG1	VAL	A	121	160.859	52.912	117.063	1.00	0.00
ATOM	1894	2HG1	VAL	A	121	162.103	53.548	118.140	1.00	0.00
ATOM	1895	3HG1	VAL	A	121	160.400	53.587	118.636	1.00	0.00
ATOM	1896	CG2	VAL	A	121	159.512	55.217	116.619	1.00	0.00
ATOM	1897	1HG2	VAL	A	121	159.186	54.290	116.152	1.00	0.00
ATOM	1898	2HG2	VAL	A	121	158.834	55.463	117.428	1.00	0.00
ATOM	1899	3HG2	VAL	A	121	159.478	56.014	115.875	1.00	0.00
ATOM	1900	C	VAL	A	121	162.793	56.171	118.499	1.00	0.00
ATOM	1901	O	VAL	A	121	163.582	55.985	117.566	1.00	0.00
ATOM	1902	N	LEU	A	122	163.212	56.464	119.756	1.00	0.00
ATOM	1903	H	LEU	A	122	162.557	56.524	120.522	1.00	0.00
ATOM	1904	CA	LEU	A	122	164.668	56.624	119.926	1.00	0.00
ATOM	1905	HA	LEU	A	122	165.091	56.988	118.992	1.00	0.00
ATOM	1906	CB	LEU	A	122	165.002	57.664	121.007	1.00	0.00
ATOM	1907	HB1	LEU	A	122	164.948	57.164	121.963	1.00	0.00
ATOM	1908	HB2	LEU	A	122	164.259	58.463	120.973	1.00	0.00
ATOM	1909	CG	LEU	A	122	166.399	58.313	120.918	1.00	0.00
ATOM	1910	HG	LEU	A	122	167.165	57.550	121.048	1.00	0.00
ATOM	1911	CD1	LEU	A	122	166.640	59.029	119.588	1.00	0.00
ATOM	1912	1HD1	LEU	A	122	167.546	59.632	119.650	1.00	0.00
ATOM	1913	2HD1	LEU	A	122	166.782	58.305	118.787	1.00	0.00
ATOM	1914	3HD1	LEU	A	122	165.797	59.678	119.351	1.00	0.00
ATOM	1915	CD2	LEU	A	122	166.555	59.341	122.031	1.00	0.00
ATOM	1916	1HD2	LEU	A	122	167.570	59.736	122.031	1.00	0.00
ATOM	1917	2HD2	LEU	A	122	165.849	60.158	121.889	1.00	0.00
ATOM	1918	3HD2	LEU	A	122	166.370	58.879	122.998	1.00	0.00
ATOM	1919	C	LEU	A	122	165.283	55.259	120.195	1.00	0.00
ATOM	1920	O	LEU	A	122	164.552	54.351	120.553	1.00	0.00
ATOM	1921	N	SER	A	123	166.600	55.130	120.068	1.00	0.00
ATOM	1922	H	SER	A	123	167.125	55.938	119.763	1.00	0.00
ATOM	1923	CA	SER	A	123	167.400	53.904	120.193	1.00	0.00
ATOM	1924	HA	SER	A	123	167.273	53.352	119.275	1.00	0.00
ATOM	1925	CB	SER	A	123	168.879	54.291	120.229	1.00	0.00
ATOM	1926	HB1	SER	A	123	169.501	53.397	120.289	1.00	0.00
ATOM	1927	HB2	SER	A	123	169.078	54.933	121.089	1.00	0.00
ATOM	1928	OG	SER	A	123	169.156	54.982	119.029	1.00	0.00
ATOM	1929	HG	SER	A	123	169.140	54.310	118.321	1.00	0.00
ATOM	1930	C	SER	A	123	167.029	52.939	121.326	1.00	0.00
ATOM	1931	O	SER	A	123	167.721	52.859	122.332	1.00	0.00
ATOM	1932	N	ASN	A	124	165.933	52.194	121.171	1.00	0.00
ATOM	1933	H	ASN	A	124	165.343	52.443	120.389	1.00	0.00
ATOM	1934	CA	ASN	A	124	165.440	51.129	122.039	1.00	0.00
ATOM	1935	HA	ASN	A	124	165.348	51.486	123.059	1.00	0.00
ATOM	1936	CB	ASN	A	124	164.047	50.689	121.546	1.00	0.00
ATOM	1937	HB1	ASN	A	124	163.706	49.883	122.185	1.00	0.00
ATOM	1938	HB2	ASN	A	124	164.123	50.288	120.537	1.00	0.00
ATOM	1939	CG	ASN	A	124	162.997	51.784	121.564	1.00	0.00
ATOM	1940	OD1	ASN	A	124	162.727	52.410	122.569	1.00	0.00
ATOM	1941	ND2	ASN	A	124	162.375	52.075	120.453	1.00	0.00
ATOM	1942	1HD2	ASN	A	124	162.577	51.576	119.593	1.00	0.00
ATOM	1943	2HD2	ASN	A	124	161.812	52.916	120.469	1.00	0.00
ATOM	1944	C	ASN	A	124	166.441	49.966	122.027	1.00	0.00
ATOM	1945	O	ASN	A	124	166.287	49.039	121.235	1.00	0.00
ATOM	1946	N	TYR	A	125	167.526	50.086	122.792	1.00	0.00
ATOM	1947	H	TYR	A	125	167.625	50.923	123.355	1.00	0.00
ATOM	1948	CA	TYR	A	125	168.760	49.343	122.543	1.00	0.00
ATOM	1949	HA	TYR	A	125	168.525	48.306	122.315	1.00	0.00
ATOM	1950	CB	TYR	A	125	169.440	49.991	121.318	1.00	0.00

ATOM	1951	HB1	TYR	A	125	169.583	51.050	121.538	1.00	0.00
ATOM	1952	HB2	TYR	A	125	168.757	49.932	120.470	1.00	0.00
ATOM	1953	CG	TYR	A	125	170.765	49.410	120.862	1.00	0.00
ATOM	1954	CD1	TYR	A	125	170.908	48.027	120.635	1.00	0.00
ATOM	1955	HD1	TYR	A	125	170.078	47.361	120.832	1.00	0.00
ATOM	1956	CE1	TYR	A	125	172.118	47.521	120.124	1.00	0.00
ATOM	1957	HE1	TYR	A	125	172.220	46.470	119.917	1.00	0.00
ATOM	1958	CZ	TYR	A	125	173.187	48.393	119.833	1.00	0.00
ATOM	1959	OH	TYR	A	125	174.354	47.895	119.345	1.00	0.00
ATOM	1960	HH	TYR	A	125	174.935	48.595	119.041	1.00	0.00
ATOM	1961	CE2	TYR	A	125	173.045	49.777	120.062	1.00	0.00
ATOM	1962	HE2	TYR	A	125	173.861	50.451	119.847	1.00	0.00
ATOM	1963	CD2	TYR	A	125	171.836	50.280	120.577	1.00	0.00
ATOM	1964	HD2	TYR	A	125	171.727	51.346	120.744	1.00	0.00
ATOM	1965	C	TYR	A	125	169.679	49.392	123.769	1.00	0.00
ATOM	1966	O	TYR	A	125	170.131	50.470	124.159	1.00	0.00
ATOM	1967	N	ASP	A	126	169.934	48.244	124.395	1.00	0.00
ATOM	1968	H	ASP	A	126	169.539	47.384	124.026	1.00	0.00
ATOM	1969	CA	ASP	A	126	170.607	48.178	125.696	1.00	0.00
ATOM	1970	HA	ASP	A	126	170.624	49.180	126.125	1.00	0.00
ATOM	1971	CB	ASP	A	126	169.789	47.325	126.668	1.00	0.00
ATOM	1972	HB1	ASP	A	126	168.737	47.553	126.592	1.00	0.00
ATOM	1973	HB2	ASP	A	126	170.088	47.587	127.685	1.00	0.00
ATOM	1974	CG	ASP	A	126	170.047	45.840	126.447	1.00	0.00
ATOM	1975	OD1	ASP	A	126	170.345	45.186	127.465	1.00	0.00
ATOM	1976	OD2	ASP	A	126	170.192	45.412	125.277	1.00	0.00
ATOM	1977	C	ASP	A	126	172.081	47.714	125.614	1.00	0.00
ATOM	1978	O	ASP	A	126	172.724	47.718	124.562	1.00	0.00
ATOM	1979	N	ALA	A	127	172.647	47.298	126.752	1.00	0.00
ATOM	1980	H	ALA	A	127	172.081	47.286	127.588	1.00	0.00
ATOM	1981	CA	ALA	A	127	173.928	46.608	126.804	1.00	0.00
ATOM	1982	HA	ALA	A	127	174.669	47.192	126.260	1.00	0.00
ATOM	1983	CB	ALA	A	127	174.359	46.556	128.274	1.00	0.00
ATOM	1984	HB1	ALA	A	127	175.332	46.071	128.357	1.00	0.00
ATOM	1985	HB2	ALA	A	127	174.436	47.567	128.679	1.00	0.00
ATOM	1986	HB3	ALA	A	127	173.631	45.995	128.862	1.00	0.00
ATOM	1987	C	ALA	A	127	173.890	45.182	126.209	1.00	0.00
ATOM	1988	O	ALA	A	127	174.831	44.825	125.497	1.00	0.00
ATOM	1989	N	ASN	A	128	172.821	44.405	126.418	1.00	0.00
ATOM	1990	H	ASN	A	128	172.044	44.783	126.962	1.00	0.00
ATOM	1991	CA	ASN	A	128	172.603	43.061	125.863	1.00	0.00
ATOM	1992	HA	ASN	A	128	173.423	42.425	126.201	1.00	0.00
ATOM	1993	CB	ASN	A	128	171.276	42.512	126.460	1.00	0.00
ATOM	1994	HB1	ASN	A	128	170.450	43.090	126.060	1.00	0.00
ATOM	1995	HB2	ASN	A	128	171.291	42.656	127.540	1.00	0.00
ATOM	1996	CG	ASN	A	128	170.884	41.057	126.227	1.00	0.00
ATOM	1997	OD1	ASN	A	128	169.838	40.605	126.677	1.00	0.00
ATOM	1998	ND2	ASN	A	128	171.639	40.266	125.508	1.00	0.00
ATOM	1999	1HD2	ASN	A	128	172.400	40.653	124.959	1.00	0.00
ATOM	2000	2HD2	ASN	A	128	171.332	39.322	125.386	1.00	0.00
ATOM	2001	C	ASN	A	128	172.601	43.026	124.324	1.00	0.00
ATOM	2002	O	ASN	A	128	172.942	41.981	123.776	1.00	0.00
ATOM	2003	N	LYS	A	129	172.240	44.136	123.652	1.00	0.00
ATOM	2004	H	LYS	A	129	171.886	44.880	124.253	1.00	0.00
ATOM	2005	CA	LYS	A	129	171.811	44.202	122.229	1.00	0.00
ATOM	2006	HA	LYS	A	129	171.641	45.247	121.973	1.00	0.00
ATOM	2007	CB	LYS	A	129	172.830	43.611	121.210	1.00	0.00
ATOM	2008	HB1	LYS	A	129	172.323	43.551	120.251	1.00	0.00
ATOM	2009	HB2	LYS	A	129	173.056	42.579	121.466	1.00	0.00
ATOM	2010	CG	LYS	A	129	174.159	44.317	120.872	1.00	0.00
ATOM	2011	HG1	LYS	A	129	173.972	45.110	120.150	1.00	0.00

```
ATOM   2012  HG2 LYS A 129     174.786  43.586 120.361  1.00  0.00
ATOM   2013  CD  LYS A 129     174.973  44.892 122.031  1.00  0.00
ATOM   2014  HD1 LYS A 129     176.006  45.014 121.703  1.00  0.00
ATOM   2015  HD2 LYS A 129     174.949  44.196 122.872  1.00  0.00
ATOM   2016  CE  LYS A 129     174.407  46.256 122.419  1.00  0.00
ATOM   2017  HE1 LYS A 129     173.355  46.138 122.684  1.00  0.00
ATOM   2018  HE2 LYS A 129     174.460  46.934 121.564  1.00  0.00
ATOM   2019  NZ  LYS A 129     175.119  46.827 123.573  1.00  0.00
ATOM   2020  HZ1 LYS A 129     174.505  47.527 123.990  1.00  0.00
ATOM   2021  HZ2 LYS A 129     175.249  46.097 124.269  1.00  0.00
ATOM   2022  HZ3 LYS A 129     176.010  47.222 123.319  1.00  0.00
ATOM   2023  C   LYS A 129     170.422  43.550 122.085  1.00  0.00
ATOM   2024  O   LYS A 129     170.161  42.776 121.168  1.00  0.00
ATOM   2025  N   THR A 130     169.528  43.878 123.010  1.00  0.00
ATOM   2026  H   THR A 130     169.822  44.503 123.759  1.00  0.00
ATOM   2027  CA  THR A 130     168.084  43.652 122.887  1.00  0.00
ATOM   2028  HA  THR A 130     167.884  43.182 121.924  1.00  0.00
ATOM   2029  CB  THR A 130     167.605  42.665 123.953  1.00  0.00
ATOM   2030  HB  THR A 130     168.399  41.932 124.093  1.00  0.00
ATOM   2031  CG2 THR A 130     167.303  43.291 125.310  1.00  0.00
ATOM   2032 1HG2 THR A 130     166.339  43.789 125.302  1.00  0.00
ATOM   2033 2HG2 THR A 130     167.300  42.520 126.072  1.00  0.00
ATOM   2034 3HG2 THR A 130     168.074  44.010 125.571  1.00  0.00
ATOM   2035  OG1 THR A 130     166.490  41.962 123.455  1.00  0.00
ATOM   2036  HG1 THR A 130     165.660  42.399 123.785  1.00  0.00
ATOM   2037  C   THR A 130     167.358  45.003 122.869  1.00  0.00
ATOM   2038  O   THR A 130     168.015  46.045 122.914  1.00  0.00
ATOM   2039  N   GLY A 131     166.036  45.036 122.678  1.00  0.00
ATOM   2040  H   GLY A 131     165.466  44.201 122.814  1.00  0.00
ATOM   2041  CA  GLY A 131     165.400  46.248 122.159  1.00  0.00
ATOM   2042  HA1 GLY A 131     165.837  46.484 121.192  1.00  0.00
ATOM   2043  HA2 GLY A 131     165.618  47.084 122.811  1.00  0.00
ATOM   2044  C   GLY A 131     163.880  46.154 121.998  1.00  0.00
ATOM   2045  O   GLY A 131     163.209  45.279 122.549  1.00  0.00
ATOM   2046  N   LEU A 132     163.303  47.086 121.247  1.00  0.00
ATOM   2047  H   LEU A 132     163.898  47.778 120.815  1.00  0.00
ATOM   2048  CA  LEU A 132     161.902  47.019 120.834  1.00  0.00
ATOM   2049  HA  LEU A 132     161.300  46.602 121.645  1.00  0.00
ATOM   2050  CB  LEU A 132     161.436  48.468 120.569  1.00  0.00
ATOM   2051  HB1 LEU A 132     161.936  48.854 119.680  1.00  0.00
ATOM   2052  HB2 LEU A 132     161.760  49.067 121.415  1.00  0.00
ATOM   2053  CG  LEU A 132     159.923  48.717 120.441  1.00  0.00
ATOM   2054  HG  LEU A 132     159.435  48.404 121.364  1.00  0.00
ATOM   2055  CD1 LEU A 132     159.662  50.214 120.242  1.00  0.00
ATOM   2056 1HD1 LEU A 132     158.590  50.397 120.188  1.00  0.00
ATOM   2057 2HD1 LEU A 132     160.073  50.774 121.073  1.00  0.00
ATOM   2058 3HD1 LEU A 132     160.140  50.552 119.323  1.00  0.00
ATOM   2059  CD2 LEU A 132     159.274  48.012 119.261  1.00  0.00
ATOM   2060 1HD2 LEU A 132     158.223  48.288 119.224  1.00  0.00
ATOM   2061 2HD2 LEU A 132     159.775  48.269 118.328  1.00  0.00
ATOM   2062 3HD2 LEU A 132     159.298  46.945 119.419  1.00  0.00
ATOM   2063  C   LEU A 132     161.772  46.110 119.600  1.00  0.00
ATOM   2064  O   LEU A 132     162.364  46.441 118.582  1.00  0.00
ATOM   2065  N   LYS A 133     161.000  45.014 119.681  1.00  0.00
ATOM   2066  H   LYS A 133     160.528  44.823 120.550  1.00  0.00
ATOM   2067  CA  LYS A 133     160.800  44.067 118.568  1.00  0.00
ATOM   2068  HA  LYS A 133     161.440  44.381 117.749  1.00  0.00
ATOM   2069  CB  LYS A 133     161.234  42.636 118.976  1.00  0.00
ATOM   2070  HB1 LYS A 133     160.347  42.014 119.110  1.00  0.00
ATOM   2071  HB2 LYS A 133     161.738  42.676 119.942  1.00  0.00
ATOM   2072  CG  LYS A 133     162.197  41.928 117.995  1.00  0.00
```

ATOM	2073	HG1	LYS	A	133	162.378	40.921	118.374	1.00	0.00
ATOM	2074	HG2	LYS	A	133	163.152	42.449	118.011	1.00	0.00
ATOM	2075	CD	LYS	A	133	161.687	41.810	116.544	1.00	0.00
ATOM	2076	HD1	LYS	A	133	161.454	42.795	116.142	1.00	0.00
ATOM	2077	HD2	LYS	A	133	160.767	41.223	116.556	1.00	0.00
ATOM	2078	CE	LYS	A	133	162.682	41.138	115.584	1.00	0.00
ATOM	2079	HE1	LYS	A	133	162.135	40.832	114.691	1.00	0.00
ATOM	2080	HE2	LYS	A	133	163.085	40.232	116.044	1.00	0.00
ATOM	2081	NZ	LYS	A	133	163.781	42.046	115.180	1.00	0.00
ATOM	2082	HZ1	LYS	A	133	164.311	41.648	114.407	1.00	0.00
ATOM	2083	HZ2	LYS	A	133	163.432	42.969	114.919	1.00	0.00
ATOM	2084	HZ3	LYS	A	133	164.433	42.171	115.947	1.00	0.00
ATOM	2085	C	LYS	A	133	159.378	44.090	117.979	1.00	0.00
ATOM	2086	O	LYS	A	133	159.213	43.598	116.871	1.00	0.00
ATOM	2087	N	GLU	A	134	158.354	44.672	118.620	1.00	0.00
ATOM	2088	H	GLU	A	134	158.465	45.059	119.544	1.00	0.00
ATOM	2089	CA	GLU	A	134	156.989	44.470	118.120	1.00	0.00
ATOM	2090	HA	GLU	A	134	156.982	44.228	117.055	1.00	0.00
ATOM	2091	CB	GLU	A	134	156.399	43.256	118.876	1.00	0.00
ATOM	2092	HB1	GLU	A	134	156.457	43.483	119.929	1.00	0.00
ATOM	2093	HB2	GLU	A	134	157.051	42.398	118.704	1.00	0.00
ATOM	2094	CG	GLU	A	134	154.959	42.818	118.570	1.00	0.00
ATOM	2095	HG1	GLU	A	134	154.935	42.294	117.611	1.00	0.00
ATOM	2096	HG2	GLU	A	134	154.309	43.686	118.499	1.00	0.00
ATOM	2097	CD	GLU	A	134	154.439	41.903	119.698	1.00	0.00
ATOM	2098	OE1	GLU	A	134	153.785	42.414	120.632	1.00	0.00
ATOM	2099	OE2	GLU	A	134	154.799	40.698	119.716	1.00	0.00
ATOM	2100	C	GLU	A	134	156.175	45.749	118.285	1.00	0.00
ATOM	2101	O	GLU	A	134	155.557	45.945	119.320	1.00	0.00
ATOM	2102	N	LEU	A	135	156.216	46.649	117.300	1.00	0.00
ATOM	2103	H	LEU	A	135	156.716	46.406	116.458	1.00	0.00
ATOM	2104	CA	LEU	A	135	155.702	48.025	117.397	1.00	0.00
ATOM	2105	HA	LEU	A	135	155.562	48.257	118.448	1.00	0.00
ATOM	2106	CB	LEU	A	135	156.804	48.977	116.844	1.00	0.00
ATOM	2107	HB1	LEU	A	135	156.734	49.025	115.760	1.00	0.00
ATOM	2108	HB2	LEU	A	135	157.772	48.526	117.050	1.00	0.00
ATOM	2109	CG	LEU	A	135	156.837	50.407	117.426	1.00	0.00
ATOM	2110	HG	LEU	A	135	156.843	50.352	118.515	1.00	0.00
ATOM	2111	CD1	LEU	A	135	158.118	51.114	116.977	1.00	0.00
ATOM	2112	1HD1	LEU	A	135	158.173	52.110	117.413	1.00	0.00
ATOM	2113	2HD1	LEU	A	135	158.980	50.543	117.311	1.00	0.00
ATOM	2114	3HD1	LEU	A	135	158.142	51.193	115.892	1.00	0.00
ATOM	2115	CD2	LEU	A	135	155.672	51.307	117.002	1.00	0.00
ATOM	2116	1HD2	LEU	A	135	155.811	52.312	117.392	1.00	0.00
ATOM	2117	2HD2	LEU	A	135	155.607	51.344	115.915	1.00	0.00
ATOM	2118	3HD2	LEU	A	135	154.741	50.915	117.404	1.00	0.00
ATOM	2119	C	LEU	A	135	154.336	48.190	116.669	1.00	0.00
ATOM	2120	O	LEU	A	135	154.241	48.996	115.745	1.00	0.00
ATOM	2121	N	PRO	A	136	153.260	47.434	116.999	1.00	0.00
ATOM	2122	CD	PRO	A	136	153.089	46.427	118.035	1.00	0.00
ATOM	2123	HD1	PRO	A	136	153.400	46.789	119.010	1.00	0.00
ATOM	2124	HD2	PRO	A	136	153.639	45.539	117.739	1.00	0.00
ATOM	2125	CG	PRO	A	136	151.610	46.062	118.101	1.00	0.00
ATOM	2126	HG1	PRO	A	136	151.082	46.741	118.772	1.00	0.00
ATOM	2127	HG2	PRO	A	136	151.464	45.024	118.403	1.00	0.00
ATOM	2128	CB	PRO	A	136	151.194	46.302	116.662	1.00	0.00
ATOM	2129	HB1	PRO	A	136	150.122	46.464	116.559	1.00	0.00
ATOM	2130	HB2	PRO	A	136	151.508	45.450	116.067	1.00	0.00
ATOM	2131	CA	PRO	A	136	152.017	47.524	116.251	1.00	0.00
ATOM	2132	HA	PRO	A	136	152.223	47.452	115.189	1.00	0.00
ATOM	2133	C	PRO	A	136	151.260	48.818	116.564	1.00	0.00

```
ATOM   2134  O   PRO A 136     150.986  49.147 117.721  1.00  0.00
ATOM   2135  N   MET A 137     150.874  49.512 115.495  1.00  0.00
ATOM   2136  H   MET A 137     151.215  49.215 114.590  1.00  0.00
ATOM   2137  CA  MET A 137     150.043  50.712 115.516  1.00  0.00
ATOM   2138  HA  MET A 137     149.483  50.743 116.442  1.00  0.00
ATOM   2139  CB  MET A 137     150.889  51.999 115.521  1.00  0.00
ATOM   2140  HB1 MET A 137     150.211  52.839 115.642  1.00  0.00
ATOM   2141  HB2 MET A 137     151.410  52.112 114.576  1.00  0.00
ATOM   2142  CG  MET A 137     151.905  52.059 116.669  1.00  0.00
ATOM   2143  HG1 MET A 137     152.673  51.311 116.477  1.00  0.00
ATOM   2144  HG2 MET A 137     151.388  51.781 117.585  1.00  0.00
ATOM   2145  SD  MET A 137     152.726  53.657 116.939  1.00  0.00
ATOM   2146  CE  MET A 137     151.330  54.660 117.523  1.00  0.00
ATOM   2147  HE1 MET A 137     151.696  55.631 117.857  1.00  0.00
ATOM   2148  HE2 MET A 137     150.613  54.808 116.717  1.00  0.00
ATOM   2149  HE3 MET A 137     150.835  54.156 118.351  1.00  0.00
ATOM   2150  C   MET A 137     149.020  50.676 114.372  1.00  0.00
ATOM   2151  O   MET A 137     148.863  51.641 113.641  1.00  0.00
ATOM   2152  N   ARG A 138     148.343  49.532 114.174  1.00  0.00
ATOM   2153  H   ARG A 138     148.512  48.767 114.814  1.00  0.00
ATOM   2154  CA  ARG A 138     147.507  49.266 112.982  1.00  0.00
ATOM   2155  HA  ARG A 138     148.166  49.420 112.129  1.00  0.00
ATOM   2156  CB  ARG A 138     147.096  47.776 112.911  1.00  0.00
ATOM   2157  HB1 ARG A 138     147.600  47.217 113.699  1.00  0.00
ATOM   2158  HB2 ARG A 138     147.478  47.389 111.966  1.00  0.00
ATOM   2159  CG  ARG A 138     145.598  47.454 112.951  1.00  0.00
ATOM   2160  HG1 ARG A 138     145.463  46.408 112.695  1.00  0.00
ATOM   2161  HG2 ARG A 138     145.074  48.024 112.186  1.00  0.00
ATOM   2162  CD  ARG A 138     144.957  47.713 114.323  1.00  0.00
ATOM   2163  HD1 ARG A 138     145.262  48.694 114.681  1.00  0.00
ATOM   2164  HD2 ARG A 138     145.301  46.967 115.043  1.00  0.00
ATOM   2165  NE  ARG A 138     143.492  47.699 114.219  1.00  0.00
ATOM   2166  HE  ARG A 138     143.035  48.606 114.192  1.00  0.00
ATOM   2167  CZ  ARG A 138     142.731  46.628 114.115  1.00  0.00
ATOM   2168  NH1 ARG A 138     143.175  45.416 114.235  1.00  0.00
ATOM   2169 1HH1 ARG A 138     144.160  45.191 114.217  1.00  0.00
ATOM   2170 2HH1 ARG A 138     142.498  44.679 114.058  1.00  0.00
ATOM   2171  NH2 ARG A 138     141.461  46.707 113.862  1.00  0.00
ATOM   2172 1HH2 ARG A 138     141.006  47.590 113.762  1.00  0.00
ATOM   2173 2HH2 ARG A 138     141.006  45.822 113.611  1.00  0.00
ATOM   2174  C   ARG A 138     146.381  50.277 112.710  1.00  0.00
ATOM   2175  O   ARG A 138     145.896  50.368 111.585  1.00  0.00
ATOM   2176  N   ASN A 139     145.995  51.044 113.729  1.00  0.00
ATOM   2177  H   ASN A 139     146.409  50.868 114.629  1.00  0.00
ATOM   2178  CA  ASN A 139     145.035  52.141 113.646  1.00  0.00
ATOM   2179  HA  ASN A 139     144.288  51.924 112.879  1.00  0.00
ATOM   2180  CB  ASN A 139     144.334  52.261 115.018  1.00  0.00
ATOM   2181  HB1 ASN A 139     144.020  53.290 115.161  1.00  0.00
ATOM   2182  HB2 ASN A 139     145.034  52.046 115.823  1.00  0.00
ATOM   2183  CG  ASN A 139     143.092  51.407 115.174  1.00  0.00
ATOM   2184  OD1 ASN A 139     142.788  50.519 114.382  1.00  0.00
ATOM   2185  ND2 ASN A 139     142.296  51.686 116.178  1.00  0.00
ATOM   2186 1HD2 ASN A 139     142.571  52.368 116.880  1.00  0.00
ATOM   2187 2HD2 ASN A 139     141.486  51.111 116.303  1.00  0.00
ATOM   2188  C   ASN A 139     145.626  53.512 113.281  1.00  0.00
ATOM   2189  O   ASN A 139     144.845  54.398 112.948  1.00  0.00
ATOM   2190  N   LEU A 140     146.952  53.715 113.376  1.00  0.00
ATOM   2191  H   LEU A 140     147.569  52.933 113.562  1.00  0.00
ATOM   2192  CA  LEU A 140     147.556  55.045 113.250  1.00  0.00
ATOM   2193  HA  LEU A 140     147.079  55.682 113.991  1.00  0.00
ATOM   2194  CB  LEU A 140     149.064  55.027 113.536  1.00  0.00
```

ATOM	2195	HB1	LEU	A	140	149.615	54.864	112.608	1.00	0.00
ATOM	2196	HB2	LEU	A	140	149.283	54.194	114.196	1.00	0.00
ATOM	2197	CG	LEU	A	140	149.576	56.303	114.235	1.00	0.00
ATOM	2198	HG	LEU	A	140	149.248	56.275	115.272	1.00	0.00
ATOM	2199	CD1	LEU	A	140	151.103	56.306	114.218	1.00	0.00
ATOM	2200	1HD1	LEU	A	140	151.477	57.115	114.843	1.00	0.00
ATOM	2201	2HD1	LEU	A	140	151.487	55.361	114.599	1.00	0.00
ATOM	2202	3HD1	LEU	A	140	151.460	56.443	113.198	1.00	0.00
ATOM	2203	CD2	LEU	A	140	149.094	57.626	113.625	1.00	0.00
ATOM	2204	1HD2	LEU	A	140	149.620	58.465	114.079	1.00	0.00
ATOM	2205	2HD2	LEU	A	140	149.273	57.628	112.554	1.00	0.00
ATOM	2206	3HD2	LEU	A	140	148.031	57.758	113.825	1.00	0.00
ATOM	2207	C	LEU	A	140	147.233	55.616	111.868	1.00	0.00
ATOM	2208	O	LEU	A	140	147.712	55.121	110.847	1.00	0.00
ATOM	2209	N	GLN	A	141	146.363	56.628	111.835	1.00	0.00
ATOM	2210	H	GLN	A	141	145.910	56.937	112.691	1.00	0.00
ATOM	2211	CA	GLN	A	141	145.917	57.148	110.556	1.00	0.00
ATOM	2212	HA	GLN	A	141	146.801	57.102	109.919	1.00	0.00
ATOM	2213	CB	GLN	A	141	144.870	56.224	109.920	1.00	0.00
ATOM	2214	HB1	GLN	A	141	143.873	56.531	110.222	1.00	0.00
ATOM	2215	HB2	GLN	A	141	145.013	55.200	110.265	1.00	0.00
ATOM	2216	CG	GLN	A	141	145.012	56.289	108.394	1.00	0.00
ATOM	2217	HG1	GLN	A	141	145.794	56.982	108.082	1.00	0.00
ATOM	2218	HG2	GLN	A	141	144.076	56.678	108.009	1.00	0.00
ATOM	2219	CD	GLN	A	141	145.322	54.950	107.741	1.00	0.00
ATOM	2220	OE1	GLN	A	141	144.602	54.524	106.840	1.00	0.00
ATOM	2221	NE2	GLN	A	141	146.346	54.235	108.155	1.00	0.00
ATOM	2222	1HE2	GLN	A	141	146.847	54.490	109.001	1.00	0.00
ATOM	2223	2HE2	GLN	A	141	146.465	53.322	107.723	1.00	0.00
ATOM	2224	C	GLN	A	141	145.575	58.639	110.511	1.00	0.00
ATOM	2225	O	GLN	A	141	144.851	59.076	109.626	1.00	0.00
ATOM	2226	N	GLU	A	142	146.192	59.425	111.396	1.00	0.00
ATOM	2227	H	GLU	A	142	146.654	58.997	112.184	1.00	0.00
ATOM	2228	CA	GLU	A	142	146.550	60.817	111.097	1.00	0.00
ATOM	2229	HA	GLU	A	142	146.925	60.856	110.073	1.00	0.00
ATOM	2230	CB	GLU	A	142	145.307	61.731	111.192	1.00	0.00
ATOM	2231	HB1	GLU	A	142	145.128	61.946	112.240	1.00	0.00
ATOM	2232	HB2	GLU	A	142	144.419	61.225	110.826	1.00	0.00
ATOM	2233	CG	GLU	A	142	145.450	63.069	110.450	1.00	0.00
ATOM	2234	HG1	GLU	A	142	146.459	63.442	110.583	1.00	0.00
ATOM	2235	HG2	GLU	A	142	144.775	63.792	110.915	1.00	0.00
ATOM	2236	CD	GLU	A	142	145.163	63.017	108.944	1.00	0.00
ATOM	2237	OE1	GLU	A	142	145.771	62.184	108.234	1.00	0.00
ATOM	2238	OE2	GLU	A	142	144.454	63.927	108.454	1.00	0.00
ATOM	2239	C	GLU	A	142	147.657	61.329	112.044	1.00	0.00
ATOM	2240	O	GLU	A	142	147.493	61.327	113.268	1.00	0.00
ATOM	2241	N	ILE	A	143	148.773	61.820	111.487	1.00	0.00
ATOM	2242	H	ILE	A	143	148.887	61.723	110.482	1.00	0.00
ATOM	2243	CA	ILE	A	143	149.814	62.571	112.236	1.00	0.00
ATOM	2244	HA	ILE	A	143	149.987	62.033	113.166	1.00	0.00
ATOM	2245	CB	ILE	A	143	151.147	62.619	111.433	1.00	0.00
ATOM	2246	HB	ILE	A	143	151.163	61.786	110.733	1.00	0.00
ATOM	2247	CG2	ILE	A	143	151.321	63.902	110.585	1.00	0.00
ATOM	2248	1HG2	ILE	A	143	152.240	63.844	110.008	1.00	0.00
ATOM	2249	2HG2	ILE	A	143	150.466	64.034	109.921	1.00	0.00
ATOM	2250	3HG2	ILE	A	143	151.424	64.774	111.230	1.00	0.00
ATOM	2251	CG1	ILE	A	143	152.368	62.459	112.358	1.00	0.00
ATOM	2252	1HG1	ILE	A	143	152.230	63.067	113.251	1.00	0.00
ATOM	2253	2HG1	ILE	A	143	153.272	62.802	111.854	1.00	0.00
ATOM	2254	CD	ILE	A	143	152.610	61.001	112.782	1.00	0.00
ATOM	2255	HD1	ILE	A	143	153.411	60.970	113.520	1.00	0.00

ATOM	2256	HD2	ILE	A	143	151.704	60.578	113.213	1.00	0.00
ATOM	2257	HD3	ILE	A	143	152.903	60.413	111.912	1.00	0.00
ATOM	2258	C	ILE	A	143	149.379	64.000	112.642	1.00	0.00
ATOM	2259	O	ILE	A	143	150.094	64.696	113.357	1.00	0.00
ATOM	2260	N	LEU	A	144	148.247	64.431	112.085	1.00	0.00
ATOM	2261	H	LEU	A	144	147.714	63.696	111.658	1.00	0.00
ATOM	2262	CA	LEU	A	144	147.850	65.790	111.701	1.00	0.00
ATOM	2263	HA	LEU	A	144	147.172	65.657	110.858	1.00	0.00
ATOM	2264	CB	LEU	A	144	147.011	66.444	112.811	1.00	0.00
ATOM	2265	HB1	LEU	A	144	147.674	66.844	113.574	1.00	0.00
ATOM	2266	HB2	LEU	A	144	146.400	65.667	113.273	1.00	0.00
ATOM	2267	CG	LEU	A	144	146.063	67.543	112.291	1.00	0.00
ATOM	2268	HG	LEU	A	144	145.453	67.143	111.482	1.00	0.00
ATOM	2269	CD1	LEU	A	144	145.125	67.971	113.415	1.00	0.00
ATOM	2270	1HD1	LEU	A	144	144.458	68.764	113.075	1.00	0.00
ATOM	2271	2HD1	LEU	A	144	144.519	67.118	113.709	1.00	0.00
ATOM	2272	3HD1	LEU	A	144	145.719	68.334	114.254	1.00	0.00
ATOM	2273	CD2	LEU	A	144	146.774	68.803	111.797	1.00	0.00
ATOM	2274	1HD2	LEU	A	144	146.057	69.604	111.622	1.00	0.00
ATOM	2275	2HD2	LEU	A	144	147.499	69.137	112.541	1.00	0.00
ATOM	2276	3HD2	LEU	A	144	147.290	68.609	110.859	1.00	0.00
ATOM	2277	C	LEU	A	144	148.988	66.660	111.156	1.00	0.00
ATOM	2278	O	LEU	A	144	149.005	66.874	109.943	1.00	0.00
ATOM	2279	N	HIS	A	145	149.915	67.142	111.985	1.00	0.00
ATOM	2280	H	HIS	A	145	149.908	66.799	112.943	1.00	0.00
ATOM	2281	CA	HIS	A	145	151.070	67.947	111.573	1.00	0.00
ATOM	2282	HA	HIS	A	145	151.370	67.605	110.582	1.00	0.00
ATOM	2283	CB	HIS	A	145	150.675	69.437	111.470	1.00	0.00
ATOM	2284	HB1	HIS	A	145	151.551	70.052	111.678	1.00	0.00
ATOM	2285	HB2	HIS	A	145	149.921	69.677	112.220	1.00	0.00
ATOM	2286	CG	HIS	A	145	150.178	69.824	110.095	1.00	0.00
ATOM	2287	ND1	HIS	A	145	149.006	70.525	109.779	1.00	0.00
ATOM	2288	CE1	HIS	A	145	149.033	70.705	108.448	1.00	0.00
ATOM	2289	HE1	HIS	A	145	148.287	71.251	107.887	1.00	0.00
ATOM	2290	NE2	HIS	A	145	150.134	70.153	107.918	1.00	0.00
ATOM	2291	HE2	HIS	A	145	150.426	70.182	106.934	1.00	0.00
ATOM	2292	CD2	HIS	A	145	150.864	69.600	108.941	1.00	0.00
ATOM	2293	HD2	HIS	A	145	151.815	69.103	108.846	1.00	0.00
ATOM	2294	C	HIS	A	145	152.290	67.712	112.471	1.00	0.00
ATOM	2295	O	HIS	A	145	152.179	67.345	113.632	1.00	0.00
ATOM	2296	N	GLY	A	146	153.488	67.876	111.907	1.00	0.00
ATOM	2297	H	GLY	A	146	153.526	68.217	110.962	1.00	0.00
ATOM	2298	CA	GLY	A	146	154.737	67.317	112.445	1.00	0.00
ATOM	2299	HA1	GLY	A	146	154.642	67.101	113.505	1.00	0.00
ATOM	2300	HA2	GLY	A	146	155.523	68.062	112.337	1.00	0.00
ATOM	2301	C	GLY	A	146	155.160	66.045	111.693	1.00	0.00
ATOM	2302	O	GLY	A	146	154.355	65.436	110.987	1.00	0.00
ATOM	2303	N	ALA	A	147	156.421	65.624	111.847	1.00	0.00
ATOM	2304	H	ALA	A	147	157.051	66.179	112.415	1.00	0.00
ATOM	2305	CA	ALA	A	147	156.946	64.408	111.209	1.00	0.00
ATOM	2306	HA	ALA	A	147	156.117	63.725	111.013	1.00	0.00
ATOM	2307	CB	ALA	A	147	157.597	64.720	109.864	1.00	0.00
ATOM	2308	HB1	ALA	A	147	157.957	63.798	109.412	1.00	0.00
ATOM	2309	HB2	ALA	A	147	156.871	65.147	109.195	1.00	0.00
ATOM	2310	HB3	ALA	A	147	158.440	65.400	109.985	1.00	0.00
ATOM	2311	C	ALA	A	147	157.967	63.655	112.088	1.00	0.00
ATOM	2312	O	ALA	A	147	159.091	64.110	112.270	1.00	0.00
ATOM	2313	N	VAL	A	148	157.569	62.468	112.566	1.00	0.00
ATOM	2314	H	VAL	A	148	156.574	62.322	112.553	1.00	0.00
ATOM	2315	CA	VAL	A	148	158.339	61.241	112.306	1.00	0.00
ATOM	2316	HA	VAL	A	148	158.074	60.507	113.058	1.00	0.00

ATOM	2317	CB	VAL	A	148	157.889	60.590	110.973	1.00	0.00
ATOM	2318	HB	VAL	A	148	158.173	61.231	110.138	1.00	0.00
ATOM	2319	CG1	VAL	A	148	158.519	59.205	110.770	1.00	0.00
ATOM	2320	1HG1	VAL	A	148	158.180	58.777	109.826	1.00	0.00
ATOM	2321	2HG1	VAL	A	148	159.604	59.284	110.725	1.00	0.00
ATOM	2322	3HG1	VAL	A	148	158.234	58.537	111.582	1.00	0.00
ATOM	2323	CG2	VAL	A	148	156.363	60.387	110.920	1.00	0.00
ATOM	2324	1HG2	VAL	A	148	156.096	59.855	110.006	1.00	0.00
ATOM	2325	2HG2	VAL	A	148	156.031	59.801	111.777	1.00	0.00
ATOM	2326	3HG2	VAL	A	148	155.849	61.346	110.904	1.00	0.00
ATOM	2327	C	VAL	A	148	159.872	61.413	112.338	1.00	0.00
ATOM	2328	O	VAL	A	148	160.485	61.791	111.343	1.00	0.00
ATOM	2329	N	ARG	A	149	160.479	61.064	113.483	1.00	0.00
ATOM	2330	H	ARG	A	149	159.870	60.908	114.277	1.00	0.00
ATOM	2331	CA	ARG	A	149	161.891	60.650	113.588	1.00	0.00
ATOM	2332	HA	ARG	A	149	162.330	60.702	112.599	1.00	0.00
ATOM	2333	CB	ARG	A	149	162.765	61.585	114.455	1.00	0.00
ATOM	2334	HB1	ARG	A	149	162.451	61.525	115.494	1.00	0.00
ATOM	2335	HB2	ARG	A	149	162.652	62.617	114.118	1.00	0.00
ATOM	2336	CG	ARG	A	149	164.257	61.172	114.348	1.00	0.00
ATOM	2337	HG1	ARG	A	149	164.643	61.422	113.362	1.00	0.00
ATOM	2338	HG2	ARG	A	149	164.341	60.093	114.454	1.00	0.00
ATOM	2339	CD	ARG	A	149	165.168	61.774	115.420	1.00	0.00
ATOM	2340	HD1	ARG	A	149	166.108	61.218	115.410	1.00	0.00
ATOM	2341	HD2	ARG	A	149	164.699	61.629	116.396	1.00	0.00
ATOM	2342	NE	ARG	A	149	165.474	63.203	115.215	1.00	0.00
ATOM	2343	HE	ARG	A	149	165.174	63.633	114.355	1.00	0.00
ATOM	2344	CZ	ARG	A	149	166.079	63.974	116.105	1.00	0.00
ATOM	2345	NH1	ARG	A	149	166.502	63.510	117.243	1.00	0.00
ATOM	2346	1HH1	ARG	A	149	166.408	62.542	117.462	1.00	0.00
ATOM	2347	2HH1	ARG	A	149	166.682	64.173	118.005	1.00	0.00
ATOM	2348	NH2	ARG	A	149	166.220	65.251	115.932	1.00	0.00
ATOM	2349	1HH2	ARG	A	149	165.885	65.771	115.134	1.00	0.00
ATOM	2350	2HH2	ARG	A	149	166.554	65.781	116.742	1.00	0.00
ATOM	2351	C	ARG	A	149	161.982	59.188	114.032	1.00	0.00
ATOM	2352	O	ARG	A	149	162.123	58.882	115.222	1.00	0.00
ATOM	2353	N	PHE	A	150	161.876	58.264	113.083	1.00	0.00
ATOM	2354	H	PHE	A	150	161.797	58.594	112.127	1.00	0.00
ATOM	2355	CA	PHE	A	150	162.435	56.924	113.315	1.00	0.00
ATOM	2356	HA	PHE	A	150	162.110	56.544	114.285	1.00	0.00
ATOM	2357	CB	PHE	A	150	161.958	55.957	112.223	1.00	0.00
ATOM	2358	HB1	PHE	A	150	162.786	55.312	111.926	1.00	0.00
ATOM	2359	HB2	PHE	A	150	161.645	56.513	111.340	1.00	0.00
ATOM	2360	CG	PHE	A	150	160.815	55.081	112.685	1.00	0.00
ATOM	2361	CD1	PHE	A	150	161.068	53.765	113.113	1.00	0.00
ATOM	2362	HD1	PHE	A	150	162.075	53.367	113.061	1.00	0.00
ATOM	2363	CE1	PHE	A	150	160.019	52.974	113.607	1.00	0.00
ATOM	2364	HE1	PHE	A	150	160.219	51.973	113.961	1.00	0.00
ATOM	2365	CZ	PHE	A	150	158.711	53.484	113.654	1.00	0.00
ATOM	2366	HZ	PHE	A	150	157.901	52.877	114.027	1.00	0.00
ATOM	2367	CE2	PHE	A	150	158.457	54.800	113.229	1.00	0.00
ATOM	2368	HE2	PHE	A	150	157.455	55.199	113.273	1.00	0.00
ATOM	2369	CD2	PHE	A	150	159.507	55.597	112.741	1.00	0.00
ATOM	2370	HD2	PHE	A	150	159.313	56.609	112.415	1.00	0.00
ATOM	2371	C	PHE	A	150	163.959	57.041	113.344	1.00	0.00
ATOM	2372	O	PHE	A	150	164.499	57.788	112.535	1.00	0.00
ATOM	2373	N	SER	A	151	164.656	56.345	114.256	1.00	0.00
ATOM	2374	H	SER	A	151	164.197	55.717	114.898	1.00	0.00
ATOM	2375	CA	SER	A	151	166.115	56.283	114.113	1.00	0.00
ATOM	2376	HA	SER	A	151	166.353	56.423	113.062	1.00	0.00
ATOM	2377	CB	SER	A	151	166.762	57.446	114.877	1.00	0.00

ATOM	2378	HB1	SER	A	151	166.674	57.278	115.952	1.00	0.00
ATOM	2379	HB2	SER	A	151	166.234	58.368	114.628	1.00	0.00
ATOM	2380	OG	SER	A	151	168.128	57.630	114.542	1.00	0.00
ATOM	2381	HG	SER	A	151	168.313	57.215	113.654	1.00	0.00
ATOM	2382	C	SER	A	151	166.651	54.900	114.450	1.00	0.00
ATOM	2383	O	SER	A	151	165.926	53.922	114.262	1.00	0.00
ATOM	2384	N	ASN	A	152	167.901	54.821	114.912	1.00	0.00
ATOM	2385	H	ASN	A	152	168.430	55.685	114.926	1.00	0.00
ATOM	2386	CA	ASN	A	152	168.645	53.585	115.040	1.00	0.00
ATOM	2387	HA	ASN	A	152	168.412	53.003	114.144	1.00	0.00
ATOM	2388	CB	ASN	A	152	170.158	53.874	114.983	1.00	0.00
ATOM	2389	HB1	ASN	A	152	170.572	54.014	115.979	1.00	0.00
ATOM	2390	HB2	ASN	A	152	170.335	54.783	114.408	1.00	0.00
ATOM	2391	CG	ASN	A	152	170.887	52.747	114.277	1.00	0.00
ATOM	2392	OD1	ASN	A	152	171.410	51.825	114.894	1.00	0.00
ATOM	2393	ND2	ASN	A	152	170.875	52.761	112.969	1.00	0.00
ATOM	2394	1HD2	ASN	A	152	170.240	53.409	112.490	1.00	0.00
ATOM	2395	2HD2	ASN	A	152	171.386	52.049	112.461	1.00	0.00
ATOM	2396	C	ASN	A	152	168.185	52.762	116.238	1.00	0.00
ATOM	2397	O	ASN	A	152	168.721	52.896	117.341	1.00	0.00
ATOM	2398	N	ASN	A	153	167.172	51.914	116.031	1.00	0.00
ATOM	2399	H	ASN	A	153	166.746	51.924	115.112	1.00	0.00
ATOM	2400	CA	ASN	A	153	166.809	50.847	116.966	1.00	0.00
ATOM	2401	HA	ASN	A	153	167.628	50.786	117.682	1.00	0.00
ATOM	2402	CB	ASN	A	153	165.642	51.218	117.910	1.00	0.00
ATOM	2403	HB1	ASN	A	153	165.919	52.122	118.422	1.00	0.00
ATOM	2404	HB2	ASN	A	153	165.550	50.424	118.653	1.00	0.00
ATOM	2405	CG	ASN	A	153	164.260	51.500	117.362	1.00	0.00
ATOM	2406	OD1	ASN	A	153	163.275	51.017	117.898	1.00	0.00
ATOM	2407	ND2	ASN	A	153	164.123	52.365	116.386	1.00	0.00
ATOM	2408	1HD2	ASN	A	153	164.919	52.782	115.920	1.00	0.00
ATOM	2409	2HD2	ASN	A	153	163.188	52.562	116.083	1.00	0.00
ATOM	2410	C	ASN	A	153	166.802	49.452	116.314	1.00	0.00
ATOM	2411	O	ASN	A	153	165.736	48.925	116.004	1.00	0.00
ATOM	2412	N	PRO	A	154	167.994	48.841	116.103	1.00	0.00
ATOM	2413	CD	PRO	A	154	169.302	49.376	116.489	1.00	0.00
ATOM	2414	HD1	PRO	A	154	169.492	49.144	117.538	1.00	0.00
ATOM	2415	HD2	PRO	A	154	169.383	50.447	116.328	1.00	0.00
ATOM	2416	CG	PRO	A	154	170.334	48.681	115.615	1.00	0.00
ATOM	2417	HG1	PRO	A	154	171.303	48.609	116.109	1.00	0.00
ATOM	2418	HG2	PRO	A	154	170.423	49.211	114.668	1.00	0.00
ATOM	2419	CB	PRO	A	154	169.704	47.316	115.391	1.00	0.00
ATOM	2420	HB1	PRO	A	154	169.963	46.672	116.230	1.00	0.00
ATOM	2421	HB2	PRO	A	154	170.042	46.880	114.451	1.00	0.00
ATOM	2422	CA	PRO	A	154	168.190	47.580	115.373	1.00	0.00
ATOM	2423	HA	PRO	A	154	167.895	47.767	114.345	1.00	0.00
ATOM	2424	C	PRO	A	154	167.434	46.324	115.848	1.00	0.00
ATOM	2425	O	PRO	A	154	167.699	45.251	115.311	1.00	0.00
ATOM	2426	N	ALA	A	155	166.529	46.416	116.828	1.00	0.00
ATOM	2427	H	ALA	A	155	166.331	47.333	117.202	1.00	0.00
ATOM	2428	CA	ALA	A	155	165.611	45.345	117.206	1.00	0.00
ATOM	2429	HA	ALA	A	155	166.019	44.384	116.906	1.00	0.00
ATOM	2430	CB	ALA	A	155	165.466	45.342	118.727	1.00	0.00
ATOM	2431	HB1	ALA	A	155	164.785	44.549	119.039	1.00	0.00
ATOM	2432	HB2	ALA	A	155	166.434	45.174	119.198	1.00	0.00
ATOM	2433	HB3	ALA	A	155	165.060	46.297	119.057	1.00	0.00
ATOM	2434	C	ALA	A	155	164.241	45.415	116.514	1.00	0.00
ATOM	2435	O	ALA	A	155	163.529	44.421	116.553	1.00	0.00
ATOM	2436	N	LEU	A	156	163.886	46.520	115.847	1.00	0.00
ATOM	2437	H	LEU	A	156	164.511	47.316	115.850	1.00	0.00
ATOM	2438	CA	LEU	A	156	162.615	46.660	115.130	1.00	0.00

```
ATOM   2439  HA  LEU A 156     161.811  46.719 115.864  1.00  0.00
ATOM   2440  CB  LEU A 156     162.631  47.946 114.280  1.00  0.00
ATOM   2441  HB1 LEU A 156     161.794  47.899 113.586  1.00  0.00
ATOM   2442  HB2 LEU A 156     163.548  47.955 113.687  1.00  0.00
ATOM   2443  CG  LEU A 156     162.507  49.277 115.032  1.00  0.00
ATOM   2444  HG  LEU A 156     163.284  49.344 115.785  1.00  0.00
ATOM   2445  CD1 LEU A 156     162.657  50.435 114.044  1.00  0.00
ATOM   2446 1HD1 LEU A 156     162.586  51.385 114.561  1.00  0.00
ATOM   2447 2HD1 LEU A 156     163.629  50.374 113.551  1.00  0.00
ATOM   2448 3HD1 LEU A 156     161.871  50.394 113.293  1.00  0.00
ATOM   2449  CD2 LEU A 156     161.148  49.432 115.709  1.00  0.00
ATOM   2450 1HD2 LEU A 156     161.093  50.410 116.185  1.00  0.00
ATOM   2451 2HD2 LEU A 156     160.341  49.320 114.988  1.00  0.00
ATOM   2452 3HD2 LEU A 156     161.050  48.682 116.491  1.00  0.00
ATOM   2453  C   LEU A 156     162.296  45.462 114.213  1.00  0.00
ATOM   2454  O   LEU A 156     163.165  44.693 113.796  1.00  0.00
ATOM   2455  N   CYS A 157     161.028  45.363 113.833  1.00  0.00
ATOM   2456  H   CYS A 157     160.333  45.951 114.268  1.00  0.00
ATOM   2457  CA  CYS A 157     160.685  44.821 112.535  1.00  0.00
ATOM   2458  HA  CYS A 157     161.511  45.013 111.851  1.00  0.00
ATOM   2459  CB  CYS A 157     160.556  43.312 112.620  1.00  0.00
ATOM   2460  HB1 CYS A 157     161.476  42.916 113.047  1.00  0.00
ATOM   2461  HB2 CYS A 157     160.490  42.943 111.599  1.00  0.00
ATOM   2462  SG  CYS A 157     159.158  42.633 113.536  1.00  0.00
ATOM   2463  C   CYS A 157     159.451  45.492 111.930  1.00  0.00
ATOM   2464  O   CYS A 157     158.747  46.226 112.624  1.00  0.00
ATOM   2465  N   ASN A 158     159.233  45.240 110.640  1.00  0.00
ATOM   2466  H   ASN A 158     159.914  44.636 110.197  1.00  0.00
ATOM   2467  CA  ASN A 158     158.034  45.494 109.831  1.00  0.00
ATOM   2468  HA  ASN A 158     158.268  45.149 108.822  1.00  0.00
ATOM   2469  CB  ASN A 158     156.874  44.600 110.318  1.00  0.00
ATOM   2470  HB1 ASN A 158     155.985  44.871 109.746  1.00  0.00
ATOM   2471  HB2 ASN A 158     156.665  44.803 111.368  1.00  0.00
ATOM   2472  CG  ASN A 158     157.144  43.118 110.140  1.00  0.00
ATOM   2473  OD1 ASN A 158     158.226  42.599 110.382  1.00  0.00
ATOM   2474  ND2 ASN A 158     156.167  42.365 109.705  1.00  0.00
ATOM   2475 1HD2 ASN A 158     155.292  42.760 109.408  1.00  0.00
ATOM   2476 2HD2 ASN A 158     156.365  41.370 109.636  1.00  0.00
ATOM   2477  C   ASN A 158     157.603  46.954 109.629  1.00  0.00
ATOM   2478  O   ASN A 158     157.230  47.303 108.517  1.00  0.00
ATOM   2479  N   VAL A 159     157.709  47.826 110.637  1.00  0.00
ATOM   2480  H   VAL A 159     158.009  47.476 111.541  1.00  0.00
ATOM   2481  CA  VAL A 159     157.354  49.258 110.544  1.00  0.00
ATOM   2482  HA  VAL A 159     156.331  49.278 110.182  1.00  0.00
ATOM   2483  CB  VAL A 159     157.329  49.952 111.911  1.00  0.00
ATOM   2484  HB  VAL A 159     157.073  51.002 111.763  1.00  0.00
ATOM   2485  CG1 VAL A 159     156.253  49.332 112.802  1.00  0.00
ATOM   2486 1HG1 VAL A 159     156.126  49.938 113.698  1.00  0.00
ATOM   2487 2HG1 VAL A 159     155.306  49.302 112.265  1.00  0.00
ATOM   2488 3HG1 VAL A 159     156.520  48.316 113.093  1.00  0.00
ATOM   2489  CG2 VAL A 159     158.678  49.887 112.633  1.00  0.00
ATOM   2490 1HG2 VAL A 159     158.586  50.384 113.596  1.00  0.00
ATOM   2491 2HG2 VAL A 159     158.971  48.852 112.799  1.00  0.00
ATOM   2492 3HG2 VAL A 159     159.438  50.395 112.038  1.00  0.00
ATOM   2493  C   VAL A 159     158.158  50.070 109.516  1.00  0.00
ATOM   2494  O   VAL A 159     157.709  51.125 109.070  1.00  0.00
ATOM   2495  N   GLU A 160     159.301  49.545 109.073  1.00  0.00
ATOM   2496  H   GLU A 160     159.611  48.690 109.501  1.00  0.00
ATOM   2497  CA  GLU A 160     160.001  49.952 107.845  1.00  0.00
ATOM   2498  HA  GLU A 160     160.515  50.896 107.996  1.00  0.00
ATOM   2499  CB  GLU A 160     161.043  48.863 107.539  1.00  0.00
```

```
ATOM   2500  HB1 GLU A 160     160.517  47.928 107.339  1.00  0.00
ATOM   2501  HB2 GLU A 160     161.642  48.704 108.421  1.00  0.00
ATOM   2502  CG  GLU A 160     161.986  49.178 106.375  1.00  0.00
ATOM   2503  HG1 GLU A 160     162.534  50.096 106.602  1.00  0.00
ATOM   2504  HG2 GLU A 160     161.405  49.341 105.465  1.00  0.00
ATOM   2505  CD  GLU A 160     162.959  48.012 106.148  1.00  0.00
ATOM   2506  OE1 GLU A 160     162.465  46.873 106.008  1.00  0.00
ATOM   2507  OE2 GLU A 160     164.185  48.264 106.125  1.00  0.00
ATOM   2508  C   GLU A 160     159.055  50.072 106.648  1.00  0.00
ATOM   2509  O   GLU A 160     159.021  51.090 105.959  1.00  0.00
ATOM   2510  N   SER A 161     158.238  49.038 106.442  1.00  0.00
ATOM   2511  H   SER A 161     158.235  48.297 107.134  1.00  0.00
ATOM   2512  CA  SER A 161     157.317  48.915 105.312  1.00  0.00
ATOM   2513  HA  SER A 161     157.844  49.252 104.419  1.00  0.00
ATOM   2514  CB  SER A 161     156.951  47.442 105.097  1.00  0.00
ATOM   2515  HB1 SER A 161     156.178  47.368 104.333  1.00  0.00
ATOM   2516  HB2 SER A 161     156.573  47.007 106.023  1.00  0.00
ATOM   2517  OG  SER A 161     158.082  46.725 104.642  1.00  0.00
ATOM   2518  HG  SER A 161     158.836  46.880 105.220  1.00  0.00
ATOM   2519  C   SER A 161     156.066  49.796 105.420  1.00  0.00
ATOM   2520  O   SER A 161     155.177  49.677 104.573  1.00  0.00
ATOM   2521  N   ILE A 162     155.936  50.674 106.424  1.00  0.00
ATOM   2522  H   ILE A 162     156.718  50.797 107.059  1.00  0.00
ATOM   2523  CA  ILE A 162     154.781  51.583 106.538  1.00  0.00
ATOM   2524  HA  ILE A 162     153.892  51.029 106.250  1.00  0.00
ATOM   2525  CB  ILE A 162     154.586  52.052 107.995  1.00  0.00
ATOM   2526  HB  ILE A 162     155.533  52.450 108.361  1.00  0.00
ATOM   2527  CG2 ILE A 162     153.544  53.183 108.134  1.00  0.00
ATOM   2528 1HG2 ILE A 162     153.367  53.384 109.191  1.00  0.00
ATOM   2529 2HG2 ILE A 162     153.929  54.097 107.685  1.00  0.00
ATOM   2530 3HG2 ILE A 162     152.603  52.906 107.662  1.00  0.00
ATOM   2531  CG1 ILE A 162     154.192  50.858 108.891  1.00  0.00
ATOM   2532 1HG1 ILE A 162     154.112  51.204 109.922  1.00  0.00
ATOM   2533 2HG1 ILE A 162     154.991  50.123 108.854  1.00  0.00
ATOM   2534  CD  ILE A 162     152.895  50.125 108.517  1.00  0.00
ATOM   2535  HD1 ILE A 162     152.688  49.374 109.275  1.00  0.00
ATOM   2536  HD2 ILE A 162     152.059  50.821 108.474  1.00  0.00
ATOM   2537  HD3 ILE A 162     153.007  49.611 107.563  1.00  0.00
ATOM   2538  C   ILE A 162     154.883  52.760 105.555  1.00  0.00
ATOM   2539  O   ILE A 162     155.839  53.535 105.538  1.00  0.00
ATOM   2540  N   GLN A 163     153.839  52.921 104.743  1.00  0.00
ATOM   2541  H   GLN A 163     153.121  52.217 104.771  1.00  0.00
ATOM   2542  CA  GLN A 163     153.702  54.005 103.774  1.00  0.00
ATOM   2543  HA  GLN A 163     154.669  54.148 103.291  1.00  0.00
ATOM   2544  CB  GLN A 163     152.703  53.587 102.676  1.00  0.00
ATOM   2545  HB1 GLN A 163     152.495  54.443 102.033  1.00  0.00
ATOM   2546  HB2 GLN A 163     151.768  53.260 103.135  1.00  0.00
ATOM   2547  CG  GLN A 163     153.274  52.459 101.802  1.00  0.00
ATOM   2548  HG1 GLN A 163     153.603  51.636 102.431  1.00  0.00
ATOM   2549  HG2 GLN A 163     154.149  52.825 101.263  1.00  0.00
ATOM   2550  CD  GLN A 163     152.269  51.936 100.777  1.00  0.00
ATOM   2551  OE1 GLN A 163     151.796  52.664  99.915  1.00  0.00
ATOM   2552  NE2 GLN A 163     151.914  50.671 100.819  1.00  0.00
ATOM   2553 1HE2 GLN A 163     152.279  50.036 101.530  1.00  0.00
ATOM   2554 2HE2 GLN A 163     151.299  50.348 100.100  1.00  0.00
ATOM   2555  C   GLN A 163     153.347  55.353 104.429  1.00  0.00
ATOM   2556  O   GLN A 163     152.235  55.862 104.275  1.00  0.00
ATOM   2557  N   TRP A 164     154.302  55.971 105.133  1.00  0.00
ATOM   2558  H   TRP A 164     155.143  55.431 105.322  1.00  0.00
ATOM   2559  CA  TRP A 164     154.149  57.240 105.871  1.00  0.00
ATOM   2560  HA  TRP A 164     153.506  57.023 106.725  1.00  0.00
```

```
ATOM   2561  CB  TRP A 164     155.508  57.644 106.468  1.00  0.00
ATOM   2562  HB1 TRP A 164     155.399  58.619 106.945  1.00  0.00
ATOM   2563  HB2 TRP A 164     156.236  57.751 105.663  1.00  0.00
ATOM   2564  CG  TRP A 164     156.031  56.682 107.494  1.00  0.00
ATOM   2565  CD1 TRP A 164     156.969  55.729 107.281  1.00  0.00
ATOM   2566  HD1 TRP A 164     157.484  55.537 106.343  1.00  0.00
ATOM   2567  NE1 TRP A 164     157.132  54.973 108.421  1.00  0.00
ATOM   2568  HE1 TRP A 164     157.756  54.176 108.484  1.00  0.00
ATOM   2569  CE2 TRP A 164     156.297  55.387 109.435  1.00  0.00
ATOM   2570  CZ2 TRP A 164     156.059  54.911 110.733  1.00  0.00
ATOM   2571  HZ2 TRP A 164     156.586  54.034 111.083  1.00  0.00
ATOM   2572  CH2 TRP A 164     155.137  55.586 111.548  1.00  0.00
ATOM   2573  HH2 TRP A 164     154.937  55.231 112.549  1.00  0.00
ATOM   2574  CZ3 TRP A 164     154.474  56.725 111.061  1.00  0.00
ATOM   2575  HZ3 TRP A 164     153.774  57.253 111.701  1.00  0.00
ATOM   2576  CE3 TRP A 164     154.703  57.179 109.746  1.00  0.00
ATOM   2577  HE3 TRP A 164     154.172  58.043 109.374  1.00  0.00
ATOM   2578  CD2 TRP A 164     155.613  56.514 108.889  1.00  0.00
ATOM   2579  C   TRP A 164     153.460  58.408 105.145  1.00  0.00
ATOM   2580  O   TRP A 164     152.873  59.254 105.814  1.00  0.00
ATOM   2581  N   ARG A 165     153.428  58.434 103.801  1.00  0.00
ATOM   2582  H   ARG A 165     153.917  57.690 103.328  1.00  0.00
ATOM   2583  CA  ARG A 165     152.623  59.382 102.992  1.00  0.00
ATOM   2584  HA  ARG A 165     152.945  60.389 103.257  1.00  0.00
ATOM   2585  CB  ARG A 165     152.934  59.170 101.499  1.00  0.00
ATOM   2586  HB1 ARG A 165     152.506  58.223 101.168  1.00  0.00
ATOM   2587  HB2 ARG A 165     154.018  59.121 101.372  1.00  0.00
ATOM   2588  CG  ARG A 165     152.402  60.315 100.620  1.00  0.00
ATOM   2589  HG1 ARG A 165     152.796  61.260 100.993  1.00  0.00
ATOM   2590  HG2 ARG A 165     151.316  60.361 100.688  1.00  0.00
ATOM   2591  CD  ARG A 165     152.812  60.168  99.144  1.00  0.00
ATOM   2592  HD1 ARG A 165     153.490  59.324  99.020  1.00  0.00
ATOM   2593  HD2 ARG A 165     153.362  61.065  98.855  1.00  0.00
ATOM   2594  NE  ARG A 165     151.634  60.040  98.260  1.00  0.00
ATOM   2595  HE  ARG A 165     151.170  60.901  98.024  1.00  0.00
ATOM   2596  CZ  ARG A 165     151.114  58.936  97.750  1.00  0.00
ATOM   2597  NH1 ARG A 165     151.615  57.751  97.987  1.00  0.00
ATOM   2598 1HH1 ARG A 165     152.415  57.669  98.586  1.00  0.00
ATOM   2599 2HH1 ARG A 165     151.196  56.933  97.580  1.00  0.00
ATOM   2600  NH2 ARG A 165     150.047  59.002  96.998  1.00  0.00
ATOM   2601 1HH2 ARG A 165     149.617  59.887  96.791  1.00  0.00
ATOM   2602 2HH2 ARG A 165     149.630  58.160  96.637  1.00  0.00
ATOM   2603  C   ARG A 165     151.118  59.338 103.283  1.00  0.00
ATOM   2604  O   ARG A 165     150.440  60.337 103.081  1.00  0.00
ATOM   2605  N   ASP A 166     150.588  58.211 103.752  1.00  0.00
ATOM   2606  H   ASP A 166     151.187  57.401 103.865  1.00  0.00
ATOM   2607  CA  ASP A 166     149.201  58.129 104.225  1.00  0.00
ATOM   2608  HA  ASP A 166     148.560  58.703 103.562  1.00  0.00
ATOM   2609  CB  ASP A 166     148.738  56.672 104.218  1.00  0.00
ATOM   2610  HB1 ASP A 166     149.172  56.130 105.061  1.00  0.00
ATOM   2611  HB2 ASP A 166     149.080  56.200 103.301  1.00  0.00
ATOM   2612  CG  ASP A 166     147.215  56.610 104.276  1.00  0.00
ATOM   2613  OD1 ASP A 166     146.639  56.753 105.372  1.00  0.00
ATOM   2614  OD2 ASP A 166     146.579  56.518 103.205  1.00  0.00
ATOM   2615  C   ASP A 166     149.034  58.732 105.620  1.00  0.00
ATOM   2616  O   ASP A 166     148.075  59.453 105.877  1.00  0.00
ATOM   2617  N   ILE A 167     149.992  58.486 106.508  1.00  0.00
ATOM   2618  H   ILE A 167     150.784  57.944 106.205  1.00  0.00
ATOM   2619  CA  ILE A 167     149.959  58.929 107.901  1.00  0.00
ATOM   2620  HA  ILE A 167     148.997  58.656 108.335  1.00  0.00
ATOM   2621  CB  ILE A 167     151.096  58.219 108.686  1.00  0.00
```

ATOM	2622	HB	ILE	A	167	152.045	58.643	108.354	1.00	0.00
ATOM	2623	CG2	ILE	A	167	150.970	58.506	110.188	1.00	0.00
ATOM	2624	1HG2	ILE	A	167	151.684	57.911	110.753	1.00	0.00
ATOM	2625	2HG2	ILE	A	167	151.166	59.555	110.388	1.00	0.00
ATOM	2626	3HG2	ILE	A	167	149.963	58.272	110.528	1.00	0.00
ATOM	2627	CG1	ILE	A	167	151.207	56.691	108.442	1.00	0.00
ATOM	2628	1HG1	ILE	A	167	152.089	56.319	108.963	1.00	0.00
ATOM	2629	2HG1	ILE	A	167	151.364	56.493	107.382	1.00	0.00
ATOM	2630	CD	ILE	A	167	150.008	55.871	108.913	1.00	0.00
ATOM	2631	HD1	ILE	A	167	149.925	55.945	109.994	1.00	0.00
ATOM	2632	HD2	ILE	A	167	149.091	56.221	108.440	1.00	0.00
ATOM	2633	HD3	ILE	A	167	150.163	54.826	108.656	1.00	0.00
ATOM	2634	C	ILE	A	167	150.117	60.454	108.010	1.00	0.00
ATOM	2635	O	ILE	A	167	149.412	61.103	108.782	1.00	0.00
ATOM	2636	N	VAL	A	168	151.080	60.991	107.258	1.00	0.00
ATOM	2637	H	VAL	A	168	151.556	60.375	106.609	1.00	0.00
ATOM	2638	CA	VAL	A	168	151.621	62.350	107.399	1.00	0.00
ATOM	2639	HA	VAL	A	168	151.608	62.578	108.455	1.00	0.00
ATOM	2640	CB	VAL	A	168	153.111	62.332	106.947	1.00	0.00
ATOM	2641	HB	VAL	A	168	153.526	61.384	107.286	1.00	0.00
ATOM	2642	CG1	VAL	A	168	153.275	62.371	105.422	1.00	0.00
ATOM	2643	1HG1	VAL	A	168	154.285	62.056	105.158	1.00	0.00
ATOM	2644	2HG1	VAL	A	168	152.553	61.701	104.958	1.00	0.00
ATOM	2645	3HG1	VAL	A	168	153.108	63.374	105.043	1.00	0.00
ATOM	2646	CG2	VAL	A	168	154.012	63.402	107.577	1.00	0.00
ATOM	2647	1HG2	VAL	A	168	155.043	63.237	107.262	1.00	0.00
ATOM	2648	2HG2	VAL	A	168	153.724	64.403	107.268	1.00	0.00
ATOM	2649	3HG2	VAL	A	168	153.977	63.335	108.662	1.00	0.00
ATOM	2650	C	VAL	A	168	150.745	63.399	106.689	1.00	0.00
ATOM	2651	O	VAL	A	168	149.750	63.065	106.029	1.00	0.00
ATOM	2652	N	SER	A	169	151.145	64.667	106.783	1.00	0.00
ATOM	2653	H	SER	A	169	151.961	64.863	107.342	1.00	0.00
ATOM	2654	CA	SER	A	169	150.729	65.736	105.863	1.00	0.00
ATOM	2655	HA	SER	A	169	149.871	65.405	105.280	1.00	0.00
ATOM	2656	CB	SER	A	169	150.300	66.984	106.640	1.00	0.00
ATOM	2657	HB1	SER	A	169	150.190	67.820	105.950	1.00	0.00
ATOM	2658	HB2	SER	A	169	151.055	67.231	107.386	1.00	0.00
ATOM	2659	OG	SER	A	169	149.051	66.733	107.262	1.00	0.00
ATOM	2660	HG	SER	A	169	149.156	66.901	108.221	1.00	0.00
ATOM	2661	C	SER	A	169	151.871	66.040	104.894	1.00	0.00
ATOM	2662	O	SER	A	169	153.044	65.901	105.251	1.00	0.00
ATOM	2663	N	SER	A	170	151.559	66.355	103.632	1.00	0.00
ATOM	2664	H	SER	A	170	150.598	66.522	103.382	1.00	0.00
ATOM	2665	CA	SER	A	170	152.585	66.322	102.576	1.00	0.00
ATOM	2666	HA	SER	A	170	153.147	65.399	102.723	1.00	0.00
ATOM	2667	CB	SER	A	170	151.957	66.240	101.186	1.00	0.00
ATOM	2668	HB1	SER	A	170	151.693	67.237	100.830	1.00	0.00
ATOM	2669	HB2	SER	A	170	151.062	65.618	101.221	1.00	0.00
ATOM	2670	OG	SER	A	170	152.895	65.634	100.322	1.00	0.00
ATOM	2671	HG	SER	A	170	153.715	66.148	100.378	1.00	0.00
ATOM	2672	C	SER	A	170	153.608	67.457	102.645	1.00	0.00
ATOM	2673	O	SER	A	170	154.666	67.345	102.030	1.00	0.00
ATOM	2674	N	ASP	A	171	153.325	68.504	103.420	1.00	0.00
ATOM	2675	H	ASP	A	171	152.407	68.546	103.840	1.00	0.00
ATOM	2676	CA	ASP	A	171	154.315	69.450	103.942	1.00	0.00
ATOM	2677	HA	ASP	A	171	154.803	69.948	103.103	1.00	0.00
ATOM	2678	CB	ASP	A	171	153.584	70.527	104.771	1.00	0.00
ATOM	2679	HB1	ASP	A	171	153.171	71.265	104.082	1.00	0.00
ATOM	2680	HB2	ASP	A	171	154.296	71.040	105.419	1.00	0.00
ATOM	2681	CG	ASP	A	171	152.435	69.956	105.617	1.00	0.00
ATOM	2682	OD1	ASP	A	171	152.712	69.244	106.602	1.00	0.00

ATOM	2683	OD2	ASP	A	171	151.258	70.113	105.228	1.00	0.00
ATOM	2684	C	ASP	A	171	155.421	68.729	104.730	1.00	0.00
ATOM	2685	O	ASP	A	171	156.581	68.702	104.313	1.00	0.00
ATOM	2686	N	PHE	A	172	155.049	68.084	105.830	1.00	0.00
ATOM	2687	H	PHE	A	172	154.074	68.193	106.106	1.00	0.00
ATOM	2688	CA	PHE	A	172	155.940	67.465	106.805	1.00	0.00
ATOM	2689	HA	PHE	A	172	156.749	68.177	106.973	1.00	0.00
ATOM	2690	CB	PHE	A	172	155.175	67.328	108.128	1.00	0.00
ATOM	2691	HB1	PHE	A	172	155.325	66.345	108.550	1.00	0.00
ATOM	2692	HB2	PHE	A	172	154.100	67.407	107.958	1.00	0.00
ATOM	2693	CG	PHE	A	172	155.610	68.386	109.124	1.00	0.00
ATOM	2694	CD1	PHE	A	172	156.858	68.273	109.764	1.00	0.00
ATOM	2695	HD1	PHE	A	172	157.494	67.435	109.551	1.00	0.00
ATOM	2696	CE1	PHE	A	172	157.300	69.273	110.645	1.00	0.00
ATOM	2697	HE1	PHE	A	172	158.271	69.186	111.112	1.00	0.00
ATOM	2698	CZ	PHE	A	172	156.494	70.398	110.888	1.00	0.00
ATOM	2699	HZ	PHE	A	172	156.836	71.181	111.547	1.00	0.00
ATOM	2700	CE2	PHE	A	172	155.257	70.531	110.232	1.00	0.00
ATOM	2701	HE2	PHE	A	172	154.655	71.416	110.380	1.00	0.00
ATOM	2702	CD2	PHE	A	172	154.825	69.533	109.340	1.00	0.00
ATOM	2703	HD2	PHE	A	172	153.905	69.670	108.787	1.00	0.00
ATOM	2704	C	PHE	A	172	156.646	66.195	106.324	1.00	0.00
ATOM	2705	O	PHE	A	172	157.686	65.839	106.876	1.00	0.00
ATOM	2706	N	LEU	A	173	156.189	65.583	105.229	1.00	0.00
ATOM	2707	H	LEU	A	173	155.324	65.935	104.840	1.00	0.00
ATOM	2708	CA	LEU	A	173	156.966	64.608	104.448	1.00	0.00
ATOM	2709	HA	LEU	A	173	157.104	63.713	105.057	1.00	0.00
ATOM	2710	CB	LEU	A	173	156.123	64.248	103.203	1.00	0.00
ATOM	2711	HB1	LEU	A	173	156.267	65.018	102.443	1.00	0.00
ATOM	2712	HB2	LEU	A	173	155.073	64.275	103.496	1.00	0.00
ATOM	2713	CG	LEU	A	173	156.373	62.861	102.588	1.00	0.00
ATOM	2714	HG	LEU	A	173	156.185	62.097	103.343	1.00	0.00
ATOM	2715	CD1	LEU	A	173	155.391	62.644	101.433	1.00	0.00
ATOM	2716	1HD1	LEU	A	173	155.487	61.628	101.053	1.00	0.00
ATOM	2717	2HD1	LEU	A	173	154.373	62.804	101.786	1.00	0.00
ATOM	2718	3HD1	LEU	A	173	155.596	63.356	100.633	1.00	0.00
ATOM	2719	CD2	LEU	A	173	157.782	62.660	102.033	1.00	0.00
ATOM	2720	1HD2	LEU	A	173	157.836	61.731	101.467	1.00	0.00
ATOM	2721	2HD2	LEU	A	173	158.058	63.492	101.385	1.00	0.00
ATOM	2722	3HD2	LEU	A	173	158.493	62.587	102.853	1.00	0.00
ATOM	2723	C	LEU	A	173	158.360	65.169	104.098	1.00	0.00
ATOM	2724	O	LEU	A	173	159.365	64.479	104.239	1.00	0.00
ATOM	2725	N	SER	A	174	158.438	66.455	103.749	1.00	0.00
ATOM	2726	H	SER	A	174	157.575	66.983	103.702	1.00	0.00
ATOM	2727	CA	SER	A	174	159.678	67.176	103.403	1.00	0.00
ATOM	2728	HA	SER	A	174	160.216	66.603	102.648	1.00	0.00
ATOM	2729	CB	SER	A	174	159.344	68.560	102.818	1.00	0.00
ATOM	2730	HB1	SER	A	174	160.177	68.892	102.198	1.00	0.00
ATOM	2731	HB2	SER	A	174	159.218	69.276	103.632	1.00	0.00
ATOM	2732	OG	SER	A	174	158.155	68.559	102.050	1.00	0.00
ATOM	2733	HG	SER	A	174	157.417	68.685	102.673	1.00	0.00
ATOM	2734	C	SER	A	174	160.634	67.417	104.576	1.00	0.00
ATOM	2735	O	SER	A	174	161.761	67.860	104.373	1.00	0.00
ATOM	2736	N	ASN	A	175	160.182	67.177	105.809	1.00	0.00
ATOM	2737	H	ASN	A	175	159.245	66.806	105.906	1.00	0.00
ATOM	2738	CA	ASN	A	175	160.953	67.354	107.044	1.00	0.00
ATOM	2739	HA	ASN	A	175	161.996	67.541	106.793	1.00	0.00
ATOM	2740	CB	ASN	A	175	160.416	68.573	107.824	1.00	0.00
ATOM	2741	HB1	ASN	A	175	161.026	68.699	108.718	1.00	0.00
ATOM	2742	HB2	ASN	A	175	159.396	68.363	108.142	1.00	0.00
ATOM	2743	CG	ASN	A	175	160.410	69.909	107.088	1.00	0.00

ATOM	2744	OD1	ASN	A	175	159.644	70.801	107.412	1.00	0.00
ATOM	2745	ND2	ASN	A	175	161.226	70.135	106.080	1.00	0.00
ATOM	2746	1HD2	ASN	A	175	161.766	69.377	105.683	1.00	0.00
ATOM	2747	2HD2	ASN	A	175	161.129	71.025	105.631	1.00	0.00
ATOM	2748	C	ASN	A	175	160.970	66.059	107.878	1.00	0.00
ATOM	2749	O	ASN	A	175	161.368	66.073	109.042	1.00	0.00
ATOM	2750	N	MET	A	176	160.524	64.946	107.281	1.00	0.00
ATOM	2751	H	MET	A	176	160.225	65.003	106.317	1.00	0.00
ATOM	2752	CA	MET	A	176	160.596	63.622	107.886	1.00	0.00
ATOM	2753	HA	MET	A	176	160.122	63.691	108.864	1.00	0.00
ATOM	2754	CB	MET	A	176	159.801	62.600	107.054	1.00	0.00
ATOM	2755	HB1	MET	A	176	158.860	63.055	106.747	1.00	0.00
ATOM	2756	HB2	MET	A	176	159.559	61.742	107.681	1.00	0.00
ATOM	2757	CG	MET	A	176	160.536	62.090	105.802	1.00	0.00
ATOM	2758	HG1	MET	A	176	161.130	62.905	105.386	1.00	0.00
ATOM	2759	HG2	MET	A	176	159.786	61.815	105.060	1.00	0.00
ATOM	2760	SD	MET	A	176	161.604	60.644	106.067	1.00	0.00
ATOM	2761	CE	MET	A	176	162.475	60.627	104.481	1.00	0.00
ATOM	2762	HE1	MET	A	176	163.141	59.766	104.441	1.00	0.00
ATOM	2763	HE2	MET	A	176	161.753	60.566	103.668	1.00	0.00
ATOM	2764	HE3	MET	A	176	163.059	61.542	104.381	1.00	0.00
ATOM	2765	C	MET	A	176	162.040	63.184	108.123	1.00	0.00
ATOM	2766	O	MET	A	176	162.981	63.616	107.455	1.00	0.00
ATOM	2767	N	SER	A	177	162.200	62.261	109.060	1.00	0.00
ATOM	2768	H	SER	A	177	161.395	61.966	109.605	1.00	0.00
ATOM	2769	CA	SER	A	177	163.456	61.578	109.315	1.00	0.00
ATOM	2770	HA	SER	A	177	164.048	61.568	108.399	1.00	0.00
ATOM	2771	CB	SER	A	177	164.260	62.334	110.381	1.00	0.00
ATOM	2772	HB1	SER	A	177	164.656	63.244	109.927	1.00	0.00
ATOM	2773	HB2	SER	A	177	165.099	61.721	110.713	1.00	0.00
ATOM	2774	OG	SER	A	177	163.465	62.706	111.492	1.00	0.00
ATOM	2775	HG	SER	A	177	162.566	62.878	111.174	1.00	0.00
ATOM	2776	C	SER	A	177	163.166	60.133	109.701	1.00	0.00
ATOM	2777	O	SER	A	177	162.279	59.805	110.497	1.00	0.00
ATOM	2778	N	MET	A	178	163.906	59.220	109.097	1.00	0.00
ATOM	2779	H	MET	A	178	164.609	59.512	108.436	1.00	0.00
ATOM	2780	CA	MET	A	178	163.733	57.795	109.301	1.00	0.00
ATOM	2781	HA	MET	A	178	163.413	57.608	110.324	1.00	0.00
ATOM	2782	CB	MET	A	178	162.650	57.267	108.345	1.00	0.00
ATOM	2783	HB1	MET	A	178	162.917	57.498	107.314	1.00	0.00
ATOM	2784	HB2	MET	A	178	161.720	57.785	108.577	1.00	0.00
ATOM	2785	CG	MET	A	178	162.412	55.761	108.479	1.00	0.00
ATOM	2786	HG1	MET	A	178	162.509	55.467	109.523	1.00	0.00
ATOM	2787	HG2	MET	A	178	163.173	55.227	107.908	1.00	0.00
ATOM	2788	SD	MET	A	178	160.767	55.269	107.901	1.00	0.00
ATOM	2789	CE	MET	A	178	160.842	53.489	108.197	1.00	0.00
ATOM	2790	HE1	MET	A	178	159.898	53.039	107.891	1.00	0.00
ATOM	2791	HE2	MET	A	178	161.651	53.054	107.612	1.00	0.00
ATOM	2792	HE3	MET	A	178	161.011	53.295	109.255	1.00	0.00
ATOM	2793	C	MET	A	178	165.081	57.136	109.086	1.00	0.00
ATOM	2794	O	MET	A	178	165.616	57.244	107.985	1.00	0.00
ATOM	2795	N	ASP	A	179	165.603	56.437	110.100	1.00	0.00
ATOM	2796	H	ASP	A	179	165.165	56.447	111.014	1.00	0.00
ATOM	2797	CA	ASP	A	179	166.512	55.351	109.776	1.00	0.00
ATOM	2798	HA	ASP	A	179	166.528	55.234	108.693	1.00	0.00
ATOM	2799	CB	ASP	A	179	167.985	55.650	110.140	1.00	0.00
ATOM	2800	HB1	ASP	A	179	168.200	56.696	109.915	1.00	0.00
ATOM	2801	HB2	ASP	A	179	168.609	55.053	109.472	1.00	0.00
ATOM	2802	CG	ASP	A	179	168.436	55.339	111.571	1.00	0.00
ATOM	2803	OD1	ASP	A	179	168.709	54.156	111.862	1.00	0.00
ATOM	2804	OD2	ASP	A	179	168.594	56.287	112.376	1.00	0.00

ATOM	2805	C	ASP	A	179	165.914	54.056	110.289	1.00	0.00
ATOM	2806	O	ASP	A	179	165.146	54.027	111.258	1.00	0.00
ATOM	2807	N	PHE	A	180	166.186	53.001	109.539	1.00	0.00
ATOM	2808	H	PHE	A	180	166.800	53.106	108.746	1.00	0.00
ATOM	2809	CA	PHE	A	180	165.734	51.668	109.840	1.00	0.00
ATOM	2810	HA	PHE	A	180	165.542	51.596	110.912	1.00	0.00
ATOM	2811	CB	PHE	A	180	164.424	51.340	109.109	1.00	0.00
ATOM	2812	HB1	PHE	A	180	164.611	51.274	108.036	1.00	0.00
ATOM	2813	HB2	PHE	A	180	163.713	52.150	109.277	1.00	0.00
ATOM	2814	CG	PHE	A	180	163.825	50.041	109.603	1.00	0.00
ATOM	2815	CD1	PHE	A	180	164.377	48.819	109.176	1.00	0.00
ATOM	2816	HD1	PHE	A	180	165.166	48.807	108.435	1.00	0.00
ATOM	2817	CE1	PHE	A	180	163.883	47.605	109.675	1.00	0.00
ATOM	2818	HE1	PHE	A	180	164.298	46.677	109.312	1.00	0.00
ATOM	2819	CZ	PHE	A	180	162.815	47.606	110.584	1.00	0.00
ATOM	2820	HZ	PHE	A	180	162.436	46.665	110.930	1.00	0.00
ATOM	2821	CE2	PHE	A	180	162.227	48.820	110.980	1.00	0.00
ATOM	2822	HE2	PHE	A	180	161.371	48.813	111.632	1.00	0.00
ATOM	2823	CD2	PHE	A	180	162.746	50.041	110.509	1.00	0.00
ATOM	2824	HD2	PHE	A	180	162.304	50.972	110.827	1.00	0.00
ATOM	2825	C	PHE	A	180	166.870	50.715	109.484	1.00	0.00
ATOM	2826	O	PHE	A	180	167.398	50.713	108.376	1.00	0.00
ATOM	2827	N	GLN	A	181	167.218	49.886	110.458	1.00	0.00
ATOM	2828	H	GLN	A	181	166.741	49.973	111.345	1.00	0.00
ATOM	2829	CA	GLN	A	181	167.915	48.633	110.243	1.00	0.00
ATOM	2830	HA	GLN	A	181	167.691	48.273	109.237	1.00	0.00
ATOM	2831	CB	GLN	A	181	169.442	48.813	110.359	1.00	0.00
ATOM	2832	HB1	GLN	A	181	169.765	49.555	109.627	1.00	0.00
ATOM	2833	HB2	GLN	A	181	169.923	47.866	110.107	1.00	0.00
ATOM	2834	CG	GLN	A	181	169.930	49.251	111.752	1.00	0.00
ATOM	2835	HG1	GLN	A	181	169.643	48.497	112.483	1.00	0.00
ATOM	2836	HG2	GLN	A	181	169.448	50.190	112.026	1.00	0.00
ATOM	2837	CD	GLN	A	181	171.441	49.447	111.802	1.00	0.00
ATOM	2838	OE1	GLN	A	181	171.973	50.524	111.606	1.00	0.00
ATOM	2839	NE2	GLN	A	181	172.222	48.422	112.066	1.00	0.00
ATOM	2840	1HE2	GLN	A	181	171.856	47.479	112.054	1.00	0.00
ATOM	2841	2HE2	GLN	A	181	173.204	48.623	112.106	1.00	0.00
ATOM	2842	C	GLN	A	181	167.348	47.625	111.245	1.00	0.00
ATOM	2843	O	GLN	A	181	166.784	48.026	112.265	1.00	0.00
ATOM	2844	N	ASN	A	182	167.533	46.328	111.000	1.00	0.00
ATOM	2845	H	ASN	A	182	167.945	46.050	110.121	1.00	0.00
ATOM	2846	CA	ASN	A	182	167.248	45.298	111.996	1.00	0.00
ATOM	2847	HA	ASN	A	182	167.277	45.782	112.969	1.00	0.00
ATOM	2848	CB	ASN	A	182	165.806	44.735	111.902	1.00	0.00
ATOM	2849	HB1	ASN	A	182	165.130	45.527	112.223	1.00	0.00
ATOM	2850	HB2	ASN	A	182	165.707	43.917	112.613	1.00	0.00
ATOM	2851	CG	ASN	A	182	165.267	44.237	110.565	1.00	0.00
ATOM	2852	OD1	ASN	A	182	164.079	44.308	110.319	1.00	0.00
ATOM	2853	ND2	ASN	A	182	166.077	43.710	109.675	1.00	0.00
ATOM	2854	1HD2	ASN	A	182	167.030	43.483	109.928	1.00	0.00
ATOM	2855	2HD2	ASN	A	182	165.662	43.454	108.795	1.00	0.00
ATOM	2856	C	ASN	A	182	168.348	44.234	112.048	1.00	0.00
ATOM	2857	O	ASN	A	182	168.314	43.237	111.332	1.00	0.00
ATOM	2858	N	HIS	A	183	169.338	44.481	112.912	1.00	0.00
ATOM	2859	H	HIS	A	183	169.279	45.318	113.470	1.00	0.00
ATOM	2860	CA	HIS	A	183	170.461	43.566	113.166	1.00	0.00
ATOM	2861	HA	HIS	A	183	170.655	42.990	112.258	1.00	0.00
ATOM	2862	CB	HIS	A	183	171.738	44.357	113.489	1.00	0.00
ATOM	2863	HB1	HIS	A	183	172.478	43.678	113.913	1.00	0.00
ATOM	2864	HB2	HIS	A	183	171.532	45.125	114.233	1.00	0.00
ATOM	2865	CG	HIS	A	183	172.358	44.990	112.266	1.00	0.00

ATOM	2866	ND1	HIS	A	183	171.659	45.622	111.234	1.00	0.00
ATOM	2867	CE1	HIS	A	183	172.569	45.925	110.296	1.00	0.00
ATOM	2868	HE1	HIS	A	183	172.346	46.415	109.355	1.00	0.00
ATOM	2869	NE2	HIS	A	183	173.796	45.563	110.706	1.00	0.00
ATOM	2870	HE2	HIS	A	183	174.651	45.676	110.176	1.00	0.00
ATOM	2871	CD2	HIS	A	183	173.682	44.964	111.941	1.00	0.00
ATOM	2872	HD2	HIS	A	183	174.471	44.507	112.527	1.00	0.00
ATOM	2873	C	HIS	A	183	170.152	42.522	114.247	1.00	0.00
ATOM	2874	O	HIS	A	183	170.943	41.606	114.448	1.00	0.00
ATOM	2875	N	LEU	A	184	169.009	42.631	114.926	1.00	0.00
ATOM	2876	H	LEU	A	184	168.426	43.435	114.729	1.00	0.00
ATOM	2877	CA	LEU	A	184	168.700	41.882	116.141	1.00	0.00
ATOM	2878	HA	LEU	A	184	169.486	41.150	116.332	1.00	0.00
ATOM	2879	CB	LEU	A	184	168.685	42.839	117.355	1.00	0.00
ATOM	2880	HB1	LEU	A	184	168.585	42.245	118.265	1.00	0.00
ATOM	2881	HB2	LEU	A	184	167.808	43.466	117.282	1.00	0.00
ATOM	2882	CG	LEU	A	184	169.908	43.765	117.493	1.00	0.00
ATOM	2883	HG	LEU	A	184	170.065	44.317	116.567	1.00	0.00
ATOM	2884	CD1	LEU	A	184	169.650	44.812	118.582	1.00	0.00
ATOM	2885	1HD1	LEU	A	184	170.549	45.396	118.760	1.00	0.00
ATOM	2886	2HD1	LEU	A	184	168.855	45.482	118.263	1.00	0.00
ATOM	2887	3HD1	LEU	A	184	169.338	44.329	119.503	1.00	0.00
ATOM	2888	CD2	LEU	A	184	171.195	42.988	117.783	1.00	0.00
ATOM	2889	1HD2	LEU	A	184	172.022	43.683	117.914	1.00	0.00
ATOM	2890	2HD2	LEU	A	184	171.064	42.392	118.686	1.00	0.00
ATOM	2891	3HD2	LEU	A	184	171.428	42.328	116.950	1.00	0.00
ATOM	2892	C	LEU	A	184	167.400	41.094	115.966	1.00	0.00
ATOM	2893	O	LEU	A	184	166.311	41.515	116.362	1.00	0.00
ATOM	2894	N	GLY	A	185	167.519	39.959	115.279	1.00	0.00
ATOM	2895	H	GLY	A	185	168.428	39.747	114.893	1.00	0.00
ATOM	2896	CA	GLY	A	185	166.433	39.006	115.042	1.00	0.00
ATOM	2897	HA1	GLY	A	185	165.721	39.036	115.867	1.00	0.00
ATOM	2898	HA2	GLY	A	185	166.856	38.002	115.004	1.00	0.00
ATOM	2899	C	GLY	A	185	165.666	39.273	113.746	1.00	0.00
ATOM	2900	O	GLY	A	185	165.260	40.399	113.453	1.00	0.00
ATOM	2901	N	SER	A	186	165.410	38.188	113.017	1.00	0.00
ATOM	2902	H	SER	A	186	165.840	37.316	113.279	1.00	0.00
ATOM	2903	CA	SER	A	186	164.412	38.114	111.945	1.00	0.00
ATOM	2904	HA	SER	A	186	164.709	38.784	111.137	1.00	0.00
ATOM	2905	CB	SER	A	186	164.345	36.675	111.403	1.00	0.00
ATOM	2906	HB1	SER	A	186	163.765	36.667	110.480	1.00	0.00
ATOM	2907	HB2	SER	A	186	163.846	36.041	112.138	1.00	0.00
ATOM	2908	OG	SER	A	186	165.640	36.154	111.157	1.00	0.00
ATOM	2909	HG	SER	A	186	165.558	35.302	110.716	1.00	0.00
ATOM	2910	C	SER	A	186	163.023	38.532	112.448	1.00	0.00
ATOM	2911	O	SER	A	186	162.769	38.568	113.655	1.00	0.00
ATOM	2912	N	CYS	A	187	162.094	38.763	111.522	1.00	0.00
ATOM	2913	H	CYS	A	187	162.346	38.728	110.546	1.00	0.00
ATOM	2914	CA	CYS	A	187	160.671	38.850	111.837	1.00	0.00
ATOM	2915	HA	CYS	A	187	160.493	38.331	112.782	1.00	0.00
ATOM	2916	CB	CYS	A	187	160.223	40.295	111.999	1.00	0.00
ATOM	2917	HB1	CYS	A	187	159.365	40.502	111.356	1.00	0.00
ATOM	2918	HB2	CYS	A	187	161.032	40.947	111.676	1.00	0.00
ATOM	2919	SG	CYS	A	187	159.762	40.707	113.700	1.00	0.00
ATOM	2920	C	CYS	A	187	159.808	38.143	110.798	1.00	0.00
ATOM	2921	O	CYS	A	187	160.242	37.842	109.685	1.00	0.00
ATOM	2922	N	GLN	A	188	158.564	37.886	111.191	1.00	0.00
ATOM	2923	H	GLN	A	188	158.279	38.209	112.101	1.00	0.00
ATOM	2924	CA	GLN	A	188	157.539	37.390	110.292	1.00	0.00
ATOM	2925	HA	GLN	A	188	157.984	36.590	109.699	1.00	0.00
ATOM	2926	CB	GLN	A	188	156.401	36.777	111.125	1.00	0.00

ATOM	2927	HB1	GLN	A	188	156.060	37.524	111.839	1.00	0.00
ATOM	2928	HB2	GLN	A	188	156.805	35.941	111.698	1.00	0.00
ATOM	2929	CG	GLN	A	188	155.189	36.267	110.322	1.00	0.00
ATOM	2930	HG1	GLN	A	188	154.729	37.091	109.780	1.00	0.00
ATOM	2931	HG2	GLN	A	188	154.446	35.903	111.032	1.00	0.00
ATOM	2932	CD	GLN	A	188	155.529	35.153	109.334	1.00	0.00
ATOM	2933	OE1	GLN	A	188	156.265	35.326	108.375	1.00	0.00
ATOM	2934	NE2	GLN	A	188	155.022	33.958	109.536	1.00	0.00
ATOM	2935	1HE2	GLN	A	188	154.419	33.776	110.318	1.00	0.00
ATOM	2936	2HE2	GLN	A	188	155.284	33.246	108.880	1.00	0.00
ATOM	2937	C	GLN	A	188	157.069	38.484	109.329	1.00	0.00
ATOM	2938	O	GLN	A	188	156.865	39.634	109.712	1.00	0.00
ATOM	2939	N	LYS	A	189	156.894	38.088	108.066	1.00	0.00
ATOM	2940	H	LYS	A	189	156.972	37.092	107.890	1.00	0.00
ATOM	2941	CA	LYS	A	189	156.423	38.935	106.967	1.00	0.00
ATOM	2942	HA	LYS	A	189	157.065	39.814	106.893	1.00	0.00
ATOM	2943	CB	LYS	A	189	156.471	38.139	105.652	1.00	0.00
ATOM	2944	HB1	LYS	A	189	156.088	38.761	104.841	1.00	0.00
ATOM	2945	HB2	LYS	A	189	155.817	37.270	105.746	1.00	0.00
ATOM	2946	CG	LYS	A	189	157.884	37.667	105.272	1.00	0.00
ATOM	2947	HG1	LYS	A	189	158.305	37.071	106.081	1.00	0.00
ATOM	2948	HG2	LYS	A	189	158.521	38.536	105.102	1.00	0.00
ATOM	2949	CD	LYS	A	189	157.829	36.808	104.006	1.00	0.00
ATOM	2950	HD1	LYS	A	189	157.439	37.403	103.179	1.00	0.00
ATOM	2951	HD2	LYS	A	189	157.163	35.962	104.181	1.00	0.00
ATOM	2952	CE	LYS	A	189	159.229	36.293	103.657	1.00	0.00
ATOM	2953	HE1	LYS	A	189	159.648	35.803	104.540	1.00	0.00
ATOM	2954	HE2	LYS	A	189	159.862	37.152	103.415	1.00	0.00
ATOM	2955	NZ	LYS	A	189	159.187	35.342	102.519	1.00	0.00
ATOM	2956	HZ1	LYS	A	189	160.122	35.038	102.275	1.00	0.00
ATOM	2957	HZ2	LYS	A	189	158.638	34.528	102.763	1.00	0.00
ATOM	2958	HZ3	LYS	A	189	158.770	35.781	101.708	1.00	0.00
ATOM	2959	C	LYS	A	189	154.992	39.424	107.195	1.00	0.00
ATOM	2960	O	LYS	A	189	154.214	38.804	107.919	1.00	0.00
ATOM	2961	N	CYS	A	190	154.642	40.501	106.506	1.00	0.00
ATOM	2962	H	CYS	A	190	155.345	40.982	105.968	1.00	0.00
ATOM	2963	CA	CYS	A	190	153.251	40.879	106.288	1.00	0.00
ATOM	2964	HA	CYS	A	190	152.779	41.041	107.258	1.00	0.00
ATOM	2965	CB	CYS	A	190	153.237	42.200	105.514	1.00	0.00
ATOM	2966	HB1	CYS	A	190	152.206	42.544	105.441	1.00	0.00
ATOM	2967	HB2	CYS	A	190	153.604	42.026	104.501	1.00	0.00
ATOM	2968	SG	CYS	A	190	154.238	43.500	106.287	1.00	0.00
ATOM	2969	C	CYS	A	190	152.479	39.765	105.564	1.00	0.00
ATOM	2970	O	CYS	A	190	153.037	39.051	104.728	1.00	0.00
ATOM	2971	N	ASP	A	191	151.206	39.599	105.913	1.00	0.00
ATOM	2972	H	ASP	A	191	150.776	40.243	106.574	1.00	0.00
ATOM	2973	CA	ASP	A	191	150.301	38.642	105.271	1.00	0.00
ATOM	2974	HA	ASP	A	191	150.789	37.667	105.276	1.00	0.00
ATOM	2975	CB	ASP	A	191	149.023	38.525	106.113	1.00	0.00
ATOM	2976	HB1	ASP	A	191	149.269	38.032	107.053	1.00	0.00
ATOM	2977	HB2	ASP	A	191	148.289	37.906	105.601	1.00	0.00
ATOM	2978	CG	ASP	A	191	148.425	39.890	106.409	1.00	0.00
ATOM	2979	OD1	ASP	A	191	148.927	40.515	107.369	1.00	0.00
ATOM	2980	OD2	ASP	A	191	147.484	40.276	105.683	1.00	0.00
ATOM	2981	C	ASP	A	191	150.005	39.002	103.798	1.00	0.00
ATOM	2982	O	ASP	A	191	150.140	40.159	103.400	1.00	0.00
ATOM	2983	N	PRO	A	192	149.595	38.042	102.946	1.00	0.00
ATOM	2984	CD	PRO	A	192	149.420	36.623	103.220	1.00	0.00
ATOM	2985	HD1	PRO	A	192	148.815	36.447	104.108	1.00	0.00
ATOM	2986	HD2	PRO	A	192	150.397	36.152	103.327	1.00	0.00
ATOM	2987	CG	PRO	A	192	148.708	36.057	101.992	1.00	0.00

ATOM	2988	HG1	PRO	A	192	147.629	36.177	102.106	1.00	0.00
ATOM	2989	HG2	PRO	A	192	148.963	35.011	101.822	1.00	0.00
ATOM	2990	CB	PRO	A	192	149.208	36.954	100.862	1.00	0.00
ATOM	2991	HB1	PRO	A	192	148.516	36.967	100.018	1.00	0.00
ATOM	2992	HB2	PRO	A	192	150.192	36.615	100.537	1.00	0.00
ATOM	2993	CA	PRO	A	192	149.340	38.326	101.531	1.00	0.00
ATOM	2994	HA	PRO	A	192	150.200	38.847	101.105	1.00	0.00
ATOM	2995	C	PRO	A	192	148.105	39.207	101.272	1.00	0.00
ATOM	2996	O	PRO	A	192	147.963	39.745	100.179	1.00	0.00
ATOM	2997	N	SER	A	193	147.223	39.367	102.265	1.00	0.00
ATOM	2998	H	SER	A	193	147.370	38.878	103.137	1.00	0.00
ATOM	2999	CA	SER	A	193	146.143	40.364	102.271	1.00	0.00
ATOM	3000	HA	SER	A	193	145.612	40.293	101.322	1.00	0.00
ATOM	3001	CB	SER	A	193	145.123	40.074	103.389	1.00	0.00
ATOM	3002	HB1	SER	A	193	144.241	39.622	102.935	1.00	0.00
ATOM	3003	HB2	SER	A	193	144.812	41.003	103.869	1.00	0.00
ATOM	3004	OG	SER	A	193	145.614	39.172	104.368	1.00	0.00
ATOM	3005	HG	SER	A	193	146.268	39.675	104.927	1.00	0.00
ATOM	3006	C	SER	A	193	146.649	41.809	102.368	1.00	0.00
ATOM	3007	O	SER	A	193	145.897	42.740	102.067	1.00	0.00
ATOM	3008	N	CYS	A	194	147.908	42.027	102.760	1.00	0.00
ATOM	3009	H	CYS	A	194	148.484	41.241	103.036	1.00	0.00
ATOM	3010	CA	CYS	A	194	148.389	43.369	103.039	1.00	0.00
ATOM	3011	HA	CYS	A	194	147.613	43.798	103.653	1.00	0.00
ATOM	3012	CB	CYS	A	194	149.714	43.341	103.804	1.00	0.00
ATOM	3013	HB1	CYS	A	194	150.235	44.283	103.632	1.00	0.00
ATOM	3014	HB2	CYS	A	194	150.352	42.541	103.431	1.00	0.00
ATOM	3015	SG	CYS	A	194	149.494	43.197	105.577	1.00	0.00
ATOM	3016	C	CYS	A	194	148.550	44.284	101.816	1.00	0.00
ATOM	3017	O	CYS	A	194	149.253	43.916	100.872	1.00	0.00
ATOM	3018	N	PRO	A	195	148.038	45.535	101.882	1.00	0.00
ATOM	3019	CD	PRO	A	195	147.053	46.031	102.837	1.00	0.00
ATOM	3020	HD1	PRO	A	195	147.547	46.265	103.777	1.00	0.00
ATOM	3021	HD2	PRO	A	195	146.242	45.318	102.993	1.00	0.00
ATOM	3022	CG	PRO	A	195	146.499	47.324	102.243	1.00	0.00
ATOM	3023	HG1	PRO	A	195	146.114	47.996	103.009	1.00	0.00
ATOM	3024	HG2	PRO	A	195	145.729	47.091	101.505	1.00	0.00
ATOM	3025	CB	PRO	A	195	147.733	47.887	101.550	1.00	0.00
ATOM	3026	HB1	PRO	A	195	148.385	48.346	102.296	1.00	0.00
ATOM	3027	HB2	PRO	A	195	147.469	48.603	100.771	1.00	0.00
ATOM	3028	CA	PRO	A	195	148.372	46.618	100.966	1.00	0.00
ATOM	3029	HA	PRO	A	195	147.863	46.401	100.028	1.00	0.00
ATOM	3030	C	PRO	A	195	149.864	46.778	100.658	1.00	0.00
ATOM	3031	O	PRO	A	195	150.599	47.472	101.359	1.00	0.00
ATOM	3032	N	ASN	A	196	150.300	46.186	99.546	1.00	0.00
ATOM	3033	H	ASN	A	196	149.667	45.538	99.095	1.00	0.00
ATOM	3034	CA	ASN	A	196	151.617	46.387	98.940	1.00	0.00
ATOM	3035	HA	ASN	A	196	151.703	45.662	98.129	1.00	0.00
ATOM	3036	CB	ASN	A	196	151.621	47.794	98.302	1.00	0.00
ATOM	3037	HB1	ASN	A	196	151.869	48.536	99.057	1.00	0.00
ATOM	3038	HB2	ASN	A	196	150.629	48.022	97.911	1.00	0.00
ATOM	3039	CG	ASN	A	196	152.582	47.928	97.138	1.00	0.00
ATOM	3040	OD1	ASN	A	196	152.192	47.951	95.987	1.00	0.00
ATOM	3041	ND2	ASN	A	196	153.863	48.010	97.392	1.00	0.00
ATOM	3042	1HD2	ASN	A	196	154.185	47.860	98.343	1.00	0.00
ATOM	3043	2HD2	ASN	A	196	154.480	48.087	96.605	1.00	0.00
ATOM	3044	C	ASN	A	196	152.809	46.096	99.885	1.00	0.00
ATOM	3045	O	ASN	A	196	153.799	46.823	99.898	1.00	0.00
ATOM	3046	N	GLY	A	197	152.697	45.053	100.714	1.00	0.00
ATOM	3047	H	GLY	A	197	151.836	44.523	100.706	1.00	0.00
ATOM	3048	CA	GLY	A	197	153.774	44.634	101.624	1.00	0.00

ATOM	3049	HA1	GLY	A	197	154.738	44.732	101.124	1.00	0.00
ATOM	3050	HA2	GLY	A	197	153.628	43.587	101.886	1.00	0.00
ATOM	3051	C	GLY	A	197	153.837	45.441	102.927	1.00	0.00
ATOM	3052	O	GLY	A	197	154.740	45.222	103.726	1.00	0.00
ATOM	3053	N	SER	A	198	152.893	46.355	103.172	1.00	0.00
ATOM	3054	H	SER	A	198	152.206	46.555	102.456	1.00	0.00
ATOM	3055	CA	SER	A	198	152.888	47.198	104.366	1.00	0.00
ATOM	3056	HA	SER	A	198	153.911	47.360	104.702	1.00	0.00
ATOM	3057	CB	SER	A	198	152.272	48.558	104.035	1.00	0.00
ATOM	3058	HB1	SER	A	198	152.230	49.163	104.940	1.00	0.00
ATOM	3059	HB2	SER	A	198	151.260	48.428	103.648	1.00	0.00
ATOM	3060	OG	SER	A	198	153.065	49.232	103.080	1.00	0.00
ATOM	3061	HG	SER	A	198	153.964	49.304	103.465	1.00	0.00
ATOM	3062	C	SER	A	198	152.093	46.579	105.521	1.00	0.00
ATOM	3063	O	SER	A	198	150.889	46.350	105.420	1.00	0.00
ATOM	3064	N	CYS	A	199	152.746	46.392	106.667	1.00	0.00
ATOM	3065	H	CYS	A	199	153.740	46.581	106.701	1.00	0.00
ATOM	3066	CA	CYS	A	199	152.121	46.009	107.931	1.00	0.00
ATOM	3067	HA	CYS	A	199	151.126	46.450	107.985	1.00	0.00
ATOM	3068	CB	CYS	A	199	151.978	44.487	108.037	1.00	0.00
ATOM	3069	HB1	CYS	A	199	151.440	44.114	107.170	1.00	0.00
ATOM	3070	HB2	CYS	A	199	151.375	44.247	108.916	1.00	0.00
ATOM	3071	SG	CYS	A	199	153.529	43.571	108.186	1.00	0.00
ATOM	3072	C	CYS	A	199	152.927	46.538	109.124	1.00	0.00
ATOM	3073	O	CYS	A	199	154.090	46.919	108.980	1.00	0.00
ATOM	3074	N	TRP	A	200	152.327	46.551	110.318	1.00	0.00
ATOM	3075	H	TRP	A	200	151.365	46.235	110.397	1.00	0.00
ATOM	3076	CA	TRP	A	200	152.995	47.006	111.548	1.00	0.00
ATOM	3077	HA	TRP	A	200	153.826	47.663	111.288	1.00	0.00
ATOM	3078	CB	TRP	A	200	152.004	47.836	112.380	1.00	0.00
ATOM	3079	HB1	TRP	A	200	152.533	48.153	113.275	1.00	0.00
ATOM	3080	HB2	TRP	A	200	151.175	47.199	112.683	1.00	0.00
ATOM	3081	CG	TRP	A	200	151.450	49.083	111.760	1.00	0.00
ATOM	3082	CD1	TRP	A	200	150.384	49.157	110.928	1.00	0.00
ATOM	3083	HD1	TRP	A	200	149.794	48.311	110.598	1.00	0.00
ATOM	3084	NE1	TRP	A	200	150.139	50.480	110.600	1.00	0.00
ATOM	3085	HE1	TRP	A	200	149.399	50.797	109.968	1.00	0.00
ATOM	3086	CE2	TRP	A	200	151.054	51.324	111.194	1.00	0.00
ATOM	3087	CZ2	TRP	A	200	151.233	52.714	111.165	1.00	0.00
ATOM	3088	HZ2	TRP	A	200	150.548	53.330	110.605	1.00	0.00
ATOM	3089	CH2	TRP	A	200	152.303	53.280	111.877	1.00	0.00
ATOM	3090	HH2	TRP	A	200	152.455	54.349	111.864	1.00	0.00
ATOM	3091	CZ3	TRP	A	200	153.171	52.453	112.612	1.00	0.00
ATOM	3092	HZ3	TRP	A	200	153.987	52.891	113.175	1.00	0.00
ATOM	3093	CE3	TRP	A	200	152.967	51.061	112.658	1.00	0.00
ATOM	3094	HE3	TRP	A	200	153.614	50.443	113.264	1.00	0.00
ATOM	3095	CD2	TRP	A	200	151.910	50.458	111.936	1.00	0.00
ATOM	3096	C	TRP	A	200	153.578	45.856	112.394	1.00	0.00
ATOM	3097	O	TRP	A	200	154.216	46.076	113.421	1.00	0.00
ATOM	3098	N	GLY	A	201	153.351	44.625	111.953	1.00	0.00
ATOM	3099	H	GLY	A	201	152.749	44.538	111.146	1.00	0.00
ATOM	3100	CA	GLY	A	201	153.768	43.359	112.550	1.00	0.00
ATOM	3101	HA1	GLY	A	201	153.581	43.360	113.622	1.00	0.00
ATOM	3102	HA2	GLY	A	201	154.834	43.205	112.385	1.00	0.00
ATOM	3103	C	GLY	A	201	152.985	42.227	111.878	1.00	0.00
ATOM	3104	O	GLY	A	201	152.232	42.476	110.933	1.00	0.00
ATOM	3105	N	ALA	A	202	153.154	40.994	112.345	1.00	0.00
ATOM	3106	H	ALA	A	202	153.752	40.842	113.140	1.00	0.00
ATOM	3107	CA	ALA	A	202	152.478	39.834	111.767	1.00	0.00
ATOM	3108	HA	ALA	A	202	152.607	39.863	110.683	1.00	0.00
ATOM	3109	CB	ALA	A	202	153.172	38.582	112.303	1.00	0.00

ATOM	3110	HB1	ALA	A	202	152.751	37.697	111.826	1.00	0.00
ATOM	3111	HB2	ALA	A	202	154.233	38.646	112.078	1.00	0.00
ATOM	3112	HB3	ALA	A	202	153.034	38.510	113.383	1.00	0.00
ATOM	3113	C	ALA	A	202	150.965	39.792	112.083	1.00	0.00
ATOM	3114	O	ALA	A	202	150.560	40.077	113.209	1.00	0.00
ATOM	3115	N	GLY	A	203	150.173	39.356	111.097	1.00	0.00
ATOM	3116	H	GLY	A	203	150.577	39.267	110.177	1.00	0.00
ATOM	3117	CA	GLY	A	203	148.727	39.112	111.194	1.00	0.00
ATOM	3118	HA1	GLY	A	203	148.396	39.162	112.231	1.00	0.00
ATOM	3119	HA2	GLY	A	203	148.506	38.115	110.815	1.00	0.00
ATOM	3120	C	GLY	A	203	147.912	40.130	110.385	1.00	0.00
ATOM	3121	O	GLY	A	203	148.231	41.317	110.459	1.00	0.00
ATOM	3122	N	GLU	A	204	146.853	39.712	109.675	1.00	0.00
ATOM	3123	H	GLU	A	204	146.673	38.728	109.589	1.00	0.00
ATOM	3124	CA	GLU	A	204	146.035	40.637	108.853	1.00	0.00
ATOM	3125	HA	GLU	A	204	146.670	41.083	108.089	1.00	0.00
ATOM	3126	CB	GLU	A	204	144.871	39.924	108.131	1.00	0.00
ATOM	3127	HB1	GLU	A	204	144.120	39.625	108.863	1.00	0.00
ATOM	3128	HB2	GLU	A	204	145.270	39.014	107.682	1.00	0.00
ATOM	3129	CG	GLU	A	204	144.207	40.750	107.003	1.00	0.00
ATOM	3130	HG1	GLU	A	204	143.499	40.100	106.484	1.00	0.00
ATOM	3131	HG2	GLU	A	204	144.980	41.027	106.287	1.00	0.00
ATOM	3132	CD	GLU	A	204	143.460	42.029	107.431	1.00	0.00
ATOM	3133	OE1	GLU	A	204	143.600	43.084	106.772	1.00	0.00
ATOM	3134	OE2	GLU	A	204	142.764	42.037	108.470	1.00	0.00
ATOM	3135	C	GLU	A	204	145.489	41.803	109.692	1.00	0.00
ATOM	3136	O	GLU	A	204	145.601	42.979	109.338	1.00	0.00
ATOM	3137	N	GLU	A	205	144.992	41.515	110.893	1.00	0.00
ATOM	3138	H	GLU	A	205	144.875	40.549	111.147	1.00	0.00
ATOM	3139	CA	GLU	A	205	144.529	42.558	111.810	1.00	0.00
ATOM	3140	HA	GLU	A	205	144.066	43.320	111.191	1.00	0.00
ATOM	3141	CB	GLU	A	205	143.437	41.995	112.734	1.00	0.00
ATOM	3142	HB1	GLU	A	205	143.461	42.474	113.713	1.00	0.00
ATOM	3143	HB2	GLU	A	205	143.593	40.925	112.885	1.00	0.00
ATOM	3144	CG	GLU	A	205	142.048	42.230	112.111	1.00	0.00
ATOM	3145	HG1	GLU	A	205	141.320	41.613	112.643	1.00	0.00
ATOM	3146	HG2	GLU	A	205	142.045	41.915	111.068	1.00	0.00
ATOM	3147	CD	GLU	A	205	141.644	43.707	112.232	1.00	0.00
ATOM	3148	OE1	GLU	A	205	141.015	44.045	113.260	1.00	0.00
ATOM	3149	OE2	GLU	A	205	142.063	44.568	111.423	1.00	0.00
ATOM	3150	C	GLU	A	205	145.663	43.342	112.521	1.00	0.00
ATOM	3151	O	GLU	A	205	145.455	44.034	113.517	1.00	0.00
ATOM	3152	N	ASN	A	206	146.868	43.292	111.956	1.00	0.00
ATOM	3153	H	ASN	A	206	146.993	42.634	111.193	1.00	0.00
ATOM	3154	CA	ASN	A	206	147.997	44.178	112.229	1.00	0.00
ATOM	3155	HA	ASN	A	206	147.644	45.010	112.836	1.00	0.00
ATOM	3156	CB	ASN	A	206	149.010	43.375	113.050	1.00	0.00
ATOM	3157	HB1	ASN	A	206	149.540	42.689	112.390	1.00	0.00
ATOM	3158	HB2	ASN	A	206	148.490	42.790	113.808	1.00	0.00
ATOM	3159	CG	ASN	A	206	150.006	44.255	113.763	1.00	0.00
ATOM	3160	OD1	ASN	A	206	149.837	45.451	113.945	1.00	0.00
ATOM	3161	ND2	ASN	A	206	151.063	43.646	114.240	1.00	0.00
ATOM	3162	1HD2	ASN	A	206	151.124	42.643	114.168	1.00	0.00
ATOM	3163	2HD2	ASN	A	206	151.756	44.211	114.701	1.00	0.00
ATOM	3164	C	ASN	A	206	148.600	44.783	110.940	1.00	0.00
ATOM	3165	O	ASN	A	206	149.628	45.475	110.944	1.00	0.00
ATOM	3166	N	CYS	A	207	147.935	44.535	109.814	1.00	0.00
ATOM	3167	H	CYS	A	207	147.125	43.930	109.858	1.00	0.00
ATOM	3168	CA	CYS	A	207	148.237	45.090	108.514	1.00	0.00
ATOM	3169	HA	CYS	A	207	149.299	44.942	108.327	1.00	0.00
ATOM	3170	CB	CYS	A	207	147.466	44.301	107.472	1.00	0.00

ATOM	3171	HB1	CYS	A	207	146.410	44.568	107.492	1.00	0.00
ATOM	3172	HB2	CYS	A	207	147.568	43.227	107.648	1.00	0.00
ATOM	3173	SG	CYS	A	207	148.145	44.676	105.876	1.00	0.00
ATOM	3174	C	CYS	A	207	147.929	46.598	108.415	1.00	0.00
ATOM	3175	O	CYS	A	207	147.084	47.136	109.129	1.00	0.00
ATOM	3176	N	GLN	A	208	148.606	47.285	107.487	1.00	0.00
ATOM	3177	H	GLN	A	208	149.215	46.770	106.859	1.00	0.00
ATOM	3178	CA	GLN	A	208	148.413	48.704	107.193	1.00	0.00
ATOM	3179	HA	GLN	A	208	148.261	49.215	108.144	1.00	0.00
ATOM	3180	CB	GLN	A	208	149.697	49.273	106.557	1.00	0.00
ATOM	3181	HB1	GLN	A	208	149.937	48.665	105.689	1.00	0.00
ATOM	3182	HB2	GLN	A	208	150.508	49.164	107.274	1.00	0.00
ATOM	3183	CG	GLN	A	208	149.670	50.736	106.062	1.00	0.00
ATOM	3184	HG1	GLN	A	208	148.998	50.802	105.211	1.00	0.00
ATOM	3185	HG2	GLN	A	208	150.668	50.995	105.707	1.00	0.00
ATOM	3186	CD	GLN	A	208	149.245	51.789	107.082	1.00	0.00
ATOM	3187	OE1	GLN	A	208	148.911	51.507	108.218	1.00	0.00
ATOM	3188	NE2	GLN	A	208	149.247	53.053	106.713	1.00	0.00
ATOM	3189	1HE2	GLN	A	208	149.474	53.320	105.774	1.00	0.00
ATOM	3190	2HE2	GLN	A	208	148.866	53.703	107.381	1.00	0.00
ATOM	3191	C	GLN	A	208	147.157	48.955	106.347	1.00	0.00
ATOM	3192	O	GLN	A	208	147.197	49.032	105.118	1.00	0.00
ATOM	3193	N	LYS	A	209	146.026	49.116	107.043	1.00	0.00
ATOM	3194	H	LYS	A	209	146.085	48.969	108.046	1.00	0.00
ATOM	3195	CA	LYS	A	209	144.841	49.798	106.511	1.00	0.00
ATOM	3196	HA	LYS	A	209	144.527	49.272	105.611	1.00	0.00
ATOM	3197	CB	LYS	A	209	143.700	49.711	107.546	1.00	0.00
ATOM	3198	HB1	LYS	A	209	142.947	50.453	107.275	1.00	0.00
ATOM	3199	HB2	LYS	A	209	144.069	49.965	108.542	1.00	0.00
ATOM	3200	CG	LYS	A	209	142.993	48.328	107.579	1.00	0.00
ATOM	3201	HG1	LYS	A	209	142.789	48.026	106.551	1.00	0.00
ATOM	3202	HG2	LYS	A	209	142.028	48.475	108.065	1.00	0.00
ATOM	3203	CD	LYS	A	209	143.699	47.163	108.309	1.00	0.00
ATOM	3204	HD1	LYS	A	209	143.768	47.392	109.373	1.00	0.00
ATOM	3205	HD2	LYS	A	209	144.701	47.013	107.913	1.00	0.00
ATOM	3206	CE	LYS	A	209	142.901	45.859	108.103	1.00	0.00
ATOM	3207	HE1	LYS	A	209	143.017	45.543	107.061	1.00	0.00
ATOM	3208	HE2	LYS	A	209	141.841	46.048	108.280	1.00	0.00
ATOM	3209	NZ	LYS	A	209	143.334	44.741	108.973	1.00	0.00
ATOM	3210	HZ1	LYS	A	209	144.331	44.572	108.948	1.00	0.00
ATOM	3211	HZ2	LYS	A	209	142.976	44.798	109.928	1.00	0.00
ATOM	3212	HZ3	LYS	A	209	142.992	43.850	108.590	1.00	0.00
ATOM	3213	C	LYS	A	209	145.222	51.221	106.061	1.00	0.00
ATOM	3214	O	LYS	A	209	146.102	51.849	106.649	1.00	0.00
ATOM	3215	N	LEU	A	210	144.618	51.707	104.976	1.00	0.00
ATOM	3216	H	LEU	A	210	143.870	51.169	104.570	1.00	0.00
ATOM	3217	CA	LEU	A	210	145.169	52.804	104.166	1.00	0.00
ATOM	3218	HA	LEU	A	210	145.780	53.451	104.796	1.00	0.00
ATOM	3219	CB	LEU	A	210	146.036	52.130	103.076	1.00	0.00
ATOM	3220	HB1	LEU	A	210	145.380	51.753	102.291	1.00	0.00
ATOM	3221	HB2	LEU	A	210	146.543	51.263	103.503	1.00	0.00
ATOM	3222	CG	LEU	A	210	147.134	52.990	102.423	1.00	0.00
ATOM	3223	HG	LEU	A	210	146.689	53.902	102.027	1.00	0.00
ATOM	3224	CD1	LEU	A	210	148.245	53.340	103.416	1.00	0.00
ATOM	3225	1HD1	LEU	A	210	148.994	53.954	102.922	1.00	0.00
ATOM	3226	2HD1	LEU	A	210	147.836	53.889	104.261	1.00	0.00
ATOM	3227	3HD1	LEU	A	210	148.717	52.429	103.770	1.00	0.00
ATOM	3228	CD2	LEU	A	210	147.779	52.197	101.281	1.00	0.00
ATOM	3229	1HD2	LEU	A	210	148.252	51.296	101.669	1.00	0.00
ATOM	3230	2HD2	LEU	A	210	147.028	51.911	100.549	1.00	0.00
ATOM	3231	3HD2	LEU	A	210	148.530	52.804	100.781	1.00	0.00

ATOM	3232	C	LEU	A	210	144.011	53.632	103.593	1.00	0.00
ATOM	3233	O	LEU	A	210	143.117	53.042	102.981	1.00	0.00
ATOM	3234	N	THR	A	211	143.997	54.955	103.815	1.00	0.00
ATOM	3235	H	THR	A	211	144.792	55.365	104.299	1.00	0.00
ATOM	3236	CA	THR	A	211	142.756	55.756	103.673	1.00	0.00
ATOM	3237	HA	THR	A	211	142.135	55.288	102.914	1.00	0.00
ATOM	3238	CB	THR	A	211	141.918	55.727	104.977	1.00	0.00
ATOM	3239	HB	THR	A	211	141.064	56.389	104.862	1.00	0.00
ATOM	3240	CG2	THR	A	211	141.378	54.355	105.375	1.00	0.00
ATOM	3241	1HG2	THR	A	211	140.715	54.464	106.232	1.00	0.00
ATOM	3242	2HG2	THR	A	211	140.817	53.927	104.545	1.00	0.00
ATOM	3243	3HG2	THR	A	211	142.191	53.684	105.645	1.00	0.00
ATOM	3244	OG1	THR	A	211	142.653	56.194	106.072	1.00	0.00
ATOM	3245	HG1	THR	A	211	143.308	55.495	106.291	1.00	0.00
ATOM	3246	C	THR	A	211	142.910	57.214	103.200	1.00	0.00
ATOM	3247	O	THR	A	211	141.914	57.933	103.197	1.00	0.00
ATOM	3248	N	LYS	A	212	144.089	57.674	102.747	1.00	0.00
ATOM	3249	H	LYS	A	212	144.918	57.096	102.883	1.00	0.00
ATOM	3250	CA	LYS	A	212	144.203	58.904	101.927	1.00	0.00
ATOM	3251	HA	LYS	A	212	143.201	59.156	101.579	1.00	0.00
ATOM	3252	CB	LYS	A	212	144.669	60.145	102.727	1.00	0.00
ATOM	3253	HB1	LYS	A	212	144.056	60.248	103.620	1.00	0.00
ATOM	3254	HB2	LYS	A	212	144.456	61.013	102.101	1.00	0.00
ATOM	3255	CG	LYS	A	212	146.157	60.190	103.124	1.00	0.00
ATOM	3256	HG1	LYS	A	212	146.761	59.798	102.312	1.00	0.00
ATOM	3257	HG2	LYS	A	212	146.316	59.564	103.997	1.00	0.00
ATOM	3258	CD	LYS	A	212	146.678	61.618	103.387	1.00	0.00
ATOM	3259	HD1	LYS	A	212	146.400	62.245	102.539	1.00	0.00
ATOM	3260	HD2	LYS	A	212	147.768	61.597	103.422	1.00	0.00
ATOM	3261	CE	LYS	A	212	146.145	62.280	104.667	1.00	0.00
ATOM	3262	HE1	LYS	A	212	145.155	61.871	104.895	1.00	0.00
ATOM	3263	HE2	LYS	A	212	146.026	63.352	104.487	1.00	0.00
ATOM	3264	NZ	LYS	A	212	147.064	62.079	105.814	1.00	0.00
ATOM	3265	HZ1	LYS	A	212	146.612	62.359	106.688	1.00	0.00
ATOM	3266	HZ2	LYS	A	212	147.295	61.098	105.921	1.00	0.00
ATOM	3267	HZ3	LYS	A	212	147.948	62.574	105.721	1.00	0.00
ATOM	3268	C	LYS	A	212	145.005	58.709	100.639	1.00	0.00
ATOM	3269	O	LYS	A	212	144.629	59.248	99.602	1.00	0.00
ATOM	3270	N	ILE	A	213	146.062	57.883	100.639	1.00	0.00
ATOM	3271	H	ILE	A	213	146.332	57.437	101.516	1.00	0.00
ATOM	3272	CA	ILE	A	213	146.885	57.659	99.422	1.00	0.00
ATOM	3273	HA	ILE	A	213	146.927	58.612	98.892	1.00	0.00
ATOM	3274	CB	ILE	A	213	148.354	57.286	99.722	1.00	0.00
ATOM	3275	HB	ILE	A	213	148.875	57.264	98.766	1.00	0.00
ATOM	3276	CG2	ILE	A	213	148.992	58.411	100.540	1.00	0.00
ATOM	3277	1HG2	ILE	A	213	150.063	58.260	100.635	1.00	0.00
ATOM	3278	2HG2	ILE	A	213	148.817	59.376	100.065	1.00	0.00
ATOM	3279	3HG2	ILE	A	213	148.553	58.427	101.529	1.00	0.00
ATOM	3280	CG1	ILE	A	213	148.540	55.891	100.350	1.00	0.00
ATOM	3281	1HG1	ILE	A	213	148.095	55.140	99.696	1.00	0.00
ATOM	3282	2HG1	ILE	A	213	148.026	55.844	101.303	1.00	0.00
ATOM	3283	CD	ILE	A	213	150.015	55.512	100.556	1.00	0.00
ATOM	3284	HD1	ILE	A	213	150.079	54.486	100.911	1.00	0.00
ATOM	3285	HD2	ILE	A	213	150.552	55.583	99.611	1.00	0.00
ATOM	3286	HD3	ILE	A	213	150.479	56.158	101.299	1.00	0.00
ATOM	3287	C	ILE	A	213	146.267	56.693	98.405	1.00	0.00
ATOM	3288	O	ILE	A	213	146.909	56.344	97.417	1.00	0.00
ATOM	3289	N	ILE	A	214	145.027	56.283	98.670	1.00	0.00
ATOM	3290	H	ILE	A	214	144.629	56.614	99.533	1.00	0.00
ATOM	3291	CA	ILE	A	214	144.189	55.392	97.866	1.00	0.00
ATOM	3292	HA	ILE	A	214	144.763	55.038	97.007	1.00	0.00

ATOM	3293	CB	ILE	A	214	143.770	54.178	98.714	1.00	0.00
ATOM	3294	HB	ILE	A	214	143.085	53.577	98.122	1.00	0.00
ATOM	3295	CG2	ILE	A	214	144.997	53.297	98.995	1.00	0.00
ATOM	3296	1HG2	ILE	A	214	144.701	52.377	99.498	1.00	0.00
ATOM	3297	2HG2	ILE	A	214	145.490	53.037	98.057	1.00	0.00
ATOM	3298	3HG2	ILE	A	214	145.710	53.828	99.626	1.00	0.00
ATOM	3299	CG1	ILE	A	214	143.095	54.499	100.068	1.00	0.00
ATOM	3300	1HG1	ILE	A	214	143.765	55.081	100.700	1.00	0.00
ATOM	3301	2HG1	ILE	A	214	142.917	53.558	100.576	1.00	0.00
ATOM	3302	CD	ILE	A	214	141.744	55.227	99.991	1.00	0.00
ATOM	3303	HD1	ILE	A	214	141.215	55.119	100.937	1.00	0.00
ATOM	3304	HD2	ILE	A	214	141.901	56.287	99.805	1.00	0.00
ATOM	3305	HD3	ILE	A	214	141.128	54.798	99.202	1.00	0.00
ATOM	3306	C	ILE	A	214	142.963	56.121	97.280	1.00	0.00
ATOM	3307	O	ILE	A	214	142.087	55.493	96.694	1.00	0.00
ATOM	3308	N	CYS	A	215	142.861	57.434	97.517	1.00	0.00
ATOM	3309	H	CYS	A	215	143.619	57.905	97.990	1.00	0.00
ATOM	3310	CA	CYS	A	215	141.685	58.230	97.198	1.00	0.00
ATOM	3311	HA	CYS	A	215	140.806	57.776	97.657	1.00	0.00
ATOM	3312	CB	CYS	A	215	141.875	59.639	97.790	1.00	0.00
ATOM	3313	HB1	CYS	A	215	141.229	60.343	97.263	1.00	0.00
ATOM	3314	HB2	CYS	A	215	142.906	59.967	97.646	1.00	0.00
ATOM	3315	SG	CYS	A	215	141.445	59.653	99.549	1.00	0.00
ATOM	3316	HG	CYS	A	215	141.772	60.934	99.770	1.00	0.00
ATOM	3317	C	CYS	A	215	141.425	58.372	95.691	1.00	0.00
ATOM	3318	O	CYS	A	215	142.310	58.715	94.900	1.00	0.00
ATOM	3319	N	ALA	A	216	140.149	58.277	95.315	1.00	0.00
ATOM	3320	H	ALA	A	216	139.458	57.959	95.976	1.00	0.00
ATOM	3321	CA	ALA	A	216	139.673	58.924	94.103	1.00	0.00
ATOM	3322	HA	ALA	A	216	140.188	58.477	93.250	1.00	0.00
ATOM	3323	CB	ALA	A	216	138.168	58.653	93.952	1.00	0.00
ATOM	3324	HB1	ALA	A	216	137.804	59.097	93.026	1.00	0.00
ATOM	3325	HB2	ALA	A	216	137.988	57.578	93.925	1.00	0.00
ATOM	3326	HB3	ALA	A	216	137.624	59.085	94.793	1.00	0.00
ATOM	3327	C	ALA	A	216	139.988	60.425	94.130	1.00	0.00
ATOM	3328	O	ALA	A	216	139.899	61.054	95.178	1.00	0.00
ATOM	3329	N	GLN	A	217	140.292	61.020	92.974	1.00	0.00
ATOM	3330	H	GLN	A	217	140.336	60.461	92.136	1.00	0.00
ATOM	3331	CA	GLN	A	217	140.640	62.450	92.870	1.00	0.00
ATOM	3332	HA	GLN	A	217	141.402	62.668	93.620	1.00	0.00
ATOM	3333	CB	GLN	A	217	141.248	62.725	91.484	1.00	0.00
ATOM	3334	HB1	GLN	A	217	141.625	63.748	91.464	1.00	0.00
ATOM	3335	HB2	GLN	A	217	140.466	62.643	90.729	1.00	0.00
ATOM	3336	CG	GLN	A	217	142.397	61.769	91.102	1.00	0.00
ATOM	3337	HG1	GLN	A	217	142.795	62.075	90.133	1.00	0.00
ATOM	3338	HG2	GLN	A	217	142.024	60.751	90.993	1.00	0.00
ATOM	3339	CD	GLN	A	217	143.549	61.776	92.108	1.00	0.00
ATOM	3340	OE1	GLN	A	217	144.458	62.582	92.028	1.00	0.00
ATOM	3341	NE2	GLN	A	217	143.581	60.898	93.087	1.00	0.00
ATOM	3342	1HE2	GLN	A	217	142.877	60.191	93.229	1.00	0.00
ATOM	3343	2HE2	GLN	A	217	144.354	60.988	93.718	1.00	0.00
ATOM	3344	C	GLN	A	217	139.455	63.394	93.187	1.00	0.00
ATOM	3345	O	GLN	A	217	139.626	64.606	93.252	1.00	0.00
ATOM	3346	N	GLN	A	218	138.255	62.840	93.401	1.00	0.00
ATOM	3347	H	GLN	A	218	138.193	61.843	93.306	1.00	0.00
ATOM	3348	CA	GLN	A	218	137.085	63.539	93.942	1.00	0.00
ATOM	3349	HA	GLN	A	218	137.075	64.558	93.550	1.00	0.00
ATOM	3350	CB	GLN	A	218	135.819	62.813	93.432	1.00	0.00
ATOM	3351	HB1	GLN	A	218	135.817	61.784	93.797	1.00	0.00
ATOM	3352	HB2	GLN	A	218	135.870	62.786	92.343	1.00	0.00
ATOM	3353	CG	GLN	A	218	134.497	63.487	93.836	1.00	0.00

ATOM	3354	HG1	GLN	A	218	134.597	64.569	93.738	1.00	0.00
ATOM	3355	HG2	GLN	A	218	134.268	63.257	94.876	1.00	0.00
ATOM	3356	CD	GLN	A	218	133.326	63.054	92.958	1.00	0.00
ATOM	3357	OE1	GLN	A	218	133.149	63.525	91.848	1.00	0.00
ATOM	3358	NE2	GLN	A	218	132.484	62.141	93.388	1.00	0.00
ATOM	3359	1HE2	GLN	A	218	132.588	61.678	94.288	1.00	0.00
ATOM	3360	2HE2	GLN	A	218	131.707	61.952	92.784	1.00	0.00
ATOM	3361	C	GLN	A	218	137.108	63.655	95.489	1.00	0.00
ATOM	3362	O	GLN	A	218	136.319	64.408	96.052	1.00	0.00
ATOM	3363	N	CYS	A	219	137.992	62.932	96.191	1.00	0.00
ATOM	3364	H	CYS	A	219	138.675	62.369	95.701	1.00	0.00
ATOM	3365	CA	CYS	A	219	138.031	62.889	97.654	1.00	0.00
ATOM	3366	HA	CYS	A	219	137.057	63.185	98.042	1.00	0.00
ATOM	3367	CB	CYS	A	219	138.311	61.457	98.137	1.00	0.00
ATOM	3368	HB1	CYS	A	219	138.276	61.465	99.227	1.00	0.00
ATOM	3369	HB2	CYS	A	219	139.329	61.195	97.851	1.00	0.00
ATOM	3370	SG	CYS	A	219	137.221	60.121	97.570	1.00	0.00
ATOM	3371	C	CYS	A	219	139.087	63.837	98.247	1.00	0.00
ATOM	3372	O	CYS	A	219	140.280	63.720	97.965	1.00	0.00
ATOM	3373	N	SER	A	220	138.649	64.744	99.121	1.00	0.00
ATOM	3374	H	SER	A	220	137.665	64.770	99.351	1.00	0.00
ATOM	3375	CA	SER	A	220	139.432	65.896	99.586	1.00	0.00
ATOM	3376	HA	SER	A	220	140.070	66.238	98.771	1.00	0.00
ATOM	3377	CB	SER	A	220	138.471	67.039	99.932	1.00	0.00
ATOM	3378	HB1	SER	A	220	137.988	67.393	99.019	1.00	0.00
ATOM	3379	HB2	SER	A	220	139.017	67.871	100.382	1.00	0.00
ATOM	3380	OG	SER	A	220	137.481	66.568	100.826	1.00	0.00
ATOM	3381	HG	SER	A	220	137.029	67.339	101.223	1.00	0.00
ATOM	3382	C	SER	A	220	140.368	65.669	100.788	1.00	0.00
ATOM	3383	O	SER	A	220	141.172	66.553	101.068	1.00	0.00
ATOM	3384	N	GLY	A	221	140.304	64.543	101.519	1.00	0.00
ATOM	3385	H	GLY	A	221	139.639	63.834	101.257	1.00	0.00
ATOM	3386	CA	GLY	A	221	141.118	64.408	102.745	1.00	0.00
ATOM	3387	HA1	GLY	A	221	140.666	65.033	103.516	1.00	0.00
ATOM	3388	HA2	GLY	A	221	142.115	64.810	102.550	1.00	0.00
ATOM	3389	C	GLY	A	221	141.312	63.020	103.347	1.00	0.00
ATOM	3390	O	GLY	A	221	142.432	62.630	103.677	1.00	0.00
ATOM	3391	N	ARG	A	222	140.221	62.256	103.424	1.00	0.00
ATOM	3392	H	ARG	A	222	139.337	62.673	103.165	1.00	0.00
ATOM	3393	CA	ARG	A	222	140.185	60.840	103.805	1.00	0.00
ATOM	3394	HA	ARG	A	222	141.140	60.380	103.555	1.00	0.00
ATOM	3395	CB	ARG	A	222	139.859	60.707	105.309	1.00	0.00
ATOM	3396	HB1	ARG	A	222	139.478	59.705	105.494	1.00	0.00
ATOM	3397	HB2	ARG	A	222	139.070	61.412	105.576	1.00	0.00
ATOM	3398	CG	ARG	A	222	141.051	60.914	106.253	1.00	0.00
ATOM	3399	HG1	ARG	A	222	140.683	60.901	107.278	1.00	0.00
ATOM	3400	HG2	ARG	A	222	141.524	61.877	106.059	1.00	0.00
ATOM	3401	CD	ARG	A	222	142.053	59.775	106.068	1.00	0.00
ATOM	3402	HD1	ARG	A	222	142.334	59.722	105.021	1.00	0.00
ATOM	3403	HD2	ARG	A	222	141.589	58.828	106.338	1.00	0.00
ATOM	3404	NE	ARG	A	222	143.289	59.961	106.834	1.00	0.00
ATOM	3405	HE	ARG	A	222	143.337	60.705	107.531	1.00	0.00
ATOM	3406	CZ	ARG	A	222	144.412	59.352	106.524	1.00	0.00
ATOM	3407	NH1	ARG	A	222	144.449	58.298	105.773	1.00	0.00
ATOM	3408	1HH1	ARG	A	222	143.610	57.752	105.685	1.00	0.00
ATOM	3409	2HH1	ARG	A	222	145.337	57.821	105.614	1.00	0.00
ATOM	3410	NH2	ARG	A	222	145.550	59.791	106.928	1.00	0.00
ATOM	3411	1HH2	ARG	A	222	145.551	60.559	107.603	1.00	0.00
ATOM	3412	2HH2	ARG	A	222	146.398	59.294	106.692	1.00	0.00
ATOM	3413	C	ARG	A	222	139.119	60.125	102.973	1.00	0.00
ATOM	3414	O	ARG	A	222	138.233	60.755	102.377	1.00	0.00

ATOM	3415	N	CYS	A	223	139.220	58.804	102.938	1.00	0.00
ATOM	3416	H	CYS	A	223	140.053	58.396	103.346	1.00	0.00
ATOM	3417	CA	CYS	A	223	138.334	57.900	102.219	1.00	0.00
ATOM	3418	HA	CYS	A	223	137.333	58.327	102.173	1.00	0.00
ATOM	3419	CB	CYS	A	223	138.875	57.782	100.786	1.00	0.00
ATOM	3420	HB1	CYS	A	223	139.918	57.462	100.818	1.00	0.00
ATOM	3421	HB2	CYS	A	223	138.832	58.762	100.309	1.00	0.00
ATOM	3422	SG	CYS	A	223	137.920	56.608	99.786	1.00	0.00
ATOM	3423	HG	CYS	A	223	136.718	57.172	99.991	1.00	0.00
ATOM	3424	C	CYS	A	223	138.263	56.534	102.921	1.00	0.00
ATOM	3425	O	CYS	A	223	139.278	55.957	103.301	1.00	0.00
ATOM	3426	N	ARG	A	224	137.053	55.987	103.061	1.00	0.00
ATOM	3427	H	ARG	A	224	136.265	56.499	102.660	1.00	0.00
ATOM	3428	CA	ARG	A	224	136.772	54.726	103.774	1.00	0.00
ATOM	3429	HA	ARG	A	224	137.644	54.459	104.378	1.00	0.00
ATOM	3430	CB	ARG	A	224	135.601	54.991	104.747	1.00	0.00
ATOM	3431	HB1	ARG	A	224	135.968	55.707	105.484	1.00	0.00
ATOM	3432	HB2	ARG	A	224	135.377	54.073	105.294	1.00	0.00
ATOM	3433	CG	ARG	A	224	134.284	55.582	104.182	1.00	0.00
ATOM	3434	HG1	ARG	A	224	134.485	56.547	103.722	1.00	0.00
ATOM	3435	HG2	ARG	A	224	133.614	55.764	105.023	1.00	0.00
ATOM	3436	CD	ARG	A	224	133.533	54.720	103.154	1.00	0.00
ATOM	3437	HD1	ARG	A	224	134.146	54.635	102.258	1.00	0.00
ATOM	3438	HD2	ARG	A	224	132.609	55.223	102.867	1.00	0.00
ATOM	3439	NE	ARG	A	224	133.260	53.372	103.684	1.00	0.00
ATOM	3440	HE	ARG	A	224	134.032	52.722	103.581	1.00	0.00
ATOM	3441	CZ	ARG	A	224	132.249	52.996	104.446	1.00	0.00
ATOM	3442	NH1	ARG	A	224	131.201	53.741	104.632	1.00	0.00
ATOM	3443	1HH1	ARG	A	224	131.126	54.594	104.112	1.00	0.00
ATOM	3444	2HH1	ARG	A	224	130.570	53.561	105.401	1.00	0.00
ATOM	3445	NH2	ARG	A	224	132.299	51.861	105.076	1.00	0.00
ATOM	3446	1HH2	ARG	A	224	133.110	51.279	104.965	1.00	0.00
ATOM	3447	2HH2	ARG	A	224	131.540	51.584	105.669	1.00	0.00
ATOM	3448	C	ARG	A	224	136.547	53.536	102.831	1.00	0.00
ATOM	3449	O	ARG	A	224	135.947	52.533	103.211	1.00	0.00
ATOM	3450	N	GLY	A	225	136.970	53.692	101.578	1.00	0.00
ATOM	3451	H	GLY	A	225	137.443	54.557	101.360	1.00	0.00
ATOM	3452	CA	GLY	A	225	136.740	52.774	100.467	1.00	0.00
ATOM	3453	HA1	GLY	A	225	135.725	52.878	100.104	1.00	0.00
ATOM	3454	HA2	GLY	A	225	136.850	51.746	100.812	1.00	0.00
ATOM	3455	C	GLY	A	225	137.750	53.001	99.342	1.00	0.00
ATOM	3456	O	GLY	A	225	138.951	53.046	99.611	1.00	0.00
ATOM	3457	N	LYS	A	226	137.284	53.105	98.098	1.00	0.00
ATOM	3458	H	LYS	A	226	136.272	53.014	98.004	1.00	0.00
ATOM	3459	CA	LYS	A	226	138.063	53.481	96.893	1.00	0.00
ATOM	3460	HA	LYS	A	226	138.892	54.132	97.172	1.00	0.00
ATOM	3461	CB	LYS	A	226	138.592	52.218	96.177	1.00	0.00
ATOM	3462	HB1	LYS	A	226	139.106	52.521	95.261	1.00	0.00
ATOM	3463	HB2	LYS	A	226	137.736	51.607	95.880	1.00	0.00
ATOM	3464	CG	LYS	A	226	139.550	51.324	96.985	1.00	0.00
ATOM	3465	HG1	LYS	A	226	139.718	50.407	96.417	1.00	0.00
ATOM	3466	HG2	LYS	A	226	139.072	51.037	97.921	1.00	0.00
ATOM	3467	CD	LYS	A	226	140.915	51.976	97.251	1.00	0.00
ATOM	3468	HD1	LYS	A	226	140.794	53.020	97.544	1.00	0.00
ATOM	3469	HD2	LYS	A	226	141.495	51.958	96.327	1.00	0.00
ATOM	3470	CE	LYS	A	226	141.675	51.209	98.344	1.00	0.00
ATOM	3471	HE1	LYS	A	226	142.748	51.378	98.218	1.00	0.00
ATOM	3472	HE2	LYS	A	226	141.481	50.142	98.210	1.00	0.00
ATOM	3473	NZ	LYS	A	226	141.259	51.652	99.695	1.00	0.00
ATOM	3474	HZ1	LYS	A	226	141.634	52.566	99.902	1.00	0.00
ATOM	3475	HZ2	LYS	A	226	140.251	51.800	99.728	1.00	0.00

ATOM	3476	HZ3	LYS	A	226	141.523	51.012	100.426	1.00	0.00
ATOM	3477	C	LYS	A	226	137.215	54.281	95.884	1.00	0.00
ATOM	3478	O	LYS	A	226	137.767	54.989	95.044	1.00	0.00
ATOM	3479	N	SER	A	227	135.890	54.135	95.935	1.00	0.00
ATOM	3480	H	SER	A	227	135.491	53.599	96.697	1.00	0.00
ATOM	3481	CA	SER	A	227	134.931	54.727	95.005	1.00	0.00
ATOM	3482	HA	SER	A	227	135.293	54.532	93.997	1.00	0.00
ATOM	3483	CB	SER	A	227	133.550	54.041	95.152	1.00	0.00
ATOM	3484	HB1	SER	A	227	133.658	53.080	95.656	1.00	0.00
ATOM	3485	HB2	SER	A	227	133.159	53.837	94.155	1.00	0.00
ATOM	3486	OG	SER	A	227	132.604	54.843	95.839	1.00	0.00
ATOM	3487	HG	SER	A	227	132.452	54.443	96.737	1.00	0.00
ATOM	3488	C	SER	A	227	134.788	56.252	95.186	1.00	0.00
ATOM	3489	O	SER	A	227	135.071	56.783	96.260	1.00	0.00
ATOM	3490	N	PRO	A	228	134.256	56.964	94.175	1.00	0.00
ATOM	3491	CD	PRO	A	228	133.936	56.465	92.843	1.00	0.00
ATOM	3492	HD1	PRO	A	228	133.308	55.577	92.881	1.00	0.00
ATOM	3493	HD2	PRO	A	228	134.861	56.249	92.307	1.00	0.00
ATOM	3494	CG	PRO	A	228	133.183	57.589	92.137	1.00	0.00
ATOM	3495	HG1	PRO	A	228	132.116	57.499	92.345	1.00	0.00
ATOM	3496	HG2	PRO	A	228	133.371	57.590	91.063	1.00	0.00
ATOM	3497	CB	PRO	A	228	133.737	58.837	92.815	1.00	0.00
ATOM	3498	HB1	PRO	A	228	133.039	59.665	92.725	1.00	0.00
ATOM	3499	HB2	PRO	A	228	134.694	59.101	92.362	1.00	0.00
ATOM	3500	CA	PRO	A	228	133.966	58.396	94.267	1.00	0.00
ATOM	3501	HA	PRO	A	228	134.848	58.909	94.652	1.00	0.00
ATOM	3502	C	PRO	A	228	132.793	58.787	95.187	1.00	0.00
ATOM	3503	O	PRO	A	228	132.477	59.972	95.272	1.00	0.00
ATOM	3504	N	SER	A	229	132.164	57.836	95.887	1.00	0.00
ATOM	3505	H	SER	A	229	132.422	56.866	95.726	1.00	0.00
ATOM	3506	CA	SER	A	229	131.208	58.086	96.984	1.00	0.00
ATOM	3507	HA	SER	A	229	131.084	59.162	97.116	1.00	0.00
ATOM	3508	CB	SER	A	229	129.831	57.506	96.637	1.00	0.00
ATOM	3509	HB1	SER	A	229	129.892	56.417	96.605	1.00	0.00
ATOM	3510	HB2	SER	A	229	129.514	57.874	95.660	1.00	0.00
ATOM	3511	OG	SER	A	229	128.888	57.904	97.612	1.00	0.00
ATOM	3512	HG	SER	A	229	129.326	57.822	98.476	1.00	0.00
ATOM	3513	C	SER	A	229	131.710	57.524	98.322	1.00	0.00
ATOM	3514	O	SER	A	229	131.013	57.623	99.330	1.00	0.00
ATOM	3515	N	ASP	A	230	132.930	56.981	98.353	1.00	0.00
ATOM	3516	H	ASP	A	230	133.454	56.910	97.489	1.00	0.00
ATOM	3517	CA	ASP	A	230	133.583	56.479	99.566	1.00	0.00
ATOM	3518	HA	ASP	A	230	132.818	56.189	100.287	1.00	0.00
ATOM	3519	CB	ASP	A	230	134.430	55.240	99.238	1.00	0.00
ATOM	3520	HB1	ASP	A	230	134.837	54.863	100.172	1.00	0.00
ATOM	3521	HB2	ASP	A	230	135.275	55.551	98.622	1.00	0.00
ATOM	3522	CG	ASP	A	230	133.737	54.066	98.530	1.00	0.00
ATOM	3523	OD1	ASP	A	230	132.505	54.061	98.321	1.00	0.00
ATOM	3524	OD2	ASP	A	230	134.500	53.164	98.117	1.00	0.00
ATOM	3525	C	ASP	A	230	134.480	57.544	100.231	1.00	0.00
ATOM	3526	O	ASP	A	230	135.189	57.250	101.203	1.00	0.00
ATOM	3527	N	CYS	A	231	134.499	58.757	99.668	1.00	0.00
ATOM	3528	H	CYS	A	231	133.930	58.885	98.844	1.00	0.00
ATOM	3529	CA	CYS	A	231	135.080	59.954	100.262	1.00	0.00
ATOM	3530	HA	CYS	A	231	136.146	59.784	100.415	1.00	0.00
ATOM	3531	CB	CYS	A	231	134.892	61.143	99.310	1.00	0.00
ATOM	3532	HB1	CYS	A	231	135.470	61.984	99.695	1.00	0.00
ATOM	3533	HB2	CYS	A	231	133.841	61.437	99.335	1.00	0.00
ATOM	3534	SG	CYS	A	231	135.339	60.906	97.562	1.00	0.00
ATOM	3535	C	CYS	A	231	134.430	60.264	101.621	1.00	0.00
ATOM	3536	O	CYS	A	231	133.267	59.946	101.871	1.00	0.00

```
ATOM   3537  N   CYS A 232     135.182  60.954 102.471  1.00  0.00
ATOM   3538  H   CYS A 232     136.127  61.192 102.208  1.00  0.00
ATOM   3539  CA  CYS A 232     134.781  61.263 103.838  1.00  0.00
ATOM   3540  HA  CYS A 232     133.816  60.805 104.064  1.00  0.00
ATOM   3541  CB  CYS A 232     135.828  60.651 104.767  1.00  0.00
ATOM   3542  HB1 CYS A 232     135.532  60.801 105.799  1.00  0.00
ATOM   3543  HB2 CYS A 232     136.797  61.127 104.607  1.00  0.00
ATOM   3544  SG  CYS A 232     135.941  58.869 104.441  1.00  0.00
ATOM   3545  HG  CYS A 232     136.745  58.622 105.482  1.00  0.00
ATOM   3546  C   CYS A 232     134.616  62.780 104.013  1.00  0.00
ATOM   3547  O   CYS A 232     134.997  63.557 103.138  1.00  0.00
ATOM   3548  N   HIS A 233     134.049  63.224 105.142  1.00  0.00
ATOM   3549  H   HIS A 233     133.736  62.569 105.841  1.00  0.00
ATOM   3550  CA  HIS A 233     133.932  64.656 105.421  1.00  0.00
ATOM   3551  HA  HIS A 233     133.367  65.118 104.610  1.00  0.00
ATOM   3552  CB  HIS A 233     133.156  64.894 106.722  1.00  0.00
ATOM   3553  HB1 HIS A 233     133.623  64.324 107.526  1.00  0.00
ATOM   3554  HB2 HIS A 233     132.133  64.539 106.596  1.00  0.00
ATOM   3555  CG  HIS A 233     133.121  66.352 107.117  1.00  0.00
ATOM   3556  ND1 HIS A 233     132.681  67.412 106.315  1.00  0.00
ATOM   3557  CE1 HIS A 233     132.778  68.510 107.085  1.00  0.00
ATOM   3558  HE1 HIS A 233     132.377  69.480 106.826  1.00  0.00
ATOM   3559  NE2 HIS A 233     133.326  68.207 108.271  1.00  0.00
ATOM   3560  HE2 HIS A 233     133.237  68.811 109.101  1.00  0.00
ATOM   3561  CD2 HIS A 233     133.549  66.850 108.308  1.00  0.00
ATOM   3562  HD2 HIS A 233     133.900  66.273 109.149  1.00  0.00
ATOM   3563  C   HIS A 233     135.305  65.343 105.469  1.00  0.00
ATOM   3564  O   HIS A 233     136.293  64.772 105.926  1.00  0.00
ATOM   3565  N   ASN A 234     135.349  66.619 105.107  1.00  0.00
ATOM   3566  H   ASN A 234     134.474  67.043 104.839  1.00  0.00
ATOM   3567  CA  ASN A 234     136.545  67.462 105.102  1.00  0.00
ATOM   3568  HA  ASN A 234     137.282  67.004 104.438  1.00  0.00
ATOM   3569  CB  ASN A 234     136.163  68.837 104.521  1.00  0.00
ATOM   3570  HB1 ASN A 234     137.057  69.456 104.452  1.00  0.00
ATOM   3571  HB2 ASN A 234     135.454  69.332 105.185  1.00  0.00
ATOM   3572  CG  ASN A 234     135.574  68.706 103.136  1.00  0.00
ATOM   3573  OD1 ASN A 234     136.270  68.561 102.144  1.00  0.00
ATOM   3574  ND2 ASN A 234     134.262  68.697 103.021  1.00  0.00
ATOM   3575 1HD2 ASN A 234     133.667  68.837 103.821  1.00  0.00
ATOM   3576 2HD2 ASN A 234     133.888  68.568 102.098  1.00  0.00
ATOM   3577  C   ASN A 234     137.226  67.634 106.467  1.00  0.00
ATOM   3578  O   ASN A 234     138.350  68.123 106.522  1.00  0.00
ATOM   3579  N   GLN A 235     136.557  67.242 107.559  1.00  0.00
ATOM   3580  H   GLN A 235     135.621  66.885 107.429  1.00  0.00
ATOM   3581  CA  GLN A 235     137.129  67.204 108.902  1.00  0.00
ATOM   3582  HA  GLN A 235     138.201  67.346 108.763  1.00  0.00
ATOM   3583  CB  GLN A 235     136.722  68.430 109.734  1.00  0.00
ATOM   3584  HB1 GLN A 235     137.265  68.373 110.675  1.00  0.00
ATOM   3585  HB2 GLN A 235     135.647  68.425 109.907  1.00  0.00
ATOM   3586  CG  GLN A 235     137.130  69.743 109.030  1.00  0.00
ATOM   3587  HG1 GLN A 235     136.452  69.913 108.193  1.00  0.00
ATOM   3588  HG2 GLN A 235     138.137  69.634 108.629  1.00  0.00
ATOM   3589  CD  GLN A 235     137.150  70.990 109.909  1.00  0.00
ATOM   3590  OE1 GLN A 235     137.336  70.948 111.114  1.00  0.00
ATOM   3591  NE2 GLN A 235     137.026  72.148 109.297  1.00  0.00
ATOM   3592 1HE2 GLN A 235     136.860  72.196 108.312  1.00  0.00
ATOM   3593 2HE2 GLN A 235     137.070  73.011 109.845  1.00  0.00
ATOM   3594  C   GLN A 235     137.118  65.807 109.564  1.00  0.00
ATOM   3595  O   GLN A 235     137.382  65.680 110.763  1.00  0.00
ATOM   3596  N   CYS A 236     136.967  64.750 108.751  1.00  0.00
ATOM   3597  H   CYS A 236     136.755  64.946 107.781  1.00  0.00
```

ATOM	3598	CA	CYS	A	236	137.497	63.411	109.027	1.00	0.00
ATOM	3599	HA	CYS	A	236	137.171	63.077	110.013	1.00	0.00
ATOM	3600	CB	CYS	A	236	136.998	62.415	107.968	1.00	0.00
ATOM	3601	HB1	CYS	A	236	137.661	61.549	107.965	1.00	0.00
ATOM	3602	HB2	CYS	A	236	137.081	62.850	106.977	1.00	0.00
ATOM	3603	SG	CYS	A	236	135.326	61.782	108.214	1.00	0.00
ATOM	3604	C	CYS	A	236	139.042	63.388	108.981	1.00	0.00
ATOM	3605	O	CYS	A	236	139.643	63.479	107.911	1.00	0.00
ATOM	3606	N	ALA	A	237	139.683	63.240	110.142	1.00	0.00
ATOM	3607	H	ALA	A	237	139.121	63.189	110.987	1.00	0.00
ATOM	3608	CA	ALA	A	237	141.135	63.100	110.260	1.00	0.00
ATOM	3609	HA	ALA	A	237	141.617	63.827	109.600	1.00	0.00
ATOM	3610	CB	ALA	A	237	141.533	63.435	111.706	1.00	0.00
ATOM	3611	HB1	ALA	A	237	141.163	64.424	111.969	1.00	0.00
ATOM	3612	HB2	ALA	A	237	141.108	62.711	112.396	1.00	0.00
ATOM	3613	HB3	ALA	A	237	142.620	63.414	111.799	1.00	0.00
ATOM	3614	C	ALA	A	237	141.650	61.709	109.841	1.00	0.00
ATOM	3615	O	ALA	A	237	142.603	61.598	109.082	1.00	0.00
ATOM	3616	N	ALA	A	238	140.975	60.643	110.275	1.00	0.00
ATOM	3617	H	ALA	A	238	140.143	60.791	110.823	1.00	0.00
ATOM	3618	CA	ALA	A	238	141.331	59.257	109.982	1.00	0.00
ATOM	3619	HA	ALA	A	238	142.112	59.228	109.220	1.00	0.00
ATOM	3620	CB	ALA	A	238	141.892	58.603	111.243	1.00	0.00
ATOM	3621	HB1	ALA	A	238	141.122	58.555	112.003	1.00	0.00
ATOM	3622	HB2	ALA	A	238	142.192	57.584	111.023	1.00	0.00
ATOM	3623	HB3	ALA	A	238	142.752	59.161	111.616	1.00	0.00
ATOM	3624	C	ALA	A	238	140.103	58.518	109.437	1.00	0.00
ATOM	3625	O	ALA	A	238	139.006	58.664	109.974	1.00	0.00
ATOM	3626	N	GLY	A	239	140.309	57.771	108.346	1.00	0.00
ATOM	3627	H	GLY	A	239	141.268	57.671	108.049	1.00	0.00
ATOM	3628	CA	GLY	A	239	139.293	57.176	107.464	1.00	0.00
ATOM	3629	HA1	GLY	A	239	139.225	56.112	107.688	1.00	0.00
ATOM	3630	HA2	GLY	A	239	139.626	57.272	106.432	1.00	0.00
ATOM	3631	C	GLY	A	239	137.886	57.781	107.538	1.00	0.00
ATOM	3632	O	GLY	A	239	137.676	58.835	106.958	1.00	0.00
ATOM	3633	N	CYS	A	240	136.958	57.060	108.187	1.00	0.00
ATOM	3634	H	CYS	A	240	137.282	56.181	108.553	1.00	0.00
ATOM	3635	CA	CYS	A	240	135.545	57.347	108.531	1.00	0.00
ATOM	3636	HA	CYS	A	240	135.524	57.433	109.618	1.00	0.00
ATOM	3637	CB	CYS	A	240	135.023	58.701	108.031	1.00	0.00
ATOM	3638	HB1	CYS	A	240	133.942	58.765	108.156	1.00	0.00
ATOM	3639	HB2	CYS	A	240	135.250	58.842	106.983	1.00	0.00
ATOM	3640	SG	CYS	A	240	135.778	59.990	109.039	1.00	0.00
ATOM	3641	C	CYS	A	240	134.638	56.131	108.264	1.00	0.00
ATOM	3642	O	CYS	A	240	135.127	55.091	107.827	1.00	0.00
ATOM	3643	N	THR	A	241	133.335	56.206	108.555	1.00	0.00
ATOM	3644	H	THR	A	241	132.991	57.036	109.032	1.00	0.00
ATOM	3645	CA	THR	A	241	132.427	55.045	108.395	1.00	0.00
ATOM	3646	HA	THR	A	241	132.961	54.254	107.873	1.00	0.00
ATOM	3647	CB	THR	A	241	132.063	54.459	109.766	1.00	0.00
ATOM	3648	HB	THR	A	241	132.979	54.337	110.347	1.00	0.00
ATOM	3649	CG2	THR	A	241	131.090	55.321	110.572	1.00	0.00
ATOM	3650	1HG2	THR	A	241	130.915	54.850	111.539	1.00	0.00
ATOM	3651	2HG2	THR	A	241	131.528	56.302	110.747	1.00	0.00
ATOM	3652	3HG2	THR	A	241	130.141	55.430	110.050	1.00	0.00
ATOM	3653	OG1	THR	A	241	131.488	53.186	109.594	1.00	0.00
ATOM	3654	HG1	THR	A	241	130.698	53.304	109.057	1.00	0.00
ATOM	3655	C	THR	A	241	131.194	55.319	107.529	1.00	0.00
ATOM	3656	O	THR	A	241	130.399	54.416	107.252	1.00	0.00
ATOM	3657	N	GLY	A	242	131.073	56.546	107.031	1.00	0.00
ATOM	3658	H	GLY	A	242	131.770	57.235	107.281	1.00	0.00

ATOM	3659	CA	GLY	A	242	130.018	57.033	106.151	1.00	0.00
ATOM	3660	HA1	GLY	A	242	129.070	57.043	106.688	1.00	0.00
ATOM	3661	HA2	GLY	A	242	129.923	56.374	105.289	1.00	0.00
ATOM	3662	C	GLY	A	242	130.343	58.450	105.661	1.00	0.00
ATOM	3663	O	GLY	A	242	131.406	58.975	106.001	1.00	0.00
ATOM	3664	N	PRO	A	243	129.474	59.044	104.823	1.00	0.00
ATOM	3665	CD	PRO	A	243	128.238	58.462	104.317	1.00	0.00
ATOM	3666	HD1	PRO	A	243	127.561	58.204	105.133	1.00	0.00
ATOM	3667	HD2	PRO	A	243	128.468	57.578	103.721	1.00	0.00
ATOM	3668	CG	PRO	A	243	127.606	59.527	103.421	1.00	0.00
ATOM	3669	HG1	PRO	A	243	126.931	60.152	104.007	1.00	0.00
ATOM	3670	HG2	PRO	A	243	127.079	59.084	102.576	1.00	0.00
ATOM	3671	CB	PRO	A	243	128.809	60.354	102.968	1.00	0.00
ATOM	3672	HB1	PRO	A	243	128.523	61.365	102.675	1.00	0.00
ATOM	3673	HB2	PRO	A	243	129.307	59.849	102.138	1.00	0.00
ATOM	3674	CA	PRO	A	243	129.726	60.335	104.196	1.00	0.00
ATOM	3675	HA	PRO	A	243	130.763	60.394	103.861	1.00	0.00
ATOM	3676	C	PRO	A	243	129.456	61.554	105.099	1.00	0.00
ATOM	3677	O	PRO	A	243	129.928	62.645	104.790	1.00	0.00
ATOM	3678	N	ARG	A	244	128.673	61.414	106.180	1.00	0.00
ATOM	3679	H	ARG	A	244	128.457	60.473	106.502	1.00	0.00
ATOM	3680	CA	ARG	A	244	128.190	62.559	106.982	1.00	0.00
ATOM	3681	HA	ARG	A	244	127.897	63.361	106.304	1.00	0.00
ATOM	3682	CB	ARG	A	244	126.980	62.148	107.836	1.00	0.00
ATOM	3683	HB1	ARG	A	244	126.595	63.027	108.357	1.00	0.00
ATOM	3684	HB2	ARG	A	244	127.341	61.462	108.596	1.00	0.00
ATOM	3685	CG	ARG	A	244	125.826	61.497	107.048	1.00	0.00
ATOM	3686	HG1	ARG	A	244	126.208	60.760	106.341	1.00	0.00
ATOM	3687	HG2	ARG	A	244	125.301	62.270	106.484	1.00	0.00
ATOM	3688	CD	ARG	A	244	124.834	60.780	107.974	1.00	0.00
ATOM	3689	HD1	ARG	A	244	124.001	60.414	107.371	1.00	0.00
ATOM	3690	HD2	ARG	A	244	124.454	61.490	108.712	1.00	0.00
ATOM	3691	NE	ARG	A	244	125.487	59.653	108.665	1.00	0.00
ATOM	3692	HE	ARG	A	244	126.508	59.676	108.719	1.00	0.00
ATOM	3693	CZ	ARG	A	244	124.954	58.632	109.295	1.00	0.00
ATOM	3694	NH1	ARG	A	244	123.669	58.392	109.290	1.00	0.00
ATOM	3695	1HH1	ARG	A	244	123.068	59.028	108.801	1.00	0.00
ATOM	3696	2HH1	ARG	A	244	123.301	57.603	109.785	1.00	0.00
ATOM	3697	NH2	ARG	A	244	125.739	57.840	109.960	1.00	0.00
ATOM	3698	1HH2	ARG	A	244	126.704	58.171	110.061	1.00	0.00
ATOM	3699	2HH2	ARG	A	244	125.393	57.089	110.516	1.00	0.00
ATOM	3700	C	ARG	A	244	129.299	63.113	107.893	1.00	0.00
ATOM	3701	O	ARG	A	244	130.158	62.369	108.356	1.00	0.00
ATOM	3702	N	GLU	A	245	129.235	64.395	108.267	1.00	0.00
ATOM	3703	H	GLU	A	245	128.540	64.991	107.852	1.00	0.00
ATOM	3704	CA	GLU	A	245	130.155	64.984	109.267	1.00	0.00
ATOM	3705	HA	GLU	A	245	131.173	64.816	108.914	1.00	0.00
ATOM	3706	CB	GLU	A	245	129.966	66.519	109.346	1.00	0.00
ATOM	3707	HB1	GLU	A	245	128.933	66.743	109.614	1.00	0.00
ATOM	3708	HB2	GLU	A	245	130.164	66.928	108.354	1.00	0.00
ATOM	3709	CG	GLU	A	245	130.913	67.194	110.365	1.00	0.00
ATOM	3710	HG1	GLU	A	245	131.885	66.710	110.294	1.00	0.00
ATOM	3711	HG2	GLU	A	245	130.544	66.996	111.366	1.00	0.00
ATOM	3712	CD	GLU	A	245	131.095	68.713	110.196	1.00	0.00
ATOM	3713	OE1	GLU	A	245	132.191	69.216	110.562	1.00	0.00
ATOM	3714	OE2	GLU	A	245	130.190	69.349	109.616	1.00	0.00
ATOM	3715	C	GLU	A	245	130.083	64.318	110.667	1.00	0.00
ATOM	3716	O	GLU	A	245	131.009	64.435	111.476	1.00	0.00
ATOM	3717	N	SER	A	246	129.042	63.542	110.968	1.00	0.00
ATOM	3718	H	SER	A	246	128.291	63.468	110.301	1.00	0.00
ATOM	3719	CA	SER	A	246	128.956	62.690	112.166	1.00	0.00

ATOM	3720	HA	SER	A	246	129.299	63.238	113.039	1.00	0.00
ATOM	3721	CB	SER	A	246	127.487	62.304	112.380	1.00	0.00
ATOM	3722	HB1	SER	A	246	126.918	63.206	112.612	1.00	0.00
ATOM	3723	HB2	SER	A	246	127.401	61.621	113.226	1.00	0.00
ATOM	3724	OG	SER	A	246	126.929	61.700	111.219	1.00	0.00
ATOM	3725	HG	SER	A	246	127.420	60.884	111.000	1.00	0.00
ATOM	3726	C	SER	A	246	129.745	61.381	112.096	1.00	0.00
ATOM	3727	O	SER	A	246	129.903	60.703	113.107	1.00	0.00
ATOM	3728	N	ASP	A	247	130.187	60.989	110.900	1.00	0.00
ATOM	3729	H	ASP	A	247	130.037	61.596	110.101	1.00	0.00
ATOM	3730	CA	ASP	A	247	130.776	59.669	110.630	1.00	0.00
ATOM	3731	HA	ASP	A	247	130.391	58.962	111.368	1.00	0.00
ATOM	3732	CB	ASP	A	247	130.307	59.184	109.244	1.00	0.00
ATOM	3733	HB1	ASP	A	247	130.647	58.159	109.113	1.00	0.00
ATOM	3734	HB2	ASP	A	247	130.784	59.795	108.480	1.00	0.00
ATOM	3735	CG	ASP	A	247	128.776	59.219	109.032	1.00	0.00
ATOM	3736	OD1	ASP	A	247	128.001	59.384	110.008	1.00	0.00
ATOM	3737	OD2	ASP	A	247	128.310	59.074	107.881	1.00	0.00
ATOM	3738	C	ASP	A	247	132.318	59.694	110.806	1.00	0.00
ATOM	3739	O	ASP	A	247	133.015	58.701	110.567	1.00	0.00
ATOM	3740	N	CYS	A	248	132.811	60.856	111.257	1.00	0.00
ATOM	3741	H	CYS	A	248	132.127	61.590	111.360	1.00	0.00
ATOM	3742	CA	CYS	A	248	134.142	61.182	111.750	1.00	0.00
ATOM	3743	HA	CYS	A	248	134.839	61.141	110.943	1.00	0.00
ATOM	3744	CB	CYS	A	248	134.164	62.655	112.196	1.00	0.00
ATOM	3745	HB1	CYS	A	248	135.139	62.854	112.644	1.00	0.00
ATOM	3746	HB2	CYS	A	248	133.391	62.829	112.949	1.00	0.00
ATOM	3747	SG	CYS	A	248	133.954	63.779	110.785	1.00	0.00
ATOM	3748	HG	CYS	A	248	132.618	63.871	110.849	1.00	0.00
ATOM	3749	C	CYS	A	248	134.650	60.255	112.862	1.00	0.00
ATOM	3750	O	CYS	A	248	134.382	60.467	114.047	1.00	0.00
ATOM	3751	N	LEU	A	249	135.497	59.290	112.483	1.00	0.00
ATOM	3752	H	LEU	A	249	135.636	59.156	111.493	1.00	0.00
ATOM	3753	CA	LEU	A	249	136.267	58.448	113.414	1.00	0.00
ATOM	3754	HA	LEU	A	249	135.550	57.828	113.950	1.00	0.00
ATOM	3755	CB	LEU	A	249	137.232	57.549	112.609	1.00	0.00
ATOM	3756	HB1	LEU	A	249	138.258	57.877	112.777	1.00	0.00
ATOM	3757	HB2	LEU	A	249	137.053	57.664	111.539	1.00	0.00
ATOM	3758	CG	LEU	A	249	137.167	56.041	112.924	1.00	0.00
ATOM	3759	HG	LEU	A	249	138.013	55.566	112.430	1.00	0.00
ATOM	3760	CD1	LEU	A	249	137.250	55.728	114.419	1.00	0.00
ATOM	3761	1HD1	LEU	A	249	137.417	54.660	114.561	1.00	0.00
ATOM	3762	2HD1	LEU	A	249	138.092	56.264	114.857	1.00	0.00
ATOM	3763	3HD1	LEU	A	249	136.334	56.012	114.936	1.00	0.00
ATOM	3764	CD2	LEU	A	249	135.897	55.407	112.350	1.00	0.00
ATOM	3765	1HD2	LEU	A	249	135.883	55.533	111.268	1.00	0.00
ATOM	3766	2HD2	LEU	A	249	135.886	54.341	112.578	1.00	0.00
ATOM	3767	3HD2	LEU	A	249	135.009	55.872	112.779	1.00	0.00
ATOM	3768	C	LEU	A	249	136.999	59.298	114.477	1.00	0.00
ATOM	3769	O	LEU	A	249	136.851	59.088	115.681	1.00	0.00
ATOM	3770	N	VAL	A	250	137.696	60.332	113.999	1.00	0.00
ATOM	3771	H	VAL	A	250	137.757	60.395	112.992	1.00	0.00
ATOM	3772	CA	VAL	A	250	138.372	61.399	114.754	1.00	0.00
ATOM	3773	HA	VAL	A	250	137.846	61.566	115.689	1.00	0.00
ATOM	3774	CB	VAL	A	250	139.849	61.045	115.065	1.00	0.00
ATOM	3775	HB	VAL	A	250	140.401	61.977	115.199	1.00	0.00
ATOM	3776	CG1	VAL	A	250	139.972	60.251	116.362	1.00	0.00
ATOM	3777	1HG1	VAL	A	250	141.015	60.017	116.566	1.00	0.00
ATOM	3778	2HG1	VAL	A	250	139.588	60.842	117.189	1.00	0.00
ATOM	3779	3HG1	VAL	A	250	139.418	59.315	116.294	1.00	0.00
ATOM	3780	CG2	VAL	A	250	140.540	60.238	113.956	1.00	0.00

```
ATOM   3781 1HG2 VAL A 250     141.611  60.190 114.144  1.00  0.00
ATOM   3782 2HG2 VAL A 250     140.143  59.222 113.945  1.00  0.00
ATOM   3783 3HG2 VAL A 250     140.360  60.704 112.993  1.00  0.00
ATOM   3784  C   VAL A 250     138.287  62.705 113.954  1.00  0.00
ATOM   3785  O   VAL A 250     138.338  62.687 112.721  1.00  0.00
ATOM   3786  N   CYS A 251     138.145  63.836 114.650  1.00  0.00
ATOM   3787  H   CYS A 251     138.143  63.755 115.652  1.00  0.00
ATOM   3788  CA  CYS A 251     137.489  65.026 114.101  1.00  0.00
ATOM   3789  HA  CYS A 251     137.216  64.851 113.059  1.00  0.00
ATOM   3790  CB  CYS A 251     136.186  65.278 114.875  1.00  0.00
ATOM   3791  HB1 CYS A 251     135.591  65.956 114.282  1.00  0.00
ATOM   3792  HB2 CYS A 251     136.402  65.789 115.810  1.00  0.00
ATOM   3793  SG  CYS A 251     135.203  63.795 115.229  1.00  0.00
ATOM   3794  HG  CYS A 251     134.064  64.406 115.596  1.00  0.00
ATOM   3795  C   CYS A 251     138.356  66.281 114.183  1.00  0.00
ATOM   3796  O   CYS A 251     138.640  66.746 115.281  1.00  0.00
ATOM   3797  N   ARG A 252     138.816  66.775 113.031  1.00  0.00
ATOM   3798  H   ARG A 252     138.326  66.426 112.209  1.00  0.00
ATOM   3799  CA  ARG A 252     140.213  67.265 112.798  1.00  0.00
ATOM   3800  HA  ARG A 252     140.900  66.545 113.242  1.00  0.00
ATOM   3801  CB  ARG A 252     140.474  67.298 111.269  1.00  0.00
ATOM   3802  HB1 ARG A 252     140.049  68.214 110.855  1.00  0.00
ATOM   3803  HB2 ARG A 252     139.946  66.455 110.824  1.00  0.00
ATOM   3804  CG  ARG A 252     141.955  67.174 110.851  1.00  0.00
ATOM   3805  HG1 ARG A 252     142.382  66.287 111.316  1.00  0.00
ATOM   3806  HG2 ARG A 252     142.514  68.045 111.191  1.00  0.00
ATOM   3807  CD  ARG A 252     142.082  67.047 109.319  1.00  0.00
ATOM   3808  HD1 ARG A 252     141.923  68.020 108.852  1.00  0.00
ATOM   3809  HD2 ARG A 252     141.302  66.378 108.951  1.00  0.00
ATOM   3810  NE  ARG A 252     143.378  66.480 108.883  1.00  0.00
ATOM   3811  HE  ARG A 252     143.410  65.480 108.657  1.00  0.00
ATOM   3812  CZ  ARG A 252     144.536  67.082 108.680  1.00  0.00
ATOM   3813  NH1 ARG A 252     144.745  68.347 108.901  1.00  0.00
ATOM   3814 1HH1 ARG A 252     143.985  68.907 109.227  1.00  0.00
ATOM   3815 2HH1 ARG A 252     145.674  68.716 108.785  1.00  0.00
ATOM   3816  NH2 ARG A 252     145.538  66.378 108.253  1.00  0.00
ATOM   3817 1HH2 ARG A 252     145.369  65.376 108.137  1.00  0.00
ATOM   3818 2HH2 ARG A 252     146.451  66.765 108.084  1.00  0.00
ATOM   3819  C   ARG A 252     140.536  68.601 113.470  1.00  0.00
ATOM   3820  O   ARG A 252     141.710  68.869 113.701  1.00  0.00
ATOM   3821  N   LYS A 253     139.524  69.379 113.872  1.00  0.00
ATOM   3822  H   LYS A 253     138.586  69.088 113.626  1.00  0.00
ATOM   3823  CA  LYS A 253     139.702  70.606 114.672  1.00  0.00
ATOM   3824  HA  LYS A 253     140.759  70.760 114.889  1.00  0.00
ATOM   3825  CB  LYS A 253     139.214  71.838 113.897  1.00  0.00
ATOM   3826  HB1 LYS A 253     139.230  72.697 114.568  1.00  0.00
ATOM   3827  HB2 LYS A 253     138.182  71.677 113.583  1.00  0.00
ATOM   3828  CG  LYS A 253     140.089  72.146 112.671  1.00  0.00
ATOM   3829  HG1 LYS A 253     140.064  71.294 111.991  1.00  0.00
ATOM   3830  HG2 LYS A 253     141.121  72.319 112.978  1.00  0.00
ATOM   3831  CD  LYS A 253     139.550  73.373 111.932  1.00  0.00
ATOM   3832  HD1 LYS A 253     138.472  73.256 111.856  1.00  0.00
ATOM   3833  HD2 LYS A 253     139.956  73.395 110.921  1.00  0.00
ATOM   3834  CE  LYS A 253     139.885  74.697 112.633  1.00  0.00
ATOM   3835  HE1 LYS A 253     140.904  74.983 112.362  1.00  0.00
ATOM   3836  HE2 LYS A 253     139.841  74.559 113.715  1.00  0.00
ATOM   3837  NZ  LYS A 253     138.926  75.756 112.246  1.00  0.00
ATOM   3838  HZ1 LYS A 253     139.269  76.697 112.330  1.00  0.00
ATOM   3839  HZ2 LYS A 253     138.560  75.601 111.303  1.00  0.00
ATOM   3840  HZ3 LYS A 253     138.064  75.688 112.792  1.00  0.00
ATOM   3841  C   LYS A 253     139.013  70.525 116.022  1.00  0.00
```

ATOM	3842	O	LYS	A	253	139.704	70.498	117.043	1.00	0.00
ATOM	3843	N	PHE	A	254	137.711	70.279	116.046	1.00	0.00
ATOM	3844	H	PHE	A	254	137.160	70.352	115.197	1.00	0.00
ATOM	3845	CA	PHE	A	254	137.026	69.792	117.236	1.00	0.00
ATOM	3846	HA	PHE	A	254	137.748	69.546	118.016	1.00	0.00
ATOM	3847	CB	PHE	A	254	136.111	70.880	117.814	1.00	0.00
ATOM	3848	HB1	PHE	A	254	135.536	70.448	118.633	1.00	0.00
ATOM	3849	HB2	PHE	A	254	135.399	71.213	117.061	1.00	0.00
ATOM	3850	CG	PHE	A	254	136.885	72.069	118.350	1.00	0.00
ATOM	3851	CD1	PHE	A	254	137.220	73.142	117.502	1.00	0.00
ATOM	3852	HD1	PHE	A	254	136.901	73.129	116.471	1.00	0.00
ATOM	3853	CE1	PHE	A	254	137.998	74.210	117.988	1.00	0.00
ATOM	3854	HE1	PHE	A	254	138.278	75.017	117.331	1.00	0.00
ATOM	3855	CZ	PHE	A	254	138.439	74.205	119.323	1.00	0.00
ATOM	3856	HZ	PHE	A	254	139.050	75.017	119.691	1.00	0.00
ATOM	3857	CE2	PHE	A	254	138.111	73.133	120.171	1.00	0.00
ATOM	3858	HE2	PHE	A	254	138.470	73.117	121.192	1.00	0.00
ATOM	3859	CD2	PHE	A	254	137.337	72.066	119.683	1.00	0.00
ATOM	3860	HD2	PHE	A	254	137.086	71.238	120.335	1.00	0.00
ATOM	3861	C	PHE	A	254	136.290	68.501	116.893	1.00	0.00
ATOM	3862	O	PHE	A	254	135.831	68.302	115.760	1.00	0.00
ATOM	3863	N	ARG	A	255	136.200	67.629	117.903	1.00	0.00
ATOM	3864	H	ARG	A	255	136.780	67.806	118.718	1.00	0.00
ATOM	3865	CA	ARG	A	255	135.002	66.851	118.186	1.00	0.00
ATOM	3866	HA	ARG	A	255	134.504	66.551	117.262	1.00	0.00
ATOM	3867	CB	ARG	A	255	135.319	65.596	119.034	1.00	0.00
ATOM	3868	HB1	ARG	A	255	134.443	64.952	118.967	1.00	0.00
ATOM	3869	HB2	ARG	A	255	135.419	65.888	120.080	1.00	0.00
ATOM	3870	CG	ARG	A	255	136.581	64.803	118.662	1.00	0.00
ATOM	3871	HG1	ARG	A	255	137.465	65.344	119.001	1.00	0.00
ATOM	3872	HG2	ARG	A	255	136.650	64.680	117.587	1.00	0.00
ATOM	3873	CD	ARG	A	255	136.575	63.410	119.307	1.00	0.00
ATOM	3874	HD1	ARG	A	255	136.369	63.508	120.374	1.00	0.00
ATOM	3875	HD2	ARG	A	255	137.567	62.969	119.195	1.00	0.00
ATOM	3876	NE	ARG	A	255	135.548	62.561	118.672	1.00	0.00
ATOM	3877	HE	ARG	A	255	134.604	62.913	118.687	1.00	0.00
ATOM	3878	CZ	ARG	A	255	135.740	61.487	117.931	1.00	0.00
ATOM	3879	NH1	ARG	A	255	136.872	60.840	117.897	1.00	0.00
ATOM	3880	1HH1	ARG	A	255	137.626	61.055	118.553	1.00	0.00
ATOM	3881	2HH1	ARG	A	255	136.949	60.015	117.306	1.00	0.00
ATOM	3882	NH2	ARG	A	255	134.772	61.055	117.176	1.00	0.00
ATOM	3883	1HH2	ARG	A	255	133.938	61.600	117.040	1.00	0.00
ATOM	3884	2HH2	ARG	A	255	134.940	60.296	116.531	1.00	0.00
ATOM	3885	C	ARG	A	255	134.080	67.740	119.012	1.00	0.00
ATOM	3886	O	ARG	A	255	134.517	68.312	120.023	1.00	0.00
ATOM	3887	N	ASP	A	256	132.842	67.817	118.547	1.00	0.00
ATOM	3888	H	ASP	A	256	132.679	67.344	117.664	1.00	0.00
ATOM	3889	CA	ASP	A	256	131.621	67.954	119.340	1.00	0.00
ATOM	3890	HA	ASP	A	256	131.787	68.648	120.162	1.00	0.00
ATOM	3891	CB	ASP	A	256	130.546	68.546	118.397	1.00	0.00
ATOM	3892	HB1	ASP	A	256	130.213	67.769	117.709	1.00	0.00
ATOM	3893	HB2	ASP	A	256	130.999	69.343	117.805	1.00	0.00
ATOM	3894	CG	ASP	A	256	129.362	69.118	119.149	1.00	0.00
ATOM	3895	OD1	ASP	A	256	128.340	68.411	119.298	1.00	0.00
ATOM	3896	OD2	ASP	A	256	129.453	70.253	119.676	1.00	0.00
ATOM	3897	C	ASP	A	256	131.243	66.572	119.927	1.00	0.00
ATOM	3898	O	ASP	A	256	132.031	65.626	119.888	1.00	0.00
ATOM	3899	N	GLU	A	257	130.032	66.417	120.463	1.00	0.00
ATOM	3900	H	GLU	A	257	129.334	67.141	120.319	1.00	0.00
ATOM	3901	CA	GLU	A	257	129.649	65.266	121.298	1.00	0.00
ATOM	3902	HA	GLU	A	257	130.538	64.973	121.859	1.00	0.00

ATOM	3903	CB	GLU	A	257	128.632	65.754	122.363	1.00	0.00
ATOM	3904	HB1	GLU	A	257	128.183	64.900	122.870	1.00	0.00
ATOM	3905	HB2	GLU	A	257	127.844	66.325	121.871	1.00	0.00
ATOM	3906	CG	GLU	A	257	129.362	66.641	123.426	1.00	0.00
ATOM	3907	HG1	GLU	A	257	130.146	67.210	122.926	1.00	0.00
ATOM	3908	HG2	GLU	A	257	129.865	65.972	124.128	1.00	0.00
ATOM	3909	CD	GLU	A	257	128.508	67.667	124.203	1.00	0.00
ATOM	3910	OE1	GLU	A	257	127.727	68.402	123.553	1.00	0.00
ATOM	3911	OE2	GLU	A	257	128.694	67.822	125.437	1.00	0.00
ATOM	3912	C	GLU	A	257	129.337	63.991	120.477	1.00	0.00
ATOM	3913	O	GLU	A	257	129.164	62.917	121.046	1.00	0.00
ATOM	3914	N	ALA	A	258	129.436	64.087	119.142	1.00	0.00
ATOM	3915	H	ALA	A	258	129.587	65.028	118.817	1.00	0.00
ATOM	3916	CA	ALA	A	258	129.902	63.018	118.242	1.00	0.00
ATOM	3917	HA	ALA	A	258	130.645	62.412	118.762	1.00	0.00
ATOM	3918	CB	ALA	A	258	128.714	62.121	117.860	1.00	0.00
ATOM	3919	HB1	ALA	A	258	129.044	61.345	117.170	1.00	0.00
ATOM	3920	HB2	ALA	A	258	128.309	61.649	118.755	1.00	0.00
ATOM	3921	HB3	ALA	A	258	127.930	62.713	117.388	1.00	0.00
ATOM	3922	C	ALA	A	258	130.579	63.617	116.995	1.00	0.00
ATOM	3923	O	ALA	A	258	131.709	63.268	116.637	1.00	0.00
ATOM	3924	N	THR	A	259	129.850	64.551	116.384	1.00	0.00
ATOM	3925	H	THR	A	259	128.929	64.723	116.774	1.00	0.00
ATOM	3926	CA	THR	A	259	130.141	65.357	115.189	1.00	0.00
ATOM	3927	HA	THR	A	259	130.096	64.705	114.321	1.00	0.00
ATOM	3928	CB	THR	A	259	129.034	66.424	115.055	1.00	0.00
ATOM	3929	HB	THR	A	259	129.341	67.339	115.565	1.00	0.00
ATOM	3930	CG2	THR	A	259	128.644	66.743	113.619	1.00	0.00
ATOM	3931	1HG2	THR	A	259	127.773	67.394	113.605	1.00	0.00
ATOM	3932	2HG2	THR	A	259	129.456	67.268	113.137	1.00	0.00
ATOM	3933	3HG2	THR	A	259	128.421	65.831	113.072	1.00	0.00
ATOM	3934	OG1	THR	A	259	127.827	65.968	115.642	1.00	0.00
ATOM	3935	HG1	THR	A	259	127.366	66.749	115.955	1.00	0.00
ATOM	3936	C	THR	A	259	131.546	65.984	115.221	1.00	0.00
ATOM	3937	O	THR	A	259	131.993	66.446	116.267	1.00	0.00
ATOM	3938	N	CYS	A	260	132.263	66.078	114.095	1.00	0.00
ATOM	3939	H	CYS	A	260	131.868	65.703	113.241	1.00	0.00
ATOM	3940	CA	CYS	A	260	133.224	67.164	113.966	1.00	0.00
ATOM	3941	HA	CYS	A	260	133.938	67.081	114.781	1.00	0.00
ATOM	3942	CB	CYS	A	260	133.955	67.027	112.627	1.00	0.00
ATOM	3943	HB1	CYS	A	260	133.299	67.344	111.819	1.00	0.00
ATOM	3944	HB2	CYS	A	260	134.244	65.990	112.469	1.00	0.00
ATOM	3945	SG	CYS	A	260	135.443	68.062	112.645	1.00	0.00
ATOM	3946	HG	CYS	A	260	134.828	69.201	112.295	1.00	0.00
ATOM	3947	C	CYS	A	260	132.545	68.535	114.103	1.00	0.00
ATOM	3948	O	CYS	A	260	131.367	68.679	113.781	1.00	0.00
ATOM	3949	N	LYS	A	261	133.304	69.556	114.514	1.00	0.00
ATOM	3950	H	LYS	A	261	134.256	69.348	114.802	1.00	0.00
ATOM	3951	CA	LYS	A	261	133.006	70.927	114.066	1.00	0.00
ATOM	3952	HA	LYS	A	261	132.532	70.840	113.084	1.00	0.00
ATOM	3953	CB	LYS	A	261	132.003	71.626	115.017	1.00	0.00
ATOM	3954	HB1	LYS	A	261	132.494	71.892	115.952	1.00	0.00
ATOM	3955	HB2	LYS	A	261	131.208	70.918	115.254	1.00	0.00
ATOM	3956	CG	LYS	A	261	131.350	72.867	114.362	1.00	0.00
ATOM	3957	HG1	LYS	A	261	131.100	72.602	113.334	1.00	0.00
ATOM	3958	HG2	LYS	A	261	132.062	73.691	114.324	1.00	0.00
ATOM	3959	CD	LYS	A	261	130.036	73.349	115.020	1.00	0.00
ATOM	3960	HD1	LYS	A	261	129.439	72.468	115.260	1.00	0.00
ATOM	3961	HD2	LYS	A	261	129.475	73.914	114.275	1.00	0.00
ATOM	3962	CE	LYS	A	261	130.174	74.208	116.287	1.00	0.00
ATOM	3963	HE1	LYS	A	261	130.853	73.697	116.976	1.00	0.00

```
ATOM   3964  HE2 LYS A 261     129.193  74.259 116.769  1.00  0.00
ATOM   3965  NZ  LYS A 261     130.663  75.588 116.009  1.00  0.00
ATOM   3966  HZ1 LYS A 261     130.642  76.173 116.832  1.00  0.00
ATOM   3967  HZ2 LYS A 261     131.636  75.581 115.705  1.00  0.00
ATOM   3968  HZ3 LYS A 261     130.121  76.062 115.297  1.00  0.00
ATOM   3969  C   LYS A 261     134.315  71.685 113.856  1.00  0.00
ATOM   3970  O   LYS A 261     135.321  71.417 114.515  1.00  0.00
ATOM   3971  N   ASP A 262     134.296  72.666 112.961  1.00  0.00
ATOM   3972  H   ASP A 262     133.475  72.757 112.383  1.00  0.00
ATOM   3973  CA  ASP A 262     135.436  73.555 112.707  1.00  0.00
ATOM   3974  HA  ASP A 262     136.290  72.937 112.427  1.00  0.00
ATOM   3975  CB  ASP A 262     135.068  74.440 111.503  1.00  0.00
ATOM   3976  HB1 ASP A 262     134.232  75.088 111.773  1.00  0.00
ATOM   3977  HB2 ASP A 262     134.752  73.804 110.674  1.00  0.00
ATOM   3978  CG  ASP A 262     136.253  75.282 111.057  1.00  0.00
ATOM   3979  OD1 ASP A 262     137.168  74.724 110.405  1.00  0.00
ATOM   3980  OD2 ASP A 262     136.370  76.437 111.516  1.00  0.00
ATOM   3981  C   ASP A 262     135.853  74.401 113.938  1.00  0.00
ATOM   3982  O   ASP A 262     137.005  74.824 114.082  1.00  0.00
ATOM   3983  N   THR A 263     134.906  74.605 114.854  1.00  0.00
ATOM   3984  H   THR A 263     134.013  74.169 114.668  1.00  0.00
ATOM   3985  CA  THR A 263     134.957  75.441 116.062  1.00  0.00
ATOM   3986  HA  THR A 263     135.976  75.531 116.433  1.00  0.00
ATOM   3987  CB  THR A 263     134.373  76.839 115.759  1.00  0.00
ATOM   3988  HB  THR A 263     134.223  77.368 116.702  1.00  0.00
ATOM   3989  CG2 THR A 263     135.283  77.687 114.879  1.00  0.00
ATOM   3990 1HG2 THR A 263     134.868  78.688 114.765  1.00  0.00
ATOM   3991 2HG2 THR A 263     136.265  77.775 115.341  1.00  0.00
ATOM   3992 3HG2 THR A 263     135.400  77.234 113.895  1.00  0.00
ATOM   3993  OG1 THR A 263     133.130  76.742 115.080  1.00  0.00
ATOM   3994  HG1 THR A 263     133.211  77.316 114.308  1.00  0.00
ATOM   3995  C   THR A 263     134.065  74.821 117.132  1.00  0.00
ATOM   3996  O   THR A 263     133.062  74.207 116.773  1.00  0.00
ATOM   3997  N   CYS A 264     134.307  75.043 118.429  1.00  0.00
ATOM   3998  H   CYS A 264     135.144  75.525 118.717  1.00  0.00
ATOM   3999  CA  CYS A 264     133.225  74.837 119.404  1.00  0.00
ATOM   4000  HA  CYS A 264     132.796  73.860 119.206  1.00  0.00
ATOM   4001  CB  CYS A 264     133.745  74.864 120.850  1.00  0.00
ATOM   4002  HB1 CYS A 264     132.889  74.889 121.526  1.00  0.00
ATOM   4003  HB2 CYS A 264     134.326  75.771 121.015  1.00  0.00
ATOM   4004  SG  CYS A 264     134.738  73.418 121.300  1.00  0.00
ATOM   4005  C   CYS A 264     132.085  75.849 119.191  1.00  0.00
ATOM   4006  O   CYS A 264     132.320  76.941 118.663  1.00  0.00
ATOM   4007  N   PRO A 265     130.829  75.516 119.532  1.00  0.00
ATOM   4008  CD  PRO A 265     130.350  74.208 119.966  1.00  0.00
ATOM   4009  HD1 PRO A 265     130.933  73.823 120.802  1.00  0.00
ATOM   4010  HD2 PRO A 265     130.384  73.504 119.132  1.00  0.00
ATOM   4011  CG  PRO A 265     128.901  74.421 120.395  1.00  0.00
ATOM   4012  HG1 PRO A 265     128.874  74.654 121.454  1.00  0.00
ATOM   4013  HG2 PRO A 265     128.272  73.561 120.166  1.00  0.00
ATOM   4014  CB  PRO A 265     128.462  75.665 119.638  1.00  0.00
ATOM   4015  HB1 PRO A 265     127.701  76.208 120.192  1.00  0.00
ATOM   4016  HB2 PRO A 265     128.096  75.385 118.649  1.00  0.00
ATOM   4017  CA  PRO A 265     129.740  76.484 119.501  1.00  0.00
ATOM   4018  HA  PRO A 265     129.724  76.981 118.534  1.00  0.00
ATOM   4019  C   PRO A 265     129.890  77.521 120.628  1.00  0.00
ATOM   4020  O   PRO A 265     130.117  77.133 121.779  1.00  0.00
ATOM   4021  N   PRO A 266     129.821  78.829 120.319  1.00  0.00
ATOM   4022  CD  PRO A 266     129.382  79.413 119.058  1.00  0.00
ATOM   4023  HD1 PRO A 266     128.411  79.019 118.754  1.00  0.00
ATOM   4024  HD2 PRO A 266     130.129  79.222 118.287  1.00  0.00
```

```
ATOM   4025  CG  PRO A 266     129.279  80.917 119.310  1.00  0.00
ATOM   4026  HG1 PRO A 266     128.290  81.157 119.702  1.00  0.00
ATOM   4027  HG2 PRO A 266     129.486  81.499 118.411  1.00  0.00
ATOM   4028  CB  PRO A 266     130.328  81.150 120.395  1.00  0.00
ATOM   4029  HB1 PRO A 266     130.120  82.047 120.978  1.00  0.00
ATOM   4030  HB2 PRO A 266     131.315  81.216 119.936  1.00  0.00
ATOM   4031  CA  PRO A 266     130.247  79.875 121.243  1.00  0.00
ATOM   4032  HA  PRO A 266     131.237  79.636 121.631  1.00  0.00
ATOM   4033  C   PRO A 266     129.275  80.035 122.416  1.00  0.00
ATOM   4034  O   PRO A 266     128.063  79.904 122.272  1.00  0.00
ATOM   4035  N   LEU A 267     129.810  80.380 123.591  1.00  0.00
ATOM   4036  H   LEU A 267     130.811  80.468 123.657  1.00  0.00
ATOM   4037  CA  LEU A 267     129.015  80.585 124.811  1.00  0.00
ATOM   4038  HA  LEU A 267     128.280  79.782 124.875  1.00  0.00
ATOM   4039  CB  LEU A 267     129.935  80.487 126.043  1.00  0.00
ATOM   4040  HB1 LEU A 267     129.405  80.845 126.927  1.00  0.00
ATOM   4041  HB2 LEU A 267     130.789  81.146 125.881  1.00  0.00
ATOM   4042  CG  LEU A 267     130.415  79.047 126.309  1.00  0.00
ATOM   4043  HG  LEU A 267     130.683  78.578 125.365  1.00  0.00
ATOM   4044  CD1 LEU A 267     131.669  79.078 127.176  1.00  0.00
ATOM   4045 1HD1 LEU A 267     132.061  78.069 127.273  1.00  0.00
ATOM   4046 2HD1 LEU A 267     132.416  79.707 126.695  1.00  0.00
ATOM   4047 3HD1 LEU A 267     131.438  79.485 128.160  1.00  0.00
ATOM   4048  CD2 LEU A 267     129.355  78.191 127.007  1.00  0.00
ATOM   4049 1HD2 LEU A 267     129.737  77.183 127.163  1.00  0.00
ATOM   4050 2HD2 LEU A 267     129.102  78.627 127.973  1.00  0.00
ATOM   4051 3HD2 LEU A 267     128.458  78.132 126.394  1.00  0.00
ATOM   4052  C   LEU A 267     128.184  81.879 124.810  1.00  0.00
ATOM   4053  O   LEU A 267     127.416  82.101 125.743  1.00  0.00
ATOM   4054  N   MET A 268     128.298  82.700 123.763  1.00  0.00
ATOM   4055  H   MET A 268     128.905  82.432 123.007  1.00  0.00
ATOM   4056  CA  MET A 268     127.383  83.809 123.509  1.00  0.00
ATOM   4057  HA  MET A 268     126.421  83.530 123.928  1.00  0.00
ATOM   4058  CB  MET A 268     127.854  85.088 124.220  1.00  0.00
ATOM   4059  HB1 MET A 268     127.888  84.905 125.295  1.00  0.00
ATOM   4060  HB2 MET A 268     127.126  85.880 124.039  1.00  0.00
ATOM   4061  CG  MET A 268     129.236  85.570 123.761  1.00  0.00
ATOM   4062  HG1 MET A 268     129.228  85.715 122.681  1.00  0.00
ATOM   4063  HG2 MET A 268     129.969  84.800 123.999  1.00  0.00
ATOM   4064  SD  MET A 268     129.764  87.121 124.538  1.00  0.00
ATOM   4065  CE  MET A 268     131.500  87.141 124.023  1.00  0.00
ATOM   4066  HE1 MET A 268     131.976  88.049 124.393  1.00  0.00
ATOM   4067  HE2 MET A 268     132.013  86.274 124.438  1.00  0.00
ATOM   4068  HE3 MET A 268     131.560  87.115 122.936  1.00  0.00
ATOM   4069  C   MET A 268     127.157  84.049 122.017  1.00  0.00
ATOM   4070  O   MET A 268     128.014  83.742 121.184  1.00  0.00
ATOM   4071  N   LEU A 269     126.004  84.649 121.720  1.00  0.00
ATOM   4072  H   LEU A 269     125.396  84.900 122.493  1.00  0.00
ATOM   4073  CA  LEU A 269     125.505  84.980 120.383  1.00  0.00
ATOM   4074  HA  LEU A 269     126.351  85.111 119.709  1.00  0.00
ATOM   4075  CB  LEU A 269     124.593  83.823 119.912  1.00  0.00
ATOM   4076  HB1 LEU A 269     123.552  84.108 120.078  1.00  0.00
ATOM   4077  HB2 LEU A 269     124.784  82.933 120.510  1.00  0.00
ATOM   4078  CG  LEU A 269     124.779  83.434 118.441  1.00  0.00
ATOM   4079  HG  LEU A 269     124.686  84.319 117.818  1.00  0.00
ATOM   4080  CD1 LEU A 269     126.141  82.775 118.211  1.00  0.00
ATOM   4081 1HD1 LEU A 269     126.242  82.501 117.162  1.00  0.00
ATOM   4082 2HD1 LEU A 269     126.943  83.464 118.464  1.00  0.00
ATOM   4083 3HD1 LEU A 269     126.226  81.877 118.822  1.00  0.00
ATOM   4084  CD2 LEU A 269     123.710  82.419 118.036  1.00  0.00
ATOM   4085 1HD2 LEU A 269     123.866  82.115 117.005  1.00  0.00
```

ATOM	4086	2HD2	LEU	A	269	123.760	81.540	118.678	1.00	0.00
ATOM	4087	3HD2	LEU	A	269	122.726	82.879	118.122	1.00	0.00
ATOM	4088	C	LEU	A	269	124.735	86.305	120.451	1.00	0.00
ATOM	4089	O	LEU	A	269	124.098	86.554	121.479	1.00	0.00
ATOM	4090	N	TYR	A	270	124.747	87.153	119.406	1.00	0.00
ATOM	4091	H	TYR	A	270	125.268	86.920	118.570	1.00	0.00
ATOM	4092	CA	TYR	A	270	123.751	88.229	119.375	1.00	0.00
ATOM	4093	HA	TYR	A	270	123.816	88.759	120.327	1.00	0.00
ATOM	4094	CB	TYR	A	270	124.061	89.232	118.255	1.00	0.00
ATOM	4095	HB1	TYR	A	270	123.335	89.126	117.448	1.00	0.00
ATOM	4096	HB2	TYR	A	270	125.048	89.031	117.834	1.00	0.00
ATOM	4097	CG	TYR	A	270	124.045	90.654	118.781	1.00	0.00
ATOM	4098	CD1	TYR	A	270	125.191	91.151	119.433	1.00	0.00
ATOM	4099	HD1	TYR	A	270	126.071	90.535	119.515	1.00	0.00
ATOM	4100	CE1	TYR	A	270	125.173	92.434	120.010	1.00	0.00
ATOM	4101	HE1	TYR	A	270	126.038	92.827	120.526	1.00	0.00
ATOM	4102	CZ	TYR	A	270	123.994	93.205	119.967	1.00	0.00
ATOM	4103	OH	TYR	A	270	123.981	94.419	120.570	1.00	0.00
ATOM	4104	HH	TYR	A	270	123.079	94.759	120.684	1.00	0.00
ATOM	4105	CE2	TYR	A	270	122.850	92.710	119.310	1.00	0.00
ATOM	4106	HE2	TYR	A	270	121.960	93.321	119.291	1.00	0.00
ATOM	4107	CD2	TYR	A	270	122.873	91.432	118.717	1.00	0.00
ATOM	4108	HD2	TYR	A	270	121.985	91.025	118.256	1.00	0.00
ATOM	4109	C	TYR	A	270	122.336	87.641	119.261	1.00	0.00
ATOM	4110	O	TYR	A	270	122.116	86.674	118.535	1.00	0.00
ATOM	4111	N	ASN	A	271	121.373	88.242	119.960	1.00	0.00
ATOM	4112	H	ASN	A	271	121.648	88.974	120.610	1.00	0.00
ATOM	4113	CA	ASN	A	271	119.960	87.862	119.910	1.00	0.00
ATOM	4114	HA	ASN	A	271	119.820	87.027	119.223	1.00	0.00
ATOM	4115	CB	ASN	A	271	119.516	87.373	121.301	1.00	0.00
ATOM	4116	HB1	ASN	A	271	119.714	88.139	122.046	1.00	0.00
ATOM	4117	HB2	ASN	A	271	120.112	86.501	121.565	1.00	0.00
ATOM	4118	CG	ASN	A	271	118.047	86.981	121.388	1.00	0.00
ATOM	4119	OD1	ASN	A	271	117.198	87.407	120.608	1.00	0.00
ATOM	4120	ND2	ASN	A	271	117.696	86.183	122.362	1.00	0.00
ATOM	4121	1HD2	ASN	A	271	118.343	86.087	123.151	1.00	0.00
ATOM	4122	2HD2	ASN	A	271	116.717	86.064	122.529	1.00	0.00
ATOM	4123	C	ASN	A	271	119.175	89.081	119.392	1.00	0.00
ATOM	4124	O	ASN	A	271	118.967	90.031	120.149	1.00	0.00
ATOM	4125	N	PRO	A	272	118.756	89.113	118.113	1.00	0.00
ATOM	4126	CD	PRO	A	272	119.154	88.166	117.074	1.00	0.00
ATOM	4127	HD1	PRO	A	272	118.528	87.275	117.135	1.00	0.00
ATOM	4128	HD2	PRO	A	272	120.207	87.890	117.149	1.00	0.00
ATOM	4129	CG	PRO	A	272	118.912	88.873	115.745	1.00	0.00
ATOM	4130	HG1	PRO	A	272	118.687	88.168	114.944	1.00	0.00
ATOM	4131	HG2	PRO	A	272	119.781	89.480	115.488	1.00	0.00
ATOM	4132	CB	PRO	A	272	117.725	89.774	116.067	1.00	0.00
ATOM	4133	HB1	PRO	A	272	116.806	89.190	115.987	1.00	0.00
ATOM	4134	HB2	PRO	A	272	117.682	90.638	115.404	1.00	0.00
ATOM	4135	CA	PRO	A	272	117.947	90.192	117.530	1.00	0.00
ATOM	4136	HA	PRO	A	272	118.550	91.101	117.546	1.00	0.00
ATOM	4137	C	PRO	A	272	116.591	90.513	118.192	1.00	0.00
ATOM	4138	O	PRO	A	272	115.862	91.353	117.668	1.00	0.00
ATOM	4139	N	THR	A	273	116.242	89.890	119.325	1.00	0.00
ATOM	4140	H	THR	A	273	116.834	89.140	119.658	1.00	0.00
ATOM	4141	CA	THR	A	273	115.102	90.288	120.172	1.00	0.00
ATOM	4142	HA	THR	A	273	114.597	91.130	119.699	1.00	0.00
ATOM	4143	CB	THR	A	273	114.047	89.181	120.306	1.00	0.00
ATOM	4144	HB	THR	A	273	113.184	89.597	120.827	1.00	0.00
ATOM	4145	CG2	THR	A	273	113.563	88.654	118.954	1.00	0.00
ATOM	4146	1HG2	THR	A	273	112.748	87.950	119.114	1.00	0.00

ATOM	4147	2HG2	THR	A	273	113.204	89.484	118.346	1.00	0.00
ATOM	4148	3HG2	THR	A	273	114.373	88.147	118.431	1.00	0.00
ATOM	4149	OG1	THR	A	273	114.510	88.083	121.053	1.00	0.00
ATOM	4150	HG1	THR	A	273	115.363	87.790	120.696	1.00	0.00
ATOM	4151	C	THR	A	273	115.533	90.799	121.555	1.00	0.00
ATOM	4152	O	THR	A	273	114.927	91.750	122.041	1.00	0.00
ATOM	4153	N	THR	A	274	116.618	90.281	122.143	1.00	0.00
ATOM	4154	H	THR	A	274	116.996	89.415	121.787	1.00	0.00
ATOM	4155	CA	THR	A	274	117.292	90.880	123.319	1.00	0.00
ATOM	4156	HA	THR	A	274	116.533	91.176	124.043	1.00	0.00
ATOM	4157	CB	THR	A	274	118.231	89.856	124.004	1.00	0.00
ATOM	4158	HB	THR	A	274	119.142	89.764	123.414	1.00	0.00
ATOM	4159	CG2	THR	A	274	118.596	90.196	125.444	1.00	0.00
ATOM	4160	1HG2	THR	A	274	119.205	89.395	125.867	1.00	0.00
ATOM	4161	2HG2	THR	A	274	119.173	91.117	125.484	1.00	0.00
ATOM	4162	3HG2	THR	A	274	117.692	90.310	126.042	1.00	0.00
ATOM	4163	OG1	THR	A	274	117.600	88.602	124.063	1.00	0.00
ATOM	4164	HG1	THR	A	274	118.241	87.945	124.420	1.00	0.00
ATOM	4165	C	THR	A	274	118.107	92.134	122.965	1.00	0.00
ATOM	4166	O	THR	A	274	118.411	92.948	123.829	1.00	0.00
ATOM	4167	N	TYR	A	275	118.486	92.273	121.686	1.00	0.00
ATOM	4168	H	TYR	A	275	118.204	91.530	121.058	1.00	0.00
ATOM	4169	CA	TYR	A	275	119.425	93.260	121.126	1.00	0.00
ATOM	4170	HA	TYR	A	275	119.692	92.896	120.136	1.00	0.00
ATOM	4171	CB	TYR	A	275	118.716	94.606	120.928	1.00	0.00
ATOM	4172	HB1	TYR	A	275	119.444	95.369	120.651	1.00	0.00
ATOM	4173	HB2	TYR	A	275	118.266	94.923	121.869	1.00	0.00
ATOM	4174	CG	TYR	A	275	117.654	94.567	119.846	1.00	0.00
ATOM	4175	CD1	TYR	A	275	117.957	95.039	118.555	1.00	0.00
ATOM	4176	HD1	TYR	A	275	118.934	95.458	118.362	1.00	0.00
ATOM	4177	CE1	TYR	A	275	116.971	95.047	117.553	1.00	0.00
ATOM	4178	HE1	TYR	A	275	117.184	95.450	116.569	1.00	0.00
ATOM	4179	CZ	TYR	A	275	115.679	94.564	117.836	1.00	0.00
ATOM	4180	OH	TYR	A	275	114.735	94.565	116.859	1.00	0.00
ATOM	4181	HH	TYR	A	275	113.927	94.147	117.155	1.00	0.00
ATOM	4182	CE2	TYR	A	275	115.371	94.097	119.133	1.00	0.00
ATOM	4183	HE2	TYR	A	275	114.381	93.733	119.360	1.00	0.00
ATOM	4184	CD2	TYR	A	275	116.356	94.110	120.137	1.00	0.00
ATOM	4185	HD2	TYR	A	275	116.117	93.781	121.139	1.00	0.00
ATOM	4186	C	TYR	A	275	120.767	93.372	121.870	1.00	0.00
ATOM	4187	O	TYR	A	275	121.468	94.375	121.762	1.00	0.00
ATOM	4188	N	GLN	A	276	121.174	92.294	122.535	1.00	0.00
ATOM	4189	H	GLN	A	276	120.594	91.471	122.482	1.00	0.00
ATOM	4190	CA	GLN	A	276	122.461	92.142	123.213	1.00	0.00
ATOM	4191	HA	GLN	A	276	123.170	92.878	122.834	1.00	0.00
ATOM	4192	CB	GLN	A	276	122.282	92.355	124.732	1.00	0.00
ATOM	4193	HB1	GLN	A	276	123.210	92.116	125.252	1.00	0.00
ATOM	4194	HB2	GLN	A	276	121.516	91.675	125.106	1.00	0.00
ATOM	4195	CG	GLN	A	276	121.902	93.797	125.116	1.00	0.00
ATOM	4196	HG1	GLN	A	276	121.829	93.870	126.200	1.00	0.00
ATOM	4197	HG2	GLN	A	276	120.920	94.028	124.701	1.00	0.00
ATOM	4198	CD	GLN	A	276	122.915	94.836	124.629	1.00	0.00
ATOM	4199	OE1	GLN	A	276	124.104	94.594	124.507	1.00	0.00
ATOM	4200	NE2	GLN	A	276	122.471	96.027	124.306	1.00	0.00
ATOM	4201	1HE2	GLN	A	276	121.482	96.221	124.384	1.00	0.00
ATOM	4202	2HE2	GLN	A	276	123.124	96.635	123.846	1.00	0.00
ATOM	4203	C	GLN	A	276	123.062	90.763	122.888	1.00	0.00
ATOM	4204	O	GLN	A	276	122.489	89.982	122.118	1.00	0.00
ATOM	4205	N	MET	A	277	124.218	90.474	123.485	1.00	0.00
ATOM	4206	H	MET	A	277	124.598	91.176	124.102	1.00	0.00
ATOM	4207	CA	MET	A	277	124.719	89.106	123.644	1.00	0.00

```
ATOM   4208  HA  MET A 277     124.661  88.595 122.686  1.00  0.00
ATOM   4209  CB  MET A 277     126.181  89.136 124.114  1.00  0.00
ATOM   4210  HB1 MET A 277     126.466  88.152 124.489  1.00  0.00
ATOM   4211  HB2 MET A 277     126.287  89.848 124.934  1.00  0.00
ATOM   4212  CG  MET A 277     127.160  89.510 122.993  1.00  0.00
ATOM   4213  HG1 MET A 277     128.142  89.661 123.443  1.00  0.00
ATOM   4214  HG2 MET A 277     126.842  90.449 122.541  1.00  0.00
ATOM   4215  SD  MET A 277     127.322  88.246 121.702  1.00  0.00
ATOM   4216  CE  MET A 277     128.617  88.995 120.685  1.00  0.00
ATOM   4217  HE1 MET A 277     128.804  88.359 119.821  1.00  0.00
ATOM   4218  HE2 MET A 277     128.294  89.978 120.347  1.00  0.00
ATOM   4219  HE3 MET A 277     129.529  89.093 121.273  1.00  0.00
ATOM   4220  C   MET A 277     123.881  88.321 124.651  1.00  0.00
ATOM   4221  O   MET A 277     123.394  88.883 125.633  1.00  0.00
ATOM   4222  N   ASP A 278     123.758  87.020 124.420  1.00  0.00
ATOM   4223  H   ASP A 278     124.123  86.662 123.545  1.00  0.00
ATOM   4224  CA  ASP A 278     122.851  86.147 125.165  1.00  0.00
ATOM   4225  HA  ASP A 278     122.587  86.618 126.114  1.00  0.00
ATOM   4226  CB  ASP A 278     121.581  85.992 124.303  1.00  0.00
ATOM   4227  HB1 ASP A 278     121.635  85.067 123.726  1.00  0.00
ATOM   4228  HB2 ASP A 278     121.516  86.818 123.591  1.00  0.00
ATOM   4229  CG  ASP A 278     120.307  86.002 125.136  1.00  0.00
ATOM   4230  OD1 ASP A 278     120.355  85.443 126.254  1.00  0.00
ATOM   4231  OD2 ASP A 278     119.299  86.564 124.634  1.00  0.00
ATOM   4232  C   ASP A 278     123.513  84.793 125.455  1.00  0.00
ATOM   4233  O   ASP A 278     124.406  84.362 124.722  1.00  0.00
ATOM   4234  N   VAL A 279     123.088  84.117 126.527  1.00  0.00
ATOM   4235  H   VAL A 279     122.247  84.464 126.984  1.00  0.00
ATOM   4236  CA  VAL A 279     123.666  82.837 126.986  1.00  0.00
ATOM   4237  HA  VAL A 279     124.747  82.878 126.841  1.00  0.00
ATOM   4238  CB  VAL A 279     123.413  82.595 128.490  1.00  0.00
ATOM   4239  HB  VAL A 279     122.344  82.483 128.675  1.00  0.00
ATOM   4240  CG1 VAL A 279     124.146  81.339 128.982  1.00  0.00
ATOM   4241 1HG1 VAL A 279     123.954  81.192 130.046  1.00  0.00
ATOM   4242 2HG1 VAL A 279     123.790  80.458 128.449  1.00  0.00
ATOM   4243 3HG1 VAL A 279     125.220  81.441 128.820  1.00  0.00
ATOM   4244  CG2 VAL A 279     123.927  83.769 129.344  1.00  0.00
ATOM   4245 1HG2 VAL A 279     123.780  83.551 130.401  1.00  0.00
ATOM   4246 2HG2 VAL A 279     124.988  83.934 129.155  1.00  0.00
ATOM   4247 3HG2 VAL A 279     123.371  84.675 129.103  1.00  0.00
ATOM   4248  C   VAL A 279     123.129  81.706 126.107  1.00  0.00
ATOM   4249  O   VAL A 279     122.205  80.984 126.468  1.00  0.00
ATOM   4250  N   ASN A 280     123.719  81.611 124.914  1.00  0.00
ATOM   4251  H   ASN A 280     124.386  82.344 124.702  1.00  0.00
ATOM   4252  CA  ASN A 280     123.436  80.635 123.869  1.00  0.00
ATOM   4253  HA  ASN A 280     122.553  80.965 123.328  1.00  0.00
ATOM   4254  CB  ASN A 280     124.624  80.672 122.891  1.00  0.00
ATOM   4255  HB1 ASN A 280     125.547  80.466 123.430  1.00  0.00
ATOM   4256  HB2 ASN A 280     124.704  81.659 122.442  1.00  0.00
ATOM   4257  CG  ASN A 280     124.495  79.652 121.776  1.00  0.00
ATOM   4258  OD1 ASN A 280     123.451  79.041 121.594  1.00  0.00
ATOM   4259  ND2 ASN A 280     125.557  79.396 121.058  1.00  0.00
ATOM   4260 1HD2 ASN A 280     126.460  79.761 121.337  1.00  0.00
ATOM   4261 2HD2 ASN A 280     125.498  78.654 120.350  1.00  0.00
ATOM   4262  C   ASN A 280     123.199  79.222 124.448  1.00  0.00
ATOM   4263  O   ASN A 280     124.155  78.586 124.905  1.00  0.00
ATOM   4264  N   PRO A 281     121.957  78.704 124.429  1.00  0.00
ATOM   4265  CD  PRO A 281     120.757  79.332 123.893  1.00  0.00
ATOM   4266  HD1 PRO A 281     120.889  79.621 122.849  1.00  0.00
ATOM   4267  HD2 PRO A 281     120.502  80.204 124.500  1.00  0.00
ATOM   4268  CG  PRO A 281     119.649  78.286 124.005  1.00  0.00
```

ATOM	4269	HG1	PRO	A	281	119.633	77.673	123.102	1.00	0.00
ATOM	4270	HG2	PRO	A	281	118.677	78.746	124.179	1.00	0.00
ATOM	4271	CB	PRO	A	281	120.100	77.441	125.195	1.00	0.00
ATOM	4272	HB1	PRO	A	281	119.673	76.438	125.163	1.00	0.00
ATOM	4273	HB2	PRO	A	281	119.817	77.944	126.122	1.00	0.00
ATOM	4274	CA	PRO	A	281	121.627	77.436	125.067	1.00	0.00
ATOM	4275	HA	PRO	A	281	122.067	77.405	126.064	1.00	0.00
ATOM	4276	C	PRO	A	281	122.125	76.215	124.282	1.00	0.00
ATOM	4277	O	PRO	A	281	122.128	75.125	124.858	1.00	0.00
ATOM	4278	N	GLU	A	282	122.612	76.348	123.038	1.00	0.00
ATOM	4279	H	GLU	A	282	122.581	77.259	122.588	1.00	0.00
ATOM	4280	CA	GLU	A	282	123.414	75.287	122.404	1.00	0.00
ATOM	4281	HA	GLU	A	282	123.125	74.345	122.869	1.00	0.00
ATOM	4282	CB	GLU	A	282	123.052	75.049	120.922	1.00	0.00
ATOM	4283	HB1	GLU	A	282	121.966	74.941	120.876	1.00	0.00
ATOM	4284	HB2	GLU	A	282	123.459	74.081	120.628	1.00	0.00
ATOM	4285	CG	GLU	A	282	123.446	76.083	119.855	1.00	0.00
ATOM	4286	HG1	GLU	A	282	123.122	77.067	120.180	1.00	0.00
ATOM	4287	HG2	GLU	A	282	122.881	75.855	118.950	1.00	0.00
ATOM	4288	CD	GLU	A	282	124.937	76.085	119.492	1.00	0.00
ATOM	4289	OE1	GLU	A	282	125.492	74.988	119.260	1.00	0.00
ATOM	4290	OE2	GLU	A	282	125.527	77.188	119.447	1.00	0.00
ATOM	4291	C	GLU	A	282	124.910	75.417	122.711	1.00	0.00
ATOM	4292	O	GLU	A	282	125.504	74.396	123.059	1.00	0.00
ATOM	4293	N	GLY	A	283	125.440	76.649	122.776	1.00	0.00
ATOM	4294	H	GLY	A	283	124.845	77.405	122.463	1.00	0.00
ATOM	4295	CA	GLY	A	283	126.784	77.045	123.243	1.00	0.00
ATOM	4296	HA1	GLY	A	283	126.717	78.036	123.693	1.00	0.00
ATOM	4297	HA2	GLY	A	283	127.426	77.132	122.367	1.00	0.00
ATOM	4298	C	GLY	A	283	127.452	76.114	124.258	1.00	0.00
ATOM	4299	O	GLY	A	283	126.857	75.738	125.275	1.00	0.00
ATOM	4300	N	LYS	A	284	128.718	75.759	124.005	1.00	0.00
ATOM	4301	H	LYS	A	284	129.191	76.200	123.219	1.00	0.00
ATOM	4302	CA	LYS	A	284	129.458	74.737	124.763	1.00	0.00
ATOM	4303	HA	LYS	A	284	128.876	74.462	125.642	1.00	0.00
ATOM	4304	CB	LYS	A	284	129.693	73.461	123.935	1.00	0.00
ATOM	4305	HB1	LYS	A	284	130.274	72.750	124.525	1.00	0.00
ATOM	4306	HB2	LYS	A	284	130.270	73.712	123.045	1.00	0.00
ATOM	4307	CG	LYS	A	284	128.372	72.799	123.540	1.00	0.00
ATOM	4308	HG1	LYS	A	284	127.780	73.507	122.973	1.00	0.00
ATOM	4309	HG2	LYS	A	284	127.822	72.529	124.440	1.00	0.00
ATOM	4310	CD	LYS	A	284	128.566	71.551	122.680	1.00	0.00
ATOM	4311	HD1	LYS	A	284	128.933	70.735	123.304	1.00	0.00
ATOM	4312	HD2	LYS	A	284	129.287	71.749	121.886	1.00	0.00
ATOM	4313	CE	LYS	A	284	127.214	71.192	122.056	1.00	0.00
ATOM	4314	HE1	LYS	A	284	126.929	71.976	121.349	1.00	0.00
ATOM	4315	HE2	LYS	A	284	126.465	71.141	122.847	1.00	0.00
ATOM	4316	NZ	LYS	A	284	127.281	69.890	121.371	1.00	0.00
ATOM	4317	HZ1	LYS	A	284	126.439	69.642	120.886	1.00	0.00
ATOM	4318	HZ2	LYS	A	284	128.031	69.870	120.676	1.00	0.00
ATOM	4319	HZ3	LYS	A	284	127.495	69.153	122.047	1.00	0.00
ATOM	4320	C	LYS	A	284	130.785	75.272	125.274	1.00	0.00
ATOM	4321	O	LYS	A	284	131.410	76.147	124.684	1.00	0.00
ATOM	4322	N	TYR	A	285	131.230	74.682	126.376	1.00	0.00
ATOM	4323	H	TYR	A	285	130.710	73.900	126.745	1.00	0.00
ATOM	4324	CA	TYR	A	285	132.579	74.871	126.887	1.00	0.00
ATOM	4325	HA	TYR	A	285	132.839	75.927	126.815	1.00	0.00
ATOM	4326	CB	TYR	A	285	132.594	74.480	128.377	1.00	0.00
ATOM	4327	HB1	TYR	A	285	133.572	74.707	128.799	1.00	0.00
ATOM	4328	HB2	TYR	A	285	132.447	73.403	128.469	1.00	0.00
ATOM	4329	CG	TYR	A	285	131.525	75.204	129.191	1.00	0.00

ATOM	4330	CD1	TYR	A	285	131.713	76.545	129.581	1.00	0.00
ATOM	4331	HD1	TYR	A	285	132.648	77.042	129.362	1.00	0.00
ATOM	4332	CE1	TYR	A	285	130.677	77.248	130.231	1.00	0.00
ATOM	4333	HE1	TYR	A	285	130.804	78.287	130.497	1.00	0.00
ATOM	4334	CZ	TYR	A	285	129.453	76.612	130.517	1.00	0.00
ATOM	4335	OH	TYR	A	285	128.457	77.302	131.138	1.00	0.00
ATOM	4336	HH	TYR	A	285	127.649	76.783	131.167	1.00	0.00
ATOM	4337	CE2	TYR	A	285	129.267	75.265	130.136	1.00	0.00
ATOM	4338	HE2	TYR	A	285	128.333	74.769	130.344	1.00	0.00
ATOM	4339	CD2	TYR	A	285	130.301	74.570	129.478	1.00	0.00
ATOM	4340	HD2	TYR	A	285	130.149	73.547	129.171	1.00	0.00
ATOM	4341	C	TYR	A	285	133.577	74.086	126.031	1.00	0.00
ATOM	4342	O	TYR	A	285	133.210	73.147	125.318	1.00	0.00
ATOM	4343	N	SER	A	286	134.843	74.486	126.079	1.00	0.00
ATOM	4344	H	SER	A	286	135.095	75.195	126.760	1.00	0.00
ATOM	4345	CA	SER	A	286	135.892	73.959	125.206	1.00	0.00
ATOM	4346	HA	SER	A	286	135.475	73.249	124.493	1.00	0.00
ATOM	4347	CB	SER	A	286	136.517	75.116	124.422	1.00	0.00
ATOM	4348	HB1	SER	A	286	136.947	75.836	125.120	1.00	0.00
ATOM	4349	HB2	SER	A	286	135.745	75.616	123.836	1.00	0.00
ATOM	4350	OG	SER	A	286	137.532	74.651	123.558	1.00	0.00
ATOM	4351	HG	SER	A	286	137.122	74.258	122.781	1.00	0.00
ATOM	4352	C	SER	A	286	136.928	73.250	126.060	1.00	0.00
ATOM	4353	O	SER	A	286	137.378	73.800	127.067	1.00	0.00
ATOM	4354	N	PHE	A	287	137.291	72.036	125.642	1.00	0.00
ATOM	4355	H	PHE	A	287	136.861	71.658	124.804	1.00	0.00
ATOM	4356	CA	PHE	A	287	138.336	71.221	126.247	1.00	0.00
ATOM	4357	HA	PHE	A	287	139.018	71.883	126.782	1.00	0.00
ATOM	4358	CB	PHE	A	287	137.684	70.255	127.253	1.00	0.00
ATOM	4359	HB1	PHE	A	287	137.108	69.507	126.707	1.00	0.00
ATOM	4360	HB2	PHE	A	287	136.974	70.817	127.863	1.00	0.00
ATOM	4361	CG	PHE	A	287	138.631	69.558	128.215	1.00	0.00
ATOM	4362	CD1	PHE	A	287	138.569	68.162	128.382	1.00	0.00
ATOM	4363	HD1	PHE	A	287	137.856	67.581	127.818	1.00	0.00
ATOM	4364	CE1	PHE	A	287	139.428	67.521	129.291	1.00	0.00
ATOM	4365	HE1	PHE	A	287	139.382	66.452	129.408	1.00	0.00
ATOM	4366	CZ	PHE	A	287	140.350	68.270	130.042	1.00	0.00
ATOM	4367	HZ	PHE	A	287	141.013	67.776	130.740	1.00	0.00
ATOM	4368	CE2	PHE	A	287	140.413	69.664	129.881	1.00	0.00
ATOM	4369	HE2	PHE	A	287	141.113	70.253	130.460	1.00	0.00
ATOM	4370	CD2	PHE	A	287	139.557	70.308	128.971	1.00	0.00
ATOM	4371	HD2	PHE	A	287	139.616	71.382	128.864	1.00	0.00
ATOM	4372	C	PHE	A	287	139.150	70.532	125.136	1.00	0.00
ATOM	4373	O	PHE	A	287	138.789	70.624	123.961	1.00	0.00
ATOM	4374	N	GLY	A	288	140.243	69.873	125.535	1.00	0.00
ATOM	4375	H	GLY	A	288	140.367	69.827	126.535	1.00	0.00
ATOM	4376	CA	GLY	A	288	141.371	69.351	124.740	1.00	0.00
ATOM	4377	HA1	GLY	A	288	141.564	68.342	125.096	1.00	0.00
ATOM	4378	HA2	GLY	A	288	142.252	69.952	124.964	1.00	0.00
ATOM	4379	C	GLY	A	288	141.262	69.256	123.208	1.00	0.00
ATOM	4380	O	GLY	A	288	142.116	69.777	122.497	1.00	0.00
ATOM	4381	N	ALA	A	289	140.220	68.615	122.683	1.00	0.00
ATOM	4382	H	ALA	A	289	139.546	68.216	123.326	1.00	0.00
ATOM	4383	CA	ALA	A	289	139.902	68.609	121.250	1.00	0.00
ATOM	4384	HA	ALA	A	289	140.166	69.576	120.815	1.00	0.00
ATOM	4385	CB	ALA	A	289	140.745	67.502	120.597	1.00	0.00
ATOM	4386	HB1	ALA	A	289	140.550	67.468	119.530	1.00	0.00
ATOM	4387	HB2	ALA	A	289	141.805	67.696	120.754	1.00	0.00
ATOM	4388	HB3	ALA	A	289	140.491	66.534	121.027	1.00	0.00
ATOM	4389	C	ALA	A	289	138.387	68.385	121.030	1.00	0.00
ATOM	4390	O	ALA	A	289	137.970	67.666	120.129	1.00	0.00

ATOM	4391	N	THR	A	290	137.574	68.920	121.942	1.00	0.00
ATOM	4392	H	THR	A	290	138.018	69.601	122.553	1.00	0.00
ATOM	4393	CA	THR	A	290	136.415	68.266	122.548	1.00	0.00
ATOM	4394	HA	THR	A	290	135.838	67.738	121.798	1.00	0.00
ATOM	4395	CB	THR	A	290	136.819	67.221	123.614	1.00	0.00
ATOM	4396	HB	THR	A	290	135.927	66.954	124.184	1.00	0.00
ATOM	4397	CG2	THR	A	290	137.396	65.939	123.019	1.00	0.00
ATOM	4398	1HG2	THR	A	290	137.537	65.204	123.811	1.00	0.00
ATOM	4399	2HG2	THR	A	290	136.706	65.532	122.281	1.00	0.00
ATOM	4400	3HG2	THR	A	290	138.356	66.132	122.545	1.00	0.00
ATOM	4401	OG1	THR	A	290	137.779	67.751	124.504	1.00	0.00
ATOM	4402	HG1	THR	A	290	137.354	68.541	124.863	1.00	0.00
ATOM	4403	C	THR	A	290	135.543	69.334	123.199	1.00	0.00
ATOM	4404	O	THR	A	290	135.796	69.764	124.332	1.00	0.00
ATOM	4405	N	CYS	A	291	134.544	69.794	122.454	1.00	0.00
ATOM	4406	H	CYS	A	291	134.415	69.388	121.531	1.00	0.00
ATOM	4407	CA	CYS	A	291	133.472	70.618	122.995	1.00	0.00
ATOM	4408	HA	CYS	A	291	133.917	71.438	123.558	1.00	0.00
ATOM	4409	CB	CYS	A	291	132.660	71.203	121.838	1.00	0.00
ATOM	4410	HB1	CYS	A	291	132.021	72.000	122.216	1.00	0.00
ATOM	4411	HB2	CYS	A	291	132.018	70.422	121.426	1.00	0.00
ATOM	4412	SG	CYS	A	291	133.702	71.844	120.500	1.00	0.00
ATOM	4413	C	CYS	A	291	132.627	69.771	123.948	1.00	0.00
ATOM	4414	O	CYS	A	291	132.376	68.597	123.669	1.00	0.00
ATOM	4415	N	VAL	A	292	132.214	70.348	125.076	1.00	0.00
ATOM	4416	H	VAL	A	292	132.472	71.314	125.258	1.00	0.00
ATOM	4417	CA	VAL	A	292	131.473	69.622	126.114	1.00	0.00
ATOM	4418	HA	VAL	A	292	130.864	68.866	125.620	1.00	0.00
ATOM	4419	CB	VAL	A	292	132.441	68.867	127.047	1.00	0.00
ATOM	4420	HB	VAL	A	292	133.046	68.201	126.432	1.00	0.00
ATOM	4421	CG1	VAL	A	292	133.413	69.784	127.807	1.00	0.00
ATOM	4422	1HG1	VAL	A	292	134.067	69.182	128.436	1.00	0.00
ATOM	4423	2HG1	VAL	A	292	134.031	70.331	127.094	1.00	0.00
ATOM	4424	3HG1	VAL	A	292	132.868	70.499	128.420	1.00	0.00
ATOM	4425	CG2	VAL	A	292	131.688	67.982	128.047	1.00	0.00
ATOM	4426	1HG2	VAL	A	292	132.395	67.343	128.574	1.00	0.00
ATOM	4427	2HG2	VAL	A	292	131.150	68.586	128.772	1.00	0.00
ATOM	4428	3HG2	VAL	A	292	130.985	67.344	127.506	1.00	0.00
ATOM	4429	C	VAL	A	292	130.517	70.542	126.864	1.00	0.00
ATOM	4430	O	VAL	A	292	130.855	71.681	127.200	1.00	0.00
ATOM	4431	N	LYS	A	293	129.296	70.071	127.125	1.00	0.00
ATOM	4432	H	LYS	A	293	129.040	69.174	126.692	1.00	0.00
ATOM	4433	CA	LYS	A	293	128.243	70.917	127.704	1.00	0.00
ATOM	4434	HA	LYS	A	293	128.266	71.835	127.125	1.00	0.00
ATOM	4435	CB	LYS	A	293	126.882	70.227	127.483	1.00	0.00
ATOM	4436	HB1	LYS	A	293	126.778	69.406	128.194	1.00	0.00
ATOM	4437	HB2	LYS	A	293	126.871	69.791	126.481	1.00	0.00
ATOM	4438	CG	LYS	A	293	125.658	71.156	127.597	1.00	0.00
ATOM	4439	HG1	LYS	A	293	125.557	71.516	128.620	1.00	0.00
ATOM	4440	HG2	LYS	A	293	124.769	70.570	127.359	1.00	0.00
ATOM	4441	CD	LYS	A	293	125.742	72.350	126.635	1.00	0.00
ATOM	4442	HD1	LYS	A	293	125.931	71.978	125.628	1.00	0.00
ATOM	4443	HD2	LYS	A	293	126.553	73.013	126.933	1.00	0.00
ATOM	4444	CE	LYS	A	293	124.451	73.162	126.623	1.00	0.00
ATOM	4445	HE1	LYS	A	293	124.237	73.518	127.634	1.00	0.00
ATOM	4446	HE2	LYS	A	293	123.631	72.517	126.296	1.00	0.00
ATOM	4447	NZ	LYS	A	293	124.583	74.304	125.696	1.00	0.00
ATOM	4448	HZ1	LYS	A	293	123.701	74.794	125.585	1.00	0.00
ATOM	4449	HZ2	LYS	A	293	125.313	74.965	125.960	1.00	0.00
ATOM	4450	HZ3	LYS	A	293	124.850	74.002	124.763	1.00	0.00
ATOM	4451	C	LYS	A	293	128.461	71.349	129.155	1.00	0.00

```
ATOM   4452  O   LYS A 293     127.830  72.327 129.565  1.00  0.00
ATOM   4453  N   LYS A 294     129.330  70.675 129.928  1.00  0.00
ATOM   4454  H   LYS A 294     129.776  69.863 129.529  1.00  0.00
ATOM   4455  CA  LYS A 294     129.506  70.990 131.364  1.00  0.00
ATOM   4456  HA  LYS A 294     129.401  72.074 131.427  1.00  0.00
ATOM   4457  CB  LYS A 294     128.332  70.386 132.177  1.00  0.00
ATOM   4458  HB1 LYS A 294     128.636  69.445 132.639  1.00  0.00
ATOM   4459  HB2 LYS A 294     127.490  70.178 131.516  1.00  0.00
ATOM   4460  CG  LYS A 294     127.828  71.366 133.264  1.00  0.00
ATOM   4461  HG1 LYS A 294     127.688  72.351 132.818  1.00  0.00
ATOM   4462  HG2 LYS A 294     128.589  71.426 134.039  1.00  0.00
ATOM   4463  CD  LYS A 294     126.504  70.939 133.914  1.00  0.00
ATOM   4464  HD1 LYS A 294     126.641  69.935 134.319  1.00  0.00
ATOM   4465  HD2 LYS A 294     125.715  70.896 133.162  1.00  0.00
ATOM   4466  CE  LYS A 294     126.077  71.860 135.074  1.00  0.00
ATOM   4467  HE1 LYS A 294     126.951  72.074 135.697  1.00  0.00
ATOM   4468  HE2 LYS A 294     125.368  71.311 135.701  1.00  0.00
ATOM   4469  NZ  LYS A 294     125.435  73.133 134.644  1.00  0.00
ATOM   4470  HZ1 LYS A 294     125.068  73.656 135.442  1.00  0.00
ATOM   4471  HZ2 LYS A 294     126.073  73.815 134.238  1.00  0.00
ATOM   4472  HZ3 LYS A 294     124.665  73.008 134.010  1.00  0.00
ATOM   4473  C   LYS A 294     130.885  70.787 132.011  1.00  0.00
ATOM   4474  O   LYS A 294     130.966  70.880 133.229  1.00  0.00
ATOM   4475  N   CYS A 295     131.960  70.611 131.231  1.00  0.00
ATOM   4476  H   CYS A 295     131.814  70.668 130.240  1.00  0.00
ATOM   4477  CA  CYS A 295     133.318  70.251 131.701  1.00  0.00
ATOM   4478  HA  CYS A 295     133.914  70.086 130.806  1.00  0.00
ATOM   4479  CB  CYS A 295     133.968  71.427 132.462  1.00  0.00
ATOM   4480  HB1 CYS A 295     134.919  71.111 132.888  1.00  0.00
ATOM   4481  HB2 CYS A 295     133.333  71.695 133.307  1.00  0.00
ATOM   4482  SG  CYS A 295     134.277  72.928 131.494  1.00  0.00
ATOM   4483  C   CYS A 295     133.366  68.926 132.522  1.00  0.00
ATOM   4484  O   CYS A 295     132.333  68.364 132.899  1.00  0.00
ATOM   4485  N   PRO A 296     134.566  68.367 132.775  1.00  0.00
ATOM   4486  CD  PRO A 296     135.806  68.620 132.052  1.00  0.00
ATOM   4487  HD1 PRO A 296     136.362  69.422 132.538  1.00  0.00
ATOM   4488  HD2 PRO A 296     135.634  68.859 131.002  1.00  0.00
ATOM   4489  CG  PRO A 296     136.587  67.314 132.159  1.00  0.00
ATOM   4490  HG1 PRO A 296     137.659  67.463 132.038  1.00  0.00
ATOM   4491  HG2 PRO A 296     136.216  66.605 131.417  1.00  0.00
ATOM   4492  CB  PRO A 296     136.218  66.837 133.562  1.00  0.00
ATOM   4493  HB1 PRO A 296     136.831  67.364 134.294  1.00  0.00
ATOM   4494  HB2 PRO A 296     136.344  65.759 133.667  1.00  0.00
ATOM   4495  CA  PRO A 296     134.746  67.255 133.712  1.00  0.00
ATOM   4496  HA  PRO A 296     134.111  66.434 133.375  1.00  0.00
ATOM   4497  C   PRO A 296     134.418  67.568 135.173  1.00  0.00
ATOM   4498  O   PRO A 296     134.415  68.723 135.590  1.00  0.00
ATOM   4499  N   ARG A 297     134.246  66.505 135.978  1.00  0.00
ATOM   4500  H   ARG A 297     134.325  65.599 135.538  1.00  0.00
ATOM   4501  CA  ARG A 297     133.760  66.533 137.379  1.00  0.00
ATOM   4502  HA  ARG A 297     132.721  66.864 137.339  1.00  0.00
ATOM   4503  CB  ARG A 297     133.801  65.114 137.996  1.00  0.00
ATOM   4504  HB1 ARG A 297     134.287  65.170 138.974  1.00  0.00
ATOM   4505  HB2 ARG A 297     134.408  64.444 137.384  1.00  0.00
ATOM   4506  CG  ARG A 297     132.404  64.503 138.221  1.00  0.00
ATOM   4507  HG1 ARG A 297     131.754  65.261 138.661  1.00  0.00
ATOM   4508  HG2 ARG A 297     132.493  63.693 138.947  1.00  0.00
ATOM   4509  CD  ARG A 297     131.753  63.954 136.946  1.00  0.00
ATOM   4510  HD1 ARG A 297     131.802  64.710 136.160  1.00  0.00
ATOM   4511  HD2 ARG A 297     130.703  63.742 137.160  1.00  0.00
ATOM   4512  NE  ARG A 297     132.405  62.704 136.499  1.00  0.00
```

```
ATOM   4513  HE  ARG A 297     133.044  62.277 137.144  1.00  0.00
ATOM   4514  CZ  ARG A 297     132.247  62.098 135.328  1.00  0.00
ATOM   4515  NH1 ARG A 297     131.428  62.540 134.423  1.00  0.00
ATOM   4516 1HH1 ARG A 297     130.810  63.300 134.592  1.00  0.00
ATOM   4517 2HH1 ARG A 297     131.422  62.037 133.527  1.00  0.00
ATOM   4518  NH2 ARG A 297     132.930  61.035 135.028  1.00  0.00
ATOM   4519 1HH2 ARG A 297     133.499  60.552 135.689  1.00  0.00
ATOM   4520 2HH2 ARG A 297     132.806  60.631 134.089  1.00  0.00
ATOM   4521  C   ARG A 297     134.397  67.545 138.350  1.00  0.00
ATOM   4522  O   ARG A 297     133.787  67.817 139.375  1.00  0.00
ATOM   4523  N   ASN A 298     135.602  68.046 138.081  1.00  0.00
ATOM   4524  H   ASN A 298     135.986  67.866 137.171  1.00  0.00
ATOM   4525  CA  ASN A 298     136.411  68.800 139.047  1.00  0.00
ATOM   4526  HA  ASN A 298     135.734  69.343 139.712  1.00  0.00
ATOM   4527  CB  ASN A 298     137.209  67.776 139.892  1.00  0.00
ATOM   4528  HB1 ASN A 298     138.149  67.532 139.398  1.00  0.00
ATOM   4529  HB2 ASN A 298     136.633  66.856 139.992  1.00  0.00
ATOM   4530  CG  ASN A 298     137.498  68.249 141.307  1.00  0.00
ATOM   4531  OD1 ASN A 298     136.928  67.764 142.270  1.00  0.00
ATOM   4532  ND2 ASN A 298     138.373  69.202 141.497  1.00  0.00
ATOM   4533 1HD2 ASN A 298     138.759  69.691 140.695  1.00  0.00
ATOM   4534 2HD2 ASN A 298     138.482  69.523 142.442  1.00  0.00
ATOM   4535  C   ASN A 298     137.312  69.864 138.375  1.00  0.00
ATOM   4536  O   ASN A 298     138.376  70.190 138.902  1.00  0.00
ATOM   4537  N   TYR A 299     136.924  70.375 137.198  1.00  0.00
ATOM   4538  H   TYR A 299     136.021  70.111 136.820  1.00  0.00
ATOM   4539  CA  TYR A 299     137.691  71.386 136.452  1.00  0.00
ATOM   4540  HA  TYR A 299     138.524  71.727 137.063  1.00  0.00
ATOM   4541  CB  TYR A 299     138.286  70.777 135.165  1.00  0.00
ATOM   4542  HB1 TYR A 299     138.709  71.592 134.576  1.00  0.00
ATOM   4543  HB2 TYR A 299     137.463  70.362 134.584  1.00  0.00
ATOM   4544  CG  TYR A 299     139.379  69.715 135.304  1.00  0.00
ATOM   4545  CD1 TYR A 299     140.289  69.707 136.386  1.00  0.00
ATOM   4546  HD1 TYR A 299     140.214  70.447 137.175  1.00  0.00
ATOM   4547  CE1 TYR A 299     141.313  68.741 136.458  1.00  0.00
ATOM   4548  HE1 TYR A 299     141.988  68.748 137.301  1.00  0.00
ATOM   4549  CZ  TYR A 299     141.437  67.767 135.446  1.00  0.00
ATOM   4550  OH  TYR A 299     142.444  66.849 135.503  1.00  0.00
ATOM   4551  HH  TYR A 299     142.343  66.178 134.819  1.00  0.00
ATOM   4552  CE2 TYR A 299     140.542  67.776 134.354  1.00  0.00
ATOM   4553  HE2 TYR A 299     140.640  67.050 133.562  1.00  0.00
ATOM   4554  CD2 TYR A 299     139.528  68.753 134.284  1.00  0.00
ATOM   4555  HD2 TYR A 299     138.871  68.779 133.436  1.00  0.00
ATOM   4556  C   TYR A 299     136.864  72.641 136.147  1.00  0.00
ATOM   4557  O   TYR A 299     135.723  72.561 135.688  1.00  0.00
ATOM   4558  N   VAL A 300     137.464  73.797 136.427  1.00  0.00
ATOM   4559  H   VAL A 300     138.445  73.766 136.668  1.00  0.00
ATOM   4560  CA  VAL A 300     136.863  75.132 136.337  1.00  0.00
ATOM   4561  HA  VAL A 300     135.852  75.085 136.742  1.00  0.00
ATOM   4562  CB  VAL A 300     137.668  76.138 137.194  1.00  0.00
ATOM   4563  HB  VAL A 300     137.442  75.932 138.238  1.00  0.00
ATOM   4564  CG1 VAL A 300     139.185  76.004 137.010  1.00  0.00
ATOM   4565 1HG1 VAL A 300     139.688  76.833 137.501  1.00  0.00
ATOM   4566 2HG1 VAL A 300     139.543  75.084 137.468  1.00  0.00
ATOM   4567 3HG1 VAL A 300     139.426  76.015 135.952  1.00  0.00
ATOM   4568  CG2 VAL A 300     137.310  77.602 136.918  1.00  0.00
ATOM   4569 1HG2 VAL A 300     137.616  77.887 135.912  1.00  0.00
ATOM   4570 2HG2 VAL A 300     136.243  77.756 137.053  1.00  0.00
ATOM   4571 3HG2 VAL A 300     137.858  78.231 137.607  1.00  0.00
ATOM   4572  C   VAL A 300     136.757  75.604 134.885  1.00  0.00
ATOM   4573  O   VAL A 300     137.670  75.437 134.082  1.00  0.00
```

ATOM	4574	N	VAL	A	301	135.658	76.276	134.547	1.00	0.00
ATOM	4575	H	VAL	A	301	134.925	76.365	135.243	1.00	0.00
ATOM	4576	CA	VAL	A	301	135.529	77.056	133.309	1.00	0.00
ATOM	4577	HA	VAL	A	301	135.983	76.519	132.475	1.00	0.00
ATOM	4578	CB	VAL	A	301	134.041	77.286	133.002	1.00	0.00
ATOM	4579	HB	VAL	A	301	133.550	77.628	133.905	1.00	0.00
ATOM	4580	CG1	VAL	A	301	133.785	78.322	131.907	1.00	0.00
ATOM	4581	1HG1	VAL	A	301	132.727	78.362	131.673	1.00	0.00
ATOM	4582	2HG1	VAL	A	301	134.070	79.312	132.255	1.00	0.00
ATOM	4583	3HG1	VAL	A	301	134.349	78.075	131.008	1.00	0.00
ATOM	4584	CG2	VAL	A	301	133.377	75.984	132.557	1.00	0.00
ATOM	4585	1HG2	VAL	A	301	132.314	76.147	132.399	1.00	0.00
ATOM	4586	2HG2	VAL	A	301	133.826	75.665	131.618	1.00	0.00
ATOM	4587	3HG2	VAL	A	301	133.511	75.213	133.316	1.00	0.00
ATOM	4588	C	VAL	A	301	136.226	78.409	133.455	1.00	0.00
ATOM	4589	O	VAL	A	301	135.917	79.166	134.374	1.00	0.00
ATOM	4590	N	THR	A	302	137.120	78.736	132.526	1.00	0.00
ATOM	4591	H	THR	A	302	137.368	78.049	131.820	1.00	0.00
ATOM	4592	CA	THR	A	302	137.681	80.080	132.402	1.00	0.00
ATOM	4593	HA	THR	A	302	137.849	80.432	133.416	1.00	0.00
ATOM	4594	CB	THR	A	302	139.072	80.045	131.741	1.00	0.00
ATOM	4595	HB	THR	A	302	139.511	79.071	131.959	1.00	0.00
ATOM	4596	CG2	THR	A	302	139.113	80.239	130.229	1.00	0.00
ATOM	4597	1HG2	THR	A	302	140.094	79.940	129.862	1.00	0.00
ATOM	4598	2HG2	THR	A	302	138.350	79.623	129.754	1.00	0.00
ATOM	4599	3HG2	THR	A	302	138.960	81.285	129.981	1.00	0.00
ATOM	4600	OG1	THR	A	302	139.908	81.006	132.349	1.00	0.00
ATOM	4601	HG1	THR	A	302	139.724	81.912	132.018	1.00	0.00
ATOM	4602	C	THR	A	302	136.709	81.067	131.746	1.00	0.00
ATOM	4603	O	THR	A	302	135.825	80.692	130.971	1.00	0.00
ATOM	4604	N	ASP	A	303	136.947	82.344	132.004	1.00	0.00
ATOM	4605	H	ASP	A	303	137.776	82.526	132.553	1.00	0.00
ATOM	4606	CA	ASP	A	303	136.381	83.557	131.399	1.00	0.00
ATOM	4607	HA	ASP	A	303	135.353	83.650	131.748	1.00	0.00
ATOM	4608	CB	ASP	A	303	137.174	84.761	131.988	1.00	0.00
ATOM	4609	HB1	ASP	A	303	136.879	84.862	133.034	1.00	0.00
ATOM	4610	HB2	ASP	A	303	136.850	85.674	131.485	1.00	0.00
ATOM	4611	CG	ASP	A	303	138.723	84.692	131.957	1.00	0.00
ATOM	4612	OD1	ASP	A	303	139.361	85.756	132.151	1.00	0.00
ATOM	4613	OD2	ASP	A	303	139.296	83.581	131.832	1.00	0.00
ATOM	4614	C	ASP	A	303	136.299	83.622	129.857	1.00	0.00
ATOM	4615	O	ASP	A	303	135.700	84.549	129.324	1.00	0.00
ATOM	4616	N	HIS	A	304	136.828	82.625	129.136	1.00	0.00
ATOM	4617	H	HIS	A	304	137.306	81.909	129.657	1.00	0.00
ATOM	4618	CA	HIS	A	304	136.815	82.502	127.664	1.00	0.00
ATOM	4619	HA	HIS	A	304	136.171	83.282	127.253	1.00	0.00
ATOM	4620	CB	HIS	A	304	138.235	82.738	127.109	1.00	0.00
ATOM	4621	HB1	HIS	A	304	138.195	82.762	126.020	1.00	0.00
ATOM	4622	HB2	HIS	A	304	138.881	81.911	127.404	1.00	0.00
ATOM	4623	CG	HIS	A	304	138.858	84.029	127.582	1.00	0.00
ATOM	4624	ND1	HIS	A	304	138.645	85.292	127.024	1.00	0.00
ATOM	4625	CE1	HIS	A	304	139.191	86.163	127.888	1.00	0.00
ATOM	4626	HE1	HIS	A	304	139.138	87.235	127.786	1.00	0.00
ATOM	4627	NE2	HIS	A	304	139.750	85.518	128.928	1.00	0.00
ATOM	4628	HE2	HIS	A	304	140.090	85.940	129.785	1.00	0.00
ATOM	4629	CD2	HIS	A	304	139.572	84.170	128.735	1.00	0.00
ATOM	4630	HD2	HIS	A	304	139.840	83.381	129.425	1.00	0.00
ATOM	4631	C	HIS	A	304	136.240	81.153	127.180	1.00	0.00
ATOM	4632	O	HIS	A	304	136.274	80.859	125.988	1.00	0.00
ATOM	4633	N	GLY	A	305	135.724	80.312	128.088	1.00	0.00
ATOM	4634	H	GLY	A	305	135.715	80.621	129.056	1.00	0.00

ATOM	4635	CA	GLY	A	305	134.964	79.090	127.772	1.00	0.00
ATOM	4636	HA1	GLY	A	305	134.594	79.158	126.750	1.00	0.00
ATOM	4637	HA2	GLY	A	305	134.112	79.041	128.448	1.00	0.00
ATOM	4638	C	GLY	A	305	135.727	77.761	127.891	1.00	0.00
ATOM	4639	O	GLY	A	305	135.110	76.714	128.087	1.00	0.00
ATOM	4640	N	SER	A	306	137.056	77.786	127.787	1.00	0.00
ATOM	4641	H	SER	A	306	137.473	78.675	127.553	1.00	0.00
ATOM	4642	CA	SER	A	306	137.979	76.710	128.101	1.00	0.00
ATOM	4643	HA	SER	A	306	137.774	75.922	127.404	1.00	0.00
ATOM	4644	CB	SER	A	306	139.419	77.154	127.849	1.00	0.00
ATOM	4645	HB1	SER	A	306	140.102	76.329	128.059	1.00	0.00
ATOM	4646	HB2	SER	A	306	139.671	77.993	128.497	1.00	0.00
ATOM	4647	OG	SER	A	306	139.556	77.543	126.495	1.00	0.00
ATOM	4648	HG	SER	A	306	139.522	76.759	125.939	1.00	0.00
ATOM	4649	C	SER	A	306	137.856	76.175	129.538	1.00	0.00
ATOM	4650	O	SER	A	306	138.030	76.924	130.498	1.00	0.00
ATOM	4651	N	CYS	A	307	137.641	74.866	129.689	1.00	0.00
ATOM	4652	H	CYS	A	307	137.468	74.318	128.855	1.00	0.00
ATOM	4653	CA	CYS	A	307	137.883	74.149	130.940	1.00	0.00
ATOM	4654	HA	CYS	A	307	137.392	74.683	131.752	1.00	0.00
ATOM	4655	CB	CYS	A	307	137.302	72.720	130.868	1.00	0.00
ATOM	4656	HB1	CYS	A	307	137.277	72.316	131.881	1.00	0.00
ATOM	4657	HB2	CYS	A	307	138.008	72.116	130.301	1.00	0.00
ATOM	4658	SG	CYS	A	307	135.676	72.449	130.094	1.00	0.00
ATOM	4659	C	CYS	A	307	139.398	74.053	131.217	1.00	0.00
ATOM	4660	O	CYS	A	307	140.158	73.619	130.350	1.00	0.00
ATOM	4661	N	VAL	A	308	139.837	74.374	132.434	1.00	0.00
ATOM	4662	H	VAL	A	308	139.151	74.706	133.111	1.00	0.00
ATOM	4663	CA	VAL	A	308	141.238	74.363	132.879	1.00	0.00
ATOM	4664	HA	VAL	A	308	141.814	73.736	132.200	1.00	0.00
ATOM	4665	CB	VAL	A	308	141.868	75.775	132.846	1.00	0.00
ATOM	4666	HB	VAL	A	308	142.883	75.696	133.236	1.00	0.00
ATOM	4667	CG1	VAL	A	308	141.972	76.314	131.416	1.00	0.00
ATOM	4668	1HG1	VAL	A	308	142.541	77.243	131.413	1.00	0.00
ATOM	4669	2HG1	VAL	A	308	142.476	75.587	130.781	1.00	0.00
ATOM	4670	3HG1	VAL	A	308	140.980	76.516	131.013	1.00	0.00
ATOM	4671	CG2	VAL	A	308	141.117	76.810	133.686	1.00	0.00
ATOM	4672	1HG2	VAL	A	308	141.604	77.781	133.602	1.00	0.00
ATOM	4673	2HG2	VAL	A	308	140.081	76.905	133.361	1.00	0.00
ATOM	4674	3HG2	VAL	A	308	141.146	76.517	134.731	1.00	0.00
ATOM	4675	C	VAL	A	308	141.351	73.733	134.270	1.00	0.00
ATOM	4676	O	VAL	A	308	140.378	73.663	135.020	1.00	0.00
ATOM	4677	N	ARG	A	309	142.528	73.201	134.617	1.00	0.00
ATOM	4678	H	ARG	A	309	143.299	73.292	133.974	1.00	0.00
ATOM	4679	CA	ARG	A	309	142.704	72.410	135.854	1.00	0.00
ATOM	4680	HA	ARG	A	309	141.851	71.741	135.955	1.00	0.00
ATOM	4681	CB	ARG	A	309	143.987	71.553	135.779	1.00	0.00
ATOM	4682	HB1	ARG	A	309	144.001	70.900	136.653	1.00	0.00
ATOM	4683	HB2	ARG	A	309	144.855	72.212	135.835	1.00	0.00
ATOM	4684	CG	ARG	A	309	144.094	70.695	134.505	1.00	0.00
ATOM	4685	HG1	ARG	A	309	144.271	71.350	133.652	1.00	0.00
ATOM	4686	HG2	ARG	A	309	143.158	70.161	134.336	1.00	0.00
ATOM	4687	CD	ARG	A	309	145.251	69.684	134.575	1.00	0.00
ATOM	4688	HD1	ARG	A	309	146.147	70.188	134.945	1.00	0.00
ATOM	4689	HD2	ARG	A	309	145.459	69.320	133.566	1.00	0.00
ATOM	4690	NE	ARG	A	309	144.908	68.531	135.428	1.00	0.00
ATOM	4691	HE	ARG	A	309	143.929	68.292	135.495	1.00	0.00
ATOM	4692	CZ	ARG	A	309	145.731	67.761	136.115	1.00	0.00
ATOM	4693	NH1	ARG	A	309	147.026	67.887	136.104	1.00	0.00
ATOM	4694	1HH1	ARG	A	309	147.458	68.428	135.358	1.00	0.00
ATOM	4695	2HH1	ARG	A	309	147.611	67.279	136.641	1.00	0.00

```
ATOM   4696  NH2 ARG A 309     145.241  66.802 136.844  1.00  0.00
ATOM   4697 1HH2 ARG A 309     144.246  66.655 136.848  1.00  0.00
ATOM   4698 2HH2 ARG A 309     145.851  66.198 137.365  1.00  0.00
ATOM   4699  C   ARG A 309     142.704  73.246 137.139  1.00  0.00
ATOM   4700  O   ARG A 309     142.511  72.682 138.209  1.00  0.00
ATOM   4701  N   ALA A 310     142.964  74.549 137.015  1.00  0.00
ATOM   4702  H   ALA A 310     143.123  74.907 136.090  1.00  0.00
ATOM   4703  CA  ALA A 310     142.935  75.559 138.069  1.00  0.00
ATOM   4704  HA  ALA A 310     142.093  75.363 138.739  1.00  0.00
ATOM   4705  CB  ALA A 310     144.246  75.489 138.866  1.00  0.00
ATOM   4706  HB1 ALA A 310     144.239  76.246 139.651  1.00  0.00
ATOM   4707  HB2 ALA A 310     144.345  74.506 139.328  1.00  0.00
ATOM   4708  HB3 ALA A 310     145.095  75.670 138.206  1.00  0.00
ATOM   4709  C   ALA A 310     142.757  76.960 137.455  1.00  0.00
ATOM   4710  O   ALA A 310     143.195  77.202 136.328  1.00  0.00
ATOM   4711  N   CYS A 311     142.118  77.863 138.195  1.00  0.00
ATOM   4712  H   CYS A 311     141.669  77.549 139.051  1.00  0.00
ATOM   4713  CA  CYS A 311     141.906  79.249 137.783  1.00  0.00
ATOM   4714  HA  CYS A 311     141.524  79.226 136.763  1.00  0.00
ATOM   4715  CB  CYS A 311     140.823  79.823 138.697  1.00  0.00
ATOM   4716  HB1 CYS A 311     141.260  80.047 139.672  1.00  0.00
ATOM   4717  HB2 CYS A 311     140.065  79.056 138.835  1.00  0.00
ATOM   4718  SG  CYS A 311     139.983  81.290 138.067  1.00  0.00
ATOM   4719  C   CYS A 311     143.187  80.112 137.787  1.00  0.00
ATOM   4720  O   CYS A 311     144.201  79.761 138.389  1.00  0.00
ATOM   4721  N   GLY A 312     143.137  81.259 137.100  1.00  0.00
ATOM   4722  H   GLY A 312     142.264  81.531 136.678  1.00  0.00
ATOM   4723  CA  GLY A 312     144.269  82.180 136.992  1.00  0.00
ATOM   4724  HA1 GLY A 312     144.082  82.906 136.200  1.00  0.00
ATOM   4725  HA2 GLY A 312     145.165  81.616 136.733  1.00  0.00
ATOM   4726  C   GLY A 312     144.529  82.952 138.288  1.00  0.00
ATOM   4727  O   GLY A 312     143.608  83.282 139.031  1.00  0.00
ATOM   4728  N   ALA A 313     145.792  83.302 138.527  1.00  0.00
ATOM   4729  H   ALA A 313     146.490  83.005 137.868  1.00  0.00
ATOM   4730  CA  ALA A 313     146.252  84.082 139.684  1.00  0.00
ATOM   4731  HA  ALA A 313     145.835  83.631 140.585  1.00  0.00
ATOM   4732  CB  ALA A 313     147.782  83.955 139.742  1.00  0.00
ATOM   4733  HB1 ALA A 313     148.156  84.455 140.637  1.00  0.00
ATOM   4734  HB2 ALA A 313     148.066  82.903 139.782  1.00  0.00
ATOM   4735  HB3 ALA A 313     148.228  84.423 138.863  1.00  0.00
ATOM   4736  C   ALA A 313     145.799  85.568 139.680  1.00  0.00
ATOM   4737  O   ALA A 313     146.290  86.373 140.462  1.00  0.00
ATOM   4738  N   ASP A 314     144.868  85.925 138.796  1.00  0.00
ATOM   4739  H   ASP A 314     144.515  85.203 138.182  1.00  0.00
ATOM   4740  CA  ASP A 314     144.201  87.224 138.643  1.00  0.00
ATOM   4741  HA  ASP A 314     144.643  87.963 139.312  1.00  0.00
ATOM   4742  CB  ASP A 314     144.407  87.658 137.175  1.00  0.00
ATOM   4743  HB1 ASP A 314     145.474  87.658 136.946  1.00  0.00
ATOM   4744  HB2 ASP A 314     144.049  88.683 137.065  1.00  0.00
ATOM   4745  CG  ASP A 314     143.677  86.776 136.129  1.00  0.00
ATOM   4746  OD1 ASP A 314     143.264  85.630 136.429  1.00  0.00
ATOM   4747  OD2 ASP A 314     143.456  87.251 134.996  1.00  0.00
ATOM   4748  C   ASP A 314     142.687  87.135 138.929  1.00  0.00
ATOM   4749  O   ASP A 314     141.942  88.116 138.842  1.00  0.00
ATOM   4750  N   SER A 315     142.224  85.922 139.209  1.00  0.00
ATOM   4751  H   SER A 315     142.900  85.175 139.322  1.00  0.00
ATOM   4752  CA  SER A 315     140.834  85.511 139.211  1.00  0.00
ATOM   4753  HA  SER A 315     140.183  86.380 139.205  1.00  0.00
ATOM   4754  CB  SER A 315     140.529  84.641 137.989  1.00  0.00
ATOM   4755  HB1 SER A 315     139.488  84.318 138.023  1.00  0.00
ATOM   4756  HB2 SER A 315     141.175  83.764 138.009  1.00  0.00
```

ATOM	4757	OG	SER	A	315	140.750	85.331	136.778	1.00	0.00
ATOM	4758	HG	SER	A	315	141.741	85.407	136.698	1.00	0.00
ATOM	4759	C	SER	A	315	140.555	84.715	140.482	1.00	0.00
ATOM	4760	O	SER	A	315	141.449	84.207	141.158	1.00	0.00
ATOM	4761	N	TYR	A	316	139.277	84.567	140.791	1.00	0.00
ATOM	4762	H	TYR	A	316	138.595	85.100	140.261	1.00	0.00
ATOM	4763	CA	TYR	A	316	138.779	83.632	141.774	1.00	0.00
ATOM	4764	HA	TYR	A	316	139.579	82.958	142.085	1.00	0.00
ATOM	4765	CB	TYR	A	316	138.288	84.405	143.004	1.00	0.00
ATOM	4766	HB1	TYR	A	316	137.360	84.926	142.766	1.00	0.00
ATOM	4767	HB2	TYR	A	316	139.028	85.165	143.262	1.00	0.00
ATOM	4768	CG	TYR	A	316	138.073	83.536	144.230	1.00	0.00
ATOM	4769	CD1	TYR	A	316	139.113	83.390	145.170	1.00	0.00
ATOM	4770	HD1	TYR	A	316	140.065	83.880	144.996	1.00	0.00
ATOM	4771	CE1	TYR	A	316	138.922	82.590	146.314	1.00	0.00
ATOM	4772	HE1	TYR	A	316	139.715	82.453	147.034	1.00	0.00
ATOM	4773	CZ	TYR	A	316	137.696	81.925	146.513	1.00	0.00
ATOM	4774	OH	TYR	A	316	137.525	81.157	147.622	1.00	0.00
ATOM	4775	HH	TYR	A	316	136.700	80.668	147.585	1.00	0.00
ATOM	4776	CE2	TYR	A	316	136.658	82.057	145.562	1.00	0.00
ATOM	4777	HE2	TYR	A	316	135.723	81.532	145.705	1.00	0.00
ATOM	4778	CD2	TYR	A	316	136.849	82.865	144.424	1.00	0.00
ATOM	4779	HD2	TYR	A	316	136.057	82.966	143.693	1.00	0.00
ATOM	4780	C	TYR	A	316	137.673	82.815	141.117	1.00	0.00
ATOM	4781	O	TYR	A	316	136.748	83.358	140.507	1.00	0.00
ATOM	4782	N	GLU	A	317	137.793	81.495	141.177	1.00	0.00
ATOM	4783	H	GLU	A	317	138.569	81.085	141.673	1.00	0.00
ATOM	4784	CA	GLU	A	317	136.783	80.601	140.622	1.00	0.00
ATOM	4785	HA	GLU	A	317	136.397	81.051	139.710	1.00	0.00
ATOM	4786	CB	GLU	A	317	137.397	79.269	140.193	1.00	0.00
ATOM	4787	HB1	GLU	A	317	137.963	79.477	139.293	1.00	0.00
ATOM	4788	HB2	GLU	A	317	136.581	78.595	139.925	1.00	0.00
ATOM	4789	CG	GLU	A	317	138.342	78.580	141.184	1.00	0.00
ATOM	4790	HG1	GLU	A	317	137.896	78.550	142.178	1.00	0.00
ATOM	4791	HG2	GLU	A	317	139.272	79.148	141.256	1.00	0.00
ATOM	4792	CD	GLU	A	317	138.620	77.155	140.693	1.00	0.00
ATOM	4793	OE1	GLU	A	317	137.704	76.316	140.886	1.00	0.00
ATOM	4794	OE2	GLU	A	317	139.686	76.957	140.064	1.00	0.00
ATOM	4795	C	GLU	A	317	135.589	80.424	141.565	1.00	0.00
ATOM	4796	O	GLU	A	317	135.704	79.964	142.702	1.00	0.00
ATOM	4797	N	MET	A	318	134.418	80.782	141.051	1.00	0.00
ATOM	4798	H	MET	A	318	134.399	81.110	140.090	1.00	0.00
ATOM	4799	CA	MET	A	318	133.147	80.817	141.757	1.00	0.00
ATOM	4800	HA	MET	A	318	133.193	80.207	142.658	1.00	0.00
ATOM	4801	CB	MET	A	318	132.872	82.274	142.171	1.00	0.00
ATOM	4802	HB1	MET	A	318	132.939	82.910	141.287	1.00	0.00
ATOM	4803	HB2	MET	A	318	133.659	82.587	142.859	1.00	0.00
ATOM	4804	CG	MET	A	318	131.518	82.493	142.855	1.00	0.00
ATOM	4805	HG1	MET	A	318	131.689	82.960	143.826	1.00	0.00
ATOM	4806	HG2	MET	A	318	131.027	81.537	143.039	1.00	0.00
ATOM	4807	SD	MET	A	318	130.392	83.562	141.915	1.00	0.00
ATOM	4808	CE	MET	A	318	128.901	83.384	142.929	1.00	0.00
ATOM	4809	HE1	MET	A	318	128.098	83.991	142.511	1.00	0.00
ATOM	4810	HE2	MET	A	318	128.586	82.340	142.941	1.00	0.00
ATOM	4811	HE3	MET	A	318	129.110	83.708	143.949	1.00	0.00
ATOM	4812	C	MET	A	318	132.075	80.249	140.836	1.00	0.00
ATOM	4813	O	MET	A	318	132.076	80.505	139.633	1.00	0.00
ATOM	4814	N	GLU	A	319	131.189	79.438	141.385	1.00	0.00
ATOM	4815	H	GLU	A	319	131.232	79.306	142.394	1.00	0.00
ATOM	4816	CA	GLU	A	319	130.127	78.742	140.665	1.00	0.00
ATOM	4817	HA	GLU	A	319	130.493	78.557	139.664	1.00	0.00

ATOM	4818	CB	GLU	A	319	129.813	77.380	141.325	1.00	0.00
ATOM	4819	HB1	GLU	A	319	130.661	76.718	141.163	1.00	0.00
ATOM	4820	HB2	GLU	A	319	128.967	76.927	140.809	1.00	0.00
ATOM	4821	CG	GLU	A	319	129.499	77.418	142.830	1.00	0.00
ATOM	4822	HG1	GLU	A	319	128.933	76.520	143.087	1.00	0.00
ATOM	4823	HG2	GLU	A	319	128.874	78.283	143.062	1.00	0.00
ATOM	4824	CD	GLU	A	319	130.792	77.442	143.649	1.00	0.00
ATOM	4825	OE1	GLU	A	319	131.404	76.372	143.846	1.00	0.00
ATOM	4826	OE2	GLU	A	319	131.307	78.538	143.959	1.00	0.00
ATOM	4827	C	GLU	A	319	128.865	79.583	140.453	1.00	0.00
ATOM	4828	O	GLU	A	319	128.732	80.694	140.964	1.00	0.00
ATOM	4829	N	GLU	A	320	127.941	79.053	139.649	1.00	0.00
ATOM	4830	H	GLU	A	320	128.156	78.174	139.205	1.00	0.00
ATOM	4831	CA	GLU	A	320	126.709	79.752	139.272	1.00	0.00
ATOM	4832	HA	GLU	A	320	126.354	80.313	140.139	1.00	0.00
ATOM	4833	CB	GLU	A	320	127.027	80.780	138.169	1.00	0.00
ATOM	4834	HB1	GLU	A	320	127.703	81.525	138.589	1.00	0.00
ATOM	4835	HB2	GLU	A	320	126.112	81.299	137.881	1.00	0.00
ATOM	4836	CG	GLU	A	320	127.674	80.175	136.914	1.00	0.00
ATOM	4837	HG1	GLU	A	320	126.880	79.831	136.250	1.00	0.00
ATOM	4838	HG2	GLU	A	320	128.288	79.308	137.165	1.00	0.00
ATOM	4839	CD	GLU	A	320	128.563	81.184	136.191	1.00	0.00
ATOM	4840	OE1	GLU	A	320	129.642	81.524	136.729	1.00	0.00
ATOM	4841	OE2	GLU	A	320	128.212	81.530	135.043	1.00	0.00
ATOM	4842	C	GLU	A	320	125.552	78.813	138.879	1.00	0.00
ATOM	4843	O	GLU	A	320	124.477	78.913	139.461	1.00	0.00
ATOM	4844	N	ASP	A	321	125.758	77.853	137.968	1.00	0.00
ATOM	4845	H	ASP	A	321	126.615	77.896	137.431	1.00	0.00
ATOM	4846	CA	ASP	A	321	124.686	76.986	137.425	1.00	0.00
ATOM	4847	HA	ASP	A	321	123.747	77.210	137.934	1.00	0.00
ATOM	4848	CB	ASP	A	321	124.489	77.306	135.924	1.00	0.00
ATOM	4849	HB1	ASP	A	321	124.374	78.384	135.802	1.00	0.00
ATOM	4850	HB2	ASP	A	321	123.556	76.846	135.593	1.00	0.00
ATOM	4851	CG	ASP	A	321	125.636	76.812	135.027	1.00	0.00
ATOM	4852	OD1	ASP	A	321	125.461	75.802	134.301	1.00	0.00
ATOM	4853	OD2	ASP	A	321	126.725	77.432	135.068	1.00	0.00
ATOM	4854	C	ASP	A	321	124.960	75.478	137.641	1.00	0.00
ATOM	4855	O	ASP	A	321	124.545	74.613	136.863	1.00	0.00
ATOM	4856	N	GLY	A	322	125.743	75.145	138.668	1.00	0.00
ATOM	4857	H	GLY	A	322	125.986	75.874	139.324	1.00	0.00
ATOM	4858	CA	GLY	A	322	126.336	73.812	138.820	1.00	0.00
ATOM	4859	HA1	GLY	A	322	125.637	73.045	138.487	1.00	0.00
ATOM	4860	HA2	GLY	A	322	126.547	73.641	139.875	1.00	0.00
ATOM	4861	C	GLY	A	322	127.650	73.651	138.044	1.00	0.00
ATOM	4862	O	GLY	A	322	128.018	72.542	137.665	1.00	0.00
ATOM	4863	N	VAL	A	323	128.323	74.770	137.768	1.00	0.00
ATOM	4864	H	VAL	A	323	127.906	75.638	138.062	1.00	0.00
ATOM	4865	CA	VAL	A	323	129.620	74.876	137.090	1.00	0.00
ATOM	4866	HA	VAL	A	323	130.140	73.918	137.137	1.00	0.00
ATOM	4867	CB	VAL	A	323	129.475	75.301	135.613	1.00	0.00
ATOM	4868	HB	VAL	A	323	129.140	76.333	135.578	1.00	0.00
ATOM	4869	CG1	VAL	A	323	130.794	75.182	134.849	1.00	0.00
ATOM	4870	1HG1	VAL	A	323	130.640	75.471	133.810	1.00	0.00
ATOM	4871	2HG1	VAL	A	323	131.549	75.837	135.278	1.00	0.00
ATOM	4872	3HG1	VAL	A	323	131.157	74.154	134.877	1.00	0.00
ATOM	4873	CG2	VAL	A	323	128.448	74.457	134.869	1.00	0.00
ATOM	4874	1HG2	VAL	A	323	128.403	74.759	133.823	1.00	0.00
ATOM	4875	2HG2	VAL	A	323	128.726	73.412	134.955	1.00	0.00
ATOM	4876	3HG2	VAL	A	323	127.476	74.624	135.320	1.00	0.00
ATOM	4877	C	VAL	A	323	130.424	75.937	137.821	1.00	0.00
ATOM	4878	O	VAL	A	323	129.878	77.009	138.094	1.00	0.00

ATOM	4879	N	ARG	A	324	131.690	75.653	138.143	1.00	0.00
ATOM	4880	H	ARG	A	324	132.063	74.764	137.845	1.00	0.00
ATOM	4881	CA	ARG	A	324	132.636	76.641	138.682	1.00	0.00
ATOM	4882	HA	ARG	A	324	132.116	77.344	139.325	1.00	0.00
ATOM	4883	CB	ARG	A	324	133.750	75.965	139.515	1.00	0.00
ATOM	4884	HB1	ARG	A	324	134.253	76.753	140.075	1.00	0.00
ATOM	4885	HB2	ARG	A	324	134.493	75.523	138.850	1.00	0.00
ATOM	4886	CG	ARG	A	324	133.264	74.867	140.477	1.00	0.00
ATOM	4887	HG1	ARG	A	324	133.256	73.915	139.944	1.00	0.00
ATOM	4888	HG2	ARG	A	324	132.248	75.072	140.809	1.00	0.00
ATOM	4889	CD	ARG	A	324	134.161	74.730	141.716	1.00	0.00
ATOM	4890	HD1	ARG	A	324	135.211	74.771	141.419	1.00	0.00
ATOM	4891	HD2	ARG	A	324	133.974	73.759	142.175	1.00	0.00
ATOM	4892	NE	ARG	A	324	133.832	75.778	142.699	1.00	0.00
ATOM	4893	HE	ARG	A	324	132.917	75.734	143.155	1.00	0.00
ATOM	4894	CZ	ARG	A	324	134.506	76.872	142.974	1.00	0.00
ATOM	4895	NH1	ARG	A	324	135.700	77.129	142.538	1.00	0.00
ATOM	4896	1HH1	ARG	A	324	136.241	76.486	141.954	1.00	0.00
ATOM	4897	2HH1	ARG	A	324	136.087	78.041	142.717	1.00	0.00
ATOM	4898	NH2	ARG	A	324	133.964	77.818	143.675	1.00	0.00
ATOM	4899	1HH2	ARG	A	324	132.983	77.732	143.974	1.00	0.00
ATOM	4900	2HH2	ARG	A	324	134.456	78.688	143.780	1.00	0.00
ATOM	4901	C	ARG	A	324	133.270	77.401	137.522	1.00	0.00
ATOM	4902	O	ARG	A	324	133.739	76.758	136.587	1.00	0.00
ATOM	4903	N	LYS	A	325	133.319	78.736	137.575	1.00	0.00
ATOM	4904	H	LYS	A	325	132.880	79.197	138.366	1.00	0.00
ATOM	4905	CA	LYS	A	325	133.816	79.601	136.516	1.00	0.00
ATOM	4906	HA	LYS	A	325	134.390	78.960	135.881	1.00	0.00
ATOM	4907	CB	LYS	A	325	132.673	80.191	135.658	1.00	0.00
ATOM	4908	HB1	LYS	A	325	133.106	80.541	134.718	1.00	0.00
ATOM	4909	HB2	LYS	A	325	132.243	81.053	136.172	1.00	0.00
ATOM	4910	CG	LYS	A	325	131.548	79.184	135.363	1.00	0.00
ATOM	4911	HG1	LYS	A	325	130.918	79.112	136.251	1.00	0.00
ATOM	4912	HG2	LYS	A	325	131.956	78.197	135.174	1.00	0.00
ATOM	4913	CD	LYS	A	325	130.666	79.553	134.161	1.00	0.00
ATOM	4914	HD1	LYS	A	325	131.215	79.422	133.230	1.00	0.00
ATOM	4915	HD2	LYS	A	325	130.373	80.598	134.248	1.00	0.00
ATOM	4916	CE	LYS	A	325	129.445	78.625	134.196	1.00	0.00
ATOM	4917	HE1	LYS	A	325	129.211	78.420	135.244	1.00	0.00
ATOM	4918	HE2	LYS	A	325	129.679	77.671	133.721	1.00	0.00
ATOM	4919	NZ	LYS	A	325	128.230	79.216	133.597	1.00	0.00
ATOM	4920	HZ1	LYS	A	325	127.435	78.676	133.947	1.00	0.00
ATOM	4921	HZ2	LYS	A	325	128.109	80.178	133.928	1.00	0.00
ATOM	4922	HZ3	LYS	A	325	128.248	79.202	132.591	1.00	0.00
ATOM	4923	C	LYS	A	325	134.731	80.682	137.100	1.00	0.00
ATOM	4924	O	LYS	A	325	134.403	81.296	138.115	1.00	0.00
ATOM	4925	N	CYS	A	326	135.890	80.902	136.491	1.00	0.00
ATOM	4926	H	CYS	A	326	136.091	80.355	135.663	1.00	0.00
ATOM	4927	CA	CYS	A	326	136.789	81.992	136.840	1.00	0.00
ATOM	4928	HA	CYS	A	326	137.113	81.895	137.875	1.00	0.00
ATOM	4929	CB	CYS	A	326	138.016	81.970	135.926	1.00	0.00
ATOM	4930	HB1	CYS	A	326	138.538	82.924	136.012	1.00	0.00
ATOM	4931	HB2	CYS	A	326	137.686	81.882	134.894	1.00	0.00
ATOM	4932	SG	CYS	A	326	139.228	80.684	136.277	1.00	0.00
ATOM	4933	C	CYS	A	326	136.094	83.339	136.659	1.00	0.00
ATOM	4934	O	CYS	A	326	135.573	83.628	135.581	1.00	0.00
ATOM	4935	N	LYS	A	327	136.166	84.193	137.679	1.00	0.00
ATOM	4936	H	LYS	A	327	136.531	83.859	138.567	1.00	0.00
ATOM	4937	CA	LYS	A	327	135.715	85.586	137.621	1.00	0.00
ATOM	4938	HA	LYS	A	327	135.482	85.864	136.592	1.00	0.00
ATOM	4939	CB	LYS	A	327	134.437	85.746	138.472	1.00	0.00

ATOM	4940	HB1	LYS	A	327	134.118	86.789	138.424	1.00	0.00
ATOM	4941	HB2	LYS	A	327	134.663	85.500	139.511	1.00	0.00
ATOM	4942	CG	LYS	A	327	133.296	84.841	137.973	1.00	0.00
ATOM	4943	HG1	LYS	A	327	133.572	83.797	138.112	1.00	0.00
ATOM	4944	HG2	LYS	A	327	133.138	85.024	136.909	1.00	0.00
ATOM	4945	CD	LYS	A	327	131.977	85.088	138.715	1.00	0.00
ATOM	4946	HD1	LYS	A	327	131.681	86.128	138.565	1.00	0.00
ATOM	4947	HD2	LYS	A	327	132.101	84.910	139.784	1.00	0.00
ATOM	4948	CE	LYS	A	327	130.868	84.185	138.149	1.00	0.00
ATOM	4949	HE1	LYS	A	327	131.000	84.112	137.065	1.00	0.00
ATOM	4950	HE2	LYS	A	327	129.902	84.659	138.334	1.00	0.00
ATOM	4951	NZ	LYS	A	327	130.859	82.827	138.739	1.00	0.00
ATOM	4952	HZ1	LYS	A	327	130.272	82.223	138.156	1.00	0.00
ATOM	4953	HZ2	LYS	A	327	131.765	82.376	138.730	1.00	0.00
ATOM	4954	HZ3	LYS	A	327	130.496	82.813	139.683	1.00	0.00
ATOM	4955	C	LYS	A	327	136.855	86.466	138.114	1.00	0.00
ATOM	4956	O	LYS	A	327	137.450	86.155	139.143	1.00	0.00
ATOM	4957	N	LYS	A	328	137.228	87.502	137.364	1.00	0.00
ATOM	4958	H	LYS	A	328	136.685	87.722	136.546	1.00	0.00
ATOM	4959	CA	LYS	A	328	138.354	88.379	137.733	1.00	0.00
ATOM	4960	HA	LYS	A	328	139.256	87.770	137.766	1.00	0.00
ATOM	4961	CB	LYS	A	328	138.503	89.480	136.661	1.00	0.00
ATOM	4962	HB1	LYS	A	328	137.993	90.382	137.004	1.00	0.00
ATOM	4963	HB2	LYS	A	328	138.004	89.172	135.742	1.00	0.00
ATOM	4964	CG	LYS	A	328	139.955	89.844	136.306	1.00	0.00
ATOM	4965	HG1	LYS	A	328	140.452	90.222	137.200	1.00	0.00
ATOM	4966	HG2	LYS	A	328	139.934	90.652	135.573	1.00	0.00
ATOM	4967	CD	LYS	A	328	140.806	88.697	135.728	1.00	0.00
ATOM	4968	HD1	LYS	A	328	140.985	87.967	136.512	1.00	0.00
ATOM	4969	HD2	LYS	A	328	141.775	89.112	135.449	1.00	0.00
ATOM	4970	CE	LYS	A	328	140.208	87.978	134.507	1.00	0.00
ATOM	4971	HE1	LYS	A	328	140.280	88.635	133.637	1.00	0.00
ATOM	4972	HE2	LYS	A	328	139.153	87.766	134.688	1.00	0.00
ATOM	4973	NZ	LYS	A	328	140.907	86.701	134.228	1.00	0.00
ATOM	4974	HZ1	LYS	A	328	140.545	86.260	133.379	1.00	0.00
ATOM	4975	HZ2	LYS	A	328	141.920	86.839	134.172	1.00	0.00
ATOM	4976	HZ3	LYS	A	328	140.782	86.058	135.002	1.00	0.00
ATOM	4977	C	LYS	A	328	138.140	88.949	139.143	1.00	0.00
ATOM	4978	O	LYS	A	328	137.005	89.309	139.465	1.00	0.00
ATOM	4979	N	CYS	A	329	139.174	88.990	139.982	1.00	0.00
ATOM	4980	H	CYS	A	329	140.093	88.724	139.650	1.00	0.00
ATOM	4981	CA	CYS	A	329	139.052	89.568	141.325	1.00	0.00
ATOM	4982	HA	CYS	A	329	138.247	89.064	141.860	1.00	0.00
ATOM	4983	CB	CYS	A	329	140.364	89.372	142.097	1.00	0.00
ATOM	4984	HB1	CYS	A	329	140.299	89.893	143.053	1.00	0.00
ATOM	4985	HB2	CYS	A	329	141.172	89.839	141.530	1.00	0.00
ATOM	4986	SG	CYS	A	329	140.808	87.643	142.416	1.00	0.00
ATOM	4987	C	CYS	A	329	138.715	91.070	141.238	1.00	0.00
ATOM	4988	O	CYS	A	329	139.272	91.786	140.407	1.00	0.00
ATOM	4989	N	GLU	A	330	137.847	91.557	142.126	1.00	0.00
ATOM	4990	H	GLU	A	330	137.392	90.908	142.744	1.00	0.00
ATOM	4991	CA	GLU	A	330	137.931	92.941	142.610	1.00	0.00
ATOM	4992	HA	GLU	A	330	137.967	93.636	141.768	1.00	0.00
ATOM	4993	CB	GLU	A	330	136.670	93.263	143.460	1.00	0.00
ATOM	4994	HB1	GLU	A	330	136.150	92.328	143.675	1.00	0.00
ATOM	4995	HB2	GLU	A	330	135.991	93.850	142.842	1.00	0.00
ATOM	4996	CG	GLU	A	330	136.842	93.981	144.822	1.00	0.00
ATOM	4997	HG1	GLU	A	330	137.406	93.324	145.488	1.00	0.00
ATOM	4998	HG2	GLU	A	330	135.848	94.092	145.260	1.00	0.00
ATOM	4999	CD	GLU	A	330	137.512	95.361	144.769	1.00	0.00
ATOM	5000	OE1	GLU	A	330	137.905	95.863	145.847	1.00	0.00

ATOM	5001	OE2	GLU	A	330	137.668	95.899	143.646	1.00	0.00
ATOM	5002	C	GLU	A	330	139.238	93.083	143.404	1.00	0.00
ATOM	5003	O	GLU	A	330	139.593	92.172	144.155	1.00	0.00
ATOM	5004	N	GLY	A	331	139.909	94.229	143.263	1.00	0.00
ATOM	5005	H	GLY	A	331	139.423	94.964	142.759	1.00	0.00
ATOM	5006	CA	GLY	A	331	141.030	94.647	144.107	1.00	0.00
ATOM	5007	HA1	GLY	A	331	140.603	95.026	145.034	1.00	0.00
ATOM	5008	HA2	GLY	A	331	141.545	95.473	143.621	1.00	0.00
ATOM	5009	C	GLY	A	331	142.053	93.537	144.388	1.00	0.00
ATOM	5010	O	GLY	A	331	142.521	92.868	143.460	1.00	0.00
ATOM	5011	N	PRO	A	332	142.456	93.323	145.653	1.00	0.00
ATOM	5012	CD	PRO	A	332	142.174	94.146	146.825	1.00	0.00
ATOM	5013	HD1	PRO	A	332	141.100	94.207	147.007	1.00	0.00
ATOM	5014	HD2	PRO	A	332	142.588	95.143	146.675	1.00	0.00
ATOM	5015	CG	PRO	A	332	142.865	93.468	148.010	1.00	0.00
ATOM	5016	HG1	PRO	A	332	142.139	92.888	148.581	1.00	0.00
ATOM	5017	HG2	PRO	A	332	143.359	94.191	148.660	1.00	0.00
ATOM	5018	CB	PRO	A	332	143.878	92.534	147.345	1.00	0.00
ATOM	5019	HB1	PRO	A	332	144.111	91.670	147.969	1.00	0.00
ATOM	5020	HB2	PRO	A	332	144.778	93.105	147.124	1.00	0.00
ATOM	5021	CA	PRO	A	332	143.213	92.138	146.021	1.00	0.00
ATOM	5022	HA	PRO	A	332	143.980	91.918	145.277	1.00	0.00
ATOM	5023	C	PRO	A	332	142.285	90.920	146.201	1.00	0.00
ATOM	5024	O	PRO	A	332	141.384	90.927	147.034	1.00	0.00
ATOM	5025	N	CYS	A	333	142.576	89.817	145.501	1.00	0.00
ATOM	5026	H	CYS	A	333	143.230	89.883	144.736	1.00	0.00
ATOM	5027	CA	CYS	A	333	141.945	88.520	145.782	1.00	0.00
ATOM	5028	HA	CYS	A	333	140.893	88.597	145.499	1.00	0.00
ATOM	5029	CB	CYS	A	333	142.611	87.442	144.914	1.00	0.00
ATOM	5030	HB1	CYS	A	333	142.087	86.497	145.056	1.00	0.00
ATOM	5031	HB2	CYS	A	333	143.630	87.304	145.276	1.00	0.00
ATOM	5032	SG	CYS	A	333	142.719	87.734	143.124	1.00	0.00
ATOM	5033	C	CYS	A	333	142.022	88.119	147.279	1.00	0.00
ATOM	5034	O	CYS	A	333	142.981	88.446	147.979	1.00	0.00
ATOM	5035	N	ARG	A	334	141.026	87.356	147.764	1.00	0.00
ATOM	5036	H	ARG	A	334	140.323	87.099	147.086	1.00	0.00
ATOM	5037	CA	ARG	A	334	140.664	87.126	149.190	1.00	0.00
ATOM	5038	HA	ARG	A	334	140.579	88.121	149.613	1.00	0.00
ATOM	5039	CB	ARG	A	334	139.253	86.491	149.235	1.00	0.00
ATOM	5040	HB1	ARG	A	334	139.351	85.405	149.201	1.00	0.00
ATOM	5041	HB2	ARG	A	334	138.674	86.795	148.362	1.00	0.00
ATOM	5042	CG	ARG	A	334	138.469	86.927	150.489	1.00	0.00
ATOM	5043	HG1	ARG	A	334	137.889	87.821	150.257	1.00	0.00
ATOM	5044	HG2	ARG	A	334	139.165	87.192	151.282	1.00	0.00
ATOM	5045	CD	ARG	A	334	137.522	85.828	151.008	1.00	0.00
ATOM	5046	HD1	ARG	A	334	138.090	84.903	151.137	1.00	0.00
ATOM	5047	HD2	ARG	A	334	136.747	85.648	150.260	1.00	0.00
ATOM	5048	NE	ARG	A	334	136.891	86.197	152.296	1.00	0.00
ATOM	5049	HE	ARG	A	334	135.913	86.418	152.283	1.00	0.00
ATOM	5050	CZ	ARG	A	334	137.490	86.269	153.474	1.00	0.00
ATOM	5051	NH1	ARG	A	334	138.752	85.988	153.610	1.00	0.00
ATOM	5052	1HH1	ARG	A	334	139.310	85.736	152.813	1.00	0.00
ATOM	5053	2HH1	ARG	A	334	139.210	86.253	154.474	1.00	0.00
ATOM	5054	NH2	ARG	A	334	136.827	86.647	154.532	1.00	0.00
ATOM	5055	1HH2	ARG	A	334	135.853	86.876	154.502	1.00	0.00
ATOM	5056	2HH2	ARG	A	334	137.300	86.765	155.437	1.00	0.00
ATOM	5057	C	ARG	A	334	141.663	86.368	150.119	1.00	0.00
ATOM	5058	O	ARG	A	334	141.233	85.678	151.049	1.00	0.00
ATOM	5059	N	LYS	A	335	142.976	86.456	149.879	1.00	0.00
ATOM	5060	H	LYS	A	335	143.251	87.123	149.166	1.00	0.00
ATOM	5061	CA	LYS	A	335	144.046	85.699	150.563	1.00	0.00

ATOM	5062	HA	LYS	A	335	143.914	84.650	150.295	1.00	0.00
ATOM	5063	CB	LYS	A	335	145.428	86.157	150.039	1.00	0.00
ATOM	5064	HB1	LYS	A	335	145.682	87.102	150.517	1.00	0.00
ATOM	5065	HB2	LYS	A	335	145.367	86.335	148.965	1.00	0.00
ATOM	5066	CG	LYS	A	335	146.537	85.116	150.292	1.00	0.00
ATOM	5067	HG1	LYS	A	335	146.351	84.263	149.639	1.00	0.00
ATOM	5068	HG2	LYS	A	335	146.487	84.752	151.312	1.00	0.00
ATOM	5069	CD	LYS	A	335	147.964	85.627	150.018	1.00	0.00
ATOM	5070	HD1	LYS	A	335	147.978	86.088	149.028	1.00	0.00
ATOM	5071	HD2	LYS	A	335	148.651	84.779	149.989	1.00	0.00
ATOM	5072	CE	LYS	A	335	148.491	86.659	151.033	1.00	0.00
ATOM	5073	HE1	LYS	A	335	147.728	87.426	151.167	1.00	0.00
ATOM	5074	HE2	LYS	A	335	149.363	87.155	150.594	1.00	0.00
ATOM	5075	NZ	LYS	A	335	148.869	86.084	152.346	1.00	0.00
ATOM	5076	HZ1	LYS	A	335	149.190	86.817	152.966	1.00	0.00
ATOM	5077	HZ2	LYS	A	335	148.079	85.631	152.801	1.00	0.00
ATOM	5078	HZ3	LYS	A	335	149.630	85.423	152.257	1.00	0.00
ATOM	5079	C	LYS	A	335	144.000	85.805	152.088	1.00	0.00
ATOM	5080	O	LYS	A	335	144.006	86.903	152.648	1.00	0.00
ATOM	5081	N	VAL	A	336	144.026	84.669	152.775	1.00	0.00
ATOM	5082	H	VAL	A	336	144.010	83.804	152.261	1.00	0.00
ATOM	5083	CA	VAL	A	336	144.264	84.601	154.226	1.00	0.00
ATOM	5084	HA	VAL	A	336	143.553	85.262	154.721	1.00	0.00
ATOM	5085	CB	VAL	A	336	143.995	83.154	154.703	1.00	0.00
ATOM	5086	HB	VAL	A	336	143.004	82.884	154.337	1.00	0.00
ATOM	5087	CG1	VAL	A	336	144.986	82.126	154.140	1.00	0.00
ATOM	5088	1HG1	VAL	A	336	144.683	81.124	154.448	1.00	0.00
ATOM	5089	2HG1	VAL	A	336	144.998	82.154	153.052	1.00	0.00
ATOM	5090	3HG1	VAL	A	336	145.994	82.314	154.510	1.00	0.00
ATOM	5091	CG2	VAL	A	336	143.944	83.017	156.227	1.00	0.00
ATOM	5092	1HG2	VAL	A	336	143.506	82.053	156.489	1.00	0.00
ATOM	5093	2HG2	VAL	A	336	144.944	83.059	156.652	1.00	0.00
ATOM	5094	3HG2	VAL	A	336	143.326	83.806	156.655	1.00	0.00
ATOM	5095	C	VAL	A	336	145.688	85.084	154.578	1.00	0.00
ATOM	5096	O	VAL	A	336	146.616	84.985	153.763	1.00	0.00
ATOM	5097	N	CYS	A	337	145.911	85.516	155.819	1.00	0.00
ATOM	5098	H	CYS	A	337	145.128	85.761	156.406	1.00	0.00
ATOM	5099	CA	CYS	A	337	147.090	85.012	156.532	1.00	0.00
ATOM	5100	HA	CYS	A	337	147.361	84.060	156.071	1.00	0.00
ATOM	5101	CB	CYS	A	337	148.332	85.904	156.352	1.00	0.00
ATOM	5102	HB1	CYS	A	337	148.710	85.739	155.346	1.00	0.00
ATOM	5103	HB2	CYS	A	337	149.113	85.548	157.024	1.00	0.00
ATOM	5104	SG	CYS	A	337	148.199	87.686	156.597	1.00	0.00
ATOM	5105	C	CYS	A	337	146.805	84.644	157.991	1.00	0.00
ATOM	5106	O	CYS	A	337	145.734	84.943	158.522	1.00	0.00
ATOM	5107	N	ASN	A	338	147.747	83.934	158.609	1.00	0.00
ATOM	5108	H	ASN	A	338	148.586	83.708	158.096	1.00	0.00
ATOM	5109	CA	ASN	A	338	147.756	83.644	160.039	1.00	0.00
ATOM	5110	HA	ASN	A	338	146.844	83.113	160.322	1.00	0.00
ATOM	5111	CB	ASN	A	338	148.986	82.757	160.315	1.00	0.00
ATOM	5112	HB1	ASN	A	338	149.134	82.633	161.385	1.00	0.00
ATOM	5113	HB2	ASN	A	338	149.884	83.239	159.925	1.00	0.00
ATOM	5114	CG	ASN	A	338	148.870	81.370	159.716	1.00	0.00
ATOM	5115	OD1	ASN	A	338	147.808	80.773	159.679	1.00	0.00
ATOM	5116	ND2	ASN	A	338	149.948	80.815	159.225	1.00	0.00
ATOM	5117	1HD2	ASN	A	338	150.801	81.370	159.051	1.00	0.00
ATOM	5118	2HD2	ASN	A	338	149.844	79.922	158.786	1.00	0.00
ATOM	5119	C	ASN	A	338	147.858	84.922	160.881	1.00	0.00
ATOM	5120	O	ASN	A	338	148.279	85.966	160.383	1.00	0.00
ATOM	5121	N	GLY	A	339	147.545	84.807	162.173	1.00	0.00
ATOM	5122	H	GLY	A	339	147.122	83.945	162.490	1.00	0.00

```
ATOM   5123  CA  GLY A 339     147.873  85.828 163.166  1.00  0.00
ATOM   5124  HA1 GLY A 339     147.179  85.789 163.995  1.00  0.00
ATOM   5125  HA2 GLY A 339     147.767  86.812 162.715  1.00  0.00
ATOM   5126  C   GLY A 339     149.281  85.712 163.745  1.00  0.00
ATOM   5127  O   GLY A 339     149.943  84.666 163.696  1.00  0.00
ATOM   5128  N   ILE A 340     149.720  86.825 164.323  1.00  0.00
ATOM   5129  H   ILE A 340     149.052  87.588 164.394  1.00  0.00
ATOM   5130  CA  ILE A 340     151.059  87.063 164.876  1.00  0.00
ATOM   5131  HA  ILE A 340     151.788  86.662 164.173  1.00  0.00
ATOM   5132  CB  ILE A 340     151.317  88.588 164.969  1.00  0.00
ATOM   5133  HB  ILE A 340     150.582  89.032 165.643  1.00  0.00
ATOM   5134  CG2 ILE A 340     152.719  88.905 165.512  1.00  0.00
ATOM   5135 1HG2 ILE A 340     152.843  89.977 165.627  1.00  0.00
ATOM   5136 2HG2 ILE A 340     152.825  88.481 166.504  1.00  0.00
ATOM   5137 3HG2 ILE A 340     153.488  88.510 164.853  1.00  0.00
ATOM   5138  CG1 ILE A 340     151.129  89.196 163.560  1.00  0.00
ATOM   5139 1HG1 ILE A 340     150.071  89.169 163.302  1.00  0.00
ATOM   5140 2HG1 ILE A 340     151.672  88.594 162.829  1.00  0.00
ATOM   5141  CD  ILE A 340     151.592  90.642 163.399  1.00  0.00
ATOM   5142  HD1 ILE A 340     151.287  91.019 162.425  1.00  0.00
ATOM   5143  HD2 ILE A 340     151.152  91.259 164.178  1.00  0.00
ATOM   5144  HD3 ILE A 340     152.677  90.680 163.459  1.00  0.00
ATOM   5145  C   ILE A 340     151.241  86.291 166.191  1.00  0.00
ATOM   5146  O   ILE A 340     151.139  86.838 167.284  1.00  0.00
ATOM   5147  N   GLY A 341     151.421  84.977 166.050  1.00  0.00
ATOM   5148  H   GLY A 341     151.431  84.617 165.107  1.00  0.00
ATOM   5149  CA  GLY A 341     151.490  84.026 167.155  1.00  0.00
ATOM   5150  HA1 GLY A 341     150.746  84.307 167.902  1.00  0.00
ATOM   5151  HA2 GLY A 341     152.474  84.091 167.619  1.00  0.00
ATOM   5152  C   GLY A 341     151.232  82.562 166.773  1.00  0.00
ATOM   5153  O   GLY A 341     151.569  81.698 167.577  1.00  0.00
ATOM   5154  N   ILE A 342     150.666  82.240 165.590  1.00  0.00
ATOM   5155  H   ILE A 342     150.393  82.997 164.975  1.00  0.00
ATOM   5156  CA  ILE A 342     150.046  80.908 165.376  1.00  0.00
ATOM   5157  HA  ILE A 342     150.187  80.355 166.304  1.00  0.00
ATOM   5158  CB  ILE A 342     148.508  81.046 165.243  1.00  0.00
ATOM   5159  HB  ILE A 342     148.175  81.742 166.017  1.00  0.00
ATOM   5160  CG2 ILE A 342     148.082  81.623 163.883  1.00  0.00
ATOM   5161 1HG2 ILE A 342     147.024  81.887 163.901  1.00  0.00
ATOM   5162 2HG2 ILE A 342     148.648  82.523 163.671  1.00  0.00
ATOM   5163 3HG2 ILE A 342     148.247  80.894 163.088  1.00  0.00
ATOM   5164  CG1 ILE A 342     147.809  79.695 165.519  1.00  0.00
ATOM   5165 1HG1 ILE A 342     148.180  79.293 166.462  1.00  0.00
ATOM   5166 2HG1 ILE A 342     148.052  78.984 164.728  1.00  0.00
ATOM   5167  CD  ILE A 342     146.281  79.792 165.627  1.00  0.00
ATOM   5168  HD1 ILE A 342     145.881  78.823 165.927  1.00  0.00
ATOM   5169  HD2 ILE A 342     146.007  80.536 166.375  1.00  0.00
ATOM   5170  HD3 ILE A 342     145.848  80.064 164.665  1.00  0.00
ATOM   5171  C   ILE A 342     150.658  79.971 164.319  1.00  0.00
ATOM   5172  O   ILE A 342     150.787  78.773 164.572  1.00  0.00
ATOM   5173  N   GLY A 343     151.089  80.479 163.168  1.00  0.00
ATOM   5174  H   GLY A 343     150.998  81.473 163.013  1.00  0.00
ATOM   5175  CA  GLY A 343     151.726  79.708 162.090  1.00  0.00
ATOM   5176  HA1 GLY A 343     150.981  79.386 161.364  1.00  0.00
ATOM   5177  HA2 GLY A 343     152.234  78.833 162.497  1.00  0.00
ATOM   5178  C   GLY A 343     152.735  80.617 161.410  1.00  0.00
ATOM   5179  O   GLY A 343     152.456  81.807 161.330  1.00  0.00
ATOM   5180  N   GLU A 344     153.908  80.078 161.050  1.00  0.00
ATOM   5181  H   GLU A 344     153.949  79.072 161.069  1.00  0.00
ATOM   5182  CA  GLU A 344     155.211  80.753 160.835  1.00  0.00
ATOM   5183  HA  GLU A 344     155.969  79.976 160.922  1.00  0.00
```

ATOM	5184	CB	GLU	A	344	155.348	81.274	159.384	1.00	0.00
ATOM	5185	HB1	GLU	A	344	155.178	80.437	158.706	1.00	0.00
ATOM	5186	HB2	GLU	A	344	156.393	81.561	159.257	1.00	0.00
ATOM	5187	CG	GLU	A	344	154.485	82.475	158.944	1.00	0.00
ATOM	5188	HG1	GLU	A	344	155.150	83.225	158.519	1.00	0.00
ATOM	5189	HG2	GLU	A	344	154.009	82.957	159.790	1.00	0.00
ATOM	5190	CD	GLU	A	344	153.426	82.108	157.895	1.00	0.00
ATOM	5191	OE1	GLU	A	344	152.222	82.023	158.247	1.00	0.00
ATOM	5192	OE2	GLU	A	344	153.840	81.923	156.729	1.00	0.00
ATOM	5193	C	GLU	A	344	155.623	81.797	161.895	1.00	0.00
ATOM	5194	O	GLU	A	344	156.656	81.646	162.539	1.00	0.00
ATOM	5195	N	PHE	A	345	154.764	82.768	162.190	1.00	0.00
ATOM	5196	H	PHE	A	345	153.937	82.804	161.596	1.00	0.00
ATOM	5197	CA	PHE	A	345	154.770	83.724	163.296	1.00	0.00
ATOM	5198	HA	PHE	A	345	155.718	84.259	163.265	1.00	0.00
ATOM	5199	CB	PHE	A	345	153.621	84.726	163.074	1.00	0.00
ATOM	5200	HB1	PHE	A	345	153.791	85.597	163.704	1.00	0.00
ATOM	5201	HB2	PHE	A	345	152.699	84.251	163.414	1.00	0.00
ATOM	5202	CG	PHE	A	345	153.401	85.212	161.649	1.00	0.00
ATOM	5203	CD1	PHE	A	345	154.480	85.641	160.854	1.00	0.00
ATOM	5204	HD1	PHE	A	345	155.482	85.627	161.250	1.00	0.00
ATOM	5205	CE1	PHE	A	345	154.261	86.039	159.522	1.00	0.00
ATOM	5206	HE1	PHE	A	345	155.093	86.343	158.901	1.00	0.00
ATOM	5207	CZ	PHE	A	345	152.962	86.021	158.986	1.00	0.00
ATOM	5208	HZ	PHE	A	345	152.785	86.311	157.960	1.00	0.00
ATOM	5209	CE2	PHE	A	345	151.881	85.610	159.781	1.00	0.00
ATOM	5210	HE2	PHE	A	345	150.882	85.597	159.374	1.00	0.00
ATOM	5211	CD2	PHE	A	345	152.103	85.200	161.104	1.00	0.00
ATOM	5212	HD2	PHE	A	345	151.263	84.849	161.686	1.00	0.00
ATOM	5213	C	PHE	A	345	154.648	83.080	164.693	1.00	0.00
ATOM	5214	O	PHE	A	345	154.335	83.772	165.661	1.00	0.00
ATOM	5215	N	LYS	A	346	154.869	81.765	164.818	1.00	0.00
ATOM	5216	H	LYS	A	346	155.266	81.307	164.007	1.00	0.00
ATOM	5217	CA	LYS	A	346	154.572	80.941	166.006	1.00	0.00
ATOM	5218	HA	LYS	A	346	153.504	81.026	166.198	1.00	0.00
ATOM	5219	CB	LYS	A	346	154.890	79.465	165.696	1.00	0.00
ATOM	5220	HB1	LYS	A	346	155.948	79.292	165.889	1.00	0.00
ATOM	5221	HB2	LYS	A	346	154.719	79.269	164.636	1.00	0.00
ATOM	5222	CG	LYS	A	346	154.016	78.477	166.498	1.00	0.00
ATOM	5223	HG1	LYS	A	346	153.168	78.203	165.870	1.00	0.00
ATOM	5224	HG2	LYS	A	346	153.605	78.945	167.393	1.00	0.00
ATOM	5225	CD	LYS	A	346	154.766	77.190	166.895	1.00	0.00
ATOM	5226	HD1	LYS	A	346	155.584	77.006	166.196	1.00	0.00
ATOM	5227	HD2	LYS	A	346	154.073	76.352	166.804	1.00	0.00
ATOM	5228	CE	LYS	A	346	155.306	77.186	168.337	1.00	0.00
ATOM	5229	HE1	LYS	A	346	155.929	76.298	168.473	1.00	0.00
ATOM	5230	HE2	LYS	A	346	154.459	77.121	169.026	1.00	0.00
ATOM	5231	NZ	LYS	A	346	156.094	78.396	168.657	1.00	0.00
ATOM	5232	HZ1	LYS	A	346	156.847	78.599	167.992	1.00	0.00
ATOM	5233	HZ2	LYS	A	346	155.532	79.243	168.668	1.00	0.00
ATOM	5234	HZ3	LYS	A	346	156.587	78.386	169.543	1.00	0.00
ATOM	5235	C	LYS	A	346	155.272	81.391	167.300	1.00	0.00
ATOM	5236	O	LYS	A	346	154.937	80.907	168.377	1.00	0.00
ATOM	5237	N	ASP	A	347	156.265	82.269	167.197	1.00	0.00
ATOM	5238	H	ASP	A	347	156.514	82.544	166.264	1.00	0.00
ATOM	5239	CA	ASP	A	347	157.122	82.743	168.297	1.00	0.00
ATOM	5240	HA	ASP	A	347	156.642	82.523	169.252	1.00	0.00
ATOM	5241	CB	ASP	A	347	158.458	81.966	168.256	1.00	0.00
ATOM	5242	HB1	ASP	A	347	159.095	82.306	169.073	1.00	0.00
ATOM	5243	HB2	ASP	A	347	158.966	82.173	167.314	1.00	0.00
ATOM	5244	CG	ASP	A	347	158.228	80.461	168.399	1.00	0.00

ATOM	5245	OD1	ASP	A	347	158.067	79.976	169.541	1.00	0.00
ATOM	5246	OD2	ASP	A	347	158.034	79.770	167.370	1.00	0.00
ATOM	5247	C	ASP	A	347	157.314	84.277	168.234	1.00	0.00
ATOM	5248	O	ASP	A	347	158.285	84.838	168.729	1.00	0.00
ATOM	5249	N	SER	A	348	156.383	84.967	167.562	1.00	0.00
ATOM	5250	H	SER	A	348	155.565	84.458	167.243	1.00	0.00
ATOM	5251	CA	SER	A	348	156.554	86.324	167.038	1.00	0.00
ATOM	5252	HA	SER	A	348	157.623	86.526	166.945	1.00	0.00
ATOM	5253	CB	SER	A	348	155.951	86.365	165.637	1.00	0.00
ATOM	5254	HB1	SER	A	348	154.867	86.251	165.686	1.00	0.00
ATOM	5255	HB2	SER	A	348	156.368	85.552	165.043	1.00	0.00
ATOM	5256	OG	SER	A	348	156.255	87.576	164.986	1.00	0.00
ATOM	5257	HG	SER	A	348	155.710	88.268	165.389	1.00	0.00
ATOM	5258	C	SER	A	348	155.941	87.413	167.926	1.00	0.00
ATOM	5259	O	SER	A	348	154.811	87.833	167.718	1.00	0.00
ATOM	5260	N	LEU	A	349	156.717	87.943	168.866	1.00	0.00
ATOM	5261	H	LEU	A	349	157.607	87.480	168.991	1.00	0.00
ATOM	5262	CA	LEU	A	349	156.392	89.007	169.835	1.00	0.00
ATOM	5263	HA	LEU	A	349	155.681	88.588	170.548	1.00	0.00
ATOM	5264	CB	LEU	A	349	157.735	89.252	170.568	1.00	0.00
ATOM	5265	HB1	LEU	A	349	158.461	89.599	169.830	1.00	0.00
ATOM	5266	HB2	LEU	A	349	158.091	88.288	170.941	1.00	0.00
ATOM	5267	CG	LEU	A	349	157.764	90.236	171.746	1.00	0.00
ATOM	5268	HG	LEU	A	349	157.552	91.235	171.382	1.00	0.00
ATOM	5269	CD1	LEU	A	349	156.774	89.875	172.852	1.00	0.00
ATOM	5270	1HD1	LEU	A	349	156.856	90.595	173.665	1.00	0.00
ATOM	5271	2HD1	LEU	A	349	155.757	89.908	172.465	1.00	0.00
ATOM	5272	3HD1	LEU	A	349	156.984	88.875	173.234	1.00	0.00
ATOM	5273	CD2	LEU	A	349	159.172	90.258	172.349	1.00	0.00
ATOM	5274	1HD2	LEU	A	349	159.221	91.007	173.139	1.00	0.00
ATOM	5275	2HD2	LEU	A	349	159.421	89.281	172.764	1.00	0.00
ATOM	5276	3HD2	LEU	A	349	159.904	90.515	171.582	1.00	0.00
ATOM	5277	C	LEU	A	349	155.744	90.322	169.303	1.00	0.00
ATOM	5278	O	LEU	A	349	155.347	91.182	170.085	1.00	0.00
ATOM	5279	N	SER	A	350	155.677	90.522	167.987	1.00	0.00
ATOM	5280	H	SER	A	350	155.836	89.712	167.408	1.00	0.00
ATOM	5281	CA	SER	A	350	155.283	91.752	167.280	1.00	0.00
ATOM	5282	HA	SER	A	350	154.339	92.134	167.664	1.00	0.00
ATOM	5283	CB	SER	A	350	156.397	92.795	167.502	1.00	0.00
ATOM	5284	HB1	SER	A	350	157.327	92.432	167.062	1.00	0.00
ATOM	5285	HB2	SER	A	350	156.556	92.909	168.574	1.00	0.00
ATOM	5286	OG	SER	A	350	156.089	94.068	166.968	1.00	0.00
ATOM	5287	HG	SER	A	350	156.291	94.064	166.007	1.00	0.00
ATOM	5288	C	SER	A	350	155.176	91.465	165.784	1.00	0.00
ATOM	5289	O	SER	A	350	155.746	90.468	165.319	1.00	0.00
ATOM	5290	N	ILE	A	351	154.564	92.349	164.985	1.00	0.00
ATOM	5291	H	ILE	A	351	153.957	93.060	165.375	1.00	0.00
ATOM	5292	CA	ILE	A	351	154.962	92.428	163.566	1.00	0.00
ATOM	5293	HA	ILE	A	351	155.060	91.413	163.186	1.00	0.00
ATOM	5294	CB	ILE	A	351	153.911	93.150	162.688	1.00	0.00
ATOM	5295	HB	ILE	A	351	152.927	92.975	163.119	1.00	0.00
ATOM	5296	CG2	ILE	A	351	154.107	94.668	162.608	1.00	0.00
ATOM	5297	1HG2	ILE	A	351	153.222	95.137	162.182	1.00	0.00
ATOM	5298	2HG2	ILE	A	351	154.257	95.058	163.609	1.00	0.00
ATOM	5299	3HG2	ILE	A	351	154.974	94.909	161.997	1.00	0.00
ATOM	5300	CG1	ILE	A	351	153.919	92.513	161.282	1.00	0.00
ATOM	5301	1HG1	ILE	A	351	153.829	91.430	161.378	1.00	0.00
ATOM	5302	2HG1	ILE	A	351	154.868	92.732	160.790	1.00	0.00
ATOM	5303	CD	ILE	A	351	152.777	92.987	160.389	1.00	0.00
ATOM	5304	HD1	ILE	A	351	152.805	92.436	159.449	1.00	0.00
ATOM	5305	HD2	ILE	A	351	151.817	92.811	160.873	1.00	0.00

```
ATOM   5306  HD3 ILE A 351     152.891  94.047 160.181  1.00  0.00
ATOM   5307  C   ILE A 351     156.351  93.083 163.551  1.00  0.00
ATOM   5308  O   ILE A 351     156.617  93.983 164.359  1.00  0.00
ATOM   5309  N   ASN A 352     157.264  92.624 162.704  1.00  0.00
ATOM   5310  H   ASN A 352     156.996  91.903 162.042  1.00  0.00
ATOM   5311  CA  ASN A 352     158.604  93.178 162.590  1.00  0.00
ATOM   5312  HA  ASN A 352     158.511  94.259 162.681  1.00  0.00
ATOM   5313  CB  ASN A 352     159.504  92.691 163.758  1.00  0.00
ATOM   5314  HB1 ASN A 352     159.890  91.696 163.543  1.00  0.00
ATOM   5315  HB2 ASN A 352     158.925  92.642 164.679  1.00  0.00
ATOM   5316  CG  ASN A 352     160.660  93.650 164.011  1.00  0.00
ATOM   5317  OD1 ASN A 352     160.713  94.735 163.467  1.00  0.00
ATOM   5318  ND2 ASN A 352     161.654  93.311 164.781  1.00  0.00
ATOM   5319 1HD2 ASN A 352     161.716  92.421 165.247  1.00  0.00
ATOM   5320 2HD2 ASN A 352     162.401  93.986 164.835  1.00  0.00
ATOM   5321  C   ASN A 352     159.231  92.910 161.202  1.00  0.00
ATOM   5322  O   ASN A 352     158.710  92.151 160.380  1.00  0.00
ATOM   5323  N   ALA A 353     160.390  93.523 160.958  1.00  0.00
ATOM   5324  H   ALA A 353     160.743  94.139 161.687  1.00  0.00
ATOM   5325  CA  ALA A 353     161.112  93.554 159.695  1.00  0.00
ATOM   5326  HA  ALA A 353     160.519  94.138 158.989  1.00  0.00
ATOM   5327  CB  ALA A 353     162.425  94.303 159.945  1.00  0.00
ATOM   5328  HB1 ALA A 353     162.923  94.517 159.000  1.00  0.00
ATOM   5329  HB2 ALA A 353     162.223  95.243 160.454  1.00  0.00
ATOM   5330  HB3 ALA A 353     163.086  93.704 160.572  1.00  0.00
ATOM   5331  C   ALA A 353     161.395  92.189 159.037  1.00  0.00
ATOM   5332  O   ALA A 353     161.661  92.159 157.834  1.00  0.00
ATOM   5333  N   THR A 354     161.332  91.078 159.777  1.00  0.00
ATOM   5334  H   THR A 354     161.125  91.173 160.759  1.00  0.00
ATOM   5335  CA  THR A 354     161.559  89.716 159.265  1.00  0.00
ATOM   5336  HA  THR A 354     162.068  89.775 158.303  1.00  0.00
ATOM   5337  CB  THR A 354     162.464  88.895 160.199  1.00  0.00
ATOM   5338  HB  THR A 354     162.590  87.894 159.783  1.00  0.00
ATOM   5339  CG2 THR A 354     163.850  89.524 160.363  1.00  0.00
ATOM   5340 1HG2 THR A 354     164.492  88.855 160.938  1.00  0.00
ATOM   5341 2HG2 THR A 354     164.302  89.678 159.382  1.00  0.00
ATOM   5342 3HG2 THR A 354     163.777  90.480 160.881  1.00  0.00
ATOM   5343  OG1 THR A 354     161.875  88.793 161.475  1.00  0.00
ATOM   5344  HG1 THR A 354     162.265  88.032 161.918  1.00  0.00
ATOM   5345  C   THR A 354     160.270  88.921 159.016  1.00  0.00
ATOM   5346  O   THR A 354     160.343  87.852 158.417  1.00  0.00
ATOM   5347  N   ASN A 355     159.091  89.429 159.410  1.00  0.00
ATOM   5348  H   ASN A 355     159.090  90.325 159.888  1.00  0.00
ATOM   5349  CA  ASN A 355     157.802  88.726 159.273  1.00  0.00
ATOM   5350  HA  ASN A 355     157.967  87.798 158.723  1.00  0.00
ATOM   5351  CB  ASN A 355     157.273  88.309 160.664  1.00  0.00
ATOM   5352  HB1 ASN A 355     158.053  87.750 161.181  1.00  0.00
ATOM   5353  HB2 ASN A 355     156.428  87.643 160.519  1.00  0.00
ATOM   5354  CG  ASN A 355     156.795  89.440 161.563  1.00  0.00
ATOM   5355  OD1 ASN A 355     156.416  90.511 161.131  1.00  0.00
ATOM   5356  ND2 ASN A 355     156.809  89.258 162.858  1.00  0.00
ATOM   5357 1HD2 ASN A 355     157.014  88.356 163.271  1.00  0.00
ATOM   5358 2HD2 ASN A 355     156.415  89.962 163.468  1.00  0.00
ATOM   5359  C   ASN A 355     156.758  89.490 158.433  1.00  0.00
ATOM   5360  O   ASN A 355     155.865  88.893 157.837  1.00  0.00
ATOM   5361  N   ILE A 356     156.921  90.806 158.278  1.00  0.00
ATOM   5362  H   ILE A 356     157.569  91.274 158.901  1.00  0.00
ATOM   5363  CA  ILE A 356     156.069  91.631 157.410  1.00  0.00
ATOM   5364  HA  ILE A 356     155.038  91.378 157.661  1.00  0.00
ATOM   5365  CB  ILE A 356     156.285  93.116 157.759  1.00  0.00
ATOM   5366  HB  ILE A 356     156.248  93.207 158.845  1.00  0.00
```

```
ATOM   5367  CG2 ILE A 356     157.667  93.597 157.289  1.00  0.00
ATOM   5368 1HG2 ILE A 356     157.786  94.660 157.477  1.00  0.00
ATOM   5369 2HG2 ILE A 356     158.455  93.064 157.816  1.00  0.00
ATOM   5370 3HG2 ILE A 356     157.778  93.439 156.222  1.00  0.00
ATOM   5371  CG1 ILE A 356     155.131  93.965 157.192  1.00  0.00
ATOM   5372 1HG1 ILE A 356     154.194  93.440 157.375  1.00  0.00
ATOM   5373 2HG1 ILE A 356     155.248  94.076 156.117  1.00  0.00
ATOM   5374  CD  ILE A 356     155.023  95.352 157.827  1.00  0.00
ATOM   5375  HD1 ILE A 356     154.103  95.824 157.493  1.00  0.00
ATOM   5376  HD2 ILE A 356     155.001  95.270 158.913  1.00  0.00
ATOM   5377  HD3 ILE A 356     155.860  95.972 157.516  1.00  0.00
ATOM   5378  C   ILE A 356     156.240  91.319 155.901  1.00  0.00
ATOM   5379  O   ILE A 356     155.424  91.693 155.058  1.00  0.00
ATOM   5380  N   LYS A 357     157.307  90.601 155.531  1.00  0.00
ATOM   5381  H   LYS A 357     157.941  90.303 156.262  1.00  0.00
ATOM   5382  CA  LYS A 357     157.498  90.079 154.173  1.00  0.00
ATOM   5383  HA  LYS A 357     157.358  90.872 153.444  1.00  0.00
ATOM   5384  CB  LYS A 357     158.925  89.518 154.009  1.00  0.00
ATOM   5385  HB1 LYS A 357     158.924  88.821 153.169  1.00  0.00
ATOM   5386  HB2 LYS A 357     159.214  88.952 154.896  1.00  0.00
ATOM   5387  CG  LYS A 357     159.993  90.582 153.691  1.00  0.00
ATOM   5388  HG1 LYS A 357     159.669  91.158 152.824  1.00  0.00
ATOM   5389  HG2 LYS A 357     160.915  90.065 153.421  1.00  0.00
ATOM   5390  CD  LYS A 357     160.309  91.548 154.842  1.00  0.00
ATOM   5391  HD1 LYS A 357     160.652  90.980 155.707  1.00  0.00
ATOM   5392  HD2 LYS A 357     159.415  92.108 155.110  1.00  0.00
ATOM   5393  CE  LYS A 357     161.393  92.544 154.412  1.00  0.00
ATOM   5394  HE1 LYS A 357     161.082  93.002 153.468  1.00  0.00
ATOM   5395  HE2 LYS A 357     162.328  92.003 154.249  1.00  0.00
ATOM   5396  NZ  LYS A 357     161.578  93.609 155.422  1.00  0.00
ATOM   5397  HZ1 LYS A 357     162.300  94.258 155.157  1.00  0.00
ATOM   5398  HZ2 LYS A 357     160.732  94.187 155.458  1.00  0.00
ATOM   5399  HZ3 LYS A 357     161.738  93.223 156.348  1.00  0.00
ATOM   5400  C   LYS A 357     156.460  89.003 153.832  1.00  0.00
ATOM   5401  O   LYS A 357     156.025  88.954 152.693  1.00  0.00
ATOM   5402  N   HIS A 358     155.998  88.222 154.808  1.00  0.00
ATOM   5403  H   HIS A 358     156.451  88.248 155.714  1.00  0.00
ATOM   5404  CA  HIS A 358     154.942  87.218 154.649  1.00  0.00
ATOM   5405  HA  HIS A 358     155.125  86.618 153.757  1.00  0.00
ATOM   5406  CB  HIS A 358     154.959  86.294 155.873  1.00  0.00
ATOM   5407  HB1 HIS A 358     154.238  85.491 155.719  1.00  0.00
ATOM   5408  HB2 HIS A 358     154.631  86.867 156.737  1.00  0.00
ATOM   5409  CG  HIS A 358     156.297  85.683 156.219  1.00  0.00
ATOM   5410  ND1 HIS A 358     157.472  86.385 156.528  1.00  0.00
ATOM   5411  CE1 HIS A 358     158.371  85.453 156.874  1.00  0.00
ATOM   5412  HE1 HIS A 358     159.386  85.667 157.183  1.00  0.00
ATOM   5413  NE2 HIS A 358     157.828  84.225 156.794  1.00  0.00
ATOM   5414  HE2 HIS A 358     158.293  83.360 157.023  1.00  0.00
ATOM   5415  CD2 HIS A 358     156.523  84.349 156.381  1.00  0.00
ATOM   5416  HD2 HIS A 358     155.790  83.556 156.259  1.00  0.00
ATOM   5417  C   HIS A 358     153.543  87.843 154.524  1.00  0.00
ATOM   5418  O   HIS A 358     152.675  87.311 153.836  1.00  0.00
ATOM   5419  N   PHE A 359     153.321  88.998 155.162  1.00  0.00
ATOM   5420  H   PHE A 359     154.059  89.353 155.753  1.00  0.00
ATOM   5421  CA  PHE A 359     152.033  89.706 155.179  1.00  0.00
ATOM   5422  HA  PHE A 359     151.261  89.006 155.499  1.00  0.00
ATOM   5423  CB  PHE A 359     152.118  90.834 156.222  1.00  0.00
ATOM   5424  HB1 PHE A 359     151.533  91.689 155.880  1.00  0.00
ATOM   5425  HB2 PHE A 359     153.145  91.182 156.323  1.00  0.00
ATOM   5426  CG  PHE A 359     151.597  90.447 157.585  1.00  0.00
ATOM   5427  CD1 PHE A 359     152.323  89.571 158.414  1.00  0.00
```

ATOM	5428	HD1	PHE	A	359	153.275	89.175	158.095	1.00	0.00
ATOM	5429	CE1	PHE	A	359	151.794	89.190	159.658	1.00	0.00
ATOM	5430	HE1	PHE	A	359	152.336	88.498	160.288	1.00	0.00
ATOM	5431	CZ	PHE	A	359	150.552	89.695	160.077	1.00	0.00
ATOM	5432	HZ	PHE	A	359	150.133	89.384	161.017	1.00	0.00
ATOM	5433	CE2	PHE	A	359	149.844	90.596	159.267	1.00	0.00
ATOM	5434	HE2	PHE	A	359	148.894	90.998	159.592	1.00	0.00
ATOM	5435	CD2	PHE	A	359	150.357	90.955	158.014	1.00	0.00
ATOM	5436	HD2	PHE	A	359	149.788	91.619	157.374	1.00	0.00
ATOM	5437	C	PHE	A	359	151.576	90.268	153.818	1.00	0.00
ATOM	5438	O	PHE	A	359	150.476	90.821	153.719	1.00	0.00
ATOM	5439	N	LYS	A	360	152.397	90.188	152.770	1.00	0.00
ATOM	5440	H	LYS	A	360	153.230	89.630	152.889	1.00	0.00
ATOM	5441	CA	LYS	A	360	152.131	90.795	151.455	1.00	0.00
ATOM	5442	HA	LYS	A	360	152.070	91.875	151.584	1.00	0.00
ATOM	5443	CB	LYS	A	360	153.286	90.469	150.486	1.00	0.00
ATOM	5444	HB1	LYS	A	360	153.037	90.835	149.489	1.00	0.00
ATOM	5445	HB2	LYS	A	360	153.379	89.383	150.425	1.00	0.00
ATOM	5446	CG	LYS	A	360	154.635	91.052	150.926	1.00	0.00
ATOM	5447	HG1	LYS	A	360	155.426	90.484	150.437	1.00	0.00
ATOM	5448	HG2	LYS	A	360	154.745	90.931	152.000	1.00	0.00
ATOM	5449	CD	LYS	A	360	154.841	92.534	150.587	1.00	0.00
ATOM	5450	HD1	LYS	A	360	154.019	93.137	150.966	1.00	0.00
ATOM	5451	HD2	LYS	A	360	154.870	92.643	149.502	1.00	0.00
ATOM	5452	CE	LYS	A	360	156.176	93.013	151.174	1.00	0.00
ATOM	5453	HE1	LYS	A	360	156.435	93.980	150.730	1.00	0.00
ATOM	5454	HE2	LYS	A	360	156.957	92.301	150.887	1.00	0.00
ATOM	5455	NZ	LYS	A	360	156.144	93.149	152.651	1.00	0.00
ATOM	5456	HZ1	LYS	A	360	157.083	93.339	153.010	1.00	0.00
ATOM	5457	HZ2	LYS	A	360	155.582	93.931	152.947	1.00	0.00
ATOM	5458	HZ3	LYS	A	360	155.806	92.324	153.139	1.00	0.00
ATOM	5459	C	LYS	A	360	150.789	90.327	150.884	1.00	0.00
ATOM	5460	O	LYS	A	360	150.524	89.127	150.771	1.00	0.00
ATOM	5461	N	ASN	A	361	149.935	91.279	150.512	1.00	0.00
ATOM	5462	H	ASN	A	361	150.172	92.235	150.723	1.00	0.00
ATOM	5463	CA	ASN	A	361	148.606	91.058	149.945	1.00	0.00
ATOM	5464	HA	ASN	A	361	148.084	92.010	150.054	1.00	0.00
ATOM	5465	CB	ASN	A	361	148.721	90.794	148.428	1.00	0.00
ATOM	5466	HB1	ASN	A	361	148.914	89.738	148.246	1.00	0.00
ATOM	5467	HB2	ASN	A	361	149.547	91.370	148.014	1.00	0.00
ATOM	5468	CG	ASN	A	361	147.470	91.240	147.689	1.00	0.00
ATOM	5469	OD1	ASN	A	361	147.147	92.415	147.658	1.00	0.00
ATOM	5470	ND2	ASN	A	361	146.744	90.354	147.047	1.00	0.00
ATOM	5471	1HD2	ASN	A	361	146.935	89.377	147.154	1.00	0.00
ATOM	5472	2HD2	ASN	A	361	145.938	90.703	146.562	1.00	0.00
ATOM	5473	C	ASN	A	361	147.732	90.037	150.713	1.00	0.00
ATOM	5474	O	ASN	A	361	147.017	89.250	150.093	1.00	0.00
ATOM	5475	N	CYS	A	362	147.799	89.983	152.052	1.00	0.00
ATOM	5476	H	CYS	A	362	148.422	90.620	152.538	1.00	0.00
ATOM	5477	CA	CYS	A	362	146.716	89.372	152.837	1.00	0.00
ATOM	5478	HA	CYS	A	362	146.504	88.368	152.472	1.00	0.00
ATOM	5479	CB	CYS	A	362	147.091	89.292	154.320	1.00	0.00
ATOM	5480	HB1	CYS	A	362	146.267	88.820	154.858	1.00	0.00
ATOM	5481	HB2	CYS	A	362	147.197	90.302	154.718	1.00	0.00
ATOM	5482	SG	CYS	A	362	148.590	88.384	154.727	1.00	0.00
ATOM	5483	C	CYS	A	362	145.430	90.215	152.715	1.00	0.00
ATOM	5484	O	CYS	A	362	145.517	91.412	152.458	1.00	0.00
ATOM	5485	N	THR	A	363	144.250	89.653	152.974	1.00	0.00
ATOM	5486	H	THR	A	363	144.200	88.648	153.099	1.00	0.00
ATOM	5487	CA	THR	A	363	143.055	90.474	153.227	1.00	0.00
ATOM	5488	HA	THR	A	363	143.420	91.444	153.545	1.00	0.00

ATOM	5489	CB	THR	A	363	142.240	90.760	151.963	1.00	0.00
ATOM	5490	HB	THR	A	363	142.920	90.926	151.126	1.00	0.00
ATOM	5491	CG2	THR	A	363	141.257	89.658	151.601	1.00	0.00
ATOM	5492	1HG2	THR	A	363	140.775	89.938	150.666	1.00	0.00
ATOM	5493	2HG2	THR	A	363	141.800	88.724	151.489	1.00	0.00
ATOM	5494	3HG2	THR	A	363	140.493	89.557	152.370	1.00	0.00
ATOM	5495	OG1	THR	A	363	141.496	91.931	152.167	1.00	0.00
ATOM	5496	HG1	THR	A	363	142.136	92.667	152.182	1.00	0.00
ATOM	5497	C	THR	A	363	142.174	90.013	154.398	1.00	0.00
ATOM	5498	O	THR	A	363	141.482	90.835	154.993	1.00	0.00
ATOM	5499	N	SER	A	364	142.292	88.755	154.828	1.00	0.00
ATOM	5500	H	SER	A	364	142.889	88.117	154.322	1.00	0.00
ATOM	5501	CA	SER	A	364	141.988	88.415	156.228	1.00	0.00
ATOM	5502	HA	SER	A	364	141.390	89.192	156.695	1.00	0.00
ATOM	5503	CB	SER	A	364	141.215	87.099	156.367	1.00	0.00
ATOM	5504	HB1	SER	A	364	141.060	86.866	157.422	1.00	0.00
ATOM	5505	HB2	SER	A	364	141.789	86.292	155.911	1.00	0.00
ATOM	5506	OG	SER	A	364	139.966	87.199	155.737	1.00	0.00
ATOM	5507	HG	SER	A	364	139.257	87.355	156.412	1.00	0.00
ATOM	5508	C	SER	A	364	143.272	88.269	157.017	1.00	0.00
ATOM	5509	O	SER	A	364	144.323	88.085	156.418	1.00	0.00
ATOM	5510	N	ILE	A	365	143.170	88.246	158.340	1.00	0.00
ATOM	5511	H	ILE	A	365	142.321	88.630	158.739	1.00	0.00
ATOM	5512	CA	ILE	A	365	144.184	87.759	159.271	1.00	0.00
ATOM	5513	HA	ILE	A	365	144.788	87.047	158.721	1.00	0.00
ATOM	5514	CB	ILE	A	365	145.178	88.869	159.704	1.00	0.00
ATOM	5515	HB	ILE	A	365	145.929	88.917	158.914	1.00	0.00
ATOM	5516	CG2	ILE	A	365	144.550	90.270	159.789	1.00	0.00
ATOM	5517	1HG2	ILE	A	365	145.258	90.996	160.176	1.00	0.00
ATOM	5518	2HG2	ILE	A	365	144.239	90.606	158.801	1.00	0.00
ATOM	5519	3HG2	ILE	A	365	143.685	90.237	160.437	1.00	0.00
ATOM	5520	CG1	ILE	A	365	145.905	88.507	161.016	1.00	0.00
ATOM	5521	1HG1	ILE	A	365	146.094	87.435	161.051	1.00	0.00
ATOM	5522	2HG1	ILE	A	365	145.291	88.777	161.877	1.00	0.00
ATOM	5523	CD	ILE	A	365	147.266	89.195	161.125	1.00	0.00
ATOM	5524	HD1	ILE	A	365	147.736	88.942	162.071	1.00	0.00
ATOM	5525	HD2	ILE	A	365	147.895	88.840	160.310	1.00	0.00
ATOM	5526	HD3	ILE	A	365	147.159	90.275	161.064	1.00	0.00
ATOM	5527	C	ILE	A	365	143.504	86.986	160.414	1.00	0.00
ATOM	5528	O	ILE	A	365	142.590	87.461	161.099	1.00	0.00
ATOM	5529	N	SER	A	366	143.949	85.740	160.568	1.00	0.00
ATOM	5530	H	SER	A	366	144.677	85.451	159.923	1.00	0.00
ATOM	5531	CA	SER	A	366	143.298	84.652	161.307	1.00	0.00
ATOM	5532	HA	SER	A	366	142.303	84.967	161.615	1.00	0.00
ATOM	5533	CB	SER	A	366	143.110	83.446	160.392	1.00	0.00
ATOM	5534	HB1	SER	A	366	142.688	82.616	160.958	1.00	0.00
ATOM	5535	HB2	SER	A	366	144.062	83.142	159.957	1.00	0.00
ATOM	5536	OG	SER	A	366	142.208	83.821	159.368	1.00	0.00
ATOM	5537	HG	SER	A	366	141.992	83.044	158.848	1.00	0.00
ATOM	5538	C	SER	A	366	144.084	84.296	162.563	1.00	0.00
ATOM	5539	O	SER	A	366	144.952	83.426	162.589	1.00	0.00
ATOM	5540	N	GLY	A	367	143.791	85.097	163.579	1.00	0.00
ATOM	5541	H	GLY	A	367	143.122	85.834	163.396	1.00	0.00
ATOM	5542	CA	GLY	A	367	144.590	85.298	164.780	1.00	0.00
ATOM	5543	HA1	GLY	A	367	145.575	84.848	164.680	1.00	0.00
ATOM	5544	HA2	GLY	A	367	144.094	84.849	165.640	1.00	0.00
ATOM	5545	C	GLY	A	367	144.691	86.804	165.003	1.00	0.00
ATOM	5546	O	GLY	A	367	143.915	87.582	164.440	1.00	0.00
ATOM	5547	N	ASP	A	368	145.611	87.207	165.852	1.00	0.00
ATOM	5548	H	ASP	A	368	146.147	86.503	166.353	1.00	0.00
ATOM	5549	CA	ASP	A	368	145.823	88.588	166.300	1.00	0.00

ATOM	5550	HA	ASP	A	368	144.881	89.126	166.250	1.00	0.00
ATOM	5551	CB	ASP	A	368	146.301	88.543	167.768	1.00	0.00
ATOM	5552	HB1	ASP	A	368	145.836	89.370	168.294	1.00	0.00
ATOM	5553	HB2	ASP	A	368	147.382	88.690	167.802	1.00	0.00
ATOM	5554	CG	ASP	A	368	145.968	87.212	168.453	1.00	0.00
ATOM	5555	OD1	ASP	A	368	144.794	87.016	168.843	1.00	0.00
ATOM	5556	OD2	ASP	A	368	146.815	86.298	168.367	1.00	0.00
ATOM	5557	C	ASP	A	368	146.840	89.318	165.422	1.00	0.00
ATOM	5558	O	ASP	A	368	147.492	88.679	164.613	1.00	0.00
ATOM	5559	N	LEU	A	369	147.029	90.627	165.594	1.00	0.00
ATOM	5560	H	LEU	A	369	146.398	91.109	166.221	1.00	0.00
ATOM	5561	CA	LEU	A	369	148.069	91.431	164.937	1.00	0.00
ATOM	5562	HA	LEU	A	369	148.879	90.776	164.621	1.00	0.00
ATOM	5563	CB	LEU	A	369	147.493	92.130	163.689	1.00	0.00
ATOM	5564	HB1	LEU	A	369	146.790	92.903	164.005	1.00	0.00
ATOM	5565	HB2	LEU	A	369	146.933	91.381	163.131	1.00	0.00
ATOM	5566	CG	LEU	A	369	148.504	92.751	162.699	1.00	0.00
ATOM	5567	HG	LEU	A	369	149.130	91.954	162.299	1.00	0.00
ATOM	5568	CD1	LEU	A	369	147.732	93.379	161.533	1.00	0.00
ATOM	5569	1HD1	LEU	A	369	148.437	93.746	160.785	1.00	0.00
ATOM	5570	2HD1	LEU	A	369	147.087	92.634	161.067	1.00	0.00
ATOM	5571	3HD1	LEU	A	369	147.115	94.203	161.894	1.00	0.00
ATOM	5572	CD2	LEU	A	369	149.397	93.854	163.267	1.00	0.00
ATOM	5573	1HD2	LEU	A	369	149.981	94.317	162.472	1.00	0.00
ATOM	5574	2HD2	LEU	A	369	148.790	94.611	163.763	1.00	0.00
ATOM	5575	3HD2	LEU	A	369	150.100	93.433	163.979	1.00	0.00
ATOM	5576	C	LEU	A	369	148.608	92.419	165.971	1.00	0.00
ATOM	5577	O	LEU	A	369	147.975	93.430	166.298	1.00	0.00
ATOM	5578	N	HIS	A	370	149.775	92.077	166.502	1.00	0.00
ATOM	5579	H	HIS	A	370	150.200	91.213	166.204	1.00	0.00
ATOM	5580	CA	HIS	A	370	150.552	92.929	167.384	1.00	0.00
ATOM	5581	HA	HIS	A	370	149.895	93.600	167.935	1.00	0.00
ATOM	5582	CB	HIS	A	370	151.300	92.017	168.374	1.00	0.00
ATOM	5583	HB1	HIS	A	370	152.157	91.559	167.882	1.00	0.00
ATOM	5584	HB2	HIS	A	370	150.628	91.214	168.678	1.00	0.00
ATOM	5585	CG	HIS	A	370	151.747	92.706	169.633	1.00	0.00
ATOM	5586	ND1	HIS	A	370	151.654	94.075	169.886	1.00	0.00
ATOM	5587	CE1	HIS	A	370	151.935	94.223	171.186	1.00	0.00
ATOM	5588	HE1	HIS	A	370	151.900	95.165	171.714	1.00	0.00
ATOM	5589	NE2	HIS	A	370	152.236	93.040	171.746	1.00	0.00
ATOM	5590	HE2	HIS	A	370	152.459	92.905	172.720	1.00	0.00
ATOM	5591	CD2	HIS	A	370	152.126	92.068	170.777	1.00	0.00
ATOM	5592	HD2	HIS	A	370	152.257	91.001	170.898	1.00	0.00
ATOM	5593	C	HIS	A	370	151.518	93.768	166.562	1.00	0.00
ATOM	5594	O	HIS	A	370	152.123	93.268	165.624	1.00	0.00
ATOM	5595	N	ILE	A	371	151.746	95.014	166.945	1.00	0.00
ATOM	5596	H	ILE	A	371	151.177	95.383	167.700	1.00	0.00
ATOM	5597	CA	ILE	A	371	152.906	95.804	166.532	1.00	0.00
ATOM	5598	HA	ILE	A	371	153.697	95.125	166.214	1.00	0.00
ATOM	5599	CB	ILE	A	371	152.584	96.725	165.336	1.00	0.00
ATOM	5600	HB	ILE	A	371	152.351	96.066	164.498	1.00	0.00
ATOM	5601	CG2	ILE	A	371	151.361	97.645	165.508	1.00	0.00
ATOM	5602	1HG2	ILE	A	371	151.133	98.137	164.565	1.00	0.00
ATOM	5603	2HG2	ILE	A	371	150.495	97.054	165.802	1.00	0.00
ATOM	5604	3HG2	ILE	A	371	151.559	98.397	166.268	1.00	0.00
ATOM	5605	CG1	ILE	A	371	153.854	97.525	164.980	1.00	0.00
ATOM	5606	1HG1	ILE	A	371	154.707	96.846	164.961	1.00	0.00
ATOM	5607	2HG1	ILE	A	371	154.048	98.280	165.743	1.00	0.00
ATOM	5608	CD	ILE	A	371	153.766	98.206	163.615	1.00	0.00
ATOM	5609	HD1	ILE	A	371	154.770	98.475	163.301	1.00	0.00
ATOM	5610	HD2	ILE	A	371	153.345	97.523	162.878	1.00	0.00

```
ATOM   5611  HD3 ILE A 371     153.151  99.101 163.679  1.00  0.00
ATOM   5612  C   ILE A 371     153.398  96.520 167.789  1.00  0.00
ATOM   5613  O   ILE A 371     152.686  97.355 168.327  1.00  0.00
ATOM   5614  N   LEU A 372     154.580  96.169 168.303  1.00  0.00
ATOM   5615  H   LEU A 372     155.118  95.459 167.815  1.00  0.00
ATOM   5616  CA  LEU A 372     155.159  96.755 169.522  1.00  0.00
ATOM   5617  HA  LEU A 372     154.355  97.096 170.170  1.00  0.00
ATOM   5618  CB  LEU A 372     156.021  95.690 170.245  1.00  0.00
ATOM   5619  HB1 LEU A 372     156.767  96.179 170.864  1.00  0.00
ATOM   5620  HB2 LEU A 372     156.565  95.115 169.497  1.00  0.00
ATOM   5621  CG  LEU A 372     155.288  94.721 171.184  1.00  0.00
ATOM   5622  HG  LEU A 372     154.545  94.171 170.615  1.00  0.00
ATOM   5623  CD1 LEU A 372     156.301  93.743 171.768  1.00  0.00
ATOM   5624 1HD1 LEU A 372     155.776  92.954 172.308  1.00  0.00
ATOM   5625 2HD1 LEU A 372     156.860  93.285 170.954  1.00  0.00
ATOM   5626 3HD1 LEU A 372     156.994  94.247 172.439  1.00  0.00
ATOM   5627  CD2 LEU A 372     154.665  95.419 172.395  1.00  0.00
ATOM   5628 1HD2 LEU A 372     154.181  94.682 173.034  1.00  0.00
ATOM   5629 2HD2 LEU A 372     155.425  95.931 172.983  1.00  0.00
ATOM   5630 3HD2 LEU A 372     153.912  96.130 172.074  1.00  0.00
ATOM   5631  C   LEU A 372     156.087  97.955 169.226  1.00  0.00
ATOM   5632  O   LEU A 372     156.634  98.054 168.131  1.00  0.00
ATOM   5633  N   PRO A 373     156.489  98.730 170.257  1.00  0.00
ATOM   5634  CD  PRO A 373     155.884  98.842 171.576  1.00  0.00
ATOM   5635  HD1 PRO A 373     155.559  97.884 171.969  1.00  0.00
ATOM   5636  HD2 PRO A 373     155.036  99.520 171.518  1.00  0.00
ATOM   5637  CG  PRO A 373     156.954  99.446 172.478  1.00  0.00
ATOM   5638  HG1 PRO A 373     157.604  98.657 172.859  1.00  0.00
ATOM   5639  HG2 PRO A 373     156.517 100.013 173.300  1.00  0.00
ATOM   5640  CB  PRO A 373     157.733 100.341 171.521  1.00  0.00
ATOM   5641  HB1 PRO A 373     158.755 100.506 171.865  1.00  0.00
ATOM   5642  HB2 PRO A 373     157.214 101.294 171.414  1.00  0.00
ATOM   5643  CA  PRO A 373     157.678  99.581 170.190  1.00  0.00
ATOM   5644  HA  PRO A 373     157.537 100.310 169.391  1.00  0.00
ATOM   5645  C   PRO A 373     158.998  98.848 169.890  1.00  0.00
ATOM   5646  O   PRO A 373     159.960  99.472 169.453  1.00  0.00
ATOM   5647  N   VAL A 374     159.049  97.524 170.075  1.00  0.00
ATOM   5648  H   VAL A 374     158.230  97.068 170.441  1.00  0.00
ATOM   5649  CA  VAL A 374     160.156  96.662 169.617  1.00  0.00
ATOM   5650  HA  VAL A 374     161.091  97.032 170.037  1.00  0.00
ATOM   5651  CB  VAL A 374     159.955  95.212 170.106  1.00  0.00
ATOM   5652  HB  VAL A 374     159.063  94.794 169.638  1.00  0.00
ATOM   5653  CG1 VAL A 374     161.156  94.334 169.763  1.00  0.00
ATOM   5654 1HG1 VAL A 374     161.017  93.327 170.159  1.00  0.00
ATOM   5655 2HG1 VAL A 374     161.278  94.252 168.684  1.00  0.00
ATOM   5656 3HG1 VAL A 374     162.067  94.757 170.189  1.00  0.00
ATOM   5657  CG2 VAL A 374     159.776  95.155 171.633  1.00  0.00
ATOM   5658 1HG2 VAL A 374     159.659  94.117 171.946  1.00  0.00
ATOM   5659 2HG2 VAL A 374     160.654  95.572 172.126  1.00  0.00
ATOM   5660 3HG2 VAL A 374     158.893  95.705 171.950  1.00  0.00
ATOM   5661  C   VAL A 374     160.307  96.718 168.087  1.00  0.00
ATOM   5662  O   VAL A 374     161.427  96.775 167.584  1.00  0.00
ATOM   5663  N   ALA A 375     159.197  96.817 167.343  1.00  0.00
ATOM   5664  H   ALA A 375     158.290  96.849 167.789  1.00  0.00
ATOM   5665  CA  ALA A 375     159.231  96.972 165.890  1.00  0.00
ATOM   5666  HA  ALA A 375     159.674  96.074 165.458  1.00  0.00
ATOM   5667  CB  ALA A 375     157.799  97.095 165.361  1.00  0.00
ATOM   5668  HB1 ALA A 375     157.820  97.037 164.276  1.00  0.00
ATOM   5669  HB2 ALA A 375     157.186  96.283 165.748  1.00  0.00
ATOM   5670  HB3 ALA A 375     157.368  98.053 165.652  1.00  0.00
ATOM   5671  C   ALA A 375     160.097  98.167 165.437  1.00  0.00
```

ATOM	5672	O	ALA	A	375	160.846	98.090	164.472	1.00	0.00
ATOM	5673	N	PHE	A	376	160.067	99.274	166.184	1.00	0.00
ATOM	5674	H	PHE	A	376	159.465	99.286	166.995	1.00	0.00
ATOM	5675	CA	PHE	A	376	160.754	100.515	165.809	1.00	0.00
ATOM	5676	HA	PHE	A	376	160.998	100.469	164.746	1.00	0.00
ATOM	5677	CB	PHE	A	376	159.787	101.690	165.981	1.00	0.00
ATOM	5678	HB1	PHE	A	376	160.268	102.608	165.644	1.00	0.00
ATOM	5679	HB2	PHE	A	376	159.537	101.804	167.037	1.00	0.00
ATOM	5680	CG	PHE	A	376	158.524	101.479	165.170	1.00	0.00
ATOM	5681	CD1	PHE	A	376	157.332	101.108	165.812	1.00	0.00
ATOM	5682	HD1	PHE	A	376	157.304	101.038	166.888	1.00	0.00
ATOM	5683	CE1	PHE	A	376	156.196	100.785	165.055	1.00	0.00
ATOM	5684	HE1	PHE	A	376	155.278	100.475	165.545	1.00	0.00
ATOM	5685	CZ	PHE	A	376	156.255	100.823	163.654	1.00	0.00
ATOM	5686	HZ	PHE	A	376	155.386	100.562	163.076	1.00	0.00
ATOM	5687	CE2	PHE	A	376	157.449	101.180	163.003	1.00	0.00
ATOM	5688	HE2	PHE	A	376	157.518	101.162	161.924	1.00	0.00
ATOM	5689	CD2	PHE	A	376	158.582	101.514	163.762	1.00	0.00
ATOM	5690	HD2	PHE	A	376	159.512	101.752	163.257	1.00	0.00
ATOM	5691	C	PHE	A	376	162.100	100.707	166.512	1.00	0.00
ATOM	5692	O	PHE	A	376	162.646	101.808	166.543	1.00	0.00
ATOM	5693	N	ARG	A	377	162.656	99.619	167.048	1.00	0.00
ATOM	5694	H	ARG	A	377	162.092	98.776	167.052	1.00	0.00
ATOM	5695	CA	ARG	A	377	163.984	99.552	167.671	1.00	0.00
ATOM	5696	HA	ARG	A	377	164.523	100.492	167.540	1.00	0.00
ATOM	5697	CB	ARG	A	377	163.787	99.266	169.163	1.00	0.00
ATOM	5698	HB1	ARG	A	377	164.751	98.993	169.592	1.00	0.00
ATOM	5699	HB2	ARG	A	377	163.116	98.412	169.271	1.00	0.00
ATOM	5700	CG	ARG	A	377	163.225	100.458	169.959	1.00	0.00
ATOM	5701	HG1	ARG	A	377	162.379	100.896	169.433	1.00	0.00
ATOM	5702	HG2	ARG	A	377	164.003	101.216	170.045	1.00	0.00
ATOM	5703	CD	ARG	A	377	162.752	100.036	171.360	1.00	0.00
ATOM	5704	HD1	ARG	A	377	161.779	99.550	171.256	1.00	0.00
ATOM	5705	HD2	ARG	A	377	162.610	100.922	171.984	1.00	0.00
ATOM	5706	NE	ARG	A	377	163.669	99.077	172.009	1.00	0.00
ATOM	5707	HE	ARG	A	377	163.419	98.105	171.915	1.00	0.00
ATOM	5708	CZ	ARG	A	377	164.870	99.313	172.497	1.00	0.00
ATOM	5709	NH1	ARG	A	377	165.335	100.525	172.651	1.00	0.00
ATOM	5710	1HH1	ARG	A	377	164.756	101.303	172.391	1.00	0.00
ATOM	5711	2HH1	ARG	A	377	166.261	100.680	173.002	1.00	0.00
ATOM	5712	NH2	ARG	A	377	165.627	98.308	172.829	1.00	0.00
ATOM	5713	1HH2	ARG	A	377	165.305	97.367	172.664	1.00	0.00
ATOM	5714	2HH2	ARG	A	377	166.559	98.457	173.162	1.00	0.00
ATOM	5715	C	ARG	A	377	164.868	98.457	167.075	1.00	0.00
ATOM	5716	O	ARG	A	377	166.075	98.475	167.292	1.00	0.00
ATOM	5717	N	GLY	A	378	164.266	97.501	166.364	1.00	0.00
ATOM	5718	H	GLY	A	378	163.263	97.562	166.282	1.00	0.00
ATOM	5719	CA	GLY	A	378	164.838	96.167	166.230	1.00	0.00
ATOM	5720	HA1	GLY	A	378	165.880	96.227	165.916	1.00	0.00
ATOM	5721	HA2	GLY	A	378	164.279	95.608	165.481	1.00	0.00
ATOM	5722	C	GLY	A	378	164.754	95.410	167.559	1.00	0.00
ATOM	5723	O	GLY	A	378	164.254	95.924	168.561	1.00	0.00
ATOM	5724	N	ASP	A	379	165.299	94.201	167.580	1.00	0.00
ATOM	5725	H	ASP	A	379	165.715	93.842	166.724	1.00	0.00
ATOM	5726	CA	ASP	A	379	165.624	93.518	168.825	1.00	0.00
ATOM	5727	HA	ASP	A	379	165.779	94.268	169.603	1.00	0.00
ATOM	5728	CB	ASP	A	379	164.492	92.608	169.294	1.00	0.00
ATOM	5729	HB1	ASP	A	379	164.323	91.819	168.560	1.00	0.00
ATOM	5730	HB2	ASP	A	379	163.579	93.184	169.410	1.00	0.00
ATOM	5731	CG	ASP	A	379	164.910	92.025	170.635	1.00	0.00
ATOM	5732	OD1	ASP	A	379	165.322	90.843	170.631	1.00	0.00

ATOM	5733	OD2	ASP	A	379	164.997	92.806	171.608	1.00	0.00
ATOM	5734	C	ASP	A	379	166.930	92.735	168.667	1.00	0.00
ATOM	5735	O	ASP	A	379	167.123	91.996	167.702	1.00	0.00
ATOM	5736	N	SER	A	380	167.839	92.921	169.619	1.00	0.00
ATOM	5737	H	SER	A	380	167.558	93.445	170.437	1.00	0.00
ATOM	5738	CA	SER	A	380	169.218	92.427	169.543	1.00	0.00
ATOM	5739	HA	SER	A	380	169.518	92.376	168.496	1.00	0.00
ATOM	5740	CB	SER	A	380	170.131	93.430	170.252	1.00	0.00
ATOM	5741	HB1	SER	A	380	169.967	93.381	171.330	1.00	0.00
ATOM	5742	HB2	SER	A	380	169.906	94.439	169.904	1.00	0.00
ATOM	5743	OG	SER	A	380	171.479	93.144	169.963	1.00	0.00
ATOM	5744	HG	SER	A	380	171.591	92.187	170.084	1.00	0.00
ATOM	5745	C	SER	A	380	169.405	91.021	170.146	1.00	0.00
ATOM	5746	O	SER	A	380	170.536	90.559	170.296	1.00	0.00
ATOM	5747	N	PHE	A	381	168.305	90.350	170.501	1.00	0.00
ATOM	5748	H	PHE	A	381	167.409	90.825	170.447	1.00	0.00
ATOM	5749	CA	PHE	A	381	168.290	88.973	171.001	1.00	0.00
ATOM	5750	HA	PHE	A	381	169.307	88.582	171.032	1.00	0.00
ATOM	5751	CB	PHE	A	381	167.726	88.953	172.428	1.00	0.00
ATOM	5752	HB1	PHE	A	381	167.849	87.950	172.838	1.00	0.00
ATOM	5753	HB2	PHE	A	381	166.656	89.159	172.388	1.00	0.00
ATOM	5754	CG	PHE	A	381	168.385	89.949	173.365	1.00	0.00
ATOM	5755	CD1	PHE	A	381	169.701	89.736	173.813	1.00	0.00
ATOM	5756	HD1	PHE	A	381	170.240	88.853	173.500	1.00	0.00
ATOM	5757	CE1	PHE	A	381	170.322	90.675	174.654	1.00	0.00
ATOM	5758	HE1	PHE	A	381	171.335	90.513	174.991	1.00	0.00
ATOM	5759	CZ	PHE	A	381	169.628	91.832	175.048	1.00	0.00
ATOM	5760	HZ	PHE	A	381	170.105	92.557	175.690	1.00	0.00
ATOM	5761	CE2	PHE	A	381	168.314	92.049	174.603	1.00	0.00
ATOM	5762	HE2	PHE	A	381	167.775	92.936	174.904	1.00	0.00
ATOM	5763	CD2	PHE	A	381	167.694	91.112	173.757	1.00	0.00
ATOM	5764	HD2	PHE	A	381	166.687	91.292	173.400	1.00	0.00
ATOM	5765	C	PHE	A	381	167.485	88.057	170.068	1.00	0.00
ATOM	5766	O	PHE	A	381	167.869	86.916	169.824	1.00	0.00
ATOM	5767	N	THR	A	382	166.433	88.611	169.466	1.00	0.00
ATOM	5768	H	THR	A	382	166.118	89.501	169.840	1.00	0.00
ATOM	5769	CA	THR	A	382	165.718	88.099	168.288	1.00	0.00
ATOM	5770	HA	THR	A	382	165.575	87.028	168.416	1.00	0.00
ATOM	5771	CB	THR	A	382	164.330	88.766	168.166	1.00	0.00
ATOM	5772	HB	THR	A	382	164.427	89.702	167.614	1.00	0.00
ATOM	5773	CG2	THR	A	382	163.319	87.861	167.462	1.00	0.00
ATOM	5774	1HG2	THR	A	382	162.385	88.404	167.326	1.00	0.00
ATOM	5775	2HG2	THR	A	382	163.689	87.540	166.491	1.00	0.00
ATOM	5776	3HG2	THR	A	382	163.132	86.979	168.075	1.00	0.00
ATOM	5777	OG1	THR	A	382	163.742	89.036	169.423	1.00	0.00
ATOM	5778	HG1	THR	A	382	164.298	89.676	169.910	1.00	0.00
ATOM	5779	C	THR	A	382	166.511	88.314	166.985	1.00	0.00
ATOM	5780	O	THR	A	382	166.193	87.720	165.955	1.00	0.00
ATOM	5781	N	HIS	A	383	167.562	89.144	167.019	1.00	0.00
ATOM	5782	H	HIS	A	383	167.744	89.599	167.902	1.00	0.00
ATOM	5783	CA	HIS	A	383	168.422	89.555	165.889	1.00	0.00
ATOM	5784	HA	HIS	A	383	169.046	90.369	166.260	1.00	0.00
ATOM	5785	CB	HIS	A	383	169.384	88.421	165.509	1.00	0.00
ATOM	5786	HB1	HIS	A	383	170.054	88.757	164.717	1.00	0.00
ATOM	5787	HB2	HIS	A	383	168.823	87.559	165.145	1.00	0.00
ATOM	5788	CG	HIS	A	383	170.224	88.018	166.691	1.00	0.00
ATOM	5789	ND1	HIS	A	383	171.226	88.804	167.257	1.00	0.00
ATOM	5790	CE1	HIS	A	383	171.532	88.221	168.424	1.00	0.00
ATOM	5791	HE1	HIS	A	383	172.232	88.628	169.142	1.00	0.00
ATOM	5792	NE2	HIS	A	383	170.782	87.121	168.612	1.00	0.00
ATOM	5793	HE2	HIS	A	383	170.731	86.582	169.464	1.00	0.00

ATOM	5794	CD2	HIS	A	383	169.946	86.981	167.530	1.00	0.00
ATOM	5795	HD2	HIS	A	383	169.128	86.279	167.422	1.00	0.00
ATOM	5796	C	HIS	A	383	167.671	90.174	164.701	1.00	0.00
ATOM	5797	O	HIS	A	383	168.163	90.212	163.574	1.00	0.00
ATOM	5798	N	THR	A	384	166.468	90.686	164.945	1.00	0.00
ATOM	5799	H	THR	A	384	166.145	90.679	165.902	1.00	0.00
ATOM	5800	CA	THR	A	384	165.631	91.336	163.936	1.00	0.00
ATOM	5801	HA	THR	A	384	165.793	90.851	162.975	1.00	0.00
ATOM	5802	CB	THR	A	384	164.146	91.179	164.290	1.00	0.00
ATOM	5803	HB	THR	A	384	163.559	91.776	163.592	1.00	0.00
ATOM	5804	CG2	THR	A	384	163.681	89.731	164.203	1.00	0.00
ATOM	5805	1HG2	THR	A	384	162.611	89.676	164.395	1.00	0.00
ATOM	5806	2HG2	THR	A	384	163.894	89.335	163.216	1.00	0.00
ATOM	5807	3HG2	THR	A	384	164.209	89.129	164.937	1.00	0.00
ATOM	5808	OG1	THR	A	384	163.881	91.605	165.608	1.00	0.00
ATOM	5809	HG1	THR	A	384	164.505	92.318	165.799	1.00	0.00
ATOM	5810	C	THR	A	384	165.961	92.830	163.835	1.00	0.00
ATOM	5811	O	THR	A	384	165.850	93.515	164.856	1.00	0.00
ATOM	5812	N	PRO	A	385	166.319	93.375	162.654	1.00	0.00
ATOM	5813	CD	PRO	A	385	166.665	92.648	161.442	1.00	0.00
ATOM	5814	HD1	PRO	A	385	165.754	92.340	160.930	1.00	0.00
ATOM	5815	HD2	PRO	A	385	167.296	91.785	161.656	1.00	0.00
ATOM	5816	CG	PRO	A	385	167.433	93.641	160.575	1.00	0.00
ATOM	5817	HG1	PRO	A	385	167.324	93.421	159.513	1.00	0.00
ATOM	5818	HG2	PRO	A	385	168.486	93.638	160.862	1.00	0.00
ATOM	5819	CB	PRO	A	385	166.805	94.978	160.950	1.00	0.00
ATOM	5820	HB1	PRO	A	385	165.895	95.123	160.364	1.00	0.00
ATOM	5821	HB2	PRO	A	385	167.494	95.806	160.782	1.00	0.00
ATOM	5822	CA	PRO	A	385	166.451	94.823	162.436	1.00	0.00
ATOM	5823	HA	PRO	A	385	167.285	95.175	163.043	1.00	0.00
ATOM	5824	C	PRO	A	385	165.179	95.639	162.765	1.00	0.00
ATOM	5825	O	PRO	A	385	164.101	95.047	162.867	1.00	0.00
ATOM	5826	N	PRO	A	386	165.268	96.981	162.888	1.00	0.00
ATOM	5827	CD	PRO	A	386	166.504	97.746	162.978	1.00	0.00
ATOM	5828	HD1	PRO	A	386	166.937	97.858	161.983	1.00	0.00
ATOM	5829	HD2	PRO	A	386	167.220	97.277	163.653	1.00	0.00
ATOM	5830	CG	PRO	A	386	166.108	99.116	163.521	1.00	0.00
ATOM	5831	HG1	PRO	A	386	166.777	99.900	163.168	1.00	0.00
ATOM	5832	HG2	PRO	A	386	166.099	99.087	164.609	1.00	0.00
ATOM	5833	CB	PRO	A	386	164.685	99.307	163.001	1.00	0.00
ATOM	5834	HB1	PRO	A	386	164.733	99.748	162.003	1.00	0.00
ATOM	5835	HB2	PRO	A	386	164.098	99.942	163.665	1.00	0.00
ATOM	5836	CA	PRO	A	386	164.111	97.887	162.920	1.00	0.00
ATOM	5837	HA	PRO	A	386	163.532	97.680	163.818	1.00	0.00
ATOM	5838	C	PRO	A	386	163.167	97.767	161.699	1.00	0.00
ATOM	5839	O	PRO	A	386	163.604	97.407	160.603	1.00	0.00
ATOM	5840	N	LEU	A	387	161.890	98.136	161.858	1.00	0.00
ATOM	5841	H	LEU	A	387	161.593	98.380	162.795	1.00	0.00
ATOM	5842	CA	LEU	A	387	160.824	97.938	160.867	1.00	0.00
ATOM	5843	HA	LEU	A	387	161.134	97.150	160.184	1.00	0.00
ATOM	5844	CB	LEU	A	387	159.546	97.450	161.589	1.00	0.00
ATOM	5845	HB1	LEU	A	387	159.307	98.160	162.380	1.00	0.00
ATOM	5846	HB2	LEU	A	387	159.757	96.493	162.055	1.00	0.00
ATOM	5847	CG	LEU	A	387	158.279	97.299	160.715	1.00	0.00
ATOM	5848	HG	LEU	A	387	157.996	98.265	160.303	1.00	0.00
ATOM	5849	CD1	LEU	A	387	158.447	96.324	159.553	1.00	0.00
ATOM	5850	1HD1	LEU	A	387	157.557	96.390	158.935	1.00	0.00
ATOM	5851	2HD1	LEU	A	387	159.295	96.611	158.933	1.00	0.00
ATOM	5852	3HD1	LEU	A	387	158.562	95.308	159.916	1.00	0.00
ATOM	5853	CD2	LEU	A	387	157.114	96.814	161.574	1.00	0.00
ATOM	5854	1HD2	LEU	A	387	156.263	96.580	160.940	1.00	0.00

```
ATOM   5855 2HD2 LEU A 387     157.384  95.929 162.138  1.00  0.00
ATOM   5856 3HD2 LEU A 387     156.829  97.605 162.264  1.00  0.00
ATOM   5857  C   LEU A 387     160.497  99.170 160.013  1.00  0.00
ATOM   5858  O   LEU A 387     159.783 100.072 160.452  1.00  0.00
ATOM   5859  N   ASP A 388     160.911  99.170 158.745  1.00  0.00
ATOM   5860  H   ASP A 388     161.496  98.424 158.413  1.00  0.00
ATOM   5861  CA  ASP A 388     160.505 100.221 157.804  1.00  0.00
ATOM   5862  HA  ASP A 388     161.073 101.106 158.082  1.00  0.00
ATOM   5863  CB  ASP A 388     160.941  99.916 156.365  1.00  0.00
ATOM   5864  HB1 ASP A 388     160.318  99.124 155.950  1.00  0.00
ATOM   5865  HB2 ASP A 388     161.976  99.568 156.375  1.00  0.00
ATOM   5866  CG  ASP A 388     160.842 101.175 155.484  1.00  0.00
ATOM   5867  OD1 ASP A 388     161.738 101.402 154.653  1.00  0.00
ATOM   5868  OD2 ASP A 388     159.871 101.948 155.628  1.00  0.00
ATOM   5869  C   ASP A 388     158.964 100.539 157.880  1.00  0.00
ATOM   5870  O   ASP A 388     158.087  99.710 157.556  1.00  0.00
ATOM   5871  N   PRO A 389     158.605 101.763 158.331  1.00  0.00
ATOM   5872  CD  PRO A 389     159.499 102.790 158.860  1.00  0.00
ATOM   5873  HD1 PRO A 389     160.026 103.272 158.036  1.00  0.00
ATOM   5874  HD2 PRO A 389     160.213 102.386 159.577  1.00  0.00
ATOM   5875  CG  PRO A 389     158.606 103.804 159.564  1.00  0.00
ATOM   5876  HG1 PRO A 389     159.047 104.801 159.568  1.00  0.00
ATOM   5877  HG2 PRO A 389     158.393 103.465 160.578  1.00  0.00
ATOM   5878  CB  PRO A 389     157.361 103.730 158.697  1.00  0.00
ATOM   5879  HB1 PRO A 389     157.547 104.297 157.785  1.00  0.00
ATOM   5880  HB2 PRO A 389     156.471 104.130 159.167  1.00  0.00
ATOM   5881  CA  PRO A 389     157.226 102.232 158.375  1.00  0.00
ATOM   5882  HA  PRO A 389     156.694 101.721 159.174  1.00  0.00
ATOM   5883  C   PRO A 389     156.459 102.132 157.044  1.00  0.00
ATOM   5884  O   PRO A 389     155.243 101.896 156.981  1.00  0.00
ATOM   5885  N   GLN A 390     157.166 102.374 155.946  1.00  0.00
ATOM   5886  H   GLN A 390     158.181 102.458 156.017  1.00  0.00
ATOM   5887  CA  GLN A 390     156.625 102.221 154.608  1.00  0.00
ATOM   5888  HA  GLN A 390     155.737 102.841 154.497  1.00  0.00
ATOM   5889  CB  GLN A 390     157.658 102.644 153.547  1.00  0.00
ATOM   5890  HB1 GLN A 390     157.181 102.614 152.565  1.00  0.00
ATOM   5891  HB2 GLN A 390     158.462 101.908 153.533  1.00  0.00
ATOM   5892  CG  GLN A 390     158.250 104.047 153.766  1.00  0.00
ATOM   5893  HG1 GLN A 390     158.564 104.178 154.799  1.00  0.00
ATOM   5894  HG2 GLN A 390     157.497 104.799 153.537  1.00  0.00
ATOM   5895  CD  GLN A 390     159.478 104.290 152.893  1.00  0.00
ATOM   5896  OE1 GLN A 390     159.459 105.064 151.954  1.00  0.00
ATOM   5897  NE2 GLN A 390     160.588 103.648 153.182  1.00  0.00
ATOM   5898 1HE2 GLN A 390     160.639 102.940 153.925  1.00  0.00
ATOM   5899 2HE2 GLN A 390     161.380 103.804 152.596  1.00  0.00
ATOM   5900  C   GLN A 390     156.222 100.746 154.383  1.00  0.00
ATOM   5901  O   GLN A 390     155.194 100.442 153.757  1.00  0.00
ATOM   5902  N   GLU A 391     156.960  99.795 154.986  1.00  0.00
ATOM   5903  H   GLU A 391     157.732 100.032 155.601  1.00  0.00
ATOM   5904  CA  GLU A 391     156.527  98.409 154.839  1.00  0.00
ATOM   5905  HA  GLU A 391     156.274  98.282 153.787  1.00  0.00
ATOM   5906  CB  GLU A 391     157.658  97.402 155.120  1.00  0.00
ATOM   5907  HB1 GLU A 391     157.742  97.218 156.191  1.00  0.00
ATOM   5908  HB2 GLU A 391     158.594  97.825 154.755  1.00  0.00
ATOM   5909  CG  GLU A 391     157.398  96.084 154.363  1.00  0.00
ATOM   5910  HG1 GLU A 391     157.138  96.306 153.327  1.00  0.00
ATOM   5911  HG2 GLU A 391     156.545  95.584 154.816  1.00  0.00
ATOM   5912  CD  GLU A 391     158.578  95.103 154.345  1.00  0.00
ATOM   5913  OE1 GLU A 391     158.457  94.042 153.677  1.00  0.00
ATOM   5914  OE2 GLU A 391     159.579  95.318 155.055  1.00  0.00
ATOM   5915  C   GLU A 391     155.213  98.146 155.611  1.00  0.00
```

```
ATOM   5916  O   GLU A 391      154.393  97.310 155.204  1.00  0.00
ATOM   5917  N   LEU A 392      154.898  98.965 156.625  1.00  0.00
ATOM   5918  H   LEU A 392      155.556  99.681 156.908  1.00  0.00
ATOM   5919  CA  LEU A 392      153.509  98.984 157.142  1.00  0.00
ATOM   5920  HA  LEU A 392      153.290  97.980 157.499  1.00  0.00
ATOM   5921  CB  LEU A 392      153.322  99.938 158.334  1.00  0.00
ATOM   5922  HB1 LEU A 392      152.367  99.697 158.798  1.00  0.00
ATOM   5923  HB2 LEU A 392      153.229 100.951 157.950  1.00  0.00
ATOM   5924  CG  LEU A 392      154.411  99.894 159.410  1.00  0.00
ATOM   5925  HG  LEU A 392      155.392 100.003 158.956  1.00  0.00
ATOM   5926  CD1 LEU A 392      154.214 101.063 160.381  1.00  0.00
ATOM   5927 1HD1 LEU A 392      155.102 101.145 160.999  1.00  0.00
ATOM   5928 2HD1 LEU A 392      154.091 101.992 159.825  1.00  0.00
ATOM   5929 3HD1 LEU A 392      153.338 100.885 161.002  1.00  0.00
ATOM   5930  CD2 LEU A 392      154.377  98.586 160.186  1.00  0.00
ATOM   5931 1HD2 LEU A 392      155.114  98.628 160.984  1.00  0.00
ATOM   5932 2HD2 LEU A 392      153.387  98.420 160.607  1.00  0.00
ATOM   5933 3HD2 LEU A 392      154.630  97.759 159.523  1.00  0.00
ATOM   5934  C   LEU A 392      152.433  99.327 156.086  1.00  0.00
ATOM   5935  O   LEU A 392      151.335  98.756 156.125  1.00  0.00
ATOM   5936  N   ASP A 393      152.715 100.207 155.113  1.00  0.00
ATOM   5937  H   ASP A 393      153.650 100.584 155.015  1.00  0.00
ATOM   5938  CA  ASP A 393      151.751 100.341 153.983  1.00  0.00
ATOM   5939  HA  ASP A 393      150.798 100.654 154.415  1.00  0.00
ATOM   5940  CB  ASP A 393      152.139 101.434 152.962  1.00  0.00
ATOM   5941  HB1 ASP A 393      151.835 101.117 151.964  1.00  0.00
ATOM   5942  HB2 ASP A 393      153.216 101.574 152.935  1.00  0.00
ATOM   5943  CG  ASP A 393      151.458 102.776 153.240  1.00  0.00
ATOM   5944  OD1 ASP A 393      152.178 103.736 153.602  1.00  0.00
ATOM   5945  OD2 ASP A 393      150.215 102.853 153.139  1.00  0.00
ATOM   5946  C   ASP A 393      151.428  99.022 153.262  1.00  0.00
ATOM   5947  O   ASP A 393      150.304  98.875 152.780  1.00  0.00
ATOM   5948  N   ILE A 394      152.321  98.019 153.258  1.00  0.00
ATOM   5949  H   ILE A 394      153.201  98.125 153.748  1.00  0.00
ATOM   5950  CA  ILE A 394      151.893  96.673 152.762  1.00  0.00
ATOM   5951  HA  ILE A 394      151.476  96.842 151.768  1.00  0.00
ATOM   5952  CB  ILE A 394      153.055  95.671 152.566  1.00  0.00
ATOM   5953  HB  ILE A 394      152.692  94.932 151.850  1.00  0.00
ATOM   5954  CG2 ILE A 394      154.267  96.354 151.907  1.00  0.00
ATOM   5955 1HG2 ILE A 394      155.009  95.619 151.610  1.00  0.00
ATOM   5956 2HG2 ILE A 394      153.947  96.901 151.020  1.00  0.00
ATOM   5957 3HG2 ILE A 394      154.724  97.064 152.594  1.00  0.00
ATOM   5958  CG1 ILE A 394      153.450  94.910 153.853  1.00  0.00
ATOM   5959 1HG1 ILE A 394      153.061  95.412 154.734  1.00  0.00
ATOM   5960 2HG1 ILE A 394      154.530  94.891 153.957  1.00  0.00
ATOM   5961  CD  ILE A 394      152.935  93.475 153.921  1.00  0.00
ATOM   5962  HD1 ILE A 394      153.187  93.065 154.896  1.00  0.00
ATOM   5963  HD2 ILE A 394      151.853  93.445 153.801  1.00  0.00
ATOM   5964  HD3 ILE A 394      153.404  92.869 153.154  1.00  0.00
ATOM   5965  C   ILE A 394      150.763  96.030 153.568  1.00  0.00
ATOM   5966  O   ILE A 394      150.026  95.210 153.016  1.00  0.00
ATOM   5967  N   LEU A 395      150.596  96.402 154.844  1.00  0.00
ATOM   5968  H   LEU A 395      151.180  97.144 155.214  1.00  0.00
ATOM   5969  CA  LEU A 395      149.440  95.946 155.651  1.00  0.00
ATOM   5970  HA  LEU A 395      149.388  94.860 155.586  1.00  0.00
ATOM   5971  CB  LEU A 395      149.593  96.332 157.136  1.00  0.00
ATOM   5972  HB1 LEU A 395      148.800  95.834 157.694  1.00  0.00
ATOM   5973  HB2 LEU A 395      149.436  97.404 157.241  1.00  0.00
ATOM   5974  CG  LEU A 395      150.941  95.969 157.769  1.00  0.00
ATOM   5975  HG  LEU A 395      151.731  96.542 157.299  1.00  0.00
ATOM   5976  CD1 LEU A 395      150.927  96.311 159.262  1.00  0.00
```

```
ATOM   5977 1HD1 LEU A 395     151.919  96.154 159.683  1.00  0.00
ATOM   5978 2HD1 LEU A 395     150.650  97.357 159.393  1.00  0.00
ATOM   5979 3HD1 LEU A 395     150.204  95.680 159.780  1.00  0.00
ATOM   5980  CD2 LEU A 395     151.272  94.492 157.592  1.00  0.00
ATOM   5981 1HD2 LEU A 395     152.222  94.284 158.066  1.00  0.00
ATOM   5982 2HD2 LEU A 395     150.499  93.877 158.049  1.00  0.00
ATOM   5983 3HD2 LEU A 395     151.368  94.244 156.537  1.00  0.00
ATOM   5984  C   LEU A 395     148.102  96.465 155.147  1.00  0.00
ATOM   5985  O   LEU A 395     147.092  95.811 155.392  1.00  0.00
ATOM   5986  N   LYS A 396     148.064  97.588 154.418  1.00  0.00
ATOM   5987  H   LYS A 396     148.934  98.043 154.166  1.00  0.00
ATOM   5988  CA  LYS A 396     146.773  98.186 153.995  1.00  0.00
ATOM   5989  HA  LYS A 396     146.214  98.349 154.915  1.00  0.00
ATOM   5990  CB  LYS A 396     147.017  99.578 153.401  1.00  0.00
ATOM   5991  HB1 LYS A 396     147.864 100.025 153.921  1.00  0.00
ATOM   5992  HB2 LYS A 396     146.139 100.195 153.600  1.00  0.00
ATOM   5993  CG  LYS A 396     147.295  99.616 151.891  1.00  0.00
ATOM   5994  HG1 LYS A 396     146.349  99.518 151.355  1.00  0.00
ATOM   5995  HG2 LYS A 396     147.931  98.772 151.621  1.00  0.00
ATOM   5996  CD  LYS A 396     147.996 100.914 151.440  1.00  0.00
ATOM   5997  HD1 LYS A 396     148.088 100.869 150.355  1.00  0.00
ATOM   5998  HD2 LYS A 396     149.010 100.926 151.840  1.00  0.00
ATOM   5999  CE  LYS A 396     147.284 102.222 151.809  1.00  0.00
ATOM   6000  HE1 LYS A 396     146.212 102.126 151.617  1.00  0.00
ATOM   6001  HE2 LYS A 396     147.677 103.013 151.164  1.00  0.00
ATOM   6002  NZ  LYS A 396     147.507 102.630 153.214  1.00  0.00
ATOM   6003  HZ1 LYS A 396     146.991 103.476 153.453  1.00  0.00
ATOM   6004  HZ2 LYS A 396     147.139 101.969 153.876  1.00  0.00
ATOM   6005  HZ3 LYS A 396     148.498 102.789 153.417  1.00  0.00
ATOM   6006  C   LYS A 396     145.816  97.296 153.178  1.00  0.00
ATOM   6007  O   LYS A 396     144.657  97.665 153.021  1.00  0.00
ATOM   6008  N   THR A 397     146.218  96.125 152.682  1.00  0.00
ATOM   6009  H   THR A 397     147.160  95.810 152.876  1.00  0.00
ATOM   6010  CA  THR A 397     145.295  95.189 152.007  1.00  0.00
ATOM   6011  HA  THR A 397     144.637  95.772 151.362  1.00  0.00
ATOM   6012  CB  THR A 397     146.066  94.230 151.089  1.00  0.00
ATOM   6013  HB  THR A 397     145.366  93.564 150.581  1.00  0.00
ATOM   6014  CG2 THR A 397     146.866  94.995 150.030  1.00  0.00
ATOM   6015 1HG2 THR A 397     147.360  94.285 149.370  1.00  0.00
ATOM   6016 2HG2 THR A 397     146.192  95.616 149.440  1.00  0.00
ATOM   6017 3HG2 THR A 397     147.627  95.618 150.500  1.00  0.00
ATOM   6018  OG1 THR A 397     146.994  93.464 151.811  1.00  0.00
ATOM   6019  HG1 THR A 397     146.490  92.703 152.160  1.00  0.00
ATOM   6020  C   THR A 397     144.359  94.432 152.970  1.00  0.00
ATOM   6021  O   THR A 397     143.370  93.854 152.511  1.00  0.00
ATOM   6022  N   VAL A 398     144.592  94.466 154.294  1.00  0.00
ATOM   6023  H   VAL A 398     145.409  94.966 154.638  1.00  0.00
ATOM   6024  CA  VAL A 398     143.695  93.823 155.282  1.00  0.00
ATOM   6025  HA  VAL A 398     143.541  92.805 154.941  1.00  0.00
ATOM   6026  CB  VAL A 398     144.323  93.647 156.685  1.00  0.00
ATOM   6027  HB  VAL A 398     143.612  93.087 157.291  1.00  0.00
ATOM   6028  CG1 VAL A 398     145.600  92.800 156.598  1.00  0.00
ATOM   6029 1HG1 VAL A 398     145.972  92.586 157.600  1.00  0.00
ATOM   6030 2HG1 VAL A 398     145.386  91.856 156.100  1.00  0.00
ATOM   6031 3HG1 VAL A 398     146.374  93.328 156.043  1.00  0.00
ATOM   6032  CG2 VAL A 398     144.627  94.947 157.442  1.00  0.00
ATOM   6033 1HG2 VAL A 398     145.146  94.718 158.373  1.00  0.00
ATOM   6034 2HG2 VAL A 398     145.232  95.616 156.844  1.00  0.00
ATOM   6035 3HG2 VAL A 398     143.703  95.458 157.675  1.00  0.00
ATOM   6036  C   VAL A 398     142.295  94.456 155.342  1.00  0.00
ATOM   6037  O   VAL A 398     142.120  95.618 155.721  1.00  0.00
```

ATOM	6038	N	LYS	A	399	141.285	93.659	154.982	1.00	0.00
ATOM	6039	H	LYS	A	399	141.519	92.740	154.623	1.00	0.00
ATOM	6040	CA	LYS	A	399	139.866	93.900	155.272	1.00	0.00
ATOM	6041	HA	LYS	A	399	139.668	94.972	155.279	1.00	0.00
ATOM	6042	CB	LYS	A	399	138.987	93.194	154.219	1.00	0.00
ATOM	6043	HB1	LYS	A	399	137.965	93.123	154.599	1.00	0.00
ATOM	6044	HB2	LYS	A	399	139.346	92.174	154.074	1.00	0.00
ATOM	6045	CG	LYS	A	399	138.936	93.896	152.855	1.00	0.00
ATOM	6046	HG1	LYS	A	399	138.401	93.253	152.155	1.00	0.00
ATOM	6047	HG2	LYS	A	399	139.947	94.060	152.481	1.00	0.00
ATOM	6048	CD	LYS	A	399	138.210	95.238	152.956	1.00	0.00
ATOM	6049	HD1	LYS	A	399	138.808	95.915	153.566	1.00	0.00
ATOM	6050	HD2	LYS	A	399	137.237	95.100	153.429	1.00	0.00
ATOM	6051	CE	LYS	A	399	138.008	95.861	151.579	1.00	0.00
ATOM	6052	HE1	LYS	A	399	137.242	95.301	151.035	1.00	0.00
ATOM	6053	HE2	LYS	A	399	138.945	95.809	151.020	1.00	0.00
ATOM	6054	NZ	LYS	A	399	137.612	97.271	151.751	1.00	0.00
ATOM	6055	HZ1	LYS	A	399	137.362	97.695	150.874	1.00	0.00
ATOM	6056	HZ2	LYS	A	399	136.827	97.333	152.407	1.00	0.00
ATOM	6057	HZ3	LYS	A	399	138.406	97.770	152.155	1.00	0.00
ATOM	6058	C	LYS	A	399	139.467	93.383	156.644	1.00	0.00
ATOM	6059	O	LYS	A	399	138.612	93.992	157.280	1.00	0.00
ATOM	6060	N	GLU	A	400	140.049	92.261	157.078	1.00	0.00
ATOM	6061	H	GLU	A	400	140.721	91.799	156.475	1.00	0.00
ATOM	6062	CA	GLU	A	400	139.439	91.503	158.169	1.00	0.00
ATOM	6063	HA	GLU	A	400	138.738	92.125	158.725	1.00	0.00
ATOM	6064	CB	GLU	A	400	138.649	90.341	157.542	1.00	0.00
ATOM	6065	HB1	GLU	A	400	139.254	89.862	156.771	1.00	0.00
ATOM	6066	HB2	GLU	A	400	137.764	90.756	157.055	1.00	0.00
ATOM	6067	CG	GLU	A	400	138.192	89.251	158.522	1.00	0.00
ATOM	6068	HG1	GLU	A	400	137.694	89.715	159.374	1.00	0.00
ATOM	6069	HG2	GLU	A	400	139.066	88.712	158.893	1.00	0.00
ATOM	6070	CD	GLU	A	400	137.251	88.276	157.814	1.00	0.00
ATOM	6071	OE1	GLU	A	400	136.021	88.387	158.021	1.00	0.00
ATOM	6072	OE2	GLU	A	400	137.748	87.429	157.031	1.00	0.00
ATOM	6073	C	GLU	A	400	140.494	91.039	159.157	1.00	0.00
ATOM	6074	O	GLU	A	400	141.459	90.382	158.786	1.00	0.00
ATOM	6075	N	ILE	A	401	140.258	91.329	160.433	1.00	0.00
ATOM	6076	H	ILE	A	401	139.455	91.895	160.669	1.00	0.00
ATOM	6077	CA	ILE	A	401	141.019	90.746	161.525	1.00	0.00
ATOM	6078	HA	ILE	A	401	141.633	89.950	161.108	1.00	0.00
ATOM	6079	CB	ILE	A	401	141.999	91.769	162.138	1.00	0.00
ATOM	6080	HB	ILE	A	401	142.619	92.142	161.322	1.00	0.00
ATOM	6081	CG2	ILE	A	401	141.307	92.982	162.772	1.00	0.00
ATOM	6082	1HG2	ILE	A	401	142.042	93.732	163.057	1.00	0.00
ATOM	6083	2HG2	ILE	A	401	140.624	93.439	162.057	1.00	0.00
ATOM	6084	3HG2	ILE	A	401	140.750	92.672	163.652	1.00	0.00
ATOM	6085	CG1	ILE	A	401	142.916	91.053	163.149	1.00	0.00
ATOM	6086	1HG1	ILE	A	401	143.254	90.106	162.728	1.00	0.00
ATOM	6087	2HG1	ILE	A	401	142.370	90.840	164.068	1.00	0.00
ATOM	6088	CD	ILE	A	401	144.159	91.863	163.493	1.00	0.00
ATOM	6089	HD1	ILE	A	401	144.818	91.236	164.087	1.00	0.00
ATOM	6090	HD2	ILE	A	401	144.673	92.166	162.582	1.00	0.00
ATOM	6091	HD3	ILE	A	401	143.892	92.740	164.074	1.00	0.00
ATOM	6092	C	ILE	A	401	140.074	90.066	162.501	1.00	0.00
ATOM	6093	O	ILE	A	401	138.944	90.515	162.726	1.00	0.00
ATOM	6094	N	THR	A	402	140.573	88.933	163.003	1.00	0.00
ATOM	6095	H	THR	A	402	141.502	88.672	162.692	1.00	0.00
ATOM	6096	CA	THR	A	402	139.773	87.883	163.631	1.00	0.00
ATOM	6097	HA	THR	A	402	138.722	88.092	163.456	1.00	0.00
ATOM	6098	CB	THR	A	402	140.098	86.521	162.993	1.00	0.00

ATOM	6099	HB	THR	A	402	141.114	86.228	163.260	1.00	0.00
ATOM	6100	CG2	THR	A	402	139.139	85.413	163.435	1.00	0.00
ATOM	6101	1HG2	THR	A	402	139.400	84.492	162.913	1.00	0.00
ATOM	6102	2HG2	THR	A	402	139.224	85.251	164.507	1.00	0.00
ATOM	6103	3HG2	THR	A	402	138.116	85.691	163.183	1.00	0.00
ATOM	6104	OG1	THR	A	402	140.013	86.596	161.594	1.00	0.00
ATOM	6105	HG1	THR	A	402	140.875	86.900	161.265	1.00	0.00
ATOM	6106	C	THR	A	402	140.022	87.811	165.125	1.00	0.00
ATOM	6107	O	THR	A	402	139.050	87.800	165.873	1.00	0.00
ATOM	6108	N	GLY	A	403	141.279	87.771	165.577	1.00	0.00
ATOM	6109	H	GLY	A	403	142.048	87.873	164.922	1.00	0.00
ATOM	6110	CA	GLY	A	403	141.642	87.453	166.958	1.00	0.00
ATOM	6111	HA1	GLY	A	403	142.634	87.003	166.979	1.00	0.00
ATOM	6112	HA2	GLY	A	403	140.942	86.733	167.379	1.00	0.00
ATOM	6113	C	GLY	A	403	141.671	88.680	167.859	1.00	0.00
ATOM	6114	O	GLY	A	403	140.768	88.809	168.699	1.00	0.00
ATOM	6115	N	PHE	A	404	142.637	89.588	167.625	1.00	0.00
ATOM	6116	H	PHE	A	404	143.365	89.370	166.960	1.00	0.00
ATOM	6117	CA	PHE	A	404	142.583	90.963	168.161	1.00	0.00
ATOM	6118	HA	PHE	A	404	141.573	91.350	168.015	1.00	0.00
ATOM	6119	CB	PHE	A	404	142.871	90.986	169.677	1.00	0.00
ATOM	6120	HB1	PHE	A	404	142.132	90.354	170.152	1.00	0.00
ATOM	6121	HB2	PHE	A	404	142.692	91.995	170.047	1.00	0.00
ATOM	6122	CG	PHE	A	404	144.250	90.558	170.161	1.00	0.00
ATOM	6123	CD1	PHE	A	404	145.357	91.416	170.031	1.00	0.00
ATOM	6124	HD1	PHE	A	404	145.235	92.379	169.565	1.00	0.00
ATOM	6125	CE1	PHE	A	404	146.632	91.005	170.466	1.00	0.00
ATOM	6126	HE1	PHE	A	404	147.495	91.637	170.301	1.00	0.00
ATOM	6127	CZ	PHE	A	404	146.799	89.752	171.080	1.00	0.00
ATOM	6128	HZ	PHE	A	404	147.785	89.428	171.389	1.00	0.00
ATOM	6129	CE2	PHE	A	404	145.690	88.906	171.246	1.00	0.00
ATOM	6130	HE2	PHE	A	404	145.823	87.921	171.677	1.00	0.00
ATOM	6131	CD2	PHE	A	404	144.427	89.306	170.777	1.00	0.00
ATOM	6132	HD2	PHE	A	404	143.605	88.621	170.861	1.00	0.00
ATOM	6133	C	PHE	A	404	143.530	91.920	167.432	1.00	0.00
ATOM	6134	O	PHE	A	404	144.365	91.490	166.637	1.00	0.00
ATOM	6135	N	LEU	A	405	143.416	93.222	167.726	1.00	0.00
ATOM	6136	H	LEU	A	405	142.686	93.531	168.360	1.00	0.00
ATOM	6137	CA	LEU	A	405	144.176	94.257	166.999	1.00	0.00
ATOM	6138	HA	LEU	A	405	144.942	93.782	166.383	1.00	0.00
ATOM	6139	CB	LEU	A	405	143.229	95.046	166.073	1.00	0.00
ATOM	6140	HB1	LEU	A	405	142.424	95.469	166.677	1.00	0.00
ATOM	6141	HB2	LEU	A	405	142.779	94.355	165.360	1.00	0.00
ATOM	6142	CG	LEU	A	405	143.922	96.194	165.302	1.00	0.00
ATOM	6143	HG	LEU	A	405	144.310	96.922	166.014	1.00	0.00
ATOM	6144	CD1	LEU	A	405	145.072	95.702	164.419	1.00	0.00
ATOM	6145	1HD1	LEU	A	405	145.464	96.527	163.824	1.00	0.00
ATOM	6146	2HD1	LEU	A	405	145.891	95.312	165.023	1.00	0.00
ATOM	6147	3HD1	LEU	A	405	144.721	94.924	163.745	1.00	0.00
ATOM	6148	CD2	LEU	A	405	142.914	96.905	164.406	1.00	0.00
ATOM	6149	1HD2	LEU	A	405	143.391	97.747	163.902	1.00	0.00
ATOM	6150	2HD2	LEU	A	405	142.533	96.203	163.671	1.00	0.00
ATOM	6151	3HD2	LEU	A	405	142.080	97.274	164.993	1.00	0.00
ATOM	6152	C	LEU	A	405	144.884	95.179	167.992	1.00	0.00
ATOM	6153	O	LEU	A	405	144.242	95.882	168.776	1.00	0.00
ATOM	6154	N	LEU	A	406	146.216	95.179	167.956	1.00	0.00
ATOM	6155	H	LEU	A	406	146.690	94.600	167.267	1.00	0.00
ATOM	6156	CA	LEU	A	406	147.043	95.736	169.020	1.00	0.00
ATOM	6157	HA	LEU	A	406	146.418	96.353	169.670	1.00	0.00
ATOM	6158	CB	LEU	A	406	147.576	94.576	169.875	1.00	0.00
ATOM	6159	HB1	LEU	A	406	148.377	94.076	169.336	1.00	0.00

```
ATOM   6160  HB2 LEU A 406     146.769  93.862 169.998  1.00  0.00
ATOM   6161  CG  LEU A 406     148.042  94.939 171.290  1.00  0.00
ATOM   6162  HG  LEU A 406     147.175  95.246 171.872  1.00  0.00
ATOM   6163  CD1 LEU A 406     148.658  93.695 171.937  1.00  0.00
ATOM   6164 1HD1 LEU A 406     149.148  93.965 172.870  1.00  0.00
ATOM   6165 2HD1 LEU A 406     147.870  92.975 172.145  1.00  0.00
ATOM   6166 3HD1 LEU A 406     149.383  93.246 171.262  1.00  0.00
ATOM   6167  CD2 LEU A 406     149.050  96.082 171.335  1.00  0.00
ATOM   6168 1HD2 LEU A 406     149.487  96.150 172.328  1.00  0.00
ATOM   6169 2HD2 LEU A 406     149.836  95.921 170.600  1.00  0.00
ATOM   6170 3HD2 LEU A 406     148.549  97.028 171.134  1.00  0.00
ATOM   6171  C   LEU A 406     148.143  96.642 168.452  1.00  0.00
ATOM   6172  O   LEU A 406     149.203  96.200 168.001  1.00  0.00
ATOM   6173  N   ILE A 407     147.869  97.941 168.528  1.00  0.00
ATOM   6174  H   ILE A 407     146.970  98.201 168.922  1.00  0.00
ATOM   6175  CA  ILE A 407     148.772  99.018 168.129  1.00  0.00
ATOM   6176  HA  ILE A 407     149.555  98.594 167.501  1.00  0.00
ATOM   6177  CB  ILE A 407     148.032 100.077 167.283  1.00  0.00
ATOM   6178  HB  ILE A 407     147.369 100.628 167.944  1.00  0.00
ATOM   6179  CG2 ILE A 407     149.050 101.070 166.697  1.00  0.00
ATOM   6180 1HG2 ILE A 407     148.541 101.801 166.071  1.00  0.00
ATOM   6181 2HG2 ILE A 407     149.560 101.605 167.497  1.00  0.00
ATOM   6182 3HG2 ILE A 407     149.795 100.543 166.103  1.00  0.00
ATOM   6183  CG1 ILE A 407     147.129  99.506 166.165  1.00  0.00
ATOM   6184 1HG1 ILE A 407     146.698 100.336 165.604  1.00  0.00
ATOM   6185 2HG1 ILE A 407     146.296  98.968 166.620  1.00  0.00
ATOM   6186  CD  ILE A 407     147.817  98.562 165.171  1.00  0.00
ATOM   6187  HD1 ILE A 407     148.173  97.669 165.683  1.00  0.00
ATOM   6188  HD2 ILE A 407     147.093  98.258 164.417  1.00  0.00
ATOM   6189  HD3 ILE A 407     148.652  99.067 164.687  1.00  0.00
ATOM   6190  C   ILE A 407     149.439  99.620 169.373  1.00  0.00
ATOM   6191  O   ILE A 407     148.912 100.519 170.042  1.00  0.00
ATOM   6192  N   GLN A 408     150.628  99.097 169.682  1.00  0.00
ATOM   6193  H   GLN A 408     150.982  98.331 169.114  1.00  0.00
ATOM   6194  CA  GLN A 408     151.457  99.564 170.799  1.00  0.00
ATOM   6195  HA  GLN A 408     150.837 100.179 171.447  1.00  0.00
ATOM   6196  CB  GLN A 408     151.954  98.361 171.617  1.00  0.00
ATOM   6197  HB1 GLN A 408     153.042  98.362 171.651  1.00  0.00
ATOM   6198  HB2 GLN A 408     151.639  97.423 171.162  1.00  0.00
ATOM   6199  CG  GLN A 408     151.426  98.425 173.058  1.00  0.00
ATOM   6200  HG1 GLN A 408     150.339  98.351 173.051  1.00  0.00
ATOM   6201  HG2 GLN A 408     151.702  99.389 173.478  1.00  0.00
ATOM   6202  CD  GLN A 408     152.006  97.325 173.926  1.00  0.00
ATOM   6203  OE1 GLN A 408     151.611  96.172 173.891  1.00  0.00
ATOM   6204  NE2 GLN A 408     153.002  97.624 174.729  1.00  0.00
ATOM   6205 1HE2 GLN A 408     153.330  98.569 174.807  1.00  0.00
ATOM   6206 2HE2 GLN A 408     153.367  96.876 175.291  1.00  0.00
ATOM   6207  C   GLN A 408     152.610 100.469 170.363  1.00  0.00
ATOM   6208  O   GLN A 408     153.192 101.151 171.203  1.00  0.00
ATOM   6209  N   ALA A 409     152.883 100.552 169.060  1.00  0.00
ATOM   6210  H   ALA A 409     152.449  99.881 168.440  1.00  0.00
ATOM   6211  CA  ALA A 409     153.518 101.703 168.425  1.00  0.00
ATOM   6212  HA  ALA A 409     153.224 102.611 168.953  1.00  0.00
ATOM   6213  CB  ALA A 409     155.041 101.552 168.524  1.00  0.00
ATOM   6214  HB1 ALA A 409     155.529 102.380 168.011  1.00  0.00
ATOM   6215  HB2 ALA A 409     155.344 101.559 169.571  1.00  0.00
ATOM   6216  HB3 ALA A 409     155.345 100.612 168.065  1.00  0.00
ATOM   6217  C   ALA A 409     153.068 101.824 166.964  1.00  0.00
ATOM   6218  O   ALA A 409     152.786 100.819 166.305  1.00  0.00
ATOM   6219  N   TRP A 410     153.046 103.056 166.461  1.00  0.00
ATOM   6220  H   TRP A 410     153.256 103.828 167.081  1.00  0.00
```

ATOM	6221	CA	TRP	A	410	152.952	103.382	165.037	1.00	0.00
ATOM	6222	HA	TRP	A	410	153.449	102.581	164.493	1.00	0.00
ATOM	6223	CB	TRP	A	410	151.476	103.430	164.588	1.00	0.00
ATOM	6224	HB1	TRP	A	410	151.213	104.452	164.309	1.00	0.00
ATOM	6225	HB2	TRP	A	410	150.831	103.142	165.418	1.00	0.00
ATOM	6226	CG	TRP	A	410	151.128	102.525	163.450	1.00	0.00
ATOM	6227	CD1	TRP	A	410	151.118	101.176	163.504	1.00	0.00
ATOM	6228	HD1	TRP	A	410	151.346	100.591	164.394	1.00	0.00
ATOM	6229	NE1	TRP	A	410	150.824	100.659	162.262	1.00	0.00
ATOM	6230	HE1	TRP	A	410	150.779	99.668	162.065	1.00	0.00
ATOM	6231	CE2	TRP	A	410	150.670	101.660	161.332	1.00	0.00
ATOM	6232	CZ2	TRP	A	410	150.475	101.651	159.947	1.00	0.00
ATOM	6233	HZ2	TRP	A	410	150.445	100.712	159.418	1.00	0.00
ATOM	6234	CH2	TRP	A	410	150.320	102.870	159.270	1.00	0.00
ATOM	6235	HH2	TRP	A	410	150.185	102.886	158.200	1.00	0.00
ATOM	6236	CZ3	TRP	A	410	150.304	104.070	159.997	1.00	0.00
ATOM	6237	HZ3	TRP	A	410	150.114	104.999	159.481	1.00	0.00
ATOM	6238	CE3	TRP	A	410	150.536	104.072	161.385	1.00	0.00
ATOM	6239	HE3	TRP	A	410	150.536	105.012	161.924	1.00	0.00
ATOM	6240	CD2	TRP	A	410	150.768	102.868	162.079	1.00	0.00
ATOM	6241	C	TRP	A	410	153.705	104.704	164.797	1.00	0.00
ATOM	6242	O	TRP	A	410	153.608	105.595	165.638	1.00	0.00
ATOM	6243	N	PRO	A	411	154.513	104.795	163.724	1.00	0.00
ATOM	6244	CD	PRO	A	411	154.546	103.860	162.619	1.00	0.00
ATOM	6245	HD1	PRO	A	411	153.572	103.779	162.134	1.00	0.00
ATOM	6246	HD2	PRO	A	411	154.871	102.885	162.973	1.00	0.00
ATOM	6247	CG	PRO	A	411	155.575	104.391	161.638	1.00	0.00
ATOM	6248	HG1	PRO	A	411	155.072	104.939	160.843	1.00	0.00
ATOM	6249	HG2	PRO	A	411	156.154	103.558	161.247	1.00	0.00
ATOM	6250	CB	PRO	A	411	156.460	105.345	162.435	1.00	0.00
ATOM	6251	HB1	PRO	A	411	156.777	106.195	161.831	1.00	0.00
ATOM	6252	HB2	PRO	A	411	157.335	104.804	162.799	1.00	0.00
ATOM	6253	CA	PRO	A	411	155.602	105.770	163.630	1.00	0.00
ATOM	6254	HA	PRO	A	411	156.215	105.676	164.527	1.00	0.00
ATOM	6255	C	PRO	A	411	155.096	107.206	163.552	1.00	0.00
ATOM	6256	O	PRO	A	411	154.251	107.507	162.716	1.00	0.00
ATOM	6257	N	GLU	A	412	155.646	108.069	164.417	1.00	0.00
ATOM	6258	H	GLU	A	412	156.294	107.686	165.088	1.00	0.00
ATOM	6259	CA	GLU	A	412	155.238	109.461	164.683	1.00	0.00
ATOM	6260	HA	GLU	A	412	154.400	109.450	165.383	1.00	0.00
ATOM	6261	CB	GLU	A	412	156.436	110.155	165.364	1.00	0.00
ATOM	6262	HB1	GLU	A	412	157.218	110.293	164.617	1.00	0.00
ATOM	6263	HB2	GLU	A	412	156.849	109.504	166.135	1.00	0.00
ATOM	6264	CG	GLU	A	412	156.129	111.524	165.991	1.00	0.00
ATOM	6265	HG1	GLU	A	412	155.549	112.125	165.290	1.00	0.00
ATOM	6266	HG2	GLU	A	412	157.082	112.035	166.143	1.00	0.00
ATOM	6267	CD	GLU	A	412	155.403	111.446	167.343	1.00	0.00
ATOM	6268	OE1	GLU	A	412	155.299	112.501	168.006	1.00	0.00
ATOM	6269	OE2	GLU	A	412	154.938	110.353	167.744	1.00	0.00
ATOM	6270	C	GLU	A	412	154.796	110.240	163.431	1.00	0.00
ATOM	6271	O	GLU	A	412	153.748	110.882	163.422	1.00	0.00
ATOM	6272	N	ASN	A	413	155.548	110.131	162.329	1.00	0.00
ATOM	6273	H	ASN	A	413	156.353	109.523	162.361	1.00	0.00
ATOM	6274	CA	ASN	A	413	155.266	110.827	161.071	1.00	0.00
ATOM	6275	HA	ASN	A	413	155.147	111.887	161.304	1.00	0.00
ATOM	6276	CB	ASN	A	413	156.487	110.663	160.140	1.00	0.00
ATOM	6277	HB1	ASN	A	413	157.404	110.849	160.697	1.00	0.00
ATOM	6278	HB2	ASN	A	413	156.417	111.422	159.362	1.00	0.00
ATOM	6279	CG	ASN	A	413	156.576	109.289	159.488	1.00	0.00
ATOM	6280	OD1	ASN	A	413	156.834	108.282	160.118	1.00	0.00
ATOM	6281	ND2	ASN	A	413	156.377	109.194	158.190	1.00	0.00

```
ATOM   6282 1HD2 ASN A 413     156.107 110.004 157.657  1.00  0.00
ATOM   6283 2HD2 ASN A 413     156.479 108.285 157.775  1.00  0.00
ATOM   6284  C   ASN A 413     153.982 110.400 160.336  1.00  0.00
ATOM   6285  O   ASN A 413     153.692 110.952 159.273  1.00  0.00
ATOM   6286  N   ARG A 414     153.251 109.394 160.829  1.00  0.00
ATOM   6287  H   ARG A 414     153.561 108.965 161.699  1.00  0.00
ATOM   6288  CA  ARG A 414     152.069 108.845 160.155  1.00  0.00
ATOM   6289  HA  ARG A 414     152.262 108.869 159.084  1.00  0.00
ATOM   6290  CB  ARG A 414     151.823 107.398 160.598  1.00  0.00
ATOM   6291  HB1 ARG A 414     150.810 107.107 160.325  1.00  0.00
ATOM   6292  HB2 ARG A 414     151.905 107.318 161.682  1.00  0.00
ATOM   6293  CG  ARG A 414     152.801 106.421 159.918  1.00  0.00
ATOM   6294  HG1 ARG A 414     152.678 105.439 160.370  1.00  0.00
ATOM   6295  HG2 ARG A 414     153.827 106.751 160.081  1.00  0.00
ATOM   6296  CD  ARG A 414     152.512 106.343 158.414  1.00  0.00
ATOM   6297  HD1 ARG A 414     152.922 107.225 157.923  1.00  0.00
ATOM   6298  HD2 ARG A 414     151.433 106.341 158.260  1.00  0.00
ATOM   6299  NE  ARG A 414     153.078 105.143 157.773  1.00  0.00
ATOM   6300  HE  ARG A 414     153.675 104.541 158.309  1.00  0.00
ATOM   6301  CZ  ARG A 414     152.834 104.775 156.525  1.00  0.00
ATOM   6302  NH1 ARG A 414     152.015 105.412 155.748  1.00  0.00
ATOM   6303 1HH1 ARG A 414     151.467 106.183 156.069  1.00  0.00
ATOM   6304 2HH1 ARG A 414     151.847 105.005 154.822  1.00  0.00
ATOM   6305  NH2 ARG A 414     153.403 103.743 155.997  1.00  0.00
ATOM   6306 1HH2 ARG A 414     154.026 103.122 156.495  1.00  0.00
ATOM   6307 2HH2 ARG A 414     153.139 103.550 155.028  1.00  0.00
ATOM   6308  C   ARG A 414     150.808 109.699 160.276  1.00  0.00
ATOM   6309  O   ARG A 414     149.952 109.540 159.416  1.00  0.00
ATOM   6310  N   THR A 415     150.702 110.567 161.288  1.00  0.00
ATOM   6311  H   THR A 415     151.522 110.645 161.872  1.00  0.00
ATOM   6312  CA  THR A 415     149.473 111.227 161.815  1.00  0.00
ATOM   6313  HA  THR A 415     149.757 111.584 162.805  1.00  0.00
ATOM   6314  CB  THR A 415     149.069 112.518 161.061  1.00  0.00
ATOM   6315  HB  THR A 415     149.857 113.252 161.238  1.00  0.00
ATOM   6316  CG2 THR A 415     148.891 112.401 159.549  1.00  0.00
ATOM   6317 1HG2 THR A 415     149.851 112.210 159.074  1.00  0.00
ATOM   6318 2HG2 THR A 415     148.208 111.590 159.304  1.00  0.00
ATOM   6319 3HG2 THR A 415     148.492 113.333 159.153  1.00  0.00
ATOM   6320  OG1 THR A 415     147.872 113.058 161.586  1.00  0.00
ATOM   6321  HG1 THR A 415     147.137 112.506 161.216  1.00  0.00
ATOM   6322  C   THR A 415     148.262 110.331 162.102  1.00  0.00
ATOM   6323  O   THR A 415     147.542 110.603 163.053  1.00  0.00
ATOM   6324  N   ASP A 416     148.062 109.239 161.376  1.00  0.00
ATOM   6325  H   ASP A 416     148.656 109.102 160.570  1.00  0.00
ATOM   6326  CA  ASP A 416     146.850 108.427 161.387  1.00  0.00
ATOM   6327  HA  ASP A 416     146.316 108.558 162.322  1.00  0.00
ATOM   6328  CB  ASP A 416     145.955 108.931 160.231  1.00  0.00
ATOM   6329  HB1 ASP A 416     145.149 108.208 160.092  1.00  0.00
ATOM   6330  HB2 ASP A 416     146.539 108.947 159.307  1.00  0.00
ATOM   6331  CG  ASP A 416     145.330 110.324 160.468  1.00  0.00
ATOM   6332  OD1 ASP A 416     144.084 110.387 160.528  1.00  0.00
ATOM   6333  OD2 ASP A 416     146.035 111.351 160.613  1.00  0.00
ATOM   6334  C   ASP A 416     147.231 106.934 161.250  1.00  0.00
ATOM   6335  O   ASP A 416     148.385 106.598 160.964  1.00  0.00
ATOM   6336  N   LEU A 417     146.279 106.004 161.432  1.00  0.00
ATOM   6337  H   LEU A 417     145.343 106.315 161.656  1.00  0.00
ATOM   6338  CA  LEU A 417     146.499 104.567 161.160  1.00  0.00
ATOM   6339  HA  LEU A 417     147.507 104.317 161.495  1.00  0.00
ATOM   6340  CB  LEU A 417     145.520 103.647 161.920  1.00  0.00
ATOM   6341  HB1 LEU A 417     145.769 102.628 161.636  1.00  0.00
ATOM   6342  HB2 LEU A 417     144.516 103.824 161.548  1.00  0.00
```

ATOM	6343	CG	LEU	A	417	145.514	103.688	163.456	1.00	0.00
ATOM	6344	HG	LEU	A	417	145.082	104.625	163.791	1.00	0.00
ATOM	6345	CD1	LEU	A	417	144.648	102.550	164.002	1.00	0.00
ATOM	6346	1HD1	LEU	A	417	144.598	102.608	165.087	1.00	0.00
ATOM	6347	2HD1	LEU	A	417	143.643	102.647	163.597	1.00	0.00
ATOM	6348	3HD1	LEU	A	417	145.066	101.584	163.717	1.00	0.00
ATOM	6349	CD2	LEU	A	417	146.907	103.516	164.064	1.00	0.00
ATOM	6350	1HD2	LEU	A	417	147.345	102.571	163.742	1.00	0.00
ATOM	6351	2HD2	LEU	A	417	147.556	104.329	163.743	1.00	0.00
ATOM	6352	3HD2	LEU	A	417	146.840	103.541	165.150	1.00	0.00
ATOM	6353	C	LEU	A	417	146.486	104.261	159.649	1.00	0.00
ATOM	6354	O	LEU	A	417	145.720	103.427	159.171	1.00	0.00
ATOM	6355	N	HIS	A	418	147.335	104.942	158.884	1.00	0.00
ATOM	6356	H	HIS	A	418	147.912	105.613	159.384	1.00	0.00
ATOM	6357	CA	HIS	A	418	147.464	104.935	157.415	1.00	0.00
ATOM	6358	HA	HIS	A	418	146.492	105.193	156.993	1.00	0.00
ATOM	6359	CB	HIS	A	418	148.457	106.067	157.058	1.00	0.00
ATOM	6360	HB1	HIS	A	418	149.458	105.656	156.943	1.00	0.00
ATOM	6361	HB2	HIS	A	418	148.507	106.782	157.882	1.00	0.00
ATOM	6362	CG	HIS	A	418	148.104	106.870	155.831	1.00	0.00
ATOM	6363	ND1	HIS	A	418	147.894	108.247	155.841	1.00	0.00
ATOM	6364	CE1	HIS	A	418	147.525	108.581	154.598	1.00	0.00
ATOM	6365	HE1	HIS	A	418	147.239	109.580	154.299	1.00	0.00
ATOM	6366	NE2	HIS	A	418	147.533	107.496	153.805	1.00	0.00
ATOM	6367	HE2	HIS	A	418	147.231	107.477	152.844	1.00	0.00
ATOM	6368	CD2	HIS	A	418	147.899	106.408	154.565	1.00	0.00
ATOM	6369	HD2	HIS	A	418	147.945	105.384	154.239	1.00	0.00
ATOM	6370	C	HIS	A	418	147.903	103.588	156.777	1.00	0.00
ATOM	6371	O	HIS	A	418	148.508	103.566	155.710	1.00	0.00
ATOM	6372	N	ALA	A	419	147.653	102.453	157.430	1.00	0.00
ATOM	6373	H	ALA	A	419	147.195	102.542	158.325	1.00	0.00
ATOM	6374	CA	ALA	A	419	147.631	101.133	156.791	1.00	0.00
ATOM	6375	HA	ALA	A	419	147.418	101.284	155.736	1.00	0.00
ATOM	6376	CB	ALA	A	419	148.987	100.449	156.891	1.00	0.00
ATOM	6377	HB1	ALA	A	419	148.994	99.609	156.201	1.00	0.00
ATOM	6378	HB2	ALA	A	419	149.787	101.135	156.618	1.00	0.00
ATOM	6379	HB3	ALA	A	419	149.131	100.065	157.897	1.00	0.00
ATOM	6380	C	ALA	A	419	146.497	100.244	157.328	1.00	0.00
ATOM	6381	O	ALA	A	419	146.508	99.026	157.173	1.00	0.00
ATOM	6382	N	PHE	A	420	145.504	100.855	157.972	1.00	0.00
ATOM	6383	H	PHE	A	420	145.560	101.862	158.071	1.00	0.00
ATOM	6384	CA	PHE	A	420	144.297	100.212	158.468	1.00	0.00
ATOM	6385	HA	PHE	A	420	144.218	99.218	158.025	1.00	0.00
ATOM	6386	CB	PHE	A	420	144.398	100.053	159.991	1.00	0.00
ATOM	6387	HB1	PHE	A	420	143.460	99.647	160.364	1.00	0.00
ATOM	6388	HB2	PHE	A	420	144.523	101.037	160.440	1.00	0.00
ATOM	6389	CG	PHE	A	420	145.521	99.130	160.450	1.00	0.00
ATOM	6390	CD1	PHE	A	420	145.530	97.778	160.055	1.00	0.00
ATOM	6391	HD1	PHE	A	420	144.759	97.402	159.399	1.00	0.00
ATOM	6392	CE1	PHE	A	420	146.552	96.912	160.482	1.00	0.00
ATOM	6393	HE1	PHE	A	420	146.556	95.881	160.167	1.00	0.00
ATOM	6394	CZ	PHE	A	420	147.583	97.397	161.302	1.00	0.00
ATOM	6395	HZ	PHE	A	420	148.370	96.736	161.634	1.00	0.00
ATOM	6396	CE2	PHE	A	420	147.594	98.749	161.684	1.00	0.00
ATOM	6397	HE2	PHE	A	420	148.387	99.120	162.306	1.00	0.00
ATOM	6398	CD2	PHE	A	420	146.569	99.613	161.259	1.00	0.00
ATOM	6399	HD2	PHE	A	420	146.595	100.645	161.559	1.00	0.00
ATOM	6400	C	PHE	A	420	143.043	100.971	157.998	1.00	0.00
ATOM	6401	O	PHE	A	420	141.931	100.602	158.370	1.00	0.00
ATOM	6402	N	GLU	A	421	143.188	101.952	157.096	1.00	0.00
ATOM	6403	H	GLU	A	421	144.124	102.157	156.752	1.00	0.00

ATOM	6404	CA	GLU	A	421	142.084	102.726	156.502	1.00	0.00
ATOM	6405	HA	GLU	A	421	141.574	103.224	157.321	1.00	0.00
ATOM	6406	CB	GLU	A	421	142.557	103.810	155.504	1.00	0.00
ATOM	6407	HB1	GLU	A	421	141.790	104.586	155.496	1.00	0.00
ATOM	6408	HB2	GLU	A	421	142.576	103.384	154.500	1.00	0.00
ATOM	6409	CG	GLU	A	421	143.912	104.490	155.755	1.00	0.00
ATOM	6410	HG1	GLU	A	421	144.127	104.524	156.825	1.00	0.00
ATOM	6411	HG2	GLU	A	421	143.854	105.520	155.397	1.00	0.00
ATOM	6412	CD	GLU	A	421	145.030	103.764	154.999	1.00	0.00
ATOM	6413	OE1	GLU	A	421	145.328	102.601	155.350	1.00	0.00
ATOM	6414	OE2	GLU	A	421	145.598	104.322	154.034	1.00	0.00
ATOM	6415	C	GLU	A	421	141.040	101.849	155.793	1.00	0.00
ATOM	6416	O	GLU	A	421	139.908	102.276	155.538	1.00	0.00
ATOM	6417	N	ASN	A	422	141.452	100.623	155.470	1.00	0.00
ATOM	6418	H	ASN	A	422	142.407	100.420	155.728	1.00	0.00
ATOM	6419	CA	ASN	A	422	140.747	99.556	154.784	1.00	0.00
ATOM	6420	HA	ASN	A	422	139.973	99.974	154.143	1.00	0.00
ATOM	6421	CB	ASN	A	422	141.812	98.898	153.895	1.00	0.00
ATOM	6422	HB1	ASN	A	422	142.542	98.403	154.537	1.00	0.00
ATOM	6423	HB2	ASN	A	422	142.334	99.655	153.310	1.00	0.00
ATOM	6424	CG	ASN	A	422	141.261	97.883	152.921	1.00	0.00
ATOM	6425	OD1	ASN	A	422	140.191	98.042	152.343	1.00	0.00
ATOM	6426	ND2	ASN	A	422	141.997	96.837	152.661	1.00	0.00
ATOM	6427	1HD2	ASN	A	422	142.924	96.821	153.068	1.00	0.00
ATOM	6428	2HD2	ASN	A	422	141.660	96.124	152.042	1.00	0.00
ATOM	6429	C	ASN	A	422	140.088	98.522	155.723	1.00	0.00
ATOM	6430	O	ASN	A	422	139.176	97.833	155.267	1.00	0.00
ATOM	6431	N	LEU	A	423	140.489	98.440	157.004	1.00	0.00
ATOM	6432	H	LEU	A	423	141.132	99.132	157.370	1.00	0.00
ATOM	6433	CA	LEU	A	423	139.922	97.471	157.950	1.00	0.00
ATOM	6434	HA	LEU	A	423	140.078	96.481	157.523	1.00	0.00
ATOM	6435	CB	LEU	A	423	140.560	97.526	159.351	1.00	0.00
ATOM	6436	HB1	LEU	A	423	139.808	97.210	160.078	1.00	0.00
ATOM	6437	HB2	LEU	A	423	140.817	98.559	159.585	1.00	0.00
ATOM	6438	CG	LEU	A	423	141.784	96.624	159.540	1.00	0.00
ATOM	6439	HG	LEU	A	423	142.574	96.959	158.871	1.00	0.00
ATOM	6440	CD1	LEU	A	423	142.272	96.747	160.981	1.00	0.00
ATOM	6441	1HD1	LEU	A	423	143.192	96.180	161.120	1.00	0.00
ATOM	6442	2HD1	LEU	A	423	142.459	97.792	161.223	1.00	0.00
ATOM	6443	3HD1	LEU	A	423	141.501	96.363	161.649	1.00	0.00
ATOM	6444	CD2	LEU	A	423	141.504	95.130	159.310	1.00	0.00
ATOM	6445	1HD2	LEU	A	423	142.371	94.541	159.609	1.00	0.00
ATOM	6446	2HD2	LEU	A	423	140.634	94.820	159.893	1.00	0.00
ATOM	6447	3HD2	LEU	A	423	141.319	94.941	158.256	1.00	0.00
ATOM	6448	C	LEU	A	423	138.423	97.667	158.061	1.00	0.00
ATOM	6449	O	LEU	A	423	137.953	98.757	158.363	1.00	0.00
ATOM	6450	N	GLU	A	424	137.680	96.605	157.802	1.00	0.00
ATOM	6451	H	GLU	A	424	138.162	95.752	157.538	1.00	0.00
ATOM	6452	CA	GLU	A	424	136.227	96.607	157.666	1.00	0.00
ATOM	6453	HA	GLU	A	424	135.875	97.636	157.593	1.00	0.00
ATOM	6454	CB	GLU	A	424	135.893	95.889	156.334	1.00	0.00
ATOM	6455	HB1	GLU	A	424	135.802	94.825	156.548	1.00	0.00
ATOM	6456	HB2	GLU	A	424	136.732	95.997	155.645	1.00	0.00
ATOM	6457	CG	GLU	A	424	134.620	96.349	155.599	1.00	0.00
ATOM	6458	HG1	GLU	A	424	133.861	96.627	156.332	1.00	0.00
ATOM	6459	HG2	GLU	A	424	134.228	95.492	155.046	1.00	0.00
ATOM	6460	CD	GLU	A	424	134.836	97.496	154.589	1.00	0.00
ATOM	6461	OE1	GLU	A	424	134.125	98.530	154.674	1.00	0.00
ATOM	6462	OE2	GLU	A	424	135.651	97.392	153.643	1.00	0.00
ATOM	6463	C	GLU	A	424	135.545	95.969	158.896	1.00	0.00
ATOM	6464	O	GLU	A	424	134.384	96.260	159.174	1.00	0.00

```
ATOM   6465  N   ILE A 425     136.298  95.144 159.635  1.00  0.00
ATOM   6466  H   ILE A 425     137.279  95.111 159.397  1.00  0.00
ATOM   6467  CA  ILE A 425     135.899  94.098 160.599  1.00  0.00
ATOM   6468  HA  ILE A 425     134.967  94.355 161.089  1.00  0.00
ATOM   6469  CB  ILE A 425     135.753  92.791 159.768  1.00  0.00
ATOM   6470  HB  ILE A 425     136.632  92.740 159.123  1.00  0.00
ATOM   6471  CG2 ILE A 425     135.756  91.502 160.598  1.00  0.00
ATOM   6472 1HG2 ILE A 425     135.630  90.646 159.944  1.00  0.00
ATOM   6473 2HG2 ILE A 425     136.706  91.366 161.111  1.00  0.00
ATOM   6474 3HG2 ILE A 425     134.941  91.529 161.324  1.00  0.00
ATOM   6475  CG1 ILE A 425     134.504  92.764 158.852  1.00  0.00
ATOM   6476 1HG1 ILE A 425     134.182  93.768 158.596  1.00  0.00
ATOM   6477 2HG1 ILE A 425     133.673  92.286 159.369  1.00  0.00
ATOM   6478  CD  ILE A 425     134.771  92.022 157.535  1.00  0.00
ATOM   6479  HD1 ILE A 425     133.856  91.987 156.946  1.00  0.00
ATOM   6480  HD2 ILE A 425     135.539  92.546 156.967  1.00  0.00
ATOM   6481  HD3 ILE A 425     135.116  91.008 157.725  1.00  0.00
ATOM   6482  C   ILE A 425     137.020  93.928 161.646  1.00  0.00
ATOM   6483  O   ILE A 425     138.147  94.274 161.320  1.00  0.00
ATOM   6484  N   ILE A 426     136.737  93.369 162.838  1.00  0.00
ATOM   6485  H   ILE A 426     135.744  93.275 163.014  1.00  0.00
ATOM   6486  CA  ILE A 426     137.610  93.050 163.994  1.00  0.00
ATOM   6487  HA  ILE A 426     138.525  92.604 163.624  1.00  0.00
ATOM   6488  CB  ILE A 426     138.027  94.301 164.838  1.00  0.00
ATOM   6489  HB  ILE A 426     137.132  94.710 165.305  1.00  0.00
ATOM   6490  CG2 ILE A 426     139.013  93.881 165.953  1.00  0.00
ATOM   6491 1HG2 ILE A 426     138.649  93.025 166.516  1.00  0.00
ATOM   6492 2HG2 ILE A 426     139.991  93.642 165.540  1.00  0.00
ATOM   6493 3HG2 ILE A 426     139.137  94.687 166.673  1.00  0.00
ATOM   6494  CG1 ILE A 426     138.676  95.452 164.030  1.00  0.00
ATOM   6495 1HG1 ILE A 426     137.975  95.777 163.272  1.00  0.00
ATOM   6496 2HG1 ILE A 426     139.573  95.088 163.534  1.00  0.00
ATOM   6497  CD  ILE A 426     139.041  96.720 164.814  1.00  0.00
ATOM   6498  HD1 ILE A 426     139.439  97.463 164.122  1.00  0.00
ATOM   6499  HD2 ILE A 426     138.153  97.131 165.288  1.00  0.00
ATOM   6500  HD3 ILE A 426     139.799  96.510 165.566  1.00  0.00
ATOM   6501  C   ILE A 426     136.856  92.023 164.879  1.00  0.00
ATOM   6502  O   ILE A 426     136.234  92.381 165.876  1.00  0.00
ATOM   6503  N   ARG A 427     136.852  90.732 164.520  1.00  0.00
ATOM   6504  H   ARG A 427     137.406  90.489 163.706  1.00  0.00
ATOM   6505  CA  ARG A 427     135.975  89.691 165.118  1.00  0.00
ATOM   6506  HA  ARG A 427     134.986  90.136 165.154  1.00  0.00
ATOM   6507  CB  ARG A 427     135.869  88.449 164.204  1.00  0.00
ATOM   6508  HB1 ARG A 427     135.046  87.831 164.563  1.00  0.00
ATOM   6509  HB2 ARG A 427     136.779  87.857 164.266  1.00  0.00
ATOM   6510  CG  ARG A 427     135.583  88.812 162.735  1.00  0.00
ATOM   6511  HG1 ARG A 427     136.475  89.255 162.295  1.00  0.00
ATOM   6512  HG2 ARG A 427     134.785  89.554 162.728  1.00  0.00
ATOM   6513  CD  ARG A 427     135.167  87.603 161.880  1.00  0.00
ATOM   6514  HD1 ARG A 427     134.343  87.078 162.364  1.00  0.00
ATOM   6515  HD2 ARG A 427     136.014  86.917 161.804  1.00  0.00
ATOM   6516  NE  ARG A 427     134.755  88.008 160.517  1.00  0.00
ATOM   6517  HE  ARG A 427     135.404  87.878 159.743  1.00  0.00
ATOM   6518  CZ  ARG A 427     133.697  88.719 160.184  1.00  0.00
ATOM   6519  NH1 ARG A 427     132.770  89.026 161.040  1.00  0.00
ATOM   6520 1HH1 ARG A 427     132.896  88.759 161.993  1.00  0.00
ATOM   6521 2HH1 ARG A 427     132.225  89.886 160.893  1.00  0.00
ATOM   6522  NH2 ARG A 427     133.579  89.178 158.979  1.00  0.00
ATOM   6523 1HH2 ARG A 427     134.353  88.969 158.340  1.00  0.00
ATOM   6524 2HH2 ARG A 427     132.826  89.795 158.737  1.00  0.00
ATOM   6525  C   ARG A 427     136.239  89.307 166.584  1.00  0.00
```

```
ATOM   6526  O   ARG A 427     135.566  88.396 167.078  1.00  0.00
ATOM   6527  N   GLY A 428     137.197  89.961 167.257  1.00  0.00
ATOM   6528  H   GLY A 428     137.588  90.758 166.778  1.00  0.00
ATOM   6529  CA  GLY A 428     137.556  89.788 168.678  1.00  0.00
ATOM   6530  HA1 GLY A 428     136.864  90.371 169.281  1.00  0.00
ATOM   6531  HA2 GLY A 428     138.558  90.185 168.831  1.00  0.00
ATOM   6532  C   GLY A 428     137.538  88.349 169.210  1.00  0.00
ATOM   6533  O   GLY A 428     137.078  88.094 170.329  1.00  0.00
ATOM   6534  N   ARG A 429     137.956  87.385 168.389  1.00  0.00
ATOM   6535  H   ARG A 429     138.321  87.682 167.488  1.00  0.00
ATOM   6536  CA  ARG A 429     137.813  85.949 168.640  1.00  0.00
ATOM   6537  HA  ARG A 429     136.865  85.815 169.141  1.00  0.00
ATOM   6538  CB  ARG A 429     137.816  85.214 167.285  1.00  0.00
ATOM   6539  HB1 ARG A 429     138.838  85.187 166.902  1.00  0.00
ATOM   6540  HB2 ARG A 429     137.220  85.797 166.579  1.00  0.00
ATOM   6541  CG  ARG A 429     137.251  83.778 167.274  1.00  0.00
ATOM   6542  HG1 ARG A 429     137.995  83.101 167.693  1.00  0.00
ATOM   6543  HG2 ARG A 429     137.101  83.488 166.232  1.00  0.00
ATOM   6544  CD  ARG A 429     135.930  83.575 168.034  1.00  0.00
ATOM   6545  HD1 ARG A 429     136.178  83.448 169.089  1.00  0.00
ATOM   6546  HD2 ARG A 429     135.457  82.654 167.688  1.00  0.00
ATOM   6547  NE  ARG A 429     134.993  84.709 167.866  1.00  0.00
ATOM   6548  HE  ARG A 429     134.960  85.161 166.968  1.00  0.00
ATOM   6549  CZ  ARG A 429     134.406  85.367 168.848  1.00  0.00
ATOM   6550  NH1 ARG A 429     134.330  84.927 170.071  1.00  0.00
ATOM   6551 1HH1 ARG A 429     134.591  83.989 170.306  1.00  0.00
ATOM   6552 2HH1 ARG A 429     133.987  85.581 170.782  1.00  0.00
ATOM   6553  NH2 ARG A 429     133.924  86.547 168.661  1.00  0.00
ATOM   6554 1HH2 ARG A 429     134.196  87.105 167.863  1.00  0.00
ATOM   6555 2HH2 ARG A 429     133.593  87.020 169.495  1.00  0.00
ATOM   6556  C   ARG A 429     138.832  85.397 169.627  1.00  0.00
ATOM   6557  O   ARG A 429     138.651  84.262 170.062  1.00  0.00
ATOM   6558  N   THR A 430     139.806  86.201 170.050  1.00  0.00
ATOM   6559  H   THR A 430     139.951  87.082 169.575  1.00  0.00
ATOM   6560  CA  THR A 430     140.732  85.846 171.142  1.00  0.00
ATOM   6561  HA  THR A 430     140.388  84.942 171.644  1.00  0.00
ATOM   6562  CB  THR A 430     142.149  85.569 170.615  1.00  0.00
ATOM   6563  HB  THR A 430     142.856  85.538 171.445  1.00  0.00
ATOM   6564  CG2 THR A 430     142.237  84.268 169.820  1.00  0.00
ATOM   6565 1HG2 THR A 430     143.271  84.105 169.518  1.00  0.00
ATOM   6566 2HG2 THR A 430     141.914  83.435 170.443  1.00  0.00
ATOM   6567 3HG2 THR A 430     141.606  84.324 168.935  1.00  0.00
ATOM   6568  OG1 THR A 430     142.462  86.622 169.751  1.00  0.00
ATOM   6569  HG1 THR A 430     143.423  86.607 169.489  1.00  0.00
ATOM   6570  C   THR A 430     140.788  86.931 172.208  1.00  0.00
ATOM   6571  O   THR A 430     140.487  86.625 173.360  1.00  0.00
ATOM   6572  N   LYS A 431     141.064  88.183 171.800  1.00  0.00
ATOM   6573  H   LYS A 431     141.305  88.268 170.815  1.00  0.00
ATOM   6574  CA  LYS A 431     141.207  89.396 172.631  1.00  0.00
ATOM   6575  HA  LYS A 431     141.247  90.276 171.997  1.00  0.00
ATOM   6576  CB  LYS A 431     139.980  89.577 173.533  1.00  0.00
ATOM   6577  HB1 LYS A 431     140.140  90.468 174.114  1.00  0.00
ATOM   6578  HB2 LYS A 431     139.915  88.755 174.236  1.00  0.00
ATOM   6579  CG  LYS A 431     138.653  89.735 172.788  1.00  0.00
ATOM   6580  HG1 LYS A 431     138.596  89.022 171.975  1.00  0.00
ATOM   6581  HG2 LYS A 431     138.605  90.740 172.373  1.00  0.00
ATOM   6582  CD  LYS A 431     137.458  89.516 173.728  1.00  0.00
ATOM   6583  HD1 LYS A 431     136.571  89.936 173.266  1.00  0.00
ATOM   6584  HD2 LYS A 431     137.636  90.048 174.661  1.00  0.00
ATOM   6585  CE  LYS A 431     137.187  88.040 174.019  1.00  0.00
ATOM   6586  HE1 LYS A 431     136.722  87.962 175.006  1.00  0.00
```

ATOM	6587	HE2	LYS	A	431	138.133	87.490	174.052	1.00	0.00
ATOM	6588	NZ	LYS	A	431	136.279	87.482	172.993	1.00	0.00
ATOM	6589	HZ1	LYS	A	431	136.648	87.603	172.055	1.00	0.00
ATOM	6590	HZ2	LYS	A	431	136.079	86.510	173.128	1.00	0.00
ATOM	6591	HZ3	LYS	A	431	135.380	87.974	172.992	1.00	0.00
ATOM	6592	C	LYS	A	431	142.484	89.400	173.481	1.00	0.00
ATOM	6593	O	LYS	A	431	142.777	88.432	174.184	1.00	0.00
ATOM	6594	N	GLN	A	432	143.231	90.504	173.483	1.00	0.00
ATOM	6595	H	GLN	A	432	142.971	91.291	172.899	1.00	0.00
ATOM	6596	CA	GLN	A	432	144.389	90.643	174.376	1.00	0.00
ATOM	6597	HA	GLN	A	432	145.092	89.839	174.141	1.00	0.00
ATOM	6598	CB	GLN	A	432	145.090	91.983	174.117	1.00	0.00
ATOM	6599	HB1	GLN	A	432	144.441	92.790	174.447	1.00	0.00
ATOM	6600	HB2	GLN	A	432	145.279	92.097	173.049	1.00	0.00
ATOM	6601	CG	GLN	A	432	146.424	92.066	174.868	1.00	0.00
ATOM	6602	HG1	GLN	A	432	147.117	91.343	174.443	1.00	0.00
ATOM	6603	HG2	GLN	A	432	146.277	91.822	175.916	1.00	0.00
ATOM	6604	CD	GLN	A	432	147.067	93.446	174.824	1.00	0.00
ATOM	6605	OE1	GLN	A	432	146.576	94.399	174.256	1.00	0.00
ATOM	6606	NE2	GLN	A	432	148.214	93.596	175.456	1.00	0.00
ATOM	6607	1HE2	GLN	A	432	148.662	92.834	175.923	1.00	0.00
ATOM	6608	2HE2	GLN	A	432	148.633	94.509	175.404	1.00	0.00
ATOM	6609	C	GLN	A	432	143.942	90.511	175.843	1.00	0.00
ATOM	6610	O	GLN	A	432	142.924	91.095	176.229	1.00	0.00
ATOM	6611	N	HIS	A	433	144.663	89.719	176.646	1.00	0.00
ATOM	6612	H	HIS	A	433	145.469	89.252	176.259	1.00	0.00
ATOM	6613	CA	HIS	A	433	144.284	89.379	178.030	1.00	0.00
ATOM	6614	HA	HIS	A	433	144.950	88.588	178.376	1.00	0.00
ATOM	6615	CB	HIS	A	433	144.534	90.604	178.934	1.00	0.00
ATOM	6616	HB1	HIS	A	433	143.679	91.280	178.886	1.00	0.00
ATOM	6617	HB2	HIS	A	433	145.406	91.147	178.569	1.00	0.00
ATOM	6618	CG	HIS	A	433	144.810	90.260	180.377	1.00	0.00
ATOM	6619	ND1	HIS	A	433	146.064	89.938	180.897	1.00	0.00
ATOM	6620	CE1	HIS	A	433	145.870	89.716	182.206	1.00	0.00
ATOM	6621	HE1	HIS	A	433	146.651	89.440	182.901	1.00	0.00
ATOM	6622	NE2	HIS	A	433	144.576	89.885	182.527	1.00	0.00
ATOM	6623	HE2	HIS	A	433	144.175	89.764	183.445	1.00	0.00
ATOM	6624	CD2	HIS	A	433	143.892	90.232	181.385	1.00	0.00
ATOM	6625	HD2	HIS	A	433	142.836	90.420	181.283	1.00	0.00
ATOM	6626	C	HIS	A	433	142.853	88.795	178.162	1.00	0.00
ATOM	6627	O	HIS	A	433	142.242	88.865	179.217	1.00	0.00
ATOM	6628	N	GLY	A	434	142.288	88.256	177.075	1.00	0.00
ATOM	6629	H	GLY	A	434	142.798	88.240	176.199	1.00	0.00
ATOM	6630	CA	GLY	A	434	140.918	87.745	177.037	1.00	0.00
ATOM	6631	HA1	GLY	A	434	140.714	87.178	177.945	1.00	0.00
ATOM	6632	HA2	GLY	A	434	140.823	87.069	176.188	1.00	0.00
ATOM	6633	C	GLY	A	434	139.825	88.820	176.900	1.00	0.00
ATOM	6634	O	GLY	A	434	138.662	88.459	176.741	1.00	0.00
ATOM	6635	N	GLN	A	435	140.157	90.122	176.901	1.00	0.00
ATOM	6636	H	GLN	A	435	141.137	90.367	176.990	1.00	0.00
ATOM	6637	CA	GLN	A	435	139.158	91.202	176.924	1.00	0.00
ATOM	6638	HA	GLN	A	435	138.166	90.766	176.812	1.00	0.00
ATOM	6639	CB	GLN	A	435	139.172	91.891	178.303	1.00	0.00
ATOM	6640	HB1	GLN	A	435	140.138	92.372	178.460	1.00	0.00
ATOM	6641	HB2	GLN	A	435	139.029	91.132	179.074	1.00	0.00
ATOM	6642	CG	GLN	A	435	138.049	92.942	178.425	1.00	0.00
ATOM	6643	HG1	GLN	A	435	137.092	92.482	178.182	1.00	0.00
ATOM	6644	HG2	GLN	A	435	138.232	93.739	177.712	1.00	0.00
ATOM	6645	CD	GLN	A	435	137.929	93.593	179.795	1.00	0.00
ATOM	6646	OE1	GLN	A	435	138.459	93.151	180.797	1.00	0.00
ATOM	6647	NE2	GLN	A	435	137.209	94.688	179.927	1.00	0.00

ATOM	6648	1HE2	GLN	A	435	136.783	95.158	179.136	1.00	0.00
ATOM	6649	2HE2	GLN	A	435	137.175	95.042	180.864	1.00	0.00
ATOM	6650	C	GLN	A	435	139.292	92.238	175.788	1.00	0.00
ATOM	6651	O	GLN	A	435	138.263	92.698	175.297	1.00	0.00
ATOM	6652	N	PHE	A	436	140.493	92.586	175.310	1.00	0.00
ATOM	6653	H	PHE	A	436	141.324	92.179	175.731	1.00	0.00
ATOM	6654	CA	PHE	A	436	140.683	93.718	174.382	1.00	0.00
ATOM	6655	HA	PHE	A	436	139.796	94.349	174.433	1.00	0.00
ATOM	6656	CB	PHE	A	436	141.830	94.605	174.888	1.00	0.00
ATOM	6657	HB1	PHE	A	436	141.748	95.588	174.424	1.00	0.00
ATOM	6658	HB2	PHE	A	436	142.777	94.177	174.567	1.00	0.00
ATOM	6659	CG	PHE	A	436	141.848	94.769	176.402	1.00	0.00
ATOM	6660	CD1	PHE	A	436	142.946	94.303	177.151	1.00	0.00
ATOM	6661	HD1	PHE	A	436	143.797	93.874	176.653	1.00	0.00
ATOM	6662	CE1	PHE	A	436	142.925	94.374	178.555	1.00	0.00
ATOM	6663	HE1	PHE	A	436	143.765	94.004	179.124	1.00	0.00
ATOM	6664	CZ	PHE	A	436	141.807	94.909	179.216	1.00	0.00
ATOM	6665	HZ	PHE	A	436	141.793	94.950	180.296	1.00	0.00
ATOM	6666	CE2	PHE	A	436	140.709	95.375	178.474	1.00	0.00
ATOM	6667	HE2	PHE	A	436	139.851	95.774	178.992	1.00	0.00
ATOM	6668	CD2	PHE	A	436	140.728	95.301	177.070	1.00	0.00
ATOM	6669	HD2	PHE	A	436	139.886	95.643	176.503	1.00	0.00
ATOM	6670	C	PHE	A	436	140.823	93.317	172.884	1.00	0.00
ATOM	6671	O	PHE	A	436	141.892	92.946	172.360	1.00	0.00
ATOM	6672	N	SER	A	437	139.700	93.385	172.167	1.00	0.00
ATOM	6673	H	SER	A	437	138.869	93.782	172.592	1.00	0.00
ATOM	6674	CA	SER	A	437	139.670	93.174	170.715	1.00	0.00
ATOM	6675	HA	SER	A	437	140.249	92.280	170.486	1.00	0.00
ATOM	6676	CB	SER	A	437	138.258	92.937	170.198	1.00	0.00
ATOM	6677	HB1	SER	A	437	137.901	91.988	170.591	1.00	0.00
ATOM	6678	HB2	SER	A	437	138.269	92.881	169.109	1.00	0.00
ATOM	6679	OG	SER	A	437	137.409	93.978	170.610	1.00	0.00
ATOM	6680	HG	SER	A	437	136.501	93.688	170.405	1.00	0.00
ATOM	6681	C	SER	A	437	140.277	94.315	169.906	1.00	0.00
ATOM	6682	O	SER	A	437	140.762	94.055	168.804	1.00	0.00
ATOM	6683	N	LEU	A	438	140.317	95.526	170.465	1.00	0.00
ATOM	6684	H	LEU	A	438	139.838	95.650	171.349	1.00	0.00
ATOM	6685	CA	LEU	A	438	141.109	96.640	169.956	1.00	0.00
ATOM	6686	HA	LEU	A	438	141.875	96.246	169.290	1.00	0.00
ATOM	6687	CB	LEU	A	438	140.224	97.588	169.135	1.00	0.00
ATOM	6688	HB1	LEU	A	438	139.347	97.804	169.737	1.00	0.00
ATOM	6689	HB2	LEU	A	438	139.903	97.070	168.229	1.00	0.00
ATOM	6690	CG	LEU	A	438	140.891	98.926	168.751	1.00	0.00
ATOM	6691	HG	LEU	A	438	141.169	99.473	169.650	1.00	0.00
ATOM	6692	CD1	LEU	A	438	142.128	98.766	167.866	1.00	0.00
ATOM	6693	1HD1	LEU	A	438	142.524	99.746	167.602	1.00	0.00
ATOM	6694	2HD1	LEU	A	438	142.910	98.214	168.382	1.00	0.00
ATOM	6695	3HD1	LEU	A	438	141.858	98.230	166.964	1.00	0.00
ATOM	6696	CD2	LEU	A	438	139.892	99.788	167.983	1.00	0.00
ATOM	6697	1HD2	LEU	A	438	140.339	100.756	167.755	1.00	0.00
ATOM	6698	2HD2	LEU	A	438	139.612	99.295	167.051	1.00	0.00
ATOM	6699	3HD2	LEU	A	438	138.996	99.948	168.578	1.00	0.00
ATOM	6700	C	LEU	A	438	141.814	97.368	171.102	1.00	0.00
ATOM	6701	O	LEU	A	438	141.180	97.809	172.062	1.00	0.00
ATOM	6702	N	ALA	A	439	143.106	97.611	170.935	1.00	0.00
ATOM	6703	H	ALA	A	439	143.579	97.187	170.142	1.00	0.00
ATOM	6704	CA	ALA	A	439	143.861	98.567	171.722	1.00	0.00
ATOM	6705	HA	ALA	A	439	143.168	99.250	172.211	1.00	0.00
ATOM	6706	CB	ALA	A	439	144.638	97.810	172.807	1.00	0.00
ATOM	6707	HB1	ALA	A	439	145.168	98.518	173.442	1.00	0.00
ATOM	6708	HB2	ALA	A	439	143.948	97.230	173.422	1.00	0.00

ATOM	6709	HB3	ALA	A	439	145.357	97.132	172.345	1.00	0.00
ATOM	6710	C	ALA	A	439	144.779	99.399	170.817	1.00	0.00
ATOM	6711	O	ALA	A	439	145.413	98.882	169.903	1.00	0.00
ATOM	6712	N	VAL	A	440	144.846	100.710	171.073	1.00	0.00
ATOM	6713	H	VAL	A	440	144.310	101.080	171.854	1.00	0.00
ATOM	6714	CA	VAL	A	440	145.665	101.647	170.285	1.00	0.00
ATOM	6715	HA	VAL	A	440	146.535	101.103	169.930	1.00	0.00
ATOM	6716	CB	VAL	A	440	144.930	102.125	169.008	1.00	0.00
ATOM	6717	HB	VAL	A	440	144.826	101.256	168.357	1.00	0.00
ATOM	6718	CG1	VAL	A	440	143.525	102.671	169.248	1.00	0.00
ATOM	6719	1HG1	VAL	A	440	143.055	102.932	168.301	1.00	0.00
ATOM	6720	2HG1	VAL	A	440	142.903	101.921	169.733	1.00	0.00
ATOM	6721	3HG1	VAL	A	440	143.577	103.554	169.873	1.00	0.00
ATOM	6722	CG2	VAL	A	440	145.734	103.170	168.227	1.00	0.00
ATOM	6723	1HG2	VAL	A	440	145.232	103.386	167.286	1.00	0.00
ATOM	6724	2HG2	VAL	A	440	145.826	104.094	168.799	1.00	0.00
ATOM	6725	3HG2	VAL	A	440	146.725	102.784	168.005	1.00	0.00
ATOM	6726	C	VAL	A	440	146.213	102.747	171.188	1.00	0.00
ATOM	6727	O	VAL	A	440	145.553	103.749	171.465	1.00	0.00
ATOM	6728	N	VAL	A	441	147.350	102.442	171.824	1.00	0.00
ATOM	6729	H	VAL	A	441	147.869	101.635	171.493	1.00	0.00
ATOM	6730	CA	VAL	A	441	147.525	102.863	173.224	1.00	0.00
ATOM	6731	HA	VAL	A	441	146.541	102.763	173.683	1.00	0.00
ATOM	6732	CB	VAL	A	441	148.422	101.912	174.046	1.00	0.00
ATOM	6733	HB	VAL	A	441	148.340	102.196	175.094	1.00	0.00
ATOM	6734	CG1	VAL	A	441	147.907	100.469	173.922	1.00	0.00
ATOM	6735	1HG1	VAL	A	441	148.441	99.826	174.621	1.00	0.00
ATOM	6736	2HG1	VAL	A	441	146.847	100.436	174.170	1.00	0.00
ATOM	6737	3HG1	VAL	A	441	148.060	100.089	172.911	1.00	0.00
ATOM	6738	CG2	VAL	A	441	149.909	101.911	173.680	1.00	0.00
ATOM	6739	1HG2	VAL	A	441	150.403	101.074	174.168	1.00	0.00
ATOM	6740	2HG2	VAL	A	441	150.034	101.836	172.602	1.00	0.00
ATOM	6741	3HG2	VAL	A	441	150.396	102.812	174.044	1.00	0.00
ATOM	6742	C	VAL	A	441	147.864	104.338	173.402	1.00	0.00
ATOM	6743	O	VAL	A	441	147.032	105.082	173.934	1.00	0.00
ATOM	6744	N	SER	A	442	149.069	104.710	172.967	1.00	0.00
ATOM	6745	H	SER	A	442	149.575	104.017	172.440	1.00	0.00
ATOM	6746	CA	SER	A	442	149.844	105.895	173.352	1.00	0.00
ATOM	6747	HA	SER	A	442	149.170	106.741	173.457	1.00	0.00
ATOM	6748	CB	SER	A	442	150.579	105.677	174.680	1.00	0.00
ATOM	6749	HB1	SER	A	442	151.121	106.585	174.946	1.00	0.00
ATOM	6750	HB2	SER	A	442	151.300	104.865	174.573	1.00	0.00
ATOM	6751	OG	SER	A	442	149.680	105.353	175.736	1.00	0.00
ATOM	6752	HG	SER	A	442	150.192	104.987	176.468	1.00	0.00
ATOM	6753	C	SER	A	442	150.825	106.219	172.223	1.00	0.00
ATOM	6754	O	SER	A	442	152.025	105.989	172.347	1.00	0.00
ATOM	6755	N	LEU	A	443	150.290	106.709	171.107	1.00	0.00
ATOM	6756	H	LEU	A	443	149.279	106.811	171.071	1.00	0.00
ATOM	6757	CA	LEU	A	443	151.041	107.293	169.999	1.00	0.00
ATOM	6758	HA	LEU	A	443	152.062	107.477	170.339	1.00	0.00
ATOM	6759	CB	LEU	A	443	151.149	106.322	168.814	1.00	0.00
ATOM	6760	HB1	LEU	A	443	151.701	105.436	169.129	1.00	0.00
ATOM	6761	HB2	LEU	A	443	151.754	106.812	168.050	1.00	0.00
ATOM	6762	CG	LEU	A	443	149.803	105.897	168.202	1.00	0.00
ATOM	6763	HG	LEU	A	443	149.059	106.674	168.351	1.00	0.00
ATOM	6764	CD1	LEU	A	443	149.986	105.702	166.706	1.00	0.00
ATOM	6765	1HD1	LEU	A	443	149.086	105.282	166.257	1.00	0.00
ATOM	6766	2HD1	LEU	A	443	150.206	106.659	166.233	1.00	0.00
ATOM	6767	3HD1	LEU	A	443	150.834	105.047	166.548	1.00	0.00
ATOM	6768	CD2	LEU	A	443	149.273	104.581	168.773	1.00	0.00
ATOM	6769	1HD2	LEU	A	443	148.345	104.332	168.271	1.00	0.00

```
ATOM   6770 2HD2 LEU A 443     149.993 103.778 168.625  1.00  0.00
ATOM   6771 3HD2 LEU A 443     149.062 104.694 169.834  1.00  0.00
ATOM   6772  C   LEU A 443     150.478 108.674 169.629  1.00  0.00
ATOM   6773  O   LEU A 443     149.362 109.006 170.032  1.00  0.00
ATOM   6774  N   ASN A 444     151.213 109.487 168.857  1.00  0.00
ATOM   6775  H   ASN A 444     152.132 109.195 168.549  1.00  0.00
ATOM   6776  CA  ASN A 444     150.808 110.874 168.565  1.00  0.00
ATOM   6777  HA  ASN A 444     150.133 111.177 169.367  1.00  0.00
ATOM   6778  CB  ASN A 444     151.999 111.838 168.677  1.00  0.00
ATOM   6779  HB1 ASN A 444     151.706 112.834 168.352  1.00  0.00
ATOM   6780  HB2 ASN A 444     152.792 111.507 168.019  1.00  0.00
ATOM   6781  CG  ASN A 444     152.519 111.976 170.108  1.00  0.00
ATOM   6782  OD1 ASN A 444     151.772 111.859 171.075  1.00  0.00
ATOM   6783  ND2 ASN A 444     153.785 112.245 170.296  1.00  0.00
ATOM   6784 1HD2 ASN A 444     154.398 112.290 169.470  1.00  0.00
ATOM   6785 2HD2 ASN A 444     154.147 112.305 171.220  1.00  0.00
ATOM   6786  C   ASN A 444     149.917 111.051 167.318  1.00  0.00
ATOM   6787  O   ASN A 444     149.919 112.073 166.637  1.00  0.00
ATOM   6788  N   ILE A 445     149.106 110.022 167.091  1.00  0.00
ATOM   6789  H   ILE A 445     149.149 109.279 167.771  1.00  0.00
ATOM   6790  CA  ILE A 445     147.983 109.976 166.163  1.00  0.00
ATOM   6791  HA  ILE A 445     148.393 110.064 165.161  1.00  0.00
ATOM   6792  CB  ILE A 445     147.344 108.575 166.312  1.00  0.00
ATOM   6793  HB  ILE A 445     148.150 107.867 166.123  1.00  0.00
ATOM   6794  CG2 ILE A 445     146.775 108.244 167.707  1.00  0.00
ATOM   6795 1HG2 ILE A 445     147.518 108.402 168.481  1.00  0.00
ATOM   6796 2HG2 ILE A 445     145.924 108.880 167.925  1.00  0.00
ATOM   6797 3HG2 ILE A 445     146.459 107.202 167.750  1.00  0.00
ATOM   6798  CG1 ILE A 445     146.253 108.319 165.267  1.00  0.00
ATOM   6799 1HG1 ILE A 445     146.399 108.979 164.422  1.00  0.00
ATOM   6800 2HG1 ILE A 445     145.266 108.526 165.679  1.00  0.00
ATOM   6801  CD  ILE A 445     146.314 106.882 164.758  1.00  0.00
ATOM   6802  HD1 ILE A 445     145.545 106.748 164.003  1.00  0.00
ATOM   6803  HD2 ILE A 445     147.285 106.692 164.300  1.00  0.00
ATOM   6804  HD3 ILE A 445     146.156 106.177 165.573  1.00  0.00
ATOM   6805  C   ILE A 445     146.996 111.136 166.409  1.00  0.00
ATOM   6806  O   ILE A 445     146.581 111.380 167.539  1.00  0.00
ATOM   6807  N   THR A 446     146.611 111.852 165.348  1.00  0.00
ATOM   6808  H   THR A 446     147.018 111.620 164.447  1.00  0.00
ATOM   6809  CA  THR A 446     145.561 112.888 165.374  1.00  0.00
ATOM   6810  HA  THR A 446     145.625 113.453 166.304  1.00  0.00
ATOM   6811  CB  THR A 446     145.688 113.859 164.180  1.00  0.00
ATOM   6812  HB  THR A 446     145.384 113.359 163.260  1.00  0.00
ATOM   6813  CG2 THR A 446     144.840 115.123 164.355  1.00  0.00
ATOM   6814 1HG2 THR A 446     145.006 115.787 163.508  1.00  0.00
ATOM   6815 2HG2 THR A 446     143.784 114.860 164.383  1.00  0.00
ATOM   6816 3HG2 THR A 446     145.119 115.628 165.279  1.00  0.00
ATOM   6817  OG1 THR A 446     147.013 114.302 164.006  1.00  0.00
ATOM   6818  HG1 THR A 446     147.354 113.826 163.223  1.00  0.00
ATOM   6819  C   THR A 446     144.178 112.263 165.275  1.00  0.00
ATOM   6820  O   THR A 446     143.246 112.695 165.947  1.00  0.00
ATOM   6821  N   SER A 447     144.071 111.232 164.437  1.00  0.00
ATOM   6822  H   SER A 447     144.884 110.984 163.882  1.00  0.00
ATOM   6823  CA  SER A 447     142.824 110.641 163.973  1.00  0.00
ATOM   6824  HA  SER A 447     142.078 110.697 164.767  1.00  0.00
ATOM   6825  CB  SER A 447     142.287 111.442 162.771  1.00  0.00
ATOM   6826  HB1 SER A 447     141.520 112.110 163.160  1.00  0.00
ATOM   6827  HB2 SER A 447     141.832 110.762 162.051  1.00  0.00
ATOM   6828  OG  SER A 447     143.233 112.251 162.096  1.00  0.00
ATOM   6829  HG  SER A 447     143.676 111.659 161.430  1.00  0.00
ATOM   6830  C   SER A 447     143.053 109.166 163.644  1.00  0.00
```

```
ATOM   6831  O   SER A 447     144.089 108.783 163.109  1.00  0.00
ATOM   6832  N   LEU A 448     142.126 108.280 164.023  1.00  0.00
ATOM   6833  H   LEU A 448     141.266 108.632 164.415  1.00  0.00
ATOM   6834  CA  LEU A 448     142.353 106.830 163.907  1.00  0.00
ATOM   6835  HA  LEU A 448     143.373 106.637 164.230  1.00  0.00
ATOM   6836  CB  LEU A 448     141.414 106.035 164.843  1.00  0.00
ATOM   6837  HB1 LEU A 448     141.428 104.989 164.536  1.00  0.00
ATOM   6838  HB2 LEU A 448     140.394 106.395 164.712  1.00  0.00
ATOM   6839  CG  LEU A 448     141.778 106.069 166.344  1.00  0.00
ATOM   6840  HG  LEU A 448     141.661 107.083 166.719  1.00  0.00
ATOM   6841  CD1 LEU A 448     140.829 105.161 167.128  1.00  0.00
ATOM   6842 1HD1 LEU A 448     141.079 105.198 168.185  1.00  0.00
ATOM   6843 2HD1 LEU A 448     139.809 105.511 166.999  1.00  0.00
ATOM   6844 3HD1 LEU A 448     140.906 104.135 166.768  1.00  0.00
ATOM   6845  CD2 LEU A 448     143.209 105.600 166.641  1.00  0.00
ATOM   6846 1HD2 LEU A 448     143.349 105.493 167.716  1.00  0.00
ATOM   6847 2HD2 LEU A 448     143.398 104.650 166.142  1.00  0.00
ATOM   6848 3HD2 LEU A 448     143.915 106.346 166.286  1.00  0.00
ATOM   6849  C   LEU A 448     142.328 106.295 162.465  1.00  0.00
ATOM   6850  O   LEU A 448     142.637 105.128 162.287  1.00  0.00
ATOM   6851  N   GLY A 449     141.945 107.099 161.462  1.00  0.00
ATOM   6852  H   GLY A 449     141.791 108.073 161.682  1.00  0.00
ATOM   6853  CA  GLY A 449     141.882 106.774 160.022  1.00  0.00
ATOM   6854  HA1 GLY A 449     142.899 106.643 159.656  1.00  0.00
ATOM   6855  HA2 GLY A 449     141.448 107.630 159.503  1.00  0.00
ATOM   6856  C   GLY A 449     141.074 105.527 159.604  1.00  0.00
ATOM   6857  O   GLY A 449     140.970 105.230 158.419  1.00  0.00
ATOM   6858  N   LEU A 450     140.492 104.804 160.571  1.00  0.00
ATOM   6859  H   LEU A 450     140.720 105.104 161.507  1.00  0.00
ATOM   6860  CA  LEU A 450     139.739 103.544 160.483  1.00  0.00
ATOM   6861  HA  LEU A 450     140.356 102.825 159.942  1.00  0.00
ATOM   6862  CB  LEU A 450     139.499 103.019 161.929  1.00  0.00
ATOM   6863  HB1 LEU A 450     138.625 102.372 161.961  1.00  0.00
ATOM   6864  HB2 LEU A 450     139.284 103.870 162.575  1.00  0.00
ATOM   6865  CG  LEU A 450     140.648 102.202 162.539  1.00  0.00
ATOM   6866  HG  LEU A 450     141.580 102.755 162.452  1.00  0.00
ATOM   6867  CD1 LEU A 450     140.370 101.944 164.024  1.00  0.00
ATOM   6868 1HD1 LEU A 450     141.234 101.462 164.480  1.00  0.00
ATOM   6869 2HD1 LEU A 450     140.186 102.886 164.538  1.00  0.00
ATOM   6870 3HD1 LEU A 450     139.493 101.308 164.138  1.00  0.00
ATOM   6871  CD2 LEU A 450     140.813 100.841 161.855  1.00  0.00
ATOM   6872 1HD2 LEU A 450     141.619 100.294 162.340  1.00  0.00
ATOM   6873 2HD2 LEU A 450     139.889 100.267 161.924  1.00  0.00
ATOM   6874 3HD2 LEU A 450     141.073 100.977 160.807  1.00  0.00
ATOM   6875  C   LEU A 450     138.423 103.640 159.678  1.00  0.00
ATOM   6876  O   LEU A 450     137.462 102.944 159.973  1.00  0.00
ATOM   6877  N   ARG A 451     138.320 104.549 158.699  1.00  0.00
ATOM   6878  H   ARG A 451     139.143 105.111 158.499  1.00  0.00
ATOM   6879  CA  ARG A 451     137.070 104.864 157.990  1.00  0.00
ATOM   6880  HA  ARG A 451     136.408 105.312 158.712  1.00  0.00
ATOM   6881  CB  ARG A 451     137.348 105.867 156.857  1.00  0.00
ATOM   6882  HB1 ARG A 451     138.143 105.472 156.223  1.00  0.00
ATOM   6883  HB2 ARG A 451     137.731 106.788 157.293  1.00  0.00
ATOM   6884  CG  ARG A 451     136.151 106.211 155.941  1.00  0.00
ATOM   6885  HG1 ARG A 451     136.014 105.405 155.217  1.00  0.00
ATOM   6886  HG2 ARG A 451     136.436 107.090 155.367  1.00  0.00
ATOM   6887  CD  ARG A 451     134.773 106.473 156.599  1.00  0.00
ATOM   6888  HD1 ARG A 451     134.206 105.548 156.623  1.00  0.00
ATOM   6889  HD2 ARG A 451     134.187 107.141 155.970  1.00  0.00
ATOM   6890  NE  ARG A 451     134.879 107.052 157.949  1.00  0.00
ATOM   6891  HE  ARG A 451     135.631 107.718 158.120  1.00  0.00
```

```
ATOM   6892  CZ  ARG A 451     134.311 106.664 159.063  1.00  0.00
ATOM   6893  NH1 ARG A 451     133.580 105.595 159.146  1.00  0.00
ATOM   6894 1HH1 ARG A 451     133.607 104.951 158.370  1.00  0.00
ATOM   6895 2HH1 ARG A 451     133.135 105.369 160.014  1.00  0.00
ATOM   6896  NH2 ARG A 451     134.577 107.328 160.140  1.00  0.00
ATOM   6897 1HH2 ARG A 451     135.306 108.040 160.041  1.00  0.00
ATOM   6898 2HH2 ARG A 451     134.258 107.026 161.038  1.00  0.00
ATOM   6899  C   ARG A 451     136.341 103.642 157.455  1.00  0.00
ATOM   6900  O   ARG A 451     135.110 103.592 157.479  1.00  0.00
ATOM   6901  N   SER A 452     137.063 102.633 156.987  1.00  0.00
ATOM   6902  H   SER A 452     138.072 102.697 157.003  1.00  0.00
ATOM   6903  CA  SER A 452     136.413 101.400 156.554  1.00  0.00
ATOM   6904  HA  SER A 452     135.656 101.659 155.815  1.00  0.00
ATOM   6905  CB  SER A 452     137.413 100.473 155.878  1.00  0.00
ATOM   6906  HB1 SER A 452     136.949  99.504 155.683  1.00  0.00
ATOM   6907  HB2 SER A 452     138.281 100.340 156.520  1.00  0.00
ATOM   6908  OG  SER A 452     137.810 101.067 154.653  1.00  0.00
ATOM   6909  HG  SER A 452     138.612 101.593 154.884  1.00  0.00
ATOM   6910  C   SER A 452     135.679 100.650 157.669  1.00  0.00
ATOM   6911  O   SER A 452     134.708  99.966 157.355  1.00  0.00
ATOM   6912  N   LEU A 453     136.076 100.791 158.933  1.00  0.00
ATOM   6913  H   LEU A 453     136.833 101.434 159.123  1.00  0.00
ATOM   6914  CA  LEU A 453     135.657  99.935 160.039  1.00  0.00
ATOM   6915  HA  LEU A 453     135.888  98.915 159.735  1.00  0.00
ATOM   6916  CB  LEU A 453     136.512 100.238 161.284  1.00  0.00
ATOM   6917  HB1 LEU A 453     136.303 101.249 161.627  1.00  0.00
ATOM   6918  HB2 LEU A 453     137.555 100.210 160.967  1.00  0.00
ATOM   6919  CG  LEU A 453     136.384  99.265 162.472  1.00  0.00
ATOM   6920  HG  LEU A 453     137.269  99.406 163.095  1.00  0.00
ATOM   6921  CD1 LEU A 453     135.174  99.555 163.361  1.00  0.00
ATOM   6922 1HD1 LEU A 453     135.242  98.957 164.269  1.00  0.00
ATOM   6923 2HD1 LEU A 453     135.159 100.610 163.621  1.00  0.00
ATOM   6924 3HD1 LEU A 453     134.251  99.295 162.851  1.00  0.00
ATOM   6925  CD2 LEU A 453     136.329  97.793 162.070  1.00  0.00
ATOM   6926 1HD2 LEU A 453     136.308  97.179 162.969  1.00  0.00
ATOM   6927 2HD2 LEU A 453     135.429  97.572 161.501  1.00  0.00
ATOM   6928 3HD2 LEU A 453     137.209  97.535 161.482  1.00  0.00
ATOM   6929  C   LEU A 453     134.149 100.010 160.267  1.00  0.00
ATOM   6930  O   LEU A 453     133.629 101.004 160.790  1.00  0.00
ATOM   6931  N   LYS A 454     133.458  98.953 159.832  1.00  0.00
ATOM   6932  H   LYS A 454     133.970  98.194 159.401  1.00  0.00
ATOM   6933  CA  LYS A 454     132.051  98.727 160.136  1.00  0.00
ATOM   6934  HA  LYS A 454     131.532  99.682 160.231  1.00  0.00
ATOM   6935  CB  LYS A 454     131.363  97.849 159.075  1.00  0.00
ATOM   6936  HB1 LYS A 454     130.307  97.753 159.330  1.00  0.00
ATOM   6937  HB2 LYS A 454     131.791  96.846 159.088  1.00  0.00
ATOM   6938  CG  LYS A 454     131.465  98.376 157.638  1.00  0.00
ATOM   6939  HG1 LYS A 454     130.912  97.702 156.981  1.00  0.00
ATOM   6940  HG2 LYS A 454     132.513  98.350 157.345  1.00  0.00
ATOM   6941  CD  LYS A 454     130.900  99.796 157.493  1.00  0.00
ATOM   6942  HD1 LYS A 454     131.398 100.476 158.185  1.00  0.00
ATOM   6943  HD2 LYS A 454     129.833  99.787 157.725  1.00  0.00
ATOM   6944  CE  LYS A 454     131.099 100.342 156.076  1.00  0.00
ATOM   6945  HE1 LYS A 454     130.669 101.346 156.043  1.00  0.00
ATOM   6946  HE2 LYS A 454     130.558  99.713 155.365  1.00  0.00
ATOM   6947  NZ  LYS A 454     132.539 100.396 155.739  1.00  0.00
ATOM   6948  HZ1 LYS A 454     133.097 100.627 156.556  1.00  0.00
ATOM   6949  HZ2 LYS A 454     132.911  99.478 155.474  1.00  0.00
ATOM   6950  HZ3 LYS A 454     132.736 101.029 154.985  1.00  0.00
ATOM   6951  C   LYS A 454     131.917  98.036 161.480  1.00  0.00
ATOM   6952  O   LYS A 454     131.095  98.499 162.269  1.00  0.00
```

ATOM	6953	N	GLU	A	455	132.674	96.962	161.758	1.00	0.00
ATOM	6954	H	GLU	A	455	133.324	96.619	161.066	1.00	0.00
ATOM	6955	CA	GLU	A	455	132.271	96.085	162.871	1.00	0.00
ATOM	6956	HA	GLU	A	455	131.678	96.667	163.578	1.00	0.00
ATOM	6957	CB	GLU	A	455	131.329	94.952	162.385	1.00	0.00
ATOM	6958	HB1	GLU	A	455	130.478	95.433	161.899	1.00	0.00
ATOM	6959	HB2	GLU	A	455	130.928	94.436	163.259	1.00	0.00
ATOM	6960	CG	GLU	A	455	131.925	93.928	161.400	1.00	0.00
ATOM	6961	HG1	GLU	A	455	132.874	94.294	161.016	1.00	0.00
ATOM	6962	HG2	GLU	A	455	131.252	93.875	160.541	1.00	0.00
ATOM	6963	CD	GLU	A	455	132.104	92.499	161.959	1.00	0.00
ATOM	6964	OE1	GLU	A	455	131.826	91.537	161.200	1.00	0.00
ATOM	6965	OE2	GLU	A	455	132.565	92.319	163.116	1.00	0.00
ATOM	6966	C	GLU	A	455	133.406	95.517	163.720	1.00	0.00
ATOM	6967	O	GLU	A	455	134.519	95.254	163.260	1.00	0.00
ATOM	6968	N	ILE	A	456	133.087	95.315	164.994	1.00	0.00
ATOM	6969	H	ILE	A	456	132.154	95.589	165.288	1.00	0.00
ATOM	6970	CA	ILE	A	456	133.975	94.777	166.021	1.00	0.00
ATOM	6971	HA	ILE	A	456	134.687	94.135	165.511	1.00	0.00
ATOM	6972	CB	ILE	A	456	134.810	95.891	166.693	1.00	0.00
ATOM	6973	HB	ILE	A	456	135.520	96.231	165.938	1.00	0.00
ATOM	6974	CG2	ILE	A	456	133.960	97.116	167.053	1.00	0.00
ATOM	6975	1HG2	ILE	A	456	133.627	97.574	166.127	1.00	0.00
ATOM	6976	2HG2	ILE	A	456	133.093	96.828	167.640	1.00	0.00
ATOM	6977	3HG2	ILE	A	456	134.536	97.856	167.599	1.00	0.00
ATOM	6978	CG1	ILE	A	456	135.649	95.390	167.890	1.00	0.00
ATOM	6979	1HG1	ILE	A	456	136.039	94.398	167.664	1.00	0.00
ATOM	6980	2HG1	ILE	A	456	135.017	95.318	168.776	1.00	0.00
ATOM	6981	CD	ILE	A	456	136.838	96.314	168.182	1.00	0.00
ATOM	6982	HD1	ILE	A	456	137.510	96.334	167.328	1.00	0.00
ATOM	6983	HD2	ILE	A	456	136.500	97.326	168.384	1.00	0.00
ATOM	6984	HD3	ILE	A	456	137.388	95.955	169.048	1.00	0.00
ATOM	6985	C	ILE	A	456	133.162	93.859	166.945	1.00	0.00
ATOM	6986	O	ILE	A	456	132.664	94.240	168.009	1.00	0.00
ATOM	6987	N	SER	A	457	132.914	92.657	166.427	1.00	0.00
ATOM	6988	H	SER	A	457	133.317	92.465	165.521	1.00	0.00
ATOM	6989	CA	SER	A	457	131.875	91.722	166.873	1.00	0.00
ATOM	6990	HA	SER	A	457	130.951	92.297	166.892	1.00	0.00
ATOM	6991	CB	SER	A	457	131.636	90.603	165.835	1.00	0.00
ATOM	6992	HB1	SER	A	457	130.726	90.838	165.282	1.00	0.00
ATOM	6993	HB2	SER	A	457	131.471	89.656	166.348	1.00	0.00
ATOM	6994	OG	SER	A	457	132.686	90.431	164.894	1.00	0.00
ATOM	6995	HG	SER	A	457	132.598	91.138	164.199	1.00	0.00
ATOM	6996	C	SER	A	457	131.984	91.158	168.302	1.00	0.00
ATOM	6997	O	SER	A	457	131.026	90.556	168.784	1.00	0.00
ATOM	6998	N	ASP	A	458	133.110	91.359	168.988	1.00	0.00
ATOM	6999	H	ASP	A	458	133.827	91.918	168.548	1.00	0.00
ATOM	7000	CA	ASP	A	458	133.407	90.876	170.348	1.00	0.00
ATOM	7001	HA	ASP	A	458	132.540	91.014	170.994	1.00	0.00
ATOM	7002	CB	ASP	A	458	133.714	89.369	170.206	1.00	0.00
ATOM	7003	HB1	ASP	A	458	134.639	89.258	169.641	1.00	0.00
ATOM	7004	HB2	ASP	A	458	132.920	88.912	169.614	1.00	0.00
ATOM	7005	CG	ASP	A	458	133.827	88.540	171.485	1.00	0.00
ATOM	7006	OD1	ASP	A	458	133.861	87.294	171.356	1.00	0.00
ATOM	7007	OD2	ASP	A	458	134.033	89.085	172.587	1.00	0.00
ATOM	7008	C	ASP	A	458	134.613	91.662	170.917	1.00	0.00
ATOM	7009	O	ASP	A	458	135.378	92.240	170.150	1.00	0.00
ATOM	7010	N	GLY	A	459	134.791	91.715	172.238	1.00	0.00
ATOM	7011	H	GLY	A	459	134.226	91.074	172.787	1.00	0.00
ATOM	7012	CA	GLY	A	459	135.874	92.438	172.928	1.00	0.00
ATOM	7013	HA1	GLY	A	459	136.809	92.334	172.391	1.00	0.00

ATOM	7014	HA2	GLY	A	459	136.018	91.965	173.897	1.00	0.00
ATOM	7015	C	GLY	A	459	135.631	93.927	173.216	1.00	0.00
ATOM	7016	O	GLY	A	459	134.811	94.590	172.583	1.00	0.00
ATOM	7017	N	ASP	A	460	136.354	94.443	174.204	1.00	0.00
ATOM	7018	H	ASP	A	460	137.022	93.833	174.660	1.00	0.00
ATOM	7019	CA	ASP	A	460	136.437	95.868	174.517	1.00	0.00
ATOM	7020	HA	ASP	A	460	135.449	96.321	174.422	1.00	0.00
ATOM	7021	CB	ASP	A	460	136.949	96.047	175.960	1.00	0.00
ATOM	7022	HB1	ASP	A	460	137.527	96.971	176.031	1.00	0.00
ATOM	7023	HB2	ASP	A	460	137.637	95.239	176.198	1.00	0.00
ATOM	7024	CG	ASP	A	460	135.873	96.131	177.041	1.00	0.00
ATOM	7025	OD1	ASP	A	460	136.207	96.671	178.126	1.00	0.00
ATOM	7026	OD2	ASP	A	460	134.725	95.665	176.868	1.00	0.00
ATOM	7027	C	ASP	A	460	137.391	96.623	173.580	1.00	0.00
ATOM	7028	O	ASP	A	460	138.279	96.041	172.951	1.00	0.00
ATOM	7029	N	VAL	A	461	137.259	97.952	173.602	1.00	0.00
ATOM	7030	H	VAL	A	461	136.511	98.336	174.165	1.00	0.00
ATOM	7031	CA	VAL	A	461	138.131	98.896	172.894	1.00	0.00
ATOM	7032	HA	VAL	A	461	138.873	98.344	172.321	1.00	0.00
ATOM	7033	CB	VAL	A	461	137.295	99.715	171.899	1.00	0.00
ATOM	7034	HB	VAL	A	461	136.457	100.159	172.429	1.00	0.00
ATOM	7035	CG1	VAL	A	461	138.131	100.834	171.273	1.00	0.00
ATOM	7036	1HG1	VAL	A	461	137.606	101.284	170.439	1.00	0.00
ATOM	7037	2HG1	VAL	A	461	138.308	101.609	172.018	1.00	0.00
ATOM	7038	3HG1	VAL	A	461	139.085	100.442	170.920	1.00	0.00
ATOM	7039	CG2	VAL	A	461	136.720	98.859	170.764	1.00	0.00
ATOM	7040	1HG2	VAL	A	461	135.865	99.363	170.323	1.00	0.00
ATOM	7041	2HG2	VAL	A	461	137.462	98.706	169.989	1.00	0.00
ATOM	7042	3HG2	VAL	A	461	136.394	97.891	171.138	1.00	0.00
ATOM	7043	C	VAL	A	461	138.854	99.803	173.908	1.00	0.00
ATOM	7044	O	VAL	A	461	138.282	100.231	174.914	1.00	0.00
ATOM	7045	N	ILE	A	462	140.131	100.080	173.633	1.00	0.00
ATOM	7046	H	ILE	A	462	140.511	99.612	172.818	1.00	0.00
ATOM	7047	CA	ILE	A	462	141.113	100.678	174.549	1.00	0.00
ATOM	7048	HA	ILE	A	462	140.585	101.201	175.346	1.00	0.00
ATOM	7049	CB	ILE	A	462	141.976	99.553	175.194	1.00	0.00
ATOM	7050	HB	ILE	A	462	142.532	99.065	174.395	1.00	0.00
ATOM	7051	CG2	ILE	A	462	143.011	100.091	176.198	1.00	0.00
ATOM	7052	1HG2	ILE	A	462	143.460	99.268	176.756	1.00	0.00
ATOM	7053	2HG2	ILE	A	462	143.817	100.588	175.667	1.00	0.00
ATOM	7054	3HG2	ILE	A	462	142.551	100.793	176.894	1.00	0.00
ATOM	7055	CG1	ILE	A	462	141.176	98.420	175.882	1.00	0.00
ATOM	7056	1HG1	ILE	A	462	141.886	97.643	176.164	1.00	0.00
ATOM	7057	2HG1	ILE	A	462	140.492	97.966	175.166	1.00	0.00
ATOM	7058	CD	ILE	A	462	140.395	98.820	177.139	1.00	0.00
ATOM	7059	HD1	ILE	A	462	139.825	97.962	177.489	1.00	0.00
ATOM	7060	HD2	ILE	A	462	141.077	99.132	177.930	1.00	0.00
ATOM	7061	HD3	ILE	A	462	139.710	99.629	176.915	1.00	0.00
ATOM	7062	C	ILE	A	462	141.986	101.729	173.817	1.00	0.00
ATOM	7063	O	ILE	A	462	143.131	101.491	173.422	1.00	0.00
ATOM	7064	N	ILE	A	463	141.442	102.937	173.637	1.00	0.00
ATOM	7065	H	ILE	A	463	140.503	103.095	173.993	1.00	0.00
ATOM	7066	CA	ILE	A	463	142.217	104.147	173.296	1.00	0.00
ATOM	7067	HA	ILE	A	463	143.074	103.898	172.668	1.00	0.00
ATOM	7068	CB	ILE	A	463	141.287	105.139	172.543	1.00	0.00
ATOM	7069	HB	ILE	A	463	140.304	105.127	173.009	1.00	0.00
ATOM	7070	CG2	ILE	A	463	141.804	106.585	172.599	1.00	0.00
ATOM	7071	1HG2	ILE	A	463	141.131	107.237	172.044	1.00	0.00
ATOM	7072	2HG2	ILE	A	463	141.847	106.960	173.620	1.00	0.00
ATOM	7073	3HG2	ILE	A	463	142.794	106.629	172.162	1.00	0.00
ATOM	7074	CG1	ILE	A	463	141.064	104.831	171.046	1.00	0.00

```
ATOM   7075 1HG1 ILE A 463     142.013 104.880 170.521  1.00  0.00
ATOM   7076 2HG1 ILE A 463     140.415 105.599 170.622  1.00  0.00
ATOM   7077  CD  ILE A 463     140.391 103.486 170.751  1.00  0.00
ATOM   7078  HD1 ILE A 463     140.194 103.401 169.683  1.00  0.00
ATOM   7079  HD2 ILE A 463     141.031 102.659 171.051  1.00  0.00
ATOM   7080  HD3 ILE A 463     139.446 103.441 171.289  1.00  0.00
ATOM   7081  C   ILE A 463     142.712 104.745 174.627  1.00  0.00
ATOM   7082  O   ILE A 463     141.887 104.894 175.536  1.00  0.00
ATOM   7083  N   SER A 464     144.016 105.068 174.786  1.00  0.00
ATOM   7084  H   SER A 464     144.676 104.965 174.024  1.00  0.00
ATOM   7085  CA  SER A 464     144.519 105.291 176.159  1.00  0.00
ATOM   7086  HA  SER A 464     143.690 105.284 176.865  1.00  0.00
ATOM   7087  CB  SER A 464     145.392 104.110 176.617  1.00  0.00
ATOM   7088  HB1 SER A 464     145.649 104.246 177.668  1.00  0.00
ATOM   7089  HB2 SER A 464     146.310 104.059 176.036  1.00  0.00
ATOM   7090  OG  SER A 464     144.688 102.895 176.472  1.00  0.00
ATOM   7091  HG  SER A 464     144.567 102.710 175.535  1.00  0.00
ATOM   7092  C   SER A 464     145.186 106.634 176.425  1.00  0.00
ATOM   7093  O   SER A 464     144.550 107.535 176.986  1.00  0.00
ATOM   7094  N   GLY A 465     146.459 106.735 176.045  1.00  0.00
ATOM   7095  H   GLY A 465     146.825 105.962 175.496  1.00  0.00
ATOM   7096  CA  GLY A 465     147.433 107.758 176.415  1.00  0.00
ATOM   7097  HA1 GLY A 465     148.331 107.267 176.786  1.00  0.00
ATOM   7098  HA2 GLY A 465     147.038 108.372 177.212  1.00  0.00
ATOM   7099  C   GLY A 465     147.841 108.662 175.260  1.00  0.00
ATOM   7100  O   GLY A 465     148.662 109.545 175.481  1.00  0.00
ATOM   7101  N   ASN A 466     147.261 108.469 174.068  1.00  0.00
ATOM   7102  H   ASN A 466     146.616 107.694 174.007  1.00  0.00
ATOM   7103  CA  ASN A 466     147.504 109.216 172.826  1.00  0.00
ATOM   7104  HA  ASN A 466     148.519 109.000 172.485  1.00  0.00
ATOM   7105  CB  ASN A 466     146.524 108.721 171.741  1.00  0.00
ATOM   7106  HB1 ASN A 466     146.789 109.200 170.801  1.00  0.00
ATOM   7107  HB2 ASN A 466     145.518 109.037 172.008  1.00  0.00
ATOM   7108  CG  ASN A 466     146.500 107.232 171.498  1.00  0.00
ATOM   7109  OD1 ASN A 466     147.452 106.633 171.023  1.00  0.00
ATOM   7110  ND2 ASN A 466     145.399 106.588 171.806  1.00  0.00
ATOM   7111 1HD2 ASN A 466     144.599 107.084 172.157  1.00  0.00
ATOM   7112 2HD2 ASN A 466     145.401 105.587 171.640  1.00  0.00
ATOM   7113  C   ASN A 466     147.388 110.748 172.987  1.00  0.00
ATOM   7114  O   ASN A 466     146.321 111.311 172.739  1.00  0.00
ATOM   7115  N   LYS A 467     148.438 111.453 173.419  1.00  0.00
ATOM   7116  H   LYS A 467     149.295 110.956 173.622  1.00  0.00
ATOM   7117  CA  LYS A 467     148.338 112.878 173.801  1.00  0.00
ATOM   7118  HA  LYS A 467     147.531 112.968 174.526  1.00  0.00
ATOM   7119  CB  LYS A 467     149.657 113.363 174.452  1.00  0.00
ATOM   7120  HB1 LYS A 467     149.647 114.453 174.508  1.00  0.00
ATOM   7121  HB2 LYS A 467     150.490 113.076 173.807  1.00  0.00
ATOM   7122  CG  LYS A 467     149.889 112.806 175.865  1.00  0.00
ATOM   7123  HG1 LYS A 467     150.910 113.043 176.171  1.00  0.00
ATOM   7124  HG2 LYS A 467     149.813 111.723 175.833  1.00  0.00
ATOM   7125  CD  LYS A 467     148.924 113.387 176.923  1.00  0.00
ATOM   7126  HD1 LYS A 467     147.991 113.712 176.461  1.00  0.00
ATOM   7127  HD2 LYS A 467     149.387 114.276 177.356  1.00  0.00
ATOM   7128  CE  LYS A 467     148.593 112.397 178.049  1.00  0.00
ATOM   7129  HE1 LYS A 467     148.039 112.935 178.824  1.00  0.00
ATOM   7130  HE2 LYS A 467     149.523 112.025 178.488  1.00  0.00
ATOM   7131  NZ  LYS A 467     147.770 111.272 177.549  1.00  0.00
ATOM   7132  HZ1 LYS A 467     147.395 110.686 178.290  1.00  0.00
ATOM   7133  HZ2 LYS A 467     146.951 111.612 177.049  1.00  0.00
ATOM   7134  HZ3 LYS A 467     148.280 110.692 176.890  1.00  0.00
ATOM   7135  C   LYS A 467     147.917 113.821 172.672  1.00  0.00
```

```
ATOM   7136  O   LYS A 467     147.456 114.907 172.988  1.00  0.00
ATOM   7137  N   ASN A 468     148.005 113.423 171.400  1.00  0.00
ATOM   7138  H   ASN A 468     148.425 112.528 171.209  1.00  0.00
ATOM   7139  CA  ASN A 468     147.472 114.202 170.275  1.00  0.00
ATOM   7140  HA  ASN A 468     147.468 115.255 170.551  1.00  0.00
ATOM   7141  CB  ASN A 468     148.428 114.052 169.082  1.00  0.00
ATOM   7142  HB1 ASN A 468     148.432 113.011 168.766  1.00  0.00
ATOM   7143  HB2 ASN A 468     149.440 114.330 169.371  1.00  0.00
ATOM   7144  CG  ASN A 468     148.029 114.918 167.893  1.00  0.00
ATOM   7145  OD1 ASN A 468     147.525 116.026 168.033  1.00  0.00
ATOM   7146  ND2 ASN A 468     148.253 114.431 166.697  1.00  0.00
ATOM   7147 1HD2 ASN A 468     148.751 113.552 166.584  1.00  0.00
ATOM   7148 2HD2 ASN A 468     147.913 114.911 165.875  1.00  0.00
ATOM   7149  C   ASN A 468     146.022 113.833 169.870  1.00  0.00
ATOM   7150  O   ASN A 468     145.348 114.650 169.246  1.00  0.00
ATOM   7151  N   LEU A 469     145.522 112.642 170.217  1.00  0.00
ATOM   7152  H   LEU A 469     146.061 112.044 170.825  1.00  0.00
ATOM   7153  CA  LEU A 469     144.319 112.088 169.577  1.00  0.00
ATOM   7154  HA  LEU A 469     144.489 112.131 168.499  1.00  0.00
ATOM   7155  CB  LEU A 469     144.149 110.613 169.954  1.00  0.00
ATOM   7156  HB1 LEU A 469     144.066 110.554 171.038  1.00  0.00
ATOM   7157  HB2 LEU A 469     145.051 110.088 169.650  1.00  0.00
ATOM   7158  CG  LEU A 469     142.937 109.874 169.335  1.00  0.00
ATOM   7159  HG  LEU A 469     142.015 110.310 169.718  1.00  0.00
ATOM   7160  CD1 LEU A 469     142.888 109.871 167.808  1.00  0.00
ATOM   7161 1HD1 LEU A 469     142.654 110.858 167.436  1.00  0.00
ATOM   7162 2HD1 LEU A 469     143.840 109.561 167.393  1.00  0.00
ATOM   7163 3HD1 LEU A 469     142.105 109.209 167.457  1.00  0.00
ATOM   7164  CD2 LEU A 469     142.987 108.410 169.759  1.00  0.00
ATOM   7165 1HD2 LEU A 469     142.070 107.907 169.459  1.00  0.00
ATOM   7166 2HD2 LEU A 469     143.840 107.915 169.297  1.00  0.00
ATOM   7167 3HD2 LEU A 469     143.096 108.363 170.838  1.00  0.00
ATOM   7168  C   LEU A 469     143.047 112.902 169.841  1.00  0.00
ATOM   7169  O   LEU A 469     142.781 113.358 170.956  1.00  0.00
ATOM   7170  N   CYS A 470     142.231 113.024 168.799  1.00  0.00
ATOM   7171  H   CYS A 470     142.543 112.659 167.901  1.00  0.00
ATOM   7172  CA  CYS A 470     141.063 113.880 168.738  1.00  0.00
ATOM   7173  HA  CYS A 470     140.668 114.048 169.742  1.00  0.00
ATOM   7174  CB  CYS A 470     141.591 115.219 168.183  1.00  0.00
ATOM   7175  HB1 CYS A 470     142.330 114.984 167.415  1.00  0.00
ATOM   7176  HB2 CYS A 470     142.132 115.714 168.988  1.00  0.00
ATOM   7177  SG  CYS A 470     140.485 116.437 167.425  1.00  0.00
ATOM   7178  C   CYS A 470     139.958 113.226 167.887  1.00  0.00
ATOM   7179  O   CYS A 470     140.211 112.352 167.059  1.00  0.00
ATOM   7180  N   TYR A 471     138.708 113.570 168.195  1.00  0.00
ATOM   7181  H   TYR A 471     138.609 114.308 168.876  1.00  0.00
ATOM   7182  CA  TYR A 471     137.463 113.016 167.642  1.00  0.00
ATOM   7183  HA  TYR A 471     136.740 113.213 168.429  1.00  0.00
ATOM   7184  CB  TYR A 471     136.985 113.856 166.435  1.00  0.00
ATOM   7185  HB1 TYR A 471     136.650 113.182 165.648  1.00  0.00
ATOM   7186  HB2 TYR A 471     137.821 114.424 166.024  1.00  0.00
ATOM   7187  CG  TYR A 471     135.854 114.835 166.768  1.00  0.00
ATOM   7188  CD1 TYR A 471     135.774 115.454 168.035  1.00  0.00
ATOM   7189  HD1 TYR A 471     136.543 115.299 168.772  1.00  0.00
ATOM   7190  CE1 TYR A 471     134.668 116.242 168.384  1.00  0.00
ATOM   7191  HE1 TYR A 471     134.600 116.659 169.374  1.00  0.00
ATOM   7192  CZ  TYR A 471     133.613 116.421 167.472  1.00  0.00
ATOM   7193  OH  TYR A 471     132.518 117.141 167.827  1.00  0.00
ATOM   7194  HH  TYR A 471     131.830 117.042 167.165  1.00  0.00
ATOM   7195  CE2 TYR A 471     133.701 115.845 166.189  1.00  0.00
ATOM   7196  HE2 TYR A 471     132.880 115.959 165.499  1.00  0.00
```

```
ATOM   7197  CD2 TYR A 471     134.827 115.075 165.834  1.00  0.00
ATOM   7198  HD2 TYR A 471     134.874 114.617 164.854  1.00  0.00
ATOM   7199  C   TYR A 471     137.275 111.481 167.577  1.00  0.00
ATOM   7200  O   TYR A 471     136.203 111.008 167.190  1.00  0.00
ATOM   7201  N   ALA A 472     138.162 110.697 168.199  1.00  0.00
ATOM   7202  H   ALA A 472     139.070 111.096 168.382  1.00  0.00
ATOM   7203  CA  ALA A 472     137.864 109.356 168.731  1.00  0.00
ATOM   7204  HA  ALA A 472     137.318 108.812 167.960  1.00  0.00
ATOM   7205  CB  ALA A 472     139.194 108.620 168.954  1.00  0.00
ATOM   7206  HB1 ALA A 472     138.995 107.593 169.262  1.00  0.00
ATOM   7207  HB2 ALA A 472     139.769 108.605 168.028  1.00  0.00
ATOM   7208  HB3 ALA A 472     139.767 109.123 169.731  1.00  0.00
ATOM   7209  C   ALA A 472     136.951 109.385 169.980  1.00  0.00
ATOM   7210  O   ALA A 472     137.218 108.740 170.997  1.00  0.00
ATOM   7211  N   ASN A 473     135.875 110.162 169.884  1.00  0.00
ATOM   7212  H   ASN A 473     135.779 110.644 169.002  1.00  0.00
ATOM   7213  CA  ASN A 473     134.805 110.346 170.864  1.00  0.00
ATOM   7214  HA  ASN A 473     134.606 109.389 171.349  1.00  0.00
ATOM   7215  CB  ASN A 473     135.294 111.345 171.934  1.00  0.00
ATOM   7216  HB1 ASN A 473     136.287 111.069 172.283  1.00  0.00
ATOM   7217  HB2 ASN A 473     134.618 111.306 172.787  1.00  0.00
ATOM   7218  CG  ASN A 473     135.331 112.777 171.422  1.00  0.00
ATOM   7219  OD1 ASN A 473     136.346 113.272 170.958  1.00  0.00
ATOM   7220  ND2 ASN A 473     134.191 113.425 171.446  1.00  0.00
ATOM   7221 1HD2 ASN A 473     133.349 112.969 171.762  1.00  0.00
ATOM   7222 2HD2 ASN A 473     134.114 114.391 171.141  1.00  0.00
ATOM   7223  C   ASN A 473     133.494 110.820 170.208  1.00  0.00
ATOM   7224  O   ASN A 473     132.505 110.974 170.915  1.00  0.00
ATOM   7225  N   THR A 474     133.482 111.096 168.891  1.00  0.00
ATOM   7226  H   THR A 474     134.328 110.983 168.345  1.00  0.00
ATOM   7227  CA  THR A 474     132.282 111.575 168.175  1.00  0.00
ATOM   7228  HA  THR A 474     131.612 112.029 168.907  1.00  0.00
ATOM   7229  CB  THR A 474     132.625 112.686 167.167  1.00  0.00
ATOM   7230  HB  THR A 474     133.205 113.454 167.682  1.00  0.00
ATOM   7231  CG2 THR A 474     133.429 112.192 165.964  1.00  0.00
ATOM   7232 1HG2 THR A 474     133.200 112.796 165.092  1.00  0.00
ATOM   7233 2HG2 THR A 474     134.483 112.313 166.175  1.00  0.00
ATOM   7234 3HG2 THR A 474     133.233 111.145 165.753  1.00  0.00
ATOM   7235  OG1 THR A 474     131.451 113.297 166.685  1.00  0.00
ATOM   7236  HG1 THR A 474     130.826 112.577 166.509  1.00  0.00
ATOM   7237  C   THR A 474     131.478 110.444 167.518  1.00  0.00
ATOM   7238  O   THR A 474     130.544 110.726 166.769  1.00  0.00
ATOM   7239  N   ILE A 475     131.871 109.185 167.735  1.00  0.00
ATOM   7240  H   ILE A 475     132.558 109.038 168.455  1.00  0.00
ATOM   7241  CA  ILE A 475     131.507 108.008 166.931  1.00  0.00
ATOM   7242  HA  ILE A 475     130.735 108.313 166.220  1.00  0.00
ATOM   7243  CB  ILE A 475     132.717 107.528 166.083  1.00  0.00
ATOM   7244  HB  ILE A 475     132.901 108.300 165.333  1.00  0.00
ATOM   7245  CG2 ILE A 475     134.012 107.367 166.904  1.00  0.00
ATOM   7246 1HG2 ILE A 475     134.846 107.114 166.251  1.00  0.00
ATOM   7247 2HG2 ILE A 475     134.279 108.299 167.397  1.00  0.00
ATOM   7248 3HG2 ILE A 475     133.899 106.585 167.655  1.00  0.00
ATOM   7249  CG1 ILE A 475     132.360 106.220 165.342  1.00  0.00
ATOM   7250 1HG1 ILE A 475     131.402 106.347 164.840  1.00  0.00
ATOM   7251 2HG1 ILE A 475     132.260 105.400 166.053  1.00  0.00
ATOM   7252  CD  ILE A 475     133.400 105.802 164.298  1.00  0.00
ATOM   7253  HD1 ILE A 475     133.005 104.987 163.693  1.00  0.00
ATOM   7254  HD2 ILE A 475     133.653 106.647 163.659  1.00  0.00
ATOM   7255  HD3 ILE A 475     134.293 105.458 164.808  1.00  0.00
ATOM   7256  C   ILE A 475     130.900 106.897 167.795  1.00  0.00
ATOM   7257  O   ILE A 475     131.434 106.520 168.845  1.00  0.00
```

ATOM	7258	N	ASN	A	476	129.778	106.326	167.348	1.00	0.00
ATOM	7259	H	ASN	A	476	129.372	106.675	166.485	1.00	0.00
ATOM	7260	CA	ASN	A	476	129.002	105.371	168.145	1.00	0.00
ATOM	7261	HA	ASN	A	476	129.095	105.668	169.194	1.00	0.00
ATOM	7262	CB	ASN	A	476	127.508	105.491	167.809	1.00	0.00
ATOM	7263	HB1	ASN	A	476	126.939	104.798	168.428	1.00	0.00
ATOM	7264	HB2	ASN	A	476	127.327	105.236	166.771	1.00	0.00
ATOM	7265	CG	ASN	A	476	126.997	106.888	168.102	1.00	0.00
ATOM	7266	OD1	ASN	A	476	126.685	107.201	169.233	1.00	0.00
ATOM	7267	ND2	ASN	A	476	126.950	107.746	167.107	1.00	0.00
ATOM	7268	1HD2	ASN	A	476	127.199	107.438	166.165	1.00	0.00
ATOM	7269	2HD2	ASN	A	476	126.666	108.689	167.304	1.00	0.00
ATOM	7270	C	ASN	A	476	129.528	103.926	168.038	1.00	0.00
ATOM	7271	O	ASN	A	476	129.026	103.061	167.285	1.00	0.00
ATOM	7272	N	TRP	A	477	130.492	103.638	168.912	1.00	0.00
ATOM	7273	H	TRP	A	477	130.940	104.420	169.380	1.00	0.00
ATOM	7274	CA	TRP	A	477	130.919	102.284	169.261	1.00	0.00
ATOM	7275	HA	TRP	A	477	131.479	101.883	168.419	1.00	0.00
ATOM	7276	CB	TRP	A	477	131.863	102.320	170.468	1.00	0.00
ATOM	7277	HB1	TRP	A	477	131.757	101.394	171.032	1.00	0.00
ATOM	7278	HB2	TRP	A	477	131.590	103.135	171.140	1.00	0.00
ATOM	7279	CG	TRP	A	477	133.312	102.433	170.113	1.00	0.00
ATOM	7280	CD1	TRP	A	477	134.046	101.436	169.567	1.00	0.00
ATOM	7281	HD1	TRP	A	477	133.669	100.445	169.340	1.00	0.00
ATOM	7282	NE1	TRP	A	477	135.334	101.880	169.351	1.00	0.00
ATOM	7283	HE1	TRP	A	477	136.066	101.297	168.964	1.00	0.00
ATOM	7284	CE2	TRP	A	477	135.511	103.171	169.795	1.00	0.00
ATOM	7285	CZ2	TRP	A	477	136.626	104.017	169.844	1.00	0.00
ATOM	7286	HZ2	TRP	A	477	137.585	103.676	169.483	1.00	0.00
ATOM	7287	CH2	TRP	A	477	136.477	105.312	170.370	1.00	0.00
ATOM	7288	HH2	TRP	A	477	137.323	105.985	170.426	1.00	0.00
ATOM	7289	CZ3	TRP	A	477	135.222	105.739	170.833	1.00	0.00
ATOM	7290	HZ3	TRP	A	477	135.115	106.749	171.227	1.00	0.00
ATOM	7291	CE3	TRP	A	477	134.108	104.879	170.782	1.00	0.00
ATOM	7292	HE3	TRP	A	477	133.152	105.233	171.136	1.00	0.00
ATOM	7293	CD2	TRP	A	477	134.222	103.567	170.269	1.00	0.00
ATOM	7294	C	TRP	A	477	129.761	101.316	169.518	1.00	0.00
ATOM	7295	O	TRP	A	477	129.904	100.144	169.214	1.00	0.00
ATOM	7296	N	LYS	A	478	128.589	101.754	170.007	1.00	0.00
ATOM	7297	H	LYS	A	478	128.511	102.732	170.241	1.00	0.00
ATOM	7298	CA	LYS	A	478	127.433	100.863	170.251	1.00	0.00
ATOM	7299	HA	LYS	A	478	127.850	99.874	170.401	1.00	0.00
ATOM	7300	CB	LYS	A	478	126.701	101.290	171.539	1.00	0.00
ATOM	7301	HB1	LYS	A	478	125.861	101.940	171.287	1.00	0.00
ATOM	7302	HB2	LYS	A	478	127.377	101.867	172.172	1.00	0.00
ATOM	7303	CG	LYS	A	478	126.184	100.109	172.387	1.00	0.00
ATOM	7304	HG1	LYS	A	478	125.440	99.544	171.824	1.00	0.00
ATOM	7305	HG2	LYS	A	478	125.704	100.512	173.279	1.00	0.00
ATOM	7306	CD	LYS	A	478	127.314	99.155	172.821	1.00	0.00
ATOM	7307	HD1	LYS	A	478	128.131	99.744	173.241	1.00	0.00
ATOM	7308	HD2	LYS	A	478	127.663	98.599	171.958	1.00	0.00
ATOM	7309	CE	LYS	A	478	126.853	98.133	173.848	1.00	0.00
ATOM	7310	HE1	LYS	A	478	125.981	97.600	173.454	1.00	0.00
ATOM	7311	HE2	LYS	A	478	126.553	98.654	174.762	1.00	0.00
ATOM	7312	NZ	LYS	A	478	127.937	97.167	174.147	1.00	0.00
ATOM	7313	HZ1	LYS	A	478	127.669	96.621	174.959	1.00	0.00
ATOM	7314	HZ2	LYS	A	478	128.116	96.526	173.375	1.00	0.00
ATOM	7315	HZ3	LYS	A	478	128.804	97.620	174.428	1.00	0.00
ATOM	7316	C	LYS	A	478	126.500	100.637	169.053	1.00	0.00
ATOM	7317	O	LYS	A	478	125.448	100.028	169.201	1.00	0.00
ATOM	7318	N	LYS	A	479	126.959	101.037	167.865	1.00	0.00

```
ATOM   7319  H   LYS A 479     127.763 101.652 167.876  1.00  0.00
ATOM   7320  CA  LYS A 479     126.600 100.442 166.565  1.00  0.00
ATOM   7321  HA  LYS A 479     126.172  99.456 166.769  1.00  0.00
ATOM   7322  CB  LYS A 479     125.479 101.241 165.864  1.00  0.00
ATOM   7323  HB1 LYS A 479     124.569 101.069 166.442  1.00  0.00
ATOM   7324  HB2 LYS A 479     125.292 100.814 164.877  1.00  0.00
ATOM   7325  CG  LYS A 479     125.651 102.769 165.753  1.00  0.00
ATOM   7326  HG1 LYS A 479     126.473 103.108 166.375  1.00  0.00
ATOM   7327  HG2 LYS A 479     124.746 103.229 166.150  1.00  0.00
ATOM   7328  CD  LYS A 479     125.829 103.290 164.319  1.00  0.00
ATOM   7329  HD1 LYS A 479     125.667 104.369 164.318  1.00  0.00
ATOM   7330  HD2 LYS A 479     125.066 102.848 163.676  1.00  0.00
ATOM   7331  CE  LYS A 479     127.208 103.005 163.716  1.00  0.00
ATOM   7332  HE1 LYS A 479     127.206 103.365 162.681  1.00  0.00
ATOM   7333  HE2 LYS A 479     127.389 101.933 163.697  1.00  0.00
ATOM   7334  NZ  LYS A 479     128.280 103.715 164.446  1.00  0.00
ATOM   7335  HZ1 LYS A 479     128.254 103.542 165.442  1.00  0.00
ATOM   7336  HZ2 LYS A 479     129.183 103.456 164.082  1.00  0.00
ATOM   7337  HZ3 LYS A 479     128.154 104.722 164.274  1.00  0.00
ATOM   7338  C   LYS A 479     127.839 100.115 165.723  1.00  0.00
ATOM   7339  O   LYS A 479     127.733  99.979 164.513  1.00  0.00
ATOM   7340  N   LEU A 480     129.004  99.965 166.372  1.00  0.00
ATOM   7341  H   LEU A 480     129.026 100.173 167.357  1.00  0.00
ATOM   7342  CA  LEU A 480     130.097  99.097 165.860  1.00  0.00
ATOM   7343  HA  LEU A 480     129.947  98.889 164.796  1.00  0.00
ATOM   7344  CB  LEU A 480     131.431  99.859 166.019  1.00  0.00
ATOM   7345  HB1 LEU A 480     132.224  99.125 166.100  1.00  0.00
ATOM   7346  HB2 LEU A 480     131.413 100.387 166.966  1.00  0.00
ATOM   7347  CG  LEU A 480     131.867 100.824 164.899  1.00  0.00
ATOM   7348  HG  LEU A 480     132.200 100.245 164.041  1.00  0.00
ATOM   7349  CD1 LEU A 480     130.791 101.803 164.439  1.00  0.00
ATOM   7350 1HD1 LEU A 480     131.205 102.459 163.676  1.00  0.00
ATOM   7351 2HD1 LEU A 480     129.968 101.248 163.997  1.00  0.00
ATOM   7352 3HD1 LEU A 480     130.450 102.378 165.293  1.00  0.00
ATOM   7353  CD2 LEU A 480     133.033 101.681 165.406  1.00  0.00
ATOM   7354 1HD2 LEU A 480     133.386 102.329 164.606  1.00  0.00
ATOM   7355 2HD2 LEU A 480     132.720 102.294 166.252  1.00  0.00
ATOM   7356 3HD2 LEU A 480     133.850 101.030 165.716  1.00  0.00
ATOM   7357  C   LEU A 480     130.179  97.716 166.570  1.00  0.00
ATOM   7358  O   LEU A 480     130.713  96.764 166.006  1.00  0.00
ATOM   7359  N   PHE A 481     129.678  97.608 167.804  1.00  0.00
ATOM   7360  H   PHE A 481     129.343  98.455 168.236  1.00  0.00
ATOM   7361  CA  PHE A 481     129.729  96.425 168.669  1.00  0.00
ATOM   7362  HA  PHE A 481     130.649  95.878 168.473  1.00  0.00
ATOM   7363  CB  PHE A 481     129.711  96.878 170.146  1.00  0.00
ATOM   7364  HB1 PHE A 481     129.482  96.007 170.761  1.00  0.00
ATOM   7365  HB2 PHE A 481     128.886  97.577 170.275  1.00  0.00
ATOM   7366  CG  PHE A 481     130.983  97.496 170.722  1.00  0.00
ATOM   7367  CD1 PHE A 481     132.246  96.981 170.380  1.00  0.00
ATOM   7368  HD1 PHE A 481     132.336  96.188 169.659  1.00  0.00
ATOM   7369  CE1 PHE A 481     133.408  97.440 171.017  1.00  0.00
ATOM   7370  HE1 PHE A 481     134.357  96.986 170.770  1.00  0.00
ATOM   7371  CZ  PHE A 481     133.330  98.446 171.992  1.00  0.00
ATOM   7372  HZ  PHE A 481     134.230  98.784 172.483  1.00  0.00
ATOM   7373  CE2 PHE A 481     132.079  98.988 172.330  1.00  0.00
ATOM   7374  HE2 PHE A 481     132.021  99.764 173.079  1.00  0.00
ATOM   7375  CD2 PHE A 481     130.913  98.512 171.699  1.00  0.00
ATOM   7376  HD2 PHE A 481     129.961  98.926 171.971  1.00  0.00
ATOM   7377  C   PHE A 481     128.569  95.465 168.401  1.00  0.00
ATOM   7378  O   PHE A 481     127.410  95.848 168.528  1.00  0.00
ATOM   7379  N   GLY A 482     128.907  94.205 168.108  1.00  0.00
```

ATOM	7380	H	GLY	A	482	129.892	93.988	168.056	1.00	0.00
ATOM	7381	CA	GLY	A	482	127.952	93.104	167.927	1.00	0.00
ATOM	7382	HA1	GLY	A	482	128.485	92.234	167.543	1.00	0.00
ATOM	7383	HA2	GLY	A	482	127.196	93.397	167.199	1.00	0.00
ATOM	7384	C	GLY	A	482	127.234	92.678	169.211	1.00	0.00
ATOM	7385	O	GLY	A	482	126.151	92.108	169.137	1.00	0.00
ATOM	7386	N	THR	A	483	127.809	92.961	170.392	1.00	0.00
ATOM	7387	H	THR	A	483	128.723	93.405	170.392	1.00	0.00
ATOM	7388	CA	THR	A	483	127.268	92.496	171.680	1.00	0.00
ATOM	7389	HA	THR	A	483	126.245	92.165	171.502	1.00	0.00
ATOM	7390	CB	THR	A	483	128.001	91.252	172.216	1.00	0.00
ATOM	7391	HB	THR	A	483	127.471	90.910	173.106	1.00	0.00
ATOM	7392	CG2	THR	A	483	128.050	90.092	171.225	1.00	0.00
ATOM	7393	1HG2	THR	A	483	128.466	89.212	171.716	1.00	0.00
ATOM	7394	2HG2	THR	A	483	127.044	89.861	170.874	1.00	0.00
ATOM	7395	3HG2	THR	A	483	128.674	90.344	170.367	1.00	0.00
ATOM	7396	OG1	THR	A	483	129.322	91.536	172.582	1.00	0.00
ATOM	7397	HG1	THR	A	483	129.767	91.843	171.778	1.00	0.00
ATOM	7398	C	THR	A	483	127.166	93.587	172.758	1.00	0.00
ATOM	7399	O	THR	A	483	128.015	94.472	172.937	1.00	0.00
ATOM	7400	N	SER	A	484	126.071	93.527	173.520	1.00	0.00
ATOM	7401	H	SER	A	484	125.352	92.866	173.257	1.00	0.00
ATOM	7402	CA	SER	A	484	125.689	94.532	174.521	1.00	0.00
ATOM	7403	HA	SER	A	484	125.593	95.492	174.017	1.00	0.00
ATOM	7404	CB	SER	A	484	124.317	94.188	175.120	1.00	0.00
ATOM	7405	HB1	SER	A	484	123.999	94.992	175.785	1.00	0.00
ATOM	7406	HB2	SER	A	484	124.385	93.262	175.690	1.00	0.00
ATOM	7407	OG	SER	A	484	123.364	94.028	174.089	1.00	0.00
ATOM	7408	HG	SER	A	484	122.487	93.904	174.473	1.00	0.00
ATOM	7409	C	SER	A	484	126.679	94.689	175.676	1.00	0.00
ATOM	7410	O	SER	A	484	126.747	95.780	176.240	1.00	0.00
ATOM	7411	N	GLY	A	485	127.499	93.688	175.999	1.00	0.00
ATOM	7412	H	GLY	A	485	127.453	92.838	175.456	1.00	0.00
ATOM	7413	CA	GLY	A	485	128.447	93.756	177.122	1.00	0.00
ATOM	7414	HA1	GLY	A	485	128.631	92.744	177.481	1.00	0.00
ATOM	7415	HA2	GLY	A	485	128.007	94.323	177.943	1.00	0.00
ATOM	7416	C	GLY	A	485	129.810	94.380	176.792	1.00	0.00
ATOM	7417	O	GLY	A	485	130.600	94.635	177.703	1.00	0.00
ATOM	7418	N	GLN	A	486	130.101	94.637	175.512	1.00	0.00
ATOM	7419	H	GLN	A	486	129.458	94.330	174.793	1.00	0.00
ATOM	7420	CA	GLN	A	486	131.319	95.332	175.081	1.00	0.00
ATOM	7421	HA	GLN	A	486	132.190	94.827	175.502	1.00	0.00
ATOM	7422	CB	GLN	A	486	131.432	95.284	173.553	1.00	0.00
ATOM	7423	HB1	GLN	A	486	132.287	95.883	173.241	1.00	0.00
ATOM	7424	HB2	GLN	A	486	130.530	95.708	173.110	1.00	0.00
ATOM	7425	CG	GLN	A	486	131.629	93.853	173.053	1.00	0.00
ATOM	7426	HG1	GLN	A	486	130.852	93.216	173.457	1.00	0.00
ATOM	7427	HG2	GLN	A	486	132.587	93.468	173.401	1.00	0.00
ATOM	7428	CD	GLN	A	486	131.575	93.786	171.534	1.00	0.00
ATOM	7429	OE1	GLN	A	486	130.536	93.577	170.925	1.00	0.00
ATOM	7430	NE2	GLN	A	486	132.687	93.969	170.877	1.00	0.00
ATOM	7431	1HE2	GLN	A	486	133.550	94.166	171.376	1.00	0.00
ATOM	7432	2HE2	GLN	A	486	132.661	93.938	169.864	1.00	0.00
ATOM	7433	C	GLN	A	486	131.335	96.792	175.549	1.00	0.00
ATOM	7434	O	GLN	A	486	130.323	97.498	175.471	1.00	0.00
ATOM	7435	N	LYS	A	487	132.497	97.249	176.010	1.00	0.00
ATOM	7436	H	LYS	A	487	133.297	96.615	176.006	1.00	0.00
ATOM	7437	CA	LYS	A	487	132.721	98.578	176.594	1.00	0.00
ATOM	7438	HA	LYS	A	487	131.829	99.195	176.475	1.00	0.00
ATOM	7439	CB	LYS	A	487	133.026	98.428	178.099	1.00	0.00
ATOM	7440	HB1	LYS	A	487	133.220	99.412	178.529	1.00	0.00

```
ATOM   7441  HB2 LYS A 487     133.935  97.844 178.202  1.00  0.00
ATOM   7442  CG  LYS A 487     131.880  97.766 178.891  1.00  0.00
ATOM   7443  HG1 LYS A 487     131.361  97.035 178.279  1.00  0.00
ATOM   7444  HG2 LYS A 487     131.149  98.534 179.144  1.00  0.00
ATOM   7445  CD  LYS A 487     132.320  97.058 180.181  1.00  0.00
ATOM   7446  HD1 LYS A 487     131.435  96.620 180.645  1.00  0.00
ATOM   7447  HD2 LYS A 487     132.714  97.802 180.875  1.00  0.00
ATOM   7448  CE  LYS A 487     133.378  95.943 180.026  1.00  0.00
ATOM   7449  HE1 LYS A 487     133.443  95.410 180.978  1.00  0.00
ATOM   7450  HE2 LYS A 487     134.353  96.400 179.834  1.00  0.00
ATOM   7451  NZ  LYS A 487     133.081  94.981 178.933  1.00  0.00
ATOM   7452  HZ1 LYS A 487     133.458  94.070 179.144  1.00  0.00
ATOM   7453  HZ2 LYS A 487     132.084  94.906 178.740  1.00  0.00
ATOM   7454  HZ3 LYS A 487     133.548  95.295 178.077  1.00  0.00
ATOM   7455  C   LYS A 487     133.868  99.261 175.841  1.00  0.00
ATOM   7456  O   LYS A 487     134.618  98.617 175.110  1.00  0.00
ATOM   7457  N   THR A 488     134.030 100.567 176.032  1.00  0.00
ATOM   7458  H   THR A 488     133.417 101.060 176.664  1.00  0.00
ATOM   7459  CA  THR A 488     135.128 101.322 175.415  1.00  0.00
ATOM   7460  HA  THR A 488     135.935 100.632 175.181  1.00  0.00
ATOM   7461  CB  THR A 488     134.705 101.947 174.072  1.00  0.00
ATOM   7462  HB  THR A 488     134.304 101.156 173.436  1.00  0.00
ATOM   7463  CG2 THR A 488     133.657 103.048 174.190  1.00  0.00
ATOM   7464 1HG2 THR A 488     133.386 103.401 173.197  1.00  0.00
ATOM   7465 2HG2 THR A 488     132.760 102.668 174.678  1.00  0.00
ATOM   7466 3HG2 THR A 488     134.049 103.884 174.770  1.00  0.00
ATOM   7467  OG1 THR A 488     135.840 102.479 173.434  1.00  0.00
ATOM   7468  HG1 THR A 488     135.564 102.909 172.617  1.00  0.00
ATOM   7469  C   THR A 488     135.698 102.347 176.397  1.00  0.00
ATOM   7470  O   THR A 488     135.044 102.705 177.374  1.00  0.00
ATOM   7471  N   LYS A 489     136.965 102.714 176.174  1.00  0.00
ATOM   7472  H   LYS A 489     137.351 102.420 175.277  1.00  0.00
ATOM   7473  CA  LYS A 489     137.876 103.520 177.011  1.00  0.00
ATOM   7474  HA  LYS A 489     137.317 104.134 177.719  1.00  0.00
ATOM   7475  CB  LYS A 489     138.886 102.597 177.737  1.00  0.00
ATOM   7476  HB1 LYS A 489     139.827 103.141 177.841  1.00  0.00
ATOM   7477  HB2 LYS A 489     139.100 101.748 177.092  1.00  0.00
ATOM   7478  CG  LYS A 489     138.546 102.082 179.149  1.00  0.00
ATOM   7479  HG1 LYS A 489     138.574 102.933 179.831  1.00  0.00
ATOM   7480  HG2 LYS A 489     139.342 101.402 179.454  1.00  0.00
ATOM   7481  CD  LYS A 489     137.203 101.367 179.353  1.00  0.00
ATOM   7482  HD1 LYS A 489     136.420 102.111 179.251  1.00  0.00
ATOM   7483  HD2 LYS A 489     137.147 101.019 180.384  1.00  0.00
ATOM   7484  CE  LYS A 489     136.918 100.196 178.399  1.00  0.00
ATOM   7485  HE1 LYS A 489     137.248 100.449 177.387  1.00  0.00
ATOM   7486  HE2 LYS A 489     135.836 100.050 178.369  1.00  0.00
ATOM   7487  NZ  LYS A 489     137.557  98.933 178.840  1.00  0.00
ATOM   7488  HZ1 LYS A 489     137.127  98.131 178.370  1.00  0.00
ATOM   7489  HZ2 LYS A 489     138.541  98.922 178.611  1.00  0.00
ATOM   7490  HZ3 LYS A 489     137.424  98.795 179.831  1.00  0.00
ATOM   7491  C   LYS A 489     138.679 104.433 176.051  1.00  0.00
ATOM   7492  O   LYS A 489     138.961 104.016 174.928  1.00  0.00
ATOM   7493  N   ILE A 490     139.046 105.652 176.475  1.00  0.00
ATOM   7494  H   ILE A 490     138.805 105.895 177.421  1.00  0.00
ATOM   7495  CA  ILE A 490     139.459 106.750 175.565  1.00  0.00
ATOM   7496  HA  ILE A 490     139.901 106.297 174.686  1.00  0.00
ATOM   7497  CB  ILE A 490     138.226 107.561 175.066  1.00  0.00
ATOM   7498  HB  ILE A 490     138.604 108.343 174.405  1.00  0.00
ATOM   7499  CG2 ILE A 490     137.287 106.703 174.200  1.00  0.00
ATOM   7500 1HG2 ILE A 490     136.579 107.341 173.672  1.00  0.00
ATOM   7501 2HG2 ILE A 490     137.866 106.164 173.450  1.00  0.00
```

```
ATOM   7502 3HG2 ILE A 490     136.736 105.986 174.806  1.00  0.00
ATOM   7503  CG1 ILE A 490     137.465 108.238 176.226  1.00  0.00
ATOM   7504 1HG1 ILE A 490     138.171 108.739 176.888  1.00  0.00
ATOM   7505 2HG1 ILE A 490     136.932 107.489 176.812  1.00  0.00
ATOM   7506  CD  ILE A 490     136.461 109.298 175.753  1.00  0.00
ATOM   7507  HD1 ILE A 490     136.019 109.790 176.619  1.00  0.00
ATOM   7508  HD2 ILE A 490     136.970 110.042 175.142  1.00  0.00
ATOM   7509  HD3 ILE A 490     135.663 108.836 175.176  1.00  0.00
ATOM   7510  C   ILE A 490     140.570 107.706 176.112  1.00  0.00
ATOM   7511  O   ILE A 490     140.634 108.878 175.737  1.00  0.00
ATOM   7512  N   ILE A 491     141.365 107.308 177.114  1.00  0.00
ATOM   7513  H   ILE A 491     141.518 106.309 177.147  1.00  0.00
ATOM   7514  CA  ILE A 491     141.106 107.952 178.417  1.00  0.00
ATOM   7515  HA  ILE A 491     140.055 108.241 178.423  1.00  0.00
ATOM   7516  CB  ILE A 491     141.273 106.889 179.534  1.00  0.00
ATOM   7517  HB  ILE A 491     140.849 105.956 179.157  1.00  0.00
ATOM   7518  CG2 ILE A 491     142.754 106.617 179.858  1.00  0.00
ATOM   7519 1HG2 ILE A 491     142.840 105.756 180.519  1.00  0.00
ATOM   7520 2HG2 ILE A 491     143.301 106.397 178.945  1.00  0.00
ATOM   7521 3HG2 ILE A 491     143.198 107.482 180.350  1.00  0.00
ATOM   7522  CG1 ILE A 491     140.462 107.252 180.797  1.00  0.00
ATOM   7523 1HG1 ILE A 491     139.451 107.531 180.498  1.00  0.00
ATOM   7524 2HG1 ILE A 491     140.915 108.104 181.303  1.00  0.00
ATOM   7525  CD  ILE A 491     140.346 106.101 181.805  1.00  0.00
ATOM   7526  HD1 ILE A 491     139.672 106.395 182.609  1.00  0.00
ATOM   7527  HD2 ILE A 491     139.949 105.213 181.316  1.00  0.00
ATOM   7528  HD3 ILE A 491     141.319 105.874 182.235  1.00  0.00
ATOM   7529  C   ILE A 491     141.871 109.252 178.712  1.00  0.00
ATOM   7530  O   ILE A 491     141.527 109.969 179.649  1.00  0.00
ATOM   7531  N   SER A 492     142.889 109.592 177.927  1.00  0.00
ATOM   7532  H   SER A 492     143.153 108.929 177.208  1.00  0.00
ATOM   7533  CA  SER A 492     143.671 110.832 178.064  1.00  0.00
ATOM   7534  HA  SER A 492     143.003 111.602 178.456  1.00  0.00
ATOM   7535  CB  SER A 492     144.785 110.645 179.099  1.00  0.00
ATOM   7536  HB1 SER A 492     144.340 110.351 180.051  1.00  0.00
ATOM   7537  HB2 SER A 492     145.309 111.590 179.243  1.00  0.00
ATOM   7538  OG  SER A 492     145.705 109.648 178.696  1.00  0.00
ATOM   7539  HG  SER A 492     145.185 108.869 178.428  1.00  0.00
ATOM   7540  C   SER A 492     144.139 111.359 176.697  1.00  0.00
ATOM   7541  O   SER A 492     145.269 111.826 176.535  1.00  0.00
ATOM   7542  N   ASN A 493     143.243 111.254 175.714  1.00  0.00
ATOM   7543  H   ASN A 493     142.400 110.738 175.918  1.00  0.00
ATOM   7544  CA  ASN A 493     143.246 112.016 174.463  1.00  0.00
ATOM   7545  HA  ASN A 493     144.083 111.700 173.836  1.00  0.00
ATOM   7546  CB  ASN A 493     141.914 111.689 173.753  1.00  0.00
ATOM   7547  HB1 ASN A 493     141.706 112.436 172.992  1.00  0.00
ATOM   7548  HB2 ASN A 493     141.095 111.728 174.471  1.00  0.00
ATOM   7549  CG  ASN A 493     141.880 110.340 173.063  1.00  0.00
ATOM   7550  OD1 ASN A 493     142.822 109.564 173.057  1.00  0.00
ATOM   7551  ND2 ASN A 493     140.784 110.045 172.407  1.00  0.00
ATOM   7552 1HD2 ASN A 493     139.990 110.653 172.444  1.00  0.00
ATOM   7553 2HD2 ASN A 493     140.765 109.163 171.933  1.00  0.00
ATOM   7554  C   ASN A 493     143.379 113.542 174.688  1.00  0.00
ATOM   7555  O   ASN A 493     143.278 114.028 175.818  1.00  0.00
ATOM   7556  N   ARG A 494     143.527 114.328 173.610  1.00  0.00
ATOM   7557  H   ARG A 494     143.493 113.884 172.695  1.00  0.00
ATOM   7558  CA  ARG A 494     143.811 115.783 173.642  1.00  0.00
ATOM   7559  HA  ARG A 494     144.627 115.901 174.357  1.00  0.00
ATOM   7560  CB  ARG A 494     144.341 116.218 172.269  1.00  0.00
ATOM   7561  HB1 ARG A 494     143.510 116.537 171.637  1.00  0.00
ATOM   7562  HB2 ARG A 494     144.824 115.370 171.785  1.00  0.00
```

```
ATOM   7563  CG  ARG A 494     145.400 117.336 172.381  1.00  0.00
ATOM   7564  HG1 ARG A 494     146.243 116.986 172.968  1.00  0.00
ATOM   7565  HG2 ARG A 494     144.992 118.197 172.904  1.00  0.00
ATOM   7566  CD  ARG A 494     145.901 117.773 171.003  1.00  0.00
ATOM   7567  HD1 ARG A 494     146.263 116.905 170.459  1.00  0.00
ATOM   7568  HD2 ARG A 494     146.731 118.468 171.134  1.00  0.00
ATOM   7569  NE  ARG A 494     144.824 118.435 170.262  1.00  0.00
ATOM   7570  HE  ARG A 494     144.268 119.096 170.791  1.00  0.00
ATOM   7571  CZ  ARG A 494     144.423 118.173 169.034  1.00  0.00
ATOM   7572  NH1 ARG A 494     144.976 117.330 168.215  1.00  0.00
ATOM   7573 1HH1 ARG A 494     145.757 116.741 168.492  1.00  0.00
ATOM   7574 2HH1 ARG A 494     144.582 117.212 167.295  1.00  0.00
ATOM   7575  NH2 ARG A 494     143.398 118.815 168.570  1.00  0.00
ATOM   7576 1HH2 ARG A 494     142.974 119.552 169.102  1.00  0.00
ATOM   7577 2HH2 ARG A 494     143.153 118.642 167.601  1.00  0.00
ATOM   7578  C   ARG A 494     142.696 116.715 174.185  1.00  0.00
ATOM   7579  O   ARG A 494     142.693 117.927 173.946  1.00  0.00
ATOM   7580  N   GLY A 495     141.741 116.166 174.929  1.00  0.00
ATOM   7581  H   GLY A 495     141.872 115.201 175.196  1.00  0.00
ATOM   7582  CA  GLY A 495     140.574 116.885 175.426  1.00  0.00
ATOM   7583  HA1 GLY A 495     140.898 117.774 175.967  1.00  0.00
ATOM   7584  HA2 GLY A 495     140.030 116.242 176.117  1.00  0.00
ATOM   7585  C   GLY A 495     139.661 117.312 174.286  1.00  0.00
ATOM   7586  O   GLY A 495     140.062 118.001 173.350  1.00  0.00
ATOM   7587  N   GLU A 496     138.395 116.903 174.329  1.00  0.00
ATOM   7588  H   GLU A 496     138.083 116.278 175.057  1.00  0.00
ATOM   7589  CA  GLU A 496     137.465 117.306 173.273  1.00  0.00
ATOM   7590  HA  GLU A 496     137.861 116.929 172.331  1.00  0.00
ATOM   7591  CB  GLU A 496     136.108 116.651 173.502  1.00  0.00
ATOM   7592  HB1 GLU A 496     135.576 117.213 174.268  1.00  0.00
ATOM   7593  HB2 GLU A 496     136.233 115.622 173.843  1.00  0.00
ATOM   7594  CG  GLU A 496     135.359 116.635 172.168  1.00  0.00
ATOM   7595  HG1 GLU A 496     135.667 115.760 171.597  1.00  0.00
ATOM   7596  HG2 GLU A 496     135.613 117.506 171.565  1.00  0.00
ATOM   7597  CD  GLU A 496     133.851 116.661 172.341  1.00  0.00
ATOM   7598  OE1 GLU A 496     133.363 117.463 173.165  1.00  0.00
ATOM   7599  OE2 GLU A 496     133.150 116.096 171.477  1.00  0.00
ATOM   7600  C   GLU A 496     137.342 118.841 173.160  1.00  0.00
ATOM   7601  O   GLU A 496     137.182 119.382 172.063  1.00  0.00
ATOM   7602  N   ASN A 497     137.537 119.561 174.269  1.00  0.00
ATOM   7603  H   ASN A 497     137.720 119.067 175.130  1.00  0.00
ATOM   7604  CA  ASN A 497     137.737 121.004 174.250  1.00  0.00
ATOM   7605  HA  ASN A 497     136.862 121.439 173.763  1.00  0.00
ATOM   7606  CB  ASN A 497     137.774 121.550 175.693  1.00  0.00
ATOM   7607  HB1 ASN A 497     136.843 121.289 176.197  1.00  0.00
ATOM   7608  HB2 ASN A 497     137.832 122.637 175.639  1.00  0.00
ATOM   7609  CG  ASN A 497     138.929 121.049 176.543  1.00  0.00
ATOM   7610  OD1 ASN A 497     139.571 120.050 176.261  1.00  0.00
ATOM   7611  ND2 ASN A 497     139.223 121.716 177.632  1.00  0.00
ATOM   7612 1HD2 ASN A 497     138.686 122.513 177.922  1.00  0.00
ATOM   7613 2HD2 ASN A 497     139.975 121.353 178.193  1.00  0.00
ATOM   7614  C   ASN A 497     138.953 121.453 173.401  1.00  0.00
ATOM   7615  O   ASN A 497     138.753 122.365 172.597  1.00  0.00
ATOM   7616  N   SER A 498     140.171 120.867 173.484  1.00  0.00
ATOM   7617  H   SER A 498     140.328 120.100 174.124  1.00  0.00
ATOM   7618  CA  SER A 498     141.245 121.364 172.597  1.00  0.00
ATOM   7619  HA  SER A 498     141.215 122.451 172.617  1.00  0.00
ATOM   7620  CB  SER A 498     142.660 120.987 173.054  1.00  0.00
ATOM   7621  HB1 SER A 498     142.672 120.805 174.130  1.00  0.00
ATOM   7622  HB2 SER A 498     143.323 121.825 172.833  1.00  0.00
ATOM   7623  OG  SER A 498     143.140 119.858 172.353  1.00  0.00
```

ATOM	7624	HG	SER	A	498	142.941	119.084	172.931	1.00	0.00
ATOM	7625	C	SER	A	498	141.032	120.942	171.136	1.00	0.00
ATOM	7626	O	SER	A	498	141.359	121.671	170.201	1.00	0.00
ATOM	7627	N	CYS	A	499	140.452	119.759	170.940	1.00	0.00
ATOM	7628	H	CYS	A	499	140.249	119.211	171.773	1.00	0.00
ATOM	7629	CA	CYS	A	499	140.088	119.194	169.645	1.00	0.00
ATOM	7630	HA	CYS	A	499	140.976	119.037	169.043	1.00	0.00
ATOM	7631	CB	CYS	A	499	139.488	117.823	169.980	1.00	0.00
ATOM	7632	HB1	CYS	A	499	138.642	117.983	170.640	1.00	0.00
ATOM	7633	HB2	CYS	A	499	140.234	117.254	170.537	1.00	0.00
ATOM	7634	SG	CYS	A	499	138.896	116.767	168.646	1.00	0.00
ATOM	7635	C	CYS	A	499	139.154	120.120	168.849	1.00	0.00
ATOM	7636	O	CYS	A	499	139.459	120.473	167.706	1.00	0.00
ATOM	7637	N	LYS	A	500	138.084	120.603	169.496	1.00	0.00
ATOM	7638	H	LYS	A	500	137.903	120.254	170.434	1.00	0.00
ATOM	7639	CA	LYS	A	500	137.172	121.626	168.961	1.00	0.00
ATOM	7640	HA	LYS	A	500	136.898	121.345	167.944	1.00	0.00
ATOM	7641	CB	LYS	A	500	135.891	121.669	169.817	1.00	0.00
ATOM	7642	HB1	LYS	A	500	135.312	122.557	169.552	1.00	0.00
ATOM	7643	HB2	LYS	A	500	136.160	121.736	170.874	1.00	0.00
ATOM	7644	CG	LYS	A	500	135.018	120.427	169.568	1.00	0.00
ATOM	7645	HG1	LYS	A	500	135.623	119.523	169.670	1.00	0.00
ATOM	7646	HG2	LYS	A	500	134.637	120.465	168.545	1.00	0.00
ATOM	7647	CD	LYS	A	500	133.830	120.312	170.539	1.00	0.00
ATOM	7648	HD1	LYS	A	500	133.224	121.218	170.499	1.00	0.00
ATOM	7649	HD2	LYS	A	500	134.214	120.169	171.551	1.00	0.00
ATOM	7650	CE	LYS	A	500	132.987	119.099	170.125	1.00	0.00
ATOM	7651	HE1	LYS	A	500	133.671	118.270	169.942	1.00	0.00
ATOM	7652	HE2	LYS	A	500	132.476	119.307	169.182	1.00	0.00
ATOM	7653	NZ	LYS	A	500	132.021	118.661	171.160	1.00	0.00
ATOM	7654	HZ1	LYS	A	500	132.507	118.485	172.046	1.00	0.00
ATOM	7655	HZ2	LYS	A	500	131.255	119.294	171.309	1.00	0.00
ATOM	7656	HZ3	LYS	A	500	131.698	117.722	170.939	1.00	0.00
ATOM	7657	C	LYS	A	500	137.829	123.006	168.832	1.00	0.00
ATOM	7658	O	LYS	A	500	137.613	123.675	167.829	1.00	0.00
ATOM	7659	N	ALA	A	501	138.655	123.421	169.797	1.00	0.00
ATOM	7660	H	ALA	A	501	138.770	122.847	170.621	1.00	0.00
ATOM	7661	CA	ALA	A	501	139.320	124.729	169.774	1.00	0.00
ATOM	7662	HA	ALA	A	501	138.558	125.496	169.623	1.00	0.00
ATOM	7663	CB	ALA	A	501	139.973	124.984	171.138	1.00	0.00
ATOM	7664	HB1	ALA	A	501	140.412	125.982	171.146	1.00	0.00
ATOM	7665	HB2	ALA	A	501	139.223	124.924	171.927	1.00	0.00
ATOM	7666	HB3	ALA	A	501	140.759	124.252	171.317	1.00	0.00
ATOM	7667	C	ALA	A	501	140.346	124.903	168.639	1.00	0.00
ATOM	7668	O	ALA	A	501	140.515	126.018	168.159	1.00	0.00
ATOM	7669	N	THR	A	502	140.987	123.823	168.162	1.00	0.00
ATOM	7670	H	THR	A	502	140.895	122.945	168.662	1.00	0.00
ATOM	7671	CA	THR	A	502	141.819	123.866	166.942	1.00	0.00
ATOM	7672	HA	THR	A	502	142.102	124.906	166.775	1.00	0.00
ATOM	7673	CB	THR	A	502	143.151	123.121	167.111	1.00	0.00
ATOM	7674	HB	THR	A	502	143.601	123.415	168.060	1.00	0.00
ATOM	7675	CG2	THR	A	502	143.019	121.600	167.090	1.00	0.00
ATOM	7676	1HG2	THR	A	502	142.315	121.283	167.854	1.00	0.00
ATOM	7677	2HG2	THR	A	502	142.664	121.268	166.120	1.00	0.00
ATOM	7678	3HG2	THR	A	502	144.000	121.166	167.281	1.00	0.00
ATOM	7679	OG1	THR	A	502	144.032	123.474	166.072	1.00	0.00
ATOM	7680	HG1	THR	A	502	143.529	123.416	165.245	1.00	0.00
ATOM	7681	C	THR	A	502	141.065	123.455	165.669	1.00	0.00
ATOM	7682	O	THR	A	502	141.689	123.259	164.624	1.00	0.00
ATOM	7683	N	GLY	A	503	139.740	123.302	165.737	1.00	0.00
ATOM	7684	H	GLY	A	503	139.273	123.518	166.609	1.00	0.00

ATOM	7685	CA	GLY	A	503	138.890	123.008	164.584	1.00	0.00
ATOM	7686	HA1	GLY	A	503	139.095	123.737	163.799	1.00	0.00
ATOM	7687	HA2	GLY	A	503	137.851	123.126	164.890	1.00	0.00
ATOM	7688	C	GLY	A	503	139.049	121.607	163.980	1.00	0.00
ATOM	7689	O	GLY	A	503	138.579	121.374	162.871	1.00	0.00
ATOM	7690	N	GLN	A	504	139.658	120.648	164.687	1.00	0.00
ATOM	7691	H	GLN	A	504	139.941	120.865	165.633	1.00	0.00
ATOM	7692	CA	GLN	A	504	139.780	119.248	164.244	1.00	0.00
ATOM	7693	HA	GLN	A	504	139.897	119.236	163.159	1.00	0.00
ATOM	7694	CB	GLN	A	504	141.034	118.616	164.859	1.00	0.00
ATOM	7695	HB1	GLN	A	504	140.997	117.532	164.740	1.00	0.00
ATOM	7696	HB2	GLN	A	504	141.077	118.859	165.921	1.00	0.00
ATOM	7697	CG	GLN	A	504	142.311	119.097	164.158	1.00	0.00
ATOM	7698	HG1	GLN	A	504	142.364	120.184	164.171	1.00	0.00
ATOM	7699	HG2	GLN	A	504	142.281	118.777	163.117	1.00	0.00
ATOM	7700	CD	GLN	A	504	143.574	118.536	164.797	1.00	0.00
ATOM	7701	OE1	GLN	A	504	143.582	118.007	165.903	1.00	0.00
ATOM	7702	NE2	GLN	A	504	144.703	118.643	164.135	1.00	0.00
ATOM	7703	1HE2	GLN	A	504	144.715	119.065	163.225	1.00	0.00
ATOM	7704	2HE2	GLN	A	504	145.524	118.218	164.537	1.00	0.00
ATOM	7705	C	GLN	A	504	138.493	118.438	164.525	1.00	0.00
ATOM	7706	O	GLN	A	504	138.500	117.338	165.070	1.00	0.00
ATOM	7707	N	VAL	A	505	137.358	119.045	164.183	1.00	0.00
ATOM	7708	H	VAL	A	505	137.450	119.894	163.636	1.00	0.00
ATOM	7709	CA	VAL	A	505	136.011	118.487	164.337	1.00	0.00
ATOM	7710	HA	VAL	A	505	135.983	117.896	165.253	1.00	0.00
ATOM	7711	CB	VAL	A	505	134.954	119.627	164.429	1.00	0.00
ATOM	7712	HB	VAL	A	505	134.560	119.843	163.437	1.00	0.00
ATOM	7713	CG1	VAL	A	505	133.784	119.235	165.346	1.00	0.00
ATOM	7714	1HG1	VAL	A	505	133.084	120.067	165.432	1.00	0.00
ATOM	7715	2HG1	VAL	A	505	133.234	118.395	164.934	1.00	0.00
ATOM	7716	3HG1	VAL	A	505	134.160	118.984	166.337	1.00	0.00
ATOM	7717	CG2	VAL	A	505	135.491	120.955	164.991	1.00	0.00
ATOM	7718	1HG2	VAL	A	505	134.677	121.663	165.140	1.00	0.00
ATOM	7719	2HG2	VAL	A	505	136.003	120.783	165.935	1.00	0.00
ATOM	7720	3HG2	VAL	A	505	136.174	121.402	164.271	1.00	0.00
ATOM	7721	C	VAL	A	505	135.685	117.558	163.153	1.00	0.00
ATOM	7722	O	VAL	A	505	136.461	117.421	162.208	1.00	0.00
ATOM	7723	N	CYS	A	506	134.482	116.976	163.161	1.00	0.00
ATOM	7724	H	CYS	A	506	133.922	117.058	163.991	1.00	0.00
ATOM	7725	CA	CYS	A	506	133.817	116.504	161.945	1.00	0.00
ATOM	7726	HA	CYS	A	506	134.338	115.618	161.584	1.00	0.00
ATOM	7727	CB	CYS	A	506	132.347	116.172	162.237	1.00	0.00
ATOM	7728	HB1	CYS	A	506	131.800	116.065	161.297	1.00	0.00
ATOM	7729	HB2	CYS	A	506	131.883	116.991	162.789	1.00	0.00
ATOM	7730	SG	CYS	A	506	132.191	114.628	163.166	1.00	0.00
ATOM	7731	HG	CYS	A	506	132.652	113.820	162.199	1.00	0.00
ATOM	7732	C	CYS	A	506	133.843	117.548	160.818	1.00	0.00
ATOM	7733	O	CYS	A	506	133.683	118.744	161.061	1.00	0.00
ATOM	7734	N	HIS	A	507	133.911	117.077	159.571	1.00	0.00
ATOM	7735	H	HIS	A	507	134.026	116.085	159.425	1.00	0.00
ATOM	7736	CA	HIS	A	507	133.561	117.911	158.425	1.00	0.00
ATOM	7737	HA	HIS	A	507	134.115	118.849	158.512	1.00	0.00
ATOM	7738	CB	HIS	A	507	133.989	117.213	157.128	1.00	0.00
ATOM	7739	HB1	HIS	A	507	133.240	116.479	156.830	1.00	0.00
ATOM	7740	HB2	HIS	A	507	134.931	116.688	157.288	1.00	0.00
ATOM	7741	CG	HIS	A	507	134.191	118.198	156.011	1.00	0.00
ATOM	7742	ND1	HIS	A	507	133.182	118.748	155.217	1.00	0.00
ATOM	7743	CE1	HIS	A	507	133.793	119.632	154.413	1.00	0.00
ATOM	7744	HE1	HIS	A	507	133.283	120.242	153.672	1.00	0.00
ATOM	7745	NE2	HIS	A	507	135.110	119.667	154.672	1.00	0.00

ATOM	7746	HE2	HIS	A	507	135.781	120.269	154.215	1.00	0.00
ATOM	7747	CD2	HIS	A	507	135.379	118.778	155.685	1.00	0.00
ATOM	7748	HD2	HIS	A	507	136.337	118.578	156.161	1.00	0.00
ATOM	7749	C	HIS	A	507	132.062	118.246	158.421	1.00	0.00
ATOM	7750	O	HIS	A	507	131.244	117.432	158.850	1.00	0.00
ATOM	7751	N	ALA	A	508	131.685	119.404	157.874	1.00	0.00
ATOM	7752	H	ALA	A	508	132.403	119.990	157.482	1.00	0.00
ATOM	7753	CA	ALA	A	508	130.292	119.844	157.726	1.00	0.00
ATOM	7754	HA	ALA	A	508	129.820	119.829	158.710	1.00	0.00
ATOM	7755	CB	ALA	A	508	130.301	121.286	157.206	1.00	0.00
ATOM	7756	HB1	ALA	A	508	129.278	121.658	157.151	1.00	0.00
ATOM	7757	HB2	ALA	A	508	130.865	121.924	157.886	1.00	0.00
ATOM	7758	HB3	ALA	A	508	130.748	121.323	156.212	1.00	0.00
ATOM	7759	C	ALA	A	508	129.457	118.932	156.807	1.00	0.00
ATOM	7760	O	ALA	A	508	128.230	118.892	156.894	1.00	0.00
ATOM	7761	N	LEU	A	509	130.138	118.194	155.929	1.00	0.00
ATOM	7762	H	LEU	A	509	131.137	118.353	155.870	1.00	0.00
ATOM	7763	CA	LEU	A	509	129.571	117.123	155.116	1.00	0.00
ATOM	7764	HA	LEU	A	509	128.653	117.484	154.652	1.00	0.00
ATOM	7765	CB	LEU	A	509	130.604	116.777	154.027	1.00	0.00
ATOM	7766	HB1	LEU	A	509	130.344	115.829	153.578	1.00	0.00
ATOM	7767	HB2	LEU	A	509	131.584	116.644	154.488	1.00	0.00
ATOM	7768	CG	LEU	A	509	130.702	117.822	152.900	1.00	0.00
ATOM	7769	HG	LEU	A	509	130.996	118.788	153.309	1.00	0.00
ATOM	7770	CD1	LEU	A	509	131.743	117.392	151.871	1.00	0.00
ATOM	7771	1HD1	LEU	A	509	131.790	118.107	151.050	1.00	0.00
ATOM	7772	2HD1	LEU	A	509	132.719	117.361	152.342	1.00	0.00
ATOM	7773	3HD1	LEU	A	509	131.500	116.407	151.475	1.00	0.00
ATOM	7774	CD2	LEU	A	509	129.377	117.983	152.161	1.00	0.00
ATOM	7775	1HD2	LEU	A	509	129.540	118.564	151.259	1.00	0.00
ATOM	7776	2HD2	LEU	A	509	128.970	117.006	151.903	1.00	0.00
ATOM	7777	3HD2	LEU	A	509	128.657	118.522	152.775	1.00	0.00
ATOM	7778	C	LEU	A	509	129.156	115.887	155.932	1.00	0.00
ATOM	7779	O	LEU	A	509	128.300	115.120	155.489	1.00	0.00
ATOM	7780	N	CYS	A	510	129.711	115.684	157.127	1.00	0.00
ATOM	7781	H	CYS	A	510	130.314	116.396	157.520	1.00	0.00
ATOM	7782	CA	CYS	A	510	129.362	114.556	157.982	1.00	0.00
ATOM	7783	HA	CYS	A	510	129.224	113.674	157.355	1.00	0.00
ATOM	7784	CB	CYS	A	510	130.512	114.271	158.948	1.00	0.00
ATOM	7785	HB1	CYS	A	510	130.288	113.347	159.484	1.00	0.00
ATOM	7786	HB2	CYS	A	510	130.557	115.073	159.685	1.00	0.00
ATOM	7787	SG	CYS	A	510	132.157	114.107	158.192	1.00	0.00
ATOM	7788	C	CYS	A	510	128.050	114.794	158.747	1.00	0.00
ATOM	7789	O	CYS	A	510	127.721	115.913	159.142	1.00	0.00
ATOM	7790	N	SER	A	511	127.296	113.721	158.987	1.00	0.00
ATOM	7791	H	SER	A	511	127.609	112.835	158.615	1.00	0.00
ATOM	7792	CA	SER	A	511	126.183	113.690	159.937	1.00	0.00
ATOM	7793	HA	SER	A	511	125.605	114.606	159.837	1.00	0.00
ATOM	7794	CB	SER	A	511	125.278	112.487	159.648	1.00	0.00
ATOM	7795	HB1	SER	A	511	124.722	112.206	160.541	1.00	0.00
ATOM	7796	HB2	SER	A	511	125.873	111.627	159.339	1.00	0.00
ATOM	7797	OG	SER	A	511	124.342	112.822	158.649	1.00	0.00
ATOM	7798	HG	SER	A	511	124.576	112.332	157.838	1.00	0.00
ATOM	7799	C	SER	A	511	126.687	113.572	161.388	1.00	0.00
ATOM	7800	O	SER	A	511	127.832	113.167	161.597	1.00	0.00
ATOM	7801	N	PRO	A	512	125.823	113.802	162.397	1.00	0.00
ATOM	7802	CD	PRO	A	512	124.538	114.483	162.252	1.00	0.00
ATOM	7803	HD1	PRO	A	512	123.791	113.778	161.889	1.00	0.00
ATOM	7804	HD2	PRO	A	512	124.608	115.336	161.578	1.00	0.00
ATOM	7805	CG	PRO	A	512	124.158	114.964	163.647	1.00	0.00
ATOM	7806	HG1	PRO	A	512	123.077	115.040	163.771	1.00	0.00

ATOM	7807	HG2	PRO	A	512	124.640	115.921	163.851	1.00	0.00
ATOM	7808	CB	PRO	A	512	124.761	113.887	164.539	1.00	0.00
ATOM	7809	HB1	PRO	A	512	124.081	113.033	164.574	1.00	0.00
ATOM	7810	HB2	PRO	A	512	124.954	114.258	165.547	1.00	0.00
ATOM	7811	CA	PRO	A	512	126.059	113.497	163.821	1.00	0.00
ATOM	7812	HA	PRO	A	512	126.853	114.162	164.163	1.00	0.00
ATOM	7813	C	PRO	A	512	126.470	112.058	164.221	1.00	0.00
ATOM	7814	O	PRO	A	512	126.408	111.715	165.397	1.00	0.00
ATOM	7815	N	GLU	A	513	126.879	111.204	163.279	1.00	0.00
ATOM	7816	H	GLU	A	513	126.989	111.576	162.347	1.00	0.00
ATOM	7817	CA	GLU	A	513	127.416	109.855	163.536	1.00	0.00
ATOM	7818	HA	GLU	A	513	127.053	109.518	164.507	1.00	0.00
ATOM	7819	CB	GLU	A	513	126.888	108.893	162.453	1.00	0.00
ATOM	7820	HB1	GLU	A	513	127.670	108.652	161.736	1.00	0.00
ATOM	7821	HB2	GLU	A	513	126.091	109.386	161.894	1.00	0.00
ATOM	7822	CG	GLU	A	513	126.280	107.605	163.030	1.00	0.00
ATOM	7823	HG1	GLU	A	513	125.678	107.140	162.247	1.00	0.00
ATOM	7824	HG2	GLU	A	513	125.598	107.860	163.845	1.00	0.00
ATOM	7825	CD	GLU	A	513	127.318	106.585	163.512	1.00	0.00
ATOM	7826	OE1	GLU	A	513	127.577	106.471	164.733	1.00	0.00
ATOM	7827	OE2	GLU	A	513	127.778	105.756	162.704	1.00	0.00
ATOM	7828	C	GLU	A	513	128.954	109.836	163.630	1.00	0.00
ATOM	7829	O	GLU	A	513	129.558	108.806	163.929	1.00	0.00
ATOM	7830	N	GLY	A	514	129.597	110.984	163.399	1.00	0.00
ATOM	7831	H	GLY	A	514	129.056	111.784	163.098	1.00	0.00
ATOM	7832	CA	GLY	A	514	130.993	111.199	163.751	1.00	0.00
ATOM	7833	HA1	GLY	A	514	131.278	110.492	164.526	1.00	0.00
ATOM	7834	HA2	GLY	A	514	131.103	112.205	164.147	1.00	0.00
ATOM	7835	C	GLY	A	514	131.962	111.011	162.589	1.00	0.00
ATOM	7836	O	GLY	A	514	131.592	110.889	161.412	1.00	0.00
ATOM	7837	N	CYS	A	515	133.232	110.952	162.970	1.00	0.00
ATOM	7838	H	CYS	A	515	133.421	110.954	163.961	1.00	0.00
ATOM	7839	CA	CYS	A	515	134.397	110.786	162.120	1.00	0.00
ATOM	7840	HA	CYS	A	515	134.078	110.348	161.182	1.00	0.00
ATOM	7841	CB	CYS	A	515	135.048	112.156	161.898	1.00	0.00
ATOM	7842	HB1	CYS	A	515	136.068	112.031	161.541	1.00	0.00
ATOM	7843	HB2	CYS	A	515	135.074	112.720	162.832	1.00	0.00
ATOM	7844	SG	CYS	A	515	134.124	113.061	160.640	1.00	0.00
ATOM	7845	HG	CYS	A	515	134.465	112.252	159.622	1.00	0.00
ATOM	7846	C	CYS	A	515	135.410	109.827	162.775	1.00	0.00
ATOM	7847	O	CYS	A	515	135.299	109.572	163.977	1.00	0.00
ATOM	7848	N	TRP	A	516	136.426	109.367	162.032	1.00	0.00
ATOM	7849	H	TRP	A	516	136.425	109.513	161.026	1.00	0.00
ATOM	7850	CA	TRP	A	516	137.705	109.005	162.665	1.00	0.00
ATOM	7851	HA	TRP	A	516	137.570	108.959	163.745	1.00	0.00
ATOM	7852	CB	TRP	A	516	138.228	107.620	162.257	1.00	0.00
ATOM	7853	HB1	TRP	A	516	139.208	107.484	162.715	1.00	0.00
ATOM	7854	HB2	TRP	A	516	138.396	107.600	161.183	1.00	0.00
ATOM	7855	CG	TRP	A	516	137.429	106.409	162.624	1.00	0.00
ATOM	7856	CD1	TRP	A	516	136.857	105.595	161.718	1.00	0.00
ATOM	7857	HD1	TRP	A	516	136.883	105.775	160.654	1.00	0.00
ATOM	7858	NE1	TRP	A	516	136.360	104.467	162.337	1.00	0.00
ATOM	7859	HE1	TRP	A	516	135.999	103.668	161.826	1.00	0.00
ATOM	7860	CE2	TRP	A	516	136.572	104.513	163.698	1.00	0.00
ATOM	7861	CZ2	TRP	A	516	136.273	103.621	164.739	1.00	0.00
ATOM	7862	HZ2	TRP	A	516	135.795	102.677	164.525	1.00	0.00
ATOM	7863	CH2	TRP	A	516	136.566	103.992	166.061	1.00	0.00
ATOM	7864	HH2	TRP	A	516	136.306	103.330	166.883	1.00	0.00
ATOM	7865	CZ3	TRP	A	516	137.173	105.233	166.325	1.00	0.00
ATOM	7866	HZ3	TRP	A	516	137.375	105.518	167.347	1.00	0.00
ATOM	7867	CE3	TRP	A	516	137.501	106.110	165.268	1.00	0.00

ATOM	7868	HE3	TRP	A	516	137.963	107.059	165.484	1.00	0.00
ATOM	7869	CD2	TRP	A	516	137.209	105.773	163.925	1.00	0.00
ATOM	7870	C	TRP	A	516	138.804	110.060	162.458	1.00	0.00
ATOM	7871	O	TRP	A	516	139.767	110.046	163.220	1.00	0.00
ATOM	7872	N	GLY	A	517	138.613	110.993	161.518	1.00	0.00
ATOM	7873	H	GLY	A	517	137.877	110.817	160.853	1.00	0.00
ATOM	7874	CA	GLY	A	517	139.471	112.129	161.187	1.00	0.00
ATOM	7875	HA1	GLY	A	517	140.346	111.775	160.643	1.00	0.00
ATOM	7876	HA2	GLY	A	517	139.786	112.618	162.106	1.00	0.00
ATOM	7877	C	GLY	A	517	138.734	113.163	160.320	1.00	0.00
ATOM	7878	O	GLY	A	517	137.584	112.942	159.936	1.00	0.00
ATOM	7879	N	PRO	A	518	139.350	114.321	160.029	1.00	0.00
ATOM	7880	CD	PRO	A	518	140.742	114.619	160.339	1.00	0.00
ATOM	7881	HD1	PRO	A	518	141.420	113.912	159.857	1.00	0.00
ATOM	7882	HD2	PRO	A	518	140.891	114.606	161.420	1.00	0.00
ATOM	7883	CG	PRO	A	518	140.982	116.031	159.812	1.00	0.00
ATOM	7884	HG1	PRO	A	518	141.251	115.991	158.756	1.00	0.00
ATOM	7885	HG2	PRO	A	518	141.745	116.554	160.389	1.00	0.00
ATOM	7886	CB	PRO	A	518	139.605	116.676	159.969	1.00	0.00
ATOM	7887	HB1	PRO	A	518	139.493	117.549	159.326	1.00	0.00
ATOM	7888	HB2	PRO	A	518	139.458	116.965	161.011	1.00	0.00
ATOM	7889	CA	PRO	A	518	138.637	115.537	159.618	1.00	0.00
ATOM	7890	HA	PRO	A	518	137.743	115.650	160.232	1.00	0.00
ATOM	7891	C	PRO	A	518	138.172	115.624	158.150	1.00	0.00
ATOM	7892	O	PRO	A	518	137.690	116.672	157.715	1.00	0.00
ATOM	7893	N	GLU	A	519	138.322	114.569	157.349	1.00	0.00
ATOM	7894	H	GLU	A	519	138.532	113.676	157.773	1.00	0.00
ATOM	7895	CA	GLU	A	519	138.130	114.635	155.889	1.00	0.00
ATOM	7896	HA	GLU	A	519	138.274	115.667	155.575	1.00	0.00
ATOM	7897	CB	GLU	A	519	139.230	113.787	155.226	1.00	0.00
ATOM	7898	HB1	GLU	A	519	139.042	112.726	155.403	1.00	0.00
ATOM	7899	HB2	GLU	A	519	140.198	114.052	155.655	1.00	0.00
ATOM	7900	CG	GLU	A	519	139.284	114.058	153.720	1.00	0.00
ATOM	7901	HG1	GLU	A	519	139.367	115.136	153.568	1.00	0.00
ATOM	7902	HG2	GLU	A	519	138.352	113.718	153.267	1.00	0.00
ATOM	7903	CD	GLU	A	519	140.438	113.360	153.017	1.00	0.00
ATOM	7904	OE1	GLU	A	519	141.219	114.066	152.339	1.00	0.00
ATOM	7905	OE2	GLU	A	519	140.442	112.108	152.971	1.00	0.00
ATOM	7906	C	GLU	A	519	136.719	114.162	155.470	1.00	0.00
ATOM	7907	O	GLU	A	519	136.185	113.251	156.099	1.00	0.00
ATOM	7908	N	PRO	A	520	136.104	114.690	154.385	1.00	0.00
ATOM	7909	CD	PRO	A	520	136.488	115.909	153.685	1.00	0.00
ATOM	7910	HD1	PRO	A	520	137.306	115.709	152.995	1.00	0.00
ATOM	7911	HD2	PRO	A	520	136.762	116.705	154.378	1.00	0.00
ATOM	7912	CG	PRO	A	520	135.264	116.321	152.874	1.00	0.00
ATOM	7913	HG1	PRO	A	520	135.537	116.873	151.975	1.00	0.00
ATOM	7914	HG2	PRO	A	520	134.601	116.908	153.506	1.00	0.00
ATOM	7915	CB	PRO	A	520	134.605	114.986	152.548	1.00	0.00
ATOM	7916	HB1	PRO	A	520	135.119	114.524	151.703	1.00	0.00
ATOM	7917	HB2	PRO	A	520	133.542	115.092	152.335	1.00	0.00
ATOM	7918	CA	PRO	A	520	134.842	114.178	153.824	1.00	0.00
ATOM	7919	HA	PRO	A	520	134.050	114.413	154.536	1.00	0.00
ATOM	7920	C	PRO	A	520	134.762	112.674	153.525	1.00	0.00
ATOM	7921	O	PRO	A	520	133.663	112.110	153.474	1.00	0.00
ATOM	7922	N	ARG	A	521	135.904	112.003	153.342	1.00	0.00
ATOM	7923	H	ARG	A	521	136.764	112.530	153.358	1.00	0.00
ATOM	7924	CA	ARG	A	521	135.981	110.533	153.286	1.00	0.00
ATOM	7925	HA	ARG	A	521	135.259	110.144	152.576	1.00	0.00
ATOM	7926	CB	ARG	A	521	137.392	110.072	152.878	1.00	0.00
ATOM	7927	HB1	ARG	A	521	137.360	108.995	152.708	1.00	0.00
ATOM	7928	HB2	ARG	A	521	138.105	110.275	153.678	1.00	0.00

ATOM	7929	CG	ARG	A	521	137.887	110.769	151.612	1.00	0.00
ATOM	7930	HG1	ARG	A	521	138.193	111.785	151.852	1.00	0.00
ATOM	7931	HG2	ARG	A	521	137.072	110.850	150.907	1.00	0.00
ATOM	7932	CD	ARG	A	521	139.034	110.030	150.927	1.00	0.00
ATOM	7933	HD1	ARG	A	521	138.760	108.982	150.792	1.00	0.00
ATOM	7934	HD2	ARG	A	521	139.943	110.088	151.528	1.00	0.00
ATOM	7935	NE	ARG	A	521	139.220	110.635	149.606	1.00	0.00
ATOM	7936	HE	ARG	A	521	138.579	110.309	148.875	1.00	0.00
ATOM	7937	CZ	ARG	A	521	139.777	111.804	149.374	1.00	0.00
ATOM	7938	NH1	ARG	A	521	140.632	112.326	150.202	1.00	0.00
ATOM	7939	1HH1	ARG	A	521	140.725	111.916	151.137	1.00	0.00
ATOM	7940	2HH1	ARG	A	521	140.915	113.288	150.167	1.00	0.00
ATOM	7941	NH2	ARG	A	521	139.409	112.482	148.328	1.00	0.00
ATOM	7942	1HH2	ARG	A	521	138.515	112.205	147.901	1.00	0.00
ATOM	7943	2HH2	ARG	A	521	139.769	113.394	148.177	1.00	0.00
ATOM	7944	C	ARG	A	521	135.651	109.915	154.621	1.00	0.00
ATOM	7945	O	ARG	A	521	134.940	108.924	154.684	1.00	0.00
ATOM	7946	N	ASP	A	522	136.162	110.508	155.696	1.00	0.00
ATOM	7947	H	ASP	A	522	136.607	111.406	155.576	1.00	0.00
ATOM	7948	CA	ASP	A	522	136.184	109.939	157.035	1.00	0.00
ATOM	7949	HA	ASP	A	522	136.135	108.871	156.867	1.00	0.00
ATOM	7950	CB	ASP	A	522	137.539	110.177	157.712	1.00	0.00
ATOM	7951	HB1	ASP	A	522	137.576	111.190	158.107	1.00	0.00
ATOM	7952	HB2	ASP	A	522	138.329	110.076	156.965	1.00	0.00
ATOM	7953	CG	ASP	A	522	137.821	109.158	158.829	1.00	0.00
ATOM	7954	OD1	ASP	A	522	138.938	109.174	159.370	1.00	0.00
ATOM	7955	OD2	ASP	A	522	136.936	108.338	159.162	1.00	0.00
ATOM	7956	C	ASP	A	522	134.953	110.299	157.897	1.00	0.00
ATOM	7957	O	ASP	A	522	134.978	110.192	159.118	1.00	0.00
ATOM	7958	N	CYS	A	523	133.829	110.655	157.279	1.00	0.00
ATOM	7959	H	CYS	A	523	133.846	110.734	156.272	1.00	0.00
ATOM	7960	CA	CYS	A	523	132.528	110.612	157.944	1.00	0.00
ATOM	7961	HA	CYS	A	523	132.587	111.099	158.919	1.00	0.00
ATOM	7962	CB	CYS	A	523	131.498	111.343	157.070	1.00	0.00
ATOM	7963	HB1	CYS	A	523	130.560	111.404	157.621	1.00	0.00
ATOM	7964	HB2	CYS	A	523	131.316	110.740	156.179	1.00	0.00
ATOM	7965	SG	CYS	A	523	131.893	113.017	156.494	1.00	0.00
ATOM	7966	C	CYS	A	523	132.047	109.157	158.109	1.00	0.00
ATOM	7967	O	CYS	A	523	132.137	108.376	157.166	1.00	0.00
ATOM	7968	N	VAL	A	524	131.432	108.799	159.244	1.00	0.00
ATOM	7969	H	VAL	A	524	131.394	109.460	160.021	1.00	0.00
ATOM	7970	CA	VAL	A	524	130.726	107.495	159.337	1.00	0.00
ATOM	7971	HA	VAL	A	524	131.354	106.739	158.876	1.00	0.00
ATOM	7972	CB	VAL	A	524	130.452	107.054	160.788	1.00	0.00
ATOM	7973	HB	VAL	A	524	129.602	107.611	161.178	1.00	0.00
ATOM	7974	CG1	VAL	A	524	130.152	105.547	160.819	1.00	0.00
ATOM	7975	1HG1	VAL	A	524	130.099	105.201	161.851	1.00	0.00
ATOM	7976	2HG1	VAL	A	524	129.182	105.355	160.359	1.00	0.00
ATOM	7977	3HG1	VAL	A	524	130.916	104.979	160.290	1.00	0.00
ATOM	7978	CG2	VAL	A	524	131.635	107.256	161.739	1.00	0.00
ATOM	7979	1HG2	VAL	A	524	132.001	108.278	161.701	1.00	0.00
ATOM	7980	2HG2	VAL	A	524	131.278	107.079	162.751	1.00	0.00
ATOM	7981	3HG2	VAL	A	524	132.431	106.557	161.514	1.00	0.00
ATOM	7982	C	VAL	A	524	129.426	107.493	158.531	1.00	0.00
ATOM	7983	O	VAL	A	524	129.027	106.484	157.951	1.00	0.00
ATOM	7984	N	SER	A	525	128.791	108.657	158.424	1.00	0.00
ATOM	7985	H	SER	A	525	129.151	109.434	158.960	1.00	0.00
ATOM	7986	CA	SER	A	525	127.670	108.915	157.521	1.00	0.00
ATOM	7987	HA	SER	A	525	127.717	108.237	156.667	1.00	0.00
ATOM	7988	CB	SER	A	525	126.344	108.691	158.248	1.00	0.00
ATOM	7989	HB1	SER	A	525	125.515	108.962	157.592	1.00	0.00

ATOM	7990	HB2	SER	A	525	126.301	109.310	159.144	1.00	0.00
ATOM	7991	OG	SER	A	525	126.209	107.332	158.611	1.00	0.00
ATOM	7992	HG	SER	A	525	127.017	106.857	158.367	1.00	0.00
ATOM	7993	C	SER	A	525	127.730	110.339	157.001	1.00	0.00
ATOM	7994	O	SER	A	525	127.946	111.281	157.759	1.00	0.00
ATOM	7995	N	CYS	A	526	127.516	110.497	155.700	1.00	0.00
ATOM	7996	H	CYS	A	526	127.377	109.665	155.145	1.00	0.00
ATOM	7997	CA	CYS	A	526	127.260	111.779	155.052	1.00	0.00
ATOM	7998	HA	CYS	A	526	128.121	112.431	155.203	1.00	0.00
ATOM	7999	CB	CYS	A	526	127.121	111.429	153.563	1.00	0.00
ATOM	8000	HB1	CYS	A	526	126.185	110.894	153.400	1.00	0.00
ATOM	8001	HB2	CYS	A	526	127.924	110.738	153.312	1.00	0.00
ATOM	8002	SG	CYS	A	526	127.232	112.736	152.325	1.00	0.00
ATOM	8003	C	CYS	A	526	126.007	112.455	155.636	1.00	0.00
ATOM	8004	O	CYS	A	526	125.181	111.815	156.290	1.00	0.00
ATOM	8005	N	ARG	A	527	125.836	113.749	155.363	1.00	0.00
ATOM	8006	H	ARG	A	527	126.637	114.242	154.968	1.00	0.00
ATOM	8007	CA	ARG	A	527	124.650	114.558	155.721	1.00	0.00
ATOM	8008	HA	ARG	A	527	123.983	113.983	156.363	1.00	0.00
ATOM	8009	CB	ARG	A	527	125.191	115.767	156.500	1.00	0.00
ATOM	8010	HB1	ARG	A	527	125.589	116.486	155.780	1.00	0.00
ATOM	8011	HB2	ARG	A	527	126.032	115.452	157.111	1.00	0.00
ATOM	8012	CG	ARG	A	527	124.189	116.435	157.456	1.00	0.00
ATOM	8013	HG1	ARG	A	527	124.419	116.133	158.478	1.00	0.00
ATOM	8014	HG2	ARG	A	527	123.172	116.116	157.228	1.00	0.00
ATOM	8015	CD	ARG	A	527	124.286	117.959	157.347	1.00	0.00
ATOM	8016	HD1	ARG	A	527	125.332	118.263	157.444	1.00	0.00
ATOM	8017	HD2	ARG	A	527	123.728	118.420	158.161	1.00	0.00
ATOM	8018	NE	ARG	A	527	123.763	118.389	156.039	1.00	0.00
ATOM	8019	HE	ARG	A	527	124.363	118.217	155.239	1.00	0.00
ATOM	8020	CZ	ARG	A	527	122.521	118.709	155.741	1.00	0.00
ATOM	8021	NH1	ARG	A	527	121.618	118.937	156.649	1.00	0.00
ATOM	8022	1HH1	ARG	A	527	121.901	118.832	157.605	1.00	0.00
ATOM	8023	2HH1	ARG	A	527	120.689	119.198	156.389	1.00	0.00
ATOM	8024	NH2	ARG	A	527	122.187	118.799	154.494	1.00	0.00
ATOM	8025	1HH2	ARG	A	527	122.878	118.463	153.833	1.00	0.00
ATOM	8026	2HH2	ARG	A	527	121.236	118.917	154.219	1.00	0.00
ATOM	8027	C	ARG	A	527	123.841	114.969	154.483	1.00	0.00
ATOM	8028	O	ARG	A	527	122.841	115.672	154.592	1.00	0.00
ATOM	8029	N	ASN	A	528	124.312	114.535	153.309	1.00	0.00
ATOM	8030	H	ASN	A	528	125.134	113.957	153.344	1.00	0.00
ATOM	8031	CA	ASN	A	528	123.848	114.923	151.985	1.00	0.00
ATOM	8032	HA	ASN	A	528	122.819	115.286	152.056	1.00	0.00
ATOM	8033	CB	ASN	A	528	124.725	116.089	151.481	1.00	0.00
ATOM	8034	HB1	ASN	A	528	124.616	116.141	150.401	1.00	0.00
ATOM	8035	HB2	ASN	A	528	125.771	115.879	151.698	1.00	0.00
ATOM	8036	CG	ASN	A	528	124.389	117.460	152.062	1.00	0.00
ATOM	8037	OD1	ASN	A	528	124.466	117.736	153.252	1.00	0.00
ATOM	8038	ND2	ASN	A	528	123.999	118.400	151.234	1.00	0.00
ATOM	8039	1HD2	ASN	A	528	124.002	118.207	150.233	1.00	0.00
ATOM	8040	2HD2	ASN	A	528	123.918	119.336	151.576	1.00	0.00
ATOM	8041	C	ASN	A	528	123.794	113.697	151.045	1.00	0.00
ATOM	8042	O	ASN	A	528	122.806	112.962	151.077	1.00	0.00
ATOM	8043	N	VAL	A	529	124.835	113.443	150.240	1.00	0.00
ATOM	8044	H	VAL	A	529	125.624	114.083	150.235	1.00	0.00
ATOM	8045	CA	VAL	A	529	124.913	112.316	149.291	1.00	0.00
ATOM	8046	HA	VAL	A	529	124.466	111.437	149.752	1.00	0.00
ATOM	8047	CB	VAL	A	529	124.099	112.631	148.010	1.00	0.00
ATOM	8048	HB	VAL	A	529	123.045	112.559	148.276	1.00	0.00
ATOM	8049	CG1	VAL	A	529	124.312	114.043	147.443	1.00	0.00
ATOM	8050	1HG1	VAL	A	529	123.687	114.169	146.561	1.00	0.00

ATOM	8051	2HG1	VAL	A	529	124.020	114.798	148.172	1.00	0.00
ATOM	8052	3HG1	VAL	A	529	125.360	114.187	147.182	1.00	0.00
ATOM	8053	CG2	VAL	A	529	124.347	111.624	146.886	1.00	0.00
ATOM	8054	1HG2	VAL	A	529	123.603	111.759	146.107	1.00	0.00
ATOM	8055	2HG2	VAL	A	529	125.343	111.756	146.461	1.00	0.00
ATOM	8056	3HG2	VAL	A	529	124.267	110.609	147.265	1.00	0.00
ATOM	8057	C	VAL	A	529	126.373	111.991	148.977	1.00	0.00
ATOM	8058	O	VAL	A	529	127.142	112.841	148.543	1.00	0.00
ATOM	8059	N	SER	A	530	126.793	110.757	149.236	1.00	0.00
ATOM	8060	H	SER	A	530	126.121	110.056	149.534	1.00	0.00
ATOM	8061	CA	SER	A	530	128.170	110.324	148.973	1.00	0.00
ATOM	8062	HA	SER	A	530	128.855	111.127	149.214	1.00	0.00
ATOM	8063	CB	SER	A	530	128.509	109.111	149.831	1.00	0.00
ATOM	8064	HB1	SER	A	530	129.545	108.819	149.653	1.00	0.00
ATOM	8065	HB2	SER	A	530	127.843	108.290	149.563	1.00	0.00
ATOM	8066	OG	SER	A	530	128.363	109.382	151.208	1.00	0.00
ATOM	8067	HG	SER	A	530	128.450	108.520	151.627	1.00	0.00
ATOM	8068	C	SER	A	530	128.397	109.992	147.500	1.00	0.00
ATOM	8069	O	SER	A	530	127.459	109.916	146.709	1.00	0.00
ATOM	8070	N	ARG	A	531	129.666	109.784	147.144	1.00	0.00
ATOM	8071	H	ARG	A	531	130.369	109.923	147.866	1.00	0.00
ATOM	8072	CA	ARG	A	531	130.104	109.406	145.801	1.00	0.00
ATOM	8073	HA	ARG	A	531	129.332	109.700	145.090	1.00	0.00
ATOM	8074	CB	ARG	A	531	131.397	110.174	145.436	1.00	0.00
ATOM	8075	HB1	ARG	A	531	131.631	109.969	144.391	1.00	0.00
ATOM	8076	HB2	ARG	A	531	132.237	109.841	146.049	1.00	0.00
ATOM	8077	CG	ARG	A	531	131.232	111.694	145.647	1.00	0.00
ATOM	8078	HG1	ARG	A	531	131.353	111.909	146.707	1.00	0.00
ATOM	8079	HG2	ARG	A	531	130.212	111.969	145.375	1.00	0.00
ATOM	8080	CD	ARG	A	531	132.224	112.571	144.853	1.00	0.00
ATOM	8081	HD1	ARG	A	531	132.916	111.942	144.289	1.00	0.00
ATOM	8082	HD2	ARG	A	531	132.822	113.150	145.559	1.00	0.00
ATOM	8083	NE	ARG	A	531	131.516	113.500	143.935	1.00	0.00
ATOM	8084	HE	ARG	A	531	131.501	114.474	144.181	1.00	0.00
ATOM	8085	CZ	ARG	A	531	130.755	113.124	142.919	1.00	0.00
ATOM	8086	NH1	ARG	A	531	130.767	111.891	142.532	1.00	0.00
ATOM	8087	1HH1	ARG	A	531	131.304	111.223	143.037	1.00	0.00
ATOM	8088	2HH1	ARG	A	531	130.068	111.557	141.861	1.00	0.00
ATOM	8089	NH2	ARG	A	531	129.959	113.954	142.306	1.00	0.00
ATOM	8090	1HH2	ARG	A	531	129.888	114.924	142.542	1.00	0.00
ATOM	8091	2HH2	ARG	A	531	129.305	113.534	141.636	1.00	0.00
ATOM	8092	C	ARG	A	531	130.212	107.890	145.685	1.00	0.00
ATOM	8093	O	ARG	A	531	129.339	107.287	145.056	1.00	0.00
ATOM	8094	N	GLY	A	532	131.277	107.382	146.317	1.00	0.00
ATOM	8095	H	GLY	A	532	131.790	108.061	146.850	1.00	0.00
ATOM	8096	CA	GLY	A	532	131.837	106.028	146.307	1.00	0.00
ATOM	8097	HA1	GLY	A	532	132.363	105.856	145.368	1.00	0.00
ATOM	8098	HA2	GLY	A	532	131.016	105.314	146.386	1.00	0.00
ATOM	8099	C	GLY	A	532	132.799	105.779	147.472	1.00	0.00
ATOM	8100	O	GLY	A	532	133.941	105.413	147.219	1.00	0.00
ATOM	8101	N	ARG	A	533	132.358	106.066	148.714	1.00	0.00
ATOM	8102	H	ARG	A	533	131.360	106.313	148.757	1.00	0.00
ATOM	8103	CA	ARG	A	533	133.054	105.958	150.036	1.00	0.00
ATOM	8104	HA	ARG	A	533	132.322	105.524	150.717	1.00	0.00
ATOM	8105	CB	ARG	A	533	134.277	105.007	150.007	1.00	0.00
ATOM	8106	HB1	ARG	A	533	135.125	105.523	149.555	1.00	0.00
ATOM	8107	HB2	ARG	A	533	134.015	104.155	149.380	1.00	0.00
ATOM	8108	CG	ARG	A	533	134.700	104.430	151.369	1.00	0.00
ATOM	8109	HG1	ARG	A	533	133.835	103.958	151.834	1.00	0.00
ATOM	8110	HG2	ARG	A	533	135.073	105.214	152.028	1.00	0.00
ATOM	8111	CD	ARG	A	533	135.817	103.389	151.154	1.00	0.00

ATOM	8112	HD1	ARG	A	533	136.761	103.924	151.027	1.00	0.00
ATOM	8113	HD2	ARG	A	533	135.627	102.853	150.221	1.00	0.00
ATOM	8114	NE	ARG	A	533	135.940	102.407	152.253	1.00	0.00
ATOM	8115	HE	ARG	A	533	136.801	102.414	152.785	1.00	0.00
ATOM	8116	CZ	ARG	A	533	135.173	101.347	152.438	1.00	0.00
ATOM	8117	NH1	ARG	A	533	134.047	101.178	151.806	1.00	0.00
ATOM	8118	1HH1	ARG	A	533	133.726	101.884	151.166	1.00	0.00
ATOM	8119	2HH1	ARG	A	533	133.561	100.305	151.902	1.00	0.00
ATOM	8120	NH2	ARG	A	533	135.542	100.404	153.253	1.00	0.00
ATOM	8121	1HH2	ARG	A	533	136.432	100.481	153.733	1.00	0.00
ATOM	8122	2HH2	ARG	A	533	134.991	99.567	153.472	1.00	0.00
ATOM	8123	C	ARG	A	533	133.388	107.325	150.643	1.00	0.00
ATOM	8124	O	ARG	A	533	133.803	107.383	151.797	1.00	0.00
ATOM	8125	N	GLU	A	534	133.184	108.419	149.907	1.00	0.00
ATOM	8126	H	GLU	A	534	132.821	108.296	148.978	1.00	0.00
ATOM	8127	CA	GLU	A	534	133.420	109.786	150.392	1.00	0.00
ATOM	8128	HA	GLU	A	534	133.546	109.734	151.475	1.00	0.00
ATOM	8129	CB	GLU	A	534	134.734	110.340	149.837	1.00	0.00
ATOM	8130	HB1	GLU	A	534	135.502	109.619	150.115	1.00	0.00
ATOM	8131	HB2	GLU	A	534	134.939	111.280	150.350	1.00	0.00
ATOM	8132	CG	GLU	A	534	134.847	110.587	148.326	1.00	0.00
ATOM	8133	HG1	GLU	A	534	134.218	111.435	148.045	1.00	0.00
ATOM	8134	HG2	GLU	A	534	134.504	109.704	147.782	1.00	0.00
ATOM	8135	CD	GLU	A	534	136.319	110.870	147.981	1.00	0.00
ATOM	8136	OE1	GLU	A	534	136.703	112.046	147.790	1.00	0.00
ATOM	8137	OE2	GLU	A	534	137.117	109.908	147.981	1.00	0.00
ATOM	8138	C	GLU	A	534	132.226	110.710	150.170	1.00	0.00
ATOM	8139	O	GLU	A	534	131.634	110.720	149.091	1.00	0.00
ATOM	8140	N	CYS	A	535	131.833	111.451	151.210	1.00	0.00
ATOM	8141	H	CYS	A	535	132.400	111.456	152.056	1.00	0.00
ATOM	8142	CA	CYS	A	535	130.643	112.300	151.187	1.00	0.00
ATOM	8143	HA	CYS	A	535	129.803	111.710	150.843	1.00	0.00
ATOM	8144	CB	CYS	A	535	130.335	112.723	152.622	1.00	0.00
ATOM	8145	HB1	CYS	A	535	131.200	113.264	153.008	1.00	0.00
ATOM	8146	HB2	CYS	A	535	130.202	111.828	153.229	1.00	0.00
ATOM	8147	SG	CYS	A	535	128.882	113.786	152.842	1.00	0.00
ATOM	8148	C	CYS	A	535	130.798	113.530	150.270	1.00	0.00
ATOM	8149	O	CYS	A	535	131.874	114.131	150.235	1.00	0.00
ATOM	8150	N	VAL	A	536	129.712	113.988	149.628	1.00	0.00
ATOM	8151	H	VAL	A	536	128.842	113.468	149.689	1.00	0.00
ATOM	8152	CA	VAL	A	536	129.623	115.339	149.042	1.00	0.00
ATOM	8153	HA	VAL	A	536	130.353	115.971	149.543	1.00	0.00
ATOM	8154	CB	VAL	A	536	129.965	115.388	147.536	1.00	0.00
ATOM	8155	HB	VAL	A	536	129.798	116.409	147.189	1.00	0.00
ATOM	8156	CG1	VAL	A	536	131.446	115.092	147.279	1.00	0.00
ATOM	8157	1HG1	VAL	A	536	131.680	115.265	146.232	1.00	0.00
ATOM	8158	2HG1	VAL	A	536	132.063	115.752	147.887	1.00	0.00
ATOM	8159	3HG1	VAL	A	536	131.679	114.064	147.545	1.00	0.00
ATOM	8160	CG2	VAL	A	536	129.083	114.473	146.686	1.00	0.00
ATOM	8161	1HG2	VAL	A	536	129.320	114.600	145.631	1.00	0.00
ATOM	8162	2HG2	VAL	A	536	129.228	113.433	146.973	1.00	0.00
ATOM	8163	3HG2	VAL	A	536	128.034	114.730	146.830	1.00	0.00
ATOM	8164	C	VAL	A	536	128.270	116.000	149.316	1.00	0.00
ATOM	8165	O	VAL	A	536	127.329	115.358	149.779	1.00	0.00
ATOM	8166	N	ASP	A	537	128.155	117.311	149.075	1.00	0.00
ATOM	8167	H	ASP	A	537	128.934	117.828	148.688	1.00	0.00
ATOM	8168	CA	ASP	A	537	126.908	118.041	149.343	1.00	0.00
ATOM	8169	HA	ASP	A	537	126.516	117.667	150.285	1.00	0.00
ATOM	8170	CB	ASP	A	537	127.161	119.545	149.559	1.00	0.00
ATOM	8171	HB1	ASP	A	537	128.046	119.697	150.173	1.00	0.00
ATOM	8172	HB2	ASP	A	537	126.310	119.949	150.111	1.00	0.00

ATOM	8173	CG	ASP	A	537	127.314	120.348	148.268	1.00	0.00
ATOM	8174	OD1	ASP	A	537	128.036	119.902	147.348	1.00	0.00
ATOM	8175	OD2	ASP	A	537	126.651	121.396	148.120	1.00	0.00
ATOM	8176	C	ASP	A	537	125.824	117.801	148.269	1.00	0.00
ATOM	8177	O	ASP	A	537	124.632	117.880	148.561	1.00	0.00
ATOM	8178	N	LYS	A	538	126.233	117.486	147.035	1.00	0.00
ATOM	8179	H	LYS	A	538	127.233	117.486	146.881	1.00	0.00
ATOM	8180	CA	LYS	A	538	125.364	117.257	145.873	1.00	0.00
ATOM	8181	HA	LYS	A	538	124.388	116.890	146.192	1.00	0.00
ATOM	8182	CB	LYS	A	538	125.208	118.561	145.063	1.00	0.00
ATOM	8183	HB1	LYS	A	538	124.624	118.347	144.166	1.00	0.00
ATOM	8184	HB2	LYS	A	538	126.203	118.873	144.746	1.00	0.00
ATOM	8185	CG	LYS	A	538	124.535	119.727	145.804	1.00	0.00
ATOM	8186	HG1	LYS	A	538	125.051	119.914	146.738	1.00	0.00
ATOM	8187	HG2	LYS	A	538	123.508	119.447	146.041	1.00	0.00
ATOM	8188	CD	LYS	A	538	124.522	121.030	144.991	1.00	0.00
ATOM	8189	HD1	LYS	A	538	124.115	121.823	145.621	1.00	0.00
ATOM	8190	HD2	LYS	A	538	123.845	120.906	144.143	1.00	0.00
ATOM	8191	CE	LYS	A	538	125.886	121.493	144.441	1.00	0.00
ATOM	8192	HE1	LYS	A	538	125.740	122.463	143.957	1.00	0.00
ATOM	8193	HE2	LYS	A	538	126.216	120.788	143.673	1.00	0.00
ATOM	8194	NZ	LYS	A	538	126.927	121.614	145.495	1.00	0.00
ATOM	8195	HZ1	LYS	A	538	127.730	122.145	145.207	1.00	0.00
ATOM	8196	HZ2	LYS	A	538	126.563	122.001	146.367	1.00	0.00
ATOM	8197	HZ3	LYS	A	538	127.261	120.713	145.845	1.00	0.00
ATOM	8198	C	LYS	A	538	126.008	116.243	144.924	1.00	0.00
ATOM	8199	O	LYS	A	538	127.182	116.371	144.563	1.00	0.00
ATOM	8200	N	CYS	A	539	125.225	115.277	144.463	1.00	0.00
ATOM	8201	H	CYS	A	539	124.279	115.224	144.797	1.00	0.00
ATOM	8202	CA	CYS	A	539	125.612	114.435	143.340	1.00	0.00
ATOM	8203	HA	CYS	A	539	126.644	114.106	143.479	1.00	0.00
ATOM	8204	CB	CYS	A	539	124.717	113.201	143.342	1.00	0.00
ATOM	8205	HB1	CYS	A	539	123.766	113.467	142.879	1.00	0.00
ATOM	8206	HB2	CYS	A	539	124.534	112.905	144.373	1.00	0.00
ATOM	8207	SG	CYS	A	539	125.406	111.769	142.491	1.00	0.00
ATOM	8208	C	CYS	A	539	125.518	115.200	142.009	1.00	0.00
ATOM	8209	O	CYS	A	539	124.791	116.191	141.942	1.00	0.00
ATOM	8210	N	ASN	A	540	126.188	114.744	140.943	1.00	0.00
ATOM	8211	H	ASN	A	540	126.750	113.897	141.043	1.00	0.00
ATOM	8212	CA	ASN	A	540	126.131	115.345	139.596	1.00	0.00
ATOM	8213	HA	ASN	A	540	126.214	116.431	139.698	1.00	0.00
ATOM	8214	CB	ASN	A	540	127.354	114.867	138.802	1.00	0.00
ATOM	8215	HB1	ASN	A	540	127.294	113.791	138.684	1.00	0.00
ATOM	8216	HB2	ASN	A	540	128.250	115.099	139.375	1.00	0.00
ATOM	8217	CG	ASN	A	540	127.515	115.454	137.417	1.00	0.00
ATOM	8218	OD1	ASN	A	540	126.660	116.059	136.790	1.00	0.00
ATOM	8219	ND2	ASN	A	540	128.649	115.205	136.829	1.00	0.00
ATOM	8220	1HD2	ASN	A	540	129.419	114.838	137.360	1.00	0.00
ATOM	8221	2HD2	ASN	A	540	128.763	115.669	135.936	1.00	0.00
ATOM	8222	C	ASN	A	540	124.788	115.074	138.871	1.00	0.00
ATOM	8223	O	ASN	A	540	124.700	114.486	137.789	1.00	0.00
ATOM	8224	N	LEU	A	541	123.700	115.505	139.497	1.00	0.00
ATOM	8225	H	LEU	A	541	123.853	115.949	140.393	1.00	0.00
ATOM	8226	CA	LEU	A	541	122.511	115.928	138.779	1.00	0.00
ATOM	8227	HA	LEU	A	541	122.225	115.156	138.063	1.00	0.00
ATOM	8228	CB	LEU	A	541	121.369	116.150	139.794	1.00	0.00
ATOM	8229	HB1	LEU	A	541	120.444	116.326	139.245	1.00	0.00
ATOM	8230	HB2	LEU	A	541	121.591	117.057	140.358	1.00	0.00
ATOM	8231	CG	LEU	A	541	121.142	115.013	140.814	1.00	0.00
ATOM	8232	HG	LEU	A	541	122.044	114.863	141.406	1.00	0.00
ATOM	8233	CD1	LEU	A	541	120.028	115.406	141.785	1.00	0.00

```
ATOM   8234 1HD1 LEU A 541     119.877 114.610 142.512  1.00  0.00
ATOM   8235 2HD1 LEU A 541     120.308 116.318 142.313  1.00  0.00
ATOM   8236 3HD1 LEU A 541     119.099 115.575 141.244  1.00  0.00
ATOM   8237  CD2 LEU A 541     120.772 113.676 140.166  1.00  0.00
ATOM   8238 1HD2 LEU A 541     120.610 112.927 140.940  1.00  0.00
ATOM   8239 2HD2 LEU A 541     119.869 113.782 139.569  1.00  0.00
ATOM   8240 3HD2 LEU A 541     121.591 113.341 139.530  1.00  0.00
ATOM   8241  C   LEU A 541     122.859 117.227 138.040  1.00  0.00
ATOM   8242  O   LEU A 541     123.537 118.081 138.602  1.00  0.00
ATOM   8243  N   LEU A 542     122.365 117.368 136.808  1.00  0.00
ATOM   8244  H   LEU A 542     121.738 116.644 136.511  1.00  0.00
ATOM   8245  CA  LEU A 542     122.548 118.502 135.883  1.00  0.00
ATOM   8246  HA  LEU A 542     122.051 118.229 134.956  1.00  0.00
ATOM   8247  CB  LEU A 542     121.775 119.707 136.458  1.00  0.00
ATOM   8248  HB1 LEU A 542     122.350 120.119 137.290  1.00  0.00
ATOM   8249  HB2 LEU A 542     120.818 119.369 136.861  1.00  0.00
ATOM   8250  CG  LEU A 542     121.501 120.829 135.444  1.00  0.00
ATOM   8251  HG  LEU A 542     122.408 121.077 134.896  1.00  0.00
ATOM   8252  CD1 LEU A 542     120.426 120.429 134.433  1.00  0.00
ATOM   8253 1HD1 LEU A 542     120.236 121.289 133.797  1.00  0.00
ATOM   8254 2HD1 LEU A 542     120.770 119.595 133.824  1.00  0.00
ATOM   8255 3HD1 LEU A 542     119.508 120.155 134.955  1.00  0.00
ATOM   8256  CD2 LEU A 542     121.014 122.082 136.170  1.00  0.00
ATOM   8257 1HD2 LEU A 542     120.862 122.894 135.459  1.00  0.00
ATOM   8258 2HD2 LEU A 542     120.085 121.886 136.703  1.00  0.00
ATOM   8259 3HD2 LEU A 542     121.773 122.407 136.885  1.00  0.00
ATOM   8260  C   LEU A 542     123.997 118.876 135.478  1.00  0.00
ATOM   8261  O   LEU A 542     124.241 118.993 134.277  1.00  0.00
ATOM   8262  N   GLU A 543     124.936 119.035 136.414  1.00  0.00
ATOM   8263  H   GLU A 543     124.671 118.857 137.378  1.00  0.00
ATOM   8264  CA  GLU A 543     126.203 119.758 136.216  1.00  0.00
ATOM   8265  HA  GLU A 543     126.388 119.865 135.148  1.00  0.00
ATOM   8266  CB  GLU A 543     126.096 121.177 136.816  1.00  0.00
ATOM   8267  HB1 GLU A 543     127.043 121.691 136.646  1.00  0.00
ATOM   8268  HB2 GLU A 543     125.942 121.093 137.892  1.00  0.00
ATOM   8269  CG  GLU A 543     124.966 122.018 136.218  1.00  0.00
ATOM   8270  HG1 GLU A 543     124.020 121.531 136.422  1.00  0.00
ATOM   8271  HG2 GLU A 543     125.098 122.079 135.136  1.00  0.00
ATOM   8272  CD  GLU A 543     124.908 123.420 136.830  1.00  0.00
ATOM   8273  OE1 GLU A 543     124.624 123.509 138.047  1.00  0.00
ATOM   8274  OE2 GLU A 543     125.133 124.388 136.070  1.00  0.00
ATOM   8275  C   GLU A 543     127.415 119.055 136.858  1.00  0.00
ATOM   8276  O   GLU A 543     127.500 118.859 138.071  1.00  0.00
ATOM   8277  N   GLY A 544     128.390 118.715 136.013  1.00  0.00
ATOM   8278  H   GLY A 544     128.224 118.842 135.028  1.00  0.00
ATOM   8279  CA  GLY A 544     129.669 118.098 136.374  1.00  0.00
ATOM   8280  HA1 GLY A 544     129.598 117.590 137.335  1.00  0.00
ATOM   8281  HA2 GLY A 544     130.427 118.875 136.467  1.00  0.00
ATOM   8282  C   GLY A 544     130.124 117.107 135.301  1.00  0.00
ATOM   8283  O   GLY A 544     129.338 116.737 134.423  1.00  0.00
ATOM   8284  N   GLU A 545     131.328 116.565 135.399  1.00  0.00
ATOM   8285  H   GLU A 545     131.956 116.869 136.148  1.00  0.00
ATOM   8286  CA  GLU A 545     131.911 115.715 134.352  1.00  0.00
ATOM   8287  HA  GLU A 545     131.641 116.195 133.409  1.00  0.00
ATOM   8288  CB  GLU A 545     133.453 115.791 134.378  1.00  0.00
ATOM   8289  HB1 GLU A 545     133.736 116.842 134.311  1.00  0.00
ATOM   8290  HB2 GLU A 545     133.820 115.321 133.466  1.00  0.00
ATOM   8291  CG  GLU A 545     134.232 115.166 135.552  1.00  0.00
ATOM   8292  HG1 GLU A 545     135.294 115.355 135.386  1.00  0.00
ATOM   8293  HG2 GLU A 545     134.097 114.085 135.518  1.00  0.00
ATOM   8294  CD  GLU A 545     133.858 115.709 136.945  1.00  0.00
```

```
ATOM   8295  OE1 GLU A 545     133.854 114.903 137.901  1.00  0.00
ATOM   8296  OE2 GLU A 545     133.496 116.903 137.048  1.00  0.00
ATOM   8297  C   GLU A 545     131.351 114.278 134.179  1.00  0.00
ATOM   8298  O   GLU A 545     131.130 113.923 133.022  1.00  0.00
ATOM   8299  N   PRO A 546     131.037 113.463 135.215  1.00  0.00
ATOM   8300  CD  PRO A 546     131.431 113.569 136.612  1.00  0.00
ATOM   8301  HD1 PRO A 546     131.251 114.556 137.034  1.00  0.00
ATOM   8302  HD2 PRO A 546     132.485 113.313 136.692  1.00  0.00
ATOM   8303  CG  PRO A 546     130.649 112.497 137.363  1.00  0.00
ATOM   8304  HG1 PRO A 546     129.656 112.862 137.614  1.00  0.00
ATOM   8305  HG2 PRO A 546     131.176 112.165 138.259  1.00  0.00
ATOM   8306  CB  PRO A 546     130.544 111.385 136.325  1.00  0.00
ATOM   8307  HB1 PRO A 546     129.672 110.755 136.500  1.00  0.00
ATOM   8308  HB2 PRO A 546     131.453 110.785 136.346  1.00  0.00
ATOM   8309  CA  PRO A 546     130.464 112.130 134.988  1.00  0.00
ATOM   8310  HA  PRO A 546     131.084 111.598 134.265  1.00  0.00
ATOM   8311  C   PRO A 546     129.035 112.124 134.432  1.00  0.00
ATOM   8312  O   PRO A 546     128.675 111.243 133.658  1.00  0.00
ATOM   8313  N   ARG A 547     128.217 113.095 134.868  1.00  0.00
ATOM   8314  H   ARG A 547     128.670 113.819 135.404  1.00  0.00
ATOM   8315  CA  ARG A 547     126.766 112.957 135.086  1.00  0.00
ATOM   8316  HA  ARG A 547     126.425 113.821 135.649  1.00  0.00
ATOM   8317  CB  ARG A 547     125.998 112.949 133.755  1.00  0.00
ATOM   8318  HB1 ARG A 547     124.931 112.909 133.969  1.00  0.00
ATOM   8319  HB2 ARG A 547     126.247 112.048 133.195  1.00  0.00
ATOM   8320  CG  ARG A 547     126.288 114.167 132.861  1.00  0.00
ATOM   8321  HG1 ARG A 547     125.661 114.081 131.979  1.00  0.00
ATOM   8322  HG2 ARG A 547     127.325 114.134 132.527  1.00  0.00
ATOM   8323  CD  ARG A 547     126.028 115.536 133.512  1.00  0.00
ATOM   8324  HD1 ARG A 547     126.237 116.324 132.787  1.00  0.00
ATOM   8325  HD2 ARG A 547     126.727 115.671 134.338  1.00  0.00
ATOM   8326  NE  ARG A 547     124.646 115.648 134.018  1.00  0.00
ATOM   8327  HE  ARG A 547     124.530 115.437 135.000  1.00  0.00
ATOM   8328  CZ  ARG A 547     123.582 116.050 133.342  1.00  0.00
ATOM   8329  NH1 ARG A 547     123.661 116.588 132.163  1.00  0.00
ATOM   8330 1HH1 ARG A 547     124.556 116.861 131.814  1.00  0.00
ATOM   8331 2HH1 ARG A 547     122.819 116.904 131.695  1.00  0.00
ATOM   8332  NH2 ARG A 547     122.388 115.902 133.841  1.00  0.00
ATOM   8333 1HH2 ARG A 547     122.296 115.515 134.755  1.00  0.00
ATOM   8334 2HH2 ARG A 547     121.589 116.289 133.341  1.00  0.00
ATOM   8335  C   ARG A 547     126.462 111.740 135.964  1.00  0.00
ATOM   8336  O   ARG A 547     126.979 110.637 135.795  1.00  0.00
ATOM   8337  N   GLU A 548     125.593 111.929 136.934  1.00  0.00
ATOM   8338  H   GLU A 548     125.144 112.831 137.043  1.00  0.00
ATOM   8339  CA  GLU A 548     125.249 110.910 137.913  1.00  0.00
ATOM   8340  HA  GLU A 548     125.510 109.925 137.535  1.00  0.00
ATOM   8341  CB  GLU A 548     125.997 111.156 139.237  1.00  0.00
ATOM   8342  HB1 GLU A 548     125.625 110.446 139.975  1.00  0.00
ATOM   8343  HB2 GLU A 548     125.757 112.164 139.574  1.00  0.00
ATOM   8344  CG  GLU A 548     127.515 110.982 139.136  1.00  0.00
ATOM   8345  HG1 GLU A 548     127.903 111.571 138.309  1.00  0.00
ATOM   8346  HG2 GLU A 548     127.740 109.934 138.935  1.00  0.00
ATOM   8347  CD  GLU A 548     128.228 111.425 140.418  1.00  0.00
ATOM   8348  OE1 GLU A 548     128.964 110.611 141.015  1.00  0.00
ATOM   8349  OE2 GLU A 548     128.129 112.613 140.805  1.00  0.00
ATOM   8350  C   GLU A 548     123.741 110.933 138.152  1.00  0.00
ATOM   8351  O   GLU A 548     123.076 111.954 137.968  1.00  0.00
ATOM   8352  N   PHE A 549     123.217 109.811 138.619  1.00  0.00
ATOM   8353  H   PHE A 549     123.843 109.017 138.718  1.00  0.00
ATOM   8354  CA  PHE A 549     121.965 109.737 139.355  1.00  0.00
ATOM   8355  HA  PHE A 549     121.448 110.693 139.293  1.00  0.00
```

ATOM	8356	CB	PHE	A	549	121.081	108.656	138.715	1.00	0.00
ATOM	8357	HB1	PHE	A	549	121.449	107.678	139.019	1.00	0.00
ATOM	8358	HB2	PHE	A	549	121.191	108.713	137.634	1.00	0.00
ATOM	8359	CG	PHE	A	549	119.604	108.753	139.044	1.00	0.00
ATOM	8360	CD1	PHE	A	549	118.838	109.827	138.551	1.00	0.00
ATOM	8361	HD1	PHE	A	549	119.298	110.576	137.925	1.00	0.00
ATOM	8362	CE1	PHE	A	549	117.467	109.913	138.854	1.00	0.00
ATOM	8363	HE1	PHE	A	549	116.877	110.732	138.474	1.00	0.00
ATOM	8364	CZ	PHE	A	549	116.857	108.925	139.644	1.00	0.00
ATOM	8365	HZ	PHE	A	549	115.804	108.990	139.883	1.00	0.00
ATOM	8366	CE2	PHE	A	549	117.617	107.849	140.132	1.00	0.00
ATOM	8367	HE2	PHE	A	549	117.157	107.088	140.748	1.00	0.00
ATOM	8368	CD2	PHE	A	549	118.988	107.761	139.831	1.00	0.00
ATOM	8369	HD2	PHE	A	549	119.563	106.930	140.215	1.00	0.00
ATOM	8370	C	PHE	A	549	122.274	109.450	140.831	1.00	0.00
ATOM	8371	O	PHE	A	549	123.429	109.234	141.205	1.00	0.00
ATOM	8372	N	VAL	A	550	121.248	109.409	141.682	1.00	0.00
ATOM	8373	H	VAL	A	550	120.306	109.463	141.324	1.00	0.00
ATOM	8374	CA	VAL	A	550	121.405	109.133	143.119	1.00	0.00
ATOM	8375	HA	VAL	A	550	122.432	108.833	143.343	1.00	0.00
ATOM	8376	CB	VAL	A	550	121.071	110.375	143.971	1.00	0.00
ATOM	8377	HB	VAL	A	550	120.069	110.725	143.726	1.00	0.00
ATOM	8378	CG1	VAL	A	550	121.148	110.092	145.476	1.00	0.00
ATOM	8379	1HG1	VAL	A	550	121.020	111.013	146.046	1.00	0.00
ATOM	8380	2HG1	VAL	A	550	120.354	109.410	145.777	1.00	0.00
ATOM	8381	3HG1	VAL	A	550	122.113	109.648	145.724	1.00	0.00
ATOM	8382	CG2	VAL	A	550	122.066	111.493	143.680	1.00	0.00
ATOM	8383	1HG2	VAL	A	550	121.845	112.363	144.295	1.00	0.00
ATOM	8384	2HG2	VAL	A	550	123.063	111.120	143.904	1.00	0.00
ATOM	8385	3HG2	VAL	A	550	122.021	111.784	142.632	1.00	0.00
ATOM	8386	C	VAL	A	550	120.492	107.991	143.507	1.00	0.00
ATOM	8387	O	VAL	A	550	119.304	108.011	143.190	1.00	0.00
ATOM	8388	N	GLU	A	551	121.027	107.032	144.258	1.00	0.00
ATOM	8389	H	GLU	A	551	122.023	107.067	144.480	1.00	0.00
ATOM	8390	CA	GLU	A	551	120.240	105.983	144.906	1.00	0.00
ATOM	8391	HA	GLU	A	551	119.169	106.139	144.772	1.00	0.00
ATOM	8392	CB	GLU	A	551	120.639	104.594	144.376	1.00	0.00
ATOM	8393	HB1	GLU	A	551	120.102	103.858	144.974	1.00	0.00
ATOM	8394	HB2	GLU	A	551	121.707	104.462	144.563	1.00	0.00
ATOM	8395	CG	GLU	A	551	120.371	104.266	142.907	1.00	0.00
ATOM	8396	HG1	GLU	A	551	120.798	105.047	142.275	1.00	0.00
ATOM	8397	HG2	GLU	A	551	119.293	104.226	142.735	1.00	0.00
ATOM	8398	CD	GLU	A	551	121.012	102.910	142.547	1.00	0.00
ATOM	8399	OE1	GLU	A	551	121.227	102.056	143.439	1.00	0.00
ATOM	8400	OE2	GLU	A	551	121.398	102.712	141.374	1.00	0.00
ATOM	8401	C	GLU	A	551	120.597	105.984	146.392	1.00	0.00
ATOM	8402	O	GLU	A	551	121.775	105.963	146.734	1.00	0.00
ATOM	8403	N	ASN	A	552	119.621	105.963	147.301	1.00	0.00
ATOM	8404	H	ASN	A	552	118.667	105.928	146.978	1.00	0.00
ATOM	8405	CA	ASN	A	552	119.861	105.839	148.752	1.00	0.00
ATOM	8406	HA	ASN	A	552	118.894	105.941	149.245	1.00	0.00
ATOM	8407	CB	ASN	A	552	120.390	104.424	149.071	1.00	0.00
ATOM	8408	HB1	ASN	A	552	120.254	104.219	150.131	1.00	0.00
ATOM	8409	HB2	ASN	A	552	121.461	104.389	148.871	1.00	0.00
ATOM	8410	CG	ASN	A	552	119.715	103.319	148.281	1.00	0.00
ATOM	8411	OD1	ASN	A	552	118.535	103.043	148.442	1.00	0.00
ATOM	8412	ND2	ASN	A	552	120.432	102.693	147.378	1.00	0.00
ATOM	8413	1HD2	ASN	A	552	121.372	102.996	147.191	1.00	0.00
ATOM	8414	2HD2	ASN	A	552	119.964	102.006	146.814	1.00	0.00
ATOM	8415	C	ASN	A	552	120.779	106.931	149.367	1.00	0.00
ATOM	8416	O	ASN	A	552	121.206	106.791	150.516	1.00	0.00

ATOM	8417	N	SER	A	553	121.066	108.010	148.629	1.00	0.00
ATOM	8418	H	SER	A	553	120.658	108.035	147.712	1.00	0.00
ATOM	8419	CA	SER	A	553	122.139	108.983	148.890	1.00	0.00
ATOM	8420	HA	SER	A	553	122.076	109.692	148.068	1.00	0.00
ATOM	8421	CB	SER	A	553	121.887	109.814	150.164	1.00	0.00
ATOM	8422	HB1	SER	A	553	122.819	110.023	150.688	1.00	0.00
ATOM	8423	HB2	SER	A	553	121.230	109.284	150.855	1.00	0.00
ATOM	8424	OG	SER	A	553	121.298	111.048	149.801	1.00	0.00
ATOM	8425	HG	SER	A	553	121.683	111.740	150.370	1.00	0.00
ATOM	8426	C	SER	A	553	123.582	108.443	148.757	1.00	0.00
ATOM	8427	O	SER	A	553	124.496	108.960	149.404	1.00	0.00
ATOM	8428	N	GLU	A	554	123.849	107.539	147.814	1.00	0.00
ATOM	8429	H	GLU	A	554	123.079	107.075	147.341	1.00	0.00
ATOM	8430	CA	GLU	A	554	125.170	107.425	147.171	1.00	0.00
ATOM	8431	HA	GLU	A	554	125.713	108.334	147.420	1.00	0.00
ATOM	8432	CB	GLU	A	554	126.011	106.274	147.796	1.00	0.00
ATOM	8433	HB1	GLU	A	554	125.592	105.310	147.506	1.00	0.00
ATOM	8434	HB2	GLU	A	554	125.929	106.361	148.880	1.00	0.00
ATOM	8435	CG	GLU	A	554	127.514	106.322	147.429	1.00	0.00
ATOM	8436	HG1	GLU	A	554	127.756	107.350	147.166	1.00	0.00
ATOM	8437	HG2	GLU	A	554	127.670	105.712	146.538	1.00	0.00
ATOM	8438	CD	GLU	A	554	128.526	105.884	148.526	1.00	0.00
ATOM	8439	OE1	GLU	A	554	129.548	106.598	148.698	1.00	0.00
ATOM	8440	OE2	GLU	A	554	128.329	104.835	149.173	1.00	0.00
ATOM	8441	C	GLU	A	554	125.059	107.465	145.633	1.00	0.00
ATOM	8442	O	GLU	A	554	123.989	107.181	145.063	1.00	0.00
ATOM	8443	N	CYS	A	555	126.111	107.973	144.968	1.00	0.00
ATOM	8444	H	CYS	A	555	126.957	108.226	145.464	1.00	0.00
ATOM	8445	CA	CYS	A	555	125.985	108.341	143.559	1.00	0.00
ATOM	8446	HA	CYS	A	555	125.011	108.801	143.378	1.00	0.00
ATOM	8447	CB	CYS	A	555	127.074	109.348	143.176	1.00	0.00
ATOM	8448	HB1	CYS	A	555	127.127	109.378	142.089	1.00	0.00
ATOM	8449	HB2	CYS	A	555	128.030	108.972	143.530	1.00	0.00
ATOM	8450	SG	CYS	A	555	126.842	111.056	143.745	1.00	0.00
ATOM	8451	C	CYS	A	555	126.103	107.088	142.681	1.00	0.00
ATOM	8452	O	CYS	A	555	126.733	106.093	143.037	1.00	0.00
ATOM	8453	N	ILE	A	556	125.591	107.180	141.457	1.00	0.00
ATOM	8454	H	ILE	A	556	125.033	107.996	141.221	1.00	0.00
ATOM	8455	CA	ILE	A	556	125.946	106.250	140.383	1.00	0.00
ATOM	8456	HA	ILE	A	556	126.856	105.719	140.661	1.00	0.00
ATOM	8457	CB	ILE	A	556	124.847	105.180	140.142	1.00	0.00
ATOM	8458	HB	ILE	A	556	125.235	104.476	139.405	1.00	0.00
ATOM	8459	CG2	ILE	A	556	124.604	104.373	141.425	1.00	0.00
ATOM	8460	1HG2	ILE	A	556	123.915	103.561	141.215	1.00	0.00
ATOM	8461	2HG2	ILE	A	556	125.543	103.966	141.796	1.00	0.00
ATOM	8462	3HG2	ILE	A	556	124.158	105.000	142.198	1.00	0.00
ATOM	8463	CG1	ILE	A	556	123.479	105.700	139.628	1.00	0.00
ATOM	8464	1HG1	ILE	A	556	123.332	106.726	139.955	1.00	0.00
ATOM	8465	2HG1	ILE	A	556	122.674	105.103	140.057	1.00	0.00
ATOM	8466	CD	ILE	A	556	123.323	105.613	138.104	1.00	0.00
ATOM	8467	HD1	ILE	A	556	122.310	105.899	137.822	1.00	0.00
ATOM	8468	HD2	ILE	A	556	124.022	106.273	137.599	1.00	0.00
ATOM	8469	HD3	ILE	A	556	123.496	104.594	137.764	1.00	0.00
ATOM	8470	C	ILE	A	556	126.258	107.048	139.128	1.00	0.00
ATOM	8471	O	ILE	A	556	125.509	107.951	138.762	1.00	0.00
ATOM	8472	N	GLN	A	557	127.377	106.747	138.470	1.00	0.00
ATOM	8473	H	GLN	A	557	127.943	105.976	138.778	1.00	0.00
ATOM	8474	CA	GLN	A	557	127.683	107.373	137.189	1.00	0.00
ATOM	8475	HA	GLN	A	557	127.575	108.453	137.287	1.00	0.00
ATOM	8476	CB	GLN	A	557	129.115	107.074	136.744	1.00	0.00
ATOM	8477	HB1	GLN	A	557	129.247	107.468	135.735	1.00	0.00

ATOM	8478	HB2	GLN	A	557	129.285	105.997	136.717	1.00	0.00
ATOM	8479	CG	GLN	A	557	130.163	107.738	137.655	1.00	0.00
ATOM	8480	HG1	GLN	A	557	130.169	107.237	138.622	1.00	0.00
ATOM	8481	HG2	GLN	A	557	129.888	108.780	137.812	1.00	0.00
ATOM	8482	CD	GLN	A	557	131.565	107.689	137.056	1.00	0.00
ATOM	8483	OE1	GLN	A	557	131.831	107.095	136.030	1.00	0.00
ATOM	8484	NE2	GLN	A	557	132.545	108.302	137.682	1.00	0.00
ATOM	8485	1HE2	GLN	A	557	132.365	108.828	138.515	1.00	0.00
ATOM	8486	2HE2	GLN	A	557	133.442	108.255	137.231	1.00	0.00
ATOM	8487	C	GLN	A	557	126.699	106.912	136.117	1.00	0.00
ATOM	8488	O	GLN	A	557	126.254	105.759	136.084	1.00	0.00
ATOM	8489	N	CYS	A	558	126.402	107.819	135.203	1.00	0.00
ATOM	8490	H	CYS	A	558	126.860	108.722	135.276	1.00	0.00
ATOM	8491	CA	CYS	A	558	125.747	107.498	133.947	1.00	0.00
ATOM	8492	HA	CYS	A	558	124.787	107.014	134.133	1.00	0.00
ATOM	8493	CB	CYS	A	558	125.537	108.845	133.244	1.00	0.00
ATOM	8494	HB1	CYS	A	558	125.178	108.698	132.233	1.00	0.00
ATOM	8495	HB2	CYS	A	558	126.488	109.384	133.184	1.00	0.00
ATOM	8496	SG	CYS	A	558	124.333	109.833	134.189	1.00	0.00
ATOM	8497	HG	CYS	A	558	125.091	109.926	135.291	1.00	0.00
ATOM	8498	C	CYS	A	558	126.642	106.541	133.133	1.00	0.00
ATOM	8499	O	CYS	A	558	127.869	106.657	133.151	1.00	0.00
ATOM	8500	N	HIS	A	559	126.049	105.590	132.407	1.00	0.00
ATOM	8501	H	HIS	A	559	125.044	105.561	132.362	1.00	0.00
ATOM	8502	CA	HIS	A	559	126.830	104.787	131.461	1.00	0.00
ATOM	8503	HA	HIS	A	559	127.678	104.382	132.014	1.00	0.00
ATOM	8504	CB	HIS	A	559	126.016	103.594	130.948	1.00	0.00
ATOM	8505	HB1	HIS	A	559	125.777	102.945	131.791	1.00	0.00
ATOM	8506	HB2	HIS	A	559	126.619	103.018	130.248	1.00	0.00
ATOM	8507	CG	HIS	A	559	124.739	103.975	130.265	1.00	0.00
ATOM	8508	ND1	HIS	A	559	124.623	104.343	128.926	1.00	0.00
ATOM	8509	CE1	HIS	A	559	123.313	104.564	128.728	1.00	0.00
ATOM	8510	HE1	HIS	A	559	122.876	104.835	127.780	1.00	0.00
ATOM	8511	NE2	HIS	A	559	122.620	104.338	129.859	1.00	0.00
ATOM	8512	HE2	HIS	A	559	121.614	104.368	129.948	1.00	0.00
ATOM	8513	CD2	HIS	A	559	123.508	103.979	130.845	1.00	0.00
ATOM	8514	HD2	HIS	A	559	123.275	103.714	131.871	1.00	0.00
ATOM	8515	C	HIS	A	559	127.400	105.680	130.349	1.00	0.00
ATOM	8516	O	HIS	A	559	126.774	106.679	129.992	1.00	0.00
ATOM	8517	N	PRO	A	560	128.549	105.350	129.734	1.00	0.00
ATOM	8518	CD	PRO	A	560	129.400	104.205	130.022	1.00	0.00
ATOM	8519	HD1	PRO	A	560	128.839	103.273	130.012	1.00	0.00
ATOM	8520	HD2	PRO	A	560	129.882	104.349	130.989	1.00	0.00
ATOM	8521	CG	PRO	A	560	130.452	104.194	128.918	1.00	0.00
ATOM	8522	HG1	PRO	A	560	130.074	103.645	128.056	1.00	0.00
ATOM	8523	HG2	PRO	A	560	131.394	103.766	129.261	1.00	0.00
ATOM	8524	CB	PRO	A	560	130.593	105.674	128.566	1.00	0.00
ATOM	8525	HB1	PRO	A	560	130.986	105.810	127.560	1.00	0.00
ATOM	8526	HB2	PRO	A	560	131.252	106.155	129.290	1.00	0.00
ATOM	8527	CA	PRO	A	560	129.171	106.222	128.739	1.00	0.00
ATOM	8528	HA	PRO	A	560	129.229	107.235	129.140	1.00	0.00
ATOM	8529	C	PRO	A	560	128.405	106.313	127.398	1.00	0.00
ATOM	8530	O	PRO	A	560	128.901	106.944	126.459	1.00	0.00
ATOM	8531	N	GLU	A	561	127.226	105.692	127.271	1.00	0.00
ATOM	8532	H	GLU	A	561	126.855	105.210	128.079	1.00	0.00
ATOM	8533	CA	GLU	A	561	126.321	105.843	126.122	1.00	0.00
ATOM	8534	HA	GLU	A	561	126.877	106.236	125.273	1.00	0.00
ATOM	8535	CB	GLU	A	561	125.739	104.477	125.688	1.00	0.00
ATOM	8536	HB1	GLU	A	561	125.375	104.582	124.668	1.00	0.00
ATOM	8537	HB2	GLU	A	561	124.886	104.221	126.311	1.00	0.00
ATOM	8538	CG	GLU	A	561	126.735	103.309	125.713	1.00	0.00

ATOM	8539	HG1	GLU	A	561	126.284	102.435	125.245	1.00	0.00
ATOM	8540	HG2	GLU	A	561	126.956	103.049	126.751	1.00	0.00
ATOM	8541	CD	GLU	A	561	128.027	103.660	124.978	1.00	0.00
ATOM	8542	OE1	GLU	A	561	129.128	103.382	125.504	1.00	0.00
ATOM	8543	OE2	GLU	A	561	127.982	104.330	123.922	1.00	0.00
ATOM	8544	C	GLU	A	561	125.197	106.863	126.403	1.00	0.00
ATOM	8545	O	GLU	A	561	124.514	107.316	125.481	1.00	0.00
ATOM	8546	N	CYS	A	562	125.045	107.279	127.665	1.00	0.00
ATOM	8547	H	CYS	A	562	125.637	106.860	128.375	1.00	0.00
ATOM	8548	CA	CYS	A	562	124.265	108.436	128.089	1.00	0.00
ATOM	8549	HA	CYS	A	562	123.225	108.310	127.806	1.00	0.00
ATOM	8550	CB	CYS	A	562	124.367	108.563	129.616	1.00	0.00
ATOM	8551	HB1	CYS	A	562	123.903	109.496	129.884	1.00	0.00
ATOM	8552	HB2	CYS	A	562	125.421	108.659	129.873	1.00	0.00
ATOM	8553	SG	CYS	A	562	123.641	107.278	130.670	1.00	0.00
ATOM	8554	C	CYS	A	562	124.799	109.743	127.480	1.00	0.00
ATOM	8555	O	CYS	A	562	125.980	110.053	127.640	1.00	0.00
ATOM	8556	N	LEU	A	563	123.945	110.584	126.879	1.00	0.00
ATOM	8557	H	LEU	A	563	122.988	110.295	126.708	1.00	0.00
ATOM	8558	CA	LEU	A	563	124.335	111.964	126.553	1.00	0.00
ATOM	8559	HA	LEU	A	563	125.383	111.961	126.261	1.00	0.00
ATOM	8560	CB	LEU	A	563	123.517	112.500	125.363	1.00	0.00
ATOM	8561	HB1	LEU	A	563	122.481	112.610	125.680	1.00	0.00
ATOM	8562	HB2	LEU	A	563	123.538	111.786	124.540	1.00	0.00
ATOM	8563	CG	LEU	A	563	124.029	113.870	124.857	1.00	0.00
ATOM	8564	HG	LEU	A	563	124.196	114.539	125.700	1.00	0.00
ATOM	8565	CD1	LEU	A	563	125.340	113.742	124.077	1.00	0.00
ATOM	8566	1HD1	LEU	A	563	125.652	114.724	123.721	1.00	0.00
ATOM	8567	2HD1	LEU	A	563	126.128	113.338	124.708	1.00	0.00
ATOM	8568	3HD1	LEU	A	563	125.199	113.083	123.219	1.00	0.00
ATOM	8569	CD2	LEU	A	563	123.001	114.537	123.952	1.00	0.00
ATOM	8570	1HD2	LEU	A	563	123.403	115.466	123.548	1.00	0.00
ATOM	8571	2HD2	LEU	A	563	122.744	113.876	123.124	1.00	0.00
ATOM	8572	3HD2	LEU	A	563	122.110	114.775	124.527	1.00	0.00
ATOM	8573	C	LEU	A	563	124.101	112.887	127.770	1.00	0.00
ATOM	8574	O	LEU	A	563	122.987	112.899	128.307	1.00	0.00
ATOM	8575	N	PRO	A	564	125.083	113.715	128.178	1.00	0.00
ATOM	8576	CD	PRO	A	564	126.495	113.531	127.850	1.00	0.00
ATOM	8577	HD1	PRO	A	564	126.708	114.004	126.892	1.00	0.00
ATOM	8578	HD2	PRO	A	564	126.774	112.478	127.825	1.00	0.00
ATOM	8579	CG	PRO	A	564	127.281	114.227	128.953	1.00	0.00
ATOM	8580	HG1	PRO	A	564	128.264	114.552	128.611	1.00	0.00
ATOM	8581	HG2	PRO	A	564	127.370	113.570	129.819	1.00	0.00
ATOM	8582	CB	PRO	A	564	126.371	115.401	129.279	1.00	0.00
ATOM	8583	HB1	PRO	A	564	126.521	116.176	128.526	1.00	0.00
ATOM	8584	HB2	PRO	A	564	126.566	115.798	130.275	1.00	0.00
ATOM	8585	CA	PRO	A	564	124.960	114.800	129.164	1.00	0.00
ATOM	8586	HA	PRO	A	564	124.735	114.324	130.111	1.00	0.00
ATOM	8587	C	PRO	A	564	123.881	115.895	128.953	1.00	0.00
ATOM	8588	O	PRO	A	564	124.196	117.083	128.953	1.00	0.00
ATOM	8589	N	GLN	A	565	122.608	115.546	128.775	1.00	0.00
ATOM	8590	H	GLN	A	565	122.405	114.556	128.752	1.00	0.00
ATOM	8591	CA	GLN	A	565	121.491	116.468	128.556	1.00	0.00
ATOM	8592	HA	GLN	A	565	121.647	116.890	127.563	1.00	0.00
ATOM	8593	CB	GLN	A	565	120.182	115.657	128.509	1.00	0.00
ATOM	8594	HB1	GLN	A	565	119.850	115.433	129.525	1.00	0.00
ATOM	8595	HB2	GLN	A	565	120.362	114.710	127.996	1.00	0.00
ATOM	8596	CG	GLN	A	565	119.066	116.392	127.747	1.00	0.00
ATOM	8597	HG1	GLN	A	565	119.406	116.609	126.735	1.00	0.00
ATOM	8598	HG2	GLN	A	565	118.841	117.334	128.241	1.00	0.00
ATOM	8599	CD	GLN	A	565	117.782	115.574	127.682	1.00	0.00

ATOM	8600	OE1	GLN	A	565	117.075	115.400	128.661	1.00	0.00
ATOM	8601	NE2	GLN	A	565	117.422	115.019	126.548	1.00	0.00
ATOM	8602	1HE2	GLN	A	565	117.945	115.141	125.701	1.00	0.00
ATOM	8603	2HE2	GLN	A	565	116.577	114.468	126.563	1.00	0.00
ATOM	8604	C	GLN	A	565	121.420	117.643	129.544	1.00	0.00
ATOM	8605	O	GLN	A	565	121.402	117.449	130.759	1.00	0.00
ATOM	8606	N	ALA	A	566	121.367	118.869	129.010	1.00	0.00
ATOM	8607	H	ALA	A	566	121.398	118.960	128.008	1.00	0.00
ATOM	8608	CA	ALA	A	566	121.069	120.081	129.777	1.00	0.00
ATOM	8609	HA	ALA	A	566	121.757	120.132	130.625	1.00	0.00
ATOM	8610	CB	ALA	A	566	121.314	121.295	128.874	1.00	0.00
ATOM	8611	HB1	ALA	A	566	121.145	122.214	129.437	1.00	0.00
ATOM	8612	HB2	ALA	A	566	122.344	121.290	128.515	1.00	0.00
ATOM	8613	HB3	ALA	A	566	120.634	121.271	128.021	1.00	0.00
ATOM	8614	C	ALA	A	566	119.629	120.056	130.323	1.00	0.00
ATOM	8615	O	ALA	A	566	118.794	119.296	129.844	1.00	0.00
ATOM	8616	N	MET	A	567	119.332	120.897	131.317	1.00	0.00
ATOM	8617	H	MET	A	567	120.075	121.490	131.651	1.00	0.00
ATOM	8618	CA	MET	A	567	118.021	121.042	131.990	1.00	0.00
ATOM	8619	HA	MET	A	567	118.169	121.781	132.777	1.00	0.00
ATOM	8620	CB	MET	A	567	117.006	121.645	130.995	1.00	0.00
ATOM	8621	HB1	MET	A	567	116.652	120.861	130.325	1.00	0.00
ATOM	8622	HB2	MET	A	567	117.504	122.407	130.393	1.00	0.00
ATOM	8623	CG	MET	A	567	115.792	122.306	131.658	1.00	0.00
ATOM	8624	HG1	MET	A	567	115.258	121.566	132.252	1.00	0.00
ATOM	8625	HG2	MET	A	567	115.115	122.641	130.871	1.00	0.00
ATOM	8626	SD	MET	A	567	116.185	123.733	132.706	1.00	0.00
ATOM	8627	CE	MET	A	567	114.550	124.017	133.433	1.00	0.00
ATOM	8628	HE1	MET	A	567	114.595	124.868	134.113	1.00	0.00
ATOM	8629	HE2	MET	A	567	113.828	124.222	132.643	1.00	0.00
ATOM	8630	HE3	MET	A	567	114.242	123.129	133.987	1.00	0.00
ATOM	8631	C	MET	A	567	117.464	119.792	132.712	1.00	0.00
ATOM	8632	O	MET	A	567	116.556	119.916	133.530	1.00	0.00
ATOM	8633	N	ASN	A	568	118.001	118.595	132.472	1.00	0.00
ATOM	8634	H	ASN	A	568	118.744	118.550	131.790	1.00	0.00
ATOM	8635	CA	ASN	A	568	117.395	117.326	132.871	1.00	0.00
ATOM	8636	HA	ASN	A	568	116.660	117.494	133.662	1.00	0.00
ATOM	8637	CB	ASN	A	568	116.670	116.788	131.627	1.00	0.00
ATOM	8638	HB1	ASN	A	568	117.414	116.512	130.882	1.00	0.00
ATOM	8639	HB2	ASN	A	568	116.035	117.563	131.198	1.00	0.00
ATOM	8640	CG	ASN	A	568	115.782	115.594	131.915	1.00	0.00
ATOM	8641	OD1	ASN	A	568	115.299	115.410	133.016	1.00	0.00
ATOM	8642	ND2	ASN	A	568	115.580	114.738	130.950	1.00	0.00
ATOM	8643	1HD2	ASN	A	568	116.021	114.880	130.044	1.00	0.00
ATOM	8644	2HD2	ASN	A	568	114.996	113.950	131.152	1.00	0.00
ATOM	8645	C	ASN	A	568	118.443	116.335	133.417	1.00	0.00
ATOM	8646	O	ASN	A	568	119.650	116.505	133.218	1.00	0.00
ATOM	8647	N	ILE	A	569	118.003	115.281	134.112	1.00	0.00
ATOM	8648	H	ILE	A	569	116.997	115.147	134.191	1.00	0.00
ATOM	8649	CA	ILE	A	569	118.894	114.233	134.622	1.00	0.00
ATOM	8650	HA	ILE	A	569	119.864	114.696	134.800	1.00	0.00
ATOM	8651	CB	ILE	A	569	118.485	113.687	136.012	1.00	0.00
ATOM	8652	HB	ILE	A	569	119.309	113.050	136.339	1.00	0.00
ATOM	8653	CG2	ILE	A	569	118.428	114.848	137.023	1.00	0.00
ATOM	8654	1HG2	ILE	A	569	118.219	114.461	138.019	1.00	0.00
ATOM	8655	2HG2	ILE	A	569	119.382	115.374	137.047	1.00	0.00
ATOM	8656	3HG2	ILE	A	569	117.636	115.553	136.764	1.00	0.00
ATOM	8657	CG1	ILE	A	569	117.220	112.805	136.109	1.00	0.00
ATOM	8658	1HG1	ILE	A	569	117.044	112.585	137.161	1.00	0.00
ATOM	8659	2HG1	ILE	A	569	117.425	111.852	135.620	1.00	0.00
ATOM	8660	CD	ILE	A	569	115.925	113.372	135.525	1.00	0.00

```
ATOM   8661  HD1 ILE A 569     115.092 112.726 135.805  1.00  0.00
ATOM   8662  HD2 ILE A 569     115.733 114.374 135.906  1.00  0.00
ATOM   8663  HD3 ILE A 569     115.989 113.391 134.440  1.00  0.00
ATOM   8664  C   ILE A 569     119.128 113.155 133.560  1.00  0.00
ATOM   8665  O   ILE A 569     118.261 112.352 133.233  1.00  0.00
ATOM   8666  N   THR A 570     120.331 113.191 132.992  1.00  0.00
ATOM   8667  H   THR A 570     120.930 113.957 133.254  1.00  0.00
ATOM   8668  CA  THR A 570     120.821 112.363 131.884  1.00  0.00
ATOM   8669  HA  THR A 570     120.423 112.775 130.957  1.00  0.00
ATOM   8670  CB  THR A 570     122.347 112.513 131.859  1.00  0.00
ATOM   8671  HB  THR A 570     122.728 112.598 132.877  1.00  0.00
ATOM   8672  CG2 THR A 570     123.130 111.431 131.148  1.00  0.00
ATOM   8673 1HG2 THR A 570     124.172 111.730 131.033  1.00  0.00
ATOM   8674 2HG2 THR A 570     123.103 110.521 131.743  1.00  0.00
ATOM   8675 3HG2 THR A 570     122.681 111.232 130.177  1.00  0.00
ATOM   8676  OG1 THR A 570     122.638 113.686 131.158  1.00  0.00
ATOM   8677  HG1 THR A 570     122.641 113.420 130.226  1.00  0.00
ATOM   8678  C   THR A 570     120.408 110.892 131.887  1.00  0.00
ATOM   8679  O   THR A 570     120.032 110.385 130.829  1.00  0.00
ATOM   8680  N   CYS A 571     120.480 110.215 133.031  1.00  0.00
ATOM   8681  H   CYS A 571     120.724 110.702 133.879  1.00  0.00
ATOM   8682  CA  CYS A 571     120.158 108.796 133.158  1.00  0.00
ATOM   8683  HA  CYS A 571     119.368 108.547 132.447  1.00  0.00
ATOM   8684  CB  CYS A 571     121.417 107.972 132.806  1.00  0.00
ATOM   8685  HB1 CYS A 571     121.281 106.950 133.166  1.00  0.00
ATOM   8686  HB2 CYS A 571     122.274 108.393 133.323  1.00  0.00
ATOM   8687  SG  CYS A 571     121.751 107.897 131.018  1.00  0.00
ATOM   8688  C   CYS A 571     119.628 108.502 134.565  1.00  0.00
ATOM   8689  O   CYS A 571     119.998 109.168 135.532  1.00  0.00
ATOM   8690  N   THR A 572     118.746 107.504 134.672  1.00  0.00
ATOM   8691  H   THR A 572     118.489 107.027 133.811  1.00  0.00
ATOM   8692  CA  THR A 572     117.874 107.251 135.845  1.00  0.00
ATOM   8693  HA  THR A 572     118.191 107.889 136.669  1.00  0.00
ATOM   8694  CB  THR A 572     116.417 107.612 135.522  1.00  0.00
ATOM   8695  HB  THR A 572     115.774 107.330 136.357  1.00  0.00
ATOM   8696  CG2 THR A 572     116.224 109.109 135.270  1.00  0.00
ATOM   8697 1HG2 THR A 572     115.168 109.311 135.093  1.00  0.00
ATOM   8698 2HG2 THR A 572     116.552 109.667 136.143  1.00  0.00
ATOM   8699 3HG2 THR A 572     116.795 109.422 134.397  1.00  0.00
ATOM   8700  OG1 THR A 572     116.009 106.929 134.360  1.00  0.00
ATOM   8701  HG1 THR A 572     115.814 106.024 134.627  1.00  0.00
ATOM   8702  C   THR A 572     118.010 105.816 136.358  1.00  0.00
ATOM   8703  O   THR A 572     117.028 105.143 136.670  1.00  0.00
ATOM   8704  N   GLY A 573     119.241 105.309 136.349  1.00  0.00
ATOM   8705  H   GLY A 573     119.998 105.911 136.055  1.00  0.00
ATOM   8706  CA  GLY A 573     119.585 103.919 136.629  1.00  0.00
ATOM   8707  HA1 GLY A 573     118.817 103.259 136.225  1.00  0.00
ATOM   8708  HA2 GLY A 573     119.644 103.767 137.708  1.00  0.00
ATOM   8709  C   GLY A 573     120.930 103.553 136.001  1.00  0.00
ATOM   8710  O   GLY A 573     121.627 104.414 135.462  1.00  0.00
ATOM   8711  N   ARG A 574     121.313 102.276 136.091  1.00  0.00
ATOM   8712  H   ARG A 574     120.631 101.616 136.436  1.00  0.00
ATOM   8713  CA  ARG A 574     122.689 101.811 135.835  1.00  0.00
ATOM   8714  HA  ARG A 574     123.361 102.674 135.848  1.00  0.00
ATOM   8715  CB  ARG A 574     123.133 100.850 136.961  1.00  0.00
ATOM   8716  HB1 ARG A 574     124.163 100.546 136.764  1.00  0.00
ATOM   8717  HB2 ARG A 574     122.509  99.957 136.924  1.00  0.00
ATOM   8718  CG  ARG A 574     123.072 101.442 138.380  1.00  0.00
ATOM   8719  HG1 ARG A 574     122.041 101.689 138.636  1.00  0.00
ATOM   8720  HG2 ARG A 574     123.676 102.347 138.429  1.00  0.00
ATOM   8721  CD  ARG A 574     123.608 100.432 139.409  1.00  0.00
```

ATOM	8722	HD1	ARG	A	574	124.666	100.264	139.212	1.00	0.00
ATOM	8723	HD2	ARG	A	574	123.076	99.488	139.283	1.00	0.00
ATOM	8724	NE	ARG	A	574	123.396	100.906	140.787	1.00	0.00
ATOM	8725	HE	ARG	A	574	122.522	101.421	140.961	1.00	0.00
ATOM	8726	CZ	ARG	A	574	124.222	100.886	141.819	1.00	0.00
ATOM	8727	NH1	ARG	A	574	125.440	100.419	141.752	1.00	0.00
ATOM	8728	1HH1	ARG	A	574	125.771	100.075	140.873	1.00	0.00
ATOM	8729	2HH1	ARG	A	574	126.005	100.423	142.579	1.00	0.00
ATOM	8730	NH2	ARG	A	574	123.827	101.353	142.963	1.00	0.00
ATOM	8731	1HH2	ARG	A	574	122.844	101.652	143.053	1.00	0.00
ATOM	8732	2HH2	ARG	A	574	124.443	101.493	143.737	1.00	0.00
ATOM	8733	C	ARG	A	574	122.915	101.151	134.470	1.00	0.00
ATOM	8734	O	ARG	A	574	124.067	100.947	134.095	1.00	0.00
ATOM	8735	N	GLY	A	575	121.863	100.783	133.736	1.00	0.00
ATOM	8736	H	GLY	A	575	120.937	101.059	134.048	1.00	0.00
ATOM	8737	CA	GLY	A	575	121.982	100.026	132.480	1.00	0.00
ATOM	8738	HA1	GLY	A	575	121.157	99.317	132.417	1.00	0.00
ATOM	8739	HA2	GLY	A	575	122.911	99.457	132.499	1.00	0.00
ATOM	8740	C	GLY	A	575	121.942	100.918	131.230	1.00	0.00
ATOM	8741	O	GLY	A	575	121.342	101.989	131.300	1.00	0.00
ATOM	8742	N	PRO	A	576	122.498	100.479	130.074	1.00	0.00
ATOM	8743	CD	PRO	A	576	123.265	99.251	129.911	1.00	0.00
ATOM	8744	HD1	PRO	A	576	122.587	98.396	129.926	1.00	0.00
ATOM	8745	HD2	PRO	A	576	124.023	99.145	130.687	1.00	0.00
ATOM	8746	CG	PRO	A	576	123.942	99.349	128.546	1.00	0.00
ATOM	8747	HG1	PRO	A	576	124.085	98.370	128.090	1.00	0.00
ATOM	8748	HG2	PRO	A	576	124.895	99.868	128.646	1.00	0.00
ATOM	8749	CB	PRO	A	576	122.975	100.210	127.741	1.00	0.00
ATOM	8750	HB1	PRO	A	576	122.169	99.585	127.355	1.00	0.00
ATOM	8751	HB2	PRO	A	576	123.480	100.726	126.926	1.00	0.00
ATOM	8752	CA	PRO	A	576	122.434	101.192	128.784	1.00	0.00
ATOM	8753	HA	PRO	A	576	123.118	102.032	128.824	1.00	0.00
ATOM	8754	C	PRO	A	576	121.039	101.687	128.379	1.00	0.00
ATOM	8755	O	PRO	A	576	120.872	102.714	127.728	1.00	0.00
ATOM	8756	N	ASP	A	577	120.041	100.934	128.823	1.00	0.00
ATOM	8757	H	ASP	A	577	120.317	100.074	129.267	1.00	0.00
ATOM	8758	CA	ASP	A	577	118.603	101.172	128.812	1.00	0.00
ATOM	8759	HA	ASP	A	577	118.205	101.115	127.798	1.00	0.00
ATOM	8760	CB	ASP	A	577	118.114	99.981	129.650	1.00	0.00
ATOM	8761	HB1	ASP	A	577	118.464	100.090	130.679	1.00	0.00
ATOM	8762	HB2	ASP	A	577	118.563	99.059	129.276	1.00	0.00
ATOM	8763	CG	ASP	A	577	116.609	99.795	129.666	1.00	0.00
ATOM	8764	OD1	ASP	A	577	115.980	100.452	130.525	1.00	0.00
ATOM	8765	OD2	ASP	A	577	116.166	98.922	128.887	1.00	0.00
ATOM	8766	C	ASP	A	577	118.147	102.493	129.448	1.00	0.00
ATOM	8767	O	ASP	A	577	117.289	103.191	128.917	1.00	0.00
ATOM	8768	N	ASN	A	578	118.733	102.862	130.588	1.00	0.00
ATOM	8769	H	ASN	A	578	119.530	102.321	130.901	1.00	0.00
ATOM	8770	CA	ASN	A	578	118.190	103.867	131.510	1.00	0.00
ATOM	8771	HA	ASN	A	578	117.100	103.801	131.458	1.00	0.00
ATOM	8772	CB	ASN	A	578	118.613	103.500	132.948	1.00	0.00
ATOM	8773	HB1	ASN	A	578	118.079	104.156	133.634	1.00	0.00
ATOM	8774	HB2	ASN	A	578	119.681	103.690	133.053	1.00	0.00
ATOM	8775	CG	ASN	A	578	118.347	102.062	133.390	1.00	0.00
ATOM	8776	OD1	ASN	A	578	119.098	101.519	134.192	1.00	0.00
ATOM	8777	ND2	ASN	A	578	117.317	101.376	132.944	1.00	0.00
ATOM	8778	1HD2	ASN	A	578	116.749	101.665	132.151	1.00	0.00
ATOM	8779	2HD2	ASN	A	578	117.207	100.436	133.271	1.00	0.00
ATOM	8780	C	ASN	A	578	118.542	105.314	131.099	1.00	0.00
ATOM	8781	O	ASN	A	578	118.917	106.139	131.931	1.00	0.00
ATOM	8782	N	CYS	A	579	118.476	105.580	129.790	1.00	0.00

ATOM	8783	H	CYS	A	579	118.042	104.865	129.208	1.00	0.00
ATOM	8784	CA	CYS	A	579	119.122	106.671	129.072	1.00	0.00
ATOM	8785	HA	CYS	A	579	119.750	107.228	129.741	1.00	0.00
ATOM	8786	CB	CYS	A	579	119.959	106.021	127.968	1.00	0.00
ATOM	8787	HB1	CYS	A	579	119.299	105.499	127.270	1.00	0.00
ATOM	8788	HB2	CYS	A	579	120.635	105.292	128.406	1.00	0.00
ATOM	8789	SG	CYS	A	579	120.904	107.283	127.083	1.00	0.00
ATOM	8790	HG	CYS	A	579	121.670	106.452	126.370	1.00	0.00
ATOM	8791	C	CYS	A	579	118.090	107.639	128.487	1.00	0.00
ATOM	8792	O	CYS	A	579	117.331	107.280	127.589	1.00	0.00
ATOM	8793	N	ILE	A	580	118.079	108.886	128.965	1.00	0.00
ATOM	8794	H	ILE	A	580	118.742	109.147	129.688	1.00	0.00
ATOM	8795	CA	ILE	A	580	117.092	109.889	128.532	1.00	0.00
ATOM	8796	HA	ILE	A	580	116.120	109.391	128.520	1.00	0.00
ATOM	8797	CB	ILE	A	580	117.034	111.014	129.603	1.00	0.00
ATOM	8798	HB	ILE	A	580	117.434	110.593	130.527	1.00	0.00
ATOM	8799	CG2	ILE	A	580	117.927	112.226	129.280	1.00	0.00
ATOM	8800	1HG2	ILE	A	580	117.942	112.907	130.131	1.00	0.00
ATOM	8801	2HG2	ILE	A	580	118.943	111.899	129.069	1.00	0.00
ATOM	8802	3HG2	ILE	A	580	117.548	112.758	128.410	1.00	0.00
ATOM	8803	CG1	ILE	A	580	115.595	111.414	129.986	1.00	0.00
ATOM	8804	1HG1	ILE	A	580	115.648	112.064	130.858	1.00	0.00
ATOM	8805	2HG1	ILE	A	580	115.057	110.518	130.298	1.00	0.00
ATOM	8806	CD	ILE	A	580	114.756	112.117	128.910	1.00	0.00
ATOM	8807	HD1	ILE	A	580	113.811	112.436	129.350	1.00	0.00
ATOM	8808	HD2	ILE	A	580	115.276	112.992	128.524	1.00	0.00
ATOM	8809	HD3	ILE	A	580	114.539	111.428	128.097	1.00	0.00
ATOM	8810	C	ILE	A	580	117.326	110.404	127.096	1.00	0.00
ATOM	8811	O	ILE	A	580	116.434	110.968	126.466	1.00	0.00
ATOM	8812	N	GLN	A	581	118.542	110.220	126.572	1.00	0.00
ATOM	8813	H	GLN	A	581	119.219	109.764	127.164	1.00	0.00
ATOM	8814	CA	GLN	A	581	118.998	110.681	125.263	1.00	0.00
ATOM	8815	HA	GLN	A	581	118.213	110.516	124.522	1.00	0.00
ATOM	8816	CB	GLN	A	581	119.291	112.192	125.368	1.00	0.00
ATOM	8817	HB1	GLN	A	581	119.969	112.368	126.205	1.00	0.00
ATOM	8818	HB2	GLN	A	581	118.357	112.708	125.584	1.00	0.00
ATOM	8819	CG	GLN	A	581	119.906	112.814	124.108	1.00	0.00
ATOM	8820	HG1	GLN	A	581	120.876	112.363	123.903	1.00	0.00
ATOM	8821	HG2	GLN	A	581	120.057	113.875	124.291	1.00	0.00
ATOM	8822	CD	GLN	A	581	119.011	112.681	122.891	1.00	0.00
ATOM	8823	OE1	GLN	A	581	117.928	113.239	122.837	1.00	0.00
ATOM	8824	NE2	GLN	A	581	119.415	111.939	121.881	1.00	0.00
ATOM	8825	1HE2	GLN	A	581	120.319	111.475	121.882	1.00	0.00
ATOM	8826	2HE2	GLN	A	581	118.809	111.909	121.080	1.00	0.00
ATOM	8827	C	GLN	A	581	120.256	109.906	124.866	1.00	0.00
ATOM	8828	O	GLN	A	581	121.326	110.103	125.452	1.00	0.00
ATOM	8829	N	CYS	A	582	120.141	109.018	123.882	1.00	0.00
ATOM	8830	H	CYS	A	582	119.244	108.881	123.445	1.00	0.00
ATOM	8831	CA	CYS	A	582	121.284	108.267	123.376	1.00	0.00
ATOM	8832	HA	CYS	A	582	121.770	107.788	124.228	1.00	0.00
ATOM	8833	CB	CYS	A	582	120.764	107.183	122.437	1.00	0.00
ATOM	8834	HB1	CYS	A	582	120.250	107.649	121.594	1.00	0.00
ATOM	8835	HB2	CYS	A	582	120.048	106.582	122.993	1.00	0.00
ATOM	8836	SG	CYS	A	582	122.026	106.068	121.788	1.00	0.00
ATOM	8837	C	CYS	A	582	122.310	109.193	122.703	1.00	0.00
ATOM	8838	O	CYS	A	582	121.936	110.094	121.945	1.00	0.00
ATOM	8839	N	ALA	A	583	123.589	108.982	123.027	1.00	0.00
ATOM	8840	H	ALA	A	583	123.801	108.255	123.704	1.00	0.00
ATOM	8841	CA	ALA	A	583	124.695	109.834	122.594	1.00	0.00
ATOM	8842	HA	ALA	A	583	124.331	110.856	122.490	1.00	0.00
ATOM	8843	CB	ALA	A	583	125.761	109.815	123.694	1.00	0.00

```
ATOM   8844  HB1 ALA A 583     126.605 110.445 123.406  1.00  0.00
ATOM   8845  HB2 ALA A 583     125.345 110.184 124.625  1.00  0.00
ATOM   8846  HB3 ALA A 583     126.105 108.795 123.854  1.00  0.00
ATOM   8847  C   ALA A 583     125.321 109.459 121.239  1.00  0.00
ATOM   8848  O   ALA A 583     126.040 110.282 120.681  1.00  0.00
ATOM   8849  N   HIS A 584     125.059 108.252 120.716  1.00  0.00
ATOM   8850  H   HIS A 584     124.442 107.639 121.221  1.00  0.00
ATOM   8851  CA  HIS A 584     125.679 107.760 119.475  1.00  0.00
ATOM   8852  HA  HIS A 584     125.978 108.621 118.873  1.00  0.00
ATOM   8853  CB  HIS A 584     126.970 106.947 119.780  1.00  0.00
ATOM   8854  HB1 HIS A 584     127.618 106.987 118.905  1.00  0.00
ATOM   8855  HB2 HIS A 584     126.711 105.905 119.934  1.00  0.00
ATOM   8856  CG  HIS A 584     127.766 107.383 120.994  1.00  0.00
ATOM   8857  ND1 HIS A 584     128.741 108.381 121.035  1.00  0.00
ATOM   8858  CE1 HIS A 584     129.052 108.543 122.331  1.00  0.00
ATOM   8859  HE1 HIS A 584     129.722 109.308 122.706  1.00  0.00
ATOM   8860  NE2 HIS A 584     128.370 107.672 123.096  1.00  0.00
ATOM   8861  HE2 HIS A 584     128.337 107.682 124.111  1.00  0.00
ATOM   8862  CD2 HIS A 584     127.561 106.926 122.266  1.00  0.00
ATOM   8863  HD2 HIS A 584     126.841 106.170 122.564  1.00  0.00
ATOM   8864  C   HIS A 584     124.688 106.963 118.625  1.00  0.00
ATOM   8865  O   HIS A 584     124.233 107.490 117.618  1.00  0.00
ATOM   8866  N   TYR A 585     124.283 105.754 119.037  1.00  0.00
ATOM   8867  H   TYR A 585     124.723 105.327 119.844  1.00  0.00
ATOM   8868  CA  TYR A 585     123.343 104.915 118.278  1.00  0.00
ATOM   8869  HA  TYR A 585     122.703 105.548 117.664  1.00  0.00
ATOM   8870  CB  TYR A 585     124.119 103.968 117.346  1.00  0.00
ATOM   8871  HB1 TYR A 585     123.403 103.395 116.755  1.00  0.00
ATOM   8872  HB2 TYR A 585     124.683 103.258 117.952  1.00  0.00
ATOM   8873  CG  TYR A 585     125.071 104.672 116.399  1.00  0.00
ATOM   8874  CD1 TYR A 585     124.587 105.270 115.223  1.00  0.00
ATOM   8875  HD1 TYR A 585     123.532 105.212 114.998  1.00  0.00
ATOM   8876  CE1 TYR A 585     125.460 105.990 114.384  1.00  0.00
ATOM   8877  HE1 TYR A 585     125.088 106.474 113.492  1.00  0.00
ATOM   8878  CZ  TYR A 585     126.819 106.133 114.737  1.00  0.00
ATOM   8879  OH  TYR A 585     127.651 106.873 113.954  1.00  0.00
ATOM   8880  HH  TYR A 585     128.537 106.915 114.316  1.00  0.00
ATOM   8881  CE2 TYR A 585     127.306 105.531 115.918  1.00  0.00
ATOM   8882  HE2 TYR A 585     128.340 105.646 116.203  1.00  0.00
ATOM   8883  CD2 TYR A 585     126.428 104.804 116.744  1.00  0.00
ATOM   8884  HD2 TYR A 585     126.780 104.390 117.676  1.00  0.00
ATOM   8885  C   TYR A 585     122.448 104.088 119.198  1.00  0.00
ATOM   8886  O   TYR A 585     122.915 103.529 120.187  1.00  0.00
ATOM   8887  N   ILE A 586     121.164 103.994 118.857  1.00  0.00
ATOM   8888  H   ILE A 586     120.895 104.412 117.971  1.00  0.00
ATOM   8889  CA  ILE A 586     120.231 103.029 119.454  1.00  0.00
ATOM   8890  HA  ILE A 586     120.531 102.807 120.477  1.00  0.00
ATOM   8891  CB  ILE A 586     118.793 103.609 119.461  1.00  0.00
ATOM   8892  HB  ILE A 586     118.620 104.068 118.486  1.00  0.00
ATOM   8893  CG2 ILE A 586     117.699 102.546 119.654  1.00  0.00
ATOM   8894 1HG2 ILE A 586     116.724 103.013 119.788  1.00  0.00
ATOM   8895 2HG2 ILE A 586     117.636 101.898 118.782  1.00  0.00
ATOM   8896 3HG2 ILE A 586     117.912 101.927 120.525  1.00  0.00
ATOM   8897  CG1 ILE A 586     118.612 104.725 120.519  1.00  0.00
ATOM   8898 1HG1 ILE A 586     117.646 105.201 120.344  1.00  0.00
ATOM   8899 2HG1 ILE A 586     119.375 105.485 120.361  1.00  0.00
ATOM   8900  CD  ILE A 586     118.658 104.265 121.984  1.00  0.00
ATOM   8901  HD1 ILE A 586     118.457 105.114 122.635  1.00  0.00
ATOM   8902  HD2 ILE A 586     117.897 103.512 122.184  1.00  0.00
ATOM   8903  HD3 ILE A 586     119.636 103.857 122.226  1.00  0.00
ATOM   8904  C   ILE A 586     120.300 101.725 118.671  1.00  0.00
```

ATOM	8905	O	ILE	A	586	120.359	101.756	117.452	1.00	0.00
ATOM	8906	N	ASP	A	587	120.189	100.586	119.338	1.00	0.00
ATOM	8907	H	ASP	A	587	120.235	100.622	120.355	1.00	0.00
ATOM	8908	CA	ASP	A	587	119.906	99.274	118.749	1.00	0.00
ATOM	8909	HA	ASP	A	587	119.477	99.387	117.752	1.00	0.00
ATOM	8910	CB	ASP	A	587	121.223	98.478	118.662	1.00	0.00
ATOM	8911	HB1	ASP	A	587	121.627	98.330	119.665	1.00	0.00
ATOM	8912	HB2	ASP	A	587	121.955	99.050	118.091	1.00	0.00
ATOM	8913	CG	ASP	A	587	121.044	97.117	117.987	1.00	0.00
ATOM	8914	OD1	ASP	A	587	121.646	96.110	118.421	1.00	0.00
ATOM	8915	OD2	ASP	A	587	120.321	97.008	116.972	1.00	0.00
ATOM	8916	C	ASP	A	587	118.888	98.591	119.652	1.00	0.00
ATOM	8917	O	ASP	A	587	119.160	98.471	120.833	1.00	0.00
ATOM	8918	N	GLY	A	588	117.699	98.208	119.177	1.00	0.00
ATOM	8919	H	GLY	A	588	117.499	98.333	118.197	1.00	0.00
ATOM	8920	CA	GLY	A	588	116.620	97.787	120.092	1.00	0.00
ATOM	8921	HA1	GLY	A	588	116.942	96.882	120.592	1.00	0.00
ATOM	8922	HA2	GLY	A	588	115.720	97.564	119.522	1.00	0.00
ATOM	8923	C	GLY	A	588	116.288	98.862	121.142	1.00	0.00
ATOM	8924	O	GLY	A	588	116.179	100.033	120.785	1.00	0.00
ATOM	8925	N	PRO	A	589	116.105	98.506	122.428	1.00	0.00
ATOM	8926	CD	PRO	A	589	115.994	97.145	122.932	1.00	0.00
ATOM	8927	HD1	PRO	A	589	116.885	96.562	122.702	1.00	0.00
ATOM	8928	HD2	PRO	A	589	115.109	96.668	122.508	1.00	0.00
ATOM	8929	CG	PRO	A	589	115.845	97.261	124.445	1.00	0.00
ATOM	8930	HG1	PRO	A	589	116.826	97.136	124.891	1.00	0.00
ATOM	8931	HG2	PRO	A	589	115.150	96.520	124.841	1.00	0.00
ATOM	8932	CB	PRO	A	589	115.345	98.689	124.668	1.00	0.00
ATOM	8933	HB1	PRO	A	589	115.640	99.071	125.647	1.00	0.00
ATOM	8934	HB2	PRO	A	589	114.258	98.704	124.576	1.00	0.00
ATOM	8935	CA	PRO	A	589	115.959	99.484	123.510	1.00	0.00
ATOM	8936	HA	PRO	A	589	115.266	100.271	123.213	1.00	0.00
ATOM	8937	C	PRO	A	589	117.274	100.171	123.933	1.00	0.00
ATOM	8938	O	PRO	A	589	117.236	101.114	124.719	1.00	0.00
ATOM	8939	N	HIS	A	590	118.449	99.705	123.484	1.00	0.00
ATOM	8940	H	HIS	A	590	118.470	99.041	122.718	1.00	0.00
ATOM	8941	CA	HIS	A	590	119.714	99.972	124.186	1.00	0.00
ATOM	8942	HA	HIS	A	590	119.502	100.518	125.105	1.00	0.00
ATOM	8943	CB	HIS	A	590	120.354	98.634	124.601	1.00	0.00
ATOM	8944	HB1	HIS	A	590	121.344	98.835	125.011	1.00	0.00
ATOM	8945	HB2	HIS	A	590	120.472	98.013	123.712	1.00	0.00
ATOM	8946	CG	HIS	A	590	119.561	97.868	125.640	1.00	0.00
ATOM	8947	ND1	HIS	A	590	119.635	96.491	125.880	1.00	0.00
ATOM	8948	CE1	HIS	A	590	118.726	96.235	126.841	1.00	0.00
ATOM	8949	HE1	HIS	A	590	118.508	95.249	127.233	1.00	0.00
ATOM	8950	NE2	HIS	A	590	118.105	97.368	127.221	1.00	0.00
ATOM	8951	HE2	HIS	A	590	117.335	97.471	127.891	1.00	0.00
ATOM	8952	CD2	HIS	A	590	118.624	98.397	126.479	1.00	0.00
ATOM	8953	HD2	HIS	A	590	118.287	99.429	126.515	1.00	0.00
ATOM	8954	C	HIS	A	590	120.660	100.870	123.386	1.00	0.00
ATOM	8955	O	HIS	A	590	120.926	100.646	122.211	1.00	0.00
ATOM	8956	N	CYS	A	591	121.162	101.922	124.040	1.00	0.00
ATOM	8957	H	CYS	A	591	120.938	102.042	125.016	1.00	0.00
ATOM	8958	CA	CYS	A	591	122.151	102.816	123.446	1.00	0.00
ATOM	8959	HA	CYS	A	591	121.870	103.004	122.410	1.00	0.00
ATOM	8960	CB	CYS	A	591	122.123	104.149	124.198	1.00	0.00
ATOM	8961	HB1	CYS	A	591	122.516	103.979	125.201	1.00	0.00
ATOM	8962	HB2	CYS	A	591	121.090	104.486	124.286	1.00	0.00
ATOM	8963	SG	CYS	A	591	123.083	105.474	123.418	1.00	0.00
ATOM	8964	C	CYS	A	591	123.550	102.186	123.458	1.00	0.00
ATOM	8965	O	CYS	A	591	123.934	101.545	124.438	1.00	0.00

ATOM	8966	N	VAL	A	592	124.309	102.388	122.383	1.00	0.00
ATOM	8967	H	VAL	A	592	123.897	102.909	121.611	1.00	0.00
ATOM	8968	CA	VAL	A	592	125.629	101.799	122.141	1.00	0.00
ATOM	8969	HA	VAL	A	592	126.117	101.620	123.098	1.00	0.00
ATOM	8970	CB	VAL	A	592	125.499	100.443	121.405	1.00	0.00
ATOM	8971	HB	VAL	A	592	126.488	100.170	121.036	1.00	0.00
ATOM	8972	CG1	VAL	A	592	125.048	99.325	122.353	1.00	0.00
ATOM	8973	1HG1	VAL	A	592	125.093	98.367	121.835	1.00	0.00
ATOM	8974	2HG1	VAL	A	592	125.697	99.285	123.227	1.00	0.00
ATOM	8975	3HG1	VAL	A	592	124.022	99.497	122.680	1.00	0.00
ATOM	8976	CG2	VAL	A	592	124.531	100.486	120.212	1.00	0.00
ATOM	8977	1HG2	VAL	A	592	124.584	99.546	119.665	1.00	0.00
ATOM	8978	2HG2	VAL	A	592	123.505	100.630	120.553	1.00	0.00
ATOM	8979	3HG2	VAL	A	592	124.798	101.298	119.540	1.00	0.00
ATOM	8980	C	VAL	A	592	126.522	102.768	121.348	1.00	0.00
ATOM	8981	O	VAL	A	592	126.036	103.567	120.541	1.00	0.00
ATOM	8982	N	LYS	A	593	127.851	102.659	121.518	1.00	0.00
ATOM	8983	H	LYS	A	593	128.172	102.088	122.285	1.00	0.00
ATOM	8984	CA	LYS	A	593	128.863	103.451	120.775	1.00	0.00
ATOM	8985	HA	LYS	A	593	128.702	104.506	120.984	1.00	0.00
ATOM	8986	CB	LYS	A	593	130.302	103.007	121.146	1.00	0.00
ATOM	8987	HB1	LYS	A	593	130.983	103.260	120.331	1.00	0.00
ATOM	8988	HB2	LYS	A	593	130.320	101.921	121.247	1.00	0.00
ATOM	8989	CG	LYS	A	593	130.895	103.624	122.410	1.00	0.00
ATOM	8990	HG1	LYS	A	593	131.860	103.158	122.610	1.00	0.00
ATOM	8991	HG2	LYS	A	593	130.237	103.380	123.234	1.00	0.00
ATOM	8992	CD	LYS	A	593	131.107	105.147	122.279	1.00	0.00
ATOM	8993	HD1	LYS	A	593	130.217	105.621	121.868	1.00	0.00
ATOM	8994	HD2	LYS	A	593	131.930	105.335	121.590	1.00	0.00
ATOM	8995	CE	LYS	A	593	131.409	105.811	123.623	1.00	0.00
ATOM	8996	HE1	LYS	A	593	131.529	106.882	123.455	1.00	0.00
ATOM	8997	HE2	LYS	A	593	132.334	105.408	124.037	1.00	0.00
ATOM	8998	NZ	LYS	A	593	130.287	105.579	124.550	1.00	0.00
ATOM	8999	HZ1	LYS	A	593	129.398	105.499	124.051	1.00	0.00
ATOM	9000	HZ2	LYS	A	593	130.295	104.646	124.972	1.00	0.00
ATOM	9001	HZ3	LYS	A	593	130.167	106.267	125.285	1.00	0.00
ATOM	9002	C	LYS	A	593	128.781	103.308	119.261	1.00	0.00
ATOM	9003	O	LYS	A	593	129.238	104.172	118.518	1.00	0.00
ATOM	9004	N	THR	A	594	128.282	102.163	118.824	1.00	0.00
ATOM	9005	H	THR	A	594	127.893	101.537	119.513	1.00	0.00
ATOM	9006	CA	THR	A	594	128.265	101.700	117.447	1.00	0.00
ATOM	9007	HA	THR	A	594	128.053	102.531	116.775	1.00	0.00
ATOM	9008	CB	THR	A	594	129.624	101.084	117.056	1.00	0.00
ATOM	9009	HB	THR	A	594	130.367	101.882	117.068	1.00	0.00
ATOM	9010	CG2	THR	A	594	130.125	99.960	117.968	1.00	0.00
ATOM	9011	1HG2	THR	A	594	131.078	99.588	117.593	1.00	0.00
ATOM	9012	2HG2	THR	A	594	130.278	100.340	118.976	1.00	0.00
ATOM	9013	3HG2	THR	A	594	129.407	99.141	117.999	1.00	0.00
ATOM	9014	OG1	THR	A	594	129.581	100.563	115.748	1.00	0.00
ATOM	9015	HG1	THR	A	594	129.178	99.681	115.785	1.00	0.00
ATOM	9016	C	THR	A	594	127.162	100.664	117.322	1.00	0.00
ATOM	9017	O	THR	A	594	126.891	99.924	118.266	1.00	0.00
ATOM	9018	N	CYS	A	595	126.596	100.546	116.126	1.00	0.00
ATOM	9019	H	CYS	A	595	126.852	101.195	115.398	1.00	0.00
ATOM	9020	CA	CYS	A	595	125.924	99.319	115.742	1.00	0.00
ATOM	9021	HA	CYS	A	595	124.997	99.249	116.308	1.00	0.00
ATOM	9022	CB	CYS	A	595	125.589	99.386	114.247	1.00	0.00
ATOM	9023	HB1	CYS	A	595	125.513	98.365	113.873	1.00	0.00
ATOM	9024	HB2	CYS	A	595	126.412	99.862	113.713	1.00	0.00
ATOM	9025	SG	CYS	A	595	124.043	100.218	113.826	1.00	0.00
ATOM	9026	C	CYS	A	595	126.803	98.077	116.008	1.00	0.00

ATOM	9027	O	CYS	A	595	128.025	98.141	115.814	1.00	0.00
ATOM	9028	N	PRO	A	596	126.200	96.938	116.379	1.00	0.00
ATOM	9029	CD	PRO	A	596	124.865	96.831	116.948	1.00	0.00
ATOM	9030	HD1	PRO	A	596	124.116	96.928	116.162	1.00	0.00
ATOM	9031	HD2	PRO	A	596	124.699	97.575	117.730	1.00	0.00
ATOM	9032	CG	PRO	A	596	124.819	95.436	117.548	1.00	0.00
ATOM	9033	HG1	PRO	A	596	123.810	95.041	117.622	1.00	0.00
ATOM	9034	HG2	PRO	A	596	125.297	95.450	118.527	1.00	0.00
ATOM	9035	CB	PRO	A	596	125.676	94.639	116.576	1.00	0.00
ATOM	9036	HB1	PRO	A	596	125.101	94.407	115.679	1.00	0.00
ATOM	9037	HB2	PRO	A	596	126.032	93.734	117.045	1.00	0.00
ATOM	9038	CA	PRO	A	596	126.813	95.618	116.252	1.00	0.00
ATOM	9039	HA	PRO	A	596	127.592	95.526	117.010	1.00	0.00
ATOM	9040	C	PRO	A	596	127.401	95.398	114.855	1.00	0.00
ATOM	9041	O	PRO	A	596	126.693	95.471	113.853	1.00	0.00
ATOM	9042	N	ALA	A	597	128.717	95.179	114.800	1.00	0.00
ATOM	9043	H	ALA	A	597	129.242	95.180	115.658	1.00	0.00
ATOM	9044	CA	ALA	A	597	129.456	95.287	113.545	1.00	0.00
ATOM	9045	HA	ALA	A	597	129.021	96.100	112.958	1.00	0.00
ATOM	9046	CB	ALA	A	597	130.900	95.692	113.878	1.00	0.00
ATOM	9047	HB1	ALA	A	597	131.474	95.794	112.956	1.00	0.00
ATOM	9048	HB2	ALA	A	597	130.901	96.654	114.393	1.00	0.00
ATOM	9049	HB3	ALA	A	597	131.379	94.948	114.514	1.00	0.00
ATOM	9050	C	ALA	A	597	129.384	94.037	112.681	1.00	0.00
ATOM	9051	O	ALA	A	597	128.767	94.109	111.628	1.00	0.00
ATOM	9052	N	GLY	A	598	130.003	92.949	113.159	1.00	0.00
ATOM	9053	H	GLY	A	598	130.445	93.066	114.056	1.00	0.00
ATOM	9054	CA	GLY	A	598	130.160	91.623	112.544	1.00	0.00
ATOM	9055	HA1	GLY	A	598	131.097	91.566	111.991	1.00	0.00
ATOM	9056	HA2	GLY	A	598	129.326	91.426	111.868	1.00	0.00
ATOM	9057	C	GLY	A	598	130.162	90.580	113.658	1.00	0.00
ATOM	9058	O	GLY	A	598	131.132	89.844	113.824	1.00	0.00
ATOM	9059	N	VAL	A	599	129.144	90.620	114.525	1.00	0.00
ATOM	9060	H	VAL	A	599	128.323	91.167	114.276	1.00	0.00
ATOM	9061	CA	VAL	A	599	129.182	89.937	115.829	1.00	0.00
ATOM	9062	HA	VAL	A	599	130.203	89.594	115.995	1.00	0.00
ATOM	9063	CB	VAL	A	599	128.884	90.875	117.008	1.00	0.00
ATOM	9064	HB	VAL	A	599	128.887	90.275	117.919	1.00	0.00
ATOM	9065	CG1	VAL	A	599	129.999	91.922	117.147	1.00	0.00
ATOM	9066	1HG1	VAL	A	599	129.825	92.528	118.035	1.00	0.00
ATOM	9067	2HG1	VAL	A	599	130.964	91.425	117.248	1.00	0.00
ATOM	9068	3HG1	VAL	A	599	130.021	92.572	116.274	1.00	0.00
ATOM	9069	CG2	VAL	A	599	127.542	91.594	116.922	1.00	0.00
ATOM	9070	1HG2	VAL	A	599	127.415	92.210	117.810	1.00	0.00
ATOM	9071	2HG2	VAL	A	599	127.494	92.211	116.026	1.00	0.00
ATOM	9072	3HG2	VAL	A	599	126.730	90.868	116.898	1.00	0.00
ATOM	9073	C	VAL	A	599	128.307	88.684	115.845	1.00	0.00
ATOM	9074	O	VAL	A	599	127.112	88.716	115.551	1.00	0.00
ATOM	9075	N	MET	A	600	128.958	87.569	116.188	1.00	0.00
ATOM	9076	H	MET	A	600	129.934	87.669	116.413	1.00	0.00
ATOM	9077	CA	MET	A	600	128.435	86.201	116.108	1.00	0.00
ATOM	9078	HA	MET	A	600	128.617	85.834	115.099	1.00	0.00
ATOM	9079	CB	MET	A	600	129.190	85.275	117.076	1.00	0.00
ATOM	9080	HB1	MET	A	600	128.775	84.270	116.989	1.00	0.00
ATOM	9081	HB2	MET	A	600	129.035	85.626	118.097	1.00	0.00
ATOM	9082	CG	MET	A	600	130.694	85.198	116.789	1.00	0.00
ATOM	9083	HG1	MET	A	600	131.140	86.181	116.937	1.00	0.00
ATOM	9084	HG2	MET	A	600	130.832	84.920	115.743	1.00	0.00
ATOM	9085	SD	MET	A	600	131.604	84.001	117.808	1.00	0.00
ATOM	9086	CE	MET	A	600	131.286	84.634	119.480	1.00	0.00
ATOM	9087	HE1	MET	A	600	131.880	84.069	120.199	1.00	0.00

ATOM	9088	HE2	MET	A	600	131.561	85.687	119.534	1.00	0.00
ATOM	9089	HE3	MET	A	600	130.231	84.516	119.730	1.00	0.00
ATOM	9090	C	MET	A	600	126.937	86.098	116.355	1.00	0.00
ATOM	9091	O	MET	A	600	126.496	86.277	117.487	1.00	0.00
ATOM	9092	N	GLY	A	601	126.181	85.831	115.298	1.00	0.00
ATOM	9093	H	GLY	A	601	126.612	85.707	114.393	1.00	0.00
ATOM	9094	CA	GLY	A	601	124.739	85.662	115.315	1.00	0.00
ATOM	9095	HA1	GLY	A	601	124.297	86.296	114.550	1.00	0.00
ATOM	9096	HA2	GLY	A	601	124.314	85.956	116.275	1.00	0.00
ATOM	9097	C	GLY	A	601	124.347	84.220	115.014	1.00	0.00
ATOM	9098	O	GLY	A	601	125.168	83.299	114.929	1.00	0.00
ATOM	9099	N	GLU	A	602	123.049	84.043	114.859	1.00	0.00
ATOM	9100	H	GLU	A	602	122.484	84.887	114.966	1.00	0.00
ATOM	9101	CA	GLU	A	602	122.330	82.863	114.406	1.00	0.00
ATOM	9102	HA	GLU	A	602	122.136	82.242	115.279	1.00	0.00
ATOM	9103	CB	GLU	A	602	120.961	83.358	113.878	1.00	0.00
ATOM	9104	HB1	GLU	A	602	120.333	83.627	114.728	1.00	0.00
ATOM	9105	HB2	GLU	A	602	120.477	82.514	113.385	1.00	0.00
ATOM	9106	CG	GLU	A	602	120.980	84.551	112.884	1.00	0.00
ATOM	9107	HG1	GLU	A	602	120.138	84.409	112.202	1.00	0.00
ATOM	9108	HG2	GLU	A	602	121.885	84.514	112.275	1.00	0.00
ATOM	9109	CD	GLU	A	602	120.848	85.968	113.511	1.00	0.00
ATOM	9110	OE1	GLU	A	602	121.466	86.239	114.568	1.00	0.00
ATOM	9111	OE2	GLU	A	602	120.121	86.796	112.918	1.00	0.00
ATOM	9112	C	GLU	A	602	123.074	81.989	113.373	1.00	0.00
ATOM	9113	O	GLU	A	602	123.529	82.459	112.331	1.00	0.00
ATOM	9114	N	ASN	A	603	123.191	80.681	113.630	1.00	0.00
ATOM	9115	H	ASN	A	603	122.802	80.336	114.495	1.00	0.00
ATOM	9116	CA	ASN	A	603	123.920	79.723	112.775	1.00	0.00
ATOM	9117	HA	ASN	A	603	123.958	78.783	113.328	1.00	0.00
ATOM	9118	CB	ASN	A	603	123.085	79.461	111.503	1.00	0.00
ATOM	9119	HB1	ASN	A	603	123.102	80.344	110.865	1.00	0.00
ATOM	9120	HB2	ASN	A	603	122.049	79.259	111.775	1.00	0.00
ATOM	9121	CG	ASN	A	603	123.589	78.268	110.711	1.00	0.00
ATOM	9122	OD1	ASN	A	603	123.894	77.221	111.259	1.00	0.00
ATOM	9123	ND2	ASN	A	603	123.705	78.384	109.409	1.00	0.00
ATOM	9124	1HD2	ASN	A	603	123.553	79.278	108.974	1.00	0.00
ATOM	9125	2HD2	ASN	A	603	124.100	77.600	108.925	1.00	0.00
ATOM	9126	C	ASN	A	603	125.400	80.087	112.500	1.00	0.00
ATOM	9127	O	ASN	A	603	125.957	79.698	111.472	1.00	0.00
ATOM	9128	N	ASN	A	604	126.054	80.811	113.419	1.00	0.00
ATOM	9129	H	ASN	A	604	125.507	81.170	114.193	1.00	0.00
ATOM	9130	CA	ASN	A	604	127.407	81.380	113.278	1.00	0.00
ATOM	9131	HA	ASN	A	604	127.650	81.820	114.247	1.00	0.00
ATOM	9132	CB	ASN	A	604	128.491	80.302	112.975	1.00	0.00
ATOM	9133	HB1	ASN	A	604	129.463	80.710	113.250	1.00	0.00
ATOM	9134	HB2	ASN	A	604	128.522	80.146	111.896	1.00	0.00
ATOM	9135	CG	ASN	A	604	128.365	78.924	113.627	1.00	0.00
ATOM	9136	OD1	ASN	A	604	128.979	78.598	114.635	1.00	0.00
ATOM	9137	ND2	ASN	A	604	127.639	78.020	113.010	1.00	0.00
ATOM	9138	1HD2	ASN	A	604	127.108	78.307	112.191	1.00	0.00
ATOM	9139	2HD2	ASN	A	604	127.540	77.114	113.423	1.00	0.00
ATOM	9140	C	ASN	A	604	127.472	82.548	112.266	1.00	0.00
ATOM	9141	O	ASN	A	604	128.557	83.063	111.989	1.00	0.00
ATOM	9142	N	THR	A	605	126.338	82.965	111.693	1.00	0.00
ATOM	9143	H	THR	A	605	125.467	82.557	112.010	1.00	0.00
ATOM	9144	CA	THR	A	605	126.231	84.092	110.754	1.00	0.00
ATOM	9145	HA	THR	A	605	126.948	83.960	109.945	1.00	0.00
ATOM	9146	CB	THR	A	605	124.812	84.208	110.164	1.00	0.00
ATOM	9147	HB	THR	A	605	124.138	84.646	110.900	1.00	0.00
ATOM	9148	CG2	THR	A	605	124.789	85.074	108.907	1.00	0.00

```
ATOM   9149 1HG2 THR A 605     123.772  85.115 108.521  1.00  0.00
ATOM   9150 2HG2 THR A 605     125.110  86.085 109.150  1.00  0.00
ATOM   9151 3HG2 THR A 605     125.444  84.652 108.149  1.00  0.00
ATOM   9152  OG1 THR A 605     124.305  82.942 109.812  1.00  0.00
ATOM   9153  HG1 THR A 605     123.875  82.641 110.634  1.00  0.00
ATOM   9154  C   THR A 605     126.538  85.368 111.524  1.00  0.00
ATOM   9155  O   THR A 605     125.855  85.679 112.492  1.00  0.00
ATOM   9156  N   LEU A 606     127.586  86.100 111.159  1.00  0.00
ATOM   9157  H   LEU A 606     128.139  85.807 110.372  1.00  0.00
ATOM   9158  CA  LEU A 606     127.930  87.350 111.840  1.00  0.00
ATOM   9159  HA  LEU A 606     127.983  87.176 112.915  1.00  0.00
ATOM   9160  CB  LEU A 606     129.306  87.832 111.343  1.00  0.00
ATOM   9161  HB1 LEU A 606     129.540  88.755 111.863  1.00  0.00
ATOM   9162  HB2 LEU A 606     129.230  88.072 110.281  1.00  0.00
ATOM   9163  CG  LEU A 606     130.471  86.843 111.532  1.00  0.00
ATOM   9164  HG  LEU A 606     130.323  85.985 110.874  1.00  0.00
ATOM   9165  CD1 LEU A 606     131.777  87.527 111.129  1.00  0.00
ATOM   9166 1HD1 LEU A 606     132.600  86.818 111.184  1.00  0.00
ATOM   9167 2HD1 LEU A 606     131.700  87.892 110.106  1.00  0.00
ATOM   9168 3HD1 LEU A 606     131.983  88.364 111.794  1.00  0.00
ATOM   9169  CD2 LEU A 606     130.602  86.326 112.968  1.00  0.00
ATOM   9170 1HD2 LEU A 606     131.510  85.731 113.061  1.00  0.00
ATOM   9171 2HD2 LEU A 606     130.646  87.162 113.665  1.00  0.00
ATOM   9172 3HD2 LEU A 606     129.754  85.684 113.193  1.00  0.00
ATOM   9173  C   LEU A 606     126.846  88.406 111.594  1.00  0.00
ATOM   9174  O   LEU A 606     126.677  88.816 110.442  1.00  0.00
ATOM   9175  N   VAL A 607     126.109  88.865 112.620  1.00  0.00
ATOM   9176  H   VAL A 607     126.240  88.526 113.567  1.00  0.00
ATOM   9177  CA  VAL A 607     125.172  89.966 112.344  1.00  0.00
ATOM   9178  HA  VAL A 607     124.654  89.729 111.413  1.00  0.00
ATOM   9179  CB  VAL A 607     124.048  90.191 113.382  1.00  0.00
ATOM   9180  HB  VAL A 607     123.510  91.090 113.075  1.00  0.00
ATOM   9181  CG1 VAL A 607     123.039  89.043 113.347  1.00  0.00
ATOM   9182 1HG1 VAL A 607     122.231  89.214 114.057  1.00  0.00
ATOM   9183 2HG1 VAL A 607     122.600  88.950 112.354  1.00  0.00
ATOM   9184 3HG1 VAL A 607     123.526  88.102 113.599  1.00  0.00
ATOM   9185  CG2 VAL A 607     124.483  90.413 114.831  1.00  0.00
ATOM   9186 1HG2 VAL A 607     123.632  90.760 115.414  1.00  0.00
ATOM   9187 2HG2 VAL A 607     124.826  89.474 115.255  1.00  0.00
ATOM   9188 3HG2 VAL A 607     125.275  91.157 114.886  1.00  0.00
ATOM   9189  C   VAL A 607     125.958  91.243 112.104  1.00  0.00
ATOM   9190  O   VAL A 607     126.819  91.621 112.908  1.00  0.00
ATOM   9191  N   TRP A 608     125.599  91.909 111.013  1.00  0.00
ATOM   9192  H   TRP A 608     124.902  91.498 110.417  1.00  0.00
ATOM   9193  CA  TRP A 608     126.056  93.247 110.695  1.00  0.00
ATOM   9194  HA  TRP A 608     126.684  93.569 111.512  1.00  0.00
ATOM   9195  CB  TRP A 608     126.814  93.318 109.352  1.00  0.00
ATOM   9196  HB1 TRP A 608     127.518  94.140 109.432  1.00  0.00
ATOM   9197  HB2 TRP A 608     126.105  93.584 108.569  1.00  0.00
ATOM   9198  CG  TRP A 608     127.567  92.120 108.834  1.00  0.00
ATOM   9199  CD1 TRP A 608     126.999  91.109 108.139  1.00  0.00
ATOM   9200  HD1 TRP A 608     125.946  91.032 107.907  1.00  0.00
ATOM   9201  NE1 TRP A 608     127.962  90.206 107.740  1.00  0.00
ATOM   9202  HE1 TRP A 608     127.750  89.407 107.161  1.00  0.00
ATOM   9203  CE2 TRP A 608     129.217  90.595 108.144  1.00  0.00
ATOM   9204  CZ2 TRP A 608     130.488  90.031 107.970  1.00  0.00
ATOM   9205  HZ2 TRP A 608     130.604  89.095 107.442  1.00  0.00
ATOM   9206  CH2 TRP A 608     131.615  90.706 108.469  1.00  0.00
ATOM   9207  HH2 TRP A 608     132.600  90.282 108.336  1.00  0.00
ATOM   9208  CZ3 TRP A 608     131.453  91.946 109.112  1.00  0.00
ATOM   9209  HZ3 TRP A 608     132.323  92.471 109.485  1.00  0.00
```

ATOM	9210	CE3	TRP	A	608	130.170	92.506	109.268	1.00	0.00
ATOM	9211	HE3	TRP	A	608	130.067	93.469	109.743	1.00	0.00
ATOM	9212	CD2	TRP	A	608	129.008	91.836	108.820	1.00	0.00
ATOM	9213	C	TRP	A	608	124.876	94.176	110.720	1.00	0.00
ATOM	9214	O	TRP	A	608	123.812	93.815	110.197	1.00	0.00
ATOM	9215	N	LYS	A	609	125.029	95.356	111.322	1.00	0.00
ATOM	9216	H	LYS	A	609	125.904	95.575	111.791	1.00	0.00
ATOM	9217	CA	LYS	A	609	123.880	96.278	111.488	1.00	0.00
ATOM	9218	HA	LYS	A	609	123.111	96.037	110.753	1.00	0.00
ATOM	9219	CB	LYS	A	609	123.243	96.066	112.878	1.00	0.00
ATOM	9220	HB1	LYS	A	609	122.357	96.695	112.930	1.00	0.00
ATOM	9221	HB2	LYS	A	609	123.944	96.366	113.658	1.00	0.00
ATOM	9222	CG	LYS	A	609	122.806	94.602	113.112	1.00	0.00
ATOM	9223	HG1	LYS	A	609	123.662	94.006	113.435	1.00	0.00
ATOM	9224	HG2	LYS	A	609	122.453	94.190	112.178	1.00	0.00
ATOM	9225	CD	LYS	A	609	121.649	94.421	114.098	1.00	0.00
ATOM	9226	HD1	LYS	A	609	121.233	93.422	113.956	1.00	0.00
ATOM	9227	HD2	LYS	A	609	120.874	95.161	113.884	1.00	0.00
ATOM	9228	CE	LYS	A	609	122.107	94.546	115.541	1.00	0.00
ATOM	9229	HE1	LYS	A	609	122.593	95.517	115.671	1.00	0.00
ATOM	9230	HE2	LYS	A	609	122.834	93.762	115.769	1.00	0.00
ATOM	9231	NZ	LYS	A	609	120.959	94.473	116.465	1.00	0.00
ATOM	9232	HZ1	LYS	A	609	120.364	95.294	116.312	1.00	0.00
ATOM	9233	HZ2	LYS	A	609	120.440	93.613	116.446	1.00	0.00
ATOM	9234	HZ3	LYS	A	609	121.285	94.695	117.409	1.00	0.00
ATOM	9235	C	LYS	A	609	124.268	97.727	111.172	1.00	0.00
ATOM	9236	O	LYS	A	609	125.424	98.103	111.363	1.00	0.00
ATOM	9237	N	TYR	A	610	123.320	98.508	110.657	1.00	0.00
ATOM	9238	H	TYR	A	610	122.377	98.139	110.598	1.00	0.00
ATOM	9239	CA	TYR	A	610	123.535	99.880	110.180	1.00	0.00
ATOM	9240	HA	TYR	A	610	124.536	100.201	110.468	1.00	0.00
ATOM	9241	CB	TYR	A	610	123.446	99.960	108.638	1.00	0.00
ATOM	9242	HB1	TYR	A	610	123.766	99.010	108.211	1.00	0.00
ATOM	9243	HB2	TYR	A	610	124.162	100.712	108.308	1.00	0.00
ATOM	9244	CG	TYR	A	610	122.091	100.345	108.056	1.00	0.00
ATOM	9245	CD1	TYR	A	610	121.189	99.350	107.628	1.00	0.00
ATOM	9246	HD1	TYR	A	610	121.461	98.306	107.679	1.00	0.00
ATOM	9247	CE1	TYR	A	610	119.922	99.712	107.130	1.00	0.00
ATOM	9248	HE1	TYR	A	610	119.226	98.954	106.801	1.00	0.00
ATOM	9249	CZ	TYR	A	610	119.534	101.066	107.088	1.00	0.00
ATOM	9250	OH	TYR	A	610	118.287	101.397	106.662	1.00	0.00
ATOM	9251	HH	TYR	A	610	118.029	102.294	106.975	1.00	0.00
ATOM	9252	CE2	TYR	A	610	120.436	102.064	107.509	1.00	0.00
ATOM	9253	HE2	TYR	A	610	120.141	103.101	107.479	1.00	0.00
ATOM	9254	CD2	TYR	A	610	121.718	101.704	107.965	1.00	0.00
ATOM	9255	HD2	TYR	A	610	122.401	102.482	108.280	1.00	0.00
ATOM	9256	C	TYR	A	610	122.534	100.830	110.839	1.00	0.00
ATOM	9257	O	TYR	A	610	121.414	100.424	111.156	1.00	0.00
ATOM	9258	N	ALA	A	611	122.935	102.088	111.020	1.00	0.00
ATOM	9259	H	ALA	A	611	123.840	102.368	110.686	1.00	0.00
ATOM	9260	CA	ALA	A	611	122.115	103.093	111.678	1.00	0.00
ATOM	9261	HA	ALA	A	611	121.317	102.593	112.214	1.00	0.00
ATOM	9262	CB	ALA	A	611	122.965	103.823	112.715	1.00	0.00
ATOM	9263	HB1	ALA	A	611	122.340	104.561	113.213	1.00	0.00
ATOM	9264	HB2	ALA	A	611	123.338	103.116	113.457	1.00	0.00
ATOM	9265	HB3	ALA	A	611	123.800	104.329	112.231	1.00	0.00
ATOM	9266	C	ALA	A	611	121.487	104.072	110.683	1.00	0.00
ATOM	9267	O	ALA	A	611	122.170	104.612	109.814	1.00	0.00
ATOM	9268	N	ASP	A	612	120.185	104.285	110.835	1.00	0.00
ATOM	9269	H	ASP	A	612	119.703	103.750	111.550	1.00	0.00
ATOM	9270	CA	ASP	A	612	119.377	105.132	109.965	1.00	0.00

ATOM	9271	HA	ASP	A	612	119.752	105.016	108.946	1.00	0.00
ATOM	9272	CB	ASP	A	612	117.937	104.615	110.030	1.00	0.00
ATOM	9273	HB1	ASP	A	612	117.317	105.256	110.660	1.00	0.00
ATOM	9274	HB2	ASP	A	612	117.903	103.612	110.451	1.00	0.00
ATOM	9275	CG	ASP	A	612	117.400	104.579	108.610	1.00	0.00
ATOM	9276	OD1	ASP	A	612	117.556	103.511	107.974	1.00	0.00
ATOM	9277	OD2	ASP	A	612	116.955	105.652	108.156	1.00	0.00
ATOM	9278	C	ASP	A	612	119.445	106.632	110.304	1.00	0.00
ATOM	9279	O	ASP	A	612	120.147	107.049	111.226	1.00	0.00
ATOM	9280	N	ALA	A	613	118.641	107.453	109.621	1.00	0.00
ATOM	9281	H	ALA	A	613	118.047	107.043	108.898	1.00	0.00
ATOM	9282	CA	ALA	A	613	118.464	108.880	109.923	1.00	0.00
ATOM	9283	HA	ALA	A	613	119.449	109.351	109.899	1.00	0.00
ATOM	9284	CB	ALA	A	613	117.614	109.486	108.797	1.00	0.00
ATOM	9285	HB1	ALA	A	613	117.519	110.562	108.949	1.00	0.00
ATOM	9286	HB2	ALA	A	613	118.091	109.302	107.834	1.00	0.00
ATOM	9287	HB3	ALA	A	613	116.622	109.033	108.797	1.00	0.00
ATOM	9288	C	ALA	A	613	117.858	109.193	111.313	1.00	0.00
ATOM	9289	O	ALA	A	613	117.761	110.359	111.690	1.00	0.00
ATOM	9290	N	GLY	A	614	117.457	108.172	112.077	1.00	0.00
ATOM	9291	H	GLY	A	614	117.526	107.252	111.670	1.00	0.00
ATOM	9292	CA	GLY	A	614	117.097	108.278	113.498	1.00	0.00
ATOM	9293	HA1	GLY	A	614	116.187	107.709	113.682	1.00	0.00
ATOM	9294	HA2	GLY	A	614	116.909	109.320	113.759	1.00	0.00
ATOM	9295	C	GLY	A	614	118.190	107.753	114.436	1.00	0.00
ATOM	9296	O	GLY	A	614	117.910	107.518	115.608	1.00	0.00
ATOM	9297	N	HIS	A	615	119.391	107.481	113.905	1.00	0.00
ATOM	9298	H	HIS	A	615	119.547	107.761	112.944	1.00	0.00
ATOM	9299	CA	HIS	A	615	120.501	106.734	114.517	1.00	0.00
ATOM	9300	HA	HIS	A	615	121.191	106.478	113.711	1.00	0.00
ATOM	9301	CB	HIS	A	615	121.265	107.648	115.497	1.00	0.00
ATOM	9302	HB1	HIS	A	615	121.983	107.049	116.055	1.00	0.00
ATOM	9303	HB2	HIS	A	615	120.566	108.082	116.213	1.00	0.00
ATOM	9304	CG	HIS	A	615	122.036	108.757	114.828	1.00	0.00
ATOM	9305	ND1	HIS	A	615	121.487	109.852	114.159	1.00	0.00
ATOM	9306	CE1	HIS	A	615	122.526	110.590	113.744	1.00	0.00
ATOM	9307	HE1	HIS	A	615	122.433	111.518	113.195	1.00	0.00
ATOM	9308	NE2	HIS	A	615	123.685	110.022	114.117	1.00	0.00
ATOM	9309	HE2	HIS	A	615	124.606	110.403	113.962	1.00	0.00
ATOM	9310	CD2	HIS	A	615	123.394	108.867	114.805	1.00	0.00
ATOM	9311	HD2	HIS	A	615	124.095	108.188	115.273	1.00	0.00
ATOM	9312	C	HIS	A	615	120.093	105.386	115.154	1.00	0.00
ATOM	9313	O	HIS	A	615	120.835	104.815	115.953	1.00	0.00
ATOM	9314	N	VAL	A	616	118.915	104.865	114.803	1.00	0.00
ATOM	9315	H	VAL	A	616	118.366	105.384	114.141	1.00	0.00
ATOM	9316	CA	VAL	A	616	118.426	103.540	115.199	1.00	0.00
ATOM	9317	HA	VAL	A	616	118.709	103.357	116.235	1.00	0.00
ATOM	9318	CB	VAL	A	616	116.889	103.427	115.098	1.00	0.00
ATOM	9319	HB	VAL	A	616	116.616	102.385	115.265	1.00	0.00
ATOM	9320	CG1	VAL	A	616	116.217	104.252	116.200	1.00	0.00
ATOM	9321	1HG1	VAL	A	616	115.137	104.122	116.154	1.00	0.00
ATOM	9322	2HG1	VAL	A	616	116.563	103.915	117.176	1.00	0.00
ATOM	9323	3HG1	VAL	A	616	116.455	105.310	116.088	1.00	0.00
ATOM	9324	CG2	VAL	A	616	116.297	103.860	113.746	1.00	0.00
ATOM	9325	1HG2	VAL	A	616	115.220	103.699	113.750	1.00	0.00
ATOM	9326	2HG2	VAL	A	616	116.497	104.912	113.555	1.00	0.00
ATOM	9327	3HG2	VAL	A	616	116.719	103.258	112.942	1.00	0.00
ATOM	9328	C	VAL	A	616	119.066	102.454	114.352	1.00	0.00
ATOM	9329	O	VAL	A	616	119.197	102.631	113.140	1.00	0.00
ATOM	9330	N	CYS	A	617	119.456	101.341	114.976	1.00	0.00
ATOM	9331	H	CYS	A	617	119.407	101.302	115.985	1.00	0.00

ATOM	9332	CA	CYS	A	617	120.257	100.334	114.303	1.00	0.00
ATOM	9333	HA	CYS	A	617	120.714	100.760	113.415	1.00	0.00
ATOM	9334	CB	CYS	A	617	121.375	99.908	115.254	1.00	0.00
ATOM	9335	HB1	CYS	A	617	120.926	99.337	116.062	1.00	0.00
ATOM	9336	HB2	CYS	A	617	121.851	100.793	115.677	1.00	0.00
ATOM	9337	SG	CYS	A	617	122.652	98.897	114.493	1.00	0.00
ATOM	9338	C	CYS	A	617	119.367	99.155	113.904	1.00	0.00
ATOM	9339	O	CYS	A	617	118.665	98.583	114.743	1.00	0.00
ATOM	9340	N	HIS	A	618	119.428	98.756	112.635	1.00	0.00
ATOM	9341	H	HIS	A	618	120.004	99.294	111.993	1.00	0.00
ATOM	9342	CA	HIS	A	618	118.715	97.583	112.128	1.00	0.00
ATOM	9343	HA	HIS	A	618	118.456	96.963	112.987	1.00	0.00
ATOM	9344	CB	HIS	A	618	117.366	97.943	111.466	1.00	0.00
ATOM	9345	HB1	HIS	A	618	116.596	97.799	112.225	1.00	0.00
ATOM	9346	HB2	HIS	A	618	117.151	97.236	110.666	1.00	0.00
ATOM	9347	CG	HIS	A	618	117.173	99.334	110.919	1.00	0.00
ATOM	9348	ND1	HIS	A	618	116.031	100.105	111.143	1.00	0.00
ATOM	9349	CE1	HIS	A	618	116.179	101.208	110.398	1.00	0.00
ATOM	9350	HE1	HIS	A	618	115.456	102.010	110.334	1.00	0.00
ATOM	9351	NE2	HIS	A	618	117.336	101.166	109.721	1.00	0.00
ATOM	9352	HE2	HIS	A	618	117.637	101.873	109.049	1.00	0.00
ATOM	9353	CD2	HIS	A	618	117.985	99.991	110.041	1.00	0.00
ATOM	9354	HD2	HIS	A	618	118.929	99.640	109.655	1.00	0.00
ATOM	9355	C	HIS	A	618	119.612	96.684	111.282	1.00	0.00
ATOM	9356	O	HIS	A	618	120.735	97.038	110.936	1.00	0.00
ATOM	9357	N	LEU	A	619	119.141	95.454	111.049	1.00	0.00
ATOM	9358	H	LEU	A	619	118.191	95.244	111.315	1.00	0.00
ATOM	9359	CA	LEU	A	619	119.909	94.410	110.371	1.00	0.00
ATOM	9360	HA	LEU	A	619	120.863	94.297	110.874	1.00	0.00
ATOM	9361	CB	LEU	A	619	119.169	93.054	110.437	1.00	0.00
ATOM	9362	HB1	LEU	A	619	119.602	92.416	109.665	1.00	0.00
ATOM	9363	HB2	LEU	A	619	118.118	93.195	110.183	1.00	0.00
ATOM	9364	CG	LEU	A	619	119.288	92.285	111.773	1.00	0.00
ATOM	9365	HG	LEU	A	619	120.339	92.241	112.059	1.00	0.00
ATOM	9366	CD1	LEU	A	619	118.480	92.917	112.910	1.00	0.00
ATOM	9367	1HD1	LEU	A	619	118.574	92.305	113.806	1.00	0.00
ATOM	9368	2HD1	LEU	A	619	118.850	93.911	113.139	1.00	0.00
ATOM	9369	3HD1	LEU	A	619	117.429	92.977	112.630	1.00	0.00
ATOM	9370	CD2	LEU	A	619	118.782	90.853	111.591	1.00	0.00
ATOM	9371	1HD2	LEU	A	619	118.937	90.281	112.508	1.00	0.00
ATOM	9372	2HD2	LEU	A	619	117.721	90.849	111.342	1.00	0.00
ATOM	9373	3HD2	LEU	A	619	119.340	90.358	110.797	1.00	0.00
ATOM	9374	C	LEU	A	619	120.223	94.786	108.915	1.00	0.00
ATOM	9375	O	LEU	A	619	119.377	95.302	108.186	1.00	0.00
ATOM	9376	N	CYS	A	620	121.445	94.457	108.495	1.00	0.00
ATOM	9377	H	CYS	A	620	122.083	94.028	109.156	1.00	0.00
ATOM	9378	CA	CYS	A	620	121.821	94.399	107.082	1.00	0.00
ATOM	9379	HA	CYS	A	620	121.524	95.324	106.587	1.00	0.00
ATOM	9380	CB	CYS	A	620	123.346	94.247	107.003	1.00	0.00
ATOM	9381	HB1	CYS	A	620	123.660	94.208	105.958	1.00	0.00
ATOM	9382	HB2	CYS	A	620	123.650	93.315	107.485	1.00	0.00
ATOM	9383	SG	CYS	A	620	124.136	95.653	107.844	1.00	0.00
ATOM	9384	HG	CYS	A	620	123.690	95.347	109.070	1.00	0.00
ATOM	9385	C	CYS	A	620	121.118	93.221	106.379	1.00	0.00
ATOM	9386	O	CYS	A	620	120.636	92.297	107.048	1.00	0.00
ATOM	9387	N	HIS	A	621	121.079	93.190	105.040	1.00	0.00
ATOM	9388	H	HIS	A	621	121.534	93.902	104.485	1.00	0.00
ATOM	9389	CA	HIS	A	621	120.631	91.962	104.377	1.00	0.00
ATOM	9390	HA	HIS	A	621	119.684	91.696	104.846	1.00	0.00
ATOM	9391	CB	HIS	A	621	120.322	92.138	102.884	1.00	0.00
ATOM	9392	HB1	HIS	A	621	119.552	92.889	102.776	1.00	0.00

ATOM	9393	HB2	HIS	A	621	119.880	91.208	102.527	1.00	0.00
ATOM	9394	CG	HIS	A	621	121.479	92.454	101.972	1.00	0.00
ATOM	9395	ND1	HIS	A	621	122.633	93.168	102.291	1.00	0.00
ATOM	9396	CE1	HIS	A	621	123.386	93.154	101.178	1.00	0.00
ATOM	9397	HE1	HIS	A	621	124.369	93.583	101.093	1.00	0.00
ATOM	9398	NE2	HIS	A	621	122.780	92.463	100.205	1.00	0.00
ATOM	9399	HE2	HIS	A	621	123.191	92.195	99.312	1.00	0.00
ATOM	9400	CD2	HIS	A	621	121.567	92.037	100.680	1.00	0.00
ATOM	9401	HD2	HIS	A	621	120.818	91.479	100.136	1.00	0.00
ATOM	9402	C	HIS	A	621	121.611	90.811	104.635	1.00	0.00
ATOM	9403	O	HIS	A	621	122.825	91.047	104.672	1.00	0.00
ATOM	9404	N	PRO	A	622	121.139	89.556	104.775	1.00	0.00
ATOM	9405	CD	PRO	A	622	119.731	89.170	104.816	1.00	0.00
ATOM	9406	HD1	PRO	A	622	119.192	89.514	103.935	1.00	0.00
ATOM	9407	HD2	PRO	A	622	119.275	89.575	105.721	1.00	0.00
ATOM	9408	CG	PRO	A	622	119.707	87.644	104.878	1.00	0.00
ATOM	9409	HG1	PRO	A	622	119.714	87.231	103.867	1.00	0.00
ATOM	9410	HG2	PRO	A	622	118.848	87.276	105.440	1.00	0.00
ATOM	9411	CB	PRO	A	622	121.024	87.322	105.577	1.00	0.00
ATOM	9412	HB1	PRO	A	622	121.347	86.298	105.394	1.00	0.00
ATOM	9413	HB2	PRO	A	622	120.911	87.483	106.649	1.00	0.00
ATOM	9414	CA	PRO	A	622	121.979	88.378	105.002	1.00	0.00
ATOM	9415	HA	PRO	A	622	122.740	88.608	105.748	1.00	0.00
ATOM	9416	C	PRO	A	622	122.704	87.917	103.715	1.00	0.00
ATOM	9417	O	PRO	A	622	122.663	86.764	103.299	1.00	0.00
ATOM	9418	N	ASN	A	623	123.343	88.876	103.052	1.00	0.00
ATOM	9419	H	ASN	A	623	123.338	89.784	103.496	1.00	0.00
ATOM	9420	CA	ASN	A	623	124.152	88.739	101.849	1.00	0.00
ATOM	9421	HA	ASN	A	623	124.602	87.744	101.835	1.00	0.00
ATOM	9422	CB	ASN	A	623	123.228	88.874	100.622	1.00	0.00
ATOM	9423	HB1	ASN	A	623	122.499	89.655	100.807	1.00	0.00
ATOM	9424	HB2	ASN	A	623	122.683	87.941	100.489	1.00	0.00
ATOM	9425	CG	ASN	A	623	123.945	89.207	99.327	1.00	0.00
ATOM	9426	OD1	ASN	A	623	123.772	90.268	98.752	1.00	0.00
ATOM	9427	ND2	ASN	A	623	124.758	88.320	98.808	1.00	0.00
ATOM	9428	1HD2	ASN	A	623	124.983	87.481	99.307	1.00	0.00
ATOM	9429	2HD2	ASN	A	623	125.267	88.614	97.994	1.00	0.00
ATOM	9430	C	ASN	A	623	125.325	89.738	101.856	1.00	0.00
ATOM	9431	O	ASN	A	623	126.372	89.415	101.299	1.00	0.00
ATOM	9432	N	CYS	A	624	125.214	90.883	102.551	1.00	0.00
ATOM	9433	H	CYS	A	624	124.335	91.147	102.978	1.00	0.00
ATOM	9434	CA	CYS	A	624	126.387	91.712	102.823	1.00	0.00
ATOM	9435	HA	CYS	A	624	126.830	91.948	101.857	1.00	0.00
ATOM	9436	CB	CYS	A	624	126.013	93.041	103.505	1.00	0.00
ATOM	9437	HB1	CYS	A	624	126.083	92.919	104.588	1.00	0.00
ATOM	9438	HB2	CYS	A	624	124.983	93.295	103.278	1.00	0.00
ATOM	9439	SG	CYS	A	624	127.051	94.451	103.005	1.00	0.00
ATOM	9440	C	CYS	A	624	127.436	90.919	103.626	1.00	0.00
ATOM	9441	O	CYS	A	624	127.138	90.340	104.670	1.00	0.00
ATOM	9442	N	THR	A	625	128.666	90.864	103.115	1.00	0.00
ATOM	9443	H	THR	A	625	128.837	91.334	102.237	1.00	0.00
ATOM	9444	CA	THR	A	625	129.790	90.110	103.708	1.00	0.00
ATOM	9445	HA	THR	A	625	129.391	89.330	104.355	1.00	0.00
ATOM	9446	CB	THR	A	625	130.628	89.445	102.596	1.00	0.00
ATOM	9447	HB	THR	A	625	131.423	90.122	102.279	1.00	0.00
ATOM	9448	CG2	THR	A	625	131.247	88.119	103.038	1.00	0.00
ATOM	9449	1HG2	THR	A	625	131.804	87.689	102.206	1.00	0.00
ATOM	9450	2HG2	THR	A	625	131.931	88.288	103.869	1.00	0.00
ATOM	9451	3HG2	THR	A	625	130.467	87.424	103.348	1.00	0.00
ATOM	9452	OG1	THR	A	625	129.838	89.149	101.460	1.00	0.00
ATOM	9453	HG1	THR	A	625	128.946	88.946	101.759	1.00	0.00

ATOM	9454	C	THR	A	625	130.678	91.007	104.587	1.00	0.00
ATOM	9455	O	THR	A	625	131.786	90.640	104.965	1.00	0.00
ATOM	9456	N	TYR	A	626	130.179	92.203	104.872	1.00	0.00
ATOM	9457	H	TYR	A	626	129.238	92.380	104.545	1.00	0.00
ATOM	9458	CA	TYR	A	626	130.710	93.274	105.707	1.00	0.00
ATOM	9459	HA	TYR	A	626	131.196	92.847	106.582	1.00	0.00
ATOM	9460	CB	TYR	A	626	131.743	94.108	104.922	1.00	0.00
ATOM	9461	HB1	TYR	A	626	132.713	93.623	105.038	1.00	0.00
ATOM	9462	HB2	TYR	A	626	131.836	95.100	105.364	1.00	0.00
ATOM	9463	CG	TYR	A	626	131.476	94.253	103.431	1.00	0.00
ATOM	9464	CD1	TYR	A	626	130.592	95.240	102.956	1.00	0.00
ATOM	9465	HD1	TYR	A	626	130.110	95.919	103.645	1.00	0.00
ATOM	9466	CE1	TYR	A	626	130.303	95.319	101.580	1.00	0.00
ATOM	9467	HE1	TYR	A	626	129.605	96.057	101.210	1.00	0.00
ATOM	9468	CZ	TYR	A	626	130.884	94.403	100.678	1.00	0.00
ATOM	9469	OH	TYR	A	626	130.588	94.473	99.355	1.00	0.00
ATOM	9470	HH	TYR	A	626	130.995	93.770	98.849	1.00	0.00
ATOM	9471	CE2	TYR	A	626	131.789	93.429	101.153	1.00	0.00
ATOM	9472	HE2	TYR	A	626	132.245	92.735	100.465	1.00	0.00
ATOM	9473	CD2	TYR	A	626	132.085	93.359	102.527	1.00	0.00
ATOM	9474	HD2	TYR	A	626	132.766	92.602	102.899	1.00	0.00
ATOM	9475	C	TYR	A	626	129.500	94.095	106.194	1.00	0.00
ATOM	9476	O	TYR	A	626	128.370	93.823	105.792	1.00	0.00
ATOM	9477	N	GLY	A	627	129.694	95.095	107.058	1.00	0.00
ATOM	9478	H	GLY	A	627	130.630	95.313	107.358	1.00	0.00
ATOM	9479	CA	GLY	A	627	128.596	95.991	107.424	1.00	0.00
ATOM	9480	HA1	GLY	A	627	128.925	96.710	108.173	1.00	0.00
ATOM	9481	HA2	GLY	A	627	127.778	95.412	107.838	1.00	0.00
ATOM	9482	C	GLY	A	627	128.047	96.764	106.213	1.00	0.00
ATOM	9483	O	GLY	A	627	128.800	97.316	105.416	1.00	0.00
ATOM	9484	N	CYS	A	628	126.722	96.800	106.100	1.00	0.00
ATOM	9485	H	CYS	A	628	126.186	96.321	106.804	1.00	0.00
ATOM	9486	CA	CYS	A	628	125.973	97.634	105.169	1.00	0.00
ATOM	9487	HA	CYS	A	628	126.465	97.625	104.193	1.00	0.00
ATOM	9488	CB	CYS	A	628	124.575	96.995	105.055	1.00	0.00
ATOM	9489	HB1	CYS	A	628	123.904	97.447	105.787	1.00	0.00
ATOM	9490	HB2	CYS	A	628	124.680	95.933	105.262	1.00	0.00
ATOM	9491	SG	CYS	A	628	123.806	97.065	103.431	1.00	0.00
ATOM	9492	HG	CYS	A	628	122.588	97.500	103.806	1.00	0.00
ATOM	9493	C	CYS	A	628	125.896	99.082	105.698	1.00	0.00
ATOM	9494	O	CYS	A	628	126.004	99.317	106.901	1.00	0.00
ATOM	9495	N	THR	A	629	125.633	100.055	104.826	1.00	0.00
ATOM	9496	H	THR	A	629	125.660	99.821	103.837	1.00	0.00
ATOM	9497	CA	THR	A	629	125.417	101.471	105.190	1.00	0.00
ATOM	9498	HA	THR	A	629	125.340	101.549	106.273	1.00	0.00
ATOM	9499	CB	THR	A	629	126.591	102.373	104.759	1.00	0.00
ATOM	9500	HB	THR	A	629	126.443	103.358	105.202	1.00	0.00
ATOM	9501	CG2	THR	A	629	127.957	101.852	105.212	1.00	0.00
ATOM	9502	1HG2	THR	A	629	128.711	102.616	105.026	1.00	0.00
ATOM	9503	2HG2	THR	A	629	127.928	101.621	106.277	1.00	0.00
ATOM	9504	3HG2	THR	A	629	128.221	100.953	104.654	1.00	0.00
ATOM	9505	OG1	THR	A	629	126.612	102.516	103.359	1.00	0.00
ATOM	9506	HG1	THR	A	629	125.751	102.882	103.119	1.00	0.00
ATOM	9507	C	THR	A	629	124.087	101.984	104.631	1.00	0.00
ATOM	9508	O	THR	A	629	123.980	103.105	104.138	1.00	0.00
ATOM	9509	N	GLY	A	630	123.090	101.103	104.617	1.00	0.00
ATOM	9510	H	GLY	A	630	123.251	100.195	105.035	1.00	0.00
ATOM	9511	CA	GLY	A	630	121.773	101.296	104.024	1.00	0.00
ATOM	9512	HA1	GLY	A	630	121.880	101.677	103.007	1.00	0.00
ATOM	9513	HA2	GLY	A	630	121.224	102.027	104.617	1.00	0.00
ATOM	9514	C	GLY	A	630	120.985	99.976	103.989	1.00	0.00

ATOM	9515	O	GLY	A	630	121.486	98.956	104.477	1.00	0.00
ATOM	9516	N	PRO	A	631	119.769	99.989	103.415	1.00	0.00
ATOM	9517	CD	PRO	A	631	119.064	101.205	103.026	1.00	0.00
ATOM	9518	HD1	PRO	A	631	119.403	101.521	102.038	1.00	0.00
ATOM	9519	HD2	PRO	A	631	119.203	102.007	103.751	1.00	0.00
ATOM	9520	CG	PRO	A	631	117.589	100.828	102.972	1.00	0.00
ATOM	9521	HG1	PRO	A	631	117.035	101.453	102.271	1.00	0.00
ATOM	9522	HG2	PRO	A	631	117.155	100.885	103.972	1.00	0.00
ATOM	9523	CB	PRO	A	631	117.650	99.378	102.516	1.00	0.00
ATOM	9524	HB1	PRO	A	631	117.773	99.359	101.433	1.00	0.00
ATOM	9525	HB2	PRO	A	631	116.751	98.831	102.804	1.00	0.00
ATOM	9526	CA	PRO	A	631	118.908	98.820	103.205	1.00	0.00
ATOM	9527	HA	PRO	A	631	118.618	98.444	104.186	1.00	0.00
ATOM	9528	C	PRO	A	631	119.495	97.633	102.401	1.00	0.00
ATOM	9529	O	PRO	A	631	120.696	97.538	102.128	1.00	0.00
ATOM	9530	N	GLY	A	632	118.617	96.679	102.079	1.00	0.00
ATOM	9531	H	GLY	A	632	117.647	96.940	102.109	1.00	0.00
ATOM	9532	CA	GLY	A	632	118.894	95.261	101.866	1.00	0.00
ATOM	9533	HA1	GLY	A	632	117.967	94.702	101.992	1.00	0.00
ATOM	9534	HA2	GLY	A	632	119.579	94.970	102.660	1.00	0.00
ATOM	9535	C	GLY	A	632	119.535	94.797	100.555	1.00	0.00
ATOM	9536	O	GLY	A	632	119.175	93.727	100.069	1.00	0.00
ATOM	9537	N	LEU	A	633	120.498	95.579	100.071	1.00	0.00
ATOM	9538	H	LEU	A	633	120.618	96.431	100.607	1.00	0.00
ATOM	9539	CA	LEU	A	633	121.431	95.420	98.941	1.00	0.00
ATOM	9540	HA	LEU	A	633	122.282	94.824	99.261	1.00	0.00
ATOM	9541	CB	LEU	A	633	120.755	94.721	97.734	1.00	0.00
ATOM	9542	HB1	LEU	A	633	119.800	95.208	97.521	1.00	0.00
ATOM	9543	HB2	LEU	A	633	120.539	93.699	98.041	1.00	0.00
ATOM	9544	CG	LEU	A	633	121.580	94.640	96.431	1.00	0.00
ATOM	9545	HG	LEU	A	633	122.633	94.498	96.676	1.00	0.00
ATOM	9546	CD1	LEU	A	633	121.116	93.437	95.601	1.00	0.00
ATOM	9547	1HD1	LEU	A	633	121.706	93.364	94.689	1.00	0.00
ATOM	9548	2HD1	LEU	A	633	121.249	92.519	96.173	1.00	0.00
ATOM	9549	3HD1	LEU	A	633	120.062	93.548	95.345	1.00	0.00
ATOM	9550	CD2	LEU	A	633	121.425	95.868	95.524	1.00	0.00
ATOM	9551	1HD2	LEU	A	633	121.979	95.718	94.600	1.00	0.00
ATOM	9552	2HD2	LEU	A	633	120.372	96.032	95.296	1.00	0.00
ATOM	9553	3HD2	LEU	A	633	121.823	96.756	96.006	1.00	0.00
ATOM	9554	C	LEU	A	633	121.964	96.807	98.587	1.00	0.00
ATOM	9555	O	LEU	A	633	123.157	96.978	98.344	1.00	0.00
ATOM	9556	N	GLU	A	634	121.094	97.814	98.650	1.00	0.00
ATOM	9557	H	GLU	A	634	120.133	97.594	98.922	1.00	0.00
ATOM	9558	CA	GLU	A	634	121.381	99.211	98.326	1.00	0.00
ATOM	9559	HA	GLU	A	634	121.769	99.260	97.308	1.00	0.00
ATOM	9560	CB	GLU	A	634	120.093	100.058	98.398	1.00	0.00
ATOM	9561	HB1	GLU	A	634	120.344	101.057	98.040	1.00	0.00
ATOM	9562	HB2	GLU	A	634	119.782	100.163	99.439	1.00	0.00
ATOM	9563	CG	GLU	A	634	118.911	99.549	97.551	1.00	0.00
ATOM	9564	HG1	GLU	A	634	119.279	99.293	96.555	1.00	0.00
ATOM	9565	HG2	GLU	A	634	118.205	100.374	97.437	1.00	0.00
ATOM	9566	CD	GLU	A	634	118.149	98.351	98.151	1.00	0.00
ATOM	9567	OE1	GLU	A	634	117.312	97.782	97.420	1.00	0.00
ATOM	9568	OE2	GLU	A	634	118.409	98.005	99.330	1.00	0.00
ATOM	9569	C	GLU	A	634	122.435	99.844	99.247	1.00	0.00
ATOM	9570	O	GLU	A	634	123.101	100.802	98.865	1.00	0.00
ATOM	9571	N	GLY	A	635	122.604	99.303	100.457	1.00	0.00
ATOM	9572	H	GLY	A	635	121.978	98.555	100.735	1.00	0.00
ATOM	9573	CA	GLY	A	635	123.637	99.717	101.403	1.00	0.00
ATOM	9574	HA1	GLY	A	635	123.264	99.546	102.407	1.00	0.00
ATOM	9575	HA2	GLY	A	635	123.825	100.785	101.292	1.00	0.00

ATOM	9576	C	GLY	A	635	124.985	98.991	101.278	1.00	0.00
ATOM	9577	O	GLY	A	635	125.861	99.243	102.103	1.00	0.00
ATOM	9578	N	CYS	A	636	125.154	98.083	100.310	1.00	0.00
ATOM	9579	H	CYS	A	636	124.388	97.926	99.664	1.00	0.00
ATOM	9580	CA	CYS	A	636	126.285	97.150	100.242	1.00	0.00
ATOM	9581	HA	CYS	A	636	127.003	97.390	101.028	1.00	0.00
ATOM	9582	CB	CYS	A	636	125.744	95.741	100.531	1.00	0.00
ATOM	9583	HB1	CYS	A	636	125.204	95.399	99.647	1.00	0.00
ATOM	9584	HB2	CYS	A	636	125.033	95.792	101.353	1.00	0.00
ATOM	9585	SG	CYS	A	636	126.994	94.498	100.960	1.00	0.00
ATOM	9586	C	CYS	A	636	127.038	97.246	98.896	1.00	0.00
ATOM	9587	O	CYS	A	636	127.129	96.264	98.154	1.00	0.00
ATOM	9588	N	PRO	A	637	127.551	98.433	98.516	1.00	0.00
ATOM	9589	CD	PRO	A	637	127.613	99.662	99.298	1.00	0.00
ATOM	9590	HD1	PRO	A	637	127.963	99.477	100.314	1.00	0.00
ATOM	9591	HD2	PRO	A	637	126.630	100.133	99.315	1.00	0.00
ATOM	9592	CG	PRO	A	637	128.590	100.578	98.562	1.00	0.00
ATOM	9593	HG1	PRO	A	637	129.607	100.380	98.906	1.00	0.00
ATOM	9594	HG2	PRO	A	637	128.336	101.630	98.696	1.00	0.00
ATOM	9595	CB	PRO	A	637	128.435	100.132	97.109	1.00	0.00
ATOM	9596	HB1	PRO	A	637	129.319	100.373	96.516	1.00	0.00
ATOM	9597	HB2	PRO	A	637	127.551	100.604	96.678	1.00	0.00
ATOM	9598	CA	PRO	A	637	128.201	98.621	97.224	1.00	0.00
ATOM	9599	HA	PRO	A	637	127.531	98.296	96.428	1.00	0.00
ATOM	9600	C	PRO	A	637	129.520	97.845	97.120	1.00	0.00
ATOM	9601	O	PRO	A	637	130.286	97.736	98.081	1.00	0.00
ATOM	9602	N	THR	A	638	129.823	97.412	95.894	1.00	0.00
ATOM	9603	H	THR	A	638	129.116	97.469	95.177	1.00	0.00
ATOM	9604	CA	THR	A	638	131.115	96.852	95.484	1.00	0.00
ATOM	9605	HA	THR	A	638	131.262	95.916	96.020	1.00	0.00
ATOM	9606	CB	THR	A	638	131.114	96.541	93.977	1.00	0.00
ATOM	9607	HB	THR	A	638	131.122	97.473	93.412	1.00	0.00
ATOM	9608	CG2	THR	A	638	132.304	95.679	93.565	1.00	0.00
ATOM	9609	1HG2	THR	A	638	132.262	95.477	92.496	1.00	0.00
ATOM	9610	2HG2	THR	A	638	133.236	96.204	93.779	1.00	0.00
ATOM	9611	3HG2	THR	A	638	132.291	94.735	94.109	1.00	0.00
ATOM	9612	OG1	THR	A	638	129.948	95.820	93.644	1.00	0.00
ATOM	9613	HG1	THR	A	638	129.983	95.631	92.703	1.00	0.00
ATOM	9614	C	THR	A	638	132.249	97.808	95.840	1.00	0.00
ATOM	9615	O	THR	A	638	132.427	98.840	95.198	1.00	0.00
ATOM	9616	N	ASN	A	639	133.003	97.473	96.887	1.00	0.00
ATOM	9617	H	ASN	A	639	132.795	96.612	97.370	1.00	0.00
ATOM	9618	CA	ASN	A	639	134.013	98.340	97.489	1.00	0.00
ATOM	9619	HA	ASN	A	639	134.230	99.152	96.794	1.00	0.00
ATOM	9620	CB	ASN	A	639	133.443	98.976	98.768	1.00	0.00
ATOM	9621	HB1	ASN	A	639	132.433	99.342	98.589	1.00	0.00
ATOM	9622	HB2	ASN	A	639	134.056	99.828	99.051	1.00	0.00
ATOM	9623	CG	ASN	A	639	133.434	98.014	99.936	1.00	0.00
ATOM	9624	OD1	ASN	A	639	134.393	97.932	100.687	1.00	0.00
ATOM	9625	ND2	ASN	A	639	132.380	97.259	100.097	1.00	0.00
ATOM	9626	1HD2	ASN	A	639	131.585	97.348	99.468	1.00	0.00
ATOM	9627	2HD2	ASN	A	639	132.352	96.640	100.889	1.00	0.00
ATOM	9628	C	ASN	A	639	135.340	97.592	97.711	1.00	0.00
ATOM	9629	O	ASN	A	639	135.439	96.389	97.472	1.00	0.00
ATOM	9630	N	GLY	A	640	136.362	98.314	98.170	1.00	0.00
ATOM	9631	H	GLY	A	640	136.191	99.283	98.390	1.00	0.00
ATOM	9632	CA	GLY	A	640	137.720	97.805	98.364	1.00	0.00
ATOM	9633	HA1	GLY	A	640	138.384	98.305	97.660	1.00	0.00
ATOM	9634	HA2	GLY	A	640	137.767	96.736	98.161	1.00	0.00
ATOM	9635	C	GLY	A	640	138.223	98.076	99.783	1.00	0.00
ATOM	9636	O	GLY	A	640	139.014	99.005	99.932	1.00	0.00

ATOM	9637	N	PRO	A	641	137.758	97.328	100.809	1.00	0.00
ATOM	9638	CD	PRO	A	641	137.138	96.015	100.636	1.00	0.00
ATOM	9639	HD1	PRO	A	641	137.883	95.307	100.272	1.00	0.00
ATOM	9640	HD2	PRO	A	641	136.297	96.058	99.944	1.00	0.00
ATOM	9641	CG	PRO	A	641	136.635	95.566	102.011	1.00	0.00
ATOM	9642	HG1	PRO	A	641	136.827	94.507	102.184	1.00	0.00
ATOM	9643	HG2	PRO	A	641	135.568	95.776	102.093	1.00	0.00
ATOM	9644	CB	PRO	A	641	137.405	96.448	102.990	1.00	0.00
ATOM	9645	HB1	PRO	A	641	138.393	96.024	103.179	1.00	0.00
ATOM	9646	HB2	PRO	A	641	136.857	96.590	103.921	1.00	0.00
ATOM	9647	CA	PRO	A	641	137.531	97.749	102.202	1.00	0.00
ATOM	9648	HA	PRO	A	641	136.544	98.213	102.210	1.00	0.00
ATOM	9649	C	PRO	A	641	138.456	98.768	102.898	1.00	0.00
ATOM	9650	O	PRO	A	641	138.004	99.528	103.752	1.00	0.00
ATOM	9651	N	LYS	A	642	139.734	98.860	102.531	1.00	0.00
ATOM	9652	H	LYS	A	642	140.018	98.264	101.767	1.00	0.00
ATOM	9653	CA	LYS	A	642	140.606	99.996	102.868	1.00	0.00
ATOM	9654	HA	LYS	A	642	140.453	100.248	103.919	1.00	0.00
ATOM	9655	CB	LYS	A	642	142.067	99.544	102.668	1.00	0.00
ATOM	9656	HB1	LYS	A	642	142.243	99.390	101.603	1.00	0.00
ATOM	9657	HB2	LYS	A	642	142.215	98.580	103.161	1.00	0.00
ATOM	9658	CG	LYS	A	642	143.098	100.530	103.252	1.00	0.00
ATOM	9659	HG1	LYS	A	642	143.237	100.282	104.305	1.00	0.00
ATOM	9660	HG2	LYS	A	642	142.732	101.556	103.209	1.00	0.00
ATOM	9661	CD	LYS	A	642	144.460	100.453	102.538	1.00	0.00
ATOM	9662	HD1	LYS	A	642	144.634	99.441	102.167	1.00	0.00
ATOM	9663	HD2	LYS	A	642	145.233	100.660	103.282	1.00	0.00
ATOM	9664	CE	LYS	A	642	144.632	101.480	101.403	1.00	0.00
ATOM	9665	HE1	LYS	A	642	145.650	101.387	101.013	1.00	0.00
ATOM	9666	HE2	LYS	A	642	144.539	102.482	101.833	1.00	0.00
ATOM	9667	NZ	LYS	A	642	143.655	101.321	100.296	1.00	0.00
ATOM	9668	HZ1	LYS	A	642	143.820	102.003	99.567	1.00	0.00
ATOM	9669	HZ2	LYS	A	642	143.691	100.395	99.895	1.00	0.00
ATOM	9670	HZ3	LYS	A	642	142.704	101.471	100.637	1.00	0.00
ATOM	9671	C	LYS	A	642	140.247	101.249	102.034	1.00	0.00
ATOM	9672	O	LYS	A	642	141.067	101.686	101.219	1.00	0.00
ATOM	9673	N	ILE	A	643	139.042	101.808	102.199	1.00	0.00
ATOM	9674	H	ILE	A	643	138.429	101.383	102.889	1.00	0.00
ATOM	9675	CA	ILE	A	643	138.424	102.733	101.224	1.00	0.00
ATOM	9676	HA	ILE	A	643	139.162	102.898	100.440	1.00	0.00
ATOM	9677	CB	ILE	A	643	137.264	102.038	100.464	1.00	0.00
ATOM	9678	HB	ILE	A	643	137.682	101.130	100.036	1.00	0.00
ATOM	9679	CG2	ILE	A	643	136.092	101.592	101.359	1.00	0.00
ATOM	9680	1HG2	ILE	A	643	135.439	100.911	100.817	1.00	0.00
ATOM	9681	2HG2	ILE	A	643	136.454	101.068	102.241	1.00	0.00
ATOM	9682	3HG2	ILE	A	643	135.504	102.450	101.680	1.00	0.00
ATOM	9683	CG1	ILE	A	643	136.806	102.904	99.268	1.00	0.00
ATOM	9684	1HG1	ILE	A	643	137.685	103.211	98.699	1.00	0.00
ATOM	9685	2HG1	ILE	A	643	136.304	103.802	99.626	1.00	0.00
ATOM	9686	CD	ILE	A	643	135.860	102.186	98.300	1.00	0.00
ATOM	9687	HD1	ILE	A	643	135.680	102.823	97.435	1.00	0.00
ATOM	9688	HD2	ILE	A	643	136.307	101.252	97.963	1.00	0.00
ATOM	9689	HD3	ILE	A	643	134.906	101.990	98.785	1.00	0.00
ATOM	9690	C	ILE	A	643	138.109	104.157	101.739	1.00	0.00
ATOM	9691	O	ILE	A	643	136.970	104.476	102.070	1.00	0.00
ATOM	9692	N	PRO	A	644	139.112	105.052	101.793	1.00	0.00
ATOM	9693	CD	PRO	A	644	140.539	104.754	101.723	1.00	0.00
ATOM	9694	HD1	PRO	A	644	140.819	104.393	100.734	1.00	0.00
ATOM	9695	HD2	PRO	A	644	140.791	104.018	102.486	1.00	0.00
ATOM	9696	CG	PRO	A	644	141.275	106.056	102.032	1.00	0.00
ATOM	9697	HG1	PRO	A	644	141.571	106.546	101.104	1.00	0.00

```
ATOM   9698  HG2 PRO A 644     142.147 105.885 102.663  1.00  0.00
ATOM   9699  CB  PRO A 644     140.211 106.883 102.753  1.00  0.00
ATOM   9700  HB1 PRO A 644     140.391 107.949 102.653  1.00  0.00
ATOM   9701  HB2 PRO A 644     140.185 106.595 103.804  1.00  0.00
ATOM   9702  CA  PRO A 644     138.900 106.465 102.097  1.00  0.00
ATOM   9703  HA  PRO A 644     138.094 106.575 102.818  1.00  0.00
ATOM   9704  C   PRO A 644     138.600 107.328 100.852  1.00  0.00
ATOM   9705  O   PRO A 644     139.426 107.444  99.948  1.00  0.00
ATOM   9706  N   SER A 645     137.449 108.006 100.835  1.00  0.00
ATOM   9707  H   SER A 645     136.777 107.778 101.553  1.00  0.00
ATOM   9708  CA  SER A 645     136.973 108.936  99.787  1.00  0.00
ATOM   9709  HA  SER A 645     137.095 108.442  98.822  1.00  0.00
ATOM   9710  CB  SER A 645     135.468 109.205  99.981  1.00  0.00
ATOM   9711  HB1 SER A 645     135.087 109.757  99.120  1.00  0.00
ATOM   9712  HB2 SER A 645     135.319 109.807 100.878  1.00  0.00
ATOM   9713  OG  SER A 645     134.745 107.997 100.115  1.00  0.00
ATOM   9714  HG  SER A 645     133.803 108.191 100.088  1.00  0.00
ATOM   9715  C   SER A 645     137.732 110.282  99.705  1.00  0.00
ATOM   9716  O   SER A 645     137.109 111.342  99.670  1.00  0.00
ATOM   9717  N   ILE A 646     139.072 110.272  99.749  1.00  0.00
ATOM   9718  H   ILE A 646     139.515 109.359  99.734  1.00  0.00
ATOM   9719  CA  ILE A 646     139.914 111.447 100.066  1.00  0.00
ATOM   9720  HA  ILE A 646     139.749 111.657 101.114  1.00  0.00
ATOM   9721  CB  ILE A 646     141.418 111.111  99.899  1.00  0.00
ATOM   9722  HB  ILE A 646     141.564 110.712  98.893  1.00  0.00
ATOM   9723  CG2 ILE A 646     142.310 112.360 100.029  1.00  0.00
ATOM   9724 1HG2 ILE A 646     143.362 112.072 100.060  1.00  0.00
ATOM   9725 2HG2 ILE A 646     142.173 113.019  99.171  1.00  0.00
ATOM   9726 3HG2 ILE A 646     142.061 112.914 100.932  1.00  0.00
ATOM   9727  CG1 ILE A 646     141.898 110.034 100.898  1.00  0.00
ATOM   9728 1HG1 ILE A 646     142.964 109.865 100.737  1.00  0.00
ATOM   9729 2HG1 ILE A 646     141.396 109.094 100.678  1.00  0.00
ATOM   9730  CD  ILE A 646     141.684 110.362 102.384  1.00  0.00
ATOM   9731  HD1 ILE A 646     142.219 109.635 102.989  1.00  0.00
ATOM   9732  HD2 ILE A 646     142.069 111.349 102.626  1.00  0.00
ATOM   9733  HD3 ILE A 646     140.625 110.318 102.634  1.00  0.00
ATOM   9734  C   ILE A 646     139.536 112.752  99.344  1.00  0.00
ATOM   9735  O   ILE A 646     139.304 113.774  99.997  1.00  0.00
ATOM   9736  N   ALA A 647     139.424 112.723  98.015  1.00  0.00
ATOM   9737  H   ALA A 647     139.574 111.846  97.542  1.00  0.00
ATOM   9738  CA  ALA A 647     139.125 113.898  97.187  1.00  0.00
ATOM   9739  HA  ALA A 647     139.785 114.709  97.498  1.00  0.00
ATOM   9740  CB  ALA A 647     139.464 113.538  95.733  1.00  0.00
ATOM   9741  HB1 ALA A 647     139.328 114.414  95.098  1.00  0.00
ATOM   9742  HB2 ALA A 647     140.502 113.212  95.659  1.00  0.00
ATOM   9743  HB3 ALA A 647     138.805 112.744  95.380  1.00  0.00
ATOM   9744  C   ALA A 647     137.679 114.432  97.317  1.00  0.00
ATOM   9745  O   ALA A 647     137.255 115.289  96.545  1.00  0.00
ATOM   9746  N   THR A 648     136.900 113.924  98.273  1.00  0.00
ATOM   9747  H   THR A 648     137.267 113.152  98.817  1.00  0.00
ATOM   9748  CA  THR A 648     135.501 114.304  98.525  1.00  0.00
ATOM   9749  HA  THR A 648     135.326 115.297  98.109  1.00  0.00
ATOM   9750  CB  THR A 648     134.526 113.315  97.850  1.00  0.00
ATOM   9751  HB  THR A 648     134.368 112.450  98.494  1.00  0.00
ATOM   9752  CG2 THR A 648     133.178 113.955  97.510  1.00  0.00
ATOM   9753 1HG2 THR A 648     132.542 113.217  97.021  1.00  0.00
ATOM   9754 2HG2 THR A 648     132.684 114.292  98.420  1.00  0.00
ATOM   9755 3HG2 THR A 648     133.321 114.805  96.843  1.00  0.00
ATOM   9756  OG1 THR A 648     135.041 112.858  96.617  1.00  0.00
ATOM   9757  HG1 THR A 648     135.446 113.621  96.192  1.00  0.00
ATOM   9758  C   THR A 648     135.253 114.406 100.041  1.00  0.00
```

ATOM	9759	O	THR	A	648	134.149	114.167	100.519	1.00	0.00
ATOM	9760	N	GLY	A	649	136.293	114.722	100.831	1.00	0.00
ATOM	9761	H	GLY	A	649	137.210	114.768	100.407	1.00	0.00
ATOM	9762	CA	GLY	A	649	136.162	114.814	102.294	1.00	0.00
ATOM	9763	HA1	GLY	A	649	136.342	113.831	102.716	1.00	0.00
ATOM	9764	HA2	GLY	A	649	135.131	115.070	102.539	1.00	0.00
ATOM	9765	C	GLY	A	649	137.029	115.839	103.047	1.00	0.00
ATOM	9766	O	GLY	A	649	136.799	116.048	104.238	1.00	0.00
ATOM	9767	N	MET	A	650	138.008	116.458	102.371	1.00	0.00
ATOM	9768	H	MET	A	650	138.135	116.138	101.422	1.00	0.00
ATOM	9769	CA	MET	A	650	138.787	117.675	102.703	1.00	0.00
ATOM	9770	HA	MET	A	650	138.492	118.410	101.954	1.00	0.00
ATOM	9771	CB	MET	A	650	138.525	118.344	104.072	1.00	0.00
ATOM	9772	HB1	MET	A	650	139.326	119.060	104.256	1.00	0.00
ATOM	9773	HB2	MET	A	650	138.590	117.609	104.873	1.00	0.00
ATOM	9774	CG	MET	A	650	137.206	119.131	104.166	1.00	0.00
ATOM	9775	HG1	MET	A	650	137.182	119.619	105.139	1.00	0.00
ATOM	9776	HG2	MET	A	650	136.369	118.437	104.131	1.00	0.00
ATOM	9777	SD	MET	A	650	136.899	120.407	102.907	1.00	0.00
ATOM	9778	CE	MET	A	650	138.426	121.385	102.974	1.00	0.00
ATOM	9779	HE1	MET	A	650	138.323	122.255	102.327	1.00	0.00
ATOM	9780	HE2	MET	A	650	138.611	121.714	103.995	1.00	0.00
ATOM	9781	HE3	MET	A	650	139.266	120.786	102.622	1.00	0.00
ATOM	9782	C	MET	A	650	140.302	117.546	102.492	1.00	0.00
ATOM	9783	O	MET	A	650	140.889	118.452	101.915	1.00	0.00
ATOM	9784	N	VAL	A	651	140.954	116.473	102.954	1.00	0.00
ATOM	9785	H	VAL	A	651	140.438	115.721	103.387	1.00	0.00
ATOM	9786	CA	VAL	A	651	142.423	116.314	102.851	1.00	0.00
ATOM	9787	HA	VAL	A	651	142.714	116.629	101.849	1.00	0.00
ATOM	9788	CB	VAL	A	651	143.213	117.184	103.878	1.00	0.00
ATOM	9789	HB	VAL	A	651	143.936	116.552	104.388	1.00	0.00
ATOM	9790	CG1	VAL	A	651	144.041	118.250	103.152	1.00	0.00
ATOM	9791	1HG1	VAL	A	651	144.659	118.792	103.867	1.00	0.00
ATOM	9792	2HG1	VAL	A	651	144.690	117.782	102.414	1.00	0.00
ATOM	9793	3HG1	VAL	A	651	143.384	118.959	102.646	1.00	0.00
ATOM	9794	CG2	VAL	A	651	142.390	117.898	104.965	1.00	0.00
ATOM	9795	1HG2	VAL	A	651	143.066	118.334	105.699	1.00	0.00
ATOM	9796	2HG2	VAL	A	651	141.787	118.692	104.527	1.00	0.00
ATOM	9797	3HG2	VAL	A	651	141.746	117.183	105.475	1.00	0.00
ATOM	9798	C	VAL	A	651	142.840	114.848	102.968	1.00	0.00
ATOM	9799	O	VAL	A	651	142.025	114.019	103.338	1.00	0.00
ATOM	9800	N	GLY	A	652	144.099	114.501	102.668	1.00	0.00
ATOM	9801	H	GLY	A	652	144.721	115.194	102.282	1.00	0.00
ATOM	9802	CA	GLY	A	652	144.645	113.181	103.028	1.00	0.00
ATOM	9803	HA1	GLY	A	652	145.627	113.060	102.573	1.00	0.00
ATOM	9804	HA2	GLY	A	652	143.994	112.393	102.656	1.00	0.00
ATOM	9805	C	GLY	A	652	144.791	113.027	104.544	1.00	0.00
ATOM	9806	O	GLY	A	652	144.317	112.058	105.131	1.00	0.00
ATOM	9807	N	ALA	A	653	145.381	114.031	105.194	1.00	0.00
ATOM	9808	H	ALA	A	653	145.731	114.811	104.666	1.00	0.00
ATOM	9809	CA	ALA	A	653	145.330	114.162	106.647	1.00	0.00
ATOM	9810	HA	ALA	A	653	145.509	113.180	107.089	1.00	0.00
ATOM	9811	CB	ALA	A	653	146.482	115.081	107.073	1.00	0.00
ATOM	9812	HB1	ALA	A	653	146.553	115.109	108.160	1.00	0.00
ATOM	9813	HB2	ALA	A	653	147.423	114.692	106.683	1.00	0.00
ATOM	9814	HB3	ALA	A	653	146.321	116.088	106.686	1.00	0.00
ATOM	9815	C	ALA	A	653	143.953	114.656	107.133	1.00	0.00
ATOM	9816	O	ALA	A	653	143.155	115.177	106.361	1.00	0.00
ATOM	9817	N	LEU	A	654	143.668	114.503	108.431	1.00	0.00
ATOM	9818	H	LEU	A	654	144.371	114.073	109.012	1.00	0.00
ATOM	9819	CA	LEU	A	654	142.397	114.899	109.067	1.00	0.00

```
ATOM   9820  HA  LEU A 654     142.391 114.458 110.064  1.00  0.00
ATOM   9821  CB  LEU A 654     142.354 116.438 109.246  1.00  0.00
ATOM   9822  HB1 LEU A 654     141.449 116.645 109.802  1.00  0.00
ATOM   9823  HB2 LEU A 654     142.218 116.903 108.268  1.00  0.00
ATOM   9824  CG  LEU A 654     143.484 117.185 109.995  1.00  0.00
ATOM   9825  HG  LEU A 654     143.033 118.073 110.437  1.00  0.00
ATOM   9826  CD1 LEU A 654     144.125 116.391 111.138  1.00  0.00
ATOM   9827 1HD1 LEU A 654     144.771 117.052 111.717  1.00  0.00
ATOM   9828 2HD1 LEU A 654     143.356 115.984 111.791  1.00  0.00
ATOM   9829 3HD1 LEU A 654     144.726 115.572 110.745  1.00  0.00
ATOM   9830  CD2 LEU A 654     144.583 117.685 109.065  1.00  0.00
ATOM   9831 1HD2 LEU A 654     145.294 118.292 109.625  1.00  0.00
ATOM   9832 2HD2 LEU A 654     145.109 116.850 108.621  1.00  0.00
ATOM   9833 3HD2 LEU A 654     144.150 118.295 108.274  1.00  0.00
ATOM   9834  C   LEU A 654     141.118 114.354 108.374  1.00  0.00
ATOM   9835  O   LEU A 654     140.036 114.923 108.544  1.00  0.00
ATOM   9836  N   LEU A 655     141.233 113.253 107.622  1.00  0.00
ATOM   9837  H   LEU A 655     142.159 112.874 107.496  1.00  0.00
ATOM   9838  CA  LEU A 655     140.137 112.574 106.930  1.00  0.00
ATOM   9839  HA  LEU A 655     139.208 112.831 107.436  1.00  0.00
ATOM   9840  CB  LEU A 655     140.046 113.078 105.487  1.00  0.00
ATOM   9841  HB1 LEU A 655     140.955 112.747 104.993  1.00  0.00
ATOM   9842  HB2 LEU A 655     140.040 114.169 105.495  1.00  0.00
ATOM   9843  CG  LEU A 655     138.831 112.576 104.686  1.00  0.00
ATOM   9844  HG  LEU A 655     138.831 111.494 104.608  1.00  0.00
ATOM   9845  CD1 LEU A 655     137.502 112.980 105.319  1.00  0.00
ATOM   9846 1HD1 LEU A 655     136.676 112.726 104.657  1.00  0.00
ATOM   9847 2HD1 LEU A 655     137.354 112.460 106.263  1.00  0.00
ATOM   9848 3HD1 LEU A 655     137.496 114.049 105.511  1.00  0.00
ATOM   9849  CD2 LEU A 655     138.963 113.082 103.263  1.00  0.00
ATOM   9850 1HD2 LEU A 655     138.164 112.686 102.638  1.00  0.00
ATOM   9851 2HD2 LEU A 655     138.975 114.163 103.255  1.00  0.00
ATOM   9852 3HD2 LEU A 655     139.916 112.751 102.860  1.00  0.00
ATOM   9853  C   LEU A 655     140.292 111.042 107.047  1.00  0.00
ATOM   9854  O   LEU A 655     140.108 110.514 108.133  1.00  0.00
ATOM   9855  N   LEU A 656     140.533 110.316 105.947  1.00  0.00
ATOM   9856  H   LEU A 656     140.849 110.821 105.132  1.00  0.00
ATOM   9857  CA  LEU A 656     140.116 108.920 105.739  1.00  0.00
ATOM   9858  HA  LEU A 656     140.046 108.776 104.666  1.00  0.00
ATOM   9859  CB  LEU A 656     141.188 107.927 106.270  1.00  0.00
ATOM   9860  HB1 LEU A 656     140.742 106.933 106.258  1.00  0.00
ATOM   9861  HB2 LEU A 656     141.417 108.156 107.311  1.00  0.00
ATOM   9862  CG  LEU A 656     142.489 107.823 105.455  1.00  0.00
ATOM   9863  HG  LEU A 656     142.260 107.888 104.395  1.00  0.00
ATOM   9864  CD1 LEU A 656     143.494 108.914 105.811  1.00  0.00
ATOM   9865 1HD1 LEU A 656     144.401 108.800 105.219  1.00  0.00
ATOM   9866 2HD1 LEU A 656     143.075 109.892 105.594  1.00  0.00
ATOM   9867 3HD1 LEU A 656     143.749 108.866 106.868  1.00  0.00
ATOM   9868  CD2 LEU A 656     143.165 106.470 105.692  1.00  0.00
ATOM   9869 1HD2 LEU A 656     144.097 106.415 105.131  1.00  0.00
ATOM   9870 2HD2 LEU A 656     143.381 106.345 106.752  1.00  0.00
ATOM   9871 3HD2 LEU A 656     142.506 105.665 105.364  1.00  0.00
ATOM   9872  C   LEU A 656     138.689 108.587 106.271  1.00  0.00
ATOM   9873  O   LEU A 656     138.461 107.513 106.808  1.00  0.00
ATOM   9874  N   LEU A 657     137.732 109.514 106.122  1.00  0.00
ATOM   9875  H   LEU A 657     137.976 110.330 105.594  1.00  0.00
ATOM   9876  CA  LEU A 657     136.398 109.521 106.762  1.00  0.00
ATOM   9877  HA  LEU A 657     135.934 110.462 106.473  1.00  0.00
ATOM   9878  CB  LEU A 657     135.455 108.410 106.240  1.00  0.00
ATOM   9879  HB1 LEU A 657     134.539 108.462 106.829  1.00  0.00
ATOM   9880  HB2 LEU A 657     135.890 107.431 106.441  1.00  0.00
```

ATOM	9881	CG	LEU	A	657	134.993	108.471	104.771	1.00	0.00
ATOM	9882	HG	LEU	A	657	134.123	107.820	104.687	1.00	0.00
ATOM	9883	CD1	LEU	A	657	134.571	109.870	104.314	1.00	0.00
ATOM	9884	1HD1	LEU	A	657	134.119	109.805	103.325	1.00	0.00
ATOM	9885	2HD1	LEU	A	657	133.830	110.270	105.007	1.00	0.00
ATOM	9886	3HD1	LEU	A	657	135.428	110.539	104.267	1.00	0.00
ATOM	9887	CD2	LEU	A	657	136.034	107.951	103.790	1.00	0.00
ATOM	9888	1HD2	LEU	A	657	135.551	107.807	102.826	1.00	0.00
ATOM	9889	2HD2	LEU	A	657	136.865	108.646	103.705	1.00	0.00
ATOM	9890	3HD2	LEU	A	657	136.390	106.983	104.144	1.00	0.00
ATOM	9891	C	LEU	A	657	136.434	109.628	108.304	1.00	0.00
ATOM	9892	O	LEU	A	657	135.639	109.027	109.019	1.00	0.00
ATOM	9893	N	LEU	A	658	137.364	110.456	108.791	1.00	0.00
ATOM	9894	H	LEU	A	658	138.029	110.806	108.124	1.00	0.00
ATOM	9895	CA	LEU	A	658	137.734	110.635	110.195	1.00	0.00
ATOM	9896	HA	LEU	A	658	138.621	111.259	110.194	1.00	0.00
ATOM	9897	CB	LEU	A	658	136.654	111.398	110.992	1.00	0.00
ATOM	9898	HB1	LEU	A	658	137.039	111.569	111.999	1.00	0.00
ATOM	9899	HB2	LEU	A	658	135.771	110.764	111.085	1.00	0.00
ATOM	9900	CG	LEU	A	658	136.217	112.752	110.397	1.00	0.00
ATOM	9901	HG	LEU	A	658	135.709	112.585	109.447	1.00	0.00
ATOM	9902	CD1	LEU	A	658	135.244	113.439	111.357	1.00	0.00
ATOM	9903	1HD1	LEU	A	658	134.865	114.357	110.911	1.00	0.00
ATOM	9904	2HD1	LEU	A	658	134.400	112.777	111.553	1.00	0.00
ATOM	9905	3HD1	LEU	A	658	135.739	113.662	112.302	1.00	0.00
ATOM	9906	CD2	LEU	A	658	137.386	113.715	110.166	1.00	0.00
ATOM	9907	1HD2	LEU	A	658	137.005	114.681	109.841	1.00	0.00
ATOM	9908	2HD2	LEU	A	658	137.953	113.836	111.087	1.00	0.00
ATOM	9909	3HD2	LEU	A	658	138.041	113.321	109.393	1.00	0.00
ATOM	9910	C	LEU	A	658	138.191	109.311	110.836	1.00	0.00
ATOM	9911	O	LEU	A	658	137.622	108.831	111.817	1.00	0.00
ATOM	9912	N	VAL	A	659	139.236	108.713	110.252	1.00	0.00
ATOM	9913	H	VAL	A	659	139.643	109.185	109.452	1.00	0.00
ATOM	9914	CA	VAL	A	659	139.833	107.434	110.679	1.00	0.00
ATOM	9915	HA	VAL	A	659	139.334	107.136	111.600	1.00	0.00
ATOM	9916	CB	VAL	A	659	139.505	106.291	109.671	1.00	0.00
ATOM	9917	HB	VAL	A	659	139.954	106.522	108.708	1.00	0.00
ATOM	9918	CG1	VAL	A	659	139.958	104.877	110.095	1.00	0.00
ATOM	9919	1HG1	VAL	A	659	139.410	104.122	109.531	1.00	0.00
ATOM	9920	2HG1	VAL	A	659	141.006	104.702	109.872	1.00	0.00
ATOM	9921	3HG1	VAL	A	659	139.762	104.721	111.155	1.00	0.00
ATOM	9922	CG2	VAL	A	659	137.980	106.151	109.511	1.00	0.00
ATOM	9923	1HG2	VAL	A	659	137.743	105.415	108.744	1.00	0.00
ATOM	9924	2HG2	VAL	A	659	137.518	105.856	110.453	1.00	0.00
ATOM	9925	3HG2	VAL	A	659	137.532	107.089	109.192	1.00	0.00
ATOM	9926	C	VAL	A	659	141.296	107.645	111.106	1.00	0.00
ATOM	9927	O	VAL	A	659	141.568	108.636	111.772	1.00	0.00
ATOM	9928	N	VAL	A	660	142.217	106.702	110.897	1.00	0.00
ATOM	9929	H	VAL	A	660	141.931	105.936	110.312	1.00	0.00
ATOM	9930	CA	VAL	A	660	143.548	106.547	111.547	1.00	0.00
ATOM	9931	HA	VAL	A	660	143.809	105.503	111.374	1.00	0.00
ATOM	9932	CB	VAL	A	660	144.690	107.324	110.850	1.00	0.00
ATOM	9933	HB	VAL	A	660	145.631	106.971	111.273	1.00	0.00
ATOM	9934	CG1	VAL	A	660	144.730	107.017	109.348	1.00	0.00
ATOM	9935	1HG1	VAL	A	660	145.618	107.470	108.907	1.00	0.00
ATOM	9936	2HG1	VAL	A	660	144.773	105.940	109.190	1.00	0.00
ATOM	9937	3HG1	VAL	A	660	143.846	107.424	108.859	1.00	0.00
ATOM	9938	CG2	VAL	A	660	144.655	108.841	111.035	1.00	0.00
ATOM	9939	1HG2	VAL	A	660	145.529	109.285	110.561	1.00	0.00
ATOM	9940	2HG2	VAL	A	660	143.758	109.262	110.582	1.00	0.00
ATOM	9941	3HG2	VAL	A	660	144.684	109.073	112.097	1.00	0.00

ATOM	9942	C	VAL	A	660	143.612	106.644	113.095	1.00	0.00
ATOM	9943	O	VAL	A	660	144.608	106.219	113.670	1.00	0.00
ATOM	9944	N	ALA	A	661	142.576	107.133	113.798	1.00	0.00
ATOM	9945	H	ALA	A	661	141.855	107.597	113.261	1.00	0.00
ATOM	9946	CA	ALA	A	661	142.580	107.368	115.251	1.00	0.00
ATOM	9947	HA	ALA	A	661	143.066	106.519	115.732	1.00	0.00
ATOM	9948	CB	ALA	A	661	143.434	108.624	115.511	1.00	0.00
ATOM	9949	HB1	ALA	A	661	143.526	108.795	116.583	1.00	0.00
ATOM	9950	HB2	ALA	A	661	144.433	108.495	115.095	1.00	0.00
ATOM	9951	HB3	ALA	A	661	142.963	109.494	115.053	1.00	0.00
ATOM	9952	C	ALA	A	661	141.175	107.516	115.913	1.00	0.00
ATOM	9953	O	ALA	A	661	140.957	108.351	116.819	1.00	0.00
ATOM	9954	N	LEU	A	662	140.228	106.699	115.430	1.00	0.00
ATOM	9955	H	LEU	A	662	140.519	106.071	114.698	1.00	0.00
ATOM	9956	CA	LEU	A	662	138.790	106.742	115.732	1.00	0.00
ATOM	9957	HA	LEU	A	662	138.307	105.989	115.109	1.00	0.00
ATOM	9958	CB	LEU	A	662	138.562	106.347	117.214	1.00	0.00
ATOM	9959	HB1	LEU	A	662	139.095	107.053	117.851	1.00	0.00
ATOM	9960	HB2	LEU	A	662	139.007	105.365	117.377	1.00	0.00
ATOM	9961	CG	LEU	A	662	137.104	106.308	117.710	1.00	0.00
ATOM	9962	HG	LEU	A	662	136.649	107.286	117.571	1.00	0.00
ATOM	9963	CD1	LEU	A	662	136.261	105.263	116.978	1.00	0.00
ATOM	9964	1HD1	LEU	A	662	135.245	105.273	117.371	1.00	0.00
ATOM	9965	2HD1	LEU	A	662	136.220	105.467	115.912	1.00	0.00
ATOM	9966	3HD1	LEU	A	662	136.687	104.273	117.132	1.00	0.00
ATOM	9967	CD2	LEU	A	662	137.063	105.996	119.206	1.00	0.00
ATOM	9968	1HD2	LEU	A	662	136.033	106.003	119.562	1.00	0.00
ATOM	9969	2HD2	LEU	A	662	137.499	105.015	119.393	1.00	0.00
ATOM	9970	3HD2	LEU	A	662	137.628	106.746	119.758	1.00	0.00
ATOM	9971	C	LEU	A	662	138.157	108.103	115.343	1.00	0.00
ATOM	9972	O	LEU	A	662	138.801	109.151	115.368	1.00	0.00
ATOM	9973	N	GLY	A	663	136.857	108.103	115.047	1.00	0.00
ATOM	9974	H	GLY	A	663	136.376	107.224	114.953	1.00	0.00
ATOM	9975	CA	GLY	A	663	136.118	109.335	114.756	1.00	0.00
ATOM	9976	HA1	GLY	A	663	135.055	109.108	114.685	1.00	0.00
ATOM	9977	HA2	GLY	A	663	136.443	109.729	113.794	1.00	0.00
ATOM	9978	C	GLY	A	663	136.299	110.440	115.800	1.00	0.00
ATOM	9979	O	GLY	A	663	136.535	111.595	115.454	1.00	0.00
ATOM	9980	N	ILE	A	664	136.254	110.075	117.087	1.00	0.00
ATOM	9981	H	ILE	A	664	136.043	109.112	117.286	1.00	0.00
ATOM	9982	CA	ILE	A	664	136.473	110.988	118.215	1.00	0.00
ATOM	9983	HA	ILE	A	664	135.839	111.863	118.068	1.00	0.00
ATOM	9984	CB	ILE	A	664	136.071	110.316	119.555	1.00	0.00
ATOM	9985	HB	ILE	A	664	136.786	109.520	119.771	1.00	0.00
ATOM	9986	CG2	ILE	A	664	136.128	111.348	120.697	1.00	0.00
ATOM	9987	1HG2	ILE	A	664	135.938	110.873	121.658	1.00	0.00
ATOM	9988	2HG2	ILE	A	664	137.115	111.803	120.756	1.00	0.00
ATOM	9989	3HG2	ILE	A	664	135.389	112.132	120.533	1.00	0.00
ATOM	9990	CG1	ILE	A	664	134.657	109.682	119.487	1.00	0.00
ATOM	9991	1HG1	ILE	A	664	134.615	108.970	118.665	1.00	0.00
ATOM	9992	2HG1	ILE	A	664	133.920	110.461	119.293	1.00	0.00
ATOM	9993	CD	ILE	A	664	134.240	108.909	120.745	1.00	0.00
ATOM	9994	HD1	ILE	A	664	133.308	108.380	120.551	1.00	0.00
ATOM	9995	HD2	ILE	A	664	135.007	108.181	121.004	1.00	0.00
ATOM	9996	HD3	ILE	A	664	134.080	109.590	121.579	1.00	0.00
ATOM	9997	C	ILE	A	664	137.932	111.485	118.245	1.00	0.00
ATOM	9998	O	ILE	A	664	138.161	112.700	118.274	1.00	0.00
ATOM	9999	N	GLY	A	665	138.933	110.580	118.229	1.00	0.00
ATOM	10000	H	GLY	A	665	138.747	109.606	118.045	1.00	0.00
ATOM	10001	CA	GLY	A	665	140.294	111.024	118.515	1.00	0.00
ATOM	10002	HA1	GLY	A	665	140.931	110.151	118.652	1.00	0.00

```
ATOM  10003  HA2 GLY A 665     140.305 111.625 119.424  1.00  0.00
ATOM  10004  C   GLY A 665     140.845 111.854 117.358  1.00  0.00
ATOM  10005  O   GLY A 665     141.519 112.868 117.600  1.00  0.00
ATOM  10006  N   LEU A 666     140.526 111.464 116.116  1.00  0.00
ATOM  10007  H   LEU A 666     139.998 110.600 115.986  1.00  0.00
ATOM  10008  CA  LEU A 666     140.847 112.266 114.946  1.00  0.00
ATOM  10009  HA  LEU A 666     141.888 112.557 115.051  1.00  0.00
ATOM  10010  CB  LEU A 666     140.723 111.487 113.623  1.00  0.00
ATOM  10011  HB1 LEU A 666     139.683 111.504 113.292  1.00  0.00
ATOM  10012  HB2 LEU A 666     141.006 110.444 113.768  1.00  0.00
ATOM  10013  CG  LEU A 666     141.634 112.132 112.548  1.00  0.00
ATOM  10014  HG  LEU A 666     141.659 113.214 112.665  1.00  0.00
ATOM  10015  CD1 LEU A 666     143.074 111.637 112.699  1.00  0.00
ATOM  10016 1HD1 LEU A 666     143.697 112.052 111.909  1.00  0.00
ATOM  10017 2HD1 LEU A 666     143.480 111.937 113.662  1.00  0.00
ATOM  10018 3HD1 LEU A 666     143.079 110.553 112.628  1.00  0.00
ATOM  10019  CD2 LEU A 666     141.144 111.888 111.126  1.00  0.00
ATOM  10020 1HD2 LEU A 666     141.904 112.178 110.406  1.00  0.00
ATOM  10021 2HD2 LEU A 666     140.915 110.837 110.976  1.00  0.00
ATOM  10022 3HD2 LEU A 666     140.249 112.480 110.951  1.00  0.00
ATOM  10023  C   LEU A 666     140.039 113.565 114.854  1.00  0.00
ATOM  10024  O   LEU A 666     140.629 114.571 114.485  1.00  0.00
ATOM  10025  N   PHE A 667     138.753 113.627 115.217  1.00  0.00
ATOM  10026  H   PHE A 667     138.248 112.777 115.457  1.00  0.00
ATOM  10027  CA  PHE A 667     138.042 114.918 115.262  1.00  0.00
ATOM  10028  HA  PHE A 667     138.063 115.357 114.265  1.00  0.00
ATOM  10029  CB  PHE A 667     136.572 114.688 115.653  1.00  0.00
ATOM  10030  HB1 PHE A 667     136.552 114.034 116.525  1.00  0.00
ATOM  10031  HB2 PHE A 667     136.076 114.160 114.838  1.00  0.00
ATOM  10032  CG  PHE A 667     135.743 115.925 115.983  1.00  0.00
ATOM  10033  CD1 PHE A 667     134.828 115.879 117.053  1.00  0.00
ATOM  10034  HD1 PHE A 667     134.723 114.972 117.633  1.00  0.00
ATOM  10035  CE1 PHE A 667     134.003 116.983 117.332  1.00  0.00
ATOM  10036  HE1 PHE A 667     133.263 116.919 118.119  1.00  0.00
ATOM  10037  CZ  PHE A 667     134.096 118.148 116.552  1.00  0.00
ATOM  10038  HZ  PHE A 667     133.433 118.984 116.739  1.00  0.00
ATOM  10039  CE2 PHE A 667     135.017 118.208 115.491  1.00  0.00
ATOM  10040  HE2 PHE A 667     135.063 119.085 114.861  1.00  0.00
ATOM  10041  CD2 PHE A 667     135.831 117.096 115.200  1.00  0.00
ATOM  10042  HD2 PHE A 667     136.502 117.134 114.355  1.00  0.00
ATOM  10043  C   PHE A 667     138.706 115.923 116.219  1.00  0.00
ATOM  10044  O   PHE A 667     138.948 117.081 115.847  1.00  0.00
ATOM  10045  N   MET A 668     139.032 115.492 117.445  1.00  0.00
ATOM  10046  H   MET A 668     138.789 114.535 117.701  1.00  0.00
ATOM  10047  CA  MET A 668     139.722 116.346 118.426  1.00  0.00
ATOM  10048  HA  MET A 668     139.118 117.232 118.618  1.00  0.00
ATOM  10049  CB  MET A 668     139.896 115.569 119.739  1.00  0.00
ATOM  10050  HB1 MET A 668     140.522 116.151 120.416  1.00  0.00
ATOM  10051  HB2 MET A 668     140.396 114.620 119.536  1.00  0.00
ATOM  10052  CG  MET A 668     138.560 115.286 120.436  1.00  0.00
ATOM  10053  HG1 MET A 668     137.875 114.835 119.718  1.00  0.00
ATOM  10054  HG2 MET A 668     138.128 116.229 120.769  1.00  0.00
ATOM  10055  SD  MET A 668     138.676 114.161 121.851  1.00  0.00
ATOM  10056  CE  MET A 668     139.646 115.146 123.025  1.00  0.00
ATOM  10057  HE1 MET A 668     139.721 114.607 123.969  1.00  0.00
ATOM  10058  HE2 MET A 668     140.648 115.313 122.632  1.00  0.00
ATOM  10059  HE3 MET A 668     139.156 116.103 123.201  1.00  0.00
ATOM  10060  C   MET A 668     141.096 116.828 117.922  1.00  0.00
ATOM  10061  O   MET A 668     141.405 118.027 117.997  1.00  0.00
ATOM  10062  N   ARG A 669     141.888 115.906 117.343  1.00  0.00
ATOM  10063  H   ARG A 669     141.565 114.943 117.329  1.00  0.00
```

```
ATOM  10064  CA  ARG A 669     143.171 116.225 116.687  1.00  0.00
ATOM  10065  HA  ARG A 669     143.848 116.672 117.417  1.00  0.00
ATOM  10066  CB  ARG A 669     143.816 114.944 116.114  1.00  0.00
ATOM  10067  HB1 ARG A 669     144.604 115.225 115.413  1.00  0.00
ATOM  10068  HB2 ARG A 669     143.059 114.399 115.553  1.00  0.00
ATOM  10069  CG  ARG A 669     144.438 114.025 117.176  1.00  0.00
ATOM  10070  HG1 ARG A 669     143.799 113.989 118.056  1.00  0.00
ATOM  10071  HG2 ARG A 669     145.406 114.427 117.475  1.00  0.00
ATOM  10072  CD  ARG A 669     144.626 112.598 116.617  1.00  0.00
ATOM  10073  HD1 ARG A 669     145.211 112.662 115.697  1.00  0.00
ATOM  10074  HD2 ARG A 669     143.653 112.182 116.354  1.00  0.00
ATOM  10075  NE  ARG A 669     145.311 111.685 117.554  1.00  0.00
ATOM  10076  HE  ARG A 669     146.219 111.357 117.270  1.00  0.00
ATOM  10077  CZ  ARG A 669     144.843 111.193 118.686  1.00  0.00
ATOM  10078  NH1 ARG A 669     143.664 111.500 119.151  1.00  0.00
ATOM  10079 1HH1 ARG A 669     143.075 112.118 118.608  1.00  0.00
ATOM  10080 2HH1 ARG A 669     143.341 111.120 120.019  1.00  0.00
ATOM  10081  NH2 ARG A 669     145.572 110.375 119.390  1.00  0.00
ATOM  10082 1HH2 ARG A 669     146.489 110.123 119.070  1.00  0.00
ATOM  10083 2HH2 ARG A 669     145.232 110.004 120.258  1.00  0.00
ATOM  10084  C   ARG A 669     143.007 117.266 115.574  1.00  0.00
ATOM  10085  O   ARG A 669     143.676 118.288 115.621  1.00  0.00
ATOM  10086  N   ARG A 670     142.100 117.044 114.619  1.00  0.00
ATOM  10087  H   ARG A 670     141.587 116.170 114.687  1.00  0.00
ATOM  10088  CA  ARG A 670     141.769 117.924 113.484  1.00  0.00
ATOM  10089  HA  ARG A 670     142.638 118.006 112.832  1.00  0.00
ATOM  10090  CB  ARG A 670     140.592 117.296 112.692  1.00  0.00
ATOM  10091  HB1 ARG A 670     139.799 117.031 113.390  1.00  0.00
ATOM  10092  HB2 ARG A 670     140.942 116.368 112.236  1.00  0.00
ATOM  10093  CG  ARG A 670     139.962 118.182 111.596  1.00  0.00
ATOM  10094  HG1 ARG A 670     140.736 118.538 110.918  1.00  0.00
ATOM  10095  HG2 ARG A 670     139.491 119.048 112.064  1.00  0.00
ATOM  10096  CD  ARG A 670     138.889 117.414 110.793  1.00  0.00
ATOM  10097  HD1 ARG A 670     138.145 117.025 111.492  1.00  0.00
ATOM  10098  HD2 ARG A 670     139.350 116.557 110.304  1.00  0.00
ATOM  10099  NE  ARG A 670     138.202 118.278 109.805  1.00  0.00
ATOM  10100  HE  ARG A 670     137.952 119.199 110.126  1.00  0.00
ATOM  10101  CZ  ARG A 670     137.761 117.940 108.597  1.00  0.00
ATOM  10102  NH1 ARG A 670     138.057 116.832 107.977  1.00  0.00
ATOM  10103 1HH1 ARG A 670     138.726 116.178 108.381  1.00  0.00
ATOM  10104 2HH1 ARG A 670     137.666 116.610 107.080  1.00  0.00
ATOM  10105  NH2 ARG A 670     136.963 118.749 107.960  1.00  0.00
ATOM  10106 1HH2 ARG A 670     136.652 119.615 108.364  1.00  0.00
ATOM  10107 2HH2 ARG A 670     136.605 118.465 107.065  1.00  0.00
ATOM  10108  C   ARG A 670     141.464 119.341 113.945  1.00  0.00
ATOM  10109  O   ARG A 670     142.000 120.291 113.385  1.00  0.00
ATOM  10110  N   ARG A 671     140.632 119.475 114.980  1.00  0.00
ATOM  10111  H   ARG A 671     140.229 118.627 115.371  1.00  0.00
ATOM  10112  CA  ARG A 671     140.260 120.766 115.570  1.00  0.00
ATOM  10113  HA  ARG A 671     139.904 121.425 114.777  1.00  0.00
ATOM  10114  CB  ARG A 671     139.080 120.463 116.504  1.00  0.00
ATOM  10115  HB1 ARG A 671     139.392 119.752 117.271  1.00  0.00
ATOM  10116  HB2 ARG A 671     138.328 119.970 115.885  1.00  0.00
ATOM  10117  CG  ARG A 671     138.423 121.669 117.190  1.00  0.00
ATOM  10118  HG1 ARG A 671     138.552 122.560 116.574  1.00  0.00
ATOM  10119  HG2 ARG A 671     138.920 121.840 118.146  1.00  0.00
ATOM  10120  CD  ARG A 671     136.912 121.459 117.419  1.00  0.00
ATOM  10121  HD1 ARG A 671     136.393 121.693 116.487  1.00  0.00
ATOM  10122  HD2 ARG A 671     136.553 122.160 118.173  1.00  0.00
ATOM  10123  NE  ARG A 671     136.545 120.071 117.793  1.00  0.00
ATOM  10124  HE  ARG A 671     136.203 119.481 117.048  1.00  0.00
```

```
ATOM  10125  CZ  ARG A 671     136.628 119.497 118.979  1.00  0.00
ATOM  10126  NH1 ARG A 671     137.064 120.127 120.033  1.00  0.00
ATOM  10127 1HH1 ARG A 671     137.351 121.084 119.941  1.00  0.00
ATOM  10128 2HH1 ARG A 671     137.097 119.671 120.925  1.00  0.00
ATOM  10129  NH2 ARG A 671     136.253 118.258 119.126  1.00  0.00
ATOM  10130 1HH2 ARG A 671     135.827 117.771 118.348  1.00  0.00
ATOM  10131 2HH2 ARG A 671     136.238 117.836 120.034  1.00  0.00
ATOM  10132  C   ARG A 671     141.451 121.496 116.216  1.00  0.00
ATOM  10133  O   ARG A 671     141.704 122.654 115.880  1.00  0.00
ATOM  10134  N   HIS A 672     142.239 120.815 117.057  1.00  0.00
ATOM  10135  H   HIS A 672     141.994 119.855 117.278  1.00  0.00
ATOM  10136  CA  HIS A 672     143.446 121.404 117.679  1.00  0.00
ATOM  10137  HA  HIS A 672     143.148 122.298 118.229  1.00  0.00
ATOM  10138  CB  HIS A 672     144.022 120.393 118.693  1.00  0.00
ATOM  10139  HB1 HIS A 672     144.694 119.700 118.186  1.00  0.00
ATOM  10140  HB2 HIS A 672     143.205 119.799 119.105  1.00  0.00
ATOM  10141  CG  HIS A 672     144.745 121.014 119.870  1.00  0.00
ATOM  10142  ND1 HIS A 672     146.098 121.361 119.929  1.00  0.00
ATOM  10143  CE1 HIS A 672     146.304 121.824 121.176  1.00  0.00
ATOM  10144  HE1 HIS A 672     147.254 122.173 121.564  1.00  0.00
ATOM  10145  NE2 HIS A 672     145.173 121.766 121.896  1.00  0.00
ATOM  10146  HE2 HIS A 672     145.091 122.005 122.879  1.00  0.00
ATOM  10147  CD2 HIS A 672     144.180 121.254 121.090  1.00  0.00
ATOM  10148  HD2 HIS A 672     143.159 121.034 121.376  1.00  0.00
ATOM  10149  C   HIS A 672     144.513 121.847 116.654  1.00  0.00
ATOM  10150  O   HIS A 672     145.072 122.944 116.746  1.00  0.00
ATOM  10151  N   ILE A 673     144.764 121.001 115.653  1.00  0.00
ATOM  10152  H   ILE A 673     144.267 120.113 115.672  1.00  0.00
ATOM  10153  CA  ILE A 673     145.692 121.202 114.533  1.00  0.00
ATOM  10154  HA  ILE A 673     146.665 121.468 114.933  1.00  0.00
ATOM  10155  CB  ILE A 673     145.847 119.884 113.731  1.00  0.00
ATOM  10156  HB  ILE A 673     144.849 119.482 113.549  1.00  0.00
ATOM  10157  CG2 ILE A 673     146.487 120.091 112.346  1.00  0.00
ATOM  10158 1HG2 ILE A 673     146.709 119.126 111.891  1.00  0.00
ATOM  10159 2HG2 ILE A 673     145.802 120.619 111.683  1.00  0.00
ATOM  10160 3HG2 ILE A 673     147.410 120.666 112.430  1.00  0.00
ATOM  10161  CG1 ILE A 673     146.641 118.818 114.527  1.00  0.00
ATOM  10162 1HG1 ILE A 673     146.566 117.870 113.995  1.00  0.00
ATOM  10163 2HG1 ILE A 673     146.177 118.677 115.500  1.00  0.00
ATOM  10164  CD  ILE A 673     148.129 119.114 114.763  1.00  0.00
ATOM  10165  HD1 ILE A 673     148.568 118.299 115.338  1.00  0.00
ATOM  10166  HD2 ILE A 673     148.661 119.190 113.818  1.00  0.00
ATOM  10167  HD3 ILE A 673     148.254 120.035 115.323  1.00  0.00
ATOM  10168  C   ILE A 673     145.265 122.374 113.651  1.00  0.00
ATOM  10169  O   ILE A 673     146.079 123.265 113.423  1.00  0.00
ATOM  10170  N   VAL A 674     144.009 122.452 113.194  1.00  0.00
ATOM  10171  H   VAL A 674     143.355 121.696 113.385  1.00  0.00
ATOM  10172  CA  VAL A 674     143.583 123.590 112.361  1.00  0.00
ATOM  10173  HA  VAL A 674     144.338 123.696 111.584  1.00  0.00
ATOM  10174  CB  VAL A 674     142.249 123.322 111.636  1.00  0.00
ATOM  10175  HB  VAL A 674     142.289 122.313 111.225  1.00  0.00
ATOM  10176  CG1 VAL A 674     141.007 123.425 112.521  1.00  0.00
ATOM  10177 1HG1 VAL A 674     140.127 123.106 111.964  1.00  0.00
ATOM  10178 2HG1 VAL A 674     141.121 122.773 113.381  1.00  0.00
ATOM  10179 3HG1 VAL A 674     140.851 124.446 112.865  1.00  0.00
ATOM  10180  CG2 VAL A 674     142.082 124.280 110.445  1.00  0.00
ATOM  10181 1HG2 VAL A 674     141.174 124.020 109.900  1.00  0.00
ATOM  10182 2HG2 VAL A 674     142.006 125.310 110.792  1.00  0.00
ATOM  10183 3HG2 VAL A 674     142.930 124.179 109.767  1.00  0.00
ATOM  10184  C   VAL A 674     143.589 124.899 113.155  1.00  0.00
ATOM  10185  O   VAL A 674     143.973 125.943 112.609  1.00  0.00
```

```
ATOM  10186  N   ARG A 675     143.268 124.860 114.458  1.00  0.00
ATOM  10187  H   ARG A 675     142.920 123.983 114.841  1.00  0.00
ATOM  10188  CA  ARG A 675     143.356 126.039 115.335  1.00  0.00
ATOM  10189  HA  ARG A 675     142.760 126.826 114.871  1.00  0.00
ATOM  10190  CB  ARG A 675     142.725 125.692 116.701  1.00  0.00
ATOM  10191  HB1 ARG A 675     143.420 125.118 117.316  1.00  0.00
ATOM  10192  HB2 ARG A 675     141.851 125.068 116.516  1.00  0.00
ATOM  10193  CG  ARG A 675     142.205 126.911 117.470  1.00  0.00
ATOM  10194  HG1 ARG A 675     141.484 126.568 118.214  1.00  0.00
ATOM  10195  HG2 ARG A 675     141.666 127.551 116.769  1.00  0.00
ATOM  10196  CD  ARG A 675     143.276 127.732 118.205  1.00  0.00
ATOM  10197  HD1 ARG A 675     144.182 127.824 117.611  1.00  0.00
ATOM  10198  HD2 ARG A 675     143.543 127.213 119.129  1.00  0.00
ATOM  10199  NE  ARG A 675     142.758 129.073 118.518  1.00  0.00
ATOM  10200  HE  ARG A 675     142.369 129.202 119.440  1.00  0.00
ATOM  10201  CZ  ARG A 675     142.670 130.093 117.679  1.00  0.00
ATOM  10202  NH1 ARG A 675     143.232 130.096 116.501  1.00  0.00
ATOM  10203 1HH1 ARG A 675     143.793 129.305 116.216  1.00  0.00
ATOM  10204 2HH1 ARG A 675     143.143 130.895 115.901  1.00  0.00
ATOM  10205  NH2 ARG A 675     141.975 131.136 118.022  1.00  0.00
ATOM  10206 1HH2 ARG A 675     141.501 131.167 118.908  1.00  0.00
ATOM  10207 2HH2 ARG A 675     141.878 131.902 117.384  1.00  0.00
ATOM  10208  C   ARG A 675     144.787 126.573 115.454  1.00  0.00
ATOM  10209  O   ARG A 675     144.986 127.784 115.270  1.00  0.00
ATOM  10210  N   LYS A 676     145.770 125.704 115.740  1.00  0.00
ATOM  10211  H   LYS A 676     145.517 124.730 115.891  1.00  0.00
ATOM  10212  CA  LYS A 676     147.180 126.114 115.902  1.00  0.00
ATOM  10213  HA  LYS A 676     147.184 127.025 116.502  1.00  0.00
ATOM  10214  CB  LYS A 676     147.992 125.077 116.704  1.00  0.00
ATOM  10215  HB1 LYS A 676     147.392 124.770 117.561  1.00  0.00
ATOM  10216  HB2 LYS A 676     148.884 125.572 117.090  1.00  0.00
ATOM  10217  CG  LYS A 676     148.446 123.845 115.903  1.00  0.00
ATOM  10218  HG1 LYS A 676     149.162 124.156 115.142  1.00  0.00
ATOM  10219  HG2 LYS A 676     147.586 123.410 115.405  1.00  0.00
ATOM  10220  CD  LYS A 676     149.110 122.768 116.772  1.00  0.00
ATOM  10221  HD1 LYS A 676     149.901 123.216 117.375  1.00  0.00
ATOM  10222  HD2 LYS A 676     149.549 122.013 116.118  1.00  0.00
ATOM  10223  CE  LYS A 676     148.061 122.108 117.673  1.00  0.00
ATOM  10224  HE1 LYS A 676     147.266 121.697 117.044  1.00  0.00
ATOM  10225  HE2 LYS A 676     147.598 122.875 118.300  1.00  0.00
ATOM  10226  NZ  LYS A 676     148.624 121.047 118.536  1.00  0.00
ATOM  10227  HZ1 LYS A 676     147.876 120.720 119.145  1.00  0.00
ATOM  10228  HZ2 LYS A 676     148.972 120.269 117.993  1.00  0.00
ATOM  10229  HZ3 LYS A 676     149.361 121.406 119.125  1.00  0.00
ATOM  10230  C   LYS A 676     147.851 126.513 114.587  1.00  0.00
ATOM  10231  O   LYS A 676     148.567 127.504 114.571  1.00  0.00
ATOM  10232  N   ARG A 677     147.534 125.837 113.477  1.00  0.00
ATOM  10233  H   ARG A 677     146.981 124.992 113.593  1.00  0.00
ATOM  10234  CA  ARG A 677     147.893 126.225 112.099  1.00  0.00
ATOM  10235  HA  ARG A 677     148.978 126.202 111.970  1.00  0.00
ATOM  10236  CB  ARG A 677     147.238 125.192 111.162  1.00  0.00
ATOM  10237  HB1 ARG A 677     146.184 125.124 111.430  1.00  0.00
ATOM  10238  HB2 ARG A 677     147.674 124.212 111.363  1.00  0.00
ATOM  10239  CG  ARG A 677     147.344 125.463 109.650  1.00  0.00
ATOM  10240  HG1 ARG A 677     148.050 124.749 109.223  1.00  0.00
ATOM  10241  HG2 ARG A 677     147.727 126.464 109.443  1.00  0.00
ATOM  10242  CD  ARG A 677     145.984 125.285 108.953  1.00  0.00
ATOM  10243  HD1 ARG A 677     145.504 124.377 109.323  1.00  0.00
ATOM  10244  HD2 ARG A 677     146.161 125.133 107.887  1.00  0.00
ATOM  10245  NE  ARG A 677     145.084 126.446 109.168  1.00  0.00
ATOM  10246  HE  ARG A 677     144.632 126.533 110.067  1.00  0.00
```

```
ATOM  10247  CZ  ARG A 677     144.827 127.414 108.301  1.00  0.00
ATOM  10248  NH1 ARG A 677     145.338 127.427 107.100  1.00  0.00
ATOM  10249 1HH1 ARG A 677     145.895 126.645 106.807  1.00  0.00
ATOM  10250 2HH1 ARG A 677     145.171 128.196 106.476  1.00  0.00
ATOM  10251  NH2 ARG A 677     144.051 128.412 108.610  1.00  0.00
ATOM  10252 1HH2 ARG A 677     143.615 128.424 109.530  1.00  0.00
ATOM  10253 2HH2 ARG A 677     143.776 129.085 107.917  1.00  0.00
ATOM  10254  C   ARG A 677     147.433 127.644 111.774  1.00  0.00
ATOM  10255  O   ARG A 677     148.220 128.473 111.323  1.00  0.00
ATOM  10256  N   THR A 678     146.157 127.932 112.038  1.00  0.00
ATOM  10257  H   THR A 678     145.570 127.197 112.411  1.00  0.00
ATOM  10258  CA  THR A 678     145.576 129.262 111.790  1.00  0.00
ATOM  10259  HA  THR A 678     145.736 129.527 110.746  1.00  0.00
ATOM  10260  CB  THR A 678     144.063 129.267 112.064  1.00  0.00
ATOM  10261  HB  THR A 678     143.878 129.124 113.129  1.00  0.00
ATOM  10262  CG2 THR A 678     143.385 130.552 111.598  1.00  0.00
ATOM  10263 1HG2 THR A 678     142.307 130.468 111.737  1.00  0.00
ATOM  10264 2HG2 THR A 678     143.743 131.401 112.179  1.00  0.00
ATOM  10265 3HG2 THR A 678     143.595 130.733 110.544  1.00  0.00
ATOM  10266  OG1 THR A 678     143.457 128.213 111.350  1.00  0.00
ATOM  10267  HG1 THR A 678     143.476 127.424 111.926  1.00  0.00
ATOM  10268  C   THR A 678     146.248 130.333 112.638  1.00  0.00
ATOM  10269  O   THR A 678     146.598 131.382 112.111  1.00  0.00
ATOM  10270  N   LEU A 679     146.494 130.057 113.925  1.00  0.00
ATOM  10271  H   LEU A 679     146.208 129.162 114.300  1.00  0.00
ATOM  10272  CA  LEU A 679     147.212 130.988 114.804  1.00  0.00
ATOM  10273  HA  LEU A 679     146.676 131.936 114.788  1.00  0.00
ATOM  10274  CB  LEU A 679     147.199 130.413 116.237  1.00  0.00
ATOM  10275  HB1 LEU A 679     147.931 129.607 116.305  1.00  0.00
ATOM  10276  HB2 LEU A 679     146.220 129.969 116.418  1.00  0.00
ATOM  10277  CG  LEU A 679     147.452 131.400 117.397  1.00  0.00
ATOM  10278  HG  LEU A 679     147.355 130.822 118.316  1.00  0.00
ATOM  10279  CD1 LEU A 679     148.839 132.039 117.397  1.00  0.00
ATOM  10280 1HD1 LEU A 679     149.012 132.520 118.359  1.00  0.00
ATOM  10281 2HD1 LEU A 679     149.603 131.276 117.251  1.00  0.00
ATOM  10282 3HD1 LEU A 679     148.919 132.798 116.623  1.00  0.00
ATOM  10283  CD2 LEU A 679     146.397 132.507 117.441  1.00  0.00
ATOM  10284 1HD2 LEU A 679     146.509 133.075 118.364  1.00  0.00
ATOM  10285 2HD2 LEU A 679     146.517 133.192 116.602  1.00  0.00
ATOM  10286 3HD2 LEU A 679     145.398 132.078 117.418  1.00  0.00
ATOM  10287  C   LEU A 679     148.635 131.262 114.289  1.00  0.00
ATOM  10288  O   LEU A 679     149.041 132.416 114.168  1.00  0.00
ATOM  10289  N   ARG A 680     149.387 130.230 113.887  1.00  0.00
ATOM  10290  H   ARG A 680     149.034 129.286 114.018  1.00  0.00
ATOM  10291  CA  ARG A 680     150.725 130.403 113.310  1.00  0.00
ATOM  10292  HA  ARG A 680     151.129 131.240 113.856  1.00  0.00
ATOM  10293  CB  ARG A 680     151.645 129.195 113.562  1.00  0.00
ATOM  10294  HB1 ARG A 680     152.153 128.910 112.640  1.00  0.00
ATOM  10295  HB2 ARG A 680     151.055 128.333 113.873  1.00  0.00
ATOM  10296  CG  ARG A 680     152.723 129.468 114.634  1.00  0.00
ATOM  10297  HG1 ARG A 680     153.338 128.570 114.704  1.00  0.00
ATOM  10298  HG2 ARG A 680     152.240 129.609 115.602  1.00  0.00
ATOM  10299  CD  ARG A 680     153.668 130.661 114.350  1.00  0.00
ATOM  10300  HD1 ARG A 680     153.816 130.756 113.274  1.00  0.00
ATOM  10301  HD2 ARG A 680     154.641 130.425 114.784  1.00  0.00
ATOM  10302  NE  ARG A 680     153.197 131.931 114.956  1.00  0.00
ATOM  10303  HE  ARG A 680     153.166 131.961 115.962  1.00  0.00
ATOM  10304  CZ  ARG A 680     152.788 133.028 114.332  1.00  0.00
ATOM  10305  NH1 ARG A 680     152.801 133.172 113.043  1.00  0.00
ATOM  10306 1HH1 ARG A 680     152.995 132.379 112.445  1.00  0.00
ATOM  10307 2HH1 ARG A 680     152.343 133.983 112.654  1.00  0.00
```

```
ATOM  10308  NH2 ARG A 680     152.259 134.026 114.975  1.00  0.00
ATOM  10309 1HH2 ARG A 680     152.117 133.983 115.966  1.00  0.00
ATOM  10310 2HH2 ARG A 680     151.767 134.720 114.420  1.00  0.00
ATOM  10311  C   ARG A 680     150.765 130.908 111.859  1.00  0.00
ATOM  10312  O   ARG A 680     151.858 131.167 111.367  1.00  0.00
ATOM  10313  N   ARG A 681     149.610 131.191 111.243  1.00  0.00
ATOM  10314  H   ARG A 681     148.754 130.883 111.686  1.00  0.00
ATOM  10315  CA  ARG A 681     149.495 132.061 110.054  1.00  0.00
ATOM  10316  HA  ARG A 681     150.488 132.447 109.819  1.00  0.00
ATOM  10317  CB  ARG A 681     149.065 131.201 108.843  1.00  0.00
ATOM  10318  HB1 ARG A 681     148.070 130.801 109.049  1.00  0.00
ATOM  10319  HB2 ARG A 681     149.748 130.349 108.808  1.00  0.00
ATOM  10320  CG  ARG A 681     149.059 131.832 107.432  1.00  0.00
ATOM  10321  HG1 ARG A 681     148.128 132.385 107.301  1.00  0.00
ATOM  10322  HG2 ARG A 681     149.021 131.006 106.722  1.00  0.00
ATOM  10323  CD  ARG A 681     150.245 132.736 107.025  1.00  0.00
ATOM  10324  HD1 ARG A 681     150.467 132.558 105.971  1.00  0.00
ATOM  10325  HD2 ARG A 681     151.130 132.485 107.611  1.00  0.00
ATOM  10326  NE  ARG A 681     149.891 134.155 107.196  1.00  0.00
ATOM  10327  HE  ARG A 681     149.115 134.330 107.822  1.00  0.00
ATOM  10328  CZ  ARG A 681     150.400 135.243 106.639  1.00  0.00
ATOM  10329  NH1 ARG A 681     151.463 135.233 105.899  1.00  0.00
ATOM  10330 1HH1 ARG A 681     151.969 134.392 105.739  1.00  0.00
ATOM  10331 2HH1 ARG A 681     151.855 136.154 105.646  1.00  0.00
ATOM  10332  NH2 ARG A 681     149.816 136.384 106.845  1.00  0.00
ATOM  10333 1HH2 ARG A 681     148.988 136.479 107.396  1.00  0.00
ATOM  10334 2HH2 ARG A 681     150.217 137.237 106.424  1.00  0.00
ATOM  10335  C   ARG A 681     148.661 133.329 110.333  1.00  0.00
ATOM  10336  O   ARG A 681     148.337 134.073 109.408  1.00  0.00
ATOM  10337  N   LEU A 682     148.436 133.677 111.604  1.00  0.00
ATOM  10338  H   LEU A 682     148.633 133.002 112.330  1.00  0.00
ATOM  10339  CA  LEU A 682     148.436 135.079 112.010  1.00  0.00
ATOM  10340  HA  LEU A 682     147.879 135.647 111.264  1.00  0.00
ATOM  10341  CB  LEU A 682     147.761 135.287 113.375  1.00  0.00
ATOM  10342  HB1 LEU A 682     148.316 134.752 114.142  1.00  0.00
ATOM  10343  HB2 LEU A 682     146.744 134.889 113.342  1.00  0.00
ATOM  10344  CG  LEU A 682     147.706 136.772 113.802  1.00  0.00
ATOM  10345  HG  LEU A 682     148.719 137.175 113.819  1.00  0.00
ATOM  10346  CD1 LEU A 682     146.856 137.628 112.860  1.00  0.00
ATOM  10347 1HD1 LEU A 682     146.780 138.641 113.257  1.00  0.00
ATOM  10348 2HD1 LEU A 682     147.320 137.691 111.877  1.00  0.00
ATOM  10349 3HD1 LEU A 682     145.856 137.207 112.767  1.00  0.00
ATOM  10350  CD2 LEU A 682     147.125 136.890 115.207  1.00  0.00
ATOM  10351 1HD2 LEU A 682     147.142 137.933 115.525  1.00  0.00
ATOM  10352 2HD2 LEU A 682     146.098 136.526 115.222  1.00  0.00
ATOM  10353 3HD2 LEU A 682     147.720 136.307 115.909  1.00  0.00
ATOM  10354  C   LEU A 682     149.893 135.558 112.006  1.00  0.00
ATOM  10355  O   LEU A 682     150.732 135.129 112.807  1.00  0.00
ATOM  10356  N   LEU A 683     150.195 136.446 111.075  1.00  0.00
ATOM  10357  H   LEU A 683     149.458 136.775 110.463  1.00  0.00
ATOM  10358  CA  LEU A 683     151.447 137.177 110.979  1.00  0.00
ATOM  10359  HA  LEU A 683     151.987 137.135 111.925  1.00  0.00
ATOM  10360  CB  LEU A 683     152.319 136.605 109.844  1.00  0.00
ATOM  10361  HB1 LEU A 683     153.095 137.327 109.592  1.00  0.00
ATOM  10362  HB2 LEU A 683     151.700 136.474 108.957  1.00  0.00
ATOM  10363  CG  LEU A 683     153.008 135.269 110.185  1.00  0.00
ATOM  10364  HG  LEU A 683     152.275 134.578 110.599  1.00  0.00
ATOM  10365  CD1 LEU A 683     153.602 134.623 108.936  1.00  0.00
ATOM  10366 1HD1 LEU A 683     154.071 133.673 109.190  1.00  0.00
ATOM  10367 2HD1 LEU A 683     152.817 134.439 108.207  1.00  0.00
ATOM  10368 3HD1 LEU A 683     154.345 135.288 108.494  1.00  0.00
```

```
ATOM  10369  CD2 LEU A 683      154.136 135.476 111.198  1.00  0.00
ATOM  10370 1HD2 LEU A 683      153.743 135.835 112.147  1.00  0.00
ATOM  10371 2HD2 LEU A 683      154.660 134.536 111.373  1.00  0.00
ATOM  10372 3HD2 LEU A 683      154.856 136.200 110.812  1.00  0.00
ATOM  10373  C   LEU A 683      151.075 138.636 110.720  1.00  0.00
ATOM  10374  O   LEU A 683      150.089 138.907 110.038  1.00  0.00
ATOM  10375  N   GLN A 684      151.839 139.548 111.314  1.00  0.00
ATOM  10376  H   GLN A 684      152.628 139.234 111.853  1.00  0.00
ATOM  10377  CA  GLN A 684      151.728 140.981 111.040  1.00  0.00
ATOM  10378  HA  GLN A 684      150.683 141.226 110.841  1.00  0.00
ATOM  10379  CB  GLN A 684      152.174 141.776 112.282  1.00  0.00
ATOM  10380  HB1 GLN A 684      151.527 141.514 113.120  1.00  0.00
ATOM  10381  HB2 GLN A 684      152.054 142.841 112.077  1.00  0.00
ATOM  10382  CG  GLN A 684      153.636 141.501 112.666  1.00  0.00
ATOM  10383  HG1 GLN A 684      154.253 141.733 111.805  1.00  0.00
ATOM  10384  HG2 GLN A 684      153.782 140.452 112.921  1.00  0.00
ATOM  10385  CD  GLN A 684      154.140 142.343 113.829  1.00  0.00
ATOM  10386  OE1 GLN A 684      153.439 142.677 114.767  1.00  0.00
ATOM  10387  NE2 GLN A 684      155.357 142.824 113.762  1.00  0.00
ATOM  10388 1HE2 GLN A 684      155.933 142.546 112.946  1.00  0.00
ATOM  10389 2HE2 GLN A 684      155.798 143.167 114.592  1.00  0.00
ATOM  10390  C   GLN A 684      152.523 141.385 109.787  1.00  0.00
ATOM  10391  O   GLN A 684      152.198 142.393 109.171  1.00  0.00
ATOM  10392  N   GLU A 685      153.557 140.602 109.452  1.00  0.00
ATOM  10393  H   GLU A 685      153.863 139.953 110.161  1.00  0.00
ATOM  10394  CA  GLU A 685      154.422 140.578 108.263  1.00  0.00
ATOM  10395  HA  GLU A 685      155.333 140.088 108.607  1.00  0.00
ATOM  10396  CB  GLU A 685      153.873 139.596 107.221  1.00  0.00
ATOM  10397  HB1 GLU A 685      153.668 138.669 107.757  1.00  0.00
ATOM  10398  HB2 GLU A 685      154.657 139.381 106.500  1.00  0.00
ATOM  10399  CG  GLU A 685      152.613 140.030 106.468  1.00  0.00
ATOM  10400  HG1 GLU A 685      152.892 140.662 105.623  1.00  0.00
ATOM  10401  HG2 GLU A 685      151.970 140.609 107.129  1.00  0.00
ATOM  10402  CD  GLU A 685      151.836 138.803 105.980  1.00  0.00
ATOM  10403  OE1 GLU A 685      150.587 138.834 105.964  1.00  0.00
ATOM  10404  OE2 GLU A 685      152.434 137.741 105.689  1.00  0.00
ATOM  10405  C   GLU A 685      154.970 141.903 107.716  1.00  0.00
ATOM  10406  O   GLU A 685      155.713 141.897 106.739  1.00  0.00
ATOM  10407  N   ARG A 686      154.759 143.012 108.436  1.00  0.00
ATOM  10408  H   ARG A 686      154.060 142.907 109.161  1.00  0.00
ATOM  10409  CA  ARG A 686      155.437 144.324 108.351  1.00  0.00
ATOM  10410  HA  ARG A 686      155.323 144.676 107.325  1.00  0.00
ATOM  10411  CB  ARG A 686      154.710 145.318 109.277  1.00  0.00
ATOM  10412  HB1 ARG A 686      153.668 145.381 108.962  1.00  0.00
ATOM  10413  HB2 ARG A 686      155.155 146.307 109.157  1.00  0.00
ATOM  10414  CG  ARG A 686      154.760 144.912 110.759  1.00  0.00
ATOM  10415  HG1 ARG A 686      155.795 144.833 111.088  1.00  0.00
ATOM  10416  HG2 ARG A 686      154.281 143.943 110.878  1.00  0.00
ATOM  10417  CD  ARG A 686      154.030 145.936 111.632  1.00  0.00
ATOM  10418  HD1 ARG A 686      153.045 146.122 111.203  1.00  0.00
ATOM  10419  HD2 ARG A 686      154.602 146.864 111.632  1.00  0.00
ATOM  10420  NE  ARG A 686      153.871 145.435 113.009  1.00  0.00
ATOM  10421  HE  ARG A 686      154.284 144.527 113.196  1.00  0.00
ATOM  10422  CZ  ARG A 686      153.265 146.031 114.021  1.00  0.00
ATOM  10423  NH1 ARG A 686      152.842 147.261 113.945  1.00  0.00
ATOM  10424 1HH1 ARG A 686      152.994 147.758 113.089  1.00  0.00
ATOM  10425 2HH1 ARG A 686      152.399 147.698 114.728  1.00  0.00
ATOM  10426  NH2 ARG A 686      153.069 145.395 115.137  1.00  0.00
ATOM  10427 1HH2 ARG A 686      153.261 144.392 115.161  1.00  0.00
ATOM  10428 2HH2 ARG A 686      152.609 145.827 115.912  1.00  0.00
ATOM  10429  C   ARG A 686      156.959 144.299 108.586  1.00  0.00
```

```
ATOM  10430  O   ARG A 686     157.566 145.319 108.905  1.00  0.00
ATOM  10431  N   GLU A 687     157.554 143.132 108.409  1.00  0.00
ATOM  10432  H   GLU A 687     156.946 142.389 108.104  1.00  0.00
ATOM  10433  CA  GLU A 687     158.964 142.792 108.552  1.00  0.00
ATOM  10434  HA  GLU A 687     159.545 143.713 108.576  1.00  0.00
ATOM  10435  CB  GLU A 687     159.185 142.067 109.899  1.00  0.00
ATOM  10436  HB1 GLU A 687     159.094 142.793 110.706  1.00  0.00
ATOM  10437  HB2 GLU A 687     160.218 141.722 109.922  1.00  0.00
ATOM  10438  CG  GLU A 687     158.273 140.851 110.187  1.00  0.00
ATOM  10439  HG1 GLU A 687     158.832 140.171 110.834  1.00  0.00
ATOM  10440  HG2 GLU A 687     158.082 140.318 109.252  1.00  0.00
ATOM  10441  CD  GLU A 687     156.929 141.150 110.895  1.00  0.00
ATOM  10442  OE1 GLU A 687     156.719 142.263 111.431  1.00  0.00
ATOM  10443  OE2 GLU A 687     156.070 140.239 110.938  1.00  0.00
ATOM  10444  C   GLU A 687     159.494 141.995 107.336  1.00  0.00
ATOM  10445  O   GLU A 687     160.669 141.648 107.284  1.00  0.00
ATOM  10446  N   LEU A 688     158.648 141.752 106.322  1.00  0.00
ATOM  10447  H   LEU A 688     157.687 142.074 106.390  1.00  0.00
ATOM  10448  CA  LEU A 688     159.029 141.172 105.035  1.00  0.00
ATOM  10449  HA  LEU A 688     160.071 141.435 104.847  1.00  0.00
ATOM  10450  CB  LEU A 688     158.961 139.626 105.122  1.00  0.00
ATOM  10451  HB1 LEU A 688     159.710 139.325 105.857  1.00  0.00
ATOM  10452  HB2 LEU A 688     159.275 139.201 104.169  1.00  0.00
ATOM  10453  CG  LEU A 688     157.624 138.963 105.525  1.00  0.00
ATOM  10454  HG  LEU A 688     157.235 139.426 106.432  1.00  0.00
ATOM  10455  CD1 LEU A 688     156.563 139.043 104.423  1.00  0.00
ATOM  10456 1HD1 LEU A 688     155.707 138.418 104.677  1.00  0.00
ATOM  10457 2HD1 LEU A 688     156.206 140.069 104.339  1.00  0.00
ATOM  10458 3HD1 LEU A 688     156.976 138.715 103.471  1.00  0.00
ATOM  10459  CD2 LEU A 688     157.858 137.478 105.811  1.00  0.00
ATOM  10460 1HD2 LEU A 688     156.924 137.011 106.123  1.00  0.00
ATOM  10461 2HD2 LEU A 688     158.232 136.978 104.919  1.00  0.00
ATOM  10462 3HD2 LEU A 688     158.581 137.368 106.619  1.00  0.00
ATOM  10463  C   LEU A 688     158.229 141.771 103.864  1.00  0.00
ATOM  10464  O   LEU A 688     157.261 142.506 104.038  1.00  0.00
ATOM  10465  N   VAL A 689     158.652 141.406 102.658  1.00  0.00
ATOM  10466  H   VAL A 689     159.459 140.796 102.631  1.00  0.00
ATOM  10467  CA  VAL A 689     157.886 141.476 101.406  1.00  0.00
ATOM  10468  HA  VAL A 689     156.825 141.545 101.642  1.00  0.00
ATOM  10469  CB  VAL A 689     158.298 142.691 100.541  1.00  0.00
ATOM  10470  HB  VAL A 689     159.331 142.573 100.211  1.00  0.00
ATOM  10471  CG1 VAL A 689     157.390 142.781  99.308  1.00  0.00
ATOM  10472 1HG1 VAL A 689     157.486 143.755  98.827  1.00  0.00
ATOM  10473 2HG1 VAL A 689     157.650 142.014  98.585  1.00  0.00
ATOM  10474 3HG1 VAL A 689     156.359 142.637  99.608  1.00  0.00
ATOM  10475  CG2 VAL A 689     158.172 144.025 101.292  1.00  0.00
ATOM  10476 1HG2 VAL A 689     158.421 144.854 100.629  1.00  0.00
ATOM  10477 2HG2 VAL A 689     157.154 144.152 101.660  1.00  0.00
ATOM  10478 3HG2 VAL A 689     158.862 144.052 102.134  1.00  0.00
ATOM  10479  C   VAL A 689     158.151 140.139 100.706  1.00  0.00
ATOM  10480  O   VAL A 689     159.195 139.530 100.942  1.00  0.00
ATOM  10481  N   GLU A 690     157.231 139.621  99.892  1.00  0.00
ATOM  10482  H   GLU A 690     156.420 140.167  99.630  1.00  0.00
ATOM  10483  CA  GLU A 690     157.316 138.215  99.455  1.00  0.00
ATOM  10484  HA  GLU A 690     157.384 137.631 100.374  1.00  0.00
ATOM  10485  CB  GLU A 690     155.996 137.747  98.801  1.00  0.00
ATOM  10486  HB1 GLU A 690     155.198 137.828  99.541  1.00  0.00
ATOM  10487  HB2 GLU A 690     156.109 136.685  98.584  1.00  0.00
ATOM  10488  CG  GLU A 690     155.511 138.422  97.505  1.00  0.00
ATOM  10489  HG1 GLU A 690     154.871 137.706  96.987  1.00  0.00
ATOM  10490  HG2 GLU A 690     156.351 138.602  96.836  1.00  0.00
```

```
ATOM  10491  CD  GLU A 690     154.687 139.707  97.724  1.00  0.00
ATOM  10492  OE1 GLU A 690     155.240 140.821  97.566  1.00  0.00
ATOM  10493  OE2 GLU A 690     153.459 139.593  97.939  1.00  0.00
ATOM  10494  C   GLU A 690     158.591 137.774  98.684  1.00  0.00
ATOM  10495  O   GLU A 690     158.957 136.607  98.815  1.00  0.00
ATOM  10496  N   PRO A 691     159.345 138.645  97.975  1.00  0.00
ATOM  10497  CD  PRO A 691     158.881 139.853  97.327  1.00  0.00
ATOM  10498  HD1 PRO A 691     159.385 140.704  97.777  1.00  0.00
ATOM  10499  HD2 PRO A 691     157.805 139.990  97.378  1.00  0.00
ATOM  10500  CG  PRO A 691     159.318 139.682  95.878  1.00  0.00
ATOM  10501  HG1 PRO A 691     159.407 140.638  95.362  1.00  0.00
ATOM  10502  HG2 PRO A 691     158.615 139.028  95.361  1.00  0.00
ATOM  10503  CB  PRO A 691     160.670 138.976  96.011  1.00  0.00
ATOM  10504  HB1 PRO A 691     161.465 139.713  95.930  1.00  0.00
ATOM  10505  HB2 PRO A 691     160.788 138.229  95.227  1.00  0.00
ATOM  10506  CA  PRO A 691     160.661 138.315  97.403  1.00  0.00
ATOM  10507  HA  PRO A 691     160.744 137.236  97.272  1.00  0.00
ATOM  10508  C   PRO A 691     161.859 138.774  98.263  1.00  0.00
ATOM  10509  O   PRO A 691     162.999 138.707  97.802  1.00  0.00
ATOM  10510  N   LEU A 692     161.605 139.299  99.473  1.00  0.00
ATOM  10511  H   LEU A 692     160.643 139.232  99.787  1.00  0.00
ATOM  10512  CA  LEU A 692     162.543 139.882 100.455  1.00  0.00
ATOM  10513  HA  LEU A 692     161.951 140.592 101.028  1.00  0.00
ATOM  10514  CB  LEU A 692     163.007 138.759 101.406  1.00  0.00
ATOM  10515  HB1 LEU A 692     163.822 138.219 100.922  1.00  0.00
ATOM  10516  HB2 LEU A 692     162.188 138.054 101.554  1.00  0.00
ATOM  10517  CG  LEU A 692     163.469 139.244 102.795  1.00  0.00
ATOM  10518  HG  LEU A 692     164.221 140.024 102.696  1.00  0.00
ATOM  10519  CD1 LEU A 692     162.304 139.783 103.630  1.00  0.00
ATOM  10520 1HD1 LEU A 692     162.654 140.041 104.630  1.00  0.00
ATOM  10521 2HD1 LEU A 692     161.893 140.684 103.182  1.00  0.00
ATOM  10522 3HD1 LEU A 692     161.526 139.028 103.713  1.00  0.00
ATOM  10523  CD2 LEU A 692     164.091 138.072 103.556  1.00  0.00
ATOM  10524 1HD2 LEU A 692     164.430 138.411 104.534  1.00  0.00
ATOM  10525 2HD2 LEU A 692     163.357 137.277 103.683  1.00  0.00
ATOM  10526 3HD2 LEU A 692     164.948 137.694 103.001  1.00  0.00
ATOM  10527  C   LEU A 692     163.702 140.713  99.853  1.00  0.00
ATOM  10528  O   LEU A 692     164.843 140.693 100.312  1.00  0.00
ATOM  10529  N   THR A 693     163.362 141.446  98.801  1.00  0.00
ATOM  10530  H   THR A 693     162.415 141.337  98.485  1.00  0.00
ATOM  10531  CA  THR A 693     164.221 142.338  98.012  1.00  0.00
ATOM  10532  HA  THR A 693     165.058 142.651  98.636  1.00  0.00
ATOM  10533  CB  THR A 693     164.800 141.632  96.774  1.00  0.00
ATOM  10534  HB  THR A 693     165.144 142.382  96.062  1.00  0.00
ATOM  10535  CG2 THR A 693     165.979 140.725  97.117  1.00  0.00
ATOM  10536 1HG2 THR A 693     166.388 140.299  96.203  1.00  0.00
ATOM  10537 2HG2 THR A 693     166.756 141.304  97.614  1.00  0.00
ATOM  10538 3HG2 THR A 693     165.662 139.919  97.777  1.00  0.00
ATOM  10539  OG1 THR A 693     163.838 140.812  96.154  1.00  0.00
ATOM  10540  HG1 THR A 693     163.762 140.009  96.697  1.00  0.00
ATOM  10541  C   THR A 693     163.511 143.642  97.587  1.00  0.00
ATOM  10542  O   THR A 693     164.226 144.617  97.356  1.00  0.00
ATOM  10543  N   PRO A 694     162.158 143.727  97.501  1.00  0.00
ATOM  10544  CD  PRO A 694     161.204 142.646  97.285  1.00  0.00
ATOM  10545  HD1 PRO A 694     160.871 142.262  98.250  1.00  0.00
ATOM  10546  HD2 PRO A 694     161.629 141.851  96.677  1.00  0.00
ATOM  10547  CG  PRO A 694     160.018 143.269  96.556  1.00  0.00
ATOM  10548  HG1 PRO A 694     159.095 142.714  96.707  1.00  0.00
ATOM  10549  HG2 PRO A 694     160.244 143.364  95.492  1.00  0.00
ATOM  10550  CB  PRO A 694     159.958 144.645  97.192  1.00  0.00
ATOM  10551  HB1 PRO A 694     159.464 144.582  98.161  1.00  0.00
```

ATOM	10552	HB2	PRO	A	694	159.448	145.362	96.550	1.00	0.00
ATOM	10553	CA	PRO	A	694	161.432	144.993	97.388	1.00	0.00
ATOM	10554	HA	PRO	A	694	161.782	145.489	96.481	1.00	0.00
ATOM	10555	C	PRO	A	694	161.594	145.993	98.537	1.00	0.00
ATOM	10556	O	PRO	A	694	162.151	145.683	99.590	1.00	0.00
ATOM	10557	N	SER	A	695	161.062	147.195	98.317	1.00	0.00
ATOM	10558	H	SER	A	695	160.449	147.301	97.513	1.00	0.00
ATOM	10559	CA	SER	A	695	161.342	148.415	99.080	1.00	0.00
ATOM	10560	HA	SER	A	695	162.425	148.524	99.136	1.00	0.00
ATOM	10561	CB	SER	A	695	160.786	149.661	98.371	1.00	0.00
ATOM	10562	HB1	SER	A	695	161.372	150.530	98.674	1.00	0.00
ATOM	10563	HB2	SER	A	695	159.753	149.825	98.682	1.00	0.00
ATOM	10564	OG	SER	A	695	160.813	149.542	96.961	1.00	0.00
ATOM	10565	HG	SER	A	695	160.016	149.010	96.717	1.00	0.00
ATOM	10566	C	SER	A	695	160.819	148.429	100.520	1.00	0.00
ATOM	10567	O	SER	A	695	161.362	149.157	101.349	1.00	0.00
ATOM	10568	N	GLY	A	696	159.749	147.683	100.815	1.00	0.00
ATOM	10569	H	GLY	A	696	159.339	147.151	100.049	1.00	0.00
ATOM	10570	CA	GLY	A	696	159.038	147.735	102.104	1.00	0.00
ATOM	10571	HA1	GLY	A	696	159.581	148.356	102.816	1.00	0.00
ATOM	10572	HA2	GLY	A	696	158.983	146.725	102.507	1.00	0.00
ATOM	10573	C	GLY	A	696	157.606	148.279	101.986	1.00	0.00
ATOM	10574	O	GLY	A	696	157.081	148.876	102.922	1.00	0.00
ATOM	10575	N	GLU	A	697	156.983	148.115	100.822	1.00	0.00
ATOM	10576	H	GLU	A	697	157.461	147.549	100.125	1.00	0.00
ATOM	10577	CA	GLU	A	697	155.781	148.809	100.336	1.00	0.00
ATOM	10578	HA	GLU	A	697	155.930	149.881	100.471	1.00	0.00
ATOM	10579	CB	GLU	A	697	155.637	148.528	98.817	1.00	0.00
ATOM	10580	HB1	GLU	A	697	154.764	149.078	98.466	1.00	0.00
ATOM	10581	HB2	GLU	A	697	155.434	147.470	98.658	1.00	0.00
ATOM	10582	CG	GLU	A	697	156.817	148.946	97.916	1.00	0.00
ATOM	10583	HG1	GLU	A	697	157.254	149.867	98.309	1.00	0.00
ATOM	10584	HG2	GLU	A	697	156.403	149.181	96.933	1.00	0.00
ATOM	10585	CD	GLU	A	697	157.916	147.886	97.723	1.00	0.00
ATOM	10586	OE1	GLU	A	697	158.708	148.015	96.761	1.00	0.00
ATOM	10587	OE2	GLU	A	697	158.090	146.994	98.585	1.00	0.00
ATOM	10588	C	GLU	A	697	154.447	148.459	101.033	1.00	0.00
ATOM	10589	O	GLU	A	697	153.407	149.017	100.676	1.00	0.00
ATOM	10590	N	ALA	A	698	154.462	147.519	101.985	1.00	0.00
ATOM	10591	H	ALA	A	698	155.388	147.315	102.332	1.00	0.00
ATOM	10592	CA	ALA	A	698	153.469	146.439	102.122	1.00	0.00
ATOM	10593	HA	ALA	A	698	153.852	145.810	102.919	1.00	0.00
ATOM	10594	CB	ALA	A	698	152.115	146.981	102.607	1.00	0.00
ATOM	10595	HB1	ALA	A	698	151.454	146.151	102.858	1.00	0.00
ATOM	10596	HB2	ALA	A	698	152.260	147.606	103.487	1.00	0.00
ATOM	10597	HB3	ALA	A	698	151.644	147.573	101.823	1.00	0.00
ATOM	10598	C	ALA	A	698	153.369	145.581	100.827	1.00	0.00
ATOM	10599	O	ALA	A	698	153.531	146.096	99.726	1.00	0.00
ATOM	10600	N	PRO	A	699	153.191	144.247	100.910	1.00	0.00
ATOM	10601	CD	PRO	A	699	152.962	143.470	102.124	1.00	0.00
ATOM	10602	HD1	PRO	A	699	151.905	143.519	102.391	1.00	0.00
ATOM	10603	HD2	PRO	A	699	153.572	143.807	102.960	1.00	0.00
ATOM	10604	CG	PRO	A	699	153.347	142.035	101.783	1.00	0.00
ATOM	10605	HG1	PRO	A	699	152.738	141.314	102.327	1.00	0.00
ATOM	10606	HG2	PRO	A	699	154.404	141.885	102.004	1.00	0.00
ATOM	10607	CB	PRO	A	699	153.123	141.950	100.278	1.00	0.00
ATOM	10608	HB1	PRO	A	699	152.084	141.688	100.079	1.00	0.00
ATOM	10609	HB2	PRO	A	699	153.793	141.218	99.839	1.00	0.00
ATOM	10610	CA	PRO	A	699	153.423	143.361	99.758	1.00	0.00
ATOM	10611	HA	PRO	A	699	154.476	143.432	99.512	1.00	0.00
ATOM	10612	C	PRO	A	699	152.637	143.657	98.479	1.00	0.00

```
ATOM  10613  O   PRO A 699     151.516 144.165  98.537  1.00  0.00
ATOM  10614  N   ASN A 700     153.154 143.266  97.308  1.00  0.00
ATOM  10615  H   ASN A 700     154.027 142.748  97.309  1.00  0.00
ATOM  10616  CA  ASN A 700     152.450 143.526  96.037  1.00  0.00
ATOM  10617  HA  ASN A 700     152.233 144.595  96.003  1.00  0.00
ATOM  10618  CB  ASN A 700     153.363 143.204  94.839  1.00  0.00
ATOM  10619  HB1 ASN A 700     154.391 143.490  95.056  1.00  0.00
ATOM  10620  HB2 ASN A 700     153.024 143.800  93.992  1.00  0.00
ATOM  10621  CG  ASN A 700     153.320 141.754  94.390  1.00  0.00
ATOM  10622  OD1 ASN A 700     152.560 141.377  93.518  1.00  0.00
ATOM  10623  ND2 ASN A 700     154.116 140.884  94.961  1.00  0.00
ATOM  10624 1HD2 ASN A 700     154.656 141.112  95.795  1.00  0.00
ATOM  10625 2HD2 ASN A 700     154.067 139.942  94.622  1.00  0.00
ATOM  10626  C   ASN A 700     151.089 142.791  95.986  1.00  0.00
ATOM  10627  O   ASN A 700     150.071 143.344  95.554  1.00  0.00
ATOM  10628  N   GLN A 701     151.049 141.575  96.544  1.00  0.00
ATOM  10629  H   GLN A 701     151.915 141.171  96.891  1.00  0.00
ATOM  10630  CA  GLN A 701     149.817 140.816  96.712  1.00  0.00
ATOM  10631  HA  GLN A 701     149.299 140.770  95.752  1.00  0.00
ATOM  10632  CB  GLN A 701     150.141 139.384  97.160  1.00  0.00
ATOM  10633  HB1 GLN A 701     149.205 138.863  97.375  1.00  0.00
ATOM  10634  HB2 GLN A 701     150.722 139.427  98.085  1.00  0.00
ATOM  10635  CG  GLN A 701     150.908 138.566  96.109  1.00  0.00
ATOM  10636  HG1 GLN A 701     151.829 139.079  95.832  1.00  0.00
ATOM  10637  HG2 GLN A 701     150.292 138.452  95.215  1.00  0.00
ATOM  10638  CD  GLN A 701     151.263 137.188  96.653  1.00  0.00
ATOM  10639  OE1 GLN A 701     150.709 136.171  96.273  1.00  0.00
ATOM  10640  NE2 GLN A 701     152.165 137.113  97.601  1.00  0.00
ATOM  10641 1HE2 GLN A 701     152.641 137.969  97.897  1.00  0.00
ATOM  10642 2HE2 GLN A 701     152.375 136.205  97.963  1.00  0.00
ATOM  10643  C   GLN A 701     148.858 141.454  97.723  1.00  0.00
ATOM  10644  O   GLN A 701     147.648 141.301  97.587  1.00  0.00
ATOM  10645  N   ALA A 702     149.361 142.171  98.732  1.00  0.00
ATOM  10646  H   ALA A 702     150.357 142.340  98.756  1.00  0.00
ATOM  10647  CA  ALA A 702     148.519 142.893  99.681  1.00  0.00
ATOM  10648  HA  ALA A 702     147.707 142.239  99.999  1.00  0.00
ATOM  10649  CB  ALA A 702     149.338 143.247 100.931  1.00  0.00
ATOM  10650  HB1 ALA A 702     148.684 143.702 101.677  1.00  0.00
ATOM  10651  HB2 ALA A 702     149.780 142.346 101.357  1.00  0.00
ATOM  10652  HB3 ALA A 702     150.127 143.955 100.682  1.00  0.00
ATOM  10653  C   ALA A 702     147.896 144.135  99.041  1.00  0.00
ATOM  10654  O   ALA A 702     146.701 144.336  99.189  1.00  0.00
ATOM  10655  N   LEU A 703     148.664 144.916  98.277  1.00  0.00
ATOM  10656  H   LEU A 703     149.659 144.708  98.238  1.00  0.00
ATOM  10657  CA  LEU A 703     148.178 146.039  97.471  1.00  0.00
ATOM  10658  HA  LEU A 703     147.751 146.788  98.136  1.00  0.00
ATOM  10659  CB  LEU A 703     149.419 146.639  96.768  1.00  0.00
ATOM  10660  HB1 LEU A 703     149.886 145.872  96.151  1.00  0.00
ATOM  10661  HB2 LEU A 703     150.139 146.886  97.550  1.00  0.00
ATOM  10662  CG  LEU A 703     149.247 147.904  95.911  1.00  0.00
ATOM  10663  HG  LEU A 703     150.246 148.293  95.713  1.00  0.00
ATOM  10664  CD1 LEU A 703     148.598 147.642  94.551  1.00  0.00
ATOM  10665 1HD1 LEU A 703     148.689 148.533  93.932  1.00  0.00
ATOM  10666 2HD1 LEU A 703     149.098 146.813  94.051  1.00  0.00
ATOM  10667 3HD1 LEU A 703     147.538 147.418  94.660  1.00  0.00
ATOM  10668  CD2 LEU A 703     148.465 148.996  96.642  1.00  0.00
ATOM  10669 1HD2 LEU A 703     148.476 149.910  96.049  1.00  0.00
ATOM  10670 2HD2 LEU A 703     147.429 148.687  96.790  1.00  0.00
ATOM  10671 3HD2 LEU A 703     148.930 149.194  97.608  1.00  0.00
ATOM  10672  C   LEU A 703     147.067 145.586  96.513  1.00  0.00
ATOM  10673  O   LEU A 703     145.988 146.170  96.504  1.00  0.00
```

```
ATOM  10674  N   LEU A 704     147.286 144.478  95.790  1.00  0.00
ATOM  10675  H   LEU A 704     148.216 144.063  95.819  1.00  0.00
ATOM  10676  CA  LEU A 704     146.266 143.863  94.930  1.00  0.00
ATOM  10677  HA  LEU A 704     145.845 144.644  94.303  1.00  0.00
ATOM  10678  CB  LEU A 704     146.925 142.807  94.027  1.00  0.00
ATOM  10679  HB1 LEU A 704     146.144 142.211  93.554  1.00  0.00
ATOM  10680  HB2 LEU A 704     147.525 142.139  94.646  1.00  0.00
ATOM  10681  CG  LEU A 704     147.813 143.403  92.914  1.00  0.00
ATOM  10682  HG  LEU A 704     148.565 144.060  93.348  1.00  0.00
ATOM  10683  CD1 LEU A 704     148.530 142.271  92.179  1.00  0.00
ATOM  10684 1HD1 LEU A 704     149.179 142.686  91.409  1.00  0.00
ATOM  10685 2HD1 LEU A 704     149.153 141.717  92.882  1.00  0.00
ATOM  10686 3HD1 LEU A 704     147.809 141.595  91.724  1.00  0.00
ATOM  10687  CD2 LEU A 704     146.999 144.192  91.884  1.00  0.00
ATOM  10688 1HD2 LEU A 704     147.636 144.486  91.050  1.00  0.00
ATOM  10689 2HD2 LEU A 704     146.176 143.584  91.507  1.00  0.00
ATOM  10690 3HD2 LEU A 704     146.601 145.101  92.332  1.00  0.00
ATOM  10691  C   LEU A 704     145.079 143.269  95.705  1.00  0.00
ATOM  10692  O   LEU A 704     143.937 143.415  95.274  1.00  0.00
ATOM  10693  N   ARG A 705     145.282 142.661  96.877  1.00  0.00
ATOM  10694  H   ARG A 705     146.238 142.513  97.180  1.00  0.00
ATOM  10695  CA  ARG A 705     144.182 142.249  97.775  1.00  0.00
ATOM  10696  HA  ARG A 705     143.334 141.946  97.160  1.00  0.00
ATOM  10697  CB  ARG A 705     144.597 141.040  98.624  1.00  0.00
ATOM  10698  HB1 ARG A 705     143.836 140.900  99.387  1.00  0.00
ATOM  10699  HB2 ARG A 705     145.541 141.242  99.129  1.00  0.00
ATOM  10700  CG  ARG A 705     144.699 139.769  97.762  1.00  0.00
ATOM  10701  HG1 ARG A 705     145.604 139.802  97.157  1.00  0.00
ATOM  10702  HG2 ARG A 705     143.846 139.717  97.084  1.00  0.00
ATOM  10703  CD  ARG A 705     144.708 138.495  98.616  1.00  0.00
ATOM  10704  HD1 ARG A 705     145.552 138.542  99.304  1.00  0.00
ATOM  10705  HD2 ARG A 705     144.859 137.638  97.956  1.00  0.00
ATOM  10706  NE  ARG A 705     143.423 138.338  99.332  1.00  0.00
ATOM  10707  HE  ARG A 705     142.597 138.248  98.762  1.00  0.00
ATOM  10708  CZ  ARG A 705     143.197 138.517 100.617  1.00  0.00
ATOM  10709  NH1 ARG A 705     144.157 138.580 101.502  1.00  0.00
ATOM  10710 1HH1 ARG A 705     145.106 138.480 101.191  1.00  0.00
ATOM  10711 2HH1 ARG A 705     143.956 138.732 102.472  1.00  0.00
ATOM  10712  NH2 ARG A 705     141.977 138.646 101.049  1.00  0.00
ATOM  10713 1HH2 ARG A 705     141.215 138.730 100.374  1.00  0.00
ATOM  10714 2HH2 ARG A 705     141.791 138.822 102.016  1.00  0.00
ATOM  10715  C   ARG A 705     143.623 143.383  98.636  1.00  0.00
ATOM  10716  O   ARG A 705     142.724 143.150  99.438  1.00  0.00
ATOM  10717  N   ILE A 706     144.075 144.612  98.415  1.00  0.00
ATOM  10718  H   ILE A 706     144.902 144.703  97.836  1.00  0.00
ATOM  10719  CA  ILE A 706     143.501 145.870  98.890  1.00  0.00
ATOM  10720  HA  ILE A 706     142.524 145.659  99.322  1.00  0.00
ATOM  10721  CB  ILE A 706     144.368 146.522 100.009  1.00  0.00
ATOM  10722  HB  ILE A 706     145.342 146.779  99.584  1.00  0.00
ATOM  10723  CG2 ILE A 706     143.718 147.801 100.591  1.00  0.00
ATOM  10724 1HG2 ILE A 706     142.755 147.564 101.039  1.00  0.00
ATOM  10725 2HG2 ILE A 706     144.365 148.247 101.343  1.00  0.00
ATOM  10726 3HG2 ILE A 706     143.582 148.558  99.827  1.00  0.00
ATOM  10727  CG1 ILE A 706     144.570 145.549 101.203  1.00  0.00
ATOM  10728 1HG1 ILE A 706     144.843 144.560 100.847  1.00  0.00
ATOM  10729 2HG1 ILE A 706     143.631 145.435 101.744  1.00  0.00
ATOM  10730  CD  ILE A 706     145.670 145.972 102.187  1.00  0.00
ATOM  10731  HD1 ILE A 706     146.596 146.160 101.647  1.00  0.00
ATOM  10732  HD2 ILE A 706     145.381 146.865 102.734  1.00  0.00
ATOM  10733  HD3 ILE A 706     145.836 145.170 102.904  1.00  0.00
ATOM  10734  C   ILE A 706     143.253 146.710  97.618  1.00  0.00
```

ATOM	10735	O	ILE	A	706	143.525	147.898	97.563	1.00	0.00
ATOM	10736	N	LEU	A	707	142.707	146.084	96.560	1.00	0.00
ATOM	10737	H	LEU	A	707	142.689	145.074	96.550	1.00	0.00
ATOM	10738	CA	LEU	A	707	142.169	146.782	95.381	1.00	0.00
ATOM	10739	HA	LEU	A	707	142.905	147.537	95.104	1.00	0.00
ATOM	10740	CB	LEU	A	707	142.012	145.804	94.194	1.00	0.00
ATOM	10741	HB1	LEU	A	707	141.089	146.020	93.655	1.00	0.00
ATOM	10742	HB2	LEU	A	707	141.932	144.785	94.569	1.00	0.00
ATOM	10743	CG	LEU	A	707	143.179	145.893	93.197	1.00	0.00
ATOM	10744	HG	LEU	A	707	144.124	145.854	93.734	1.00	0.00
ATOM	10745	CD1	LEU	A	707	143.117	144.734	92.203	1.00	0.00
ATOM	10746	1HD1	LEU	A	707	143.956	144.798	91.512	1.00	0.00
ATOM	10747	2HD1	LEU	A	707	143.177	143.788	92.739	1.00	0.00
ATOM	10748	3HD1	LEU	A	707	142.185	144.771	91.642	1.00	0.00
ATOM	10749	CD2	LEU	A	707	143.123	147.195	92.390	1.00	0.00
ATOM	10750	1HD2	LEU	A	707	143.945	147.218	91.678	1.00	0.00
ATOM	10751	2HD2	LEU	A	707	142.179	147.258	91.850	1.00	0.00
ATOM	10752	3HD2	LEU	A	707	143.216	148.059	93.046	1.00	0.00
ATOM	10753	C	LEU	A	707	140.873	147.575	95.649	1.00	0.00
ATOM	10754	O	LEU	A	707	140.386	148.295	94.783	1.00	0.00
ATOM	10755	N	LYS	A	708	140.345	147.504	96.876	1.00	0.00
ATOM	10756	H	LYS	A	708	140.722	146.795	97.487	1.00	0.00
ATOM	10757	CA	LYS	A	708	139.528	148.563	97.490	1.00	0.00
ATOM	10758	HA	LYS	A	708	138.736	148.847	96.798	1.00	0.00
ATOM	10759	CB	LYS	A	708	138.889	148.061	98.803	1.00	0.00
ATOM	10760	HB1	LYS	A	708	138.141	147.306	98.576	1.00	0.00
ATOM	10761	HB2	LYS	A	708	138.362	148.892	99.272	1.00	0.00
ATOM	10762	CG	LYS	A	708	139.912	147.465	99.788	1.00	0.00
ATOM	10763	HG1	LYS	A	708	140.776	148.122	99.867	1.00	0.00
ATOM	10764	HG2	LYS	A	708	140.254	146.505	99.401	1.00	0.00
ATOM	10765	CD	LYS	A	708	139.333	147.228	101.193	1.00	0.00
ATOM	10766	HD1	LYS	A	708	140.056	146.661	101.781	1.00	0.00
ATOM	10767	HD2	LYS	A	708	138.430	146.621	101.098	1.00	0.00
ATOM	10768	CE	LYS	A	708	138.981	148.520	101.946	1.00	0.00
ATOM	10769	HE1	LYS	A	708	138.494	148.256	102.889	1.00	0.00
ATOM	10770	HE2	LYS	A	708	138.253	149.097	101.370	1.00	0.00
ATOM	10771	NZ	LYS	A	708	140.164	149.361	102.238	1.00	0.00
ATOM	10772	HZ1	LYS	A	708	139.869	150.162	102.807	1.00	0.00
ATOM	10773	HZ2	LYS	A	708	140.859	148.881	102.786	1.00	0.00
ATOM	10774	HZ3	LYS	A	708	140.597	149.740	101.397	1.00	0.00
ATOM	10775	C	LYS	A	708	140.321	149.841	97.757	1.00	0.00
ATOM	10776	O	LYS	A	708	139.731	150.813	98.210	1.00	0.00
ATOM	10777	N	GLU	A	709	141.630	149.834	97.525	1.00	0.00
ATOM	10778	H	GLU	A	709	142.033	148.975	97.175	1.00	0.00
ATOM	10779	CA	GLU	A	709	142.613	150.827	97.949	1.00	0.00
ATOM	10780	HA	GLU	A	709	143.600	150.364	97.936	1.00	0.00
ATOM	10781	CB	GLU	A	709	142.644	151.971	96.921	1.00	0.00
ATOM	10782	HB1	GLU	A	709	143.294	152.769	97.281	1.00	0.00
ATOM	10783	HB2	GLU	A	709	141.637	152.367	96.800	1.00	0.00
ATOM	10784	CG	GLU	A	709	143.170	151.495	95.559	1.00	0.00
ATOM	10785	HG1	GLU	A	709	142.575	150.654	95.201	1.00	0.00
ATOM	10786	HG2	GLU	A	709	144.201	151.154	95.671	1.00	0.00
ATOM	10787	CD	GLU	A	709	143.117	152.626	94.531	1.00	0.00
ATOM	10788	OE1	GLU	A	709	143.605	153.741	94.820	1.00	0.00
ATOM	10789	OE2	GLU	A	709	142.557	152.440	93.427	1.00	0.00
ATOM	10790	C	GLU	A	709	142.386	151.274	99.416	1.00	0.00
ATOM	10791	O	GLU	A	709	141.690	150.622	100.209	1.00	0.00
ATOM	10792	N	THR	A	710	142.995	152.386	99.819	1.00	0.00
ATOM	10793	H	THR	A	710	143.644	152.847	99.202	1.00	0.00
ATOM	10794	CA	THR	A	710	142.615	153.067	101.056	1.00	0.00
ATOM	10795	HA	THR	A	710	142.599	152.349	101.878	1.00	0.00

```
ATOM  10796  CB  THR A 710     143.625 154.172 101.402  1.00  0.00
ATOM  10797  HB  THR A 710     143.638 154.922 100.612  1.00  0.00
ATOM  10798  CG2 THR A 710     143.335 154.857 102.740  1.00  0.00
ATOM  10799 1HG2 THR A 710     144.132 155.562 102.976  1.00  0.00
ATOM  10800 2HG2 THR A 710     142.395 155.404 102.680  1.00  0.00
ATOM  10801 3HG2 THR A 710     143.270 154.110 103.532  1.00  0.00
ATOM  10802  OG1 THR A 710     144.908 153.610 101.511  1.00  0.00
ATOM  10803  HG1 THR A 710     145.543 154.314 101.659  1.00  0.00
ATOM  10804  C   THR A 710     141.232 153.684 100.897  1.00  0.00
ATOM  10805  O   THR A 710     141.038 154.522 100.020  1.00  0.00
ATOM  10806  N   GLU A 711     140.284 153.275 101.739  1.00  0.00
ATOM  10807  H   GLU A 711     140.587 152.639 102.475  1.00  0.00
ATOM  10808  CA  GLU A 711     138.822 153.410 101.600  1.00  0.00
ATOM  10809  HA  GLU A 711     138.466 152.383 101.538  1.00  0.00
ATOM  10810  CB  GLU A 711     138.140 153.962 102.868  1.00  0.00
ATOM  10811  HB1 GLU A 711     137.063 153.839 102.743  1.00  0.00
ATOM  10812  HB2 GLU A 711     138.340 155.024 102.969  1.00  0.00
ATOM  10813  CG  GLU A 711     138.554 153.258 104.177  1.00  0.00
ATOM  10814  HG1 GLU A 711     137.731 153.317 104.894  1.00  0.00
ATOM  10815  HG2 GLU A 711     139.403 153.803 104.600  1.00  0.00
ATOM  10816  CD  GLU A 711     138.960 151.796 103.949  1.00  0.00
ATOM  10817  OE1 GLU A 711     138.118 150.985 103.515  1.00  0.00
ATOM  10818  OE2 GLU A 711     140.181 151.517 103.934  1.00  0.00
ATOM  10819  C   GLU A 711     138.307 153.980 100.271  1.00  0.00
ATOM  10820  O   GLU A 711     137.810 155.108 100.220  1.00  0.00
ATOM  10821  N   PHE A 712     138.444 153.157  99.222  1.00  0.00
ATOM  10822  H   PHE A 712     138.896 152.267  99.406  1.00  0.00
ATOM  10823  CA  PHE A 712     138.229 153.415  97.794  1.00  0.00
ATOM  10824  HA  PHE A 712     138.743 152.616  97.279  1.00  0.00
ATOM  10825  CB  PHE A 712     136.765 153.248  97.371  1.00  0.00
ATOM  10826  HB1 PHE A 712     136.179 154.046  97.822  1.00  0.00
ATOM  10827  HB2 PHE A 712     136.411 152.291  97.748  1.00  0.00
ATOM  10828  CG  PHE A 712     136.555 153.256  95.862  1.00  0.00
ATOM  10829  CD1 PHE A 712     135.608 154.125  95.286  1.00  0.00
ATOM  10830  HD1 PHE A 712     135.000 154.759  95.915  1.00  0.00
ATOM  10831  CE1 PHE A 712     135.461 154.188  93.888  1.00  0.00
ATOM  10832  HE1 PHE A 712     134.746 154.871  93.447  1.00  0.00
ATOM  10833  CZ  PHE A 712     136.257 153.380  93.059  1.00  0.00
ATOM  10834  HZ  PHE A 712     136.159 153.445  91.982  1.00  0.00
ATOM  10835  CE2 PHE A 712     137.188 152.495  93.629  1.00  0.00
ATOM  10836  HE2 PHE A 712     137.812 151.876  92.994  1.00  0.00
ATOM  10837  CD2 PHE A 712     137.329 152.425  95.025  1.00  0.00
ATOM  10838  HD2 PHE A 712     138.058 151.746  95.443  1.00  0.00
ATOM  10839  C   PHE A 712     138.921 154.660  97.242  1.00  0.00
ATOM  10840  O   PHE A 712     138.276 155.654  96.929  1.00  0.00
ATOM  10841  N   LYS A 713     140.261 154.596  97.165  1.00  0.00
ATOM  10842  H   LYS A 713     140.675 153.750  97.530  1.00  0.00
ATOM  10843  CA  LYS A 713     141.205 155.651  96.720  1.00  0.00
ATOM  10844  HA  LYS A 713     142.213 155.249  96.838  1.00  0.00
ATOM  10845  CB  LYS A 713     140.990 155.892  95.208  1.00  0.00
ATOM  10846  HB1 LYS A 713     140.017 156.360  95.050  1.00  0.00
ATOM  10847  HB2 LYS A 713     140.976 154.918  94.718  1.00  0.00
ATOM  10848  CG  LYS A 713     142.067 156.763  94.530  1.00  0.00
ATOM  10849  HG1 LYS A 713     143.053 156.346  94.732  1.00  0.00
ATOM  10850  HG2 LYS A 713     142.035 157.768  94.948  1.00  0.00
ATOM  10851  CD  LYS A 713     141.875 156.908  93.007  1.00  0.00
ATOM  10852  HD1 LYS A 713     142.680 157.531  92.613  1.00  0.00
ATOM  10853  HD2 LYS A 713     140.933 157.429  92.828  1.00  0.00
ATOM  10854  CE  LYS A 713     141.846 155.579  92.242  1.00  0.00
ATOM  10855  HE1 LYS A 713     141.665 155.772  91.181  1.00  0.00
ATOM  10856  HE2 LYS A 713     141.013 154.981  92.619  1.00  0.00
```

ATOM	10857	NZ	LYS	A	713	143.093	154.806	92.413	1.00	0.00
ATOM	10858	HZ1	LYS	A	713	143.345	154.739	93.405	1.00	0.00
ATOM	10859	HZ2	LYS	A	713	142.918	153.807	92.269	1.00	0.00
ATOM	10860	HZ3	LYS	A	713	143.881	155.127	91.881	1.00	0.00
ATOM	10861	C	LYS	A	713	141.165	156.943	97.555	1.00	0.00
ATOM	10862	O	LYS	A	713	142.036	157.805	97.442	1.00	0.00
ATOM	10863	N	LYS	A	714	140.159	157.082	98.415	1.00	0.00
ATOM	10864	H	LYS	A	714	139.480	156.335	98.363	1.00	0.00
ATOM	10865	CA	LYS	A	714	139.884	158.104	99.422	1.00	0.00
ATOM	10866	HA	LYS	A	714	138.975	157.769	99.896	1.00	0.00
ATOM	10867	CB	LYS	A	714	141.004	158.096	100.482	1.00	0.00
ATOM	10868	HB1	LYS	A	714	141.813	158.743	100.139	1.00	0.00
ATOM	10869	HB2	LYS	A	714	141.419	157.091	100.579	1.00	0.00
ATOM	10870	CG	LYS	A	714	140.546	158.568	101.870	1.00	0.00
ATOM	10871	HG1	LYS	A	714	139.969	159.490	101.790	1.00	0.00
ATOM	10872	HG2	LYS	A	714	141.445	158.802	102.442	1.00	0.00
ATOM	10873	CD	LYS	A	714	139.760	157.501	102.653	1.00	0.00
ATOM	10874	HD1	LYS	A	714	140.221	157.420	103.638	1.00	0.00
ATOM	10875	HD2	LYS	A	714	139.864	156.524	102.178	1.00	0.00
ATOM	10876	CE	LYS	A	714	138.277	157.831	102.886	1.00	0.00
ATOM	10877	HE1	LYS	A	714	138.173	158.901	103.084	1.00	0.00
ATOM	10878	HE2	LYS	A	714	137.960	157.296	103.785	1.00	0.00
ATOM	10879	NZ	LYS	A	714	137.400	157.421	101.761	1.00	0.00
ATOM	10880	HZ1	LYS	A	714	137.594	156.470	101.452	1.00	0.00
ATOM	10881	HZ2	LYS	A	714	136.425	157.493	102.003	1.00	0.00
ATOM	10882	HZ3	LYS	A	714	137.521	157.987	100.924	1.00	0.00
ATOM	10883	C	LYS	A	714	139.593	159.517	98.935	1.00	0.00
ATOM	10884	O	LYS	A	714	138.664	160.120	99.466	1.00	0.00
ATOM	10885	N	ILE	A	715	140.347	160.006	97.945	1.00	0.00
ATOM	10886	H	ILE	A	715	141.040	159.348	97.599	1.00	0.00
ATOM	10887	CA	ILE	A	715	140.568	161.410	97.527	1.00	0.00
ATOM	10888	HA	ILE	A	715	141.319	161.801	98.213	1.00	0.00
ATOM	10889	CB	ILE	A	715	141.230	161.415	96.126	1.00	0.00
ATOM	10890	HB	ILE	A	715	142.120	160.788	96.201	1.00	0.00
ATOM	10891	CG2	ILE	A	715	140.323	160.787	95.051	1.00	0.00
ATOM	10892	1HG2	ILE	A	715	140.864	160.702	94.109	1.00	0.00
ATOM	10893	2HG2	ILE	A	715	140.028	159.779	95.337	1.00	0.00
ATOM	10894	3HG2	ILE	A	715	139.436	161.397	94.884	1.00	0.00
ATOM	10895	CG1	ILE	A	715	141.699	162.833	95.739	1.00	0.00
ATOM	10896	1HG1	ILE	A	715	142.256	163.259	96.574	1.00	0.00
ATOM	10897	2HG1	ILE	A	715	140.839	163.471	95.536	1.00	0.00
ATOM	10898	CD	ILE	A	715	142.621	162.862	94.511	1.00	0.00
ATOM	10899	HD1	ILE	A	715	143.006	163.872	94.374	1.00	0.00
ATOM	10900	HD2	ILE	A	715	143.459	162.181	94.660	1.00	0.00
ATOM	10901	HD3	ILE	A	715	142.073	162.576	93.614	1.00	0.00
ATOM	10902	C	ILE	A	715	139.380	162.385	97.650	1.00	0.00
ATOM	10903	O	ILE	A	715	139.566	163.553	98.001	1.00	0.00
ATOM	10904	N	LYS	A	716	138.164	161.893	97.412	1.00	0.00
ATOM	10905	H	LYS	A	716	138.167	160.969	97.003	1.00	0.00
ATOM	10906	CA	LYS	A	716	136.890	162.288	98.057	1.00	0.00
ATOM	10907	HA	LYS	A	716	136.997	162.160	99.136	1.00	0.00
ATOM	10908	CB	LYS	A	716	136.531	163.771	97.800	1.00	0.00
ATOM	10909	HB1	LYS	A	716	137.211	164.379	98.390	1.00	0.00
ATOM	10910	HB2	LYS	A	716	135.535	163.967	98.198	1.00	0.00
ATOM	10911	CG	LYS	A	716	136.568	164.218	96.326	1.00	0.00
ATOM	10912	HG1	LYS	A	716	135.675	163.835	95.830	1.00	0.00
ATOM	10913	HG2	LYS	A	716	137.436	163.809	95.807	1.00	0.00
ATOM	10914	CD	LYS	A	716	136.594	165.752	96.174	1.00	0.00
ATOM	10915	HD1	LYS	A	716	136.019	166.213	96.979	1.00	0.00
ATOM	10916	HD2	LYS	A	716	136.087	165.993	95.237	1.00	0.00
ATOM	10917	CE	LYS	A	716	138.008	166.351	96.099	1.00	0.00

```
ATOM  10918  HE1 LYS A 716     137.914 167.422  95.894  1.00  0.00
ATOM  10919  HE2 LYS A 716     138.532 165.904  95.248  1.00  0.00
ATOM  10920  NZ  LYS A 716     138.810 166.151  97.335  1.00  0.00
ATOM  10921  HZ1 LYS A 716     139.716 166.593  97.260  1.00  0.00
ATOM  10922  HZ2 LYS A 716     138.332 166.521  98.143  1.00  0.00
ATOM  10923  HZ3 LYS A 716     138.978 165.158  97.503  1.00  0.00
ATOM  10924  C   LYS A 716     135.795 161.309  97.632  1.00  0.00
ATOM  10925  O   LYS A 716     134.714 161.672  97.187  1.00  0.00
ATOM  10926  N   VAL A 717     136.167 160.038  97.723  1.00  0.00
ATOM  10927  H   VAL A 717     137.031 159.862  98.220  1.00  0.00
ATOM  10928  CA  VAL A 717     135.411 158.846  97.317  1.00  0.00
ATOM  10929  HA  VAL A 717     134.352 159.095  97.235  1.00  0.00
ATOM  10930  CB  VAL A 717     135.896 158.266  95.973  1.00  0.00
ATOM  10931  HB  VAL A 717     135.662 157.202  95.945  1.00  0.00
ATOM  10932  CG1 VAL A 717     135.142 158.933  94.818  1.00  0.00
ATOM  10933 1HG1 VAL A 717     135.476 158.511  93.870  1.00  0.00
ATOM  10934 2HG1 VAL A 717     134.072 158.754  94.922  1.00  0.00
ATOM  10935 3HG1 VAL A 717     135.321 160.008  94.818  1.00  0.00
ATOM  10936  CG2 VAL A 717     137.402 158.444  95.730  1.00  0.00
ATOM  10937 1HG2 VAL A 717     137.703 157.823  94.886  1.00  0.00
ATOM  10938 2HG2 VAL A 717     137.630 159.483  95.504  1.00  0.00
ATOM  10939 3HG2 VAL A 717     137.957 158.125  96.609  1.00  0.00
ATOM  10940  C   VAL A 717     135.555 157.847  98.461  1.00  0.00
ATOM  10941  O   VAL A 717     136.507 157.930  99.241  1.00  0.00
ATOM  10942  N   LEU A 718     134.579 156.957  98.631  1.00  0.00
ATOM  10943  H   LEU A 718     133.812 156.924  97.971  1.00  0.00
ATOM  10944  CA  LEU A 718     134.517 156.060  99.783  1.00  0.00
ATOM  10945  HA  LEU A 718     135.531 155.839 100.103  1.00  0.00
ATOM  10946  CB  LEU A 718     133.778 156.776 100.933  1.00  0.00
ATOM  10947  HB1 LEU A 718     132.737 156.943 100.653  1.00  0.00
ATOM  10948  HB2 LEU A 718     134.240 157.756 101.057  1.00  0.00
ATOM  10949  CG  LEU A 718     133.844 156.024 102.279  1.00  0.00
ATOM  10950  HG  LEU A 718     134.753 155.421 102.308  1.00  0.00
ATOM  10951  CD1 LEU A 718     133.911 157.023 103.436  1.00  0.00
ATOM  10952 1HD1 LEU A 718     133.964 156.486 104.384  1.00  0.00
ATOM  10953 2HD1 LEU A 718     134.788 157.660 103.349  1.00  0.00
ATOM  10954 3HD1 LEU A 718     133.017 157.648 103.440  1.00  0.00
ATOM  10955  CD2 LEU A 718     132.632 155.119 102.514  1.00  0.00
ATOM  10956 1HD2 LEU A 718     132.753 154.579 103.453  1.00  0.00
ATOM  10957 2HD2 LEU A 718     131.719 155.715 102.564  1.00  0.00
ATOM  10958 3HD2 LEU A 718     132.519 154.391 101.714  1.00  0.00
ATOM  10959  C   LEU A 718     133.860 154.738  99.395  1.00  0.00
ATOM  10960  O   LEU A 718     132.851 154.728  98.703  1.00  0.00
ATOM  10961  N   GLY A 719     134.437 153.634  99.871  1.00  0.00
ATOM  10962  H   GLY A 719     135.273 153.727 100.427  1.00  0.00
ATOM  10963  CA  GLY A 719     133.987 152.285  99.549  1.00  0.00
ATOM  10964  HA1 GLY A 719     134.591 151.858  98.753  1.00  0.00
ATOM  10965  HA2 GLY A 719     132.961 152.317  99.203  1.00  0.00
ATOM  10966  C   GLY A 719     134.058 151.373 100.759  1.00  0.00
ATOM  10967  O   GLY A 719     135.087 150.765 101.047  1.00  0.00
ATOM  10968  N   SER A 720     132.943 151.340 101.477  1.00  0.00
ATOM  10969  H   SER A 720     132.180 151.909 101.137  1.00  0.00
ATOM  10970  CA  SER A 720     132.541 150.487 102.605  1.00  0.00
ATOM  10971  HA  SER A 720     133.266 150.609 103.409  1.00  0.00
ATOM  10972  CB  SER A 720     131.183 151.034 103.096  1.00  0.00
ATOM  10973  HB1 SER A 720     131.377 151.876 103.760  1.00  0.00
ATOM  10974  HB2 SER A 720     130.636 150.276 103.659  1.00  0.00
ATOM  10975  OG  SER A 720     130.400 151.513 102.003  1.00  0.00
ATOM  10976  HG  SER A 720     129.617 151.982 102.334  1.00  0.00
ATOM  10977  C   SER A 720     132.497 148.978 102.266  1.00  0.00
ATOM  10978  O   SER A 720     131.505 148.291 102.499  1.00  0.00
```

```
ATOM  10979  N   GLY A 721     133.570 148.457 101.660  1.00  0.00
ATOM  10980  H   GLY A 721     134.365 149.073 101.524  1.00  0.00
ATOM  10981  CA  GLY A 721     133.544 147.227 100.869  1.00  0.00
ATOM  10982  HA1 GLY A 721     133.782 147.479  99.836  1.00  0.00
ATOM  10983  HA2 GLY A 721     132.543 146.796 100.866  1.00  0.00
ATOM  10984  C   GLY A 721     134.522 146.142 101.302  1.00  0.00
ATOM  10985  O   GLY A 721     135.645 146.069 100.804  1.00  0.00
ATOM  10986  N   ALA A 722     134.057 145.262 102.188  1.00  0.00
ATOM  10987  H   ALA A 722     133.129 145.404 102.559  1.00  0.00
ATOM  10988  CA  ALA A 722     134.702 143.989 102.497  1.00  0.00
ATOM  10989  HA  ALA A 722     135.784 144.110 102.419  1.00  0.00
ATOM  10990  CB  ALA A 722     134.373 143.625 103.953  1.00  0.00
ATOM  10991  HB1 ALA A 722     134.847 142.677 104.211  1.00  0.00
ATOM  10992  HB2 ALA A 722     134.746 144.401 104.622  1.00  0.00
ATOM  10993  HB3 ALA A 722     133.295 143.524 104.082  1.00  0.00
ATOM  10994  C   ALA A 722     134.254 142.889 101.517  1.00  0.00
ATOM  10995  O   ALA A 722     133.112 142.436 101.547  1.00  0.00
ATOM  10996  N   PHE A 723     135.191 142.423 100.687  1.00  0.00
ATOM  10997  H   PHE A 723     136.109 142.839 100.714  1.00  0.00
ATOM  10998  CA  PHE A 723     135.059 141.190  99.911  1.00  0.00
ATOM  10999  HA  PHE A 723     134.367 140.535 100.442  1.00  0.00
ATOM  11000  CB  PHE A 723     134.449 141.488  98.536  1.00  0.00
ATOM  11001  HB1 PHE A 723     135.173 142.064  97.967  1.00  0.00
ATOM  11002  HB2 PHE A 723     133.559 142.105  98.667  1.00  0.00
ATOM  11003  CG  PHE A 723     134.044 140.244  97.763  1.00  0.00
ATOM  11004  CD1 PHE A 723     133.074 139.368  98.286  1.00  0.00
ATOM  11005  HD1 PHE A 723     132.573 139.603  99.215  1.00  0.00
ATOM  11006  CE1 PHE A 723     132.739 138.189  97.596  1.00  0.00
ATOM  11007  HE1 PHE A 723     131.988 137.520  97.993  1.00  0.00
ATOM  11008  CZ  PHE A 723     133.373 137.882  96.379  1.00  0.00
ATOM  11009  HZ  PHE A 723     133.111 136.981  95.843  1.00  0.00
ATOM  11010  CE2 PHE A 723     134.331 138.760  95.846  1.00  0.00
ATOM  11011  HE2 PHE A 723     134.805 138.534  94.901  1.00  0.00
ATOM  11012  CD2 PHE A 723     134.657 139.941  96.535  1.00  0.00
ATOM  11013  HD2 PHE A 723     135.365 140.628  96.109  1.00  0.00
ATOM  11014  C   PHE A 723     136.402 140.451  99.835  1.00  0.00
ATOM  11015  O   PHE A 723     137.446 141.034 100.146  1.00  0.00
ATOM  11016  N   GLY A 724     136.378 139.167  99.456  1.00  0.00
ATOM  11017  H   GLY A 724     135.488 138.764  99.195  1.00  0.00
ATOM  11018  CA  GLY A 724     137.553 138.289  99.475  1.00  0.00
ATOM  11019  HA1 GLY A 724     137.268 137.317  99.075  1.00  0.00
ATOM  11020  HA2 GLY A 724     137.882 138.156 100.506  1.00  0.00
ATOM  11021  C   GLY A 724     138.747 138.799  98.652  1.00  0.00
ATOM  11022  O   GLY A 724     139.893 138.699  99.109  1.00  0.00
ATOM  11023  N   THR A 725     138.475 139.356  97.464  1.00  0.00
ATOM  11024  H   THR A 725     137.522 139.311  97.146  1.00  0.00
ATOM  11025  CA  THR A 725     139.506 139.796  96.497  1.00  0.00
ATOM  11026  HA  THR A 725     140.363 140.191  97.043  1.00  0.00
ATOM  11027  CB  THR A 725     139.980 138.604  95.630  1.00  0.00
ATOM  11028  HB  THR A 725     140.548 138.980  94.779  1.00  0.00
ATOM  11029  CG2 THR A 725     140.871 137.605  96.361  1.00  0.00
ATOM  11030 1HG2 THR A 725     141.288 136.899  95.643  1.00  0.00
ATOM  11031 2HG2 THR A 725     141.693 138.130  96.842  1.00  0.00
ATOM  11032 3HG2 THR A 725     140.301 137.051  97.105  1.00  0.00
ATOM  11033  OG1 THR A 725     138.864 137.888  95.153  1.00  0.00
ATOM  11034  HG1 THR A 725     139.181 137.273  94.489  1.00  0.00
ATOM  11035  C   THR A 725     139.111 140.880  95.483  1.00  0.00
ATOM  11036  O   THR A 725     139.993 141.370  94.781  1.00  0.00
ATOM  11037  N   VAL A 726     137.831 141.255  95.358  1.00  0.00
ATOM  11038  H   VAL A 726     137.158 140.885  96.007  1.00  0.00
ATOM  11039  CA  VAL A 726     137.316 142.121  94.269  1.00  0.00
```

ATOM	11040	HA	VAL	A	726	138.162	142.560	93.737	1.00	0.00
ATOM	11041	CB	VAL	A	726	136.483	141.313	93.245	1.00	0.00
ATOM	11042	HB	VAL	A	726	135.589	140.928	93.734	1.00	0.00
ATOM	11043	CG1	VAL	A	726	136.044	142.168	92.045	1.00	0.00
ATOM	11044	1HG1	VAL	A	726	135.497	141.548	91.334	1.00	0.00
ATOM	11045	2HG1	VAL	A	726	135.375	142.964	92.371	1.00	0.00
ATOM	11046	3HG1	VAL	A	726	136.917	142.594	91.552	1.00	0.00
ATOM	11047	CG2	VAL	A	726	137.262	140.124	92.672	1.00	0.00
ATOM	11048	1HG2	VAL	A	726	136.674	139.622	91.903	1.00	0.00
ATOM	11049	2HG2	VAL	A	726	138.203	140.465	92.237	1.00	0.00
ATOM	11050	3HG2	VAL	A	726	137.472	139.401	93.457	1.00	0.00
ATOM	11051	C	VAL	A	726	136.490	143.267	94.846	1.00	0.00
ATOM	11052	O	VAL	A	726	135.756	143.076	95.810	1.00	0.00
ATOM	11053	N	TYR	A	727	136.633	144.463	94.268	1.00	0.00
ATOM	11054	H	TYR	A	727	137.204	144.530	93.440	1.00	0.00
ATOM	11055	CA	TYR	A	727	136.248	145.717	94.924	1.00	0.00
ATOM	11056	HA	TYR	A	727	135.467	145.500	95.655	1.00	0.00
ATOM	11057	CB	TYR	A	727	137.469	146.244	95.699	1.00	0.00
ATOM	11058	HB1	TYR	A	727	137.160	147.096	96.304	1.00	0.00
ATOM	11059	HB2	TYR	A	727	138.215	146.591	94.985	1.00	0.00
ATOM	11060	CG	TYR	A	727	138.114	145.217	96.622	1.00	0.00
ATOM	11061	CD1	TYR	A	727	139.303	144.567	96.239	1.00	0.00
ATOM	11062	HD1	TYR	A	727	139.759	144.802	95.288	1.00	0.00
ATOM	11063	CE1	TYR	A	727	139.871	143.570	97.054	1.00	0.00
ATOM	11064	HE1	TYR	A	727	140.766	143.049	96.742	1.00	0.00
ATOM	11065	CZ	TYR	A	727	139.219	143.184	98.240	1.00	0.00
ATOM	11066	OH	TYR	A	727	139.683	142.136	98.963	1.00	0.00
ATOM	11067	HH	TYR	A	727	139.037	141.868	99.632	1.00	0.00
ATOM	11068	CE2	TYR	A	727	138.039	143.847	98.638	1.00	0.00
ATOM	11069	HE2	TYR	A	727	137.549	143.567	99.554	1.00	0.00
ATOM	11070	CD2	TYR	A	727	137.485	144.857	97.831	1.00	0.00
ATOM	11071	HD2	TYR	A	727	136.558	145.328	98.126	1.00	0.00
ATOM	11072	C	TYR	A	727	135.619	146.723	93.937	1.00	0.00
ATOM	11073	O	TYR	A	727	134.774	146.345	93.131	1.00	0.00
ATOM	11074	N	LYS	A	728	135.970	148.021	94.033	1.00	0.00
ATOM	11075	H	LYS	A	728	136.716	148.258	94.672	1.00	0.00
ATOM	11076	CA	LYS	A	728	135.292	149.136	93.335	1.00	0.00
ATOM	11077	HA	LYS	A	728	135.675	150.064	93.763	1.00	0.00
ATOM	11078	CB	LYS	A	728	135.679	149.114	91.839	1.00	0.00
ATOM	11079	HB1	LYS	A	728	135.124	148.319	91.341	1.00	0.00
ATOM	11080	HB2	LYS	A	728	136.739	148.871	91.748	1.00	0.00
ATOM	11081	CG	LYS	A	728	135.450	150.464	91.124	1.00	0.00
ATOM	11082	HG1	LYS	A	728	136.417	150.954	91.007	1.00	0.00
ATOM	11083	HG2	LYS	A	728	134.828	151.115	91.739	1.00	0.00
ATOM	11084	CD	LYS	A	728	134.799	150.331	89.739	1.00	0.00
ATOM	11085	HD1	LYS	A	728	135.378	149.635	89.129	1.00	0.00
ATOM	11086	HD2	LYS	A	728	134.813	151.312	89.262	1.00	0.00
ATOM	11087	CE	LYS	A	728	133.347	149.847	89.866	1.00	0.00
ATOM	11088	HE1	LYS	A	728	132.827	150.496	90.578	1.00	0.00
ATOM	11089	HE2	LYS	A	728	133.349	148.844	90.304	1.00	0.00
ATOM	11090	NZ	LYS	A	728	132.631	149.842	88.570	1.00	0.00
ATOM	11091	HZ1	LYS	A	728	131.694	149.483	88.693	1.00	0.00
ATOM	11092	HZ2	LYS	A	728	132.562	150.780	88.203	1.00	0.00
ATOM	11093	HZ3	LYS	A	728	133.108	149.260	87.898	1.00	0.00
ATOM	11094	C	LYS	A	728	133.770	149.163	93.596	1.00	0.00
ATOM	11095	O	LYS	A	728	132.983	149.540	92.727	1.00	0.00
ATOM	11096	N	GLY	A	729	133.370	148.755	94.803	1.00	0.00
ATOM	11097	H	GLY	A	729	134.085	148.500	95.464	1.00	0.00
ATOM	11098	CA	GLY	A	729	132.016	148.877	95.335	1.00	0.00
ATOM	11099	HA1	GLY	A	729	131.708	147.935	95.787	1.00	0.00
ATOM	11100	HA2	GLY	A	729	131.312	149.124	94.540	1.00	0.00

ATOM	11101	C	GLY	A	729	131.979	149.970	96.400	1.00	0.00
ATOM	11102	O	GLY	A	729	132.972	150.198	97.101	1.00	0.00
ATOM	11103	N	LEU	A	730	130.860	150.685	96.470	1.00	0.00
ATOM	11104	H	LEU	A	730	130.053	150.381	95.943	1.00	0.00
ATOM	11105	CA	LEU	A	730	130.765	151.975	97.151	1.00	0.00
ATOM	11106	HA	LEU	A	730	131.388	151.923	98.036	1.00	0.00
ATOM	11107	CB	LEU	A	730	131.324	153.115	96.270	1.00	0.00
ATOM	11108	HB1	LEU	A	730	132.362	152.878	96.033	1.00	0.00
ATOM	11109	HB2	LEU	A	730	131.338	154.006	96.892	1.00	0.00
ATOM	11110	CG	LEU	A	730	130.600	153.519	94.965	1.00	0.00
ATOM	11111	HG	LEU	A	730	129.604	153.894	95.190	1.00	0.00
ATOM	11112	CD1	LEU	A	730	131.382	154.665	94.323	1.00	0.00
ATOM	11113	1HD1	LEU	A	730	130.855	155.021	93.439	1.00	0.00
ATOM	11114	2HD1	LEU	A	730	131.466	155.493	95.028	1.00	0.00
ATOM	11115	3HD1	LEU	A	730	132.377	154.329	94.038	1.00	0.00
ATOM	11116	CD2	LEU	A	730	130.488	152.400	93.925	1.00	0.00
ATOM	11117	1HD2	LEU	A	730	130.106	152.805	92.989	1.00	0.00
ATOM	11118	2HD2	LEU	A	730	131.463	151.943	93.756	1.00	0.00
ATOM	11119	3HD2	LEU	A	730	129.778	151.651	94.270	1.00	0.00
ATOM	11120	C	LEU	A	730	129.345	152.239	97.655	1.00	0.00
ATOM	11121	O	LEU	A	730	128.431	152.462	96.861	1.00	0.00
ATOM	11122	N	TRP	A	731	129.142	152.189	98.972	1.00	0.00
ATOM	11123	H	TRP	A	731	129.926	151.986	99.576	1.00	0.00
ATOM	11124	CA	TRP	A	731	127.805	152.147	99.572	1.00	0.00
ATOM	11125	HA	TRP	A	731	127.060	152.203	98.779	1.00	0.00
ATOM	11126	CB	TRP	A	731	127.559	150.802	100.281	1.00	0.00
ATOM	11127	HB1	TRP	A	731	126.485	150.613	100.299	1.00	0.00
ATOM	11128	HB2	TRP	A	731	127.876	150.884	101.321	1.00	0.00
ATOM	11129	CG	TRP	A	731	128.247	149.603	99.693	1.00	0.00
ATOM	11130	CD1	TRP	A	731	129.098	148.804	100.370	1.00	0.00
ATOM	11131	HD1	TRP	A	731	129.361	148.929	101.412	1.00	0.00
ATOM	11132	NE1	TRP	A	731	129.601	147.834	99.530	1.00	0.00
ATOM	11133	HE1	TRP	A	731	130.258	147.132	99.841	1.00	0.00
ATOM	11134	CE2	TRP	A	731	129.098	147.959	98.253	1.00	0.00
ATOM	11135	CZ2	TRP	A	731	129.309	147.241	97.068	1.00	0.00
ATOM	11136	HZ2	TRP	A	731	129.972	146.387	97.056	1.00	0.00
ATOM	11137	CH2	TRP	A	731	128.638	147.637	95.899	1.00	0.00
ATOM	11138	HH2	TRP	A	731	128.780	147.090	94.978	1.00	0.00
ATOM	11139	CZ3	TRP	A	731	127.763	148.738	95.934	1.00	0.00
ATOM	11140	HZ3	TRP	A	731	127.235	149.037	95.038	1.00	0.00
ATOM	11141	CE3	TRP	A	731	127.551	149.446	97.134	1.00	0.00
ATOM	11142	HE3	TRP	A	731	126.876	150.290	97.144	1.00	0.00
ATOM	11143	CD2	TRP	A	731	128.216	149.080	98.326	1.00	0.00
ATOM	11144	C	TRP	A	731	127.574	153.345	100.497	1.00	0.00
ATOM	11145	O	TRP	A	731	128.151	153.440	101.584	1.00	0.00
ATOM	11146	N	ILE	A	732	126.781	154.292	99.997	1.00	0.00
ATOM	11147	H	ILE	A	732	126.386	154.119	99.086	1.00	0.00
ATOM	11148	CA	ILE	A	732	126.405	155.548	100.657	1.00	0.00
ATOM	11149	HA	ILE	A	732	127.319	156.069	100.934	1.00	0.00
ATOM	11150	CB	ILE	A	732	125.626	156.415	99.629	1.00	0.00
ATOM	11151	HB	ILE	A	732	124.909	155.766	99.122	1.00	0.00
ATOM	11152	CG2	ILE	A	732	124.818	157.568	100.256	1.00	0.00
ATOM	11153	1HG2	ILE	A	732	124.251	158.091	99.488	1.00	0.00
ATOM	11154	2HG2	ILE	A	732	124.087	157.193	100.974	1.00	0.00
ATOM	11155	3HG2	ILE	A	732	125.477	158.283	100.746	1.00	0.00
ATOM	11156	CG1	ILE	A	732	126.624	156.960	98.577	1.00	0.00
ATOM	11157	1HG1	ILE	A	732	127.228	156.140	98.188	1.00	0.00
ATOM	11158	2HG1	ILE	A	732	127.300	157.670	99.054	1.00	0.00
ATOM	11159	CD	ILE	A	732	125.972	157.643	97.367	1.00	0.00
ATOM	11160	HD1	ILE	A	732	126.741	157.878	96.632	1.00	0.00
ATOM	11161	HD2	ILE	A	732	125.238	156.976	96.915	1.00	0.00

ATOM	11162	HD3	ILE	A	732	125.487	158.571	97.664	1.00	0.00
ATOM	11163	C	ILE	A	732	125.568	155.291	101.929	1.00	0.00
ATOM	11164	O	ILE	A	732	124.698	154.416	101.913	1.00	0.00
ATOM	11165	N	PRO	A	733	125.770	156.065	103.012	1.00	0.00
ATOM	11166	CD	PRO	A	733	126.851	157.023	103.216	1.00	0.00
ATOM	11167	HD1	PRO	A	733	126.886	157.766	102.420	1.00	0.00
ATOM	11168	HD2	PRO	A	733	127.798	156.485	103.269	1.00	0.00
ATOM	11169	CG	PRO	A	733	126.574	157.709	104.552	1.00	0.00
ATOM	11170	HG1	PRO	A	733	125.975	158.605	104.387	1.00	0.00
ATOM	11171	HG2	PRO	A	733	127.496	157.959	105.076	1.00	0.00
ATOM	11172	CB	PRO	A	733	125.754	156.668	105.312	1.00	0.00
ATOM	11173	HB1	PRO	A	733	125.125	157.123	106.078	1.00	0.00
ATOM	11174	HB2	PRO	A	733	126.427	155.937	105.761	1.00	0.00
ATOM	11175	CA	PRO	A	733	124.935	155.990	104.207	1.00	0.00
ATOM	11176	HA	PRO	A	733	124.799	154.946	104.477	1.00	0.00
ATOM	11177	C	PRO	A	733	123.559	156.644	104.000	1.00	0.00
ATOM	11178	O	PRO	A	733	123.369	157.846	104.165	1.00	0.00
ATOM	11179	N	GLU	A	734	122.585	155.815	103.654	1.00	0.00
ATOM	11180	H	GLU	A	734	122.838	154.853	103.472	1.00	0.00
ATOM	11181	CA	GLU	A	734	121.158	156.126	103.522	1.00	0.00
ATOM	11182	HA	GLU	A	734	120.898	156.961	104.173	1.00	0.00
ATOM	11183	CB	GLU	A	734	120.915	156.538	102.056	1.00	0.00
ATOM	11184	HB1	GLU	A	734	121.458	155.861	101.394	1.00	0.00
ATOM	11185	HB2	GLU	A	734	121.357	157.526	101.923	1.00	0.00
ATOM	11186	CG	GLU	A	734	119.450	156.613	101.607	1.00	0.00
ATOM	11187	HG1	GLU	A	734	119.336	157.512	100.997	1.00	0.00
ATOM	11188	HG2	GLU	A	734	118.785	156.725	102.465	1.00	0.00
ATOM	11189	CD	GLU	A	734	119.055	155.394	100.762	1.00	0.00
ATOM	11190	OE1	GLU	A	734	119.305	154.235	101.165	1.00	0.00
ATOM	11191	OE2	GLU	A	734	118.562	155.587	99.633	1.00	0.00
ATOM	11192	C	GLU	A	734	120.366	154.884	103.970	1.00	0.00
ATOM	11193	O	GLU	A	734	120.903	153.778	103.920	1.00	0.00
ATOM	11194	N	GLY	A	735	119.136	155.064	104.471	1.00	0.00
ATOM	11195	H	GLY	A	735	118.746	155.990	104.424	1.00	0.00
ATOM	11196	CA	GLY	A	735	118.365	154.052	105.215	1.00	0.00
ATOM	11197	HA1	GLY	A	735	117.327	154.378	105.269	1.00	0.00
ATOM	11198	HA2	GLY	A	735	118.757	153.989	106.231	1.00	0.00
ATOM	11199	C	GLY	A	735	118.366	152.630	104.635	1.00	0.00
ATOM	11200	O	GLY	A	735	118.410	151.673	105.400	1.00	0.00
ATOM	11201	N	GLU	A	736	118.363	152.474	103.308	1.00	0.00
ATOM	11202	H	GLU	A	736	118.464	153.292	102.712	1.00	0.00
ATOM	11203	CA	GLU	A	736	118.456	151.160	102.672	1.00	0.00
ATOM	11204	HA	GLU	A	736	118.087	150.395	103.357	1.00	0.00
ATOM	11205	CB	GLU	A	736	117.539	151.139	101.433	1.00	0.00
ATOM	11206	HB1	GLU	A	736	117.759	151.997	100.796	1.00	0.00
ATOM	11207	HB2	GLU	A	736	116.514	151.259	101.791	1.00	0.00
ATOM	11208	CG	GLU	A	736	117.592	149.846	100.595	1.00	0.00
ATOM	11209	HG1	GLU	A	736	116.648	149.752	100.054	1.00	0.00
ATOM	11210	HG2	GLU	A	736	117.676	148.983	101.260	1.00	0.00
ATOM	11211	CD	GLU	A	736	118.736	149.855	99.571	1.00	0.00
ATOM	11212	OE1	GLU	A	736	118.782	150.796	98.749	1.00	0.00
ATOM	11213	OE2	GLU	A	736	119.620	148.969	99.636	1.00	0.00
ATOM	11214	C	GLU	A	736	119.900	150.784	102.343	1.00	0.00
ATOM	11215	O	GLU	A	736	120.350	149.702	102.719	1.00	0.00
ATOM	11216	N	LYS	A	737	120.658	151.667	101.681	1.00	0.00
ATOM	11217	H	LYS	A	737	120.243	152.576	101.463	1.00	0.00
ATOM	11218	CA	LYS	A	737	122.042	151.381	101.249	1.00	0.00
ATOM	11219	HA	LYS	A	737	122.031	150.506	100.604	1.00	0.00
ATOM	11220	CB	LYS	A	737	122.563	152.557	100.410	1.00	0.00
ATOM	11221	HB1	LYS	A	737	123.643	152.480	100.279	1.00	0.00
ATOM	11222	HB2	LYS	A	737	122.343	153.491	100.930	1.00	0.00

```
ATOM  11223  CG  LYS A 737     121.895 152.525  99.021  1.00  0.00
ATOM  11224  HG1 LYS A 737     120.993 151.924  99.083  1.00  0.00
ATOM  11225  HG2 LYS A 737     122.555 152.025  98.312  1.00  0.00
ATOM  11226  CD  LYS A 737     121.525 153.912  98.476  1.00  0.00
ATOM  11227  HD1 LYS A 737     122.394 154.338  97.969  1.00  0.00
ATOM  11228  HD2 LYS A 737     121.251 154.579  99.293  1.00  0.00
ATOM  11229  CE  LYS A 737     120.341 153.842  97.498  1.00  0.00
ATOM  11230  HE1 LYS A 737     120.581 153.154  96.683  1.00  0.00
ATOM  11231  HE2 LYS A 737     120.190 154.840  97.081  1.00  0.00
ATOM  11232  NZ  LYS A 737     119.095 153.412  98.177  1.00  0.00
ATOM  11233  HZ1 LYS A 737     118.925 153.999  99.001  1.00  0.00
ATOM  11234  HZ2 LYS A 737     118.280 153.504  97.595  1.00  0.00
ATOM  11235  HZ3 LYS A 737     119.144 152.440  98.493  1.00  0.00
ATOM  11236  C   LYS A 737     122.963 151.039 102.417  1.00  0.00
ATOM  11237  O   LYS A 737     123.789 150.142 102.269  1.00  0.00
ATOM  11238  N   VAL A 738     122.742 151.653 103.581  1.00  0.00
ATOM  11239  H   VAL A 738     122.007 152.356 103.590  1.00  0.00
ATOM  11240  CA  VAL A 738     123.475 151.413 104.836  1.00  0.00
ATOM  11241  HA  VAL A 738     124.522 151.276 104.569  1.00  0.00
ATOM  11242  CB  VAL A 738     123.377 152.666 105.733  1.00  0.00
ATOM  11243  HB  VAL A 738     123.325 153.536 105.083  1.00  0.00
ATOM  11244  CG1 VAL A 738     122.136 152.704 106.630  1.00  0.00
ATOM  11245 1HG1 VAL A 738     122.029 153.696 107.070  1.00  0.00
ATOM  11246 2HG1 VAL A 738     121.240 152.476 106.054  1.00  0.00
ATOM  11247 3HG1 VAL A 738     122.239 151.975 107.426  1.00  0.00
ATOM  11248  CG2 VAL A 738     124.627 152.852 106.600  1.00  0.00
ATOM  11249 1HG2 VAL A 738     124.543 153.776 107.170  1.00  0.00
ATOM  11250 2HG2 VAL A 738     124.733 152.016 107.291  1.00  0.00
ATOM  11251 3HG2 VAL A 738     125.511 152.907 105.965  1.00  0.00
ATOM  11252  C   VAL A 738     123.045 150.139 105.590  1.00  0.00
ATOM  11253  O   VAL A 738     123.750 149.720 106.502  1.00  0.00
ATOM  11254  N   LYS A 739     121.917 149.507 105.219  1.00  0.00
ATOM  11255  H   LYS A 739     121.404 149.918 104.449  1.00  0.00
ATOM  11256  CA  LYS A 739     121.406 148.212 105.738  1.00  0.00
ATOM  11257  HA  LYS A 739     120.416 148.062 105.309  1.00  0.00
ATOM  11258  CB  LYS A 739     122.321 147.056 105.248  1.00  0.00
ATOM  11259  HB1 LYS A 739     121.919 146.104 105.598  1.00  0.00
ATOM  11260  HB2 LYS A 739     123.302 147.177 105.711  1.00  0.00
ATOM  11261  CG  LYS A 739     122.518 146.950 103.728  1.00  0.00
ATOM  11262  HG1 LYS A 739     123.307 146.225 103.531  1.00  0.00
ATOM  11263  HG2 LYS A 739     122.848 147.911 103.341  1.00  0.00
ATOM  11264  CD  LYS A 739     121.246 146.486 103.002  1.00  0.00
ATOM  11265  HD1 LYS A 739     120.401 147.114 103.286  1.00  0.00
ATOM  11266  HD2 LYS A 739     121.019 145.463 103.304  1.00  0.00
ATOM  11267  CE  LYS A 739     121.393 146.532 101.475  1.00  0.00
ATOM  11268  HE1 LYS A 739     120.466 146.145 101.040  1.00  0.00
ATOM  11269  HE2 LYS A 739     122.218 145.888 101.161  1.00  0.00
ATOM  11270  NZ  LYS A 739     121.600 147.916 101.007  1.00  0.00
ATOM  11271  HZ1 LYS A 739     121.080 148.101 100.142  1.00  0.00
ATOM  11272  HZ2 LYS A 739     121.135 148.560 101.642  1.00  0.00
ATOM  11273  HZ3 LYS A 739     122.572 148.182 100.949  1.00  0.00
ATOM  11274  C   LYS A 739     121.168 148.107 107.264  1.00  0.00
ATOM  11275  O   LYS A 739     121.156 147.009 107.815  1.00  0.00
ATOM  11276  N   ILE A 740     120.999 149.231 107.959  1.00  0.00
ATOM  11277  H   ILE A 740     120.995 150.094 107.441  1.00  0.00
ATOM  11278  CA  ILE A 740     120.880 149.295 109.429  1.00  0.00
ATOM  11279  HA  ILE A 740     121.760 148.788 109.819  1.00  0.00
ATOM  11280  CB  ILE A 740     120.976 150.780 109.862  1.00  0.00
ATOM  11281  HB  ILE A 740     121.868 151.181 109.385  1.00  0.00
ATOM  11282  CG2 ILE A 740     119.774 151.622 109.388  1.00  0.00
ATOM  11283 1HG2 ILE A 740     119.984 152.684 109.505  1.00  0.00
```

```
ATOM  11284 2HG2 ILE A 740     119.561 151.439 108.335  1.00  0.00
ATOM  11285 3HG2 ILE A 740     118.886 151.371 109.969  1.00  0.00
ATOM  11286  CG1 ILE A 740     121.227 150.929 111.378  1.00  0.00
ATOM  11287 1HG1 ILE A 740     122.097 150.328 111.641  1.00  0.00
ATOM  11288 2HG1 ILE A 740     120.368 150.557 111.930  1.00  0.00
ATOM  11289  CD  ILE A 740     121.511 152.367 111.827  1.00  0.00
ATOM  11290  HD1 ILE A 740     121.799 152.365 112.878  1.00  0.00
ATOM  11291  HD2 ILE A 740     122.325 152.790 111.237  1.00  0.00
ATOM  11292  HD3 ILE A 740     120.620 152.985 111.717  1.00  0.00
ATOM  11293  C   ILE A 740     119.619 148.590 110.006  1.00  0.00
ATOM  11294  O   ILE A 740     118.523 148.764 109.470  1.00  0.00
ATOM  11295  N   PRO A 741     119.727 147.828 111.117  1.00  0.00
ATOM  11296  CD  PRO A 741     120.960 147.368 111.743  1.00  0.00
ATOM  11297  HD1 PRO A 741     121.634 148.191 111.970  1.00  0.00
ATOM  11298  HD2 PRO A 741     121.451 146.652 111.081  1.00  0.00
ATOM  11299  CG  PRO A 741     120.545 146.672 113.037  1.00  0.00
ATOM  11300  HG1 PRO A 741     120.513 147.401 113.849  1.00  0.00
ATOM  11301  HG2 PRO A 741     121.216 145.851 113.287  1.00  0.00
ATOM  11302  CB  PRO A 741     119.133 146.182 112.729  1.00  0.00
ATOM  11303  HB1 PRO A 741     118.548 146.034 113.636  1.00  0.00
ATOM  11304  HB2 PRO A 741     119.190 145.253 112.159  1.00  0.00
ATOM  11305  CA  PRO A 741     118.567 147.289 111.835  1.00  0.00
ATOM  11306  HA  PRO A 741     117.864 146.852 111.126  1.00  0.00
ATOM  11307  C   PRO A 741     117.821 148.356 112.660  1.00  0.00
ATOM  11308  O   PRO A 741     118.373 149.398 113.008  1.00  0.00
ATOM  11309  N   VAL A 742     116.570 148.072 113.043  1.00  0.00
ATOM  11310  H   VAL A 742     116.167 147.196 112.747  1.00  0.00
ATOM  11311  CA  VAL A 742     115.682 149.039 113.714  1.00  0.00
ATOM  11312  HA  VAL A 742     116.292 149.874 114.055  1.00  0.00
ATOM  11313  CB  VAL A 742     114.679 149.653 112.711  1.00  0.00
ATOM  11314  HB  VAL A 742     115.265 150.190 111.966  1.00  0.00
ATOM  11315  CG1 VAL A 742     113.811 148.639 111.955  1.00  0.00
ATOM  11316 1HG1 VAL A 742     113.192 149.160 111.226  1.00  0.00
ATOM  11317 2HG1 VAL A 742     114.436 147.930 111.414  1.00  0.00
ATOM  11318 3HG1 VAL A 742     113.161 148.096 112.640  1.00  0.00
ATOM  11319  CG2 VAL A 742     113.768 150.684 113.392  1.00  0.00
ATOM  11320 1HG2 VAL A 742     113.160 151.182 112.637  1.00  0.00
ATOM  11321 2HG2 VAL A 742     113.105 150.196 114.106  1.00  0.00
ATOM  11322 3HG2 VAL A 742     114.377 151.433 113.896  1.00  0.00
ATOM  11323  C   VAL A 742     115.018 148.453 114.967  1.00  0.00
ATOM  11324  O   VAL A 742     114.179 147.559 114.912  1.00  0.00
ATOM  11325  N   ALA A 743     115.415 148.968 116.132  1.00  0.00
ATOM  11326  H   ALA A 743     116.143 149.665 116.121  1.00  0.00
ATOM  11327  CA  ALA A 743     114.888 148.543 117.426  1.00  0.00
ATOM  11328  HA  ALA A 743     114.975 147.456 117.488  1.00  0.00
ATOM  11329  CB  ALA A 743     115.774 149.140 118.529  1.00  0.00
ATOM  11330  HB1 ALA A 743     115.408 148.815 119.504  1.00  0.00
ATOM  11331  HB2 ALA A 743     116.801 148.792 118.409  1.00  0.00
ATOM  11332  HB3 ALA A 743     115.754 150.229 118.483  1.00  0.00
ATOM  11333  C   ALA A 743     113.409 148.921 117.623  1.00  0.00
ATOM  11334  O   ALA A 743     113.061 150.096 117.780  1.00  0.00
ATOM  11335  N   ILE A 744     112.541 147.909 117.662  1.00  0.00
ATOM  11336  H   ILE A 744     112.891 146.987 117.443  1.00  0.00
ATOM  11337  CA  ILE A 744     111.154 148.010 118.138  1.00  0.00
ATOM  11338  HA  ILE A 744     110.648 148.790 117.570  1.00  0.00
ATOM  11339  CB  ILE A 744     110.420 146.666 117.877  1.00  0.00
ATOM  11340  HB  ILE A 744     110.978 145.866 118.367  1.00  0.00
ATOM  11341  CG2 ILE A 744     108.986 146.658 118.447  1.00  0.00
ATOM  11342 1HG2 ILE A 744     108.489 145.715 118.227  1.00  0.00
ATOM  11343 2HG2 ILE A 744     109.002 146.741 119.533  1.00  0.00
ATOM  11344 3HG2 ILE A 744     108.397 147.470 118.019  1.00  0.00
```

ATOM	11345	CG1	ILE	A	744	110.386	146.371	116.352	1.00	0.00
ATOM	11346	1HG1	ILE	A	744	111.400	146.400	115.959	1.00	0.00
ATOM	11347	2HG1	ILE	A	744	109.814	147.150	115.850	1.00	0.00
ATOM	11348	CD	ILE	A	744	109.804	145.007	115.968	1.00	0.00
ATOM	11349	HD1	ILE	A	744	109.979	144.828	114.907	1.00	0.00
ATOM	11350	HD2	ILE	A	744	110.288	144.220	116.542	1.00	0.00
ATOM	11351	HD3	ILE	A	744	108.732	144.983	116.142	1.00	0.00
ATOM	11352	C	ILE	A	744	111.126	148.419	119.623	1.00	0.00
ATOM	11353	O	ILE	A	744	112.033	148.108	120.391	1.00	0.00
ATOM	11354	N	LYS	A	745	110.062	149.116	120.018	1.00	0.00
ATOM	11355	H	LYS	A	745	109.368	149.334	119.312	1.00	0.00
ATOM	11356	CA	LYS	A	745	109.586	149.272	121.398	1.00	0.00
ATOM	11357	HA	LYS	A	745	109.783	148.349	121.944	1.00	0.00
ATOM	11358	CB	LYS	A	745	110.278	150.437	122.140	1.00	0.00
ATOM	11359	HB1	LYS	A	745	111.355	150.278	122.128	1.00	0.00
ATOM	11360	HB2	LYS	A	745	109.967	150.380	123.185	1.00	0.00
ATOM	11361	CG	LYS	A	745	109.958	151.875	121.666	1.00	0.00
ATOM	11362	HG1	LYS	A	745	110.364	152.563	122.409	1.00	0.00
ATOM	11363	HG2	LYS	A	745	108.877	152.018	121.664	1.00	0.00
ATOM	11364	CD	LYS	A	745	110.509	152.283	120.292	1.00	0.00
ATOM	11365	HD1	LYS	A	745	110.184	153.305	120.091	1.00	0.00
ATOM	11366	HD2	LYS	A	745	110.085	151.646	119.518	1.00	0.00
ATOM	11367	CE	LYS	A	745	112.041	152.225	120.261	1.00	0.00
ATOM	11368	HE1	LYS	A	745	112.366	151.225	120.561	1.00	0.00
ATOM	11369	HE2	LYS	A	745	112.426	152.940	120.992	1.00	0.00
ATOM	11370	NZ	LYS	A	745	112.579	152.525	118.913	1.00	0.00
ATOM	11371	HZ1	LYS	A	745	112.160	153.352	118.520	1.00	0.00
ATOM	11372	HZ2	LYS	A	745	113.582	152.630	118.949	1.00	0.00
ATOM	11373	HZ3	LYS	A	745	112.412	151.728	118.301	1.00	0.00
ATOM	11374	C	LYS	A	745	108.073	149.462	121.336	1.00	0.00
ATOM	11375	O	LYS	A	745	107.566	149.921	120.314	1.00	0.00
ATOM	11376	N	GLU	A	746	107.373	149.126	122.407	1.00	0.00
ATOM	11377	H	GLU	A	746	107.863	148.795	123.220	1.00	0.00
ATOM	11378	CA	GLU	A	746	105.989	149.559	122.585	1.00	0.00
ATOM	11379	HA	GLU	A	746	105.419	149.324	121.683	1.00	0.00
ATOM	11380	CB	GLU	A	746	105.401	148.767	123.770	1.00	0.00
ATOM	11381	HB1	GLU	A	746	106.007	148.961	124.657	1.00	0.00
ATOM	11382	HB2	GLU	A	746	105.479	147.703	123.541	1.00	0.00
ATOM	11383	CG	GLU	A	746	103.940	149.072	124.113	1.00	0.00
ATOM	11384	HG1	GLU	A	746	103.866	150.097	124.482	1.00	0.00
ATOM	11385	HG2	GLU	A	746	103.624	148.413	124.925	1.00	0.00
ATOM	11386	CD	GLU	A	746	103.011	148.894	122.912	1.00	0.00
ATOM	11387	OE1	GLU	A	746	102.896	149.859	122.128	1.00	0.00
ATOM	11388	OE2	GLU	A	746	102.326	147.854	122.795	1.00	0.00
ATOM	11389	C	GLU	A	746	105.953	151.083	122.807	1.00	0.00
ATOM	11390	O	GLU	A	746	106.819	151.645	123.480	1.00	0.00
ATOM	11391	N	LEU	A	747	104.942	151.737	122.240	1.00	0.00
ATOM	11392	H	LEU	A	747	104.263	151.172	121.732	1.00	0.00
ATOM	11393	CA	LEU	A	747	104.697	153.185	122.302	1.00	0.00
ATOM	11394	HA	LEU	A	747	105.460	153.645	122.931	1.00	0.00
ATOM	11395	CB	LEU	A	747	104.842	153.724	120.862	1.00	0.00
ATOM	11396	HB1	LEU	A	747	104.065	153.269	120.245	1.00	0.00
ATOM	11397	HB2	LEU	A	747	105.806	153.385	120.478	1.00	0.00
ATOM	11398	CG	LEU	A	747	104.773	155.252	120.669	1.00	0.00
ATOM	11399	HG	LEU	A	747	103.772	155.608	120.905	1.00	0.00
ATOM	11400	CD1	LEU	A	747	105.786	156.004	121.533	1.00	0.00
ATOM	11401	1HD1	LEU	A	747	105.752	157.066	121.291	1.00	0.00
ATOM	11402	2HD1	LEU	A	747	105.516	155.897	122.583	1.00	0.00
ATOM	11403	3HD1	LEU	A	747	106.791	155.620	121.368	1.00	0.00
ATOM	11404	CD2	LEU	A	747	105.062	155.583	119.203	1.00	0.00
ATOM	11405	1HD2	LEU	A	747	104.938	156.653	119.035	1.00	0.00

```
ATOM  11406 2HD2 LEU A 747     106.079 155.294 118.940  1.00  0.00
ATOM  11407 3HD2 LEU A 747     104.360 155.056 118.558  1.00  0.00
ATOM  11408  C   LEU A 747     103.328 153.497 122.955  1.00  0.00
ATOM  11409  O   LEU A 747     102.946 154.648 123.125  1.00  0.00
ATOM  11410  N   ARG A 748     102.558 152.441 123.247  1.00  0.00
ATOM  11411  H   ARG A 748     102.996 151.546 123.046  1.00  0.00
ATOM  11412  CA  ARG A 748     101.095 152.348 123.369  1.00  0.00
ATOM  11413  HA  ARG A 748     100.958 151.308 123.127  1.00  0.00
ATOM  11414  CB  ARG A 748     100.574 152.538 124.808  1.00  0.00
ATOM  11415  HB1 ARG A 748      99.843 153.347 124.829  1.00  0.00
ATOM  11416  HB2 ARG A 748     101.407 152.832 125.449  1.00  0.00
ATOM  11417  CG  ARG A 748      99.934 151.259 125.402  1.00  0.00
ATOM  11418  HG1 ARG A 748      99.742 151.463 126.456  1.00  0.00
ATOM  11419  HG2 ARG A 748     100.671 150.456 125.367  1.00  0.00
ATOM  11420  CD  ARG A 748      98.601 150.765 124.783  1.00  0.00
ATOM  11421  HD1 ARG A 748      97.878 151.582 124.827  1.00  0.00
ATOM  11422  HD2 ARG A 748      98.212 149.946 125.391  1.00  0.00
ATOM  11423  NE  ARG A 748      98.718 150.315 123.379  1.00  0.00
ATOM  11424  HE  ARG A 748      98.510 151.030 122.686  1.00  0.00
ATOM  11425  CZ  ARG A 748      99.486 149.332 122.939  1.00  0.00
ATOM  11426  NH1 ARG A 748      99.846 148.342 123.697  1.00  0.00
ATOM  11427 1HH1 ARG A 748      99.481 148.238 124.619  1.00  0.00
ATOM  11428 2HH1 ARG A 748     100.658 147.809 123.369  1.00  0.00
ATOM  11429  NH2 ARG A 748     100.005 149.371 121.748  1.00  0.00
ATOM  11430 1HH2 ARG A 748      99.866 150.165 121.135  1.00  0.00
ATOM  11431 2HH2 ARG A 748     100.875 148.853 121.622  1.00  0.00
ATOM  11432  C   ARG A 748     100.240 153.053 122.296  1.00  0.00
ATOM  11433  O   ARG A 748      99.047 152.769 122.200  1.00  0.00
ATOM  11434  N   GLU A 749     100.830 153.872 121.434  1.00  0.00
ATOM  11435  H   GLU A 749     101.752 154.199 121.686  1.00  0.00
ATOM  11436  CA  GLU A 749     100.379 154.121 120.061  1.00  0.00
ATOM  11437  HA  GLU A 749      99.303 154.303 120.081  1.00  0.00
ATOM  11438  CB  GLU A 749     101.082 155.412 119.559  1.00  0.00
ATOM  11439  HB1 GLU A 749     102.087 155.163 119.222  1.00  0.00
ATOM  11440  HB2 GLU A 749     101.194 156.095 120.402  1.00  0.00
ATOM  11441  CG  GLU A 749     100.334 156.194 118.457  1.00  0.00
ATOM  11442  HG1 GLU A 749     100.047 157.171 118.851  1.00  0.00
ATOM  11443  HG2 GLU A 749      99.413 155.665 118.204  1.00  0.00
ATOM  11444  CD  GLU A 749     101.173 156.381 117.181  1.00  0.00
ATOM  11445  OE1 GLU A 749     101.853 157.418 117.009  1.00  0.00
ATOM  11446  OE2 GLU A 749     101.172 155.430 116.368  1.00  0.00
ATOM  11447  C   GLU A 749     100.622 152.874 119.166  1.00  0.00
ATOM  11448  O   GLU A 749     100.430 151.749 119.637  1.00  0.00
ATOM  11449  N   ALA A 750     101.028 153.042 117.895  1.00  0.00
ATOM  11450  H   ALA A 750     101.115 153.981 117.516  1.00  0.00
ATOM  11451  CA  ALA A 750     101.284 151.929 116.976  1.00  0.00
ATOM  11452  HA  ALA A 750     101.498 151.032 117.560  1.00  0.00
ATOM  11453  CB  ALA A 750      99.993 151.674 116.184  1.00  0.00
ATOM  11454  HB1 ALA A 750     100.138 150.827 115.513  1.00  0.00
ATOM  11455  HB2 ALA A 750      99.179 151.444 116.872  1.00  0.00
ATOM  11456  HB3 ALA A 750      99.734 152.556 115.599  1.00  0.00
ATOM  11457  C   ALA A 750     102.478 152.096 116.006  1.00  0.00
ATOM  11458  O   ALA A 750     103.211 151.131 115.769  1.00  0.00
ATOM  11459  N   THR A 751     102.668 153.262 115.375  1.00  0.00
ATOM  11460  H   THR A 751     102.035 154.025 115.603  1.00  0.00
ATOM  11461  CA  THR A 751     103.742 153.482 114.361  1.00  0.00
ATOM  11462  HA  THR A 751     104.681 153.149 114.803  1.00  0.00
ATOM  11463  CB  THR A 751     103.508 152.700 113.035  1.00  0.00
ATOM  11464  HB  THR A 751     103.163 153.385 112.261  1.00  0.00
ATOM  11465  CG2 THR A 751     104.791 152.027 112.549  1.00  0.00
ATOM  11466 1HG2 THR A 751     104.596 151.496 111.619  1.00  0.00
```

```
ATOM  11467 2HG2 THR A 751     105.557 152.778 112.367  1.00  0.00
ATOM  11468 3HG2 THR A 751     105.147 151.320 113.296  1.00  0.00
ATOM  11469  OG1 THR A 751     102.549 151.674 113.116  1.00  0.00
ATOM  11470  HG1 THR A 751     102.792 151.159 113.900  1.00  0.00
ATOM  11471  C   THR A 751     103.957 154.945 113.935  1.00  0.00
ATOM  11472  O   THR A 751     104.822 155.220 113.107  1.00  0.00
ATOM  11473  N   SER A 752     103.171 155.888 114.449  1.00  0.00
ATOM  11474  H   SER A 752     102.509 155.620 115.174  1.00  0.00
ATOM  11475  CA  SER A 752     103.216 157.317 114.121  1.00  0.00
ATOM  11476  HA  SER A 752     102.373 157.738 114.664  1.00  0.00
ATOM  11477  CB  SER A 752     104.427 157.997 114.761  1.00  0.00
ATOM  11478  HB1 SER A 752     104.391 159.067 114.559  1.00  0.00
ATOM  11479  HB2 SER A 752     105.350 157.586 114.349  1.00  0.00
ATOM  11480  OG  SER A 752     104.400 157.803 116.171  1.00  0.00
ATOM  11481  HG  SER A 752     103.471 157.677 116.480  1.00  0.00
ATOM  11482  C   SER A 752     102.908 157.742 112.679  1.00  0.00
ATOM  11483  O   SER A 752     103.642 158.552 112.099  1.00  0.00
ATOM  11484  N   PRO A 753     101.749 157.337 112.114  1.00  0.00
ATOM  11485  CD  PRO A 753     100.720 156.509 112.737  1.00  0.00
ATOM  11486  HD1 PRO A 753     100.558 156.772 113.784  1.00  0.00
ATOM  11487  HD2 PRO A 753     100.999 155.458 112.653  1.00  0.00
ATOM  11488  CG  PRO A 753      99.442 156.764 111.939  1.00  0.00
ATOM  11489  HG1 PRO A 753      98.926 157.636 112.344  1.00  0.00
ATOM  11490  HG2 PRO A 753      98.786 155.892 111.942  1.00  0.00
ATOM  11491  CB  PRO A 753      99.967 157.078 110.541  1.00  0.00
ATOM  11492  HB1 PRO A 753      99.262 157.688 109.976  1.00  0.00
ATOM  11493  HB2 PRO A 753     100.162 156.145 110.012  1.00  0.00
ATOM  11494  CA  PRO A 753     101.292 157.803 110.801  1.00  0.00
ATOM  11495  HA  PRO A 753     102.009 157.481 110.050  1.00  0.00
ATOM  11496  C   PRO A 753     101.143 159.328 110.717  1.00  0.00
ATOM  11497  O   PRO A 753     101.339 159.865 109.631  1.00  0.00
ATOM  11498  N   LYS A 754     100.912 160.022 111.841  1.00  0.00
ATOM  11499  H   LYS A 754     100.734 159.473 112.666  1.00  0.00
ATOM  11500  CA  LYS A 754     100.827 161.489 112.001  1.00  0.00
ATOM  11501  HA  LYS A 754      99.815 161.813 111.740  1.00  0.00
ATOM  11502  CB  LYS A 754     101.082 161.811 113.493  1.00  0.00
ATOM  11503  HB1 LYS A 754     101.144 162.890 113.614  1.00  0.00
ATOM  11504  HB2 LYS A 754     102.030 161.380 113.820  1.00  0.00
ATOM  11505  CG  LYS A 754      99.943 161.270 114.371  1.00  0.00
ATOM  11506  HG1 LYS A 754     100.049 160.191 114.494  1.00  0.00
ATOM  11507  HG2 LYS A 754      99.029 161.454 113.826  1.00  0.00
ATOM  11508  CD  LYS A 754      99.831 161.935 115.749  1.00  0.00
ATOM  11509  HD1 LYS A 754      99.965 163.015 115.661  1.00  0.00
ATOM  11510  HD2 LYS A 754     100.613 161.546 116.403  1.00  0.00
ATOM  11511  CE  LYS A 754      98.456 161.671 116.389  1.00  0.00
ATOM  11512  HE1 LYS A 754      98.482 162.032 117.420  1.00  0.00
ATOM  11513  HE2 LYS A 754      98.251 160.599 116.395  1.00  0.00
ATOM  11514  NZ  LYS A 754      97.376 162.389 115.676  1.00  0.00
ATOM  11515  HZ1 LYS A 754      97.232 162.082 114.713  1.00  0.00
ATOM  11516  HZ2 LYS A 754      96.482 162.379 116.165  1.00  0.00
ATOM  11517  HZ3 LYS A 754      97.552 163.395 115.699  1.00  0.00
ATOM  11518  C   LYS A 754     101.767 162.298 111.092  1.00  0.00
ATOM  11519  O   LYS A 754     101.358 163.176 110.321  1.00  0.00
ATOM  11520  N   ALA A 755     103.052 161.939 111.128  1.00  0.00
ATOM  11521  H   ALA A 755     103.323 161.163 111.713  1.00  0.00
ATOM  11522  CA  ALA A 755     104.069 162.606 110.324  1.00  0.00
ATOM  11523  HA  ALA A 755     104.240 163.604 110.732  1.00  0.00
ATOM  11524  CB  ALA A 755     105.371 161.804 110.434  1.00  0.00
ATOM  11525  HB1 ALA A 755     106.149 162.280 109.837  1.00  0.00
ATOM  11526  HB2 ALA A 755     105.701 161.772 111.472  1.00  0.00
ATOM  11527  HB3 ALA A 755     105.224 160.785 110.077  1.00  0.00
```

```
ATOM  11528  C   ALA A 755     103.603 162.781 108.860  1.00  0.00
ATOM  11529  O   ALA A 755     103.614 163.890 108.329  1.00  0.00
ATOM  11530  N   ASN A 756     103.057 161.701 108.281  1.00  0.00
ATOM  11531  H   ASN A 756     102.885 160.918 108.894  1.00  0.00
ATOM  11532  CA  ASN A 756     102.853 161.445 106.855  1.00  0.00
ATOM  11533  HA  ASN A 756     103.855 161.476 106.419  1.00  0.00
ATOM  11534  CB  ASN A 756     102.340 160.015 106.615  1.00  0.00
ATOM  11535  HB1 ASN A 756     102.122 159.877 105.556  1.00  0.00
ATOM  11536  HB2 ASN A 756     101.421 159.828 107.167  1.00  0.00
ATOM  11537  CG  ASN A 756     103.405 159.001 106.969  1.00  0.00
ATOM  11538  OD1 ASN A 756     104.236 158.643 106.162  1.00  0.00
ATOM  11539  ND2 ASN A 756     103.448 158.571 108.203  1.00  0.00
ATOM  11540 1HD2 ASN A 756     102.743 158.877 108.856  1.00  0.00
ATOM  11541 2HD2 ASN A 756     104.175 157.917 108.438  1.00  0.00
ATOM  11542  C   ASN A 756     102.081 162.446 106.009  1.00  0.00
ATOM  11543  O   ASN A 756     102.208 162.434 104.779  1.00  0.00
ATOM  11544  N   LYS A 757     101.320 163.344 106.617  1.00  0.00
ATOM  11545  H   LYS A 757     101.261 163.300 107.631  1.00  0.00
ATOM  11546  CA  LYS A 757     100.811 164.532 105.929  1.00  0.00
ATOM  11547  HA  LYS A 757     101.567 164.844 105.215  1.00  0.00
ATOM  11548  CB  LYS A 757      99.516 164.171 105.169  1.00  0.00
ATOM  11549  HB1 LYS A 757      98.726 163.987 105.893  1.00  0.00
ATOM  11550  HB2 LYS A 757      99.665 163.232 104.636  1.00  0.00
ATOM  11551  CG  LYS A 757      99.029 165.208 104.139  1.00  0.00
ATOM  11552  HG1 LYS A 757      98.721 166.117 104.653  1.00  0.00
ATOM  11553  HG2 LYS A 757      98.142 164.805 103.651  1.00  0.00
ATOM  11554  CD  LYS A 757     100.023 165.604 103.030  1.00  0.00
ATOM  11555  HD1 LYS A 757     100.918 166.056 103.461  1.00  0.00
ATOM  11556  HD2 LYS A 757      99.533 166.380 102.439  1.00  0.00
ATOM  11557  CE  LYS A 757     100.383 164.450 102.081  1.00  0.00
ATOM  11558  HE1 LYS A 757     100.504 164.863 101.076  1.00  0.00
ATOM  11559  HE2 LYS A 757      99.544 163.750 102.052  1.00  0.00
ATOM  11560  NZ  LYS A 757     101.633 163.748 102.465  1.00  0.00
ATOM  11561  HZ1 LYS A 757     101.828 162.976 101.843  1.00  0.00
ATOM  11562  HZ2 LYS A 757     102.431 164.369 102.453  1.00  0.00
ATOM  11563  HZ3 LYS A 757     101.573 163.355 103.404  1.00  0.00
ATOM  11564  C   LYS A 757     100.730 165.691 106.912  1.00  0.00
ATOM  11565  O   LYS A 757      99.746 166.407 106.904  1.00  0.00
ATOM  11566  N   GLU A 758     101.774 165.857 107.738  1.00  0.00
ATOM  11567  H   GLU A 758     102.541 165.199 107.703  1.00  0.00
ATOM  11568  CA  GLU A 758     101.909 166.993 108.667  1.00  0.00
ATOM  11569  HA  GLU A 758     102.783 166.819 109.298  1.00  0.00
ATOM  11570  CB  GLU A 758     102.165 168.317 107.893  1.00  0.00
ATOM  11571  HB1 GLU A 758     101.488 169.105 108.209  1.00  0.00
ATOM  11572  HB2 GLU A 758     101.946 168.151 106.837  1.00  0.00
ATOM  11573  CG  GLU A 758     103.619 168.825 107.980  1.00  0.00
ATOM  11574  HG1 GLU A 758     103.782 169.476 107.118  1.00  0.00
ATOM  11575  HG2 GLU A 758     104.292 167.971 107.864  1.00  0.00
ATOM  11576  CD  GLU A 758     103.990 169.619 109.249  1.00  0.00
ATOM  11577  OE1 GLU A 758     105.185 169.960 109.390  1.00  0.00
ATOM  11578  OE2 GLU A 758     103.097 169.957 110.062  1.00  0.00
ATOM  11579  C   GLU A 758     100.711 166.970 109.616  1.00  0.00
ATOM  11580  O   GLU A 758      99.958 167.922 109.776  1.00  0.00
ATOM  11581  N   ILE A 759     100.495 165.770 110.157  1.00  0.00
ATOM  11582  H   ILE A 759     101.148 165.027 109.955  1.00  0.00
ATOM  11583  CA  ILE A 759      99.344 165.413 111.003  1.00  0.00
ATOM  11584  HA  ILE A 759      99.303 164.325 110.993  1.00  0.00
ATOM  11585  CB  ILE A 759      99.552 165.821 112.488  1.00  0.00
ATOM  11586  HB  ILE A 759      99.151 166.825 112.638  1.00  0.00
ATOM  11587  CG2 ILE A 759      98.749 164.830 113.347  1.00  0.00
ATOM  11588 1HG2 ILE A 759      98.754 165.136 114.391  1.00  0.00
```

```
ATOM  11589 2HG2 ILE A 759      97.715 164.794 113.018  1.00  0.00
ATOM  11590 3HG2 ILE A 759      99.149 163.828 113.248  1.00  0.00
ATOM  11591  CG1 ILE A 759     101.041 165.860 112.924  1.00  0.00
ATOM  11592 1HG1 ILE A 759     101.539 166.658 112.373  1.00  0.00
ATOM  11593 2HG1 ILE A 759     101.523 164.920 112.660  1.00  0.00
ATOM  11594  CD  ILE A 759     101.274 166.137 114.413  1.00  0.00
ATOM  11595  HD1 ILE A 759     102.338 166.297 114.590  1.00  0.00
ATOM  11596  HD2 ILE A 759     100.727 167.031 114.716  1.00  0.00
ATOM  11597  HD3 ILE A 759     100.950 165.292 115.020  1.00  0.00
ATOM  11598  C   ILE A 759      97.984 165.848 110.421  1.00  0.00
ATOM  11599  O   ILE A 759      97.166 166.482 111.085  1.00  0.00
ATOM  11600  N   LEU A 760      97.712 165.484 109.162  1.00  0.00
ATOM  11601  H   LEU A 760      98.461 165.084 108.625  1.00  0.00
ATOM  11602  CA  LEU A 760      96.389 165.620 108.526  1.00  0.00
ATOM  11603  HA  LEU A 760      96.201 166.684 108.381  1.00  0.00
ATOM  11604  CB  LEU A 760      96.439 164.942 107.143  1.00  0.00
ATOM  11605  HB1 LEU A 760      96.786 163.918 107.292  1.00  0.00
ATOM  11606  HB2 LEU A 760      97.172 165.478 106.543  1.00  0.00
ATOM  11607  CG  LEU A 760      95.115 164.887 106.358  1.00  0.00
ATOM  11608  HG  LEU A 760      94.360 164.357 106.938  1.00  0.00
ATOM  11609  CD1 LEU A 760      94.581 166.279 105.993  1.00  0.00
ATOM  11610 1HD1 LEU A 760      93.671 166.178 105.407  1.00  0.00
ATOM  11611 2HD1 LEU A 760      94.340 166.825 106.904  1.00  0.00
ATOM  11612 3HD1 LEU A 760      95.326 166.830 105.423  1.00  0.00
ATOM  11613  CD2 LEU A 760      95.328 164.113 105.054  1.00  0.00
ATOM  11614 1HD2 LEU A 760      94.367 163.966 104.564  1.00  0.00
ATOM  11615 2HD2 LEU A 760      95.981 164.672 104.389  1.00  0.00
ATOM  11616 3HD2 LEU A 760      95.759 163.136 105.270  1.00  0.00
ATOM  11617  C   LEU A 760      95.230 165.082 109.393  1.00  0.00
ATOM  11618  O   LEU A 760      94.118 165.594 109.316  1.00  0.00
ATOM  11619  N   ASP A 761      95.504 164.085 110.234  1.00  0.00
ATOM  11620  H   ASP A 761      96.460 163.746 110.273  1.00  0.00
ATOM  11621  CA  ASP A 761      94.607 163.500 111.228  1.00  0.00
ATOM  11622  HA  ASP A 761      93.629 163.381 110.760  1.00  0.00
ATOM  11623  CB  ASP A 761      95.113 162.087 111.577  1.00  0.00
ATOM  11624  HB1 ASP A 761      95.040 161.468 110.681  1.00  0.00
ATOM  11625  HB2 ASP A 761      94.449 161.651 112.325  1.00  0.00
ATOM  11626  CG  ASP A 761      96.560 162.034 112.077  1.00  0.00
ATOM  11627  OD1 ASP A 761      96.782 161.469 113.170  1.00  0.00
ATOM  11628  OD2 ASP A 761      97.467 162.507 111.351  1.00  0.00
ATOM  11629  C   ASP A 761      94.343 164.367 112.486  1.00  0.00
ATOM  11630  O   ASP A 761      93.365 164.116 113.178  1.00  0.00
ATOM  11631  N   GLU A 762      95.120 165.434 112.734  1.00  0.00
ATOM  11632  H   GLU A 762      95.926 165.572 112.139  1.00  0.00
ATOM  11633  CA  GLU A 762      94.759 166.547 113.644  1.00  0.00
ATOM  11634  HA  GLU A 762      93.857 166.263 114.188  1.00  0.00
ATOM  11635  CB  GLU A 762      95.858 166.851 114.689  1.00  0.00
ATOM  11636  HB1 GLU A 762      95.994 167.931 114.746  1.00  0.00
ATOM  11637  HB2 GLU A 762      96.816 166.455 114.366  1.00  0.00
ATOM  11638  CG  GLU A 762      95.542 166.385 116.126  1.00  0.00
ATOM  11639  HG1 GLU A 762      94.462 166.374 116.291  1.00  0.00
ATOM  11640  HG2 GLU A 762      95.956 167.123 116.819  1.00  0.00
ATOM  11641  CD  GLU A 762      96.158 165.024 116.474  1.00  0.00
ATOM  11642  OE1 GLU A 762      95.434 164.017 116.633  1.00  0.00
ATOM  11643  OE2 GLU A 762      97.403 164.930 116.572  1.00  0.00
ATOM  11644  C   GLU A 762      94.363 167.845 112.902  1.00  0.00
ATOM  11645  O   GLU A 762      93.813 168.762 113.511  1.00  0.00
ATOM  11646  N   ALA A 763      94.587 167.903 111.585  1.00  0.00
ATOM  11647  H   ALA A 763      95.078 167.117 111.185  1.00  0.00
ATOM  11648  CA  ALA A 763      94.093 168.915 110.652  1.00  0.00
ATOM  11649  HA  ALA A 763      94.649 168.773 109.724  1.00  0.00
```

```
ATOM  11650  CB  ALA A 763      92.624 168.603 110.313  1.00  0.00
ATOM  11651  HB1 ALA A 763      92.532 167.579 109.954  1.00  0.00
ATOM  11652  HB2 ALA A 763      92.009 168.723 111.206  1.00  0.00
ATOM  11653  HB3 ALA A 763      92.273 169.286 109.540  1.00  0.00
ATOM  11654  C   ALA A 763      94.304 170.396 111.040  1.00  0.00
ATOM  11655  O   ALA A 763      93.391 171.067 111.515  1.00  0.00
ATOM  11656  N   TYR A 764      95.455 170.990 110.698  1.00  0.00
ATOM  11657  H   TYR A 764      96.217 170.416 110.370  1.00  0.00
ATOM  11658  CA  TYR A 764      95.480 172.432 110.380  1.00  0.00
ATOM  11659  HA  TYR A 764      95.307 173.002 111.291  1.00  0.00
ATOM  11660  CB  TYR A 764      96.825 172.849 109.776  1.00  0.00
ATOM  11661  HB1 TYR A 764      96.862 173.936 109.755  1.00  0.00
ATOM  11662  HB2 TYR A 764      96.851 172.520 108.736  1.00  0.00
ATOM  11663  CG  TYR A 764      98.079 172.344 110.451  1.00  0.00
ATOM  11664  CD1 TYR A 764      98.489 172.860 111.695  1.00  0.00
ATOM  11665  HD1 TYR A 764      97.880 173.592 112.210  1.00  0.00
ATOM  11666  CE1 TYR A 764      99.703 172.428 112.269  1.00  0.00
ATOM  11667  HE1 TYR A 764     100.016 172.795 113.234  1.00  0.00
ATOM  11668  CZ  TYR A 764     100.541 171.537 111.567  1.00  0.00
ATOM  11669  OH  TYR A 764     101.788 171.250 112.023  1.00  0.00
ATOM  11670  HH  TYR A 764     102.261 170.685 111.370  1.00  0.00
ATOM  11671  CE2 TYR A 764     100.105 171.010 110.341  1.00  0.00
ATOM  11672  HE2 TYR A 764     100.728 170.296 109.827  1.00  0.00
ATOM  11673  CD2 TYR A 764      98.877 171.398 109.787  1.00  0.00
ATOM  11674  HD2 TYR A 764      98.561 170.974 108.846  1.00  0.00
ATOM  11675  C   TYR A 764      94.399 172.761 109.328  1.00  0.00
ATOM  11676  O   TYR A 764      94.128 171.952 108.442  1.00  0.00
ATOM  11677  N   VAL A 765      93.768 173.934 109.305  1.00  0.00
ATOM  11678  H   VAL A 765      93.129 174.005 108.529  1.00  0.00
ATOM  11679  CA  VAL A 765      93.849 175.114 110.196  1.00  0.00
ATOM  11680  HA  VAL A 765      94.869 175.500 110.230  1.00  0.00
ATOM  11681  CB  VAL A 765      92.901 176.191 109.607  1.00  0.00
ATOM  11682  HB  VAL A 765      91.882 175.810 109.661  1.00  0.00
ATOM  11683  CG1 VAL A 765      92.904 177.525 110.370  1.00  0.00
ATOM  11684 1HG1 VAL A 765      92.371 178.288 109.804  1.00  0.00
ATOM  11685 2HG1 VAL A 765      92.396 177.397 111.326  1.00  0.00
ATOM  11686 3HG1 VAL A 765      93.923 177.851 110.564  1.00  0.00
ATOM  11687  CG2 VAL A 765      93.192 176.425 108.114  1.00  0.00
ATOM  11688 1HG2 VAL A 765      92.673 177.312 107.758  1.00  0.00
ATOM  11689 2HG2 VAL A 765      94.263 176.522 107.962  1.00  0.00
ATOM  11690 3HG2 VAL A 765      92.832 175.588 107.520  1.00  0.00
ATOM  11691  C   VAL A 765      93.383 174.816 111.618  1.00  0.00
ATOM  11692  O   VAL A 765      93.798 175.511 112.536  1.00  0.00
ATOM  11693  N   MET A 766      92.546 173.793 111.821  1.00  0.00
ATOM  11694  H   MET A 766      92.322 173.175 111.055  1.00  0.00
ATOM  11695  CA  MET A 766      91.917 173.510 113.117  1.00  0.00
ATOM  11696  HA  MET A 766      91.313 174.377 113.390  1.00  0.00
ATOM  11697  CB  MET A 766      90.978 172.281 113.082  1.00  0.00
ATOM  11698  HB1 MET A 766      90.209 172.433 113.840  1.00  0.00
ATOM  11699  HB2 MET A 766      91.534 171.390 113.381  1.00  0.00
ATOM  11700  CG  MET A 766      90.283 171.946 111.758  1.00  0.00
ATOM  11701  HG1 MET A 766      89.550 171.166 111.965  1.00  0.00
ATOM  11702  HG2 MET A 766      91.007 171.526 111.062  1.00  0.00
ATOM  11703  SD  MET A 766      89.443 173.313 110.925  1.00  0.00
ATOM  11704  CE  MET A 766      87.996 172.428 110.295  1.00  0.00
ATOM  11705  HE1 MET A 766      87.395 172.065 111.128  1.00  0.00
ATOM  11706  HE2 MET A 766      87.401 173.110 109.695  1.00  0.00
ATOM  11707  HE3 MET A 766      88.306 171.586 109.678  1.00  0.00
ATOM  11708  C   MET A 766      92.963 173.300 114.219  1.00  0.00
ATOM  11709  O   MET A 766      92.863 173.897 115.288  1.00  0.00
ATOM  11710  N   ALA A 767      94.036 172.552 113.946  1.00  0.00
```

```
ATOM  11711  H   ALA A 767      94.014 171.993 113.099  1.00  0.00
ATOM  11712  CA  ALA A 767      95.143 172.345 114.888  1.00  0.00
ATOM  11713  HA  ALA A 767      94.739 171.818 115.755  1.00  0.00
ATOM  11714  CB  ALA A 767      96.177 171.424 114.236  1.00  0.00
ATOM  11715  HB1 ALA A 767      95.712 170.483 113.949  1.00  0.00
ATOM  11716  HB2 ALA A 767      96.595 171.897 113.351  1.00  0.00
ATOM  11717  HB3 ALA A 767      96.980 171.209 114.940  1.00  0.00
ATOM  11718  C   ALA A 767      95.802 173.640 115.446  1.00  0.00
ATOM  11719  O   ALA A 767      96.487 173.606 116.469  1.00  0.00
ATOM  11720  N   SER A 768      95.550 174.789 114.817  1.00  0.00
ATOM  11721  H   SER A 768      94.975 174.753 113.981  1.00  0.00
ATOM  11722  CA  SER A 768      96.067 176.113 115.189  1.00  0.00
ATOM  11723  HA  SER A 768      96.884 176.005 115.901  1.00  0.00
ATOM  11724  CB  SER A 768      96.611 176.788 113.908  1.00  0.00
ATOM  11725  HB1 SER A 768      97.150 177.697 114.174  1.00  0.00
ATOM  11726  HB2 SER A 768      95.770 177.066 113.273  1.00  0.00
ATOM  11727  OG  SER A 768      97.474 175.935 113.163  1.00  0.00
ATOM  11728  HG  SER A 768      97.404 176.172 112.215  1.00  0.00
ATOM  11729  C   SER A 768      95.010 177.044 115.830  1.00  0.00
ATOM  11730  O   SER A 768      95.276 178.233 116.019  1.00  0.00
ATOM  11731  N   VAL A 769      93.802 176.517 116.114  1.00  0.00
ATOM  11732  H   VAL A 769      93.664 175.534 115.906  1.00  0.00
ATOM  11733  CA  VAL A 769      92.630 177.275 116.616  1.00  0.00
ATOM  11734  HA  VAL A 769      93.006 178.123 117.188  1.00  0.00
ATOM  11735  CB  VAL A 769      91.756 177.842 115.465  1.00  0.00
ATOM  11736  HB  VAL A 769      91.089 177.054 115.113  1.00  0.00
ATOM  11737  CG1 VAL A 769      90.917 179.033 115.952  1.00  0.00
ATOM  11738 1HG1 VAL A 769      90.397 179.501 115.120  1.00  0.00
ATOM  11739 2HG1 VAL A 769      90.174 178.698 116.676  1.00  0.00
ATOM  11740 3HG1 VAL A 769      91.560 179.777 116.426  1.00  0.00
ATOM  11741  CG2 VAL A 769      92.524 178.364 114.241  1.00  0.00
ATOM  11742 1HG2 VAL A 769      91.822 178.715 113.484  1.00  0.00
ATOM  11743 2HG2 VAL A 769      93.180 179.183 114.529  1.00  0.00
ATOM  11744 3HG2 VAL A 769      93.117 177.568 113.802  1.00  0.00
ATOM  11745  C   VAL A 769      91.713 176.485 117.577  1.00  0.00
ATOM  11746  O   VAL A 769      91.048 177.087 118.417  1.00  0.00
ATOM  11747  N   ASP A 770      91.644 175.155 117.477  1.00  0.00
ATOM  11748  H   ASP A 770      92.231 174.698 116.794  1.00  0.00
ATOM  11749  CA  ASP A 770      90.505 174.369 117.977  1.00  0.00
ATOM  11750  HA  ASP A 770      89.627 175.000 117.820  1.00  0.00
ATOM  11751  CB  ASP A 770      90.275 173.134 117.080  1.00  0.00
ATOM  11752  HB1 ASP A 770      90.845 172.277 117.441  1.00  0.00
ATOM  11753  HB2 ASP A 770      90.607 173.357 116.069  1.00  0.00
ATOM  11754  CG  ASP A 770      88.793 172.786 116.951  1.00  0.00
ATOM  11755  OD1 ASP A 770      88.100 173.446 116.139  1.00  0.00
ATOM  11756  OD2 ASP A 770      88.308 171.865 117.645  1.00  0.00
ATOM  11757  C   ASP A 770      90.527 174.083 119.489  1.00  0.00
ATOM  11758  O   ASP A 770      90.846 172.973 119.926  1.00  0.00
ATOM  11759  N   ASN A 771      90.188 175.084 120.314  1.00  0.00
ATOM  11760  H   ASN A 771      90.093 176.012 119.910  1.00  0.00
ATOM  11761  CA  ASN A 771      90.210 174.957 121.781  1.00  0.00
ATOM  11762  HA  ASN A 771      90.120 173.893 122.003  1.00  0.00
ATOM  11763  CB  ASN A 771      91.596 175.418 122.266  1.00  0.00
ATOM  11764  HB1 ASN A 771      91.613 176.505 122.331  1.00  0.00
ATOM  11765  HB2 ASN A 771      92.367 175.105 121.563  1.00  0.00
ATOM  11766  CG  ASN A 771      91.972 174.847 123.629  1.00  0.00
ATOM  11767  OD1 ASN A 771      92.158 175.533 124.622  1.00  0.00
ATOM  11768  ND2 ASN A 771      92.112 173.541 123.713  1.00  0.00
ATOM  11769 1HD2 ASN A 771      91.943 172.968 122.901  1.00  0.00
ATOM  11770 2HD2 ASN A 771      92.399 173.182 124.603  1.00  0.00
ATOM  11771  C   ASN A 771      89.096 175.583 122.667  1.00  0.00
```

ATOM	11772	O	ASN	A	771	89.102	175.243	123.852	1.00	0.00
ATOM	11773	N	PRO	A	772	88.106	176.381	122.190	1.00	0.00
ATOM	11774	CD	PRO	A	772	87.935	176.878	120.823	1.00	0.00
ATOM	11775	HD1	PRO	A	772	87.608	176.095	120.153	1.00	0.00
ATOM	11776	HD2	PRO	A	772	88.855	177.317	120.439	1.00	0.00
ATOM	11777	CG	PRO	A	772	86.855	177.950	120.886	1.00	0.00
ATOM	11778	HG1	PRO	A	772	86.242	177.979	119.988	1.00	0.00
ATOM	11779	HG2	PRO	A	772	87.336	178.901	121.050	1.00	0.00
ATOM	11780	CB	PRO	A	772	86.051	177.567	122.121	1.00	0.00
ATOM	11781	HB1	PRO	A	772	85.372	176.751	121.865	1.00	0.00
ATOM	11782	HB2	PRO	A	772	85.506	178.403	122.547	1.00	0.00
ATOM	11783	CA	PRO	A	772	87.151	177.089	123.060	1.00	0.00
ATOM	11784	HA	PRO	A	772	87.664	177.966	123.462	1.00	0.00
ATOM	11785	C	PRO	A	772	86.582	176.284	124.240	1.00	0.00
ATOM	11786	O	PRO	A	772	85.734	175.409	124.046	1.00	0.00
ATOM	11787	N	HIS	A	773	87.037	176.575	125.470	1.00	0.00
ATOM	11788	H	HIS	A	773	87.758	177.284	125.522	1.00	0.00
ATOM	11789	CA	HIS	A	773	86.954	175.623	126.594	1.00	0.00
ATOM	11790	HA	HIS	A	773	87.591	174.771	126.345	1.00	0.00
ATOM	11791	CB	HIS	A	773	87.501	176.243	127.888	1.00	0.00
ATOM	11792	HB1	HIS	A	773	87.362	175.531	128.702	1.00	0.00
ATOM	11793	HB2	HIS	A	773	86.923	177.135	128.128	1.00	0.00
ATOM	11794	CG	HIS	A	773	88.963	176.613	127.875	1.00	0.00
ATOM	11795	ND1	HIS	A	773	89.523	177.591	128.694	1.00	0.00
ATOM	11796	CE1	HIS	A	773	90.827	177.649	128.358	1.00	0.00
ATOM	11797	HE1	HIS	A	773	91.556	178.319	128.795	1.00	0.00
ATOM	11798	NE2	HIS	A	773	91.100	176.757	127.390	1.00	0.00
ATOM	11799	HE2	HIS	A	773	91.995	176.641	126.925	1.00	0.00
ATOM	11800	CD2	HIS	A	773	89.939	176.090	127.074	1.00	0.00
ATOM	11801	HD2	HIS	A	773	89.826	175.342	126.304	1.00	0.00
ATOM	11802	C	HIS	A	773	85.573	175.027	126.865	1.00	0.00
ATOM	11803	O	HIS	A	773	85.454	173.823	127.068	1.00	0.00
ATOM	11804	N	VAL	A	774	84.527	175.853	126.821	1.00	0.00
ATOM	11805	H	VAL	A	774	84.731	176.822	126.632	1.00	0.00
ATOM	11806	CA	VAL	A	774	83.108	175.480	126.993	1.00	0.00
ATOM	11807	HA	VAL	A	774	83.002	175.046	127.988	1.00	0.00
ATOM	11808	CB	VAL	A	774	82.270	176.778	126.954	1.00	0.00
ATOM	11809	HB	VAL	A	774	82.680	177.442	127.712	1.00	0.00
ATOM	11810	CG1	VAL	A	774	82.349	177.527	125.614	1.00	0.00
ATOM	11811	1HG1	VAL	A	774	81.776	178.449	125.670	1.00	0.00
ATOM	11812	2HG1	VAL	A	774	83.381	177.785	125.383	1.00	0.00
ATOM	11813	3HG1	VAL	A	774	81.950	176.908	124.812	1.00	0.00
ATOM	11814	CG2	VAL	A	774	80.797	176.573	127.317	1.00	0.00
ATOM	11815	1HG2	VAL	A	774	80.323	177.525	127.521	1.00	0.00
ATOM	11816	2HG2	VAL	A	774	80.266	176.079	126.502	1.00	0.00
ATOM	11817	3HG2	VAL	A	774	80.725	175.953	128.211	1.00	0.00
ATOM	11818	C	VAL	A	774	82.575	174.425	126.018	1.00	0.00
ATOM	11819	O	VAL	A	774	81.528	173.834	126.286	1.00	0.00
ATOM	11820	N	CYS	A	775	83.280	174.133	124.923	1.00	0.00
ATOM	11821	H	CYS	A	775	84.121	174.664	124.735	1.00	0.00
ATOM	11822	CA	CYS	A	775	82.891	173.091	123.972	1.00	0.00
ATOM	11823	HA	CYS	A	775	82.100	172.478	124.414	1.00	0.00
ATOM	11824	CB	CYS	A	775	82.310	173.743	122.708	1.00	0.00
ATOM	11825	HB1	CYS	A	775	82.032	172.956	122.006	1.00	0.00
ATOM	11826	HB2	CYS	A	775	83.060	174.380	122.238	1.00	0.00
ATOM	11827	SG	CYS	A	775	80.832	174.725	123.120	1.00	0.00
ATOM	11828	HG	CYS	A	775	80.420	173.978	124.152	1.00	0.00
ATOM	11829	C	CYS	A	775	84.018	172.094	123.666	1.00	0.00
ATOM	11830	O	CYS	A	775	83.746	171.118	122.964	1.00	0.00
ATOM	11831	N	ARG	A	776	85.260	172.295	124.143	1.00	0.00
ATOM	11832	H	ARG	A	776	85.431	173.125	124.700	1.00	0.00

```
ATOM  11833  CA  ARG A 776      86.431 171.592 123.579  1.00  0.00
ATOM  11834  HA  ARG A 776      86.094 170.635 123.177  1.00  0.00
ATOM  11835  CB  ARG A 776      86.996 172.417 122.387  1.00  0.00
ATOM  11836  HB1 ARG A 776      87.729 171.793 121.871  1.00  0.00
ATOM  11837  HB2 ARG A 776      87.527 173.278 122.792  1.00  0.00
ATOM  11838  CG  ARG A 776      86.003 172.940 121.322  1.00  0.00
ATOM  11839  HG1 ARG A 776      85.403 173.743 121.749  1.00  0.00
ATOM  11840  HG2 ARG A 776      85.350 172.130 120.994  1.00  0.00
ATOM  11841  CD  ARG A 776      86.775 173.476 120.107  1.00  0.00
ATOM  11842  HD1 ARG A 776      87.267 172.631 119.629  1.00  0.00
ATOM  11843  HD2 ARG A 776      87.569 174.121 120.467  1.00  0.00
ATOM  11844  NE  ARG A 776      85.934 174.220 119.130  1.00  0.00
ATOM  11845  HE  ARG A 776      85.803 175.217 119.251  1.00  0.00
ATOM  11846  CZ  ARG A 776      85.484 173.770 117.972  1.00  0.00
ATOM  11847  NH1 ARG A 776      85.701 172.554 117.566  1.00  0.00
ATOM  11848 1HH1 ARG A 776      86.573 172.150 117.918  1.00  0.00
ATOM  11849 2HH1 ARG A 776      85.602 172.385 116.592  1.00  0.00
ATOM  11850  NH2 ARG A 776      84.812 174.537 117.168  1.00  0.00
ATOM  11851 1HH2 ARG A 776      84.827 175.548 117.367  1.00  0.00
ATOM  11852 2HH2 ARG A 776      84.637 174.267 116.225  1.00  0.00
ATOM  11853  C   ARG A 776      87.583 171.216 124.534  1.00  0.00
ATOM  11854  O   ARG A 776      88.713 171.036 124.094  1.00  0.00
ATOM  11855  N   LEU A 777      87.337 171.065 125.837  1.00  0.00
ATOM  11856  H   LEU A 777      86.401 171.240 126.174  1.00  0.00
ATOM  11857  CA  LEU A 777      88.293 170.377 126.731  1.00  0.00
ATOM  11858  HA  LEU A 777      89.265 170.862 126.628  1.00  0.00
ATOM  11859  CB  LEU A 777      87.791 170.567 128.177  1.00  0.00
ATOM  11860  HB1 LEU A 777      86.869 169.999 128.307  1.00  0.00
ATOM  11861  HB2 LEU A 777      87.530 171.618 128.308  1.00  0.00
ATOM  11862  CG  LEU A 777      88.746 170.205 129.331  1.00  0.00
ATOM  11863  HG  LEU A 777      88.230 170.462 130.257  1.00  0.00
ATOM  11864  CD1 LEU A 777      89.086 168.719 129.404  1.00  0.00
ATOM  11865 1HD1 LEU A 777      89.498 168.497 130.385  1.00  0.00
ATOM  11866 2HD1 LEU A 777      88.181 168.127 129.267  1.00  0.00
ATOM  11867 3HD1 LEU A 777      89.822 168.450 128.648  1.00  0.00
ATOM  11868  CD2 LEU A 777      90.045 171.014 129.295  1.00  0.00
ATOM  11869 1HD2 LEU A 777      90.610 170.825 130.207  1.00  0.00
ATOM  11870 2HD2 LEU A 777      90.656 170.722 128.440  1.00  0.00
ATOM  11871 3HD2 LEU A 777      89.813 172.077 129.236  1.00  0.00
ATOM  11872  C   LEU A 777      88.431 168.895 126.307  1.00  0.00
ATOM  11873  O   LEU A 777      87.466 168.139 126.364  1.00  0.00
ATOM  11874  N   LEU A 778      89.637 168.481 125.888  1.00  0.00
ATOM  11875  H   LEU A 778      90.407 169.131 125.928  1.00  0.00
ATOM  11876  CA  LEU A 778      89.930 167.155 125.315  1.00  0.00
ATOM  11877  HA  LEU A 778      89.093 166.486 125.509  1.00  0.00
ATOM  11878  CB  LEU A 778      90.107 167.296 123.782  1.00  0.00
ATOM  11879  HB1 LEU A 778      90.461 166.350 123.374  1.00  0.00
ATOM  11880  HB2 LEU A 778      90.885 168.040 123.600  1.00  0.00
ATOM  11881  CG  LEU A 778      88.844 167.705 122.997  1.00  0.00
ATOM  11882  HG  LEU A 778      88.452 168.637 123.390  1.00  0.00
ATOM  11883  CD1 LEU A 778      89.192 167.917 121.524  1.00  0.00
ATOM  11884 1HD1 LEU A 778      88.313 168.262 120.982  1.00  0.00
ATOM  11885 2HD1 LEU A 778      89.964 168.680 121.435  1.00  0.00
ATOM  11886 3HD1 LEU A 778      89.548 166.990 121.077  1.00  0.00
ATOM  11887  CD2 LEU A 778      87.739 166.651 123.077  1.00  0.00
ATOM  11888 1HD2 LEU A 778      86.900 166.953 122.451  1.00  0.00
ATOM  11889 2HD2 LEU A 778      88.109 165.686 122.736  1.00  0.00
ATOM  11890 3HD2 LEU A 778      87.375 166.562 124.100  1.00  0.00
ATOM  11891  C   LEU A 778      91.163 166.505 125.972  1.00  0.00
ATOM  11892  O   LEU A 778      91.879 167.121 126.761  1.00  0.00
ATOM  11893  N   GLY A 779      91.411 165.232 125.656  1.00  0.00
```

ATOM	11894	H	GLY	A	779	90.830	164.798	124.958	1.00	0.00
ATOM	11895	CA	GLY	A	779	92.487	164.435	126.255	1.00	0.00
ATOM	11896	HA1	GLY	A	779	92.305	163.381	126.046	1.00	0.00
ATOM	11897	HA2	GLY	A	779	92.466	164.568	127.337	1.00	0.00
ATOM	11898	C	GLY	A	779	93.880	164.783	125.729	1.00	0.00
ATOM	11899	O	GLY	A	779	94.179	164.560	124.556	1.00	0.00
ATOM	11900	N	ILE	A	780	94.756	165.259	126.619	1.00	0.00
ATOM	11901	H	ILE	A	780	94.414	165.462	127.545	1.00	0.00
ATOM	11902	CA	ILE	A	780	96.203	165.391	126.385	1.00	0.00
ATOM	11903	HA	ILE	A	780	96.346	165.968	125.471	1.00	0.00
ATOM	11904	CB	ILE	A	780	96.910	166.146	127.538	1.00	0.00
ATOM	11905	HB	ILE	A	780	96.847	165.527	128.437	1.00	0.00
ATOM	11906	CG2	ILE	A	780	98.407	166.344	127.214	1.00	0.00
ATOM	11907	1HG2	ILE	A	780	98.874	166.951	127.991	1.00	0.00
ATOM	11908	2HG2	ILE	A	780	98.920	165.382	127.181	1.00	0.00
ATOM	11909	3HG2	ILE	A	780	98.526	166.843	126.252	1.00	0.00
ATOM	11910	CG1	ILE	A	780	96.254	167.498	127.886	1.00	0.00
ATOM	11911	1HG1	ILE	A	780	96.774	167.905	128.751	1.00	0.00
ATOM	11912	2HG1	ILE	A	780	95.221	167.332	128.192	1.00	0.00
ATOM	11913	CD	ILE	A	780	96.259	168.560	126.778	1.00	0.00
ATOM	11914	HD1	ILE	A	780	97.280	168.825	126.508	1.00	0.00
ATOM	11915	HD2	ILE	A	780	95.729	168.193	125.900	1.00	0.00
ATOM	11916	HD3	ILE	A	780	95.754	169.456	127.138	1.00	0.00
ATOM	11917	C	ILE	A	780	96.800	163.998	126.172	1.00	0.00
ATOM	11918	O	ILE	A	780	97.138	163.293	127.122	1.00	0.00
ATOM	11919	N	CYS	A	781	96.872	163.574	124.914	1.00	0.00
ATOM	11920	H	CYS	A	781	96.473	164.167	124.203	1.00	0.00
ATOM	11921	CA	CYS	A	781	97.376	162.272	124.488	1.00	0.00
ATOM	11922	HA	CYS	A	781	96.948	161.504	125.135	1.00	0.00
ATOM	11923	CB	CYS	A	781	96.895	162.006	123.054	1.00	0.00
ATOM	11924	HB1	CYS	A	781	97.313	161.063	122.702	1.00	0.00
ATOM	11925	HB2	CYS	A	781	97.238	162.799	122.387	1.00	0.00
ATOM	11926	SG	CYS	A	781	95.078	161.892	123.015	1.00	0.00
ATOM	11927	HG	CYS	A	781	94.799	163.110	123.511	1.00	0.00
ATOM	11928	C	CYS	A	781	98.906	162.186	124.645	1.00	0.00
ATOM	11929	O	CYS	A	781	99.623	162.064	123.659	1.00	0.00
ATOM	11930	N	LEU	A	782	99.399	162.255	125.886	1.00	0.00
ATOM	11931	H	LEU	A	782	98.718	162.425	126.619	1.00	0.00
ATOM	11932	CA	LEU	A	782	100.812	162.227	126.302	1.00	0.00
ATOM	11933	HA	LEU	A	782	101.318	163.061	125.812	1.00	0.00
ATOM	11934	CB	LEU	A	782	100.849	162.438	127.837	1.00	0.00
ATOM	11935	HB1	LEU	A	782	101.177	161.516	128.321	1.00	0.00
ATOM	11936	HB2	LEU	A	782	99.839	162.640	128.197	1.00	0.00
ATOM	11937	CG	LEU	A	782	101.744	163.595	128.306	1.00	0.00
ATOM	11938	HG	LEU	A	782	101.429	164.513	127.813	1.00	0.00
ATOM	11939	CD1	LEU	A	782	101.583	163.779	129.816	1.00	0.00
ATOM	11940	1HD1	LEU	A	782	102.178	164.630	130.149	1.00	0.00
ATOM	11941	2HD1	LEU	A	782	100.538	163.973	130.055	1.00	0.00
ATOM	11942	3HD1	LEU	A	782	101.917	162.884	130.340	1.00	0.00
ATOM	11943	CD2	LEU	A	782	103.226	163.357	128.013	1.00	0.00
ATOM	11944	1HD2	LEU	A	782	103.822	164.173	128.418	1.00	0.00
ATOM	11945	2HD2	LEU	A	782	103.546	162.414	128.458	1.00	0.00
ATOM	11946	3HD2	LEU	A	782	103.385	163.309	126.936	1.00	0.00
ATOM	11947	C	LEU	A	782	101.583	160.944	125.898	1.00	0.00
ATOM	11948	O	LEU	A	782	102.791	160.854	126.090	1.00	0.00
ATOM	11949	N	THR	A	783	100.898	159.953	125.324	1.00	0.00
ATOM	11950	H	THR	A	783	99.897	160.062	125.258	1.00	0.00
ATOM	11951	CA	THR	A	783	101.475	158.863	124.509	1.00	0.00
ATOM	11952	HA	THR	A	783	102.338	158.445	125.027	1.00	0.00
ATOM	11953	CB	THR	A	783	100.436	157.752	124.305	1.00	0.00
ATOM	11954	HB	THR	A	783	100.774	157.050	123.542	1.00	0.00

```
ATOM  11955  CG2 THR A 783     100.187 156.967 125.590  1.00  0.00
ATOM  11956 1HG2 THR A 783      99.453 156.186 125.400  1.00  0.00
ATOM  11957 2HG2 THR A 783     101.114 156.502 125.917  1.00  0.00
ATOM  11958 3HG2 THR A 783      99.816 157.626 126.373  1.00  0.00
ATOM  11959  OG1 THR A 783      99.201 158.316 123.907  1.00  0.00
ATOM  11960  HG1 THR A 783      99.166 158.277 122.964  1.00  0.00
ATOM  11961  C   THR A 783     101.973 159.319 123.126  1.00  0.00
ATOM  11962  O   THR A 783     102.440 158.519 122.319  1.00  0.00
ATOM  11963  N   SER A 784     101.869 160.613 122.852  1.00  0.00
ATOM  11964  H   SER A 784     101.385 161.198 123.524  1.00  0.00
ATOM  11965  CA  SER A 784     102.549 161.343 121.797  1.00  0.00
ATOM  11966  HA  SER A 784     103.438 160.782 121.503  1.00  0.00
ATOM  11967  CB  SER A 784     101.628 161.488 120.585  1.00  0.00
ATOM  11968  HB1 SER A 784     101.004 162.376 120.698  1.00  0.00
ATOM  11969  HB2 SER A 784     100.984 160.611 120.508  1.00  0.00
ATOM  11970  OG  SER A 784     102.413 161.578 119.412  1.00  0.00
ATOM  11971  HG  SER A 784     102.507 160.690 119.041  1.00  0.00
ATOM  11972  C   SER A 784     102.996 162.693 122.369  1.00  0.00
ATOM  11973  O   SER A 784     102.649 163.069 123.487  1.00  0.00
ATOM  11974  N   THR A 785     103.858 163.403 121.645  1.00  0.00
ATOM  11975  H   THR A 785     104.062 163.100 120.705  1.00  0.00
ATOM  11976  CA  THR A 785     104.576 164.551 122.219  1.00  0.00
ATOM  11977  HA  THR A 785     105.035 164.210 123.146  1.00  0.00
ATOM  11978  CB  THR A 785     105.714 164.998 121.303  1.00  0.00
ATOM  11979  HB  THR A 785     106.373 164.146 121.138  1.00  0.00
ATOM  11980  CG2 THR A 785     105.291 165.557 119.942  1.00  0.00
ATOM  11981 1HG2 THR A 785     106.177 165.727 119.332  1.00  0.00
ATOM  11982 2HG2 THR A 785     104.651 164.843 119.427  1.00  0.00
ATOM  11983 3HG2 THR A 785     104.757 166.498 120.061  1.00  0.00
ATOM  11984  OG1 THR A 785     106.424 166.002 121.978  1.00  0.00
ATOM  11985  HG1 THR A 785     107.249 166.123 121.463  1.00  0.00
ATOM  11986  C   THR A 785     103.677 165.742 122.558  1.00  0.00
ATOM  11987  O   THR A 785     102.820 166.140 121.773  1.00  0.00
ATOM  11988  N   VAL A 786     103.968 166.410 123.680  1.00  0.00
ATOM  11989  H   VAL A 786     104.682 166.031 124.283  1.00  0.00
ATOM  11990  CA  VAL A 786     103.363 167.709 124.042  1.00  0.00
ATOM  11991  HA  VAL A 786     102.360 167.718 123.612  1.00  0.00
ATOM  11992  CB  VAL A 786     103.181 167.875 125.574  1.00  0.00
ATOM  11993  HB  VAL A 786     104.094 168.279 126.009  1.00  0.00
ATOM  11994  CG1 VAL A 786     102.019 168.828 125.887  1.00  0.00
ATOM  11995 1HG1 VAL A 786     101.929 168.971 126.963  1.00  0.00
ATOM  11996 2HG1 VAL A 786     102.197 169.802 125.435  1.00  0.00
ATOM  11997 3HG1 VAL A 786     101.083 168.424 125.502  1.00  0.00
ATOM  11998  CG2 VAL A 786     102.861 166.556 126.296  1.00  0.00
ATOM  11999 1HG2 VAL A 786     102.672 166.744 127.352  1.00  0.00
ATOM  12000 2HG2 VAL A 786     101.977 166.095 125.852  1.00  0.00
ATOM  12001 3HG2 VAL A 786     103.700 165.868 126.227  1.00  0.00
ATOM  12002  C   VAL A 786     104.112 168.885 123.379  1.00  0.00
ATOM  12003  O   VAL A 786     104.288 169.934 123.986  1.00  0.00
ATOM  12004  N   GLN A 787     104.619 168.685 122.151  1.00  0.00
ATOM  12005  H   GLN A 787     104.351 167.809 121.723  1.00  0.00
ATOM  12006  CA  GLN A 787     105.647 169.469 121.420  1.00  0.00
ATOM  12007  HA  GLN A 787     105.949 168.886 120.550  1.00  0.00
ATOM  12008  CB  GLN A 787     105.047 170.803 120.890  1.00  0.00
ATOM  12009  HB1 GLN A 787     105.865 171.447 120.564  1.00  0.00
ATOM  12010  HB2 GLN A 787     104.539 171.312 121.709  1.00  0.00
ATOM  12011  CG  GLN A 787     104.065 170.666 119.708  1.00  0.00
ATOM  12012  HG1 GLN A 787     103.370 169.851 119.915  1.00  0.00
ATOM  12013  HG2 GLN A 787     104.622 170.418 118.804  1.00  0.00
ATOM  12014  CD  GLN A 787     103.232 171.933 119.454  1.00  0.00
ATOM  12015  OE1 GLN A 787     102.040 171.967 119.720  1.00  0.00
```

```
ATOM  12016  NE2 GLN A 787     103.801 173.013 118.953  1.00  0.00
ATOM  12017 1HE2 GLN A 787     104.745 173.000 118.609  1.00  0.00
ATOM  12018 2HE2 GLN A 787     103.208 173.834 118.846  1.00  0.00
ATOM  12019  C   GLN A 787     106.940 169.721 122.221  1.00  0.00
ATOM  12020  O   GLN A 787     107.992 169.183 121.883  1.00  0.00
ATOM  12021  N   LEU A 788     106.846 170.490 123.308  1.00  0.00
ATOM  12022  H   LEU A 788     105.912 170.814 123.538  1.00  0.00
ATOM  12023  CA  LEU A 788     107.904 170.821 124.269  1.00  0.00
ATOM  12024  HA  LEU A 788     108.529 171.629 123.884  1.00  0.00
ATOM  12025  CB  LEU A 788     107.221 171.259 125.573  1.00  0.00
ATOM  12026  HB1 LEU A 788     107.972 171.361 126.359  1.00  0.00
ATOM  12027  HB2 LEU A 788     106.542 170.464 125.883  1.00  0.00
ATOM  12028  CG  LEU A 788     106.433 172.565 125.441  1.00  0.00
ATOM  12029  HG  LEU A 788     106.167 172.753 124.402  1.00  0.00
ATOM  12030  CD1 LEU A 788     105.144 172.460 126.247  1.00  0.00
ATOM  12031 1HD1 LEU A 788     104.616 173.414 126.233  1.00  0.00
ATOM  12032 2HD1 LEU A 788     104.488 171.710 125.811  1.00  0.00
ATOM  12033 3HD1 LEU A 788     105.352 172.174 127.276  1.00  0.00
ATOM  12034  CD2 LEU A 788     107.269 173.730 125.941  1.00  0.00
ATOM  12035 1HD2 LEU A 788     106.658 174.625 125.876  1.00  0.00
ATOM  12036 2HD2 LEU A 788     107.576 173.576 126.975  1.00  0.00
ATOM  12037 3HD2 LEU A 788     108.150 173.855 125.311  1.00  0.00
ATOM  12038  C   LEU A 788     108.796 169.620 124.593  1.00  0.00
ATOM  12039  O   LEU A 788     110.010 169.661 124.438  1.00  0.00
ATOM  12040  N   ILE A 789     108.159 168.522 125.004  1.00  0.00
ATOM  12041  H   ILE A 789     107.158 168.586 125.086  1.00  0.00
ATOM  12042  CA  ILE A 789     108.804 167.305 125.495  1.00  0.00
ATOM  12043  HA  ILE A 789     109.414 167.561 126.362  1.00  0.00
ATOM  12044  CB  ILE A 789     107.714 166.296 125.941  1.00  0.00
ATOM  12045  HB  ILE A 789     107.125 166.033 125.059  1.00  0.00
ATOM  12046  CG2 ILE A 789     108.302 164.992 126.501  1.00  0.00
ATOM  12047 1HG2 ILE A 789     107.513 164.358 126.907  1.00  0.00
ATOM  12048 2HG2 ILE A 789     108.800 164.431 125.710  1.00  0.00
ATOM  12049 3HG2 ILE A 789     109.026 165.203 127.287  1.00  0.00
ATOM  12050  CG1 ILE A 789     106.729 166.894 126.978  1.00  0.00
ATOM  12051 1HG1 ILE A 789     105.996 166.130 127.242  1.00  0.00
ATOM  12052 2HG1 ILE A 789     106.183 167.712 126.513  1.00  0.00
ATOM  12053  CD  ILE A 789     107.361 167.425 128.271  1.00  0.00
ATOM  12054  HD1 ILE A 789     106.574 167.799 128.925  1.00  0.00
ATOM  12055  HD2 ILE A 789     107.895 166.632 128.792  1.00  0.00
ATOM  12056  HD3 ILE A 789     108.044 168.246 128.057  1.00  0.00
ATOM  12057  C   ILE A 789     109.769 166.678 124.473  1.00  0.00
ATOM  12058  O   ILE A 789     110.732 166.033 124.865  1.00  0.00
ATOM  12059  N   THR A 790     109.554 166.909 123.172  1.00  0.00
ATOM  12060  H   THR A 790     108.762 167.476 122.899  1.00  0.00
ATOM  12061  CA  THR A 790     110.434 166.409 122.105  1.00  0.00
ATOM  12062  HA  THR A 790     111.226 165.811 122.555  1.00  0.00
ATOM  12063  CB  THR A 790     109.712 165.479 121.123  1.00  0.00
ATOM  12064  HB  THR A 790     110.408 165.160 120.348  1.00  0.00
ATOM  12065  CG2 THR A 790     109.196 164.220 121.819  1.00  0.00
ATOM  12066 1HG2 THR A 790     108.690 163.584 121.094  1.00  0.00
ATOM  12067 2HG2 THR A 790     110.038 163.668 122.237  1.00  0.00
ATOM  12068 3HG2 THR A 790     108.507 164.470 122.625  1.00  0.00
ATOM  12069  OG1 THR A 790     108.636 166.154 120.508  1.00  0.00
ATOM  12070  HG1 THR A 790     109.021 166.845 119.954  1.00  0.00
ATOM  12071  C   THR A 790     111.178 167.512 121.352  1.00  0.00
ATOM  12072  O   THR A 790     111.823 167.212 120.350  1.00  0.00
ATOM  12073  N   GLN A 791     111.111 168.775 121.787  1.00  0.00
ATOM  12074  H   GLN A 791     110.537 169.000 122.593  1.00  0.00
ATOM  12075  CA  GLN A 791     111.857 169.857 121.134  1.00  0.00
ATOM  12076  HA  GLN A 791     112.806 169.420 120.819  1.00  0.00
```

ATOM	12077	CB	GLN	A	791	111.122	170.317	119.847	1.00	0.00
ATOM	12078	HB1	GLN	A	791	110.398	171.096	120.088	1.00	0.00
ATOM	12079	HB2	GLN	A	791	110.578	169.470	119.429	1.00	0.00
ATOM	12080	CG	GLN	A	791	112.119	170.816	118.780	1.00	0.00
ATOM	12081	HG1	GLN	A	791	112.902	170.070	118.640	1.00	0.00
ATOM	12082	HG2	GLN	A	791	112.595	171.733	119.122	1.00	0.00
ATOM	12083	CD	GLN	A	791	111.497	171.076	117.409	1.00	0.00
ATOM	12084	OE1	GLN	A	791	110.558	170.429	116.982	1.00	0.00
ATOM	12085	NE2	GLN	A	791	112.017	172.028	116.657	1.00	0.00
ATOM	12086	1HE2	GLN	A	791	112.767	172.594	117.005	1.00	0.00
ATOM	12087	2HE2	GLN	A	791	111.653	172.154	115.719	1.00	0.00
ATOM	12088	C	GLN	A	791	112.261	171.028	122.052	1.00	0.00
ATOM	12089	O	GLN	A	791	112.457	172.148	121.586	1.00	0.00
ATOM	12090	N	LEU	A	792	112.397	170.791	123.363	1.00	0.00
ATOM	12091	H	LEU	A	792	112.141	169.886	123.731	1.00	0.00
ATOM	12092	CA	LEU	A	792	113.070	171.727	124.267	1.00	0.00
ATOM	12093	HA	LEU	A	792	112.633	172.711	124.100	1.00	0.00
ATOM	12094	CB	LEU	A	792	112.836	171.335	125.739	1.00	0.00
ATOM	12095	HB1	LEU	A	792	113.672	171.694	126.341	1.00	0.00
ATOM	12096	HB2	LEU	A	792	112.818	170.248	125.818	1.00	0.00
ATOM	12097	CG	LEU	A	792	111.538	171.927	126.324	1.00	0.00
ATOM	12098	HG	LEU	A	792	110.698	171.714	125.670	1.00	0.00
ATOM	12099	CD1	LEU	A	792	111.257	171.300	127.691	1.00	0.00
ATOM	12100	1HD1	LEU	A	792	110.320	171.690	128.087	1.00	0.00
ATOM	12101	2HD1	LEU	A	792	111.170	170.220	127.584	1.00	0.00
ATOM	12102	3HD1	LEU	A	792	112.065	171.536	128.383	1.00	0.00
ATOM	12103	CD2	LEU	A	792	111.612	173.443	126.518	1.00	0.00
ATOM	12104	1HD2	LEU	A	792	110.702	173.802	127.001	1.00	0.00
ATOM	12105	2HD2	LEU	A	792	112.460	173.710	127.145	1.00	0.00
ATOM	12106	3HD2	LEU	A	792	111.700	173.956	125.564	1.00	0.00
ATOM	12107	C	LEU	A	792	114.559	171.839	123.919	1.00	0.00
ATOM	12108	O	LEU	A	792	115.275	170.847	123.824	1.00	0.00
ATOM	12109	N	MET	A	793	115.002	173.076	123.713	1.00	0.00
ATOM	12110	H	MET	A	793	114.359	173.838	123.881	1.00	0.00
ATOM	12111	CA	MET	A	793	116.346	173.468	123.282	1.00	0.00
ATOM	12112	HA	MET	A	793	117.066	172.728	123.633	1.00	0.00
ATOM	12113	CB	MET	A	793	116.385	173.549	121.743	1.00	0.00
ATOM	12114	HB1	MET	A	793	117.333	173.980	121.432	1.00	0.00
ATOM	12115	HB2	MET	A	793	115.586	174.205	121.396	1.00	0.00
ATOM	12116	CG	MET	A	793	116.233	172.180	121.061	1.00	0.00
ATOM	12117	HG1	MET	A	793	115.239	171.794	121.284	1.00	0.00
ATOM	12118	HG2	MET	A	793	116.961	171.489	121.486	1.00	0.00
ATOM	12119	SD	MET	A	793	116.420	172.188	119.256	1.00	0.00
ATOM	12120	CE	MET	A	793	118.208	172.441	119.083	1.00	0.00
ATOM	12121	HE1	MET	A	793	118.740	171.625	119.567	1.00	0.00
ATOM	12122	HE2	MET	A	793	118.471	172.460	118.027	1.00	0.00
ATOM	12123	HE3	MET	A	793	118.501	173.386	119.533	1.00	0.00
ATOM	12124	C	MET	A	793	116.676	174.835	123.903	1.00	0.00
ATOM	12125	O	MET	A	793	115.741	175.521	124.310	1.00	0.00
ATOM	12126	N	PRO	A	794	117.948	175.284	123.966	1.00	0.00
ATOM	12127	CD	PRO	A	794	119.141	174.543	123.578	1.00	0.00
ATOM	12128	HD1	PRO	A	794	119.258	174.581	122.495	1.00	0.00
ATOM	12129	HD2	PRO	A	794	119.105	173.509	123.920	1.00	0.00
ATOM	12130	CG	PRO	A	794	120.314	175.265	124.240	1.00	0.00
ATOM	12131	HG1	PRO	A	794	121.227	175.181	123.651	1.00	0.00
ATOM	12132	HG2	PRO	A	794	120.467	174.867	125.244	1.00	0.00
ATOM	12133	CB	PRO	A	794	119.826	176.708	124.330	1.00	0.00
ATOM	12134	HB1	PRO	A	794	119.984	177.203	123.370	1.00	0.00
ATOM	12135	HB2	PRO	A	794	120.324	177.258	125.129	1.00	0.00
ATOM	12136	CA	PRO	A	794	118.324	176.556	124.600	1.00	0.00
ATOM	12137	HA	PRO	A	794	118.180	176.448	125.676	1.00	0.00

ATOM	12138	C	PRO	A	794	117.560	177.814	124.157	1.00	0.00
ATOM	12139	O	PRO	A	794	117.476	178.771	124.919	1.00	0.00
ATOM	12140	N	PHE	A	795	117.019	177.826	122.932	1.00	0.00
ATOM	12141	H	PHE	A	795	117.124	177.009	122.356	1.00	0.00
ATOM	12142	CA	PHE	A	795	116.180	178.916	122.430	1.00	0.00
ATOM	12143	HA	PHE	A	795	116.568	179.849	122.835	1.00	0.00
ATOM	12144	CB	PHE	A	795	116.297	179.002	120.898	1.00	0.00
ATOM	12145	HB1	PHE	A	795	117.336	179.221	120.652	1.00	0.00
ATOM	12146	HB2	PHE	A	795	115.698	179.844	120.551	1.00	0.00
ATOM	12147	CG	PHE	A	795	115.864	177.757	120.146	1.00	0.00
ATOM	12148	CD1	PHE	A	795	116.828	176.927	119.542	1.00	0.00
ATOM	12149	HD1	PHE	A	795	117.878	177.194	119.574	1.00	0.00
ATOM	12150	CE1	PHE	A	795	116.431	175.763	118.864	1.00	0.00
ATOM	12151	HE1	PHE	A	795	117.174	175.138	118.385	1.00	0.00
ATOM	12152	CZ	PHE	A	795	115.070	175.414	118.801	1.00	0.00
ATOM	12153	HZ	PHE	A	795	114.766	174.508	118.291	1.00	0.00
ATOM	12154	CE2	PHE	A	795	114.104	176.247	119.390	1.00	0.00
ATOM	12155	HE2	PHE	A	795	113.058	175.982	119.330	1.00	0.00
ATOM	12156	CD2	PHE	A	795	114.499	177.426	120.044	1.00	0.00
ATOM	12157	HD2	PHE	A	795	113.743	178.077	120.465	1.00	0.00
ATOM	12158	C	PHE	A	795	114.713	178.840	122.877	1.00	0.00
ATOM	12159	O	PHE	A	795	114.012	179.836	122.765	1.00	0.00
ATOM	12160	N	GLY	A	796	114.236	177.711	123.402	1.00	0.00
ATOM	12161	H	GLY	A	796	114.890	176.951	123.534	1.00	0.00
ATOM	12162	CA	GLY	A	796	112.937	177.571	124.075	1.00	0.00
ATOM	12163	HA1	GLY	A	796	112.673	176.516	124.158	1.00	0.00
ATOM	12164	HA2	GLY	A	796	112.161	178.083	123.504	1.00	0.00
ATOM	12165	C	GLY	A	796	112.972	178.164	125.478	1.00	0.00
ATOM	12166	O	GLY	A	796	112.768	177.458	126.452	1.00	0.00
ATOM	12167	N	CYS	A	797	113.394	179.424	125.555	1.00	0.00
ATOM	12168	H	CYS	A	797	113.536	179.918	124.680	1.00	0.00
ATOM	12169	CA	CYS	A	797	113.673	180.173	126.764	1.00	0.00
ATOM	12170	HA	CYS	A	797	112.829	180.057	127.449	1.00	0.00
ATOM	12171	CB	CYS	A	797	114.951	179.602	127.405	1.00	0.00
ATOM	12172	HB1	CYS	A	797	115.825	179.908	126.828	1.00	0.00
ATOM	12173	HB2	CYS	A	797	114.904	178.512	127.405	1.00	0.00
ATOM	12174	SG	CYS	A	797	115.093	180.162	129.122	1.00	0.00
ATOM	12175	HG	CYS	A	797	114.027	179.492	129.578	1.00	0.00
ATOM	12176	C	CYS	A	797	113.851	181.650	126.407	1.00	0.00
ATOM	12177	O	CYS	A	797	114.089	182.026	125.255	1.00	0.00
ATOM	12178	N	LEU	A	798	113.700	182.491	127.416	1.00	0.00
ATOM	12179	H	LEU	A	798	113.682	182.072	128.333	1.00	0.00
ATOM	12180	CA	LEU	A	798	113.171	183.844	127.311	1.00	0.00
ATOM	12181	HA	LEU	A	798	113.364	184.262	126.323	1.00	0.00
ATOM	12182	CB	LEU	A	798	111.645	183.750	127.564	1.00	0.00
ATOM	12183	HB1	LEU	A	798	111.234	184.738	127.368	1.00	0.00
ATOM	12184	HB2	LEU	A	798	111.494	183.504	128.616	1.00	0.00
ATOM	12185	CG	LEU	A	798	110.819	182.726	126.750	1.00	0.00
ATOM	12186	HG	LEU	A	798	111.253	181.734	126.832	1.00	0.00
ATOM	12187	CD1	LEU	A	798	109.383	182.614	127.260	1.00	0.00
ATOM	12188	1HD1	LEU	A	798	108.810	181.966	126.598	1.00	0.00
ATOM	12189	2HD1	LEU	A	798	109.423	182.135	128.236	1.00	0.00
ATOM	12190	3HD1	LEU	A	798	108.927	183.593	127.342	1.00	0.00
ATOM	12191	CD2	LEU	A	798	110.783	183.117	125.283	1.00	0.00
ATOM	12192	1HD2	LEU	A	798	110.162	182.422	124.723	1.00	0.00
ATOM	12193	2HD2	LEU	A	798	110.410	184.131	125.165	1.00	0.00
ATOM	12194	3HD2	LEU	A	798	111.796	183.059	124.899	1.00	0.00
ATOM	12195	C	LEU	A	798	113.906	184.680	128.364	1.00	0.00
ATOM	12196	O	LEU	A	798	113.291	185.219	129.283	1.00	0.00
ATOM	12197	N	LEU	A	799	115.239	184.645	128.310	1.00	0.00
ATOM	12198	H	LEU	A	799	115.666	184.175	127.528	1.00	0.00

```
ATOM  12199  CA  LEU A 799     116.133 185.248 129.298  1.00  0.00
ATOM  12200  HA  LEU A 799     115.570 186.027 129.814  1.00  0.00
ATOM  12201  CB  LEU A 799     116.532 184.214 130.374  1.00  0.00
ATOM  12202  HB1 LEU A 799     115.601 183.810 130.776  1.00  0.00
ATOM  12203  HB2 LEU A 799     117.019 184.742 131.195  1.00  0.00
ATOM  12204  CG  LEU A 799     117.429 183.033 129.945  1.00  0.00
ATOM  12205  HG  LEU A 799     117.010 182.552 129.062  1.00  0.00
ATOM  12206  CD1 LEU A 799     118.887 183.418 129.671  1.00  0.00
ATOM  12207 1HD1 LEU A 799     119.491 182.522 129.537  1.00  0.00
ATOM  12208 2HD1 LEU A 799     118.958 183.989 128.749  1.00  0.00
ATOM  12209 3HD1 LEU A 799     119.286 184.000 130.501  1.00  0.00
ATOM  12210  CD2 LEU A 799     117.460 182.020 131.095  1.00  0.00
ATOM  12211 1HD2 LEU A 799     118.035 181.146 130.794  1.00  0.00
ATOM  12212 2HD2 LEU A 799     117.910 182.466 131.982  1.00  0.00
ATOM  12213 3HD2 LEU A 799     116.447 181.694 131.332  1.00  0.00
ATOM  12214  C   LEU A 799     117.305 185.986 128.653  1.00  0.00
ATOM  12215  O   LEU A 799     117.492 185.878 127.441  1.00  0.00
ATOM  12216  N   ASP A 800     118.066 186.700 129.499  1.00  0.00
ATOM  12217  H   ASP A 800     117.701 186.765 130.441  1.00  0.00
ATOM  12218  CA  ASP A 800     119.241 187.547 129.219  1.00  0.00
ATOM  12219  HA  ASP A 800     118.961 188.569 129.464  1.00  0.00
ATOM  12220  CB  ASP A 800     120.408 187.158 130.151  1.00  0.00
ATOM  12221  HB1 ASP A 800     120.571 186.081 130.082  1.00  0.00
ATOM  12222  HB2 ASP A 800     120.128 187.387 131.181  1.00  0.00
ATOM  12223  CG  ASP A 800     121.728 187.881 129.818  1.00  0.00
ATOM  12224  OD1 ASP A 800     122.795 187.234 129.936  1.00  0.00
ATOM  12225  OD2 ASP A 800     121.670 189.078 129.438  1.00  0.00
ATOM  12226  C   ASP A 800     119.705 187.565 127.748  1.00  0.00
ATOM  12227  O   ASP A 800     119.498 188.563 127.048  1.00  0.00
ATOM  12228  N   TYR A 801     120.265 186.449 127.267  1.00  0.00
ATOM  12229  H   TYR A 801     120.314 185.646 127.875  1.00  0.00
ATOM  12230  CA  TYR A 801     120.547 186.241 125.854  1.00  0.00
ATOM  12231  HA  TYR A 801     120.099 187.059 125.292  1.00  0.00
ATOM  12232  CB  TYR A 801     122.050 186.237 125.567  1.00  0.00
ATOM  12233  HB1 TYR A 801     122.214 185.953 124.529  1.00  0.00
ATOM  12234  HB2 TYR A 801     122.524 185.476 126.189  1.00  0.00
ATOM  12235  CG  TYR A 801     122.734 187.559 125.781  1.00  0.00
ATOM  12236  CD1 TYR A 801     122.624 188.599 124.836  1.00  0.00
ATOM  12237  HD1 TYR A 801     122.012 188.487 123.950  1.00  0.00
ATOM  12238  CE1 TYR A 801     123.337 189.797 125.040  1.00  0.00
ATOM  12239  HE1 TYR A 801     123.313 190.596 124.321  1.00  0.00
ATOM  12240  CZ  TYR A 801     124.091 189.983 126.215  1.00  0.00
ATOM  12241  OH  TYR A 801     124.751 191.154 126.416  1.00  0.00
ATOM  12242  HH  TYR A 801     125.419 191.085 127.115  1.00  0.00
ATOM  12243  CE2 TYR A 801     124.132 188.959 127.173  1.00  0.00
ATOM  12244  HE2 TYR A 801     124.601 189.115 128.122  1.00  0.00
ATOM  12245  CD2 TYR A 801     123.493 187.738 126.938  1.00  0.00
ATOM  12246  HD2 TYR A 801     123.537 186.954 127.681  1.00  0.00
ATOM  12247  C   TYR A 801     119.964 184.947 125.318  1.00  0.00
ATOM  12248  O   TYR A 801     119.696 184.002 126.058  1.00  0.00
ATOM  12249  N   VAL A 802     119.825 184.951 123.990  1.00  0.00
ATOM  12250  H   VAL A 802     120.222 185.779 123.568  1.00  0.00
ATOM  12251  CA  VAL A 802     118.875 184.241 123.106  1.00  0.00
ATOM  12252  HA  VAL A 802     119.438 183.771 122.304  1.00  0.00
ATOM  12253  CB  VAL A 802     118.027 183.139 123.805  1.00  0.00
ATOM  12254  HB  VAL A 802     117.685 183.497 124.775  1.00  0.00
ATOM  12255  CG1 VAL A 802     116.769 182.713 123.042  1.00  0.00
ATOM  12256 1HG1 VAL A 802     116.271 181.908 123.586  1.00  0.00
ATOM  12257 2HG1 VAL A 802     116.060 183.537 122.997  1.00  0.00
ATOM  12258 3HG1 VAL A 802     117.023 182.376 122.039  1.00  0.00
ATOM  12259  CG2 VAL A 802     118.874 181.869 123.970  1.00  0.00
```

```
ATOM  12260 1HG2 VAL A 802     118.349 181.178 124.628  1.00  0.00
ATOM  12261 2HG2 VAL A 802     119.050 181.394 123.005  1.00  0.00
ATOM  12262 3HG2 VAL A 802     119.832 182.102 124.430  1.00  0.00
ATOM  12263  C   VAL A 802     117.997 185.302 122.455  1.00  0.00
ATOM  12264  O   VAL A 802     117.515 185.121 121.339  1.00  0.00
ATOM  12265  N   ARG A 803     117.857 186.427 123.153  1.00  0.00
ATOM  12266  H   ARG A 803     118.293 186.413 124.067  1.00  0.00
ATOM  12267  CA  ARG A 803     117.220 187.700 122.829  1.00  0.00
ATOM  12268  HA  ARG A 803     116.964 187.760 121.779  1.00  0.00
ATOM  12269  CB  ARG A 803     115.960 187.800 123.723  1.00  0.00
ATOM  12270  HB1 ARG A 803     115.326 188.597 123.335  1.00  0.00
ATOM  12271  HB2 ARG A 803     116.308 188.092 124.717  1.00  0.00
ATOM  12272  CG  ARG A 803     115.103 186.519 123.916  1.00  0.00
ATOM  12273  HG1 ARG A 803     114.456 186.663 124.777  1.00  0.00
ATOM  12274  HG2 ARG A 803     115.733 185.675 124.193  1.00  0.00
ATOM  12275  CD  ARG A 803     114.206 186.113 122.741  1.00  0.00
ATOM  12276  HD1 ARG A 803     113.375 186.813 122.678  1.00  0.00
ATOM  12277  HD2 ARG A 803     113.800 185.131 122.970  1.00  0.00
ATOM  12278  NE  ARG A 803     114.944 186.042 121.467  1.00  0.00
ATOM  12279  HE  ARG A 803     115.804 185.512 121.466  1.00  0.00
ATOM  12280  CZ  ARG A 803     114.894 186.920 120.493  1.00  0.00
ATOM  12281  NH1 ARG A 803     113.924 187.754 120.315  1.00  0.00
ATOM  12282 1HH1 ARG A 803     113.034 187.491 120.713  1.00  0.00
ATOM  12283 2HH1 ARG A 803     113.975 188.387 119.518  1.00  0.00
ATOM  12284  NH2 ARG A 803     115.868 187.056 119.658  1.00  0.00
ATOM  12285 1HH2 ARG A 803     116.716 186.500 119.715  1.00  0.00
ATOM  12286 2HH2 ARG A 803     115.815 187.864 119.051  1.00  0.00
ATOM  12287  C   ARG A 803     118.264 188.793 123.107  1.00  0.00
ATOM  12288  O   ARG A 803     119.422 188.471 123.362  1.00  0.00
ATOM  12289  N   GLU A 804     117.876 190.066 123.057  1.00  0.00
ATOM  12290  H   GLU A 804     116.915 190.249 122.829  1.00  0.00
ATOM  12291  CA  GLU A 804     118.698 191.194 123.568  1.00  0.00
ATOM  12292  HA  GLU A 804     119.570 190.806 124.095  1.00  0.00
ATOM  12293  CB  GLU A 804     119.140 192.118 122.418  1.00  0.00
ATOM  12294  HB1 GLU A 804     118.273 192.689 122.079  1.00  0.00
ATOM  12295  HB2 GLU A 804     119.427 191.520 121.567  1.00  0.00
ATOM  12296  CG  GLU A 804     120.263 193.118 122.765  1.00  0.00
ATOM  12297  HG1 GLU A 804     119.876 193.909 123.408  1.00  0.00
ATOM  12298  HG2 GLU A 804     120.563 193.606 121.835  1.00  0.00
ATOM  12299  CD  GLU A 804     121.505 192.484 123.417  1.00  0.00
ATOM  12300  OE1 GLU A 804     122.477 192.258 122.661  1.00  0.00
ATOM  12301  OE2 GLU A 804     121.487 192.178 124.636  1.00  0.00
ATOM  12302  C   GLU A 804     117.924 192.071 124.543  1.00  0.00
ATOM  12303  O   GLU A 804     118.475 192.919 125.233  1.00  0.00
ATOM  12304  N   HIS A 805     116.611 191.901 124.615  1.00  0.00
ATOM  12305  H   HIS A 805     116.177 191.109 124.167  1.00  0.00
ATOM  12306  CA  HIS A 805     115.730 192.784 125.372  1.00  0.00
ATOM  12307  HA  HIS A 805     116.147 193.792 125.332  1.00  0.00
ATOM  12308  CB  HIS A 805     114.351 192.826 124.706  1.00  0.00
ATOM  12309  HB1 HIS A 805     113.894 193.784 124.944  1.00  0.00
ATOM  12310  HB2 HIS A 805     113.707 192.047 125.115  1.00  0.00
ATOM  12311  CG  HIS A 805     114.419 192.652 123.201  1.00  0.00
ATOM  12312  ND1 HIS A 805     114.717 193.641 122.263  1.00  0.00
ATOM  12313  CE1 HIS A 805     114.860 193.009 121.087  1.00  0.00
ATOM  12314  HE1 HIS A 805     115.105 193.494 120.148  1.00  0.00
ATOM  12315  NE2 HIS A 805     114.672 191.685 121.236  1.00  0.00
ATOM  12316  HE2 HIS A 805     114.732 190.997 120.486  1.00  0.00
ATOM  12317  CD2 HIS A 805     114.404 191.445 122.568  1.00  0.00
ATOM  12318  HD2 HIS A 805     114.245 190.494 123.064  1.00  0.00
ATOM  12319  C   HIS A 805     115.706 192.379 126.856  1.00  0.00
ATOM  12320  O   HIS A 805     114.851 192.829 127.613  1.00  0.00
```

```
ATOM  12321  N   LYS A 806     116.659 191.528 127.253  1.00  0.00
ATOM  12322  H   LYS A 806     117.274 191.257 126.504  1.00  0.00
ATOM  12323  CA  LYS A 806     116.827 190.742 128.479  1.00  0.00
ATOM  12324  HA  LYS A 806     117.626 190.033 128.266  1.00  0.00
ATOM  12325  CB  LYS A 806     117.316 191.597 129.657  1.00  0.00
ATOM  12326  HB1 LYS A 806     117.572 190.926 130.474  1.00  0.00
ATOM  12327  HB2 LYS A 806     116.497 192.220 130.001  1.00  0.00
ATOM  12328  CG  LYS A 806     118.550 192.491 129.409  1.00  0.00
ATOM  12329  HG1 LYS A 806     118.754 193.013 130.345  1.00  0.00
ATOM  12330  HG2 LYS A 806     118.317 193.249 128.659  1.00  0.00
ATOM  12331  CD  LYS A 806     119.817 191.714 129.017  1.00  0.00
ATOM  12332  HD1 LYS A 806     119.713 190.675 129.321  1.00  0.00
ATOM  12333  HD2 LYS A 806     120.672 192.133 129.551  1.00  0.00
ATOM  12334  CE  LYS A 806     120.102 191.800 127.514  1.00  0.00
ATOM  12335  HE1 LYS A 806     120.428 192.814 127.267  1.00  0.00
ATOM  12336  HE2 LYS A 806     119.179 191.613 126.966  1.00  0.00
ATOM  12337  NZ  LYS A 806     121.122 190.828 127.071  1.00  0.00
ATOM  12338  HZ1 LYS A 806     121.502 191.114 126.161  1.00  0.00
ATOM  12339  HZ2 LYS A 806     121.863 190.706 127.749  1.00  0.00
ATOM  12340  HZ3 LYS A 806     120.706 189.904 126.980  1.00  0.00
ATOM  12341  C   LYS A 806     115.663 189.815 128.849  1.00  0.00
ATOM  12342  O   LYS A 806     115.912 188.789 129.465  1.00  0.00
ATOM  12343  N   ASP A 807     114.435 190.139 128.439  1.00  0.00
ATOM  12344  H   ASP A 807     114.361 191.018 127.944  1.00  0.00
ATOM  12345  CA  ASP A 807     113.271 189.241 128.344  1.00  0.00
ATOM  12346  HA  ASP A 807     112.413 189.848 128.052  1.00  0.00
ATOM  12347  CB  ASP A 807     113.547 188.259 127.177  1.00  0.00
ATOM  12348  HB1 ASP A 807     112.758 187.512 127.144  1.00  0.00
ATOM  12349  HB2 ASP A 807     114.477 187.723 127.369  1.00  0.00
ATOM  12350  CG  ASP A 807     113.637 188.939 125.798  1.00  0.00
ATOM  12351  OD1 ASP A 807     112.802 188.604 124.929  1.00  0.00
ATOM  12352  OD2 ASP A 807     114.560 189.758 125.559  1.00  0.00
ATOM  12353  C   ASP A 807     112.855 188.543 129.678  1.00  0.00
ATOM  12354  O   ASP A 807     111.987 187.677 129.710  1.00  0.00
ATOM  12355  N   ASN A 808     113.467 188.912 130.804  1.00  0.00
ATOM  12356  H   ASN A 808     114.207 189.592 130.706  1.00  0.00
ATOM  12357  CA  ASN A 808     113.490 188.069 131.998  1.00  0.00
ATOM  12358  HA  ASN A 808     113.839 187.080 131.694  1.00  0.00
ATOM  12359  CB  ASN A 808     114.501 188.670 132.986  1.00  0.00
ATOM  12360  HB1 ASN A 808     114.422 188.188 133.958  1.00  0.00
ATOM  12361  HB2 ASN A 808     114.268 189.718 133.116  1.00  0.00
ATOM  12362  CG  ASN A 808     115.940 188.533 132.547  1.00  0.00
ATOM  12363  OD1 ASN A 808     116.428 187.447 132.269  1.00  0.00
ATOM  12364  ND2 ASN A 808     116.666 189.622 132.529  1.00  0.00
ATOM  12365 1HD2 ASN A 808     116.245 190.521 132.750  1.00  0.00
ATOM  12366 2HD2 ASN A 808     117.592 189.530 132.161  1.00  0.00
ATOM  12367  C   ASN A 808     112.155 187.874 132.738  1.00  0.00
ATOM  12368  O   ASN A 808     111.229 188.676 132.679  1.00  0.00
ATOM  12369  N   ILE A 809     112.111 186.841 133.579  1.00  0.00
ATOM  12370  H   ILE A 809     112.877 186.191 133.543  1.00  0.00
ATOM  12371  CA  ILE A 809     111.026 186.569 134.525  1.00  0.00
ATOM  12372  HA  ILE A 809     110.158 186.322 133.923  1.00  0.00
ATOM  12373  CB  ILE A 809     111.355 185.286 135.332  1.00  0.00
ATOM  12374  HB  ILE A 809     111.474 184.478 134.606  1.00  0.00
ATOM  12375  CG2 ILE A 809     112.693 185.401 136.098  1.00  0.00
ATOM  12376 1HG2 ILE A 809     112.681 186.259 136.761  1.00  0.00
ATOM  12377 2HG2 ILE A 809     112.884 184.488 136.661  1.00  0.00
ATOM  12378 3HG2 ILE A 809     113.518 185.523 135.397  1.00  0.00
ATOM  12379  CG1 ILE A 809     110.178 184.889 136.253  1.00  0.00
ATOM  12380 1HG1 ILE A 809     109.261 184.906 135.663  1.00  0.00
ATOM  12381 2HG1 ILE A 809     110.077 185.610 137.063  1.00  0.00
```

```
ATOM  12382  CD  ILE A 809     110.296 183.490 136.872  1.00  0.00
ATOM  12383  HD1 ILE A 809     109.367 183.247 137.388  1.00  0.00
ATOM  12384  HD2 ILE A 809     110.468 182.753 136.088  1.00  0.00
ATOM  12385  HD3 ILE A 809     111.108 183.457 137.595  1.00  0.00
ATOM  12386  C   ILE A 809     110.652 187.765 135.427  1.00  0.00
ATOM  12387  O   ILE A 809     111.497 188.510 135.942  1.00  0.00
ATOM  12388  N   GLY A 810     109.359 187.880 135.702  1.00  0.00
ATOM  12389  H   GLY A 810     108.746 187.197 135.278  1.00  0.00
ATOM  12390  CA  GLY A 810     108.716 188.870 136.560  1.00  0.00
ATOM  12391  HA1 GLY A 810     108.876 189.864 136.159  1.00  0.00
ATOM  12392  HA2 GLY A 810     109.115 188.804 137.554  1.00  0.00
ATOM  12393  C   GLY A 810     107.236 188.545 136.651  1.00  0.00
ATOM  12394  O   GLY A 810     106.948 187.495 137.216  1.00  0.00
ATOM  12395  N   SER A 811     106.339 189.319 136.019  1.00  0.00
ATOM  12396  H   SER A 811     106.662 190.228 135.683  1.00  0.00
ATOM  12397  CA  SER A 811     105.201 188.699 135.298  1.00  0.00
ATOM  12398  HA  SER A 811     105.581 187.926 134.635  1.00  0.00
ATOM  12399  CB  SER A 811     104.213 188.006 136.266  1.00  0.00
ATOM  12400  HB1 SER A 811     103.735 188.747 136.907  1.00  0.00
ATOM  12401  HB2 SER A 811     104.710 187.276 136.896  1.00  0.00
ATOM  12402  OG  SER A 811     103.238 187.312 135.522  1.00  0.00
ATOM  12403  HG  SER A 811     103.718 186.670 134.959  1.00  0.00
ATOM  12404  C   SER A 811     104.337 189.639 134.453  1.00  0.00
ATOM  12405  O   SER A 811     104.046 189.360 133.290  1.00  0.00
ATOM  12406  N   GLN A 812     103.877 190.725 135.064  1.00  0.00
ATOM  12407  H   GLN A 812     104.254 190.943 135.975  1.00  0.00
ATOM  12408  CA  GLN A 812     102.872 191.654 134.543  1.00  0.00
ATOM  12409  HA  GLN A 812     103.134 192.005 133.563  1.00  0.00
ATOM  12410  CB  GLN A 812     101.494 190.995 134.358  1.00  0.00
ATOM  12411  HB1 GLN A 812     101.573 190.278 133.541  1.00  0.00
ATOM  12412  HB2 GLN A 812     100.787 191.760 134.035  1.00  0.00
ATOM  12413  CG  GLN A 812     100.895 190.272 135.576  1.00  0.00
ATOM  12414  HG1 GLN A 812     100.692 190.988 136.369  1.00  0.00
ATOM  12415  HG2 GLN A 812     101.591 189.521 135.941  1.00  0.00
ATOM  12416  CD  GLN A 812      99.592 189.585 135.194  1.00  0.00
ATOM  12417  OE1 GLN A 812      98.507 189.989 135.576  1.00  0.00
ATOM  12418  NE2 GLN A 812      99.646 188.522 134.418  1.00  0.00
ATOM  12419 1HE2 GLN A 812     100.529 188.208 134.054  1.00  0.00
ATOM  12420 2HE2 GLN A 812      98.769 188.157 134.087  1.00  0.00
ATOM  12421  C   GLN A 812     102.818 192.869 135.473  1.00  0.00
ATOM  12422  O   GLN A 812     102.560 192.738 136.668  1.00  0.00
ATOM  12423  N   TYR A 813     103.071 194.037 134.885  1.00  0.00
ATOM  12424  H   TYR A 813     103.502 193.978 133.974  1.00  0.00
ATOM  12425  CA  TYR A 813     102.887 195.387 135.438  1.00  0.00
ATOM  12426  HA  TYR A 813     102.202 195.312 136.283  1.00  0.00
ATOM  12427  CB  TYR A 813     104.230 195.916 135.975  1.00  0.00
ATOM  12428  HB1 TYR A 813     104.654 196.633 135.270  1.00  0.00
ATOM  12429  HB2 TYR A 813     104.925 195.083 136.043  1.00  0.00
ATOM  12430  CG  TYR A 813     104.171 196.545 137.350  1.00  0.00
ATOM  12431  CD1 TYR A 813     103.600 197.818 137.529  1.00  0.00
ATOM  12432  HD1 TYR A 813     103.176 198.349 136.684  1.00  0.00
ATOM  12433  CE1 TYR A 813     103.572 198.403 138.809  1.00  0.00
ATOM  12434  HE1 TYR A 813     103.125 199.371 138.959  1.00  0.00
ATOM  12435  CZ  TYR A 813     104.115 197.717 139.913  1.00  0.00
ATOM  12436  OH  TYR A 813     104.083 198.288 141.144  1.00  0.00
ATOM  12437  HH  TYR A 813     104.322 197.626 141.813  1.00  0.00
ATOM  12438  CE2 TYR A 813     104.683 196.437 139.738  1.00  0.00
ATOM  12439  HE2 TYR A 813     105.106 195.912 140.584  1.00  0.00
ATOM  12440  CD2 TYR A 813     104.711 195.856 138.457  1.00  0.00
ATOM  12441  HD2 TYR A 813     105.148 194.878 138.324  1.00  0.00
ATOM  12442  C   TYR A 813     102.228 196.334 134.399  1.00  0.00
```

```
ATOM  12443  O   TYR A 813     101.968 197.508 134.654  1.00  0.00
ATOM  12444  N   LEU A 814     101.899 195.775 133.234  1.00  0.00
ATOM  12445  H   LEU A 814     102.234 194.835 133.082  1.00  0.00
ATOM  12446  CA  LEU A 814     100.786 196.115 132.356  1.00  0.00
ATOM  12447  HA  LEU A 814     100.052 196.699 132.912  1.00  0.00
ATOM  12448  CB  LEU A 814     101.302 196.888 131.128  1.00  0.00
ATOM  12449  HB1 LEU A 814     101.547 196.179 130.340  1.00  0.00
ATOM  12450  HB2 LEU A 814     102.229 197.400 131.386  1.00  0.00
ATOM  12451  CG  LEU A 814     100.350 197.948 130.531  1.00  0.00
ATOM  12452  HG  LEU A 814     100.774 198.261 129.578  1.00  0.00
ATOM  12453  CD1 LEU A 814      98.921 197.477 130.267  1.00  0.00
ATOM  12454 1HD1 LEU A 814      98.384 198.253 129.720  1.00  0.00
ATOM  12455 2HD1 LEU A 814      98.934 196.582 129.647  1.00  0.00
ATOM  12456 3HD1 LEU A 814      98.395 197.290 131.199  1.00  0.00
ATOM  12457  CD2 LEU A 814     100.292 199.180 131.434  1.00  0.00
ATOM  12458 1HD2 LEU A 814      99.665 199.942 130.971  1.00  0.00
ATOM  12459 2HD2 LEU A 814      99.884 198.919 132.411  1.00  0.00
ATOM  12460 3HD2 LEU A 814     101.294 199.587 131.571  1.00  0.00
ATOM  12461  C   LEU A 814     100.174 194.755 131.964  1.00  0.00
ATOM  12462  O   LEU A 814     100.915 193.770 131.888  1.00  0.00
ATOM  12463  N   LEU A 815      98.862 194.665 131.738  1.00  0.00
ATOM  12464  H   LEU A 815      98.309 195.500 131.834  1.00  0.00
ATOM  12465  CA  LEU A 815      98.183 193.391 131.429  1.00  0.00
ATOM  12466  HA  LEU A 815      98.471 192.663 132.188  1.00  0.00
ATOM  12467  CB  LEU A 815      96.656 193.572 131.495  1.00  0.00
ATOM  12468  HB1 LEU A 815      96.189 192.625 131.215  1.00  0.00
ATOM  12469  HB2 LEU A 815      96.359 194.317 130.755  1.00  0.00
ATOM  12470  CG  LEU A 815      96.111 193.987 132.876  1.00  0.00
ATOM  12471  HG  LEU A 815      96.536 194.952 133.154  1.00  0.00
ATOM  12472  CD1 LEU A 815      94.594 194.140 132.792  1.00  0.00
ATOM  12473 1HD1 LEU A 815      94.202 194.469 133.754  1.00  0.00
ATOM  12474 2HD1 LEU A 815      94.338 194.881 132.035  1.00  0.00
ATOM  12475 3HD1 LEU A 815      94.135 193.187 132.531  1.00  0.00
ATOM  12476  CD2 LEU A 815      96.435 192.972 133.977  1.00  0.00
ATOM  12477 1HD2 LEU A 815      95.950 193.264 134.909  1.00  0.00
ATOM  12478 2HD2 LEU A 815      96.087 191.981 133.687  1.00  0.00
ATOM  12479 3HD2 LEU A 815      97.508 192.934 134.159  1.00  0.00
ATOM  12480  C   LEU A 815      98.591 192.748 130.097  1.00  0.00
ATOM  12481  O   LEU A 815      98.486 191.535 129.921  1.00  0.00
ATOM  12482  N   ASN A 816      99.101 193.582 129.205  1.00  0.00
ATOM  12483  H   ASN A 816      99.160 194.546 129.486  1.00  0.00
ATOM  12484  CA  ASN A 816      99.643 193.287 127.893  1.00  0.00
ATOM  12485  HA  ASN A 816      99.956 192.244 127.800  1.00  0.00
ATOM  12486  CB  ASN A 816      98.578 193.652 126.843  1.00  0.00
ATOM  12487  HB1 ASN A 816      99.022 193.582 125.852  1.00  0.00
ATOM  12488  HB2 ASN A 816      98.249 194.680 126.990  1.00  0.00
ATOM  12489  CG  ASN A 816      97.366 192.757 126.903  1.00  0.00
ATOM  12490  OD1 ASN A 816      96.399 193.010 127.608  1.00  0.00
ATOM  12491  ND2 ASN A 816      97.368 191.713 126.116  1.00  0.00
ATOM  12492 1HD2 ASN A 816      98.159 191.562 125.485  1.00  0.00
ATOM  12493 2HD2 ASN A 816      96.568 191.111 126.129  1.00  0.00
ATOM  12494  C   ASN A 816     100.809 194.243 127.769  1.00  0.00
ATOM  12495  O   ASN A 816     100.651 195.456 127.960  1.00  0.00
ATOM  12496  N   TRP A 817     102.003 193.711 127.636  1.00  0.00
ATOM  12497  H   TRP A 817     102.090 192.717 127.484  1.00  0.00
ATOM  12498  CA  TRP A 817     103.153 194.446 128.123  1.00  0.00
ATOM  12499  HA  TRP A 817     102.893 194.878 129.086  1.00  0.00
ATOM  12500  CB  TRP A 817     104.241 193.440 128.335  1.00  0.00
ATOM  12501  HB1 TRP A 817     105.207 193.945 128.279  1.00  0.00
ATOM  12502  HB2 TRP A 817     104.205 192.666 127.569  1.00  0.00
ATOM  12503  CG  TRP A 817     104.131 192.858 129.689  1.00  0.00
```

ATOM	12504	CD1	TRP	A	817	103.763	191.607	130.042	1.00	0.00
ATOM	12505	HD1	TRP	A	817	103.443	190.831	129.358	1.00	0.00
ATOM	12506	NE1	TRP	A	817	104.071	191.439	131.382	1.00	0.00
ATOM	12507	HE1	TRP	A	817	103.990	190.548	131.876	1.00	0.00
ATOM	12508	CE2	TRP	A	817	104.693	192.569	131.887	1.00	0.00
ATOM	12509	CZ2	TRP	A	817	105.392	192.876	133.058	1.00	0.00
ATOM	12510	HZ2	TRP	A	817	105.715	192.105	133.737	1.00	0.00
ATOM	12511	CH2	TRP	A	817	105.591	194.223	133.381	1.00	0.00
ATOM	12512	HH2	TRP	A	817	106.050	194.495	134.313	1.00	0.00
ATOM	12513	CZ3	TRP	A	817	105.058	195.225	132.564	1.00	0.00
ATOM	12514	HZ3	TRP	A	817	105.041	196.245	132.905	1.00	0.00
ATOM	12515	CE3	TRP	A	817	104.576	194.904	131.289	1.00	0.00
ATOM	12516	HE3	TRP	A	817	104.294	195.686	130.609	1.00	0.00
ATOM	12517	CD2	TRP	A	817	104.434	193.554	130.915	1.00	0.00
ATOM	12518	C	TRP	A	817	103.599	195.593	127.220	1.00	0.00
ATOM	12519	O	TRP	A	817	103.610	195.506	125.993	1.00	0.00
ATOM	12520	N	CYS	A	818	104.107	196.649	127.857	1.00	0.00
ATOM	12521	H	CYS	A	818	104.047	196.668	128.859	1.00	0.00
ATOM	12522	CA	CYS	A	818	104.754	197.791	127.208	1.00	0.00
ATOM	12523	HA	CYS	A	818	104.045	198.206	126.489	1.00	0.00
ATOM	12524	CB	CYS	A	818	105.062	198.861	128.258	1.00	0.00
ATOM	12525	HB1	CYS	A	818	105.464	199.735	127.743	1.00	0.00
ATOM	12526	HB2	CYS	A	818	105.819	198.483	128.942	1.00	0.00
ATOM	12527	SG	CYS	A	818	103.559	199.329	129.167	1.00	0.00
ATOM	12528	HG	CYS	A	818	102.804	199.660	128.109	1.00	0.00
ATOM	12529	C	CYS	A	818	106.035	197.459	126.416	1.00	0.00
ATOM	12530	O	CYS	A	818	106.670	198.366	125.878	1.00	0.00
ATOM	12531	N	VAL	A	819	106.414	196.180	126.337	1.00	0.00
ATOM	12532	H	VAL	A	819	105.824	195.522	126.824	1.00	0.00
ATOM	12533	CA	VAL	A	819	107.533	195.589	125.580	1.00	0.00
ATOM	12534	HA	VAL	A	819	108.465	195.983	125.970	1.00	0.00
ATOM	12535	CB	VAL	A	819	107.526	194.075	125.865	1.00	0.00
ATOM	12536	HB	VAL	A	819	107.352	193.996	126.934	1.00	0.00
ATOM	12537	CG1	VAL	A	819	106.401	193.366	125.108	1.00	0.00
ATOM	12538	1HG1	VAL	A	819	106.491	193.529	124.040	1.00	0.00
ATOM	12539	2HG1	VAL	A	819	106.471	192.299	125.288	1.00	0.00
ATOM	12540	3HG1	VAL	A	819	105.430	193.715	125.449	1.00	0.00
ATOM	12541	CG2	VAL	A	819	108.826	193.324	125.597	1.00	0.00
ATOM	12542	1HG2	VAL	A	819	108.779	192.342	126.068	1.00	0.00
ATOM	12543	2HG2	VAL	A	819	108.960	193.170	124.530	1.00	0.00
ATOM	12544	3HG2	VAL	A	819	109.669	193.867	126.025	1.00	0.00
ATOM	12545	C	VAL	A	819	107.501	195.916	124.070	1.00	0.00
ATOM	12546	O	VAL	A	819	108.445	195.620	123.348	1.00	0.00
ATOM	12547	N	GLN	A	820	106.454	196.579	123.570	1.00	0.00
ATOM	12548	H	GLN	A	820	105.654	196.708	124.172	1.00	0.00
ATOM	12549	CA	GLN	A	820	106.477	197.283	122.279	1.00	0.00
ATOM	12550	HA	GLN	A	820	106.730	196.568	121.494	1.00	0.00
ATOM	12551	CB	GLN	A	820	105.069	197.852	121.987	1.00	0.00
ATOM	12552	HB1	GLN	A	820	105.152	198.669	121.269	1.00	0.00
ATOM	12553	HB2	GLN	A	820	104.673	198.281	122.908	1.00	0.00
ATOM	12554	CG	GLN	A	820	104.058	196.819	121.440	1.00	0.00
ATOM	12555	HG1	GLN	A	820	103.055	197.157	121.701	1.00	0.00
ATOM	12556	HG2	GLN	A	820	104.209	195.846	121.906	1.00	0.00
ATOM	12557	CD	GLN	A	820	104.113	196.658	119.912	1.00	0.00
ATOM	12558	OE1	GLN	A	820	105.149	196.828	119.291	1.00	0.00
ATOM	12559	NE2	GLN	A	820	103.003	196.388	119.249	1.00	0.00
ATOM	12560	1HE2	GLN	A	820	102.124	196.309	119.727	1.00	0.00
ATOM	12561	2HE2	GLN	A	820	103.007	196.431	118.226	1.00	0.00
ATOM	12562	C	GLN	A	820	107.547	198.411	122.190	1.00	0.00
ATOM	12563	O	GLN	A	820	108.028	198.721	121.105	1.00	0.00
ATOM	12564	N	ILE	A	821	107.950	198.978	123.334	1.00	0.00

```
ATOM  12565  H   ILE A 821     107.452 198.697 124.165  1.00  0.00
ATOM  12566  CA  ILE A 821     109.035 199.969 123.500  1.00  0.00
ATOM  12567  HA  ILE A 821     109.643 199.961 122.600  1.00  0.00
ATOM  12568  CB  ILE A 821     108.476 201.399 123.686  1.00  0.00
ATOM  12569  HB  ILE A 821     108.227 201.757 122.692  1.00  0.00
ATOM  12570  CG2 ILE A 821     107.183 201.449 124.532  1.00  0.00
ATOM  12571 1HG2 ILE A 821     106.834 202.475 124.632  1.00  0.00
ATOM  12572 2HG2 ILE A 821     106.385 200.897 124.035  1.00  0.00
ATOM  12573 3HG2 ILE A 821     107.358 201.015 125.515  1.00  0.00
ATOM  12574  CG1 ILE A 821     109.464 202.395 124.342  1.00  0.00
ATOM  12575 1HG1 ILE A 821     109.731 202.055 125.343  1.00  0.00
ATOM  12576 2HG1 ILE A 821     108.949 203.331 124.461  1.00  0.00
ATOM  12577  CD  ILE A 821     110.742 202.750 123.586  1.00  0.00
ATOM  12578  HD1 ILE A 821     111.404 203.319 124.236  1.00  0.00
ATOM  12579  HD2 ILE A 821     111.275 201.860 123.292  1.00  0.00
ATOM  12580  HD3 ILE A 821     110.501 203.361 122.719  1.00  0.00
ATOM  12581  C   ILE A 821     109.960 199.582 124.655  1.00  0.00
ATOM  12582  O   ILE A 821     111.176 199.735 124.578  1.00  0.00
ATOM  12583  N   ALA A 822     109.414 198.946 125.689  1.00  0.00
ATOM  12584  H   ALA A 822     108.406 198.879 125.738  1.00  0.00
ATOM  12585  CA  ALA A 822     110.196 198.230 126.681  1.00  0.00
ATOM  12586  HA  ALA A 822     110.984 198.903 127.005  1.00  0.00
ATOM  12587  CB  ALA A 822     109.327 197.963 127.911  1.00  0.00
ATOM  12588  HB1 ALA A 822     109.960 197.541 128.690  1.00  0.00
ATOM  12589  HB2 ALA A 822     108.897 198.902 128.254  1.00  0.00
ATOM  12590  HB3 ALA A 822     108.535 197.258 127.684  1.00  0.00
ATOM  12591  C   ALA A 822     110.896 196.965 126.118  1.00  0.00
ATOM  12592  O   ALA A 822     111.317 196.106 126.891  1.00  0.00
ATOM  12593  N   LYS A 823     111.034 196.851 124.785  1.00  0.00
ATOM  12594  H   LYS A 823     110.704 197.629 124.231  1.00  0.00
ATOM  12595  CA  LYS A 823     112.016 196.010 124.094  1.00  0.00
ATOM  12596  HA  LYS A 823     112.734 195.690 124.850  1.00  0.00
ATOM  12597  CB  LYS A 823     111.411 194.726 123.486  1.00  0.00
ATOM  12598  HB1 LYS A 823     110.486 194.537 124.004  1.00  0.00
ATOM  12599  HB2 LYS A 823     112.061 193.903 123.753  1.00  0.00
ATOM  12600  CG  LYS A 823     111.163 194.625 121.965  1.00  0.00
ATOM  12601  HG1 LYS A 823     112.109 194.742 121.434  1.00  0.00
ATOM  12602  HG2 LYS A 823     110.477 195.408 121.642  1.00  0.00
ATOM  12603  CD  LYS A 823     110.573 193.235 121.633  1.00  0.00
ATOM  12604  HD1 LYS A 823     109.581 193.158 122.084  1.00  0.00
ATOM  12605  HD2 LYS A 823     111.212 192.477 122.084  1.00  0.00
ATOM  12606  CE  LYS A 823     110.465 192.948 120.125  1.00  0.00
ATOM  12607  HE1 LYS A 823     111.453 193.071 119.674  1.00  0.00
ATOM  12608  HE2 LYS A 823     109.794 193.690 119.682  1.00  0.00
ATOM  12609  NZ  LYS A 823     109.950 191.577 119.842  1.00  0.00
ATOM  12610  HZ1 LYS A 823     109.734 191.400 118.866  1.00  0.00
ATOM  12611  HZ2 LYS A 823     110.575 190.826 120.124  1.00  0.00
ATOM  12612  HZ3 LYS A 823     109.076 191.380 120.325  1.00  0.00
ATOM  12613  C   LYS A 823     112.795 196.877 123.110  1.00  0.00
ATOM  12614  O   LYS A 823     112.324 197.909 122.654  1.00  0.00
ATOM  12615  N   GLY A 824     114.063 196.516 122.909  1.00  0.00
ATOM  12616  H   GLY A 824     114.360 195.586 123.180  1.00  0.00
ATOM  12617  CA  GLY A 824     115.090 197.454 122.454  1.00  0.00
ATOM  12618  HA1 GLY A 824     114.699 198.040 121.621  1.00  0.00
ATOM  12619  HA2 GLY A 824     115.952 196.892 122.092  1.00  0.00
ATOM  12620  C   GLY A 824     115.574 198.426 123.545  1.00  0.00
ATOM  12621  O   GLY A 824     116.777 198.625 123.683  1.00  0.00
ATOM  12622  N   MET A 825     114.664 198.957 124.372  1.00  0.00
ATOM  12623  H   MET A 825     113.689 198.828 124.128  1.00  0.00
ATOM  12624  CA  MET A 825     114.937 199.749 125.582  1.00  0.00
ATOM  12625  HA  MET A 825     115.971 199.576 125.881  1.00  0.00
```

```
ATOM  12626  CB  MET A 825     114.773 201.255 125.332  1.00  0.00
ATOM  12627  HB1 MET A 825     113.721 201.526 125.246  1.00  0.00
ATOM  12628  HB2 MET A 825     115.259 201.489 124.385  1.00  0.00
ATOM  12629  CG  MET A 825     115.463 202.117 126.410  1.00  0.00
ATOM  12630  HG1 MET A 825     115.539 203.133 126.023  1.00  0.00
ATOM  12631  HG2 MET A 825     116.478 201.739 126.522  1.00  0.00
ATOM  12632  SD  MET A 825     114.749 202.221 128.082  1.00  0.00
ATOM  12633  CE  MET A 825     113.175 203.047 127.732  1.00  0.00
ATOM  12634  HE1 MET A 825     112.557 202.417 127.093  1.00  0.00
ATOM  12635  HE2 MET A 825     112.644 203.222 128.666  1.00  0.00
ATOM  12636  HE3 MET A 825     113.367 203.997 127.240  1.00  0.00
ATOM  12637  C   MET A 825     114.051 199.209 126.713  1.00  0.00
ATOM  12638  O   MET A 825     113.097 199.821 127.173  1.00  0.00
ATOM  12639  N   ASN A 826     114.345 197.973 127.117  1.00  0.00
ATOM  12640  H   ASN A 826     115.127 197.520 126.670  1.00  0.00
ATOM  12641  CA  ASN A 826     113.862 197.418 128.376  1.00  0.00
ATOM  12642  HA  ASN A 826     112.775 197.368 128.378  1.00  0.00
ATOM  12643  CB  ASN A 826     114.453 195.993 128.489  1.00  0.00
ATOM  12644  HB1 ASN A 826     115.527 196.031 128.316  1.00  0.00
ATOM  12645  HB2 ASN A 826     114.004 195.357 127.730  1.00  0.00
ATOM  12646  CG  ASN A 826     114.253 195.303 129.837  1.00  0.00
ATOM  12647  OD1 ASN A 826     113.434 195.687 130.653  1.00  0.00
ATOM  12648  ND2 ASN A 826     114.983 194.254 130.101  1.00  0.00
ATOM  12649 1HD2 ASN A 826     115.485 193.817 129.344  1.00  0.00
ATOM  12650 2HD2 ASN A 826     114.777 193.692 130.922  1.00  0.00
ATOM  12651  C   ASN A 826     114.306 198.309 129.550  1.00  0.00
ATOM  12652  O   ASN A 826     115.437 198.809 129.545  1.00  0.00
ATOM  12653  N   TYR A 827     113.490 198.390 130.612  1.00  0.00
ATOM  12654  H   TYR A 827     112.587 197.939 130.518  1.00  0.00
ATOM  12655  CA  TYR A 827     114.063 198.437 131.959  1.00  0.00
ATOM  12656  HA  TYR A 827     114.878 197.718 131.988  1.00  0.00
ATOM  12657  CB  TYR A 827     114.676 199.842 132.181  1.00  0.00
ATOM  12658  HB1 TYR A 827     114.042 200.588 131.699  1.00  0.00
ATOM  12659  HB2 TYR A 827     115.654 199.890 131.707  1.00  0.00
ATOM  12660  CG  TYR A 827     114.873 200.238 133.621  1.00  0.00
ATOM  12661  CD1 TYR A 827     115.530 199.361 134.500  1.00  0.00
ATOM  12662  HD1 TYR A 827     115.933 198.425 134.139  1.00  0.00
ATOM  12663  CE1 TYR A 827     115.623 199.678 135.858  1.00  0.00
ATOM  12664  HE1 TYR A 827     116.123 199.008 136.532  1.00  0.00
ATOM  12665  CZ  TYR A 827     115.097 200.891 136.331  1.00  0.00
ATOM  12666  OH  TYR A 827     115.278 201.226 137.628  1.00  0.00
ATOM  12667  HH  TYR A 827     114.861 202.067 137.829  1.00  0.00
ATOM  12668  CE2 TYR A 827     114.432 201.770 135.452  1.00  0.00
ATOM  12669  HE2 TYR A 827     114.013 202.694 135.805  1.00  0.00
ATOM  12670  CD2 TYR A 827     114.332 201.447 134.091  1.00  0.00
ATOM  12671  HD2 TYR A 827     113.848 202.134 133.408  1.00  0.00
ATOM  12672  C   TYR A 827     113.149 198.060 133.143  1.00  0.00
ATOM  12673  O   TYR A 827     113.505 197.197 133.945  1.00  0.00
ATOM  12674  N   LEU A 828     112.060 198.786 133.365  1.00  0.00
ATOM  12675  H   LEU A 828     111.740 199.352 132.596  1.00  0.00
ATOM  12676  CA  LEU A 828     111.546 199.130 134.696  1.00  0.00
ATOM  12677  HA  LEU A 828     112.395 199.392 135.327  1.00  0.00
ATOM  12678  CB  LEU A 828     110.671 200.393 134.510  1.00  0.00
ATOM  12679  HB1 LEU A 828     109.935 200.187 133.739  1.00  0.00
ATOM  12680  HB2 LEU A 828     111.308 201.190 134.127  1.00  0.00
ATOM  12681  CG  LEU A 828     109.920 200.943 135.741  1.00  0.00
ATOM  12682  HG  LEU A 828     109.257 200.178 136.144  1.00  0.00
ATOM  12683  CD1 LEU A 828     110.894 201.406 136.823  1.00  0.00
ATOM  12684 1HD1 LEU A 828     110.334 201.796 137.668  1.00  0.00
ATOM  12685 2HD1 LEU A 828     111.488 200.568 137.184  1.00  0.00
ATOM  12686 3HD1 LEU A 828     111.555 202.179 136.435  1.00  0.00
```

```
ATOM  12687  CD2 LEU A 828     109.059 202.144 135.335  1.00  0.00
ATOM  12688 1HD2 LEU A 828     108.527 202.531 136.205  1.00  0.00
ATOM  12689 2HD2 LEU A 828     109.685 202.931 134.912  1.00  0.00
ATOM  12690 3HD2 LEU A 828     108.324 201.831 134.594  1.00  0.00
ATOM  12691  C   LEU A 828     110.807 197.994 135.438  1.00  0.00
ATOM  12692  O   LEU A 828     110.713 198.080 136.654  1.00  0.00
ATOM  12693  N   GLU A 829     110.341 196.922 134.777  1.00  0.00
ATOM  12694  H   GLU A 829     110.479 196.878 133.782  1.00  0.00
ATOM  12695  CA  GLU A 829     109.982 195.687 135.506  1.00  0.00
ATOM  12696  HA  GLU A 829     109.787 195.958 136.546  1.00  0.00
ATOM  12697  CB  GLU A 829     108.702 194.964 135.016  1.00  0.00
ATOM  12698  HB1 GLU A 829     108.964 194.260 134.225  1.00  0.00
ATOM  12699  HB2 GLU A 829     108.012 195.701 134.604  1.00  0.00
ATOM  12700  CG  GLU A 829     108.008 194.235 136.201  1.00  0.00
ATOM  12701  HG1 GLU A 829     107.248 194.903 136.611  1.00  0.00
ATOM  12702  HG2 GLU A 829     108.718 194.066 137.012  1.00  0.00
ATOM  12703  CD  GLU A 829     107.368 192.861 135.898  1.00  0.00
ATOM  12704  OE1 GLU A 829     108.089 191.932 135.475  1.00  0.00
ATOM  12705  OE2 GLU A 829     106.169 192.651 136.220  1.00  0.00
ATOM  12706  C   GLU A 829     111.155 194.692 135.563  1.00  0.00
ATOM  12707  O   GLU A 829     111.209 193.875 136.476  1.00  0.00
ATOM  12708  N   ASP A 830     112.163 194.795 134.685  1.00  0.00
ATOM  12709  H   ASP A 830     112.179 195.543 134.003  1.00  0.00
ATOM  12710  CA  ASP A 830     113.435 194.087 134.906  1.00  0.00
ATOM  12711  HA  ASP A 830     113.203 193.086 135.249  1.00  0.00
ATOM  12712  CB  ASP A 830     114.264 193.998 133.609  1.00  0.00
ATOM  12713  HB1 ASP A 830     115.089 194.712 133.635  1.00  0.00
ATOM  12714  HB2 ASP A 830     113.624 194.267 132.772  1.00  0.00
ATOM  12715  CG  ASP A 830     114.811 192.591 133.338  1.00  0.00
ATOM  12716  OD1 ASP A 830     115.014 191.796 134.279  1.00  0.00
ATOM  12717  OD2 ASP A 830     114.857 192.194 132.155  1.00  0.00
ATOM  12718  C   ASP A 830     114.273 194.715 136.047  1.00  0.00
ATOM  12719  O   ASP A 830     115.263 194.152 136.516  1.00  0.00
ATOM  12720  N   ARG A 831     113.825 195.859 136.587  1.00  0.00
ATOM  12721  H   ARG A 831     113.088 196.324 136.075  1.00  0.00
ATOM  12722  CA  ARG A 831     114.242 196.395 137.891  1.00  0.00
ATOM  12723  HA  ARG A 831     115.326 196.464 137.884  1.00  0.00
ATOM  12724  CB  ARG A 831     113.636 197.800 138.104  1.00  0.00
ATOM  12725  HB1 ARG A 831     112.561 197.684 138.221  1.00  0.00
ATOM  12726  HB2 ARG A 831     113.797 198.385 137.204  1.00  0.00
ATOM  12727  CG  ARG A 831     114.121 198.638 139.316  1.00  0.00
ATOM  12728  HG1 ARG A 831     113.633 198.281 140.224  1.00  0.00
ATOM  12729  HG2 ARG A 831     113.774 199.661 139.163  1.00  0.00
ATOM  12730  CD  ARG A 831     115.645 198.646 139.550  1.00  0.00
ATOM  12731  HD1 ARG A 831     116.139 198.620 138.582  1.00  0.00
ATOM  12732  HD2 ARG A 831     115.927 197.749 140.102  1.00  0.00
ATOM  12733  NE  ARG A 831     116.096 199.848 140.293  1.00  0.00
ATOM  12734  HE  ARG A 831     115.407 200.286 140.878  1.00  0.00
ATOM  12735  CZ  ARG A 831     117.259 200.473 140.145  1.00  0.00
ATOM  12736  NH1 ARG A 831     118.231 199.950 139.460  1.00  0.00
ATOM  12737 1HH1 ARG A 831     118.083 199.059 139.036  1.00  0.00
ATOM  12738 2HH1 ARG A 831     119.102 200.460 139.325  1.00  0.00
ATOM  12739  NH2 ARG A 831     117.467 201.644 140.677  1.00  0.00
ATOM  12740 1HH2 ARG A 831     116.772 202.089 141.242  1.00  0.00
ATOM  12741 2HH2 ARG A 831     118.313 202.165 140.443  1.00  0.00
ATOM  12742  C   ARG A 831     113.873 195.500 139.077  1.00  0.00
ATOM  12743  O   ARG A 831     114.312 195.817 140.180  1.00  0.00
ATOM  12744  N   ARG A 832     113.147 194.391 138.851  1.00  0.00
ATOM  12745  H   ARG A 832     112.791 194.286 137.907  1.00  0.00
ATOM  12746  CA  ARG A 832     112.781 193.320 139.807  1.00  0.00
ATOM  12747  HA  ARG A 832     111.961 193.689 140.424  1.00  0.00
```

```
ATOM  12748  CB  ARG A 832     112.273 192.125 138.993  1.00  0.00
ATOM  12749  HB1 ARG A 832     113.057 191.797 138.309  1.00  0.00
ATOM  12750  HB2 ARG A 832     111.430 192.474 138.407  1.00  0.00
ATOM  12751  CG  ARG A 832     111.798 190.932 139.825  1.00  0.00
ATOM  12752  HG1 ARG A 832     111.128 191.268 140.616  1.00  0.00
ATOM  12753  HG2 ARG A 832     112.650 190.420 140.265  1.00  0.00
ATOM  12754  CD  ARG A 832     111.077 189.977 138.881  1.00  0.00
ATOM  12755  HD1 ARG A 832     111.799 189.567 138.179  1.00  0.00
ATOM  12756  HD2 ARG A 832     110.345 190.540 138.302  1.00  0.00
ATOM  12757  NE  ARG A 832     110.360 188.902 139.590  1.00  0.00
ATOM  12758  HE  ARG A 832     109.465 189.130 140.023  1.00  0.00
ATOM  12759  CZ  ARG A 832     110.602 187.612 139.509  1.00  0.00
ATOM  12760  NH1 ARG A 832     111.695 187.113 139.017  1.00  0.00
ATOM  12761 1HH1 ARG A 832     112.336 187.736 138.556  1.00  0.00
ATOM  12762 2HH1 ARG A 832     111.788 186.121 138.941  1.00  0.00
ATOM  12763  NH2 ARG A 832     109.691 186.788 139.916  1.00  0.00
ATOM  12764 1HH2 ARG A 832     108.786 187.202 140.144  1.00  0.00
ATOM  12765 2HH2 ARG A 832     109.812 185.797 139.890  1.00  0.00
ATOM  12766  C   ARG A 832     113.880 192.914 140.800  1.00  0.00
ATOM  12767  O   ARG A 832     113.576 192.343 141.840  1.00  0.00
ATOM  12768  N   LEU A 833     115.144 193.204 140.494  1.00  0.00
ATOM  12769  H   LEU A 833     115.265 193.610 139.582  1.00  0.00
ATOM  12770  CA  LEU A 833     116.298 193.296 141.406  1.00  0.00
ATOM  12771  HA  LEU A 833     117.118 193.648 140.781  1.00  0.00
ATOM  12772  CB  LEU A 833     116.082 194.371 142.500  1.00  0.00
ATOM  12773  HB1 LEU A 833     116.127 193.907 143.486  1.00  0.00
ATOM  12774  HB2 LEU A 833     115.085 194.800 142.405  1.00  0.00
ATOM  12775  CG  LEU A 833     117.113 195.521 142.436  1.00  0.00
ATOM  12776  HG  LEU A 833     117.177 195.882 141.409  1.00  0.00
ATOM  12777  CD1 LEU A 833     116.685 196.690 143.322  1.00  0.00
ATOM  12778 1HD1 LEU A 833     117.417 197.494 143.261  1.00  0.00
ATOM  12779 2HD1 LEU A 833     115.723 197.070 142.981  1.00  0.00
ATOM  12780 3HD1 LEU A 833     116.598 196.362 144.358  1.00  0.00
ATOM  12781  CD2 LEU A 833     118.509 195.083 142.890  1.00  0.00
ATOM  12782 1HD2 LEU A 833     119.200 195.924 142.846  1.00  0.00
ATOM  12783 2HD2 LEU A 833     118.472 194.709 143.912  1.00  0.00
ATOM  12784 3HD2 LEU A 833     118.888 194.295 142.243  1.00  0.00
ATOM  12785  C   LEU A 833     116.817 191.960 141.966  1.00  0.00
ATOM  12786  O   LEU A 833     118.016 191.823 142.197  1.00  0.00
ATOM  12787  N   VAL A 834     115.949 190.967 142.129  1.00  0.00
ATOM  12788  H   VAL A 834     114.971 191.173 141.948  1.00  0.00
ATOM  12789  CA  VAL A 834     116.264 189.605 142.558  1.00  0.00
ATOM  12790  HA  VAL A 834     117.130 189.264 141.992  1.00  0.00
ATOM  12791  CB  VAL A 834     116.633 189.601 144.061  1.00  0.00
ATOM  12792  HB  VAL A 834     117.407 190.353 144.218  1.00  0.00
ATOM  12793  CG1 VAL A 834     115.464 189.973 144.978  1.00  0.00
ATOM  12794 1HG1 VAL A 834     115.787 189.951 146.016  1.00  0.00
ATOM  12795 2HG1 VAL A 834     115.140 190.986 144.743  1.00  0.00
ATOM  12796 3HG1 VAL A 834     114.627 189.295 144.835  1.00  0.00
ATOM  12797  CG2 VAL A 834     117.254 188.278 144.525  1.00  0.00
ATOM  12798 1HG2 VAL A 834     117.654 188.405 145.530  1.00  0.00
ATOM  12799 2HG2 VAL A 834     116.520 187.478 144.536  1.00  0.00
ATOM  12800 3HG2 VAL A 834     118.078 188.012 143.862  1.00  0.00
ATOM  12801  C   VAL A 834     115.088 188.680 142.221  1.00  0.00
ATOM  12802  O   VAL A 834     114.003 189.144 141.883  1.00  0.00
ATOM  12803  N   HIS A 835     115.254 187.359 142.266  1.00  0.00
ATOM  12804  H   HIS A 835     116.150 186.986 142.539  1.00  0.00
ATOM  12805  CA  HIS A 835     114.197 186.420 141.854  1.00  0.00
ATOM  12806  HA  HIS A 835     113.858 186.727 140.866  1.00  0.00
ATOM  12807  CB  HIS A 835     114.792 185.007 141.724  1.00  0.00
ATOM  12808  HB1 HIS A 835     115.719 185.056 141.150  1.00  0.00
```

```
ATOM  12809  HB2 HIS A 835     114.090 184.400 141.151  1.00  0.00
ATOM  12810  CG  HIS A 835     115.069 184.296 143.030  1.00  0.00
ATOM  12811  ND1 HIS A 835     115.686 184.845 144.159  1.00  0.00
ATOM  12812  CE1 HIS A 835     115.765 183.844 145.050  1.00  0.00
ATOM  12813  HE1 HIS A 835     116.214 183.927 146.028  1.00  0.00
ATOM  12814  NE2 HIS A 835     115.225 182.720 144.552  1.00  0.00
ATOM  12815  HE2 HIS A 835     115.184 181.823 145.023  1.00  0.00
ATOM  12816  CD2 HIS A 835     114.784 182.984 143.278  1.00  0.00
ATOM  12817  HD2 HIS A 835     114.324 182.291 142.595  1.00  0.00
ATOM  12818  C   HIS A 835     112.937 186.397 142.752  1.00  0.00
ATOM  12819  O   HIS A 835     111.932 185.801 142.354  1.00  0.00
ATOM  12820  N   ARG A 836     112.993 187.018 143.941  1.00  0.00
ATOM  12821  H   ARG A 836     113.847 187.526 144.095  1.00  0.00
ATOM  12822  CA  ARG A 836     112.072 186.912 145.104  1.00  0.00
ATOM  12823  HA  ARG A 836     111.882 185.852 145.277  1.00  0.00
ATOM  12824  CB  ARG A 836     112.752 187.499 146.358  1.00  0.00
ATOM  12825  HB1 ARG A 836     112.084 187.366 147.210  1.00  0.00
ATOM  12826  HB2 ARG A 836     112.888 188.570 146.207  1.00  0.00
ATOM  12827  CG  ARG A 836     114.090 186.854 146.724  1.00  0.00
ATOM  12828  HG1 ARG A 836     114.790 186.946 145.896  1.00  0.00
ATOM  12829  HG2 ARG A 836     113.944 185.799 146.954  1.00  0.00
ATOM  12830  CD  ARG A 836     114.678 187.573 147.946  1.00  0.00
ATOM  12831  HD1 ARG A 836     114.008 187.423 148.796  1.00  0.00
ATOM  12832  HD2 ARG A 836     114.717 188.640 147.722  1.00  0.00
ATOM  12833  NE  ARG A 836     116.020 187.063 148.276  1.00  0.00
ATOM  12834  HE  ARG A 836     116.169 186.083 148.113  1.00  0.00
ATOM  12835  CZ  ARG A 836     117.034 187.751 148.767  1.00  0.00
ATOM  12836  NH1 ARG A 836     116.963 189.029 149.004  1.00  0.00
ATOM  12837 1HH1 ARG A 836     116.098 189.506 148.822  1.00  0.00
ATOM  12838 2HH1 ARG A 836     117.755 189.537 149.352  1.00  0.00
ATOM  12839  NH2 ARG A 836     118.162 187.154 149.041  1.00  0.00
ATOM  12840 1HH2 ARG A 836     118.266 186.166 148.893  1.00  0.00
ATOM  12841 2HH2 ARG A 836     118.934 187.678 149.416  1.00  0.00
ATOM  12842  C   ARG A 836     110.661 187.548 144.936  1.00  0.00
ATOM  12843  O   ARG A 836     110.119 188.131 145.870  1.00  0.00
ATOM  12844  N   ASP A 837     110.105 187.470 143.738  1.00  0.00
ATOM  12845  H   ASP A 837     110.609 186.897 143.077  1.00  0.00
ATOM  12846  CA  ASP A 837     108.999 188.266 143.195  1.00  0.00
ATOM  12847  HA  ASP A 837     109.443 188.682 142.293  1.00  0.00
ATOM  12848  CB  ASP A 837     107.832 187.389 142.685  1.00  0.00
ATOM  12849  HB1 ASP A 837     107.191 187.083 143.513  1.00  0.00
ATOM  12850  HB2 ASP A 837     108.236 186.486 142.227  1.00  0.00
ATOM  12851  CG  ASP A 837     107.046 188.176 141.633  1.00  0.00
ATOM  12852  OD1 ASP A 837     105.847 188.483 141.807  1.00  0.00
ATOM  12853  OD2 ASP A 837     107.678 188.546 140.614  1.00  0.00
ATOM  12854  C   ASP A 837     108.504 189.531 143.920  1.00  0.00
ATOM  12855  O   ASP A 837     109.131 190.572 143.771  1.00  0.00
ATOM  12856  N   LEU A 838     107.306 189.511 144.522  1.00  0.00
ATOM  12857  H   LEU A 838     106.874 188.603 144.629  1.00  0.00
ATOM  12858  CA  LEU A 838     106.383 190.654 144.424  1.00  0.00
ATOM  12859  HA  LEU A 838     106.234 190.831 143.359  1.00  0.00
ATOM  12860  CB  LEU A 838     105.024 190.235 145.028  1.00  0.00
ATOM  12861  HB1 LEU A 838     105.136 190.059 146.098  1.00  0.00
ATOM  12862  HB2 LEU A 838     104.751 189.280 144.577  1.00  0.00
ATOM  12863  CG  LEU A 838     103.821 191.178 144.799  1.00  0.00
ATOM  12864  HG  LEU A 838     102.926 190.583 144.966  1.00  0.00
ATOM  12865  CD1 LEU A 838     103.757 192.343 145.787  1.00  0.00
ATOM  12866 1HD1 LEU A 838     102.811 192.867 145.654  1.00  0.00
ATOM  12867 2HD1 LEU A 838     103.804 191.955 146.803  1.00  0.00
ATOM  12868 3HD1 LEU A 838     104.573 193.042 145.619  1.00  0.00
ATOM  12869  CD2 LEU A 838     103.777 191.720 143.369  1.00  0.00
```

```
ATOM  12870 1HD2 LEU A 838     103.849 190.897 142.662  1.00  0.00
ATOM  12871 2HD2 LEU A 838     102.845 192.257 143.207  1.00  0.00
ATOM  12872 3HD2 LEU A 838     104.595 192.418 143.199  1.00  0.00
ATOM  12873  C   LEU A 838     106.935 191.956 145.013  1.00  0.00
ATOM  12874  O   LEU A 838     106.933 192.982 144.330  1.00  0.00
ATOM  12875  N   ALA A 839     107.469 191.919 146.236  1.00  0.00
ATOM  12876  H   ALA A 839     107.494 191.033 146.715  1.00  0.00
ATOM  12877  CA  ALA A 839     108.122 193.075 146.855  1.00  0.00
ATOM  12878  HA  ALA A 839     107.400 193.891 146.921  1.00  0.00
ATOM  12879  CB  ALA A 839     108.547 192.683 148.276  1.00  0.00
ATOM  12880  HB1 ALA A 839     107.674 192.364 148.846  1.00  0.00
ATOM  12881  HB2 ALA A 839     109.271 191.868 148.238  1.00  0.00
ATOM  12882  HB3 ALA A 839     109.000 193.546 148.766  1.00  0.00
ATOM  12883  C   ALA A 839     109.321 193.593 146.033  1.00  0.00
ATOM  12884  O   ALA A 839     109.532 194.801 145.914  1.00  0.00
ATOM  12885  N   ALA A 840     110.066 192.667 145.426  1.00  0.00
ATOM  12886  H   ALA A 840     109.758 191.707 145.452  1.00  0.00
ATOM  12887  CA  ALA A 840     111.203 192.975 144.578  1.00  0.00
ATOM  12888  HA  ALA A 840     111.815 193.711 145.101  1.00  0.00
ATOM  12889  CB  ALA A 840     112.051 191.703 144.428  1.00  0.00
ATOM  12890  HB1 ALA A 840     111.656 191.074 143.630  1.00  0.00
ATOM  12891  HB2 ALA A 840     113.070 191.994 144.190  1.00  0.00
ATOM  12892  HB3 ALA A 840     112.069 191.146 145.365  1.00  0.00
ATOM  12893  C   ALA A 840     110.787 193.604 143.235  1.00  0.00
ATOM  12894  O   ALA A 840     111.507 194.462 142.734  1.00  0.00
ATOM  12895  N   ARG A 841     109.608 193.294 142.671  1.00  0.00
ATOM  12896  H   ARG A 841     109.056 192.563 143.107  1.00  0.00
ATOM  12897  CA  ARG A 841     109.034 194.071 141.551  1.00  0.00
ATOM  12898  HA  ARG A 841     109.819 194.267 140.820  1.00  0.00
ATOM  12899  CB  ARG A 841     107.900 193.301 140.850  1.00  0.00
ATOM  12900  HB1 ARG A 841     107.377 193.999 140.193  1.00  0.00
ATOM  12901  HB2 ARG A 841     107.189 192.911 141.580  1.00  0.00
ATOM  12902  CG  ARG A 841     108.474 192.160 140.000  1.00  0.00
ATOM  12903  HG1 ARG A 841     108.775 191.330 140.640  1.00  0.00
ATOM  12904  HG2 ARG A 841     109.347 192.536 139.471  1.00  0.00
ATOM  12905  CD  ARG A 841     107.496 191.670 138.930  1.00  0.00
ATOM  12906  HD1 ARG A 841     108.051 191.149 138.151  1.00  0.00
ATOM  12907  HD2 ARG A 841     107.035 192.551 138.490  1.00  0.00
ATOM  12908  NE  ARG A 841     106.470 190.768 139.469  1.00  0.00
ATOM  12909  HE  ARG A 841     106.782 190.034 140.104  1.00  0.00
ATOM  12910  CZ  ARG A 841     105.181 190.804 139.222  1.00  0.00
ATOM  12911  NH1 ARG A 841     104.667 191.589 138.305  1.00  0.00
ATOM  12912 1HH1 ARG A 841     105.283 192.111 137.675  1.00  0.00
ATOM  12913 2HH1 ARG A 841     103.676 191.685 138.143  1.00  0.00
ATOM  12914  NH2 ARG A 841     104.402 190.040 139.927  1.00  0.00
ATOM  12915 1HH2 ARG A 841     104.838 189.431 140.634  1.00  0.00
ATOM  12916 2HH2 ARG A 841     103.412 190.089 139.819  1.00  0.00
ATOM  12917  C   ARG A 841     108.578 195.466 141.984  1.00  0.00
ATOM  12918  O   ARG A 841     108.977 196.457 141.383  1.00  0.00
ATOM  12919  N   ASN A 842     107.800 195.545 143.067  1.00  0.00
ATOM  12920  H   ASN A 842     107.524 194.662 143.481  1.00  0.00
ATOM  12921  CA  ASN A 842     107.345 196.771 143.752  1.00  0.00
ATOM  12922  HA  ASN A 842     107.016 197.480 142.991  1.00  0.00
ATOM  12923  CB  ASN A 842     106.137 196.397 144.662  1.00  0.00
ATOM  12924  HB1 ASN A 842     106.207 196.879 145.634  1.00  0.00
ATOM  12925  HB2 ASN A 842     106.122 195.330 144.868  1.00  0.00
ATOM  12926  CG  ASN A 842     104.788 196.772 144.073  1.00  0.00
ATOM  12927  OD1 ASN A 842     104.414 196.336 143.000  1.00  0.00
ATOM  12928  ND2 ASN A 842     104.014 197.609 144.719  1.00  0.00
ATOM  12929 1HD2 ASN A 842     104.346 198.056 145.572  1.00  0.00
ATOM  12930 2HD2 ASN A 842     103.130 197.828 144.298  1.00  0.00
```

```
ATOM  12931  C   ASN A 842     108.469 197.490 144.552  1.00  0.00
ATOM  12932  O   ASN A 842     108.189 198.175 145.542  1.00  0.00
ATOM  12933  N   VAL A 843     109.741 197.338 144.165  1.00  0.00
ATOM  12934  H   VAL A 843     109.919 196.771 143.349  1.00  0.00
ATOM  12935  CA  VAL A 843     110.894 197.871 144.907  1.00  0.00
ATOM  12936  HA  VAL A 843     110.695 197.675 145.962  1.00  0.00
ATOM  12937  CB  VAL A 843     112.171 197.079 144.563  1.00  0.00
ATOM  12938  HB  VAL A 843     111.940 196.025 144.702  1.00  0.00
ATOM  12939  CG1 VAL A 843     112.654 197.271 143.116  1.00  0.00
ATOM  12940 1HG1 VAL A 843     113.417 196.526 142.894  1.00  0.00
ATOM  12941 2HG1 VAL A 843     111.831 197.117 142.418  1.00  0.00
ATOM  12942 3HG1 VAL A 843     113.077 198.262 142.973  1.00  0.00
ATOM  12943  CG2 VAL A 843     113.330 197.401 145.512  1.00  0.00
ATOM  12944 1HG2 VAL A 843     114.164 196.732 145.306  1.00  0.00
ATOM  12945 2HG2 VAL A 843     113.658 198.432 145.386  1.00  0.00
ATOM  12946 3HG2 VAL A 843     113.012 197.248 146.543  1.00  0.00
ATOM  12947  C   VAL A 843     111.120 199.386 144.791  1.00  0.00
ATOM  12948  O   VAL A 843     111.345 200.025 145.822  1.00  0.00
ATOM  12949  N   LEU A 844     111.105 199.998 143.591  1.00  0.00
ATOM  12950  H   LEU A 844     110.900 199.449 142.768  1.00  0.00
ATOM  12951  CA  LEU A 844     111.542 201.400 143.431  1.00  0.00
ATOM  12952  HA  LEU A 844     111.196 201.944 144.311  1.00  0.00
ATOM  12953  CB  LEU A 844     113.087 201.459 143.442  1.00  0.00
ATOM  12954  HB1 LEU A 844     113.457 200.868 142.604  1.00  0.00
ATOM  12955  HB2 LEU A 844     113.451 201.004 144.364  1.00  0.00
ATOM  12956  CG  LEU A 844     113.679 202.884 143.339  1.00  0.00
ATOM  12957  HG  LEU A 844     113.409 203.328 142.381  1.00  0.00
ATOM  12958  CD1 LEU A 844     113.222 203.816 144.461  1.00  0.00
ATOM  12959 1HD1 LEU A 844     113.762 204.761 144.405  1.00  0.00
ATOM  12960 2HD1 LEU A 844     112.161 204.034 144.387  1.00  0.00
ATOM  12961 3HD1 LEU A 844     113.421 203.359 145.432  1.00  0.00
ATOM  12962  CD2 LEU A 844     115.203 202.802 143.394  1.00  0.00
ATOM  12963 1HD2 LEU A 844     115.519 202.442 144.375  1.00  0.00
ATOM  12964 2HD2 LEU A 844     115.560 202.113 142.637  1.00  0.00
ATOM  12965 3HD2 LEU A 844     115.638 203.790 143.225  1.00  0.00
ATOM  12966  C   LEU A 844     111.006 202.152 142.197  1.00  0.00
ATOM  12967  O   LEU A 844     111.329 201.816 141.060  1.00  0.00
ATOM  12968  N   VAL A 845     110.344 203.276 142.492  1.00  0.00
ATOM  12969  H   VAL A 845     110.142 203.382 143.473  1.00  0.00
ATOM  12970  CA  VAL A 845     110.368 204.532 141.708  1.00  0.00
ATOM  12971  HA  VAL A 845     111.303 204.579 141.151  1.00  0.00
ATOM  12972  CB  VAL A 845     109.207 204.654 140.684  1.00  0.00
ATOM  12973  HB  VAL A 845     109.293 205.631 140.210  1.00  0.00
ATOM  12974  CG1 VAL A 845     109.316 203.607 139.573  1.00  0.00
ATOM  12975 1HG1 VAL A 845     108.627 203.850 138.765  1.00  0.00
ATOM  12976 2HG1 VAL A 845     110.329 203.586 139.183  1.00  0.00
ATOM  12977 3HG1 VAL A 845     109.067 202.615 139.953  1.00  0.00
ATOM  12978  CG2 VAL A 845     107.794 204.533 141.276  1.00  0.00
ATOM  12979 1HG2 VAL A 845     107.061 204.500 140.469  1.00  0.00
ATOM  12980 2HG2 VAL A 845     107.694 203.620 141.861  1.00  0.00
ATOM  12981 3HG2 VAL A 845     107.550 205.404 141.879  1.00  0.00
ATOM  12982  C   VAL A 845     110.385 205.718 142.676  1.00  0.00
ATOM  12983  O   VAL A 845     111.332 206.503 142.650  1.00  0.00
ATOM  12984  N   LYS A 846     109.440 205.718 143.628  1.00  0.00
ATOM  12985  H   LYS A 846     108.684 205.064 143.495  1.00  0.00
ATOM  12986  CA  LYS A 846     109.472 206.451 144.902  1.00  0.00
ATOM  12987  HA  LYS A 846     110.474 206.860 145.041  1.00  0.00
ATOM  12988  CB  LYS A 846     108.494 207.648 144.918  1.00  0.00
ATOM  12989  HB1 LYS A 846     108.438 208.020 145.942  1.00  0.00
ATOM  12990  HB2 LYS A 846     107.498 207.323 144.615  1.00  0.00
ATOM  12991  CG  LYS A 846     108.958 208.803 144.014  1.00  0.00
```

ATOM	12992	HG1	LYS	A	846	108.867	208.510	142.967	1.00	0.00
ATOM	12993	HG2	LYS	A	846	110.006	209.023	144.221	1.00	0.00
ATOM	12994	CD	LYS	A	846	108.134	210.078	144.260	1.00	0.00
ATOM	12995	HD1	LYS	A	846	108.200	210.355	145.312	1.00	0.00
ATOM	12996	HD2	LYS	A	846	107.091	209.887	144.000	1.00	0.00
ATOM	12997	CE	LYS	A	846	108.659	211.239	143.411	1.00	0.00
ATOM	12998	HE1	LYS	A	846	108.586	210.965	142.355	1.00	0.00
ATOM	12999	HE2	LYS	A	846	109.710	211.414	143.655	1.00	0.00
ATOM	13000	NZ	LYS	A	846	107.894	212.483	143.662	1.00	0.00
ATOM	13001	HZ1	LYS	A	846	108.172	213.220	143.023	1.00	0.00
ATOM	13002	HZ2	LYS	A	846	106.903	212.368	143.450	1.00	0.00
ATOM	13003	HZ3	LYS	A	846	108.091	212.844	144.592	1.00	0.00
ATOM	13004	C	LYS	A	846	109.304	205.472	146.065	1.00	0.00
ATOM	13005	O	LYS	A	846	110.289	204.855	146.458	1.00	0.00
ATOM	13006	N	THR	A	847	108.081	205.199	146.516	1.00	0.00
ATOM	13007	H	THR	A	847	107.325	205.804	146.228	1.00	0.00
ATOM	13008	CA	THR	A	847	107.824	204.300	147.664	1.00	0.00
ATOM	13009	HA	THR	A	847	108.739	203.769	147.900	1.00	0.00
ATOM	13010	CB	THR	A	847	107.476	205.136	148.906	1.00	0.00
ATOM	13011	HB	THR	A	847	107.124	204.487	149.711	1.00	0.00
ATOM	13012	CG2	THR	A	847	108.672	205.932	149.425	1.00	0.00
ATOM	13013	1HG2	THR	A	847	108.388	206.440	150.348	1.00	0.00
ATOM	13014	2HG2	THR	A	847	109.499	205.255	149.638	1.00	0.00
ATOM	13015	3HG2	THR	A	847	108.985	206.673	148.691	1.00	0.00
ATOM	13016	OG1	THR	A	847	106.474	206.071	148.569	1.00	0.00
ATOM	13017	HG1	THR	A	847	105.629	205.647	148.752	1.00	0.00
ATOM	13018	C	THR	A	847	106.770	203.206	147.390	1.00	0.00
ATOM	13019	O	THR	A	847	105.769	203.115	148.108	1.00	0.00
ATOM	13020	N	PRO	A	848	106.928	202.379	146.333	1.00	0.00
ATOM	13021	CD	PRO	A	848	107.976	202.462	145.321	1.00	0.00
ATOM	13022	HD1	PRO	A	848	108.969	202.461	145.772	1.00	0.00
ATOM	13023	HD2	PRO	A	848	107.828	203.357	144.719	1.00	0.00
ATOM	13024	CG	PRO	A	848	107.814	201.247	144.419	1.00	0.00
ATOM	13025	HG1	PRO	A	848	108.447	200.452	144.799	1.00	0.00
ATOM	13026	HG2	PRO	A	848	108.067	201.473	143.385	1.00	0.00
ATOM	13027	CB	PRO	A	848	106.340	200.882	144.571	1.00	0.00
ATOM	13028	HB1	PRO	A	848	106.167	199.821	144.380	1.00	0.00
ATOM	13029	HB2	PRO	A	848	105.751	201.482	143.875	1.00	0.00
ATOM	13030	CA	PRO	A	848	105.995	201.295	146.009	1.00	0.00
ATOM	13031	HA	PRO	A	848	104.978	201.689	146.017	1.00	0.00
ATOM	13032	C	PRO	A	848	106.007	200.087	146.970	1.00	0.00
ATOM	13033	O	PRO	A	848	105.199	199.177	146.789	1.00	0.00
ATOM	13034	N	GLN	A	849	106.869	200.057	147.994	1.00	0.00
ATOM	13035	H	GLN	A	849	107.434	200.877	148.166	1.00	0.00
ATOM	13036	CA	GLN	A	849	107.161	198.867	148.816	1.00	0.00
ATOM	13037	HA	GLN	A	849	107.324	198.039	148.125	1.00	0.00
ATOM	13038	CB	GLN	A	849	108.462	199.047	149.621	1.00	0.00
ATOM	13039	HB1	GLN	A	849	108.755	198.070	150.008	1.00	0.00
ATOM	13040	HB2	GLN	A	849	108.282	199.689	150.485	1.00	0.00
ATOM	13041	CG	GLN	A	849	109.662	199.594	148.818	1.00	0.00
ATOM	13042	HG1	GLN	A	849	109.681	199.128	147.834	1.00	0.00
ATOM	13043	HG2	GLN	A	849	110.578	199.317	149.337	1.00	0.00
ATOM	13044	CD	GLN	A	849	109.667	201.113	148.670	1.00	0.00
ATOM	13045	OE1	GLN	A	849	108.824	201.826	149.198	1.00	0.00
ATOM	13046	NE2	GLN	A	849	110.586	201.674	147.916	1.00	0.00
ATOM	13047	1HE2	GLN	A	849	111.172	201.071	147.343	1.00	0.00
ATOM	13048	2HE2	GLN	A	849	110.638	202.677	147.845	1.00	0.00
ATOM	13049	C	GLN	A	849	106.017	198.413	149.753	1.00	0.00
ATOM	13050	O	GLN	A	849	106.172	197.448	150.502	1.00	0.00
ATOM	13051	N	HIS	A	850	104.870	199.098	149.751	1.00	0.00
ATOM	13052	H	HIS	A	850	104.774	199.827	149.060	1.00	0.00

```
ATOM  13053  CA  HIS A 850     103.784 198.904 150.717  1.00  0.00
ATOM  13054  HA  HIS A 850     104.205 198.441 151.612  1.00  0.00
ATOM  13055  CB  HIS A 850     103.231 200.273 151.152  1.00  0.00
ATOM  13056  HB1 HIS A 850     102.605 200.690 150.363  1.00  0.00
ATOM  13057  HB2 HIS A 850     104.068 200.955 151.309  1.00  0.00
ATOM  13058  CG  HIS A 850     102.449 200.189 152.436  1.00  0.00
ATOM  13059  ND1 HIS A 850     101.086 199.908 152.552  1.00  0.00
ATOM  13060  CE1 HIS A 850     100.839 199.856 153.869  1.00  0.00
ATOM  13061  HE1 HIS A 850      99.864 199.641 154.305  1.00  0.00
ATOM  13062  NE2 HIS A 850     101.958 200.090 154.573  1.00  0.00
ATOM  13063  HE2 HIS A 850     102.033 200.081 155.581  1.00  0.00
ATOM  13064  CD2 HIS A 850     102.986 200.303 153.684  1.00  0.00
ATOM  13065  HD2 HIS A 850     104.023 200.493 153.916  1.00  0.00
ATOM  13066  C   HIS A 850     102.701 197.945 150.208  1.00  0.00
ATOM  13067  O   HIS A 850     101.613 198.346 149.780  1.00  0.00
ATOM  13068  N   VAL A 851     103.002 196.650 150.217  1.00  0.00
ATOM  13069  H   VAL A 851     103.916 196.396 150.567  1.00  0.00
ATOM  13070  CA  VAL A 851     102.065 195.561 149.886  1.00  0.00
ATOM  13071  HA  VAL A 851     101.549 195.804 148.957  1.00  0.00
ATOM  13072  CB  VAL A 851     102.855 194.254 149.698  1.00  0.00
ATOM  13073  HB  VAL A 851     103.326 193.976 150.641  1.00  0.00
ATOM  13074  CG1 VAL A 851     101.951 193.098 149.258  1.00  0.00
ATOM  13075 1HG1 VAL A 851     102.561 192.210 149.103  1.00  0.00
ATOM  13076 2HG1 VAL A 851     101.222 192.870 150.033  1.00  0.00
ATOM  13077 3HG1 VAL A 851     101.437 193.349 148.329  1.00  0.00
ATOM  13078  CG2 VAL A 851     103.960 194.396 148.639  1.00  0.00
ATOM  13079 1HG2 VAL A 851     104.486 193.450 148.524  1.00  0.00
ATOM  13080 2HG2 VAL A 851     103.529 194.694 147.683  1.00  0.00
ATOM  13081 3HG2 VAL A 851     104.694 195.143 148.942  1.00  0.00
ATOM  13082  C   VAL A 851     101.020 195.397 150.999  1.00  0.00
ATOM  13083  O   VAL A 851     101.372 195.449 152.181  1.00  0.00
ATOM  13084  N   LYS A 852      99.745 195.155 150.664  1.00  0.00
ATOM  13085  H   LYS A 852      99.492 195.121 149.682  1.00  0.00
ATOM  13086  CA  LYS A 852      98.658 195.048 151.655  1.00  0.00
ATOM  13087  HA  LYS A 852      99.084 194.937 152.653  1.00  0.00
ATOM  13088  CB  LYS A 852      97.797 196.328 151.654  1.00  0.00
ATOM  13089  HB1 LYS A 852      97.065 196.257 152.455  1.00  0.00
ATOM  13090  HB2 LYS A 852      97.249 196.351 150.719  1.00  0.00
ATOM  13091  CG  LYS A 852      98.558 197.655 151.823  1.00  0.00
ATOM  13092  HG1 LYS A 852      99.459 197.633 151.219  1.00  0.00
ATOM  13093  HG2 LYS A 852      98.867 197.763 152.861  1.00  0.00
ATOM  13094  CD  LYS A 852      97.686 198.858 151.409  1.00  0.00
ATOM  13095  HD1 LYS A 852      97.261 199.310 152.306  1.00  0.00
ATOM  13096  HD2 LYS A 852      96.851 198.527 150.789  1.00  0.00
ATOM  13097  CE  LYS A 852      98.446 199.937 150.615  1.00  0.00
ATOM  13098  HE1 LYS A 852      99.202 200.393 151.258  1.00  0.00
ATOM  13099  HE2 LYS A 852      97.730 200.711 150.328  1.00  0.00
ATOM  13100  NZ  LYS A 852      99.087 199.377 149.403  1.00  0.00
ATOM  13101  HZ1 LYS A 852      99.916 198.841 149.655  1.00  0.00
ATOM  13102  HZ2 LYS A 852      98.466 198.710 148.932  1.00  0.00
ATOM  13103  HZ3 LYS A 852      99.384 200.084 148.751  1.00  0.00
ATOM  13104  C   LYS A 852      97.784 193.824 151.362  1.00  0.00
ATOM  13105  O   LYS A 852      97.075 193.779 150.361  1.00  0.00
ATOM  13106  N   ILE A 853      97.750 192.858 152.284  1.00  0.00
ATOM  13107  H   ILE A 853      98.373 192.944 153.071  1.00  0.00
ATOM  13108  CA  ILE A 853      96.796 191.724 152.312  1.00  0.00
ATOM  13109  HA  ILE A 853      96.739 191.329 151.296  1.00  0.00
ATOM  13110  CB  ILE A 853      97.381 190.596 153.213  1.00  0.00
ATOM  13111  HB  ILE A 853      98.396 190.412 152.853  1.00  0.00
ATOM  13112  CG2 ILE A 853      97.527 191.012 154.689  1.00  0.00
ATOM  13113 1HG2 ILE A 853      98.073 190.241 155.233  1.00  0.00
```

```
ATOM  13114 2HG2 ILE A 853      98.079 191.944 154.782  1.00  0.00
ATOM  13115 3HG2 ILE A 853      96.549 191.125 155.156  1.00  0.00
ATOM  13116  CG1 ILE A 853      96.638 189.243 153.128  1.00  0.00
ATOM  13117 1HG1 ILE A 853      95.570 189.375 153.285  1.00  0.00
ATOM  13118 2HG1 ILE A 853      97.001 188.586 153.918  1.00  0.00
ATOM  13119  CD  ILE A 853      96.854 188.509 151.802  1.00  0.00
ATOM  13120  HD1 ILE A 853      96.265 187.592 151.794  1.00  0.00
ATOM  13121  HD2 ILE A 853      96.552 189.131 150.960  1.00  0.00
ATOM  13122  HD3 ILE A 853      97.905 188.243 151.705  1.00  0.00
ATOM  13123  C   ILE A 853      95.354 192.180 152.670  1.00  0.00
ATOM  13124  O   ILE A 853      94.678 191.667 153.555  1.00  0.00
ATOM  13125  N   THR A 854      94.927 193.248 152.002  1.00  0.00
ATOM  13126  H   THR A 854      95.549 193.547 151.270  1.00  0.00
ATOM  13127  CA  THR A 854      93.621 193.908 152.052  1.00  0.00
ATOM  13128  HA  THR A 854      92.850 193.143 152.049  1.00  0.00
ATOM  13129  CB  THR A 854      93.433 194.723 153.347  1.00  0.00
ATOM  13130  HB  THR A 854      93.464 194.039 154.195  1.00  0.00
ATOM  13131  CG2 THR A 854      94.491 195.800 153.579  1.00  0.00
ATOM  13132 1HG2 THR A 854      94.228 196.375 154.467  1.00  0.00
ATOM  13133 2HG2 THR A 854      95.455 195.328 153.754  1.00  0.00
ATOM  13134 3HG2 THR A 854      94.548 196.472 152.724  1.00  0.00
ATOM  13135  OG1 THR A 854      92.178 195.360 153.352  1.00  0.00
ATOM  13136  HG1 THR A 854      91.893 195.469 152.432  1.00  0.00
ATOM  13137  C   THR A 854      93.412 194.795 150.813  1.00  0.00
ATOM  13138  O   THR A 854      92.268 195.166 150.556  1.00  0.00
ATOM  13139  N   ASP A 855      94.456 195.117 150.027  1.00  0.00
ATOM  13140  H   ASP A 855      95.385 194.825 150.288  1.00  0.00
ATOM  13141  CA  ASP A 855      94.302 195.591 148.640  1.00  0.00
ATOM  13142  HA  ASP A 855      93.288 195.979 148.522  1.00  0.00
ATOM  13143  CB  ASP A 855      95.246 196.770 148.311  1.00  0.00
ATOM  13144  HB1 ASP A 855      95.128 197.518 149.095  1.00  0.00
ATOM  13145  HB2 ASP A 855      94.890 197.222 147.385  1.00  0.00
ATOM  13146  CG  ASP A 855      96.746 196.472 148.127  1.00  0.00
ATOM  13147  OD1 ASP A 855      97.134 195.365 147.693  1.00  0.00
ATOM  13148  OD2 ASP A 855      97.537 197.395 148.440  1.00  0.00
ATOM  13149  C   ASP A 855      94.431 194.459 147.604  1.00  0.00
ATOM  13150  O   ASP A 855      93.865 194.557 146.518  1.00  0.00
ATOM  13151  N   PHE A 856      95.115 193.369 147.970  1.00  0.00
ATOM  13152  H   PHE A 856      95.655 193.445 148.818  1.00  0.00
ATOM  13153  CA  PHE A 856      95.300 192.153 147.176  1.00  0.00
ATOM  13154  HA  PHE A 856      96.054 191.562 147.692  1.00  0.00
ATOM  13155  CB  PHE A 856      94.003 191.326 147.190  1.00  0.00
ATOM  13156  HB1 PHE A 856      94.168 190.387 146.659  1.00  0.00
ATOM  13157  HB2 PHE A 856      93.225 191.868 146.650  1.00  0.00
ATOM  13158  CG  PHE A 856      93.498 190.977 148.575  1.00  0.00
ATOM  13159  CD1 PHE A 856      94.060 189.895 149.277  1.00  0.00
ATOM  13160  HD1 PHE A 856      94.842 189.306 148.821  1.00  0.00
ATOM  13161  CE1 PHE A 856      93.583 189.563 150.556  1.00  0.00
ATOM  13162  HE1 PHE A 856      94.004 188.719 151.082  1.00  0.00
ATOM  13163  CZ  PHE A 856      92.545 190.309 151.136  1.00  0.00
ATOM  13164  HZ  PHE A 856      92.184 190.054 152.123  1.00  0.00
ATOM  13165  CE2 PHE A 856      91.972 191.379 150.430  1.00  0.00
ATOM  13166  HE2 PHE A 856      91.163 191.947 150.865  1.00  0.00
ATOM  13167  CD2 PHE A 856      92.455 191.723 149.157  1.00  0.00
ATOM  13168  HD2 PHE A 856      92.019 192.556 148.620  1.00  0.00
ATOM  13169  C   PHE A 856      95.860 192.351 145.758  1.00  0.00
ATOM  13170  O   PHE A 856      95.663 191.482 144.903  1.00  0.00
ATOM  13171  N   GLY A 857      96.606 193.436 145.512  1.00  0.00
ATOM  13172  H   GLY A 857      96.729 194.105 146.273  1.00  0.00
ATOM  13173  CA  GLY A 857      97.132 193.803 144.190  1.00  0.00
ATOM  13174  HA1 GLY A 857      97.755 194.688 144.301  1.00  0.00
```

ATOM	13175	HA2	GLY	A	857	96.295	194.060	143.545	1.00	0.00
ATOM	13176	C	GLY	A	857	97.963	192.737	143.469	1.00	0.00
ATOM	13177	O	GLY	A	857	98.212	192.904	142.281	1.00	0.00
ATOM	13178	N	LEU	A	858	98.374	191.663	144.157	1.00	0.00
ATOM	13179	H	LEU	A	858	98.094	191.621	145.121	1.00	0.00
ATOM	13180	CA	LEU	A	858	98.923	190.438	143.576	1.00	0.00
ATOM	13181	HA	LEU	A	858	98.328	190.214	142.690	1.00	0.00
ATOM	13182	CB	LEU	A	858	100.391	190.701	143.117	1.00	0.00
ATOM	13183	HB1	LEU	A	858	101.083	190.214	143.795	1.00	0.00
ATOM	13184	HB2	LEU	A	858	100.602	191.765	143.212	1.00	0.00
ATOM	13185	CG	LEU	A	858	100.798	190.298	141.681	1.00	0.00
ATOM	13186	HG	LEU	A	858	101.876	190.422	141.596	1.00	0.00
ATOM	13187	CD1	LEU	A	858	100.499	188.843	141.339	1.00	0.00
ATOM	13188	1HD1	LEU	A	858	100.844	188.613	140.332	1.00	0.00
ATOM	13189	2HD1	LEU	A	858	101.016	188.200	142.044	1.00	0.00
ATOM	13190	3HD1	LEU	A	858	99.428	188.653	141.397	1.00	0.00
ATOM	13191	CD2	LEU	A	858	100.175	191.186	140.607	1.00	0.00
ATOM	13192	1HD2	LEU	A	858	99.093	191.063	140.613	1.00	0.00
ATOM	13193	2HD2	LEU	A	858	100.408	192.233	140.809	1.00	0.00
ATOM	13194	3HD2	LEU	A	858	100.552	190.923	139.621	1.00	0.00
ATOM	13195	C	LEU	A	858	98.764	189.236	144.530	1.00	0.00
ATOM	13196	O	LEU	A	858	99.738	188.721	145.070	1.00	0.00
ATOM	13197	N	ALA	A	859	97.527	188.795	144.781	1.00	0.00
ATOM	13198	H	ALA	A	859	96.752	189.333	144.417	1.00	0.00
ATOM	13199	CA	ALA	A	859	97.249	187.444	145.316	1.00	0.00
ATOM	13200	HA	ALA	A	859	97.553	187.422	146.362	1.00	0.00
ATOM	13201	CB	ALA	A	859	95.734	187.211	145.263	1.00	0.00
ATOM	13202	HB1	ALA	A	859	95.355	187.343	144.256	1.00	0.00
ATOM	13203	HB2	ALA	A	859	95.503	186.199	145.575	1.00	0.00
ATOM	13204	HB3	ALA	A	859	95.233	187.908	145.932	1.00	0.00
ATOM	13205	C	ALA	A	859	98.072	186.389	144.543	1.00	0.00
ATOM	13206	O	ALA	A	859	97.995	186.337	143.313	1.00	0.00
ATOM	13207	N	LYS	A	860	98.849	185.579	145.291	1.00	0.00
ATOM	13208	H	LYS	A	860	98.375	185.247	146.128	1.00	0.00
ATOM	13209	CA	LYS	A	860	100.334	185.587	145.478	1.00	0.00
ATOM	13210	HA	LYS	A	860	100.596	184.577	145.772	1.00	0.00
ATOM	13211	CB	LYS	A	860	101.119	185.921	144.188	1.00	0.00
ATOM	13212	HB1	LYS	A	860	102.181	185.990	144.426	1.00	0.00
ATOM	13213	HB2	LYS	A	860	100.807	186.891	143.818	1.00	0.00
ATOM	13214	CG	LYS	A	860	100.969	184.888	143.065	1.00	0.00
ATOM	13215	HG1	LYS	A	860	101.602	185.197	142.231	1.00	0.00
ATOM	13216	HG2	LYS	A	860	99.939	184.888	142.716	1.00	0.00
ATOM	13217	CD	LYS	A	860	101.368	183.467	143.498	1.00	0.00
ATOM	13218	HD1	LYS	A	860	100.717	183.114	144.296	1.00	0.00
ATOM	13219	HD2	LYS	A	860	102.399	183.471	143.851	1.00	0.00
ATOM	13220	CE	LYS	A	860	101.244	182.481	142.338	1.00	0.00
ATOM	13221	HE1	LYS	A	860	101.581	181.496	142.667	1.00	0.00
ATOM	13222	HE2	LYS	A	860	101.893	182.821	141.525	1.00	0.00
ATOM	13223	NZ	LYS	A	860	99.850	182.407	141.862	1.00	0.00
ATOM	13224	HZ1	LYS	A	860	99.767	181.850	141.011	1.00	0.00
ATOM	13225	HZ2	LYS	A	860	99.165	182.087	142.541	1.00	0.00
ATOM	13226	HZ3	LYS	A	860	99.523	183.314	141.522	1.00	0.00
ATOM	13227	C	LYS	A	860	100.807	186.383	146.711	1.00	0.00
ATOM	13228	O	LYS	A	860	101.996	186.672	146.832	1.00	0.00
ATOM	13229	N	LEU	A	861	99.897	186.645	147.663	1.00	0.00
ATOM	13230	H	LEU	A	861	98.946	186.346	147.495	1.00	0.00
ATOM	13231	CA	LEU	A	861	100.202	187.190	149.001	1.00	0.00
ATOM	13232	HA	LEU	A	861	101.265	187.431	149.063	1.00	0.00
ATOM	13233	CB	LEU	A	861	99.397	188.482	149.254	1.00	0.00
ATOM	13234	HB1	LEU	A	861	99.686	188.842	150.242	1.00	0.00
ATOM	13235	HB2	LEU	A	861	98.340	188.216	149.291	1.00	0.00

```
ATOM  13236  CG  LEU A 861      99.549 189.644 148.259  1.00  0.00
ATOM  13237  HG  LEU A 861      99.025 189.395 147.343  1.00  0.00
ATOM  13238  CD1 LEU A 861      98.881 190.894 148.844  1.00  0.00
ATOM  13239 1HD1 LEU A 861      98.935 191.714 148.128  1.00  0.00
ATOM  13240 2HD1 LEU A 861      97.835 190.677 149.058  1.00  0.00
ATOM  13241 3HD1 LEU A 861      99.380 191.186 149.766  1.00  0.00
ATOM  13242  CD2 LEU A 861     101.005 189.987 147.931  1.00  0.00
ATOM  13243 1HD2 LEU A 861     101.048 190.889 147.323  1.00  0.00
ATOM  13244 2HD2 LEU A 861     101.571 190.121 148.851  1.00  0.00
ATOM  13245 3HD2 LEU A 861     101.441 189.177 147.350  1.00  0.00
ATOM  13246  C   LEU A 861      99.916 186.185 150.133  1.00  0.00
ATOM  13247  O   LEU A 861     100.570 186.257 151.170  1.00  0.00
ATOM  13248  N   LEU A 862      98.999 185.229 149.923  1.00  0.00
ATOM  13249  H   LEU A 862      98.423 185.288 149.088  1.00  0.00
ATOM  13250  CA  LEU A 862      98.742 184.096 150.826  1.00  0.00
ATOM  13251  HA  LEU A 862      99.300 184.252 151.750  1.00  0.00
ATOM  13252  CB  LEU A 862      97.241 184.075 151.169  1.00  0.00
ATOM  13253  HB1 LEU A 862      96.677 183.952 150.249  1.00  0.00
ATOM  13254  HB2 LEU A 862      96.966 185.037 151.598  1.00  0.00
ATOM  13255  CG  LEU A 862      96.815 182.960 152.141  1.00  0.00
ATOM  13256  HG  LEU A 862      97.083 181.987 151.730  1.00  0.00
ATOM  13257  CD1 LEU A 862      97.464 183.119 153.517  1.00  0.00
ATOM  13258 1HD1 LEU A 862      97.087 182.350 154.189  1.00  0.00
ATOM  13259 2HD1 LEU A 862      98.542 182.991 153.434  1.00  0.00
ATOM  13260 3HD1 LEU A 862      97.245 184.105 153.924  1.00  0.00
ATOM  13261  CD2 LEU A 862      95.297 182.994 152.334  1.00  0.00
ATOM  13262 1HD2 LEU A 862      94.991 182.195 153.009  1.00  0.00
ATOM  13263 2HD2 LEU A 862      94.992 183.955 152.743  1.00  0.00
ATOM  13264 3HD2 LEU A 862      94.806 182.839 151.375  1.00  0.00
ATOM  13265  C   LEU A 862      99.247 182.769 150.228  1.00  0.00
ATOM  13266  O   LEU A 862      99.857 181.963 150.926  1.00  0.00
ATOM  13267  N   GLY A 863      99.073 182.599 148.917  1.00  0.00
ATOM  13268  H   GLY A 863      98.516 183.294 148.431  1.00  0.00
ATOM  13269  CA  GLY A 863      99.595 181.515 148.095  1.00  0.00
ATOM  13270  HA1 GLY A 863     100.568 181.204 148.473  1.00  0.00
ATOM  13271  HA2 GLY A 863      99.721 181.893 147.082  1.00  0.00
ATOM  13272  C   GLY A 863      98.686 180.281 148.037  1.00  0.00
ATOM  13273  O   GLY A 863      97.889 180.035 148.941  1.00  0.00
ATOM  13274  N   ALA A 864      98.887 179.494 146.972  1.00  0.00
ATOM  13275  H   ALA A 864      99.533 179.858 146.299  1.00  0.00
ATOM  13276  CA  ALA A 864      98.236 178.237 146.578  1.00  0.00
ATOM  13277  HA  ALA A 864      98.339 178.149 145.497  1.00  0.00
ATOM  13278  CB  ALA A 864      99.034 177.081 147.199  1.00  0.00
ATOM  13279  HB1 ALA A 864      98.611 176.128 146.879  1.00  0.00
ATOM  13280  HB2 ALA A 864     100.076 177.127 146.886  1.00  0.00
ATOM  13281  HB3 ALA A 864      98.987 177.129 148.287  1.00  0.00
ATOM  13282  C   ALA A 864      96.720 178.085 146.847  1.00  0.00
ATOM  13283  O   ALA A 864      95.947 178.027 145.896  1.00  0.00
ATOM  13284  N   GLU A 865      96.300 178.013 148.111  1.00  0.00
ATOM  13285  H   GLU A 865      96.995 178.127 148.837  1.00  0.00
ATOM  13286  CA  GLU A 865      94.896 177.953 148.537  1.00  0.00
ATOM  13287  HA  GLU A 865      94.394 177.172 147.963  1.00  0.00
ATOM  13288  CB  GLU A 865      94.801 177.587 150.027  1.00  0.00
ATOM  13289  HB1 GLU A 865      93.749 177.467 150.286  1.00  0.00
ATOM  13290  HB2 GLU A 865      95.203 178.408 150.622  1.00  0.00
ATOM  13291  CG  GLU A 865      95.555 176.296 150.399  1.00  0.00
ATOM  13292  HG1 GLU A 865      95.287 176.023 151.422  1.00  0.00
ATOM  13293  HG2 GLU A 865      96.627 176.508 150.393  1.00  0.00
ATOM  13294  CD  GLU A 865      95.254 175.120 149.458  1.00  0.00
ATOM  13295  OE1 GLU A 865      96.219 174.410 149.095  1.00  0.00
ATOM  13296  OE2 GLU A 865      94.067 174.951 149.091  1.00  0.00
```

ATOM	13297	C	GLU	A	865	94.131	179.264	148.248	1.00	0.00
ATOM	13298	O	GLU	A	865	92.927	179.275	147.992	1.00	0.00
ATOM	13299	N	GLU	A	866	94.865	180.373	148.171	1.00	0.00
ATOM	13300	H	GLU	A	866	95.847	180.272	148.420	1.00	0.00
ATOM	13301	CA	GLU	A	866	94.558	181.553	147.361	1.00	0.00
ATOM	13302	HA	GLU	A	866	93.487	181.680	147.203	1.00	0.00
ATOM	13303	CB	GLU	A	866	95.142	182.764	148.125	1.00	0.00
ATOM	13304	HB1	GLU	A	866	96.146	182.492	148.453	1.00	0.00
ATOM	13305	HB2	GLU	A	866	94.547	182.930	149.022	1.00	0.00
ATOM	13306	CG	GLU	A	866	95.236	184.098	147.356	1.00	0.00
ATOM	13307	HG1	GLU	A	866	94.361	184.706	147.598	1.00	0.00
ATOM	13308	HG2	GLU	A	866	95.216	183.917	146.280	1.00	0.00
ATOM	13309	CD	GLU	A	866	96.532	184.862	147.690	1.00	0.00
ATOM	13310	OE1	GLU	A	866	96.499	185.994	148.208	1.00	0.00
ATOM	13311	OE2	GLU	A	866	97.635	184.357	147.396	1.00	0.00
ATOM	13312	C	GLU	A	866	95.298	181.360	146.019	1.00	0.00
ATOM	13313	O	GLU	A	866	96.523	181.284	146.012	1.00	0.00
ATOM	13314	N	LYS	A	867	94.594	181.232	144.879	1.00	0.00
ATOM	13315	H	LYS	A	867	93.592	181.425	144.862	1.00	0.00
ATOM	13316	CA	LYS	A	867	95.257	180.782	143.641	1.00	0.00
ATOM	13317	HA	LYS	A	867	96.010	180.044	143.920	1.00	0.00
ATOM	13318	CB	LYS	A	867	94.295	180.099	142.639	1.00	0.00
ATOM	13319	HB1	LYS	A	867	94.674	180.297	141.636	1.00	0.00
ATOM	13320	HB2	LYS	A	867	93.305	180.542	142.680	1.00	0.00
ATOM	13321	CG	LYS	A	867	94.216	178.562	142.752	1.00	0.00
ATOM	13322	HG1	LYS	A	867	95.154	178.170	143.144	1.00	0.00
ATOM	13323	HG2	LYS	A	867	94.111	178.169	141.741	1.00	0.00
ATOM	13324	CD	LYS	A	867	93.046	177.989	143.565	1.00	0.00
ATOM	13325	HD1	LYS	A	867	93.109	176.901	143.510	1.00	0.00
ATOM	13326	HD2	LYS	A	867	92.106	178.290	143.100	1.00	0.00
ATOM	13327	CE	LYS	A	867	93.077	178.385	145.042	1.00	0.00
ATOM	13328	HE1	LYS	A	867	94.020	178.891	145.251	1.00	0.00
ATOM	13329	HE2	LYS	A	867	93.063	177.480	145.657	1.00	0.00
ATOM	13330	NZ	LYS	A	867	91.946	179.259	145.416	1.00	0.00
ATOM	13331	HZ1	LYS	A	867	91.059	178.817	145.241	1.00	0.00
ATOM	13332	HZ2	LYS	A	867	92.012	179.523	146.397	1.00	0.00
ATOM	13333	HZ3	LYS	A	867	91.910	180.103	144.835	1.00	0.00
ATOM	13334	C	LYS	A	867	96.059	181.890	142.952	1.00	0.00
ATOM	13335	O	LYS	A	867	97.283	181.817	142.825	1.00	0.00
ATOM	13336	N	GLU	A	868	95.355	182.893	142.447	1.00	0.00
ATOM	13337	H	GLU	A	868	94.360	182.913	142.642	1.00	0.00
ATOM	13338	CA	GLU	A	868	95.868	183.890	141.504	1.00	0.00
ATOM	13339	HA	GLU	A	868	96.882	184.206	141.753	1.00	0.00
ATOM	13340	CB	GLU	A	868	95.784	183.345	140.059	1.00	0.00
ATOM	13341	HB1	GLU	A	868	95.807	184.177	139.365	1.00	0.00
ATOM	13342	HB2	GLU	A	868	94.808	182.877	139.923	1.00	0.00
ATOM	13343	CG	GLU	A	868	96.833	182.329	139.581	1.00	0.00
ATOM	13344	HG1	GLU	A	868	96.562	182.021	138.569	1.00	0.00
ATOM	13345	HG2	GLU	A	868	96.778	181.441	140.213	1.00	0.00
ATOM	13346	CD	GLU	A	868	98.283	182.838	139.550	1.00	0.00
ATOM	13347	OE1	GLU	A	868	99.182	182.008	139.279	1.00	0.00
ATOM	13348	OE2	GLU	A	868	98.599	183.957	140.007	1.00	0.00
ATOM	13349	C	GLU	A	868	94.944	185.107	141.527	1.00	0.00
ATOM	13350	O	GLU	A	868	93.722	184.954	141.409	1.00	0.00
ATOM	13351	N	TYR	A	869	95.492	186.320	141.590	1.00	0.00
ATOM	13352	H	TYR	A	869	96.494	186.403	141.735	1.00	0.00
ATOM	13353	CA	TYR	A	869	94.662	187.533	141.516	1.00	0.00
ATOM	13354	HA	TYR	A	869	93.892	187.432	142.281	1.00	0.00
ATOM	13355	CB	TYR	A	869	95.511	188.762	141.891	1.00	0.00
ATOM	13356	HB1	TYR	A	869	96.337	188.457	142.526	1.00	0.00
ATOM	13357	HB2	TYR	A	869	94.885	189.439	142.474	1.00	0.00

ATOM	13358	CG	TYR	A	869	96.113	189.554	140.746	1.00	0.00
ATOM	13359	CD1	TYR	A	869	95.918	190.947	140.683	1.00	0.00
ATOM	13360	HD1	TYR	A	869	95.390	191.460	141.481	1.00	0.00
ATOM	13361	CE1	TYR	A	869	96.409	191.671	139.582	1.00	0.00
ATOM	13362	HE1	TYR	A	869	96.262	192.740	139.520	1.00	0.00
ATOM	13363	CZ	TYR	A	869	97.072	191.001	138.536	1.00	0.00
ATOM	13364	OH	TYR	A	869	97.473	191.684	137.437	1.00	0.00
ATOM	13365	HH	TYR	A	869	97.825	191.079	136.761	1.00	0.00
ATOM	13366	CE2	TYR	A	869	97.307	189.616	138.626	1.00	0.00
ATOM	13367	HE2	TYR	A	869	97.828	189.107	137.829	1.00	0.00
ATOM	13368	CD2	TYR	A	869	96.852	188.899	139.744	1.00	0.00
ATOM	13369	HD2	TYR	A	869	97.043	187.843	139.814	1.00	0.00
ATOM	13370	C	TYR	A	869	93.898	187.736	140.181	1.00	0.00
ATOM	13371	O	TYR	A	869	92.960	188.529	140.126	1.00	0.00
ATOM	13372	N	HIS	A	870	94.280	187.028	139.105	1.00	0.00
ATOM	13373	H	HIS	A	870	95.043	186.377	139.241	1.00	0.00
ATOM	13374	CA	HIS	A	870	93.941	187.339	137.702	1.00	0.00
ATOM	13375	HA	HIS	A	870	94.281	188.357	137.499	1.00	0.00
ATOM	13376	CB	HIS	A	870	94.703	186.387	136.768	1.00	0.00
ATOM	13377	HB1	HIS	A	870	94.459	186.632	135.734	1.00	0.00
ATOM	13378	HB2	HIS	A	870	94.374	185.364	136.952	1.00	0.00
ATOM	13379	CG	HIS	A	870	96.205	186.433	136.913	1.00	0.00
ATOM	13380	ND1	HIS	A	870	96.918	185.960	138.011	1.00	0.00
ATOM	13381	CE1	HIS	A	870	98.213	186.166	137.744	1.00	0.00
ATOM	13382	HE1	HIS	A	870	99.021	185.915	138.417	1.00	0.00
ATOM	13383	NE2	HIS	A	870	98.346	186.738	136.536	1.00	0.00
ATOM	13384	HE2	HIS	A	870	99.222	187.042	136.127	1.00	0.00
ATOM	13385	CD2	HIS	A	870	97.089	186.921	135.999	1.00	0.00
ATOM	13386	HD2	HIS	A	870	96.835	187.375	135.053	1.00	0.00
ATOM	13387	C	HIS	A	870	92.450	187.320	137.337	1.00	0.00
ATOM	13388	O	HIS	A	870	92.070	187.847	136.292	1.00	0.00
ATOM	13389	N	ALA	A	871	91.615	186.703	138.169	1.00	0.00
ATOM	13390	H	ALA	A	871	92.029	186.390	139.030	1.00	0.00
ATOM	13391	CA	ALA	A	871	90.155	186.759	138.087	1.00	0.00
ATOM	13392	HA	ALA	A	871	89.843	187.797	137.957	1.00	0.00
ATOM	13393	CB	ALA	A	871	89.675	185.934	136.876	1.00	0.00
ATOM	13394	HB1	ALA	A	871	88.593	186.016	136.792	1.00	0.00
ATOM	13395	HB2	ALA	A	871	90.117	186.305	135.954	1.00	0.00
ATOM	13396	HB3	ALA	A	871	89.942	184.886	137.012	1.00	0.00
ATOM	13397	C	ALA	A	871	89.475	186.208	139.357	1.00	0.00
ATOM	13398	O	ALA	A	871	88.299	186.513	139.569	1.00	0.00
ATOM	13399	N	GLU	A	872	90.184	185.403	140.180	1.00	0.00
ATOM	13400	H	GLU	A	872	91.160	185.253	139.978	1.00	0.00
ATOM	13401	CA	GLU	A	872	89.585	184.478	141.164	1.00	0.00
ATOM	13402	HA	GLU	A	872	89.034	183.728	140.598	1.00	0.00
ATOM	13403	CB	GLU	A	872	90.669	183.711	141.974	1.00	0.00
ATOM	13404	HB1	GLU	A	872	91.300	184.426	142.500	1.00	0.00
ATOM	13405	HB2	GLU	A	872	91.284	183.144	141.275	1.00	0.00
ATOM	13406	CG	GLU	A	872	90.052	182.738	143.006	1.00	0.00
ATOM	13407	HG1	GLU	A	872	89.209	182.232	142.531	1.00	0.00
ATOM	13408	HG2	GLU	A	872	89.664	183.317	143.846	1.00	0.00
ATOM	13409	CD	GLU	A	872	90.987	181.654	143.572	1.00	0.00
ATOM	13410	OE1	GLU	A	872	91.903	181.929	144.381	1.00	0.00
ATOM	13411	OE2	GLU	A	872	90.754	180.457	143.297	1.00	0.00
ATOM	13412	C	GLU	A	872	88.527	185.110	142.101	1.00	0.00
ATOM	13413	O	GLU	A	872	87.624	184.422	142.565	1.00	0.00
ATOM	13414	N	GLY	A	873	88.592	186.422	142.345	1.00	0.00
ATOM	13415	H	GLY	A	873	89.306	186.949	141.863	1.00	0.00
ATOM	13416	CA	GLY	A	873	87.726	187.122	143.292	1.00	0.00
ATOM	13417	HA1	GLY	A	873	88.357	187.743	143.927	1.00	0.00
ATOM	13418	HA2	GLY	A	873	87.238	186.396	143.942	1.00	0.00

```
ATOM  13419  C   GLY A 873      86.627 188.031 142.735  1.00  0.00
ATOM  13420  O   GLY A 873      86.133 188.888 143.463  1.00  0.00
ATOM  13421  N   GLY A 874      86.229 187.868 141.469  1.00  0.00
ATOM  13422  H   GLY A 874      86.707 187.169 140.913  1.00  0.00
ATOM  13423  CA  GLY A 874      85.106 188.638 140.908  1.00  0.00
ATOM  13424  HA1 GLY A 874      85.448 189.655 140.725  1.00  0.00
ATOM  13425  HA2 GLY A 874      84.304 188.686 141.646  1.00  0.00
ATOM  13426  C   GLY A 874      84.501 188.107 139.607  1.00  0.00
ATOM  13427  O   GLY A 874      83.415 188.563 139.238  1.00  0.00
ATOM  13428  N   LYS A 875      85.152 187.175 138.896  1.00  0.00
ATOM  13429  H   LYS A 875      86.041 186.820 139.235  1.00  0.00
ATOM  13430  CA  LYS A 875      84.697 186.712 137.574  1.00  0.00
ATOM  13431  HA  LYS A 875      83.611 186.609 137.590  1.00  0.00
ATOM  13432  CB  LYS A 875      85.114 187.792 136.550  1.00  0.00
ATOM  13433  HB1 LYS A 875      86.178 187.680 136.329  1.00  0.00
ATOM  13434  HB2 LYS A 875      84.979 188.782 136.993  1.00  0.00
ATOM  13435  CG  LYS A 875      84.316 187.782 135.238  1.00  0.00
ATOM  13436  HG1 LYS A 875      83.261 187.937 135.466  1.00  0.00
ATOM  13437  HG2 LYS A 875      84.426 186.823 134.737  1.00  0.00
ATOM  13438  CD  LYS A 875      84.817 188.897 134.303  1.00  0.00
ATOM  13439  HD1 LYS A 875      85.901 188.807 134.200  1.00  0.00
ATOM  13440  HD2 LYS A 875      84.596 189.869 134.748  1.00  0.00
ATOM  13441  CE  LYS A 875      84.199 188.815 132.901  1.00  0.00
ATOM  13442  HE1 LYS A 875      84.490 187.856 132.466  1.00  0.00
ATOM  13443  HE2 LYS A 875      84.608 189.616 132.280  1.00  0.00
ATOM  13444  NZ  LYS A 875      82.725 188.894 132.945  1.00  0.00
ATOM  13445  HZ1 LYS A 875      82.369 189.747 133.342  1.00  0.00
ATOM  13446  HZ2 LYS A 875      82.282 188.668 132.057  1.00  0.00
ATOM  13447  HZ3 LYS A 875      82.376 188.096 133.486  1.00  0.00
ATOM  13448  C   LYS A 875      85.320 185.361 137.185  1.00  0.00
ATOM  13449  O   LYS A 875      86.377 184.990 137.680  1.00  0.00
ATOM  13450  N   VAL A 876      84.704 184.657 136.237  1.00  0.00
ATOM  13451  H   VAL A 876      83.849 185.029 135.858  1.00  0.00
ATOM  13452  CA  VAL A 876      85.376 183.589 135.473  1.00  0.00
ATOM  13453  HA  VAL A 876      85.877 182.917 136.170  1.00  0.00
ATOM  13454  CB  VAL A 876      84.328 182.774 134.666  1.00  0.00
ATOM  13455  HB  VAL A 876      84.843 182.076 134.012  1.00  0.00
ATOM  13456  CG1 VAL A 876      83.450 181.945 135.605  1.00  0.00
ATOM  13457 1HG1 VAL A 876      82.770 181.323 135.027  1.00  0.00
ATOM  13458 2HG1 VAL A 876      84.071 181.295 136.216  1.00  0.00
ATOM  13459 3HG1 VAL A 876      82.867 182.589 136.258  1.00  0.00
ATOM  13460  CG2 VAL A 876      83.412 183.622 133.773  1.00  0.00
ATOM  13461 1HG2 VAL A 876      82.819 182.969 133.134  1.00  0.00
ATOM  13462 2HG2 VAL A 876      82.738 184.223 134.379  1.00  0.00
ATOM  13463 3HG2 VAL A 876      84.006 184.281 133.141  1.00  0.00
ATOM  13464  C   VAL A 876      86.444 184.153 134.512  1.00  0.00
ATOM  13465  O   VAL A 876      86.458 185.364 134.261  1.00  0.00
ATOM  13466  N   PRO A 877      87.317 183.313 133.915  1.00  0.00
ATOM  13467  CD  PRO A 877      87.614 181.937 134.293  1.00  0.00
ATOM  13468  HD1 PRO A 877      86.793 181.277 134.018  1.00  0.00
ATOM  13469  HD2 PRO A 877      87.826 181.852 135.360  1.00  0.00
ATOM  13470  CG  PRO A 877      88.849 181.541 133.481  1.00  0.00
ATOM  13471  HG1 PRO A 877      88.860 180.475 133.253  1.00  0.00
ATOM  13472  HG2 PRO A 877      89.751 181.831 134.023  1.00  0.00
ATOM  13473  CB  PRO A 877      88.715 182.390 132.219  1.00  0.00
ATOM  13474  HB1 PRO A 877      88.010 181.916 131.534  1.00  0.00
ATOM  13475  HB2 PRO A 877      89.677 182.550 131.732  1.00  0.00
ATOM  13476  CA  PRO A 877      88.127 183.699 132.756  1.00  0.00
ATOM  13477  HA  PRO A 877      88.942 184.324 133.120  1.00  0.00
ATOM  13478  C   PRO A 877      87.345 184.463 131.686  1.00  0.00
ATOM  13479  O   PRO A 877      86.176 184.167 131.444  1.00  0.00
```

```
ATOM  13480  N   ILE A 878      88.001 185.463 131.085  1.00  0.00
ATOM  13481  H   ILE A 878      88.986 185.512 131.298  1.00  0.00
ATOM  13482  CA  ILE A 878      87.467 186.622 130.339  1.00  0.00
ATOM  13483  HA  ILE A 878      87.206 187.342 131.115  1.00  0.00
ATOM  13484  CB  ILE A 878      88.618 187.300 129.551  1.00  0.00
ATOM  13485  HB  ILE A 878      89.484 187.267 130.216  1.00  0.00
ATOM  13486  CG2 ILE A 878      89.069 186.541 128.293  1.00  0.00
ATOM  13487 1HG2 ILE A 878      90.031 186.928 127.957  1.00  0.00
ATOM  13488 2HG2 ILE A 878      89.181 185.478 128.508  1.00  0.00
ATOM  13489 3HG2 ILE A 878      88.359 186.669 127.479  1.00  0.00
ATOM  13490  CG1 ILE A 878      88.418 188.810 129.288  1.00  0.00
ATOM  13491 1HG1 ILE A 878      89.319 189.190 128.804  1.00  0.00
ATOM  13492 2HG1 ILE A 878      88.333 189.317 130.249  1.00  0.00
ATOM  13493  CD  ILE A 878      87.216 189.228 128.430  1.00  0.00
ATOM  13494  HD1 ILE A 878      87.272 190.298 128.237  1.00  0.00
ATOM  13495  HD2 ILE A 878      87.227 188.705 127.475  1.00  0.00
ATOM  13496  HD3 ILE A 878      86.286 189.021 128.956  1.00  0.00
ATOM  13497  C   ILE A 878      86.135 186.392 129.584  1.00  0.00
ATOM  13498  O   ILE A 878      86.095 186.259 128.367  1.00  0.00
ATOM  13499  N   LYS A 879      85.001 186.352 130.307  1.00  0.00
ATOM  13500  H   LYS A 879      85.117 186.269 131.306  1.00  0.00
ATOM  13501  CA  LYS A 879      83.648 186.125 129.753  1.00  0.00
ATOM  13502  HA  LYS A 879      82.961 186.032 130.596  1.00  0.00
ATOM  13503  CB  LYS A 879      83.231 187.426 129.004  1.00  0.00
ATOM  13504  HB1 LYS A 879      84.126 187.894 128.596  1.00  0.00
ATOM  13505  HB2 LYS A 879      82.835 188.128 129.739  1.00  0.00
ATOM  13506  CG  LYS A 879      82.260 187.370 127.819  1.00  0.00
ATOM  13507  HG1 LYS A 879      82.646 186.660 127.092  1.00  0.00
ATOM  13508  HG2 LYS A 879      82.284 188.341 127.329  1.00  0.00
ATOM  13509  CD  LYS A 879      80.795 187.044 128.139  1.00  0.00
ATOM  13510  HD1 LYS A 879      80.726 186.213 128.840  1.00  0.00
ATOM  13511  HD2 LYS A 879      80.324 186.752 127.201  1.00  0.00
ATOM  13512  CE  LYS A 879      80.035 188.245 128.701  1.00  0.00
ATOM  13513  HE1 LYS A 879      80.282 189.120 128.096  1.00  0.00
ATOM  13514  HE2 LYS A 879      80.357 188.418 129.731  1.00  0.00
ATOM  13515  NZ  LYS A 879      78.578 188.003 128.648  1.00  0.00
ATOM  13516  HZ1 LYS A 879      78.036 188.829 128.861  1.00  0.00
ATOM  13517  HZ2 LYS A 879      78.296 187.720 127.711  1.00  0.00
ATOM  13518  HZ3 LYS A 879      78.303 187.266 129.295  1.00  0.00
ATOM  13519  C   LYS A 879      83.482 184.758 129.017  1.00  0.00
ATOM  13520  O   LYS A 879      82.448 184.495 128.418  1.00  0.00
ATOM  13521  N   TRP A 880      84.449 183.846 129.118  1.00  0.00
ATOM  13522  H   TRP A 880      85.176 184.047 129.794  1.00  0.00
ATOM  13523  CA  TRP A 880      84.762 182.797 128.129  1.00  0.00
ATOM  13524  HA  TRP A 880      84.754 183.289 127.171  1.00  0.00
ATOM  13525  CB  TRP A 880      86.217 182.314 128.373  1.00  0.00
ATOM  13526  HB1 TRP A 880      86.214 181.259 128.649  1.00  0.00
ATOM  13527  HB2 TRP A 880      86.629 182.837 129.236  1.00  0.00
ATOM  13528  CG  TRP A 880      87.193 182.516 127.250  1.00  0.00
ATOM  13529  CD1 TRP A 880      87.554 183.717 126.744  1.00  0.00
ATOM  13530  HD1 TRP A 880      87.158 184.666 127.054  1.00  0.00
ATOM  13531  NE1 TRP A 880      88.563 183.558 125.816  1.00  0.00
ATOM  13532  HE1 TRP A 880      89.051 184.331 125.381  1.00  0.00
ATOM  13533  CE2 TRP A 880      88.912 182.241 125.673  1.00  0.00
ATOM  13534  CZ2 TRP A 880      89.894 181.610 124.905  1.00  0.00
ATOM  13535  HZ2 TRP A 880      90.561 182.187 124.287  1.00  0.00
ATOM  13536  CH2 TRP A 880      90.017 180.216 124.976  1.00  0.00
ATOM  13537  HH2 TRP A 880      90.773 179.700 124.402  1.00  0.00
ATOM  13538  CZ3 TRP A 880      89.161 179.485 125.813  1.00  0.00
ATOM  13539  HZ3 TRP A 880      89.296 178.413 125.869  1.00  0.00
ATOM  13540  CE3 TRP A 880      88.190 180.133 126.605  1.00  0.00
```

```
ATOM  13541  HE3 TRP A 880      87.575 179.554 127.286  1.00  0.00
ATOM  13542  CD2 TRP A 880      88.036 181.536 126.557  1.00  0.00
ATOM  13543  C   TRP A 880      83.809 181.592 127.968  1.00  0.00
ATOM  13544  O   TRP A 880      84.171 180.614 127.313  1.00  0.00
ATOM  13545  N   MET A 881      82.616 181.601 128.567  1.00  0.00
ATOM  13546  H   MET A 881      82.287 182.468 128.972  1.00  0.00
ATOM  13547  CA  MET A 881      81.734 180.427 128.584  1.00  0.00
ATOM  13548  HA  MET A 881      82.017 179.751 127.777  1.00  0.00
ATOM  13549  CB  MET A 881      81.885 179.665 129.914  1.00  0.00
ATOM  13550  HB1 MET A 881      81.212 178.805 129.897  1.00  0.00
ATOM  13551  HB2 MET A 881      81.589 180.309 130.744  1.00  0.00
ATOM  13552  CG  MET A 881      83.309 179.154 130.166  1.00  0.00
ATOM  13553  HG1 MET A 881      83.962 180.006 130.359  1.00  0.00
ATOM  13554  HG2 MET A 881      83.670 178.653 129.269  1.00  0.00
ATOM  13555  SD  MET A 881      83.441 178.001 131.559  1.00  0.00
ATOM  13556  CE  MET A 881      85.194 177.560 131.414  1.00  0.00
ATOM  13557  HE1 MET A 881      85.466 176.874 132.218  1.00  0.00
ATOM  13558  HE2 MET A 881      85.375 177.072 130.456  1.00  0.00
ATOM  13559  HE3 MET A 881      85.808 178.458 131.483  1.00  0.00
ATOM  13560  C   MET A 881      80.279 180.801 128.370  1.00  0.00
ATOM  13561  O   MET A 881      79.784 181.693 129.046  1.00  0.00
ATOM  13562  N   ALA A 882      79.594 180.057 127.492  1.00  0.00
ATOM  13563  H   ALA A 882      80.100 179.330 127.012  1.00  0.00
ATOM  13564  CA  ALA A 882      78.197 180.275 127.081  1.00  0.00
ATOM  13565  HA  ALA A 882      78.182 181.130 126.408  1.00  0.00
ATOM  13566  CB  ALA A 882      77.739 179.056 126.270  1.00  0.00
ATOM  13567  HB1 ALA A 882      76.726 179.226 125.902  1.00  0.00
ATOM  13568  HB2 ALA A 882      78.401 178.900 125.417  1.00  0.00
ATOM  13569  HB3 ALA A 882      77.739 178.164 126.898  1.00  0.00
ATOM  13570  C   ALA A 882      77.221 180.599 128.236  1.00  0.00
ATOM  13571  O   ALA A 882      76.286 181.372 128.044  1.00  0.00
ATOM  13572  N   LEU A 883      77.471 180.067 129.438  1.00  0.00
ATOM  13573  H   LEU A 883      78.273 179.465 129.519  1.00  0.00
ATOM  13574  CA  LEU A 883      76.796 180.463 130.673  1.00  0.00
ATOM  13575  HA  LEU A 883      75.728 180.267 130.559  1.00  0.00
ATOM  13576  CB  LEU A 883      77.331 179.559 131.808  1.00  0.00
ATOM  13577  HB1 LEU A 883      78.403 179.726 131.925  1.00  0.00
ATOM  13578  HB2 LEU A 883      77.196 178.529 131.475  1.00  0.00
ATOM  13579  CG  LEU A 883      76.644 179.706 133.181  1.00  0.00
ATOM  13580  HG  LEU A 883      75.562 179.718 133.050  1.00  0.00
ATOM  13581  CD1 LEU A 883      77.010 178.491 134.040  1.00  0.00
ATOM  13582 1HD1 LEU A 883      76.501 178.549 135.002  1.00  0.00
ATOM  13583 2HD1 LEU A 883      76.690 177.573 133.546  1.00  0.00
ATOM  13584 3HD1 LEU A 883      78.087 178.454 134.203  1.00  0.00
ATOM  13585  CD2 LEU A 883      77.069 180.956 133.965  1.00  0.00
ATOM  13586 1HD2 LEU A 883      76.704 180.889 134.990  1.00  0.00
ATOM  13587 2HD2 LEU A 883      78.155 181.046 133.978  1.00  0.00
ATOM  13588 3HD2 LEU A 883      76.626 181.850 133.532  1.00  0.00
ATOM  13589  C   LEU A 883      76.945 181.959 130.984  1.00  0.00
ATOM  13590  O   LEU A 883      75.936 182.650 131.026  1.00  0.00
ATOM  13591  N   GLU A 884      78.159 182.487 131.189  1.00  0.00
ATOM  13592  H   GLU A 884      78.974 181.912 131.034  1.00  0.00
ATOM  13593  CA  GLU A 884      78.339 183.926 131.444  1.00  0.00
ATOM  13594  HA  GLU A 884      77.625 184.210 132.216  1.00  0.00
ATOM  13595  CB  GLU A 884      79.747 184.263 131.964  1.00  0.00
ATOM  13596  HB1 GLU A 884      80.429 184.372 131.119  1.00  0.00
ATOM  13597  HB2 GLU A 884      80.113 183.455 132.600  1.00  0.00
ATOM  13598  CG  GLU A 884      79.700 185.561 132.795  1.00  0.00
ATOM  13599  HG1 GLU A 884      79.613 185.292 133.849  1.00  0.00
ATOM  13600  HG2 GLU A 884      78.808 186.140 132.554  1.00  0.00
ATOM  13601  CD  GLU A 884      80.916 186.465 132.586  1.00  0.00
```

```
ATOM  13602  OE1 GLU A 884      81.842 186.445 133.425  1.00  0.00
ATOM  13603  OE2 GLU A 884      80.926 187.299 131.645  1.00  0.00
ATOM  13604  C   GLU A 884      78.040 184.778 130.198  1.00  0.00
ATOM  13605  O   GLU A 884      77.627 185.935 130.302  1.00  0.00
ATOM  13606  N   SER A 885      78.222 184.222 129.000  1.00  0.00
ATOM  13607  H   SER A 885      78.645 183.299 128.957  1.00  0.00
ATOM  13608  CA  SER A 885      77.819 184.847 127.743  1.00  0.00
ATOM  13609  HA  SER A 885      78.394 185.748 127.594  1.00  0.00
ATOM  13610  CB  SER A 885      78.107 183.949 126.539  1.00  0.00
ATOM  13611  HB1 SER A 885      77.999 184.525 125.621  1.00  0.00
ATOM  13612  HB2 SER A 885      77.388 183.132 126.513  1.00  0.00
ATOM  13613  OG  SER A 885      79.408 183.405 126.603  1.00  0.00
ATOM  13614  HG  SER A 885      80.069 184.106 126.414  1.00  0.00
ATOM  13615  C   SER A 885      76.341 185.220 127.772  1.00  0.00
ATOM  13616  O   SER A 885      76.033 186.406 127.710  1.00  0.00
ATOM  13617  N   ILE A 886      75.445 184.241 127.955  1.00  0.00
ATOM  13618  H   ILE A 886      75.784 183.291 128.072  1.00  0.00
ATOM  13619  CA  ILE A 886      73.992 184.463 127.998  1.00  0.00
ATOM  13620  HA  ILE A 886      73.720 185.104 127.159  1.00  0.00
ATOM  13621  CB  ILE A 886      73.242 183.122 127.841  1.00  0.00
ATOM  13622  HB  ILE A 886      73.593 182.439 128.617  1.00  0.00
ATOM  13623  CG2 ILE A 886      71.724 183.295 128.044  1.00  0.00
ATOM  13624 1HG2 ILE A 886      71.209 182.362 127.814  1.00  0.00
ATOM  13625 2HG2 ILE A 886      71.510 183.551 129.082  1.00  0.00
ATOM  13626 3HG2 ILE A 886      71.341 184.088 127.400  1.00  0.00
ATOM  13627  CG1 ILE A 886      73.510 182.447 126.471  1.00  0.00
ATOM  13628 1HG1 ILE A 886      72.976 181.497 126.446  1.00  0.00
ATOM  13629 2HG1 ILE A 886      74.570 182.214 126.390  1.00  0.00
ATOM  13630  CD  ILE A 886      73.117 183.255 125.227  1.00  0.00
ATOM  13631  HD1 ILE A 886      73.303 182.655 124.336  1.00  0.00
ATOM  13632  HD2 ILE A 886      72.059 183.513 125.252  1.00  0.00
ATOM  13633  HD3 ILE A 886      73.710 184.166 125.155  1.00  0.00
ATOM  13634  C   ILE A 886      73.559 185.233 129.255  1.00  0.00
ATOM  13635  O   ILE A 886      72.829 186.222 129.140  1.00  0.00
ATOM  13636  N   LEU A 887      74.075 184.869 130.435  1.00  0.00
ATOM  13637  H   LEU A 887      74.673 184.049 130.459  1.00  0.00
ATOM  13638  CA  LEU A 887      73.829 185.557 131.712  1.00  0.00
ATOM  13639  HA  LEU A 887      72.756 185.515 131.900  1.00  0.00
ATOM  13640  CB  LEU A 887      74.552 184.790 132.834  1.00  0.00
ATOM  13641  HB1 LEU A 887      75.619 184.843 132.640  1.00  0.00
ATOM  13642  HB2 LEU A 887      74.248 183.743 132.788  1.00  0.00
ATOM  13643  CG  LEU A 887      74.314 185.290 134.272  1.00  0.00
ATOM  13644  HG  LEU A 887      74.663 186.317 134.370  1.00  0.00
ATOM  13645  CD1 LEU A 887      72.842 185.226 134.687  1.00  0.00
ATOM  13646 1HD1 LEU A 887      72.744 185.507 135.734  1.00  0.00
ATOM  13647 2HD1 LEU A 887      72.259 185.924 134.089  1.00  0.00
ATOM  13648 3HD1 LEU A 887      72.457 184.216 134.551  1.00  0.00
ATOM  13649  CD2 LEU A 887      75.128 184.431 135.240  1.00  0.00
ATOM  13650 1HD2 LEU A 887      75.018 184.816 136.253  1.00  0.00
ATOM  13651 2HD2 LEU A 887      74.783 183.398 135.207  1.00  0.00
ATOM  13652 3HD2 LEU A 887      76.182 184.465 134.967  1.00  0.00
ATOM  13653  C   LEU A 887      74.218 187.047 131.680  1.00  0.00
ATOM  13654  O   LEU A 887      73.737 187.838 132.493  1.00  0.00
ATOM  13655  N   HIS A 888      75.060 187.454 130.727  1.00  0.00
ATOM  13656  H   HIS A 888      75.445 186.736 130.125  1.00  0.00
ATOM  13657  CA  HIS A 888      75.474 188.838 130.516  1.00  0.00
ATOM  13658  HA  HIS A 888      74.726 189.477 130.988  1.00  0.00
ATOM  13659  CB  HIS A 888      76.810 189.093 131.263  1.00  0.00
ATOM  13660  HB1 HIS A 888      77.449 189.775 130.709  1.00  0.00
ATOM  13661  HB2 HIS A 888      77.367 188.164 131.366  1.00  0.00
ATOM  13662  CG  HIS A 888      76.637 189.687 132.637  1.00  0.00
```

```
ATOM  13663  ND1 HIS A 888      76.703 191.052 132.946  1.00  0.00
ATOM  13664  CE1 HIS A 888      76.548 191.124 134.282  1.00  0.00
ATOM  13665  HE1 HIS A 888      76.566 192.040 134.863  1.00  0.00
ATOM  13666  NE2 HIS A 888      76.374 189.900 134.807  1.00  0.00
ATOM  13667  HE2 HIS A 888      76.226 189.691 135.786  1.00  0.00
ATOM  13668  CD2 HIS A 888      76.433 188.983 133.785  1.00  0.00
ATOM  13669  HD2 HIS A 888      76.327 187.907 133.868  1.00  0.00
ATOM  13670  C   HIS A 888      75.450 189.254 129.036  1.00  0.00
ATOM  13671  O   HIS A 888      76.409 189.861 128.544  1.00  0.00
ATOM  13672  N   ARG A 889      74.334 188.960 128.355  1.00  0.00
ATOM  13673  H   ARG A 889      73.714 188.291 128.810  1.00  0.00
ATOM  13674  CA  ARG A 889      73.827 189.534 127.080  1.00  0.00
ATOM  13675  HA  ARG A 889      72.737 189.484 127.120  1.00  0.00
ATOM  13676  CB  ARG A 889      74.210 191.009 126.838  1.00  0.00
ATOM  13677  HB1 ARG A 889      73.784 191.340 125.890  1.00  0.00
ATOM  13678  HB2 ARG A 889      75.291 191.105 126.749  1.00  0.00
ATOM  13679  CG  ARG A 889      73.680 191.931 127.953  1.00  0.00
ATOM  13680  HG1 ARG A 889      73.673 191.388 128.897  1.00  0.00
ATOM  13681  HG2 ARG A 889      72.646 192.204 127.734  1.00  0.00
ATOM  13682  CD  ARG A 889      74.524 193.205 128.124  1.00  0.00
ATOM  13683  HD1 ARG A 889      74.100 193.997 127.505  1.00  0.00
ATOM  13684  HD2 ARG A 889      75.545 193.028 127.784  1.00  0.00
ATOM  13685  NE  ARG A 889      74.551 193.626 129.540  1.00  0.00
ATOM  13686  HE  ARG A 889      73.918 194.359 129.810  1.00  0.00
ATOM  13687  CZ  ARG A 889      75.263 193.052 130.498  1.00  0.00
ATOM  13688  NH1 ARG A 889      76.156 192.137 130.275  1.00  0.00
ATOM  13689 1HH1 ARG A 889      76.306 191.770 129.345  1.00  0.00
ATOM  13690 2HH1 ARG A 889      76.626 191.726 131.074  1.00  0.00
ATOM  13691  NH2 ARG A 889      75.087 193.355 131.747  1.00  0.00
ATOM  13692 1HH2 ARG A 889      74.393 194.023 132.025  1.00  0.00
ATOM  13693 2HH2 ARG A 889      75.587 192.813 132.439  1.00  0.00
ATOM  13694  C   ARG A 889      74.190 188.647 125.902  1.00  0.00
ATOM  13695  O   ARG A 889      73.308 188.137 125.222  1.00  0.00
ATOM  13696  N   ILE A 890      75.485 188.488 125.675  1.00  0.00
ATOM  13697  H   ILE A 890      76.118 188.853 126.369  1.00  0.00
ATOM  13698  CA  ILE A 890      76.069 187.811 124.521  1.00  0.00
ATOM  13699  HA  ILE A 890      75.472 186.933 124.270  1.00  0.00
ATOM  13700  CB  ILE A 890      76.111 188.772 123.288  1.00  0.00
ATOM  13701  HB  ILE A 890      76.667 188.270 122.493  1.00  0.00
ATOM  13702  CG2 ILE A 890      74.716 189.037 122.694  1.00  0.00
ATOM  13703 1HG2 ILE A 890      74.812 189.456 121.693  1.00  0.00
ATOM  13704 2HG2 ILE A 890      74.163 188.101 122.615  1.00  0.00
ATOM  13705 3HG2 ILE A 890      74.156 189.736 123.314  1.00  0.00
ATOM  13706  CG1 ILE A 890      76.843 190.103 123.599  1.00  0.00
ATOM  13707 1HG1 ILE A 890      77.818 189.884 124.034  1.00  0.00
ATOM  13708 2HG1 ILE A 890      76.271 190.677 124.329  1.00  0.00
ATOM  13709  CD  ILE A 890      77.080 190.993 122.374  1.00  0.00
ATOM  13710  HD1 ILE A 890      77.694 191.846 122.662  1.00  0.00
ATOM  13711  HD2 ILE A 890      77.597 190.433 121.596  1.00  0.00
ATOM  13712  HD3 ILE A 890      76.134 191.364 121.981  1.00  0.00
ATOM  13713  C   ILE A 890      77.491 187.355 124.838  1.00  0.00
ATOM  13714  O   ILE A 890      78.085 187.705 125.869  1.00  0.00
ATOM  13715  N   TYR A 891      78.058 186.622 123.893  1.00  0.00
ATOM  13716  H   TYR A 891      77.493 186.381 123.091  1.00  0.00
ATOM  13717  CA  TYR A 891      79.492 186.516 123.685  1.00  0.00
ATOM  13718  HA  TYR A 891      79.962 186.426 124.655  1.00  0.00
ATOM  13719  CB  TYR A 891      79.772 185.252 122.851  1.00  0.00
ATOM  13720  HB1 TYR A 891      79.851 184.412 123.538  1.00  0.00
ATOM  13721  HB2 TYR A 891      80.735 185.348 122.346  1.00  0.00
ATOM  13722  CG  TYR A 891      78.699 184.888 121.844  1.00  0.00
ATOM  13723  CD1 TYR A 891      78.736 185.411 120.539  1.00  0.00
```

```
ATOM  13724  HD1 TYR A 891      79.519 186.097 120.240  1.00  0.00
ATOM  13725  CE1 TYR A 891      77.756 185.024 119.604  1.00  0.00
ATOM  13726  HE1 TYR A 891      77.794 185.387 118.584  1.00  0.00
ATOM  13727  CZ  TYR A 891      76.744 184.116 119.977  1.00  0.00
ATOM  13728  OH  TYR A 891      75.855 183.698 119.047  1.00  0.00
ATOM  13729  HH  TYR A 891      75.331 182.941 119.325  1.00  0.00
ATOM  13730  CE2 TYR A 891      76.680 183.624 121.297  1.00  0.00
ATOM  13731  HE2 TYR A 891      75.902 182.933 121.577  1.00  0.00
ATOM  13732  CD2 TYR A 891      77.662 184.012 122.227  1.00  0.00
ATOM  13733  HD2 TYR A 891      77.635 183.611 123.232  1.00  0.00
ATOM  13734  C   TYR A 891      80.073 187.779 123.030  1.00  0.00
ATOM  13735  O   TYR A 891      79.431 188.454 122.229  1.00  0.00
ATOM  13736  N   THR A 892      81.318 188.079 123.393  1.00  0.00
ATOM  13737  H   THR A 892      81.777 187.436 124.020  1.00  0.00
ATOM  13738  CA  THR A 892      82.147 189.158 122.834  1.00  0.00
ATOM  13739  HA  THR A 892      81.765 189.405 121.843  1.00  0.00
ATOM  13740  CB  THR A 892      82.034 190.449 123.673  1.00  0.00
ATOM  13741  HB  THR A 892      81.016 190.828 123.567  1.00  0.00
ATOM  13742  CG2 THR A 892      82.294 190.251 125.167  1.00  0.00
ATOM  13743 1HG2 THR A 892      82.306 191.222 125.664  1.00  0.00
ATOM  13744 2HG2 THR A 892      81.490 189.653 125.592  1.00  0.00
ATOM  13745 3HG2 THR A 892      83.251 189.758 125.327  1.00  0.00
ATOM  13746  OG1 THR A 892      82.913 191.457 123.230  1.00  0.00
ATOM  13747  HG1 THR A 892      82.524 191.894 122.465  1.00  0.00
ATOM  13748  C   THR A 892      83.550 188.583 122.588  1.00  0.00
ATOM  13749  O   THR A 892      83.626 187.501 122.016  1.00  0.00
ATOM  13750  N   HIS A 893      84.641 189.234 123.004  1.00  0.00
ATOM  13751  H   HIS A 893      84.501 190.159 123.397  1.00  0.00
ATOM  13752  CA  HIS A 893      86.017 188.708 122.966  1.00  0.00
ATOM  13753  HA  HIS A 893      86.231 188.416 121.936  1.00  0.00
ATOM  13754  CB  HIS A 893      87.004 189.831 123.330  1.00  0.00
ATOM  13755  HB1 HIS A 893      87.023 189.988 124.409  1.00  0.00
ATOM  13756  HB2 HIS A 893      86.661 190.757 122.869  1.00  0.00
ATOM  13757  CG  HIS A 893      88.403 189.588 122.819  1.00  0.00
ATOM  13758  ND1 HIS A 893      88.771 189.684 121.480  1.00  0.00
ATOM  13759  CE1 HIS A 893      90.094 189.465 121.444  1.00  0.00
ATOM  13760  HE1 HIS A 893      90.688 189.447 120.538  1.00  0.00
ATOM  13761  NE2 HIS A 893      90.569 189.241 122.680  1.00  0.00
ATOM  13762  HE2 HIS A 893      91.545 189.059 122.895  1.00  0.00
ATOM  13763  CD2 HIS A 893      89.511 189.303 123.564  1.00  0.00
ATOM  13764  HD2 HIS A 893      89.545 189.162 124.632  1.00  0.00
ATOM  13765  C   HIS A 893      86.205 187.437 123.828  1.00  0.00
ATOM  13766  O   HIS A 893      86.761 187.490 124.920  1.00  0.00
ATOM  13767  N   GLN A 894      85.689 186.298 123.356  1.00  0.00
ATOM  13768  H   GLN A 894      85.212 186.336 122.460  1.00  0.00
ATOM  13769  CA  GLN A 894      85.747 184.985 124.010  1.00  0.00
ATOM  13770  HA  GLN A 894      86.613 184.959 124.663  1.00  0.00
ATOM  13771  CB  GLN A 894      84.512 184.759 124.887  1.00  0.00
ATOM  13772  HB1 GLN A 894      84.521 185.462 125.721  1.00  0.00
ATOM  13773  HB2 GLN A 894      84.599 183.751 125.276  1.00  0.00
ATOM  13774  CG  GLN A 894      83.171 184.837 124.159  1.00  0.00
ATOM  13775  HG1 GLN A 894      83.206 184.279 123.225  1.00  0.00
ATOM  13776  HG2 GLN A 894      82.940 185.875 123.937  1.00  0.00
ATOM  13777  CD  GLN A 894      82.070 184.257 125.032  1.00  0.00
ATOM  13778  OE1 GLN A 894      81.332 184.965 125.696  1.00  0.00
ATOM  13779  NE2 GLN A 894      81.883 182.960 125.061  1.00  0.00
ATOM  13780 1HE2 GLN A 894      82.471 182.348 124.502  1.00  0.00
ATOM  13781 2HE2 GLN A 894      81.113 182.622 125.616  1.00  0.00
ATOM  13782  C   GLN A 894      85.884 183.825 123.011  1.00  0.00
ATOM  13783  O   GLN A 894      85.639 184.017 121.830  1.00  0.00
ATOM  13784  N   SER A 895      86.199 182.616 123.497  1.00  0.00
```

```
ATOM  13785  H   SER A 895      86.378 182.523 124.485  1.00  0.00
ATOM  13786  CA  SER A 895      86.154 181.390 122.681  1.00  0.00
ATOM  13787  HA  SER A 895      86.365 180.550 123.341  1.00  0.00
ATOM  13788  CB  SER A 895      84.745 181.163 122.100  1.00  0.00
ATOM  13789  HB1 SER A 895      84.639 180.138 121.756  1.00  0.00
ATOM  13790  HB2 SER A 895      84.643 181.794 121.222  1.00  0.00
ATOM  13791  OG  SER A 895      83.682 181.489 122.989  1.00  0.00
ATOM  13792  HG  SER A 895      83.031 181.905 122.381  1.00  0.00
ATOM  13793  C   SER A 895      87.222 181.364 121.576  1.00  0.00
ATOM  13794  O   SER A 895      86.899 181.397 120.389  1.00  0.00
ATOM  13795  N   ASP A 896      88.484 181.278 122.011  1.00  0.00
ATOM  13796  H   ASP A 896      88.621 181.084 122.993  1.00  0.00
ATOM  13797  CA  ASP A 896      89.725 181.432 121.230  1.00  0.00
ATOM  13798  HA  ASP A 896      90.501 181.529 121.988  1.00  0.00
ATOM  13799  CB  ASP A 896      90.120 180.147 120.499  1.00  0.00
ATOM  13800  HB1 ASP A 896      90.880 180.376 119.751  1.00  0.00
ATOM  13801  HB2 ASP A 896      89.262 179.701 119.999  1.00  0.00
ATOM  13802  CG  ASP A 896      90.717 179.189 121.539  1.00  0.00
ATOM  13803  OD1 ASP A 896      89.953 178.571 122.309  1.00  0.00
ATOM  13804  OD2 ASP A 896      91.953 179.181 121.689  1.00  0.00
ATOM  13805  C   ASP A 896      89.824 182.759 120.449  1.00  0.00
ATOM  13806  O   ASP A 896      89.169 183.011 119.439  1.00  0.00
ATOM  13807  N   VAL A 897      90.613 183.676 121.030  1.00  0.00
ATOM  13808  H   VAL A 897      91.170 183.366 121.811  1.00  0.00
ATOM  13809  CA  VAL A 897      90.390 185.128 120.901  1.00  0.00
ATOM  13810  HA  VAL A 897      89.583 185.294 120.188  1.00  0.00
ATOM  13811  CB  VAL A 897      89.948 185.754 122.233  1.00  0.00
ATOM  13812  HB  VAL A 897      89.664 186.785 122.045  1.00  0.00
ATOM  13813  CG1 VAL A 897      88.720 185.072 122.793  1.00  0.00
ATOM  13814 1HG1 VAL A 897      88.410 185.614 123.682  1.00  0.00
ATOM  13815 2HG1 VAL A 897      87.948 185.129 122.028  1.00  0.00
ATOM  13816 3HG1 VAL A 897      88.926 184.031 123.037  1.00  0.00
ATOM  13817  CG2 VAL A 897      91.022 185.743 123.330  1.00  0.00
ATOM  13818 1HG2 VAL A 897      90.616 186.159 124.251  1.00  0.00
ATOM  13819 2HG2 VAL A 897      91.382 184.731 123.514  1.00  0.00
ATOM  13820 3HG2 VAL A 897      91.852 186.370 123.015  1.00  0.00
ATOM  13821  C   VAL A 897      91.601 185.916 120.396  1.00  0.00
ATOM  13822  O   VAL A 897      91.500 187.130 120.228  1.00  0.00
ATOM  13823  N   TRP A 898      92.736 185.267 120.131  1.00  0.00
ATOM  13824  H   TRP A 898      92.718 184.260 120.198  1.00  0.00
ATOM  13825  CA  TRP A 898      93.912 185.950 119.571  1.00  0.00
ATOM  13826  HA  TRP A 898      93.625 186.946 119.233  1.00  0.00
ATOM  13827  CB  TRP A 898      95.021 186.117 120.609  1.00  0.00
ATOM  13828  HB1 TRP A 898      95.805 186.740 120.177  1.00  0.00
ATOM  13829  HB2 TRP A 898      95.465 185.139 120.804  1.00  0.00
ATOM  13830  CG  TRP A 898      94.628 186.714 121.920  1.00  0.00
ATOM  13831  CD1 TRP A 898      94.566 186.020 123.069  1.00  0.00
ATOM  13832  HD1 TRP A 898      94.767 184.960 123.160  1.00  0.00
ATOM  13833  NE1 TRP A 898      94.294 186.866 124.119  1.00  0.00
ATOM  13834  HE1 TRP A 898      94.335 186.528 125.090  1.00  0.00
ATOM  13835  CE2 TRP A 898      94.064 188.152 123.684  1.00  0.00
ATOM  13836  CZ2 TRP A 898      93.704 189.339 124.341  1.00  0.00
ATOM  13837  HZ2 TRP A 898      93.582 189.351 125.415  1.00  0.00
ATOM  13838  CH2 TRP A 898      93.519 190.509 123.584  1.00  0.00
ATOM  13839  HH2 TRP A 898      93.249 191.431 124.075  1.00  0.00
ATOM  13840  CZ3 TRP A 898      93.683 190.473 122.186  1.00  0.00
ATOM  13841  HZ3 TRP A 898      93.532 191.376 121.609  1.00  0.00
ATOM  13842  CE3 TRP A 898      94.043 189.277 121.537  1.00  0.00
ATOM  13843  HE3 TRP A 898      94.151 189.249 120.466  1.00  0.00
ATOM  13844  CD2 TRP A 898      94.257 188.087 122.270  1.00  0.00
ATOM  13845  C   TRP A 898      94.453 185.224 118.340  1.00  0.00
```

```
ATOM  13846  O   TRP A 898      94.663 185.863 117.307  1.00  0.00
ATOM  13847  N   SER A 899      94.565 183.893 118.404  1.00  0.00
ATOM  13848  H   SER A 899      94.454 183.459 119.306  1.00  0.00
ATOM  13849  CA  SER A 899      94.162 183.086 117.250  1.00  0.00
ATOM  13850  HA  SER A 899      94.663 183.468 116.360  1.00  0.00
ATOM  13851  CB  SER A 899      94.562 181.616 117.406  1.00  0.00
ATOM  13852  HB1 SER A 899      94.008 181.152 118.223  1.00  0.00
ATOM  13853  HB2 SER A 899      95.629 181.557 117.615  1.00  0.00
ATOM  13854  OG  SER A 899      94.295 180.940 116.190  1.00  0.00
ATOM  13855  HG  SER A 899      94.691 180.050 116.242  1.00  0.00
ATOM  13856  C   SER A 899      92.660 183.247 117.102  1.00  0.00
ATOM  13857  O   SER A 899      91.923 183.104 118.073  1.00  0.00
ATOM  13858  N   TYR A 900      92.220 183.698 115.930  1.00  0.00
ATOM  13859  H   TYR A 900      92.888 183.772 115.175  1.00  0.00
ATOM  13860  CA  TYR A 900      90.948 184.405 115.777  1.00  0.00
ATOM  13861  HA  TYR A 900      90.227 184.057 116.523  1.00  0.00
ATOM  13862  CB  TYR A 900      91.242 185.897 116.021  1.00  0.00
ATOM  13863  HB1 TYR A 900      91.865 186.283 115.214  1.00  0.00
ATOM  13864  HB2 TYR A 900      91.837 185.986 116.931  1.00  0.00
ATOM  13865  CG  TYR A 900      90.049 186.812 116.208  1.00  0.00
ATOM  13866  CD1 TYR A 900      89.495 186.971 117.490  1.00  0.00
ATOM  13867  HD1 TYR A 900      89.861 186.367 118.306  1.00  0.00
ATOM  13868  CE1 TYR A 900      88.529 187.965 117.734  1.00  0.00
ATOM  13869  HE1 TYR A 900      88.157 188.141 118.728  1.00  0.00
ATOM  13870  CZ  TYR A 900      88.104 188.796 116.681  1.00  0.00
ATOM  13871  OH  TYR A 900      87.249 189.820 116.923  1.00  0.00
ATOM  13872  HH  TYR A 900      87.237 190.448 116.183  1.00  0.00
ATOM  13873  CE2 TYR A 900      88.613 188.591 115.379  1.00  0.00
ATOM  13874  HE2 TYR A 900      88.269 189.212 114.569  1.00  0.00
ATOM  13875  CD2 TYR A 900      89.590 187.606 115.143  1.00  0.00
ATOM  13876  HD2 TYR A 900      90.021 187.491 114.159  1.00  0.00
ATOM  13877  C   TYR A 900      90.415 184.122 114.377  1.00  0.00
ATOM  13878  O   TYR A 900      90.930 184.650 113.386  1.00  0.00
ATOM  13879  N   GLY A 901      89.434 183.220 114.270  1.00  0.00
ATOM  13880  H   GLY A 901      89.084 182.798 115.121  1.00  0.00
ATOM  13881  CA  GLY A 901      88.760 182.913 113.003  1.00  0.00
ATOM  13882  HA1 GLY A 901      87.942 182.215 113.179  1.00  0.00
ATOM  13883  HA2 GLY A 901      89.467 182.458 112.311  1.00  0.00
ATOM  13884  C   GLY A 901      88.188 184.191 112.383  1.00  0.00
ATOM  13885  O   GLY A 901      87.197 184.733 112.875  1.00  0.00
ATOM  13886  N   VAL A 902      88.855 184.710 111.351  1.00  0.00
ATOM  13887  H   VAL A 902      89.684 184.200 111.091  1.00  0.00
ATOM  13888  CA  VAL A 902      88.736 186.095 110.842  1.00  0.00
ATOM  13889  HA  VAL A 902      89.051 186.757 111.650  1.00  0.00
ATOM  13890  CB  VAL A 902      89.738 186.262 109.677  1.00  0.00
ATOM  13891  HB  VAL A 902      89.897 185.286 109.215  1.00  0.00
ATOM  13892  CG1 VAL A 902      89.354 187.209 108.534  1.00  0.00
ATOM  13893 1HG1 VAL A 902      90.148 187.219 107.790  1.00  0.00
ATOM  13894 2HG1 VAL A 902      88.443 186.849 108.056  1.00  0.00
ATOM  13895 3HG1 VAL A 902      89.181 188.213 108.916  1.00  0.00
ATOM  13896  CG2 VAL A 902      91.075 186.736 110.259  1.00  0.00
ATOM  13897 1HG2 VAL A 902      91.851 186.701 109.500  1.00  0.00
ATOM  13898 2HG2 VAL A 902      90.982 187.750 110.647  1.00  0.00
ATOM  13899 3HG2 VAL A 902      91.371 186.084 111.082  1.00  0.00
ATOM  13900  C   VAL A 902      87.319 186.574 110.499  1.00  0.00
ATOM  13901  O   VAL A 902      87.086 187.778 110.456  1.00  0.00
ATOM  13902  N   THR A 903      86.357 185.665 110.340  1.00  0.00
ATOM  13903  H   THR A 903      86.606 184.692 110.456  1.00  0.00
ATOM  13904  CA  THR A 903      84.909 185.921 110.316  1.00  0.00
ATOM  13905  HA  THR A 903      84.687 186.640 109.532  1.00  0.00
ATOM  13906  CB  THR A 903      84.170 184.602 109.995  1.00  0.00
```

```
ATOM  13907  HB  THR A 903      83.139 184.636 110.339  1.00  0.00
ATOM  13908  CG2 THR A 903      84.158 184.350 108.489  1.00  0.00
ATOM  13909 1HG2 THR A 903      83.653 183.406 108.286  1.00  0.00
ATOM  13910 2HG2 THR A 903      83.615 185.154 107.992  1.00  0.00
ATOM  13911 3HG2 THR A 903      85.177 184.304 108.105  1.00  0.00
ATOM  13912  OG1 THR A 903      84.823 183.480 110.564  1.00  0.00
ATOM  13913  HG1 THR A 903      84.418 183.242 111.416  1.00  0.00
ATOM  13914  C   THR A 903      84.305 186.507 111.599  1.00  0.00
ATOM  13915  O   THR A 903      83.099 186.701 111.623  1.00  0.00
ATOM  13916  N   VAL A 904      85.070 186.747 112.671  1.00  0.00
ATOM  13917  H   VAL A 904      86.052 186.520 112.591  1.00  0.00
ATOM  13918  CA  VAL A 904      84.566 186.972 114.050  1.00  0.00
ATOM  13919  HA  VAL A 904      85.437 186.842 114.690  1.00  0.00
ATOM  13920  CB  VAL A 904      84.073 188.409 114.342  1.00  0.00
ATOM  13921  HB  VAL A 904      83.910 188.471 115.418  1.00  0.00
ATOM  13922  CG1 VAL A 904      85.171 189.420 114.013  1.00  0.00
ATOM  13923 1HG1 VAL A 904      84.902 190.391 114.424  1.00  0.00
ATOM  13924 2HG1 VAL A 904      86.092 189.101 114.487  1.00  0.00
ATOM  13925 3HG1 VAL A 904      85.327 189.492 112.938  1.00  0.00
ATOM  13926  CG2 VAL A 904      82.767 188.846 113.664  1.00  0.00
ATOM  13927 1HG2 VAL A 904      82.426 189.777 114.110  1.00  0.00
ATOM  13928 2HG2 VAL A 904      82.913 188.981 112.595  1.00  0.00
ATOM  13929 3HG2 VAL A 904      81.995 188.094 113.824  1.00  0.00
ATOM  13930  C   VAL A 904      83.589 185.893 114.551  1.00  0.00
ATOM  13931  O   VAL A 904      82.743 186.141 115.408  1.00  0.00
ATOM  13932  N   TRP A 905      83.754 184.669 114.049  1.00  0.00
ATOM  13933  H   TRP A 905      84.464 184.559 113.341  1.00  0.00
ATOM  13934  CA  TRP A 905      83.234 183.438 114.648  1.00  0.00
ATOM  13935  HA  TRP A 905      83.283 183.559 115.720  1.00  0.00
ATOM  13936  CB  TRP A 905      81.759 183.237 114.254  1.00  0.00
ATOM  13937  HB1 TRP A 905      81.168 184.012 114.744  1.00  0.00
ATOM  13938  HB2 TRP A 905      81.419 182.284 114.658  1.00  0.00
ATOM  13939  CG  TRP A 905      81.419 183.256 112.793  1.00  0.00
ATOM  13940  CD1 TRP A 905      81.662 182.244 111.939  1.00  0.00
ATOM  13941  HD1 TRP A 905      82.149 181.323 112.232  1.00  0.00
ATOM  13942  NE1 TRP A 905      81.209 182.565 110.676  1.00  0.00
ATOM  13943  HE1 TRP A 905      81.336 181.953 109.872  1.00  0.00
ATOM  13944  CE2 TRP A 905      80.607 183.804 110.661  1.00  0.00
ATOM  13945  CZ2 TRP A 905      79.992 184.553 109.648  1.00  0.00
ATOM  13946  HZ2 TRP A 905      79.917 184.165 108.641  1.00  0.00
ATOM  13947  CH2 TRP A 905      79.480 185.823 109.960  1.00  0.00
ATOM  13948  HH2 TRP A 905      79.006 186.426 109.197  1.00  0.00
ATOM  13949  CZ3 TRP A 905      79.601 186.327 111.268  1.00  0.00
ATOM  13950  HZ3 TRP A 905      79.229 187.313 111.499  1.00  0.00
ATOM  13951  CE3 TRP A 905      80.225 185.566 112.277  1.00  0.00
ATOM  13952  HE3 TRP A 905      80.343 185.980 113.270  1.00  0.00
ATOM  13953  CD2 TRP A 905      80.742 184.283 112.002  1.00  0.00
ATOM  13954  C   TRP A 905      84.158 182.263 114.216  1.00  0.00
ATOM  13955  O   TRP A 905      84.518 182.216 113.041  1.00  0.00
ATOM  13956  N   GLU A 906      84.642 181.293 115.007  1.00  0.00
ATOM  13957  H   GLU A 906      85.310 180.711 114.526  1.00  0.00
ATOM  13958  CA  GLU A 906      84.288 180.771 116.346  1.00  0.00
ATOM  13959  HA  GLU A 906      83.650 179.900 116.181  1.00  0.00
ATOM  13960  CB  GLU A 906      85.540 180.238 117.083  1.00  0.00
ATOM  13961  HB1 GLU A 906      85.284 180.014 118.120  1.00  0.00
ATOM  13962  HB2 GLU A 906      86.302 181.019 117.086  1.00  0.00
ATOM  13963  CG  GLU A 906      86.142 178.970 116.470  1.00  0.00
ATOM  13964  HG1 GLU A 906      87.128 178.808 116.909  1.00  0.00
ATOM  13965  HG2 GLU A 906      86.264 179.126 115.398  1.00  0.00
ATOM  13966  CD  GLU A 906      85.273 177.726 116.720  1.00  0.00
ATOM  13967  OE1 GLU A 906      85.400 177.090 117.795  1.00  0.00
```

ATOM	13968	OE2	GLU	A	906	84.430	177.421	115.846	1.00	0.00
ATOM	13969	C	GLU	A	906	83.440	181.673	117.243	1.00	0.00
ATOM	13970	O	GLU	A	906	82.237	181.730	117.013	1.00	0.00
ATOM	13971	N	LEU	A	907	84.059	182.398	118.189	1.00	0.00
ATOM	13972	H	LEU	A	907	85.053	182.232	118.293	1.00	0.00
ATOM	13973	CA	LEU	A	907	83.491	183.368	119.146	1.00	0.00
ATOM	13974	HA	LEU	A	907	84.234	183.510	119.927	1.00	0.00
ATOM	13975	CB	LEU	A	907	83.321	184.744	118.454	1.00	0.00
ATOM	13976	HB1	LEU	A	907	82.744	185.384	119.121	1.00	0.00
ATOM	13977	HB2	LEU	A	907	82.706	184.606	117.566	1.00	0.00
ATOM	13978	CG	LEU	A	907	84.587	185.550	118.041	1.00	0.00
ATOM	13979	HG	LEU	A	907	84.257	186.363	117.400	1.00	0.00
ATOM	13980	CD1	LEU	A	907	85.272	186.216	119.230	1.00	0.00
ATOM	13981	1HD1	LEU	A	907	86.045	186.898	118.883	1.00	0.00
ATOM	13982	2HD1	LEU	A	907	84.541	186.790	119.792	1.00	0.00
ATOM	13983	3HD1	LEU	A	907	85.730	185.465	119.865	1.00	0.00
ATOM	13984	CD2	LEU	A	907	85.686	184.790	117.285	1.00	0.00
ATOM	13985	1HD2	LEU	A	907	86.439	185.494	116.932	1.00	0.00
ATOM	13986	2HD2	LEU	A	907	86.174	184.074	117.949	1.00	0.00
ATOM	13987	3HD2	LEU	A	907	85.268	184.265	116.433	1.00	0.00
ATOM	13988	C	LEU	A	907	82.213	182.869	119.867	1.00	0.00
ATOM	13989	O	LEU	A	907	82.274	182.498	121.041	1.00	0.00
ATOM	13990	N	MET	A	908	81.070	182.782	119.181	1.00	0.00
ATOM	13991	H	MET	A	908	81.107	183.181	118.252	1.00	0.00
ATOM	13992	CA	MET	A	908	80.111	181.668	119.283	1.00	0.00
ATOM	13993	HA	MET	A	908	80.665	180.739	119.133	1.00	0.00
ATOM	13994	CB	MET	A	908	79.424	181.625	120.667	1.00	0.00
ATOM	13995	HB1	MET	A	908	78.348	181.730	120.555	1.00	0.00
ATOM	13996	HB2	MET	A	908	79.775	182.447	121.286	1.00	0.00
ATOM	13997	CG	MET	A	908	79.707	180.314	121.398	1.00	0.00
ATOM	13998	HG1	MET	A	908	79.417	180.427	122.441	1.00	0.00
ATOM	13999	HG2	MET	A	908	80.779	180.114	121.365	1.00	0.00
ATOM	14000	SD	MET	A	908	78.811	178.892	120.716	1.00	0.00
ATOM	14001	CE	MET	A	908	79.690	177.546	121.548	1.00	0.00
ATOM	14002	HE1	MET	A	908	79.239	176.594	121.272	1.00	0.00
ATOM	14003	HE2	MET	A	908	80.736	177.547	121.244	1.00	0.00
ATOM	14004	HE3	MET	A	908	79.628	177.672	122.627	1.00	0.00
ATOM	14005	C	MET	A	908	79.068	181.748	118.142	1.00	0.00
ATOM	14006	O	MET	A	908	79.027	182.697	117.356	1.00	0.00
ATOM	14007	N	THR	A	909	78.219	180.727	118.033	1.00	0.00
ATOM	14008	H	THR	A	909	78.328	179.959	118.678	1.00	0.00
ATOM	14009	CA	THR	A	909	77.317	180.520	116.893	1.00	0.00
ATOM	14010	HA	THR	A	909	77.855	180.782	115.987	1.00	0.00
ATOM	14011	CB	THR	A	909	76.950	179.028	116.803	1.00	0.00
ATOM	14012	HB	THR	A	909	76.375	178.743	117.684	1.00	0.00
ATOM	14013	CG2	THR	A	909	76.165	178.656	115.547	1.00	0.00
ATOM	14014	1HG2	THR	A	909	76.014	177.578	115.512	1.00	0.00
ATOM	14015	2HG2	THR	A	909	75.189	179.137	115.558	1.00	0.00
ATOM	14016	3HG2	THR	A	909	76.715	178.963	114.660	1.00	0.00
ATOM	14017	OG1	THR	A	909	78.132	178.266	116.776	1.00	0.00
ATOM	14018	HG1	THR	A	909	77.898	177.344	116.825	1.00	0.00
ATOM	14019	C	THR	A	909	76.022	181.344	116.959	1.00	0.00
ATOM	14020	O	THR	A	909	75.197	181.154	117.852	1.00	0.00
ATOM	14021	N	PHE	A	910	75.792	182.172	115.934	1.00	0.00
ATOM	14022	H	PHE	A	910	76.569	182.343	115.306	1.00	0.00
ATOM	14023	CA	PHE	A	910	74.620	183.039	115.733	1.00	0.00
ATOM	14024	HA	PHE	A	910	73.922	182.882	116.555	1.00	0.00
ATOM	14025	CB	PHE	A	910	75.085	184.510	115.793	1.00	0.00
ATOM	14026	HB1	PHE	A	910	75.576	184.770	114.853	1.00	0.00
ATOM	14027	HB2	PHE	A	910	75.842	184.608	116.571	1.00	0.00
ATOM	14028	CG	PHE	A	910	73.984	185.515	116.090	1.00	0.00

ATOM	14029	CD1	PHE	A	910	73.625	185.795	117.423	1.00	0.00
ATOM	14030	HD1	PHE	A	910	74.131	185.298	118.233	1.00	0.00
ATOM	14031	CE1	PHE	A	910	72.622	186.736	117.712	1.00	0.00
ATOM	14032	HE1	PHE	A	910	72.360	186.950	118.739	1.00	0.00
ATOM	14033	CZ	PHE	A	910	71.965	187.403	116.664	1.00	0.00
ATOM	14034	HZ	PHE	A	910	71.197	188.129	116.885	1.00	0.00
ATOM	14035	CE2	PHE	A	910	72.318	187.131	115.331	1.00	0.00
ATOM	14036	HE2	PHE	A	910	71.819	187.643	114.523	1.00	0.00
ATOM	14037	CD2	PHE	A	910	73.328	186.194	115.045	1.00	0.00
ATOM	14038	HD2	PHE	A	910	73.602	185.997	114.021	1.00	0.00
ATOM	14039	C	PHE	A	910	73.882	182.677	114.418	1.00	0.00
ATOM	14040	O	PHE	A	910	73.630	183.511	113.557	1.00	0.00
ATOM	14041	N	GLY	A	911	73.671	181.374	114.186	1.00	0.00
ATOM	14042	H	GLY	A	911	73.823	180.727	114.943	1.00	0.00
ATOM	14043	CA	GLY	A	911	73.512	180.822	112.827	1.00	0.00
ATOM	14044	HA1	GLY	A	911	72.845	181.459	112.244	1.00	0.00
ATOM	14045	HA2	GLY	A	911	73.070	179.828	112.892	1.00	0.00
ATOM	14046	C	GLY	A	911	74.845	180.701	112.067	1.00	0.00
ATOM	14047	O	GLY	A	911	74.866	180.377	110.883	1.00	0.00
ATOM	14048	N	SER	A	912	75.960	180.985	112.748	1.00	0.00
ATOM	14049	H	SER	A	912	75.866	181.305	113.695	1.00	0.00
ATOM	14050	CA	SER	A	912	77.307	181.032	112.184	1.00	0.00
ATOM	14051	HA	SER	A	912	77.322	181.804	111.414	1.00	0.00
ATOM	14052	CB	SER	A	912	78.328	181.424	113.262	1.00	0.00
ATOM	14053	HB1	SER	A	912	79.269	181.663	112.775	1.00	0.00
ATOM	14054	HB2	SER	A	912	78.497	180.578	113.927	1.00	0.00
ATOM	14055	OG	SER	A	912	77.896	182.540	114.017	1.00	0.00
ATOM	14056	HG	SER	A	912	78.679	182.999	114.356	1.00	0.00
ATOM	14057	C	SER	A	912	77.770	179.712	111.563	1.00	0.00
ATOM	14058	O	SER	A	912	77.392	178.628	112.000	1.00	0.00
ATOM	14059	N	LYS	A	913	78.742	179.820	110.648	1.00	0.00
ATOM	14060	H	LYS	A	913	78.992	180.749	110.336	1.00	0.00
ATOM	14061	CA	LYS	A	913	79.601	178.708	110.219	1.00	0.00
ATOM	14062	HA	LYS	A	913	79.538	177.930	110.978	1.00	0.00
ATOM	14063	CB	LYS	A	913	79.075	178.052	108.925	1.00	0.00
ATOM	14064	HB1	LYS	A	913	78.121	177.579	109.164	1.00	0.00
ATOM	14065	HB2	LYS	A	913	79.747	177.242	108.648	1.00	0.00
ATOM	14066	CG	LYS	A	913	78.841	178.981	107.715	1.00	0.00
ATOM	14067	HG1	LYS	A	913	79.109	180.010	107.955	1.00	0.00
ATOM	14068	HG2	LYS	A	913	77.770	178.981	107.507	1.00	0.00
ATOM	14069	CD	LYS	A	913	79.565	178.561	106.423	1.00	0.00
ATOM	14070	HD1	LYS	A	913	79.077	179.079	105.597	1.00	0.00
ATOM	14071	HD2	LYS	A	913	79.457	177.489	106.250	1.00	0.00
ATOM	14072	CE	LYS	A	913	81.048	178.967	106.393	1.00	0.00
ATOM	14073	HE1	LYS	A	913	81.143	179.943	106.875	1.00	0.00
ATOM	14074	HE2	LYS	A	913	81.362	179.073	105.353	1.00	0.00
ATOM	14075	NZ	LYS	A	913	81.932	177.996	107.072	1.00	0.00
ATOM	14076	HZ1	LYS	A	913	82.081	177.112	106.579	1.00	0.00
ATOM	14077	HZ2	LYS	A	913	82.842	178.383	107.300	1.00	0.00
ATOM	14078	HZ3	LYS	A	913	81.575	177.667	107.965	1.00	0.00
ATOM	14079	C	LYS	A	913	81.060	179.173	110.118	1.00	0.00
ATOM	14080	O	LYS	A	913	81.326	180.074	109.321	1.00	0.00
ATOM	14081	N	PRO	A	914	81.987	178.662	110.947	1.00	0.00
ATOM	14082	CD	PRO	A	914	81.765	177.622	111.948	1.00	0.00
ATOM	14083	HD1	PRO	A	914	81.456	176.685	111.481	1.00	0.00
ATOM	14084	HD2	PRO	A	914	81.010	177.955	112.662	1.00	0.00
ATOM	14085	CG	PRO	A	914	83.091	177.430	112.680	1.00	0.00
ATOM	14086	HG1	PRO	A	914	83.686	176.658	112.190	1.00	0.00
ATOM	14087	HG2	PRO	A	914	82.934	177.182	113.728	1.00	0.00
ATOM	14088	CB	PRO	A	914	83.745	178.798	112.504	1.00	0.00
ATOM	14089	HB1	PRO	A	914	84.819	178.780	112.697	1.00	0.00

ATOM	14090	HB2	PRO	A	914	83.279	179.497	113.199	1.00	0.00
ATOM	14091	CA	PRO	A	914	83.352	179.173	111.060	1.00	0.00
ATOM	14092	HA	PRO	A	914	83.377	180.256	110.960	1.00	0.00
ATOM	14093	C	PRO	A	914	84.271	178.626	109.951	1.00	0.00
ATOM	14094	O	PRO	A	914	83.992	178.755	108.757	1.00	0.00
ATOM	14095	N	TYR	A	915	85.409	178.061	110.339	1.00	0.00
ATOM	14096	H	TYR	A	915	85.578	177.980	111.332	1.00	0.00
ATOM	14097	CA	TYR	A	915	86.484	177.579	109.481	1.00	0.00
ATOM	14098	HA	TYR	A	915	86.508	178.158	108.559	1.00	0.00
ATOM	14099	CB	TYR	A	915	87.787	177.816	110.282	1.00	0.00
ATOM	14100	HB1	TYR	A	915	87.972	178.890	110.288	1.00	0.00
ATOM	14101	HB2	TYR	A	915	88.631	177.358	109.769	1.00	0.00
ATOM	14102	CG	TYR	A	915	87.821	177.320	111.723	1.00	0.00
ATOM	14103	CD1	TYR	A	915	88.078	178.236	112.764	1.00	0.00
ATOM	14104	HD1	TYR	A	915	88.219	179.287	112.559	1.00	0.00
ATOM	14105	CE1	TYR	A	915	88.197	177.776	114.088	1.00	0.00
ATOM	14106	HE1	TYR	A	915	88.410	178.456	114.894	1.00	0.00
ATOM	14107	CZ	TYR	A	915	88.061	176.403	114.386	1.00	0.00
ATOM	14108	OH	TYR	A	915	88.235	175.980	115.660	1.00	0.00
ATOM	14109	HH	TYR	A	915	88.156	175.003	115.768	1.00	0.00
ATOM	14110	CE2	TYR	A	915	87.784	175.492	113.353	1.00	0.00
ATOM	14111	HE2	TYR	A	915	87.698	174.443	113.587	1.00	0.00
ATOM	14112	CD2	TYR	A	915	87.662	175.953	112.028	1.00	0.00
ATOM	14113	HD2	TYR	A	915	87.486	175.233	111.245	1.00	0.00
ATOM	14114	C	TYR	A	915	86.347	176.098	109.090	1.00	0.00
ATOM	14115	O	TYR	A	915	87.332	175.479	108.710	1.00	0.00
ATOM	14116	N	ASP	A	916	85.152	175.520	109.213	1.00	0.00
ATOM	14117	H	ASP	A	916	84.385	176.097	109.528	1.00	0.00
ATOM	14118	CA	ASP	A	916	84.836	174.128	108.844	1.00	0.00
ATOM	14119	HA	ASP	A	916	85.196	173.469	109.634	1.00	0.00
ATOM	14120	CB	ASP	A	916	83.300	173.986	108.785	1.00	0.00
ATOM	14121	HB1	ASP	A	916	82.930	173.865	109.804	1.00	0.00
ATOM	14122	HB2	ASP	A	916	83.035	173.087	108.225	1.00	0.00
ATOM	14123	CG	ASP	A	916	82.609	175.209	108.162	1.00	0.00
ATOM	14124	OD1	ASP	A	916	82.086	176.059	108.922	1.00	0.00
ATOM	14125	OD2	ASP	A	916	82.707	175.422	106.931	1.00	0.00
ATOM	14126	C	ASP	A	916	85.515	173.649	107.540	1.00	0.00
ATOM	14127	O	ASP	A	916	86.184	172.617	107.521	1.00	0.00
ATOM	14128	N	GLY	A	917	85.422	174.451	106.477	1.00	0.00
ATOM	14129	H	GLY	A	917	84.757	175.215	106.542	1.00	0.00
ATOM	14130	CA	GLY	A	917	86.099	174.235	105.198	1.00	0.00
ATOM	14131	HA1	GLY	A	917	85.512	174.702	104.407	1.00	0.00
ATOM	14132	HA2	GLY	A	917	86.139	173.166	104.992	1.00	0.00
ATOM	14133	C	GLY	A	917	87.531	174.788	105.132	1.00	0.00
ATOM	14134	O	GLY	A	917	87.780	175.672	104.314	1.00	0.00
ATOM	14135	N	ILE	A	918	88.442	174.300	105.990	1.00	0.00
ATOM	14136	H	ILE	A	918	88.074	173.666	106.692	1.00	0.00
ATOM	14137	CA	ILE	A	918	89.920	174.482	105.963	1.00	0.00
ATOM	14138	HA	ILE	A	918	90.238	174.572	107.001	1.00	0.00
ATOM	14139	CB	ILE	A	918	90.615	173.213	105.395	1.00	0.00
ATOM	14140	HB	ILE	A	918	90.393	173.152	104.328	1.00	0.00
ATOM	14141	CG2	ILE	A	918	92.153	173.265	105.547	1.00	0.00
ATOM	14142	1HG2	ILE	A	918	92.593	172.345	105.160	1.00	0.00
ATOM	14143	2HG2	ILE	A	918	92.570	174.096	104.978	1.00	0.00
ATOM	14144	3HG2	ILE	A	918	92.431	173.373	106.593	1.00	0.00
ATOM	14145	CG1	ILE	A	918	90.109	171.892	106.027	1.00	0.00
ATOM	14146	1HG1	ILE	A	918	90.634	171.055	105.566	1.00	0.00
ATOM	14147	2HG1	ILE	A	918	89.052	171.760	105.789	1.00	0.00
ATOM	14148	CD	ILE	A	918	90.279	171.781	107.549	1.00	0.00
ATOM	14149	HD1	ILE	A	918	91.332	171.784	107.821	1.00	0.00
ATOM	14150	HD2	ILE	A	918	89.771	172.606	108.043	1.00	0.00

ATOM	14151	HD3	ILE	A	918	89.839	170.844	107.892	1.00	0.00
ATOM	14152	C	ILE	A	918	90.399	175.786	105.256	1.00	0.00
ATOM	14153	O	ILE	A	918	90.868	175.743	104.115	1.00	0.00
ATOM	14154	N	PRO	A	919	90.212	176.974	105.864	1.00	0.00
ATOM	14155	CD	PRO	A	919	89.723	177.183	107.219	1.00	0.00
ATOM	14156	HD1	PRO	A	919	90.471	176.944	107.971	1.00	0.00
ATOM	14157	HD2	PRO	A	919	88.838	176.578	107.362	1.00	0.00
ATOM	14158	CG	PRO	A	919	89.324	178.656	107.289	1.00	0.00
ATOM	14159	HG1	PRO	A	919	90.200	179.281	107.470	1.00	0.00
ATOM	14160	HG2	PRO	A	919	88.556	178.863	108.022	1.00	0.00
ATOM	14161	CB	PRO	A	919	88.791	178.861	105.881	1.00	0.00
ATOM	14162	HB1	PRO	A	919	88.681	179.919	105.639	1.00	0.00
ATOM	14163	HB2	PRO	A	919	87.835	178.346	105.764	1.00	0.00
ATOM	14164	CA	PRO	A	919	89.875	178.148	105.068	1.00	0.00
ATOM	14165	HA	PRO	A	919	89.439	177.837	104.116	1.00	0.00
ATOM	14166	C	PRO	A	919	90.986	179.148	104.775	1.00	0.00
ATOM	14167	O	PRO	A	919	92.016	179.195	105.438	1.00	0.00
ATOM	14168	N	ALA	A	920	90.653	180.056	103.847	1.00	0.00
ATOM	14169	H	ALA	A	920	89.786	179.901	103.357	1.00	0.00
ATOM	14170	CA	ALA	A	920	91.259	181.375	103.674	1.00	0.00
ATOM	14171	HA	ALA	A	920	90.959	181.746	102.693	1.00	0.00
ATOM	14172	CB	ALA	A	920	90.645	182.316	104.725	1.00	0.00
ATOM	14173	HB1	ALA	A	920	91.022	183.328	104.579	1.00	0.00
ATOM	14174	HB2	ALA	A	920	89.560	182.327	104.628	1.00	0.00
ATOM	14175	HB3	ALA	A	920	90.914	181.979	105.726	1.00	0.00
ATOM	14176	C	ALA	A	920	92.803	181.383	103.676	1.00	0.00
ATOM	14177	O	ALA	A	920	93.422	182.317	104.174	1.00	0.00
ATOM	14178	N	SER	A	921	93.421	180.335	103.116	1.00	0.00
ATOM	14179	H	SER	A	921	92.836	179.599	102.750	1.00	0.00
ATOM	14180	CA	SER	A	921	94.869	180.087	103.127	1.00	0.00
ATOM	14181	HA	SER	A	921	95.002	179.116	102.651	1.00	0.00
ATOM	14182	CB	SER	A	921	95.611	181.086	102.211	1.00	0.00
ATOM	14183	HB1	SER	A	921	95.104	181.113	101.245	1.00	0.00
ATOM	14184	HB2	SER	A	921	96.627	180.731	102.039	1.00	0.00
ATOM	14185	OG	SER	A	921	95.652	182.394	102.741	1.00	0.00
ATOM	14186	HG	SER	A	921	94.867	182.499	103.312	1.00	0.00
ATOM	14187	C	SER	A	921	95.500	179.909	104.520	1.00	0.00
ATOM	14188	O	SER	A	921	96.601	180.388	104.763	1.00	0.00
ATOM	14189	N	GLU	A	922	94.811	179.216	105.432	1.00	0.00
ATOM	14190	H	GLU	A	922	93.894	178.885	105.156	1.00	0.00
ATOM	14191	CA	GLU	A	922	95.103	179.164	106.877	1.00	0.00
ATOM	14192	HA	GLU	A	922	94.280	178.621	107.321	1.00	0.00
ATOM	14193	CB	GLU	A	922	96.375	178.330	107.212	1.00	0.00
ATOM	14194	HB1	GLU	A	922	97.263	178.820	106.814	1.00	0.00
ATOM	14195	HB2	GLU	A	922	96.267	177.368	106.709	1.00	0.00
ATOM	14196	CG	GLU	A	922	96.573	178.074	108.728	1.00	0.00
ATOM	14197	HG1	GLU	A	922	95.590	178.017	109.187	1.00	0.00
ATOM	14198	HG2	GLU	A	922	97.080	178.936	109.165	1.00	0.00
ATOM	14199	CD	GLU	A	922	97.351	176.795	109.117	1.00	0.00
ATOM	14200	OE1	GLU	A	922	97.319	176.419	110.315	1.00	0.00
ATOM	14201	OE2	GLU	A	922	98.003	176.149	108.260	1.00	0.00
ATOM	14202	C	GLU	A	922	94.976	180.556	107.516	1.00	0.00
ATOM	14203	O	GLU	A	922	95.891	181.087	108.131	1.00	0.00
ATOM	14204	N	ILE	A	923	93.806	181.183	107.300	1.00	0.00
ATOM	14205	H	ILE	A	923	93.132	180.671	106.743	1.00	0.00
ATOM	14206	CA	ILE	A	923	93.409	182.550	107.731	1.00	0.00
ATOM	14207	HA	ILE	A	923	92.352	182.648	107.499	1.00	0.00
ATOM	14208	CB	ILE	A	923	93.559	182.729	109.269	1.00	0.00
ATOM	14209	HB	ILE	A	923	94.619	182.733	109.524	1.00	0.00
ATOM	14210	CG2	ILE	A	923	93.002	184.084	109.729	1.00	0.00
ATOM	14211	1HG2	ILE	A	923	92.995	184.137	110.819	1.00	0.00

```
ATOM  14212 2HG2 ILE A 923      93.645 184.885 109.361  1.00  0.00
ATOM  14213 3HG2 ILE A 923      91.995 184.219 109.344  1.00  0.00
ATOM  14214  CG1 ILE A 923      92.896 181.600 110.099  1.00  0.00
ATOM  14215 1HG1 ILE A 923      93.046 181.810 111.159  1.00  0.00
ATOM  14216 2HG1 ILE A 923      93.406 180.659 109.896  1.00  0.00
ATOM  14217  CD  ILE A 923      91.391 181.392 109.861  1.00  0.00
ATOM  14218  HD1 ILE A 923      91.038 180.584 110.501  1.00  0.00
ATOM  14219  HD2 ILE A 923      90.838 182.297 110.108  1.00  0.00
ATOM  14220  HD3 ILE A 923      91.206 181.124 108.822  1.00  0.00
ATOM  14221  C   ILE A 923      94.095 183.683 106.936  1.00  0.00
ATOM  14222  O   ILE A 923      93.498 184.737 106.697  1.00  0.00
ATOM  14223  N   SER A 924      95.318 183.400 106.502  1.00  0.00
ATOM  14224  H   SER A 924      95.677 182.522 106.867  1.00  0.00
ATOM  14225  CA  SER A 924      96.316 184.170 105.749  1.00  0.00
ATOM  14226  HA  SER A 924      96.144 184.041 104.683  1.00  0.00
ATOM  14227  CB  SER A 924      96.325 185.670 106.084  1.00  0.00
ATOM  14228  HB1 SER A 924      95.415 186.140 105.708  1.00  0.00
ATOM  14229  HB2 SER A 924      97.181 186.140 105.597  1.00  0.00
ATOM  14230  OG  SER A 924      96.416 185.877 107.478  1.00  0.00
ATOM  14231  HG  SER A 924      96.955 185.138 107.809  1.00  0.00
ATOM  14232  C   SER A 924      97.637 183.497 106.137  1.00  0.00
ATOM  14233  O   SER A 924      97.940 183.473 107.327  1.00  0.00
ATOM  14234  N   SER A 925      98.341 182.861 105.196  1.00  0.00
ATOM  14235  H   SER A 925      97.988 182.893 104.249  1.00  0.00
ATOM  14236  CA  SER A 925      99.263 181.739 105.496  1.00  0.00
ATOM  14237  HA  SER A 925      98.672 180.946 105.957  1.00  0.00
ATOM  14238  CB  SER A 925      99.849 181.169 104.201  1.00  0.00
ATOM  14239  HB1 SER A 925     100.378 180.243 104.427  1.00  0.00
ATOM  14240  HB2 SER A 925     100.547 181.885 103.766  1.00  0.00
ATOM  14241  OG  SER A 925      98.812 180.907 103.282  1.00  0.00
ATOM  14242  HG  SER A 925      98.030 180.631 103.802  1.00  0.00
ATOM  14243  C   SER A 925     100.405 182.002 106.484  1.00  0.00
ATOM  14244  O   SER A 925     100.840 181.063 107.141  1.00  0.00
ATOM  14245  N   ILE A 926     100.831 183.258 106.650  1.00  0.00
ATOM  14246  H   ILE A 926     100.467 183.930 105.988  1.00  0.00
ATOM  14247  CA  ILE A 926     100.955 183.828 108.001  1.00  0.00
ATOM  14248  HA  ILE A 926     100.383 183.175 108.663  1.00  0.00
ATOM  14249  CB  ILE A 926     102.389 183.873 108.590  1.00  0.00
ATOM  14250  HB  ILE A 926     102.279 184.067 109.657  1.00  0.00
ATOM  14251  CG2 ILE A 926     103.117 182.522 108.481  1.00  0.00
ATOM  14252 1HG2 ILE A 926     104.031 182.523 109.073  1.00  0.00
ATOM  14253 2HG2 ILE A 926     102.478 181.739 108.881  1.00  0.00
ATOM  14254 3HG2 ILE A 926     103.353 182.299 107.441  1.00  0.00
ATOM  14255  CG1 ILE A 926     103.213 185.034 108.020  1.00  0.00
ATOM  14256 1HG1 ILE A 926     102.706 185.959 108.283  1.00  0.00
ATOM  14257 2HG1 ILE A 926     103.237 184.967 106.934  1.00  0.00
ATOM  14258  CD  ILE A 926     104.644 185.132 108.573  1.00  0.00
ATOM  14259  HD1 ILE A 926     105.142 185.986 108.121  1.00  0.00
ATOM  14260  HD2 ILE A 926     104.614 185.262 109.655  1.00  0.00
ATOM  14261  HD3 ILE A 926     105.223 184.243 108.331  1.00  0.00
ATOM  14262  C   ILE A 926     100.220 185.181 108.027  1.00  0.00
ATOM  14263  O   ILE A 926      99.803 185.693 106.988  1.00  0.00
ATOM  14264  N   LEU A 927     100.002 185.708 109.233  1.00  0.00
ATOM  14265  H   LEU A 927     100.400 185.220 110.019  1.00  0.00
ATOM  14266  CA  LEU A 927      99.019 186.752 109.535  1.00  0.00
ATOM  14267  HA  LEU A 927      98.086 186.481 109.058  1.00  0.00
ATOM  14268  CB  LEU A 927      98.752 186.792 111.059  1.00  0.00
ATOM  14269  HB1 LEU A 927      98.105 187.648 111.254  1.00  0.00
ATOM  14270  HB2 LEU A 927      99.690 186.982 111.582  1.00  0.00
ATOM  14271  CG  LEU A 927      98.060 185.570 111.701  1.00  0.00
ATOM  14272  HG  LEU A 927      97.805 185.856 112.721  1.00  0.00
```

```
ATOM  14273  CD1 LEU A 927      96.760 185.187 110.987  1.00  0.00
ATOM  14274 1HD1 LEU A 927      96.250 184.413 111.562  1.00  0.00
ATOM  14275 2HD1 LEU A 927      96.113 186.059 110.900  1.00  0.00
ATOM  14276 3HD1 LEU A 927      96.979 184.785 109.998  1.00  0.00
ATOM  14277  CD2 LEU A 927      98.945 184.321 111.790  1.00  0.00
ATOM  14278 1HD2 LEU A 927      98.459 183.581 112.422  1.00  0.00
ATOM  14279 2HD2 LEU A 927      99.090 183.868 110.811  1.00  0.00
ATOM  14280 3HD2 LEU A 927      99.906 184.574 112.238  1.00  0.00
ATOM  14281  C   LEU A 927      99.372 188.141 108.967  1.00  0.00
ATOM  14282  O   LEU A 927      99.742 189.062 109.697  1.00  0.00
ATOM  14283  N   GLU A 928      99.189 188.300 107.659  1.00  0.00
ATOM  14284  H   GLU A 928      99.022 187.441 107.143  1.00  0.00
ATOM  14285  CA  GLU A 928      99.278 189.524 106.844  1.00  0.00
ATOM  14286  HA  GLU A 928     100.245 189.975 107.046  1.00  0.00
ATOM  14287  CB  GLU A 928      99.237 189.061 105.365  1.00  0.00
ATOM  14288  HB1 GLU A 928      98.228 188.706 105.148  1.00  0.00
ATOM  14289  HB2 GLU A 928      99.904 188.204 105.247  1.00  0.00
ATOM  14290  CG  GLU A 928      99.629 190.089 104.287  1.00  0.00
ATOM  14291  HG1 GLU A 928      99.067 191.013 104.416  1.00  0.00
ATOM  14292  HG2 GLU A 928      99.350 189.676 103.315  1.00  0.00
ATOM  14293  CD  GLU A 928     101.128 190.404 104.281  1.00  0.00
ATOM  14294  OE1 GLU A 928     101.765 190.295 103.206  1.00  0.00
ATOM  14295  OE2 GLU A 928     101.637 190.796 105.352  1.00  0.00
ATOM  14296  C   GLU A 928      98.165 190.551 107.187  1.00  0.00
ATOM  14297  O   GLU A 928      97.452 191.084 106.337  1.00  0.00
ATOM  14298  N   LYS A 929      97.951 190.776 108.485  1.00  0.00
ATOM  14299  H   LYS A 929      98.596 190.281 109.088  1.00  0.00
ATOM  14300  CA  LYS A 929      96.885 191.579 109.116  1.00  0.00
ATOM  14301  HA  LYS A 929      96.552 192.369 108.440  1.00  0.00
ATOM  14302  CB  LYS A 929      95.717 190.643 109.485  1.00  0.00
ATOM  14303  HB1 LYS A 929      95.053 191.168 110.174  1.00  0.00
ATOM  14304  HB2 LYS A 929      96.119 189.773 110.011  1.00  0.00
ATOM  14305  CG  LYS A 929      94.861 190.163 108.303  1.00  0.00
ATOM  14306  HG1 LYS A 929      95.494 189.793 107.499  1.00  0.00
ATOM  14307  HG2 LYS A 929      94.275 191.005 107.934  1.00  0.00
ATOM  14308  CD  LYS A 929      93.937 189.020 108.748  1.00  0.00
ATOM  14309  HD1 LYS A 929      93.484 189.248 109.716  1.00  0.00
ATOM  14310  HD2 LYS A 929      94.542 188.115 108.855  1.00  0.00
ATOM  14311  CE  LYS A 929      92.834 188.748 107.719  1.00  0.00
ATOM  14312  HE1 LYS A 929      92.449 187.739 107.887  1.00  0.00
ATOM  14313  HE2 LYS A 929      93.272 188.768 106.716  1.00  0.00
ATOM  14314  NZ  LYS A 929      91.728 189.734 107.840  1.00  0.00
ATOM  14315  HZ1 LYS A 929      92.067 190.670 107.659  1.00  0.00
ATOM  14316  HZ2 LYS A 929      90.979 189.510 107.201  1.00  0.00
ATOM  14317  HZ3 LYS A 929      91.326 189.731 108.782  1.00  0.00
ATOM  14318  C   LYS A 929      97.353 192.243 110.419  1.00  0.00
ATOM  14319  O   LYS A 929      96.836 193.290 110.793  1.00  0.00
ATOM  14320  N   GLY A 930      98.280 191.591 111.120  1.00  0.00
ATOM  14321  H   GLY A 930      98.634 190.727 110.738  1.00  0.00
ATOM  14322  CA  GLY A 930      98.678 191.894 112.488  1.00  0.00
ATOM  14323  HA1 GLY A 930      97.964 192.563 112.971  1.00  0.00
ATOM  14324  HA2 GLY A 930      99.655 192.375 112.481  1.00  0.00
ATOM  14325  C   GLY A 930      98.758 190.600 113.303  1.00  0.00
ATOM  14326  O   GLY A 930      97.940 189.697 113.118  1.00  0.00
ATOM  14327  N   GLU A 931      99.769 190.490 114.158  1.00  0.00
ATOM  14328  H   GLU A 931     100.418 191.257 114.223  1.00  0.00
ATOM  14329  CA  GLU A 931     100.121 189.231 114.825  1.00  0.00
ATOM  14330  HA  GLU A 931     100.031 188.409 114.110  1.00  0.00
ATOM  14331  CB  GLU A 931     101.585 189.277 115.299  1.00  0.00
ATOM  14332  HB1 GLU A 931     102.233 189.171 114.428  1.00  0.00
ATOM  14333  HB2 GLU A 931     101.768 188.425 115.956  1.00  0.00
```

ATOM	14334	CG	GLU	A	931	101.971	190.576	116.030	1.00	0.00
ATOM	14335	HG1	GLU	A	931	101.208	190.829	116.765	1.00	0.00
ATOM	14336	HG2	GLU	A	931	102.020	191.394	115.308	1.00	0.00
ATOM	14337	CD	GLU	A	931	103.319	190.447	116.737	1.00	0.00
ATOM	14338	OE1	GLU	A	931	103.352	190.651	117.970	1.00	0.00
ATOM	14339	OE2	GLU	A	931	104.327	190.181	116.046	1.00	0.00
ATOM	14340	C	GLU	A	931	99.201	188.867	115.993	1.00	0.00
ATOM	14341	O	GLU	A	931	98.777	187.726	116.079	1.00	0.00
ATOM	14342	N	ARG	A	932	98.830	189.828	116.848	1.00	0.00
ATOM	14343	H	ARG	A	932	99.198	190.759	116.708	1.00	0.00
ATOM	14344	CA	ARG	A	932	97.852	189.629	117.926	1.00	0.00
ATOM	14345	HA	ARG	A	932	97.505	188.593	117.894	1.00	0.00
ATOM	14346	CB	ARG	A	932	98.461	189.846	119.309	1.00	0.00
ATOM	14347	HB1	ARG	A	932	97.668	189.787	120.058	1.00	0.00
ATOM	14348	HB2	ARG	A	932	98.895	190.846	119.359	1.00	0.00
ATOM	14349	CG	ARG	A	932	99.511	188.756	119.638	1.00	0.00
ATOM	14350	HG1	ARG	A	932	99.543	187.998	118.856	1.00	0.00
ATOM	14351	HG2	ARG	A	932	99.226	188.258	120.565	1.00	0.00
ATOM	14352	CD	ARG	A	932	100.905	189.355	119.809	1.00	0.00
ATOM	14353	HD1	ARG	A	932	101.183	189.876	118.894	1.00	0.00
ATOM	14354	HD2	ARG	A	932	101.623	188.555	119.995	1.00	0.00
ATOM	14355	NE	ARG	A	932	100.888	190.282	120.944	1.00	0.00
ATOM	14356	HE	ARG	A	932	100.191	190.088	121.665	1.00	0.00
ATOM	14357	CZ	ARG	A	932	101.628	191.343	121.154	1.00	0.00
ATOM	14358	NH1	ARG	A	932	102.589	191.707	120.355	1.00	0.00
ATOM	14359	1HH1	ARG	A	932	102.782	191.152	119.508	1.00	0.00
ATOM	14360	2HH1	ARG	A	932	103.100	192.540	120.536	1.00	0.00
ATOM	14361	NH2	ARG	A	932	101.384	192.090	122.191	1.00	0.00
ATOM	14362	1HH2	ARG	A	932	100.617	191.835	122.820	1.00	0.00
ATOM	14363	2HH2	ARG	A	932	102.118	192.680	122.550	1.00	0.00
ATOM	14364	C	ARG	A	932	96.648	190.506	117.619	1.00	0.00
ATOM	14365	O	ARG	A	932	96.782	191.703	117.393	1.00	0.00
ATOM	14366	N	LEU	A	933	95.498	189.850	117.453	1.00	0.00
ATOM	14367	H	LEU	A	933	95.489	188.887	117.754	1.00	0.00
ATOM	14368	CA	LEU	A	933	94.609	190.157	116.330	1.00	0.00
ATOM	14369	HA	LEU	A	933	94.909	191.117	115.908	1.00	0.00
ATOM	14370	CB	LEU	A	933	94.879	189.068	115.258	1.00	0.00
ATOM	14371	HB1	LEU	A	933	94.582	188.100	115.667	1.00	0.00
ATOM	14372	HB2	LEU	A	933	95.956	189.030	115.083	1.00	0.00
ATOM	14373	CG	LEU	A	933	94.195	189.224	113.882	1.00	0.00
ATOM	14374	HG	LEU	A	933	93.121	189.080	113.974	1.00	0.00
ATOM	14375	CD1	LEU	A	933	94.438	190.603	113.258	1.00	0.00
ATOM	14376	1HD1	LEU	A	933	93.978	190.634	112.274	1.00	0.00
ATOM	14377	2HD1	LEU	A	933	93.982	191.375	113.875	1.00	0.00
ATOM	14378	3HD1	LEU	A	933	95.508	190.785	113.166	1.00	0.00
ATOM	14379	CD2	LEU	A	933	94.730	188.153	112.926	1.00	0.00
ATOM	14380	1HD2	LEU	A	933	94.227	188.233	111.963	1.00	0.00
ATOM	14381	2HD2	LEU	A	933	95.805	188.272	112.784	1.00	0.00
ATOM	14382	3HD2	LEU	A	933	94.527	187.166	113.342	1.00	0.00
ATOM	14383	C	LEU	A	933	93.122	190.298	116.733	1.00	0.00
ATOM	14384	O	LEU	A	933	92.264	189.553	116.251	1.00	0.00
ATOM	14385	N	PRO	A	934	92.779	191.271	117.607	1.00	0.00
ATOM	14386	CD	PRO	A	934	93.686	192.184	118.295	1.00	0.00
ATOM	14387	HD1	PRO	A	934	94.350	192.692	117.596	1.00	0.00
ATOM	14388	HD2	PRO	A	934	94.264	191.634	119.038	1.00	0.00
ATOM	14389	CG	PRO	A	934	92.812	193.214	119.015	1.00	0.00
ATOM	14390	HG1	PRO	A	934	92.656	194.077	118.366	1.00	0.00
ATOM	14391	HG2	PRO	A	934	93.249	193.519	119.966	1.00	0.00
ATOM	14392	CB	PRO	A	934	91.497	192.464	119.209	1.00	0.00
ATOM	14393	HB1	PRO	A	934	90.653	193.141	119.346	1.00	0.00
ATOM	14394	HB2	PRO	A	934	91.584	191.797	120.068	1.00	0.00

ATOM	14395	CA	PRO	A	934	91.399	191.629	117.926	1.00	0.00
ATOM	14396	HA	PRO	A	934	90.820	190.730	118.138	1.00	0.00
ATOM	14397	C	PRO	A	934	90.709	192.362	116.754	1.00	0.00
ATOM	14398	O	PRO	A	934	90.380	193.546	116.841	1.00	0.00
ATOM	14399	N	GLN	A	935	90.545	191.682	115.619	1.00	0.00
ATOM	14400	H	GLN	A	935	90.818	190.705	115.623	1.00	0.00
ATOM	14401	CA	GLN	A	935	89.960	192.246	114.402	1.00	0.00
ATOM	14402	HA	GLN	A	935	90.493	193.172	114.194	1.00	0.00
ATOM	14403	CB	GLN	A	935	90.237	191.286	113.228	1.00	0.00
ATOM	14404	HB1	GLN	A	935	89.680	190.368	113.404	1.00	0.00
ATOM	14405	HB2	GLN	A	935	91.297	191.027	113.220	1.00	0.00
ATOM	14406	CG	GLN	A	935	89.865	191.854	111.847	1.00	0.00
ATOM	14407	HG1	GLN	A	935	90.720	192.380	111.423	1.00	0.00
ATOM	14408	HG2	GLN	A	935	89.036	192.555	111.915	1.00	0.00
ATOM	14409	CD	GLN	A	935	89.436	190.735	110.906	1.00	0.00
ATOM	14410	OE1	GLN	A	935	90.234	190.187	110.149	1.00	0.00
ATOM	14411	NE2	GLN	A	935	88.183	190.347	110.972	1.00	0.00
ATOM	14412	1HE2	GLN	A	935	87.519	190.876	111.535	1.00	0.00
ATOM	14413	2HE2	GLN	A	935	87.872	189.506	110.497	1.00	0.00
ATOM	14414	C	GLN	A	935	88.448	192.528	114.546	1.00	0.00
ATOM	14415	O	GLN	A	935	87.711	191.647	114.999	1.00	0.00
ATOM	14416	N	PRO	A	936	87.950	193.696	114.094	1.00	0.00
ATOM	14417	CD	PRO	A	936	88.721	194.882	113.737	1.00	0.00
ATOM	14418	HD1	PRO	A	936	89.189	194.740	112.762	1.00	0.00
ATOM	14419	HD2	PRO	A	936	89.477	195.106	114.491	1.00	0.00
ATOM	14420	CG	PRO	A	936	87.717	196.029	113.656	1.00	0.00
ATOM	14421	HG1	PRO	A	936	88.024	196.786	112.934	1.00	0.00
ATOM	14422	HG2	PRO	A	936	87.583	196.470	114.645	1.00	0.00
ATOM	14423	CB	PRO	A	936	86.434	195.323	113.233	1.00	0.00
ATOM	14424	HB1	PRO	A	936	86.441	195.181	112.149	1.00	0.00
ATOM	14425	HB2	PRO	A	936	85.549	195.884	113.535	1.00	0.00
ATOM	14426	CA	PRO	A	936	86.518	193.962	113.940	1.00	0.00
ATOM	14427	HA	PRO	A	936	86.115	194.064	114.945	1.00	0.00
ATOM	14428	C	PRO	A	936	85.711	192.909	113.155	1.00	0.00
ATOM	14429	O	PRO	A	936	86.282	192.166	112.343	1.00	0.00
ATOM	14430	N	PRO	A	937	84.372	192.889	113.340	1.00	0.00
ATOM	14431	CD	PRO	A	937	83.636	193.591	114.384	1.00	0.00
ATOM	14432	HD1	PRO	A	937	83.569	194.651	114.131	1.00	0.00
ATOM	14433	HD2	PRO	A	937	84.101	193.459	115.361	1.00	0.00
ATOM	14434	CG	PRO	A	937	82.239	192.979	114.398	1.00	0.00
ATOM	14435	HG1	PRO	A	937	81.486	193.691	114.737	1.00	0.00
ATOM	14436	HG2	PRO	A	937	82.236	192.087	115.026	1.00	0.00
ATOM	14437	CB	PRO	A	937	82.032	192.585	112.939	1.00	0.00
ATOM	14438	HB1	PRO	A	937	81.689	193.458	112.381	1.00	0.00
ATOM	14439	HB2	PRO	A	937	81.317	191.768	112.838	1.00	0.00
ATOM	14440	CA	PRO	A	937	83.434	192.174	112.478	1.00	0.00
ATOM	14441	HA	PRO	A	937	83.572	191.112	112.635	1.00	0.00
ATOM	14442	C	PRO	A	937	83.624	192.466	110.989	1.00	0.00
ATOM	14443	O	PRO	A	937	83.856	193.607	110.606	1.00	0.00
ATOM	14444	N	ILE	A	938	83.421	191.445	110.151	1.00	0.00
ATOM	14445	H	ILE	A	938	83.185	190.544	110.535	1.00	0.00
ATOM	14446	CA	ILE	A	938	83.428	191.573	108.691	1.00	0.00
ATOM	14447	HA	ILE	A	938	83.531	192.631	108.446	1.00	0.00
ATOM	14448	CB	ILE	A	938	84.637	190.870	108.032	1.00	0.00
ATOM	14449	HB	ILE	A	938	84.502	190.957	106.952	1.00	0.00
ATOM	14450	CG2	ILE	A	938	85.918	191.640	108.395	1.00	0.00
ATOM	14451	1HG2	ILE	A	938	86.768	191.266	107.830	1.00	0.00
ATOM	14452	2HG2	ILE	A	938	85.798	192.698	108.157	1.00	0.00
ATOM	14453	3HG2	ILE	A	938	86.127	191.551	109.461	1.00	0.00
ATOM	14454	CG1	ILE	A	938	84.745	189.365	108.372	1.00	0.00
ATOM	14455	1HG1	ILE	A	938	83.794	188.879	108.155	1.00	0.00

```
ATOM  14456 2HG1 ILE A 938      84.952 189.243 109.436  1.00  0.00
ATOM  14457  CD  ILE A 938      85.827 188.640 107.561  1.00  0.00
ATOM  14458  HD1 ILE A 938      85.772 187.570 107.754  1.00  0.00
ATOM  14459  HD2 ILE A 938      85.672 188.809 106.497  1.00  0.00
ATOM  14460  HD3 ILE A 938      86.816 188.992 107.847  1.00  0.00
ATOM  14461  C   ILE A 938      82.091 191.136 108.107  1.00  0.00
ATOM  14462  O   ILE A 938      81.561 190.090 108.477  1.00  0.00
ATOM  14463  N   CYS A 939      81.507 191.979 107.243  1.00  0.00
ATOM  14464  H   CYS A 939      81.993 192.823 106.972  1.00  0.00
ATOM  14465  CA  CYS A 939      80.143 191.750 106.764  1.00  0.00
ATOM  14466  HA  CYS A 939      80.035 190.679 106.572  1.00  0.00
ATOM  14467  CB  CYS A 939      79.156 192.102 107.900  1.00  0.00
ATOM  14468  HB1 CYS A 939      79.312 191.407 108.727  1.00  0.00
ATOM  14469  HB2 CYS A 939      78.135 191.962 107.543  1.00  0.00
ATOM  14470  SG  CYS A 939      79.353 193.804 108.525  1.00  0.00
ATOM  14471  HG  CYS A 939      80.576 193.674 109.062  1.00  0.00
ATOM  14472  C   CYS A 939      79.745 192.422 105.437  1.00  0.00
ATOM  14473  O   CYS A 939      79.402 191.746 104.472  1.00  0.00
ATOM  14474  N   THR A 940      79.709 193.754 105.417  1.00  0.00
ATOM  14475  H   THR A 940      79.990 194.228 106.264  1.00  0.00
ATOM  14476  CA  THR A 940      79.061 194.557 104.360  1.00  0.00
ATOM  14477  HA  THR A 940      79.477 194.272 103.396  1.00  0.00
ATOM  14478  CB  THR A 940      77.541 194.268 104.345  1.00  0.00
ATOM  14479  HB  THR A 940      77.373 193.217 104.114  1.00  0.00
ATOM  14480  CG2 THR A 940      76.818 194.596 105.653  1.00  0.00
ATOM  14481 1HG2 THR A 940      76.940 195.648 105.910  1.00  0.00
ATOM  14482 2HG2 THR A 940      75.757 194.375 105.549  1.00  0.00
ATOM  14483 3HG2 THR A 940      77.213 193.986 106.461  1.00  0.00
ATOM  14484  OG1 THR A 940      76.896 195.034 103.360  1.00  0.00
ATOM  14485  HG1 THR A 940      77.452 195.836 103.280  1.00  0.00
ATOM  14486  C   THR A 940      79.298 196.065 104.524  1.00  0.00
ATOM  14487  O   THR A 940      78.745 196.845 103.750  1.00  0.00
ATOM  14488  N   ILE A 941      80.036 196.491 105.546  1.00  0.00
ATOM  14489  H   ILE A 941      80.582 195.817 106.060  1.00  0.00
ATOM  14490  CA  ILE A 941      80.437 197.878 105.780  1.00  0.00
ATOM  14491  HA  ILE A 941      80.463 198.398 104.821  1.00  0.00
ATOM  14492  CB  ILE A 941      79.485 198.654 106.726  1.00  0.00
ATOM  14493  HB  ILE A 941      80.028 199.522 107.106  1.00  0.00
ATOM  14494  CG2 ILE A 941      78.300 199.201 105.913  1.00  0.00
ATOM  14495 1HG2 ILE A 941      77.711 199.879 106.530  1.00  0.00
ATOM  14496 2HG2 ILE A 941      78.667 199.755 105.048  1.00  0.00
ATOM  14497 3HG2 ILE A 941      77.664 198.385 105.576  1.00  0.00
ATOM  14498  CG1 ILE A 941      78.908 197.893 107.945  1.00  0.00
ATOM  14499 1HG1 ILE A 941      78.348 197.021 107.607  1.00  0.00
ATOM  14500 2HG1 ILE A 941      78.206 198.556 108.452  1.00  0.00
ATOM  14501  CD  ILE A 941      79.936 197.451 108.988  1.00  0.00
ATOM  14502  HD1 ILE A 941      79.418 197.134 109.894  1.00  0.00
ATOM  14503  HD2 ILE A 941      80.512 196.607 108.617  1.00  0.00
ATOM  14504  HD3 ILE A 941      80.606 198.276 109.230  1.00  0.00
ATOM  14505  C   ILE A 941      81.885 197.872 106.277  1.00  0.00
ATOM  14506  O   ILE A 941      82.286 196.894 106.906  1.00  0.00
ATOM  14507  N   ASP A 942      82.648 198.922 105.947  1.00  0.00
ATOM  14508  H   ASP A 942      82.237 199.651 105.385  1.00  0.00
ATOM  14509  CA  ASP A 942      84.126 198.907 105.958  1.00  0.00
ATOM  14510  HA  ASP A 942      84.441 199.699 105.278  1.00  0.00
ATOM  14511  CB  ASP A 942      84.684 199.283 107.353  1.00  0.00
ATOM  14512  HB1 ASP A 942      84.635 198.416 108.014  1.00  0.00
ATOM  14513  HB2 ASP A 942      84.044 200.054 107.787  1.00  0.00
ATOM  14514  CG  ASP A 942      86.124 199.852 107.340  1.00  0.00
ATOM  14515  OD1 ASP A 942      86.367 200.827 106.588  1.00  0.00
ATOM  14516  OD2 ASP A 942      86.961 199.412 108.160  1.00  0.00
```

```
ATOM  14517  C   ASP A 942      84.696 197.599 105.355  1.00  0.00
ATOM  14518  O   ASP A 942      85.521 196.902 105.946  1.00  0.00
ATOM  14519  N   VAL A 943      84.146 197.278 104.173  1.00  0.00
ATOM  14520  H   VAL A 943      83.605 198.038 103.765  1.00  0.00
ATOM  14521  CA  VAL A 943      84.341 196.138 103.262  1.00  0.00
ATOM  14522  HA  VAL A 943      84.996 196.529 102.484  1.00  0.00
ATOM  14523  CB  VAL A 943      85.097 194.908 103.832  1.00  0.00
ATOM  14524  HB  VAL A 943      86.055 195.251 104.217  1.00  0.00
ATOM  14525  CG1 VAL A 943      84.380 194.120 104.942  1.00  0.00
ATOM  14526 1HG1 VAL A 943      85.073 193.410 105.387  1.00  0.00
ATOM  14527 2HG1 VAL A 943      84.042 194.805 105.720  1.00  0.00
ATOM  14528 3HG1 VAL A 943      83.518 193.588 104.546  1.00  0.00
ATOM  14529  CG2 VAL A 943      85.436 193.924 102.700  1.00  0.00
ATOM  14530 1HG2 VAL A 943      86.110 193.157 103.079  1.00  0.00
ATOM  14531 2HG2 VAL A 943      84.533 193.448 102.323  1.00  0.00
ATOM  14532 3HG2 VAL A 943      85.933 194.457 101.889  1.00  0.00
ATOM  14533  C   VAL A 943      83.038 195.743 102.559  1.00  0.00
ATOM  14534  O   VAL A 943      82.126 195.157 103.156  1.00  0.00
ATOM  14535  N   TYR A 944      82.995 195.930 101.241  1.00  0.00
ATOM  14536  H   TYR A 944      83.624 196.644 100.859  1.00  0.00
ATOM  14537  CA  TYR A 944      82.447 194.929 100.328  1.00  0.00
ATOM  14538  HA  TYR A 944      82.710 193.955 100.742  1.00  0.00
ATOM  14539  CB  TYR A 944      80.908 195.006 100.271  1.00  0.00
ATOM  14540  HB1 TYR A 944      80.600 195.642  99.439  1.00  0.00
ATOM  14541  HB2 TYR A 944      80.521 195.468 101.181  1.00  0.00
ATOM  14542  CG  TYR A 944      80.287 193.628 100.152  1.00  0.00
ATOM  14543  CD1 TYR A 944      79.737 193.194  98.935  1.00  0.00
ATOM  14544  HD1 TYR A 944      79.734 193.865  98.090  1.00  0.00
ATOM  14545  CE1 TYR A 944      79.201 191.898  98.822  1.00  0.00
ATOM  14546  HE1 TYR A 944      78.777 191.545  97.897  1.00  0.00
ATOM  14547  CZ  TYR A 944      79.222 191.026  99.925  1.00  0.00
ATOM  14548  OH  TYR A 944      78.706 189.777  99.799  1.00  0.00
ATOM  14549  HH  TYR A 944      78.805 189.258 100.600  1.00  0.00
ATOM  14550  CE2 TYR A 944      79.789 191.449 101.146  1.00  0.00
ATOM  14551  HE2 TYR A 944      79.820 190.789 102.002  1.00  0.00
ATOM  14552  CD2 TYR A 944      80.327 192.746 101.252  1.00  0.00
ATOM  14553  HD2 TYR A 944      80.793 193.058 102.181  1.00  0.00
ATOM  14554  C   TYR A 944      83.102 194.992  98.940  1.00  0.00
ATOM  14555  O   TYR A 944      84.014 195.763  98.688  1.00  0.00
ATOM  14556  N   MET A 945      82.614 194.189  97.993  1.00  0.00
ATOM  14557  H   MET A 945      81.888 193.539  98.257  1.00  0.00
ATOM  14558  CA  MET A 945      83.022 194.238  96.578  1.00  0.00
ATOM  14559  HA  MET A 945      84.113 194.216  96.537  1.00  0.00
ATOM  14560  CB  MET A 945      82.489 192.979  95.866  1.00  0.00
ATOM  14561  HB1 MET A 945      82.745 193.021  94.807  1.00  0.00
ATOM  14562  HB2 MET A 945      81.402 192.949  95.956  1.00  0.00
ATOM  14563  CG  MET A 945      83.091 191.689  96.434  1.00  0.00
ATOM  14564  HG1 MET A 945      82.853 191.603  97.495  1.00  0.00
ATOM  14565  HG2 MET A 945      84.175 191.748  96.337  1.00  0.00
ATOM  14566  SD  MET A 945      82.541 190.174  95.603  1.00  0.00
ATOM  14567  CE  MET A 945      80.858 190.026  96.256  1.00  0.00
ATOM  14568  HE1 MET A 945      80.389 189.132  95.846  1.00  0.00
ATOM  14569  HE2 MET A 945      80.892 189.941  97.341  1.00  0.00
ATOM  14570  HE3 MET A 945      80.273 190.899  95.976  1.00  0.00
ATOM  14571  C   MET A 945      82.587 195.517  95.833  1.00  0.00
ATOM  14572  O   MET A 945      82.607 195.519  94.606  1.00  0.00
ATOM  14573  N   ILE A 946      82.191 196.576  96.559  1.00  0.00
ATOM  14574  H   ILE A 946      82.345 196.496  97.555  1.00  0.00
ATOM  14575  CA  ILE A 946      81.817 197.934  96.116  1.00  0.00
ATOM  14576  HA  ILE A 946      81.576 198.513  97.010  1.00  0.00
ATOM  14577  CB  ILE A 946      83.027 198.621  95.419  1.00  0.00
```

```
ATOM  14578  HB  ILE A 946      83.134 198.176  94.428  1.00  0.00
ATOM  14579  CG2 ILE A 946      82.754 200.118  95.205  1.00  0.00
ATOM  14580 1HG2 ILE A 946      83.620 200.596  94.748  1.00  0.00
ATOM  14581 2HG2 ILE A 946      81.899 200.257  94.544  1.00  0.00
ATOM  14582 3HG2 ILE A 946      82.559 200.602  96.164  1.00  0.00
ATOM  14583  CG1 ILE A 946      84.394 198.430  96.123  1.00  0.00
ATOM  14584 1HG1 ILE A 946      85.165 198.920  95.528  1.00  0.00
ATOM  14585 2HG1 ILE A 946      84.650 197.371  96.142  1.00  0.00
ATOM  14586  CD  ILE A 946      84.486 198.968  97.555  1.00  0.00
ATOM  14587  HD1 ILE A 946      85.429 198.650  98.000  1.00  0.00
ATOM  14588  HD2 ILE A 946      84.462 200.055  97.539  1.00  0.00
ATOM  14589  HD3 ILE A 946      83.666 198.590  98.164  1.00  0.00
ATOM  14590  C   ILE A 946      80.582 197.986  95.208  1.00  0.00
ATOM  14591  O   ILE A 946      79.658 198.741  95.465  1.00  0.00
ATOM  14592  N   MET A 947      80.518 197.161  94.170  1.00  0.00
ATOM  14593  H   MET A 947      81.326 196.571  94.006  1.00  0.00
ATOM  14594  CA  MET A 947      79.409 197.087  93.220  1.00  0.00
ATOM  14595  HA  MET A 947      78.960 198.078  93.143  1.00  0.00
ATOM  14596  CB  MET A 947      79.941 196.706  91.825  1.00  0.00
ATOM  14597  HB1 MET A 947      79.145 196.871  91.097  1.00  0.00
ATOM  14598  HB2 MET A 947      80.189 195.643  91.814  1.00  0.00
ATOM  14599  CG  MET A 947      81.186 197.496  91.382  1.00  0.00
ATOM  14600  HG1 MET A 947      81.347 197.308  90.319  1.00  0.00
ATOM  14601  HG2 MET A 947      82.047 197.091  91.914  1.00  0.00
ATOM  14602  SD  MET A 947      81.183 199.296  91.651  1.00  0.00
ATOM  14603  CE  MET A 947      79.733 199.795  90.690  1.00  0.00
ATOM  14604  HE1 MET A 947      78.845 199.324  91.104  1.00  0.00
ATOM  14605  HE2 MET A 947      79.618 200.878  90.742  1.00  0.00
ATOM  14606  HE3 MET A 947      79.856 199.492  89.651  1.00  0.00
ATOM  14607  C   MET A 947      78.293 196.139  93.683  1.00  0.00
ATOM  14608  O   MET A 947      77.422 195.767  92.902  1.00  0.00
ATOM  14609  N   VAL A 948      78.321 195.704  94.948  1.00  0.00
ATOM  14610  H   VAL A 948      79.025 196.092  95.561  1.00  0.00
ATOM  14611  CA  VAL A 948      77.431 194.668  95.487  1.00  0.00
ATOM  14612  HA  VAL A 948      76.524 194.631  94.883  1.00  0.00
ATOM  14613  CB  VAL A 948      78.102 193.273  95.423  1.00  0.00
ATOM  14614  HB  VAL A 948      78.897 193.231  96.162  1.00  0.00
ATOM  14615  CG1 VAL A 948      77.086 192.165  95.731  1.00  0.00
ATOM  14616 1HG1 VAL A 948      77.572 191.191  95.669  1.00  0.00
ATOM  14617 2HG1 VAL A 948      76.688 192.271  96.738  1.00  0.00
ATOM  14618 3HG1 VAL A 948      76.270 192.194  95.009  1.00  0.00
ATOM  14619  CG2 VAL A 948      78.730 192.948  94.062  1.00  0.00
ATOM  14620 1HG2 VAL A 948      79.109 191.927  94.051  1.00  0.00
ATOM  14621 2HG2 VAL A 948      77.989 193.058  93.269  1.00  0.00
ATOM  14622 3HG2 VAL A 948      79.570 193.612  93.861  1.00  0.00
ATOM  14623  C   VAL A 948      77.049 195.016  96.923  1.00  0.00
ATOM  14624  O   VAL A 948      77.911 195.417  97.699  1.00  0.00
ATOM  14625  N   LYS A 949      75.771 194.820  97.282  1.00  0.00
ATOM  14626  H   LYS A 949      75.161 194.511  96.540  1.00  0.00
ATOM  14627  CA  LYS A 949      75.178 194.841  98.643  1.00  0.00
ATOM  14628  HA  LYS A 949      74.101 194.754  98.498  1.00  0.00
ATOM  14629  CB  LYS A 949      75.624 193.538  99.356  1.00  0.00
ATOM  14630  HB1 LYS A 949      76.706 193.435  99.250  1.00  0.00
ATOM  14631  HB2 LYS A 949      75.163 192.683  98.860  1.00  0.00
ATOM  14632  CG  LYS A 949      75.331 193.450 100.858  1.00  0.00
ATOM  14633  HG1 LYS A 949      75.868 194.271 101.312  1.00  0.00
ATOM  14634  HG2 LYS A 949      75.761 192.527 101.252  1.00  0.00
ATOM  14635  CD  LYS A 949      73.841 193.506 101.248  1.00  0.00
ATOM  14636  HD1 LYS A 949      73.426 192.500 101.177  1.00  0.00
ATOM  14637  HD2 LYS A 949      73.283 194.137 100.558  1.00  0.00
ATOM  14638  CE  LYS A 949      73.626 194.038 102.675  1.00  0.00
```

```
ATOM  14639  HE1 LYS A 949      74.122 193.371 103.386  1.00  0.00
ATOM  14640  HE2 LYS A 949      72.554 194.017 102.885  1.00  0.00
ATOM  14641  NZ  LYS A 949      74.140 195.423 102.835  1.00  0.00
ATOM  14642  HZ1 LYS A 949      73.797 195.867 103.671  1.00  0.00
ATOM  14643  HZ2 LYS A 949      75.159 195.416 102.888  1.00  0.00
ATOM  14644  HZ3 LYS A 949      73.912 195.994 102.021  1.00  0.00
ATOM  14645  C   LYS A 949      75.314 196.123  99.501  1.00  0.00
ATOM  14646  O   LYS A 949      74.393 196.428 100.256  1.00  0.00
ATOM  14647  N   CYS A 950      76.384 196.895  99.402  1.00  0.00
ATOM  14648  H   CYS A 950      77.146 196.553  98.827  1.00  0.00
ATOM  14649  CA  CYS A 950      76.560 198.228  99.982  1.00  0.00
ATOM  14650  HA  CYS A 950      75.596 198.702 100.170  1.00  0.00
ATOM  14651  CB  CYS A 950      77.329 198.148 101.305  1.00  0.00
ATOM  14652  HB1 CYS A 950      77.943 199.041 101.428  1.00  0.00
ATOM  14653  HB2 CYS A 950      78.004 197.290 101.306  1.00  0.00
ATOM  14654  SG  CYS A 950      76.148 198.065 102.680  1.00  0.00
ATOM  14655  HG  CYS A 950      77.101 198.176 103.622  1.00  0.00
ATOM  14656  C   CYS A 950      77.287 199.062  98.921  1.00  0.00
ATOM  14657  O   CYS A 950      78.507 199.184  98.896  1.00  0.00
ATOM  14658  N   TRP A 951      76.489 199.488  97.945  1.00  0.00
ATOM  14659  H   TRP A 951      75.496 199.387  98.061  1.00  0.00
ATOM  14660  CA  TRP A 951      76.949 200.019  96.671  1.00  0.00
ATOM  14661  HA  TRP A 951      77.547 199.260  96.179  1.00  0.00
ATOM  14662  CB  TRP A 951      75.705 200.257  95.804  1.00  0.00
ATOM  14663  HB1 TRP A 951      75.052 200.968  96.314  1.00  0.00
ATOM  14664  HB2 TRP A 951      75.157 199.318  95.714  1.00  0.00
ATOM  14665  CG  TRP A 951      75.958 200.772  94.423  1.00  0.00
ATOM  14666  CD1 TRP A 951      76.163 202.071  94.109  1.00  0.00
ATOM  14667  HD1 TRP A 951      76.183 202.883  94.825  1.00  0.00
ATOM  14668  NE1 TRP A 951      76.373 202.192  92.752  1.00  0.00
ATOM  14669  HE1 TRP A 951      76.629 203.066  92.310  1.00  0.00
ATOM  14670  CE2 TRP A 951      76.280 200.977  92.110  1.00  0.00
ATOM  14671  CZ2 TRP A 951      76.390 200.595  90.766  1.00  0.00
ATOM  14672  HZ2 TRP A 951      76.582 201.336  90.006  1.00  0.00
ATOM  14673  CH2 TRP A 951      76.244 199.238  90.427  1.00  0.00
ATOM  14674  HH2 TRP A 951      76.321 198.926  89.393  1.00  0.00
ATOM  14675  CZ3 TRP A 951      76.006 198.288  91.436  1.00  0.00
ATOM  14676  HZ3 TRP A 951      75.903 197.244  91.173  1.00  0.00
ATOM  14677  CE3 TRP A 951      75.897 198.684  92.783  1.00  0.00
ATOM  14678  HE3 TRP A 951      75.709 197.943  93.542  1.00  0.00
ATOM  14679  CD2 TRP A 951      76.026 200.039  93.157  1.00  0.00
ATOM  14680  C   TRP A 951      77.777 201.306  96.814  1.00  0.00
ATOM  14681  O   TRP A 951      77.332 202.258  97.448  1.00  0.00
ATOM  14682  N   MET A 952      78.944 201.343  96.164  1.00  0.00
ATOM  14683  H   MET A 952      79.219 200.494  95.683  1.00  0.00
ATOM  14684  CA  MET A 952      79.925 202.442  96.183  1.00  0.00
ATOM  14685  HA  MET A 952      80.829 202.059  95.717  1.00  0.00
ATOM  14686  CB  MET A 952      79.444 203.607  95.293  1.00  0.00
ATOM  14687  HB1 MET A 952      80.108 204.460  95.423  1.00  0.00
ATOM  14688  HB2 MET A 952      78.439 203.909  95.589  1.00  0.00
ATOM  14689  CG  MET A 952      79.456 203.238  93.803  1.00  0.00
ATOM  14690  HG1 MET A 952      78.908 202.308  93.667  1.00  0.00
ATOM  14691  HG2 MET A 952      80.487 203.055  93.498  1.00  0.00
ATOM  14692  SD  MET A 952      78.736 204.488  92.692  1.00  0.00
ATOM  14693  CE  MET A 952      79.906 205.853  92.917  1.00  0.00
ATOM  14694  HE1 MET A 952      79.633 206.674  92.257  1.00  0.00
ATOM  14695  HE2 MET A 952      79.879 206.201  93.949  1.00  0.00
ATOM  14696  HE3 MET A 952      80.913 205.519  92.673  1.00  0.00
ATOM  14697  C   MET A 952      80.386 202.882  97.590  1.00  0.00
ATOM  14698  O   MET A 952      80.801 204.025  97.771  1.00  0.00
ATOM  14699  N   ILE A 953      80.333 201.981  98.578  1.00  0.00
```

```
ATOM  14700  H   ILE A 953      79.968 201.066  98.361  1.00  0.00
ATOM  14701  CA  ILE A 953      80.884 202.183  99.931  1.00  0.00
ATOM  14702  HA  ILE A 953      80.789 203.237 100.185  1.00  0.00
ATOM  14703  CB  ILE A 953      80.013 201.389 100.949  1.00  0.00
ATOM  14704  HB  ILE A 953      80.057 200.334 100.673  1.00  0.00
ATOM  14705  CG2 ILE A 953      80.451 201.474 102.424  1.00  0.00
ATOM  14706 1HG2 ILE A 953      79.681 201.061 103.074  1.00  0.00
ATOM  14707 2HG2 ILE A 953      81.344 200.870 102.581  1.00  0.00
ATOM  14708 3HG2 ILE A 953      80.647 202.507 102.714  1.00  0.00
ATOM  14709  CG1 ILE A 953      78.525 201.832 100.888  1.00  0.00
ATOM  14710 1HG1 ILE A 953      77.948 201.253 101.609  1.00  0.00
ATOM  14711 2HG1 ILE A 953      78.115 201.599  99.908  1.00  0.00
ATOM  14712  CD  ILE A 953      78.249 203.318 101.162  1.00  0.00
ATOM  14713  HD1 ILE A 953      77.172 203.489 101.141  1.00  0.00
ATOM  14714  HD2 ILE A 953      78.632 203.605 102.141  1.00  0.00
ATOM  14715  HD3 ILE A 953      78.709 203.938 100.393  1.00  0.00
ATOM  14716  C   ILE A 953      82.399 201.913  99.969  1.00  0.00
ATOM  14717  O   ILE A 953      82.986 201.572  98.942  1.00  0.00
ATOM  14718  N   ASP A 954      83.039 202.140 101.120  1.00  0.00
ATOM  14719  H   ASP A 954      82.479 202.341 101.936  1.00  0.00
ATOM  14720  CA  ASP A 954      84.493 202.098 101.328  1.00  0.00
ATOM  14721  HA  ASP A 954      84.668 202.329 102.379  1.00  0.00
ATOM  14722  CB  ASP A 954      85.060 200.677 101.081  1.00  0.00
ATOM  14723  HB1 ASP A 954      86.052 200.620 101.532  1.00  0.00
ATOM  14724  HB2 ASP A 954      85.187 200.531 100.007  1.00  0.00
ATOM  14725  CG  ASP A 954      84.212 199.506 101.621  1.00  0.00
ATOM  14726  OD1 ASP A 954      83.559 199.646 102.680  1.00  0.00
ATOM  14727  OD2 ASP A 954      84.236 198.430 100.977  1.00  0.00
ATOM  14728  C   ASP A 954      85.176 203.204 100.503  1.00  0.00
ATOM  14729  O   ASP A 954      86.174 202.987  99.809  1.00  0.00
ATOM  14730  N   ALA A 955      84.593 204.404 100.524  1.00  0.00
ATOM  14731  H   ALA A 955      83.794 204.494 101.142  1.00  0.00
ATOM  14732  CA  ALA A 955      84.964 205.558  99.689  1.00  0.00
ATOM  14733  HA  ALA A 955      85.877 205.339  99.143  1.00  0.00
ATOM  14734  CB  ALA A 955      83.845 205.780  98.664  1.00  0.00
ATOM  14735  HB1 ALA A 955      84.109 206.606  98.005  1.00  0.00
ATOM  14736  HB2 ALA A 955      83.705 204.875  98.072  1.00  0.00
ATOM  14737  HB3 ALA A 955      82.910 206.014  99.174  1.00  0.00
ATOM  14738  C   ALA A 955      85.230 206.810 100.540  1.00  0.00
ATOM  14739  O   ALA A 955      86.162 207.564 100.265  1.00  0.00
ATOM  14740  N   ASP A 956      84.463 206.950 101.618  1.00  0.00
ATOM  14741  H   ASP A 956      83.719 206.270 101.745  1.00  0.00
ATOM  14742  CA  ASP A 956      84.818 207.639 102.853  1.00  0.00
ATOM  14743  HA  ASP A 956      85.106 208.662 102.613  1.00  0.00
ATOM  14744  CB  ASP A 956      83.566 207.687 103.758  1.00  0.00
ATOM  14745  HB1 ASP A 956      82.821 208.315 103.267  1.00  0.00
ATOM  14746  HB2 ASP A 956      83.844 208.183 104.688  1.00  0.00
ATOM  14747  CG  ASP A 956      82.912 206.330 104.081  1.00  0.00
ATOM  14748  OD1 ASP A 956      82.272 206.262 105.148  1.00  0.00
ATOM  14749  OD2 ASP A 956      83.043 205.382 103.262  1.00  0.00
ATOM  14750  C   ASP A 956      86.025 206.978 103.550  1.00  0.00
ATOM  14751  O   ASP A 956      87.060 207.614 103.757  1.00  0.00
ATOM  14752  N   SER A 957      85.937 205.677 103.842  1.00  0.00
ATOM  14753  H   SER A 957      85.046 205.214 103.673  1.00  0.00
ATOM  14754  CA  SER A 957      87.041 204.896 104.412  1.00  0.00
ATOM  14755  HA  SER A 957      87.571 205.515 105.137  1.00  0.00
ATOM  14756  CB  SER A 957      86.503 203.678 105.174  1.00  0.00
ATOM  14757  HB1 SER A 957      86.055 202.965 104.480  1.00  0.00
ATOM  14758  HB2 SER A 957      85.742 203.998 105.886  1.00  0.00
ATOM  14759  OG  SER A 957      87.558 203.055 105.880  1.00  0.00
ATOM  14760  HG  SER A 957      87.195 202.180 106.188  1.00  0.00
```

```
ATOM  14761  C   SER A 957      88.043 204.464 103.328  1.00  0.00
ATOM  14762  O   SER A 957      87.967 203.357 102.798  1.00  0.00
ATOM  14763  N   ARG A 958      89.017 205.328 102.976  1.00  0.00
ATOM  14764  H   ARG A 958      88.944 206.274 103.334  1.00  0.00
ATOM  14765  CA  ARG A 958      90.157 204.944 102.101  1.00  0.00
ATOM  14766  HA  ARG A 958      90.167 203.854 102.072  1.00  0.00
ATOM  14767  CB  ARG A 958      89.933 205.386 100.640  1.00  0.00
ATOM  14768  HB1 ARG A 958      90.794 205.075 100.046  1.00  0.00
ATOM  14769  HB2 ARG A 958      89.841 206.473 100.584  1.00  0.00
ATOM  14770  CG  ARG A 958      88.671 204.724 100.063  1.00  0.00
ATOM  14771  HG1 ARG A 958      87.802 205.272 100.426  1.00  0.00
ATOM  14772  HG2 ARG A 958      88.606 203.703 100.433  1.00  0.00
ATOM  14773  CD  ARG A 958      88.611 204.653  98.531  1.00  0.00
ATOM  14774  HD1 ARG A 958      88.875 205.628  98.121  1.00  0.00
ATOM  14775  HD2 ARG A 958      87.584 204.438  98.234  1.00  0.00
ATOM  14776  NE  ARG A 958      89.494 203.603  97.973  1.00  0.00
ATOM  14777  HE  ARG A 958      90.313 203.934  97.500  1.00  0.00
ATOM  14778  CZ  ARG A 958      89.279 202.291  97.983  1.00  0.00
ATOM  14779  NH1 ARG A 958      88.256 201.726  98.558  1.00  0.00
ATOM  14780 1HH1 ARG A 958      87.564 202.301  99.049  1.00  0.00
ATOM  14781 2HH1 ARG A 958      88.081 200.740  98.546  1.00  0.00
ATOM  14782  NH2 ARG A 958      90.125 201.497  97.381  1.00  0.00
ATOM  14783 1HH2 ARG A 958      90.871 201.873  96.834  1.00  0.00
ATOM  14784 2HH2 ARG A 958      89.942 200.511  97.389  1.00  0.00
ATOM  14785  C   ARG A 958      91.570 205.265 102.665  1.00  0.00
ATOM  14786  O   ARG A 958      92.362 205.907 101.978  1.00  0.00
ATOM  14787  N   PRO A 959      91.941 204.809 103.882  1.00  0.00
ATOM  14788  CD  PRO A 959      91.158 203.906 104.731  1.00  0.00
ATOM  14789  HD1 PRO A 959      91.038 202.923 104.275  1.00  0.00
ATOM  14790  HD2 PRO A 959      90.184 204.339 104.951  1.00  0.00
ATOM  14791  CG  PRO A 959      91.912 203.795 106.052  1.00  0.00
ATOM  14792  HG1 PRO A 959      92.659 203.004 105.992  1.00  0.00
ATOM  14793  HG2 PRO A 959      91.235 203.636 106.891  1.00  0.00
ATOM  14794  CB  PRO A 959      92.594 205.159 106.119  1.00  0.00
ATOM  14795  HB1 PRO A 959      93.406 205.162 106.845  1.00  0.00
ATOM  14796  HB2 PRO A 959      91.859 205.921 106.382  1.00  0.00
ATOM  14797  CA  PRO A 959      93.059 205.367 104.667  1.00  0.00
ATOM  14798  HA  PRO A 959      93.145 206.438 104.476  1.00  0.00
ATOM  14799  C   PRO A 959      94.479 204.753 104.498  1.00  0.00
ATOM  14800  O   PRO A 959      95.377 205.023 105.298  1.00  0.00
ATOM  14801  N   LYS A 960      94.712 203.931 103.464  1.00  0.00
ATOM  14802  H   LYS A 960      93.938 203.761 102.843  1.00  0.00
ATOM  14803  CA  LYS A 960      96.072 203.569 102.995  1.00  0.00
ATOM  14804  HA  LYS A 960      96.001 202.654 102.409  1.00  0.00
ATOM  14805  CB  LYS A 960      96.487 204.702 102.032  1.00  0.00
ATOM  14806  HB1 LYS A 960      96.732 205.590 102.620  1.00  0.00
ATOM  14807  HB2 LYS A 960      95.628 204.947 101.403  1.00  0.00
ATOM  14808  CG  LYS A 960      97.656 204.373 101.100  1.00  0.00
ATOM  14809  HG1 LYS A 960      97.477 203.427 100.589  1.00  0.00
ATOM  14810  HG2 LYS A 960      98.550 204.278 101.700  1.00  0.00
ATOM  14811  CD  LYS A 960      97.827 205.494 100.055  1.00  0.00
ATOM  14812  HD1 LYS A 960      97.577 206.451 100.517  1.00  0.00
ATOM  14813  HD2 LYS A 960      97.133 205.325  99.230  1.00  0.00
ATOM  14814  CE  LYS A 960      99.254 205.632  99.510  1.00  0.00
ATOM  14815  HE1 LYS A 960      99.903 205.918 100.346  1.00  0.00
ATOM  14816  HE2 LYS A 960      99.289 206.442  98.776  1.00  0.00
ATOM  14817  NZ  LYS A 960      99.793 204.386  98.926  1.00  0.00
ATOM  14818  HZ1 LYS A 960      99.815 203.655  99.645  1.00  0.00
ATOM  14819  HZ2 LYS A 960      99.351 204.068  98.084  1.00  0.00
ATOM  14820  HZ3 LYS A 960     100.806 204.498  98.827  1.00  0.00
ATOM  14821  C   LYS A 960      97.064 203.271 104.148  1.00  0.00
```

ATOM	14822	O	LYS	A	960	98.005	204.017	104.409	1.00	0.00
ATOM	14823	N	PHE	A	961	96.728	202.221	104.913	1.00	0.00
ATOM	14824	H	PHE	A	961	95.899	201.724	104.627	1.00	0.00
ATOM	14825	CA	PHE	A	961	97.120	202.019	106.320	1.00	0.00
ATOM	14826	HA	PHE	A	961	96.489	202.682	106.914	1.00	0.00
ATOM	14827	CB	PHE	A	961	96.791	200.584	106.750	1.00	0.00
ATOM	14828	HB1	PHE	A	961	97.117	200.444	107.782	1.00	0.00
ATOM	14829	HB2	PHE	A	961	97.348	199.880	106.129	1.00	0.00
ATOM	14830	CG	PHE	A	961	95.311	200.266	106.673	1.00	0.00
ATOM	14831	CD1	PHE	A	961	94.448	200.724	107.685	1.00	0.00
ATOM	14832	HD1	PHE	A	961	94.839	201.283	108.524	1.00	0.00
ATOM	14833	CE1	PHE	A	961	93.072	200.449	107.614	1.00	0.00
ATOM	14834	HE1	PHE	A	961	92.402	200.789	108.398	1.00	0.00
ATOM	14835	CZ	PHE	A	961	92.553	199.729	106.526	1.00	0.00
ATOM	14836	HZ	PHE	A	961	91.492	199.514	106.478	1.00	0.00
ATOM	14837	CE2	PHE	A	961	93.412	199.280	105.507	1.00	0.00
ATOM	14838	HE2	PHE	A	961	93.008	198.717	104.680	1.00	0.00
ATOM	14839	CD2	PHE	A	961	94.792	199.543	105.582	1.00	0.00
ATOM	14840	HD2	PHE	A	961	95.451	199.176	104.810	1.00	0.00
ATOM	14841	C	PHE	A	961	98.549	202.405	106.696	1.00	0.00
ATOM	14842	O	PHE	A	961	98.717	203.355	107.452	1.00	0.00
ATOM	14843	N	ARG	A	962	99.564	201.725	106.147	1.00	0.00
ATOM	14844	H	ARG	A	962	99.344	200.998	105.487	1.00	0.00
ATOM	14845	CA	ARG	A	962	100.990	201.998	106.432	1.00	0.00
ATOM	14846	HA	ARG	A	962	101.189	201.831	107.492	1.00	0.00
ATOM	14847	CB	ARG	A	962	101.883	201.067	105.589	1.00	0.00
ATOM	14848	HB1	ARG	A	962	102.928	201.308	105.796	1.00	0.00
ATOM	14849	HB2	ARG	A	962	101.694	201.284	104.536	1.00	0.00
ATOM	14850	CG	ARG	A	962	101.674	199.566	105.838	1.00	0.00
ATOM	14851	HG1	ARG	A	962	100.619	199.309	105.734	1.00	0.00
ATOM	14852	HG2	ARG	A	962	102.013	199.302	106.843	1.00	0.00
ATOM	14853	CD	ARG	A	962	102.474	198.783	104.792	1.00	0.00
ATOM	14854	HD1	ARG	A	962	103.539	198.954	104.955	1.00	0.00
ATOM	14855	HD2	ARG	A	962	102.213	199.174	103.807	1.00	0.00
ATOM	14856	NE	ARG	A	962	102.199	197.335	104.820	1.00	0.00
ATOM	14857	HE	ARG	A	962	101.921	196.910	105.690	1.00	0.00
ATOM	14858	CZ	ARG	A	962	102.379	196.502	103.811	1.00	0.00
ATOM	14859	NH1	ARG	A	962	102.790	196.882	102.639	1.00	0.00
ATOM	14860	1HH1	ARG	A	962	103.173	197.831	102.536	1.00	0.00
ATOM	14861	2HH1	ARG	A	962	102.884	196.234	101.889	1.00	0.00
ATOM	14862	NH2	ARG	A	962	102.120	195.239	103.954	1.00	0.00
ATOM	14863	1HH2	ARG	A	962	101.837	194.933	104.874	1.00	0.00
ATOM	14864	2HH2	ARG	A	962	102.438	194.555	103.292	1.00	0.00
ATOM	14865	C	ARG	A	962	101.397	203.451	106.160	1.00	0.00
ATOM	14866	O	ARG	A	962	102.422	203.886	106.666	1.00	0.00
ATOM	14867	N	GLU	A	963	100.605	204.180	105.373	1.00	0.00
ATOM	14868	H	GLU	A	963	99.739	203.787	105.036	1.00	0.00
ATOM	14869	CA	GLU	A	963	101.002	205.465	104.802	1.00	0.00
ATOM	14870	HA	GLU	A	963	102.010	205.721	105.128	1.00	0.00
ATOM	14871	CB	GLU	A	963	101.025	205.360	103.265	1.00	0.00
ATOM	14872	HB1	GLU	A	963	101.341	206.317	102.848	1.00	0.00
ATOM	14873	HB2	GLU	A	963	100.006	205.184	102.943	1.00	0.00
ATOM	14874	CG	GLU	A	963	101.947	204.252	102.720	1.00	0.00
ATOM	14875	HG1	GLU	A	963	101.857	203.341	103.310	1.00	0.00
ATOM	14876	HG2	GLU	A	963	102.986	204.582	102.774	1.00	0.00
ATOM	14877	CD	GLU	A	963	101.574	203.908	101.274	1.00	0.00
ATOM	14878	OE1	GLU	A	963	100.666	203.068	101.058	1.00	0.00
ATOM	14879	OE2	GLU	A	963	102.051	204.576	100.329	1.00	0.00
ATOM	14880	C	GLU	A	963	100.093	206.590	105.308	1.00	0.00
ATOM	14881	O	GLU	A	963	100.289	207.741	104.941	1.00	0.00
ATOM	14882	N	LEU	A	964	99.174	206.267	106.235	1.00	0.00

```
ATOM  14883  H   LEU A 964      98.986 205.288 106.400  1.00  0.00
ATOM  14884  CA  LEU A 964      98.787 207.229 107.279  1.00  0.00
ATOM  14885  HA  LEU A 964      99.180 208.209 107.013  1.00  0.00
ATOM  14886  CB  LEU A 964      97.256 207.348 107.354  1.00  0.00
ATOM  14887  HB1 LEU A 964      97.001 207.930 108.241  1.00  0.00
ATOM  14888  HB2 LEU A 964      96.831 206.352 107.471  1.00  0.00
ATOM  14889  CG  LEU A 964      96.583 208.023 106.140  1.00  0.00
ATOM  14890  HG  LEU A 964      96.719 207.400 105.258  1.00  0.00
ATOM  14891  CD1 LEU A 964      95.083 208.171 106.416  1.00  0.00
ATOM  14892 1HD1 LEU A 964      94.581 208.542 105.525  1.00  0.00
ATOM  14893 2HD1 LEU A 964      94.667 207.200 106.673  1.00  0.00
ATOM  14894 3HD1 LEU A 964      94.920 208.860 107.243  1.00  0.00
ATOM  14895  CD2 LEU A 964      97.117 209.426 105.847  1.00  0.00
ATOM  14896 1HD2 LEU A 964      96.517 209.899 105.072  1.00  0.00
ATOM  14897 2HD2 LEU A 964      97.096 210.036 106.749  1.00  0.00
ATOM  14898 3HD2 LEU A 964      98.141 209.361 105.481  1.00  0.00
ATOM  14899  C   LEU A 964      99.411 206.947 108.654  1.00  0.00
ATOM  14900  O   LEU A 964      99.668 207.907 109.383  1.00  0.00
ATOM  14901  N   ILE A 965      99.643 205.688 109.042  1.00  0.00
ATOM  14902  H   ILE A 965      99.389 204.939 108.404  1.00  0.00
ATOM  14903  CA  ILE A 965     100.075 205.310 110.402  1.00  0.00
ATOM  14904  HA  ILE A 965     100.885 205.978 110.676  1.00  0.00
ATOM  14905  CB  ILE A 965      98.910 205.513 111.438  1.00  0.00
ATOM  14906  HB  ILE A 965      98.822 204.607 112.030  1.00  0.00
ATOM  14907  CG2 ILE A 965      99.281 206.620 112.443  1.00  0.00
ATOM  14908 1HG2 ILE A 965      98.465 206.777 113.147  1.00  0.00
ATOM  14909 2HG2 ILE A 965     100.151 206.329 113.010  1.00  0.00
ATOM  14910 3HG2 ILE A 965      99.480 207.555 111.917  1.00  0.00
ATOM  14911  CG1 ILE A 965      97.473 205.888 110.953  1.00  0.00
ATOM  14912 1HG1 ILE A 965      97.507 206.852 110.451  1.00  0.00
ATOM  14913 2HG1 ILE A 965      96.841 206.036 111.830  1.00  0.00
ATOM  14914  CD  ILE A 965      96.719 204.902 110.053  1.00  0.00
ATOM  14915  HD1 ILE A 965      97.390 204.389 109.372  1.00  0.00
ATOM  14916  HD2 ILE A 965      96.193 204.172 110.662  1.00  0.00
ATOM  14917  HD3 ILE A 965      95.969 205.446 109.480  1.00  0.00
ATOM  14918  C   ILE A 965     100.617 203.855 110.481  1.00  0.00
ATOM  14919  O   ILE A 965      99.876 202.902 110.248  1.00  0.00
ATOM  14920  N   ILE A 966     101.883 203.667 110.903  1.00  0.00
ATOM  14921  H   ILE A 966     102.477 204.480 110.982  1.00  0.00
ATOM  14922  CA  ILE A 966     102.426 202.367 111.369  1.00  0.00
ATOM  14923  HA  ILE A 966     102.418 201.704 110.503  1.00  0.00
ATOM  14924  CB  ILE A 966     103.895 202.445 111.858  1.00  0.00
ATOM  14925  HB  ILE A 966     103.919 203.013 112.785  1.00  0.00
ATOM  14926  CG2 ILE A 966     104.438 201.050 112.224  1.00  0.00
ATOM  14927 1HG2 ILE A 966     105.496 201.121 112.473  1.00  0.00
ATOM  14928 2HG2 ILE A 966     103.912 200.649 113.090  1.00  0.00
ATOM  14929 3HG2 ILE A 966     104.314 200.370 111.386  1.00  0.00
ATOM  14930  CG1 ILE A 966     104.847 203.122 110.847  1.00  0.00
ATOM  14931 1HG1 ILE A 966     105.848 203.164 111.280  1.00  0.00
ATOM  14932 2HG1 ILE A 966     104.532 204.151 110.691  1.00  0.00
ATOM  14933  CD  ILE A 966     104.950 202.448 109.470  1.00  0.00
ATOM  14934  HD1 ILE A 966     103.972 202.406 108.991  1.00  0.00
ATOM  14935  HD2 ILE A 966     105.620 203.028 108.837  1.00  0.00
ATOM  14936  HD3 ILE A 966     105.352 201.441 109.567  1.00  0.00
ATOM  14937  C   ILE A 966     101.519 201.729 112.425  1.00  0.00
ATOM  14938  O   ILE A 966     101.058 202.404 113.353  1.00  0.00
ATOM  14939  N   GLU A 967     101.260 200.425 112.263  1.00  0.00
ATOM  14940  H   GLU A 967     101.763 199.951 111.524  1.00  0.00
ATOM  14941  CA  GLU A 967     100.253 199.631 113.000  1.00  0.00
ATOM  14942  HA  GLU A 967      99.970 198.790 112.365  1.00  0.00
ATOM  14943  CB  GLU A 967     100.876 199.016 114.282  1.00  0.00
```

ATOM	14944	HB1	GLU	A	967	100.097	198.689	114.969	1.00	0.00
ATOM	14945	HB2	GLU	A	967	101.468	199.775	114.782	1.00	0.00
ATOM	14946	CG	GLU	A	967	101.761	197.792	113.984	1.00	0.00
ATOM	14947	HG1	GLU	A	967	102.230	197.934	113.007	1.00	0.00
ATOM	14948	HG2	GLU	A	967	101.127	196.906	113.914	1.00	0.00
ATOM	14949	CD	GLU	A	967	102.868	197.570	115.028	1.00	0.00
ATOM	14950	OE1	GLU	A	967	104.052	197.708	114.635	1.00	0.00
ATOM	14951	OE2	GLU	A	967	102.599	197.313	116.229	1.00	0.00
ATOM	14952	C	GLU	A	967	98.919	200.385	113.232	1.00	0.00
ATOM	14953	O	GLU	A	967	98.287	200.260	114.273	1.00	0.00
ATOM	14954	N	PHE	A	968	98.540	201.235	112.264	1.00	0.00
ATOM	14955	H	PHE	A	968	99.157	201.323	111.466	1.00	0.00
ATOM	14956	CA	PHE	A	968	97.405	202.171	112.253	1.00	0.00
ATOM	14957	HA	PHE	A	968	97.643	202.862	111.454	1.00	0.00
ATOM	14958	CB	PHE	A	968	96.126	201.432	111.794	1.00	0.00
ATOM	14959	HB1	PHE	A	968	96.317	201.006	110.808	1.00	0.00
ATOM	14960	HB2	PHE	A	968	95.325	202.161	111.669	1.00	0.00
ATOM	14961	CG	PHE	A	968	95.624	200.326	112.709	1.00	0.00
ATOM	14962	CD1	PHE	A	968	95.954	198.982	112.442	1.00	0.00
ATOM	14963	HD1	PHE	A	968	96.561	198.732	111.586	1.00	0.00
ATOM	14964	CE1	PHE	A	968	95.508	197.959	113.298	1.00	0.00
ATOM	14965	HE1	PHE	A	968	95.777	196.935	113.098	1.00	0.00
ATOM	14966	CZ	PHE	A	968	94.729	198.275	114.424	1.00	0.00
ATOM	14967	HZ	PHE	A	968	94.399	197.494	115.092	1.00	0.00
ATOM	14968	CE2	PHE	A	968	94.383	199.612	114.686	1.00	0.00
ATOM	14969	HE2	PHE	A	968	93.781	199.860	115.548	1.00	0.00
ATOM	14970	CD2	PHE	A	968	94.823	200.634	113.826	1.00	0.00
ATOM	14971	HD2	PHE	A	968	94.547	201.657	114.028	1.00	0.00
ATOM	14972	C	PHE	A	968	97.200	203.058	113.495	1.00	0.00
ATOM	14973	O	PHE	A	968	96.171	203.715	113.628	1.00	0.00
ATOM	14974	N	SER	A	969	98.177	203.092	114.404	1.00	0.00
ATOM	14975	H	SER	A	969	99.010	202.562	114.187	1.00	0.00
ATOM	14976	CA	SER	A	969	98.023	203.635	115.762	1.00	0.00
ATOM	14977	HA	SER	A	969	97.552	204.617	115.709	1.00	0.00
ATOM	14978	CB	SER	A	969	97.108	202.703	116.573	1.00	0.00
ATOM	14979	HB1	SER	A	969	96.119	202.672	116.113	1.00	0.00
ATOM	14980	HB2	SER	A	969	97.003	203.100	117.584	1.00	0.00
ATOM	14981	OG	SER	A	969	97.630	201.387	116.644	1.00	0.00
ATOM	14982	HG	SER	A	969	97.626	200.967	115.758	1.00	0.00
ATOM	14983	C	SER	A	969	99.357	203.787	116.495	1.00	0.00
ATOM	14984	O	SER	A	969	99.581	204.800	117.151	1.00	0.00
ATOM	14985	N	LYS	A	970	100.306	202.858	116.310	1.00	0.00
ATOM	14986	H	LYS	A	970	100.051	202.016	115.810	1.00	0.00
ATOM	14987	CA	LYS	A	970	101.683	202.980	116.818	1.00	0.00
ATOM	14988	HA	LYS	A	970	101.654	203.004	117.908	1.00	0.00
ATOM	14989	CB	LYS	A	970	102.495	201.750	116.376	1.00	0.00
ATOM	14990	HB1	LYS	A	970	102.455	201.665	115.294	1.00	0.00
ATOM	14991	HB2	LYS	A	970	102.050	200.858	116.818	1.00	0.00
ATOM	14992	CG	LYS	A	970	103.970	201.839	116.774	1.00	0.00
ATOM	14993	HG1	LYS	A	970	104.030	201.995	117.847	1.00	0.00
ATOM	14994	HG2	LYS	A	970	104.429	202.690	116.272	1.00	0.00
ATOM	14995	CD	LYS	A	970	104.768	200.587	116.379	1.00	0.00
ATOM	14996	HD1	LYS	A	970	105.830	200.833	116.400	1.00	0.00
ATOM	14997	HD2	LYS	A	970	104.508	200.298	115.360	1.00	0.00
ATOM	14998	CE	LYS	A	970	104.513	199.426	117.341	1.00	0.00
ATOM	14999	HE1	LYS	A	970	103.454	199.153	117.311	1.00	0.00
ATOM	15000	HE2	LYS	A	970	104.762	199.733	118.358	1.00	0.00
ATOM	15001	NZ	LYS	A	970	105.326	198.252	116.971	1.00	0.00
ATOM	15002	HZ1	LYS	A	970	105.172	197.502	117.639	1.00	0.00
ATOM	15003	HZ2	LYS	A	970	104.998	197.901	116.067	1.00	0.00
ATOM	15004	HZ3	LYS	A	970	106.309	198.459	116.964	1.00	0.00

```
ATOM  15005  C   LYS A 970     102.347 204.284 116.368  1.00  0.00
ATOM  15006  O   LYS A 970     102.845 205.043 117.203  1.00  0.00
ATOM  15007  N   MET A 971     102.312 204.590 115.071  1.00  0.00
ATOM  15008  H   MET A 971     101.908 203.910 114.435  1.00  0.00
ATOM  15009  CA  MET A 971     102.852 205.851 114.537  1.00  0.00
ATOM  15010  HA  MET A 971     103.869 205.954 114.911  1.00  0.00
ATOM  15011  CB  MET A 971     102.919 205.783 113.010  1.00  0.00
ATOM  15012  HB1 MET A 971     101.986 205.365 112.668  1.00  0.00
ATOM  15013  HB2 MET A 971     103.723 205.105 112.746  1.00  0.00
ATOM  15014  CG  MET A 971     103.167 207.072 112.217  1.00  0.00
ATOM  15015  HG1 MET A 971     104.060 207.561 112.596  1.00  0.00
ATOM  15016  HG2 MET A 971     102.312 207.735 112.344  1.00  0.00
ATOM  15017  SD  MET A 971     103.404 206.760 110.447  1.00  0.00
ATOM  15018  CE  MET A 971     103.055 208.401 109.767  1.00  0.00
ATOM  15019  HE1 MET A 971     103.109 208.357 108.680  1.00  0.00
ATOM  15020  HE2 MET A 971     102.052 208.714 110.056  1.00  0.00
ATOM  15021  HE3 MET A 971     103.787 209.116 110.140  1.00  0.00
ATOM  15022  C   MET A 971     102.108 207.104 115.041  1.00  0.00
ATOM  15023  O   MET A 971     102.647 208.209 114.961  1.00  0.00
ATOM  15024  N   ALA A 972     100.908 206.956 115.612  1.00  0.00
ATOM  15025  H   ALA A 972     100.525 206.029 115.736  1.00  0.00
ATOM  15026  CA  ALA A 972     100.202 208.051 116.263  1.00  0.00
ATOM  15027  HA  ALA A 972     100.304 208.935 115.631  1.00  0.00
ATOM  15028  CB  ALA A 972      98.703 207.738 116.353  1.00  0.00
ATOM  15029  HB1 ALA A 972      98.161 208.650 116.607  1.00  0.00
ATOM  15030  HB2 ALA A 972      98.332 207.366 115.400  1.00  0.00
ATOM  15031  HB3 ALA A 972      98.508 207.009 117.138  1.00  0.00
ATOM  15032  C   ALA A 972     100.793 208.446 117.651  1.00  0.00
ATOM  15033  O   ALA A 972     100.256 209.344 118.285  1.00  0.00
ATOM  15034  N   ARG A 973     101.887 207.797 118.110  1.00  0.00
ATOM  15035  H   ARG A 973     102.150 206.972 117.579  1.00  0.00
ATOM  15036  CA  ARG A 973     102.860 208.197 119.175  1.00  0.00
ATOM  15037  HA  ARG A 973     103.845 207.872 118.842  1.00  0.00
ATOM  15038  CB  ARG A 973     102.950 209.722 119.421  1.00  0.00
ATOM  15039  HB1 ARG A 973     103.696 209.897 120.193  1.00  0.00
ATOM  15040  HB2 ARG A 973     102.008 210.089 119.834  1.00  0.00
ATOM  15041  CG  ARG A 973     103.354 210.616 118.231  1.00  0.00
ATOM  15042  HG1 ARG A 973     103.322 211.649 118.580  1.00  0.00
ATOM  15043  HG2 ARG A 973     102.621 210.526 117.431  1.00  0.00
ATOM  15044  CD  ARG A 973     104.772 210.341 117.700  1.00  0.00
ATOM  15045  HD1 ARG A 973     105.312 209.707 118.403  1.00  0.00
ATOM  15046  HD2 ARG A 973     105.299 211.292 117.621  1.00  0.00
ATOM  15047  NE  ARG A 973     104.740 209.713 116.369  1.00  0.00
ATOM  15048  HE  ARG A 973     103.856 209.696 115.882  1.00  0.00
ATOM  15049  CZ  ARG A 973     105.708 209.102 115.717  1.00  0.00
ATOM  15050  NH1 ARG A 973     106.904 208.918 116.199  1.00  0.00
ATOM  15051 1HH1 ARG A 973     107.173 209.265 117.092  1.00  0.00
ATOM  15052 2HH1 ARG A 973     107.519 208.292 115.661  1.00  0.00
ATOM  15053  NH2 ARG A 973     105.463 208.613 114.544  1.00  0.00
ATOM  15054 1HH2 ARG A 973     104.519 208.603 114.200  1.00  0.00
ATOM  15055 2HH2 ARG A 973     106.120 207.924 114.157  1.00  0.00
ATOM  15056  C   ARG A 973     102.702 207.485 120.542  1.00  0.00
ATOM  15057  O   ARG A 973     103.619 207.468 121.369  1.00  0.00
ATOM  15058  N   ASP A 974     101.536 206.876 120.726  1.00  0.00
ATOM  15059  H   ASP A 974     100.886 206.974 119.967  1.00  0.00
ATOM  15060  CA  ASP A 974     101.033 206.290 121.973  1.00  0.00
ATOM  15061  HA  ASP A 974      99.985 206.088 121.750  1.00  0.00
ATOM  15062  CB  ASP A 974     101.645 204.881 122.191  1.00  0.00
ATOM  15063  HB1 ASP A 974     102.270 204.624 121.334  1.00  0.00
ATOM  15064  HB2 ASP A 974     100.827 204.160 122.187  1.00  0.00
ATOM  15065  CG  ASP A 974     102.452 204.652 123.471  1.00  0.00
```

```
ATOM  15066  OD1 ASP A 974     103.636 204.261 123.351  1.00  0.00
ATOM  15067  OD2 ASP A 974     101.881 204.773 124.569  1.00  0.00
ATOM  15068  C   ASP A 974     100.970 207.282 123.174  1.00  0.00
ATOM  15069  O   ASP A 974     101.684 208.295 123.222  1.00  0.00
ATOM  15070  N   PRO A 975     100.067 207.055 124.149  1.00  0.00
ATOM  15071  CD  PRO A 975      99.082 205.985 124.227  1.00  0.00
ATOM  15072  HD1 PRO A 975      99.471 205.029 123.883  1.00  0.00
ATOM  15073  HD2 PRO A 975      98.204 206.263 123.642  1.00  0.00
ATOM  15074  CG  PRO A 975      98.713 205.894 125.702  1.00  0.00
ATOM  15075  HG1 PRO A 975      99.446 205.276 126.224  1.00  0.00
ATOM  15076  HG2 PRO A 975      97.707 205.499 125.847  1.00  0.00
ATOM  15077  CB  PRO A 975      98.831 207.341 126.173  1.00  0.00
ATOM  15078  HB1 PRO A 975      99.056 207.390 127.239  1.00  0.00
ATOM  15079  HB2 PRO A 975      97.899 207.867 125.958  1.00  0.00
ATOM  15080  CA  PRO A 975      99.954 207.929 125.312  1.00  0.00
ATOM  15081  HA  PRO A 975      99.656 208.921 124.975  1.00  0.00
ATOM  15082  C   PRO A 975     101.271 208.086 126.090  1.00  0.00
ATOM  15083  O   PRO A 975     101.549 209.173 126.587  1.00  0.00
ATOM  15084  N   GLN A 976     102.130 207.067 126.110  1.00  0.00
ATOM  15085  H   GLN A 976     101.863 206.203 125.638  1.00  0.00
ATOM  15086  CA  GLN A 976     103.483 207.121 126.661  1.00  0.00
ATOM  15087  HA  GLN A 976     103.556 208.003 127.299  1.00  0.00
ATOM  15088  CB  GLN A 976     103.755 205.881 127.540  1.00  0.00
ATOM  15089  HB1 GLN A 976     104.813 205.632 127.533  1.00  0.00
ATOM  15090  HB2 GLN A 976     103.213 205.025 127.134  1.00  0.00
ATOM  15091  CG  GLN A 976     103.354 206.092 129.007  1.00  0.00
ATOM  15092  HG1 GLN A 976     103.489 205.153 129.537  1.00  0.00
ATOM  15093  HG2 GLN A 976     102.295 206.345 129.048  1.00  0.00
ATOM  15094  CD  GLN A 976     104.154 207.176 129.736  1.00  0.00
ATOM  15095  OE1 GLN A 976     103.603 208.170 130.180  1.00  0.00
ATOM  15096  NE2 GLN A 976     105.452 207.064 129.934  1.00  0.00
ATOM  15097 1HE2 GLN A 976     106.008 206.356 129.461  1.00  0.00
ATOM  15098 2HE2 GLN A 976     105.862 207.755 130.548  1.00  0.00
ATOM  15099  C   GLN A 976     104.580 207.338 125.588  1.00  0.00
ATOM  15100  O   GLN A 976     105.750 207.083 125.888  1.00  0.00
ATOM  15101  N   ARG A 977     104.299 207.975 124.430  1.00  0.00
ATOM  15102  H   ARG A 977     103.337 207.991 124.102  1.00  0.00
ATOM  15103  CA  ARG A 977     105.119 209.185 124.196  1.00  0.00
ATOM  15104  HA  ARG A 977     106.061 209.076 124.730  1.00  0.00
ATOM  15105  CB  ARG A 977     105.438 209.431 122.714  1.00  0.00
ATOM  15106  HB1 ARG A 977     104.528 209.734 122.205  1.00  0.00
ATOM  15107  HB2 ARG A 977     105.744 208.501 122.257  1.00  0.00
ATOM  15108  CG  ARG A 977     106.520 210.511 122.446  1.00  0.00
ATOM  15109  HG1 ARG A 977     106.064 211.497 122.543  1.00  0.00
ATOM  15110  HG2 ARG A 977     106.867 210.399 121.419  1.00  0.00
ATOM  15111  CD  ARG A 977     107.723 210.445 123.401  1.00  0.00
ATOM  15112  HD1 ARG A 977     108.101 209.423 123.457  1.00  0.00
ATOM  15113  HD2 ARG A 977     107.400 210.714 124.406  1.00  0.00
ATOM  15114  NE  ARG A 977     108.821 211.368 123.050  1.00  0.00
ATOM  15115  HE  ARG A 977     108.908 212.208 123.604  1.00  0.00
ATOM  15116  CZ  ARG A 977     109.869 211.057 122.306  1.00  0.00
ATOM  15117  NH1 ARG A 977     109.926 209.947 121.613  1.00  0.00
ATOM  15118 1HH1 ARG A 977     109.210 209.249 121.726  1.00  0.00
ATOM  15119 2HH1 ARG A 977     110.709 209.733 121.013  1.00  0.00
ATOM  15120  NH2 ARG A 977     110.892 211.861 122.286  1.00  0.00
ATOM  15121 1HH2 ARG A 977     110.924 212.666 122.912  1.00  0.00
ATOM  15122 2HH2 ARG A 977     111.729 211.597 121.775  1.00  0.00
ATOM  15123  C   ARG A 977     104.481 210.466 124.731  1.00  0.00
ATOM  15124  O   ARG A 977     105.194 211.339 125.208  1.00  0.00
ATOM  15125  N   TYR A 978     103.179 210.644 124.498  1.00  0.00
ATOM  15126  H   TYR A 978     102.645 209.858 124.149  1.00  0.00
```

ATOM	15127	CA	TYR	A	978	102.705	211.985	124.138	1.00	0.00
ATOM	15128	HA	TYR	A	978	103.571	212.642	124.027	1.00	0.00
ATOM	15129	CB	TYR	A	978	102.031	211.912	122.760	1.00	0.00
ATOM	15130	HB1	TYR	A	978	100.969	211.693	122.879	1.00	0.00
ATOM	15131	HB2	TYR	A	978	102.462	211.094	122.182	1.00	0.00
ATOM	15132	CG	TYR	A	978	102.210	213.180	121.946	1.00	0.00
ATOM	15133	CD1	TYR	A	978	103.435	213.419	121.291	1.00	0.00
ATOM	15134	HD1	TYR	A	978	104.229	212.685	121.349	1.00	0.00
ATOM	15135	CE1	TYR	A	978	103.641	214.627	120.598	1.00	0.00
ATOM	15136	HE1	TYR	A	978	104.586	214.834	120.116	1.00	0.00
ATOM	15137	CZ	TYR	A	978	102.618	215.600	120.558	1.00	0.00
ATOM	15138	OH	TYR	A	978	102.827	216.783	119.925	1.00	0.00
ATOM	15139	HH	TYR	A	978	102.068	217.364	119.985	1.00	0.00
ATOM	15140	CE2	TYR	A	978	101.387	215.351	121.202	1.00	0.00
ATOM	15141	HE2	TYR	A	978	100.608	216.093	121.187	1.00	0.00
ATOM	15142	CD2	TYR	A	978	101.186	214.142	121.895	1.00	0.00
ATOM	15143	HD2	TYR	A	978	100.260	213.969	122.424	1.00	0.00
ATOM	15144	C	TYR	A	978	101.823	212.681	125.191	1.00	0.00
ATOM	15145	O	TYR	A	978	101.552	213.872	125.059	1.00	0.00
ATOM	15146	N	LEU	A	979	101.439	212.007	126.281	1.00	0.00
ATOM	15147	H	LEU	A	979	101.686	211.023	126.354	1.00	0.00
ATOM	15148	CA	LEU	A	979	100.832	212.630	127.469	1.00	0.00
ATOM	15149	HA	LEU	A	979	100.143	213.403	127.133	1.00	0.00
ATOM	15150	CB	LEU	A	979	100.021	211.587	128.266	1.00	0.00
ATOM	15151	HB1	LEU	A	979	99.624	212.067	129.160	1.00	0.00
ATOM	15152	HB2	LEU	A	979	100.697	210.795	128.594	1.00	0.00
ATOM	15153	CG	LEU	A	979	98.835	210.954	127.510	1.00	0.00
ATOM	15154	HG	LEU	A	979	99.204	210.399	126.652	1.00	0.00
ATOM	15155	CD1	LEU	A	979	98.102	209.980	128.437	1.00	0.00
ATOM	15156	1HD1	LEU	A	979	97.299	209.485	127.894	1.00	0.00
ATOM	15157	2HD1	LEU	A	979	98.808	209.234	128.802	1.00	0.00
ATOM	15158	3HD1	LEU	A	979	97.688	210.522	129.287	1.00	0.00
ATOM	15159	CD2	LEU	A	979	97.815	211.981	127.019	1.00	0.00
ATOM	15160	1HD2	LEU	A	979	96.966	211.473	126.565	1.00	0.00
ATOM	15161	2HD2	LEU	A	979	97.467	212.592	127.851	1.00	0.00
ATOM	15162	3HD2	LEU	A	979	98.266	212.622	126.263	1.00	0.00
ATOM	15163	C	LEU	A	979	101.870	213.379	128.349	1.00	0.00
ATOM	15164	O	LEU	A	979	101.814	213.343	129.573	1.00	0.00
ATOM	15165	N	VAL	A	980	102.785	214.089	127.679	1.00	0.00
ATOM	15166	H	VAL	A	980	102.635	214.098	126.679	1.00	0.00
ATOM	15167	CA	VAL	A	980	103.891	214.928	128.181	1.00	0.00
ATOM	15168	HA	VAL	A	980	104.492	215.121	127.294	1.00	0.00
ATOM	15169	CB	VAL	A	980	103.407	216.330	128.632	1.00	0.00
ATOM	15170	HB	VAL	A	980	104.287	216.957	128.771	1.00	0.00
ATOM	15171	CG1	VAL	A	980	102.564	217.029	127.557	1.00	0.00
ATOM	15172	1HG1	VAL	A	980	102.349	218.052	127.867	1.00	0.00
ATOM	15173	2HG1	VAL	A	980	103.109	217.050	126.614	1.00	0.00
ATOM	15174	3HG1	VAL	A	980	101.620	216.505	127.406	1.00	0.00
ATOM	15175	CG2	VAL	A	980	102.636	216.352	129.958	1.00	0.00
ATOM	15176	1HG2	VAL	A	980	102.488	217.382	130.280	1.00	0.00
ATOM	15177	2HG2	VAL	A	980	101.664	215.877	129.838	1.00	0.00
ATOM	15178	3HG2	VAL	A	980	103.195	215.825	130.729	1.00	0.00
ATOM	15179	C	VAL	A	980	104.900	214.319	129.175	1.00	0.00
ATOM	15180	O	VAL	A	980	104.791	213.197	129.665	1.00	0.00
ATOM	15181	N	ILE	A	981	105.957	215.103	129.431	1.00	0.00
ATOM	15182	H	ILE	A	981	105.978	216.010	128.997	1.00	0.00
ATOM	15183	CA	ILE	A	981	106.973	214.860	130.459	1.00	0.00
ATOM	15184	HA	ILE	A	981	107.375	213.864	130.280	1.00	0.00
ATOM	15185	CB	ILE	A	981	108.126	215.887	130.302	1.00	0.00
ATOM	15186	HB	ILE	A	981	108.496	215.800	129.280	1.00	0.00
ATOM	15187	CG2	ILE	A	981	107.654	217.340	130.497	1.00	0.00

```
ATOM  15188 1HG2 ILE A 981     108.476 218.028 130.301  1.00  0.00
ATOM  15189 2HG2 ILE A 981     106.845 217.588 129.811  1.00  0.00
ATOM  15190 3HG2 ILE A 981     107.306 217.502 131.518  1.00  0.00
ATOM  15191  CG1 ILE A 981     109.333 215.642 131.237  1.00  0.00
ATOM  15192 1HG1 ILE A 981     109.035 215.769 132.277  1.00  0.00
ATOM  15193 2HG1 ILE A 981     110.093 216.394 131.022  1.00  0.00
ATOM  15194  CD  ILE A 981     109.995 214.268 131.073  1.00  0.00
ATOM  15195  HD1 ILE A 981     109.317 213.473 131.379  1.00  0.00
ATOM  15196  HD2 ILE A 981     110.289 214.117 130.034  1.00  0.00
ATOM  15197  HD3 ILE A 981     110.885 214.224 131.700  1.00  0.00
ATOM  15198  C   ILE A 981     106.371 214.876 131.870  1.00  0.00
ATOM  15199  O   ILE A 981     105.435 215.613 132.171  1.00  0.00
ATOM  15200  N   GLN A 982     106.932 214.065 132.759  1.00  0.00
ATOM  15201  H   GLN A 982     107.740 213.547 132.451  1.00  0.00
ATOM  15202  CA  GLN A 982     106.290 213.588 133.988  1.00  0.00
ATOM  15203  HA  GLN A 982     105.517 214.298 134.283  1.00  0.00
ATOM  15204  CB  GLN A 982     105.586 212.249 133.674  1.00  0.00
ATOM  15205  HB1 GLN A 982     104.790 212.477 132.964  1.00  0.00
ATOM  15206  HB2 GLN A 982     105.112 211.863 134.577  1.00  0.00
ATOM  15207  CG  GLN A 982     106.514 211.168 133.079  1.00  0.00
ATOM  15208  HG1 GLN A 982     107.099 210.717 133.881  1.00  0.00
ATOM  15209  HG2 GLN A 982     107.212 211.609 132.374  1.00  0.00
ATOM  15210  CD  GLN A 982     105.788 210.054 132.323  1.00  0.00
ATOM  15211  OE1 GLN A 982     106.155 208.897 132.416  1.00  0.00
ATOM  15212  NE2 GLN A 982     104.831 210.356 131.472  1.00  0.00
ATOM  15213 1HE2 GLN A 982     104.579 211.306 131.240  1.00  0.00
ATOM  15214 2HE2 GLN A 982     104.368 209.588 130.985  1.00  0.00
ATOM  15215  C   GLN A 982     107.283 213.536 135.159  1.00  0.00
ATOM  15216  O   GLN A 982     107.402 212.532 135.859  1.00  0.00
ATOM  15217  N   GLY A 983     108.042 214.624 135.326  1.00  0.00
ATOM  15218  H   GLY A 983     107.847 215.412 134.729  1.00  0.00
ATOM  15219  CA  GLY A 983     109.019 214.825 136.399  1.00  0.00
ATOM  15220  HA1 GLY A 983     109.616 215.713 136.193  1.00  0.00
ATOM  15221  HA2 GLY A 983     109.691 213.967 136.448  1.00  0.00
ATOM  15222  C   GLY A 983     108.358 214.999 137.769  1.00  0.00
ATOM  15223  O   GLY A 983     108.306 216.102 138.302  1.00  0.00
ATOM  15224  N   ASP A 984     107.850 213.887 138.300  1.00  0.00
ATOM  15225  H   ASP A 984     107.866 213.061 137.712  1.00  0.00
ATOM  15226  CA  ASP A 984     107.329 213.700 139.662  1.00  0.00
ATOM  15227  HA  ASP A 984     106.392 214.248 139.769  1.00  0.00
ATOM  15228  CB  ASP A 984     107.032 212.191 139.776  1.00  0.00
ATOM  15229  HB1 ASP A 984     107.977 211.660 139.649  1.00  0.00
ATOM  15230  HB2 ASP A 984     106.376 211.904 138.951  1.00  0.00
ATOM  15231  CG  ASP A 984     106.382 211.685 141.070  1.00  0.00
ATOM  15232  OD1 ASP A 984     106.537 210.472 141.338  1.00  0.00
ATOM  15233  OD2 ASP A 984     105.805 212.483 141.837  1.00  0.00
ATOM  15234  C   ASP A 984     108.293 214.214 140.755  1.00  0.00
ATOM  15235  O   ASP A 984     107.873 214.585 141.851  1.00  0.00
ATOM  15236  N   GLU A 985     109.596 214.288 140.474  1.00  0.00
ATOM  15237  H   GLU A 985     109.917 213.978 139.572  1.00  0.00
ATOM  15238  CA  GLU A 985     110.560 214.996 141.323  1.00  0.00
ATOM  15239  HA  GLU A 985     110.104 215.934 141.644  1.00  0.00
ATOM  15240  CB  GLU A 985     110.870 214.130 142.561  1.00  0.00
ATOM  15241  HB1 GLU A 985     111.896 213.763 142.537  1.00  0.00
ATOM  15242  HB2 GLU A 985     110.228 213.255 142.570  1.00  0.00
ATOM  15243  CG  GLU A 985     110.660 214.909 143.859  1.00  0.00
ATOM  15244  HG1 GLU A 985     109.654 215.329 143.879  1.00  0.00
ATOM  15245  HG2 GLU A 985     111.375 215.733 143.903  1.00  0.00
ATOM  15246  CD  GLU A 985     110.853 213.958 145.034  1.00  0.00
ATOM  15247  OE1 GLU A 985     112.022 213.825 145.458  1.00  0.00
ATOM  15248  OE2 GLU A 985     109.844 213.324 145.431  1.00  0.00
```

```
ATOM  15249  C   GLU A 985      111.865 215.348 140.580  1.00  0.00
ATOM  15250  O   GLU A 985      112.017 215.113 139.380  1.00  0.00
ATOM  15251  N   ARG A 986      112.859 215.861 141.324  1.00  0.00
ATOM  15252  H   ARG A 986      112.667 216.006 142.306  1.00  0.00
ATOM  15253  CA  ARG A 986      114.262 215.961 140.870  1.00  0.00
ATOM  15254  HA  ARG A 986      114.249 216.482 139.913  1.00  0.00
ATOM  15255  CB  ARG A 986      115.056 216.813 141.886  1.00  0.00
ATOM  15256  HB1 ARG A 986      115.159 216.273 142.829  1.00  0.00
ATOM  15257  HB2 ARG A 986      114.463 217.706 142.090  1.00  0.00
ATOM  15258  CG  ARG A 986      116.439 217.303 141.404  1.00  0.00
ATOM  15259  HG1 ARG A 986      116.631 218.274 141.864  1.00  0.00
ATOM  15260  HG2 ARG A 986      116.403 217.468 140.326  1.00  0.00
ATOM  15261  CD  ARG A 986      117.623 216.385 141.770  1.00  0.00
ATOM  15262  HD1 ARG A 986      117.242 215.468 142.223  1.00  0.00
ATOM  15263  HD2 ARG A 986      118.245 216.885 142.513  1.00  0.00
ATOM  15264  NE  ARG A 986      118.420 216.023 140.582  1.00  0.00
ATOM  15265  HE  ARG A 986      117.992 215.347 139.966  1.00  0.00
ATOM  15266  CZ  ARG A 986      119.590 216.501 140.187  1.00  0.00
ATOM  15267  NH1 ARG A 986      120.310 217.336 140.882  1.00  0.00
ATOM  15268 1HH1 ARG A 986      120.030 217.662 141.779  1.00  0.00
ATOM  15269 2HH1 ARG A 986      121.242 217.600 140.517  1.00  0.00
ATOM  15270  NH2 ARG A 986      120.080 216.134 139.049  1.00  0.00
ATOM  15271 1HH2 ARG A 986      119.594 215.522 138.426  1.00  0.00
ATOM  15272 2HH2 ARG A 986      120.902 216.653 138.699  1.00  0.00
ATOM  15273  C   ARG A 986      114.912 214.597 140.579  1.00  0.00
ATOM  15274  O   ARG A 986      115.979 214.553 139.971  1.00  0.00
ATOM  15275  N   MET A 987      114.257 213.510 140.979  1.00  0.00
ATOM  15276  H   MET A 987      113.375 213.649 141.454  1.00  0.00
ATOM  15277  CA  MET A 987      114.542 212.132 140.588  1.00  0.00
ATOM  15278  HA  MET A 987      115.135 212.138 139.672  1.00  0.00
ATOM  15279  CB  MET A 987      115.367 211.427 141.687  1.00  0.00
ATOM  15280  HB1 MET A 987      116.294 211.985 141.821  1.00  0.00
ATOM  15281  HB2 MET A 987      115.639 210.427 141.353  1.00  0.00
ATOM  15282  CG  MET A 987      114.686 211.310 143.062  1.00  0.00
ATOM  15283  HG1 MET A 987      114.206 212.258 143.305  1.00  0.00
ATOM  15284  HG2 MET A 987      115.464 211.139 143.805  1.00  0.00
ATOM  15285  SD  MET A 987      113.472 209.973 143.245  1.00  0.00
ATOM  15286  CE  MET A 987      112.737 210.483 144.821  1.00  0.00
ATOM  15287  HE1 MET A 987      112.051 209.715 145.176  1.00  0.00
ATOM  15288  HE2 MET A 987      113.520 210.639 145.562  1.00  0.00
ATOM  15289  HE3 MET A 987      112.191 211.415 144.685  1.00  0.00
ATOM  15290  C   MET A 987      113.222 211.414 140.290  1.00  0.00
ATOM  15291  O   MET A 987      112.154 211.808 140.749  1.00  0.00
ATOM  15292  N   HIS A 988      113.312 210.360 139.487  1.00  0.00
ATOM  15293  H   HIS A 988      114.230 210.072 139.183  1.00  0.00
ATOM  15294  CA  HIS A 988      112.206 209.482 139.101  1.00  0.00
ATOM  15295  HA  HIS A 988      111.629 209.228 139.993  1.00  0.00
ATOM  15296  CB  HIS A 988      111.290 210.205 138.087  1.00  0.00
ATOM  15297  HB1 HIS A 988      111.710 210.134 137.083  1.00  0.00
ATOM  15298  HB2 HIS A 988      111.231 211.265 138.337  1.00  0.00
ATOM  15299  CG  HIS A 988      109.864 209.704 138.072  1.00  0.00
ATOM  15300  ND1 HIS A 988      109.258 208.940 139.072  1.00  0.00
ATOM  15301  CE1 HIS A 988      107.949 208.910 138.774  1.00  0.00
ATOM  15302  HE1 HIS A 988      107.180 208.492 139.417  1.00  0.00
ATOM  15303  NE2 HIS A 988      107.725 209.536 137.607  1.00  0.00
ATOM  15304  HE2 HIS A 988      106.815 209.722 137.209  1.00  0.00
ATOM  15305  CD2 HIS A 988      108.920 210.045 137.151  1.00  0.00
ATOM  15306  HD2 HIS A 988      109.074 210.666 136.278  1.00  0.00
ATOM  15307  C   HIS A 988      112.822 208.205 138.513  1.00  0.00
ATOM  15308  O   HIS A 988      114.016 207.957 138.687  1.00  0.00
ATOM  15309  N   LEU A 989      112.032 207.422 137.778  1.00  0.00
```

```
ATOM  15310  H   LEU A 989     111.060 207.694 137.715  1.00  0.00
ATOM  15311  CA  LEU A 989     112.533 206.443 136.805  1.00  0.00
ATOM  15312  HA  LEU A 989     113.540 206.734 136.501  1.00  0.00
ATOM  15313  CB  LEU A 989     112.597 205.042 137.443  1.00  0.00
ATOM  15314  HB1 LEU A 989     112.684 204.313 136.640  1.00  0.00
ATOM  15315  HB2 LEU A 989     111.659 204.858 137.957  1.00  0.00
ATOM  15316  CG  LEU A 989     113.753 204.805 138.437  1.00  0.00
ATOM  15317  HG  LEU A 989     113.723 205.553 139.222  1.00  0.00
ATOM  15318  CD1 LEU A 989     113.566 203.453 139.117  1.00  0.00
ATOM  15319 1HD1 LEU A 989     114.443 203.186 139.704  1.00  0.00
ATOM  15320 2HD1 LEU A 989     112.729 203.524 139.803  1.00  0.00
ATOM  15321 3HD1 LEU A 989     113.323 202.676 138.398  1.00  0.00
ATOM  15322  CD2 LEU A 989     115.133 204.840 137.773  1.00  0.00
ATOM  15323 1HD2 LEU A 989     115.904 204.575 138.495  1.00  0.00
ATOM  15324 2HD2 LEU A 989     115.191 204.160 136.930  1.00  0.00
ATOM  15325 3HD2 LEU A 989     115.348 205.847 137.414  1.00  0.00
ATOM  15326  C   LEU A 989     111.713 206.436 135.497  1.00  0.00
ATOM  15327  O   LEU A 989     112.332 206.312 134.445  1.00  0.00
ATOM  15328  N   PRO A 990     110.374 206.612 135.501  1.00  0.00
ATOM  15329  CD  PRO A 990     109.448 206.279 136.574  1.00  0.00
ATOM  15330  HD1 PRO A 990     109.453 207.064 137.324  1.00  0.00
ATOM  15331  HD2 PRO A 990     109.690 205.318 137.026  1.00  0.00
ATOM  15332  CG  PRO A 990     108.066 206.210 135.929  1.00  0.00
ATOM  15333  HG1 PRO A 990     107.280 206.506 136.623  1.00  0.00
ATOM  15334  HG2 PRO A 990     107.888 205.200 135.557  1.00  0.00
ATOM  15335  CB  PRO A 990     108.177 207.176 134.752  1.00  0.00
ATOM  15336  HB1 PRO A 990     107.981 208.191 135.095  1.00  0.00
ATOM  15337  HB2 PRO A 990     107.486 206.911 133.951  1.00  0.00
ATOM  15338  CA  PRO A 990     109.637 207.031 134.313  1.00  0.00
ATOM  15339  HA  PRO A 990     109.701 206.227 133.579  1.00  0.00
ATOM  15340  C   PRO A 990     110.152 208.328 133.663  1.00  0.00
ATOM  15341  O   PRO A 990     110.404 209.322 134.349  1.00  0.00
ATOM  15342  N   SER A 991     110.212 208.327 132.330  1.00  0.00
ATOM  15343  H   SER A 991     110.104 207.444 131.851  1.00  0.00
ATOM  15344  CA  SER A 991     110.193 209.506 131.459  1.00  0.00
ATOM  15345  HA  SER A 991     109.446 210.212 131.815  1.00  0.00
ATOM  15346  CB  SER A 991     111.562 210.199 131.424  1.00  0.00
ATOM  15347  HB1 SER A 991     112.308 209.512 131.020  1.00  0.00
ATOM  15348  HB2 SER A 991     111.854 210.496 132.432  1.00  0.00
ATOM  15349  OG  SER A 991     111.464 211.347 130.602  1.00  0.00
ATOM  15350  HG  SER A 991     112.331 211.537 130.214  1.00  0.00
ATOM  15351  C   SER A 991     109.886 209.045 130.026  1.00  0.00
ATOM  15352  O   SER A 991     110.422 208.014 129.613  1.00  0.00
ATOM  15353  N   PRO A 992     109.045 209.759 129.256  1.00  0.00
ATOM  15354  CD  PRO A 992     108.101 210.766 129.710  1.00  0.00
ATOM  15355  HD1 PRO A 992     108.609 211.686 129.995  1.00  0.00
ATOM  15356  HD2 PRO A 992     107.544 210.356 130.544  1.00  0.00
ATOM  15357  CG  PRO A 992     107.136 211.017 128.548  1.00  0.00
ATOM  15358  HG1 PRO A 992     107.448 211.901 127.988  1.00  0.00
ATOM  15359  HG2 PRO A 992     106.108 211.116 128.895  1.00  0.00
ATOM  15360  CB  PRO A 992     107.300 209.766 127.685  1.00  0.00
ATOM  15361  HB1 PRO A 992     107.029 209.951 126.648  1.00  0.00
ATOM  15362  HB2 PRO A 992     106.695 208.955 128.096  1.00  0.00
ATOM  15363  CA  PRO A 992     108.784 209.445 127.858  1.00  0.00
ATOM  15364  HA  PRO A 992     108.951 208.388 127.652  1.00  0.00
ATOM  15365  C   PRO A 992     109.629 210.270 126.886  1.00  0.00
ATOM  15366  O   PRO A 992     109.523 210.069 125.679  1.00  0.00
ATOM  15367  N   THR A 993     110.434 211.237 127.332  1.00  0.00
ATOM  15368  H   THR A 993     110.573 211.370 128.326  1.00  0.00
ATOM  15369  CA  THR A 993     111.171 212.096 126.397  1.00  0.00
ATOM  15370  HA  THR A 993     111.433 211.484 125.534  1.00  0.00
```

ATOM	15371	CB	THR	A	993	110.277	213.227	125.866	1.00	0.00
ATOM	15372	HB	THR	A	993	109.304	212.792	125.638	1.00	0.00
ATOM	15373	CG2	THR	A	993	110.026	214.392	126.825	1.00	0.00
ATOM	15374	1HG2	THR	A	993	109.397	215.135	126.338	1.00	0.00
ATOM	15375	2HG2	THR	A	993	109.519	214.018	127.713	1.00	0.00
ATOM	15376	3HG2	THR	A	993	110.965	214.860	127.119	1.00	0.00
ATOM	15377	OG1	THR	A	993	110.794	213.683	124.637	1.00	0.00
ATOM	15378	HG1	THR	A	993	111.454	214.400	124.850	1.00	0.00
ATOM	15379	C	THR	A	993	112.497	212.573	126.970	1.00	0.00
ATOM	15380	O	THR	A	993	112.617	212.838	128.166	1.00	0.00
ATOM	15381	N	ASP	A	994	113.487	212.650	126.071	1.00	0.00
ATOM	15382	H	ASP	A	994	113.254	212.489	125.104	1.00	0.00
ATOM	15383	CA	ASP	A	994	114.817	213.226	126.323	1.00	0.00
ATOM	15384	HA	ASP	A	994	115.430	213.010	125.447	1.00	0.00
ATOM	15385	CB	ASP	A	994	114.671	214.756	126.371	1.00	0.00
ATOM	15386	HB1	ASP	A	994	115.660	215.217	126.374	1.00	0.00
ATOM	15387	HB2	ASP	A	994	114.141	215.052	127.278	1.00	0.00
ATOM	15388	CG	ASP	A	994	113.886	215.213	125.131	1.00	0.00
ATOM	15389	OD1	ASP	A	994	114.473	215.152	124.031	1.00	0.00
ATOM	15390	OD2	ASP	A	994	112.655	215.438	125.249	1.00	0.00
ATOM	15391	C	ASP	A	994	115.541	212.533	127.504	1.00	0.00
ATOM	15392	O	ASP	A	994	116.235	213.122	128.329	1.00	0.00
ATOM	15393	N	SER	A	995	115.315	211.219	127.577	1.00	0.00
ATOM	15394	H	SER	A	995	114.645	210.861	126.914	1.00	0.00
ATOM	15395	CA	SER	A	995	115.632	210.314	128.684	1.00	0.00
ATOM	15396	HA	SER	A	995	115.320	210.814	129.600	1.00	0.00
ATOM	15397	CB	SER	A	995	114.758	209.055	128.541	1.00	0.00
ATOM	15398	HB1	SER	A	995	114.912	208.395	129.395	1.00	0.00
ATOM	15399	HB2	SER	A	995	115.029	208.522	127.628	1.00	0.00
ATOM	15400	OG	SER	A	995	113.386	209.425	128.482	1.00	0.00
ATOM	15401	HG	SER	A	995	112.900	208.732	127.990	1.00	0.00
ATOM	15402	C	SER	A	995	117.122	209.965	128.884	1.00	0.00
ATOM	15403	O	SER	A	995	117.445	208.823	129.214	1.00	0.00
ATOM	15404	N	ASN	A	996	118.042	210.928	128.757	1.00	0.00
ATOM	15405	H	ASN	A	996	117.723	211.844	128.452	1.00	0.00
ATOM	15406	CA	ASN	A	996	119.379	210.845	129.381	1.00	0.00
ATOM	15407	HA	ASN	A	996	119.772	209.846	129.186	1.00	0.00
ATOM	15408	CB	ASN	A	996	120.361	211.849	128.728	1.00	0.00
ATOM	15409	HB1	ASN	A	996	120.715	211.415	127.794	1.00	0.00
ATOM	15410	HB2	ASN	A	996	121.231	211.996	129.371	1.00	0.00
ATOM	15411	CG	ASN	A	996	119.759	213.199	128.389	1.00	0.00
ATOM	15412	OD1	ASN	A	996	119.233	213.375	127.306	1.00	0.00
ATOM	15413	ND2	ASN	A	996	119.813	214.191	129.248	1.00	0.00
ATOM	15414	1HD2	ASN	A	996	120.389	214.189	130.099	1.00	0.00
ATOM	15415	2HD2	ASN	A	996	119.390	215.047	128.949	1.00	0.00
ATOM	15416	C	ASN	A	996	119.363	210.962	130.914	1.00	0.00
ATOM	15417	O	ASN	A	996	120.409	210.839	131.540	1.00	0.00
ATOM	15418	N	PHE	A	997	118.187	211.182	131.510	1.00	0.00
ATOM	15419	H	PHE	A	997	117.399	211.287	130.893	1.00	0.00
ATOM	15420	CA	PHE	A	997	117.923	211.413	132.938	1.00	0.00
ATOM	15421	HA	PHE	A	997	118.561	212.244	133.236	1.00	0.00
ATOM	15422	CB	PHE	A	997	116.458	211.890	133.041	1.00	0.00
ATOM	15423	HB1	PHE	A	997	115.788	211.032	132.958	1.00	0.00
ATOM	15424	HB2	PHE	A	997	116.238	212.540	132.191	1.00	0.00
ATOM	15425	CG	PHE	A	997	116.124	212.685	134.289	1.00	0.00
ATOM	15426	CD1	PHE	A	997	115.379	212.108	135.335	1.00	0.00
ATOM	15427	HD1	PHE	A	997	115.066	211.075	135.260	1.00	0.00
ATOM	15428	CE1	PHE	A	997	115.035	212.874	136.463	1.00	0.00
ATOM	15429	HE1	PHE	A	997	114.454	212.435	137.255	1.00	0.00
ATOM	15430	CZ	PHE	A	997	115.438	214.217	136.553	1.00	0.00
ATOM	15431	HZ	PHE	A	997	115.168	214.802	137.411	1.00	0.00

ATOM	15432	CE2	PHE	A	997	116.187	214.796	135.512	1.00	0.00
ATOM	15433	HE2	PHE	A	997	116.503	215.828	135.575	1.00	0.00
ATOM	15434	CD2	PHE	A	997	116.524	214.034	134.380	1.00	0.00
ATOM	15435	HD2	PHE	A	997	117.096	214.482	133.579	1.00	0.00
ATOM	15436	C	PHE	A	997	118.262	210.237	133.871	1.00	0.00
ATOM	15437	O	PHE	A	997	117.387	209.739	134.580	1.00	0.00
ATOM	15438	N	TYR	A	998	119.515	209.758	133.879	1.00	0.00
ATOM	15439	H	TYR	A	998	120.182	210.228	133.273	1.00	0.00
ATOM	15440	CA	TYR	A	998	119.969	208.512	134.526	1.00	0.00
ATOM	15441	HA	TYR	A	998	119.207	207.782	134.251	1.00	0.00
ATOM	15442	CB	TYR	A	998	121.258	207.980	133.884	1.00	0.00
ATOM	15443	HB1	TYR	A	998	121.185	208.113	132.804	1.00	0.00
ATOM	15444	HB2	TYR	A	998	121.275	206.903	134.053	1.00	0.00
ATOM	15445	CG	TYR	A	998	122.600	208.533	134.341	1.00	0.00
ATOM	15446	CD1	TYR	A	998	123.110	209.718	133.775	1.00	0.00
ATOM	15447	HD1	TYR	A	998	122.518	210.294	133.073	1.00	0.00
ATOM	15448	CE1	TYR	A	998	124.403	210.158	134.114	1.00	0.00
ATOM	15449	HE1	TYR	A	998	124.793	211.073	133.677	1.00	0.00
ATOM	15450	CZ	TYR	A	998	125.204	209.416	135.006	1.00	0.00
ATOM	15451	OH	TYR	A	998	126.471	209.817	135.298	1.00	0.00
ATOM	15452	HH	TYR	A	998	126.940	209.220	135.883	1.00	0.00
ATOM	15453	CE2	TYR	A	998	124.697	208.229	135.566	1.00	0.00
ATOM	15454	HE2	TYR	A	998	125.317	207.656	136.240	1.00	0.00
ATOM	15455	CD2	TYR	A	998	123.397	207.795	135.238	1.00	0.00
ATOM	15456	HD2	TYR	A	998	123.012	206.878	135.663	1.00	0.00
ATOM	15457	C	TYR	A	998	119.938	208.480	136.074	1.00	0.00
ATOM	15458	O	TYR	A	998	120.564	207.625	136.693	1.00	0.00
ATOM	15459	N	ARG	A	999	119.150	209.376	136.687	1.00	0.00
ATOM	15460	H	ARG	A	999	118.638	209.964	136.044	1.00	0.00
ATOM	15461	CA	ARG	A	999	118.820	209.530	138.114	1.00	0.00
ATOM	15462	HA	ARG	A	999	118.053	210.301	138.183	1.00	0.00
ATOM	15463	CB	ARG	A	999	118.183	208.207	138.602	1.00	0.00
ATOM	15464	HB1	ARG	A	999	118.978	207.476	138.747	1.00	0.00
ATOM	15465	HB2	ARG	A	999	117.522	207.831	137.818	1.00	0.00
ATOM	15466	CG	ARG	A	999	117.369	208.307	139.891	1.00	0.00
ATOM	15467	HG1	ARG	A	999	116.514	208.964	139.738	1.00	0.00
ATOM	15468	HG2	ARG	A	999	117.991	208.716	140.677	1.00	0.00
ATOM	15469	CD	ARG	A	999	116.875	206.907	140.286	1.00	0.00
ATOM	15470	HD1	ARG	A	999	117.697	206.197	140.178	1.00	0.00
ATOM	15471	HD2	ARG	A	999	116.076	206.607	139.614	1.00	0.00
ATOM	15472	NE	ARG	A	999	116.425	206.848	141.683	1.00	0.00
ATOM	15473	HE	ARG	A	999	117.155	206.680	142.381	1.00	0.00
ATOM	15474	CZ	ARG	A	999	115.217	207.022	142.182	1.00	0.00
ATOM	15475	NH1	ARG	A	999	114.154	207.178	141.444	1.00	0.00
ATOM	15476	1HH1	ARG	A	999	114.267	207.331	140.450	1.00	0.00
ATOM	15477	2HH1	ARG	A	999	113.264	207.334	141.895	1.00	0.00
ATOM	15478	NH2	ARG	A	999	115.061	207.036	143.467	1.00	0.00
ATOM	15479	1HH2	ARG	A	999	115.916	207.260	143.997	1.00	0.00
ATOM	15480	2HH2	ARG	A	999	114.198	207.353	143.856	1.00	0.00
ATOM	15481	C	ARG	A	999	119.972	210.027	138.994	1.00	0.00
ATOM	15482	O	ARG	A	999	119.768	210.945	139.787	1.00	0.00
ATOM	15483	N	ALA	A	1000	121.185	209.530	138.768	1.00	0.00
ATOM	15484	H	ALA	A	1000	121.207	208.778	138.088	1.00	0.00
ATOM	15485	CA	ALA	A	1000	122.335	210.433	138.720	1.00	0.00
ATOM	15486	HA	ALA	A	1000	122.289	211.138	139.552	1.00	0.00
ATOM	15487	CB	ALA	A	1000	123.638	209.638	138.820	1.00	0.00
ATOM	15488	HB1	ALA	A	1000	124.485	210.324	138.771	1.00	0.00
ATOM	15489	HB2	ALA	A	1000	123.668	209.089	139.762	1.00	0.00
ATOM	15490	HB3	ALA	A	1000	123.707	208.938	137.991	1.00	0.00
ATOM	15491	C	ALA	A	1000	122.231	211.225	137.400	1.00	0.00
ATOM	15492	O	ALA	A	1000	121.751	210.703	136.404	1.00	0.00

```
ATOM  15493  N   LEU A1001     122.678 212.475 137.389  1.00  0.00
ATOM  15494  H   LEU A1001     123.132 212.843 138.210  1.00  0.00
ATOM  15495  CA  LEU A1001     123.014 213.147 136.137  1.00  0.00
ATOM  15496  HA  LEU A1001     122.682 212.572 135.270  1.00  0.00
ATOM  15497  CB  LEU A1001     122.327 214.524 136.067  1.00  0.00
ATOM  15498  HB1 LEU A1001     122.651 215.026 135.159  1.00  0.00
ATOM  15499  HB2 LEU A1001     122.657 215.132 136.909  1.00  0.00
ATOM  15500  CG  LEU A1001     120.784 214.454 136.063  1.00  0.00
ATOM  15501  HG  LEU A1001     120.450 214.139 137.051  1.00  0.00
ATOM  15502  CD1 LEU A1001     120.187 215.833 135.784  1.00  0.00
ATOM  15503 1HD1 LEU A1001     119.117 215.830 135.974  1.00  0.00
ATOM  15504 2HD1 LEU A1001     120.676 216.589 136.397  1.00  0.00
ATOM  15505 3HD1 LEU A1001     120.361 216.102 134.742  1.00  0.00
ATOM  15506  CD2 LEU A1001     120.168 213.502 135.033  1.00  0.00
ATOM  15507 1HD2 LEU A1001     119.084 213.543 135.096  1.00  0.00
ATOM  15508 2HD2 LEU A1001     120.495 213.781 134.030  1.00  0.00
ATOM  15509 3HD2 LEU A1001     120.476 212.480 135.218  1.00  0.00
ATOM  15510  C   LEU A1001     124.541 213.179 136.027  1.00  0.00
ATOM  15511  O   LEU A1001     125.230 212.854 137.004  1.00  0.00
ATOM  15512  N   MET A1002     125.058 213.522 134.843  1.00  0.00
ATOM  15513  H   MET A1002     124.425 213.801 134.091  1.00  0.00
ATOM  15514  CA  MET A1002     126.492 213.793 134.698  1.00  0.00
ATOM  15515  HA  MET A1002     127.054 212.917 135.019  1.00  0.00
ATOM  15516  CB  MET A1002     126.867 214.121 133.243  1.00  0.00
ATOM  15517  HB1 MET A1002     127.946 214.255 133.182  1.00  0.00
ATOM  15518  HB2 MET A1002     126.407 215.065 132.963  1.00  0.00
ATOM  15519  CG  MET A1002     126.447 213.071 132.200  1.00  0.00
ATOM  15520  HG1 MET A1002     126.698 213.466 131.215  1.00  0.00
ATOM  15521  HG2 MET A1002     125.362 212.970 132.239  1.00  0.00
ATOM  15522  SD  MET A1002     127.186 211.418 132.352  1.00  0.00
ATOM  15523  CE  MET A1002     128.908 211.768 131.930  1.00  0.00
ATOM  15524  HE1 MET A1002     128.955 212.195 130.930  1.00  0.00
ATOM  15525  HE2 MET A1002     129.328 212.470 132.647  1.00  0.00
ATOM  15526  HE3 MET A1002     129.488 210.848 131.958  1.00  0.00
ATOM  15527  C   MET A1002     126.866 214.977 135.599  1.00  0.00
ATOM  15528  O   MET A1002     126.051 215.861 135.835  1.00  0.00
ATOM  15529  N   ASP A1003     128.104 215.038 136.081  1.00  0.00
ATOM  15530  H   ASP A1003     128.806 214.393 135.739  1.00  0.00
ATOM  15531  CA  ASP A1003     128.594 216.249 136.747  1.00  0.00
ATOM  15532  HA  ASP A1003     127.933 216.479 137.584  1.00  0.00
ATOM  15533  CB  ASP A1003     129.998 215.998 137.314  1.00  0.00
ATOM  15534  HB1 ASP A1003     129.890 215.339 138.178  1.00  0.00
ATOM  15535  HB2 ASP A1003     130.396 216.943 137.682  1.00  0.00
ATOM  15536  CG  ASP A1003     131.005 215.360 136.352  1.00  0.00
ATOM  15537  OD1 ASP A1003     132.072 214.958 136.855  1.00  0.00
ATOM  15538  OD2 ASP A1003     130.711 215.188 135.140  1.00  0.00
ATOM  15539  C   ASP A1003     128.541 217.471 135.803  1.00  0.00
ATOM  15540  O   ASP A1003     128.025 218.518 136.185  1.00  0.00
ATOM  15541  N   GLU A1004     128.921 217.269 134.538  1.00  0.00
ATOM  15542  H   GLU A1004     129.446 216.409 134.385  1.00  0.00
ATOM  15543  CA  GLU A1004     128.706 218.136 133.361  1.00  0.00
ATOM  15544  HA  GLU A1004     129.331 219.024 133.453  1.00  0.00
ATOM  15545  CB  GLU A1004     129.143 217.306 132.125  1.00  0.00
ATOM  15546  HB1 GLU A1004     128.337 216.612 131.883  1.00  0.00
ATOM  15547  HB2 GLU A1004     130.004 216.696 132.389  1.00  0.00
ATOM  15548  CG  GLU A1004     129.512 218.088 130.853  1.00  0.00
ATOM  15549  HG1 GLU A1004     130.453 218.615 131.014  1.00  0.00
ATOM  15550  HG2 GLU A1004     128.753 218.837 130.633  1.00  0.00
ATOM  15551  CD  GLU A1004     129.636 217.152 129.641  1.00  0.00
ATOM  15552  OE1 GLU A1004     128.728 217.115 128.780  1.00  0.00
ATOM  15553  OE2 GLU A1004     130.658 216.442 129.484  1.00  0.00
```

ATOM	15554	C	GLU	A1004	127.246	218.603	133.112	1.00	0.00
ATOM	15555	O	GLU	A1004	126.988	219.443	132.255	1.00	0.00
ATOM	15556	N	GLU	A1005	126.269	218.021	133.812	1.00	0.00
ATOM	15557	H	GLU	A1005	126.542	217.351	134.521	1.00	0.00
ATOM	15558	CA	GLU	A1005	124.821	218.226	133.627	1.00	0.00
ATOM	15559	HA	GLU	A1005	124.651	218.986	132.866	1.00	0.00
ATOM	15560	CB	GLU	A1005	124.248	216.868	133.123	1.00	0.00
ATOM	15561	HB1	GLU	A1005	124.395	216.143	133.919	1.00	0.00
ATOM	15562	HB2	GLU	A1005	124.857	216.555	132.273	1.00	0.00
ATOM	15563	CG	GLU	A1005	122.778	216.742	132.655	1.00	0.00
ATOM	15564	HG1	GLU	A1005	122.631	217.407	131.800	1.00	0.00
ATOM	15565	HG2	GLU	A1005	122.121	217.071	133.460	1.00	0.00
ATOM	15566	CD	GLU	A1005	122.377	215.290	132.255	1.00	0.00
ATOM	15567	OE1	GLU	A1005	121.392	215.121	131.486	1.00	0.00
ATOM	15568	OE2	GLU	A1005	123.027	214.327	132.734	1.00	0.00
ATOM	15569	C	GLU	A1005	124.153	218.730	134.931	1.00	0.00
ATOM	15570	O	GLU	A1005	122.978	219.093	134.917	1.00	0.00
ATOM	15571	N	ASP	A1006	124.916	218.827	136.033	1.00	0.00
ATOM	15572	H	ASP	A1006	125.875	218.509	135.955	1.00	0.00
ATOM	15573	CA	ASP	A1006	124.483	219.327	137.350	1.00	0.00
ATOM	15574	HA	ASP	A1006	123.454	219.676	137.251	1.00	0.00
ATOM	15575	CB	ASP	A1006	124.498	218.182	138.388	1.00	0.00
ATOM	15576	HB1	ASP	A1006	125.230	218.395	139.169	1.00	0.00
ATOM	15577	HB2	ASP	A1006	124.802	217.247	137.914	1.00	0.00
ATOM	15578	CG	ASP	A1006	123.123	217.946	139.037	1.00	0.00
ATOM	15579	OD1	ASP	A1006	122.999	217.875	140.282	1.00	0.00
ATOM	15580	OD2	ASP	A1006	122.127	217.731	138.315	1.00	0.00
ATOM	15581	C	ASP	A1006	125.284	220.560	137.834	1.00	0.00
ATOM	15582	O	ASP	A1006	124.945	221.154	138.857	1.00	0.00
ATOM	15583	N	MET	A1007	126.316	220.969	137.085	1.00	0.00
ATOM	15584	H	MET	A1007	126.552	220.415	136.269	1.00	0.00
ATOM	15585	CA	MET	A1007	127.141	222.167	137.296	1.00	0.00
ATOM	15586	HA	MET	A1007	126.576	222.922	137.841	1.00	0.00
ATOM	15587	CB	MET	A1007	128.413	221.793	138.076	1.00	0.00
ATOM	15588	HB1	MET	A1007	129.017	222.690	138.215	1.00	0.00
ATOM	15589	HB2	MET	A1007	128.987	221.088	137.476	1.00	0.00
ATOM	15590	CG	MET	A1007	128.156	221.174	139.459	1.00	0.00
ATOM	15591	HG1	MET	A1007	127.532	220.288	139.341	1.00	0.00
ATOM	15592	HG2	MET	A1007	127.602	221.897	140.057	1.00	0.00
ATOM	15593	SD	MET	A1007	129.651	220.685	140.369	1.00	0.00
ATOM	15594	CE	MET	A1007	130.181	219.292	139.330	1.00	0.00
ATOM	15595	HE1	MET	A1007	130.427	219.644	138.328	1.00	0.00
ATOM	15596	HE2	MET	A1007	131.063	218.821	139.758	1.00	0.00
ATOM	15597	HE3	MET	A1007	129.380	218.559	139.260	1.00	0.00
ATOM	15598	C	MET	A1007	127.559	222.738	135.926	1.00	0.00
ATOM	15599	O	MET	A1007	127.724	221.983	134.971	1.00	0.00
ATOM	15600	N	ASP	A1008	127.741	224.058	135.822	1.00	0.00
ATOM	15601	H	ASP	A1008	127.469	224.653	136.601	1.00	0.00
ATOM	15602	CA	ASP	A1008	128.061	224.759	134.563	1.00	0.00
ATOM	15603	HA	ASP	A1008	127.237	224.597	133.867	1.00	0.00
ATOM	15604	CB	ASP	A1008	128.162	226.274	134.842	1.00	0.00
ATOM	15605	HB1	ASP	A1008	128.320	226.790	133.893	1.00	0.00
ATOM	15606	HB2	ASP	A1008	129.041	226.457	135.462	1.00	0.00
ATOM	15607	CG	ASP	A1008	126.934	226.871	135.546	1.00	0.00
ATOM	15608	OD1	ASP	A1008	126.252	227.719	134.926	1.00	0.00
ATOM	15609	OD2	ASP	A1008	126.709	226.505	136.726	1.00	0.00
ATOM	15610	C	ASP	A1008	129.346	224.259	133.860	1.00	0.00
ATOM	15611	O	ASP	A1008	129.482	224.349	132.639	1.00	0.00
ATOM	15612	N	ASP	A1009	130.270	223.726	134.656	1.00	0.00
ATOM	15613	H	ASP	A1009	130.040	223.721	135.639	1.00	0.00
ATOM	15614	CA	ASP	A1009	131.399	222.847	134.333	1.00	0.00

```
ATOM  15615  HA  ASP A1009     131.084 222.076 133.630  1.00  0.00
ATOM  15616  CB  ASP A1009     132.550 223.683 133.707  1.00  0.00
ATOM  15617  HB1 ASP A1009     132.836 224.456 134.422  1.00  0.00
ATOM  15618  HB2 ASP A1009     132.172 224.185 132.817  1.00  0.00
ATOM  15619  CG  ASP A1009     133.809 222.896 133.299  1.00  0.00
ATOM  15620  OD1 ASP A1009     133.707 221.668 133.064  1.00  0.00
ATOM  15621  OD2 ASP A1009     134.899 223.512 133.276  1.00  0.00
ATOM  15622  C   ASP A1009     131.825 222.187 135.671  1.00  0.00
ATOM  15623  O   ASP A1009     131.294 222.527 136.734  1.00  0.00
ATOM  15624  N   VAL A1010     132.792 221.268 135.610  1.00  0.00
ATOM  15625  H   VAL A1010     133.121 221.097 134.660  1.00  0.00
ATOM  15626  CA  VAL A1010     133.559 220.566 136.662  1.00  0.00
ATOM  15627  HA  VAL A1010     134.605 220.748 136.428  1.00  0.00
ATOM  15628  CB  VAL A1010     133.391 221.043 138.129  1.00  0.00
ATOM  15629  HB  VAL A1010     132.343 221.050 138.419  1.00  0.00
ATOM  15630  CG1 VAL A1010     134.154 220.154 139.127  1.00  0.00
ATOM  15631 1HG1 VAL A1010     134.091 220.576 140.129  1.00  0.00
ATOM  15632 2HG1 VAL A1010     133.708 219.161 139.164  1.00  0.00
ATOM  15633 3HG1 VAL A1010     135.200 220.062 138.835  1.00  0.00
ATOM  15634  CG2 VAL A1010     134.003 222.444 138.315  1.00  0.00
ATOM  15635 1HG2 VAL A1010     133.833 222.787 139.335  1.00  0.00
ATOM  15636 2HG2 VAL A1010     135.072 222.420 138.108  1.00  0.00
ATOM  15637 3HG2 VAL A1010     133.537 223.155 137.637  1.00  0.00
ATOM  15638  C   VAL A1010     133.361 219.064 136.484  1.00  0.00
ATOM  15639  O   VAL A1010     132.452 218.466 137.056  1.00  0.00
ATOM  15640  N   VAL A1011     134.217 218.466 135.653  1.00  0.00
ATOM  15641  H   VAL A1011     134.903 219.040 135.189  1.00  0.00
ATOM  15642  CA  VAL A1011     134.050 217.101 135.142  1.00  0.00
ATOM  15643  HA  VAL A1011     133.135 216.694 135.561  1.00  0.00
ATOM  15644  CB  VAL A1011     133.846 217.094 133.612  1.00  0.00
ATOM  15645  HB  VAL A1011     133.831 216.056 133.279  1.00  0.00
ATOM  15646  CG1 VAL A1011     132.495 217.719 133.256  1.00  0.00
ATOM  15647 1HG1 VAL A1011     132.294 217.572 132.196  1.00  0.00
ATOM  15648 2HG1 VAL A1011     131.713 217.237 133.838  1.00  0.00
ATOM  15649 3HG1 VAL A1011     132.491 218.786 133.480  1.00  0.00
ATOM  15650  CG2 VAL A1011     134.915 217.846 132.801  1.00  0.00
ATOM  15651 1HG2 VAL A1011     134.703 217.734 131.738  1.00  0.00
ATOM  15652 2HG2 VAL A1011     134.902 218.910 133.043  1.00  0.00
ATOM  15653 3HG2 VAL A1011     135.904 217.443 133.000  1.00  0.00
ATOM  15654  C   VAL A1011     135.165 216.155 135.579  1.00  0.00
ATOM  15655  O   VAL A1011     136.319 216.291 135.163  1.00  0.00
ATOM  15656  N   ASP A1012     134.841 215.192 136.441  1.00  0.00
ATOM  15657  H   ASP A1012     133.858 215.093 136.715  1.00  0.00
ATOM  15658  CA  ASP A1012     135.815 214.292 137.065  1.00  0.00
ATOM  15659  HA  ASP A1012     136.735 214.868 137.172  1.00  0.00
ATOM  15660  CB  ASP A1012     135.403 213.903 138.493  1.00  0.00
ATOM  15661  HB1 ASP A1012     134.823 212.979 138.496  1.00  0.00
ATOM  15662  HB2 ASP A1012     134.774 214.694 138.907  1.00  0.00
ATOM  15663  CG  ASP A1012     136.644 213.758 139.383  1.00  0.00
ATOM  15664  OD1 ASP A1012     136.552 214.177 140.560  1.00  0.00
ATOM  15665  OD2 ASP A1012     137.697 213.319 138.863  1.00  0.00
ATOM  15666  C   ASP A1012     136.171 213.102 136.155  1.00  0.00
ATOM  15667  O   ASP A1012     135.669 211.985 136.288  1.00  0.00
ATOM  15668  N   ALA A1013     136.990 213.389 135.141  1.00  0.00
ATOM  15669  H   ALA A1013     137.316 214.345 135.107  1.00  0.00
ATOM  15670  CA  ALA A1013     137.180 212.577 133.939  1.00  0.00
ATOM  15671  HA  ALA A1013     136.267 212.658 133.350  1.00  0.00
ATOM  15672  CB  ALA A1013     138.302 213.218 133.111  1.00  0.00
ATOM  15673  HB1 ALA A1013     138.406 212.683 132.168  1.00  0.00
ATOM  15674  HB2 ALA A1013     138.065 214.262 132.911  1.00  0.00
ATOM  15675  HB3 ALA A1013     139.243 213.152 133.656  1.00  0.00
```

```
ATOM  15676  C   ALA A1013     137.449 211.068 134.147  1.00  0.00
ATOM  15677  O   ALA A1013     137.069 210.276 133.286  1.00  0.00
ATOM  15678  N   ASP A1014     138.060 210.663 135.266  1.00  0.00
ATOM  15679  H   ASP A1014     138.287 211.355 135.966  1.00  0.00
ATOM  15680  CA  ASP A1014     138.428 209.264 135.546  1.00  0.00
ATOM  15681  HA  ASP A1014     138.482 208.745 134.589  1.00  0.00
ATOM  15682  CB  ASP A1014     139.846 209.202 136.153  1.00  0.00
ATOM  15683  HB1 ASP A1014     139.820 208.617 137.074  1.00  0.00
ATOM  15684  HB2 ASP A1014     140.187 210.205 136.417  1.00  0.00
ATOM  15685  CG  ASP A1014     140.849 208.570 135.180  1.00  0.00
ATOM  15686  OD1 ASP A1014     141.504 207.574 135.563  1.00  0.00
ATOM  15687  OD2 ASP A1014     140.960 209.055 134.030  1.00  0.00
ATOM  15688  C   ASP A1014     137.389 208.465 136.358  1.00  0.00
ATOM  15689  O   ASP A1014     137.506 207.244 136.463  1.00  0.00
ATOM  15690  N   GLU A1015     136.333 209.140 136.829  1.00  0.00
ATOM  15691  H   GLU A1015     136.357 210.152 136.760  1.00  0.00
ATOM  15692  CA  GLU A1015     135.074 208.548 137.327  1.00  0.00
ATOM  15693  HA  GLU A1015     135.169 207.461 137.338  1.00  0.00
ATOM  15694  CB  GLU A1015     134.787 209.036 138.760  1.00  0.00
ATOM  15695  HB1 GLU A1015     133.780 208.737 139.053  1.00  0.00
ATOM  15696  HB2 GLU A1015     134.816 210.126 138.762  1.00  0.00
ATOM  15697  CG  GLU A1015     135.772 208.525 139.832  1.00  0.00
ATOM  15698  HG1 GLU A1015     135.679 209.180 140.702  1.00  0.00
ATOM  15699  HG2 GLU A1015     136.799 208.610 139.471  1.00  0.00
ATOM  15700  CD  GLU A1015     135.497 207.078 140.280  1.00  0.00
ATOM  15701  OE1 GLU A1015     135.504 206.814 141.509  1.00  0.00
ATOM  15702  OE2 GLU A1015     135.258 206.213 139.409  1.00  0.00
ATOM  15703  C   GLU A1015     133.890 208.861 136.378  1.00  0.00
ATOM  15704  O   GLU A1015     132.823 208.255 136.450  1.00  0.00
ATOM  15705  N   TYR A1016     134.084 209.771 135.422  1.00  0.00
ATOM  15706  H   TYR A1016     134.902 210.361 135.522  1.00  0.00
ATOM  15707  CA  TYR A1016     133.285 209.896 134.197  1.00  0.00
ATOM  15708  HA  TYR A1016     132.225 209.883 134.454  1.00  0.00
ATOM  15709  CB  TYR A1016     133.660 211.267 133.602  1.00  0.00
ATOM  15710  HB1 TYR A1016     134.664 211.192 133.193  1.00  0.00
ATOM  15711  HB2 TYR A1016     133.703 211.990 134.419  1.00  0.00
ATOM  15712  CG  TYR A1016     132.794 211.888 132.518  1.00  0.00
ATOM  15713  CD1 TYR A1016     132.660 211.261 131.262  1.00  0.00
ATOM  15714  HD1 TYR A1016     133.103 210.287 131.089  1.00  0.00
ATOM  15715  CE1 TYR A1016     131.969 211.910 130.223  1.00  0.00
ATOM  15716  HE1 TYR A1016     131.841 211.432 129.266  1.00  0.00
ATOM  15717  CZ  TYR A1016     131.450 213.207 130.417  1.00  0.00
ATOM  15718  OH  TYR A1016     130.806 213.815 129.388  1.00  0.00
ATOM  15719  HH  TYR A1016     130.679 214.776 129.543  1.00  0.00
ATOM  15720  CE2 TYR A1016     131.588 213.842 131.668  1.00  0.00
ATOM  15721  HE2 TYR A1016     131.183 214.830 131.820  1.00  0.00
ATOM  15722  CD2 TYR A1016     132.243 213.173 132.720  1.00  0.00
ATOM  15723  HD2 TYR A1016     132.341 213.664 133.687  1.00  0.00
ATOM  15724  C   TYR A1016     133.590 208.730 133.233  1.00  0.00
ATOM  15725  O   TYR A1016     132.689 208.187 132.591  1.00  0.00
ATOM  15726  N   LEU A1017     134.858 208.294 133.141  1.00  0.00
ATOM  15727  H   LEU A1017     135.564 208.829 133.635  1.00  0.00
ATOM  15728  CA  LEU A1017     135.195 206.916 132.762  1.00  0.00
ATOM  15729  HA  LEU A1017     134.806 206.699 131.770  1.00  0.00
ATOM  15730  CB  LEU A1017     136.722 206.689 132.798  1.00  0.00
ATOM  15731  HB1 LEU A1017     136.907 205.614 132.828  1.00  0.00
ATOM  15732  HB2 LEU A1017     137.101 207.091 133.738  1.00  0.00
ATOM  15733  CG  LEU A1017     137.553 207.278 131.646  1.00  0.00
ATOM  15734  HG  LEU A1017     137.382 208.348 131.572  1.00  0.00
ATOM  15735  CD1 LEU A1017     139.034 207.012 131.891  1.00  0.00
ATOM  15736 1HD1 LEU A1017     139.639 207.506 131.134  1.00  0.00
```

```
ATOM  15737 2HD1 LEU A1017     139.320 207.427 132.857  1.00  0.00
ATOM  15738 3HD1 LEU A1017     139.239 205.943 131.896  1.00  0.00
ATOM  15739  CD2 LEU A1017     137.224 206.624 130.297  1.00  0.00
ATOM  15740 1HD2 LEU A1017     137.924 206.968 129.538  1.00  0.00
ATOM  15741 2HD2 LEU A1017     137.287 205.542 130.387  1.00  0.00
ATOM  15742 3HD2 LEU A1017     136.224 206.913 129.997  1.00  0.00
ATOM  15743  C   LEU A1017     134.533 205.973 133.779  1.00  0.00
ATOM  15744  O   LEU A1017     134.790 206.054 134.968  1.00  0.00
ATOM  15745  N   ILE A1018     133.697 205.058 133.293  1.00  0.00
ATOM  15746  H   ILE A1018     133.528 205.078 132.306  1.00  0.00
ATOM  15747  CA  ILE A1018     132.737 204.311 134.118  1.00  0.00
ATOM  15748  HA  ILE A1018     132.082 205.095 134.504  1.00  0.00
ATOM  15749  CB  ILE A1018     131.851 203.441 133.192  1.00  0.00
ATOM  15750  HB  ILE A1018     131.661 204.032 132.293  1.00  0.00
ATOM  15751  CG2 ILE A1018     132.558 202.144 132.757  1.00  0.00
ATOM  15752 1HG2 ILE A1018     132.690 201.485 133.616  1.00  0.00
ATOM  15753 2HG2 ILE A1018     131.960 201.625 132.010  1.00  0.00
ATOM  15754 3HG2 ILE A1018     133.530 202.373 132.322  1.00  0.00
ATOM  15755  CG1 ILE A1018     130.473 203.128 133.816  1.00  0.00
ATOM  15756 1HG1 ILE A1018     130.146 203.976 134.421  1.00  0.00
ATOM  15757 2HG1 ILE A1018     130.557 202.258 134.469  1.00  0.00
ATOM  15758  CD  ILE A1018     129.385 202.870 132.769  1.00  0.00
ATOM  15759  HD1 ILE A1018     128.457 202.603 133.275  1.00  0.00
ATOM  15760  HD2 ILE A1018     129.219 203.769 132.177  1.00  0.00
ATOM  15761  HD3 ILE A1018     129.671 202.057 132.107  1.00  0.00
ATOM  15762  C   ILE A1018     133.178 203.567 135.423  1.00  0.00
ATOM  15763  O   ILE A1018     132.280 203.328 136.228  1.00  0.00
ATOM  15764  N   PRO A1019     134.433 203.122 135.693  1.00  0.00
ATOM  15765  CD  PRO A1019     135.551 203.072 134.756  1.00  0.00
ATOM  15766  HD1 PRO A1019     136.344 203.747 135.080  1.00  0.00
ATOM  15767  HD2 PRO A1019     135.258 203.308 133.740  1.00  0.00
ATOM  15768  CG  PRO A1019     136.041 201.629 134.758  1.00  0.00
ATOM  15769  HG1 PRO A1019     137.063 201.577 135.135  1.00  0.00
ATOM  15770  HG2 PRO A1019     135.966 201.223 133.757  1.00  0.00
ATOM  15771  CB  PRO A1019     135.082 200.890 135.686  1.00  0.00
ATOM  15772  HB1 PRO A1019     135.572 200.057 136.184  1.00  0.00
ATOM  15773  HB2 PRO A1019     134.227 200.522 135.117  1.00  0.00
ATOM  15774  CA  PRO A1019     134.627 202.003 136.633  1.00  0.00
ATOM  15775  HA  PRO A1019     133.693 201.695 137.100  1.00  0.00
ATOM  15776  C   PRO A1019     135.648 202.190 137.778  1.00  0.00
ATOM  15777  O   PRO A1019     136.214 201.195 138.238  1.00  0.00
ATOM  15778  N   GLN A1020     135.979 203.412 138.208  1.00  0.00
ATOM  15779  H   GLN A1020     135.449 204.221 137.896  1.00  0.00
ATOM  15780  CA  GLN A1020     137.097 203.682 139.139  1.00  0.00
ATOM  15781  HA  GLN A1020     137.214 204.767 139.151  1.00  0.00
ATOM  15782  CB  GLN A1020     136.767 203.255 140.594  1.00  0.00
ATOM  15783  HB1 GLN A1020     137.375 203.870 141.258  1.00  0.00
ATOM  15784  HB2 GLN A1020     137.074 202.218 140.740  1.00  0.00
ATOM  15785  CG  GLN A1020     135.296 203.358 141.038  1.00  0.00
ATOM  15786  HG1 GLN A1020     134.748 202.503 140.655  1.00  0.00
ATOM  15787  HG2 GLN A1020     134.831 204.243 140.623  1.00  0.00
ATOM  15788  CD  GLN A1020     135.144 203.423 142.557  1.00  0.00
ATOM  15789  OE1 GLN A1020     134.958 202.428 143.240  1.00  0.00
ATOM  15790  NE2 GLN A1020     135.194 204.609 143.124  1.00  0.00
ATOM  15791 1HE2 GLN A1020     135.302 205.446 142.525  1.00  0.00
ATOM  15792 2HE2 GLN A1020     135.035 204.705 144.111  1.00  0.00
ATOM  15793  C   GLN A1020     138.462 203.125 138.677  1.00  0.00
ATOM  15794  O   GLN A1020     139.366 202.942 139.492  1.00  0.00
ATOM  15795  N   GLN A1021     138.604 202.799 137.383  1.00  0.00
ATOM  15796  H   GLN A1021     137.824 203.075 136.806  1.00  0.00
ATOM  15797  CA  GLN A1021     139.711 202.098 136.694  1.00  0.00
```

```
ATOM  15798  HA  GLN A1021     139.389 201.931 135.665  1.00  0.00
ATOM  15799  CB  GLN A1021     140.945 203.018 136.622  1.00  0.00
ATOM  15800  HB1 GLN A1021     141.742 202.510 136.078  1.00  0.00
ATOM  15801  HB2 GLN A1021     141.308 203.185 137.637  1.00  0.00
ATOM  15802  CG  GLN A1021     140.722 204.398 135.976  1.00  0.00
ATOM  15803  HG1 GLN A1021     141.534 205.043 136.311  1.00  0.00
ATOM  15804  HG2 GLN A1021     139.795 204.859 136.318  1.00  0.00
ATOM  15805  CD  GLN A1021     140.740 204.398 134.445  1.00  0.00
ATOM  15806  OE1 GLN A1021     140.296 203.477 133.771  1.00  0.00
ATOM  15807  NE2 GLN A1021     141.278 205.431 133.848  1.00  0.00
ATOM  15808 1HE2 GLN A1021     141.496 206.259 134.424  1.00  0.00
ATOM  15809 2HE2 GLN A1021     141.245 205.493 132.854  1.00  0.00
ATOM  15810  C   GLN A1021     140.122 200.713 137.230  1.00  0.00
ATOM  15811  O   GLN A1021     140.460 199.844 136.426  1.00  0.00
ATOM  15812  N   GLY A1022     140.041 200.465 138.544  1.00  0.00
ATOM  15813  H   GLY A1022     139.792 201.259 139.123  1.00  0.00
ATOM  15814  CA  GLY A1022     140.366 199.210 139.241  1.00  0.00
ATOM  15815  HA1 GLY A1022     140.021 199.289 140.272  1.00  0.00
ATOM  15816  HA2 GLY A1022     141.448 199.085 139.250  1.00  0.00
ATOM  15817  C   GLY A1022     139.751 197.943 138.643  1.00  0.00
ATOM  15818  O   GLY A1022     140.259 196.851 138.877  1.00  0.00
ATOM  15819  N   PHE A1023     138.690 198.085 137.851  1.00  0.00
ATOM  15820  H   PHE A1023     138.349 199.025 137.713  1.00  0.00
ATOM  15821  CA  PHE A1023     138.027 196.998 137.143  1.00  0.00
ATOM  15822  HA  PHE A1023     138.803 196.419 136.640  1.00  0.00
ATOM  15823  CB  PHE A1023     137.326 196.023 138.125  1.00  0.00
ATOM  15824  HB1 PHE A1023     138.110 195.430 138.597  1.00  0.00
ATOM  15825  HB2 PHE A1023     136.730 195.328 137.535  1.00  0.00
ATOM  15826  CG  PHE A1023     136.435 196.520 139.257  1.00  0.00
ATOM  15827  CD1 PHE A1023     136.159 197.884 139.497  1.00  0.00
ATOM  15828  HD1 PHE A1023     136.507 198.662 138.838  1.00  0.00
ATOM  15829  CE1 PHE A1023     135.415 198.272 140.620  1.00  0.00
ATOM  15830  HE1 PHE A1023     135.260 199.325 140.807  1.00  0.00
ATOM  15831  CZ  PHE A1023     134.916 197.306 141.509  1.00  0.00
ATOM  15832  HZ  PHE A1023     134.364 197.608 142.388  1.00  0.00
ATOM  15833  CE2 PHE A1023     135.158 195.946 141.260  1.00  0.00
ATOM  15834  HE2 PHE A1023     134.796 195.192 141.947  1.00  0.00
ATOM  15835  CD2 PHE A1023     135.908 195.558 140.138  1.00  0.00
ATOM  15836  HD2 PHE A1023     136.099 194.503 139.978  1.00  0.00
ATOM  15837  C   PHE A1023     137.086 197.481 136.039  1.00  0.00
ATOM  15838  O   PHE A1023     135.879 197.326 136.152  1.00  0.00
ATOM  15839  N   PHE A1024     137.648 198.048 134.960  1.00  0.00
ATOM  15840  H   PHE A1024     138.645 198.226 135.002  1.00  0.00
ATOM  15841  CA  PHE A1024     136.982 198.368 133.675  1.00  0.00
ATOM  15842  HA  PHE A1024     136.148 199.030 133.896  1.00  0.00
ATOM  15843  CB  PHE A1024     137.987 199.130 132.782  1.00  0.00
ATOM  15844  HB1 PHE A1024     138.576 198.431 132.187  1.00  0.00
ATOM  15845  HB2 PHE A1024     138.697 199.646 133.431  1.00  0.00
ATOM  15846  CG  PHE A1024     137.393 200.188 131.860  1.00  0.00
ATOM  15847  CD1 PHE A1024     137.942 201.484 131.858  1.00  0.00
ATOM  15848  HD1 PHE A1024     138.802 201.713 132.478  1.00  0.00
ATOM  15849  CE1 PHE A1024     137.368 202.502 131.081  1.00  0.00
ATOM  15850  HE1 PHE A1024     137.799 203.492 131.109  1.00  0.00
ATOM  15851  CZ  PHE A1024     136.244 202.235 130.283  1.00  0.00
ATOM  15852  HZ  PHE A1024     135.798 203.018 129.685  1.00  0.00
ATOM  15853  CE2 PHE A1024     135.702 200.938 130.260  1.00  0.00
ATOM  15854  HE2 PHE A1024     134.846 200.720 129.642  1.00  0.00
ATOM  15855  CD2 PHE A1024     136.272 199.916 131.044  1.00  0.00
ATOM  15856  HD2 PHE A1024     135.836 198.932 131.012  1.00  0.00
ATOM  15857  C   PHE A1024     136.360 197.120 132.978  1.00  0.00
ATOM  15858  O   PHE A1024     136.741 196.737 131.876  1.00  0.00
```

```
ATOM  15859  N   SER A1025     135.444 196.439 133.669  1.00  0.00
ATOM  15860  H   SER A1025     135.204 196.805 134.583  1.00  0.00
ATOM  15861  CA  SER A1025     134.722 195.249 133.219  1.00  0.00
ATOM  15862  HA  SER A1025     135.454 194.523 132.866  1.00  0.00
ATOM  15863  CB  SER A1025     133.975 194.634 134.413  1.00  0.00
ATOM  15864  HB1 SER A1025     133.107 195.249 134.660  1.00  0.00
ATOM  15865  HB2 SER A1025     134.637 194.598 135.280  1.00  0.00
ATOM  15866  OG  SER A1025     133.560 193.313 134.102  1.00  0.00
ATOM  15867  HG  SER A1025     133.002 192.998 134.848  1.00  0.00
ATOM  15868  C   SER A1025     133.752 195.544 132.071  1.00  0.00
ATOM  15869  O   SER A1025     133.600 196.685 131.653  1.00  0.00
ATOM  15870  N   SER A1026     133.114 194.498 131.539  1.00  0.00
ATOM  15871  H   SER A1026     133.229 193.623 132.031  1.00  0.00
ATOM  15872  CA  SER A1026     132.485 194.468 130.210  1.00  0.00
ATOM  15873  HA  SER A1026     133.269 194.715 129.496  1.00  0.00
ATOM  15874  CB  SER A1026     132.071 193.027 129.880  1.00  0.00
ATOM  15875  HB1 SER A1026     131.132 192.784 130.382  1.00  0.00
ATOM  15876  HB2 SER A1026     132.841 192.344 130.235  1.00  0.00
ATOM  15877  OG  SER A1026     131.934 192.841 128.485  1.00  0.00
ATOM  15878  HG  SER A1026     132.594 192.200 128.177  1.00  0.00
ATOM  15879  C   SER A1026     131.311 195.436 129.972  1.00  0.00
ATOM  15880  O   SER A1026     130.214 195.233 130.519  1.00  0.00
ATOM  15881  N   PRO A1027     131.482 196.461 129.113  1.00  0.00
ATOM  15882  CD  PRO A1027     132.725 197.067 128.656  1.00  0.00
ATOM  15883  HD1 PRO A1027     133.070 196.576 127.744  1.00  0.00
ATOM  15884  HD2 PRO A1027     133.526 197.038 129.386  1.00  0.00
ATOM  15885  CG  PRO A1027     132.346 198.515 128.353  1.00  0.00
ATOM  15886  HG1 PRO A1027     132.976 198.940 127.573  1.00  0.00
ATOM  15887  HG2 PRO A1027     132.428 199.103 129.268  1.00  0.00
ATOM  15888  CB  PRO A1027     130.873 198.442 127.927  1.00  0.00
ATOM  15889  HB1 PRO A1027     130.775 198.607 126.855  1.00  0.00
ATOM  15890  HB2 PRO A1027     130.294 199.204 128.443  1.00  0.00
ATOM  15891  CA  PRO A1027     130.399 197.031 128.321  1.00  0.00
ATOM  15892  HA  PRO A1027     129.474 197.080 128.892  1.00  0.00
ATOM  15893  C   PRO A1027     130.175 196.190 127.052  1.00  0.00
ATOM  15894  O   PRO A1027     131.128 195.782 126.394  1.00  0.00
ATOM  15895  N   SER A1028     128.930 196.066 126.594  1.00  0.00
ATOM  15896  H   SER A1028     128.171 196.432 127.148  1.00  0.00
ATOM  15897  CA  SER A1028     128.647 195.640 125.208  1.00  0.00
ATOM  15898  HA  SER A1028     129.359 194.865 124.919  1.00  0.00
ATOM  15899  CB  SER A1028     127.242 195.028 125.123  1.00  0.00
ATOM  15900  HB1 SER A1028     126.486 195.814 125.149  1.00  0.00
ATOM  15901  HB2 SER A1028     127.087 194.364 125.974  1.00  0.00
ATOM  15902  OG  SER A1028     127.118 194.267 123.941  1.00  0.00
ATOM  15903  HG  SER A1028     126.191 194.022 123.836  1.00  0.00
ATOM  15904  C   SER A1028     128.831 196.831 124.240  1.00  0.00
ATOM  15905  O   SER A1028     127.901 197.350 123.624  1.00  0.00
ATOM  15906  N   THR A1029     130.041 197.398 124.263  1.00  0.00
ATOM  15907  H   THR A1029     130.751 196.915 124.803  1.00  0.00
ATOM  15908  CA  THR A1029     130.407 198.654 123.594  1.00  0.00
ATOM  15909  HA  THR A1029     129.766 199.435 124.002  1.00  0.00
ATOM  15910  CB  THR A1029     131.867 199.092 123.901  1.00  0.00
ATOM  15911  HB  THR A1029     131.812 199.917 124.612  1.00  0.00
ATOM  15912  CG2 THR A1029     132.761 198.021 124.533  1.00  0.00
ATOM  15913 1HG2 THR A1029     133.786 198.387 124.601  1.00  0.00
ATOM  15914 2HG2 THR A1029     132.428 197.806 125.541  1.00  0.00
ATOM  15915 3HG2 THR A1029     132.723 197.101 123.953  1.00  0.00
ATOM  15916  OG1 THR A1029     132.554 199.564 122.761  1.00  0.00
ATOM  15917  HG1 THR A1029     133.294 198.947 122.608  1.00  0.00
ATOM  15918  C   THR A1029     130.119 198.584 122.094  1.00  0.00
ATOM  15919  O   THR A1029     130.649 197.726 121.384  1.00  0.00
```

```
ATOM  15920  N   SER A1030     129.315 199.521 121.584  1.00  0.00
ATOM  15921  H   SER A1030     128.865 200.167 122.211  1.00  0.00
ATOM  15922  CA  SER A1030     128.952 199.541 120.164  1.00  0.00
ATOM  15923  HA  SER A1030     128.578 198.547 119.911  1.00  0.00
ATOM  15924  CB  SER A1030     127.822 200.532 119.876  1.00  0.00
ATOM  15925  HB1 SER A1030     128.197 201.554 119.953  1.00  0.00
ATOM  15926  HB2 SER A1030     127.015 200.386 120.596  1.00  0.00
ATOM  15927  OG  SER A1030     127.329 200.290 118.575  1.00  0.00
ATOM  15928  HG  SER A1030     128.080 200.105 117.992  1.00  0.00
ATOM  15929  C   SER A1030     130.133 199.819 119.227  1.00  0.00
ATOM  15930  O   SER A1030     129.998 199.584 118.027  1.00  0.00
ATOM  15931  N   ARG A1031     131.284 200.289 119.737  1.00  0.00
ATOM  15932  H   ARG A1031     131.350 200.415 120.739  1.00  0.00
ATOM  15933  CA  ARG A1031     132.511 200.401 118.934  1.00  0.00
ATOM  15934  HA  ARG A1031     132.213 200.635 117.909  1.00  0.00
ATOM  15935  CB  ARG A1031     133.412 201.536 119.453  1.00  0.00
ATOM  15936  HB1 ARG A1031     134.145 201.136 120.156  1.00  0.00
ATOM  15937  HB2 ARG A1031     132.806 202.282 119.972  1.00  0.00
ATOM  15938  CG  ARG A1031     134.120 202.231 118.277  1.00  0.00
ATOM  15939  HG1 ARG A1031     133.373 202.781 117.704  1.00  0.00
ATOM  15940  HG2 ARG A1031     134.574 201.487 117.629  1.00  0.00
ATOM  15941  CD  ARG A1031     135.211 203.193 118.754  1.00  0.00
ATOM  15942  HD1 ARG A1031     136.091 202.620 119.054  1.00  0.00
ATOM  15943  HD2 ARG A1031     134.844 203.726 119.634  1.00  0.00
ATOM  15944  NE  ARG A1031     135.578 204.196 117.729  1.00  0.00
ATOM  15945  HE  ARG A1031     135.279 205.140 117.917  1.00  0.00
ATOM  15946  CZ  ARG A1031     136.308 204.023 116.640  1.00  0.00
ATOM  15947  NH1 ARG A1031     136.710 202.852 116.237  1.00  0.00
ATOM  15948 1HH1 ARG A1031     136.555 202.035 116.813  1.00  0.00
ATOM  15949 2HH1 ARG A1031     137.266 202.748 115.403  1.00  0.00
ATOM  15950  NH2 ARG A1031     136.694 205.064 115.958  1.00  0.00
ATOM  15951 1HH2 ARG A1031     136.439 205.987 116.260  1.00  0.00
ATOM  15952 2HH2 ARG A1031     137.484 204.964 115.331  1.00  0.00
ATOM  15953  C   ARG A1031     133.275 199.075 118.844  1.00  0.00
ATOM  15954  O   ARG A1031     134.126 198.891 117.971  1.00  0.00
ATOM  15955  N   THR A1032     132.991 198.138 119.747  1.00  0.00
ATOM  15956  H   THR A1032     132.242 198.321 120.405  1.00  0.00
ATOM  15957  CA  THR A1032     133.758 196.906 119.938  1.00  0.00
ATOM  15958  HA  THR A1032     134.186 196.620 118.983  1.00  0.00
ATOM  15959  CB  THR A1032     134.940 197.124 120.910  1.00  0.00
ATOM  15960  HB  THR A1032     135.428 196.164 121.088  1.00  0.00
ATOM  15961  CG2 THR A1032     135.996 198.107 120.421  1.00  0.00
ATOM  15962 1HG2 THR A1032     136.845 198.079 121.105  1.00  0.00
ATOM  15963 2HG2 THR A1032     136.333 197.825 119.428  1.00  0.00
ATOM  15964 3HG2 THR A1032     135.597 199.119 120.396  1.00  0.00
ATOM  15965  OG1 THR A1032     134.487 197.636 122.134  1.00  0.00
ATOM  15966  HG1 THR A1032     134.054 196.888 122.570  1.00  0.00
ATOM  15967  C   THR A1032     132.890 195.735 120.415  1.00  0.00
ATOM  15968  O   THR A1032     132.921 195.381 121.600  1.00  0.00
ATOM  15969  N   PRO A1033     132.135 195.086 119.509  1.00  0.00
ATOM  15970  CD  PRO A1033     131.904 195.474 118.122  1.00  0.00
ATOM  15971  HD1 PRO A1033     132.831 195.709 117.602  1.00  0.00
ATOM  15972  HD2 PRO A1033     131.231 196.333 118.097  1.00  0.00
ATOM  15973  CG  PRO A1033     131.233 194.276 117.452  1.00  0.00
ATOM  15974  HG1 PRO A1033     131.994 193.616 117.032  1.00  0.00
ATOM  15975  HG2 PRO A1033     130.528 194.587 116.680  1.00  0.00
ATOM  15976  CB  PRO A1033     130.530 193.576 118.613  1.00  0.00
ATOM  15977  HB1 PRO A1033     130.376 192.515 118.413  1.00  0.00
ATOM  15978  HB2 PRO A1033     129.573 194.065 118.802  1.00  0.00
ATOM  15979  CA  PRO A1033     131.467 193.824 119.804  1.00  0.00
ATOM  15980  HA  PRO A1033     130.863 193.930 120.705  1.00  0.00
```

```
ATOM  15981  C   PRO A1033     132.502 192.714 120.034  1.00  0.00
ATOM  15982  O   PRO A1033     132.848 191.959 119.123  1.00  0.00
ATOM  15983  N   LEU A1034     132.999 192.595 121.271  1.00  0.00
ATOM  15984  H   LEU A1034     132.751 193.330 121.924  1.00  0.00
ATOM  15985  CA  LEU A1034     133.797 191.466 121.780  1.00  0.00
ATOM  15986  HA  LEU A1034     134.530 191.209 121.020  1.00  0.00
ATOM  15987  CB  LEU A1034     134.563 191.882 123.055  1.00  0.00
ATOM  15988  HB1 LEU A1034     135.167 191.032 123.374  1.00  0.00
ATOM  15989  HB2 LEU A1034     133.841 192.091 123.846  1.00  0.00
ATOM  15990  CG  LEU A1034     135.499 193.104 122.908  1.00  0.00
ATOM  15991  HG  LEU A1034     134.898 193.996 122.736  1.00  0.00
ATOM  15992  CD1 LEU A1034     136.282 193.303 124.205  1.00  0.00
ATOM  15993 1HD1 LEU A1034     136.887 194.205 124.136  1.00  0.00
ATOM  15994 2HD1 LEU A1034     135.590 193.414 125.040  1.00  0.00
ATOM  15995 3HD1 LEU A1034     136.931 192.448 124.393  1.00  0.00
ATOM  15996  CD2 LEU A1034     136.502 192.966 121.761  1.00  0.00
ATOM  15997 1HD2 LEU A1034     137.186 193.814 121.761  1.00  0.00
ATOM  15998 2HD2 LEU A1034     137.075 192.046 121.874  1.00  0.00
ATOM  15999 3HD2 LEU A1034     135.972 192.958 120.811  1.00  0.00
ATOM  16000  C   LEU A1034     132.942 190.187 121.966  1.00  0.00
ATOM  16001  O   LEU A1034     133.013 189.486 122.978  1.00  0.00
ATOM  16002  N   LEU A1035     132.064 189.932 120.993  1.00  0.00
ATOM  16003  H   LEU A1035     132.143 190.532 120.183  1.00  0.00
ATOM  16004  CA  LEU A1035     131.315 188.695 120.809  1.00  0.00
ATOM  16005  HA  LEU A1035     130.865 188.397 121.757  1.00  0.00
ATOM  16006  CB  LEU A1035     130.212 188.933 119.752  1.00  0.00
ATOM  16007  HB1 LEU A1035     129.665 188.002 119.601  1.00  0.00
ATOM  16008  HB2 LEU A1035     130.697 189.186 118.808  1.00  0.00
ATOM  16009  CG  LEU A1035     129.195 190.042 120.089  1.00  0.00
ATOM  16010  HG  LEU A1035     129.716 190.989 120.225  1.00  0.00
ATOM  16011  CD1 LEU A1035     128.205 190.204 118.931  1.00  0.00
ATOM  16012 1HD1 LEU A1035     127.490 190.993 119.159  1.00  0.00
ATOM  16013 2HD1 LEU A1035     128.743 190.470 118.021  1.00  0.00
ATOM  16014 3HD1 LEU A1035     127.666 189.271 118.763  1.00  0.00
ATOM  16015  CD2 LEU A1035     128.395 189.735 121.356  1.00  0.00
ATOM  16016 1HD2 LEU A1035     127.684 190.541 121.537  1.00  0.00
ATOM  16017 2HD2 LEU A1035     127.848 188.802 121.233  1.00  0.00
ATOM  16018 3HD2 LEU A1035     129.060 189.673 122.216  1.00  0.00
ATOM  16019  C   LEU A1035     132.261 187.567 120.351  1.00  0.00
ATOM  16020  O   LEU A1035     133.400 187.819 119.970  1.00  0.00
ATOM  16021  N   SER A1036     131.772 186.324 120.324  1.00  0.00
ATOM  16022  H   SER A1036     130.817 186.157 120.607  1.00  0.00
ATOM  16023  CA  SER A1036     132.476 185.214 119.664  1.00  0.00
ATOM  16024  HA  SER A1036     133.445 185.081 120.143  1.00  0.00
ATOM  16025  CB  SER A1036     131.675 183.913 119.839  1.00  0.00
ATOM  16026  HB1 SER A1036     131.781 183.572 120.870  1.00  0.00
ATOM  16027  HB2 SER A1036     132.080 183.149 119.173  1.00  0.00
ATOM  16028  OG  SER A1036     130.298 184.123 119.565  1.00  0.00
ATOM  16029  HG  SER A1036     129.856 183.269 119.486  1.00  0.00
ATOM  16030  C   SER A1036     132.748 185.471 118.176  1.00  0.00
ATOM  16031  O   SER A1036     133.844 185.214 117.687  1.00  0.00
ATOM  16032  N   SER A1037     131.774 186.042 117.469  1.00  0.00
ATOM  16033  H   SER A1037     130.884 186.175 117.926  1.00  0.00
ATOM  16034  CA  SER A1037     131.890 186.591 116.112  1.00  0.00
ATOM  16035  HA  SER A1037     132.483 185.910 115.500  1.00  0.00
ATOM  16036  CB  SER A1037     130.484 186.686 115.510  1.00  0.00
ATOM  16037  HB1 SER A1037     130.031 185.694 115.483  1.00  0.00
ATOM  16038  HB2 SER A1037     130.542 187.074 114.492  1.00  0.00
ATOM  16039  OG  SER A1037     129.687 187.542 116.306  1.00  0.00
ATOM  16040  HG  SER A1037     130.080 188.420 116.189  1.00  0.00
ATOM  16041  C   SER A1037     132.607 187.961 116.109  1.00  0.00
```

```
ATOM  16042  O   SER A1037     132.012 188.986 115.771  1.00  0.00
ATOM  16043  N   LEU A1038     133.847 188.000 116.598  1.00  0.00
ATOM  16044  H   LEU A1038     134.245 187.126 116.923  1.00  0.00
ATOM  16045  CA  LEU A1038     134.643 189.217 116.783  1.00  0.00
ATOM  16046  HA  LEU A1038     134.050 189.933 117.354  1.00  0.00
ATOM  16047  CB  LEU A1038     135.880 188.807 117.616  1.00  0.00
ATOM  16048  HB1 LEU A1038     136.373 187.980 117.103  1.00  0.00
ATOM  16049  HB2 LEU A1038     135.544 188.437 118.585  1.00  0.00
ATOM  16050  CG  LEU A1038     136.941 189.901 117.850  1.00  0.00
ATOM  16051  HG  LEU A1038     137.300 190.285 116.895  1.00  0.00
ATOM  16052  CD1 LEU A1038     136.396 191.063 118.676  1.00  0.00
ATOM  16053 1HD1 LEU A1038     137.173 191.812 118.812  1.00  0.00
ATOM  16054 2HD1 LEU A1038     135.536 191.516 118.189  1.00  0.00
ATOM  16055 3HD1 LEU A1038     136.091 190.687 119.650  1.00  0.00
ATOM  16056  CD2 LEU A1038     138.136 189.322 118.608  1.00  0.00
ATOM  16057 1HD2 LEU A1038     138.901 190.086 118.742  1.00  0.00
ATOM  16058 2HD2 LEU A1038     137.821 188.950 119.582  1.00  0.00
ATOM  16059 3HD2 LEU A1038     138.567 188.499 118.038  1.00  0.00
ATOM  16060  C   LEU A1038     135.100 189.888 115.469  1.00  0.00
ATOM  16061  O   LEU A1038     135.719 189.255 114.619  1.00  0.00
ATOM  16062  N   SER A1039     134.929 191.211 115.378  1.00  0.00
ATOM  16063  H   SER A1039     134.298 191.642 116.036  1.00  0.00
ATOM  16064  CA  SER A1039     135.539 192.068 114.340  1.00  0.00
ATOM  16065  HA  SER A1039     136.526 191.673 114.098  1.00  0.00
ATOM  16066  CB  SER A1039     134.679 192.018 113.066  1.00  0.00
ATOM  16067  HB1 SER A1039     134.467 190.980 112.806  1.00  0.00
ATOM  16068  HB2 SER A1039     135.229 192.474 112.242  1.00  0.00
ATOM  16069  OG  SER A1039     133.464 192.719 113.254  1.00  0.00
ATOM  16070  HG  SER A1039     133.700 193.655 113.298  1.00  0.00
ATOM  16071  C   SER A1039     135.727 193.526 114.822  1.00  0.00
ATOM  16072  O   SER A1039     135.508 194.490 114.088  1.00  0.00
ATOM  16073  N   ALA A1040     136.004 193.703 116.113  1.00  0.00
ATOM  16074  H   ALA A1040     136.216 192.888 116.666  1.00  0.00
ATOM  16075  CA  ALA A1040     135.963 194.994 116.796  1.00  0.00
ATOM  16076  HA  ALA A1040     134.989 195.449 116.618  1.00  0.00
ATOM  16077  CB  ALA A1040     136.106 194.693 118.294  1.00  0.00
ATOM  16078  HB1 ALA A1040     136.160 195.616 118.860  1.00  0.00
ATOM  16079  HB2 ALA A1040     135.257 194.102 118.642  1.00  0.00
ATOM  16080  HB3 ALA A1040     137.032 194.146 118.472  1.00  0.00
ATOM  16081  C   ALA A1040     137.036 196.016 116.367  1.00  0.00
ATOM  16082  O   ALA A1040     138.137 195.659 115.953  1.00  0.00
ATOM  16083  N   THR A1041     136.757 197.305 116.611  1.00  0.00
ATOM  16084  H   THR A1041     135.834 197.543 116.953  1.00  0.00
ATOM  16085  CA  THR A1041     137.684 198.424 116.353  1.00  0.00
ATOM  16086  HA  THR A1041     138.645 197.998 116.066  1.00  0.00
ATOM  16087  CB  THR A1041     137.253 199.251 115.124  1.00  0.00
ATOM  16088  HB  THR A1041     137.380 198.619 114.245  1.00  0.00
ATOM  16089  CG2 THR A1041     135.798 199.717 115.134  1.00  0.00
ATOM  16090 1HG2 THR A1041     135.618 200.383 114.290  1.00  0.00
ATOM  16091 2HG2 THR A1041     135.136 198.856 115.040  1.00  0.00
ATOM  16092 3HG2 THR A1041     135.572 200.232 116.059  1.00  0.00
ATOM  16093  OG1 THR A1041     138.083 200.383 114.949  1.00  0.00
ATOM  16094  HG1 THR A1041     138.668 200.185 114.209  1.00  0.00
ATOM  16095  C   THR A1041     137.974 199.239 117.618  1.00  0.00
ATOM  16096  O   THR A1041     137.445 200.332 117.862  1.00  0.00
ATOM  16097  N   SER A1042     138.840 198.643 118.443  1.00  0.00
ATOM  16098  H   SER A1042     139.124 197.706 118.198  1.00  0.00
ATOM  16099  CA  SER A1042     139.405 199.133 119.713  1.00  0.00
ATOM  16100  HA  SER A1042     138.580 199.449 120.349  1.00  0.00
ATOM  16101  CB  SER A1042     140.107 197.962 120.429  1.00  0.00
ATOM  16102  HB1 SER A1042     140.326 198.254 121.458  1.00  0.00
```

```
ATOM  16103  HB2 SER A1042     141.047 197.739 119.920  1.00  0.00
ATOM  16104  OG  SER A1042     139.292 196.794 120.424  1.00  0.00
ATOM  16105  HG  SER A1042     139.718 196.136 120.979  1.00  0.00
ATOM  16106  C   SER A1042     140.334 200.345 119.548  1.00  0.00
ATOM  16107  O   SER A1042     141.481 200.343 119.973  1.00  0.00
ATOM  16108  N   ASN A1043     139.828 201.404 118.905  1.00  0.00
ATOM  16109  H   ASN A1043     138.899 201.281 118.518  1.00  0.00
ATOM  16110  CA  ASN A1043     140.487 202.704 118.696  1.00  0.00
ATOM  16111  HA  ASN A1043     141.413 202.528 118.146  1.00  0.00
ATOM  16112  CB  ASN A1043     139.520 203.529 117.823  1.00  0.00
ATOM  16113  HB1 ASN A1043     138.709 203.909 118.442  1.00  0.00
ATOM  16114  HB2 ASN A1043     139.106 202.850 117.083  1.00  0.00
ATOM  16115  CG  ASN A1043     140.065 204.699 117.001  1.00  0.00
ATOM  16116  OD1 ASN A1043     139.585 204.934 115.898  1.00  0.00
ATOM  16117  ND2 ASN A1043     140.975 205.496 117.500  1.00  0.00
ATOM  16118 1HD2 ASN A1043     141.369 205.314 118.419  1.00  0.00
ATOM  16119 2HD2 ASN A1043     141.299 206.256 116.930  1.00  0.00
ATOM  16120  C   ASN A1043     140.867 203.406 120.022  1.00  0.00
ATOM  16121  O   ASN A1043     141.559 204.419 119.980  1.00  0.00
ATOM  16122  N   ASN A1044     140.392 202.907 121.171  1.00  0.00
ATOM  16123  H   ASN A1044     139.937 202.008 121.105  1.00  0.00
ATOM  16124  CA  ASN A1044     140.666 203.345 122.547  1.00  0.00
ATOM  16125  HA  ASN A1044     139.815 203.014 123.142  1.00  0.00
ATOM  16126  CB  ASN A1044     141.881 202.554 123.083  1.00  0.00
ATOM  16127  HB1 ASN A1044     142.197 202.987 124.032  1.00  0.00
ATOM  16128  HB2 ASN A1044     142.713 202.629 122.383  1.00  0.00
ATOM  16129  CG  ASN A1044     141.568 201.084 123.339  1.00  0.00
ATOM  16130  OD1 ASN A1044     140.521 200.558 122.975  1.00  0.00
ATOM  16131  ND2 ASN A1044     142.447 200.378 124.014  1.00  0.00
ATOM  16132 1HD2 ASN A1044     143.314 200.795 124.296  1.00  0.00
ATOM  16133 2HD2 ASN A1044     142.219 199.415 124.188  1.00  0.00
ATOM  16134  C   ASN A1044     140.684 204.871 122.806  1.00  0.00
ATOM  16135  O   ASN A1044     141.294 205.348 123.756  1.00  0.00
ATOM  16136  N   SER A1045     139.930 205.633 122.001  1.00  0.00
ATOM  16137  H   SER A1045     139.507 205.164 121.220  1.00  0.00
ATOM  16138  CA  SER A1045     139.677 207.080 122.121  1.00  0.00
ATOM  16139  HA  SER A1045     140.637 207.585 122.235  1.00  0.00
ATOM  16140  CB  SER A1045     139.037 207.562 120.811  1.00  0.00
ATOM  16141  HB1 SER A1045     138.013 207.191 120.740  1.00  0.00
ATOM  16142  HB2 SER A1045     139.612 207.168 119.972  1.00  0.00
ATOM  16143  OG  SER A1045     139.039 208.968 120.720  1.00  0.00
ATOM  16144  HG  SER A1045     138.400 209.316 121.361  1.00  0.00
ATOM  16145  C   SER A1045     138.807 207.464 123.345  1.00  0.00
ATOM  16146  O   SER A1045     138.020 208.406 123.296  1.00  0.00
ATOM  16147  N   THR A1046     138.861 206.664 124.413  1.00  0.00
ATOM  16148  H   THR A1046     139.625 205.995 124.444  1.00  0.00
ATOM  16149  CA  THR A1046     137.710 206.337 125.269  1.00  0.00
ATOM  16150  HA  THR A1046     136.972 205.840 124.642  1.00  0.00
ATOM  16151  CB  THR A1046     138.121 205.319 126.343  1.00  0.00
ATOM  16152  HB  THR A1046     138.835 205.784 127.024  1.00  0.00
ATOM  16153  CG2 THR A1046     136.948 204.760 127.149  1.00  0.00
ATOM  16154 1HG2 THR A1046     137.312 204.030 127.872  1.00  0.00
ATOM  16155 2HG2 THR A1046     136.445 205.560 127.692  1.00  0.00
ATOM  16156 3HG2 THR A1046     136.232 204.277 126.483  1.00  0.00
ATOM  16157  OG1 THR A1046     138.740 204.227 125.699  1.00  0.00
ATOM  16158  HG1 THR A1046     139.122 203.669 126.381  1.00  0.00
ATOM  16159  C   THR A1046     136.997 207.532 125.896  1.00  0.00
ATOM  16160  O   THR A1046     135.772 207.571 125.811  1.00  0.00
ATOM  16161  N   VAL A1047     137.714 208.518 126.459  1.00  0.00
ATOM  16162  H   VAL A1047     138.722 208.460 126.446  1.00  0.00
ATOM  16163  CA  VAL A1047     137.068 209.722 127.028  1.00  0.00
```

```
ATOM  16164  HA  VAL A1047     136.328 209.388 127.757  1.00  0.00
ATOM  16165  CB  VAL A1047     138.048 210.647 127.791  1.00  0.00
ATOM  16166  HB  VAL A1047     137.540 211.593 127.976  1.00  0.00
ATOM  16167  CG1 VAL A1047     138.396 210.040 129.149  1.00  0.00
ATOM  16168 1HG1 VAL A1047     139.038 210.715 129.716  1.00  0.00
ATOM  16169 2HG1 VAL A1047     137.487 209.884 129.730  1.00  0.00
ATOM  16170 3HG1 VAL A1047     138.912 209.089 129.030  1.00  0.00
ATOM  16171  CG2 VAL A1047     139.366 210.950 127.062  1.00  0.00
ATOM  16172 1HG2 VAL A1047     139.955 211.646 127.662  1.00  0.00
ATOM  16173 2HG2 VAL A1047     139.948 210.041 126.912  1.00  0.00
ATOM  16174 3HG2 VAL A1047     139.174 211.424 126.101  1.00  0.00
ATOM  16175  C   VAL A1047     136.277 210.508 125.976  1.00  0.00
ATOM  16176  O   VAL A1047     135.069 210.677 126.125  1.00  0.00
ATOM  16177  N   ALA A1048     136.914 210.867 124.858  1.00  0.00
ATOM  16178  H   ALA A1048     137.877 210.590 124.759  1.00  0.00
ATOM  16179  CA  ALA A1048     136.299 211.541 123.706  1.00  0.00
ATOM  16180  HA  ALA A1048     135.570 212.264 124.070  1.00  0.00
ATOM  16181  CB  ALA A1048     137.411 212.310 122.981  1.00  0.00
ATOM  16182  HB1 ALA A1048     136.988 212.851 122.135  1.00  0.00
ATOM  16183  HB2 ALA A1048     137.868 213.031 123.659  1.00  0.00
ATOM  16184  HB3 ALA A1048     138.171 211.619 122.613  1.00  0.00
ATOM  16185  C   ALA A1048     135.558 210.550 122.777  1.00  0.00
ATOM  16186  O   ALA A1048     135.760 210.512 121.559  1.00  0.00
ATOM  16187  N   CYS A1049     134.795 209.650 123.385  1.00  0.00
ATOM  16188  H   CYS A1049     134.670 209.805 124.377  1.00  0.00
ATOM  16189  CA  CYS A1049     134.046 208.567 122.747  1.00  0.00
ATOM  16190  HA  CYS A1049     133.572 208.948 121.841  1.00  0.00
ATOM  16191  CB  CYS A1049     135.032 207.445 122.372  1.00  0.00
ATOM  16192  HB1 CYS A1049     135.416 206.992 123.287  1.00  0.00
ATOM  16193  HB2 CYS A1049     135.865 207.881 121.818  1.00  0.00
ATOM  16194  SG  CYS A1049     134.230 206.181 121.339  1.00  0.00
ATOM  16195  HG  CYS A1049     133.052 206.215 121.988  1.00  0.00
ATOM  16196  C   CYS A1049     132.938 208.036 123.664  1.00  0.00
ATOM  16197  O   CYS A1049     131.978 207.451 123.167  1.00  0.00
ATOM  16198  N   ILE A1050     133.048 208.250 124.983  1.00  0.00
ATOM  16199  H   ILE A1050     133.939 208.551 125.355  1.00  0.00
ATOM  16200  CA  ILE A1050     131.903 208.210 125.894  1.00  0.00
ATOM  16201  HA  ILE A1050     131.165 207.531 125.470  1.00  0.00
ATOM  16202  CB  ILE A1050     132.250 207.635 127.286  1.00  0.00
ATOM  16203  HB  ILE A1050     131.325 207.631 127.866  1.00  0.00
ATOM  16204  CG2 ILE A1050     132.687 206.168 127.111  1.00  0.00
ATOM  16205 1HG2 ILE A1050     132.730 205.667 128.076  1.00  0.00
ATOM  16206 2HG2 ILE A1050     131.958 205.639 126.498  1.00  0.00
ATOM  16207 3HG2 ILE A1050     133.664 206.117 126.630  1.00  0.00
ATOM  16208  CG1 ILE A1050     133.285 208.469 128.070  1.00  0.00
ATOM  16209 1HG1 ILE A1050     132.906 209.480 128.209  1.00  0.00
ATOM  16210 2HG1 ILE A1050     134.213 208.526 127.514  1.00  0.00
ATOM  16211  CD  ILE A1050     133.591 207.909 129.460  1.00  0.00
ATOM  16212  HD1 ILE A1050     134.277 208.579 129.979  1.00  0.00
ATOM  16213  HD2 ILE A1050     132.666 207.829 130.030  1.00  0.00
ATOM  16214  HD3 ILE A1050     134.054 206.928 129.382  1.00  0.00
ATOM  16215  C   ILE A1050     131.197 209.562 125.966  1.00  0.00
ATOM  16216  O   ILE A1050     129.982 209.575 125.825  1.00  0.00
ATOM  16217  N   ASP A1051     131.910 210.687 126.079  1.00  0.00
ATOM  16218  H   ASP A1051     132.924 210.650 126.061  1.00  0.00
ATOM  16219  CA  ASP A1051     131.296 211.999 126.322  1.00  0.00
ATOM  16220  HA  ASP A1051     130.738 211.924 127.256  1.00  0.00
ATOM  16221  CB  ASP A1051     132.409 213.034 126.556  1.00  0.00
ATOM  16222  HB1 ASP A1051     133.181 212.602 127.195  1.00  0.00
ATOM  16223  HB2 ASP A1051     131.973 213.865 127.101  1.00  0.00
ATOM  16224  CG  ASP A1051     133.050 213.579 125.285  1.00  0.00
```

```
ATOM  16225  OD1 ASP A1051     133.213 214.823 125.228  1.00  0.00
ATOM  16226  OD2 ASP A1051     133.277 212.785 124.344  1.00  0.00
ATOM  16227  C   ASP A1051     130.260 212.407 125.256  1.00  0.00
ATOM  16228  O   ASP A1051     129.147 212.824 125.589  1.00  0.00
ATOM  16229  N   ARG A1052     130.550 212.150 123.974  1.00  0.00
ATOM  16230  H   ARG A1052     131.531 211.955 123.775  1.00  0.00
ATOM  16231  CA  ARG A1052     129.610 212.369 122.856  1.00  0.00
ATOM  16232  HA  ARG A1052     128.761 212.905 123.272  1.00  0.00
ATOM  16233  CB  ARG A1052     130.185 213.352 121.819  1.00  0.00
ATOM  16234  HB1 ARG A1052     129.387 213.642 121.135  1.00  0.00
ATOM  16235  HB2 ARG A1052     130.959 212.844 121.241  1.00  0.00
ATOM  16236  CG  ARG A1052     130.791 214.635 122.450  1.00  0.00
ATOM  16237  HG1 ARG A1052     131.049 215.321 121.644  1.00  0.00
ATOM  16238  HG2 ARG A1052     131.736 214.353 122.912  1.00  0.00
ATOM  16239  CD  ARG A1052     129.933 215.411 123.488  1.00  0.00
ATOM  16240  HD1 ARG A1052     129.096 214.809 123.828  1.00  0.00
ATOM  16241  HD2 ARG A1052     129.527 216.302 123.012  1.00  0.00
ATOM  16242  NE  ARG A1052     130.731 215.807 124.668  1.00  0.00
ATOM  16243  HE  ARG A1052     131.734 215.622 124.618  1.00  0.00
ATOM  16244  CZ  ARG A1052     130.338 216.186 125.876  1.00  0.00
ATOM  16245  NH1 ARG A1052     129.095 216.353 126.207  1.00  0.00
ATOM  16246 1HH1 ARG A1052     128.354 216.110 125.593  1.00  0.00
ATOM  16247 2HH1 ARG A1052     128.900 216.614 127.182  1.00  0.00
ATOM  16248  NH2 ARG A1052     131.240 216.370 126.788  1.00  0.00
ATOM  16249 1HH2 ARG A1052     132.166 216.018 126.573  1.00  0.00
ATOM  16250 2HH2 ARG A1052     130.956 216.510 127.763  1.00  0.00
ATOM  16251  C   ARG A1052     128.870 211.123 122.362  1.00  0.00
ATOM  16252  O   ARG A1052     128.243 211.130 121.308  1.00  0.00
ATOM  16253  N   ASN A1053     128.813 210.121 123.241  1.00  0.00
ATOM  16254  H   ASN A1053     129.411 210.208 124.055  1.00  0.00
ATOM  16255  CA  ASN A1053     127.730 209.133 123.352  1.00  0.00
ATOM  16256  HA  ASN A1053     127.104 209.207 122.461  1.00  0.00
ATOM  16257  CB  ASN A1053     128.360 207.714 123.346  1.00  0.00
ATOM  16258  HB1 ASN A1053     129.443 207.790 123.308  1.00  0.00
ATOM  16259  HB2 ASN A1053     128.052 207.205 122.434  1.00  0.00
ATOM  16260  CG  ASN A1053     128.033 206.789 124.513  1.00  0.00
ATOM  16261  OD1 ASN A1053     127.495 205.702 124.344  1.00  0.00
ATOM  16262  ND2 ASN A1053     128.386 207.159 125.723  1.00  0.00
ATOM  16263 1HD2 ASN A1053     128.797 208.078 125.889  1.00  0.00
ATOM  16264 2HD2 ASN A1053     128.180 206.544 126.481  1.00  0.00
ATOM  16265  C   ASN A1053     126.784 209.467 124.543  1.00  0.00
ATOM  16266  O   ASN A1053     125.798 208.761 124.751  1.00  0.00
ATOM  16267  N   GLY A1054     127.056 210.528 125.319  1.00  0.00
ATOM  16268  H   GLY A1054     127.895 211.066 125.145  1.00  0.00
ATOM  16269  CA  GLY A1054     126.419 210.771 126.616  1.00  0.00
ATOM  16270  HA1 GLY A1054     125.335 210.797 126.499  1.00  0.00
ATOM  16271  HA2 GLY A1054     126.754 211.725 127.026  1.00  0.00
ATOM  16272  C   GLY A1054     126.805 209.662 127.599  1.00  0.00
ATOM  16273  O   GLY A1054     127.985 209.385 127.777  1.00  0.00
ATOM  16274  N   LEU A1055     125.834 208.957 128.179  1.00  0.00
ATOM  16275  H   LEU A1055     124.874 209.233 128.048  1.00  0.00
ATOM  16276  CA  LEU A1055     126.065 207.590 128.648  1.00  0.00
ATOM  16277  HA  LEU A1055     126.888 207.159 128.083  1.00  0.00
ATOM  16278  CB  LEU A1055     126.426 207.545 130.150  1.00  0.00
ATOM  16279  HB1 LEU A1055     126.260 206.520 130.484  1.00  0.00
ATOM  16280  HB2 LEU A1055     125.722 208.187 130.681  1.00  0.00
ATOM  16281  CG  LEU A1055     127.854 207.924 130.585  1.00  0.00
ATOM  16282  HG  LEU A1055     128.043 208.976 130.373  1.00  0.00
ATOM  16283  CD1 LEU A1055     127.978 207.701 132.095  1.00  0.00
ATOM  16284 1HD1 LEU A1055     128.974 207.981 132.435  1.00  0.00
ATOM  16285 2HD1 LEU A1055     127.255 208.328 132.614  1.00  0.00
```

```
ATOM  16286 3HD1 LEU A1055     127.787 206.660 132.346  1.00  0.00
ATOM  16287  CD2 LEU A1055     128.943 207.070 129.918  1.00  0.00
ATOM  16288 1HD2 LEU A1055     129.914 207.348 130.327  1.00  0.00
ATOM  16289 2HD2 LEU A1055     128.756 206.016 130.111  1.00  0.00
ATOM  16290 3HD2 LEU A1055     128.957 207.264 128.849  1.00  0.00
ATOM  16291  C   LEU A1055     124.845 206.698 128.387  1.00  0.00
ATOM  16292  O   LEU A1055     123.692 207.102 128.564  1.00  0.00
ATOM  16293  N   GLN A1056     125.129 205.445 128.029  1.00  0.00
ATOM  16294  H   GLN A1056     126.100 205.202 127.957  1.00  0.00
ATOM  16295  CA  GLN A1056     124.181 204.328 128.032  1.00  0.00
ATOM  16296  HA  GLN A1056     123.193 204.704 127.763  1.00  0.00
ATOM  16297  CB  GLN A1056     124.578 203.246 126.998  1.00  0.00
ATOM  16298  HB1 GLN A1056     123.734 202.568 126.867  1.00  0.00
ATOM  16299  HB2 GLN A1056     125.429 202.668 127.360  1.00  0.00
ATOM  16300  CG  GLN A1056     124.967 203.858 125.636  1.00  0.00
ATOM  16301  HG1 GLN A1056     125.960 204.287 125.753  1.00  0.00
ATOM  16302  HG2 GLN A1056     124.291 204.676 125.395  1.00  0.00
ATOM  16303  CD  GLN A1056     124.999 202.899 124.447  1.00  0.00
ATOM  16304  OE1 GLN A1056     124.201 201.979 124.289  1.00  0.00
ATOM  16305  NE2 GLN A1056     125.898 203.124 123.515  1.00  0.00
ATOM  16306 1HE2 GLN A1056     126.501 203.935 123.611  1.00  0.00
ATOM  16307 2HE2 GLN A1056     125.847 202.546 122.697  1.00  0.00
ATOM  16308  C   GLN A1056     124.086 203.791 129.472  1.00  0.00
ATOM  16309  O   GLN A1056     124.601 202.723 129.803  1.00  0.00
ATOM  16310  N   SER A1057     123.621 204.660 130.370  1.00  0.00
ATOM  16311  H   SER A1057     123.252 205.543 130.039  1.00  0.00
ATOM  16312  CA  SER A1057     123.701 204.450 131.808  1.00  0.00
ATOM  16313  HA  SER A1057     124.684 204.055 132.065  1.00  0.00
ATOM  16314  CB  SER A1057     123.511 205.770 132.567  1.00  0.00
ATOM  16315  HB1 SER A1057     123.324 205.536 133.615  1.00  0.00
ATOM  16316  HB2 SER A1057     122.643 206.289 132.160  1.00  0.00
ATOM  16317  OG  SER A1057     124.644 206.610 132.495  1.00  0.00
ATOM  16318  HG  SER A1057     124.464 207.411 133.010  1.00  0.00
ATOM  16319  C   SER A1057     122.638 203.474 132.275  1.00  0.00
ATOM  16320  O   SER A1057     121.480 203.857 132.459  1.00  0.00
ATOM  16321  N   CYS A1058     123.082 202.256 132.582  1.00  0.00
ATOM  16322  H   CYS A1058     123.984 201.999 132.208  1.00  0.00
ATOM  16323  CA  CYS A1058     122.388 201.264 133.397  1.00  0.00
ATOM  16324  HA  CYS A1058     121.661 200.781 132.759  1.00  0.00
ATOM  16325  CB  CYS A1058     123.439 200.262 133.872  1.00  0.00
ATOM  16326  HB1 CYS A1058     122.998 199.585 134.599  1.00  0.00
ATOM  16327  HB2 CYS A1058     124.242 200.822 134.349  1.00  0.00
ATOM  16328  SG  CYS A1058     124.138 199.281 132.520  1.00  0.00
ATOM  16329  HG  CYS A1058     125.311 199.035 133.095  1.00  0.00
ATOM  16330  C   CYS A1058     121.642 201.877 134.602  1.00  0.00
ATOM  16331  O   CYS A1058     122.139 202.840 135.189  1.00  0.00
ATOM  16332  N   PRO A1059     120.490 201.315 135.034  1.00  0.00
ATOM  16333  CD  PRO A1059     119.972 200.018 134.674  1.00  0.00
ATOM  16334  HD1 PRO A1059     120.768 199.292 134.665  1.00  0.00
ATOM  16335  HD2 PRO A1059     119.480 200.077 133.709  1.00  0.00
ATOM  16336  CG  PRO A1059     118.954 199.635 135.734  1.00  0.00
ATOM  16337  HG1 PRO A1059     119.458 199.203 136.599  1.00  0.00
ATOM  16338  HG2 PRO A1059     118.206 198.953 135.343  1.00  0.00
ATOM  16339  CB  PRO A1059     118.372 200.998 136.075  1.00  0.00
ATOM  16340  HB1 PRO A1059     117.906 201.021 137.057  1.00  0.00
ATOM  16341  HB2 PRO A1059     117.669 201.295 135.300  1.00  0.00
ATOM  16342  CA  PRO A1059     119.584 201.913 135.999  1.00  0.00
ATOM  16343  HA  PRO A1059     119.255 202.886 135.636  1.00  0.00
ATOM  16344  C   PRO A1059     120.256 202.101 137.340  1.00  0.00
ATOM  16345  O   PRO A1059     120.415 201.164 138.142  1.00  0.00
ATOM  16346  N   ILE A1060     120.689 203.335 137.557  1.00  0.00
```

ATOM	16347	H	ILE	A1060	120.627	204.017	136.813	1.00	0.00
ATOM	16348	CA	ILE	A1060	121.503	203.663	138.704	1.00	0.00
ATOM	16349	HA	ILE	A1060	122.162	202.806	138.834	1.00	0.00
ATOM	16350	CB	ILE	A1060	122.455	204.851	138.395	1.00	0.00
ATOM	16351	HB	ILE	A1060	122.518	204.933	137.308	1.00	0.00
ATOM	16352	CG2	ILE	A1060	121.978	206.198	138.927	1.00	0.00
ATOM	16353	1HG2	ILE	A1060	122.436	206.991	138.343	1.00	0.00
ATOM	16354	2HG2	ILE	A1060	120.898	206.277	138.833	1.00	0.00
ATOM	16355	3HG2	ILE	A1060	122.269	206.333	139.965	1.00	0.00
ATOM	16356	CG1	ILE	A1060	123.881	204.535	138.899	1.00	0.00
ATOM	16357	1HG1	ILE	A1060	124.186	203.560	138.522	1.00	0.00
ATOM	16358	2HG1	ILE	A1060	123.894	204.494	139.987	1.00	0.00
ATOM	16359	CD	ILE	A1060	124.943	205.543	138.436	1.00	0.00
ATOM	16360	HD1	ILE	A1060	125.922	205.219	138.789	1.00	0.00
ATOM	16361	HD2	ILE	A1060	124.954	205.581	137.347	1.00	0.00
ATOM	16362	HD3	ILE	A1060	124.735	206.532	138.841	1.00	0.00
ATOM	16363	C	ILE	A1060	120.676	203.692	139.997	1.00	0.00
ATOM	16364	O	ILE	A1060	119.452	203.538	140.000	1.00	0.00
ATOM	16365	N	LYS	A1061	121.380	203.781	141.123	1.00	0.00
ATOM	16366	H	LYS	A1061	122.386	203.795	141.033	1.00	0.00
ATOM	16367	CA	LYS	A1061	120.827	204.332	142.357	1.00	0.00
ATOM	16368	HA	LYS	A1061	119.776	204.062	142.477	1.00	0.00
ATOM	16369	CB	LYS	A1061	121.627	203.707	143.530	1.00	0.00
ATOM	16370	HB1	LYS	A1061	122.663	203.592	143.203	1.00	0.00
ATOM	16371	HB2	LYS	A1061	121.259	202.688	143.663	1.00	0.00
ATOM	16372	CG	LYS	A1061	121.605	204.385	144.921	1.00	0.00
ATOM	16373	HG1	LYS	A1061	121.138	203.716	145.644	1.00	0.00
ATOM	16374	HG2	LYS	A1061	121.017	205.302	144.903	1.00	0.00
ATOM	16375	CD	LYS	A1061	123.053	204.679	145.365	1.00	0.00
ATOM	16376	HD1	LYS	A1061	123.620	205.025	144.502	1.00	0.00
ATOM	16377	HD2	LYS	A1061	123.514	203.752	145.711	1.00	0.00
ATOM	16378	CE	LYS	A1061	123.184	205.742	146.458	1.00	0.00
ATOM	16379	HE1	LYS	A1061	122.933	205.292	147.420	1.00	0.00
ATOM	16380	HE2	LYS	A1061	122.479	206.553	146.251	1.00	0.00
ATOM	16381	NZ	LYS	A1061	124.567	206.284	146.457	1.00	0.00
ATOM	16382	HZ1	LYS	A1061	125.258	205.554	146.545	1.00	0.00
ATOM	16383	HZ2	LYS	A1061	124.731	206.775	145.578	1.00	0.00
ATOM	16384	HZ3	LYS	A1061	124.763	206.988	147.170	1.00	0.00
ATOM	16385	C	LYS	A1061	120.829	205.860	142.173	1.00	0.00
ATOM	16386	O	LYS	A1061	120.057	206.384	141.379	1.00	0.00
ATOM	16387	N	GLU	A1062	121.776	206.547	142.798	1.00	0.00
ATOM	16388	H	GLU	A1062	122.455	206.033	143.333	1.00	0.00
ATOM	16389	CA	GLU	A1062	121.869	207.999	142.913	1.00	0.00
ATOM	16390	HA	GLU	A1062	121.754	208.497	141.949	1.00	0.00
ATOM	16391	CB	GLU	A1062	120.781	208.472	143.930	1.00	0.00
ATOM	16392	HB1	GLU	A1062	121.082	209.431	144.351	1.00	0.00
ATOM	16393	HB2	GLU	A1062	120.740	207.778	144.770	1.00	0.00
ATOM	16394	CG	GLU	A1062	119.370	208.704	143.370	1.00	0.00
ATOM	16395	HG1	GLU	A1062	119.400	208.637	142.288	1.00	0.00
ATOM	16396	HG2	GLU	A1062	119.087	209.736	143.595	1.00	0.00
ATOM	16397	CD	GLU	A1062	118.279	207.771	143.938	1.00	0.00
ATOM	16398	OE1	GLU	A1062	118.326	206.538	143.735	1.00	0.00
ATOM	16399	OE2	GLU	A1062	117.258	208.276	144.446	1.00	0.00
ATOM	16400	C	GLU	A1062	123.232	208.353	143.529	1.00	0.00
ATOM	16401	O	GLU	A1062	123.957	207.459	143.962	1.00	0.00
ATOM	16402	N	ASP	A1063	123.503	209.649	143.700	1.00	0.00
ATOM	16403	H	ASP	A1063	122.850	210.315	143.324	1.00	0.00
ATOM	16404	CA	ASP	A1063	124.597	210.188	144.515	1.00	0.00
ATOM	16405	HA	ASP	A1063	124.463	211.270	144.498	1.00	0.00
ATOM	16406	CB	ASP	A1063	124.420	209.821	146.012	1.00	0.00
ATOM	16407	HB1	ASP	A1063	123.809	208.925	146.114	1.00	0.00

```
ATOM  16408  HB2 ASP A1063     123.872 210.626 146.500  1.00  0.00
ATOM  16409  CG  ASP A1063     125.744 209.606 146.741  1.00  0.00
ATOM  16410  OD1 ASP A1063     125.970 208.455 147.183  1.00  0.00
ATOM  16411  OD2 ASP A1063     126.553 210.558 146.775  1.00  0.00
ATOM  16412  C   ASP A1063     126.003 209.958 143.934  1.00  0.00
ATOM  16413  O   ASP A1063     126.401 208.853 143.583  1.00  0.00
ATOM  16414  N   SER A1064     126.772 211.049 143.850  1.00  0.00
ATOM  16415  H   SER A1064     126.444 211.907 144.272  1.00  0.00
ATOM  16416  CA  SER A1064     128.141 211.081 143.317  1.00  0.00
ATOM  16417  HA  SER A1064     128.104 210.734 142.283  1.00  0.00
ATOM  16418  CB  SER A1064     128.669 212.523 143.305  1.00  0.00
ATOM  16419  HB1 SER A1064     128.154 213.075 142.519  1.00  0.00
ATOM  16420  HB2 SER A1064     129.738 212.523 143.086  1.00  0.00
ATOM  16421  OG  SER A1064     128.422 213.163 144.543  1.00  0.00
ATOM  16422  HG  SER A1064     128.943 212.738 145.239  1.00  0.00
ATOM  16423  C   SER A1064     129.135 210.164 144.032  1.00  0.00
ATOM  16424  O   SER A1064     130.189 209.888 143.472  1.00  0.00
ATOM  16425  N   PHE A1065     128.798 209.655 145.216  1.00  0.00
ATOM  16426  H   PHE A1065     127.940 209.983 145.660  1.00  0.00
ATOM  16427  CA  PHE A1065     129.637 208.753 146.006  1.00  0.00
ATOM  16428  HA  PHE A1065     130.619 208.679 145.538  1.00  0.00
ATOM  16429  CB  PHE A1065     129.829 209.409 147.375  1.00  0.00
ATOM  16430  HB1 PHE A1065     130.596 208.875 147.932  1.00  0.00
ATOM  16431  HB2 PHE A1065     128.890 209.347 147.922  1.00  0.00
ATOM  16432  CG  PHE A1065     130.241 210.874 147.273  1.00  0.00
ATOM  16433  CD1 PHE A1065     131.446 211.226 146.631  1.00  0.00
ATOM  16434  HD1 PHE A1065     132.114 210.450 146.271  1.00  0.00
ATOM  16435  CE1 PHE A1065     131.773 212.578 146.428  1.00  0.00
ATOM  16436  HE1 PHE A1065     132.694 212.831 145.922  1.00  0.00
ATOM  16437  CZ  PHE A1065     130.905 213.589 146.876  1.00  0.00
ATOM  16438  HZ  PHE A1065     131.160 214.624 146.713  1.00  0.00
ATOM  16439  CE2 PHE A1065     129.707 213.244 147.524  1.00  0.00
ATOM  16440  HE2 PHE A1065     129.036 214.015 147.868  1.00  0.00
ATOM  16441  CD2 PHE A1065     129.372 211.891 147.711  1.00  0.00
ATOM  16442  HD2 PHE A1065     128.429 211.628 148.177  1.00  0.00
ATOM  16443  C   PHE A1065     129.083 207.301 146.011  1.00  0.00
ATOM  16444  O   PHE A1065     129.628 206.393 146.624  1.00  0.00
ATOM  16445  N   LEU A1066     128.050 207.058 145.190  1.00  0.00
ATOM  16446  H   LEU A1066     127.628 207.885 144.777  1.00  0.00
ATOM  16447  CA  LEU A1066     127.734 205.802 144.501  1.00  0.00
ATOM  16448  HA  LEU A1066     126.960 206.039 143.768  1.00  0.00
ATOM  16449  CB  LEU A1066     128.965 205.300 143.701  1.00  0.00
ATOM  16450  HB1 LEU A1066     128.664 204.423 143.132  1.00  0.00
ATOM  16451  HB2 LEU A1066     129.730 204.973 144.408  1.00  0.00
ATOM  16452  CG  LEU A1066     129.625 206.293 142.721  1.00  0.00
ATOM  16453  HG  LEU A1066     130.003 207.150 143.274  1.00  0.00
ATOM  16454  CD1 LEU A1066     130.804 205.613 142.026  1.00  0.00
ATOM  16455 1HD1 LEU A1066     131.314 206.328 141.382  1.00  0.00
ATOM  16456 2HD1 LEU A1066     131.520 205.265 142.767  1.00  0.00
ATOM  16457 3HD1 LEU A1066     130.469 204.774 141.423  1.00  0.00
ATOM  16458  CD2 LEU A1066     128.665 206.788 141.638  1.00  0.00
ATOM  16459 1HD2 LEU A1066     128.265 205.945 141.076  1.00  0.00
ATOM  16460 2HD2 LEU A1066     127.852 207.348 142.092  1.00  0.00
ATOM  16461 3HD2 LEU A1066     129.201 207.452 140.957  1.00  0.00
ATOM  16462  C   LEU A1066     127.163 204.649 145.337  1.00  0.00
ATOM  16463  O   LEU A1066     126.167 204.059 144.927  1.00  0.00
ATOM  16464  N   GLN A1067     127.747 204.336 146.496  1.00  0.00
ATOM  16465  H   GLN A1067     128.506 204.947 146.790  1.00  0.00
ATOM  16466  CA  GLN A1067     127.644 203.019 147.150  1.00  0.00
ATOM  16467  HA  GLN A1067     126.865 202.450 146.642  1.00  0.00
ATOM  16468  CB  GLN A1067     128.984 202.264 146.944  1.00  0.00
```

```
ATOM  16469  HB1 GLN A1067     129.616 202.385 147.822  1.00  0.00
ATOM  16470  HB2 GLN A1067     129.538 202.701 146.112  1.00  0.00
ATOM  16471  CG  GLN A1067     128.804 200.758 146.652  1.00  0.00
ATOM  16472  HG1 GLN A1067     127.850 200.399 147.033  1.00  0.00
ATOM  16473  HG2 GLN A1067     129.583 200.207 147.176  1.00  0.00
ATOM  16474  CD  GLN A1067     128.903 200.434 145.160  1.00  0.00
ATOM  16475  OE1 GLN A1067     128.257 201.052 144.323  1.00  0.00
ATOM  16476  NE2 GLN A1067     129.715 199.475 144.771  1.00  0.00
ATOM  16477 1HE2 GLN A1067     130.221 198.931 145.454  1.00  0.00
ATOM  16478 2HE2 GLN A1067     129.868 199.301 143.777  1.00  0.00
ATOM  16479  C   GLN A1067     127.201 203.147 148.621  1.00  0.00
ATOM  16480  O   GLN A1067     126.409 204.031 148.949  1.00  0.00
ATOM  16481  N   ARG A1068     127.627 202.231 149.501  1.00  0.00
ATOM  16482  H   ARG A1068     128.241 201.512 149.144  1.00  0.00
ATOM  16483  CA  ARG A1068     127.192 202.120 150.908  1.00  0.00
ATOM  16484  HA  ARG A1068     127.118 203.124 151.330  1.00  0.00
ATOM  16485  CB  ARG A1068     125.789 201.477 150.900  1.00  0.00
ATOM  16486  HB1 ARG A1068     125.814 200.630 150.214  1.00  0.00
ATOM  16487  HB2 ARG A1068     125.075 202.200 150.500  1.00  0.00
ATOM  16488  CG  ARG A1068     125.248 200.970 152.242  1.00  0.00
ATOM  16489  HG1 ARG A1068     125.815 200.086 152.526  1.00  0.00
ATOM  16490  HG2 ARG A1068     124.216 200.659 152.102  1.00  0.00
ATOM  16491  CD  ARG A1068     125.298 201.995 153.376  1.00  0.00
ATOM  16492  HD1 ARG A1068     124.279 202.280 153.646  1.00  0.00
ATOM  16493  HD2 ARG A1068     125.818 202.898 153.056  1.00  0.00
ATOM  16494  NE  ARG A1068     125.969 201.422 154.551  1.00  0.00
ATOM  16495  HE  ARG A1068     126.020 200.409 154.635  1.00  0.00
ATOM  16496  CZ  ARG A1068     126.419 202.097 155.586  1.00  0.00
ATOM  16497  NH1 ARG A1068     126.466 203.397 155.632  1.00  0.00
ATOM  16498 1HH1 ARG A1068     125.996 203.981 154.968  1.00  0.00
ATOM  16499 2HH1 ARG A1068     126.686 203.803 156.547  1.00  0.00
ATOM  16500  NH2 ARG A1068     126.824 201.469 156.641  1.00  0.00
ATOM  16501 1HH2 ARG A1068     126.728 200.471 156.687  1.00  0.00
ATOM  16502 2HH2 ARG A1068     126.807 202.028 157.505  1.00  0.00
ATOM  16503  C   ARG A1068     128.235 201.327 151.712  1.00  0.00
ATOM  16504  O   ARG A1068     128.248 200.106 151.691  1.00  0.00
ATOM  16505  N   TYR A1069     129.190 202.036 152.317  1.00  0.00
ATOM  16506  H   TYR A1069     129.103 203.043 152.308  1.00  0.00
ATOM  16507  CA  TYR A1069     130.549 201.518 152.563  1.00  0.00
ATOM  16508  HA  TYR A1069     130.867 201.011 151.653  1.00  0.00
ATOM  16509  CB  TYR A1069     131.492 202.696 152.776  1.00  0.00
ATOM  16510  HB1 TYR A1069     132.518 202.330 152.810  1.00  0.00
ATOM  16511  HB2 TYR A1069     131.273 203.150 153.745  1.00  0.00
ATOM  16512  CG  TYR A1069     131.427 203.771 151.705  1.00  0.00
ATOM  16513  CD1 TYR A1069     131.179 203.465 150.346  1.00  0.00
ATOM  16514  HD1 TYR A1069     130.995 202.449 150.035  1.00  0.00
ATOM  16515  CE1 TYR A1069     131.212 204.487 149.375  1.00  0.00
ATOM  16516  HE1 TYR A1069     131.020 204.294 148.331  1.00  0.00
ATOM  16517  CZ  TYR A1069     131.555 205.795 149.751  1.00  0.00
ATOM  16518  OH  TYR A1069     131.717 206.760 148.820  1.00  0.00
ATOM  16519  HH  TYR A1069     131.831 207.626 149.228  1.00  0.00
ATOM  16520  CE2 TYR A1069     131.754 206.093 151.103  1.00  0.00
ATOM  16521  HE2 TYR A1069     132.034 207.091 151.374  1.00  0.00
ATOM  16522  CD2 TYR A1069     131.666 205.100 152.085  1.00  0.00
ATOM  16523  HD2 TYR A1069     131.879 205.348 153.118  1.00  0.00
ATOM  16524  C   TYR A1069     130.729 200.453 153.658  1.00  0.00
ATOM  16525  O   TYR A1069     131.849 200.033 153.934  1.00  0.00
ATOM  16526  N   SER A1070     129.654 199.974 154.273  1.00  0.00
ATOM  16527  H   SER A1070     128.748 200.297 153.966  1.00  0.00
ATOM  16528  CA  SER A1070     129.686 198.816 155.167  1.00  0.00
ATOM  16529  HA  SER A1070     130.387 198.073 154.784  1.00  0.00
```

```
ATOM  16530  CB  SER A1070     130.132 199.245 156.567  1.00  0.00
ATOM  16531  HB1 SER A1070     129.871 198.477 157.296  1.00  0.00
ATOM  16532  HB2 SER A1070     129.647 200.181 156.848  1.00  0.00
ATOM  16533  OG  SER A1070     131.534 199.405 156.565  1.00  0.00
ATOM  16534  HG  SER A1070     131.812 199.737 155.691  1.00  0.00
ATOM  16535  C   SER A1070     128.301 198.182 155.243  1.00  0.00
ATOM  16536  O   SER A1070     127.285 198.875 155.143  1.00  0.00
ATOM  16537  N   SER A1071     128.298 196.861 155.450  1.00  0.00
ATOM  16538  H   SER A1071     129.186 196.408 155.602  1.00  0.00
ATOM  16539  CA  SER A1071     127.111 195.995 155.523  1.00  0.00
ATOM  16540  HA  SER A1071     127.468 194.979 155.681  1.00  0.00
ATOM  16541  CB  SER A1071     126.250 196.348 156.743  1.00  0.00
ATOM  16542  HB1 SER A1071     125.482 195.585 156.879  1.00  0.00
ATOM  16543  HB2 SER A1071     125.761 197.311 156.589  1.00  0.00
ATOM  16544  OG  SER A1071     127.054 196.409 157.911  1.00  0.00
ATOM  16545  HG  SER A1071     126.485 196.584 158.662  1.00  0.00
ATOM  16546  C   SER A1071     126.266 195.933 154.238  1.00  0.00
ATOM  16547  O   SER A1071     125.069 195.668 154.313  1.00  0.00
ATOM  16548  N   ASP A1072     126.903 196.128 153.079  1.00  0.00
ATOM  16549  H   ASP A1072     127.887 196.319 153.114  1.00  0.00
ATOM  16550  CA  ASP A1072     126.308 196.005 151.737  1.00  0.00
ATOM  16551  HA  ASP A1072     127.089 196.288 151.030  1.00  0.00
ATOM  16552  CB  ASP A1072     125.964 194.515 151.470  1.00  0.00
ATOM  16553  HB1 ASP A1072     124.884 194.409 151.354  1.00  0.00
ATOM  16554  HB2 ASP A1072     126.253 193.897 152.322  1.00  0.00
ATOM  16555  CG  ASP A1072     126.683 193.950 150.236  1.00  0.00
ATOM  16556  OD1 ASP A1072     126.002 193.294 149.418  1.00  0.00
ATOM  16557  OD2 ASP A1072     127.897 194.220 150.111  1.00  0.00
ATOM  16558  C   ASP A1072     125.146 197.007 151.472  1.00  0.00
ATOM  16559  O   ASP A1072     124.757 197.792 152.354  1.00  0.00
ATOM  16560  N   PRO A1073     124.625 197.129 150.233  1.00  0.00
ATOM  16561  CD  PRO A1073     124.952 196.333 149.055  1.00  0.00
ATOM  16562  HD1 PRO A1073     124.601 195.310 149.169  1.00  0.00
ATOM  16563  HD2 PRO A1073     126.027 196.357 148.870  1.00  0.00
ATOM  16564  CG  PRO A1073     124.212 196.966 147.877  1.00  0.00
ATOM  16565  HG1 PRO A1073     123.229 196.504 147.771  1.00  0.00
ATOM  16566  HG2 PRO A1073     124.783 196.883 146.952  1.00  0.00
ATOM  16567  CB  PRO A1073     124.055 198.416 148.319  1.00  0.00
ATOM  16568  HB1 PRO A1073     123.228 198.912 147.812  1.00  0.00
ATOM  16569  HB2 PRO A1073     124.984 198.951 148.121  1.00  0.00
ATOM  16570  CA  PRO A1073     123.850 198.295 149.836  1.00  0.00
ATOM  16571  HA  PRO A1073     124.290 199.175 150.289  1.00  0.00
ATOM  16572  C   PRO A1073     122.373 198.292 150.264  1.00  0.00
ATOM  16573  O   PRO A1073     121.471 198.033 149.464  1.00  0.00
ATOM  16574  N   THR A1074     122.117 198.729 151.498  1.00  0.00
ATOM  16575  H   THR A1074     122.907 198.736 152.136  1.00  0.00
ATOM  16576  CA  THR A1074     120.800 199.188 151.998  1.00  0.00
ATOM  16577  HA  THR A1074     120.084 198.406 151.740  1.00  0.00
ATOM  16578  CB  THR A1074     120.809 199.235 153.540  1.00  0.00
ATOM  16579  HB  THR A1074     121.119 198.250 153.887  1.00  0.00
ATOM  16580  CG2 THR A1074     121.757 200.256 154.174  1.00  0.00
ATOM  16581 1HG2 THR A1074     121.654 200.215 155.258  1.00  0.00
ATOM  16582 2HG2 THR A1074     122.785 200.010 153.921  1.00  0.00
ATOM  16583 3HG2 THR A1074     121.527 201.266 153.845  1.00  0.00
ATOM  16584  OG1 THR A1074     119.511 199.444 154.030  1.00  0.00
ATOM  16585  HG1 THR A1074     119.266 200.364 153.858  1.00  0.00
ATOM  16586  C   THR A1074     120.266 200.469 151.321  1.00  0.00
ATOM  16587  O   THR A1074     119.818 201.402 151.980  1.00  0.00
ATOM  16588  N   GLY A1075     120.360 200.563 149.987  1.00  0.00
ATOM  16589  H   GLY A1075     120.679 199.726 149.506  1.00  0.00
ATOM  16590  CA  GLY A1075     119.990 201.727 149.157  1.00  0.00
```

```
ATOM  16591  HA1 GLY A1075     119.006 202.084 149.466  1.00  0.00
ATOM  16592  HA2 GLY A1075     119.933 201.423 148.112  1.00  0.00
ATOM  16593  C   GLY A1075     120.976 202.899 149.265  1.00  0.00
ATOM  16594  O   GLY A1075     121.566 203.299 148.264  1.00  0.00
ATOM  16595  N   ALA A1076     121.146 203.386 150.498  1.00  0.00
ATOM  16596  H   ALA A1076     120.569 202.921 151.193  1.00  0.00
ATOM  16597  CA  ALA A1076     121.987 204.464 151.049  1.00  0.00
ATOM  16598  HA  ALA A1076     122.752 203.999 151.671  1.00  0.00
ATOM  16599  CB  ALA A1076     122.717 205.309 149.994  1.00  0.00
ATOM  16600  HB1 ALA A1076     121.998 205.762 149.311  1.00  0.00
ATOM  16601  HB2 ALA A1076     123.275 206.102 150.497  1.00  0.00
ATOM  16602  HB3 ALA A1076     123.423 204.684 149.447  1.00  0.00
ATOM  16603  C   ALA A1076     121.140 205.335 151.990  1.00  0.00
ATOM  16604  O   ALA A1076     121.481 205.462 153.165  1.00  0.00
ATOM  16605  N   LEU A1077     119.989 205.803 151.492  1.00  0.00
ATOM  16606  H   LEU A1077     119.811 205.661 150.511  1.00  0.00
ATOM  16607  CA  LEU A1077     118.898 206.383 152.281  1.00  0.00
ATOM  16608  HA  LEU A1077     119.283 207.272 152.780  1.00  0.00
ATOM  16609  CB  LEU A1077     117.780 206.828 151.311  1.00  0.00
ATOM  16610  HB1 LEU A1077     117.365 205.943 150.827  1.00  0.00
ATOM  16611  HB2 LEU A1077     118.249 207.430 150.532  1.00  0.00
ATOM  16612  CG  LEU A1077     116.624 207.671 151.882  1.00  0.00
ATOM  16613  HG  LEU A1077     116.021 207.984 151.030  1.00  0.00
ATOM  16614  CD1 LEU A1077     115.686 206.895 152.812  1.00  0.00
ATOM  16615 1HD1 LEU A1077     114.782 207.479 152.980  1.00  0.00
ATOM  16616 2HD1 LEU A1077     115.418 205.944 152.351  1.00  0.00
ATOM  16617 3HD1 LEU A1077     116.168 206.714 153.771  1.00  0.00
ATOM  16618  CD2 LEU A1077     117.087 208.936 152.599  1.00  0.00
ATOM  16619 1HD2 LEU A1077     116.234 209.574 152.823  1.00  0.00
ATOM  16620 2HD2 LEU A1077     117.598 208.687 153.530  1.00  0.00
ATOM  16621 3HD2 LEU A1077     117.784 209.486 151.966  1.00  0.00
ATOM  16622  C   LEU A1077     118.450 205.387 153.355  1.00  0.00
ATOM  16623  O   LEU A1077     117.792 204.401 153.022  1.00  0.00
ATOM  16624  N   THR A1078     118.863 205.620 154.605  1.00  0.00
ATOM  16625  H   THR A1078     119.316 206.529 154.749  1.00  0.00
ATOM  16626  CA  THR A1078     118.896 204.698 155.772  1.00  0.00
ATOM  16627  HA  THR A1078     117.967 204.786 156.335  1.00  0.00
ATOM  16628  CB  THR A1078     119.118 203.205 155.397  1.00  0.00
ATOM  16629  HB  THR A1078     119.898 203.141 154.636  1.00  0.00
ATOM  16630  CG2 THR A1078     119.497 202.278 156.557  1.00  0.00
ATOM  16631 1HG2 THR A1078     120.516 202.477 156.884  1.00  0.00
ATOM  16632 2HG2 THR A1078     118.809 202.426 157.389  1.00  0.00
ATOM  16633 3HG2 THR A1078     119.442 201.238 156.234  1.00  0.00
ATOM  16634  OG1 THR A1078     117.942 202.633 154.888  1.00  0.00
ATOM  16635  HG1 THR A1078     117.712 203.176 154.109  1.00  0.00
ATOM  16636  C   THR A1078     120.046 205.101 156.698  1.00  0.00
ATOM  16637  O   THR A1078     119.895 205.092 157.920  1.00  0.00
ATOM  16638  N   GLU A1079     121.203 205.442 156.129  1.00  0.00
ATOM  16639  H   GLU A1079     121.244 205.494 155.113  1.00  0.00
ATOM  16640  CA  GLU A1079     122.426 205.773 156.861  1.00  0.00
ATOM  16641  HA  GLU A1079     122.143 206.233 157.809  1.00  0.00
ATOM  16642  CB  GLU A1079     123.233 204.494 157.198  1.00  0.00
ATOM  16643  HB1 GLU A1079     123.722 204.122 156.298  1.00  0.00
ATOM  16644  HB2 GLU A1079     122.546 203.723 157.546  1.00  0.00
ATOM  16645  CG  GLU A1079     124.278 204.728 158.310  1.00  0.00
ATOM  16646  HG1 GLU A1079     123.754 204.907 159.251  1.00  0.00
ATOM  16647  HG2 GLU A1079     124.849 205.629 158.074  1.00  0.00
ATOM  16648  CD  GLU A1079     125.245 203.545 158.472  1.00  0.00
ATOM  16649  OE1 GLU A1079     124.805 202.393 158.677  1.00  0.00
ATOM  16650  OE2 GLU A1079     126.473 203.732 158.282  1.00  0.00
ATOM  16651  C   GLU A1079     123.238 206.813 156.078  1.00  0.00
```

```
ATOM  16652  O   GLU A1079     124.270 206.527 155.470  1.00  0.00
ATOM  16653  N   ASP A1080     122.667 208.017 156.052  1.00  0.00
ATOM  16654  H   ASP A1080     121.756 208.075 156.490  1.00  0.00
ATOM  16655  CA  ASP A1080     122.969 209.140 155.165  1.00  0.00
ATOM  16656  HA  ASP A1080     123.114 208.714 154.171  1.00  0.00
ATOM  16657  CB  ASP A1080     121.704 210.007 155.053  1.00  0.00
ATOM  16658  HB1 ASP A1080     121.924 210.887 154.449  1.00  0.00
ATOM  16659  HB2 ASP A1080     121.392 210.340 156.045  1.00  0.00
ATOM  16660  CG  ASP A1080     120.567 209.213 154.382  1.00  0.00
ATOM  16661  OD1 ASP A1080     120.377 209.398 153.161  1.00  0.00
ATOM  16662  OD2 ASP A1080     119.901 208.405 155.078  1.00  0.00
ATOM  16663  C   ASP A1080     124.286 209.890 155.459  1.00  0.00
ATOM  16664  O   ASP A1080     124.308 211.099 155.684  1.00  0.00
ATOM  16665  N   SER A1081     125.402 209.154 155.400  1.00  0.00
ATOM  16666  H   SER A1081     125.287 208.149 155.272  1.00  0.00
ATOM  16667  CA  SER A1081     126.746 209.689 155.126  1.00  0.00
ATOM  16668  HA  SER A1081     126.631 210.614 154.559  1.00  0.00
ATOM  16669  CB  SER A1081     127.507 210.021 156.412  1.00  0.00
ATOM  16670  HB1 SER A1081     127.901 209.111 156.868  1.00  0.00
ATOM  16671  HB2 SER A1081     126.824 210.500 157.115  1.00  0.00
ATOM  16672  OG  SER A1081     128.560 210.930 156.130  1.00  0.00
ATOM  16673  HG  SER A1081     129.422 210.448 156.054  1.00  0.00
ATOM  16674  C   SER A1081     127.546 208.706 154.268  1.00  0.00
ATOM  16675  O   SER A1081     127.262 207.503 154.243  1.00  0.00
ATOM  16676  N   ILE A1082     128.501 209.220 153.495  1.00  0.00
ATOM  16677  H   ILE A1082     128.725 210.198 153.628  1.00  0.00
ATOM  16678  CA  ILE A1082     129.091 208.504 152.351  1.00  0.00
ATOM  16679  HA  ILE A1082     129.181 207.463 152.651  1.00  0.00
ATOM  16680  CB  ILE A1082     128.097 208.582 151.160  1.00  0.00
ATOM  16681  HB  ILE A1082     127.110 208.324 151.547  1.00  0.00
ATOM  16682  CG2 ILE A1082     127.947 210.012 150.598  1.00  0.00
ATOM  16683 1HG2 ILE A1082     127.625 210.694 151.384  1.00  0.00
ATOM  16684 2HG2 ILE A1082     128.892 210.361 150.184  1.00  0.00
ATOM  16685 3HG2 ILE A1082     127.195 210.016 149.807  1.00  0.00
ATOM  16686  CG1 ILE A1082     128.376 207.566 150.040  1.00  0.00
ATOM  16687 1HG1 ILE A1082     129.374 207.708 149.642  1.00  0.00
ATOM  16688 2HG1 ILE A1082     127.669 207.726 149.227  1.00  0.00
ATOM  16689  CD  ILE A1082     128.231 206.105 150.479  1.00  0.00
ATOM  16690  HD1 ILE A1082     128.460 205.466 149.629  1.00  0.00
ATOM  16691  HD2 ILE A1082     128.923 205.863 151.282  1.00  0.00
ATOM  16692  HD3 ILE A1082     127.211 205.918 150.813  1.00  0.00
ATOM  16693  C   ILE A1082     130.536 208.963 152.060  1.00  0.00
ATOM  16694  O   ILE A1082     130.939 209.193 150.922  1.00  0.00
ATOM  16695  N   ASP A1083     131.320 209.088 153.127  1.00  0.00
ATOM  16696  H   ASP A1083     130.882 208.931 154.031  1.00  0.00
ATOM  16697  CA  ASP A1083     132.610 209.783 153.203  1.00  0.00
ATOM  16698  HA  ASP A1083     132.426 210.830 152.960  1.00  0.00
ATOM  16699  CB  ASP A1083     133.052 209.744 154.682  1.00  0.00
ATOM  16700  HB1 ASP A1083     134.002 210.273 154.772  1.00  0.00
ATOM  16701  HB2 ASP A1083     133.223 208.706 154.973  1.00  0.00
ATOM  16702  CG  ASP A1083     132.013 210.374 155.633  1.00  0.00
ATOM  16703  OD1 ASP A1083     132.312 211.451 156.194  1.00  0.00
ATOM  16704  OD2 ASP A1083     130.907 209.785 155.786  1.00  0.00
ATOM  16705  C   ASP A1083     133.714 209.319 152.219  1.00  0.00
ATOM  16706  O   ASP A1083     134.181 210.118 151.418  1.00  0.00
ATOM  16707  N   ASP A1084     134.103 208.036 152.242  1.00  0.00
ATOM  16708  H   ASP A1084     133.677 207.432 152.929  1.00  0.00
ATOM  16709  CA  ASP A1084     134.985 207.360 151.255  1.00  0.00
ATOM  16710  HA  ASP A1084     134.629 207.546 150.241  1.00  0.00
ATOM  16711  CB  ASP A1084     136.426 207.923 151.395  1.00  0.00
ATOM  16712  HB1 ASP A1084     136.725 207.855 152.444  1.00  0.00
```

```
ATOM  16713  HB2 ASP A1084     136.438 208.977 151.119  1.00  0.00
ATOM  16714  CG  ASP A1084     137.495 207.215 150.539  1.00  0.00
ATOM  16715  OD1 ASP A1084     137.219 206.943 149.348  1.00  0.00
ATOM  16716  OD2 ASP A1084     138.559 206.872 151.095  1.00  0.00
ATOM  16717  C   ASP A1084     135.006 205.832 151.518  1.00  0.00
ATOM  16718  O   ASP A1084     134.548 205.364 152.564  1.00  0.00
ATOM  16719  N   THR A1085     135.567 205.075 150.566  1.00  0.00
ATOM  16720  H   THR A1085     135.934 205.642 149.800  1.00  0.00
ATOM  16721  CA  THR A1085     136.182 203.718 150.604  1.00  0.00
ATOM  16722  HA  THR A1085     137.175 203.850 150.172  1.00  0.00
ATOM  16723  CB  THR A1085     136.428 203.069 151.983  1.00  0.00
ATOM  16724  HB  THR A1085     136.879 202.089 151.816  1.00  0.00
ATOM  16725  CG2 THR A1085     137.412 203.852 152.856  1.00  0.00
ATOM  16726 1HG2 THR A1085     137.579 203.310 153.786  1.00  0.00
ATOM  16727 2HG2 THR A1085     138.359 203.957 152.325  1.00  0.00
ATOM  16728 3HG2 THR A1085     137.032 204.846 153.084  1.00  0.00
ATOM  16729  OG1 THR A1085     135.235 202.858 152.696  1.00  0.00
ATOM  16730  HG1 THR A1085     134.862 203.756 152.792  1.00  0.00
ATOM  16731  C   THR A1085     135.504 202.697 149.684  1.00  0.00
ATOM  16732  O   THR A1085     136.114 201.680 149.365  1.00  0.00
ATOM  16733  N   PHE A1086     134.285 202.996 149.211  1.00  0.00
ATOM  16734  H   PHE A1086     133.894 203.857 149.567  1.00  0.00
ATOM  16735  CA  PHE A1086     133.533 202.370 148.104  1.00  0.00
ATOM  16736  HA  PHE A1086     132.591 202.895 148.017  1.00  0.00
ATOM  16737  CB  PHE A1086     134.259 202.646 146.775  1.00  0.00
ATOM  16738  HB1 PHE A1086     133.719 202.151 145.968  1.00  0.00
ATOM  16739  HB2 PHE A1086     135.257 202.210 146.802  1.00  0.00
ATOM  16740  CG  PHE A1086     134.362 204.121 146.437  1.00  0.00
ATOM  16741  CD1 PHE A1086     133.286 204.763 145.793  1.00  0.00
ATOM  16742  HD1 PHE A1086     132.407 204.206 145.508  1.00  0.00
ATOM  16743  CE1 PHE A1086     133.355 206.139 145.510  1.00  0.00
ATOM  16744  HE1 PHE A1086     132.534 206.637 145.015  1.00  0.00
ATOM  16745  CZ  PHE A1086     134.500 206.873 145.868  1.00  0.00
ATOM  16746  HZ  PHE A1086     134.554 207.928 145.648  1.00  0.00
ATOM  16747  CE2 PHE A1086     135.577 206.231 146.506  1.00  0.00
ATOM  16748  HE2 PHE A1086     136.452 206.804 146.792  1.00  0.00
ATOM  16749  CD2 PHE A1086     135.511 204.857 146.785  1.00  0.00
ATOM  16750  HD2 PHE A1086     136.343 204.381 147.277  1.00  0.00
ATOM  16751  C   PHE A1086     133.140 200.884 148.198  1.00  0.00
ATOM  16752  O   PHE A1086     132.049 200.534 147.758  1.00  0.00
ATOM  16753  N   LEU A1087     133.998 200.032 148.754  1.00  0.00
ATOM  16754  H   LEU A1087     134.801 200.474 149.184  1.00  0.00
ATOM  16755  CA  LEU A1087     134.264 198.663 148.297  1.00  0.00
ATOM  16756  HA  LEU A1087     134.615 198.747 147.267  1.00  0.00
ATOM  16757  CB  LEU A1087     135.461 198.181 149.145  1.00  0.00
ATOM  16758  HB1 LEU A1087     135.157 198.100 150.189  1.00  0.00
ATOM  16759  HB2 LEU A1087     136.214 198.968 149.098  1.00  0.00
ATOM  16760  CG  LEU A1087     136.136 196.867 148.701  1.00  0.00
ATOM  16761  HG  LEU A1087     136.028 196.741 147.624  1.00  0.00
ATOM  16762  CD1 LEU A1087     137.630 196.923 149.035  1.00  0.00
ATOM  16763 1HD1 LEU A1087     138.108 195.986 148.751  1.00  0.00
ATOM  16764 2HD1 LEU A1087     138.097 197.732 148.472  1.00  0.00
ATOM  16765 3HD1 LEU A1087     137.773 197.096 150.101  1.00  0.00
ATOM  16766  CD2 LEU A1087     135.569 195.643 149.426  1.00  0.00
ATOM  16767 1HD2 LEU A1087     136.090 194.746 149.090  1.00  0.00
ATOM  16768 2HD2 LEU A1087     135.693 195.752 150.502  1.00  0.00
ATOM  16769 3HD2 LEU A1087     134.516 195.520 149.186  1.00  0.00
ATOM  16770  C   LEU A1087     133.120 197.611 148.245  1.00  0.00
ATOM  16771  O   LEU A1087     133.177 196.799 147.323  1.00  0.00
ATOM  16772  N   PRO A1088     132.157 197.513 149.182  1.00  0.00
ATOM  16773  CD  PRO A1088     131.923 198.385 150.327  1.00  0.00
```

```
ATOM  16774  HD1 PRO A1088     131.744 199.411 150.018  1.00  0.00
ATOM  16775  HD2 PRO A1088     132.775 198.335 151.005  1.00  0.00
ATOM  16776  CG  PRO A1088     130.675 197.840 151.016  1.00  0.00
ATOM  16777  HG1 PRO A1088     129.787 198.255 150.541  1.00  0.00
ATOM  16778  HG2 PRO A1088     130.676 198.041 152.085  1.00  0.00
ATOM  16779  CB  PRO A1088     130.751 196.345 150.721  1.00  0.00
ATOM  16780  HB1 PRO A1088     129.770 195.873 150.780  1.00  0.00
ATOM  16781  HB2 PRO A1088     131.441 195.872 151.422  1.00  0.00
ATOM  16782  CA  PRO A1088     131.344 196.292 149.308  1.00  0.00
ATOM  16783  HA  PRO A1088     132.006 195.426 149.260  1.00  0.00
ATOM  16784  C   PRO A1088     130.251 196.113 148.255  1.00  0.00
ATOM  16785  O   PRO A1088     129.408 196.994 148.050  1.00  0.00
ATOM  16786  N   VAL A1089     130.321 194.955 147.589  1.00  0.00
ATOM  16787  H   VAL A1089     131.056 194.313 147.859  1.00  0.00
ATOM  16788  CA  VAL A1089     129.472 194.449 146.496  1.00  0.00
ATOM  16789  HA  VAL A1089     128.435 194.517 146.828  1.00  0.00
ATOM  16790  CB  VAL A1089     129.658 195.275 145.193  1.00  0.00
ATOM  16791  HB  VAL A1089     129.703 194.576 144.367  1.00  0.00
ATOM  16792  CG1 VAL A1089     128.437 196.171 144.935  1.00  0.00
ATOM  16793 1HG1 VAL A1089     128.555 196.686 143.983  1.00  0.00
ATOM  16794 2HG1 VAL A1089     127.531 195.568 144.906  1.00  0.00
ATOM  16795 3HG1 VAL A1089     128.342 196.906 145.734  1.00  0.00
ATOM  16796  CG2 VAL A1089     130.924 196.149 145.122  1.00  0.00
ATOM  16797 1HG2 VAL A1089     131.002 196.654 144.167  1.00  0.00
ATOM  16798 2HG2 VAL A1089     130.909 196.929 145.878  1.00  0.00
ATOM  16799 3HG2 VAL A1089     131.807 195.523 145.264  1.00  0.00
ATOM  16800  C   VAL A1089     129.806 192.951 146.275  1.00  0.00
ATOM  16801  O   VAL A1089     130.811 192.469 146.810  1.00  0.00
ATOM  16802  N   PRO A1090     129.001 192.196 145.490  1.00  0.00
ATOM  16803  CD  PRO A1090     127.624 192.529 145.161  1.00  0.00
ATOM  16804  HD1 PRO A1090     127.605 193.263 144.354  1.00  0.00
ATOM  16805  HD2 PRO A1090     127.081 192.901 146.032  1.00  0.00
ATOM  16806  CG  PRO A1090     127.004 191.224 144.677  1.00  0.00
ATOM  16807  HG1 PRO A1090     126.170 191.401 143.998  1.00  0.00
ATOM  16808  HG2 PRO A1090     126.688 190.629 145.535  1.00  0.00
ATOM  16809  CB  PRO A1090     128.175 190.542 143.977  1.00  0.00
ATOM  16810  HB1 PRO A1090     128.243 190.934 142.962  1.00  0.00
ATOM  16811  HB2 PRO A1090     128.055 189.459 143.955  1.00  0.00
ATOM  16812  CA  PRO A1090     129.412 190.959 144.794  1.00  0.00
ATOM  16813  HA  PRO A1090     129.640 190.192 145.534  1.00  0.00
ATOM  16814  C   PRO A1090     130.646 191.169 143.886  1.00  0.00
ATOM  16815  O   PRO A1090     131.366 192.149 144.029  1.00  0.00
ATOM  16816  N   GLU A1091     130.881 190.317 142.882  1.00  0.00
ATOM  16817  H   GLU A1091     130.279 189.523 142.728  1.00  0.00
ATOM  16818  CA  GLU A1091     131.938 190.571 141.888  1.00  0.00
ATOM  16819  HA  GLU A1091     132.216 191.623 141.951  1.00  0.00
ATOM  16820  CB  GLU A1091     133.192 189.743 142.245  1.00  0.00
ATOM  16821  HB1 GLU A1091     133.259 188.878 141.585  1.00  0.00
ATOM  16822  HB2 GLU A1091     133.118 189.377 143.269  1.00  0.00
ATOM  16823  CG  GLU A1091     134.467 190.591 142.146  1.00  0.00
ATOM  16824  HG1 GLU A1091     135.333 189.966 142.371  1.00  0.00
ATOM  16825  HG2 GLU A1091     134.428 191.397 142.881  1.00  0.00
ATOM  16826  CD  GLU A1091     134.593 191.169 140.739  1.00  0.00
ATOM  16827  OE1 GLU A1091     134.856 190.371 139.823  1.00  0.00
ATOM  16828  OE2 GLU A1091     134.233 192.350 140.525  1.00  0.00
ATOM  16829  C   GLU A1091     131.433 190.358 140.449  1.00  0.00
ATOM  16830  O   GLU A1091     130.643 189.446 140.188  1.00  0.00
ATOM  16831  N   TYR A1092     131.804 191.268 139.545  1.00  0.00
ATOM  16832  H   TYR A1092     132.560 191.893 139.828  1.00  0.00
ATOM  16833  CA  TYR A1092     131.285 191.423 138.179  1.00  0.00
ATOM  16834  HA  TYR A1092     130.796 190.493 137.889  1.00  0.00
```

```
ATOM  16835  CB  TYR A1092     130.238 192.561 138.106  1.00  0.00
ATOM  16836  HB1 TYR A1092     130.217 192.941 137.087  1.00  0.00
ATOM  16837  HB2 TYR A1092     130.590 193.389 138.716  1.00  0.00
ATOM  16838  CG  TYR A1092     128.777 192.282 138.447  1.00  0.00
ATOM  16839  CD1 TYR A1092     128.387 191.766 139.698  1.00  0.00
ATOM  16840  HD1 TYR A1092     129.125 191.463 140.418  1.00  0.00
ATOM  16841  CE1 TYR A1092     127.028 191.698 140.056  1.00  0.00
ATOM  16842  HE1 TYR A1092     126.732 191.343 141.029  1.00  0.00
ATOM  16843  CZ  TYR A1092     126.040 192.136 139.156  1.00  0.00
ATOM  16844  OH  TYR A1092     124.739 192.158 139.542  1.00  0.00
ATOM  16845  HH  TYR A1092     124.153 192.597 138.900  1.00  0.00
ATOM  16846  CE2 TYR A1092     126.415 192.595 137.879  1.00  0.00
ATOM  16847  HE2 TYR A1092     125.652 192.925 137.188  1.00  0.00
ATOM  16848  CD2 TYR A1092     127.776 192.672 137.531  1.00  0.00
ATOM  16849  HD2 TYR A1092     128.049 193.078 136.561  1.00  0.00
ATOM  16850  C   TYR A1092     132.433 191.647 137.157  1.00  0.00
ATOM  16851  O   TYR A1092     132.190 192.157 136.055  1.00  0.00
ATOM  16852  N   ILE A1093     133.688 191.283 137.452  1.00  0.00
ATOM  16853  H   ILE A1093     133.911 190.959 138.397  1.00  0.00
ATOM  16854  CA  ILE A1093     134.689 191.022 136.403  1.00  0.00
ATOM  16855  HA  ILE A1093     134.875 191.951 135.869  1.00  0.00
ATOM  16856  CB  ILE A1093     136.046 190.532 136.965  1.00  0.00
ATOM  16857  HB  ILE A1093     135.852 189.728 137.674  1.00  0.00
ATOM  16858  CG2 ILE A1093     136.943 189.918 135.865  1.00  0.00
ATOM  16859 1HG2 ILE A1093     137.935 189.723 136.276  1.00  0.00
ATOM  16860 2HG2 ILE A1093     136.528 188.970 135.531  1.00  0.00
ATOM  16861 3HG2 ILE A1093     137.027 190.598 135.017  1.00  0.00
ATOM  16862  CG1 ILE A1093     136.819 191.652 137.699  1.00  0.00
ATOM  16863 1HG1 ILE A1093     137.609 191.186 138.291  1.00  0.00
ATOM  16864 2HG1 ILE A1093     136.150 192.156 138.397  1.00  0.00
ATOM  16865  CD  ILE A1093     137.467 192.718 136.804  1.00  0.00
ATOM  16866  HD1 ILE A1093     138.027 193.407 137.432  1.00  0.00
ATOM  16867  HD2 ILE A1093     138.174 192.271 136.108  1.00  0.00
ATOM  16868  HD3 ILE A1093     136.704 193.264 136.250  1.00  0.00
ATOM  16869  C   ILE A1093     134.111 190.001 135.421  1.00  0.00
ATOM  16870  O   ILE A1093     133.762 188.873 135.778  1.00  0.00
ATOM  16871  N   ASN A1094     133.993 190.395 134.155  1.00  0.00
ATOM  16872  H   ASN A1094     134.270 191.343 133.941  1.00  0.00
ATOM  16873  CA  ASN A1094     133.174 189.696 133.172  1.00  0.00
ATOM  16874  HA  ASN A1094     132.864 188.733 133.583  1.00  0.00
ATOM  16875  CB  ASN A1094     131.903 190.539 132.935  1.00  0.00
ATOM  16876  HB1 ASN A1094     132.152 191.450 132.393  1.00  0.00
ATOM  16877  HB2 ASN A1094     131.463 190.823 133.890  1.00  0.00
ATOM  16878  CG  ASN A1094     130.827 189.794 132.162  1.00  0.00
ATOM  16879  OD1 ASN A1094     130.899 188.599 131.927  1.00  0.00
ATOM  16880  ND2 ASN A1094     129.746 190.437 131.798  1.00  0.00
ATOM  16881 1HD2 ASN A1094     129.579 191.406 132.050  1.00  0.00
ATOM  16882 2HD2 ASN A1094     129.002 189.887 131.409  1.00  0.00
ATOM  16883  C   ASN A1094     133.992 189.418 131.905  1.00  0.00
ATOM  16884  O   ASN A1094     133.989 190.174 130.924  1.00  0.00
ATOM  16885  N   GLN A1095     134.737 188.320 132.003  1.00  0.00
ATOM  16886  H   GLN A1095     134.587 187.793 132.850  1.00  0.00
ATOM  16887  CA  GLN A1095     135.552 187.681 130.972  1.00  0.00
ATOM  16888  HA  GLN A1095     136.506 188.206 130.900  1.00  0.00
ATOM  16889  CB  GLN A1095     135.809 186.197 131.349  1.00  0.00
ATOM  16890  HB1 GLN A1095     136.618 185.834 130.716  1.00  0.00
ATOM  16891  HB2 GLN A1095     134.927 185.608 131.096  1.00  0.00
ATOM  16892  CG  GLN A1095     136.175 185.875 132.817  1.00  0.00
ATOM  16893  HG1 GLN A1095     137.015 186.499 133.119  1.00  0.00
ATOM  16894  HG2 GLN A1095     136.495 184.835 132.885  1.00  0.00
ATOM  16895  CD  GLN A1095     135.020 186.066 133.816  1.00  0.00
```

```
ATOM  16896  OE1 GLN A1095     133.846 186.011 133.476  1.00  0.00
ATOM  16897  NE2 GLN A1095     135.309 186.455 135.035  1.00  0.00
ATOM  16898 1HE2 GLN A1095     136.254 186.499 135.367  1.00  0.00
ATOM  16899 2HE2 GLN A1095     134.540 186.813 135.593  1.00  0.00
ATOM  16900  C   GLN A1095     134.816 187.758 129.623  1.00  0.00
ATOM  16901  O   GLN A1095     133.669 187.320 129.518  1.00  0.00
ATOM  16902  N   SER A1096     135.449 188.322 128.599  1.00  0.00
ATOM  16903  H   SER A1096     136.412 188.597 128.702  1.00  0.00
ATOM  16904  CA  SER A1096     134.804 188.563 127.308  1.00  0.00
ATOM  16905  HA  SER A1096     133.755 188.273 127.356  1.00  0.00
ATOM  16906  CB  SER A1096     134.813 190.061 126.985  1.00  0.00
ATOM  16907  HB1 SER A1096     134.465 190.218 125.963  1.00  0.00
ATOM  16908  HB2 SER A1096     135.827 190.453 127.080  1.00  0.00
ATOM  16909  OG  SER A1096     133.938 190.739 127.880  1.00  0.00
ATOM  16910  HG  SER A1096     134.159 190.472 128.786  1.00  0.00
ATOM  16911  C   SER A1096     135.446 187.701 126.222  1.00  0.00
ATOM  16912  O   SER A1096     136.661 187.530 126.205  1.00  0.00
ATOM  16913  N   VAL A1097     134.581 187.144 125.364  1.00  0.00
ATOM  16914  H   VAL A1097     133.646 187.514 125.397  1.00  0.00
ATOM  16915  CA  VAL A1097     134.801 185.966 124.502  1.00  0.00
ATOM  16916  HA  VAL A1097     133.892 185.861 123.916  1.00  0.00
ATOM  16917  CB  VAL A1097     135.933 186.209 123.473  1.00  0.00
ATOM  16918  HB  VAL A1097     136.899 186.208 123.974  1.00  0.00
ATOM  16919  CG1 VAL A1097     135.948 185.097 122.413  1.00  0.00
ATOM  16920 1HG1 VAL A1097     136.743 185.298 121.693  1.00  0.00
ATOM  16921 2HG1 VAL A1097     136.152 184.134 122.879  1.00  0.00
ATOM  16922 3HG1 VAL A1097     134.996 185.065 121.886  1.00  0.00
ATOM  16923  CG2 VAL A1097     135.770 187.537 122.716  1.00  0.00
ATOM  16924 1HG2 VAL A1097     136.553 187.638 121.965  1.00  0.00
ATOM  16925 2HG2 VAL A1097     134.799 187.572 122.224  1.00  0.00
ATOM  16926 3HG2 VAL A1097     135.855 188.380 123.401  1.00  0.00
ATOM  16927  C   VAL A1097     135.027 184.669 125.310  1.00  0.00
ATOM  16928  O   VAL A1097     135.787 184.663 126.275  1.00  0.00
ATOM  16929  N   PRO A1098     134.340 183.548 124.993  1.00  0.00
ATOM  16930  CD  PRO A1098     134.659 182.250 125.577  1.00  0.00
ATOM  16931  HD1 PRO A1098     134.725 182.305 126.665  1.00  0.00
ATOM  16932  HD2 PRO A1098     135.607 181.896 125.170  1.00  0.00
ATOM  16933  CG  PRO A1098     133.536 181.296 125.167  1.00  0.00
ATOM  16934  HG1 PRO A1098     132.757 181.298 125.930  1.00  0.00
ATOM  16935  HG2 PRO A1098     133.906 180.285 124.997  1.00  0.00
ATOM  16936  CB  PRO A1098     133.004 181.929 123.884  1.00  0.00
ATOM  16937  HB1 PRO A1098     131.976 181.628 123.680  1.00  0.00
ATOM  16938  HB2 PRO A1098     133.650 181.654 123.050  1.00  0.00
ATOM  16939  CA  PRO A1098     133.137 183.433 124.155  1.00  0.00
ATOM  16940  HA  PRO A1098     133.277 183.943 123.203  1.00  0.00
ATOM  16941  C   PRO A1098     131.870 183.990 124.832  1.00  0.00
ATOM  16942  O   PRO A1098     130.807 183.984 124.219  1.00  0.00
ATOM  16943  N   LYS A1099     131.966 184.480 126.079  1.00  0.00
ATOM  16944  H   LYS A1099     132.887 184.447 126.496  1.00  0.00
ATOM  16945  CA  LYS A1099     130.852 184.775 127.006  1.00  0.00
ATOM  16946  HA  LYS A1099     130.331 183.834 127.190  1.00  0.00
ATOM  16947  CB  LYS A1099     131.467 185.245 128.342  1.00  0.00
ATOM  16948  HB1 LYS A1099     131.612 186.325 128.302  1.00  0.00
ATOM  16949  HB2 LYS A1099     132.452 184.789 128.463  1.00  0.00
ATOM  16950  CG  LYS A1099     130.652 184.887 129.603  1.00  0.00
ATOM  16951  HG1 LYS A1099     130.524 183.804 129.653  1.00  0.00
ATOM  16952  HG2 LYS A1099     129.668 185.351 129.561  1.00  0.00
ATOM  16953  CD  LYS A1099     131.404 185.363 130.860  1.00  0.00
ATOM  16954  HD1 LYS A1099     131.656 186.417 130.753  1.00  0.00
ATOM  16955  HD2 LYS A1099     132.342 184.810 130.928  1.00  0.00
ATOM  16956  CE  LYS A1099     130.642 185.173 132.180  1.00  0.00
```

```
ATOM  16957  HE1 LYS A1099     131.376 185.251 132.990  1.00  0.00
ATOM  16958  HE2 LYS A1099     130.208 184.170 132.210  1.00  0.00
ATOM  16959  NZ  LYS A1099     129.605 186.217 132.376  1.00  0.00
ATOM  16960  HZ1 LYS A1099     128.883 186.186 131.672  1.00  0.00
ATOM  16961  HZ2 LYS A1099     129.179 186.169 133.291  1.00  0.00
ATOM  16962  HZ3 LYS A1099     130.049 187.131 132.301  1.00  0.00
ATOM  16963  C   LYS A1099     129.761 185.748 126.517  1.00  0.00
ATOM  16964  O   LYS A1099     128.675 185.729 127.083  1.00  0.00
ATOM  16965  N   ARG A1100     130.008 186.567 125.475  1.00  0.00
ATOM  16966  H   ARG A1100     130.912 186.452 125.045  1.00  0.00
ATOM  16967  CA  ARG A1100     129.057 187.501 124.804  1.00  0.00
ATOM  16968  HA  ARG A1100     129.655 188.294 124.353  1.00  0.00
ATOM  16969  CB  ARG A1100     128.369 186.758 123.625  1.00  0.00
ATOM  16970  HB1 ARG A1100     129.159 186.524 122.909  1.00  0.00
ATOM  16971  HB2 ARG A1100     127.695 187.435 123.109  1.00  0.00
ATOM  16972  CG  ARG A1100     127.630 185.419 123.895  1.00  0.00
ATOM  16973  HG1 ARG A1100     128.259 184.789 124.519  1.00  0.00
ATOM  16974  HG2 ARG A1100     127.551 184.892 122.943  1.00  0.00
ATOM  16975  CD  ARG A1100     126.218 185.439 124.523  1.00  0.00
ATOM  16976  HD1 ARG A1100     126.141 186.167 125.326  1.00  0.00
ATOM  16977  HD2 ARG A1100     126.073 184.469 124.998  1.00  0.00
ATOM  16978  NE  ARG A1100     125.138 185.603 123.525  1.00  0.00
ATOM  16979  HE  ARG A1100     124.672 184.758 123.237  1.00  0.00
ATOM  16980  CZ  ARG A1100     124.688 186.711 122.966  1.00  0.00
ATOM  16981  NH1 ARG A1100     125.114 187.899 123.284  1.00  0.00
ATOM  16982 1HH1 ARG A1100     125.669 188.002 124.133  1.00  0.00
ATOM  16983 2HH1 ARG A1100     124.591 188.699 122.966  1.00  0.00
ATOM  16984  NH2 ARG A1100     123.787 186.624 122.030  1.00  0.00
ATOM  16985 1HH2 ARG A1100     123.429 185.739 121.731  1.00  0.00
ATOM  16986 2HH2 ARG A1100     123.574 187.447 121.476  1.00  0.00
ATOM  16987  C   ARG A1100     128.081 188.297 125.724  1.00  0.00
ATOM  16988  O   ARG A1100     126.891 188.320 125.434  1.00  0.00
ATOM  16989  N   PRO A1101     128.529 188.881 126.847  1.00  0.00
ATOM  16990  CD  PRO A1101     129.884 189.395 127.057  1.00  0.00
ATOM  16991  HD1 PRO A1101     129.997 190.377 126.598  1.00  0.00
ATOM  16992  HD2 PRO A1101     130.634 188.716 126.659  1.00  0.00
ATOM  16993  CG  PRO A1101     130.065 189.500 128.568  1.00  0.00
ATOM  16994  HG1 PRO A1101     129.765 190.491 128.910  1.00  0.00
ATOM  16995  HG2 PRO A1101     131.089 189.279 128.872  1.00  0.00
ATOM  16996  CB  PRO A1101     129.080 188.443 129.065  1.00  0.00
ATOM  16997  HB1 PRO A1101     128.836 188.563 130.115  1.00  0.00
ATOM  16998  HB2 PRO A1101     129.512 187.460 128.910  1.00  0.00
ATOM  16999  CA  PRO A1101     127.875 188.634 128.135  1.00  0.00
ATOM  17000  HA  PRO A1101     127.303 187.705 128.093  1.00  0.00
ATOM  17001  C   PRO A1101     126.956 189.728 128.708  1.00  0.00
ATOM  17002  O   PRO A1101     126.845 190.845 128.204  1.00  0.00
ATOM  17003  N   ALA A1102     126.345 189.368 129.837  1.00  0.00
ATOM  17004  H   ALA A1102     126.381 188.389 130.069  1.00  0.00
ATOM  17005  CA  ALA A1102     125.845 190.214 130.916  1.00  0.00
ATOM  17006  HA  ALA A1102     126.120 191.253 130.730  1.00  0.00
ATOM  17007  CB  ALA A1102     124.317 190.107 130.966  1.00  0.00
ATOM  17008  HB1 ALA A1102     123.899 190.755 131.733  1.00  0.00
ATOM  17009  HB2 ALA A1102     123.890 190.401 130.007  1.00  0.00
ATOM  17010  HB3 ALA A1102     124.027 189.079 131.185  1.00  0.00
ATOM  17011  C   ALA A1102     126.540 189.719 132.223  1.00  0.00
ATOM  17012  O   ALA A1102     127.220 188.685 132.216  1.00  0.00
ATOM  17013  N   GLY A1103     126.484 190.405 133.370  1.00  0.00
ATOM  17014  H   GLY A1103     126.965 189.978 134.147  1.00  0.00
ATOM  17015  CA  GLY A1103     125.999 191.773 133.567  1.00  0.00
ATOM  17016  HA1 GLY A1103     125.999 192.003 134.633  1.00  0.00
ATOM  17017  HA2 GLY A1103     124.972 191.866 133.219  1.00  0.00
```

```
ATOM  17018  C   GLY A1103     126.851 192.842 132.861  1.00  0.00
ATOM  17019  O   GLY A1103     127.907 192.543 132.299  1.00  0.00
ATOM  17020  N   SER A1104     126.376 194.087 132.886  1.00  0.00
ATOM  17021  H   SER A1104     125.559 194.287 133.445  1.00  0.00
ATOM  17022  CA  SER A1104     126.818 195.164 131.987  1.00  0.00
ATOM  17023  HA  SER A1104     127.699 194.861 131.418  1.00  0.00
ATOM  17024  CB  SER A1104     125.684 195.545 131.014  1.00  0.00
ATOM  17025  HB1 SER A1104     126.091 196.156 130.207  1.00  0.00
ATOM  17026  HB2 SER A1104     124.948 196.146 131.551  1.00  0.00
ATOM  17027  OG  SER A1104     125.005 194.428 130.477  1.00  0.00
ATOM  17028  HG  SER A1104     125.531 194.046 129.766  1.00  0.00
ATOM  17029  C   SER A1104     127.127 196.435 132.781  1.00  0.00
ATOM  17030  O   SER A1104     126.330 196.824 133.632  1.00  0.00
ATOM  17031  N   VAL A1105     128.262 197.083 132.493  1.00  0.00
ATOM  17032  H   VAL A1105     128.875 196.666 131.803  1.00  0.00
ATOM  17033  CA  VAL A1105     128.786 198.233 133.258  1.00  0.00
ATOM  17034  HA  VAL A1105     129.235 197.806 134.139  1.00  0.00
ATOM  17035  CB  VAL A1105     129.934 198.986 132.561  1.00  0.00
ATOM  17036  HB  VAL A1105     130.137 199.896 133.126  1.00  0.00
ATOM  17037  CG1 VAL A1105     131.209 198.155 132.596  1.00  0.00
ATOM  17038 1HG1 VAL A1105     132.027 198.724 132.153  1.00  0.00
ATOM  17039 2HG1 VAL A1105     131.479 197.913 133.622  1.00  0.00
ATOM  17040 3HG1 VAL A1105     131.068 197.234 132.039  1.00  0.00
ATOM  17041  CG2 VAL A1105     129.643 199.381 131.112  1.00  0.00
ATOM  17042 1HG2 VAL A1105     130.505 199.901 130.698  1.00  0.00
ATOM  17043 2HG2 VAL A1105     129.427 198.495 130.525  1.00  0.00
ATOM  17044 3HG2 VAL A1105     128.785 200.050 131.069  1.00  0.00
ATOM  17045  C   VAL A1105     127.750 199.264 133.730  1.00  0.00
ATOM  17046  O   VAL A1105     127.125 199.980 132.945  1.00  0.00
ATOM  17047  N   GLN A1106     127.651 199.371 135.046  1.00  0.00
ATOM  17048  H   GLN A1106     128.184 198.709 135.578  1.00  0.00
ATOM  17049  CA  GLN A1106     126.933 200.359 135.831  1.00  0.00
ATOM  17050  HA  GLN A1106     126.604 201.181 135.192  1.00  0.00
ATOM  17051  CB  GLN A1106     125.702 199.639 136.406  1.00  0.00
ATOM  17052  HB1 GLN A1106     126.061 198.781 136.967  1.00  0.00
ATOM  17053  HB2 GLN A1106     125.137 199.202 135.587  1.00  0.00
ATOM  17054  CG  GLN A1106     124.751 200.501 137.261  1.00  0.00
ATOM  17055  HG1 GLN A1106     123.785 200.578 136.763  1.00  0.00
ATOM  17056  HG2 GLN A1106     125.145 201.511 137.379  1.00  0.00
ATOM  17057  CD  GLN A1106     124.554 199.893 138.645  1.00  0.00
ATOM  17058  OE1 GLN A1106     125.469 199.331 139.221  1.00  0.00
ATOM  17059  NE2 GLN A1106     123.374 199.932 139.222  1.00  0.00
ATOM  17060 1HE2 GLN A1106     122.583 200.369 138.780  1.00  0.00
ATOM  17061 2HE2 GLN A1106     123.339 199.494 140.138  1.00  0.00
ATOM  17062  C   GLN A1106     127.941 200.933 136.859  1.00  0.00
ATOM  17063  O   GLN A1106     128.892 201.582 136.451  1.00  0.00
ATOM  17064  N   ASN A1107     127.791 200.672 138.161  1.00  0.00
ATOM  17065  H   ASN A1107     127.001 200.119 138.464  1.00  0.00
ATOM  17066  CA  ASN A1107     128.741 201.077 139.211  1.00  0.00
ATOM  17067  HA  ASN A1107     129.053 202.110 139.046  1.00  0.00
ATOM  17068  CB  ASN A1107     128.005 200.945 140.572  1.00  0.00
ATOM  17069  HB1 ASN A1107     128.737 200.863 141.372  1.00  0.00
ATOM  17070  HB2 ASN A1107     127.446 200.012 140.603  1.00  0.00
ATOM  17071  CG  ASN A1107     127.078 202.091 140.923  1.00  0.00
ATOM  17072  OD1 ASN A1107     126.329 202.622 140.116  1.00  0.00
ATOM  17073  ND2 ASN A1107     127.110 202.514 142.167  1.00  0.00
ATOM  17074 1HD2 ASN A1107     127.682 202.042 142.864  1.00  0.00
ATOM  17075 2HD2 ASN A1107     126.505 203.272 142.426  1.00  0.00
ATOM  17076  C   ASN A1107     129.990 200.183 139.287  1.00  0.00
ATOM  17077  O   ASN A1107     129.954 199.085 138.749  1.00  0.00
ATOM  17078  N   PRO A1108     131.035 200.563 140.060  1.00  0.00
```

```
ATOM  17079  CD  PRO A1108     131.232 201.872 140.675  1.00  0.00
ATOM  17080  HD1 PRO A1108     130.310 202.438 140.803  1.00  0.00
ATOM  17081  HD2 PRO A1108     131.931 202.452 140.069  1.00  0.00
ATOM  17082  CG  PRO A1108     131.849 201.579 142.035  1.00  0.00
ATOM  17083  HG1 PRO A1108     131.070 201.284 142.738  1.00  0.00
ATOM  17084  HG2 PRO A1108     132.405 202.433 142.419  1.00  0.00
ATOM  17085  CB  PRO A1108     132.752 200.385 141.726  1.00  0.00
ATOM  17086  HB1 PRO A1108     132.929 199.778 142.614  1.00  0.00
ATOM  17087  HB2 PRO A1108     133.698 200.749 141.336  1.00  0.00
ATOM  17088  CA  PRO A1108     132.013 199.616 140.619  1.00  0.00
ATOM  17089  HA  PRO A1108     132.725 199.332 139.846  1.00  0.00
ATOM  17090  C   PRO A1108     131.308 198.359 141.153  1.00  0.00
ATOM  17091  O   PRO A1108     130.625 198.423 142.175  1.00  0.00
ATOM  17092  N   VAL A1109     131.472 197.249 140.419  1.00  0.00
ATOM  17093  H   VAL A1109     132.076 197.348 139.620  1.00  0.00
ATOM  17094  CA  VAL A1109     130.570 196.079 140.378  1.00  0.00
ATOM  17095  HA  VAL A1109     130.979 195.408 139.629  1.00  0.00
ATOM  17096  CB  VAL A1109     130.554 195.287 141.694  1.00  0.00
ATOM  17097  HB  VAL A1109     130.034 195.878 142.435  1.00  0.00
ATOM  17098  CG1 VAL A1109     129.809 193.962 141.547  1.00  0.00
ATOM  17099 1HG1 VAL A1109     129.715 193.476 142.513  1.00  0.00
ATOM  17100 2HG1 VAL A1109     128.806 194.117 141.155  1.00  0.00
ATOM  17101 3HG1 VAL A1109     130.375 193.299 140.902  1.00  0.00
ATOM  17102  CG2 VAL A1109     131.976 194.969 142.168  1.00  0.00
ATOM  17103 1HG2 VAL A1109     131.944 194.361 143.069  1.00  0.00
ATOM  17104 2HG2 VAL A1109     132.521 194.423 141.398  1.00  0.00
ATOM  17105 3HG2 VAL A1109     132.504 195.886 142.413  1.00  0.00
ATOM  17106  C   VAL A1109     129.145 196.470 139.938  1.00  0.00
ATOM  17107  O   VAL A1109     128.458 197.276 140.557  1.00  0.00
ATOM  17108  N   TYR A1110     128.675 195.860 138.846  1.00  0.00
ATOM  17109  H   TYR A1110     129.178 195.048 138.538  1.00  0.00
ATOM  17110  CA  TYR A1110     127.965 196.583 137.782  1.00  0.00
ATOM  17111  HA  TYR A1110     127.947 197.646 138.025  1.00  0.00
ATOM  17112  CB  TYR A1110     128.813 196.399 136.494  1.00  0.00
ATOM  17113  HB1 TYR A1110     128.280 196.878 135.685  1.00  0.00
ATOM  17114  HB2 TYR A1110     128.841 195.337 136.247  1.00  0.00
ATOM  17115  CG  TYR A1110     130.265 196.909 136.545  1.00  0.00
ATOM  17116  CD1 TYR A1110     130.589 198.249 136.243  1.00  0.00
ATOM  17117  HD1 TYR A1110     129.842 198.962 135.951  1.00  0.00
ATOM  17118  CE1 TYR A1110     131.855 198.776 136.537  1.00  0.00
ATOM  17119  HE1 TYR A1110     132.045 199.831 136.433  1.00  0.00
ATOM  17120  CZ  TYR A1110     132.820 197.960 137.138  1.00  0.00
ATOM  17121  OH  TYR A1110     133.933 198.541 137.630  1.00  0.00
ATOM  17122  HH  TYR A1110     134.553 197.896 137.981  1.00  0.00
ATOM  17123  CE2 TYR A1110     132.578 196.579 137.299  1.00  0.00
ATOM  17124  HE2 TYR A1110     133.343 195.937 137.718  1.00  0.00
ATOM  17125  CD2 TYR A1110     131.304 196.057 136.987  1.00  0.00
ATOM  17126  HD2 TYR A1110     131.107 195.012 137.162  1.00  0.00
ATOM  17127  C   TYR A1110     126.490 196.121 137.603  1.00  0.00
ATOM  17128  O   TYR A1110     126.169 195.370 136.678  1.00  0.00
ATOM  17129  N   HIS A1111     125.604 196.522 138.530  1.00  0.00
ATOM  17130  H   HIS A1111     125.928 197.255 139.159  1.00  0.00
ATOM  17131  CA  HIS A1111     124.417 195.764 138.993  1.00  0.00
ATOM  17132  HA  HIS A1111     124.831 194.919 139.549  1.00  0.00
ATOM  17133  CB  HIS A1111     123.587 196.560 140.018  1.00  0.00
ATOM  17134  HB1 HIS A1111     122.846 195.889 140.455  1.00  0.00
ATOM  17135  HB2 HIS A1111     123.043 197.339 139.491  1.00  0.00
ATOM  17136  CG  HIS A1111     124.357 197.190 141.158  1.00  0.00
ATOM  17137  ND1 HIS A1111     123.968 198.345 141.846  1.00  0.00
ATOM  17138  CE1 HIS A1111     124.992 198.642 142.666  1.00  0.00
ATOM  17139  HE1 HIS A1111     125.040 199.513 143.309  1.00  0.00
```

```
ATOM  17140  NE2 HIS A1111     125.973 197.732 142.548  1.00  0.00
ATOM  17141  HE2 HIS A1111     126.889 197.785 142.980  1.00  0.00
ATOM  17142  CD2 HIS A1111     125.578 196.795 141.624  1.00  0.00
ATOM  17143  HD2 HIS A1111     126.166 195.960 141.270  1.00  0.00
ATOM  17144  C   HIS A1111     123.496 195.097 137.948  1.00  0.00
ATOM  17145  O   HIS A1111     123.287 193.890 138.062  1.00  0.00
ATOM  17146  N   ASN A1112     122.923 195.816 136.968  1.00  0.00
ATOM  17147  H   ASN A1112     123.164 196.792 136.879  1.00  0.00
ATOM  17148  CA  ASN A1112     122.068 195.238 135.906  1.00  0.00
ATOM  17149  HA  ASN A1112     122.610 194.395 135.472  1.00  0.00
ATOM  17150  CB  ASN A1112     120.731 194.712 136.518  1.00  0.00
ATOM  17151  HB1 ASN A1112     120.085 195.558 136.743  1.00  0.00
ATOM  17152  HB2 ASN A1112     120.915 194.180 137.448  1.00  0.00
ATOM  17153  CG  ASN A1112     119.965 193.737 135.620  1.00  0.00
ATOM  17154  OD1 ASN A1112     120.494 193.192 134.667  1.00  0.00
ATOM  17155  ND2 ASN A1112     118.691 193.517 135.859  1.00  0.00
ATOM  17156 1HD2 ASN A1112     118.218 193.923 136.644  1.00  0.00
ATOM  17157 2HD2 ASN A1112     118.210 192.846 135.280  1.00  0.00
ATOM  17158  C   ASN A1112     121.766 196.241 134.768  1.00  0.00
ATOM  17159  O   ASN A1112     122.039 197.418 134.927  1.00  0.00
ATOM  17160  N   GLN A1113     121.104 195.745 133.706  1.00  0.00
ATOM  17161  H   GLN A1113     120.983 194.737 133.721  1.00  0.00
ATOM  17162  CA  GLN A1113     120.318 196.403 132.643  1.00  0.00
ATOM  17163  HA  GLN A1113     120.187 195.611 131.911  1.00  0.00
ATOM  17164  CB  GLN A1113     118.880 196.661 133.138  1.00  0.00
ATOM  17165  HB1 GLN A1113     118.927 197.258 134.045  1.00  0.00
ATOM  17166  HB2 GLN A1113     118.443 195.696 133.404  1.00  0.00
ATOM  17167  CG  GLN A1113     117.931 197.356 132.137  1.00  0.00
ATOM  17168  HG1 GLN A1113     118.155 198.424 132.132  1.00  0.00
ATOM  17169  HG2 GLN A1113     116.914 197.231 132.492  1.00  0.00
ATOM  17170  CD  GLN A1113     117.999 196.826 130.705  1.00  0.00
ATOM  17171  OE1 GLN A1113     118.183 195.653 130.445  1.00  0.00
ATOM  17172  NE2 GLN A1113     117.928 197.687 129.719  1.00  0.00
ATOM  17173 1HE2 GLN A1113     117.436 198.563 129.854  1.00  0.00
ATOM  17174 2HE2 GLN A1113     118.013 197.321 128.785  1.00  0.00
ATOM  17175  C   GLN A1113     120.960 197.576 131.886  1.00  0.00
ATOM  17176  O   GLN A1113     120.931 198.677 132.395  1.00  0.00
ATOM  17177  N   PRO A1114     121.439 197.412 130.636  1.00  0.00
ATOM  17178  CD  PRO A1114     121.570 196.143 129.922  1.00  0.00
ATOM  17179  HD1 PRO A1114     120.584 195.789 129.621  1.00  0.00
ATOM  17180  HD2 PRO A1114     122.067 195.392 130.540  1.00  0.00
ATOM  17181  CG  PRO A1114     122.402 196.433 128.677  1.00  0.00
ATOM  17182  HG1 PRO A1114     122.111 195.799 127.838  1.00  0.00
ATOM  17183  HG2 PRO A1114     123.463 196.311 128.902  1.00  0.00
ATOM  17184  CB  PRO A1114     122.105 197.904 128.406  1.00  0.00
ATOM  17185  HB1 PRO A1114     121.148 197.992 127.890  1.00  0.00
ATOM  17186  HB2 PRO A1114     122.898 198.379 127.828  1.00  0.00
ATOM  17187  CA  PRO A1114     122.000 198.494 129.813  1.00  0.00
ATOM  17188  HA  PRO A1114     123.016 198.671 130.160  1.00  0.00
ATOM  17189  C   PRO A1114     121.304 199.874 129.760  1.00  0.00
ATOM  17190  O   PRO A1114     121.996 200.841 129.471  1.00  0.00
ATOM  17191  N   LEU A1115     119.984 199.979 130.000  1.00  0.00
ATOM  17192  H   LEU A1115     119.512 199.133 130.272  1.00  0.00
ATOM  17193  CA  LEU A1115     119.163 201.216 129.939  1.00  0.00
ATOM  17194  HA  LEU A1115     118.152 200.895 129.687  1.00  0.00
ATOM  17195  CB  LEU A1115     119.087 201.826 131.351  1.00  0.00
ATOM  17196  HB1 LEU A1115     120.101 202.010 131.688  1.00  0.00
ATOM  17197  HB2 LEU A1115     118.657 201.063 131.996  1.00  0.00
ATOM  17198  CG  LEU A1115     118.265 203.117 131.547  1.00  0.00
ATOM  17199  HG  LEU A1115     118.785 203.971 131.119  1.00  0.00
ATOM  17200  CD1 LEU A1115     116.872 203.014 130.921  1.00  0.00
```

```
ATOM  17201 1HD1 LEU A1115     116.308 203.920 131.137  1.00  0.00
ATOM  17202 2HD1 LEU A1115     116.971 202.933 129.839  1.00  0.00
ATOM  17203 3HD1 LEU A1115     116.340 202.149 131.308  1.00  0.00
ATOM  17204  CD2 LEU A1115     118.085 203.394 133.041  1.00  0.00
ATOM  17205 1HD2 LEU A1115     117.561 204.341 133.167  1.00  0.00
ATOM  17206 2HD2 LEU A1115     117.511 202.593 133.497  1.00  0.00
ATOM  17207 3HD2 LEU A1115     119.073 203.477 133.495  1.00  0.00
ATOM  17208  C   LEU A1115     119.552 202.168 128.785  1.00  0.00
ATOM  17209  O   LEU A1115     119.663 203.385 128.898  1.00  0.00
ATOM  17210  N   ASN A1116     119.805 201.554 127.639  1.00  0.00
ATOM  17211  H   ASN A1116     119.649 200.557 127.608  1.00  0.00
ATOM  17212  CA  ASN A1116     120.468 202.146 126.486  1.00  0.00
ATOM  17213  HA  ASN A1116     121.353 202.662 126.854  1.00  0.00
ATOM  17214  CB  ASN A1116     120.905 200.961 125.604  1.00  0.00
ATOM  17215  HB1 ASN A1116     121.678 200.398 126.126  1.00  0.00
ATOM  17216  HB2 ASN A1116     121.331 201.326 124.671  1.00  0.00
ATOM  17217  CG  ASN A1116     119.750 200.021 125.306  1.00  0.00
ATOM  17218  OD1 ASN A1116     119.446 199.122 126.082  1.00  0.00
ATOM  17219  ND2 ASN A1116     119.064 200.211 124.210  1.00  0.00
ATOM  17220 1HD2 ASN A1116     119.211 201.043 123.648  1.00  0.00
ATOM  17221 2HD2 ASN A1116     118.267 199.603 124.067  1.00  0.00
ATOM  17222  C   ASN A1116     119.592 203.133 125.687  1.00  0.00
ATOM  17223  O   ASN A1116     118.359 203.043 125.703  1.00  0.00
ATOM  17224  N   PRO A1117     120.221 204.007 124.875  1.00  0.00
ATOM  17225  CD  PRO A1117     121.635 204.339 124.904  1.00  0.00
ATOM  17226  HD1 PRO A1117     122.206 203.489 124.539  1.00  0.00
ATOM  17227  HD2 PRO A1117     121.950 204.610 125.911  1.00  0.00
ATOM  17228  CG  PRO A1117     121.813 205.524 123.960  1.00  0.00
ATOM  17229  HG1 PRO A1117     122.802 205.532 123.503  1.00  0.00
ATOM  17230  HG2 PRO A1117     121.630 206.453 124.499  1.00  0.00
ATOM  17231  CB  PRO A1117     120.713 205.294 122.929  1.00  0.00
ATOM  17232  HB1 PRO A1117     121.050 204.557 122.199  1.00  0.00
ATOM  17233  HB2 PRO A1117     120.416 206.221 122.439  1.00  0.00
ATOM  17234  CA  PRO A1117     119.573 204.711 123.772  1.00  0.00
ATOM  17235  HA  PRO A1117     118.997 205.530 124.204  1.00  0.00
ATOM  17236  C   PRO A1117     118.640 203.820 122.948  1.00  0.00
ATOM  17237  O   PRO A1117     118.917 202.638 122.736  1.00  0.00
ATOM  17238  N   ALA A1118     117.561 204.405 122.416  1.00  0.00
ATOM  17239  H   ALA A1118     117.387 205.367 122.649  1.00  0.00
ATOM  17240  CA  ALA A1118     116.470 203.679 121.757  1.00  0.00
ATOM  17241  HA  ALA A1118     116.644 202.607 121.860  1.00  0.00
ATOM  17242  CB  ALA A1118     115.177 203.995 122.527  1.00  0.00
ATOM  17243  HB1 ALA A1118     114.344 203.434 122.101  1.00  0.00
ATOM  17244  HB2 ALA A1118     115.291 203.717 123.574  1.00  0.00
ATOM  17245  HB3 ALA A1118     114.947 205.059 122.464  1.00  0.00
ATOM  17246  C   ALA A1118     116.348 203.978 120.237  1.00  0.00
ATOM  17247  O   ALA A1118     115.517 204.805 119.836  1.00  0.00
ATOM  17248  N   PRO A1119     117.213 203.387 119.382  1.00  0.00
ATOM  17249  CD  PRO A1119     118.482 202.763 119.747  1.00  0.00
ATOM  17250  HD1 PRO A1119     118.324 201.804 120.243  1.00  0.00
ATOM  17251  HD2 PRO A1119     119.044 203.438 120.387  1.00  0.00
ATOM  17252  CG  PRO A1119     119.288 202.576 118.462  1.00  0.00
ATOM  17253  HG1 PRO A1119     119.186 201.554 118.100  1.00  0.00
ATOM  17254  HG2 PRO A1119     120.339 202.822 118.611  1.00  0.00
ATOM  17255  CB  PRO A1119     118.616 203.554 117.496  1.00  0.00
ATOM  17256  HB1 PRO A1119     118.753 203.255 116.456  1.00  0.00
ATOM  17257  HB2 PRO A1119     119.018 204.556 117.656  1.00  0.00
ATOM  17258  CA  PRO A1119     117.147 203.519 117.930  1.00  0.00
ATOM  17259  HA  PRO A1119     116.671 204.460 117.652  1.00  0.00
ATOM  17260  C   PRO A1119     116.428 202.368 117.212  1.00  0.00
ATOM  17261  O   PRO A1119     116.754 201.191 117.409  1.00  0.00
```

```
ATOM  17262  N   SER A1120     115.575 202.700 116.241  1.00  0.00
ATOM  17263  H   SER A1120     115.242 203.649 116.189  1.00  0.00
ATOM  17264  CA  SER A1120     115.069 201.755 115.230  1.00  0.00
ATOM  17265  HA  SER A1120     114.789 200.838 115.751  1.00  0.00
ATOM  17266  CB  SER A1120     113.781 202.285 114.583  1.00  0.00
ATOM  17267  HB1 SER A1120     113.421 201.562 113.849  1.00  0.00
ATOM  17268  HB2 SER A1120     113.972 203.238 114.089  1.00  0.00
ATOM  17269  OG  SER A1120     112.805 202.445 115.597  1.00  0.00
ATOM  17270  HG  SER A1120     111.934 202.558 115.202  1.00  0.00
ATOM  17271  C   SER A1120     116.139 201.349 114.199  1.00  0.00
ATOM  17272  O   SER A1120     115.943 201.510 112.998  1.00  0.00
ATOM  17273  N   ARG A1121     117.307 200.889 114.685  1.00  0.00
ATOM  17274  H   ARG A1121     117.364 200.808 115.692  1.00  0.00
ATOM  17275  CA  ARG A1121     118.495 200.531 113.892  1.00  0.00
ATOM  17276  HA  ARG A1121     118.746 201.389 113.265  1.00  0.00
ATOM  17277  CB  ARG A1121     119.721 200.235 114.791  1.00  0.00
ATOM  17278  HB1 ARG A1121     119.996 201.155 115.309  1.00  0.00
ATOM  17279  HB2 ARG A1121     120.547 199.986 114.123  1.00  0.00
ATOM  17280  CG  ARG A1121     119.606 199.109 115.853  1.00  0.00
ATOM  17281  HG1 ARG A1121     119.154 198.219 115.428  1.00  0.00
ATOM  17282  HG2 ARG A1121     118.979 199.451 116.673  1.00  0.00
ATOM  17283  CD  ARG A1121     121.001 198.733 116.387  1.00  0.00
ATOM  17284  HD1 ARG A1121     121.369 199.563 116.993  1.00  0.00
ATOM  17285  HD2 ARG A1121     121.679 198.635 115.538  1.00  0.00
ATOM  17286  NE  ARG A1121     121.035 197.491 117.202  1.00  0.00
ATOM  17287  HE  ARG A1121     120.482 197.474 118.044  1.00  0.00
ATOM  17288  CZ  ARG A1121     121.841 196.456 117.009  1.00  0.00
ATOM  17289  NH1 ARG A1121     122.411 196.217 115.861  1.00  0.00
ATOM  17290 1HH1 ARG A1121     121.950 196.638 115.043  1.00  0.00
ATOM  17291 2HH1 ARG A1121     122.990 195.424 115.714  1.00  0.00
ATOM  17292  NH2 ARG A1121     122.154 195.649 117.986  1.00  0.00
ATOM  17293 1HH2 ARG A1121     121.791 195.803 118.907  1.00  0.00
ATOM  17294 2HH2 ARG A1121     122.794 194.894 117.831  1.00  0.00
ATOM  17295  C   ARG A1121     118.255 199.400 112.894  1.00  0.00
ATOM  17296  O   ARG A1121     118.599 199.532 111.728  1.00  0.00
ATOM  17297  N   ASP A1122     117.675 198.300 113.370  1.00  0.00
ATOM  17298  H   ASP A1122     117.398 198.285 114.338  1.00  0.00
ATOM  17299  CA  ASP A1122     117.578 197.027 112.667  1.00  0.00
ATOM  17300  HA  ASP A1122     117.111 197.207 111.698  1.00  0.00
ATOM  17301  CB  ASP A1122     118.994 196.448 112.419  1.00  0.00
ATOM  17302  HB1 ASP A1122     119.507 197.074 111.688  1.00  0.00
ATOM  17303  HB2 ASP A1122     118.907 195.461 111.967  1.00  0.00
ATOM  17304  CG  ASP A1122     119.861 196.333 113.674  1.00  0.00
ATOM  17305  OD1 ASP A1122     119.516 195.528 114.568  1.00  0.00
ATOM  17306  OD2 ASP A1122     120.910 197.009 113.754  1.00  0.00
ATOM  17307  C   ASP A1122     116.706 196.032 113.454  1.00  0.00
ATOM  17308  O   ASP A1122     116.431 196.251 114.637  1.00  0.00
ATOM  17309  N   PRO A1123     116.283 194.919 112.820  1.00  0.00
ATOM  17310  CD  PRO A1123     116.128 194.761 111.378  1.00  0.00
ATOM  17311  HD1 PRO A1123     117.079 194.847 110.854  1.00  0.00
ATOM  17312  HD2 PRO A1123     115.432 195.513 111.007  1.00  0.00
ATOM  17313  CG  PRO A1123     115.536 193.367 111.161  1.00  0.00
ATOM  17314  HG1 PRO A1123     116.338 192.649 110.979  1.00  0.00
ATOM  17315  HG2 PRO A1123     114.817 193.356 110.340  1.00  0.00
ATOM  17316  CB  PRO A1123     114.869 193.064 112.504  1.00  0.00
ATOM  17317  HB1 PRO A1123     114.751 191.993 112.665  1.00  0.00
ATOM  17318  HB2 PRO A1123     113.902 193.567 112.553  1.00  0.00
ATOM  17319  CA  PRO A1123     115.830 193.713 113.508  1.00  0.00
ATOM  17320  HA  PRO A1123     115.291 193.968 114.421  1.00  0.00
ATOM  17321  C   PRO A1123     116.976 192.754 113.860  1.00  0.00
ATOM  17322  O   PRO A1123     116.709 191.678 114.392  1.00  0.00
```

```
ATOM  17323  N   HIS A1124     118.234 193.092 113.546  1.00  0.00
ATOM  17324  H   HIS A1124     118.412 194.038 113.241  1.00  0.00
ATOM  17325  CA  HIS A1124     119.397 192.239 113.809  1.00  0.00
ATOM  17326  HA  HIS A1124     119.215 191.265 113.351  1.00  0.00
ATOM  17327  CB  HIS A1124     120.663 192.829 113.166  1.00  0.00
ATOM  17328  HB1 HIS A1124     120.981 193.714 113.713  1.00  0.00
ATOM  17329  HB2 HIS A1124     120.431 193.131 112.143  1.00  0.00
ATOM  17330  CG  HIS A1124     121.832 191.875 113.115  1.00  0.00
ATOM  17331  ND1 HIS A1124     122.246 191.182 111.978  1.00  0.00
ATOM  17332  CE1 HIS A1124     123.321 190.471 112.350  1.00  0.00
ATOM  17333  HE1 HIS A1124     123.872 189.812 111.692  1.00  0.00
ATOM  17334  NE2 HIS A1124     123.599 190.682 113.649  1.00  0.00
ATOM  17335  HE2 HIS A1124     124.348 190.243 114.162  1.00  0.00
ATOM  17336  CD2 HIS A1124     122.665 191.563 114.150  1.00  0.00
ATOM  17337  HD2 HIS A1124     122.578 191.916 115.168  1.00  0.00
ATOM  17338  C   HIS A1124     119.583 191.991 115.299  1.00  0.00
ATOM  17339  O   HIS A1124     119.856 190.845 115.642  1.00  0.00
ATOM  17340  N   TYR A1125     119.404 193.033 116.141  1.00  0.00
ATOM  17341  H   TYR A1125     119.212 193.929 115.692  1.00  0.00
ATOM  17342  CA  TYR A1125     119.670 193.076 117.596  1.00  0.00
ATOM  17343  HA  TYR A1125     120.408 193.856 117.745  1.00  0.00
ATOM  17344  CB  TYR A1125     118.427 193.502 118.401  1.00  0.00
ATOM  17345  HB1 TYR A1125     118.484 193.067 119.399  1.00  0.00
ATOM  17346  HB2 TYR A1125     117.528 193.104 117.928  1.00  0.00
ATOM  17347  CG  TYR A1125     118.290 195.009 118.579  1.00  0.00
ATOM  17348  CD1 TYR A1125     118.895 195.658 119.677  1.00  0.00
ATOM  17349  HD1 TYR A1125     119.454 195.087 120.406  1.00  0.00
ATOM  17350  CE1 TYR A1125     118.740 197.050 119.849  1.00  0.00
ATOM  17351  HE1 TYR A1125     119.166 197.547 120.707  1.00  0.00
ATOM  17352  CZ  TYR A1125     117.978 197.806 118.931  1.00  0.00
ATOM  17353  OH  TYR A1125     117.845 199.147 119.114  1.00  0.00
ATOM  17354  HH  TYR A1125     117.273 199.574 118.464  1.00  0.00
ATOM  17355  CE2 TYR A1125     117.381 197.154 117.829  1.00  0.00
ATOM  17356  HE2 TYR A1125     116.795 197.713 117.113  1.00  0.00
ATOM  17357  CD2 TYR A1125     117.544 195.764 117.658  1.00  0.00
ATOM  17358  HD2 TYR A1125     117.100 195.273 116.809  1.00  0.00
ATOM  17359  C   TYR A1125     120.344 191.806 118.150  1.00  0.00
ATOM  17360  O   TYR A1125     121.570 191.788 118.259  1.00  0.00
ATOM  17361  N   GLN A1126     119.569 190.747 118.404  1.00  0.00
ATOM  17362  H   GLN A1126     118.593 190.858 118.181  1.00  0.00
ATOM  17363  CA  GLN A1126     120.035 189.373 118.640  1.00  0.00
ATOM  17364  HA  GLN A1126     120.980 189.215 118.116  1.00  0.00
ATOM  17365  CB  GLN A1126     120.232 189.120 120.151  1.00  0.00
ATOM  17366  HB1 GLN A1126     120.462 188.070 120.328  1.00  0.00
ATOM  17367  HB2 GLN A1126     119.280 189.323 120.637  1.00  0.00
ATOM  17368  CG  GLN A1126     121.305 189.944 120.887  1.00  0.00
ATOM  17369  HG1 GLN A1126     121.102 189.883 121.955  1.00  0.00
ATOM  17370  HG2 GLN A1126     121.242 190.988 120.601  1.00  0.00
ATOM  17371  CD  GLN A1126     122.739 189.503 120.697  1.00  0.00
ATOM  17372  OE1 GLN A1126     123.064 188.485 120.102  1.00  0.00
ATOM  17373  NE2 GLN A1126     123.671 190.202 121.300  1.00  0.00
ATOM  17374 1HE2 GLN A1126     123.351 191.055 121.791  1.00  0.00
ATOM  17375 2HE2 GLN A1126     124.611 190.128 120.959  1.00  0.00
ATOM  17376  C   GLN A1126     118.996 188.371 118.076  1.00  0.00
ATOM  17377  O   GLN A1126     118.591 187.439 118.770  1.00  0.00
ATOM  17378  N   ASP A1127     118.457 188.618 116.871  1.00  0.00
ATOM  17379  H   ASP A1127     118.864 189.377 116.328  1.00  0.00
ATOM  17380  CA  ASP A1127     117.146 188.091 116.432  1.00  0.00
ATOM  17381  HA  ASP A1127     116.806 187.398 117.199  1.00  0.00
ATOM  17382  CB  ASP A1127     116.135 189.267 116.417  1.00  0.00
ATOM  17383  HB1 ASP A1127     115.727 189.425 115.421  1.00  0.00
```

```
ATOM  17384  HB2 ASP A1127     116.633 190.193 116.709  1.00  0.00
ATOM  17385  CG  ASP A1127     114.991 189.008 117.388  1.00  0.00
ATOM  17386  OD1 ASP A1127     115.005 189.579 118.504  1.00  0.00
ATOM  17387  OD2 ASP A1127     114.165 188.107 117.127  1.00  0.00
ATOM  17388  C   ASP A1127     117.126 187.203 115.153  1.00  0.00
ATOM  17389  O   ASP A1127     116.158 187.257 114.380  1.00  0.00
ATOM  17390  N   PRO A1128     118.149 186.355 114.900  1.00  0.00
ATOM  17391  CD  PRO A1128     119.175 185.925 115.842  1.00  0.00
ATOM  17392  HD1 PRO A1128     118.756 185.718 116.824  1.00  0.00
ATOM  17393  HD2 PRO A1128     119.950 186.687 115.914  1.00  0.00
ATOM  17394  CG  PRO A1128     119.784 184.658 115.253  1.00  0.00
ATOM  17395  HG1 PRO A1128     119.160 183.798 115.501  1.00  0.00
ATOM  17396  HG2 PRO A1128     120.810 184.507 115.587  1.00  0.00
ATOM  17397  CB  PRO A1128     119.705 184.947 113.757  1.00  0.00
ATOM  17398  HB1 PRO A1128     119.728 184.028 113.169  1.00  0.00
ATOM  17399  HB2 PRO A1128     120.537 185.593 113.472  1.00  0.00
ATOM  17400  CA  PRO A1128     118.386 185.714 113.608  1.00  0.00
ATOM  17401  HA  PRO A1128     118.529 186.493 112.857  1.00  0.00
ATOM  17402  C   PRO A1128     117.260 184.788 113.122  1.00  0.00
ATOM  17403  O   PRO A1128     116.965 183.766 113.729  1.00  0.00
ATOM  17404  N   HIS A1129     116.689 185.141 111.966  1.00  0.00
ATOM  17405  H   HIS A1129     116.937 186.048 111.608  1.00  0.00
ATOM  17406  CA  HIS A1129     115.680 184.370 111.216  1.00  0.00
ATOM  17407  HA  HIS A1129     115.903 183.302 111.291  1.00  0.00
ATOM  17408  CB  HIS A1129     114.305 184.645 111.854  1.00  0.00
ATOM  17409  HB1 HIS A1129     113.825 185.502 111.380  1.00  0.00
ATOM  17410  HB2 HIS A1129     114.448 184.907 112.895  1.00  0.00
ATOM  17411  CG  HIS A1129     113.378 183.460 111.829  1.00  0.00
ATOM  17412  ND1 HIS A1129     113.558 182.306 112.589  1.00  0.00
ATOM  17413  CE1 HIS A1129     112.479 181.541 112.358  1.00  0.00
ATOM  17414  HE1 HIS A1129     112.291 180.573 112.814  1.00  0.00
ATOM  17415  NE2 HIS A1129     111.645 182.156 111.504  1.00  0.00
ATOM  17416  HE2 HIS A1129     110.736 181.790 111.217  1.00  0.00
ATOM  17417  CD2 HIS A1129     112.194 183.370 111.155  1.00  0.00
ATOM  17418  HD2 HIS A1129     111.759 184.117 110.502  1.00  0.00
ATOM  17419  C   HIS A1129     115.666 184.776 109.722  1.00  0.00
ATOM  17420  O   HIS A1129     114.636 184.737 109.051  1.00  0.00
ATOM  17421  N   SER A1130     116.798 185.289 109.236  1.00  0.00
ATOM  17422  H   SER A1130     117.611 185.182 109.822  1.00  0.00
ATOM  17423  CA  SER A1130     117.059 185.851 107.897  1.00  0.00
ATOM  17424  HA  SER A1130     118.109 186.144 107.892  1.00  0.00
ATOM  17425  CB  SER A1130     116.933 184.767 106.818  1.00  0.00
ATOM  17426  HB1 SER A1130     117.273 185.175 105.864  1.00  0.00
ATOM  17427  HB2 SER A1130     115.892 184.457 106.720  1.00  0.00
ATOM  17428  OG  SER A1130     117.733 183.651 107.167  1.00  0.00
ATOM  17429  HG  SER A1130     117.819 183.076 106.402  1.00  0.00
ATOM  17430  C   SER A1130     116.296 187.122 107.484  1.00  0.00
ATOM  17431  O   SER A1130     116.920 188.022 106.942  1.00  0.00
ATOM  17432  N   THR A1131     114.994 187.228 107.768  1.00  0.00
ATOM  17433  H   THR A1131     114.571 186.404 108.182  1.00  0.00
ATOM  17434  CA  THR A1131     114.164 188.455 107.734  1.00  0.00
ATOM  17435  HA  THR A1131     113.182 188.133 107.390  1.00  0.00
ATOM  17436  CB  THR A1131     113.918 188.969 109.167  1.00  0.00
ATOM  17437  HB  THR A1131     113.359 188.204 109.706  1.00  0.00
ATOM  17438  CG2 THR A1131     115.175 189.283 109.988  1.00  0.00
ATOM  17439 1HG2 THR A1131     114.884 189.709 110.947  1.00  0.00
ATOM  17440 2HG2 THR A1131     115.734 188.367 110.169  1.00  0.00
ATOM  17441 3HG2 THR A1131     115.811 189.993 109.462  1.00  0.00
ATOM  17442  OG1 THR A1131     113.120 190.124 109.107  1.00  0.00
ATOM  17443  HG1 THR A1131     113.661 190.776 108.637  1.00  0.00
ATOM  17444  C   THR A1131     114.549 189.586 106.753  1.00  0.00
```

```
ATOM  17445  O   THR A1131     114.919 190.688 107.158  1.00  0.00
ATOM  17446  N   ALA A1132     114.337 189.359 105.454  1.00  0.00
ATOM  17447  H   ALA A1132     114.047 188.441 105.150  1.00  0.00
ATOM  17448  CA  ALA A1132     114.317 190.425 104.452  1.00  0.00
ATOM  17449  HA  ALA A1132     114.020 191.361 104.929  1.00  0.00
ATOM  17450  CB  ALA A1132     115.734 190.607 103.888  1.00  0.00
ATOM  17451  HB1 ALA A1132     115.741 191.417 103.159  1.00  0.00
ATOM  17452  HB2 ALA A1132     116.423 190.853 104.698  1.00  0.00
ATOM  17453  HB3 ALA A1132     116.061 189.685 103.406  1.00  0.00
ATOM  17454  C   ALA A1132     113.305 190.121 103.337  1.00  0.00
ATOM  17455  O   ALA A1132     113.065 188.960 103.004  1.00  0.00
ATOM  17456  N   VAL A1133     112.763 191.179 102.738  1.00  0.00
ATOM  17457  H   VAL A1133     113.038 192.089 103.082  1.00  0.00
ATOM  17458  CA  VAL A1133     112.053 191.191 101.448  1.00  0.00
ATOM  17459  HA  VAL A1133     112.384 190.337 100.856  1.00  0.00
ATOM  17460  CB  VAL A1133     110.510 191.096 101.598  1.00  0.00
ATOM  17461  HB  VAL A1133     110.095 191.003 100.593  1.00  0.00
ATOM  17462  CG1 VAL A1133     110.066 189.853 102.376  1.00  0.00
ATOM  17463 1HG1 VAL A1133     108.981 189.756 102.328  1.00  0.00
ATOM  17464 2HG1 VAL A1133     110.514 188.958 101.943  1.00  0.00
ATOM  17465 3HG1 VAL A1133     110.363 189.930 103.421  1.00  0.00
ATOM  17466  CG2 VAL A1133     109.882 192.338 102.237  1.00  0.00
ATOM  17467 1HG2 VAL A1133     108.815 192.173 102.399  1.00  0.00
ATOM  17468 2HG2 VAL A1133     110.356 192.553 103.191  1.00  0.00
ATOM  17469 3HG2 VAL A1133     109.982 193.201 101.584  1.00  0.00
ATOM  17470  C   VAL A1133     112.457 192.468 100.703  1.00  0.00
ATOM  17471  O   VAL A1133     113.149 193.316 101.268  1.00  0.00
ATOM  17472  N   GLY A1134     112.032 192.645  99.451  1.00  0.00
ATOM  17473  H   GLY A1134     111.468 191.924  99.029  1.00  0.00
ATOM  17474  CA  GLY A1134     112.443 193.781  98.608  1.00  0.00
ATOM  17475  HA1 GLY A1134     111.996 193.658  97.621  1.00  0.00
ATOM  17476  HA2 GLY A1134     113.527 193.747  98.487  1.00  0.00
ATOM  17477  C   GLY A1134     112.089 195.201  99.091  1.00  0.00
ATOM  17478  O   GLY A1134     112.458 196.155  98.415  1.00  0.00
ATOM  17479  N   ASN A1135     111.376 195.368 100.212  1.00  0.00
ATOM  17480  H   ASN A1135     111.164 194.552 100.762  1.00  0.00
ATOM  17481  CA  ASN A1135     110.986 196.669 100.762  1.00  0.00
ATOM  17482  HA  ASN A1135     111.785 197.386 100.579  1.00  0.00
ATOM  17483  CB  ASN A1135     109.741 197.146  99.981  1.00  0.00
ATOM  17484  HB1 ASN A1135     108.983 196.365 100.012  1.00  0.00
ATOM  17485  HB2 ASN A1135     110.006 197.314  98.937  1.00  0.00
ATOM  17486  CG  ASN A1135     109.120 198.428 100.509  1.00  0.00
ATOM  17487  OD1 ASN A1135     109.695 199.137 101.320  1.00  0.00
ATOM  17488  ND2 ASN A1135     107.899 198.695 100.122  1.00  0.00
ATOM  17489 1HD2 ASN A1135     107.387 198.062  99.533  1.00  0.00
ATOM  17490 2HD2 ASN A1135     107.376 199.489 100.505  1.00  0.00
ATOM  17491  C   ASN A1135     110.699 196.580 102.282  1.00  0.00
ATOM  17492  O   ASN A1135     109.847 195.781 102.673  1.00  0.00
ATOM  17493  N   PRO A1136     111.345 197.389 103.150  1.00  0.00
ATOM  17494  CD  PRO A1136     112.491 198.234 102.846  1.00  0.00
ATOM  17495  HD1 PRO A1136     112.151 199.160 102.380  1.00  0.00
ATOM  17496  HD2 PRO A1136     113.207 197.726 102.200  1.00  0.00
ATOM  17497  CG  PRO A1136     113.142 198.544 104.191  1.00  0.00
ATOM  17498  HG1 PRO A1136     113.656 199.506 104.181  1.00  0.00
ATOM  17499  HG2 PRO A1136     113.832 197.742 104.458  1.00  0.00
ATOM  17500  CB  PRO A1136     111.958 198.542 105.153  1.00  0.00
ATOM  17501  HB1 PRO A1136     111.477 199.522 105.126  1.00  0.00
ATOM  17502  HB2 PRO A1136     112.263 198.296 106.170  1.00  0.00
ATOM  17503  CA  PRO A1136     111.014 197.476 104.578  1.00  0.00
ATOM  17504  HA  PRO A1136     111.249 196.517 105.039  1.00  0.00
ATOM  17505  C   PRO A1136     109.558 197.825 104.918  1.00  0.00
```

ATOM	17506	O	PRO	A1136	109.029	197.326	105.905	1.00	0.00
ATOM	17507	N	GLU	A1137	108.894	198.673	104.124	1.00	0.00
ATOM	17508	H	GLU	A1137	109.357	199.046	103.299	1.00	0.00
ATOM	17509	CA	GLU	A1137	107.479	199.025	104.325	1.00	0.00
ATOM	17510	HA	GLU	A1137	107.313	199.265	105.375	1.00	0.00
ATOM	17511	CB	GLU	A1137	107.178	200.298	103.504	1.00	0.00
ATOM	17512	HB1	GLU	A1137	107.660	200.229	102.531	1.00	0.00
ATOM	17513	HB2	GLU	A1137	107.654	201.131	104.026	1.00	0.00
ATOM	17514	CG	GLU	A1137	105.698	200.674	103.290	1.00	0.00
ATOM	17515	HG1	GLU	A1137	105.641	201.754	103.140	1.00	0.00
ATOM	17516	HG2	GLU	A1137	105.134	200.441	104.195	1.00	0.00
ATOM	17517	CD	GLU	A1137	105.095	199.982	102.060	1.00	0.00
ATOM	17518	OE1	GLU	A1137	105.657	200.142	100.953	1.00	0.00
ATOM	17519	OE2	GLU	A1137	104.114	199.220	102.226	1.00	0.00
ATOM	17520	C	GLU	A1137	106.566	197.833	104.003	1.00	0.00
ATOM	17521	O	GLU	A1137	105.631	197.576	104.762	1.00	0.00
ATOM	17522	N	TYR	A1138	106.883	197.014	102.992	1.00	0.00
ATOM	17523	H	TYR	A1138	107.659	197.253	102.397	1.00	0.00
ATOM	17524	CA	TYR	A1138	106.161	195.755	102.772	1.00	0.00
ATOM	17525	HA	TYR	A1138	105.103	195.975	102.680	1.00	0.00
ATOM	17526	CB	TYR	A1138	106.610	195.030	101.496	1.00	0.00
ATOM	17527	HB1	TYR	A1138	106.180	194.027	101.522	1.00	0.00
ATOM	17528	HB2	TYR	A1138	107.692	194.908	101.519	1.00	0.00
ATOM	17529	CG	TYR	A1138	106.217	195.635	100.160	1.00	0.00
ATOM	17530	CD1	TYR	A1138	106.584	194.949	98.987	1.00	0.00
ATOM	17531	HD1	TYR	A1138	107.107	194.006	99.059	1.00	0.00
ATOM	17532	CE1	TYR	A1138	106.267	195.482	97.724	1.00	0.00
ATOM	17533	HE1	TYR	A1138	106.546	194.964	96.823	1.00	0.00
ATOM	17534	CZ	TYR	A1138	105.582	196.710	97.630	1.00	0.00
ATOM	17535	OH	TYR	A1138	105.312	197.238	96.411	1.00	0.00
ATOM	17536	HH	TYR	A1138	104.884	198.093	96.483	1.00	0.00
ATOM	17537	CE2	TYR	A1138	105.198	197.394	98.802	1.00	0.00
ATOM	17538	HE2	TYR	A1138	104.683	198.346	98.750	1.00	0.00
ATOM	17539	CD2	TYR	A1138	105.504	196.848	100.060	1.00	0.00
ATOM	17540	HD2	TYR	A1138	105.213	197.403	100.942	1.00	0.00
ATOM	17541	C	TYR	A1138	106.301	194.819	103.971	1.00	0.00
ATOM	17542	O	TYR	A1138	105.292	194.302	104.444	1.00	0.00
ATOM	17543	N	LEU	A1139	107.516	194.675	104.510	1.00	0.00
ATOM	17544	H	LEU	A1139	108.290	195.154	104.065	1.00	0.00
ATOM	17545	CA	LEU	A1139	107.777	193.917	105.737	1.00	0.00
ATOM	17546	HA	LEU	A1139	107.287	192.950	105.615	1.00	0.00
ATOM	17547	CB	LEU	A1139	109.301	193.679	105.902	1.00	0.00
ATOM	17548	HB1	LEU	A1139	109.657	194.229	106.774	1.00	0.00
ATOM	17549	HB2	LEU	A1139	109.824	194.088	105.038	1.00	0.00
ATOM	17550	CG	LEU	A1139	109.696	192.192	106.042	1.00	0.00
ATOM	17551	HG	LEU	A1139	109.366	191.659	105.152	1.00	0.00
ATOM	17552	CD1	LEU	A1139	111.219	192.059	106.150	1.00	0.00
ATOM	17553	1HD1	LEU	A1139	111.494	191.005	106.173	1.00	0.00
ATOM	17554	2HD1	LEU	A1139	111.694	192.528	105.291	1.00	0.00
ATOM	17555	3HD1	LEU	A1139	111.570	192.543	107.060	1.00	0.00
ATOM	17556	CD2	LEU	A1139	109.089	191.496	107.261	1.00	0.00
ATOM	17557	1HD2	LEU	A1139	109.442	190.466	107.318	1.00	0.00
ATOM	17558	2HD2	LEU	A1139	109.366	192.025	108.172	1.00	0.00
ATOM	17559	3HD2	LEU	A1139	108.003	191.483	107.185	1.00	0.00
ATOM	17560	C	LEU	A1139	107.154	194.551	107.004	1.00	0.00
ATOM	17561	O	LEU	A1139	107.176	193.926	108.062	1.00	0.00
ATOM	17562	N	ASN	A1140	106.561	195.756	106.944	1.00	0.00
ATOM	17563	H	ASN	A1140	106.578	196.267	106.070	1.00	0.00
ATOM	17564	CA	ASN	A1140	105.696	196.256	108.020	1.00	0.00
ATOM	17565	HA	ASN	A1140	106.170	195.975	108.961	1.00	0.00
ATOM	17566	CB	ASN	A1140	105.580	197.792	107.984	1.00	0.00

```
ATOM  17567  HB1 ASN A1140     104.938 198.099 107.160  1.00  0.00
ATOM  17568  HB2 ASN A1140     106.568 198.229 107.847  1.00  0.00
ATOM  17569  CG  ASN A1140     104.988 198.340 109.278  1.00  0.00
ATOM  17570  OD1 ASN A1140     103.950 198.981 109.318  1.00  0.00
ATOM  17571  ND2 ASN A1140     105.640 198.084 110.396  1.00  0.00
ATOM  17572 1HD2 ASN A1140     106.468 197.522 110.381  1.00  0.00
ATOM  17573 2HD2 ASN A1140     105.206 198.376 111.258  1.00  0.00
ATOM  17574  C   ASN A1140     104.311 195.572 108.041  1.00  0.00
ATOM  17575  O   ASN A1140     103.498 195.816 108.934  1.00  0.00
ATOM  17576  N   THR A1141     104.042 194.650 107.114  1.00  0.00
ATOM  17577  H   THR A1141     104.705 194.510 106.352  1.00  0.00
ATOM  17578  CA  THR A1141     103.145 193.525 107.405  1.00  0.00
ATOM  17579  HA  THR A1141     102.955 193.524 108.478  1.00  0.00
ATOM  17580  CB  THR A1141     101.780 193.639 106.720  1.00  0.00
ATOM  17581  HB  THR A1141     101.826 193.247 105.705  1.00  0.00
ATOM  17582  CG2 THR A1141     100.677 192.952 107.520  1.00  0.00
ATOM  17583 1HG2 THR A1141      99.748 192.994 106.952  1.00  0.00
ATOM  17584 2HG2 THR A1141     100.937 191.910 107.680  1.00  0.00
ATOM  17585 3HG2 THR A1141     100.538 193.439 108.485  1.00  0.00
ATOM  17586  OG1 THR A1141     101.389 194.998 106.689  1.00  0.00
ATOM  17587  HG1 THR A1141     101.604 195.333 107.572  1.00  0.00
ATOM  17588  C   THR A1141     103.866 192.208 107.120  1.00  0.00
ATOM  17589  O   THR A1141     104.777 192.139 106.295  1.00  0.00
ATOM  17590  N   VAL A1142     103.595 191.206 107.958  1.00  0.00
ATOM  17591  H   VAL A1142     102.813 191.284 108.587  1.00  0.00
ATOM  17592  CA  VAL A1142     104.527 190.099 108.152  1.00  0.00
ATOM  17593  HA  VAL A1142     105.527 190.490 107.966  1.00  0.00
ATOM  17594  CB  VAL A1142     104.555 189.660 109.630  1.00  0.00
ATOM  17595  HB  VAL A1142     104.759 190.559 110.216  1.00  0.00
ATOM  17596  CG1 VAL A1142     103.240 189.093 110.193  1.00  0.00
ATOM  17597 1HG1 VAL A1142     103.339 188.958 111.270  1.00  0.00
ATOM  17598 2HG1 VAL A1142     102.422 189.783 109.996  1.00  0.00
ATOM  17599 3HG1 VAL A1142     103.017 188.131 109.736  1.00  0.00
ATOM  17600  CG2 VAL A1142     105.717 188.705 109.904  1.00  0.00
ATOM  17601 1HG2 VAL A1142     105.778 188.488 110.969  1.00  0.00
ATOM  17602 2HG2 VAL A1142     105.571 187.772 109.367  1.00  0.00
ATOM  17603 3HG2 VAL A1142     106.653 189.159 109.582  1.00  0.00
ATOM  17604  C   VAL A1142     104.329 188.994 107.115  1.00  0.00
ATOM  17605  O   VAL A1142     103.597 188.031 107.325  1.00  0.00
ATOM  17606  N   GLN A1143     104.972 189.159 105.967  1.00  0.00
ATOM  17607  H   GLN A1143     105.418 190.056 105.816  1.00  0.00
ATOM  17608  CA  GLN A1143     104.871 188.230 104.836  1.00  0.00
ATOM  17609  HA  GLN A1143     103.806 188.131 104.616  1.00  0.00
ATOM  17610  CB  GLN A1143     105.546 188.885 103.608  1.00  0.00
ATOM  17611  HB1 GLN A1143     105.210 188.354 102.717  1.00  0.00
ATOM  17612  HB2 GLN A1143     106.629 188.774 103.684  1.00  0.00
ATOM  17613  CG  GLN A1143     105.211 190.389 103.466  1.00  0.00
ATOM  17614  HG1 GLN A1143     105.973 190.982 103.972  1.00  0.00
ATOM  17615  HG2 GLN A1143     104.260 190.600 103.955  1.00  0.00
ATOM  17616  CD  GLN A1143     105.100 190.853 102.025  1.00  0.00
ATOM  17617  OE1 GLN A1143     106.077 191.094 101.330  1.00  0.00
ATOM  17618  NE2 GLN A1143     103.893 190.985 101.525  1.00  0.00
ATOM  17619 1HE2 GLN A1143     103.087 190.749 102.123  1.00  0.00
ATOM  17620 2HE2 GLN A1143     103.796 191.246 100.569  1.00  0.00
ATOM  17621  C   GLN A1143     105.462 186.841 105.175  1.00  0.00
ATOM  17622  O   GLN A1143     106.460 186.762 105.905  1.00  0.00
ATOM  17623  N   PRO A1144     104.894 185.730 104.657  1.00  0.00
ATOM  17624  CD  PRO A1144     103.784 185.697 103.717  1.00  0.00
ATOM  17625  HD1 PRO A1144     103.906 186.431 102.920  1.00  0.00
ATOM  17626  HD2 PRO A1144     102.851 185.879 104.255  1.00  0.00
ATOM  17627  CG  PRO A1144     103.785 184.287 103.134  1.00  0.00
```

```
ATOM  17628  HG1 PRO A1144     104.484 184.243 102.297  1.00  0.00
ATOM  17629  HG2 PRO A1144     102.787 183.981 102.821  1.00  0.00
ATOM  17630  CB  PRO A1144     104.311 183.430 104.283  1.00  0.00
ATOM  17631  HB1 PRO A1144     104.815 182.536 103.915  1.00  0.00
ATOM  17632  HB2 PRO A1144     103.480 183.154 104.934  1.00  0.00
ATOM  17633  CA  PRO A1144     105.261 184.363 105.046  1.00  0.00
ATOM  17634  HA  PRO A1144     105.058 184.233 106.103  1.00  0.00
ATOM  17635  C   PRO A1144     106.743 184.044 104.822  1.00  0.00
ATOM  17636  O   PRO A1144     107.256 184.063 103.707  1.00  0.00
ATOM  17637  N   THR A1145     107.404 183.674 105.917  1.00  0.00
ATOM  17638  H   THR A1145     106.881 183.629 106.777  1.00  0.00
ATOM  17639  CA  THR A1145     108.790 183.187 105.994  1.00  0.00
ATOM  17640  HA  THR A1145     109.112 182.874 105.001  1.00  0.00
ATOM  17641  CB  THR A1145     109.751 184.301 106.465  1.00  0.00
ATOM  17642  HB  THR A1145     110.700 183.859 106.765  1.00  0.00
ATOM  17643  CG2 THR A1145     110.030 185.317 105.361  1.00  0.00
ATOM  17644 1HG2 THR A1145     110.743 186.060 105.720  1.00  0.00
ATOM  17645 2HG2 THR A1145     110.454 184.817 104.492  1.00  0.00
ATOM  17646 3HG2 THR A1145     109.110 185.821 105.062  1.00  0.00
ATOM  17647  OG1 THR A1145     109.218 185.026 107.544  1.00  0.00
ATOM  17648  HG1 THR A1145     108.493 185.562 107.192  1.00  0.00
ATOM  17649  C   THR A1145     108.839 181.937 106.879  1.00  0.00
ATOM  17650  O   THR A1145     107.786 181.428 107.261  1.00  0.00
ATOM  17651  N   CYS A1146     110.024 181.378 107.156  1.00  0.00
ATOM  17652  H   CYS A1146     110.865 181.798 106.797  1.00  0.00
ATOM  17653  CA  CYS A1146     110.178 180.186 107.997  1.00  0.00
ATOM  17654  HA  CYS A1146     109.789 179.324 107.453  1.00  0.00
ATOM  17655  CB  CYS A1146     111.669 179.968 108.286  1.00  0.00
ATOM  17656  HB1 CYS A1146     111.783 179.118 108.960  1.00  0.00
ATOM  17657  HB2 CYS A1146     112.079 180.854 108.777  1.00  0.00
ATOM  17658  SG  CYS A1146     112.565 179.635 106.742  1.00  0.00
ATOM  17659  HG  CYS A1146     113.770 179.424 107.286  1.00  0.00
ATOM  17660  C   CYS A1146     109.402 180.306 109.319  1.00  0.00
ATOM  17661  O   CYS A1146     109.382 181.383 109.921  1.00  0.00
ATOM  17662  N   VAL A1147     108.825 179.189 109.775  1.00  0.00
ATOM  17663  H   VAL A1147     108.883 178.352 109.218  1.00  0.00
ATOM  17664  CA  VAL A1147     108.016 179.124 111.002  1.00  0.00
ATOM  17665  HA  VAL A1147     107.139 179.743 110.820  1.00  0.00
ATOM  17666  CB  VAL A1147     107.500 177.713 111.319  1.00  0.00
ATOM  17667  HB  VAL A1147     107.052 177.725 112.312  1.00  0.00
ATOM  17668  CG1 VAL A1147     106.399 177.336 110.329  1.00  0.00
ATOM  17669 1HG1 VAL A1147     105.924 176.415 110.660  1.00  0.00
ATOM  17670 2HG1 VAL A1147     105.635 178.112 110.296  1.00  0.00
ATOM  17671 3HG1 VAL A1147     106.807 177.181 109.331  1.00  0.00
ATOM  17672  CG2 VAL A1147     108.620 176.665 111.330  1.00  0.00
ATOM  17673 1HG2 VAL A1147     108.193 175.729 111.677  1.00  0.00
ATOM  17674 2HG2 VAL A1147     109.026 176.523 110.330  1.00  0.00
ATOM  17675 3HG2 VAL A1147     109.410 176.972 112.014  1.00  0.00
ATOM  17676  C   VAL A1147     108.682 179.740 112.225  1.00  0.00
ATOM  17677  O   VAL A1147     109.915 179.736 112.340  1.00  0.00
ATOM  17678  N   ASN A1148     107.859 180.268 113.134  1.00  0.00
ATOM  17679  H   ASN A1148     106.866 180.099 113.046  1.00  0.00
ATOM  17680  CA  ASN A1148     108.331 180.751 114.417  1.00  0.00
ATOM  17681  HA  ASN A1148     109.361 181.093 114.279  1.00  0.00
ATOM  17682  CB  ASN A1148     107.538 182.000 114.858  1.00  0.00
ATOM  17683  HB1 ASN A1148     106.559 181.709 115.223  1.00  0.00
ATOM  17684  HB2 ASN A1148     107.398 182.657 114.005  1.00  0.00
ATOM  17685  CG  ASN A1148     108.296 182.799 115.913  1.00  0.00
ATOM  17686  OD1 ASN A1148     109.274 183.473 115.618  1.00  0.00
ATOM  17687  ND2 ASN A1148     107.934 182.673 117.169  1.00  0.00
ATOM  17688 1HD2 ASN A1148     107.172 182.041 117.431  1.00  0.00
```

```
ATOM  17689 2HD2 ASN A1148     108.510 183.063 117.893  1.00  0.00
ATOM  17690  C   ASN A1148     108.427 179.601 115.445  1.00  0.00
ATOM  17691  O   ASN A1148     108.133 178.443 115.147  1.00  0.00
ATOM  17692  N   SER A1149     108.961 179.918 116.621  1.00  0.00
ATOM  17693  H   SER A1149     109.085 180.894 116.853  1.00  0.00
ATOM  17694  CA  SER A1149     109.437 178.970 117.620  1.00  0.00
ATOM  17695  HA  SER A1149     110.145 178.301 117.131  1.00  0.00
ATOM  17696  CB  SER A1149     110.224 179.719 118.724  1.00  0.00
ATOM  17697  HB1 SER A1149     111.268 179.767 118.412  1.00  0.00
ATOM  17698  HB2 SER A1149     110.184 179.160 119.660  1.00  0.00
ATOM  17699  OG  SER A1149     109.791 181.050 118.954  1.00  0.00
ATOM  17700  HG  SER A1149     108.838 181.044 119.244  1.00  0.00
ATOM  17701  C   SER A1149     108.366 178.079 118.255  1.00  0.00
ATOM  17702  O   SER A1149     107.160 178.315 118.205  1.00  0.00
ATOM  17703  N   THR A1150     108.826 177.007 118.897  1.00  0.00
ATOM  17704  H   THR A1150     109.809 176.789 118.874  1.00  0.00
ATOM  17705  CA  THR A1150     108.018 176.247 119.847  1.00  0.00
ATOM  17706  HA  THR A1150     107.182 175.798 119.315  1.00  0.00
ATOM  17707  CB  THR A1150     108.857 175.146 120.526  1.00  0.00
ATOM  17708  HB  THR A1150     109.272 175.519 121.462  1.00  0.00
ATOM  17709  CG2 THR A1150     108.030 173.890 120.824  1.00  0.00
ATOM  17710 1HG2 THR A1150     108.652 173.161 121.346  1.00  0.00
ATOM  17711 2HG2 THR A1150     107.189 174.149 121.466  1.00  0.00
ATOM  17712 3HG2 THR A1150     107.660 173.464 119.892  1.00  0.00
ATOM  17713  OG1 THR A1150     109.941 174.717 119.721  1.00  0.00
ATOM  17714  HG1 THR A1150     110.387 174.021 120.214  1.00  0.00
ATOM  17715  C   THR A1150     107.482 177.169 120.935  1.00  0.00
ATOM  17716  O   THR A1150     108.222 178.025 121.431  1.00  0.00
ATOM  17717  N   PHE A1151     106.245 176.950 121.386  1.00  0.00
ATOM  17718  H   PHE A1151     105.685 176.228 120.969  1.00  0.00
ATOM  17719  CA  PHE A1151     105.869 177.433 122.710  1.00  0.00
ATOM  17720  HA  PHE A1151     106.106 178.500 122.733  1.00  0.00
ATOM  17721  CB  PHE A1151     104.348 177.346 122.920  1.00  0.00
ATOM  17722  HB1 PHE A1151     103.903 178.011 122.179  1.00  0.00
ATOM  17723  HB2 PHE A1151     104.133 177.800 123.883  1.00  0.00
ATOM  17724  CG  PHE A1151     103.624 176.007 122.825  1.00  0.00
ATOM  17725  CD1 PHE A1151     104.174 174.800 123.311  1.00  0.00
ATOM  17726  HD1 PHE A1151     105.142 174.786 123.776  1.00  0.00
ATOM  17727  CE1 PHE A1151     103.438 173.601 123.238  1.00  0.00
ATOM  17728  HE1 PHE A1151     103.850 172.681 123.629  1.00  0.00
ATOM  17729  CZ  PHE A1151     102.150 173.596 122.680  1.00  0.00
ATOM  17730  HZ  PHE A1151     101.599 172.669 122.615  1.00  0.00
ATOM  17731  CE2 PHE A1151     101.580 174.798 122.237  1.00  0.00
ATOM  17732  HE2 PHE A1151     100.570 174.808 121.842  1.00  0.00
ATOM  17733  CD2 PHE A1151     102.312 175.994 122.317  1.00  0.00
ATOM  17734  HD2 PHE A1151     101.845 176.916 122.006  1.00  0.00
ATOM  17735  C   PHE A1151     106.759 176.808 123.809  1.00  0.00
ATOM  17736  O   PHE A1151     107.451 175.809 123.589  1.00  0.00
ATOM  17737  N   ASP A1152     106.707 177.409 124.993  1.00  0.00
ATOM  17738  H   ASP A1152     105.983 178.098 125.133  1.00  0.00
ATOM  17739  CA  ASP A1152     107.582 177.173 126.148  1.00  0.00
ATOM  17740  HA  ASP A1152     108.212 176.300 125.977  1.00  0.00
ATOM  17741  CB  ASP A1152     108.521 178.399 126.275  1.00  0.00
ATOM  17742  HB1 ASP A1152     107.917 179.305 126.339  1.00  0.00
ATOM  17743  HB2 ASP A1152     109.119 178.471 125.365  1.00  0.00
ATOM  17744  CG  ASP A1152     109.492 178.368 127.468  1.00  0.00
ATOM  17745  OD1 ASP A1152     109.648 177.265 128.037  1.00  0.00
ATOM  17746  OD2 ASP A1152     109.994 179.459 127.832  1.00  0.00
ATOM  17747  C   ASP A1152     106.676 176.905 127.372  1.00  0.00
ATOM  17748  O   ASP A1152     105.519 177.330 127.395  1.00  0.00
ATOM  17749  N   SER A1153     107.110 176.120 128.361  1.00  0.00
```

```
ATOM  17750  H   SER A1153     108.112 175.937 128.407  1.00  0.00
ATOM  17751  CA  SER A1153     106.255 175.693 129.483  1.00  0.00
ATOM  17752  HA  SER A1153     105.635 176.527 129.776  1.00  0.00
ATOM  17753  CB  SER A1153     105.283 174.594 129.048  1.00  0.00
ATOM  17754  HB1 SER A1153     104.850 174.121 129.931  1.00  0.00
ATOM  17755  HB2 SER A1153     105.835 173.845 128.489  1.00  0.00
ATOM  17756  OG  SER A1153     104.227 175.130 128.274  1.00  0.00
ATOM  17757  HG  SER A1153     104.578 175.908 127.788  1.00  0.00
ATOM  17758  C   SER A1153     107.035 175.150 130.693  1.00  0.00
ATOM  17759  O   SER A1153     107.770 174.166 130.546  1.00  0.00
ATOM  17760  N   PRO A1154     106.782 175.678 131.911  1.00  0.00
ATOM  17761  CD  PRO A1154     107.306 175.109 133.143  1.00  0.00
ATOM  17762  HD1 PRO A1154     108.322 175.475 133.302  1.00  0.00
ATOM  17763  HD2 PRO A1154     107.302 174.019 133.114  1.00  0.00
ATOM  17764  CG  PRO A1154     106.386 175.618 134.246  1.00  0.00
ATOM  17765  HG1 PRO A1154     106.891 175.660 135.210  1.00  0.00
ATOM  17766  HG2 PRO A1154     105.499 174.985 134.303  1.00  0.00
ATOM  17767  CB  PRO A1154     105.992 177.007 133.750  1.00  0.00
ATOM  17768  HB1 PRO A1154     106.761 177.719 134.053  1.00  0.00
ATOM  17769  HB2 PRO A1154     105.020 177.313 134.140  1.00  0.00
ATOM  17770  CA  PRO A1154     105.960 176.860 132.221  1.00  0.00
ATOM  17771  HA  PRO A1154     104.932 176.662 131.911  1.00  0.00
ATOM  17772  C   PRO A1154     106.475 178.134 131.543  1.00  0.00
ATOM  17773  O   PRO A1154     107.639 178.475 131.709  1.00  0.00
ATOM  17774  N   ALA A1155     105.620 178.835 130.790  1.00  0.00
ATOM  17775  H   ALA A1155     104.683 178.486 130.653  1.00  0.00
ATOM  17776  CA  ALA A1155     105.989 180.093 130.137  1.00  0.00
ATOM  17777  HA  ALA A1155     106.587 180.663 130.850  1.00  0.00
ATOM  17778  CB  ALA A1155     106.891 179.831 128.928  1.00  0.00
ATOM  17779  HB1 ALA A1155     107.449 180.733 128.683  1.00  0.00
ATOM  17780  HB2 ALA A1155     107.607 179.049 129.172  1.00  0.00
ATOM  17781  HB3 ALA A1155     106.296 179.543 128.067  1.00  0.00
ATOM  17782  C   ALA A1155     104.781 180.974 129.794  1.00  0.00
ATOM  17783  O   ALA A1155     103.829 181.027 130.563  1.00  0.00
ATOM  17784  N   HIS A1156     104.898 181.802 128.746  1.00  0.00
ATOM  17785  H   HIS A1156     105.686 181.629 128.140  1.00  0.00
ATOM  17786  CA  HIS A1156     104.227 183.103 128.570  1.00  0.00
ATOM  17787  HA  HIS A1156     104.592 183.522 127.631  1.00  0.00
ATOM  17788  CB  HIS A1156     102.706 182.931 128.421  1.00  0.00
ATOM  17789  HB1 HIS A1156     102.249 182.890 129.406  1.00  0.00
ATOM  17790  HB2 HIS A1156     102.494 181.986 127.919  1.00  0.00
ATOM  17791  CG  HIS A1156     102.030 184.020 127.621  1.00  0.00
ATOM  17792  ND1 HIS A1156     102.551 184.612 126.470  1.00  0.00
ATOM  17793  CE1 HIS A1156     101.587 185.427 126.010  1.00  0.00
ATOM  17794  HE1 HIS A1156     101.652 186.018 125.103  1.00  0.00
ATOM  17795  NE2 HIS A1156     100.511 185.385 126.812  1.00  0.00
ATOM  17796  HE2 HIS A1156      99.650 185.893 126.646  1.00  0.00
ATOM  17797  CD2 HIS A1156     100.771 184.501 127.837  1.00  0.00
ATOM  17798  HD2 HIS A1156     100.103 184.208 128.640  1.00  0.00
ATOM  17799  C   HIS A1156     104.598 184.135 129.645  1.00  0.00
ATOM  17800  O   HIS A1156     104.553 185.325 129.376  1.00  0.00
ATOM  17801  N   TRP A1157     105.020 183.686 130.832  1.00  0.00
ATOM  17802  H   TRP A1157     104.910 182.689 130.963  1.00  0.00
ATOM  17803  CA  TRP A1157     105.489 184.402 132.028  1.00  0.00
ATOM  17804  HA  TRP A1157     105.393 183.651 132.814  1.00  0.00
ATOM  17805  CB  TRP A1157     107.000 184.664 131.997  1.00  0.00
ATOM  17806  HB1 TRP A1157     107.263 185.172 132.921  1.00  0.00
ATOM  17807  HB2 TRP A1157     107.248 185.328 131.175  1.00  0.00
ATOM  17808  CG  TRP A1157     107.830 183.412 131.953  1.00  0.00
ATOM  17809  CD1 TRP A1157     108.524 182.925 130.897  1.00  0.00
ATOM  17810  HD1 TRP A1157     108.591 183.389 129.927  1.00  0.00
```

```
ATOM  17811  NE1 TRP A1157     109.133 181.733 131.244  1.00  0.00
ATOM  17812  HE1 TRP A1157     109.632 181.136 130.588  1.00  0.00
ATOM  17813  CE2 TRP A1157     108.892 181.410 132.560  1.00  0.00
ATOM  17814  CZ2 TRP A1157     109.284 180.338 133.372  1.00  0.00
ATOM  17815  HZ2 TRP A1157     109.898 179.549 132.960  1.00  0.00
ATOM  17816  CH2 TRP A1157     108.802 180.257 134.688  1.00  0.00
ATOM  17817  HH2 TRP A1157     109.069 179.410 135.308  1.00  0.00
ATOM  17818  CZ3 TRP A1157     107.947 181.258 135.181  1.00  0.00
ATOM  17819  HZ3 TRP A1157     107.559 181.184 136.186  1.00  0.00
ATOM  17820  CE3 TRP A1157     107.574 182.343 134.365  1.00  0.00
ATOM  17821  HE3 TRP A1157     106.911 183.100 134.752  1.00  0.00
ATOM  17822  CD2 TRP A1157     108.038 182.449 133.034  1.00  0.00
ATOM  17823  C   TRP A1157     104.586 185.516 132.588  1.00  0.00
ATOM  17824  O   TRP A1157     104.594 185.744 133.792  1.00  0.00
ATOM  17825  N   ALA A1158     103.638 186.031 131.807  1.00  0.00
ATOM  17826  H   ALA A1158     103.731 185.858 130.813  1.00  0.00
ATOM  17827  CA  ALA A1158     102.350 186.572 132.236  1.00  0.00
ATOM  17828  HA  ALA A1158     102.534 187.257 133.062  1.00  0.00
ATOM  17829  CB  ALA A1158     101.790 187.405 131.070  1.00  0.00
ATOM  17830  HB1 ALA A1158     100.875 187.911 131.379  1.00  0.00
ATOM  17831  HB2 ALA A1158     102.516 188.161 130.770  1.00  0.00
ATOM  17832  HB3 ALA A1158     101.573 186.760 130.218  1.00  0.00
ATOM  17833  C   ALA A1158     101.347 185.505 132.748  1.00  0.00
ATOM  17834  O   ALA A1158     100.138 185.645 132.563  1.00  0.00
ATOM  17835  N   GLN A1159     101.827 184.427 133.381  1.00  0.00
ATOM  17836  H   GLN A1159     102.816 184.416 133.596  1.00  0.00
ATOM  17837  CA  GLN A1159     101.058 183.218 133.722  1.00  0.00
ATOM  17838  HA  GLN A1159     101.779 182.452 134.010  1.00  0.00
ATOM  17839  CB  GLN A1159     100.155 183.467 134.963  1.00  0.00
ATOM  17840  HB1 GLN A1159      99.662 182.531 135.231  1.00  0.00
ATOM  17841  HB2 GLN A1159      99.379 184.188 134.703  1.00  0.00
ATOM  17842  CG  GLN A1159     100.837 183.975 136.250  1.00  0.00
ATOM  17843  HG1 GLN A1159     100.069 184.118 137.009  1.00  0.00
ATOM  17844  HG2 GLN A1159     101.311 184.938 136.061  1.00  0.00
ATOM  17845  CD  GLN A1159     101.878 183.006 136.794  1.00  0.00
ATOM  17846  OE1 GLN A1159     103.005 182.969 136.320  1.00  0.00
ATOM  17847  NE2 GLN A1159     101.572 182.194 137.787  1.00  0.00
ATOM  17848 1HE2 GLN A1159     100.638 182.172 138.223  1.00  0.00
ATOM  17849 2HE2 GLN A1159     102.284 181.546 138.050  1.00  0.00
ATOM  17850  C   GLN A1159     100.292 182.620 132.516  1.00  0.00
ATOM  17851  O   GLN A1159     100.895 182.386 131.465  1.00  0.00
ATOM  17852  N   LYS A1160      99.018 182.237 132.704  1.00  0.00
ATOM  17853  H   LYS A1160      98.562 182.653 133.502  1.00  0.00
ATOM  17854  CA  LYS A1160      98.404 180.985 132.210  1.00  0.00
ATOM  17855  HA  LYS A1160      97.459 180.811 132.729  1.00  0.00
ATOM  17856  CB  LYS A1160      98.097 181.054 130.698  1.00  0.00
ATOM  17857  HB1 LYS A1160      97.726 180.073 130.415  1.00  0.00
ATOM  17858  HB2 LYS A1160      99.012 181.229 130.131  1.00  0.00
ATOM  17859  CG  LYS A1160      97.061 182.103 130.255  1.00  0.00
ATOM  17860  HG1 LYS A1160      97.422 183.096 130.527  1.00  0.00
ATOM  17861  HG2 LYS A1160      96.118 181.919 130.771  1.00  0.00
ATOM  17862  CD  LYS A1160      96.827 182.063 128.729  1.00  0.00
ATOM  17863  HD1 LYS A1160      97.777 182.223 128.217  1.00  0.00
ATOM  17864  HD2 LYS A1160      96.144 182.870 128.458  1.00  0.00
ATOM  17865  CE  LYS A1160      96.219 180.725 128.293  1.00  0.00
ATOM  17866  HE1 LYS A1160      95.219 180.622 128.726  1.00  0.00
ATOM  17867  HE2 LYS A1160      96.831 179.913 128.686  1.00  0.00
ATOM  17868  NZ  LYS A1160      96.143 180.569 126.824  1.00  0.00
ATOM  17869  HZ1 LYS A1160      97.084 180.524 126.439  1.00  0.00
ATOM  17870  HZ2 LYS A1160      95.638 179.733 126.574  1.00  0.00
ATOM  17871  HZ3 LYS A1160      95.630 181.361 126.430  1.00  0.00
```

ATOM	17872	C	LYS	A1160	99.281	179.768	132.509	1.00	0.00
ATOM	17873	O	LYS	A1160	98.944	178.985	133.393	1.00	0.00
ATOM	17874	N	GLY	A1161	100.418	179.653	131.821	1.00	0.00
ATOM	17875	H	GLY	A1161	100.593	180.425	131.188	1.00	0.00
ATOM	17876	CA	GLY	A1161	101.530	178.718	132.010	1.00	0.00
ATOM	17877	HA1	GLY	A1161	101.926	178.864	133.015	1.00	0.00
ATOM	17878	HA2	GLY	A1161	102.318	178.986	131.306	1.00	0.00
ATOM	17879	C	GLY	A1161	101.269	177.219	131.854	1.00	0.00
ATOM	17880	O	GLY	A1161	102.212	176.513	131.499	1.00	0.00
ATOM	17881	N	SER	A1162	100.041	176.761	132.120	1.00	0.00
ATOM	17882	H	SER	A1162	99.412	177.461	132.496	1.00	0.00
ATOM	17883	CA	SER	A1162	99.420	175.469	131.786	1.00	0.00
ATOM	17884	HA	SER	A1162	98.571	175.707	131.149	1.00	0.00
ATOM	17885	CB	SER	A1162	100.312	174.527	130.974	1.00	0.00
ATOM	17886	HB1	SER	A1162	101.100	174.102	131.596	1.00	0.00
ATOM	17887	HB2	SER	A1162	100.761	175.061	130.136	1.00	0.00
ATOM	17888	OG	SER	A1162	99.497	173.502	130.448	1.00	0.00
ATOM	17889	HG	SER	A1162	99.346	173.786	129.531	1.00	0.00
ATOM	17890	C	SER	A1162	98.884	174.701	132.999	1.00	0.00
ATOM	17891	O	SER	A1162	99.618	174.031	133.728	1.00	0.00
ATOM	17892	N	HIS	A1163	97.555	174.640	133.092	1.00	0.00
ATOM	17893	H	HIS	A1163	97.026	175.321	132.565	1.00	0.00
ATOM	17894	CA	HIS	A1163	96.776	173.707	133.935	1.00	0.00
ATOM	17895	HA	HIS	A1163	97.177	173.766	134.950	1.00	0.00
ATOM	17896	CB	HIS	A1163	95.304	174.179	133.974	1.00	0.00
ATOM	17897	HB1	HIS	A1163	94.822	173.791	134.871	1.00	0.00
ATOM	17898	HB2	HIS	A1163	94.776	173.781	133.107	1.00	0.00
ATOM	17899	CG	HIS	A1163	95.182	175.687	133.959	1.00	0.00
ATOM	17900	ND1	HIS	A1163	95.881	176.546	134.803	1.00	0.00
ATOM	17901	CE1	HIS	A1163	95.842	177.755	134.225	1.00	0.00
ATOM	17902	HE1	HIS	A1163	96.366	178.632	134.590	1.00	0.00
ATOM	17903	NE2	HIS	A1163	95.151	177.698	133.075	1.00	0.00
ATOM	17904	HE2	HIS	A1163	95.131	178.433	132.356	1.00	0.00
ATOM	17905	CD2	HIS	A1163	94.718	176.405	132.895	1.00	0.00
ATOM	17906	HD2	HIS	A1163	94.226	176.014	132.017	1.00	0.00
ATOM	17907	C	HIS	A1163	96.902	172.241	133.472	1.00	0.00
ATOM	17908	O	HIS	A1163	96.135	171.383	133.907	1.00	0.00
ATOM	17909	N	GLN	A1164	97.834	171.964	132.552	1.00	0.00
ATOM	17910	H	GLN	A1164	98.442	172.731	132.297	1.00	0.00
ATOM	17911	CA	GLN	A1164	98.103	170.725	131.819	1.00	0.00
ATOM	17912	HA	GLN	A1164	98.925	170.932	131.132	1.00	0.00
ATOM	17913	CB	GLN	A1164	98.597	169.613	132.772	1.00	0.00
ATOM	17914	HB1	GLN	A1164	98.932	168.770	132.167	1.00	0.00
ATOM	17915	HB2	GLN	A1164	97.762	169.271	133.383	1.00	0.00
ATOM	17916	CG	GLN	A1164	99.739	170.033	133.713	1.00	0.00
ATOM	17917	HG1	GLN	A1164	100.019	169.174	134.321	1.00	0.00
ATOM	17918	HG2	GLN	A1164	99.391	170.811	134.392	1.00	0.00
ATOM	17919	CD	GLN	A1164	100.981	170.507	132.972	1.00	0.00
ATOM	17920	OE1	GLN	A1164	101.654	169.750	132.294	1.00	0.00
ATOM	17921	NE2	GLN	A1164	101.340	171.769	133.076	1.00	0.00
ATOM	17922	1HE2	GLN	A1164	100.759	172.459	133.543	1.00	0.00
ATOM	17923	2HE2	GLN	A1164	102.168	172.036	132.578	1.00	0.00
ATOM	17924	C	GLN	A1164	96.935	170.254	130.942	1.00	0.00
ATOM	17925	O	GLN	A1164	97.057	170.226	129.724	1.00	0.00
ATOM	17926	N	ILE	A1165	95.771	169.961	131.530	1.00	0.00
ATOM	17927	H	ILE	A1165	95.707	170.094	132.534	1.00	0.00
ATOM	17928	CA	ILE	A1165	94.590	169.477	130.799	1.00	0.00
ATOM	17929	HA	ILE	A1165	94.923	168.636	130.193	1.00	0.00
ATOM	17930	CB	ILE	A1165	93.550	168.923	131.799	1.00	0.00
ATOM	17931	HB	ILE	A1165	94.089	168.267	132.487	1.00	0.00
ATOM	17932	CG2	ILE	A1165	92.890	170.029	132.646	1.00	0.00

```
ATOM  17933 1HG2 ILE A1165      92.298 169.583 133.446  1.00  0.00
ATOM  17934 2HG2 ILE A1165      93.648 170.664 133.104  1.00  0.00
ATOM  17935 3HG2 ILE A1165      92.241 170.649 132.028  1.00  0.00
ATOM  17936  CG1 ILE A1165      92.516 168.047 131.063  1.00  0.00
ATOM  17937 1HG1 ILE A1165      93.044 167.348 130.414  1.00  0.00
ATOM  17938 2HG1 ILE A1165      91.885 168.673 130.436  1.00  0.00
ATOM  17939  CD  ILE A1165      91.625 167.225 132.001  1.00  0.00
ATOM  17940  HD1 ILE A1165      90.992 166.564 131.410  1.00  0.00
ATOM  17941  HD2 ILE A1165      92.240 166.621 132.667  1.00  0.00
ATOM  17942  HD3 ILE A1165      90.986 167.882 132.590  1.00  0.00
ATOM  17943  C   ILE A1165      93.999 170.500 129.806  1.00  0.00
ATOM  17944  O   ILE A1165      93.472 170.113 128.767  1.00  0.00
ATOM  17945  N   SER A1166      94.152 171.799 130.085  1.00  0.00
ATOM  17946  H   SER A1166      94.506 172.042 130.994  1.00  0.00
ATOM  17947  CA  SER A1166      93.816 172.891 129.161  1.00  0.00
ATOM  17948  HA  SER A1166      92.947 172.607 128.568  1.00  0.00
ATOM  17949  CB  SER A1166      93.472 174.162 129.941  1.00  0.00
ATOM  17950  HB1 SER A1166      93.180 174.958 129.254  1.00  0.00
ATOM  17951  HB2 SER A1166      94.355 174.472 130.497  1.00  0.00
ATOM  17952  OG  SER A1166      92.428 173.925 130.860  1.00  0.00
ATOM  17953  HG  SER A1166      91.599 174.021 130.404  1.00  0.00
ATOM  17954  C   SER A1166      94.940 173.241 128.180  1.00  0.00
ATOM  17955  O   SER A1166      94.683 173.980 127.238  1.00  0.00
ATOM  17956  N   LEU A1167      96.160 172.733 128.404  1.00  0.00
ATOM  17957  H   LEU A1167      96.206 171.984 129.084  1.00  0.00
ATOM  17958  CA  LEU A1167      97.453 173.078 127.783  1.00  0.00
ATOM  17959  HA  LEU A1167      98.204 172.534 128.354  1.00  0.00
ATOM  17960  CB  LEU A1167      97.476 172.486 126.358  1.00  0.00
ATOM  17961  HB1 LEU A1167      96.723 172.999 125.758  1.00  0.00
ATOM  17962  HB2 LEU A1167      97.190 171.437 126.426  1.00  0.00
ATOM  17963  CG  LEU A1167      98.821 172.574 125.615  1.00  0.00
ATOM  17964  HG  LEU A1167      99.097 173.619 125.480  1.00  0.00
ATOM  17965  CD1 LEU A1167      99.952 171.859 126.356  1.00  0.00
ATOM  17966 1HD1 LEU A1167     100.862 171.899 125.760  1.00  0.00
ATOM  17967 2HD1 LEU A1167     100.155 172.351 127.305  1.00  0.00
ATOM  17968 3HD1 LEU A1167      99.690 170.817 126.542  1.00  0.00
ATOM  17969  CD2 LEU A1167      98.667 171.933 124.234  1.00  0.00
ATOM  17970 1HD2 LEU A1167      99.605 172.010 123.686  1.00  0.00
ATOM  17971 2HD2 LEU A1167      98.399 170.882 124.335  1.00  0.00
ATOM  17972 3HD2 LEU A1167      97.889 172.453 123.675  1.00  0.00
ATOM  17973  C   LEU A1167      97.871 174.559 127.824  1.00  0.00
ATOM  17974  O   LEU A1167      99.044 174.820 128.093  1.00  0.00
ATOM  17975  N   ASP A1168      96.933 175.489 127.642  1.00  0.00
ATOM  17976  H   ASP A1168      96.039 175.133 127.321  1.00  0.00
ATOM  17977  CA  ASP A1168      96.901 176.889 128.095  1.00  0.00
ATOM  17978  HA  ASP A1168      96.258 177.414 127.386  1.00  0.00
ATOM  17979  CB  ASP A1168      96.192 176.923 129.476  1.00  0.00
ATOM  17980  HB1 ASP A1168      96.920 176.871 130.286  1.00  0.00
ATOM  17981  HB2 ASP A1168      95.552 176.047 129.568  1.00  0.00
ATOM  17982  CG  ASP A1168      95.286 178.142 129.657  1.00  0.00
ATOM  17983  OD1 ASP A1168      95.475 178.906 130.632  1.00  0.00
ATOM  17984  OD2 ASP A1168      94.402 178.315 128.791  1.00  0.00
ATOM  17985  C   ASP A1168      98.248 177.621 128.068  1.00  0.00
ATOM  17986  O   ASP A1168      98.736 178.165 129.057  1.00  0.00
ATOM  17987  N   ASN A1169      98.886 177.556 126.905  1.00  0.00
ATOM  17988  H   ASN A1169      98.400 177.103 126.144  1.00  0.00
ATOM  17989  CA  ASN A1169     100.180 178.166 126.600  1.00  0.00
ATOM  17990  HA  ASN A1169     100.819 178.106 127.485  1.00  0.00
ATOM  17991  CB  ASN A1169     100.769 177.257 125.492  1.00  0.00
ATOM  17992  HB1 ASN A1169     101.086 177.831 124.627  1.00  0.00
ATOM  17993  HB2 ASN A1169     100.032 176.544 125.118  1.00  0.00
```

```
ATOM  17994  CG  ASN A1169     101.934 176.470 126.055  1.00  0.00
ATOM  17995  OD1 ASN A1169     103.080 176.768 125.795  1.00  0.00
ATOM  17996  ND2 ASN A1169     101.692 175.482 126.887  1.00  0.00
ATOM  17997 1HD2 ASN A1169     100.754 175.275 127.209  1.00  0.00
ATOM  17998 2HD2 ASN A1169     102.514 175.072 127.325  1.00  0.00
ATOM  17999  C   ASN A1169      99.993 179.678 126.292  1.00  0.00
ATOM  18000  O   ASN A1169      98.933 180.224 126.629  1.00  0.00
ATOM  18001  N   PRO A1170     100.972 180.398 125.692  1.00  0.00
ATOM  18002  CD  PRO A1170     102.384 180.055 125.542  1.00  0.00
ATOM  18003  HD1 PRO A1170     102.516 179.230 124.851  1.00  0.00
ATOM  18004  HD2 PRO A1170     102.838 179.814 126.503  1.00  0.00
ATOM  18005  CG  PRO A1170     103.058 181.276 124.919  1.00  0.00
ATOM  18006  HG1 PRO A1170     103.874 180.990 124.254  1.00  0.00
ATOM  18007  HG2 PRO A1170     103.421 181.942 125.701  1.00  0.00
ATOM  18008  CB  PRO A1170     101.923 181.950 124.156  1.00  0.00
ATOM  18009  HB1 PRO A1170     101.779 181.461 123.190  1.00  0.00
ATOM  18010  HB2 PRO A1170     102.118 183.012 124.030  1.00  0.00
ATOM  18011  CA  PRO A1170     100.714 181.699 125.059  1.00  0.00
ATOM  18012  HA  PRO A1170     100.713 182.455 125.841  1.00  0.00
ATOM  18013  C   PRO A1170      99.372 181.767 124.330  1.00  0.00
ATOM  18014  O   PRO A1170      98.916 180.780 123.773  1.00  0.00
ATOM  18015  N   ASP A1171      98.677 182.893 124.437  1.00  0.00
ATOM  18016  H   ASP A1171      99.211 183.752 124.553  1.00  0.00
ATOM  18017  CA  ASP A1171      97.223 183.005 124.242  1.00  0.00
ATOM  18018  HA  ASP A1171      96.778 182.100 124.648  1.00  0.00
ATOM  18019  CB  ASP A1171      96.736 184.221 125.067  1.00  0.00
ATOM  18020  HB1 ASP A1171      96.721 185.105 124.430  1.00  0.00
ATOM  18021  HB2 ASP A1171      97.462 184.423 125.859  1.00  0.00
ATOM  18022  CG  ASP A1171      95.386 184.027 125.774  1.00  0.00
ATOM  18023  OD1 ASP A1171      94.997 182.857 125.990  1.00  0.00
ATOM  18024  OD2 ASP A1171      94.777 185.047 126.172  1.00  0.00
ATOM  18025  C   ASP A1171      96.841 183.022 122.750  1.00  0.00
ATOM  18026  O   ASP A1171      95.677 182.817 122.398  1.00  0.00
ATOM  18027  N   TYR A1172      97.832 183.112 121.849  1.00  0.00
ATOM  18028  H   TYR A1172      98.767 183.359 122.173  1.00  0.00
ATOM  18029  CA  TYR A1172      97.699 182.616 120.473  1.00  0.00
ATOM  18030  HA  TYR A1172      96.788 183.057 120.069  1.00  0.00
ATOM  18031  CB  TYR A1172      98.863 183.116 119.600  1.00  0.00
ATOM  18032  HB1 TYR A1172      99.692 182.408 119.641  1.00  0.00
ATOM  18033  HB2 TYR A1172      99.221 184.066 119.996  1.00  0.00
ATOM  18034  CG  TYR A1172      98.453 183.360 118.159  1.00  0.00
ATOM  18035  CD1 TYR A1172      98.478 182.320 117.207  1.00  0.00
ATOM  18036  HD1 TYR A1172      98.866 181.348 117.482  1.00  0.00
ATOM  18037  CE1 TYR A1172      97.937 182.521 115.923  1.00  0.00
ATOM  18038  HE1 TYR A1172      97.883 181.713 115.212  1.00  0.00
ATOM  18039  CZ  TYR A1172      97.412 183.783 115.578  1.00  0.00
ATOM  18040  OH  TYR A1172      96.819 183.975 114.372  1.00  0.00
ATOM  18041  HH  TYR A1172      96.525 184.884 114.297  1.00  0.00
ATOM  18042  CE2 TYR A1172      97.464 184.846 116.500  1.00  0.00
ATOM  18043  HE2 TYR A1172      97.088 185.818 116.225  1.00  0.00
ATOM  18044  CD2 TYR A1172      97.969 184.629 117.792  1.00  0.00
ATOM  18045  HD2 TYR A1172      97.965 185.434 118.513  1.00  0.00
ATOM  18046  C   TYR A1172      97.505 181.079 120.359  1.00  0.00
ATOM  18047  O   TYR A1172      97.281 180.562 119.276  1.00  0.00
ATOM  18048  N   GLN A1173      97.605 180.323 121.458  1.00  0.00
ATOM  18049  H   GLN A1173      97.809 180.815 122.321  1.00  0.00
ATOM  18050  CA  GLN A1173      97.640 178.853 121.580  1.00  0.00
ATOM  18051  HA  GLN A1173      97.778 178.646 122.641  1.00  0.00
ATOM  18052  CB  GLN A1173      96.291 178.221 121.191  1.00  0.00
ATOM  18053  HB1 GLN A1173      96.327 177.151 121.397  1.00  0.00
ATOM  18054  HB2 GLN A1173      96.163 178.336 120.114  1.00  0.00
```

```
ATOM  18055  CG  GLN A1173      95.033 178.799 121.873  1.00  0.00
ATOM  18056  HG1 GLN A1173      94.205 178.478 121.248  1.00  0.00
ATOM  18057  HG2 GLN A1173      95.037 179.888 121.865  1.00  0.00
ATOM  18058  CD  GLN A1173      94.763 178.301 123.301  1.00  0.00
ATOM  18059  OE1 GLN A1173      95.647 178.192 124.138  1.00  0.00
ATOM  18060  NE2 GLN A1173      93.530 177.963 123.616  1.00  0.00
ATOM  18061 1HE2 GLN A1173      92.774 178.221 122.968  1.00  0.00
ATOM  18062 2HE2 GLN A1173      93.346 177.455 124.463  1.00  0.00
ATOM  18063  C   GLN A1173      98.827 178.142 120.888  1.00  0.00
ATOM  18064  O   GLN A1173      99.077 176.974 121.163  1.00  0.00
ATOM  18065  N   GLN A1174      99.600 178.832 120.038  1.00  0.00
ATOM  18066  H   GLN A1174      99.315 179.780 119.824  1.00  0.00
ATOM  18067  CA  GLN A1174     100.840 178.364 119.401  1.00  0.00
ATOM  18068  HA  GLN A1174     101.367 177.665 120.046  1.00  0.00
ATOM  18069  CB  GLN A1174     100.525 177.692 118.045  1.00  0.00
ATOM  18070  HB1 GLN A1174     101.451 177.590 117.478  1.00  0.00
ATOM  18071  HB2 GLN A1174      99.872 178.352 117.473  1.00  0.00
ATOM  18072  CG  GLN A1174      99.856 176.301 118.099  1.00  0.00
ATOM  18073  HG1 GLN A1174      99.569 176.025 117.085  1.00  0.00
ATOM  18074  HG2 GLN A1174      98.939 176.364 118.680  1.00  0.00
ATOM  18075  CD  GLN A1174     100.748 175.191 118.649  1.00  0.00
ATOM  18076  OE1 GLN A1174     101.971 175.234 118.583  1.00  0.00
ATOM  18077  NE2 GLN A1174     100.178 174.116 119.149  1.00  0.00
ATOM  18078 1HE2 GLN A1174      99.175 174.027 119.142  1.00  0.00
ATOM  18079 2HE2 GLN A1174     100.787 173.354 119.449  1.00  0.00
ATOM  18080  C   GLN A1174     101.743 179.579 119.130  1.00  0.00
ATOM  18081  O   GLN A1174     101.303 180.512 118.458  1.00  0.00
ATOM  18082  N   ASP A1175     103.019 179.541 119.532  1.00  0.00
ATOM  18083  H   ASP A1175     103.301 178.766 120.106  1.00  0.00
ATOM  18084  CA  ASP A1175     104.048 180.570 119.222  1.00  0.00
ATOM  18085  HA  ASP A1175     103.605 181.547 119.422  1.00  0.00
ATOM  18086  CB  ASP A1175     105.216 180.393 120.209  1.00  0.00
ATOM  18087  HB1 ASP A1175     105.719 179.451 120.000  1.00  0.00
ATOM  18088  HB2 ASP A1175     104.811 180.325 121.219  1.00  0.00
ATOM  18089  CG  ASP A1175     106.238 181.541 120.197  1.00  0.00
ATOM  18090  OD1 ASP A1175     107.348 181.345 119.662  1.00  0.00
ATOM  18091  OD2 ASP A1175     105.958 182.615 120.782  1.00  0.00
ATOM  18092  C   ASP A1175     104.469 180.579 117.712  1.00  0.00
ATOM  18093  O   ASP A1175     105.596 180.874 117.323  1.00  0.00
ATOM  18094  N   PHE A1176     103.515 180.247 116.841  1.00  0.00
ATOM  18095  H   PHE A1176     102.593 180.191 117.251  1.00  0.00
ATOM  18096  CA  PHE A1176     103.565 179.987 115.405  1.00  0.00
ATOM  18097  HA  PHE A1176     102.612 179.492 115.227  1.00  0.00
ATOM  18098  CB  PHE A1176     103.482 181.275 114.573  1.00  0.00
ATOM  18099  HB1 PHE A1176     104.456 181.540 114.163  1.00  0.00
ATOM  18100  HB2 PHE A1176     103.159 182.105 115.205  1.00  0.00
ATOM  18101  CG  PHE A1176     102.474 181.121 113.445  1.00  0.00
ATOM  18102  CD1 PHE A1176     101.115 181.403 113.688  1.00  0.00
ATOM  18103  HD1 PHE A1176     100.806 181.811 114.644  1.00  0.00
ATOM  18104  CE1 PHE A1176     100.151 181.140 112.699  1.00  0.00
ATOM  18105  HE1 PHE A1176      99.107 181.350 112.889  1.00  0.00
ATOM  18106  CZ  PHE A1176     100.543 180.601 111.460  1.00  0.00
ATOM  18107  HZ  PHE A1176      99.800 180.409 110.698  1.00  0.00
ATOM  18108  CE2 PHE A1176     101.899 180.315 111.219  1.00  0.00
ATOM  18109  HE2 PHE A1176     102.192 179.884 110.272  1.00  0.00
ATOM  18110  CD2 PHE A1176     102.863 180.580 112.206  1.00  0.00
ATOM  18111  HD2 PHE A1176     103.902 180.343 112.021  1.00  0.00
ATOM  18112  C   PHE A1176     104.576 178.928 114.909  1.00  0.00
ATOM  18113  O   PHE A1176     105.190 179.052 113.852  1.00  0.00
ATOM  18114  N   PHE A1177     104.597 177.800 115.626  1.00  0.00
ATOM  18115  H   PHE A1177     104.172 177.840 116.536  1.00  0.00
```

ATOM	18116	CA	PHE	A1177	105.188	176.514	115.232	1.00	0.00
ATOM	18117	HA	PHE	A1177	106.256	176.693	115.078	1.00	0.00
ATOM	18118	CB	PHE	A1177	105.035	175.589	116.450	1.00	0.00
ATOM	18119	HB1	PHE	A1177	103.986	175.314	116.561	1.00	0.00
ATOM	18120	HB2	PHE	A1177	105.321	176.137	117.350	1.00	0.00
ATOM	18121	CG	PHE	A1177	105.873	174.323	116.421	1.00	0.00
ATOM	18122	CD1	PHE	A1177	107.252	174.393	116.701	1.00	0.00
ATOM	18123	HD1	PHE	A1177	107.715	175.351	116.897	1.00	0.00
ATOM	18124	CE1	PHE	A1177	108.030	173.223	116.707	1.00	0.00
ATOM	18125	HE1	PHE	A1177	109.091	173.277	116.917	1.00	0.00
ATOM	18126	CZ	PHE	A1177	107.438	171.982	116.411	1.00	0.00
ATOM	18127	HZ	PHE	A1177	108.050	171.090	116.387	1.00	0.00
ATOM	18128	CE2	PHE	A1177	106.064	171.912	116.118	1.00	0.00
ATOM	18129	HE2	PHE	A1177	105.616	170.957	115.864	1.00	0.00
ATOM	18130	CD2	PHE	A1177	105.283	173.079	116.126	1.00	0.00
ATOM	18131	HD2	PHE	A1177	104.230	173.022	115.883	1.00	0.00
ATOM	18132	C	PHE	A1177	104.693	175.794	113.936	1.00	0.00
ATOM	18133	O	PHE	A1177	105.436	174.940	113.451	1.00	0.00
ATOM	18134	N	PRO	A1178	103.468	175.991	113.381	1.00	0.00
ATOM	18135	CD	PRO	A1178	102.491	177.041	113.663	1.00	0.00
ATOM	18136	HD1	PRO	A1178	102.866	178.013	113.355	1.00	0.00
ATOM	18137	HD2	PRO	A1178	102.231	177.035	114.723	1.00	0.00
ATOM	18138	CG	PRO	A1178	101.248	176.743	112.836	1.00	0.00
ATOM	18139	HG1	PRO	A1178	101.336	177.200	111.850	1.00	0.00
ATOM	18140	HG2	PRO	A1178	100.338	177.078	113.334	1.00	0.00
ATOM	18141	CB	PRO	A1178	101.314	175.230	112.723	1.00	0.00
ATOM	18142	HB1	PRO	A1178	100.725	174.869	111.884	1.00	0.00
ATOM	18143	HB2	PRO	A1178	100.949	174.798	113.654	1.00	0.00
ATOM	18144	CA	PRO	A1178	102.816	174.943	112.581	1.00	0.00
ATOM	18145	HA	PRO	A1178	103.005	173.974	113.047	1.00	0.00
ATOM	18146	C	PRO	A1178	103.276	174.826	111.118	1.00	0.00
ATOM	18147	O	PRO	A1178	102.621	175.385	110.240	1.00	0.00
ATOM	18148	N	LYS	A1179	104.338	174.035	110.898	1.00	0.00
ATOM	18149	H	LYS	A1179	104.837	173.815	111.751	1.00	0.00
ATOM	18150	CA	LYS	A1179	104.862	173.342	109.688	1.00	0.00
ATOM	18151	HA	LYS	A1179	104.530	172.305	109.765	1.00	0.00
ATOM	18152	CB	LYS	A1179	104.363	173.879	108.320	1.00	0.00
ATOM	18153	HB1	LYS	A1179	105.203	173.845	107.625	1.00	0.00
ATOM	18154	HB2	LYS	A1179	104.073	174.926	108.374	1.00	0.00
ATOM	18155	CG	LYS	A1179	103.251	173.022	107.673	1.00	0.00
ATOM	18156	HG1	LYS	A1179	103.622	171.998	107.615	1.00	0.00
ATOM	18157	HG2	LYS	A1179	103.107	173.361	106.645	1.00	0.00
ATOM	18158	CD	LYS	A1179	101.892	173.003	108.388	1.00	0.00
ATOM	18159	HD1	LYS	A1179	102.054	172.925	109.462	1.00	0.00
ATOM	18160	HD2	LYS	A1179	101.350	172.112	108.074	1.00	0.00
ATOM	18161	CE	LYS	A1179	101.032	174.232	108.043	1.00	0.00
ATOM	18162	HE1	LYS	A1179	100.492	174.037	107.114	1.00	0.00
ATOM	18163	HE2	LYS	A1179	101.696	175.086	107.885	1.00	0.00
ATOM	18164	NZ	LYS	A1179	100.073	174.571	109.124	1.00	0.00
ATOM	18165	HZ1	LYS	A1179	99.347	175.211	108.776	1.00	0.00
ATOM	18166	HZ2	LYS	A1179	99.627	173.744	109.494	1.00	0.00
ATOM	18167	HZ3	LYS	A1179	100.579	175.040	109.866	1.00	0.00
ATOM	18168	C	LYS	A1179	106.399	173.275	109.723	1.00	0.00
ATOM	18169	O	LYS	A1179	107.070	174.293	109.859	1.00	0.00
ATOM	18170	N	GLU	A1180	106.988	172.101	109.511	1.00	0.00
ATOM	18171	H	GLU	A1180	106.377	171.279	109.463	1.00	0.00
ATOM	18172	CA	GLU	A1180	108.367	171.941	108.998	1.00	0.00
ATOM	18173	HA	GLU	A1180	108.510	170.863	108.914	1.00	0.00
ATOM	18174	CB	GLU	A1180	108.429	172.514	107.554	1.00	0.00
ATOM	18175	HB1	GLU	A1180	109.423	172.911	107.346	1.00	0.00
ATOM	18176	HB2	GLU	A1180	107.736	173.350	107.451	1.00	0.00

ATOM	18177	CG	GLU	A1180	108.094	171.464	106.488	1.00	0.00
ATOM	18178	HG1	GLU	A1180	107.865	171.974	105.550	1.00	0.00
ATOM	18179	HG2	GLU	A1180	107.215	170.888	106.785	1.00	0.00
ATOM	18180	CD	GLU	A1180	109.304	170.555	106.292	1.00	0.00
ATOM	18181	OE1	GLU	A1180	110.224	170.947	105.537	1.00	0.00
ATOM	18182	OE2	GLU	A1180	109.478	169.577	107.058	1.00	0.00
ATOM	18183	C	GLU	A1180	109.570	172.384	109.875	1.00	0.00
ATOM	18184	O	GLU	A1180	110.711	172.326	109.409	1.00	0.00
ATOM	18185	N	ALA	A1181	109.329	172.766	111.139	1.00	0.00
ATOM	18186	H	ALA	A1181	108.347	172.769	111.377	1.00	0.00
ATOM	18187	CA	ALA	A1181	110.191	173.431	112.146	1.00	0.00
ATOM	18188	HA	ALA	A1181	110.388	174.428	111.754	1.00	0.00
ATOM	18189	CB	ALA	A1181	109.314	173.614	113.400	1.00	0.00
ATOM	18190	HB1	ALA	A1181	109.804	174.294	114.097	1.00	0.00
ATOM	18191	HB2	ALA	A1181	108.344	174.034	113.140	1.00	0.00
ATOM	18192	HB3	ALA	A1181	109.159	172.651	113.887	1.00	0.00
ATOM	18193	C	ALA	A1181	111.589	172.856	112.515	1.00	0.00
ATOM	18194	O	ALA	A1181	111.961	172.816	113.694	1.00	0.00
ATOM	18195	N	LYS	A1182	112.391	172.417	111.542	1.00	0.00
ATOM	18196	H	LYS	A1182	112.014	172.479	110.604	1.00	0.00
ATOM	18197	CA	LYS	A1182	113.745	171.852	111.718	1.00	0.00
ATOM	18198	HA	LYS	A1182	113.649	171.037	112.430	1.00	0.00
ATOM	18199	CB	LYS	A1182	114.217	171.238	110.387	1.00	0.00
ATOM	18200	HB1	LYS	A1182	115.278	170.995	110.459	1.00	0.00
ATOM	18201	HB2	LYS	A1182	114.076	171.962	109.585	1.00	0.00
ATOM	18202	CG	LYS	A1182	113.431	169.945	110.095	1.00	0.00
ATOM	18203	HG1	LYS	A1182	112.360	170.138	110.154	1.00	0.00
ATOM	18204	HG2	LYS	A1182	113.670	169.216	110.871	1.00	0.00
ATOM	18205	CD	LYS	A1182	113.738	169.301	108.732	1.00	0.00
ATOM	18206	HD1	LYS	A1182	113.187	168.361	108.669	1.00	0.00
ATOM	18207	HD2	LYS	A1182	114.802	169.065	108.686	1.00	0.00
ATOM	18208	CE	LYS	A1182	113.369	170.164	107.518	1.00	0.00
ATOM	18209	HE1	LYS	A1182	113.569	169.588	106.609	1.00	0.00
ATOM	18210	HE2	LYS	A1182	113.999	171.056	107.497	1.00	0.00
ATOM	18211	NZ	LYS	A1182	111.947	170.569	107.526	1.00	0.00
ATOM	18212	HZ1	LYS	A1182	111.635	170.963	106.633	1.00	0.00
ATOM	18213	HZ2	LYS	A1182	111.283	169.794	107.586	1.00	0.00
ATOM	18214	HZ3	LYS	A1182	111.700	171.229	108.256	1.00	0.00
ATOM	18215	C	LYS	A1182	114.743	172.877	112.293	1.00	0.00
ATOM	18216	O	LYS	A1182	115.083	173.834	111.596	1.00	0.00
ATOM	18217	N	PRO	A1183	115.221	172.705	113.541	1.00	0.00
ATOM	18218	CD	PRO	A1183	114.961	171.584	114.439	1.00	0.00
ATOM	18219	HD1	PRO	A1183	115.218	170.636	113.968	1.00	0.00
ATOM	18220	HD2	PRO	A1183	113.914	171.582	114.735	1.00	0.00
ATOM	18221	CG	PRO	A1183	115.838	171.803	115.670	1.00	0.00
ATOM	18222	HG1	PRO	A1183	116.795	171.296	115.537	1.00	0.00
ATOM	18223	HG2	PRO	A1183	115.343	171.459	116.578	1.00	0.00
ATOM	18224	CB	PRO	A1183	116.051	173.315	115.671	1.00	0.00
ATOM	18225	HB1	PRO	A1183	116.948	173.597	116.222	1.00	0.00
ATOM	18226	HB2	PRO	A1183	115.177	173.802	116.102	1.00	0.00
ATOM	18227	CA	PRO	A1183	116.130	173.653	114.179	1.00	0.00
ATOM	18228	HA	PRO	A1183	115.783	174.674	114.019	1.00	0.00
ATOM	18229	C	PRO	A1183	117.563	173.535	113.637	1.00	0.00
ATOM	18230	O	PRO	A1183	117.907	172.613	112.899	1.00	0.00
ATOM	18231	N	ASN	A1184	118.409	174.465	114.068	1.00	0.00
ATOM	18232	H	ASN	A1184	118.030	175.154	114.702	1.00	0.00
ATOM	18233	CA	ASN	A1184	119.869	174.362	114.045	1.00	0.00
ATOM	18234	HA	ASN	A1184	120.136	173.310	114.164	1.00	0.00
ATOM	18235	CB	ASN	A1184	120.423	174.818	112.678	1.00	0.00
ATOM	18236	HB1	ASN	A1184	119.963	174.216	111.894	1.00	0.00
ATOM	18237	HB2	ASN	A1184	121.494	174.622	112.643	1.00	0.00

```
ATOM  18238  CG  ASN A1184     120.203 176.284 112.371  1.00  0.00
ATOM  18239  OD1 ASN A1184     120.974 177.132 112.777  1.00  0.00
ATOM  18240  ND2 ASN A1184     119.145 176.638 111.682  1.00  0.00
ATOM  18241 1HD2 ASN A1184     118.475 175.951 111.377  1.00  0.00
ATOM  18242 2HD2 ASN A1184     119.030 177.619 111.500  1.00  0.00
ATOM  18243  C   ASN A1184     120.400 175.077 115.312  1.00  0.00
ATOM  18244  O   ASN A1184     119.783 174.934 116.363  1.00  0.00
ATOM  18245  N   GLY A1185     121.489 175.845 115.243  1.00  0.00
ATOM  18246  H   GLY A1185     121.908 176.010 114.336  1.00  0.00
ATOM  18247  CA  GLY A1185     121.963 176.687 116.353  1.00  0.00
ATOM  18248  HA1 GLY A1185     123.048 176.762 116.298  1.00  0.00
ATOM  18249  HA2 GLY A1185     121.705 176.225 117.306  1.00  0.00
ATOM  18250  C   GLY A1185     121.400 178.121 116.362  1.00  0.00
ATOM  18251  O   GLY A1185     121.863 178.946 117.145  1.00  0.00
ATOM  18252  N   ILE A1186     120.418 178.429 115.502  1.00  0.00
ATOM  18253  H   ILE A1186     120.195 177.759 114.781  1.00  0.00
ATOM  18254  CA  ILE A1186     119.606 179.656 115.594  1.00  0.00
ATOM  18255  HA  ILE A1186     120.250 180.493 115.325  1.00  0.00
ATOM  18256  CB  ILE A1186     118.406 179.638 114.615  1.00  0.00
ATOM  18257  HB  ILE A1186     117.711 180.421 114.926  1.00  0.00
ATOM  18258  CG2 ILE A1186     118.889 180.031 113.207  1.00  0.00
ATOM  18259 1HG2 ILE A1186     118.041 180.105 112.527  1.00  0.00
ATOM  18260 2HG2 ILE A1186     119.366 181.011 113.249  1.00  0.00
ATOM  18261 3HG2 ILE A1186     119.606 179.304 112.834  1.00  0.00
ATOM  18262  CG1 ILE A1186     117.642 178.293 114.647  1.00  0.00
ATOM  18263 1HG1 ILE A1186     117.394 178.055 115.682  1.00  0.00
ATOM  18264 2HG1 ILE A1186     118.274 177.495 114.260  1.00  0.00
ATOM  18265  CD  ILE A1186     116.333 178.294 113.848  1.00  0.00
ATOM  18266  HD1 ILE A1186     115.809 177.353 114.020  1.00  0.00
ATOM  18267  HD2 ILE A1186     115.699 179.119 114.175  1.00  0.00
ATOM  18268  HD3 ILE A1186     116.536 178.388 112.783  1.00  0.00
ATOM  18269  C   ILE A1186     119.112 179.934 117.026  1.00  0.00
ATOM  18270  O   ILE A1186     118.777 179.022 117.780  1.00  0.00
ATOM  18271  N   PHE A1187     119.012 181.213 117.387  1.00  0.00
ATOM  18272  H   PHE A1187     119.252 181.939 116.727  1.00  0.00
ATOM  18273  CA  PHE A1187     118.619 181.635 118.728  1.00  0.00
ATOM  18274  HA  PHE A1187     118.132 180.806 119.238  1.00  0.00
ATOM  18275  CB  PHE A1187     119.858 181.997 119.566  1.00  0.00
ATOM  18276  HB1 PHE A1187     120.420 181.081 119.752  1.00  0.00
ATOM  18277  HB2 PHE A1187     119.525 182.360 120.537  1.00  0.00
ATOM  18278  CG  PHE A1187     120.821 183.021 118.975  1.00  0.00
ATOM  18279  CD1 PHE A1187     121.919 182.585 118.208  1.00  0.00
ATOM  18280  HD1 PHE A1187     122.065 181.526 118.027  1.00  0.00
ATOM  18281  CE1 PHE A1187     122.823 183.518 117.668  1.00  0.00
ATOM  18282  HE1 PHE A1187     123.654 183.173 117.067  1.00  0.00
ATOM  18283  CZ  PHE A1187     122.636 184.892 117.896  1.00  0.00
ATOM  18284  HZ  PHE A1187     123.321 185.613 117.469  1.00  0.00
ATOM  18285  CE2 PHE A1187     121.548 185.334 118.667  1.00  0.00
ATOM  18286  HE2 PHE A1187     121.392 186.391 118.833  1.00  0.00
ATOM  18287  CD2 PHE A1187     120.644 184.402 119.209  1.00  0.00
ATOM  18288  HD2 PHE A1187     119.794 184.754 119.773  1.00  0.00
ATOM  18289  C   PHE A1187     117.602 182.774 118.661  1.00  0.00
ATOM  18290  O   PHE A1187     117.885 183.876 118.200  1.00  0.00
ATOM  18291  N   LYS A1188     116.372 182.450 119.052  1.00  0.00
ATOM  18292  H   LYS A1188     116.226 181.500 119.362  1.00  0.00
ATOM  18293  CA  LYS A1188     115.191 183.294 118.882  1.00  0.00
ATOM  18294  HA  LYS A1188     115.407 184.297 119.250  1.00  0.00
ATOM  18295  CB  LYS A1188     114.859 183.366 117.376  1.00  0.00
ATOM  18296  HB1 LYS A1188     114.495 182.395 117.031  1.00  0.00
ATOM  18297  HB2 LYS A1188     115.762 183.587 116.805  1.00  0.00
ATOM  18298  CG  LYS A1188     113.836 184.455 117.031  1.00  0.00
```

ATOM	18299	HG1	LYS	A1188	114.284	185.428	117.228	1.00	0.00
ATOM	18300	HG2	LYS	A1188	112.933	184.344	117.630	1.00	0.00
ATOM	18301	CD	LYS	A1188	113.485	184.316	115.545	1.00	0.00
ATOM	18302	HD1	LYS	A1188	112.925	183.390	115.405	1.00	0.00
ATOM	18303	HD2	LYS	A1188	114.405	184.235	114.968	1.00	0.00
ATOM	18304	CE	LYS	A1188	112.639	185.470	114.998	1.00	0.00
ATOM	18305	HE1	LYS	A1188	111.745	185.573	115.621	1.00	0.00
ATOM	18306	HE2	LYS	A1188	112.314	185.187	113.993	1.00	0.00
ATOM	18307	NZ	LYS	A1188	113.398	186.745	114.950	1.00	0.00
ATOM	18308	HZ1	LYS	A1188	112.925	187.437	114.392	1.00	0.00
ATOM	18309	HZ2	LYS	A1188	113.507	187.130	115.892	1.00	0.00
ATOM	18310	HZ3	LYS	A1188	114.347	186.633	114.592	1.00	0.00
ATOM	18311	C	LYS	A1188	114.040	182.706	119.691	1.00	0.00
ATOM	18312	O	LYS	A1188	113.121	182.116	119.133	1.00	0.00
ATOM	18313	N	GLY	A1189	114.143	182.765	121.012	1.00	0.00
ATOM	18314	H	GLY	A1189	114.937	183.230	121.420	1.00	0.00
ATOM	18315	CA	GLY	A1189	113.048	182.363	121.891	1.00	0.00
ATOM	18316	HA1	GLY	A1189	113.389	182.319	122.923	1.00	0.00
ATOM	18317	HA2	GLY	A1189	112.667	181.389	121.587	1.00	0.00
ATOM	18318	C	GLY	A1189	111.894	183.352	121.797	1.00	0.00
ATOM	18319	O	GLY	A1189	111.675	184.121	122.721	1.00	0.00
ATOM	18320	N	SER	A1190	111.197	183.415	120.661	1.00	0.00
ATOM	18321	H	SER	A1190	111.397	182.731	119.936	1.00	0.00
ATOM	18322	CA	SER	A1190	110.147	184.400	120.421	1.00	0.00
ATOM	18323	HA	SER	A1190	109.954	184.441	119.349	1.00	0.00
ATOM	18324	CB	SER	A1190	108.886	183.837	121.085	1.00	0.00
ATOM	18325	HB1	SER	A1190	109.007	183.828	122.166	1.00	0.00
ATOM	18326	HB2	SER	A1190	108.789	182.801	120.780	1.00	0.00
ATOM	18327	OG	SER	A1190	107.726	184.539	120.706	1.00	0.00
ATOM	18328	HG	SER	A1190	107.027	183.835	120.637	1.00	0.00
ATOM	18329	C	SER	A1190	110.559	185.819	120.876	1.00	0.00
ATOM	18330	O	SER	A1190	111.684	186.235	120.598	1.00	0.00
ATOM	18331	N	THR	A1191	109.733	186.595	121.588	1.00	0.00
ATOM	18332	H	THR	A1191	108.779	186.281	121.709	1.00	0.00
ATOM	18333	CA	THR	A1191	110.250	187.660	122.492	1.00	0.00
ATOM	18334	HA	THR	A1191	111.184	187.279	122.907	1.00	0.00
ATOM	18335	CB	THR	A1191	110.594	188.979	121.759	1.00	0.00
ATOM	18336	HB	THR	A1191	109.791	189.687	121.903	1.00	0.00
ATOM	18337	CG2	THR	A1191	111.850	189.664	122.266	1.00	0.00
ATOM	18338	1HG2	THR	A1191	112.655	188.940	122.364	1.00	0.00
ATOM	18339	2HG2	THR	A1191	112.146	190.446	121.573	1.00	0.00
ATOM	18340	3HG2	THR	A1191	111.654	190.112	123.241	1.00	0.00
ATOM	18341	OG1	THR	A1191	110.809	188.843	120.371	1.00	0.00
ATOM	18342	HG1	THR	A1191	110.982	187.900	120.222	1.00	0.00
ATOM	18343	C	THR	A1191	109.407	187.977	123.762	1.00	0.00
ATOM	18344	O	THR	A1191	109.414	189.114	124.226	1.00	0.00
ATOM	18345	N	ALA	A1192	108.659	187.083	124.417	1.00	0.00
ATOM	18346	H	ALA	A1192	108.383	187.433	125.321	1.00	0.00
ATOM	18347	CA	ALA	A1192	107.807	186.003	123.935	1.00	0.00
ATOM	18348	HA	ALA	A1192	108.359	185.422	123.211	1.00	0.00
ATOM	18349	CB	ALA	A1192	107.486	185.037	125.081	1.00	0.00
ATOM	18350	HB1	ALA	A1192	106.611	185.387	125.623	1.00	0.00
ATOM	18351	HB2	ALA	A1192	107.273	184.048	124.675	1.00	0.00
ATOM	18352	HB3	ALA	A1192	108.338	184.969	125.752	1.00	0.00
ATOM	18353	C	ALA	A1192	106.508	186.524	123.284	1.00	0.00
ATOM	18354	O	ALA	A1192	106.200	187.720	123.345	1.00	0.00
ATOM	18355	N	GLU	A1193	105.746	185.611	122.679	1.00	0.00
ATOM	18356	H	GLU	A1193	106.039	184.645	122.715	1.00	0.00
ATOM	18357	CA	GLU	A1193	104.560	185.902	121.857	1.00	0.00
ATOM	18358	HA	GLU	A1193	104.417	185.047	121.189	1.00	0.00
ATOM	18359	CB	GLU	A1193	103.316	185.984	122.756	1.00	0.00

```
ATOM  18360  HB1 GLU A1193     103.393 186.873 123.379  1.00  0.00
ATOM  18361  HB2 GLU A1193     103.311 185.121 123.425  1.00  0.00
ATOM  18362  CG  GLU A1193     101.983 186.026 121.980  1.00  0.00
ATOM  18363  HG1 GLU A1193     102.174 186.063 120.906  1.00  0.00
ATOM  18364  HG2 GLU A1193     101.441 186.934 122.243  1.00  0.00
ATOM  18365  CD  GLU A1193     101.100 184.812 122.286  1.00  0.00
ATOM  18366  OE1 GLU A1193     101.238 183.800 121.572  1.00  0.00
ATOM  18367  OE2 GLU A1193     100.300 184.871 123.250  1.00  0.00
ATOM  18368  C   GLU A1193     104.771 187.134 120.936  1.00  0.00
ATOM  18369  O   GLU A1193     103.958 188.058 120.896  1.00  0.00
ATOM  18370  N   ASN A1194     105.952 187.180 120.307  1.00  0.00
ATOM  18371  H   ASN A1194     106.466 186.310 120.359  1.00  0.00
ATOM  18372  CA  ASN A1194     106.720 188.349 119.837  1.00  0.00
ATOM  18373  HA  ASN A1194     107.751 187.998 119.832  1.00  0.00
ATOM  18374  CB  ASN A1194     106.405 188.690 118.370  1.00  0.00
ATOM  18375  HB1 ASN A1194     105.392 189.086 118.315  1.00  0.00
ATOM  18376  HB2 ASN A1194     106.445 187.784 117.767  1.00  0.00
ATOM  18377  CG  ASN A1194     107.379 189.704 117.760  1.00  0.00
ATOM  18378  OD1 ASN A1194     108.552 189.807 118.105  1.00  0.00
ATOM  18379  ND2 ASN A1194     106.931 190.511 116.836  1.00  0.00
ATOM  18380 1HD2 ASN A1194     105.955 190.404 116.523  1.00  0.00
ATOM  18381 2HD2 ASN A1194     107.563 191.120 116.366  1.00  0.00
ATOM  18382  C   ASN A1194     106.782 189.609 120.730  1.00  0.00
ATOM  18383  O   ASN A1194     107.861 190.180 120.864  1.00  0.00
ATOM  18384  N   ALA A1195     105.708 190.102 121.332  1.00  0.00
ATOM  18385  H   ALA A1195     104.828 189.611 121.182  1.00  0.00
ATOM  18386  CA  ALA A1195     105.737 191.298 122.186  1.00  0.00
ATOM  18387  HA  ALA A1195     106.574 191.209 122.882  1.00  0.00
ATOM  18388  CB  ALA A1195     105.947 192.542 121.292  1.00  0.00
ATOM  18389  HB1 ALA A1195     105.893 193.459 121.875  1.00  0.00
ATOM  18390  HB2 ALA A1195     106.924 192.523 120.818  1.00  0.00
ATOM  18391  HB3 ALA A1195     105.191 192.583 120.511  1.00  0.00
ATOM  18392  C   ALA A1195     104.452 191.414 123.037  1.00  0.00
ATOM  18393  O   ALA A1195     103.932 192.516 123.215  1.00  0.00
ATOM  18394  N   GLU A1196     103.916 190.293 123.539  1.00  0.00
ATOM  18395  H   GLU A1196     104.323 189.402 123.270  1.00  0.00
ATOM  18396  CA  GLU A1196     102.901 190.282 124.617  1.00  0.00
ATOM  18397  HA  GLU A1196     102.307 191.193 124.556  1.00  0.00
ATOM  18398  CB  GLU A1196     101.951 189.082 124.419  1.00  0.00
ATOM  18399  HB1 GLU A1196     102.397 188.209 124.894  1.00  0.00
ATOM  18400  HB2 GLU A1196     101.870 188.873 123.354  1.00  0.00
ATOM  18401  CG  GLU A1196     100.517 189.247 124.964  1.00  0.00
ATOM  18402  HG1 GLU A1196     100.545 189.560 126.009  1.00  0.00
ATOM  18403  HG2 GLU A1196     100.017 188.277 124.921  1.00  0.00
ATOM  18404  CD  GLU A1196      99.706 190.258 124.137  1.00  0.00
ATOM  18405  OE1 GLU A1196      99.208 189.904 123.051  1.00  0.00
ATOM  18406  OE2 GLU A1196      99.701 191.458 124.494  1.00  0.00
ATOM  18407  C   GLU A1196     103.543 190.281 126.034  1.00  0.00
ATOM  18408  O   GLU A1196     102.860 190.133 127.046  1.00  0.00
ATOM  18409  N   TYR A1197     104.866 190.435 126.125  1.00  0.00
ATOM  18410  H   TYR A1197     105.341 190.681 125.274  1.00  0.00
ATOM  18411  CA  TYR A1197     105.707 189.777 127.136  1.00  0.00
ATOM  18412  HA  TYR A1197     105.059 189.229 127.821  1.00  0.00
ATOM  18413  CB  TYR A1197     106.512 188.741 126.356  1.00  0.00
ATOM  18414  HB1 TYR A1197     107.090 189.264 125.593  1.00  0.00
ATOM  18415  HB2 TYR A1197     105.804 188.090 125.843  1.00  0.00
ATOM  18416  CG  TYR A1197     107.423 187.862 127.166  1.00  0.00
ATOM  18417  CD1 TYR A1197     106.927 186.723 127.813  1.00  0.00
ATOM  18418  HD1 TYR A1197     105.872 186.505 127.758  1.00  0.00
ATOM  18419  CE1 TYR A1197     107.801 185.871 128.512  1.00  0.00
ATOM  18420  HE1 TYR A1197     107.417 185.013 129.039  1.00  0.00
```

ATOM	18421	CZ	TYR	A1197	109.180	186.167	128.572	1.00	0.00
ATOM	18422	OH	TYR	A1197	110.006	185.491	129.412	1.00	0.00
ATOM	18423	HH	TYR	A1197	110.886	185.902	129.453	1.00	0.00
ATOM	18424	CE2	TYR	A1197	109.674	187.269	127.843	1.00	0.00
ATOM	18425	HE2	TYR	A1197	110.717	187.482	127.816	1.00	0.00
ATOM	18426	CD2	TYR	A1197	108.798	188.144	127.194	1.00	0.00
ATOM	18427	HD2	TYR	A1197	109.208	189.016	126.696	1.00	0.00
ATOM	18428	C	TYR	A1197	106.581	190.705	128.014	1.00	0.00
ATOM	18429	O	TYR	A1197	106.632	191.903	127.814	1.00	0.00
ATOM	18430	N	LEU	A1198	107.229	190.203	129.068	1.00	0.00
ATOM	18431	H	LEU	A1198	107.402	189.209	129.070	1.00	0.00
ATOM	18432	CA	LEU	A1198	107.042	190.801	130.402	1.00	0.00
ATOM	18433	HA	LEU	A1198	106.101	191.281	130.286	1.00	0.00
ATOM	18434	CB	LEU	A1198	106.847	189.699	131.457	1.00	0.00
ATOM	18435	HB1	LEU	A1198	106.136	188.972	131.062	1.00	0.00
ATOM	18436	HB2	LEU	A1198	106.393	190.153	132.335	1.00	0.00
ATOM	18437	CG	LEU	A1198	108.129	188.984	131.918	1.00	0.00
ATOM	18438	HG	LEU	A1198	108.811	189.671	132.408	1.00	0.00
ATOM	18439	CD1	LEU	A1198	107.714	187.919	132.918	1.00	0.00
ATOM	18440	1HD1	LEU	A1198	108.506	187.192	133.032	1.00	0.00
ATOM	18441	2HD1	LEU	A1198	107.526	188.418	133.858	1.00	0.00
ATOM	18442	3HD1	LEU	A1198	106.810	187.414	132.582	1.00	0.00
ATOM	18443	CD2	LEU	A1198	108.838	188.317	130.766	1.00	0.00
ATOM	18444	1HD2	LEU	A1198	109.351	189.056	130.153	1.00	0.00
ATOM	18445	2HD2	LEU	A1198	109.582	187.607	131.123	1.00	0.00
ATOM	18446	3HD2	LEU	A1198	108.089	187.784	130.197	1.00	0.00
ATOM	18447	C	LEU	A1198	107.883	191.975	130.914	1.00	0.00
ATOM	18448	O	LEU	A1198	108.177	192.047	132.109	1.00	0.00
ATOM	18449	N	ARG	A1199	108.245	192.937	130.072	1.00	0.00
ATOM	18450	H	ARG	A1199	107.908	192.887	129.115	1.00	0.00
ATOM	18451	CA	ARG	A1199	109.041	194.095	130.510	1.00	0.00
ATOM	18452	HA	ARG	A1199	109.197	194.051	131.589	1.00	0.00
ATOM	18453	CB	ARG	A1199	110.429	194.015	129.835	1.00	0.00
ATOM	18454	HB1	ARG	A1199	110.771	195.028	129.615	1.00	0.00
ATOM	18455	HB2	ARG	A1199	110.362	193.482	128.885	1.00	0.00
ATOM	18456	CG	ARG	A1199	111.500	193.360	130.728	1.00	0.00
ATOM	18457	HG1	ARG	A1199	111.570	193.953	131.641	1.00	0.00
ATOM	18458	HG2	ARG	A1199	112.445	193.427	130.201	1.00	0.00
ATOM	18459	CD	ARG	A1199	111.360	191.881	131.113	1.00	0.00
ATOM	18460	HD1	ARG	A1199	111.780	191.270	130.310	1.00	0.00
ATOM	18461	HD2	ARG	A1199	110.327	191.592	131.210	1.00	0.00
ATOM	18462	NE	ARG	A1199	112.085	191.589	132.365	1.00	0.00
ATOM	18463	HE	ARG	A1199	113.093	191.511	132.286	1.00	0.00
ATOM	18464	CZ	ARG	A1199	111.571	191.355	133.563	1.00	0.00
ATOM	18465	NH1	ARG	A1199	110.307	191.529	133.814	1.00	0.00
ATOM	18466	1HH1	ARG	A1199	109.681	191.754	133.052	1.00	0.00
ATOM	18467	2HH1	ARG	A1199	109.878	191.439	134.727	1.00	0.00
ATOM	18468	NH2	ARG	A1199	112.329	190.949	134.543	1.00	0.00
ATOM	18469	1HH2	ARG	A1199	113.343	190.979	134.427	1.00	0.00
ATOM	18470	2HH2	ARG	A1199	111.900	190.510	135.338	1.00	0.00
ATOM	18471	C	ARG	A1199	108.305	195.420	130.286	1.00	0.00
ATOM	18472	O	ARG	A1199	107.393	195.543	129.464	1.00	0.00
ATOM	18473	N	VAL	A1200	108.716	196.425	131.053	1.00	0.00
ATOM	18474	H	VAL	A1200	109.530	196.263	131.624	1.00	0.00
ATOM	18475	CA	VAL	A1200	108.134	197.779	131.075	1.00	0.00
ATOM	18476	HA	VAL	A1200	107.591	197.920	130.142	1.00	0.00
ATOM	18477	CB	VAL	A1200	107.131	197.937	132.245	1.00	0.00
ATOM	18478	HB	VAL	A1200	106.967	196.945	132.647	1.00	0.00
ATOM	18479	CG1	VAL	A1200	107.538	198.786	133.454	1.00	0.00
ATOM	18480	1HG1	VAL	A1200	106.815	198.632	134.256	1.00	0.00
ATOM	18481	2HG1	VAL	A1200	108.505	198.457	133.813	1.00	0.00

```
ATOM  18482 3HG1 VAL A1200     107.559 199.846 133.209  1.00  0.00
ATOM  18483  CG2 VAL A1200     105.799 198.497 131.737  1.00  0.00
ATOM  18484 1HG2 VAL A1200     105.366 197.797 131.026  1.00  0.00
ATOM  18485 2HG2 VAL A1200     105.097 198.610 132.564  1.00  0.00
ATOM  18486 3HG2 VAL A1200     105.934 199.465 131.263  1.00  0.00
ATOM  18487  C   VAL A1200     109.265 198.815 131.091  1.00  0.00
ATOM  18488  O   VAL A1200     110.429 198.451 131.174  1.00  0.00
ATOM  18489  N   ALA A1201     108.880 200.088 130.963  1.00  0.00
ATOM  18490  H   ALA A1201     107.881 200.206 130.887  1.00  0.00
ATOM  18491  CA  ALA A1201     109.638 201.342 130.828  1.00  0.00
ATOM  18492  HA  ALA A1201     109.445 201.960 131.705  1.00  0.00
ATOM  18493  CB  ALA A1201     111.167 201.179 130.728  1.00  0.00
ATOM  18494  HB1 ALA A1201     111.622 202.156 130.556  1.00  0.00
ATOM  18495  HB2 ALA A1201     111.568 200.781 131.653  1.00  0.00
ATOM  18496  HB3 ALA A1201     111.431 200.528 129.891  1.00  0.00
ATOM  18497  C   ALA A1201     109.065 202.091 129.594  1.00  0.00
ATOM  18498  O   ALA A1201     109.722 202.220 128.561  1.00  0.00
ATOM  18499  N   PRO A1202     107.762 202.430 129.614  1.00  0.00
ATOM  18500  CD  PRO A1202     106.889 202.530 130.779  1.00  0.00
ATOM  18501  HD1 PRO A1202     107.320 203.191 131.531  1.00  0.00
ATOM  18502  HD2 PRO A1202     106.706 201.548 131.204  1.00  0.00
ATOM  18503  CG  PRO A1202     105.568 203.108 130.274  1.00  0.00
ATOM  18504  HG1 PRO A1202     105.573 204.191 130.398  1.00  0.00
ATOM  18505  HG2 PRO A1202     104.712 202.670 130.788  1.00  0.00
ATOM  18506  CB  PRO A1202     105.576 202.751 128.786  1.00  0.00
ATOM  18507  HB1 PRO A1202     104.962 203.431 128.197  1.00  0.00
ATOM  18508  HB2 PRO A1202     105.206 201.738 128.668  1.00  0.00
ATOM  18509  CA  PRO A1202     107.056 202.802 128.399  1.00  0.00
ATOM  18510  HA  PRO A1202     107.248 202.075 127.610  1.00  0.00
ATOM  18511  C   PRO A1202     107.483 204.203 127.907  1.00  0.00
ATOM  18512  O   PRO A1202     107.300 205.178 128.631  1.00  0.00
ATOM  18513  N   GLN A1203     108.040 204.294 126.695  1.00  0.00
ATOM  18514  H   GLN A1203     108.214 203.429 126.207  1.00  0.00
ATOM  18515  CA  GLN A1203     108.766 205.488 126.218  1.00  0.00
ATOM  18516  HA  GLN A1203     108.499 206.322 126.869  1.00  0.00
ATOM  18517  CB  GLN A1203     110.288 205.246 126.442  1.00  0.00
ATOM  18518  HB1 GLN A1203     110.464 204.171 126.412  1.00  0.00
ATOM  18519  HB2 GLN A1203     110.534 205.551 127.460  1.00  0.00
ATOM  18520  CG  GLN A1203     111.352 205.849 125.492  1.00  0.00
ATOM  18521  HG1 GLN A1203     111.073 205.642 124.461  1.00  0.00
ATOM  18522  HG2 GLN A1203     112.290 205.328 125.667  1.00  0.00
ATOM  18523  CD  GLN A1203     111.644 207.342 125.655  1.00  0.00
ATOM  18524  OE1 GLN A1203     112.209 207.809 126.636  1.00  0.00
ATOM  18525  NE2 GLN A1203     111.302 208.136 124.669  1.00  0.00
ATOM  18526 1HE2 GLN A1203     110.589 207.760 124.053  1.00  0.00
ATOM  18527 2HE2 GLN A1203     111.210 209.099 124.943  1.00  0.00
ATOM  18528  C   GLN A1203     108.367 206.016 124.811  1.00  0.00
ATOM  18529  O   GLN A1203     108.814 207.090 124.436  1.00  0.00
ATOM  18530  N   SER A1204     107.508 205.289 124.080  1.00  0.00
ATOM  18531  H   SER A1204     107.146 204.474 124.547  1.00  0.00
ATOM  18532  CA  SER A1204     106.931 205.546 122.743  1.00  0.00
ATOM  18533  HA  SER A1204     105.896 205.849 122.913  1.00  0.00
ATOM  18534  CB  SER A1204     107.573 206.677 121.920  1.00  0.00
ATOM  18535  HB1 SER A1204     107.558 207.597 122.492  1.00  0.00
ATOM  18536  HB2 SER A1204     106.979 206.825 121.017  1.00  0.00
ATOM  18537  OG  SER A1204     108.913 206.450 121.529  1.00  0.00
ATOM  18538  HG  SER A1204     109.003 205.502 121.348  1.00  0.00
ATOM  18539  C   SER A1204     106.905 204.282 121.913  1.00  0.00
ATOM  18540  O   SER A1204     107.958 203.831 121.486  1.00  0.00
ATOM  18541  N   SER A1205     105.731 203.714 121.665  1.00  0.00
ATOM  18542  H   SER A1205     104.918 204.104 122.141  1.00  0.00
```

```
ATOM  18543  CA  SER A1205     105.557 202.421 120.982  1.00  0.00
ATOM  18544  HA  SER A1205     106.167 201.692 121.516  1.00  0.00
ATOM  18545  CB  SER A1205     104.112 201.940 121.116  1.00  0.00
ATOM  18546  HB1 SER A1205     104.002 200.974 120.619  1.00  0.00
ATOM  18547  HB2 SER A1205     103.432 202.659 120.657  1.00  0.00
ATOM  18548  OG  SER A1205     103.815 201.791 122.493  1.00  0.00
ATOM  18549  HG  SER A1205     103.780 202.689 122.902  1.00  0.00
ATOM  18550  C   SER A1205     106.061 202.373 119.537  1.00  0.00
ATOM  18551  O   SER A1205     106.231 201.280 119.010  1.00  0.00
ATOM  18552  N   GLU A1206     106.380 203.510 118.900  1.00  0.00
ATOM  18553  H   GLU A1206     106.229 204.380 119.380  1.00  0.00
ATOM  18554  CA  GLU A1206     107.199 203.535 117.667  1.00  0.00
ATOM  18555  HA  GLU A1206     106.895 202.687 117.059  1.00  0.00
ATOM  18556  CB  GLU A1206     106.915 204.805 116.841  1.00  0.00
ATOM  18557  HB1 GLU A1206     107.539 205.623 117.207  1.00  0.00
ATOM  18558  HB2 GLU A1206     105.868 205.091 116.969  1.00  0.00
ATOM  18559  CG  GLU A1206     107.156 204.574 115.334  1.00  0.00
ATOM  18560  HG1 GLU A1206     106.478 203.789 114.992  1.00  0.00
ATOM  18561  HG2 GLU A1206     108.171 204.227 115.154  1.00  0.00
ATOM  18562  CD  GLU A1206     106.931 205.849 114.523  1.00  0.00
ATOM  18563  OE1 GLU A1206     105.940 205.920 113.769  1.00  0.00
ATOM  18564  OE2 GLU A1206     107.678 206.837 114.705  1.00  0.00
ATOM  18565  C   GLU A1206     108.715 203.331 117.926  1.00  0.00
ATOM  18566  O   GLU A1206     109.538 203.460 117.028  1.00  0.00
ATOM  18567  N   PHE A1207     109.094 203.029 119.169  1.00  0.00
ATOM  18568  H   PHE A1207     108.349 202.980 119.852  1.00  0.00
ATOM  18569  CA  PHE A1207     110.434 202.856 119.739  1.00  0.00
ATOM  18570  HA  PHE A1207     110.286 202.586 120.780  1.00  0.00
ATOM  18571  CB  PHE A1207     111.110 201.646 119.055  1.00  0.00
ATOM  18572  HB1 PHE A1207     111.354 201.899 118.022  1.00  0.00
ATOM  18573  HB2 PHE A1207     110.413 200.807 119.043  1.00  0.00
ATOM  18574  CG  PHE A1207     112.362 201.215 119.776  1.00  0.00
ATOM  18575  CD1 PHE A1207     112.239 200.789 121.101  1.00  0.00
ATOM  18576  HD1 PHE A1207     111.264 200.649 121.522  1.00  0.00
ATOM  18577  CE1 PHE A1207     113.372 200.617 121.901  1.00  0.00
ATOM  18578  HE1 PHE A1207     113.229 200.381 122.936  1.00  0.00
ATOM  18579  CZ  PHE A1207     114.650 200.740 121.339  1.00  0.00
ATOM  18580  HZ  PHE A1207     115.531 200.592 121.950  1.00  0.00
ATOM  18581  CE2 PHE A1207     114.777 201.057 119.982  1.00  0.00
ATOM  18582  HE2 PHE A1207     115.764 201.113 119.563  1.00  0.00
ATOM  18583  CD2 PHE A1207     113.639 201.334 119.199  1.00  0.00
ATOM  18584  HD2 PHE A1207     113.739 201.651 118.170  1.00  0.00
ATOM  18585  C   PHE A1207     111.371 204.079 119.738  1.00  0.00
ATOM  18586  O   PHE A1207     112.174 204.270 120.653  1.00  0.00
ATOM  18587  N   ILE A1208     111.265 204.934 118.726  1.00  0.00
ATOM  18588  H   ILE A1208     110.597 204.694 118.002  1.00  0.00
ATOM  18589  CA  ILE A1208     112.188 206.037 118.459  1.00  0.00
ATOM  18590  HA  ILE A1208     113.199 205.626 118.468  1.00  0.00
ATOM  18591  CB  ILE A1208     111.928 206.614 117.043  1.00  0.00
ATOM  18592  HB  ILE A1208     110.895 206.961 116.990  1.00  0.00
ATOM  18593  CG2 ILE A1208     112.859 207.804 116.754  1.00  0.00
ATOM  18594 1HG2 ILE A1208     112.673 208.203 115.759  1.00  0.00
ATOM  18595 2HG2 ILE A1208     112.648 208.611 117.449  1.00  0.00
ATOM  18596 3HG2 ILE A1208     113.901 207.497 116.833  1.00  0.00
ATOM  18597  CG1 ILE A1208     112.141 205.541 115.948  1.00  0.00
ATOM  18598 1HG1 ILE A1208     111.589 204.648 116.221  1.00  0.00
ATOM  18599 2HG1 ILE A1208     113.199 205.279 115.904  1.00  0.00
ATOM  18600  CD  ILE A1208     111.658 205.938 114.550  1.00  0.00
ATOM  18601  HD1 ILE A1208     111.736 205.080 113.884  1.00  0.00
ATOM  18602  HD2 ILE A1208     110.615 206.251 114.591  1.00  0.00
ATOM  18603  HD3 ILE A1208     112.264 206.744 114.138  1.00  0.00
```

```
ATOM  18604  C   ILE A1208     112.121 207.115 119.550  1.00  0.00
ATOM  18605  O   ILE A1208     111.193 207.930 119.599  1.00  0.00
ATOM  18606  N   GLY A1209     113.158 207.215 120.382  1.00  0.00
ATOM  18607  H   GLY A1209     113.821 206.447 120.407  1.00  0.00
ATOM  18608  CA  GLY A1209     113.314 208.364 121.285  1.00  0.00
ATOM  18609  HA1 GLY A1209     114.153 208.174 121.954  1.00  0.00
ATOM  18610  HA2 GLY A1209     112.419 208.476 121.897  1.00  0.00
ATOM  18611  C   GLY A1209     113.583 209.686 120.555  1.00  0.00
ATOM  18612  O   GLY A1209     113.113 210.731 120.996  1.00  0.00
ATOM  18613  N   ALA A1210     114.282 209.640 119.411  1.00  0.00
ATOM  18614  H   ALA A1210     114.595 208.737 119.091  1.00  0.00
ATOM  18615  CA  ALA A1210     114.619 210.804 118.580  1.00  0.00
ATOM  18616  HA  ALA A1210     115.264 211.452 119.177  1.00  0.00
ATOM  18617  CB  ALA A1210     115.426 210.352 117.358  1.00  0.00
ATOM  18618  HB1 ALA A1210     115.798 211.227 116.826  1.00  0.00
ATOM  18619  HB2 ALA A1210     116.273 209.740 117.666  1.00  0.00
ATOM  18620  HB3 ALA A1210     114.797 209.782 116.676  1.00  0.00
ATOM  18621  C   ALA A1210     113.393 211.659 118.199  1.00  0.00
TER
END
```
