# Supplementary material for: Structural analysis of hubs in human NR-RTK network
Source: Biol Direct. 2011 Oct 5;6:49. doi: 10.1186/1745-6150-6-49 (PMC3220635; doi:10.1186/1745-6150-6-49)
Supplement: Additional file 2 — ESR1. ESR1 structure. [file 1745-6150-6-49-S2.PDF]

```
HEADER ESR1
REMARK PARENT 3dzy_A 3dzy 3dzu_A 1qku_A
ATOM      1  N   PRO A   1      84.152  76.466  45.930  1.00  0.00
ATOM      2  CD  PRO A   1      83.008  76.200  45.078  1.00  0.00
ATOM      3  HD1 PRO A   1      83.004  75.163  44.739  1.00  0.00
ATOM      4  HD2 PRO A   1      83.031  76.875  44.222  1.00  0.00
ATOM      5  CG  PRO A   1      81.776  76.487  45.938  1.00  0.00
ATOM      6  HG1 PRO A   1      81.478  75.581  46.466  1.00  0.00
ATOM      7  HG2 PRO A   1      80.949  76.875  45.342  1.00  0.00
ATOM      8  CB  PRO A   1      82.293  77.524  46.938  1.00  0.00
ATOM      9  HB1 PRO A   1      81.692  77.544  47.848  1.00  0.00
ATOM     10  HB2 PRO A   1      82.304  78.509  46.469  1.00  0.00
ATOM     11  CA  PRO A   1      83.733  77.065  47.194  1.00  0.00
ATOM     12  HA  PRO A   1      84.357  77.929  47.419  1.00  0.00
ATOM     13  C   PRO A   1      83.804  76.091  48.382  1.00  0.00
ATOM     14  O   PRO A   1      83.636  76.501  49.532  1.00  0.00
ATOM     15  N   ASN A   2      84.067  74.805  48.120  1.00  0.00
ATOM     16  H   ASN A   2      84.183  74.555  47.147  1.00  0.00
ATOM     17  CA  ASN A   2      83.983  73.705  49.089  1.00  0.00
ATOM     18  HA  ASN A   2      82.965  73.709  49.486  1.00  0.00
ATOM     19  CB  ASN A   2      84.180  72.373  48.355  1.00  0.00
ATOM     20  HB1 ASN A   2      84.248  71.564  49.083  1.00  0.00
ATOM     21  HB2 ASN A   2      85.097  72.391  47.766  1.00  0.00
ATOM     22  CG  ASN A   2      83.004  72.096  47.442  1.00  0.00
ATOM     23  OD1 ASN A   2      83.025  72.402  46.265  1.00  0.00
ATOM     24  ND2 ASN A   2      81.927  71.571  47.976  1.00  0.00
ATOM     25 1HD2 ASN A   2      81.879  71.418  48.982  1.00  0.00
ATOM     26 2HD2 ASN A   2      81.144  71.416  47.374  1.00  0.00
ATOM     27  C   ASN A   2      84.880  73.829  50.335  1.00  0.00
ATOM     28  O   ASN A   2      84.693  73.093  51.304  1.00  0.00
ATOM     29  N   SER A   3      85.796  74.794  50.369  1.00  0.00
ATOM     30  H   SER A   3      85.931  75.348  49.537  1.00  0.00
ATOM     31  CA  SER A   3      86.520  75.203  51.575  1.00  0.00
ATOM     32  HA  SER A   3      87.118  74.359  51.919  1.00  0.00
ATOM     33  CB  SER A   3      87.469  76.358  51.247  1.00  0.00
ATOM     34  HB1 SER A   3      88.085  76.579  52.120  1.00  0.00
ATOM     35  HB2 SER A   3      86.887  77.243  50.986  1.00  0.00
ATOM     36  OG  SER A   3      88.302  76.008  50.157  1.00  0.00
ATOM     37  HG  SER A   3      88.907  76.732  49.976  1.00  0.00
ATOM     38  C   SER A   3      85.611  75.629  52.737  1.00  0.00
ATOM     39  O   SER A   3      85.989  75.398  53.889  1.00  0.00
ATOM     40  N   ASP A   4      84.408  76.175  52.469  1.00  0.00
ATOM     41  H   ASP A   4      84.149  76.338  51.501  1.00  0.00
ATOM     42  CA  ASP A   4      83.364  76.369  53.498  1.00  0.00
ATOM     43  HA  ASP A   4      83.641  77.186  54.165  1.00  0.00
ATOM     44  CB  ASP A   4      82.001  76.710  52.852  1.00  0.00
ATOM     45  HB1 ASP A   4      81.873  76.103  51.954  1.00  0.00
ATOM     46  HB2 ASP A   4      82.004  77.758  52.548  1.00  0.00
ATOM     47  CG  ASP A   4      80.814  76.436  53.801  1.00  0.00
ATOM     48  OD1 ASP A   4      80.460  77.335  54.593  1.00  0.00
ATOM     49  OD2 ASP A   4      80.306  75.287  53.770  1.00  0.00
ATOM     50  C   ASP A   4      83.220  75.100  54.345  1.00  0.00
ATOM     51  O   ASP A   4      83.343  75.121  55.570  1.00  0.00
ATOM     52  N   ASN A   5      83.077  73.962  53.669  1.00  0.00
ATOM     53  H   ASN A   5      83.102  74.010  52.659  1.00  0.00
ATOM     54  CA  ASN A   5      82.702  72.696  54.273  1.00  0.00
ATOM     55  HA  ASN A   5      81.810  72.864  54.880  1.00  0.00
ATOM     56  CB  ASN A   5      82.370  71.677  53.159  1.00  0.00
ATOM     57  HB1 ASN A   5      81.795  70.861  53.594  1.00  0.00
ATOM     58  HB2 ASN A   5      83.303  71.262  52.780  1.00  0.00
ATOM     59  CG  ASN A   5      81.598  72.173  51.932  1.00  0.00
```

ATOM	60	OD1	ASN	A	5	81.776	71.670	50.835	1.00	0.00
ATOM	61	ND2	ASN	A	5	80.721	73.145	52.029	1.00	0.00
ATOM	62	1HD2	ASN	A	5	80.594	73.705	52.880	1.00	0.00
ATOM	63	2HD2	ASN	A	5	80.234	73.407	51.195	1.00	0.00
ATOM	64	C	ASN	A	5	83.805	72.123	55.203	1.00	0.00
ATOM	65	O	ASN	A	5	83.565	71.146	55.912	1.00	0.00
ATOM	66	N	ARG	A	6	85.017	72.712	55.200	1.00	0.00
ATOM	67	H	ARG	A	6	85.129	73.507	54.575	1.00	0.00
ATOM	68	CA	ARG	A	6	86.195	72.211	55.933	1.00	0.00
ATOM	69	HA	ARG	A	6	85.835	71.499	56.651	1.00	0.00
ATOM	70	CB	ARG	A	6	87.115	71.462	54.943	1.00	0.00
ATOM	71	HB1	ARG	A	6	87.688	72.204	54.386	1.00	0.00
ATOM	72	HB2	ARG	A	6	86.488	70.932	54.223	1.00	0.00
ATOM	73	CG	ARG	A	6	88.093	70.431	55.542	1.00	0.00
ATOM	74	HG1	ARG	A	6	88.783	70.911	56.236	1.00	0.00
ATOM	75	HG2	ARG	A	6	88.692	70.054	54.713	1.00	0.00
ATOM	76	CD	ARG	A	6	87.440	69.204	56.217	1.00	0.00
ATOM	77	HD1	ARG	A	6	88.084	68.344	56.030	1.00	0.00
ATOM	78	HD2	ARG	A	6	86.481	68.990	55.742	1.00	0.00
ATOM	79	NE	ARG	A	6	87.305	69.356	57.682	1.00	0.00
ATOM	80	HE	ARG	A	6	88.156	69.335	58.220	1.00	0.00
ATOM	81	CZ	ARG	A	6	86.198	69.557	58.370	1.00	0.00
ATOM	82	NH1	ARG	A	6	85.006	69.578	57.839	1.00	0.00
ATOM	83	1HH1	ARG	A	6	84.866	69.570	56.842	1.00	0.00
ATOM	84	2HH1	ARG	A	6	84.233	69.898	58.410	1.00	0.00
ATOM	85	NH2	ARG	A	6	86.230	69.781	59.651	1.00	0.00
ATOM	86	1HH2	ARG	A	6	87.092	69.743	60.155	1.00	0.00
ATOM	87	2HH2	ARG	A	6	85.386	70.132	60.091	1.00	0.00
ATOM	88	C	ARG	A	6	86.933	73.236	56.805	1.00	0.00
ATOM	89	O	ARG	A	6	87.792	72.840	57.595	1.00	0.00
ATOM	90	N	ARG	A	7	86.523	74.512	56.767	1.00	0.00
ATOM	91	H	ARG	A	7	85.884	74.766	56.022	1.00	0.00
ATOM	92	CA	ARG	A	7	86.849	75.527	57.796	1.00	0.00
ATOM	93	HA	ARG	A	7	87.524	75.058	58.508	1.00	0.00
ATOM	94	CB	ARG	A	7	87.567	76.722	57.152	1.00	0.00
ATOM	95	HB1	ARG	A	7	86.879	77.235	56.477	1.00	0.00
ATOM	96	HB2	ARG	A	7	88.404	76.358	56.556	1.00	0.00
ATOM	97	CG	ARG	A	7	88.110	77.753	58.165	1.00	0.00
ATOM	98	HG1	ARG	A	7	87.279	78.176	58.733	1.00	0.00
ATOM	99	HG2	ARG	A	7	88.584	78.563	57.610	1.00	0.00
ATOM	100	CD	ARG	A	7	89.140	77.153	59.138	1.00	0.00
ATOM	101	HD1	ARG	A	7	89.970	76.748	58.555	1.00	0.00
ATOM	102	HD2	ARG	A	7	88.678	76.343	59.703	1.00	0.00
ATOM	103	NE	ARG	A	7	89.629	78.158	60.102	1.00	0.00
ATOM	104	HE	ARG	A	7	89.005	78.926	60.305	1.00	0.00
ATOM	105	CZ	ARG	A	7	90.755	78.128	60.791	1.00	0.00
ATOM	106	NH1	ARG	A	7	91.641	77.177	60.644	1.00	0.00
ATOM	107	1HH1	ARG	A	7	91.457	76.454	59.971	1.00	0.00
ATOM	108	2HH1	ARG	A	7	92.490	77.160	61.172	1.00	0.00
ATOM	109	NH2	ARG	A	7	91.023	79.070	61.652	1.00	0.00
ATOM	110	1HH2	ARG	A	7	90.369	79.824	61.808	1.00	0.00
ATOM	111	2HH2	ARG	A	7	91.880	79.050	62.172	1.00	0.00
ATOM	112	C	ARG	A	7	85.634	75.946	58.635	1.00	0.00
ATOM	113	O	ARG	A	7	85.785	76.185	59.825	1.00	0.00
ATOM	114	N	GLN	A	8	84.451	75.890	58.026	1.00	0.00
ATOM	115	H	GLN	A	8	84.446	75.738	57.027	1.00	0.00
ATOM	116	CA	GLN	A	8	83.124	75.873	58.633	1.00	0.00
ATOM	117	HA	GLN	A	8	82.430	75.784	57.794	1.00	0.00
ATOM	118	CB	GLN	A	8	82.930	74.574	59.456	1.00	0.00
ATOM	119	HB1	GLN	A	8	81.930	74.564	59.890	1.00	0.00
ATOM	120	HB2	GLN	A	8	83.650	74.547	60.276	1.00	0.00

ATOM	121	CG	GLN	A	8	83.086	73.313	58.583	1.00	0.00
ATOM	122	HG1	GLN	A	8	84.096	73.262	58.180	1.00	0.00
ATOM	123	HG2	GLN	A	8	82.389	73.363	57.747	1.00	0.00
ATOM	124	CD	GLN	A	8	82.820	72.017	59.349	1.00	0.00
ATOM	125	OE1	GLN	A	8	83.724	71.258	59.682	1.00	0.00
ATOM	126	NE2	GLN	A	8	81.585	71.685	59.640	1.00	0.00
ATOM	127	1HE2	GLN	A	8	80.817	72.252	59.332	1.00	0.00
ATOM	128	2HE2	GLN	A	8	81.462	70.827	60.146	1.00	0.00
ATOM	129	C	GLN	A	8	82.648	77.168	59.321	1.00	0.00
ATOM	130	O	GLN	A	8	83.340	78.175	59.413	1.00	0.00
ATOM	131	N	GLY	A	9	81.369	77.122	59.688	1.00	0.00
ATOM	132	H	GLY	A	9	80.928	76.222	59.614	1.00	0.00
ATOM	133	CA	GLY	A	9	80.483	78.248	59.988	1.00	0.00
ATOM	134	HA1	GLY	A	9	80.733	79.094	59.346	1.00	0.00
ATOM	135	HA2	GLY	A	9	80.594	78.542	61.031	1.00	0.00
ATOM	136	C	GLY	A	9	79.029	77.839	59.723	1.00	0.00
ATOM	137	O	GLY	A	9	78.145	78.184	60.501	1.00	0.00
ATOM	138	N	GLY	A	10	78.824	76.986	58.701	1.00	0.00
ATOM	139	H	GLY	A	10	79.580	76.887	58.033	1.00	0.00
ATOM	140	CA	GLY	A	10	77.701	76.043	58.570	1.00	0.00
ATOM	141	HA1	GLY	A	10	77.865	75.200	59.240	1.00	0.00
ATOM	142	HA2	GLY	A	10	77.675	75.668	57.547	1.00	0.00
ATOM	143	C	GLY	A	10	76.338	76.656	58.900	1.00	0.00
ATOM	144	O	GLY	A	10	75.649	76.254	59.842	1.00	0.00
ATOM	145	N	ARG	A	11	75.986	77.704	58.153	1.00	0.00
ATOM	146	H	ARG	A	11	76.607	77.976	57.403	1.00	0.00
ATOM	147	CA	ARG	A	11	74.928	78.648	58.528	1.00	0.00
ATOM	148	HA	ARG	A	11	74.453	78.283	59.430	1.00	0.00
ATOM	149	CB	ARG	A	11	75.582	80.016	58.827	1.00	0.00
ATOM	150	HB1	ARG	A	11	75.480	80.674	57.962	1.00	0.00
ATOM	151	HB2	ARG	A	11	76.649	79.895	59.016	1.00	0.00
ATOM	152	CG	ARG	A	11	74.964	80.686	60.057	1.00	0.00
ATOM	153	HG1	ARG	A	11	73.889	80.782	59.896	1.00	0.00
ATOM	154	HG2	ARG	A	11	75.377	81.689	60.161	1.00	0.00
ATOM	155	CD	ARG	A	11	75.244	79.899	61.355	1.00	0.00
ATOM	156	HD1	ARG	A	11	76.002	80.434	61.931	1.00	0.00
ATOM	157	HD2	ARG	A	11	75.656	78.915	61.128	1.00	0.00
ATOM	158	NE	ARG	A	11	74.015	79.772	62.156	1.00	0.00
ATOM	159	HE	ARG	A	11	73.714	80.599	62.640	1.00	0.00
ATOM	160	CZ	ARG	A	11	73.156	78.763	62.150	1.00	0.00
ATOM	161	NH1	ARG	A	11	73.391	77.590	61.636	1.00	0.00
ATOM	162	1HH1	ARG	A	11	74.290	77.352	61.238	1.00	0.00
ATOM	163	2HH1	ARG	A	11	72.632	76.927	61.625	1.00	0.00
ATOM	164	NH2	ARG	A	11	71.978	78.937	62.668	1.00	0.00
ATOM	165	1HH2	ARG	A	11	71.642	79.851	62.914	1.00	0.00
ATOM	166	2HH2	ARG	A	11	71.300	78.191	62.546	1.00	0.00
ATOM	167	C	ARG	A	11	73.766	78.701	57.535	1.00	0.00
ATOM	168	O	ARG	A	11	73.121	79.729	57.372	1.00	0.00
ATOM	169	N	GLU	A	12	73.532	77.568	56.873	1.00	0.00
ATOM	170	H	GLU	A	12	74.122	76.778	57.076	1.00	0.00
ATOM	171	CA	GLU	A	12	72.418	77.373	55.940	1.00	0.00
ATOM	172	HA	GLU	A	12	72.595	78.048	55.099	1.00	0.00
ATOM	173	CB	GLU	A	12	72.449	75.928	55.398	1.00	0.00
ATOM	174	HB1	GLU	A	12	71.765	75.302	55.973	1.00	0.00
ATOM	175	HB2	GLU	A	12	73.451	75.515	55.531	1.00	0.00
ATOM	176	CG	GLU	A	12	72.124	75.826	53.898	1.00	0.00
ATOM	177	HG1	GLU	A	12	72.203	74.781	53.590	1.00	0.00
ATOM	178	HG2	GLU	A	12	72.862	76.401	53.333	1.00	0.00
ATOM	179	CD	GLU	A	12	70.715	76.341	53.588	1.00	0.00
ATOM	180	OE1	GLU	A	12	69.754	75.704	54.061	1.00	0.00
ATOM	181	OE2	GLU	A	12	70.588	77.470	53.060	1.00	0.00

ATOM	182	C	GLU	A	12	71.049	77.748	56.543	1.00	0.00
ATOM	183	O	GLU	A	12	70.791	77.537	57.728	1.00	0.00
ATOM	184	N	ARG	A	13	70.153	78.276	55.697	1.00	0.00
ATOM	185	H	ARG	A	13	70.400	78.282	54.705	1.00	0.00
ATOM	186	CA	ARG	A	13	68.776	78.654	56.024	1.00	0.00
ATOM	187	HA	ARG	A	13	68.807	79.496	56.714	1.00	0.00
ATOM	188	CB	ARG	A	13	68.063	79.078	54.733	1.00	0.00
ATOM	189	HB1	ARG	A	13	67.870	78.200	54.113	1.00	0.00
ATOM	190	HB2	ARG	A	13	68.707	79.758	54.174	1.00	0.00
ATOM	191	CG	ARG	A	13	66.740	79.795	55.051	1.00	0.00
ATOM	192	HG1	ARG	A	13	66.975	80.717	55.578	1.00	0.00
ATOM	193	HG2	ARG	A	13	66.132	79.174	55.709	1.00	0.00
ATOM	194	CD	ARG	A	13	65.906	80.103	53.800	1.00	0.00
ATOM	195	HD1	ARG	A	13	65.003	80.639	54.101	1.00	0.00
ATOM	196	HD2	ARG	A	13	65.601	79.155	53.352	1.00	0.00
ATOM	197	NE	ARG	A	13	66.643	80.890	52.793	1.00	0.00
ATOM	198	HE	ARG	A	13	67.005	80.370	52.009	1.00	0.00
ATOM	199	CZ	ARG	A	13	66.940	82.173	52.841	1.00	0.00
ATOM	200	NH1	ARG	A	13	66.570	82.944	53.831	1.00	0.00
ATOM	201	1HH1	ARG	A	13	66.068	82.550	54.626	1.00	0.00
ATOM	202	2HH1	ARG	A	13	66.828	83.908	53.866	1.00	0.00
ATOM	203	NH2	ARG	A	13	67.630	82.714	51.872	1.00	0.00
ATOM	204	1HH2	ARG	A	13	67.960	82.139	51.115	1.00	0.00
ATOM	205	2HH2	ARG	A	13	67.899	83.679	51.909	1.00	0.00
ATOM	206	C	ARG	A	13	68.004	77.539	56.728	1.00	0.00
ATOM	207	O	ARG	A	13	67.330	77.817	57.715	1.00	0.00
ATOM	208	N	LEU	A	14	68.109	76.293	56.271	1.00	0.00
ATOM	209	H	LEU	A	14	68.687	76.132	55.442	1.00	0.00
ATOM	210	CA	LEU	A	14	67.449	75.131	56.881	1.00	0.00
ATOM	211	HA	LEU	A	14	66.395	75.366	57.014	1.00	0.00
ATOM	212	CB	LEU	A	14	67.576	73.925	55.934	1.00	0.00
ATOM	213	HB1	LEU	A	14	67.230	73.028	56.448	1.00	0.00
ATOM	214	HB2	LEU	A	14	68.632	73.783	55.695	1.00	0.00
ATOM	215	CG	LEU	A	14	66.783	74.069	54.619	1.00	0.00
ATOM	216	HG	LEU	A	14	67.017	75.018	54.139	1.00	0.00
ATOM	217	CD1	LEU	A	14	67.161	72.940	53.662	1.00	0.00
ATOM	218	1HD1	LEU	A	14	66.620	73.048	52.724	1.00	0.00
ATOM	219	2HD1	LEU	A	14	68.229	72.997	53.449	1.00	0.00
ATOM	220	3HD1	LEU	A	14	66.935	71.972	54.107	1.00	0.00
ATOM	221	CD2	LEU	A	14	65.268	74.001	54.851	1.00	0.00
ATOM	222	1HD2	LEU	A	14	64.752	74.053	53.893	1.00	0.00
ATOM	223	2HD2	LEU	A	14	65.015	73.068	55.354	1.00	0.00
ATOM	224	3HD2	LEU	A	14	64.945	74.840	55.464	1.00	0.00
ATOM	225	C	LEU	A	14	67.967	74.779	58.289	1.00	0.00
ATOM	226	O	LEU	A	14	67.315	74.036	59.012	1.00	0.00
ATOM	227	N	ALA	A	15	69.092	75.364	58.703	1.00	0.00
ATOM	228	H	ALA	A	15	69.569	75.970	58.047	1.00	0.00
ATOM	229	CA	ALA	A	15	69.673	75.268	60.042	1.00	0.00
ATOM	230	HA	ALA	A	15	69.091	74.559	60.633	1.00	0.00
ATOM	231	CB	ALA	A	15	71.098	74.718	59.900	1.00	0.00
ATOM	232	HB1	ALA	A	15	71.534	74.552	60.885	1.00	0.00
ATOM	233	HB2	ALA	A	15	71.073	73.766	59.370	1.00	0.00
ATOM	234	HB3	ALA	A	15	71.718	75.419	59.340	1.00	0.00
ATOM	235	C	ALA	A	15	69.617	76.618	60.788	1.00	0.00
ATOM	236	O	ALA	A	15	70.402	76.868	61.711	1.00	0.00
ATOM	237	N	SER	A	16	68.719	77.504	60.353	1.00	0.00
ATOM	238	H	SER	A	16	68.158	77.226	59.553	1.00	0.00
ATOM	239	CA	SER	A	16	68.492	78.860	60.859	1.00	0.00
ATOM	240	HA	SER	A	16	68.514	78.838	61.949	1.00	0.00
ATOM	241	CB	SER	A	16	69.621	79.764	60.341	1.00	0.00
ATOM	242	HB1	SER	A	16	69.425	80.072	59.312	1.00	0.00

ATOM	243	HB2	SER	A	16	70.565	79.221	60.353	1.00	0.00
ATOM	244	OG	SER	A	16	69.745	80.892	61.185	1.00	0.00
ATOM	245	HG	SER	A	16	68.956	81.428	61.033	1.00	0.00
ATOM	246	C	SER	A	16	67.110	79.357	60.416	1.00	0.00
ATOM	247	O	SER	A	16	66.978	80.465	59.888	1.00	0.00
ATOM	248	N	THR	A	17	66.129	78.457	60.529	1.00	0.00
ATOM	249	H	THR	A	17	66.329	77.599	61.029	1.00	0.00
ATOM	250	CA	THR	A	17	64.879	78.404	59.762	1.00	0.00
ATOM	251	HA	THR	A	17	65.159	78.241	58.726	1.00	0.00
ATOM	252	CB	THR	A	17	64.035	77.188	60.210	1.00	0.00
ATOM	253	HB	THR	A	17	63.222	77.529	60.846	1.00	0.00
ATOM	254	CG2	THR	A	17	63.501	76.448	58.983	1.00	0.00
ATOM	255	1HG2	THR	A	17	62.849	75.638	59.309	1.00	0.00
ATOM	256	2HG2	THR	A	17	62.910	77.119	58.363	1.00	0.00
ATOM	257	3HG2	THR	A	17	64.325	76.038	58.399	1.00	0.00
ATOM	258	OG1	THR	A	17	64.792	76.228	60.931	1.00	0.00
ATOM	259	HG1	THR	A	17	64.188	75.506	61.135	1.00	0.00
ATOM	260	C	THR	A	17	64.072	79.698	59.804	1.00	0.00
ATOM	261	O	THR	A	17	64.118	80.482	60.752	1.00	0.00
ATOM	262	N	ASN	A	18	63.333	79.956	58.724	1.00	0.00
ATOM	263	H	ASN	A	18	63.311	79.267	57.991	1.00	0.00
ATOM	264	CA	ASN	A	18	62.529	81.173	58.558	1.00	0.00
ATOM	265	HA	ASN	A	18	62.591	81.782	59.461	1.00	0.00
ATOM	266	CB	ASN	A	18	63.053	82.008	57.369	1.00	0.00
ATOM	267	HB1	ASN	A	18	62.560	82.979	57.380	1.00	0.00
ATOM	268	HB2	ASN	A	18	62.788	81.507	56.438	1.00	0.00
ATOM	269	CG	ASN	A	18	64.555	82.267	57.333	1.00	0.00
ATOM	270	OD1	ASN	A	18	65.146	82.364	56.264	1.00	0.00
ATOM	271	ND2	ASN	A	18	65.203	82.388	58.467	1.00	0.00
ATOM	272	1HD2	ASN	A	18	64.742	82.142	59.333	1.00	0.00
ATOM	273	2HD2	ASN	A	18	66.207	82.360	58.450	1.00	0.00
ATOM	274	C	ASN	A	18	61.038	80.851	58.395	1.00	0.00
ATOM	275	O	ASN	A	18	60.272	81.780	58.165	1.00	0.00
ATOM	276	N	ASP	A	19	60.678	79.558	58.454	1.00	0.00
ATOM	277	H	ASP	A	19	61.338	78.922	58.896	1.00	0.00
ATOM	278	CA	ASP	A	19	59.347	78.954	58.296	1.00	0.00
ATOM	279	HA	ASP	A	19	59.478	78.043	57.713	1.00	0.00
ATOM	280	CB	ASP	A	19	58.818	78.496	59.665	1.00	0.00
ATOM	281	HB1	ASP	A	19	57.775	78.190	59.566	1.00	0.00
ATOM	282	HB2	ASP	A	19	58.877	79.315	60.384	1.00	0.00
ATOM	283	CG	ASP	A	19	59.638	77.297	60.172	1.00	0.00
ATOM	284	OD1	ASP	A	19	60.883	77.428	60.188	1.00	0.00
ATOM	285	OD2	ASP	A	19	59.026	76.244	60.452	1.00	0.00
ATOM	286	C	ASP	A	19	58.390	79.828	57.474	1.00	0.00
ATOM	287	O	ASP	A	19	57.321	80.248	57.917	1.00	0.00
ATOM	288	N	LYS	A	20	58.845	80.182	56.261	1.00	0.00
ATOM	289	H	LYS	A	20	59.731	79.788	55.992	1.00	0.00
ATOM	290	CA	LYS	A	20	58.352	81.334	55.488	1.00	0.00
ATOM	291	HA	LYS	A	20	58.154	82.109	56.234	1.00	0.00
ATOM	292	CB	LYS	A	20	59.470	81.861	54.561	1.00	0.00
ATOM	293	HB1	LYS	A	20	59.589	81.200	53.700	1.00	0.00
ATOM	294	HB2	LYS	A	20	60.405	81.838	55.123	1.00	0.00
ATOM	295	CG	LYS	A	20	59.249	83.324	54.116	1.00	0.00
ATOM	296	HG1	LYS	A	20	60.170	83.882	54.290	1.00	0.00
ATOM	297	HG2	LYS	A	20	58.489	83.782	54.749	1.00	0.00
ATOM	298	CD	LYS	A	20	58.875	83.492	52.625	1.00	0.00
ATOM	299	HD1	LYS	A	20	58.497	82.554	52.220	1.00	0.00
ATOM	300	HD2	LYS	A	20	59.780	83.737	52.070	1.00	0.00
ATOM	301	CE	LYS	A	20	57.829	84.588	52.377	1.00	0.00
ATOM	302	HE1	LYS	A	20	57.689	84.688	51.298	1.00	0.00
ATOM	303	HE2	LYS	A	20	58.192	85.543	52.763	1.00	0.00

ATOM	304	NZ	LYS	A	20	56.530	84.240	53.001	1.00	0.00
ATOM	305	HZ1	LYS	A	20	56.523	84.415	54.001	1.00	0.00
ATOM	306	HZ2	LYS	A	20	55.733	84.756	52.635	1.00	0.00
ATOM	307	HZ3	LYS	A	20	56.320	83.252	52.886	1.00	0.00
ATOM	308	C	LYS	A	20	56.997	81.074	54.806	1.00	0.00
ATOM	309	O	LYS	A	20	56.709	81.600	53.735	1.00	0.00
ATOM	310	N	GLY	A	21	56.126	80.311	55.462	1.00	0.00
ATOM	311	H	GLY	A	21	56.426	79.970	56.371	1.00	0.00
ATOM	312	CA	GLY	A	21	54.718	80.129	55.127	1.00	0.00
ATOM	313	HA1	GLY	A	21	54.262	79.481	55.877	1.00	0.00
ATOM	314	HA2	GLY	A	21	54.643	79.635	54.157	1.00	0.00
ATOM	315	C	GLY	A	21	53.884	81.418	55.062	1.00	0.00
ATOM	316	O	GLY	A	21	52.750	81.385	54.610	1.00	0.00
ATOM	317	N	SER	A	22	54.420	82.557	55.519	1.00	0.00
ATOM	318	H	SER	A	22	55.348	82.522	55.905	1.00	0.00
ATOM	319	CA	SER	A	22	53.719	83.840	55.609	1.00	0.00
ATOM	320	HA	SER	A	22	52.867	83.691	56.273	1.00	0.00
ATOM	321	CB	SER	A	22	54.614	84.892	56.280	1.00	0.00
ATOM	322	HB1	SER	A	22	54.683	84.663	57.345	1.00	0.00
ATOM	323	HB2	SER	A	22	54.155	85.877	56.174	1.00	0.00
ATOM	324	OG	SER	A	22	55.932	84.915	55.742	1.00	0.00
ATOM	325	HG	SER	A	22	56.306	85.781	55.962	1.00	0.00
ATOM	326	C	SER	A	22	53.158	84.396	54.300	1.00	0.00
ATOM	327	O	SER	A	22	53.903	84.713	53.365	1.00	0.00
ATOM	328	N	MET	A	23	51.843	84.589	54.270	1.00	0.00
ATOM	329	H	MET	A	23	51.300	84.280	55.057	1.00	0.00
ATOM	330	CA	MET	A	23	51.181	85.400	53.253	1.00	0.00
ATOM	331	HA	MET	A	23	51.490	85.006	52.284	1.00	0.00
ATOM	332	CB	MET	A	23	49.647	85.226	53.320	1.00	0.00
ATOM	333	HB1	MET	A	23	49.418	84.231	52.936	1.00	0.00
ATOM	334	HB2	MET	A	23	49.174	85.942	52.647	1.00	0.00
ATOM	335	CG	MET	A	23	48.985	85.340	54.699	1.00	0.00
ATOM	336	HG1	MET	A	23	49.377	84.565	55.357	1.00	0.00
ATOM	337	HG2	MET	A	23	47.922	85.138	54.568	1.00	0.00
ATOM	338	SD	MET	A	23	49.131	86.934	55.543	1.00	0.00
ATOM	339	CE	MET	A	23	47.938	86.659	56.882	1.00	0.00
ATOM	340	HE1	MET	A	23	48.266	85.826	57.503	1.00	0.00
ATOM	341	HE2	MET	A	23	47.865	87.554	57.496	1.00	0.00
ATOM	342	HE3	MET	A	23	46.958	86.439	56.459	1.00	0.00
ATOM	343	C	MET	A	23	51.626	86.872	53.265	1.00	0.00
ATOM	344	O	MET	A	23	52.133	87.403	54.252	1.00	0.00
ATOM	345	N	ALA	A	24	51.371	87.521	52.135	1.00	0.00
ATOM	346	H	ALA	A	24	50.957	86.994	51.381	1.00	0.00
ATOM	347	CA	ALA	A	24	51.162	88.954	52.026	1.00	0.00
ATOM	348	HA	ALA	A	24	51.144	89.383	53.026	1.00	0.00
ATOM	349	CB	ALA	A	24	52.298	89.583	51.216	1.00	0.00
ATOM	350	HB1	ALA	A	24	52.128	90.656	51.116	1.00	0.00
ATOM	351	HB2	ALA	A	24	53.246	89.424	51.729	1.00	0.00
ATOM	352	HB3	ALA	A	24	52.340	89.137	50.222	1.00	0.00
ATOM	353	C	ALA	A	24	49.778	89.136	51.383	1.00	0.00
ATOM	354	O	ALA	A	24	49.326	88.248	50.656	1.00	0.00
ATOM	355	N	MET	A	25	49.096	90.241	51.682	1.00	0.00
ATOM	356	H	MET	A	25	49.547	90.953	52.248	1.00	0.00
ATOM	357	CA	MET	A	25	47.682	90.416	51.350	1.00	0.00
ATOM	358	HA	MET	A	25	47.382	89.640	50.645	1.00	0.00
ATOM	359	CB	MET	A	25	46.792	90.241	52.591	1.00	0.00
ATOM	360	HB1	MET	A	25	45.760	90.268	52.255	1.00	0.00
ATOM	361	HB2	MET	A	25	46.963	91.060	53.290	1.00	0.00
ATOM	362	CG	MET	A	25	46.985	88.901	53.311	1.00	0.00
ATOM	363	HG1	MET	A	25	47.901	88.945	53.895	1.00	0.00
ATOM	364	HG2	MET	A	25	47.108	88.120	52.562	1.00	0.00

ATOM	365	SD	MET	A	25	45.610	88.397	54.392	1.00	0.00
ATOM	366	CE	MET	A	25	45.431	89.833	55.483	1.00	0.00
ATOM	367	HE1	MET	A	25	46.360	90.004	56.018	1.00	0.00
ATOM	368	HE2	MET	A	25	45.174	90.716	54.900	1.00	0.00
ATOM	369	HE3	MET	A	25	44.636	89.640	56.201	1.00	0.00
ATOM	370	C	MET	A	25	47.434	91.753	50.652	1.00	0.00
ATOM	371	O	MET	A	25	47.659	92.826	51.215	1.00	0.00
ATOM	372	N	GLU	A	26	46.964	91.661	49.412	1.00	0.00
ATOM	373	H	GLU	A	26	46.751	90.741	49.060	1.00	0.00
ATOM	374	CA	GLU	A	26	46.655	92.780	48.525	1.00	0.00
ATOM	375	HA	GLU	A	26	47.056	93.698	48.956	1.00	0.00
ATOM	376	CB	GLU	A	26	47.341	92.534	47.161	1.00	0.00
ATOM	377	HB1	GLU	A	26	46.663	91.981	46.511	1.00	0.00
ATOM	378	HB2	GLU	A	26	48.225	91.912	47.317	1.00	0.00
ATOM	379	CG	GLU	A	26	47.819	93.807	46.443	1.00	0.00
ATOM	380	HG1	GLU	A	26	48.241	93.527	45.476	1.00	0.00
ATOM	381	HG2	GLU	A	26	48.618	94.261	47.034	1.00	0.00
ATOM	382	CD	GLU	A	26	46.683	94.806	46.248	1.00	0.00
ATOM	383	OE1	GLU	A	26	46.621	95.770	47.050	1.00	0.00
ATOM	384	OE2	GLU	A	26	45.768	94.531	45.445	1.00	0.00
ATOM	385	C	GLU	A	26	45.125	92.917	48.396	1.00	0.00
ATOM	386	O	GLU	A	26	44.388	91.935	48.487	1.00	0.00
ATOM	387	N	SER	A	27	44.639	94.147	48.236	1.00	0.00
ATOM	388	H	SER	A	27	45.297	94.903	48.060	1.00	0.00
ATOM	389	CA	SER	A	27	43.220	94.485	48.284	1.00	0.00
ATOM	390	HA	SER	A	27	42.664	93.654	47.850	1.00	0.00
ATOM	391	CB	SER	A	27	42.773	94.634	49.751	1.00	0.00
ATOM	392	HB1	SER	A	27	42.984	93.710	50.282	1.00	0.00
ATOM	393	HB2	SER	A	27	41.696	94.796	49.786	1.00	0.00
ATOM	394	OG	SER	A	27	43.419	95.710	50.414	1.00	0.00
ATOM	395	HG	SER	A	27	43.171	96.514	49.949	1.00	0.00
ATOM	396	C	SER	A	27	42.872	95.728	47.452	1.00	0.00
ATOM	397	O	SER	A	27	42.160	96.609	47.950	1.00	0.00
ATOM	398	N	ALA	A	28	43.312	95.801	46.190	1.00	0.00
ATOM	399	H	ALA	A	28	44.047	95.141	45.918	1.00	0.00
ATOM	400	CA	ALA	A	28	42.994	96.835	45.184	1.00	0.00
ATOM	401	HA	ALA	A	28	43.422	97.769	45.549	1.00	0.00
ATOM	402	CB	ALA	A	28	43.738	96.481	43.888	1.00	0.00
ATOM	403	HB1	ALA	A	28	43.560	97.242	43.128	1.00	0.00
ATOM	404	HB2	ALA	A	28	44.811	96.438	44.075	1.00	0.00
ATOM	405	HB3	ALA	A	28	43.403	95.513	43.514	1.00	0.00
ATOM	406	C	ALA	A	28	41.493	97.141	44.912	1.00	0.00
ATOM	407	O	ALA	A	28	41.157	97.804	43.932	1.00	0.00
ATOM	408	N	LYS	A	29	40.572	96.688	45.772	1.00	0.00
ATOM	409	H	LYS	A	29	40.939	96.182	46.566	1.00	0.00
ATOM	410	CA	LYS	A	29	39.129	96.972	45.743	1.00	0.00
ATOM	411	HA	LYS	A	29	38.966	97.819	45.073	1.00	0.00
ATOM	412	CB	LYS	A	29	38.402	95.740	45.156	1.00	0.00
ATOM	413	HB1	LYS	A	29	38.308	94.998	45.948	1.00	0.00
ATOM	414	HB2	LYS	A	29	39.023	95.297	44.374	1.00	0.00
ATOM	415	CG	LYS	A	29	37.033	96.073	44.528	1.00	0.00
ATOM	416	HG1	LYS	A	29	37.178	96.233	43.460	1.00	0.00
ATOM	417	HG2	LYS	A	29	36.637	97.005	44.922	1.00	0.00
ATOM	418	CD	LYS	A	29	35.975	94.969	44.715	1.00	0.00
ATOM	419	HD1	LYS	A	29	36.342	94.054	44.249	1.00	0.00
ATOM	420	HD2	LYS	A	29	35.064	95.267	44.194	1.00	0.00
ATOM	421	CE	LYS	A	29	35.630	94.661	46.184	1.00	0.00
ATOM	422	HE1	LYS	A	29	36.523	94.271	46.681	1.00	0.00
ATOM	423	HE2	LYS	A	29	34.875	93.872	46.215	1.00	0.00
ATOM	424	NZ	LYS	A	29	35.147	95.848	46.927	1.00	0.00
ATOM	425	HZ1	LYS	A	29	34.255	96.183	46.604	1.00	0.00

ATOM	426	HZ2	LYS	A	29	35.091	95.613	47.924	1.00	0.00
ATOM	427	HZ3	LYS	A	29	35.836	96.597	46.901	1.00	0.00
ATOM	428	C	LYS	A	29	38.548	97.382	47.110	1.00	0.00
ATOM	429	O	LYS	A	29	37.332	97.541	47.219	1.00	0.00
ATOM	430	N	GLU	A	30	39.350	97.480	48.178	1.00	0.00
ATOM	431	H	GLU	A	30	40.349	97.357	48.041	1.00	0.00
ATOM	432	CA	GLU	A	30	38.831	97.396	49.558	1.00	0.00
ATOM	433	HA	GLU	A	30	37.858	97.889	49.577	1.00	0.00
ATOM	434	CB	GLU	A	30	38.610	95.904	49.900	1.00	0.00
ATOM	435	HB1	GLU	A	30	39.545	95.463	50.242	1.00	0.00
ATOM	436	HB2	GLU	A	30	38.320	95.352	49.005	1.00	0.00
ATOM	437	CG	GLU	A	30	37.525	95.662	50.964	1.00	0.00
ATOM	438	HG1	GLU	A	30	37.709	96.303	51.828	1.00	0.00
ATOM	439	HG2	GLU	A	30	37.592	94.624	51.299	1.00	0.00
ATOM	440	CD	GLU	A	30	36.108	95.909	50.422	1.00	0.00
ATOM	441	OE1	GLU	A	30	35.404	96.785	50.972	1.00	0.00
ATOM	442	OE2	GLU	A	30	35.698	95.231	49.457	1.00	0.00
ATOM	443	C	GLU	A	30	39.687	98.129	50.612	1.00	0.00
ATOM	444	O	GLU	A	30	40.090	97.557	51.626	1.00	0.00
ATOM	445	N	THR	A	31	39.990	99.408	50.379	1.00	0.00
ATOM	446	H	THR	A	31	39.602	99.822	49.542	1.00	0.00
ATOM	447	CA	THR	A	31	40.907	100.284	51.151	1.00	0.00
ATOM	448	HA	THR	A	31	41.860	99.757	51.223	1.00	0.00
ATOM	449	CB	THR	A	31	41.170	101.578	50.355	1.00	0.00
ATOM	450	HB	THR	A	31	41.866	102.208	50.907	1.00	0.00
ATOM	451	CG2	THR	A	31	41.765	101.305	48.975	1.00	0.00
ATOM	452	1HG2	THR	A	31	42.044	102.250	48.514	1.00	0.00
ATOM	453	2HG2	THR	A	31	42.653	100.683	49.067	1.00	0.00
ATOM	454	3HG2	THR	A	31	41.044	100.804	48.331	1.00	0.00
ATOM	455	OG1	THR	A	31	39.961	102.270	50.154	1.00	0.00
ATOM	456	HG1	THR	A	31	39.725	102.661	51.003	1.00	0.00
ATOM	457	C	THR	A	31	40.509	100.611	52.607	1.00	0.00
ATOM	458	O	THR	A	31	40.542	101.782	52.988	1.00	0.00
ATOM	459	N	ARG	A	32	40.054	99.646	53.430	1.00	0.00
ATOM	460	H	ARG	A	32	40.043	98.697	53.064	1.00	0.00
ATOM	461	CA	ARG	A	32	39.352	99.965	54.700	1.00	0.00
ATOM	462	HA	ARG	A	32	39.885	100.807	55.147	1.00	0.00
ATOM	463	CB	ARG	A	32	37.938	100.504	54.360	1.00	0.00
ATOM	464	HB1	ARG	A	32	38.055	101.417	53.775	1.00	0.00
ATOM	465	HB2	ARG	A	32	37.431	100.805	55.276	1.00	0.00
ATOM	466	CG	ARG	A	32	37.018	99.567	53.559	1.00	0.00
ATOM	467	HG1	ARG	A	32	37.514	99.251	52.640	1.00	0.00
ATOM	468	HG2	ARG	A	32	36.130	100.135	53.279	1.00	0.00
ATOM	469	CD	ARG	A	32	36.570	98.334	54.346	1.00	0.00
ATOM	470	HD1	ARG	A	32	36.145	98.664	55.295	1.00	0.00
ATOM	471	HD2	ARG	A	32	37.428	97.690	54.540	1.00	0.00
ATOM	472	NE	ARG	A	32	35.580	97.568	53.583	1.00	0.00
ATOM	473	HE	ARG	A	32	35.670	97.560	52.567	1.00	0.00
ATOM	474	CZ	ARG	A	32	34.572	96.852	54.026	1.00	0.00
ATOM	475	NH1	ARG	A	32	34.309	96.703	55.295	1.00	0.00
ATOM	476	1HH1	ARG	A	32	34.912	97.144	55.966	1.00	0.00
ATOM	477	2HH1	ARG	A	32	33.514	96.168	55.578	1.00	0.00
ATOM	478	NH2	ARG	A	32	33.814	96.269	53.148	1.00	0.00
ATOM	479	1HH2	ARG	A	32	34.115	96.373	52.172	1.00	0.00
ATOM	480	2HH2	ARG	A	32	33.015	95.720	53.392	1.00	0.00
ATOM	481	C	ARG	A	32	39.335	98.929	55.834	1.00	0.00
ATOM	482	O	ARG	A	32	38.395	98.906	56.632	1.00	0.00
ATOM	483	N	TYR	A	33	40.339	98.065	55.941	1.00	0.00
ATOM	484	H	TYR	A	33	41.098	98.106	55.272	1.00	0.00
ATOM	485	CA	TYR	A	33	40.447	97.099	57.048	1.00	0.00
ATOM	486	HA	TYR	A	33	39.859	97.461	57.892	1.00	0.00

ATOM	487	CB	TYR	A	33	39.848	95.763	56.564	1.00	0.00
ATOM	488	HB1	TYR	A	33	40.439	95.425	55.710	1.00	0.00
ATOM	489	HB2	TYR	A	33	38.836	95.954	56.202	1.00	0.00
ATOM	490	CG	TYR	A	33	39.763	94.639	57.580	1.00	0.00
ATOM	491	CD1	TYR	A	33	39.429	94.900	58.925	1.00	0.00
ATOM	492	HD1	TYR	A	33	39.203	95.906	59.258	1.00	0.00
ATOM	493	CE1	TYR	A	33	39.371	93.846	59.854	1.00	0.00
ATOM	494	HE1	TYR	A	33	39.136	94.028	60.894	1.00	0.00
ATOM	495	CZ	TYR	A	33	39.611	92.521	59.438	1.00	0.00
ATOM	496	OH	TYR	A	33	39.555	91.523	60.353	1.00	0.00
ATOM	497	HH	TYR	A	33	39.822	90.676	59.993	1.00	0.00
ATOM	498	CE2	TYR	A	33	39.914	92.252	58.086	1.00	0.00
ATOM	499	HE2	TYR	A	33	40.066	91.232	57.766	1.00	0.00
ATOM	500	CD2	TYR	A	33	39.977	93.312	57.160	1.00	0.00
ATOM	501	HD2	TYR	A	33	40.150	93.099	56.112	1.00	0.00
ATOM	502	C	TYR	A	33	41.897	96.975	57.531	1.00	0.00
ATOM	503	O	TYR	A	33	42.810	97.354	56.805	1.00	0.00
ATOM	504	N	CYS	A	34	42.108	96.432	58.735	1.00	0.00
ATOM	505	H	CYS	A	34	41.288	96.174	59.262	1.00	0.00
ATOM	506	CA	CYS	A	34	43.397	96.391	59.447	1.00	0.00
ATOM	507	HA	CYS	A	34	44.176	96.809	58.812	1.00	0.00
ATOM	508	CB	CYS	A	34	43.272	97.229	60.719	1.00	0.00
ATOM	509	HB1	CYS	A	34	44.017	96.941	61.460	1.00	0.00
ATOM	510	HB2	CYS	A	34	42.274	97.144	61.151	1.00	0.00
ATOM	511	SG	CYS	A	34	43.598	98.910	60.231	1.00	0.00
ATOM	512	HG	CYS	A	34	42.425	99.076	59.599	1.00	0.00
ATOM	513	C	CYS	A	34	43.893	94.995	59.809	1.00	0.00
ATOM	514	O	CYS	A	34	44.864	94.907	60.564	1.00	0.00
ATOM	515	N	ALA	A	35	43.244	93.956	59.291	1.00	0.00
ATOM	516	H	ALA	A	35	42.475	94.213	58.703	1.00	0.00
ATOM	517	CA	ALA	A	35	43.370	92.507	59.524	1.00	0.00
ATOM	518	HA	ALA	A	35	42.448	92.061	59.152	1.00	0.00
ATOM	519	CB	ALA	A	35	44.491	91.981	58.616	1.00	0.00
ATOM	520	HB1	ALA	A	35	44.403	90.902	58.520	1.00	0.00
ATOM	521	HB2	ALA	A	35	44.423	92.413	57.623	1.00	0.00
ATOM	522	HB3	ALA	A	35	45.460	92.241	59.042	1.00	0.00
ATOM	523	C	ALA	A	35	43.509	91.957	60.970	1.00	0.00
ATOM	524	O	ALA	A	35	43.160	90.811	61.226	1.00	0.00
ATOM	525	N	VAL	A	36	44.010	92.763	61.904	1.00	0.00
ATOM	526	H	VAL	A	36	44.297	93.667	61.563	1.00	0.00
ATOM	527	CA	VAL	A	36	44.533	92.393	63.225	1.00	0.00
ATOM	528	HA	VAL	A	36	44.220	91.369	63.434	1.00	0.00
ATOM	529	CB	VAL	A	36	46.076	92.419	63.140	1.00	0.00
ATOM	530	HB	VAL	A	36	46.377	92.013	62.172	1.00	0.00
ATOM	531	CG1	VAL	A	36	46.700	93.813	63.255	1.00	0.00
ATOM	532	1HG1	VAL	A	36	47.781	93.746	63.146	1.00	0.00
ATOM	533	2HG1	VAL	A	36	46.327	94.457	62.467	1.00	0.00
ATOM	534	3HG1	VAL	A	36	46.451	94.271	64.206	1.00	0.00
ATOM	535	CG2	VAL	A	36	46.667	91.495	64.191	1.00	0.00
ATOM	536	1HG2	VAL	A	36	47.715	91.725	64.334	1.00	0.00
ATOM	537	2HG2	VAL	A	36	46.143	91.586	65.135	1.00	0.00
ATOM	538	3HG2	VAL	A	36	46.575	90.475	63.829	1.00	0.00
ATOM	539	C	VAL	A	36	43.980	93.265	64.361	1.00	0.00
ATOM	540	O	VAL	A	36	44.019	92.899	65.536	1.00	0.00
ATOM	541	N	CYS	A	37	43.379	94.383	63.979	1.00	0.00
ATOM	542	H	CYS	A	37	43.441	94.584	62.992	1.00	0.00
ATOM	543	CA	CYS	A	37	42.279	95.071	64.648	1.00	0.00
ATOM	544	HA	CYS	A	37	41.782	94.393	65.346	1.00	0.00
ATOM	545	CB	CYS	A	37	42.806	96.286	65.428	1.00	0.00
ATOM	546	HB1	CYS	A	37	43.586	95.963	66.114	1.00	0.00
ATOM	547	HB2	CYS	A	37	41.997	96.727	66.013	1.00	0.00

```
ATOM    548  SG  CYS A  37      43.470  97.523  64.277  1.00  0.00
ATOM    549  HG  CYS A  37      43.212  98.620  65.026  1.00  0.00
ATOM    550  C   CYS A  37      41.282  95.451  63.530  1.00  0.00
ATOM    551  O   CYS A  37      41.446  95.067  62.362  1.00  0.00
ATOM    552  N   ASN A  38      40.258  96.233  63.850  1.00  0.00
ATOM    553  H   ASN A  38      40.193  96.566  64.801  1.00  0.00
ATOM    554  CA  ASN A  38      39.192  96.627  62.921  1.00  0.00
ATOM    555  HA  ASN A  38      39.303  96.091  61.980  1.00  0.00
ATOM    556  CB  ASN A  38      37.852  96.213  63.571  1.00  0.00
ATOM    557  HB1 ASN A  38      37.870  95.147  63.796  1.00  0.00
ATOM    558  HB2 ASN A  38      37.051  96.386  62.854  1.00  0.00
ATOM    559  CG  ASN A  38      37.507  96.965  64.859  1.00  0.00
ATOM    560  OD1 ASN A  38      38.324  97.614  65.503  1.00  0.00
ATOM    561  ND2 ASN A  38      36.280  96.898  65.310  1.00  0.00
ATOM    562 1HD2 ASN A  38      35.576  96.406  64.787  1.00  0.00
ATOM    563 2HD2 ASN A  38      36.077  97.350  66.181  1.00  0.00
ATOM    564  C   ASN A  38      39.215  98.136  62.583  1.00  0.00
ATOM    565  O   ASN A  38      38.179  98.749  62.303  1.00  0.00
ATOM    566  N   ASP A  39      40.371  98.773  62.734  1.00  0.00
ATOM    567  H   ASP A  39      41.168  98.225  63.023  1.00  0.00
ATOM    568  CA  ASP A  39      40.643 100.124  62.236  1.00  0.00
ATOM    569  HA  ASP A  39      39.824 100.786  62.509  1.00  0.00
ATOM    570  CB  ASP A  39      41.924 100.673  62.892  1.00  0.00
ATOM    571  HB1 ASP A  39      42.204 101.623  62.438  1.00  0.00
ATOM    572  HB2 ASP A  39      42.738  99.968  62.743  1.00  0.00
ATOM    573  CG  ASP A  39      41.699 100.905  64.375  1.00  0.00
ATOM    574  OD1 ASP A  39      40.690 101.580  64.687  1.00  0.00
ATOM    575  OD2 ASP A  39      42.435 100.292  65.176  1.00  0.00
ATOM    576  C   ASP A  39      40.718 100.178  60.703  1.00  0.00
ATOM    577  O   ASP A  39      40.579  99.166  60.006  1.00  0.00
ATOM    578  N   TYR A  40      40.972 101.371  60.164  1.00  0.00
ATOM    579  H   TYR A  40      41.144 102.157  60.774  1.00  0.00
ATOM    580  CA  TYR A  40      41.280 101.564  58.751  1.00  0.00
ATOM    581  HA  TYR A  40      40.909 100.719  58.172  1.00  0.00
ATOM    582  CB  TYR A  40      40.585 102.827  58.219  1.00  0.00
ATOM    583  HB1 TYR A  40      40.856 102.939  57.168  1.00  0.00
ATOM    584  HB2 TYR A  40      40.994 103.687  58.753  1.00  0.00
ATOM    585  CG  TYR A  40      39.064 102.885  58.315  1.00  0.00
ATOM    586  CD1 TYR A  40      38.275 101.726  58.487  1.00  0.00
ATOM    587  HD1 TYR A  40      38.728 100.748  58.553  1.00  0.00
ATOM    588  CE1 TYR A  40      36.875 101.831  58.586  1.00  0.00
ATOM    589  HE1 TYR A  40      36.270 100.948  58.727  1.00  0.00
ATOM    590  CZ  TYR A  40      36.249 103.090  58.497  1.00  0.00
ATOM    591  OH  TYR A  40      34.897 103.183  58.602  1.00  0.00
ATOM    592  HH  TYR A  40      34.587 104.087  58.519  1.00  0.00
ATOM    593  CE2 TYR A  40      37.030 104.250  58.307  1.00  0.00
ATOM    594  HE2 TYR A  40      36.553 105.221  58.233  1.00  0.00
ATOM    595  CD2 TYR A  40      38.432 104.141  58.215  1.00  0.00
ATOM    596  HD2 TYR A  40      39.029 105.032  58.067  1.00  0.00
ATOM    597  C   TYR A  40      42.797 101.627  58.559  1.00  0.00
ATOM    598  O   TYR A  40      43.468 102.436  59.209  1.00  0.00
ATOM    599  N   ALA A  41      43.349 100.739  57.729  1.00  0.00
ATOM    600  H   ALA A  41      42.767 100.098  57.215  1.00  0.00
ATOM    601  CA  ALA A  41      44.792 100.640  57.537  1.00  0.00
ATOM    602  HA  ALA A  41      45.301 100.792  58.488  1.00  0.00
ATOM    603  CB  ALA A  41      45.172  99.263  56.986  1.00  0.00
ATOM    604  HB1 ALA A  41      46.243  99.234  56.792  1.00  0.00
ATOM    605  HB2 ALA A  41      44.937  98.494  57.710  1.00  0.00
ATOM    606  HB3 ALA A  41      44.642  99.072  56.054  1.00  0.00
ATOM    607  C   ALA A  41      45.281 101.703  56.555  1.00  0.00
ATOM    608  O   ALA A  41      44.711 101.832  55.470  1.00  0.00
```

ATOM	609	N	SER	A	42	46.405	102.361	56.844	1.00	0.00
ATOM	610	H	SER	A	42	46.857	102.230	57.741	1.00	0.00
ATOM	611	CA	SER	A	42	47.101	103.192	55.857	1.00	0.00
ATOM	612	HA	SER	A	42	46.355	103.664	55.216	1.00	0.00
ATOM	613	CB	SER	A	42	47.906	104.338	56.498	1.00	0.00
ATOM	614	HB1	SER	A	42	47.979	105.165	55.791	1.00	0.00
ATOM	615	HB2	SER	A	42	48.916	104.009	56.741	1.00	0.00
ATOM	616	OG	SER	A	42	47.287	104.788	57.687	1.00	0.00
ATOM	617	HG	SER	A	42	47.453	104.088	58.338	1.00	0.00
ATOM	618	C	SER	A	42	47.982	102.305	54.963	1.00	0.00
ATOM	619	O	SER	A	42	49.206	102.371	54.972	1.00	0.00
ATOM	620	N	GLY	A	43	47.345	101.367	54.262	1.00	0.00
ATOM	621	H	GLY	A	43	46.335	101.328	54.334	1.00	0.00
ATOM	622	CA	GLY	A	43	48.031	100.353	53.466	1.00	0.00
ATOM	623	HA1	GLY	A	43	48.795	100.830	52.853	1.00	0.00
ATOM	624	HA2	GLY	A	43	47.314	99.883	52.794	1.00	0.00
ATOM	625	C	GLY	A	43	48.703	99.250	54.289	1.00	0.00
ATOM	626	O	GLY	A	43	48.625	99.192	55.522	1.00	0.00
ATOM	627	N	TYR	A	44	49.356	98.343	53.573	1.00	0.00
ATOM	628	H	TYR	A	44	49.363	98.449	52.569	1.00	0.00
ATOM	629	CA	TYR	A	44	50.122	97.227	54.122	1.00	0.00
ATOM	630	HA	TYR	A	44	49.473	96.675	54.794	1.00	0.00
ATOM	631	CB	TYR	A	44	50.561	96.264	52.996	1.00	0.00
ATOM	632	HB1	TYR	A	44	50.294	95.246	53.283	1.00	0.00
ATOM	633	HB2	TYR	A	44	51.648	96.283	52.904	1.00	0.00
ATOM	634	CG	TYR	A	44	49.980	96.554	51.621	1.00	0.00
ATOM	635	CD1	TYR	A	44	50.627	97.479	50.776	1.00	0.00
ATOM	636	HD1	TYR	A	44	51.557	97.939	51.088	1.00	0.00
ATOM	637	CE1	TYR	A	44	50.061	97.811	49.531	1.00	0.00
ATOM	638	HE1	TYR	A	44	50.553	98.509	48.868	1.00	0.00
ATOM	639	CZ	TYR	A	44	48.855	97.210	49.115	1.00	0.00
ATOM	640	OH	TYR	A	44	48.337	97.533	47.903	1.00	0.00
ATOM	641	HH	TYR	A	44	47.615	96.923	47.622	1.00	0.00
ATOM	642	CE2	TYR	A	44	48.204	96.293	49.968	1.00	0.00
ATOM	643	HE2	TYR	A	44	47.271	95.847	49.652	1.00	0.00
ATOM	644	CD2	TYR	A	44	48.763	95.968	51.220	1.00	0.00
ATOM	645	HD2	TYR	A	44	48.260	95.254	51.860	1.00	0.00
ATOM	646	C	TYR	A	44	51.333	97.706	54.938	1.00	0.00
ATOM	647	O	TYR	A	44	51.962	98.705	54.608	1.00	0.00
ATOM	648	N	HIS	A	45	51.723	96.920	55.945	1.00	0.00
ATOM	649	H	HIS	A	45	51.114	96.153	56.219	1.00	0.00
ATOM	650	CA	HIS	A	45	53.143	96.747	56.275	1.00	0.00
ATOM	651	HA	HIS	A	45	53.702	96.801	55.338	1.00	0.00
ATOM	652	CB	HIS	A	45	53.715	97.861	57.177	1.00	0.00
ATOM	653	HB1	HIS	A	45	53.322	97.743	58.180	1.00	0.00
ATOM	654	HB2	HIS	A	45	53.419	98.834	56.790	1.00	0.00
ATOM	655	CG	HIS	A	45	55.226	97.842	57.232	1.00	0.00
ATOM	656	ND1	HIS	A	45	56.056	97.442	56.182	1.00	0.00
ATOM	657	CE1	HIS	A	45	57.304	97.438	56.670	1.00	0.00
ATOM	658	HE1	HIS	A	45	58.185	97.147	56.107	1.00	0.00
ATOM	659	NE2	HIS	A	45	57.299	97.811	57.959	1.00	0.00
ATOM	660	HE2	HIS	A	45	58.109	97.842	58.558	1.00	0.00
ATOM	661	CD2	HIS	A	45	55.998	98.071	58.332	1.00	0.00
ATOM	662	HD2	HIS	A	45	55.656	98.350	59.316	1.00	0.00
ATOM	663	C	HIS	A	45	53.401	95.346	56.843	1.00	0.00
ATOM	664	O	HIS	A	45	52.468	94.633	57.221	1.00	0.00
ATOM	665	N	TYR	A	46	54.666	94.927	56.842	1.00	0.00
ATOM	666	H	TYR	A	46	55.349	95.621	56.555	1.00	0.00
ATOM	667	CA	TYR	A	46	55.198	93.590	57.155	1.00	0.00
ATOM	668	HA	TYR	A	46	56.284	93.642	57.062	1.00	0.00
ATOM	669	CB	TYR	A	46	54.886	93.180	58.600	1.00	0.00

ATOM	670	HB1	TYR	A	46	55.392	92.237	58.806	1.00	0.00
ATOM	671	HB2	TYR	A	46	53.818	93.004	58.721	1.00	0.00
ATOM	672	CG	TYR	A	46	55.327	94.182	59.640	1.00	0.00
ATOM	673	CD1	TYR	A	46	56.590	94.048	60.240	1.00	0.00
ATOM	674	HD1	TYR	A	46	57.228	93.218	59.973	1.00	0.00
ATOM	675	CE1	TYR	A	46	57.014	94.989	61.188	1.00	0.00
ATOM	676	HE1	TYR	A	46	57.962	94.872	61.685	1.00	0.00
ATOM	677	CZ	TYR	A	46	56.185	96.077	61.509	1.00	0.00
ATOM	678	OH	TYR	A	46	56.614	96.988	62.403	1.00	0.00
ATOM	679	HH	TYR	A	46	55.898	97.579	62.687	1.00	0.00
ATOM	680	CE2	TYR	A	46	54.918	96.212	60.912	1.00	0.00
ATOM	681	HE2	TYR	A	46	54.297	97.061	61.136	1.00	0.00
ATOM	682	CD2	TYR	A	46	54.484	95.253	59.988	1.00	0.00
ATOM	683	HD2	TYR	A	46	53.513	95.344	59.532	1.00	0.00
ATOM	684	C	TYR	A	46	54.732	92.467	56.212	1.00	0.00
ATOM	685	O	TYR	A	46	55.440	91.478	56.036	1.00	0.00
ATOM	686	N	GLY	A	47	53.547	92.611	55.623	1.00	0.00
ATOM	687	H	GLY	A	47	53.033	93.447	55.869	1.00	0.00
ATOM	688	CA	GLY	A	47	52.884	91.646	54.756	1.00	0.00
ATOM	689	HA1	GLY	A	47	53.021	90.642	55.159	1.00	0.00
ATOM	690	HA2	GLY	A	47	53.328	91.687	53.761	1.00	0.00
ATOM	691	C	GLY	A	47	51.377	91.916	54.642	1.00	0.00
ATOM	692	O	GLY	A	47	50.776	91.643	53.601	1.00	0.00
ATOM	693	N	VAL	A	48	50.765	92.512	55.675	1.00	0.00
ATOM	694	H	VAL	A	48	51.317	92.793	56.476	1.00	0.00
ATOM	695	CA	VAL	A	48	49.303	92.664	55.783	1.00	0.00
ATOM	696	HA	VAL	A	48	48.851	92.308	54.856	1.00	0.00
ATOM	697	CB	VAL	A	48	48.700	91.801	56.909	1.00	0.00
ATOM	698	HB	VAL	A	48	47.617	91.932	56.884	1.00	0.00
ATOM	699	CG1	VAL	A	48	49.004	90.324	56.672	1.00	0.00
ATOM	700	1HG1	VAL	A	48	48.400	89.720	57.341	1.00	0.00
ATOM	701	2HG1	VAL	A	48	48.765	90.047	55.649	1.00	0.00
ATOM	702	3HG1	VAL	A	48	50.055	90.106	56.855	1.00	0.00
ATOM	703	CG2	VAL	A	48	49.204	92.204	58.292	1.00	0.00
ATOM	704	1HG2	VAL	A	48	48.719	91.598	59.055	1.00	0.00
ATOM	705	2HG2	VAL	A	48	50.275	92.048	58.351	1.00	0.00
ATOM	706	3HG2	VAL	A	48	48.978	93.245	58.511	1.00	0.00
ATOM	707	C	VAL	A	48	48.839	94.107	55.950	1.00	0.00
ATOM	708	O	VAL	A	48	49.516	94.945	56.564	1.00	0.00
ATOM	709	N	TRP	A	49	47.639	94.396	55.450	1.00	0.00
ATOM	710	H	TRP	A	49	47.130	93.677	54.956	1.00	0.00
ATOM	711	CA	TRP	A	49	46.917	95.619	55.793	1.00	0.00
ATOM	712	HA	TRP	A	49	47.541	96.474	55.537	1.00	0.00
ATOM	713	CB	TRP	A	49	45.648	95.754	54.922	1.00	0.00
ATOM	714	HB1	TRP	A	49	45.969	95.858	53.884	1.00	0.00
ATOM	715	HB2	TRP	A	49	45.163	96.694	55.188	1.00	0.00
ATOM	716	CG	TRP	A	49	44.609	94.669	54.977	1.00	0.00
ATOM	717	CD1	TRP	A	49	43.733	94.484	55.983	1.00	0.00
ATOM	718	HD1	TRP	A	49	43.726	95.079	56.877	1.00	0.00
ATOM	719	NE1	TRP	A	49	42.901	93.421	55.715	1.00	0.00
ATOM	720	HE1	TRP	A	49	42.175	93.110	56.343	1.00	0.00
ATOM	721	CE2	TRP	A	49	43.187	92.862	54.494	1.00	0.00
ATOM	722	CZ2	TRP	A	49	42.634	91.782	53.792	1.00	0.00
ATOM	723	HZ2	TRP	A	49	41.828	91.206	54.218	1.00	0.00
ATOM	724	CH2	TRP	A	49	43.133	91.477	52.519	1.00	0.00
ATOM	725	HH2	TRP	A	49	42.716	90.659	51.946	1.00	0.00
ATOM	726	CZ3	TRP	A	49	44.175	92.247	51.978	1.00	0.00
ATOM	727	HZ3	TRP	A	49	44.547	92.016	50.988	1.00	0.00
ATOM	728	CE3	TRP	A	49	44.741	93.315	52.699	1.00	0.00
ATOM	729	HE3	TRP	A	49	45.542	93.897	52.264	1.00	0.00
ATOM	730	CD2	TRP	A	49	44.263	93.652	53.982	1.00	0.00

ATOM	731	C	TRP	A	49	46.651	95.702	57.306	1.00	0.00
ATOM	732	O	TRP	A	49	45.922	94.888	57.876	1.00	0.00
ATOM	733	N	SER	A	50	47.283	96.681	57.962	1.00	0.00
ATOM	734	H	SER	A	50	47.834	97.336	57.424	1.00	0.00
ATOM	735	CA	SER	A	50	47.277	96.883	59.418	1.00	0.00
ATOM	736	HA	SER	A	50	46.329	96.540	59.811	1.00	0.00
ATOM	737	CB	SER	A	50	48.390	96.056	60.079	1.00	0.00
ATOM	738	HB1	SER	A	50	48.151	94.996	59.987	1.00	0.00
ATOM	739	HB2	SER	A	50	48.439	96.300	61.140	1.00	0.00
ATOM	740	OG	SER	A	50	49.664	96.287	59.498	1.00	0.00
ATOM	741	HG	SER	A	50	49.747	95.721	58.716	1.00	0.00
ATOM	742	C	SER	A	50	47.374	98.371	59.770	1.00	0.00
ATOM	743	O	SER	A	50	47.911	99.147	58.979	1.00	0.00
ATOM	744	N	CYS	A	51	46.811	98.819	60.904	1.00	0.00
ATOM	745	H	CYS	A	51	46.326	98.180	61.516	1.00	0.00
ATOM	746	CA	CYS	A	51	46.858	100.246	61.259	1.00	0.00
ATOM	747	HA	CYS	A	51	46.700	100.816	60.341	1.00	0.00
ATOM	748	CB	CYS	A	51	45.754	100.683	62.243	1.00	0.00
ATOM	749	HB1	CYS	A	51	44.782	100.345	61.908	1.00	0.00
ATOM	750	HB2	CYS	A	51	45.707	101.774	62.253	1.00	0.00
ATOM	751	SG	CYS	A	51	46.005	100.120	63.948	1.00	0.00
ATOM	752	HG	CYS	A	51	44.790	100.462	64.415	1.00	0.00
ATOM	753	C	CYS	A	51	48.232	100.653	61.791	1.00	0.00
ATOM	754	O	CYS	A	51	48.974	99.839	62.337	1.00	0.00
ATOM	755	N	GLU	A	52	48.530	101.947	61.693	1.00	0.00
ATOM	756	H	GLU	A	52	47.916	102.525	61.137	1.00	0.00
ATOM	757	CA	GLU	A	52	49.841	102.510	62.030	1.00	0.00
ATOM	758	HA	GLU	A	52	50.606	101.997	61.453	1.00	0.00
ATOM	759	CB	GLU	A	52	49.925	103.997	61.651	1.00	0.00
ATOM	760	HB1	GLU	A	52	50.975	104.294	61.675	1.00	0.00
ATOM	761	HB2	GLU	A	52	49.393	104.595	62.393	1.00	0.00
ATOM	762	CG	GLU	A	52	49.354	104.340	60.263	1.00	0.00
ATOM	763	HG1	GLU	A	52	49.807	105.266	59.906	1.00	0.00
ATOM	764	HG2	GLU	A	52	48.283	104.517	60.376	1.00	0.00
ATOM	765	CD	GLU	A	52	49.561	103.221	59.233	1.00	0.00
ATOM	766	OE1	GLU	A	52	50.715	102.925	58.861	1.00	0.00
ATOM	767	OE2	GLU	A	52	48.545	102.583	58.855	1.00	0.00
ATOM	768	C	GLU	A	52	50.207	102.296	63.502	1.00	0.00
ATOM	769	O	GLU	A	52	51.356	102.002	63.814	1.00	0.00
ATOM	770	N	GLY	A	53	49.216	102.320	64.400	1.00	0.00
ATOM	771	H	GLY	A	53	48.296	102.590	64.079	1.00	0.00
ATOM	772	CA	GLY	A	53	49.391	101.912	65.791	1.00	0.00
ATOM	773	HA1	GLY	A	53	48.448	102.048	66.317	1.00	0.00
ATOM	774	HA2	GLY	A	53	50.139	102.552	66.256	1.00	0.00
ATOM	775	C	GLY	A	53	49.820	100.451	65.952	1.00	0.00
ATOM	776	O	GLY	A	53	50.742	100.148	66.718	1.00	0.00
ATOM	777	N	CYS	A	54	49.203	99.528	65.207	1.00	0.00
ATOM	778	H	CYS	A	54	48.544	99.841	64.503	1.00	0.00
ATOM	779	CA	CYS	A	54	49.601	98.118	65.197	1.00	0.00
ATOM	780	HA	CYS	A	54	49.629	97.754	66.225	1.00	0.00
ATOM	781	CB	CYS	A	54	48.577	97.287	64.410	1.00	0.00
ATOM	782	HB1	CYS	A	54	48.965	96.278	64.266	1.00	0.00
ATOM	783	HB2	CYS	A	54	48.398	97.729	63.427	1.00	0.00
ATOM	784	SG	CYS	A	54	47.043	97.219	65.362	1.00	0.00
ATOM	785	HG	CYS	A	54	46.687	98.500	65.179	1.00	0.00
ATOM	786	C	CYS	A	54	51.007	97.929	64.629	1.00	0.00
ATOM	787	O	CYS	A	54	51.833	97.253	65.246	1.00	0.00
ATOM	788	N	LYS	A	55	51.272	98.536	63.467	1.00	0.00
ATOM	789	H	LYS	A	55	50.520	99.064	63.033	1.00	0.00
ATOM	790	CA	LYS	A	55	52.565	98.493	62.776	1.00	0.00
ATOM	791	HA	LYS	A	55	52.769	97.460	62.506	1.00	0.00

ATOM	792	CB	LYS	A	55	52.505	99.360	61.508	1.00	0.00
ATOM	793	HB1	LYS	A	55	53.513	99.459	61.100	1.00	0.00
ATOM	794	HB2	LYS	A	55	52.169	100.358	61.792	1.00	0.00
ATOM	795	CG	LYS	A	55	51.594	98.814	60.394	1.00	0.00
ATOM	796	HG1	LYS	A	55	50.642	98.489	60.811	1.00	0.00
ATOM	797	HG2	LYS	A	55	52.073	97.960	59.921	1.00	0.00
ATOM	798	CD	LYS	A	55	51.336	99.939	59.385	1.00	0.00
ATOM	799	HD1	LYS	A	55	52.261	100.476	59.168	1.00	0.00
ATOM	800	HD2	LYS	A	55	50.648	100.626	59.871	1.00	0.00
ATOM	801	CE	LYS	A	55	50.700	99.521	58.058	1.00	0.00
ATOM	802	HE1	LYS	A	55	49.956	98.745	58.248	1.00	0.00
ATOM	803	HE2	LYS	A	55	51.463	99.110	57.397	1.00	0.00
ATOM	804	NZ	LYS	A	55	50.053	100.693	57.420	1.00	0.00
ATOM	805	HZ1	LYS	A	55	50.682	101.491	57.355	1.00	0.00
ATOM	806	HZ2	LYS	A	55	49.642	100.460	56.521	1.00	0.00
ATOM	807	HZ3	LYS	A	55	49.323	101.069	58.023	1.00	0.00
ATOM	808	C	LYS	A	55	53.698	98.972	63.684	1.00	0.00
ATOM	809	O	LYS	A	55	54.730	98.299	63.765	1.00	0.00
ATOM	810	N	ALA	A	56	53.496	100.077	64.394	1.00	0.00
ATOM	811	H	ALA	A	56	52.646	100.602	64.204	1.00	0.00
ATOM	812	CA	ALA	A	56	54.425	100.634	65.372	1.00	0.00
ATOM	813	HA	ALA	A	56	55.391	100.775	64.885	1.00	0.00
ATOM	814	CB	ALA	A	56	53.913	102.013	65.807	1.00	0.00
ATOM	815	HB1	ALA	A	56	54.601	102.452	66.530	1.00	0.00
ATOM	816	HB2	ALA	A	56	53.848	102.674	64.943	1.00	0.00
ATOM	817	HB3	ALA	A	56	52.926	101.925	66.261	1.00	0.00
ATOM	818	C	ALA	A	56	54.647	99.715	66.583	1.00	0.00
ATOM	819	O	ALA	A	56	55.793	99.454	66.962	1.00	0.00
ATOM	820	N	PHE	A	57	53.572	99.174	67.177	1.00	0.00
ATOM	821	H	PHE	A	57	52.644	99.433	66.859	1.00	0.00
ATOM	822	CA	PHE	A	57	53.737	98.246	68.306	1.00	0.00
ATOM	823	HA	PHE	A	57	54.364	98.764	69.030	1.00	0.00
ATOM	824	CB	PHE	A	57	52.406	97.936	69.010	1.00	0.00
ATOM	825	HB1	PHE	A	57	52.050	96.949	68.712	1.00	0.00
ATOM	826	HB2	PHE	A	57	51.652	98.662	68.701	1.00	0.00
ATOM	827	CG	PHE	A	57	52.530	97.993	70.527	1.00	0.00
ATOM	828	CD1	PHE	A	57	53.235	96.995	71.229	1.00	0.00
ATOM	829	HD1	PHE	A	57	53.656	96.156	70.699	1.00	0.00
ATOM	830	CE1	PHE	A	57	53.417	97.099	72.621	1.00	0.00
ATOM	831	HE1	PHE	A	57	53.971	96.333	73.155	1.00	0.00
ATOM	832	CZ	PHE	A	57	52.885	98.197	73.322	1.00	0.00
ATOM	833	HZ	PHE	A	57	53.023	98.272	74.398	1.00	0.00
ATOM	834	CE2	PHE	A	57	52.162	99.185	72.628	1.00	0.00
ATOM	835	HE2	PHE	A	57	51.735	100.021	73.169	1.00	0.00
ATOM	836	CD2	PHE	A	57	51.994	99.086	71.235	1.00	0.00
ATOM	837	HD2	PHE	A	57	51.452	99.857	70.709	1.00	0.00
ATOM	838	C	PHE	A	57	54.505	96.978	67.916	1.00	0.00
ATOM	839	O	PHE	A	57	55.339	96.497	68.692	1.00	0.00
ATOM	840	N	PHE	A	58	54.283	96.467	66.706	1.00	0.00
ATOM	841	H	PHE	A	58	53.561	96.895	66.130	1.00	0.00
ATOM	842	CA	PHE	A	58	54.997	95.309	66.183	1.00	0.00
ATOM	843	HA	PHE	A	58	54.989	94.547	66.964	1.00	0.00
ATOM	844	CB	PHE	A	58	54.249	94.743	64.980	1.00	0.00
ATOM	845	HB1	PHE	A	58	54.475	95.325	64.085	1.00	0.00
ATOM	846	HB2	PHE	A	58	53.174	94.796	65.153	1.00	0.00
ATOM	847	CG	PHE	A	58	54.614	93.294	64.773	1.00	0.00
ATOM	848	CD1	PHE	A	58	53.954	92.292	65.505	1.00	0.00
ATOM	849	HD1	PHE	A	58	53.147	92.561	66.162	1.00	0.00
ATOM	850	CE1	PHE	A	58	54.324	90.946	65.360	1.00	0.00
ATOM	851	HE1	PHE	A	58	53.793	90.168	65.887	1.00	0.00
ATOM	852	CZ	PHE	A	58	55.365	90.610	64.481	1.00	0.00

ATOM	853	HZ	PHE	A	58	55.617	89.581	64.318	1.00	0.00
ATOM	854	CE2	PHE	A	58	56.053	91.616	63.780	1.00	0.00
ATOM	855	HE2	PHE	A	58	56.825	91.363	63.069	1.00	0.00
ATOM	856	CD2	PHE	A	58	55.671	92.957	63.916	1.00	0.00
ATOM	857	HD2	PHE	A	58	56.175	93.722	63.344	1.00	0.00
ATOM	858	C	PHE	A	58	56.477	95.617	65.887	1.00	0.00
ATOM	859	O	PHE	A	58	57.339	94.835	66.297	1.00	0.00
ATOM	860	N	LYS	A	59	56.782	96.796	65.307	1.00	0.00
ATOM	861	H	LYS	A	59	56.005	97.358	64.964	1.00	0.00
ATOM	862	CA	LYS	A	59	58.151	97.338	65.121	1.00	0.00
ATOM	863	HA	LYS	A	59	58.698	96.701	64.424	1.00	0.00
ATOM	864	CB	LYS	A	59	58.107	98.785	64.541	1.00	0.00
ATOM	865	HB1	LYS	A	59	57.180	99.247	64.868	1.00	0.00
ATOM	866	HB2	LYS	A	59	58.046	98.734	63.457	1.00	0.00
ATOM	867	CG	LYS	A	59	59.236	99.785	64.898	1.00	0.00
ATOM	868	HG1	LYS	A	59	59.324	99.874	65.980	1.00	0.00
ATOM	869	HG2	LYS	A	59	58.924	100.765	64.535	1.00	0.00
ATOM	870	CD	LYS	A	59	60.632	99.509	64.307	1.00	0.00
ATOM	871	HD1	LYS	A	59	60.574	99.584	63.223	1.00	0.00
ATOM	872	HD2	LYS	A	59	60.965	98.510	64.582	1.00	0.00
ATOM	873	CE	LYS	A	59	61.630	100.560	64.825	1.00	0.00
ATOM	874	HE1	LYS	A	59	61.759	100.425	65.901	1.00	0.00
ATOM	875	HE2	LYS	A	59	61.209	101.555	64.667	1.00	0.00
ATOM	876	NZ	LYS	A	59	62.956	100.491	64.161	1.00	0.00
ATOM	877	HZ1	LYS	A	59	63.639	101.113	64.611	1.00	0.00
ATOM	878	HZ2	LYS	A	59	63.405	99.591	64.237	1.00	0.00
ATOM	879	HZ3	LYS	A	59	62.965	100.787	63.200	1.00	0.00
ATOM	880	C	LYS	A	59	58.894	97.308	66.446	1.00	0.00
ATOM	881	O	LYS	A	59	59.973	96.728	66.529	1.00	0.00
ATOM	882	N	ARG	A	60	58.311	97.902	67.491	1.00	0.00
ATOM	883	H	ARG	A	60	57.422	98.375	67.323	1.00	0.00
ATOM	884	CA	ARG	A	60	58.897	97.950	68.835	1.00	0.00
ATOM	885	HA	ARG	A	60	59.881	98.422	68.778	1.00	0.00
ATOM	886	CB	ARG	A	60	57.993	98.806	69.744	1.00	0.00
ATOM	887	HB1	ARG	A	60	56.975	98.418	69.708	1.00	0.00
ATOM	888	HB2	ARG	A	60	57.963	99.818	69.336	1.00	0.00
ATOM	889	CG	ARG	A	60	58.478	98.883	71.206	1.00	0.00
ATOM	890	HG1	ARG	A	60	58.575	99.932	71.488	1.00	0.00
ATOM	891	HG2	ARG	A	60	59.469	98.441	71.305	1.00	0.00
ATOM	892	CD	ARG	A	60	57.523	98.182	72.191	1.00	0.00
ATOM	893	HD1	ARG	A	60	58.022	98.070	73.156	1.00	0.00
ATOM	894	HD2	ARG	A	60	57.299	97.178	71.824	1.00	0.00
ATOM	895	NE	ARG	A	60	56.264	98.936	72.369	1.00	0.00
ATOM	896	HE	ARG	A	60	55.437	98.539	71.945	1.00	0.00
ATOM	897	CZ	ARG	A	60	56.114	100.060	73.063	1.00	0.00
ATOM	898	NH1	ARG	A	60	57.070	100.605	73.760	1.00	0.00
ATOM	899	1HH1	ARG	A	60	57.945	100.116	73.830	1.00	0.00
ATOM	900	2HH1	ARG	A	60	56.920	101.476	74.237	1.00	0.00
ATOM	901	NH2	ARG	A	60	54.963	100.669	73.038	1.00	0.00
ATOM	902	1HH2	ARG	A	60	54.198	100.294	72.499	1.00	0.00
ATOM	903	2HH2	ARG	A	60	54.830	101.506	73.571	1.00	0.00
ATOM	904	C	ARG	A	60	59.130	96.550	69.416	1.00	0.00
ATOM	905	O	ARG	A	60	60.204	96.297	69.949	1.00	0.00
ATOM	906	N	SER	A	61	58.151	95.650	69.279	1.00	0.00
ATOM	907	H	SER	A	61	57.298	95.942	68.819	1.00	0.00
ATOM	908	CA	SER	A	61	58.248	94.261	69.747	1.00	0.00
ATOM	909	HA	SER	A	61	58.405	94.264	70.825	1.00	0.00
ATOM	910	CB	SER	A	61	56.936	93.514	69.459	1.00	0.00
ATOM	911	HB1	SER	A	61	56.604	93.744	68.448	1.00	0.00
ATOM	912	HB2	SER	A	61	56.170	93.859	70.152	1.00	0.00
ATOM	913	OG	SER	A	61	57.080	92.102	69.560	1.00	0.00

ATOM	914	HG	SER	A	61	57.343	91.893	70.473	1.00	0.00
ATOM	915	C	SER	A	61	59.421	93.489	69.127	1.00	0.00
ATOM	916	O	SER	A	61	60.136	92.795	69.844	1.00	0.00
ATOM	917	N	ILE	A	62	59.653	93.595	67.814	1.00	0.00
ATOM	918	H	ILE	A	62	59.046	94.179	67.245	1.00	0.00
ATOM	919	CA	ILE	A	62	60.817	92.928	67.200	1.00	0.00
ATOM	920	HA	ILE	A	62	60.951	91.963	67.692	1.00	0.00
ATOM	921	CB	ILE	A	62	60.564	92.611	65.710	1.00	0.00
ATOM	922	HB	ILE	A	62	59.656	92.007	65.709	1.00	0.00
ATOM	923	CG2	ILE	A	62	60.256	93.838	64.850	1.00	0.00
ATOM	924	1HG2	ILE	A	62	59.926	93.495	63.872	1.00	0.00
ATOM	925	2HG2	ILE	A	62	59.453	94.424	65.276	1.00	0.00
ATOM	926	3HG2	ILE	A	62	61.129	94.480	64.757	1.00	0.00
ATOM	927	CG1	ILE	A	62	61.666	91.740	65.063	1.00	0.00
ATOM	928	1HG1	ILE	A	62	61.192	91.188	64.263	1.00	0.00
ATOM	929	2HG1	ILE	A	62	62.021	91.012	65.794	1.00	0.00
ATOM	930	CD	ILE	A	62	62.877	92.438	64.423	1.00	0.00
ATOM	931	HD1	ILE	A	62	63.606	91.687	64.119	1.00	0.00
ATOM	932	HD2	ILE	A	62	62.571	92.987	63.534	1.00	0.00
ATOM	933	HD3	ILE	A	62	63.356	93.129	65.109	1.00	0.00
ATOM	934	C	ILE	A	62	62.135	93.683	67.429	1.00	0.00
ATOM	935	O	ILE	A	62	63.175	93.061	67.641	1.00	0.00
ATOM	936	N	GLN	A	63	62.112	95.017	67.421	1.00	0.00
ATOM	937	H	GLN	A	63	61.229	95.484	67.246	1.00	0.00
ATOM	938	CA	GLN	A	63	63.305	95.846	67.609	1.00	0.00
ATOM	939	HA	GLN	A	63	64.054	95.555	66.873	1.00	0.00
ATOM	940	CB	GLN	A	63	62.916	97.317	67.382	1.00	0.00
ATOM	941	HB1	GLN	A	63	62.355	97.689	68.240	1.00	0.00
ATOM	942	HB2	GLN	A	63	62.263	97.391	66.520	1.00	0.00
ATOM	943	CG	GLN	A	63	64.107	98.250	67.128	1.00	0.00
ATOM	944	HG1	GLN	A	63	64.833	98.154	67.936	1.00	0.00
ATOM	945	HG2	GLN	A	63	63.745	99.278	67.120	1.00	0.00
ATOM	946	CD	GLN	A	63	64.796	97.983	65.792	1.00	0.00
ATOM	947	OE1	GLN	A	63	66.009	97.957	65.699	1.00	0.00
ATOM	948	NE2	GLN	A	63	64.053	97.726	64.720	1.00	0.00
ATOM	949	1HE2	GLN	A	63	63.078	97.517	64.832	1.00	0.00
ATOM	950	2HE2	GLN	A	63	64.518	97.526	63.839	1.00	0.00
ATOM	951	C	GLN	A	63	63.917	95.676	69.005	1.00	0.00
ATOM	952	O	GLN	A	63	65.103	95.383	69.122	1.00	0.00
ATOM	953	N	GLY	A	64	63.082	95.778	70.046	1.00	0.00
ATOM	954	H	GLY	A	64	62.103	95.980	69.864	1.00	0.00
ATOM	955	CA	GLY	A	64	63.477	95.595	71.448	1.00	0.00
ATOM	956	HA1	GLY	A	64	62.865	96.242	72.073	1.00	0.00
ATOM	957	HA2	GLY	A	64	64.520	95.888	71.576	1.00	0.00
ATOM	958	C	GLY	A	64	63.328	94.157	71.948	1.00	0.00
ATOM	959	O	GLY	A	64	63.523	93.907	73.136	1.00	0.00
ATOM	960	N	HIS	A	65	62.977	93.227	71.049	1.00	0.00
ATOM	961	H	HIS	A	65	62.818	93.539	70.104	1.00	0.00
ATOM	962	CA	HIS	A	65	62.881	91.781	71.287	1.00	0.00
ATOM	963	HA	HIS	A	65	62.390	91.347	70.417	1.00	0.00
ATOM	964	CB	HIS	A	65	64.315	91.209	71.359	1.00	0.00
ATOM	965	HB1	HIS	A	65	64.485	90.710	72.313	1.00	0.00
ATOM	966	HB2	HIS	A	65	65.054	92.009	71.303	1.00	0.00
ATOM	967	CG	HIS	A	65	64.618	90.235	70.252	1.00	0.00
ATOM	968	ND1	HIS	A	65	65.214	90.551	69.026	1.00	0.00
ATOM	969	CE1	HIS	A	65	65.300	89.386	68.364	1.00	0.00
ATOM	970	HE1	HIS	A	65	65.733	89.267	67.375	1.00	0.00
ATOM	971	NE2	HIS	A	65	64.801	88.380	69.100	1.00	0.00
ATOM	972	HE2	HIS	A	65	64.800	87.403	68.842	1.00	0.00
ATOM	973	CD2	HIS	A	65	64.357	88.901	70.294	1.00	0.00
ATOM	974	HD2	HIS	A	65	63.909	88.374	71.126	1.00	0.00

ATOM	975	C	HIS	A	65	61.979	91.368	72.474	1.00	0.00
ATOM	976	O	HIS	A	65	62.131	90.277	73.016	1.00	0.00
ATOM	977	N	ASN	A	66	61.062	92.252	72.888	1.00	0.00
ATOM	978	H	ASN	A	66	60.935	93.087	72.341	1.00	0.00
ATOM	979	CA	ASN	A	66	60.329	92.100	74.147	1.00	0.00
ATOM	980	HA	ASN	A	66	61.066	91.840	74.906	1.00	0.00
ATOM	981	CB	ASN	A	66	59.633	93.403	74.623	1.00	0.00
ATOM	982	HB1	ASN	A	66	59.993	93.621	75.627	1.00	0.00
ATOM	983	HB2	ASN	A	66	58.567	93.220	74.732	1.00	0.00
ATOM	984	CG	ASN	A	66	59.817	94.655	73.787	1.00	0.00
ATOM	985	OD1	ASN	A	66	58.919	95.147	73.108	1.00	0.00
ATOM	986	ND2	ASN	A	66	60.984	95.250	73.840	1.00	0.00
ATOM	987	1HD2	ASN	A	66	61.745	94.798	74.334	1.00	0.00
ATOM	988	2HD2	ASN	A	66	61.108	96.080	73.299	1.00	0.00
ATOM	989	C	ASN	A	66	59.316	90.946	74.110	1.00	0.00
ATOM	990	O	ASN	A	66	58.431	90.918	73.241	1.00	0.00
ATOM	991	N	ASP	A	67	59.417	90.061	75.100	1.00	0.00
ATOM	992	H	ASP	A	67	60.195	90.145	75.732	1.00	0.00
ATOM	993	CA	ASP	A	67	58.336	89.169	75.511	1.00	0.00
ATOM	994	HA	ASP	A	67	57.955	88.658	74.626	1.00	0.00
ATOM	995	CB	ASP	A	67	58.897	88.105	76.463	1.00	0.00
ATOM	996	HB1	ASP	A	67	59.326	88.597	77.338	1.00	0.00
ATOM	997	HB2	ASP	A	67	59.687	87.548	75.959	1.00	0.00
ATOM	998	CG	ASP	A	67	57.810	87.124	76.910	1.00	0.00
ATOM	999	OD1	ASP	A	67	57.755	86.840	78.124	1.00	0.00
ATOM	1000	OD2	ASP	A	67	56.995	86.722	76.048	1.00	0.00
ATOM	1001	C	ASP	A	67	57.152	89.926	76.150	1.00	0.00
ATOM	1002	O	ASP	A	67	57.310	91.020	76.697	1.00	0.00
ATOM	1003	N	TYR	A	68	55.968	89.314	76.088	1.00	0.00
ATOM	1004	H	TYR	A	68	55.985	88.348	75.760	1.00	0.00
ATOM	1005	CA	TYR	A	68	54.725	89.816	76.656	1.00	0.00
ATOM	1006	HA	TYR	A	68	54.958	90.381	77.560	1.00	0.00
ATOM	1007	CB	TYR	A	68	54.038	90.770	75.661	1.00	0.00
ATOM	1008	HB1	TYR	A	68	53.064	91.033	76.072	1.00	0.00
ATOM	1009	HB2	TYR	A	68	53.854	90.232	74.731	1.00	0.00
ATOM	1010	CG	TYR	A	68	54.764	92.068	75.337	1.00	0.00
ATOM	1011	CD1	TYR	A	68	54.659	93.177	76.199	1.00	0.00
ATOM	1012	HD1	TYR	A	68	54.157	93.065	77.145	1.00	0.00
ATOM	1013	CE1	TYR	A	68	55.240	94.413	75.847	1.00	0.00
ATOM	1014	HE1	TYR	A	68	55.188	95.257	76.512	1.00	0.00
ATOM	1015	CZ	TYR	A	68	55.974	94.529	74.651	1.00	0.00
ATOM	1016	OH	TYR	A	68	56.572	95.705	74.321	1.00	0.00
ATOM	1017	HH	TYR	A	68	57.331	95.526	73.738	1.00	0.00
ATOM	1018	CE2	TYR	A	68	56.122	93.407	73.807	1.00	0.00
ATOM	1019	HE2	TYR	A	68	56.724	93.465	72.916	1.00	0.00
ATOM	1020	CD2	TYR	A	68	55.521	92.183	74.154	1.00	0.00
ATOM	1021	HD2	TYR	A	68	55.673	91.312	73.533	1.00	0.00
ATOM	1022	C	TYR	A	68	53.781	88.666	77.047	1.00	0.00
ATOM	1023	O	TYR	A	68	52.986	88.193	76.239	1.00	0.00
ATOM	1024	N	MET	A	69	53.847	88.232	78.307	1.00	0.00
ATOM	1025	H	MET	A	69	54.597	88.581	78.881	1.00	0.00
ATOM	1026	CA	MET	A	69	52.962	87.201	78.871	1.00	0.00
ATOM	1027	HA	MET	A	69	52.960	86.351	78.187	1.00	0.00
ATOM	1028	CB	MET	A	69	53.534	86.722	80.216	1.00	0.00
ATOM	1029	HB1	MET	A	69	52.892	85.939	80.620	1.00	0.00
ATOM	1030	HB2	MET	A	69	53.549	87.552	80.921	1.00	0.00
ATOM	1031	CG	MET	A	69	54.952	86.162	80.072	1.00	0.00
ATOM	1032	HG1	MET	A	69	55.611	86.945	79.709	1.00	0.00
ATOM	1033	HG2	MET	A	69	54.948	85.365	79.334	1.00	0.00
ATOM	1034	SD	MET	A	69	55.660	85.517	81.606	1.00	0.00
ATOM	1035	CE	MET	A	69	57.349	85.254	81.005	1.00	0.00

ATOM	1036	HE1	MET	A	69	57.963	84.835	81.801	1.00	0.00
ATOM	1037	HE2	MET	A	69	57.335	84.573	80.154	1.00	0.00
ATOM	1038	HE3	MET	A	69	57.771	86.205	80.679	1.00	0.00
ATOM	1039	C	MET	A	69	51.501	87.664	79.042	1.00	0.00
ATOM	1040	O	MET	A	69	51.203	88.855	79.159	1.00	0.00
ATOM	1041	N	CYS	A	70	50.565	86.712	79.092	1.00	0.00
ATOM	1042	H	CYS	A	70	50.864	85.747	79.044	1.00	0.00
ATOM	1043	CA	CYS	A	70	49.121	86.957	79.073	1.00	0.00
ATOM	1044	HA	CYS	A	70	48.938	87.975	78.732	1.00	0.00
ATOM	1045	CB	CYS	A	70	48.498	86.016	78.026	1.00	0.00
ATOM	1046	HB1	CYS	A	70	48.823	86.327	77.035	1.00	0.00
ATOM	1047	HB2	CYS	A	70	47.412	86.086	78.077	1.00	0.00
ATOM	1048	SG	CYS	A	70	49.002	84.284	78.288	1.00	0.00
ATOM	1049	HG	CYS	A	70	48.618	84.211	79.578	1.00	0.00
ATOM	1050	C	CYS	A	70	48.473	86.757	80.464	1.00	0.00
ATOM	1051	O	CYS	A	70	48.350	85.618	80.915	1.00	0.00
ATOM	1052	N	PRO	A	71	47.949	87.808	81.124	1.00	0.00
ATOM	1053	CD	PRO	A	71	48.117	89.216	80.785	1.00	0.00
ATOM	1054	HD1	PRO	A	71	47.759	89.430	79.780	1.00	0.00
ATOM	1055	HD2	PRO	A	71	49.169	89.487	80.879	1.00	0.00
ATOM	1056	CG	PRO	A	71	47.291	90.004	81.802	1.00	0.00
ATOM	1057	HG1	PRO	A	71	46.285	90.169	81.413	1.00	0.00
ATOM	1058	HG2	PRO	A	71	47.762	90.953	82.056	1.00	0.00
ATOM	1059	CB	PRO	A	71	47.233	89.062	83.001	1.00	0.00
ATOM	1060	HB1	PRO	A	71	46.397	89.285	83.665	1.00	0.00
ATOM	1061	HB2	PRO	A	71	48.170	89.128	83.558	1.00	0.00
ATOM	1062	CA	PRO	A	71	47.134	87.679	82.341	1.00	0.00
ATOM	1063	HA	PRO	A	71	47.572	86.935	83.007	1.00	0.00
ATOM	1064	C	PRO	A	71	45.683	87.244	82.028	1.00	0.00
ATOM	1065	O	PRO	A	71	44.716	87.859	82.473	1.00	0.00
ATOM	1066	N	ALA	A	72	45.533	86.231	81.175	1.00	0.00
ATOM	1067	H	ALA	A	72	46.378	85.733	80.912	1.00	0.00
ATOM	1068	CA	ALA	A	72	44.279	85.623	80.734	1.00	0.00
ATOM	1069	HA	ALA	A	72	43.624	85.456	81.590	1.00	0.00
ATOM	1070	CB	ALA	A	72	43.593	86.571	79.732	1.00	0.00
ATOM	1071	HB1	ALA	A	72	42.649	86.142	79.397	1.00	0.00
ATOM	1072	HB2	ALA	A	72	43.380	87.526	80.212	1.00	0.00
ATOM	1073	HB3	ALA	A	72	44.240	86.740	78.873	1.00	0.00
ATOM	1074	C	ALA	A	72	44.601	84.271	80.063	1.00	0.00
ATOM	1075	O	ALA	A	72	45.763	83.924	79.876	1.00	0.00
ATOM	1076	N	THR	A	73	43.584	83.572	79.559	1.00	0.00
ATOM	1077	H	THR	A	73	42.641	83.907	79.713	1.00	0.00
ATOM	1078	CA	THR	A	73	43.675	82.397	78.663	1.00	0.00
ATOM	1079	HA	THR	A	73	44.291	81.640	79.146	1.00	0.00
ATOM	1080	CB	THR	A	73	42.259	81.819	78.468	1.00	0.00
ATOM	1081	HB	THR	A	73	42.229	81.125	77.628	1.00	0.00
ATOM	1082	CG2	THR	A	73	41.772	81.077	79.714	1.00	0.00
ATOM	1083	1HG2	THR	A	73	40.770	80.688	79.534	1.00	0.00
ATOM	1084	2HG2	THR	A	73	42.439	80.243	79.924	1.00	0.00
ATOM	1085	3HG2	THR	A	73	41.745	81.748	80.571	1.00	0.00
ATOM	1086	OG1	THR	A	73	41.349	82.873	78.251	1.00	0.00
ATOM	1087	HG1	THR	A	73	40.612	82.513	77.720	1.00	0.00
ATOM	1088	C	THR	A	73	44.337	82.668	77.287	1.00	0.00
ATOM	1089	O	THR	A	73	44.085	81.959	76.322	1.00	0.00
ATOM	1090	N	ASN	A	74	45.151	83.728	77.153	1.00	0.00
ATOM	1091	H	ASN	A	74	45.471	84.141	78.019	1.00	0.00
ATOM	1092	CA	ASN	A	74	45.626	84.335	75.893	1.00	0.00
ATOM	1093	HA	ASN	A	74	45.922	85.350	76.148	1.00	0.00
ATOM	1094	CB	ASN	A	74	46.898	83.609	75.401	1.00	0.00
ATOM	1095	HB1	ASN	A	74	46.624	82.770	74.762	1.00	0.00
ATOM	1096	HB2	ASN	A	74	47.426	83.191	76.255	1.00	0.00

ATOM	1097	CG	ASN	A	74	47.843	84.533	74.638	1.00	0.00
ATOM	1098	OD1	ASN	A	74	47.455	85.290	73.763	1.00	0.00
ATOM	1099	ND2	ASN	A	74	49.110	84.532	74.972	1.00	0.00
ATOM	1100	1HD2	ASN	A	74	49.436	83.928	75.707	1.00	0.00
ATOM	1101	2HD2	ASN	A	74	49.725	85.154	74.477	1.00	0.00
ATOM	1102	C	ASN	A	74	44.524	84.474	74.816	1.00	0.00
ATOM	1103	O	ASN	A	74	44.735	84.276	73.618	1.00	0.00
ATOM	1104	N	GLN	A	75	43.320	84.798	75.287	1.00	0.00
ATOM	1105	H	GLN	A	75	43.231	84.841	76.290	1.00	0.00
ATOM	1106	CA	GLN	A	75	42.063	84.836	74.539	1.00	0.00
ATOM	1107	HA	GLN	A	75	42.224	84.463	73.529	1.00	0.00
ATOM	1108	CB	GLN	A	75	41.108	83.893	75.280	1.00	0.00
ATOM	1109	HB1	GLN	A	75	40.813	84.374	76.213	1.00	0.00
ATOM	1110	HB2	GLN	A	75	41.661	82.984	75.519	1.00	0.00
ATOM	1111	CG	GLN	A	75	39.834	83.446	74.550	1.00	0.00
ATOM	1112	HG1	GLN	A	75	40.107	83.011	73.589	1.00	0.00
ATOM	1113	HG2	GLN	A	75	39.184	84.301	74.377	1.00	0.00
ATOM	1114	CD	GLN	A	75	39.064	82.405	75.363	1.00	0.00
ATOM	1115	OE1	GLN	A	75	39.434	82.020	76.465	1.00	0.00
ATOM	1116	NE2	GLN	A	75	37.969	81.891	74.849	1.00	0.00
ATOM	1117	1HE2	GLN	A	75	37.618	82.205	73.963	1.00	0.00
ATOM	1118	2HE2	GLN	A	75	37.495	81.207	75.410	1.00	0.00
ATOM	1119	C	GLN	A	75	41.522	86.277	74.438	1.00	0.00
ATOM	1120	O	GLN	A	75	40.407	86.496	73.977	1.00	0.00
ATOM	1121	N	CYS	A	76	42.296	87.274	74.895	1.00	0.00
ATOM	1122	H	CYS	A	76	43.239	87.063	75.180	1.00	0.00
ATOM	1123	CA	CYS	A	76	41.867	88.666	74.905	1.00	0.00
ATOM	1124	HA	CYS	A	76	40.935	88.726	75.470	1.00	0.00
ATOM	1125	CB	CYS	A	76	42.899	89.551	75.616	1.00	0.00
ATOM	1126	HB1	CYS	A	76	43.825	89.548	75.047	1.00	0.00
ATOM	1127	HB2	CYS	A	76	43.077	89.166	76.622	1.00	0.00
ATOM	1128	SG	CYS	A	76	42.294	91.262	75.722	1.00	0.00
ATOM	1129	HG	CYS	A	76	42.301	91.533	74.403	1.00	0.00
ATOM	1130	C	CYS	A	76	41.582	89.172	73.481	1.00	0.00
ATOM	1131	O	CYS	A	76	42.419	89.095	72.580	1.00	0.00
ATOM	1132	N	THR	A	77	40.382	89.716	73.316	1.00	0.00
ATOM	1133	H	THR	A	77	39.758	89.726	74.112	1.00	0.00
ATOM	1134	CA	THR	A	77	39.895	90.361	72.100	1.00	0.00
ATOM	1135	HA	THR	A	77	39.857	89.634	71.289	1.00	0.00
ATOM	1136	CB	THR	A	77	38.494	90.922	72.368	1.00	0.00
ATOM	1137	HB	THR	A	77	38.176	91.544	71.532	1.00	0.00
ATOM	1138	CG2	THR	A	77	37.447	89.829	72.567	1.00	0.00
ATOM	1139	1HG2	THR	A	77	36.468	90.287	72.699	1.00	0.00
ATOM	1140	2HG2	THR	A	77	37.417	89.183	71.691	1.00	0.00
ATOM	1141	3HG2	THR	A	77	37.685	89.229	73.444	1.00	0.00
ATOM	1142	OG1	THR	A	77	38.535	91.711	73.540	1.00	0.00
ATOM	1143	HG1	THR	A	77	39.344	92.238	73.493	1.00	0.00
ATOM	1144	C	THR	A	77	40.751	91.552	71.654	1.00	0.00
ATOM	1145	O	THR	A	77	41.467	92.154	72.447	1.00	0.00
ATOM	1146	N	ILE	A	78	40.560	91.967	70.404	1.00	0.00
ATOM	1147	H	ILE	A	78	39.867	91.473	69.861	1.00	0.00
ATOM	1148	CA	ILE	A	78	41.542	92.747	69.638	1.00	0.00
ATOM	1149	HA	ILE	A	78	42.326	93.107	70.304	1.00	0.00
ATOM	1150	CB	ILE	A	78	42.175	91.804	68.579	1.00	0.00
ATOM	1151	HB	ILE	A	78	42.892	92.374	67.986	1.00	0.00
ATOM	1152	CG2	ILE	A	78	42.981	90.679	69.263	1.00	0.00
ATOM	1153	1HG2	ILE	A	78	43.581	90.153	68.525	1.00	0.00
ATOM	1154	2HG2	ILE	A	78	43.647	91.103	70.015	1.00	0.00
ATOM	1155	3HG2	ILE	A	78	42.314	89.968	69.748	1.00	0.00
ATOM	1156	CG1	ILE	A	78	41.082	91.252	67.618	1.00	0.00
ATOM	1157	1HG1	ILE	A	78	40.557	92.092	67.166	1.00	0.00

ATOM	1158	2HG1	ILE	A	78	40.354	90.669	68.180	1.00	0.00
ATOM	1159	CD	ILE	A	78	41.576	90.371	66.474	1.00	0.00
ATOM	1160	HD1	ILE	A	78	40.746	90.157	65.801	1.00	0.00
ATOM	1161	HD2	ILE	A	78	42.355	90.887	65.916	1.00	0.00
ATOM	1162	HD3	ILE	A	78	41.946	89.426	66.868	1.00	0.00
ATOM	1163	C	ILE	A	78	40.958	93.993	68.932	1.00	0.00
ATOM	1164	O	ILE	A	78	41.622	94.565	68.073	1.00	0.00
ATOM	1165	N	ASP	A	79	39.709	94.389	69.206	1.00	0.00
ATOM	1166	H	ASP	A	79	39.197	93.927	69.939	1.00	0.00
ATOM	1167	CA	ASP	A	79	39.144	95.601	68.595	1.00	0.00
ATOM	1168	HA	ASP	A	79	39.199	95.480	67.513	1.00	0.00
ATOM	1169	CB	ASP	A	79	37.656	95.783	68.950	1.00	0.00
ATOM	1170	HB1	ASP	A	79	37.117	94.891	68.619	1.00	0.00
ATOM	1171	HB2	ASP	A	79	37.264	96.627	68.378	1.00	0.00
ATOM	1172	CG	ASP	A	79	37.370	96.029	70.435	1.00	0.00
ATOM	1173	OD1	ASP	A	79	37.905	96.996	71.029	1.00	0.00
ATOM	1174	OD2	ASP	A	79	36.589	95.228	70.997	1.00	0.00
ATOM	1175	C	ASP	A	79	39.937	96.867	68.928	1.00	0.00
ATOM	1176	O	ASP	A	79	40.630	96.909	69.934	1.00	0.00
ATOM	1177	N	LYS	A	80	39.792	97.918	68.120	1.00	0.00
ATOM	1178	H	LYS	A	80	39.187	97.805	67.316	1.00	0.00
ATOM	1179	CA	LYS	A	80	40.536	99.187	68.231	1.00	0.00
ATOM	1180	HA	LYS	A	80	41.550	99.018	67.864	1.00	0.00
ATOM	1181	CB	LYS	A	80	39.893	100.215	67.299	1.00	0.00
ATOM	1182	HB1	LYS	A	80	39.927	99.784	66.306	1.00	0.00
ATOM	1183	HB2	LYS	A	80	40.505	101.118	67.302	1.00	0.00
ATOM	1184	CG	LYS	A	80	38.439	100.599	67.658	1.00	0.00
ATOM	1185	HG1	LYS	A	80	38.439	101.027	68.662	1.00	0.00
ATOM	1186	HG2	LYS	A	80	37.804	99.711	67.669	1.00	0.00
ATOM	1187	CD	LYS	A	80	37.822	101.648	66.713	1.00	0.00
ATOM	1188	HD1	LYS	A	80	38.576	102.399	66.468	1.00	0.00
ATOM	1189	HD2	LYS	A	80	37.023	102.153	67.258	1.00	0.00
ATOM	1190	CE	LYS	A	80	37.210	101.084	65.421	1.00	0.00
ATOM	1191	HE1	LYS	A	80	36.765	101.912	64.866	1.00	0.00
ATOM	1192	HE2	LYS	A	80	36.419	100.377	65.675	1.00	0.00
ATOM	1193	NZ	LYS	A	80	38.226	100.425	64.584	1.00	0.00
ATOM	1194	HZ1	LYS	A	80	37.914	100.195	63.643	1.00	0.00
ATOM	1195	HZ2	LYS	A	80	39.068	101.006	64.533	1.00	0.00
ATOM	1196	HZ3	LYS	A	80	38.537	99.560	65.008	1.00	0.00
ATOM	1197	C	LYS	A	80	40.714	99.771	69.637	1.00	0.00
ATOM	1198	O	LYS	A	80	41.704	100.443	69.891	1.00	0.00
ATOM	1199	N	ASN	A	81	39.796	99.501	70.571	1.00	0.00
ATOM	1200	H	ASN	A	81	39.068	98.836	70.343	1.00	0.00
ATOM	1201	CA	ASN	A	81	39.959	99.885	71.969	1.00	0.00
ATOM	1202	HA	ASN	A	81	40.580	100.781	72.037	1.00	0.00
ATOM	1203	CB	ASN	A	81	38.562	100.212	72.508	1.00	0.00
ATOM	1204	HB1	ASN	A	81	37.931	99.323	72.508	1.00	0.00
ATOM	1205	HB2	ASN	A	81	38.099	100.944	71.846	1.00	0.00
ATOM	1206	CG	ASN	A	81	38.571	100.788	73.910	1.00	0.00
ATOM	1207	OD1	ASN	A	81	39.409	100.489	74.754	1.00	0.00
ATOM	1208	ND2	ASN	A	81	37.625	101.637	74.210	1.00	0.00
ATOM	1209	1HD2	ASN	A	81	36.938	101.887	73.521	1.00	0.00
ATOM	1210	2HD2	ASN	A	81	37.592	101.993	75.147	1.00	0.00
ATOM	1211	C	ASN	A	81	40.665	98.757	72.781	1.00	0.00
ATOM	1212	O	ASN	A	81	41.744	98.916	73.385	1.00	0.00
ATOM	1213	N	ARG	A	82	40.071	97.565	72.810	1.00	0.00
ATOM	1214	H	ARG	A	82	39.251	97.411	72.215	1.00	0.00
ATOM	1215	CA	ARG	A	82	40.617	96.433	73.569	1.00	0.00
ATOM	1216	HA	ARG	A	82	40.829	96.758	74.587	1.00	0.00
ATOM	1217	CB	ARG	A	82	39.584	95.297	73.648	1.00	0.00
ATOM	1218	HB1	ARG	A	82	40.056	94.412	74.076	1.00	0.00

ATOM	1219	HB2	ARG	A	82	39.226	95.048	72.648	1.00	0.00
ATOM	1220	CG	ARG	A	82	38.410	95.715	74.554	1.00	0.00
ATOM	1221	HG1	ARG	A	82	37.892	96.572	74.121	1.00	0.00
ATOM	1222	HG2	ARG	A	82	38.808	96.011	75.526	1.00	0.00
ATOM	1223	CD	ARG	A	82	37.397	94.591	74.785	1.00	0.00
ATOM	1224	HD1	ARG	A	82	36.705	94.909	75.567	1.00	0.00
ATOM	1225	HD2	ARG	A	82	37.920	93.697	75.129	1.00	0.00
ATOM	1226	NE	ARG	A	82	36.635	94.301	73.563	1.00	0.00
ATOM	1227	HE	ARG	A	82	36.814	94.885	72.743	1.00	0.00
ATOM	1228	CZ	ARG	A	82	35.692	93.395	73.387	1.00	0.00
ATOM	1229	NH1	ARG	A	82	35.336	92.561	74.315	1.00	0.00
ATOM	1230	1HH1	ARG	A	82	35.868	92.564	75.162	1.00	0.00
ATOM	1231	2HH1	ARG	A	82	34.634	91.876	74.129	1.00	0.00
ATOM	1232	NH2	ARG	A	82	35.096	93.345	72.235	1.00	0.00
ATOM	1233	1HH2	ARG	A	82	35.423	94.017	71.534	1.00	0.00
ATOM	1234	2HH2	ARG	A	82	34.347	92.717	72.052	1.00	0.00
ATOM	1235	C	ARG	A	82	41.975	95.935	73.045	1.00	0.00
ATOM	1236	O	ARG	A	82	42.668	95.230	73.756	1.00	0.00
ATOM	1237	N	ARG	A	83	42.429	96.396	71.879	1.00	0.00
ATOM	1238	H	ARG	A	83	41.756	96.901	71.310	1.00	0.00
ATOM	1239	CA	ARG	A	83	43.750	96.165	71.280	1.00	0.00
ATOM	1240	HA	ARG	A	83	43.878	95.091	71.150	1.00	0.00
ATOM	1241	CB	ARG	A	83	43.743	96.851	69.898	1.00	0.00
ATOM	1242	HB1	ARG	A	83	43.645	97.928	70.043	1.00	0.00
ATOM	1243	HB2	ARG	A	83	42.879	96.514	69.338	1.00	0.00
ATOM	1244	CG	ARG	A	83	44.941	96.595	68.985	1.00	0.00
ATOM	1245	HG1	ARG	A	83	45.872	96.821	69.505	1.00	0.00
ATOM	1246	HG2	ARG	A	83	44.856	97.280	68.141	1.00	0.00
ATOM	1247	CD	ARG	A	83	44.974	95.161	68.443	1.00	0.00
ATOM	1248	HD1	ARG	A	83	43.998	94.894	68.040	1.00	0.00
ATOM	1249	HD2	ARG	A	83	45.226	94.474	69.254	1.00	0.00
ATOM	1250	NE	ARG	A	83	45.969	95.090	67.368	1.00	0.00
ATOM	1251	HE	ARG	A	83	46.201	95.963	66.923	1.00	0.00
ATOM	1252	CZ	ARG	A	83	46.739	94.076	67.053	1.00	0.00
ATOM	1253	NH1	ARG	A	83	46.457	92.850	67.377	1.00	0.00
ATOM	1254	1HH1	ARG	A	83	45.496	92.646	67.601	1.00	0.00
ATOM	1255	2HH1	ARG	A	83	47.127	92.113	67.199	1.00	0.00
ATOM	1256	NH2	ARG	A	83	47.852	94.306	66.427	1.00	0.00
ATOM	1257	1HH2	ARG	A	83	48.011	95.215	66.030	1.00	0.00
ATOM	1258	2HH2	ARG	A	83	48.476	93.542	66.230	1.00	0.00
ATOM	1259	C	ARG	A	83	44.929	96.659	72.112	1.00	0.00
ATOM	1260	O	ARG	A	83	45.986	96.033	72.055	1.00	0.00
ATOM	1261	N	LYS	A	84	44.787	97.750	72.881	1.00	0.00
ATOM	1262	H	LYS	A	84	43.918	98.266	72.811	1.00	0.00
ATOM	1263	CA	LYS	A	84	45.780	98.124	73.912	1.00	0.00
ATOM	1264	HA	LYS	A	84	46.764	97.891	73.512	1.00	0.00
ATOM	1265	CB	LYS	A	84	45.816	99.648	74.185	1.00	0.00
ATOM	1266	HB1	LYS	A	84	46.078	100.130	73.242	1.00	0.00
ATOM	1267	HB2	LYS	A	84	46.633	99.855	74.878	1.00	0.00
ATOM	1268	CG	LYS	A	84	44.545	100.331	74.708	1.00	0.00
ATOM	1269	HG1	LYS	A	84	43.714	100.028	74.088	1.00	0.00
ATOM	1270	HG2	LYS	A	84	44.659	101.406	74.562	1.00	0.00
ATOM	1271	CD	LYS	A	84	44.209	100.109	76.198	1.00	0.00
ATOM	1272	HD1	LYS	A	84	44.945	100.646	76.797	1.00	0.00
ATOM	1273	HD2	LYS	A	84	44.263	99.057	76.465	1.00	0.00
ATOM	1274	CE	LYS	A	84	42.810	100.639	76.534	1.00	0.00
ATOM	1275	HE1	LYS	A	84	42.704	101.637	76.102	1.00	0.00
ATOM	1276	HE2	LYS	A	84	42.713	100.727	77.618	1.00	0.00
ATOM	1277	NZ	LYS	A	84	41.751	99.741	76.010	1.00	0.00
ATOM	1278	HZ1	LYS	A	84	41.736	98.848	76.483	1.00	0.00
ATOM	1279	HZ2	LYS	A	84	40.822	100.157	76.041	1.00	0.00

ATOM	1280	HZ3	LYS	A	84	41.900	99.558	75.020	1.00	0.00
ATOM	1281	C	LYS	A	84	45.652	97.290	75.177	1.00	0.00
ATOM	1282	O	LYS	A	84	46.619	97.230	75.932	1.00	0.00
ATOM	1283	N	SER	A	85	44.534	96.585	75.393	1.00	0.00
ATOM	1284	H	SER	A	85	43.816	96.533	74.684	1.00	0.00
ATOM	1285	CA	SER	A	85	44.612	95.468	76.357	1.00	0.00
ATOM	1286	HA	SER	A	85	44.996	95.861	77.298	1.00	0.00
ATOM	1287	CB	SER	A	85	43.236	94.857	76.674	1.00	0.00
ATOM	1288	HB1	SER	A	85	43.340	94.221	77.554	1.00	0.00
ATOM	1289	HB2	SER	A	85	42.913	94.218	75.855	1.00	0.00
ATOM	1290	OG	SER	A	85	42.264	95.853	76.934	1.00	0.00
ATOM	1291	HG	SER	A	85	41.468	95.383	77.197	1.00	0.00
ATOM	1292	C	SER	A	85	45.587	94.366	75.851	1.00	0.00
ATOM	1293	O	SER	A	85	45.832	94.158	74.660	1.00	0.00
ATOM	1294	N	CYS	A	86	46.162	93.632	76.799	1.00	0.00
ATOM	1295	H	CYS	A	86	45.951	93.858	77.758	1.00	0.00
ATOM	1296	CA	CYS	A	86	46.986	92.444	76.554	1.00	0.00
ATOM	1297	HA	CYS	A	86	47.629	92.347	77.428	1.00	0.00
ATOM	1298	CB	CYS	A	86	46.067	91.216	76.575	1.00	0.00
ATOM	1299	HB1	CYS	A	86	45.583	91.103	75.602	1.00	0.00
ATOM	1300	HB2	CYS	A	86	45.295	91.328	77.340	1.00	0.00
ATOM	1301	SG	CYS	A	86	47.067	89.758	76.972	1.00	0.00
ATOM	1302	HG	CYS	A	86	46.102	88.859	76.744	1.00	0.00
ATOM	1303	C	CYS	A	86	47.935	92.464	75.335	1.00	0.00
ATOM	1304	O	CYS	A	86	47.639	91.930	74.258	1.00	0.00
ATOM	1305	N	GLN	A	87	49.123	93.044	75.512	1.00	0.00
ATOM	1306	H	GLN	A	87	49.279	93.507	76.393	1.00	0.00
ATOM	1307	CA	GLN	A	87	50.200	93.081	74.500	1.00	0.00
ATOM	1308	HA	GLN	A	87	49.824	93.644	73.647	1.00	0.00
ATOM	1309	CB	GLN	A	87	51.472	93.803	75.008	1.00	0.00
ATOM	1310	HB1	GLN	A	87	51.667	94.656	74.362	1.00	0.00
ATOM	1311	HB2	GLN	A	87	52.319	93.139	74.859	1.00	0.00
ATOM	1312	CG	GLN	A	87	51.516	94.286	76.470	1.00	0.00
ATOM	1313	HG1	GLN	A	87	52.475	94.762	76.666	1.00	0.00
ATOM	1314	HG2	GLN	A	87	51.428	93.428	77.138	1.00	0.00
ATOM	1315	CD	GLN	A	87	50.439	95.309	76.819	1.00	0.00
ATOM	1316	OE1	GLN	A	87	50.093	96.179	76.026	1.00	0.00
ATOM	1317	NE2	GLN	A	87	49.786	95.176	77.949	1.00	0.00
ATOM	1318	1HE2	GLN	A	87	50.133	94.554	78.664	1.00	0.00
ATOM	1319	2HE2	GLN	A	87	49.131	95.910	78.162	1.00	0.00
ATOM	1320	C	GLN	A	87	50.550	91.686	73.947	1.00	0.00
ATOM	1321	O	GLN	A	87	50.793	91.547	72.747	1.00	0.00
ATOM	1322	N	ALA	A	88	50.470	90.657	74.800	1.00	0.00
ATOM	1323	H	ALA	A	88	50.318	90.844	75.776	1.00	0.00
ATOM	1324	CA	ALA	A	88	50.511	89.263	74.382	1.00	0.00
ATOM	1325	HA	ALA	A	88	51.518	89.021	74.035	1.00	0.00
ATOM	1326	CB	ALA	A	88	50.161	88.371	75.587	1.00	0.00
ATOM	1327	HB1	ALA	A	88	50.546	87.366	75.412	1.00	0.00
ATOM	1328	HB2	ALA	A	88	50.602	88.768	76.499	1.00	0.00
ATOM	1329	HB3	ALA	A	88	49.087	88.303	75.732	1.00	0.00
ATOM	1330	C	ALA	A	88	49.511	88.996	73.251	1.00	0.00
ATOM	1331	O	ALA	A	88	49.908	88.541	72.181	1.00	0.00
ATOM	1332	N	CYS	A	89	48.220	89.308	73.458	1.00	0.00
ATOM	1333	H	CYS	A	89	47.945	89.726	74.336	1.00	0.00
ATOM	1334	CA	CYS	A	89	47.193	88.894	72.504	1.00	0.00
ATOM	1335	HA	CYS	A	89	47.375	87.859	72.209	1.00	0.00
ATOM	1336	CB	CYS	A	89	45.807	88.965	73.156	1.00	0.00
ATOM	1337	HB1	CYS	A	89	45.045	88.727	72.408	1.00	0.00
ATOM	1338	HB2	CYS	A	89	45.619	89.971	73.535	1.00	0.00
ATOM	1339	SG	CYS	A	89	45.717	87.749	74.502	1.00	0.00
ATOM	1340	HG	CYS	A	89	46.208	86.701	73.812	1.00	0.00

ATOM	1341	C	CYS	A	89	47.249	89.751	71.235	1.00	0.00
ATOM	1342	O	CYS	A	89	47.043	89.244	70.131	1.00	0.00
ATOM	1343	N	ARG	A	90	47.629	91.027	71.373	1.00	0.00
ATOM	1344	H	ARG	A	90	47.723	91.385	72.320	1.00	0.00
ATOM	1345	CA	ARG	A	90	47.975	91.905	70.252	1.00	0.00
ATOM	1346	HA	ARG	A	90	47.073	92.084	69.674	1.00	0.00
ATOM	1347	CB	ARG	A	90	48.469	93.238	70.840	1.00	0.00
ATOM	1348	HB1	ARG	A	90	49.343	93.043	71.455	1.00	0.00
ATOM	1349	HB2	ARG	A	90	47.683	93.616	71.494	1.00	0.00
ATOM	1350	CG	ARG	A	90	48.852	94.337	69.826	1.00	0.00
ATOM	1351	HG1	ARG	A	90	47.944	94.699	69.350	1.00	0.00
ATOM	1352	HG2	ARG	A	90	49.521	93.938	69.064	1.00	0.00
ATOM	1353	CD	ARG	A	90	49.545	95.525	70.515	1.00	0.00
ATOM	1354	HD1	ARG	A	90	49.672	96.334	69.793	1.00	0.00
ATOM	1355	HD2	ARG	A	90	50.528	95.204	70.866	1.00	0.00
ATOM	1356	NE	ARG	A	90	48.722	95.985	71.638	1.00	0.00
ATOM	1357	HE	ARG	A	90	47.716	95.883	71.534	1.00	0.00
ATOM	1358	CZ	ARG	A	90	49.094	96.140	72.885	1.00	0.00
ATOM	1359	NH1	ARG	A	90	50.266	96.563	73.242	1.00	0.00
ATOM	1360	1HH1	ARG	A	90	50.946	96.859	72.566	1.00	0.00
ATOM	1361	2HH1	ARG	A	90	50.450	96.618	74.241	1.00	0.00
ATOM	1362	NH2	ARG	A	90	48.253	95.878	73.836	1.00	0.00
ATOM	1363	1HH2	ARG	A	90	47.336	95.491	73.615	1.00	0.00
ATOM	1364	2HH2	ARG	A	90	48.498	96.115	74.789	1.00	0.00
ATOM	1365	C	ARG	A	90	49.007	91.296	69.302	1.00	0.00
ATOM	1366	O	ARG	A	90	48.765	91.264	68.095	1.00	0.00
ATOM	1367	N	LEU	A	91	50.144	90.841	69.845	1.00	0.00
ATOM	1368	H	LEU	A	91	50.246	90.895	70.855	1.00	0.00
ATOM	1369	CA	LEU	A	91	51.302	90.398	69.056	1.00	0.00
ATOM	1370	HA	LEU	A	91	51.371	91.017	68.162	1.00	0.00
ATOM	1371	CB	LEU	A	91	52.568	90.613	69.898	1.00	0.00
ATOM	1372	HB1	LEU	A	91	53.432	90.209	69.369	1.00	0.00
ATOM	1373	HB2	LEU	A	91	52.464	90.067	70.838	1.00	0.00
ATOM	1374	CG	LEU	A	91	52.838	92.105	70.219	1.00	0.00
ATOM	1375	HG	LEU	A	91	51.936	92.571	70.615	1.00	0.00
ATOM	1376	CD1	LEU	A	91	53.924	92.218	71.278	1.00	0.00
ATOM	1377	1HD1	LEU	A	91	54.121	93.262	71.516	1.00	0.00
ATOM	1378	2HD1	LEU	A	91	53.585	91.723	72.189	1.00	0.00
ATOM	1379	3HD1	LEU	A	91	54.841	91.740	70.942	1.00	0.00
ATOM	1380	CD2	LEU	A	91	53.277	92.900	68.987	1.00	0.00
ATOM	1381	1HD2	LEU	A	91	53.552	93.912	69.283	1.00	0.00
ATOM	1382	2HD2	LEU	A	91	54.140	92.423	68.524	1.00	0.00
ATOM	1383	3HD2	LEU	A	91	52.455	92.961	68.276	1.00	0.00
ATOM	1384	C	LEU	A	91	51.156	88.953	68.544	1.00	0.00
ATOM	1385	O	LEU	A	91	51.474	88.682	67.384	1.00	0.00
ATOM	1386	N	ARG	A	92	50.549	88.076	69.350	1.00	0.00
ATOM	1387	H	ARG	A	92	50.383	88.366	70.313	1.00	0.00
ATOM	1388	CA	ARG	A	92	49.998	86.782	68.924	1.00	0.00
ATOM	1389	HA	ARG	A	92	50.815	86.106	68.660	1.00	0.00
ATOM	1390	CB	ARG	A	92	49.195	86.192	70.098	1.00	0.00
ATOM	1391	HB1	ARG	A	92	48.433	86.915	70.384	1.00	0.00
ATOM	1392	HB2	ARG	A	92	49.852	86.045	70.958	1.00	0.00
ATOM	1393	CG	ARG	A	92	48.514	84.853	69.770	1.00	0.00
ATOM	1394	HG1	ARG	A	92	49.201	84.033	69.979	1.00	0.00
ATOM	1395	HG2	ARG	A	92	48.282	84.814	68.710	1.00	0.00
ATOM	1396	CD	ARG	A	92	47.208	84.652	70.555	1.00	0.00
ATOM	1397	HD1	ARG	A	92	46.715	85.612	70.710	1.00	0.00
ATOM	1398	HD2	ARG	A	92	47.446	84.217	71.526	1.00	0.00
ATOM	1399	NE	ARG	A	92	46.299	83.787	69.791	1.00	0.00
ATOM	1400	HE	ARG	A	92	46.298	83.952	68.786	1.00	0.00
ATOM	1401	CZ	ARG	A	92	45.585	82.768	70.234	1.00	0.00

ATOM	1402	NH1	ARG	A	92	45.386	82.557	71.502	1.00	0.00
ATOM	1403	1HH1	ARG	A	92	45.602	83.303	72.158	1.00	0.00
ATOM	1404	2HH1	ARG	A	92	44.840	81.782	71.816	1.00	0.00
ATOM	1405	NH2	ARG	A	92	45.043	81.951	69.376	1.00	0.00
ATOM	1406	1HH2	ARG	A	92	45.205	82.125	68.376	1.00	0.00
ATOM	1407	2HH2	ARG	A	92	44.555	81.134	69.678	1.00	0.00
ATOM	1408	C	ARG	A	92	49.138	86.957	67.680	1.00	0.00
ATOM	1409	O	ARG	A	92	49.434	86.332	66.666	1.00	0.00
ATOM	1410	N	LYS	A	93	48.142	87.849	67.729	1.00	0.00
ATOM	1411	H	LYS	A	93	47.953	88.323	68.605	1.00	0.00
ATOM	1412	CA	LYS	A	93	47.260	88.083	66.585	1.00	0.00
ATOM	1413	HA	LYS	A	93	46.927	87.099	66.264	1.00	0.00
ATOM	1414	CB	LYS	A	93	46.026	88.902	66.986	1.00	0.00
ATOM	1415	HB1	LYS	A	93	46.311	89.951	67.073	1.00	0.00
ATOM	1416	HB2	LYS	A	93	45.680	88.571	67.964	1.00	0.00
ATOM	1417	CG	LYS	A	93	44.874	88.763	65.977	1.00	0.00
ATOM	1418	HG1	LYS	A	93	45.207	89.106	65.000	1.00	0.00
ATOM	1419	HG2	LYS	A	93	44.088	89.433	66.296	1.00	0.00
ATOM	1420	CD	LYS	A	93	44.287	87.348	65.802	1.00	0.00
ATOM	1421	HD1	LYS	A	93	45.048	86.705	65.362	1.00	0.00
ATOM	1422	HD2	LYS	A	93	43.465	87.405	65.088	1.00	0.00
ATOM	1423	CE	LYS	A	93	43.765	86.718	67.105	1.00	0.00
ATOM	1424	HE1	LYS	A	93	42.829	87.208	67.379	1.00	0.00
ATOM	1425	HE2	LYS	A	93	44.496	86.892	67.898	1.00	0.00
ATOM	1426	NZ	LYS	A	93	43.575	85.255	66.956	1.00	0.00
ATOM	1427	HZ1	LYS	A	93	43.084	84.842	67.732	1.00	0.00
ATOM	1428	HZ2	LYS	A	93	43.121	85.018	66.089	1.00	0.00
ATOM	1429	HZ3	LYS	A	93	44.499	84.810	66.934	1.00	0.00
ATOM	1430	C	LYS	A	93	48.015	88.659	65.392	1.00	0.00
ATOM	1431	O	LYS	A	93	47.760	88.225	64.280	1.00	0.00
ATOM	1432	N	CYS	A	94	48.993	89.551	65.592	1.00	0.00
ATOM	1433	H	CYS	A	94	49.162	89.891	66.527	1.00	0.00
ATOM	1434	CA	CYS	A	94	49.881	89.978	64.497	1.00	0.00
ATOM	1435	HA	CYS	A	94	49.310	90.484	63.720	1.00	0.00
ATOM	1436	CB	CYS	A	94	50.950	90.937	65.036	1.00	0.00
ATOM	1437	HB1	CYS	A	94	51.683	91.119	64.246	1.00	0.00
ATOM	1438	HB2	CYS	A	94	51.471	90.476	65.869	1.00	0.00
ATOM	1439	SG	CYS	A	94	50.263	92.525	65.562	1.00	0.00
ATOM	1440	HG	CYS	A	94	50.058	92.962	64.308	1.00	0.00
ATOM	1441	C	CYS	A	94	50.586	88.809	63.786	1.00	0.00
ATOM	1442	O	CYS	A	94	50.764	88.870	62.573	1.00	0.00
ATOM	1443	N	TYR	A	95	50.989	87.752	64.505	1.00	0.00
ATOM	1444	H	TYR	A	95	50.840	87.722	65.507	1.00	0.00
ATOM	1445	CA	TYR	A	95	51.636	86.609	63.851	1.00	0.00
ATOM	1446	HA	TYR	A	95	52.151	86.990	62.971	1.00	0.00
ATOM	1447	CB	TYR	A	95	52.719	85.984	64.748	1.00	0.00
ATOM	1448	HB1	TYR	A	95	52.418	84.977	65.036	1.00	0.00
ATOM	1449	HB2	TYR	A	95	52.828	86.566	65.663	1.00	0.00
ATOM	1450	CG	TYR	A	95	54.064	85.947	64.044	1.00	0.00
ATOM	1451	CD1	TYR	A	95	54.606	87.149	63.550	1.00	0.00
ATOM	1452	HD1	TYR	A	95	54.063	88.074	63.687	1.00	0.00
ATOM	1453	CE1	TYR	A	95	55.788	87.134	62.790	1.00	0.00
ATOM	1454	HE1	TYR	A	95	56.162	88.048	62.354	1.00	0.00
ATOM	1455	CZ	TYR	A	95	56.446	85.915	62.532	1.00	0.00
ATOM	1456	OH	TYR	A	95	57.570	85.902	61.768	1.00	0.00
ATOM	1457	HH	TYR	A	95	57.865	84.997	61.627	1.00	0.00
ATOM	1458	CE2	TYR	A	95	55.917	84.709	63.044	1.00	0.00
ATOM	1459	HE2	TYR	A	95	56.403	83.765	62.835	1.00	0.00
ATOM	1460	CD2	TYR	A	95	54.723	84.726	63.794	1.00	0.00
ATOM	1461	HD2	TYR	A	95	54.295	83.800	64.144	1.00	0.00
ATOM	1462	C	TYR	A	95	50.665	85.570	63.263	1.00	0.00

ATOM	1463	O	TYR	A	95	50.923	85.042	62.186	1.00	0.00
ATOM	1464	N	GLU	A	96	49.502	85.352	63.882	1.00	0.00
ATOM	1465	H	GLU	A	96	49.364	85.769	64.797	1.00	0.00
ATOM	1466	CA	GLU	A	96	48.360	84.655	63.261	1.00	0.00
ATOM	1467	HA	GLU	A	96	48.659	83.646	62.978	1.00	0.00
ATOM	1468	CB	GLU	A	96	47.191	84.563	64.263	1.00	0.00
ATOM	1469	HB1	GLU	A	96	46.300	84.204	63.745	1.00	0.00
ATOM	1470	HB2	GLU	A	96	46.976	85.567	64.625	1.00	0.00
ATOM	1471	CG	GLU	A	96	47.436	83.635	65.467	1.00	0.00
ATOM	1472	HG1	GLU	A	96	48.406	83.859	65.913	1.00	0.00
ATOM	1473	HG2	GLU	A	96	47.456	82.602	65.122	1.00	0.00
ATOM	1474	CD	GLU	A	96	46.349	83.819	66.536	1.00	0.00
ATOM	1475	OE1	GLU	A	96	46.266	84.892	67.169	1.00	0.00
ATOM	1476	OE2	GLU	A	96	45.510	82.932	66.814	1.00	0.00
ATOM	1477	C	GLU	A	96	47.865	85.345	61.973	1.00	0.00
ATOM	1478	O	GLU	A	96	47.229	84.720	61.132	1.00	0.00
ATOM	1479	N	VAL	A	97	48.183	86.632	61.814	1.00	0.00
ATOM	1480	H	VAL	A	97	48.603	87.092	62.613	1.00	0.00
ATOM	1481	CA	VAL	A	97	47.829	87.484	60.676	1.00	0.00
ATOM	1482	HA	VAL	A	97	47.346	86.869	59.921	1.00	0.00
ATOM	1483	CB	VAL	A	97	46.812	88.560	61.115	1.00	0.00
ATOM	1484	HB	VAL	A	97	47.297	89.269	61.786	1.00	0.00
ATOM	1485	CG1	VAL	A	97	46.214	89.325	59.933	1.00	0.00
ATOM	1486	1HG1	VAL	A	97	45.466	90.010	60.313	1.00	0.00
ATOM	1487	2HG1	VAL	A	97	46.967	89.915	59.423	1.00	0.00
ATOM	1488	3HG1	VAL	A	97	45.753	88.633	59.232	1.00	0.00
ATOM	1489	CG2	VAL	A	97	45.602	87.935	61.837	1.00	0.00
ATOM	1490	1HG2	VAL	A	97	45.124	87.200	61.189	1.00	0.00
ATOM	1491	2HG2	VAL	A	97	45.905	87.437	62.753	1.00	0.00
ATOM	1492	3HG2	VAL	A	97	44.880	88.706	62.102	1.00	0.00
ATOM	1493	C	VAL	A	97	49.127	88.023	60.040	1.00	0.00
ATOM	1494	O	VAL	A	97	49.248	89.198	59.701	1.00	0.00
ATOM	1495	N	GLY	A	98	50.127	87.149	59.877	1.00	0.00
ATOM	1496	H	GLY	A	98	50.029	86.252	60.336	1.00	0.00
ATOM	1497	CA	GLY	A	98	51.124	87.247	58.805	1.00	0.00
ATOM	1498	HA1	GLY	A	98	50.613	87.578	57.902	1.00	0.00
ATOM	1499	HA2	GLY	A	98	51.515	86.250	58.615	1.00	0.00
ATOM	1500	C	GLY	A	98	52.334	88.186	58.987	1.00	0.00
ATOM	1501	O	GLY	A	98	53.212	88.185	58.125	1.00	0.00
ATOM	1502	N	MET	A	99	52.413	88.994	60.055	1.00	0.00
ATOM	1503	H	MET	A	99	51.659	88.915	60.735	1.00	0.00
ATOM	1504	CA	MET	A	99	53.289	90.187	60.160	1.00	0.00
ATOM	1505	HA	MET	A	99	53.161	90.739	59.229	1.00	0.00
ATOM	1506	CB	MET	A	99	52.801	91.120	61.291	1.00	0.00
ATOM	1507	HB1	MET	A	99	53.510	91.940	61.405	1.00	0.00
ATOM	1508	HB2	MET	A	99	52.765	90.575	62.232	1.00	0.00
ATOM	1509	CG	MET	A	99	51.432	91.749	60.995	1.00	0.00
ATOM	1510	HG1	MET	A	99	50.699	90.950	60.899	1.00	0.00
ATOM	1511	HG2	MET	A	99	51.506	92.262	60.041	1.00	0.00
ATOM	1512	SD	MET	A	99	50.802	92.943	62.213	1.00	0.00
ATOM	1513	CE	MET	A	99	51.911	94.346	61.930	1.00	0.00
ATOM	1514	HE1	MET	A	99	52.945	94.042	62.091	1.00	0.00
ATOM	1515	HE2	MET	A	99	51.789	94.696	60.906	1.00	0.00
ATOM	1516	HE3	MET	A	99	51.665	95.152	62.618	1.00	0.00
ATOM	1517	C	MET	A	99	54.827	89.959	60.272	1.00	0.00
ATOM	1518	O	MET	A	99	55.475	90.320	61.253	1.00	0.00
ATOM	1519	N	MET	A	100	55.469	89.376	59.265	1.00	0.00
ATOM	1520	H	MET	A	100	54.893	89.065	58.489	1.00	0.00
ATOM	1521	CA	MET	A	100	56.900	89.012	59.259	1.00	0.00
ATOM	1522	HA	MET	A	100	56.951	87.985	59.624	1.00	0.00
ATOM	1523	CB	MET	A	100	57.419	88.990	57.811	1.00	0.00

ATOM	1524	HB1	MET	A	100	58.483	88.754	57.828	1.00	0.00
ATOM	1525	HB2	MET	A	100	57.291	89.975	57.362	1.00	0.00
ATOM	1526	CG	MET	A	100	56.714	87.934	56.953	1.00	0.00
ATOM	1527	HG1	MET	A	100	55.678	88.235	56.788	1.00	0.00
ATOM	1528	HG2	MET	A	100	56.710	86.997	57.511	1.00	0.00
ATOM	1529	SD	MET	A	100	57.495	87.622	55.342	1.00	0.00
ATOM	1530	CE	MET	A	100	57.043	89.151	54.480	1.00	0.00
ATOM	1531	HE1	MET	A	100	57.405	89.116	53.453	1.00	0.00
ATOM	1532	HE2	MET	A	100	55.958	89.262	54.473	1.00	0.00
ATOM	1533	HE3	MET	A	100	57.485	90.007	54.985	1.00	0.00
ATOM	1534	C	MET	A	100	57.860	89.851	60.151	1.00	0.00
ATOM	1535	O	MET	A	100	58.179	91.021	59.894	1.00	0.00
ATOM	1536	N	LYS	A	101	58.414	89.204	61.191	1.00	0.00
ATOM	1537	H	LYS	A	101	58.117	88.256	61.384	1.00	0.00
ATOM	1538	CA	LYS	A	101	59.452	89.812	62.046	1.00	0.00
ATOM	1539	HA	LYS	A	101	59.095	90.803	62.334	1.00	0.00
ATOM	1540	CB	LYS	A	101	59.664	88.980	63.332	1.00	0.00
ATOM	1541	HB1	LYS	A	101	60.646	89.191	63.751	1.00	0.00
ATOM	1542	HB2	LYS	A	101	59.627	87.914	63.104	1.00	0.00
ATOM	1543	CG	LYS	A	101	58.579	89.334	64.371	1.00	0.00
ATOM	1544	HG1	LYS	A	101	57.646	88.904	64.021	1.00	0.00
ATOM	1545	HG2	LYS	A	101	58.477	90.415	64.413	1.00	0.00
ATOM	1546	CD	LYS	A	101	58.837	88.840	65.803	1.00	0.00
ATOM	1547	HD1	LYS	A	101	59.727	89.325	66.205	1.00	0.00
ATOM	1548	HD2	LYS	A	101	59.027	87.766	65.760	1.00	0.00
ATOM	1549	CE	LYS	A	101	57.636	89.075	66.744	1.00	0.00
ATOM	1550	HE1	LYS	A	101	57.828	88.550	67.685	1.00	0.00
ATOM	1551	HE2	LYS	A	101	56.751	88.613	66.298	1.00	0.00
ATOM	1552	NZ	LYS	A	101	57.351	90.508	67.046	1.00	0.00
ATOM	1553	HZ1	LYS	A	101	56.560	90.587	67.673	1.00	0.00
ATOM	1554	HZ2	LYS	A	101	58.110	90.944	67.553	1.00	0.00
ATOM	1555	HZ3	LYS	A	101	57.135	91.038	66.209	1.00	0.00
ATOM	1556	C	LYS	A	101	60.753	90.116	61.289	1.00	0.00
ATOM	1557	O	LYS	A	101	61.391	91.126	61.565	1.00	0.00
ATOM	1558	N	GLY	A	102	61.070	89.331	60.258	1.00	0.00
ATOM	1559	H	GLY	A	102	60.515	88.504	60.123	1.00	0.00
ATOM	1560	CA	GLY	A	102	62.066	89.692	59.238	1.00	0.00
ATOM	1561	HA1	GLY	A	102	62.334	88.799	58.673	1.00	0.00
ATOM	1562	HA2	GLY	A	102	62.963	90.072	59.731	1.00	0.00
ATOM	1563	C	GLY	A	102	61.575	90.761	58.247	1.00	0.00
ATOM	1564	O	GLY	A	102	62.382	91.316	57.501	1.00	0.00
ATOM	1565	N	GLY	A	103	60.276	91.087	58.249	1.00	0.00
ATOM	1566	H	GLY	A	103	59.665	90.660	58.930	1.00	0.00
ATOM	1567	CA	GLY	A	103	59.700	92.186	57.472	1.00	0.00
ATOM	1568	HA1	GLY	A	103	58.629	92.015	57.365	1.00	0.00
ATOM	1569	HA2	GLY	A	103	60.148	92.210	56.479	1.00	0.00
ATOM	1570	C	GLY	A	103	59.882	93.557	58.120	1.00	0.00
ATOM	1571	O	GLY	A	103	59.458	94.556	57.544	1.00	0.00
ATOM	1572	N	ILE	A	104	60.598	93.631	59.249	1.00	0.00
ATOM	1573	H	ILE	A	104	60.895	92.778	59.700	1.00	0.00
ATOM	1574	CA	ILE	A	104	61.363	94.867	59.546	1.00	0.00
ATOM	1575	HA	ILE	A	104	61.360	95.455	58.628	1.00	0.00
ATOM	1576	CB	ILE	A	104	60.616	95.730	60.578	1.00	0.00
ATOM	1577	HB	ILE	A	104	59.559	95.534	60.416	1.00	0.00
ATOM	1578	CG2	ILE	A	104	60.974	95.307	62.003	1.00	0.00
ATOM	1579	1HG2	ILE	A	104	60.246	95.718	62.700	1.00	0.00
ATOM	1580	2HG2	ILE	A	104	60.957	94.220	62.059	1.00	0.00
ATOM	1581	3HG2	ILE	A	104	61.968	95.659	62.274	1.00	0.00
ATOM	1582	CG1	ILE	A	104	60.807	97.244	60.359	1.00	0.00
ATOM	1583	1HG1	ILE	A	104	60.801	97.461	59.292	1.00	0.00
ATOM	1584	2HG1	ILE	A	104	61.759	97.569	60.773	1.00	0.00

ATOM	1585	CD	ILE	A	104	59.679	98.055	61.001	1.00	0.00
ATOM	1586	HD1	ILE	A	104	59.862	99.118	60.851	1.00	0.00
ATOM	1587	HD2	ILE	A	104	58.727	97.794	60.544	1.00	0.00
ATOM	1588	HD3	ILE	A	104	59.632	97.841	62.062	1.00	0.00
ATOM	1589	C	ILE	A	104	62.863	94.646	59.789	1.00	0.00
ATOM	1590	O	ILE	A	104	63.349	93.515	59.733	1.00	0.00
ATOM	1591	N	ARG	A	105	63.622	95.733	59.968	1.00	0.00
ATOM	1592	H	ARG	A	105	63.145	96.620	60.040	1.00	0.00
ATOM	1593	CA	ARG	A	105	65.091	95.748	60.096	1.00	0.00
ATOM	1594	HA	ARG	A	105	65.414	94.724	60.277	1.00	0.00
ATOM	1595	CB	ARG	A	105	65.723	96.249	58.780	1.00	0.00
ATOM	1596	HB1	ARG	A	105	66.123	97.254	58.929	1.00	0.00
ATOM	1597	HB2	ARG	A	105	64.950	96.332	58.014	1.00	0.00
ATOM	1598	CG	ARG	A	105	66.838	95.344	58.220	1.00	0.00
ATOM	1599	HG1	ARG	A	105	67.709	95.422	58.872	1.00	0.00
ATOM	1600	HG2	ARG	A	105	67.130	95.742	57.248	1.00	0.00
ATOM	1601	CD	ARG	A	105	66.497	93.847	58.056	1.00	0.00
ATOM	1602	HD1	ARG	A	105	66.589	93.360	59.028	1.00	0.00
ATOM	1603	HD2	ARG	A	105	67.237	93.381	57.403	1.00	0.00
ATOM	1604	NE	ARG	A	105	65.137	93.576	57.541	1.00	0.00
ATOM	1605	HE	ARG	A	105	64.472	93.190	58.210	1.00	0.00
ATOM	1606	CZ	ARG	A	105	64.676	93.751	56.317	1.00	0.00
ATOM	1607	NH1	ARG	A	105	65.389	94.311	55.384	1.00	0.00
ATOM	1608	1HH1	ARG	A	105	66.327	94.583	55.601	1.00	0.00
ATOM	1609	2HH1	ARG	A	105	65.024	94.410	54.455	1.00	0.00
ATOM	1610	NH2	ARG	A	105	63.479	93.365	56.000	1.00	0.00
ATOM	1611	1HH2	ARG	A	105	62.982	92.763	56.654	1.00	0.00
ATOM	1612	2HH2	ARG	A	105	63.122	93.473	55.072	1.00	0.00
ATOM	1613	C	ARG	A	105	65.529	96.517	61.349	1.00	0.00
ATOM	1614	O	ARG	A	105	64.705	97.200	61.965	1.00	0.00
ATOM	1615	N	LYS	A	106	66.798	96.381	61.759	1.00	0.00
ATOM	1616	H	LYS	A	106	67.446	95.859	61.193	1.00	0.00
ATOM	1617	CA	LYS	A	106	67.278	96.910	63.049	1.00	0.00
ATOM	1618	HA	LYS	A	106	66.406	97.274	63.572	1.00	0.00
ATOM	1619	CB	LYS	A	106	67.868	95.819	63.971	1.00	0.00
ATOM	1620	HB1	LYS	A	106	68.335	96.326	64.817	1.00	0.00
ATOM	1621	HB2	LYS	A	106	68.648	95.266	63.445	1.00	0.00
ATOM	1622	CG	LYS	A	106	66.809	94.836	64.527	1.00	0.00
ATOM	1623	HG1	LYS	A	106	66.768	93.959	63.880	1.00	0.00
ATOM	1624	HG2	LYS	A	106	65.827	95.308	64.520	1.00	0.00
ATOM	1625	CD	LYS	A	106	67.116	94.401	65.976	1.00	0.00
ATOM	1626	HD1	LYS	A	106	67.068	95.281	66.619	1.00	0.00
ATOM	1627	HD2	LYS	A	106	68.122	93.983	66.025	1.00	0.00
ATOM	1628	CE	LYS	A	106	66.109	93.355	66.486	1.00	0.00
ATOM	1629	HE1	LYS	A	106	66.251	92.423	65.932	1.00	0.00
ATOM	1630	HE2	LYS	A	106	65.098	93.711	66.277	1.00	0.00
ATOM	1631	NZ	LYS	A	106	66.232	93.102	67.944	1.00	0.00
ATOM	1632	HZ1	LYS	A	106	67.157	92.796	68.201	1.00	0.00
ATOM	1633	HZ2	LYS	A	106	66.001	93.941	68.473	1.00	0.00
ATOM	1634	HZ3	LYS	A	106	65.566	92.386	68.242	1.00	0.00
ATOM	1635	C	LYS	A	106	68.160	98.154	62.957	1.00	0.00
ATOM	1636	O	LYS	A	106	68.985	98.290	62.056	1.00	0.00
ATOM	1637	N	ASP	A	107	67.913	99.077	63.883	1.00	0.00
ATOM	1638	H	ASP	A	107	67.322	98.787	64.657	1.00	0.00
ATOM	1639	CA	ASP	A	107	67.901	100.509	63.594	1.00	0.00
ATOM	1640	HA	ASP	A	107	67.185	100.621	62.778	1.00	0.00
ATOM	1641	CB	ASP	A	107	67.362	101.323	64.781	1.00	0.00
ATOM	1642	HB1	ASP	A	107	67.771	102.334	64.746	1.00	0.00
ATOM	1643	HB2	ASP	A	107	67.666	100.862	65.723	1.00	0.00
ATOM	1644	CG	ASP	A	107	65.840	101.433	64.693	1.00	0.00
ATOM	1645	OD1	ASP	A	107	65.133	101.076	65.657	1.00	0.00

```
ATOM   1646  OD2 ASP A 107      65.319 101.774  63.606  1.00  0.00
ATOM   1647  C   ASP A 107      69.198 101.098  63.046  1.00  0.00
ATOM   1648  O   ASP A 107      70.287 100.961  63.605  1.00  0.00
ATOM   1649  N   ARG A 108      69.037 101.835  61.941  1.00  0.00
ATOM   1650  H   ARG A 108      68.089 101.931  61.596  1.00  0.00
ATOM   1651  CA  ARG A 108      70.106 102.441  61.137  1.00  0.00
ATOM   1652  HA  ARG A 108      70.884 101.683  61.039  1.00  0.00
ATOM   1653  CB  ARG A 108      69.543 102.739  59.728  1.00  0.00
ATOM   1654  HB1 ARG A 108      69.160 103.761  59.702  1.00  0.00
ATOM   1655  HB2 ARG A 108      68.697 102.079  59.525  1.00  0.00
ATOM   1656  CG  ARG A 108      70.586 102.496  58.621  1.00  0.00
ATOM   1657  HG1 ARG A 108      70.307 101.596  58.072  1.00  0.00
ATOM   1658  HG2 ARG A 108      71.576 102.328  59.049  1.00  0.00
ATOM   1659  CD  ARG A 108      70.686 103.660  57.634  1.00  0.00
ATOM   1660  HD1 ARG A 108      69.687 103.925  57.277  1.00  0.00
ATOM   1661  HD2 ARG A 108      71.288 103.327  56.785  1.00  0.00
ATOM   1662  NE  ARG A 108      71.321 104.823  58.277  1.00  0.00
ATOM   1663  HE  ARG A 108      71.248 104.873  59.288  1.00  0.00
ATOM   1664  CZ  ARG A 108      72.056 105.746  57.692  1.00  0.00
ATOM   1665  NH1 ARG A 108      72.259 105.771  56.404  1.00  0.00
ATOM   1666 1HH1 ARG A 108      71.821 105.073  55.833  1.00  0.00
ATOM   1667 2HH1 ARG A 108      72.831 106.481  55.987  1.00  0.00
ATOM   1668  NH2 ARG A 108      72.614 106.679  58.408  1.00  0.00
ATOM   1669 1HH2 ARG A 108      72.491 106.662  59.406  1.00  0.00
ATOM   1670 2HH2 ARG A 108      73.177 107.386  57.970  1.00  0.00
ATOM   1671  C   ARG A 108      70.772 103.666  61.795  1.00  0.00
ATOM   1672  O   ARG A 108      71.318 104.528  61.099  1.00  0.00
ATOM   1673  N   ARG A 109      70.717 103.749  63.129  1.00  0.00
ATOM   1674  H   ARG A 109      70.222 102.991  63.580  1.00  0.00
ATOM   1675  CA  ARG A 109      71.344 104.775  63.986  1.00  0.00
ATOM   1676  HA  ARG A 109      71.957 105.439  63.373  1.00  0.00
ATOM   1677  CB  ARG A 109      70.270 105.619  64.702  1.00  0.00
ATOM   1678  HB1 ARG A 109      70.755 106.180  65.503  1.00  0.00
ATOM   1679  HB2 ARG A 109      69.533 104.958  65.163  1.00  0.00
ATOM   1680  CG  ARG A 109      69.547 106.633  63.799  1.00  0.00
ATOM   1681  HG1 ARG A 109      68.974 106.090  63.047  1.00  0.00
ATOM   1682  HG2 ARG A 109      70.282 107.258  63.291  1.00  0.00
ATOM   1683  CD  ARG A 109      68.589 107.532  64.603  1.00  0.00
ATOM   1684  HD1 ARG A 109      67.890 106.893  65.147  1.00  0.00
ATOM   1685  HD2 ARG A 109      68.019 108.136  63.895  1.00  0.00
ATOM   1686  NE  ARG A 109      69.297 108.428  65.548  1.00  0.00
ATOM   1687  HE  ARG A 109      70.303 108.404  65.521  1.00  0.00
ATOM   1688  CZ  ARG A 109      68.738 109.240  66.433  1.00  0.00
ATOM   1689  NH1 ARG A 109      67.446 109.341  66.541  1.00  0.00
ATOM   1690 1HH1 ARG A 109      66.869 108.806  65.917  1.00  0.00
ATOM   1691 2HH1 ARG A 109      67.033 109.949  67.223  1.00  0.00
ATOM   1692  NH2 ARG A 109      69.471 109.971  67.228  1.00  0.00
ATOM   1693 1HH2 ARG A 109      70.473 109.938  67.174  1.00  0.00
ATOM   1694 2HH2 ARG A 109      69.033 110.589  67.888  1.00  0.00
ATOM   1695  C   ARG A 109      72.293 104.213  65.047  1.00  0.00
ATOM   1696  O   ARG A 109      73.149 104.951  65.517  1.00  0.00
ATOM   1697  N   GLY A 110      72.149 102.943  65.438  1.00  0.00
ATOM   1698  H   GLY A 110      71.416 102.386  65.029  1.00  0.00
ATOM   1699  CA  GLY A 110      72.922 102.362  66.541  1.00  0.00
ATOM   1700  HA1 GLY A 110      72.561 101.353  66.748  1.00  0.00
ATOM   1701  HA2 GLY A 110      72.762 102.962  67.438  1.00  0.00
ATOM   1702  C   GLY A 110      74.422 102.283  66.267  1.00  0.00
ATOM   1703  O   GLY A 110      74.819 101.758  65.220  1.00  0.00
ATOM   1704  N   GLY A 111      75.240 102.744  67.221  1.00  0.00
ATOM   1705  H   GLY A 111      74.824 103.191  68.025  1.00  0.00
ATOM   1706  CA  GLY A 111      76.700 102.581  67.234  1.00  0.00
```

ATOM	1707	HA1	GLY	A	111	77.096	102.918	68.192	1.00	0.00
ATOM	1708	HA2	GLY	A	111	77.138	103.193	66.447	1.00	0.00
ATOM	1709	C	GLY	A	111	77.107	101.121	67.034	1.00	0.00
ATOM	1710	O	GLY	A	111	77.009	100.310	67.954	1.00	0.00
ATOM	1711	N	ARG	A	112	77.514	100.759	65.812	1.00	0.00
ATOM	1712	H	ARG	A	112	77.514	101.475	65.097	1.00	0.00
ATOM	1713	CA	ARG	A	112	77.558	99.351	65.383	1.00	0.00
ATOM	1714	HA	ARG	A	112	76.600	98.928	65.685	1.00	0.00
ATOM	1715	CB	ARG	A	112	77.648	99.275	63.842	1.00	0.00
ATOM	1716	HB1	ARG	A	112	78.696	99.329	63.544	1.00	0.00
ATOM	1717	HB2	ARG	A	112	77.157	100.153	63.420	1.00	0.00
ATOM	1718	CG	ARG	A	112	76.994	98.031	63.192	1.00	0.00
ATOM	1719	HG1	ARG	A	112	77.603	97.153	63.408	1.00	0.00
ATOM	1720	HG2	ARG	A	112	77.023	98.178	62.112	1.00	0.00
ATOM	1721	CD	ARG	A	112	75.538	97.723	63.597	1.00	0.00
ATOM	1722	HD1	ARG	A	112	75.543	97.218	64.565	1.00	0.00
ATOM	1723	HD2	ARG	A	112	75.111	97.042	62.861	1.00	0.00
ATOM	1724	NE	ARG	A	112	74.718	98.944	63.710	1.00	0.00
ATOM	1725	HE	ARG	A	112	75.192	99.761	64.066	1.00	0.00
ATOM	1726	CZ	ARG	A	112	73.434	99.129	63.463	1.00	0.00
ATOM	1727	NH1	ARG	A	112	72.607	98.216	63.029	1.00	0.00
ATOM	1728	1HH1	ARG	A	112	72.939	97.289	62.845	1.00	0.00
ATOM	1729	2HH1	ARG	A	112	71.643	98.456	62.845	1.00	0.00
ATOM	1730	NH2	ARG	A	112	72.957	100.308	63.675	1.00	0.00
ATOM	1731	1HH2	ARG	A	112	73.571	101.011	64.069	1.00	0.00
ATOM	1732	2HH2	ARG	A	112	71.960	100.463	63.587	1.00	0.00
ATOM	1733	C	ARG	A	112	78.579	98.462	66.094	1.00	0.00
ATOM	1734	O	ARG	A	112	78.533	97.257	65.882	1.00	0.00
ATOM	1735	N	MET	A	113	79.420	98.996	66.988	1.00	0.00
ATOM	1736	H	MET	A	113	79.369	99.994	67.123	1.00	0.00
ATOM	1737	CA	MET	A	113	80.331	98.242	67.874	1.00	0.00
ATOM	1738	HA	MET	A	113	81.098	97.786	67.246	1.00	0.00
ATOM	1739	CB	MET	A	113	81.055	99.196	68.842	1.00	0.00
ATOM	1740	HB1	MET	A	113	81.489	98.606	69.649	1.00	0.00
ATOM	1741	HB2	MET	A	113	80.340	99.887	69.288	1.00	0.00
ATOM	1742	CG	MET	A	113	82.199	99.968	68.166	1.00	0.00
ATOM	1743	HG1	MET	A	113	82.865	99.247	67.691	1.00	0.00
ATOM	1744	HG2	MET	A	113	82.777	100.474	68.941	1.00	0.00
ATOM	1745	SD	MET	A	113	81.740	101.208	66.913	1.00	0.00
ATOM	1746	CE	MET	A	113	81.132	102.536	67.987	1.00	0.00
ATOM	1747	HE1	MET	A	113	80.318	102.171	68.611	1.00	0.00
ATOM	1748	HE2	MET	A	113	81.942	102.891	68.622	1.00	0.00
ATOM	1749	HE3	MET	A	113	80.774	103.365	67.380	1.00	0.00
ATOM	1750	C	MET	A	113	79.674	97.066	68.623	1.00	0.00
ATOM	1751	O	MET	A	113	80.362	96.113	68.971	1.00	0.00
ATOM	1752	N	LEU	A	114	78.346	97.081	68.770	1.00	0.00
ATOM	1753	H	LEU	A	114	77.877	97.942	68.529	1.00	0.00
ATOM	1754	CA	LEU	A	114	77.500	95.960	69.199	1.00	0.00
ATOM	1755	HA	LEU	A	114	77.692	95.808	70.262	1.00	0.00
ATOM	1756	CB	LEU	A	114	76.030	96.428	69.003	1.00	0.00
ATOM	1757	HB1	LEU	A	114	75.732	96.220	67.975	1.00	0.00
ATOM	1758	HB2	LEU	A	114	76.002	97.514	69.120	1.00	0.00
ATOM	1759	CG	LEU	A	114	74.927	95.904	69.946	1.00	0.00
ATOM	1760	HG	LEU	A	114	74.019	96.461	69.715	1.00	0.00
ATOM	1761	CD1	LEU	A	114	74.607	94.428	69.752	1.00	0.00
ATOM	1762	1HD1	LEU	A	114	73.747	94.162	70.367	1.00	0.00
ATOM	1763	2HD1	LEU	A	114	74.360	94.243	68.707	1.00	0.00
ATOM	1764	3HD1	LEU	A	114	75.455	93.822	70.064	1.00	0.00
ATOM	1765	CD2	LEU	A	114	75.252	96.157	71.417	1.00	0.00
ATOM	1766	1HD2	LEU	A	114	74.382	95.915	72.028	1.00	0.00
ATOM	1767	2HD2	LEU	A	114	76.086	95.537	71.743	1.00	0.00

```
ATOM   1768 3HD2 LEU A 114      75.498  97.209  71.562  1.00  0.00
ATOM   1769  C   LEU A 114      77.828  94.626  68.488  1.00  0.00
ATOM   1770  O   LEU A 114      77.903  93.583  69.149  1.00  0.00
ATOM   1771  N   LYS A 115      78.066  94.667  67.163  1.00  0.00
ATOM   1772  H   LYS A 115      78.041  95.584  66.736  1.00  0.00
ATOM   1773  CA  LYS A 115      78.507  93.518  66.337  1.00  0.00
ATOM   1774  HA  LYS A 115      79.198  92.961  66.973  1.00  0.00
ATOM   1775  CB  LYS A 115      77.314  92.573  66.025  1.00  0.00
ATOM   1776  HB1 LYS A 115      77.442  92.139  65.033  1.00  0.00
ATOM   1777  HB2 LYS A 115      76.383  93.141  65.995  1.00  0.00
ATOM   1778  CG  LYS A 115      77.197  91.391  67.028  1.00  0.00
ATOM   1779  HG1 LYS A 115      78.091  91.341  67.648  1.00  0.00
ATOM   1780  HG2 LYS A 115      77.165  90.462  66.456  1.00  0.00
ATOM   1781  CD  LYS A 115      75.952  91.435  67.928  1.00  0.00
ATOM   1782  HD1 LYS A 115      75.088  91.112  67.344  1.00  0.00
ATOM   1783  HD2 LYS A 115      75.765  92.448  68.265  1.00  0.00
ATOM   1784  CE  LYS A 115      76.088  90.507  69.156  1.00  0.00
ATOM   1785  HE1 LYS A 115      76.541  89.570  68.821  1.00  0.00
ATOM   1786  HE2 LYS A 115      75.094  90.269  69.547  1.00  0.00
ATOM   1787  NZ  LYS A 115      76.907  91.100  70.241  1.00  0.00
ATOM   1788  HZ1 LYS A 115      76.364  91.535  70.984  1.00  0.00
ATOM   1789  HZ2 LYS A 115      77.361  90.375  70.803  1.00  0.00
ATOM   1790  HZ3 LYS A 115      77.567  91.797  69.917  1.00  0.00
ATOM   1791  C   LYS A 115      79.415  93.819  65.127  1.00  0.00
ATOM   1792  O   LYS A 115      79.702  92.926  64.339  1.00  0.00
ATOM   1793  N   HIS A 116      79.939  95.039  64.987  1.00  0.00
ATOM   1794  H   HIS A 116      79.666  95.752  65.650  1.00  0.00
ATOM   1795  CA  HIS A 116      80.995  95.371  64.019  1.00  0.00
ATOM   1796  HA  HIS A 116      80.666  94.976  63.059  1.00  0.00
ATOM   1797  CB  HIS A 116      81.116  96.901  63.855  1.00  0.00
ATOM   1798  HB1 HIS A 116      81.623  97.322  64.723  1.00  0.00
ATOM   1799  HB2 HIS A 116      80.110  97.312  63.837  1.00  0.00
ATOM   1800  CG  HIS A 116      81.772  97.381  62.578  1.00  0.00
ATOM   1801  ND1 HIS A 116      82.472  98.590  62.438  1.00  0.00
ATOM   1802  CE1 HIS A 116      82.775  98.696  61.132  1.00  0.00
ATOM   1803  HE1 HIS A 116      83.299  99.533  60.685  1.00  0.00
ATOM   1804  NE2 HIS A 116      82.321  97.628  60.458  1.00  0.00
ATOM   1805  HE2 HIS A 116      82.407  97.491  59.457  1.00  0.00
ATOM   1806  CD2 HIS A 116      81.673  96.799  61.345  1.00  0.00
ATOM   1807  HD2 HIS A 116      81.132  95.895  61.096  1.00  0.00
ATOM   1808  C   HIS A 116      82.301  94.636  64.372  1.00  0.00
ATOM   1809  O   HIS A 116      83.080  95.105  65.186  1.00  0.00
ATOM   1810  N   LYS A 117      82.508  93.441  63.808  1.00  0.00
ATOM   1811  H   LYS A 117      81.740  93.078  63.257  1.00  0.00
ATOM   1812  CA  LYS A 117      83.569  92.469  64.153  1.00  0.00
ATOM   1813  HA  LYS A 117      83.516  92.319  65.231  1.00  0.00
ATOM   1814  CB  LYS A 117      83.250  91.120  63.454  1.00  0.00
ATOM   1815  HB1 LYS A 117      84.046  90.856  62.755  1.00  0.00
ATOM   1816  HB2 LYS A 117      82.345  91.225  62.851  1.00  0.00
ATOM   1817  CG  LYS A 117      83.016  89.931  64.406  1.00  0.00
ATOM   1818  HG1 LYS A 117      82.680  89.087  63.802  1.00  0.00
ATOM   1819  HG2 LYS A 117      82.214  90.185  65.098  1.00  0.00
ATOM   1820  CD  LYS A 117      84.255  89.493  65.203  1.00  0.00
ATOM   1821  HD1 LYS A 117      84.521  90.280  65.908  1.00  0.00
ATOM   1822  HD2 LYS A 117      85.090  89.337  64.519  1.00  0.00
ATOM   1823  CE  LYS A 117      83.964  88.186  65.960  1.00  0.00
ATOM   1824  HE1 LYS A 117      83.750  87.395  65.235  1.00  0.00
ATOM   1825  HE2 LYS A 117      83.073  88.326  66.577  1.00  0.00
ATOM   1826  NZ  LYS A 117      85.094  87.773  66.823  1.00  0.00
ATOM   1827  HZ1 LYS A 117      85.272  88.432  67.573  1.00  0.00
ATOM   1828  HZ2 LYS A 117      84.903  86.891  67.312  1.00  0.00
```

ATOM	1829	HZ3	LYS	A	117	85.941	87.587	66.309	1.00	0.00
ATOM	1830	C	LYS	A	117	85.025	92.895	63.889	1.00	0.00
ATOM	1831	O	LYS	A	117	85.931	92.086	64.055	1.00	0.00
ATOM	1832	N	ARG	A	118	85.279	94.155	63.516	1.00	0.00
ATOM	1833	H	ARG	A	118	84.476	94.761	63.461	1.00	0.00
ATOM	1834	CA	ARG	A	118	86.602	94.803	63.601	1.00	0.00
ATOM	1835	HA	ARG	A	118	87.328	94.171	63.083	1.00	0.00
ATOM	1836	CB	ARG	A	118	86.592	96.182	62.894	1.00	0.00
ATOM	1837	HB1	ARG	A	118	86.738	96.002	61.828	1.00	0.00
ATOM	1838	HB2	ARG	A	118	87.446	96.775	63.221	1.00	0.00
ATOM	1839	CG	ARG	A	118	85.317	97.052	63.006	1.00	0.00
ATOM	1840	HG1	ARG	A	118	84.510	96.512	62.512	1.00	0.00
ATOM	1841	HG2	ARG	A	118	85.472	97.970	62.439	1.00	0.00
ATOM	1842	CD	ARG	A	118	84.825	97.436	64.417	1.00	0.00
ATOM	1843	HD1	ARG	A	118	84.841	96.567	65.062	1.00	0.00
ATOM	1844	HD2	ARG	A	118	83.777	97.710	64.370	1.00	0.00
ATOM	1845	NE	ARG	A	118	85.582	98.523	65.041	1.00	0.00
ATOM	1846	HE	ARG	A	118	86.298	98.242	65.698	1.00	0.00
ATOM	1847	CZ	ARG	A	118	85.299	99.808	64.968	1.00	0.00
ATOM	1848	NH1	ARG	A	118	84.358	100.282	64.190	1.00	0.00
ATOM	1849	1HH1	ARG	A	118	83.792	99.638	63.647	1.00	0.00
ATOM	1850	2HH1	ARG	A	118	84.097	101.248	64.234	1.00	0.00
ATOM	1851	NH2	ARG	A	118	85.963	100.648	65.706	1.00	0.00
ATOM	1852	1HH2	ARG	A	118	86.609	100.261	66.390	1.00	0.00
ATOM	1853	2HH2	ARG	A	118	85.776	101.630	65.679	1.00	0.00
ATOM	1854	C	ARG	A	118	87.069	94.873	65.068	1.00	0.00
ATOM	1855	O	ARG	A	118	86.976	95.937	65.652	1.00	0.00
ATOM	1856	N	GLN	A	119	87.534	93.768	65.675	1.00	0.00
ATOM	1857	H	GLN	A	119	87.530	92.934	65.104	1.00	0.00
ATOM	1858	CA	GLN	A	119	87.668	93.567	67.144	1.00	0.00
ATOM	1859	HA	GLN	A	119	86.657	93.516	67.551	1.00	0.00
ATOM	1860	CB	GLN	A	119	88.361	92.218	67.474	1.00	0.00
ATOM	1861	HB1	GLN	A	119	88.487	92.132	68.554	1.00	0.00
ATOM	1862	HB2	GLN	A	119	89.364	92.251	67.043	1.00	0.00
ATOM	1863	CG	GLN	A	119	87.692	90.912	66.981	1.00	0.00
ATOM	1864	HG1	GLN	A	119	88.477	90.255	66.604	1.00	0.00
ATOM	1865	HG2	GLN	A	119	87.035	91.127	66.142	1.00	0.00
ATOM	1866	CD	GLN	A	119	86.892	90.114	68.029	1.00	0.00
ATOM	1867	OE1	GLN	A	119	85.700	90.326	68.228	1.00	0.00
ATOM	1868	NE2	GLN	A	119	87.437	89.064	68.614	1.00	0.00
ATOM	1869	1HE2	GLN	A	119	88.397	88.810	68.439	1.00	0.00
ATOM	1870	2HE2	GLN	A	119	86.860	88.419	69.170	1.00	0.00
ATOM	1871	C	GLN	A	119	88.368	94.694	67.953	1.00	0.00
ATOM	1872	O	GLN	A	119	88.237	94.735	69.174	1.00	0.00
ATOM	1873	N	ARG	A	120	89.040	95.649	67.299	1.00	0.00
ATOM	1874	H	ARG	A	120	89.156	95.473	66.314	1.00	0.00
ATOM	1875	CA	ARG	A	120	89.117	97.075	67.688	1.00	0.00
ATOM	1876	HA	ARG	A	120	89.584	97.110	68.675	1.00	0.00
ATOM	1877	CB	ARG	A	120	90.079	97.808	66.736	1.00	0.00
ATOM	1878	HB1	ARG	A	120	91.065	97.348	66.815	1.00	0.00
ATOM	1879	HB2	ARG	A	120	90.186	98.828	67.096	1.00	0.00
ATOM	1880	CG	ARG	A	120	89.665	97.808	65.250	1.00	0.00
ATOM	1881	HG1	ARG	A	120	88.587	97.908	65.158	1.00	0.00
ATOM	1882	HG2	ARG	A	120	89.963	96.863	64.797	1.00	0.00
ATOM	1883	CD	ARG	A	120	90.307	98.962	64.468	1.00	0.00
ATOM	1884	HD1	ARG	A	120	90.106	98.821	63.404	1.00	0.00
ATOM	1885	HD2	ARG	A	120	91.388	98.949	64.628	1.00	0.00
ATOM	1886	NE	ARG	A	120	89.740	100.246	64.906	1.00	0.00
ATOM	1887	HE	ARG	A	120	89.111	100.212	65.693	1.00	0.00
ATOM	1888	CZ	ARG	A	120	90.149	101.460	64.601	1.00	0.00
ATOM	1889	NH1	ARG	A	120	91.106	101.697	63.755	1.00	0.00

```
ATOM   1890 1HH1 ARG A 120      91.627 100.940  63.366  1.00  0.00
ATOM   1891 2HH1 ARG A 120      91.557 102.623  63.798  1.00  0.00
ATOM   1892  NH2 ARG A 120      89.622 102.454  65.241  1.00  0.00
ATOM   1893 1HH2 ARG A 120      89.221 102.231  66.138  1.00  0.00
ATOM   1894 2HH2 ARG A 120      90.140 103.343  65.239  1.00  0.00
ATOM   1895  C   ARG A 120      87.752  97.780  67.881  1.00  0.00
ATOM   1896  O   ARG A 120      87.511  98.867  67.341  1.00  0.00
ATOM   1897  N   ASP A 121      86.836  97.174  68.643  1.00  0.00
ATOM   1898  H   ASP A 121      87.057  96.246  68.987  1.00  0.00
ATOM   1899  CA  ASP A 121      85.616  97.808  69.173  1.00  0.00
ATOM   1900  HA  ASP A 121      85.384  98.647  68.519  1.00  0.00
ATOM   1901  CB  ASP A 121      84.378  96.892  69.057  1.00  0.00
ATOM   1902  HB1 ASP A 121      84.217  96.676  67.999  1.00  0.00
ATOM   1903  HB2 ASP A 121      83.504  97.439  69.405  1.00  0.00
ATOM   1904  CG  ASP A 121      84.467  95.570  69.807  1.00  0.00
ATOM   1905  OD1 ASP A 121      83.890  95.404  70.903  1.00  0.00
ATOM   1906  OD2 ASP A 121      85.134  94.628  69.326  1.00  0.00
ATOM   1907  C   ASP A 121      85.855  98.478  70.547  1.00  0.00
ATOM   1908  O   ASP A 121      84.948  98.592  71.360  1.00  0.00
ATOM   1909  N   ASP A 122      87.085  98.972  70.757  1.00  0.00
ATOM   1910  H   ASP A 122      87.774  98.758  70.061  1.00  0.00
ATOM   1911  CA  ASP A 122      87.515  99.814  71.894  1.00  0.00
ATOM   1912  HA  ASP A 122      86.630 100.147  72.441  1.00  0.00
ATOM   1913  CB  ASP A 122      88.401  98.994  72.851  1.00  0.00
ATOM   1914  HB1 ASP A 122      89.176  99.636  73.274  1.00  0.00
ATOM   1915  HB2 ASP A 122      88.912  98.221  72.276  1.00  0.00
ATOM   1916  CG  ASP A 122      87.674  98.354  74.038  1.00  0.00
ATOM   1917  OD1 ASP A 122      86.430  98.375  74.158  1.00  0.00
ATOM   1918  OD2 ASP A 122      88.337  97.702  74.873  1.00  0.00
ATOM   1919  C   ASP A 122      88.266 101.108  71.513  1.00  0.00
ATOM   1920  O   ASP A 122      88.457 101.976  72.360  1.00  0.00
ATOM   1921  N   GLY A 123      88.705 101.250  70.261  1.00  0.00
ATOM   1922  H   GLY A 123      88.481 100.529  69.594  1.00  0.00
ATOM   1923  CA  GLY A 123      89.628 102.301  69.810  1.00  0.00
ATOM   1924  HA1 GLY A 123      90.363 102.504  70.589  1.00  0.00
ATOM   1925  HA2 GLY A 123      89.082 103.218  69.587  1.00  0.00
ATOM   1926  C   GLY A 123      90.341 101.819  68.548  1.00  0.00
ATOM   1927  O   GLY A 123      89.687 101.207  67.702  1.00  0.00
ATOM   1928  N   GLU A 124      91.651 102.058  68.427  1.00  0.00
ATOM   1929  H   GLU A 124      92.093 102.650  69.112  1.00  0.00
ATOM   1930  CA  GLU A 124      92.497 101.503  67.352  1.00  0.00
ATOM   1931  HA  GLU A 124      91.935 101.497  66.425  1.00  0.00
ATOM   1932  CB  GLU A 124      93.746 102.377  67.112  1.00  0.00
ATOM   1933  HB1 GLU A 124      94.285 101.965  66.259  1.00  0.00
ATOM   1934  HB2 GLU A 124      94.398 102.307  67.983  1.00  0.00
ATOM   1935  CG  GLU A 124      93.469 103.864  66.831  1.00  0.00
ATOM   1936  HG1 GLU A 124      94.392 104.336  66.489  1.00  0.00
ATOM   1937  HG2 GLU A 124      93.179 104.347  67.766  1.00  0.00
ATOM   1938  CD  GLU A 124      92.365 104.071  65.788  1.00  0.00
ATOM   1939  OE1 GLU A 124      91.221 104.361  66.198  1.00  0.00
ATOM   1940  OE2 GLU A 124      92.568 103.805  64.583  1.00  0.00
ATOM   1941  C   GLU A 124      92.932 100.047  67.589  1.00  0.00
ATOM   1942  O   GLU A 124      92.974  99.258  66.650  1.00  0.00
ATOM   1943  N   GLY A 125      93.171  99.677  68.852  1.00  0.00
ATOM   1944  H   GLY A 125      93.055 100.380  69.565  1.00  0.00
ATOM   1945  CA  GLY A 125      93.566  98.335  69.304  1.00  0.00
ATOM   1946  HA1 GLY A 125      94.629  98.342  69.550  1.00  0.00
ATOM   1947  HA2 GLY A 125      93.405  97.606  68.510  1.00  0.00
ATOM   1948  C   GLY A 125      92.765  97.906  70.537  1.00  0.00
ATOM   1949  O   GLY A 125      91.761  98.539  70.877  1.00  0.00
ATOM   1950  N   ARG A 126      93.174  96.815  71.203  1.00  0.00
```

```
ATOM   1951  H   ARG A 126      94.008  96.333  70.875  1.00  0.00
ATOM   1952  CA  ARG A 126      92.470  96.276  72.393  1.00  0.00
ATOM   1953  HA  ARG A 126      92.159  97.126  73.002  1.00  0.00
ATOM   1954  CB  ARG A 126      91.224  95.511  71.930  1.00  0.00
ATOM   1955  HB1 ARG A 126      91.471  94.479  71.676  1.00  0.00
ATOM   1956  HB2 ARG A 126      90.808  95.992  71.044  1.00  0.00
ATOM   1957  CG  ARG A 126      90.167  95.557  73.037  1.00  0.00
ATOM   1958  HG1 ARG A 126      89.990  96.595  73.296  1.00  0.00
ATOM   1959  HG2 ARG A 126      90.495  95.031  73.930  1.00  0.00
ATOM   1960  CD  ARG A 126      88.887  94.928  72.511  1.00  0.00
ATOM   1961  HD1 ARG A 126      88.945  93.848  72.613  1.00  0.00
ATOM   1962  HD2 ARG A 126      88.797  95.170  71.454  1.00  0.00
ATOM   1963  NE  ARG A 126      87.697  95.455  73.181  1.00  0.00
ATOM   1964  HE  ARG A 126      87.823  96.195  73.867  1.00  0.00
ATOM   1965  CZ  ARG A 126      86.480  95.231  72.742  1.00  0.00
ATOM   1966  NH1 ARG A 126      86.244  94.235  71.938  1.00  0.00
ATOM   1967 1HH1 ARG A 126      87.024  93.718  71.581  1.00  0.00
ATOM   1968 2HH1 ARG A 126      85.386  94.289  71.396  1.00  0.00
ATOM   1969  NH2 ARG A 126      85.481  96.013  73.032  1.00  0.00
ATOM   1970 1HH2 ARG A 126      85.681  96.935  73.427  1.00  0.00
ATOM   1971 2HH2 ARG A 126      84.656  95.919  72.436  1.00  0.00
ATOM   1972  C   ARG A 126      93.370  95.399  73.281  1.00  0.00
ATOM   1973  O   ARG A 126      92.948  94.393  73.845  1.00  0.00
ATOM   1974  N   GLY A 127      94.647  95.749  73.295  1.00  0.00
ATOM   1975  H   GLY A 127      94.871  96.632  72.869  1.00  0.00
ATOM   1976  CA  GLY A 127      95.742  94.788  73.353  1.00  0.00
ATOM   1977  HA1 GLY A 127      95.376  93.761  73.396  1.00  0.00
ATOM   1978  HA2 GLY A 127      96.373  94.985  74.218  1.00  0.00
ATOM   1979  C   GLY A 127      96.534  94.977  72.071  1.00  0.00
ATOM   1980  O   GLY A 127      95.927  94.929  70.999  1.00  0.00
ATOM   1981  N   GLU A 128      97.817  95.319  72.162  1.00  0.00
ATOM   1982  H   GLU A 128      98.280  95.365  73.054  1.00  0.00
ATOM   1983  CA  GLU A 128      98.566  95.710  70.967  1.00  0.00
ATOM   1984  HA  GLU A 128      97.953  96.463  70.469  1.00  0.00
ATOM   1985  CB  GLU A 128      99.910  96.362  71.349  1.00  0.00
ATOM   1986  HB1 GLU A 128     100.600  95.594  71.703  1.00  0.00
ATOM   1987  HB2 GLU A 128      99.732  97.062  72.168  1.00  0.00
ATOM   1988  CG  GLU A 128     100.550  97.141  70.189  1.00  0.00
ATOM   1989  HG1 GLU A 128     100.950  96.431  69.462  1.00  0.00
ATOM   1990  HG2 GLU A 128     101.385  97.730  70.572  1.00  0.00
ATOM   1991  CD  GLU A 128      99.522  98.058  69.512  1.00  0.00
ATOM   1992  OE1 GLU A 128      99.175  99.096  70.112  1.00  0.00
ATOM   1993  OE2 GLU A 128      98.962  97.605  68.486  1.00  0.00
ATOM   1994  C   GLU A 128      98.713  94.534  69.990  1.00  0.00
ATOM   1995  O   GLU A 128      98.841  93.375  70.408  1.00  0.00
ATOM   1996  N   VAL A 129      98.587  94.821  68.695  1.00  0.00
ATOM   1997  H   VAL A 129      98.576  95.817  68.465  1.00  0.00
ATOM   1998  CA  VAL A 129      98.347  93.890  67.572  1.00  0.00
ATOM   1999  HA  VAL A 129      97.937  94.530  66.791  1.00  0.00
ATOM   2000  CB  VAL A 129      99.690  93.364  67.003  1.00  0.00
ATOM   2001  HB  VAL A 129     100.424  94.166  67.104  1.00  0.00
ATOM   2002  CG1 VAL A 129     100.277  92.138  67.716  1.00  0.00
ATOM   2003 1HG1 VAL A 129     101.235  91.879  67.268  1.00  0.00
ATOM   2004 2HG1 VAL A 129     100.444  92.369  68.765  1.00  0.00
ATOM   2005 3HG1 VAL A 129      99.604  91.286  67.631  1.00  0.00
ATOM   2006  CG2 VAL A 129      99.605  93.081  65.495  1.00  0.00
ATOM   2007 1HG2 VAL A 129     100.592  92.812  65.118  1.00  0.00
ATOM   2008 2HG2 VAL A 129      98.912  92.265  65.300  1.00  0.00
ATOM   2009 3HG2 VAL A 129      99.267  93.976  64.974  1.00  0.00
ATOM   2010  C   VAL A 129      97.241  92.835  67.796  1.00  0.00
ATOM   2011  O   VAL A 129      97.141  91.849  67.072  1.00  0.00
```

ATOM	2012	N	GLY	A	130	96.375	93.044	68.794	1.00	0.00
ATOM	2013	H	GLY	A	130	96.509	93.862	69.377	1.00	0.00
ATOM	2014	CA	GLY	A	130	95.376	92.070	69.237	1.00	0.00
ATOM	2015	HA1	GLY	A	130	94.836	91.684	68.373	1.00	0.00
ATOM	2016	HA2	GLY	A	130	94.668	92.576	69.891	1.00	0.00
ATOM	2017	C	GLY	A	130	95.945	90.884	70.012	1.00	0.00
ATOM	2018	O	GLY	A	130	95.223	89.901	70.145	1.00	0.00
ATOM	2019	N	SER	A	131	97.197	91.005	70.496	1.00	0.00
ATOM	2020	H	SER	A	131	97.654	91.871	70.230	1.00	0.00
ATOM	2021	CA	SER	A	131	97.922	90.284	71.571	1.00	0.00
ATOM	2022	HA	SER	A	131	98.975	90.514	71.412	1.00	0.00
ATOM	2023	CB	SER	A	131	97.571	90.920	72.928	1.00	0.00
ATOM	2024	HB1	SER	A	131	98.115	91.862	73.015	1.00	0.00
ATOM	2025	HB2	SER	A	131	97.896	90.271	73.742	1.00	0.00
ATOM	2026	OG	SER	A	131	96.189	91.204	73.062	1.00	0.00
ATOM	2027	HG	SER	A	131	95.675	90.378	72.884	1.00	0.00
ATOM	2028	C	SER	A	131	97.890	88.744	71.654	1.00	0.00
ATOM	2029	O	SER	A	131	98.697	88.182	72.395	1.00	0.00
ATOM	2030	N	ALA	A	132	97.025	88.062	70.895	1.00	0.00
ATOM	2031	H	ALA	A	132	96.407	88.638	70.341	1.00	0.00
ATOM	2032	CA	ALA	A	132	96.728	86.627	70.849	1.00	0.00
ATOM	2033	HA	ALA	A	132	95.669	86.530	70.610	1.00	0.00
ATOM	2034	CB	ALA	A	132	97.507	86.015	69.675	1.00	0.00
ATOM	2035	HB1	ALA	A	132	97.196	84.981	69.531	1.00	0.00
ATOM	2036	HB2	ALA	A	132	97.296	86.568	68.760	1.00	0.00
ATOM	2037	HB3	ALA	A	132	98.578	86.046	69.878	1.00	0.00
ATOM	2038	C	ALA	A	132	96.935	85.859	72.179	1.00	0.00
ATOM	2039	O	ALA	A	132	97.429	84.732	72.215	1.00	0.00
ATOM	2040	N	GLY	A	133	96.620	86.469	73.312	1.00	0.00
ATOM	2041	H	GLY	A	133	96.086	87.327	73.227	1.00	0.00
ATOM	2042	CA	GLY	A	133	97.250	86.136	74.594	1.00	0.00
ATOM	2043	HA1	GLY	A	133	98.266	86.530	74.589	1.00	0.00
ATOM	2044	HA2	GLY	A	133	97.294	85.055	74.719	1.00	0.00
ATOM	2045	C	GLY	A	133	96.526	86.718	75.797	1.00	0.00
ATOM	2046	O	GLY	A	133	96.490	86.086	76.848	1.00	0.00
ATOM	2047	N	ASP	A	134	95.877	87.863	75.588	1.00	0.00
ATOM	2048	H	ASP	A	134	96.131	88.358	74.744	1.00	0.00
ATOM	2049	CA	ASP	A	134	94.557	88.232	76.114	1.00	0.00
ATOM	2050	HA	ASP	A	134	94.708	88.936	76.930	1.00	0.00
ATOM	2051	CB	ASP	A	134	93.813	88.990	74.998	1.00	0.00
ATOM	2052	HB1	ASP	A	134	94.172	90.022	74.994	1.00	0.00
ATOM	2053	HB2	ASP	A	134	92.751	89.022	75.196	1.00	0.00
ATOM	2054	CG	ASP	A	134	94.070	88.385	73.614	1.00	0.00
ATOM	2055	OD1	ASP	A	134	94.852	88.987	72.854	1.00	0.00
ATOM	2056	OD2	ASP	A	134	93.693	87.226	73.364	1.00	0.00
ATOM	2057	C	ASP	A	134	93.770	87.064	76.718	1.00	0.00
ATOM	2058	O	ASP	A	134	93.496	87.088	77.917	1.00	0.00
ATOM	2059	N	MET	A	135	93.499	86.012	75.941	1.00	0.00
ATOM	2060	H	MET	A	135	93.636	86.132	74.939	1.00	0.00
ATOM	2061	CA	MET	A	135	92.659	84.908	76.405	1.00	0.00
ATOM	2062	HA	MET	A	135	92.671	84.917	77.488	1.00	0.00
ATOM	2063	CB	MET	A	135	91.219	85.152	75.904	1.00	0.00
ATOM	2064	HB1	MET	A	135	90.592	84.284	76.104	1.00	0.00
ATOM	2065	HB2	MET	A	135	91.267	85.286	74.828	1.00	0.00
ATOM	2066	CG	MET	A	135	90.488	86.335	76.558	1.00	0.00
ATOM	2067	HG1	MET	A	135	90.985	86.584	77.494	1.00	0.00
ATOM	2068	HG2	MET	A	135	89.478	86.014	76.814	1.00	0.00
ATOM	2069	SD	MET	A	135	90.340	87.824	75.553	1.00	0.00
ATOM	2070	CE	MET	A	135	90.614	89.041	76.861	1.00	0.00
ATOM	2071	HE1	MET	A	135	89.834	88.961	77.616	1.00	0.00
ATOM	2072	HE2	MET	A	135	91.579	88.852	77.326	1.00	0.00

ATOM	2073	HE3	MET	A	135	90.620	90.039	76.432	1.00	0.00
ATOM	2074	C	MET	A	135	93.150	83.515	76.034	1.00	0.00
ATOM	2075	O	MET	A	135	92.308	82.677	75.718	1.00	0.00
ATOM	2076	N	ARG	A	136	94.459	83.221	76.120	1.00	0.00
ATOM	2077	H	ARG	A	136	95.094	83.954	76.418	1.00	0.00
ATOM	2078	CA	ARG	A	136	94.987	81.884	75.751	1.00	0.00
ATOM	2079	HA	ARG	A	136	94.782	81.785	74.706	1.00	0.00
ATOM	2080	CB	ARG	A	136	96.512	81.830	75.859	1.00	0.00
ATOM	2081	HB1	ARG	A	136	96.812	82.019	76.880	1.00	0.00
ATOM	2082	HB2	ARG	A	136	96.931	82.647	75.271	1.00	0.00
ATOM	2083	CG	ARG	A	136	97.093	80.494	75.384	1.00	0.00
ATOM	2084	HG1	ARG	A	136	96.673	79.660	75.943	1.00	0.00
ATOM	2085	HG2	ARG	A	136	98.132	80.507	75.656	1.00	0.00
ATOM	2086	CD	ARG	A	136	96.968	80.198	73.872	1.00	0.00
ATOM	2087	HD1	ARG	A	136	95.922	79.970	73.648	1.00	0.00
ATOM	2088	HD2	ARG	A	136	97.555	79.303	73.657	1.00	0.00
ATOM	2089	NE	ARG	A	136	97.416	81.333	73.027	1.00	0.00
ATOM	2090	HE	ARG	A	136	97.065	82.241	73.280	1.00	0.00
ATOM	2091	CZ	ARG	A	136	98.203	81.324	71.969	1.00	0.00
ATOM	2092	NH1	ARG	A	136	98.704	80.255	71.458	1.00	0.00
ATOM	2093	1HH1	ARG	A	136	98.297	79.385	71.739	1.00	0.00
ATOM	2094	2HH1	ARG	A	136	99.145	80.341	70.559	1.00	0.00
ATOM	2095	NH2	ARG	A	136	98.544	82.433	71.401	1.00	0.00
ATOM	2096	1HH2	ARG	A	136	98.151	83.309	71.722	1.00	0.00
ATOM	2097	2HH2	ARG	A	136	99.118	82.439	70.568	1.00	0.00
ATOM	2098	C	ARG	A	136	94.208	80.668	76.291	1.00	0.00
ATOM	2099	O	ARG	A	136	94.203	79.640	75.626	1.00	0.00
ATOM	2100	N	ALA	A	137	93.475	80.809	77.397	1.00	0.00
ATOM	2101	H	ALA	A	137	93.544	81.678	77.899	1.00	0.00
ATOM	2102	CA	ALA	A	137	92.377	79.919	77.775	1.00	0.00
ATOM	2103	HA	ALA	A	137	91.856	79.619	76.868	1.00	0.00
ATOM	2104	CB	ALA	A	137	92.979	78.669	78.415	1.00	0.00
ATOM	2105	HB1	ALA	A	137	92.199	77.920	78.531	1.00	0.00
ATOM	2106	HB2	ALA	A	137	93.765	78.277	77.776	1.00	0.00
ATOM	2107	HB3	ALA	A	137	93.399	78.908	79.390	1.00	0.00
ATOM	2108	C	ALA	A	137	91.334	80.592	78.705	1.00	0.00
ATOM	2109	O	ALA	A	137	90.945	79.998	79.711	1.00	0.00
ATOM	2110	N	ALA	A	138	90.956	81.851	78.429	1.00	0.00
ATOM	2111	H	ALA	A	138	91.216	82.234	77.529	1.00	0.00
ATOM	2112	CA	ALA	A	138	90.326	82.744	79.412	1.00	0.00
ATOM	2113	HA	ALA	A	138	90.474	82.304	80.397	1.00	0.00
ATOM	2114	CB	ALA	A	138	91.078	84.079	79.373	1.00	0.00
ATOM	2115	HB1	ALA	A	138	92.150	83.922	79.504	1.00	0.00
ATOM	2116	HB2	ALA	A	138	90.857	84.547	78.433	1.00	0.00
ATOM	2117	HB3	ALA	A	138	90.714	84.754	80.143	1.00	0.00
ATOM	2118	C	ALA	A	138	88.827	83.051	79.273	1.00	0.00
ATOM	2119	O	ALA	A	138	88.374	83.547	78.249	1.00	0.00
ATOM	2120	N	ASN	A	139	88.088	82.898	80.383	1.00	0.00
ATOM	2121	H	ASN	A	139	88.581	82.511	81.175	1.00	0.00
ATOM	2122	CA	ASN	A	139	86.668	82.555	80.314	1.00	0.00
ATOM	2123	HA	ASN	A	139	86.406	82.519	79.261	1.00	0.00
ATOM	2124	CB	ASN	A	139	86.520	81.126	80.892	1.00	0.00
ATOM	2125	HB1	ASN	A	139	85.481	80.815	80.842	1.00	0.00
ATOM	2126	HB2	ASN	A	139	86.798	81.177	81.933	1.00	0.00
ATOM	2127	CG	ASN	A	139	87.352	80.012	80.272	1.00	0.00
ATOM	2128	OD1	ASN	A	139	88.169	80.178	79.389	1.00	0.00
ATOM	2129	ND2	ASN	A	139	87.167	78.803	80.740	1.00	0.00
ATOM	2130	1HD2	ASN	A	139	86.493	78.637	81.464	1.00	0.00
ATOM	2131	2HD2	ASN	A	139	87.814	78.096	80.414	1.00	0.00
ATOM	2132	C	ASN	A	139	85.665	83.585	80.917	1.00	0.00
ATOM	2133	O	ASN	A	139	84.669	83.157	81.484	1.00	0.00

ATOM	2134	N	LEU	A	140	85.922	84.903	80.769	1.00	0.00
ATOM	2135	H	LEU	A	140	86.848	85.128	80.429	1.00	0.00
ATOM	2136	CA	LEU	A	140	84.969	86.055	80.649	1.00	0.00
ATOM	2137	HA	LEU	A	140	84.378	85.916	79.751	1.00	0.00
ATOM	2138	CB	LEU	A	140	83.979	86.119	81.831	1.00	0.00
ATOM	2139	HB1	LEU	A	140	84.530	86.288	82.755	1.00	0.00
ATOM	2140	HB2	LEU	A	140	83.510	85.146	81.909	1.00	0.00
ATOM	2141	CG	LEU	A	140	82.770	87.080	81.783	1.00	0.00
ATOM	2142	HG	LEU	A	140	82.026	86.677	82.444	1.00	0.00
ATOM	2143	CD1	LEU	A	140	83.062	88.432	82.393	1.00	0.00
ATOM	2144	1HD1	LEU	A	140	82.127	88.987	82.487	1.00	0.00
ATOM	2145	2HD1	LEU	A	140	83.483	88.313	83.393	1.00	0.00
ATOM	2146	3HD1	LEU	A	140	83.765	88.975	81.788	1.00	0.00
ATOM	2147	CD2	LEU	A	140	82.084	87.223	80.420	1.00	0.00
ATOM	2148	1HD2	LEU	A	140	81.121	87.713	80.569	1.00	0.00
ATOM	2149	2HD2	LEU	A	140	82.659	87.819	79.724	1.00	0.00
ATOM	2150	3HD2	LEU	A	140	81.905	86.237	80.000	1.00	0.00
ATOM	2151	C	LEU	A	140	85.781	87.373	80.452	1.00	0.00
ATOM	2152	O	LEU	A	140	86.968	87.414	80.778	1.00	0.00
ATOM	2153	N	TRP	A	141	85.195	88.405	79.823	1.00	0.00
ATOM	2154	H	TRP	A	141	84.227	88.302	79.584	1.00	0.00
ATOM	2155	CA	TRP	A	141	85.867	89.653	79.409	1.00	0.00
ATOM	2156	HA	TRP	A	141	86.589	89.902	80.189	1.00	0.00
ATOM	2157	CB	TRP	A	141	86.680	89.358	78.138	1.00	0.00
ATOM	2158	HB1	TRP	A	141	86.011	88.967	77.379	1.00	0.00
ATOM	2159	HB2	TRP	A	141	87.388	88.562	78.367	1.00	0.00
ATOM	2160	CG	TRP	A	141	87.476	90.465	77.526	1.00	0.00
ATOM	2161	CD1	TRP	A	141	88.164	91.426	78.187	1.00	0.00
ATOM	2162	HD1	TRP	A	141	88.209	91.532	79.269	1.00	0.00
ATOM	2163	NE1	TRP	A	141	88.858	92.201	77.277	1.00	0.00
ATOM	2164	HE1	TRP	A	141	89.545	92.885	77.555	1.00	0.00
ATOM	2165	CE2	TRP	A	141	88.667	91.767	75.983	1.00	0.00
ATOM	2166	CZ2	TRP	A	141	89.228	92.140	74.753	1.00	0.00
ATOM	2167	HZ2	TRP	A	141	89.961	92.937	74.705	1.00	0.00
ATOM	2168	CH2	TRP	A	141	88.895	91.408	73.600	1.00	0.00
ATOM	2169	HH2	TRP	A	141	89.368	91.639	72.652	1.00	0.00
ATOM	2170	CZ3	TRP	A	141	87.999	90.330	73.687	1.00	0.00
ATOM	2171	HZ3	TRP	A	141	87.776	89.738	72.802	1.00	0.00
ATOM	2172	CE3	TRP	A	141	87.451	89.953	74.928	1.00	0.00
ATOM	2173	HE3	TRP	A	141	86.842	89.061	74.992	1.00	0.00
ATOM	2174	CD2	TRP	A	141	87.775	90.658	76.109	1.00	0.00
ATOM	2175	C	TRP	A	141	84.914	90.888	79.301	1.00	0.00
ATOM	2176	O	TRP	A	141	84.812	91.531	78.262	1.00	0.00
ATOM	2177	N	PRO	A	142	84.169	91.227	80.368	1.00	0.00
ATOM	2178	CD	PRO	A	142	84.779	91.429	81.676	1.00	0.00
ATOM	2179	HD1	PRO	A	142	85.444	92.294	81.640	1.00	0.00
ATOM	2180	HD2	PRO	A	142	85.343	90.552	82.003	1.00	0.00
ATOM	2181	CG	PRO	A	142	83.653	91.710	82.670	1.00	0.00
ATOM	2182	HG1	PRO	A	142	83.874	92.614	83.235	1.00	0.00
ATOM	2183	HG2	PRO	A	142	83.540	90.876	83.358	1.00	0.00
ATOM	2184	CB	PRO	A	142	82.392	91.882	81.818	1.00	0.00
ATOM	2185	HB1	PRO	A	142	82.174	92.938	81.735	1.00	0.00
ATOM	2186	HB2	PRO	A	142	81.538	91.372	82.262	1.00	0.00
ATOM	2187	CA	PRO	A	142	82.696	91.343	80.418	1.00	0.00
ATOM	2188	HA	PRO	A	142	82.291	90.335	80.352	1.00	0.00
ATOM	2189	C	PRO	A	142	81.924	92.200	79.381	1.00	0.00
ATOM	2190	O	PRO	A	142	80.713	92.017	79.293	1.00	0.00
ATOM	2191	N	SER	A	143	82.572	93.139	78.668	1.00	0.00
ATOM	2192	H	SER	A	143	83.573	93.123	78.807	1.00	0.00
ATOM	2193	CA	SER	A	143	82.116	93.981	77.525	1.00	0.00
ATOM	2194	HA	SER	A	143	82.792	93.732	76.710	1.00	0.00

ATOM	2195	CB	SER	A	143	80.701	93.649	77.001	1.00	0.00
ATOM	2196	HB1	SER	A	143	80.595	92.583	76.824	1.00	0.00
ATOM	2197	HB2	SER	A	143	80.498	94.150	76.058	1.00	0.00
ATOM	2198	OG	SER	A	143	79.715	94.040	77.917	1.00	0.00
ATOM	2199	HG	SER	A	143	79.781	93.336	78.595	1.00	0.00
ATOM	2200	C	SER	A	143	82.233	95.510	77.728	1.00	0.00
ATOM	2201	O	SER	A	143	82.366	95.978	78.854	1.00	0.00
ATOM	2202	N	PRO	A	144	82.178	96.337	76.655	1.00	0.00
ATOM	2203	CD	PRO	A	144	82.401	95.981	75.254	1.00	0.00
ATOM	2204	HD1	PRO	A	144	81.746	95.190	74.900	1.00	0.00
ATOM	2205	HD2	PRO	A	144	83.441	95.682	75.124	1.00	0.00
ATOM	2206	CG	PRO	A	144	82.138	97.250	74.446	1.00	0.00
ATOM	2207	HG1	PRO	A	144	81.075	97.322	74.211	1.00	0.00
ATOM	2208	HG2	PRO	A	144	82.734	97.284	73.533	1.00	0.00
ATOM	2209	CB	PRO	A	144	82.527	98.356	75.422	1.00	0.00
ATOM	2210	HB1	PRO	A	144	82.028	99.296	75.186	1.00	0.00
ATOM	2211	HB2	PRO	A	144	83.609	98.492	75.397	1.00	0.00
ATOM	2212	CA	PRO	A	144	82.111	97.799	76.791	1.00	0.00
ATOM	2213	HA	PRO	A	144	82.847	98.121	77.528	1.00	0.00
ATOM	2214	C	PRO	A	144	80.754	98.361	77.241	1.00	0.00
ATOM	2215	O	PRO	A	144	80.724	99.507	77.696	1.00	0.00
ATOM	2216	N	LEU	A	145	79.656	97.605	77.145	1.00	0.00
ATOM	2217	H	LEU	A	145	79.741	96.692	76.729	1.00	0.00
ATOM	2218	CA	LEU	A	145	78.396	97.942	77.821	1.00	0.00
ATOM	2219	HA	LEU	A	145	78.272	99.026	77.869	1.00	0.00
ATOM	2220	CB	LEU	A	145	77.195	97.332	77.060	1.00	0.00
ATOM	2221	HB1	LEU	A	145	76.292	97.522	77.643	1.00	0.00
ATOM	2222	HB2	LEU	A	145	77.329	96.250	77.000	1.00	0.00
ATOM	2223	CG	LEU	A	145	76.967	97.854	75.630	1.00	0.00
ATOM	2224	HG	LEU	A	145	77.846	97.642	75.022	1.00	0.00
ATOM	2225	CD1	LEU	A	145	75.768	97.122	75.022	1.00	0.00
ATOM	2226	1HD1	LEU	A	145	75.615	97.466	74.001	1.00	0.00
ATOM	2227	2HD1	LEU	A	145	75.958	96.047	75.007	1.00	0.00
ATOM	2228	3HD1	LEU	A	145	74.869	97.322	75.606	1.00	0.00
ATOM	2229	CD2	LEU	A	145	76.675	99.353	75.575	1.00	0.00
ATOM	2230	1HD2	LEU	A	145	76.459	99.647	74.547	1.00	0.00
ATOM	2231	2HD2	LEU	A	145	75.819	99.592	76.205	1.00	0.00
ATOM	2232	3HD2	LEU	A	145	77.544	99.913	75.914	1.00	0.00
ATOM	2233	C	LEU	A	145	78.452	97.429	79.266	1.00	0.00
ATOM	2234	O	LEU	A	145	78.587	98.228	80.188	1.00	0.00
ATOM	2235	N	MET	A	146	78.381	96.100	79.425	1.00	0.00
ATOM	2236	H	MET	A	146	78.436	95.559	78.570	1.00	0.00
ATOM	2237	CA	MET	A	146	78.212	95.262	80.629	1.00	0.00
ATOM	2238	HA	MET	A	146	77.767	94.336	80.261	1.00	0.00
ATOM	2239	CB	MET	A	146	79.583	94.835	81.199	1.00	0.00
ATOM	2240	HB1	MET	A	146	80.156	94.335	80.426	1.00	0.00
ATOM	2241	HB2	MET	A	146	79.426	94.119	82.005	1.00	0.00
ATOM	2242	CG	MET	A	146	80.422	96.002	81.717	1.00	0.00
ATOM	2243	HG1	MET	A	146	79.859	96.477	82.512	1.00	0.00
ATOM	2244	HG2	MET	A	146	80.541	96.722	80.909	1.00	0.00
ATOM	2245	SD	MET	A	146	82.099	95.621	82.312	1.00	0.00
ATOM	2246	CE	MET	A	146	81.771	94.640	83.801	1.00	0.00
ATOM	2247	HE1	MET	A	146	81.322	95.266	84.561	1.00	0.00
ATOM	2248	HE2	MET	A	146	81.108	93.807	83.572	1.00	0.00
ATOM	2249	HE3	MET	A	146	82.706	94.248	84.195	1.00	0.00
ATOM	2250	C	MET	A	146	77.223	95.707	81.729	1.00	0.00
ATOM	2251	O	MET	A	146	76.879	94.887	82.576	1.00	0.00
ATOM	2252	N	ILE	A	147	76.757	96.964	81.755	1.00	0.00
ATOM	2253	H	ILE	A	147	77.110	97.560	81.020	1.00	0.00
ATOM	2254	CA	ILE	A	147	76.085	97.682	82.864	1.00	0.00
ATOM	2255	HA	ILE	A	147	75.938	98.711	82.532	1.00	0.00

ATOM	2256	CB	ILE	A	147	74.671	97.115	83.173	1.00	0.00
ATOM	2257	HB	ILE	A	147	74.770	96.088	83.525	1.00	0.00
ATOM	2258	CG2	ILE	A	147	74.000	97.934	84.294	1.00	0.00
ATOM	2259	1HG2	ILE	A	147	73.042	97.496	84.571	1.00	0.00
ATOM	2260	2HG2	ILE	A	147	74.612	97.948	85.195	1.00	0.00
ATOM	2261	3HG2	ILE	A	147	73.838	98.962	83.966	1.00	0.00
ATOM	2262	CG1	ILE	A	147	73.791	97.111	81.900	1.00	0.00
ATOM	2263	1HG1	ILE	A	147	74.276	96.503	81.136	1.00	0.00
ATOM	2264	2HG1	ILE	A	147	73.704	98.129	81.519	1.00	0.00
ATOM	2265	CD	ILE	A	147	72.380	96.538	82.097	1.00	0.00
ATOM	2266	HD1	ILE	A	147	71.895	96.441	81.126	1.00	0.00
ATOM	2267	HD2	ILE	A	147	72.440	95.553	82.559	1.00	0.00
ATOM	2268	HD3	ILE	A	147	71.779	97.200	82.718	1.00	0.00
ATOM	2269	C	ILE	A	147	76.975	97.771	84.121	1.00	0.00
ATOM	2270	O	ILE	A	147	77.251	98.859	84.621	1.00	0.00
ATOM	2271	N	LYS	A	148	77.539	96.646	84.559	1.00	0.00
ATOM	2272	H	LYS	A	148	77.247	95.813	84.057	1.00	0.00
ATOM	2273	CA	LYS	A	148	78.490	96.436	85.662	1.00	0.00
ATOM	2274	HA	LYS	A	148	78.025	96.759	86.594	1.00	0.00
ATOM	2275	CB	LYS	A	148	78.741	94.905	85.719	1.00	0.00
ATOM	2276	HB1	LYS	A	148	79.452	94.681	86.517	1.00	0.00
ATOM	2277	HB2	LYS	A	148	79.203	94.621	84.775	1.00	0.00
ATOM	2278	CG	LYS	A	148	77.502	94.000	85.913	1.00	0.00
ATOM	2279	HG1	LYS	A	148	76.676	94.358	85.300	1.00	0.00
ATOM	2280	HG2	LYS	A	148	77.191	94.036	86.956	1.00	0.00
ATOM	2281	CD	LYS	A	148	77.796	92.545	85.503	1.00	0.00
ATOM	2282	HD1	LYS	A	148	78.547	92.110	86.167	1.00	0.00
ATOM	2283	HD2	LYS	A	148	78.182	92.525	84.481	1.00	0.00
ATOM	2284	CE	LYS	A	148	76.515	91.694	85.549	1.00	0.00
ATOM	2285	HE1	LYS	A	148	75.748	92.208	84.963	1.00	0.00
ATOM	2286	HE2	LYS	A	148	76.169	91.626	86.584	1.00	0.00
ATOM	2287	NZ	LYS	A	148	76.725	90.343	84.986	1.00	0.00
ATOM	2288	HZ1	LYS	A	148	75.895	89.769	85.004	1.00	0.00
ATOM	2289	HZ2	LYS	A	148	77.013	90.388	84.004	1.00	0.00
ATOM	2290	HZ3	LYS	A	148	77.459	89.807	85.448	1.00	0.00
ATOM	2291	C	LYS	A	148	79.844	97.185	85.552	1.00	0.00
ATOM	2292	O	LYS	A	148	80.707	96.971	86.406	1.00	0.00
ATOM	2293	N	ARG	A	149	80.030	97.974	84.480	1.00	0.00
ATOM	2294	H	ARG	A	149	79.143	98.162	84.029	1.00	0.00
ATOM	2295	CA	ARG	A	149	81.186	98.655	83.814	1.00	0.00
ATOM	2296	HA	ARG	A	149	81.149	98.290	82.791	1.00	0.00
ATOM	2297	CB	ARG	A	149	80.874	100.174	83.735	1.00	0.00
ATOM	2298	HB1	ARG	A	149	81.683	100.749	84.190	1.00	0.00
ATOM	2299	HB2	ARG	A	149	79.968	100.396	84.305	1.00	0.00
ATOM	2300	CG	ARG	A	149	80.652	100.669	82.290	1.00	0.00
ATOM	2301	HG1	ARG	A	149	80.323	101.707	82.328	1.00	0.00
ATOM	2302	HG2	ARG	A	149	79.853	100.082	81.837	1.00	0.00
ATOM	2303	CD	ARG	A	149	81.915	100.582	81.419	1.00	0.00
ATOM	2304	HD1	ARG	A	149	82.289	99.558	81.430	1.00	0.00
ATOM	2305	HD2	ARG	A	149	82.692	101.220	81.841	1.00	0.00
ATOM	2306	NE	ARG	A	149	81.650	100.931	80.009	1.00	0.00
ATOM	2307	HE	ARG	A	149	81.175	100.226	79.455	1.00	0.00
ATOM	2308	CZ	ARG	A	149	82.002	102.020	79.346	1.00	0.00
ATOM	2309	NH1	ARG	A	149	82.518	103.069	79.923	1.00	0.00
ATOM	2310	1HH1	ARG	A	149	82.654	103.051	80.915	1.00	0.00
ATOM	2311	2HH1	ARG	A	149	82.760	103.878	79.380	1.00	0.00
ATOM	2312	NH2	ARG	A	149	81.818	102.052	78.061	1.00	0.00
ATOM	2313	1HH2	ARG	A	149	81.374	101.244	77.632	1.00	0.00
ATOM	2314	2HH2	ARG	A	149	82.052	102.862	77.522	1.00	0.00
ATOM	2315	C	ARG	A	149	82.669	98.396	84.176	1.00	0.00
ATOM	2316	O	ARG	A	149	83.544	99.022	83.582	1.00	0.00

ATOM	2317	N	SER	A	150	83.006	97.492	85.083	1.00	0.00
ATOM	2318	H	SER	A	150	82.254	96.985	85.523	1.00	0.00
ATOM	2319	CA	SER	A	150	84.356	97.218	85.581	1.00	0.00
ATOM	2320	HA	SER	A	150	84.936	98.141	85.546	1.00	0.00
ATOM	2321	CB	SER	A	150	84.257	96.810	87.061	1.00	0.00
ATOM	2322	HB1	SER	A	150	83.951	97.676	87.650	1.00	0.00
ATOM	2323	HB2	SER	A	150	85.238	96.495	87.419	1.00	0.00
ATOM	2324	OG	SER	A	150	83.320	95.764	87.275	1.00	0.00
ATOM	2325	HG	SER	A	150	82.440	96.149	87.396	1.00	0.00
ATOM	2326	C	SER	A	150	85.116	96.170	84.740	1.00	0.00
ATOM	2327	O	SER	A	150	85.031	94.974	84.996	1.00	0.00
ATOM	2328	N	LYS	A	151	85.900	96.591	83.740	1.00	0.00
ATOM	2329	H	LYS	A	151	85.874	97.575	83.511	1.00	0.00
ATOM	2330	CA	LYS	A	151	86.691	95.680	82.873	1.00	0.00
ATOM	2331	HA	LYS	A	151	86.030	94.886	82.519	1.00	0.00
ATOM	2332	CB	LYS	A	151	87.227	96.447	81.642	1.00	0.00
ATOM	2333	HB1	LYS	A	151	88.121	95.940	81.277	1.00	0.00
ATOM	2334	HB2	LYS	A	151	87.525	97.454	81.937	1.00	0.00
ATOM	2335	CG	LYS	A	151	86.242	96.512	80.462	1.00	0.00
ATOM	2336	HG1	LYS	A	151	85.334	97.035	80.762	1.00	0.00
ATOM	2337	HG2	LYS	A	151	85.985	95.497	80.158	1.00	0.00
ATOM	2338	CD	LYS	A	151	86.890	97.246	79.271	1.00	0.00
ATOM	2339	HD1	LYS	A	151	87.872	96.813	79.075	1.00	0.00
ATOM	2340	HD2	LYS	A	151	87.028	98.292	79.542	1.00	0.00
ATOM	2341	CE	LYS	A	151	86.046	97.150	77.992	1.00	0.00
ATOM	2342	HE1	LYS	A	151	85.026	97.467	78.219	1.00	0.00
ATOM	2343	HE2	LYS	A	151	86.013	96.107	77.668	1.00	0.00
ATOM	2344	NZ	LYS	A	151	86.609	97.994	76.905	1.00	0.00
ATOM	2345	HZ1	LYS	A	151	87.492	97.646	76.528	1.00	0.00
ATOM	2346	HZ2	LYS	A	151	86.049	98.016	76.053	1.00	0.00
ATOM	2347	HZ3	LYS	A	151	86.757	98.948	77.183	1.00	0.00
ATOM	2348	C	LYS	A	151	87.858	94.928	83.543	1.00	0.00
ATOM	2349	O	LYS	A	151	88.365	93.988	82.946	1.00	0.00
ATOM	2350	N	LYS	A	152	88.317	95.368	84.724	1.00	0.00
ATOM	2351	H	LYS	A	152	87.796	96.135	85.108	1.00	0.00
ATOM	2352	CA	LYS	A	152	89.519	94.948	85.501	1.00	0.00
ATOM	2353	HA	LYS	A	152	89.608	95.635	86.344	1.00	0.00
ATOM	2354	CB	LYS	A	152	89.379	93.538	86.111	1.00	0.00
ATOM	2355	HB1	LYS	A	152	90.014	93.489	86.997	1.00	0.00
ATOM	2356	HB2	LYS	A	152	89.776	92.814	85.397	1.00	0.00
ATOM	2357	CG	LYS	A	152	87.983	93.037	86.496	1.00	0.00
ATOM	2358	HG1	LYS	A	152	88.143	92.085	86.996	1.00	0.00
ATOM	2359	HG2	LYS	A	152	87.434	92.856	85.573	1.00	0.00
ATOM	2360	CD	LYS	A	152	87.138	93.931	87.419	1.00	0.00
ATOM	2361	HD1	LYS	A	152	86.917	94.874	86.928	1.00	0.00
ATOM	2362	HD2	LYS	A	152	87.702	94.137	88.330	1.00	0.00
ATOM	2363	CE	LYS	A	152	85.808	93.258	87.815	1.00	0.00
ATOM	2364	HE1	LYS	A	152	85.203	93.962	88.393	1.00	0.00
ATOM	2365	HE2	LYS	A	152	86.042	92.399	88.454	1.00	0.00
ATOM	2366	NZ	LYS	A	152	85.044	92.763	86.643	1.00	0.00
ATOM	2367	HZ1	LYS	A	152	84.224	92.258	86.925	1.00	0.00
ATOM	2368	HZ2	LYS	A	152	84.823	93.498	85.982	1.00	0.00
ATOM	2369	HZ3	LYS	A	152	85.606	92.044	86.169	1.00	0.00
ATOM	2370	C	LYS	A	152	90.879	95.031	84.795	1.00	0.00
ATOM	2371	O	LYS	A	152	91.877	95.233	85.476	1.00	0.00
ATOM	2372	N	ASN	A	153	90.924	94.892	83.468	1.00	0.00
ATOM	2373	H	ASN	A	153	90.059	94.624	83.020	1.00	0.00
ATOM	2374	CA	ASN	A	153	92.111	94.954	82.605	1.00	0.00
ATOM	2375	HA	ASN	A	153	91.836	94.468	81.669	1.00	0.00
ATOM	2376	CB	ASN	A	153	92.413	96.435	82.288	1.00	0.00
ATOM	2377	HB1	ASN	A	153	93.475	96.579	82.101	1.00	0.00

ATOM	2378	HB2	ASN	A	153	92.147	97.073	83.130	1.00	0.00
ATOM	2379	CG	ASN	A	153	91.662	96.889	81.058	1.00	0.00
ATOM	2380	OD1	ASN	A	153	90.487	97.225	81.086	1.00	0.00
ATOM	2381	ND2	ASN	A	153	92.309	96.865	79.918	1.00	0.00
ATOM	2382	1HD2	ASN	A	153	93.276	96.588	79.899	1.00	0.00
ATOM	2383	2HD2	ASN	A	153	91.815	97.176	79.100	1.00	0.00
ATOM	2384	C	ASN	A	153	93.313	94.134	83.114	1.00	0.00
ATOM	2385	O	ASN	A	153	94.465	94.513	82.915	1.00	0.00
ATOM	2386	N	SER	A	154	93.050	93.009	83.774	1.00	0.00
ATOM	2387	H	SER	A	154	92.089	92.720	83.896	1.00	0.00
ATOM	2388	CA	SER	A	154	94.061	92.214	84.467	1.00	0.00
ATOM	2389	HA	SER	A	154	95.013	92.308	83.942	1.00	0.00
ATOM	2390	CB	SER	A	154	94.249	92.750	85.894	1.00	0.00
ATOM	2391	HB1	SER	A	154	94.779	93.703	85.849	1.00	0.00
ATOM	2392	HB2	SER	A	154	94.847	92.050	86.478	1.00	0.00
ATOM	2393	OG	SER	A	154	93.009	92.962	86.544	1.00	0.00
ATOM	2394	HG	SER	A	154	92.683	93.837	86.273	1.00	0.00
ATOM	2395	C	SER	A	154	93.692	90.731	84.474	1.00	0.00
ATOM	2396	O	SER	A	154	92.533	90.366	84.297	1.00	0.00
ATOM	2397	N	LEU	A	155	94.706	89.878	84.660	1.00	0.00
ATOM	2398	H	LEU	A	155	95.608	90.286	84.847	1.00	0.00
ATOM	2399	CA	LEU	A	155	94.703	88.434	84.385	1.00	0.00
ATOM	2400	HA	LEU	A	155	95.734	88.087	84.475	1.00	0.00
ATOM	2401	CB	LEU	A	155	93.874	87.666	85.441	1.00	0.00
ATOM	2402	HB1	LEU	A	155	94.022	86.599	85.271	1.00	0.00
ATOM	2403	HB2	LEU	A	155	92.815	87.884	85.291	1.00	0.00
ATOM	2404	CG	LEU	A	155	94.220	87.991	86.906	1.00	0.00
ATOM	2405	HG	LEU	A	155	93.985	89.033	87.115	1.00	0.00
ATOM	2406	CD1	LEU	A	155	93.380	87.120	87.844	1.00	0.00
ATOM	2407	1HD1	LEU	A	155	93.599	87.383	88.878	1.00	0.00
ATOM	2408	2HD1	LEU	A	155	92.322	87.302	87.657	1.00	0.00
ATOM	2409	3HD1	LEU	A	155	93.600	86.067	87.680	1.00	0.00
ATOM	2410	CD2	LEU	A	155	95.695	87.750	87.234	1.00	0.00
ATOM	2411	1HD2	LEU	A	155	95.868	87.934	88.294	1.00	0.00
ATOM	2412	2HD2	LEU	A	155	95.973	86.723	86.995	1.00	0.00
ATOM	2413	3HD2	LEU	A	155	96.329	88.430	86.668	1.00	0.00
ATOM	2414	C	LEU	A	155	94.307	88.110	82.936	1.00	0.00
ATOM	2415	O	LEU	A	155	95.194	87.880	82.122	1.00	0.00
ATOM	2416	N	ALA	A	156	93.020	88.195	82.588	1.00	0.00
ATOM	2417	H	ALA	A	156	92.385	88.552	83.293	1.00	0.00
ATOM	2418	CA	ALA	A	156	92.463	88.002	81.242	1.00	0.00
ATOM	2419	HA	ALA	A	156	92.880	87.090	80.817	1.00	0.00
ATOM	2420	CB	ALA	A	156	90.949	87.795	81.398	1.00	0.00
ATOM	2421	HB1	ALA	A	156	90.509	87.565	80.427	1.00	0.00
ATOM	2422	HB2	ALA	A	156	90.754	86.969	82.078	1.00	0.00
ATOM	2423	HB3	ALA	A	156	90.486	88.701	81.790	1.00	0.00
ATOM	2424	C	ALA	A	156	92.811	89.142	80.252	1.00	0.00
ATOM	2425	O	ALA	A	156	91.932	89.698	79.602	1.00	0.00
ATOM	2426	N	LEU	A	157	94.083	89.544	80.191	1.00	0.00
ATOM	2427	H	LEU	A	157	94.724	89.064	80.803	1.00	0.00
ATOM	2428	CA	LEU	A	157	94.586	90.650	79.362	1.00	0.00
ATOM	2429	HA	LEU	A	157	93.974	90.704	78.462	1.00	0.00
ATOM	2430	CB	LEU	A	157	94.439	91.967	80.174	1.00	0.00
ATOM	2431	HB1	LEU	A	157	95.429	92.345	80.429	1.00	0.00
ATOM	2432	HB2	LEU	A	157	93.953	91.764	81.129	1.00	0.00
ATOM	2433	CG	LEU	A	157	93.655	93.126	79.520	1.00	0.00
ATOM	2434	HG	LEU	A	157	93.753	93.992	80.176	1.00	0.00
ATOM	2435	CD1	LEU	A	157	94.189	93.550	78.148	1.00	0.00
ATOM	2436	1HD1	LEU	A	157	94.073	92.738	77.432	1.00	0.00
ATOM	2437	2HD1	LEU	A	157	93.646	94.424	77.792	1.00	0.00
ATOM	2438	3HD1	LEU	A	157	95.249	93.790	78.235	1.00	0.00

ATOM	2439	CD2	LEU	A	157	92.164	92.829	79.384	1.00	0.00
ATOM	2440	1HD2	LEU	A	157	91.631	93.739	79.113	1.00	0.00
ATOM	2441	2HD2	LEU	A	157	92.000	92.079	78.609	1.00	0.00
ATOM	2442	3HD2	LEU	A	157	91.776	92.442	80.326	1.00	0.00
ATOM	2443	C	LEU	A	157	96.049	90.470	78.896	1.00	0.00
ATOM	2444	O	LEU	A	157	96.583	91.348	78.217	1.00	0.00
ATOM	2445	N	SER	A	158	96.762	89.425	79.340	1.00	0.00
ATOM	2446	H	SER	A	158	96.296	88.736	79.912	1.00	0.00
ATOM	2447	CA	SER	A	158	98.244	89.399	79.284	1.00	0.00
ATOM	2448	HA	SER	A	158	98.569	89.818	78.332	1.00	0.00
ATOM	2449	CB	SER	A	158	98.822	90.273	80.416	1.00	0.00
ATOM	2450	HB1	SER	A	158	99.905	90.337	80.305	1.00	0.00
ATOM	2451	HB2	SER	A	158	98.598	89.815	81.379	1.00	0.00
ATOM	2452	OG	SER	A	158	98.293	91.586	80.394	1.00	0.00
ATOM	2453	HG	SER	A	158	97.896	91.733	79.522	1.00	0.00
ATOM	2454	C	SER	A	158	98.882	87.992	79.377	1.00	0.00
ATOM	2455	O	SER	A	158	100.073	87.895	79.642	1.00	0.00
ATOM	2456	N	LEU	A	159	98.139	86.885	79.212	1.00	0.00
ATOM	2457	H	LEU	A	159	97.188	86.956	78.869	1.00	0.00
ATOM	2458	CA	LEU	A	159	98.611	85.552	79.645	1.00	0.00
ATOM	2459	HA	LEU	A	159	98.999	85.671	80.658	1.00	0.00
ATOM	2460	CB	LEU	A	159	97.476	84.515	79.704	1.00	0.00
ATOM	2461	HB1	LEU	A	159	97.949	83.576	79.984	1.00	0.00
ATOM	2462	HB2	LEU	A	159	97.060	84.369	78.708	1.00	0.00
ATOM	2463	CG	LEU	A	159	96.343	84.675	80.725	1.00	0.00
ATOM	2464	HG	LEU	A	159	95.955	83.680	80.915	1.00	0.00
ATOM	2465	CD1	LEU	A	159	96.815	85.207	82.076	1.00	0.00
ATOM	2466	1HD1	LEU	A	159	95.983	85.198	82.777	1.00	0.00
ATOM	2467	2HD1	LEU	A	159	97.610	84.570	82.464	1.00	0.00
ATOM	2468	3HD1	LEU	A	159	97.182	86.226	81.972	1.00	0.00
ATOM	2469	CD2	LEU	A	159	95.179	85.489	80.175	1.00	0.00
ATOM	2470	1HD2	LEU	A	159	94.365	85.476	80.897	1.00	0.00
ATOM	2471	2HD2	LEU	A	159	95.481	86.513	79.977	1.00	0.00
ATOM	2472	3HD2	LEU	A	159	94.824	85.040	79.247	1.00	0.00
ATOM	2473	C	LEU	A	159	99.790	84.909	78.873	1.00	0.00
ATOM	2474	O	LEU	A	159	100.305	83.848	79.250	1.00	0.00
ATOM	2475	N	THR	A	160	100.157	85.514	77.744	1.00	0.00
ATOM	2476	H	THR	A	160	99.743	86.420	77.594	1.00	0.00
ATOM	2477	CA	THR	A	160	100.830	84.868	76.609	1.00	0.00
ATOM	2478	HA	THR	A	160	100.578	85.492	75.754	1.00	0.00
ATOM	2479	CB	THR	A	160	102.371	84.969	76.694	1.00	0.00
ATOM	2480	HB	THR	A	160	102.630	86.017	76.850	1.00	0.00
ATOM	2481	CG2	THR	A	160	103.063	84.159	77.784	1.00	0.00
ATOM	2482	1HG2	THR	A	160	102.807	83.113	77.691	1.00	0.00
ATOM	2483	2HG2	THR	A	160	104.142	84.277	77.689	1.00	0.00
ATOM	2484	3HG2	THR	A	160	102.764	84.535	78.761	1.00	0.00
ATOM	2485	OG1	THR	A	160	102.912	84.579	75.454	1.00	0.00
ATOM	2486	HG1	THR	A	160	103.640	83.930	75.602	1.00	0.00
ATOM	2487	C	THR	A	160	100.280	83.466	76.242	1.00	0.00
ATOM	2488	O	THR	A	160	99.552	82.770	76.969	1.00	0.00
ATOM	2489	N	ALA	A	161	100.643	83.021	75.040	1.00	0.00
ATOM	2490	H	ALA	A	161	101.278	83.603	74.506	1.00	0.00
ATOM	2491	CA	ALA	A	161	100.435	81.649	74.600	1.00	0.00
ATOM	2492	HA	ALA	A	161	99.389	81.498	74.416	1.00	0.00
ATOM	2493	CB	ALA	A	161	101.224	81.472	73.290	1.00	0.00
ATOM	2494	HB1	ALA	A	161	101.056	80.470	72.897	1.00	0.00
ATOM	2495	HB2	ALA	A	161	100.914	82.214	72.556	1.00	0.00
ATOM	2496	HB3	ALA	A	161	102.293	81.602	73.473	1.00	0.00
ATOM	2497	C	ALA	A	161	100.925	80.627	75.632	1.00	0.00
ATOM	2498	O	ALA	A	161	100.246	79.686	76.036	1.00	0.00
ATOM	2499	N	ASP	A	162	102.136	80.873	76.089	1.00	0.00

ATOM	2500	H	ASP	A	162	102.615	81.698	75.738	1.00	0.00
ATOM	2501	CA	ASP	A	162	102.920	79.950	76.879	1.00	0.00
ATOM	2502	HA	ASP	A	162	102.828	78.950	76.452	1.00	0.00
ATOM	2503	CB	ASP	A	162	104.396	80.402	76.785	1.00	0.00
ATOM	2504	HB1	ASP	A	162	105.043	79.527	76.839	1.00	0.00
ATOM	2505	HB2	ASP	A	162	104.628	81.043	77.637	1.00	0.00
ATOM	2506	CG	ASP	A	162	104.695	81.196	75.495	1.00	0.00
ATOM	2507	OD1	ASP	A	162	104.994	80.577	74.446	1.00	0.00
ATOM	2508	OD2	ASP	A	162	104.465	82.427	75.527	1.00	0.00
ATOM	2509	C	ASP	A	162	102.410	79.904	78.333	1.00	0.00
ATOM	2510	O	ASP	A	162	102.504	78.851	78.969	1.00	0.00
ATOM	2511	N	GLN	A	163	101.807	80.995	78.854	1.00	0.00
ATOM	2512	H	GLN	A	163	101.641	81.817	78.290	1.00	0.00
ATOM	2513	CA	GLN	A	163	101.458	81.032	80.267	1.00	0.00
ATOM	2514	HA	GLN	A	163	101.844	80.134	80.742	1.00	0.00
ATOM	2515	CB	GLN	A	163	102.179	82.146	81.043	1.00	0.00
ATOM	2516	HB1	GLN	A	163	101.752	82.210	82.045	1.00	0.00
ATOM	2517	HB2	GLN	A	163	102.065	83.105	80.561	1.00	0.00
ATOM	2518	CG	GLN	A	163	103.677	81.855	81.164	1.00	0.00
ATOM	2519	HG1	GLN	A	163	104.106	81.708	80.173	1.00	0.00
ATOM	2520	HG2	GLN	A	163	103.838	80.954	81.755	1.00	0.00
ATOM	2521	CD	GLN	A	163	104.385	83.016	81.850	1.00	0.00
ATOM	2522	OE1	GLN	A	163	104.077	83.405	82.957	1.00	0.00
ATOM	2523	NE2	GLN	A	163	105.355	83.628	81.207	1.00	0.00
ATOM	2524	1HE2	GLN	A	163	105.652	83.337	80.297	1.00	0.00
ATOM	2525	2HE2	GLN	A	163	105.787	84.388	81.699	1.00	0.00
ATOM	2526	C	GLN	A	163	99.962	80.899	80.528	1.00	0.00
ATOM	2527	O	GLN	A	163	99.653	80.426	81.625	1.00	0.00
ATOM	2528	N	MET	A	164	99.062	81.039	79.529	1.00	0.00
ATOM	2529	H	MET	A	164	99.320	81.477	78.649	1.00	0.00
ATOM	2530	CA	MET	A	164	97.810	80.270	79.663	1.00	0.00
ATOM	2531	HA	MET	A	164	97.668	80.130	80.728	1.00	0.00
ATOM	2532	CB	MET	A	164	96.528	80.991	79.252	1.00	0.00
ATOM	2533	HB1	MET	A	164	96.094	80.510	78.380	1.00	0.00
ATOM	2534	HB2	MET	A	164	96.720	82.036	79.045	1.00	0.00
ATOM	2535	CG	MET	A	164	95.534	80.894	80.418	1.00	0.00
ATOM	2536	HG1	MET	A	164	96.023	81.240	81.329	1.00	0.00
ATOM	2537	HG2	MET	A	164	95.280	79.842	80.549	1.00	0.00
ATOM	2538	SD	MET	A	164	94.016	81.835	80.258	1.00	0.00
ATOM	2539	CE	MET	A	164	93.156	81.069	81.654	1.00	0.00
ATOM	2540	HE1	MET	A	164	93.201	79.984	81.568	1.00	0.00
ATOM	2541	HE2	MET	A	164	93.616	81.384	82.590	1.00	0.00
ATOM	2542	HE3	MET	A	164	92.112	81.363	81.648	1.00	0.00
ATOM	2543	C	MET	A	164	97.864	78.808	79.214	1.00	0.00
ATOM	2544	O	MET	A	164	97.100	78.030	79.762	1.00	0.00
ATOM	2545	N	VAL	A	165	98.810	78.365	78.367	1.00	0.00
ATOM	2546	H	VAL	A	165	99.413	79.015	77.871	1.00	0.00
ATOM	2547	CA	VAL	A	165	99.108	76.910	78.300	1.00	0.00
ATOM	2548	HA	VAL	A	165	98.193	76.371	78.049	1.00	0.00
ATOM	2549	CB	VAL	A	165	100.161	76.622	77.203	1.00	0.00
ATOM	2550	HB	VAL	A	165	100.883	77.438	77.178	1.00	0.00
ATOM	2551	CG1	VAL	A	165	100.950	75.325	77.410	1.00	0.00
ATOM	2552	1HG1	VAL	A	165	101.521	75.086	76.512	1.00	0.00
ATOM	2553	2HG1	VAL	A	165	101.650	75.432	78.239	1.00	0.00
ATOM	2554	3HG1	VAL	A	165	100.261	74.509	77.625	1.00	0.00
ATOM	2555	CG2	VAL	A	165	99.454	76.532	75.850	1.00	0.00
ATOM	2556	1HG2	VAL	A	165	100.189	76.392	75.058	1.00	0.00
ATOM	2557	2HG2	VAL	A	165	98.765	75.689	75.848	1.00	0.00
ATOM	2558	3HG2	VAL	A	165	98.893	77.442	75.650	1.00	0.00
ATOM	2559	C	VAL	A	165	99.566	76.362	79.665	1.00	0.00
ATOM	2560	O	VAL	A	165	99.074	75.331	80.124	1.00	0.00

ATOM	2561	N	SER	A	166	100.469	77.074	80.346	1.00	0.00
ATOM	2562	H	SER	A	166	100.895	77.875	79.897	1.00	0.00
ATOM	2563	CA	SER	A	166	100.930	76.652	81.680	1.00	0.00
ATOM	2564	HA	SER	A	166	101.343	75.647	81.606	1.00	0.00
ATOM	2565	CB	SER	A	166	102.037	77.583	82.190	1.00	0.00
ATOM	2566	HB1	SER	A	166	102.350	77.240	83.177	1.00	0.00
ATOM	2567	HB2	SER	A	166	101.650	78.598	82.284	1.00	0.00
ATOM	2568	OG	SER	A	166	103.157	77.573	81.329	1.00	0.00
ATOM	2569	HG	SER	A	166	102.924	77.947	80.460	1.00	0.00
ATOM	2570	C	SER	A	166	99.799	76.618	82.716	1.00	0.00
ATOM	2571	O	SER	A	166	99.611	75.597	83.384	1.00	0.00
ATOM	2572	N	ALA	A	167	99.006	77.696	82.801	1.00	0.00
ATOM	2573	H	ALA	A	167	99.220	78.516	82.246	1.00	0.00
ATOM	2574	CA	ALA	A	167	97.840	77.741	83.695	1.00	0.00
ATOM	2575	HA	ALA	A	167	98.189	77.614	84.722	1.00	0.00
ATOM	2576	CB	ALA	A	167	97.173	79.118	83.587	1.00	0.00
ATOM	2577	HB1	ALA	A	167	96.357	79.179	84.309	1.00	0.00
ATOM	2578	HB2	ALA	A	167	97.898	79.903	83.801	1.00	0.00
ATOM	2579	HB3	ALA	A	167	96.765	79.254	82.588	1.00	0.00
ATOM	2580	C	ALA	A	167	96.830	76.614	83.419	1.00	0.00
ATOM	2581	O	ALA	A	167	96.273	76.024	84.346	1.00	0.00
ATOM	2582	N	LEU	A	168	96.626	76.266	82.146	1.00	0.00
ATOM	2583	H	LEU	A	168	97.070	76.814	81.415	1.00	0.00
ATOM	2584	CA	LEU	A	168	95.793	75.135	81.751	1.00	0.00
ATOM	2585	HA	LEU	A	168	94.805	75.257	82.192	1.00	0.00
ATOM	2586	CB	LEU	A	168	95.710	75.108	80.216	1.00	0.00
ATOM	2587	HB1	LEU	A	168	95.585	74.076	79.913	1.00	0.00
ATOM	2588	HB2	LEU	A	168	96.662	75.410	79.791	1.00	0.00
ATOM	2589	CG	LEU	A	168	94.613	75.976	79.584	1.00	0.00
ATOM	2590	HG	LEU	A	168	94.880	77.019	79.708	1.00	0.00
ATOM	2591	CD1	LEU	A	168	94.599	75.678	78.081	1.00	0.00
ATOM	2592	1HD1	LEU	A	168	95.450	76.171	77.613	1.00	0.00
ATOM	2593	2HD1	LEU	A	168	94.682	74.614	77.874	1.00	0.00
ATOM	2594	3HD1	LEU	A	168	93.689	76.053	77.621	1.00	0.00
ATOM	2595	CD2	LEU	A	168	93.247	75.804	80.246	1.00	0.00
ATOM	2596	1HD2	LEU	A	168	93.161	74.860	80.767	1.00	0.00
ATOM	2597	2HD2	LEU	A	168	93.130	76.598	80.976	1.00	0.00
ATOM	2598	3HD2	LEU	A	168	92.439	75.883	79.526	1.00	0.00
ATOM	2599	C	LEU	A	168	96.321	73.783	82.242	1.00	0.00
ATOM	2600	O	LEU	A	168	95.533	72.961	82.707	1.00	0.00
ATOM	2601	N	LEU	A	169	97.631	73.553	82.125	1.00	0.00
ATOM	2602	H	LEU	A	169	98.207	74.277	81.712	1.00	0.00
ATOM	2603	CA	LEU	A	169	98.269	72.308	82.554	1.00	0.00
ATOM	2604	HA	LEU	A	169	97.703	71.470	82.149	1.00	0.00
ATOM	2605	CB	LEU	A	169	99.706	72.261	82.006	1.00	0.00
ATOM	2606	HB1	LEU	A	169	100.251	71.453	82.497	1.00	0.00
ATOM	2607	HB2	LEU	A	169	100.201	73.198	82.266	1.00	0.00
ATOM	2608	CG	LEU	A	169	99.778	72.052	80.478	1.00	0.00
ATOM	2609	HG	LEU	A	169	99.045	72.679	79.975	1.00	0.00
ATOM	2610	CD1	LEU	A	169	101.167	72.432	79.965	1.00	0.00
ATOM	2611	1HD1	LEU	A	169	101.221	72.296	78.886	1.00	0.00
ATOM	2612	2HD1	LEU	A	169	101.359	73.481	80.184	1.00	0.00
ATOM	2613	3HD1	LEU	A	169	101.929	71.818	80.442	1.00	0.00
ATOM	2614	CD2	LEU	A	169	99.517	70.593	80.088	1.00	0.00
ATOM	2615	1HD2	LEU	A	169	99.613	70.492	79.010	1.00	0.00
ATOM	2616	2HD2	LEU	A	169	100.237	69.942	80.582	1.00	0.00
ATOM	2617	3HD2	LEU	A	169	98.509	70.310	80.385	1.00	0.00
ATOM	2618	C	LEU	A	169	98.241	72.139	84.082	1.00	0.00
ATOM	2619	O	LEU	A	169	97.976	71.035	84.554	1.00	0.00
ATOM	2620	N	ASP	A	170	98.434	73.217	84.852	1.00	0.00
ATOM	2621	H	ASP	A	170	98.667	74.097	84.404	1.00	0.00

ATOM	2622	CA	ASP	A	170	98.156	73.214	86.297	1.00	0.00
ATOM	2623	HA	ASP	A	170	98.746	72.435	86.782	1.00	0.00
ATOM	2624	CB	ASP	A	170	98.567	74.571	86.892	1.00	0.00
ATOM	2625	HB1	ASP	A	170	98.312	75.366	86.187	1.00	0.00
ATOM	2626	HB2	ASP	A	170	99.648	74.585	87.045	1.00	0.00
ATOM	2627	CG	ASP	A	170	97.866	74.878	88.223	1.00	0.00
ATOM	2628	OD1	ASP	A	170	98.017	74.116	89.208	1.00	0.00
ATOM	2629	OD2	ASP	A	170	97.108	75.867	88.274	1.00	0.00
ATOM	2630	C	ASP	A	170	96.670	72.897	86.589	1.00	0.00
ATOM	2631	O	ASP	A	170	96.347	72.111	87.481	1.00	0.00
ATOM	2632	N	ALA	A	171	95.743	73.461	85.809	1.00	0.00
ATOM	2633	H	ALA	A	171	96.053	74.137	85.118	1.00	0.00
ATOM	2634	CA	ALA	A	171	94.308	73.192	85.867	1.00	0.00
ATOM	2635	HA	ALA	A	171	94.025	73.107	86.914	1.00	0.00
ATOM	2636	CB	ALA	A	171	93.585	74.410	85.276	1.00	0.00
ATOM	2637	HB1	ALA	A	171	92.509	74.308	85.398	1.00	0.00
ATOM	2638	HB2	ALA	A	171	93.913	75.320	85.779	1.00	0.00
ATOM	2639	HB3	ALA	A	171	93.806	74.495	84.213	1.00	0.00
ATOM	2640	C	ALA	A	171	93.865	71.858	85.183	1.00	0.00
ATOM	2641	O	ALA	A	171	92.676	71.678	84.870	1.00	0.00
ATOM	2642	N	GLU	A	172	94.771	70.890	85.011	1.00	0.00
ATOM	2643	H	GLU	A	172	95.741	71.109	85.203	1.00	0.00
ATOM	2644	CA	GLU	A	172	94.449	69.498	84.646	1.00	0.00
ATOM	2645	HA	GLU	A	172	93.372	69.428	84.716	1.00	0.00
ATOM	2646	CB	GLU	A	172	94.849	69.203	83.173	1.00	0.00
ATOM	2647	HB1	GLU	A	172	95.537	68.365	83.106	1.00	0.00
ATOM	2648	HB2	GLU	A	172	95.398	70.060	82.780	1.00	0.00
ATOM	2649	CG	GLU	A	172	93.673	68.934	82.220	1.00	0.00
ATOM	2650	HG1	GLU	A	172	94.083	68.712	81.234	1.00	0.00
ATOM	2651	HG2	GLU	A	172	93.106	69.858	82.137	1.00	0.00
ATOM	2652	CD	GLU	A	172	92.693	67.812	82.610	1.00	0.00
ATOM	2653	OE1	GLU	A	172	92.025	67.249	81.727	1.00	0.00
ATOM	2654	OE2	GLU	A	172	92.336	67.651	83.799	1.00	0.00
ATOM	2655	C	GLU	A	172	94.947	68.422	85.656	1.00	0.00
ATOM	2656	O	GLU	A	172	95.571	67.426	85.272	1.00	0.00
ATOM	2657	N	PRO	A	173	94.709	68.606	86.970	1.00	0.00
ATOM	2658	CD	PRO	A	173	93.900	69.650	87.560	1.00	0.00
ATOM	2659	HD1	PRO	A	173	92.972	69.753	87.013	1.00	0.00
ATOM	2660	HD2	PRO	A	173	94.448	70.586	87.555	1.00	0.00
ATOM	2661	CG	PRO	A	173	93.680	69.226	89.001	1.00	0.00
ATOM	2662	HG1	PRO	A	173	92.913	68.455	89.035	1.00	0.00
ATOM	2663	HG2	PRO	A	173	93.436	70.066	89.652	1.00	0.00
ATOM	2664	CB	PRO	A	173	95.025	68.609	89.337	1.00	0.00
ATOM	2665	HB1	PRO	A	173	94.965	67.930	90.186	1.00	0.00
ATOM	2666	HB2	PRO	A	173	95.750	69.399	89.541	1.00	0.00
ATOM	2667	CA	PRO	A	173	95.398	67.890	88.035	1.00	0.00
ATOM	2668	HA	PRO	A	173	96.460	68.064	87.866	1.00	0.00
ATOM	2669	C	PRO	A	173	95.208	66.375	88.133	1.00	0.00
ATOM	2670	O	PRO	A	173	94.221	65.824	87.644	1.00	0.00
ATOM	2671	N	PRO	A	174	96.155	65.687	88.806	1.00	0.00
ATOM	2672	CD	PRO	A	174	97.349	66.237	89.444	1.00	0.00
ATOM	2673	HD1	PRO	A	174	97.097	66.999	90.182	1.00	0.00
ATOM	2674	HD2	PRO	A	174	98.009	66.655	88.682	1.00	0.00
ATOM	2675	CG	PRO	A	174	98.043	65.065	90.131	1.00	0.00
ATOM	2676	HG1	PRO	A	174	97.640	64.930	91.136	1.00	0.00
ATOM	2677	HG2	PRO	A	174	99.125	65.195	90.162	1.00	0.00
ATOM	2678	CB	PRO	A	174	97.635	63.890	89.248	1.00	0.00
ATOM	2679	HB1	PRO	A	174	97.728	62.944	89.779	1.00	0.00
ATOM	2680	HB2	PRO	A	174	98.255	63.877	88.353	1.00	0.00
ATOM	2681	CA	PRO	A	174	96.188	64.236	88.864	1.00	0.00
ATOM	2682	HA	PRO	A	174	95.986	63.832	87.870	1.00	0.00

ATOM	2683	C	PRO	A	174	95.190	63.599	89.837	1.00	0.00
ATOM	2684	O	PRO	A	174	94.562	64.269	90.656	1.00	0.00
ATOM	2685	N	ILE	A	175	95.139	62.267	89.714	1.00	0.00
ATOM	2686	H	ILE	A	175	95.638	61.911	88.917	1.00	0.00
ATOM	2687	CA	ILE	A	175	94.689	61.231	90.659	1.00	0.00
ATOM	2688	HA	ILE	A	175	93.778	60.792	90.264	1.00	0.00
ATOM	2689	CB	ILE	A	175	95.792	60.132	90.759	1.00	0.00
ATOM	2690	HB	ILE	A	175	96.658	60.578	91.252	1.00	0.00
ATOM	2691	CG2	ILE	A	175	95.328	58.954	91.644	1.00	0.00
ATOM	2692	1HG2	ILE	A	175	96.141	58.245	91.799	1.00	0.00
ATOM	2693	2HG2	ILE	A	175	95.036	59.300	92.636	1.00	0.00
ATOM	2694	3HG2	ILE	A	175	94.490	58.439	91.180	1.00	0.00
ATOM	2695	CG1	ILE	A	175	96.287	59.626	89.375	1.00	0.00
ATOM	2696	1HG1	ILE	A	175	96.682	60.465	88.811	1.00	0.00
ATOM	2697	2HG1	ILE	A	175	95.456	59.215	88.813	1.00	0.00
ATOM	2698	CD	ILE	A	175	97.400	58.578	89.424	1.00	0.00
ATOM	2699	HD1	ILE	A	175	98.216	58.928	90.052	1.00	0.00
ATOM	2700	HD2	ILE	A	175	97.026	57.633	89.804	1.00	0.00
ATOM	2701	HD3	ILE	A	175	97.784	58.408	88.419	1.00	0.00
ATOM	2702	C	ILE	A	175	94.398	61.754	92.074	1.00	0.00
ATOM	2703	O	ILE	A	175	95.272	62.341	92.708	1.00	0.00
ATOM	2704	N	LEU	A	176	93.213	61.427	92.606	1.00	0.00
ATOM	2705	H	LEU	A	176	92.550	60.945	92.022	1.00	0.00
ATOM	2706	CA	LEU	A	176	92.820	61.771	93.976	1.00	0.00
ATOM	2707	HA	LEU	A	176	93.560	62.454	94.390	1.00	0.00
ATOM	2708	CB	LEU	A	176	91.472	62.525	93.953	1.00	0.00
ATOM	2709	HB1	LEU	A	176	91.097	62.611	94.975	1.00	0.00
ATOM	2710	HB2	LEU	A	176	90.752	61.943	93.378	1.00	0.00
ATOM	2711	CG	LEU	A	176	91.558	63.942	93.351	1.00	0.00
ATOM	2712	HG	LEU	A	176	92.008	63.898	92.359	1.00	0.00
ATOM	2713	CD1	LEU	A	176	90.151	64.521	93.208	1.00	0.00
ATOM	2714	1HD1	LEU	A	176	90.209	65.528	92.798	1.00	0.00
ATOM	2715	2HD1	LEU	A	176	89.576	63.912	92.510	1.00	0.00
ATOM	2716	3HD1	LEU	A	176	89.656	64.563	94.176	1.00	0.00
ATOM	2717	CD2	LEU	A	176	92.377	64.895	94.232	1.00	0.00
ATOM	2718	1HD2	LEU	A	176	92.276	65.912	93.865	1.00	0.00
ATOM	2719	2HD2	LEU	A	176	92.022	64.854	95.260	1.00	0.00
ATOM	2720	3HD2	LEU	A	176	93.429	64.615	94.186	1.00	0.00
ATOM	2721	C	LEU	A	176	92.867	60.561	94.929	1.00	0.00
ATOM	2722	O	LEU	A	176	93.943	60.219	95.412	1.00	0.00
ATOM	2723	N	TYR	A	177	91.734	59.914	95.224	1.00	0.00
ATOM	2724	H	TYR	A	177	90.878	60.189	94.771	1.00	0.00
ATOM	2725	CA	TYR	A	177	91.698	58.745	96.109	1.00	0.00
ATOM	2726	HA	TYR	A	177	92.584	58.146	95.893	1.00	0.00
ATOM	2727	CB	TYR	A	177	91.763	59.197	97.584	1.00	0.00
ATOM	2728	HB1	TYR	A	177	90.755	59.424	97.930	1.00	0.00
ATOM	2729	HB2	TYR	A	177	92.342	60.118	97.651	1.00	0.00
ATOM	2730	CG	TYR	A	177	92.426	58.196	98.515	1.00	0.00
ATOM	2731	CD1	TYR	A	177	93.739	57.756	98.254	1.00	0.00
ATOM	2732	HD1	TYR	A	177	94.279	58.139	97.394	1.00	0.00
ATOM	2733	CE1	TYR	A	177	94.360	56.834	99.116	1.00	0.00
ATOM	2734	HE1	TYR	A	177	95.366	56.489	98.923	1.00	0.00
ATOM	2735	CZ	TYR	A	177	93.674	56.356	100.250	1.00	0.00
ATOM	2736	OH	TYR	A	177	94.275	55.468	101.083	1.00	0.00
ATOM	2737	HH	TYR	A	177	93.697	55.222	101.804	1.00	0.00
ATOM	2738	CE2	TYR	A	177	92.366	56.808	100.525	1.00	0.00
ATOM	2739	HE2	TYR	A	177	91.834	56.453	101.393	1.00	0.00
ATOM	2740	CD2	TYR	A	177	91.751	57.733	99.660	1.00	0.00
ATOM	2741	HD2	TYR	A	177	90.750	58.085	99.878	1.00	0.00
ATOM	2742	C	TYR	A	177	90.490	57.844	95.843	1.00	0.00
ATOM	2743	O	TYR	A	177	89.481	58.309	95.298	1.00	0.00

ATOM	2744	N	SER	A	178	90.600	56.562	96.178	1.00	0.00
ATOM	2745	H	SER	A	178	91.348	56.352	96.819	1.00	0.00
ATOM	2746	CA	SER	A	178	90.262	55.478	95.244	1.00	0.00
ATOM	2747	HA	SER	A	178	89.576	55.838	94.480	1.00	0.00
ATOM	2748	CB	SER	A	178	91.579	55.011	94.581	1.00	0.00
ATOM	2749	HB1	SER	A	178	91.346	54.413	93.703	1.00	0.00
ATOM	2750	HB2	SER	A	178	92.126	54.379	95.281	1.00	0.00
ATOM	2751	OG	SER	A	178	92.438	56.081	94.220	1.00	0.00
ATOM	2752	HG	SER	A	178	93.150	55.710	93.661	1.00	0.00
ATOM	2753	C	SER	A	178	89.608	54.274	95.945	1.00	0.00
ATOM	2754	O	SER	A	178	89.879	53.139	95.550	1.00	0.00
ATOM	2755	N	GLU	A	179	88.841	54.478	97.024	1.00	0.00
ATOM	2756	H	GLU	A	179	88.773	55.444	97.342	1.00	0.00
ATOM	2757	CA	GLU	A	179	88.328	53.438	97.951	1.00	0.00
ATOM	2758	HA	GLU	A	179	89.204	52.901	98.317	1.00	0.00
ATOM	2759	CB	GLU	A	179	87.632	54.038	99.194	1.00	0.00
ATOM	2760	HB1	GLU	A	179	87.360	53.205	99.845	1.00	0.00
ATOM	2761	HB2	GLU	A	179	86.706	54.534	98.894	1.00	0.00
ATOM	2762	CG	GLU	A	179	88.460	55.002	100.051	1.00	0.00
ATOM	2763	HG1	GLU	A	179	89.510	54.702	100.034	1.00	0.00
ATOM	2764	HG2	GLU	A	179	88.107	54.941	101.082	1.00	0.00
ATOM	2765	CD	GLU	A	179	88.288	56.429	99.548	1.00	0.00
ATOM	2766	OE1	GLU	A	179	89.018	56.824	98.621	1.00	0.00
ATOM	2767	OE2	GLU	A	179	87.360	57.146	99.981	1.00	0.00
ATOM	2768	C	GLU	A	179	87.401	52.346	97.335	1.00	0.00
ATOM	2769	O	GLU	A	179	86.233	52.166	97.709	1.00	0.00
ATOM	2770	N	TYR	A	180	87.940	51.552	96.417	1.00	0.00
ATOM	2771	H	TYR	A	180	88.873	51.794	96.100	1.00	0.00
ATOM	2772	CA	TYR	A	180	87.329	50.401	95.777	1.00	0.00
ATOM	2773	HA	TYR	A	180	86.439	50.129	96.330	1.00	0.00
ATOM	2774	CB	TYR	A	180	86.872	50.737	94.346	1.00	0.00
ATOM	2775	HB1	TYR	A	180	87.150	49.925	93.677	1.00	0.00
ATOM	2776	HB2	TYR	A	180	87.377	51.633	93.988	1.00	0.00
ATOM	2777	CG	TYR	A	180	85.369	50.928	94.278	1.00	0.00
ATOM	2778	CD1	TYR	A	180	84.778	52.129	94.719	1.00	0.00
ATOM	2779	HD1	TYR	A	180	85.399	52.936	95.072	1.00	0.00
ATOM	2780	CE1	TYR	A	180	83.376	52.276	94.709	1.00	0.00
ATOM	2781	HE1	TYR	A	180	82.918	53.197	95.024	1.00	0.00
ATOM	2782	CZ	TYR	A	180	82.565	51.218	94.246	1.00	0.00
ATOM	2783	OH	TYR	A	180	81.217	51.346	94.186	1.00	0.00
ATOM	2784	HH	TYR	A	180	80.837	50.630	93.670	1.00	0.00
ATOM	2785	CE2	TYR	A	180	83.160	50.008	93.836	1.00	0.00
ATOM	2786	HE2	TYR	A	180	82.542	49.192	93.475	1.00	0.00
ATOM	2787	CD2	TYR	A	180	84.555	49.862	93.852	1.00	0.00
ATOM	2788	HD2	TYR	A	180	85.003	48.936	93.523	1.00	0.00
ATOM	2789	C	TYR	A	180	88.270	49.206	95.887	1.00	0.00
ATOM	2790	O	TYR	A	180	89.156	49.004	95.058	1.00	0.00
ATOM	2791	N	ASP	A	181	88.133	48.460	96.984	1.00	0.00
ATOM	2792	H	ASP	A	181	87.374	48.628	97.631	1.00	0.00
ATOM	2793	CA	ASP	A	181	89.009	47.330	97.260	1.00	0.00
ATOM	2794	HA	ASP	A	181	90.024	47.719	97.183	1.00	0.00
ATOM	2795	CB	ASP	A	181	88.844	46.852	98.709	1.00	0.00
ATOM	2796	HB1	ASP	A	181	89.148	47.659	99.377	1.00	0.00
ATOM	2797	HB2	ASP	A	181	89.519	46.015	98.883	1.00	0.00
ATOM	2798	CG	ASP	A	181	87.413	46.446	99.053	1.00	0.00
ATOM	2799	OD1	ASP	A	181	86.616	47.369	99.348	1.00	0.00
ATOM	2800	OD2	ASP	A	181	87.105	45.237	98.995	1.00	0.00
ATOM	2801	C	ASP	A	181	88.855	46.193	96.236	1.00	0.00
ATOM	2802	O	ASP	A	181	87.728	45.814	95.913	1.00	0.00
ATOM	2803	N	PRO	A	182	89.967	45.624	95.724	1.00	0.00
ATOM	2804	CD	PRO	A	182	91.350	45.939	96.060	1.00	0.00

ATOM	2805	HD1	PRO	A	182	91.505	45.939	97.140	1.00	0.00
ATOM	2806	HD2	PRO	A	182	91.609	46.912	95.640	1.00	0.00
ATOM	2807	CG	PRO	A	182	92.201	44.851	95.407	1.00	0.00
ATOM	2808	HG1	PRO	A	182	92.297	44.003	96.086	1.00	0.00
ATOM	2809	HG2	PRO	A	182	93.184	45.223	95.120	1.00	0.00
ATOM	2810	CB	PRO	A	182	91.367	44.448	94.195	1.00	0.00
ATOM	2811	HB1	PRO	A	182	91.605	43.440	93.855	1.00	0.00
ATOM	2812	HB2	PRO	A	182	91.528	45.165	93.389	1.00	0.00
ATOM	2813	CA	PRO	A	182	89.928	44.574	94.706	1.00	0.00
ATOM	2814	HA	PRO	A	182	89.280	44.883	93.886	1.00	0.00
ATOM	2815	C	PRO	A	182	89.389	43.235	95.236	1.00	0.00
ATOM	2816	O	PRO	A	182	89.204	42.293	94.469	1.00	0.00
ATOM	2817	N	THR	A	183	89.132	43.139	96.544	1.00	0.00
ATOM	2818	H	THR	A	183	89.358	43.923	97.140	1.00	0.00
ATOM	2819	CA	THR	A	183	88.347	42.074	97.173	1.00	0.00
ATOM	2820	HA	THR	A	183	88.764	41.118	96.859	1.00	0.00
ATOM	2821	CB	THR	A	183	88.411	42.154	98.712	1.00	0.00
ATOM	2822	HB	THR	A	183	87.428	41.965	99.143	1.00	0.00
ATOM	2823	CG2	THR	A	183	89.388	41.116	99.259	1.00	0.00
ATOM	2824	1HG2	THR	A	183	89.443	41.206	100.343	1.00	0.00
ATOM	2825	2HG2	THR	A	183	89.042	40.116	99.005	1.00	0.00
ATOM	2826	3HG2	THR	A	183	90.378	41.278	98.836	1.00	0.00
ATOM	2827	OG1	THR	A	183	88.888	43.401	99.171	1.00	0.00
ATOM	2828	HG1	THR	A	183	88.132	44.041	99.152	1.00	0.00
ATOM	2829	C	THR	A	183	86.884	42.047	96.720	1.00	0.00
ATOM	2830	O	THR	A	183	86.254	40.992	96.774	1.00	0.00
ATOM	2831	N	ARG	A	184	86.345	43.168	96.218	1.00	0.00
ATOM	2832	H	ARG	A	184	86.909	44.015	96.232	1.00	0.00
ATOM	2833	CA	ARG	A	184	85.084	43.203	95.466	1.00	0.00
ATOM	2834	HA	ARG	A	184	84.352	42.574	95.966	1.00	0.00
ATOM	2835	CB	ARG	A	184	84.545	44.659	95.397	1.00	0.00
ATOM	2836	HB1	ARG	A	184	83.565	44.644	94.921	1.00	0.00
ATOM	2837	HB2	ARG	A	184	85.207	45.246	94.759	1.00	0.00
ATOM	2838	CG	ARG	A	184	84.438	45.413	96.735	1.00	0.00
ATOM	2839	HG1	ARG	A	184	85.409	45.387	97.215	1.00	0.00
ATOM	2840	HG2	ARG	A	184	83.715	44.923	97.386	1.00	0.00
ATOM	2841	CD	ARG	A	184	84.047	46.889	96.529	1.00	0.00
ATOM	2842	HD1	ARG	A	184	82.962	46.963	96.428	1.00	0.00
ATOM	2843	HD2	ARG	A	184	84.513	47.250	95.610	1.00	0.00
ATOM	2844	NE	ARG	A	184	84.521	47.729	97.648	1.00	0.00
ATOM	2845	HE	ARG	A	184	85.102	47.258	98.346	1.00	0.00
ATOM	2846	CZ	ARG	A	184	84.526	49.045	97.747	1.00	0.00
ATOM	2847	NH1	ARG	A	184	83.911	49.813	96.897	1.00	0.00
ATOM	2848	1HH1	ARG	A	184	83.499	49.412	96.075	1.00	0.00
ATOM	2849	2HH1	ARG	A	184	84.029	50.812	96.957	1.00	0.00
ATOM	2850	NH2	ARG	A	184	85.214	49.632	98.683	1.00	0.00
ATOM	2851	1HH2	ARG	A	184	85.761	49.006	99.282	1.00	0.00
ATOM	2852	2HH2	ARG	A	184	85.378	50.634	98.664	1.00	0.00
ATOM	2853	C	ARG	A	184	85.348	42.713	94.034	1.00	0.00
ATOM	2854	O	ARG	A	184	86.013	43.429	93.277	1.00	0.00
ATOM	2855	N	PRO	A	185	84.802	41.573	93.570	1.00	0.00
ATOM	2856	CD	PRO	A	185	84.015	40.597	94.311	1.00	0.00
ATOM	2857	HD1	PRO	A	185	83.284	41.073	94.963	1.00	0.00
ATOM	2858	HD2	PRO	A	185	84.683	39.956	94.888	1.00	0.00
ATOM	2859	CG	PRO	A	185	83.285	39.764	93.255	1.00	0.00
ATOM	2860	HG1	PRO	A	185	82.312	40.212	93.052	1.00	0.00
ATOM	2861	HG2	PRO	A	185	83.168	38.729	93.572	1.00	0.00
ATOM	2862	CB	PRO	A	185	84.174	39.880	92.017	1.00	0.00
ATOM	2863	HB1	PRO	A	185	83.603	39.759	91.096	1.00	0.00
ATOM	2864	HB2	PRO	A	185	84.966	39.132	92.073	1.00	0.00
ATOM	2865	CA	PRO	A	185	84.794	41.277	92.138	1.00	0.00

ATOM	2866	HA	PRO	A	185	85.813	41.264	91.751	1.00	0.00
ATOM	2867	C	PRO	A	185	83.980	42.367	91.429	1.00	0.00
ATOM	2868	O	PRO	A	185	82.891	42.718	91.889	1.00	0.00
ATOM	2869	N	PHE	A	186	84.501	42.935	90.337	1.00	0.00
ATOM	2870	H	PHE	A	186	85.406	42.599	90.045	1.00	0.00
ATOM	2871	CA	PHE	A	186	84.091	44.263	89.835	1.00	0.00
ATOM	2872	HA	PHE	A	186	84.457	44.988	90.562	1.00	0.00
ATOM	2873	CB	PHE	A	186	84.794	44.567	88.511	1.00	0.00
ATOM	2874	HB1	PHE	A	186	84.393	45.494	88.099	1.00	0.00
ATOM	2875	HB2	PHE	A	186	84.584	43.774	87.794	1.00	0.00
ATOM	2876	CG	PHE	A	186	86.294	44.741	88.661	1.00	0.00
ATOM	2877	CD1	PHE	A	186	86.811	45.954	89.155	1.00	0.00
ATOM	2878	HD1	PHE	A	186	86.145	46.764	89.429	1.00	0.00
ATOM	2879	CE1	PHE	A	186	88.197	46.113	89.323	1.00	0.00
ATOM	2880	HE1	PHE	A	186	88.594	47.040	89.718	1.00	0.00
ATOM	2881	CZ	PHE	A	186	89.070	45.060	89.000	1.00	0.00
ATOM	2882	HZ	PHE	A	186	90.135	45.182	89.135	1.00	0.00
ATOM	2883	CE2	PHE	A	186	88.556	43.848	88.505	1.00	0.00
ATOM	2884	HE2	PHE	A	186	89.229	43.039	88.256	1.00	0.00
ATOM	2885	CD2	PHE	A	186	87.170	43.688	88.336	1.00	0.00
ATOM	2886	HD2	PHE	A	186	86.782	42.754	87.958	1.00	0.00
ATOM	2887	C	PHE	A	186	82.575	44.528	89.729	1.00	0.00
ATOM	2888	O	PHE	A	186	82.119	45.614	90.117	1.00	0.00
ATOM	2889	N	SER	A	187	81.807	43.527	89.294	1.00	0.00
ATOM	2890	H	SER	A	187	82.275	42.657	89.097	1.00	0.00
ATOM	2891	CA	SER	A	187	80.333	43.502	89.223	1.00	0.00
ATOM	2892	HA	SER	A	187	80.055	42.610	88.663	1.00	0.00
ATOM	2893	CB	SER	A	187	79.727	43.329	90.627	1.00	0.00
ATOM	2894	HB1	SER	A	187	78.657	43.138	90.537	1.00	0.00
ATOM	2895	HB2	SER	A	187	79.866	44.243	91.200	1.00	0.00
ATOM	2896	OG	SER	A	187	80.325	42.247	91.313	1.00	0.00
ATOM	2897	HG	SER	A	187	81.230	42.501	91.578	1.00	0.00
ATOM	2898	C	SER	A	187	79.662	44.684	88.501	1.00	0.00
ATOM	2899	O	SER	A	187	80.223	45.773	88.324	1.00	0.00
ATOM	2900	N	GLU	A	188	78.407	44.500	88.105	1.00	0.00
ATOM	2901	H	GLU	A	188	77.917	43.652	88.368	1.00	0.00
ATOM	2902	CA	GLU	A	188	77.618	45.573	87.504	1.00	0.00
ATOM	2903	HA	GLU	A	188	78.183	45.955	86.669	1.00	0.00
ATOM	2904	CB	GLU	A	188	76.275	45.046	86.952	1.00	0.00
ATOM	2905	HB1	GLU	A	188	76.446	44.714	85.935	1.00	0.00
ATOM	2906	HB2	GLU	A	188	75.556	45.863	86.898	1.00	0.00
ATOM	2907	CG	GLU	A	188	75.599	43.847	87.649	1.00	0.00
ATOM	2908	HG1	GLU	A	188	76.233	42.962	87.543	1.00	0.00
ATOM	2909	HG2	GLU	A	188	74.650	43.638	87.149	1.00	0.00
ATOM	2910	CD	GLU	A	188	75.344	44.124	89.130	1.00	0.00
ATOM	2911	OE1	GLU	A	188	76.249	43.789	89.929	1.00	0.00
ATOM	2912	OE2	GLU	A	188	74.368	44.833	89.436	1.00	0.00
ATOM	2913	C	GLU	A	188	77.456	46.787	88.444	1.00	0.00
ATOM	2914	O	GLU	A	188	77.864	47.913	88.121	1.00	0.00
ATOM	2915	N	ALA	A	189	76.946	46.538	89.649	1.00	0.00
ATOM	2916	H	ALA	A	189	76.611	45.596	89.851	1.00	0.00
ATOM	2917	CA	ALA	A	189	76.697	47.544	90.656	1.00	0.00
ATOM	2918	HA	ALA	A	189	76.133	48.360	90.203	1.00	0.00
ATOM	2919	CB	ALA	A	189	75.842	46.925	91.765	1.00	0.00
ATOM	2920	HB1	ALA	A	189	75.633	47.668	92.533	1.00	0.00
ATOM	2921	HB2	ALA	A	189	74.896	46.575	91.351	1.00	0.00
ATOM	2922	HB3	ALA	A	189	76.362	46.077	92.210	1.00	0.00
ATOM	2923	C	ALA	A	189	77.998	48.129	91.220	1.00	0.00
ATOM	2924	O	ALA	A	189	78.042	49.333	91.511	1.00	0.00
ATOM	2925	N	SER	A	190	79.063	47.309	91.367	1.00	0.00
ATOM	2926	H	SER	A	190	79.000	46.346	91.072	1.00	0.00

ATOM	2927	CA	SER	A	190	80.282	47.862	91.944	1.00	0.00
ATOM	2928	HA	SER	A	190	79.983	48.499	92.776	1.00	0.00
ATOM	2929	CB	SER	A	190	81.179	46.789	92.585	1.00	0.00
ATOM	2930	HB1	SER	A	190	80.887	45.799	92.239	1.00	0.00
ATOM	2931	HB2	SER	A	190	81.034	46.815	93.666	1.00	0.00
ATOM	2932	OG	SER	A	190	82.547	46.992	92.307	1.00	0.00
ATOM	2933	HG	SER	A	190	82.673	46.581	91.431	1.00	0.00
ATOM	2934	C	SER	A	190	80.941	48.792	90.931	1.00	0.00
ATOM	2935	O	SER	A	190	81.293	49.911	91.316	1.00	0.00
ATOM	2936	N	MET	A	191	80.971	48.446	89.638	1.00	0.00
ATOM	2937	H	MET	A	191	80.657	47.515	89.362	1.00	0.00
ATOM	2938	CA	MET	A	191	81.427	49.371	88.594	1.00	0.00
ATOM	2939	HA	MET	A	191	82.430	49.697	88.871	1.00	0.00
ATOM	2940	CB	MET	A	191	81.553	48.601	87.281	1.00	0.00
ATOM	2941	HB1	MET	A	191	80.610	48.106	87.072	1.00	0.00
ATOM	2942	HB2	MET	A	191	82.320	47.834	87.406	1.00	0.00
ATOM	2943	CG	MET	A	191	81.940	49.491	86.090	1.00	0.00
ATOM	2944	HG1	MET	A	191	82.559	50.310	86.450	1.00	0.00
ATOM	2945	HG2	MET	A	191	81.034	49.916	85.658	1.00	0.00
ATOM	2946	SD	MET	A	191	82.862	48.612	84.804	1.00	0.00
ATOM	2947	CE	MET	A	191	83.279	49.973	83.684	1.00	0.00
ATOM	2948	HE1	MET	A	191	83.910	49.594	82.880	1.00	0.00
ATOM	2949	HE2	MET	A	191	82.370	50.393	83.262	1.00	0.00
ATOM	2950	HE3	MET	A	191	83.817	50.749	84.225	1.00	0.00
ATOM	2951	C	MET	A	191	80.589	50.658	88.448	1.00	0.00
ATOM	2952	O	MET	A	191	81.184	51.733	88.310	1.00	0.00
ATOM	2953	N	MET	A	192	79.252	50.614	88.503	1.00	0.00
ATOM	2954	H	MET	A	192	78.776	49.716	88.568	1.00	0.00
ATOM	2955	CA	MET	A	192	78.454	51.857	88.400	1.00	0.00
ATOM	2956	HA	MET	A	192	78.833	52.434	87.553	1.00	0.00
ATOM	2957	CB	MET	A	192	76.995	51.513	88.081	1.00	0.00
ATOM	2958	HB1	MET	A	192	76.403	52.422	88.143	1.00	0.00
ATOM	2959	HB2	MET	A	192	76.611	50.785	88.796	1.00	0.00
ATOM	2960	CG	MET	A	192	76.880	50.968	86.651	1.00	0.00
ATOM	2961	HG1	MET	A	192	77.457	50.050	86.558	1.00	0.00
ATOM	2962	HG2	MET	A	192	77.311	51.702	85.971	1.00	0.00
ATOM	2963	SD	MET	A	192	75.189	50.641	86.093	1.00	0.00
ATOM	2964	CE	MET	A	192	74.937	48.987	86.788	1.00	0.00
ATOM	2965	HE1	MET	A	192	73.953	48.620	86.506	1.00	0.00
ATOM	2966	HE2	MET	A	192	75.688	48.309	86.393	1.00	0.00
ATOM	2967	HE3	MET	A	192	75.020	49.020	87.872	1.00	0.00
ATOM	2968	C	MET	A	192	78.600	52.795	89.626	1.00	0.00
ATOM	2969	O	MET	A	192	78.735	54.026	89.490	1.00	0.00
ATOM	2970	N	GLY	A	193	78.691	52.206	90.827	1.00	0.00
ATOM	2971	H	GLY	A	193	78.567	51.198	90.893	1.00	0.00
ATOM	2972	CA	GLY	A	193	79.047	52.961	92.037	1.00	0.00
ATOM	2973	HA1	GLY	A	193	79.047	52.283	92.889	1.00	0.00
ATOM	2974	HA2	GLY	A	193	78.304	53.741	92.213	1.00	0.00
ATOM	2975	C	GLY	A	193	80.431	53.632	91.948	1.00	0.00
ATOM	2976	O	GLY	A	193	80.584	54.820	92.239	1.00	0.00
ATOM	2977	N	LEU	A	194	81.434	52.884	91.476	1.00	0.00
ATOM	2978	H	LEU	A	194	81.225	51.916	91.263	1.00	0.00
ATOM	2979	CA	LEU	A	194	82.826	53.323	91.294	1.00	0.00
ATOM	2980	HA	LEU	A	194	83.220	53.646	92.258	1.00	0.00
ATOM	2981	CB	LEU	A	194	83.629	52.096	90.819	1.00	0.00
ATOM	2982	HB1	LEU	A	194	83.284	51.811	89.827	1.00	0.00
ATOM	2983	HB2	LEU	A	194	83.387	51.277	91.489	1.00	0.00
ATOM	2984	CG	LEU	A	194	85.168	52.177	90.785	1.00	0.00
ATOM	2985	HG	LEU	A	194	85.548	52.468	91.764	1.00	0.00
ATOM	2986	CD1	LEU	A	194	85.672	50.763	90.463	1.00	0.00
ATOM	2987	1HD1	LEU	A	194	86.729	50.779	90.216	1.00	0.00

```
ATOM   2988 2HD1 LEU A 194      85.534  50.122  91.330  1.00  0.00
ATOM   2989 3HD1 LEU A 194      85.113  50.336  89.633  1.00  0.00
ATOM   2990  CD2 LEU A 194      85.711  53.146  89.734  1.00  0.00
ATOM   2991 1HD2 LEU A 194      86.761  52.947  89.537  1.00  0.00
ATOM   2992 2HD2 LEU A 194      85.154  53.063  88.805  1.00  0.00
ATOM   2993 3HD2 LEU A 194      85.640  54.166  90.105  1.00  0.00
ATOM   2994  C   LEU A 194      82.934  54.514  90.332  1.00  0.00
ATOM   2995  O   LEU A 194      83.569  55.517  90.660  1.00  0.00
ATOM   2996  N   LEU A 195      82.262  54.407  89.174  1.00  0.00
ATOM   2997  H   LEU A 195      81.780  53.533  89.006  1.00  0.00
ATOM   2998  CA  LEU A 195      82.071  55.485  88.197  1.00  0.00
ATOM   2999  HA  LEU A 195      83.028  55.725  87.739  1.00  0.00
ATOM   3000  CB  LEU A 195      81.087  54.987  87.116  1.00  0.00
ATOM   3001  HB1 LEU A 195      80.222  54.559  87.612  1.00  0.00
ATOM   3002  HB2 LEU A 195      81.551  54.180  86.552  1.00  0.00
ATOM   3003  CG  LEU A 195      80.556  56.049  86.130  1.00  0.00
ATOM   3004  HG  LEU A 195      80.087  56.864  86.677  1.00  0.00
ATOM   3005  CD1 LEU A 195      81.655  56.622  85.234  1.00  0.00
ATOM   3006 1HD1 LEU A 195      81.223  57.355  84.554  1.00  0.00
ATOM   3007 2HD1 LEU A 195      82.407  57.114  85.849  1.00  0.00
ATOM   3008 3HD1 LEU A 195      82.121  55.825  84.656  1.00  0.00
ATOM   3009  CD2 LEU A 195      79.493  55.408  85.237  1.00  0.00
ATOM   3010 1HD2 LEU A 195      78.677  55.035  85.858  1.00  0.00
ATOM   3011 2HD2 LEU A 195      79.094  56.145  84.541  1.00  0.00
ATOM   3012 3HD2 LEU A 195      79.922  54.579  84.674  1.00  0.00
ATOM   3013  C   LEU A 195      81.578  56.768  88.887  1.00  0.00
ATOM   3014  O   LEU A 195      82.178  57.847  88.744  1.00  0.00
ATOM   3015  N   THR A 196      80.499  56.624  89.668  1.00  0.00
ATOM   3016  H   THR A 196      80.071  55.716  89.788  1.00  0.00
ATOM   3017  CA  THR A 196      79.904  57.822  90.273  1.00  0.00
ATOM   3018  HA  THR A 196      79.826  58.553  89.467  1.00  0.00
ATOM   3019  CB  THR A 196      78.481  57.607  90.786  1.00  0.00
ATOM   3020  HB  THR A 196      78.475  56.998  91.691  1.00  0.00
ATOM   3021  CG2 THR A 196      77.855  58.974  91.045  1.00  0.00
ATOM   3022 1HG2 THR A 196      76.781  58.899  91.132  1.00  0.00
ATOM   3023 2HG2 THR A 196      78.263  59.418  91.954  1.00  0.00
ATOM   3024 3HG2 THR A 196      78.073  59.629  90.212  1.00  0.00
ATOM   3025  OG1 THR A 196      77.727  56.978  89.773  1.00  0.00
ATOM   3026  HG1 THR A 196      77.988  56.051  89.740  1.00  0.00
ATOM   3027  C   THR A 196      80.803  58.481  91.330  1.00  0.00
ATOM   3028  O   THR A 196      80.977  59.707  91.313  1.00  0.00
ATOM   3029  N   ASN A 197      81.475  57.689  92.179  1.00  0.00
ATOM   3030  H   ASN A 197      81.292  56.691  92.181  1.00  0.00
ATOM   3031  CA  ASN A 197      82.317  58.284  93.220  1.00  0.00
ATOM   3032  HA  ASN A 197      81.790  59.204  93.466  1.00  0.00
ATOM   3033  CB  ASN A 197      82.297  57.491  94.534  1.00  0.00
ATOM   3034  HB1 ASN A 197      83.029  56.685  94.498  1.00  0.00
ATOM   3035  HB2 ASN A 197      81.305  57.065  94.675  1.00  0.00
ATOM   3036  CG  ASN A 197      82.567  58.395  95.737  1.00  0.00
ATOM   3037  OD1 ASN A 197      83.432  58.113  96.561  1.00  0.00
ATOM   3038  ND2 ASN A 197      81.881  59.503  95.896  1.00  0.00
ATOM   3039 1HD2 ASN A 197      81.208  59.840  95.196  1.00  0.00
ATOM   3040 2HD2 ASN A 197      82.005  60.036  96.734  1.00  0.00
ATOM   3041  C   ASN A 197      83.689  58.849  92.759  1.00  0.00
ATOM   3042  O   ASN A 197      84.157  59.878  93.288  1.00  0.00
ATOM   3043  N   LEU A 198      84.235  58.282  91.670  1.00  0.00
ATOM   3044  H   LEU A 198      83.811  57.436  91.305  1.00  0.00
ATOM   3045  CA  LEU A 198      85.163  59.008  90.791  1.00  0.00
ATOM   3046  HA  LEU A 198      86.115  59.140  91.306  1.00  0.00
ATOM   3047  CB  LEU A 198      85.412  58.209  89.480  1.00  0.00
ATOM   3048  HB1 LEU A 198      84.554  57.566  89.306  1.00  0.00
```

ATOM	3049	HB2	LEU	A	198	86.267	57.551	89.638	1.00	0.00
ATOM	3050	CG	LEU	A	198	85.633	59.047	88.194	1.00	0.00
ATOM	3051	HG	LEU	A	198	84.834	59.767	88.045	1.00	0.00
ATOM	3052	CD1	LEU	A	198	86.960	59.793	88.215	1.00	0.00
ATOM	3053	1HD1	LEU	A	198	87.093	60.359	87.291	1.00	0.00
ATOM	3054	2HD1	LEU	A	198	86.992	60.495	89.045	1.00	0.00
ATOM	3055	3HD1	LEU	A	198	87.768	59.081	88.333	1.00	0.00
ATOM	3056	CD2	LEU	A	198	85.612	58.175	86.946	1.00	0.00
ATOM	3057	1HD2	LEU	A	198	85.652	58.819	86.070	1.00	0.00
ATOM	3058	2HD2	LEU	A	198	86.464	57.507	86.942	1.00	0.00
ATOM	3059	3HD2	LEU	A	198	84.684	57.605	86.917	1.00	0.00
ATOM	3060	C	LEU	A	198	84.613	60.412	90.502	1.00	0.00
ATOM	3061	O	LEU	A	198	85.312	61.400	90.743	1.00	0.00
ATOM	3062	N	ALA	A	199	83.384	60.485	89.976	1.00	0.00
ATOM	3063	H	ALA	A	199	82.834	59.645	89.834	1.00	0.00
ATOM	3064	CA	ALA	A	199	82.865	61.764	89.503	1.00	0.00
ATOM	3065	HA	ALA	A	199	83.604	62.175	88.814	1.00	0.00
ATOM	3066	CB	ALA	A	199	81.581	61.542	88.694	1.00	0.00
ATOM	3067	HB1	ALA	A	199	81.270	62.487	88.248	1.00	0.00
ATOM	3068	HB2	ALA	A	199	81.762	60.809	87.908	1.00	0.00
ATOM	3069	HB3	ALA	A	199	80.778	61.186	89.336	1.00	0.00
ATOM	3070	C	ALA	A	199	82.681	62.827	90.598	1.00	0.00
ATOM	3071	O	ALA	A	199	82.946	63.989	90.311	1.00	0.00
ATOM	3072	N	ASP	A	200	82.315	62.483	91.840	1.00	0.00
ATOM	3073	H	ASP	A	200	81.977	61.542	92.031	1.00	0.00
ATOM	3074	CA	ASP	A	200	82.275	63.526	92.897	1.00	0.00
ATOM	3075	HA	ASP	A	200	81.594	64.311	92.573	1.00	0.00
ATOM	3076	CB	ASP	A	200	81.713	62.936	94.200	1.00	0.00
ATOM	3077	HB1	ASP	A	200	81.628	63.738	94.934	1.00	0.00
ATOM	3078	HB2	ASP	A	200	82.425	62.208	94.593	1.00	0.00
ATOM	3079	CG	ASP	A	200	80.350	62.271	94.023	1.00	0.00
ATOM	3080	OD1	ASP	A	200	79.343	63.001	93.894	1.00	0.00
ATOM	3081	OD2	ASP	A	200	80.332	61.019	94.092	1.00	0.00
ATOM	3082	C	ASP	A	200	83.645	64.172	93.153	1.00	0.00
ATOM	3083	O	ASP	A	200	83.803	65.407	93.125	1.00	0.00
ATOM	3084	N	ARG	A	201	84.664	63.328	93.376	1.00	0.00
ATOM	3085	H	ARG	A	201	84.478	62.336	93.304	1.00	0.00
ATOM	3086	CA	ARG	A	201	86.033	63.821	93.689	1.00	0.00
ATOM	3087	HA	ARG	A	201	86.002	64.461	94.571	1.00	0.00
ATOM	3088	CB	ARG	A	201	86.906	62.581	93.966	1.00	0.00
ATOM	3089	HB1	ARG	A	201	87.928	62.887	94.174	1.00	0.00
ATOM	3090	HB2	ARG	A	201	86.917	61.957	93.072	1.00	0.00
ATOM	3091	CG	ARG	A	201	86.380	61.749	95.157	1.00	0.00
ATOM	3092	HG1	ARG	A	201	85.291	61.722	95.157	1.00	0.00
ATOM	3093	HG2	ARG	A	201	86.699	62.214	96.089	1.00	0.00
ATOM	3094	CD	ARG	A	201	86.862	60.295	95.121	1.00	0.00
ATOM	3095	HD1	ARG	A	201	87.934	60.279	95.304	1.00	0.00
ATOM	3096	HD2	ARG	A	201	86.682	59.885	94.126	1.00	0.00
ATOM	3097	NE	ARG	A	201	86.122	59.478	96.108	1.00	0.00
ATOM	3098	HE	ARG	A	201	85.142	59.301	95.927	1.00	0.00
ATOM	3099	CZ	ARG	A	201	86.585	58.937	97.217	1.00	0.00
ATOM	3100	NH1	ARG	A	201	87.836	58.984	97.533	1.00	0.00
ATOM	3101	1HH1	ARG	A	201	88.482	59.158	96.783	1.00	0.00
ATOM	3102	2HH1	ARG	A	201	88.153	58.324	98.250	1.00	0.00
ATOM	3103	NH2	ARG	A	201	85.799	58.329	98.047	1.00	0.00
ATOM	3104	1HH2	ARG	A	201	84.821	58.218	97.808	1.00	0.00
ATOM	3105	2HH2	ARG	A	201	86.207	57.915	98.892	1.00	0.00
ATOM	3106	C	ARG	A	201	86.559	64.685	92.536	1.00	0.00
ATOM	3107	O	ARG	A	201	87.002	65.829	92.725	1.00	0.00
ATOM	3108	N	GLU	A	202	86.389	64.165	91.324	1.00	0.00
ATOM	3109	H	GLU	A	202	85.993	63.236	91.242	1.00	0.00

```
ATOM   3110  CA  GLU A 202      86.770  64.859  90.101  1.00  0.00
ATOM   3111  HA  GLU A 202      87.810  65.148  90.222  1.00  0.00
ATOM   3112  CB  GLU A 202      86.715  63.860  88.933  1.00  0.00
ATOM   3113  HB1 GLU A 202      85.690  63.794  88.574  1.00  0.00
ATOM   3114  HB2 GLU A 202      86.989  62.866  89.287  1.00  0.00
ATOM   3115  CG  GLU A 202      87.668  64.203  87.769  1.00  0.00
ATOM   3116  HG1 GLU A 202      87.517  65.240  87.469  1.00  0.00
ATOM   3117  HG2 GLU A 202      87.391  63.570  86.924  1.00  0.00
ATOM   3118  CD  GLU A 202      89.154  63.970  88.086  1.00  0.00
ATOM   3119  OE1 GLU A 202      89.970  64.068  87.142  1.00  0.00
ATOM   3120  OE2 GLU A 202      89.492  63.672  89.258  1.00  0.00
ATOM   3121  C   GLU A 202      85.988  66.154  89.826  1.00  0.00
ATOM   3122  O   GLU A 202      86.521  67.022  89.141  1.00  0.00
ATOM   3123  N   LEU A 203      84.790  66.359  90.384  1.00  0.00
ATOM   3124  H   LEU A 203      84.357  65.595  90.899  1.00  0.00
ATOM   3125  CA  LEU A 203      84.031  67.609  90.235  1.00  0.00
ATOM   3126  HA  LEU A 203      84.278  68.026  89.261  1.00  0.00
ATOM   3127  CB  LEU A 203      82.540  67.268  90.202  1.00  0.00
ATOM   3128  HB1 LEU A 203      82.256  66.939  91.193  1.00  0.00
ATOM   3129  HB2 LEU A 203      82.426  66.419  89.525  1.00  0.00
ATOM   3130  CG  LEU A 203      81.621  68.399  89.695  1.00  0.00
ATOM   3131  HG  LEU A 203      82.175  69.012  88.988  1.00  0.00
ATOM   3132  CD1 LEU A 203      80.415  67.806  88.961  1.00  0.00
ATOM   3133 1HD1 LEU A 203      79.715  68.591  88.686  1.00  0.00
ATOM   3134 2HD1 LEU A 203      80.741  67.304  88.052  1.00  0.00
ATOM   3135 3HD1 LEU A 203      79.884  67.095  89.589  1.00  0.00
ATOM   3136  CD2 LEU A 203      81.101  69.288  90.827  1.00  0.00
ATOM   3137 1HD2 LEU A 203      81.916  69.804  91.322  1.00  0.00
ATOM   3138 2HD2 LEU A 203      80.414  70.026  90.428  1.00  0.00
ATOM   3139 3HD2 LEU A 203      80.541  68.701  91.541  1.00  0.00
ATOM   3140  C   LEU A 203      84.423  68.672  91.271  1.00  0.00
ATOM   3141  O   LEU A 203      84.424  69.856  90.928  1.00  0.00
ATOM   3142  N   VAL A 204      84.882  68.289  92.473  1.00  0.00
ATOM   3143  H   VAL A 204      84.848  67.304  92.714  1.00  0.00
ATOM   3144  CA  VAL A 204      85.639  69.242  93.337  1.00  0.00
ATOM   3145  HA  VAL A 204      85.017  70.118  93.517  1.00  0.00
ATOM   3146  CB  VAL A 204      85.983  68.603  94.698  1.00  0.00
ATOM   3147  HB  VAL A 204      86.669  67.769  94.548  1.00  0.00
ATOM   3148  CG1 VAL A 204      86.657  69.614  95.636  1.00  0.00
ATOM   3149 1HG1 VAL A 204      86.856  69.146  96.600  1.00  0.00
ATOM   3150 2HG1 VAL A 204      87.608  69.941  95.219  1.00  0.00
ATOM   3151 3HG1 VAL A 204      86.013  70.480  95.780  1.00  0.00
ATOM   3152  CG2 VAL A 204      84.736  68.071  95.419  1.00  0.00
ATOM   3153 1HG2 VAL A 204      85.003  67.674  96.398  1.00  0.00
ATOM   3154 2HG2 VAL A 204      84.004  68.867  95.543  1.00  0.00
ATOM   3155 3HG2 VAL A 204      84.281  67.263  94.849  1.00  0.00
ATOM   3156  C   VAL A 204      86.926  69.735  92.640  1.00  0.00
ATOM   3157  O   VAL A 204      87.251  70.940  92.624  1.00  0.00
ATOM   3158  N   HIS A 205      87.619  68.784  91.995  1.00  0.00
ATOM   3159  H   HIS A 205      87.300  67.821  92.057  1.00  0.00
ATOM   3160  CA  HIS A 205      88.730  69.103  91.091  1.00  0.00
ATOM   3161  HA  HIS A 205      89.519  69.582  91.671  1.00  0.00
ATOM   3162  CB  HIS A 205      89.280  67.796  90.484  1.00  0.00
ATOM   3163  HB1 HIS A 205      89.114  67.779  89.408  1.00  0.00
ATOM   3164  HB2 HIS A 205      88.725  66.956  90.892  1.00  0.00
ATOM   3165  CG  HIS A 205      90.733  67.479  90.743  1.00  0.00
ATOM   3166  ND1 HIS A 205      91.617  68.198  91.560  1.00  0.00
ATOM   3167  CE1 HIS A 205      92.724  67.444  91.643  1.00  0.00
ATOM   3168  HE1 HIS A 205      93.593  67.688  92.232  1.00  0.00
ATOM   3169  NE2 HIS A 205      92.603  66.335  90.893  1.00  0.00
ATOM   3170  HE2 HIS A 205      93.291  65.584  90.814  1.00  0.00
```

ATOM	3171	CD2	HIS	A	205	91.355	66.343	90.322	1.00	0.00
ATOM	3172	HD2	HIS	A	205	90.907	65.552	89.722	1.00	0.00
ATOM	3173	C	HIS	A	205	88.285	70.111	90.028	1.00	0.00
ATOM	3174	O	HIS	A	205	88.871	71.184	89.954	1.00	0.00
ATOM	3175	N	MET	A	206	87.201	69.855	89.290	1.00	0.00
ATOM	3176	H	MET	A	206	86.797	68.924	89.371	1.00	0.00
ATOM	3177	CA	MET	A	206	86.670	70.733	88.240	1.00	0.00
ATOM	3178	HA	MET	A	206	87.432	70.829	87.483	1.00	0.00
ATOM	3179	CB	MET	A	206	85.450	70.074	87.574	1.00	0.00
ATOM	3180	HB1	MET	A	206	84.584	70.141	88.232	1.00	0.00
ATOM	3181	HB2	MET	A	206	85.673	69.017	87.413	1.00	0.00
ATOM	3182	CG	MET	A	206	85.101	70.674	86.206	1.00	0.00
ATOM	3183	HG1	MET	A	206	84.381	70.006	85.732	1.00	0.00
ATOM	3184	HG2	MET	A	206	85.992	70.680	85.585	1.00	0.00
ATOM	3185	SD	MET	A	206	84.402	72.351	86.210	1.00	0.00
ATOM	3186	CE	MET	A	206	83.601	72.412	84.590	1.00	0.00
ATOM	3187	HE1	MET	A	206	83.067	71.483	84.398	1.00	0.00
ATOM	3188	HE2	MET	A	206	82.891	73.235	84.563	1.00	0.00
ATOM	3189	HE3	MET	A	206	84.356	72.569	83.823	1.00	0.00
ATOM	3190	C	MET	A	206	86.375	72.149	88.725	1.00	0.00
ATOM	3191	O	MET	A	206	86.775	73.101	88.055	1.00	0.00
ATOM	3192	N	ILE	A	207	85.782	72.325	89.907	1.00	0.00
ATOM	3193	H	ILE	A	207	85.463	71.501	90.412	1.00	0.00
ATOM	3194	CA	ILE	A	207	85.630	73.649	90.538	1.00	0.00
ATOM	3195	HA	ILE	A	207	85.022	74.274	89.883	1.00	0.00
ATOM	3196	CB	ILE	A	207	84.886	73.493	91.890	1.00	0.00
ATOM	3197	HB	ILE	A	207	85.364	72.693	92.455	1.00	0.00
ATOM	3198	CG2	ILE	A	207	84.939	74.775	92.744	1.00	0.00
ATOM	3199	1HG2	ILE	A	207	84.400	74.630	93.680	1.00	0.00
ATOM	3200	2HG2	ILE	A	207	85.969	75.013	93.009	1.00	0.00
ATOM	3201	3HG2	ILE	A	207	84.502	75.609	92.196	1.00	0.00
ATOM	3202	CG1	ILE	A	207	83.414	73.098	91.630	1.00	0.00
ATOM	3203	1HG1	ILE	A	207	83.372	72.316	90.873	1.00	0.00
ATOM	3204	2HG1	ILE	A	207	82.882	73.965	91.238	1.00	0.00
ATOM	3205	CD	ILE	A	207	82.678	72.569	92.864	1.00	0.00
ATOM	3206	HD1	ILE	A	207	81.687	72.221	92.571	1.00	0.00
ATOM	3207	HD2	ILE	A	207	83.224	71.731	93.297	1.00	0.00
ATOM	3208	HD3	ILE	A	207	82.555	73.351	93.612	1.00	0.00
ATOM	3209	C	ILE	A	207	86.989	74.346	90.691	1.00	0.00
ATOM	3210	O	ILE	A	207	87.124	75.522	90.313	1.00	0.00
ATOM	3211	N	ASN	A	208	88.017	73.636	91.173	1.00	0.00
ATOM	3212	H	ASN	A	208	87.889	72.649	91.377	1.00	0.00
ATOM	3213	CA	ASN	A	208	89.368	74.222	91.206	1.00	0.00
ATOM	3214	HA	ASN	A	208	89.299	75.182	91.719	1.00	0.00
ATOM	3215	CB	ASN	A	208	90.298	73.335	92.051	1.00	0.00
ATOM	3216	HB1	ASN	A	208	91.329	73.630	91.870	1.00	0.00
ATOM	3217	HB2	ASN	A	208	90.191	72.287	91.789	1.00	0.00
ATOM	3218	CG	ASN	A	208	90.025	73.541	93.531	1.00	0.00
ATOM	3219	OD1	ASN	A	208	90.548	74.451	94.142	1.00	0.00
ATOM	3220	ND2	ASN	A	208	89.170	72.745	94.135	1.00	0.00
ATOM	3221	1HD2	ASN	A	208	88.648	72.052	93.611	1.00	0.00
ATOM	3222	2HD2	ASN	A	208	89.015	72.902	95.114	1.00	0.00
ATOM	3223	C	ASN	A	208	89.942	74.559	89.808	1.00	0.00
ATOM	3224	O	ASN	A	208	90.531	75.625	89.639	1.00	0.00
ATOM	3225	N	TRP	A	209	89.743	73.725	88.781	1.00	0.00
ATOM	3226	H	TRP	A	209	89.285	72.837	88.972	1.00	0.00
ATOM	3227	CA	TRP	A	209	90.186	73.995	87.398	1.00	0.00
ATOM	3228	HA	TRP	A	209	91.270	74.110	87.384	1.00	0.00
ATOM	3229	CB	TRP	A	209	89.798	72.825	86.459	1.00	0.00
ATOM	3230	HB1	TRP	A	209	90.311	72.987	85.511	1.00	0.00
ATOM	3231	HB2	TRP	A	209	88.731	72.899	86.248	1.00	0.00

ATOM	3232	CG	TRP	A	209	90.045	71.402	86.879	1.00	0.00
ATOM	3233	CD1	TRP	A	209	90.840	70.974	87.883	1.00	0.00
ATOM	3234	HD1	TRP	A	209	91.407	71.613	88.542	1.00	0.00
ATOM	3235	NE1	TRP	A	209	90.749	69.601	88.001	1.00	0.00
ATOM	3236	HE1	TRP	A	209	91.203	69.064	88.731	1.00	0.00
ATOM	3237	CE2	TRP	A	209	90.004	69.052	86.993	1.00	0.00
ATOM	3238	CZ2	TRP	A	209	89.709	67.733	86.629	1.00	0.00
ATOM	3239	HZ2	TRP	A	209	90.140	66.901	87.168	1.00	0.00
ATOM	3240	CH2	TRP	A	209	88.832	67.502	85.561	1.00	0.00
ATOM	3241	HH2	TRP	A	209	88.594	66.484	85.286	1.00	0.00
ATOM	3242	CZ3	TRP	A	209	88.248	68.588	84.889	1.00	0.00
ATOM	3243	HZ3	TRP	A	209	87.562	68.379	84.081	1.00	0.00
ATOM	3244	CE3	TRP	A	209	88.544	69.912	85.272	1.00	0.00
ATOM	3245	HE3	TRP	A	209	88.065	70.737	84.785	1.00	0.00
ATOM	3246	CD2	TRP	A	209	89.477	70.182	86.300	1.00	0.00
ATOM	3247	C	TRP	A	209	89.575	75.299	86.854	1.00	0.00
ATOM	3248	O	TRP	A	209	90.271	76.201	86.391	1.00	0.00
ATOM	3249	N	ALA	A	210	88.249	75.410	86.971	1.00	0.00
ATOM	3250	H	ALA	A	210	87.742	74.623	87.360	1.00	0.00
ATOM	3251	CA	ALA	A	210	87.470	76.570	86.570	1.00	0.00
ATOM	3252	HA	ALA	A	210	87.581	76.721	85.498	1.00	0.00
ATOM	3253	CB	ALA	A	210	86.000	76.259	86.849	1.00	0.00
ATOM	3254	HB1	ALA	A	210	85.376	77.048	86.438	1.00	0.00
ATOM	3255	HB2	ALA	A	210	85.724	75.317	86.372	1.00	0.00
ATOM	3256	HB3	ALA	A	210	85.825	76.178	87.922	1.00	0.00
ATOM	3257	C	ALA	A	210	87.927	77.847	87.289	1.00	0.00
ATOM	3258	O	ALA	A	210	88.020	78.904	86.672	1.00	0.00
ATOM	3259	N	LYS	A	211	88.282	77.755	88.579	1.00	0.00
ATOM	3260	H	LYS	A	211	88.177	76.865	89.060	1.00	0.00
ATOM	3261	CA	LYS	A	211	88.865	78.902	89.295	1.00	0.00
ATOM	3262	HA	LYS	A	211	88.358	79.807	88.963	1.00	0.00
ATOM	3263	CB	LYS	A	211	88.629	78.763	90.817	1.00	0.00
ATOM	3264	HB1	LYS	A	211	89.418	79.309	91.336	1.00	0.00
ATOM	3265	HB2	LYS	A	211	88.694	77.716	91.120	1.00	0.00
ATOM	3266	CG	LYS	A	211	87.282	79.374	91.259	1.00	0.00
ATOM	3267	HG1	LYS	A	211	87.227	80.389	90.862	1.00	0.00
ATOM	3268	HG2	LYS	A	211	87.285	79.459	92.347	1.00	0.00
ATOM	3269	CD	LYS	A	211	86.016	78.596	90.846	1.00	0.00
ATOM	3270	HD1	LYS	A	211	85.727	77.914	91.646	1.00	0.00
ATOM	3271	HD2	LYS	A	211	86.215	77.990	89.969	1.00	0.00
ATOM	3272	CE	LYS	A	211	84.844	79.518	90.478	1.00	0.00
ATOM	3273	HE1	LYS	A	211	84.035	78.910	90.059	1.00	0.00
ATOM	3274	HE2	LYS	A	211	85.172	80.198	89.684	1.00	0.00
ATOM	3275	NZ	LYS	A	211	84.333	80.311	91.623	1.00	0.00
ATOM	3276	HZ1	LYS	A	211	83.632	80.957	91.255	1.00	0.00
ATOM	3277	HZ2	LYS	A	211	83.897	79.728	92.319	1.00	0.00
ATOM	3278	HZ3	LYS	A	211	85.060	80.871	92.046	1.00	0.00
ATOM	3279	C	LYS	A	211	90.336	79.182	88.931	1.00	0.00
ATOM	3280	O	LYS	A	211	90.794	80.294	89.179	1.00	0.00
ATOM	3281	N	ARG	A	212	91.052	78.257	88.272	1.00	0.00
ATOM	3282	H	ARG	A	212	90.631	77.348	88.125	1.00	0.00
ATOM	3283	CA	ARG	A	212	92.381	78.497	87.674	1.00	0.00
ATOM	3284	HA	ARG	A	212	92.788	79.415	88.099	1.00	0.00
ATOM	3285	CB	ARG	A	212	93.355	77.357	88.033	1.00	0.00
ATOM	3286	HB1	ARG	A	212	94.173	77.342	87.313	1.00	0.00
ATOM	3287	HB2	ARG	A	212	92.856	76.391	87.965	1.00	0.00
ATOM	3288	CG	ARG	A	212	94.006	77.551	89.425	1.00	0.00
ATOM	3289	HG1	ARG	A	212	93.829	78.567	89.777	1.00	0.00
ATOM	3290	HG2	ARG	A	212	95.084	77.446	89.305	1.00	0.00
ATOM	3291	CD	ARG	A	212	93.533	76.574	90.509	1.00	0.00
ATOM	3292	HD1	ARG	A	212	92.456	76.678	90.635	1.00	0.00

ATOM	3293	HD2	ARG	A	212	93.997	76.847	91.458	1.00	0.00
ATOM	3294	NE	ARG	A	212	93.877	75.178	90.179	1.00	0.00
ATOM	3295	HE	ARG	A	212	93.173	74.639	89.712	1.00	0.00
ATOM	3296	CZ	ARG	A	212	95.083	74.636	90.277	1.00	0.00
ATOM	3297	NH1	ARG	A	212	96.085	75.243	90.832	1.00	0.00
ATOM	3298	1HH1	ARG	A	212	95.995	76.223	90.988	1.00	0.00
ATOM	3299	2HH1	ARG	A	212	96.983	74.932	90.453	1.00	0.00
ATOM	3300	NH2	ARG	A	212	95.361	73.469	89.783	1.00	0.00
ATOM	3301	1HH2	ARG	A	212	94.765	73.029	89.119	1.00	0.00
ATOM	3302	2HH2	ARG	A	212	96.362	73.293	89.669	1.00	0.00
ATOM	3303	C	ARG	A	212	92.370	78.828	86.167	1.00	0.00
ATOM	3304	O	ARG	A	212	93.439	79.063	85.602	1.00	0.00
ATOM	3305	N	VAL	A	213	91.199	79.040	85.545	1.00	0.00
ATOM	3306	H	VAL	A	213	90.352	78.774	86.025	1.00	0.00
ATOM	3307	CA	VAL	A	213	91.098	79.657	84.194	1.00	0.00
ATOM	3308	HA	VAL	A	213	92.115	79.830	83.847	1.00	0.00
ATOM	3309	CB	VAL	A	213	90.514	78.681	83.147	1.00	0.00
ATOM	3310	HB	VAL	A	213	90.440	79.200	82.195	1.00	0.00
ATOM	3311	CG1	VAL	A	213	91.456	77.489	82.960	1.00	0.00
ATOM	3312	1HG1	VAL	A	213	91.109	76.882	82.124	1.00	0.00
ATOM	3313	2HG1	VAL	A	213	92.463	77.846	82.752	1.00	0.00
ATOM	3314	3HG1	VAL	A	213	91.481	76.870	83.857	1.00	0.00
ATOM	3315	CG2	VAL	A	213	89.135	78.119	83.505	1.00	0.00
ATOM	3316	1HG2	VAL	A	213	88.785	77.471	82.703	1.00	0.00
ATOM	3317	2HG2	VAL	A	213	89.211	77.543	84.421	1.00	0.00
ATOM	3318	3HG2	VAL	A	213	88.422	78.927	83.647	1.00	0.00
ATOM	3319	C	VAL	A	213	90.470	81.078	84.121	1.00	0.00
ATOM	3320	O	VAL	A	213	89.418	81.278	83.500	1.00	0.00
ATOM	3321	N	PRO	A	214	91.072	82.132	84.717	1.00	0.00
ATOM	3322	CD	PRO	A	214	92.201	82.090	85.637	1.00	0.00
ATOM	3323	HD1	PRO	A	214	93.106	81.811	85.094	1.00	0.00
ATOM	3324	HD2	PRO	A	214	92.017	81.398	86.458	1.00	0.00
ATOM	3325	CG	PRO	A	214	92.344	83.499	86.205	1.00	0.00
ATOM	3326	HG1	PRO	A	214	93.381	83.736	86.445	1.00	0.00
ATOM	3327	HG2	PRO	A	214	91.715	83.601	87.090	1.00	0.00
ATOM	3328	CB	PRO	A	214	91.804	84.384	85.087	1.00	0.00
ATOM	3329	HB1	PRO	A	214	92.601	84.566	84.364	1.00	0.00
ATOM	3330	HB2	PRO	A	214	91.421	85.331	85.471	1.00	0.00
ATOM	3331	CA	PRO	A	214	90.694	83.532	84.461	1.00	0.00
ATOM	3332	HA	PRO	A	214	89.770	83.725	85.000	1.00	0.00
ATOM	3333	C	PRO	A	214	90.511	83.881	82.988	1.00	0.00
ATOM	3334	O	PRO	A	214	91.399	83.585	82.216	1.00	0.00
ATOM	3335	N	GLY	A	215	89.424	84.514	82.534	1.00	0.00
ATOM	3336	H	GLY	A	215	89.416	84.642	81.534	1.00	0.00
ATOM	3337	CA	GLY	A	215	88.443	85.333	83.259	1.00	0.00
ATOM	3338	HA1	GLY	A	215	88.031	86.067	82.569	1.00	0.00
ATOM	3339	HA2	GLY	A	215	88.962	85.889	84.039	1.00	0.00
ATOM	3340	C	GLY	A	215	87.246	84.636	83.927	1.00	0.00
ATOM	3341	O	GLY	A	215	86.341	85.338	84.361	1.00	0.00
ATOM	3342	N	PHE	A	216	87.228	83.305	84.094	1.00	0.00
ATOM	3343	H	PHE	A	216	87.989	82.732	83.745	1.00	0.00
ATOM	3344	CA	PHE	A	216	86.092	82.629	84.755	1.00	0.00
ATOM	3345	HA	PHE	A	216	85.185	82.848	84.189	1.00	0.00
ATOM	3346	CB	PHE	A	216	86.306	81.109	84.771	1.00	0.00
ATOM	3347	HB1	PHE	A	216	86.922	80.861	85.631	1.00	0.00
ATOM	3348	HB2	PHE	A	216	86.869	80.779	83.906	1.00	0.00
ATOM	3349	CG	PHE	A	216	85.016	80.312	84.823	1.00	0.00
ATOM	3350	CD1	PHE	A	216	84.172	80.265	83.697	1.00	0.00
ATOM	3351	HD1	PHE	A	216	84.416	80.815	82.801	1.00	0.00
ATOM	3352	CE1	PHE	A	216	82.976	79.534	83.740	1.00	0.00
ATOM	3353	HE1	PHE	A	216	82.325	79.503	82.874	1.00	0.00

ATOM	3354	CZ	PHE	A	216	82.602	78.863	84.915	1.00	0.00
ATOM	3355	HZ	PHE	A	216	81.676	78.312	84.944	1.00	0.00
ATOM	3356	CE2	PHE	A	216	83.437	78.914	86.045	1.00	0.00
ATOM	3357	HE2	PHE	A	216	83.153	78.392	86.942	1.00	0.00
ATOM	3358	CD2	PHE	A	216	84.643	79.636	85.997	1.00	0.00
ATOM	3359	HD2	PHE	A	216	85.285	79.683	86.862	1.00	0.00
ATOM	3360	C	PHE	A	216	85.844	83.058	86.213	1.00	0.00
ATOM	3361	O	PHE	A	216	84.792	82.771	86.772	1.00	0.00
ATOM	3362	N	VAL	A	217	86.826	83.696	86.857	1.00	0.00
ATOM	3363	H	VAL	A	217	87.695	83.822	86.365	1.00	0.00
ATOM	3364	CA	VAL	A	217	86.691	84.285	88.200	1.00	0.00
ATOM	3365	HA	VAL	A	217	86.016	83.666	88.789	1.00	0.00
ATOM	3366	CB	VAL	A	217	88.050	84.322	88.927	1.00	0.00
ATOM	3367	HB	VAL	A	217	87.902	84.735	89.925	1.00	0.00
ATOM	3368	CG1	VAL	A	217	88.608	82.900	89.089	1.00	0.00
ATOM	3369	1HG1	VAL	A	217	89.506	82.927	89.707	1.00	0.00
ATOM	3370	2HG1	VAL	A	217	87.863	82.276	89.577	1.00	0.00
ATOM	3371	3HG1	VAL	A	217	88.863	82.470	88.121	1.00	0.00
ATOM	3372	CG2	VAL	A	217	89.110	85.173	88.206	1.00	0.00
ATOM	3373	1HG2	VAL	A	217	90.038	85.152	88.779	1.00	0.00
ATOM	3374	2HG2	VAL	A	217	89.299	84.790	87.206	1.00	0.00
ATOM	3375	3HG2	VAL	A	217	88.776	86.210	88.151	1.00	0.00
ATOM	3376	C	VAL	A	217	86.077	85.690	88.179	1.00	0.00
ATOM	3377	O	VAL	A	217	85.511	86.100	89.189	1.00	0.00
ATOM	3378	N	ASP	A	218	86.188	86.385	87.041	1.00	0.00
ATOM	3379	H	ASP	A	218	86.647	85.959	86.249	1.00	0.00
ATOM	3380	CA	ASP	A	218	85.646	87.732	86.818	1.00	0.00
ATOM	3381	HA	ASP	A	218	85.637	88.250	87.778	1.00	0.00
ATOM	3382	CB	ASP	A	218	86.562	88.487	85.845	1.00	0.00
ATOM	3383	HB1	ASP	A	218	86.374	88.159	84.821	1.00	0.00
ATOM	3384	HB2	ASP	A	218	87.607	88.284	86.089	1.00	0.00
ATOM	3385	CG	ASP	A	218	86.319	89.984	85.984	1.00	0.00
ATOM	3386	OD1	ASP	A	218	86.088	90.699	84.983	1.00	0.00
ATOM	3387	OD2	ASP	A	218	86.341	90.460	87.137	1.00	0.00
ATOM	3388	C	ASP	A	218	84.195	87.739	86.289	1.00	0.00
ATOM	3389	O	ASP	A	218	83.478	88.732	86.389	1.00	0.00
ATOM	3390	N	LEU	A	219	83.778	86.585	85.755	1.00	0.00
ATOM	3391	H	LEU	A	219	84.494	85.879	85.649	1.00	0.00
ATOM	3392	CA	LEU	A	219	82.414	86.178	85.406	1.00	0.00
ATOM	3393	HA	LEU	A	219	82.024	86.825	84.624	1.00	0.00
ATOM	3394	CB	LEU	A	219	82.564	84.728	84.912	1.00	0.00
ATOM	3395	HB1	LEU	A	219	83.052	84.160	85.697	1.00	0.00
ATOM	3396	HB2	LEU	A	219	83.226	84.700	84.052	1.00	0.00
ATOM	3397	CG	LEU	A	219	81.279	83.964	84.565	1.00	0.00
ATOM	3398	HG	LEU	A	219	80.650	83.886	85.446	1.00	0.00
ATOM	3399	CD1	LEU	A	219	80.504	84.667	83.462	1.00	0.00
ATOM	3400	1HD1	LEU	A	219	79.704	84.025	83.110	1.00	0.00
ATOM	3401	2HD1	LEU	A	219	80.107	85.610	83.835	1.00	0.00
ATOM	3402	3HD1	LEU	A	219	81.156	84.853	82.629	1.00	0.00
ATOM	3403	CD2	LEU	A	219	81.653	82.560	84.098	1.00	0.00
ATOM	3404	1HD2	LEU	A	219	80.754	81.992	83.869	1.00	0.00
ATOM	3405	2HD2	LEU	A	219	82.293	82.608	83.219	1.00	0.00
ATOM	3406	3HD2	LEU	A	219	82.203	82.041	84.876	1.00	0.00
ATOM	3407	C	LEU	A	219	81.454	86.228	86.604	1.00	0.00
ATOM	3408	O	LEU	A	219	81.707	85.545	87.594	1.00	0.00
ATOM	3409	N	THR	A	220	80.323	86.940	86.555	1.00	0.00
ATOM	3410	H	THR	A	220	80.081	87.473	85.729	1.00	0.00
ATOM	3411	CA	THR	A	220	79.434	86.990	87.740	1.00	0.00
ATOM	3412	HA	THR	A	220	80.036	87.452	88.522	1.00	0.00
ATOM	3413	CB	THR	A	220	78.183	87.879	87.570	1.00	0.00
ATOM	3414	HB	THR	A	220	77.350	87.444	88.121	1.00	0.00

```
ATOM   3415  CG2 THR A 220      78.445  89.279  88.124  1.00  0.00
ATOM   3416 1HG2 THR A 220      77.556  89.895  88.004  1.00  0.00
ATOM   3417 2HG2 THR A 220      78.683  89.217  89.185  1.00  0.00
ATOM   3418 3HG2 THR A 220      79.284  89.733  87.594  1.00  0.00
ATOM   3419  OG1 THR A 220      77.791  88.088  86.238  1.00  0.00
ATOM   3420  HG1 THR A 220      77.292  87.263  85.978  1.00  0.00
ATOM   3421  C   THR A 220      79.078  85.604  88.293  1.00  0.00
ATOM   3422  O   THR A 220      78.981  84.633  87.551  1.00  0.00
ATOM   3423  N   LEU A 221      78.981  85.474  89.625  1.00  0.00
ATOM   3424  H   LEU A 221      79.054  86.299  90.197  1.00  0.00
ATOM   3425  CA  LEU A 221      79.002  84.157  90.289  1.00  0.00
ATOM   3426  HA  LEU A 221      79.936  83.655  90.031  1.00  0.00
ATOM   3427  CB  LEU A 221      78.944  84.324  91.820  1.00  0.00
ATOM   3428  HB1 LEU A 221      78.999  83.330  92.264  1.00  0.00
ATOM   3429  HB2 LEU A 221      77.975  84.745  92.091  1.00  0.00
ATOM   3430  CG  LEU A 221      80.046  85.200  92.442  1.00  0.00
ATOM   3431  HG  LEU A 221      79.904  86.235  92.126  1.00  0.00
ATOM   3432  CD1 LEU A 221      79.951  85.142  93.962  1.00  0.00
ATOM   3433 1HD1 LEU A 221      80.706  85.784  94.413  1.00  0.00
ATOM   3434 2HD1 LEU A 221      78.969  85.496  94.281  1.00  0.00
ATOM   3435 3HD1 LEU A 221      80.079  84.120  94.317  1.00  0.00
ATOM   3436  CD2 LEU A 221      81.459  84.768  92.044  1.00  0.00
ATOM   3437 1HD2 LEU A 221      82.196  85.339  92.609  1.00  0.00
ATOM   3438 2HD2 LEU A 221      81.593  83.704  92.232  1.00  0.00
ATOM   3439 3HD2 LEU A 221      81.611  84.973  90.986  1.00  0.00
ATOM   3440  C   LEU A 221      77.868  83.239  89.818  1.00  0.00
ATOM   3441  O   LEU A 221      78.085  82.042  89.657  1.00  0.00
ATOM   3442  N   HIS A 222      76.700  83.816  89.533  1.00  0.00
ATOM   3443  H   HIS A 222      76.607  84.806  89.714  1.00  0.00
ATOM   3444  CA  HIS A 222      75.556  83.187  88.864  1.00  0.00
ATOM   3445  HA  HIS A 222      75.090  82.451  89.523  1.00  0.00
ATOM   3446  CB  HIS A 222      74.542  84.303  88.526  1.00  0.00
ATOM   3447  HB1 HIS A 222      73.584  83.851  88.271  1.00  0.00
ATOM   3448  HB2 HIS A 222      74.886  84.865  87.657  1.00  0.00
ATOM   3449  CG  HIS A 222      74.348  85.293  89.642  1.00  0.00
ATOM   3450  ND1 HIS A 222      75.173  86.395  89.909  1.00  0.00
ATOM   3451  CE1 HIS A 222      74.792  86.843  91.115  1.00  0.00
ATOM   3452  HE1 HIS A 222      75.261  87.665  91.645  1.00  0.00
ATOM   3453  NE2 HIS A 222      73.792  86.092  91.604  1.00  0.00
ATOM   3454  HE2 HIS A 222      73.422  86.140  92.541  1.00  0.00
ATOM   3455  CD2 HIS A 222      73.496  85.115  90.686  1.00  0.00
ATOM   3456  HD2 HIS A 222      72.823  84.281  90.816  1.00  0.00
ATOM   3457  C   HIS A 222      75.968  82.497  87.568  1.00  0.00
ATOM   3458  O   HIS A 222      75.576  81.365  87.267  1.00  0.00
ATOM   3459  N   ASP A 223      76.792  83.199  86.794  1.00  0.00
ATOM   3460  H   ASP A 223      77.129  84.100  87.116  1.00  0.00
ATOM   3461  CA  ASP A 223      77.097  82.817  85.434  1.00  0.00
ATOM   3462  HA  ASP A 223      76.200  82.389  84.988  1.00  0.00
ATOM   3463  CB  ASP A 223      77.472  84.095  84.672  1.00  0.00
ATOM   3464  HB1 ASP A 223      77.654  83.863  83.627  1.00  0.00
ATOM   3465  HB2 ASP A 223      78.381  84.514  85.095  1.00  0.00
ATOM   3466  CG  ASP A 223      76.384  85.182  84.732  1.00  0.00
ATOM   3467  OD1 ASP A 223      75.523  85.206  83.823  1.00  0.00
ATOM   3468  OD2 ASP A 223      76.441  86.016  85.662  1.00  0.00
ATOM   3469  C   ASP A 223      78.183  81.724  85.461  1.00  0.00
ATOM   3470  O   ASP A 223      78.085  80.725  84.733  1.00  0.00
ATOM   3471  N   GLN A 224      79.122  81.834  86.419  1.00  0.00
ATOM   3472  H   GLN A 224      79.096  82.679  86.983  1.00  0.00
ATOM   3473  CA  GLN A 224      80.035  80.751  86.803  1.00  0.00
ATOM   3474  HA  GLN A 224      80.673  80.523  85.950  1.00  0.00
ATOM   3475  CB  GLN A 224      80.934  81.112  88.013  1.00  0.00
```

ATOM	3476	HB1	GLN	A	224	81.638	80.292	88.158	1.00	0.00
ATOM	3477	HB2	GLN	A	224	80.326	81.180	88.912	1.00	0.00
ATOM	3478	CG	GLN	A	224	81.735	82.411	87.889	1.00	0.00
ATOM	3479	HG1	GLN	A	224	81.032	83.224	87.778	1.00	0.00
ATOM	3480	HG2	GLN	A	224	82.348	82.361	86.998	1.00	0.00
ATOM	3481	CD	GLN	A	224	82.656	82.720	89.081	1.00	0.00
ATOM	3482	OE1	GLN	A	224	82.868	81.912	89.974	1.00	0.00
ATOM	3483	NE2	GLN	A	224	83.225	83.903	89.146	1.00	0.00
ATOM	3484	1HE2	GLN	A	224	82.918	84.614	88.481	1.00	0.00
ATOM	3485	2HE2	GLN	A	224	83.838	84.162	89.894	1.00	0.00
ATOM	3486	C	GLN	A	224	79.277	79.469	87.164	1.00	0.00
ATOM	3487	O	GLN	A	224	79.586	78.431	86.599	1.00	0.00
ATOM	3488	N	VAL	A	225	78.299	79.514	88.082	1.00	0.00
ATOM	3489	H	VAL	A	225	78.074	80.400	88.527	1.00	0.00
ATOM	3490	CA	VAL	A	225	77.569	78.304	88.505	1.00	0.00
ATOM	3491	HA	VAL	A	225	78.294	77.551	88.799	1.00	0.00
ATOM	3492	CB	VAL	A	225	76.672	78.550	89.739	1.00	0.00
ATOM	3493	HB	VAL	A	225	75.986	79.372	89.528	1.00	0.00
ATOM	3494	CG1	VAL	A	225	75.866	77.303	90.133	1.00	0.00
ATOM	3495	1HG1	VAL	A	225	75.300	77.489	91.045	1.00	0.00
ATOM	3496	2HG1	VAL	A	225	75.164	77.034	89.345	1.00	0.00
ATOM	3497	3HG1	VAL	A	225	76.526	76.449	90.291	1.00	0.00
ATOM	3498	CG2	VAL	A	225	77.525	78.905	90.964	1.00	0.00
ATOM	3499	1HG2	VAL	A	225	76.877	79.113	91.817	1.00	0.00
ATOM	3500	2HG2	VAL	A	225	78.190	78.079	91.215	1.00	0.00
ATOM	3501	3HG2	VAL	A	225	78.123	79.792	90.772	1.00	0.00
ATOM	3502	C	VAL	A	225	76.780	77.689	87.353	1.00	0.00
ATOM	3503	O	VAL	A	225	76.844	76.486	87.152	1.00	0.00
ATOM	3504	N	HIS	A	226	76.111	78.488	86.521	1.00	0.00
ATOM	3505	H	HIS	A	226	76.093	79.487	86.705	1.00	0.00
ATOM	3506	CA	HIS	A	226	75.305	77.960	85.412	1.00	0.00
ATOM	3507	HA	HIS	A	226	74.638	77.189	85.808	1.00	0.00
ATOM	3508	CB	HIS	A	226	74.459	79.121	84.902	1.00	0.00
ATOM	3509	HB1	HIS	A	226	75.106	79.850	84.423	1.00	0.00
ATOM	3510	HB2	HIS	A	226	73.963	79.608	85.742	1.00	0.00
ATOM	3511	CG	HIS	A	226	73.390	78.679	83.952	1.00	0.00
ATOM	3512	ND1	HIS	A	226	72.244	77.977	84.314	1.00	0.00
ATOM	3513	CE1	HIS	A	226	71.611	77.705	83.171	1.00	0.00
ATOM	3514	HE1	HIS	A	226	70.692	77.138	83.099	1.00	0.00
ATOM	3515	NE2	HIS	A	226	72.275	78.228	82.125	1.00	0.00
ATOM	3516	HE2	HIS	A	226	71.964	78.179	81.160	1.00	0.00
ATOM	3517	CD2	HIS	A	226	73.410	78.841	82.601	1.00	0.00
ATOM	3518	HD2	HIS	A	226	74.192	79.328	82.041	1.00	0.00
ATOM	3519	C	HIS	A	226	76.132	77.291	84.288	1.00	0.00
ATOM	3520	O	HIS	A	226	75.777	76.214	83.777	1.00	0.00
ATOM	3521	N	LEU	A	227	77.271	77.892	83.916	1.00	0.00
ATOM	3522	H	LEU	A	227	77.521	78.770	84.361	1.00	0.00
ATOM	3523	CA	LEU	A	227	78.226	77.251	82.999	1.00	0.00
ATOM	3524	HA	LEU	A	227	77.685	76.898	82.124	1.00	0.00
ATOM	3525	CB	LEU	A	227	79.297	78.251	82.556	1.00	0.00
ATOM	3526	HB1	LEU	A	227	80.199	77.720	82.247	1.00	0.00
ATOM	3527	HB2	LEU	A	227	79.565	78.883	83.405	1.00	0.00
ATOM	3528	CG	LEU	A	227	78.816	79.106	81.380	1.00	0.00
ATOM	3529	HG	LEU	A	227	77.814	79.469	81.589	1.00	0.00
ATOM	3530	CD1	LEU	A	227	79.733	80.312	81.269	1.00	0.00
ATOM	3531	1HD1	LEU	A	227	79.376	80.954	80.480	1.00	0.00
ATOM	3532	2HD1	LEU	A	227	79.707	80.877	82.200	1.00	0.00
ATOM	3533	3HD1	LEU	A	227	80.754	79.996	81.054	1.00	0.00
ATOM	3534	CD2	LEU	A	227	78.798	78.356	80.040	1.00	0.00
ATOM	3535	1HD2	LEU	A	227	78.557	79.043	79.231	1.00	0.00
ATOM	3536	2HD2	LEU	A	227	79.774	77.920	79.845	1.00	0.00

ATOM	3537	3HD2	LEU	A	227	78.051	77.565	80.055	1.00	0.00
ATOM	3538	C	LEU	A	227	78.907	76.012	83.585	1.00	0.00
ATOM	3539	O	LEU	A	227	79.135	75.058	82.844	1.00	0.00
ATOM	3540	N	LEU	A	228	79.218	76.034	84.886	1.00	0.00
ATOM	3541	H	LEU	A	228	79.008	76.870	85.416	1.00	0.00
ATOM	3542	CA	LEU	A	228	79.734	74.877	85.613	1.00	0.00
ATOM	3543	HA	LEU	A	228	80.658	74.549	85.143	1.00	0.00
ATOM	3544	CB	LEU	A	228	80.009	75.240	87.087	1.00	0.00
ATOM	3545	HB1	LEU	A	228	80.018	74.324	87.671	1.00	0.00
ATOM	3546	HB2	LEU	A	228	79.187	75.835	87.469	1.00	0.00
ATOM	3547	CG	LEU	A	228	81.322	75.992	87.363	1.00	0.00
ATOM	3548	HG	LEU	A	228	81.448	76.791	86.641	1.00	0.00
ATOM	3549	CD1	LEU	A	228	81.345	76.575	88.778	1.00	0.00
ATOM	3550	1HD1	LEU	A	228	82.287	77.089	88.954	1.00	0.00
ATOM	3551	2HD1	LEU	A	228	80.529	77.287	88.886	1.00	0.00
ATOM	3552	3HD1	LEU	A	228	81.210	75.779	89.509	1.00	0.00
ATOM	3553	CD2	LEU	A	228	82.534	75.073	87.257	1.00	0.00
ATOM	3554	1HD2	LEU	A	228	82.617	74.703	86.242	1.00	0.00
ATOM	3555	2HD2	LEU	A	228	83.438	75.622	87.502	1.00	0.00
ATOM	3556	3HD2	LEU	A	228	82.420	74.237	87.944	1.00	0.00
ATOM	3557	C	LEU	A	228	78.754	73.720	85.514	1.00	0.00
ATOM	3558	O	LEU	A	228	79.167	72.680	85.032	1.00	0.00
ATOM	3559	N	GLU	A	229	77.479	73.913	85.845	1.00	0.00
ATOM	3560	H	GLU	A	229	77.240	74.780	86.318	1.00	0.00
ATOM	3561	CA	GLU	A	229	76.408	72.927	85.685	1.00	0.00
ATOM	3562	HA	GLU	A	229	76.576	72.117	86.394	1.00	0.00
ATOM	3563	CB	GLU	A	229	75.067	73.628	85.996	1.00	0.00
ATOM	3564	HB1	GLU	A	229	74.542	73.902	85.084	1.00	0.00
ATOM	3565	HB2	GLU	A	229	75.235	74.564	86.525	1.00	0.00
ATOM	3566	CG	GLU	A	229	74.130	72.774	86.845	1.00	0.00
ATOM	3567	HG1	GLU	A	229	73.946	71.828	86.328	1.00	0.00
ATOM	3568	HG2	GLU	A	229	73.186	73.310	86.950	1.00	0.00
ATOM	3569	CD	GLU	A	229	74.733	72.522	88.227	1.00	0.00
ATOM	3570	OE1	GLU	A	229	74.792	71.338	88.614	1.00	0.00
ATOM	3571	OE2	GLU	A	229	75.202	73.495	88.854	1.00	0.00
ATOM	3572	C	GLU	A	229	76.422	72.321	84.267	1.00	0.00
ATOM	3573	O	GLU	A	229	76.707	71.129	84.064	1.00	0.00
ATOM	3574	N	CYS	A	230	76.177	73.183	83.269	1.00	0.00
ATOM	3575	H	CYS	A	230	76.029	74.155	83.521	1.00	0.00
ATOM	3576	CA	CYS	A	230	75.977	72.741	81.889	1.00	0.00
ATOM	3577	HA	CYS	A	230	75.097	72.098	81.859	1.00	0.00
ATOM	3578	CB	CYS	A	230	75.720	74.012	81.058	1.00	0.00
ATOM	3579	HB1	CYS	A	230	76.668	74.512	80.845	1.00	0.00
ATOM	3580	HB2	CYS	A	230	75.108	74.701	81.645	1.00	0.00
ATOM	3581	SG	CYS	A	230	74.848	73.619	79.515	1.00	0.00
ATOM	3582	HG	CYS	A	230	73.845	72.927	80.078	1.00	0.00
ATOM	3583	C	CYS	A	230	77.186	71.916	81.374	1.00	0.00
ATOM	3584	O	CYS	A	230	77.072	70.808	80.818	1.00	0.00
ATOM	3585	N	ALA	A	231	78.377	72.450	81.625	1.00	0.00
ATOM	3586	H	ALA	A	231	78.418	73.293	82.190	1.00	0.00
ATOM	3587	CA	ALA	A	231	79.633	71.875	81.190	1.00	0.00
ATOM	3588	HA	ALA	A	231	79.522	71.541	80.156	1.00	0.00
ATOM	3589	CB	ALA	A	231	80.692	72.984	81.228	1.00	0.00
ATOM	3590	HB1	ALA	A	231	81.621	72.627	80.800	1.00	0.00
ATOM	3591	HB2	ALA	A	231	80.351	73.845	80.653	1.00	0.00
ATOM	3592	HB3	ALA	A	231	80.875	73.282	82.260	1.00	0.00
ATOM	3593	C	ALA	A	231	80.104	70.662	82.004	1.00	0.00
ATOM	3594	O	ALA	A	231	80.953	69.924	81.513	1.00	0.00
ATOM	3595	N	TRP	A	232	79.630	70.461	83.236	1.00	0.00
ATOM	3596	H	TRP	A	232	78.923	71.102	83.594	1.00	0.00
ATOM	3597	CA	TRP	A	232	80.361	69.665	84.232	1.00	0.00

ATOM	3598	HA	TRP	A	232	81.338	70.118	84.351	1.00	0.00
ATOM	3599	CB	TRP	A	232	79.642	69.633	85.601	1.00	0.00
ATOM	3600	HB1	TRP	A	232	79.641	68.608	85.970	1.00	0.00
ATOM	3601	HB2	TRP	A	232	78.601	69.930	85.471	1.00	0.00
ATOM	3602	CG	TRP	A	232	80.241	70.457	86.698	1.00	0.00
ATOM	3603	CD1	TRP	A	232	81.557	70.688	86.860	1.00	0.00
ATOM	3604	HD1	TRP	A	232	82.323	70.285	86.213	1.00	0.00
ATOM	3605	NE1	TRP	A	232	81.761	71.541	87.919	1.00	0.00
ATOM	3606	HE1	TRP	A	232	82.664	71.926	88.148	1.00	0.00
ATOM	3607	CE2	TRP	A	232	80.584	71.848	88.557	1.00	0.00
ATOM	3608	CZ2	TRP	A	232	80.288	72.631	89.680	1.00	0.00
ATOM	3609	HZ2	TRP	A	232	81.064	73.178	90.182	1.00	0.00
ATOM	3610	CH2	TRP	A	232	78.963	72.687	90.139	1.00	0.00
ATOM	3611	HH2	TRP	A	232	78.694	73.287	90.997	1.00	0.00
ATOM	3612	CZ3	TRP	A	232	77.969	71.953	89.474	1.00	0.00
ATOM	3613	HZ3	TRP	A	232	76.944	71.984	89.827	1.00	0.00
ATOM	3614	CE3	TRP	A	232	78.277	71.193	88.330	1.00	0.00
ATOM	3615	HE3	TRP	A	232	77.488	70.634	87.841	1.00	0.00
ATOM	3616	CD2	TRP	A	232	79.590	71.132	87.822	1.00	0.00
ATOM	3617	C	TRP	A	232	80.606	68.250	83.758	1.00	0.00
ATOM	3618	O	TRP	A	232	81.757	67.795	83.763	1.00	0.00
ATOM	3619	N	LEU	A	233	79.542	67.578	83.315	1.00	0.00
ATOM	3620	H	LEU	A	233	78.639	68.029	83.320	1.00	0.00
ATOM	3621	CA	LEU	A	233	79.710	66.203	82.811	1.00	0.00
ATOM	3622	HA	LEU	A	233	80.249	65.629	83.564	1.00	0.00
ATOM	3623	CB	LEU	A	233	78.338	65.539	82.590	1.00	0.00
ATOM	3624	HB1	LEU	A	233	78.510	64.554	82.154	1.00	0.00
ATOM	3625	HB2	LEU	A	233	77.778	66.134	81.865	1.00	0.00
ATOM	3626	CG	LEU	A	233	77.498	65.374	83.870	1.00	0.00
ATOM	3627	HG	LEU	A	233	77.277	66.356	84.285	1.00	0.00
ATOM	3628	CD1	LEU	A	233	76.170	64.698	83.528	1.00	0.00
ATOM	3629	1HD1	LEU	A	233	75.569	64.607	84.434	1.00	0.00
ATOM	3630	2HD1	LEU	A	233	75.621	65.312	82.812	1.00	0.00
ATOM	3631	3HD1	LEU	A	233	76.345	63.712	83.098	1.00	0.00
ATOM	3632	CD2	LEU	A	233	78.184	64.521	84.938	1.00	0.00
ATOM	3633	1HD2	LEU	A	233	77.505	64.357	85.774	1.00	0.00
ATOM	3634	2HD2	LEU	A	233	78.485	63.560	84.524	1.00	0.00
ATOM	3635	3HD2	LEU	A	233	79.056	65.043	85.329	1.00	0.00
ATOM	3636	C	LEU	A	233	80.585	66.135	81.550	1.00	0.00
ATOM	3637	O	LEU	A	233	81.430	65.242	81.437	1.00	0.00
ATOM	3638	N	GLU	A	234	80.423	67.092	80.629	1.00	0.00
ATOM	3639	H	GLU	A	234	79.751	67.828	80.785	1.00	0.00
ATOM	3640	CA	GLU	A	234	81.222	67.091	79.381	1.00	0.00
ATOM	3641	HA	GLU	A	234	81.084	66.140	78.882	1.00	0.00
ATOM	3642	CB	GLU	A	234	80.631	68.215	78.515	1.00	0.00
ATOM	3643	HB1	GLU	A	234	80.880	69.175	78.968	1.00	0.00
ATOM	3644	HB2	GLU	A	234	79.544	68.114	78.522	1.00	0.00
ATOM	3645	CG	GLU	A	234	81.081	68.231	77.054	1.00	0.00
ATOM	3646	HG1	GLU	A	234	80.758	67.313	76.564	1.00	0.00
ATOM	3647	HG2	GLU	A	234	82.169	68.271	77.023	1.00	0.00
ATOM	3648	CD	GLU	A	234	80.501	69.439	76.298	1.00	0.00
ATOM	3649	OE1	GLU	A	234	79.512	70.067	76.740	1.00	0.00
ATOM	3650	OE2	GLU	A	234	81.043	69.800	75.236	1.00	0.00
ATOM	3651	C	GLU	A	234	82.745	67.243	79.616	1.00	0.00
ATOM	3652	O	GLU	A	234	83.566	66.520	79.041	1.00	0.00
ATOM	3653	N	ILE	A	235	83.103	68.145	80.529	1.00	0.00
ATOM	3654	H	ILE	A	235	82.337	68.677	80.932	1.00	0.00
ATOM	3655	CA	ILE	A	235	84.454	68.468	81.011	1.00	0.00
ATOM	3656	HA	ILE	A	235	85.084	68.760	80.173	1.00	0.00
ATOM	3657	CB	ILE	A	235	84.314	69.671	81.977	1.00	0.00
ATOM	3658	HB	ILE	A	235	83.528	69.418	82.688	1.00	0.00

ATOM	3659	CG2	ILE	A	235	85.582	69.901	82.812	1.00	0.00
ATOM	3660	1HG2	ILE	A	235	85.598	70.904	83.230	1.00	0.00
ATOM	3661	2HG2	ILE	A	235	85.579	69.190	83.637	1.00	0.00
ATOM	3662	3HG2	ILE	A	235	86.470	69.747	82.204	1.00	0.00
ATOM	3663	CG1	ILE	A	235	83.831	70.951	81.262	1.00	0.00
ATOM	3664	1HG1	ILE	A	235	83.343	71.586	81.996	1.00	0.00
ATOM	3665	2HG1	ILE	A	235	83.081	70.697	80.518	1.00	0.00
ATOM	3666	CD	ILE	A	235	84.885	71.784	80.556	1.00	0.00
ATOM	3667	HD1	ILE	A	235	84.383	72.606	80.050	1.00	0.00
ATOM	3668	HD2	ILE	A	235	85.571	72.211	81.282	1.00	0.00
ATOM	3669	HD3	ILE	A	235	85.413	71.176	79.822	1.00	0.00
ATOM	3670	C	ILE	A	235	85.107	67.284	81.738	1.00	0.00
ATOM	3671	O	ILE	A	235	86.277	66.950	81.488	1.00	0.00
ATOM	3672	N	LEU	A	236	84.352	66.634	82.632	1.00	0.00
ATOM	3673	H	LEU	A	236	83.416	66.977	82.833	1.00	0.00
ATOM	3674	CA	LEU	A	236	84.806	65.405	83.289	1.00	0.00
ATOM	3675	HA	LEU	A	236	85.712	65.604	83.862	1.00	0.00
ATOM	3676	CB	LEU	A	236	83.691	64.892	84.220	1.00	0.00
ATOM	3677	HB1	LEU	A	236	83.887	63.843	84.455	1.00	0.00
ATOM	3678	HB2	LEU	A	236	82.744	64.925	83.681	1.00	0.00
ATOM	3679	CG	LEU	A	236	83.545	65.658	85.541	1.00	0.00
ATOM	3680	HG	LEU	A	236	83.471	66.730	85.362	1.00	0.00
ATOM	3681	CD1	LEU	A	236	82.274	65.183	86.250	1.00	0.00
ATOM	3682	1HD1	LEU	A	236	82.205	65.657	87.228	1.00	0.00
ATOM	3683	2HD1	LEU	A	236	81.403	65.472	85.663	1.00	0.00
ATOM	3684	3HD1	LEU	A	236	82.297	64.102	86.380	1.00	0.00
ATOM	3685	CD2	LEU	A	236	84.730	65.390	86.456	1.00	0.00
ATOM	3686	1HD2	LEU	A	236	84.544	65.825	87.437	1.00	0.00
ATOM	3687	2HD2	LEU	A	236	84.886	64.319	86.564	1.00	0.00
ATOM	3688	3HD2	LEU	A	236	85.633	65.845	86.055	1.00	0.00
ATOM	3689	C	LEU	A	236	85.145	64.316	82.276	1.00	0.00
ATOM	3690	O	LEU	A	236	86.210	63.705	82.359	1.00	0.00
ATOM	3691	N	MET	A	237	84.251	64.070	81.317	1.00	0.00
ATOM	3692	H	MET	A	237	83.395	64.621	81.286	1.00	0.00
ATOM	3693	CA	MET	A	237	84.434	62.964	80.377	1.00	0.00
ATOM	3694	HA	MET	A	237	84.722	62.083	80.955	1.00	0.00
ATOM	3695	CB	MET	A	237	83.075	62.664	79.745	1.00	0.00
ATOM	3696	HB1	MET	A	237	82.687	63.555	79.257	1.00	0.00
ATOM	3697	HB2	MET	A	237	82.382	62.407	80.545	1.00	0.00
ATOM	3698	CG	MET	A	237	83.115	61.483	78.772	1.00	0.00
ATOM	3699	HG1	MET	A	237	82.104	61.078	78.697	1.00	0.00
ATOM	3700	HG2	MET	A	237	83.754	60.698	79.180	1.00	0.00
ATOM	3701	SD	MET	A	237	83.668	61.883	77.095	1.00	0.00
ATOM	3702	CE	MET	A	237	82.383	63.077	76.691	1.00	0.00
ATOM	3703	HE1	MET	A	237	82.651	64.064	77.071	1.00	0.00
ATOM	3704	HE2	MET	A	237	82.285	63.110	75.615	1.00	0.00
ATOM	3705	HE3	MET	A	237	81.433	62.768	77.124	1.00	0.00
ATOM	3706	C	MET	A	237	85.581	63.215	79.383	1.00	0.00
ATOM	3707	O	MET	A	237	86.396	62.312	79.185	1.00	0.00
ATOM	3708	N	ILE	A	238	85.729	64.434	78.845	1.00	0.00
ATOM	3709	H	ILE	A	238	85.013	65.134	79.019	1.00	0.00
ATOM	3710	CA	ILE	A	238	86.884	64.774	77.991	1.00	0.00
ATOM	3711	HA	ILE	A	238	86.919	64.011	77.213	1.00	0.00
ATOM	3712	CB	ILE	A	238	86.657	66.128	77.277	1.00	0.00
ATOM	3713	HB	ILE	A	238	85.653	66.111	76.848	1.00	0.00
ATOM	3714	CG2	ILE	A	238	86.742	67.333	78.223	1.00	0.00
ATOM	3715	1HG2	ILE	A	238	86.419	68.235	77.708	1.00	0.00
ATOM	3716	2HG2	ILE	A	238	86.065	67.164	79.048	1.00	0.00
ATOM	3717	3HG2	ILE	A	238	87.756	67.468	78.595	1.00	0.00
ATOM	3718	CG1	ILE	A	238	87.654	66.291	76.118	1.00	0.00
ATOM	3719	1HG1	ILE	A	238	87.611	65.392	75.507	1.00	0.00

ATOM	3720	2HG1	ILE	A	238	88.653	66.380	76.536	1.00	0.00
ATOM	3721	CD	ILE	A	238	87.394	67.502	75.217	1.00	0.00
ATOM	3722	HD1	ILE	A	238	86.356	67.516	74.893	1.00	0.00
ATOM	3723	HD2	ILE	A	238	87.612	68.428	75.749	1.00	0.00
ATOM	3724	HD3	ILE	A	238	88.044	67.446	74.344	1.00	0.00
ATOM	3725	C	ILE	A	238	88.222	64.687	78.749	1.00	0.00
ATOM	3726	O	ILE	A	238	89.193	64.135	78.212	1.00	0.00
ATOM	3727	N	GLY	A	239	88.274	65.131	80.012	1.00	0.00
ATOM	3728	H	GLY	A	239	87.457	65.577	80.415	1.00	0.00
ATOM	3729	CA	GLY	A	239	89.477	64.996	80.849	1.00	0.00
ATOM	3730	HA1	GLY	A	239	89.313	65.528	81.785	1.00	0.00
ATOM	3731	HA2	GLY	A	239	90.325	65.461	80.343	1.00	0.00
ATOM	3732	C	GLY	A	239	89.839	63.545	81.185	1.00	0.00
ATOM	3733	O	GLY	A	239	91.015	63.185	81.180	1.00	0.00
ATOM	3734	N	LEU	A	240	88.856	62.680	81.459	1.00	0.00
ATOM	3735	H	LEU	A	240	87.908	63.038	81.500	1.00	0.00
ATOM	3736	CA	LEU	A	240	89.047	61.238	81.699	1.00	0.00
ATOM	3737	HA	LEU	A	240	89.756	61.094	82.508	1.00	0.00
ATOM	3738	CB	LEU	A	240	87.688	60.651	82.154	1.00	0.00
ATOM	3739	HB1	LEU	A	240	87.053	60.499	81.281	1.00	0.00
ATOM	3740	HB2	LEU	A	240	87.196	61.395	82.780	1.00	0.00
ATOM	3741	CG	LEU	A	240	87.724	59.361	82.996	1.00	0.00
ATOM	3742	HG	LEU	A	240	88.237	59.558	83.939	1.00	0.00
ATOM	3743	CD1	LEU	A	240	86.288	58.936	83.298	1.00	0.00
ATOM	3744	1HD1	LEU	A	240	86.288	58.053	83.936	1.00	0.00
ATOM	3745	2HD1	LEU	A	240	85.770	59.743	83.815	1.00	0.00
ATOM	3746	3HD1	LEU	A	240	85.760	58.705	82.373	1.00	0.00
ATOM	3747	CD2	LEU	A	240	88.421	58.187	82.310	1.00	0.00
ATOM	3748	1HD2	LEU	A	240	88.241	57.270	82.871	1.00	0.00
ATOM	3749	2HD2	LEU	A	240	88.056	58.083	81.291	1.00	0.00
ATOM	3750	3HD2	LEU	A	240	89.495	58.363	82.308	1.00	0.00
ATOM	3751	C	LEU	A	240	89.607	60.537	80.455	1.00	0.00
ATOM	3752	O	LEU	A	240	90.595	59.798	80.527	1.00	0.00
ATOM	3753	N	VAL	A	241	89.001	60.820	79.299	1.00	0.00
ATOM	3754	H	VAL	A	241	88.205	61.450	79.325	1.00	0.00
ATOM	3755	CA	VAL	A	241	89.411	60.298	77.988	1.00	0.00
ATOM	3756	HA	VAL	A	241	89.418	59.210	78.043	1.00	0.00
ATOM	3757	CB	VAL	A	241	88.371	60.717	76.931	1.00	0.00
ATOM	3758	HB	VAL	A	241	88.055	61.743	77.111	1.00	0.00
ATOM	3759	CG1	VAL	A	241	88.883	60.645	75.492	1.00	0.00
ATOM	3760	1HG1	VAL	A	241	89.380	59.693	75.341	1.00	0.00
ATOM	3761	2HG1	VAL	A	241	88.047	60.738	74.800	1.00	0.00
ATOM	3762	3HG1	VAL	A	241	89.589	61.458	75.314	1.00	0.00
ATOM	3763	CG2	VAL	A	241	87.152	59.792	77.045	1.00	0.00
ATOM	3764	1HG2	VAL	A	241	86.376	60.101	76.345	1.00	0.00
ATOM	3765	2HG2	VAL	A	241	87.430	58.759	76.833	1.00	0.00
ATOM	3766	3HG2	VAL	A	241	86.732	59.847	78.049	1.00	0.00
ATOM	3767	C	VAL	A	241	90.837	60.709	77.615	1.00	0.00
ATOM	3768	O	VAL	A	241	91.564	59.853	77.108	1.00	0.00
ATOM	3769	N	TRP	A	242	91.256	61.953	77.903	1.00	0.00
ATOM	3770	H	TRP	A	242	90.584	62.643	78.232	1.00	0.00
ATOM	3771	CA	TRP	A	242	92.670	62.355	77.850	1.00	0.00
ATOM	3772	HA	TRP	A	242	93.064	62.190	76.850	1.00	0.00
ATOM	3773	CB	TRP	A	242	92.811	63.845	78.191	1.00	0.00
ATOM	3774	HB1	TRP	A	242	92.294	64.046	79.129	1.00	0.00
ATOM	3775	HB2	TRP	A	242	92.311	64.438	77.443	1.00	0.00
ATOM	3776	CG	TRP	A	242	94.225	64.339	78.333	1.00	0.00
ATOM	3777	CD1	TRP	A	242	95.211	64.222	77.414	1.00	0.00
ATOM	3778	HD1	TRP	A	242	95.090	63.759	76.446	1.00	0.00
ATOM	3779	NE1	TRP	A	242	96.377	64.779	77.898	1.00	0.00
ATOM	3780	HE1	TRP	A	242	97.243	64.800	77.380	1.00	0.00

ATOM	3781	CE2	TRP	A	242	96.230	65.196	79.202	1.00	0.00
ATOM	3782	CZ2	TRP	A	242	97.121	65.744	80.139	1.00	0.00
ATOM	3783	HZ2	TRP	A	242	98.151	65.919	79.878	1.00	0.00
ATOM	3784	CH2	TRP	A	242	96.654	66.040	81.433	1.00	0.00
ATOM	3785	HH2	TRP	A	242	97.326	66.454	82.171	1.00	0.00
ATOM	3786	CZ3	TRP	A	242	95.313	65.783	81.766	1.00	0.00
ATOM	3787	HZ3	TRP	A	242	94.977	65.992	82.773	1.00	0.00
ATOM	3788	CE3	TRP	A	242	94.427	65.259	80.808	1.00	0.00
ATOM	3789	HE3	TRP	A	242	93.392	65.096	81.070	1.00	0.00
ATOM	3790	CD2	TRP	A	242	94.860	64.941	79.506	1.00	0.00
ATOM	3791	C	TRP	A	242	93.544	61.586	78.840	1.00	0.00
ATOM	3792	O	TRP	A	242	94.533	60.994	78.435	1.00	0.00
ATOM	3793	N	ARG	A	243	93.206	61.535	80.131	1.00	0.00
ATOM	3794	H	ARG	A	243	92.376	62.034	80.427	1.00	0.00
ATOM	3795	CA	ARG	A	243	94.023	60.859	81.158	1.00	0.00
ATOM	3796	HA	ARG	A	243	95.067	61.051	80.899	1.00	0.00
ATOM	3797	CB	ARG	A	243	93.778	61.543	82.538	1.00	0.00
ATOM	3798	HB1	ARG	A	243	93.617	62.606	82.362	1.00	0.00
ATOM	3799	HB2	ARG	A	243	94.704	61.468	83.108	1.00	0.00
ATOM	3800	CG	ARG	A	243	92.648	60.992	83.436	1.00	0.00
ATOM	3801	HG1	ARG	A	243	93.100	60.340	84.182	1.00	0.00
ATOM	3802	HG2	ARG	A	243	91.996	60.362	82.832	1.00	0.00
ATOM	3803	CD	ARG	A	243	91.731	62.004	84.176	1.00	0.00
ATOM	3804	HD1	ARG	A	243	91.137	61.436	84.893	1.00	0.00
ATOM	3805	HD2	ARG	A	243	91.023	62.415	83.466	1.00	0.00
ATOM	3806	NE	ARG	A	243	92.379	63.126	84.899	1.00	0.00
ATOM	3807	HE	ARG	A	243	92.506	63.022	85.891	1.00	0.00
ATOM	3808	CZ	ARG	A	243	92.392	64.394	84.503	1.00	0.00
ATOM	3809	NH1	ARG	A	243	92.061	64.758	83.301	1.00	0.00
ATOM	3810	1HH1	ARG	A	243	91.862	64.062	82.602	1.00	0.00
ATOM	3811	2HH1	ARG	A	243	92.063	65.748	83.047	1.00	0.00
ATOM	3812	NH2	ARG	A	243	92.753	65.355	85.297	1.00	0.00
ATOM	3813	1HH2	ARG	A	243	93.081	65.219	86.246	1.00	0.00
ATOM	3814	2HH2	ARG	A	243	92.689	66.298	84.927	1.00	0.00
ATOM	3815	C	ARG	A	243	93.920	59.318	81.119	1.00	0.00
ATOM	3816	O	ARG	A	243	94.292	58.641	82.076	1.00	0.00
ATOM	3817	N	SER	A	244	93.428	58.786	79.994	1.00	0.00
ATOM	3818	H	SER	A	244	93.149	59.453	79.295	1.00	0.00
ATOM	3819	CA	SER	A	244	93.451	57.372	79.576	1.00	0.00
ATOM	3820	HA	SER	A	244	94.028	56.807	80.298	1.00	0.00
ATOM	3821	CB	SER	A	244	92.029	56.794	79.540	1.00	0.00
ATOM	3822	HB1	SER	A	244	92.087	55.716	79.386	1.00	0.00
ATOM	3823	HB2	SER	A	244	91.481	57.238	78.708	1.00	0.00
ATOM	3824	OG	SER	A	244	91.320	57.052	80.736	1.00	0.00
ATOM	3825	HG	SER	A	244	91.241	58.019	80.805	1.00	0.00
ATOM	3826	C	SER	A	244	94.117	57.176	78.190	1.00	0.00
ATOM	3827	O	SER	A	244	93.919	56.131	77.565	1.00	0.00
ATOM	3828	N	MET	A	245	94.826	58.185	77.656	1.00	0.00
ATOM	3829	H	MET	A	245	95.017	58.994	78.239	1.00	0.00
ATOM	3830	CA	MET	A	245	95.340	58.235	76.271	1.00	0.00
ATOM	3831	HA	MET	A	245	94.489	58.215	75.595	1.00	0.00
ATOM	3832	CB	MET	A	245	96.159	59.529	76.041	1.00	0.00
ATOM	3833	HB1	MET	A	245	97.175	59.375	76.408	1.00	0.00
ATOM	3834	HB2	MET	A	245	95.755	60.356	76.600	1.00	0.00
ATOM	3835	CG	MET	A	245	96.224	59.986	74.576	1.00	0.00
ATOM	3836	HG1	MET	A	245	96.056	59.134	73.922	1.00	0.00
ATOM	3837	HG2	MET	A	245	97.233	60.352	74.385	1.00	0.00
ATOM	3838	SD	MET	A	245	95.068	61.320	74.127	1.00	0.00
ATOM	3839	CE	MET	A	245	93.501	60.555	74.623	1.00	0.00
ATOM	3840	HE1	MET	A	245	92.686	61.263	74.506	1.00	0.00
ATOM	3841	HE2	MET	A	245	93.313	59.668	74.018	1.00	0.00

ATOM	3842	HE3	MET	A	245	93.550	60.276	75.674	1.00	0.00
ATOM	3843	C	MET	A	245	96.266	57.083	75.867	1.00	0.00
ATOM	3844	O	MET	A	245	96.380	56.782	74.686	1.00	0.00
ATOM	3845	N	GLU	A	246	96.942	56.457	76.821	1.00	0.00
ATOM	3846	H	GLU	A	246	96.871	56.858	77.759	1.00	0.00
ATOM	3847	CA	GLU	A	246	97.999	55.481	76.563	1.00	0.00
ATOM	3848	HA	GLU	A	246	98.341	55.577	75.532	1.00	0.00
ATOM	3849	CB	GLU	A	246	99.218	55.726	77.484	1.00	0.00
ATOM	3850	HB1	GLU	A	246	100.106	55.396	76.942	1.00	0.00
ATOM	3851	HB2	GLU	A	246	99.141	55.093	78.370	1.00	0.00
ATOM	3852	CG	GLU	A	246	99.459	57.174	77.958	1.00	0.00
ATOM	3853	HG1	GLU	A	246	99.237	57.876	77.151	1.00	0.00
ATOM	3854	HG2	GLU	A	246	100.518	57.280	78.202	1.00	0.00
ATOM	3855	CD	GLU	A	246	98.629	57.488	79.214	1.00	0.00
ATOM	3856	OE1	GLU	A	246	97.456	57.906	79.068	1.00	0.00
ATOM	3857	OE2	GLU	A	246	99.122	57.184	80.324	1.00	0.00
ATOM	3858	C	GLU	A	246	97.477	54.056	76.760	1.00	0.00
ATOM	3859	O	GLU	A	246	97.837	53.142	76.015	1.00	0.00
ATOM	3860	N	HIS	A	247	96.584	53.851	77.731	1.00	0.00
ATOM	3861	H	HIS	A	247	96.285	54.660	78.267	1.00	0.00
ATOM	3862	CA	HIS	A	247	96.123	52.530	78.165	1.00	0.00
ATOM	3863	HA	HIS	A	247	96.999	51.888	78.224	1.00	0.00
ATOM	3864	CB	HIS	A	247	95.492	52.598	79.573	1.00	0.00
ATOM	3865	HB1	HIS	A	247	95.658	51.652	80.083	1.00	0.00
ATOM	3866	HB2	HIS	A	247	94.415	52.743	79.484	1.00	0.00
ATOM	3867	CG	HIS	A	247	96.010	53.706	80.460	1.00	0.00
ATOM	3868	ND1	HIS	A	247	95.200	54.680	81.028	1.00	0.00
ATOM	3869	CE1	HIS	A	247	96.034	55.635	81.478	1.00	0.00
ATOM	3870	HE1	HIS	A	247	95.721	56.589	81.885	1.00	0.00
ATOM	3871	NE2	HIS	A	247	97.306	55.299	81.236	1.00	0.00
ATOM	3872	HE2	HIS	A	247	98.074	55.980	81.239	1.00	0.00
ATOM	3873	CD2	HIS	A	247	97.317	54.072	80.615	1.00	0.00
ATOM	3874	HD2	HIS	A	247	98.182	53.587	80.184	1.00	0.00
ATOM	3875	C	HIS	A	247	95.131	51.963	77.130	1.00	0.00
ATOM	3876	O	HIS	A	247	94.093	52.581	76.894	1.00	0.00
ATOM	3877	N	PRO	A	248	95.412	50.844	76.442	1.00	0.00
ATOM	3878	CD	PRO	A	248	96.645	50.069	76.484	1.00	0.00
ATOM	3879	HD1	PRO	A	248	96.793	49.623	77.469	1.00	0.00
ATOM	3880	HD2	PRO	A	248	97.493	50.705	76.229	1.00	0.00
ATOM	3881	CG	PRO	A	248	96.502	48.977	75.423	1.00	0.00
ATOM	3882	HG1	PRO	A	248	96.141	48.057	75.884	1.00	0.00
ATOM	3883	HG2	PRO	A	248	97.445	48.794	74.909	1.00	0.00
ATOM	3884	CB	PRO	A	248	95.440	49.538	74.476	1.00	0.00
ATOM	3885	HB1	PRO	A	248	94.910	48.746	73.946	1.00	0.00
ATOM	3886	HB2	PRO	A	248	95.914	50.210	73.759	1.00	0.00
ATOM	3887	CA	PRO	A	248	94.520	50.349	75.396	1.00	0.00
ATOM	3888	HA	PRO	A	248	94.077	51.172	74.830	1.00	0.00
ATOM	3889	C	PRO	A	248	93.411	49.461	75.978	1.00	0.00
ATOM	3890	O	PRO	A	248	93.674	48.549	76.760	1.00	0.00
ATOM	3891	N	GLY	A	249	92.157	49.676	75.575	1.00	0.00
ATOM	3892	H	GLY	A	249	91.974	50.449	74.951	1.00	0.00
ATOM	3893	CA	GLY	A	249	91.042	48.784	75.925	1.00	0.00
ATOM	3894	HA1	GLY	A	249	91.336	47.749	75.747	1.00	0.00
ATOM	3895	HA2	GLY	A	249	90.205	49.013	75.268	1.00	0.00
ATOM	3896	C	GLY	A	249	90.528	48.884	77.366	1.00	0.00
ATOM	3897	O	GLY	A	249	89.668	48.095	77.744	1.00	0.00
ATOM	3898	N	LYS	A	250	91.008	49.846	78.157	1.00	0.00
ATOM	3899	H	LYS	A	250	91.640	50.526	77.750	1.00	0.00
ATOM	3900	CA	LYS	A	250	90.493	50.173	79.496	1.00	0.00
ATOM	3901	HA	LYS	A	250	89.427	49.930	79.537	1.00	0.00
ATOM	3902	CB	LYS	A	250	91.217	49.367	80.605	1.00	0.00

ATOM	3903	HB1	LYS	A	250	91.157	48.306	80.376	1.00	0.00
ATOM	3904	HB2	LYS	A	250	90.648	49.515	81.520	1.00	0.00
ATOM	3905	CG	LYS	A	250	92.668	49.727	80.957	1.00	0.00
ATOM	3906	HG1	LYS	A	250	92.867	49.314	81.946	1.00	0.00
ATOM	3907	HG2	LYS	A	250	92.768	50.810	81.042	1.00	0.00
ATOM	3908	CD	LYS	A	250	93.767	49.202	80.024	1.00	0.00
ATOM	3909	HD1	LYS	A	250	94.709	49.605	80.397	1.00	0.00
ATOM	3910	HD2	LYS	A	250	93.622	49.599	79.022	1.00	0.00
ATOM	3911	CE	LYS	A	250	93.918	47.670	80.010	1.00	0.00
ATOM	3912	HE1	LYS	A	250	93.715	47.278	81.011	1.00	0.00
ATOM	3913	HE2	LYS	A	250	94.962	47.445	79.776	1.00	0.00
ATOM	3914	NZ	LYS	A	250	93.060	47.001	79.005	1.00	0.00
ATOM	3915	HZ1	LYS	A	250	93.262	46.016	78.937	1.00	0.00
ATOM	3916	HZ2	LYS	A	250	92.067	46.994	79.256	1.00	0.00
ATOM	3917	HZ3	LYS	A	250	93.165	47.426	78.089	1.00	0.00
ATOM	3918	C	LYS	A	250	90.602	51.664	79.744	1.00	0.00
ATOM	3919	O	LYS	A	250	91.368	52.316	79.037	1.00	0.00
ATOM	3920	N	LEU	A	251	89.903	52.187	80.748	1.00	0.00
ATOM	3921	H	LEU	A	251	89.328	51.577	81.321	1.00	0.00
ATOM	3922	CA	LEU	A	251	90.037	53.609	81.131	1.00	0.00
ATOM	3923	HA	LEU	A	251	90.769	54.117	80.499	1.00	0.00
ATOM	3924	CB	LEU	A	251	88.663	54.304	80.949	1.00	0.00
ATOM	3925	HB1	LEU	A	251	88.627	55.141	81.647	1.00	0.00
ATOM	3926	HB2	LEU	A	251	87.880	53.607	81.251	1.00	0.00
ATOM	3927	CG	LEU	A	251	88.318	54.876	79.559	1.00	0.00
ATOM	3928	HG	LEU	A	251	89.021	55.671	79.310	1.00	0.00
ATOM	3929	CD1	LEU	A	251	88.310	53.862	78.412	1.00	0.00
ATOM	3930	1HD1	LEU	A	251	89.332	53.571	78.186	1.00	0.00
ATOM	3931	2HD1	LEU	A	251	87.721	52.991	78.694	1.00	0.00
ATOM	3932	3HD1	LEU	A	251	87.879	54.308	77.517	1.00	0.00
ATOM	3933	CD2	LEU	A	251	86.910	55.482	79.614	1.00	0.00
ATOM	3934	1HD2	LEU	A	251	86.680	55.973	78.667	1.00	0.00
ATOM	3935	2HD2	LEU	A	251	86.172	54.700	79.800	1.00	0.00
ATOM	3936	3HD2	LEU	A	251	86.856	56.216	80.417	1.00	0.00
ATOM	3937	C	LEU	A	251	90.522	53.731	82.585	1.00	0.00
ATOM	3938	O	LEU	A	251	90.304	52.818	83.387	1.00	0.00
ATOM	3939	N	LEU	A	252	91.136	54.881	82.904	1.00	0.00
ATOM	3940	H	LEU	A	252	91.244	55.583	82.176	1.00	0.00
ATOM	3941	CA	LEU	A	252	91.691	55.232	84.218	1.00	0.00
ATOM	3942	HA	LEU	A	252	91.752	54.333	84.831	1.00	0.00
ATOM	3943	CB	LEU	A	252	93.126	55.769	84.023	1.00	0.00
ATOM	3944	HB1	LEU	A	252	93.083	56.698	83.454	1.00	0.00
ATOM	3945	HB2	LEU	A	252	93.649	55.037	83.406	1.00	0.00
ATOM	3946	CG	LEU	A	252	93.959	55.984	85.304	1.00	0.00
ATOM	3947	HG	LEU	A	252	93.925	55.081	85.913	1.00	0.00
ATOM	3948	CD1	LEU	A	252	95.417	56.227	84.905	1.00	0.00
ATOM	3949	1HD1	LEU	A	252	95.792	55.375	84.336	1.00	0.00
ATOM	3950	2HD1	LEU	A	252	95.484	57.124	84.288	1.00	0.00
ATOM	3951	3HD1	LEU	A	252	96.035	56.353	85.793	1.00	0.00
ATOM	3952	CD2	LEU	A	252	93.510	57.161	86.179	1.00	0.00
ATOM	3953	1HD2	LEU	A	252	94.265	57.383	86.932	1.00	0.00
ATOM	3954	2HD2	LEU	A	252	93.354	58.042	85.555	1.00	0.00
ATOM	3955	3HD2	LEU	A	252	92.591	56.906	86.700	1.00	0.00
ATOM	3956	C	LEU	A	252	90.760	56.244	84.900	1.00	0.00
ATOM	3957	O	LEU	A	252	90.652	57.418	84.541	1.00	0.00
ATOM	3958	N	PHE	A	253	90.075	55.754	85.921	1.00	0.00
ATOM	3959	H	PHE	A	253	90.250	54.787	86.176	1.00	0.00
ATOM	3960	CA	PHE	A	253	89.059	56.447	86.691	1.00	0.00
ATOM	3961	HA	PHE	A	253	88.469	57.068	86.016	1.00	0.00
ATOM	3962	CB	PHE	A	253	88.128	55.371	87.298	1.00	0.00
ATOM	3963	HB1	PHE	A	253	87.573	55.819	88.124	1.00	0.00

ATOM	3964	HB2	PHE	A	253	88.726	54.567	87.729	1.00	0.00
ATOM	3965	CG	PHE	A	253	87.119	54.766	86.322	1.00	0.00
ATOM	3966	CD1	PHE	A	253	87.529	54.122	85.136	1.00	0.00
ATOM	3967	HD1	PHE	A	253	88.577	53.979	84.928	1.00	0.00
ATOM	3968	CE1	PHE	A	253	86.580	53.659	84.207	1.00	0.00
ATOM	3969	HE1	PHE	A	253	86.907	53.173	83.297	1.00	0.00
ATOM	3970	CZ	PHE	A	253	85.208	53.809	84.470	1.00	0.00
ATOM	3971	HZ	PHE	A	253	84.471	53.442	83.762	1.00	0.00
ATOM	3972	CE2	PHE	A	253	84.789	54.389	85.679	1.00	0.00
ATOM	3973	HE2	PHE	A	253	83.742	54.462	85.900	1.00	0.00
ATOM	3974	CD2	PHE	A	253	85.740	54.867	86.597	1.00	0.00
ATOM	3975	HD2	PHE	A	253	85.409	55.326	87.519	1.00	0.00
ATOM	3976	C	PHE	A	253	89.705	57.381	87.719	1.00	0.00
ATOM	3977	O	PHE	A	253	89.710	57.044	88.901	1.00	0.00
ATOM	3978	N	ALA	A	254	90.265	58.503	87.234	1.00	0.00
ATOM	3979	H	ALA	A	254	90.240	58.565	86.224	1.00	0.00
ATOM	3980	CA	ALA	A	254	91.072	59.540	87.911	1.00	0.00
ATOM	3981	HA	ALA	A	254	91.884	59.740	87.211	1.00	0.00
ATOM	3982	CB	ALA	A	254	90.331	60.881	87.971	1.00	0.00
ATOM	3983	HB1	ALA	A	254	90.977	61.636	88.422	1.00	0.00
ATOM	3984	HB2	ALA	A	254	90.077	61.211	86.965	1.00	0.00
ATOM	3985	HB3	ALA	A	254	89.412	60.822	88.539	1.00	0.00
ATOM	3986	C	ALA	A	254	91.825	59.088	89.184	1.00	0.00
ATOM	3987	O	ALA	A	254	93.029	58.866	89.058	1.00	0.00
ATOM	3988	N	PRO	A	255	91.194	58.859	90.353	1.00	0.00
ATOM	3989	CD	PRO	A	255	89.966	59.544	90.754	1.00	0.00
ATOM	3990	HD1	PRO	A	255	89.147	59.238	90.107	1.00	0.00
ATOM	3991	HD2	PRO	A	255	90.092	60.626	90.724	1.00	0.00
ATOM	3992	CG	PRO	A	255	89.660	59.099	92.172	1.00	0.00
ATOM	3993	HG1	PRO	A	255	88.591	59.115	92.385	1.00	0.00
ATOM	3994	HG2	PRO	A	255	90.207	59.713	92.883	1.00	0.00
ATOM	3995	CB	PRO	A	255	90.219	57.688	92.167	1.00	0.00
ATOM	3996	HB1	PRO	A	255	89.504	57.019	91.685	1.00	0.00
ATOM	3997	HB2	PRO	A	255	90.443	57.346	93.165	1.00	0.00
ATOM	3998	CA	PRO	A	255	91.501	57.790	91.332	1.00	0.00
ATOM	3999	HA	PRO	A	255	92.288	58.169	91.983	1.00	0.00
ATOM	4000	C	PRO	A	255	91.957	56.382	90.846	1.00	0.00
ATOM	4001	O	PRO	A	255	91.527	55.370	91.395	1.00	0.00
ATOM	4002	N	ASN	A	256	92.836	56.272	89.850	1.00	0.00
ATOM	4003	H	ASN	A	256	93.080	57.146	89.406	1.00	0.00
ATOM	4004	CA	ASN	A	256	93.680	55.120	89.475	1.00	0.00
ATOM	4005	HA	ASN	A	256	94.038	55.371	88.475	1.00	0.00
ATOM	4006	CB	ASN	A	256	94.935	55.107	90.363	1.00	0.00
ATOM	4007	HB1	ASN	A	256	95.272	56.124	90.553	1.00	0.00
ATOM	4008	HB2	ASN	A	256	95.738	54.585	89.841	1.00	0.00
ATOM	4009	CG	ASN	A	256	94.711	54.417	91.698	1.00	0.00
ATOM	4010	OD1	ASN	A	256	94.371	55.031	92.692	1.00	0.00
ATOM	4011	ND2	ASN	A	256	94.905	53.120	91.758	1.00	0.00
ATOM	4012	1HD2	ASN	A	256	95.056	52.599	90.909	1.00	0.00
ATOM	4013	2HD2	ASN	A	256	94.751	52.684	92.650	1.00	0.00
ATOM	4014	C	ASN	A	256	93.090	53.702	89.261	1.00	0.00
ATOM	4015	O	ASN	A	256	93.855	52.805	88.908	1.00	0.00
ATOM	4016	N	LEU	A	257	91.790	53.472	89.438	1.00	0.00
ATOM	4017	H	LEU	A	257	91.230	54.210	89.850	1.00	0.00
ATOM	4018	CA	LEU	A	257	91.141	52.227	89.020	1.00	0.00
ATOM	4019	HA	LEU	A	257	91.706	51.390	89.428	1.00	0.00
ATOM	4020	CB	LEU	A	257	89.701	52.178	89.581	1.00	0.00
ATOM	4021	HB1	LEU	A	257	89.057	51.686	88.851	1.00	0.00
ATOM	4022	HB2	LEU	A	257	89.326	53.196	89.697	1.00	0.00
ATOM	4023	CG	LEU	A	257	89.547	51.435	90.925	1.00	0.00
ATOM	4024	HG	LEU	A	257	88.527	51.590	91.271	1.00	0.00

```
ATOM   4025  CD1 LEU A 257      89.743  49.924  90.752  1.00  0.00
ATOM   4026 1HD1 LEU A 257      89.515  49.424  91.693  1.00  0.00
ATOM   4027 2HD1 LEU A 257      89.068  49.552  89.983  1.00  0.00
ATOM   4028 3HD1 LEU A 257      90.770  49.700  90.474  1.00  0.00
ATOM   4029  CD2 LEU A 257      90.476  51.942  92.026  1.00  0.00
ATOM   4030 1HD2 LEU A 257      90.274  51.409  92.957  1.00  0.00
ATOM   4031 2HD2 LEU A 257      91.521  51.805  91.754  1.00  0.00
ATOM   4032 3HD2 LEU A 257      90.289  53.002  92.189  1.00  0.00
ATOM   4033  C   LEU A 257      91.185  52.107  87.493  1.00  0.00
ATOM   4034  O   LEU A 257      90.759  53.018  86.786  1.00  0.00
ATOM   4035  N   LEU A 258      91.682  50.978  86.989  1.00  0.00
ATOM   4036  H   LEU A 258      92.046  50.286  87.626  1.00  0.00
ATOM   4037  CA  LEU A 258      91.598  50.621  85.574  1.00  0.00
ATOM   4038  HA  LEU A 258      91.414  51.516  84.987  1.00  0.00
ATOM   4039  CB  LEU A 258      92.918  49.993  85.092  1.00  0.00
ATOM   4040  HB1 LEU A 258      92.689  49.200  84.381  1.00  0.00
ATOM   4041  HB2 LEU A 258      93.454  49.544  85.927  1.00  0.00
ATOM   4042  CG  LEU A 258      93.829  51.009  84.371  1.00  0.00
ATOM   4043  HG  LEU A 258      93.297  51.403  83.505  1.00  0.00
ATOM   4044  CD1 LEU A 258      94.246  52.176  85.260  1.00  0.00
ATOM   4045 1HD1 LEU A 258      94.909  52.839  84.705  1.00  0.00
ATOM   4046 2HD1 LEU A 258      93.372  52.749  85.563  1.00  0.00
ATOM   4047 3HD1 LEU A 258      94.755  51.813  86.152  1.00  0.00
ATOM   4048  CD2 LEU A 258      95.102  50.319  83.884  1.00  0.00
ATOM   4049 1HD2 LEU A 258      95.681  49.959  84.733  1.00  0.00
ATOM   4050 2HD2 LEU A 258      94.851  49.476  83.243  1.00  0.00
ATOM   4051 3HD2 LEU A 258      95.708  51.024  83.315  1.00  0.00
ATOM   4052  C   LEU A 258      90.397  49.697  85.385  1.00  0.00
ATOM   4053  O   LEU A 258      90.375  48.595  85.932  1.00  0.00
ATOM   4054  N   LEU A 259      89.409  50.150  84.606  1.00  0.00
ATOM   4055  H   LEU A 259      89.498  51.074  84.190  1.00  0.00
ATOM   4056  CA  LEU A 259      88.227  49.332  84.296  1.00  0.00
ATOM   4057  HA  LEU A 259      88.296  48.376  84.812  1.00  0.00
ATOM   4058  CB  LEU A 259      86.932  50.003  84.771  1.00  0.00
ATOM   4059  HB1 LEU A 259      86.101  49.356  84.489  1.00  0.00
ATOM   4060  HB2 LEU A 259      86.815  50.945  84.239  1.00  0.00
ATOM   4061  CG  LEU A 259      86.848  50.271  86.286  1.00  0.00
ATOM   4062  HG  LEU A 259      87.598  51.007  86.572  1.00  0.00
ATOM   4063  CD1 LEU A 259      85.468  50.827  86.603  1.00  0.00
ATOM   4064 1HD1 LEU A 259      85.256  51.674  85.962  1.00  0.00
ATOM   4065 2HD1 LEU A 259      84.719  50.056  86.454  1.00  0.00
ATOM   4066 3HD1 LEU A 259      85.443  51.162  87.631  1.00  0.00
ATOM   4067  CD2 LEU A 259      87.002  49.020  87.154  1.00  0.00
ATOM   4068 1HD2 LEU A 259      86.843  49.271  88.201  1.00  0.00
ATOM   4069 2HD2 LEU A 259      86.288  48.258  86.845  1.00  0.00
ATOM   4070 3HD2 LEU A 259      88.011  48.626  87.062  1.00  0.00
ATOM   4071  C   LEU A 259      88.205  49.017  82.802  1.00  0.00
ATOM   4072  O   LEU A 259      88.156  49.904  81.953  1.00  0.00
ATOM   4073  N   ASP A 260      88.319  47.744  82.461  1.00  0.00
ATOM   4074  H   ASP A 260      88.389  47.029  83.178  1.00  0.00
ATOM   4075  CA  ASP A 260      88.378  47.321  81.076  1.00  0.00
ATOM   4076  HA  ASP A 260      89.060  47.971  80.541  1.00  0.00
ATOM   4077  CB  ASP A 260      88.943  45.899  81.023  1.00  0.00
ATOM   4078  HB1 ASP A 260      88.160  45.176  81.262  1.00  0.00
ATOM   4079  HB2 ASP A 260      89.751  45.800  81.750  1.00  0.00
ATOM   4080  CG  ASP A 260      89.526  45.592  79.652  1.00  0.00
ATOM   4081  OD1 ASP A 260      88.742  45.140  78.790  1.00  0.00
ATOM   4082  OD2 ASP A 260      90.736  45.854  79.459  1.00  0.00
ATOM   4083  C   ASP A 260      87.018  47.451  80.381  1.00  0.00
ATOM   4084  O   ASP A 260      85.963  47.423  81.022  1.00  0.00
ATOM   4085  N   ARG A 261      87.045  47.493  79.042  1.00  0.00
```

ATOM	4086	H	ARG	A	261	87.958	47.443	78.591	1.00	0.00
ATOM	4087	CA	ARG	A	261	85.852	47.262	78.217	1.00	0.00
ATOM	4088	HA	ARG	A	261	85.151	48.088	78.324	1.00	0.00
ATOM	4089	CB	ARG	A	261	86.253	47.094	76.746	1.00	0.00
ATOM	4090	HB1	ARG	A	261	85.371	46.774	76.189	1.00	0.00
ATOM	4091	HB2	ARG	A	261	86.990	46.292	76.693	1.00	0.00
ATOM	4092	CG	ARG	A	261	86.822	48.343	76.064	1.00	0.00
ATOM	4093	HG1	ARG	A	261	87.539	48.843	76.710	1.00	0.00
ATOM	4094	HG2	ARG	A	261	86.019	49.045	75.837	1.00	0.00
ATOM	4095	CD	ARG	A	261	87.529	47.926	74.766	1.00	0.00
ATOM	4096	HD1	ARG	A	261	88.291	47.180	74.992	1.00	0.00
ATOM	4097	HD2	ARG	A	261	88.030	48.784	74.331	1.00	0.00
ATOM	4098	NE	ARG	A	261	86.559	47.426	73.785	1.00	0.00
ATOM	4099	HE	ARG	A	261	85.912	48.113	73.386	1.00	0.00
ATOM	4100	CZ	ARG	A	261	86.330	46.196	73.393	1.00	0.00
ATOM	4101	NH1	ARG	A	261	87.085	45.192	73.758	1.00	0.00
ATOM	4102	1HH1	ARG	A	261	87.883	45.395	74.331	1.00	0.00
ATOM	4103	2HH1	ARG	A	261	86.905	44.264	73.426	1.00	0.00
ATOM	4104	NH2	ARG	A	261	85.300	45.965	72.645	1.00	0.00
ATOM	4105	1HH2	ARG	A	261	84.621	46.736	72.585	1.00	0.00
ATOM	4106	2HH2	ARG	A	261	85.047	45.054	72.334	1.00	0.00
ATOM	4107	C	ARG	A	261	85.123	46.003	78.650	1.00	0.00
ATOM	4108	O	ARG	A	261	83.905	45.997	78.728	1.00	0.00
ATOM	4109	N	ASN	A	262	85.858	44.940	78.968	1.00	0.00
ATOM	4110	H	ASN	A	262	86.870	45.011	78.891	1.00	0.00
ATOM	4111	CA	ASN	A	262	85.258	43.701	79.446	1.00	0.00
ATOM	4112	HA	ASN	A	262	84.488	43.392	78.736	1.00	0.00
ATOM	4113	CB	ASN	A	262	86.340	42.610	79.469	1.00	0.00
ATOM	4114	HB1	ASN	A	262	85.920	41.708	79.910	1.00	0.00
ATOM	4115	HB2	ASN	A	262	87.186	42.927	80.076	1.00	0.00
ATOM	4116	CG	ASN	A	262	86.816	42.255	78.066	1.00	0.00
ATOM	4117	OD1	ASN	A	262	86.213	41.462	77.364	1.00	0.00
ATOM	4118	ND2	ASN	A	262	87.891	42.834	77.592	1.00	0.00
ATOM	4119	1HD2	ASN	A	262	88.354	43.581	78.114	1.00	0.00
ATOM	4120	2HD2	ASN	A	262	88.257	42.459	76.741	1.00	0.00
ATOM	4121	C	ASN	A	262	84.548	43.895	80.800	1.00	0.00
ATOM	4122	O	ASN	A	262	83.483	43.304	80.968	1.00	0.00
ATOM	4123	N	GLN	A	263	85.020	44.767	81.716	1.00	0.00
ATOM	4124	H	GLN	A	263	85.828	45.343	81.518	1.00	0.00
ATOM	4125	CA	GLN	A	263	84.251	44.986	82.959	1.00	0.00
ATOM	4126	HA	GLN	A	263	83.931	44.027	83.368	1.00	0.00
ATOM	4127	CB	GLN	A	263	85.146	45.683	84.012	1.00	0.00
ATOM	4128	HB1	GLN	A	263	84.514	46.085	84.803	1.00	0.00
ATOM	4129	HB2	GLN	A	263	85.659	46.521	83.540	1.00	0.00
ATOM	4130	CG	GLN	A	263	86.171	44.725	84.659	1.00	0.00
ATOM	4131	HG1	GLN	A	263	86.297	43.839	84.036	1.00	0.00
ATOM	4132	HG2	GLN	A	263	85.795	44.392	85.625	1.00	0.00
ATOM	4133	CD	GLN	A	263	87.553	45.354	84.838	1.00	0.00
ATOM	4134	OE1	GLN	A	263	88.364	45.349	83.936	1.00	0.00
ATOM	4135	NE2	GLN	A	263	87.897	45.937	85.962	1.00	0.00
ATOM	4136	1HE2	GLN	A	263	87.323	45.862	86.786	1.00	0.00
ATOM	4137	2HE2	GLN	A	263	88.824	46.333	86.018	1.00	0.00
ATOM	4138	C	GLN	A	263	82.965	45.791	82.648	1.00	0.00
ATOM	4139	O	GLN	A	263	81.847	45.385	82.987	1.00	0.00
ATOM	4140	N	GLY	A	264	83.124	46.855	81.853	1.00	0.00
ATOM	4141	H	GLY	A	264	84.068	47.097	81.570	1.00	0.00
ATOM	4142	CA	GLY	A	264	82.037	47.719	81.385	1.00	0.00
ATOM	4143	HA1	GLY	A	264	82.472	48.555	80.838	1.00	0.00
ATOM	4144	HA2	GLY	A	264	81.505	48.122	82.244	1.00	0.00
ATOM	4145	C	GLY	A	264	81.002	47.064	80.467	1.00	0.00
ATOM	4146	O	GLY	A	264	79.988	47.687	80.173	1.00	0.00

```
ATOM   4147  N   LYS A 265      81.247  45.815  80.049  1.00  0.00
ATOM   4148  H   LYS A 265      82.176  45.456  80.227  1.00  0.00
ATOM   4149  CA  LYS A 265      80.326  44.943  79.304  1.00  0.00
ATOM   4150  HA  LYS A 265      79.416  45.505  79.099  1.00  0.00
ATOM   4151  CB  LYS A 265      80.981  44.511  77.980  1.00  0.00
ATOM   4152  HB1 LYS A 265      80.336  43.777  77.495  1.00  0.00
ATOM   4153  HB2 LYS A 265      81.940  44.035  78.191  1.00  0.00
ATOM   4154  CG  LYS A 265      81.195  45.677  76.999  1.00  0.00
ATOM   4155  HG1 LYS A 265      81.836  46.436  77.438  1.00  0.00
ATOM   4156  HG2 LYS A 265      80.237  46.150  76.812  1.00  0.00
ATOM   4157  CD  LYS A 265      81.803  45.218  75.662  1.00  0.00
ATOM   4158  HD1 LYS A 265      82.006  44.145  75.698  1.00  0.00
ATOM   4159  HD2 LYS A 265      82.754  45.731  75.499  1.00  0.00
ATOM   4160  CE  LYS A 265      80.860  45.496  74.486  1.00  0.00
ATOM   4161  HE1 LYS A 265      79.853  45.155  74.736  1.00  0.00
ATOM   4162  HE2 LYS A 265      81.197  44.935  73.613  1.00  0.00
ATOM   4163  NZ  LYS A 265      80.828  46.928  74.135  1.00  0.00
ATOM   4164  HZ1 LYS A 265      80.143  47.121  73.394  1.00  0.00
ATOM   4165  HZ2 LYS A 265      80.582  47.539  74.903  1.00  0.00
ATOM   4166  HZ3 LYS A 265      81.709  47.243  73.741  1.00  0.00
ATOM   4167  C   LYS A 265      79.869  43.723  80.115  1.00  0.00
ATOM   4168  O   LYS A 265      78.922  43.050  79.729  1.00  0.00
ATOM   4169  N   CYS A 266      80.487  43.483  81.271  1.00  0.00
ATOM   4170  H   CYS A 266      81.293  44.046  81.495  1.00  0.00
ATOM   4171  CA  CYS A 266      79.874  42.757  82.383  1.00  0.00
ATOM   4172  HA  CYS A 266      79.215  41.980  81.989  1.00  0.00
ATOM   4173  CB  CYS A 266      80.970  42.077  83.218  1.00  0.00
ATOM   4174  HB1 CYS A 266      80.504  41.544  84.048  1.00  0.00
ATOM   4175  HB2 CYS A 266      81.649  42.826  83.625  1.00  0.00
ATOM   4176  SG  CYS A 266      81.900  40.882  82.211  1.00  0.00
ATOM   4177  HG  CYS A 266      82.483  41.788  81.412  1.00  0.00
ATOM   4178  C   CYS A 266      78.998  43.676  83.258  1.00  0.00
ATOM   4179  O   CYS A 266      78.570  43.255  84.330  1.00  0.00
ATOM   4180  N   VAL A 267      78.713  44.909  82.804  1.00  0.00
ATOM   4181  H   VAL A 267      79.103  45.209  81.924  1.00  0.00
ATOM   4182  CA  VAL A 267      77.551  45.683  83.300  1.00  0.00
ATOM   4183  HA  VAL A 267      76.977  45.038  83.956  1.00  0.00
ATOM   4184  CB  VAL A 267      77.954  46.944  84.116  1.00  0.00
ATOM   4185  HB  VAL A 267      77.285  46.989  84.973  1.00  0.00
ATOM   4186  CG1 VAL A 267      79.398  46.920  84.635  1.00  0.00
ATOM   4187 1HG1 VAL A 267      79.632  45.976  85.124  1.00  0.00
ATOM   4188 2HG1 VAL A 267      80.092  47.067  83.813  1.00  0.00
ATOM   4189 3HG1 VAL A 267      79.520  47.733  85.340  1.00  0.00
ATOM   4190  CG2 VAL A 267      77.814  48.287  83.395  1.00  0.00
ATOM   4191 1HG2 VAL A 267      78.136  49.102  84.041  1.00  0.00
ATOM   4192 2HG2 VAL A 267      78.411  48.284  82.485  1.00  0.00
ATOM   4193 3HG2 VAL A 267      76.770  48.477  83.149  1.00  0.00
ATOM   4194  C   VAL A 267      76.600  46.051  82.158  1.00  0.00
ATOM   4195  O   VAL A 267      77.048  46.284  81.034  1.00  0.00
ATOM   4196  N   GLU A 268      75.310  46.195  82.471  1.00  0.00
ATOM   4197  H   GLU A 268      75.015  45.998  83.415  1.00  0.00
ATOM   4198  CA  GLU A 268      74.323  46.868  81.621  1.00  0.00
ATOM   4199  HA  GLU A 268      74.868  47.478  80.912  1.00  0.00
ATOM   4200  CB  GLU A 268      73.468  45.846  80.845  1.00  0.00
ATOM   4201  HB1 GLU A 268      72.530  46.314  80.542  1.00  0.00
ATOM   4202  HB2 GLU A 268      73.215  45.020  81.514  1.00  0.00
ATOM   4203  CG  GLU A 268      74.145  45.282  79.585  1.00  0.00
ATOM   4204  HG1 GLU A 268      73.576  44.405  79.267  1.00  0.00
ATOM   4205  HG2 GLU A 268      75.153  44.939  79.821  1.00  0.00
ATOM   4206  CD  GLU A 268      74.184  46.298  78.431  1.00  0.00
ATOM   4207  OE1 GLU A 268      73.580  46.005  77.379  1.00  0.00
```

ATOM	4208	OE2	GLU	A	268	74.792	47.384	78.604	1.00	0.00
ATOM	4209	C	GLU	A	268	73.441	47.831	82.448	1.00	0.00
ATOM	4210	O	GLU	A	268	73.308	47.669	83.659	1.00	0.00
ATOM	4211	N	GLY	A	269	72.833	48.873	81.872	1.00	0.00
ATOM	4212	H	GLY	A	269	72.305	49.458	82.498	1.00	0.00
ATOM	4213	CA	GLY	A	269	73.037	49.389	80.510	1.00	0.00
ATOM	4214	HA1	GLY	A	269	72.182	50.005	80.234	1.00	0.00
ATOM	4215	HA2	GLY	A	269	73.097	48.577	79.791	1.00	0.00
ATOM	4216	C	GLY	A	269	74.285	50.275	80.430	1.00	0.00
ATOM	4217	O	GLY	A	269	74.273	51.389	80.942	1.00	0.00
ATOM	4218	N	MET	A	270	75.367	49.784	79.816	1.00	0.00
ATOM	4219	H	MET	A	270	75.287	48.862	79.388	1.00	0.00
ATOM	4220	CA	MET	A	270	76.607	50.555	79.642	1.00	0.00
ATOM	4221	HA	MET	A	270	76.290	51.551	79.328	1.00	0.00
ATOM	4222	CB	MET	A	270	77.341	50.703	80.991	1.00	0.00
ATOM	4223	HB1	MET	A	270	78.205	50.038	81.015	1.00	0.00
ATOM	4224	HB2	MET	A	270	76.674	50.432	81.809	1.00	0.00
ATOM	4225	CG	MET	A	270	77.788	52.152	81.220	1.00	0.00
ATOM	4226	HG1	MET	A	270	76.939	52.805	81.013	1.00	0.00
ATOM	4227	HG2	MET	A	270	78.583	52.394	80.516	1.00	0.00
ATOM	4228	SD	MET	A	270	78.347	52.533	82.907	1.00	0.00
ATOM	4229	CE	MET	A	270	79.877	51.567	82.972	1.00	0.00
ATOM	4230	HE1	MET	A	270	80.339	51.688	83.951	1.00	0.00
ATOM	4231	HE2	MET	A	270	79.661	50.512	82.804	1.00	0.00
ATOM	4232	HE3	MET	A	270	80.561	51.924	82.204	1.00	0.00
ATOM	4233	C	MET	A	270	77.570	50.075	78.544	1.00	0.00
ATOM	4234	O	MET	A	270	78.467	50.851	78.189	1.00	0.00
ATOM	4235	N	VAL	A	271	77.386	48.883	77.962	1.00	0.00
ATOM	4236	H	VAL	A	271	76.602	48.312	78.281	1.00	0.00
ATOM	4237	CA	VAL	A	271	78.334	48.229	77.023	1.00	0.00
ATOM	4238	HA	VAL	A	271	79.176	47.871	77.612	1.00	0.00
ATOM	4239	CB	VAL	A	271	77.646	46.995	76.353	1.00	0.00
ATOM	4240	HB	VAL	A	271	78.332	46.600	75.606	1.00	0.00
ATOM	4241	CG1	VAL	A	271	77.380	45.854	77.346	1.00	0.00
ATOM	4242	1HG1	VAL	A	271	76.524	45.251	77.040	1.00	0.00
ATOM	4243	2HG1	VAL	A	271	78.230	45.183	77.380	1.00	0.00
ATOM	4244	3HG1	VAL	A	271	77.183	46.235	78.348	1.00	0.00
ATOM	4245	CG2	VAL	A	271	76.332	47.306	75.611	1.00	0.00
ATOM	4246	1HG2	VAL	A	271	76.518	47.887	74.714	1.00	0.00
ATOM	4247	2HG2	VAL	A	271	75.862	46.379	75.279	1.00	0.00
ATOM	4248	3HG2	VAL	A	271	75.624	47.826	76.254	1.00	0.00
ATOM	4249	C	VAL	A	271	78.910	49.143	75.929	1.00	0.00
ATOM	4250	O	VAL	A	271	80.129	49.209	75.733	1.00	0.00
ATOM	4251	N	GLU	A	272	78.039	49.878	75.235	1.00	0.00
ATOM	4252	H	GLU	A	272	77.063	49.710	75.427	1.00	0.00
ATOM	4253	CA	GLU	A	272	78.383	50.621	74.017	1.00	0.00
ATOM	4254	HA	GLU	A	272	79.386	50.322	73.712	1.00	0.00
ATOM	4255	CB	GLU	A	272	77.430	50.278	72.854	1.00	0.00
ATOM	4256	HB1	GLU	A	272	76.971	51.190	72.472	1.00	0.00
ATOM	4257	HB2	GLU	A	272	76.621	49.637	73.200	1.00	0.00
ATOM	4258	CG	GLU	A	272	78.161	49.582	71.688	1.00	0.00
ATOM	4259	HG1	GLU	A	272	78.842	50.305	71.229	1.00	0.00
ATOM	4260	HG2	GLU	A	272	77.426	49.289	70.933	1.00	0.00
ATOM	4261	CD	GLU	A	272	78.953	48.346	72.138	1.00	0.00
ATOM	4262	OE1	GLU	A	272	78.472	47.591	73.009	1.00	0.00
ATOM	4263	OE2	GLU	A	272	80.122	48.166	71.737	1.00	0.00
ATOM	4264	C	GLU	A	272	78.521	52.129	74.248	1.00	0.00
ATOM	4265	O	GLU	A	272	79.095	52.821	73.416	1.00	0.00
ATOM	4266	N	ILE	A	273	78.119	52.629	75.420	1.00	0.00
ATOM	4267	H	ILE	A	273	77.692	51.990	76.078	1.00	0.00
ATOM	4268	CA	ILE	A	273	78.662	53.889	75.936	1.00	0.00

```
ATOM   4269  HA  ILE A 273      78.579  54.663  75.171  1.00  0.00
ATOM   4270  CB  ILE A 273      77.874  54.340  77.191  1.00  0.00
ATOM   4271  HB  ILE A 273      77.786  53.493  77.873  1.00  0.00
ATOM   4272  CG2 ILE A 273      78.608  55.468  77.946  1.00  0.00
ATOM   4273 1HG2 ILE A 273      78.030  55.788  78.811  1.00  0.00
ATOM   4274 2HG2 ILE A 273      79.570  55.115  78.319  1.00  0.00
ATOM   4275 3HG2 ILE A 273      78.772  56.319  77.283  1.00  0.00
ATOM   4276  CG1 ILE A 273      76.453  54.785  76.770  1.00  0.00
ATOM   4277 1HG1 ILE A 273      75.975  53.976  76.217  1.00  0.00
ATOM   4278 2HG1 ILE A 273      76.523  55.648  76.108  1.00  0.00
ATOM   4279  CD  ILE A 273      75.515  55.141  77.936  1.00  0.00
ATOM   4280  HD1 ILE A 273      74.509  55.300  77.548  1.00  0.00
ATOM   4281  HD2 ILE A 273      75.495  54.323  78.656  1.00  0.00
ATOM   4282  HD3 ILE A 273      75.844  56.058  78.423  1.00  0.00
ATOM   4283  C   ILE A 273      80.159  53.707  76.231  1.00  0.00
ATOM   4284  O   ILE A 273      80.967  54.533  75.809  1.00  0.00
ATOM   4285  N   PHE A 274      80.531  52.619  76.917  1.00  0.00
ATOM   4286  H   PHE A 274      79.827  51.962  77.240  1.00  0.00
ATOM   4287  CA  PHE A 274      81.931  52.345  77.219  1.00  0.00
ATOM   4288  HA  PHE A 274      82.345  53.206  77.744  1.00  0.00
ATOM   4289  CB  PHE A 274      82.029  51.123  78.147  1.00  0.00
ATOM   4290  HB1 PHE A 274      81.879  50.207  77.575  1.00  0.00
ATOM   4291  HB2 PHE A 274      81.234  51.178  78.893  1.00  0.00
ATOM   4292  CG  PHE A 274      83.361  51.063  78.863  1.00  0.00
ATOM   4293  CD1 PHE A 274      83.457  51.480  80.204  1.00  0.00
ATOM   4294  HD1 PHE A 274      82.566  51.776  80.746  1.00  0.00
ATOM   4295  CE1 PHE A 274      84.713  51.556  80.828  1.00  0.00
ATOM   4296  HE1 PHE A 274      84.795  51.892  81.848  1.00  0.00
ATOM   4297  CZ  PHE A 274      85.871  51.221  80.111  1.00  0.00
ATOM   4298  HZ  PHE A 274      86.839  51.326  80.573  1.00  0.00
ATOM   4299  CE2 PHE A 274      85.780  50.775  78.784  1.00  0.00
ATOM   4300  HE2 PHE A 274      86.674  50.522  78.232  1.00  0.00
ATOM   4301  CD2 PHE A 274      84.524  50.684  78.162  1.00  0.00
ATOM   4302  HD2 PHE A 274      84.453  50.368  77.133  1.00  0.00
ATOM   4303  C   PHE A 274      82.756  52.153  75.937  1.00  0.00
ATOM   4304  O   PHE A 274      83.818  52.763  75.788  1.00  0.00
ATOM   4305  N   ASP A 275      82.273  51.333  74.992  1.00  0.00
ATOM   4306  H   ASP A 275      81.437  50.788  75.175  1.00  0.00
ATOM   4307  CA  ASP A 275      82.998  51.175  73.721  1.00  0.00
ATOM   4308  HA  ASP A 275      84.045  51.003  73.979  1.00  0.00
ATOM   4309  CB  ASP A 275      82.518  49.938  72.950  1.00  0.00
ATOM   4310  HB1 ASP A 275      82.293  50.203  71.917  1.00  0.00
ATOM   4311  HB2 ASP A 275      81.605  49.556  73.402  1.00  0.00
ATOM   4312  CG  ASP A 275      83.587  48.840  72.942  1.00  0.00
ATOM   4313  OD1 ASP A 275      84.752  49.089  72.574  1.00  0.00
ATOM   4314  OD2 ASP A 275      83.312  47.703  73.385  1.00  0.00
ATOM   4315  C   ASP A 275      83.021  52.424  72.818  1.00  0.00
ATOM   4316  O   ASP A 275      84.051  52.685  72.202  1.00  0.00
ATOM   4317  N   MET A 276      81.971  53.252  72.750  1.00  0.00
ATOM   4318  H   MET A 276      81.108  53.001  73.218  1.00  0.00
ATOM   4319  CA  MET A 276      82.017  54.483  71.937  1.00  0.00
ATOM   4320  HA  MET A 276      82.435  54.212  70.969  1.00  0.00
ATOM   4321  CB  MET A 276      80.595  55.017  71.692  1.00  0.00
ATOM   4322  HB1 MET A 276      80.231  55.533  72.583  1.00  0.00
ATOM   4323  HB2 MET A 276      79.932  54.181  71.475  1.00  0.00
ATOM   4324  CG  MET A 276      80.528  55.953  70.479  1.00  0.00
ATOM   4325  HG1 MET A 276      80.819  55.385  69.597  1.00  0.00
ATOM   4326  HG2 MET A 276      81.250  56.753  70.614  1.00  0.00
ATOM   4327  SD  MET A 276      78.884  56.676  70.185  1.00  0.00
ATOM   4328  CE  MET A 276      79.133  57.438  68.561  1.00  0.00
ATOM   4329  HE1 MET A 276      78.216  57.941  68.253  1.00  0.00
```

```
ATOM   4330  HE2 MET A 276      79.941  58.164  68.610  1.00  0.00
ATOM   4331  HE3 MET A 276      79.381  56.671  67.829  1.00  0.00
ATOM   4332  C   MET A 276      82.945  55.553  72.545  1.00  0.00
ATOM   4333  O   MET A 276      83.670  56.245  71.810  1.00  0.00
ATOM   4334  N   LEU A 277      82.997  55.647  73.883  1.00  0.00
ATOM   4335  H   LEU A 277      82.361  55.067  74.424  1.00  0.00
ATOM   4336  CA  LEU A 277      84.010  56.422  74.612  1.00  0.00
ATOM   4337  HA  LEU A 277      83.953  57.464  74.302  1.00  0.00
ATOM   4338  CB  LEU A 277      83.769  56.310  76.130  1.00  0.00
ATOM   4339  HB1 LEU A 277      84.683  56.579  76.659  1.00  0.00
ATOM   4340  HB2 LEU A 277      83.560  55.271  76.372  1.00  0.00
ATOM   4341  CG  LEU A 277      82.633  57.185  76.680  1.00  0.00
ATOM   4342  HG  LEU A 277      81.737  57.059  76.073  1.00  0.00
ATOM   4343  CD1 LEU A 277      82.327  56.782  78.122  1.00  0.00
ATOM   4344 1HD1 LEU A 277      81.491  57.367  78.501  1.00  0.00
ATOM   4345 2HD1 LEU A 277      82.049  55.730  78.151  1.00  0.00
ATOM   4346 3HD1 LEU A 277      83.198  56.939  78.757  1.00  0.00
ATOM   4347  CD2 LEU A 277      83.026  58.661  76.676  1.00  0.00
ATOM   4348 1HD2 LEU A 277      82.204  59.247  77.083  1.00  0.00
ATOM   4349 2HD2 LEU A 277      83.920  58.813  77.281  1.00  0.00
ATOM   4350 3HD2 LEU A 277      83.225  59.001  75.663  1.00  0.00
ATOM   4351  C   LEU A 277      85.433  55.945  74.304  1.00  0.00
ATOM   4352  O   LEU A 277      86.319  56.774  74.105  1.00  0.00
ATOM   4353  N   LEU A 278      85.657  54.623  74.252  1.00  0.00
ATOM   4354  H   LEU A 278      84.892  53.996  74.484  1.00  0.00
ATOM   4355  CA  LEU A 278      86.956  54.032  73.908  1.00  0.00
ATOM   4356  HA  LEU A 278      87.713  54.506  74.533  1.00  0.00
ATOM   4357  CB  LEU A 278      86.895  52.540  74.287  1.00  0.00
ATOM   4358  HB1 LEU A 278      86.094  52.063  73.722  1.00  0.00
ATOM   4359  HB2 LEU A 278      86.606  52.489  75.339  1.00  0.00
ATOM   4360  CG  LEU A 278      88.164  51.682  74.138  1.00  0.00
ATOM   4361  HG  LEU A 278      88.008  50.814  74.771  1.00  0.00
ATOM   4362  CD1 LEU A 278      88.379  51.168  72.713  1.00  0.00
ATOM   4363 1HD1 LEU A 278      89.173  50.424  72.706  1.00  0.00
ATOM   4364 2HD1 LEU A 278      87.462  50.699  72.352  1.00  0.00
ATOM   4365 3HD1 LEU A 278      88.650  51.977  72.039  1.00  0.00
ATOM   4366  CD2 LEU A 278      89.450  52.340  74.640  1.00  0.00
ATOM   4367 1HD2 LEU A 278      90.265  51.623  74.605  1.00  0.00
ATOM   4368 2HD2 LEU A 278      89.711  53.193  74.016  1.00  0.00
ATOM   4369 3HD2 LEU A 278      89.309  52.678  75.665  1.00  0.00
ATOM   4370  C   LEU A 278      87.363  54.287  72.441  1.00  0.00
ATOM   4371  O   LEU A 278      88.520  54.614  72.173  1.00  0.00
ATOM   4372  N   ALA A 279      86.430  54.232  71.488  1.00  0.00
ATOM   4373  H   ALA A 279      85.512  53.866  71.738  1.00  0.00
ATOM   4374  CA  ALA A 279      86.666  54.605  70.091  1.00  0.00
ATOM   4375  HA  ALA A 279      87.487  54.001  69.703  1.00  0.00
ATOM   4376  CB  ALA A 279      85.405  54.260  69.282  1.00  0.00
ATOM   4377  HB1 ALA A 279      85.582  54.465  68.227  1.00  0.00
ATOM   4378  HB2 ALA A 279      85.168  53.203  69.406  1.00  0.00
ATOM   4379  HB3 ALA A 279      84.558  54.854  69.626  1.00  0.00
ATOM   4380  C   ALA A 279      87.053  56.086  69.949  1.00  0.00
ATOM   4381  O   ALA A 279      88.038  56.432  69.283  1.00  0.00
ATOM   4382  N   THR A 280      86.349  56.976  70.649  1.00  0.00
ATOM   4383  H   THR A 280      85.535  56.645  71.159  1.00  0.00
ATOM   4384  CA  THR A 280      86.664  58.415  70.657  1.00  0.00
ATOM   4385  HA  THR A 280      86.714  58.754  69.623  1.00  0.00
ATOM   4386  CB  THR A 280      85.544  59.203  71.344  1.00  0.00
ATOM   4387  HB  THR A 280      85.486  58.941  72.402  1.00  0.00
ATOM   4388  CG2 THR A 280      85.711  60.711  71.182  1.00  0.00
ATOM   4389 1HG2 THR A 280      84.886  61.226  71.675  1.00  0.00
ATOM   4390 2HG2 THR A 280      86.645  61.031  71.633  1.00  0.00
```

ATOM	4391	3HG2	THR	A	280	85.712	60.976	70.125	1.00	0.00
ATOM	4392	OG1	THR	A	280	84.331	58.894	70.705	1.00	0.00
ATOM	4393	HG1	THR	A	280	84.013	58.041	71.039	1.00	0.00
ATOM	4394	C	THR	A	280	88.038	58.689	71.281	1.00	0.00
ATOM	4395	O	THR	A	280	88.857	59.392	70.695	1.00	0.00
ATOM	4396	N	SER	A	281	88.357	58.043	72.410	1.00	0.00
ATOM	4397	H	SER	A	281	87.628	57.487	72.853	1.00	0.00
ATOM	4398	CA	SER	A	281	89.698	58.012	73.018	1.00	0.00
ATOM	4399	HA	SER	A	281	89.970	59.025	73.308	1.00	0.00
ATOM	4400	CB	SER	A	281	89.678	57.131	74.271	1.00	0.00
ATOM	4401	HB1	SER	A	281	89.437	56.106	73.993	1.00	0.00
ATOM	4402	HB2	SER	A	281	88.922	57.485	74.974	1.00	0.00
ATOM	4403	OG	SER	A	281	90.946	57.134	74.904	1.00	0.00
ATOM	4404	HG	SER	A	281	90.994	57.888	75.509	1.00	0.00
ATOM	4405	C	SER	A	281	90.781	57.508	72.066	1.00	0.00
ATOM	4406	O	SER	A	281	91.930	57.945	72.151	1.00	0.00
ATOM	4407	N	SER	A	282	90.444	56.615	71.138	1.00	0.00
ATOM	4408	H	SER	A	282	89.489	56.283	71.119	1.00	0.00
ATOM	4409	CA	SER	A	282	91.373	56.109	70.127	1.00	0.00
ATOM	4410	HA	SER	A	282	92.326	55.901	70.610	1.00	0.00
ATOM	4411	CB	SER	A	282	90.880	54.775	69.555	1.00	0.00
ATOM	4412	HB1	SER	A	282	91.625	54.397	68.852	1.00	0.00
ATOM	4413	HB2	SER	A	282	89.938	54.908	69.026	1.00	0.00
ATOM	4414	OG	SER	A	282	90.719	53.838	70.607	1.00	0.00
ATOM	4415	HG	SER	A	282	89.947	54.084	71.145	1.00	0.00
ATOM	4416	C	SER	A	282	91.628	57.155	69.036	1.00	0.00
ATOM	4417	O	SER	A	282	92.792	57.378	68.690	1.00	0.00
ATOM	4418	N	ARG	A	283	90.605	57.898	68.570	1.00	0.00
ATOM	4419	H	ARG	A	283	89.663	57.664	68.871	1.00	0.00
ATOM	4420	CA	ARG	A	283	90.821	59.076	67.693	1.00	0.00
ATOM	4421	HA	ARG	A	283	91.407	58.739	66.836	1.00	0.00
ATOM	4422	CB	ARG	A	283	89.491	59.659	67.150	1.00	0.00
ATOM	4423	HB1	ARG	A	283	88.851	59.964	67.978	1.00	0.00
ATOM	4424	HB2	ARG	A	283	88.980	58.874	66.590	1.00	0.00
ATOM	4425	CG	ARG	A	283	89.737	60.872	66.219	1.00	0.00
ATOM	4426	HG1	ARG	A	283	90.599	60.642	65.590	1.00	0.00
ATOM	4427	HG2	ARG	A	283	89.994	61.745	66.819	1.00	0.00
ATOM	4428	CD	ARG	A	283	88.587	61.247	65.263	1.00	0.00
ATOM	4429	HD1	ARG	A	283	88.286	60.356	64.710	1.00	0.00
ATOM	4430	HD2	ARG	A	283	88.977	61.954	64.528	1.00	0.00
ATOM	4431	NE	ARG	A	283	87.402	61.827	65.934	1.00	0.00
ATOM	4432	HE	ARG	A	283	86.788	61.182	66.403	1.00	0.00
ATOM	4433	CZ	ARG	A	283	87.005	63.095	65.940	1.00	0.00
ATOM	4434	NH1	ARG	A	283	87.687	64.065	65.402	1.00	0.00
ATOM	4435	1HH1	ARG	A	283	88.439	63.859	64.752	1.00	0.00
ATOM	4436	2HH1	ARG	A	283	87.420	65.024	65.530	1.00	0.00
ATOM	4437	NH2	ARG	A	283	85.869	63.396	66.511	1.00	0.00
ATOM	4438	1HH2	ARG	A	283	85.281	62.664	66.900	1.00	0.00
ATOM	4439	2HH2	ARG	A	283	85.505	64.335	66.489	1.00	0.00
ATOM	4440	C	ARG	A	283	91.692	60.161	68.352	1.00	0.00
ATOM	4441	O	ARG	A	283	92.645	60.635	67.743	1.00	0.00
ATOM	4442	N	PHE	A	284	91.407	60.521	69.606	1.00	0.00
ATOM	4443	H	PHE	A	284	90.583	60.114	70.034	1.00	0.00
ATOM	4444	CA	PHE	A	284	92.200	61.486	70.386	1.00	0.00
ATOM	4445	HA	PHE	A	284	92.170	62.466	69.907	1.00	0.00
ATOM	4446	CB	PHE	A	284	91.604	61.584	71.800	1.00	0.00
ATOM	4447	HB1	PHE	A	284	92.391	61.965	72.448	1.00	0.00
ATOM	4448	HB2	PHE	A	284	91.362	60.586	72.160	1.00	0.00
ATOM	4449	CG	PHE	A	284	90.431	62.509	72.013	1.00	0.00
ATOM	4450	CD1	PHE	A	284	89.199	62.029	72.493	1.00	0.00
ATOM	4451	HD1	PHE	A	284	89.028	60.970	72.592	1.00	0.00

ATOM	4452	CE1	PHE	A	284	88.207	62.929	72.922	1.00	0.00
ATOM	4453	HE1	PHE	A	284	87.270	62.566	73.313	1.00	0.00
ATOM	4454	CZ	PHE	A	284	88.458	64.308	72.898	1.00	0.00
ATOM	4455	HZ	PHE	A	284	87.714	64.998	73.268	1.00	0.00
ATOM	4456	CE2	PHE	A	284	89.681	64.790	72.410	1.00	0.00
ATOM	4457	HE2	PHE	A	284	89.898	65.841	72.439	1.00	0.00
ATOM	4458	CD2	PHE	A	284	90.651	63.889	71.946	1.00	0.00
ATOM	4459	HD2	PHE	A	284	91.602	64.252	71.613	1.00	0.00
ATOM	4460	C	PHE	A	284	93.678	61.097	70.540	1.00	0.00
ATOM	4461	O	PHE	A	284	94.572	61.912	70.323	1.00	0.00
ATOM	4462	N	ARG	A	285	93.905	59.827	70.892	1.00	0.00
ATOM	4463	H	ARG	A	285	93.088	59.266	71.107	1.00	0.00
ATOM	4464	CA	ARG	A	285	95.205	59.155	71.015	1.00	0.00
ATOM	4465	HA	ARG	A	285	95.810	59.652	71.773	1.00	0.00
ATOM	4466	CB	ARG	A	285	94.870	57.734	71.475	1.00	0.00
ATOM	4467	HB1	ARG	A	285	94.088	57.346	70.826	1.00	0.00
ATOM	4468	HB2	ARG	A	285	94.464	57.853	72.480	1.00	0.00
ATOM	4469	CG	ARG	A	285	95.949	56.646	71.536	1.00	0.00
ATOM	4470	HG1	ARG	A	285	96.715	56.938	72.252	1.00	0.00
ATOM	4471	HG2	ARG	A	285	96.425	56.543	70.560	1.00	0.00
ATOM	4472	CD	ARG	A	285	95.317	55.288	71.931	1.00	0.00
ATOM	4473	HD1	ARG	A	285	96.106	54.634	72.304	1.00	0.00
ATOM	4474	HD2	ARG	A	285	94.905	54.833	71.029	1.00	0.00
ATOM	4475	NE	ARG	A	285	94.212	55.416	72.919	1.00	0.00
ATOM	4476	HE	ARG	A	285	93.499	56.092	72.701	1.00	0.00
ATOM	4477	CZ	ARG	A	285	94.160	54.957	74.156	1.00	0.00
ATOM	4478	NH1	ARG	A	285	95.014	54.105	74.641	1.00	0.00
ATOM	4479	1HH1	ARG	A	285	95.872	53.929	74.155	1.00	0.00
ATOM	4480	2HH1	ARG	A	285	94.889	53.807	75.599	1.00	0.00
ATOM	4481	NH2	ARG	A	285	93.235	55.360	74.977	1.00	0.00
ATOM	4482	1HH2	ARG	A	285	92.513	56.017	74.708	1.00	0.00
ATOM	4483	2HH2	ARG	A	285	93.394	55.233	75.973	1.00	0.00
ATOM	4484	C	ARG	A	285	96.003	59.223	69.725	1.00	0.00
ATOM	4485	O	ARG	A	285	97.137	59.697	69.752	1.00	0.00
ATOM	4486	N	MET	A	286	95.399	58.865	68.591	1.00	0.00
ATOM	4487	H	MET	A	286	94.462	58.484	68.640	1.00	0.00
ATOM	4488	CA	MET	A	286	96.041	58.969	67.275	1.00	0.00
ATOM	4489	HA	MET	A	286	97.047	58.559	67.361	1.00	0.00
ATOM	4490	CB	MET	A	286	95.270	58.134	66.240	1.00	0.00
ATOM	4491	HB1	MET	A	286	95.712	58.298	65.257	1.00	0.00
ATOM	4492	HB2	MET	A	286	94.232	58.470	66.203	1.00	0.00
ATOM	4493	CG	MET	A	286	95.305	56.627	66.543	1.00	0.00
ATOM	4494	HG1	MET	A	286	94.845	56.104	65.704	1.00	0.00
ATOM	4495	HG2	MET	A	286	94.689	56.433	67.420	1.00	0.00
ATOM	4496	SD	MET	A	286	96.935	55.885	66.858	1.00	0.00
ATOM	4497	CE	MET	A	286	97.804	56.299	65.320	1.00	0.00
ATOM	4498	HE1	MET	A	286	98.796	55.849	65.335	1.00	0.00
ATOM	4499	HE2	MET	A	286	97.244	55.914	64.468	1.00	0.00
ATOM	4500	HE3	MET	A	286	97.912	57.380	65.232	1.00	0.00
ATOM	4501	C	MET	A	286	96.240	60.408	66.778	1.00	0.00
ATOM	4502	O	MET	A	286	97.041	60.622	65.869	1.00	0.00
ATOM	4503	N	MET	A	287	95.579	61.396	67.390	1.00	0.00
ATOM	4504	H	MET	A	287	94.880	61.164	68.081	1.00	0.00
ATOM	4505	CA	MET	A	287	95.867	62.817	67.161	1.00	0.00
ATOM	4506	HA	MET	A	287	96.262	62.937	66.153	1.00	0.00
ATOM	4507	CB	MET	A	287	94.561	63.632	67.236	1.00	0.00
ATOM	4508	HB1	MET	A	287	94.804	64.695	67.231	1.00	0.00
ATOM	4509	HB2	MET	A	287	94.068	63.407	68.178	1.00	0.00
ATOM	4510	CG	MET	A	287	93.575	63.347	66.096	1.00	0.00
ATOM	4511	HG1	MET	A	287	92.696	63.973	66.247	1.00	0.00
ATOM	4512	HG2	MET	A	287	93.249	62.314	66.161	1.00	0.00

ATOM	4513	SD	MET	A	287	94.144	63.596	64.387	1.00	0.00
ATOM	4514	CE	MET	A	287	94.746	65.297	64.474	1.00	0.00
ATOM	4515	HE1	MET	A	287	95.018	65.638	63.476	1.00	0.00
ATOM	4516	HE2	MET	A	287	93.969	65.943	64.883	1.00	0.00
ATOM	4517	HE3	MET	A	287	95.629	65.339	65.113	1.00	0.00
ATOM	4518	C	MET	A	287	96.940	63.398	68.099	1.00	0.00
ATOM	4519	O	MET	A	287	97.358	64.531	67.863	1.00	0.00
ATOM	4520	N	ASN	A	288	97.420	62.655	69.115	1.00	0.00
ATOM	4521	H	ASN	A	288	97.067	61.711	69.226	1.00	0.00
ATOM	4522	CA	ASN	A	288	98.413	63.099	70.112	1.00	0.00
ATOM	4523	HA	ASN	A	288	98.247	62.494	71.004	1.00	0.00
ATOM	4524	CB	ASN	A	288	99.827	62.745	69.599	1.00	0.00
ATOM	4525	HB1	ASN	A	288	100.120	63.448	68.821	1.00	0.00
ATOM	4526	HB2	ASN	A	288	99.806	61.746	69.163	1.00	0.00
ATOM	4527	CG	ASN	A	288	100.884	62.734	70.696	1.00	0.00
ATOM	4528	OD1	ASN	A	288	101.421	61.708	71.067	1.00	0.00
ATOM	4529	ND2	ASN	A	288	101.203	63.877	71.256	1.00	0.00
ATOM	4530	1HD2	ASN	A	288	100.769	64.748	70.972	1.00	0.00
ATOM	4531	2HD2	ASN	A	288	101.934	63.867	71.944	1.00	0.00
ATOM	4532	C	ASN	A	288	98.208	64.581	70.529	1.00	0.00
ATOM	4533	O	ASN	A	288	99.014	65.471	70.240	1.00	0.00
ATOM	4534	N	LEU	A	289	97.059	64.830	71.160	1.00	0.00
ATOM	4535	H	LEU	A	289	96.482	64.019	71.327	1.00	0.00
ATOM	4536	CA	LEU	A	289	96.371	66.122	71.362	1.00	0.00
ATOM	4537	HA	LEU	A	289	96.145	66.540	70.381	1.00	0.00
ATOM	4538	CB	LEU	A	289	95.060	65.784	72.106	1.00	0.00
ATOM	4539	HB1	LEU	A	289	95.092	66.282	73.069	1.00	0.00
ATOM	4540	HB2	LEU	A	289	95.012	64.714	72.314	1.00	0.00
ATOM	4541	CG	LEU	A	289	93.743	66.179	71.441	1.00	0.00
ATOM	4542	HG	LEU	A	289	92.946	65.820	72.086	1.00	0.00
ATOM	4543	CD1	LEU	A	289	93.558	67.683	71.344	1.00	0.00
ATOM	4544	1HD1	LEU	A	289	92.551	67.912	71.005	1.00	0.00
ATOM	4545	2HD1	LEU	A	289	93.695	68.129	72.331	1.00	0.00
ATOM	4546	3HD1	LEU	A	289	94.293	68.113	70.664	1.00	0.00
ATOM	4547	CD2	LEU	A	289	93.585	65.510	70.076	1.00	0.00
ATOM	4548	1HD2	LEU	A	289	92.583	65.687	69.701	1.00	0.00
ATOM	4549	2HD2	LEU	A	289	94.310	65.916	69.371	1.00	0.00
ATOM	4550	3HD2	LEU	A	289	93.740	64.439	70.184	1.00	0.00
ATOM	4551	C	LEU	A	289	97.077	67.254	72.139	1.00	0.00
ATOM	4552	O	LEU	A	289	96.574	68.375	72.217	1.00	0.00
ATOM	4553	N	GLN	A	290	98.186	66.938	72.801	1.00	0.00
ATOM	4554	H	GLN	A	290	98.634	66.063	72.573	1.00	0.00
ATOM	4555	CA	GLN	A	290	98.721	67.731	73.907	1.00	0.00
ATOM	4556	HA	GLN	A	290	99.543	67.149	74.324	1.00	0.00
ATOM	4557	CB	GLN	A	290	99.321	69.046	73.392	1.00	0.00
ATOM	4558	HB1	GLN	A	290	99.849	69.517	74.217	1.00	0.00
ATOM	4559	HB2	GLN	A	290	98.529	69.720	73.077	1.00	0.00
ATOM	4560	CG	GLN	A	290	100.302	68.853	72.222	1.00	0.00
ATOM	4561	HG1	GLN	A	290	100.706	69.825	71.936	1.00	0.00
ATOM	4562	HG2	GLN	A	290	99.786	68.441	71.355	1.00	0.00
ATOM	4563	CD	GLN	A	290	101.438	67.926	72.625	1.00	0.00
ATOM	4564	OE1	GLN	A	290	101.410	66.721	72.418	1.00	0.00
ATOM	4565	NE2	GLN	A	290	102.439	68.455	73.292	1.00	0.00
ATOM	4566	1HE2	GLN	A	290	102.408	69.467	73.491	1.00	0.00
ATOM	4567	2HE2	GLN	A	290	103.201	67.879	73.577	1.00	0.00
ATOM	4568	C	GLN	A	290	97.722	67.949	75.067	1.00	0.00
ATOM	4569	O	GLN	A	290	96.510	68.115	74.909	1.00	0.00
ATOM	4570	N	GLY	A	291	98.268	68.004	76.289	1.00	0.00
ATOM	4571	H	GLY	A	291	99.260	67.862	76.387	1.00	0.00
ATOM	4572	CA	GLY	A	291	97.501	68.538	77.419	1.00	0.00
ATOM	4573	HA1	GLY	A	291	98.129	68.579	78.307	1.00	0.00

ATOM	4574	HA2	GLY	A	291	96.639	67.902	77.618	1.00	0.00
ATOM	4575	C	GLY	A	291	97.008	69.944	77.094	1.00	0.00
ATOM	4576	O	GLY	A	291	95.829	70.223	77.231	1.00	0.00
ATOM	4577	N	GLU	A	292	97.900	70.768	76.540	1.00	0.00
ATOM	4578	H	GLU	A	292	98.846	70.432	76.493	1.00	0.00
ATOM	4579	CA	GLU	A	292	97.680	72.102	75.965	1.00	0.00
ATOM	4580	HA	GLU	A	292	97.681	72.838	76.769	1.00	0.00
ATOM	4581	CB	GLU	A	292	98.869	72.406	75.005	1.00	0.00
ATOM	4582	HB1	GLU	A	292	98.849	73.466	74.752	1.00	0.00
ATOM	4583	HB2	GLU	A	292	98.725	71.852	74.078	1.00	0.00
ATOM	4584	CG	GLU	A	292	100.290	72.050	75.523	1.00	0.00
ATOM	4585	HG1	GLU	A	292	100.305	71.030	75.907	1.00	0.00
ATOM	4586	HG2	GLU	A	292	100.532	72.698	76.362	1.00	0.00
ATOM	4587	CD	GLU	A	292	101.390	72.159	74.446	1.00	0.00
ATOM	4588	OE1	GLU	A	292	101.795	73.301	74.147	1.00	0.00
ATOM	4589	OE2	GLU	A	292	101.856	71.093	73.971	1.00	0.00
ATOM	4590	C	GLU	A	292	96.323	72.222	75.226	1.00	0.00
ATOM	4591	O	GLU	A	292	95.457	73.020	75.636	1.00	0.00
ATOM	4592	N	GLU	A	293	96.097	71.402	74.181	1.00	0.00
ATOM	4593	H	GLU	A	293	96.794	70.734	73.886	1.00	0.00
ATOM	4594	CA	GLU	A	293	94.891	71.582	73.373	1.00	0.00
ATOM	4595	HA	GLU	A	293	94.651	72.644	73.352	1.00	0.00
ATOM	4596	CB	GLU	A	293	95.091	71.178	71.907	1.00	0.00
ATOM	4597	HB1	GLU	A	293	94.306	71.670	71.335	1.00	0.00
ATOM	4598	HB2	GLU	A	293	94.944	70.110	71.804	1.00	0.00
ATOM	4599	CG	GLU	A	293	96.452	71.578	71.319	1.00	0.00
ATOM	4600	HG1	GLU	A	293	97.255	71.035	71.816	1.00	0.00
ATOM	4601	HG2	GLU	A	293	96.614	72.646	71.486	1.00	0.00
ATOM	4602	CD	GLU	A	293	96.496	71.280	69.819	1.00	0.00
ATOM	4603	OE1	GLU	A	293	96.912	70.160	69.423	1.00	0.00
ATOM	4604	OE2	GLU	A	293	96.080	72.182	69.061	1.00	0.00
ATOM	4605	C	GLU	A	293	93.688	70.867	73.999	1.00	0.00
ATOM	4606	O	GLU	A	293	92.562	71.356	73.874	1.00	0.00
ATOM	4607	N	PHE	A	294	93.910	69.796	74.781	1.00	0.00
ATOM	4608	H	PHE	A	294	94.852	69.418	74.855	1.00	0.00
ATOM	4609	CA	PHE	A	294	92.843	69.239	75.623	1.00	0.00
ATOM	4610	HA	PHE	A	294	92.034	68.936	74.956	1.00	0.00
ATOM	4611	CB	PHE	A	294	93.326	67.989	76.377	1.00	0.00
ATOM	4612	HB1	PHE	A	294	92.963	68.025	77.405	1.00	0.00
ATOM	4613	HB2	PHE	A	294	94.412	67.980	76.434	1.00	0.00
ATOM	4614	CG	PHE	A	294	92.816	66.700	75.774	1.00	0.00
ATOM	4615	CD1	PHE	A	294	93.712	65.782	75.200	1.00	0.00
ATOM	4616	HD1	PHE	A	294	94.767	66.008	75.177	1.00	0.00
ATOM	4617	CE1	PHE	A	294	93.243	64.555	74.694	1.00	0.00
ATOM	4618	HE1	PHE	A	294	93.939	63.850	74.262	1.00	0.00
ATOM	4619	CZ	PHE	A	294	91.881	64.234	74.794	1.00	0.00
ATOM	4620	HZ	PHE	A	294	91.526	63.270	74.465	1.00	0.00
ATOM	4621	CE2	PHE	A	294	90.982	65.160	75.341	1.00	0.00
ATOM	4622	HE2	PHE	A	294	89.939	64.884	75.419	1.00	0.00
ATOM	4623	CD2	PHE	A	294	91.447	66.395	75.824	1.00	0.00
ATOM	4624	HD2	PHE	A	294	90.764	67.096	76.279	1.00	0.00
ATOM	4625	C	PHE	A	294	92.219	70.241	76.602	1.00	0.00
ATOM	4626	O	PHE	A	294	90.994	70.242	76.767	1.00	0.00
ATOM	4627	N	VAL	A	295	93.011	71.110	77.238	1.00	0.00
ATOM	4628	H	VAL	A	295	94.018	71.034	77.109	1.00	0.00
ATOM	4629	CA	VAL	A	295	92.477	72.083	78.200	1.00	0.00
ATOM	4630	HA	VAL	A	295	91.559	71.643	78.575	1.00	0.00
ATOM	4631	CB	VAL	A	295	93.342	72.305	79.456	1.00	0.00
ATOM	4632	HB	VAL	A	295	93.744	73.295	79.374	1.00	0.00
ATOM	4633	CG1	VAL	A	295	92.460	72.258	80.703	1.00	0.00
ATOM	4634	1HG1	VAL	A	295	93.058	72.452	81.595	1.00	0.00

ATOM	4635	2HG1	VAL	A	295	91.693	73.028	80.630	1.00	0.00
ATOM	4636	3HG1	VAL	A	295	92.003	71.277	80.777	1.00	0.00
ATOM	4637	CG2	VAL	A	295	94.512	71.353	79.695	1.00	0.00
ATOM	4638	1HG2	VAL	A	295	94.869	71.428	80.720	1.00	0.00
ATOM	4639	2HG2	VAL	A	295	94.226	70.321	79.499	1.00	0.00
ATOM	4640	3HG2	VAL	A	295	95.334	71.650	79.054	1.00	0.00
ATOM	4641	C	VAL	A	295	92.037	73.395	77.546	1.00	0.00
ATOM	4642	O	VAL	A	295	91.117	74.028	78.062	1.00	0.00
ATOM	4643	N	CYS	A	296	92.553	73.746	76.355	1.00	0.00
ATOM	4644	H	CYS	A	296	93.361	73.256	75.990	1.00	0.00
ATOM	4645	CA	CYS	A	296	91.877	74.743	75.508	1.00	0.00
ATOM	4646	HA	CYS	A	296	91.813	75.689	76.050	1.00	0.00
ATOM	4647	CB	CYS	A	296	92.666	74.979	74.214	1.00	0.00
ATOM	4648	HB1	CYS	A	296	92.074	75.605	73.543	1.00	0.00
ATOM	4649	HB2	CYS	A	296	92.873	74.031	73.715	1.00	0.00
ATOM	4650	SG	CYS	A	296	94.219	75.851	74.563	1.00	0.00
ATOM	4651	HG	CYS	A	296	94.805	74.878	75.280	1.00	0.00
ATOM	4652	C	CYS	A	296	90.440	74.309	75.184	1.00	0.00
ATOM	4653	O	CYS	A	296	89.493	75.063	75.418	1.00	0.00
ATOM	4654	N	LEU	A	297	90.253	73.063	74.732	1.00	0.00
ATOM	4655	H	LEU	A	297	91.074	72.498	74.530	1.00	0.00
ATOM	4656	CA	LEU	A	297	88.926	72.481	74.486	1.00	0.00
ATOM	4657	HA	LEU	A	297	88.396	73.118	73.777	1.00	0.00
ATOM	4658	CB	LEU	A	297	89.082	71.088	73.850	1.00	0.00
ATOM	4659	HB1	LEU	A	297	88.267	70.459	74.204	1.00	0.00
ATOM	4660	HB2	LEU	A	297	90.012	70.622	74.175	1.00	0.00
ATOM	4661	CG	LEU	A	297	89.018	71.106	72.309	1.00	0.00
ATOM	4662	HG	LEU	A	297	88.061	71.524	72.013	1.00	0.00
ATOM	4663	CD1	LEU	A	297	90.104	71.922	71.614	1.00	0.00
ATOM	4664	1HD1	LEU	A	297	89.944	71.908	70.538	1.00	0.00
ATOM	4665	2HD1	LEU	A	297	90.064	72.956	71.946	1.00	0.00
ATOM	4666	3HD1	LEU	A	297	91.090	71.516	71.834	1.00	0.00
ATOM	4667	CD2	LEU	A	297	89.079	69.665	71.811	1.00	0.00
ATOM	4668	1HD2	LEU	A	297	88.967	69.656	70.730	1.00	0.00
ATOM	4669	2HD2	LEU	A	297	90.039	69.221	72.079	1.00	0.00
ATOM	4670	3HD2	LEU	A	297	88.274	69.087	72.263	1.00	0.00
ATOM	4671	C	LEU	A	297	88.052	72.443	75.758	1.00	0.00
ATOM	4672	O	LEU	A	297	86.882	72.821	75.697	1.00	0.00
ATOM	4673	N	LYS	A	298	88.633	72.090	76.920	1.00	0.00
ATOM	4674	H	LYS	A	298	89.584	71.734	76.887	1.00	0.00
ATOM	4675	CA	LYS	A	298	87.979	72.219	78.233	1.00	0.00
ATOM	4676	HA	LYS	A	298	87.126	71.542	78.244	1.00	0.00
ATOM	4677	CB	LYS	A	298	88.962	71.794	79.353	1.00	0.00
ATOM	4678	HB1	LYS	A	298	89.739	72.546	79.446	1.00	0.00
ATOM	4679	HB2	LYS	A	298	89.422	70.839	79.090	1.00	0.00
ATOM	4680	CG	LYS	A	298	88.305	71.645	80.735	1.00	0.00
ATOM	4681	HG1	LYS	A	298	87.598	70.820	80.677	1.00	0.00
ATOM	4682	HG2	LYS	A	298	87.752	72.562	80.934	1.00	0.00
ATOM	4683	CD	LYS	A	298	89.245	71.420	81.939	1.00	0.00
ATOM	4684	HD1	LYS	A	298	88.644	71.578	82.831	1.00	0.00
ATOM	4685	HD2	LYS	A	298	90.012	72.192	81.940	1.00	0.00
ATOM	4686	CE	LYS	A	298	89.893	70.029	82.029	1.00	0.00
ATOM	4687	HE1	LYS	A	298	90.536	69.863	81.160	1.00	0.00
ATOM	4688	HE2	LYS	A	298	89.112	69.263	81.996	1.00	0.00
ATOM	4689	NZ	LYS	A	298	90.684	69.854	83.282	1.00	0.00
ATOM	4690	HZ1	LYS	A	298	91.086	68.913	83.333	1.00	0.00
ATOM	4691	HZ2	LYS	A	298	91.454	70.512	83.380	1.00	0.00
ATOM	4692	HZ3	LYS	A	298	90.104	69.892	84.109	1.00	0.00
ATOM	4693	C	LYS	A	298	87.410	73.636	78.451	1.00	0.00
ATOM	4694	O	LYS	A	298	86.222	73.761	78.716	1.00	0.00
ATOM	4695	N	SER	A	299	88.211	74.700	78.287	1.00	0.00

ATOM	4696	H	SER	A	299	89.190	74.548	78.062	1.00	0.00
ATOM	4697	CA	SER	A	299	87.734	76.092	78.416	1.00	0.00
ATOM	4698	HA	SER	A	299	87.302	76.209	79.410	1.00	0.00
ATOM	4699	CB	SER	A	299	88.880	77.112	78.287	1.00	0.00
ATOM	4700	HB1	SER	A	299	88.487	78.040	77.870	1.00	0.00
ATOM	4701	HB2	SER	A	299	89.657	76.734	77.621	1.00	0.00
ATOM	4702	OG	SER	A	299	89.420	77.400	79.565	1.00	0.00
ATOM	4703	HG	SER	A	299	89.827	78.287	79.533	1.00	0.00
ATOM	4704	C	SER	A	299	86.619	76.460	77.428	1.00	0.00
ATOM	4705	O	SER	A	299	85.613	77.053	77.823	1.00	0.00
ATOM	4706	N	ILE	A	300	86.754	76.053	76.158	1.00	0.00
ATOM	4707	H	ILE	A	300	87.607	75.567	75.901	1.00	0.00
ATOM	4708	CA	ILE	A	300	85.708	76.226	75.131	1.00	0.00
ATOM	4709	HA	ILE	A	300	85.548	77.294	74.974	1.00	0.00
ATOM	4710	CB	ILE	A	300	86.179	75.597	73.789	1.00	0.00
ATOM	4711	HB	ILE	A	300	86.743	74.695	74.013	1.00	0.00
ATOM	4712	CG2	ILE	A	300	85.015	75.176	72.874	1.00	0.00
ATOM	4713	1HG2	ILE	A	300	84.331	76.006	72.727	1.00	0.00
ATOM	4714	2HG2	ILE	A	300	85.382	74.851	71.905	1.00	0.00
ATOM	4715	3HG2	ILE	A	300	84.470	74.338	73.309	1.00	0.00
ATOM	4716	CG1	ILE	A	300	87.131	76.580	73.079	1.00	0.00
ATOM	4717	1HG1	ILE	A	300	87.886	76.889	73.795	1.00	0.00
ATOM	4718	2HG1	ILE	A	300	86.578	77.466	72.769	1.00	0.00
ATOM	4719	CD	ILE	A	300	87.875	76.010	71.864	1.00	0.00
ATOM	4720	HD1	ILE	A	300	88.616	76.736	71.527	1.00	0.00
ATOM	4721	HD2	ILE	A	300	88.385	75.086	72.136	1.00	0.00
ATOM	4722	HD3	ILE	A	300	87.188	75.817	71.042	1.00	0.00
ATOM	4723	C	ILE	A	300	84.363	75.655	75.605	1.00	0.00
ATOM	4724	O	ILE	A	300	83.341	76.341	75.551	1.00	0.00
ATOM	4725	N	ILE	A	301	84.365	74.422	76.119	1.00	0.00
ATOM	4726	H	ILE	A	301	85.243	73.913	76.136	1.00	0.00
ATOM	4727	CA	ILE	A	301	83.168	73.751	76.639	1.00	0.00
ATOM	4728	HA	ILE	A	301	82.405	73.721	75.862	1.00	0.00
ATOM	4729	CB	ILE	A	301	83.535	72.304	77.036	1.00	0.00
ATOM	4730	HB	ILE	A	301	84.387	72.339	77.710	1.00	0.00
ATOM	4731	CG2	ILE	A	301	82.371	71.646	77.790	1.00	0.00
ATOM	4732	1HG2	ILE	A	301	82.578	70.595	77.975	1.00	0.00
ATOM	4733	2HG2	ILE	A	301	82.217	72.125	78.749	1.00	0.00
ATOM	4734	3HG2	ILE	A	301	81.459	71.747	77.203	1.00	0.00
ATOM	4735	CG1	ILE	A	301	83.943	71.456	75.809	1.00	0.00
ATOM	4736	1HG1	ILE	A	301	84.568	72.045	75.142	1.00	0.00
ATOM	4737	2HG1	ILE	A	301	83.058	71.182	75.240	1.00	0.00
ATOM	4738	CD	ILE	A	301	84.731	70.198	76.196	1.00	0.00
ATOM	4739	HD1	ILE	A	301	85.047	69.675	75.296	1.00	0.00
ATOM	4740	HD2	ILE	A	301	85.618	70.477	76.765	1.00	0.00
ATOM	4741	HD3	ILE	A	301	84.120	69.523	76.790	1.00	0.00
ATOM	4742	C	ILE	A	301	82.583	74.521	77.837	1.00	0.00
ATOM	4743	O	ILE	A	301	81.382	74.798	77.881	1.00	0.00
ATOM	4744	N	LEU	A	302	83.446	74.934	78.772	1.00	0.00
ATOM	4745	H	LEU	A	302	84.424	74.705	78.643	1.00	0.00
ATOM	4746	CA	LEU	A	302	83.091	75.736	79.953	1.00	0.00
ATOM	4747	HA	LEU	A	302	82.256	75.240	80.446	1.00	0.00
ATOM	4748	CB	LEU	A	302	84.301	75.786	80.911	1.00	0.00
ATOM	4749	HB1	LEU	A	302	84.627	76.821	81.034	1.00	0.00
ATOM	4750	HB2	LEU	A	302	85.141	75.245	80.478	1.00	0.00
ATOM	4751	CG	LEU	A	302	84.030	75.194	82.303	1.00	0.00
ATOM	4752	HG	LEU	A	302	83.792	74.136	82.205	1.00	0.00
ATOM	4753	CD1	LEU	A	302	85.308	75.346	83.133	1.00	0.00
ATOM	4754	1HD1	LEU	A	302	85.171	74.866	84.100	1.00	0.00
ATOM	4755	2HD1	LEU	A	302	86.143	74.871	82.618	1.00	0.00
ATOM	4756	3HD1	LEU	A	302	85.523	76.404	83.290	1.00	0.00

```
ATOM   4757  CD2 LEU A 302      82.886  75.894  83.041  1.00  0.00
ATOM   4758 1HD2 LEU A 302      82.867  75.597  84.086  1.00  0.00
ATOM   4759 2HD2 LEU A 302      83.023  76.972  82.974  1.00  0.00
ATOM   4760 3HD2 LEU A 302      81.930  75.637  82.588  1.00  0.00
ATOM   4761  C   LEU A 302      82.615  77.171  79.641  1.00  0.00
ATOM   4762  O   LEU A 302      82.216  77.904  80.542  1.00  0.00
ATOM   4763  N   LEU A 303      82.648  77.559  78.366  1.00  0.00
ATOM   4764  H   LEU A 303      83.074  76.928  77.700  1.00  0.00
ATOM   4765  CA  LEU A 303      82.232  78.859  77.855  1.00  0.00
ATOM   4766  HA  LEU A 303      81.880  79.485  78.671  1.00  0.00
ATOM   4767  CB  LEU A 303      83.431  79.477  77.149  1.00  0.00
ATOM   4768  HB1 LEU A 303      83.108  80.263  76.470  1.00  0.00
ATOM   4769  HB2 LEU A 303      83.939  78.720  76.551  1.00  0.00
ATOM   4770  CG  LEU A 303      84.393  80.118  78.134  1.00  0.00
ATOM   4771  HG  LEU A 303      84.768  79.388  78.849  1.00  0.00
ATOM   4772  CD1 LEU A 303      85.547  80.621  77.284  1.00  0.00
ATOM   4773 1HD1 LEU A 303      86.339  81.016  77.900  1.00  0.00
ATOM   4774 2HD1 LEU A 303      85.949  79.776  76.732  1.00  0.00
ATOM   4775 3HD1 LEU A 303      85.196  81.382  76.600  1.00  0.00
ATOM   4776  CD2 LEU A 303      83.668  81.242  78.862  1.00  0.00
ATOM   4777 1HD2 LEU A 303      84.370  81.966  79.228  1.00  0.00
ATOM   4778 2HD2 LEU A 303      82.956  81.730  78.201  1.00  0.00
ATOM   4779 3HD2 LEU A 303      83.147  80.835  79.726  1.00  0.00
ATOM   4780  C   LEU A 303      81.066  78.790  76.872  1.00  0.00
ATOM   4781  O   LEU A 303      80.722  79.792  76.247  1.00  0.00
ATOM   4782  N   ASN A 304      80.466  77.615  76.692  1.00  0.00
ATOM   4783  H   ASN A 304      80.826  76.816  77.192  1.00  0.00
ATOM   4784  CA  ASN A 304      79.430  77.439  75.694  1.00  0.00
ATOM   4785  HA  ASN A 304      79.849  77.838  74.772  1.00  0.00
ATOM   4786  CB  ASN A 304      79.200  75.936  75.405  1.00  0.00
ATOM   4787  HB1 ASN A 304      80.154  75.407  75.432  1.00  0.00
ATOM   4788  HB2 ASN A 304      78.823  75.852  74.386  1.00  0.00
ATOM   4789  CG  ASN A 304      78.212  75.203  76.300  1.00  0.00
ATOM   4790  OD1 ASN A 304      77.119  74.849  75.870  1.00  0.00
ATOM   4791  ND2 ASN A 304      78.574  74.843  77.506  1.00  0.00
ATOM   4792 1HD2 ASN A 304      79.533  74.988  77.812  1.00  0.00
ATOM   4793 2HD2 ASN A 304      77.897  74.404  78.101  1.00  0.00
ATOM   4794  C   ASN A 304      78.176  78.293  75.977  1.00  0.00
ATOM   4795  O   ASN A 304      77.257  77.850  76.662  1.00  0.00
ATOM   4796  N   SER A 305      78.110  79.517  75.431  1.00  0.00
ATOM   4797  H   SER A 305      78.971  79.862  75.027  1.00  0.00
ATOM   4798  CA  SER A 305      77.031  80.507  75.636  1.00  0.00
ATOM   4799  HA  SER A 305      76.866  80.556  76.710  1.00  0.00
ATOM   4800  CB  SER A 305      77.429  81.898  75.160  1.00  0.00
ATOM   4801  HB1 SER A 305      76.935  82.155  74.221  1.00  0.00
ATOM   4802  HB2 SER A 305      78.506  81.966  75.008  1.00  0.00
ATOM   4803  OG  SER A 305      77.032  82.786  76.185  1.00  0.00
ATOM   4804  HG  SER A 305      77.120  83.709  75.867  1.00  0.00
ATOM   4805  C   SER A 305      75.673  80.121  75.014  1.00  0.00
ATOM   4806  O   SER A 305      74.790  80.955  74.813  1.00  0.00
ATOM   4807  N   GLY A 306      75.486  78.833  74.742  1.00  0.00
ATOM   4808  H   GLY A 306      76.255  78.210  74.933  1.00  0.00
ATOM   4809  CA  GLY A 306      74.173  78.231  74.614  1.00  0.00
ATOM   4810  HA1 GLY A 306      74.267  77.284  74.085  1.00  0.00
ATOM   4811  HA2 GLY A 306      73.512  78.880  74.040  1.00  0.00
ATOM   4812  C   GLY A 306      73.513  77.950  75.969  1.00  0.00
ATOM   4813  O   GLY A 306      72.305  77.841  76.023  1.00  0.00
ATOM   4814  N   VAL A 307      74.284  77.900  77.066  1.00  0.00
ATOM   4815  H   VAL A 307      75.286  77.918  76.913  1.00  0.00
ATOM   4816  CA  VAL A 307      73.855  78.108  78.469  1.00  0.00
ATOM   4817  HA  VAL A 307      74.543  77.517  79.077  1.00  0.00
```

ATOM	4818	CB	VAL	A	307	74.081	79.566	78.913	1.00	0.00
ATOM	4819	HB	VAL	A	307	73.683	79.679	79.918	1.00	0.00
ATOM	4820	CG1	VAL	A	307	75.593	79.811	78.996	1.00	0.00
ATOM	4821	1HG1	VAL	A	307	75.830	80.831	78.705	1.00	0.00
ATOM	4822	2HG1	VAL	A	307	75.939	79.624	80.012	1.00	0.00
ATOM	4823	3HG1	VAL	A	307	76.147	79.157	78.341	1.00	0.00
ATOM	4824	CG2	VAL	A	307	73.399	80.633	78.049	1.00	0.00
ATOM	4825	1HG2	VAL	A	307	73.704	80.550	77.012	1.00	0.00
ATOM	4826	2HG2	VAL	A	307	72.320	80.528	78.111	1.00	0.00
ATOM	4827	3HG2	VAL	A	307	73.659	81.629	78.403	1.00	0.00
ATOM	4828	C	VAL	A	307	72.463	77.574	78.855	1.00	0.00
ATOM	4829	O	VAL	A	307	71.533	78.325	79.119	1.00	0.00
ATOM	4830	N	TYR	A	308	72.338	76.242	78.913	1.00	0.00
ATOM	4831	H	TYR	A	308	73.157	75.695	78.702	1.00	0.00
ATOM	4832	CA	TYR	A	308	71.096	75.474	79.145	1.00	0.00
ATOM	4833	HA	TYR	A	308	71.393	74.425	79.169	1.00	0.00
ATOM	4834	CB	TYR	A	308	70.443	75.762	80.509	1.00	0.00
ATOM	4835	HB1	TYR	A	308	69.359	75.735	80.398	1.00	0.00
ATOM	4836	HB2	TYR	A	308	70.654	76.771	80.832	1.00	0.00
ATOM	4837	CG	TYR	A	308	70.809	74.778	81.597	1.00	0.00
ATOM	4838	CD1	TYR	A	308	72.157	74.528	81.922	1.00	0.00
ATOM	4839	HD1	TYR	A	308	72.947	75.080	81.424	1.00	0.00
ATOM	4840	CE1	TYR	A	308	72.476	73.586	82.917	1.00	0.00
ATOM	4841	HE1	TYR	A	308	73.501	73.386	83.189	1.00	0.00
ATOM	4842	CZ	TYR	A	308	71.453	72.918	83.615	1.00	0.00
ATOM	4843	OH	TYR	A	308	71.759	72.052	84.610	1.00	0.00
ATOM	4844	HH	TYR	A	308	70.967	71.827	85.120	1.00	0.00
ATOM	4845	CE2	TYR	A	308	70.103	73.177	83.290	1.00	0.00
ATOM	4846	HE2	TYR	A	308	69.315	72.666	83.808	1.00	0.00
ATOM	4847	CD2	TYR	A	308	69.786	74.097	82.279	1.00	0.00
ATOM	4848	HD2	TYR	A	308	68.750	74.278	82.029	1.00	0.00
ATOM	4849	C	TYR	A	308	70.039	75.524	78.023	1.00	0.00
ATOM	4850	O	TYR	A	308	69.033	74.821	78.100	1.00	0.00
ATOM	4851	N	THR	A	309	70.256	76.303	76.964	1.00	0.00
ATOM	4852	H	THR	A	309	71.142	76.791	76.925	1.00	0.00
ATOM	4853	CA	THR	A	309	69.287	76.704	75.920	1.00	0.00
ATOM	4854	HA	THR	A	309	69.872	77.161	75.123	1.00	0.00
ATOM	4855	CB	THR	A	309	68.515	75.527	75.268	1.00	0.00
ATOM	4856	HB	THR	A	309	67.591	75.357	75.822	1.00	0.00
ATOM	4857	CG2	THR	A	309	68.183	75.760	73.794	1.00	0.00
ATOM	4858	1HG2	THR	A	309	67.658	74.892	73.400	1.00	0.00
ATOM	4859	2HG2	THR	A	309	67.540	76.631	73.679	1.00	0.00
ATOM	4860	3HG2	THR	A	309	69.098	75.911	73.224	1.00	0.00
ATOM	4861	OG1	THR	A	309	69.274	74.343	75.304	1.00	0.00
ATOM	4862	HG1	THR	A	309	69.235	74.057	76.225	1.00	0.00
ATOM	4863	C	THR	A	309	68.319	77.804	76.401	1.00	0.00
ATOM	4864	O	THR	A	309	67.310	78.084	75.758	1.00	0.00
ATOM	4865	N	PHE	A	310	68.626	78.426	77.545	1.00	0.00
ATOM	4866	H	PHE	A	310	69.476	78.109	77.989	1.00	0.00
ATOM	4867	CA	PHE	A	310	68.039	79.644	78.118	1.00	0.00
ATOM	4868	HA	PHE	A	310	68.023	79.465	79.193	1.00	0.00
ATOM	4869	CB	PHE	A	310	69.001	80.836	77.921	1.00	0.00
ATOM	4870	HB1	PHE	A	310	69.925	80.606	78.451	1.00	0.00
ATOM	4871	HB2	PHE	A	310	68.575	81.712	78.410	1.00	0.00
ATOM	4872	CG	PHE	A	310	69.374	81.226	76.496	1.00	0.00
ATOM	4873	CD1	PHE	A	310	70.462	80.596	75.868	1.00	0.00
ATOM	4874	HD1	PHE	A	310	70.993	79.815	76.388	1.00	0.00
ATOM	4875	CE1	PHE	A	310	70.867	80.980	74.579	1.00	0.00
ATOM	4876	HE1	PHE	A	310	71.709	80.491	74.109	1.00	0.00
ATOM	4877	CZ	PHE	A	310	70.180	82.003	73.906	1.00	0.00
ATOM	4878	HZ	PHE	A	310	70.490	82.293	72.909	1.00	0.00

ATOM	4879	CE2	PHE	A	310	69.092	82.640	74.525	1.00	0.00
ATOM	4880	HE2	PHE	A	310	68.553	83.419	74.001	1.00	0.00
ATOM	4881	CD2	PHE	A	310	68.696	82.259	75.819	1.00	0.00
ATOM	4882	HD2	PHE	A	310	67.863	82.757	76.290	1.00	0.00
ATOM	4883	C	PHE	A	310	66.570	80.007	77.790	1.00	0.00
ATOM	4884	O	PHE	A	310	66.272	81.074	77.252	1.00	0.00
ATOM	4885	N	LEU	A	311	65.624	79.217	78.302	1.00	0.00
ATOM	4886	H	LEU	A	311	65.902	78.312	78.648	1.00	0.00
ATOM	4887	CA	LEU	A	311	64.337	79.768	78.755	1.00	0.00
ATOM	4888	HA	LEU	A	311	63.871	80.280	77.911	1.00	0.00
ATOM	4889	CB	LEU	A	311	63.431	78.596	79.192	1.00	0.00
ATOM	4890	HB1	LEU	A	311	63.858	78.129	80.080	1.00	0.00
ATOM	4891	HB2	LEU	A	311	63.455	77.848	78.398	1.00	0.00
ATOM	4892	CG	LEU	A	311	61.943	78.901	79.468	1.00	0.00
ATOM	4893	HG	LEU	A	311	61.459	77.943	79.653	1.00	0.00
ATOM	4894	CD1	LEU	A	311	61.704	79.772	80.700	1.00	0.00
ATOM	4895	1HD1	LEU	A	311	60.640	79.774	80.938	1.00	0.00
ATOM	4896	2HD1	LEU	A	311	62.248	79.367	81.550	1.00	0.00
ATOM	4897	3HD1	LEU	A	311	62.010	80.799	80.516	1.00	0.00
ATOM	4898	CD2	LEU	A	311	61.247	79.543	78.269	1.00	0.00
ATOM	4899	1HD2	LEU	A	311	60.176	79.602	78.458	1.00	0.00
ATOM	4900	2HD2	LEU	A	311	61.621	80.549	78.093	1.00	0.00
ATOM	4901	3HD2	LEU	A	311	61.410	78.938	77.379	1.00	0.00
ATOM	4902	C	LEU	A	311	64.630	80.796	79.872	1.00	0.00
ATOM	4903	O	LEU	A	311	64.861	80.429	81.025	1.00	0.00
ATOM	4904	N	SER	A	312	64.745	82.069	79.475	1.00	0.00
ATOM	4905	H	SER	A	312	64.513	82.220	78.502	1.00	0.00
ATOM	4906	CA	SER	A	312	65.666	83.073	80.051	1.00	0.00
ATOM	4907	HA	SER	A	312	66.616	82.561	80.199	1.00	0.00
ATOM	4908	CB	SER	A	312	65.929	84.165	78.996	1.00	0.00
ATOM	4909	HB1	SER	A	312	65.001	84.702	78.788	1.00	0.00
ATOM	4910	HB2	SER	A	312	66.279	83.700	78.073	1.00	0.00
ATOM	4911	OG	SER	A	312	66.908	85.080	79.454	1.00	0.00
ATOM	4912	HG	SER	A	312	66.514	85.480	80.254	1.00	0.00
ATOM	4913	C	SER	A	312	65.287	83.679	81.417	1.00	0.00
ATOM	4914	O	SER	A	312	65.284	84.900	81.590	1.00	0.00
ATOM	4915	N	SER	A	313	64.987	82.850	82.412	1.00	0.00
ATOM	4916	H	SER	A	313	64.974	81.861	82.186	1.00	0.00
ATOM	4917	CA	SER	A	313	65.028	83.247	83.826	1.00	0.00
ATOM	4918	HA	SER	A	313	64.596	84.244	83.936	1.00	0.00
ATOM	4919	CB	SER	A	313	64.205	82.246	84.643	1.00	0.00
ATOM	4920	HB1	SER	A	313	64.629	81.249	84.520	1.00	0.00
ATOM	4921	HB2	SER	A	313	63.178	82.233	84.278	1.00	0.00
ATOM	4922	OG	SER	A	313	64.206	82.577	86.020	1.00	0.00
ATOM	4923	HG	SER	A	313	63.618	83.327	86.160	1.00	0.00
ATOM	4924	C	SER	A	313	66.474	83.270	84.340	1.00	0.00
ATOM	4925	O	SER	A	313	67.257	82.378	84.005	1.00	0.00
ATOM	4926	N	THR	A	314	66.821	84.193	85.235	1.00	0.00
ATOM	4927	H	THR	A	314	66.239	85.009	85.359	1.00	0.00
ATOM	4928	CA	THR	A	314	68.115	84.215	85.955	1.00	0.00
ATOM	4929	HA	THR	A	314	68.906	84.096	85.213	1.00	0.00
ATOM	4930	CB	THR	A	314	68.337	85.594	86.619	1.00	0.00
ATOM	4931	HB	THR	A	314	67.831	85.597	87.585	1.00	0.00
ATOM	4932	CG2	THR	A	314	69.803	85.972	86.826	1.00	0.00
ATOM	4933	1HG2	THR	A	314	69.869	86.997	87.188	1.00	0.00
ATOM	4934	2HG2	THR	A	314	70.270	85.317	87.557	1.00	0.00
ATOM	4935	3HG2	THR	A	314	70.344	85.899	85.883	1.00	0.00
ATOM	4936	OG1	THR	A	314	67.756	86.616	85.833	1.00	0.00
ATOM	4937	HG1	THR	A	314	68.421	86.925	85.170	1.00	0.00
ATOM	4938	C	THR	A	314	68.286	83.066	86.962	1.00	0.00
ATOM	4939	O	THR	A	314	69.283	82.998	87.670	1.00	0.00

ATOM	4940	N	LEU	A	315	67.343	82.114	87.008	1.00	0.00
ATOM	4941	H	LEU	A	315	66.503	82.282	86.477	1.00	0.00
ATOM	4942	CA	LEU	A	315	67.483	80.823	87.693	1.00	0.00
ATOM	4943	HA	LEU	A	315	68.468	80.781	88.161	1.00	0.00
ATOM	4944	CB	LEU	A	315	66.429	80.722	88.804	1.00	0.00
ATOM	4945	HB1	LEU	A	315	66.652	79.830	89.383	1.00	0.00
ATOM	4946	HB2	LEU	A	315	65.446	80.625	88.342	1.00	0.00
ATOM	4947	CG	LEU	A	315	66.392	81.890	89.809	1.00	0.00
ATOM	4948	HG	LEU	A	315	66.260	82.838	89.288	1.00	0.00
ATOM	4949	CD1	LEU	A	315	65.194	81.691	90.736	1.00	0.00
ATOM	4950	1HD1	LEU	A	315	65.167	82.483	91.485	1.00	0.00
ATOM	4951	2HD1	LEU	A	315	64.273	81.738	90.156	1.00	0.00
ATOM	4952	3HD1	LEU	A	315	65.257	80.727	91.237	1.00	0.00
ATOM	4953	CD2	LEU	A	315	67.673	81.942	90.644	1.00	0.00
ATOM	4954	1HD2	LEU	A	315	67.595	82.740	91.380	1.00	0.00
ATOM	4955	2HD2	LEU	A	315	67.846	80.988	91.141	1.00	0.00
ATOM	4956	3HD2	LEU	A	315	68.522	82.162	89.998	1.00	0.00
ATOM	4957	C	LEU	A	315	67.460	79.636	86.707	1.00	0.00
ATOM	4958	O	LEU	A	315	67.308	78.491	87.125	1.00	0.00
ATOM	4959	N	LYS	A	316	67.583	79.903	85.396	1.00	0.00
ATOM	4960	H	LYS	A	316	67.615	80.882	85.134	1.00	0.00
ATOM	4961	CA	LYS	A	316	67.697	78.912	84.306	1.00	0.00
ATOM	4962	HA	LYS	A	316	68.143	78.005	84.716	1.00	0.00
ATOM	4963	CB	LYS	A	316	66.295	78.561	83.755	1.00	0.00
ATOM	4964	HB1	LYS	A	316	66.411	77.819	82.964	1.00	0.00
ATOM	4965	HB2	LYS	A	316	65.851	79.455	83.317	1.00	0.00
ATOM	4966	CG	LYS	A	316	65.325	77.984	84.804	1.00	0.00
ATOM	4967	HG1	LYS	A	316	65.042	78.774	85.499	1.00	0.00
ATOM	4968	HG2	LYS	A	316	65.832	77.190	85.352	1.00	0.00
ATOM	4969	CD	LYS	A	316	64.056	77.406	84.165	1.00	0.00
ATOM	4970	HD1	LYS	A	316	64.315	76.529	83.569	1.00	0.00
ATOM	4971	HD2	LYS	A	316	63.624	78.152	83.495	1.00	0.00
ATOM	4972	CE	LYS	A	316	62.987	77.022	85.200	1.00	0.00
ATOM	4973	HE1	LYS	A	316	62.112	76.633	84.667	1.00	0.00
ATOM	4974	HE2	LYS	A	316	62.668	77.921	85.735	1.00	0.00
ATOM	4975	NZ	LYS	A	316	63.454	76.008	86.178	1.00	0.00
ATOM	4976	HZ1	LYS	A	316	62.673	75.715	86.765	1.00	0.00
ATOM	4977	HZ2	LYS	A	316	63.813	75.186	85.720	1.00	0.00
ATOM	4978	HZ3	LYS	A	316	64.163	76.375	86.800	1.00	0.00
ATOM	4979	C	LYS	A	316	68.633	79.349	83.159	1.00	0.00
ATOM	4980	O	LYS	A	316	68.690	78.679	82.127	1.00	0.00
ATOM	4981	N	SER	A	317	69.348	80.469	83.302	1.00	0.00
ATOM	4982	H	SER	A	317	69.332	80.940	84.199	1.00	0.00
ATOM	4983	CA	SER	A	317	70.232	81.054	82.282	1.00	0.00
ATOM	4984	HA	SER	A	317	70.790	80.251	81.810	1.00	0.00
ATOM	4985	CB	SER	A	317	69.412	81.742	81.186	1.00	0.00
ATOM	4986	HB1	SER	A	317	68.727	81.013	80.754	1.00	0.00
ATOM	4987	HB2	SER	A	317	70.083	82.091	80.401	1.00	0.00
ATOM	4988	OG	SER	A	317	68.653	82.840	81.645	1.00	0.00
ATOM	4989	HG	SER	A	317	68.374	82.665	82.561	1.00	0.00
ATOM	4990	C	SER	A	317	71.253	82.023	82.895	1.00	0.00
ATOM	4991	O	SER	A	317	71.069	82.462	84.028	1.00	0.00
ATOM	4992	N	LEU	A	318	72.296	82.359	82.123	1.00	0.00
ATOM	4993	H	LEU	A	318	72.376	81.926	81.222	1.00	0.00
ATOM	4994	CA	LEU	A	318	73.159	83.523	82.380	1.00	0.00
ATOM	4995	HA	LEU	A	318	73.606	83.427	83.371	1.00	0.00
ATOM	4996	CB	LEU	A	318	74.277	83.618	81.321	1.00	0.00
ATOM	4997	HB1	LEU	A	318	74.912	84.461	81.573	1.00	0.00
ATOM	4998	HB2	LEU	A	318	73.827	83.844	80.355	1.00	0.00
ATOM	4999	CG	LEU	A	318	75.193	82.405	81.135	1.00	0.00
ATOM	5000	HG	LEU	A	318	74.618	81.679	80.578	1.00	0.00

```
ATOM   5001  CD1 LEU A 318      76.415  82.839  80.331  1.00  0.00
ATOM   5002 1HD1 LEU A 318      77.100  82.003  80.208  1.00  0.00
ATOM   5003 2HD1 LEU A 318      76.119  83.204  79.349  1.00  0.00
ATOM   5004 3HD1 LEU A 318      76.938  83.633  80.860  1.00  0.00
ATOM   5005  CD2 LEU A 318      75.680  81.712  82.401  1.00  0.00
ATOM   5006 1HD2 LEU A 318      76.467  82.302  82.838  1.00  0.00
ATOM   5007 2HD2 LEU A 318      74.867  81.600  83.114  1.00  0.00
ATOM   5008 3HD2 LEU A 318      76.087  80.733  82.161  1.00  0.00
ATOM   5009  C   LEU A 318      72.370  84.832  82.365  1.00  0.00
ATOM   5010  O   LEU A 318      71.283  84.868  81.788  1.00  0.00
ATOM   5011  N   GLU A 319      73.000  85.932  82.769  1.00  0.00
ATOM   5012  H   GLU A 319      73.868  85.802  83.290  1.00  0.00
ATOM   5013  CA  GLU A 319      72.746  87.239  82.142  1.00  0.00
ATOM   5014  HA  GLU A 319      71.867  87.151  81.512  1.00  0.00
ATOM   5015  CB  GLU A 319      72.454  88.307  83.212  1.00  0.00
ATOM   5016  HB1 GLU A 319      73.260  89.043  83.224  1.00  0.00
ATOM   5017  HB2 GLU A 319      72.417  87.847  84.201  1.00  0.00
ATOM   5018  CG  GLU A 319      71.116  89.034  82.977  1.00  0.00
ATOM   5019  HG1 GLU A 319      71.202  89.618  82.058  1.00  0.00
ATOM   5020  HG2 GLU A 319      70.951  89.734  83.798  1.00  0.00
ATOM   5021  CD  GLU A 319      69.914  88.075  82.867  1.00  0.00
ATOM   5022  OE1 GLU A 319      69.532  87.769  81.707  1.00  0.00
ATOM   5023  OE2 GLU A 319      69.391  87.633  83.918  1.00  0.00
ATOM   5024  C   GLU A 319      73.876  87.600  81.131  1.00  0.00
ATOM   5025  O   GLU A 319      73.671  88.168  80.055  1.00  0.00
ATOM   5026  N   GLU A 320      75.087  87.101  81.361  1.00  0.00
ATOM   5027  H   GLU A 320      75.238  86.580  82.225  1.00  0.00
ATOM   5028  CA  GLU A 320      76.259  87.396  80.527  1.00  0.00
ATOM   5029  HA  GLU A 320      76.304  88.476  80.370  1.00  0.00
ATOM   5030  CB  GLU A 320      77.534  86.970  81.305  1.00  0.00
ATOM   5031  HB1 GLU A 320      78.427  87.265  80.750  1.00  0.00
ATOM   5032  HB2 GLU A 320      77.537  85.880  81.367  1.00  0.00
ATOM   5033  CG  GLU A 320      77.722  87.490  82.741  1.00  0.00
ATOM   5034  HG1 GLU A 320      78.451  86.853  83.241  1.00  0.00
ATOM   5035  HG2 GLU A 320      76.786  87.405  83.287  1.00  0.00
ATOM   5036  CD  GLU A 320      78.183  88.938  82.824  1.00  0.00
ATOM   5037  OE1 GLU A 320      79.234  89.229  83.436  1.00  0.00
ATOM   5038  OE2 GLU A 320      77.377  89.814  82.436  1.00  0.00
ATOM   5039  C   GLU A 320      76.311  86.721  79.126  1.00  0.00
ATOM   5040  O   GLU A 320      77.409  86.497  78.607  1.00  0.00
ATOM   5041  N   LYS A 321      75.176  86.345  78.512  1.00  0.00
ATOM   5042  H   LYS A 321      74.322  86.630  78.979  1.00  0.00
ATOM   5043  CA  LYS A 321      75.101  85.597  77.230  1.00  0.00
ATOM   5044  HA  LYS A 321      75.350  84.552  77.419  1.00  0.00
ATOM   5045  CB  LYS A 321      73.663  85.668  76.653  1.00  0.00
ATOM   5046  HB1 LYS A 321      73.705  85.194  75.671  1.00  0.00
ATOM   5047  HB2 LYS A 321      73.396  86.712  76.483  1.00  0.00
ATOM   5048  CG  LYS A 321      72.517  84.974  77.421  1.00  0.00
ATOM   5049  HG1 LYS A 321      72.873  84.031  77.833  1.00  0.00
ATOM   5050  HG2 LYS A 321      71.761  84.732  76.672  1.00  0.00
ATOM   5051  CD  LYS A 321      71.821  85.794  78.530  1.00  0.00
ATOM   5052  HD1 LYS A 321      71.805  86.852  78.272  1.00  0.00
ATOM   5053  HD2 LYS A 321      72.361  85.664  79.465  1.00  0.00
ATOM   5054  CE  LYS A 321      70.364  85.305  78.681  1.00  0.00
ATOM   5055  HE1 LYS A 321      70.375  84.213  78.728  1.00  0.00
ATOM   5056  HE2 LYS A 321      69.814  85.592  77.781  1.00  0.00
ATOM   5057  NZ  LYS A 321      69.653  85.818  79.879  1.00  0.00
ATOM   5058  HZ1 LYS A 321      69.708  86.822  80.054  1.00  0.00
ATOM   5059  HZ2 LYS A 321      68.672  85.573  79.857  1.00  0.00
ATOM   5060  HZ3 LYS A 321      70.026  85.419  80.739  1.00  0.00
ATOM   5061  C   LYS A 321      76.110  86.085  76.172  1.00  0.00
```

ATOM	5062	O	LYS	A	321	76.957	85.325	75.686	1.00	0.00
ATOM	5063	N	ASP	A	322	76.045	87.373	75.840	1.00	0.00
ATOM	5064	H	ASP	A	322	75.359	87.949	76.296	1.00	0.00
ATOM	5065	CA	ASP	A	322	76.905	87.976	74.816	1.00	0.00
ATOM	5066	HA	ASP	A	322	76.780	87.400	73.897	1.00	0.00
ATOM	5067	CB	ASP	A	322	76.435	89.418	74.532	1.00	0.00
ATOM	5068	HB1	ASP	A	322	76.790	90.076	75.329	1.00	0.00
ATOM	5069	HB2	ASP	A	322	75.343	89.437	74.556	1.00	0.00
ATOM	5070	CG	ASP	A	322	76.857	90.000	73.166	1.00	0.00
ATOM	5071	OD1	ASP	A	322	76.707	91.225	72.958	1.00	0.00
ATOM	5072	OD2	ASP	A	322	77.253	89.258	72.240	1.00	0.00
ATOM	5073	C	ASP	A	322	78.389	87.902	75.185	1.00	0.00
ATOM	5074	O	ASP	A	322	79.190	87.510	74.330	1.00	0.00
ATOM	5075	N	HIS	A	323	78.783	88.168	76.450	1.00	0.00
ATOM	5076	H	HIS	A	323	78.117	88.351	77.190	1.00	0.00
ATOM	5077	CA	HIS	A	323	80.244	88.186	76.697	1.00	0.00
ATOM	5078	HA	HIS	A	323	80.694	88.353	75.718	1.00	0.00
ATOM	5079	CB	HIS	A	323	80.778	89.427	77.423	1.00	0.00
ATOM	5080	HB1	HIS	A	323	80.973	89.196	78.470	1.00	0.00
ATOM	5081	HB2	HIS	A	323	80.028	90.219	77.377	1.00	0.00
ATOM	5082	CG	HIS	A	323	82.046	89.927	76.743	1.00	0.00
ATOM	5083	ND1	HIS	A	323	82.130	90.973	75.809	1.00	0.00
ATOM	5084	CE1	HIS	A	323	83.407	91.016	75.406	1.00	0.00
ATOM	5085	HE1	HIS	A	323	83.820	91.755	74.740	1.00	0.00
ATOM	5086	NE2	HIS	A	323	84.104	90.013	75.954	1.00	0.00
ATOM	5087	HE2	HIS	A	323	85.106	89.893	75.880	1.00	0.00
ATOM	5088	CD2	HIS	A	323	83.267	89.324	76.802	1.00	0.00
ATOM	5089	HD2	HIS	A	323	83.511	88.452	77.382	1.00	0.00
ATOM	5090	C	HIS	A	323	80.868	86.839	77.053	1.00	0.00
ATOM	5091	O	HIS	A	323	82.032	86.628	76.719	1.00	0.00
ATOM	5092	N	ILE	A	324	80.107	85.865	77.556	1.00	0.00
ATOM	5093	H	ILE	A	324	79.157	86.081	77.847	1.00	0.00
ATOM	5094	CA	ILE	A	324	80.515	84.453	77.501	1.00	0.00
ATOM	5095	HA	ILE	A	324	81.503	84.380	77.940	1.00	0.00
ATOM	5096	CB	ILE	A	324	79.567	83.582	78.351	1.00	0.00
ATOM	5097	HB	ILE	A	324	78.549	83.939	78.186	1.00	0.00
ATOM	5098	CG2	ILE	A	324	79.578	82.088	77.972	1.00	0.00
ATOM	5099	1HG2	ILE	A	324	78.787	81.564	78.499	1.00	0.00
ATOM	5100	2HG2	ILE	A	324	79.393	81.956	76.915	1.00	0.00
ATOM	5101	3HG2	ILE	A	324	80.537	81.624	78.169	1.00	0.00
ATOM	5102	CG1	ILE	A	324	79.902	83.765	79.849	1.00	0.00
ATOM	5103	1HG1	ILE	A	324	79.155	83.239	80.438	1.00	0.00
ATOM	5104	2HG1	ILE	A	324	79.825	84.820	80.113	1.00	0.00
ATOM	5105	CD	ILE	A	324	81.282	83.243	80.280	1.00	0.00
ATOM	5106	HD1	ILE	A	324	82.088	83.861	79.891	1.00	0.00
ATOM	5107	HD2	ILE	A	324	81.344	83.227	81.356	1.00	0.00
ATOM	5108	HD3	ILE	A	324	81.421	82.219	79.973	1.00	0.00
ATOM	5109	C	ILE	A	324	80.696	83.971	76.051	1.00	0.00
ATOM	5110	O	ILE	A	324	81.687	83.288	75.768	1.00	0.00
ATOM	5111	N	HIS	A	325	79.850	84.393	75.096	1.00	0.00
ATOM	5112	H	HIS	A	325	79.073	84.999	75.334	1.00	0.00
ATOM	5113	CA	HIS	A	325	80.126	84.074	73.684	1.00	0.00
ATOM	5114	HA	HIS	A	325	80.283	82.996	73.596	1.00	0.00
ATOM	5115	CB	HIS	A	325	78.920	84.431	72.804	1.00	0.00
ATOM	5116	HB1	HIS	A	325	78.766	85.509	72.799	1.00	0.00
ATOM	5117	HB2	HIS	A	325	78.023	83.965	73.206	1.00	0.00
ATOM	5118	CG	HIS	A	325	79.104	83.957	71.386	1.00	0.00
ATOM	5119	ND1	HIS	A	325	79.262	82.624	71.002	1.00	0.00
ATOM	5120	CE1	HIS	A	325	79.524	82.648	69.689	1.00	0.00
ATOM	5121	HE1	HIS	A	325	79.726	81.774	69.086	1.00	0.00
ATOM	5122	NE2	HIS	A	325	79.516	83.913	69.230	1.00	0.00

```
ATOM   5123  HE2 HIS A 325      79.720  84.190  68.282  1.00  0.00
ATOM   5124  CD2 HIS A 325      79.258  84.755  70.290  1.00  0.00
ATOM   5125  HD2 HIS A 325      79.223  85.831  70.283  1.00  0.00
ATOM   5126  C   HIS A 325      81.412  84.734  73.150  1.00  0.00
ATOM   5127  O   HIS A 325      82.178  84.117  72.404  1.00  0.00
ATOM   5128  N   ARG A 326      81.710  85.983  73.548  1.00  0.00
ATOM   5129  H   ARG A 326      81.028  86.488  74.106  1.00  0.00
ATOM   5130  CA  ARG A 326      82.977  86.625  73.133  1.00  0.00
ATOM   5131  HA  ARG A 326      83.101  86.408  72.071  1.00  0.00
ATOM   5132  CB  ARG A 326      82.905  88.154  73.245  1.00  0.00
ATOM   5133  HB1 ARG A 326      83.886  88.571  73.013  1.00  0.00
ATOM   5134  HB2 ARG A 326      82.631  88.432  74.260  1.00  0.00
ATOM   5135  CG  ARG A 326      81.870  88.740  72.260  1.00  0.00
ATOM   5136  HG1 ARG A 326      80.874  88.511  72.634  1.00  0.00
ATOM   5137  HG2 ARG A 326      81.977  88.260  71.286  1.00  0.00
ATOM   5138  CD  ARG A 326      81.974  90.261  72.075  1.00  0.00
ATOM   5139  HD1 ARG A 326      82.122  90.732  73.046  1.00  0.00
ATOM   5140  HD2 ARG A 326      81.021  90.613  71.674  1.00  0.00
ATOM   5141  NE  ARG A 326      83.055  90.648  71.137  1.00  0.00
ATOM   5142  HE  ARG A 326      83.609  89.902  70.750  1.00  0.00
ATOM   5143  CZ  ARG A 326      83.351  91.882  70.747  1.00  0.00
ATOM   5144  NH1 ARG A 326      82.762  92.939  71.217  1.00  0.00
ATOM   5145 1HH1 ARG A 326      81.992  92.851  71.849  1.00  0.00
ATOM   5146 2HH1 ARG A 326      83.066  93.875  70.934  1.00  0.00
ATOM   5147  NH2 ARG A 326      84.260  92.087  69.849  1.00  0.00
ATOM   5148 1HH2 ARG A 326      84.739  91.324  69.384  1.00  0.00
ATOM   5149 2HH2 ARG A 326      84.492  93.049  69.590  1.00  0.00
ATOM   5150  C   ARG A 326      84.244  86.019  73.755  1.00  0.00
ATOM   5151  O   ARG A 326      85.289  86.019  73.099  1.00  0.00
ATOM   5152  N   VAL A 327      84.195  85.458  74.964  1.00  0.00
ATOM   5153  H   VAL A 327      83.338  85.507  75.503  1.00  0.00
ATOM   5154  CA  VAL A 327      85.355  84.667  75.436  1.00  0.00
ATOM   5155  HA  VAL A 327      86.257  85.160  75.073  1.00  0.00
ATOM   5156  CB  VAL A 327      85.537  84.594  76.943  1.00  0.00
ATOM   5157  HB  VAL A 327      86.204  83.766  77.160  1.00  0.00
ATOM   5158  CG1 VAL A 327      86.195  85.893  77.413  1.00  0.00
ATOM   5159 1HG1 VAL A 327      86.574  85.770  78.420  1.00  0.00
ATOM   5160 2HG1 VAL A 327      87.057  86.127  76.788  1.00  0.00
ATOM   5161 3HG1 VAL A 327      85.480  86.710  77.367  1.00  0.00
ATOM   5162  CG2 VAL A 327      84.228  84.321  77.669  1.00  0.00
ATOM   5163 1HG2 VAL A 327      83.699  83.522  77.159  1.00  0.00
ATOM   5164 2HG2 VAL A 327      84.426  84.032  78.687  1.00  0.00
ATOM   5165 3HG2 VAL A 327      83.626  85.216  77.697  1.00  0.00
ATOM   5166  C   VAL A 327      85.446  83.282  74.818  1.00  0.00
ATOM   5167  O   VAL A 327      86.566  82.857  74.562  1.00  0.00
ATOM   5168  N   LEU A 328      84.348  82.614  74.458  1.00  0.00
ATOM   5169  H   LEU A 328      83.439  82.968  74.734  1.00  0.00
ATOM   5170  CA  LEU A 328      84.407  81.413  73.607  1.00  0.00
ATOM   5171  HA  LEU A 328      84.955  80.628  74.129  1.00  0.00
ATOM   5172  CB  LEU A 328      82.947  80.949  73.398  1.00  0.00
ATOM   5173  HB1 LEU A 328      82.345  81.806  73.113  1.00  0.00
ATOM   5174  HB2 LEU A 328      82.558  80.598  74.349  1.00  0.00
ATOM   5175  CG  LEU A 328      82.705  79.864  72.338  1.00  0.00
ATOM   5176  HG  LEU A 328      83.015  80.228  71.360  1.00  0.00
ATOM   5177  CD1 LEU A 328      83.468  78.583  72.660  1.00  0.00
ATOM   5178 1HD1 LEU A 328      83.258  77.844  71.889  1.00  0.00
ATOM   5179 2HD1 LEU A 328      84.540  78.776  72.671  1.00  0.00
ATOM   5180 3HD1 LEU A 328      83.161  78.203  73.634  1.00  0.00
ATOM   5181  CD2 LEU A 328      81.209  79.539  72.287  1.00  0.00
ATOM   5182 1HD2 LEU A 328      81.019  78.781  71.530  1.00  0.00
ATOM   5183 2HD2 LEU A 328      80.880  79.177  73.257  1.00  0.00
```

ATOM	5184	3HD2	LEU	A	328	80.649	80.440	72.033	1.00	0.00
ATOM	5185	C	LEU	A	328	85.143	81.674	72.261	1.00	0.00
ATOM	5186	O	LEU	A	328	86.040	80.928	71.849	1.00	0.00
ATOM	5187	N	ASP	A	329	84.801	82.795	71.615	1.00	0.00
ATOM	5188	H	ASP	A	329	84.015	83.325	71.984	1.00	0.00
ATOM	5189	CA	ASP	A	329	85.499	83.349	70.442	1.00	0.00
ATOM	5190	HA	ASP	A	329	85.409	82.646	69.612	1.00	0.00
ATOM	5191	CB	ASP	A	329	84.764	84.654	70.059	1.00	0.00
ATOM	5192	HB1	ASP	A	329	84.604	85.246	70.948	1.00	0.00
ATOM	5193	HB2	ASP	A	329	83.773	84.385	69.690	1.00	0.00
ATOM	5194	CG	ASP	A	329	85.416	85.579	69.012	1.00	0.00
ATOM	5195	OD1	ASP	A	329	85.762	86.747	69.329	1.00	0.00
ATOM	5196	OD2	ASP	A	329	85.328	85.270	67.805	1.00	0.00
ATOM	5197	C	ASP	A	329	86.996	83.564	70.688	1.00	0.00
ATOM	5198	O	ASP	A	329	87.829	83.155	69.870	1.00	0.00
ATOM	5199	N	LYS	A	330	87.361	84.142	71.837	1.00	0.00
ATOM	5200	H	LYS	A	330	86.641	84.507	72.450	1.00	0.00
ATOM	5201	CA	LYS	A	330	88.773	84.416	72.126	1.00	0.00
ATOM	5202	HA	LYS	A	330	89.203	84.670	71.160	1.00	0.00
ATOM	5203	CB	LYS	A	330	88.880	85.685	72.985	1.00	0.00
ATOM	5204	HB1	LYS	A	330	89.441	85.479	73.882	1.00	0.00
ATOM	5205	HB2	LYS	A	330	87.891	86.006	73.309	1.00	0.00
ATOM	5206	CG	LYS	A	330	89.515	86.837	72.182	1.00	0.00
ATOM	5207	HG1	LYS	A	330	89.445	87.756	72.764	1.00	0.00
ATOM	5208	HG2	LYS	A	330	88.960	86.982	71.254	1.00	0.00
ATOM	5209	CD	LYS	A	330	90.992	86.570	71.857	1.00	0.00
ATOM	5210	HD1	LYS	A	330	91.103	85.625	71.338	1.00	0.00
ATOM	5211	HD2	LYS	A	330	91.513	86.490	72.803	1.00	0.00
ATOM	5212	CE	LYS	A	330	91.618	87.664	70.985	1.00	0.00
ATOM	5213	HE1	LYS	A	330	91.535	88.619	71.508	1.00	0.00
ATOM	5214	HE2	LYS	A	330	91.072	87.716	70.041	1.00	0.00
ATOM	5215	NZ	LYS	A	330	93.041	87.365	70.701	1.00	0.00
ATOM	5216	HZ1	LYS	A	330	93.490	88.124	70.213	1.00	0.00
ATOM	5217	HZ2	LYS	A	330	93.530	87.228	71.584	1.00	0.00
ATOM	5218	HZ3	LYS	A	330	93.126	86.515	70.131	1.00	0.00
ATOM	5219	C	LYS	A	330	89.616	83.193	72.553	1.00	0.00
ATOM	5220	O	LYS	A	330	90.805	83.136	72.219	1.00	0.00
ATOM	5221	N	ILE	A	331	89.013	82.143	73.122	1.00	0.00
ATOM	5222	H	ILE	A	331	88.056	82.250	73.436	1.00	0.00
ATOM	5223	CA	ILE	A	331	89.680	80.831	73.252	1.00	0.00
ATOM	5224	HA	ILE	A	331	90.650	80.986	73.726	1.00	0.00
ATOM	5225	CB	ILE	A	331	88.930	79.735	74.049	1.00	0.00
ATOM	5226	HB	ILE	A	331	88.151	79.311	73.414	1.00	0.00
ATOM	5227	CG2	ILE	A	331	89.928	78.605	74.397	1.00	0.00
ATOM	5228	1HG2	ILE	A	331	89.465	77.871	75.056	1.00	0.00
ATOM	5229	2HG2	ILE	A	331	90.254	78.091	73.493	1.00	0.00
ATOM	5230	3HG2	ILE	A	331	90.812	79.015	74.887	1.00	0.00
ATOM	5231	CG1	ILE	A	331	88.227	80.186	75.326	1.00	0.00
ATOM	5232	1HG1	ILE	A	331	88.005	79.311	75.933	1.00	0.00
ATOM	5233	2HG1	ILE	A	331	87.275	80.626	75.050	1.00	0.00
ATOM	5234	CD	ILE	A	331	89.024	81.173	76.161	1.00	0.00
ATOM	5235	HD1	ILE	A	331	88.595	81.209	77.155	1.00	0.00
ATOM	5236	HD2	ILE	A	331	90.056	80.844	76.216	1.00	0.00
ATOM	5237	HD3	ILE	A	331	88.984	82.174	75.736	1.00	0.00
ATOM	5238	C	ILE	A	331	89.906	80.246	71.867	1.00	0.00
ATOM	5239	O	ILE	A	331	90.994	79.750	71.595	1.00	0.00
ATOM	5240	N	THR	A	332	88.909	80.309	70.977	1.00	0.00
ATOM	5241	H	THR	A	332	88.027	80.723	71.252	1.00	0.00
ATOM	5242	CA	THR	A	332	89.084	79.754	69.621	1.00	0.00
ATOM	5243	HA	THR	A	332	89.351	78.702	69.724	1.00	0.00
ATOM	5244	CB	THR	A	332	87.781	79.819	68.799	1.00	0.00

```
ATOM   5245  HB  THR A 332      87.482  80.856  68.660  1.00  0.00
ATOM   5246  CG2 THR A 332      87.916  79.147  67.432  1.00  0.00
ATOM   5247 1HG2 THR A 332      86.944  79.139  66.939  1.00  0.00
ATOM   5248 2HG2 THR A 332      88.616  79.704  66.812  1.00  0.00
ATOM   5249 3HG2 THR A 332      88.268  78.123  67.557  1.00  0.00
ATOM   5250  OG1 THR A 332      86.753  79.120  69.461  1.00  0.00
ATOM   5251  HG1 THR A 332      86.448  79.676  70.192  1.00  0.00
ATOM   5252  C   THR A 332      90.229  80.437  68.859  1.00  0.00
ATOM   5253  O   THR A 332      91.073  79.747  68.279  1.00  0.00
ATOM   5254  N   ASP A 333      90.313  81.769  68.906  1.00  0.00
ATOM   5255  H   ASP A 333      89.559  82.270  69.365  1.00  0.00
ATOM   5256  CA  ASP A 333      91.444  82.577  68.407  1.00  0.00
ATOM   5257  HA  ASP A 333      91.488  82.514  67.321  1.00  0.00
ATOM   5258  CB  ASP A 333      91.138  84.038  68.784  1.00  0.00
ATOM   5259  HB1 ASP A 333      90.761  84.047  69.800  1.00  0.00
ATOM   5260  HB2 ASP A 333      90.327  84.388  68.140  1.00  0.00
ATOM   5261  CG  ASP A 333      92.288  85.059  68.740  1.00  0.00
ATOM   5262  OD1 ASP A 333      93.319  84.886  69.430  1.00  0.00
ATOM   5263  OD2 ASP A 333      92.048  86.175  68.232  1.00  0.00
ATOM   5264  C   ASP A 333      92.800  82.116  68.953  1.00  0.00
ATOM   5265  O   ASP A 333      93.759  81.869  68.211  1.00  0.00
ATOM   5266  N   THR A 334      92.881  81.975  70.271  1.00  0.00
ATOM   5267  H   THR A 334      92.075  82.184  70.847  1.00  0.00
ATOM   5268  CA  THR A 334      94.176  81.729  70.896  1.00  0.00
ATOM   5269  HA  THR A 334      94.918  82.336  70.375  1.00  0.00
ATOM   5270  CB  THR A 334      94.149  82.225  72.327  1.00  0.00
ATOM   5271  HB  THR A 334      95.066  81.891  72.787  1.00  0.00
ATOM   5272  CG2 THR A 334      94.098  83.748  72.418  1.00  0.00
ATOM   5273 1HG2 THR A 334      94.276  84.077  73.438  1.00  0.00
ATOM   5274 2HG2 THR A 334      94.849  84.179  71.762  1.00  0.00
ATOM   5275 3HG2 THR A 334      93.131  84.125  72.092  1.00  0.00
ATOM   5276  OG1 THR A 334      93.046  81.709  73.024  1.00  0.00
ATOM   5277  HG1 THR A 334      92.281  82.270  72.812  1.00  0.00
ATOM   5278  C   THR A 334      94.652  80.282  70.749  1.00  0.00
ATOM   5279  O   THR A 334      95.849  80.049  70.594  1.00  0.00
ATOM   5280  N   LEU A 335      93.743  79.312  70.661  1.00  0.00
ATOM   5281  H   LEU A 335      92.776  79.572  70.857  1.00  0.00
ATOM   5282  CA  LEU A 335      94.004  77.942  70.202  1.00  0.00
ATOM   5283  HA  LEU A 335      94.788  77.512  70.828  1.00  0.00
ATOM   5284  CB  LEU A 335      92.721  77.113  70.398  1.00  0.00
ATOM   5285  HB1 LEU A 335      91.893  77.644  69.930  1.00  0.00
ATOM   5286  HB2 LEU A 335      92.514  77.050  71.467  1.00  0.00
ATOM   5287  CG  LEU A 335      92.752  75.689  69.810  1.00  0.00
ATOM   5288  HG  LEU A 335      92.858  75.754  68.727  1.00  0.00
ATOM   5289  CD1 LEU A 335      93.883  74.815  70.354  1.00  0.00
ATOM   5290 1HD1 LEU A 335      93.819  73.814  69.924  1.00  0.00
ATOM   5291 2HD1 LEU A 335      94.850  75.227  70.069  1.00  0.00
ATOM   5292 3HD1 LEU A 335      93.826  74.748  71.439  1.00  0.00
ATOM   5293  CD2 LEU A 335      91.415  75.010  70.109  1.00  0.00
ATOM   5294 1HD2 LEU A 335      91.396  74.024  69.647  1.00  0.00
ATOM   5295 2HD2 LEU A 335      91.283  74.906  71.185  1.00  0.00
ATOM   5296 3HD2 LEU A 335      90.595  75.602  69.703  1.00  0.00
ATOM   5297  C   LEU A 335      94.506  77.909  68.743  1.00  0.00
ATOM   5298  O   LEU A 335      95.556  77.300  68.485  1.00  0.00
ATOM   5299  N   ILE A 336      93.841  78.585  67.787  1.00  0.00
ATOM   5300  H   ILE A 336      92.998  79.101  68.024  1.00  0.00
ATOM   5301  CA  ILE A 336      94.383  78.612  66.413  1.00  0.00
ATOM   5302  HA  ILE A 336      94.523  77.574  66.137  1.00  0.00
ATOM   5303  CB  ILE A 336      93.425  79.183  65.339  1.00  0.00
ATOM   5304  HB  ILE A 336      92.527  78.564  65.393  1.00  0.00
ATOM   5305  CG2 ILE A 336      92.963  80.627  65.546  1.00  0.00
```

```
ATOM   5306 1HG2 ILE A 336      92.310  80.945  64.735  1.00  0.00
ATOM   5307 2HG2 ILE A 336      92.394  80.684  66.465  1.00  0.00
ATOM   5308 3HG2 ILE A 336      93.810  81.308  65.616  1.00  0.00
ATOM   5309  CG1 ILE A 336      94.001  78.984  63.910  1.00  0.00
ATOM   5310 1HG1 ILE A 336      93.168  78.785  63.245  1.00  0.00
ATOM   5311 2HG1 ILE A 336      94.623  78.093  63.902  1.00  0.00
ATOM   5312  CD  ILE A 336      94.817  80.134  63.300  1.00  0.00
ATOM   5313  HD1 ILE A 336      95.628  80.431  63.962  1.00  0.00
ATOM   5314  HD2 ILE A 336      95.245  79.807  62.353  1.00  0.00
ATOM   5315  HD3 ILE A 336      94.179  80.997  63.110  1.00  0.00
ATOM   5316  C   ILE A 336      95.794  79.200  66.359  1.00  0.00
ATOM   5317  O   ILE A 336      96.657  78.644  65.676  1.00  0.00
ATOM   5318  N   HIS A 337      96.077  80.251  67.135  1.00  0.00
ATOM   5319  H   HIS A 337      95.324  80.715  67.639  1.00  0.00
ATOM   5320  CA  HIS A 337      97.446  80.757  67.263  1.00  0.00
ATOM   5321  HA  HIS A 337      97.814  80.999  66.267  1.00  0.00
ATOM   5322  CB  HIS A 337      97.445  82.049  68.087  1.00  0.00
ATOM   5323  HB1 HIS A 337      97.188  81.821  69.119  1.00  0.00
ATOM   5324  HB2 HIS A 337      96.670  82.715  67.700  1.00  0.00
ATOM   5325  CG  HIS A 337      98.765  82.781  68.048  1.00  0.00
ATOM   5326  ND1 HIS A 337      99.875  82.527  68.863  1.00  0.00
ATOM   5327  CE1 HIS A 337     100.803  83.428  68.493  1.00  0.00
ATOM   5328  HE1 HIS A 337     101.786  83.514  68.933  1.00  0.00
ATOM   5329  NE2 HIS A 337     100.337  84.216  67.510  1.00  0.00
ATOM   5330  HE2 HIS A 337     100.845  84.972  67.065  1.00  0.00
ATOM   5331  CD2 HIS A 337      99.052  83.824  67.215  1.00  0.00
ATOM   5332  HD2 HIS A 337      98.391  84.261  66.476  1.00  0.00
ATOM   5333  C   HIS A 337      98.431  79.715  67.835  1.00  0.00
ATOM   5334  O   HIS A 337      99.545  79.597  67.342  1.00  0.00
ATOM   5335  N   LEU A 338      98.014  78.945  68.853  1.00  0.00
ATOM   5336  H   LEU A 338      97.058  79.071  69.177  1.00  0.00
ATOM   5337  CA  LEU A 338      98.818  77.894  69.500  1.00  0.00
ATOM   5338  HA  LEU A 338      99.701  78.346  69.948  1.00  0.00
ATOM   5339  CB  LEU A 338      97.975  77.209  70.608  1.00  0.00
ATOM   5340  HB1 LEU A 338      97.274  76.511  70.150  1.00  0.00
ATOM   5341  HB2 LEU A 338      97.369  77.965  71.102  1.00  0.00
ATOM   5342  CG  LEU A 338      98.754  76.451  71.702  1.00  0.00
ATOM   5343  HG  LEU A 338      99.505  77.114  72.132  1.00  0.00
ATOM   5344  CD1 LEU A 338      97.762  76.062  72.798  1.00  0.00
ATOM   5345 1HD1 LEU A 338      98.267  75.483  73.566  1.00  0.00
ATOM   5346 2HD1 LEU A 338      97.324  76.945  73.257  1.00  0.00
ATOM   5347 3HD1 LEU A 338      96.966  75.446  72.381  1.00  0.00
ATOM   5348  CD2 LEU A 338      99.439  75.174  71.209  1.00  0.00
ATOM   5349 1HD2 LEU A 338      99.863  74.625  72.053  1.00  0.00
ATOM   5350 2HD2 LEU A 338      98.719  74.534  70.695  1.00  0.00
ATOM   5351 3HD2 LEU A 338     100.257  75.423  70.541  1.00  0.00
ATOM   5352  C   LEU A 338      99.318  76.885  68.471  1.00  0.00
ATOM   5353  O   LEU A 338     100.531  76.680  68.348  1.00  0.00
ATOM   5354  N   MET A 339      98.386  76.327  67.697  1.00  0.00
ATOM   5355  H   MET A 339      97.413  76.553  67.881  1.00  0.00
ATOM   5356  CA  MET A 339      98.695  75.388  66.613  1.00  0.00
ATOM   5357  HA  MET A 339      99.274  74.563  67.025  1.00  0.00
ATOM   5358  CB  MET A 339      97.382  74.837  66.072  1.00  0.00
ATOM   5359  HB1 MET A 339      96.804  75.691  65.732  1.00  0.00
ATOM   5360  HB2 MET A 339      96.843  74.363  66.891  1.00  0.00
ATOM   5361  CG  MET A 339      97.483  73.840  64.903  1.00  0.00
ATOM   5362  HG1 MET A 339      97.819  74.371  64.013  1.00  0.00
ATOM   5363  HG2 MET A 339      96.476  73.482  64.722  1.00  0.00
ATOM   5364  SD  MET A 339      98.481  72.348  65.083  1.00  0.00
ATOM   5365  CE  MET A 339      98.106  71.934  66.795  1.00  0.00
ATOM   5366  HE1 MET A 339      98.422  70.918  67.013  1.00  0.00
```

ATOM	5367	HE2	MET	A	339	98.607	72.608	67.485	1.00	0.00
ATOM	5368	HE3	MET	A	339	97.033	72.017	66.937	1.00	0.00
ATOM	5369	C	MET	A	339	99.542	76.015	65.498	1.00	0.00
ATOM	5370	O	MET	A	339	100.469	75.387	64.994	1.00	0.00
ATOM	5371	N	ALA	A	340	99.299	77.285	65.177	1.00	0.00
ATOM	5372	H	ALA	A	340	98.502	77.754	65.596	1.00	0.00
ATOM	5373	CA	ALA	A	340	100.170	78.067	64.302	1.00	0.00
ATOM	5374	HA	ALA	A	340	100.431	77.448	63.442	1.00	0.00
ATOM	5375	CB	ALA	A	340	99.363	79.269	63.789	1.00	0.00
ATOM	5376	HB1	ALA	A	340	99.927	79.787	63.013	1.00	0.00
ATOM	5377	HB2	ALA	A	340	98.412	78.935	63.374	1.00	0.00
ATOM	5378	HB3	ALA	A	340	99.173	79.963	64.606	1.00	0.00
ATOM	5379	C	ALA	A	340	101.510	78.518	64.953	1.00	0.00
ATOM	5380	O	ALA	A	340	102.209	79.339	64.357	1.00	0.00
ATOM	5381	N	LYS	A	341	101.897	78.040	66.155	1.00	0.00
ATOM	5382	H	LYS	A	341	101.267	77.404	66.630	1.00	0.00
ATOM	5383	CA	LYS	A	341	103.100	78.536	66.869	1.00	0.00
ATOM	5384	HA	LYS	A	341	103.738	79.046	66.144	1.00	0.00
ATOM	5385	CB	LYS	A	341	102.641	79.597	67.902	1.00	0.00
ATOM	5386	HB1	LYS	A	341	101.937	79.146	68.601	1.00	0.00
ATOM	5387	HB2	LYS	A	341	102.101	80.367	67.350	1.00	0.00
ATOM	5388	CG	LYS	A	341	103.754	80.316	68.701	1.00	0.00
ATOM	5389	HG1	LYS	A	341	103.431	81.342	68.878	1.00	0.00
ATOM	5390	HG2	LYS	A	341	104.658	80.364	68.093	1.00	0.00
ATOM	5391	CD	LYS	A	341	104.085	79.698	70.080	1.00	0.00
ATOM	5392	HD1	LYS	A	341	104.272	78.633	69.974	1.00	0.00
ATOM	5393	HD2	LYS	A	341	103.232	79.837	70.747	1.00	0.00
ATOM	5394	CE	LYS	A	341	105.332	80.366	70.682	1.00	0.00
ATOM	5395	HE1	LYS	A	341	105.140	81.437	70.790	1.00	0.00
ATOM	5396	HE2	LYS	A	341	106.156	80.241	69.974	1.00	0.00
ATOM	5397	NZ	LYS	A	341	105.726	79.796	72.000	1.00	0.00
ATOM	5398	HZ1	LYS	A	341	105.820	78.791	71.974	1.00	0.00
ATOM	5399	HZ2	LYS	A	341	106.585	80.208	72.337	1.00	0.00
ATOM	5400	HZ3	LYS	A	341	105.076	80.045	72.753	1.00	0.00
ATOM	5401	C	LYS	A	341	104.023	77.518	67.540	1.00	0.00
ATOM	5402	O	LYS	A	341	105.224	77.778	67.586	1.00	0.00
ATOM	5403	N	ALA	A	342	103.534	76.426	68.128	1.00	0.00
ATOM	5404	H	ALA	A	342	102.532	76.266	68.082	1.00	0.00
ATOM	5405	CA	ALA	A	342	104.308	75.742	69.181	1.00	0.00
ATOM	5406	HA	ALA	A	342	105.033	76.451	69.582	1.00	0.00
ATOM	5407	CB	ALA	A	342	103.369	75.435	70.362	1.00	0.00
ATOM	5408	HB1	ALA	A	342	102.914	76.352	70.724	1.00	0.00
ATOM	5409	HB2	ALA	A	342	102.582	74.751	70.074	1.00	0.00
ATOM	5410	HB3	ALA	A	342	103.933	74.982	71.174	1.00	0.00
ATOM	5411	C	ALA	A	342	105.179	74.547	68.736	1.00	0.00
ATOM	5412	O	ALA	A	342	105.909	74.003	69.557	1.00	0.00
ATOM	5413	N	GLY	A	343	105.210	74.193	67.446	1.00	0.00
ATOM	5414	H	GLY	A	343	104.610	74.671	66.791	1.00	0.00
ATOM	5415	CA	GLY	A	343	106.195	73.233	66.933	1.00	0.00
ATOM	5416	HA1	GLY	A	343	106.103	72.299	67.486	1.00	0.00
ATOM	5417	HA2	GLY	A	343	107.192	73.633	67.115	1.00	0.00
ATOM	5418	C	GLY	A	343	106.072	72.922	65.446	1.00	0.00
ATOM	5419	O	GLY	A	343	105.057	73.208	64.809	1.00	0.00
ATOM	5420	N	LEU	A	344	107.101	72.278	64.884	1.00	0.00
ATOM	5421	H	LEU	A	344	107.928	72.116	65.437	1.00	0.00
ATOM	5422	CA	LEU	A	344	107.087	71.785	63.498	1.00	0.00
ATOM	5423	HA	LEU	A	344	106.938	72.639	62.836	1.00	0.00
ATOM	5424	CB	LEU	A	344	108.454	71.140	63.178	1.00	0.00
ATOM	5425	HB1	LEU	A	344	108.428	70.082	63.447	1.00	0.00
ATOM	5426	HB2	LEU	A	344	109.213	71.616	63.800	1.00	0.00
ATOM	5427	CG	LEU	A	344	108.924	71.283	61.718	1.00	0.00

ATOM	5428	HG	LEU	A	344	109.024	72.342	61.480	1.00	0.00
ATOM	5429	CD1	LEU	A	344	110.297	70.622	61.573	1.00	0.00
ATOM	5430	1HD1	LEU	A	344	110.674	70.771	60.561	1.00	0.00
ATOM	5431	2HD1	LEU	A	344	111.004	71.070	62.271	1.00	0.00
ATOM	5432	3HD1	LEU	A	344	110.219	69.553	61.772	1.00	0.00
ATOM	5433	CD2	LEU	A	344	107.995	70.642	60.688	1.00	0.00
ATOM	5434	1HD2	LEU	A	344	108.440	70.706	59.696	1.00	0.00
ATOM	5435	2HD2	LEU	A	344	107.836	69.594	60.937	1.00	0.00
ATOM	5436	3HD2	LEU	A	344	107.042	71.163	60.667	1.00	0.00
ATOM	5437	C	LEU	A	344	105.912	70.818	63.279	1.00	0.00
ATOM	5438	O	LEU	A	344	105.138	70.991	62.345	1.00	0.00
ATOM	5439	N	THR	A	345	105.706	69.901	64.234	1.00	0.00
ATOM	5440	H	THR	A	345	106.410	69.812	64.946	1.00	0.00
ATOM	5441	CA	THR	A	345	104.547	68.992	64.298	1.00	0.00
ATOM	5442	HA	THR	A	345	104.526	68.429	63.372	1.00	0.00
ATOM	5443	CB	THR	A	345	104.725	67.940	65.409	1.00	0.00
ATOM	5444	HB	THR	A	345	105.678	67.434	65.256	1.00	0.00
ATOM	5445	CG2	THR	A	345	104.720	68.525	66.820	1.00	0.00
ATOM	5446	1HG2	THR	A	345	104.855	67.721	67.545	1.00	0.00
ATOM	5447	2HG2	THR	A	345	105.530	69.242	66.941	1.00	0.00
ATOM	5448	3HG2	THR	A	345	103.774	69.019	67.030	1.00	0.00
ATOM	5449	OG1	THR	A	345	103.704	66.974	65.337	1.00	0.00
ATOM	5450	HG1	THR	A	345	102.882	67.454	65.154	1.00	0.00
ATOM	5451	C	THR	A	345	103.180	69.677	64.378	1.00	0.00
ATOM	5452	O	THR	A	345	102.175	68.980	64.330	1.00	0.00
ATOM	5453	N	LEU	A	346	103.110	71.004	64.484	1.00	0.00
ATOM	5454	H	LEU	A	346	103.968	71.536	64.502	1.00	0.00
ATOM	5455	CA	LEU	A	346	101.860	71.764	64.559	1.00	0.00
ATOM	5456	HA	LEU	A	346	101.005	71.089	64.601	1.00	0.00
ATOM	5457	CB	LEU	A	346	101.876	72.598	65.853	1.00	0.00
ATOM	5458	HB1	LEU	A	346	100.894	73.034	65.997	1.00	0.00
ATOM	5459	HB2	LEU	A	346	102.570	73.427	65.724	1.00	0.00
ATOM	5460	CG	LEU	A	346	102.267	71.843	67.140	1.00	0.00
ATOM	5461	HG	LEU	A	346	103.322	71.576	67.086	1.00	0.00
ATOM	5462	CD1	LEU	A	346	102.073	72.801	68.305	1.00	0.00
ATOM	5463	1HD1	LEU	A	346	102.555	73.746	68.081	1.00	0.00
ATOM	5464	2HD1	LEU	A	346	101.012	72.988	68.472	1.00	0.00
ATOM	5465	3HD1	LEU	A	346	102.505	72.375	69.210	1.00	0.00
ATOM	5466	CD2	LEU	A	346	101.457	70.588	67.471	1.00	0.00
ATOM	5467	1HD2	LEU	A	346	101.895	70.091	68.335	1.00	0.00
ATOM	5468	2HD2	LEU	A	346	100.438	70.855	67.728	1.00	0.00
ATOM	5469	3HD2	LEU	A	346	101.436	69.901	66.632	1.00	0.00
ATOM	5470	C	LEU	A	346	101.704	72.614	63.285	1.00	0.00
ATOM	5471	O	LEU	A	346	100.717	72.493	62.562	1.00	0.00
ATOM	5472	N	GLN	A	347	102.774	73.321	62.899	1.00	0.00
ATOM	5473	H	GLN	A	347	103.552	73.391	63.542	1.00	0.00
ATOM	5474	CA	GLN	A	347	102.879	74.015	61.611	1.00	0.00
ATOM	5475	HA	GLN	A	347	102.045	74.717	61.571	1.00	0.00
ATOM	5476	CB	GLN	A	347	104.191	74.820	61.568	1.00	0.00
ATOM	5477	HB1	GLN	A	347	105.024	74.140	61.751	1.00	0.00
ATOM	5478	HB2	GLN	A	347	104.174	75.548	62.381	1.00	0.00
ATOM	5479	CG	GLN	A	347	104.466	75.565	60.247	1.00	0.00
ATOM	5480	HG1	GLN	A	347	104.639	74.841	59.451	1.00	0.00
ATOM	5481	HG2	GLN	A	347	105.381	76.144	60.358	1.00	0.00
ATOM	5482	CD	GLN	A	347	103.364	76.528	59.788	1.00	0.00
ATOM	5483	OE1	GLN	A	347	102.455	76.895	60.517	1.00	0.00
ATOM	5484	NE2	GLN	A	347	103.400	76.995	58.559	1.00	0.00
ATOM	5485	1HE2	GLN	A	347	104.123	76.708	57.927	1.00	0.00
ATOM	5486	2HE2	GLN	A	347	102.647	77.611	58.308	1.00	0.00
ATOM	5487	C	GLN	A	347	102.720	73.086	60.391	1.00	0.00
ATOM	5488	O	GLN	A	347	102.248	73.524	59.343	1.00	0.00

ATOM	5489	N	GLN	A	348	103.060	71.798	60.509	1.00	0.00
ATOM	5490	H	GLN	A	348	103.520	71.499	61.362	1.00	0.00
ATOM	5491	CA	GLN	A	348	102.811	70.801	59.461	1.00	0.00
ATOM	5492	HA	GLN	A	348	102.812	71.330	58.508	1.00	0.00
ATOM	5493	CB	GLN	A	348	103.982	69.807	59.377	1.00	0.00
ATOM	5494	HB1	GLN	A	348	104.910	70.376	59.442	1.00	0.00
ATOM	5495	HB2	GLN	A	348	103.953	69.338	58.394	1.00	0.00
ATOM	5496	CG	GLN	A	348	103.997	68.698	60.439	1.00	0.00
ATOM	5497	HG1	GLN	A	348	103.256	67.943	60.182	1.00	0.00
ATOM	5498	HG2	GLN	A	348	103.736	69.114	61.407	1.00	0.00
ATOM	5499	CD	GLN	A	348	105.364	68.025	60.523	1.00	0.00
ATOM	5500	OE1	GLN	A	348	105.966	67.885	61.578	1.00	0.00
ATOM	5501	NE2	GLN	A	348	105.922	67.569	59.423	1.00	0.00
ATOM	5502	1HE2	GLN	A	348	105.451	67.671	58.544	1.00	0.00
ATOM	5503	2HE2	GLN	A	348	106.817	67.124	59.527	1.00	0.00
ATOM	5504	C	GLN	A	348	101.403	70.149	59.556	1.00	0.00
ATOM	5505	O	GLN	A	348	101.170	69.097	58.967	1.00	0.00
ATOM	5506	N	GLN	A	349	100.459	70.737	60.310	1.00	0.00
ATOM	5507	H	GLN	A	349	100.715	71.572	60.828	1.00	0.00
ATOM	5508	CA	GLN	A	349	99.141	70.159	60.629	1.00	0.00
ATOM	5509	HA	GLN	A	349	98.854	69.497	59.812	1.00	0.00
ATOM	5510	CB	GLN	A	349	99.227	69.300	61.920	1.00	0.00
ATOM	5511	HB1	GLN	A	349	98.238	68.874	62.084	1.00	0.00
ATOM	5512	HB2	GLN	A	349	99.463	69.946	62.769	1.00	0.00
ATOM	5513	CG	GLN	A	349	100.207	68.112	61.919	1.00	0.00
ATOM	5514	HG1	GLN	A	349	101.227	68.486	61.861	1.00	0.00
ATOM	5515	HG2	GLN	A	349	100.026	67.493	61.041	1.00	0.00
ATOM	5516	CD	GLN	A	349	100.094	67.213	63.164	1.00	0.00
ATOM	5517	OE1	GLN	A	349	99.156	67.275	63.957	1.00	0.00
ATOM	5518	NE2	GLN	A	349	101.041	66.326	63.400	1.00	0.00
ATOM	5519	1HE2	GLN	A	349	101.859	66.287	62.818	1.00	0.00
ATOM	5520	2HE2	GLN	A	349	100.947	65.747	64.214	1.00	0.00
ATOM	5521	C	GLN	A	349	97.981	71.209	60.768	1.00	0.00
ATOM	5522	O	GLN	A	349	97.148	71.155	61.682	1.00	0.00
ATOM	5523	N	HIS	A	350	97.865	72.181	59.847	1.00	0.00
ATOM	5524	H	HIS	A	350	98.591	72.274	59.151	1.00	0.00
ATOM	5525	CA	HIS	A	350	96.812	73.229	59.932	1.00	0.00
ATOM	5526	HA	HIS	A	350	96.961	73.727	60.895	1.00	0.00
ATOM	5527	CB	HIS	A	350	97.016	74.316	58.868	1.00	0.00
ATOM	5528	HB1	HIS	A	350	96.057	74.780	58.639	1.00	0.00
ATOM	5529	HB2	HIS	A	350	97.414	73.885	57.949	1.00	0.00
ATOM	5530	CG	HIS	A	350	97.938	75.389	59.386	1.00	0.00
ATOM	5531	ND1	HIS	A	350	97.518	76.496	60.136	1.00	0.00
ATOM	5532	CE1	HIS	A	350	98.643	77.115	60.524	1.00	0.00
ATOM	5533	HE1	HIS	A	350	98.684	78.004	61.127	1.00	0.00
ATOM	5534	NE2	HIS	A	350	99.722	76.456	60.075	1.00	0.00
ATOM	5535	HE2	HIS	A	350	100.689	76.700	60.290	1.00	0.00
ATOM	5536	CD2	HIS	A	350	99.300	75.359	59.361	1.00	0.00
ATOM	5537	HD2	HIS	A	350	99.927	74.596	58.920	1.00	0.00
ATOM	5538	C	HIS	A	350	95.339	72.722	60.039	1.00	0.00
ATOM	5539	O	HIS	A	350	94.481	73.215	60.798	1.00	0.00
ATOM	5540	N	GLN	A	351	95.063	71.614	59.348	1.00	0.00
ATOM	5541	H	GLN	A	351	95.779	71.253	58.737	1.00	0.00
ATOM	5542	CA	GLN	A	351	93.813	70.867	59.510	1.00	0.00
ATOM	5543	HA	GLN	A	351	92.996	71.543	59.254	1.00	0.00
ATOM	5544	CB	GLN	A	351	93.814	69.707	58.497	1.00	0.00
ATOM	5545	HB1	GLN	A	351	94.535	68.948	58.800	1.00	0.00
ATOM	5546	HB2	GLN	A	351	94.151	70.105	57.538	1.00	0.00
ATOM	5547	CG	GLN	A	351	92.440	69.051	58.222	1.00	0.00
ATOM	5548	HG1	GLN	A	351	92.508	68.535	57.265	1.00	0.00
ATOM	5549	HG2	GLN	A	351	91.686	69.832	58.125	1.00	0.00

ATOM	5550	CD	GLN	A	351	91.975	68.022	59.253	1.00	0.00
ATOM	5551	OE1	GLN	A	351	90.835	68.012	59.695	1.00	0.00
ATOM	5552	NE2	GLN	A	351	92.805	67.082	59.649	1.00	0.00
ATOM	5553	1HE2	GLN	A	351	93.727	66.999	59.258	1.00	0.00
ATOM	5554	2HE2	GLN	A	351	92.449	66.384	60.278	1.00	0.00
ATOM	5555	C	GLN	A	351	93.556	70.393	60.962	1.00	0.00
ATOM	5556	O	GLN	A	351	92.391	70.218	61.329	1.00	0.00
ATOM	5557	N	ARG	A	352	94.597	70.231	61.803	1.00	0.00
ATOM	5558	H	ARG	A	352	95.522	70.507	61.491	1.00	0.00
ATOM	5559	CA	ARG	A	352	94.455	69.816	63.208	1.00	0.00
ATOM	5560	HA	ARG	A	352	93.990	68.831	63.186	1.00	0.00
ATOM	5561	CB	ARG	A	352	95.765	69.666	64.014	1.00	0.00
ATOM	5562	HB1	ARG	A	352	96.120	70.651	64.320	1.00	0.00
ATOM	5563	HB2	ARG	A	352	96.526	69.191	63.404	1.00	0.00
ATOM	5564	CG	ARG	A	352	95.506	68.777	65.253	1.00	0.00
ATOM	5565	HG1	ARG	A	352	95.588	67.737	64.954	1.00	0.00
ATOM	5566	HG2	ARG	A	352	94.497	68.915	65.621	1.00	0.00
ATOM	5567	CD	ARG	A	352	96.399	69.005	66.472	1.00	0.00
ATOM	5568	HD1	ARG	A	352	95.912	68.543	67.333	1.00	0.00
ATOM	5569	HD2	ARG	A	352	96.464	70.064	66.679	1.00	0.00
ATOM	5570	NE	ARG	A	352	97.729	68.398	66.307	1.00	0.00
ATOM	5571	HE	ARG	A	352	98.098	68.293	65.371	1.00	0.00
ATOM	5572	CZ	ARG	A	352	98.371	67.736	67.247	1.00	0.00
ATOM	5573	NH1	ARG	A	352	98.153	67.870	68.522	1.00	0.00
ATOM	5574	1HH1	ARG	A	352	97.592	68.673	68.840	1.00	0.00
ATOM	5575	2HH1	ARG	A	352	98.623	67.288	69.191	1.00	0.00
ATOM	5576	NH2	ARG	A	352	99.289	66.904	66.877	1.00	0.00
ATOM	5577	1HH2	ARG	A	352	99.439	66.812	65.882	1.00	0.00
ATOM	5578	2HH2	ARG	A	352	99.643	66.255	67.552	1.00	0.00
ATOM	5579	C	ARG	A	352	93.491	70.696	63.970	1.00	0.00
ATOM	5580	O	ARG	A	352	92.623	70.128	64.626	1.00	0.00
ATOM	5581	N	LEU	A	353	93.544	72.025	63.814	1.00	0.00
ATOM	5582	H	LEU	A	353	94.268	72.434	63.243	1.00	0.00
ATOM	5583	CA	LEU	A	353	92.529	72.876	64.495	1.00	0.00
ATOM	5584	HA	LEU	A	353	92.668	72.810	65.574	1.00	0.00
ATOM	5585	CB	LEU	A	353	92.564	74.349	64.028	1.00	0.00
ATOM	5586	HB1	LEU	A	353	91.635	74.808	64.370	1.00	0.00
ATOM	5587	HB2	LEU	A	353	92.544	74.389	62.938	1.00	0.00
ATOM	5588	CG	LEU	A	353	93.679	75.252	64.558	1.00	0.00
ATOM	5589	HG	LEU	A	353	93.268	76.258	64.607	1.00	0.00
ATOM	5590	CD1	LEU	A	353	94.070	74.861	65.984	1.00	0.00
ATOM	5591	1HD1	LEU	A	353	94.734	75.592	66.430	1.00	0.00
ATOM	5592	2HD1	LEU	A	353	93.181	74.801	66.609	1.00	0.00
ATOM	5593	3HD1	LEU	A	353	94.572	73.895	66.000	1.00	0.00
ATOM	5594	CD2	LEU	A	353	94.832	75.304	63.554	1.00	0.00
ATOM	5595	1HD2	LEU	A	353	95.636	75.931	63.932	1.00	0.00
ATOM	5596	2HD2	LEU	A	353	95.201	74.308	63.335	1.00	0.00
ATOM	5597	3HD2	LEU	A	353	94.490	75.739	62.617	1.00	0.00
ATOM	5598	C	LEU	A	353	91.101	72.369	64.238	1.00	0.00
ATOM	5599	O	LEU	A	353	90.325	72.075	65.161	1.00	0.00
ATOM	5600	N	ALA	A	354	90.790	72.234	62.943	1.00	0.00
ATOM	5601	H	ALA	A	354	91.495	72.371	62.236	1.00	0.00
ATOM	5602	CA	ALA	A	354	89.446	71.785	62.572	1.00	0.00
ATOM	5603	HA	ALA	A	354	88.719	72.482	62.998	1.00	0.00
ATOM	5604	CB	ALA	A	354	89.314	71.839	61.043	1.00	0.00
ATOM	5605	HB1	ALA	A	354	88.295	71.591	60.758	1.00	0.00
ATOM	5606	HB2	ALA	A	354	89.536	72.842	60.686	1.00	0.00
ATOM	5607	HB3	ALA	A	354	89.995	71.126	60.579	1.00	0.00
ATOM	5608	C	ALA	A	354	89.102	70.381	63.134	1.00	0.00
ATOM	5609	O	ALA	A	354	87.959	70.104	63.509	1.00	0.00
ATOM	5610	N	GLN	A	355	90.112	69.506	63.208	1.00	0.00

ATOM	5611	H	GLN	A	355	91.033	69.799	62.901	1.00	0.00
ATOM	5612	CA	GLN	A	355	89.972	68.148	63.728	1.00	0.00
ATOM	5613	HA	GLN	A	355	89.118	67.708	63.216	1.00	0.00
ATOM	5614	CB	GLN	A	355	91.246	67.372	63.345	1.00	0.00
ATOM	5615	HB1	GLN	A	355	92.045	67.627	64.040	1.00	0.00
ATOM	5616	HB2	GLN	A	355	91.564	67.689	62.352	1.00	0.00
ATOM	5617	CG	GLN	A	355	91.081	65.850	63.321	1.00	0.00
ATOM	5618	HG1	GLN	A	355	90.958	65.493	64.343	1.00	0.00
ATOM	5619	HG2	GLN	A	355	91.987	65.409	62.910	1.00	0.00
ATOM	5620	CD	GLN	A	355	89.894	65.352	62.493	1.00	0.00
ATOM	5621	OE1	GLN	A	355	89.160	64.486	62.943	1.00	0.00
ATOM	5622	NE2	GLN	A	355	89.630	65.869	61.305	1.00	0.00
ATOM	5623	1HE2	GLN	A	355	90.180	66.622	60.900	1.00	0.00
ATOM	5624	2HE2	GLN	A	355	88.853	65.476	60.803	1.00	0.00
ATOM	5625	C	GLN	A	355	89.653	68.088	65.238	1.00	0.00
ATOM	5626	O	GLN	A	355	88.806	67.288	65.658	1.00	0.00
ATOM	5627	N	LEU	A	356	90.270	68.972	66.032	1.00	0.00
ATOM	5628	H	LEU	A	356	90.954	69.595	65.611	1.00	0.00
ATOM	5629	CA	LEU	A	356	89.979	69.129	67.458	1.00	0.00
ATOM	5630	HA	LEU	A	356	90.047	68.153	67.939	1.00	0.00
ATOM	5631	CB	LEU	A	356	90.991	70.072	68.129	1.00	0.00
ATOM	5632	HB1	LEU	A	356	90.757	70.084	69.192	1.00	0.00
ATOM	5633	HB2	LEU	A	356	90.854	71.087	67.756	1.00	0.00
ATOM	5634	CG	LEU	A	356	92.474	69.696	67.967	1.00	0.00
ATOM	5635	HG	LEU	A	356	92.840	70.094	67.030	1.00	0.00
ATOM	5636	CD1	LEU	A	356	93.286	70.355	69.068	1.00	0.00
ATOM	5637	1HD1	LEU	A	356	94.329	70.060	68.965	1.00	0.00
ATOM	5638	2HD1	LEU	A	356	93.230	71.440	68.974	1.00	0.00
ATOM	5639	3HD1	LEU	A	356	92.917	70.061	70.047	1.00	0.00
ATOM	5640	CD2	LEU	A	356	92.740	68.187	67.972	1.00	0.00
ATOM	5641	1HD2	LEU	A	356	93.806	68.003	68.089	1.00	0.00
ATOM	5642	2HD2	LEU	A	356	92.196	67.733	68.793	1.00	0.00
ATOM	5643	3HD2	LEU	A	356	92.410	67.748	67.033	1.00	0.00
ATOM	5644	C	LEU	A	356	88.538	69.631	67.677	1.00	0.00
ATOM	5645	O	LEU	A	356	87.775	69.073	68.475	1.00	0.00
ATOM	5646	N	LEU	A	357	88.105	70.635	66.900	1.00	0.00
ATOM	5647	H	LEU	A	357	88.722	71.065	66.218	1.00	0.00
ATOM	5648	CA	LEU	A	357	86.707	71.085	67.047	1.00	0.00
ATOM	5649	HA	LEU	A	357	86.535	71.314	68.102	1.00	0.00
ATOM	5650	CB	LEU	A	357	86.475	72.389	66.254	1.00	0.00
ATOM	5651	HB1	LEU	A	357	85.463	72.731	66.477	1.00	0.00
ATOM	5652	HB2	LEU	A	357	86.517	72.161	65.189	1.00	0.00
ATOM	5653	CG	LEU	A	357	87.462	73.543	66.548	1.00	0.00
ATOM	5654	HG	LEU	A	357	88.425	73.300	66.114	1.00	0.00
ATOM	5655	CD1	LEU	A	357	86.977	74.838	65.890	1.00	0.00
ATOM	5656	1HD1	LEU	A	357	87.724	75.619	66.025	1.00	0.00
ATOM	5657	2HD1	LEU	A	357	86.828	74.672	64.824	1.00	0.00
ATOM	5658	3HD1	LEU	A	357	86.039	75.155	66.344	1.00	0.00
ATOM	5659	CD2	LEU	A	357	87.680	73.831	68.037	1.00	0.00
ATOM	5660	1HD2	LEU	A	357	88.356	74.678	68.156	1.00	0.00
ATOM	5661	2HD2	LEU	A	357	86.728	74.061	68.516	1.00	0.00
ATOM	5662	3HD2	LEU	A	357	88.135	72.971	68.522	1.00	0.00
ATOM	5663	C	LEU	A	357	85.671	69.964	66.683	1.00	0.00
ATOM	5664	O	LEU	A	357	84.658	69.729	67.365	1.00	0.00
ATOM	5665	N	LEU	A	358	85.973	69.148	65.661	1.00	0.00
ATOM	5666	H	LEU	A	358	86.767	69.373	65.070	1.00	0.00
ATOM	5667	CA	LEU	A	358	85.192	67.927	65.382	1.00	0.00
ATOM	5668	HA	LEU	A	358	84.157	68.235	65.229	1.00	0.00
ATOM	5669	CB	LEU	A	358	85.711	67.332	64.059	1.00	0.00
ATOM	5670	HB1	LEU	A	358	86.758	67.060	64.184	1.00	0.00
ATOM	5671	HB2	LEU	A	358	85.665	68.112	63.297	1.00	0.00

ATOM	5672	CG	LEU	A	358	84.959	66.096	63.530	1.00	0.00
ATOM	5673	HG	LEU	A	358	85.041	65.284	64.251	1.00	0.00
ATOM	5674	CD1	LEU	A	358	83.480	66.369	63.256	1.00	0.00
ATOM	5675	1HD1	LEU	A	358	83.014	65.489	62.813	1.00	0.00
ATOM	5676	2HD1	LEU	A	358	82.961	66.597	64.186	1.00	0.00
ATOM	5677	3HD1	LEU	A	358	83.378	67.212	62.573	1.00	0.00
ATOM	5678	CD2	LEU	A	358	85.620	65.647	62.227	1.00	0.00
ATOM	5679	1HD2	LEU	A	358	85.113	64.768	61.835	1.00	0.00
ATOM	5680	2HD2	LEU	A	358	85.572	66.440	61.483	1.00	0.00
ATOM	5681	3HD2	LEU	A	358	86.661	65.389	62.405	1.00	0.00
ATOM	5682	C	LEU	A	358	85.166	66.875	66.537	1.00	0.00
ATOM	5683	O	LEU	A	358	84.176	66.160	66.779	1.00	0.00
ATOM	5684	N	ILE	A	359	86.278	66.777	67.276	1.00	0.00
ATOM	5685	H	ILE	A	359	87.057	67.390	67.050	1.00	0.00
ATOM	5686	CA	ILE	A	359	86.334	66.061	68.563	1.00	0.00
ATOM	5687	HA	ILE	A	359	86.027	65.035	68.387	1.00	0.00
ATOM	5688	CB	ILE	A	359	87.799	66.032	69.066	1.00	0.00
ATOM	5689	HB	ILE	A	359	88.403	66.597	68.358	1.00	0.00
ATOM	5690	CG2	ILE	A	359	87.991	66.686	70.436	1.00	0.00
ATOM	5691	1HG2	ILE	A	359	89.051	66.790	70.648	1.00	0.00
ATOM	5692	2HG2	ILE	A	359	87.561	67.682	70.458	1.00	0.00
ATOM	5693	3HG2	ILE	A	359	87.502	66.098	71.208	1.00	0.00
ATOM	5694	CG1	ILE	A	359	88.367	64.592	69.059	1.00	0.00
ATOM	5695	1HG1	ILE	A	359	87.900	64.012	68.272	1.00	0.00
ATOM	5696	2HG1	ILE	A	359	88.142	64.105	70.008	1.00	0.00
ATOM	5697	CD	ILE	A	359	89.874	64.557	68.798	1.00	0.00
ATOM	5698	HD1	ILE	A	359	90.087	64.927	67.795	1.00	0.00
ATOM	5699	HD2	ILE	A	359	90.394	65.181	69.518	1.00	0.00
ATOM	5700	HD3	ILE	A	359	90.234	63.533	68.883	1.00	0.00
ATOM	5701	C	ILE	A	359	85.279	66.574	69.565	1.00	0.00
ATOM	5702	O	ILE	A	359	84.592	65.757	70.201	1.00	0.00
ATOM	5703	N	LEU	A	360	85.044	67.895	69.604	1.00	0.00
ATOM	5704	H	LEU	A	360	85.605	68.515	69.032	1.00	0.00
ATOM	5705	CA	LEU	A	360	83.959	68.445	70.450	1.00	0.00
ATOM	5706	HA	LEU	A	360	84.163	68.125	71.465	1.00	0.00
ATOM	5707	CB	LEU	A	360	83.975	69.985	70.482	1.00	0.00
ATOM	5708	HB1	LEU	A	360	83.143	70.320	71.103	1.00	0.00
ATOM	5709	HB2	LEU	A	360	83.789	70.380	69.489	1.00	0.00
ATOM	5710	CG	LEU	A	360	85.269	70.606	71.048	1.00	0.00
ATOM	5711	HG	LEU	A	360	86.141	70.225	70.515	1.00	0.00
ATOM	5712	CD1	LEU	A	360	85.224	72.124	70.877	1.00	0.00
ATOM	5713	1HD1	LEU	A	360	85.133	72.368	69.820	1.00	0.00
ATOM	5714	2HD1	LEU	A	360	84.373	72.536	71.417	1.00	0.00
ATOM	5715	3HD1	LEU	A	360	86.140	72.571	71.256	1.00	0.00
ATOM	5716	CD2	LEU	A	360	85.400	70.286	72.540	1.00	0.00
ATOM	5717	1HD2	LEU	A	360	86.179	70.892	72.991	1.00	0.00
ATOM	5718	2HD2	LEU	A	360	84.460	70.504	73.047	1.00	0.00
ATOM	5719	3HD2	LEU	A	360	85.644	69.234	72.675	1.00	0.00
ATOM	5720	C	LEU	A	360	82.547	67.918	70.118	1.00	0.00
ATOM	5721	O	LEU	A	360	81.757	67.659	71.032	1.00	0.00
ATOM	5722	N	SER	A	361	82.229	67.704	68.834	1.00	0.00
ATOM	5723	H	SER	A	361	82.923	67.888	68.120	1.00	0.00
ATOM	5724	CA	SER	A	361	80.913	67.076	68.505	1.00	0.00
ATOM	5725	HA	SER	A	361	80.116	67.706	68.901	1.00	0.00
ATOM	5726	CB	SER	A	361	80.731	66.997	66.986	1.00	0.00
ATOM	5727	HB1	SER	A	361	79.778	66.515	66.761	1.00	0.00
ATOM	5728	HB2	SER	A	361	81.538	66.410	66.546	1.00	0.00
ATOM	5729	OG	SER	A	361	80.730	68.298	66.434	1.00	0.00
ATOM	5730	HG	SER	A	361	81.500	68.782	66.744	1.00	0.00
ATOM	5731	C	SER	A	361	80.730	65.678	69.118	1.00	0.00
ATOM	5732	O	SER	A	361	79.726	65.398	69.806	1.00	0.00

ATOM	5733	N	HIS	A	362	81.719	64.794	68.898	1.00	0.00
ATOM	5734	H	HIS	A	362	82.546	65.076	68.388	1.00	0.00
ATOM	5735	CA	HIS	A	362	81.640	63.434	69.478	1.00	0.00
ATOM	5736	HA	HIS	A	362	80.752	62.926	69.101	1.00	0.00
ATOM	5737	CB	HIS	A	362	82.888	62.623	69.075	1.00	0.00
ATOM	5738	HB1	HIS	A	362	82.931	61.731	69.699	1.00	0.00
ATOM	5739	HB2	HIS	A	362	83.782	63.209	69.291	1.00	0.00
ATOM	5740	CG	HIS	A	362	82.948	62.159	67.637	1.00	0.00
ATOM	5741	ND1	HIS	A	362	83.945	61.321	67.120	1.00	0.00
ATOM	5742	CE1	HIS	A	362	83.587	61.057	65.854	1.00	0.00
ATOM	5743	HE1	HIS	A	362	84.126	60.398	65.187	1.00	0.00
ATOM	5744	NE2	HIS	A	362	82.463	61.717	65.538	1.00	0.00
ATOM	5745	HE2	HIS	A	362	81.983	61.662	64.649	1.00	0.00
ATOM	5746	CD2	HIS	A	362	82.043	62.415	66.648	1.00	0.00
ATOM	5747	HD2	HIS	A	362	81.147	63.008	66.732	1.00	0.00
ATOM	5748	C	HIS	A	362	81.496	63.469	71.003	1.00	0.00
ATOM	5749	O	HIS	A	362	80.583	62.830	71.545	1.00	0.00
ATOM	5750	N	ILE	A	363	82.340	64.241	71.701	1.00	0.00
ATOM	5751	H	ILE	A	363	83.041	64.800	71.226	1.00	0.00
ATOM	5752	CA	ILE	A	363	82.239	64.291	73.174	1.00	0.00
ATOM	5753	HA	ILE	A	363	82.205	63.253	73.499	1.00	0.00
ATOM	5754	CB	ILE	A	363	83.498	64.891	73.847	1.00	0.00
ATOM	5755	HB	ILE	A	363	83.363	64.880	74.926	1.00	0.00
ATOM	5756	CG2	ILE	A	363	84.707	63.984	73.548	1.00	0.00
ATOM	5757	1HG2	ILE	A	363	85.559	64.322	74.139	1.00	0.00
ATOM	5758	2HG2	ILE	A	363	84.480	62.955	73.834	1.00	0.00
ATOM	5759	3HG2	ILE	A	363	84.965	64.029	72.490	1.00	0.00
ATOM	5760	CG1	ILE	A	363	83.815	66.334	73.434	1.00	0.00
ATOM	5761	1HG1	ILE	A	363	83.460	66.476	72.426	1.00	0.00
ATOM	5762	2HG1	ILE	A	363	84.891	66.499	73.436	1.00	0.00
ATOM	5763	CD	ILE	A	363	83.177	67.383	74.347	1.00	0.00
ATOM	5764	HD1	ILE	A	363	83.405	68.379	73.972	1.00	0.00
ATOM	5765	HD2	ILE	A	363	82.098	67.266	74.371	1.00	0.00
ATOM	5766	HD3	ILE	A	363	83.578	67.284	75.354	1.00	0.00
ATOM	5767	C	ILE	A	363	80.914	64.882	73.694	1.00	0.00
ATOM	5768	O	ILE	A	363	80.416	64.413	74.724	1.00	0.00
ATOM	5769	N	ARG	A	364	80.261	65.807	72.968	1.00	0.00
ATOM	5770	H	ARG	A	364	80.707	66.213	72.151	1.00	0.00
ATOM	5771	CA	ARG	A	364	78.881	66.213	73.318	1.00	0.00
ATOM	5772	HA	ARG	A	364	78.871	66.486	74.374	1.00	0.00
ATOM	5773	CB	ARG	A	364	78.451	67.454	72.506	1.00	0.00
ATOM	5774	HB1	ARG	A	364	77.369	67.573	72.579	1.00	0.00
ATOM	5775	HB2	ARG	A	364	78.736	67.352	71.458	1.00	0.00
ATOM	5776	CG	ARG	A	364	79.128	68.673	73.141	1.00	0.00
ATOM	5777	HG1	ARG	A	364	80.210	68.569	73.060	1.00	0.00
ATOM	5778	HG2	ARG	A	364	78.866	68.660	74.199	1.00	0.00
ATOM	5779	CD	ARG	A	364	78.741	70.061	72.611	1.00	0.00
ATOM	5780	HD1	ARG	A	364	77.656	70.112	72.513	1.00	0.00
ATOM	5781	HD2	ARG	A	364	79.209	70.226	71.639	1.00	0.00
ATOM	5782	NE	ARG	A	364	79.218	71.051	73.592	1.00	0.00
ATOM	5783	HE	ARG	A	364	79.981	70.701	74.183	1.00	0.00
ATOM	5784	CZ	ARG	A	364	78.526	72.027	74.142	1.00	0.00
ATOM	5785	NH1	ARG	A	364	77.634	72.731	73.509	1.00	0.00
ATOM	5786	1HH1	ARG	A	364	77.496	72.542	72.535	1.00	0.00
ATOM	5787	2HH1	ARG	A	364	77.164	73.471	74.008	1.00	0.00
ATOM	5788	NH2	ARG	A	364	78.707	72.304	75.389	1.00	0.00
ATOM	5789	1HH2	ARG	A	364	79.183	71.578	75.948	1.00	0.00
ATOM	5790	2HH2	ARG	A	364	78.105	72.962	75.848	1.00	0.00
ATOM	5791	C	ARG	A	364	77.882	65.055	73.239	1.00	0.00
ATOM	5792	O	ARG	A	364	77.179	64.815	74.225	1.00	0.00
ATOM	5793	N	HIS	A	365	77.904	64.256	72.162	1.00	0.00

ATOM	5794	H	HIS	A	365	78.536	64.464	71.400	1.00	0.00
ATOM	5795	CA	HIS	A	365	77.040	63.046	72.123	1.00	0.00
ATOM	5796	HA	HIS	A	365	75.999	63.365	72.201	1.00	0.00
ATOM	5797	CB	HIS	A	365	77.210	62.341	70.769	1.00	0.00
ATOM	5798	HB1	HIS	A	365	78.270	62.179	70.577	1.00	0.00
ATOM	5799	HB2	HIS	A	365	76.826	62.990	69.982	1.00	0.00
ATOM	5800	CG	HIS	A	365	76.514	61.005	70.680	1.00	0.00
ATOM	5801	ND1	HIS	A	365	75.155	60.799	70.424	1.00	0.00
ATOM	5802	CE1	HIS	A	365	74.987	59.469	70.402	1.00	0.00
ATOM	5803	HE1	HIS	A	365	74.038	58.971	70.206	1.00	0.00
ATOM	5804	NE2	HIS	A	365	76.149	58.840	70.644	1.00	0.00
ATOM	5805	HE2	HIS	A	365	76.294	57.840	70.669	1.00	0.00
ATOM	5806	CD2	HIS	A	365	77.121	59.794	70.823	1.00	0.00
ATOM	5807	HD2	HIS	A	365	78.171	59.624	71.035	1.00	0.00
ATOM	5808	C	HIS	A	365	77.269	62.071	73.301	1.00	0.00
ATOM	5809	O	HIS	A	365	76.305	61.675	73.980	1.00	0.00
ATOM	5810	N	MET	A	366	78.532	61.719	73.595	1.00	0.00
ATOM	5811	H	MET	A	366	79.287	62.053	73.008	1.00	0.00
ATOM	5812	CA	MET	A	366	78.812	60.803	74.728	1.00	0.00
ATOM	5813	HA	MET	A	366	78.296	59.861	74.537	1.00	0.00
ATOM	5814	CB	MET	A	366	80.311	60.486	74.906	1.00	0.00
ATOM	5815	HB1	MET	A	366	80.389	59.606	75.543	1.00	0.00
ATOM	5816	HB2	MET	A	366	80.778	61.316	75.430	1.00	0.00
ATOM	5817	CG	MET	A	366	81.118	60.245	73.639	1.00	0.00
ATOM	5818	HG1	MET	A	366	82.147	60.023	73.915	1.00	0.00
ATOM	5819	HG2	MET	A	366	81.141	61.173	73.080	1.00	0.00
ATOM	5820	SD	MET	A	366	80.535	58.926	72.554	1.00	0.00
ATOM	5821	CE	MET	A	366	81.158	59.615	71.002	1.00	0.00
ATOM	5822	HE1	MET	A	366	80.496	60.405	70.661	1.00	0.00
ATOM	5823	HE2	MET	A	366	82.148	60.031	71.164	1.00	0.00
ATOM	5824	HE3	MET	A	366	81.216	58.843	70.243	1.00	0.00
ATOM	5825	C	MET	A	366	78.302	61.356	76.066	1.00	0.00
ATOM	5826	O	MET	A	366	77.759	60.613	76.888	1.00	0.00
ATOM	5827	N	SER	A	367	78.472	62.670	76.264	1.00	0.00
ATOM	5828	H	SER	A	367	78.919	63.214	75.537	1.00	0.00
ATOM	5829	CA	SER	A	367	78.025	63.368	77.477	1.00	0.00
ATOM	5830	HA	SER	A	367	78.488	62.898	78.344	1.00	0.00
ATOM	5831	CB	SER	A	367	78.458	64.836	77.457	1.00	0.00
ATOM	5832	HB1	SER	A	367	78.167	65.311	78.395	1.00	0.00
ATOM	5833	HB2	SER	A	367	77.980	65.363	76.630	1.00	0.00
ATOM	5834	OG	SER	A	367	79.858	64.905	77.317	1.00	0.00
ATOM	5835	HG	SER	A	367	80.082	64.753	76.381	1.00	0.00
ATOM	5836	C	SER	A	367	76.516	63.290	77.656	1.00	0.00
ATOM	5837	O	SER	A	367	76.057	62.966	78.755	1.00	0.00
ATOM	5838	N	ASN	A	368	75.744	63.503	76.579	1.00	0.00
ATOM	5839	H	ASN	A	368	76.178	63.784	75.703	1.00	0.00
ATOM	5840	CA	ASN	A	368	74.301	63.258	76.610	1.00	0.00
ATOM	5841	HA	ASN	A	368	73.858	63.909	77.364	1.00	0.00
ATOM	5842	CB	ASN	A	368	73.650	63.560	75.250	1.00	0.00
ATOM	5843	HB1	ASN	A	368	72.622	63.194	75.266	1.00	0.00
ATOM	5844	HB2	ASN	A	368	74.180	63.039	74.454	1.00	0.00
ATOM	5845	CG	ASN	A	368	73.595	65.032	74.905	1.00	0.00
ATOM	5846	OD1	ASN	A	368	74.182	65.486	73.943	1.00	0.00
ATOM	5847	ND2	ASN	A	368	72.851	65.826	75.641	1.00	0.00
ATOM	5848	1HD2	ASN	A	368	72.253	65.454	76.356	1.00	0.00
ATOM	5849	2HD2	ASN	A	368	72.817	66.776	75.318	1.00	0.00
ATOM	5850	C	ASN	A	368	73.984	61.826	77.042	1.00	0.00
ATOM	5851	O	ASN	A	368	73.247	61.653	78.015	1.00	0.00
ATOM	5852	N	LYS	A	369	74.541	60.800	76.374	1.00	0.00
ATOM	5853	H	LYS	A	369	75.163	60.990	75.594	1.00	0.00
ATOM	5854	CA	LYS	A	369	74.108	59.419	76.687	1.00	0.00

ATOM	5855	HA	LYS	A	369	73.016	59.427	76.689	1.00	0.00
ATOM	5856	CB	LYS	A	369	74.540	58.415	75.600	1.00	0.00
ATOM	5857	HB1	LYS	A	369	74.005	57.482	75.784	1.00	0.00
ATOM	5858	HB2	LYS	A	369	75.604	58.205	75.727	1.00	0.00
ATOM	5859	CG	LYS	A	369	74.306	58.831	74.127	1.00	0.00
ATOM	5860	HG1	LYS	A	369	74.370	57.935	73.511	1.00	0.00
ATOM	5861	HG2	LYS	A	369	75.141	59.459	73.829	1.00	0.00
ATOM	5862	CD	LYS	A	369	73.008	59.586	73.761	1.00	0.00
ATOM	5863	HD1	LYS	A	369	73.086	59.896	72.720	1.00	0.00
ATOM	5864	HD2	LYS	A	369	72.932	60.493	74.356	1.00	0.00
ATOM	5865	CE	LYS	A	369	71.725	58.764	73.931	1.00	0.00
ATOM	5866	HE1	LYS	A	369	71.698	58.364	74.949	1.00	0.00
ATOM	5867	HE2	LYS	A	369	71.731	57.926	73.231	1.00	0.00
ATOM	5868	NZ	LYS	A	369	70.516	59.602	73.719	1.00	0.00
ATOM	5869	HZ1	LYS	A	369	69.667	59.083	73.884	1.00	0.00
ATOM	5870	HZ2	LYS	A	369	70.489	60.337	74.431	1.00	0.00
ATOM	5871	HZ3	LYS	A	369	70.455	60.032	72.805	1.00	0.00
ATOM	5872	C	LYS	A	369	74.489	58.974	78.115	1.00	0.00
ATOM	5873	O	LYS	A	369	73.719	58.263	78.765	1.00	0.00
ATOM	5874	N	GLY	A	370	75.619	59.463	78.641	1.00	0.00
ATOM	5875	H	GLY	A	370	76.220	60.013	78.036	1.00	0.00
ATOM	5876	CA	GLY	A	370	76.015	59.295	80.043	1.00	0.00
ATOM	5877	HA1	GLY	A	370	77.003	59.733	80.181	1.00	0.00
ATOM	5878	HA2	GLY	A	370	76.073	58.231	80.275	1.00	0.00
ATOM	5879	C	GLY	A	370	75.056	59.963	81.030	1.00	0.00
ATOM	5880	O	GLY	A	370	74.636	59.327	81.998	1.00	0.00
ATOM	5881	N	MET	A	371	74.655	61.211	80.761	1.00	0.00
ATOM	5882	H	MET	A	371	75.067	61.695	79.968	1.00	0.00
ATOM	5883	CA	MET	A	371	73.630	61.912	81.546	1.00	0.00
ATOM	5884	HA	MET	A	371	73.970	61.961	82.580	1.00	0.00
ATOM	5885	CB	MET	A	371	73.503	63.355	81.028	1.00	0.00
ATOM	5886	HB1	MET	A	371	73.264	63.339	79.966	1.00	0.00
ATOM	5887	HB2	MET	A	371	74.461	63.859	81.146	1.00	0.00
ATOM	5888	CG	MET	A	371	72.424	64.171	81.744	1.00	0.00
ATOM	5889	HG1	MET	A	371	71.462	63.699	81.557	1.00	0.00
ATOM	5890	HG2	MET	A	371	72.400	65.166	81.302	1.00	0.00
ATOM	5891	SD	MET	A	371	72.615	64.372	83.529	1.00	0.00
ATOM	5892	CE	MET	A	371	70.922	64.852	83.946	1.00	0.00
ATOM	5893	HE1	MET	A	371	70.646	65.739	83.378	1.00	0.00
ATOM	5894	HE2	MET	A	371	70.244	64.036	83.706	1.00	0.00
ATOM	5895	HE3	MET	A	371	70.864	65.074	85.010	1.00	0.00
ATOM	5896	C	MET	A	371	72.282	61.169	81.563	1.00	0.00
ATOM	5897	O	MET	A	371	71.659	61.067	82.617	1.00	0.00
ATOM	5898	N	GLU	A	372	71.843	60.602	80.437	1.00	0.00
ATOM	5899	H	GLU	A	372	72.373	60.758	79.585	1.00	0.00
ATOM	5900	CA	GLU	A	372	70.631	59.772	80.366	1.00	0.00
ATOM	5901	HA	GLU	A	372	69.800	60.334	80.796	1.00	0.00
ATOM	5902	CB	GLU	A	372	70.297	59.502	78.885	1.00	0.00
ATOM	5903	HB1	GLU	A	372	69.490	58.771	78.826	1.00	0.00
ATOM	5904	HB2	GLU	A	372	71.173	59.086	78.388	1.00	0.00
ATOM	5905	CG	GLU	A	372	69.851	60.785	78.149	1.00	0.00
ATOM	5906	HG1	GLU	A	372	70.519	61.609	78.400	1.00	0.00
ATOM	5907	HG2	GLU	A	372	68.856	61.061	78.501	1.00	0.00
ATOM	5908	CD	GLU	A	372	69.830	60.608	76.626	1.00	0.00
ATOM	5909	OE1	GLU	A	372	70.698	61.168	75.918	1.00	0.00
ATOM	5910	OE2	GLU	A	372	68.963	59.869	76.101	1.00	0.00
ATOM	5911	C	GLU	A	372	70.749	58.480	81.209	1.00	0.00
ATOM	5912	O	GLU	A	372	69.820	58.148	81.958	1.00	0.00
ATOM	5913	N	HIS	A	373	71.905	57.796	81.184	1.00	0.00
ATOM	5914	H	HIS	A	373	72.630	58.088	80.540	1.00	0.00
ATOM	5915	CA	HIS	A	373	72.147	56.646	82.079	1.00	0.00

ATOM	5916	HA	HIS	A	373	71.358	55.911	81.918	1.00	0.00
ATOM	5917	CB	HIS	A	373	73.477	55.959	81.748	1.00	0.00
ATOM	5918	HB1	HIS	A	373	74.301	56.657	81.888	1.00	0.00
ATOM	5919	HB2	HIS	A	373	73.469	55.640	80.706	1.00	0.00
ATOM	5920	CG	HIS	A	373	73.706	54.746	82.618	1.00	0.00
ATOM	5921	ND1	HIS	A	373	72.786	53.711	82.796	1.00	0.00
ATOM	5922	CE1	HIS	A	373	73.345	52.881	83.692	1.00	0.00
ATOM	5923	HE1	HIS	A	373	72.888	51.966	84.047	1.00	0.00
ATOM	5924	NE2	HIS	A	373	74.537	53.350	84.097	1.00	0.00
ATOM	5925	HE2	HIS	A	373	75.127	52.904	84.786	1.00	0.00
ATOM	5926	CD2	HIS	A	373	74.782	54.529	83.428	1.00	0.00
ATOM	5927	HD2	HIS	A	373	75.638	55.178	83.532	1.00	0.00
ATOM	5928	C	HIS	A	373	72.084	57.007	83.573	1.00	0.00
ATOM	5929	O	HIS	A	373	71.371	56.364	84.346	1.00	0.00
ATOM	5930	N	LEU	A	374	72.794	58.055	83.999	1.00	0.00
ATOM	5931	H	LEU	A	374	73.376	58.551	83.333	1.00	0.00
ATOM	5932	CA	LEU	A	374	72.826	58.454	85.409	1.00	0.00
ATOM	5933	HA	LEU	A	374	73.024	57.563	86.004	1.00	0.00
ATOM	5934	CB	LEU	A	374	73.980	59.448	85.646	1.00	0.00
ATOM	5935	HB1	LEU	A	374	73.925	59.789	86.681	1.00	0.00
ATOM	5936	HB2	LEU	A	374	73.828	60.313	84.998	1.00	0.00
ATOM	5937	CG	LEU	A	374	75.378	58.853	85.388	1.00	0.00
ATOM	5938	HG	LEU	A	374	75.468	58.553	84.346	1.00	0.00
ATOM	5939	CD1	LEU	A	374	76.434	59.927	85.663	1.00	0.00
ATOM	5940	1HD1	LEU	A	374	77.427	59.529	85.456	1.00	0.00
ATOM	5941	2HD1	LEU	A	374	76.263	60.789	85.020	1.00	0.00
ATOM	5942	3HD1	LEU	A	374	76.391	60.240	86.705	1.00	0.00
ATOM	5943	CD2	LEU	A	374	75.684	57.631	86.264	1.00	0.00
ATOM	5944	1HD2	LEU	A	374	76.719	57.325	86.121	1.00	0.00
ATOM	5945	2HD2	LEU	A	374	75.529	57.870	87.315	1.00	0.00
ATOM	5946	3HD2	LEU	A	374	75.050	56.792	85.980	1.00	0.00
ATOM	5947	C	LEU	A	374	71.476	58.981	85.914	1.00	0.00
ATOM	5948	O	LEU	A	374	71.111	58.745	87.066	1.00	0.00
ATOM	5949	N	TYR	A	375	70.692	59.647	85.054	1.00	0.00
ATOM	5950	H	TYR	A	375	71.045	59.849	84.123	1.00	0.00
ATOM	5951	CA	TYR	A	375	69.304	59.996	85.378	1.00	0.00
ATOM	5952	HA	TYR	A	375	69.303	60.505	86.341	1.00	0.00
ATOM	5953	CB	TYR	A	375	68.701	60.965	84.354	1.00	0.00
ATOM	5954	HB1	TYR	A	375	68.593	60.466	83.389	1.00	0.00
ATOM	5955	HB2	TYR	A	375	69.367	61.819	84.227	1.00	0.00
ATOM	5956	CG	TYR	A	375	67.349	61.463	84.838	1.00	0.00
ATOM	5957	CD1	TYR	A	375	67.284	62.512	85.777	1.00	0.00
ATOM	5958	HD1	TYR	A	375	68.184	63.039	86.061	1.00	0.00
ATOM	5959	CE1	TYR	A	375	66.065	62.812	86.415	1.00	0.00
ATOM	5960	HE1	TYR	A	375	66.018	63.587	87.165	1.00	0.00
ATOM	5961	CZ	TYR	A	375	64.910	62.057	86.127	1.00	0.00
ATOM	5962	OH	TYR	A	375	63.739	62.324	86.769	1.00	0.00
ATOM	5963	HH	TYR	A	375	63.018	61.822	86.359	1.00	0.00
ATOM	5964	CE2	TYR	A	375	64.967	61.038	85.153	1.00	0.00
ATOM	5965	HE2	TYR	A	375	64.084	60.450	84.936	1.00	0.00
ATOM	5966	CD2	TYR	A	375	66.182	60.750	84.504	1.00	0.00
ATOM	5967	HD2	TYR	A	375	66.237	59.929	83.797	1.00	0.00
ATOM	5968	C	TYR	A	375	68.428	58.751	85.565	1.00	0.00
ATOM	5969	O	TYR	A	375	67.657	58.707	86.526	1.00	0.00
ATOM	5970	N	SER	A	376	68.585	57.719	84.723	1.00	0.00
ATOM	5971	H	SER	A	376	69.218	57.813	83.934	1.00	0.00
ATOM	5972	CA	SER	A	376	67.903	56.433	84.932	1.00	0.00
ATOM	5973	HA	SER	A	376	66.828	56.611	84.925	1.00	0.00
ATOM	5974	CB	SER	A	376	68.223	55.485	83.773	1.00	0.00
ATOM	5975	HB1	SER	A	376	69.254	55.139	83.844	1.00	0.00
ATOM	5976	HB2	SER	A	376	68.089	56.008	82.826	1.00	0.00

ATOM	5977	OG	SER	A	376	67.351	54.374	83.812	1.00	0.00
ATOM	5978	HG	SER	A	376	67.690	53.706	83.211	1.00	0.00
ATOM	5979	C	SER	A	376	68.269	55.796	86.283	1.00	0.00
ATOM	5980	O	SER	A	376	67.377	55.446	87.065	1.00	0.00
ATOM	5981	N	MET	A	377	69.555	55.755	86.649	1.00	0.00
ATOM	5982	H	MET	A	377	70.250	56.037	85.961	1.00	0.00
ATOM	5983	CA	MET	A	377	70.013	55.259	87.955	1.00	0.00
ATOM	5984	HA	MET	A	377	69.723	54.213	88.050	1.00	0.00
ATOM	5985	CB	MET	A	377	71.542	55.357	88.060	1.00	0.00
ATOM	5986	HB1	MET	A	377	71.818	55.231	89.104	1.00	0.00
ATOM	5987	HB2	MET	A	377	71.883	56.342	87.751	1.00	0.00
ATOM	5988	CG	MET	A	377	72.281	54.295	87.239	1.00	0.00
ATOM	5989	HG1	MET	A	377	72.190	54.544	86.182	1.00	0.00
ATOM	5990	HG2	MET	A	377	71.811	53.325	87.401	1.00	0.00
ATOM	5991	SD	MET	A	377	74.045	54.148	87.650	1.00	0.00
ATOM	5992	CE	MET	A	377	73.942	53.369	89.288	1.00	0.00
ATOM	5993	HE1	MET	A	377	74.943	53.221	89.684	1.00	0.00
ATOM	5994	HE2	MET	A	377	73.391	54.006	89.975	1.00	0.00
ATOM	5995	HE3	MET	A	377	73.439	52.407	89.204	1.00	0.00
ATOM	5996	C	MET	A	377	69.385	56.003	89.149	1.00	0.00
ATOM	5997	O	MET	A	377	68.814	55.371	90.044	1.00	0.00
ATOM	5998	N	LYS	A	378	69.457	57.342	89.148	1.00	0.00
ATOM	5999	H	LYS	A	378	69.937	57.789	88.371	1.00	0.00
ATOM	6000	CA	LYS	A	378	68.894	58.193	90.208	1.00	0.00
ATOM	6001	HA	LYS	A	378	69.363	57.931	91.156	1.00	0.00
ATOM	6002	CB	LYS	A	378	69.216	59.667	89.867	1.00	0.00
ATOM	6003	HB1	LYS	A	378	68.737	59.903	88.915	1.00	0.00
ATOM	6004	HB2	LYS	A	378	70.292	59.761	89.717	1.00	0.00
ATOM	6005	CG	LYS	A	378	68.756	60.717	90.904	1.00	0.00
ATOM	6006	HG1	LYS	A	378	67.777	60.435	91.291	1.00	0.00
ATOM	6007	HG2	LYS	A	378	68.629	61.673	90.393	1.00	0.00
ATOM	6008	CD	LYS	A	378	69.722	60.919	92.084	1.00	0.00
ATOM	6009	HD1	LYS	A	378	70.030	59.942	92.453	1.00	0.00
ATOM	6010	HD2	LYS	A	378	69.192	61.422	92.894	1.00	0.00
ATOM	6011	CE	LYS	A	378	70.970	61.736	91.709	1.00	0.00
ATOM	6012	HE1	LYS	A	378	71.315	61.437	90.716	1.00	0.00
ATOM	6013	HE2	LYS	A	378	71.771	61.502	92.416	1.00	0.00
ATOM	6014	NZ	LYS	A	378	70.733	63.202	91.745	1.00	0.00
ATOM	6015	HZ1	LYS	A	378	69.929	63.528	91.201	1.00	0.00
ATOM	6016	HZ2	LYS	A	378	70.532	63.534	92.677	1.00	0.00
ATOM	6017	HZ3	LYS	A	378	71.555	63.720	91.437	1.00	0.00
ATOM	6018	C	LYS	A	378	67.390	57.959	90.358	1.00	0.00
ATOM	6019	O	LYS	A	378	66.913	57.694	91.463	1.00	0.00
ATOM	6020	N	CYS	A	379	66.654	58.008	89.247	1.00	0.00
ATOM	6021	H	CYS	A	379	67.133	58.189	88.368	1.00	0.00
ATOM	6022	CA	CYS	A	379	65.203	57.836	89.213	1.00	0.00
ATOM	6023	HA	CYS	A	379	64.761	58.491	89.966	1.00	0.00
ATOM	6024	CB	CYS	A	379	64.698	58.284	87.834	1.00	0.00
ATOM	6025	HB1	CYS	A	379	64.889	57.501	87.097	1.00	0.00
ATOM	6026	HB2	CYS	A	379	65.220	59.188	87.517	1.00	0.00
ATOM	6027	SG	CYS	A	379	62.922	58.645	87.938	1.00	0.00
ATOM	6028	HG	CYS	A	379	62.578	57.522	88.586	1.00	0.00
ATOM	6029	C	CYS	A	379	64.747	56.402	89.543	1.00	0.00
ATOM	6030	O	CYS	A	379	63.642	56.213	90.054	1.00	0.00
ATOM	6031	N	LYS	A	380	65.607	55.396	89.329	1.00	0.00
ATOM	6032	H	LYS	A	380	66.456	55.604	88.805	1.00	0.00
ATOM	6033	CA	LYS	A	380	65.380	54.000	89.739	1.00	0.00
ATOM	6034	HA	LYS	A	380	64.306	53.897	89.856	1.00	0.00
ATOM	6035	CB	LYS	A	380	65.835	53.054	88.605	1.00	0.00
ATOM	6036	HB1	LYS	A	380	66.926	53.033	88.582	1.00	0.00
ATOM	6037	HB2	LYS	A	380	65.498	53.475	87.656	1.00	0.00

ATOM	6038	CG	LYS	A	380	65.308	51.606	88.675	1.00	0.00
ATOM	6039	HG1	LYS	A	380	65.778	51.075	89.503	1.00	0.00
ATOM	6040	HG2	LYS	A	380	65.641	51.095	87.771	1.00	0.00
ATOM	6041	CD	LYS	A	380	63.770	51.464	88.760	1.00	0.00
ATOM	6042	HD1	LYS	A	380	63.437	50.948	87.857	1.00	0.00
ATOM	6043	HD2	LYS	A	380	63.278	52.437	88.747	1.00	0.00
ATOM	6044	CE	LYS	A	380	63.279	50.645	89.967	1.00	0.00
ATOM	6045	HE1	LYS	A	380	63.750	49.659	89.922	1.00	0.00
ATOM	6046	HE2	LYS	A	380	62.199	50.502	89.876	1.00	0.00
ATOM	6047	NZ	LYS	A	380	63.586	51.281	91.274	1.00	0.00
ATOM	6048	HZ1	LYS	A	380	63.333	50.699	92.056	1.00	0.00
ATOM	6049	HZ2	LYS	A	380	64.581	51.479	91.359	1.00	0.00
ATOM	6050	HZ3	LYS	A	380	63.125	52.178	91.413	1.00	0.00
ATOM	6051	C	LYS	A	380	65.953	53.676	91.121	1.00	0.00
ATOM	6052	O	LYS	A	380	66.021	52.504	91.495	1.00	0.00
ATOM	6053	N	ASN	A	381	66.262	54.696	91.922	1.00	0.00
ATOM	6054	H	ASN	A	381	66.232	55.627	91.524	1.00	0.00
ATOM	6055	CA	ASN	A	381	66.591	54.601	93.347	1.00	0.00
ATOM	6056	HA	ASN	A	381	66.717	55.623	93.703	1.00	0.00
ATOM	6057	CB	ASN	A	381	65.428	53.966	94.151	1.00	0.00
ATOM	6058	HB1	ASN	A	381	65.507	54.275	95.193	1.00	0.00
ATOM	6059	HB2	ASN	A	381	65.539	52.882	94.134	1.00	0.00
ATOM	6060	CG	ASN	A	381	64.035	54.288	93.645	1.00	0.00
ATOM	6061	OD1	ASN	A	381	63.410	53.493	92.947	1.00	0.00
ATOM	6062	ND2	ASN	A	381	63.508	55.445	93.951	1.00	0.00
ATOM	6063	1HD2	ASN	A	381	64.027	56.116	94.489	1.00	0.00
ATOM	6064	2HD2	ASN	A	381	62.601	55.649	93.569	1.00	0.00
ATOM	6065	C	ASN	A	381	67.912	53.865	93.644	1.00	0.00
ATOM	6066	O	ASN	A	381	68.250	53.693	94.813	1.00	0.00
ATOM	6067	N	VAL	A	382	68.675	53.449	92.626	1.00	0.00
ATOM	6068	H	VAL	A	382	68.395	53.693	91.684	1.00	0.00
ATOM	6069	CA	VAL	A	382	70.048	52.972	92.812	1.00	0.00
ATOM	6070	HA	VAL	A	382	70.085	52.409	93.746	1.00	0.00
ATOM	6071	CB	VAL	A	382	70.493	51.960	91.729	1.00	0.00
ATOM	6072	HB	VAL	A	382	69.838	51.094	91.840	1.00	0.00
ATOM	6073	CG1	VAL	A	382	70.351	52.426	90.278	1.00	0.00
ATOM	6074	1HG1	VAL	A	382	70.656	51.625	89.606	1.00	0.00
ATOM	6075	2HG1	VAL	A	382	69.314	52.678	90.062	1.00	0.00
ATOM	6076	3HG1	VAL	A	382	70.979	53.294	90.098	1.00	0.00
ATOM	6077	CG2	VAL	A	382	71.920	51.464	91.982	1.00	0.00
ATOM	6078	1HG2	VAL	A	382	72.145	50.636	91.310	1.00	0.00
ATOM	6079	2HG2	VAL	A	382	72.642	52.258	91.800	1.00	0.00
ATOM	6080	3HG2	VAL	A	382	72.021	51.112	93.008	1.00	0.00
ATOM	6081	C	VAL	A	382	70.947	54.202	93.012	1.00	0.00
ATOM	6082	O	VAL	A	382	71.634	54.679	92.114	1.00	0.00
ATOM	6083	N	VAL	A	383	70.866	54.756	94.229	1.00	0.00
ATOM	6084	H	VAL	A	383	70.208	54.335	94.875	1.00	0.00
ATOM	6085	CA	VAL	A	383	71.477	56.029	94.631	1.00	0.00
ATOM	6086	HA	VAL	A	383	71.660	56.571	93.707	1.00	0.00
ATOM	6087	CB	VAL	A	383	70.473	56.917	95.406	1.00	0.00
ATOM	6088	HB	VAL	A	383	70.956	57.861	95.654	1.00	0.00
ATOM	6089	CG1	VAL	A	383	69.269	57.286	94.533	1.00	0.00
ATOM	6090	1HG1	VAL	A	383	69.607	57.742	93.606	1.00	0.00
ATOM	6091	2HG1	VAL	A	383	68.689	56.402	94.306	1.00	0.00
ATOM	6092	3HG1	VAL	A	383	68.637	57.997	95.061	1.00	0.00
ATOM	6093	CG2	VAL	A	383	69.979	56.276	96.705	1.00	0.00
ATOM	6094	1HG2	VAL	A	383	69.340	56.979	97.239	1.00	0.00
ATOM	6095	2HG2	VAL	A	383	69.413	55.368	96.504	1.00	0.00
ATOM	6096	3HG2	VAL	A	383	70.827	56.030	97.345	1.00	0.00
ATOM	6097	C	VAL	A	383	72.879	55.983	95.309	1.00	0.00
ATOM	6098	O	VAL	A	383	73.155	56.883	96.104	1.00	0.00

ATOM	6099	N	PRO	A	384	73.812	55.043	95.034	1.00	0.00
ATOM	6100	CD	PRO	A	384	73.592	53.678	94.564	1.00	0.00
ATOM	6101	HD1	PRO	A	384	73.541	53.676	93.477	1.00	0.00
ATOM	6102	HD2	PRO	A	384	72.699	53.239	95.003	1.00	0.00
ATOM	6103	CG	PRO	A	384	74.805	52.872	95.006	1.00	0.00
ATOM	6104	HG1	PRO	A	384	74.958	51.992	94.382	1.00	0.00
ATOM	6105	HG2	PRO	A	384	74.706	52.593	96.055	1.00	0.00
ATOM	6106	CB	PRO	A	384	75.918	53.892	94.839	1.00	0.00
ATOM	6107	HB1	PRO	A	384	76.194	53.927	93.783	1.00	0.00
ATOM	6108	HB2	PRO	A	384	76.787	53.650	95.451	1.00	0.00
ATOM	6109	CA	PRO	A	384	75.260	55.211	95.279	1.00	0.00
ATOM	6110	HA	PRO	A	384	75.388	55.306	96.358	1.00	0.00
ATOM	6111	C	PRO	A	384	75.960	56.448	94.631	1.00	0.00
ATOM	6112	O	PRO	A	384	76.975	56.289	93.959	1.00	0.00
ATOM	6113	N	LEU	A	385	75.426	57.673	94.771	1.00	0.00
ATOM	6114	H	LEU	A	385	74.622	57.753	95.385	1.00	0.00
ATOM	6115	CA	LEU	A	385	75.697	58.804	93.857	1.00	0.00
ATOM	6116	HA	LEU	A	385	76.582	58.564	93.275	1.00	0.00
ATOM	6117	CB	LEU	A	385	74.487	58.958	92.891	1.00	0.00
ATOM	6118	HB1	LEU	A	385	74.759	59.670	92.111	1.00	0.00
ATOM	6119	HB2	LEU	A	385	73.686	59.418	93.471	1.00	0.00
ATOM	6120	CG	LEU	A	385	73.902	57.699	92.212	1.00	0.00
ATOM	6121	HG	LEU	A	385	73.640	56.975	92.973	1.00	0.00
ATOM	6122	CD1	LEU	A	385	72.614	58.074	91.475	1.00	0.00
ATOM	6123	1HD1	LEU	A	385	72.182	57.178	91.027	1.00	0.00
ATOM	6124	2HD1	LEU	A	385	71.899	58.490	92.180	1.00	0.00
ATOM	6125	3HD1	LEU	A	385	72.832	58.797	90.688	1.00	0.00
ATOM	6126	CD2	LEU	A	385	74.817	56.999	91.216	1.00	0.00
ATOM	6127	1HD2	LEU	A	385	74.334	56.098	90.840	1.00	0.00
ATOM	6128	2HD2	LEU	A	385	75.038	57.653	90.373	1.00	0.00
ATOM	6129	3HD2	LEU	A	385	75.747	56.709	91.699	1.00	0.00
ATOM	6130	C	LEU	A	385	75.996	60.186	94.528	1.00	0.00
ATOM	6131	O	LEU	A	385	75.960	61.226	93.860	1.00	0.00
ATOM	6132	N	TYR	A	386	76.262	60.169	95.841	1.00	0.00
ATOM	6133	H	TYR	A	386	76.310	59.259	96.266	1.00	0.00
ATOM	6134	CA	TYR	A	386	76.646	61.301	96.712	1.00	0.00
ATOM	6135	HA	TYR	A	386	76.378	60.979	97.719	1.00	0.00
ATOM	6136	CB	TYR	A	386	78.175	61.435	96.736	1.00	0.00
ATOM	6137	HB1	TYR	A	386	78.473	62.475	96.876	1.00	0.00
ATOM	6138	HB2	TYR	A	386	78.565	61.122	95.772	1.00	0.00
ATOM	6139	CG	TYR	A	386	78.817	60.604	97.837	1.00	0.00
ATOM	6140	CD1	TYR	A	386	79.259	59.291	97.571	1.00	0.00
ATOM	6141	HD1	TYR	A	386	79.165	58.892	96.567	1.00	0.00
ATOM	6142	CE1	TYR	A	386	79.838	58.518	98.599	1.00	0.00
ATOM	6143	HE1	TYR	A	386	80.199	57.520	98.400	1.00	0.00
ATOM	6144	CZ	TYR	A	386	79.945	59.050	99.905	1.00	0.00
ATOM	6145	OH	TYR	A	386	80.481	58.307	100.908	1.00	0.00
ATOM	6146	HH	TYR	A	386	80.460	58.792	101.732	1.00	0.00
ATOM	6147	CE2	TYR	A	386	79.485	60.356	100.171	1.00	0.00
ATOM	6148	HE2	TYR	A	386	79.546	60.763	101.166	1.00	0.00
ATOM	6149	CD2	TYR	A	386	78.928	61.128	99.137	1.00	0.00
ATOM	6150	HD2	TYR	A	386	78.572	62.127	99.348	1.00	0.00
ATOM	6151	C	TYR	A	386	75.876	62.658	96.604	1.00	0.00
ATOM	6152	O	TYR	A	386	74.972	62.878	95.793	1.00	0.00
ATOM	6153	N	ASP	A	387	76.179	63.578	97.526	1.00	0.00
ATOM	6154	H	ASP	A	387	76.803	63.319	98.290	1.00	0.00
ATOM	6155	CA	ASP	A	387	75.540	64.897	97.657	1.00	0.00
ATOM	6156	HA	ASP	A	387	74.468	64.757	97.791	1.00	0.00
ATOM	6157	CB	ASP	A	387	76.098	65.620	98.904	1.00	0.00
ATOM	6158	HB1	ASP	A	387	75.489	66.505	99.090	1.00	0.00
ATOM	6159	HB2	ASP	A	387	77.112	65.960	98.691	1.00	0.00

ATOM	6160	CG	ASP	A	387	76.145	64.748	100.158	1.00	0.00
ATOM	6161	OD1	ASP	A	387	75.319	64.980	101.066	1.00	0.00
ATOM	6162	OD2	ASP	A	387	77.029	63.858	100.186	1.00	0.00
ATOM	6163	C	ASP	A	387	75.754	65.807	96.431	1.00	0.00
ATOM	6164	O	ASP	A	387	74.872	66.504	95.910	1.00	0.00
ATOM	6165	N	LEU	A	388	76.993	65.764	95.957	1.00	0.00
ATOM	6166	H	LEU	A	388	77.643	65.136	96.407	1.00	0.00
ATOM	6167	CA	LEU	A	388	77.429	66.493	94.786	1.00	0.00
ATOM	6168	HA	LEU	A	388	77.152	67.541	94.910	1.00	0.00
ATOM	6169	CB	LEU	A	388	78.957	66.396	94.795	1.00	0.00
ATOM	6170	HB1	LEU	A	388	79.266	65.352	94.818	1.00	0.00
ATOM	6171	HB2	LEU	A	388	79.299	66.852	95.724	1.00	0.00
ATOM	6172	CG	LEU	A	388	79.665	67.102	93.637	1.00	0.00
ATOM	6173	HG	LEU	A	388	79.254	68.103	93.509	1.00	0.00
ATOM	6174	CD1	LEU	A	388	81.148	67.237	94.007	1.00	0.00
ATOM	6175	1HD1	LEU	A	388	81.702	67.741	93.221	1.00	0.00
ATOM	6176	2HD1	LEU	A	388	81.251	67.821	94.921	1.00	0.00
ATOM	6177	3HD1	LEU	A	388	81.577	66.249	94.172	1.00	0.00
ATOM	6178	CD2	LEU	A	388	79.483	66.298	92.346	1.00	0.00
ATOM	6179	1HD2	LEU	A	388	80.296	66.471	91.668	1.00	0.00
ATOM	6180	2HD2	LEU	A	388	79.454	65.227	92.553	1.00	0.00
ATOM	6181	3HD2	LEU	A	388	78.565	66.603	91.844	1.00	0.00
ATOM	6182	C	LEU	A	388	76.684	65.984	93.540	1.00	0.00
ATOM	6183	O	LEU	A	388	76.125	66.833	92.833	1.00	0.00
ATOM	6184	N	LEU	A	389	76.548	64.658	93.295	1.00	0.00
ATOM	6185	H	LEU	A	389	77.080	63.968	93.819	1.00	0.00
ATOM	6186	CA	LEU	A	389	75.729	64.256	92.148	1.00	0.00
ATOM	6187	HA	LEU	A	389	75.871	65.032	91.408	1.00	0.00
ATOM	6188	CB	LEU	A	389	76.283	62.983	91.467	1.00	0.00
ATOM	6189	HB1	LEU	A	389	75.513	62.215	91.411	1.00	0.00
ATOM	6190	HB2	LEU	A	389	77.112	62.595	92.060	1.00	0.00
ATOM	6191	CG	LEU	A	389	76.820	63.304	90.055	1.00	0.00
ATOM	6192	HG	LEU	A	389	77.413	64.217	90.093	1.00	0.00
ATOM	6193	CD1	LEU	A	389	77.721	62.214	89.484	1.00	0.00
ATOM	6194	1HD1	LEU	A	389	78.183	62.574	88.565	1.00	0.00
ATOM	6195	2HD1	LEU	A	389	78.508	61.983	90.203	1.00	0.00
ATOM	6196	3HD1	LEU	A	389	77.131	61.326	89.276	1.00	0.00
ATOM	6197	CD2	LEU	A	389	75.663	63.496	89.069	1.00	0.00
ATOM	6198	1HD2	LEU	A	389	76.057	63.688	88.072	1.00	0.00
ATOM	6199	2HD2	LEU	A	389	75.048	62.597	89.052	1.00	0.00
ATOM	6200	3HD2	LEU	A	389	75.052	64.342	89.374	1.00	0.00
ATOM	6201	C	LEU	A	389	74.209	64.356	92.410	1.00	0.00
ATOM	6202	O	LEU	A	389	73.414	64.293	91.458	1.00	0.00
ATOM	6203	N	LEU	A	390	73.771	64.633	93.650	1.00	0.00
ATOM	6204	H	LEU	A	390	74.448	64.619	94.403	1.00	0.00
ATOM	6205	CA	LEU	A	390	72.440	65.193	93.909	1.00	0.00
ATOM	6206	HA	LEU	A	390	71.751	64.521	93.433	1.00	0.00
ATOM	6207	CB	LEU	A	390	72.046	65.158	95.399	1.00	0.00
ATOM	6208	HB1	LEU	A	390	72.669	65.824	95.973	1.00	0.00
ATOM	6209	HB2	LEU	A	390	72.202	64.145	95.774	1.00	0.00
ATOM	6210	CG	LEU	A	390	70.592	65.582	95.691	1.00	0.00
ATOM	6211	HG	LEU	A	390	70.471	66.637	95.447	1.00	0.00
ATOM	6212	CD1	LEU	A	390	69.558	64.775	94.899	1.00	0.00
ATOM	6213	1HD1	LEU	A	390	68.558	65.025	95.253	1.00	0.00
ATOM	6214	2HD1	LEU	A	390	69.605	65.049	93.845	1.00	0.00
ATOM	6215	3HD1	LEU	A	390	69.732	63.709	95.032	1.00	0.00
ATOM	6216	CD2	LEU	A	390	70.306	65.391	97.181	1.00	0.00
ATOM	6217	1HD2	LEU	A	390	69.312	65.770	97.413	1.00	0.00
ATOM	6218	2HD2	LEU	A	390	70.371	64.339	97.450	1.00	0.00
ATOM	6219	3HD2	LEU	A	390	71.034	65.950	97.769	1.00	0.00
ATOM	6220	C	LEU	A	390	72.237	66.540	93.212	1.00	0.00

ATOM	6221	O	LEU	A	390	71.457	66.559	92.253	1.00	0.00
ATOM	6222	N	GLU	A	391	72.951	67.605	93.617	1.00	0.00
ATOM	6223	H	GLU	A	391	73.642	67.504	94.355	1.00	0.00
ATOM	6224	CA	GLU	A	391	72.739	68.921	92.941	1.00	0.00
ATOM	6225	HA	GLU	A	391	71.686	69.194	93.033	1.00	0.00
ATOM	6226	CB	GLU	A	391	73.575	70.045	93.583	1.00	0.00
ATOM	6227	HB1	GLU	A	391	73.934	70.732	92.815	1.00	0.00
ATOM	6228	HB2	GLU	A	391	74.453	69.608	94.064	1.00	0.00
ATOM	6229	CG	GLU	A	391	72.787	70.861	94.622	1.00	0.00
ATOM	6230	HG1	GLU	A	391	73.511	71.348	95.278	1.00	0.00
ATOM	6231	HG2	GLU	A	391	72.193	70.184	95.240	1.00	0.00
ATOM	6232	CD	GLU	A	391	71.874	71.942	94.004	1.00	0.00
ATOM	6233	OE1	GLU	A	391	71.990	73.125	94.399	1.00	0.00
ATOM	6234	OE2	GLU	A	391	70.982	71.616	93.198	1.00	0.00
ATOM	6235	C	GLU	A	391	73.005	68.838	91.427	1.00	0.00
ATOM	6236	O	GLU	A	391	72.138	69.205	90.635	1.00	0.00
ATOM	6237	N	MET	A	392	74.125	68.237	91.014	1.00	0.00
ATOM	6238	H	MET	A	392	74.797	67.938	91.712	1.00	0.00
ATOM	6239	CA	MET	A	392	74.487	68.139	89.589	1.00	0.00
ATOM	6240	HA	MET	A	392	74.547	69.142	89.188	1.00	0.00
ATOM	6241	CB	MET	A	392	75.897	67.506	89.501	1.00	0.00
ATOM	6242	HB1	MET	A	392	75.967	66.773	90.297	1.00	0.00
ATOM	6243	HB2	MET	A	392	76.643	68.274	89.720	1.00	0.00
ATOM	6244	CG	MET	A	392	76.321	66.748	88.230	1.00	0.00
ATOM	6245	HG1	MET	A	392	75.475	66.214	87.798	1.00	0.00
ATOM	6246	HG2	MET	A	392	77.032	65.989	88.555	1.00	0.00
ATOM	6247	SD	MET	A	392	77.172	67.664	86.927	1.00	0.00
ATOM	6248	CE	MET	A	392	75.835	68.690	86.274	1.00	0.00
ATOM	6249	HE1	MET	A	392	75.449	69.345	87.055	1.00	0.00
ATOM	6250	HE2	MET	A	392	76.220	69.301	85.466	1.00	0.00
ATOM	6251	HE3	MET	A	392	75.040	68.058	85.883	1.00	0.00
ATOM	6252	C	MET	A	392	73.453	67.405	88.728	1.00	0.00
ATOM	6253	O	MET	A	392	73.263	67.765	87.565	1.00	0.00
ATOM	6254	N	LEU	A	393	72.760	66.378	89.244	1.00	0.00
ATOM	6255	H	LEU	A	393	72.898	66.118	90.212	1.00	0.00
ATOM	6256	CA	LEU	A	393	71.714	65.751	88.417	1.00	0.00
ATOM	6257	HA	LEU	A	393	72.034	65.817	87.377	1.00	0.00
ATOM	6258	CB	LEU	A	393	71.599	64.263	88.751	1.00	0.00
ATOM	6259	HB1	LEU	A	393	70.715	64.126	89.369	1.00	0.00
ATOM	6260	HB2	LEU	A	393	72.480	63.930	89.292	1.00	0.00
ATOM	6261	CG	LEU	A	393	71.485	63.365	87.516	1.00	0.00
ATOM	6262	HG	LEU	A	393	70.678	63.714	86.873	1.00	0.00
ATOM	6263	CD1	LEU	A	393	72.782	63.246	86.713	1.00	0.00
ATOM	6264	1HD1	LEU	A	393	73.555	62.770	87.311	1.00	0.00
ATOM	6265	2HD1	LEU	A	393	72.604	62.640	85.824	1.00	0.00
ATOM	6266	3HD1	LEU	A	393	73.137	64.229	86.404	1.00	0.00
ATOM	6267	CD2	LEU	A	393	71.157	61.965	88.006	1.00	0.00
ATOM	6268	1HD2	LEU	A	393	71.120	61.308	87.149	1.00	0.00
ATOM	6269	2HD2	LEU	A	393	71.927	61.602	88.689	1.00	0.00
ATOM	6270	3HD2	LEU	A	393	70.183	61.968	88.492	1.00	0.00
ATOM	6271	C	LEU	A	393	70.370	66.462	88.459	1.00	0.00
ATOM	6272	O	LEU	A	393	69.633	66.412	87.465	1.00	0.00
ATOM	6273	N	ASP	A	394	70.043	67.134	89.558	1.00	0.00
ATOM	6274	H	ASP	A	394	70.722	67.222	90.312	1.00	0.00
ATOM	6275	CA	ASP	A	394	68.704	67.687	89.752	1.00	0.00
ATOM	6276	HA	ASP	A	394	68.050	67.207	89.029	1.00	0.00
ATOM	6277	CB	ASP	A	394	68.171	67.194	91.103	1.00	0.00
ATOM	6278	HB1	ASP	A	394	67.259	67.736	91.352	1.00	0.00
ATOM	6279	HB2	ASP	A	394	68.901	67.367	91.895	1.00	0.00
ATOM	6280	CG	ASP	A	394	67.838	65.686	90.986	1.00	0.00
ATOM	6281	OD1	ASP	A	394	68.654	64.797	91.335	1.00	0.00

ATOM	6282	OD2	ASP	A	394	66.733	65.386	90.477	1.00	0.00
ATOM	6283	C	ASP	A	394	68.589	69.163	89.363	1.00	0.00
ATOM	6284	O	ASP	A	394	67.477	69.601	89.090	1.00	0.00
ATOM	6285	N	ALA	A	395	69.708	69.848	89.105	1.00	0.00
ATOM	6286	H	ALA	A	395	70.580	69.501	89.502	1.00	0.00
ATOM	6287	CA	ALA	A	395	69.832	71.011	88.222	1.00	0.00
ATOM	6288	HA	ALA	A	395	69.061	71.731	88.490	1.00	0.00
ATOM	6289	CB	ALA	A	395	71.198	71.640	88.494	1.00	0.00
ATOM	6290	HB1	ALA	A	395	71.305	72.556	87.917	1.00	0.00
ATOM	6291	HB2	ALA	A	395	71.305	71.878	89.555	1.00	0.00
ATOM	6292	HB3	ALA	A	395	72.000	70.953	88.213	1.00	0.00
ATOM	6293	C	ALA	A	395	69.669	70.694	86.725	1.00	0.00
ATOM	6294	O	ALA	A	395	69.414	71.608	85.944	1.00	0.00
ATOM	6295	N	HIS	A	396	69.819	69.434	86.295	1.00	0.00
ATOM	6296	H	HIS	A	396	70.035	68.744	87.001	1.00	0.00
ATOM	6297	CA	HIS	A	396	70.059	69.078	84.881	1.00	0.00
ATOM	6298	HA	HIS	A	396	70.143	69.986	84.282	1.00	0.00
ATOM	6299	CB	HIS	A	396	71.431	68.385	84.819	1.00	0.00
ATOM	6300	HB1	HIS	A	396	71.332	67.334	85.079	1.00	0.00
ATOM	6301	HB2	HIS	A	396	72.072	68.839	85.577	1.00	0.00
ATOM	6302	CG	HIS	A	396	72.177	68.527	83.518	1.00	0.00
ATOM	6303	ND1	HIS	A	396	71.859	67.910	82.304	1.00	0.00
ATOM	6304	CE1	HIS	A	396	72.843	68.260	81.457	1.00	0.00
ATOM	6305	HE1	HIS	A	396	72.901	67.942	80.422	1.00	0.00
ATOM	6306	NE2	HIS	A	396	73.747	69.052	82.063	1.00	0.00
ATOM	6307	HE2	HIS	A	396	74.597	69.426	81.653	1.00	0.00
ATOM	6308	CD2	HIS	A	396	73.335	69.233	83.363	1.00	0.00
ATOM	6309	HD2	HIS	A	396	73.839	69.798	84.140	1.00	0.00
ATOM	6310	C	HIS	A	396	68.896	68.280	84.268	1.00	0.00
ATOM	6311	O	HIS	A	396	68.367	68.660	83.228	1.00	0.00
ATOM	6312	N	ARG	A	397	68.408	67.246	84.972	1.00	0.00
ATOM	6313	H	ARG	A	397	68.894	67.036	85.841	1.00	0.00
ATOM	6314	CA	ARG	A	397	67.183	66.465	84.690	1.00	0.00
ATOM	6315	HA	ARG	A	397	67.361	65.464	85.083	1.00	0.00
ATOM	6316	CB	ARG	A	397	65.973	67.038	85.452	1.00	0.00
ATOM	6317	HB1	ARG	A	397	65.096	66.435	85.215	1.00	0.00
ATOM	6318	HB2	ARG	A	397	65.783	68.067	85.142	1.00	0.00
ATOM	6319	CG	ARG	A	397	66.152	67.005	86.974	1.00	0.00
ATOM	6320	HG1	ARG	A	397	66.838	67.797	87.261	1.00	0.00
ATOM	6321	HG2	ARG	A	397	66.592	66.047	87.248	1.00	0.00
ATOM	6322	CD	ARG	A	397	64.817	67.194	87.714	1.00	0.00
ATOM	6323	HD1	ARG	A	397	63.984	67.087	87.021	1.00	0.00
ATOM	6324	HD2	ARG	A	397	64.799	68.201	88.122	1.00	0.00
ATOM	6325	NE	ARG	A	397	64.697	66.222	88.811	1.00	0.00
ATOM	6326	HE	ARG	A	397	65.460	66.228	89.486	1.00	0.00
ATOM	6327	CZ	ARG	A	397	64.064	65.068	88.787	1.00	0.00
ATOM	6328	NH1	ARG	A	397	62.998	64.835	88.075	1.00	0.00
ATOM	6329	1HH1	ARG	A	397	62.436	65.596	87.743	1.00	0.00
ATOM	6330	2HH1	ARG	A	397	62.704	63.878	87.956	1.00	0.00
ATOM	6331	NH2	ARG	A	397	64.559	64.096	89.489	1.00	0.00
ATOM	6332	1HH2	ARG	A	397	65.414	64.310	90.019	1.00	0.00
ATOM	6333	2HH2	ARG	A	397	64.148	63.183	89.473	1.00	0.00
ATOM	6334	C	ARG	A	397	66.845	66.234	83.205	1.00	0.00
ATOM	6335	O	ARG	A	397	65.764	66.615	82.742	1.00	0.00
ATOM	6336	N	LEU	A	398	67.678	65.476	82.487	1.00	0.00
ATOM	6337	H	LEU	A	398	68.571	65.234	82.892	1.00	0.00
ATOM	6338	CA	LEU	A	398	67.312	64.910	81.176	1.00	0.00
ATOM	6339	HA	LEU	A	398	66.838	65.706	80.603	1.00	0.00
ATOM	6340	CB	LEU	A	398	68.572	64.490	80.385	1.00	0.00
ATOM	6341	HB1	LEU	A	398	68.244	64.103	79.419	1.00	0.00
ATOM	6342	HB2	LEU	A	398	69.069	63.673	80.908	1.00	0.00

ATOM	6343	CG	LEU	A	398	69.609	65.600	80.101	1.00	0.00
ATOM	6344	HG	LEU	A	398	70.145	65.817	81.023	1.00	0.00
ATOM	6345	CD1	LEU	A	398	70.609	65.106	79.047	1.00	0.00
ATOM	6346	1HD1	LEU	A	398	71.447	65.802	78.989	1.00	0.00
ATOM	6347	2HD1	LEU	A	398	70.981	64.117	79.313	1.00	0.00
ATOM	6348	3HD1	LEU	A	398	70.123	65.044	78.073	1.00	0.00
ATOM	6349	CD2	LEU	A	398	68.994	66.903	79.584	1.00	0.00
ATOM	6350	1HD2	LEU	A	398	69.789	67.600	79.319	1.00	0.00
ATOM	6351	2HD2	LEU	A	398	68.374	66.714	78.708	1.00	0.00
ATOM	6352	3HD2	LEU	A	398	68.403	67.374	80.370	1.00	0.00
ATOM	6353	C	LEU	A	398	66.243	63.785	81.240	1.00	0.00
ATOM	6354	O	LEU	A	398	66.247	62.860	80.432	1.00	0.00
ATOM	6355	N	HIS	A	399	65.266	63.882	82.147	1.00	0.00
ATOM	6356	H	HIS	A	399	65.334	64.648	82.797	1.00	0.00
ATOM	6357	CA	HIS	A	399	63.972	63.199	82.028	1.00	0.00
ATOM	6358	HA	HIS	A	399	64.142	62.126	81.917	1.00	0.00
ATOM	6359	CB	HIS	A	399	63.150	63.445	83.305	1.00	0.00
ATOM	6360	HB1	HIS	A	399	62.778	64.464	83.326	1.00	0.00
ATOM	6361	HB2	HIS	A	399	63.810	63.347	84.162	1.00	0.00
ATOM	6362	CG	HIS	A	399	61.983	62.511	83.521	1.00	0.00
ATOM	6363	ND1	HIS	A	399	61.758	61.790	84.694	1.00	0.00
ATOM	6364	CE1	HIS	A	399	60.638	61.080	84.489	1.00	0.00
ATOM	6365	HE1	HIS	A	399	60.197	60.398	85.212	1.00	0.00
ATOM	6366	NE2	HIS	A	399	60.144	61.338	83.268	1.00	0.00
ATOM	6367	HE2	HIS	A	399	59.302	60.922	82.888	1.00	0.00
ATOM	6368	CD2	HIS	A	399	60.973	62.243	82.641	1.00	0.00
ATOM	6369	HD2	HIS	A	399	60.846	62.659	81.652	1.00	0.00
ATOM	6370	C	HIS	A	399	63.266	63.733	80.768	1.00	0.00
ATOM	6371	O	HIS	A	399	62.565	64.747	80.819	1.00	0.00
ATOM	6372	N	ALA	A	400	63.530	63.122	79.620	1.00	0.00
ATOM	6373	H	ALA	A	400	64.220	62.377	79.624	1.00	0.00
ATOM	6374	CA	ALA	A	400	63.048	63.552	78.313	1.00	0.00
ATOM	6375	HA	ALA	A	400	62.102	64.082	78.426	1.00	0.00
ATOM	6376	CB	ALA	A	400	64.095	64.498	77.704	1.00	0.00
ATOM	6377	HB1	ALA	A	400	63.762	64.855	76.731	1.00	0.00
ATOM	6378	HB2	ALA	A	400	64.252	65.352	78.359	1.00	0.00
ATOM	6379	HB3	ALA	A	400	65.040	63.971	77.574	1.00	0.00
ATOM	6380	C	ALA	A	400	62.836	62.310	77.423	1.00	0.00
ATOM	6381	O	ALA	A	400	63.495	61.280	77.619	1.00	0.00
ATOM	6382	N	PRO	A	401	61.943	62.375	76.422	1.00	0.00
ATOM	6383	CD	PRO	A	401	61.120	63.516	76.035	1.00	0.00
ATOM	6384	HD1	PRO	A	401	61.723	64.404	75.844	1.00	0.00
ATOM	6385	HD2	PRO	A	401	60.385	63.715	76.817	1.00	0.00
ATOM	6386	CG	PRO	A	401	60.400	63.093	74.756	1.00	0.00
ATOM	6387	HG1	PRO	A	401	61.020	63.318	73.886	1.00	0.00
ATOM	6388	HG2	PRO	A	401	59.423	63.564	74.657	1.00	0.00
ATOM	6389	CB	PRO	A	401	60.287	61.581	74.936	1.00	0.00
ATOM	6390	HB1	PRO	A	401	60.121	61.074	73.984	1.00	0.00
ATOM	6391	HB2	PRO	A	401	59.468	61.358	75.623	1.00	0.00
ATOM	6392	CA	PRO	A	401	61.627	61.219	75.590	1.00	0.00
ATOM	6393	HA	PRO	A	401	61.498	60.329	76.207	1.00	0.00
ATOM	6394	C	PRO	A	401	62.686	60.911	74.517	1.00	0.00
ATOM	6395	O	PRO	A	401	62.461	60.033	73.695	1.00	0.00
ATOM	6396	N	THR	A	402	63.812	61.629	74.496	1.00	0.00
ATOM	6397	H	THR	A	402	64.005	62.250	75.268	1.00	0.00
ATOM	6398	CA	THR	A	402	64.706	61.758	73.338	1.00	0.00
ATOM	6399	HA	THR	A	402	64.633	60.860	72.728	1.00	0.00
ATOM	6400	CB	THR	A	402	64.204	62.934	72.467	1.00	0.00
ATOM	6401	HB	THR	A	402	63.148	62.757	72.257	1.00	0.00
ATOM	6402	CG2	THR	A	402	64.321	64.308	73.128	1.00	0.00
ATOM	6403	1HG2	THR	A	402	63.896	65.061	72.466	1.00	0.00

```
ATOM   6404 2HG2 THR A 402      63.770  64.328  74.065  1.00  0.00
ATOM   6405 3HG2 THR A 402      65.365  64.555  73.314  1.00  0.00
ATOM   6406  OG1 THR A 402      64.864  62.998  71.234  1.00  0.00
ATOM   6407  HG1 THR A 402      64.580  62.206  70.723  1.00  0.00
ATOM   6408  C   THR A 402      66.160  61.926  73.802  1.00  0.00
ATOM   6409  O   THR A 402      66.382  62.456  74.887  1.00  0.00
ATOM   6410  N   SER A 403      67.182  61.449  73.085  1.00  0.00
ATOM   6411  H   SER A 403      68.091  61.594  73.504  1.00  0.00
ATOM   6412  CA  SER A 403      67.151  60.838  71.742  1.00  0.00
ATOM   6413  HA  SER A 403      66.233  61.098  71.239  1.00  0.00
ATOM   6414  CB  SER A 403      68.277  61.422  70.882  1.00  0.00
ATOM   6415  HB1 SER A 403      68.108  62.495  70.781  1.00  0.00
ATOM   6416  HB2 SER A 403      68.236  60.976  69.886  1.00  0.00
ATOM   6417  OG  SER A 403      69.569  61.210  71.442  1.00  0.00
ATOM   6418  HG  SER A 403      70.164  61.803  70.966  1.00  0.00
ATOM   6419  C   SER A 403      67.219  59.309  71.749  1.00  0.00
ATOM   6420  O   SER A 403      68.003  58.698  72.480  1.00  0.00
ATOM   6421  N   ARG A 404      66.371  58.690  70.917  1.00  0.00
ATOM   6422  H   ARG A 404      65.705  59.286  70.423  1.00  0.00
ATOM   6423  CA  ARG A 404      66.354  57.232  70.653  1.00  0.00
ATOM   6424  HA  ARG A 404      67.328  56.804  70.900  1.00  0.00
ATOM   6425  CB  ARG A 404      65.275  56.496  71.492  1.00  0.00
ATOM   6426  HB1 ARG A 404      65.378  55.430  71.281  1.00  0.00
ATOM   6427  HB2 ARG A 404      64.286  56.790  71.135  1.00  0.00
ATOM   6428  CG  ARG A 404      65.329  56.648  73.020  1.00  0.00
ATOM   6429  HG1 ARG A 404      66.364  56.726  73.344  1.00  0.00
ATOM   6430  HG2 ARG A 404      64.901  55.754  73.477  1.00  0.00
ATOM   6431  CD  ARG A 404      64.496  57.854  73.484  1.00  0.00
ATOM   6432  HD1 ARG A 404      63.454  57.663  73.226  1.00  0.00
ATOM   6433  HD2 ARG A 404      64.803  58.739  72.935  1.00  0.00
ATOM   6434  NE  ARG A 404      64.577  58.141  74.934  1.00  0.00
ATOM   6435  HE  ARG A 404      63.708  58.099  75.437  1.00  0.00
ATOM   6436  CZ  ARG A 404      65.610  58.673  75.567  1.00  0.00
ATOM   6437  NH1 ARG A 404      66.781  58.745  75.022  1.00  0.00
ATOM   6438 1HH1 ARG A 404      66.952  58.385  74.098  1.00  0.00
ATOM   6439 2HH1 ARG A 404      67.549  59.211  75.512  1.00  0.00
ATOM   6440  NH2 ARG A 404      65.498  59.176  76.761  1.00  0.00
ATOM   6441 1HH2 ARG A 404      64.597  59.377  77.162  1.00  0.00
ATOM   6442 2HH2 ARG A 404      66.318  59.590  77.170  1.00  0.00
ATOM   6443  C   ARG A 404      66.108  56.877  69.184  1.00  0.00
ATOM   6444  O   ARG A 404      66.276  55.717  68.836  1.00  0.00
ATOM   6445  N   GLY A 405      65.717  57.837  68.340  1.00  0.00
ATOM   6446  H   GLY A 405      65.633  58.788  68.677  1.00  0.00
ATOM   6447  CA  GLY A 405      65.341  57.590  66.942  1.00  0.00
ATOM   6448  HA1 GLY A 405      64.358  58.024  66.764  1.00  0.00
ATOM   6449  HA2 GLY A 405      65.275  56.519  66.743  1.00  0.00
ATOM   6450  C   GLY A 405      66.321  58.207  65.946  1.00  0.00
ATOM   6451  O   GLY A 405      66.834  57.505  65.080  1.00  0.00
ATOM   6452  N   GLY A 406      66.646  59.494  66.102  1.00  0.00
ATOM   6453  H   GLY A 406      66.212  60.009  66.862  1.00  0.00
ATOM   6454  CA  GLY A 406      67.585  60.187  65.217  1.00  0.00
ATOM   6455  HA1 GLY A 406      67.474  59.799  64.202  1.00  0.00
ATOM   6456  HA2 GLY A 406      68.597  59.957  65.545  1.00  0.00
ATOM   6457  C   GLY A 406      67.470  61.714  65.124  1.00  0.00
ATOM   6458  O   GLY A 406      67.994  62.272  64.167  1.00  0.00
ATOM   6459  N   ALA A 407      66.798  62.384  66.070  1.00  0.00
ATOM   6460  H   ALA A 407      66.412  61.856  66.847  1.00  0.00
ATOM   6461  CA  ALA A 407      66.399  63.800  65.983  1.00  0.00
ATOM   6462  HA  ALA A 407      65.787  64.010  66.862  1.00  0.00
ATOM   6463  CB  ALA A 407      67.621  64.729  66.098  1.00  0.00
ATOM   6464  HB1 ALA A 407      67.277  65.764  66.135  1.00  0.00
```

ATOM	6465	HB2	ALA	A	407	68.172	64.504	67.010	1.00	0.00
ATOM	6466	HB3	ALA	A	407	68.280	64.612	65.238	1.00	0.00
ATOM	6467	C	ALA	A	407	65.488	64.083	64.774	1.00	0.00
ATOM	6468	O	ALA	A	407	64.267	64.023	64.934	1.00	0.00
ATOM	6469	N	SER	A	408	66.002	64.211	63.552	1.00	0.00
ATOM	6470	H	SER	A	408	67.009	64.244	63.469	1.00	0.00
ATOM	6471	CA	SER	A	408	65.209	64.391	62.319	1.00	0.00
ATOM	6472	HA	SER	A	408	64.391	65.058	62.569	1.00	0.00
ATOM	6473	CB	SER	A	408	66.028	65.128	61.253	1.00	0.00
ATOM	6474	HB1	SER	A	408	65.387	65.348	60.398	1.00	0.00
ATOM	6475	HB2	SER	A	408	66.857	64.500	60.927	1.00	0.00
ATOM	6476	OG	SER	A	408	66.523	66.341	61.785	1.00	0.00
ATOM	6477	HG	SER	A	408	65.790	66.795	62.277	1.00	0.00
ATOM	6478	C	SER	A	408	64.524	63.120	61.792	1.00	0.00
ATOM	6479	O	SER	A	408	64.382	62.912	60.589	1.00	0.00
ATOM	6480	N	VAL	A	409	64.059	62.296	62.734	1.00	0.00
ATOM	6481	H	VAL	A	409	64.244	62.612	63.674	1.00	0.00
ATOM	6482	CA	VAL	A	409	62.962	61.325	62.587	1.00	0.00
ATOM	6483	HA	VAL	A	409	62.429	61.575	61.675	1.00	0.00
ATOM	6484	CB	VAL	A	409	63.476	59.886	62.402	1.00	0.00
ATOM	6485	HB	VAL	A	409	64.182	59.894	61.569	1.00	0.00
ATOM	6486	CG1	VAL	A	409	64.218	59.343	63.629	1.00	0.00
ATOM	6487	1HG1	VAL	A	409	64.647	58.371	63.386	1.00	0.00
ATOM	6488	2HG1	VAL	A	409	65.024	60.024	63.892	1.00	0.00
ATOM	6489	3HG1	VAL	A	409	63.532	59.230	64.468	1.00	0.00
ATOM	6490	CG2	VAL	A	409	62.341	58.929	62.036	1.00	0.00
ATOM	6491	1HG2	VAL	A	409	62.738	57.943	61.802	1.00	0.00
ATOM	6492	2HG2	VAL	A	409	61.628	58.838	62.856	1.00	0.00
ATOM	6493	3HG2	VAL	A	409	61.802	59.307	61.166	1.00	0.00
ATOM	6494	C	VAL	A	409	61.973	61.474	63.752	1.00	0.00
ATOM	6495	O	VAL	A	409	60.766	61.504	63.555	1.00	0.00
ATOM	6496	N	GLU	A	410	62.465	61.736	64.966	1.00	0.00
ATOM	6497	H	GLU	A	410	63.463	61.765	65.080	1.00	0.00
ATOM	6498	CA	GLU	A	410	61.649	62.173	66.113	1.00	0.00
ATOM	6499	HA	GLU	A	410	60.876	61.422	66.291	1.00	0.00
ATOM	6500	CB	GLU	A	410	62.559	62.241	67.371	1.00	0.00
ATOM	6501	HB1	GLU	A	410	62.012	62.711	68.188	1.00	0.00
ATOM	6502	HB2	GLU	A	410	63.426	62.862	67.151	1.00	0.00
ATOM	6503	CG	GLU	A	410	63.040	60.847	67.840	1.00	0.00
ATOM	6504	HG1	GLU	A	410	63.280	60.234	66.969	1.00	0.00
ATOM	6505	HG2	GLU	A	410	62.219	60.348	68.361	1.00	0.00
ATOM	6506	CD	GLU	A	410	64.293	60.854	68.750	1.00	0.00
ATOM	6507	OE1	GLU	A	410	65.424	60.781	68.222	1.00	0.00
ATOM	6508	OE2	GLU	A	410	64.197	60.774	69.993	1.00	0.00
ATOM	6509	C	GLU	A	410	60.888	63.495	65.840	1.00	0.00
ATOM	6510	O	GLU	A	410	59.786	63.687	66.346	1.00	0.00
ATOM	6511	N	GLU	A	411	61.444	64.373	64.997	1.00	0.00
ATOM	6512	H	GLU	A	411	62.405	64.195	64.731	1.00	0.00
ATOM	6513	CA	GLU	A	411	60.819	65.596	64.468	1.00	0.00
ATOM	6514	HA	GLU	A	411	60.333	66.143	65.277	1.00	0.00
ATOM	6515	CB	GLU	A	411	61.914	66.473	63.836	1.00	0.00
ATOM	6516	HB1	GLU	A	411	61.449	67.292	63.287	1.00	0.00
ATOM	6517	HB2	GLU	A	411	62.448	65.854	63.117	1.00	0.00
ATOM	6518	CG	GLU	A	411	62.908	67.077	64.844	1.00	0.00
ATOM	6519	HG1	GLU	A	411	62.986	66.422	65.714	1.00	0.00
ATOM	6520	HG2	GLU	A	411	62.514	68.033	65.195	1.00	0.00
ATOM	6521	CD	GLU	A	411	64.317	67.277	64.254	1.00	0.00
ATOM	6522	OE1	GLU	A	411	64.446	67.460	63.019	1.00	0.00
ATOM	6523	OE2	GLU	A	411	65.282	67.175	65.039	1.00	0.00
ATOM	6524	C	GLU	A	411	59.772	65.314	63.381	1.00	0.00
ATOM	6525	O	GLU	A	411	58.708	65.935	63.404	1.00	0.00

ATOM	6526	N	THR	A	412	60.039	64.410	62.425	1.00	0.00
ATOM	6527	H	THR	A	412	60.875	63.845	62.507	1.00	0.00
ATOM	6528	CA	THR	A	412	59.110	64.110	61.309	1.00	0.00
ATOM	6529	HA	THR	A	412	58.764	65.047	60.877	1.00	0.00
ATOM	6530	CB	THR	A	412	59.751	63.271	60.193	1.00	0.00
ATOM	6531	HB	THR	A	412	58.985	62.998	59.465	1.00	0.00
ATOM	6532	CG2	THR	A	412	60.837	64.064	59.471	1.00	0.00
ATOM	6533	1HG2	THR	A	412	60.422	64.984	59.068	1.00	0.00
ATOM	6534	2HG2	THR	A	412	61.650	64.299	60.154	1.00	0.00
ATOM	6535	3HG2	THR	A	412	61.233	63.469	58.652	1.00	0.00
ATOM	6536	OG1	THR	A	412	60.367	62.099	60.670	1.00	0.00
ATOM	6537	HG1	THR	A	412	59.703	61.437	60.982	1.00	0.00
ATOM	6538	C	THR	A	412	57.883	63.344	61.758	1.00	0.00
ATOM	6539	O	THR	A	412	56.790	63.545	61.222	1.00	0.00
ATOM	6540	N	ASP	A	413	58.068	62.520	62.784	1.00	0.00
ATOM	6541	H	ASP	A	413	59.030	62.319	63.052	1.00	0.00
ATOM	6542	CA	ASP	A	413	57.047	61.676	63.389	1.00	0.00
ATOM	6543	HA	ASP	A	413	56.287	61.453	62.638	1.00	0.00
ATOM	6544	CB	ASP	A	413	57.687	60.341	63.803	1.00	0.00
ATOM	6545	HB1	ASP	A	413	56.942	59.749	64.338	1.00	0.00
ATOM	6546	HB2	ASP	A	413	58.516	60.525	64.490	1.00	0.00
ATOM	6547	CG	ASP	A	413	58.182	59.522	62.593	1.00	0.00
ATOM	6548	OD1	ASP	A	413	57.962	58.292	62.621	1.00	0.00
ATOM	6549	OD2	ASP	A	413	58.746	60.116	61.639	1.00	0.00
ATOM	6550	C	ASP	A	413	56.329	62.424	64.531	1.00	0.00
ATOM	6551	O	ASP	A	413	55.525	61.848	65.261	1.00	0.00
ATOM	6552	N	GLN	A	414	56.584	63.740	64.629	1.00	0.00
ATOM	6553	H	GLN	A	414	57.362	64.066	64.071	1.00	0.00
ATOM	6554	CA	GLN	A	414	55.694	64.787	65.146	1.00	0.00
ATOM	6555	HA	GLN	A	414	56.330	65.638	65.388	1.00	0.00
ATOM	6556	CB	GLN	A	414	54.752	65.242	64.018	1.00	0.00
ATOM	6557	HB1	GLN	A	414	53.957	65.871	64.415	1.00	0.00
ATOM	6558	HB2	GLN	A	414	54.297	64.370	63.546	1.00	0.00
ATOM	6559	CG	GLN	A	414	55.527	66.059	62.974	1.00	0.00
ATOM	6560	HG1	GLN	A	414	56.393	65.503	62.641	1.00	0.00
ATOM	6561	HG2	GLN	A	414	55.894	66.984	63.415	1.00	0.00
ATOM	6562	CD	GLN	A	414	54.678	66.388	61.764	1.00	0.00
ATOM	6563	OE1	GLN	A	414	53.962	67.377	61.732	1.00	0.00
ATOM	6564	NE2	GLN	A	414	54.735	65.569	60.736	1.00	0.00
ATOM	6565	1HE2	GLN	A	414	55.349	64.756	60.789	1.00	0.00
ATOM	6566	2HE2	GLN	A	414	54.151	65.766	59.949	1.00	0.00
ATOM	6567	C	GLN	A	414	54.987	64.456	66.462	1.00	0.00
ATOM	6568	O	GLN	A	414	53.817	64.783	66.684	1.00	0.00
ATOM	6569	N	SER	A	415	55.740	63.837	67.368	1.00	0.00
ATOM	6570	H	SER	A	415	56.678	63.593	67.080	1.00	0.00
ATOM	6571	CA	SER	A	415	55.334	63.521	68.737	1.00	0.00
ATOM	6572	HA	SER	A	415	54.309	63.148	68.716	1.00	0.00
ATOM	6573	CB	SER	A	415	56.218	62.391	69.269	1.00	0.00
ATOM	6574	HB1	SER	A	415	56.003	62.220	70.325	1.00	0.00
ATOM	6575	HB2	SER	A	415	57.271	62.652	69.155	1.00	0.00
ATOM	6576	OG	SER	A	415	55.925	61.207	68.547	1.00	0.00
ATOM	6577	HG	SER	A	415	55.987	61.381	67.594	1.00	0.00
ATOM	6578	C	SER	A	415	55.337	64.779	69.621	1.00	0.00
ATOM	6579	O	SER	A	415	56.064	64.864	70.611	1.00	0.00
ATOM	6580	N	HIS	A	416	54.557	65.783	69.197	1.00	0.00
ATOM	6581	H	HIS	A	416	54.040	65.581	68.347	1.00	0.00
ATOM	6582	CA	HIS	A	416	54.469	67.175	69.666	1.00	0.00
ATOM	6583	HA	HIS	A	416	55.474	67.596	69.640	1.00	0.00
ATOM	6584	CB	HIS	A	416	53.616	67.993	68.672	1.00	0.00
ATOM	6585	HB1	HIS	A	416	53.453	68.988	69.086	1.00	0.00
ATOM	6586	HB2	HIS	A	416	52.643	67.515	68.557	1.00	0.00

ATOM	6587	CG	HIS	A	416	54.238	68.196	67.310	1.00	0.00
ATOM	6588	ND1	HIS	A	416	55.571	68.533	67.070	1.00	0.00
ATOM	6589	CE1	HIS	A	416	55.683	68.694	65.742	1.00	0.00
ATOM	6590	HE1	HIS	A	416	56.606	68.950	65.231	1.00	0.00
ATOM	6591	NE2	HIS	A	416	54.496	68.494	65.143	1.00	0.00
ATOM	6592	HE2	HIS	A	416	54.325	68.542	64.142	1.00	0.00
ATOM	6593	CD2	HIS	A	416	53.575	68.172	66.117	1.00	0.00
ATOM	6594	HD2	HIS	A	416	52.526	67.951	65.975	1.00	0.00
ATOM	6595	C	HIS	A	416	53.985	67.356	71.124	1.00	0.00
ATOM	6596	O	HIS	A	416	52.958	67.984	71.383	1.00	0.00
ATOM	6597	N	LEU	A	417	54.698	66.785	72.097	1.00	0.00
ATOM	6598	H	LEU	A	417	55.502	66.224	71.827	1.00	0.00
ATOM	6599	CA	LEU	A	417	54.349	66.880	73.514	1.00	0.00
ATOM	6600	HA	LEU	A	417	53.287	66.634	73.567	1.00	0.00
ATOM	6601	CB	LEU	A	417	55.057	65.778	74.343	1.00	0.00
ATOM	6602	HB1	LEU	A	417	54.979	64.839	73.791	1.00	0.00
ATOM	6603	HB2	LEU	A	417	54.468	65.655	75.252	1.00	0.00
ATOM	6604	CG	LEU	A	417	56.527	65.969	74.781	1.00	0.00
ATOM	6605	HG	LEU	A	417	56.607	66.854	75.407	1.00	0.00
ATOM	6606	CD1	LEU	A	417	56.958	64.781	75.647	1.00	0.00
ATOM	6607	1HD1	LEU	A	417	57.967	64.949	76.016	1.00	0.00
ATOM	6608	2HD1	LEU	A	417	56.287	64.687	76.499	1.00	0.00
ATOM	6609	3HD1	LEU	A	417	56.935	63.864	75.059	1.00	0.00
ATOM	6610	CD2	LEU	A	417	57.504	66.086	73.614	1.00	0.00
ATOM	6611	1HD2	LEU	A	417	58.523	66.165	73.987	1.00	0.00
ATOM	6612	2HD2	LEU	A	417	57.421	65.215	72.963	1.00	0.00
ATOM	6613	3HD2	LEU	A	417	57.287	66.986	73.042	1.00	0.00
ATOM	6614	C	LEU	A	417	54.497	68.306	74.093	1.00	0.00
ATOM	6615	O	LEU	A	417	55.594	68.821	74.300	1.00	0.00
ATOM	6616	N	ALA	A	418	53.363	68.937	74.402	1.00	0.00
ATOM	6617	H	ALA	A	418	52.503	68.500	74.110	1.00	0.00
ATOM	6618	CA	ALA	A	418	53.276	70.331	74.853	1.00	0.00
ATOM	6619	HA	ALA	A	418	53.697	70.956	74.063	1.00	0.00
ATOM	6620	CB	ALA	A	418	51.789	70.687	74.981	1.00	0.00
ATOM	6621	HB1	ALA	A	418	51.684	71.744	75.223	1.00	0.00
ATOM	6622	HB2	ALA	A	418	51.279	70.499	74.037	1.00	0.00
ATOM	6623	HB3	ALA	A	418	51.323	70.092	75.764	1.00	0.00
ATOM	6624	C	ALA	A	418	54.049	70.696	76.142	1.00	0.00
ATOM	6625	O	ALA	A	418	54.230	71.877	76.426	1.00	0.00
ATOM	6626	N	THR	A	419	54.526	69.698	76.900	1.00	0.00
ATOM	6627	H	THR	A	419	54.332	68.769	76.560	1.00	0.00
ATOM	6628	CA	THR	A	419	55.497	69.785	78.015	1.00	0.00
ATOM	6629	HA	THR	A	419	55.684	68.764	78.345	1.00	0.00
ATOM	6630	CB	THR	A	419	56.844	70.308	77.485	1.00	0.00
ATOM	6631	HB	THR	A	419	56.711	71.261	76.975	1.00	0.00
ATOM	6632	CG2	THR	A	419	57.919	70.478	78.557	1.00	0.00
ATOM	6633	1HG2	THR	A	419	58.866	70.730	78.082	1.00	0.00
ATOM	6634	2HG2	THR	A	419	57.643	71.293	79.224	1.00	0.00
ATOM	6635	3HG2	THR	A	419	58.034	69.549	79.111	1.00	0.00
ATOM	6636	OG1	THR	A	419	57.367	69.363	76.578	1.00	0.00
ATOM	6637	HG1	THR	A	419	56.831	69.376	75.771	1.00	0.00
ATOM	6638	C	THR	A	419	55.033	70.525	79.279	1.00	0.00
ATOM	6639	O	THR	A	419	55.120	69.931	80.349	1.00	0.00
ATOM	6640	N	ALA	A	420	54.548	71.762	79.141	1.00	0.00
ATOM	6641	H	ALA	A	420	54.579	72.104	78.188	1.00	0.00
ATOM	6642	CA	ALA	A	420	53.947	72.711	80.095	1.00	0.00
ATOM	6643	HA	ALA	A	420	52.967	72.959	79.685	1.00	0.00
ATOM	6644	CB	ALA	A	420	53.705	72.137	81.497	1.00	0.00
ATOM	6645	HB1	ALA	A	420	53.175	72.859	82.118	1.00	0.00
ATOM	6646	HB2	ALA	A	420	53.105	71.229	81.434	1.00	0.00
ATOM	6647	HB3	ALA	A	420	54.660	71.910	81.962	1.00	0.00

ATOM	6648	C	ALA	A	420	54.740	74.028	80.139	1.00	0.00
ATOM	6649	O	ALA	A	420	55.919	74.071	79.796	1.00	0.00
ATOM	6650	N	GLY	A	421	54.090	75.101	80.607	1.00	0.00
ATOM	6651	H	GLY	A	421	53.137	74.963	80.903	1.00	0.00
ATOM	6652	CA	GLY	A	421	54.574	76.491	80.544	1.00	0.00
ATOM	6653	HA1	GLY	A	421	53.766	77.161	80.835	1.00	0.00
ATOM	6654	HA2	GLY	A	421	54.828	76.710	79.507	1.00	0.00
ATOM	6655	C	GLY	A	421	55.807	76.844	81.387	1.00	0.00
ATOM	6656	O	GLY	A	421	55.909	77.986	81.821	1.00	0.00
ATOM	6657	N	SER	A	422	56.660	75.877	81.751	1.00	0.00
ATOM	6658	H	SER	A	422	56.583	74.986	81.271	1.00	0.00
ATOM	6659	CA	SER	A	422	57.596	75.944	82.890	1.00	0.00
ATOM	6660	HA	SER	A	422	57.932	74.935	83.099	1.00	0.00
ATOM	6661	CB	SER	A	422	58.845	76.755	82.500	1.00	0.00
ATOM	6662	HB1	SER	A	422	58.569	77.800	82.347	1.00	0.00
ATOM	6663	HB2	SER	A	422	59.256	76.359	81.571	1.00	0.00
ATOM	6664	OG	SER	A	422	59.838	76.679	83.510	1.00	0.00
ATOM	6665	HG	SER	A	422	59.385	76.919	84.336	1.00	0.00
ATOM	6666	C	SER	A	422	56.946	76.457	84.192	1.00	0.00
ATOM	6667	O	SER	A	422	57.633	76.921	85.101	1.00	0.00
ATOM	6668	N	THR	A	423	55.612	76.374	84.266	1.00	0.00
ATOM	6669	H	THR	A	423	55.150	75.976	83.468	1.00	0.00
ATOM	6670	CA	THR	A	423	54.773	76.839	85.374	1.00	0.00
ATOM	6671	HA	THR	A	423	54.928	77.912	85.488	1.00	0.00
ATOM	6672	CB	THR	A	423	53.291	76.614	85.038	1.00	0.00
ATOM	6673	HB	THR	A	423	53.064	77.147	84.114	1.00	0.00
ATOM	6674	CG2	THR	A	423	52.906	75.141	84.854	1.00	0.00
ATOM	6675	1HG2	THR	A	423	51.852	75.069	84.591	1.00	0.00
ATOM	6676	2HG2	THR	A	423	53.495	74.695	84.054	1.00	0.00
ATOM	6677	3HG2	THR	A	423	53.080	74.587	85.775	1.00	0.00
ATOM	6678	OG1	THR	A	423	52.469	77.132	86.055	1.00	0.00
ATOM	6679	HG1	THR	A	423	51.576	77.219	85.708	1.00	0.00
ATOM	6680	C	THR	A	423	55.172	76.178	86.692	1.00	0.00
ATOM	6681	O	THR	A	423	55.748	75.089	86.680	1.00	0.00
ATOM	6682	N	SER	A	424	54.850	76.794	87.829	1.00	0.00
ATOM	6683	H	SER	A	424	54.361	77.679	87.779	1.00	0.00
ATOM	6684	CA	SER	A	424	55.347	76.391	89.153	1.00	0.00
ATOM	6685	HA	SER	A	424	56.426	76.546	89.162	1.00	0.00
ATOM	6686	CB	SER	A	424	54.750	77.285	90.248	1.00	0.00
ATOM	6687	HB1	SER	A	424	55.303	77.131	91.176	1.00	0.00
ATOM	6688	HB2	SER	A	424	53.707	77.008	90.410	1.00	0.00
ATOM	6689	OG	SER	A	424	54.808	78.652	89.877	1.00	0.00
ATOM	6690	HG	SER	A	424	54.616	79.191	90.649	1.00	0.00
ATOM	6691	C	SER	A	424	55.106	74.923	89.537	1.00	0.00
ATOM	6692	O	SER	A	424	55.828	74.405	90.378	1.00	0.00
ATOM	6693	N	SER	A	425	54.129	74.237	88.927	1.00	0.00
ATOM	6694	H	SER	A	425	53.561	74.729	88.256	1.00	0.00
ATOM	6695	CA	SER	A	425	54.002	72.774	89.039	1.00	0.00
ATOM	6696	HA	SER	A	425	54.089	72.493	90.090	1.00	0.00
ATOM	6697	CB	SER	A	425	52.625	72.317	88.542	1.00	0.00
ATOM	6698	HB1	SER	A	425	52.549	72.456	87.463	1.00	0.00
ATOM	6699	HB2	SER	A	425	51.861	72.928	89.023	1.00	0.00
ATOM	6700	OG	SER	A	425	52.375	70.964	88.867	1.00	0.00
ATOM	6701	HG	SER	A	425	52.953	70.361	88.357	1.00	0.00
ATOM	6702	C	SER	A	425	55.097	72.030	88.268	1.00	0.00
ATOM	6703	O	SER	A	425	55.824	71.217	88.823	1.00	0.00
ATOM	6704	N	HIS	A	426	55.245	72.328	86.979	1.00	0.00
ATOM	6705	H	HIS	A	426	54.744	73.127	86.626	1.00	0.00
ATOM	6706	CA	HIS	A	426	56.184	71.668	86.073	1.00	0.00
ATOM	6707	HA	HIS	A	426	56.030	70.588	86.105	1.00	0.00
ATOM	6708	CB	HIS	A	426	55.872	72.172	84.662	1.00	0.00

ATOM	6709	HB1	HIS	A	426	55.861	73.262	84.665	1.00	0.00
ATOM	6710	HB2	HIS	A	426	54.877	71.825	84.383	1.00	0.00
ATOM	6711	CG	HIS	A	426	56.857	71.723	83.626	1.00	0.00
ATOM	6712	ND1	HIS	A	426	57.918	72.495	83.156	1.00	0.00
ATOM	6713	CE1	HIS	A	426	58.449	71.801	82.143	1.00	0.00
ATOM	6714	HE1	HIS	A	426	59.258	72.158	81.518	1.00	0.00
ATOM	6715	NE2	HIS	A	426	57.799	70.637	81.981	1.00	0.00
ATOM	6716	HE2	HIS	A	426	57.939	69.984	81.227	1.00	0.00
ATOM	6717	CD2	HIS	A	426	56.791	70.569	82.911	1.00	0.00
ATOM	6718	HD2	HIS	A	426	56.030	69.804	82.994	1.00	0.00
ATOM	6719	C	HIS	A	426	57.645	71.932	86.445	1.00	0.00
ATOM	6720	O	HIS	A	426	58.426	70.987	86.525	1.00	0.00
ATOM	6721	N	SER	A	427	57.996	73.181	86.761	1.00	0.00
ATOM	6722	H	SER	A	427	57.280	73.899	86.715	1.00	0.00
ATOM	6723	CA	SER	A	427	59.334	73.557	87.246	1.00	0.00
ATOM	6724	HA	SER	A	427	60.064	72.950	86.709	1.00	0.00
ATOM	6725	CB	SER	A	427	59.603	75.025	86.892	1.00	0.00
ATOM	6726	HB1	SER	A	427	58.908	75.669	87.433	1.00	0.00
ATOM	6727	HB2	SER	A	427	59.471	75.169	85.820	1.00	0.00
ATOM	6728	OG	SER	A	427	60.933	75.349	87.246	1.00	0.00
ATOM	6729	HG	SER	A	427	60.997	75.043	88.174	1.00	0.00
ATOM	6730	C	SER	A	427	59.548	73.269	88.748	1.00	0.00
ATOM	6731	O	SER	A	427	60.424	73.866	89.377	1.00	0.00
ATOM	6732	N	LEU	A	428	58.746	72.356	89.303	1.00	0.00
ATOM	6733	H	LEU	A	428	58.009	71.989	88.723	1.00	0.00
ATOM	6734	CA	LEU	A	428	58.949	71.698	90.592	1.00	0.00
ATOM	6735	HA	LEU	A	428	59.924	71.983	90.990	1.00	0.00
ATOM	6736	CB	LEU	A	428	57.852	72.188	91.556	1.00	0.00
ATOM	6737	HB1	LEU	A	428	56.875	71.920	91.155	1.00	0.00
ATOM	6738	HB2	LEU	A	428	57.900	73.277	91.577	1.00	0.00
ATOM	6739	CG	LEU	A	428	57.907	71.724	93.025	1.00	0.00
ATOM	6740	HG	LEU	A	428	57.151	72.303	93.558	1.00	0.00
ATOM	6741	CD1	LEU	A	428	57.549	70.250	93.221	1.00	0.00
ATOM	6742	1HD1	LEU	A	428	57.351	70.072	94.277	1.00	0.00
ATOM	6743	2HD1	LEU	A	428	56.655	70.004	92.649	1.00	0.00
ATOM	6744	3HD1	LEU	A	428	58.369	69.604	92.917	1.00	0.00
ATOM	6745	CD2	LEU	A	428	59.253	72.007	93.682	1.00	0.00
ATOM	6746	1HD2	LEU	A	428	59.195	71.796	94.749	1.00	0.00
ATOM	6747	2HD2	LEU	A	428	60.032	71.385	93.243	1.00	0.00
ATOM	6748	3HD2	LEU	A	428	59.518	73.056	93.552	1.00	0.00
ATOM	6749	C	LEU	A	428	58.969	70.172	90.372	1.00	0.00
ATOM	6750	O	LEU	A	428	59.864	69.490	90.862	1.00	0.00
ATOM	6751	N	GLN	A	429	58.063	69.631	89.550	1.00	0.00
ATOM	6752	H	GLN	A	429	57.314	70.230	89.220	1.00	0.00
ATOM	6753	CA	GLN	A	429	58.057	68.225	89.110	1.00	0.00
ATOM	6754	HA	GLN	A	429	57.995	67.589	89.995	1.00	0.00
ATOM	6755	CB	GLN	A	429	56.815	67.978	88.234	1.00	0.00
ATOM	6756	HB1	GLN	A	429	56.914	67.012	87.738	1.00	0.00
ATOM	6757	HB2	GLN	A	429	56.768	68.746	87.461	1.00	0.00
ATOM	6758	CG	GLN	A	429	55.498	67.968	89.028	1.00	0.00
ATOM	6759	HG1	GLN	A	429	55.441	68.837	89.681	1.00	0.00
ATOM	6760	HG2	GLN	A	429	55.464	67.077	89.655	1.00	0.00
ATOM	6761	CD	GLN	A	429	54.284	67.987	88.101	1.00	0.00
ATOM	6762	OE1	GLN	A	429	53.845	69.027	87.629	1.00	0.00
ATOM	6763	NE2	GLN	A	429	53.701	66.851	87.784	1.00	0.00
ATOM	6764	1HE2	GLN	A	429	54.046	65.984	88.150	1.00	0.00
ATOM	6765	2HE2	GLN	A	429	52.891	66.913	87.191	1.00	0.00
ATOM	6766	C	GLN	A	429	59.326	67.796	88.347	1.00	0.00
ATOM	6767	O	GLN	A	429	59.774	66.649	88.469	1.00	0.00
ATOM	6768	N	LYS	A	430	59.965	68.710	87.613	1.00	0.00
ATOM	6769	H	LYS	A	430	59.488	69.595	87.461	1.00	0.00

ATOM	6770	CA	LYS	A	430	61.428	68.719	87.500	1.00	0.00
ATOM	6771	HA	LYS	A	430	61.832	67.737	87.729	1.00	0.00
ATOM	6772	CB	LYS	A	430	61.897	69.164	86.106	1.00	0.00
ATOM	6773	HB1	LYS	A	430	62.964	69.390	86.172	1.00	0.00
ATOM	6774	HB2	LYS	A	430	61.374	70.078	85.814	1.00	0.00
ATOM	6775	CG	LYS	A	430	61.687	68.082	85.046	1.00	0.00
ATOM	6776	HG1	LYS	A	430	60.624	68.001	84.811	1.00	0.00
ATOM	6777	HG2	LYS	A	430	62.040	67.123	85.431	1.00	0.00
ATOM	6778	CD	LYS	A	430	62.481	68.429	83.779	1.00	0.00
ATOM	6779	HD1	LYS	A	430	63.539	68.499	84.033	1.00	0.00
ATOM	6780	HD2	LYS	A	430	62.140	69.385	83.381	1.00	0.00
ATOM	6781	CE	LYS	A	430	62.273	67.326	82.744	1.00	0.00
ATOM	6782	HE1	LYS	A	430	61.259	67.400	82.344	1.00	0.00
ATOM	6783	HE2	LYS	A	430	62.354	66.368	83.257	1.00	0.00
ATOM	6784	NZ	LYS	A	430	63.269	67.380	81.649	1.00	0.00
ATOM	6785	HZ1	LYS	A	430	63.130	66.578	81.038	1.00	0.00
ATOM	6786	HZ2	LYS	A	430	63.184	68.225	81.102	1.00	0.00
ATOM	6787	HZ3	LYS	A	430	64.219	67.315	82.015	1.00	0.00
ATOM	6788	C	LYS	A	430	61.952	69.703	88.540	1.00	0.00
ATOM	6789	O	LYS	A	430	61.721	70.903	88.414	1.00	0.00
ATOM	6790	N	TYR	A	431	62.699	69.195	89.518	1.00	0.00
ATOM	6791	H	TYR	A	431	62.745	68.198	89.641	1.00	0.00
ATOM	6792	CA	TYR	A	431	63.555	70.019	90.370	1.00	0.00
ATOM	6793	HA	TYR	A	431	62.921	70.696	90.942	1.00	0.00
ATOM	6794	CB	TYR	A	431	64.339	69.123	91.344	1.00	0.00
ATOM	6795	HB1	TYR	A	431	64.825	69.753	92.090	1.00	0.00
ATOM	6796	HB2	TYR	A	431	65.127	68.624	90.779	1.00	0.00
ATOM	6797	CG	TYR	A	431	63.540	68.057	92.085	1.00	0.00
ATOM	6798	CD1	TYR	A	431	62.254	68.337	92.587	1.00	0.00
ATOM	6799	HD1	TYR	A	431	61.824	69.322	92.467	1.00	0.00
ATOM	6800	CE1	TYR	A	431	61.511	67.335	93.239	1.00	0.00
ATOM	6801	HE1	TYR	A	431	60.518	67.548	93.610	1.00	0.00
ATOM	6802	CZ	TYR	A	431	62.060	66.052	93.417	1.00	0.00
ATOM	6803	OH	TYR	A	431	61.334	65.099	94.059	1.00	0.00
ATOM	6804	HH	TYR	A	431	61.820	64.281	94.141	1.00	0.00
ATOM	6805	CE2	TYR	A	431	63.359	65.770	92.940	1.00	0.00
ATOM	6806	HE2	TYR	A	431	63.800	64.797	93.086	1.00	0.00
ATOM	6807	CD2	TYR	A	431	64.092	66.773	92.276	1.00	0.00
ATOM	6808	HD2	TYR	A	431	65.087	66.563	91.922	1.00	0.00
ATOM	6809	C	TYR	A	431	64.524	70.871	89.529	1.00	0.00
ATOM	6810	O	TYR	A	431	64.748	70.590	88.346	1.00	0.00
ATOM	6811	N	TYR	A	432	65.111	71.894	90.148	1.00	0.00
ATOM	6812	H	TYR	A	432	64.916	72.057	91.126	1.00	0.00
ATOM	6813	CA	TYR	A	432	66.260	72.612	89.603	1.00	0.00
ATOM	6814	HA	TYR	A	432	66.935	71.879	89.174	1.00	0.00
ATOM	6815	CB	TYR	A	432	65.836	73.543	88.443	1.00	0.00
ATOM	6816	HB1	TYR	A	432	65.247	72.980	87.721	1.00	0.00
ATOM	6817	HB2	TYR	A	432	66.743	73.844	87.916	1.00	0.00
ATOM	6818	CG	TYR	A	432	65.058	74.791	88.811	1.00	0.00
ATOM	6819	CD1	TYR	A	432	63.704	74.720	89.197	1.00	0.00
ATOM	6820	HD1	TYR	A	432	63.205	73.759	89.254	1.00	0.00
ATOM	6821	CE1	TYR	A	432	63.016	75.889	89.584	1.00	0.00
ATOM	6822	HE1	TYR	A	432	62.007	75.835	89.956	1.00	0.00
ATOM	6823	CZ	TYR	A	432	63.676	77.133	89.572	1.00	0.00
ATOM	6824	OH	TYR	A	432	63.049	78.258	90.006	1.00	0.00
ATOM	6825	HH	TYR	A	432	63.638	79.013	90.057	1.00	0.00
ATOM	6826	CE2	TYR	A	432	65.007	77.203	89.124	1.00	0.00
ATOM	6827	HE2	TYR	A	432	65.523	78.142	89.119	1.00	0.00
ATOM	6828	CD2	TYR	A	432	65.697	76.041	88.748	1.00	0.00
ATOM	6829	HD2	TYR	A	432	66.741	76.114	88.456	1.00	0.00
ATOM	6830	C	TYR	A	432	67.049	73.330	90.709	1.00	0.00

ATOM	6831	O	TYR	A	432	66.633	73.353	91.867	1.00	0.00
ATOM	6832	N	ILE	A	433	68.200	73.883	90.321	1.00	0.00
ATOM	6833	H	ILE	A	433	68.402	73.853	89.338	1.00	0.00
ATOM	6834	CA	ILE	A	433	69.344	74.235	91.171	1.00	0.00
ATOM	6835	HA	ILE	A	433	69.752	73.285	91.529	1.00	0.00
ATOM	6836	CB	ILE	A	433	70.423	74.882	90.269	1.00	0.00
ATOM	6837	HB	ILE	A	433	70.519	74.247	89.390	1.00	0.00
ATOM	6838	CG2	ILE	A	433	70.034	76.286	89.775	1.00	0.00
ATOM	6839	1HG2	ILE	A	433	70.690	76.604	88.965	1.00	0.00
ATOM	6840	2HG2	ILE	A	433	69.012	76.297	89.405	1.00	0.00
ATOM	6841	3HG2	ILE	A	433	70.114	77.011	90.585	1.00	0.00
ATOM	6842	CG1	ILE	A	433	71.797	74.890	90.965	1.00	0.00
ATOM	6843	1HG1	ILE	A	433	72.007	73.893	91.353	1.00	0.00
ATOM	6844	2HG1	ILE	A	433	71.782	75.594	91.791	1.00	0.00
ATOM	6845	CD	ILE	A	433	72.934	75.279	90.023	1.00	0.00
ATOM	6846	HD1	ILE	A	433	73.885	75.134	90.535	1.00	0.00
ATOM	6847	HD2	ILE	A	433	72.907	74.654	89.132	1.00	0.00
ATOM	6848	HD3	ILE	A	433	72.850	76.321	89.726	1.00	0.00
ATOM	6849	C	ILE	A	433	69.074	75.078	92.430	1.00	0.00
ATOM	6850	O	ILE	A	433	68.360	76.082	92.386	1.00	0.00
ATOM	6851	N	THR	A	434	69.758	74.710	93.524	1.00	0.00
ATOM	6852	H	THR	A	434	70.311	73.848	93.493	1.00	0.00
ATOM	6853	CA	THR	A	434	69.785	75.490	94.778	1.00	0.00
ATOM	6854	HA	THR	A	434	68.972	76.214	94.739	1.00	0.00
ATOM	6855	CB	THR	A	434	69.520	74.624	96.021	1.00	0.00
ATOM	6856	HB	THR	A	434	69.183	75.291	96.813	1.00	0.00
ATOM	6857	CG2	THR	A	434	68.436	73.568	95.814	1.00	0.00
ATOM	6858	1HG2	THR	A	434	68.174	73.126	96.773	1.00	0.00
ATOM	6859	2HG2	THR	A	434	67.547	74.030	95.386	1.00	0.00
ATOM	6860	3HG2	THR	A	434	68.795	72.785	95.148	1.00	0.00
ATOM	6861	OG1	THR	A	434	70.677	73.979	96.493	1.00	0.00
ATOM	6862	HG1	THR	A	434	71.175	73.623	95.720	1.00	0.00
ATOM	6863	C	THR	A	434	71.059	76.310	94.975	1.00	0.00
ATOM	6864	O	THR	A	434	70.988	77.349	95.614	1.00	0.00
ATOM	6865	N	GLY	A	435	72.190	75.901	94.389	1.00	0.00
ATOM	6866	H	GLY	A	435	72.194	74.932	94.066	1.00	0.00
ATOM	6867	CA	GLY	A	435	73.388	76.695	94.066	1.00	0.00
ATOM	6868	HA1	GLY	A	435	73.189	77.267	93.162	1.00	0.00
ATOM	6869	HA2	GLY	A	435	74.187	75.992	93.837	1.00	0.00
ATOM	6870	C	GLY	A	435	73.956	77.666	95.114	1.00	0.00
ATOM	6871	O	GLY	A	435	74.657	78.598	94.718	1.00	0.00
ATOM	6872	N	GLU	A	436	73.705	77.429	96.402	1.00	0.00
ATOM	6873	H	GLU	A	436	73.001	76.734	96.593	1.00	0.00
ATOM	6874	CA	GLU	A	436	74.424	77.994	97.555	1.00	0.00
ATOM	6875	HA	GLU	A	436	74.023	77.503	98.443	1.00	0.00
ATOM	6876	CB	GLU	A	436	75.910	77.549	97.425	1.00	0.00
ATOM	6877	HB1	GLU	A	436	76.363	78.085	96.592	1.00	0.00
ATOM	6878	HB2	GLU	A	436	75.921	76.488	97.165	1.00	0.00
ATOM	6879	CG	GLU	A	436	76.854	77.716	98.627	1.00	0.00
ATOM	6880	HG1	GLU	A	436	77.003	78.775	98.838	1.00	0.00
ATOM	6881	HG2	GLU	A	436	77.830	77.310	98.349	1.00	0.00
ATOM	6882	CD	GLU	A	436	76.337	76.996	99.874	1.00	0.00
ATOM	6883	OE1	GLU	A	436	75.445	77.588	100.527	1.00	0.00
ATOM	6884	OE2	GLU	A	436	76.805	75.868	100.141	1.00	0.00
ATOM	6885	C	GLU	A	436	74.266	79.513	97.800	1.00	0.00
ATOM	6886	O	GLU	A	436	73.799	80.293	96.959	1.00	0.00
ATOM	6887	N	ALA	A	437	74.665	79.934	99.005	1.00	0.00
ATOM	6888	H	ALA	A	437	75.006	79.231	99.660	1.00	0.00
ATOM	6889	CA	ALA	A	437	74.844	81.334	99.384	1.00	0.00
ATOM	6890	HA	ALA	A	437	73.858	81.799	99.422	1.00	0.00
ATOM	6891	CB	ALA	A	437	75.448	81.381	100.791	1.00	0.00

ATOM	6892	HB1	ALA	A	437	75.549	82.417	101.114	1.00	0.00
ATOM	6893	HB2	ALA	A	437	74.804	80.847	101.490	1.00	0.00
ATOM	6894	HB3	ALA	A	437	76.434	80.913	100.783	1.00	0.00
ATOM	6895	C	ALA	A	437	75.698	82.118	98.370	1.00	0.00
ATOM	6896	O	ALA	A	437	76.664	81.610	97.806	1.00	0.00
ATOM	6897	N	GLU	A	438	75.325	83.380	98.151	1.00	0.00
ATOM	6898	H	GLU	A	438	74.669	83.796	98.808	1.00	0.00
ATOM	6899	CA	GLU	A	438	75.862	84.302	97.138	1.00	0.00
ATOM	6900	HA	GLU	A	438	75.095	85.067	97.021	1.00	0.00
ATOM	6901	CB	GLU	A	438	77.095	85.049	97.683	1.00	0.00
ATOM	6902	HB1	GLU	A	438	77.335	85.861	96.997	1.00	0.00
ATOM	6903	HB2	GLU	A	438	77.948	84.371	97.714	1.00	0.00
ATOM	6904	CG	GLU	A	438	76.879	85.649	99.087	1.00	0.00
ATOM	6905	HG1	GLU	A	438	77.652	86.398	99.278	1.00	0.00
ATOM	6906	HG2	GLU	A	438	77.006	84.859	99.831	1.00	0.00
ATOM	6907	CD	GLU	A	438	75.482	86.282	99.256	1.00	0.00
ATOM	6908	OE1	GLU	A	438	74.612	85.600	99.846	1.00	0.00
ATOM	6909	OE2	GLU	A	438	75.270	87.391	98.723	1.00	0.00
ATOM	6910	C	GLU	A	438	76.007	83.741	95.702	1.00	0.00
ATOM	6911	O	GLU	A	438	76.659	84.355	94.854	1.00	0.00
ATOM	6912	N	GLY	A	439	75.323	82.633	95.383	1.00	0.00
ATOM	6913	H	GLY	A	439	74.916	82.099	96.142	1.00	0.00
ATOM	6914	CA	GLY	A	439	75.186	82.087	94.031	1.00	0.00
ATOM	6915	HA1	GLY	A	439	75.595	81.076	94.024	1.00	0.00
ATOM	6916	HA2	GLY	A	439	75.754	82.684	93.318	1.00	0.00
ATOM	6917	C	GLY	A	439	73.740	82.029	93.556	1.00	0.00
ATOM	6918	O	GLY	A	439	73.295	82.934	92.856	1.00	0.00
ATOM	6919	N	PHE	A	440	72.960	81.041	93.990	1.00	0.00
ATOM	6920	H	PHE	A	440	73.369	80.335	94.603	1.00	0.00
ATOM	6921	CA	PHE	A	440	71.528	80.952	93.662	1.00	0.00
ATOM	6922	HA	PHE	A	440	71.199	81.900	93.241	1.00	0.00
ATOM	6923	CB	PHE	A	440	71.277	79.874	92.586	1.00	0.00
ATOM	6924	HB1	PHE	A	440	70.200	79.742	92.470	1.00	0.00
ATOM	6925	HB2	PHE	A	440	71.670	78.924	92.946	1.00	0.00
ATOM	6926	CG	PHE	A	440	71.814	80.162	91.195	1.00	0.00
ATOM	6927	CD1	PHE	A	440	71.446	81.342	90.517	1.00	0.00
ATOM	6928	HD1	PHE	A	440	70.820	82.077	91.001	1.00	0.00
ATOM	6929	CE1	PHE	A	440	71.876	81.565	89.198	1.00	0.00
ATOM	6930	HE1	PHE	A	440	71.582	82.464	88.676	1.00	0.00
ATOM	6931	CZ	PHE	A	440	72.665	80.607	88.543	1.00	0.00
ATOM	6932	HZ	PHE	A	440	72.980	80.781	87.525	1.00	0.00
ATOM	6933	CE2	PHE	A	440	73.024	79.424	89.208	1.00	0.00
ATOM	6934	HE2	PHE	A	440	73.609	78.678	88.690	1.00	0.00
ATOM	6935	CD2	PHE	A	440	72.603	79.203	90.531	1.00	0.00
ATOM	6936	HD2	PHE	A	440	72.868	78.285	91.031	1.00	0.00
ATOM	6937	C	PHE	A	440	70.660	80.751	94.918	1.00	0.00
ATOM	6938	O	PHE	A	440	69.887	79.799	94.965	1.00	0.00
ATOM	6939	N	PRO	A	441	70.655	81.697	95.884	1.00	0.00
ATOM	6940	CD	PRO	A	441	71.475	82.902	95.927	1.00	0.00
ATOM	6941	HD1	PRO	A	441	71.505	83.407	94.961	1.00	0.00
ATOM	6942	HD2	PRO	A	441	72.484	82.635	96.243	1.00	0.00
ATOM	6943	CG	PRO	A	441	70.837	83.811	96.971	1.00	0.00
ATOM	6944	HG1	PRO	A	441	70.048	84.405	96.507	1.00	0.00
ATOM	6945	HG2	PRO	A	441	71.573	84.460	97.448	1.00	0.00
ATOM	6946	CB	PRO	A	441	70.222	82.826	97.961	1.00	0.00
ATOM	6947	HB1	PRO	A	441	69.374	83.265	98.488	1.00	0.00
ATOM	6948	HB2	PRO	A	441	70.986	82.512	98.675	1.00	0.00
ATOM	6949	CA	PRO	A	441	69.822	81.623	97.094	1.00	0.00
ATOM	6950	HA	PRO	A	441	70.116	80.723	97.635	1.00	0.00
ATOM	6951	C	PRO	A	441	68.296	81.502	96.871	1.00	0.00
ATOM	6952	O	PRO	A	441	67.538	81.457	97.832	1.00	0.00

```
ATOM   6953  N   ALA A 442      67.834  81.366  95.621  1.00  0.00
ATOM   6954  H   ALA A 442      68.535  81.313  94.898  1.00  0.00
ATOM   6955  CA  ALA A 442      66.529  80.819  95.270  1.00  0.00
ATOM   6956  HA  ALA A 442      65.785  81.599  95.444  1.00  0.00
ATOM   6957  CB  ALA A 442      66.522  80.491  93.778  1.00  0.00
ATOM   6958  HB1 ALA A 442      65.543  80.100  93.499  1.00  0.00
ATOM   6959  HB2 ALA A 442      66.724  81.400  93.218  1.00  0.00
ATOM   6960  HB3 ALA A 442      67.281  79.739  93.553  1.00  0.00
ATOM   6961  C   ALA A 442      66.089  79.610  96.105  1.00  0.00
ATOM   6962  O   ALA A 442      64.930  79.563  96.504  1.00  0.00
ATOM   6963  N   THR A 443      66.988  78.654  96.384  1.00  0.00
ATOM   6964  H   THR A 443      67.924  78.772  96.011  1.00  0.00
ATOM   6965  CA  THR A 443      66.741  77.439  97.208  1.00  0.00
ATOM   6966  HA  THR A 443      67.677  76.882  97.240  1.00  0.00
ATOM   6967  CB  THR A 443      66.414  77.827  98.666  1.00  0.00
ATOM   6968  HB  THR A 443      65.403  78.231  98.721  1.00  0.00
ATOM   6969  CG2 THR A 443      66.535  76.669  99.658  1.00  0.00
ATOM   6970 1HG2 THR A 443      66.397  77.044 100.672  1.00  0.00
ATOM   6971 2HG2 THR A 443      65.769  75.921  99.461  1.00  0.00
ATOM   6972 3HG2 THR A 443      67.520  76.211  99.577  1.00  0.00
ATOM   6973  OG1 THR A 443      67.317  78.807  99.131  1.00  0.00
ATOM   6974  HG1 THR A 443      67.183  79.627  98.634  1.00  0.00
ATOM   6975  C   THR A 443      65.690  76.471  96.632  1.00  0.00
ATOM   6976  O   THR A 443      65.699  75.291  96.968  1.00  0.00
ATOM   6977  N   VAL A 444      64.832  76.929  95.711  1.00  0.00
ATOM   6978  H   VAL A 444      64.826  77.933  95.593  1.00  0.00
ATOM   6979  CA  VAL A 444      63.859  76.169  94.906  1.00  0.00
ATOM   6980  HA  VAL A 444      63.078  76.852  94.575  1.00  0.00
ATOM   6981  CB  VAL A 444      64.543  75.576  93.656  1.00  0.00
ATOM   6982  HB  VAL A 444      65.343  74.906  93.974  1.00  0.00
ATOM   6983  CG1 VAL A 444      63.564  74.773  92.789  1.00  0.00
ATOM   6984 1HG1 VAL A 444      64.090  74.397  91.914  1.00  0.00
ATOM   6985 2HG1 VAL A 444      63.181  73.916  93.339  1.00  0.00
ATOM   6986 3HG1 VAL A 444      62.734  75.407  92.475  1.00  0.00
ATOM   6987  CG2 VAL A 444      65.149  76.679  92.779  1.00  0.00
ATOM   6988 1HG2 VAL A 444      65.611  76.228  91.901  1.00  0.00
ATOM   6989 2HG2 VAL A 444      64.379  77.384  92.470  1.00  0.00
ATOM   6990 3HG2 VAL A 444      65.934  77.204  93.320  1.00  0.00
ATOM   6991  C   VAL A 444      63.178  75.060  95.712  1.00  0.00
TER
END
```
