# Supplementary material for: Structural analysis of hubs in human NR-RTK network
Source: Biol Direct. 2011 Oct 5;6:49. doi: 10.1186/1745-6150-6-49 (PMC3220635; doi:10.1186/1745-6150-6-49)
Supplement: Additional file 3 — PGR. PGR structure. [file 1745-6150-6-49-S3.PDF]

HEADER PGR

ATOM	1	N	PRO	563	26.644	38.847	78.366	1.00	50.00
ATOM	2	CA	PRO	563	25.869	40.074	78.611	1.00	50.00
ATOM	3	C	PRO	563	24.901	40.476	77.490	1.00	50.00
ATOM	4	O	PRO	563	23.829	41.004	77.784	1.00	50.00
ATOM	5	CB	PRO	563	26.902	41.163	78.922	1.00	50.00
ATOM	6	CG	PRO	563	28.228	40.587	78.422	1.00	50.00
ATOM	7	CD	PRO	563	28.073	39.078	78.623	1.00	50.00
ATOM	8	N	GLN	564	25.255	40.233	76.227	1.00	50.00
ATOM	9	CA	GLN	564	24.349	40.546	75.102	1.00	50.00
ATOM	10	C	GLN	564	23.248	39.475	75.033	1.00	50.00
ATOM	11	O	GLN	564	23.527	38.277	75.065	1.00	50.00
ATOM	12	CB	GLN	564	25.111	40.581	73.775	1.00	50.00
ATOM	13	CG	GLN	564	24.579	41.705	72.877	1.00	50.00
ATOM	14	CD	GLN	564	24.902	41.519	71.389	1.00	50.00
ATOM	15	OE1	GLN	564	25.541	40.582	70.934	1.00	50.00
ATOM	16	NE2	GLN	564	24.385	42.410	70.574	1.00	50.00
ATOM	17	N	LYS	565	22.001	39.932	75.024	1.00	50.00
ATOM	18	CA	LYS	565	20.842	39.020	74.938	1.00	50.00
ATOM	19	C	LYS	565	20.554	38.728	73.461	1.00	50.00
ATOM	20	O	LYS	565	20.518	39.644	72.642	1.00	50.00
ATOM	21	CB	LYS	565	19.614	39.640	75.614	1.00	50.00
ATOM	22	CG	LYS	565	19.895	40.102	77.048	1.00	50.00
ATOM	23	CD	LYS	565	18.906	39.491	78.042	1.00	50.00
ATOM	24	CE	LYS	565	18.631	40.443	79.209	1.00	50.00
ATOM	25	NZ	LYS	565	17.858	41.612	78.757	1.00	50.00
ATOM	26	N	ILE	566	20.330	37.457	73.149	1.00	50.00
ATOM	27	CA	ILE	566	20.171	36.991	71.756	1.00	50.00
ATOM	28	C	ILE	566	18.698	36.647	71.494	1.00	50.00
ATOM	29	O	ILE	566	18.084	35.876	72.229	1.00	50.00
ATOM	30	CB	ILE	566	21.093	35.772	71.525	1.00	50.00
ATOM	31	CG1	ILE	566	22.566	36.151	71.717	1.00	50.00
ATOM	32	CG2	ILE	566	20.926	35.137	70.139	1.00	50.00
ATOM	33	CD1	ILE	566	23.210	35.334	72.840	1.00	50.00
ATOM	34	N	CYS	567	18.163	37.233	70.424	1.00	50.00
ATOM	35	CA	CYS	567	16.826	36.889	69.903	1.00	50.00
ATOM	36	C	CYS	567	16.765	35.375	69.655	1.00	50.00
ATOM	37	O	CYS	567	17.527	34.848	68.845	1.00	50.00
ATOM	38	CB	CYS	567	16.577	37.627	68.582	1.00	50.00
ATOM	39	SG	CYS	567	14.981	37.198	67.790	1.00	50.00
ATOM	40	N	LEU	568	15.758	34.746	70.247	1.00	50.00
ATOM	41	CA	LEU	568	15.546	33.296	70.077	1.00	50.00
ATOM	42	C	LEU	568	15.027	32.881	68.693	1.00	50.00
ATOM	43	O	LEU	568	15.092	31.713	68.327	1.00	50.00
ATOM	44	CB	LEU	568	14.668	32.723	71.197	1.00	50.00
ATOM	45	CG	LEU	568	15.410	32.614	72.538	1.00	50.00
ATOM	46	CD1	LEU	568	14.440	32.132	73.618	1.00	50.00
ATOM	47	CD2	LEU	568	16.601	31.650	72.457	1.00	50.00
ATOM	48	N	ILE	569	14.569	33.865	67.921	1.00	50.00
ATOM	49	CA	ILE	569	14.007	33.610	66.578	1.00	50.00
ATOM	50	C	ILE	569	15.074	33.721	65.477	1.00	50.00
ATOM	51	O	ILE	569	15.163	32.875	64.597	1.00	50.00
ATOM	52	CB	ILE	569	12.820	34.555	66.283	1.00	50.00
ATOM	53	CG1	ILE	569	11.813	34.647	67.445	1.00	50.00
ATOM	54	CG2	ILE	569	12.108	34.186	64.971	1.00	50.00
ATOM	55	CD1	ILE	569	11.271	33.308	67.965	1.00	50.00
ATOM	56	N	CYS	570	15.862	34.788	65.521	1.00	50.00
ATOM	57	CA	CYS	570	16.791	35.082	64.412	1.00	50.00
ATOM	58	C	CYS	570	18.272	35.159	64.810	1.00	50.00
ATOM	59	O	CYS	570	19.128	35.375	63.952	1.00	50.00
ATOM	60	CB	CYS	570	16.357	36.362	63.693	1.00	50.00

ATOM	61	SG	CYS	570	16.643	37.881	64.670	1.00	50.00
ATOM	62	N	GLY	571	18.521	35.225	66.126	1.00	50.00
ATOM	63	CA	GLY	571	19.893	35.351	66.650	1.00	50.00
ATOM	64	C	GLY	571	20.492	36.766	66.615	1.00	50.00
ATOM	65	O	GLY	571	21.664	36.934	66.938	1.00	50.00
ATOM	66	N	ASP	572	19.711	37.770	66.211	1.00	50.00
ATOM	67	CA	ASP	572	20.117	39.183	66.381	1.00	50.00
ATOM	68	C	ASP	572	20.172	39.500	67.892	1.00	50.00
ATOM	69	O	ASP	572	19.759	38.695	68.725	1.00	50.00
ATOM	70	CB	ASP	572	19.118	40.100	65.655	1.00	50.00
ATOM	71	CG	ASP	572	19.559	41.562	65.459	1.00	50.00
ATOM	72	OD1	ASP	572	20.695	41.910	65.848	1.00	50.00
ATOM	73	OD2	ASP	572	18.728	42.323	64.922	1.00	50.00
ATOM	74	N	GLU	573	20.681	40.676	68.240	1.00	50.00
ATOM	75	CA	GLU	573	20.557	41.217	69.605	1.00	50.00
ATOM	76	C	GLU	573	19.065	41.372	69.942	1.00	50.00
ATOM	77	O	GLU	573	18.302	41.986	69.195	1.00	50.00
ATOM	78	CB	GLU	573	21.227	42.590	69.680	1.00	50.00
ATOM	79	CG	GLU	573	21.165	43.147	71.108	1.00	50.00
ATOM	80	CD	GLU	573	21.671	44.585	71.180	1.00	50.00
ATOM	81	OE1	GLU	573	22.910	44.740	71.104	1.00	50.00
ATOM	82	OE2	GLU	573	20.819	45.491	71.263	1.00	50.00
ATOM	83	N	ALA	574	18.700	40.826	71.090	1.00	50.00
ATOM	84	CA	ALA	574	17.333	40.920	71.622	1.00	50.00
ATOM	85	C	ALA	574	17.186	42.211	72.434	1.00	50.00
ATOM	86	O	ALA	574	18.106	42.641	73.128	1.00	50.00
ATOM	87	CB	ALA	574	17.041	39.704	72.498	1.00	50.00
ATOM	88	N	SER	575	16.005	42.799	72.310	1.00	50.00
ATOM	89	CA	SER	575	15.672	44.067	72.992	1.00	50.00
ATOM	90	C	SER	575	14.909	43.879	74.308	1.00	50.00
ATOM	91	O	SER	575	15.051	44.685	75.226	1.00	50.00
ATOM	92	CB	SER	575	14.890	45.007	72.071	1.00	50.00
ATOM	93	OG	SER	575	13.771	44.333	71.483	1.00	50.00
ATOM	94	N	GLY	576	14.154	42.776	74.388	1.00	50.00
ATOM	95	CA	GLY	576	13.375	42.441	75.590	1.00	50.00
ATOM	96	C	GLY	576	12.647	41.108	75.409	1.00	50.00
ATOM	97	O	GLY	576	12.925	40.334	74.489	1.00	50.00
ATOM	98	N	CYS	577	11.738	40.869	76.342	1.00	50.00
ATOM	99	CA	CYS	577	10.844	39.701	76.304	1.00	50.00
ATOM	100	C	CYS	577	9.506	40.133	75.689	1.00	50.00
ATOM	101	O	CYS	577	8.753	40.909	76.276	1.00	50.00
ATOM	102	CB	CYS	577	10.668	39.173	77.729	1.00	50.00
ATOM	103	SG	CYS	577	9.606	37.688	77.829	1.00	50.00
ATOM	104	N	HIS	578	9.283	39.695	74.456	1.00	50.00
ATOM	105	CA	HIS	578	8.123	40.143	73.659	1.00	50.00
ATOM	106	C	HIS	578	7.206	38.971	73.325	1.00	50.00
ATOM	107	O	HIS	578	7.676	37.911	72.913	1.00	50.00
ATOM	108	CB	HIS	578	8.584	40.788	72.353	1.00	50.00
ATOM	109	CG	HIS	578	9.522	41.968	72.605	1.00	50.00
ATOM	110	ND1	HIS	578	9.193	43.128	73.166	1.00	50.00
ATOM	111	CD2	HIS	578	10.814	42.003	72.295	1.00	50.00
ATOM	112	CE1	HIS	578	10.289	43.881	73.198	1.00	50.00
ATOM	113	NE2	HIS	578	11.294	43.185	72.665	1.00	50.00
ATOM	114	N	TYR	579	5.918	39.185	73.595	1.00	50.00
ATOM	115	CA	TYR	579	4.858	38.158	73.464	1.00	50.00
ATOM	116	C	TYR	579	5.217	36.803	74.111	1.00	50.00
ATOM	117	O	TYR	579	4.846	35.741	73.611	1.00	50.00
ATOM	118	CB	TYR	579	4.458	37.971	71.992	1.00	50.00
ATOM	119	CG	TYR	579	3.983	39.275	71.359	1.00	50.00
ATOM	120	CD1	TYR	579	2.787	39.863	71.752	1.00	50.00
ATOM	121	CD2	TYR	579	4.801	39.891	70.423	1.00	50.00

ATOM	122	CE1	TYR	579	2.412	41.084	71.208	1.00	50.00
ATOM	123	CE2	TYR	579	4.423	41.103	69.869	1.00	50.00
ATOM	124	CZ	TYR	579	3.228	41.692	70.260	1.00	50.00
ATOM	125	OH	TYR	579	2.871	42.882	69.722	1.00	50.00
ATOM	126	N	GLY	580	6.064	36.877	75.149	1.00	50.00
ATOM	127	CA	GLY	580	6.443	35.688	75.933	1.00	50.00
ATOM	128	C	GLY	580	7.879	35.167	75.757	1.00	50.00
ATOM	129	O	GLY	580	8.289	34.312	76.537	1.00	50.00
ATOM	130	N	VAL	581	8.616	35.617	74.738	1.00	50.00
ATOM	131	CA	VAL	581	9.985	35.095	74.499	1.00	50.00
ATOM	132	C	VAL	581	11.001	36.224	74.253	1.00	50.00
ATOM	133	O	VAL	581	10.641	37.337	73.867	1.00	50.00
ATOM	134	CB	VAL	581	10.076	33.894	73.501	1.00	50.00
ATOM	135	CG1	VAL	581	8.730	33.292	73.091	1.00	50.00
ATOM	136	CG2	VAL	581	10.932	34.077	72.241	1.00	50.00
ATOM	137	N	LEU	582	12.267	35.895	74.494	1.00	50.00
ATOM	138	CA	LEU	582	13.416	36.774	74.203	1.00	50.00
ATOM	139	C	LEU	582	13.567	37.031	72.693	1.00	50.00
ATOM	140	O	LEU	582	13.949	36.137	71.932	1.00	50.00
ATOM	141	CB	LEU	582	14.715	36.120	74.688	1.00	50.00
ATOM	142	CG	LEU	582	15.589	36.926	75.662	1.00	50.00
ATOM	143	CD1	LEU	582	17.038	36.474	75.481	1.00	50.00
ATOM	144	CD2	LEU	582	15.478	38.451	75.549	1.00	50.00
ATOM	145	N	THR	583	13.177	38.223	72.248	1.00	50.00
ATOM	146	CA	THR	583	13.292	38.567	70.813	1.00	50.00
ATOM	147	C	THR	583	13.869	39.971	70.584	1.00	50.00
ATOM	148	O	THR	583	13.968	40.808	71.484	1.00	50.00
ATOM	149	CB	THR	583	11.987	38.421	69.995	1.00	50.00
ATOM	150	OG1	THR	583	11.157	39.573	70.131	1.00	50.00
ATOM	151	CG2	THR	583	11.169	37.171	70.330	1.00	50.00
ATOM	152	N	CYS	584	14.390	40.140	69.374	1.00	50.00
ATOM	153	CA	CYS	584	14.688	41.473	68.823	1.00	50.00
ATOM	154	C	CYS	584	13.363	42.181	68.474	1.00	50.00
ATOM	155	O	CYS	584	12.356	41.530	68.177	1.00	50.00
ATOM	156	CB	CYS	584	15.569	41.339	67.575	1.00	50.00
ATOM	157	SG	CYS	584	14.710	40.614	66.132	1.00	50.00
ATOM	158	N	GLY	585	13.484	43.494	68.252	1.00	50.00
ATOM	159	CA	GLY	585	12.352	44.351	67.844	1.00	50.00
ATOM	160	C	GLY	585	11.740	43.985	66.482	1.00	50.00
ATOM	161	O	GLY	585	10.526	44.064	66.321	1.00	50.00
ATOM	162	N	SER	586	12.556	43.456	65.566	1.00	50.00
ATOM	163	CA	SER	586	12.085	43.142	64.198	1.00	50.00
ATOM	164	C	SER	586	11.185	41.901	64.170	1.00	50.00
ATOM	165	O	SER	586	10.101	41.937	63.596	1.00	50.00
ATOM	166	CB	SER	586	13.230	42.981	63.193	1.00	50.00
ATOM	167	OG	SER	586	14.032	41.851	63.533	1.00	50.00
ATOM	168	N	CYS	587	11.579	40.884	64.941	1.00	50.00
ATOM	169	CA	CYS	587	10.777	39.659	65.111	1.00	50.00
ATOM	170	C	CYS	587	9.506	39.933	65.926	1.00	50.00
ATOM	171	O	CYS	587	8.437	39.466	65.546	1.00	50.00
ATOM	172	CB	CYS	587	11.606	38.552	65.761	1.00	50.00
ATOM	173	SG	CYS	587	12.982	37.993	64.692	1.00	50.00
ATOM	174	N	LYS	588	9.614	40.823	66.918	1.00	50.00
ATOM	175	CA	LYS	588	8.453	41.299	67.700	1.00	50.00
ATOM	176	C	LYS	588	7.314	41.799	66.782	1.00	50.00
ATOM	177	O	LYS	588	6.213	41.248	66.816	1.00	50.00
ATOM	178	CB	LYS	588	8.889	42.408	68.668	1.00	50.00
ATOM	179	CG	LYS	588	7.701	42.934	69.480	1.00	50.00
ATOM	180	CD	LYS	588	7.831	44.434	69.722	1.00	50.00
ATOM	181	CE	LYS	588	6.459	45.023	70.044	1.00	50.00
ATOM	182	NZ	LYS	588	6.412	46.434	69.636	1.00	50.00

ATOM	183	N	VAL	589	7.602	42.823	65.974	1.00	50.00
ATOM	184	CA	VAL	589	6.623	43.380	65.011	1.00	50.00
ATOM	185	C	VAL	589	6.229	42.393	63.917	1.00	50.00
ATOM	186	O	VAL	589	5.049	42.307	63.583	1.00	50.00
ATOM	187	CB	VAL	589	7.019	44.658	64.252	1.00	50.00
ATOM	188	CG1	VAL	589	6.118	45.811	64.687	1.00	50.00
ATOM	189	CG2	VAL	589	8.499	45.039	64.316	1.00	50.00
ATOM	190	N	PHE	590	7.211	41.634	63.428	1.00	50.00
ATOM	191	CA	PHE	590	6.958	40.649	62.364	1.00	50.00
ATOM	192	C	PHE	590	5.864	39.684	62.823	1.00	50.00
ATOM	193	O	PHE	590	4.876	39.493	62.124	1.00	50.00
ATOM	194	CB	PHE	590	8.228	39.864	62.012	1.00	50.00
ATOM	195	CG	PHE	590	7.886	38.623	61.180	1.00	50.00
ATOM	196	CD1	PHE	590	7.606	38.753	59.827	1.00	50.00
ATOM	197	CD2	PHE	590	7.669	37.411	61.827	1.00	50.00
ATOM	198	CE1	PHE	590	7.123	37.664	59.114	1.00	50.00
ATOM	199	CE2	PHE	590	7.192	36.323	61.112	1.00	50.00
ATOM	200	CZ	PHE	590	6.931	36.446	59.753	1.00	50.00
ATOM	201	N	PHE	591	6.004	39.231	64.063	1.00	50.00
ATOM	202	CA	PHE	591	5.065	38.268	64.643	1.00	50.00
ATOM	203	C	PHE	591	3.643	38.838	64.736	1.00	50.00
ATOM	204	O	PHE	591	2.718	38.216	64.218	1.00	50.00
ATOM	205	CB	PHE	591	5.602	37.820	65.998	1.00	50.00
ATOM	206	CG	PHE	591	4.580	36.973	66.751	1.00	50.00
ATOM	207	CD1	PHE	591	4.353	35.655	66.383	1.00	50.00
ATOM	208	CD2	PHE	591	3.844	37.560	67.771	1.00	50.00
ATOM	209	CE1	PHE	591	3.382	34.916	67.044	1.00	50.00
ATOM	210	CE2	PHE	591	2.878	36.818	68.435	1.00	50.00
ATOM	211	CZ	PHE	591	2.651	35.495	68.075	1.00	50.00
ATOM	212	N	LYS	592	3.521	40.069	65.238	1.00	50.00
ATOM	213	CA	LYS	592	2.211	40.745	65.327	1.00	50.00
ATOM	214	C	LYS	592	1.595	40.998	63.939	1.00	50.00
ATOM	215	O	LYS	592	0.448	40.627	63.698	1.00	50.00
ATOM	216	CB	LYS	592	2.301	42.036	66.153	1.00	50.00
ATOM	217	CG	LYS	592	0.960	42.783	66.186	1.00	50.00
ATOM	218	CD	LYS	592	0.476	43.071	67.607	1.00	50.00
ATOM	219	CE	LYS	592	-0.884	43.775	67.644	1.00	50.00
ATOM	220	NZ	LYS	592	-0.872	45.105	67.011	1.00	50.00
ATOM	221	N	ARG	593	2.371	41.600	63.038	1.00	50.00
ATOM	222	CA	ARG	593	1.939	41.806	61.642	1.00	50.00
ATOM	223	C	ARG	593	1.542	40.502	60.939	1.00	50.00
ATOM	224	O	ARG	593	0.466	40.436	60.357	1.00	50.00
ATOM	225	CB	ARG	593	3.038	42.487	60.831	1.00	50.00
ATOM	226	CG	ARG	593	3.263	43.921	61.302	1.00	50.00
ATOM	227	CD	ARG	593	4.394	44.543	60.494	1.00	50.00
ATOM	228	NE	ARG	593	4.474	45.976	60.818	1.00	50.00
ATOM	229	CZ	ARG	593	5.579	46.721	60.795	1.00	50.00
ATOM	230	NH1	ARG	593	6.759	46.201	60.484	1.00	50.00
ATOM	231	NH2	ARG	593	5.505	48.018	61.057	1.00	50.00
ATOM	232	N	ALA	594	2.308	39.435	61.157	1.00	50.00
ATOM	233	CA	ALA	594	2.067	38.130	60.510	1.00	50.00
ATOM	234	C	ALA	594	0.772	37.477	61.007	1.00	50.00
ATOM	235	O	ALA	594	-0.086	37.130	60.200	1.00	50.00
ATOM	236	CB	ALA	594	3.249	37.189	60.750	1.00	50.00
ATOM	237	N	MET	595	0.587	37.502	62.326	1.00	50.00
ATOM	238	CA	MET	595	-0.609	36.940	62.982	1.00	50.00
ATOM	239	C	MET	595	-1.908	37.702	62.683	1.00	50.00
ATOM	240	O	MET	595	-2.982	37.108	62.653	1.00	50.00
ATOM	241	CB	MET	595	-0.388	36.853	64.493	1.00	50.00
ATOM	242	CG	MET	595	0.637	35.775	64.858	1.00	50.00
ATOM	243	SD	MET	595	0.124	34.075	64.412	1.00	50.00

ATOM	244	CE	MET	595	-1.001	33.720	65.746	1.00	50.00
ATOM	245	N	GLU	596	-1.778	38.999	62.421	1.00	50.00
ATOM	246	CA	GLU	596	-2.934	39.880	62.169	1.00	50.00
ATOM	247	C	GLU	596	-3.218	40.171	60.690	1.00	50.00
ATOM	248	O	GLU	596	-4.298	40.653	60.346	1.00	50.00
ATOM	249	CB	GLU	596	-2.745	41.181	62.942	1.00	50.00
ATOM	250	CG	GLU	596	-3.587	41.141	64.218	1.00	50.00
ATOM	251	CD	GLU	596	-3.014	42.018	65.332	1.00	50.00
ATOM	252	OE1	GLU	596	-2.350	43.030	65.008	1.00	50.00
ATOM	253	OE2	GLU	596	-3.261	41.650	66.501	1.00	50.00
ATOM	254	N	GLY	597	-2.190	39.969	59.858	1.00	50.00
ATOM	255	CA	GLY	597	-2.258	40.191	58.404	1.00	50.00
ATOM	256	C	GLY	597	-3.247	39.231	57.735	1.00	50.00
ATOM	257	O	GLY	597	-3.734	38.274	58.331	1.00	50.00
ATOM	258	N	GLN	598	-3.445	39.469	56.445	1.00	50.00
ATOM	259	CA	GLN	598	-4.325	38.609	55.628	1.00	50.00
ATOM	260	C	GLN	598	-3.587	37.462	54.912	1.00	50.00
ATOM	261	O	GLN	598	-4.189	36.731	54.127	1.00	50.00
ATOM	262	CB	GLN	598	-5.099	39.438	54.590	1.00	50.00
ATOM	263	CG	GLN	598	-5.979	40.553	55.172	1.00	50.00
ATOM	264	CD	GLN	598	-6.837	40.101	56.357	1.00	50.00
ATOM	265	OE1	GLN	598	-7.680	39.218	56.284	1.00	50.00
ATOM	266	NE2	GLN	598	-6.605	40.739	57.482	1.00	50.00
ATOM	267	N	HIS	599	-2.334	37.210	55.290	1.00	50.00
ATOM	268	CA	HIS	599	-1.500	36.239	54.556	1.00	50.00
ATOM	269	C	HIS	599	-1.078	35.043	55.410	1.00	50.00
ATOM	270	O	HIS	599	-0.394	35.193	56.421	1.00	50.00
ATOM	271	CB	HIS	599	-0.235	36.884	53.977	1.00	50.00
ATOM	272	CG	HIS	599	-0.518	37.957	52.923	1.00	50.00
ATOM	273	ND1	HIS	599	-0.389	39.266	53.106	1.00	50.00
ATOM	274	CD2	HIS	599	-0.870	37.749	51.657	1.00	50.00
ATOM	275	CE1	HIS	599	-0.656	39.870	51.953	1.00	50.00
ATOM	276	NE2	HIS	599	-0.952	38.933	51.057	1.00	50.00
ATOM	277	N	ASN	600	-1.493	33.866	54.957	1.00	50.00
ATOM	278	CA	ASN	600	-0.930	32.605	55.464	1.00	50.00
ATOM	279	C	ASN	600	0.222	32.212	54.532	1.00	50.00
ATOM	280	O	ASN	600	0.014	31.768	53.402	1.00	50.00
ATOM	281	CB	ASN	600	-2.009	31.520	55.534	1.00	50.00
ATOM	282	CG	ASN	600	-1.473	30.205	56.110	1.00	50.00
ATOM	283	OD1	ASN	600	-0.481	30.136	56.824	1.00	50.00
ATOM	284	ND2	ASN	600	-2.134	29.125	55.764	1.00	50.00
ATOM	285	N	TYR	601	1.425	32.556	54.980	1.00	50.00
ATOM	286	CA	TYR	601	2.639	32.423	54.155	1.00	50.00
ATOM	287	C	TYR	601	2.891	30.951	53.803	1.00	50.00
ATOM	288	O	TYR	601	2.793	30.068	54.654	1.00	50.00
ATOM	289	CB	TYR	601	3.859	32.990	54.889	1.00	50.00
ATOM	290	CG	TYR	601	3.700	34.447	55.328	1.00	50.00
ATOM	291	CD1	TYR	601	3.686	35.465	54.383	1.00	50.00
ATOM	292	CD2	TYR	601	3.686	34.757	56.682	1.00	50.00
ATOM	293	CE1	TYR	601	3.653	36.792	54.789	1.00	50.00
ATOM	294	CE2	TYR	601	3.653	36.084	57.090	1.00	50.00
ATOM	295	CZ	TYR	601	3.624	37.101	56.143	1.00	50.00
ATOM	296	OH	TYR	601	3.551	38.399	56.533	1.00	50.00
ATOM	297	N	LEU	602	3.293	30.731	52.558	1.00	50.00
ATOM	298	CA	LEU	602	3.621	29.382	52.073	1.00	50.00
ATOM	299	C	LEU	602	5.134	29.315	51.847	1.00	50.00
ATOM	300	O	LEU	602	5.679	30.068	51.039	1.00	50.00
ATOM	301	CB	LEU	602	2.914	29.094	50.740	1.00	50.00
ATOM	302	CG	LEU	602	2.324	27.684	50.538	1.00	50.00
ATOM	303	CD1	LEU	602	2.413	27.322	49.055	1.00	50.00
ATOM	304	CD2	LEU	602	2.920	26.566	51.405	1.00	50.00

ATOM	305	N	CYS	603	5.819	28.548	52.692	1.00	50.00
ATOM	306	CA	CYS	603	7.267	28.314	52.521	1.00	50.00
ATOM	307	C	CYS	603	7.527	27.731	51.123	1.00	50.00
ATOM	308	O	CYS	603	7.002	26.675	50.783	1.00	50.00
ATOM	309	CB	CYS	603	7.780	27.342	53.589	1.00	50.00
ATOM	310	SG	CYS	603	9.555	26.936	53.398	1.00	50.00
ATOM	311	N	ALA	604	8.409	28.395	50.380	1.00	50.00
ATOM	312	CA	ALA	604	8.772	27.947	49.019	1.00	50.00
ATOM	313	C	ALA	604	9.768	26.769	49.006	1.00	50.00
ATOM	314	O	ALA	604	10.023	26.174	47.963	1.00	50.00
ATOM	315	CB	ALA	604	9.287	29.133	48.199	1.00	50.00
ATOM	316	N	GLY	605	10.372	26.515	50.178	1.00	50.00
ATOM	317	CA	GLY	605	11.313	25.399	50.386	1.00	50.00
ATOM	318	C	GLY	605	10.594	24.175	50.966	1.00	50.00
ATOM	319	O	GLY	605	9.639	23.663	50.391	1.00	50.00
ATOM	320	N	ARG	606	11.028	23.784	52.162	1.00	50.00
ATOM	321	CA	ARG	606	10.496	22.578	52.838	1.00	50.00
ATOM	322	C	ARG	606	10.016	22.799	54.282	1.00	50.00
ATOM	323	O	ARG	606	10.038	21.875	55.093	1.00	50.00
ATOM	324	CB	ARG	606	11.559	21.476	52.846	1.00	50.00
ATOM	325	CG	ARG	606	11.980	21.063	51.443	1.00	50.00
ATOM	326	CD	ARG	606	13.020	19.968	51.584	1.00	50.00
ATOM	327	NE	ARG	606	13.827	19.855	50.368	1.00	50.00
ATOM	328	CZ	ARG	606	14.701	18.826	50.142	1.00	50.00
ATOM	329	NH1	ARG	606	14.665	17.951	51.119	1.00	50.00
ATOM	330	NH2	ARG	606	15.429	18.800	49.043	1.00	50.00
ATOM	331	N	ASN	607	9.641	24.035	54.626	1.00	50.00
ATOM	332	CA	ASN	607	9.245	24.397	56.013	1.00	50.00
ATOM	333	C	ASN	607	10.385	24.119	57.013	1.00	50.00
ATOM	334	O	ASN	607	10.195	23.798	58.181	1.00	50.00
ATOM	335	CB	ASN	607	7.967	23.653	56.434	1.00	50.00
ATOM	336	CG	ASN	607	6.753	24.135	55.646	1.00	50.00
ATOM	337	OD1	ASN	607	6.364	25.292	55.718	1.00	50.00
ATOM	338	ND2	ASN	607	6.199	23.277	54.817	1.00	50.00
ATOM	339	N	ASP	608	11.579	24.386	56.500	1.00	50.00
ATOM	340	CA	ASP	608	12.875	24.003	57.079	1.00	50.00
ATOM	341	C	ASP	608	13.818	25.192	57.300	1.00	50.00
ATOM	342	O	ASP	608	14.907	25.024	57.843	1.00	50.00
ATOM	343	CB	ASP	608	13.522	23.099	56.029	1.00	50.00
ATOM	344	CG	ASP	608	14.003	21.748	56.559	1.00	50.00
ATOM	345	OD1	ASP	608	13.657	21.402	57.708	1.00	50.00
ATOM	346	OD2	ASP	608	14.647	21.056	55.742	1.00	50.00
ATOM	347	N	CYS	609	13.442	26.333	56.723	1.00	50.00
ATOM	348	CA	CYS	609	14.395	27.421	56.437	1.00	50.00
ATOM	349	C	CYS	609	15.119	27.896	57.698	1.00	50.00
ATOM	350	O	CYS	609	14.562	27.887	58.795	1.00	50.00
ATOM	351	CB	CYS	609	13.683	28.612	55.792	1.00	50.00
ATOM	352	SG	CYS	609	12.870	28.220	54.201	1.00	50.00
ATOM	353	N	ILE	610	16.391	28.223	57.512	1.00	50.00
ATOM	354	CA	ILE	610	17.180	28.867	58.576	1.00	50.00
ATOM	355	C	ILE	610	16.679	30.310	58.739	1.00	50.00
ATOM	356	O	ILE	610	16.544	31.057	57.770	1.00	50.00
ATOM	357	CB	ILE	610	18.680	28.752	58.249	1.00	50.00
ATOM	358	CG1	ILE	610	19.095	27.285	58.421	1.00	50.00
ATOM	359	CG2	ILE	610	19.562	29.658	59.127	1.00	50.00
ATOM	360	CD1	ILE	610	20.196	26.864	57.444	1.00	50.00
ATOM	361	N	VAL	611	16.490	30.680	59.997	1.00	50.00
ATOM	362	CA	VAL	611	16.053	32.041	60.352	1.00	50.00
ATOM	363	C	VAL	611	17.189	32.671	61.163	1.00	50.00
ATOM	364	O	VAL	611	17.382	32.382	62.340	1.00	50.00
ATOM	365	CB	VAL	611	14.727	32.046	61.138	1.00	50.00

ATOM	366	CG1	VAL	611	14.268	33.480	61.430	1.00	50.00
ATOM	367	CG2	VAL	611	13.608	31.316	60.388	1.00	50.00
ATOM	368	N	ASP	612	18.031	33.388	60.436	1.00	50.00
ATOM	369	CA	ASP	612	19.140	34.123	61.066	1.00	50.00
ATOM	370	C	ASP	612	18.997	35.619	60.758	1.00	50.00
ATOM	371	O	ASP	612	18.158	36.004	59.944	1.00	50.00
ATOM	372	CB	ASP	612	20.496	33.536	60.640	1.00	50.00
ATOM	373	CG	ASP	612	20.741	33.518	59.128	1.00	50.00
ATOM	374	OD1	ASP	612	20.107	34.334	58.418	1.00	50.00
ATOM	375	OD2	ASP	612	21.624	32.742	58.718	1.00	50.00
ATOM	376	N	LYS	613	19.958	36.406	61.220	1.00	50.00
ATOM	377	CA	LYS	613	19.894	37.874	61.098	1.00	50.00
ATOM	378	C	LYS	613	19.786	38.371	59.642	1.00	50.00
ATOM	379	O	LYS	613	18.829	39.064	59.308	1.00	50.00
ATOM	380	CB	LYS	613	21.128	38.468	61.767	1.00	50.00
ATOM	381	CG	LYS	613	20.953	39.963	62.020	1.00	50.00
ATOM	382	CD	LYS	613	22.336	40.607	62.102	1.00	50.00
ATOM	383	CE	LYS	613	22.397	41.720	63.148	1.00	50.00
ATOM	384	NZ	LYS	613	21.445	42.810	62.885	1.00	50.00
ATOM	385	N	ILE	614	20.688	37.908	58.773	1.00	50.00
ATOM	386	CA	ILE	614	20.718	38.345	57.357	1.00	50.00
ATOM	387	C	ILE	614	19.461	37.911	56.574	1.00	50.00
ATOM	388	O	ILE	614	18.955	38.661	55.741	1.00	50.00
ATOM	389	CB	ILE	614	22.027	37.891	56.669	1.00	50.00
ATOM	390	CG1	ILE	614	23.223	38.604	57.322	1.00	50.00
ATOM	391	CG2	ILE	614	22.025	38.149	55.148	1.00	50.00
ATOM	392	CD1	ILE	614	24.585	37.984	56.982	1.00	50.00
ATOM	393	N	ARG	615	18.925	36.744	56.913	1.00	50.00
ATOM	394	CA	ARG	615	17.918	36.090	56.058	1.00	50.00
ATOM	395	C	ARG	615	16.540	35.916	56.699	1.00	50.00
ATOM	396	O	ARG	615	15.645	35.359	56.071	1.00	50.00
ATOM	397	CB	ARG	615	18.425	34.715	55.624	1.00	50.00
ATOM	398	CG	ARG	615	19.709	34.831	54.805	1.00	50.00
ATOM	399	CD	ARG	615	20.223	33.456	54.398	1.00	50.00
ATOM	400	NE	ARG	615	20.655	32.644	55.553	1.00	50.00
ATOM	401	CZ	ARG	615	21.238	31.448	55.449	1.00	50.00
ATOM	402	NH1	ARG	615	21.431	30.903	54.256	1.00	50.00
ATOM	403	NH2	ARG	615	21.688	30.803	56.517	1.00	50.00
ATOM	404	N	ARG	616	16.316	36.524	57.860	1.00	50.00
ATOM	405	CA	ARG	616	15.013	36.416	58.551	1.00	50.00
ATOM	406	C	ARG	616	13.819	36.872	57.692	1.00	50.00
ATOM	407	O	ARG	616	12.778	36.219	57.700	1.00	50.00
ATOM	408	CB	ARG	616	15.051	37.143	59.897	1.00	50.00
ATOM	409	CG	ARG	616	15.368	38.632	59.757	1.00	50.00
ATOM	410	CD	ARG	616	15.713	39.205	61.122	1.00	50.00
ATOM	411	NE	ARG	616	16.019	40.634	60.968	1.00	50.00
ATOM	412	CZ	ARG	616	16.607	41.401	61.883	1.00	50.00
ATOM	413	NH1	ARG	616	17.005	40.905	63.045	1.00	50.00
ATOM	414	NH2	ARG	616	16.782	42.693	61.649	1.00	50.00
ATOM	415	N	LYS	617	14.069	37.841	56.806	1.00	50.00
ATOM	416	CA	LYS	617	13.038	38.338	55.873	1.00	50.00
ATOM	417	C	LYS	617	12.714	37.336	54.746	1.00	50.00
ATOM	418	O	LYS	617	11.635	37.397	54.163	1.00	50.00
ATOM	419	CB	LYS	617	13.399	39.728	55.317	1.00	50.00
ATOM	420	CG	LYS	617	14.193	39.735	54.004	1.00	50.00
ATOM	421	CD	LYS	617	14.491	41.155	53.525	1.00	50.00
ATOM	422	CE	LYS	617	15.237	41.150	52.187	1.00	50.00
ATOM	423	NZ	LYS	617	14.374	40.781	51.052	1.00	50.00
ATOM	424	N	ASN	618	13.674	36.462	54.426	1.00	50.00
ATOM	425	CA	ASN	618	13.580	35.518	53.292	1.00	50.00
ATOM	426	C	ASN	618	12.302	34.674	53.279	1.00	50.00

ATOM	427	O	ASN	618	11.583	34.668	52.284	1.00	50.00
ATOM	428	CB	ASN	618	14.795	34.581	53.250	1.00	50.00
ATOM	429	CG	ASN	618	16.064	35.231	52.690	1.00	50.00
ATOM	430	OD1	ASN	618	16.971	34.564	52.217	1.00	50.00
ATOM	431	ND2	ASN	618	16.154	36.544	52.744	1.00	50.00
ATOM	432	N	CYS	619	12.012	34.030	54.407	1.00	50.00
ATOM	433	CA	CYS	619	10.846	33.139	54.493	1.00	50.00
ATOM	434	C	CYS	619	9.976	33.476	55.712	1.00	50.00
ATOM	435	O	CYS	619	10.100	32.837	56.764	1.00	50.00
ATOM	436	CB	CYS	619	11.282	31.671	54.463	1.00	50.00
ATOM	437	SG	CYS	619	9.854	30.527	54.457	1.00	50.00
ATOM	438	N	PRO	620	8.991	34.354	55.479	1.00	50.00
ATOM	439	CA	PRO	620	8.025	34.669	56.552	1.00	50.00
ATOM	440	C	PRO	620	7.280	33.460	57.138	1.00	50.00
ATOM	441	O	PRO	620	7.143	33.385	58.356	1.00	50.00
ATOM	442	CB	PRO	620	7.078	35.712	55.962	1.00	50.00
ATOM	443	CG	PRO	620	7.150	35.459	54.459	1.00	50.00
ATOM	444	CD	PRO	620	8.612	35.076	54.248	1.00	50.00
ATOM	445	N	ALA	621	7.045	32.424	56.328	1.00	50.00
ATOM	446	CA	ALA	621	6.376	31.195	56.809	1.00	50.00
ATOM	447	C	ALA	621	7.178	30.508	57.928	1.00	50.00
ATOM	448	O	ALA	621	6.711	30.432	59.063	1.00	50.00
ATOM	449	CB	ALA	621	6.143	30.215	55.658	1.00	50.00
ATOM	450	N	CYS	622	8.470	30.310	57.664	1.00	50.00
ATOM	451	CA	CYS	622	9.393	29.673	58.624	1.00	50.00
ATOM	452	C	CYS	622	9.729	30.545	59.838	1.00	50.00
ATOM	453	O	CYS	622	9.821	30.043	60.954	1.00	50.00
ATOM	454	CB	CYS	622	10.681	29.227	57.936	1.00	50.00
ATOM	455	SG	CYS	622	10.409	27.794	56.832	1.00	50.00
ATOM	456	N	ARG	623	9.774	31.859	59.624	1.00	50.00
ATOM	457	CA	ARG	623	10.026	32.819	60.713	1.00	50.00
ATOM	458	C	ARG	623	8.848	32.829	61.699	1.00	50.00
ATOM	459	O	ARG	623	9.045	32.736	62.909	1.00	50.00
ATOM	460	CB	ARG	623	10.233	34.212	60.123	1.00	50.00
ATOM	461	CG	ARG	623	10.658	35.213	61.196	1.00	50.00
ATOM	462	CD	ARG	623	10.767	36.582	60.543	1.00	50.00
ATOM	463	NE	ARG	623	11.381	37.545	61.467	1.00	50.00
ATOM	464	CZ	ARG	623	11.591	38.831	61.185	1.00	50.00
ATOM	465	NH1	ARG	623	11.201	39.351	60.028	1.00	50.00
ATOM	466	NH2	ARG	623	12.254	39.598	62.033	1.00	50.00
ATOM	467	N	LEU	624	7.637	32.792	61.144	1.00	50.00
ATOM	468	CA	LEU	624	6.402	32.745	61.943	1.00	50.00
ATOM	469	C	LEU	624	6.282	31.410	62.684	1.00	50.00
ATOM	470	O	LEU	624	6.064	31.399	63.894	1.00	50.00
ATOM	471	CB	LEU	624	5.177	33.002	61.058	1.00	50.00
ATOM	472	CG	LEU	624	3.839	32.853	61.800	1.00	50.00
ATOM	473	CD1	LEU	624	3.687	33.853	62.951	1.00	50.00
ATOM	474	CD2	LEU	624	2.687	32.987	60.806	1.00	50.00
ATOM	475	N	ARG	625	6.609	30.330	61.978	1.00	50.00
ATOM	476	CA	ARG	625	6.621	28.980	62.566	1.00	50.00
ATOM	477	C	ARG	625	7.593	28.922	63.758	1.00	50.00
ATOM	478	O	ARG	625	7.185	28.561	64.861	1.00	50.00
ATOM	479	CB	ARG	625	7.039	27.970	61.500	1.00	50.00
ATOM	480	CG	ARG	625	6.835	26.541	62.011	1.00	50.00
ATOM	481	CD	ARG	625	7.755	25.550	61.302	1.00	50.00
ATOM	482	NE	ARG	625	9.159	25.867	61.631	1.00	50.00
ATOM	483	CZ	ARG	625	10.096	26.231	60.754	1.00	50.00
ATOM	484	NH1	ARG	625	9.800	26.378	59.473	1.00	50.00
ATOM	485	NH2	ARG	625	11.346	26.433	61.148	1.00	50.00
ATOM	486	N	LYS	626	8.774	29.511	63.567	1.00	50.00
ATOM	487	CA	LYS	626	9.804	29.591	64.616	1.00	50.00

ATOM	488	C	LYS	626	9.353	30.467	65.799	1.00	50.00
ATOM	489	O	LYS	626	9.499	30.053	66.946	1.00	50.00
ATOM	490	CB	LYS	626	11.123	30.062	63.996	1.00	50.00
ATOM	491	CG	LYS	626	12.300	29.851	64.951	1.00	50.00
ATOM	492	CD	LYS	626	13.626	29.919	64.195	1.00	50.00
ATOM	493	CE	LYS	626	14.809	29.824	65.160	1.00	50.00
ATOM	494	NZ	LYS	626	16.080	30.069	64.463	1.00	50.00
ATOM	495	N	CYS	627	8.609	31.538	65.511	1.00	50.00
ATOM	496	CA	CYS	627	8.005	32.394	66.553	1.00	50.00
ATOM	497	C	CYS	627	7.016	31.613	67.430	1.00	50.00
ATOM	498	O	CYS	627	7.192	31.538	68.647	1.00	50.00
ATOM	499	CB	CYS	627	7.254	33.585	65.955	1.00	50.00
ATOM	500	SG	CYS	627	8.292	34.907	65.241	1.00	50.00
ATOM	501	N	CYS	628	6.103	30.900	66.771	1.00	50.00
ATOM	502	CA	CYS	628	5.045	30.152	67.473	1.00	50.00
ATOM	503	C	CYS	628	5.612	28.955	68.246	1.00	50.00
ATOM	504	O	CYS	628	5.378	28.853	69.448	1.00	50.00
ATOM	505	CB	CYS	628	3.931	29.730	66.511	1.00	50.00
ATOM	506	SG	CYS	628	3.014	31.150	65.807	1.00	50.00
ATOM	507	N	GLN	629	6.579	28.263	67.640	1.00	50.00
ATOM	508	CA	GLN	629	7.266	27.128	68.292	1.00	50.00
ATOM	509	C	GLN	629	8.113	27.537	69.505	1.00	50.00
ATOM	510	O	GLN	629	8.250	26.761	70.447	1.00	50.00
ATOM	511	CB	GLN	629	8.121	26.344	67.297	1.00	50.00
ATOM	512	CG	GLN	629	7.235	25.480	66.396	1.00	50.00
ATOM	513	CD	GLN	629	8.000	24.835	65.236	1.00	50.00
ATOM	514	OE1	GLN	629	9.072	25.245	64.812	1.00	50.00
ATOM	515	NE2	GLN	629	7.402	23.820	64.653	1.00	50.00
ATOM	516	N	ALA	630	8.598	28.778	69.496	1.00	50.00
ATOM	517	CA	ALA	630	9.392	29.332	70.609	1.00	50.00
ATOM	518	C	ALA	630	8.541	29.692	71.840	1.00	50.00
ATOM	519	O	ALA	630	9.085	29.912	72.922	1.00	50.00
ATOM	520	CB	ALA	630	10.190	30.544	70.129	1.00	50.00
ATOM	521	N	GLY	631	7.226	29.837	71.618	1.00	50.00
ATOM	522	CA	GLY	631	6.269	30.162	72.692	1.00	50.00
ATOM	523	C	GLY	631	5.601	31.536	72.541	1.00	50.00
ATOM	524	O	GLY	631	4.820	31.923	73.408	1.00	50.00
ATOM	525	N	MET	632	5.923	32.267	71.470	1.00	50.00
ATOM	526	CA	MET	632	5.294	33.576	71.196	1.00	50.00
ATOM	527	C	MET	632	3.783	33.415	71.007	1.00	50.00
ATOM	528	O	MET	632	3.311	32.539	70.282	1.00	50.00
ATOM	529	CB	MET	632	5.887	34.259	69.961	1.00	50.00
ATOM	530	CG	MET	632	7.312	34.745	70.226	1.00	50.00
ATOM	531	SD	MET	632	8.109	35.567	68.803	1.00	50.00
ATOM	532	CE	MET	632	7.312	37.153	68.906	1.00	50.00
ATOM	533	N	VAL	633	3.061	34.269	71.716	1.00	50.00
ATOM	534	CA	VAL	633	1.587	34.258	71.751	1.00	50.00
ATOM	535	C	VAL	633	1.118	35.714	71.693	1.00	50.00
ATOM	536	O	VAL	633	1.634	36.577	72.405	1.00	50.00
ATOM	537	CB	VAL	633	1.095	33.539	73.031	1.00	50.00
ATOM	538	CG1	VAL	633	-0.399	33.730	73.323	1.00	50.00
ATOM	539	CG2	VAL	633	1.331	32.028	72.929	1.00	50.00
ATOM	540	N	LEU	634	0.104	35.939	70.867	1.00	50.00
ATOM	541	CA	LEU	634	-0.589	37.236	70.872	1.00	50.00
ATOM	542	C	LEU	634	-1.290	37.503	72.206	1.00	50.00
ATOM	543	O	LEU	634	-1.836	36.600	72.837	1.00	50.00
ATOM	544	CB	LEU	634	-1.616	37.370	69.744	1.00	50.00
ATOM	545	CG	LEU	634	-1.026	37.757	68.382	1.00	50.00
ATOM	546	CD1	LEU	634	-2.182	38.137	67.457	1.00	50.00
ATOM	547	CD2	LEU	634	-0.033	38.925	68.458	1.00	50.00
ATOM	548	N	GLY	635	-1.215	38.772	72.601	1.00	50.00

ATOM	549	CA	GLY	635	-1.922	39.268	73.792	1.00	50.00
ATOM	550	C	GLY	635	-1.844	40.793	73.835	1.00	50.00
ATOM	551	O	GLY	635	-0.991	41.396	73.180	1.00	50.00
ATOM	552	N	GLY	636	-2.864	41.374	74.476	1.00	50.00
ATOM	553	CA	GLY	636	-2.868	42.818	74.774	1.00	50.00
ATOM	554	C	GLY	636	-1.780	43.149	75.808	1.00	50.00
ATOM	555	O	GLY	636	-0.984	42.298	76.211	1.00	50.00
ATOM	556	N	ARG	637	-1.829	44.382	76.295	1.00	50.00
ATOM	557	CA	ARG	637	-0.904	44.821	77.349	1.00	50.00
ATOM	558	C	ARG	637	-1.366	44.319	78.727	1.00	50.00
ATOM	559	O	ARG	637	-2.344	44.816	79.282	1.00	50.00
ATOM	560	CB	ARG	637	-0.726	46.346	77.358	1.00	50.00
ATOM	561	CG	ARG	637	0.744	46.795	77.409	1.00	50.00
ATOM	562	CD	ARG	637	1.638	45.816	78.175	1.00	50.00
ATOM	563	NE	ARG	637	2.945	46.377	78.530	1.00	50.00
ATOM	564	CZ	ARG	637	3.664	45.968	79.577	1.00	50.00
ATOM	565	NH1	ARG	637	3.237	44.988	80.366	1.00	50.00
ATOM	566	NH2	ARG	637	4.807	46.562	79.877	1.00	50.00
ATOM	567	N	LYS	638	-0.710	43.255	79.182	1.00	50.00
ATOM	568	CA	LYS	638	-1.041	42.574	80.451	1.00	50.00
ATOM	569	C	LYS	638	-0.390	43.265	81.663	1.00	50.00
ATOM	570	O	LYS	638	0.619	43.956	81.529	1.00	50.00
ATOM	571	CB	LYS	638	-0.671	41.081	80.397	1.00	50.00
ATOM	572	CG	LYS	638	0.834	40.799	80.296	1.00	50.00
ATOM	573	CD	LYS	638	1.333	40.698	78.853	1.00	50.00
ATOM	574	CE	LYS	638	2.669	41.428	78.709	1.00	50.00
ATOM	575	NZ	LYS	638	3.382	41.017	77.493	1.00	50.00
ATOM	576	N	PHE	639	-0.927	42.984	82.845	1.00	50.00
ATOM	577	CA	PHE	639	-0.485	43.645	84.090	1.00	50.00
ATOM	578	C	PHE	639	0.052	42.609	85.088	1.00	50.00
ATOM	579	O	PHE	639	-0.375	41.456	85.098	1.00	50.00
ATOM	580	CB	PHE	639	-1.645	44.418	84.737	1.00	50.00
ATOM	581	CG	PHE	639	-2.688	44.939	83.740	1.00	50.00
ATOM	582	CD1	PHE	639	-2.408	45.994	82.879	1.00	50.00
ATOM	583	CD2	PHE	639	-3.925	44.310	83.692	1.00	50.00
ATOM	584	CE1	PHE	639	-3.373	46.420	81.973	1.00	50.00
ATOM	585	CE2	PHE	639	-4.892	44.742	82.793	1.00	50.00
ATOM	586	CZ	PHE	639	-4.616	45.799	81.934	1.00	50.00
ATOM	587	N	LYS	640	0.967	43.061	85.940	1.00	50.00
ATOM	588	CA	LYS	640	1.571	42.213	86.995	1.00	50.00
ATOM	589	C	LYS	640	0.947	42.419	88.395	1.00	50.00
ATOM	590	O	LYS	640	0.134	43.362	88.526	1.00	50.00
ATOM	591	CB	LYS	640	3.092	42.426	87.029	1.00	50.00
ATOM	592	CG	LYS	640	3.491	43.859	87.394	1.00	50.00
ATOM	593	CD	LYS	640	5.011	44.012	87.449	1.00	50.00
ATOM	594	CE	LYS	640	5.414	45.438	87.835	1.00	50.00
ATOM	595	NZ	LYS	640	4.995	45.776	89.205	1.00	50.00
ATOM	596	OXT	LYS	640	1.339	41.670	89.317	1.00	99.99
TER	597		LYS	640					
