# Supplementary material for: Structural analysis of hubs in human NR-RTK network
Source: Biol Direct. 2011 Oct 5;6:49. doi: 10.1186/1745-6150-6-49 (PMC3220635; doi:10.1186/1745-6150-6-49)
Supplement: Additional file 4 — IGF1R. IGF1R structure. [file 1745-6150-6-49-S4.PDF]

```
HEADER    IGF1R
ATOM      1  N   ASP   985      -16.836 -21.889   4.997  1.00 50.00
ATOM      2  CA  ASP   985      -18.057 -22.060   4.183  1.00 50.00
ATOM      3  C   ASP   985      -19.289 -22.218   5.110  1.00 50.00
ATOM      4  O   ASP   985      -19.197 -22.892   6.126  1.00 50.00
ATOM      5  CB  ASP   985      -17.901 -23.299   3.291  1.00 50.00
ATOM      6  CG  ASP   985      -19.058 -23.575   2.315  1.00 50.00
ATOM      7  OD1 ASP   985      -19.983 -22.731   2.218  1.00 50.00
ATOM      8  OD2 ASP   985      -18.961 -24.586   1.599  1.00 50.00
ATOM      9  N   VAL   986      -20.402 -21.658   4.651  1.00 50.00
ATOM     10  CA  VAL   986      -21.725 -21.766   5.332  1.00 50.00
ATOM     11  C   VAL   986      -21.709 -20.914   6.593  1.00 50.00
ATOM     12  O   VAL   986      -20.973 -21.166   7.549  1.00 50.00
ATOM     13  CB  VAL   986      -22.086 -23.238   5.643  1.00 50.00
ATOM     14  CG1 VAL   986      -23.385 -23.381   6.448  1.00 50.00
ATOM     15  CG2 VAL   986      -22.195 -24.089   4.374  1.00 50.00
ATOM     16  N   TYR   987      -22.527 -19.867   6.547  1.00 50.00
ATOM     17  CA  TYR   987      -22.810 -19.071   7.740  1.00 50.00
ATOM     18  C   TYR   987      -24.201 -19.459   8.242  1.00 50.00
ATOM     19  O   TYR   987      -25.202 -19.307   7.553  1.00 50.00
ATOM     20  CB  TYR   987      -22.733 -17.569   7.432  1.00 50.00
ATOM     21  CG  TYR   987      -23.159 -16.749   8.652  1.00 50.00
ATOM     22  CD1 TYR   987      -22.257 -16.517   9.684  1.00 50.00
ATOM     23  CD2 TYR   987      -24.480 -16.339   8.781  1.00 50.00
ATOM     24  CE1 TYR   987      -22.676 -15.880  10.841  1.00 50.00
ATOM     25  CE2 TYR   987      -24.902 -15.709   9.942  1.00 50.00
ATOM     26  CZ  TYR   987      -23.999 -15.480  10.966  1.00 50.00
ATOM     27  OH  TYR   987      -24.416 -14.848  12.087  1.00 50.00
ATOM     28  N   VAL   988      -24.197 -19.917   9.495  1.00 50.00
ATOM     29  CA  VAL   988      -25.413 -20.336  10.198  1.00 50.00
ATOM     30  C   VAL   988      -25.885 -19.216  11.130  1.00 50.00
ATOM     31  O   VAL   988      -25.145 -18.798  12.030  1.00 50.00
ATOM     32  CB  VAL   988      -25.219 -21.713  10.889  1.00 50.00
ATOM     33  CG1 VAL   988      -23.941 -21.793  11.729  1.00 50.00
ATOM     34  CG2 VAL   988      -26.433 -22.112  11.737  1.00 50.00
ATOM     35  N   PRO   989      -27.114 -18.717  10.888  1.00 50.00
ATOM     36  CA  PRO   989      -27.738 -17.665  11.698  1.00 50.00
ATOM     37  C   PRO   989      -27.834 -18.130  13.152  1.00 50.00
ATOM     38  O   PRO   989      -28.041 -19.302  13.444  1.00 50.00
ATOM     39  CB  PRO   989      -29.150 -17.515  11.127  1.00 50.00
ATOM     40  CG  PRO   989      -28.988 -17.955   9.677  1.00 50.00
ATOM     41  CD  PRO   989      -27.984 -19.101   9.760  1.00 50.00
ATOM     42  N   ASP   990      -27.608 -17.177  14.052  1.00 50.00
ATOM     43  CA  ASP   990      -27.683 -17.438  15.496  1.00 50.00
ATOM     44  C   ASP   990      -28.527 -16.363  16.216  1.00 50.00
ATOM     45  O   ASP   990      -29.236 -15.596  15.589  1.00 50.00
ATOM     46  CB  ASP   990      -26.244 -17.578  16.031  1.00 50.00
ATOM     47  CG  ASP   990      -25.343 -16.371  15.754  1.00 50.00
ATOM     48  OD1 ASP   990      -25.874 -15.294  15.416  1.00 50.00
ATOM     49  OD2 ASP   990      -24.145 -16.516  16.084  1.00 50.00
ATOM     50  N   GLU   991      -28.257 -16.211  17.516  1.00 50.00
ATOM     51  CA  GLU   991      -28.897 -15.206  18.397  1.00 50.00
ATOM     52  C   GLU   991      -28.593 -13.759  18.017  1.00 50.00
ATOM     53  O   GLU   991      -29.340 -12.851  18.393  1.00 50.00
ATOM     54  CB  GLU   991      -28.545 -15.453  19.877  1.00 50.00
ATOM     55  CG  GLU   991      -27.043 -15.464  20.213  1.00 50.00
ATOM     56  CD  GLU   991      -26.305 -16.779  19.908  1.00 50.00
ATOM     57  OE1 GLU   991      -26.895 -17.685  19.280  1.00 50.00
ATOM     58  OE2 GLU   991      -25.098 -16.822  20.223  1.00 50.00
ATOM     59  N   TRP   992      -27.478 -13.542  17.319  1.00 50.00
ATOM     60  CA  TRP   992      -27.061 -12.219  16.838  1.00 50.00
```

ATOM	61	C	TRP	992	-27.860	-11.729	15.612	1.00	50.00
ATOM	62	O	TRP	992	-27.724	-10.579	15.199	1.00	50.00
ATOM	63	CB	TRP	992	-25.564	-12.218	16.522	1.00	50.00
ATOM	64	CG	TRP	992	-24.739	-12.416	17.795	1.00	50.00
ATOM	65	CD1	TRP	992	-24.261	-13.573	18.249	1.00	50.00
ATOM	66	CD2	TRP	992	-24.264	-11.412	18.624	1.00	50.00
ATOM	67	NE1	TRP	992	-23.490	-13.344	19.313	1.00	50.00
ATOM	68	CE2	TRP	992	-23.463	-12.039	19.566	1.00	50.00
ATOM	69	CE3	TRP	992	-24.447	-10.035	18.658	1.00	50.00
ATOM	70	CZ2	TRP	992	-22.818	-11.284	20.538	1.00	50.00
ATOM	71	CZ3	TRP	992	-23.807	-9.282	19.636	1.00	50.00
ATOM	72	CH2	TRP	992	-22.992	-9.905	20.573	1.00	50.00
ATOM	73	N	GLU	993	-28.613	-12.638	15.001	1.00	50.00
ATOM	74	CA	GLU	993	-29.466	-12.304	13.839	1.00	50.00
ATOM	75	C	GLU	993	-30.497	-11.249	14.241	1.00	50.00
ATOM	76	O	GLU	993	-31.211	-11.391	15.228	1.00	50.00
ATOM	77	CB	GLU	993	-30.185	-13.565	13.351	1.00	50.00
ATOM	78	CG	GLU	993	-30.808	-13.369	11.962	1.00	50.00
ATOM	79	CD	GLU	993	-29.753	-13.078	10.888	1.00	50.00
ATOM	80	OE1	GLU	993	-28.621	-13.591	11.018	1.00	50.00
ATOM	81	OE2	GLU	993	-30.101	-12.438	9.874	1.00	50.00
ATOM	82	N	VAL	994	-30.579	-10.209	13.409	1.00	50.00
ATOM	83	CA	VAL	994	-31.525	-9.103	13.602	1.00	50.00
ATOM	84	C	VAL	994	-32.409	-9.012	12.354	1.00	50.00
ATOM	85	O	VAL	994	-31.930	-9.122	11.223	1.00	50.00
ATOM	86	CB	VAL	994	-30.762	-7.781	13.857	1.00	50.00
ATOM	87	CG1	VAL	994	-31.691	-6.568	13.981	1.00	50.00
ATOM	88	CG2	VAL	994	-29.940	-7.865	15.147	1.00	50.00
ATOM	89	N	ALA	995	-33.692	-8.766	12.598	1.00	50.00
ATOM	90	CA	ALA	995	-34.656	-8.509	11.517	1.00	50.00
ATOM	91	C	ALA	995	-34.249	-7.234	10.759	1.00	50.00
ATOM	92	O	ALA	995	-34.009	-6.183	11.353	1.00	50.00
ATOM	93	CB	ALA	995	-36.050	-8.373	12.131	1.00	50.00
ATOM	94	N	ARG	996	-34.284	-7.338	9.433	1.00	50.00
ATOM	95	CA	ARG	996	-33.871	-6.244	8.521	1.00	50.00
ATOM	96	C	ARG	996	-34.665	-4.950	8.724	1.00	50.00
ATOM	97	O	ARG	996	-34.119	-3.853	8.779	1.00	50.00
ATOM	98	CB	ARG	996	-34.048	-6.755	7.090	1.00	50.00
ATOM	99	CG	ARG	996	-33.613	-5.731	6.039	1.00	50.00
ATOM	100	CD	ARG	996	-33.757	-6.328	4.639	1.00	50.00
ATOM	101	NE	ARG	996	-32.810	-7.452	4.472	1.00	50.00
ATOM	102	CZ	ARG	996	-31.494	-7.343	4.350	1.00	50.00
ATOM	103	NH1	ARG	996	-30.887	-6.169	4.364	1.00	50.00
ATOM	104	NH2	ARG	996	-30.745	-8.438	4.247	1.00	50.00
ATOM	105	N	GLU	997	-35.962	-5.145	8.993	1.00	50.00
ATOM	106	CA	GLU	997	-36.949	-4.104	9.316	1.00	50.00
ATOM	107	C	GLU	997	-36.523	-3.168	10.458	1.00	50.00
ATOM	108	O	GLU	997	-36.840	-1.976	10.442	1.00	50.00
ATOM	109	CB	GLU	997	-38.297	-4.769	9.670	1.00	50.00
ATOM	110	CG	GLU	997	-38.107	-6.129	10.350	1.00	50.00
ATOM	111	CD	GLU	997	-39.285	-6.609	11.185	1.00	50.00
ATOM	112	OE1	GLU	997	-39.459	-6.017	12.269	1.00	50.00
ATOM	113	OE2	GLU	997	-39.884	-7.619	10.767	1.00	50.00
ATOM	114	N	LYS	998	-35.790	-3.715	11.422	1.00	50.00
ATOM	115	CA	LYS	998	-35.285	-2.974	12.591	1.00	50.00
ATOM	116	C	LYS	998	-34.169	-1.962	12.278	1.00	50.00
ATOM	117	O	LYS	998	-33.911	-1.060	13.070	1.00	50.00
ATOM	118	CB	LYS	998	-34.810	-3.967	13.653	1.00	50.00
ATOM	119	CG	LYS	998	-35.972	-4.814	14.175	1.00	50.00
ATOM	120	CD	LYS	998	-35.499	-5.673	15.342	1.00	50.00
ATOM	121	CE	LYS	998	-36.675	-6.381	16.010	1.00	50.00

ATOM	122	NZ	LYS	998	-36.300	-6.712	17.390	1.00	50.00
ATOM	123	N	ILE	999	-33.484	-2.157	11.148	1.00	50.00
ATOM	124	CA	ILE	999	-32.320	-1.333	10.768	1.00	50.00
ATOM	125	C	ILE	999	-32.734	-0.274	9.737	1.00	50.00
ATOM	126	O	ILE	999	-33.446	-0.548	8.776	1.00	50.00
ATOM	127	CB	ILE	999	-31.202	-2.229	10.190	1.00	50.00
ATOM	128	CG1	ILE	999	-30.864	-3.429	11.095	1.00	50.00
ATOM	129	CG2	ILE	999	-29.937	-1.429	9.835	1.00	50.00
ATOM	130	CD1	ILE	999	-30.422	-3.083	12.524	1.00	50.00
ATOM	131	N	THR	1000	-32.219	0.933	9.957	1.00	50.00
ATOM	132	CA	THR	1000	-32.393	2.059	9.018	1.00	50.00
ATOM	133	C	THR	1000	-31.029	2.691	8.723	1.00	50.00
ATOM	134	O	THR	1000	-30.389	3.243	9.616	1.00	50.00
ATOM	135	CB	THR	1000	-33.342	3.116	9.606	1.00	50.00
ATOM	136	OG1	THR	1000	-34.536	2.484	10.075	1.00	50.00
ATOM	137	CG2	THR	1000	-33.717	4.177	8.564	1.00	50.00
ATOM	138	N	MET	1001	-30.584	2.531	7.473	1.00	50.00
ATOM	139	CA	MET	1001	-29.332	3.139	6.988	1.00	50.00
ATOM	140	C	MET	1001	-29.584	4.584	6.541	1.00	50.00
ATOM	141	O	MET	1001	-30.550	4.866	5.839	1.00	50.00
ATOM	142	CB	MET	1001	-28.766	2.330	5.812	1.00	50.00
ATOM	143	CG	MET	1001	-28.345	0.930	6.260	1.00	50.00
ATOM	144	SD	MET	1001	-27.675	-0.141	4.939	1.00	50.00
ATOM	145	CE	MET	1001	-29.178	-0.963	4.457	1.00	50.00
ATOM	146	N	SER	1002	-28.683	5.475	6.957	1.00	50.00
ATOM	147	CA	SER	1002	-28.813	6.902	6.631	1.00	50.00
ATOM	148	C	SER	1002	-27.642	7.400	5.765	1.00	50.00
ATOM	149	O	SER	1002	-27.666	7.266	4.550	1.00	50.00
ATOM	150	CB	SER	1002	-29.008	7.719	7.915	1.00	50.00
ATOM	151	OG	SER	1002	-29.223	9.087	7.566	1.00	50.00
ATOM	152	N	ARG	1003	-26.603	7.917	6.413	1.00	50.00
ATOM	153	CA	ARG	1003	-25.462	8.535	5.711	1.00	50.00
ATOM	154	C	ARG	1003	-24.196	7.696	5.820	1.00	50.00
ATOM	155	O	ARG	1003	-23.999	6.946	6.771	1.00	50.00
ATOM	156	CB	ARG	1003	-25.257	9.984	6.180	1.00	50.00
ATOM	157	CG	ARG	1003	-25.424	10.189	7.689	1.00	50.00
ATOM	158	CD	ARG	1003	-25.497	11.680	8.025	1.00	50.00
ATOM	159	NE	ARG	1003	-25.990	11.868	9.400	1.00	50.00
ATOM	160	CZ	ARG	1003	-27.236	11.666	9.844	1.00	50.00
ATOM	161	NH1	ARG	1003	-28.211	11.290	9.026	1.00	50.00
ATOM	162	NH2	ARG	1003	-27.532	11.837	11.118	1.00	50.00
ATOM	163	N	GLU	1004	-23.438	7.719	4.728	1.00	50.00
ATOM	164	CA	GLU	1004	-22.188	6.949	4.609	1.00	50.00
ATOM	165	C	GLU	1004	-21.173	7.382	5.670	1.00	50.00
ATOM	166	O	GLU	1004	-21.080	8.546	6.036	1.00	50.00
ATOM	167	CB	GLU	1004	-21.525	7.193	3.251	1.00	50.00
ATOM	168	CG	GLU	1004	-22.372	6.800	2.039	1.00	50.00
ATOM	169	CD	GLU	1004	-21.611	7.028	0.726	1.00	50.00
ATOM	170	OE1	GLU	1004	-20.792	7.973	0.669	1.00	50.00
ATOM	171	OE2	GLU	1004	-21.882	6.244	-0.208	1.00	50.00
ATOM	172	N	LEU	1005	-20.410	6.388	6.121	1.00	50.00
ATOM	173	CA	LEU	1005	-19.208	6.615	6.932	1.00	50.00
ATOM	174	C	LEU	1005	-17.938	6.505	6.073	1.00	50.00
ATOM	175	O	LEU	1005	-16.947	7.188	6.334	1.00	50.00
ATOM	176	CB	LEU	1005	-19.150	5.621	8.098	1.00	50.00
ATOM	177	CG	LEU	1005	-20.275	5.841	9.116	1.00	50.00
ATOM	178	CD1	LEU	1005	-20.207	4.753	10.186	1.00	50.00
ATOM	179	CD2	LEU	1005	-20.186	7.221	9.780	1.00	50.00
ATOM	180	N	GLY	1006	-18.043	5.697	5.018	1.00	50.00
ATOM	181	CA	GLY	1006	-16.942	5.498	4.057	1.00	50.00
ATOM	182	C	GLY	1006	-16.855	4.045	3.586	1.00	50.00

ATOM	183	O	GLY	1006	-17.601	3.174	4.025	1.00	50.00
ATOM	184	N	GLN	1007	-16.056	3.888	2.537	1.00	50.00
ATOM	185	CA	GLN	1007	-15.784	2.580	1.913	1.00	50.00
ATOM	186	C	GLN	1007	-15.103	1.646	2.915	1.00	50.00
ATOM	187	O	GLN	1007	-14.086	1.986	3.517	1.00	50.00
ATOM	188	CB	GLN	1007	-14.869	2.822	0.703	1.00	50.00
ATOM	189	CG	GLN	1007	-14.508	1.553	-0.080	1.00	50.00
ATOM	190	CD	GLN	1007	-15.733	0.905	-0.734	1.00	50.00
ATOM	191	OE1	GLN	1007	-16.380	1.455	-1.609	1.00	50.00
ATOM	192	NE2	GLN	1007	-16.088	-0.272	-0.270	1.00	50.00
ATOM	193	N	GLY	1008	-15.695	0.450	3.051	1.00	50.00
ATOM	194	CA	GLY	1008	-15.112	-0.625	3.867	1.00	50.00
ATOM	195	C	GLY	1008	-14.448	-1.665	2.954	1.00	50.00
ATOM	196	O	GLY	1008	-14.407	-1.507	1.736	1.00	50.00
ATOM	197	N	SER	1009	-14.128	-2.809	3.556	1.00	50.00
ATOM	198	CA	SER	1009	-13.451	-3.907	2.833	1.00	50.00
ATOM	199	C	SER	1009	-14.358	-4.685	1.887	1.00	50.00
ATOM	200	O	SER	1009	-14.015	-4.889	0.725	1.00	50.00
ATOM	201	CB	SER	1009	-12.799	-4.886	3.803	1.00	50.00
ATOM	202	OG	SER	1009	-11.829	-4.100	4.472	1.00	50.00
ATOM	203	N	PHE	1010	-15.578	-4.923	2.341	1.00	50.00
ATOM	204	CA	PHE	1010	-16.579	-5.681	1.564	1.00	50.00
ATOM	205	C	PHE	1010	-17.645	-4.805	0.887	1.00	50.00
ATOM	206	O	PHE	1010	-18.411	-5.285	0.052	1.00	50.00
ATOM	207	CB	PHE	1010	-17.241	-6.742	2.446	1.00	50.00
ATOM	208	CG	PHE	1010	-16.267	-7.872	2.790	1.00	50.00
ATOM	209	CD1	PHE	1010	-16.214	-9.008	1.991	1.00	50.00
ATOM	210	CD2	PHE	1010	-15.487	-7.794	3.937	1.00	50.00
ATOM	211	CE1	PHE	1010	-15.391	-10.071	2.344	1.00	50.00
ATOM	212	CE2	PHE	1010	-14.669	-8.858	4.292	1.00	50.00
ATOM	213	CZ	PHE	1010	-14.619	-9.997	3.498	1.00	50.00
ATOM	214	N	GLY	1011	-17.702	-3.535	1.298	1.00	50.00
ATOM	215	CA	GLY	1011	-18.660	-2.569	0.730	1.00	50.00
ATOM	216	C	GLY	1011	-18.692	-1.288	1.559	1.00	50.00
ATOM	217	O	GLY	1011	-17.880	-1.090	2.457	1.00	50.00
ATOM	218	N	MET	1012	-19.638	-0.428	1.206	1.00	50.00
ATOM	219	CA	MET	1012	-19.847	0.850	1.911	1.00	50.00
ATOM	220	C	MET	1012	-20.250	0.620	3.368	1.00	50.00
ATOM	221	O	MET	1012	-20.949	-0.330	3.725	1.00	50.00
ATOM	222	CB	MET	1012	-20.930	1.647	1.171	1.00	50.00
ATOM	223	CG	MET	1012	-21.082	3.078	1.704	1.00	50.00
ATOM	224	SD	MET	1012	-19.545	4.072	1.666	1.00	50.00
ATOM	225	CE	MET	1012	-19.179	4.069	-0.077	1.00	50.00
ATOM	226	N	VAL	1013	-19.788	1.504	4.236	1.00	50.00
ATOM	227	CA	VAL	1013	-20.194	1.494	5.645	1.00	50.00
ATOM	228	C	VAL	1013	-21.084	2.728	5.835	1.00	50.00
ATOM	229	O	VAL	1013	-20.775	3.811	5.357	1.00	50.00
ATOM	230	CB	VAL	1013	-18.960	1.533	6.569	1.00	50.00
ATOM	231	CG1	VAL	1013	-19.359	1.470	8.047	1.00	50.00
ATOM	232	CG2	VAL	1013	-17.997	0.377	6.276	1.00	50.00
ATOM	233	N	TYR	1014	-22.185	2.506	6.549	1.00	50.00
ATOM	234	CA	TYR	1014	-23.174	3.561	6.819	1.00	50.00
ATOM	235	C	TYR	1014	-23.332	3.805	8.319	1.00	50.00
ATOM	236	O	TYR	1014	-22.948	3.016	9.163	1.00	50.00
ATOM	237	CB	TYR	1014	-24.543	3.183	6.244	1.00	50.00
ATOM	238	CG	TYR	1014	-24.520	3.073	4.721	1.00	50.00
ATOM	239	CD1	TYR	1014	-24.778	4.188	3.932	1.00	50.00
ATOM	240	CD2	TYR	1014	-24.282	1.839	4.133	1.00	50.00
ATOM	241	CE1	TYR	1014	-24.805	4.066	2.551	1.00	50.00
ATOM	242	CE2	TYR	1014	-24.310	1.715	2.752	1.00	50.00
ATOM	243	CZ	TYR	1014	-24.566	2.828	1.963	1.00	50.00

ATOM	244	OH	TYR	1014	-24.511	2.712	0.612	1.00	50.00
ATOM	245	N	GLU	1015	-23.831	5.004	8.586	1.00	50.00
ATOM	246	CA	GLU	1015	-24.371	5.382	9.889	1.00	50.00
ATOM	247	C	GLU	1015	-25.892	5.206	9.773	1.00	50.00
ATOM	248	O	GLU	1015	-26.461	5.275	8.682	1.00	50.00
ATOM	249	CB	GLU	1015	-24.041	6.858	10.063	1.00	50.00
ATOM	250	CG	GLU	1015	-24.291	7.331	11.488	1.00	50.00
ATOM	251	CD	GLU	1015	-24.434	8.849	11.537	1.00	50.00
ATOM	252	OE1	GLU	1015	-23.760	9.562	10.746	1.00	50.00
ATOM	253	OE2	GLU	1015	-25.277	9.257	12.339	1.00	50.00
ATOM	254	N	GLY	1016	-26.522	4.955	10.924	1.00	50.00
ATOM	255	CA	GLY	1016	-27.983	4.810	10.968	1.00	50.00
ATOM	256	C	GLY	1016	-28.471	4.581	12.393	1.00	50.00
ATOM	257	O	GLY	1016	-27.830	4.973	13.361	1.00	50.00
ATOM	258	N	VAL	1017	-29.643	3.952	12.449	1.00	50.00
ATOM	259	CA	VAL	1017	-30.336	3.645	13.709	1.00	50.00
ATOM	260	C	VAL	1017	-30.924	2.230	13.649	1.00	50.00
ATOM	261	O	VAL	1017	-31.262	1.729	12.578	1.00	50.00
ATOM	262	CB	VAL	1017	-31.422	4.694	14.043	1.00	50.00
ATOM	263	CG1	VAL	1017	-30.819	6.086	14.266	1.00	50.00
ATOM	264	CG2	VAL	1017	-32.533	4.765	12.988	1.00	50.00
ATOM	265	N	ALA	1018	-30.994	1.604	14.823	1.00	50.00
ATOM	266	CA	ALA	1018	-31.521	0.244	14.959	1.00	50.00
ATOM	267	C	ALA	1018	-32.538	0.138	16.105	1.00	50.00
ATOM	268	O	ALA	1018	-32.443	0.854	17.097	1.00	50.00
ATOM	269	CB	ALA	1018	-30.348	-0.706	15.190	1.00	50.00
ATOM	270	N	LYS	1019	-33.416	-0.852	15.947	1.00	50.00
ATOM	271	CA	LYS	1019	-34.465	-1.183	16.925	1.00	50.00
ATOM	272	C	LYS	1019	-34.087	-2.495	17.622	1.00	50.00
ATOM	273	O	LYS	1019	-33.742	-3.478	16.966	1.00	50.00
ATOM	274	CB	LYS	1019	-35.781	-1.392	16.168	1.00	50.00
ATOM	275	CG	LYS	1019	-37.005	-1.559	17.079	1.00	50.00
ATOM	276	CD	LYS	1019	-37.540	-0.214	17.572	1.00	50.00
ATOM	277	CE	LYS	1019	-38.158	0.605	16.435	1.00	50.00
ATOM	278	NZ	LYS	1019	-38.233	2.033	16.770	1.00	50.00
ATOM	279	N	GLY	1020	-34.066	-2.427	18.957	1.00	50.00
ATOM	280	CA	GLY	1020	-33.839	-3.608	19.823	1.00	50.00
ATOM	281	C	GLY	1020	-32.510	-4.340	19.611	1.00	50.00
ATOM	282	O	GLY	1020	-32.474	-5.569	19.548	1.00	50.00
ATOM	283	N	VAL	1021	-31.416	-3.579	19.519	1.00	50.00
ATOM	284	CA	VAL	1021	-30.058	-4.148	19.423	1.00	50.00
ATOM	285	C	VAL	1021	-29.234	-3.910	20.704	1.00	50.00
ATOM	286	O	VAL	1021	-28.247	-4.596	20.980	1.00	50.00
ATOM	287	CB	VAL	1021	-29.294	-3.689	18.162	1.00	50.00
ATOM	288	CG1	VAL	1021	-29.998	-4.174	16.890	1.00	50.00
ATOM	289	CG2	VAL	1021	-29.057	-2.175	18.129	1.00	50.00
ATOM	290	N	VAL	1022	-29.606	-2.860	21.420	1.00	50.00
ATOM	291	CA	VAL	1022	-29.084	-2.553	22.766	1.00	50.00
ATOM	292	C	VAL	1022	-30.276	-2.715	23.716	1.00	50.00
ATOM	293	O	VAL	1022	-31.071	-1.802	23.917	1.00	50.00
ATOM	294	CB	VAL	1022	-28.494	-1.131	22.823	1.00	50.00
ATOM	295	CG1	VAL	1022	-28.009	-0.773	24.234	1.00	50.00
ATOM	296	CG2	VAL	1022	-27.311	-0.994	21.861	1.00	50.00
ATOM	297	N	LYS	1023	-30.431	-3.965	24.166	1.00	50.00
ATOM	298	CA	LYS	1023	-31.598	-4.402	24.958	1.00	50.00
ATOM	299	C	LYS	1023	-32.883	-4.119	24.146	1.00	50.00
ATOM	300	O	LYS	1023	-32.943	-4.446	22.971	1.00	50.00
ATOM	301	CB	LYS	1023	-31.569	-3.766	26.365	1.00	50.00
ATOM	302	CG	LYS	1023	-30.261	-4.031	27.119	1.00	50.00
ATOM	303	CD	LYS	1023	-30.014	-5.528	27.310	1.00	50.00
ATOM	304	CE	LYS	1023	-28.600	-5.757	27.833	1.00	50.00

ATOM	305	NZ	LYS	1023	-28.335	-7.189	28.018	1.00	50.00
ATOM	306	N	ASP	1024	-33.773	-3.295	24.697	1.00	50.00
ATOM	307	CA	ASP	1024	-35.025	-2.921	24.014	1.00	50.00
ATOM	308	C	ASP	1024	-35.081	-1.436	23.616	1.00	50.00
ATOM	309	O	ASP	1024	-36.167	-0.882	23.453	1.00	50.00
ATOM	310	CB	ASP	1024	-36.200	-3.311	24.926	1.00	50.00
ATOM	311	CG	ASP	1024	-36.256	-2.538	26.254	1.00	50.00
ATOM	312	OD1	ASP	1024	-35.223	-1.957	26.651	1.00	50.00
ATOM	313	OD2	ASP	1024	-37.324	-2.625	26.900	1.00	50.00
ATOM	314	N	GLU	1025	-33.915	-0.801	23.469	1.00	50.00
ATOM	315	CA	GLU	1025	-33.861	0.583	22.956	1.00	50.00
ATOM	316	C	GLU	1025	-34.440	0.647	21.541	1.00	50.00
ATOM	317	O	GLU	1025	-33.986	-0.087	20.654	1.00	50.00
ATOM	318	CB	GLU	1025	-32.452	1.171	22.934	1.00	50.00
ATOM	319	CG	GLU	1025	-31.898	1.454	24.331	1.00	50.00
ATOM	320	CD	GLU	1025	-30.607	2.282	24.274	1.00	50.00
ATOM	321	OE1	GLU	1025	-29.902	2.225	23.241	1.00	50.00
ATOM	322	OE2	GLU	1025	-30.345	2.967	25.283	1.00	50.00
ATOM	323	N	PRO	1026	-35.485	1.476	21.362	1.00	50.00
ATOM	324	CA	PRO	1026	-36.161	1.656	20.070	1.00	50.00
ATOM	325	C	PRO	1026	-35.237	2.248	18.997	1.00	50.00
ATOM	326	O	PRO	1026	-35.365	1.945	17.811	1.00	50.00
ATOM	327	CB	PRO	1026	-37.311	2.627	20.361	1.00	50.00
ATOM	328	CG	PRO	1026	-37.591	2.419	21.846	1.00	50.00
ATOM	329	CD	PRO	1026	-36.193	2.213	22.426	1.00	50.00
ATOM	330	N	GLU	1027	-34.335	3.122	19.418	1.00	50.00
ATOM	331	CA	GLU	1027	-33.489	3.885	18.488	1.00	50.00
ATOM	332	C	GLU	1027	-32.078	4.089	19.037	1.00	50.00
ATOM	333	O	GLU	1027	-31.823	4.952	19.871	1.00	50.00
ATOM	334	CB	GLU	1027	-34.196	5.217	18.225	1.00	50.00
ATOM	335	CG	GLU	1027	-33.458	6.106	17.222	1.00	50.00
ATOM	336	CD	GLU	1027	-34.127	7.473	17.031	1.00	50.00
ATOM	337	OE1	GLU	1027	-35.069	7.798	17.788	1.00	50.00
ATOM	338	OE2	GLU	1027	-33.668	8.176	16.106	1.00	50.00
ATOM	339	N	THR	1028	-31.173	3.257	18.524	1.00	50.00
ATOM	340	CA	THR	1028	-29.746	3.371	18.853	1.00	50.00
ATOM	341	C	THR	1028	-28.997	3.770	17.581	1.00	50.00
ATOM	342	O	THR	1028	-29.189	3.155	16.534	1.00	50.00
ATOM	343	CB	THR	1028	-29.209	2.022	19.365	1.00	50.00
ATOM	344	OG1	THR	1028	-30.060	1.549	20.413	1.00	50.00
ATOM	345	CG2	THR	1028	-27.762	2.125	19.858	1.00	50.00
ATOM	346	N	ARG	1029	-28.145	4.789	17.707	1.00	50.00
ATOM	347	CA	ARG	1029	-27.243	5.192	16.613	1.00	50.00
ATOM	348	C	ARG	1029	-26.163	4.126	16.418	1.00	50.00
ATOM	349	O	ARG	1029	-25.468	3.733	17.347	1.00	50.00
ATOM	350	CB	ARG	1029	-26.566	6.530	16.907	1.00	50.00
ATOM	351	CG	ARG	1029	-27.571	7.682	16.938	1.00	50.00
ATOM	352	CD	ARG	1029	-26.838	9.019	17.049	1.00	50.00
ATOM	353	NE	ARG	1029	-26.059	9.301	15.862	1.00	50.00
ATOM	354	CZ	ARG	1029	-25.074	10.178	15.694	1.00	50.00
ATOM	355	NH1	ARG	1029	-24.694	10.951	16.780	1.00	50.00
ATOM	356	NH2	ARG	1029	-24.440	10.380	14.633	1.00	50.00
ATOM	357	N	VAL	1030	-26.139	3.602	15.193	1.00	50.00
ATOM	358	CA	VAL	1030	-25.298	2.447	14.841	1.00	50.00
ATOM	359	C	VAL	1030	-24.480	2.697	13.566	1.00	50.00
ATOM	360	O	VAL	1030	-24.822	3.544	12.747	1.00	50.00
ATOM	361	CB	VAL	1030	-26.150	1.163	14.705	1.00	50.00
ATOM	362	CG1	VAL	1030	-26.792	0.772	16.040	1.00	50.00
ATOM	363	CG2	VAL	1030	-27.226	1.267	13.616	1.00	50.00
ATOM	364	N	ALA	1031	-23.406	1.915	13.446	1.00	50.00
ATOM	365	CA	ALA	1031	-22.626	1.804	12.205	1.00	50.00

ATOM	366	C	ALA	1031	-23.011	0.474	11.531	1.00	50.00
ATOM	367	O	ALA	1031	-23.243	-0.531	12.186	1.00	50.00
ATOM	368	CB	ALA	1031	-21.133	1.803	12.539	1.00	50.00
ATOM	369	N	ILE	1032	-23.159	0.553	10.209	1.00	50.00
ATOM	370	CA	ILE	1032	-23.689	-0.560	9.395	1.00	50.00
ATOM	371	C	ILE	1032	-22.699	-0.894	8.279	1.00	50.00
ATOM	372	O	ILE	1032	-22.515	-0.112	7.349	1.00	50.00
ATOM	373	CB	ILE	1032	-25.066	-0.158	8.821	1.00	50.00
ATOM	374	CG1	ILE	1032	-26.007	0.296	9.948	1.00	50.00
ATOM	375	CG2	ILE	1032	-25.689	-1.342	8.064	1.00	50.00
ATOM	376	CD1	ILE	1032	-27.162	1.192	9.498	1.00	50.00
ATOM	377	N	LYS	1033	-22.005	-2.024	8.444	1.00	50.00
ATOM	378	CA	LYS	1033	-21.147	-2.551	7.372	1.00	50.00
ATOM	379	C	LYS	1033	-22.004	-3.343	6.384	1.00	50.00
ATOM	380	O	LYS	1033	-22.900	-4.084	6.781	1.00	50.00
ATOM	381	CB	LYS	1033	-20.057	-3.481	7.910	1.00	50.00
ATOM	382	CG	LYS	1033	-19.116	-2.794	8.902	1.00	50.00
ATOM	383	CD	LYS	1033	-17.858	-3.624	9.180	1.00	50.00
ATOM	384	CE	LYS	1033	-18.171	-5.007	9.755	1.00	50.00
ATOM	385	NZ	LYS	1033	-16.943	-5.745	10.072	1.00	50.00
ATOM	386	N	THR	1034	-21.719	-3.129	5.095	1.00	50.00
ATOM	387	CA	THR	1034	-22.461	-3.793	4.017	1.00	50.00
ATOM	388	C	THR	1034	-21.499	-4.553	3.094	1.00	50.00
ATOM	389	O	THR	1034	-20.291	-4.331	3.083	1.00	50.00
ATOM	390	CB	THR	1034	-23.288	-2.803	3.166	1.00	50.00
ATOM	391	OG1	THR	1034	-22.430	-2.036	2.317	1.00	50.00
ATOM	392	CG2	THR	1034	-24.189	-1.898	4.013	1.00	50.00
ATOM	393	N	VAL	1035	-22.098	-5.466	2.336	1.00	50.00
ATOM	394	CA	VAL	1035	-21.409	-6.173	1.248	1.00	50.00
ATOM	395	C	VAL	1035	-21.997	-5.604	-0.050	1.00	50.00
ATOM	396	O	VAL	1035	-23.215	-5.546	-0.207	1.00	50.00
ATOM	397	CB	VAL	1035	-21.681	-7.686	1.348	1.00	50.00
ATOM	398	CG1	VAL	1035	-20.970	-8.469	0.248	1.00	50.00
ATOM	399	CG2	VAL	1035	-21.192	-8.250	2.678	1.00	50.00
ATOM	400	N	ASN	1036	-21.107	-5.208	-0.961	1.00	50.00
ATOM	401	CA	ASN	1036	-21.538	-4.667	-2.265	1.00	50.00
ATOM	402	C	ASN	1036	-22.477	-5.653	-2.968	1.00	50.00
ATOM	403	O	ASN	1036	-22.329	-6.871	-2.893	1.00	50.00
ATOM	404	CB	ASN	1036	-20.318	-4.318	-3.134	1.00	50.00
ATOM	405	CG	ASN	1036	-19.499	-5.534	-3.550	1.00	50.00
ATOM	406	OD1	ASN	1036	-18.694	-6.082	-2.795	1.00	50.00
ATOM	407	ND2	ASN	1036	-19.728	-6.018	-4.743	1.00	50.00
ATOM	408	N	GLU	1037	-23.406	-5.078	-3.733	1.00	50.00
ATOM	409	CA	GLU	1037	-24.470	-5.827	-4.424	1.00	50.00
ATOM	410	C	GLU	1037	-23.990	-6.943	-5.369	1.00	50.00
ATOM	411	O	GLU	1037	-24.652	-7.976	-5.502	1.00	50.00
ATOM	412	CB	GLU	1037	-25.378	-4.843	-5.178	1.00	50.00
ATOM	413	CG	GLU	1037	-24.591	-3.865	-6.060	1.00	50.00
ATOM	414	CD	GLU	1037	-25.121	-3.797	-7.493	1.00	50.00
ATOM	415	OE1	GLU	1037	-25.349	-4.880	-8.075	1.00	50.00
ATOM	416	OE2	GLU	1037	-25.153	-2.661	-8.005	1.00	50.00
ATOM	417	N	ALA	1038	-22.848	-6.721	-6.004	1.00	50.00
ATOM	418	CA	ALA	1038	-22.252	-7.657	-6.975	1.00	50.00
ATOM	419	C	ALA	1038	-21.593	-8.898	-6.348	1.00	50.00
ATOM	420	O	ALA	1038	-21.501	-9.935	-7.003	1.00	50.00
ATOM	421	CB	ALA	1038	-21.241	-6.919	-7.854	1.00	50.00
ATOM	422	N	ALA	1039	-21.106	-8.758	-5.114	1.00	50.00
ATOM	423	CA	ALA	1039	-20.330	-9.812	-4.426	1.00	50.00
ATOM	424	C	ALA	1039	-20.970	-11.205	-4.490	1.00	50.00
ATOM	425	O	ALA	1039	-22.186	-11.381	-4.524	1.00	50.00
ATOM	426	CB	ALA	1039	-20.129	-9.413	-2.968	1.00	50.00

ATOM	427	N	SER	1040	-20.086	-12.201	-4.554	1.00	50.00
ATOM	428	CA	SER	1040	-20.474	-13.618	-4.634	1.00	50.00
ATOM	429	C	SER	1040	-21.021	-14.130	-3.288	1.00	50.00
ATOM	430	O	SER	1040	-20.918	-13.469	-2.257	1.00	50.00
ATOM	431	CB	SER	1040	-19.265	-14.452	-5.081	1.00	50.00
ATOM	432	OG	SER	1040	-18.285	-14.550	-4.039	1.00	50.00
ATOM	433	N	MET	1041	-21.440	-15.397	-3.289	1.00	50.00
ATOM	434	CA	MET	1041	-21.910	-16.084	-2.067	1.00	50.00
ATOM	435	C	MET	1041	-20.784	-16.286	-1.030	1.00	50.00
ATOM	436	O	MET	1041	-20.972	-16.062	0.169	1.00	50.00
ATOM	437	CB	MET	1041	-22.575	-17.427	-2.415	1.00	50.00
ATOM	438	CG	MET	1041	-21.645	-18.568	-2.855	1.00	50.00
ATOM	439	SD	MET	1041	-20.627	-18.212	-4.333	1.00	50.00
ATOM	440	CE	MET	1041	-20.828	-19.772	-5.162	1.00	50.00
ATOM	441	N	ARG	1042	-19.601	-16.605	-1.540	1.00	50.00
ATOM	442	CA	ARG	1042	-18.391	-16.870	-0.733	1.00	50.00
ATOM	443	C	ARG	1042	-17.958	-15.625	0.042	1.00	50.00
ATOM	444	O	ARG	1042	-17.690	-15.706	1.239	1.00	50.00
ATOM	445	CB	ARG	1042	-17.266	-17.302	-1.671	1.00	50.00
ATOM	446	CG	ARG	1042	-16.700	-18.675	-1.310	1.00	50.00
ATOM	447	CD	ARG	1042	-15.339	-18.581	-0.610	1.00	50.00
ATOM	448	NE	ARG	1042	-15.470	-18.281	0.825	1.00	50.00
ATOM	449	CZ	ARG	1042	-15.333	-19.180	1.815	1.00	50.00
ATOM	450	NH1	ARG	1042	-15.097	-20.455	1.541	1.00	50.00
ATOM	451	NH2	ARG	1042	-15.384	-18.799	3.079	1.00	50.00
ATOM	452	N	GLU	1043	-18.039	-14.475	-0.627	1.00	50.00
ATOM	453	CA	GLU	1043	-17.665	-13.171	-0.043	1.00	50.00
ATOM	454	C	GLU	1043	-18.570	-12.751	1.116	1.00	50.00
ATOM	455	O	GLU	1043	-18.072	-12.330	2.157	1.00	50.00
ATOM	456	CB	GLU	1043	-17.661	-12.090	-1.124	1.00	50.00
ATOM	457	CG	GLU	1043	-16.521	-12.322	-2.117	1.00	50.00
ATOM	458	CD	GLU	1043	-16.596	-11.326	-3.273	1.00	50.00
ATOM	459	OE1	GLU	1043	-17.270	-11.670	-4.268	1.00	50.00
ATOM	460	OE2	GLU	1043	-15.992	-10.242	-3.130	1.00	50.00
ATOM	461	N	ARG	1044	-19.865	-13.051	0.991	1.00	50.00
ATOM	462	CA	ARG	1044	-20.862	-12.781	2.044	1.00	50.00
ATOM	463	C	ARG	1044	-20.629	-13.669	3.272	1.00	50.00
ATOM	464	O	ARG	1044	-20.561	-13.171	4.395	1.00	50.00
ATOM	465	CB	ARG	1044	-22.274	-13.043	1.515	1.00	50.00
ATOM	466	CG	ARG	1044	-22.552	-12.201	0.274	1.00	50.00
ATOM	467	CD	ARG	1044	-23.871	-12.614	-0.373	1.00	50.00
ATOM	468	NE	ARG	1044	-23.997	-11.889	-1.648	1.00	50.00
ATOM	469	CZ	ARG	1044	-24.449	-10.642	-1.814	1.00	50.00
ATOM	470	NH1	ARG	1044	-24.867	-9.906	-0.801	1.00	50.00
ATOM	471	NH2	ARG	1044	-24.429	-10.104	-3.027	1.00	50.00
ATOM	472	N	ILE	1045	-20.348	-14.946	3.014	1.00	50.00
ATOM	473	CA	ILE	1045	-20.003	-15.926	4.069	1.00	50.00
ATOM	474	C	ILE	1045	-18.688	-15.521	4.756	1.00	50.00
ATOM	475	O	ILE	1045	-18.571	-15.572	5.977	1.00	50.00
ATOM	476	CB	ILE	1045	-19.948	-17.355	3.483	1.00	50.00
ATOM	477	CG1	ILE	1045	-21.354	-17.779	3.024	1.00	50.00
ATOM	478	CG2	ILE	1045	-19.361	-18.367	4.483	1.00	50.00
ATOM	479	CD1	ILE	1045	-21.417	-19.119	2.275	1.00	50.00
ATOM	480	N	GLU	1046	-17.719	-15.101	3.942	1.00	50.00
ATOM	481	CA	GLU	1046	-16.406	-14.647	4.423	1.00	50.00
ATOM	482	C	GLU	1046	-16.560	-13.407	5.318	1.00	50.00
ATOM	483	O	GLU	1046	-16.097	-13.414	6.456	1.00	50.00
ATOM	484	CB	GLU	1046	-15.544	-14.323	3.206	1.00	50.00
ATOM	485	CG	GLU	1046	-14.055	-14.518	3.498	1.00	50.00
ATOM	486	CD	GLU	1046	-13.172	-14.158	2.296	1.00	50.00
ATOM	487	OE1	GLU	1046	-13.677	-14.194	1.152	1.00	50.00

ATOM	488	OE2	GLU	1046	-11.991	-13.846	2.560	1.00	50.00
ATOM	489	N	PHE	1047	-17.392	-12.471	4.856	1.00	50.00
ATOM	490	CA	PHE	1047	-17.757	-11.249	5.596	1.00	50.00
ATOM	491	C	PHE	1047	-18.453	-11.563	6.929	1.00	50.00
ATOM	492	O	PHE	1047	-18.033	-11.080	7.980	1.00	50.00
ATOM	493	CB	PHE	1047	-18.648	-10.388	4.693	1.00	50.00
ATOM	494	CG	PHE	1047	-19.290	-9.208	5.427	1.00	50.00
ATOM	495	CD1	PHE	1047	-18.581	-8.032	5.623	1.00	50.00
ATOM	496	CD2	PHE	1047	-20.628	-9.278	5.786	1.00	50.00
ATOM	497	CE1	PHE	1047	-19.210	-6.919	6.160	1.00	50.00
ATOM	498	CE2	PHE	1047	-21.266	-8.161	6.311	1.00	50.00
ATOM	499	CZ	PHE	1047	-20.556	-6.982	6.499	1.00	50.00
ATOM	500	N	LEU	1048	-19.429	-12.469	6.869	1.00	50.00
ATOM	501	CA	LEU	1048	-20.188	-12.900	8.053	1.00	50.00
ATOM	502	C	LEU	1048	-19.327	-13.580	9.121	1.00	50.00
ATOM	503	O	LEU	1048	-19.210	-13.062	10.226	1.00	50.00
ATOM	504	CB	LEU	1048	-21.350	-13.804	7.639	1.00	50.00
ATOM	505	CG	LEU	1048	-22.718	-13.124	7.438	1.00	50.00
ATOM	506	CD1	LEU	1048	-22.735	-11.591	7.501	1.00	50.00
ATOM	507	CD2	LEU	1048	-23.296	-13.590	6.104	1.00	50.00
ATOM	508	N	ASN	1049	-18.571	-14.570	8.678	1.00	50.00
ATOM	509	CA	ASN	1049	-17.671	-15.334	9.568	1.00	50.00
ATOM	510	C	ASN	1049	-16.533	-14.500	10.165	1.00	50.00
ATOM	511	O	ASN	1049	-16.197	-14.681	11.333	1.00	50.00
ATOM	512	CB	ASN	1049	-17.123	-16.562	8.847	1.00	50.00
ATOM	513	CG	ASN	1049	-18.213	-17.612	8.638	1.00	50.00
ATOM	514	OD1	ASN	1049	-19.106	-17.830	9.452	1.00	50.00
ATOM	515	ND2	ASN	1049	-18.143	-18.301	7.529	1.00	50.00
ATOM	516	N	GLU	1050	-16.026	-13.534	9.398	1.00	50.00
ATOM	517	CA	GLU	1050	-14.968	-12.632	9.897	1.00	50.00
ATOM	518	C	GLU	1050	-15.493	-11.736	11.027	1.00	50.00
ATOM	519	O	GLU	1050	-14.900	-11.669	12.105	1.00	50.00
ATOM	520	CB	GLU	1050	-14.407	-11.777	8.759	1.00	50.00
ATOM	521	CG	GLU	1050	-13.258	-10.897	9.267	1.00	50.00
ATOM	522	CD	GLU	1050	-12.736	-9.913	8.221	1.00	50.00
ATOM	523	OE1	GLU	1050	-13.542	-9.465	7.378	1.00	50.00
ATOM	524	OE2	GLU	1050	-11.517	-9.653	8.288	1.00	50.00
ATOM	525	N	ALA	1051	-16.630	-11.086	10.774	1.00	50.00
ATOM	526	CA	ALA	1051	-17.280	-10.196	11.751	1.00	50.00
ATOM	527	C	ALA	1051	-17.725	-10.933	13.027	1.00	50.00
ATOM	528	O	ALA	1051	-17.644	-10.389	14.126	1.00	50.00
ATOM	529	CB	ALA	1051	-18.489	-9.535	11.096	1.00	50.00
ATOM	530	N	SER	1052	-18.029	-12.219	12.862	1.00	50.00
ATOM	531	CA	SER	1052	-18.465	-13.114	13.953	1.00	50.00
ATOM	532	C	SER	1052	-17.413	-13.337	15.047	1.00	50.00
ATOM	533	O	SER	1052	-17.749	-13.762	16.148	1.00	50.00
ATOM	534	CB	SER	1052	-18.916	-14.463	13.397	1.00	50.00
ATOM	535	OG	SER	1052	-20.094	-14.265	12.614	1.00	50.00
ATOM	536	N	VAL	1053	-16.162	-12.978	14.762	1.00	50.00
ATOM	537	CA	VAL	1053	-15.072	-13.008	15.771	1.00	50.00
ATOM	538	C	VAL	1053	-15.376	-12.033	16.925	1.00	50.00
ATOM	539	O	VAL	1053	-15.104	-12.311	18.092	1.00	50.00
ATOM	540	CB	VAL	1053	-13.713	-12.690	15.112	1.00	50.00
ATOM	541	CG1	VAL	1053	-12.560	-12.715	16.124	1.00	50.00
ATOM	542	CG2	VAL	1053	-13.385	-13.693	14.002	1.00	50.00
ATOM	543	N	MET	1054	-16.021	-10.916	16.574	1.00	50.00
ATOM	544	CA	MET	1054	-16.413	-9.860	17.520	1.00	50.00
ATOM	545	C	MET	1054	-17.532	-10.281	18.484	1.00	50.00
ATOM	546	O	MET	1054	-17.644	-9.711	19.569	1.00	50.00
ATOM	547	CB	MET	1054	-16.839	-8.600	16.760	1.00	50.00
ATOM	548	CG	MET	1054	-15.755	-8.092	15.801	1.00	50.00

ATOM	549	SD	MET	1054	-14.139	-7.746	16.589	1.00	50.00
ATOM	550	CE	MET	1054	-14.547	-6.311	17.560	1.00	50.00
ATOM	551	N	LYS	1055	-18.276	-11.328	18.117	1.00	50.00
ATOM	552	CA	LYS	1055	-19.369	-11.887	18.945	1.00	50.00
ATOM	553	C	LYS	1055	-18.909	-12.276	20.357	1.00	50.00
ATOM	554	O	LYS	1055	-19.703	-12.253	21.298	1.00	50.00
ATOM	555	CB	LYS	1055	-19.967	-13.140	18.298	1.00	50.00
ATOM	556	CG	LYS	1055	-20.780	-12.824	17.046	1.00	50.00
ATOM	557	CD	LYS	1055	-21.298	-14.094	16.380	1.00	50.00
ATOM	558	CE	LYS	1055	-22.206	-13.736	15.205	1.00	50.00
ATOM	559	NZ	LYS	1055	-22.571	-14.952	14.471	1.00	50.00
ATOM	560	N	GLU	1056	-17.635	-12.635	20.471	1.00	50.00
ATOM	561	CA	GLU	1056	-17.038	-13.085	21.743	1.00	50.00
ATOM	562	C	GLU	1056	-16.762	-11.947	22.743	1.00	50.00
ATOM	563	O	GLU	1056	-16.640	-12.192	23.941	1.00	50.00
ATOM	564	CB	GLU	1056	-15.749	-13.848	21.435	1.00	50.00
ATOM	565	CG	GLU	1056	-15.348	-14.721	22.628	1.00	50.00
ATOM	566	CD	GLU	1056	-13.948	-15.321	22.488	1.00	50.00
ATOM	567	OE1	GLU	1056	-13.595	-15.740	21.365	1.00	50.00
ATOM	568	OE2	GLU	1056	-13.270	-15.392	23.536	1.00	50.00
ATOM	569	N	PHE	1057	-16.674	-10.712	22.245	1.00	50.00
ATOM	570	CA	PHE	1057	-16.206	-9.574	23.048	1.00	50.00
ATOM	571	C	PHE	1057	-17.345	-8.673	23.548	1.00	50.00
ATOM	572	O	PHE	1057	-18.326	-8.419	22.860	1.00	50.00
ATOM	573	CB	PHE	1057	-15.207	-8.755	22.224	1.00	50.00
ATOM	574	CG	PHE	1057	-14.041	-9.614	21.723	1.00	50.00
ATOM	575	CD1	PHE	1057	-13.331	-10.439	22.590	1.00	50.00
ATOM	576	CD2	PHE	1057	-13.791	-9.660	20.357	1.00	50.00
ATOM	577	CE1	PHE	1057	-12.361	-11.301	22.093	1.00	50.00
ATOM	578	CE2	PHE	1057	-12.831	-10.529	19.860	1.00	50.00
ATOM	579	CZ	PHE	1057	-12.113	-11.340	20.729	1.00	50.00
ATOM	580	N	ASN	1058	-17.105	-8.160	24.751	1.00	50.00
ATOM	581	CA	ASN	1058	-18.004	-7.229	25.463	1.00	50.00
ATOM	582	C	ASN	1058	-17.177	-6.412	26.463	1.00	50.00
ATOM	583	O	ASN	1058	-16.928	-6.811	27.598	1.00	50.00
ATOM	584	CB	ASN	1058	-19.116	-8.017	26.174	1.00	50.00
ATOM	585	CG	ASN	1058	-20.029	-7.106	27.000	1.00	50.00
ATOM	586	OD1	ASN	1058	-19.793	-6.807	28.162	1.00	50.00
ATOM	587	ND2	ASN	1058	-21.061	-6.590	26.375	1.00	50.00
ATOM	588	N	CYS	1059	-16.610	-5.332	25.933	1.00	50.00
ATOM	589	CA	CYS	1059	-15.775	-4.418	26.723	1.00	50.00
ATOM	590	C	CYS	1059	-16.111	-2.981	26.324	1.00	50.00
ATOM	591	O	CYS	1059	-16.229	-2.676	25.140	1.00	50.00
ATOM	592	CB	CYS	1059	-14.301	-4.732	26.446	1.00	50.00
ATOM	593	SG	CYS	1059	-13.105	-3.720	27.393	1.00	50.00
ATOM	594	N	HIS	1060	-16.086	-2.097	27.318	1.00	50.00
ATOM	595	CA	HIS	1060	-16.341	-0.659	27.089	1.00	50.00
ATOM	596	C	HIS	1060	-15.274	-0.021	26.195	1.00	50.00
ATOM	597	O	HIS	1060	-15.536	1.007	25.580	1.00	50.00
ATOM	598	CB	HIS	1060	-16.449	0.097	28.419	1.00	50.00
ATOM	599	CG	HIS	1060	-16.787	1.582	28.211	1.00	50.00
ATOM	600	ND1	HIS	1060	-17.993	2.083	27.978	1.00	50.00
ATOM	601	CD2	HIS	1060	-15.925	2.597	28.240	1.00	50.00
ATOM	602	CE1	HIS	1060	-17.877	3.405	27.871	1.00	50.00
ATOM	603	NE2	HIS	1060	-16.599	3.726	28.037	1.00	50.00
ATOM	604	N	HIS	1061	-14.085	-0.602	26.138	1.00	50.00
ATOM	605	CA	HIS	1061	-12.977	-0.048	25.344	1.00	50.00
ATOM	606	C	HIS	1061	-12.639	-0.875	24.088	1.00	50.00
ATOM	607	O	HIS	1061	-11.532	-0.818	23.559	1.00	50.00
ATOM	608	CB	HIS	1061	-11.753	0.131	26.245	1.00	50.00
ATOM	609	CG	HIS	1061	-12.038	1.102	27.397	1.00	50.00

ATOM	610	ND1	HIS	1061	-12.016	0.782	28.682	1.00	50.00
ATOM	611	CD2	HIS	1061	-12.354	2.387	27.297	1.00	50.00
ATOM	612	CE1	HIS	1061	-12.309	1.873	29.388	1.00	50.00
ATOM	613	NE2	HIS	1061	-12.501	2.867	28.531	1.00	50.00
ATOM	614	N	VAL	1062	-13.606	-1.685	23.666	1.00	50.00
ATOM	615	CA	VAL	1062	-13.527	-2.420	22.387	1.00	50.00
ATOM	616	C	VAL	1062	-14.867	-2.205	21.678	1.00	50.00
ATOM	617	O	VAL	1062	-15.923	-2.493	22.237	1.00	50.00
ATOM	618	CB	VAL	1062	-13.255	-3.923	22.612	1.00	50.00
ATOM	619	CG1	VAL	1062	-13.098	-4.665	21.280	1.00	50.00
ATOM	620	CG2	VAL	1062	-11.994	-4.176	23.447	1.00	50.00
ATOM	621	N	VAL	1063	-14.784	-1.728	20.433	1.00	50.00
ATOM	622	CA	VAL	1063	-15.969	-1.502	19.579	1.00	50.00
ATOM	623	C	VAL	1063	-16.813	-2.784	19.515	1.00	50.00
ATOM	624	O	VAL	1063	-16.331	-3.861	19.163	1.00	50.00
ATOM	625	CB	VAL	1063	-15.531	-1.041	18.170	1.00	50.00
ATOM	626	CG1	VAL	1063	-16.725	-0.798	17.238	1.00	50.00
ATOM	627	CG2	VAL	1063	-14.710	0.248	18.245	1.00	50.00
ATOM	628	N	ARG	1064	-18.092	-2.612	19.845	1.00	50.00
ATOM	629	CA	ARG	1064	-19.062	-3.706	19.970	1.00	50.00
ATOM	630	C	ARG	1064	-19.699	-4.089	18.627	1.00	50.00
ATOM	631	O	ARG	1064	-20.021	-3.232	17.808	1.00	50.00
ATOM	632	CB	ARG	1064	-20.178	-3.267	20.927	1.00	50.00
ATOM	633	CG	ARG	1064	-19.738	-3.216	22.395	1.00	50.00
ATOM	634	CD	ARG	1064	-20.020	-4.526	23.140	1.00	50.00
ATOM	635	NE	ARG	1064	-21.475	-4.728	23.287	1.00	50.00
ATOM	636	CZ	ARG	1064	-22.201	-5.687	22.709	1.00	50.00
ATOM	637	NH1	ARG	1064	-21.629	-6.587	21.919	1.00	50.00
ATOM	638	NH2	ARG	1064	-23.505	-5.774	22.922	1.00	50.00
ATOM	639	N	LEU	1065	-19.885	-5.399	18.471	1.00	50.00
ATOM	640	CA	LEU	1065	-20.795	-5.956	17.457	1.00	50.00
ATOM	641	C	LEU	1065	-22.195	-5.945	18.084	1.00	50.00
ATOM	642	O	LEU	1065	-22.390	-6.438	19.183	1.00	50.00
ATOM	643	CB	LEU	1065	-20.402	-7.409	17.167	1.00	50.00
ATOM	644	CG	LEU	1065	-21.301	-8.029	16.096	1.00	50.00
ATOM	645	CD1	LEU	1065	-20.805	-7.676	14.701	1.00	50.00
ATOM	646	CD2	LEU	1065	-21.389	-9.537	16.266	1.00	50.00
ATOM	647	N	LEU	1066	-23.176	-5.458	17.329	1.00	50.00
ATOM	648	CA	LEU	1066	-24.550	-5.330	17.843	1.00	50.00
ATOM	649	C	LEU	1066	-25.567	-6.278	17.200	1.00	50.00
ATOM	650	O	LEU	1066	-26.600	-6.569	17.805	1.00	50.00
ATOM	651	CB	LEU	1066	-25.026	-3.877	17.725	1.00	50.00
ATOM	652	CG	LEU	1066	-24.211	-2.915	18.599	1.00	50.00
ATOM	653	CD1	LEU	1066	-24.684	-1.484	18.353	1.00	50.00
ATOM	654	CD2	LEU	1066	-24.330	-3.252	20.090	1.00	50.00
ATOM	655	N	GLY	1067	-25.265	-6.715	15.977	1.00	50.00
ATOM	656	CA	GLY	1067	-26.180	-7.595	15.231	1.00	50.00
ATOM	657	C	GLY	1067	-25.672	-7.935	13.830	1.00	50.00
ATOM	658	O	GLY	1067	-24.731	-7.345	13.318	1.00	50.00
ATOM	659	N	VAL	1068	-26.310	-8.970	13.291	1.00	50.00
ATOM	660	CA	VAL	1068	-26.049	-9.482	11.931	1.00	50.00
ATOM	661	C	VAL	1068	-27.385	-9.616	11.195	1.00	50.00
ATOM	662	O	VAL	1068	-28.364	-10.145	11.725	1.00	50.00
ATOM	663	CB	VAL	1068	-25.307	-10.838	12.021	1.00	50.00
ATOM	664	CG1	VAL	1068	-25.204	-11.578	10.680	1.00	50.00
ATOM	665	CG2	VAL	1068	-23.880	-10.642	12.543	1.00	50.00
ATOM	666	N	VAL	1069	-27.359	-9.232	9.922	1.00	50.00
ATOM	667	CA	VAL	1069	-28.487	-9.468	9.009	1.00	50.00
ATOM	668	C	VAL	1069	-27.948	-10.305	7.843	1.00	50.00
ATOM	669	O	VAL	1069	-27.394	-9.791	6.876	1.00	50.00
ATOM	670	CB	VAL	1069	-29.122	-8.153	8.509	1.00	50.00

ATOM	671	CG1	VAL	1069	-30.417	-8.444	7.745	1.00	50.00
ATOM	672	CG2	VAL	1069	-29.418	-7.164	9.643	1.00	50.00
ATOM	673	N	SER	1070	-28.061	-11.620	8.024	1.00	50.00
ATOM	674	CA	SER	1070	-27.567	-12.608	7.045	1.00	50.00
ATOM	675	C	SER	1070	-28.689	-13.230	6.193	1.00	50.00
ATOM	676	O	SER	1070	-28.455	-14.136	5.393	1.00	50.00
ATOM	677	CB	SER	1070	-26.796	-13.717	7.767	1.00	50.00
ATOM	678	OG	SER	1070	-27.694	-14.469	8.588	1.00	50.00
ATOM	679	N	GLN	1071	-29.906	-12.752	6.425	1.00	50.00
ATOM	680	CA	GLN	1071	-31.099	-13.188	5.679	1.00	50.00
ATOM	681	C	GLN	1071	-31.465	-12.114	4.662	1.00	50.00
ATOM	682	O	GLN	1071	-31.699	-10.961	5.025	1.00	50.00
ATOM	683	CB	GLN	1071	-32.277	-13.424	6.631	1.00	50.00
ATOM	684	CG	GLN	1071	-31.985	-14.509	7.675	1.00	50.00
ATOM	685	CD	GLN	1071	-31.513	-15.811	7.030	1.00	50.00
ATOM	686	OE1	GLN	1071	-32.176	-16.420	6.195	1.00	50.00
ATOM	687	NE2	GLN	1071	-30.314	-16.225	7.365	1.00	50.00
ATOM	688	N	GLY	1072	-31.517	-12.552	3.401	1.00	50.00
ATOM	689	CA	GLY	1072	-31.706	-11.649	2.252	1.00	50.00
ATOM	690	C	GLY	1072	-30.497	-10.717	2.085	1.00	50.00
ATOM	691	O	GLY	1072	-29.426	-10.924	2.651	1.00	50.00
ATOM	692	N	GLN	1073	-30.703	-9.723	1.225	1.00	50.00
ATOM	693	CA	GLN	1073	-29.662	-8.753	0.852	1.00	50.00
ATOM	694	C	GLN	1073	-30.239	-7.329	0.892	1.00	50.00
ATOM	695	O	GLN	1073	-31.443	-7.172	0.677	1.00	50.00
ATOM	696	CB	GLN	1073	-29.146	-9.088	-0.553	1.00	50.00
ATOM	697	CG	GLN	1073	-28.568	-10.506	-0.617	1.00	50.00
ATOM	698	CD	GLN	1073	-28.324	-10.989	-2.045	1.00	50.00
ATOM	699	OE1	GLN	1073	-28.761	-10.426	-3.041	1.00	50.00
ATOM	700	NE2	GLN	1073	-27.635	-12.098	-2.146	1.00	50.00
ATOM	701	N	PRO	1074	-29.383	-6.316	1.135	1.00	50.00
ATOM	702	CA	PRO	1074	-27.925	-6.451	1.369	1.00	50.00
ATOM	703	C	PRO	1074	-27.639	-7.094	2.714	1.00	50.00
ATOM	704	O	PRO	1074	-28.364	-6.936	3.685	1.00	50.00
ATOM	705	CB	PRO	1074	-27.405	-5.010	1.323	1.00	50.00
ATOM	706	CG	PRO	1074	-28.584	-4.203	1.862	1.00	50.00
ATOM	707	CD	PRO	1074	-29.802	-4.914	1.265	1.00	50.00
ATOM	708	N	THR	1075	-26.519	-7.824	2.800	1.00	50.00
ATOM	709	CA	THR	1075	-26.127	-8.465	4.052	1.00	50.00
ATOM	710	C	THR	1075	-25.383	-7.422	4.897	1.00	50.00
ATOM	711	O	THR	1075	-24.558	-6.668	4.386	1.00	50.00
ATOM	712	CB	THR	1075	-25.310	-9.778	3.865	1.00	50.00
ATOM	713	OG1	THR	1075	-23.954	-9.616	4.273	1.00	50.00
ATOM	714	CG2	THR	1075	-25.342	-10.338	2.440	1.00	50.00
ATOM	715	N	LEU	1076	-25.774	-7.357	6.173	1.00	50.00
ATOM	716	CA	LEU	1076	-25.315	-6.297	7.076	1.00	50.00
ATOM	717	C	LEU	1076	-24.650	-6.867	8.335	1.00	50.00
ATOM	718	O	LEU	1076	-24.962	-7.966	8.780	1.00	50.00
ATOM	719	CB	LEU	1076	-26.503	-5.426	7.516	1.00	50.00
ATOM	720	CG	LEU	1076	-27.391	-4.935	6.365	1.00	50.00
ATOM	721	CD1	LEU	1076	-28.604	-4.193	6.925	1.00	50.00
ATOM	722	CD2	LEU	1076	-26.630	-4.028	5.400	1.00	50.00
ATOM	723	N	VAL	1077	-23.742	-6.052	8.862	1.00	50.00
ATOM	724	CA	VAL	1077	-23.152	-6.246	10.200	1.00	50.00
ATOM	725	C	VAL	1077	-23.305	-4.919	10.948	1.00	50.00
ATOM	726	O	VAL	1077	-22.774	-3.889	10.547	1.00	50.00
ATOM	727	CB	VAL	1077	-21.685	-6.703	10.080	1.00	50.00
ATOM	728	CG1	VAL	1077	-20.958	-6.655	11.421	1.00	50.00
ATOM	729	CG2	VAL	1077	-21.620	-8.155	9.607	1.00	50.00
ATOM	730	N	ILE	1078	-24.034	-5.004	12.066	1.00	50.00
ATOM	731	CA	ILE	1078	-24.366	-3.831	12.882	1.00	50.00

ATOM	732	C	ILE	1078	-23.353	-3.748	14.027	1.00	50.00
ATOM	733	O	ILE	1078	-23.131	-4.713	14.747	1.00	50.00
ATOM	734	CB	ILE	1078	-25.802	-3.956	13.438	1.00	50.00
ATOM	735	CG1	ILE	1078	-26.824	-4.364	12.360	1.00	50.00
ATOM	736	CG2	ILE	1078	-26.240	-2.674	14.161	1.00	50.00
ATOM	737	CD1	ILE	1078	-26.874	-3.462	11.117	1.00	50.00
ATOM	738	N	MET	1079	-22.744	-2.569	14.135	1.00	50.00
ATOM	739	CA	MET	1079	-21.725	-2.301	15.157	1.00	50.00
ATOM	740	C	MET	1079	-21.980	-0.963	15.863	1.00	50.00
ATOM	741	O	MET	1079	-22.690	-0.094	15.368	1.00	50.00
ATOM	742	CB	MET	1079	-20.320	-2.310	14.544	1.00	50.00
ATOM	743	CG	MET	1079	-19.896	-3.722	14.136	1.00	50.00
ATOM	744	SD	MET	1079	-18.165	-3.809	13.555	1.00	50.00
ATOM	745	CE	MET	1079	-18.003	-5.574	13.407	1.00	50.00
ATOM	746	N	GLU	1080	-21.372	-0.858	17.040	1.00	50.00
ATOM	747	CA	GLU	1080	-21.343	0.403	17.806	1.00	50.00
ATOM	748	C	GLU	1080	-20.820	1.536	16.915	1.00	50.00
ATOM	749	O	GLU	1080	-19.889	1.361	16.127	1.00	50.00
ATOM	750	CB	GLU	1080	-20.416	0.164	18.996	1.00	50.00
ATOM	751	CG	GLU	1080	-20.138	1.425	19.816	1.00	50.00
ATOM	752	CD	GLU	1080	-19.218	1.164	21.006	1.00	50.00
ATOM	753	OE1	GLU	1080	-18.666	0.049	21.124	1.00	50.00
ATOM	754	OE2	GLU	1080	-19.147	2.112	21.812	1.00	50.00
ATOM	755	N	LEU	1081	-21.420	2.709	17.100	1.00	50.00
ATOM	756	CA	LEU	1081	-21.034	3.896	16.333	1.00	50.00
ATOM	757	C	LEU	1081	-19.947	4.702	17.051	1.00	50.00
ATOM	758	O	LEU	1081	-20.054	5.037	18.229	1.00	50.00
ATOM	759	CB	LEU	1081	-22.273	4.756	16.053	1.00	50.00
ATOM	760	CG	LEU	1081	-21.952	6.024	15.250	1.00	50.00
ATOM	761	CD1	LEU	1081	-21.386	5.710	13.861	1.00	50.00
ATOM	762	CD2	LEU	1081	-23.207	6.883	15.136	1.00	50.00
ATOM	763	N	MET	1082	-18.921	5.018	16.265	1.00	50.00
ATOM	764	CA	MET	1082	-17.786	5.851	16.692	1.00	50.00
ATOM	765	C	MET	1082	-17.715	7.031	15.718	1.00	50.00
ATOM	766	O	MET	1082	-17.149	6.941	14.631	1.00	50.00
ATOM	767	CB	MET	1082	-16.489	5.030	16.622	1.00	50.00
ATOM	768	CG	MET	1082	-16.567	3.709	17.397	1.00	50.00
ATOM	769	SD	MET	1082	-16.739	3.901	19.208	1.00	50.00
ATOM	770	CE	MET	1082	-15.073	4.372	19.611	1.00	50.00
ATOM	771	N	THR	1083	-18.416	8.103	16.086	1.00	50.00
ATOM	772	CA	THR	1083	-18.631	9.258	15.190	1.00	50.00
ATOM	773	C	THR	1083	-17.353	9.988	14.754	1.00	50.00
ATOM	774	O	THR	1083	-17.325	10.559	13.665	1.00	50.00
ATOM	775	CB	THR	1083	-19.592	10.281	15.808	1.00	50.00
ATOM	776	OG1	THR	1083	-19.076	10.671	17.076	1.00	50.00
ATOM	777	CG2	THR	1083	-21.002	9.719	15.993	1.00	50.00
ATOM	778	N	ARG	1084	-16.288	9.886	15.550	1.00	50.00
ATOM	779	CA	ARG	1084	-15.036	10.618	15.275	1.00	50.00
ATOM	780	C	ARG	1084	-13.956	9.783	14.553	1.00	50.00
ATOM	781	O	ARG	1084	-12.806	10.208	14.429	1.00	50.00
ATOM	782	CB	ARG	1084	-14.507	11.224	16.580	1.00	50.00
ATOM	783	CG	ARG	1084	-15.523	12.189	17.196	1.00	50.00
ATOM	784	CD	ARG	1084	-14.987	12.819	18.480	1.00	50.00
ATOM	785	NE	ARG	1084	-16.036	13.661	19.078	1.00	50.00
ATOM	786	CZ	ARG	1084	-16.302	14.942	18.788	1.00	50.00
ATOM	787	NH1	ARG	1084	-15.593	15.610	17.894	1.00	50.00
ATOM	788	NH2	ARG	1084	-17.280	15.566	19.428	1.00	50.00
ATOM	789	N	GLY	1085	-14.383	8.633	14.015	1.00	50.00
ATOM	790	CA	GLY	1085	-13.550	7.761	13.156	1.00	50.00
ATOM	791	C	GLY	1085	-12.310	7.205	13.865	1.00	50.00
ATOM	792	O	GLY	1085	-12.265	7.093	15.089	1.00	50.00

ATOM	793	N	ASP	1086	-11.335	6.794	13.052	1.00	50.00
ATOM	794	CA	ASP	1086	-10.101	6.176	13.575	1.00	50.00
ATOM	795	C	ASP	1086	-9.198	7.193	14.289	1.00	50.00
ATOM	796	O	ASP	1086	-9.097	8.342	13.879	1.00	50.00
ATOM	797	CB	ASP	1086	-9.329	5.381	12.511	1.00	50.00
ATOM	798	CG	ASP	1086	-8.584	6.236	11.497	1.00	50.00
ATOM	799	OD1	ASP	1086	-9.204	6.560	10.464	1.00	50.00
ATOM	800	OD2	ASP	1086	-7.409	6.537	11.783	1.00	50.00
ATOM	801	N	LEU	1087	-8.373	6.644	15.174	1.00	50.00
ATOM	802	CA	LEU	1087	-7.488	7.464	16.018	1.00	50.00
ATOM	803	C	LEU	1087	-6.387	8.194	15.245	1.00	50.00
ATOM	804	O	LEU	1087	-6.110	9.356	15.541	1.00	50.00
ATOM	805	CB	LEU	1087	-6.918	6.612	17.157	1.00	50.00
ATOM	806	CG	LEU	1087	-6.086	7.420	18.159	1.00	50.00
ATOM	807	CD1	LEU	1087	-6.910	8.519	18.838	1.00	50.00
ATOM	808	CD2	LEU	1087	-5.496	6.482	19.209	1.00	50.00
ATOM	809	N	LYS	1088	-5.836	7.558	14.213	1.00	50.00
ATOM	810	CA	LYS	1088	-4.818	8.196	13.355	1.00	50.00
ATOM	811	C	LYS	1088	-5.364	9.434	12.624	1.00	50.00
ATOM	812	O	LYS	1088	-4.785	10.515	12.721	1.00	50.00
ATOM	813	CB	LYS	1088	-4.235	7.167	12.380	1.00	50.00
ATOM	814	CG	LYS	1088	-3.155	7.788	11.495	1.00	50.00
ATOM	815	CD	LYS	1088	-2.262	6.712	10.894	1.00	50.00
ATOM	816	CE	LYS	1088	-1.209	7.374	10.017	1.00	50.00
ATOM	817	NZ	LYS	1088	-0.046	6.491	9.903	1.00	50.00
ATOM	818	N	SER	1089	-6.562	9.294	12.052	1.00	50.00
ATOM	819	CA	SER	1089	-7.262	10.405	11.380	1.00	50.00
ATOM	820	C	SER	1089	-7.651	11.519	12.355	1.00	50.00
ATOM	821	O	SER	1089	-7.470	12.694	12.043	1.00	50.00
ATOM	822	CB	SER	1089	-8.550	9.962	10.689	1.00	50.00
ATOM	823	OG	SER	1089	-8.256	9.026	9.658	1.00	50.00
ATOM	824	N	TYR	1090	-8.080	11.125	13.551	1.00	50.00
ATOM	825	CA	TYR	1090	-8.434	12.090	14.612	1.00	50.00
ATOM	826	C	TYR	1090	-7.202	12.870	15.083	1.00	50.00
ATOM	827	O	TYR	1090	-7.239	14.087	15.252	1.00	50.00
ATOM	828	CB	TYR	1090	-9.113	11.365	15.782	1.00	50.00
ATOM	829	CG	TYR	1090	-9.311	12.298	16.981	1.00	50.00
ATOM	830	CD1	TYR	1090	-10.329	13.245	16.975	1.00	50.00
ATOM	831	CD2	TYR	1090	-8.405	12.257	18.033	1.00	50.00
ATOM	832	CE1	TYR	1090	-10.436	14.150	18.020	1.00	50.00
ATOM	833	CE2	TYR	1090	-8.515	13.157	19.084	1.00	50.00
ATOM	834	CZ	TYR	1090	-9.532	14.105	19.074	1.00	50.00
ATOM	835	OH	TYR	1090	-9.653	14.982	20.102	1.00	50.00
ATOM	836	N	LEU	1091	-6.097	12.166	15.282	1.00	50.00
ATOM	837	CA	LEU	1091	-4.832	12.794	15.698	1.00	50.00
ATOM	838	C	LEU	1091	-4.285	13.768	14.649	1.00	50.00
ATOM	839	O	LEU	1091	-3.939	14.901	14.977	1.00	50.00
ATOM	840	CB	LEU	1091	-3.784	11.729	16.018	1.00	50.00
ATOM	841	CG	LEU	1091	-4.128	10.924	17.274	1.00	50.00
ATOM	842	CD1	LEU	1091	-3.144	9.762	17.401	1.00	50.00
ATOM	843	CD2	LEU	1091	-4.091	11.795	18.535	1.00	50.00
ATOM	844	N	ARG	1092	-4.376	13.358	13.387	1.00	50.00
ATOM	845	CA	ARG	1092	-3.954	14.206	12.256	1.00	50.00
ATOM	846	C	ARG	1092	-4.837	15.436	12.032	1.00	50.00
ATOM	847	O	ARG	1092	-4.331	16.489	11.648	1.00	50.00
ATOM	848	CB	ARG	1092	-3.813	13.398	10.968	1.00	50.00
ATOM	849	CG	ARG	1092	-2.539	12.558	11.035	1.00	50.00
ATOM	850	CD	ARG	1092	-2.238	11.922	9.682	1.00	50.00
ATOM	851	NE	ARG	1092	-0.933	11.246	9.762	1.00	50.00
ATOM	852	CZ	ARG	1092	-0.314	10.604	8.765	1.00	50.00
ATOM	853	NH1	ARG	1092	-0.833	10.526	7.560	1.00	50.00

ATOM	854	NH2	ARG	1092	0.860	10.031	9.002	1.00	50.00
ATOM	855	N	SER	1093	-6.119	15.337	12.397	1.00	50.00
ATOM	856	CA	SER	1093	-7.048	16.482	12.317	1.00	50.00
ATOM	857	C	SER	1093	-6.737	17.567	13.366	1.00	50.00
ATOM	858	O	SER	1093	-7.235	18.689	13.270	1.00	50.00
ATOM	859	CB	SER	1093	-8.516	16.051	12.441	1.00	50.00
ATOM	860	OG	SER	1093	-8.824	15.685	13.786	1.00	50.00
ATOM	861	N	LEU	1094	-5.951	17.194	14.374	1.00	50.00
ATOM	862	CA	LEU	1094	-5.536	18.100	15.460	1.00	50.00
ATOM	863	C	LEU	1094	-4.325	18.980	15.109	1.00	50.00
ATOM	864	O	LEU	1094	-4.060	19.970	15.785	1.00	50.00
ATOM	865	CB	LEU	1094	-5.268	17.318	16.748	1.00	50.00
ATOM	866	CG	LEU	1094	-6.506	16.566	17.257	1.00	50.00
ATOM	867	CD1	LEU	1094	-6.151	15.827	18.544	1.00	50.00
ATOM	868	CD2	LEU	1094	-7.703	17.494	17.496	1.00	50.00
ATOM	869	N	ARG	1095	-3.604	18.574	14.070	1.00	50.00
ATOM	870	CA	ARG	1095	-2.439	19.335	13.569	1.00	50.00
ATOM	871	C	ARG	1095	-2.857	20.755	13.169	1.00	50.00
ATOM	872	O	ARG	1095	-3.926	20.953	12.580	1.00	50.00
ATOM	873	CB	ARG	1095	-1.835	18.642	12.348	1.00	50.00
ATOM	874	CG	ARG	1095	-1.267	17.258	12.655	1.00	50.00
ATOM	875	CD	ARG	1095	-0.726	16.667	11.355	1.00	50.00
ATOM	876	NE	ARG	1095	-0.098	15.361	11.599	1.00	50.00
ATOM	877	CZ	ARG	1095	1.207	15.151	11.836	1.00	50.00
ATOM	878	NH1	ARG	1095	2.058	16.154	11.925	1.00	50.00
ATOM	879	NH2	ARG	1095	1.666	13.912	11.906	1.00	50.00
ATOM	880	N	PRO	1096	-2.014	21.754	13.485	1.00	50.00
ATOM	881	CA	PRO	1096	-2.247	23.143	13.068	1.00	50.00
ATOM	882	C	PRO	1096	-2.171	23.238	11.544	1.00	50.00
ATOM	883	O	PRO	1096	-1.182	22.852	10.922	1.00	50.00
ATOM	884	CB	PRO	1096	-1.147	23.947	13.770	1.00	50.00
ATOM	885	CG	PRO	1096	-0.012	22.939	13.940	1.00	50.00
ATOM	886	CD	PRO	1096	-0.744	21.627	14.227	1.00	50.00
ATOM	887	N	GLU	1097	-3.239	23.786	10.967	1.00	50.00
ATOM	888	CA	GLU	1097	-3.389	23.901	9.508	1.00	50.00
ATOM	889	C	GLU	1097	-2.344	24.853	8.891	1.00	50.00
ATOM	890	O	GLU	1097	-1.963	24.686	7.733	1.00	50.00
ATOM	891	CB	GLU	1097	-4.800	24.403	9.197	1.00	50.00
ATOM	892	CG	GLU	1097	-5.140	24.323	7.704	1.00	50.00
ATOM	893	CD	GLU	1097	-6.479	24.995	7.392	1.00	50.00
ATOM	894	OE1	GLU	1097	-6.725	26.091	7.945	1.00	50.00
ATOM	895	OE2	GLU	1097	-7.221	24.414	6.569	1.00	50.00
ATOM	896	N	MET	1098	-1.975	25.858	9.668	1.00	50.00
ATOM	897	CA	MET	1098	-1.055	26.942	9.252	1.00	50.00
ATOM	898	C	MET	1098	-1.666	27.734	8.096	1.00	50.00
ATOM	899	O	MET	1098	-1.467	27.488	6.908	1.00	50.00
ATOM	900	CB	MET	1098	0.335	26.385	8.904	1.00	50.00
ATOM	901	CG	MET	1098	1.393	27.485	8.778	1.00	50.00
ATOM	902	SD	MET	1098	1.753	28.321	10.368	1.00	50.00
ATOM	903	CE	MET	1098	0.584	29.666	10.373	1.00	50.00
ATOM	904	N	GLU	1099	-2.472	28.710	8.511	1.00	50.00
ATOM	905	CA	GLU	1099	-3.214	29.603	7.612	1.00	50.00
ATOM	906	C	GLU	1099	-3.551	30.912	8.367	1.00	50.00
ATOM	907	O	GLU	1099	-4.529	31.595	8.076	1.00	50.00
ATOM	908	CB	GLU	1099	-4.485	28.860	7.170	1.00	50.00
ATOM	909	CG	GLU	1099	-5.097	29.396	5.871	1.00	50.00
ATOM	910	CD	GLU	1099	-4.156	29.220	4.674	1.00	50.00
ATOM	911	OE1	GLU	1099	-4.001	28.057	4.242	1.00	50.00
ATOM	912	OE2	GLU	1099	-3.609	30.252	4.229	1.00	50.00
ATOM	913	N	ASN	1100	-2.710	31.192	9.354	1.00	50.00
ATOM	914	CA	ASN	1100	-2.814	32.339	10.297	1.00	50.00

ATOM	915	C	ASN	1100	-4.063	32.360	11.191	1.00	50.00
ATOM	916	O	ASN	1100	-4.290	33.311	11.932	1.00	50.00
ATOM	917	CB	ASN	1100	-2.677	33.672	9.556	1.00	50.00
ATOM	918	CG	ASN	1100	-1.256	33.881	9.042	1.00	50.00
ATOM	919	OD1	ASN	1100	-0.310	34.060	9.799	1.00	50.00
ATOM	920	ND2	ASN	1100	-1.077	33.798	7.746	1.00	50.00
ATOM	921	N	ASN	1101	-4.791	31.244	11.212	1.00	50.00
ATOM	922	CA	ASN	1101	-5.957	31.084	12.092	1.00	50.00
ATOM	923	C	ASN	1101	-5.562	30.480	13.447	1.00	50.00
ATOM	924	O	ASN	1101	-4.606	29.701	13.525	1.00	50.00
ATOM	925	CB	ASN	1101	-7.044	30.250	11.398	1.00	50.00
ATOM	926	CG	ASN	1101	-6.548	28.922	10.825	1.00	50.00
ATOM	927	OD1	ASN	1101	-5.520	28.360	11.172	1.00	50.00
ATOM	928	ND2	ASN	1101	-7.229	28.484	9.791	1.00	50.00
ATOM	929	N	PRO	1102	-6.259	30.900	14.520	1.00	50.00
ATOM	930	CA	PRO	1102	-6.051	30.372	15.880	1.00	50.00
ATOM	931	C	PRO	1102	-6.257	28.856	15.872	1.00	50.00
ATOM	932	O	PRO	1102	-7.060	28.329	15.108	1.00	50.00
ATOM	933	CB	PRO	1102	-7.144	31.039	16.720	1.00	50.00
ATOM	934	CG	PRO	1102	-7.385	32.364	16.003	1.00	50.00
ATOM	935	CD	PRO	1102	-7.254	31.989	14.528	1.00	50.00
ATOM	936	N	VAL	1103	-5.507	28.172	16.739	1.00	50.00
ATOM	937	CA	VAL	1103	-5.536	26.706	16.797	1.00	50.00
ATOM	938	C	VAL	1103	-5.531	26.219	18.251	1.00	50.00
ATOM	939	O	VAL	1103	-4.863	26.786	19.114	1.00	50.00
ATOM	940	CB	VAL	1103	-4.371	26.122	15.964	1.00	50.00
ATOM	941	CG1	VAL	1103	-2.981	26.429	16.538	1.00	50.00
ATOM	942	CG2	VAL	1103	-4.558	24.623	15.709	1.00	50.00
ATOM	943	N	LEU	1104	-6.282	25.145	18.465	1.00	50.00
ATOM	944	CA	LEU	1104	-6.263	24.424	19.742	1.00	50.00
ATOM	945	C	LEU	1104	-4.858	23.833	19.950	1.00	50.00
ATOM	946	O	LEU	1104	-4.263	23.273	19.031	1.00	50.00
ATOM	947	CB	LEU	1104	-7.303	23.297	19.676	1.00	50.00
ATOM	948	CG	LEU	1104	-8.040	23.008	20.995	1.00	50.00
ATOM	949	CD1	LEU	1104	-7.114	22.558	22.129	1.00	50.00
ATOM	950	CD2	LEU	1104	-8.887	24.211	21.421	1.00	50.00
ATOM	951	N	ALA	1105	-4.337	24.035	21.153	1.00	50.00
ATOM	952	CA	ALA	1105	-3.052	23.437	21.566	1.00	50.00
ATOM	953	C	ALA	1105	-3.120	21.901	21.448	1.00	50.00
ATOM	954	O	ALA	1105	-4.206	21.325	21.558	1.00	50.00
ATOM	955	CB	ALA	1105	-2.755	23.862	23.009	1.00	50.00
ATOM	956	N	PRO	1106	-1.974	21.238	21.208	1.00	50.00
ATOM	957	CA	PRO	1106	-1.923	19.768	21.146	1.00	50.00
ATOM	958	C	PRO	1106	-2.415	19.166	22.473	1.00	50.00
ATOM	959	O	PRO	1106	-2.301	19.817	23.516	1.00	50.00
ATOM	960	CB	PRO	1106	-0.452	19.443	20.865	1.00	50.00
ATOM	961	CG	PRO	1106	0.295	20.621	21.484	1.00	50.00
ATOM	962	CD	PRO	1106	-0.614	21.804	21.151	1.00	50.00
ATOM	963	N	PRO	1107	-2.999	17.954	22.424	1.00	50.00
ATOM	964	CA	PRO	1107	-3.592	17.286	23.596	1.00	50.00
ATOM	965	C	PRO	1107	-2.657	17.335	24.803	1.00	50.00
ATOM	966	O	PRO	1107	-1.450	17.121	24.703	1.00	50.00
ATOM	967	CB	PRO	1107	-3.791	15.839	23.142	1.00	50.00
ATOM	968	CG	PRO	1107	-4.091	15.988	21.655	1.00	50.00
ATOM	969	CD	PRO	1107	-3.163	17.122	21.218	1.00	50.00
ATOM	970	N	SER	1108	-3.254	17.699	25.938	1.00	50.00
ATOM	971	CA	SER	1108	-2.549	17.760	27.226	1.00	50.00
ATOM	972	C	SER	1108	-2.088	16.350	27.634	1.00	50.00
ATOM	973	O	SER	1108	-2.649	15.349	27.192	1.00	50.00
ATOM	974	CB	SER	1108	-3.473	18.330	28.311	1.00	50.00
ATOM	975	OG	SER	1108	-4.554	17.431	28.587	1.00	50.00

ATOM	976	N	LEU	1109	-1.171	16.309	28.595	1.00	50.00
ATOM	977	CA	LEU	1109	-0.713	15.022	29.157	1.00	50.00
ATOM	978	C	LEU	1109	-1.868	14.204	29.746	1.00	50.00
ATOM	979	O	LEU	1109	-1.988	13.020	29.435	1.00	50.00
ATOM	980	CB	LEU	1109	0.394	15.245	30.195	1.00	50.00
ATOM	981	CG	LEU	1109	0.928	13.933	30.792	1.00	50.00
ATOM	982	CD1	LEU	1109	1.498	12.991	29.724	1.00	50.00
ATOM	983	CD2	LEU	1109	1.987	14.244	31.849	1.00	50.00
ATOM	984	N	SER	1110	-2.811	14.878	30.404	1.00	50.00
ATOM	985	CA	SER	1110	-4.016	14.228	30.957	1.00	50.00
ATOM	986	C	SER	1110	-4.909	13.604	29.876	1.00	50.00
ATOM	987	O	SER	1110	-5.255	12.428	29.971	1.00	50.00
ATOM	988	CB	SER	1110	-4.872	15.182	31.792	1.00	50.00
ATOM	989	OG	SER	1110	-4.183	15.519	32.995	1.00	50.00
ATOM	990	N	LYS	1111	-5.127	14.346	28.790	1.00	50.00
ATOM	991	CA	LYS	1111	-5.890	13.842	27.627	1.00	50.00
ATOM	992	C	LYS	1111	-5.185	12.669	26.926	1.00	50.00
ATOM	993	O	LYS	1111	-5.815	11.673	26.578	1.00	50.00
ATOM	994	CB	LYS	1111	-6.156	14.967	26.621	1.00	50.00
ATOM	995	CG	LYS	1111	-7.126	16.024	27.161	1.00	50.00
ATOM	996	CD	LYS	1111	-8.548	15.482	27.329	1.00	50.00
ATOM	997	CE	LYS	1111	-9.454	16.545	27.949	1.00	50.00
ATOM	998	NZ	LYS	1111	-10.846	16.077	28.013	1.00	50.00
ATOM	999	N	MET	1112	-3.856	12.751	26.874	1.00	50.00
ATOM	1000	CA	MET	1112	-3.014	11.675	26.313	1.00	50.00
ATOM	1001	C	MET	1112	-2.990	10.412	27.189	1.00	50.00
ATOM	1002	O	MET	1112	-3.084	9.299	26.680	1.00	50.00
ATOM	1003	CB	MET	1112	-1.588	12.161	26.045	1.00	50.00
ATOM	1004	CG	MET	1112	-1.559	13.220	24.938	1.00	50.00
ATOM	1005	SD	MET	1112	0.114	13.556	24.277	1.00	50.00
ATOM	1006	CE	MET	1112	0.886	14.323	25.686	1.00	50.00
ATOM	1007	N	ILE	1113	-3.000	10.612	28.510	1.00	50.00
ATOM	1008	CA	ILE	1113	-3.060	9.503	29.488	1.00	50.00
ATOM	1009	C	ILE	1113	-4.422	8.798	29.457	1.00	50.00
ATOM	1010	O	ILE	1113	-4.491	7.572	29.494	1.00	50.00
ATOM	1011	CB	ILE	1113	-2.683	9.975	30.910	1.00	50.00
ATOM	1012	CG1	ILE	1113	-1.232	10.479	30.964	1.00	50.00
ATOM	1013	CG2	ILE	1113	-2.918	8.913	31.999	1.00	50.00
ATOM	1014	CD1	ILE	1113	-0.184	9.505	30.404	1.00	50.00
ATOM	1015	N	GLN	1114	-5.472	9.604	29.306	1.00	50.00
ATOM	1016	CA	GLN	1114	-6.847	9.087	29.200	1.00	50.00
ATOM	1017	C	GLN	1114	-6.978	8.136	28.003	1.00	50.00
ATOM	1018	O	GLN	1114	-7.441	7.005	28.155	1.00	50.00
ATOM	1019	CB	GLN	1114	-7.822	10.260	29.071	1.00	50.00
ATOM	1020	CG	GLN	1114	-9.287	9.807	29.129	1.00	50.00
ATOM	1021	CD	GLN	1114	-9.664	9.144	30.464	1.00	50.00
ATOM	1022	OE1	GLN	1114	-9.185	9.455	31.541	1.00	50.00
ATOM	1023	NE2	GLN	1114	-10.616	8.238	30.405	1.00	50.00
ATOM	1024	N	MET	1115	-6.403	8.552	26.872	1.00	50.00
ATOM	1025	CA	MET	1115	-6.368	7.726	25.653	1.00	50.00
ATOM	1026	C	MET	1115	-5.528	6.449	25.833	1.00	50.00
ATOM	1027	O	MET	1115	-6.001	5.353	25.534	1.00	50.00
ATOM	1028	CB	MET	1115	-5.848	8.523	24.454	1.00	50.00
ATOM	1029	CG	MET	1115	-6.854	9.589	24.013	1.00	50.00
ATOM	1030	SD	MET	1115	-6.448	10.332	22.391	1.00	50.00
ATOM	1031	CE	MET	1115	-5.377	11.658	22.899	1.00	50.00
ATOM	1032	N	ALA	1116	-4.397	6.601	26.521	1.00	50.00
ATOM	1033	CA	ALA	1116	-3.475	5.486	26.830	1.00	50.00
ATOM	1034	C	ALA	1116	-4.148	4.378	27.654	1.00	50.00
ATOM	1035	O	ALA	1116	-4.152	3.218	27.238	1.00	50.00
ATOM	1036	CB	ALA	1116	-2.265	6.024	27.596	1.00	50.00

ATOM	1037	N	GLY	1117	-4.876	4.802	28.697	1.00	50.00
ATOM	1038	CA	GLY	1117	-5.569	3.884	29.622	1.00	50.00
ATOM	1039	C	GLY	1117	-6.723	3.130	28.953	1.00	50.00
ATOM	1040	O	GLY	1117	-6.873	1.927	29.139	1.00	50.00
ATOM	1041	N	GLU	1118	-7.463	3.837	28.101	1.00	50.00
ATOM	1042	CA	GLU	1118	-8.593	3.254	27.346	1.00	50.00
ATOM	1043	C	GLU	1118	-8.145	2.184	26.342	1.00	50.00
ATOM	1044	O	GLU	1118	-8.597	1.041	26.418	1.00	50.00
ATOM	1045	CB	GLU	1118	-9.357	4.364	26.628	1.00	50.00
ATOM	1046	CG	GLU	1118	-9.976	5.338	27.634	1.00	50.00
ATOM	1047	CD	GLU	1118	-10.652	6.537	26.967	1.00	50.00
ATOM	1048	OE1	GLU	1118	-10.208	6.942	25.867	1.00	50.00
ATOM	1049	OE2	GLU	1118	-11.569	7.077	27.623	1.00	50.00
ATOM	1050	N	ILE	1119	-7.071	2.503	25.605	1.00	50.00
ATOM	1051	CA	ILE	1119	-6.436	1.562	24.655	1.00	50.00
ATOM	1052	C	ILE	1119	-5.878	0.343	25.410	1.00	50.00
ATOM	1053	O	ILE	1119	-6.183	-0.802	25.076	1.00	50.00
ATOM	1054	CB	ILE	1119	-5.317	2.252	23.842	1.00	50.00
ATOM	1055	CG1	ILE	1119	-5.893	3.423	23.033	1.00	50.00
ATOM	1056	CG2	ILE	1119	-4.602	1.253	22.912	1.00	50.00
ATOM	1057	CD1	ILE	1119	-4.836	4.323	22.381	1.00	50.00
ATOM	1058	N	ALA	1120	-5.108	0.630	26.461	1.00	50.00
ATOM	1059	CA	ALA	1120	-4.482	-0.403	27.302	1.00	50.00
ATOM	1060	C	ALA	1120	-5.521	-1.309	27.983	1.00	50.00
ATOM	1061	O	ALA	1120	-5.343	-2.521	28.042	1.00	50.00
ATOM	1062	CB	ALA	1120	-3.591	0.257	28.355	1.00	50.00
ATOM	1063	N	ASP	1121	-6.662	-0.725	28.345	1.00	50.00
ATOM	1064	CA	ASP	1121	-7.765	-1.474	28.977	1.00	50.00
ATOM	1065	C	ASP	1121	-8.436	-2.431	27.989	1.00	50.00
ATOM	1066	O	ASP	1121	-8.682	-3.587	28.337	1.00	50.00
ATOM	1067	CB	ASP	1121	-8.778	-0.511	29.593	1.00	50.00
ATOM	1068	CG	ASP	1121	-9.795	-1.294	30.417	1.00	50.00
ATOM	1069	OD1	ASP	1121	-9.441	-1.694	31.547	1.00	50.00
ATOM	1070	OD2	ASP	1121	-10.927	-1.460	29.920	1.00	50.00
ATOM	1071	N	GLY	1122	-8.668	-1.929	26.767	1.00	50.00
ATOM	1072	CA	GLY	1122	-9.201	-2.748	25.661	1.00	50.00
ATOM	1073	C	GLY	1122	-8.272	-3.928	25.334	1.00	50.00
ATOM	1074	O	GLY	1122	-8.706	-5.078	25.281	1.00	50.00
ATOM	1075	N	MET	1123	-6.973	-3.637	25.334	1.00	50.00
ATOM	1076	CA	MET	1123	-5.921	-4.642	25.094	1.00	50.00
ATOM	1077	C	MET	1123	-5.751	-5.674	26.215	1.00	50.00
ATOM	1078	O	MET	1123	-5.636	-6.867	25.944	1.00	50.00
ATOM	1079	CB	MET	1123	-4.593	-3.943	24.818	1.00	50.00
ATOM	1080	CG	MET	1123	-4.579	-3.273	23.441	1.00	50.00
ATOM	1081	SD	MET	1123	-4.938	-4.403	22.043	1.00	50.00
ATOM	1082	CE	MET	1123	-3.653	-5.618	22.244	1.00	50.00
ATOM	1083	N	ALA	1124	-5.858	-5.209	27.461	1.00	50.00
ATOM	1084	CA	ALA	1124	-5.817	-6.080	28.652	1.00	50.00
ATOM	1085	C	ALA	1124	-6.999	-7.060	28.662	1.00	50.00
ATOM	1086	O	ALA	1124	-6.808	-8.258	28.870	1.00	50.00
ATOM	1087	CB	ALA	1124	-5.842	-5.234	29.925	1.00	50.00
ATOM	1088	N	TYR	1125	-8.150	-6.555	28.228	1.00	50.00
ATOM	1089	CA	TYR	1125	-9.372	-7.365	28.050	1.00	50.00
ATOM	1090	C	TYR	1125	-9.177	-8.437	26.972	1.00	50.00
ATOM	1091	O	TYR	1125	-9.491	-9.605	27.191	1.00	50.00
ATOM	1092	CB	TYR	1125	-10.542	-6.439	27.700	1.00	50.00
ATOM	1093	CG	TYR	1125	-11.804	-7.223	27.333	1.00	50.00
ATOM	1094	CD1	TYR	1125	-12.673	-7.676	28.319	1.00	50.00
ATOM	1095	CD2	TYR	1125	-12.030	-7.554	26.006	1.00	50.00
ATOM	1096	CE1	TYR	1125	-13.767	-8.457	27.981	1.00	50.00
ATOM	1097	CE2	TYR	1125	-13.124	-8.333	25.663	1.00	50.00

ATOM	1098	CZ	TYR	1125	-13.993	-8.780	26.649	1.00	50.00
ATOM	1099	OH	TYR	1125	-15.119	-9.446	26.295	1.00	50.00
ATOM	1100	N	LEU	1126	-8.607	-8.042	25.834	1.00	50.00
ATOM	1101	CA	LEU	1126	-8.371	-8.961	24.708	1.00	50.00
ATOM	1102	C	LEU	1126	-7.342	-10.053	25.033	1.00	50.00
ATOM	1103	O	LEU	1126	-7.591	-11.233	24.799	1.00	50.00
ATOM	1104	CB	LEU	1126	-7.939	-8.187	23.461	1.00	50.00
ATOM	1105	CG	LEU	1126	-9.048	-7.268	22.937	1.00	50.00
ATOM	1106	CD1	LEU	1126	-8.502	-6.441	21.775	1.00	50.00
ATOM	1107	CD2	LEU	1126	-10.291	-8.057	22.506	1.00	50.00
ATOM	1108	N	ASN	1127	-6.293	-9.639	25.735	1.00	50.00
ATOM	1109	CA	ASN	1127	-5.256	-10.563	26.235	1.00	50.00
ATOM	1110	C	ASN	1127	-5.848	-11.582	27.215	1.00	50.00
ATOM	1111	O	ASN	1127	-5.605	-12.780	27.077	1.00	50.00
ATOM	1112	CB	ASN	1127	-4.126	-9.775	26.910	1.00	50.00
ATOM	1113	CG	ASN	1127	-3.127	-10.718	27.585	1.00	50.00
ATOM	1114	OD1	ASN	1127	-2.850	-10.626	28.775	1.00	50.00
ATOM	1115	ND2	ASN	1127	-2.619	-11.674	26.844	1.00	50.00
ATOM	1116	N	ALA	1128	-6.666	-11.083	28.144	1.00	50.00
ATOM	1117	CA	ALA	1128	-7.362	-11.915	29.142	1.00	50.00
ATOM	1118	C	ALA	1128	-8.273	-12.958	28.483	1.00	50.00
ATOM	1119	O	ALA	1128	-8.326	-14.105	28.929	1.00	50.00
ATOM	1120	CB	ALA	1128	-8.195	-11.023	30.062	1.00	50.00
ATOM	1121	N	ASN	1129	-8.830	-12.585	27.333	1.00	50.00
ATOM	1122	CA	ASN	1129	-9.666	-13.476	26.513	1.00	50.00
ATOM	1123	C	ASN	1129	-8.862	-14.260	25.457	1.00	50.00
ATOM	1124	O	ASN	1129	-9.426	-14.781	24.500	1.00	50.00
ATOM	1125	CB	ASN	1129	-10.806	-12.679	25.873	1.00	50.00
ATOM	1126	CG	ASN	1129	-11.869	-12.340	26.921	1.00	50.00
ATOM	1127	OD1	ASN	1129	-12.773	-13.103	27.215	1.00	50.00
ATOM	1128	ND2	ASN	1129	-11.737	-11.187	27.536	1.00	50.00
ATOM	1129	N	LYS	1130	-7.565	-14.406	25.723	1.00	50.00
ATOM	1130	CA	LYS	1130	-6.622	-15.283	24.976	1.00	50.00
ATOM	1131	C	LYS	1130	-6.331	-14.818	23.536	1.00	50.00
ATOM	1132	O	LYS	1130	-5.897	-15.601	22.692	1.00	50.00
ATOM	1133	CB	LYS	1130	-7.135	-16.733	24.945	1.00	50.00
ATOM	1134	CG	LYS	1130	-7.556	-17.266	26.314	1.00	50.00
ATOM	1135	CD	LYS	1130	-8.229	-18.629	26.161	1.00	50.00
ATOM	1136	CE	LYS	1130	-8.887	-19.068	27.470	1.00	50.00
ATOM	1137	NZ	LYS	1130	-9.996	-18.169	27.827	1.00	50.00
ATOM	1138	N	PHE	1131	-6.404	-13.507	23.312	1.00	50.00
ATOM	1139	CA	PHE	1131	-6.114	-12.933	21.984	1.00	50.00
ATOM	1140	C	PHE	1131	-4.852	-12.079	21.983	1.00	50.00
ATOM	1141	O	PHE	1131	-4.501	-11.393	22.932	1.00	50.00
ATOM	1142	CB	PHE	1131	-7.287	-12.095	21.464	1.00	50.00
ATOM	1143	CG	PHE	1131	-8.390	-12.959	20.853	1.00	50.00
ATOM	1144	CD1	PHE	1131	-9.395	-13.472	21.662	1.00	50.00
ATOM	1145	CD2	PHE	1131	-8.413	-13.197	19.483	1.00	50.00
ATOM	1146	CE1	PHE	1131	-10.418	-14.230	21.107	1.00	50.00
ATOM	1147	CE2	PHE	1131	-9.441	-13.948	18.925	1.00	50.00
ATOM	1148	CZ	PHE	1131	-10.445	-14.464	19.737	1.00	50.00
ATOM	1149	N	VAL	1132	-4.151	-12.220	20.850	1.00	50.00
ATOM	1150	CA	VAL	1132	-3.023	-11.375	20.461	1.00	50.00
ATOM	1151	C	VAL	1132	-3.461	-10.638	19.197	1.00	50.00
ATOM	1152	O	VAL	1132	-3.783	-11.246	18.177	1.00	50.00
ATOM	1153	CB	VAL	1132	-1.679	-12.170	20.376	1.00	50.00
ATOM	1154	CG1	VAL	1132	-1.833	-13.679	20.154	1.00	50.00
ATOM	1155	CG2	VAL	1132	-0.704	-11.627	19.328	1.00	50.00
ATOM	1156	N	HIS	1133	-3.467	-9.307	19.311	1.00	50.00
ATOM	1157	CA	HIS	1133	-4.008	-8.406	18.293	1.00	50.00
ATOM	1158	C	HIS	1133	-3.239	-8.475	16.959	1.00	50.00

ATOM	1159	O	HIS	1133	-3.855	-8.571	15.897	1.00	50.00
ATOM	1160	CB	HIS	1133	-3.985	-6.974	18.838	1.00	50.00
ATOM	1161	CG	HIS	1133	-4.778	-6.023	17.951	1.00	50.00
ATOM	1162	ND1	HIS	1133	-4.360	-5.513	16.790	1.00	50.00
ATOM	1163	CD2	HIS	1133	-5.956	-5.486	18.242	1.00	50.00
ATOM	1164	CE1	HIS	1133	-5.292	-4.666	16.372	1.00	50.00
ATOM	1165	NE2	HIS	1133	-6.278	-4.634	17.273	1.00	50.00
ATOM	1166	N	ARG	1134	-1.918	-8.353	17.054	1.00	50.00
ATOM	1167	CA	ARG	1134	-0.960	-8.410	15.922	1.00	50.00
ATOM	1168	C	ARG	1134	-0.892	-7.147	15.048	1.00	50.00
ATOM	1169	O	ARG	1134	0.034	-7.003	14.257	1.00	50.00
ATOM	1170	CB	ARG	1134	-1.159	-9.628	15.010	1.00	50.00
ATOM	1171	CG	ARG	1134	-1.061	-10.924	15.811	1.00	50.00
ATOM	1172	CD	ARG	1134	-0.757	-12.125	14.924	1.00	50.00
ATOM	1173	NE	ARG	1134	-1.875	-12.432	14.024	1.00	50.00
ATOM	1174	CZ	ARG	1134	-1.807	-13.297	13.002	1.00	50.00
ATOM	1175	NH1	ARG	1134	-0.679	-13.933	12.728	1.00	50.00
ATOM	1176	NH2	ARG	1134	-2.873	-13.527	12.257	1.00	50.00
ATOM	1177	N	ASP	1135	-1.859	-6.242	15.198	1.00	50.00
ATOM	1178	CA	ASP	1135	-1.825	-4.987	14.429	1.00	50.00
ATOM	1179	C	ASP	1135	-2.318	-3.759	15.215	1.00	50.00
ATOM	1180	O	ASP	1135	-3.061	-2.922	14.706	1.00	50.00
ATOM	1181	CB	ASP	1135	-2.572	-5.203	13.112	1.00	50.00
ATOM	1182	CG	ASP	1135	-2.304	-4.075	12.116	1.00	50.00
ATOM	1183	OD1	ASP	1135	-1.304	-3.341	12.273	1.00	50.00
ATOM	1184	OD2	ASP	1135	-3.103	-3.993	11.163	1.00	50.00
ATOM	1185	N	LEU	1136	-1.918	-3.696	16.476	1.00	50.00
ATOM	1186	CA	LEU	1136	-2.251	-2.537	17.323	1.00	50.00
ATOM	1187	C	LEU	1136	-1.549	-1.294	16.783	1.00	50.00
ATOM	1188	O	LEU	1136	-0.332	-1.261	16.621	1.00	50.00
ATOM	1189	CB	LEU	1136	-1.886	-2.801	18.788	1.00	50.00
ATOM	1190	CG	LEU	1136	-2.247	-1.621	19.701	1.00	50.00
ATOM	1191	CD1	LEU	1136	-3.742	-1.284	19.662	1.00	50.00
ATOM	1192	CD2	LEU	1136	-1.818	-1.939	21.131	1.00	50.00
ATOM	1193	N	ALA	1137	-2.378	-0.294	16.484	1.00	50.00
ATOM	1194	CA	ALA	1137	-1.959	0.968	15.866	1.00	50.00
ATOM	1195	C	ALA	1137	-3.124	1.959	15.983	1.00	50.00
ATOM	1196	O	ALA	1137	-4.275	1.549	16.095	1.00	50.00
ATOM	1197	CB	ALA	1137	-1.624	0.744	14.386	1.00	50.00
ATOM	1198	N	ALA	1138	-2.799	3.250	15.922	1.00	50.00
ATOM	1199	CA	ALA	1138	-3.816	4.323	16.005	1.00	50.00
ATOM	1200	C	ALA	1138	-4.914	4.181	14.937	1.00	50.00
ATOM	1201	O	ALA	1138	-6.101	4.334	15.225	1.00	50.00
ATOM	1202	CB	ALA	1138	-3.144	5.690	15.875	1.00	50.00
ATOM	1203	N	ARG	1139	-4.517	3.712	13.755	1.00	50.00
ATOM	1204	CA	ARG	1139	-5.452	3.429	12.646	1.00	50.00
ATOM	1205	C	ARG	1139	-6.475	2.325	12.966	1.00	50.00
ATOM	1206	O	ARG	1139	-7.584	2.344	12.434	1.00	50.00
ATOM	1207	CB	ARG	1139	-4.688	3.079	11.366	1.00	50.00
ATOM	1208	CG	ARG	1139	-3.839	1.816	11.500	1.00	50.00
ATOM	1209	CD	ARG	1139	-3.231	1.411	10.160	1.00	50.00
ATOM	1210	NE	ARG	1139	-2.300	0.298	10.397	1.00	50.00
ATOM	1211	CZ	ARG	1139	-1.061	0.397	10.899	1.00	50.00
ATOM	1212	NH1	ARG	1139	-0.522	1.567	11.197	1.00	50.00
ATOM	1213	NH2	ARG	1139	-0.377	-0.692	11.188	1.00	50.00
ATOM	1214	N	ASN	1140	-6.115	1.445	13.899	1.00	50.00
ATOM	1215	CA	ASN	1140	-6.976	0.334	14.338	1.00	50.00
ATOM	1216	C	ASN	1140	-7.618	0.572	15.718	1.00	50.00
ATOM	1217	O	ASN	1140	-8.030	-0.350	16.418	1.00	50.00
ATOM	1218	CB	ASN	1140	-6.169	-0.968	14.303	1.00	50.00
ATOM	1219	CG	ASN	1140	-5.802	-1.340	12.861	1.00	50.00

ATOM	1220	OD1	ASN	1140	-6.489	-1.077	11.894	1.00	50.00
ATOM	1221	ND2	ASN	1140	-4.663	-1.974	12.715	1.00	50.00
ATOM	1222	N	CYS	1141	-7.695	1.850	16.069	1.00	50.00
ATOM	1223	CA	CYS	1141	-8.466	2.342	17.224	1.00	50.00
ATOM	1224	C	CYS	1141	-9.488	3.356	16.698	1.00	50.00
ATOM	1225	O	CYS	1141	-9.257	4.038	15.707	1.00	50.00
ATOM	1226	CB	CYS	1141	-7.533	3.011	18.234	1.00	50.00
ATOM	1227	SG	CYS	1141	-6.284	1.883	18.952	1.00	50.00
ATOM	1228	N	MET	1142	-10.625	3.402	17.386	1.00	50.00
ATOM	1229	CA	MET	1142	-11.753	4.279	17.028	1.00	50.00
ATOM	1230	C	MET	1142	-12.004	5.286	18.156	1.00	50.00
ATOM	1231	O	MET	1142	-11.767	4.992	19.321	1.00	50.00
ATOM	1232	CB	MET	1142	-12.995	3.405	16.846	1.00	50.00
ATOM	1233	CG	MET	1142	-12.977	2.506	15.609	1.00	50.00
ATOM	1234	SD	MET	1142	-13.560	3.355	14.100	1.00	50.00
ATOM	1235	CE	MET	1142	-12.039	3.361	13.187	1.00	50.00
ATOM	1236	N	VAL	1143	-12.515	6.459	17.765	1.00	50.00
ATOM	1237	CA	VAL	1143	-12.840	7.544	18.706	1.00	50.00
ATOM	1238	C	VAL	1143	-14.362	7.769	18.672	1.00	50.00
ATOM	1239	O	VAL	1143	-14.974	7.951	17.622	1.00	50.00
ATOM	1240	CB	VAL	1143	-12.095	8.846	18.338	1.00	50.00
ATOM	1241	CG1	VAL	1143	-12.280	9.920	19.416	1.00	50.00
ATOM	1242	CG2	VAL	1143	-10.595	8.625	18.121	1.00	50.00
ATOM	1243	N	ALA	1144	-14.945	7.711	19.867	1.00	50.00
ATOM	1244	CA	ALA	1144	-16.393	7.892	20.062	1.00	50.00
ATOM	1245	C	ALA	1144	-16.755	9.385	20.101	1.00	50.00
ATOM	1246	O	ALA	1144	-15.892	10.238	20.288	1.00	50.00
ATOM	1247	CB	ALA	1144	-16.822	7.193	21.357	1.00	50.00
ATOM	1248	N	GLU	1145	-18.055	9.657	20.066	1.00	50.00
ATOM	1249	CA	GLU	1145	-18.591	11.037	20.166	1.00	50.00
ATOM	1250	C	GLU	1145	-18.081	11.781	21.403	1.00	50.00
ATOM	1251	O	GLU	1145	-17.770	12.969	21.326	1.00	50.00
ATOM	1252	CB	GLU	1145	-20.125	10.986	20.169	1.00	50.00
ATOM	1253	CG	GLU	1145	-20.807	12.364	20.253	1.00	50.00
ATOM	1254	CD	GLU	1145	-20.455	13.361	19.138	1.00	50.00
ATOM	1255	OE1	GLU	1145	-19.952	12.914	18.082	1.00	50.00
ATOM	1256	OE2	GLU	1145	-20.776	14.551	19.334	1.00	50.00
ATOM	1257	N	ASP	1146	-17.985	11.071	22.524	1.00	50.00
ATOM	1258	CA	ASP	1146	-17.491	11.654	23.781	1.00	50.00
ATOM	1259	C	ASP	1146	-15.958	11.568	23.941	1.00	50.00
ATOM	1260	O	ASP	1146	-15.463	11.645	25.062	1.00	50.00
ATOM	1261	CB	ASP	1146	-18.205	10.975	24.966	1.00	50.00
ATOM	1262	CG	ASP	1146	-17.778	9.520	25.213	1.00	50.00
ATOM	1263	OD1	ASP	1146	-17.094	8.958	24.332	1.00	50.00
ATOM	1264	OD2	ASP	1146	-18.301	8.947	26.198	1.00	50.00
ATOM	1265	N	PHE	1147	-15.256	11.267	22.844	1.00	50.00
ATOM	1266	CA	PHE	1147	-13.781	11.175	22.777	1.00	50.00
ATOM	1267	C	PHE	1147	-13.152	9.916	23.400	1.00	50.00
ATOM	1268	O	PHE	1147	-11.932	9.766	23.378	1.00	50.00
ATOM	1269	CB	PHE	1147	-13.104	12.420	23.366	1.00	50.00
ATOM	1270	CG	PHE	1147	-13.484	13.669	22.575	1.00	50.00
ATOM	1271	CD1	PHE	1147	-12.797	13.920	21.400	1.00	50.00
ATOM	1272	CD2	PHE	1147	-14.478	14.534	23.018	1.00	50.00
ATOM	1273	CE1	PHE	1147	-13.104	15.045	20.652	1.00	50.00
ATOM	1274	CE2	PHE	1147	-14.783	15.664	22.267	1.00	50.00
ATOM	1275	CZ	PHE	1147	-14.093	15.917	21.086	1.00	50.00
ATOM	1276	N	THR	1148	-13.984	8.984	23.864	1.00	50.00
ATOM	1277	CA	THR	1148	-13.493	7.664	24.326	1.00	50.00
ATOM	1278	C	THR	1148	-12.846	6.964	23.132	1.00	50.00
ATOM	1279	O	THR	1148	-13.431	6.865	22.055	1.00	50.00
ATOM	1280	CB	THR	1148	-14.641	6.793	24.870	1.00	50.00

ATOM	1281	OG1	THR	1148	-15.294	7.478	25.939	1.00	50.00
ATOM	1282	CG2	THR	1148	-14.160	5.425	25.372	1.00	50.00
ATOM	1283	N	VAL	1149	-11.622	6.486	23.364	1.00	50.00
ATOM	1284	CA	VAL	1149	-10.893	5.691	22.370	1.00	50.00
ATOM	1285	C	VAL	1149	-11.115	4.213	22.711	1.00	50.00
ATOM	1286	O	VAL	1149	-11.081	3.804	23.868	1.00	50.00
ATOM	1287	CB	VAL	1149	-9.390	6.041	22.370	1.00	50.00
ATOM	1288	CG1	VAL	1149	-8.620	5.232	21.321	1.00	50.00
ATOM	1289	CG2	VAL	1149	-9.178	7.525	22.066	1.00	50.00
ATOM	1290	N	LYS	1150	-11.366	3.446	21.653	1.00	50.00
ATOM	1291	CA	LYS	1150	-11.620	2.002	21.764	1.00	50.00
ATOM	1292	C	LYS	1150	-10.828	1.245	20.693	1.00	50.00
ATOM	1293	O	LYS	1150	-10.479	1.794	19.655	1.00	50.00
ATOM	1294	CB	LYS	1150	-13.115	1.698	21.604	1.00	50.00
ATOM	1295	CG	LYS	1150	-13.970	2.498	22.590	1.00	50.00
ATOM	1296	CD	LYS	1150	-15.418	2.023	22.612	1.00	50.00
ATOM	1297	CE	LYS	1150	-16.253	2.976	23.467	1.00	50.00
ATOM	1298	NZ	LYS	1150	-17.468	2.299	23.935	1.00	50.00
ATOM	1299	N	ILE	1151	-10.545	-0.022	20.995	1.00	50.00
ATOM	1300	CA	ILE	1151	-9.928	-0.941	20.018	1.00	50.00
ATOM	1301	C	ILE	1151	-10.950	-1.206	18.905	1.00	50.00
ATOM	1302	O	ILE	1151	-12.072	-1.646	19.166	1.00	50.00
ATOM	1303	CB	ILE	1151	-9.468	-2.248	20.702	1.00	50.00
ATOM	1304	CG1	ILE	1151	-8.520	-1.985	21.887	1.00	50.00
ATOM	1305	CG2	ILE	1151	-8.850	-3.234	19.697	1.00	50.00
ATOM	1306	CD1	ILE	1151	-7.230	-1.218	21.554	1.00	50.00
ATOM	1307	N	GLY	1152	-10.548	-0.791	17.694	1.00	50.00
ATOM	1308	CA	GLY	1152	-11.372	-0.909	16.482	1.00	50.00
ATOM	1309	C	GLY	1152	-11.700	-2.362	16.107	1.00	50.00
ATOM	1310	O	GLY	1152	-11.088	-3.318	16.581	1.00	50.00
ATOM	1311	N	ASP	1153	-12.687	-2.460	15.223	1.00	50.00
ATOM	1312	CA	ASP	1153	-13.119	-3.730	14.604	1.00	50.00
ATOM	1313	C	ASP	1153	-11.927	-4.504	14.026	1.00	50.00
ATOM	1314	O	ASP	1153	-11.061	-3.946	13.351	1.00	50.00
ATOM	1315	CB	ASP	1153	-14.123	-3.374	13.498	1.00	50.00
ATOM	1316	CG	ASP	1153	-14.653	-4.584	12.724	1.00	50.00
ATOM	1317	OD1	ASP	1153	-14.782	-5.662	13.339	1.00	50.00
ATOM	1318	OD2	ASP	1153	-14.927	-4.383	11.521	1.00	50.00
ATOM	1319	N	PHE	1154	-11.950	-5.819	14.255	1.00	50.00
ATOM	1320	CA	PHE	1154	-10.896	-6.740	13.816	1.00	50.00
ATOM	1321	C	PHE	1154	-10.836	-6.978	12.307	1.00	50.00
ATOM	1322	O	PHE	1154	-11.815	-6.755	11.600	1.00	50.00
ATOM	1323	CB	PHE	1154	-11.038	-8.091	14.523	1.00	50.00
ATOM	1324	CG	PHE	1154	-10.672	-8.053	16.012	1.00	50.00
ATOM	1325	CD1	PHE	1154	-10.328	-6.876	16.669	1.00	50.00
ATOM	1326	CD2	PHE	1154	-10.696	-9.254	16.712	1.00	50.00
ATOM	1327	CE1	PHE	1154	-10.015	-6.894	18.021	1.00	50.00
ATOM	1328	CE2	PHE	1154	-10.373	-9.273	18.061	1.00	50.00
ATOM	1329	CZ	PHE	1154	-10.038	-8.092	18.711	1.00	50.00
ATOM	1330	N	GLY	1155	-9.609	-7.315	11.893	1.00	50.00
ATOM	1331	CA	GLY	1155	-9.275	-7.723	10.510	1.00	50.00
ATOM	1332	C	GLY	1155	-9.869	-6.843	9.413	1.00	50.00
ATOM	1333	O	GLY	1155	-10.063	-7.329	8.295	1.00	50.00
ATOM	1334	N	MET	1156	-10.033	-5.546	9.693	1.00	50.00
ATOM	1335	CA	MET	1156	-10.644	-4.618	8.747	1.00	50.00
ATOM	1336	C	MET	1156	-9.988	-4.585	7.370	1.00	50.00
ATOM	1337	O	MET	1156	-10.665	-4.950	6.478	1.00	50.00
ATOM	1338	CB	MET	1156	-10.871	-3.199	9.259	1.00	50.00
ATOM	1339	CG	MET	1156	-12.296	-3.088	9.806	1.00	50.00
ATOM	1340	SD	MET	1156	-13.620	-3.657	8.667	1.00	50.00
ATOM	1341	CE	MET	1156	-13.393	-2.601	7.250	1.00	50.00

ATOM	1342	N	THR	1157	-8.643	-4.365	7.367	1.00	50.00
ATOM	1343	CA	THR	1157	-7.771	-4.473	6.166	1.00	50.00
ATOM	1344	C	THR	1157	-7.861	-3.274	5.177	1.00	50.00
ATOM	1345	O	THR	1157	-7.991	-2.128	5.578	1.00	50.00
ATOM	1346	CB	THR	1157	-7.911	-5.818	5.416	1.00	50.00
ATOM	1347	OG1	THR	1157	-9.100	-5.824	4.607	1.00	50.00
ATOM	1348	CG2	THR	1157	-7.734	-7.054	6.302	1.00	50.00
ATOM	1349	N	ARG	1158	-7.513	-3.621	3.939	1.00	50.00
ATOM	1350	CA	ARG	1158	-7.440	-2.842	2.679	1.00	50.00
ATOM	1351	C	ARG	1158	-7.923	-1.396	2.614	1.00	50.00
ATOM	1352	O	ARG	1158	-8.852	-0.950	3.288	1.00	50.00
ATOM	1353	CB	ARG	1158	-8.071	-3.679	1.555	1.00	50.00
ATOM	1354	CG	ARG	1158	-9.543	-4.055	1.761	1.00	50.00
ATOM	1355	CD	ARG	1158	-9.711	-5.551	1.486	1.00	50.00
ATOM	1356	NE	ARG	1158	-10.775	-5.799	0.504	1.00	50.00
ATOM	1357	CZ	ARG	1158	-11.610	-6.848	0.498	1.00	50.00
ATOM	1358	NH1	ARG	1158	-11.583	-7.761	1.454	1.00	50.00
ATOM	1359	NH2	ARG	1158	-12.433	-7.027	-0.524	1.00	50.00
ATOM	1360	N	ASP	1159	-7.246	-0.688	1.711	1.00	50.00
ATOM	1361	CA	ASP	1159	-7.577	0.670	1.224	1.00	50.00
ATOM	1362	C	ASP	1159	-7.283	1.889	2.102	1.00	50.00
ATOM	1363	O	ASP	1159	-7.304	3.004	1.582	1.00	50.00
ATOM	1364	CB	ASP	1159	-9.034	0.777	0.737	1.00	50.00
ATOM	1365	CG	ASP	1159	-9.291	0.047	-0.584	1.00	50.00
ATOM	1366	OD1	ASP	1159	-8.303	-0.338	-1.245	1.00	50.00
ATOM	1367	OD2	ASP	1159	-10.486	-0.057	-0.941	1.00	50.00
ATOM	1368	N	ILE	1160	-6.875	1.691	3.351	1.00	50.00
ATOM	1369	CA	ILE	1160	-6.851	2.820	4.298	1.00	50.00
ATOM	1370	C	ILE	1160	-5.489	3.517	4.443	1.00	50.00
ATOM	1371	O	ILE	1160	-5.329	4.655	3.996	1.00	50.00
ATOM	1372	CB	ILE	1160	-7.573	2.428	5.607	1.00	50.00
ATOM	1373	CG1	ILE	1160	-9.047	2.080	5.328	1.00	50.00
ATOM	1374	CG2	ILE	1160	-7.532	3.511	6.686	1.00	50.00
ATOM	1375	CD1	ILE	1160	-9.856	3.167	4.593	1.00	50.00
ATOM	1376	N	TYR	1161	-4.563	2.875	5.131	1.00	50.00
ATOM	1377	CA	TYR	1161	-3.205	3.424	5.301	1.00	50.00
ATOM	1378	C	TYR	1161	-2.196	2.436	4.706	1.00	50.00
ATOM	1379	O	TYR	1161	-1.429	1.782	5.402	1.00	50.00
ATOM	1380	CB	TYR	1161	-2.897	3.701	6.780	1.00	50.00
ATOM	1381	CG	TYR	1161	-3.821	4.732	7.418	1.00	50.00
ATOM	1382	CD1	TYR	1161	-3.585	6.088	7.253	1.00	50.00
ATOM	1383	CD2	TYR	1161	-4.882	4.298	8.207	1.00	50.00
ATOM	1384	CE1	TYR	1161	-4.422	7.013	7.866	1.00	50.00
ATOM	1385	CE2	TYR	1161	-5.721	5.218	8.814	1.00	50.00
ATOM	1386	CZ	TYR	1161	-5.482	6.570	8.632	1.00	50.00
ATOM	1387	OH	TYR	1161	-6.251	7.476	9.281	1.00	50.00
ATOM	1388	N	GLU	1162	-2.194	2.385	3.374	1.00	50.00
ATOM	1389	CA	GLU	1162	-1.358	1.414	2.624	1.00	50.00
ATOM	1390	C	GLU	1162	0.136	1.531	2.935	1.00	50.00
ATOM	1391	O	GLU	1162	0.801	0.524	3.171	1.00	50.00
ATOM	1392	CB	GLU	1162	-1.537	1.520	1.105	1.00	50.00
ATOM	1393	CG	GLU	1162	-2.939	1.138	0.616	1.00	50.00
ATOM	1394	CD	GLU	1162	-3.879	2.337	0.438	1.00	50.00
ATOM	1395	OE1	GLU	1162	-3.823	3.257	1.281	1.00	50.00
ATOM	1396	OE2	GLU	1162	-4.668	2.268	-0.529	1.00	50.00
ATOM	1397	N	THR	1163	0.602	2.771	3.109	1.00	50.00
ATOM	1398	CA	THR	1163	2.009	3.066	3.438	1.00	50.00
ATOM	1399	C	THR	1163	2.434	2.583	4.838	1.00	50.00
ATOM	1400	O	THR	1163	3.625	2.484	5.128	1.00	50.00
ATOM	1401	CB	THR	1163	2.323	4.561	3.283	1.00	50.00
ATOM	1402	OG1	THR	1163	1.461	5.319	4.134	1.00	50.00

ATOM	1403	CG2	THR	1163	2.171	5.014	1.827	1.00	50.00
ATOM	1404	N	ASP	1164	1.449	2.273	5.678	1.00	50.00
ATOM	1405	CA	ASP	1164	1.670	1.689	7.020	1.00	50.00
ATOM	1406	C	ASP	1164	2.081	0.206	6.999	1.00	50.00
ATOM	1407	O	ASP	1164	2.466	-0.351	8.025	1.00	50.00
ATOM	1408	CB	ASP	1164	0.433	1.904	7.901	1.00	50.00
ATOM	1409	CG	ASP	1164	0.273	3.362	8.355	1.00	50.00
ATOM	1410	OD1	ASP	1164	0.967	4.241	7.800	1.00	50.00
ATOM	1411	OD2	ASP	1164	-0.575	3.599	9.243	1.00	50.00
ATOM	1412	N	TYR	1165	1.992	-0.400	5.817	1.00	50.00
ATOM	1413	CA	TYR	1165	2.316	-1.822	5.620	1.00	50.00
ATOM	1414	C	TYR	1165	3.432	-1.981	4.581	1.00	50.00
ATOM	1415	O	TYR	1165	3.537	-1.235	3.616	1.00	50.00
ATOM	1416	CB	TYR	1165	1.050	-2.558	5.173	1.00	50.00
ATOM	1417	CG	TYR	1165	-0.029	-2.520	6.256	1.00	50.00
ATOM	1418	CD1	TYR	1165	-0.863	-1.414	6.363	1.00	50.00
ATOM	1419	CD2	TYR	1165	-0.141	-3.572	7.159	1.00	50.00
ATOM	1420	CE1	TYR	1165	-1.801	-1.349	7.383	1.00	50.00
ATOM	1421	CE2	TYR	1165	-1.087	-3.515	8.174	1.00	50.00
ATOM	1422	CZ	TYR	1165	-1.910	-2.397	8.277	1.00	50.00
ATOM	1423	OH	TYR	1165	-2.854	-2.310	9.246	1.00	50.00
ATOM	1424	N	TYR	1166	4.284	-2.962	4.861	1.00	50.00
ATOM	1425	CA	TYR	1166	5.434	-3.296	4.007	1.00	50.00
ATOM	1426	C	TYR	1166	5.450	-4.793	3.704	1.00	50.00
ATOM	1427	O	TYR	1166	5.241	-5.628	4.583	1.00	50.00
ATOM	1428	CB	TYR	1166	6.727	-2.874	4.714	1.00	50.00
ATOM	1429	CG	TYR	1166	7.995	-3.260	3.947	1.00	50.00
ATOM	1430	CD1	TYR	1166	8.198	-2.826	2.643	1.00	50.00
ATOM	1431	CD2	TYR	1166	8.956	-4.044	4.579	1.00	50.00
ATOM	1432	CE1	TYR	1166	9.367	-3.168	1.976	1.00	50.00
ATOM	1433	CE2	TYR	1166	10.125	-4.384	3.916	1.00	50.00
ATOM	1434	CZ	TYR	1166	10.321	-3.944	2.613	1.00	50.00
ATOM	1435	OH	TYR	1166	11.461	-4.242	1.945	1.00	50.00
ATOM	1436	N	ARG	1167	5.791	-5.073	2.451	1.00	50.00
ATOM	1437	CA	ARG	1167	5.956	-6.428	1.918	1.00	50.00
ATOM	1438	C	ARG	1167	7.365	-6.944	2.302	1.00	50.00
ATOM	1439	O	ARG	1167	8.289	-6.975	1.491	1.00	50.00
ATOM	1440	CB	ARG	1167	5.745	-6.244	0.409	1.00	50.00
ATOM	1441	CG	ARG	1167	5.195	-7.504	-0.244	1.00	50.00
ATOM	1442	CD	ARG	1167	4.617	-7.340	-1.652	1.00	50.00
ATOM	1443	NE	ARG	1167	3.936	-8.578	-2.089	1.00	50.00
ATOM	1444	CZ	ARG	1167	2.786	-9.097	-1.616	1.00	50.00
ATOM	1445	NH1	ARG	1167	2.102	-8.515	-0.645	1.00	50.00
ATOM	1446	NH2	ARG	1167	2.305	-10.215	-2.144	1.00	50.00
ATOM	1447	N	LYS	1168	7.537	-7.240	3.591	1.00	50.00
ATOM	1448	CA	LYS	1168	8.842	-7.668	4.151	1.00	50.00
ATOM	1449	C	LYS	1168	9.291	-9.000	3.545	1.00	50.00
ATOM	1450	O	LYS	1168	8.484	-9.915	3.362	1.00	50.00
ATOM	1451	CB	LYS	1168	8.753	-7.751	5.679	1.00	50.00
ATOM	1452	CG	LYS	1168	10.076	-8.168	6.334	1.00	50.00
ATOM	1453	CD	LYS	1168	9.961	-8.066	7.853	1.00	50.00
ATOM	1454	CE	LYS	1168	10.424	-9.343	8.557	1.00	50.00
ATOM	1455	NZ	LYS	1168	11.877	-9.567	8.492	1.00	50.00
ATOM	1456	N	GLY	1169	10.568	-9.019	3.142	1.00	50.00
ATOM	1457	CA	GLY	1169	11.173	-10.151	2.411	1.00	50.00
ATOM	1458	C	GLY	1169	10.449	-10.422	1.080	1.00	50.00
ATOM	1459	O	GLY	1169	10.467	-11.552	0.592	1.00	50.00
ATOM	1460	N	GLY	1170	9.811	-9.376	0.547	1.00	50.00
ATOM	1461	CA	GLY	1170	8.952	-9.449	-0.657	1.00	50.00
ATOM	1462	C	GLY	1170	7.769	-10.423	-0.485	1.00	50.00
ATOM	1463	O	GLY	1170	7.218	-10.899	-1.476	1.00	50.00

ATOM	1464	N	LYS	1171	7.375	-10.634	0.766	1.00	50.00
ATOM	1465	CA	LYS	1171	6.320	-11.600	1.154	1.00	50.00
ATOM	1466	C	LYS	1171	5.062	-10.870	1.624	1.00	50.00
ATOM	1467	O	LYS	1171	4.640	-9.901	0.992	1.00	50.00
ATOM	1468	CB	LYS	1171	6.884	-12.546	2.235	1.00	50.00
ATOM	1469	CG	LYS	1171	8.129	-13.312	1.787	1.00	50.00
ATOM	1470	CD	LYS	1171	7.839	-14.281	0.636	1.00	50.00
ATOM	1471	CE	LYS	1171	8.979	-14.320	-0.387	1.00	50.00
ATOM	1472	NZ	LYS	1171	10.281	-14.645	0.215	1.00	50.00
ATOM	1473	N	GLY	1172	4.379	-11.342	2.666	1.00	50.00
ATOM	1474	CA	GLY	1172	3.148	-10.718	3.188	1.00	50.00
ATOM	1475	C	GLY	1172	3.343	-9.281	3.699	1.00	50.00
ATOM	1476	O	GLY	1172	4.377	-8.912	4.260	1.00	50.00
ATOM	1477	N	LEU	1173	2.269	-8.515	3.541	1.00	50.00
ATOM	1478	CA	LEU	1173	2.134	-7.135	4.036	1.00	50.00
ATOM	1479	C	LEU	1173	2.078	-7.088	5.574	1.00	50.00
ATOM	1480	O	LEU	1173	1.162	-7.611	6.194	1.00	50.00
ATOM	1481	CB	LEU	1173	0.848	-6.563	3.433	1.00	50.00
ATOM	1482	CG	LEU	1173	1.032	-5.206	2.744	1.00	50.00
ATOM	1483	CD1	LEU	1173	2.099	-5.251	1.645	1.00	50.00
ATOM	1484	CD2	LEU	1173	-0.304	-4.780	2.133	1.00	50.00
ATOM	1485	N	LEU	1174	3.109	-6.472	6.145	1.00	50.00
ATOM	1486	CA	LEU	1174	3.245	-6.345	7.608	1.00	50.00
ATOM	1487	C	LEU	1174	3.404	-4.884	8.050	1.00	50.00
ATOM	1488	O	LEU	1174	4.077	-4.117	7.358	1.00	50.00
ATOM	1489	CB	LEU	1174	4.441	-7.168	8.104	1.00	50.00
ATOM	1490	CG	LEU	1174	4.271	-8.680	7.912	1.00	50.00
ATOM	1491	CD1	LEU	1174	5.557	-9.386	8.338	1.00	50.00
ATOM	1492	CD2	LEU	1174	3.082	-9.235	8.706	1.00	50.00
ATOM	1493	N	PRO	1175	2.816	-4.519	9.201	1.00	50.00
ATOM	1494	CA	PRO	1175	2.966	-3.175	9.793	1.00	50.00
ATOM	1495	C	PRO	1175	4.314	-3.061	10.522	1.00	50.00
ATOM	1496	O	PRO	1175	4.395	-2.827	11.725	1.00	50.00
ATOM	1497	CB	PRO	1175	1.770	-3.086	10.745	1.00	50.00
ATOM	1498	CG	PRO	1175	1.626	-4.517	11.261	1.00	50.00
ATOM	1499	CD	PRO	1175	1.899	-5.349	10.009	1.00	50.00
ATOM	1500	N	VAL	1176	5.389	-3.088	9.731	1.00	50.00
ATOM	1501	CA	VAL	1176	6.776	-3.166	10.239	1.00	50.00
ATOM	1502	C	VAL	1176	7.196	-2.027	11.178	1.00	50.00
ATOM	1503	O	VAL	1176	7.931	-2.258	12.142	1.00	50.00
ATOM	1504	CB	VAL	1176	7.791	-3.323	9.089	1.00	50.00
ATOM	1505	CG1	VAL	1176	7.523	-4.605	8.295	1.00	50.00
ATOM	1506	CG2	VAL	1176	7.846	-2.102	8.162	1.00	50.00
ATOM	1507	N	ARG	1177	6.658	-0.833	10.942	1.00	50.00
ATOM	1508	CA	ARG	1177	6.931	0.351	11.781	1.00	50.00
ATOM	1509	C	ARG	1177	6.320	0.251	13.190	1.00	50.00
ATOM	1510	O	ARG	1177	6.698	0.988	14.098	1.00	50.00
ATOM	1511	CB	ARG	1177	6.472	1.633	11.079	1.00	50.00
ATOM	1512	CG	ARG	1177	7.215	1.844	9.759	1.00	50.00
ATOM	1513	CD	ARG	1177	6.798	3.154	9.091	1.00	50.00
ATOM	1514	NE	ARG	1177	7.516	3.283	7.814	1.00	50.00
ATOM	1515	CZ	ARG	1177	8.658	3.930	7.594	1.00	50.00
ATOM	1516	NH1	ARG	1177	9.298	4.558	8.584	1.00	50.00
ATOM	1517	NH2	ARG	1177	9.209	3.956	6.403	1.00	50.00
ATOM	1518	N	TRP	1178	5.402	-0.703	13.351	1.00	50.00
ATOM	1519	CA	TRP	1178	4.730	-1.001	14.626	1.00	50.00
ATOM	1520	C	TRP	1178	5.153	-2.347	15.234	1.00	50.00
ATOM	1521	O	TRP	1178	4.756	-2.666	16.352	1.00	50.00
ATOM	1522	CB	TRP	1178	3.213	-0.994	14.398	1.00	50.00
ATOM	1523	CG	TRP	1178	2.657	0.422	14.306	1.00	50.00
ATOM	1524	CD1	TRP	1178	2.066	1.085	15.291	1.00	50.00

ATOM	1525	CD2	TRP	1178	2.667	1.265	13.193	1.00	50.00
ATOM	1526	NE1	TRP	1178	1.721	2.301	14.877	1.00	50.00
ATOM	1527	CE2	TRP	1178	2.082	2.444	13.594	1.00	50.00
ATOM	1528	CE3	TRP	1178	3.112	1.109	11.886	1.00	50.00
ATOM	1529	CZ2	TRP	1178	1.949	3.496	12.692	1.00	50.00
ATOM	1530	CZ3	TRP	1178	2.976	2.147	10.981	1.00	50.00
ATOM	1531	CH2	TRP	1178	2.391	3.352	11.390	1.00	50.00
ATOM	1532	N	MET	1179	6.043	-3.074	14.569	1.00	50.00
ATOM	1533	CA	MET	1179	6.435	-4.426	15.004	1.00	50.00
ATOM	1534	C	MET	1179	7.662	-4.433	15.920	1.00	50.00
ATOM	1535	O	MET	1179	8.600	-3.655	15.765	1.00	50.00
ATOM	1536	CB	MET	1179	6.696	-5.296	13.777	1.00	50.00
ATOM	1537	CG	MET	1179	5.400	-5.593	13.018	1.00	50.00
ATOM	1538	SD	MET	1179	5.664	-6.510	11.458	1.00	50.00
ATOM	1539	CE	MET	1179	6.398	-8.012	12.071	1.00	50.00
ATOM	1540	N	SER	1180	7.602	-5.360	16.872	1.00	50.00
ATOM	1541	CA	SER	1180	8.710	-5.641	17.801	1.00	50.00
ATOM	1542	C	SER	1180	9.907	-6.273	17.058	1.00	50.00
ATOM	1543	O	SER	1180	9.718	-6.911	16.017	1.00	50.00
ATOM	1544	CB	SER	1180	8.217	-6.574	18.915	1.00	50.00
ATOM	1545	OG	SER	1180	8.038	-7.896	18.401	1.00	50.00
ATOM	1546	N	PRO	1181	11.112	-6.215	17.656	1.00	50.00
ATOM	1547	CA	PRO	1181	12.327	-6.846	17.105	1.00	50.00
ATOM	1548	C	PRO	1181	12.163	-8.357	16.871	1.00	50.00
ATOM	1549	O	PRO	1181	12.449	-8.847	15.783	1.00	50.00
ATOM	1550	CB	PRO	1181	13.402	-6.605	18.164	1.00	50.00
ATOM	1551	CG	PRO	1181	12.962	-5.309	18.837	1.00	50.00
ATOM	1552	CD	PRO	1181	11.442	-5.431	18.861	1.00	50.00
ATOM	1553	N	GLU	1182	11.537	-9.052	17.825	1.00	50.00
ATOM	1554	CA	GLU	1182	11.329	-10.517	17.723	1.00	50.00
ATOM	1555	C	GLU	1182	10.327	-10.906	16.630	1.00	50.00
ATOM	1556	O	GLU	1182	10.533	-11.885	15.909	1.00	50.00
ATOM	1557	CB	GLU	1182	10.940	-11.191	19.050	1.00	50.00
ATOM	1558	CG	GLU	1182	9.547	-10.842	19.590	1.00	50.00
ATOM	1559	CD	GLU	1182	9.474	-9.563	20.425	1.00	50.00
ATOM	1560	OE1	GLU	1182	10.400	-8.724	20.355	1.00	50.00
ATOM	1561	OE2	GLU	1182	8.386	-9.387	21.009	1.00	50.00
ATOM	1562	N	SER	1183	9.278	-10.091	16.488	1.00	50.00
ATOM	1563	CA	SER	1183	8.262	-10.282	15.438	1.00	50.00
ATOM	1564	C	SER	1183	8.863	-10.009	14.057	1.00	50.00
ATOM	1565	O	SER	1183	8.516	-10.679	13.090	1.00	50.00
ATOM	1566	CB	SER	1183	7.046	-9.377	15.647	1.00	50.00
ATOM	1567	OG	SER	1183	6.469	-9.641	16.927	1.00	50.00
ATOM	1568	N	LEU	1184	9.839	-9.106	14.012	1.00	50.00
ATOM	1569	CA	LEU	1184	10.630	-8.856	12.795	1.00	50.00
ATOM	1570	C	LEU	1184	11.574	-10.017	12.459	1.00	50.00
ATOM	1571	O	LEU	1184	11.676	-10.414	11.302	1.00	50.00
ATOM	1572	CB	LEU	1184	11.410	-7.544	12.914	1.00	50.00
ATOM	1573	CG	LEU	1184	10.511	-6.302	12.889	1.00	50.00
ATOM	1574	CD1	LEU	1184	11.362	-5.050	13.096	1.00	50.00
ATOM	1575	CD2	LEU	1184	9.752	-6.186	11.564	1.00	50.00
ATOM	1576	N	LYS	1185	12.165	-10.619	13.485	1.00	50.00
ATOM	1577	CA	LYS	1185	13.089	-11.757	13.296	1.00	50.00
ATOM	1578	C	LYS	1185	12.400	-13.066	12.902	1.00	50.00
ATOM	1579	O	LYS	1185	12.763	-13.685	11.904	1.00	50.00
ATOM	1580	CB	LYS	1185	13.960	-12.005	14.529	1.00	50.00
ATOM	1581	CG	LYS	1185	14.903	-10.838	14.802	1.00	50.00
ATOM	1582	CD	LYS	1185	16.000	-11.262	15.772	1.00	50.00
ATOM	1583	CE	LYS	1185	16.135	-10.228	16.882	1.00	50.00
ATOM	1584	NZ	LYS	1185	17.404	-10.386	17.605	1.00	50.00
ATOM	1585	N	ASP	1186	11.402	-13.470	13.688	1.00	50.00

```
ATOM   1586  CA  ASP  1186      10.775 -14.796  13.517  1.00 50.00
ATOM   1587  C   ASP  1186       9.287 -14.809  13.133  1.00 50.00
ATOM   1588  O   ASP  1186       8.682 -15.880  13.064  1.00 50.00
ATOM   1589  CB  ASP  1186      11.058 -15.667  14.754  1.00 50.00
ATOM   1590  CG  ASP  1186      10.611 -15.014  16.064  1.00 50.00
ATOM   1591  OD1 ASP  1186       9.393 -14.771  16.194  1.00 50.00
ATOM   1592  OD2 ASP  1186      11.510 -14.694  16.874  1.00 50.00
ATOM   1593  N   GLY  1187       8.700 -13.616  12.975  1.00 50.00
ATOM   1594  CA  GLY  1187       7.292 -13.456  12.556  1.00 50.00
ATOM   1595  C   GLY  1187       6.255 -14.029  13.534  1.00 50.00
ATOM   1596  O   GLY  1187       5.128 -14.323  13.141  1.00 50.00
ATOM   1597  N   VAL  1188       6.626 -14.115  14.811  1.00 50.00
ATOM   1598  CA  VAL  1188       5.701 -14.560  15.869  1.00 50.00
ATOM   1599  C   VAL  1188       5.296 -13.322  16.673  1.00 50.00
ATOM   1600  O   VAL  1188       6.134 -12.504  17.054  1.00 50.00
ATOM   1601  CB  VAL  1188       6.350 -15.631  16.774  1.00 50.00
ATOM   1602  CG1 VAL  1188       5.377 -16.146  17.842  1.00 50.00
ATOM   1603  CG2 VAL  1188       6.850 -16.833  15.965  1.00 50.00
ATOM   1604  N   PHE  1189       4.012 -13.283  17.009  1.00 50.00
ATOM   1605  CA  PHE  1189       3.414 -12.168  17.753  1.00 50.00
ATOM   1606  C   PHE  1189       2.798 -12.686  19.055  1.00 50.00
ATOM   1607  O   PHE  1189       2.133 -13.710  19.097  1.00 50.00
ATOM   1608  CB  PHE  1189       2.304 -11.528  16.918  1.00 50.00
ATOM   1609  CG  PHE  1189       2.779 -10.985  15.570  1.00 50.00
ATOM   1610  CD1 PHE  1189       2.900 -11.834  14.475  1.00 50.00
ATOM   1611  CD2 PHE  1189       2.970  -9.617  15.416  1.00 50.00
ATOM   1612  CE1 PHE  1189       3.218 -11.316  13.227  1.00 50.00
ATOM   1613  CE2 PHE  1189       3.280  -9.099  14.166  1.00 50.00
ATOM   1614  CZ  PHE  1189       3.407  -9.949  13.072  1.00 50.00
ATOM   1615  N   THR  1190       3.080 -11.943  20.122  1.00 50.00
ATOM   1616  CA  THR  1190       2.521 -12.219  21.456  1.00 50.00
ATOM   1617  C   THR  1190       1.876 -10.943  22.031  1.00 50.00
ATOM   1618  O   THR  1190       1.957  -9.854  21.463  1.00 50.00
ATOM   1619  CB  THR  1190       3.610 -12.746  22.409  1.00 50.00
ATOM   1620  OG1 THR  1190       4.585 -11.722  22.634  1.00 50.00
ATOM   1621  CG2 THR  1190       4.245 -14.054  21.919  1.00 50.00
ATOM   1622  N   THR  1191       1.354 -11.073  23.252  1.00 50.00
ATOM   1623  CA  THR  1191       0.868  -9.909  24.026  1.00 50.00
ATOM   1624  C   THR  1191       2.015  -8.926  24.339  1.00 50.00
ATOM   1625  O   THR  1191       1.816  -7.711  24.344  1.00 50.00
ATOM   1626  CB  THR  1191       0.145 -10.361  25.306  1.00 50.00
ATOM   1627  OG1 THR  1191      -0.606  -9.261  25.818  1.00 50.00
ATOM   1628  CG2 THR  1191       1.074 -10.931  26.390  1.00 50.00
ATOM   1629  N   TYR  1192       3.225  -9.471  24.416  1.00 50.00
ATOM   1630  CA  TYR  1192       4.476  -8.709  24.624  1.00 50.00
ATOM   1631  C   TYR  1192       4.785  -7.810  23.425  1.00 50.00
ATOM   1632  O   TYR  1192       5.086  -6.628  23.582  1.00 50.00
ATOM   1633  CB  TYR  1192       5.661  -9.658  24.806  1.00 50.00
ATOM   1634  CG  TYR  1192       5.565 -10.590  26.014  1.00 50.00
ATOM   1635  CD1 TYR  1192       5.225 -10.099  27.267  1.00 50.00
ATOM   1636  CD2 TYR  1192       5.908 -11.929  25.859  1.00 50.00
ATOM   1637  CE1 TYR  1192       5.236 -10.944  28.367  1.00 50.00
ATOM   1638  CE2 TYR  1192       5.920 -12.775  26.957  1.00 50.00
ATOM   1639  CZ  TYR  1192       5.584 -12.279  28.210  1.00 50.00
ATOM   1640  OH  TYR  1192       5.554 -13.111  29.281  1.00 50.00
ATOM   1641  N   SER  1193       4.587  -8.353  22.224  1.00 50.00
ATOM   1642  CA  SER  1193       4.752  -7.579  20.979  1.00 50.00
ATOM   1643  C   SER  1193       3.589  -6.604  20.748  1.00 50.00
ATOM   1644  O   SER  1193       3.778  -5.538  20.166  1.00 50.00
ATOM   1645  CB  SER  1193       4.950  -8.470  19.754  1.00 50.00
ATOM   1646  OG  SER  1193       3.845  -9.355  19.581  1.00 50.00
```

ATOM	1647	N	ASP	1194	2.412	-6.960	21.254	1.00	50.00
ATOM	1648	CA	ASP	1194	1.275	-6.014	21.321	1.00	50.00
ATOM	1649	C	ASP	1194	1.608	-4.809	22.207	1.00	50.00
ATOM	1650	O	ASP	1194	1.295	-3.677	21.847	1.00	50.00
ATOM	1651	CB	ASP	1194	0.000	-6.685	21.836	1.00	50.00
ATOM	1652	CG	ASP	1194	-0.653	-7.640	20.833	1.00	50.00
ATOM	1653	OD1	ASP	1194	-0.320	-7.581	19.628	1.00	50.00
ATOM	1654	OD2	ASP	1194	-1.589	-8.346	21.266	1.00	50.00
ATOM	1655	N	VAL	1195	2.341	-5.070	23.295	1.00	50.00
ATOM	1656	CA	VAL	1195	2.846	-4.025	24.205	1.00	50.00
ATOM	1657	C	VAL	1195	3.854	-3.102	23.498	1.00	50.00
ATOM	1658	O	VAL	1195	3.747	-1.882	23.628	1.00	50.00
ATOM	1659	CB	VAL	1195	3.430	-4.648	25.491	1.00	50.00
ATOM	1660	CG1	VAL	1195	4.099	-3.609	26.398	1.00	50.00
ATOM	1661	CG2	VAL	1195	2.328	-5.327	26.309	1.00	50.00
ATOM	1662	N	TRP	1196	4.712	-3.676	22.649	1.00	50.00
ATOM	1663	CA	TRP	1196	5.656	-2.881	21.842	1.00	50.00
ATOM	1664	C	TRP	1196	4.900	-1.897	20.936	1.00	50.00
ATOM	1665	O	TRP	1196	5.159	-0.691	20.940	1.00	50.00
ATOM	1666	CB	TRP	1196	6.548	-3.807	20.993	1.00	50.00
ATOM	1667	CG	TRP	1196	7.447	-3.031	20.048	1.00	50.00
ATOM	1668	CD1	TRP	1196	7.100	-2.441	18.913	1.00	50.00
ATOM	1669	CD2	TRP	1196	8.807	-2.702	20.247	1.00	50.00
ATOM	1670	NE1	TRP	1196	8.106	-1.766	18.383	1.00	50.00
ATOM	1671	CE2	TRP	1196	9.178	-1.925	19.197	1.00	50.00
ATOM	1672	CE3	TRP	1196	9.735	-3.091	21.208	1.00	50.00
ATOM	1673	CZ2	TRP	1196	10.503	-1.507	19.066	1.00	50.00
ATOM	1674	CZ3	TRP	1196	11.043	-2.705	21.089	1.00	50.00
ATOM	1675	CH2	TRP	1196	11.436	-1.888	19.990	1.00	50.00
ATOM	1676	N	SER	1197	3.855	-2.417	20.284	1.00	50.00
ATOM	1677	CA	SER	1197	2.989	-1.638	19.381	1.00	50.00
ATOM	1678	C	SER	1197	2.190	-0.562	20.132	1.00	50.00
ATOM	1679	O	SER	1197	2.022	0.547	19.631	1.00	50.00
ATOM	1680	CB	SER	1197	2.003	-2.534	18.631	1.00	50.00
ATOM	1681	OG	SER	1197	2.711	-3.594	17.991	1.00	50.00
ATOM	1682	N	PHE	1198	1.821	-0.865	21.375	1.00	50.00
ATOM	1683	CA	PHE	1198	1.159	0.111	22.271	1.00	50.00
ATOM	1684	C	PHE	1198	2.067	1.319	22.536	1.00	50.00
ATOM	1685	O	PHE	1198	1.614	2.462	22.447	1.00	50.00
ATOM	1686	CB	PHE	1198	0.783	-0.552	23.596	1.00	50.00
ATOM	1687	CG	PHE	1198	0.176	0.454	24.580	1.00	50.00
ATOM	1688	CD1	PHE	1198	-1.159	0.828	24.473	1.00	50.00
ATOM	1689	CD2	PHE	1198	0.974	1.004	25.574	1.00	50.00
ATOM	1690	CE1	PHE	1198	-1.692	1.748	25.366	1.00	50.00
ATOM	1691	CE2	PHE	1198	0.440	1.921	26.467	1.00	50.00
ATOM	1692	CZ	PHE	1198	-0.896	2.290	26.366	1.00	50.00
ATOM	1693	N	GLY	1199	3.361	1.024	22.724	1.00	50.00
ATOM	1694	CA	GLY	1199	4.398	2.062	22.898	1.00	50.00
ATOM	1695	C	GLY	1199	4.458	2.996	21.681	1.00	50.00
ATOM	1696	O	GLY	1199	4.464	4.217	21.820	1.00	50.00
ATOM	1697	N	VAL	1200	4.305	2.399	20.496	1.00	50.00
ATOM	1698	CA	VAL	1200	4.270	3.139	19.217	1.00	50.00
ATOM	1699	C	VAL	1200	2.984	3.981	19.091	1.00	50.00
ATOM	1700	O	VAL	1200	3.017	5.106	18.604	1.00	50.00
ATOM	1701	CB	VAL	1200	4.434	2.189	18.013	1.00	50.00
ATOM	1702	CG1	VAL	1200	4.555	2.976	16.704	1.00	50.00
ATOM	1703	CG2	VAL	1200	5.672	1.295	18.145	1.00	50.00
ATOM	1704	N	VAL	1201	1.857	3.449	19.574	1.00	50.00
ATOM	1705	CA	VAL	1201	0.576	4.191	19.601	1.00	50.00
ATOM	1706	C	VAL	1201	0.697	5.439	20.488	1.00	50.00
ATOM	1707	O	VAL	1201	0.292	6.523	20.075	1.00	50.00

ATOM	1708	CB	VAL	1201	-0.599	3.305	20.060	1.00	50.00
ATOM	1709	CG1	VAL	1201	-1.919	4.084	20.102	1.00	50.00
ATOM	1710	CG2	VAL	1201	-0.790	2.118	19.115	1.00	50.00
ATOM	1711	N	LEU	1202	1.349	5.286	21.644	1.00	50.00
ATOM	1712	CA	LEU	1202	1.647	6.429	22.525	1.00	50.00
ATOM	1713	C	LEU	1202	2.477	7.500	21.808	1.00	50.00
ATOM	1714	O	LEU	1202	2.144	8.682	21.855	1.00	50.00
ATOM	1715	CB	LEU	1202	2.418	5.996	23.773	1.00	50.00
ATOM	1716	CG	LEU	1202	1.651	5.048	24.694	1.00	50.00
ATOM	1717	CD1	LEU	1202	2.519	4.770	25.919	1.00	50.00
ATOM	1718	CD2	LEU	1202	0.300	5.630	25.117	1.00	50.00
ATOM	1719	N	TRP	1203	3.411	7.017	20.997	1.00	50.00
ATOM	1720	CA	TRP	1203	4.266	7.871	20.153	1.00	50.00
ATOM	1721	C	TRP	1203	3.451	8.578	19.055	1.00	50.00
ATOM	1722	O	TRP	1203	3.635	9.769	18.822	1.00	50.00
ATOM	1723	CB	TRP	1203	5.385	7.021	19.554	1.00	50.00
ATOM	1724	CG	TRP	1203	6.445	7.901	18.893	1.00	50.00
ATOM	1725	CD1	TRP	1203	7.547	8.358	19.479	1.00	50.00
ATOM	1726	CD2	TRP	1203	6.457	8.335	17.578	1.00	50.00
ATOM	1727	NE1	TRP	1203	8.260	9.056	18.595	1.00	50.00
ATOM	1728	CE2	TRP	1203	7.628	9.061	17.422	1.00	50.00
ATOM	1729	CE3	TRP	1203	5.598	8.141	16.503	1.00	50.00
ATOM	1730	CZ2	TRP	1203	7.949	9.593	16.179	1.00	50.00
ATOM	1731	CZ3	TRP	1203	5.929	8.661	15.258	1.00	50.00
ATOM	1732	CH2	TRP	1203	7.103	9.384	15.095	1.00	50.00
ATOM	1733	N	GLU	1204	2.435	7.893	18.534	1.00	50.00
ATOM	1734	CA	GLU	1204	1.501	8.466	17.541	1.00	50.00
ATOM	1735	C	GLU	1204	0.645	9.563	18.170	1.00	50.00
ATOM	1736	O	GLU	1204	0.643	10.681	17.665	1.00	50.00
ATOM	1737	CB	GLU	1204	0.583	7.404	16.936	1.00	50.00
ATOM	1738	CG	GLU	1204	1.359	6.393	16.094	1.00	50.00
ATOM	1739	CD	GLU	1204	0.386	5.421	15.432	1.00	50.00
ATOM	1740	OE1	GLU	1204	-0.094	5.753	14.326	1.00	50.00
ATOM	1741	OE2	GLU	1204	0.083	4.378	16.052	1.00	50.00
ATOM	1742	N	ILE	1205	0.144	9.307	19.377	1.00	50.00
ATOM	1743	CA	ILE	1205	-0.620	10.297	20.167	1.00	50.00
ATOM	1744	C	ILE	1205	0.239	11.540	20.452	1.00	50.00
ATOM	1745	O	ILE	1205	-0.191	12.670	20.219	1.00	50.00
ATOM	1746	CB	ILE	1205	-1.126	9.642	21.471	1.00	50.00
ATOM	1747	CG1	ILE	1205	-2.090	8.485	21.158	1.00	50.00
ATOM	1748	CG2	ILE	1205	-1.799	10.679	22.385	1.00	50.00
ATOM	1749	CD1	ILE	1205	-2.494	7.644	22.378	1.00	50.00
ATOM	1750	N	ALA	1206	1.483	11.291	20.858	1.00	50.00
ATOM	1751	CA	ALA	1206	2.448	12.342	21.221	1.00	50.00
ATOM	1752	C	ALA	1206	2.912	13.203	20.042	1.00	50.00
ATOM	1753	O	ALA	1206	3.239	14.377	20.206	1.00	50.00
ATOM	1754	CB	ALA	1206	3.649	11.689	21.905	1.00	50.00
ATOM	1755	N	THR	1207	2.904	12.615	18.845	1.00	50.00
ATOM	1756	CA	THR	1207	3.381	13.289	17.626	1.00	50.00
ATOM	1757	C	THR	1207	2.242	13.737	16.695	1.00	50.00
ATOM	1758	O	THR	1207	2.507	14.201	15.588	1.00	50.00
ATOM	1759	CB	THR	1207	4.319	12.384	16.809	1.00	50.00
ATOM	1760	OG1	THR	1207	3.608	11.228	16.343	1.00	50.00
ATOM	1761	CG2	THR	1207	5.593	12.040	17.580	1.00	50.00
ATOM	1762	N	LEU	1208	1.011	13.470	17.117	1.00	50.00
ATOM	1763	CA	LEU	1208	-0.201	13.646	16.288	1.00	50.00
ATOM	1764	C	LEU	1208	-0.153	12.816	14.987	1.00	50.00
ATOM	1765	O	LEU	1208	-0.398	13.287	13.889	1.00	50.00
ATOM	1766	CB	LEU	1208	-0.493	15.125	16.014	1.00	50.00
ATOM	1767	CG	LEU	1208	-0.828	15.919	17.281	1.00	50.00
ATOM	1768	CD1	LEU	1208	-1.163	17.357	16.890	1.00	50.00

ATOM	1769	CD2	LEU	1208	-1.994	15.294	18.056	1.00	50.00
ATOM	1770	N	ALA	1209	0.186	11.552	15.191	1.00	50.00
ATOM	1771	CA	ALA	1209	0.223	10.497	14.148	1.00	50.00
ATOM	1772	C	ALA	1209	1.218	10.793	13.024	1.00	50.00
ATOM	1773	O	ALA	1209	0.878	10.864	11.845	1.00	50.00
ATOM	1774	CB	ALA	1209	-1.205	10.237	13.642	1.00	50.00
ATOM	1775	N	GLU	1210	2.486	11.008	13.390	1.00	50.00
ATOM	1776	CA	GLU	1210	3.558	11.021	12.385	1.00	50.00
ATOM	1777	C	GLU	1210	3.886	9.567	12.015	1.00	50.00
ATOM	1778	O	GLU	1210	3.538	8.641	12.742	1.00	50.00
ATOM	1779	CB	GLU	1210	4.824	11.701	12.914	1.00	50.00
ATOM	1780	CG	GLU	1210	4.617	13.187	13.216	1.00	50.00
ATOM	1781	CD	GLU	1210	5.918	13.892	13.615	1.00	50.00
ATOM	1782	OE1	GLU	1210	6.850	13.210	14.098	1.00	50.00
ATOM	1783	OE2	GLU	1210	5.994	15.111	13.347	1.00	50.00
ATOM	1784	N	GLN	1211	4.531	9.387	10.861	1.00	50.00
ATOM	1785	CA	GLN	1211	5.032	8.053	10.485	1.00	50.00
ATOM	1786	C	GLN	1211	6.200	7.672	11.406	1.00	50.00
ATOM	1787	O	GLN	1211	7.135	8.462	11.566	1.00	50.00
ATOM	1788	CB	GLN	1211	5.482	8.006	9.021	1.00	50.00
ATOM	1789	CG	GLN	1211	4.326	8.203	8.033	1.00	50.00
ATOM	1790	CD	GLN	1211	3.239	7.147	8.189	1.00	50.00
ATOM	1791	OE1	GLN	1211	2.123	7.436	8.613	1.00	50.00
ATOM	1792	NE2	GLN	1211	3.501	5.925	7.813	1.00	50.00
ATOM	1793	N	PRO	1212	6.090	6.541	12.124	1.00	50.00
ATOM	1794	CA	PRO	1212	7.166	6.054	13.005	1.00	50.00
ATOM	1795	C	PRO	1212	8.410	5.837	12.152	1.00	50.00
ATOM	1796	O	PRO	1212	8.307	5.269	11.066	1.00	50.00
ATOM	1797	CB	PRO	1212	6.661	4.708	13.524	1.00	50.00
ATOM	1798	CG	PRO	1212	5.147	4.887	13.516	1.00	50.00
ATOM	1799	CD	PRO	1212	4.890	5.696	12.245	1.00	50.00
ATOM	1800	N	TYR	1213	9.548	6.350	12.612	1.00	50.00
ATOM	1801	CA	TYR	1213	10.843	6.255	11.897	1.00	50.00
ATOM	1802	C	TYR	1213	10.726	6.932	10.513	1.00	50.00
ATOM	1803	O	TYR	1213	11.299	6.494	9.517	1.00	50.00
ATOM	1804	CB	TYR	1213	11.286	4.788	11.735	1.00	50.00
ATOM	1805	CG	TYR	1213	11.110	3.959	13.008	1.00	50.00
ATOM	1806	CD1	TYR	1213	12.102	3.980	13.976	1.00	50.00
ATOM	1807	CD2	TYR	1213	9.957	3.206	13.198	1.00	50.00
ATOM	1808	CE1	TYR	1213	11.941	3.246	15.143	1.00	50.00
ATOM	1809	CE2	TYR	1213	9.792	2.474	14.365	1.00	50.00
ATOM	1810	CZ	TYR	1213	10.787	2.497	15.335	1.00	50.00
ATOM	1811	OH	TYR	1213	10.620	1.811	16.492	1.00	50.00
ATOM	1812	N	GLN	1214	10.108	8.108	10.539	1.00	50.00
ATOM	1813	CA	GLN	1214	9.692	8.871	9.340	1.00	50.00
ATOM	1814	C	GLN	1214	10.828	9.138	8.343	1.00	50.00
ATOM	1815	O	GLN	1214	10.612	9.066	7.129	1.00	50.00
ATOM	1816	CB	GLN	1214	9.060	10.168	9.854	1.00	50.00
ATOM	1817	CG	GLN	1214	8.324	10.974	8.782	1.00	50.00
ATOM	1818	CD	GLN	1214	7.322	11.920	9.447	1.00	50.00
ATOM	1819	OE1	GLN	1214	6.130	11.648	9.533	1.00	50.00
ATOM	1820	NE2	GLN	1214	7.810	13.019	9.980	1.00	50.00
ATOM	1821	N	GLY	1215	12.030	9.380	8.876	1.00	50.00
ATOM	1822	CA	GLY	1215	13.251	9.611	8.078	1.00	50.00
ATOM	1823	C	GLY	1215	13.762	8.370	7.326	1.00	50.00
ATOM	1824	O	GLY	1215	14.470	8.492	6.330	1.00	50.00
ATOM	1825	N	LEU	1216	13.435	7.189	7.853	1.00	50.00
ATOM	1826	CA	LEU	1216	13.947	5.914	7.335	1.00	50.00
ATOM	1827	C	LEU	1216	12.951	5.218	6.402	1.00	50.00
ATOM	1828	O	LEU	1216	11.742	5.336	6.541	1.00	50.00
ATOM	1829	CB	LEU	1216	14.273	4.966	8.497	1.00	50.00

ATOM	1830	CG	LEU	1216	15.287	5.518	9.509	1.00	50.00
ATOM	1831	CD1	LEU	1216	15.600	4.436	10.542	1.00	50.00
ATOM	1832	CD2	LEU	1216	16.580	6.016	8.850	1.00	50.00
ATOM	1833	N	SER	1217	13.524	4.508	5.435	1.00	50.00
ATOM	1834	CA	SER	1217	12.759	3.576	4.581	1.00	50.00
ATOM	1835	C	SER	1217	12.371	2.343	5.414	1.00	50.00
ATOM	1836	O	SER	1217	13.011	2.035	6.419	1.00	50.00
ATOM	1837	CB	SER	1217	13.599	3.154	3.369	1.00	50.00
ATOM	1838	OG	SER	1217	14.666	2.278	3.754	1.00	50.00
ATOM	1839	N	ASN	1218	11.461	1.532	4.877	1.00	50.00
ATOM	1840	CA	ASN	1218	11.018	0.291	5.549	1.00	50.00
ATOM	1841	C	ASN	1218	12.154	-0.704	5.844	1.00	50.00
ATOM	1842	O	ASN	1218	12.271	-1.202	6.965	1.00	50.00
ATOM	1843	CB	ASN	1218	9.933	-0.396	4.723	1.00	50.00
ATOM	1844	CG	ASN	1218	8.617	0.373	4.764	1.00	50.00
ATOM	1845	OD1	ASN	1218	8.126	0.812	5.801	1.00	50.00
ATOM	1846	ND2	ASN	1218	8.005	0.532	3.617	1.00	50.00
ATOM	1847	N	GLU	1219	13.069	-0.851	4.890	1.00	50.00
ATOM	1848	CA	GLU	1219	14.282	-1.679	5.066	1.00	50.00
ATOM	1849	C	GLU	1219	15.204	-1.149	6.173	1.00	50.00
ATOM	1850	O	GLU	1219	15.660	-1.907	7.029	1.00	50.00
ATOM	1851	CB	GLU	1219	15.060	-1.773	3.751	1.00	50.00
ATOM	1852	CG	GLU	1219	14.478	-2.814	2.785	1.00	50.00
ATOM	1853	CD	GLU	1219	14.615	-4.274	3.253	1.00	50.00
ATOM	1854	OE1	GLU	1219	15.396	-4.536	4.194	1.00	50.00
ATOM	1855	OE2	GLU	1219	13.870	-5.112	2.701	1.00	50.00
ATOM	1856	N	GLN	1220	15.349	0.176	6.214	1.00	50.00
ATOM	1857	CA	GLN	1220	16.129	0.859	7.263	1.00	50.00
ATOM	1858	C	GLN	1220	15.499	0.724	8.657	1.00	50.00
ATOM	1859	O	GLN	1220	16.218	0.537	9.633	1.00	50.00
ATOM	1860	CB	GLN	1220	16.310	2.337	6.924	1.00	50.00
ATOM	1861	CG	GLN	1220	17.169	2.537	5.675	1.00	50.00
ATOM	1862	CD	GLN	1220	17.262	4.008	5.275	1.00	50.00
ATOM	1863	OE1	GLN	1220	16.380	4.829	5.502	1.00	50.00
ATOM	1864	NE2	GLN	1220	18.331	4.349	4.598	1.00	50.00
ATOM	1865	N	VAL	1221	14.163	0.669	8.704	1.00	50.00
ATOM	1866	CA	VAL	1221	13.398	0.457	9.954	1.00	50.00
ATOM	1867	C	VAL	1221	13.680	-0.943	10.523	1.00	50.00
ATOM	1868	O	VAL	1221	13.941	-1.096	11.716	1.00	50.00
ATOM	1869	CB	VAL	1221	11.884	0.658	9.720	1.00	50.00
ATOM	1870	CG1	VAL	1221	11.062	0.444	10.997	1.00	50.00
ATOM	1871	CG2	VAL	1221	11.579	2.063	9.197	1.00	50.00
ATOM	1872	N	LEU	1222	13.727	-1.931	9.627	1.00	50.00
ATOM	1873	CA	LEU	1222	14.021	-3.332	9.973	1.00	50.00
ATOM	1874	C	LEU	1222	15.376	-3.493	10.673	1.00	50.00
ATOM	1875	O	LEU	1222	15.438	-3.944	11.815	1.00	50.00
ATOM	1876	CB	LEU	1222	13.993	-4.180	8.699	1.00	50.00
ATOM	1877	CG	LEU	1222	12.663	-4.897	8.447	1.00	50.00
ATOM	1878	CD1	LEU	1222	11.449	-3.963	8.459	1.00	50.00
ATOM	1879	CD2	LEU	1222	12.757	-5.585	7.086	1.00	50.00
ATOM	1880	N	ARG	1223	16.393	-2.932	10.027	1.00	50.00
ATOM	1881	CA	ARG	1223	17.776	-2.927	10.546	1.00	50.00
ATOM	1882	C	ARG	1223	17.877	-2.152	11.868	1.00	50.00
ATOM	1883	O	ARG	1223	18.277	-2.720	12.882	1.00	50.00
ATOM	1884	CB	ARG	1223	18.704	-2.310	9.498	1.00	50.00
ATOM	1885	CG	ARG	1223	18.838	-3.219	8.275	1.00	50.00
ATOM	1886	CD	ARG	1223	18.914	-2.379	7.001	1.00	50.00
ATOM	1887	NE	ARG	1223	20.188	-2.575	6.293	1.00	50.00
ATOM	1888	CZ	ARG	1223	20.826	-1.627	5.587	1.00	50.00
ATOM	1889	NH1	ARG	1223	20.329	-0.405	5.498	1.00	50.00
ATOM	1890	NH2	ARG	1223	21.943	-1.913	4.934	1.00	50.00

ATOM	1891	N	PHE	1224	17.281	-0.961	11.879	1.00	50.00
ATOM	1892	CA	PHE	1224	17.322	-0.059	13.047	1.00	50.00
ATOM	1893	C	PHE	1224	16.673	-0.663	14.295	1.00	50.00
ATOM	1894	O	PHE	1224	17.339	-0.785	15.322	1.00	50.00
ATOM	1895	CB	PHE	1224	16.677	1.281	12.677	1.00	50.00
ATOM	1896	CG	PHE	1224	16.822	2.316	13.794	1.00	50.00
ATOM	1897	CD1	PHE	1224	18.063	2.888	14.049	1.00	50.00
ATOM	1898	CD2	PHE	1224	15.699	2.739	14.495	1.00	50.00
ATOM	1899	CE1	PHE	1224	18.183	3.885	15.008	1.00	50.00
ATOM	1900	CE2	PHE	1224	15.819	3.745	15.446	1.00	50.00
ATOM	1901	CZ	PHE	1224	17.059	4.317	15.704	1.00	50.00
ATOM	1902	N	VAL	1225	15.467	-1.220	14.150	1.00	50.00
ATOM	1903	CA	VAL	1225	14.719	-1.799	15.285	1.00	50.00
ATOM	1904	C	VAL	1225	15.347	-3.102	15.791	1.00	50.00
ATOM	1905	O	VAL	1225	15.600	-3.247	16.988	1.00	50.00
ATOM	1906	CB	VAL	1225	13.225	-1.974	14.933	1.00	50.00
ATOM	1907	CG1	VAL	1225	12.427	-2.695	16.028	1.00	50.00
ATOM	1908	CG2	VAL	1225	12.577	-0.605	14.720	1.00	50.00
ATOM	1909	N	MET	1226	15.641	-4.024	14.868	1.00	50.00
ATOM	1910	CA	MET	1226	16.267	-5.307	15.232	1.00	50.00
ATOM	1911	C	MET	1226	17.645	-5.144	15.881	1.00	50.00
ATOM	1912	O	MET	1226	18.037	-5.979	16.693	1.00	50.00
ATOM	1913	CB	MET	1226	16.375	-6.262	14.042	1.00	50.00
ATOM	1914	CG	MET	1226	14.995	-6.752	13.602	1.00	50.00
ATOM	1915	SD	MET	1226	15.051	-8.090	12.355	1.00	50.00
ATOM	1916	CE	MET	1226	15.800	-7.260	10.969	1.00	50.00
ATOM	1917	N	GLU	1227	18.310	-4.026	15.607	1.00	50.00
ATOM	1918	CA	GLU	1227	19.609	-3.713	16.226	1.00	50.00
ATOM	1919	C	GLU	1227	19.526	-2.894	17.527	1.00	50.00
ATOM	1920	O	GLU	1227	20.553	-2.508	18.085	1.00	50.00
ATOM	1921	CB	GLU	1227	20.532	-3.053	15.194	1.00	50.00
ATOM	1922	CG	GLU	1227	20.904	-4.034	14.074	1.00	50.00
ATOM	1923	CD	GLU	1227	21.482	-5.345	14.619	1.00	50.00
ATOM	1924	OE1	GLU	1227	22.498	-5.267	15.344	1.00	50.00
ATOM	1925	OE2	GLU	1227	20.892	-6.398	14.293	1.00	50.00
ATOM	1926	N	GLY	1228	18.290	-2.670	17.984	1.00	50.00
ATOM	1927	CA	GLY	1228	18.007	-1.999	19.271	1.00	50.00
ATOM	1928	C	GLY	1228	17.812	-0.480	19.158	1.00	50.00
ATOM	1929	O	GLY	1228	18.000	0.246	20.129	1.00	50.00
ATOM	1930	N	GLY	1229	17.432	-0.030	17.952	1.00	50.00
ATOM	1931	CA	GLY	1229	17.096	1.379	17.690	1.00	50.00
ATOM	1932	C	GLY	1229	15.701	1.702	18.225	1.00	50.00
ATOM	1933	O	GLY	1229	14.779	0.891	18.162	1.00	50.00
ATOM	1934	N	LEU	1230	15.582	2.930	18.727	1.00	50.00
ATOM	1935	CA	LEU	1230	14.341	3.432	19.330	1.00	50.00
ATOM	1936	C	LEU	1230	13.889	4.748	18.681	1.00	50.00
ATOM	1937	O	LEU	1230	14.690	5.491	18.124	1.00	50.00
ATOM	1938	CB	LEU	1230	14.542	3.651	20.836	1.00	50.00
ATOM	1939	CG	LEU	1230	14.944	2.377	21.593	1.00	50.00
ATOM	1940	CD1	LEU	1230	15.257	2.720	23.049	1.00	50.00
ATOM	1941	CD2	LEU	1230	13.852	1.304	21.528	1.00	50.00
ATOM	1942	N	LEU	1231	12.579	4.975	18.745	1.00	50.00
ATOM	1943	CA	LEU	1231	11.979	6.262	18.341	1.00	50.00
ATOM	1944	C	LEU	1231	12.455	7.390	19.264	1.00	50.00
ATOM	1945	O	LEU	1231	12.678	7.195	20.458	1.00	50.00
ATOM	1946	CB	LEU	1231	10.451	6.160	18.404	1.00	50.00
ATOM	1947	CG	LEU	1231	9.871	5.312	17.269	1.00	50.00
ATOM	1948	CD1	LEU	1231	8.431	4.914	17.592	1.00	50.00
ATOM	1949	CD2	LEU	1231	9.890	6.099	15.957	1.00	50.00
ATOM	1950	N	ASP	1232	12.606	8.576	18.676	1.00	50.00
ATOM	1951	CA	ASP	1232	12.983	9.777	19.442	1.00	50.00

ATOM	1952	C	ASP	1232	11.822	10.235	20.334	1.00	50.00
ATOM	1953	O	ASP	1232	10.660	10.052	19.982	1.00	50.00
ATOM	1954	CB	ASP	1232	13.392	10.929	18.515	1.00	50.00
ATOM	1955	CG	ASP	1232	14.638	10.632	17.672	1.00	50.00
ATOM	1956	OD1	ASP	1232	15.408	9.723	18.050	1.00	50.00
ATOM	1957	OD2	ASP	1232	14.788	11.333	16.649	1.00	50.00
ATOM	1958	N	LYS	1233	12.187	10.849	21.460	1.00	50.00
ATOM	1959	CA	LYS	1233	11.204	11.520	22.327	1.00	50.00
ATOM	1960	C	LYS	1233	10.592	12.685	21.541	1.00	50.00
ATOM	1961	O	LYS	1233	11.329	13.551	21.060	1.00	50.00
ATOM	1962	CB	LYS	1233	11.856	12.011	23.626	1.00	50.00
ATOM	1963	CG	LYS	1233	10.799	12.566	24.591	1.00	50.00
ATOM	1964	CD	LYS	1233	11.365	12.910	25.971	1.00	50.00
ATOM	1965	CE	LYS	1233	12.293	14.124	25.941	1.00	50.00
ATOM	1966	NZ	LYS	1233	12.793	14.408	27.292	1.00	50.00
ATOM	1967	N	PRO	1234	9.271	12.621	21.288	1.00	50.00
ATOM	1968	CA	PRO	1234	8.548	13.669	20.552	1.00	50.00
ATOM	1969	C	PRO	1234	8.814	15.020	21.227	1.00	50.00
ATOM	1970	O	PRO	1234	9.068	15.094	22.427	1.00	50.00
ATOM	1971	CB	PRO	1234	7.075	13.293	20.710	1.00	50.00
ATOM	1972	CG	PRO	1234	7.115	11.772	20.847	1.00	50.00
ATOM	1973	CD	PRO	1234	8.368	11.522	21.680	1.00	50.00
ATOM	1974	N	ASP	1235	8.805	16.083	20.423	1.00	50.00
ATOM	1975	CA	ASP	1235	9.007	17.435	20.971	1.00	50.00
ATOM	1976	C	ASP	1235	7.877	17.770	21.945	1.00	50.00
ATOM	1977	O	ASP	1235	6.718	17.466	21.678	1.00	50.00
ATOM	1978	CB	ASP	1235	9.057	18.495	19.866	1.00	50.00
ATOM	1979	CG	ASP	1235	10.269	18.331	18.943	1.00	50.00
ATOM	1980	OD1	ASP	1235	11.276	17.741	19.389	1.00	50.00
ATOM	1981	OD2	ASP	1235	10.151	18.808	17.795	1.00	50.00
ATOM	1982	N	ASN	1236	8.274	18.301	23.102	1.00	50.00
ATOM	1983	CA	ASN	1236	7.344	18.695	24.184	1.00	50.00
ATOM	1984	C	ASN	1236	6.571	17.492	24.775	1.00	50.00
ATOM	1985	O	ASN	1236	5.543	17.655	25.430	1.00	50.00
ATOM	1986	CB	ASN	1236	6.341	19.755	23.691	1.00	50.00
ATOM	1987	CG	ASN	1236	7.015	20.898	22.931	1.00	50.00
ATOM	1988	OD1	ASN	1236	7.780	21.681	23.476	1.00	50.00
ATOM	1989	ND2	ASN	1236	6.840	20.918	21.627	1.00	50.00
ATOM	1990	N	CYS	1237	7.132	16.298	24.600	1.00	50.00
ATOM	1991	CA	CYS	1237	6.560	15.069	25.179	1.00	50.00
ATOM	1992	C	CYS	1237	7.071	14.918	26.611	1.00	50.00
ATOM	1993	O	CYS	1237	8.283	14.854	26.837	1.00	50.00
ATOM	1994	CB	CYS	1237	6.929	13.839	24.350	1.00	50.00
ATOM	1995	SG	CYS	1237	6.236	12.276	25.002	1.00	50.00
ATOM	1996	N	PRO	1238	6.146	14.876	27.585	1.00	50.00
ATOM	1997	CA	PRO	1238	6.495	14.711	29.002	1.00	50.00
ATOM	1998	C	PRO	1238	7.315	13.425	29.170	1.00	50.00
ATOM	1999	O	PRO	1238	6.972	12.379	28.623	1.00	50.00
ATOM	2000	CB	PRO	1238	5.145	14.578	29.708	1.00	50.00
ATOM	2001	CG	PRO	1238	4.212	15.402	28.824	1.00	50.00
ATOM	2002	CD	PRO	1238	4.693	15.070	27.414	1.00	50.00
ATOM	2003	N	ASP	1239	8.325	13.506	30.037	1.00	50.00
ATOM	2004	CA	ASP	1239	9.215	12.358	30.321	1.00	50.00
ATOM	2005	C	ASP	1239	8.462	11.132	30.832	1.00	50.00
ATOM	2006	O	ASP	1239	8.708	10.027	30.355	1.00	50.00
ATOM	2007	CB	ASP	1239	10.292	12.716	31.348	1.00	50.00
ATOM	2008	CG	ASP	1239	11.487	13.447	30.736	1.00	50.00
ATOM	2009	OD1	ASP	1239	11.306	14.045	29.656	1.00	50.00
ATOM	2010	OD2	ASP	1239	12.533	13.462	31.416	1.00	50.00
ATOM	2011	N	MET	1240	7.439	11.383	31.635	1.00	50.00
ATOM	2012	CA	MET	1240	6.544	10.341	32.175	1.00	50.00

ATOM	2013	C	MET	1240	5.937	9.481	31.052	1.00	50.00
ATOM	2014	O	MET	1240	5.997	8.251	31.105	1.00	50.00
ATOM	2015	CB	MET	1240	5.491	11.061	33.026	1.00	50.00
ATOM	2016	CG	MET	1240	4.345	10.185	33.540	1.00	50.00
ATOM	2017	SD	MET	1240	3.050	9.888	32.282	1.00	50.00
ATOM	2018	CE	MET	1240	1.750	9.381	33.384	1.00	50.00
ATOM	2019	N	LEU	1241	5.508	10.147	29.984	1.00	50.00
ATOM	2020	CA	LEU	1241	4.948	9.469	28.805	1.00	50.00
ATOM	2021	C	LEU	1241	6.009	8.747	27.958	1.00	50.00
ATOM	2022	O	LEU	1241	5.799	7.605	27.551	1.00	50.00
ATOM	2023	CB	LEU	1241	4.142	10.465	27.965	1.00	50.00
ATOM	2024	CG	LEU	1241	3.291	9.769	26.895	1.00	50.00
ATOM	2025	CD1	LEU	1241	2.281	8.791	27.507	1.00	50.00
ATOM	2026	CD2	LEU	1241	2.561	10.816	26.057	1.00	50.00
ATOM	2027	N	PHE	1242	7.182	9.363	27.821	1.00	50.00
ATOM	2028	CA	PHE	1242	8.302	8.751	27.079	1.00	50.00
ATOM	2029	C	PHE	1242	8.915	7.549	27.818	1.00	50.00
ATOM	2030	O	PHE	1242	9.283	6.554	27.204	1.00	50.00
ATOM	2031	CB	PHE	1242	9.389	9.782	26.771	1.00	50.00
ATOM	2032	CG	PHE	1242	10.427	9.204	25.801	1.00	50.00
ATOM	2033	CD1	PHE	1242	10.041	8.826	24.520	1.00	50.00
ATOM	2034	CD2	PHE	1242	11.750	9.045	26.201	1.00	50.00
ATOM	2035	CE1	PHE	1242	10.970	8.284	23.641	1.00	50.00
ATOM	2036	CE2	PHE	1242	12.680	8.511	25.317	1.00	50.00
ATOM	2037	CZ	PHE	1242	12.291	8.127	24.038	1.00	50.00
ATOM	2038	N	GLU	1243	8.922	7.634	29.147	1.00	50.00
ATOM	2039	CA	GLU	1243	9.405	6.553	30.028	1.00	50.00
ATOM	2040	C	GLU	1243	8.522	5.313	29.854	1.00	50.00
ATOM	2041	O	GLU	1243	9.031	4.203	29.701	1.00	50.00
ATOM	2042	CB	GLU	1243	9.347	7.041	31.475	1.00	50.00
ATOM	2043	CG	GLU	1243	10.074	6.091	32.433	1.00	50.00
ATOM	2044	CD	GLU	1243	10.042	6.590	33.882	1.00	50.00
ATOM	2045	OE1	GLU	1243	10.004	7.824	34.082	1.00	50.00
ATOM	2046	OE2	GLU	1243	10.078	5.712	34.771	1.00	50.00
ATOM	2047	N	LEU	1244	7.220	5.559	29.730	1.00	50.00
ATOM	2048	CA	LEU	1244	6.230	4.505	29.460	1.00	50.00
ATOM	2049	C	LEU	1244	6.436	3.849	28.086	1.00	50.00
ATOM	2050	O	LEU	1244	6.442	2.623	27.984	1.00	50.00
ATOM	2051	CB	LEU	1244	4.821	5.089	29.608	1.00	50.00
ATOM	2052	CG	LEU	1244	3.709	4.036	29.490	1.00	50.00
ATOM	2053	CD1	LEU	1244	3.872	2.899	30.506	1.00	50.00
ATOM	2054	CD2	LEU	1244	2.354	4.714	29.694	1.00	50.00
ATOM	2055	N	MET	1245	6.743	4.673	27.082	1.00	50.00
ATOM	2056	CA	MET	1245	7.089	4.192	25.729	1.00	50.00
ATOM	2057	C	MET	1245	8.339	3.301	25.760	1.00	50.00
ATOM	2058	O	MET	1245	8.284	2.148	25.342	1.00	50.00
ATOM	2059	CB	MET	1245	7.367	5.355	24.776	1.00	50.00
ATOM	2060	CG	MET	1245	6.169	6.280	24.586	1.00	50.00
ATOM	2061	SD	MET	1245	6.538	7.615	23.395	1.00	50.00
ATOM	2062	CE	MET	1245	5.196	8.714	23.779	1.00	50.00
ATOM	2063	N	ARG	1246	9.367	3.776	26.461	1.00	50.00
ATOM	2064	CA	ARG	1246	10.624	3.031	26.668	1.00	50.00
ATOM	2065	C	ARG	1246	10.450	1.685	27.380	1.00	50.00
ATOM	2066	O	ARG	1246	11.113	0.710	27.032	1.00	50.00
ATOM	2067	CB	ARG	1246	11.605	3.903	27.441	1.00	50.00
ATOM	2068	CG	ARG	1246	12.467	4.722	26.479	1.00	50.00
ATOM	2069	CD	ARG	1246	13.365	5.724	27.211	1.00	50.00
ATOM	2070	NE	ARG	1246	14.105	5.105	28.323	1.00	50.00
ATOM	2071	CZ	ARG	1246	14.045	5.505	29.605	1.00	50.00
ATOM	2072	NH1	ARG	1246	13.300	6.542	29.954	1.00	50.00
ATOM	2073	NH2	ARG	1246	14.712	4.849	30.540	1.00	50.00

ATOM	2074	N	MET	1247	9.498	1.627	28.310	1.00	50.00
ATOM	2075	CA	MET	1247	9.119	0.371	28.990	1.00	50.00
ATOM	2076	C	MET	1247	8.561	-0.655	27.994	1.00	50.00
ATOM	2077	O	MET	1247	9.026	-1.793	27.933	1.00	50.00
ATOM	2078	CB	MET	1247	8.063	0.665	30.052	1.00	50.00
ATOM	2079	CG	MET	1247	8.628	1.451	31.235	1.00	50.00
ATOM	2080	SD	MET	1247	7.332	2.042	32.384	1.00	50.00
ATOM	2081	CE	MET	1247	6.575	0.509	32.884	1.00	50.00
ATOM	2082	N	CYS	1248	7.680	-0.170	27.121	1.00	50.00
ATOM	2083	CA	CYS	1248	7.093	-0.964	26.025	1.00	50.00
ATOM	2084	C	CYS	1248	8.124	-1.371	24.962	1.00	50.00
ATOM	2085	O	CYS	1248	7.956	-2.387	24.292	1.00	50.00
ATOM	2086	CB	CYS	1248	5.973	-0.165	25.359	1.00	50.00
ATOM	2087	SG	CYS	1248	4.623	0.295	26.503	1.00	50.00
ATOM	2088	N	TRP	1249	9.202	-0.595	24.860	1.00	50.00
ATOM	2089	CA	TRP	1249	10.252	-0.803	23.852	1.00	50.00
ATOM	2090	C	TRP	1249	11.521	-1.482	24.392	1.00	50.00
ATOM	2091	O	TRP	1249	12.559	-1.489	23.730	1.00	50.00
ATOM	2092	CB	TRP	1249	10.616	0.538	23.208	1.00	50.00
ATOM	2093	CG	TRP	1249	9.452	1.185	22.459	1.00	50.00
ATOM	2094	CD1	TRP	1249	8.390	0.580	21.941	1.00	50.00
ATOM	2095	CD2	TRP	1249	9.305	2.548	22.239	1.00	50.00
ATOM	2096	NE1	TRP	1249	7.574	1.485	21.406	1.00	50.00
ATOM	2097	CE2	TRP	1249	8.106	2.701	21.575	1.00	50.00
ATOM	2098	CE3	TRP	1249	10.114	3.644	22.516	1.00	50.00
ATOM	2099	CZ2	TRP	1249	7.698	3.970	21.182	1.00	50.00
ATOM	2100	CZ3	TRP	1249	9.716	4.908	22.110	1.00	50.00
ATOM	2101	CH2	TRP	1249	8.501	5.069	21.440	1.00	50.00
ATOM	2102	N	GLN	1250	11.391	-2.144	25.538	1.00	50.00
ATOM	2103	CA	GLN	1250	12.472	-3.014	26.053	1.00	50.00
ATOM	2104	C	GLN	1250	12.734	-4.107	25.018	1.00	50.00
ATOM	2105	O	GLN	1250	11.799	-4.705	24.490	1.00	50.00
ATOM	2106	CB	GLN	1250	12.054	-3.640	27.385	1.00	50.00
ATOM	2107	CG	GLN	1250	12.177	-2.657	28.555	1.00	50.00
ATOM	2108	CD	GLN	1250	13.635	-2.335	28.912	1.00	50.00
ATOM	2109	OE1	GLN	1250	14.559	-3.122	28.749	1.00	50.00
ATOM	2110	NE2	GLN	1250	13.851	-1.152	29.441	1.00	50.00
ATOM	2111	N	TYR	1251	14.003	-4.333	24.689	1.00	50.00
ATOM	2112	CA	TYR	1251	14.364	-5.273	23.611	1.00	50.00
ATOM	2113	C	TYR	1251	13.883	-6.705	23.913	1.00	50.00
ATOM	2114	O	TYR	1251	13.384	-7.408	23.033	1.00	50.00
ATOM	2115	CB	TYR	1251	15.878	-5.253	23.386	1.00	50.00
ATOM	2116	CG	TYR	1251	16.237	-5.893	22.044	1.00	50.00
ATOM	2117	CD1	TYR	1251	16.236	-5.107	20.896	1.00	50.00
ATOM	2118	CD2	TYR	1251	16.520	-7.251	21.965	1.00	50.00
ATOM	2119	CE1	TYR	1251	16.516	-5.677	19.664	1.00	50.00
ATOM	2120	CE2	TYR	1251	16.799	-7.824	20.732	1.00	50.00
ATOM	2121	CZ	TYR	1251	16.789	-7.035	19.596	1.00	50.00
ATOM	2122	OH	TYR	1251	16.938	-7.634	18.394	1.00	50.00
ATOM	2123	N	ASN	1252	14.001	-7.080	25.179	1.00	50.00
ATOM	2124	CA	ASN	1252	13.491	-8.371	25.667	1.00	50.00
ATOM	2125	C	ASN	1252	11.990	-8.221	25.951	1.00	50.00
ATOM	2126	O	ASN	1252	11.616	-7.496	26.880	1.00	50.00
ATOM	2127	CB	ASN	1252	14.257	-8.808	26.921	1.00	50.00
ATOM	2128	CG	ASN	1252	13.935	-10.247	27.343	1.00	50.00
ATOM	2129	OD1	ASN	1252	12.973	-10.884	26.933	1.00	50.00
ATOM	2130	ND2	ASN	1252	14.736	-10.767	28.242	1.00	50.00
ATOM	2131	N	PRO	1253	11.152	-9.004	25.246	1.00	50.00
ATOM	2132	CA	PRO	1253	9.690	-8.979	25.414	1.00	50.00
ATOM	2133	C	PRO	1253	9.214	-9.258	26.845	1.00	50.00
ATOM	2134	O	PRO	1253	8.213	-8.695	27.283	1.00	50.00

ATOM	2135	CB	PRO	1253	9.170	-10.026	24.424	1.00	50.00
ATOM	2136	CG	PRO	1253	10.333	-11.001	24.271	1.00	50.00
ATOM	2137	CD	PRO	1253	11.546	-10.073	24.306	1.00	50.00
ATOM	2138	N	LYS	1254	9.988	-10.049	27.587	1.00	50.00
ATOM	2139	CA	LYS	1254	9.663	-10.412	28.982	1.00	50.00
ATOM	2140	C	LYS	1254	9.889	-9.268	29.984	1.00	50.00
ATOM	2141	O	LYS	1254	9.348	-9.288	31.088	1.00	50.00
ATOM	2142	CB	LYS	1254	10.446	-11.662	29.398	1.00	50.00
ATOM	2143	CG	LYS	1254	10.150	-12.832	28.456	1.00	50.00
ATOM	2144	CD	LYS	1254	10.834	-14.112	28.927	1.00	50.00
ATOM	2145	CE	LYS	1254	10.717	-15.198	27.859	1.00	50.00
ATOM	2146	NZ	LYS	1254	11.298	-16.457	28.344	1.00	50.00
ATOM	2147	N	MET	1255	10.750	-8.323	29.608	1.00	50.00
ATOM	2148	CA	MET	1255	11.036	-7.113	30.404	1.00	50.00
ATOM	2149	C	MET	1255	9.973	-6.012	30.244	1.00	50.00
ATOM	2150	O	MET	1255	9.899	-5.087	31.050	1.00	50.00
ATOM	2151	CB	MET	1255	12.423	-6.571	30.052	1.00	50.00
ATOM	2152	CG	MET	1255	13.536	-7.514	30.523	1.00	50.00
ATOM	2153	SD	MET	1255	13.634	-7.712	32.342	1.00	50.00
ATOM	2154	CE	MET	1255	14.240	-6.105	32.813	1.00	50.00
ATOM	2155	N	ARG	1256	9.220	-6.097	29.149	1.00	50.00
ATOM	2156	CA	ARG	1256	8.109	-5.171	28.860	1.00	50.00
ATOM	2157	C	ARG	1256	6.959	-5.420	29.846	1.00	50.00
ATOM	2158	O	ARG	1256	6.671	-6.572	30.187	1.00	50.00
ATOM	2159	CB	ARG	1256	7.598	-5.381	27.430	1.00	50.00
ATOM	2160	CG	ARG	1256	8.708	-5.248	26.388	1.00	50.00
ATOM	2161	CD	ARG	1256	8.121	-5.416	24.991	1.00	50.00
ATOM	2162	NE	ARG	1256	9.223	-5.480	24.016	1.00	50.00
ATOM	2163	CZ	ARG	1256	9.324	-6.346	23.004	1.00	50.00
ATOM	2164	NH1	ARG	1256	8.365	-7.207	22.763	1.00	50.00
ATOM	2165	NH2	ARG	1256	10.433	-6.373	22.272	1.00	50.00
ATOM	2166	N	PRO	1257	6.302	-4.344	30.312	1.00	50.00
ATOM	2167	CA	PRO	1257	5.150	-4.451	31.217	1.00	50.00
ATOM	2168	C	PRO	1257	3.932	-5.013	30.477	1.00	50.00
ATOM	2169	O	PRO	1257	3.749	-4.799	29.283	1.00	50.00
ATOM	2170	CB	PRO	1257	4.914	-3.014	31.688	1.00	50.00
ATOM	2171	CG	PRO	1257	5.356	-2.175	30.491	1.00	50.00
ATOM	2172	CD	PRO	1257	6.575	-2.935	29.971	1.00	50.00
ATOM	2173	N	SER	1258	3.101	-5.749	31.213	1.00	50.00
ATOM	2174	CA	SER	1258	1.794	-6.198	30.697	1.00	50.00
ATOM	2175	C	SER	1258	0.871	-4.976	30.533	1.00	50.00
ATOM	2176	O	SER	1258	1.102	-3.926	31.131	1.00	50.00
ATOM	2177	CB	SER	1258	1.159	-7.208	31.663	1.00	50.00
ATOM	2178	OG	SER	1258	0.753	-6.569	32.880	1.00	50.00
ATOM	2179	N	PHE	1259	-0.256	-5.166	29.843	1.00	50.00
ATOM	2180	CA	PHE	1259	-1.263	-4.092	29.730	1.00	50.00
ATOM	2181	C	PHE	1259	-1.888	-3.713	31.080	1.00	50.00
ATOM	2182	O	PHE	1259	-2.016	-2.529	31.388	1.00	50.00
ATOM	2183	CB	PHE	1259	-2.354	-4.430	28.715	1.00	50.00
ATOM	2184	CG	PHE	1259	-1.837	-4.245	27.291	1.00	50.00
ATOM	2185	CD1	PHE	1259	-1.639	-2.961	26.803	1.00	50.00
ATOM	2186	CD2	PHE	1259	-1.629	-5.346	26.470	1.00	50.00
ATOM	2187	CE1	PHE	1259	-1.232	-2.781	25.490	1.00	50.00
ATOM	2188	CE2	PHE	1259	-1.223	-5.160	25.154	1.00	50.00
ATOM	2189	CZ	PHE	1259	-1.021	-3.875	24.662	1.00	50.00
ATOM	2190	N	LEU	1260	-2.064	-4.715	31.936	1.00	50.00
ATOM	2191	CA	LEU	1260	-2.521	-4.511	33.327	1.00	50.00
ATOM	2192	C	LEU	1260	-1.534	-3.664	34.141	1.00	50.00
ATOM	2193	O	LEU	1260	-1.926	-2.684	34.771	1.00	50.00
ATOM	2194	CB	LEU	1260	-2.724	-5.850	34.041	1.00	50.00
ATOM	2195	CG	LEU	1260	-3.896	-6.664	33.484	1.00	50.00

ATOM	2196	CD1	LEU	1260	-3.953	-8.014	34.197	1.00	50.00
ATOM	2197	CD2	LEU	1260	-5.229	-5.931	33.668	1.00	50.00
ATOM	2198	N	GLU	1261	-0.253	-3.950	33.941	1.00	50.00
ATOM	2199	CA	GLU	1261	0.840	-3.218	34.607	1.00	50.00
ATOM	2200	C	GLU	1261	0.930	-1.762	34.125	1.00	50.00
ATOM	2201	O	GLU	1261	1.107	-0.856	34.939	1.00	50.00
ATOM	2202	CB	GLU	1261	2.128	-3.993	34.349	1.00	50.00
ATOM	2203	CG	GLU	1261	3.279	-3.558	35.258	1.00	50.00
ATOM	2204	CD	GLU	1261	4.428	-4.573	35.235	1.00	50.00
ATOM	2205	OE1	GLU	1261	4.587	-5.264	34.204	1.00	50.00
ATOM	2206	OE2	GLU	1261	5.095	-4.671	36.288	1.00	50.00
ATOM	2207	N	ILE	1262	0.662	-1.550	32.832	1.00	50.00
ATOM	2208	CA	ILE	1262	0.577	-0.200	32.230	1.00	50.00
ATOM	2209	C	ILE	1262	-0.580	0.594	32.847	1.00	50.00
ATOM	2210	O	ILE	1262	-0.442	1.769	33.186	1.00	50.00
ATOM	2211	CB	ILE	1262	0.452	-0.297	30.688	1.00	50.00
ATOM	2212	CG1	ILE	1262	1.773	-0.813	30.094	1.00	50.00
ATOM	2213	CG2	ILE	1262	0.022	1.035	30.041	1.00	50.00
ATOM	2214	CD1	ILE	1262	1.738	-1.107	28.587	1.00	50.00
ATOM	2215	N	ILE	1263	-1.762	-0.018	32.894	1.00	50.00
ATOM	2216	CA	ILE	1263	-2.965	0.638	33.437	1.00	50.00
ATOM	2217	C	ILE	1263	-2.748	0.997	34.910	1.00	50.00
ATOM	2218	O	ILE	1263	-3.005	2.128	35.314	1.00	50.00
ATOM	2219	CB	ILE	1263	-4.212	-0.252	33.283	1.00	50.00
ATOM	2220	CG1	ILE	1263	-4.432	-0.603	31.808	1.00	50.00
ATOM	2221	CG2	ILE	1263	-5.454	0.463	33.844	1.00	50.00
ATOM	2222	CD1	ILE	1263	-5.413	-1.763	31.606	1.00	50.00
ATOM	2223	N	SER	1264	-2.191	0.053	35.665	1.00	50.00
ATOM	2224	CA	SER	1264	-1.906	0.234	37.098	1.00	50.00
ATOM	2225	C	SER	1264	-1.015	1.450	37.363	1.00	50.00
ATOM	2226	O	SER	1264	-1.315	2.235	38.260	1.00	50.00
ATOM	2227	CB	SER	1264	-1.227	-1.011	37.672	1.00	50.00
ATOM	2228	OG	SER	1264	-0.992	-0.819	39.069	1.00	50.00
ATOM	2229	N	SER	1265	-0.020	1.668	36.502	1.00	50.00
ATOM	2230	CA	SER	1265	0.898	2.813	36.642	1.00	50.00
ATOM	2231	C	SER	1265	0.224	4.174	36.398	1.00	50.00
ATOM	2232	O	SER	1265	0.704	5.202	36.870	1.00	50.00
ATOM	2233	CB	SER	1265	2.128	2.665	35.736	1.00	50.00
ATOM	2234	OG	SER	1265	1.753	2.762	34.363	1.00	50.00
ATOM	2235	N	ILE	1266	-0.897	4.163	35.671	1.00	50.00
ATOM	2236	CA	ILE	1266	-1.612	5.394	35.281	1.00	50.00
ATOM	2237	C	ILE	1266	-3.075	5.484	35.763	1.00	50.00
ATOM	2238	O	ILE	1266	-3.754	6.467	35.479	1.00	50.00
ATOM	2239	CB	ILE	1266	-1.559	5.590	33.752	1.00	50.00
ATOM	2240	CG1	ILE	1266	-2.255	4.431	33.013	1.00	50.00
ATOM	2241	CG2	ILE	1266	-0.107	5.825	33.301	1.00	50.00
ATOM	2242	CD1	ILE	1266	-2.423	4.638	31.505	1.00	50.00
ATOM	2243	N	LYS	1267	-3.511	4.519	36.576	1.00	50.00
ATOM	2244	CA	LYS	1267	-4.920	4.449	37.025	1.00	50.00
ATOM	2245	C	LYS	1267	-5.347	5.676	37.841	1.00	50.00
ATOM	2246	O	LYS	1267	-6.465	6.162	37.680	1.00	50.00
ATOM	2247	CB	LYS	1267	-5.205	3.144	37.781	1.00	50.00
ATOM	2248	CG	LYS	1267	-4.443	3.049	39.102	1.00	50.00
ATOM	2249	CD	LYS	1267	-4.623	1.685	39.763	1.00	50.00
ATOM	2250	CE	LYS	1267	-3.836	1.595	41.074	1.00	50.00
ATOM	2251	NZ	LYS	1267	-2.419	1.938	40.877	1.00	50.00
ATOM	2252	N	GLU	1268	-4.386	6.260	38.560	1.00	50.00
ATOM	2253	CA	GLU	1268	-4.609	7.495	39.344	1.00	50.00
ATOM	2254	C	GLU	1268	-4.975	8.702	38.472	1.00	50.00
ATOM	2255	O	GLU	1268	-5.630	9.629	38.945	1.00	50.00
ATOM	2256	CB	GLU	1268	-3.399	7.884	40.202	1.00	50.00

ATOM	2257	CG	GLU	1268	-3.113	6.924	41.363	1.00	50.00
ATOM	2258	CD	GLU	1268	-2.520	5.578	40.933	1.00	50.00
ATOM	2259	OE1	GLU	1268	-1.899	5.505	39.851	1.00	50.00
ATOM	2260	OE2	GLU	1268	-2.762	4.619	41.694	1.00	50.00
ATOM	2261	N	GLU	1269	-4.542	8.680	37.211	1.00	50.00
ATOM	2262	CA	GLU	1269	-4.729	9.810	36.286	1.00	50.00
ATOM	2263	C	GLU	1269	-5.943	9.727	35.351	1.00	50.00
ATOM	2264	O	GLU	1269	-6.229	10.662	34.607	1.00	50.00
ATOM	2265	CB	GLU	1269	-3.439	10.034	35.491	1.00	50.00
ATOM	2266	CG	GLU	1269	-2.295	10.474	36.412	1.00	50.00
ATOM	2267	CD	GLU	1269	-2.681	11.691	37.263	1.00	50.00
ATOM	2268	OE1	GLU	1269	-3.131	12.695	36.668	1.00	50.00
ATOM	2269	OE2	GLU	1269	-2.497	11.589	38.495	1.00	50.00
ATOM	2270	N	MET	1270	-6.613	8.580	35.382	1.00	50.00
ATOM	2271	CA	MET	1270	-7.824	8.345	34.584	1.00	50.00
ATOM	2272	C	MET	1270	-9.014	9.113	35.180	1.00	50.00
ATOM	2273	O	MET	1270	-9.088	9.366	36.378	1.00	50.00
ATOM	2274	CB	MET	1270	-8.132	6.846	34.557	1.00	50.00
ATOM	2275	CG	MET	1270	-6.973	6.014	33.990	1.00	50.00
ATOM	2276	SD	MET	1270	-6.463	6.471	32.292	1.00	50.00
ATOM	2277	CE	MET	1270	-7.911	5.961	31.390	1.00	50.00
ATOM	2278	N	GLU	1271	-9.951	9.447	34.303	1.00	50.00
ATOM	2279	CA	GLU	1271	-11.232	10.041	34.736	1.00	50.00
ATOM	2280	C	GLU	1271	-11.961	9.083	35.689	1.00	50.00
ATOM	2281	O	GLU	1271	-11.930	7.869	35.466	1.00	50.00
ATOM	2282	CB	GLU	1271	-12.070	10.373	33.500	1.00	50.00
ATOM	2283	CG	GLU	1271	-11.520	11.629	32.813	1.00	50.00
ATOM	2284	CD	GLU	1271	-12.017	11.810	31.374	1.00	50.00
ATOM	2285	OE1	GLU	1271	-13.041	11.186	31.017	1.00	50.00
ATOM	2286	OE2	GLU	1271	-11.333	12.561	30.645	1.00	50.00
ATOM	2287	N	PRO	1272	-12.668	9.615	36.706	1.00	50.00
ATOM	2288	CA	PRO	1272	-13.336	8.826	37.759	1.00	50.00
ATOM	2289	C	PRO	1272	-14.240	7.696	37.231	1.00	50.00
ATOM	2290	O	PRO	1272	-14.258	6.600	37.800	1.00	50.00
ATOM	2291	CB	PRO	1272	-14.172	9.849	38.527	1.00	50.00
ATOM	2292	CG	PRO	1272	-13.352	11.130	38.397	1.00	50.00
ATOM	2293	CD	PRO	1272	-12.826	11.060	36.964	1.00	50.00
ATOM	2294	N	GLY	1273	-14.861	7.940	36.074	1.00	50.00
ATOM	2295	CA	GLY	1273	-15.719	6.955	35.374	1.00	50.00
ATOM	2296	C	GLY	1273	-14.993	5.650	34.997	1.00	50.00
ATOM	2297	O	GLY	1273	-15.610	4.589	34.964	1.00	50.00
ATOM	2298	N	PHE	1274	-13.673	5.729	34.822	1.00	50.00
ATOM	2299	CA	PHE	1274	-12.836	4.570	34.434	1.00	50.00
ATOM	2300	C	PHE	1274	-12.945	3.401	35.411	1.00	50.00
ATOM	2301	O	PHE	1274	-13.112	2.264	34.966	1.00	50.00
ATOM	2302	CB	PHE	1274	-11.372	4.996	34.296	1.00	50.00
ATOM	2303	CG	PHE	1274	-10.516	3.875	33.700	1.00	50.00
ATOM	2304	CD1	PHE	1274	-10.504	3.694	32.322	1.00	50.00
ATOM	2305	CD2	PHE	1274	-9.725	3.077	34.518	1.00	50.00
ATOM	2306	CE1	PHE	1274	-9.697	2.717	31.758	1.00	50.00
ATOM	2307	CE2	PHE	1274	-8.913	2.100	33.952	1.00	50.00
ATOM	2308	CZ	PHE	1274	-8.899	1.921	32.573	1.00	50.00
ATOM	2309	N	ARG	1275	-13.055	3.703	36.707	1.00	50.00
ATOM	2310	CA	ARG	1275	-13.227	2.687	37.762	1.00	50.00
ATOM	2311	C	ARG	1275	-14.545	1.902	37.624	1.00	50.00
ATOM	2312	O	ARG	1275	-14.677	0.785	38.113	1.00	50.00
ATOM	2313	CB	ARG	1275	-13.173	3.355	39.140	1.00	50.00
ATOM	2314	CG	ARG	1275	-11.850	4.095	39.364	1.00	50.00
ATOM	2315	CD	ARG	1275	-11.818	4.852	40.696	1.00	50.00
ATOM	2316	NE	ARG	1275	-11.570	3.946	41.831	1.00	50.00
ATOM	2317	CZ	ARG	1275	-12.491	3.403	42.639	1.00	50.00

ATOM	2318	NH1	ARG	1275	-13.785	3.612	42.444	1.00	50.00
ATOM	2319	NH2	ARG	1275	-12.114	2.708	43.701	1.00	50.00
ATOM	2320	N	GLU	1276	-15.490	2.490	36.898	1.00	50.00
ATOM	2321	CA	GLU	1276	-16.846	1.940	36.718	1.00	50.00
ATOM	2322	C	GLU	1276	-17.060	1.186	35.401	1.00	50.00
ATOM	2323	O	GLU	1276	-17.967	0.361	35.302	1.00	50.00
ATOM	2324	CB	GLU	1276	-17.846	3.094	36.799	1.00	50.00
ATOM	2325	CG	GLU	1276	-17.836	3.789	38.165	1.00	50.00
ATOM	2326	CD	GLU	1276	-18.772	5.003	38.225	1.00	50.00
ATOM	2327	OE1	GLU	1276	-19.663	5.118	37.354	1.00	50.00
ATOM	2328	OE2	GLU	1276	-18.545	5.831	39.133	1.00	50.00
ATOM	2329	N	VAL	1277	-16.270	1.517	34.375	1.00	50.00
ATOM	2330	CA	VAL	1277	-16.486	0.990	33.014	1.00	50.00
ATOM	2331	C	VAL	1277	-15.382	0.053	32.492	1.00	50.00
ATOM	2332	O	VAL	1277	-15.604	-0.743	31.578	1.00	50.00
ATOM	2333	CB	VAL	1277	-16.748	2.137	32.011	1.00	50.00
ATOM	2334	CG1	VAL	1277	-17.990	2.947	32.401	1.00	50.00
ATOM	2335	CG2	VAL	1277	-15.543	3.070	31.829	1.00	50.00
ATOM	2336	N	SER	1278	-14.162	0.264	32.981	1.00	50.00
ATOM	2337	CA	SER	1278	-12.980	-0.478	32.509	1.00	50.00
ATOM	2338	C	SER	1278	-13.051	-1.975	32.833	1.00	50.00
ATOM	2339	O	SER	1278	-13.676	-2.411	33.798	1.00	50.00
ATOM	2340	CB	SER	1278	-11.717	0.112	33.137	1.00	50.00
ATOM	2341	OG	SER	1278	-11.663	-0.187	34.530	1.00	50.00
ATOM	2342	N	PHE	1279	-12.349	-2.746	32.007	1.00	50.00
ATOM	2343	CA	PHE	1279	-12.049	-4.162	32.287	1.00	50.00
ATOM	2344	C	PHE	1279	-11.156	-4.301	33.531	1.00	50.00
ATOM	2345	O	PHE	1279	-11.290	-5.247	34.306	1.00	50.00
ATOM	2346	CB	PHE	1279	-11.365	-4.795	31.065	1.00	50.00
ATOM	2347	CG	PHE	1279	-10.668	-6.121	31.392	1.00	50.00
ATOM	2348	CD1	PHE	1279	-11.412	-7.270	31.640	1.00	50.00
ATOM	2349	CD2	PHE	1279	-9.284	-6.146	31.527	1.00	50.00
ATOM	2350	CE1	PHE	1279	-10.768	-8.443	32.009	1.00	50.00
ATOM	2351	CE2	PHE	1279	-8.644	-7.320	31.901	1.00	50.00
ATOM	2352	CZ	PHE	1279	-9.387	-8.469	32.138	1.00	50.00
ATOM	2353	N	TYR	1280	-10.250	-3.352	33.704	1.00	50.00
ATOM	2354	CA	TYR	1280	-9.271	-3.362	34.808	1.00	50.00
ATOM	2355	C	TYR	1280	-9.955	-3.436	36.176	1.00	50.00
ATOM	2356	O	TYR	1280	-9.523	-4.195	37.046	1.00	50.00
ATOM	2357	CB	TYR	1280	-8.409	-2.101	34.714	1.00	50.00
ATOM	2358	CG	TYR	1280	-7.249	-2.158	35.707	1.00	50.00
ATOM	2359	CD1	TYR	1280	-7.406	-1.676	37.000	1.00	50.00
ATOM	2360	CD2	TYR	1280	-6.033	-2.695	35.307	1.00	50.00
ATOM	2361	CE1	TYR	1280	-6.348	-1.720	37.895	1.00	50.00
ATOM	2362	CE2	TYR	1280	-4.970	-2.732	36.196	1.00	50.00
ATOM	2363	CZ	TYR	1280	-5.129	-2.242	37.484	1.00	50.00
ATOM	2364	OH	TYR	1280	-4.074	-2.245	38.333	1.00	50.00
ATOM	2365	N	TYR	1281	-11.051	-2.694	36.332	1.00	50.00
ATOM	2366	CA	TYR	1281	-11.816	-2.675	37.586	1.00	50.00
ATOM	2367	C	TYR	1281	-13.035	-3.617	37.590	1.00	50.00
ATOM	2368	O	TYR	1281	-13.824	-3.610	38.533	1.00	50.00
ATOM	2369	CB	TYR	1281	-12.272	-1.242	37.875	1.00	50.00
ATOM	2370	CG	TYR	1281	-11.144	-0.345	38.387	1.00	50.00
ATOM	2371	CD1	TYR	1281	-10.357	0.378	37.504	1.00	50.00
ATOM	2372	CD2	TYR	1281	-10.952	-0.197	39.755	1.00	50.00
ATOM	2373	CE1	TYR	1281	-9.379	1.243	37.969	1.00	50.00
ATOM	2374	CE2	TYR	1281	-9.973	0.664	40.230	1.00	50.00
ATOM	2375	CZ	TYR	1281	-9.187	1.381	39.335	1.00	50.00
ATOM	2376	OH	TYR	1281	-8.211	2.206	39.786	1.00	50.00
ATOM	2377	N	SER	1282	-13.119	-4.496	36.591	1.00	50.00
ATOM	2378	CA	SER	1282	-14.247	-5.440	36.490	1.00	50.00

```
ATOM   2379  C   SER  1282     -13.914  -6.742  37.227  1.00 50.00
ATOM   2380  O   SER  1282     -12.751  -7.115  37.387  1.00 50.00
ATOM   2381  CB  SER  1282     -14.605  -5.722  35.024  1.00 50.00
ATOM   2382  OG  SER  1282     -13.688  -6.644  34.427  1.00 50.00
ATOM   2383  N   GLU  1283     -14.968  -7.490  37.544  1.00 50.00
ATOM   2384  CA  GLU  1283     -14.835  -8.812  38.189  1.00 50.00
ATOM   2385  C   GLU  1283     -14.060  -9.796  37.293  1.00 50.00
ATOM   2386  O   GLU  1283     -13.340 -10.666  37.786  1.00 50.00
ATOM   2387  CB  GLU  1283     -16.225  -9.375  38.485  1.00 50.00
ATOM   2388  CG  GLU  1283     -16.156 -10.568  39.446  1.00 50.00
ATOM   2389  CD  GLU  1283     -17.518 -11.228  39.691  1.00 50.00
ATOM   2390  OE1 GLU  1283     -18.548 -10.533  39.548  1.00 50.00
ATOM   2391  OE2 GLU  1283     -17.500 -12.437  40.010  1.00 50.00
ATOM   2392  N   GLU  1284     -14.210  -9.622  35.984  1.00 50.00
ATOM   2393  CA  GLU  1284     -13.529 -10.455  34.974  1.00 50.00
ATOM   2394  C   GLU  1284     -11.996 -10.374  35.077  1.00 50.00
ATOM   2395  O   GLU  1284     -11.316 -11.317  34.672  1.00 50.00
ATOM   2396  CB  GLU  1284     -13.986 -10.051  33.573  1.00 50.00
ATOM   2397  CG  GLU  1284     -13.603 -11.117  32.536  1.00 50.00
ATOM   2398  CD  GLU  1284     -13.978 -10.730  31.102  1.00 50.00
ATOM   2399  OE1 GLU  1284     -14.964  -9.981  30.933  1.00 50.00
ATOM   2400  OE2 GLU  1284     -13.224 -11.135  30.190  1.00 50.00
ATOM   2401  N   ASN  1285     -11.471  -9.230  35.529  1.00 50.00
ATOM   2402  CA  ASN  1285     -10.023  -9.083  35.755  1.00 50.00
ATOM   2403  C   ASN  1285      -9.600  -9.860  37.013  1.00 50.00
ATOM   2404  O   ASN  1285      -9.375  -9.299  38.080  1.00 50.00
ATOM   2405  CB  ASN  1285      -9.648  -7.598  35.856  1.00 50.00
ATOM   2406  CG  ASN  1285      -8.135  -7.424  36.073  1.00 50.00
ATOM   2407  OD1 ASN  1285      -7.307  -8.206  35.649  1.00 50.00
ATOM   2408  ND2 ASN  1285      -7.773  -6.355  36.753  1.00 50.00
ATOM   2409  N   LYS  1286      -9.530 -11.180  36.847  1.00 50.00
ATOM   2410  CA  LYS  1286      -9.185 -12.147  37.913  1.00 50.00
ATOM   2411  C   LYS  1286      -9.688 -11.794  39.328  1.00 50.00
ATOM   2412  O   LYS  1286      -8.959 -11.100  40.069  1.00 50.00
ATOM   2413  CB  LYS  1286      -7.677 -12.450  37.873  1.00 50.00
ATOM   2414  CG  LYS  1286      -7.356 -13.626  36.941  1.00 50.00
ATOM   2415  CD  LYS  1286      -7.828 -13.393  35.502  1.00 50.00
ATOM   2416  CE  LYS  1286      -7.870 -14.695  34.710  1.00 50.00
ATOM   2417  NZ  LYS  1286      -8.491 -14.454  33.403  1.00 50.00
ATOM   2418  OXT LYS  1286     -10.806 -12.262  39.641  1.00 99.99
TER    2419      LYS  1286
```
