# Supplementary material for: Structural analysis of hubs in human NR-RTK network
Source: Biol Direct. 2011 Oct 5;6:49. doi: 10.1186/1745-6150-6-49 (PMC3220635; doi:10.1186/1745-6150-6-49)
Supplement: Additional file 5 — Erbb2. Erbb2 structure. [file 1745-6150-6-49-S5.PDF]

```
HEADER    ERBB2
ATOM      1  N   SER    22        3.966 -24.060 -39.712  1.00 50.00
ATOM      2  CA  SER    22        5.082 -23.302 -40.339  1.00 50.00
ATOM      3  C   SER    22        6.268 -23.065 -39.375  1.00 50.00
ATOM      4  O   SER    22        7.404 -22.814 -39.773  1.00 50.00
ATOM      5  CB  SER    22        4.569 -21.957 -40.879  1.00 50.00
ATOM      6  OG  SER    22        5.528 -21.338 -41.744  1.00 50.00
ATOM      7  N   THR    23        5.958 -23.187 -38.089  1.00 50.00
ATOM      8  CA  THR    23        6.920 -23.037 -36.984  1.00 50.00
ATOM      9  C   THR    23        6.795 -24.255 -36.063  1.00 50.00
ATOM     10  O   THR    23        5.737 -24.890 -35.998  1.00 50.00
ATOM     11  CB  THR    23        6.622 -21.767 -36.170  1.00 50.00
ATOM     12  OG1 THR    23        5.732 -20.878 -36.859  1.00 50.00
ATOM     13  CG2 THR    23        7.926 -21.024 -35.900  1.00 50.00
ATOM     14  N   GLN    24        7.882 -24.576 -35.375  1.00 50.00
ATOM     15  CA  GLN    24        7.887 -25.647 -34.365  1.00 50.00
ATOM     16  C   GLN    24        7.228 -25.138 -33.077  1.00 50.00
ATOM     17  O   GLN    24        7.705 -24.189 -32.445  1.00 50.00
ATOM     18  CB  GLN    24        9.316 -26.149 -34.114  1.00 50.00
ATOM     19  CG  GLN    24        9.397 -27.222 -33.019  1.00 50.00
ATOM     20  CD  GLN    24        8.624 -28.494 -33.363  1.00 50.00
ATOM     21  OE1 GLN    24        7.420 -28.608 -33.186  1.00 50.00
ATOM     22  NE2 GLN    24        9.334 -29.452 -33.913  1.00 50.00
ATOM     23  N   VAL    25        6.190 -25.852 -32.679  1.00 50.00
ATOM     24  CA  VAL    25        5.388 -25.531 -31.484  1.00 50.00
ATOM     25  C   VAL    25        5.734 -26.483 -30.328  1.00 50.00
ATOM     26  O   VAL    25        5.957 -27.672 -30.520  1.00 50.00
ATOM     27  CB  VAL    25        3.901 -25.554 -31.899  1.00 50.00
ATOM     28  CG1 VAL    25        2.902 -25.957 -30.805  1.00 50.00
ATOM     29  CG2 VAL    25        3.511 -24.164 -32.407  1.00 50.00
ATOM     30  N   CYS    26        5.594 -25.925 -29.130  1.00 50.00
ATOM     31  CA  CYS    26        5.662 -26.678 -27.866  1.00 50.00
ATOM     32  C   CYS    26        4.870 -25.936 -26.787  1.00 50.00
ATOM     33  O   CYS    26        4.607 -24.736 -26.910  1.00 50.00
ATOM     34  CB  CYS    26        7.109 -26.857 -27.393  1.00 50.00
ATOM     35  SG  CYS    26        7.952 -25.301 -26.925  1.00 50.00
ATOM     36  N   THR    27        4.447 -26.681 -25.783  1.00 50.00
ATOM     37  CA  THR    27        3.752 -26.109 -24.612  1.00 50.00
ATOM     38  C   THR    27        4.790 -25.520 -23.640  1.00 50.00
ATOM     39  O   THR    27        6.000 -25.679 -23.814  1.00 50.00
ATOM     40  CB  THR    27        2.894 -27.167 -23.897  1.00 50.00
ATOM     41  OG1 THR    27        3.740 -28.135 -23.274  1.00 50.00
ATOM     42  CG2 THR    27        1.919 -27.867 -24.853  1.00 50.00
ATOM     43  N   GLY    28        4.269 -24.789 -22.651  1.00 50.00
ATOM     44  CA  GLY    28        5.105 -24.239 -21.571  1.00 50.00
ATOM     45  C   GLY    28        4.865 -25.008 -20.270  1.00 50.00
ATOM     46  O   GLY    28        4.403 -26.153 -20.257  1.00 50.00
ATOM     47  N   THR    29        5.144 -24.322 -19.178  1.00 50.00
ATOM     48  CA  THR    29        4.946 -24.862 -17.819  1.00 50.00
ATOM     49  C   THR    29        4.212 -23.856 -16.925  1.00 50.00
ATOM     50  O   THR    29        4.059 -22.679 -17.260  1.00 50.00
ATOM     51  CB  THR    29        6.282 -25.281 -17.185  1.00 50.00
ATOM     52  OG1 THR    29        7.193 -24.181 -17.201  1.00 50.00
ATOM     53  CG2 THR    29        6.900 -26.501 -17.875  1.00 50.00
ATOM     54  N   ASP    30        3.662 -24.383 -15.837  1.00 50.00
ATOM     55  CA  ASP    30        2.935 -23.584 -14.828  1.00 50.00
ATOM     56  C   ASP    30        3.413 -23.889 -13.398  1.00 50.00
ATOM     57  O   ASP    30        2.640 -23.889 -12.444  1.00 50.00
ATOM     58  CB  ASP    30        1.427 -23.831 -14.969  1.00 50.00
ATOM     59  CG  ASP    30        0.881 -23.339 -16.310  1.00 50.00
ATOM     60  OD1 ASP    30        1.071 -22.139 -16.595  1.00 50.00
```

ATOM	61	OD2	ASP	30	0.357	-24.196	-17.052	1.00	50.00
ATOM	62	N	MET	31	4.703	-24.192	-13.286	1.00	50.00
ATOM	63	CA	MET	31	5.333	-24.522	-11.993	1.00	50.00
ATOM	64	C	MET	31	5.588	-23.289	-11.111	1.00	50.00
ATOM	65	O	MET	31	5.690	-23.386	-9.893	1.00	50.00
ATOM	66	CB	MET	31	6.637	-25.304	-12.207	1.00	50.00
ATOM	67	CG	MET	31	7.712	-24.517	-12.967	1.00	50.00
ATOM	68	SD	MET	31	9.299	-25.401	-13.159	1.00	50.00
ATOM	69	CE	MET	31	8.788	-26.725	-14.233	1.00	50.00
ATOM	70	N	LYS	32	5.789	-22.141	-11.763	1.00	50.00
ATOM	71	CA	LYS	32	6.202	-20.883	-11.111	1.00	50.00
ATOM	72	C	LYS	32	7.482	-21.087	-10.273	1.00	50.00
ATOM	73	O	LYS	32	8.414	-21.734	-10.750	1.00	50.00
ATOM	74	CB	LYS	32	5.026	-20.245	-10.351	1.00	50.00
ATOM	75	CG	LYS	32	3.943	-19.795	-11.329	1.00	50.00
ATOM	76	CD	LYS	32	3.042	-18.727	-10.713	1.00	50.00
ATOM	77	CE	LYS	32	2.066	-18.227	-11.777	1.00	50.00
ATOM	78	NZ	LYS	32	1.339	-17.033	-11.329	1.00	50.00
ATOM	79	N	LEU	33	7.486	-20.634	-9.022	1.00	50.00
ATOM	80	CA	LEU	33	8.668	-20.726	-8.141	1.00	50.00
ATOM	81	C	LEU	33	8.756	-22.033	-7.334	1.00	50.00
ATOM	82	O	LEU	33	9.527	-22.135	-6.379	1.00	50.00
ATOM	83	CB	LEU	33	8.697	-19.509	-7.205	1.00	50.00
ATOM	84	CG	LEU	33	8.762	-18.160	-7.932	1.00	50.00
ATOM	85	CD1	LEU	33	8.805	-17.026	-6.908	1.00	50.00
ATOM	86	CD2	LEU	33	9.977	-18.066	-8.854	1.00	50.00
ATOM	87	N	ARG	34	8.060	-23.061	-7.812	1.00	50.00
ATOM	88	CA	ARG	34	8.032	-24.391	-7.178	1.00	50.00
ATOM	89	C	ARG	34	9.387	-25.105	-7.296	1.00	50.00
ATOM	90	O	ARG	34	9.943	-25.233	-8.389	1.00	50.00
ATOM	91	CB	ARG	34	6.913	-25.202	-7.839	1.00	50.00
ATOM	92	CG	ARG	34	6.862	-26.668	-7.401	1.00	50.00
ATOM	93	CD	ARG	34	5.625	-27.388	-7.933	1.00	50.00
ATOM	94	NE	ARG	34	5.590	-27.489	-9.406	1.00	50.00
ATOM	95	CZ	ARG	34	4.472	-27.614	-10.126	1.00	50.00
ATOM	96	NH1	ARG	34	3.281	-27.590	-9.540	1.00	50.00
ATOM	97	NH2	ARG	34	4.522	-27.861	-11.428	1.00	50.00
ATOM	98	N	LEU	35	9.823	-25.659	-6.170	1.00	50.00
ATOM	99	CA	LEU	35	11.044	-26.482	-6.116	1.00	50.00
ATOM	100	C	LEU	35	10.839	-27.856	-6.776	1.00	50.00
ATOM	101	O	LEU	35	9.885	-28.566	-6.444	1.00	50.00
ATOM	102	CB	LEU	35	11.599	-26.665	-4.699	1.00	50.00
ATOM	103	CG	LEU	35	12.145	-25.365	-4.099	1.00	50.00
ATOM	104	CD1	LEU	35	11.028	-24.529	-3.468	1.00	50.00
ATOM	105	CD2	LEU	35	13.237	-25.681	-3.078	1.00	50.00
ATOM	106	N	PRO	36	11.720	-28.191	-7.725	1.00	50.00
ATOM	107	CA	PRO	36	11.683	-29.453	-8.493	1.00	50.00
ATOM	108	C	PRO	36	11.922	-30.669	-7.589	1.00	50.00
ATOM	109	O	PRO	36	12.558	-30.562	-6.544	1.00	50.00
ATOM	110	CB	PRO	36	12.832	-29.333	-9.496	1.00	50.00
ATOM	111	CG	PRO	36	13.038	-27.828	-9.638	1.00	50.00
ATOM	112	CD	PRO	36	12.801	-27.318	-8.226	1.00	50.00
ATOM	113	N	ALA	37	11.514	-31.836	-8.091	1.00	50.00
ATOM	114	CA	ALA	37	11.750	-33.132	-7.416	1.00	50.00
ATOM	115	C	ALA	37	13.252	-33.443	-7.301	1.00	50.00
ATOM	116	O	ALA	37	13.767	-33.774	-6.239	1.00	50.00
ATOM	117	CB	ALA	37	11.045	-34.252	-8.187	1.00	50.00
ATOM	118	N	SER	38	13.940	-33.282	-8.430	1.00	50.00
ATOM	119	CA	SER	38	15.398	-33.461	-8.510	1.00	50.00
ATOM	120	C	SER	38	15.960	-32.635	-9.677	1.00	50.00
ATOM	121	O	SER	38	15.311	-32.597	-10.737	1.00	50.00

ATOM	122	CB	SER	38	15.766	-34.947	-8.669	1.00	50.00
ATOM	123	OG	SER	38	15.405	-35.416	-9.970	1.00	50.00
ATOM	124	N	PRO	39	17.202	-32.150	-9.544	1.00	50.00
ATOM	125	CA	PRO	39	17.931	-31.425	-10.609	1.00	50.00
ATOM	126	C	PRO	39	18.075	-32.271	-11.886	1.00	50.00
ATOM	127	O	PRO	39	18.008	-31.754	-13.000	1.00	50.00
ATOM	128	CB	PRO	39	19.318	-31.161	-10.021	1.00	50.00
ATOM	129	CG	PRO	39	19.074	-31.123	-8.515	1.00	50.00
ATOM	130	CD	PRO	39	18.015	-32.200	-8.309	1.00	50.00
ATOM	131	N	GLU	40	18.125	-33.589	-11.688	1.00	50.00
ATOM	132	CA	GLU	40	18.296	-34.595	-12.755	1.00	50.00
ATOM	133	C	GLU	40	17.093	-34.666	-13.711	1.00	50.00
ATOM	134	O	GLU	40	17.232	-34.527	-14.923	1.00	50.00
ATOM	135	CB	GLU	40	18.536	-35.953	-12.090	1.00	50.00
ATOM	136	CG	GLU	40	19.868	-35.967	-11.333	1.00	50.00
ATOM	137	CD	GLU	40	19.741	-36.741	-10.021	1.00	50.00
ATOM	138	OE1	GLU	40	19.819	-37.985	-10.084	1.00	50.00
ATOM	139	OE2	GLU	40	19.499	-36.055	-9.003	1.00	50.00
ATOM	140	N	THR	41	15.905	-34.707	-13.115	1.00	50.00
ATOM	141	CA	THR	41	14.630	-34.736	-13.865	1.00	50.00
ATOM	142	C	THR	41	14.212	-33.355	-14.387	1.00	50.00
ATOM	143	O	THR	41	13.679	-33.243	-15.485	1.00	50.00
ATOM	144	CB	THR	41	13.490	-35.334	-13.031	1.00	50.00
ATOM	145	OG1	THR	41	13.371	-34.615	-11.795	1.00	50.00
ATOM	146	CG2	THR	41	13.710	-36.831	-12.789	1.00	50.00
ATOM	147	N	HIS	42	14.614	-32.312	-13.656	1.00	50.00
ATOM	148	CA	HIS	42	14.235	-30.923	-13.966	1.00	50.00
ATOM	149	C	HIS	42	14.830	-30.439	-15.297	1.00	50.00
ATOM	150	O	HIS	42	14.102	-30.063	-16.216	1.00	50.00
ATOM	151	CB	HIS	42	14.684	-30.014	-12.820	1.00	50.00
ATOM	152	CG	HIS	42	14.050	-28.633	-12.970	1.00	50.00
ATOM	153	ND1	HIS	42	12.745	-28.381	-12.966	1.00	50.00
ATOM	154	CD2	HIS	42	14.702	-27.487	-13.121	1.00	50.00
ATOM	155	CE1	HIS	42	12.584	-27.068	-13.107	1.00	50.00
ATOM	156	NE2	HIS	42	13.792	-26.525	-13.200	1.00	50.00
ATOM	157	N	LEU	43	16.150	-30.584	-15.418	1.00	50.00
ATOM	158	CA	LEU	43	16.874	-30.166	-16.631	1.00	50.00
ATOM	159	C	LEU	43	16.553	-31.048	-17.847	1.00	50.00
ATOM	160	O	LEU	43	16.332	-30.544	-18.946	1.00	50.00
ATOM	161	CB	LEU	43	18.382	-30.135	-16.353	1.00	50.00
ATOM	162	CG	LEU	43	19.202	-29.615	-17.543	1.00	50.00
ATOM	163	CD1	LEU	43	18.822	-28.180	-17.930	1.00	50.00
ATOM	164	CD2	LEU	43	20.691	-29.696	-17.214	1.00	50.00
ATOM	165	N	ASP	44	16.399	-32.343	-17.585	1.00	50.00
ATOM	166	CA	ASP	44	16.066	-33.332	-18.629	1.00	50.00
ATOM	167	C	ASP	44	14.669	-33.115	-19.221	1.00	50.00
ATOM	168	O	ASP	44	14.490	-33.202	-20.434	1.00	50.00
ATOM	169	CB	ASP	44	16.198	-34.752	-18.070	1.00	50.00
ATOM	170	CG	ASP	44	17.647	-35.153	-17.754	1.00	50.00
ATOM	171	OD1	ASP	44	18.548	-34.287	-17.857	1.00	50.00
ATOM	172	OD2	ASP	44	17.817	-36.326	-17.363	1.00	50.00
ATOM	173	N	MET	45	13.735	-32.718	-18.359	1.00	50.00
ATOM	174	CA	MET	45	12.361	-32.391	-18.775	1.00	50.00
ATOM	175	C	MET	45	12.325	-31.106	-19.616	1.00	50.00
ATOM	176	O	MET	45	11.699	-31.081	-20.672	1.00	50.00
ATOM	177	CB	MET	45	11.465	-32.318	-17.532	1.00	50.00
ATOM	178	CG	MET	45	10.012	-31.924	-17.827	1.00	50.00
ATOM	179	SD	MET	45	9.772	-30.125	-18.078	1.00	50.00
ATOM	180	CE	MET	45	10.063	-29.555	-16.417	1.00	50.00
ATOM	181	N	LEU	46	13.069	-30.090	-19.180	1.00	50.00
ATOM	182	CA	LEU	46	13.169	-28.813	-19.916	1.00	50.00

ATOM	183	C	LEU	46	13.813	-28.968	-21.298	1.00	50.00
ATOM	184	O	LEU	46	13.309	-28.431	-22.284	1.00	50.00
ATOM	185	CB	LEU	46	13.952	-27.777	-19.106	1.00	50.00
ATOM	186	CG	LEU	46	13.215	-27.324	-17.842	1.00	50.00
ATOM	187	CD1	LEU	46	14.118	-26.387	-17.042	1.00	50.00
ATOM	188	CD2	LEU	46	11.900	-26.610	-18.174	1.00	50.00
ATOM	189	N	ARG	47	14.847	-29.805	-21.350	1.00	50.00
ATOM	190	CA	ARG	47	15.554	-30.124	-22.602	1.00	50.00
ATOM	191	C	ARG	47	14.640	-30.879	-23.579	1.00	50.00
ATOM	192	O	ARG	47	14.539	-30.497	-24.741	1.00	50.00
ATOM	193	CB	ARG	47	16.810	-30.941	-22.288	1.00	50.00
ATOM	194	CG	ARG	47	17.597	-31.282	-23.559	1.00	50.00
ATOM	195	CD	ARG	47	18.877	-32.057	-23.252	1.00	50.00
ATOM	196	NE	ARG	47	19.872	-31.160	-22.637	1.00	50.00
ATOM	197	CZ	ARG	47	20.245	-31.156	-21.356	1.00	50.00
ATOM	198	NH1	ARG	47	19.695	-31.980	-20.473	1.00	50.00
ATOM	199	NH2	ARG	47	21.210	-30.343	-20.951	1.00	50.00
ATOM	200	N	HIS	48	13.891	-31.841	-23.039	1.00	50.00
ATOM	201	CA	HIS	48	12.913	-32.625	-23.815	1.00	50.00
ATOM	202	C	HIS	48	11.792	-31.729	-24.371	1.00	50.00
ATOM	203	O	HIS	48	11.321	-31.942	-25.483	1.00	50.00
ATOM	204	CB	HIS	48	12.318	-33.709	-22.908	1.00	50.00
ATOM	205	CG	HIS	48	11.393	-34.670	-23.664	1.00	50.00
ATOM	206	ND1	HIS	48	11.751	-35.829	-24.200	1.00	50.00
ATOM	207	CD2	HIS	48	10.088	-34.504	-23.878	1.00	50.00
ATOM	208	CE1	HIS	48	10.672	-36.382	-24.746	1.00	50.00
ATOM	209	NE2	HIS	48	9.643	-35.566	-24.542	1.00	50.00
ATOM	210	N	LEU	49	11.400	-30.740	-23.569	1.00	50.00
ATOM	211	CA	LEU	49	10.270	-29.851	-23.880	1.00	50.00
ATOM	212	C	LEU	49	10.600	-28.785	-24.937	1.00	50.00
ATOM	213	O	LEU	49	9.862	-28.616	-25.904	1.00	50.00
ATOM	214	CB	LEU	49	9.794	-29.180	-22.586	1.00	50.00
ATOM	215	CG	LEU	49	8.436	-28.488	-22.757	1.00	50.00
ATOM	216	CD1	LEU	49	7.310	-29.514	-22.917	1.00	50.00
ATOM	217	CD2	LEU	49	8.163	-27.559	-21.576	1.00	50.00
ATOM	218	N	TYR	50	11.740	-28.126	-24.752	1.00	50.00
ATOM	219	CA	TYR	50	12.087	-26.925	-25.534	1.00	50.00
ATOM	220	C	TYR	50	13.015	-27.133	-26.732	1.00	50.00
ATOM	221	O	TYR	50	13.027	-26.297	-27.637	1.00	50.00
ATOM	222	CB	TYR	50	12.655	-25.839	-24.617	1.00	50.00
ATOM	223	CG	TYR	50	11.596	-25.292	-23.656	1.00	50.00
ATOM	224	CD1	TYR	50	10.407	-24.760	-24.142	1.00	50.00
ATOM	225	CD2	TYR	50	11.824	-25.345	-22.288	1.00	50.00
ATOM	226	CE1	TYR	50	9.447	-24.283	-23.261	1.00	50.00
ATOM	227	CE2	TYR	50	10.865	-24.865	-21.406	1.00	50.00
ATOM	228	CZ	TYR	50	9.677	-24.332	-21.893	1.00	50.00
ATOM	229	OH	TYR	50	8.778	-23.792	-21.031	1.00	50.00
ATOM	230	N	GLN	51	13.795	-28.213	-26.740	1.00	50.00
ATOM	231	CA	GLN	51	14.729	-28.474	-27.852	1.00	50.00
ATOM	232	C	GLN	51	13.979	-28.577	-29.189	1.00	50.00
ATOM	233	O	GLN	51	13.042	-29.356	-29.340	1.00	50.00
ATOM	234	CB	GLN	51	15.515	-29.760	-27.612	1.00	50.00
ATOM	235	CG	GLN	51	16.685	-29.858	-28.594	1.00	50.00
ATOM	236	CD	GLN	51	17.495	-31.141	-28.420	1.00	50.00
ATOM	237	OE1	GLN	51	17.601	-31.741	-27.360	1.00	50.00
ATOM	238	NE2	GLN	51	18.147	-31.543	-29.488	1.00	50.00
ATOM	239	N	GLY	52	14.393	-27.698	-30.108	1.00	50.00
ATOM	240	CA	GLY	52	13.776	-27.612	-31.446	1.00	50.00
ATOM	241	C	GLY	52	12.610	-26.615	-31.506	1.00	50.00
ATOM	242	O	GLY	52	12.333	-26.069	-32.574	1.00	50.00
ATOM	243	N	CYS	53	11.982	-26.352	-30.360	1.00	50.00

ATOM	244	CA	CYS	53	10.817	-25.457	-30.265	1.00	50.00
ATOM	245	C	CYS	53	11.177	-24.018	-30.647	1.00	50.00
ATOM	246	O	CYS	53	12.200	-23.464	-30.241	1.00	50.00
ATOM	247	CB	CYS	53	10.230	-25.498	-28.855	1.00	50.00
ATOM	248	SG	CYS	53	8.647	-24.595	-28.704	1.00	50.00
ATOM	249	N	GLN	54	10.258	-23.437	-31.398	1.00	50.00
ATOM	250	CA	GLN	54	10.361	-22.045	-31.872	1.00	50.00
ATOM	251	C	GLN	54	9.282	-21.168	-31.221	1.00	50.00
ATOM	252	O	GLN	54	9.504	-19.994	-30.920	1.00	50.00
ATOM	253	CB	GLN	54	10.192	-22.024	-33.391	1.00	50.00
ATOM	254	CG	GLN	54	11.281	-22.802	-34.137	1.00	50.00
ATOM	255	CD	GLN	54	10.989	-22.838	-35.638	1.00	50.00
ATOM	256	OE1	GLN	54	10.453	-23.787	-36.188	1.00	50.00
ATOM	257	NE2	GLN	54	11.276	-21.742	-36.300	1.00	50.00
ATOM	258	N	VAL	55	8.112	-21.763	-31.011	1.00	50.00
ATOM	259	CA	VAL	55	6.943	-21.094	-30.415	1.00	50.00
ATOM	260	C	VAL	55	6.456	-21.905	-29.206	1.00	50.00
ATOM	261	O	VAL	55	5.869	-22.983	-29.349	1.00	50.00
ATOM	262	CB	VAL	55	5.816	-20.929	-31.456	1.00	50.00
ATOM	263	CG1	VAL	55	4.579	-20.242	-30.862	1.00	50.00
ATOM	264	CG2	VAL	55	6.276	-20.130	-32.679	1.00	50.00
ATOM	265	N	VAL	56	6.747	-21.369	-28.035	1.00	50.00
ATOM	266	CA	VAL	56	6.193	-21.876	-26.763	1.00	50.00
ATOM	267	C	VAL	56	4.800	-21.279	-26.516	1.00	50.00
ATOM	268	O	VAL	56	4.638	-20.102	-26.157	1.00	50.00
ATOM	269	CB	VAL	56	7.181	-21.710	-25.587	1.00	50.00
ATOM	270	CG1	VAL	56	7.825	-20.328	-25.556	1.00	50.00
ATOM	271	CG2	VAL	56	6.543	-21.983	-24.219	1.00	50.00
ATOM	272	N	GLN	57	3.814	-22.136	-26.696	1.00	50.00
ATOM	273	CA	GLN	57	2.396	-21.804	-26.472	1.00	50.00
ATOM	274	C	GLN	57	2.052	-21.976	-24.985	1.00	50.00
ATOM	275	O	GLN	57	1.530	-22.998	-24.533	1.00	50.00
ATOM	276	CB	GLN	57	1.516	-22.690	-27.350	1.00	50.00
ATOM	277	CG	GLN	57	1.865	-22.546	-28.836	1.00	50.00
ATOM	278	CD	GLN	57	0.926	-23.343	-29.742	1.00	50.00
ATOM	279	OE1	GLN	57	0.602	-22.932	-30.845	1.00	50.00
ATOM	280	NE2	GLN	57	0.505	-24.515	-29.307	1.00	50.00
ATOM	281	N	GLY	58	2.418	-20.933	-24.238	1.00	50.00
ATOM	282	CA	GLY	58	2.316	-20.918	-22.768	1.00	50.00
ATOM	283	C	GLY	58	3.468	-20.111	-22.167	1.00	50.00
ATOM	284	O	GLY	58	4.002	-19.207	-22.815	1.00	50.00
ATOM	285	N	ASN	59	3.918	-20.555	-21.005	1.00	50.00
ATOM	286	CA	ASN	59	4.926	-19.815	-20.222	1.00	50.00
ATOM	287	C	ASN	59	6.278	-20.528	-20.258	1.00	50.00
ATOM	288	O	ASN	59	6.376	-21.720	-19.952	1.00	50.00
ATOM	289	CB	ASN	59	4.458	-19.698	-18.768	1.00	50.00
ATOM	290	CG	ASN	59	3.032	-19.155	-18.666	1.00	50.00
ATOM	291	OD1	ASN	59	2.647	-18.189	-19.313	1.00	50.00
ATOM	292	ND2	ASN	59	2.227	-19.837	-17.888	1.00	50.00
ATOM	293	N	LEU	60	7.300	-19.735	-20.546	1.00	50.00
ATOM	294	CA	LEU	60	8.705	-20.177	-20.529	1.00	50.00
ATOM	295	C	LEU	60	9.305	-19.959	-19.132	1.00	50.00
ATOM	296	O	LEU	60	9.627	-18.839	-18.733	1.00	50.00
ATOM	297	CB	LEU	60	9.499	-19.419	-21.600	1.00	50.00
ATOM	298	CG	LEU	60	10.999	-19.743	-21.593	1.00	50.00
ATOM	299	CD1	LEU	60	11.276	-21.204	-21.956	1.00	50.00
ATOM	300	CD2	LEU	60	11.738	-18.804	-22.544	1.00	50.00
ATOM	301	N	GLU	61	9.383	-21.050	-18.384	1.00	50.00
ATOM	302	CA	GLU	61	9.888	-21.019	-16.996	1.00	50.00
ATOM	303	C	GLU	61	11.202	-21.792	-16.857	1.00	50.00
ATOM	304	O	GLU	61	11.250	-23.007	-17.070	1.00	50.00

ATOM	305	CB	GLU	61	8.844	-21.588	-16.033	1.00	50.00
ATOM	306	CG	GLU	61	7.528	-20.805	-16.074	1.00	50.00
ATOM	307	CD	GLU	61	6.470	-21.405	-15.147	1.00	50.00
ATOM	308	OE1	GLU	61	6.357	-22.646	-15.054	1.00	50.00
ATOM	309	OE2	GLU	61	5.820	-20.600	-14.450	1.00	50.00
ATOM	310	N	LEU	62	12.247	-21.043	-16.533	1.00	50.00
ATOM	311	CA	LEU	62	13.619	-21.561	-16.353	1.00	50.00
ATOM	312	C	LEU	62	14.107	-21.263	-14.934	1.00	50.00
ATOM	313	O	LEU	62	14.477	-20.133	-14.604	1.00	50.00
ATOM	314	CB	LEU	62	14.560	-20.908	-17.371	1.00	50.00
ATOM	315	CG	LEU	62	14.155	-21.167	-18.825	1.00	50.00
ATOM	316	CD1	LEU	62	15.005	-20.290	-19.741	1.00	50.00
ATOM	317	CD2	LEU	62	14.314	-22.644	-19.201	1.00	50.00
ATOM	318	N	THR	63	13.931	-22.254	-14.075	1.00	50.00
ATOM	319	CA	THR	63	14.173	-22.075	-12.627	1.00	50.00
ATOM	320	C	THR	63	15.127	-23.123	-12.057	1.00	50.00
ATOM	321	O	THR	63	15.166	-24.243	-12.570	1.00	50.00
ATOM	322	CB	THR	63	12.857	-22.110	-11.836	1.00	50.00
ATOM	323	OG1	THR	63	12.212	-23.372	-12.035	1.00	50.00
ATOM	324	CG2	THR	63	11.923	-20.959	-12.228	1.00	50.00
ATOM	325	N	TYR	64	15.857	-22.734	-11.018	1.00	50.00
ATOM	326	CA	TYR	64	16.732	-23.629	-10.221	1.00	50.00
ATOM	327	C	TYR	64	17.779	-24.397	-11.052	1.00	50.00
ATOM	328	O	TYR	64	18.204	-25.506	-10.712	1.00	50.00
ATOM	329	CB	TYR	64	15.883	-24.607	-9.392	1.00	50.00
ATOM	330	CG	TYR	64	14.894	-23.899	-8.467	1.00	50.00
ATOM	331	CD1	TYR	64	15.327	-23.397	-7.247	1.00	50.00
ATOM	332	CD2	TYR	64	13.550	-23.826	-8.813	1.00	50.00
ATOM	333	CE1	TYR	64	14.415	-22.821	-6.376	1.00	50.00
ATOM	334	CE2	TYR	64	12.635	-23.252	-7.943	1.00	50.00
ATOM	335	CZ	TYR	64	13.072	-22.745	-6.726	1.00	50.00
ATOM	336	OH	TYR	64	12.206	-22.137	-5.880	1.00	50.00
ATOM	337	N	LEU	65	18.197	-23.787	-12.157	1.00	50.00
ATOM	338	CA	LEU	65	19.213	-24.371	-13.046	1.00	50.00
ATOM	339	C	LEU	65	20.616	-24.207	-12.445	1.00	50.00
ATOM	340	O	LEU	65	20.965	-23.103	-12.015	1.00	50.00
ATOM	341	CB	LEU	65	19.146	-23.746	-14.443	1.00	50.00
ATOM	342	CG	LEU	65	17.799	-24.000	-15.131	1.00	50.00
ATOM	343	CD1	LEU	65	17.723	-23.178	-16.416	1.00	50.00
ATOM	344	CD2	LEU	65	17.589	-25.485	-15.442	1.00	50.00
ATOM	345	N	PRO	66	21.393	-25.294	-12.403	1.00	50.00
ATOM	346	CA	PRO	66	22.767	-25.285	-11.864	1.00	50.00
ATOM	347	C	PRO	66	23.723	-24.456	-12.734	1.00	50.00
ATOM	348	O	PRO	66	23.441	-24.183	-13.900	1.00	50.00
ATOM	349	CB	PRO	66	23.159	-26.762	-11.796	1.00	50.00
ATOM	350	CG	PRO	66	22.364	-27.401	-12.933	1.00	50.00
ATOM	351	CD	PRO	66	21.039	-26.640	-12.904	1.00	50.00
ATOM	352	N	THR	67	24.902	-24.192	-12.180	1.00	50.00
ATOM	353	CA	THR	67	25.945	-23.342	-12.805	1.00	50.00
ATOM	354	C	THR	67	26.352	-23.811	-14.212	1.00	50.00
ATOM	355	O	THR	67	26.347	-23.019	-15.150	1.00	50.00
ATOM	356	CB	THR	67	27.191	-23.301	-11.905	1.00	50.00
ATOM	357	OG1	THR	67	26.813	-22.838	-10.608	1.00	50.00
ATOM	358	CG2	THR	67	28.338	-22.453	-12.467	1.00	50.00
ATOM	359	N	ASN	68	26.632	-25.107	-14.333	1.00	50.00
ATOM	360	CA	ASN	68	27.181	-25.675	-15.581	1.00	50.00
ATOM	361	C	ASN	68	26.129	-26.404	-16.432	1.00	50.00
ATOM	362	O	ASN	68	26.444	-27.295	-17.224	1.00	50.00
ATOM	363	CB	ASN	68	28.345	-26.611	-15.229	1.00	50.00
ATOM	364	CG	ASN	68	29.489	-25.883	-14.518	1.00	50.00
ATOM	365	OD1	ASN	68	29.870	-24.766	-14.826	1.00	50.00

ATOM	366	ND2	ASN	68	30.035	-26.537	-13.518	1.00	50.00
ATOM	367	N	ALA	69	24.868	-26.013	-16.254	1.00	50.00
ATOM	368	CA	ALA	69	23.752	-26.558	-17.046	1.00	50.00
ATOM	369	C	ALA	69	23.813	-26.046	-18.492	1.00	50.00
ATOM	370	O	ALA	69	23.867	-24.842	-18.743	1.00	50.00
ATOM	371	CB	ALA	69	22.422	-26.161	-16.407	1.00	50.00
ATOM	372	N	SER	70	23.861	-26.994	-19.422	1.00	50.00
ATOM	373	CA	SER	70	23.837	-26.693	-20.868	1.00	50.00
ATOM	374	C	SER	70	22.416	-26.326	-21.312	1.00	50.00
ATOM	375	O	SER	70	21.494	-27.137	-21.226	1.00	50.00
ATOM	376	CB	SER	70	24.344	-27.885	-21.681	1.00	50.00
ATOM	377	OG	SER	70	24.357	-27.546	-23.071	1.00	50.00
ATOM	378	N	LEU	71	22.285	-25.096	-21.792	1.00	50.00
ATOM	379	CA	LEU	71	20.988	-24.511	-22.196	1.00	50.00
ATOM	380	C	LEU	71	20.903	-24.224	-23.701	1.00	50.00
ATOM	381	O	LEU	71	20.054	-23.464	-24.170	1.00	50.00
ATOM	382	CB	LEU	71	20.780	-23.219	-21.396	1.00	50.00
ATOM	383	CG	LEU	71	20.009	-23.369	-20.078	1.00	50.00
ATOM	384	CD1	LEU	71	20.575	-24.439	-19.142	1.00	50.00
ATOM	385	CD2	LEU	71	20.040	-22.024	-19.353	1.00	50.00
ATOM	386	N	SER	72	21.701	-24.965	-24.465	1.00	50.00
ATOM	387	CA	SER	72	21.747	-24.825	-25.934	1.00	50.00
ATOM	388	C	SER	72	20.448	-25.248	-26.637	1.00	50.00
ATOM	389	O	SER	72	20.170	-24.801	-27.743	1.00	50.00
ATOM	390	CB	SER	72	22.942	-25.572	-26.526	1.00	50.00
ATOM	391	OG	SER	72	22.927	-26.937	-26.102	1.00	50.00
ATOM	392	N	PHE	73	19.609	-26.020	-25.946	1.00	50.00
ATOM	393	CA	PHE	73	18.283	-26.425	-26.458	1.00	50.00
ATOM	394	C	PHE	73	17.272	-25.269	-26.602	1.00	50.00
ATOM	395	O	PHE	73	16.246	-25.416	-27.261	1.00	50.00
ATOM	396	CB	PHE	73	17.702	-27.556	-25.602	1.00	50.00
ATOM	397	CG	PHE	73	17.551	-27.192	-24.125	1.00	50.00
ATOM	398	CD1	PHE	73	16.435	-26.487	-23.692	1.00	50.00
ATOM	399	CD2	PHE	73	18.500	-27.637	-23.215	1.00	50.00
ATOM	400	CE1	PHE	73	16.269	-26.227	-22.338	1.00	50.00
ATOM	401	CE2	PHE	73	18.329	-27.380	-21.861	1.00	50.00
ATOM	402	CZ	PHE	73	17.215	-26.674	-21.423	1.00	50.00
ATOM	403	N	LEU	74	17.591	-24.129	-25.988	1.00	50.00
ATOM	404	CA	LEU	74	16.756	-22.911	-26.028	1.00	50.00
ATOM	405	C	LEU	74	16.944	-22.060	-27.290	1.00	50.00
ATOM	406	O	LEU	74	16.110	-21.209	-27.601	1.00	50.00
ATOM	407	CB	LEU	74	17.080	-22.025	-24.824	1.00	50.00
ATOM	408	CG	LEU	74	16.763	-22.671	-23.473	1.00	50.00
ATOM	409	CD1	LEU	74	17.279	-21.756	-22.365	1.00	50.00
ATOM	410	CD2	LEU	74	15.261	-22.906	-23.292	1.00	50.00
ATOM	411	N	GLN	75	17.988	-22.373	-28.053	1.00	50.00
ATOM	412	CA	GLN	75	18.477	-21.506	-29.140	1.00	50.00
ATOM	413	C	GLN	75	17.482	-21.197	-30.272	1.00	50.00
ATOM	414	O	GLN	75	17.628	-20.182	-30.941	1.00	50.00
ATOM	415	CB	GLN	75	19.799	-22.050	-29.688	1.00	50.00
ATOM	416	CG	GLN	75	19.687	-23.408	-30.392	1.00	50.00
ATOM	417	CD	GLN	75	21.068	-23.993	-30.704	1.00	50.00
ATOM	418	OE1	GLN	75	22.095	-23.330	-30.750	1.00	50.00
ATOM	419	NE2	GLN	75	21.113	-25.291	-30.905	1.00	50.00
ATOM	420	N	ASP	76	16.478	-22.057	-30.447	1.00	50.00
ATOM	421	CA	ASP	76	15.479	-21.895	-31.523	1.00	50.00
ATOM	422	C	ASP	76	14.217	-21.105	-31.146	1.00	50.00
ATOM	423	O	ASP	76	13.477	-20.697	-32.043	1.00	50.00
ATOM	424	CB	ASP	76	15.086	-23.253	-32.111	1.00	50.00
ATOM	425	CG	ASP	76	16.273	-23.914	-32.813	1.00	50.00
ATOM	426	OD1	ASP	76	16.749	-23.336	-33.815	1.00	50.00

ATOM	427	OD2	ASP	76	16.706	-24.969	-32.298	1.00	50.00
ATOM	428	N	ILE	77	13.985	-20.904	-29.848	1.00	50.00
ATOM	429	CA	ILE	77	12.794	-20.184	-29.351	1.00	50.00
ATOM	430	C	ILE	77	12.797	-18.752	-29.909	1.00	50.00
ATOM	431	O	ILE	77	13.694	-17.959	-29.627	1.00	50.00
ATOM	432	CB	ILE	77	12.724	-20.204	-27.809	1.00	50.00
ATOM	433	CG1	ILE	77	12.640	-21.658	-27.307	1.00	50.00
ATOM	434	CG2	ILE	77	11.540	-19.362	-27.293	1.00	50.00
ATOM	435	CD1	ILE	77	12.716	-21.820	-25.783	1.00	50.00
ATOM	436	N	GLN	78	11.703	-18.441	-30.581	1.00	50.00
ATOM	437	CA	GLN	78	11.493	-17.149	-31.263	1.00	50.00
ATOM	438	C	GLN	78	10.377	-16.314	-30.624	1.00	50.00
ATOM	439	O	GLN	78	10.420	-15.085	-30.659	1.00	50.00
ATOM	440	CB	GLN	78	11.189	-17.416	-32.736	1.00	50.00
ATOM	441	CG	GLN	78	12.430	-17.967	-33.444	1.00	50.00
ATOM	442	CD	GLN	78	12.118	-18.663	-34.771	1.00	50.00
ATOM	443	OE1	GLN	78	11.108	-18.458	-35.431	1.00	50.00
ATOM	444	NE2	GLN	78	12.988	-19.575	-35.147	1.00	50.00
ATOM	445	N	GLU	79	9.382	-16.986	-30.049	1.00	50.00
ATOM	446	CA	GLU	79	8.323	-16.304	-29.292	1.00	50.00
ATOM	447	C	GLU	79	7.791	-17.143	-28.129	1.00	50.00
ATOM	448	O	GLU	79	7.747	-18.377	-28.196	1.00	50.00
ATOM	449	CB	GLU	79	7.157	-15.867	-30.184	1.00	50.00
ATOM	450	CG	GLU	79	6.270	-17.000	-30.689	1.00	50.00
ATOM	451	CD	GLU	79	4.889	-16.461	-31.046	1.00	50.00
ATOM	452	OE1	GLU	79	4.046	-16.392	-30.124	1.00	50.00
ATOM	453	OE2	GLU	79	4.686	-16.164	-32.241	1.00	50.00
ATOM	454	N	VAL	80	7.312	-16.410	-27.139	1.00	50.00
ATOM	455	CA	VAL	80	6.612	-16.971	-25.970	1.00	50.00
ATOM	456	C	VAL	80	5.234	-16.299	-25.923	1.00	50.00
ATOM	457	O	VAL	80	5.136	-15.071	-25.882	1.00	50.00
ATOM	458	CB	VAL	80	7.402	-16.688	-24.677	1.00	50.00
ATOM	459	CG1	VAL	80	6.743	-17.354	-23.464	1.00	50.00
ATOM	460	CG2	VAL	80	8.868	-17.131	-24.770	1.00	50.00
ATOM	461	N	GLN	81	4.193	-17.120	-25.887	1.00	50.00
ATOM	462	CA	GLN	81	2.809	-16.600	-25.884	1.00	50.00
ATOM	463	C	GLN	81	2.335	-16.121	-24.505	1.00	50.00
ATOM	464	O	GLN	81	1.593	-15.149	-24.399	1.00	50.00
ATOM	465	CB	GLN	81	1.836	-17.615	-26.481	1.00	50.00
ATOM	466	CG	GLN	81	2.122	-17.812	-27.973	1.00	50.00
ATOM	467	CD	GLN	81	1.099	-18.725	-28.648	1.00	50.00
ATOM	468	OE1	GLN	81	0.555	-19.656	-28.074	1.00	50.00
ATOM	469	NE2	GLN	81	0.827	-18.450	-29.904	1.00	50.00
ATOM	470	N	GLY	82	2.835	-16.783	-23.458	1.00	50.00
ATOM	471	CA	GLY	82	2.549	-16.409	-22.062	1.00	50.00
ATOM	472	C	GLY	82	3.610	-15.432	-21.552	1.00	50.00
ATOM	473	O	GLY	82	3.947	-14.457	-22.230	1.00	50.00
ATOM	474	N	TYR	83	4.140	-15.760	-20.385	1.00	50.00
ATOM	475	CA	TYR	83	5.221	-14.982	-19.754	1.00	50.00
ATOM	476	C	TYR	83	6.536	-15.775	-19.727	1.00	50.00
ATOM	477	O	TYR	83	6.562	-16.998	-19.908	1.00	50.00
ATOM	478	CB	TYR	83	4.815	-14.546	-18.337	1.00	50.00
ATOM	479	CG	TYR	83	4.600	-15.718	-17.376	1.00	50.00
ATOM	480	CD1	TYR	83	5.689	-16.333	-16.767	1.00	50.00
ATOM	481	CD2	TYR	83	3.310	-16.116	-17.061	1.00	50.00
ATOM	482	CE1	TYR	83	5.488	-17.349	-15.845	1.00	50.00
ATOM	483	CE2	TYR	83	3.105	-17.123	-16.131	1.00	50.00
ATOM	484	CZ	TYR	83	4.193	-17.737	-15.529	1.00	50.00
ATOM	485	OH	TYR	83	3.972	-18.718	-14.624	1.00	50.00
ATOM	486	N	VAL	84	7.614	-15.052	-19.469	1.00	50.00
ATOM	487	CA	VAL	84	8.959	-15.624	-19.272	1.00	50.00

ATOM	488	C	VAL	84	9.368	-15.412	-17.808	1.00	50.00
ATOM	489	O	VAL	84	9.449	-14.284	-17.313	1.00	50.00
ATOM	490	CB	VAL	84	9.978	-14.984	-20.235	1.00	50.00
ATOM	491	CG1	VAL	84	11.394	-15.535	-20.032	1.00	50.00
ATOM	492	CG2	VAL	84	9.589	-15.225	-21.694	1.00	50.00
ATOM	493	N	LEU	85	9.652	-16.535	-17.166	1.00	50.00
ATOM	494	CA	LEU	85	10.125	-16.570	-15.774	1.00	50.00
ATOM	495	C	LEU	85	11.520	-17.198	-15.687	1.00	50.00
ATOM	496	O	LEU	85	11.732	-18.342	-16.096	1.00	50.00
ATOM	497	CB	LEU	85	9.124	-17.351	-14.916	1.00	50.00
ATOM	498	CG	LEU	85	9.533	-17.445	-13.440	1.00	50.00
ATOM	499	CD1	LEU	85	9.545	-16.072	-12.762	1.00	50.00
ATOM	500	CD2	LEU	85	8.604	-18.412	-12.710	1.00	50.00
ATOM	501	N	ILE	86	12.443	-16.411	-15.152	1.00	50.00
ATOM	502	CA	ILE	86	13.848	-16.819	-14.942	1.00	50.00
ATOM	503	C	ILE	86	14.209	-16.543	-13.476	1.00	50.00
ATOM	504	O	ILE	86	14.477	-15.398	-13.094	1.00	50.00
ATOM	505	CB	ILE	86	14.773	-16.073	-15.926	1.00	50.00
ATOM	506	CG1	ILE	86	14.373	-16.395	-17.376	1.00	50.00
ATOM	507	CG2	ILE	86	16.250	-16.425	-15.672	1.00	50.00
ATOM	508	CD1	ILE	86	15.043	-15.512	-18.431	1.00	50.00
ATOM	509	N	ALA	87	14.146	-17.589	-12.667	1.00	50.00
ATOM	510	CA	ALA	87	14.328	-17.441	-11.213	1.00	50.00
ATOM	511	C	ALA	87	15.194	-18.521	-10.567	1.00	50.00
ATOM	512	O	ALA	87	15.238	-19.664	-11.030	1.00	50.00
ATOM	513	CB	ALA	87	12.958	-17.410	-10.543	1.00	50.00
ATOM	514	N	HIS	88	15.882	-18.109	-9.506	1.00	50.00
ATOM	515	CA	HIS	88	16.686	-19.002	-8.642	1.00	50.00
ATOM	516	C	HIS	88	17.705	-19.879	-9.388	1.00	50.00
ATOM	517	O	HIS	88	17.999	-21.012	-9.002	1.00	50.00
ATOM	518	CB	HIS	88	15.768	-19.855	-7.758	1.00	50.00
ATOM	519	CG	HIS	88	15.186	-19.037	-6.608	1.00	50.00
ATOM	520	ND1	HIS	88	15.871	-18.614	-5.554	1.00	50.00
ATOM	521	CD2	HIS	88	13.909	-18.709	-6.439	1.00	50.00
ATOM	522	CE1	HIS	88	15.017	-18.030	-4.721	1.00	50.00
ATOM	523	NE2	HIS	88	13.808	-18.071	-5.276	1.00	50.00
ATOM	524	N	ASN	89	18.246	-19.328	-10.467	1.00	50.00
ATOM	525	CA	ASN	89	19.245	-20.035	-11.279	1.00	50.00
ATOM	526	C	ASN	89	20.653	-19.670	-10.814	1.00	50.00
ATOM	527	O	ASN	89	20.937	-18.527	-10.455	1.00	50.00
ATOM	528	CB	ASN	89	19.095	-19.660	-12.754	1.00	50.00
ATOM	529	CG	ASN	89	17.754	-20.111	-13.332	1.00	50.00
ATOM	530	OD1	ASN	89	17.375	-21.275	-13.304	1.00	50.00
ATOM	531	ND2	ASN	89	17.028	-19.168	-13.876	1.00	50.00
ATOM	532	N	GLN	90	21.497	-20.684	-10.819	1.00	50.00
ATOM	533	CA	GLN	90	22.949	-20.503	-10.666	1.00	50.00
ATOM	534	C	GLN	90	23.663	-20.412	-12.017	1.00	50.00
ATOM	535	O	GLN	90	24.805	-19.957	-12.083	1.00	50.00
ATOM	536	CB	GLN	90	23.560	-21.652	-9.881	1.00	50.00
ATOM	537	CG	GLN	90	23.295	-21.553	-8.376	1.00	50.00
ATOM	538	CD	GLN	90	24.533	-21.931	-7.551	1.00	50.00
ATOM	539	OE1	GLN	90	24.715	-21.518	-6.418	1.00	50.00
ATOM	540	NE2	GLN	90	25.438	-22.702	-8.118	1.00	50.00
ATOM	541	N	VAL	91	22.998	-20.890	-13.072	1.00	50.00
ATOM	542	CA	VAL	91	23.530	-20.819	-14.444	1.00	50.00
ATOM	543	C	VAL	91	23.943	-19.368	-14.736	1.00	50.00
ATOM	544	O	VAL	91	23.185	-18.423	-14.499	1.00	50.00
ATOM	545	CB	VAL	91	22.511	-21.370	-15.464	1.00	50.00
ATOM	546	CG1	VAL	91	21.265	-20.496	-15.667	1.00	50.00
ATOM	547	CG2	VAL	91	23.202	-21.669	-16.797	1.00	50.00
ATOM	548	N	ARG	92	25.153	-19.238	-15.246	1.00	50.00

ATOM	549	CA	ARG	92	25.767	-17.909	-15.424	1.00	50.00
ATOM	550	C	ARG	92	25.101	-17.098	-16.543	1.00	50.00
ATOM	551	O	ARG	92	25.020	-15.872	-16.486	1.00	50.00
ATOM	552	CB	ARG	92	27.272	-18.034	-15.657	1.00	50.00
ATOM	553	CG	ARG	92	27.951	-18.803	-14.520	1.00	50.00
ATOM	554	CD	ARG	92	29.467	-18.655	-14.622	1.00	50.00
ATOM	555	NE	ARG	92	30.168	-19.602	-13.735	1.00	50.00
ATOM	556	CZ	ARG	92	30.254	-19.582	-12.400	1.00	50.00
ATOM	557	NH1	ARG	92	29.662	-18.652	-11.661	1.00	50.00
ATOM	558	NH2	ARG	92	30.990	-20.500	-11.788	1.00	50.00
ATOM	559	N	GLN	93	24.631	-17.801	-17.571	1.00	50.00
ATOM	560	CA	GLN	93	23.981	-17.171	-18.732	1.00	50.00
ATOM	561	C	GLN	93	22.773	-17.975	-19.218	1.00	50.00
ATOM	562	O	GLN	93	22.818	-19.204	-19.297	1.00	50.00
ATOM	563	CB	GLN	93	25.003	-17.014	-19.861	1.00	50.00
ATOM	564	CG	GLN	93	26.088	-15.995	-19.489	1.00	50.00
ATOM	565	CD	GLN	93	27.504	-16.453	-19.848	1.00	50.00
ATOM	566	OE1	GLN	93	28.435	-16.364	-19.065	1.00	50.00
ATOM	567	NE2	GLN	93	27.674	-16.973	-21.044	1.00	50.00
ATOM	568	N	VAL	94	21.723	-17.230	-19.543	1.00	50.00
ATOM	569	CA	VAL	94	20.499	-17.768	-20.173	1.00	50.00
ATOM	570	C	VAL	94	20.452	-17.282	-21.636	1.00	50.00
ATOM	571	O	VAL	94	20.254	-16.085	-21.882	1.00	50.00
ATOM	572	CB	VAL	94	19.222	-17.421	-19.385	1.00	50.00
ATOM	573	CG1	VAL	94	17.953	-17.868	-20.121	1.00	50.00
ATOM	574	CG2	VAL	94	19.226	-18.124	-18.025	1.00	50.00
ATOM	575	N	PRO	95	20.796	-18.182	-22.563	1.00	50.00
ATOM	576	CA	PRO	95	20.852	-17.878	-24.002	1.00	50.00
ATOM	577	C	PRO	95	19.499	-18.038	-24.713	1.00	50.00
ATOM	578	O	PRO	95	19.056	-19.144	-25.019	1.00	50.00
ATOM	579	CB	PRO	95	21.940	-18.819	-24.526	1.00	50.00
ATOM	580	CG	PRO	95	21.823	-20.063	-23.647	1.00	50.00
ATOM	581	CD	PRO	95	21.385	-19.512	-22.291	1.00	50.00
ATOM	582	N	LEU	96	18.817	-16.907	-24.851	1.00	50.00
ATOM	583	CA	LEU	96	17.561	-16.802	-25.619	1.00	50.00
ATOM	584	C	LEU	96	17.731	-15.764	-26.736	1.00	50.00
ATOM	585	O	LEU	96	16.871	-14.916	-26.991	1.00	50.00
ATOM	586	CB	LEU	96	16.418	-16.411	-24.676	1.00	50.00
ATOM	587	CG	LEU	96	16.069	-17.480	-23.640	1.00	50.00
ATOM	588	CD1	LEU	96	15.133	-16.872	-22.595	1.00	50.00
ATOM	589	CD2	LEU	96	15.393	-18.680	-24.308	1.00	50.00
ATOM	590	N	GLN	97	18.837	-15.909	-27.459	1.00	50.00
ATOM	591	CA	GLN	97	19.265	-14.940	-28.486	1.00	50.00
ATOM	592	C	GLN	97	18.276	-14.797	-29.651	1.00	50.00
ATOM	593	O	GLN	97	18.323	-13.803	-30.355	1.00	50.00
ATOM	594	CB	GLN	97	20.646	-15.261	-29.069	1.00	50.00
ATOM	595	CG	GLN	97	21.755	-15.304	-28.015	1.00	50.00
ATOM	596	CD	GLN	97	21.996	-16.724	-27.496	1.00	50.00
ATOM	597	OE1	GLN	97	21.113	-17.562	-27.363	1.00	50.00
ATOM	598	NE2	GLN	97	23.231	-16.989	-27.140	1.00	50.00
ATOM	599	N	ARG	98	17.395	-15.779	-29.822	1.00	50.00
ATOM	600	CA	ARG	98	16.399	-15.779	-30.908	1.00	50.00
ATOM	601	C	ARG	98	15.000	-15.301	-30.503	1.00	50.00
ATOM	602	O	ARG	98	14.176	-15.011	-31.370	1.00	50.00
ATOM	603	CB	ARG	98	16.323	-17.167	-31.536	1.00	50.00
ATOM	604	CG	ARG	98	17.085	-17.158	-32.857	1.00	50.00
ATOM	605	CD	ARG	98	16.982	-18.515	-33.556	1.00	50.00
ATOM	606	NE	ARG	98	17.237	-18.352	-35.000	1.00	50.00
ATOM	607	CZ	ARG	98	16.407	-17.780	-35.879	1.00	50.00
ATOM	608	NH1	ARG	98	15.226	-17.305	-35.502	1.00	50.00
ATOM	609	NH2	ARG	98	16.758	-17.655	-37.151	1.00	50.00

```
ATOM    610  N   LEU    99      14.769 -15.186 -29.195  1.00 50.00
ATOM    611  CA  LEU    99      13.489 -14.701 -28.662  1.00 50.00
ATOM    612  C   LEU    99      13.233 -13.264 -29.127  1.00 50.00
ATOM    613  O   LEU    99      13.917 -12.329 -28.714  1.00 50.00
ATOM    614  CB  LEU    99      13.465 -14.804 -27.131  1.00 50.00
ATOM    615  CG  LEU    99      12.132 -14.331 -26.534  1.00 50.00
ATOM    616  CD1 LEU    99      10.951 -15.149 -27.064  1.00 50.00
ATOM    617  CD2 LEU    99      12.181 -14.413 -25.009  1.00 50.00
ATOM    618  N   ARG   100      12.211 -13.136 -29.955  1.00 50.00
ATOM    619  CA  ARG   100      11.853 -11.857 -30.594  1.00 50.00
ATOM    620  C   ARG   100      10.639 -11.176 -29.942  1.00 50.00
ATOM    621  O   ARG   100      10.581  -9.941 -29.868  1.00 50.00
ATOM    622  CB  ARG   100      11.635 -12.145 -32.081  1.00 50.00
ATOM    623  CG  ARG   100      11.138 -10.943 -32.895  1.00 50.00
ATOM    624  CD  ARG   100      11.095 -11.260 -34.393  1.00 50.00
ATOM    625  NE  ARG   100      10.328 -12.496 -34.652  1.00 50.00
ATOM    626  CZ  ARG   100       9.002 -12.625 -34.699  1.00 50.00
ATOM    627  NH1 ARG   100       8.198 -11.581 -34.539  1.00 50.00
ATOM    628  NH2 ARG   100       8.465 -13.826 -34.862  1.00 50.00
ATOM    629  N   ILE   101       9.718 -11.988 -29.432  1.00 50.00
ATOM    630  CA  ILE   101       8.439 -11.490 -28.891  1.00 50.00
ATOM    631  C   ILE   101       7.891 -12.349 -27.743  1.00 50.00
ATOM    632  O   ILE   101       7.857 -13.581 -27.800  1.00 50.00
ATOM    633  CB  ILE   101       7.411 -11.301 -30.035  1.00 50.00
ATOM    634  CG1 ILE   101       6.062 -10.776 -29.507  1.00 50.00
ATOM    635  CG2 ILE   101       7.273 -12.559 -30.908  1.00 50.00
ATOM    636  CD1 ILE   101       5.006 -10.475 -30.578  1.00 50.00
ATOM    637  N   VAL   102       7.419 -11.627 -26.738  1.00 50.00
ATOM    638  CA  VAL   102       6.634 -12.184 -25.620  1.00 50.00
ATOM    639  C   VAL   102       5.252 -11.515 -25.683  1.00 50.00
ATOM    640  O   VAL   102       5.142 -10.285 -25.654  1.00 50.00
ATOM    641  CB  VAL   102       7.337 -11.904 -24.278  1.00 50.00
ATOM    642  CG1 VAL   102       6.561 -12.497 -23.096  1.00 50.00
ATOM    643  CG2 VAL   102       8.764 -12.462 -24.257  1.00 50.00
ATOM    644  N   ARG   103       4.216 -12.341 -25.739  1.00 50.00
ATOM    645  CA  ARG   103       2.837 -11.845 -25.925  1.00 50.00
ATOM    646  C   ARG   103       2.144 -11.413 -24.629  1.00 50.00
ATOM    647  O   ARG   103       1.355 -10.471 -24.643  1.00 50.00
ATOM    648  CB  ARG   103       1.956 -12.861 -26.654  1.00 50.00
ATOM    649  CG  ARG   103       2.418 -13.103 -28.090  1.00 50.00
ATOM    650  CD  ARG   103       1.392 -13.973 -28.817  1.00 50.00
ATOM    651  NE  ARG   103       1.845 -14.265 -30.188  1.00 50.00
ATOM    652  CZ  ARG   103       1.786 -13.457 -31.251  1.00 50.00
ATOM    653  NH1 ARG   103       1.270 -12.236 -31.179  1.00 50.00
ATOM    654  NH2 ARG   103       2.250 -13.878 -32.419  1.00 50.00
ATOM    655  N   GLY   104       2.491 -12.097 -23.528  1.00 50.00
ATOM    656  CA  GLY   104       1.848 -11.860 -22.225  1.00 50.00
ATOM    657  C   GLY   104       0.349 -12.191 -22.262  1.00 50.00
ATOM    658  O   GLY   104      -0.453 -11.487 -21.651  1.00 50.00
ATOM    659  N   THR   105      -0.001 -13.272 -22.964  1.00 50.00
ATOM    660  CA  THR   105      -1.384 -13.811 -22.975  1.00 50.00
ATOM    661  C   THR   105      -1.794 -14.156 -21.532  1.00 50.00
ATOM    662  O   THR   105      -2.958 -14.071 -21.148  1.00 50.00
ATOM    663  CB  THR   105      -1.484 -15.040 -23.901  1.00 50.00
ATOM    664  OG1 THR   105      -1.198 -14.627 -25.240  1.00 50.00
ATOM    665  CG2 THR   105      -2.843 -15.752 -23.865  1.00 50.00
ATOM    666  N   GLN   106      -0.817 -14.673 -20.801  1.00 50.00
ATOM    667  CA  GLN   106      -0.883 -14.844 -19.344  1.00 50.00
ATOM    668  C   GLN   106       0.351 -14.214 -18.694  1.00 50.00
ATOM    669  O   GLN   106       1.428 -14.157 -19.293  1.00 50.00
ATOM    670  CB  GLN   106      -1.102 -16.313 -18.958  1.00 50.00
```

ATOM	671	CG	GLN	106	-0.371	-17.323	-19.848	1.00	50.00
ATOM	672	CD	GLN	106	-0.712	-18.755	-19.445	1.00	50.00
ATOM	673	OE1	GLN	106	-0.678	-19.141	-18.285	1.00	50.00
ATOM	674	NE2	GLN	106	-0.987	-19.585	-20.428	1.00	50.00
ATOM	675	N	LEU	107	0.144	-13.730	-17.479	1.00	50.00
ATOM	676	CA	LEU	107	1.133	-12.895	-16.778	1.00	50.00
ATOM	677	C	LEU	107	1.578	-13.503	-15.450	1.00	50.00
ATOM	678	O	LEU	107	0.837	-14.239	-14.792	1.00	50.00
ATOM	679	CB	LEU	107	0.560	-11.487	-16.556	1.00	50.00
ATOM	680	CG	LEU	107	0.246	-10.756	-17.869	1.00	50.00
ATOM	681	CD1	LEU	107	-0.442	-9.425	-17.576	1.00	50.00
ATOM	682	CD2	LEU	107	1.510	-10.513	-18.697	1.00	50.00
ATOM	683	N	PHE	108	2.769	-13.102	-15.042	1.00	50.00
ATOM	684	CA	PHE	108	3.363	-13.505	-13.761	1.00	50.00
ATOM	685	C	PHE	108	3.072	-12.422	-12.722	1.00	50.00
ATOM	686	O	PHE	108	3.263	-11.226	-12.966	1.00	50.00
ATOM	687	CB	PHE	108	4.869	-13.698	-13.935	1.00	50.00
ATOM	688	CG	PHE	108	5.470	-14.329	-12.679	1.00	50.00
ATOM	689	CD1	PHE	108	5.308	-15.690	-12.459	1.00	50.00
ATOM	690	CD2	PHE	108	6.179	-13.549	-11.774	1.00	50.00
ATOM	691	CE1	PHE	108	5.859	-16.275	-11.327	1.00	50.00
ATOM	692	CE2	PHE	108	6.731	-14.138	-10.644	1.00	50.00
ATOM	693	CZ	PHE	108	6.573	-15.501	-10.420	1.00	50.00
ATOM	694	N	GLU	109	2.451	-12.873	-11.635	1.00	50.00
ATOM	695	CA	GLU	109	1.932	-11.999	-10.560	1.00	50.00
ATOM	696	C	GLU	109	0.944	-10.954	-11.105	1.00	50.00
ATOM	697	O	GLU	109	0.897	-9.804	-10.673	1.00	50.00
ATOM	698	CB	GLU	109	3.068	-11.326	-9.782	1.00	50.00
ATOM	699	CG	GLU	109	3.976	-12.365	-9.123	1.00	50.00
ATOM	700	CD	GLU	109	5.005	-11.756	-8.167	1.00	50.00
ATOM	701	OE1	GLU	109	5.271	-10.536	-8.260	1.00	50.00
ATOM	702	OE2	GLU	109	5.509	-12.550	-7.347	1.00	50.00
ATOM	703	N	ASP	110	0.219	-11.383	-12.145	1.00	50.00
ATOM	704	CA	ASP	110	-0.918	-10.660	-12.754	1.00	50.00
ATOM	705	C	ASP	110	-0.526	-9.454	-13.631	1.00	50.00
ATOM	706	O	ASP	110	-1.358	-8.965	-14.385	1.00	50.00
ATOM	707	CB	ASP	110	-1.929	-10.196	-11.686	1.00	50.00
ATOM	708	CG	ASP	110	-2.183	-11.265	-10.615	1.00	50.00
ATOM	709	OD1	ASP	110	-2.912	-12.226	-10.937	1.00	50.00
ATOM	710	OD2	ASP	110	-1.556	-11.164	-9.535	1.00	50.00
ATOM	711	N	ASN	111	0.732	-9.022	-13.549	1.00	50.00
ATOM	712	CA	ASN	111	1.183	-7.794	-14.235	1.00	50.00
ATOM	713	C	ASN	111	2.405	-7.914	-15.154	1.00	50.00
ATOM	714	O	ASN	111	2.664	-7.024	-15.963	1.00	50.00
ATOM	715	CB	ASN	111	1.412	-6.680	-13.208	1.00	50.00
ATOM	716	CG	ASN	111	0.099	-6.149	-12.623	1.00	50.00
ATOM	717	OD1	ASN	111	-0.942	-6.073	-13.256	1.00	50.00
ATOM	718	ND2	ASN	111	0.153	-5.739	-11.375	1.00	50.00
ATOM	719	N	TYR	112	3.170	-8.994	-15.018	1.00	50.00
ATOM	720	CA	TYR	112	4.471	-9.077	-15.702	1.00	50.00
ATOM	721	C	TYR	112	4.559	-10.177	-16.757	1.00	50.00
ATOM	722	O	TYR	112	4.204	-11.330	-16.512	1.00	50.00
ATOM	723	CB	TYR	112	5.592	-9.225	-14.668	1.00	50.00
ATOM	724	CG	TYR	112	5.616	-8.012	-13.734	1.00	50.00
ATOM	725	CD1	TYR	112	6.191	-6.826	-14.167	1.00	50.00
ATOM	726	CD2	TYR	112	5.045	-8.086	-12.469	1.00	50.00
ATOM	727	CE1	TYR	112	6.202	-5.710	-13.345	1.00	50.00
ATOM	728	CE2	TYR	112	5.056	-6.972	-11.640	1.00	50.00
ATOM	729	CZ	TYR	112	5.632	-5.786	-12.082	1.00	50.00
ATOM	730	OH	TYR	112	5.624	-4.686	-11.291	1.00	50.00
ATOM	731	N	ALA	113	5.113	-9.781	-17.893	1.00	50.00

```
ATOM    732  CA  ALA   113       5.409 -10.693 -19.015  1.00 50.00
ATOM    733  C   ALA   113       6.828 -11.270 -18.909  1.00 50.00
ATOM    734  O   ALA   113       7.101 -12.374 -19.375  1.00 50.00
ATOM    735  CB  ALA   113       5.265  -9.940 -20.335  1.00 50.00
ATOM    736  N   LEU   114       7.730 -10.476 -18.338  1.00 50.00
ATOM    737  CA  LEU   114       9.113 -10.894 -18.069  1.00 50.00
ATOM    738  C   LEU   114       9.466 -10.667 -16.596  1.00 50.00
ATOM    739  O   LEU   114       9.382  -9.544 -16.095  1.00 50.00
ATOM    740  CB  LEU   114      10.074 -10.134 -18.994  1.00 50.00
ATOM    741  CG  LEU   114      11.551 -10.460 -18.729  1.00 50.00
ATOM    742  CD1 LEU   114      11.865 -11.943 -18.944  1.00 50.00
ATOM    743  CD2 LEU   114      12.447  -9.596 -19.615  1.00 50.00
ATOM    744  N   ALA   115       9.867 -11.750 -15.943  1.00 50.00
ATOM    745  CA  ALA   115      10.285 -11.728 -14.529  1.00 50.00
ATOM    746  C   ALA   115      11.593 -12.496 -14.303  1.00 50.00
ATOM    747  O   ALA   115      11.691 -13.699 -14.571  1.00 50.00
ATOM    748  CB  ALA   115       9.165 -12.287 -13.648  1.00 50.00
ATOM    749  N   VAL   116      12.598 -11.758 -13.856  1.00 50.00
ATOM    750  CA  VAL   116      13.949 -12.281 -13.565  1.00 50.00
ATOM    751  C   VAL   116      14.216 -12.067 -12.069  1.00 50.00
ATOM    752  O   VAL   116      14.442 -10.938 -11.617  1.00 50.00
ATOM    753  CB  VAL   116      15.018 -11.572 -14.416  1.00 50.00
ATOM    754  CG1 VAL   116      16.381 -12.254 -14.266  1.00 50.00
ATOM    755  CG2 VAL   116      14.638 -11.516 -15.898  1.00 50.00
ATOM    756  N   LEU   117      14.182 -13.164 -11.328  1.00 50.00
ATOM    757  CA  LEU   117      14.138 -13.106  -9.854  1.00 50.00
ATOM    758  C   LEU   117      15.166 -13.996  -9.161  1.00 50.00
ATOM    759  O   LEU   117      15.322 -15.168  -9.506  1.00 50.00
ATOM    760  CB  LEU   117      12.741 -13.523  -9.380  1.00 50.00
ATOM    761  CG  LEU   117      11.621 -12.655  -9.964  1.00 50.00
ATOM    762  CD1 LEU   117      10.265 -13.272  -9.631  1.00 50.00
ATOM    763  CD2 LEU   117      11.692 -11.222  -9.431  1.00 50.00
ATOM    764  N   ASP   118      15.811 -13.423  -8.150  1.00 50.00
ATOM    765  CA  ASP   118      16.640 -14.171  -7.174  1.00 50.00
ATOM    766  C   ASP   118      17.595 -15.200  -7.814  1.00 50.00
ATOM    767  O   ASP   118      17.620 -16.377  -7.441  1.00 50.00
ATOM    768  CB  ASP   118      15.727 -14.880  -6.164  1.00 50.00
ATOM    769  CG  ASP   118      14.817 -13.929  -5.404  1.00 50.00
ATOM    770  OD1 ASP   118      15.301 -13.397  -4.385  1.00 50.00
ATOM    771  OD2 ASP   118      13.690 -13.710  -5.897  1.00 50.00
ATOM    772  N   ASN   119      18.346 -14.763  -8.818  1.00 50.00
ATOM    773  CA  ASN   119      19.199 -15.686  -9.592  1.00 50.00
ATOM    774  C   ASN   119      20.649 -15.768  -9.097  1.00 50.00
ATOM    775  O   ASN   119      21.549 -15.127  -9.647  1.00 50.00
ATOM    776  CB  ASN   119      19.168 -15.355 -11.087  1.00 50.00
ATOM    777  CG  ASN   119      17.829 -15.667 -11.745  1.00 50.00
ATOM    778  OD1 ASN   119      17.537 -16.786 -12.148  1.00 50.00
ATOM    779  ND2 ASN   119      17.004 -14.661 -11.872  1.00 50.00
ATOM    780  N   GLY   120      20.806 -16.484  -7.979  1.00 50.00
ATOM    781  CA  GLY   120      22.132 -16.942  -7.527  1.00 50.00
ATOM    782  C   GLY   120      22.693 -16.214  -6.304  1.00 50.00
ATOM    783  O   GLY   120      21.963 -15.680  -5.465  1.00 50.00
ATOM    784  N   ASP   121      24.017 -16.225  -6.263  1.00 50.00
ATOM    785  CA  ASP   121      24.827 -15.717  -5.146  1.00 50.00
ATOM    786  C   ASP   121      25.739 -14.559  -5.582  1.00 50.00
ATOM    787  O   ASP   121      26.051 -14.443  -6.768  1.00 50.00
ATOM    788  CB  ASP   121      25.682 -16.853  -4.576  1.00 50.00
ATOM    789  CG  ASP   121      24.824 -17.962  -3.968  1.00 50.00
ATOM    790  OD1 ASP   121      24.021 -17.645  -3.061  1.00 50.00
ATOM    791  OD2 ASP   121      25.004 -19.109  -4.425  1.00 50.00
ATOM    792  N   PRO   122      26.083 -13.673  -4.639  1.00 50.00
```

ATOM	793	CA	PRO	122	27.005	-12.547	-4.879	1.00	50.00
ATOM	794	C	PRO	122	28.371	-13.059	-5.347	1.00	50.00
ATOM	795	O	PRO	122	28.835	-14.116	-4.910	1.00	50.00
ATOM	796	CB	PRO	122	27.152	-11.873	-3.515	1.00	50.00
ATOM	797	CG	PRO	122	25.827	-12.171	-2.821	1.00	50.00
ATOM	798	CD	PRO	122	25.517	-13.593	-3.275	1.00	50.00
ATOM	799	N	LEU	123	28.994	-12.264	-6.204	1.00	50.00
ATOM	800	CA	LEU	123	30.363	-12.509	-6.692	1.00	50.00
ATOM	801	C	LEU	123	31.011	-11.172	-7.057	1.00	50.00
ATOM	802	O	LEU	123	30.339	-10.260	-7.541	1.00	50.00
ATOM	803	CB	LEU	123	30.321	-13.436	-7.915	1.00	50.00
ATOM	804	CG	LEU	123	31.702	-13.828	-8.456	1.00	50.00
ATOM	805	CD1	LEU	123	32.503	-14.629	-7.426	1.00	50.00
ATOM	806	CD2	LEU	123	31.553	-14.622	-9.753	1.00	50.00
ATOM	807	N	ASN	124	32.291	-11.051	-6.724	1.00	50.00
ATOM	808	CA	ASN	124	33.059	-9.839	-7.066	1.00	50.00
ATOM	809	C	ASN	124	33.361	-9.758	-8.576	1.00	50.00
ATOM	810	O	ASN	124	33.007	-10.646	-9.349	1.00	50.00
ATOM	811	CB	ASN	124	34.327	-9.753	-6.205	1.00	50.00
ATOM	812	CG	ASN	124	35.289	-10.932	-6.371	1.00	50.00
ATOM	813	OD1	ASN	124	35.276	-11.688	-7.331	1.00	50.00
ATOM	814	ND2	ASN	124	36.134	-11.109	-5.384	1.00	50.00
ATOM	815	N	ASN	125	34.166	-8.761	-8.929	1.00	50.00
ATOM	816	CA	ASN	125	34.543	-8.452	-10.323	1.00	50.00
ATOM	817	C	ASN	125	35.285	-9.566	-11.074	1.00	50.00
ATOM	818	O	ASN	125	35.390	-9.512	-12.299	1.00	50.00
ATOM	819	CB	ASN	125	35.379	-7.171	-10.356	1.00	50.00
ATOM	820	CG	ASN	125	34.571	-5.922	-9.987	1.00	50.00
ATOM	821	OD1	ASN	125	33.411	-5.939	-9.600	1.00	50.00
ATOM	822	ND2	ASN	125	35.221	-4.786	-10.073	1.00	50.00
ATOM	823	N	THR	126	35.865	-10.513	-10.337	1.00	50.00
ATOM	824	CA	THR	126	36.632	-11.619	-10.940	1.00	50.00
ATOM	825	C	THR	126	35.735	-12.403	-11.906	1.00	50.00
ATOM	826	O	THR	126	34.634	-12.835	-11.564	1.00	50.00
ATOM	827	CB	THR	126	37.170	-12.570	-9.858	1.00	50.00
ATOM	828	OG1	THR	126	37.914	-11.819	-8.894	1.00	50.00
ATOM	829	CG2	THR	126	38.085	-13.660	-10.433	1.00	50.00
ATOM	830	N	THR	127	36.310	-12.661	-13.073	1.00	50.00
ATOM	831	CA	THR	127	35.725	-13.592	-14.055	1.00	50.00
ATOM	832	C	THR	127	35.658	-15.001	-13.440	1.00	50.00
ATOM	833	O	THR	127	36.674	-15.510	-12.947	1.00	50.00
ATOM	834	CB	THR	127	36.475	-13.594	-15.398	1.00	50.00
ATOM	835	OG1	THR	127	35.893	-14.588	-16.246	1.00	50.00
ATOM	836	CG2	THR	127	37.992	-13.790	-15.271	1.00	50.00
ATOM	837	N	PRO	128	34.460	-15.586	-13.413	1.00	50.00
ATOM	838	CA	PRO	128	34.198	-16.849	-12.705	1.00	50.00
ATOM	839	C	PRO	128	35.087	-18.012	-13.160	1.00	50.00
ATOM	840	O	PRO	128	35.430	-18.161	-14.332	1.00	50.00
ATOM	841	CB	PRO	128	32.716	-17.129	-12.953	1.00	50.00
ATOM	842	CG	PRO	128	32.428	-16.429	-14.279	1.00	50.00
ATOM	843	CD	PRO	128	33.271	-15.162	-14.180	1.00	50.00
ATOM	844	N	VAL	129	35.462	-18.816	-12.176	1.00	50.00
ATOM	845	CA	VAL	129	36.192	-20.078	-12.386	1.00	50.00
ATOM	846	C	VAL	129	35.097	-21.151	-12.330	1.00	50.00
ATOM	847	O	VAL	129	34.431	-21.285	-11.296	1.00	50.00
ATOM	848	CB	VAL	129	37.240	-20.285	-11.273	1.00	50.00
ATOM	849	CG1	VAL	129	38.077	-21.543	-11.528	1.00	50.00
ATOM	850	CG2	VAL	129	38.178	-19.081	-11.123	1.00	50.00
ATOM	851	N	THR	130	35.073	-22.000	-13.354	1.00	50.00
ATOM	852	CA	THR	130	33.963	-22.954	-13.612	1.00	50.00
ATOM	853	C	THR	130	33.513	-23.791	-12.400	1.00	50.00

ATOM	854	O	THR	130	32.332	-24.124	-12.295	1.00	50.00
ATOM	855	CB	THR	130	34.285	-23.830	-14.845	1.00	50.00
ATOM	856	OG1	THR	130	34.023	-23.052	-16.014	1.00	50.00
ATOM	857	CG2	THR	130	33.580	-25.192	-14.958	1.00	50.00
ATOM	858	N	GLY	131	34.467	-24.149	-11.535	1.00	50.00
ATOM	859	CA	GLY	131	34.166	-25.052	-10.407	1.00	50.00
ATOM	860	C	GLY	131	34.374	-24.458	-9.009	1.00	50.00
ATOM	861	O	GLY	131	34.300	-25.194	-8.030	1.00	50.00
ATOM	862	N	ALA	132	34.573	-23.144	-8.928	1.00	50.00
ATOM	863	CA	ALA	132	34.881	-22.486	-7.642	1.00	50.00
ATOM	864	C	ALA	132	33.983	-21.281	-7.346	1.00	50.00
ATOM	865	O	ALA	132	33.458	-21.141	-6.243	1.00	50.00
ATOM	866	CB	ALA	132	36.352	-22.059	-7.619	1.00	50.00
ATOM	867	N	SER	133	33.776	-20.463	-8.376	1.00	50.00
ATOM	868	CA	SER	133	33.009	-19.216	-8.253	1.00	50.00
ATOM	869	C	SER	133	31.522	-19.480	-7.985	1.00	50.00
ATOM	870	O	SER	133	30.921	-20.323	-8.663	1.00	50.00
ATOM	871	CB	SER	133	33.150	-18.345	-9.499	1.00	50.00
ATOM	872	OG	SER	133	34.522	-17.982	-9.682	1.00	50.00
ATOM	873	N	PRO	134	30.962	-18.759	-7.005	1.00	50.00
ATOM	874	CA	PRO	134	29.525	-18.798	-6.676	1.00	50.00
ATOM	875	C	PRO	134	28.695	-18.534	-7.937	1.00	50.00
ATOM	876	O	PRO	134	29.070	-17.717	-8.776	1.00	50.00
ATOM	877	CB	PRO	134	29.343	-17.661	-5.671	1.00	50.00
ATOM	878	CG	PRO	134	30.674	-17.641	-4.924	1.00	50.00
ATOM	879	CD	PRO	134	31.693	-17.931	-6.023	1.00	50.00
ATOM	880	N	GLY	135	27.694	-19.405	-8.113	1.00	50.00
ATOM	881	CA	GLY	135	26.781	-19.352	-9.269	1.00	50.00
ATOM	882	C	GLY	135	25.825	-18.156	-9.174	1.00	50.00
ATOM	883	O	GLY	135	25.506	-17.684	-8.081	1.00	50.00
ATOM	884	N	GLY	136	25.328	-17.760	-10.343	1.00	50.00
ATOM	885	CA	GLY	136	24.374	-16.646	-10.472	1.00	50.00
ATOM	886	C	GLY	136	24.289	-16.187	-11.926	1.00	50.00
ATOM	887	O	GLY	136	25.258	-16.286	-12.681	1.00	50.00
ATOM	888	N	LEU	137	23.110	-15.702	-12.291	1.00	50.00
ATOM	889	CA	LEU	137	22.888	-15.149	-13.638	1.00	50.00
ATOM	890	C	LEU	137	23.677	-13.844	-13.790	1.00	50.00
ATOM	891	O	LEU	137	23.516	-12.920	-12.992	1.00	50.00
ATOM	892	CB	LEU	137	21.392	-14.913	-13.867	1.00	50.00
ATOM	893	CG	LEU	137	21.056	-14.502	-15.306	1.00	50.00
ATOM	894	CD1	LEU	137	21.405	-15.614	-16.299	1.00	50.00
ATOM	895	CD2	LEU	137	19.575	-14.143	-15.408	1.00	50.00
ATOM	896	N	ARG	138	24.453	-13.779	-14.859	1.00	50.00
ATOM	897	CA	ARG	138	25.326	-12.623	-15.146	1.00	50.00
ATOM	898	C	ARG	138	24.763	-11.763	-16.281	1.00	50.00
ATOM	899	O	ARG	138	24.837	-10.536	-16.239	1.00	50.00
ATOM	900	CB	ARG	138	26.734	-13.099	-15.510	1.00	50.00
ATOM	901	CG	ARG	138	27.394	-13.847	-14.348	1.00	50.00
ATOM	902	CD	ARG	138	28.726	-14.478	-14.761	1.00	50.00
ATOM	903	NE	ARG	138	29.760	-13.457	-15.022	1.00	50.00
ATOM	904	CZ	ARG	138	30.441	-12.760	-14.109	1.00	50.00
ATOM	905	NH1	ARG	138	30.238	-12.933	-12.810	1.00	50.00
ATOM	906	NH2	ARG	138	31.399	-11.927	-14.487	1.00	50.00
ATOM	907	N	GLU	139	24.132	-12.423	-17.251	1.00	50.00
ATOM	908	CA	GLU	139	23.514	-11.738	-18.400	1.00	50.00
ATOM	909	C	GLU	139	22.403	-12.562	-19.055	1.00	50.00
ATOM	910	O	GLU	139	22.529	-13.770	-19.290	1.00	50.00
ATOM	911	CB	GLU	139	24.584	-11.321	-19.418	1.00	50.00
ATOM	912	CG	GLU	139	25.467	-12.486	-19.875	1.00	50.00
ATOM	913	CD	GLU	139	26.627	-12.032	-20.758	1.00	50.00
ATOM	914	OE1	GLU	139	27.329	-11.067	-20.374	1.00	50.00

ATOM	915	OE2	GLU	139	26.811	-12.684	-21.806	1.00	50.00
ATOM	916	N	LEU	140	21.314	-11.855	-19.309	1.00	50.00
ATOM	917	CA	LEU	140	20.137	-12.420	-19.980	1.00	50.00
ATOM	918	C	LEU	140	20.216	-12.050	-21.468	1.00	50.00
ATOM	919	O	LEU	140	19.888	-10.939	-21.882	1.00	50.00
ATOM	920	CB	LEU	140	18.864	-11.839	-19.356	1.00	50.00
ATOM	921	CG	LEU	140	17.683	-12.819	-19.311	1.00	50.00
ATOM	922	CD1	LEU	140	16.437	-12.040	-18.904	1.00	50.00
ATOM	923	CD2	LEU	140	17.427	-13.584	-20.615	1.00	50.00
ATOM	924	N	GLN	141	20.714	-13.005	-22.241	1.00	50.00
ATOM	925	CA	GLN	141	21.009	-12.806	-23.674	1.00	50.00
ATOM	926	C	GLN	141	19.741	-12.817	-24.540	1.00	50.00
ATOM	927	O	GLN	141	19.260	-13.879	-24.944	1.00	50.00
ATOM	928	CB	GLN	141	22.014	-13.860	-24.140	1.00	50.00
ATOM	929	CG	GLN	141	23.323	-13.777	-23.347	1.00	50.00
ATOM	930	CD	GLN	141	24.328	-14.848	-23.772	1.00	50.00
ATOM	931	OE1	GLN	141	25.483	-14.580	-24.051	1.00	50.00
ATOM	932	NE2	GLN	141	23.897	-16.092	-23.830	1.00	50.00
ATOM	933	N	LEU	142	19.221	-11.616	-24.767	1.00	50.00
ATOM	934	CA	LEU	142	17.980	-11.384	-25.534	1.00	50.00
ATOM	935	C	LEU	142	18.206	-10.401	-26.693	1.00	50.00
ATOM	936	O	LEU	142	17.441	-9.455	-26.892	1.00	50.00
ATOM	937	CB	LEU	142	16.901	-10.830	-24.593	1.00	50.00
ATOM	938	CG	LEU	142	16.420	-11.795	-23.514	1.00	50.00
ATOM	939	CD1	LEU	142	15.534	-11.029	-22.533	1.00	50.00
ATOM	940	CD2	LEU	142	15.625	-12.947	-24.128	1.00	50.00
ATOM	941	N	ARG	143	19.197	-10.699	-27.531	1.00	50.00
ATOM	942	CA	ARG	143	19.581	-9.787	-28.631	1.00	50.00
ATOM	943	C	ARG	143	18.420	-9.450	-29.591	1.00	50.00
ATOM	944	O	ARG	143	18.297	-8.311	-30.023	1.00	50.00
ATOM	945	CB	ARG	143	20.775	-10.319	-29.437	1.00	50.00
ATOM	946	CG	ARG	143	20.429	-11.591	-30.216	1.00	50.00
ATOM	947	CD	ARG	143	21.018	-11.590	-31.620	1.00	50.00
ATOM	948	NE	ARG	143	22.391	-12.120	-31.605	1.00	50.00
ATOM	949	CZ	ARG	143	23.200	-12.156	-32.663	1.00	50.00
ATOM	950	NH1	ARG	143	22.826	-11.624	-33.820	1.00	50.00
ATOM	951	NH2	ARG	143	24.344	-12.824	-32.619	1.00	50.00
ATOM	952	N	SER	144	17.549	-10.436	-29.824	1.00	50.00
ATOM	953	CA	SER	144	16.445	-10.328	-30.800	1.00	50.00
ATOM	954	C	SER	144	15.137	-9.762	-30.241	1.00	50.00
ATOM	955	O	SER	144	14.221	-9.461	-31.005	1.00	50.00
ATOM	956	CB	SER	144	16.165	-11.686	-31.446	1.00	50.00
ATOM	957	OG	SER	144	17.302	-12.072	-32.222	1.00	50.00
ATOM	958	N	LEU	145	15.065	-9.613	-28.918	1.00	50.00
ATOM	959	CA	LEU	145	13.853	-9.129	-28.239	1.00	50.00
ATOM	960	C	LEU	145	13.508	-7.694	-28.656	1.00	50.00
ATOM	961	O	LEU	145	14.251	-6.750	-28.385	1.00	50.00
ATOM	962	CB	LEU	145	14.014	-9.227	-26.719	1.00	50.00
ATOM	963	CG	LEU	145	12.724	-8.859	-25.973	1.00	50.00
ATOM	964	CD1	LEU	145	11.593	-9.851	-26.269	1.00	50.00
ATOM	965	CD2	LEU	145	12.995	-8.768	-24.473	1.00	50.00
ATOM	966	N	THR	146	12.361	-7.589	-29.310	1.00	50.00
ATOM	967	CA	THR	146	11.867	-6.294	-29.828	1.00	50.00
ATOM	968	C	THR	146	10.448	-5.945	-29.363	1.00	50.00
ATOM	969	O	THR	146	10.059	-4.781	-29.380	1.00	50.00
ATOM	970	CB	THR	146	11.936	-6.216	-31.363	1.00	50.00
ATOM	971	OG1	THR	146	11.109	-7.223	-31.955	1.00	50.00
ATOM	972	CG2	THR	146	13.368	-6.336	-31.890	1.00	50.00
ATOM	973	N	GLU	147	9.655	-6.975	-29.053	1.00	50.00
ATOM	974	CA	GLU	147	8.243	-6.778	-28.681	1.00	50.00
ATOM	975	C	GLU	147	7.801	-7.528	-27.422	1.00	50.00

ATOM	976	O	GLU	147	8.029	-8.731	-27.264	1.00	50.00
ATOM	977	CB	GLU	147	7.297	-7.160	-29.823	1.00	50.00
ATOM	978	CG	GLU	147	7.467	-6.289	-31.071	1.00	50.00
ATOM	979	CD	GLU	147	6.253	-6.422	-31.991	1.00	50.00
ATOM	980	OE1	GLU	147	5.980	-7.559	-32.434	1.00	50.00
ATOM	981	OE2	GLU	147	5.542	-5.400	-32.128	1.00	50.00
ATOM	982	N	ILE	148	7.153	-6.760	-26.559	1.00	50.00
ATOM	983	CA	ILE	148	6.371	-7.268	-25.415	1.00	50.00
ATOM	984	C	ILE	148	4.967	-6.670	-25.555	1.00	50.00
ATOM	985	O	ILE	148	4.770	-5.471	-25.325	1.00	50.00
ATOM	986	CB	ILE	148	7.006	-6.917	-24.053	1.00	50.00
ATOM	987	CG1	ILE	148	8.392	-7.571	-23.919	1.00	50.00
ATOM	988	CG2	ILE	148	6.081	-7.347	-22.899	1.00	50.00
ATOM	989	CD1	ILE	148	9.177	-7.174	-22.659	1.00	50.00
ATOM	990	N	LEU	149	4.036	-7.521	-25.963	1.00	50.00
ATOM	991	CA	LEU	149	2.661	-7.078	-26.252	1.00	50.00
ATOM	992	C	LEU	149	1.905	-6.591	-25.015	1.00	50.00
ATOM	993	O	LEU	149	1.350	-5.494	-25.032	1.00	50.00
ATOM	994	CB	LEU	149	1.861	-8.159	-26.985	1.00	50.00
ATOM	995	CG	LEU	149	2.475	-8.545	-28.337	1.00	50.00
ATOM	996	CD1	LEU	149	1.562	-9.556	-29.026	1.00	50.00
ATOM	997	CD2	LEU	149	2.696	-7.339	-29.257	1.00	50.00
ATOM	998	N	LYS	150	1.897	-7.430	-23.983	1.00	50.00
ATOM	999	CA	LYS	150	1.198	-7.137	-22.719	1.00	50.00
ATOM	1000	C	LYS	150	2.026	-7.582	-21.517	1.00	50.00
ATOM	1001	O	LYS	150	2.752	-8.574	-21.586	1.00	50.00
ATOM	1002	CB	LYS	150	-0.173	-7.820	-22.684	1.00	50.00
ATOM	1003	CG	LYS	150	-1.046	-7.263	-23.806	1.00	50.00
ATOM	1004	CD	LYS	150	-2.528	-7.593	-23.659	1.00	50.00
ATOM	1005	CE	LYS	150	-3.336	-6.756	-24.654	1.00	50.00
ATOM	1006	NZ	LYS	150	-3.045	-5.323	-24.481	1.00	50.00
ATOM	1007	N	GLY	151	1.910	-6.784	-20.452	1.00	50.00
ATOM	1008	CA	GLY	151	2.670	-7.015	-19.213	1.00	50.00
ATOM	1009	C	GLY	151	3.977	-6.218	-19.225	1.00	50.00
ATOM	1010	O	GLY	151	4.473	-5.808	-20.279	1.00	50.00
ATOM	1011	N	GLY	152	4.541	-6.086	-18.025	1.00	50.00
ATOM	1012	CA	GLY	152	5.782	-5.317	-17.839	1.00	50.00
ATOM	1013	C	GLY	152	6.992	-6.222	-17.601	1.00	50.00
ATOM	1014	O	GLY	152	6.921	-7.452	-17.737	1.00	50.00
ATOM	1015	N	VAL	153	8.038	-5.595	-17.088	1.00	50.00
ATOM	1016	CA	VAL	153	9.318	-6.257	-16.773	1.00	50.00
ATOM	1017	C	VAL	153	9.618	-6.103	-15.273	1.00	50.00
ATOM	1018	O	VAL	153	9.655	-4.997	-14.726	1.00	50.00
ATOM	1019	CB	VAL	153	10.449	-5.681	-17.648	1.00	50.00
ATOM	1020	CG1	VAL	153	11.780	-6.391	-17.393	1.00	50.00
ATOM	1021	CG2	VAL	153	10.134	-5.806	-19.142	1.00	50.00
ATOM	1022	N	LEU	154	9.921	-7.244	-14.673	1.00	50.00
ATOM	1023	CA	LEU	154	10.219	-7.377	-13.239	1.00	50.00
ATOM	1024	C	LEU	154	11.574	-8.063	-13.028	1.00	50.00
ATOM	1025	O	LEU	154	11.707	-9.275	-13.191	1.00	50.00
ATOM	1026	CB	LEU	154	9.086	-8.176	-12.582	1.00	50.00
ATOM	1027	CG	LEU	154	9.293	-8.421	-11.083	1.00	50.00
ATOM	1028	CD1	LEU	154	9.244	-7.117	-10.287	1.00	50.00
ATOM	1029	CD2	LEU	154	8.247	-9.409	-10.567	1.00	50.00
ATOM	1030	N	ILE	155	12.585	-7.261	-12.729	1.00	50.00
ATOM	1031	CA	ILE	155	13.954	-7.772	-12.503	1.00	50.00
ATOM	1032	C	ILE	155	14.381	-7.420	-11.074	1.00	50.00
ATOM	1033	O	ILE	155	14.681	-6.264	-10.786	1.00	50.00
ATOM	1034	CB	ILE	155	14.943	-7.203	-13.538	1.00	50.00
ATOM	1035	CG1	ILE	155	14.463	-7.499	-14.969	1.00	50.00
ATOM	1036	CG2	ILE	155	16.345	-7.790	-13.299	1.00	50.00

ATOM	1037	CD1	ILE	155	15.265	-6.801	-16.074	1.00	50.00
ATOM	1038	N	GLN	156	14.396	-8.421	-10.207	1.00	50.00
ATOM	1039	CA	GLN	156	14.705	-8.216	-8.780	1.00	50.00
ATOM	1040	C	GLN	156	15.717	-9.227	-8.243	1.00	50.00
ATOM	1041	O	GLN	156	15.644	-10.421	-8.549	1.00	50.00
ATOM	1042	CB	GLN	156	13.417	-8.314	-7.956	1.00	50.00
ATOM	1043	CG	GLN	156	12.430	-7.180	-8.258	1.00	50.00
ATOM	1044	CD	GLN	156	12.943	-5.821	-7.776	1.00	50.00
ATOM	1045	OE1	GLN	156	13.518	-5.667	-6.708	1.00	50.00
ATOM	1046	NE2	GLN	156	12.753	-4.803	-8.585	1.00	50.00
ATOM	1047	N	ARG	157	16.661	-8.703	-7.464	1.00	50.00
ATOM	1048	CA	ARG	157	17.593	-9.505	-6.638	1.00	50.00
ATOM	1049	C	ARG	157	18.457	-10.480	-7.459	1.00	50.00
ATOM	1050	O	ARG	157	18.417	-11.702	-7.308	1.00	50.00
ATOM	1051	CB	ARG	157	16.811	-10.247	-5.543	1.00	50.00
ATOM	1052	CG	ARG	157	16.000	-9.294	-4.660	1.00	50.00
ATOM	1053	CD	ARG	157	15.114	-10.057	-3.674	1.00	50.00
ATOM	1054	NE	ARG	157	14.130	-10.882	-4.401	1.00	50.00
ATOM	1055	CZ	ARG	157	12.897	-10.535	-4.762	1.00	50.00
ATOM	1056	NH1	ARG	157	12.426	-9.328	-4.489	1.00	50.00
ATOM	1057	NH2	ARG	157	12.110	-11.399	-5.388	1.00	50.00
ATOM	1058	N	ASN	158	19.184	-9.898	-8.405	1.00	50.00
ATOM	1059	CA	ASN	158	20.141	-10.654	-9.230	1.00	50.00
ATOM	1060	C	ASN	158	21.537	-10.035	-9.054	1.00	50.00
ATOM	1061	O	ASN	158	21.909	-9.130	-9.804	1.00	50.00
ATOM	1062	CB	ASN	158	19.718	-10.680	-10.699	1.00	50.00
ATOM	1063	CG	ASN	158	18.286	-11.166	-10.891	1.00	50.00
ATOM	1064	OD1	ASN	158	17.961	-12.335	-10.773	1.00	50.00
ATOM	1065	ND2	ASN	158	17.397	-10.225	-11.109	1.00	50.00
ATOM	1066	N	PRO	159	22.294	-10.543	-8.075	1.00	50.00
ATOM	1067	CA	PRO	159	23.575	-9.952	-7.640	1.00	50.00
ATOM	1068	C	PRO	159	24.662	-9.861	-8.721	1.00	50.00
ATOM	1069	O	PRO	159	25.415	-8.887	-8.752	1.00	50.00
ATOM	1070	CB	PRO	159	24.017	-10.818	-6.459	1.00	50.00
ATOM	1071	CG	PRO	159	23.393	-12.181	-6.755	1.00	50.00
ATOM	1072	CD	PRO	159	22.046	-11.823	-7.376	1.00	50.00
ATOM	1073	N	GLN	160	24.693	-10.844	-9.616	1.00	50.00
ATOM	1074	CA	GLN	160	25.707	-10.893	-10.686	1.00	50.00
ATOM	1075	C	GLN	160	25.198	-10.374	-12.037	1.00	50.00
ATOM	1076	O	GLN	160	25.966	-10.228	-12.979	1.00	50.00
ATOM	1077	CB	GLN	160	26.213	-12.323	-10.858	1.00	50.00
ATOM	1078	CG	GLN	160	26.948	-12.841	-9.616	1.00	50.00
ATOM	1079	CD	GLN	160	27.636	-14.185	-9.881	1.00	50.00
ATOM	1080	OE1	GLN	160	27.615	-15.106	-9.082	1.00	50.00
ATOM	1081	NE2	GLN	160	28.279	-14.297	-11.024	1.00	50.00
ATOM	1082	N	LEU	161	23.899	-10.114	-12.132	1.00	50.00
ATOM	1083	CA	LEU	161	23.290	-9.671	-13.392	1.00	50.00
ATOM	1084	C	LEU	161	23.699	-8.240	-13.755	1.00	50.00
ATOM	1085	O	LEU	161	23.607	-7.319	-12.945	1.00	50.00
ATOM	1086	CB	LEU	161	21.770	-9.795	-13.294	1.00	50.00
ATOM	1087	CG	LEU	161	21.024	-9.422	-14.579	1.00	50.00
ATOM	1088	CD1	LEU	161	21.400	-10.339	-15.744	1.00	50.00
ATOM	1089	CD2	LEU	161	19.521	-9.504	-14.331	1.00	50.00
ATOM	1090	N	CYS	162	24.040	-8.091	-15.021	1.00	50.00
ATOM	1091	CA	CYS	162	24.370	-6.793	-15.626	1.00	50.00
ATOM	1092	C	CYS	162	23.501	-6.522	-16.855	1.00	50.00
ATOM	1093	O	CYS	162	22.885	-7.433	-17.415	1.00	50.00
ATOM	1094	CB	CYS	162	25.847	-6.790	-16.024	1.00	50.00
ATOM	1095	SG	CYS	162	26.997	-6.505	-14.633	1.00	50.00
ATOM	1096	N	TYR	163	23.430	-5.238	-17.202	1.00	50.00
ATOM	1097	CA	TYR	163	22.890	-4.728	-18.489	1.00	50.00

ATOM	1098	C	TYR	163	21.361	-4.602	-18.598	1.00	50.00
ATOM	1099	O	TYR	163	20.845	-4.168	-19.628	1.00	50.00
ATOM	1100	CB	TYR	163	23.397	-5.552	-19.688	1.00	50.00
ATOM	1101	CG	TYR	163	24.915	-5.727	-19.675	1.00	50.00
ATOM	1102	CD1	TYR	163	25.709	-4.615	-19.909	1.00	50.00
ATOM	1103	CD2	TYR	163	25.496	-6.955	-19.369	1.00	50.00
ATOM	1104	CE1	TYR	163	27.088	-4.721	-19.837	1.00	50.00
ATOM	1105	CE2	TYR	163	26.876	-7.065	-19.295	1.00	50.00
ATOM	1106	CZ	TYR	163	27.666	-5.951	-19.537	1.00	50.00
ATOM	1107	OH	TYR	163	29.008	-6.107	-19.541	1.00	50.00
ATOM	1108	N	GLN	164	20.651	-4.729	-17.484	1.00	50.00
ATOM	1109	CA	GLN	164	19.180	-4.502	-17.455	1.00	50.00
ATOM	1110	C	GLN	164	18.858	-3.008	-17.600	1.00	50.00
ATOM	1111	O	GLN	164	17.795	-2.606	-18.104	1.00	50.00
ATOM	1112	CB	GLN	164	18.502	-4.957	-16.157	1.00	50.00
ATOM	1113	CG	GLN	164	19.140	-6.145	-15.440	1.00	50.00
ATOM	1114	CD	GLN	164	20.257	-5.655	-14.521	1.00	50.00
ATOM	1115	OE1	GLN	164	21.377	-5.440	-14.944	1.00	50.00
ATOM	1116	NE2	GLN	164	19.917	-5.410	-13.275	1.00	50.00
ATOM	1117	N	ASP	165	19.793	-2.205	-17.151	1.00	50.00
ATOM	1118	CA	ASP	165	19.741	-0.734	-17.042	1.00	50.00
ATOM	1119	C	ASP	165	20.461	-0.003	-18.191	1.00	50.00
ATOM	1120	O	ASP	165	20.652	1.209	-18.127	1.00	50.00
ATOM	1121	CB	ASP	165	20.323	-0.329	-15.677	1.00	50.00
ATOM	1122	CG	ASP	165	21.756	-0.825	-15.417	1.00	50.00
ATOM	1123	OD1	ASP	165	22.146	-1.833	-16.051	1.00	50.00
ATOM	1124	OD2	ASP	165	22.425	-0.240	-14.544	1.00	50.00
ATOM	1125	N	THR	166	20.892	-0.760	-19.195	1.00	50.00
ATOM	1126	CA	THR	166	21.392	-0.182	-20.461	1.00	50.00
ATOM	1127	C	THR	166	20.303	-0.294	-21.540	1.00	50.00
ATOM	1128	O	THR	166	20.275	0.481	-22.489	1.00	50.00
ATOM	1129	CB	THR	166	22.674	-0.871	-20.958	1.00	50.00
ATOM	1130	OG1	THR	166	22.373	-2.213	-21.345	1.00	50.00
ATOM	1131	CG2	THR	166	23.778	-0.875	-19.894	1.00	50.00
ATOM	1132	N	ILE	167	19.398	-1.255	-21.351	1.00	50.00
ATOM	1133	CA	ILE	167	18.281	-1.521	-22.270	1.00	50.00
ATOM	1134	C	ILE	167	17.239	-0.406	-22.158	1.00	50.00
ATOM	1135	O	ILE	167	16.776	-0.050	-21.071	1.00	50.00
ATOM	1136	CB	ILE	167	17.681	-2.916	-21.983	1.00	50.00
ATOM	1137	CG1	ILE	167	18.725	-4.030	-22.177	1.00	50.00
ATOM	1138	CG2	ILE	167	16.411	-3.217	-22.794	1.00	50.00
ATOM	1139	CD1	ILE	167	19.376	-4.080	-23.568	1.00	50.00
ATOM	1140	N	LEU	168	16.759	-0.039	-23.336	1.00	50.00
ATOM	1141	CA	LEU	168	15.630	0.884	-23.455	1.00	50.00
ATOM	1142	C	LEU	168	14.353	0.079	-23.713	1.00	50.00
ATOM	1143	O	LEU	168	13.944	-0.168	-24.853	1.00	50.00
ATOM	1144	CB	LEU	168	15.919	1.932	-24.537	1.00	50.00
ATOM	1145	CG	LEU	168	14.845	3.026	-24.605	1.00	50.00
ATOM	1146	CD1	LEU	168	14.631	3.718	-23.254	1.00	50.00
ATOM	1147	CD2	LEU	168	15.253	4.070	-25.643	1.00	50.00
ATOM	1148	N	TRP	169	13.702	-0.248	-22.603	1.00	50.00
ATOM	1149	CA	TRP	169	12.477	-1.076	-22.588	1.00	50.00
ATOM	1150	C	TRP	169	11.283	-0.392	-23.269	1.00	50.00
ATOM	1151	O	TRP	169	10.385	-1.063	-23.770	1.00	50.00
ATOM	1152	CB	TRP	169	12.097	-1.462	-21.156	1.00	50.00
ATOM	1153	CG	TRP	169	13.228	-2.214	-20.450	1.00	50.00
ATOM	1154	CD1	TRP	169	14.171	-1.664	-19.690	1.00	50.00
ATOM	1155	CD2	TRP	169	13.463	-3.581	-20.479	1.00	50.00
ATOM	1156	NE1	TRP	169	14.988	-2.614	-19.236	1.00	50.00
ATOM	1157	CE2	TRP	169	14.582	-3.796	-19.690	1.00	50.00
ATOM	1158	CE3	TRP	169	12.842	-4.643	-21.124	1.00	50.00

ATOM	1159	CZ2	TRP	169	15.081	-5.084	-19.531	1.00	50.00
ATOM	1160	CZ3	TRP	169	13.350	-5.929	-20.977	1.00	50.00
ATOM	1161	CH2	TRP	169	14.467	-6.149	-20.179	1.00	50.00
ATOM	1162	N	LYS	170	11.365	0.936	-23.384	1.00	50.00
ATOM	1163	CA	LYS	170	10.359	1.767	-24.073	1.00	50.00
ATOM	1164	C	LYS	170	10.205	1.350	-25.547	1.00	50.00
ATOM	1165	O	LYS	170	9.100	1.313	-26.070	1.00	50.00
ATOM	1166	CB	LYS	170	10.798	3.230	-24.004	1.00	50.00
ATOM	1167	CG	LYS	170	9.612	4.184	-24.141	1.00	50.00
ATOM	1168	CD	LYS	170	10.099	5.634	-24.114	1.00	50.00
ATOM	1169	CE	LYS	170	8.994	6.618	-23.714	1.00	50.00
ATOM	1170	NZ	LYS	170	7.862	6.639	-24.653	1.00	50.00
ATOM	1171	N	ASP	171	11.328	0.952	-26.150	1.00	50.00
ATOM	1172	CA	ASP	171	11.349	0.444	-27.535	1.00	50.00
ATOM	1173	C	ASP	171	10.810	-0.988	-27.671	1.00	50.00
ATOM	1174	O	ASP	171	10.419	-1.401	-28.758	1.00	50.00
ATOM	1175	CB	ASP	171	12.759	0.547	-28.129	1.00	50.00
ATOM	1176	CG	ASP	171	13.253	1.995	-28.260	1.00	50.00
ATOM	1177	OD1	ASP	171	12.419	2.925	-28.178	1.00	50.00
ATOM	1178	OD2	ASP	171	14.483	2.146	-28.406	1.00	50.00
ATOM	1179	N	ILE	172	10.879	-1.737	-26.573	1.00	50.00
ATOM	1180	CA	ILE	172	10.436	-3.145	-26.532	1.00	50.00
ATOM	1181	C	ILE	172	8.919	-3.251	-26.279	1.00	50.00
ATOM	1182	O	ILE	172	8.248	-4.129	-26.828	1.00	50.00
ATOM	1183	CB	ILE	172	11.265	-3.939	-25.502	1.00	50.00
ATOM	1184	CG1	ILE	172	12.767	-3.833	-25.827	1.00	50.00
ATOM	1185	CG2	ILE	172	10.828	-5.412	-25.496	1.00	50.00
ATOM	1186	CD1	ILE	172	13.698	-4.488	-24.800	1.00	50.00
ATOM	1187	N	PHE	173	8.415	-2.443	-25.351	1.00	50.00
ATOM	1188	CA	PHE	173	6.973	-2.395	-25.054	1.00	50.00
ATOM	1189	C	PHE	173	6.179	-1.936	-26.276	1.00	50.00
ATOM	1190	O	PHE	173	6.529	-0.964	-26.945	1.00	50.00
ATOM	1191	CB	PHE	173	6.688	-1.445	-23.889	1.00	50.00
ATOM	1192	CG	PHE	173	7.196	-1.988	-22.553	1.00	50.00
ATOM	1193	CD1	PHE	173	6.893	-3.283	-22.144	1.00	50.00
ATOM	1194	CD2	PHE	173	7.933	-1.154	-21.727	1.00	50.00
ATOM	1195	CE1	PHE	173	7.320	-3.742	-20.906	1.00	50.00
ATOM	1196	CE2	PHE	173	8.359	-1.614	-20.491	1.00	50.00
ATOM	1197	CZ	PHE	173	8.055	-2.905	-20.076	1.00	50.00
ATOM	1198	N	HIS	174	5.143	-2.709	-26.581	1.00	50.00
ATOM	1199	CA	HIS	174	4.196	-2.341	-27.641	1.00	50.00
ATOM	1200	C	HIS	174	3.450	-1.082	-27.196	1.00	50.00
ATOM	1201	O	HIS	174	3.114	-0.920	-26.003	1.00	50.00
ATOM	1202	CB	HIS	174	3.229	-3.507	-27.874	1.00	50.00
ATOM	1203	CG	HIS	174	2.537	-3.420	-29.239	1.00	50.00
ATOM	1204	ND1	HIS	174	2.953	-3.991	-30.364	1.00	50.00
ATOM	1205	CD2	HIS	174	1.404	-2.779	-29.504	1.00	50.00
ATOM	1206	CE1	HIS	174	2.075	-3.703	-31.322	1.00	50.00
ATOM	1207	NE2	HIS	174	1.120	-2.943	-30.790	1.00	50.00
ATOM	1208	N	LYS	175	3.104	-0.252	-28.136	1.00	50.00
ATOM	1209	CA	LYS	175	2.355	1.014	-27.958	1.00	50.00
ATOM	1210	C	LYS	175	0.961	0.821	-27.334	1.00	50.00
ATOM	1211	O	LYS	175	0.459	1.703	-26.647	1.00	50.00
ATOM	1212	CB	LYS	175	2.267	1.827	-29.262	1.00	50.00
ATOM	1213	CG	LYS	175	2.323	1.016	-30.570	1.00	50.00
ATOM	1214	CD	LYS	175	3.763	0.576	-30.871	1.00	50.00
ATOM	1215	CE	LYS	175	3.812	-0.683	-31.733	1.00	50.00
ATOM	1216	NZ	LYS	175	5.170	-1.243	-31.744	1.00	50.00
ATOM	1217	N	ASN	176	0.397	-0.366	-27.545	1.00	50.00
ATOM	1218	CA	ASN	176	-0.886	-0.759	-26.929	1.00	50.00
ATOM	1219	C	ASN	176	-0.753	-1.408	-25.545	1.00	50.00

ATOM	1220	O	ASN	176	-1.764	-1.656	-24.889	1.00	50.00
ATOM	1221	CB	ASN	176	-1.684	-1.679	-27.855	1.00	50.00
ATOM	1222	CG	ASN	176	-2.246	-0.937	-29.070	1.00	50.00
ATOM	1223	OD1	ASN	176	-2.474	0.263	-29.089	1.00	50.00
ATOM	1224	ND2	ASN	176	-2.493	-1.682	-30.123	1.00	50.00
ATOM	1225	N	ASN	177	0.479	-1.717	-25.139	1.00	50.00
ATOM	1226	CA	ASN	177	0.751	-2.353	-23.838	1.00	50.00
ATOM	1227	C	ASN	177	0.361	-1.410	-22.688	1.00	50.00
ATOM	1228	O	ASN	177	1.108	-0.504	-22.308	1.00	50.00
ATOM	1229	CB	ASN	177	2.229	-2.739	-23.728	1.00	50.00
ATOM	1230	CG	ASN	177	2.495	-3.671	-22.545	1.00	50.00
ATOM	1231	OD1	ASN	177	1.706	-3.845	-21.618	1.00	50.00
ATOM	1232	ND2	ASN	177	3.619	-4.342	-22.608	1.00	50.00
ATOM	1233	N	GLN	178	-0.812	-1.665	-22.130	1.00	50.00
ATOM	1234	CA	GLN	178	-1.368	-0.860	-21.020	1.00	50.00
ATOM	1235	C	GLN	178	-0.692	-1.147	-19.669	1.00	50.00
ATOM	1236	O	GLN	178	-0.830	-0.382	-18.718	1.00	50.00
ATOM	1237	CB	GLN	178	-2.895	-0.998	-20.917	1.00	50.00
ATOM	1238	CG	GLN	178	-3.424	-2.328	-20.355	1.00	50.00
ATOM	1239	CD	GLN	178	-3.102	-3.555	-21.212	1.00	50.00
ATOM	1240	OE1	GLN	178	-2.854	-3.495	-22.403	1.00	50.00
ATOM	1241	NE2	GLN	178	-3.058	-4.707	-20.587	1.00	50.00
ATOM	1242	N	LEU	179	-0.035	-2.303	-19.593	1.00	50.00
ATOM	1243	CA	LEU	179	0.692	-2.744	-18.392	1.00	50.00
ATOM	1244	C	LEU	179	2.213	-2.677	-18.588	1.00	50.00
ATOM	1245	O	LEU	179	2.952	-3.531	-18.094	1.00	50.00
ATOM	1246	CB	LEU	179	0.250	-4.168	-18.030	1.00	50.00
ATOM	1247	CG	LEU	179	-1.146	-4.233	-17.408	1.00	50.00
ATOM	1248	CD1	LEU	179	-1.580	-5.695	-17.317	1.00	50.00
ATOM	1249	CD2	LEU	179	-1.155	-3.608	-16.008	1.00	50.00
ATOM	1250	N	ALA	180	2.663	-1.639	-19.292	1.00	50.00
ATOM	1251	CA	ALA	180	4.097	-1.409	-19.560	1.00	50.00
ATOM	1252	C	ALA	180	4.821	-0.911	-18.294	1.00	50.00
ATOM	1253	O	ALA	180	5.222	0.245	-18.151	1.00	50.00
ATOM	1254	CB	ALA	180	4.230	-0.439	-20.740	1.00	50.00
ATOM	1255	N	LEU	181	4.992	-1.845	-17.367	1.00	50.00
ATOM	1256	CA	LEU	181	5.597	-1.586	-16.049	1.00	50.00
ATOM	1257	C	LEU	181	7.068	-1.994	-16.067	1.00	50.00
ATOM	1258	O	LEU	181	7.452	-2.986	-16.687	1.00	50.00
ATOM	1259	CB	LEU	181	4.880	-2.410	-14.973	1.00	50.00
ATOM	1260	CG	LEU	181	3.389	-2.084	-14.836	1.00	50.00
ATOM	1261	CD1	LEU	181	2.715	-3.161	-13.986	1.00	50.00
ATOM	1262	CD2	LEU	181	3.173	-0.709	-14.197	1.00	50.00
ATOM	1263	N	THR	182	7.885	-1.196	-15.404	1.00	50.00
ATOM	1264	CA	THR	182	9.307	-1.528	-15.197	1.00	50.00
ATOM	1265	C	THR	182	9.658	-1.443	-13.715	1.00	50.00
ATOM	1266	O	THR	182	9.640	-0.378	-13.099	1.00	50.00
ATOM	1267	CB	THR	182	10.260	-0.646	-16.012	1.00	50.00
ATOM	1268	OG1	THR	182	9.967	0.735	-15.792	1.00	50.00
ATOM	1269	CG2	THR	182	10.221	-0.999	-17.498	1.00	50.00
ATOM	1270	N	LEU	183	9.804	-2.621	-13.130	1.00	50.00
ATOM	1271	CA	LEU	183	10.242	-2.748	-11.733	1.00	50.00
ATOM	1272	C	LEU	183	11.597	-3.467	-11.749	1.00	50.00
ATOM	1273	O	LEU	183	11.707	-4.689	-11.599	1.00	50.00
ATOM	1274	CB	LEU	183	9.179	-3.523	-10.946	1.00	50.00
ATOM	1275	CG	LEU	183	8.873	-2.932	-9.563	1.00	50.00
ATOM	1276	CD1	LEU	183	7.838	-3.809	-8.857	1.00	50.00
ATOM	1277	CD2	LEU	183	10.112	-2.778	-8.675	1.00	50.00
ATOM	1278	N	ILE	184	12.631	-2.667	-11.978	1.00	50.00
ATOM	1279	CA	ILE	184	13.981	-3.186	-12.274	1.00	50.00
ATOM	1280	C	ILE	184	14.987	-2.736	-11.209	1.00	50.00

ATOM	1281	O	ILE	184	15.378	-1.571	-11.138	1.00	50.00
ATOM	1282	CB	ILE	184	14.420	-2.786	-13.700	1.00	50.00
ATOM	1283	CG1	ILE	184	13.435	-3.351	-14.740	1.00	50.00
ATOM	1284	CG2	ILE	184	15.861	-3.247	-13.992	1.00	50.00
ATOM	1285	CD1	ILE	184	13.648	-2.835	-16.168	1.00	50.00
ATOM	1286	N	ASP	185	15.375	-3.702	-10.392	1.00	50.00
ATOM	1287	CA	ASP	185	16.473	-3.550	-9.429	1.00	50.00
ATOM	1288	C	ASP	185	17.808	-3.558	-10.190	1.00	50.00
ATOM	1289	O	ASP	185	18.052	-4.437	-11.021	1.00	50.00
ATOM	1290	CB	ASP	185	16.393	-4.705	-8.424	1.00	50.00
ATOM	1291	CG	ASP	185	17.456	-4.653	-7.324	1.00	50.00
ATOM	1292	OD1	ASP	185	18.000	-3.550	-7.090	1.00	50.00
ATOM	1293	OD2	ASP	185	17.738	-5.740	-6.775	1.00	50.00
ATOM	1294	N	THR	186	18.678	-2.639	-9.801	1.00	50.00
ATOM	1295	CA	THR	186	20.011	-2.491	-10.426	1.00	50.00
ATOM	1296	C	THR	186	21.166	-2.778	-9.456	1.00	50.00
ATOM	1297	O	THR	186	22.330	-2.713	-9.843	1.00	50.00
ATOM	1298	CB	THR	186	20.184	-1.083	-11.010	1.00	50.00
ATOM	1299	OG1	THR	186	20.022	-0.115	-9.969	1.00	50.00
ATOM	1300	CG2	THR	186	19.192	-0.817	-12.147	1.00	50.00
ATOM	1301	N	ASN	187	20.827	-3.135	-8.215	1.00	50.00
ATOM	1302	CA	ASN	187	21.820	-3.481	-7.183	1.00	50.00
ATOM	1303	C	ASN	187	22.632	-4.704	-7.613	1.00	50.00
ATOM	1304	O	ASN	187	22.077	-5.697	-8.092	1.00	50.00
ATOM	1305	CB	ASN	187	21.138	-3.758	-5.840	1.00	50.00
ATOM	1306	CG	ASN	187	20.419	-2.527	-5.279	1.00	50.00
ATOM	1307	OD1	ASN	187	20.665	-1.382	-5.627	1.00	50.00
ATOM	1308	ND2	ASN	187	19.460	-2.769	-4.415	1.00	50.00
ATOM	1309	N	ARG	188	23.939	-4.565	-7.455	1.00	50.00
ATOM	1310	CA	ARG	188	24.912	-5.577	-7.898	1.00	50.00
ATOM	1311	C	ARG	188	26.020	-5.792	-6.873	1.00	50.00
ATOM	1312	O	ARG	188	26.359	-4.897	-6.096	1.00	50.00
ATOM	1313	CB	ARG	188	25.567	-5.149	-9.209	1.00	50.00
ATOM	1314	CG	ARG	188	24.563	-5.147	-10.352	1.00	50.00
ATOM	1315	CD	ARG	188	25.264	-4.626	-11.592	1.00	50.00
ATOM	1316	NE	ARG	188	24.268	-4.507	-12.655	1.00	50.00
ATOM	1317	CZ	ARG	188	23.800	-3.361	-13.140	1.00	50.00
ATOM	1318	NH1	ARG	188	24.208	-2.175	-12.704	1.00	50.00
ATOM	1319	NH2	ARG	188	22.841	-3.402	-14.013	1.00	50.00
ATOM	1320	N	SER	189	26.571	-6.993	-6.932	1.00	50.00
ATOM	1321	CA	SER	189	27.773	-7.367	-6.159	1.00	50.00
ATOM	1322	C	SER	189	29.072	-7.109	-6.941	1.00	50.00
ATOM	1323	O	SER	189	30.138	-6.957	-6.350	1.00	50.00
ATOM	1324	CB	SER	189	27.713	-8.834	-5.730	1.00	50.00
ATOM	1325	OG	SER	189	27.561	-9.691	-6.867	1.00	50.00
ATOM	1326	N	ARG	190	28.951	-7.109	-8.268	1.00	50.00
ATOM	1327	CA	ARG	190	30.081	-6.868	-9.180	1.00	50.00
ATOM	1328	C	ARG	190	29.811	-5.656	-10.084	1.00	50.00
ATOM	1329	O	ARG	190	28.661	-5.344	-10.410	1.00	50.00
ATOM	1330	CB	ARG	190	30.347	-8.114	-10.038	1.00	50.00
ATOM	1331	CG	ARG	190	29.213	-8.436	-11.017	1.00	50.00
ATOM	1332	CD	ARG	190	29.645	-9.529	-11.986	1.00	50.00
ATOM	1333	NE	ARG	190	28.641	-9.640	-13.056	1.00	50.00
ATOM	1334	CZ	ARG	190	28.808	-9.328	-14.340	1.00	50.00
ATOM	1335	NH1	ARG	190	29.954	-8.827	-14.779	1.00	50.00
ATOM	1336	NH2	ARG	190	27.820	-9.512	-15.205	1.00	50.00
ATOM	1337	N	ALA	191	30.893	-5.001	-10.474	1.00	50.00
ATOM	1338	CA	ALA	191	30.855	-3.922	-11.477	1.00	50.00
ATOM	1339	C	ALA	191	30.674	-4.522	-12.880	1.00	50.00
ATOM	1340	O	ALA	191	31.266	-5.549	-13.217	1.00	50.00
ATOM	1341	CB	ALA	191	32.151	-3.112	-11.405	1.00	50.00

ATOM	1342	N	CYS	192	29.912	-3.815	-13.704	1.00	50.00
ATOM	1343	CA	CYS	192	29.628	-4.256	-15.082	1.00	50.00
ATOM	1344	C	CYS	192	30.631	-3.654	-16.064	1.00	50.00
ATOM	1345	O	CYS	192	30.867	-2.443	-16.079	1.00	50.00
ATOM	1346	CB	CYS	192	28.224	-3.824	-15.502	1.00	50.00
ATOM	1347	SG	CYS	192	26.914	-4.481	-14.415	1.00	50.00
ATOM	1348	N	HIS	193	31.219	-4.532	-16.866	1.00	50.00
ATOM	1349	CA	HIS	193	32.043	-4.108	-18.011	1.00	50.00
ATOM	1350	C	HIS	193	31.157	-3.372	-19.032	1.00	50.00
ATOM	1351	O	HIS	193	30.026	-3.791	-19.277	1.00	50.00
ATOM	1352	CB	HIS	193	32.732	-5.299	-18.681	1.00	50.00
ATOM	1353	CG	HIS	193	33.728	-5.970	-17.733	1.00	50.00
ATOM	1354	ND1	HIS	193	34.920	-5.499	-17.384	1.00	50.00
ATOM	1355	CD2	HIS	193	33.595	-7.188	-17.215	1.00	50.00
ATOM	1356	CE1	HIS	193	35.531	-6.428	-16.654	1.00	50.00
ATOM	1357	NE2	HIS	193	34.713	-7.471	-16.555	1.00	50.00
ATOM	1358	N	PRO	194	31.631	-2.255	-19.589	1.00	50.00
ATOM	1359	CA	PRO	194	30.866	-1.485	-20.588	1.00	50.00
ATOM	1360	C	PRO	194	30.511	-2.381	-21.782	1.00	50.00
ATOM	1361	O	PRO	194	31.238	-3.327	-22.089	1.00	50.00
ATOM	1362	CB	PRO	194	31.831	-0.382	-21.023	1.00	50.00
ATOM	1363	CG	PRO	194	32.693	-0.159	-19.782	1.00	50.00
ATOM	1364	CD	PRO	194	32.885	-1.569	-19.229	1.00	50.00
ATOM	1365	N	CYS	195	29.360	-2.107	-22.397	1.00	50.00
ATOM	1366	CA	CYS	195	28.985	-2.746	-23.677	1.00	50.00
ATOM	1367	C	CYS	195	30.121	-2.527	-24.686	1.00	50.00
ATOM	1368	O	CYS	195	30.870	-1.548	-24.590	1.00	50.00
ATOM	1369	CB	CYS	195	27.717	-2.105	-24.256	1.00	50.00
ATOM	1370	SG	CYS	195	26.182	-2.355	-23.290	1.00	50.00
ATOM	1371	N	SER	196	30.289	-3.469	-25.605	1.00	50.00
ATOM	1372	CA	SER	196	31.276	-3.285	-26.683	1.00	50.00
ATOM	1373	C	SER	196	30.876	-2.044	-27.511	1.00	50.00
ATOM	1374	O	SER	196	29.676	-1.798	-27.696	1.00	50.00
ATOM	1375	CB	SER	196	31.385	-4.534	-27.569	1.00	50.00
ATOM	1376	OG	SER	196	30.346	-4.565	-28.550	1.00	50.00
ATOM	1377	N	PRO	197	31.855	-1.329	-28.079	1.00	50.00
ATOM	1378	CA	PRO	197	31.607	-0.133	-28.911	1.00	50.00
ATOM	1379	C	PRO	197	30.708	-0.409	-30.127	1.00	50.00
ATOM	1380	O	PRO	197	30.067	0.498	-30.645	1.00	50.00
ATOM	1381	CB	PRO	197	32.998	0.343	-29.332	1.00	50.00
ATOM	1382	CG	PRO	197	33.825	-0.941	-29.340	1.00	50.00
ATOM	1383	CD	PRO	197	33.288	-1.692	-28.124	1.00	50.00
ATOM	1384	N	MET	198	30.647	-1.680	-30.533	1.00	50.00
ATOM	1385	CA	MET	198	29.777	-2.156	-31.627	1.00	50.00
ATOM	1386	C	MET	198	28.292	-1.860	-31.366	1.00	50.00
ATOM	1387	O	MET	198	27.538	-1.592	-32.298	1.00	50.00
ATOM	1388	CB	MET	198	29.966	-3.664	-31.822	1.00	50.00
ATOM	1389	CG	MET	198	31.406	-4.064	-32.172	1.00	50.00
ATOM	1390	SD	MET	198	31.980	-3.561	-33.836	1.00	50.00
ATOM	1391	CE	MET	198	32.544	-1.896	-33.554	1.00	50.00
ATOM	1392	N	CYS	199	27.914	-1.901	-30.089	1.00	50.00
ATOM	1393	CA	CYS	199	26.545	-1.588	-29.649	1.00	50.00
ATOM	1394	C	CYS	199	26.339	-0.072	-29.565	1.00	50.00
ATOM	1395	O	CYS	199	26.639	0.570	-28.552	1.00	50.00
ATOM	1396	CB	CYS	199	26.254	-2.259	-28.301	1.00	50.00
ATOM	1397	SG	CYS	199	26.440	-4.079	-28.315	1.00	50.00
ATOM	1398	N	LYS	200	25.845	0.491	-30.663	1.00	50.00
ATOM	1399	CA	LYS	200	25.579	1.940	-30.757	1.00	50.00
ATOM	1400	C	LYS	200	24.552	2.372	-29.699	1.00	50.00
ATOM	1401	O	LYS	200	23.553	1.695	-29.458	1.00	50.00
ATOM	1402	CB	LYS	200	25.097	2.332	-32.159	1.00	50.00

ATOM	1403	CG	LYS	200	23.722	1.752	-32.507	1.00	50.00
ATOM	1404	CD	LYS	200	23.244	2.235	-33.869	1.00	50.00
ATOM	1405	CE	LYS	200	21.814	1.759	-34.109	1.00	50.00
ATOM	1406	NZ	LYS	200	21.384	2.149	-35.456	1.00	50.00
ATOM	1407	N	GLY	201	24.911	3.457	-29.011	1.00	50.00
ATOM	1408	CA	GLY	201	24.090	3.990	-27.911	1.00	50.00
ATOM	1409	C	GLY	201	24.314	3.229	-26.598	1.00	50.00
ATOM	1410	O	GLY	201	23.544	3.437	-25.670	1.00	50.00
ATOM	1411	N	SER	202	25.318	2.334	-26.591	1.00	50.00
ATOM	1412	CA	SER	202	25.790	1.578	-25.406	1.00	50.00
ATOM	1413	C	SER	202	24.679	0.744	-24.738	1.00	50.00
ATOM	1414	O	SER	202	24.648	0.549	-23.521	1.00	50.00
ATOM	1415	CB	SER	202	26.485	2.511	-24.396	1.00	50.00
ATOM	1416	OG	SER	202	25.537	3.445	-23.868	1.00	50.00
ATOM	1417	N	ARG	203	23.824	0.162	-25.577	1.00	50.00
ATOM	1418	CA	ARG	203	22.705	-0.672	-25.103	1.00	50.00
ATOM	1419	C	ARG	203	22.928	-2.113	-25.563	1.00	50.00
ATOM	1420	O	ARG	203	23.046	-2.402	-26.758	1.00	50.00
ATOM	1421	CB	ARG	203	21.359	-0.166	-25.633	1.00	50.00
ATOM	1422	CG	ARG	203	21.146	1.325	-25.364	1.00	50.00
ATOM	1423	CD	ARG	203	19.691	1.711	-25.600	1.00	50.00
ATOM	1424	NE	ARG	203	19.553	3.177	-25.551	1.00	50.00
ATOM	1425	CZ	ARG	203	18.842	3.910	-26.409	1.00	50.00
ATOM	1426	NH1	ARG	203	18.155	3.335	-27.389	1.00	50.00
ATOM	1427	NH2	ARG	203	18.814	5.233	-26.306	1.00	50.00
ATOM	1428	N	CYS	204	23.090	-2.986	-24.580	1.00	50.00
ATOM	1429	CA	CYS	204	23.387	-4.410	-24.819	1.00	50.00
ATOM	1430	C	CYS	204	22.789	-5.303	-23.725	1.00	50.00
ATOM	1431	O	CYS	204	22.428	-4.821	-22.651	1.00	50.00
ATOM	1432	CB	CYS	204	24.904	-4.630	-24.933	1.00	50.00
ATOM	1433	SG	CYS	204	25.862	-4.366	-23.394	1.00	50.00
ATOM	1434	N	TRP	205	22.813	-6.597	-23.988	1.00	50.00
ATOM	1435	CA	TRP	205	22.340	-7.660	-23.076	1.00	50.00
ATOM	1436	C	TRP	205	23.515	-8.408	-22.423	1.00	50.00
ATOM	1437	O	TRP	205	23.357	-9.097	-21.417	1.00	50.00
ATOM	1438	CB	TRP	205	21.466	-8.660	-23.843	1.00	50.00
ATOM	1439	CG	TRP	205	20.225	-8.007	-24.459	1.00	50.00
ATOM	1440	CD1	TRP	205	20.109	-7.562	-25.708	1.00	50.00
ATOM	1441	CD2	TRP	205	19.018	-7.760	-23.819	1.00	50.00
ATOM	1442	NE1	TRP	205	18.895	-7.042	-25.886	1.00	50.00
ATOM	1443	CE2	TRP	205	18.201	-7.146	-24.756	1.00	50.00
ATOM	1444	CE3	TRP	205	18.557	-7.988	-22.529	1.00	50.00
ATOM	1445	CZ2	TRP	205	16.913	-6.757	-24.405	1.00	50.00
ATOM	1446	CZ3	TRP	205	17.270	-7.597	-22.177	1.00	50.00
ATOM	1447	CH2	TRP	205	16.448	-6.985	-23.117	1.00	50.00
ATOM	1448	N	GLY	206	24.675	-8.314	-23.085	1.00	50.00
ATOM	1449	CA	GLY	206	25.932	-8.926	-22.634	1.00	50.00
ATOM	1450	C	GLY	206	27.102	-8.050	-23.086	1.00	50.00
ATOM	1451	O	GLY	206	26.947	-7.157	-23.922	1.00	50.00
ATOM	1452	N	GLU	207	28.280	-8.430	-22.631	1.00	50.00
ATOM	1453	CA	GLU	207	29.535	-7.722	-22.965	1.00	50.00
ATOM	1454	C	GLU	207	30.135	-8.052	-24.347	1.00	50.00
ATOM	1455	O	GLU	207	31.147	-7.484	-24.752	1.00	50.00
ATOM	1456	CB	GLU	207	30.547	-7.869	-21.821	1.00	50.00
ATOM	1457	CG	GLU	207	30.606	-9.280	-21.225	1.00	50.00
ATOM	1458	CD	GLU	207	31.578	-9.337	-20.050	1.00	50.00
ATOM	1459	OE1	GLU	207	31.123	-9.078	-18.914	1.00	50.00
ATOM	1460	OE2	GLU	207	32.749	-9.669	-20.327	1.00	50.00
ATOM	1461	N	SER	208	29.451	-8.921	-25.091	1.00	50.00
ATOM	1462	CA	SER	208	29.824	-9.273	-26.476	1.00	50.00
ATOM	1463	C	SER	208	29.206	-8.297	-27.488	1.00	50.00

ATOM	1464	O	SER	208	28.118	-7.755	-27.282	1.00	50.00
ATOM	1465	CB	SER	208	29.393	-10.712	-26.793	1.00	50.00
ATOM	1466	OG	SER	208	29.606	-11.031	-28.174	1.00	50.00
ATOM	1467	N	SER	209	29.870	-8.195	-28.636	1.00	50.00
ATOM	1468	CA	SER	209	29.371	-7.429	-29.798	1.00	50.00
ATOM	1469	C	SER	209	28.146	-8.055	-30.486	1.00	50.00
ATOM	1470	O	SER	209	27.486	-7.404	-31.290	1.00	50.00
ATOM	1471	CB	SER	209	30.476	-7.215	-30.830	1.00	50.00
ATOM	1472	OG	SER	209	30.978	-8.457	-31.320	1.00	50.00
ATOM	1473	N	GLU	210	27.843	-9.305	-30.137	1.00	50.00
ATOM	1474	CA	GLU	210	26.620	-9.987	-30.614	1.00	50.00
ATOM	1475	C	GLU	210	25.498	-9.971	-29.561	1.00	50.00
ATOM	1476	O	GLU	210	24.591	-10.803	-29.561	1.00	50.00
ATOM	1477	CB	GLU	210	26.934	-11.441	-30.962	1.00	50.00
ATOM	1478	CG	GLU	210	27.865	-11.632	-32.161	1.00	50.00
ATOM	1479	CD	GLU	210	28.042	-13.120	-32.498	1.00	50.00
ATOM	1480	OE1	GLU	210	27.804	-13.969	-31.607	1.00	50.00
ATOM	1481	OE2	GLU	210	28.426	-13.385	-33.656	1.00	50.00
ATOM	1482	N	ASP	211	25.602	-9.026	-28.631	1.00	50.00
ATOM	1483	CA	ASP	211	24.612	-8.889	-27.553	1.00	50.00
ATOM	1484	C	ASP	211	23.929	-7.520	-27.513	1.00	50.00
ATOM	1485	O	ASP	211	23.276	-7.200	-26.527	1.00	50.00
ATOM	1486	CB	ASP	211	25.282	-9.182	-26.210	1.00	50.00
ATOM	1487	CG	ASP	211	25.697	-10.643	-26.027	1.00	50.00
ATOM	1488	OD1	ASP	211	25.021	-11.532	-26.589	1.00	50.00
ATOM	1489	OD2	ASP	211	26.705	-10.829	-25.311	1.00	50.00
ATOM	1490	N	CYS	212	24.066	-6.742	-28.580	1.00	50.00
ATOM	1491	CA	CYS	212	23.439	-5.411	-28.673	1.00	50.00
ATOM	1492	C	CYS	212	21.910	-5.531	-28.698	1.00	50.00
ATOM	1493	O	CYS	212	21.365	-6.556	-29.118	1.00	50.00
ATOM	1494	CB	CYS	212	23.910	-4.709	-29.948	1.00	50.00
ATOM	1495	SG	CYS	212	25.730	-4.646	-30.143	1.00	50.00
ATOM	1496	N	GLN	213	21.245	-4.522	-28.150	1.00	50.00
ATOM	1497	CA	GLN	213	19.775	-4.433	-28.244	1.00	50.00
ATOM	1498	C	GLN	213	19.387	-4.206	-29.716	1.00	50.00
ATOM	1499	O	GLN	213	19.950	-3.343	-30.389	1.00	50.00
ATOM	1500	CB	GLN	213	19.267	-3.273	-27.384	1.00	50.00
ATOM	1501	CG	GLN	213	17.740	-3.305	-27.257	1.00	50.00
ATOM	1502	CD	GLN	213	17.167	-2.096	-26.511	1.00	50.00
ATOM	1503	OE1	GLN	213	17.779	-1.459	-25.664	1.00	50.00
ATOM	1504	NE2	GLN	213	15.918	-1.801	-26.795	1.00	50.00
ATOM	1505	N	SER	214	18.398	-4.969	-30.167	1.00	50.00
ATOM	1506	CA	SER	214	17.894	-4.868	-31.555	1.00	50.00
ATOM	1507	C	SER	214	16.637	-3.990	-31.649	1.00	50.00
ATOM	1508	O	SER	214	16.795	-2.806	-32.017	1.00	50.00
ATOM	1509	CB	SER	214	17.606	-6.261	-32.117	1.00	50.00
ATOM	1510	OG	SER	214	17.143	-6.162	-33.463	1.00	50.00
ATOM	1511	OXT	SER	214	15.580	-4.448	-31.164	1.00	99.99
TER	1512		SER	214					
