# Supplementary material for: Structural analysis of hubs in human NR-RTK network
Source: Biol Direct. 2011 Oct 5;6:49. doi: 10.1186/1745-6150-6-49 (PMC3220635; doi:10.1186/1745-6150-6-49)
Supplement: Additional file 6 — ESR1-EGFR. ESR1-EGFR complex structure. [file 1745-6150-6-49-S6.PDF]

```
HEADER ESR1-EGFR
REMARK original generated coordinate pdb file
ATOM      1  N   ALA   156      10.627  12.174   8.322  1.00  0.00      RX0  N
ATOM      2  H   ALA   156      11.198  11.354   8.274  0.00  0.00      RX0  H
ATOM      3  CA  ALA   156       9.864  12.527   9.538  1.00  0.00      RX0  C
ATOM      4  CB  ALA   156      10.757  12.403  10.765  1.00  0.00      RX0  C
ATOM      5  C   ALA   156       9.377  13.991   9.496  1.00  0.00      RX0  C
ATOM      6  O   ALA   156       9.121  14.644  10.500  1.00  0.00      RX0  O
ATOM      7  N   LEU   157       9.039  14.416   8.289  1.00  0.00      RX0  N
ATOM      8  H   LEU   157       9.137  13.769   7.530  0.00  0.00      RX0  H
ATOM      9  CA  LEU   157       8.850  15.849   7.979  1.00  0.00      RX0  C
ATOM     10  CB  LEU   157       9.722  16.250   6.797  1.00  0.00      RX0  C
ATOM     11  CG  LEU   157      11.139  15.706   6.916  1.00  0.00      RX0  C
ATOM     12  CD1 LEU   157      11.939  15.993   5.653  1.00  0.00      RX0  C
ATOM     13  CD2 LEU   157      11.834  16.192   8.184  1.00  0.00      RX0  C
ATOM     14  C   LEU   157       7.387  16.184   7.642  1.00  0.00      RX0  C
ATOM     15  O   LEU   157       7.075  17.216   7.039  1.00  0.00      RX0  O
ATOM     16  N   SER   158       6.522  15.244   7.955  1.00  0.00      RX0  N
ATOM     17  H   SER   158       6.875  14.327   8.103  0.00  0.00      RX0  H
ATOM     18  CA  SER   158       5.051  15.362   7.847  1.00  0.00      RX0  C
ATOM     19  CB  SER   158       4.720  14.909   6.434  1.00  0.00      RX0  C
ATOM     20  OG  SER   158       5.924  15.060   5.672  1.00  0.00      RX0  O
ATOM     21  HG  SER   158       6.093  16.000   5.644  0.00  0.00      RX0  H
ATOM     22  C   SER   158       4.335  14.560   8.949  1.00  0.00      RX0  C
ATOM     23  O   SER   158       3.148  14.670   9.188  1.00  0.00      RX0  O
ATOM     24  N   LEU   159       5.132  13.681   9.591  1.00  0.00      RX0  N
ATOM     25  H   LEU   159       6.083  13.585   9.324  0.00  0.00      RX0  H
ATOM     26  CA  LEU   159       4.759  12.951  10.797  1.00  0.00      RX0  C
ATOM     27  CB  LEU   159       5.861  11.984  11.245  1.00  0.00      RX0  C
ATOM     28  CG  LEU   159       5.866  10.607  10.573  1.00  0.00      RX0  C
ATOM     29  CD1 LEU   159       6.257  10.647   9.094  1.00  0.00      RX0  C
ATOM     30  CD2 LEU   159       6.740   9.628  11.359  1.00  0.00      RX0  C
ATOM     31  C   LEU   159       4.518  13.965  11.920  1.00  0.00      RX0  C
ATOM     32  O   LEU   159       5.291  14.932  12.058  1.00  0.00      RX0  O
ATOM     33  N   THR   160       3.434  13.807  12.646  1.00  0.00      RX0  N
ATOM     34  H   THR   160       2.847  13.019  12.460  0.00  0.00      RX0  H
ATOM     35  CA  THR   160       3.156  14.665  13.825  1.00  0.00      RX0  C
ATOM     36  CB  THR   160       1.666  14.646  14.225  1.00  0.00      RX0  C
ATOM     37  OG1 THR   160       1.372  15.740  15.098  1.00  0.00      RX0  O
ATOM     38  HG1 THR   160       0.423  15.806  15.139  0.00  0.00      RX0  H
ATOM     39  CG2 THR   160       1.177  13.334  14.825  1.00  0.00      RX0  C
ATOM     40  C   THR   160       4.203  14.411  14.921  1.00  0.00      RX0  C
ATOM     41  O   THR   160       4.902  13.383  14.913  1.00  0.00      RX0  O
ATOM     42  N   ALA   161       4.153  15.229  15.953  1.00  0.00      RX0  N
ATOM     43  H   ALA   161       3.462  15.954  15.915  0.00  0.00      RX0  H
ATOM     44  CA  ALA   161       4.942  15.044  17.184  1.00  0.00      RX0  C
ATOM     45  CB  ALA   161       4.754  16.235  18.116  1.00  0.00      RX0  C
ATOM     46  C   ALA   161       4.543  13.746  17.920  1.00  0.00      RX0  C
ATOM     47  O   ALA   161       5.387  12.950  18.288  1.00  0.00      RX0  O
ATOM     48  N   ASP   162       3.226  13.461  17.917  1.00  0.00      RX0  N
ATOM     49  H   ASP   162       2.572  14.186  17.712  0.00  0.00      RX0  H
ATOM     50  CA  ASP   162       2.690  12.196  18.469  1.00  0.00      RX0  C
ATOM     51  CB  ASP   162       1.208  12.325  18.833  1.00  0.00      RX0  C
ATOM     52  CG  ASP   162       1.079  13.188  20.095  1.00  0.00      RX0  C
ATOM     53  OD1 ASP   162       1.882  14.092  20.314  1.00  0.00      RX0  O
ATOM     54  OD2 ASP   162       0.185  12.964  20.907  1.00  0.00      RX0  O
ATOM     55  C   ASP   162       3.088  10.948  17.668  1.00  0.00      RX0  C
ATOM     56  O   ASP   162       3.397   9.903  18.257  1.00  0.00      RX0  O
ATOM     57  N   GLN   163       3.164  11.087  16.353  1.00  0.00      RX0  N
ATOM     58  H   GLN   163       3.078  12.014  15.998  0.00  0.00      RX0  H
ATOM     59  CA  GLN   163       3.593   9.998  15.449  1.00  0.00      RX0  C
```

ATOM	60	CB	GLN	163	3.253	10.288	13.995	1.00	0.00	RX0	C
ATOM	61	CG	GLN	163	1.829	9.879	13.628	1.00	0.00	RX0	C
ATOM	62	CD	GLN	163	1.565	10.376	12.228	1.00	0.00	RX0	C
ATOM	63	OE1	GLN	163	2.080	11.413	11.824	1.00	0.00	RX0	O
ATOM	64	NE2	GLN	163	0.747	9.588	11.512	1.00	0.00	RX0	N
ATOM	65	HE21	GLN	163	0.365	8.752	11.910	0.00	0.00	RX0	H
ATOM	66	HE22	GLN	163	0.496	9.808	10.568	0.00	0.00	RX0	H
ATOM	67	C	GLN	163	5.089	9.698	15.572	1.00	0.00	RX0	C
ATOM	68	O	GLN	163	5.477	8.537	15.545	1.00	0.00	RX0	O
ATOM	69	N	MET	164	5.882	10.740	15.840	1.00	0.00	RX0	N
ATOM	70	H	MET	164	5.492	11.661	15.894	0.00	0.00	RX0	H
ATOM	71	CA	MET	164	7.331	10.587	16.060	1.00	0.00	RX0	C
ATOM	72	CB	MET	164	8.014	11.956	16.081	1.00	0.00	RX0	C
ATOM	73	CG	MET	164	9.451	11.881	16.604	1.00	0.00	RX0	C
ATOM	74	SD	MET	164	10.538	10.867	15.592	1.00	0.00	RX0	S
ATOM	75	CE	MET	164	11.023	12.137	14.421	1.00	0.00	RX0	C
ATOM	76	C	MET	164	7.610	9.825	17.366	1.00	0.00	RX0	C
ATOM	77	O	MET	164	8.404	8.887	17.381	1.00	0.00	RX0	O
ATOM	78	N	VAL	165	6.828	10.145	18.396	1.00	0.00	RX0	N
ATOM	79	H	VAL	165	6.165	10.888	18.278	0.00	0.00	RX0	H
ATOM	80	CA	VAL	165	6.992	9.563	19.744	1.00	0.00	RX0	C
ATOM	81	CB	VAL	165	6.102	10.260	20.779	1.00	0.00	RX0	C
ATOM	82	CG1	VAL	165	6.182	9.566	22.138	1.00	0.00	RX0	C
ATOM	83	CG2	VAL	165	6.455	11.732	20.920	1.00	0.00	RX0	C
ATOM	84	C	VAL	165	6.649	8.067	19.731	1.00	0.00	RX0	C
ATOM	85	O	VAL	165	7.442	7.255	20.191	1.00	0.00	RX0	O
ATOM	86	N	SER	166	5.467	7.742	19.205	1.00	0.00	RX0	N
ATOM	87	H	SER	166	4.815	8.431	18.871	0.00	0.00	RX0	H
ATOM	88	CA	SER	166	5.029	6.335	19.106	1.00	0.00	RX0	C
ATOM	89	CB	SER	166	3.571	6.389	18.708	1.00	0.00	RX0	C
ATOM	90	OG	SER	166	3.013	7.457	19.476	1.00	0.00	RX0	O
ATOM	91	HG	SER	166	3.357	7.370	20.355	0.00	0.00	RX0	H
ATOM	92	C	SER	166	5.941	5.501	18.195	1.00	0.00	RX0	C
ATOM	93	O	SER	166	6.295	4.379	18.542	1.00	0.00	RX0	O
ATOM	94	N	ALA	167	6.456	6.129	17.133	1.00	0.00	RX0	N
ATOM	95	H	ALA	167	6.171	7.064	16.912	0.00	0.00	RX0	H
ATOM	96	CA	ALA	167	7.397	5.466	16.208	1.00	0.00	RX0	C
ATOM	97	CB	ALA	167	7.731	6.357	15.013	1.00	0.00	RX0	C
ATOM	98	C	ALA	167	8.706	5.103	16.927	1.00	0.00	RX0	C
ATOM	99	O	ALA	167	9.113	3.946	16.932	1.00	0.00	RX0	O
ATOM	100	N	LEU	168	9.179	6.054	17.734	1.00	0.00	RX0	N
ATOM	101	H	LEU	168	8.733	6.951	17.738	0.00	0.00	RX0	H
ATOM	102	CA	LEU	168	10.385	5.875	18.562	1.00	0.00	RX0	C
ATOM	103	CB	LEU	168	10.907	7.215	19.074	1.00	0.00	RX0	C
ATOM	104	CG	LEU	168	11.572	8.040	17.976	1.00	0.00	RX0	C
ATOM	105	CD1	LEU	168	12.103	9.368	18.516	1.00	0.00	RX0	C
ATOM	106	CD2	LEU	168	12.654	7.236	17.254	1.00	0.00	RX0	C
ATOM	107	C	LEU	168	10.197	4.896	19.724	1.00	0.00	RX0	C
ATOM	108	O	LEU	168	11.077	4.078	19.994	1.00	0.00	RX0	O
ATOM	109	N	LEU	169	9.007	4.918	20.317	1.00	0.00	RX0	N
ATOM	110	H	LEU	169	8.333	5.589	20.011	0.00	0.00	RX0	H
ATOM	111	CA	LEU	169	8.640	3.970	21.384	1.00	0.00	RX0	C
ATOM	112	CB	LEU	169	7.357	4.394	22.102	1.00	0.00	RX0	C
ATOM	113	CG	LEU	169	7.539	5.639	22.971	1.00	0.00	RX0	C
ATOM	114	CD1	LEU	169	6.228	6.046	23.641	1.00	0.00	RX0	C
ATOM	115	CD2	LEU	169	8.668	5.470	23.989	1.00	0.00	RX0	C
ATOM	116	C	LEU	169	8.505	2.536	20.864	1.00	0.00	RX0	C
ATOM	117	O	LEU	169	9.003	1.602	21.486	1.00	0.00	RX0	O
ATOM	118	N	ASP	170	7.977	2.423	19.645	1.00	0.00	RX0	N
ATOM	119	H	ASP	170	7.668	3.241	19.162	0.00	0.00	RX0	H
ATOM	120	CA	ASP	170	7.822	1.133	18.952	1.00	0.00	RX0	C

ATOM	121	CB	ASP	170	6.875	1.403	17.770	1.00	0.00	RX0	C
ATOM	122	CG	ASP	170	6.620	0.254	16.800	1.00	0.00	RX0	C
ATOM	123	OD1	ASP	170	7.095	-0.861	16.975	1.00	0.00	RX0	O
ATOM	124	OD2	ASP	170	5.989	0.484	15.772	1.00	0.00	RX0	O
ATOM	125	C	ASP	170	9.164	0.506	18.541	1.00	0.00	RX0	C
ATOM	126	O	ASP	170	9.313	-0.704	18.571	1.00	0.00	RX0	O
ATOM	127	N	ALA	171	10.119	1.387	18.228	1.00	0.00	RX0	N
ATOM	128	H	ALA	171	9.896	2.361	18.292	0.00	0.00	RX0	H
ATOM	129	CA	ALA	171	11.447	1.008	17.717	1.00	0.00	RX0	C
ATOM	130	CB	ALA	171	12.103	2.219	17.054	1.00	0.00	RX0	C
ATOM	131	C	ALA	171	12.418	0.479	18.779	1.00	0.00	RX0	C
ATOM	132	O	ALA	171	13.427	-0.136	18.431	1.00	0.00	RX0	O
ATOM	133	N	GLU	172	12.125	0.739	20.058	1.00	0.00	RX0	N
ATOM	134	H	GLU	172	11.282	1.230	20.278	0.00	0.00	RX0	H
ATOM	135	CA	GLU	172	13.017	0.374	21.170	1.00	0.00	RX0	C
ATOM	136	CB	GLU	172	12.365	0.709	22.511	1.00	0.00	RX0	C
ATOM	137	CG	GLU	172	12.323	2.228	22.682	1.00	0.00	RX0	C
ATOM	138	CD	GLU	172	13.728	2.779	22.509	1.00	0.00	RX0	C
ATOM	139	OE1	GLU	172	14.559	2.616	23.403	1.00	0.00	RX0	O
ATOM	140	OE2	GLU	172	14.028	3.387	21.480	1.00	0.00	RX0	O
ATOM	141	C	GLU	172	13.554	-1.065	21.099	1.00	0.00	RX0	C
ATOM	142	O	GLU	172	12.785	-2.004	20.837	1.00	0.00	RX0	O
ATOM	143	N	PRO	173	14.865	-1.209	21.269	1.00	0.00	RX0	N
ATOM	144	CD	PRO	173	15.790	-0.096	21.453	1.00	0.00	RX0	C
ATOM	145	CA	PRO	173	15.538	-2.517	21.328	1.00	0.00	RX0	C
ATOM	146	CB	PRO	173	17.014	-2.109	21.197	1.00	0.00	RX0	C
ATOM	147	CG	PRO	173	17.104	-0.742	21.867	1.00	0.00	RX0	C
ATOM	148	C	PRO	173	15.206	-3.249	22.640	1.00	0.00	RX0	C
ATOM	149	O	PRO	173	14.829	-2.595	23.631	1.00	0.00	RX0	O
ATOM	150	N	PRO	174	15.294	-4.574	22.646	1.00	0.00	RX0	N
ATOM	151	CD	PRO	174	15.606	-5.388	21.476	1.00	0.00	RX0	C
ATOM	152	CA	PRO	174	15.084	-5.400	23.852	1.00	0.00	RX0	C
ATOM	153	CB	PRO	174	14.970	-6.812	23.273	1.00	0.00	RX0	C
ATOM	154	CG	PRO	174	15.857	-6.784	22.034	1.00	0.00	RX0	C
ATOM	155	C	PRO	174	16.250	-5.248	24.838	1.00	0.00	RX0	C
ATOM	156	O	PRO	174	17.379	-4.922	24.444	1.00	0.00	RX0	O
ATOM	157	N	ILE	175	15.956	-5.464	26.106	1.00	0.00	RX0	N
ATOM	158	H	ILE	175	15.038	-5.786	26.328	0.00	0.00	RX0	H
ATOM	159	CA	ILE	175	16.988	-5.556	27.159	1.00	0.00	RX0	C
ATOM	160	CB	ILE	175	16.447	-5.211	28.551	1.00	0.00	RX0	C
ATOM	161	CG2	ILE	175	17.624	-5.096	29.520	1.00	0.00	RX0	C
ATOM	162	CG1	ILE	175	15.624	-3.917	28.561	1.00	0.00	RX0	C
ATOM	163	CD1	ILE	175	14.111	-4.136	28.452	1.00	0.00	RX0	C
ATOM	164	C	ILE	175	17.586	-6.969	27.112	1.00	0.00	RX0	C
ATOM	165	O	ILE	175	16.886	-7.963	27.343	1.00	0.00	RX0	O
ATOM	166	N	LEU	176	18.884	-7.017	26.884	1.00	0.00	RX0	N
ATOM	167	H	LEU	176	19.405	-6.164	26.840	0.00	0.00	RX0	H
ATOM	168	CA	LEU	176	19.617	-8.291	26.770	1.00	0.00	RX0	C
ATOM	169	CB	LEU	176	20.630	-8.175	25.638	1.00	0.00	RX0	C
ATOM	170	CG	LEU	176	19.954	-7.962	24.282	1.00	0.00	RX0	C
ATOM	171	CD1	LEU	176	20.977	-7.824	23.158	1.00	0.00	RX0	C
ATOM	172	CD2	LEU	176	18.930	-9.056	23.975	1.00	0.00	RX0	C
ATOM	173	C	LEU	176	20.277	-8.690	28.089	1.00	0.00	RX0	C
ATOM	174	O	LEU	176	20.563	-7.852	28.952	1.00	0.00	RX0	O
ATOM	175	N	TYR	177	20.459	-9.989	28.237	1.00	0.00	RX0	N
ATOM	176	H	TYR	177	20.214	-10.612	27.497	0.00	0.00	RX0	H
ATOM	177	CA	TYR	177	21.114	-10.573	29.420	1.00	0.00	RX0	C
ATOM	178	CB	TYR	177	20.420	-11.864	29.854	1.00	0.00	RX0	C
ATOM	179	CG	TYR	177	19.151	-11.561	30.614	1.00	0.00	RX0	C
ATOM	180	CD1	TYR	177	18.030	-11.068	29.957	1.00	0.00	RX0	C
ATOM	181	CE1	TYR	177	16.865	-10.809	30.670	1.00	0.00	RX0	C

ATOM	182	CD2	TYR	177	19.109	-11.790	31.984	1.00	0.00	RX0	C
ATOM	183	CE2	TYR	177	17.943	-11.538	32.696	1.00	0.00	RX0	C
ATOM	184	CZ	TYR	177	16.822	-11.045	32.039	1.00	0.00	RX0	C
ATOM	185	OH	TYR	177	15.668	-10.786	32.748	1.00	0.00	RX0	O
ATOM	186	HH	TYR	177	15.774	-11.069	33.647	0.00	0.00	RX0	H
ATOM	187	C	TYR	177	22.589	-10.858	29.163	1.00	0.00	RX0	C
ATOM	188	O	TYR	177	22.985	-11.163	28.046	1.00	0.00	RX0	O
ATOM	189	N	SER	178	23.381	-10.750	30.220	1.00	0.00	RX0	N
ATOM	190	H	SER	178	23.018	-10.512	31.124	0.00	0.00	RX0	H
ATOM	191	CA	SER	178	24.788	-11.188	30.183	1.00	0.00	RX0	C
ATOM	192	CB	SER	178	25.476	-10.629	31.410	1.00	0.00	RX0	C
ATOM	193	OG	SER	178	25.159	-9.239	31.444	1.00	0.00	RX0	O
ATOM	194	HG	SER	178	25.434	-8.878	30.610	0.00	0.00	RX0	H
ATOM	195	C	SER	178	24.834	-12.718	30.070	1.00	0.00	RX0	C
ATOM	196	O	SER	178	23.999	-13.413	30.674	1.00	0.00	RX0	O
ATOM	197	N	GLU	179	25.827	-13.218	29.362	1.00	0.00	RX0	N
ATOM	198	H	GLU	179	26.490	-12.650	28.870	0.00	0.00	RX0	H
ATOM	199	CA	GLU	179	26.033	-14.670	29.175	1.00	0.00	RX0	C
ATOM	200	CB	GLU	179	26.378	-15.132	27.762	1.00	0.00	RX0	C
ATOM	201	CG	GLU	179	25.396	-14.789	26.663	1.00	0.00	RX0	C
ATOM	202	CD	GLU	179	26.069	-13.748	25.811	1.00	0.00	RX0	C
ATOM	203	OE1	GLU	179	25.864	-13.747	24.604	1.00	0.00	RX0	O
ATOM	204	OE2	GLU	179	26.734	-12.870	26.363	1.00	0.00	RX0	O
ATOM	205	C	GLU	179	27.192	-15.208	30.012	1.00	0.00	RX0	C
ATOM	206	O	GLU	179	28.361	-15.227	29.589	1.00	0.00	RX0	O
ATOM	207	N	TYR	180	26.873	-15.475	31.254	1.00	0.00	RX0	N
ATOM	208	H	TYR	180	25.922	-15.400	31.562	0.00	0.00	RX0	H
ATOM	209	CA	TYR	180	27.735	-16.233	32.177	1.00	0.00	RX0	C
ATOM	210	CB	TYR	180	28.495	-15.324	33.155	1.00	0.00	RX0	C
ATOM	211	CG	TYR	180	27.553	-14.677	34.143	1.00	0.00	RX0	C
ATOM	212	CD1	TYR	180	26.929	-13.476	33.829	1.00	0.00	RX0	C
ATOM	213	CE1	TYR	180	26.018	-12.918	34.716	1.00	0.00	RX0	C
ATOM	214	CD2	TYR	180	27.305	-15.290	35.367	1.00	0.00	RX0	C
ATOM	215	CE2	TYR	180	26.381	-14.741	36.246	1.00	0.00	RX0	C
ATOM	216	CZ	TYR	180	25.721	-13.567	35.909	1.00	0.00	RX0	C
ATOM	217	OH	TYR	180	24.761	-13.053	36.754	1.00	0.00	RX0	O
ATOM	218	HH	TYR	180	24.672	-12.118	36.586	0.00	0.00	RX0	H
ATOM	219	C	TYR	180	26.838	-17.226	32.909	1.00	0.00	RX0	C
ATOM	220	O	TYR	180	25.642	-16.953	33.094	1.00	0.00	RX0	O
ATOM	221	N	ASP	181	27.404	-18.345	33.318	1.00	0.00	RX0	N
ATOM	222	H	ASP	181	28.390	-18.468	33.250	0.00	0.00	RX0	H
ATOM	223	CA	ASP	181	26.630	-19.347	34.059	1.00	0.00	RX0	C
ATOM	224	CB	ASP	181	27.311	-20.705	34.112	1.00	0.00	RX0	C
ATOM	225	CG	ASP	181	26.498	-21.551	35.068	1.00	0.00	RX0	C
ATOM	226	OD1	ASP	181	25.284	-21.612	34.918	1.00	0.00	RX0	O
ATOM	227	OD2	ASP	181	27.062	-22.115	35.993	1.00	0.00	RX0	O
ATOM	228	C	ASP	181	26.420	-18.851	35.504	1.00	0.00	RX0	C
ATOM	229	O	ASP	181	27.391	-18.832	36.273	1.00	0.00	RX0	O
ATOM	230	N	PRO	182	25.185	-18.489	35.856	1.00	0.00	RX0	N
ATOM	231	CD	PRO	182	24.009	-18.601	34.996	1.00	0.00	RX0	C
ATOM	232	CA	PRO	182	24.825	-17.989	37.201	1.00	0.00	RX0	C
ATOM	233	CB	PRO	182	23.393	-17.488	37.001	1.00	0.00	RX0	C
ATOM	234	CG	PRO	182	22.821	-18.410	35.929	1.00	0.00	RX0	C
ATOM	235	C	PRO	182	24.941	-19.052	38.308	1.00	0.00	RX0	C
ATOM	236	O	PRO	182	24.654	-18.763	39.474	1.00	0.00	RX0	O
ATOM	237	N	THR	183	25.345	-20.259	37.948	1.00	0.00	RX0	N
ATOM	238	H	THR	183	25.566	-20.521	37.006	0.00	0.00	RX0	H
ATOM	239	CA	THR	183	25.568	-21.363	38.913	1.00	0.00	RX0	C
ATOM	240	CB	THR	183	24.916	-22.567	38.260	1.00	0.00	RX0	C
ATOM	241	OG1	THR	183	23.899	-22.075	37.372	1.00	0.00	RX0	O
ATOM	242	HG1	THR	183	24.338	-21.995	36.522	0.00	0.00	RX0	H

ATOM	243	CG2	THR	183	24.354	-23.563	39.281	1.00	0.00	RX0	C
ATOM	244	C	THR	183	27.063	-21.532	39.218	1.00	0.00	RX0	C
ATOM	245	O	THR	183	27.455	-22.345	40.058	1.00	0.00	RX0	O
ATOM	246	N	ARG	184	27.887	-20.699	38.573	1.00	0.00	RX0	N
ATOM	247	H	ARG	184	27.519	-20.004	37.953	0.00	0.00	RX0	H
ATOM	248	CA	ARG	184	29.343	-20.701	38.681	1.00	0.00	RX0	C
ATOM	249	CB	ARG	184	29.907	-20.804	37.277	1.00	0.00	RX0	C
ATOM	250	CG	ARG	184	30.268	-22.229	36.861	1.00	0.00	RX0	C
ATOM	251	CD	ARG	184	30.858	-22.200	35.453	1.00	0.00	RX0	C
ATOM	252	NE	ARG	184	31.569	-20.939	35.270	1.00	0.00	RX0	N
ATOM	253	HE	ARG	184	30.997	-20.189	34.930	0.00	0.00	RX0	H
ATOM	254	CZ	ARG	184	32.824	-20.795	35.778	1.00	0.00	RX0	C
ATOM	255	NH1	ARG	184	33.531	-21.880	36.145	1.00	0.00	RX0	N
ATOM	256	HH11	ARG	184	34.474	-21.749	36.509	0.00	0.00	RX0	H
ATOM	257	HH12	ARG	184	33.210	-22.822	36.082	0.00	0.00	RX0	H
ATOM	258	NH2	ARG	184	33.337	-19.562	35.937	1.00	0.00	RX0	N
ATOM	259	HH21	ARG	184	34.253	-19.491	36.358	0.00	0.00	RX0	H
ATOM	260	HH22	ARG	184	32.884	-18.703	35.703	0.00	0.00	RX0	H
ATOM	261	C	ARG	184	29.836	-19.410	39.407	1.00	0.00	RX0	C
ATOM	262	O	ARG	184	29.116	-18.390	39.334	1.00	0.00	RX0	O
ATOM	263	N	PRO	185	30.940	-19.460	40.113	1.00	0.00	RX0	N
ATOM	264	CD	PRO	185	31.744	-20.664	40.290	1.00	0.00	RX0	C
ATOM	265	CA	PRO	185	31.574	-18.285	40.781	1.00	0.00	RX0	C
ATOM	266	CB	PRO	185	32.859	-18.870	41.380	1.00	0.00	RX0	C
ATOM	267	CG	PRO	185	33.143	-20.135	40.576	1.00	0.00	RX0	C
ATOM	268	C	PRO	185	31.820	-17.125	39.813	1.00	0.00	RX0	C
ATOM	269	O	PRO	185	31.836	-17.275	38.592	1.00	0.00	RX0	O
ATOM	270	N	PHE	186	32.164	-15.998	40.422	1.00	0.00	RX0	N
ATOM	271	H	PHE	186	32.236	-15.993	41.418	0.00	0.00	RX0	H
ATOM	272	CA	PHE	186	32.333	-14.726	39.697	1.00	0.00	RX0	C
ATOM	273	CB	PHE	186	31.517	-13.614	40.358	1.00	0.00	RX0	C
ATOM	274	CG	PHE	186	31.423	-12.436	39.416	1.00	0.00	RX0	C
ATOM	275	CD1	PHE	186	30.879	-12.607	38.148	1.00	0.00	RX0	C
ATOM	276	CD2	PHE	186	31.880	-11.185	39.813	1.00	0.00	RX0	C
ATOM	277	CE1	PHE	186	30.796	-11.529	37.275	1.00	0.00	RX0	C
ATOM	278	CE2	PHE	186	31.797	-10.106	38.939	1.00	0.00	RX0	C
ATOM	279	CZ	PHE	186	31.256	-10.278	37.670	1.00	0.00	RX0	C
ATOM	280	C	PHE	186	33.791	-14.305	39.507	1.00	0.00	RX0	C
ATOM	281	O	PHE	186	34.127	-13.678	38.496	1.00	0.00	RX0	O
ATOM	282	N	SER	187	34.655	-14.802	40.380	1.00	0.00	RX0	N
ATOM	283	H	SER	187	34.308	-15.345	41.140	0.00	0.00	RX0	H
ATOM	284	CA	SER	187	36.113	-14.542	40.380	1.00	0.00	RX0	C
ATOM	285	CB	SER	187	36.618	-15.275	41.605	1.00	0.00	RX0	C
ATOM	286	OG	SER	187	35.501	-15.378	42.506	1.00	0.00	RX0	O
ATOM	287	HG	SER	187	35.873	-15.435	43.379	0.00	0.00	RX0	H
ATOM	288	C	SER	187	36.764	-14.980	39.057	1.00	0.00	RX0	C
ATOM	289	O	SER	187	37.834	-14.531	38.683	1.00	0.00	RX0	O
ATOM	290	N	GLU	188	36.054	-15.878	38.369	1.00	0.00	RX0	N
ATOM	291	H	GLU	188	35.128	-16.096	38.657	0.00	0.00	RX0	H
ATOM	292	CA	GLU	188	36.561	-16.586	37.191	1.00	0.00	RX0	C
ATOM	293	CB	GLU	188	36.195	-18.049	37.402	1.00	0.00	RX0	C
ATOM	294	CG	GLU	188	36.880	-19.096	36.530	1.00	0.00	RX0	C
ATOM	295	CD	GLU	188	36.100	-20.367	36.772	1.00	0.00	RX0	C
ATOM	296	OE1	GLU	188	35.281	-20.375	37.686	1.00	0.00	RX0	O
ATOM	297	OE2	GLU	188	36.197	-21.310	35.998	1.00	0.00	RX0	O
ATOM	298	C	GLU	188	36.028	-16.018	35.856	1.00	0.00	RX0	C
ATOM	299	O	GLU	188	36.494	-16.416	34.788	1.00	0.00	RX0	O
ATOM	300	N	ALA	189	35.058	-15.107	35.914	1.00	0.00	RX0	N
ATOM	301	H	ALA	189	34.838	-14.674	36.791	0.00	0.00	RX0	H
ATOM	302	CA	ALA	189	34.543	-14.432	34.708	1.00	0.00	RX0	C
ATOM	303	CB	ALA	189	33.067	-14.081	34.892	1.00	0.00	RX0	C

ATOM	304	C	ALA	189	35.336	-13.151	34.407	1.00	0.00	RX0	C
ATOM	305	O	ALA	189	35.533	-12.292	35.270	1.00	0.00	RX0	O
ATOM	306	N	SER	190	35.819	-13.065	33.173	1.00	0.00	RX0	N
ATOM	307	H	SER	190	35.632	-13.813	32.539	0.00	0.00	RX0	H
ATOM	308	CA	SER	190	36.430	-11.825	32.646	1.00	0.00	RX0	C
ATOM	309	CB	SER	190	37.142	-12.124	31.327	1.00	0.00	RX0	C
ATOM	310	OG	SER	190	38.565	-12.153	31.522	1.00	0.00	RX0	O
ATOM	311	HG	SER	190	38.730	-12.673	32.303	0.00	0.00	RX0	H
ATOM	312	C	SER	190	35.341	-10.761	32.513	1.00	0.00	RX0	C
ATOM	313	O	SER	190	34.465	-10.869	31.639	1.00	0.00	RX0	O
ATOM	314	N	MET	191	35.401	-9.751	33.361	1.00	0.00	RX0	N
ATOM	315	H	MET	191	36.064	-9.810	34.109	0.00	0.00	RX0	H
ATOM	316	CA	MET	191	34.414	-8.652	33.337	1.00	0.00	RX0	C
ATOM	317	CB	MET	191	34.645	-7.665	34.478	1.00	0.00	RX0	C
ATOM	318	CG	MET	191	33.553	-6.595	34.497	1.00	0.00	RX0	C
ATOM	319	SD	MET	191	33.648	-5.541	35.947	1.00	0.00	RX0	S
ATOM	320	CE	MET	191	33.305	-6.808	37.180	1.00	0.00	RX0	C
ATOM	321	C	MET	191	34.384	-7.939	31.976	1.00	0.00	RX0	C
ATOM	322	O	MET	191	33.329	-7.841	31.363	1.00	0.00	RX0	O
ATOM	323	N	MET	192	35.577	-7.654	31.438	1.00	0.00	RX0	N
ATOM	324	H	MET	192	36.392	-7.773	32.002	0.00	0.00	RX0	H
ATOM	325	CA	MET	192	35.697	-7.082	30.089	1.00	0.00	RX0	C
ATOM	326	CB	MET	192	37.142	-6.662	29.808	1.00	0.00	RX0	C
ATOM	327	CG	MET	192	37.309	-5.965	28.454	1.00	0.00	RX0	C
ATOM	328	SD	MET	192	36.238	-4.529	28.256	1.00	0.00	RX0	S
ATOM	329	CE	MET	192	36.848	-3.537	29.629	1.00	0.00	RX0	C
ATOM	330	C	MET	192	35.151	-8.021	28.999	1.00	0.00	RX0	C
ATOM	331	O	MET	192	34.484	-7.587	28.093	1.00	0.00	RX0	O
ATOM	332	N	GLY	193	35.358	-9.342	29.220	1.00	0.00	RX0	N
ATOM	333	H	GLY	193	35.775	-9.619	30.081	0.00	0.00	RX0	H
ATOM	334	CA	GLY	193	34.804	-10.378	28.330	1.00	0.00	RX0	C
ATOM	335	C	GLY	193	33.267	-10.338	28.334	1.00	0.00	RX0	C
ATOM	336	O	GLY	193	32.637	-10.184	27.296	1.00	0.00	RX0	O
ATOM	337	N	LEU	194	32.696	-10.293	29.537	1.00	0.00	RX0	N
ATOM	338	H	LEU	194	33.271	-10.304	30.352	0.00	0.00	RX0	H
ATOM	339	CA	LEU	194	31.235	-10.169	29.722	1.00	0.00	RX0	C
ATOM	340	CB	LEU	194	30.847	-10.222	31.200	1.00	0.00	RX0	C
ATOM	341	CG	LEU	194	31.143	-11.560	31.869	1.00	0.00	RX0	C
ATOM	342	CD1	LEU	194	30.704	-11.544	33.333	1.00	0.00	RX0	C
ATOM	343	CD2	LEU	194	30.535	-12.730	31.096	1.00	0.00	RX0	C
ATOM	344	C	LEU	194	30.647	-8.891	29.116	1.00	0.00	RX0	C
ATOM	345	O	LEU	194	29.706	-8.959	28.317	1.00	0.00	RX0	O
ATOM	346	N	LEU	195	31.327	-7.782	29.364	1.00	0.00	RX0	N
ATOM	347	H	LEU	195	32.140	-7.844	29.940	0.00	0.00	RX0	H
ATOM	348	CA	LEU	195	30.920	-6.462	28.846	1.00	0.00	RX0	C
ATOM	349	CB	LEU	195	31.731	-5.337	29.493	1.00	0.00	RX0	C
ATOM	350	CG	LEU	195	31.535	-5.240	31.007	1.00	0.00	RX0	C
ATOM	351	CD1	LEU	195	32.368	-4.109	31.611	1.00	0.00	RX0	C
ATOM	352	CD2	LEU	195	30.060	-5.134	31.390	1.00	0.00	RX0	C
ATOM	353	C	LEU	195	31.020	-6.357	27.321	1.00	0.00	RX0	C
ATOM	354	O	LEU	195	30.051	-5.942	26.671	1.00	0.00	RX0	O
ATOM	355	N	THR	196	32.075	-6.931	26.767	1.00	0.00	RX0	N
ATOM	356	H	THR	196	32.804	-7.321	27.328	0.00	0.00	RX0	H
ATOM	357	CA	THR	196	32.335	-6.901	25.309	1.00	0.00	RX0	C
ATOM	358	CB	THR	196	33.785	-7.291	25.033	1.00	0.00	RX0	C
ATOM	359	OG1	THR	196	34.654	-6.364	25.697	1.00	0.00	RX0	O
ATOM	360	HG1	THR	196	34.329	-5.499	25.480	0.00	0.00	RX0	H
ATOM	361	CG2	THR	196	34.093	-7.340	23.535	1.00	0.00	RX0	C
ATOM	362	C	THR	196	31.317	-7.765	24.552	1.00	0.00	RX0	C
ATOM	363	O	THR	196	30.772	-7.327	23.532	1.00	0.00	RX0	O
ATOM	364	N	ASN	197	31.003	-8.928	25.107	1.00	0.00	RX0	N

ATOM	365	H	ASN	197	31.429	-9.180	25.978	0.00	0.00	RX0	H
ATOM	366	CA	ASN	197	30.010	-9.840	24.504	1.00	0.00	RX0	C
ATOM	367	CB	ASN	197	29.983	-11.212	25.177	1.00	0.00	RX0	C
ATOM	368	CG	ASN	197	31.020	-12.113	24.542	1.00	0.00	RX0	C
ATOM	369	OD1	ASN	197	30.850	-12.661	23.459	1.00	0.00	RX0	O
ATOM	370	ND2	ASN	197	32.131	-12.239	25.289	1.00	0.00	RX0	N
ATOM	371	HD21	ASN	197	32.210	-11.715	26.141	0.00	0.00	RX0	H
ATOM	372	HD22	ASN	197	32.877	-12.840	25.004	0.00	0.00	RX0	H
ATOM	373	C	ASN	197	28.594	-9.255	24.528	1.00	0.00	RX0	C
ATOM	374	O	ASN	197	27.900	-9.272	23.514	1.00	0.00	RX0	O
ATOM	375	N	LEU	198	28.277	-8.575	25.633	1.00	0.00	RX0	N
ATOM	376	H	LEU	198	28.915	-8.577	26.406	0.00	0.00	RX0	H
ATOM	377	CA	LEU	198	27.002	-7.850	25.760	1.00	0.00	RX0	C
ATOM	378	CB	LEU	198	26.852	-7.337	27.189	1.00	0.00	RX0	C
ATOM	379	CG	LEU	198	25.457	-6.806	27.502	1.00	0.00	RX0	C
ATOM	380	CD1	LEU	198	24.364	-7.845	27.239	1.00	0.00	RX0	C
ATOM	381	CD2	LEU	198	25.402	-6.273	28.929	1.00	0.00	RX0	C
ATOM	382	C	LEU	198	26.885	-6.719	24.724	1.00	0.00	RX0	C
ATOM	383	O	LEU	198	25.930	-6.676	23.947	1.00	0.00	RX0	O
ATOM	384	N	ALA	199	27.942	-5.914	24.641	1.00	0.00	RX0	N
ATOM	385	H	ALA	199	28.699	-6.060	25.279	0.00	0.00	RX0	H
ATOM	386	CA	ALA	199	28.029	-4.784	23.694	1.00	0.00	RX0	C
ATOM	387	CB	ALA	199	29.343	-4.025	23.882	1.00	0.00	RX0	C
ATOM	388	C	ALA	199	27.921	-5.230	22.227	1.00	0.00	RX0	C
ATOM	389	O	ALA	199	27.138	-4.660	21.467	1.00	0.00	RX0	O
ATOM	390	N	ASP	200	28.555	-6.360	21.908	1.00	0.00	RX0	N
ATOM	391	H	ASP	200	29.141	-6.810	22.584	0.00	0.00	RX0	H
ATOM	392	CA	ASP	200	28.494	-6.940	20.550	1.00	0.00	RX0	C
ATOM	393	CB	ASP	200	29.539	-8.050	20.381	1.00	0.00	RX0	C
ATOM	394	CG	ASP	200	30.224	-7.962	19.026	1.00	0.00	RX0	C
ATOM	395	OD1	ASP	200	30.660	-6.879	18.633	1.00	0.00	RX0	O
ATOM	396	OD2	ASP	200	30.375	-8.985	18.360	1.00	0.00	RX0	O
ATOM	397	C	ASP	200	27.084	-7.410	20.171	1.00	0.00	RX0	C
ATOM	398	O	ASP	200	26.604	-7.102	19.080	1.00	0.00	RX0	O
ATOM	399	N	ARG	201	26.390	-7.998	21.143	1.00	0.00	RX0	N
ATOM	400	H	ARG	201	26.848	-8.160	22.021	0.00	0.00	RX0	H
ATOM	401	CA	ARG	201	24.992	-8.434	20.957	1.00	0.00	RX0	C
ATOM	402	CB	ARG	201	24.553	-9.414	22.031	1.00	0.00	RX0	C
ATOM	403	CG	ARG	201	24.064	-10.712	21.388	1.00	0.00	RX0	C
ATOM	404	CD	ARG	201	23.344	-11.615	22.384	1.00	0.00	RX0	C
ATOM	405	NE	ARG	201	24.157	-11.798	23.579	1.00	0.00	RX0	N
ATOM	406	HE	ARG	201	25.011	-12.342	23.517	0.00	0.00	RX0	H
ATOM	407	CZ	ARG	201	23.743	-11.272	24.763	1.00	0.00	RX0	C
ATOM	408	NH1	ARG	201	22.565	-10.628	24.826	1.00	0.00	RX0	N
ATOM	409	HH11	ARG	201	22.243	-10.255	25.697	0.00	0.00	RX0	H
ATOM	410	HH12	ARG	201	21.990	-10.506	24.013	0.00	0.00	RX0	H
ATOM	411	NH2	ARG	201	24.496	-11.398	25.860	1.00	0.00	RX0	N
ATOM	412	HH21	ARG	201	24.215	-11.107	26.780	0.00	0.00	RX0	H
ATOM	413	HH22	ARG	201	25.395	-11.865	25.792	0.00	0.00	RX0	H
ATOM	414	C	ARG	201	23.991	-7.279	20.827	1.00	0.00	RX0	C
ATOM	415	O	ARG	201	23.123	-7.308	19.955	1.00	0.00	RX0	O
ATOM	416	N	GLU	202	24.240	-6.201	21.568	1.00	0.00	RX0	N
ATOM	417	H	GLU	202	25.009	-6.216	22.213	0.00	0.00	RX0	H
ATOM	418	CA	GLU	202	23.401	-4.988	21.493	1.00	0.00	RX0	C
ATOM	419	CB	GLU	202	23.555	-4.078	22.734	1.00	0.00	RX0	C
ATOM	420	CG	GLU	202	22.916	-4.693	23.998	1.00	0.00	RX0	C
ATOM	421	CD	GLU	202	22.873	-3.744	25.199	1.00	0.00	RX0	C
ATOM	422	OE1	GLU	202	21.855	-3.086	25.419	1.00	0.00	RX0	O
ATOM	423	OE2	GLU	202	23.821	-3.706	25.978	1.00	0.00	RX0	O
ATOM	424	C	GLU	202	23.526	-4.262	20.149	1.00	0.00	RX0	C
ATOM	425	O	GLU	202	22.539	-3.754	19.625	1.00	0.00	RX0	O

ATOM	426	N	LEU	203	24.712	-4.358	19.546	1.00	0.00	RX0	N
ATOM	427	H	LEU	203	25.463	-4.810	20.035	0.00	0.00	RX0	H
ATOM	428	CA	LEU	203	25.004	-3.680	18.270	1.00	0.00	RX0	C
ATOM	429	CB	LEU	203	26.481	-3.871	17.917	1.00	0.00	RX0	C
ATOM	430	CG	LEU	203	26.921	-3.124	16.656	1.00	0.00	RX0	C
ATOM	431	CD1	LEU	203	26.719	-1.613	16.780	1.00	0.00	RX0	C
ATOM	432	CD2	LEU	203	28.358	-3.477	16.271	1.00	0.00	RX0	C
ATOM	433	C	LEU	203	24.099	-4.160	17.127	1.00	0.00	RX0	C
ATOM	434	O	LEU	203	23.593	-3.349	16.346	1.00	0.00	RX0	O
ATOM	435	N	VAL	204	23.782	-5.447	17.151	1.00	0.00	RX0	N
ATOM	436	H	VAL	204	24.177	-6.003	17.885	0.00	0.00	RX0	H
ATOM	437	CA	VAL	204	22.925	-6.083	16.127	1.00	0.00	RX0	C
ATOM	438	CB	VAL	204	22.898	-7.597	16.334	1.00	0.00	RX0	C
ATOM	439	CG1	VAL	204	22.010	-8.279	15.293	1.00	0.00	RX0	C
ATOM	440	CG2	VAL	204	24.319	-8.164	16.352	1.00	0.00	RX0	C
ATOM	441	C	VAL	204	21.502	-5.497	16.213	1.00	0.00	RX0	C
ATOM	442	O	VAL	204	20.938	-5.041	15.221	1.00	0.00	RX0	O
ATOM	443	N	HIS	205	21.015	-5.393	17.448	1.00	0.00	RX0	N
ATOM	444	H	HIS	205	21.604	-5.674	18.208	0.00	0.00	RX0	H
ATOM	445	CA	HIS	205	19.703	-4.786	17.746	1.00	0.00	RX0	C
ATOM	446	CB	HIS	205	19.275	-5.102	19.180	1.00	0.00	RX0	C
ATOM	447	CG	HIS	205	19.002	-6.582	19.307	1.00	0.00	RX0	C
ATOM	448	ND1	HIS	205	17.799	-7.142	19.077	1.00	0.00	RX0	N
ATOM	449	HD1	HIS	205	16.977	-6.674	18.823	0.00	0.00	RX0	H
ATOM	450	CD2	HIS	205	19.903	-7.590	19.661	1.00	0.00	RX0	C
ATOM	451	NE2	HIS	205	19.228	-8.766	19.641	1.00	0.00	RX0	N
ATOM	452	CE1	HIS	205	17.935	-8.491	19.281	1.00	0.00	RX0	C
ATOM	453	C	HIS	205	19.668	-3.277	17.476	1.00	0.00	RX0	C
ATOM	454	O	HIS	205	18.642	-2.756	17.030	1.00	0.00	RX0	O
ATOM	455	N	MET	206	20.820	-2.627	17.609	1.00	0.00	RX0	N
ATOM	456	H	MET	206	21.611	-3.124	17.972	0.00	0.00	RX0	H
ATOM	457	CA	MET	206	20.969	-1.185	17.340	1.00	0.00	RX0	C
ATOM	458	CB	MET	206	22.357	-0.694	17.746	1.00	0.00	RX0	C
ATOM	459	CG	MET	206	22.541	0.806	17.510	1.00	0.00	RX0	C
ATOM	460	SD	MET	206	24.240	1.329	17.774	1.00	0.00	RX0	S
ATOM	461	CE	MET	206	24.495	0.522	19.359	1.00	0.00	RX0	C
ATOM	462	C	MET	206	20.721	-0.870	15.856	1.00	0.00	RX0	C
ATOM	463	O	MET	206	20.035	0.103	15.544	1.00	0.00	RX0	O
ATOM	464	N	ILE	207	21.183	-1.758	14.977	1.00	0.00	RX0	N
ATOM	465	H	ILE	207	21.730	-2.518	15.339	0.00	0.00	RX0	H
ATOM	466	CA	ILE	207	20.975	-1.628	13.516	1.00	0.00	RX0	C
ATOM	467	CB	ILE	207	21.673	-2.789	12.801	1.00	0.00	RX0	C
ATOM	468	CG2	ILE	207	21.431	-2.763	11.294	1.00	0.00	RX0	C
ATOM	469	CG1	ILE	207	23.165	-2.814	13.131	1.00	0.00	RX0	C
ATOM	470	CD1	ILE	207	23.917	-1.612	12.564	1.00	0.00	RX0	C
ATOM	471	C	ILE	207	19.470	-1.621	13.197	1.00	0.00	RX0	C
ATOM	472	O	ILE	207	18.988	-0.755	12.467	1.00	0.00	RX0	O
ATOM	473	N	ASN	208	18.761	-2.558	13.816	1.00	0.00	RX0	N
ATOM	474	H	ASN	208	19.239	-3.129	14.487	0.00	0.00	RX0	H
ATOM	475	CA	ASN	208	17.313	-2.734	13.586	1.00	0.00	RX0	C
ATOM	476	CB	ASN	208	16.799	-4.076	14.102	1.00	0.00	RX0	C
ATOM	477	CG	ASN	208	17.108	-5.149	13.072	1.00	0.00	RX0	C
ATOM	478	OD1	ASN	208	17.550	-4.881	11.951	1.00	0.00	RX0	O
ATOM	479	ND2	ASN	208	16.864	-6.390	13.524	1.00	0.00	RX0	N
ATOM	480	HD21	ASN	208	16.498	-6.515	14.448	0.00	0.00	RX0	H
ATOM	481	HD22	ASN	208	17.032	-7.221	12.990	0.00	0.00	RX0	H
ATOM	482	C	ASN	208	16.516	-1.532	14.103	1.00	0.00	RX0	C
ATOM	483	O	ASN	208	15.637	-1.017	13.417	1.00	0.00	RX0	O
ATOM	484	N	TRP	209	16.982	-1.008	15.238	1.00	0.00	RX0	N
ATOM	485	H	TRP	209	17.725	-1.474	15.722	0.00	0.00	RX0	H
ATOM	486	CA	TRP	209	16.425	0.201	15.864	1.00	0.00	RX0	C

ATOM	487	CB	TRP	209	17.092	0.421	17.232	1.00	0.00	RX0	C
ATOM	488	CG	TRP	209	16.693	1.750	17.835	1.00	0.00	RX0	C
ATOM	489	CD2	TRP	209	17.411	3.001	17.807	1.00	0.00	RX0	C
ATOM	490	CE2	TRP	209	16.629	3.959	18.493	1.00	0.00	RX0	C
ATOM	491	CE3	TRP	209	18.627	3.375	17.253	1.00	0.00	RX0	C
ATOM	492	CD1	TRP	209	15.526	2.031	18.552	1.00	0.00	RX0	C
ATOM	493	NE1	TRP	209	15.476	3.327	18.949	1.00	0.00	RX0	N
ATOM	494	HE1	TRP	209	14.720	3.717	19.453	0.00	0.00	RX0	H
ATOM	495	CZ2	TRP	209	17.096	5.262	18.598	1.00	0.00	RX0	C
ATOM	496	CZ3	TRP	209	19.083	4.681	17.370	1.00	0.00	RX0	C
ATOM	497	CH2	TRP	209	18.313	5.626	18.036	1.00	0.00	RX0	C
ATOM	498	C	TRP	209	16.619	1.438	14.972	1.00	0.00	RX0	C
ATOM	499	O	TRP	209	15.652	2.120	14.634	1.00	0.00	RX0	O
ATOM	500	N	ALA	210	17.853	1.624	14.503	1.00	0.00	RX0	N
ATOM	501	H	ALA	210	18.567	0.987	14.790	0.00	0.00	RX0	H
ATOM	502	CA	ALA	210	18.233	2.756	13.635	1.00	0.00	RX0	C
ATOM	503	CB	ALA	210	19.715	2.665	13.273	1.00	0.00	RX0	C
ATOM	504	C	ALA	210	17.400	2.800	12.347	1.00	0.00	RX0	C
ATOM	505	O	ALA	210	16.892	3.855	11.979	1.00	0.00	RX0	O
ATOM	506	N	LYS	211	17.095	1.613	11.820	1.00	0.00	RX0	N
ATOM	507	H	LYS	211	17.502	0.799	12.237	0.00	0.00	RX0	H
ATOM	508	CA	LYS	211	16.258	1.472	10.614	1.00	0.00	RX0	C
ATOM	509	CB	LYS	211	16.404	0.067	10.027	1.00	0.00	RX0	C
ATOM	510	CG	LYS	211	17.795	-0.099	9.401	1.00	0.00	RX0	C
ATOM	511	CD	LYS	211	18.075	-1.506	8.871	1.00	0.00	RX0	C
ATOM	512	CE	LYS	211	17.871	-2.526	9.985	1.00	0.00	RX0	C
ATOM	513	NZ	LYS	211	18.378	-3.859	9.630	1.00	0.00	RX0	N
ATOM	514	HZ1	LYS	211	18.199	-4.484	10.448	0.00	0.00	RX0	H
ATOM	515	HZ2	LYS	211	17.880	-4.231	8.801	0.00	0.00	RX0	H
ATOM	516	HZ3	LYS	211	19.401	-3.829	9.441	0.00	0.00	RX0	H
ATOM	517	C	LYS	211	14.795	1.898	10.823	1.00	0.00	RX0	C
ATOM	518	O	LYS	211	14.129	2.337	9.881	1.00	0.00	RX0	O
ATOM	519	N	ARG	212	14.357	1.870	12.071	1.00	0.00	RX0	N
ATOM	520	H	ARG	212	14.980	1.608	12.810	0.00	0.00	RX0	H
ATOM	521	CA	ARG	212	13.005	2.320	12.466	1.00	0.00	RX0	C
ATOM	522	CB	ARG	212	12.387	1.384	13.513	1.00	0.00	RX0	C
ATOM	523	CG	ARG	212	12.512	-0.090	13.124	1.00	0.00	RX0	C
ATOM	524	CD	ARG	212	11.637	-1.005	13.983	1.00	0.00	RX0	C
ATOM	525	NE	ARG	212	10.225	-0.783	13.676	1.00	0.00	RX0	N
ATOM	526	HE	ARG	212	10.016	-0.575	12.718	0.00	0.00	RX0	H
ATOM	527	CZ	ARG	212	9.272	-0.870	14.651	1.00	0.00	RX0	C
ATOM	528	NH1	ARG	212	9.621	-1.188	15.914	1.00	0.00	RX0	N
ATOM	529	HH11	ARG	212	8.917	-1.192	16.652	0.00	0.00	RX0	H
ATOM	530	HH12	ARG	212	10.552	-1.403	16.211	0.00	0.00	RX0	H
ATOM	531	NH2	ARG	212	7.990	-0.608	14.339	1.00	0.00	RX0	N
ATOM	532	HH21	ARG	212	7.289	-0.599	15.082	0.00	0.00	RX0	H
ATOM	533	HH22	ARG	212	7.652	-0.374	13.428	0.00	0.00	RX0	H
ATOM	534	C	ARG	212	12.933	3.790	12.894	1.00	0.00	RX0	C
ATOM	535	O	ARG	212	11.827	4.344	12.989	1.00	0.00	RX0	O
ATOM	536	N	VAL	213	14.074	4.417	13.148	1.00	0.00	RX0	N
ATOM	537	H	VAL	213	14.933	3.928	12.994	0.00	0.00	RX0	H
ATOM	538	CA	VAL	213	14.155	5.874	13.374	1.00	0.00	RX0	C
ATOM	539	CB	VAL	213	15.581	6.282	13.747	1.00	0.00	RX0	C
ATOM	540	CG1	VAL	213	15.749	7.801	13.838	1.00	0.00	RX0	C
ATOM	541	CG2	VAL	213	15.981	5.585	15.046	1.00	0.00	RX0	C
ATOM	542	C	VAL	213	13.672	6.590	12.095	1.00	0.00	RX0	C
ATOM	543	O	VAL	213	14.288	6.431	11.023	1.00	0.00	RX0	O
ATOM	544	N	PRO	214	12.622	7.395	12.213	1.00	0.00	RX0	N
ATOM	545	CD	PRO	214	11.896	7.609	13.458	1.00	0.00	RX0	C
ATOM	546	CA	PRO	214	12.035	8.145	11.084	1.00	0.00	RX0	C
ATOM	547	CB	PRO	214	10.926	8.954	11.761	1.00	0.00	RX0	C

ATOM	548	CG	PRO	214	10.552	8.163	13.010	1.00	0.00	RX0	C
ATOM	549	C	PRO	214	13.102	9.001	10.387	1.00	0.00	RX0	C
ATOM	550	O	PRO	214	13.853	9.727	11.025	1.00	0.00	RX0	O
ATOM	551	N	GLY	215	13.244	8.730	9.080	1.00	0.00	RX0	N
ATOM	552	H	GLY	215	12.750	7.982	8.635	0.00	0.00	RX0	H
ATOM	553	CA	GLY	215	14.194	9.473	8.227	1.00	0.00	RX0	C
ATOM	554	C	GLY	215	15.511	8.732	7.950	1.00	0.00	RX0	C
ATOM	555	O	GLY	215	16.085	8.889	6.862	1.00	0.00	RX0	O
ATOM	556	N	PHE	216	15.917	7.845	8.848	1.00	0.00	RX0	N
ATOM	557	H	PHE	216	15.330	7.653	9.637	0.00	0.00	RX0	H
ATOM	558	CA	PHE	216	17.224	7.160	8.764	1.00	0.00	RX0	C
ATOM	559	CB	PHE	216	17.451	6.293	9.997	1.00	0.00	RX0	C
ATOM	560	CG	PHE	216	18.889	5.839	10.056	1.00	0.00	RX0	C
ATOM	561	CD1	PHE	216	19.896	6.763	10.305	1.00	0.00	RX0	C
ATOM	562	CD2	PHE	216	19.206	4.497	9.876	1.00	0.00	RX0	C
ATOM	563	CE1	PHE	216	21.216	6.342	10.412	1.00	0.00	RX0	C
ATOM	564	CE2	PHE	216	20.525	4.075	9.985	1.00	0.00	RX0	C
ATOM	565	CZ	PHE	216	21.527	4.995	10.274	1.00	0.00	RX0	C
ATOM	566	C	PHE	216	17.435	6.347	7.474	1.00	0.00	RX0	C
ATOM	567	O	PHE	216	18.358	6.641	6.711	1.00	0.00	RX0	O
ATOM	568	N	VAL	217	16.482	5.483	7.151	1.00	0.00	RX0	N
ATOM	569	H	VAL	217	15.673	5.452	7.736	0.00	0.00	RX0	H
ATOM	570	CA	VAL	217	16.570	4.608	5.958	1.00	0.00	RX0	C
ATOM	571	CB	VAL	217	15.528	3.494	6.009	1.00	0.00	RX0	C
ATOM	572	CG1	VAL	217	15.905	2.475	7.075	1.00	0.00	RX0	C
ATOM	573	CG2	VAL	217	14.112	4.050	6.182	1.00	0.00	RX0	C
ATOM	574	C	VAL	217	16.469	5.342	4.608	1.00	0.00	RX0	C
ATOM	575	O	VAL	217	16.660	4.747	3.556	1.00	0.00	RX0	O
ATOM	576	N	ASP	218	16.058	6.613	4.671	1.00	0.00	RX0	N
ATOM	577	H	ASP	218	15.773	7.065	5.519	0.00	0.00	RX0	H
ATOM	578	CA	ASP	218	16.006	7.470	3.472	1.00	0.00	RX0	C
ATOM	579	CB	ASP	218	15.072	8.655	3.757	1.00	0.00	RX0	C
ATOM	580	CG	ASP	218	13.735	8.191	4.336	1.00	0.00	RX0	C
ATOM	581	OD1	ASP	218	12.742	8.225	3.613	1.00	0.00	RX0	O
ATOM	582	OD2	ASP	218	13.671	7.821	5.513	1.00	0.00	RX0	O
ATOM	583	C	ASP	218	17.401	7.924	3.023	1.00	0.00	RX0	C
ATOM	584	O	ASP	218	17.595	8.369	1.896	1.00	0.00	RX0	O
ATOM	585	N	LEU	219	18.344	7.857	3.967	1.00	0.00	RX0	N
ATOM	586	H	LEU	219	18.096	7.498	4.867	0.00	0.00	RX0	H
ATOM	587	CA	LEU	219	19.767	8.083	3.697	1.00	0.00	RX0	C
ATOM	588	CB	LEU	219	20.535	8.263	5.008	1.00	0.00	RX0	C
ATOM	589	CG	LEU	219	19.891	9.315	5.920	1.00	0.00	RX0	C
ATOM	590	CD1	LEU	219	20.548	9.333	7.297	1.00	0.00	RX0	C
ATOM	591	CD2	LEU	219	19.834	10.708	5.290	1.00	0.00	RX0	C
ATOM	592	C	LEU	219	20.350	6.970	2.832	1.00	0.00	RX0	C
ATOM	593	O	LEU	219	19.843	5.836	2.789	1.00	0.00	RX0	O
ATOM	594	N	THR	220	21.442	7.293	2.191	1.00	0.00	RX0	N
ATOM	595	H	THR	220	21.749	8.235	2.378	0.00	0.00	RX0	H
ATOM	596	CA	THR	220	22.263	6.312	1.453	1.00	0.00	RX0	C
ATOM	597	CB	THR	220	23.237	6.967	0.448	1.00	0.00	RX0	C
ATOM	598	OG1	THR	220	24.620	6.654	0.640	1.00	0.00	RX0	O
ATOM	599	HG1	THR	220	24.920	7.295	1.295	0.00	0.00	RX0	H
ATOM	600	CG2	THR	220	23.027	8.472	0.341	1.00	0.00	RX0	C
ATOM	601	C	THR	220	22.829	5.303	2.454	1.00	0.00	RX0	C
ATOM	602	O	THR	220	23.112	5.646	3.611	1.00	0.00	RX0	O
ATOM	603	N	LEU	221	23.130	4.116	1.957	1.00	0.00	RX0	N
ATOM	604	H	LEU	221	22.848	3.922	1.021	0.00	0.00	RX0	H
ATOM	605	CA	LEU	221	23.750	3.065	2.781	1.00	0.00	RX0	C
ATOM	606	CB	LEU	221	23.950	1.861	1.860	1.00	0.00	RX0	C
ATOM	607	CG	LEU	221	24.778	0.712	2.427	1.00	0.00	RX0	C
ATOM	608	CD1	LEU	221	24.068	-0.000	3.575	1.00	0.00	RX0	C

ATOM	609	CD2	LEU	221	25.226	-0.227	1.310	1.00	0.00	RX0	C
ATOM	610	C	LEU	221	25.092	3.509	3.394	1.00	0.00	RX0	C
ATOM	611	O	LEU	221	25.324	3.347	4.578	1.00	0.00	RX0	O
ATOM	612	N	HIS	222	25.854	4.270	2.593	1.00	0.00	RX0	N
ATOM	613	H	HIS	222	25.513	4.478	1.678	0.00	0.00	RX0	H
ATOM	614	CA	HIS	222	27.131	4.847	3.045	1.00	0.00	RX0	C
ATOM	615	CB	HIS	222	27.848	5.632	1.927	1.00	0.00	RX0	C
ATOM	616	CG	HIS	222	28.929	6.526	2.518	1.00	0.00	RX0	C
ATOM	617	ND1	HIS	222	29.872	6.102	3.381	1.00	0.00	RX0	N
ATOM	618	HD1	HIS	222	29.985	5.195	3.744	0.00	0.00	RX0	H
ATOM	619	CD2	HIS	222	29.076	7.905	2.347	1.00	0.00	RX0	C
ATOM	620	NE2	HIS	222	30.116	8.306	3.118	1.00	0.00	RX0	N
ATOM	621	CE1	HIS	222	30.604	7.197	3.756	1.00	0.00	RX0	C
ATOM	622	C	HIS	222	26.942	5.765	4.264	1.00	0.00	RX0	C
ATOM	623	O	HIS	222	27.673	5.642	5.246	1.00	0.00	RX0	O
ATOM	624	N	ASP	223	25.958	6.658	4.167	1.00	0.00	RX0	N
ATOM	625	H	ASP	223	25.455	6.786	3.314	0.00	0.00	RX0	H
ATOM	626	CA	ASP	223	25.694	7.636	5.242	1.00	0.00	RX0	C
ATOM	627	CB	ASP	223	24.872	8.781	4.619	1.00	0.00	RX0	C
ATOM	628	CG	ASP	223	25.600	9.324	3.372	1.00	0.00	RX0	C
ATOM	629	OD1	ASP	223	26.195	10.397	3.444	1.00	0.00	RX0	O
ATOM	630	OD2	ASP	223	25.604	8.659	2.328	1.00	0.00	RX0	O
ATOM	631	C	ASP	223	25.148	6.996	6.516	1.00	0.00	RX0	C
ATOM	632	O	ASP	223	25.558	7.375	7.616	1.00	0.00	RX0	O
ATOM	633	N	GLN	224	24.393	5.921	6.332	1.00	0.00	RX0	N
ATOM	634	H	GLN	224	24.142	5.695	5.388	0.00	0.00	RX0	H
ATOM	635	CA	GLN	224	23.868	5.118	7.452	1.00	0.00	RX0	C
ATOM	636	CB	GLN	224	22.846	4.094	6.958	1.00	0.00	RX0	C
ATOM	637	CG	GLN	224	21.592	4.770	6.398	1.00	0.00	RX0	C
ATOM	638	CD	GLN	224	20.549	3.721	6.076	1.00	0.00	RX0	C
ATOM	639	OE1	GLN	224	20.405	2.720	6.772	1.00	0.00	RX0	O
ATOM	640	NE2	GLN	224	19.829	4.001	4.976	1.00	0.00	RX0	N
ATOM	641	HE21	GLN	224	19.982	4.842	4.448	0.00	0.00	RX0	H
ATOM	642	HE22	GLN	224	19.098	3.423	4.617	0.00	0.00	RX0	H
ATOM	643	C	GLN	224	25.003	4.453	8.243	1.00	0.00	RX0	C
ATOM	644	O	GLN	224	25.073	4.591	9.468	1.00	0.00	RX0	O
ATOM	645	N	VAL	225	25.993	3.956	7.505	1.00	0.00	RX0	N
ATOM	646	H	VAL	225	25.898	3.986	6.507	0.00	0.00	RX0	H
ATOM	647	CA	VAL	225	27.191	3.319	8.093	1.00	0.00	RX0	C
ATOM	648	CB	VAL	225	28.037	2.533	7.097	1.00	0.00	RX0	C
ATOM	649	CG1	VAL	225	29.093	1.756	7.878	1.00	0.00	RX0	C
ATOM	650	CG2	VAL	225	27.204	1.563	6.263	1.00	0.00	RX0	C
ATOM	651	C	VAL	225	28.021	4.368	8.852	1.00	0.00	RX0	C
ATOM	652	O	VAL	225	28.415	4.141	9.995	1.00	0.00	RX0	O
ATOM	653	N	HIS	226	28.182	5.534	8.231	1.00	0.00	RX0	N
ATOM	654	H	HIS	226	27.810	5.642	7.306	0.00	0.00	RX0	H
ATOM	655	CA	HIS	226	28.959	6.641	8.815	1.00	0.00	RX0	C
ATOM	656	CB	HIS	226	29.138	7.825	7.869	1.00	0.00	RX0	C
ATOM	657	CG	HIS	226	30.094	8.812	8.507	1.00	0.00	RX0	C
ATOM	658	ND1	HIS	226	31.305	8.474	8.988	1.00	0.00	RX0	N
ATOM	659	HD1	HIS	226	31.699	7.576	9.017	0.00	0.00	RX0	H
ATOM	660	CD2	HIS	226	29.911	10.185	8.702	1.00	0.00	RX0	C
ATOM	661	NE2	HIS	226	31.026	10.671	9.304	1.00	0.00	RX0	N
ATOM	662	CE1	HIS	226	31.882	9.614	9.479	1.00	0.00	RX0	C
ATOM	663	C	HIS	226	28.363	7.118	10.150	1.00	0.00	RX0	C
ATOM	664	O	HIS	226	29.071	7.189	11.155	1.00	0.00	RX0	O
ATOM	665	N	LEU	227	27.047	7.317	10.166	1.00	0.00	RX0	N
ATOM	666	H	LEU	227	26.529	7.184	9.317	0.00	0.00	RX0	H
ATOM	667	CA	LEU	227	26.344	7.787	11.375	1.00	0.00	RX0	C
ATOM	668	CB	LEU	227	24.876	8.090	11.077	1.00	0.00	RX0	C
ATOM	669	CG	LEU	227	24.683	9.366	10.259	1.00	0.00	RX0	C

ATOM	670	CD1	LEU	227	23.212	9.595	9.925	1.00	0.00	RX0	C
ATOM	671	CD2	LEU	227	25.286	10.584	10.957	1.00	0.00	RX0	C
ATOM	672	C	LEU	227	26.435	6.799	12.540	1.00	0.00	RX0	C
ATOM	673	O	LEU	227	26.853	7.165	13.635	1.00	0.00	RX0	O
ATOM	674	N	LEU	228	26.270	5.522	12.200	1.00	0.00	RX0	N
ATOM	675	H	LEU	228	26.041	5.298	11.249	0.00	0.00	RX0	H
ATOM	676	CA	LEU	228	26.384	4.431	13.181	1.00	0.00	RX0	C
ATOM	677	CB	LEU	228	25.764	3.154	12.619	1.00	0.00	RX0	C
ATOM	678	CG	LEU	228	24.258	3.143	12.875	1.00	0.00	RX0	C
ATOM	679	CD1	LEU	228	23.519	2.130	12.008	1.00	0.00	RX0	C
ATOM	680	CD2	LEU	228	23.960	2.941	14.361	1.00	0.00	RX0	C
ATOM	681	C	LEU	228	27.805	4.188	13.685	1.00	0.00	RX0	C
ATOM	682	O	LEU	228	28.004	4.019	14.891	1.00	0.00	RX0	O
ATOM	683	N	GLU	229	28.784	4.376	12.809	1.00	0.00	RX0	N
ATOM	684	H	GLU	229	28.582	4.585	11.851	0.00	0.00	RX0	H
ATOM	685	CA	GLU	229	30.199	4.229	13.197	1.00	0.00	RX0	C
ATOM	686	CB	GLU	229	31.136	4.116	11.990	1.00	0.00	RX0	C
ATOM	687	CG	GLU	229	32.479	3.507	12.408	1.00	0.00	RX0	C
ATOM	688	CD	GLU	229	33.307	3.149	11.193	1.00	0.00	RX0	C
ATOM	689	OE1	GLU	229	33.193	3.838	10.182	1.00	0.00	RX0	O
ATOM	690	OE2	GLU	229	34.056	2.172	11.258	1.00	0.00	RX0	O
ATOM	691	C	GLU	229	30.618	5.338	14.175	1.00	0.00	RX0	C
ATOM	692	O	GLU	229	31.393	5.088	15.099	1.00	0.00	RX0	O
ATOM	693	N	CYS	230	30.060	6.523	13.970	1.00	0.00	RX0	N
ATOM	694	H	CYS	230	29.474	6.650	13.166	0.00	0.00	RX0	H
ATOM	695	CA	CYS	230	30.321	7.692	14.829	1.00	0.00	RX0	C
ATOM	696	CB	CYS	230	30.003	8.979	14.080	1.00	0.00	RX0	C
ATOM	697	SG	CYS	230	31.098	9.245	12.667	1.00	0.00	RX0	S
ATOM	698	C	CYS	230	29.592	7.653	16.182	1.00	0.00	RX0	C
ATOM	699	O	CYS	230	30.123	8.119	17.188	1.00	0.00	RX0	O
ATOM	700	N	ALA	231	28.434	7.000	16.215	1.00	0.00	RX0	N
ATOM	701	H	ALA	231	28.124	6.535	15.386	0.00	0.00	RX0	H
ATOM	702	CA	ALA	231	27.494	7.141	17.345	1.00	0.00	RX0	C
ATOM	703	CB	ALA	231	26.144	7.667	16.853	1.00	0.00	RX0	C
ATOM	704	C	ALA	231	27.249	5.885	18.186	1.00	0.00	RX0	C
ATOM	705	O	ALA	231	26.768	6.021	19.321	1.00	0.00	RX0	O
ATOM	706	N	TRP	232	27.687	4.719	17.731	1.00	0.00	RX0	N
ATOM	707	H	TRP	232	28.111	4.686	16.823	0.00	0.00	RX0	H
ATOM	708	CA	TRP	232	27.348	3.433	18.379	1.00	0.00	RX0	C
ATOM	709	CB	TRP	232	27.970	2.236	17.646	1.00	0.00	RX0	C
ATOM	710	CG	TRP	232	29.473	2.270	17.767	1.00	0.00	RX0	C
ATOM	711	CD2	TRP	232	30.319	1.525	18.666	1.00	0.00	RX0	C
ATOM	712	CE2	TRP	232	31.653	1.923	18.426	1.00	0.00	RX0	C
ATOM	713	CE3	TRP	232	30.049	0.574	19.642	1.00	0.00	RX0	C
ATOM	714	CD1	TRP	232	30.350	3.071	17.030	1.00	0.00	RX0	C
ATOM	715	NE1	TRP	232	31.635	2.875	17.411	1.00	0.00	RX0	N
ATOM	716	HE1	TRP	232	32.405	3.335	17.007	0.00	0.00	RX0	H
ATOM	717	CZ2	TRP	232	32.681	1.356	19.168	1.00	0.00	RX0	C
ATOM	718	CZ3	TRP	232	31.087	0.016	20.378	1.00	0.00	RX0	C
ATOM	719	CH2	TRP	232	32.400	0.406	20.142	1.00	0.00	RX0	C
ATOM	720	C	TRP	232	27.676	3.373	19.884	1.00	0.00	RX0	C
ATOM	721	O	TRP	232	26.862	2.914	20.672	1.00	0.00	RX0	O
ATOM	722	N	LEU	233	28.801	3.989	20.277	1.00	0.00	RX0	N
ATOM	723	H	LEU	233	29.368	4.444	19.591	0.00	0.00	RX0	H
ATOM	724	CA	LEU	233	29.211	3.960	21.691	1.00	0.00	RX0	C
ATOM	725	CB	LEU	233	30.720	4.160	21.833	1.00	0.00	RX0	C
ATOM	726	CG	LEU	233	31.199	3.782	23.236	1.00	0.00	RX0	C
ATOM	727	CD1	LEU	233	30.804	2.350	23.601	1.00	0.00	RX0	C
ATOM	728	CD2	LEU	233	32.696	4.018	23.416	1.00	0.00	RX0	C
ATOM	729	C	LEU	233	28.415	4.936	22.566	1.00	0.00	RX0	C
ATOM	730	O	LEU	233	27.943	4.566	23.634	1.00	0.00	RX0	O

ATOM	731	N	GLU	234	28.150	6.122	22.016	1.00	0.00	RX0	N
ATOM	732	H	GLU	234	28.475	6.315	21.092	0.00	0.00	RX0	H
ATOM	733	CA	GLU	234	27.227	7.090	22.644	1.00	0.00	RX0	C
ATOM	734	CB	GLU	234	27.038	8.333	21.766	1.00	0.00	RX0	C
ATOM	735	CG	GLU	234	28.062	9.461	21.890	1.00	0.00	RX0	C
ATOM	736	CD	GLU	234	27.750	10.573	20.900	1.00	0.00	RX0	C
ATOM	737	OE1	GLU	234	27.421	11.684	21.302	1.00	0.00	RX0	O
ATOM	738	OE2	GLU	234	27.913	10.372	19.707	1.00	0.00	RX0	O
ATOM	739	C	GLU	234	25.830	6.482	22.841	1.00	0.00	RX0	C
ATOM	740	O	GLU	234	25.253	6.598	23.926	1.00	0.00	RX0	O
ATOM	741	N	ILE	235	25.389	5.711	21.848	1.00	0.00	RX0	N
ATOM	742	H	ILE	235	25.961	5.634	21.030	0.00	0.00	RX0	H
ATOM	743	CA	ILE	235	24.069	5.045	21.863	1.00	0.00	RX0	C
ATOM	744	CB	ILE	235	23.695	4.488	20.486	1.00	0.00	RX0	C
ATOM	745	CG2	ILE	235	22.410	3.661	20.552	1.00	0.00	RX0	C
ATOM	746	CG1	ILE	235	23.557	5.620	19.468	1.00	0.00	RX0	C
ATOM	747	CD1	ILE	235	23.212	5.106	18.070	1.00	0.00	RX0	C
ATOM	748	C	ILE	235	24.018	3.945	22.939	1.00	0.00	RX0	C
ATOM	749	O	ILE	235	23.068	3.902	23.724	1.00	0.00	RX0	O
ATOM	750	N	LEU	236	25.072	3.140	23.020	1.00	0.00	RX0	N
ATOM	751	H	LEU	236	25.810	3.232	22.348	0.00	0.00	RX0	H
ATOM	752	CA	LEU	236	25.176	2.114	24.078	1.00	0.00	RX0	C
ATOM	753	CB	LEU	236	26.441	1.276	23.892	1.00	0.00	RX0	C
ATOM	754	CG	LEU	236	26.332	0.303	22.722	1.00	0.00	RX0	C
ATOM	755	CD1	LEU	236	27.658	-0.400	22.434	1.00	0.00	RX0	C
ATOM	756	CD2	LEU	236	25.192	-0.690	22.940	1.00	0.00	RX0	C
ATOM	757	C	LEU	236	25.182	2.733	25.479	1.00	0.00	RX0	C
ATOM	758	O	LEU	236	24.381	2.362	26.336	1.00	0.00	RX0	O
ATOM	759	N	MET	237	25.933	3.823	25.600	1.00	0.00	RX0	N
ATOM	760	H	MET	237	26.455	4.134	24.803	0.00	0.00	RX0	H
ATOM	761	CA	MET	237	26.132	4.523	26.881	1.00	0.00	RX0	C
ATOM	762	CB	MET	237	27.279	5.530	26.799	1.00	0.00	RX0	C
ATOM	763	CG	MET	237	28.651	4.856	26.814	1.00	0.00	RX0	C
ATOM	764	SD	MET	237	29.998	6.047	26.769	1.00	0.00	RX0	S
ATOM	765	CE	MET	237	31.346	4.909	27.123	1.00	0.00	RX0	C
ATOM	766	C	MET	237	24.875	5.215	27.409	1.00	0.00	RX0	C
ATOM	767	O	MET	237	24.517	5.003	28.572	1.00	0.00	RX0	O
ATOM	768	N	ILE	238	24.128	5.878	26.531	1.00	0.00	RX0	N
ATOM	769	H	ILE	238	24.455	5.959	25.586	0.00	0.00	RX0	H
ATOM	770	CA	ILE	238	22.871	6.546	26.925	1.00	0.00	RX0	C
ATOM	771	CB	ILE	238	22.349	7.525	25.858	1.00	0.00	RX0	C
ATOM	772	CG2	ILE	238	21.921	6.839	24.561	1.00	0.00	RX0	C
ATOM	773	CG1	ILE	238	21.224	8.383	26.442	1.00	0.00	RX0	C
ATOM	774	CD1	ILE	238	20.608	9.339	25.421	1.00	0.00	RX0	C
ATOM	775	C	ILE	238	21.800	5.514	27.357	1.00	0.00	RX0	C
ATOM	776	O	ILE	238	21.031	5.731	28.268	1.00	0.00	RX0	O
ATOM	777	N	GLY	239	21.845	4.355	26.660	1.00	0.00	RX0	N
ATOM	778	H	GLY	239	22.520	4.244	25.926	0.00	0.00	RX0	H
ATOM	779	CA	GLY	239	20.969	3.213	26.975	1.00	0.00	RX0	C
ATOM	780	C	GLY	239	21.301	2.634	28.356	1.00	0.00	RX0	C
ATOM	781	O	GLY	239	20.417	2.460	29.193	1.00	0.00	RX0	O
ATOM	782	N	LEU	240	22.605	2.583	28.639	1.00	0.00	RX0	N
ATOM	783	H	LEU	240	23.260	2.804	27.913	0.00	0.00	RX0	H
ATOM	784	CA	LEU	240	23.124	2.082	29.919	1.00	0.00	RX0	C
ATOM	785	CB	LEU	240	24.645	1.942	29.849	1.00	0.00	RX0	C
ATOM	786	CG	LEU	240	25.286	1.611	31.196	1.00	0.00	RX0	C
ATOM	787	CD1	LEU	240	24.819	0.267	31.753	1.00	0.00	RX0	C
ATOM	788	CD2	LEU	240	26.807	1.704	31.123	1.00	0.00	RX0	C
ATOM	789	C	LEU	240	22.728	2.995	31.086	1.00	0.00	RX0	C
ATOM	790	O	LEU	240	22.214	2.535	32.097	1.00	0.00	RX0	O
ATOM	791	N	VAL	241	22.901	4.295	30.880	1.00	0.00	RX0	N

ATOM	792	H	VAL	241	23.279	4.586	29.998	0.00	0.00	RX0	H
ATOM	793	CA	VAL	241	22.596	5.307	31.912	1.00	0.00	RX0	C
ATOM	794	CB	VAL	241	23.250	6.671	31.659	1.00	0.00	RX0	C
ATOM	795	CG1	VAL	241	24.770	6.511	31.608	1.00	0.00	RX0	C
ATOM	796	CG2	VAL	241	22.705	7.390	30.432	1.00	0.00	RX0	C
ATOM	797	C	VAL	241	21.084	5.392	32.193	1.00	0.00	RX0	C
ATOM	798	O	VAL	241	20.670	5.516	33.338	1.00	0.00	RX0	O
ATOM	799	N	TRP	242	20.290	5.181	31.134	1.00	0.00	RX0	N
ATOM	800	H	TRP	242	20.699	5.072	30.225	0.00	0.00	RX0	H
ATOM	801	CA	TRP	242	18.822	5.192	31.222	1.00	0.00	RX0	C
ATOM	802	CB	TRP	242	18.254	5.153	29.800	1.00	0.00	RX0	C
ATOM	803	CG	TRP	242	16.836	4.636	29.768	1.00	0.00	RX0	C
ATOM	804	CD2	TRP	242	15.632	5.287	30.221	1.00	0.00	RX0	C
ATOM	805	CE2	TRP	242	14.556	4.400	29.992	1.00	0.00	RX0	C
ATOM	806	CE3	TRP	242	15.390	6.524	30.800	1.00	0.00	RX0	C
ATOM	807	CD1	TRP	242	16.413	3.391	29.277	1.00	0.00	RX0	C
ATOM	808	NE1	TRP	242	15.069	3.249	29.406	1.00	0.00	RX0	N
ATOM	809	HE1	TRP	242	14.543	2.467	29.136	0.00	0.00	RX0	H
ATOM	810	CZ2	TRP	242	13.269	4.778	30.349	1.00	0.00	RX0	C
ATOM	811	CZ3	TRP	242	14.098	6.894	31.152	1.00	0.00	RX0	C
ATOM	812	CH2	TRP	242	13.043	6.018	30.933	1.00	0.00	RX0	C
ATOM	813	C	TRP	242	18.281	4.038	32.076	1.00	0.00	RX0	C
ATOM	814	O	TRP	242	17.477	4.269	32.979	1.00	0.00	RX0	O
ATOM	815	N	ARG	243	18.818	2.841	31.865	1.00	0.00	RX0	N
ATOM	816	H	ARG	243	19.515	2.737	31.151	0.00	0.00	RX0	H
ATOM	817	CA	ARG	243	18.359	1.661	32.627	1.00	0.00	RX0	C
ATOM	818	CB	ARG	243	18.485	0.403	31.736	1.00	0.00	RX0	C
ATOM	819	CG	ARG	243	19.889	-0.066	31.295	1.00	0.00	RX0	C
ATOM	820	CD	ARG	243	19.827	-1.096	30.145	1.00	0.00	RX0	C
ATOM	821	NE	ARG	243	21.109	-1.759	29.859	1.00	0.00	RX0	N
ATOM	822	HE	ARG	243	21.654	-2.019	30.670	0.00	0.00	RX0	H
ATOM	823	CZ	ARG	243	21.433	-2.128	28.567	1.00	0.00	RX0	C
ATOM	824	NH1	ARG	243	20.675	-1.721	27.529	1.00	0.00	RX0	N
ATOM	825	HH11	ARG	243	20.926	-2.031	26.594	0.00	0.00	RX0	H
ATOM	826	HH12	ARG	243	19.874	-1.131	27.627	0.00	0.00	RX0	H
ATOM	827	NH2	ARG	243	22.500	-2.910	28.307	1.00	0.00	RX0	N
ATOM	828	HH21	ARG	243	22.783	-3.131	27.352	0.00	0.00	RX0	H
ATOM	829	HH22	ARG	243	23.058	-3.337	29.028	0.00	0.00	RX0	H
ATOM	830	C	ARG	243	19.043	1.510	33.998	1.00	0.00	RX0	C
ATOM	831	O	ARG	243	18.610	0.722	34.836	1.00	0.00	RX0	O
ATOM	832	N	SER	244	20.027	2.366	34.245	1.00	0.00	RX0	N
ATOM	833	H	SER	244	20.316	3.010	33.538	0.00	0.00	RX0	H
ATOM	834	CA	SER	244	20.722	2.464	35.548	1.00	0.00	RX0	C
ATOM	835	CB	SER	244	22.206	2.697	35.312	1.00	0.00	RX0	C
ATOM	836	OG	SER	244	22.700	1.648	34.478	1.00	0.00	RX0	O
ATOM	837	HG	SER	244	22.229	1.719	33.654	0.00	0.00	RX0	H
ATOM	838	C	SER	244	20.112	3.547	36.442	1.00	0.00	RX0	C
ATOM	839	O	SER	244	20.448	3.642	37.630	1.00	0.00	RX0	O
ATOM	840	N	MET	245	19.184	4.322	35.895	1.00	0.00	RX0	N
ATOM	841	H	MET	245	18.884	4.151	34.954	0.00	0.00	RX0	H
ATOM	842	CA	MET	245	18.600	5.504	36.550	1.00	0.00	RX0	C
ATOM	843	CB	MET	245	17.599	6.196	35.629	1.00	0.00	RX0	C
ATOM	844	CG	MET	245	17.054	7.480	36.255	1.00	0.00	RX0	C
ATOM	845	SD	MET	245	15.767	8.249	35.268	1.00	0.00	RX0	S
ATOM	846	CE	MET	245	16.577	8.063	33.678	1.00	0.00	RX0	C
ATOM	847	C	MET	245	17.925	5.204	37.895	1.00	0.00	RX0	C
ATOM	848	O	MET	245	18.105	5.946	38.853	1.00	0.00	RX0	O
ATOM	849	N	GLU	246	17.212	4.082	37.945	1.00	0.00	RX0	N
ATOM	850	H	GLU	246	17.124	3.483	37.148	0.00	0.00	RX0	H
ATOM	851	CA	GLU	246	16.494	3.692	39.178	1.00	0.00	RX0	C
ATOM	852	CB	GLU	246	15.245	2.900	38.819	1.00	0.00	RX0	C

ATOM	853	CG	GLU	246	14.331	3.658	37.860	1.00	0.00	RX0	C
ATOM	854	CD	GLU	246	13.166	2.762	37.515	1.00	0.00	RX0	C
ATOM	855	OE1	GLU	246	13.248	1.570	37.800	1.00	0.00	RX0	O
ATOM	856	OE2	GLU	246	12.182	3.258	36.971	1.00	0.00	RX0	O
ATOM	857	C	GLU	246	17.379	2.867	40.123	1.00	0.00	RX0	C
ATOM	858	O	GLU	246	16.897	2.295	41.108	1.00	0.00	RX0	O
ATOM	859	N	HIS	247	18.674	2.844	39.836	1.00	0.00	RX0	N
ATOM	860	H	HIS	247	19.044	3.322	39.040	0.00	0.00	RX0	H
ATOM	861	CA	HIS	247	19.658	2.055	40.601	1.00	0.00	RX0	C
ATOM	862	CB	HIS	247	20.179	0.868	39.786	1.00	0.00	RX0	C
ATOM	863	CG	HIS	247	19.085	-0.129	39.474	1.00	0.00	RX0	C
ATOM	864	ND1	HIS	247	17.868	-0.159	40.053	1.00	0.00	RX0	N
ATOM	865	HD1	HIS	247	17.501	0.460	40.726	0.00	0.00	RX0	H
ATOM	866	CD2	HIS	247	19.162	-1.175	38.552	1.00	0.00	RX0	C
ATOM	867	NE2	HIS	247	17.982	-1.838	38.580	1.00	0.00	RX0	N
ATOM	868	CE1	HIS	247	17.183	-1.212	39.502	1.00	0.00	RX0	C
ATOM	869	C	HIS	247	20.841	2.947	41.015	1.00	0.00	RX0	C
ATOM	870	O	HIS	247	21.962	2.788	40.490	1.00	0.00	RX0	O
ATOM	871	N	PRO	248	20.632	3.827	41.991	1.00	0.00	RX0	N
ATOM	872	CD	PRO	248	19.384	3.974	42.732	1.00	0.00	RX0	C
ATOM	873	CA	PRO	248	21.659	4.767	42.484	1.00	0.00	RX0	C
ATOM	874	CB	PRO	248	20.979	5.450	43.675	1.00	0.00	RX0	C
ATOM	875	CG	PRO	248	19.487	5.345	43.383	1.00	0.00	RX0	C
ATOM	876	C	PRO	248	22.939	4.013	42.877	1.00	0.00	RX0	C
ATOM	877	O	PRO	248	22.892	2.963	43.503	1.00	0.00	RX0	O
ATOM	878	N	GLY	249	24.055	4.541	42.350	1.00	0.00	RX0	N
ATOM	879	H	GLY	249	23.974	5.300	41.710	0.00	0.00	RX0	H
ATOM	880	CA	GLY	249	25.407	3.996	42.610	1.00	0.00	RX0	C
ATOM	881	C	GLY	249	25.783	2.749	41.794	1.00	0.00	RX0	C
ATOM	882	O	GLY	249	26.914	2.250	41.927	1.00	0.00	RX0	O
ATOM	883	N	LYS	250	24.877	2.267	40.961	1.00	0.00	RX0	N
ATOM	884	H	LYS	250	23.983	2.697	40.813	0.00	0.00	RX0	H
ATOM	885	CA	LYS	250	25.097	1.050	40.158	1.00	0.00	RX0	C
ATOM	886	CB	LYS	250	24.366	-0.147	40.771	1.00	0.00	RX0	C
ATOM	887	CG	LYS	250	25.226	-0.877	41.812	1.00	0.00	RX0	C
ATOM	888	CD	LYS	250	24.594	-2.169	42.337	1.00	0.00	RX0	C
ATOM	889	CE	LYS	250	25.526	-3.045	43.186	1.00	0.00	RX0	C
ATOM	890	NZ	LYS	250	26.659	-3.541	42.389	1.00	0.00	RX0	N
ATOM	891	HZ1	LYS	250	27.364	-3.978	43.025	0.00	0.00	RX0	H
ATOM	892	HZ2	LYS	250	26.389	-4.216	41.637	0.00	0.00	RX0	H
ATOM	893	HZ3	LYS	250	27.185	-2.735	41.999	0.00	0.00	RX0	H
ATOM	894	C	LYS	250	24.802	1.266	38.671	1.00	0.00	RX0	C
ATOM	895	O	LYS	250	24.040	2.166	38.282	1.00	0.00	RX0	O
ATOM	896	N	LEU	251	25.472	0.476	37.863	1.00	0.00	RX0	N
ATOM	897	H	LEU	251	26.053	-0.236	38.244	0.00	0.00	RX0	H
ATOM	898	CA	LEU	251	25.292	0.430	36.401	1.00	0.00	RX0	C
ATOM	899	CB	LEU	251	26.626	0.592	35.683	1.00	0.00	RX0	C
ATOM	900	CG	LEU	251	27.159	2.018	35.762	1.00	0.00	RX0	C
ATOM	901	CD1	LEU	251	28.582	2.118	35.218	1.00	0.00	RX0	C
ATOM	902	CD2	LEU	251	26.205	3.009	35.093	1.00	0.00	RX0	C
ATOM	903	C	LEU	251	24.646	-0.892	36.017	1.00	0.00	RX0	C
ATOM	904	O	LEU	251	25.224	-1.976	36.286	1.00	0.00	RX0	O
ATOM	905	N	LEU	252	23.437	-0.808	35.533	1.00	0.00	RX0	N
ATOM	906	H	LEU	252	23.084	0.097	35.311	0.00	0.00	RX0	H
ATOM	907	CA	LEU	252	22.659	-1.981	35.114	1.00	0.00	RX0	C
ATOM	908	CB	LEU	252	21.157	-1.713	35.229	1.00	0.00	RX0	C
ATOM	909	CG	LEU	252	20.301	-2.958	34.969	1.00	0.00	RX0	C
ATOM	910	CD1	LEU	252	20.448	-3.995	36.080	1.00	0.00	RX0	C
ATOM	911	CD2	LEU	252	18.836	-2.610	34.714	1.00	0.00	RX0	C
ATOM	912	C	LEU	252	23.007	-2.330	33.663	1.00	0.00	RX0	C
ATOM	913	O	LEU	252	22.274	-2.025	32.731	1.00	0.00	RX0	O

ATOM	914	N	PHE	253	24.144	-3.005	33.506	1.00	0.00	RX0	N
ATOM	915	H	PHE	253	24.631	-3.307	34.330	0.00	0.00	RX0	H
ATOM	916	CA	PHE	253	24.599	-3.457	32.174	1.00	0.00	RX0	C
ATOM	917	CB	PHE	253	25.967	-4.126	32.267	1.00	0.00	RX0	C
ATOM	918	CG	PHE	253	27.031	-3.101	32.561	1.00	0.00	RX0	C
ATOM	919	CD1	PHE	253	27.565	-2.349	31.522	1.00	0.00	RX0	C
ATOM	920	CD2	PHE	253	27.484	-2.917	33.861	1.00	0.00	RX0	C
ATOM	921	CE1	PHE	253	28.563	-1.418	31.780	1.00	0.00	RX0	C
ATOM	922	CE2	PHE	253	28.480	-1.985	34.118	1.00	0.00	RX0	C
ATOM	923	CZ	PHE	253	29.021	-1.237	33.078	1.00	0.00	RX0	C
ATOM	924	C	PHE	253	23.603	-4.446	31.564	1.00	0.00	RX0	C
ATOM	925	O	PHE	253	23.259	-4.379	30.390	1.00	0.00	RX0	O
ATOM	926	N	ALA	254	23.094	-5.300	32.445	1.00	0.00	RX0	N
ATOM	927	H	ALA	254	23.412	-5.314	33.398	0.00	0.00	RX0	H
ATOM	928	CA	ALA	254	22.050	-6.280	32.141	1.00	0.00	RX0	C
ATOM	929	CB	ALA	254	22.690	-7.554	31.605	1.00	0.00	RX0	C
ATOM	930	C	ALA	254	21.288	-6.584	33.440	1.00	0.00	RX0	C
ATOM	931	O	ALA	254	21.887	-6.418	34.526	1.00	0.00	RX0	O
ATOM	932	N	PRO	255	20.056	-7.064	33.372	1.00	0.00	RX0	N
ATOM	933	CD	PRO	255	19.308	-7.244	32.130	1.00	0.00	RX0	C
ATOM	934	CA	PRO	255	19.236	-7.444	34.545	1.00	0.00	RX0	C
ATOM	935	CB	PRO	255	17.989	-8.072	33.923	1.00	0.00	RX0	C
ATOM	936	CG	PRO	255	17.858	-7.395	32.566	1.00	0.00	RX0	C
ATOM	937	C	PRO	255	19.972	-8.395	35.506	1.00	0.00	RX0	C
ATOM	938	O	PRO	255	19.756	-8.342	36.714	1.00	0.00	RX0	O
ATOM	939	N	ASN	256	20.900	-9.179	34.970	1.00	0.00	RX0	N
ATOM	940	H	ASN	256	21.121	-9.131	33.995	0.00	0.00	RX0	H
ATOM	941	CA	ASN	256	21.722	-10.125	35.761	1.00	0.00	RX0	C
ATOM	942	CB	ASN	256	21.706	-11.531	35.158	1.00	0.00	RX0	C
ATOM	943	CG	ASN	256	22.399	-11.537	33.805	1.00	0.00	RX0	C
ATOM	944	OD1	ASN	256	22.273	-10.607	33.009	1.00	0.00	RX0	O
ATOM	945	ND2	ASN	256	23.050	-12.684	33.539	1.00	0.00	RX0	N
ATOM	946	HD21	ASN	256	23.191	-13.357	34.269	0.00	0.00	RX0	H
ATOM	947	HD22	ASN	256	23.403	-12.929	32.631	0.00	0.00	RX0	H
ATOM	948	C	ASN	256	23.191	-9.669	35.876	1.00	0.00	RX0	C
ATOM	949	O	ASN	256	24.101	-10.505	36.011	1.00	0.00	RX0	O
ATOM	950	N	LEU	257	23.439	-8.384	35.742	1.00	0.00	RX0	N
ATOM	951	H	LEU	257	22.693	-7.727	35.623	0.00	0.00	RX0	H
ATOM	952	CA	LEU	257	24.796	-7.805	35.783	1.00	0.00	RX0	C
ATOM	953	CB	LEU	257	25.513	-7.967	34.444	1.00	0.00	RX0	C
ATOM	954	CG	LEU	257	27.023	-7.750	34.564	1.00	0.00	RX0	C
ATOM	955	CD1	LEU	257	27.659	-8.772	35.508	1.00	0.00	RX0	C
ATOM	956	CD2	LEU	257	27.712	-7.732	33.200	1.00	0.00	RX0	C
ATOM	957	C	LEU	257	24.720	-6.327	36.168	1.00	0.00	RX0	C
ATOM	958	O	LEU	257	24.738	-5.412	35.328	1.00	0.00	RX0	O
ATOM	959	N	LEU	258	24.604	-6.148	37.469	1.00	0.00	RX0	N
ATOM	960	H	LEU	258	24.690	-6.942	38.069	0.00	0.00	RX0	H
ATOM	961	CA	LEU	258	24.482	-4.834	38.118	1.00	0.00	RX0	C
ATOM	962	CB	LEU	258	23.218	-4.907	38.977	1.00	0.00	RX0	C
ATOM	963	CG	LEU	258	22.801	-3.617	39.670	1.00	0.00	RX0	C
ATOM	964	CD1	LEU	258	22.648	-2.465	38.688	1.00	0.00	RX0	C
ATOM	965	CD2	LEU	258	21.538	-3.815	40.509	1.00	0.00	RX0	C
ATOM	966	C	LEU	258	25.743	-4.564	38.938	1.00	0.00	RX0	C
ATOM	967	O	LEU	258	26.013	-5.237	39.948	1.00	0.00	RX0	O
ATOM	968	N	LEU	259	26.528	-3.622	38.460	1.00	0.00	RX0	N
ATOM	969	H	LEU	259	26.220	-3.063	37.683	0.00	0.00	RX0	H
ATOM	970	CA	LEU	259	27.862	-3.349	39.027	1.00	0.00	RX0	C
ATOM	971	CB	LEU	259	28.938	-3.496	37.947	1.00	0.00	RX0	C
ATOM	972	CG	LEU	259	28.883	-4.821	37.182	1.00	0.00	RX0	C
ATOM	973	CD1	LEU	259	29.891	-4.850	36.033	1.00	0.00	RX0	C
ATOM	974	CD2	LEU	259	29.045	-6.032	38.100	1.00	0.00	RX0	C

ATOM	975	C	LEU	259	27.958	-1.956	39.652	1.00	0.00	RX0	C
ATOM	976	O	LEU	259	27.419	-0.984	39.137	1.00	0.00	RX0	O
ATOM	977	N	ASP	260	28.645	-1.912	40.785	1.00	0.00	RX0	N
ATOM	978	H	ASP	260	29.238	-2.681	41.037	0.00	0.00	RX0	H
ATOM	979	CA	ASP	260	29.043	-0.657	41.454	1.00	0.00	RX0	C
ATOM	980	CB	ASP	260	29.048	-0.861	42.958	1.00	0.00	RX0	C
ATOM	981	CG	ASP	260	29.707	-2.202	43.206	1.00	0.00	RX0	C
ATOM	982	OD1	ASP	260	30.929	-2.286	43.206	1.00	0.00	RX0	O
ATOM	983	OD2	ASP	260	28.989	-3.188	43.355	1.00	0.00	RX0	O
ATOM	984	C	ASP	260	30.443	-0.222	40.970	1.00	0.00	RX0	C
ATOM	985	O	ASP	260	31.127	-1.008	40.295	1.00	0.00	RX0	O
ATOM	986	N	ARG	261	30.963	0.873	41.504	1.00	0.00	RX0	N
ATOM	987	H	ARG	261	30.398	1.405	42.134	0.00	0.00	RX0	H
ATOM	988	CA	ARG	261	32.263	1.408	41.044	1.00	0.00	RX0	C
ATOM	989	CB	ARG	261	32.422	2.890	41.426	1.00	0.00	RX0	C
ATOM	990	CG	ARG	261	32.656	3.193	42.910	1.00	0.00	RX0	C
ATOM	991	CD	ARG	261	32.564	4.684	43.260	1.00	0.00	RX0	C
ATOM	992	NE	ARG	261	33.468	5.512	42.462	1.00	0.00	RX0	N
ATOM	993	HE	ARG	261	33.313	5.625	41.471	0.00	0.00	RX0	H
ATOM	994	CZ	ARG	261	34.449	6.262	43.003	1.00	0.00	RX0	C
ATOM	995	NH1	ARG	261	34.684	6.254	44.310	1.00	0.00	RX0	N
ATOM	996	HH11	ARG	261	35.476	6.724	44.694	0.00	0.00	RX0	H
ATOM	997	HH12	ARG	261	34.043	5.793	44.937	0.00	0.00	RX0	H
ATOM	998	NH2	ARG	261	35.173	7.034	42.212	1.00	0.00	RX0	N
ATOM	999	HH21	ARG	261	35.898	7.654	42.524	0.00	0.00	RX0	H
ATOM	1000	HH22	ARG	261	34.958	7.028	41.221	0.00	0.00	RX0	H
ATOM	1001	C	ARG	261	33.476	0.540	41.436	1.00	0.00	RX0	C
ATOM	1002	O	ARG	261	34.378	0.347	40.637	1.00	0.00	RX0	O
ATOM	1003	N	ASN	262	33.410	-0.067	42.632	1.00	0.00	RX0	N
ATOM	1004	H	ASN	262	32.546	-0.023	43.135	0.00	0.00	RX0	H
ATOM	1005	CA	ASN	262	34.456	-1.000	43.094	1.00	0.00	RX0	C
ATOM	1006	CB	ASN	262	34.235	-1.469	44.531	1.00	0.00	RX0	C
ATOM	1007	CG	ASN	262	35.402	-2.356	44.939	1.00	0.00	RX0	C
ATOM	1008	OD1	ASN	262	36.476	-1.874	45.303	1.00	0.00	RX0	O
ATOM	1009	ND2	ASN	262	35.125	-3.674	44.901	1.00	0.00	RX0	N
ATOM	1010	HD21	ASN	262	34.218	-4.008	44.634	0.00	0.00	RX0	H
ATOM	1011	HD22	ASN	262	35.822	-4.359	45.122	0.00	0.00	RX0	H
ATOM	1012	C	ASN	262	34.635	-2.233	42.204	1.00	0.00	RX0	C
ATOM	1013	O	ASN	262	35.755	-2.665	41.964	1.00	0.00	RX0	O
ATOM	1014	N	GLN	263	33.530	-2.679	41.603	1.00	0.00	RX0	N
ATOM	1015	H	GLN	263	32.644	-2.275	41.844	0.00	0.00	RX0	H
ATOM	1016	CA	GLN	263	33.559	-3.756	40.599	1.00	0.00	RX0	C
ATOM	1017	CB	GLN	263	32.195	-4.426	40.458	1.00	0.00	RX0	C
ATOM	1018	CG	GLN	263	31.955	-5.184	41.762	1.00	0.00	RX0	C
ATOM	1019	CD	GLN	263	30.710	-6.029	41.689	1.00	0.00	RX0	C
ATOM	1020	OE1	GLN	263	30.561	-6.901	40.842	1.00	0.00	RX0	O
ATOM	1021	NE2	GLN	263	29.845	-5.766	42.679	1.00	0.00	RX0	N
ATOM	1022	HE21	GLN	263	29.993	-4.932	43.229	0.00	0.00	RX0	H
ATOM	1023	HE22	GLN	263	29.081	-6.372	42.887	0.00	0.00	RX0	H
ATOM	1024	C	GLN	263	34.189	-3.308	39.265	1.00	0.00	RX0	C
ATOM	1025	O	GLN	263	34.644	-4.116	38.479	1.00	0.00	RX0	O
ATOM	1026	N	GLY	264	34.180	-1.978	39.039	1.00	0.00	RX0	N
ATOM	1027	H	GLY	264	33.771	-1.379	39.727	0.00	0.00	RX0	H
ATOM	1028	CA	GLY	264	34.831	-1.337	37.881	1.00	0.00	RX0	C
ATOM	1029	C	GLY	264	36.364	-1.310	37.978	1.00	0.00	RX0	C
ATOM	1030	O	GLY	264	37.050	-1.510	36.977	1.00	0.00	RX0	O
ATOM	1031	N	LYS	265	36.881	-1.155	39.202	1.00	0.00	RX0	N
ATOM	1032	H	LYS	265	36.231	-0.993	39.950	0.00	0.00	RX0	H
ATOM	1033	CA	LYS	265	38.336	-1.184	39.469	1.00	0.00	RX0	C
ATOM	1034	CB	LYS	265	38.633	-1.137	40.947	1.00	0.00	RX0	C
ATOM	1035	CG	LYS	265	38.264	0.051	41.817	1.00	0.00	RX0	C

ATOM	1036	CD	LYS	265	38.477	-0.619	43.159	1.00	0.00	RX0	C
ATOM	1037	CE	LYS	265	38.677	0.166	44.441	1.00	0.00	RX0	C
ATOM	1038	NZ	LYS	265	38.997	-0.885	45.413	1.00	0.00	RX0	N
ATOM	1039	HZ1	LYS	265	39.179	-0.541	46.367	0.00	0.00	RX0	H
ATOM	1040	HZ2	LYS	265	38.234	-1.598	45.421	0.00	0.00	RX0	H
ATOM	1041	HZ3	LYS	265	39.852	-1.398	45.081	0.00	0.00	RX0	H
ATOM	1042	C	LYS	265	38.994	-2.500	39.030	1.00	0.00	RX0	C
ATOM	1043	O	LYS	265	40.184	-2.535	38.765	1.00	0.00	RX0	O
ATOM	1044	N	CYS	266	38.153	-3.548	38.917	1.00	0.00	RX0	N
ATOM	1045	H	CYS	266	37.220	-3.444	39.260	0.00	0.00	RX0	H
ATOM	1046	CA	CYS	266	38.538	-4.854	38.355	1.00	0.00	RX0	C
ATOM	1047	CB	CYS	266	37.333	-5.778	38.203	1.00	0.00	RX0	C
ATOM	1048	SG	CYS	266	36.548	-6.114	39.801	1.00	0.00	RX0	S
ATOM	1049	C	CYS	266	39.318	-4.732	37.033	1.00	0.00	RX0	C
ATOM	1050	O	CYS	266	40.108	-5.603	36.695	1.00	0.00	RX0	O
ATOM	1051	N	VAL	267	39.075	-3.636	36.304	1.00	0.00	RX0	N
ATOM	1052	H	VAL	267	38.413	-2.953	36.618	0.00	0.00	RX0	H
ATOM	1053	CA	VAL	267	39.804	-3.345	35.058	1.00	0.00	RX0	C
ATOM	1054	CB	VAL	267	38.875	-3.384	33.831	1.00	0.00	RX0	C
ATOM	1055	CG1	VAL	267	39.618	-3.083	32.524	1.00	0.00	RX0	C
ATOM	1056	CG2	VAL	267	38.160	-4.736	33.733	1.00	0.00	RX0	C
ATOM	1057	C	VAL	267	40.557	-2.013	35.191	1.00	0.00	RX0	C
ATOM	1058	O	VAL	267	39.969	-0.956	35.468	1.00	0.00	RX0	O
ATOM	1059	N	GLU	268	41.829	-2.078	34.821	1.00	0.00	RX0	N
ATOM	1060	H	GLU	268	42.176	-2.961	34.534	0.00	0.00	RX0	H
ATOM	1061	CA	GLU	268	42.726	-0.908	34.727	1.00	0.00	RX0	C
ATOM	1062	CB	GLU	268	44.146	-1.287	34.349	1.00	0.00	RX0	C
ATOM	1063	CG	GLU	268	45.155	-0.136	34.266	1.00	0.00	RX0	C
ATOM	1064	CD	GLU	268	45.087	0.513	32.903	1.00	0.00	RX0	C
ATOM	1065	OE1	GLU	268	45.191	1.733	32.814	1.00	0.00	RX0	O
ATOM	1066	OE2	GLU	268	44.944	-0.205	31.919	1.00	0.00	RX0	O
ATOM	1067	C	GLU	268	42.079	0.194	33.866	1.00	0.00	RX0	C
ATOM	1068	O	GLU	268	41.697	-0.027	32.727	1.00	0.00	RX0	O
ATOM	1069	N	GLY	269	41.924	1.355	34.524	1.00	0.00	RX0	N
ATOM	1070	H	GLY	269	42.350	1.348	35.418	0.00	0.00	RX0	H
ATOM	1071	CA	GLY	269	41.377	2.576	33.902	1.00	0.00	RX0	C
ATOM	1072	C	GLY	269	39.898	2.490	33.494	1.00	0.00	RX0	C
ATOM	1073	O	GLY	269	39.424	3.343	32.745	1.00	0.00	RX0	O
ATOM	1074	N	MET	270	39.146	1.598	34.134	1.00	0.00	RX0	N
ATOM	1075	H	MET	270	39.598	0.903	34.695	0.00	0.00	RX0	H
ATOM	1076	CA	MET	270	37.704	1.465	33.841	1.00	0.00	RX0	C
ATOM	1077	CB	MET	270	37.289	0.006	33.653	1.00	0.00	RX0	C
ATOM	1078	CG	MET	270	35.937	-0.106	32.943	1.00	0.00	RX0	C
ATOM	1079	SD	MET	270	35.445	-1.798	32.575	1.00	0.00	RX0	S
ATOM	1080	CE	MET	270	35.190	-2.372	34.260	1.00	0.00	RX0	C
ATOM	1081	C	MET	270	36.825	2.181	34.877	1.00	0.00	RX0	C
ATOM	1082	O	MET	270	35.781	2.734	34.514	1.00	0.00	RX0	O
ATOM	1083	N	VAL	271	37.310	2.290	36.107	1.00	0.00	RX0	N
ATOM	1084	H	VAL	271	38.180	1.836	36.284	0.00	0.00	RX0	H
ATOM	1085	CA	VAL	271	36.591	3.043	37.171	1.00	0.00	RX0	C
ATOM	1086	CB	VAL	271	37.215	2.861	38.570	1.00	0.00	RX0	C
ATOM	1087	CG1	VAL	271	38.661	3.335	38.681	1.00	0.00	RX0	C
ATOM	1088	CG2	VAL	271	36.314	3.466	39.649	1.00	0.00	RX0	C
ATOM	1089	C	VAL	271	36.343	4.502	36.749	1.00	0.00	RX0	C
ATOM	1090	O	VAL	271	35.261	5.071	37.095	1.00	0.00	RX0	O
ATOM	1091	N	GLU	272	37.228	5.074	36.011	1.00	0.00	RX0	N
ATOM	1092	H	GLU	272	38.065	4.558	35.836	0.00	0.00	RX0	H
ATOM	1093	CA	GLU	272	37.141	6.453	35.460	1.00	0.00	RX0	C
ATOM	1094	CB	GLU	272	38.464	6.676	34.709	1.00	0.00	RX0	C
ATOM	1095	CG	GLU	272	39.790	6.780	35.510	1.00	0.00	RX0	C
ATOM	1096	CD	GLU	272	40.275	5.547	36.296	1.00	0.00	RX0	C

ATOM	1097	OE1	GLU	272	40.000	4.397	35.955	1.00	0.00	RX0	O
ATOM	1098	OE2	GLU	272	40.996	5.722	37.273	1.00	0.00	RX0	O
ATOM	1099	C	GLU	272	35.927	6.588	34.526	1.00	0.00	RX0	C
ATOM	1100	O	GLU	272	35.142	7.521	34.681	1.00	0.00	RX0	O
ATOM	1101	N	ILE	273	35.702	5.552	33.729	1.00	0.00	RX0	N
ATOM	1102	H	ILE	273	36.296	4.754	33.819	0.00	0.00	RX0	H
ATOM	1103	CA	ILE	273	34.533	5.484	32.820	1.00	0.00	RX0	C
ATOM	1104	CB	ILE	273	34.733	4.377	31.780	1.00	0.00	RX0	C
ATOM	1105	CG2	ILE	273	33.624	4.413	30.727	1.00	0.00	RX0	C
ATOM	1106	CG1	ILE	273	36.129	4.424	31.150	1.00	0.00	RX0	C
ATOM	1107	CD1	ILE	273	36.355	5.647	30.261	1.00	0.00	RX0	C
ATOM	1108	C	ILE	273	33.249	5.235	33.628	1.00	0.00	RX0	C
ATOM	1109	O	ILE	273	32.257	5.951	33.452	1.00	0.00	RX0	O
ATOM	1110	N	PHE	274	33.320	4.302	34.575	1.00	0.00	RX0	N
ATOM	1111	H	PHE	274	34.183	3.806	34.677	0.00	0.00	RX0	H
ATOM	1112	CA	PHE	274	32.191	3.976	35.472	1.00	0.00	RX0	C
ATOM	1113	CB	PHE	274	32.607	2.931	36.511	1.00	0.00	RX0	C
ATOM	1114	CG	PHE	274	32.279	1.532	36.054	1.00	0.00	RX0	C
ATOM	1115	CD1	PHE	274	32.558	1.125	34.756	1.00	0.00	RX0	C
ATOM	1116	CD2	PHE	274	31.693	0.647	36.952	1.00	0.00	RX0	C
ATOM	1117	CE1	PHE	274	32.258	-0.173	34.360	1.00	0.00	RX0	C
ATOM	1118	CE2	PHE	274	31.394	-0.651	36.557	1.00	0.00	RX0	C
ATOM	1119	CZ	PHE	274	31.686	-1.063	35.262	1.00	0.00	RX0	C
ATOM	1120	C	PHE	274	31.669	5.203	36.222	1.00	0.00	RX0	C
ATOM	1121	O	PHE	274	30.484	5.521	36.143	1.00	0.00	RX0	O
ATOM	1122	N	ASP	275	32.607	5.983	36.757	1.00	0.00	RX0	N
ATOM	1123	H	ASP	275	33.558	5.677	36.771	0.00	0.00	RX0	H
ATOM	1124	CA	ASP	275	32.273	7.222	37.483	1.00	0.00	RX0	C
ATOM	1125	CB	ASP	275	33.558	7.873	37.995	1.00	0.00	RX0	C
ATOM	1126	CG	ASP	275	34.013	7.314	39.326	1.00	0.00	RX0	C
ATOM	1127	OD1	ASP	275	33.209	7.211	40.247	1.00	0.00	RX0	O
ATOM	1128	OD2	ASP	275	35.198	7.039	39.480	1.00	0.00	RX0	O
ATOM	1129	C	ASP	275	31.555	8.270	36.629	1.00	0.00	RX0	C
ATOM	1130	O	ASP	275	30.604	8.890	37.102	1.00	0.00	RX0	O
ATOM	1131	N	MET	276	31.921	8.336	35.353	1.00	0.00	RX0	N
ATOM	1132	H	MET	276	32.681	7.753	35.056	0.00	0.00	RX0	H
ATOM	1133	CA	MET	276	31.257	9.254	34.408	1.00	0.00	RX0	C
ATOM	1134	CB	MET	276	32.110	9.446	33.155	1.00	0.00	RX0	C
ATOM	1135	CG	MET	276	33.494	10.025	33.462	1.00	0.00	RX0	C
ATOM	1136	SD	MET	276	34.416	10.489	31.985	1.00	0.00	RX0	S
ATOM	1137	CE	MET	276	34.420	8.888	31.167	1.00	0.00	RX0	C
ATOM	1138	C	MET	276	29.833	8.798	34.061	1.00	0.00	RX0	C
ATOM	1139	O	MET	276	28.893	9.589	34.158	1.00	0.00	RX0	O
ATOM	1140	N	LEU	277	29.673	7.488	33.891	1.00	0.00	RX0	N
ATOM	1141	H	LEU	277	30.502	6.921	33.912	0.00	0.00	RX0	H
ATOM	1142	CA	LEU	277	28.362	6.866	33.606	1.00	0.00	RX0	C
ATOM	1143	CB	LEU	277	28.562	5.401	33.222	1.00	0.00	RX0	C
ATOM	1144	CG	LEU	277	29.416	5.215	31.968	1.00	0.00	RX0	C
ATOM	1145	CD1	LEU	277	29.915	3.779	31.824	1.00	0.00	RX0	C
ATOM	1146	CD2	LEU	277	28.694	5.705	30.714	1.00	0.00	RX0	C
ATOM	1147	C	LEU	277	27.393	6.992	34.787	1.00	0.00	RX0	C
ATOM	1148	O	LEU	277	26.257	7.447	34.627	1.00	0.00	RX0	O
ATOM	1149	N	LEU	278	27.939	6.795	35.983	1.00	0.00	RX0	N
ATOM	1150	H	LEU	278	28.890	6.477	36.002	0.00	0.00	RX0	H
ATOM	1151	CA	LEU	278	27.196	6.926	37.250	1.00	0.00	RX0	C
ATOM	1152	CB	LEU	278	28.067	6.468	38.419	1.00	0.00	RX0	C
ATOM	1153	CG	LEU	278	28.215	4.951	38.468	1.00	0.00	RX0	C
ATOM	1154	CD1	LEU	278	29.263	4.498	39.484	1.00	0.00	RX0	C
ATOM	1155	CD2	LEU	278	26.859	4.294	38.707	1.00	0.00	RX0	C
ATOM	1156	C	LEU	278	26.716	8.359	37.508	1.00	0.00	RX0	C
ATOM	1157	O	LEU	278	25.554	8.575	37.840	1.00	0.00	RX0	O

ATOM	1158	N	ALA	279	27.582	9.317	37.175	1.00	0.00	RX0	N
ATOM	1159	H	ALA	279	28.511	9.034	36.921	0.00	0.00	RX0	H
ATOM	1160	CA	ALA	279	27.275	10.754	37.296	1.00	0.00	RX0	C
ATOM	1161	CB	ALA	279	28.514	11.578	37.009	1.00	0.00	RX0	C
ATOM	1162	C	ALA	279	26.182	11.196	36.310	1.00	0.00	RX0	C
ATOM	1163	O	ALA	279	25.263	11.922	36.684	1.00	0.00	RX0	O
ATOM	1164	N	THR	280	26.210	10.618	35.109	1.00	0.00	RX0	N
ATOM	1165	H	THR	280	26.995	10.034	34.898	0.00	0.00	RX0	H
ATOM	1166	CA	THR	280	25.203	10.899	34.059	1.00	0.00	RX0	C
ATOM	1167	CB	THR	280	25.700	10.357	32.714	1.00	0.00	RX0	C
ATOM	1168	OG1	THR	280	27.029	10.831	32.461	1.00	0.00	RX0	O
ATOM	1169	HG1	THR	280	27.631	10.360	33.029	0.00	0.00	RX0	H
ATOM	1170	CG2	THR	280	24.779	10.752	31.556	1.00	0.00	RX0	C
ATOM	1171	C	THR	280	23.835	10.327	34.462	1.00	0.00	RX0	C
ATOM	1172	O	THR	280	22.822	11.023	34.397	1.00	0.00	RX0	O
ATOM	1173	N	SER	281	23.868	9.112	35.003	1.00	0.00	RX0	N
ATOM	1174	H	SER	281	24.740	8.624	35.046	0.00	0.00	RX0	H
ATOM	1175	CA	SER	281	22.669	8.413	35.507	1.00	0.00	RX0	C
ATOM	1176	CB	SER	281	23.143	6.988	35.885	1.00	0.00	RX0	C
ATOM	1177	OG	SER	281	22.370	6.352	36.921	1.00	0.00	RX0	O
ATOM	1178	HG	SER	281	22.540	5.418	36.815	0.00	0.00	RX0	H
ATOM	1179	C	SER	281	22.019	9.180	36.675	1.00	0.00	RX0	C
ATOM	1180	O	SER	281	20.814	9.399	36.693	1.00	0.00	RX0	O
ATOM	1181	N	SER	282	22.889	9.788	37.491	1.00	0.00	RX0	N
ATOM	1182	H	SER	282	23.861	9.594	37.353	0.00	0.00	RX0	H
ATOM	1183	CA	SER	282	22.489	10.660	38.613	1.00	0.00	RX0	C
ATOM	1184	CB	SER	282	23.662	10.783	39.573	1.00	0.00	RX0	C
ATOM	1185	OG	SER	282	23.978	9.432	39.964	1.00	0.00	RX0	O
ATOM	1186	HG	SER	282	24.737	9.188	39.431	0.00	0.00	RX0	H
ATOM	1187	C	SER	282	21.828	11.963	38.130	1.00	0.00	RX0	C
ATOM	1188	O	SER	282	20.788	12.371	38.639	1.00	0.00	RX0	O
ATOM	1189	N	ARG	283	22.365	12.516	37.039	1.00	0.00	RX0	N
ATOM	1190	H	ARG	283	23.210	12.123	36.670	0.00	0.00	RX0	H
ATOM	1191	CA	ARG	283	21.822	13.735	36.412	1.00	0.00	RX0	C
ATOM	1192	CB	ARG	283	22.815	14.202	35.351	1.00	0.00	RX0	C
ATOM	1193	CG	ARG	283	22.266	15.097	34.240	1.00	0.00	RX0	C
ATOM	1194	CD	ARG	283	21.686	16.435	34.698	1.00	0.00	RX0	C
ATOM	1195	NE	ARG	283	21.925	17.389	33.627	1.00	0.00	RX0	N
ATOM	1196	HE	ARG	283	22.606	17.131	32.932	0.00	0.00	RX0	H
ATOM	1197	CZ	ARG	283	21.463	18.645	33.588	1.00	0.00	RX0	C
ATOM	1198	NH1	ARG	283	20.598	19.082	34.495	1.00	0.00	RX0	N
ATOM	1199	HH11	ARG	283	20.236	20.020	34.458	0.00	0.00	RX0	H
ATOM	1200	HH12	ARG	283	20.292	18.480	35.228	0.00	0.00	RX0	H
ATOM	1201	NH2	ARG	283	21.905	19.432	32.617	1.00	0.00	RX0	N
ATOM	1202	HH21	ARG	283	21.586	20.366	32.490	0.00	0.00	RX0	H
ATOM	1203	HH22	ARG	283	22.622	19.064	32.008	0.00	0.00	RX0	H
ATOM	1204	C	ARG	283	20.432	13.477	35.812	1.00	0.00	RX0	C
ATOM	1205	O	ARG	283	19.498	14.246	36.035	1.00	0.00	RX0	O
ATOM	1206	N	PHE	284	20.293	12.325	35.169	1.00	0.00	RX0	N
ATOM	1207	H	PHE	284	21.114	11.770	35.022	0.00	0.00	RX0	H
ATOM	1208	CA	PHE	284	19.003	11.899	34.606	1.00	0.00	RX0	C
ATOM	1209	CB	PHE	284	19.186	10.661	33.725	1.00	0.00	RX0	C
ATOM	1210	CG	PHE	284	19.701	11.031	32.351	1.00	0.00	RX0	C
ATOM	1211	CD1	PHE	284	19.077	12.063	31.613	1.00	0.00	RX0	C
ATOM	1212	CD2	PHE	284	20.785	10.308	31.812	1.00	0.00	RX0	C
ATOM	1213	CE1	PHE	284	19.515	12.345	30.305	1.00	0.00	RX0	C
ATOM	1214	CE2	PHE	284	21.225	10.594	30.505	1.00	0.00	RX0	C
ATOM	1215	CZ	PHE	284	20.576	11.596	29.757	1.00	0.00	RX0	C
ATOM	1216	C	PHE	284	17.921	11.680	35.654	1.00	0.00	RX0	C
ATOM	1217	O	PHE	284	16.817	12.216	35.524	1.00	0.00	RX0	O
ATOM	1218	N	ARG	285	18.348	11.087	36.760	1.00	0.00	RX0	N

ATOM	1219	H	ARG	285	19.281	10.723	36.763	0.00	0.00	RX0	H
ATOM	1220	CA	ARG	285	17.485	10.841	37.923	1.00	0.00	RX0	C
ATOM	1221	CB	ARG	285	18.333	10.060	38.928	1.00	0.00	RX0	C
ATOM	1222	CG	ARG	285	17.762	9.791	40.322	1.00	0.00	RX0	C
ATOM	1223	CD	ARG	285	18.786	9.035	41.181	1.00	0.00	RX0	C
ATOM	1224	NE	ARG	285	19.214	7.827	40.479	1.00	0.00	RX0	N
ATOM	1225	HE	ARG	285	18.465	7.222	40.176	0.00	0.00	RX0	H
ATOM	1226	CZ	ARG	285	20.507	7.663	40.075	1.00	0.00	RX0	C
ATOM	1227	NH1	ARG	285	21.464	8.445	40.606	1.00	0.00	RX0	N
ATOM	1228	HH11	ARG	285	22.420	8.426	40.285	0.00	0.00	RX0	H
ATOM	1229	HH12	ARG	285	21.264	9.099	41.339	0.00	0.00	RX0	H
ATOM	1230	NH2	ARG	285	20.791	6.738	39.137	1.00	0.00	RX0	N
ATOM	1231	HH21	ARG	285	21.682	6.618	38.677	0.00	0.00	RX0	H
ATOM	1232	HH22	ARG	285	20.060	6.122	38.821	0.00	0.00	RX0	H
ATOM	1233	C	ARG	285	17.003	12.164	38.534	1.00	0.00	RX0	C
ATOM	1234	O	ARG	285	15.822	12.321	38.816	1.00	0.00	RX0	O
ATOM	1235	N	MET	286	17.922	13.130	38.591	1.00	0.00	RX0	N
ATOM	1236	H	MET	286	18.856	12.906	38.308	0.00	0.00	RX0	H
ATOM	1237	CA	MET	286	17.643	14.467	39.144	1.00	0.00	RX0	C
ATOM	1238	CB	MET	286	18.957	15.216	39.376	1.00	0.00	RX0	C
ATOM	1239	CG	MET	286	18.807	16.474	40.234	1.00	0.00	RX0	C
ATOM	1240	SD	MET	286	20.390	17.269	40.539	1.00	0.00	RX0	S
ATOM	1241	CE	MET	286	19.815	18.595	41.609	1.00	0.00	RX0	C
ATOM	1242	C	MET	286	16.681	15.257	38.243	1.00	0.00	RX0	C
ATOM	1243	O	MET	286	15.799	15.962	38.735	1.00	0.00	RX0	O
ATOM	1244	N	MET	287	16.911	15.161	36.942	1.00	0.00	RX0	N
ATOM	1245	H	MET	287	17.643	14.551	36.631	0.00	0.00	RX0	H
ATOM	1246	CA	MET	287	16.049	15.814	35.941	1.00	0.00	RX0	C
ATOM	1247	CB	MET	287	16.691	15.872	34.559	1.00	0.00	RX0	C
ATOM	1248	CG	MET	287	17.835	16.878	34.520	1.00	0.00	RX0	C
ATOM	1249	SD	MET	287	18.260	17.326	32.834	1.00	0.00	RX0	S
ATOM	1250	CE	MET	287	16.644	17.967	32.370	1.00	0.00	RX0	C
ATOM	1251	C	MET	287	14.674	15.156	35.849	1.00	0.00	RX0	C
ATOM	1252	O	MET	287	13.755	15.729	35.264	1.00	0.00	RX0	O
ATOM	1253	N	ASN	288	14.583	13.920	36.346	1.00	0.00	RX0	N
ATOM	1254	H	ASN	288	15.394	13.492	36.746	0.00	0.00	RX0	H
ATOM	1255	CA	ASN	288	13.391	13.066	36.242	1.00	0.00	RX0	C
ATOM	1256	CB	ASN	288	12.160	13.612	36.967	1.00	0.00	RX0	C
ATOM	1257	CG	ASN	288	11.009	12.652	36.725	1.00	0.00	RX0	C
ATOM	1258	OD1	ASN	288	11.181	11.440	36.643	1.00	0.00	RX0	O
ATOM	1259	ND2	ASN	288	9.816	13.262	36.593	1.00	0.00	RX0	N
ATOM	1260	HD21	ASN	288	9.734	14.253	36.685	0.00	0.00	RX0	H
ATOM	1261	HD22	ASN	288	8.991	12.738	36.378	0.00	0.00	RX0	H
ATOM	1262	C	ASN	288	13.076	12.836	34.753	1.00	0.00	RX0	C
ATOM	1263	O	ASN	288	11.986	13.121	34.256	1.00	0.00	RX0	O
ATOM	1264	N	LEU	289	14.125	12.439	34.028	1.00	0.00	RX0	N
ATOM	1265	H	LEU	289	14.957	12.176	34.521	0.00	0.00	RX0	H
ATOM	1266	CA	LEU	289	14.030	12.183	32.583	1.00	0.00	RX0	C
ATOM	1267	CB	LEU	289	15.366	11.622	32.090	1.00	0.00	RX0	C
ATOM	1268	CG	LEU	289	15.376	11.200	30.617	1.00	0.00	RX0	C
ATOM	1269	CD1	LEU	289	15.554	12.394	29.686	1.00	0.00	RX0	C
ATOM	1270	CD2	LEU	289	16.405	10.107	30.331	1.00	0.00	RX0	C
ATOM	1271	C	LEU	289	12.926	11.152	32.321	1.00	0.00	RX0	C
ATOM	1272	O	LEU	289	12.814	10.137	33.014	1.00	0.00	RX0	O
ATOM	1273	N	GLN	290	12.165	11.420	31.281	1.00	0.00	RX0	N
ATOM	1274	H	GLN	290	12.393	12.179	30.665	0.00	0.00	RX0	H
ATOM	1275	CA	GLN	290	11.011	10.584	30.916	1.00	0.00	RX0	C
ATOM	1276	CB	GLN	290	9.776	11.452	30.673	1.00	0.00	RX0	C
ATOM	1277	CG	GLN	290	9.405	12.295	31.901	1.00	0.00	RX0	C
ATOM	1278	CD	GLN	290	8.984	11.417	33.071	1.00	0.00	RX0	C
ATOM	1279	OE1	GLN	290	7.829	11.030	33.206	1.00	0.00	RX0	O

ATOM	1280	NE2	GLN	290	9.971	11.156	33.944	1.00	0.00	RX0	N
ATOM	1281	HE21	GLN	290	10.891	11.526	33.806	0.00	0.00	RX0	H
ATOM	1282	HE22	GLN	290	9.882	10.602	34.773	0.00	0.00	RX0	H
ATOM	1283	C	GLN	290	11.379	9.701	29.727	1.00	0.00	RX0	C
ATOM	1284	O	GLN	290	12.115	10.141	28.832	1.00	0.00	RX0	O
ATOM	1285	N	GLY	291	10.739	8.533	29.672	1.00	0.00	RX0	N
ATOM	1286	H	GLY	291	10.027	8.353	30.351	0.00	0.00	RX0	H
ATOM	1287	CA	GLY	291	10.952	7.542	28.589	1.00	0.00	RX0	C
ATOM	1288	C	GLY	291	10.769	8.134	27.181	1.00	0.00	RX0	C
ATOM	1289	O	GLY	291	11.559	7.865	26.269	1.00	0.00	RX0	O
ATOM	1290	N	GLU	292	9.854	9.086	27.078	1.00	0.00	RX0	N
ATOM	1291	H	GLU	292	9.255	9.248	27.866	0.00	0.00	RX0	H
ATOM	1292	CA	GLU	292	9.541	9.789	25.813	1.00	0.00	RX0	C
ATOM	1293	CB	GLU	292	8.214	10.552	25.942	1.00	0.00	RX0	C
ATOM	1294	CG	GLU	292	6.975	9.705	26.278	1.00	0.00	RX0	C
ATOM	1295	CD	GLU	292	7.096	9.114	27.669	1.00	0.00	RX0	C
ATOM	1296	OE1	GLU	292	7.471	9.841	28.587	1.00	0.00	RX0	O
ATOM	1297	OE2	GLU	292	6.914	7.910	27.819	1.00	0.00	RX0	O
ATOM	1298	C	GLU	292	10.671	10.744	25.400	1.00	0.00	RX0	C
ATOM	1299	O	GLU	292	11.065	10.802	24.241	1.00	0.00	RX0	O
ATOM	1300	N	GLU	293	11.241	11.417	26.395	1.00	0.00	RX0	N
ATOM	1301	H	GLU	293	10.950	11.211	27.330	0.00	0.00	RX0	H
ATOM	1302	CA	GLU	293	12.389	12.320	26.184	1.00	0.00	RX0	C
ATOM	1303	CB	GLU	293	12.639	13.154	27.436	1.00	0.00	RX0	C
ATOM	1304	CG	GLU	293	11.411	13.909	27.935	1.00	0.00	RX0	C
ATOM	1305	CD	GLU	293	11.729	14.502	29.290	1.00	0.00	RX0	C
ATOM	1306	OE1	GLU	293	12.649	14.033	29.952	1.00	0.00	RX0	O
ATOM	1307	OE2	GLU	293	11.055	15.433	29.709	1.00	0.00	RX0	O
ATOM	1308	C	GLU	293	13.669	11.544	25.843	1.00	0.00	RX0	C
ATOM	1309	O	GLU	293	14.364	11.888	24.886	1.00	0.00	RX0	O
ATOM	1310	N	PHE	294	13.855	10.409	26.517	1.00	0.00	RX0	N
ATOM	1311	H	PHE	294	13.195	10.200	27.241	0.00	0.00	RX0	H
ATOM	1312	CA	PHE	294	14.999	9.503	26.300	1.00	0.00	RX0	C
ATOM	1313	CB	PHE	294	14.902	8.305	27.248	1.00	0.00	RX0	C
ATOM	1314	CG	PHE	294	15.867	7.227	26.813	1.00	0.00	RX0	C
ATOM	1315	CD1	PHE	294	17.238	7.455	26.841	1.00	0.00	RX0	C
ATOM	1316	CD2	PHE	294	15.376	6.004	26.369	1.00	0.00	RX0	C
ATOM	1317	CE1	PHE	294	18.113	6.470	26.397	1.00	0.00	RX0	C
ATOM	1318	CE2	PHE	294	16.252	5.019	25.929	1.00	0.00	RX0	C
ATOM	1319	CZ	PHE	294	17.621	5.256	25.934	1.00	0.00	RX0	C
ATOM	1320	C	PHE	294	15.115	9.012	24.847	1.00	0.00	RX0	C
ATOM	1321	O	PHE	294	16.186	9.132	24.238	1.00	0.00	RX0	O
ATOM	1322	N	VAL	295	14.000	8.581	24.280	1.00	0.00	RX0	N
ATOM	1323	H	VAL	295	13.158	8.569	24.828	0.00	0.00	RX0	H
ATOM	1324	CA	VAL	295	13.976	8.026	22.907	1.00	0.00	RX0	C
ATOM	1325	CB	VAL	295	12.685	7.252	22.615	1.00	0.00	RX0	C
ATOM	1326	CG1	VAL	295	12.585	6.062	23.567	1.00	0.00	RX0	C
ATOM	1327	CG2	VAL	295	11.430	8.120	22.661	1.00	0.00	RX0	C
ATOM	1328	C	VAL	295	14.286	9.097	21.847	1.00	0.00	RX0	C
ATOM	1329	O	VAL	295	14.999	8.845	20.884	1.00	0.00	RX0	O
ATOM	1330	N	CYS	296	13.866	10.330	22.156	1.00	0.00	RX0	N
ATOM	1331	H	CYS	296	13.306	10.480	22.974	0.00	0.00	RX0	H
ATOM	1332	CA	CYS	296	14.167	11.498	21.314	1.00	0.00	RX0	C
ATOM	1333	CB	CYS	296	13.264	12.653	21.731	1.00	0.00	RX0	C
ATOM	1334	SG	CYS	296	11.519	12.261	21.463	1.00	0.00	RX0	S
ATOM	1335	C	CYS	296	15.661	11.843	21.352	1.00	0.00	RX0	C
ATOM	1336	O	CYS	296	16.290	11.969	20.305	1.00	0.00	RX0	O
ATOM	1337	N	LEU	297	16.247	11.750	22.547	1.00	0.00	RX0	N
ATOM	1338	H	LEU	297	15.681	11.548	23.351	0.00	0.00	RX0	H
ATOM	1339	CA	LEU	297	17.681	12.031	22.752	1.00	0.00	RX0	C
ATOM	1340	CB	LEU	297	18.019	12.109	24.240	1.00	0.00	RX0	C

ATOM	1341	CG	LEU	297	17.350	13.284	24.950	1.00	0.00	RX0	C
ATOM	1342	CD1	LEU	297	17.648	13.263	26.448	1.00	0.00	RX0	C
ATOM	1343	CD2	LEU	297	17.714	14.625	24.309	1.00	0.00	RX0	C
ATOM	1344	C	LEU	297	18.589	11.009	22.068	1.00	0.00	RX0	C
ATOM	1345	O	LEU	297	19.526	11.385	21.359	1.00	0.00	RX0	O
ATOM	1346	N	LYS	298	18.173	9.750	22.123	1.00	0.00	RX0	N
ATOM	1347	H	LYS	298	17.359	9.545	22.671	0.00	0.00	RX0	H
ATOM	1348	CA	LYS	298	18.945	8.654	21.515	1.00	0.00	RX0	C
ATOM	1349	CB	LYS	298	18.430	7.320	22.045	1.00	0.00	RX0	C
ATOM	1350	CG	LYS	298	19.253	6.122	21.581	1.00	0.00	RX0	C
ATOM	1351	CD	LYS	298	18.613	4.835	22.087	1.00	0.00	RX0	C
ATOM	1352	CE	LYS	298	17.097	5.004	22.048	1.00	0.00	RX0	C
ATOM	1353	NZ	LYS	298	16.429	3.710	22.147	1.00	0.00	RX0	N
ATOM	1354	HZ1	LYS	298	15.433	3.867	22.436	0.00	0.00	RX0	H
ATOM	1355	HZ2	LYS	298	16.299	3.232	21.235	0.00	0.00	RX0	H
ATOM	1356	HZ3	LYS	298	16.780	3.056	22.870	0.00	0.00	RX0	H
ATOM	1357	C	LYS	298	18.925	8.733	19.978	1.00	0.00	RX0	C
ATOM	1358	O	LYS	298	19.964	8.557	19.332	1.00	0.00	RX0	O
ATOM	1359	N	SER	299	17.791	9.152	19.431	1.00	0.00	RX0	N
ATOM	1360	H	SER	299	16.963	9.265	19.981	0.00	0.00	RX0	H
ATOM	1361	CA	SER	299	17.645	9.383	17.977	1.00	0.00	RX0	C
ATOM	1362	CB	SER	299	16.155	9.359	17.705	1.00	0.00	RX0	C
ATOM	1363	OG	SER	299	15.662	8.202	18.388	1.00	0.00	RX0	O
ATOM	1364	HG	SER	299	15.121	8.510	19.109	0.00	0.00	RX0	H
ATOM	1365	C	SER	299	18.416	10.621	17.504	1.00	0.00	RX0	C
ATOM	1366	O	SER	299	19.051	10.583	16.444	1.00	0.00	RX0	O
ATOM	1367	N	ILE	300	18.478	11.644	18.354	1.00	0.00	RX0	N
ATOM	1368	H	ILE	300	17.952	11.595	19.206	0.00	0.00	RX0	H
ATOM	1369	CA	ILE	300	19.283	12.860	18.096	1.00	0.00	RX0	C
ATOM	1370	CB	ILE	300	19.082	13.934	19.169	1.00	0.00	RX0	C
ATOM	1371	CG2	ILE	300	20.125	15.047	19.045	1.00	0.00	RX0	C
ATOM	1372	CG1	ILE	300	17.677	14.518	19.098	1.00	0.00	RX0	C
ATOM	1373	CD1	ILE	300	17.421	15.534	20.210	1.00	0.00	RX0	C
ATOM	1374	C	ILE	300	20.773	12.490	18.013	1.00	0.00	RX0	C
ATOM	1375	O	ILE	300	21.456	12.924	17.087	1.00	0.00	RX0	O
ATOM	1376	N	ILE	301	21.227	11.630	18.921	1.00	0.00	RX0	N
ATOM	1377	H	ILE	301	20.604	11.322	19.643	0.00	0.00	RX0	H
ATOM	1378	CA	ILE	301	22.629	11.156	18.925	1.00	0.00	RX0	C
ATOM	1379	CB	ILE	301	22.882	10.197	20.083	1.00	0.00	RX0	C
ATOM	1380	CG2	ILE	301	24.210	9.472	19.893	1.00	0.00	RX0	C
ATOM	1381	CG1	ILE	301	22.837	10.935	21.418	1.00	0.00	RX0	C
ATOM	1382	CD1	ILE	301	23.196	10.014	22.583	1.00	0.00	RX0	C
ATOM	1383	C	ILE	301	22.948	10.474	17.584	1.00	0.00	RX0	C
ATOM	1384	O	ILE	301	23.927	10.823	16.926	1.00	0.00	RX0	O
ATOM	1385	N	LEU	302	22.047	9.582	17.175	1.00	0.00	RX0	N
ATOM	1386	H	LEU	302	21.273	9.369	17.775	0.00	0.00	RX0	H
ATOM	1387	CA	LEU	302	22.205	8.836	15.917	1.00	0.00	RX0	C
ATOM	1388	CB	LEU	302	20.991	7.929	15.709	1.00	0.00	RX0	C
ATOM	1389	CG	LEU	302	20.999	7.210	14.360	1.00	0.00	RX0	C
ATOM	1390	CD1	LEU	302	22.182	6.252	14.222	1.00	0.00	RX0	C
ATOM	1391	CD2	LEU	302	19.665	6.524	14.082	1.00	0.00	RX0	C
ATOM	1392	C	LEU	302	22.391	9.757	14.699	1.00	0.00	RX0	C
ATOM	1393	O	LEU	302	23.285	9.544	13.882	1.00	0.00	RX0	O
ATOM	1394	N	LEU	303	21.581	10.806	14.658	1.00	0.00	RX0	N
ATOM	1395	H	LEU	303	20.934	10.945	15.410	0.00	0.00	RX0	H
ATOM	1396	CA	LEU	303	21.546	11.719	13.502	1.00	0.00	RX0	C
ATOM	1397	CB	LEU	303	20.124	12.232	13.311	1.00	0.00	RX0	C
ATOM	1398	CG	LEU	303	19.162	11.066	13.083	1.00	0.00	RX0	C
ATOM	1399	CD1	LEU	303	17.706	11.490	13.250	1.00	0.00	RX0	C
ATOM	1400	CD2	LEU	303	19.418	10.366	11.747	1.00	0.00	RX0	C
ATOM	1401	C	LEU	303	22.576	12.849	13.543	1.00	0.00	RX0	C

ATOM	1402	O	LEU	303	23.073	13.268	12.494	1.00	0.00	RX0	O
ATOM	1403	N	ASN	304	22.971	13.240	14.745	1.00	0.00	RX0	N
ATOM	1404	H	ASN	304	22.642	12.731	15.541	0.00	0.00	RX0	H
ATOM	1405	CA	ASN	304	23.832	14.419	14.940	1.00	0.00	RX0	C
ATOM	1406	CB	ASN	304	23.496	15.281	16.160	1.00	0.00	RX0	C
ATOM	1407	CG	ASN	304	24.456	14.986	17.296	1.00	0.00	RX0	C
ATOM	1408	OD1	ASN	304	25.508	15.585	17.470	1.00	0.00	RX0	O
ATOM	1409	ND2	ASN	304	24.051	13.975	18.058	1.00	0.00	RX0	N
ATOM	1410	HD21	ASN	304	23.165	13.551	17.878	0.00	0.00	RX0	H
ATOM	1411	HD22	ASN	304	24.651	13.594	18.759	0.00	0.00	RX0	H
ATOM	1412	C	ASN	304	25.330	14.097	14.987	1.00	0.00	RX0	C
ATOM	1413	O	ASN	304	26.132	14.783	14.344	1.00	0.00	RX0	O
ATOM	1414	N	SER	305	25.693	13.015	15.656	1.00	0.00	RX0	N
ATOM	1415	H	SER	305	25.019	12.430	16.121	0.00	0.00	RX0	H
ATOM	1416	CA	SER	305	27.104	12.765	16.012	1.00	0.00	RX0	C
ATOM	1417	CB	SER	305	27.116	11.499	16.835	1.00	0.00	RX0	C
ATOM	1418	OG	SER	305	26.242	11.764	17.935	1.00	0.00	RX0	O
ATOM	1419	HG	SER	305	26.492	11.115	18.601	0.00	0.00	RX0	H
ATOM	1420	C	SER	305	28.103	12.695	14.846	1.00	0.00	RX0	C
ATOM	1421	O	SER	305	29.198	13.228	14.946	1.00	0.00	RX0	O
ATOM	1422	N	GLY	306	27.634	12.177	13.693	1.00	0.00	RX0	N
ATOM	1423	H	GLY	306	26.686	11.870	13.639	0.00	0.00	RX0	H
ATOM	1424	CA	GLY	306	28.500	12.079	12.501	1.00	0.00	RX0	C
ATOM	1425	C	GLY	306	28.091	12.961	11.315	1.00	0.00	RX0	C
ATOM	1426	O	GLY	306	28.756	12.897	10.274	1.00	0.00	RX0	O
ATOM	1427	N	VAL	307	27.242	13.954	11.536	1.00	0.00	RX0	N
ATOM	1428	H	VAL	307	26.877	14.101	12.459	0.00	0.00	RX0	H
ATOM	1429	CA	VAL	307	26.760	14.815	10.435	1.00	0.00	RX0	C
ATOM	1430	CB	VAL	307	25.390	15.446	10.730	1.00	0.00	RX0	C
ATOM	1431	CG1	VAL	307	25.453	16.525	11.804	1.00	0.00	RX0	C
ATOM	1432	CG2	VAL	307	24.739	15.959	9.444	1.00	0.00	RX0	C
ATOM	1433	C	VAL	307	27.809	15.841	9.948	1.00	0.00	RX0	C
ATOM	1434	O	VAL	307	27.790	16.280	8.811	1.00	0.00	RX0	O
ATOM	1435	N	TYR	308	28.719	16.199	10.859	1.00	0.00	RX0	N
ATOM	1436	H	TYR	308	28.730	15.721	11.736	0.00	0.00	RX0	H
ATOM	1437	CA	TYR	308	29.761	17.211	10.584	1.00	0.00	RX0	C
ATOM	1438	CB	TYR	308	30.020	18.114	11.794	1.00	0.00	RX0	C
ATOM	1439	CG	TYR	308	28.711	18.744	12.227	1.00	0.00	RX0	C
ATOM	1440	CD1	TYR	308	27.917	19.407	11.267	1.00	0.00	RX0	C
ATOM	1441	CE1	TYR	308	26.671	19.920	11.658	1.00	0.00	RX0	C
ATOM	1442	CD2	TYR	308	28.325	18.629	13.578	1.00	0.00	RX0	C
ATOM	1443	CE2	TYR	308	27.082	19.146	13.972	1.00	0.00	RX0	C
ATOM	1444	CZ	TYR	308	26.265	19.754	12.998	1.00	0.00	RX0	C
ATOM	1445	OH	TYR	308	25.009	20.184	13.375	1.00	0.00	RX0	O
ATOM	1446	HH	TYR	308	24.412	20.135	12.632	0.00	0.00	RX0	H
ATOM	1447	C	TYR	308	31.032	16.660	9.940	1.00	0.00	RX0	C
ATOM	1448	O	TYR	308	31.912	17.411	9.537	1.00	0.00	RX0	O
ATOM	1449	N	THR	309	31.091	15.334	9.861	1.00	0.00	RX0	N
ATOM	1450	H	THR	309	30.383	14.719	10.211	0.00	0.00	RX0	H
ATOM	1451	CA	THR	309	32.303	14.643	9.386	1.00	0.00	RX0	C
ATOM	1452	CB	THR	309	32.706	13.720	10.534	1.00	0.00	RX0	C
ATOM	1453	OG1	THR	309	31.525	13.150	11.127	1.00	0.00	RX0	O
ATOM	1454	HG1	THR	309	31.212	12.465	10.535	0.00	0.00	RX0	H
ATOM	1455	CG2	THR	309	33.509	14.468	11.601	1.00	0.00	RX0	C
ATOM	1456	C	THR	309	32.183	13.866	8.071	1.00	0.00	RX0	C
ATOM	1457	O	THR	309	33.137	13.195	7.681	1.00	0.00	RX0	O
ATOM	1458	N	PHE	310	31.041	13.953	7.382	1.00	0.00	RX0	N
ATOM	1459	H	PHE	310	30.299	14.506	7.754	0.00	0.00	RX0	H
ATOM	1460	CA	PHE	310	30.972	13.467	5.988	1.00	0.00	RX0	C
ATOM	1461	CB	PHE	310	29.595	13.719	5.372	1.00	0.00	RX0	C
ATOM	1462	CG	PHE	310	28.522	12.912	6.060	1.00	0.00	RX0	C

ATOM	1463	CD1	PHE	310	28.412	11.550	5.812	1.00	0.00	RX0	C
ATOM	1464	CD2	PHE	310	27.632	13.534	6.928	1.00	0.00	RX0	C
ATOM	1465	CE1	PHE	310	27.406	10.813	6.426	1.00	0.00	RX0	C
ATOM	1466	CE2	PHE	310	26.626	12.795	7.539	1.00	0.00	RX0	C
ATOM	1467	CZ	PHE	310	26.510	11.434	7.286	1.00	0.00	RX0	C
ATOM	1468	C	PHE	310	32.019	14.245	5.180	1.00	0.00	RX0	C
ATOM	1469	O	PHE	310	32.102	15.468	5.301	1.00	0.00	RX0	O
ATOM	1470	N	LEU	311	32.854	13.508	4.462	1.00	0.00	RX0	N
ATOM	1471	H	LEU	311	32.732	12.519	4.522	0.00	0.00	RX0	H
ATOM	1472	CA	LEU	311	33.988	14.087	3.701	1.00	0.00	RX0	C
ATOM	1473	CB	LEU	311	34.726	13.040	2.864	1.00	0.00	RX0	C
ATOM	1474	CG	LEU	311	35.114	11.740	3.549	1.00	0.00	RX0	C
ATOM	1475	CD1	LEU	311	35.531	10.733	2.481	1.00	0.00	RX0	C
ATOM	1476	CD2	LEU	311	36.165	11.932	4.646	1.00	0.00	RX0	C
ATOM	1477	C	LEU	311	33.468	15.166	2.739	1.00	0.00	RX0	C
ATOM	1478	O	LEU	311	33.554	16.351	2.969	1.00	0.00	RX0	O
ATOM	1479	N	SER	312	32.784	14.619	1.713	1.00	0.00	RX0	N
ATOM	1480	H	SER	312	32.597	13.640	1.724	0.00	0.00	RX0	H
ATOM	1481	CA	SER	312	32.345	15.333	0.526	1.00	0.00	RX0	C
ATOM	1482	CB	SER	312	31.785	14.218	-0.338	1.00	0.00	RX0	C
ATOM	1483	OG	SER	312	32.560	13.058	-0.010	1.00	0.00	RX0	O
ATOM	1484	HG	SER	312	32.659	12.537	-0.799	0.00	0.00	RX0	H
ATOM	1485	C	SER	312	31.423	16.517	0.816	1.00	0.00	RX0	C
ATOM	1486	O	SER	312	31.042	16.847	1.948	1.00	0.00	RX0	O
ATOM	1487	N	SER	313	30.837	16.923	-0.276	1.00	0.00	RX0	N
ATOM	1488	H	SER	313	31.196	16.610	-1.156	0.00	0.00	RX0	H
ATOM	1489	CA	SER	313	29.838	17.983	-0.455	1.00	0.00	RX0	C
ATOM	1490	CB	SER	313	30.335	19.322	0.095	1.00	0.00	RX0	C
ATOM	1491	OG	SER	313	30.310	19.220	1.535	1.00	0.00	RX0	O
ATOM	1492	HG	SER	313	31.129	18.786	1.770	0.00	0.00	RX0	H
ATOM	1493	C	SER	313	29.353	17.880	-1.909	1.00	0.00	RX0	C
ATOM	1494	O	SER	313	29.168	18.836	-2.628	1.00	0.00	RX0	O
ATOM	1495	N	THR	314	29.278	16.602	-2.358	1.00	0.00	RX0	N
ATOM	1496	H	THR	314	29.459	15.826	-1.760	0.00	0.00	RX0	H
ATOM	1497	CA	THR	314	28.609	16.250	-3.614	1.00	0.00	RX0	C
ATOM	1498	CB	THR	314	28.742	14.740	-3.676	1.00	0.00	RX0	C
ATOM	1499	OG1	THR	314	29.881	14.375	-2.881	1.00	0.00	RX0	O
ATOM	1500	HG1	THR	314	30.155	13.523	-3.208	0.00	0.00	RX0	H
ATOM	1501	CG2	THR	314	28.857	14.206	-5.107	1.00	0.00	RX0	C
ATOM	1502	C	THR	314	27.167	16.747	-3.490	1.00	0.00	RX0	C
ATOM	1503	O	THR	314	26.675	16.978	-2.368	1.00	0.00	RX0	O
ATOM	1504	N	LEU	315	26.451	16.801	-4.589	1.00	0.00	RX0	N
ATOM	1505	H	LEU	315	26.871	16.634	-5.479	0.00	0.00	RX0	H
ATOM	1506	CA	LEU	315	25.050	17.256	-4.537	1.00	0.00	RX0	C
ATOM	1507	CB	LEU	315	24.458	17.340	-5.944	1.00	0.00	RX0	C
ATOM	1508	CG	LEU	315	23.042	17.920	-5.940	1.00	0.00	RX0	C
ATOM	1509	CD1	LEU	315	23.000	19.325	-5.335	1.00	0.00	RX0	C
ATOM	1510	CD2	LEU	315	22.403	17.867	-7.328	1.00	0.00	RX0	C
ATOM	1511	C	LEU	315	24.190	16.343	-3.639	1.00	0.00	RX0	C
ATOM	1512	O	LEU	315	23.484	16.800	-2.757	1.00	0.00	RX0	O
ATOM	1513	N	LYS	316	24.524	15.049	-3.730	1.00	0.00	RX0	N
ATOM	1514	H	LYS	316	25.049	14.789	-4.533	0.00	0.00	RX0	H
ATOM	1515	CA	LYS	316	23.912	13.990	-2.918	1.00	0.00	RX0	C
ATOM	1516	CB	LYS	316	24.520	12.670	-3.378	1.00	0.00	RX0	C
ATOM	1517	CG	LYS	316	23.790	11.412	-2.924	1.00	0.00	RX0	C
ATOM	1518	CD	LYS	316	24.712	10.198	-3.033	1.00	0.00	RX0	C
ATOM	1519	CE	LYS	316	23.951	8.879	-2.938	1.00	0.00	RX0	C
ATOM	1520	NZ	LYS	316	24.815	7.855	-2.334	1.00	0.00	RX0	N
ATOM	1521	HZ1	LYS	316	24.370	6.915	-2.391	0.00	0.00	RX0	H
ATOM	1522	HZ2	LYS	316	25.718	7.797	-2.844	0.00	0.00	RX0	H
ATOM	1523	HZ3	LYS	316	25.023	8.065	-1.336	0.00	0.00	RX0	H

ATOM	1524	C	LYS	316	24.213	14.187	-1.422	1.00	0.00	RX0	C
ATOM	1525	O	LYS	316	23.297	14.194	-0.611	1.00	0.00	RX0	O
ATOM	1526	N	SER	317	25.467	14.540	-1.122	1.00	0.00	RX0	N
ATOM	1527	H	SER	317	26.081	14.754	-1.875	0.00	0.00	RX0	H
ATOM	1528	CA	SER	317	25.934	14.787	0.260	1.00	0.00	RX0	C
ATOM	1529	CB	SER	317	27.466	14.863	0.207	1.00	0.00	RX0	C
ATOM	1530	OG	SER	317	28.082	14.559	1.464	1.00	0.00	RX0	O
ATOM	1531	HG	SER	317	27.702	13.728	1.754	0.00	0.00	RX0	H
ATOM	1532	C	SER	317	25.242	16.001	0.898	1.00	0.00	RX0	C
ATOM	1533	O	SER	317	24.716	15.915	2.007	1.00	0.00	RX0	O
ATOM	1534	N	LEU	318	25.067	17.043	0.088	1.00	0.00	RX0	N
ATOM	1535	H	LEU	318	25.331	16.973	-0.876	0.00	0.00	RX0	H
ATOM	1536	CA	LEU	318	24.405	18.288	0.525	1.00	0.00	RX0	C
ATOM	1537	CB	LEU	318	24.579	19.374	-0.533	1.00	0.00	RX0	C
ATOM	1538	CG	LEU	318	26.044	19.770	-0.695	1.00	0.00	RX0	C
ATOM	1539	CD1	LEU	318	26.254	20.715	-1.877	1.00	0.00	RX0	C
ATOM	1540	CD2	LEU	318	26.618	20.326	0.609	1.00	0.00	RX0	C
ATOM	1541	C	LEU	318	22.918	18.061	0.823	1.00	0.00	RX0	C
ATOM	1542	O	LEU	318	22.412	18.453	1.877	1.00	0.00	RX0	O
ATOM	1543	N	GLU	319	22.299	17.244	-0.024	1.00	0.00	RX0	N
ATOM	1544	H	GLU	319	22.759	16.921	-0.855	0.00	0.00	RX0	H
ATOM	1545	CA	GLU	319	20.900	16.808	0.157	1.00	0.00	RX0	C
ATOM	1546	CB	GLU	319	20.422	16.053	-1.088	1.00	0.00	RX0	C
ATOM	1547	CG	GLU	319	20.414	16.909	-2.359	1.00	0.00	RX0	C
ATOM	1548	CD	GLU	319	20.167	16.037	-3.578	1.00	0.00	RX0	C
ATOM	1549	OE1	GLU	319	21.134	15.589	-4.200	1.00	0.00	RX0	O
ATOM	1550	OE2	GLU	319	19.005	15.820	-3.913	1.00	0.00	RX0	O
ATOM	1551	C	GLU	319	20.725	15.924	1.402	1.00	0.00	RX0	C
ATOM	1552	O	GLU	319	19.808	16.153	2.196	1.00	0.00	RX0	O
ATOM	1553	N	GLU	320	21.706	15.058	1.645	1.00	0.00	RX0	N
ATOM	1554	H	GLU	320	22.375	14.898	0.919	0.00	0.00	RX0	H
ATOM	1555	CA	GLU	320	21.731	14.149	2.812	1.00	0.00	RX0	C
ATOM	1556	CB	GLU	320	22.928	13.193	2.785	1.00	0.00	RX0	C
ATOM	1557	CG	GLU	320	22.874	12.129	1.690	1.00	0.00	RX0	C
ATOM	1558	CD	GLU	320	21.704	11.194	1.914	1.00	0.00	RX0	C
ATOM	1559	OE1	GLU	320	20.673	11.370	1.273	1.00	0.00	RX0	O
ATOM	1560	OE2	GLU	320	21.841	10.262	2.701	1.00	0.00	RX0	O
ATOM	1561	C	GLU	320	21.765	14.931	4.131	1.00	0.00	RX0	C
ATOM	1562	O	GLU	320	20.881	14.754	4.973	1.00	0.00	RX0	O
ATOM	1563	N	LYS	321	22.647	15.926	4.188	1.00	0.00	RX0	N
ATOM	1564	H	LYS	321	23.230	16.048	3.381	0.00	0.00	RX0	H
ATOM	1565	CA	LYS	321	22.801	16.771	5.391	1.00	0.00	RX0	C
ATOM	1566	CB	LYS	321	24.079	17.634	5.229	1.00	0.00	RX0	C
ATOM	1567	CG	LYS	321	25.363	16.839	4.879	1.00	0.00	RX0	C
ATOM	1568	CD	LYS	321	26.624	17.646	4.470	1.00	0.00	RX0	C
ATOM	1569	CE	LYS	321	27.752	16.761	3.881	1.00	0.00	RX0	C
ATOM	1570	NZ	LYS	321	29.003	17.485	3.546	1.00	0.00	RX0	N
ATOM	1571	HZ1	LYS	321	29.689	16.840	3.088	0.00	0.00	RX0	H
ATOM	1572	HZ2	LYS	321	28.866	18.273	2.879	0.00	0.00	RX0	H
ATOM	1573	HZ3	LYS	321	29.461	17.842	4.407	0.00	0.00	RX0	H
ATOM	1574	C	LYS	321	21.549	17.612	5.656	1.00	0.00	RX0	C
ATOM	1575	O	LYS	321	21.102	17.724	6.798	1.00	0.00	RX0	O
ATOM	1576	N	ASP	322	20.935	18.099	4.575	1.00	0.00	RX0	N
ATOM	1577	H	ASP	322	21.313	17.934	3.660	0.00	0.00	RX0	H
ATOM	1578	CA	ASP	322	19.715	18.915	4.684	1.00	0.00	RX0	C
ATOM	1579	CB	ASP	322	19.393	19.472	3.295	1.00	0.00	RX0	C
ATOM	1580	CG	ASP	322	18.081	20.223	3.287	1.00	0.00	RX0	C
ATOM	1581	OD1	ASP	322	17.681	20.753	4.312	1.00	0.00	RX0	O
ATOM	1582	OD2	ASP	322	17.418	20.238	2.255	1.00	0.00	RX0	O
ATOM	1583	C	ASP	322	18.561	18.097	5.282	1.00	0.00	RX0	C
ATOM	1584	O	ASP	322	17.955	18.512	6.263	1.00	0.00	RX0	O

ATOM	1585	N	HIS	323	18.423	16.872	4.772	1.00	0.00	RX0	N
ATOM	1586	H	HIS	323	19.043	16.597	4.031	0.00	0.00	RX0	H
ATOM	1587	CA	HIS	323	17.423	15.916	5.274	1.00	0.00	RX0	C
ATOM	1588	CB	HIS	323	17.468	14.622	4.463	1.00	0.00	RX0	C
ATOM	1589	CG	HIS	323	16.326	13.728	4.883	1.00	0.00	RX0	C
ATOM	1590	ND1	HIS	323	16.387	12.385	4.940	1.00	0.00	RX0	N
ATOM	1591	HD1	HIS	323	17.155	11.811	4.717	0.00	0.00	RX0	H
ATOM	1592	CD2	HIS	323	15.044	14.131	5.255	1.00	0.00	RX0	C
ATOM	1593	NE2	HIS	323	14.326	13.023	5.541	1.00	0.00	RX0	N
ATOM	1594	CE1	HIS	323	15.153	11.942	5.347	1.00	0.00	RX0	C
ATOM	1595	C	HIS	323	17.630	15.607	6.766	1.00	0.00	RX0	C
ATOM	1596	O	HIS	323	16.677	15.663	7.540	1.00	0.00	RX0	O
ATOM	1597	N	ILE	324	18.888	15.420	7.164	1.00	0.00	RX0	N
ATOM	1598	H	ILE	324	19.610	15.419	6.467	0.00	0.00	RX0	H
ATOM	1599	CA	ILE	324	19.235	15.107	8.570	1.00	0.00	RX0	C
ATOM	1600	CB	ILE	324	20.719	14.764	8.702	1.00	0.00	RX0	C
ATOM	1601	CG2	ILE	324	21.128	14.629	10.168	1.00	0.00	RX0	C
ATOM	1602	CG1	ILE	324	21.037	13.490	7.918	1.00	0.00	RX0	C
ATOM	1603	CD1	ILE	324	22.526	13.141	7.916	1.00	0.00	RX0	C
ATOM	1604	C	ILE	324	18.844	16.271	9.496	1.00	0.00	RX0	C
ATOM	1605	O	ILE	324	18.200	16.053	10.527	1.00	0.00	RX0	O
ATOM	1606	N	HIS	325	19.159	17.485	9.071	1.00	0.00	RX0	N
ATOM	1607	H	HIS	325	19.584	17.591	8.169	0.00	0.00	RX0	H
ATOM	1608	CA	HIS	325	18.840	18.694	9.856	1.00	0.00	RX0	C
ATOM	1609	CB	HIS	325	19.576	19.930	9.326	1.00	0.00	RX0	C
ATOM	1610	CG	HIS	325	21.029	19.881	9.735	1.00	0.00	RX0	C
ATOM	1611	ND1	HIS	325	21.989	19.309	8.992	1.00	0.00	RX0	N
ATOM	1612	HD1	HIS	325	21.850	18.860	8.130	0.00	0.00	RX0	H
ATOM	1613	CD2	HIS	325	21.612	20.394	10.899	1.00	0.00	RX0	C
ATOM	1614	NE2	HIS	325	22.943	20.125	10.848	1.00	0.00	RX0	N
ATOM	1615	CE1	HIS	325	23.169	19.457	9.671	1.00	0.00	RX0	C
ATOM	1616	C	HIS	325	17.335	18.955	9.948	1.00	0.00	RX0	C
ATOM	1617	O	HIS	325	16.820	19.290	11.021	1.00	0.00	RX0	O
ATOM	1618	N	ARG	326	16.631	18.584	8.887	1.00	0.00	RX0	N
ATOM	1619	H	ARG	326	17.125	18.357	8.046	0.00	0.00	RX0	H
ATOM	1620	CA	ARG	326	15.160	18.594	8.873	1.00	0.00	RX0	C
ATOM	1621	CB	ARG	326	14.650	18.358	7.448	1.00	0.00	RX0	C
ATOM	1622	CG	ARG	326	14.818	19.544	6.495	1.00	0.00	RX0	C
ATOM	1623	CD	ARG	326	14.251	19.255	5.099	1.00	0.00	RX0	C
ATOM	1624	NE	ARG	326	15.305	19.036	4.108	1.00	0.00	RX0	N
ATOM	1625	HE	ARG	326	15.992	19.781	4.017	0.00	0.00	RX0	H
ATOM	1626	CZ	ARG	326	15.349	17.916	3.327	1.00	0.00	RX0	C
ATOM	1627	NH1	ARG	326	14.433	16.942	3.516	1.00	0.00	RX0	N
ATOM	1628	HH11	ARG	326	14.418	16.102	2.969	0.00	0.00	RX0	H
ATOM	1629	HH12	ARG	326	13.733	17.034	4.228	0.00	0.00	RX0	H
ATOM	1630	NH2	ARG	326	16.302	17.788	2.381	1.00	0.00	RX0	N
ATOM	1631	HH21	ARG	326	16.453	16.987	1.798	0.00	0.00	RX0	H
ATOM	1632	HH22	ARG	326	16.933	18.568	2.229	0.00	0.00	RX0	H
ATOM	1633	C	ARG	326	14.537	17.576	9.843	1.00	0.00	RX0	C
ATOM	1634	O	ARG	326	13.617	17.928	10.589	1.00	0.00	RX0	O
ATOM	1635	N	VAL	327	15.171	16.419	9.987	1.00	0.00	RX0	N
ATOM	1636	H	VAL	327	15.980	16.248	9.420	0.00	0.00	RX0	H
ATOM	1637	CA	VAL	327	14.709	15.376	10.935	1.00	0.00	RX0	C
ATOM	1638	CB	VAL	327	15.269	13.993	10.599	1.00	0.00	RX0	C
ATOM	1639	CG1	VAL	327	14.751	12.956	11.596	1.00	0.00	RX0	C
ATOM	1640	CG2	VAL	327	14.908	13.586	9.171	1.00	0.00	RX0	C
ATOM	1641	C	VAL	327	15.041	15.781	12.381	1.00	0.00	RX0	C
ATOM	1642	O	VAL	327	14.187	15.661	13.270	1.00	0.00	RX0	O
ATOM	1643	N	LEU	328	16.212	16.371	12.574	1.00	0.00	RX0	N
ATOM	1644	H	LEU	328	16.817	16.507	11.788	0.00	0.00	RX0	H
ATOM	1645	CA	LEU	328	16.631	16.899	13.887	1.00	0.00	RX0	C

ATOM	1646	CB	LEU	328	18.069	17.411	13.827	1.00	0.00	RX0	C
ATOM	1647	CG	LEU	328	19.084	16.271	13.757	1.00	0.00	RX0	C
ATOM	1648	CD1	LEU	328	20.500	16.777	13.484	1.00	0.00	RX0	C
ATOM	1649	CD2	LEU	328	19.015	15.398	15.010	1.00	0.00	RX0	C
ATOM	1650	C	LEU	328	15.692	18.005	14.386	1.00	0.00	RX0	C
ATOM	1651	O	LEU	328	15.231	17.955	15.519	1.00	0.00	RX0	O
ATOM	1652	N	ASP	329	15.222	18.821	13.436	1.00	0.00	RX0	N
ATOM	1653	H	ASP	329	15.673	18.893	12.543	0.00	0.00	RX0	H
ATOM	1654	CA	ASP	329	14.223	19.870	13.722	1.00	0.00	RX0	C
ATOM	1655	CB	ASP	329	14.090	20.860	12.558	1.00	0.00	RX0	C
ATOM	1656	CG	ASP	329	15.314	21.765	12.452	1.00	0.00	RX0	C
ATOM	1657	OD1	ASP	329	16.053	21.907	13.430	1.00	0.00	RX0	O
ATOM	1658	OD2	ASP	329	15.519	22.341	11.384	1.00	0.00	RX0	O
ATOM	1659	C	ASP	329	12.864	19.312	14.154	1.00	0.00	RX0	C
ATOM	1660	O	ASP	329	12.272	19.788	15.128	1.00	0.00	RX0	O
ATOM	1661	N	LYS	330	12.463	18.218	13.512	1.00	0.00	RX0	N
ATOM	1662	H	LYS	330	13.019	17.922	12.733	0.00	0.00	RX0	H
ATOM	1663	CA	LYS	330	11.217	17.525	13.871	1.00	0.00	RX0	C
ATOM	1664	CB	LYS	330	10.799	16.458	12.860	1.00	0.00	RX0	C
ATOM	1665	CG	LYS	330	9.619	15.609	13.368	1.00	0.00	RX0	C
ATOM	1666	CD	LYS	330	8.368	16.394	13.793	1.00	0.00	RX0	C
ATOM	1667	CE	LYS	330	7.829	17.315	12.707	1.00	0.00	RX0	C
ATOM	1668	NZ	LYS	330	7.436	16.478	11.574	1.00	0.00	RX0	N
ATOM	1669	HZ1	LYS	330	7.123	17.092	10.801	0.00	0.00	RX0	H
ATOM	1670	HZ2	LYS	330	8.243	15.888	11.278	0.00	0.00	RX0	H
ATOM	1671	HZ3	LYS	330	6.654	15.863	11.883	0.00	0.00	RX0	H
ATOM	1672	C	LYS	330	11.283	16.915	15.278	1.00	0.00	RX0	C
ATOM	1673	O	LYS	330	10.354	17.095	16.067	1.00	0.00	RX0	O
ATOM	1674	N	ILE	331	12.441	16.375	15.625	1.00	0.00	RX0	N
ATOM	1675	H	ILE	331	13.183	16.374	14.949	0.00	0.00	RX0	H
ATOM	1676	CA	ILE	331	12.656	15.802	16.972	1.00	0.00	RX0	C
ATOM	1677	CB	ILE	331	13.953	14.996	17.053	1.00	0.00	RX0	C
ATOM	1678	CG2	ILE	331	14.064	14.332	18.422	1.00	0.00	RX0	C
ATOM	1679	CG1	ILE	331	14.041	13.950	15.944	1.00	0.00	RX0	C
ATOM	1680	CD1	ILE	331	15.355	13.170	15.986	1.00	0.00	RX0	C
ATOM	1681	C	ILE	331	12.642	16.922	18.027	1.00	0.00	RX0	C
ATOM	1682	O	ILE	331	12.078	16.732	19.120	1.00	0.00	RX0	O
ATOM	1683	N	THR	332	13.158	18.084	17.675	1.00	0.00	RX0	N
ATOM	1684	H	THR	332	13.556	18.205	16.764	0.00	0.00	RX0	H
ATOM	1685	CA	THR	332	13.155	19.265	18.570	1.00	0.00	RX0	C
ATOM	1686	CB	THR	332	14.027	20.347	17.928	1.00	0.00	RX0	C
ATOM	1687	OG1	THR	332	15.324	19.800	17.648	1.00	0.00	RX0	O
ATOM	1688	HG1	THR	332	15.268	19.379	16.792	0.00	0.00	RX0	H
ATOM	1689	CG2	THR	332	14.155	21.589	18.815	1.00	0.00	RX0	C
ATOM	1690	C	THR	332	11.706	19.696	18.836	1.00	0.00	RX0	C
ATOM	1691	O	THR	332	11.302	19.832	19.995	1.00	0.00	RX0	O
ATOM	1692	N	ASP	333	10.912	19.714	17.772	1.00	0.00	RX0	N
ATOM	1693	H	ASP	333	11.279	19.674	16.837	0.00	0.00	RX0	H
ATOM	1694	CA	ASP	333	9.466	20.017	17.863	1.00	0.00	RX0	C
ATOM	1695	CB	ASP	333	8.672	19.794	16.566	1.00	0.00	RX0	C
ATOM	1696	CG	ASP	333	9.106	20.580	15.355	1.00	0.00	RX0	C
ATOM	1697	OD1	ASP	333	9.518	21.725	15.510	1.00	0.00	RX0	O
ATOM	1698	OD2	ASP	333	8.976	20.043	14.251	1.00	0.00	RX0	O
ATOM	1699	C	ASP	333	8.732	19.000	18.747	1.00	0.00	RX0	C
ATOM	1700	O	ASP	333	7.880	19.374	19.559	1.00	0.00	RX0	O
ATOM	1701	N	THR	334	9.187	17.759	18.682	1.00	0.00	RX0	N
ATOM	1702	H	THR	334	9.940	17.583	18.047	0.00	0.00	RX0	H
ATOM	1703	CA	THR	334	8.631	16.638	19.462	1.00	0.00	RX0	C
ATOM	1704	CB	THR	334	9.175	15.373	18.821	1.00	0.00	RX0	C
ATOM	1705	OG1	THR	334	8.793	15.340	17.437	1.00	0.00	RX0	O
ATOM	1706	HG1	THR	334	9.150	16.125	17.031	0.00	0.00	RX0	H

ATOM	1707	CG2	THR	334	8.723	14.119	19.556	1.00	0.00	RX0	C
ATOM	1708	C	THR	334	8.961	16.782	20.953	1.00	0.00	RX0	C
ATOM	1709	O	THR	334	8.059	16.689	21.789	1.00	0.00	RX0	O
ATOM	1710	N	LEU	335	10.217	17.081	21.263	1.00	0.00	RX0	N
ATOM	1711	H	LEU	335	10.880	17.204	20.523	0.00	0.00	RX0	H
ATOM	1712	CA	LEU	335	10.648	17.349	22.650	1.00	0.00	RX0	C
ATOM	1713	CB	LEU	335	12.150	17.613	22.696	1.00	0.00	RX0	C
ATOM	1714	CG	LEU	335	12.962	16.325	22.780	1.00	0.00	RX0	C
ATOM	1715	CD1	LEU	335	14.460	16.579	22.613	1.00	0.00	RX0	C
ATOM	1716	CD2	LEU	335	12.652	15.573	24.074	1.00	0.00	RX0	C
ATOM	1717	C	LEU	335	9.903	18.522	23.297	1.00	0.00	RX0	C
ATOM	1718	O	LEU	335	9.384	18.379	24.399	1.00	0.00	RX0	O
ATOM	1719	N	ILE	336	9.668	19.567	22.501	1.00	0.00	RX0	N
ATOM	1720	H	ILE	336	10.073	19.580	21.583	0.00	0.00	RX0	H
ATOM	1721	CA	ILE	336	8.897	20.743	22.962	1.00	0.00	RX0	C
ATOM	1722	CB	ILE	336	9.049	21.913	21.990	1.00	0.00	RX0	C
ATOM	1723	CG2	ILE	336	8.094	23.060	22.330	1.00	0.00	RX0	C
ATOM	1724	CG1	ILE	336	10.504	22.385	21.991	1.00	0.00	RX0	C
ATOM	1725	CD1	ILE	336	10.944	22.866	23.376	1.00	0.00	RX0	C
ATOM	1726	C	ILE	336	7.427	20.365	23.180	1.00	0.00	RX0	C
ATOM	1727	O	ILE	336	6.836	20.730	24.203	1.00	0.00	RX0	O
ATOM	1728	N	HIS	337	6.891	19.571	22.265	1.00	0.00	RX0	N
ATOM	1729	H	HIS	337	7.437	19.323	21.462	0.00	0.00	RX0	H
ATOM	1730	CA	HIS	337	5.501	19.096	22.359	1.00	0.00	RX0	C
ATOM	1731	CB	HIS	337	5.076	18.340	21.113	1.00	0.00	RX0	C
ATOM	1732	CG	HIS	337	3.596	18.078	21.217	1.00	0.00	RX0	C
ATOM	1733	ND1	HIS	337	2.654	19.003	20.958	1.00	0.00	RX0	N
ATOM	1734	HD1	HIS	337	2.808	19.927	20.661	0.00	0.00	RX0	H
ATOM	1735	CD2	HIS	337	2.973	16.894	21.612	1.00	0.00	RX0	C
ATOM	1736	NE2	HIS	337	1.634	17.113	21.598	1.00	0.00	RX0	N
ATOM	1737	CE1	HIS	337	1.439	18.411	21.193	1.00	0.00	RX0	C
ATOM	1738	C	HIS	337	5.301	18.263	23.631	1.00	0.00	RX0	C
ATOM	1739	O	HIS	337	4.339	18.490	24.365	1.00	0.00	RX0	O
ATOM	1740	N	LEU	338	6.274	17.405	23.914	1.00	0.00	RX0	N
ATOM	1741	H	LEU	338	7.052	17.338	23.287	0.00	0.00	RX0	H
ATOM	1742	CA	LEU	338	6.246	16.526	25.097	1.00	0.00	RX0	C
ATOM	1743	CB	LEU	338	7.442	15.576	25.090	1.00	0.00	RX0	C
ATOM	1744	CG	LEU	338	7.342	14.531	23.982	1.00	0.00	RX0	C
ATOM	1745	CD1	LEU	338	8.646	13.749	23.815	1.00	0.00	RX0	C
ATOM	1746	CD2	LEU	338	6.124	13.628	24.183	1.00	0.00	RX0	C
ATOM	1747	C	LEU	338	6.240	17.331	26.400	1.00	0.00	RX0	C
ATOM	1748	O	LEU	338	5.410	17.098	27.277	1.00	0.00	RX0	O
ATOM	1749	N	MET	339	7.027	18.402	26.394	1.00	0.00	RX0	N
ATOM	1750	H	MET	339	7.615	18.548	25.595	0.00	0.00	RX0	H
ATOM	1751	CA	MET	339	7.168	19.308	27.550	1.00	0.00	RX0	C
ATOM	1752	CB	MET	339	8.404	20.192	27.399	1.00	0.00	RX0	C
ATOM	1753	CG	MET	339	9.706	19.396	27.369	1.00	0.00	RX0	C
ATOM	1754	SD	MET	339	11.133	20.441	27.050	1.00	0.00	RX0	S
ATOM	1755	CE	MET	339	12.269	19.135	26.563	1.00	0.00	RX0	C
ATOM	1756	C	MET	339	5.924	20.183	27.753	1.00	0.00	RX0	C
ATOM	1757	O	MET	339	5.433	20.322	28.878	1.00	0.00	RX0	O
ATOM	1758	N	ALA	340	5.370	20.672	26.646	1.00	0.00	RX0	N
ATOM	1759	H	ALA	340	5.814	20.472	25.772	0.00	0.00	RX0	H
ATOM	1760	CA	ALA	340	4.120	21.456	26.636	1.00	0.00	RX0	C
ATOM	1761	CB	ALA	340	3.835	21.993	25.233	1.00	0.00	RX0	C
ATOM	1762	C	ALA	340	2.921	20.619	27.100	1.00	0.00	RX0	C
ATOM	1763	O	ALA	340	2.176	21.064	27.967	1.00	0.00	RX0	O
ATOM	1764	N	LYS	341	2.865	19.360	26.655	1.00	0.00	RX0	N
ATOM	1765	H	LYS	341	3.555	19.057	25.997	0.00	0.00	RX0	H
ATOM	1766	CA	LYS	341	1.846	18.397	27.117	1.00	0.00	RX0	C
ATOM	1767	CB	LYS	341	1.986	17.110	26.280	1.00	0.00	RX0	C

ATOM	1768	CG	LYS	341	0.726	16.240	26.169	1.00	0.00	RX0	C
ATOM	1769	CD	LYS	341	0.396	15.776	24.732	1.00	0.00	RX0	C
ATOM	1770	CE	LYS	341	1.359	14.769	24.073	1.00	0.00	RX0	C
ATOM	1771	NZ	LYS	341	1.026	14.577	22.652	1.00	0.00	RX0	N
ATOM	1772	HZ1	LYS	341	1.744	14.038	22.110	0.00	0.00	RX0	H
ATOM	1773	HZ2	LYS	341	0.157	14.035	22.456	0.00	0.00	RX0	H
ATOM	1774	HZ3	LYS	341	0.952	15.475	22.135	0.00	0.00	RX0	H
ATOM	1775	C	LYS	341	1.927	18.179	28.637	1.00	0.00	RX0	C
ATOM	1776	O	LYS	341	0.908	18.032	29.304	1.00	0.00	RX0	O
ATOM	1777	N	ALA	342	3.166	18.103	29.120	1.00	0.00	RX0	N
ATOM	1778	H	ALA	342	3.940	18.170	28.488	0.00	0.00	RX0	H
ATOM	1779	CA	ALA	342	3.459	17.942	30.556	1.00	0.00	RX0	C
ATOM	1780	CB	ALA	342	4.953	17.706	30.786	1.00	0.00	RX0	C
ATOM	1781	C	ALA	342	3.007	19.166	31.371	1.00	0.00	RX0	C
ATOM	1782	O	ALA	342	2.879	19.099	32.588	1.00	0.00	RX0	O
ATOM	1783	N	GLY	343	2.900	20.305	30.669	1.00	0.00	RX0	N
ATOM	1784	H	GLY	343	3.153	20.317	29.702	0.00	0.00	RX0	H
ATOM	1785	CA	GLY	343	2.393	21.560	31.244	1.00	0.00	RX0	C
ATOM	1786	C	GLY	343	3.505	22.433	31.829	1.00	0.00	RX0	C
ATOM	1787	O	GLY	343	3.244	23.282	32.678	1.00	0.00	RX0	O
ATOM	1788	N	LEU	344	4.736	22.226	31.356	1.00	0.00	RX0	N
ATOM	1789	H	LEU	344	4.853	21.555	30.623	0.00	0.00	RX0	H
ATOM	1790	CA	LEU	344	5.823	23.174	31.634	1.00	0.00	RX0	C
ATOM	1791	CB	LEU	344	7.152	22.611	31.138	1.00	0.00	RX0	C
ATOM	1792	CG	LEU	344	7.555	21.338	31.877	1.00	0.00	RX0	C
ATOM	1793	CD1	LEU	344	8.838	20.744	31.301	1.00	0.00	RX0	C
ATOM	1794	CD2	LEU	344	7.656	21.564	33.387	1.00	0.00	RX0	C
ATOM	1795	C	LEU	344	5.508	24.488	30.929	1.00	0.00	RX0	C
ATOM	1796	O	LEU	344	4.977	24.500	29.792	1.00	0.00	RX0	O
ATOM	1797	N	THR	345	5.822	25.577	31.575	1.00	0.00	RX0	N
ATOM	1798	H	THR	345	6.292	25.479	32.450	0.00	0.00	RX0	H
ATOM	1799	CA	THR	345	5.705	26.912	30.948	1.00	0.00	RX0	C
ATOM	1800	CB	THR	345	5.996	27.967	32.009	1.00	0.00	RX0	C
ATOM	1801	OG1	THR	345	7.177	27.617	32.728	1.00	0.00	RX0	O
ATOM	1802	HG1	THR	345	6.913	26.969	33.378	0.00	0.00	RX0	H
ATOM	1803	CG2	THR	345	4.821	28.115	32.978	1.00	0.00	RX0	C
ATOM	1804	C	THR	345	6.639	26.971	29.731	1.00	0.00	RX0	C
ATOM	1805	O	THR	345	7.615	26.233	29.623	1.00	0.00	RX0	O
ATOM	1806	N	LEU	346	6.390	27.971	28.898	1.00	0.00	RX0	N
ATOM	1807	H	LEU	346	5.565	28.513	29.045	0.00	0.00	RX0	H
ATOM	1808	CA	LEU	346	7.214	28.242	27.708	1.00	0.00	RX0	C
ATOM	1809	CB	LEU	346	6.672	29.453	26.949	1.00	0.00	RX0	C
ATOM	1810	CG	LEU	346	7.389	29.669	25.614	1.00	0.00	RX0	C
ATOM	1811	CD1	LEU	346	7.269	28.448	24.699	1.00	0.00	RX0	C
ATOM	1812	CD2	LEU	346	6.926	30.953	24.925	1.00	0.00	RX0	C
ATOM	1813	C	LEU	346	8.705	28.433	28.051	1.00	0.00	RX0	C
ATOM	1814	O	LEU	346	9.594	27.857	27.432	1.00	0.00	RX0	O
ATOM	1815	N	GLN	347	8.927	29.083	29.198	1.00	0.00	RX0	N
ATOM	1816	H	GLN	347	8.146	29.437	29.708	0.00	0.00	RX0	H
ATOM	1817	CA	GLN	347	10.275	29.275	29.754	1.00	0.00	RX0	C
ATOM	1818	CB	GLN	347	10.221	30.285	30.895	1.00	0.00	RX0	C
ATOM	1819	CG	GLN	347	11.603	30.618	31.455	1.00	0.00	RX0	C
ATOM	1820	CD	GLN	347	11.437	31.598	32.592	1.00	0.00	RX0	C
ATOM	1821	OE1	GLN	347	10.395	31.663	33.232	1.00	0.00	RX0	O
ATOM	1822	NE2	GLN	347	12.519	32.369	32.800	1.00	0.00	RX0	N
ATOM	1823	HE21	GLN	347	13.335	32.275	32.230	0.00	0.00	RX0	H
ATOM	1824	HE22	GLN	347	12.520	33.058	33.525	0.00	0.00	RX0	H
ATOM	1825	C	GLN	347	10.911	27.956	30.229	1.00	0.00	RX0	C
ATOM	1826	O	GLN	347	12.052	27.652	29.875	1.00	0.00	RX0	O
ATOM	1827	N	GLN	348	10.120	27.140	30.905	1.00	0.00	RX0	N
ATOM	1828	H	GLN	348	9.171	27.396	31.088	0.00	0.00	RX0	H

ATOM	1829	CA	GLN	348	10.582	25.823	31.394	1.00	0.00	RX0	C
ATOM	1830	CB	GLN	348	9.588	25.216	32.374	1.00	0.00	RX0	C
ATOM	1831	CG	GLN	348	9.647	25.876	33.749	1.00	0.00	RX0	C
ATOM	1832	CD	GLN	348	8.475	25.372	34.556	1.00	0.00	RX0	C
ATOM	1833	OE1	GLN	348	7.349	25.309	34.067	1.00	0.00	RX0	O
ATOM	1834	NE2	GLN	348	8.799	25.008	35.809	1.00	0.00	RX0	N
ATOM	1835	HE21	GLN	348	9.741	25.110	36.135	0.00	0.00	RX0	H
ATOM	1836	HE22	GLN	348	8.115	24.641	36.441	0.00	0.00	RX0	H
ATOM	1837	C	GLN	348	10.871	24.847	30.248	1.00	0.00	RX0	C
ATOM	1838	O	GLN	348	11.861	24.115	30.296	1.00	0.00	RX0	O
ATOM	1839	N	GLN	349	10.121	24.983	29.160	1.00	0.00	RX0	N
ATOM	1840	H	GLN	349	9.377	25.650	29.186	0.00	0.00	RX0	H
ATOM	1841	CA	GLN	349	10.299	24.172	27.940	1.00	0.00	RX0	C
ATOM	1842	CB	GLN	349	9.206	24.482	26.921	1.00	0.00	RX0	C
ATOM	1843	CG	GLN	349	7.822	24.034	27.384	1.00	0.00	RX0	C
ATOM	1844	CD	GLN	349	6.788	24.597	26.437	1.00	0.00	RX0	C
ATOM	1845	OE1	GLN	349	7.077	24.934	25.294	1.00	0.00	RX0	O
ATOM	1846	NE2	GLN	349	5.564	24.698	26.978	1.00	0.00	RX0	N
ATOM	1847	HE21	GLN	349	5.399	24.428	27.933	0.00	0.00	RX0	H
ATOM	1848	HE22	GLN	349	4.779	25.034	26.460	0.00	0.00	RX0	H
ATOM	1849	C	GLN	349	11.682	24.372	27.306	1.00	0.00	RX0	C
ATOM	1850	O	GLN	349	12.447	23.422	27.181	1.00	0.00	RX0	O
ATOM	1851	N	HIS	350	12.056	25.641	27.113	1.00	0.00	RX0	N
ATOM	1852	H	HIS	350	11.424	26.376	27.368	0.00	0.00	RX0	H
ATOM	1853	CA	HIS	350	13.344	25.953	26.463	1.00	0.00	RX0	C
ATOM	1854	CB	HIS	350	13.367	27.316	25.761	1.00	0.00	RX0	C
ATOM	1855	CG	HIS	350	13.449	28.502	26.692	1.00	0.00	RX0	C
ATOM	1856	ND1	HIS	350	12.385	29.253	27.027	1.00	0.00	RX0	N
ATOM	1857	HD1	HIS	350	11.448	29.077	26.776	0.00	0.00	RX0	H
ATOM	1858	CD2	HIS	350	14.595	29.049	27.280	1.00	0.00	RX0	C
ATOM	1859	NE2	HIS	350	14.211	30.150	27.969	1.00	0.00	RX0	N
ATOM	1860	CE1	HIS	350	12.855	30.276	27.812	1.00	0.00	RX0	C
ATOM	1861	C	HIS	350	14.540	25.670	27.386	1.00	0.00	RX0	C
ATOM	1862	O	HIS	350	15.573	25.182	26.934	1.00	0.00	RX0	O
ATOM	1863	N	GLN	351	14.320	25.854	28.691	1.00	0.00	RX0	N
ATOM	1864	H	GLN	351	13.438	26.225	28.992	0.00	0.00	RX0	H
ATOM	1865	CA	GLN	351	15.341	25.538	29.706	1.00	0.00	RX0	C
ATOM	1866	CB	GLN	351	14.921	26.076	31.069	1.00	0.00	RX0	C
ATOM	1867	CG	GLN	351	14.907	27.601	31.116	1.00	0.00	RX0	C
ATOM	1868	CD	GLN	351	14.242	28.037	32.402	1.00	0.00	RX0	C
ATOM	1869	OE1	GLN	351	13.337	27.391	32.918	1.00	0.00	RX0	O
ATOM	1870	NE2	GLN	351	14.758	29.173	32.905	1.00	0.00	RX0	N
ATOM	1871	HE21	GLN	351	15.500	29.640	32.423	0.00	0.00	RX0	H
ATOM	1872	HE22	GLN	351	14.419	29.560	33.763	0.00	0.00	RX0	H
ATOM	1873	C	GLN	351	15.597	24.030	29.805	1.00	0.00	RX0	C
ATOM	1874	O	GLN	351	16.752	23.608	29.740	1.00	0.00	RX0	O
ATOM	1875	N	ARG	352	14.527	23.242	29.744	1.00	0.00	RX0	N
ATOM	1876	H	ARG	352	13.618	23.654	29.657	0.00	0.00	RX0	H
ATOM	1877	CA	ARG	352	14.627	21.772	29.816	1.00	0.00	RX0	C
ATOM	1878	CB	ARG	352	13.311	21.108	30.246	1.00	0.00	RX0	C
ATOM	1879	CG	ARG	352	13.424	19.586	30.434	1.00	0.00	RX0	C
ATOM	1880	CD	ARG	352	12.235	18.991	31.199	1.00	0.00	RX0	C
ATOM	1881	NE	ARG	352	12.219	17.526	31.179	1.00	0.00	RX0	N
ATOM	1882	HE	ARG	352	12.010	17.058	30.304	0.00	0.00	RX0	H
ATOM	1883	CZ	ARG	352	12.351	16.774	32.315	1.00	0.00	RX0	C
ATOM	1884	NH1	ARG	352	12.648	17.382	33.482	1.00	0.00	RX0	N
ATOM	1885	HH11	ARG	352	12.826	16.839	34.315	0.00	0.00	RX0	H
ATOM	1886	HH12	ARG	352	12.715	18.377	33.558	0.00	0.00	RX0	H
ATOM	1887	NH2	ARG	352	12.182	15.438	32.257	1.00	0.00	RX0	N
ATOM	1888	HH21	ARG	352	12.220	14.805	33.038	0.00	0.00	RX0	H
ATOM	1889	HH22	ARG	352	11.991	15.016	31.348	0.00	0.00	RX0	H

ATOM	1890	C	ARG	352	15.192	21.188	28.514	1.00	0.00	RX0	C
ATOM	1891	O	ARG	352	16.048	20.305	28.551	1.00	0.00	RX0	O
ATOM	1892	N	LEU	353	14.843	21.816	27.391	1.00	0.00	RX0	N
ATOM	1893	H	LEU	353	14.136	22.524	27.429	0.00	0.00	RX0	H
ATOM	1894	CA	LEU	353	15.400	21.446	26.079	1.00	0.00	RX0	C
ATOM	1895	CB	LEU	353	14.770	22.310	24.988	1.00	0.00	RX0	C
ATOM	1896	CG	LEU	353	15.253	21.953	23.583	1.00	0.00	RX0	C
ATOM	1897	CD1	LEU	353	14.801	20.554	23.164	1.00	0.00	RX0	C
ATOM	1898	CD2	LEU	353	14.861	23.019	22.561	1.00	0.00	RX0	C
ATOM	1899	C	LEU	353	16.930	21.610	26.063	1.00	0.00	RX0	C
ATOM	1900	O	LEU	353	17.658	20.692	25.694	1.00	0.00	RX0	O
ATOM	1901	N	ALA	354	17.375	22.739	26.614	1.00	0.00	RX0	N
ATOM	1902	H	ALA	354	16.709	23.430	26.904	0.00	0.00	RX0	H
ATOM	1903	CA	ALA	354	18.808	23.073	26.716	1.00	0.00	RX0	C
ATOM	1904	CB	ALA	354	18.997	24.523	27.167	1.00	0.00	RX0	C
ATOM	1905	C	ALA	354	19.540	22.141	27.690	1.00	0.00	RX0	C
ATOM	1906	O	ALA	354	20.574	21.567	27.334	1.00	0.00	RX0	O
ATOM	1907	N	GLN	355	18.893	21.844	28.811	1.00	0.00	RX0	N
ATOM	1908	H	GLN	355	18.021	22.311	28.969	0.00	0.00	RX0	H
ATOM	1909	CA	GLN	355	19.427	20.920	29.833	1.00	0.00	RX0	C
ATOM	1910	CB	GLN	355	18.618	20.928	31.118	1.00	0.00	RX0	C
ATOM	1911	CG	GLN	355	18.834	22.244	31.859	1.00	0.00	RX0	C
ATOM	1912	CD	GLN	355	18.491	22.036	33.315	1.00	0.00	RX0	C
ATOM	1913	OE1	GLN	355	19.168	21.299	34.029	1.00	0.00	RX0	O
ATOM	1914	NE2	GLN	355	17.408	22.719	33.717	1.00	0.00	RX0	N
ATOM	1915	HE21	GLN	355	16.922	23.297	33.060	0.00	0.00	RX0	H
ATOM	1916	HE22	GLN	355	17.059	22.689	34.655	0.00	0.00	RX0	H
ATOM	1917	C	GLN	355	19.631	19.504	29.273	1.00	0.00	RX0	C
ATOM	1918	O	GLN	355	20.705	18.919	29.430	1.00	0.00	RX0	O
ATOM	1919	N	LEU	356	18.681	19.086	28.443	1.00	0.00	RX0	N
ATOM	1920	H	LEU	356	17.892	19.679	28.282	0.00	0.00	RX0	H
ATOM	1921	CA	LEU	356	18.716	17.770	27.778	1.00	0.00	RX0	C
ATOM	1922	CB	LEU	356	17.349	17.404	27.198	1.00	0.00	RX0	C
ATOM	1923	CG	LEU	356	16.340	17.040	28.286	1.00	0.00	RX0	C
ATOM	1924	CD1	LEU	356	14.983	16.654	27.699	1.00	0.00	RX0	C
ATOM	1925	CD2	LEU	356	16.885	15.953	29.210	1.00	0.00	RX0	C
ATOM	1926	C	LEU	356	19.795	17.661	26.699	1.00	0.00	RX0	C
ATOM	1927	O	LEU	356	20.593	16.720	26.700	1.00	0.00	RX0	O
ATOM	1928	N	LEU	357	19.916	18.722	25.913	1.00	0.00	RX0	N
ATOM	1929	H	LEU	357	19.287	19.494	26.033	0.00	0.00	RX0	H
ATOM	1930	CA	LEU	357	20.885	18.765	24.803	1.00	0.00	RX0	C
ATOM	1931	CB	LEU	357	20.542	19.883	23.817	1.00	0.00	RX0	C
ATOM	1932	CG	LEU	357	19.174	19.724	23.153	1.00	0.00	RX0	C
ATOM	1933	CD1	LEU	357	18.839	20.930	22.277	1.00	0.00	RX0	C
ATOM	1934	CD2	LEU	357	19.044	18.404	22.395	1.00	0.00	RX0	C
ATOM	1935	C	LEU	357	22.335	18.932	25.264	1.00	0.00	RX0	C
ATOM	1936	O	LEU	357	23.247	18.345	24.683	1.00	0.00	RX0	O
ATOM	1937	N	LEU	358	22.501	19.595	26.404	1.00	0.00	RX0	N
ATOM	1938	H	LEU	358	21.708	20.037	26.830	0.00	0.00	RX0	H
ATOM	1939	CA	LEU	358	23.826	19.754	27.029	1.00	0.00	RX0	C
ATOM	1940	CB	LEU	358	23.810	20.847	28.099	1.00	0.00	RX0	C
ATOM	1941	CG	LEU	358	23.684	22.260	27.521	1.00	0.00	RX0	C
ATOM	1942	CD1	LEU	358	23.481	23.301	28.623	1.00	0.00	RX0	C
ATOM	1943	CD2	LEU	358	24.856	22.619	26.603	1.00	0.00	RX0	C
ATOM	1944	C	LEU	358	24.390	18.455	27.609	1.00	0.00	RX0	C
ATOM	1945	O	LEU	358	25.603	18.227	27.557	1.00	0.00	RX0	O
ATOM	1946	N	ILE	359	23.510	17.559	28.043	1.00	0.00	RX0	N
ATOM	1947	H	ILE	359	22.544	17.824	28.070	0.00	0.00	RX0	H
ATOM	1948	CA	ILE	359	23.928	16.212	28.495	1.00	0.00	RX0	C
ATOM	1949	CB	ILE	359	22.756	15.428	29.105	1.00	0.00	RX0	C
ATOM	1950	CG2	ILE	359	23.207	14.075	29.664	1.00	0.00	RX0	C

ATOM	1951	CG1	ILE	359	22.056	16.244	30.187	1.00	0.00	RX0	C
ATOM	1952	CD1	ILE	359	20.715	15.656	30.624	1.00	0.00	RX0	C
ATOM	1953	C	ILE	359	24.559	15.428	27.334	1.00	0.00	RX0	C
ATOM	1954	O	ILE	359	25.552	14.723	27.543	1.00	0.00	RX0	O
ATOM	1955	N	LEU	360	24.038	15.620	26.133	1.00	0.00	RX0	N
ATOM	1956	H	LEU	360	23.254	16.236	26.033	0.00	0.00	RX0	H
ATOM	1957	CA	LEU	360	24.601	14.974	24.928	1.00	0.00	RX0	C
ATOM	1958	CB	LEU	360	23.755	15.280	23.690	1.00	0.00	RX0	C
ATOM	1959	CG	LEU	360	22.266	14.971	23.887	1.00	0.00	RX0	C
ATOM	1960	CD1	LEU	360	21.453	15.347	22.652	1.00	0.00	RX0	C
ATOM	1961	CD2	LEU	360	22.009	13.524	24.305	1.00	0.00	RX0	C
ATOM	1962	C	LEU	360	26.079	15.308	24.694	1.00	0.00	RX0	C
ATOM	1963	O	LEU	360	26.843	14.455	24.246	1.00	0.00	RX0	O
ATOM	1964	N	SER	361	26.491	16.472	25.202	1.00	0.00	RX0	N
ATOM	1965	H	SER	361	25.794	17.075	25.592	0.00	0.00	RX0	H
ATOM	1966	CA	SER	361	27.906	16.894	25.202	1.00	0.00	RX0	C
ATOM	1967	CB	SER	361	28.018	18.370	25.607	1.00	0.00	RX0	C
ATOM	1968	OG	SER	361	29.344	18.866	25.389	1.00	0.00	RX0	O
ATOM	1969	HG	SER	361	29.331	19.762	25.712	0.00	0.00	RX0	H
ATOM	1970	C	SER	361	28.769	15.985	26.099	1.00	0.00	RX0	C
ATOM	1971	O	SER	361	29.797	15.456	25.681	1.00	0.00	RX0	O
ATOM	1972	N	HIS	362	28.237	15.704	27.286	1.00	0.00	RX0	N
ATOM	1973	H	HIS	362	27.341	16.098	27.502	0.00	0.00	RX0	H
ATOM	1974	CA	HIS	362	28.854	14.793	28.272	1.00	0.00	RX0	C
ATOM	1975	CB	HIS	362	28.294	15.091	29.662	1.00	0.00	RX0	C
ATOM	1976	CG	HIS	362	28.582	16.567	29.852	1.00	0.00	RX0	C
ATOM	1977	ND1	HIS	362	27.647	17.535	29.793	1.00	0.00	RX0	N
ATOM	1978	HD1	HIS	362	26.671	17.429	29.779	0.00	0.00	RX0	H
ATOM	1979	CD2	HIS	362	29.836	17.175	29.991	1.00	0.00	RX0	C
ATOM	1980	NE2	HIS	362	29.639	18.517	30.000	1.00	0.00	RX0	N
ATOM	1981	CE1	HIS	362	28.292	18.739	29.878	1.00	0.00	RX0	C
ATOM	1982	C	HIS	362	28.890	13.339	27.777	1.00	0.00	RX0	C
ATOM	1983	O	HIS	362	29.902	12.656	27.936	1.00	0.00	RX0	O
ATOM	1984	N	ILE	363	27.856	12.940	27.039	1.00	0.00	RX0	N
ATOM	1985	H	ILE	363	27.090	13.574	26.938	0.00	0.00	RX0	H
ATOM	1986	CA	ILE	363	27.773	11.584	26.446	1.00	0.00	RX0	C
ATOM	1987	CB	ILE	363	26.354	11.295	25.956	1.00	0.00	RX0	C
ATOM	1988	CG2	ILE	363	26.235	9.896	25.350	1.00	0.00	RX0	C
ATOM	1989	CG1	ILE	363	25.378	11.468	27.121	1.00	0.00	RX0	C
ATOM	1990	CD1	ILE	363	23.925	11.233	26.720	1.00	0.00	RX0	C
ATOM	1991	C	ILE	363	28.830	11.416	25.343	1.00	0.00	RX0	C
ATOM	1992	O	ILE	363	29.487	10.370	25.263	1.00	0.00	RX0	O
ATOM	1993	N	ARG	364	29.035	12.469	24.564	1.00	0.00	RX0	N
ATOM	1994	H	ARG	364	28.356	13.206	24.603	0.00	0.00	RX0	H
ATOM	1995	CA	ARG	364	30.102	12.498	23.547	1.00	0.00	RX0	C
ATOM	1996	CB	ARG	364	30.008	13.808	22.762	1.00	0.00	RX0	C
ATOM	1997	CG	ARG	364	31.100	14.021	21.711	1.00	0.00	RX0	C
ATOM	1998	CD	ARG	364	31.244	12.902	20.672	1.00	0.00	RX0	C
ATOM	1999	NE	ARG	364	29.943	12.633	20.092	1.00	0.00	RX0	N
ATOM	2000	HE	ARG	364	29.324	11.980	20.543	0.00	0.00	RX0	H
ATOM	2001	CZ	ARG	364	29.393	13.418	19.160	1.00	0.00	RX0	C
ATOM	2002	NH1	ARG	364	30.139	14.155	18.334	1.00	0.00	RX0	N
ATOM	2003	HH11	ARG	364	29.732	14.726	17.624	0.00	0.00	RX0	H
ATOM	2004	HH12	ARG	364	31.149	14.140	18.377	0.00	0.00	RX0	H
ATOM	2005	NH2	ARG	364	28.070	13.426	19.123	1.00	0.00	RX0	N
ATOM	2006	HH21	ARG	364	27.513	13.994	18.518	0.00	0.00	RX0	H
ATOM	2007	HH22	ARG	364	27.591	12.781	19.740	0.00	0.00	RX0	H
ATOM	2008	C	ARG	364	31.469	12.319	24.223	1.00	0.00	RX0	C
ATOM	2009	O	ARG	364	32.264	11.465	23.831	1.00	0.00	RX0	O
ATOM	2010	N	HIS	365	31.645	13.058	25.316	1.00	0.00	RX0	N
ATOM	2011	H	HIS	365	30.878	13.635	25.604	0.00	0.00	RX0	H

ATOM	2012	CA	HIS	365	32.879	13.027	26.115	1.00	0.00	RX0	C
ATOM	2013	CB	HIS	365	32.934	14.097	27.196	1.00	0.00	RX0	C
ATOM	2014	CG	HIS	365	34.329	14.169	27.795	1.00	0.00	RX0	C
ATOM	2015	ND1	HIS	365	35.384	14.781	27.211	1.00	0.00	RX0	N
ATOM	2016	HD1	HIS	365	35.431	15.281	26.365	0.00	0.00	RX0	H
ATOM	2017	CD2	HIS	365	34.763	13.602	29.000	1.00	0.00	RX0	C
ATOM	2018	NE2	HIS	365	36.086	13.873	29.129	1.00	0.00	RX0	N
ATOM	2019	CE1	HIS	365	36.467	14.596	28.030	1.00	0.00	RX0	C
ATOM	2020	C	HIS	365	33.175	11.617	26.649	1.00	0.00	RX0	C
ATOM	2021	O	HIS	365	34.274	11.105	26.425	1.00	0.00	RX0	O
ATOM	2022	N	MET	366	32.138	10.946	27.137	1.00	0.00	RX0	N
ATOM	2023	H	MET	366	31.274	11.441	27.249	0.00	0.00	RX0	H
ATOM	2024	CA	MET	366	32.267	9.572	27.662	1.00	0.00	RX0	C
ATOM	2025	CB	MET	366	31.019	9.141	28.434	1.00	0.00	RX0	C
ATOM	2026	CG	MET	366	30.728	10.005	29.661	1.00	0.00	RX0	C
ATOM	2027	SD	MET	366	29.388	9.353	30.672	1.00	0.00	RX0	S
ATOM	2028	CE	MET	366	28.079	9.391	29.440	1.00	0.00	RX0	C
ATOM	2029	C	MET	366	32.567	8.557	26.555	1.00	0.00	RX0	C
ATOM	2030	O	MET	366	33.398	7.674	26.741	1.00	0.00	RX0	O
ATOM	2031	N	SER	367	31.985	8.782	25.378	1.00	0.00	RX0	N
ATOM	2032	H	SER	367	31.301	9.513	25.326	0.00	0.00	RX0	H
ATOM	2033	CA	SER	367	32.231	7.934	24.196	1.00	0.00	RX0	C
ATOM	2034	CB	SER	367	31.239	8.352	23.106	1.00	0.00	RX0	C
ATOM	2035	OG	SER	367	31.241	7.460	21.988	1.00	0.00	RX0	O
ATOM	2036	HG	SER	367	30.737	7.897	21.308	0.00	0.00	RX0	H
ATOM	2037	C	SER	367	33.681	8.046	23.711	1.00	0.00	RX0	C
ATOM	2038	O	SER	367	34.361	7.038	23.567	1.00	0.00	RX0	O
ATOM	2039	N	ASN	368	34.193	9.278	23.688	1.00	0.00	RX0	N
ATOM	2040	H	ASN	368	33.582	10.028	23.951	0.00	0.00	RX0	H
ATOM	2041	CA	ASN	368	35.583	9.549	23.266	1.00	0.00	RX0	C
ATOM	2042	CB	ASN	368	35.805	11.025	23.002	1.00	0.00	RX0	C
ATOM	2043	CG	ASN	368	35.617	11.339	21.528	1.00	0.00	RX0	C
ATOM	2044	OD1	ASN	368	35.999	12.415	21.093	1.00	0.00	RX0	O
ATOM	2045	ND2	ASN	368	34.972	10.437	20.767	1.00	0.00	RX0	N
ATOM	2046	HD21	ASN	368	34.613	9.547	21.056	0.00	0.00	RX0	H
ATOM	2047	HD22	ASN	368	34.873	10.669	19.793	0.00	0.00	RX0	H
ATOM	2048	C	ASN	368	36.615	8.948	24.226	1.00	0.00	RX0	C
ATOM	2049	O	ASN	368	37.514	8.215	23.806	1.00	0.00	RX0	O
ATOM	2050	N	LYS	369	36.353	9.119	25.515	1.00	0.00	RX0	N
ATOM	2051	H	LYS	369	35.574	9.699	25.765	0.00	0.00	RX0	H
ATOM	2052	CA	LYS	369	37.205	8.554	26.580	1.00	0.00	RX0	C
ATOM	2053	CB	LYS	369	36.876	9.116	27.968	1.00	0.00	RX0	C
ATOM	2054	CG	LYS	369	37.120	10.618	28.150	1.00	0.00	RX0	C
ATOM	2055	CD	LYS	369	38.502	11.082	27.682	1.00	0.00	RX0	C
ATOM	2056	CE	LYS	369	39.690	10.374	28.341	1.00	0.00	RX0	C
ATOM	2057	NZ	LYS	369	40.867	10.627	27.503	1.00	0.00	RX0	N
ATOM	2058	HZ1	LYS	369	41.535	11.307	27.900	0.00	0.00	RX0	H
ATOM	2059	HZ2	LYS	369	41.367	9.750	27.239	0.00	0.00	RX0	H
ATOM	2060	HZ3	LYS	369	40.561	10.951	26.558	0.00	0.00	RX0	H
ATOM	2061	C	LYS	369	37.117	7.021	26.620	1.00	0.00	RX0	C
ATOM	2062	O	LYS	369	38.123	6.337	26.771	1.00	0.00	RX0	O
ATOM	2063	N	GLY	370	35.908	6.527	26.299	1.00	0.00	RX0	N
ATOM	2064	H	GLY	370	35.159	7.177	26.171	0.00	0.00	RX0	H
ATOM	2065	CA	GLY	370	35.597	5.090	26.225	1.00	0.00	RX0	C
ATOM	2066	C	GLY	370	36.324	4.429	25.048	1.00	0.00	RX0	C
ATOM	2067	O	GLY	370	36.946	3.386	25.214	1.00	0.00	RX0	O
ATOM	2068	N	MET	371	36.380	5.143	23.928	1.00	0.00	RX0	N
ATOM	2069	H	MET	371	35.879	6.007	23.916	0.00	0.00	RX0	H
ATOM	2070	CA	MET	371	37.103	4.709	22.719	1.00	0.00	RX0	C
ATOM	2071	CB	MET	371	36.782	5.588	21.507	1.00	0.00	RX0	C
ATOM	2072	CG	MET	371	35.357	5.404	20.982	1.00	0.00	RX0	C

ATOM	2073	SD	MET	371	35.002	3.710	20.483	1.00	0.00	RX0	S
ATOM	2074	CE	MET	371	36.069	3.630	19.036	1.00	0.00	RX0	C
ATOM	2075	C	MET	371	38.619	4.671	22.937	1.00	0.00	RX0	C
ATOM	2076	O	MET	371	39.258	3.669	22.631	1.00	0.00	RX0	O
ATOM	2077	N	GLU	372	39.117	5.692	23.643	1.00	0.00	RX0	N
ATOM	2078	H	GLU	372	38.521	6.478	23.821	0.00	0.00	RX0	H
ATOM	2079	CA	GLU	372	40.525	5.740	24.082	1.00	0.00	RX0	C
ATOM	2080	CB	GLU	372	40.827	7.027	24.858	1.00	0.00	RX0	C
ATOM	2081	CG	GLU	372	40.665	8.308	24.037	1.00	0.00	RX0	C
ATOM	2082	CD	GLU	372	41.003	9.524	24.882	1.00	0.00	RX0	C
ATOM	2083	OE1	GLU	372	40.215	10.461	24.937	1.00	0.00	RX0	O
ATOM	2084	OE2	GLU	372	42.050	9.551	25.525	1.00	0.00	RX0	O
ATOM	2085	C	GLU	372	40.884	4.541	24.968	1.00	0.00	RX0	C
ATOM	2086	O	GLU	372	41.865	3.853	24.723	1.00	0.00	RX0	O
ATOM	2087	N	HIS	373	40.006	4.291	25.945	1.00	0.00	RX0	N
ATOM	2088	H	HIS	373	39.212	4.893	26.044	0.00	0.00	RX0	H
ATOM	2089	CA	HIS	373	40.194	3.212	26.919	1.00	0.00	RX0	C
ATOM	2090	CB	HIS	373	39.253	3.441	28.106	1.00	0.00	RX0	C
ATOM	2091	CG	HIS	373	38.419	2.228	28.452	1.00	0.00	RX0	C
ATOM	2092	ND1	HIS	373	37.313	1.866	27.769	1.00	0.00	RX0	N
ATOM	2093	HD1	HIS	373	36.952	2.308	26.966	0.00	0.00	RX0	H
ATOM	2094	CD2	HIS	373	38.609	1.336	29.513	1.00	0.00	RX0	C
ATOM	2095	NE2	HIS	373	37.600	0.429	29.462	1.00	0.00	RX0	N
ATOM	2096	CE1	HIS	373	36.805	0.754	28.393	1.00	0.00	RX0	C
ATOM	2097	C	HIS	373	40.078	1.832	26.262	1.00	0.00	RX0	C
ATOM	2098	O	HIS	373	40.934	1.001	26.470	1.00	0.00	RX0	O
ATOM	2099	N	LEU	374	39.111	1.679	25.351	1.00	0.00	RX0	N
ATOM	2100	H	LEU	374	38.546	2.468	25.122	0.00	0.00	RX0	H
ATOM	2101	CA	LEU	374	38.919	0.408	24.629	1.00	0.00	RX0	C
ATOM	2102	CB	LEU	374	37.653	0.446	23.771	1.00	0.00	RX0	C
ATOM	2103	CG	LEU	374	36.369	0.277	24.580	1.00	0.00	RX0	C
ATOM	2104	CD1	LEU	374	35.126	0.588	23.745	1.00	0.00	RX0	C
ATOM	2105	CD2	LEU	374	36.297	-1.105	25.231	1.00	0.00	RX0	C
ATOM	2106	C	LEU	374	40.113	0.051	23.744	1.00	0.00	RX0	C
ATOM	2107	O	LEU	374	40.555	-1.109	23.754	1.00	0.00	RX0	O
ATOM	2108	N	TYR	375	40.664	1.068	23.103	1.00	0.00	RX0	N
ATOM	2109	H	TYR	375	40.243	1.970	23.229	0.00	0.00	RX0	H
ATOM	2110	CA	TYR	375	41.854	0.983	22.222	1.00	0.00	RX0	C
ATOM	2111	CB	TYR	375	41.928	2.180	21.260	1.00	0.00	RX0	C
ATOM	2112	CG	TYR	375	40.962	2.046	20.092	1.00	0.00	RX0	C
ATOM	2113	CD1	TYR	375	40.365	0.802	19.783	1.00	0.00	RX0	C
ATOM	2114	CE1	TYR	375	39.519	0.697	18.668	1.00	0.00	RX0	C
ATOM	2115	CD2	TYR	375	40.703	3.192	19.312	1.00	0.00	RX0	C
ATOM	2116	CE2	TYR	375	39.861	3.088	18.190	1.00	0.00	RX0	C
ATOM	2117	CZ	TYR	375	39.287	1.838	17.878	1.00	0.00	RX0	C
ATOM	2118	OH	TYR	375	38.474	1.711	16.769	1.00	0.00	RX0	O
ATOM	2119	HH	TYR	375	38.502	2.514	16.263	0.00	0.00	RX0	H
ATOM	2120	C	TYR	375	43.165	0.750	22.977	1.00	0.00	RX0	C
ATOM	2121	O	TYR	375	44.176	1.452	22.753	1.00	0.00	RX0	O
ATOM	2122	N	SER	376	43.162	-0.226	23.845	1.00	0.00	RX0	N
ATOM	2123	H	SER	376	42.283	-0.629	24.072	0.00	0.00	RX0	H
ATOM	2124	CA	SER	376	44.345	-0.646	24.634	1.00	0.00	RX0	C
ATOM	2125	CB	SER	376	44.655	0.466	25.654	1.00	0.00	RX0	C
ATOM	2126	OG	SER	376	43.718	0.464	26.743	1.00	0.00	RX0	O
ATOM	2127	HG	SER	376	42.892	0.840	26.433	0.00	0.00	RX0	H
ATOM	2128	C	SER	376	44.183	-1.992	25.350	1.00	0.00	RX0	C
ATOM	2129	O	SER	376	44.985	-2.369	26.199	1.00	0.00	RX0	O
ATOM	2130	N	MET	377	43.156	-2.746	24.938	1.00	0.00	RX0	N
ATOM	2131	H	MET	377	42.534	-2.391	24.240	0.00	0.00	RX0	H
ATOM	2132	CA	MET	377	42.915	-4.112	25.422	1.00	0.00	RX0	C
ATOM	2133	CB	MET	377	41.774	-4.065	26.447	1.00	0.00	RX0	C

ATOM	2134	CG	MET	377	42.191	-3.507	27.809	1.00	0.00	RX0	C
ATOM	2135	SD	MET	377	40.777	-3.300	28.897	1.00	0.00	RX0	S
ATOM	2136	CE	MET	377	39.982	-1.974	27.975	1.00	0.00	RX0	C
ATOM	2137	C	MET	377	42.647	-5.080	24.257	1.00	0.00	RX0	C
ATOM	2138	O	MET	377	43.092	-4.854	23.130	1.00	0.00	RX0	O
ATOM	2139	N	LYS	378	41.856	-6.107	24.527	1.00	0.00	RX0	N
ATOM	2140	H	LYS	378	41.495	-6.209	25.448	0.00	0.00	RX0	H
ATOM	2141	CA	LYS	378	41.570	-7.191	23.576	1.00	0.00	RX0	C
ATOM	2142	CB	LYS	378	41.520	-8.555	24.307	1.00	0.00	RX0	C
ATOM	2143	CG	LYS	378	40.765	-8.626	25.649	1.00	0.00	RX0	C
ATOM	2144	CD	LYS	378	41.624	-9.249	26.768	1.00	0.00	RX0	C
ATOM	2145	CE	LYS	378	41.024	-9.117	28.178	1.00	0.00	RX0	C
ATOM	2146	NZ	LYS	378	41.849	-9.780	29.210	1.00	0.00	RX0	N
ATOM	2147	HZ1	LYS	378	41.291	-9.818	30.088	0.00	0.00	RX0	H
ATOM	2148	HZ2	LYS	378	42.147	-10.754	29.005	0.00	0.00	RX0	H
ATOM	2149	HZ3	LYS	378	42.647	-9.215	29.586	0.00	0.00	RX0	H
ATOM	2150	C	LYS	378	40.316	-6.920	22.720	1.00	0.00	RX0	C
ATOM	2151	O	LYS	378	40.245	-7.352	21.611	1.00	0.00	RX0	O
ATOM	2152	N	CYS	379	39.320	-6.266	23.410	1.00	0.00	RX0	N
ATOM	2153	H	CYS	379	39.575	-5.930	24.311	0.00	0.00	RX0	H
ATOM	2154	CA	CYS	379	37.939	-6.053	22.985	1.00	0.00	RX0	C
ATOM	2155	CB	CYS	379	37.587	-4.563	23.089	1.00	0.00	RX0	C
ATOM	2156	SG	CYS	379	35.818	-4.211	22.938	1.00	0.00	RX0	S
ATOM	2157	C	CYS	379	37.599	-6.654	21.614	1.00	0.00	RX0	C
ATOM	2158	O	CYS	379	37.115	-7.773	21.559	1.00	0.00	RX0	O
ATOM	2159	N	LYS	380	38.012	-5.930	20.563	1.00	0.00	RX0	N
ATOM	2160	H	LYS	380	38.368	-5.001	20.646	0.00	0.00	RX0	H
ATOM	2161	CA	LYS	380	37.949	-6.383	19.165	1.00	0.00	RX0	C
ATOM	2162	CB	LYS	380	36.475	-6.757	18.889	1.00	0.00	RX0	C
ATOM	2163	CG	LYS	380	35.914	-7.230	17.545	1.00	0.00	RX0	C
ATOM	2164	CD	LYS	380	34.399	-7.418	17.738	1.00	0.00	RX0	C
ATOM	2165	CE	LYS	380	33.593	-7.872	16.517	1.00	0.00	RX0	C
ATOM	2166	NZ	LYS	380	32.169	-7.948	16.876	1.00	0.00	RX0	N
ATOM	2167	HZ1	LYS	380	31.539	-8.197	16.094	0.00	0.00	RX0	H
ATOM	2168	HZ2	LYS	380	31.958	-8.631	17.640	0.00	0.00	RX0	H
ATOM	2169	HZ3	LYS	380	31.806	-7.069	17.307	0.00	0.00	RX0	H
ATOM	2170	C	LYS	380	38.311	-5.215	18.248	1.00	0.00	RX0	C
ATOM	2171	O	LYS	380	37.971	-4.057	18.518	1.00	0.00	RX0	O
ATOM	2172	N	ASN	381	38.881	-5.592	17.123	1.00	0.00	RX0	N
ATOM	2173	H	ASN	381	39.194	-6.541	17.100	0.00	0.00	RX0	H
ATOM	2174	CA	ASN	381	39.208	-4.700	15.996	1.00	0.00	RX0	C
ATOM	2175	CB	ASN	381	40.325	-3.734	16.419	1.00	0.00	RX0	C
ATOM	2176	CG	ASN	381	40.089	-2.299	15.975	1.00	0.00	RX0	C
ATOM	2177	OD1	ASN	381	41.015	-1.615	15.552	1.00	0.00	RX0	O
ATOM	2178	ND2	ASN	381	38.838	-1.838	16.127	1.00	0.00	RX0	N
ATOM	2179	HD21	ASN	381	38.109	-2.365	16.569	0.00	0.00	RX0	H
ATOM	2180	HD22	ASN	381	38.617	-0.909	15.821	0.00	0.00	RX0	H
ATOM	2181	C	ASN	381	39.620	-5.523	14.759	1.00	0.00	RX0	C
ATOM	2182	O	ASN	381	40.286	-5.062	13.834	1.00	0.00	RX0	O
ATOM	2183	N	VAL	382	39.176	-6.776	14.737	1.00	0.00	RX0	N
ATOM	2184	H	VAL	382	38.495	-7.053	15.409	0.00	0.00	RX0	H
ATOM	2185	CA	VAL	382	39.338	-7.659	13.572	1.00	0.00	RX0	C
ATOM	2186	CB	VAL	382	39.259	-9.127	14.002	1.00	0.00	RX0	C
ATOM	2187	CG1	VAL	382	39.372	-10.073	12.805	1.00	0.00	RX0	C
ATOM	2188	CG2	VAL	382	40.320	-9.432	15.062	1.00	0.00	RX0	C
ATOM	2189	C	VAL	382	38.195	-7.277	12.635	1.00	0.00	RX0	C
ATOM	2190	O	VAL	382	37.049	-7.276	13.059	1.00	0.00	RX0	O
ATOM	2191	N	VAL	383	38.551	-7.063	11.363	1.00	0.00	RX0	N
ATOM	2192	H	VAL	383	39.525	-7.106	11.153	0.00	0.00	RX0	H
ATOM	2193	CA	VAL	383	37.600	-6.539	10.366	1.00	0.00	RX0	C
ATOM	2194	CB	VAL	383	36.432	-7.500	10.092	1.00	0.00	RX0	C

ATOM	2195	CG1	VAL	383	35.398	-6.875	9.152	1.00	0.00	RX0	C
ATOM	2196	CG2	VAL	383	36.950	-8.842	9.571	1.00	0.00	RX0	C
ATOM	2197	C	VAL	383	37.102	-5.173	10.877	1.00	0.00	RX0	C
ATOM	2198	O	VAL	383	36.355	-5.097	11.860	1.00	0.00	RX0	O
ATOM	2199	N	PRO	384	37.530	-4.088	10.236	1.00	0.00	RX0	N
ATOM	2200	CD	PRO	384	38.374	-4.080	9.048	1.00	0.00	RX0	C
ATOM	2201	CA	PRO	384	37.142	-2.731	10.663	1.00	0.00	RX0	C
ATOM	2202	CB	PRO	384	37.735	-1.833	9.567	1.00	0.00	RX0	C
ATOM	2203	CG	PRO	384	38.068	-2.745	8.389	1.00	0.00	RX0	C
ATOM	2204	C	PRO	384	35.624	-2.628	10.828	1.00	0.00	RX0	C
ATOM	2205	O	PRO	384	34.847	-3.282	10.112	1.00	0.00	RX0	O
ATOM	2206	N	LEU	385	35.228	-1.769	11.750	1.00	0.00	RX0	N
ATOM	2207	H	LEU	385	35.914	-1.171	12.166	0.00	0.00	RX0	H
ATOM	2208	CA	LEU	385	33.812	-1.621	12.127	1.00	0.00	RX0	C
ATOM	2209	CB	LEU	385	33.680	-0.664	13.311	1.00	0.00	RX0	C
ATOM	2210	CG	LEU	385	32.288	-0.705	13.939	1.00	0.00	RX0	C
ATOM	2211	CD1	LEU	385	31.913	-2.109	14.414	1.00	0.00	RX0	C
ATOM	2212	CD2	LEU	385	32.141	0.331	15.050	1.00	0.00	RX0	C
ATOM	2213	C	LEU	385	32.896	-1.213	10.964	1.00	0.00	RX0	C
ATOM	2214	O	LEU	385	31.809	-1.761	10.815	1.00	0.00	RX0	O
ATOM	2215	N	TYR	386	33.449	-0.406	10.055	1.00	0.00	RX0	N
ATOM	2216	H	TYR	386	34.236	0.140	10.353	0.00	0.00	RX0	H
ATOM	2217	CA	TYR	386	32.718	0.069	8.867	1.00	0.00	RX0	C
ATOM	2218	CB	TYR	386	33.614	0.952	7.971	1.00	0.00	RX0	C
ATOM	2219	CG	TYR	386	32.771	1.659	6.926	1.00	0.00	RX0	C
ATOM	2220	CD1	TYR	386	32.418	3.009	7.127	1.00	0.00	RX0	C
ATOM	2221	CE1	TYR	386	31.518	3.622	6.238	1.00	0.00	RX0	C
ATOM	2222	CD2	TYR	386	32.325	0.940	5.796	1.00	0.00	RX0	C
ATOM	2223	CE2	TYR	386	31.408	1.538	4.922	1.00	0.00	RX0	C
ATOM	2224	CZ	TYR	386	30.987	2.857	5.180	1.00	0.00	RX0	C
ATOM	2225	OH	TYR	386	30.011	3.405	4.369	1.00	0.00	RX0	O
ATOM	2226	HH	TYR	386	29.515	2.695	3.978	0.00	0.00	RX0	H
ATOM	2227	C	TYR	386	32.144	-1.103	8.049	1.00	0.00	RX0	C
ATOM	2228	O	TYR	386	30.957	-1.141	7.757	1.00	0.00	RX0	O
ATOM	2229	N	ASP	387	32.969	-2.141	7.903	1.00	0.00	RX0	N
ATOM	2230	H	ASP	387	33.936	-2.050	8.150	0.00	0.00	RX0	H
ATOM	2231	CA	ASP	387	32.619	-3.323	7.093	1.00	0.00	RX0	C
ATOM	2232	CB	ASP	387	33.884	-4.124	6.763	1.00	0.00	RX0	C
ATOM	2233	CG	ASP	387	34.869	-3.279	5.967	1.00	0.00	RX0	C
ATOM	2234	OD1	ASP	387	35.415	-2.320	6.516	1.00	0.00	RX0	O
ATOM	2235	OD2	ASP	387	35.095	-3.586	4.799	1.00	0.00	RX0	O
ATOM	2236	C	ASP	387	31.563	-4.207	7.765	1.00	0.00	RX0	C
ATOM	2237	O	ASP	387	30.619	-4.649	7.114	1.00	0.00	RX0	O
ATOM	2238	N	LEU	388	31.677	-4.332	9.088	1.00	0.00	RX0	N
ATOM	2239	H	LEU	388	32.458	-3.885	9.528	0.00	0.00	RX0	H
ATOM	2240	CA	LEU	388	30.691	-5.072	9.891	1.00	0.00	RX0	C
ATOM	2241	CB	LEU	388	31.217	-5.212	11.323	1.00	0.00	RX0	C
ATOM	2242	CG	LEU	388	30.303	-6.006	12.259	1.00	0.00	RX0	C
ATOM	2243	CD1	LEU	388	30.100	-7.447	11.791	1.00	0.00	RX0	C
ATOM	2244	CD2	LEU	388	30.793	-5.945	13.707	1.00	0.00	RX0	C
ATOM	2245	C	LEU	388	29.315	-4.386	9.871	1.00	0.00	RX0	C
ATOM	2246	O	LEU	388	28.299	-5.023	9.588	1.00	0.00	RX0	O
ATOM	2247	N	LEU	389	29.339	-3.070	10.049	1.00	0.00	RX0	N
ATOM	2248	H	LEU	389	30.233	-2.634	10.170	0.00	0.00	RX0	H
ATOM	2249	CA	LEU	389	28.129	-2.229	9.970	1.00	0.00	RX0	C
ATOM	2250	CB	LEU	389	28.420	-0.790	10.381	1.00	0.00	RX0	C
ATOM	2251	CG	LEU	389	28.865	-0.647	11.833	1.00	0.00	RX0	C
ATOM	2252	CD1	LEU	389	29.254	0.794	12.137	1.00	0.00	RX0	C
ATOM	2253	CD2	LEU	389	27.818	-1.161	12.818	1.00	0.00	RX0	C
ATOM	2254	C	LEU	389	27.489	-2.268	8.583	1.00	0.00	RX0	C
ATOM	2255	O	LEU	389	26.284	-2.473	8.458	1.00	0.00	RX0	O

ATOM	2256	N	LEU	390	28.350	-2.246	7.565	1.00	0.00	RX0	N
ATOM	2257	H	LEU	390	29.328	-2.154	7.759	0.00	0.00	RX0	H
ATOM	2258	CA	LEU	390	27.929	-2.335	6.162	1.00	0.00	RX0	C
ATOM	2259	CB	LEU	390	29.171	-2.183	5.276	1.00	0.00	RX0	C
ATOM	2260	CG	LEU	390	28.939	-2.009	3.775	1.00	0.00	RX0	C
ATOM	2261	CD1	LEU	390	28.324	-0.651	3.444	1.00	0.00	RX0	C
ATOM	2262	CD2	LEU	390	30.232	-2.231	2.989	1.00	0.00	RX0	C
ATOM	2263	C	LEU	390	27.202	-3.653	5.862	1.00	0.00	RX0	C
ATOM	2264	O	LEU	390	26.123	-3.627	5.302	1.00	0.00	RX0	O
ATOM	2265	N	GLU	391	27.733	-4.754	6.404	1.00	0.00	RX0	N
ATOM	2266	H	GLU	391	28.628	-4.707	6.856	0.00	0.00	RX0	H
ATOM	2267	CA	GLU	391	27.124	-6.090	6.264	1.00	0.00	RX0	C
ATOM	2268	CB	GLU	391	27.951	-7.163	6.977	1.00	0.00	RX0	C
ATOM	2269	CG	GLU	391	29.283	-7.529	6.325	1.00	0.00	RX0	C
ATOM	2270	CD	GLU	391	29.998	-8.471	7.280	1.00	0.00	RX0	C
ATOM	2271	OE1	GLU	391	30.185	-8.098	8.441	1.00	0.00	RX0	O
ATOM	2272	OE2	GLU	391	30.352	-9.578	6.869	1.00	0.00	RX0	O
ATOM	2273	C	GLU	391	25.713	-6.159	6.867	1.00	0.00	RX0	C
ATOM	2274	O	GLU	391	24.778	-6.600	6.214	1.00	0.00	RX0	O
ATOM	2275	N	MET	392	25.589	-5.588	8.070	1.00	0.00	RX0	N
ATOM	2276	H	MET	392	26.427	-5.214	8.478	0.00	0.00	RX0	H
ATOM	2277	CA	MET	392	24.313	-5.555	8.807	1.00	0.00	RX0	C
ATOM	2278	CB	MET	392	24.543	-5.226	10.281	1.00	0.00	RX0	C
ATOM	2279	CG	MET	392	25.222	-6.386	11.013	1.00	0.00	RX0	C
ATOM	2280	SD	MET	392	25.374	-6.113	12.786	1.00	0.00	RX0	S
ATOM	2281	CE	MET	392	26.606	-4.805	12.727	1.00	0.00	RX0	C
ATOM	2282	C	MET	392	23.266	-4.630	8.173	1.00	0.00	RX0	C
ATOM	2283	O	MET	392	22.107	-5.006	8.028	1.00	0.00	RX0	O
ATOM	2284	N	LEU	393	23.721	-3.452	7.754	1.00	0.00	RX0	N
ATOM	2285	H	LEU	393	24.708	-3.285	7.789	0.00	0.00	RX0	H
ATOM	2286	CA	LEU	393	22.852	-2.457	7.099	1.00	0.00	RX0	C
ATOM	2287	CB	LEU	393	23.510	-1.081	7.149	1.00	0.00	RX0	C
ATOM	2288	CG	LEU	393	23.443	-0.452	8.538	1.00	0.00	RX0	C
ATOM	2289	CD1	LEU	393	24.504	0.628	8.723	1.00	0.00	RX0	C
ATOM	2290	CD2	LEU	393	22.039	0.068	8.848	1.00	0.00	RX0	C
ATOM	2291	C	LEU	393	22.462	-2.815	5.663	1.00	0.00	RX0	C
ATOM	2292	O	LEU	393	21.313	-2.601	5.257	1.00	0.00	RX0	O
ATOM	2293	N	ASP	394	23.400	-3.384	4.923	1.00	0.00	RX0	N
ATOM	2294	H	ASP	394	24.270	-3.665	5.323	0.00	0.00	RX0	H
ATOM	2295	CA	ASP	394	23.187	-3.768	3.516	1.00	0.00	RX0	C
ATOM	2296	CB	ASP	394	24.406	-3.718	2.599	1.00	0.00	RX0	C
ATOM	2297	CG	ASP	394	23.862	-3.468	1.201	1.00	0.00	RX0	C
ATOM	2298	OD1	ASP	394	22.714	-3.030	1.090	1.00	0.00	RX0	O
ATOM	2299	OD2	ASP	394	24.588	-3.681	0.230	1.00	0.00	RX0	O
ATOM	2300	C	ASP	394	22.492	-5.132	3.432	1.00	0.00	RX0	C
ATOM	2301	O	ASP	394	23.034	-6.108	2.888	1.00	0.00	RX0	O
ATOM	2302	N	ALA	395	21.256	-5.134	3.874	1.00	0.00	RX0	N
ATOM	2303	H	ALA	395	20.886	-4.230	4.090	0.00	0.00	RX0	H
ATOM	2304	CA	ALA	395	20.412	-6.338	3.880	1.00	0.00	RX0	C
ATOM	2305	CB	ALA	395	19.422	-6.295	5.045	1.00	0.00	RX0	C
ATOM	2306	C	ALA	395	19.639	-6.454	2.565	1.00	0.00	RX0	C
ATOM	2307	O	ALA	395	19.303	-5.463	1.911	1.00	0.00	RX0	O
ATOM	2308	N	HIS	396	19.441	-7.696	2.156	1.00	0.00	RX0	N
ATOM	2309	H	HIS	396	19.740	-8.407	2.788	0.00	0.00	RX0	H
ATOM	2310	CA	HIS	396	18.536	-7.999	1.035	1.00	0.00	RX0	C
ATOM	2311	CB	HIS	396	18.857	-9.399	0.504	1.00	0.00	RX0	C
ATOM	2312	CG	HIS	396	19.038	-10.346	1.669	1.00	0.00	RX0	C
ATOM	2313	ND1	HIS	396	20.241	-10.753	2.114	1.00	0.00	RX0	N
ATOM	2314	HD1	HIS	396	21.118	-10.503	1.747	0.00	0.00	RX0	H
ATOM	2315	CD2	HIS	396	18.050	-10.924	2.471	1.00	0.00	RX0	C
ATOM	2316	NE2	HIS	396	18.669	-11.683	3.405	1.00	0.00	RX0	N

```
ATOM   2317  CE1 HIS   396      20.020 -11.579   3.186  1.00  0.00      RX0  C
ATOM   2318  C   HIS   396      17.077  -7.909   1.523  1.00  0.00      RX0  C
ATOM   2319  O   HIS   396      16.821  -7.755   2.721  1.00  0.00      RX0  O
ATOM   2320  N   ARG   397      16.142  -8.174   0.629  1.00  0.00      RX0  N
ATOM   2321  H   ARG   397      16.391  -8.364  -0.320  0.00  0.00      RX0  H
ATOM   2322  CA  ARG   397      14.704  -8.151   0.982  1.00  0.00      RX0  C
ATOM   2323  CB  ARG   397      14.050  -6.988   0.222  1.00  0.00      RX0  C
ATOM   2324  CG  ARG   397      14.842  -5.735   0.627  1.00  0.00      RX0  C
ATOM   2325  CD  ARG   397      14.713  -4.453  -0.193  1.00  0.00      RX0  C
ATOM   2326  NE  ARG   397      15.834  -3.575   0.158  1.00  0.00      RX0  N
ATOM   2327  HE  ARG   397      16.722  -4.038   0.278  0.00  0.00      RX0  H
ATOM   2328  CZ  ARG   397      15.675  -2.226   0.304  1.00  0.00      RX0  C
ATOM   2329  NH1 ARG   397      14.451  -1.680   0.118  1.00  0.00      RX0  N
ATOM   2330 HH11 ARG   397      14.273  -0.696   0.213  0.00  0.00      RX0  H
ATOM   2331 HH12 ARG   397      13.660  -2.248  -0.127  0.00  0.00      RX0  H
ATOM   2332  NH2 ARG   397      16.747  -1.466   0.634  1.00  0.00      RX0  N
ATOM   2333 HH21 ARG   397      16.701  -0.470   0.756  0.00  0.00      RX0  H
ATOM   2334 HH22 ARG   397      17.657  -1.877   0.774  0.00  0.00      RX0  H
ATOM   2335  C   ARG   397      14.082  -9.540   0.774  1.00  0.00      RX0  C
ATOM   2336  O   ARG   397      12.875  -9.706   0.613  1.00  0.00      RX0  O
ATOM   2337  N   LEU   398      14.938 -10.556   0.835  1.00  0.00      RX0  N
ATOM   2338  H   LEU   398      15.873 -10.363   1.121  0.00  0.00      RX0  H
ATOM   2339  CA  LEU   398      14.565 -11.951   0.542  1.00  0.00      RX0  C
ATOM   2340  CB  LEU   398      15.800 -12.797   0.232  1.00  0.00      RX0  C
ATOM   2341  CG  LEU   398      16.585 -12.273  -0.972  1.00  0.00      RX0  C
ATOM   2342  CD1 LEU   398      17.871 -13.069  -1.194  1.00  0.00      RX0  C
ATOM   2343  CD2 LEU   398      15.722 -12.208  -2.234  1.00  0.00      RX0  C
ATOM   2344  C   LEU   398      13.757 -12.584   1.680  1.00  0.00      RX0  C
ATOM   2345  O   LEU   398      12.772 -13.259   1.439  1.00  0.00      RX0  O
ATOM   2346  N   HIS   399      14.100 -12.169   2.904  1.00  0.00      RX0  N
ATOM   2347  H   HIS   399      14.842 -11.512   3.010  0.00  0.00      RX0  H
ATOM   2348  CA  HIS   399      13.392 -12.616   4.118  1.00  0.00      RX0  C
ATOM   2349  CB  HIS   399      14.356 -12.612   5.307  1.00  0.00      RX0  C
ATOM   2350  CG  HIS   399      15.412 -13.665   5.063  1.00  0.00      RX0  C
ATOM   2351  ND1 HIS   399      16.709 -13.399   4.806  1.00  0.00      RX0  N
ATOM   2352  HD1 HIS   399      17.147 -12.521   4.746  0.00  0.00      RX0  H
ATOM   2353  CD2 HIS   399      15.226 -15.051   5.048  1.00  0.00      RX0  C
ATOM   2354  NE2 HIS   399      16.427 -15.618   4.779  1.00  0.00      RX0  N
ATOM   2355  CE1 HIS   399      17.339 -14.604   4.630  1.00  0.00      RX0  C
ATOM   2356  C   HIS   399      12.131 -11.786   4.401  1.00  0.00      RX0  C
ATOM   2357  O   HIS   399      11.630 -11.743   5.524  1.00  0.00      RX0  O
ATOM   2358  N   ALA   400      11.619 -11.143   3.351  1.00  0.00      RX0  N
ATOM   2359  H   ALA   400      11.998 -11.314   2.443  0.00  0.00      RX0  H
ATOM   2360  CA  ALA   400      10.356 -10.385   3.402  1.00  0.00      RX0  C
ATOM   2361  CB  ALA   400      10.145  -9.552   2.138  1.00  0.00      RX0  C
ATOM   2362  C   ALA   400       9.147 -11.323   3.579  1.00  0.00      RX0  C
ATOM   2363  O   ALA   400       8.508 -11.220   4.647  1.00  0.00      RX0  O
TER
HEADER lig.000.00.pdb
ATOM      1  N   GLU    26      48.123  22.246  20.338  1.00  0.00      LX0  N
ATOM      2  H   GLU    26      47.791  22.996  19.765  0.00  0.00      LX0  H
ATOM      3  CA  GLU    26      49.563  21.985  20.384  1.00  0.00      LX0  C
ATOM      4  CB  GLU    26      50.346  23.295  20.364  1.00  0.00      LX0  C
ATOM      5  CG  GLU    26      50.180  24.068  19.056  1.00  0.00      LX0  C
ATOM      6  CD  GLU    26      51.056  25.300  19.102  1.00  0.00      LX0  C
ATOM      7  OE1 GLU    26      52.195  25.224  18.645  1.00  0.00      LX0  O
ATOM      8  OE2 GLU    26      50.597  26.328  19.597  1.00  0.00      LX0  O
ATOM      9  C   GLU    26      49.990  21.137  21.567  1.00  0.00      LX0  C
ATOM     10  O   GLU    26      49.196  20.776  22.437  1.00  0.00      LX0  O
ATOM     11  N   GLU    27      51.289  20.806  21.561  1.00  0.00      LX0  N
ATOM     12  H   GLU    27      51.905  21.138  20.848  0.00  0.00      LX0  H
```

ATOM	13	CA	GLU	27	51.758	19.965	22.657	1.00	0.00	LX0	C
ATOM	14	CB	GLU	27	52.940	19.070	22.254	1.00	0.00	LX0	C
ATOM	15	CG	GLU	27	52.885	18.428	20.858	1.00	0.00	LX0	C
ATOM	16	CD	GLU	27	51.556	17.749	20.564	1.00	0.00	LX0	C
ATOM	17	OE1	GLU	27	51.012	17.038	21.406	1.00	0.00	LX0	O
ATOM	18	OE2	GLU	27	51.027	17.965	19.482	1.00	0.00	LX0	O
ATOM	19	C	GLU	27	52.082	20.753	23.908	1.00	0.00	LX0	C
ATOM	20	O	GLU	27	53.220	21.103	24.199	1.00	0.00	LX0	O
ATOM	21	N	LYS	28	51.000	21.021	24.653	1.00	0.00	LX0	N
ATOM	22	H	LYS	28	50.118	20.695	24.311	0.00	0.00	LX0	H
ATOM	23	CA	LYS	28	51.192	21.622	25.973	1.00	0.00	LX0	C
ATOM	24	CB	LYS	28	49.838	21.926	26.618	1.00	0.00	LX0	C
ATOM	25	CG	LYS	28	48.804	22.643	25.740	1.00	0.00	LX0	C
ATOM	26	CD	LYS	28	47.490	22.867	26.498	1.00	0.00	LX0	C
ATOM	27	CE	LYS	28	46.363	23.486	25.662	1.00	0.00	LX0	C
ATOM	28	NZ	LYS	28	45.227	23.799	26.541	1.00	0.00	LX0	N
ATOM	29	HZ1	LYS	28	44.488	24.316	26.025	0.00	0.00	LX0	H
ATOM	30	HZ2	LYS	28	44.816	22.922	26.933	0.00	0.00	LX0	H
ATOM	31	HZ3	LYS	28	45.560	24.400	27.324	0.00	0.00	LX0	H
ATOM	32	C	LYS	28	51.989	20.677	26.860	1.00	0.00	LX0	C
ATOM	33	O	LYS	28	51.841	19.464	26.759	1.00	0.00	LX0	O
ATOM	34	N	LYS	29	52.865	21.249	27.696	1.00	0.00	LX0	N
ATOM	35	H	LYS	29	52.836	22.222	27.936	0.00	0.00	LX0	H
ATOM	36	CA	LYS	29	53.744	20.316	28.398	1.00	0.00	LX0	C
ATOM	37	CB	LYS	29	55.026	20.988	28.907	1.00	0.00	LX0	C
ATOM	38	CG	LYS	29	55.535	22.085	27.963	1.00	0.00	LX0	C
ATOM	39	CD	LYS	29	56.770	22.843	28.457	1.00	0.00	LX0	C
ATOM	40	CE	LYS	29	56.721	23.354	29.904	1.00	0.00	LX0	C
ATOM	41	NZ	LYS	29	55.568	24.219	30.187	1.00	0.00	LX0	N
ATOM	42	HZ1	LYS	29	55.743	24.791	31.040	0.00	0.00	LX0	H
ATOM	43	HZ2	LYS	29	55.245	24.843	29.419	0.00	0.00	LX0	H
ATOM	44	HZ3	LYS	29	54.716	23.661	30.406	0.00	0.00	LX0	H
ATOM	45	C	LYS	29	53.017	19.595	29.512	1.00	0.00	LX0	C
ATOM	46	O	LYS	29	52.391	20.188	30.385	1.00	0.00	LX0	O
ATOM	47	N	VAL	30	53.096	18.269	29.393	1.00	0.00	LX0	N
ATOM	48	H	VAL	30	53.675	17.870	28.687	0.00	0.00	LX0	H
ATOM	49	CA	VAL	30	52.361	17.444	30.340	1.00	0.00	LX0	C
ATOM	50	CB	VAL	30	51.735	16.241	29.619	1.00	0.00	LX0	C
ATOM	51	CG1	VAL	30	50.716	16.711	28.580	1.00	0.00	LX0	C
ATOM	52	CG2	VAL	30	52.787	15.316	28.995	1.00	0.00	LX0	C
ATOM	53	C	VAL	30	53.222	16.998	31.503	1.00	0.00	LX0	C
ATOM	54	O	VAL	30	54.444	16.972	31.418	1.00	0.00	LX0	O
ATOM	55	N	CYS	31	52.530	16.620	32.579	1.00	0.00	LX0	N
ATOM	56	H	CYS	31	51.538	16.756	32.614	0.00	0.00	LX0	H
ATOM	57	CA	CYS	31	53.248	15.982	33.675	1.00	0.00	LX0	C
ATOM	58	CB	CYS	31	53.489	16.963	34.821	1.00	0.00	LX0	C
ATOM	59	SG	CYS	31	51.951	17.552	35.575	1.00	0.00	LX0	S
ATOM	60	C	CYS	31	52.474	14.781	34.159	1.00	0.00	LX0	C
ATOM	61	O	CYS	31	51.272	14.671	33.944	1.00	0.00	LX0	O
ATOM	62	N	GLN	32	53.211	13.878	34.818	1.00	0.00	LX0	N
ATOM	63	H	GLN	32	54.184	14.068	34.961	0.00	0.00	LX0	H
ATOM	64	CA	GLN	32	52.601	12.612	35.225	1.00	0.00	LX0	C
ATOM	65	CB	GLN	32	53.637	11.650	35.827	1.00	0.00	LX0	C
ATOM	66	CG	GLN	32	54.741	11.158	34.876	1.00	0.00	LX0	C
ATOM	67	CD	GLN	32	55.950	12.080	34.866	1.00	0.00	LX0	C
ATOM	68	OE1	GLN	32	55.865	13.292	34.733	1.00	0.00	LX0	O
ATOM	69	NE2	GLN	32	57.111	11.444	35.010	1.00	0.00	LX0	N
ATOM	70	HE21	GLN	32	57.152	10.449	35.119	0.00	0.00	LX0	H
ATOM	71	HE22	GLN	32	57.953	11.979	35.010	0.00	0.00	LX0	H
ATOM	72	C	GLN	32	51.422	12.779	36.171	1.00	0.00	LX0	C
ATOM	73	O	GLN	32	50.364	12.185	36.006	1.00	0.00	LX0	O

ATOM	74	N	GLY	33	51.643	13.657	37.154	1.00	0.00	LX0	N
ATOM	75	H	GLY	33	52.556	14.036	37.287	0.00	0.00	LX0	H
ATOM	76	CA	GLY	33	50.589	13.819	38.149	1.00	0.00	LX0	C
ATOM	77	C	GLY	33	50.900	13.023	39.395	1.00	0.00	LX0	C
ATOM	78	O	GLY	33	51.757	12.148	39.393	1.00	0.00	LX0	O
ATOM	79	N	THR	34	50.194	13.380	40.466	1.00	0.00	LX0	N
ATOM	80	H	THR	34	49.504	14.105	40.456	0.00	0.00	LX0	H
ATOM	81	CA	THR	34	50.445	12.650	41.700	1.00	0.00	LX0	C
ATOM	82	CB	THR	34	50.615	13.663	42.836	1.00	0.00	LX0	C
ATOM	83	OG1	THR	34	49.573	14.649	42.788	1.00	0.00	LX0	O
ATOM	84	HG1	THR	34	48.857	14.247	43.283	0.00	0.00	LX0	H
ATOM	85	CG2	THR	34	51.990	14.329	42.801	1.00	0.00	LX0	C
ATOM	86	C	THR	34	49.373	11.610	41.990	1.00	0.00	LX0	C
ATOM	87	O	THR	34	48.304	11.587	41.381	1.00	0.00	LX0	O
ATOM	88	N	SER	35	49.720	10.726	42.935	1.00	0.00	LX0	N
ATOM	89	H	SER	35	50.645	10.720	43.307	0.00	0.00	LX0	H
ATOM	90	CA	SER	35	48.747	9.749	43.421	1.00	0.00	LX0	C
ATOM	91	CB	SER	35	48.784	8.480	42.566	1.00	0.00	LX0	C
ATOM	92	OG	SER	35	48.688	8.829	41.178	1.00	0.00	LX0	O
ATOM	93	HG	SER	35	48.289	9.695	41.137	0.00	0.00	LX0	H
ATOM	94	C	SER	35	48.985	9.439	44.888	1.00	0.00	LX0	C
ATOM	95	O	SER	35	49.517	8.407	45.272	1.00	0.00	LX0	O
ATOM	96	N	ASN	36	48.606	10.434	45.688	1.00	0.00	LX0	N
ATOM	97	H	ASN	36	48.096	11.183	45.260	0.00	0.00	LX0	H
ATOM	98	CA	ASN	36	48.909	10.410	47.115	1.00	0.00	LX0	C
ATOM	99	CB	ASN	36	49.899	11.544	47.485	1.00	0.00	LX0	C
ATOM	100	CG	ASN	36	49.239	12.789	48.074	1.00	0.00	LX0	C
ATOM	101	OD1	ASN	36	48.803	12.801	49.217	1.00	0.00	LX0	O
ATOM	102	ND2	ASN	36	49.176	13.839	47.260	1.00	0.00	LX0	N
ATOM	103	HD21	ASN	36	49.353	13.755	46.277	0.00	0.00	LX0	H
ATOM	104	HD22	ASN	36	48.898	14.732	47.625	0.00	0.00	LX0	H
ATOM	105	C	ASN	36	47.669	10.387	47.991	1.00	0.00	LX0	C
ATOM	106	O	ASN	36	47.633	9.767	49.046	1.00	0.00	LX0	O
ATOM	107	N	LYS	37	46.654	11.125	47.508	1.00	0.00	LX0	N
ATOM	108	H	LYS	37	46.755	11.544	46.602	0.00	0.00	LX0	H
ATOM	109	CA	LYS	37	45.391	11.309	48.227	1.00	0.00	LX0	C
ATOM	110	CB	LYS	37	44.314	10.334	47.731	1.00	0.00	LX0	C
ATOM	111	CG	LYS	37	44.553	8.839	47.950	1.00	0.00	LX0	C
ATOM	112	CD	LYS	37	43.267	8.003	47.958	1.00	0.00	LX0	C
ATOM	113	CE	LYS	37	42.390	8.180	49.210	1.00	0.00	LX0	C
ATOM	114	NZ	LYS	37	41.549	9.380	49.147	1.00	0.00	LX0	N
ATOM	115	HZ1	LYS	37	40.868	9.399	49.937	0.00	0.00	LX0	H
ATOM	116	HZ2	LYS	37	42.069	10.282	49.137	0.00	0.00	LX0	H
ATOM	117	HZ3	LYS	37	40.903	9.353	48.329	0.00	0.00	LX0	H
ATOM	118	C	LYS	37	45.418	11.399	49.754	1.00	0.00	LX0	C
ATOM	119	O	LYS	37	44.701	10.687	50.452	1.00	0.00	LX0	O
ATOM	120	N	LEU	38	46.268	12.347	50.201	1.00	0.00	LX0	N
ATOM	121	H	LEU	38	46.845	12.781	49.507	0.00	0.00	LX0	H
ATOM	122	CA	LEU	38	46.573	12.679	51.602	1.00	0.00	LX0	C
ATOM	123	CB	LEU	38	45.354	12.685	52.539	1.00	0.00	LX0	C
ATOM	124	CG	LEU	38	44.411	13.855	52.261	1.00	0.00	LX0	C
ATOM	125	CD1	LEU	38	43.067	13.697	52.972	1.00	0.00	LX0	C
ATOM	126	CD2	LEU	38	45.078	15.188	52.594	1.00	0.00	LX0	C
ATOM	127	C	LEU	38	47.741	11.943	52.239	1.00	0.00	LX0	C
ATOM	128	O	LEU	38	48.049	12.124	53.413	1.00	0.00	LX0	O
ATOM	129	N	THR	39	48.417	11.135	51.419	1.00	0.00	LX0	N
ATOM	130	H	THR	39	48.195	11.105	50.446	0.00	0.00	LX0	H
ATOM	131	CA	THR	39	49.677	10.590	51.912	1.00	0.00	LX0	C
ATOM	132	CB	THR	39	50.008	9.285	51.185	1.00	0.00	LX0	C
ATOM	133	OG1	THR	39	48.855	8.435	51.163	1.00	0.00	LX0	O
ATOM	134	HG1	THR	39	48.249	8.828	50.539	0.00	0.00	LX0	H

ATOM	135	CG2	THR	39	51.200	8.555	51.813	1.00	0.00	LX0	C
ATOM	136	C	THR	39	50.808	11.598	51.783	1.00	0.00	LX0	C
ATOM	137	O	THR	39	51.340	11.850	50.710	1.00	0.00	LX0	O
ATOM	138	N	GLN	40	51.148	12.172	52.949	1.00	0.00	LX0	N
ATOM	139	H	GLN	40	50.630	11.919	53.764	0.00	0.00	LX0	H
ATOM	140	CA	GLN	40	52.225	13.165	52.974	1.00	0.00	LX0	C
ATOM	141	CB	GLN	40	52.408	13.689	54.403	1.00	0.00	LX0	C
ATOM	142	CG	GLN	40	52.989	15.108	54.491	1.00	0.00	LX0	C
ATOM	143	CD	GLN	40	53.021	15.589	55.936	1.00	0.00	LX0	C
ATOM	144	OE1	GLN	40	54.004	16.114	56.440	1.00	0.00	LX0	O
ATOM	145	NE2	GLN	40	51.872	15.422	56.603	1.00	0.00	LX0	N
ATOM	146	HE21	GLN	40	51.058	15.003	56.206	0.00	0.00	LX0	H
ATOM	147	HE22	GLN	40	51.856	15.742	57.548	0.00	0.00	LX0	H
ATOM	148	C	GLN	40	53.534	12.666	52.373	1.00	0.00	LX0	C
ATOM	149	O	GLN	40	54.169	11.742	52.865	1.00	0.00	LX0	O
ATOM	150	N	LEU	41	53.884	13.312	51.253	1.00	0.00	LX0	N
ATOM	151	H	LEU	41	53.271	14.010	50.892	0.00	0.00	LX0	H
ATOM	152	CA	LEU	41	55.099	12.924	50.540	1.00	0.00	LX0	C
ATOM	153	CB	LEU	41	55.006	13.337	49.068	1.00	0.00	LX0	C
ATOM	154	CG	LEU	41	53.822	12.712	48.323	1.00	0.00	LX0	C
ATOM	155	CD1	LEU	41	53.687	13.275	46.908	1.00	0.00	LX0	C
ATOM	156	CD2	LEU	41	53.877	11.182	48.325	1.00	0.00	LX0	C
ATOM	157	C	LEU	41	56.362	13.472	51.174	1.00	0.00	LX0	C
ATOM	158	O	LEU	41	56.964	14.429	50.696	1.00	0.00	LX0	O
ATOM	159	N	GLY	42	56.722	12.802	52.274	1.00	0.00	LX0	N
ATOM	160	H	GLY	42	56.096	12.110	52.638	0.00	0.00	LX0	H
ATOM	161	CA	GLY	42	57.779	13.340	53.120	1.00	0.00	LX0	C
ATOM	162	C	GLY	42	57.152	14.191	54.201	1.00	0.00	LX0	C
ATOM	163	O	GLY	42	56.016	13.965	54.599	1.00	0.00	LX0	O
ATOM	164	N	THR	43	57.926	15.185	54.642	1.00	0.00	LX0	N
ATOM	165	H	THR	43	58.838	15.310	54.250	0.00	0.00	LX0	H
ATOM	166	CA	THR	43	57.350	16.178	55.539	1.00	0.00	LX0	C
ATOM	167	CB	THR	43	58.470	17.006	56.189	1.00	0.00	LX0	C
ATOM	168	OG1	THR	43	59.110	17.860	55.230	1.00	0.00	LX0	O
ATOM	169	HG1	THR	43	59.860	17.378	54.855	0.00	0.00	LX0	H
ATOM	170	CG2	THR	43	59.489	16.134	56.925	1.00	0.00	LX0	C
ATOM	171	C	THR	43	56.381	17.086	54.796	1.00	0.00	LX0	C
ATOM	172	O	THR	43	56.148	16.938	53.602	1.00	0.00	LX0	O
ATOM	173	N	PHE	44	55.880	18.091	55.529	1.00	0.00	LX0	N
ATOM	174	H	PHE	44	55.946	18.039	56.523	0.00	0.00	LX0	H
ATOM	175	CA	PHE	44	55.132	19.148	54.848	1.00	0.00	LX0	C
ATOM	176	CB	PHE	44	54.623	20.177	55.859	1.00	0.00	LX0	C
ATOM	177	CG	PHE	44	53.684	19.543	56.860	1.00	0.00	LX0	C
ATOM	178	CD1	PHE	44	52.326	19.365	56.521	1.00	0.00	LX0	C
ATOM	179	CD2	PHE	44	54.179	19.146	58.122	1.00	0.00	LX0	C
ATOM	180	CE1	PHE	44	51.448	18.784	57.456	1.00	0.00	LX0	C
ATOM	181	CE2	PHE	44	53.304	18.561	59.057	1.00	0.00	LX0	C
ATOM	182	CZ	PHE	44	51.947	18.385	58.714	1.00	0.00	LX0	C
ATOM	183	C	PHE	44	55.927	19.850	53.757	1.00	0.00	LX0	C
ATOM	184	O	PHE	44	55.455	20.084	52.649	1.00	0.00	LX0	O
ATOM	185	N	GLU	45	57.181	20.173	54.119	1.00	0.00	LX0	N
ATOM	186	H	GLU	45	57.605	19.829	54.956	0.00	0.00	LX0	H
ATOM	187	CA	GLU	45	57.962	20.883	53.110	1.00	0.00	LX0	C
ATOM	188	CB	GLU	45	59.069	21.735	53.737	1.00	0.00	LX0	C
ATOM	189	CG	GLU	45	59.597	22.775	52.740	1.00	0.00	LX0	C
ATOM	190	CD	GLU	45	60.451	23.812	53.439	1.00	0.00	LX0	C
ATOM	191	OE1	GLU	45	59.997	24.947	53.575	1.00	0.00	LX0	O
ATOM	192	OE2	GLU	45	61.569	23.492	53.832	1.00	0.00	LX0	O
ATOM	193	C	GLU	45	58.451	19.975	51.998	1.00	0.00	LX0	C
ATOM	194	O	GLU	45	58.420	20.328	50.825	1.00	0.00	LX0	O
ATOM	195	N	ASP	46	58.802	18.737	52.386	1.00	0.00	LX0	N

ATOM	196	H	ASP	46	59.019	18.531	53.343	0.00	0.00	LX0	H
ATOM	197	CA	ASP	46	59.116	17.762	51.334	1.00	0.00	LX0	C
ATOM	198	CB	ASP	46	59.533	16.379	51.848	1.00	0.00	LX0	C
ATOM	199	CG	ASP	46	60.534	16.384	52.989	1.00	0.00	LX0	C
ATOM	200	OD1	ASP	46	61.128	17.412	53.288	1.00	0.00	LX0	O
ATOM	201	OD2	ASP	46	60.677	15.349	53.630	1.00	0.00	LX0	O
ATOM	202	C	ASP	46	57.966	17.536	50.371	1.00	0.00	LX0	C
ATOM	203	O	ASP	46	58.152	17.362	49.170	1.00	0.00	LX0	O
ATOM	204	N	HIS	47	56.752	17.595	50.944	1.00	0.00	LX0	N
ATOM	205	H	HIS	47	56.691	17.657	51.940	0.00	0.00	LX0	H
ATOM	206	CA	HIS	47	55.557	17.512	50.111	1.00	0.00	LX0	C
ATOM	207	CB	HIS	47	54.281	17.382	50.947	1.00	0.00	LX0	C
ATOM	208	CG	HIS	47	53.143	16.817	50.121	1.00	0.00	LX0	C
ATOM	209	ND1	HIS	47	53.021	16.920	48.780	1.00	0.00	LX0	N
ATOM	210	HD1	HIS	47	53.614	17.392	48.156	0.00	0.00	LX0	H
ATOM	211	CD2	HIS	47	52.053	16.093	50.605	1.00	0.00	LX0	C
ATOM	212	NE2	HIS	47	51.282	15.759	49.546	1.00	0.00	LX0	N
ATOM	213	CE1	HIS	47	51.874	16.268	48.420	1.00	0.00	LX0	C
ATOM	214	C	HIS	47	55.433	18.667	49.145	1.00	0.00	LX0	C
ATOM	215	O	HIS	47	55.242	18.461	47.952	1.00	0.00	LX0	O
ATOM	216	N	PHE	48	55.597	19.884	49.688	1.00	0.00	LX0	N
ATOM	217	H	PHE	48	55.707	19.961	50.682	0.00	0.00	LX0	H
ATOM	218	CA	PHE	48	55.651	21.071	48.828	1.00	0.00	LX0	C
ATOM	219	CB	PHE	48	55.908	22.301	49.726	1.00	0.00	LX0	C
ATOM	220	CG	PHE	48	56.661	23.423	49.038	1.00	0.00	LX0	C
ATOM	221	CD1	PHE	48	56.092	24.099	47.936	1.00	0.00	LX0	C
ATOM	222	CD2	PHE	48	57.944	23.766	49.517	1.00	0.00	LX0	C
ATOM	223	CE1	PHE	48	56.822	25.118	47.294	1.00	0.00	LX0	C
ATOM	224	CE2	PHE	48	58.675	24.787	48.880	1.00	0.00	LX0	C
ATOM	225	CZ	PHE	48	58.107	25.449	47.771	1.00	0.00	LX0	C
ATOM	226	C	PHE	48	56.645	20.918	47.678	1.00	0.00	LX0	C
ATOM	227	O	PHE	48	56.348	21.118	46.506	1.00	0.00	LX0	O
ATOM	228	N	LEU	49	57.848	20.497	48.073	1.00	0.00	LX0	N
ATOM	229	H	LEU	49	58.025	20.373	49.048	0.00	0.00	LX0	H
ATOM	230	CA	LEU	49	58.889	20.292	47.078	1.00	0.00	LX0	C
ATOM	231	CB	LEU	49	60.232	20.089	47.783	1.00	0.00	LX0	C
ATOM	232	CG	LEU	49	61.451	20.336	46.890	1.00	0.00	LX0	C
ATOM	233	CD1	LEU	49	61.420	21.719	46.235	1.00	0.00	LX0	C
ATOM	234	CD2	LEU	49	62.755	20.106	47.654	1.00	0.00	LX0	C
ATOM	235	C	LEU	49	58.578	19.184	46.086	1.00	0.00	LX0	C
ATOM	236	O	LEU	49	58.958	19.231	44.927	1.00	0.00	LX0	O
ATOM	237	N	SER	50	57.838	18.179	46.566	1.00	0.00	LX0	N
ATOM	238	H	SER	50	57.545	18.177	47.523	0.00	0.00	LX0	H
ATOM	239	CA	SER	50	57.388	17.147	45.634	1.00	0.00	LX0	C
ATOM	240	CB	SER	50	56.929	15.916	46.409	1.00	0.00	LX0	C
ATOM	241	OG	SER	50	57.993	15.534	47.292	1.00	0.00	LX0	O
ATOM	242	HG	SER	50	57.679	15.721	48.182	0.00	0.00	LX0	H
ATOM	243	C	SER	50	56.354	17.623	44.633	1.00	0.00	LX0	C
ATOM	244	O	SER	50	56.392	17.282	43.459	1.00	0.00	LX0	O
ATOM	245	N	LEU	51	55.465	18.486	45.144	1.00	0.00	LX0	N
ATOM	246	H	LEU	51	55.510	18.741	46.110	0.00	0.00	LX0	H
ATOM	247	CA	LEU	51	54.486	19.128	44.272	1.00	0.00	LX0	C
ATOM	248	CB	LEU	51	53.575	20.015	45.123	1.00	0.00	LX0	C
ATOM	249	CG	LEU	51	52.155	20.147	44.581	1.00	0.00	LX0	C
ATOM	250	CD1	LEU	51	51.430	18.807	44.631	1.00	0.00	LX0	C
ATOM	251	CD2	LEU	51	51.365	21.237	45.303	1.00	0.00	LX0	C
ATOM	252	C	LEU	51	55.164	19.921	43.166	1.00	0.00	LX0	C
ATOM	253	O	LEU	51	54.865	19.816	41.985	1.00	0.00	LX0	O
ATOM	254	N	GLN	52	56.173	20.680	43.623	1.00	0.00	LX0	N
ATOM	255	H	GLN	52	56.307	20.756	44.612	0.00	0.00	LX0	H
ATOM	256	CA	GLN	52	57.030	21.392	42.679	1.00	0.00	LX0	C

ATOM	257	CB	GLN	52	58.077	22.184	43.462	1.00	0.00	LX0	C
ATOM	258	CG	GLN	52	58.773	23.275	42.651	1.00	0.00	LX0	C
ATOM	259	CD	GLN	52	59.857	23.902	43.501	1.00	0.00	LX0	C
ATOM	260	OE1	GLN	52	61.042	23.735	43.260	1.00	0.00	LX0	O
ATOM	261	NE2	GLN	52	59.405	24.633	44.524	1.00	0.00	LX0	N
ATOM	262	HE21	GLN	52	58.434	24.759	44.715	0.00	0.00	LX0	H
ATOM	263	HE22	GLN	52	60.089	25.062	45.113	0.00	0.00	LX0	H
ATOM	264	C	GLN	52	57.671	20.493	41.631	1.00	0.00	LX0	C
ATOM	265	O	GLN	52	57.540	20.699	40.433	1.00	0.00	LX0	O
ATOM	266	N	ARG	53	58.348	19.458	42.151	1.00	0.00	LX0	N
ATOM	267	H	ARG	53	58.395	19.371	43.144	0.00	0.00	LX0	H
ATOM	268	CA	ARG	53	59.037	18.505	41.279	1.00	0.00	LX0	C
ATOM	269	CB	ARG	53	59.730	17.422	42.122	1.00	0.00	LX0	C
ATOM	270	CG	ARG	53	60.957	17.945	42.883	1.00	0.00	LX0	C
ATOM	271	CD	ARG	53	61.618	16.929	43.827	1.00	0.00	LX0	C
ATOM	272	NE	ARG	53	60.797	16.631	45.005	1.00	0.00	LX0	N
ATOM	273	HE	ARG	53	59.825	16.444	44.854	0.00	0.00	LX0	H
ATOM	274	CZ	ARG	53	61.333	16.588	46.249	1.00	0.00	LX0	C
ATOM	275	NH1	ARG	53	62.630	16.835	46.430	1.00	0.00	LX0	N
ATOM	276	HH11	ARG	53	63.047	16.811	47.339	0.00	0.00	LX0	H
ATOM	277	HH12	ARG	53	63.214	17.054	45.647	0.00	0.00	LX0	H
ATOM	278	NH2	ARG	53	60.565	16.301	47.298	1.00	0.00	LX0	N
ATOM	279	HH21	ARG	53	60.906	16.293	48.239	0.00	0.00	LX0	H
ATOM	280	HH22	ARG	53	59.589	16.076	47.181	0.00	0.00	LX0	H
ATOM	281	C	ARG	53	58.164	17.879	40.201	1.00	0.00	LX0	C
ATOM	282	O	ARG	53	58.568	17.725	39.058	1.00	0.00	LX0	O
ATOM	283	N	MET	54	56.938	17.536	40.618	1.00	0.00	LX0	N
ATOM	284	H	MET	54	56.649	17.715	41.559	0.00	0.00	LX0	H
ATOM	285	CA	MET	54	56.041	16.913	39.649	1.00	0.00	LX0	C
ATOM	286	CB	MET	54	54.986	16.087	40.395	1.00	0.00	LX0	C
ATOM	287	CG	MET	54	54.045	15.277	39.497	1.00	0.00	LX0	C
ATOM	288	SD	MET	54	54.865	14.013	38.512	1.00	0.00	LX0	S
ATOM	289	CE	MET	54	55.252	12.867	39.844	1.00	0.00	LX0	C
ATOM	290	C	MET	54	55.400	17.883	38.664	1.00	0.00	LX0	C
ATOM	291	O	MET	54	55.177	17.574	37.501	1.00	0.00	LX0	O
ATOM	292	N	PHE	55	55.078	19.074	39.184	1.00	0.00	LX0	N
ATOM	293	H	PHE	55	55.329	19.333	40.119	0.00	0.00	LX0	H
ATOM	294	CA	PHE	55	54.252	19.935	38.340	1.00	0.00	LX0	C
ATOM	295	CB	PHE	55	52.972	20.347	39.074	1.00	0.00	LX0	C
ATOM	296	CG	PHE	55	52.204	19.139	39.553	1.00	0.00	LX0	C
ATOM	297	CD1	PHE	55	51.507	18.345	38.620	1.00	0.00	LX0	C
ATOM	298	CD2	PHE	55	52.194	18.825	40.929	1.00	0.00	LX0	C
ATOM	299	CE1	PHE	55	50.787	17.223	39.069	1.00	0.00	LX0	C
ATOM	300	CE2	PHE	55	51.475	17.701	41.377	1.00	0.00	LX0	C
ATOM	301	CZ	PHE	55	50.774	16.914	40.443	1.00	0.00	LX0	C
ATOM	302	C	PHE	55	54.942	21.166	37.786	1.00	0.00	LX0	C
ATOM	303	O	PHE	55	54.300	22.098	37.308	1.00	0.00	LX0	O
ATOM	304	N	ASN	56	56.280	21.162	37.887	1.00	0.00	LX0	N
ATOM	305	H	ASN	56	56.761	20.389	38.301	0.00	0.00	LX0	H
ATOM	306	CA	ASN	56	56.976	22.399	37.531	1.00	0.00	LX0	C
ATOM	307	CB	ASN	56	58.466	22.336	37.866	1.00	0.00	LX0	C
ATOM	308	CG	ASN	56	58.948	23.745	38.151	1.00	0.00	LX0	C
ATOM	309	OD1	ASN	56	58.218	24.722	38.039	1.00	0.00	LX0	O
ATOM	310	ND2	ASN	56	60.219	23.810	38.552	1.00	0.00	LX0	N
ATOM	311	HD21	ASN	56	60.776	22.985	38.635	0.00	0.00	LX0	H
ATOM	312	HD22	ASN	56	60.609	24.700	38.777	0.00	0.00	LX0	H
ATOM	313	C	ASN	56	56.761	22.887	36.110	1.00	0.00	LX0	C
ATOM	314	O	ASN	56	57.025	22.198	35.134	1.00	0.00	LX0	O
ATOM	315	N	ASN	57	56.215	24.114	36.060	1.00	0.00	LX0	N
ATOM	316	H	ASN	57	56.209	24.612	36.926	0.00	0.00	LX0	H
ATOM	317	CA	ASN	57	55.801	24.738	34.798	1.00	0.00	LX0	C

ATOM	318	CB	ASN	57	57.015	25.375	34.099	1.00	0.00	LX0	C
ATOM	319	CG	ASN	57	56.586	26.283	32.959	1.00	0.00	LX0	C
ATOM	320	OD1	ASN	57	56.766	25.984	31.784	1.00	0.00	LX0	O
ATOM	321	ND2	ASN	57	56.022	27.428	33.350	1.00	0.00	LX0	N
ATOM	322	HD21	ASN	57	55.772	27.610	34.301	0.00	0.00	LX0	H
ATOM	323	HD22	ASN	57	55.795	28.108	32.656	0.00	0.00	LX0	H
ATOM	324	C	ASN	57	54.976	23.858	33.860	1.00	0.00	LX0	C
ATOM	325	O	ASN	57	55.079	23.923	32.639	1.00	0.00	LX0	O
ATOM	326	N	CYS	58	54.144	23.018	34.481	1.00	0.00	LX0	N
ATOM	327	H	CYS	58	54.046	23.014	35.477	0.00	0.00	LX0	H
ATOM	328	CA	CYS	58	53.319	22.171	33.627	1.00	0.00	LX0	C
ATOM	329	CB	CYS	58	52.946	20.895	34.377	1.00	0.00	LX0	C
ATOM	330	SG	CYS	58	51.976	19.741	33.380	1.00	0.00	LX0	S
ATOM	331	C	CYS	58	52.076	22.891	33.155	1.00	0.00	LX0	C
ATOM	332	O	CYS	58	51.507	23.704	33.872	1.00	0.00	LX0	O
ATOM	333	N	GLU	59	51.669	22.551	31.931	1.00	0.00	LX0	N
ATOM	334	H	GLU	59	52.229	21.978	31.328	0.00	0.00	LX0	H
ATOM	335	CA	GLU	59	50.374	23.050	31.493	1.00	0.00	LX0	C
ATOM	336	CB	GLU	59	50.386	23.554	30.043	1.00	0.00	LX0	C
ATOM	337	CG	GLU	59	51.245	24.796	29.737	1.00	0.00	LX0	C
ATOM	338	CD	GLU	59	52.722	24.478	29.544	1.00	0.00	LX0	C
ATOM	339	OE1	GLU	59	53.493	25.371	29.209	1.00	0.00	LX0	O
ATOM	340	OE2	GLU	59	53.132	23.340	29.720	1.00	0.00	LX0	O
ATOM	341	C	GLU	59	49.281	22.017	31.662	1.00	0.00	LX0	C
ATOM	342	O	GLU	59	48.160	22.347	32.032	1.00	0.00	LX0	O
ATOM	343	N	VAL	60	49.643	20.756	31.368	1.00	0.00	LX0	N
ATOM	344	H	VAL	60	50.585	20.539	31.099	0.00	0.00	LX0	H
ATOM	345	CA	VAL	60	48.641	19.699	31.507	1.00	0.00	LX0	C
ATOM	346	CB	VAL	60	48.258	19.071	30.156	1.00	0.00	LX0	C
ATOM	347	CG1	VAL	60	47.112	18.060	30.298	1.00	0.00	LX0	C
ATOM	348	CG2	VAL	60	47.905	20.132	29.120	1.00	0.00	LX0	C
ATOM	349	C	VAL	60	49.060	18.607	32.471	1.00	0.00	LX0	C
ATOM	350	O	VAL	60	49.901	17.762	32.187	1.00	0.00	LX0	O
ATOM	351	N	VAL	61	48.396	18.632	33.626	1.00	0.00	LX0	N
ATOM	352	H	VAL	61	47.683	19.320	33.758	0.00	0.00	LX0	H
ATOM	353	CA	VAL	61	48.519	17.467	34.493	1.00	0.00	LX0	C
ATOM	354	CB	VAL	61	48.024	17.814	35.900	1.00	0.00	LX0	C
ATOM	355	CG1	VAL	61	48.179	16.637	36.859	1.00	0.00	LX0	C
ATOM	356	CG2	VAL	61	48.717	19.066	36.434	1.00	0.00	LX0	C
ATOM	357	C	VAL	61	47.726	16.305	33.920	1.00	0.00	LX0	C
ATOM	358	O	VAL	61	46.531	16.418	33.675	1.00	0.00	LX0	O
ATOM	359	N	LEU	62	48.436	15.191	33.711	1.00	0.00	LX0	N
ATOM	360	H	LEU	62	49.418	15.172	33.905	0.00	0.00	LX0	H
ATOM	361	CA	LEU	62	47.713	14.008	33.250	1.00	0.00	LX0	C
ATOM	362	CB	LEU	62	48.665	13.012	32.587	1.00	0.00	LX0	C
ATOM	363	CG	LEU	62	49.369	13.574	31.351	1.00	0.00	LX0	C
ATOM	364	CD1	LEU	62	50.463	12.628	30.857	1.00	0.00	LX0	C
ATOM	365	CD2	LEU	62	48.379	13.945	30.244	1.00	0.00	LX0	C
ATOM	366	C	LEU	62	46.929	13.339	34.361	1.00	0.00	LX0	C
ATOM	367	O	LEU	62	45.711	13.222	34.322	1.00	0.00	LX0	O
ATOM	368	N	GLY	63	47.695	12.915	35.373	1.00	0.00	LX0	N
ATOM	369	H	GLY	63	48.691	13.009	35.353	0.00	0.00	LX0	H
ATOM	370	CA	GLY	63	47.024	12.349	36.536	1.00	0.00	LX0	C
ATOM	371	C	GLY	63	46.425	13.421	37.420	1.00	0.00	LX0	C
ATOM	372	O	GLY	63	45.772	14.358	36.973	1.00	0.00	LX0	O
ATOM	373	N	ASN	64	46.686	13.237	38.716	1.00	0.00	LX0	N
ATOM	374	H	ASN	64	47.324	12.540	39.042	0.00	0.00	LX0	H
ATOM	375	CA	ASN	64	46.015	14.150	39.633	1.00	0.00	LX0	C
ATOM	376	CB	ASN	64	45.661	13.478	40.951	1.00	0.00	LX0	C
ATOM	377	CG	ASN	64	44.852	12.232	40.717	1.00	0.00	LX0	C
ATOM	378	OD1	ASN	64	43.871	12.210	39.985	1.00	0.00	LX0	O

ATOM	379	ND2	ASN	64	45.321	11.182	41.392	1.00	0.00	LX0	N
ATOM	380	HD21	ASN	64	46.147	11.295	41.951	0.00	0.00	LX0	H
ATOM	381	HD22	ASN	64	44.843	10.307	41.362	0.00	0.00	LX0	H
ATOM	382	C	ASN	64	46.856	15.350	39.957	1.00	0.00	LX0	C
ATOM	383	O	ASN	64	48.080	15.297	39.959	1.00	0.00	LX0	O
ATOM	384	N	LEU	65	46.139	16.428	40.264	1.00	0.00	LX0	N
ATOM	385	H	LEU	65	45.141	16.391	40.195	0.00	0.00	LX0	H
ATOM	386	CA	LEU	65	46.812	17.552	40.892	1.00	0.00	LX0	C
ATOM	387	CB	LEU	65	46.410	18.837	40.166	1.00	0.00	LX0	C
ATOM	388	CG	LEU	65	47.091	20.115	40.656	1.00	0.00	LX0	C
ATOM	389	CD1	LEU	65	48.614	20.010	40.662	1.00	0.00	LX0	C
ATOM	390	CD2	LEU	65	46.613	21.322	39.854	1.00	0.00	LX0	C
ATOM	391	C	LEU	65	46.469	17.568	42.368	1.00	0.00	LX0	C
ATOM	392	O	LEU	65	45.474	18.137	42.806	1.00	0.00	LX0	O
ATOM	393	N	GLU	66	47.321	16.874	43.128	1.00	0.00	LX0	N
ATOM	394	H	GLU	66	48.145	16.439	42.757	0.00	0.00	LX0	H
ATOM	395	CA	GLU	66	47.011	16.847	44.554	1.00	0.00	LX0	C
ATOM	396	CB	GLU	66	47.198	15.457	45.130	1.00	0.00	LX0	C
ATOM	397	CG	GLU	66	46.341	14.405	44.430	1.00	0.00	LX0	C
ATOM	398	CD	GLU	66	46.991	13.049	44.585	1.00	0.00	LX0	C
ATOM	399	OE1	GLU	66	48.215	12.980	44.595	1.00	0.00	LX0	O
ATOM	400	OE2	GLU	66	46.289	12.051	44.694	1.00	0.00	LX0	O
ATOM	401	C	GLU	66	47.803	17.854	45.347	1.00	0.00	LX0	C
ATOM	402	O	GLU	66	48.867	17.590	45.894	1.00	0.00	LX0	O
ATOM	403	N	ILE	67	47.215	19.049	45.377	1.00	0.00	LX0	N
ATOM	404	H	ILE	67	46.279	19.099	45.025	0.00	0.00	LX0	H
ATOM	405	CA	ILE	67	47.745	20.075	46.266	1.00	0.00	LX0	C
ATOM	406	CB	ILE	67	47.158	21.437	45.881	1.00	0.00	LX0	C
ATOM	407	CG2	ILE	67	47.755	22.585	46.701	1.00	0.00	LX0	C
ATOM	408	CG1	ILE	67	47.302	21.668	44.376	1.00	0.00	LX0	C
ATOM	409	CD1	ILE	67	46.425	22.814	43.882	1.00	0.00	LX0	C
ATOM	410	C	ILE	67	47.422	19.725	47.708	1.00	0.00	LX0	C
ATOM	411	O	ILE	67	46.374	20.068	48.244	1.00	0.00	LX0	O
ATOM	412	N	THR	68	48.367	19.002	48.307	1.00	0.00	LX0	N
ATOM	413	H	THR	68	49.130	18.616	47.786	0.00	0.00	LX0	H
ATOM	414	CA	THR	68	48.135	18.633	49.693	1.00	0.00	LX0	C
ATOM	415	CB	THR	68	47.811	17.134	49.770	1.00	0.00	LX0	C
ATOM	416	OG1	THR	68	48.597	16.382	48.837	1.00	0.00	LX0	O
ATOM	417	HG1	THR	68	49.487	16.398	49.175	0.00	0.00	LX0	H
ATOM	418	CG2	THR	68	46.336	16.884	49.481	1.00	0.00	LX0	C
ATOM	419	C	THR	68	49.274	19.029	50.609	1.00	0.00	LX0	C
ATOM	420	O	THR	68	50.440	18.997	50.233	1.00	0.00	LX0	O
ATOM	421	N	TYR	69	48.872	19.400	51.839	1.00	0.00	LX0	N
ATOM	422	H	TYR	69	47.884	19.512	51.974	0.00	0.00	LX0	H
ATOM	423	CA	TYR	69	49.812	19.678	52.935	1.00	0.00	LX0	C
ATOM	424	CB	TYR	69	50.658	18.451	53.309	1.00	0.00	LX0	C
ATOM	425	CG	TYR	69	49.807	17.317	53.830	1.00	0.00	LX0	C
ATOM	426	CD1	TYR	69	49.340	17.369	55.158	1.00	0.00	LX0	C
ATOM	427	CE1	TYR	69	48.618	16.280	55.669	1.00	0.00	LX0	C
ATOM	428	CD2	TYR	69	49.528	16.226	52.984	1.00	0.00	LX0	C
ATOM	429	CE2	TYR	69	48.804	15.137	53.494	1.00	0.00	LX0	C
ATOM	430	CZ	TYR	69	48.374	15.170	54.835	1.00	0.00	LX0	C
ATOM	431	OH	TYR	69	47.702	14.087	55.363	1.00	0.00	LX0	O
ATOM	432	HH	TYR	69	47.730	13.354	54.750	0.00	0.00	LX0	H
ATOM	433	C	TYR	69	50.730	20.887	52.808	1.00	0.00	LX0	C
ATOM	434	O	TYR	69	51.596	21.118	53.647	1.00	0.00	LX0	O
ATOM	435	N	VAL	70	50.522	21.664	51.742	1.00	0.00	LX0	N
ATOM	436	H	VAL	70	49.754	21.482	51.128	0.00	0.00	LX0	H
ATOM	437	CA	VAL	70	51.399	22.814	51.539	1.00	0.00	LX0	C
ATOM	438	CB	VAL	70	51.267	23.315	50.096	1.00	0.00	LX0	C
ATOM	439	CG1	VAL	70	52.206	24.485	49.814	1.00	0.00	LX0	C

ATOM	440	CG2	VAL	70	51.499	22.173	49.102	1.00	0.00	LX0	C
ATOM	441	C	VAL	70	51.154	23.922	52.555	1.00	0.00	LX0	C
ATOM	442	O	VAL	70	50.046	24.414	52.742	1.00	0.00	LX0	O
ATOM	443	N	GLN	71	52.248	24.263	53.243	1.00	0.00	LX0	N
ATOM	444	H	GLN	71	53.140	23.894	52.986	0.00	0.00	LX0	H
ATOM	445	CA	GLN	71	52.084	25.202	54.345	1.00	0.00	LX0	C
ATOM	446	CB	GLN	71	53.059	24.872	55.479	1.00	0.00	LX0	C
ATOM	447	CG	GLN	71	52.892	23.458	56.049	1.00	0.00	LX0	C
ATOM	448	CD	GLN	71	51.525	23.291	56.687	1.00	0.00	LX0	C
ATOM	449	OE1	GLN	71	51.161	23.974	57.636	1.00	0.00	LX0	O
ATOM	450	NE2	GLN	71	50.763	22.362	56.110	1.00	0.00	LX0	N
ATOM	451	HE21	GLN	71	51.126	21.826	55.345	0.00	0.00	LX0	H
ATOM	452	HE22	GLN	71	49.833	22.185	56.427	0.00	0.00	LX0	H
ATOM	453	C	GLN	71	52.143	26.675	53.977	1.00	0.00	LX0	C
ATOM	454	O	GLN	71	52.620	27.078	52.922	1.00	0.00	LX0	O
ATOM	455	N	ARG	72	51.624	27.448	54.944	1.00	0.00	LX0	N
ATOM	456	H	ARG	72	51.233	26.941	55.713	0.00	0.00	LX0	H
ATOM	457	CA	ARG	72	51.413	28.898	54.916	1.00	0.00	LX0	C
ATOM	458	CB	ARG	72	51.816	29.494	56.267	1.00	0.00	LX0	C
ATOM	459	CG	ARG	72	50.870	30.603	56.734	1.00	0.00	LX0	C
ATOM	460	CD	ARG	72	49.519	30.051	57.195	1.00	0.00	LX0	C
ATOM	461	NE	ARG	72	48.554	31.126	57.408	1.00	0.00	LX0	N
ATOM	462	HE	ARG	72	48.576	31.932	56.804	0.00	0.00	LX0	H
ATOM	463	CZ	ARG	72	47.514	30.999	58.256	1.00	0.00	LX0	C
ATOM	464	NH1	ARG	72	47.427	29.959	59.073	1.00	0.00	LX0	N
ATOM	465	HH11	ARG	72	46.641	29.836	59.694	0.00	0.00	LX0	H
ATOM	466	HH12	ARG	72	48.130	29.256	59.115	0.00	0.00	LX0	H
ATOM	467	NH2	ARG	72	46.568	31.924	58.279	1.00	0.00	LX0	N
ATOM	468	HH21	ARG	72	45.816	31.856	58.940	0.00	0.00	LX0	H
ATOM	469	HH22	ARG	72	46.579	32.691	57.614	0.00	0.00	LX0	H
ATOM	470	C	ARG	72	51.954	29.745	53.769	1.00	0.00	LX0	C
ATOM	471	O	ARG	72	51.216	30.427	53.070	1.00	0.00	LX0	O
ATOM	472	N	ASN	73	53.286	29.707	53.633	1.00	0.00	LX0	N
ATOM	473	H	ASN	73	53.821	29.031	54.137	0.00	0.00	LX0	H
ATOM	474	CA	ASN	73	53.894	30.737	52.790	1.00	0.00	LX0	C
ATOM	475	CB	ASN	73	55.143	31.328	53.451	1.00	0.00	LX0	C
ATOM	476	CG	ASN	73	54.769	32.053	54.725	1.00	0.00	LX0	C
ATOM	477	OD1	ASN	73	54.566	31.461	55.774	1.00	0.00	LX0	O
ATOM	478	ND2	ASN	73	54.690	33.380	54.601	1.00	0.00	LX0	N
ATOM	479	HD21	ASN	73	54.863	33.843	53.735	0.00	0.00	LX0	H
ATOM	480	HD22	ASN	73	54.443	33.899	55.417	0.00	0.00	LX0	H
ATOM	481	C	ASN	73	54.242	30.339	51.372	1.00	0.00	LX0	C
ATOM	482	O	ASN	73	54.742	31.147	50.596	1.00	0.00	LX0	O
ATOM	483	N	TYR	74	54.015	29.058	51.053	1.00	0.00	LX0	N
ATOM	484	H	TYR	74	53.473	28.437	51.625	0.00	0.00	LX0	H
ATOM	485	CA	TYR	74	54.563	28.661	49.757	1.00	0.00	LX0	C
ATOM	486	CB	TYR	74	54.910	27.166	49.698	1.00	0.00	LX0	C
ATOM	487	CG	TYR	74	55.595	26.641	50.943	1.00	0.00	LX0	C
ATOM	488	CD1	TYR	74	54.979	25.569	51.614	1.00	0.00	LX0	C
ATOM	489	CE1	TYR	74	55.569	25.052	52.775	1.00	0.00	LX0	C
ATOM	490	CD2	TYR	74	56.810	27.200	51.397	1.00	0.00	LX0	C
ATOM	491	CE2	TYR	74	57.403	26.681	52.564	1.00	0.00	LX0	C
ATOM	492	CZ	TYR	74	56.766	25.621	53.244	1.00	0.00	LX0	C
ATOM	493	OH	TYR	74	57.303	25.117	54.409	1.00	0.00	LX0	O
ATOM	494	HH	TYR	74	58.258	25.058	54.302	0.00	0.00	LX0	H
ATOM	495	C	TYR	74	53.691	29.014	48.564	1.00	0.00	LX0	C
ATOM	496	O	TYR	74	52.785	28.269	48.207	1.00	0.00	LX0	O
ATOM	497	N	ASP	75	54.028	30.146	47.915	1.00	0.00	LX0	N
ATOM	498	H	ASP	75	54.689	30.775	48.325	0.00	0.00	LX0	H
ATOM	499	CA	ASP	75	53.456	30.313	46.573	1.00	0.00	LX0	C
ATOM	500	CB	ASP	75	53.689	31.730	45.988	1.00	0.00	LX0	C

ATOM	501	CG	ASP	75	53.175	31.867	44.545	1.00	0.00	LX0	C
ATOM	502	OD1	ASP	75	52.320	32.698	44.242	1.00	0.00	LX0	O
ATOM	503	OD2	ASP	75	53.647	31.142	43.682	1.00	0.00	LX0	O
ATOM	504	C	ASP	75	53.904	29.198	45.635	1.00	0.00	LX0	C
ATOM	505	O	ASP	75	55.071	28.987	45.315	1.00	0.00	LX0	O
ATOM	506	N	LEU	76	52.876	28.467	45.217	1.00	0.00	LX0	N
ATOM	507	H	LEU	76	51.973	28.703	45.571	0.00	0.00	LX0	H
ATOM	508	CA	LEU	76	53.114	27.370	44.293	1.00	0.00	LX0	C
ATOM	509	CB	LEU	76	52.024	26.322	44.494	1.00	0.00	LX0	C
ATOM	510	CG	LEU	76	51.935	25.840	45.941	1.00	0.00	LX0	C
ATOM	511	CD1	LEU	76	50.623	25.117	46.238	1.00	0.00	LX0	C
ATOM	512	CD2	LEU	76	53.157	25.011	46.321	1.00	0.00	LX0	C
ATOM	513	C	LEU	76	53.181	27.832	42.849	1.00	0.00	LX0	C
ATOM	514	O	LEU	76	52.312	27.538	42.038	1.00	0.00	LX0	O
ATOM	515	N	SER	77	54.256	28.568	42.530	1.00	0.00	LX0	N
ATOM	516	H	SER	77	54.855	28.854	43.282	0.00	0.00	LX0	H
ATOM	517	CA	SER	77	54.307	29.188	41.203	1.00	0.00	LX0	C
ATOM	518	CB	SER	77	55.564	30.042	41.046	1.00	0.00	LX0	C
ATOM	519	OG	SER	77	55.340	31.292	41.714	1.00	0.00	LX0	O
ATOM	520	HG	SER	77	55.350	31.105	42.658	0.00	0.00	LX0	H
ATOM	521	C	SER	77	54.067	28.306	39.993	1.00	0.00	LX0	C
ATOM	522	O	SER	77	53.460	28.725	39.019	1.00	0.00	LX0	O
ATOM	523	N	PHE	78	54.507	27.047	40.118	1.00	0.00	LX0	N
ATOM	524	H	PHE	78	55.013	26.796	40.940	0.00	0.00	LX0	H
ATOM	525	CA	PHE	78	54.202	26.085	39.056	1.00	0.00	LX0	C
ATOM	526	CB	PHE	78	54.808	24.712	39.377	1.00	0.00	LX0	C
ATOM	527	CG	PHE	78	54.403	24.194	40.739	1.00	0.00	LX0	C
ATOM	528	CD1	PHE	78	53.280	23.350	40.859	1.00	0.00	LX0	C
ATOM	529	CD2	PHE	78	55.165	24.559	41.871	1.00	0.00	LX0	C
ATOM	530	CE1	PHE	78	52.909	22.867	42.127	1.00	0.00	LX0	C
ATOM	531	CE2	PHE	78	54.794	24.082	43.140	1.00	0.00	LX0	C
ATOM	532	CZ	PHE	78	53.668	23.242	43.254	1.00	0.00	LX0	C
ATOM	533	C	PHE	78	52.735	25.998	38.639	1.00	0.00	LX0	C
ATOM	534	O	PHE	78	52.419	25.930	37.457	1.00	0.00	LX0	O
ATOM	535	N	LEU	79	51.853	26.079	39.657	1.00	0.00	LX0	N
ATOM	536	H	LEU	79	52.183	26.201	40.593	0.00	0.00	LX0	H
ATOM	537	CA	LEU	79	50.410	26.082	39.390	1.00	0.00	LX0	C
ATOM	538	CB	LEU	79	49.585	26.210	40.665	1.00	0.00	LX0	C
ATOM	539	CG	LEU	79	49.734	25.130	41.726	1.00	0.00	LX0	C
ATOM	540	CD1	LEU	79	48.835	25.465	42.911	1.00	0.00	LX0	C
ATOM	541	CD2	LEU	79	49.451	23.726	41.203	1.00	0.00	LX0	C
ATOM	542	C	LEU	79	49.928	27.181	38.463	1.00	0.00	LX0	C
ATOM	543	O	LEU	79	48.951	27.035	37.740	1.00	0.00	LX0	O
ATOM	544	N	LYS	80	50.675	28.295	38.486	1.00	0.00	LX0	N
ATOM	545	H	LYS	80	51.499	28.342	39.052	0.00	0.00	LX0	H
ATOM	546	CA	LYS	80	50.301	29.386	37.589	1.00	0.00	LX0	C
ATOM	547	CB	LYS	80	51.039	30.680	37.973	1.00	0.00	LX0	C
ATOM	548	CG	LYS	80	50.683	31.156	39.390	1.00	0.00	LX0	C
ATOM	549	CD	LYS	80	51.186	32.560	39.764	1.00	0.00	LX0	C
ATOM	550	CE	LYS	80	52.635	32.677	40.249	1.00	0.00	LX0	C
ATOM	551	NZ	LYS	80	52.778	32.205	41.634	1.00	0.00	LX0	N
ATOM	552	HZ1	LYS	80	53.780	32.033	41.875	0.00	0.00	LX0	H
ATOM	553	HZ2	LYS	80	52.470	32.863	42.384	0.00	0.00	LX0	H
ATOM	554	HZ3	LYS	80	52.296	31.313	41.849	0.00	0.00	LX0	H
ATOM	555	C	LYS	80	50.426	29.075	36.097	1.00	0.00	LX0	C
ATOM	556	O	LYS	80	49.932	29.813	35.254	1.00	0.00	LX0	O
ATOM	557	N	THR	81	51.084	27.940	35.810	1.00	0.00	LX0	N
ATOM	558	H	THR	81	51.481	27.380	36.533	0.00	0.00	LX0	H
ATOM	559	CA	THR	81	51.117	27.466	34.426	1.00	0.00	LX0	C
ATOM	560	CB	THR	81	52.419	26.704	34.161	1.00	0.00	LX0	C
ATOM	561	OG1	THR	81	53.465	27.114	35.061	1.00	0.00	LX0	O

ATOM	562	HG1	THR	81	53.207	26.749	35.904	0.00	0.00	LX0	H
ATOM	563	CG2	THR	81	52.843	26.819	32.696	1.00	0.00	LX0	C
ATOM	564	C	THR	81	49.937	26.583	34.025	1.00	0.00	LX0	C
ATOM	565	O	THR	81	49.553	26.482	32.863	1.00	0.00	LX0	O
ATOM	566	N	ILE	82	49.399	25.907	35.051	1.00	0.00	LX0	N
ATOM	567	H	ILE	82	49.552	26.186	35.997	0.00	0.00	LX0	H
ATOM	568	CA	ILE	82	48.530	24.775	34.743	1.00	0.00	LX0	C
ATOM	569	CB	ILE	82	48.389	23.848	35.961	1.00	0.00	LX0	C
ATOM	570	CG2	ILE	82	47.558	22.602	35.629	1.00	0.00	LX0	C
ATOM	571	CG1	ILE	82	49.776	23.475	36.497	1.00	0.00	LX0	C
ATOM	572	CD1	ILE	82	49.761	22.541	37.703	1.00	0.00	LX0	C
ATOM	573	C	ILE	82	47.184	25.182	34.181	1.00	0.00	LX0	C
ATOM	574	O	ILE	82	46.357	25.801	34.834	1.00	0.00	LX0	O
ATOM	575	N	GLN	83	47.016	24.791	32.916	1.00	0.00	LX0	N
ATOM	576	H	GLN	83	47.745	24.246	32.501	0.00	0.00	LX0	H
ATOM	577	CA	GLN	83	45.750	25.060	32.245	1.00	0.00	LX0	C
ATOM	578	CB	GLN	83	45.969	25.244	30.749	1.00	0.00	LX0	C
ATOM	579	CG	GLN	83	47.024	26.267	30.347	1.00	0.00	LX0	C
ATOM	580	CD	GLN	83	47.264	26.121	28.860	1.00	0.00	LX0	C
ATOM	581	OE1	GLN	83	46.442	25.601	28.108	1.00	0.00	LX0	O
ATOM	582	NE2	GLN	83	48.451	26.582	28.463	1.00	0.00	LX0	N
ATOM	583	HE21	GLN	83	49.091	26.957	29.137	0.00	0.00	LX0	H
ATOM	584	HE22	GLN	83	48.731	26.552	27.505	0.00	0.00	LX0	H
ATOM	585	C	GLN	83	44.753	23.932	32.416	1.00	0.00	LX0	C
ATOM	586	O	GLN	83	43.549	24.134	32.535	1.00	0.00	LX0	O
ATOM	587	N	GLU	84	45.316	22.720	32.356	1.00	0.00	LX0	N
ATOM	588	H	GLU	84	46.308	22.595	32.351	0.00	0.00	LX0	H
ATOM	589	CA	GLU	84	44.456	21.553	32.240	1.00	0.00	LX0	C
ATOM	590	CB	GLU	84	44.502	20.977	30.818	1.00	0.00	LX0	C
ATOM	591	CG	GLU	84	44.386	21.985	29.661	1.00	0.00	LX0	C
ATOM	592	CD	GLU	84	44.140	21.293	28.329	1.00	0.00	LX0	C
ATOM	593	OE1	GLU	84	44.537	20.147	28.159	1.00	0.00	LX0	O
ATOM	594	OE2	GLU	84	43.507	21.876	27.451	1.00	0.00	LX0	O
ATOM	595	C	GLU	84	44.875	20.473	33.209	1.00	0.00	LX0	C
ATOM	596	O	GLU	84	46.050	20.160	33.331	1.00	0.00	LX0	O
ATOM	597	N	VAL	85	43.873	19.896	33.874	1.00	0.00	LX0	N
ATOM	598	H	VAL	85	42.938	20.249	33.798	0.00	0.00	LX0	H
ATOM	599	CA	VAL	85	44.150	18.658	34.601	1.00	0.00	LX0	C
ATOM	600	CB	VAL	85	43.866	18.840	36.103	1.00	0.00	LX0	C
ATOM	601	CG1	VAL	85	44.204	17.591	36.927	1.00	0.00	LX0	C
ATOM	602	CG2	VAL	85	44.600	20.064	36.653	1.00	0.00	LX0	C
ATOM	603	C	VAL	85	43.266	17.584	33.999	1.00	0.00	LX0	C
ATOM	604	O	VAL	85	42.156	17.881	33.572	1.00	0.00	LX0	O
ATOM	605	N	ALA	86	43.772	16.347	33.948	1.00	0.00	LX0	N
ATOM	606	H	ALA	86	44.707	16.153	34.255	0.00	0.00	LX0	H
ATOM	607	CA	ALA	86	42.861	15.303	33.496	1.00	0.00	LX0	C
ATOM	608	CB	ALA	86	43.523	14.382	32.470	1.00	0.00	LX0	C
ATOM	609	C	ALA	86	42.243	14.500	34.624	1.00	0.00	LX0	C
ATOM	610	O	ALA	86	41.042	14.248	34.623	1.00	0.00	LX0	O
ATOM	611	N	GLY	87	43.092	14.142	35.600	1.00	0.00	LX0	N
ATOM	612	H	GLY	87	44.078	14.319	35.538	0.00	0.00	LX0	H
ATOM	613	CA	GLY	87	42.538	13.507	36.796	1.00	0.00	LX0	C
ATOM	614	C	GLY	87	41.838	14.489	37.721	1.00	0.00	LX0	C
ATOM	615	O	GLY	87	41.345	15.535	37.302	1.00	0.00	LX0	O
ATOM	616	N	TYR	88	41.811	14.105	39.004	1.00	0.00	LX0	N
ATOM	617	H	TYR	88	42.316	13.286	39.290	0.00	0.00	LX0	H
ATOM	618	CA	TYR	88	41.168	15.001	39.962	1.00	0.00	LX0	C
ATOM	619	CB	TYR	88	40.418	14.205	41.049	1.00	0.00	LX0	C
ATOM	620	CG	TYR	88	41.315	13.317	41.889	1.00	0.00	LX0	C
ATOM	621	CD1	TYR	88	42.161	13.891	42.863	1.00	0.00	LX0	C
ATOM	622	CE1	TYR	88	42.982	13.057	43.638	1.00	0.00	LX0	C

ATOM	623	CD2	TYR	88	41.263	11.923	41.686	1.00	0.00	LX0	C
ATOM	624	CE2	TYR	88	42.077	11.087	42.468	1.00	0.00	LX0	C
ATOM	625	CZ	TYR	88	42.931	11.664	43.430	1.00	0.00	LX0	C
ATOM	626	OH	TYR	88	43.736	10.836	44.188	1.00	0.00	LX0	O
ATOM	627	HH	TYR	88	44.542	11.303	44.414	0.00	0.00	LX0	H
ATOM	628	C	TYR	88	42.117	16.031	40.551	1.00	0.00	LX0	C
ATOM	629	O	TYR	88	43.333	15.910	40.464	1.00	0.00	LX0	O
ATOM	630	N	VAL	89	41.512	17.056	41.167	1.00	0.00	LX0	N
ATOM	631	H	VAL	89	40.518	17.083	41.274	0.00	0.00	LX0	H
ATOM	632	CA	VAL	89	42.347	18.026	41.870	1.00	0.00	LX0	C
ATOM	633	CB	VAL	89	42.212	19.437	41.279	1.00	0.00	LX0	C
ATOM	634	CG1	VAL	89	43.160	20.431	41.956	1.00	0.00	LX0	C
ATOM	635	CG2	VAL	89	42.448	19.428	39.773	1.00	0.00	LX0	C
ATOM	636	C	VAL	89	42.029	18.028	43.351	1.00	0.00	LX0	C
ATOM	637	O	VAL	89	41.018	18.548	43.813	1.00	0.00	LX0	O
ATOM	638	N	LEU	90	42.944	17.393	44.083	1.00	0.00	LX0	N
ATOM	639	H	LEU	90	43.822	17.156	43.663	0.00	0.00	LX0	H
ATOM	640	CA	LEU	90	42.740	17.380	45.524	1.00	0.00	LX0	C
ATOM	641	CB	LEU	90	43.189	16.034	46.098	1.00	0.00	LX0	C
ATOM	642	CG	LEU	90	43.139	15.931	47.626	1.00	0.00	LX0	C
ATOM	643	CD1	LEU	90	41.748	16.202	48.194	1.00	0.00	LX0	C
ATOM	644	CD2	LEU	90	43.716	14.605	48.110	1.00	0.00	LX0	C
ATOM	645	C	LEU	90	43.471	18.536	46.169	1.00	0.00	LX0	C
ATOM	646	O	LEU	90	44.691	18.589	46.177	1.00	0.00	LX0	O
ATOM	647	N	ILE	91	42.670	19.456	46.706	1.00	0.00	LX0	N
ATOM	648	H	ILE	91	41.677	19.329	46.702	0.00	0.00	LX0	H
ATOM	649	CA	ILE	91	43.281	20.535	47.472	1.00	0.00	LX0	C
ATOM	650	CB	ILE	91	42.777	21.898	46.971	1.00	0.00	LX0	C
ATOM	651	CG2	ILE	91	43.418	23.057	47.739	1.00	0.00	LX0	C
ATOM	652	CG1	ILE	91	42.991	22.039	45.463	1.00	0.00	LX0	C
ATOM	653	CD1	ILE	91	42.502	23.374	44.898	1.00	0.00	LX0	C
ATOM	654	C	ILE	91	42.963	20.340	48.941	1.00	0.00	LX0	C
ATOM	655	O	ILE	91	41.900	20.730	49.411	1.00	0.00	LX0	O
ATOM	656	N	ALA	92	43.902	19.702	49.646	1.00	0.00	LX0	N
ATOM	657	H	ALA	92	44.783	19.448	49.238	0.00	0.00	LX0	H
ATOM	658	CA	ALA	92	43.571	19.423	51.039	1.00	0.00	LX0	C
ATOM	659	CB	ALA	92	43.047	17.998	51.208	1.00	0.00	LX0	C
ATOM	660	C	ALA	92	44.699	19.647	52.021	1.00	0.00	LX0	C
ATOM	661	O	ALA	92	45.875	19.465	51.723	1.00	0.00	LX0	O
ATOM	662	N	LEU	93	44.267	20.045	53.233	1.00	0.00	LX0	N
ATOM	663	H	LEU	93	43.284	20.214	53.328	0.00	0.00	LX0	H
ATOM	664	CA	LEU	93	45.195	20.254	54.354	1.00	0.00	LX0	C
ATOM	665	CB	LEU	93	45.687	18.917	54.923	1.00	0.00	LX0	C
ATOM	666	CG	LEU	93	44.630	18.224	55.786	1.00	0.00	LX0	C
ATOM	667	CD1	LEU	93	44.987	16.768	56.081	1.00	0.00	LX0	C
ATOM	668	CD2	LEU	93	44.363	19.000	57.077	1.00	0.00	LX0	C
ATOM	669	C	LEU	93	46.350	21.204	54.083	1.00	0.00	LX0	C
ATOM	670	O	LEU	93	47.459	21.079	54.591	1.00	0.00	LX0	O
ATOM	671	N	ASN	94	46.025	22.184	53.238	1.00	0.00	LX0	N
ATOM	672	H	ASN	94	45.090	22.261	52.892	0.00	0.00	LX0	H
ATOM	673	CA	ASN	94	47.020	23.212	52.969	1.00	0.00	LX0	C
ATOM	674	CB	ASN	94	46.965	23.715	51.527	1.00	0.00	LX0	C
ATOM	675	CG	ASN	94	47.099	22.579	50.542	1.00	0.00	LX0	C
ATOM	676	OD1	ASN	94	48.167	22.051	50.266	1.00	0.00	LX0	O
ATOM	677	ND2	ASN	94	45.940	22.231	49.996	1.00	0.00	LX0	N
ATOM	678	HD21	ASN	94	45.078	22.641	50.295	0.00	0.00	LX0	H
ATOM	679	HD22	ASN	94	45.945	21.521	49.296	0.00	0.00	LX0	H
ATOM	680	C	ASN	94	46.763	24.384	53.873	1.00	0.00	LX0	C
ATOM	681	O	ASN	94	45.625	24.782	54.094	1.00	0.00	LX0	O
ATOM	682	N	THR	95	47.862	24.926	54.390	1.00	0.00	LX0	N
ATOM	683	H	THR	95	48.775	24.562	54.203	0.00	0.00	LX0	H

ATOM	684	CA	THR	95	47.675	26.158	55.140	1.00	0.00	LX0	C
ATOM	685	CB	THR	95	48.397	26.054	56.482	1.00	0.00	LX0	C
ATOM	686	OG1	THR	95	49.791	25.800	56.285	1.00	0.00	LX0	O
ATOM	687	HG1	THR	95	50.080	25.331	57.069	0.00	0.00	LX0	H
ATOM	688	CG2	THR	95	47.801	24.924	57.320	1.00	0.00	LX0	C
ATOM	689	C	THR	95	48.068	27.401	54.361	1.00	0.00	LX0	C
ATOM	690	O	THR	95	47.858	28.523	54.798	1.00	0.00	LX0	O
ATOM	691	N	VAL	96	48.646	27.158	53.167	1.00	0.00	LX0	N
ATOM	692	H	VAL	96	48.838	26.222	52.872	0.00	0.00	LX0	H
ATOM	693	CA	VAL	96	48.960	28.304	52.313	1.00	0.00	LX0	C
ATOM	694	CB	VAL	96	49.826	27.870	51.111	1.00	0.00	LX0	C
ATOM	695	CG1	VAL	96	49.203	26.725	50.316	1.00	0.00	LX0	C
ATOM	696	CG2	VAL	96	50.225	29.052	50.223	1.00	0.00	LX0	C
ATOM	697	C	VAL	96	47.742	29.131	51.917	1.00	0.00	LX0	C
ATOM	698	O	VAL	96	46.811	28.670	51.265	1.00	0.00	LX0	O
ATOM	699	N	GLU	97	47.799	30.388	52.379	1.00	0.00	LX0	N
ATOM	700	H	GLU	97	48.590	30.667	52.923	0.00	0.00	LX0	H
ATOM	701	CA	GLU	97	46.607	31.230	52.270	1.00	0.00	LX0	C
ATOM	702	CB	GLU	97	46.749	32.473	53.151	1.00	0.00	LX0	C
ATOM	703	CG	GLU	97	46.814	32.028	54.611	1.00	0.00	LX0	C
ATOM	704	CD	GLU	97	46.975	33.187	55.572	1.00	0.00	LX0	C
ATOM	705	OE1	GLU	97	45.985	33.623	56.153	1.00	0.00	LX0	O
ATOM	706	OE2	GLU	97	48.106	33.572	55.848	1.00	0.00	LX0	O
ATOM	707	C	GLU	97	46.173	31.579	50.861	1.00	0.00	LX0	C
ATOM	708	O	GLU	97	44.996	31.752	50.568	1.00	0.00	LX0	O
ATOM	709	N	ARG	98	47.174	31.661	49.981	1.00	0.00	LX0	N
ATOM	710	H	ARG	98	48.118	31.489	50.257	0.00	0.00	LX0	H
ATOM	711	CA	ARG	98	46.801	31.876	48.590	1.00	0.00	LX0	C
ATOM	712	CB	ARG	98	47.394	33.194	48.088	1.00	0.00	LX0	C
ATOM	713	CG	ARG	98	46.862	33.627	46.721	1.00	0.00	LX0	C
ATOM	714	CD	ARG	98	47.622	34.835	46.171	1.00	0.00	LX0	C
ATOM	715	NE	ARG	98	47.016	35.317	44.930	1.00	0.00	LX0	N
ATOM	716	HE	ARG	98	46.224	35.922	45.022	0.00	0.00	LX0	H
ATOM	717	CZ	ARG	98	47.455	34.899	43.722	1.00	0.00	LX0	C
ATOM	718	NH1	ARG	98	48.500	34.083	43.609	1.00	0.00	LX0	N
ATOM	719	HH11	ARG	98	48.750	33.761	42.686	0.00	0.00	LX0	H
ATOM	720	HH12	ARG	98	49.040	33.780	44.395	0.00	0.00	LX0	H
ATOM	721	NH2	ARG	98	46.830	35.308	42.624	1.00	0.00	LX0	N
ATOM	722	HH21	ARG	98	47.138	34.965	41.718	0.00	0.00	LX0	H
ATOM	723	HH22	ARG	98	46.059	35.938	42.643	0.00	0.00	LX0	H
ATOM	724	C	ARG	98	47.275	30.723	47.735	1.00	0.00	LX0	C
ATOM	725	O	ARG	98	48.468	30.552	47.525	1.00	0.00	LX0	O
ATOM	726	N	ILE	99	46.309	29.939	47.243	1.00	0.00	LX0	N
ATOM	727	H	ILE	99	45.347	30.188	47.379	0.00	0.00	LX0	H
ATOM	728	CA	ILE	99	46.754	28.879	46.338	1.00	0.00	LX0	C
ATOM	729	CB	ILE	99	46.033	27.552	46.630	1.00	0.00	LX0	C
ATOM	730	CG2	ILE	99	46.337	26.453	45.604	1.00	0.00	LX0	C
ATOM	731	CG1	ILE	99	46.423	27.096	48.036	1.00	0.00	LX0	C
ATOM	732	CD1	ILE	99	45.787	25.777	48.468	1.00	0.00	LX0	C
ATOM	733	C	ILE	99	46.642	29.312	44.887	1.00	0.00	LX0	C
ATOM	734	O	ILE	99	45.567	29.570	44.358	1.00	0.00	LX0	O
ATOM	735	N	PRO	100	47.829	29.428	44.252	1.00	0.00	LX0	N
ATOM	736	CD	PRO	100	49.143	29.110	44.789	1.00	0.00	LX0	C
ATOM	737	CA	PRO	100	47.909	30.045	42.926	1.00	0.00	LX0	C
ATOM	738	CB	PRO	100	49.386	30.450	42.830	1.00	0.00	LX0	C
ATOM	739	CG	PRO	100	50.008	30.209	44.202	1.00	0.00	LX0	C
ATOM	740	C	PRO	100	47.506	29.158	41.756	1.00	0.00	LX0	C
ATOM	741	O	PRO	100	48.214	29.087	40.759	1.00	0.00	LX0	O
ATOM	742	N	LEU	101	46.339	28.505	41.869	1.00	0.00	LX0	N
ATOM	743	H	LEU	101	45.743	28.669	42.656	0.00	0.00	LX0	H
ATOM	744	CA	LEU	101	45.871	27.734	40.713	1.00	0.00	LX0	C

ATOM	745	CB	LEU	101	44.953	26.603	41.173	1.00	0.00	LX0	C
ATOM	746	CG	LEU	101	45.407	25.203	40.755	1.00	0.00	LX0	C
ATOM	747	CD1	LEU	101	44.315	24.182	41.070	1.00	0.00	LX0	C
ATOM	748	CD2	LEU	101	45.870	25.118	39.297	1.00	0.00	LX0	C
ATOM	749	C	LEU	101	45.154	28.600	39.686	1.00	0.00	LX0	C
ATOM	750	O	LEU	101	44.080	28.306	39.180	1.00	0.00	LX0	O
ATOM	751	N	GLU	102	45.779	29.751	39.451	1.00	0.00	LX0	N
ATOM	752	H	GLU	102	46.752	29.838	39.662	0.00	0.00	LX0	H
ATOM	753	CA	GLU	102	44.958	30.858	38.990	1.00	0.00	LX0	C
ATOM	754	CB	GLU	102	45.503	32.146	39.607	1.00	0.00	LX0	C
ATOM	755	CG	GLU	102	46.970	32.455	39.300	1.00	0.00	LX0	C
ATOM	756	CD	GLU	102	47.381	33.693	40.070	1.00	0.00	LX0	C
ATOM	757	OE1	GLU	102	46.841	34.772	39.828	1.00	0.00	LX0	O
ATOM	758	OE2	GLU	102	48.221	33.585	40.954	1.00	0.00	LX0	O
ATOM	759	C	GLU	102	44.730	30.931	37.493	1.00	0.00	LX0	C
ATOM	760	O	GLU	102	43.797	31.553	36.999	1.00	0.00	LX0	O
ATOM	761	N	ASN	103	45.623	30.243	36.771	1.00	0.00	LX0	N
ATOM	762	H	ASN	103	46.330	29.682	37.198	0.00	0.00	LX0	H
ATOM	763	CA	ASN	103	45.402	30.262	35.325	1.00	0.00	LX0	C
ATOM	764	CB	ASN	103	46.684	30.603	34.564	1.00	0.00	LX0	C
ATOM	765	CG	ASN	103	47.112	32.031	34.851	1.00	0.00	LX0	C
ATOM	766	OD1	ASN	103	46.320	32.939	35.091	1.00	0.00	LX0	O
ATOM	767	ND2	ASN	103	48.434	32.199	34.823	1.00	0.00	LX0	N
ATOM	768	HD21	ASN	103	49.030	31.404	34.673	0.00	0.00	LX0	H
ATOM	769	HD22	ASN	103	48.848	33.096	34.951	0.00	0.00	LX0	H
ATOM	770	C	ASN	103	44.769	28.997	34.780	1.00	0.00	LX0	C
ATOM	771	O	ASN	103	44.711	28.777	33.575	1.00	0.00	LX0	O
ATOM	772	N	LEU	104	44.282	28.183	35.734	1.00	0.00	LX0	N
ATOM	773	H	LEU	104	44.293	28.450	36.695	0.00	0.00	LX0	H
ATOM	774	CA	LEU	104	43.594	26.948	35.365	1.00	0.00	LX0	C
ATOM	775	CB	LEU	104	43.217	26.215	36.656	1.00	0.00	LX0	C
ATOM	776	CG	LEU	104	42.652	24.799	36.517	1.00	0.00	LX0	C
ATOM	777	CD1	LEU	104	43.647	23.824	35.888	1.00	0.00	LX0	C
ATOM	778	CD2	LEU	104	42.147	24.285	37.864	1.00	0.00	LX0	C
ATOM	779	C	LEU	104	42.374	27.242	34.520	1.00	0.00	LX0	C
ATOM	780	O	LEU	104	41.721	28.256	34.722	1.00	0.00	LX0	O
ATOM	781	N	GLN	105	42.102	26.337	33.572	1.00	0.00	LX0	N
ATOM	782	H	GLN	105	42.734	25.577	33.432	0.00	0.00	LX0	H
ATOM	783	CA	GLN	105	40.871	26.495	32.803	1.00	0.00	LX0	C
ATOM	784	CB	GLN	105	41.126	26.927	31.362	1.00	0.00	LX0	C
ATOM	785	CG	GLN	105	41.727	28.322	31.214	1.00	0.00	LX0	C
ATOM	786	CD	GLN	105	41.667	28.719	29.757	1.00	0.00	LX0	C
ATOM	787	OE1	GLN	105	40.671	29.219	29.256	1.00	0.00	LX0	O
ATOM	788	NE2	GLN	105	42.790	28.460	29.083	1.00	0.00	LX0	N
ATOM	789	HE21	GLN	105	43.582	28.068	29.546	0.00	0.00	LX0	H
ATOM	790	HE22	GLN	105	42.819	28.669	28.107	0.00	0.00	LX0	H
ATOM	791	C	GLN	105	39.960	25.286	32.811	1.00	0.00	LX0	C
ATOM	792	O	GLN	105	38.741	25.429	32.809	1.00	0.00	LX0	O
ATOM	793	N	ILE	106	40.592	24.097	32.830	1.00	0.00	LX0	N
ATOM	794	H	ILE	106	41.591	24.026	32.821	0.00	0.00	LX0	H
ATOM	795	CA	ILE	106	39.775	22.885	32.846	1.00	0.00	LX0	C
ATOM	796	CB	ILE	106	39.608	22.311	31.423	1.00	0.00	LX0	C
ATOM	797	CG2	ILE	106	40.944	21.878	30.828	1.00	0.00	LX0	C
ATOM	798	CG1	ILE	106	38.587	21.171	31.348	1.00	0.00	LX0	C
ATOM	799	CD1	ILE	106	38.468	20.579	29.945	1.00	0.00	LX0	C
ATOM	800	C	ILE	106	40.278	21.825	33.814	1.00	0.00	LX0	C
ATOM	801	O	ILE	106	41.462	21.512	33.886	1.00	0.00	LX0	O
ATOM	802	N	ILE	107	39.306	21.259	34.536	1.00	0.00	LX0	N
ATOM	803	H	ILE	107	38.374	21.620	34.469	0.00	0.00	LX0	H
ATOM	804	CA	ILE	107	39.558	19.970	35.168	1.00	0.00	LX0	C
ATOM	805	CB	ILE	107	39.187	20.021	36.656	1.00	0.00	LX0	C

ATOM	806	CG2	ILE	107	39.465	18.696	37.376	1.00	0.00	LX0	C
ATOM	807	CG1	ILE	107	39.892	21.195	37.339	1.00	0.00	LX0	C
ATOM	808	CD1	ILE	107	39.387	21.456	38.757	1.00	0.00	LX0	C
ATOM	809	C	ILE	107	38.732	18.931	34.432	1.00	0.00	LX0	C
ATOM	810	O	ILE	107	37.547	19.119	34.182	1.00	0.00	LX0	O
ATOM	811	N	ARG	108	39.405	17.840	34.058	1.00	0.00	LX0	N
ATOM	812	H	ARG	108	40.380	17.748	34.258	0.00	0.00	LX0	H
ATOM	813	CA	ARG	108	38.641	16.795	33.385	1.00	0.00	LX0	C
ATOM	814	CB	ARG	108	39.459	16.173	32.251	1.00	0.00	LX0	C
ATOM	815	CG	ARG	108	39.754	17.212	31.166	1.00	0.00	LX0	C
ATOM	816	CD	ARG	108	40.671	16.723	30.044	1.00	0.00	LX0	C
ATOM	817	NE	ARG	108	40.771	17.733	28.987	1.00	0.00	LX0	N
ATOM	818	HE	ARG	108	39.957	17.928	28.433	0.00	0.00	LX0	H
ATOM	819	CZ	ARG	108	41.901	18.438	28.767	1.00	0.00	LX0	C
ATOM	820	NH1	ARG	108	43.009	18.228	29.469	1.00	0.00	LX0	N
ATOM	821	HH11	ARG	108	43.827	18.776	29.240	0.00	0.00	LX0	H
ATOM	822	HH12	ARG	108	43.054	17.557	30.208	0.00	0.00	LX0	H
ATOM	823	NH2	ARG	108	41.906	19.365	27.824	1.00	0.00	LX0	N
ATOM	824	HH21	ARG	108	42.737	19.923	27.663	0.00	0.00	LX0	H
ATOM	825	HH22	ARG	108	41.091	19.530	27.266	0.00	0.00	LX0	H
ATOM	826	C	ARG	108	38.057	15.757	34.324	1.00	0.00	LX0	C
ATOM	827	O	ARG	108	37.073	15.102	34.005	1.00	0.00	LX0	O
ATOM	828	N	GLY	109	38.680	15.668	35.513	1.00	0.00	LX0	N
ATOM	829	H	GLY	109	39.592	16.055	35.646	0.00	0.00	LX0	H
ATOM	830	CA	GLY	109	38.041	14.942	36.611	1.00	0.00	LX0	C
ATOM	831	C	GLY	109	37.736	13.486	36.333	1.00	0.00	LX0	C
ATOM	832	O	GLY	109	36.690	12.945	36.669	1.00	0.00	LX0	O
ATOM	833	N	ASN	110	38.718	12.868	35.672	1.00	0.00	LX0	N
ATOM	834	H	ASN	110	39.582	13.344	35.508	0.00	0.00	LX0	H
ATOM	835	CA	ASN	110	38.570	11.433	35.457	1.00	0.00	LX0	C
ATOM	836	CB	ASN	110	39.384	10.987	34.233	1.00	0.00	LX0	C
ATOM	837	CG	ASN	110	38.756	11.356	32.884	1.00	0.00	LX0	C
ATOM	838	OD1	ASN	110	39.114	10.795	31.854	1.00	0.00	LX0	O
ATOM	839	ND2	ASN	110	37.804	12.301	32.884	1.00	0.00	LX0	N
ATOM	840	HD21	ASN	110	37.516	12.845	33.674	0.00	0.00	LX0	H
ATOM	841	HD22	ASN	110	37.339	12.492	32.022	0.00	0.00	LX0	H
ATOM	842	C	ASN	110	38.999	10.713	36.720	1.00	0.00	LX0	C
ATOM	843	O	ASN	110	40.007	11.067	37.317	1.00	0.00	LX0	O
ATOM	844	N	MET	111	38.152	9.734	37.096	1.00	0.00	LX0	N
ATOM	845	H	MET	111	37.370	9.571	36.498	0.00	0.00	LX0	H
ATOM	846	CA	MET	111	38.158	9.099	38.421	1.00	0.00	LX0	C
ATOM	847	CB	MET	111	39.476	8.421	38.819	1.00	0.00	LX0	C
ATOM	848	CG	MET	111	39.277	7.177	39.700	1.00	0.00	LX0	C
ATOM	849	SD	MET	111	38.485	7.441	41.300	1.00	0.00	LX0	S
ATOM	850	CE	MET	111	39.820	8.333	42.110	1.00	0.00	LX0	C
ATOM	851	C	MET	111	37.701	10.017	39.531	1.00	0.00	LX0	C
ATOM	852	O	MET	111	38.342	10.988	39.916	1.00	0.00	LX0	O
ATOM	853	N	TYR	112	36.522	9.659	40.040	1.00	0.00	LX0	N
ATOM	854	H	TYR	112	36.063	8.819	39.744	0.00	0.00	LX0	H
ATOM	855	CA	TYR	112	35.967	10.575	41.023	1.00	0.00	LX0	C
ATOM	856	CB	TYR	112	34.441	10.536	41.021	1.00	0.00	LX0	C
ATOM	857	CG	TYR	112	33.789	10.973	39.721	1.00	0.00	LX0	C
ATOM	858	CD1	TYR	112	34.536	11.354	38.581	1.00	0.00	LX0	C
ATOM	859	CE1	TYR	112	33.865	11.656	37.386	1.00	0.00	LX0	C
ATOM	860	CD2	TYR	112	32.382	10.958	39.699	1.00	0.00	LX0	C
ATOM	861	CE2	TYR	112	31.709	11.282	38.515	1.00	0.00	LX0	C
ATOM	862	CZ	TYR	112	32.459	11.599	37.367	1.00	0.00	LX0	C
ATOM	863	OH	TYR	112	31.780	11.848	36.191	1.00	0.00	LX0	O
ATOM	864	HH	TYR	112	31.000	11.302	36.207	0.00	0.00	LX0	H
ATOM	865	C	TYR	112	36.501	10.365	42.423	1.00	0.00	LX0	C
ATOM	866	O	TYR	112	36.274	9.346	43.079	1.00	0.00	LX0	O

ATOM	867	N	TYR	113	37.219	11.409	42.851	1.00	0.00	LX0	N
ATOM	868	H	TYR	113	37.288	12.185	42.221	0.00	0.00	LX0	H
ATOM	869	CA	TYR	113	37.724	11.471	44.217	1.00	0.00	LX0	C
ATOM	870	CB	TYR	113	38.492	12.782	44.431	1.00	0.00	LX0	C
ATOM	871	CG	TYR	113	39.419	12.640	45.614	1.00	0.00	LX0	C
ATOM	872	CD1	TYR	113	40.438	11.670	45.557	1.00	0.00	LX0	C
ATOM	873	CE1	TYR	113	41.256	11.476	46.677	1.00	0.00	LX0	C
ATOM	874	CD2	TYR	113	39.232	13.460	46.744	1.00	0.00	LX0	C
ATOM	875	CE2	TYR	113	40.047	13.258	47.870	1.00	0.00	LX0	C
ATOM	876	CZ	TYR	113	41.028	12.245	47.833	1.00	0.00	LX0	C
ATOM	877	OH	TYR	113	41.782	11.974	48.963	1.00	0.00	LX0	O
ATOM	878	HH	TYR	113	41.614	12.657	49.607	0.00	0.00	LX0	H
ATOM	879	C	TYR	113	36.624	11.288	45.247	1.00	0.00	LX0	C
ATOM	880	O	TYR	113	35.522	11.802	45.098	1.00	0.00	LX0	O
ATOM	881	N	GLU	114	36.973	10.456	46.244	1.00	0.00	LX0	N
ATOM	882	H	GLU	114	37.936	10.190	46.330	0.00	0.00	LX0	H
ATOM	883	CA	GLU	114	36.077	9.951	47.293	1.00	0.00	LX0	C
ATOM	884	CB	GLU	114	36.303	10.653	48.639	1.00	0.00	LX0	C
ATOM	885	CG	GLU	114	37.749	11.032	48.988	1.00	0.00	LX0	C
ATOM	886	CD	GLU	114	38.728	9.867	48.927	1.00	0.00	LX0	C
ATOM	887	OE1	GLU	114	39.217	9.535	47.848	1.00	0.00	LX0	O
ATOM	888	OE2	GLU	114	39.100	9.346	49.972	1.00	0.00	LX0	O
ATOM	889	C	GLU	114	34.593	9.826	46.994	1.00	0.00	LX0	C
ATOM	890	O	GLU	114	33.738	10.082	47.834	1.00	0.00	LX0	O
ATOM	891	N	ASN	115	34.350	9.353	45.753	1.00	0.00	LX0	N
ATOM	892	H	ASN	115	35.144	9.346	45.148	0.00	0.00	LX0	H
ATOM	893	CA	ASN	115	33.006	9.234	45.174	1.00	0.00	LX0	C
ATOM	894	CB	ASN	115	31.950	8.528	46.050	1.00	0.00	LX0	C
ATOM	895	CG	ASN	115	32.506	7.287	46.723	1.00	0.00	LX0	C
ATOM	896	OD1	ASN	115	32.767	6.258	46.111	1.00	0.00	LX0	O
ATOM	897	ND2	ASN	115	32.687	7.433	48.037	1.00	0.00	LX0	N
ATOM	898	HD21	ASN	115	32.583	8.351	48.433	0.00	0.00	LX0	H
ATOM	899	HD22	ASN	115	32.945	6.664	48.618	0.00	0.00	LX0	H
ATOM	900	C	ASN	115	32.468	10.578	44.741	1.00	0.00	LX0	C
ATOM	901	O	ASN	115	32.398	11.537	45.491	1.00	0.00	LX0	O
ATOM	902	N	SER	116	32.099	10.602	43.456	1.00	0.00	LX0	N
ATOM	903	H	SER	116	32.230	9.786	42.895	0.00	0.00	LX0	H
ATOM	904	CA	SER	116	31.484	11.787	42.856	1.00	0.00	LX0	C
ATOM	905	CB	SER	116	30.128	12.132	43.494	1.00	0.00	LX0	C
ATOM	906	OG	SER	116	29.268	12.726	42.514	1.00	0.00	LX0	O
ATOM	907	HG	SER	116	29.760	13.452	42.139	0.00	0.00	LX0	H
ATOM	908	C	SER	116	32.314	13.049	42.639	1.00	0.00	LX0	C
ATOM	909	O	SER	116	31.908	13.884	41.839	1.00	0.00	LX0	O
ATOM	910	N	TYR	117	33.453	13.199	43.334	1.00	0.00	LX0	N
ATOM	911	H	TYR	117	33.816	12.519	43.977	0.00	0.00	LX0	H
ATOM	912	CA	TYR	117	34.091	14.509	43.193	1.00	0.00	LX0	C
ATOM	913	CB	TYR	117	34.443	15.094	44.565	1.00	0.00	LX0	C
ATOM	914	CG	TYR	117	33.240	15.030	45.474	1.00	0.00	LX0	C
ATOM	915	CD1	TYR	117	32.063	15.725	45.121	1.00	0.00	LX0	C
ATOM	916	CE1	TYR	117	30.944	15.632	45.961	1.00	0.00	LX0	C
ATOM	917	CD2	TYR	117	33.333	14.259	46.649	1.00	0.00	LX0	C
ATOM	918	CE2	TYR	117	32.215	14.170	47.492	1.00	0.00	LX0	C
ATOM	919	CZ	TYR	117	31.042	14.865	47.140	1.00	0.00	LX0	C
ATOM	920	OH	TYR	117	29.961	14.813	47.995	1.00	0.00	LX0	O
ATOM	921	HH	TYR	117	29.172	14.759	47.448	0.00	0.00	LX0	H
ATOM	922	C	TYR	117	35.297	14.553	42.280	1.00	0.00	LX0	C
ATOM	923	O	TYR	117	35.845	13.536	41.881	1.00	0.00	LX0	O
ATOM	924	N	ALA	118	35.701	15.791	41.981	1.00	0.00	LX0	N
ATOM	925	H	ALA	118	35.135	16.565	42.262	0.00	0.00	LX0	H
ATOM	926	CA	ALA	118	36.977	15.984	41.298	1.00	0.00	LX0	C
ATOM	927	CB	ALA	118	36.795	16.389	39.837	1.00	0.00	LX0	C

ATOM	928	C	ALA	118	37.783	17.067	41.970	1.00	0.00	LX0	C
ATOM	929	O	ALA	118	38.919	16.876	42.383	1.00	0.00	LX0	O
ATOM	930	N	LEU	119	37.131	18.233	42.065	1.00	0.00	LX0	N
ATOM	931	H	LEU	119	36.177	18.315	41.773	0.00	0.00	LX0	H
ATOM	932	CA	LEU	119	37.802	19.313	42.776	1.00	0.00	LX0	C
ATOM	933	CB	LEU	119	37.423	20.655	42.143	1.00	0.00	LX0	C
ATOM	934	CG	LEU	119	38.097	21.881	42.767	1.00	0.00	LX0	C
ATOM	935	CD1	LEU	119	39.622	21.798	42.735	1.00	0.00	LX0	C
ATOM	936	CD2	LEU	119	37.595	23.173	42.129	1.00	0.00	LX0	C
ATOM	937	C	LEU	119	37.445	19.254	44.245	1.00	0.00	LX0	C
ATOM	938	O	LEU	119	36.378	19.687	44.660	1.00	0.00	LX0	O
ATOM	939	N	ALA	120	38.365	18.659	45.006	1.00	0.00	LX0	N
ATOM	940	H	ALA	120	39.240	18.380	44.602	0.00	0.00	LX0	H
ATOM	941	CA	ALA	120	38.048	18.484	46.417	1.00	0.00	LX0	C
ATOM	942	CB	ALA	120	38.217	17.020	46.821	1.00	0.00	LX0	C
ATOM	943	C	ALA	120	38.882	19.374	47.315	1.00	0.00	LX0	C
ATOM	944	O	ALA	120	40.008	19.063	47.682	1.00	0.00	LX0	O
ATOM	945	N	VAL	121	38.271	20.515	47.641	1.00	0.00	LX0	N
ATOM	946	H	VAL	121	37.329	20.666	47.337	0.00	0.00	LX0	H
ATOM	947	CA	VAL	121	38.947	21.484	48.500	1.00	0.00	LX0	C
ATOM	948	CB	VAL	121	38.609	22.907	48.030	1.00	0.00	LX0	C
ATOM	949	CG1	VAL	121	39.372	23.990	48.795	1.00	0.00	LX0	C
ATOM	950	CG2	VAL	121	38.848	23.039	46.524	1.00	0.00	LX0	C
ATOM	951	C	VAL	121	38.624	21.261	49.974	1.00	0.00	LX0	C
ATOM	952	O	VAL	121	37.726	21.863	50.559	1.00	0.00	LX0	O
ATOM	953	N	LEU	122	39.398	20.329	50.539	1.00	0.00	LX0	N
ATOM	954	H	LEU	122	40.190	19.988	50.027	0.00	0.00	LX0	H
ATOM	955	CA	LEU	122	39.092	19.852	51.886	1.00	0.00	LX0	C
ATOM	956	CB	LEU	122	39.104	18.321	51.921	1.00	0.00	LX0	C
ATOM	957	CG	LEU	122	38.335	17.631	50.791	1.00	0.00	LX0	C
ATOM	958	CD1	LEU	122	38.580	16.122	50.790	1.00	0.00	LX0	C
ATOM	959	CD2	LEU	122	36.845	17.967	50.807	1.00	0.00	LX0	C
ATOM	960	C	LEU	122	40.048	20.358	52.951	1.00	0.00	LX0	C
ATOM	961	O	LEU	122	41.259	20.393	52.770	1.00	0.00	LX0	O
ATOM	962	N	SER	123	39.453	20.718	54.095	1.00	0.00	LX0	N
ATOM	963	H	SER	123	38.454	20.716	54.147	0.00	0.00	LX0	H
ATOM	964	CA	SER	123	40.235	20.831	55.331	1.00	0.00	LX0	C
ATOM	965	CB	SER	123	40.562	19.429	55.850	1.00	0.00	LX0	C
ATOM	966	OG	SER	123	39.404	18.594	55.703	1.00	0.00	LX0	O
ATOM	967	HG	SER	123	39.671	17.718	55.955	0.00	0.00	LX0	H
ATOM	968	C	SER	123	41.477	21.713	55.289	1.00	0.00	LX0	C
ATOM	969	O	SER	123	42.517	21.427	55.866	1.00	0.00	LX0	O
ATOM	970	N	ASN	124	41.330	22.810	54.542	1.00	0.00	LX0	N
ATOM	971	H	ASN	124	40.419	23.053	54.216	0.00	0.00	LX0	H
ATOM	972	CA	ASN	124	42.530	23.610	54.296	1.00	0.00	LX0	C
ATOM	973	CB	ASN	124	42.481	24.260	52.915	1.00	0.00	LX0	C
ATOM	974	CG	ASN	124	42.637	23.216	51.841	1.00	0.00	LX0	C
ATOM	975	OD1	ASN	124	43.693	22.635	51.638	1.00	0.00	LX0	O
ATOM	976	ND2	ASN	124	41.519	22.980	51.161	1.00	0.00	LX0	N
ATOM	977	HD21	ASN	124	40.689	23.516	51.292	0.00	0.00	LX0	H
ATOM	978	HD22	ASN	124	41.543	22.221	50.509	0.00	0.00	LX0	H
ATOM	979	C	ASN	124	42.742	24.681	55.335	1.00	0.00	LX0	C
ATOM	980	O	ASN	124	42.397	25.834	55.107	1.00	0.00	LX0	O
ATOM	981	N	TYR	125	43.289	24.254	56.485	1.00	0.00	LX0	N
ATOM	982	H	TYR	125	43.546	23.293	56.609	0.00	0.00	LX0	H
ATOM	983	CA	TYR	125	43.436	25.208	57.583	1.00	0.00	LX0	C
ATOM	984	CB	TYR	125	42.084	25.464	58.264	1.00	0.00	LX0	C
ATOM	985	CG	TYR	125	41.513	24.209	58.887	1.00	0.00	LX0	C
ATOM	986	CD1	TYR	125	41.851	23.899	60.217	1.00	0.00	LX0	C
ATOM	987	CE1	TYR	125	41.244	22.804	60.843	1.00	0.00	LX0	C
ATOM	988	CD2	TYR	125	40.642	23.396	58.136	1.00	0.00	LX0	C

ATOM	989	CE2	TYR	125	40.023	22.302	58.761	1.00	0.00	LX0	C
ATOM	990	CZ	TYR	125	40.291	22.062	60.122	1.00	0.00	LX0	C
ATOM	991	OH	TYR	125	39.558	21.107	60.800	1.00	0.00	LX0	O
ATOM	992	HH	TYR	125	39.489	20.336	60.238	0.00	0.00	LX0	H
ATOM	993	C	TYR	125	44.482	24.817	58.608	1.00	0.00	LX0	C
ATOM	994	O	TYR	125	44.837	23.652	58.736	1.00	0.00	LX0	O
ATOM	995	N	ASP	126	44.939	25.842	59.342	1.00	0.00	LX0	N
ATOM	996	H	ASP	126	44.669	26.790	59.171	0.00	0.00	LX0	H
ATOM	997	CA	ASP	126	45.790	25.579	60.499	1.00	0.00	LX0	C
ATOM	998	CB	ASP	126	46.869	26.677	60.628	1.00	0.00	LX0	C
ATOM	999	CG	ASP	126	46.356	28.010	61.172	1.00	0.00	LX0	C
ATOM	1000	OD1	ASP	126	47.172	28.823	61.577	1.00	0.00	LX0	O
ATOM	1001	OD2	ASP	126	45.157	28.252	61.211	1.00	0.00	LX0	O
ATOM	1002	C	ASP	126	44.988	25.405	61.781	1.00	0.00	LX0	C
ATOM	1003	O	ASP	126	43.763	25.362	61.775	1.00	0.00	LX0	O
ATOM	1004	N	ALA	127	45.729	25.348	62.903	1.00	0.00	LX0	N
ATOM	1005	H	ALA	127	46.723	25.412	62.838	0.00	0.00	LX0	H
ATOM	1006	CA	ALA	127	45.062	25.277	64.204	1.00	0.00	LX0	C
ATOM	1007	CB	ALA	127	46.096	25.257	65.330	1.00	0.00	LX0	C
ATOM	1008	C	ALA	127	44.067	26.397	64.479	1.00	0.00	LX0	C
ATOM	1009	O	ALA	127	43.054	26.217	65.142	1.00	0.00	LX0	O
ATOM	1010	N	ASN	128	44.387	27.567	63.908	1.00	0.00	LX0	N
ATOM	1011	H	ASN	128	45.142	27.635	63.251	0.00	0.00	LX0	H
ATOM	1012	CA	ASN	128	43.501	28.717	64.099	1.00	0.00	LX0	C
ATOM	1013	CB	ASN	128	44.253	30.046	63.955	1.00	0.00	LX0	C
ATOM	1014	CG	ASN	128	45.446	30.118	64.882	1.00	0.00	LX0	C
ATOM	1015	OD1	ASN	128	45.345	30.356	66.076	1.00	0.00	LX0	O
ATOM	1016	ND2	ASN	128	46.612	29.906	64.266	1.00	0.00	LX0	N
ATOM	1017	HD21	ASN	128	46.654	29.687	63.286	0.00	0.00	LX0	H
ATOM	1018	HD22	ASN	128	47.457	29.966	64.788	0.00	0.00	LX0	H
ATOM	1019	C	ASN	128	42.329	28.753	63.132	1.00	0.00	LX0	C
ATOM	1020	O	ASN	128	41.688	29.780	62.959	1.00	0.00	LX0	O
ATOM	1021	N	LYS	129	42.092	27.602	62.467	1.00	0.00	LX0	N
ATOM	1022	H	LYS	129	42.665	26.803	62.644	0.00	0.00	LX0	H
ATOM	1023	CA	LYS	129	41.087	27.536	61.403	1.00	0.00	LX0	C
ATOM	1024	CB	LYS	129	39.658	27.604	61.969	1.00	0.00	LX0	C
ATOM	1025	CG	LYS	129	39.193	26.403	62.806	1.00	0.00	LX0	C
ATOM	1026	CD	LYS	129	38.901	25.141	61.985	1.00	0.00	LX0	C
ATOM	1027	CE	LYS	129	38.263	24.015	62.811	1.00	0.00	LX0	C
ATOM	1028	NZ	LYS	129	37.979	22.838	61.971	1.00	0.00	LX0	N
ATOM	1029	HZ1	LYS	129	37.280	22.220	62.422	0.00	0.00	LX0	H
ATOM	1030	HZ2	LYS	129	37.595	23.109	61.041	0.00	0.00	LX0	H
ATOM	1031	HZ3	LYS	129	38.834	22.268	61.795	0.00	0.00	LX0	H
ATOM	1032	C	LYS	129	41.307	28.548	60.284	1.00	0.00	LX0	C
ATOM	1033	O	LYS	129	40.381	29.102	59.704	1.00	0.00	LX0	O
ATOM	1034	N	THR	130	42.598	28.752	59.994	1.00	0.00	LX0	N
ATOM	1035	H	THR	130	43.321	28.306	60.523	0.00	0.00	LX0	H
ATOM	1036	CA	THR	130	42.926	29.603	58.858	1.00	0.00	LX0	C
ATOM	1037	CB	THR	130	43.503	30.958	59.312	1.00	0.00	LX0	C
ATOM	1038	OG1	THR	130	44.759	30.813	60.002	1.00	0.00	LX0	O
ATOM	1039	HG1	THR	130	44.678	30.044	60.573	0.00	0.00	LX0	H
ATOM	1040	CG2	THR	130	42.511	31.765	60.154	1.00	0.00	LX0	C
ATOM	1041	C	THR	130	43.877	28.896	57.914	1.00	0.00	LX0	C
ATOM	1042	O	THR	130	44.992	28.540	58.274	1.00	0.00	LX0	O
ATOM	1043	N	GLY	131	43.399	28.679	56.694	1.00	0.00	LX0	N
ATOM	1044	H	GLY	131	42.433	28.816	56.464	0.00	0.00	LX0	H
ATOM	1045	CA	GLY	131	44.362	28.174	55.726	1.00	0.00	LX0	C
ATOM	1046	C	GLY	131	44.146	28.821	54.391	1.00	0.00	LX0	C
ATOM	1047	O	GLY	131	44.401	29.999	54.195	1.00	0.00	LX0	O
ATOM	1048	N	LEU	132	43.623	28.005	53.473	1.00	0.00	LX0	N
ATOM	1049	H	LEU	132	43.310	27.100	53.764	0.00	0.00	LX0	H

ATOM	1050	CA	LEU	132	43.321	28.589	52.170	1.00	0.00	LX0	C
ATOM	1051	CB	LEU	132	42.923	27.480	51.192	1.00	0.00	LX0	C
ATOM	1052	CG	LEU	132	42.429	27.936	49.816	1.00	0.00	LX0	C
ATOM	1053	CD1	LEU	132	43.400	28.881	49.113	1.00	0.00	LX0	C
ATOM	1054	CD2	LEU	132	42.051	26.738	48.949	1.00	0.00	LX0	C
ATOM	1055	C	LEU	132	42.267	29.680	52.255	1.00	0.00	LX0	C
ATOM	1056	O	LEU	132	41.098	29.428	52.507	1.00	0.00	LX0	O
ATOM	1057	N	LYS	133	42.757	30.901	52.027	1.00	0.00	LX0	N
ATOM	1058	H	LYS	133	43.741	31.003	51.885	0.00	0.00	LX0	H
ATOM	1059	CA	LYS	133	41.857	32.040	51.940	1.00	0.00	LX0	C
ATOM	1060	CB	LYS	133	42.503	33.267	52.586	1.00	0.00	LX0	C
ATOM	1061	CG	LYS	133	41.537	34.447	52.664	1.00	0.00	LX0	C
ATOM	1062	CD	LYS	133	42.201	35.745	53.107	1.00	0.00	LX0	C
ATOM	1063	CE	LYS	133	41.194	36.892	53.162	1.00	0.00	LX0	C
ATOM	1064	NZ	LYS	133	40.601	37.130	51.841	1.00	0.00	LX0	N
ATOM	1065	HZ1	LYS	133	39.859	37.864	51.868	0.00	0.00	LX0	H
ATOM	1066	HZ2	LYS	133	41.323	37.448	51.161	0.00	0.00	LX0	H
ATOM	1067	HZ3	LYS	133	40.223	36.275	51.380	0.00	0.00	LX0	H
ATOM	1068	C	LYS	133	41.443	32.326	50.510	1.00	0.00	LX0	C
ATOM	1069	O	LYS	133	40.284	32.217	50.137	1.00	0.00	LX0	O
ATOM	1070	N	GLU	134	42.448	32.704	49.713	1.00	0.00	LX0	N
ATOM	1071	H	GLU	134	43.397	32.680	50.028	0.00	0.00	LX0	H
ATOM	1072	CA	GLU	134	42.094	33.011	48.336	1.00	0.00	LX0	C
ATOM	1073	CB	GLU	134	42.623	34.375	47.895	1.00	0.00	LX0	C
ATOM	1074	CG	GLU	134	41.526	35.441	47.776	1.00	0.00	LX0	C
ATOM	1075	CD	GLU	134	41.178	36.039	49.127	1.00	0.00	LX0	C
ATOM	1076	OE1	GLU	134	40.044	35.925	49.579	1.00	0.00	LX0	O
ATOM	1077	OE2	GLU	134	42.018	36.712	49.713	1.00	0.00	LX0	O
ATOM	1078	C	GLU	134	42.534	31.946	47.362	1.00	0.00	LX0	C
ATOM	1079	O	GLU	134	43.708	31.617	47.214	1.00	0.00	LX0	O
ATOM	1080	N	LEU	135	41.508	31.435	46.674	1.00	0.00	LX0	N
ATOM	1081	H	LEU	135	40.580	31.717	46.920	0.00	0.00	LX0	H
ATOM	1082	CA	LEU	135	41.768	30.511	45.573	1.00	0.00	LX0	C
ATOM	1083	CB	LEU	135	41.100	29.176	45.917	1.00	0.00	LX0	C
ATOM	1084	CG	LEU	135	41.293	28.020	44.935	1.00	0.00	LX0	C
ATOM	1085	CD1	LEU	135	42.764	27.699	44.676	1.00	0.00	LX0	C
ATOM	1086	CD2	LEU	135	40.514	26.788	45.397	1.00	0.00	LX0	C
ATOM	1087	C	LEU	135	41.278	31.103	44.256	1.00	0.00	LX0	C
ATOM	1088	O	LEU	135	40.154	30.881	43.823	1.00	0.00	LX0	O
ATOM	1089	N	PRO	136	42.153	31.926	43.630	1.00	0.00	LX0	N
ATOM	1090	CD	PRO	136	43.534	32.209	43.992	1.00	0.00	LX0	C
ATOM	1091	CA	PRO	136	41.697	32.751	42.508	1.00	0.00	LX0	C
ATOM	1092	CB	PRO	136	42.737	33.883	42.477	1.00	0.00	LX0	C
ATOM	1093	CG	PRO	136	43.661	33.692	43.683	1.00	0.00	LX0	C
ATOM	1094	C	PRO	136	41.627	32.027	41.170	1.00	0.00	LX0	C
ATOM	1095	O	PRO	136	42.344	32.369	40.238	1.00	0.00	LX0	O
ATOM	1096	N	MET	137	40.742	31.028	41.073	1.00	0.00	LX0	N
ATOM	1097	H	MET	137	40.129	30.819	41.839	0.00	0.00	LX0	H
ATOM	1098	CA	MET	137	40.697	30.297	39.802	1.00	0.00	LX0	C
ATOM	1099	CB	MET	137	40.308	28.836	40.031	1.00	0.00	LX0	C
ATOM	1100	CG	MET	137	41.269	28.091	40.956	1.00	0.00	LX0	C
ATOM	1101	SD	MET	137	40.820	26.364	41.189	1.00	0.00	LX0	S
ATOM	1102	CE	MET	137	39.189	26.629	41.900	1.00	0.00	LX0	C
ATOM	1103	C	MET	137	39.823	30.924	38.722	1.00	0.00	LX0	C
ATOM	1104	O	MET	137	38.961	30.296	38.125	1.00	0.00	LX0	O
ATOM	1105	N	ARG	138	40.094	32.213	38.478	1.00	0.00	LX0	N
ATOM	1106	H	ARG	138	40.877	32.608	38.956	0.00	0.00	LX0	H
ATOM	1107	CA	ARG	138	39.264	33.000	37.557	1.00	0.00	LX0	C
ATOM	1108	CB	ARG	138	39.704	34.471	37.495	1.00	0.00	LX0	C
ATOM	1109	CG	ARG	138	41.002	34.805	36.738	1.00	0.00	LX0	C
ATOM	1110	CD	ARG	138	42.227	34.282	37.476	1.00	0.00	LX0	C

ATOM	1111	NE	ARG	138	43.494	34.481	36.785	1.00	0.00	LX0	N
ATOM	1112	HE	ARG	138	43.612	34.110	35.863	0.00	0.00	LX0	H
ATOM	1113	CZ	ARG	138	44.539	34.823	37.563	1.00	0.00	LX0	C
ATOM	1114	NH1	ARG	138	44.346	35.302	38.790	1.00	0.00	LX0	N
ATOM	1115	HH11	ARG	138	45.143	35.420	39.391	0.00	0.00	LX0	H
ATOM	1116	HH12	ARG	138	43.428	35.533	39.139	0.00	0.00	LX0	H
ATOM	1117	NH2	ARG	138	45.780	34.650	37.128	1.00	0.00	LX0	N
ATOM	1118	HH21	ARG	138	46.549	34.872	37.738	0.00	0.00	LX0	H
ATOM	1119	HH22	ARG	138	45.970	34.261	36.218	0.00	0.00	LX0	H
ATOM	1120	C	ARG	138	39.089	32.487	36.139	1.00	0.00	LX0	C
ATOM	1121	O	ARG	138	38.121	32.803	35.457	1.00	0.00	LX0	O
ATOM	1122	N	ASN	139	40.095	31.718	35.708	1.00	0.00	LX0	N
ATOM	1123	H	ASN	139	40.835	31.431	36.316	0.00	0.00	LX0	H
ATOM	1124	CA	ASN	139	40.012	31.268	34.322	1.00	0.00	LX0	C
ATOM	1125	CB	ASN	139	41.382	31.308	33.642	1.00	0.00	LX0	C
ATOM	1126	CG	ASN	139	41.860	32.734	33.466	1.00	0.00	LX0	C
ATOM	1127	OD1	ASN	139	41.128	33.707	33.598	1.00	0.00	LX0	O
ATOM	1128	ND2	ASN	139	43.157	32.822	33.158	1.00	0.00	LX0	N
ATOM	1129	HD21	ASN	139	43.721	31.999	33.091	0.00	0.00	LX0	H
ATOM	1130	HD22	ASN	139	43.554	33.721	32.984	0.00	0.00	LX0	H
ATOM	1131	C	ASN	139	39.386	29.899	34.172	1.00	0.00	LX0	C
ATOM	1132	O	ASN	139	39.085	29.447	33.071	1.00	0.00	LX0	O
ATOM	1133	N	LEU	140	39.195	29.258	35.342	1.00	0.00	LX0	N
ATOM	1134	H	LEU	140	39.309	29.723	36.218	0.00	0.00	LX0	H
ATOM	1135	CA	LEU	140	38.647	27.909	35.348	1.00	0.00	LX0	C
ATOM	1136	CB	LEU	140	38.853	27.273	36.729	1.00	0.00	LX0	C
ATOM	1137	CG	LEU	140	38.207	25.898	36.951	1.00	0.00	LX0	C
ATOM	1138	CD1	LEU	140	38.777	24.809	36.049	1.00	0.00	LX0	C
ATOM	1139	CD2	LEU	140	38.246	25.487	38.420	1.00	0.00	LX0	C
ATOM	1140	C	LEU	140	37.195	27.972	34.965	1.00	0.00	LX0	C
ATOM	1141	O	LEU	140	36.338	28.318	35.760	1.00	0.00	LX0	O
ATOM	1142	N	GLN	141	36.965	27.655	33.693	1.00	0.00	LX0	N
ATOM	1143	H	GLN	141	37.726	27.380	33.104	0.00	0.00	LX0	H
ATOM	1144	CA	GLN	141	35.581	27.722	33.256	1.00	0.00	LX0	C
ATOM	1145	CB	GLN	141	35.448	28.572	32.001	1.00	0.00	LX0	C
ATOM	1146	CG	GLN	141	35.907	30.018	32.163	1.00	0.00	LX0	C
ATOM	1147	CD	GLN	141	36.275	30.534	30.791	1.00	0.00	LX0	C
ATOM	1148	OE1	GLN	141	35.441	30.962	30.003	1.00	0.00	LX0	O
ATOM	1149	NE2	GLN	141	37.580	30.438	30.525	1.00	0.00	LX0	N
ATOM	1150	HE21	GLN	141	38.215	30.081	31.215	0.00	0.00	LX0	H
ATOM	1151	HE22	GLN	141	37.946	30.697	29.632	0.00	0.00	LX0	H
ATOM	1152	C	GLN	141	34.969	26.364	33.028	1.00	0.00	LX0	C
ATOM	1153	O	GLN	141	33.765	26.253	32.834	1.00	0.00	LX0	O
ATOM	1154	N	GLU	142	35.832	25.340	33.046	1.00	0.00	LX0	N
ATOM	1155	H	GLU	142	36.816	25.443	33.205	0.00	0.00	LX0	H
ATOM	1156	CA	GLU	142	35.296	24.020	32.754	1.00	0.00	LX0	C
ATOM	1157	CB	GLU	142	35.691	23.588	31.344	1.00	0.00	LX0	C
ATOM	1158	CG	GLU	142	34.806	22.473	30.780	1.00	0.00	LX0	C
ATOM	1159	CD	GLU	142	33.495	23.075	30.333	1.00	0.00	LX0	C
ATOM	1160	OE1	GLU	142	33.459	23.648	29.248	1.00	0.00	LX0	O
ATOM	1161	OE2	GLU	142	32.521	23.042	31.083	1.00	0.00	LX0	O
ATOM	1162	C	GLU	142	35.754	22.977	33.745	1.00	0.00	LX0	C
ATOM	1163	O	GLU	142	36.941	22.781	33.970	1.00	0.00	LX0	O
ATOM	1164	N	ILE	143	34.765	22.287	34.310	1.00	0.00	LX0	N
ATOM	1165	H	ILE	143	33.815	22.540	34.115	0.00	0.00	LX0	H
ATOM	1166	CA	ILE	143	35.091	20.990	34.891	1.00	0.00	LX0	C
ATOM	1167	CB	ILE	143	34.939	20.960	36.422	1.00	0.00	LX0	C
ATOM	1168	CG2	ILE	143	35.308	19.582	36.985	1.00	0.00	LX0	C
ATOM	1169	CG1	ILE	143	35.747	22.067	37.102	1.00	0.00	LX0	C
ATOM	1170	CD1	ILE	143	35.547	22.118	38.617	1.00	0.00	LX0	C
ATOM	1171	C	ILE	143	34.171	19.990	34.231	1.00	0.00	LX0	C

ATOM	1172	O	ILE	143	32.966	19.999	34.451	1.00	0.00	LX0	O
ATOM	1173	N	LEU	144	34.787	19.174	33.367	1.00	0.00	LX0	N
ATOM	1174	H	LEU	144	35.788	19.167	33.344	0.00	0.00	LX0	H
ATOM	1175	CA	LEU	144	33.955	18.248	32.603	1.00	0.00	LX0	C
ATOM	1176	CB	LEU	144	34.737	17.637	31.439	1.00	0.00	LX0	C
ATOM	1177	CG	LEU	144	34.865	18.579	30.243	1.00	0.00	LX0	C
ATOM	1178	CD1	LEU	144	35.803	18.007	29.182	1.00	0.00	LX0	C
ATOM	1179	CD2	LEU	144	33.499	18.939	29.652	1.00	0.00	LX0	C
ATOM	1180	C	LEU	144	33.346	17.157	33.453	1.00	0.00	LX0	C
ATOM	1181	O	LEU	144	32.137	16.984	33.516	1.00	0.00	LX0	O
ATOM	1182	N	HIS	145	34.250	16.424	34.110	1.00	0.00	LX0	N
ATOM	1183	H	HIS	145	35.235	16.590	34.078	0.00	0.00	LX0	H
ATOM	1184	CA	HIS	145	33.732	15.385	34.981	1.00	0.00	LX0	C
ATOM	1185	CB	HIS	145	34.027	13.995	34.412	1.00	0.00	LX0	C
ATOM	1186	CG	HIS	145	33.006	13.691	33.342	1.00	0.00	LX0	C
ATOM	1187	ND1	HIS	145	31.824	13.109	33.602	1.00	0.00	LX0	N
ATOM	1188	HD1	HIS	145	31.526	12.800	34.485	0.00	0.00	LX0	H
ATOM	1189	CD2	HIS	145	33.080	13.962	31.974	1.00	0.00	LX0	C
ATOM	1190	NE2	HIS	145	31.920	13.537	31.414	1.00	0.00	LX0	N
ATOM	1191	CE1	HIS	145	31.145	13.013	32.416	1.00	0.00	LX0	C
ATOM	1192	C	HIS	145	34.222	15.558	36.394	1.00	0.00	LX0	C
ATOM	1193	O	HIS	145	35.178	16.275	36.665	1.00	0.00	LX0	O
ATOM	1194	N	GLY	146	33.482	14.891	37.285	1.00	0.00	LX0	N
ATOM	1195	H	GLY	146	32.740	14.317	36.948	0.00	0.00	LX0	H
ATOM	1196	CA	GLY	146	33.684	15.146	38.703	1.00	0.00	LX0	C
ATOM	1197	C	GLY	146	32.978	16.400	39.179	1.00	0.00	LX0	C
ATOM	1198	O	GLY	146	32.979	17.446	38.546	1.00	0.00	LX0	O
ATOM	1199	N	ALA	147	32.340	16.233	40.337	1.00	0.00	LX0	N
ATOM	1200	H	ALA	147	32.400	15.361	40.816	0.00	0.00	LX0	H
ATOM	1201	CA	ALA	147	31.667	17.379	40.932	1.00	0.00	LX0	C
ATOM	1202	CB	ALA	147	30.460	16.914	41.748	1.00	0.00	LX0	C
ATOM	1203	C	ALA	147	32.606	18.182	41.810	1.00	0.00	LX0	C
ATOM	1204	O	ALA	147	33.765	17.828	42.018	1.00	0.00	LX0	O
ATOM	1205	N	VAL	148	32.060	19.292	42.316	1.00	0.00	LX0	N
ATOM	1206	H	VAL	148	31.087	19.490	42.211	0.00	0.00	LX0	H
ATOM	1207	CA	VAL	148	32.908	20.089	43.190	1.00	0.00	LX0	C
ATOM	1208	CB	VAL	148	32.802	21.571	42.786	1.00	0.00	LX0	C
ATOM	1209	CG1	VAL	148	31.456	22.204	43.153	1.00	0.00	LX0	C
ATOM	1210	CG2	VAL	148	34.001	22.373	43.281	1.00	0.00	LX0	C
ATOM	1211	C	VAL	148	32.593	19.827	44.657	1.00	0.00	LX0	C
ATOM	1212	O	VAL	148	31.449	19.586	45.030	1.00	0.00	LX0	O
ATOM	1213	N	ARG	149	33.666	19.858	45.452	1.00	0.00	LX0	N
ATOM	1214	H	ARG	149	34.578	20.046	45.085	0.00	0.00	LX0	H
ATOM	1215	CA	ARG	149	33.524	19.642	46.884	1.00	0.00	LX0	C
ATOM	1216	CB	ARG	149	34.055	18.256	47.267	1.00	0.00	LX0	C
ATOM	1217	CG	ARG	149	33.738	17.868	48.713	1.00	0.00	LX0	C
ATOM	1218	CD	ARG	149	32.257	17.563	48.905	1.00	0.00	LX0	C
ATOM	1219	NE	ARG	149	31.834	17.780	50.283	1.00	0.00	LX0	N
ATOM	1220	HE	ARG	149	32.107	18.633	50.744	0.00	0.00	LX0	H
ATOM	1221	CZ	ARG	149	30.968	16.984	50.937	1.00	0.00	LX0	C
ATOM	1222	NH1	ARG	149	30.490	15.871	50.383	1.00	0.00	LX0	N
ATOM	1223	HH11	ARG	149	29.871	15.266	50.876	0.00	0.00	LX0	H
ATOM	1224	HH12	ARG	149	30.703	15.616	49.433	0.00	0.00	LX0	H
ATOM	1225	NH2	ARG	149	30.575	17.343	52.152	1.00	0.00	LX0	N
ATOM	1226	HH21	ARG	149	30.007	16.789	52.751	0.00	0.00	LX0	H
ATOM	1227	HH22	ARG	149	30.834	18.270	52.477	0.00	0.00	LX0	H
ATOM	1228	C	ARG	149	34.286	20.708	47.641	1.00	0.00	LX0	C
ATOM	1229	O	ARG	149	35.471	20.923	47.416	1.00	0.00	LX0	O
ATOM	1230	N	PHE	150	33.573	21.360	48.555	1.00	0.00	LX0	N
ATOM	1231	H	PHE	150	32.606	21.136	48.706	0.00	0.00	LX0	H
ATOM	1232	CA	PHE	150	34.286	22.213	49.499	1.00	0.00	LX0	C

ATOM	1233	CB	PHE	150	33.857	23.676	49.373	1.00	0.00	LX0	C
ATOM	1234	CG	PHE	150	34.331	24.258	48.067	1.00	0.00	LX0	C
ATOM	1235	CD1	PHE	150	35.620	24.825	47.996	1.00	0.00	LX0	C
ATOM	1236	CD2	PHE	150	33.477	24.229	46.945	1.00	0.00	LX0	C
ATOM	1237	CE1	PHE	150	36.072	25.359	46.775	1.00	0.00	LX0	C
ATOM	1238	CE2	PHE	150	33.927	24.766	45.726	1.00	0.00	LX0	C
ATOM	1239	CZ	PHE	150	35.225	25.311	45.650	1.00	0.00	LX0	C
ATOM	1240	C	PHE	150	33.909	21.741	50.868	1.00	0.00	LX0	C
ATOM	1241	O	PHE	150	32.728	21.605	51.134	1.00	0.00	LX0	O
ATOM	1242	N	SER	151	34.917	21.492	51.706	1.00	0.00	LX0	N
ATOM	1243	H	SER	151	35.878	21.561	51.434	0.00	0.00	LX0	H
ATOM	1244	CA	SER	151	34.538	21.078	53.053	1.00	0.00	LX0	C
ATOM	1245	CB	SER	151	34.332	19.563	53.117	1.00	0.00	LX0	C
ATOM	1246	OG	SER	151	33.384	19.180	52.114	1.00	0.00	LX0	O
ATOM	1247	HG	SER	151	32.647	19.798	52.227	0.00	0.00	LX0	H
ATOM	1248	C	SER	151	35.538	21.546	54.080	1.00	0.00	LX0	C
ATOM	1249	O	SER	151	36.735	21.612	53.815	1.00	0.00	LX0	O
ATOM	1250	N	ASN	152	34.973	21.903	55.250	1.00	0.00	LX0	N
ATOM	1251	H	ASN	152	33.981	21.778	55.299	0.00	0.00	LX0	H
ATOM	1252	CA	ASN	152	35.685	22.610	56.327	1.00	0.00	LX0	C
ATOM	1253	CB	ASN	152	35.678	21.861	57.669	1.00	0.00	LX0	C
ATOM	1254	CG	ASN	152	35.842	22.854	58.822	1.00	0.00	LX0	C
ATOM	1255	OD1	ASN	152	36.623	22.680	59.757	1.00	0.00	LX0	O
ATOM	1256	ND2	ASN	152	35.034	23.919	58.748	1.00	0.00	LX0	N
ATOM	1257	HD21	ASN	152	34.472	24.113	57.939	0.00	0.00	LX0	H
ATOM	1258	HD22	ASN	152	34.979	24.579	59.493	0.00	0.00	LX0	H
ATOM	1259	C	ASN	152	37.056	23.195	56.041	1.00	0.00	LX0	C
ATOM	1260	O	ASN	152	38.097	22.712	56.465	1.00	0.00	LX0	O
ATOM	1261	N	ASN	153	36.979	24.296	55.302	1.00	0.00	LX0	N
ATOM	1262	H	ASN	153	36.071	24.652	55.067	0.00	0.00	LX0	H
ATOM	1263	CA	ASN	153	38.173	25.029	54.890	1.00	0.00	LX0	C
ATOM	1264	CB	ASN	153	38.314	24.911	53.362	1.00	0.00	LX0	C
ATOM	1265	CG	ASN	153	36.990	25.210	52.666	1.00	0.00	LX0	C
ATOM	1266	OD1	ASN	153	36.179	26.020	53.099	1.00	0.00	LX0	O
ATOM	1267	ND2	ASN	153	36.773	24.472	51.573	1.00	0.00	LX0	N
ATOM	1268	HD21	ASN	153	37.378	23.732	51.271	0.00	0.00	LX0	H
ATOM	1269	HD22	ASN	153	35.946	24.670	51.052	0.00	0.00	LX0	H
ATOM	1270	C	ASN	153	38.171	26.491	55.337	1.00	0.00	LX0	C
ATOM	1271	O	ASN	153	38.179	27.408	54.525	1.00	0.00	LX0	O
ATOM	1272	N	PRO	154	38.138	26.728	56.675	1.00	0.00	LX0	N
ATOM	1273	CD	PRO	154	38.151	25.814	57.806	1.00	0.00	LX0	C
ATOM	1274	CA	PRO	154	38.039	28.119	57.106	1.00	0.00	LX0	C
ATOM	1275	CB	PRO	154	37.765	28.015	58.610	1.00	0.00	LX0	C
ATOM	1276	CG	PRO	154	37.369	26.566	58.868	1.00	0.00	LX0	C
ATOM	1277	C	PRO	154	39.260	28.946	56.742	1.00	0.00	LX0	C
ATOM	1278	O	PRO	154	40.385	28.462	56.671	1.00	0.00	LX0	O
ATOM	1279	N	ALA	155	38.898	30.212	56.467	1.00	0.00	LX0	N
ATOM	1280	H	ALA	155	37.927	30.381	56.613	0.00	0.00	LX0	H
ATOM	1281	CA	ALA	155	39.661	31.258	55.776	1.00	0.00	LX0	C
ATOM	1282	CB	ALA	155	41.188	31.119	55.780	1.00	0.00	LX0	C
ATOM	1283	C	ALA	155	39.194	31.447	54.349	1.00	0.00	LX0	C
ATOM	1284	O	ALA	155	39.238	32.555	53.833	1.00	0.00	LX0	O
ATOM	1285	N	LEU	156	38.676	30.351	53.762	1.00	0.00	LX0	N
ATOM	1286	H	LEU	156	38.777	29.442	54.167	0.00	0.00	LX0	H
ATOM	1287	CA	LEU	156	38.206	30.465	52.381	1.00	0.00	LX0	C
ATOM	1288	CB	LEU	156	37.738	29.104	51.854	1.00	0.00	LX0	C
ATOM	1289	CG	LEU	156	37.657	28.989	50.326	1.00	0.00	LX0	C
ATOM	1290	CD1	LEU	156	38.948	29.409	49.627	1.00	0.00	LX0	C
ATOM	1291	CD2	LEU	156	37.270	27.577	49.896	1.00	0.00	LX0	C
ATOM	1292	C	LEU	156	37.183	31.557	52.130	1.00	0.00	LX0	C
ATOM	1293	O	LEU	156	36.055	31.560	52.616	1.00	0.00	LX0	O

ATOM	1294	N	CYS	157	37.667	32.505	51.336	1.00	0.00	LX0	N
ATOM	1295	H	CYS	157	38.592	32.410	50.964	0.00	0.00	LX0	H
ATOM	1296	CA	CYS	157	36.819	33.590	50.888	1.00	0.00	LX0	C
ATOM	1297	CB	CYS	157	37.435	34.933	51.269	1.00	0.00	LX0	C
ATOM	1298	SG	CYS	157	37.831	35.134	53.024	1.00	0.00	LX0	S
ATOM	1299	C	CYS	157	36.737	33.497	49.387	1.00	0.00	LX0	C
ATOM	1300	O	CYS	157	37.494	32.770	48.755	1.00	0.00	LX0	O
ATOM	1301	N	ASN	158	35.805	34.283	48.834	1.00	0.00	LX0	N
ATOM	1302	H	ASN	158	35.063	34.642	49.405	0.00	0.00	LX0	H
ATOM	1303	CA	ASN	158	35.785	34.598	47.402	1.00	0.00	LX0	C
ATOM	1304	CB	ASN	158	37.095	35.247	46.950	1.00	0.00	LX0	C
ATOM	1305	CG	ASN	158	36.972	36.718	47.272	1.00	0.00	LX0	C
ATOM	1306	OD1	ASN	158	35.946	37.334	47.011	1.00	0.00	LX0	O
ATOM	1307	ND2	ASN	158	38.035	37.265	47.859	1.00	0.00	LX0	N
ATOM	1308	HD21	ASN	158	38.828	36.724	48.155	0.00	0.00	LX0	H
ATOM	1309	HD22	ASN	158	38.025	38.257	48.002	0.00	0.00	LX0	H
ATOM	1310	C	ASN	158	35.258	33.540	46.453	1.00	0.00	LX0	C
ATOM	1311	O	ASN	158	34.420	33.798	45.598	1.00	0.00	LX0	O
ATOM	1312	N	VAL	159	35.720	32.306	46.687	1.00	0.00	LX0	N
ATOM	1313	H	VAL	159	36.458	32.179	47.347	0.00	0.00	LX0	H
ATOM	1314	CA	VAL	159	35.114	31.164	46.002	1.00	0.00	LX0	C
ATOM	1315	CB	VAL	159	35.973	29.909	46.221	1.00	0.00	LX0	C
ATOM	1316	CG1	VAL	159	35.548	28.767	45.299	1.00	0.00	LX0	C
ATOM	1317	CG2	VAL	159	37.460	30.213	46.027	1.00	0.00	LX0	C
ATOM	1318	C	VAL	159	33.653	30.928	46.393	1.00	0.00	LX0	C
ATOM	1319	O	VAL	159	32.868	30.319	45.675	1.00	0.00	LX0	O
ATOM	1320	N	GLU	160	33.314	31.489	47.571	1.00	0.00	LX0	N
ATOM	1321	H	GLU	160	33.985	32.031	48.070	0.00	0.00	LX0	H
ATOM	1322	CA	GLU	160	31.962	31.383	48.125	1.00	0.00	LX0	C
ATOM	1323	CB	GLU	160	31.897	32.161	49.458	1.00	0.00	LX0	C
ATOM	1324	CG	GLU	160	31.535	33.667	49.473	1.00	0.00	LX0	C
ATOM	1325	CD	GLU	160	32.529	34.566	48.747	1.00	0.00	LX0	C
ATOM	1326	OE1	GLU	160	33.203	35.379	49.374	1.00	0.00	LX0	O
ATOM	1327	OE2	GLU	160	32.606	34.519	47.529	1.00	0.00	LX0	O
ATOM	1328	C	GLU	160	30.808	31.735	47.188	1.00	0.00	LX0	C
ATOM	1329	O	GLU	160	29.683	31.274	47.325	1.00	0.00	LX0	O
ATOM	1330	N	SER	161	31.150	32.586	46.217	1.00	0.00	LX0	N
ATOM	1331	H	SER	161	32.094	32.910	46.195	0.00	0.00	LX0	H
ATOM	1332	CA	SER	161	30.109	33.146	45.370	1.00	0.00	LX0	C
ATOM	1333	CB	SER	161	30.368	34.648	45.220	1.00	0.00	LX0	C
ATOM	1334	OG	SER	161	31.735	34.897	44.839	1.00	0.00	LX0	O
ATOM	1335	HG	SER	161	32.291	34.496	45.509	0.00	0.00	LX0	H
ATOM	1336	C	SER	161	29.931	32.478	44.020	1.00	0.00	LX0	C
ATOM	1337	O	SER	161	29.047	32.835	43.250	1.00	0.00	LX0	O
ATOM	1338	N	ILE	162	30.834	31.533	43.722	1.00	0.00	LX0	N
ATOM	1339	H	ILE	162	31.429	31.133	44.423	0.00	0.00	LX0	H
ATOM	1340	CA	ILE	162	30.836	31.061	42.339	1.00	0.00	LX0	C
ATOM	1341	CB	ILE	162	32.209	30.450	41.955	1.00	0.00	LX0	C
ATOM	1342	CG2	ILE	162	32.161	29.546	40.717	1.00	0.00	LX0	C
ATOM	1343	CG1	ILE	162	33.237	31.549	41.662	1.00	0.00	LX0	C
ATOM	1344	CD1	ILE	162	33.775	32.344	42.848	1.00	0.00	LX0	C
ATOM	1345	C	ILE	162	29.670	30.139	42.031	1.00	0.00	LX0	C
ATOM	1346	O	ILE	162	29.474	29.092	42.636	1.00	0.00	LX0	O
ATOM	1347	N	GLN	163	28.907	30.557	41.010	1.00	0.00	LX0	N
ATOM	1348	H	GLN	163	29.068	31.468	40.622	0.00	0.00	LX0	H
ATOM	1349	CA	GLN	163	27.954	29.588	40.479	1.00	0.00	LX0	C
ATOM	1350	CB	GLN	163	26.828	30.276	39.695	1.00	0.00	LX0	C
ATOM	1351	CG	GLN	163	27.279	31.042	38.452	1.00	0.00	LX0	C
ATOM	1352	CD	GLN	163	26.118	31.764	37.818	1.00	0.00	LX0	C
ATOM	1353	OE1	GLN	163	24.949	31.459	38.041	1.00	0.00	LX0	O
ATOM	1354	NE2	GLN	163	26.508	32.791	37.063	1.00	0.00	LX0	N

ATOM	1355	HE21	GLN	163	27.478	32.899	36.833	0.00	0.00	LX0	H
ATOM	1356	HE22	GLN	163	25.857	33.483	36.741	0.00	0.00	LX0	H
ATOM	1357	C	GLN	163	28.641	28.472	39.703	1.00	0.00	LX0	C
ATOM	1358	O	GLN	163	28.912	28.537	38.508	1.00	0.00	LX0	O
ATOM	1359	N	TRP	164	28.925	27.410	40.467	1.00	0.00	LX0	N
ATOM	1360	H	TRP	164	28.808	27.515	41.459	0.00	0.00	LX0	H
ATOM	1361	CA	TRP	164	29.737	26.333	39.901	1.00	0.00	LX0	C
ATOM	1362	CB	TRP	164	30.047	25.280	40.968	1.00	0.00	LX0	C
ATOM	1363	CG	TRP	164	31.177	25.805	41.824	1.00	0.00	LX0	C
ATOM	1364	CD2	TRP	164	32.589	25.785	41.523	1.00	0.00	LX0	C
ATOM	1365	CE2	TRP	164	33.265	26.444	42.603	1.00	0.00	LX0	C
ATOM	1366	CE3	TRP	164	33.329	25.291	40.427	1.00	0.00	LX0	C
ATOM	1367	CD1	TRP	164	31.077	26.449	43.067	1.00	0.00	LX0	C
ATOM	1368	NE1	TRP	164	32.300	26.826	43.531	1.00	0.00	LX0	N
ATOM	1369	HE1	TRP	164	32.463	27.313	44.368	0.00	0.00	LX0	H
ATOM	1370	CZ2	TRP	164	34.669	26.577	42.572	1.00	0.00	LX0	C
ATOM	1371	CZ3	TRP	164	34.732	25.431	40.409	1.00	0.00	LX0	C
ATOM	1372	CH2	TRP	164	35.397	26.077	41.472	1.00	0.00	LX0	C
ATOM	1373	C	TRP	164	29.268	25.706	38.598	1.00	0.00	LX0	C
ATOM	1374	O	TRP	164	30.069	25.214	37.820	1.00	0.00	LX0	O
ATOM	1375	N	ARG	165	27.950	25.787	38.355	1.00	0.00	LX0	N
ATOM	1376	H	ARG	165	27.377	26.224	39.043	0.00	0.00	LX0	H
ATOM	1377	CA	ARG	165	27.416	25.239	37.101	1.00	0.00	LX0	C
ATOM	1378	CB	ARG	165	25.893	25.350	37.083	1.00	0.00	LX0	C
ATOM	1379	CG	ARG	165	25.200	24.725	38.295	1.00	0.00	LX0	C
ATOM	1380	CD	ARG	165	23.688	24.968	38.280	1.00	0.00	LX0	C
ATOM	1381	NE	ARG	165	23.377	26.399	38.318	1.00	0.00	LX0	N
ATOM	1382	HE	ARG	165	23.701	26.960	37.553	0.00	0.00	LX0	H
ATOM	1383	CZ	ARG	165	22.670	26.933	39.339	1.00	0.00	LX0	C
ATOM	1384	NH1	ARG	165	22.214	26.155	40.323	1.00	0.00	LX0	N
ATOM	1385	HH11	ARG	165	21.691	26.520	41.094	0.00	0.00	LX0	H
ATOM	1386	HH12	ARG	165	22.388	25.169	40.307	0.00	0.00	LX0	H
ATOM	1387	NH2	ARG	165	22.433	28.245	39.351	1.00	0.00	LX0	N
ATOM	1388	HH21	ARG	165	21.902	28.679	40.080	0.00	0.00	LX0	H
ATOM	1389	HH22	ARG	165	22.786	28.840	38.625	0.00	0.00	LX0	H
ATOM	1390	C	ARG	165	27.978	25.818	35.802	1.00	0.00	LX0	C
ATOM	1391	O	ARG	165	27.978	25.183	34.755	1.00	0.00	LX0	O
ATOM	1392	N	ASP	166	28.472	27.067	35.900	1.00	0.00	LX0	N
ATOM	1393	H	ASP	166	28.478	27.573	36.764	0.00	0.00	LX0	H
ATOM	1394	CA	ASP	166	29.155	27.586	34.709	1.00	0.00	LX0	C
ATOM	1395	CB	ASP	166	29.416	29.092	34.843	1.00	0.00	LX0	C
ATOM	1396	CG	ASP	166	29.694	29.736	33.493	1.00	0.00	LX0	C
ATOM	1397	OD1	ASP	166	30.797	29.598	32.974	1.00	0.00	LX0	O
ATOM	1398	OD2	ASP	166	28.805	30.385	32.952	1.00	0.00	LX0	O
ATOM	1399	C	ASP	166	30.443	26.824	34.423	1.00	0.00	LX0	C
ATOM	1400	O	ASP	166	30.807	26.473	33.300	1.00	0.00	LX0	O
ATOM	1401	N	ILE	167	31.096	26.540	35.555	1.00	0.00	LX0	N
ATOM	1402	H	ILE	167	30.643	26.694	36.430	0.00	0.00	LX0	H
ATOM	1403	CA	ILE	167	32.386	25.870	35.501	1.00	0.00	LX0	C
ATOM	1404	CB	ILE	167	33.174	26.119	36.797	1.00	0.00	LX0	C
ATOM	1405	CG2	ILE	167	34.624	25.675	36.632	1.00	0.00	LX0	C
ATOM	1406	CG1	ILE	167	33.044	27.559	37.310	1.00	0.00	LX0	C
ATOM	1407	CD1	ILE	167	33.441	28.635	36.299	1.00	0.00	LX0	C
ATOM	1408	C	ILE	167	32.222	24.387	35.223	1.00	0.00	LX0	C
ATOM	1409	O	ILE	167	32.422	23.903	34.115	1.00	0.00	LX0	O
ATOM	1410	N	VAL	168	31.801	23.689	36.290	1.00	0.00	LX0	N
ATOM	1411	H	VAL	168	31.492	24.218	37.078	0.00	0.00	LX0	H
ATOM	1412	CA	VAL	168	31.528	22.254	36.198	1.00	0.00	LX0	C
ATOM	1413	CB	VAL	168	31.102	21.687	37.572	1.00	0.00	LX0	C
ATOM	1414	CG1	VAL	168	31.424	20.198	37.682	1.00	0.00	LX0	C
ATOM	1415	CG2	VAL	168	31.751	22.409	38.755	1.00	0.00	LX0	C

ATOM	1416	C	VAL	168	30.467	22.004	35.135	1.00	0.00	LX0	C
ATOM	1417	O	VAL	168	29.859	22.952	34.647	1.00	0.00	LX0	O
ATOM	1418	N	SER	169	30.265	20.738	34.762	1.00	0.00	LX0	N
ATOM	1419	H	SER	169	30.896	20.012	35.040	0.00	0.00	LX0	H
ATOM	1420	CA	SER	169	29.227	20.508	33.765	1.00	0.00	LX0	C
ATOM	1421	CB	SER	169	29.208	19.023	33.389	1.00	0.00	LX0	C
ATOM	1422	OG	SER	169	28.239	18.302	34.163	1.00	0.00	LX0	O
ATOM	1423	HG	SER	169	27.744	17.819	33.495	0.00	0.00	LX0	H
ATOM	1424	C	SER	169	27.850	21.026	34.176	1.00	0.00	LX0	C
ATOM	1425	O	SER	169	27.594	21.377	35.325	1.00	0.00	LX0	O
ATOM	1426	N	SER	170	26.941	21.002	33.198	1.00	0.00	LX0	N
ATOM	1427	H	SER	170	27.131	20.597	32.301	0.00	0.00	LX0	H
ATOM	1428	CA	SER	170	25.551	21.342	33.500	1.00	0.00	LX0	C
ATOM	1429	CB	SER	170	24.822	21.486	32.165	1.00	0.00	LX0	C
ATOM	1430	OG	SER	170	25.419	20.619	31.185	1.00	0.00	LX0	O
ATOM	1431	HG	SER	170	25.321	19.708	31.490	0.00	0.00	LX0	H
ATOM	1432	C	SER	170	24.814	20.356	34.410	1.00	0.00	LX0	C
ATOM	1433	O	SER	170	23.624	20.482	34.678	1.00	0.00	LX0	O
ATOM	1434	N	ASP	171	25.572	19.335	34.828	1.00	0.00	LX0	N
ATOM	1435	H	ASP	171	26.553	19.331	34.649	0.00	0.00	LX0	H
ATOM	1436	CA	ASP	171	24.963	18.055	35.147	1.00	0.00	LX0	C
ATOM	1437	CB	ASP	171	25.291	17.048	34.027	1.00	0.00	LX0	C
ATOM	1438	CG	ASP	171	25.252	17.727	32.660	1.00	0.00	LX0	C
ATOM	1439	OD1	ASP	171	24.186	17.972	32.107	1.00	0.00	LX0	O
ATOM	1440	OD2	ASP	171	26.306	18.101	32.175	1.00	0.00	LX0	O
ATOM	1441	C	ASP	171	25.416	17.568	36.508	1.00	0.00	LX0	C
ATOM	1442	O	ASP	171	24.673	16.977	37.288	1.00	0.00	LX0	O
ATOM	1443	N	PHE	172	26.676	17.939	36.806	1.00	0.00	LX0	N
ATOM	1444	H	PHE	172	27.263	18.311	36.082	0.00	0.00	LX0	H
ATOM	1445	CA	PHE	172	27.210	17.702	38.151	1.00	0.00	LX0	C
ATOM	1446	CB	PHE	172	28.718	17.906	38.189	1.00	0.00	LX0	C
ATOM	1447	CG	PHE	172	29.374	16.724	37.530	1.00	0.00	LX0	C
ATOM	1448	CD1	PHE	172	29.605	15.559	38.288	1.00	0.00	LX0	C
ATOM	1449	CD2	PHE	172	29.719	16.796	36.166	1.00	0.00	LX0	C
ATOM	1450	CE1	PHE	172	30.147	14.429	37.654	1.00	0.00	LX0	C
ATOM	1451	CE2	PHE	172	30.249	15.663	35.526	1.00	0.00	LX0	C
ATOM	1452	CZ	PHE	172	30.433	14.485	36.274	1.00	0.00	LX0	C
ATOM	1453	C	PHE	172	26.576	18.458	39.302	1.00	0.00	LX0	C
ATOM	1454	O	PHE	172	26.931	18.276	40.460	1.00	0.00	LX0	O
ATOM	1455	N	LEU	173	25.564	19.274	38.953	1.00	0.00	LX0	N
ATOM	1456	H	LEU	173	25.417	19.478	37.988	0.00	0.00	LX0	H
ATOM	1457	CA	LEU	173	24.668	19.767	40.003	1.00	0.00	LX0	C
ATOM	1458	CB	LEU	173	23.559	20.660	39.416	1.00	0.00	LX0	C
ATOM	1459	CG	LEU	173	22.941	20.279	38.057	1.00	0.00	LX0	C
ATOM	1460	CD1	LEU	173	22.109	18.995	38.071	1.00	0.00	LX0	C
ATOM	1461	CD2	LEU	173	22.117	21.440	37.498	1.00	0.00	LX0	C
ATOM	1462	C	LEU	173	24.119	18.701	40.950	1.00	0.00	LX0	C
ATOM	1463	O	LEU	173	23.851	18.950	42.116	1.00	0.00	LX0	O
ATOM	1464	N	SER	174	24.032	17.482	40.393	1.00	0.00	LX0	N
ATOM	1465	H	SER	174	24.280	17.354	39.433	0.00	0.00	LX0	H
ATOM	1466	CA	SER	174	23.660	16.301	41.170	1.00	0.00	LX0	C
ATOM	1467	CB	SER	174	23.709	15.095	40.230	1.00	0.00	LX0	C
ATOM	1468	OG	SER	174	24.857	15.208	39.374	1.00	0.00	LX0	O
ATOM	1469	HG	SER	174	24.559	15.538	38.528	0.00	0.00	LX0	H
ATOM	1470	C	SER	174	24.453	16.052	42.454	1.00	0.00	LX0	C
ATOM	1471	O	SER	174	23.950	15.494	43.425	1.00	0.00	LX0	O
ATOM	1472	N	ASN	175	25.720	16.500	42.434	1.00	0.00	LX0	N
ATOM	1473	H	ASN	175	26.130	16.885	41.605	0.00	0.00	LX0	H
ATOM	1474	CA	ASN	175	26.458	16.404	43.691	1.00	0.00	LX0	C
ATOM	1475	CB	ASN	175	27.106	15.031	43.884	1.00	0.00	LX0	C
ATOM	1476	CG	ASN	175	26.970	14.664	45.349	1.00	0.00	LX0	C

```
ATOM   1477  OD1 ASN   175      27.922  14.559  46.112  1.00  0.00      LX0  O
ATOM   1478  ND2 ASN   175      25.700  14.473  45.721  1.00  0.00      LX0  N
ATOM   1479 HD21 ASN   175      24.951  14.596  45.063  0.00  0.00      LX0  H
ATOM   1480 HD22 ASN   175      25.500  14.214  46.663  0.00  0.00      LX0  H
ATOM   1481  C   ASN   175      27.463  17.505  43.936  1.00  0.00      LX0  C
ATOM   1482  O   ASN   175      28.583  17.281  44.375  1.00  0.00      LX0  O
ATOM   1483  N   MET   176      27.014  18.729  43.631  1.00  0.00      LX0  N
ATOM   1484  H   MET   176      26.074  18.849  43.310  0.00  0.00      LX0  H
ATOM   1485  CA  MET   176      27.912  19.848  43.920  1.00  0.00      LX0  C
ATOM   1486  CB  MET   176      27.641  21.025  42.969  1.00  0.00      LX0  C
ATOM   1487  CG  MET   176      26.213  21.581  42.990  1.00  0.00      LX0  C
ATOM   1488  SD  MET   176      25.978  22.960  41.857  1.00  0.00      LX0  S
ATOM   1489  CE  MET   176      24.302  23.383  42.353  1.00  0.00      LX0  C
ATOM   1490  C   MET   176      27.929  20.249  45.393  1.00  0.00      LX0  C
ATOM   1491  O   MET   176      27.337  21.229  45.830  1.00  0.00      LX0  O
ATOM   1492  N   SER   177      28.630  19.411  46.162  1.00  0.00      LX0  N
ATOM   1493  H   SER   177      29.195  18.705  45.727  0.00  0.00      LX0  H
ATOM   1494  CA  SER   177      28.652  19.654  47.599  1.00  0.00      LX0  C
ATOM   1495  CB  SER   177      28.841  18.326  48.335  1.00  0.00      LX0  C
ATOM   1496  OG  SER   177      28.457  18.411  49.715  1.00  0.00      LX0  O
ATOM   1497  HG  SER   177      29.064  19.018  50.149  0.00  0.00      LX0  H
ATOM   1498  C   SER   177      29.670  20.715  47.974  1.00  0.00      LX0  C
ATOM   1499  O   SER   177      30.817  20.472  48.328  1.00  0.00      LX0  O
ATOM   1500  N   MET   178      29.171  21.943  47.850  1.00  0.00      LX0  N
ATOM   1501  H   MET   178      28.225  22.032  47.534  0.00  0.00      LX0  H
ATOM   1502  CA  MET   178      29.995  23.083  48.226  1.00  0.00      LX0  C
ATOM   1503  CB  MET   178      29.828  24.200  47.181  1.00  0.00      LX0  C
ATOM   1504  CG  MET   178      28.377  24.563  46.835  1.00  0.00      LX0  C
ATOM   1505  SD  MET   178      28.231  25.729  45.468  1.00  0.00      LX0  S
ATOM   1506  CE  MET   178      28.869  27.197  46.292  1.00  0.00      LX0  C
ATOM   1507  C   MET   178      29.741  23.535  49.655  1.00  0.00      LX0  C
ATOM   1508  O   MET   178      29.245  24.625  49.917  1.00  0.00      LX0  O
ATOM   1509  N   ASP   179      30.098  22.634  50.580  1.00  0.00      LX0  N
ATOM   1510  H   ASP   179      30.576  21.778  50.362  0.00  0.00      LX0  H
ATOM   1511  CA  ASP   179      29.912  22.944  51.997  1.00  0.00      LX0  C
ATOM   1512  CB  ASP   179      29.597  21.693  52.856  1.00  0.00      LX0  C
ATOM   1513  CG  ASP   179      30.362  20.425  52.488  1.00  0.00      LX0  C
ATOM   1514  OD1 ASP   179      31.019  19.846  53.348  1.00  0.00      LX0  O
ATOM   1515  OD2 ASP   179      30.255  19.951  51.360  1.00  0.00      LX0  O
ATOM   1516  C   ASP   179      30.981  23.853  52.589  1.00  0.00      LX0  C
ATOM   1517  O   ASP   179      31.887  23.499  53.338  1.00  0.00      LX0  O
ATOM   1518  N   PHE   180      30.789  25.119  52.187  1.00  0.00      LX0  N
ATOM   1519  H   PHE   180      30.008  25.288  51.585  0.00  0.00      LX0  H
ATOM   1520  CA  PHE   180      31.547  26.215  52.777  1.00  0.00      LX0  C
ATOM   1521  CB  PHE   180      31.331  27.518  51.998  1.00  0.00      LX0  C
ATOM   1522  CG  PHE   180      31.997  27.529  50.642  1.00  0.00      LX0  C
ATOM   1523  CD1 PHE   180      31.295  27.054  49.514  1.00  0.00      LX0  C
ATOM   1524  CD2 PHE   180      33.302  28.054  50.522  1.00  0.00      LX0  C
ATOM   1525  CE1 PHE   180      31.896  27.131  48.242  1.00  0.00      LX0  C
ATOM   1526  CE2 PHE   180      33.901  28.133  49.250  1.00  0.00      LX0  C
ATOM   1527  CZ  PHE   180      33.187  27.682  48.122  1.00  0.00      LX0  C
ATOM   1528  C   PHE   180      31.121  26.474  54.208  1.00  0.00      LX0  C
ATOM   1529  O   PHE   180      30.089  26.023  54.688  1.00  0.00      LX0  O
ATOM   1530  N   GLN   181      31.960  27.284  54.851  1.00  0.00      LX0  N
ATOM   1531  H   GLN   181      32.789  27.603  54.391  0.00  0.00      LX0  H
ATOM   1532  CA  GLN   181      31.594  27.852  56.140  1.00  0.00      LX0  C
ATOM   1533  CB  GLN   181      32.267  27.103  57.301  1.00  0.00      LX0  C
ATOM   1534  CG  GLN   181      33.780  27.331  57.460  1.00  0.00      LX0  C
ATOM   1535  CD  GLN   181      34.553  26.690  56.329  1.00  0.00      LX0  C
ATOM   1536  OE1 GLN   181      34.805  25.496  56.357  1.00  0.00      LX0  O
ATOM   1537  NE2 GLN   181      34.942  27.522  55.353  1.00  0.00      LX0  N
```

ATOM	1538	HE21	GLN	181	34.684	28.491	55.353	0.00	0.00	LX0	H
ATOM	1539	HE22	GLN	181	35.487	27.182	54.578	0.00	0.00	LX0	H
ATOM	1540	C	GLN	181	32.041	29.294	56.125	1.00	0.00	LX0	C
ATOM	1541	O	GLN	181	32.829	29.675	55.266	1.00	0.00	LX0	O
ATOM	1542	N	ASN	182	31.563	30.063	57.117	1.00	0.00	LX0	N
ATOM	1543	H	ASN	182	30.943	29.686	57.802	0.00	0.00	LX0	H
ATOM	1544	CA	ASN	182	32.157	31.398	57.219	1.00	0.00	LX0	C
ATOM	1545	CB	ASN	182	31.440	32.272	58.258	1.00	0.00	LX0	C
ATOM	1546	CG	ASN	182	31.933	33.701	58.108	1.00	0.00	LX0	C
ATOM	1547	OD1	ASN	182	32.191	34.167	57.009	1.00	0.00	LX0	O
ATOM	1548	ND2	ASN	182	32.124	34.352	59.257	1.00	0.00	LX0	N
ATOM	1549	HD21	ASN	182	31.897	33.969	60.149	0.00	0.00	LX0	H
ATOM	1550	HD22	ASN	182	32.545	35.256	59.190	0.00	0.00	LX0	H
ATOM	1551	C	ASN	182	33.654	31.351	57.497	1.00	0.00	LX0	C
ATOM	1552	O	ASN	182	34.156	30.495	58.217	1.00	0.00	LX0	O
ATOM	1553	N	HIS	183	34.337	32.298	56.860	1.00	0.00	LX0	N
ATOM	1554	H	HIS	183	33.813	33.022	56.405	0.00	0.00	LX0	H
ATOM	1555	CA	HIS	183	35.771	32.405	57.090	1.00	0.00	LX0	C
ATOM	1556	CB	HIS	183	36.471	32.956	55.842	1.00	0.00	LX0	C
ATOM	1557	CG	HIS	183	35.649	34.020	55.153	1.00	0.00	LX0	C
ATOM	1558	ND1	HIS	183	34.912	33.763	54.062	1.00	0.00	LX0	N
ATOM	1559	HD1	HIS	183	34.869	32.900	53.589	0.00	0.00	LX0	H
ATOM	1560	CD2	HIS	183	35.505	35.369	55.493	1.00	0.00	LX0	C
ATOM	1561	NE2	HIS	183	34.665	35.921	54.584	1.00	0.00	LX0	N
ATOM	1562	CE1	HIS	183	34.296	34.932	53.706	1.00	0.00	LX0	C
ATOM	1563	C	HIS	183	36.091	33.246	58.307	1.00	0.00	LX0	C
ATOM	1564	O	HIS	183	35.306	34.080	58.734	1.00	0.00	LX0	O
ATOM	1565	N	LEU	184	37.302	33.012	58.831	1.00	0.00	LX0	N
ATOM	1566	H	LEU	184	37.905	32.325	58.435	0.00	0.00	LX0	H
ATOM	1567	CA	LEU	184	37.778	33.894	59.900	1.00	0.00	LX0	C
ATOM	1568	CB	LEU	184	38.521	33.097	60.971	1.00	0.00	LX0	C
ATOM	1569	CG	LEU	184	37.697	32.019	61.676	1.00	0.00	LX0	C
ATOM	1570	CD1	LEU	184	38.574	31.236	62.651	1.00	0.00	LX0	C
ATOM	1571	CD2	LEU	184	36.448	32.576	62.364	1.00	0.00	LX0	C
ATOM	1572	C	LEU	184	38.679	34.994	59.374	1.00	0.00	LX0	C
ATOM	1573	O	LEU	184	39.742	35.289	59.912	1.00	0.00	LX0	O
ATOM	1574	N	GLY	185	38.216	35.548	58.252	1.00	0.00	LX0	N
ATOM	1575	H	GLY	185	37.273	35.360	57.976	0.00	0.00	LX0	H
ATOM	1576	CA	GLY	185	39.010	36.535	57.540	1.00	0.00	LX0	C
ATOM	1577	C	GLY	185	38.089	37.558	56.936	1.00	0.00	LX0	C
ATOM	1578	O	GLY	185	37.180	38.070	57.587	1.00	0.00	LX0	O
ATOM	1579	N	SER	186	38.341	37.808	55.644	1.00	0.00	LX0	N
ATOM	1580	H	SER	186	39.087	37.369	55.142	0.00	0.00	LX0	H
ATOM	1581	CA	SER	186	37.446	38.724	54.951	1.00	0.00	LX0	C
ATOM	1582	CB	SER	186	37.907	40.164	55.137	1.00	0.00	LX0	C
ATOM	1583	OG	SER	186	38.160	40.384	56.526	1.00	0.00	LX0	O
ATOM	1584	HG	SER	186	37.511	39.874	57.015	0.00	0.00	LX0	H
ATOM	1585	C	SER	186	37.326	38.421	53.482	1.00	0.00	LX0	C
ATOM	1586	O	SER	186	38.306	38.180	52.783	1.00	0.00	LX0	O
ATOM	1587	N	CYS	187	36.075	38.470	53.041	1.00	0.00	LX0	N
ATOM	1588	H	CYS	187	35.334	38.599	53.701	0.00	0.00	LX0	H
ATOM	1589	CA	CYS	187	35.793	38.349	51.615	1.00	0.00	LX0	C
ATOM	1590	CB	CYS	187	34.381	37.788	51.444	1.00	0.00	LX0	C
ATOM	1591	SG	CYS	187	33.199	38.628	52.534	1.00	0.00	LX0	S
ATOM	1592	C	CYS	187	35.943	39.663	50.873	1.00	0.00	LX0	C
ATOM	1593	O	CYS	187	34.978	40.224	50.372	1.00	0.00	LX0	O
ATOM	1594	N	GLN	188	37.200	40.149	50.815	1.00	0.00	LX0	N
ATOM	1595	H	GLN	188	37.973	39.635	51.191	0.00	0.00	LX0	H
ATOM	1596	CA	GLN	188	37.399	41.356	50.012	1.00	0.00	LX0	C
ATOM	1597	CB	GLN	188	38.804	41.957	50.220	1.00	0.00	LX0	C
ATOM	1598	CG	GLN	188	40.002	41.346	49.474	1.00	0.00	LX0	C

ATOM	1599	CD	GLN	188	40.207	39.886	49.826	1.00	0.00	LX0	C
ATOM	1600	OE1	GLN	188	40.042	39.441	50.957	1.00	0.00	LX0	O
ATOM	1601	NE2	GLN	188	40.590	39.147	48.788	1.00	0.00	LX0	N
ATOM	1602	HE21	GLN	188	40.665	39.526	47.869	0.00	0.00	LX0	H
ATOM	1603	HE22	GLN	188	40.824	38.180	48.918	0.00	0.00	LX0	H
ATOM	1604	C	GLN	188	37.054	41.116	48.552	1.00	0.00	LX0	C
ATOM	1605	O	GLN	188	37.513	40.159	47.937	1.00	0.00	LX0	O
ATOM	1606	N	LYS	189	36.149	41.978	48.079	1.00	0.00	LX0	N
ATOM	1607	H	LYS	189	35.900	42.795	48.594	0.00	0.00	LX0	H
ATOM	1608	CA	LYS	189	35.421	41.561	46.891	1.00	0.00	LX0	C
ATOM	1609	CB	LYS	189	33.925	41.834	47.111	1.00	0.00	LX0	C
ATOM	1610	CG	LYS	189	33.033	40.666	46.671	1.00	0.00	LX0	C
ATOM	1611	CD	LYS	189	33.467	39.336	47.296	1.00	0.00	LX0	C
ATOM	1612	CE	LYS	189	32.621	38.145	46.843	1.00	0.00	LX0	C
ATOM	1613	NZ	LYS	189	33.359	36.904	47.083	1.00	0.00	LX0	N
ATOM	1614	HZ1	LYS	189	32.910	36.091	46.613	0.00	0.00	LX0	H
ATOM	1615	HZ2	LYS	189	34.335	36.982	46.732	0.00	0.00	LX0	H
ATOM	1616	HZ3	LYS	189	33.367	36.625	48.089	0.00	0.00	LX0	H
ATOM	1617	C	LYS	189	36.020	42.033	45.573	1.00	0.00	LX0	C
ATOM	1618	O	LYS	189	37.231	41.965	45.384	1.00	0.00	LX0	O
ATOM	1619	N	CYS	190	35.152	42.474	44.649	1.00	0.00	LX0	N
ATOM	1620	H	CYS	190	34.191	42.699	44.806	0.00	0.00	LX0	H
ATOM	1621	CA	CYS	190	35.659	42.594	43.289	1.00	0.00	LX0	C
ATOM	1622	CB	CYS	190	35.330	41.331	42.495	1.00	0.00	LX0	C
ATOM	1623	SG	CYS	190	35.689	39.817	43.418	1.00	0.00	LX0	S
ATOM	1624	C	CYS	190	35.127	43.813	42.580	1.00	0.00	LX0	C
ATOM	1625	O	CYS	190	34.271	44.519	43.099	1.00	0.00	LX0	O
ATOM	1626	N	ASP	191	35.683	44.014	41.378	1.00	0.00	LX0	N
ATOM	1627	H	ASP	191	36.330	43.360	40.991	0.00	0.00	LX0	H
ATOM	1628	CA	ASP	191	35.187	45.062	40.489	1.00	0.00	LX0	C
ATOM	1629	CB	ASP	191	36.164	45.138	39.301	1.00	0.00	LX0	C
ATOM	1630	CG	ASP	191	35.830	46.291	38.376	1.00	0.00	LX0	C
ATOM	1631	OD1	ASP	191	36.320	47.391	38.610	1.00	0.00	LX0	O
ATOM	1632	OD2	ASP	191	35.050	46.090	37.447	1.00	0.00	LX0	O
ATOM	1633	C	ASP	191	33.763	44.745	40.042	1.00	0.00	LX0	C
ATOM	1634	O	ASP	191	33.383	43.579	39.964	1.00	0.00	LX0	O
ATOM	1635	N	PRO	192	32.966	45.807	39.761	1.00	0.00	LX0	N
ATOM	1636	CD	PRO	192	33.204	47.198	40.142	1.00	0.00	LX0	C
ATOM	1637	CA	PRO	192	31.696	45.641	39.039	1.00	0.00	LX0	C
ATOM	1638	CB	PRO	192	31.279	47.086	38.752	1.00	0.00	LX0	C
ATOM	1639	CG	PRO	192	31.867	47.890	39.907	1.00	0.00	LX0	C
ATOM	1640	C	PRO	192	31.639	44.745	37.796	1.00	0.00	LX0	C
ATOM	1641	O	PRO	192	30.553	44.498	37.287	1.00	0.00	LX0	O
ATOM	1642	N	SER	193	32.794	44.227	37.337	1.00	0.00	LX0	N
ATOM	1643	H	SER	193	33.683	44.577	37.643	0.00	0.00	LX0	H
ATOM	1644	CA	SER	193	32.757	43.123	36.374	1.00	0.00	LX0	C
ATOM	1645	CB	SER	193	34.182	42.649	36.059	1.00	0.00	LX0	C
ATOM	1646	OG	SER	193	34.840	42.200	37.251	1.00	0.00	LX0	O
ATOM	1647	HG	SER	193	35.382	41.453	36.988	0.00	0.00	LX0	H
ATOM	1648	C	SER	193	31.889	41.941	36.797	1.00	0.00	LX0	C
ATOM	1649	O	SER	193	31.277	41.257	35.984	1.00	0.00	LX0	O
ATOM	1650	N	CYS	194	31.855	41.753	38.130	1.00	0.00	LX0	N
ATOM	1651	H	CYS	194	32.492	42.299	38.681	0.00	0.00	LX0	H
ATOM	1652	CA	CYS	194	30.999	40.735	38.745	1.00	0.00	LX0	C
ATOM	1653	CB	CYS	194	30.889	40.955	40.250	1.00	0.00	LX0	C
ATOM	1654	SG	CYS	194	32.483	40.941	41.093	1.00	0.00	LX0	S
ATOM	1655	C	CYS	194	29.582	40.624	38.211	1.00	0.00	LX0	C
ATOM	1656	O	CYS	194	28.756	41.516	38.363	1.00	0.00	LX0	O
ATOM	1657	N	PRO	195	29.317	39.445	37.603	1.00	0.00	LX0	N
ATOM	1658	CD	PRO	195	30.278	38.428	37.201	1.00	0.00	LX0	C
ATOM	1659	CA	PRO	195	27.937	39.093	37.268	1.00	0.00	LX0	C

ATOM	1660	CB	PRO	195	28.136	37.905	36.310	1.00	0.00	LX0	C
ATOM	1661	CG	PRO	195	29.625	37.854	35.958	1.00	0.00	LX0	C
ATOM	1662	C	PRO	195	27.158	38.756	38.541	1.00	0.00	LX0	C
ATOM	1663	O	PRO	195	27.320	39.382	39.581	1.00	0.00	LX0	O
ATOM	1664	N	ASN	196	26.321	37.709	38.461	1.00	0.00	LX0	N
ATOM	1665	H	ASN	196	26.197	37.156	37.635	0.00	0.00	LX0	H
ATOM	1666	CA	ASN	196	25.666	37.280	39.699	1.00	0.00	LX0	C
ATOM	1667	CB	ASN	196	24.384	36.484	39.400	1.00	0.00	LX0	C
ATOM	1668	CG	ASN	196	24.669	35.224	38.599	1.00	0.00	LX0	C
ATOM	1669	OD1	ASN	196	25.157	35.262	37.475	1.00	0.00	LX0	O
ATOM	1670	ND2	ASN	196	24.306	34.099	39.219	1.00	0.00	LX0	N
ATOM	1671	HD21	ASN	196	23.999	34.124	40.168	0.00	0.00	LX0	H
ATOM	1672	HD22	ASN	196	24.377	33.222	38.742	0.00	0.00	LX0	H
ATOM	1673	C	ASN	196	26.579	36.535	40.665	1.00	0.00	LX0	C
ATOM	1674	O	ASN	196	26.589	35.314	40.747	1.00	0.00	LX0	O
ATOM	1675	N	GLY	197	27.344	37.340	41.412	1.00	0.00	LX0	N
ATOM	1676	H	GLY	197	27.376	38.317	41.192	0.00	0.00	LX0	H
ATOM	1677	CA	GLY	197	28.333	36.728	42.290	1.00	0.00	LX0	C
ATOM	1678	C	GLY	197	29.571	36.332	41.515	1.00	0.00	LX0	C
ATOM	1679	O	GLY	197	30.151	37.123	40.780	1.00	0.00	LX0	O
ATOM	1680	N	SER	198	29.939	35.058	41.711	1.00	0.00	LX0	N
ATOM	1681	H	SER	198	29.315	34.457	42.211	0.00	0.00	LX0	H
ATOM	1682	CA	SER	198	31.048	34.476	40.958	1.00	0.00	LX0	C
ATOM	1683	CB	SER	198	30.557	34.132	39.563	1.00	0.00	LX0	C
ATOM	1684	OG	SER	198	29.591	33.076	39.630	1.00	0.00	LX0	O
ATOM	1685	HG	SER	198	29.311	32.965	38.723	0.00	0.00	LX0	H
ATOM	1686	C	SER	198	32.356	35.253	40.915	1.00	0.00	LX0	C
ATOM	1687	O	SER	198	32.966	35.461	39.869	1.00	0.00	LX0	O
ATOM	1688	N	CYS	199	32.762	35.673	42.117	1.00	0.00	LX0	N
ATOM	1689	H	CYS	199	32.278	35.430	42.962	0.00	0.00	LX0	H
ATOM	1690	CA	CYS	199	33.836	36.654	42.140	1.00	0.00	LX0	C
ATOM	1691	CB	CYS	199	33.214	38.018	42.424	1.00	0.00	LX0	C
ATOM	1692	SG	CYS	199	31.921	37.987	43.692	1.00	0.00	LX0	S
ATOM	1693	C	CYS	199	34.975	36.315	43.081	1.00	0.00	LX0	C
ATOM	1694	O	CYS	199	34.865	36.402	44.301	1.00	0.00	LX0	O
ATOM	1695	N	TRP	200	36.094	35.910	42.449	1.00	0.00	LX0	N
ATOM	1696	H	TRP	200	36.219	36.083	41.470	0.00	0.00	LX0	H
ATOM	1697	CA	TRP	200	37.188	35.318	43.225	1.00	0.00	LX0	C
ATOM	1698	CB	TRP	200	38.027	34.292	42.431	1.00	0.00	LX0	C
ATOM	1699	CG	TRP	200	37.307	33.443	41.400	1.00	0.00	LX0	C
ATOM	1700	CD2	TRP	200	37.123	32.010	41.403	1.00	0.00	LX0	C
ATOM	1701	CE2	TRP	200	36.476	31.657	40.169	1.00	0.00	LX0	C
ATOM	1702	CE3	TRP	200	37.436	31.004	42.340	1.00	0.00	LX0	C
ATOM	1703	CD1	TRP	200	36.771	33.865	40.173	1.00	0.00	LX0	C
ATOM	1704	NE1	TRP	200	36.287	32.826	39.444	1.00	0.00	LX0	N
ATOM	1705	HE1	TRP	200	35.884	32.882	38.552	0.00	0.00	LX0	H
ATOM	1706	CZ2	TRP	200	36.181	30.306	39.887	1.00	0.00	LX0	C
ATOM	1707	CZ3	TRP	200	37.132	29.657	42.049	1.00	0.00	LX0	C
ATOM	1708	CH2	TRP	200	36.505	29.311	40.833	1.00	0.00	LX0	C
ATOM	1709	C	TRP	200	38.173	36.329	43.815	1.00	0.00	LX0	C
ATOM	1710	O	TRP	200	39.368	36.068	43.913	1.00	0.00	LX0	O
ATOM	1711	N	GLY	201	37.641	37.501	44.191	1.00	0.00	LX0	N
ATOM	1712	H	GLY	201	36.654	37.639	44.138	0.00	0.00	LX0	H
ATOM	1713	CA	GLY	201	38.546	38.606	44.511	1.00	0.00	LX0	C
ATOM	1714	C	GLY	201	38.849	39.412	43.263	1.00	0.00	LX0	C
ATOM	1715	O	GLY	201	38.935	38.860	42.177	1.00	0.00	LX0	O
ATOM	1716	N	ALA	202	38.987	40.736	43.455	1.00	0.00	LX0	N
ATOM	1717	H	ALA	202	38.764	41.099	44.362	0.00	0.00	LX0	H
ATOM	1718	CA	ALA	202	39.102	41.642	42.303	1.00	0.00	LX0	C
ATOM	1719	CB	ALA	202	39.347	43.079	42.776	1.00	0.00	LX0	C
ATOM	1720	C	ALA	202	40.126	41.300	41.227	1.00	0.00	LX0	C

ATOM	1721	O	ALA	202	39.784	40.998	40.090	1.00	0.00	LX0	O
ATOM	1722	N	GLY	203	41.407	41.380	41.643	1.00	0.00	LX0	N
ATOM	1723	H	GLY	203	41.593	41.665	42.579	0.00	0.00	LX0	H
ATOM	1724	CA	GLY	203	42.495	41.199	40.677	1.00	0.00	LX0	C
ATOM	1725	C	GLY	203	42.370	42.105	39.461	1.00	0.00	LX0	C
ATOM	1726	O	GLY	203	41.797	43.183	39.532	1.00	0.00	LX0	O
ATOM	1727	N	GLU	204	42.896	41.587	38.346	1.00	0.00	LX0	N
ATOM	1728	H	GLU	204	43.361	40.705	38.341	0.00	0.00	LX0	H
ATOM	1729	CA	GLU	204	42.389	42.086	37.074	1.00	0.00	LX0	C
ATOM	1730	CB	GLU	204	43.539	42.403	36.122	1.00	0.00	LX0	C
ATOM	1731	CG	GLU	204	43.098	43.097	34.831	1.00	0.00	LX0	C
ATOM	1732	CD	GLU	204	44.302	43.275	33.932	1.00	0.00	LX0	C
ATOM	1733	OE1	GLU	204	44.542	42.402	33.100	1.00	0.00	LX0	O
ATOM	1734	OE2	GLU	204	44.994	44.282	34.068	1.00	0.00	LX0	O
ATOM	1735	C	GLU	204	41.529	40.957	36.555	1.00	0.00	LX0	C
ATOM	1736	O	GLU	204	41.901	39.798	36.707	1.00	0.00	LX0	O
ATOM	1737	N	GLU	205	40.343	41.346	36.046	1.00	0.00	LX0	N
ATOM	1738	H	GLU	205	40.223	42.326	35.900	0.00	0.00	LX0	H
ATOM	1739	CA	GLU	205	39.210	40.423	35.880	1.00	0.00	LX0	C
ATOM	1740	CB	GLU	205	38.714	40.282	34.433	1.00	0.00	LX0	C
ATOM	1741	CG	GLU	205	37.173	40.232	34.314	1.00	0.00	LX0	C
ATOM	1742	CD	GLU	205	36.539	39.303	35.344	1.00	0.00	LX0	C
ATOM	1743	OE1	GLU	205	36.343	38.122	35.072	1.00	0.00	LX0	O
ATOM	1744	OE2	GLU	205	36.275	39.757	36.450	1.00	0.00	LX0	O
ATOM	1745	C	GLU	205	39.309	39.046	36.516	1.00	0.00	LX0	C
ATOM	1746	O	GLU	205	39.486	38.009	35.874	1.00	0.00	LX0	O
ATOM	1747	N	ASN	206	39.185	39.083	37.839	1.00	0.00	LX0	N
ATOM	1748	H	ASN	206	39.004	39.941	38.325	0.00	0.00	LX0	H
ATOM	1749	CA	ASN	206	39.348	37.823	38.540	1.00	0.00	LX0	C
ATOM	1750	CB	ASN	206	40.445	38.046	39.589	1.00	0.00	LX0	C
ATOM	1751	CG	ASN	206	40.966	36.799	40.266	1.00	0.00	LX0	C
ATOM	1752	OD1	ASN	206	41.958	36.186	39.881	1.00	0.00	LX0	O
ATOM	1753	ND2	ASN	206	40.313	36.525	41.384	1.00	0.00	LX0	N
ATOM	1754	HD21	ASN	206	39.481	37.066	41.548	0.00	0.00	LX0	H
ATOM	1755	HD22	ASN	206	40.562	35.866	42.092	0.00	0.00	LX0	H
ATOM	1756	C	ASN	206	38.008	37.265	39.013	1.00	0.00	LX0	C
ATOM	1757	O	ASN	206	37.841	36.744	40.110	1.00	0.00	LX0	O
ATOM	1758	N	CYS	207	37.045	37.369	38.081	1.00	0.00	LX0	N
ATOM	1759	H	CYS	207	37.138	37.951	37.267	0.00	0.00	LX0	H
ATOM	1760	CA	CYS	207	35.750	36.726	38.278	1.00	0.00	LX0	C
ATOM	1761	CB	CYS	207	34.665	37.802	38.383	1.00	0.00	LX0	C
ATOM	1762	SG	CYS	207	35.010	38.939	39.750	1.00	0.00	LX0	S
ATOM	1763	C	CYS	207	35.466	35.681	37.202	1.00	0.00	LX0	C
ATOM	1764	O	CYS	207	36.349	35.319	36.426	1.00	0.00	LX0	O
ATOM	1765	N	GLN	208	34.225	35.164	37.211	1.00	0.00	LX0	N
ATOM	1766	H	GLN	208	33.547	35.538	37.846	0.00	0.00	LX0	H
ATOM	1767	CA	GLN	208	33.830	34.116	36.263	1.00	0.00	LX0	C
ATOM	1768	CB	GLN	208	32.716	33.300	36.927	1.00	0.00	LX0	C
ATOM	1769	CG	GLN	208	31.996	32.212	36.124	1.00	0.00	LX0	C
ATOM	1770	CD	GLN	208	30.789	31.738	36.920	1.00	0.00	LX0	C
ATOM	1771	OE1	GLN	208	29.749	32.380	36.994	1.00	0.00	LX0	O
ATOM	1772	NE2	GLN	208	30.956	30.567	37.532	1.00	0.00	LX0	N
ATOM	1773	HE21	GLN	208	31.840	30.108	37.530	0.00	0.00	LX0	H
ATOM	1774	HE22	GLN	208	30.174	30.129	37.976	0.00	0.00	LX0	H
ATOM	1775	C	GLN	208	33.381	34.673	34.917	1.00	0.00	LX0	C
ATOM	1776	O	GLN	208	32.543	35.560	34.838	1.00	0.00	LX0	O
ATOM	1777	N	LYS	209	33.950	34.092	33.847	1.00	0.00	LX0	N
ATOM	1778	H	LYS	209	34.581	33.329	33.983	0.00	0.00	LX0	H
ATOM	1779	CA	LYS	209	33.724	34.686	32.522	1.00	0.00	LX0	C
ATOM	1780	CB	LYS	209	34.903	34.397	31.572	1.00	0.00	LX0	C
ATOM	1781	CG	LYS	209	36.317	34.336	32.170	1.00	0.00	LX0	C

ATOM	1782	CD	LYS	209	36.751	35.589	32.932	1.00	0.00	LX0	C
ATOM	1783	CE	LYS	209	38.163	35.478	33.508	1.00	0.00	LX0	C
ATOM	1784	NZ	LYS	209	38.181	36.226	34.764	1.00	0.00	LX0	N
ATOM	1785	HZ1	LYS	209	39.133	36.494	35.091	0.00	0.00	LX0	H
ATOM	1786	HZ2	LYS	209	37.647	37.113	34.664	0.00	0.00	LX0	H
ATOM	1787	HZ3	LYS	209	37.687	35.680	35.497	0.00	0.00	LX0	H
ATOM	1788	C	LYS	209	32.417	34.329	31.803	1.00	0.00	LX0	C
ATOM	1789	O	LYS	209	32.347	34.373	30.574	1.00	0.00	LX0	O
ATOM	1790	N	LEU	210	31.401	33.947	32.609	1.00	0.00	LX0	N
ATOM	1791	H	LEU	210	31.504	34.168	33.578	0.00	0.00	LX0	H
ATOM	1792	CA	LEU	210	30.161	33.291	32.151	1.00	0.00	LX0	C
ATOM	1793	CB	LEU	210	28.913	34.150	32.386	1.00	0.00	LX0	C
ATOM	1794	CG	LEU	210	28.184	33.836	33.695	1.00	0.00	LX0	C
ATOM	1795	CD1	LEU	210	28.902	34.442	34.893	1.00	0.00	LX0	C
ATOM	1796	CD2	LEU	210	26.714	34.255	33.663	1.00	0.00	LX0	C
ATOM	1797	C	LEU	210	30.108	32.756	30.730	1.00	0.00	LX0	C
ATOM	1798	O	LEU	210	29.826	33.478	29.776	1.00	0.00	LX0	O
ATOM	1799	N	THR	211	30.398	31.463	30.618	1.00	0.00	LX0	N
ATOM	1800	H	THR	211	30.606	30.918	31.437	0.00	0.00	LX0	H
ATOM	1801	CA	THR	211	30.364	30.894	29.274	1.00	0.00	LX0	C
ATOM	1802	CB	THR	211	31.797	30.667	28.765	1.00	0.00	LX0	C
ATOM	1803	OG1	THR	211	32.605	30.115	29.810	1.00	0.00	LX0	O
ATOM	1804	HG1	THR	211	33.500	30.094	29.451	0.00	0.00	LX0	H
ATOM	1805	CG2	THR	211	32.458	31.940	28.230	1.00	0.00	LX0	C
ATOM	1806	C	THR	211	29.513	29.637	29.155	1.00	0.00	LX0	C
ATOM	1807	O	THR	211	29.570	28.917	28.165	1.00	0.00	LX0	O
ATOM	1808	N	LYS	212	28.739	29.381	30.221	1.00	0.00	LX0	N
ATOM	1809	H	LYS	212	28.816	29.958	31.038	0.00	0.00	LX0	H
ATOM	1810	CA	LYS	212	27.888	28.191	30.245	1.00	0.00	LX0	C
ATOM	1811	CB	LYS	212	28.460	27.190	31.252	1.00	0.00	LX0	C
ATOM	1812	CG	LYS	212	27.891	25.769	31.287	1.00	0.00	LX0	C
ATOM	1813	CD	LYS	212	28.747	24.766	30.508	1.00	0.00	LX0	C
ATOM	1814	CE	LYS	212	29.328	23.629	31.365	1.00	0.00	LX0	C
ATOM	1815	NZ	LYS	212	30.333	24.101	32.330	1.00	0.00	LX0	N
ATOM	1816	HZ1	LYS	212	30.027	23.879	33.298	0.00	0.00	LX0	H
ATOM	1817	HZ2	LYS	212	31.250	23.621	32.176	0.00	0.00	LX0	H
ATOM	1818	HZ3	LYS	212	30.486	25.128	32.291	0.00	0.00	LX0	H
ATOM	1819	C	LYS	212	26.450	28.540	30.599	1.00	0.00	LX0	C
ATOM	1820	O	LYS	212	25.498	28.180	29.921	1.00	0.00	LX0	O
ATOM	1821	N	ILE	213	26.327	29.293	31.710	1.00	0.00	LX0	N
ATOM	1822	H	ILE	213	27.152	29.563	32.213	0.00	0.00	LX0	H
ATOM	1823	CA	ILE	213	24.985	29.642	32.193	1.00	0.00	LX0	C
ATOM	1824	CB	ILE	213	25.080	30.435	33.515	1.00	0.00	LX0	C
ATOM	1825	CG2	ILE	213	23.732	30.979	34.008	1.00	0.00	LX0	C
ATOM	1826	CG1	ILE	213	25.739	29.582	34.601	1.00	0.00	LX0	C
ATOM	1827	CD1	ILE	213	24.926	28.341	34.978	1.00	0.00	LX0	C
ATOM	1828	C	ILE	213	24.131	30.374	31.165	1.00	0.00	LX0	C
ATOM	1829	O	ILE	213	22.964	30.079	30.951	1.00	0.00	LX0	O
ATOM	1830	N	ILE	214	24.785	31.347	30.523	1.00	0.00	LX0	N
ATOM	1831	H	ILE	214	25.762	31.476	30.679	0.00	0.00	LX0	H
ATOM	1832	CA	ILE	214	24.032	32.085	29.511	1.00	0.00	LX0	C
ATOM	1833	CB	ILE	214	24.257	33.604	29.630	1.00	0.00	LX0	C
ATOM	1834	CG2	ILE	214	23.286	34.162	30.671	1.00	0.00	LX0	C
ATOM	1835	CG1	ILE	214	25.697	34.022	29.962	1.00	0.00	LX0	C
ATOM	1836	CD1	ILE	214	26.761	33.673	28.923	1.00	0.00	LX0	C
ATOM	1837	C	ILE	214	24.199	31.587	28.086	1.00	0.00	LX0	C
ATOM	1838	O	ILE	214	24.311	32.355	27.137	1.00	0.00	LX0	O
ATOM	1839	N	CYS	215	24.213	30.251	27.971	1.00	0.00	LX0	N
ATOM	1840	H	CYS	215	24.075	29.657	28.764	0.00	0.00	LX0	H
ATOM	1841	CA	CYS	215	24.211	29.717	26.613	1.00	0.00	LX0	C
ATOM	1842	CB	CYS	215	24.580	28.233	26.609	1.00	0.00	LX0	C

ATOM	1843	SG	CYS	215	26.318	27.959	27.037	1.00	0.00	LX0	S
ATOM	1844	C	CYS	215	22.893	29.948	25.905	1.00	0.00	LX0	C
ATOM	1845	O	CYS	215	21.827	29.957	26.508	1.00	0.00	LX0	O
ATOM	1846	N	ALA	216	23.015	30.149	24.584	1.00	0.00	LX0	N
ATOM	1847	H	ALA	216	23.911	30.140	24.147	0.00	0.00	LX0	H
ATOM	1848	CA	ALA	216	21.783	30.292	23.817	1.00	0.00	LX0	C
ATOM	1849	CB	ALA	216	22.091	30.713	22.379	1.00	0.00	LX0	C
ATOM	1850	C	ALA	216	20.986	29.002	23.801	1.00	0.00	LX0	C
ATOM	1851	O	ALA	216	21.529	27.917	23.968	1.00	0.00	LX0	O
ATOM	1852	N	GLN	217	19.670	29.178	23.591	1.00	0.00	LX0	N
ATOM	1853	H	GLN	217	19.355	30.119	23.483	0.00	0.00	LX0	H
ATOM	1854	CA	GLN	217	18.735	28.048	23.682	1.00	0.00	LX0	C
ATOM	1855	CB	GLN	217	17.328	28.514	23.304	1.00	0.00	LX0	C
ATOM	1856	CG	GLN	217	16.822	29.652	24.197	1.00	0.00	LX0	C
ATOM	1857	CD	GLN	217	15.522	30.202	23.647	1.00	0.00	LX0	C
ATOM	1858	OE1	GLN	217	15.180	30.037	22.486	1.00	0.00	LX0	O
ATOM	1859	NE2	GLN	217	14.804	30.894	24.535	1.00	0.00	LX0	N
ATOM	1860	HE21	GLN	217	15.091	30.999	25.486	0.00	0.00	LX0	H
ATOM	1861	HE22	GLN	217	13.954	31.304	24.213	0.00	0.00	LX0	H
ATOM	1862	C	GLN	217	19.126	26.810	22.883	1.00	0.00	LX0	C
ATOM	1863	O	GLN	217	18.953	25.674	23.305	1.00	0.00	LX0	O
ATOM	1864	N	GLN	218	19.691	27.103	21.705	1.00	0.00	LX0	N
ATOM	1865	H	GLN	218	19.797	28.050	21.409	0.00	0.00	LX0	H
ATOM	1866	CA	GLN	218	20.459	26.060	21.042	1.00	0.00	LX0	C
ATOM	1867	CB	GLN	218	19.871	25.797	19.647	1.00	0.00	LX0	C
ATOM	1868	CG	GLN	218	20.587	24.739	18.797	1.00	0.00	LX0	C
ATOM	1869	CD	GLN	218	20.636	23.413	19.529	1.00	0.00	LX0	C
ATOM	1870	OE1	GLN	218	21.462	23.193	20.403	1.00	0.00	LX0	O
ATOM	1871	NE2	GLN	218	19.714	22.533	19.131	1.00	0.00	LX0	N
ATOM	1872	HE21	GLN	218	19.053	22.736	18.410	0.00	0.00	LX0	H
ATOM	1873	HE22	GLN	218	19.692	21.642	19.579	0.00	0.00	LX0	H
ATOM	1874	C	GLN	218	21.896	26.539	20.989	1.00	0.00	LX0	C
ATOM	1875	O	GLN	218	22.144	27.692	20.648	1.00	0.00	LX0	O
ATOM	1876	N	CYS	219	22.818	25.644	21.371	1.00	0.00	LX0	N
ATOM	1877	H	CYS	219	22.559	24.696	21.579	0.00	0.00	LX0	H
ATOM	1878	CA	CYS	219	24.204	26.094	21.501	1.00	0.00	LX0	C
ATOM	1879	CB	CYS	219	24.400	26.894	22.794	1.00	0.00	LX0	C
ATOM	1880	SG	CYS	219	25.903	27.905	22.801	1.00	0.00	LX0	S
ATOM	1881	C	CYS	219	25.189	24.950	21.454	1.00	0.00	LX0	C
ATOM	1882	O	CYS	219	24.996	23.909	22.068	1.00	0.00	LX0	O
ATOM	1883	N	SER	220	26.268	25.180	20.699	1.00	0.00	LX0	N
ATOM	1884	H	SER	220	26.413	26.029	20.189	0.00	0.00	LX0	H
ATOM	1885	CA	SER	220	27.291	24.146	20.589	1.00	0.00	LX0	C
ATOM	1886	CB	SER	220	27.990	24.313	19.241	1.00	0.00	LX0	C
ATOM	1887	OG	SER	220	27.939	25.693	18.856	1.00	0.00	LX0	O
ATOM	1888	HG	SER	220	28.842	26.006	18.889	0.00	0.00	LX0	H
ATOM	1889	C	SER	220	28.275	24.104	21.748	1.00	0.00	LX0	C
ATOM	1890	O	SER	220	29.450	24.431	21.629	1.00	0.00	LX0	O
ATOM	1891	N	GLY	221	27.737	23.665	22.891	1.00	0.00	LX0	N
ATOM	1892	H	GLY	221	26.748	23.518	22.951	0.00	0.00	LX0	H
ATOM	1893	CA	GLY	221	28.595	23.647	24.067	1.00	0.00	LX0	C
ATOM	1894	C	GLY	221	28.581	24.979	24.781	1.00	0.00	LX0	C
ATOM	1895	O	GLY	221	27.563	25.408	25.305	1.00	0.00	LX0	O
ATOM	1896	N	ARG	222	29.762	25.605	24.790	1.00	0.00	LX0	N
ATOM	1897	H	ARG	222	30.498	25.271	24.205	0.00	0.00	LX0	H
ATOM	1898	CA	ARG	222	29.832	26.889	25.485	1.00	0.00	LX0	C
ATOM	1899	CB	ARG	222	31.278	27.225	25.873	1.00	0.00	LX0	C
ATOM	1900	CG	ARG	222	32.009	26.155	26.686	1.00	0.00	LX0	C
ATOM	1901	CD	ARG	222	31.446	25.925	28.086	1.00	0.00	LX0	C
ATOM	1902	NE	ARG	222	31.619	27.091	28.953	1.00	0.00	LX0	N
ATOM	1903	HE	ARG	222	31.347	27.986	28.594	0.00	0.00	LX0	H

ATOM	1904	CZ	ARG	222	32.000	26.926	30.237	1.00	0.00	LX0	C
ATOM	1905	NH1	ARG	222	32.400	25.748	30.672	1.00	0.00	LX0	N
ATOM	1906	HH11	ARG	222	32.725	25.639	31.616	0.00	0.00	LX0	H
ATOM	1907	HH12	ARG	222	32.456	24.924	30.088	0.00	0.00	LX0	H
ATOM	1908	NH2	ARG	222	31.981	27.933	31.096	1.00	0.00	LX0	N
ATOM	1909	HH21	ARG	222	32.050	27.785	32.087	0.00	0.00	LX0	H
ATOM	1910	HH22	ARG	222	31.892	28.887	30.792	0.00	0.00	LX0	H
ATOM	1911	C	ARG	222	29.270	28.019	24.641	1.00	0.00	LX0	C
ATOM	1912	O	ARG	222	29.220	27.943	23.418	1.00	0.00	LX0	O
ATOM	1913	N	CYS	223	28.895	29.091	25.338	1.00	0.00	LX0	N
ATOM	1914	H	CYS	223	28.900	29.070	26.341	0.00	0.00	LX0	H
ATOM	1915	CA	CYS	223	28.680	30.340	24.618	1.00	0.00	LX0	C
ATOM	1916	CB	CYS	223	27.370	30.982	25.062	1.00	0.00	LX0	C
ATOM	1917	SG	CYS	223	27.261	31.107	26.866	1.00	0.00	LX0	S
ATOM	1918	C	CYS	223	29.835	31.265	24.910	1.00	0.00	LX0	C
ATOM	1919	O	CYS	223	30.153	31.534	26.063	1.00	0.00	LX0	O
ATOM	1920	N	ARG	224	30.492	31.728	23.840	1.00	0.00	LX0	N
ATOM	1921	H	ARG	224	30.135	31.629	22.910	0.00	0.00	LX0	H
ATOM	1922	CA	ARG	224	31.565	32.647	24.199	1.00	0.00	LX0	C
ATOM	1923	CB	ARG	224	32.805	32.417	23.310	1.00	0.00	LX0	C
ATOM	1924	CG	ARG	224	32.781	33.012	21.904	1.00	0.00	LX0	C
ATOM	1925	CD	ARG	224	33.590	32.246	20.848	1.00	0.00	LX0	C
ATOM	1926	NE	ARG	224	33.796	33.080	19.664	1.00	0.00	LX0	N
ATOM	1927	HE	ARG	224	34.678	33.555	19.583	0.00	0.00	LX0	H
ATOM	1928	CZ	ARG	224	32.727	33.454	18.934	1.00	0.00	LX0	C
ATOM	1929	NH1	ARG	224	31.619	32.728	18.921	1.00	0.00	LX0	N
ATOM	1930	HH11	ARG	224	30.801	33.143	18.506	0.00	0.00	LX0	H
ATOM	1931	HH12	ARG	224	31.547	31.813	19.326	0.00	0.00	LX0	H
ATOM	1932	NH2	ARG	224	32.753	34.585	18.245	1.00	0.00	LX0	N
ATOM	1933	HH21	ARG	224	31.861	34.934	17.918	0.00	0.00	LX0	H
ATOM	1934	HH22	ARG	224	33.584	35.115	18.094	0.00	0.00	LX0	H
ATOM	1935	C	ARG	224	31.038	34.072	24.318	1.00	0.00	LX0	C
ATOM	1936	O	ARG	224	31.215	34.754	25.333	1.00	0.00	LX0	O
ATOM	1937	N	GLY	225	30.284	34.426	23.255	1.00	0.00	LX0	N
ATOM	1938	H	GLY	225	30.198	33.815	22.464	0.00	0.00	LX0	H
ATOM	1939	CA	GLY	225	29.409	35.589	23.293	1.00	0.00	LX0	C
ATOM	1940	C	GLY	225	28.295	35.349	24.287	1.00	0.00	LX0	C
ATOM	1941	O	GLY	225	27.863	34.230	24.536	1.00	0.00	LX0	O
ATOM	1942	N	LYS	226	27.922	36.451	24.935	1.00	0.00	LX0	N
ATOM	1943	H	LYS	226	28.111	37.362	24.574	0.00	0.00	LX0	H
ATOM	1944	CA	LYS	226	27.487	36.211	26.305	1.00	0.00	LX0	C
ATOM	1945	CB	LYS	226	28.287	37.102	27.264	1.00	0.00	LX0	C
ATOM	1946	CG	LYS	226	29.737	37.252	26.790	1.00	0.00	LX0	C
ATOM	1947	CD	LYS	226	30.760	37.510	27.886	1.00	0.00	LX0	C
ATOM	1948	CE	LYS	226	30.787	36.374	28.905	1.00	0.00	LX0	C
ATOM	1949	NZ	LYS	226	31.112	35.071	28.295	1.00	0.00	LX0	N
ATOM	1950	HZ1	LYS	226	31.885	34.648	28.855	0.00	0.00	LX0	H
ATOM	1951	HZ2	LYS	226	30.303	34.428	28.441	0.00	0.00	LX0	H
ATOM	1952	HZ3	LYS	226	31.342	35.107	27.277	0.00	0.00	LX0	H
ATOM	1953	C	LYS	226	25.995	36.260	26.557	1.00	0.00	LX0	C
ATOM	1954	O	LYS	226	25.536	37.017	27.403	1.00	0.00	LX0	O
ATOM	1955	N	SER	227	25.308	35.418	25.759	1.00	0.00	LX0	N
ATOM	1956	H	SER	227	25.879	34.824	25.189	0.00	0.00	LX0	H
ATOM	1957	CA	SER	227	23.852	35.179	25.685	1.00	0.00	LX0	C
ATOM	1958	CB	SER	227	22.932	36.184	26.411	1.00	0.00	LX0	C
ATOM	1959	OG	SER	227	23.299	37.533	26.100	1.00	0.00	LX0	O
ATOM	1960	HG	SER	227	23.979	37.753	26.732	0.00	0.00	LX0	H
ATOM	1961	C	SER	227	23.284	34.868	24.299	1.00	0.00	LX0	C
ATOM	1962	O	SER	227	22.542	33.904	24.145	1.00	0.00	LX0	O
ATOM	1963	N	PRO	228	23.627	35.680	23.254	1.00	0.00	LX0	N
ATOM	1964	CD	PRO	228	24.438	36.900	23.198	1.00	0.00	LX0	C

ATOM	1965	CA	PRO	228	23.116	35.301	21.934	1.00	0.00	LX0	C
ATOM	1966	CB	PRO	228	23.377	36.565	21.108	1.00	0.00	LX0	C
ATOM	1967	CG	PRO	228	24.643	37.164	21.713	1.00	0.00	LX0	C
ATOM	1968	C	PRO	228	23.852	34.083	21.405	1.00	0.00	LX0	C
ATOM	1969	O	PRO	228	24.735	33.525	22.048	1.00	0.00	LX0	O
ATOM	1970	N	SER	229	23.490	33.724	20.171	1.00	0.00	LX0	N
ATOM	1971	H	SER	229	22.708	34.138	19.707	0.00	0.00	LX0	H
ATOM	1972	CA	SER	229	24.078	32.538	19.552	1.00	0.00	LX0	C
ATOM	1973	CB	SER	229	23.174	32.116	18.394	1.00	0.00	LX0	C
ATOM	1974	OG	SER	229	21.826	32.527	18.681	1.00	0.00	LX0	O
ATOM	1975	HG	SER	229	21.272	32.055	18.071	0.00	0.00	LX0	H
ATOM	1976	C	SER	229	25.538	32.604	19.108	1.00	0.00	LX0	C
ATOM	1977	O	SER	229	25.947	31.913	18.184	1.00	0.00	LX0	O
ATOM	1978	N	ASP	230	26.323	33.453	19.793	1.00	0.00	LX0	N
ATOM	1979	H	ASP	230	25.990	33.894	20.622	0.00	0.00	LX0	H
ATOM	1980	CA	ASP	230	27.753	33.470	19.502	1.00	0.00	LX0	C
ATOM	1981	CB	ASP	230	28.315	34.873	19.753	1.00	0.00	LX0	C
ATOM	1982	CG	ASP	230	29.720	34.998	19.196	1.00	0.00	LX0	C
ATOM	1983	OD1	ASP	230	30.617	35.362	19.944	1.00	0.00	LX0	O
ATOM	1984	OD2	ASP	230	29.945	34.684	18.026	1.00	0.00	LX0	O
ATOM	1985	C	ASP	230	28.461	32.360	20.269	1.00	0.00	LX0	C
ATOM	1986	O	ASP	230	29.135	32.511	21.287	1.00	0.00	LX0	O
ATOM	1987	N	CYS	231	28.186	31.174	19.726	1.00	0.00	LX0	N
ATOM	1988	H	CYS	231	27.709	31.153	18.843	0.00	0.00	LX0	H
ATOM	1989	CA	CYS	231	28.554	29.946	20.413	1.00	0.00	LX0	C
ATOM	1990	CB	CYS	231	27.679	28.814	19.878	1.00	0.00	LX0	C
ATOM	1991	SG	CYS	231	27.686	28.733	18.069	1.00	0.00	LX0	S
ATOM	1992	C	CYS	231	30.026	29.619	20.294	1.00	0.00	LX0	C
ATOM	1993	O	CYS	231	30.798	30.285	19.612	1.00	0.00	LX0	O
ATOM	1994	N	CYS	232	30.388	28.553	20.999	1.00	0.00	LX0	N
ATOM	1995	H	CYS	232	29.735	28.058	21.575	0.00	0.00	LX0	H
ATOM	1996	CA	CYS	232	31.713	28.005	20.773	1.00	0.00	LX0	C
ATOM	1997	CB	CYS	232	32.291	27.534	22.104	1.00	0.00	LX0	C
ATOM	1998	SG	CYS	232	32.613	28.924	23.218	1.00	0.00	LX0	S
ATOM	1999	C	CYS	232	31.636	26.879	19.769	1.00	0.00	LX0	C
ATOM	2000	O	CYS	232	30.560	26.472	19.347	1.00	0.00	LX0	O
ATOM	2001	N	HIS	233	32.820	26.371	19.401	1.00	0.00	LX0	N
ATOM	2002	H	HIS	233	33.673	26.727	19.783	0.00	0.00	LX0	H
ATOM	2003	CA	HIS	233	32.772	25.062	18.758	1.00	0.00	LX0	C
ATOM	2004	CB	HIS	233	34.108	24.802	18.041	1.00	0.00	LX0	C
ATOM	2005	CG	HIS	233	34.217	23.417	17.438	1.00	0.00	LX0	C
ATOM	2006	ND1	HIS	233	35.235	22.573	17.699	1.00	0.00	LX0	N
ATOM	2007	HD1	HIS	233	36.048	22.764	18.220	0.00	0.00	LX0	H
ATOM	2008	CD2	HIS	233	33.331	22.788	16.556	1.00	0.00	LX0	C
ATOM	2009	NE2	HIS	233	33.829	21.554	16.302	1.00	0.00	LX0	N
ATOM	2010	CE1	HIS	233	35.000	21.417	17.000	1.00	0.00	LX0	C
ATOM	2011	C	HIS	233	32.507	24.028	19.835	1.00	0.00	LX0	C
ATOM	2012	O	HIS	233	32.921	24.203	20.972	1.00	0.00	LX0	O
ATOM	2013	N	ASN	234	31.855	22.935	19.411	1.00	0.00	LX0	N
ATOM	2014	H	ASN	234	31.411	23.002	18.519	0.00	0.00	LX0	H
ATOM	2015	CA	ASN	234	31.745	21.704	20.207	1.00	0.00	LX0	C
ATOM	2016	CB	ASN	234	31.507	20.564	19.200	1.00	0.00	LX0	C
ATOM	2017	CG	ASN	234	31.374	19.177	19.808	1.00	0.00	LX0	C
ATOM	2018	OD1	ASN	234	31.117	18.968	20.986	1.00	0.00	LX0	O
ATOM	2019	ND2	ASN	234	31.596	18.214	18.911	1.00	0.00	LX0	N
ATOM	2020	HD21	ASN	234	31.726	18.424	17.943	0.00	0.00	LX0	H
ATOM	2021	HD22	ASN	234	31.654	17.247	19.168	0.00	0.00	LX0	H
ATOM	2022	C	ASN	234	32.930	21.441	21.142	1.00	0.00	LX0	C
ATOM	2023	O	ASN	234	32.800	21.250	22.344	1.00	0.00	LX0	O
ATOM	2024	N	GLN	235	34.124	21.470	20.536	1.00	0.00	LX0	N
ATOM	2025	H	GLN	235	34.199	21.751	19.581	0.00	0.00	LX0	H

ATOM	2026	CA	GLN	235	35.287	21.084	21.336	1.00	0.00	LX0	C
ATOM	2027	CB	GLN	235	36.344	20.475	20.426	1.00	0.00	LX0	C
ATOM	2028	CG	GLN	235	36.324	18.950	20.463	1.00	0.00	LX0	C
ATOM	2029	CD	GLN	235	34.938	18.416	20.161	1.00	0.00	LX0	C
ATOM	2030	OE1	GLN	235	34.058	18.363	21.013	1.00	0.00	LX0	O
ATOM	2031	NE2	GLN	235	34.788	17.980	18.916	1.00	0.00	LX0	N
ATOM	2032	HE21	GLN	235	35.576	18.027	18.298	0.00	0.00	LX0	H
ATOM	2033	HE22	GLN	235	33.933	17.563	18.603	0.00	0.00	LX0	H
ATOM	2034	C	GLN	235	35.894	22.098	22.281	1.00	0.00	LX0	C
ATOM	2035	O	GLN	235	36.884	21.831	22.956	1.00	0.00	LX0	O
ATOM	2036	N	CYS	236	35.270	23.271	22.291	1.00	0.00	LX0	N
ATOM	2037	H	CYS	236	34.376	23.395	21.864	0.00	0.00	LX0	H
ATOM	2038	CA	CYS	236	35.844	24.348	23.077	1.00	0.00	LX0	C
ATOM	2039	CB	CYS	236	35.477	25.681	22.458	1.00	0.00	LX0	C
ATOM	2040	SG	CYS	236	35.816	25.725	20.682	1.00	0.00	LX0	S
ATOM	2041	C	CYS	236	35.405	24.321	24.512	1.00	0.00	LX0	C
ATOM	2042	O	CYS	236	34.414	24.931	24.892	1.00	0.00	LX0	O
ATOM	2043	N	ALA	237	36.200	23.595	25.303	1.00	0.00	LX0	N
ATOM	2044	H	ALA	237	37.054	23.220	24.936	0.00	0.00	LX0	H
ATOM	2045	CA	ALA	237	35.963	23.730	26.733	1.00	0.00	LX0	C
ATOM	2046	CB	ALA	237	36.757	22.682	27.503	1.00	0.00	LX0	C
ATOM	2047	C	ALA	237	36.388	25.111	27.176	1.00	0.00	LX0	C
ATOM	2048	O	ALA	237	37.291	25.703	26.586	1.00	0.00	LX0	O
ATOM	2049	N	ALA	238	35.668	25.596	28.202	1.00	0.00	LX0	N
ATOM	2050	H	ALA	238	34.950	24.992	28.562	0.00	0.00	LX0	H
ATOM	2051	CA	ALA	238	35.775	26.982	28.671	1.00	0.00	LX0	C
ATOM	2052	CB	ALA	238	37.211	27.366	29.066	1.00	0.00	LX0	C
ATOM	2053	C	ALA	238	35.196	28.024	27.723	1.00	0.00	LX0	C
ATOM	2054	O	ALA	238	34.243	28.722	28.052	1.00	0.00	LX0	O
ATOM	2055	N	GLY	239	35.803	28.088	26.532	1.00	0.00	LX0	N
ATOM	2056	H	GLY	239	36.571	27.479	26.319	0.00	0.00	LX0	H
ATOM	2057	CA	GLY	239	35.335	29.003	25.500	1.00	0.00	LX0	C
ATOM	2058	C	GLY	239	36.248	28.885	24.298	1.00	0.00	LX0	C
ATOM	2059	O	GLY	239	37.057	27.965	24.208	1.00	0.00	LX0	O
ATOM	2060	N	CYS	240	36.108	29.851	23.382	1.00	0.00	LX0	N
ATOM	2061	H	CYS	240	35.444	30.594	23.471	0.00	0.00	LX0	H
ATOM	2062	CA	CYS	240	36.998	29.823	22.224	1.00	0.00	LX0	C
ATOM	2063	CB	CYS	240	36.485	28.873	21.141	1.00	0.00	LX0	C
ATOM	2064	SG	CYS	240	34.790	29.191	20.598	1.00	0.00	LX0	S
ATOM	2065	C	CYS	240	37.202	31.193	21.633	1.00	0.00	LX0	C
ATOM	2066	O	CYS	240	36.560	32.158	22.030	1.00	0.00	LX0	O
ATOM	2067	N	THR	241	38.107	31.221	20.652	1.00	0.00	LX0	N
ATOM	2068	H	THR	241	38.598	30.387	20.404	0.00	0.00	LX0	H
ATOM	2069	CA	THR	241	38.284	32.445	19.884	1.00	0.00	LX0	C
ATOM	2070	CB	THR	241	39.713	32.481	19.336	1.00	0.00	LX0	C
ATOM	2071	OG1	THR	241	40.625	31.925	20.294	1.00	0.00	LX0	O
ATOM	2072	HG1	THR	241	40.557	30.977	20.237	0.00	0.00	LX0	H
ATOM	2073	CG2	THR	241	40.132	33.896	18.933	1.00	0.00	LX0	C
ATOM	2074	C	THR	241	37.251	32.541	18.770	1.00	0.00	LX0	C
ATOM	2075	O	THR	241	36.415	33.437	18.707	1.00	0.00	LX0	O
ATOM	2076	N	GLY	242	37.325	31.524	17.903	1.00	0.00	LX0	N
ATOM	2077	H	GLY	242	37.982	30.776	18.001	0.00	0.00	LX0	H
ATOM	2078	CA	GLY	242	36.265	31.383	16.916	1.00	0.00	LX0	C
ATOM	2079	C	GLY	242	35.516	30.104	17.212	1.00	0.00	LX0	C
ATOM	2080	O	GLY	242	36.034	29.212	17.872	1.00	0.00	LX0	O
ATOM	2081	N	PRO	243	34.264	30.036	16.714	1.00	0.00	LX0	N
ATOM	2082	CD	PRO	243	33.548	31.089	16.000	1.00	0.00	LX0	C
ATOM	2083	CA	PRO	243	33.471	28.817	16.905	1.00	0.00	LX0	C
ATOM	2084	CB	PRO	243	32.052	29.351	16.687	1.00	0.00	LX0	C
ATOM	2085	CG	PRO	243	32.204	30.461	15.644	1.00	0.00	LX0	C
ATOM	2086	C	PRO	243	33.859	27.703	15.936	1.00	0.00	LX0	C

ATOM	2087	O	PRO	243	33.043	27.185	15.184	1.00	0.00	LX0	O
ATOM	2088	N	ARG	244	35.149	27.354	15.966	1.00	0.00	LX0	N
ATOM	2089	H	ARG	244	35.793	27.712	16.648	0.00	0.00	LX0	H
ATOM	2090	CA	ARG	244	35.651	26.458	14.935	1.00	0.00	LX0	C
ATOM	2091	CB	ARG	244	36.314	27.318	13.851	1.00	0.00	LX0	C
ATOM	2092	CG	ARG	244	36.284	26.898	12.371	1.00	0.00	LX0	C
ATOM	2093	CD	ARG	244	36.715	25.469	12.002	1.00	0.00	LX0	C
ATOM	2094	NE	ARG	244	38.142	25.100	11.881	1.00	0.00	LX0	N
ATOM	2095	HE	ARG	244	38.285	24.296	11.299	0.00	0.00	LX0	H
ATOM	2096	CZ	ARG	244	39.259	25.476	12.560	1.00	0.00	LX0	C
ATOM	2097	NH1	ARG	244	39.351	26.567	13.317	1.00	0.00	LX0	N
ATOM	2098	HH11	ARG	244	40.165	26.799	13.867	0.00	0.00	LX0	H
ATOM	2099	HH12	ARG	244	38.629	27.255	13.423	0.00	0.00	LX0	H
ATOM	2100	NH2	ARG	244	40.316	24.672	12.485	1.00	0.00	LX0	N
ATOM	2101	HH21	ARG	244	41.164	24.921	12.967	0.00	0.00	LX0	H
ATOM	2102	HH22	ARG	244	40.305	23.783	12.006	0.00	0.00	LX0	H
ATOM	2103	C	ARG	244	36.632	25.490	15.572	1.00	0.00	LX0	C
ATOM	2104	O	ARG	244	37.384	25.847	16.465	1.00	0.00	LX0	O
ATOM	2105	N	GLU	245	36.583	24.249	15.063	1.00	0.00	LX0	N
ATOM	2106	H	GLU	245	35.834	24.059	14.432	0.00	0.00	LX0	H
ATOM	2107	CA	GLU	245	37.523	23.165	15.379	1.00	0.00	LX0	C
ATOM	2108	CB	GLU	245	37.965	22.565	14.045	1.00	0.00	LX0	C
ATOM	2109	CG	GLU	245	38.948	21.402	14.054	1.00	0.00	LX0	C
ATOM	2110	CD	GLU	245	39.452	21.233	12.638	1.00	0.00	LX0	C
ATOM	2111	OE1	GLU	245	38.925	20.402	11.916	1.00	0.00	LX0	O
ATOM	2112	OE2	GLU	245	40.406	21.898	12.251	1.00	0.00	LX0	O
ATOM	2113	C	GLU	245	38.698	23.426	16.325	1.00	0.00	LX0	C
ATOM	2114	O	GLU	245	38.728	22.943	17.448	1.00	0.00	LX0	O
ATOM	2115	N	SER	246	39.689	24.170	15.815	1.00	0.00	LX0	N
ATOM	2116	H	SER	246	39.594	24.682	14.965	0.00	0.00	LX0	H
ATOM	2117	CA	SER	246	40.932	24.265	16.575	1.00	0.00	LX0	C
ATOM	2118	CB	SER	246	42.102	24.470	15.611	1.00	0.00	LX0	C
ATOM	2119	OG	SER	246	41.716	25.378	14.566	1.00	0.00	LX0	O
ATOM	2120	HG	SER	246	41.818	26.252	14.958	0.00	0.00	LX0	H
ATOM	2121	C	SER	246	40.975	25.304	17.675	1.00	0.00	LX0	C
ATOM	2122	O	SER	246	41.835	25.273	18.545	1.00	0.00	LX0	O
ATOM	2123	N	ASP	247	40.046	26.257	17.577	1.00	0.00	LX0	N
ATOM	2124	H	ASP	247	39.246	26.177	16.982	0.00	0.00	LX0	H
ATOM	2125	CA	ASP	247	40.353	27.567	18.149	1.00	0.00	LX0	C
ATOM	2126	CB	ASP	247	39.751	28.683	17.269	1.00	0.00	LX0	C
ATOM	2127	CG	ASP	247	39.821	28.375	15.770	1.00	0.00	LX0	C
ATOM	2128	OD1	ASP	247	40.813	27.824	15.289	1.00	0.00	LX0	O
ATOM	2129	OD2	ASP	247	38.854	28.654	15.069	1.00	0.00	LX0	O
ATOM	2130	C	ASP	247	39.936	27.722	19.605	1.00	0.00	LX0	C
ATOM	2131	O	ASP	247	39.497	28.777	20.061	1.00	0.00	LX0	O
ATOM	2132	N	CYS	248	40.054	26.589	20.309	1.00	0.00	LX0	N
ATOM	2133	H	CYS	248	40.625	25.863	19.926	0.00	0.00	LX0	H
ATOM	2134	CA	CYS	248	39.542	26.460	21.668	1.00	0.00	LX0	C
ATOM	2135	CB	CYS	248	39.367	24.975	21.989	1.00	0.00	LX0	C
ATOM	2136	SG	CYS	248	38.474	24.065	20.700	1.00	0.00	LX0	S
ATOM	2137	C	CYS	248	40.468	27.085	22.687	1.00	0.00	LX0	C
ATOM	2138	O	CYS	248	41.654	27.250	22.443	1.00	0.00	LX0	O
ATOM	2139	N	LEU	249	39.895	27.392	23.861	1.00	0.00	LX0	N
ATOM	2140	H	LEU	249	38.907	27.306	24.007	0.00	0.00	LX0	H
ATOM	2141	CA	LEU	249	40.813	27.724	24.952	1.00	0.00	LX0	C
ATOM	2142	CB	LEU	249	40.102	28.540	26.035	1.00	0.00	LX0	C
ATOM	2143	CG	LEU	249	39.539	29.880	25.551	1.00	0.00	LX0	C
ATOM	2144	CD1	LEU	249	38.761	30.590	26.660	1.00	0.00	LX0	C
ATOM	2145	CD2	LEU	249	40.611	30.785	24.940	1.00	0.00	LX0	C
ATOM	2146	C	LEU	249	41.436	26.471	25.548	1.00	0.00	LX0	C
ATOM	2147	O	LEU	249	42.648	26.284	25.631	1.00	0.00	LX0	O

ATOM	2148	N	VAL	250	40.522	25.579	25.946	1.00	0.00	LX0	N
ATOM	2149	H	VAL	250	39.535	25.756	25.904	0.00	0.00	LX0	H
ATOM	2150	CA	VAL	250	41.001	24.270	26.369	1.00	0.00	LX0	C
ATOM	2151	CB	VAL	250	41.005	24.138	27.898	1.00	0.00	LX0	C
ATOM	2152	CG1	VAL	250	42.219	24.842	28.507	1.00	0.00	LX0	C
ATOM	2153	CG2	VAL	250	39.707	24.634	28.528	1.00	0.00	LX0	C
ATOM	2154	C	VAL	250	40.205	23.176	25.691	1.00	0.00	LX0	C
ATOM	2155	O	VAL	250	39.110	23.394	25.187	1.00	0.00	LX0	O
ATOM	2156	N	CYS	251	40.828	21.995	25.642	1.00	0.00	LX0	N
ATOM	2157	H	CYS	251	41.691	21.854	26.132	0.00	0.00	LX0	H
ATOM	2158	CA	CYS	251	40.239	21.010	24.736	1.00	0.00	LX0	C
ATOM	2159	CB	CYS	251	41.351	20.256	24.004	1.00	0.00	LX0	C
ATOM	2160	SG	CYS	251	42.698	21.356	23.493	1.00	0.00	LX0	S
ATOM	2161	C	CYS	251	39.284	20.042	25.408	1.00	0.00	LX0	C
ATOM	2162	O	CYS	251	39.625	19.405	26.401	1.00	0.00	LX0	O
ATOM	2163	N	ARG	252	38.083	19.935	24.821	1.00	0.00	LX0	N
ATOM	2164	H	ARG	252	37.845	20.546	24.061	0.00	0.00	LX0	H
ATOM	2165	CA	ARG	252	37.157	18.917	25.327	1.00	0.00	LX0	C
ATOM	2166	CB	ARG	252	35.711	19.187	24.904	1.00	0.00	LX0	C
ATOM	2167	CG	ARG	252	34.894	19.961	25.936	1.00	0.00	LX0	C
ATOM	2168	CD	ARG	252	33.406	20.081	25.591	1.00	0.00	LX0	C
ATOM	2169	NE	ARG	252	32.722	18.784	25.618	1.00	0.00	LX0	N
ATOM	2170	HE	ARG	252	32.625	18.332	26.507	0.00	0.00	LX0	H
ATOM	2171	CZ	ARG	252	32.166	18.286	24.490	1.00	0.00	LX0	C
ATOM	2172	NH1	ARG	252	32.298	18.929	23.343	1.00	0.00	LX0	N
ATOM	2173	HH11	ARG	252	31.913	18.608	22.472	0.00	0.00	LX0	H
ATOM	2174	HH12	ARG	252	32.777	19.809	23.286	0.00	0.00	LX0	H
ATOM	2175	NH2	ARG	252	31.469	17.158	24.511	1.00	0.00	LX0	N
ATOM	2176	HH21	ARG	252	31.054	16.807	23.673	0.00	0.00	LX0	H
ATOM	2177	HH22	ARG	252	31.287	16.645	25.355	0.00	0.00	LX0	H
ATOM	2178	C	ARG	252	37.470	17.489	24.919	1.00	0.00	LX0	C
ATOM	2179	O	ARG	252	37.023	16.540	25.554	1.00	0.00	LX0	O
ATOM	2180	N	LYS	253	38.188	17.383	23.787	1.00	0.00	LX0	N
ATOM	2181	H	LYS	253	38.632	18.169	23.360	0.00	0.00	LX0	H
ATOM	2182	CA	LYS	253	38.225	16.104	23.082	1.00	0.00	LX0	C
ATOM	2183	CB	LYS	253	36.894	15.907	22.361	1.00	0.00	LX0	C
ATOM	2184	CG	LYS	253	36.024	14.837	23.011	1.00	0.00	LX0	C
ATOM	2185	CD	LYS	253	34.548	15.016	22.673	1.00	0.00	LX0	C
ATOM	2186	CE	LYS	253	34.263	15.313	21.196	1.00	0.00	LX0	C
ATOM	2187	NZ	LYS	253	34.565	14.208	20.285	1.00	0.00	LX0	N
ATOM	2188	HZ1	LYS	253	33.933	14.241	19.455	0.00	0.00	LX0	H
ATOM	2189	HZ2	LYS	253	35.550	14.252	19.948	0.00	0.00	LX0	H
ATOM	2190	HZ3	LYS	253	34.492	13.276	20.732	0.00	0.00	LX0	H
ATOM	2191	C	LYS	253	39.392	16.032	22.114	1.00	0.00	LX0	C
ATOM	2192	O	LYS	253	40.248	16.905	22.125	1.00	0.00	LX0	O
ATOM	2193	N	PHE	254	39.362	14.959	21.295	1.00	0.00	LX0	N
ATOM	2194	H	PHE	254	38.614	14.306	21.393	0.00	0.00	LX0	H
ATOM	2195	CA	PHE	254	40.411	14.619	20.326	1.00	0.00	LX0	C
ATOM	2196	CB	PHE	254	39.862	13.642	19.277	1.00	0.00	LX0	C
ATOM	2197	CG	PHE	254	39.781	12.221	19.806	1.00	0.00	LX0	C
ATOM	2198	CD1	PHE	254	40.434	11.201	19.083	1.00	0.00	LX0	C
ATOM	2199	CD2	PHE	254	39.063	11.918	20.985	1.00	0.00	LX0	C
ATOM	2200	CE1	PHE	254	40.367	9.868	19.533	1.00	0.00	LX0	C
ATOM	2201	CE2	PHE	254	38.996	10.588	21.441	1.00	0.00	LX0	C
ATOM	2202	CZ	PHE	254	39.646	9.575	20.708	1.00	0.00	LX0	C
ATOM	2203	C	PHE	254	41.147	15.763	19.648	1.00	0.00	LX0	C
ATOM	2204	O	PHE	254	40.665	16.446	18.746	1.00	0.00	LX0	O
ATOM	2205	N	ARG	255	42.373	15.924	20.146	1.00	0.00	LX0	N
ATOM	2206	H	ARG	255	42.752	15.217	20.744	0.00	0.00	LX0	H
ATOM	2207	CA	ARG	255	43.197	17.012	19.648	1.00	0.00	LX0	C
ATOM	2208	CB	ARG	255	43.991	17.611	20.813	1.00	0.00	LX0	C

ATOM	2209	CG	ARG	255	44.404	19.073	20.614	1.00	0.00	LX0	C
ATOM	2210	CD	ARG	255	45.890	19.273	20.911	1.00	0.00	LX0	C
ATOM	2211	NE	ARG	255	46.650	18.436	19.992	1.00	0.00	LX0	N
ATOM	2212	HE	ARG	255	46.237	18.163	19.117	0.00	0.00	LX0	H
ATOM	2213	CZ	ARG	255	47.914	18.037	20.189	1.00	0.00	LX0	C
ATOM	2214	NH1	ARG	255	48.617	18.432	21.236	1.00	0.00	LX0	N
ATOM	2215	HH11	ARG	255	49.550	18.060	21.346	0.00	0.00	LX0	H
ATOM	2216	HH12	ARG	255	48.288	19.106	21.899	0.00	0.00	LX0	H
ATOM	2217	NH2	ARG	255	48.472	17.237	19.298	1.00	0.00	LX0	N
ATOM	2218	HH21	ARG	255	49.456	17.022	19.394	0.00	0.00	LX0	H
ATOM	2219	HH22	ARG	255	47.944	16.885	18.526	0.00	0.00	LX0	H
ATOM	2220	C	ARG	255	44.081	16.553	18.498	1.00	0.00	LX0	C
ATOM	2221	O	ARG	255	45.300	16.441	18.602	1.00	0.00	LX0	O
ATOM	2222	N	ASP	256	43.387	16.261	17.389	1.00	0.00	LX0	N
ATOM	2223	H	ASP	256	42.415	16.490	17.387	0.00	0.00	LX0	H
ATOM	2224	CA	ASP	256	44.083	15.801	16.188	1.00	0.00	LX0	C
ATOM	2225	CB	ASP	256	43.083	15.488	15.067	1.00	0.00	LX0	C
ATOM	2226	CG	ASP	256	43.800	14.941	13.844	1.00	0.00	LX0	C
ATOM	2227	OD1	ASP	256	44.847	14.332	14.009	1.00	0.00	LX0	O
ATOM	2228	OD2	ASP	256	43.357	15.148	12.718	1.00	0.00	LX0	O
ATOM	2229	C	ASP	256	45.125	16.776	15.685	1.00	0.00	LX0	C
ATOM	2230	O	ASP	256	44.803	17.865	15.216	1.00	0.00	LX0	O
ATOM	2231	N	GLU	257	46.393	16.350	15.831	1.00	0.00	LX0	N
ATOM	2232	H	GLU	257	46.525	15.379	16.050	0.00	0.00	LX0	H
ATOM	2233	CA	GLU	257	47.510	17.254	15.543	1.00	0.00	LX0	C
ATOM	2234	CB	GLU	257	47.870	17.167	14.051	1.00	0.00	LX0	C
ATOM	2235	CG	GLU	257	48.313	15.728	13.744	1.00	0.00	LX0	C
ATOM	2236	CD	GLU	257	48.540	15.478	12.262	1.00	0.00	LX0	C
ATOM	2237	OE1	GLU	257	47.814	16.009	11.425	1.00	0.00	LX0	O
ATOM	2238	OE2	GLU	257	49.404	14.681	11.916	1.00	0.00	LX0	O
ATOM	2239	C	GLU	257	47.284	18.650	16.127	1.00	0.00	LX0	C
ATOM	2240	O	GLU	257	47.105	18.760	17.332	1.00	0.00	LX0	O
ATOM	2241	N	ALA	258	47.241	19.698	15.291	1.00	0.00	LX0	N
ATOM	2242	H	ALA	258	47.293	19.596	14.300	0.00	0.00	LX0	H
ATOM	2243	CA	ALA	258	46.999	20.994	15.934	1.00	0.00	LX0	C
ATOM	2244	CB	ALA	258	47.876	22.074	15.294	1.00	0.00	LX0	C
ATOM	2245	C	ALA	258	45.545	21.461	15.940	1.00	0.00	LX0	C
ATOM	2246	O	ALA	258	45.247	22.646	15.871	1.00	0.00	LX0	O
ATOM	2247	N	THR	259	44.625	20.486	15.977	1.00	0.00	LX0	N
ATOM	2248	H	THR	259	44.885	19.530	16.121	0.00	0.00	LX0	H
ATOM	2249	CA	THR	259	43.215	20.856	15.865	1.00	0.00	LX0	C
ATOM	2250	CB	THR	259	42.708	20.656	14.421	1.00	0.00	LX0	C
ATOM	2251	OG1	THR	259	42.745	19.276	14.018	1.00	0.00	LX0	O
ATOM	2252	HG1	THR	259	43.539	18.896	14.409	0.00	0.00	LX0	H
ATOM	2253	CG2	THR	259	43.450	21.514	13.392	1.00	0.00	LX0	C
ATOM	2254	C	THR	259	42.357	20.081	16.849	1.00	0.00	LX0	C
ATOM	2255	O	THR	259	42.701	18.972	17.231	1.00	0.00	LX0	O
ATOM	2256	N	CYS	260	41.222	20.674	17.238	1.00	0.00	LX0	N
ATOM	2257	H	CYS	260	40.931	21.583	16.948	0.00	0.00	LX0	H
ATOM	2258	CA	CYS	260	40.336	19.858	18.063	1.00	0.00	LX0	C
ATOM	2259	CB	CYS	260	39.889	20.633	19.299	1.00	0.00	LX0	C
ATOM	2260	SG	CYS	260	41.282	21.296	20.247	1.00	0.00	LX0	S
ATOM	2261	C	CYS	260	39.161	19.325	17.265	1.00	0.00	LX0	C
ATOM	2262	O	CYS	260	38.110	19.940	17.122	1.00	0.00	LX0	O
ATOM	2263	N	LYS	261	39.424	18.140	16.699	1.00	0.00	LX0	N
ATOM	2264	H	LYS	261	40.216	17.643	17.059	0.00	0.00	LX0	H
ATOM	2265	CA	LYS	261	38.394	17.487	15.892	1.00	0.00	LX0	C
ATOM	2266	CB	LYS	261	39.028	16.375	15.053	1.00	0.00	LX0	C
ATOM	2267	CG	LYS	261	40.042	16.839	14.007	1.00	0.00	LX0	C
ATOM	2268	CD	LYS	261	39.460	17.211	12.645	1.00	0.00	LX0	C
ATOM	2269	CE	LYS	261	40.555	17.582	11.634	1.00	0.00	LX0	C

ATOM	2270	NZ	LYS	261	41.175	18.865	11.994	1.00	0.00	LX0	N
ATOM	2271	HZ1	LYS	261	41.974	19.088	11.377	0.00	0.00	LX0	H
ATOM	2272	HZ2	LYS	261	40.479	19.640	11.920	0.00	0.00	LX0	H
ATOM	2273	HZ3	LYS	261	41.528	18.842	12.972	0.00	0.00	LX0	H
ATOM	2274	C	LYS	261	37.316	16.906	16.787	1.00	0.00	LX0	C
ATOM	2275	O	LYS	261	37.332	17.070	18.001	1.00	0.00	LX0	O
ATOM	2276	N	ASP	262	36.375	16.190	16.153	1.00	0.00	LX0	N
ATOM	2277	H	ASP	262	36.372	16.083	15.162	0.00	0.00	LX0	H
ATOM	2278	CA	ASP	262	35.537	15.404	17.049	1.00	0.00	LX0	C
ATOM	2279	CB	ASP	262	34.115	15.180	16.518	1.00	0.00	LX0	C
ATOM	2280	CG	ASP	262	33.235	14.767	17.689	1.00	0.00	LX0	C
ATOM	2281	OD1	ASP	262	32.720	15.622	18.396	1.00	0.00	LX0	O
ATOM	2282	OD2	ASP	262	33.137	13.586	17.993	1.00	0.00	LX0	O
ATOM	2283	C	ASP	262	36.204	14.118	17.486	1.00	0.00	LX0	C
ATOM	2284	O	ASP	262	36.540	13.959	18.653	1.00	0.00	LX0	O
ATOM	2285	N	THR	263	36.393	13.232	16.508	1.00	0.00	LX0	N
ATOM	2286	H	THR	263	36.121	13.391	15.559	0.00	0.00	LX0	H
ATOM	2287	CA	THR	263	37.048	11.965	16.797	1.00	0.00	LX0	C
ATOM	2288	CB	THR	263	35.962	10.912	17.090	1.00	0.00	LX0	C
ATOM	2289	OG1	THR	263	35.002	11.433	18.031	1.00	0.00	LX0	O
ATOM	2290	HG1	THR	263	34.390	12.000	17.557	0.00	0.00	LX0	H
ATOM	2291	CG2	THR	263	36.540	9.596	17.626	1.00	0.00	LX0	C
ATOM	2292	C	THR	263	37.900	11.636	15.581	1.00	0.00	LX0	C
ATOM	2293	O	THR	263	37.621	12.152	14.504	1.00	0.00	LX0	O
ATOM	2294	N	CYS	264	38.950	10.821	15.789	1.00	0.00	LX0	N
ATOM	2295	H	CYS	264	39.137	10.484	16.710	0.00	0.00	LX0	H
ATOM	2296	CA	CYS	264	39.869	10.479	14.692	1.00	0.00	LX0	C
ATOM	2297	CB	CYS	264	40.797	9.335	15.098	1.00	0.00	LX0	C
ATOM	2298	SG	CYS	264	41.749	9.665	16.594	1.00	0.00	LX0	S
ATOM	2299	C	CYS	264	39.229	10.064	13.375	1.00	0.00	LX0	C
ATOM	2300	O	CYS	264	38.508	9.076	13.313	1.00	0.00	LX0	O
ATOM	2301	N	PRO	265	39.549	10.832	12.304	1.00	0.00	LX0	N
ATOM	2302	CD	PRO	265	40.240	12.118	12.320	1.00	0.00	LX0	C
ATOM	2303	CA	PRO	265	39.190	10.396	10.948	1.00	0.00	LX0	C
ATOM	2304	CB	PRO	265	39.799	11.493	10.065	1.00	0.00	LX0	C
ATOM	2305	CG	PRO	265	39.925	12.724	10.959	1.00	0.00	LX0	C
ATOM	2306	C	PRO	265	39.722	9.008	10.598	1.00	0.00	LX0	C
ATOM	2307	O	PRO	265	40.927	8.775	10.571	1.00	0.00	LX0	O
ATOM	2308	N	PRO	266	38.769	8.082	10.336	1.00	0.00	LX0	N
ATOM	2309	CD	PRO	266	37.325	8.291	10.325	1.00	0.00	LX0	C
ATOM	2310	CA	PRO	266	39.138	6.686	10.067	1.00	0.00	LX0	C
ATOM	2311	CB	PRO	266	37.784	6.014	9.803	1.00	0.00	LX0	C
ATOM	2312	CG	PRO	266	36.755	6.891	10.511	1.00	0.00	LX0	C
ATOM	2313	C	PRO	266	40.115	6.515	8.916	1.00	0.00	LX0	C
ATOM	2314	O	PRO	266	40.218	7.352	8.029	1.00	0.00	LX0	O
ATOM	2315	N	LEU	267	40.843	5.387	8.974	1.00	0.00	LX0	N
ATOM	2316	H	LEU	267	40.725	4.756	9.740	0.00	0.00	LX0	H
ATOM	2317	CA	LEU	267	41.831	5.131	7.920	1.00	0.00	LX0	C
ATOM	2318	CB	LEU	267	42.717	3.941	8.292	1.00	0.00	LX0	C
ATOM	2319	CG	LEU	267	44.044	4.310	8.963	1.00	0.00	LX0	C
ATOM	2320	CD1	LEU	267	43.879	5.033	10.301	1.00	0.00	LX0	C
ATOM	2321	CD2	LEU	267	44.936	3.077	9.092	1.00	0.00	LX0	C
ATOM	2322	C	LEU	267	41.234	4.910	6.539	1.00	0.00	LX0	C
ATOM	2323	O	LEU	267	41.750	5.351	5.518	1.00	0.00	LX0	O
ATOM	2324	N	MET	268	40.105	4.193	6.560	1.00	0.00	LX0	N
ATOM	2325	H	MET	268	39.662	3.923	7.413	0.00	0.00	LX0	H
ATOM	2326	CA	MET	268	39.387	4.042	5.303	1.00	0.00	LX0	C
ATOM	2327	CB	MET	268	39.122	2.565	4.996	1.00	0.00	LX0	C
ATOM	2328	CG	MET	268	40.386	1.697	4.993	1.00	0.00	LX0	C
ATOM	2329	SD	MET	268	41.620	2.200	3.778	1.00	0.00	LX0	S
ATOM	2330	CE	MET	268	40.765	1.652	2.291	1.00	0.00	LX0	C

ATOM	2331	C	MET	268	38.088	4.800	5.418	1.00	0.00	LX0	C
ATOM	2332	O	MET	268	37.581	4.992	6.517	1.00	0.00	LX0	O
ATOM	2333	N	LEU	269	37.594	5.234	4.260	1.00	0.00	LX0	N
ATOM	2334	H	LEU	269	38.034	4.960	3.404	0.00	0.00	LX0	H
ATOM	2335	CA	LEU	269	36.271	5.846	4.227	1.00	0.00	LX0	C
ATOM	2336	CB	LEU	269	36.270	7.334	3.844	1.00	0.00	LX0	C
ATOM	2337	CG	LEU	269	37.282	8.319	4.440	1.00	0.00	LX0	C
ATOM	2338	CD1	LEU	269	37.467	8.224	5.957	1.00	0.00	LX0	C
ATOM	2339	CD2	LEU	269	38.567	8.325	3.620	1.00	0.00	LX0	C
ATOM	2340	C	LEU	269	35.475	5.094	3.187	1.00	0.00	LX0	C
ATOM	2341	O	LEU	269	35.918	4.066	2.692	1.00	0.00	LX0	O
ATOM	2342	N	TYR	270	34.312	5.644	2.839	1.00	0.00	LX0	N
ATOM	2343	H	TYR	270	33.947	6.498	3.208	0.00	0.00	LX0	H
ATOM	2344	CA	TYR	270	33.545	4.963	1.812	1.00	0.00	LX0	C
ATOM	2345	CB	TYR	270	32.536	4.083	2.537	1.00	0.00	LX0	C
ATOM	2346	CG	TYR	270	31.805	3.095	1.662	1.00	0.00	LX0	C
ATOM	2347	CD1	TYR	270	32.404	1.851	1.378	1.00	0.00	LX0	C
ATOM	2348	CE1	TYR	270	31.687	0.909	0.625	1.00	0.00	LX0	C
ATOM	2349	CD2	TYR	270	30.520	3.431	1.198	1.00	0.00	LX0	C
ATOM	2350	CE2	TYR	270	29.795	2.484	0.462	1.00	0.00	LX0	C
ATOM	2351	CZ	TYR	270	30.392	1.240	0.180	1.00	0.00	LX0	C
ATOM	2352	OH	TYR	270	29.681	0.334	-0.575	1.00	0.00	LX0	O
ATOM	2353	HH	TYR	270	30.234	0.024	-1.291	0.00	0.00	LX0	H
ATOM	2354	C	TYR	270	32.905	6.008	0.935	1.00	0.00	LX0	C
ATOM	2355	O	TYR	270	32.478	7.048	1.415	1.00	0.00	LX0	O
ATOM	2356	N	ASN	271	32.868	5.702	-0.359	1.00	0.00	LX0	N
ATOM	2357	H	ASN	271	33.314	4.863	-0.659	0.00	0.00	LX0	H
ATOM	2358	CA	ASN	271	32.446	6.742	-1.285	1.00	0.00	LX0	C
ATOM	2359	CB	ASN	271	33.000	6.462	-2.677	1.00	0.00	LX0	C
ATOM	2360	CG	ASN	271	33.143	7.782	-3.403	1.00	0.00	LX0	C
ATOM	2361	OD1	ASN	271	32.187	8.350	-3.930	1.00	0.00	LX0	O
ATOM	2362	ND2	ASN	271	34.391	8.248	-3.396	1.00	0.00	LX0	N
ATOM	2363	HD21	ASN	271	35.062	7.746	-2.823	0.00	0.00	LX0	H
ATOM	2364	HD22	ASN	271	34.701	9.060	-3.880	0.00	0.00	LX0	H
ATOM	2365	C	ASN	271	30.952	6.952	-1.359	1.00	0.00	LX0	C
ATOM	2366	O	ASN	271	30.204	6.061	-1.753	1.00	0.00	LX0	O
ATOM	2367	N	PRO	272	30.522	8.192	-1.022	1.00	0.00	LX0	N
ATOM	2368	CD	PRO	272	31.333	9.320	-0.575	1.00	0.00	LX0	C
ATOM	2369	CA	PRO	272	29.093	8.512	-1.109	1.00	0.00	LX0	C
ATOM	2370	CB	PRO	272	29.037	9.969	-0.624	1.00	0.00	LX0	C
ATOM	2371	CG	PRO	272	30.443	10.529	-0.831	1.00	0.00	LX0	C
ATOM	2372	C	PRO	272	28.509	8.314	-2.498	1.00	0.00	LX0	C
ATOM	2373	O	PRO	272	27.334	8.008	-2.676	1.00	0.00	LX0	O
ATOM	2374	N	THR	273	29.383	8.508	-3.487	1.00	0.00	LX0	N
ATOM	2375	H	THR	273	30.337	8.746	-3.307	0.00	0.00	LX0	H
ATOM	2376	CA	THR	273	28.938	8.311	-4.856	1.00	0.00	LX0	C
ATOM	2377	CB	THR	273	29.687	9.284	-5.772	1.00	0.00	LX0	C
ATOM	2378	OG1	THR	273	30.431	10.239	-4.994	1.00	0.00	LX0	O
ATOM	2379	HG1	THR	273	31.243	9.792	-4.753	0.00	0.00	LX0	H
ATOM	2380	CG2	THR	273	28.730	9.985	-6.736	1.00	0.00	LX0	C
ATOM	2381	C	THR	273	29.087	6.872	-5.320	1.00	0.00	LX0	C
ATOM	2382	O	THR	273	28.119	6.174	-5.591	1.00	0.00	LX0	O
ATOM	2383	N	THR	274	30.357	6.457	-5.392	1.00	0.00	LX0	N
ATOM	2384	H	THR	274	31.137	7.008	-5.086	0.00	0.00	LX0	H
ATOM	2385	CA	THR	274	30.651	5.194	-6.061	1.00	0.00	LX0	C
ATOM	2386	CB	THR	274	31.951	5.375	-6.834	1.00	0.00	LX0	C
ATOM	2387	OG1	THR	274	32.848	6.180	-6.058	1.00	0.00	LX0	O
ATOM	2388	HG1	THR	274	33.686	6.177	-6.508	0.00	0.00	LX0	H
ATOM	2389	CG2	THR	274	31.707	6.034	-8.192	1.00	0.00	LX0	C
ATOM	2390	C	THR	274	30.712	3.956	-5.183	1.00	0.00	LX0	C
ATOM	2391	O	THR	274	31.144	2.895	-5.619	1.00	0.00	LX0	O

ATOM	2392	N	TYR	275	30.271	4.133	-3.923	1.00	0.00	LX0	N
ATOM	2393	H	TYR	275	29.975	5.035	-3.611	0.00	0.00	LX0	H
ATOM	2394	CA	TYR	275	30.132	3.002	-2.997	1.00	0.00	LX0	C
ATOM	2395	CB	TYR	275	28.847	2.210	-3.281	1.00	0.00	LX0	C
ATOM	2396	CG	TYR	275	27.637	3.119	-3.287	1.00	0.00	LX0	C
ATOM	2397	CD1	TYR	275	27.319	3.881	-2.141	1.00	0.00	LX0	C
ATOM	2398	CE1	TYR	275	26.188	4.713	-2.168	1.00	0.00	LX0	C
ATOM	2399	CD2	TYR	275	26.852	3.170	-4.456	1.00	0.00	LX0	C
ATOM	2400	CE2	TYR	275	25.718	3.997	-4.480	1.00	0.00	LX0	C
ATOM	2401	CZ	TYR	275	25.400	4.755	-3.336	1.00	0.00	LX0	C
ATOM	2402	OH	TYR	275	24.277	5.566	-3.361	1.00	0.00	LX0	O
ATOM	2403	HH	TYR	275	23.715	5.250	-4.066	0.00	0.00	LX0	H
ATOM	2404	C	TYR	275	31.333	2.076	-2.869	1.00	0.00	LX0	C
ATOM	2405	O	TYR	275	31.232	0.858	-2.771	1.00	0.00	LX0	O
ATOM	2406	N	GLN	276	32.495	2.732	-2.878	1.00	0.00	LX0	N
ATOM	2407	H	GLN	276	32.512	3.729	-2.849	0.00	0.00	LX0	H
ATOM	2408	CA	GLN	276	33.732	1.967	-2.809	1.00	0.00	LX0	C
ATOM	2409	CB	GLN	276	34.546	2.285	-4.065	1.00	0.00	LX0	C
ATOM	2410	CG	GLN	276	35.072	1.056	-4.817	1.00	0.00	LX0	C
ATOM	2411	CD	GLN	276	33.978	0.041	-5.128	1.00	0.00	LX0	C
ATOM	2412	OE1	GLN	276	34.162	-1.151	-4.940	1.00	0.00	LX0	O
ATOM	2413	NE2	GLN	276	32.830	0.535	-5.606	1.00	0.00	LX0	N
ATOM	2414	HE21	GLN	276	32.621	1.502	-5.762	0.00	0.00	LX0	H
ATOM	2415	HE22	GLN	276	32.100	-0.122	-5.779	0.00	0.00	LX0	H
ATOM	2416	C	GLN	276	34.447	2.291	-1.519	1.00	0.00	LX0	C
ATOM	2417	O	GLN	276	34.010	3.175	-0.798	1.00	0.00	LX0	O
ATOM	2418	N	MET	277	35.521	1.551	-1.229	1.00	0.00	LX0	N
ATOM	2419	H	MET	277	35.904	0.885	-1.864	0.00	0.00	LX0	H
ATOM	2420	CA	MET	277	36.208	1.904	0.011	1.00	0.00	LX0	C
ATOM	2421	CB	MET	277	36.688	0.637	0.724	1.00	0.00	LX0	C
ATOM	2422	CG	MET	277	36.929	0.827	2.224	1.00	0.00	LX0	C
ATOM	2423	SD	MET	277	35.418	1.117	3.163	1.00	0.00	LX0	S
ATOM	2424	CE	MET	277	34.740	-0.549	3.090	1.00	0.00	LX0	C
ATOM	2425	C	MET	277	37.341	2.882	-0.244	1.00	0.00	LX0	C
ATOM	2426	O	MET	277	38.370	2.532	-0.812	1.00	0.00	LX0	O
ATOM	2427	N	ASP	278	37.081	4.127	0.164	1.00	0.00	LX0	N
ATOM	2428	H	ASP	278	36.266	4.291	0.717	0.00	0.00	LX0	H
ATOM	2429	CA	ASP	278	38.067	5.180	-0.077	1.00	0.00	LX0	C
ATOM	2430	CB	ASP	278	37.435	6.577	-0.001	1.00	0.00	LX0	C
ATOM	2431	CG	ASP	278	36.319	6.814	-1.009	1.00	0.00	LX0	C
ATOM	2432	OD1	ASP	278	36.299	6.205	-2.078	1.00	0.00	LX0	O
ATOM	2433	OD2	ASP	278	35.461	7.643	-0.727	1.00	0.00	LX0	O
ATOM	2434	C	ASP	278	39.205	5.108	0.923	1.00	0.00	LX0	C
ATOM	2435	O	ASP	278	39.110	4.463	1.961	1.00	0.00	LX0	O
ATOM	2436	N	VAL	279	40.294	5.807	0.588	1.00	0.00	LX0	N
ATOM	2437	H	VAL	279	40.296	6.386	-0.227	0.00	0.00	LX0	H
ATOM	2438	CA	VAL	279	41.414	5.793	1.527	1.00	0.00	LX0	C
ATOM	2439	CB	VAL	279	42.636	5.112	0.876	1.00	0.00	LX0	C
ATOM	2440	CG1	VAL	279	43.112	5.842	-0.385	1.00	0.00	LX0	C
ATOM	2441	CG2	VAL	279	43.766	4.854	1.879	1.00	0.00	LX0	C
ATOM	2442	C	VAL	279	41.710	7.192	2.047	1.00	0.00	LX0	C
ATOM	2443	O	VAL	279	41.611	8.171	1.317	1.00	0.00	LX0	O
ATOM	2444	N	ASN	280	42.038	7.252	3.344	1.00	0.00	LX0	N
ATOM	2445	H	ASN	280	42.134	6.415	3.889	0.00	0.00	LX0	H
ATOM	2446	CA	ASN	280	42.264	8.559	3.962	1.00	0.00	LX0	C
ATOM	2447	CB	ASN	280	41.625	8.569	5.355	1.00	0.00	LX0	C
ATOM	2448	CG	ASN	280	41.716	9.923	6.027	1.00	0.00	LX0	C
ATOM	2449	OD1	ASN	280	42.110	10.928	5.449	1.00	0.00	LX0	O
ATOM	2450	ND2	ASN	280	41.401	9.881	7.319	1.00	0.00	LX0	N
ATOM	2451	HD21	ASN	280	40.988	9.055	7.707	0.00	0.00	LX0	H
ATOM	2452	HD22	ASN	280	41.626	10.651	7.917	0.00	0.00	LX0	H

ATOM	2453	C	ASN	280	43.729	8.944	4.055	1.00	0.00	LX0	C
ATOM	2454	O	ASN	280	44.498	8.316	4.770	1.00	0.00	LX0	O
ATOM	2455	N	PRO	281	44.094	10.034	3.334	1.00	0.00	LX0	N
ATOM	2456	CD	PRO	281	43.283	10.759	2.362	1.00	0.00	LX0	C
ATOM	2457	CA	PRO	281	45.434	10.611	3.505	1.00	0.00	LX0	C
ATOM	2458	CB	PRO	281	45.377	11.861	2.619	1.00	0.00	LX0	C
ATOM	2459	CG	PRO	281	44.305	11.564	1.572	1.00	0.00	LX0	C
ATOM	2460	C	PRO	281	45.783	10.925	4.956	1.00	0.00	LX0	C
ATOM	2461	O	PRO	281	46.828	10.557	5.476	1.00	0.00	LX0	O
ATOM	2462	N	GLU	282	44.828	11.607	5.600	1.00	0.00	LX0	N
ATOM	2463	H	GLU	282	43.934	11.760	5.177	0.00	0.00	LX0	H
ATOM	2464	CA	GLU	282	44.983	11.881	7.024	1.00	0.00	LX0	C
ATOM	2465	CB	GLU	282	44.267	13.189	7.366	1.00	0.00	LX0	C
ATOM	2466	CG	GLU	282	45.026	14.453	6.935	1.00	0.00	LX0	C
ATOM	2467	CD	GLU	282	46.269	14.659	7.782	1.00	0.00	LX0	C
ATOM	2468	OE1	GLU	282	47.275	15.140	7.270	1.00	0.00	LX0	O
ATOM	2469	OE2	GLU	282	46.247	14.341	8.966	1.00	0.00	LX0	O
ATOM	2470	C	GLU	282	44.450	10.740	7.869	1.00	0.00	LX0	C
ATOM	2471	O	GLU	282	43.571	10.908	8.709	1.00	0.00	LX0	O
ATOM	2472	N	GLY	283	45.001	9.548	7.589	1.00	0.00	LX0	N
ATOM	2473	H	GLY	283	45.738	9.489	6.914	0.00	0.00	LX0	H
ATOM	2474	CA	GLY	283	44.567	8.370	8.336	1.00	0.00	LX0	C
ATOM	2475	C	GLY	283	44.944	8.471	9.798	1.00	0.00	LX0	C
ATOM	2476	O	GLY	283	46.106	8.401	10.178	1.00	0.00	LX0	O
ATOM	2477	N	LYS	284	43.910	8.697	10.612	1.00	0.00	LX0	N
ATOM	2478	H	LYS	284	42.956	8.712	10.306	0.00	0.00	LX0	H
ATOM	2479	CA	LYS	284	44.297	9.162	11.931	1.00	0.00	LX0	C
ATOM	2480	CB	LYS	284	43.455	10.363	12.329	1.00	0.00	LX0	C
ATOM	2481	CG	LYS	284	44.369	11.556	12.573	1.00	0.00	LX0	C
ATOM	2482	CD	LYS	284	45.349	11.801	11.425	1.00	0.00	LX0	C
ATOM	2483	CE	LYS	284	46.354	12.908	11.716	1.00	0.00	LX0	C
ATOM	2484	NZ	LYS	284	45.734	14.230	11.564	1.00	0.00	LX0	N
ATOM	2485	HZ1	LYS	284	46.428	14.966	11.805	0.00	0.00	LX0	H
ATOM	2486	HZ2	LYS	284	45.443	14.385	10.577	0.00	0.00	LX0	H
ATOM	2487	HZ3	LYS	284	44.922	14.349	12.214	0.00	0.00	LX0	H
ATOM	2488	C	LYS	284	44.438	8.131	13.020	1.00	0.00	LX0	C
ATOM	2489	O	LYS	284	43.517	7.792	13.759	1.00	0.00	LX0	O
ATOM	2490	N	TYR	285	45.686	7.654	13.081	1.00	0.00	LX0	N
ATOM	2491	H	TYR	285	46.373	8.084	12.490	0.00	0.00	LX0	H
ATOM	2492	CA	TYR	285	46.041	6.680	14.107	1.00	0.00	LX0	C
ATOM	2493	CB	TYR	285	47.500	6.242	13.954	1.00	0.00	LX0	C
ATOM	2494	CG	TYR	285	47.715	5.490	12.656	1.00	0.00	LX0	C
ATOM	2495	CD1	TYR	285	47.940	6.208	11.463	1.00	0.00	LX0	C
ATOM	2496	CE1	TYR	285	48.187	5.507	10.273	1.00	0.00	LX0	C
ATOM	2497	CD2	TYR	285	47.703	4.080	12.675	1.00	0.00	LX0	C
ATOM	2498	CE2	TYR	285	47.951	3.375	11.486	1.00	0.00	LX0	C
ATOM	2499	CZ	TYR	285	48.200	4.098	10.302	1.00	0.00	LX0	C
ATOM	2500	OH	TYR	285	48.469	3.411	9.135	1.00	0.00	LX0	O
ATOM	2501	HH	TYR	285	48.231	2.497	9.233	0.00	0.00	LX0	H
ATOM	2502	C	TYR	285	45.760	7.205	15.501	1.00	0.00	LX0	C
ATOM	2503	O	TYR	285	46.195	8.273	15.922	1.00	0.00	LX0	O
ATOM	2504	N	SER	286	44.928	6.422	16.177	1.00	0.00	LX0	N
ATOM	2505	H	SER	286	44.680	5.517	15.829	0.00	0.00	LX0	H
ATOM	2506	CA	SER	286	44.336	6.967	17.385	1.00	0.00	LX0	C
ATOM	2507	CB	SER	286	42.897	6.466	17.479	1.00	0.00	LX0	C
ATOM	2508	OG	SER	286	42.312	6.432	16.165	1.00	0.00	LX0	O
ATOM	2509	HG	SER	286	42.777	7.061	15.615	0.00	0.00	LX0	H
ATOM	2510	C	SER	286	45.141	6.683	18.637	1.00	0.00	LX0	C
ATOM	2511	O	SER	286	44.925	5.703	19.343	1.00	0.00	LX0	O
ATOM	2512	N	PHE	287	46.092	7.594	18.891	1.00	0.00	LX0	N
ATOM	2513	H	PHE	287	46.215	8.387	18.290	0.00	0.00	LX0	H

ATOM	2514	CA	PHE	287	46.815	7.475	20.154	1.00	0.00	LX0	C
ATOM	2515	CB	PHE	287	48.260	7.967	19.993	1.00	0.00	LX0	C
ATOM	2516	CG	PHE	287	49.121	7.524	21.156	1.00	0.00	LX0	C
ATOM	2517	CD1	PHE	287	49.369	6.148	21.363	1.00	0.00	LX0	C
ATOM	2518	CD2	PHE	287	49.669	8.499	22.016	1.00	0.00	LX0	C
ATOM	2519	CE1	PHE	287	50.171	5.741	22.446	1.00	0.00	LX0	C
ATOM	2520	CE2	PHE	287	50.473	8.094	23.100	1.00	0.00	LX0	C
ATOM	2521	CZ	PHE	287	50.715	6.719	23.305	1.00	0.00	LX0	C
ATOM	2522	C	PHE	287	46.070	8.168	21.285	1.00	0.00	LX0	C
ATOM	2523	O	PHE	287	46.431	9.225	21.792	1.00	0.00	LX0	O
ATOM	2524	N	GLY	288	44.953	7.513	21.635	1.00	0.00	LX0	N
ATOM	2525	H	GLY	288	44.721	6.665	21.157	0.00	0.00	LX0	H
ATOM	2526	CA	GLY	288	44.012	8.192	22.522	1.00	0.00	LX0	C
ATOM	2527	C	GLY	288	43.456	9.444	21.865	1.00	0.00	LX0	C
ATOM	2528	O	GLY	288	43.365	9.534	20.646	1.00	0.00	LX0	O
ATOM	2529	N	ALA	289	43.161	10.431	22.724	1.00	0.00	LX0	N
ATOM	2530	H	ALA	289	43.105	10.223	23.704	0.00	0.00	LX0	H
ATOM	2531	CA	ALA	289	42.758	11.736	22.198	1.00	0.00	LX0	C
ATOM	2532	CB	ALA	289	42.389	12.689	23.335	1.00	0.00	LX0	C
ATOM	2533	C	ALA	289	43.787	12.429	21.317	1.00	0.00	LX0	C
ATOM	2534	O	ALA	289	43.469	13.324	20.539	1.00	0.00	LX0	O
ATOM	2535	N	THR	290	45.040	11.988	21.474	1.00	0.00	LX0	N
ATOM	2536	H	THR	290	45.289	11.224	22.068	0.00	0.00	LX0	H
ATOM	2537	CA	THR	290	46.054	12.540	20.592	1.00	0.00	LX0	C
ATOM	2538	CB	THR	290	47.408	12.534	21.299	1.00	0.00	LX0	C
ATOM	2539	OG1	THR	290	47.244	12.953	22.663	1.00	0.00	LX0	O
ATOM	2540	HG1	THR	290	48.106	12.904	23.055	0.00	0.00	LX0	H
ATOM	2541	CG2	THR	290	48.429	13.428	20.586	1.00	0.00	LX0	C
ATOM	2542	C	THR	290	46.095	11.806	19.264	1.00	0.00	LX0	C
ATOM	2543	O	THR	290	46.851	10.869	19.030	1.00	0.00	LX0	O
ATOM	2544	N	CYS	291	45.217	12.289	18.382	1.00	0.00	LX0	N
ATOM	2545	H	CYS	291	44.592	13.025	18.647	0.00	0.00	LX0	H
ATOM	2546	CA	CYS	291	45.254	11.716	17.042	1.00	0.00	LX0	C
ATOM	2547	CB	CYS	291	44.025	12.131	16.243	1.00	0.00	LX0	C
ATOM	2548	SG	CYS	291	42.606	12.618	17.263	1.00	0.00	LX0	S
ATOM	2549	C	CYS	291	46.523	12.100	16.305	1.00	0.00	LX0	C
ATOM	2550	O	CYS	291	46.935	13.257	16.294	1.00	0.00	LX0	O
ATOM	2551	N	VAL	292	47.147	11.059	15.742	1.00	0.00	LX0	N
ATOM	2552	H	VAL	292	46.743	10.142	15.778	0.00	0.00	LX0	H
ATOM	2553	CA	VAL	292	48.444	11.255	15.101	1.00	0.00	LX0	C
ATOM	2554	CB	VAL	292	49.586	10.711	15.980	1.00	0.00	LX0	C
ATOM	2555	CG1	VAL	292	49.836	11.606	17.195	1.00	0.00	LX0	C
ATOM	2556	CG2	VAL	292	49.357	9.251	16.386	1.00	0.00	LX0	C
ATOM	2557	C	VAL	292	48.471	10.600	13.733	1.00	0.00	LX0	C
ATOM	2558	O	VAL	292	47.624	9.781	13.396	1.00	0.00	LX0	O
ATOM	2559	N	LYS	293	49.487	10.991	12.948	1.00	0.00	LX0	N
ATOM	2560	H	LYS	293	50.125	11.701	13.249	0.00	0.00	LX0	H
ATOM	2561	CA	LYS	293	49.538	10.390	11.616	1.00	0.00	LX0	C
ATOM	2562	CB	LYS	293	50.128	11.367	10.598	1.00	0.00	LX0	C
ATOM	2563	CG	LYS	293	49.133	11.645	9.458	1.00	0.00	LX0	C
ATOM	2564	CD	LYS	293	49.668	12.590	8.362	1.00	0.00	LX0	C
ATOM	2565	CE	LYS	293	50.025	14.010	8.849	1.00	0.00	LX0	C
ATOM	2566	NZ	LYS	293	48.812	14.717	9.290	1.00	0.00	LX0	N
ATOM	2567	HZ1	LYS	293	48.918	15.174	10.228	0.00	0.00	LX0	H
ATOM	2568	HZ2	LYS	293	48.473	15.409	8.587	0.00	0.00	LX0	H
ATOM	2569	HZ3	LYS	293	47.986	14.086	9.347	0.00	0.00	LX0	H
ATOM	2570	C	LYS	293	50.170	9.006	11.508	1.00	0.00	LX0	C
ATOM	2571	O	LYS	293	50.137	8.382	10.457	1.00	0.00	LX0	O
ATOM	2572	N	LYS	294	50.729	8.539	12.647	1.00	0.00	LX0	N
ATOM	2573	H	LYS	294	50.808	9.127	13.450	0.00	0.00	LX0	H
ATOM	2574	CA	LYS	294	51.031	7.110	12.806	1.00	0.00	LX0	C

ATOM	2575	CB	LYS	294	52.109	6.576	11.849	1.00	0.00	LX0	C
ATOM	2576	CG	LYS	294	53.513	7.165	11.985	1.00	0.00	LX0	C
ATOM	2577	CD	LYS	294	54.446	6.492	10.981	1.00	0.00	LX0	C
ATOM	2578	CE	LYS	294	55.876	7.026	11.020	1.00	0.00	LX0	C
ATOM	2579	NZ	LYS	294	56.664	6.321	10.000	1.00	0.00	LX0	N
ATOM	2580	HZ1	LYS	294	57.648	6.657	10.026	0.00	0.00	LX0	H
ATOM	2581	HZ2	LYS	294	56.636	5.299	10.190	0.00	0.00	LX0	H
ATOM	2582	HZ3	LYS	294	56.256	6.507	9.061	0.00	0.00	LX0	H
ATOM	2583	C	LYS	294	51.376	6.742	14.232	1.00	0.00	LX0	C
ATOM	2584	O	LYS	294	51.809	7.574	15.018	1.00	0.00	LX0	O
ATOM	2585	N	CYS	295	51.145	5.456	14.527	1.00	0.00	LX0	N
ATOM	2586	H	CYS	295	50.799	4.838	13.824	0.00	0.00	LX0	H
ATOM	2587	CA	CYS	295	51.380	4.970	15.888	1.00	0.00	LX0	C
ATOM	2588	CB	CYS	295	50.829	3.552	16.036	1.00	0.00	LX0	C
ATOM	2589	SG	CYS	295	49.142	3.380	15.412	1.00	0.00	LX0	S
ATOM	2590	C	CYS	295	52.838	4.970	16.322	1.00	0.00	LX0	C
ATOM	2591	O	CYS	295	53.737	4.674	15.541	1.00	0.00	LX0	O
ATOM	2592	N	PRO	296	53.048	5.295	17.622	1.00	0.00	LX0	N
ATOM	2593	CD	PRO	296	52.085	5.875	18.554	1.00	0.00	LX0	C
ATOM	2594	CA	PRO	296	54.360	5.054	18.237	1.00	0.00	LX0	C
ATOM	2595	CB	PRO	296	54.137	5.474	19.694	1.00	0.00	LX0	C
ATOM	2596	CG	PRO	296	52.957	6.444	19.665	1.00	0.00	LX0	C
ATOM	2597	C	PRO	296	54.785	3.597	18.127	1.00	0.00	LX0	C
ATOM	2598	O	PRO	296	54.009	2.692	18.404	1.00	0.00	LX0	O
ATOM	2599	N	ARG	297	56.056	3.420	17.713	1.00	0.00	LX0	N
ATOM	2600	H	ARG	297	56.570	4.246	17.498	0.00	0.00	LX0	H
ATOM	2601	CA	ARG	297	56.608	2.092	17.402	1.00	0.00	LX0	C
ATOM	2602	CB	ARG	297	58.146	2.139	17.415	1.00	0.00	LX0	C
ATOM	2603	CG	ARG	297	58.791	0.783	17.084	1.00	0.00	LX0	C
ATOM	2604	CD	ARG	297	60.013	0.454	17.944	1.00	0.00	LX0	C
ATOM	2605	NE	ARG	297	61.187	1.192	17.492	1.00	0.00	LX0	N
ATOM	2606	HE	ARG	297	61.237	1.401	16.515	0.00	0.00	LX0	H
ATOM	2607	CZ	ARG	297	62.221	1.433	18.323	1.00	0.00	LX0	C
ATOM	2608	NH1	ARG	297	62.158	1.148	19.623	1.00	0.00	LX0	N
ATOM	2609	HH11	ARG	297	62.929	1.354	20.242	0.00	0.00	LX0	H
ATOM	2610	HH12	ARG	297	61.356	0.734	20.063	0.00	0.00	LX0	H
ATOM	2611	NH2	ARG	297	63.327	1.965	17.817	1.00	0.00	LX0	N
ATOM	2612	HH21	ARG	297	64.125	2.083	18.419	0.00	0.00	LX0	H
ATOM	2613	HH22	ARG	297	63.405	2.236	16.860	0.00	0.00	LX0	H
ATOM	2614	C	ARG	297	56.156	0.918	18.266	1.00	0.00	LX0	C
ATOM	2615	O	ARG	297	55.831	-0.159	17.786	1.00	0.00	LX0	O
ATOM	2616	N	ASN	298	56.240	1.159	19.580	1.00	0.00	LX0	N
ATOM	2617	H	ASN	298	56.282	2.088	19.949	0.00	0.00	LX0	H
ATOM	2618	CA	ASN	298	56.072	0.010	20.463	1.00	0.00	LX0	C
ATOM	2619	CB	ASN	298	56.739	0.243	21.829	1.00	0.00	LX0	C
ATOM	2620	CG	ASN	298	55.980	1.280	22.637	1.00	0.00	LX0	C
ATOM	2621	OD1	ASN	298	55.353	2.181	22.096	1.00	0.00	LX0	O
ATOM	2622	ND2	ASN	298	56.047	1.096	23.954	1.00	0.00	LX0	N
ATOM	2623	HD21	ASN	298	56.577	0.348	24.361	0.00	0.00	LX0	H
ATOM	2624	HD22	ASN	298	55.622	1.771	24.551	0.00	0.00	LX0	H
ATOM	2625	C	ASN	298	54.645	-0.466	20.634	1.00	0.00	LX0	C
ATOM	2626	O	ASN	298	54.409	-1.601	21.033	1.00	0.00	LX0	O
ATOM	2627	N	TYR	299	53.699	0.440	20.360	1.00	0.00	LX0	N
ATOM	2628	H	TYR	299	53.930	1.301	19.904	0.00	0.00	LX0	H
ATOM	2629	CA	TYR	299	52.310	0.035	20.536	1.00	0.00	LX0	C
ATOM	2630	CB	TYR	299	51.410	1.270	20.666	1.00	0.00	LX0	C
ATOM	2631	CG	TYR	299	51.329	1.689	22.118	1.00	0.00	LX0	C
ATOM	2632	CD1	TYR	299	52.379	2.426	22.705	1.00	0.00	LX0	C
ATOM	2633	CE1	TYR	299	52.309	2.749	24.073	1.00	0.00	LX0	C
ATOM	2634	CD2	TYR	299	50.191	1.310	22.855	1.00	0.00	LX0	C
ATOM	2635	CE2	TYR	299	50.116	1.639	24.217	1.00	0.00	LX0	C

ATOM	2636	CZ	TYR	299	51.184	2.333	24.816	1.00	0.00	LX0	C
ATOM	2637	OH	TYR	299	51.116	2.589	26.173	1.00	0.00	LX0	O
ATOM	2638	HH	TYR	299	51.938	2.961	26.488	0.00	0.00	LX0	H
ATOM	2639	C	TYR	299	51.846	-0.891	19.434	1.00	0.00	LX0	C
ATOM	2640	O	TYR	299	52.315	-0.836	18.306	1.00	0.00	LX0	O
ATOM	2641	N	VAL	300	50.918	-1.773	19.828	1.00	0.00	LX0	N
ATOM	2642	H	VAL	300	50.457	-1.648	20.704	0.00	0.00	LX0	H
ATOM	2643	CA	VAL	300	50.330	-2.625	18.799	1.00	0.00	LX0	C
ATOM	2644	CB	VAL	300	49.465	-3.745	19.391	1.00	0.00	LX0	C
ATOM	2645	CG1	VAL	300	49.118	-4.787	18.333	1.00	0.00	LX0	C
ATOM	2646	CG2	VAL	300	50.073	-4.401	20.618	1.00	0.00	LX0	C
ATOM	2647	C	VAL	300	49.462	-1.778	17.899	1.00	0.00	LX0	C
ATOM	2648	O	VAL	300	48.564	-1.075	18.346	1.00	0.00	LX0	O
ATOM	2649	N	VAL	301	49.776	-1.857	16.614	1.00	0.00	LX0	N
ATOM	2650	H	VAL	301	50.490	-2.491	16.312	0.00	0.00	LX0	H
ATOM	2651	CA	VAL	301	48.888	-1.138	15.716	1.00	0.00	LX0	C
ATOM	2652	CB	VAL	301	49.699	-0.623	14.515	1.00	0.00	LX0	C
ATOM	2653	CG1	VAL	301	48.918	0.346	13.626	1.00	0.00	LX0	C
ATOM	2654	CG2	VAL	301	51.010	0.008	14.989	1.00	0.00	LX0	C
ATOM	2655	C	VAL	301	47.760	-2.073	15.312	1.00	0.00	LX0	C
ATOM	2656	O	VAL	301	47.937	-3.284	15.240	1.00	0.00	LX0	O
ATOM	2657	N	THR	302	46.592	-1.497	15.040	1.00	0.00	LX0	N
ATOM	2658	H	THR	302	46.418	-0.530	15.229	0.00	0.00	LX0	H
ATOM	2659	CA	THR	302	45.722	-2.319	14.215	1.00	0.00	LX0	C
ATOM	2660	CB	THR	302	44.299	-2.389	14.784	1.00	0.00	LX0	C
ATOM	2661	OG1	THR	302	43.648	-1.112	14.721	1.00	0.00	LX0	O
ATOM	2662	HG1	THR	302	42.749	-1.268	15.016	0.00	0.00	LX0	H
ATOM	2663	CG2	THR	302	44.292	-2.933	16.214	1.00	0.00	LX0	C
ATOM	2664	C	THR	302	45.721	-1.753	12.815	1.00	0.00	LX0	C
ATOM	2665	O	THR	302	45.963	-0.568	12.622	1.00	0.00	LX0	O
ATOM	2666	N	ASP	303	45.380	-2.612	11.849	1.00	0.00	LX0	N
ATOM	2667	H	ASP	303	45.374	-3.605	11.984	0.00	0.00	LX0	H
ATOM	2668	CA	ASP	303	45.184	-2.090	10.492	1.00	0.00	LX0	C
ATOM	2669	CB	ASP	303	44.934	-3.245	9.512	1.00	0.00	LX0	C
ATOM	2670	CG	ASP	303	46.026	-4.302	9.613	1.00	0.00	LX0	C
ATOM	2671	OD1	ASP	303	46.881	-4.388	8.738	1.00	0.00	LX0	O
ATOM	2672	OD2	ASP	303	46.022	-5.090	10.554	1.00	0.00	LX0	O
ATOM	2673	C	ASP	303	44.078	-1.037	10.387	1.00	0.00	LX0	C
ATOM	2674	O	ASP	303	44.025	-0.215	9.483	1.00	0.00	LX0	O
ATOM	2675	N	HIS	304	43.194	-1.086	11.401	1.00	0.00	LX0	N
ATOM	2676	H	HIS	304	43.293	-1.799	12.091	0.00	0.00	LX0	H
ATOM	2677	CA	HIS	304	42.159	-0.062	11.545	1.00	0.00	LX0	C
ATOM	2678	CB	HIS	304	41.155	-0.544	12.592	1.00	0.00	LX0	C
ATOM	2679	CG	HIS	304	39.787	0.090	12.473	1.00	0.00	LX0	C
ATOM	2680	ND1	HIS	304	39.511	1.285	11.915	1.00	0.00	LX0	N
ATOM	2681	HD1	HIS	304	40.160	1.922	11.542	0.00	0.00	LX0	H
ATOM	2682	CD2	HIS	304	38.594	-0.478	12.920	1.00	0.00	LX0	C
ATOM	2683	NE2	HIS	304	37.592	0.380	12.619	1.00	0.00	LX0	N
ATOM	2684	CE1	HIS	304	38.153	1.470	12.000	1.00	0.00	LX0	C
ATOM	2685	C	HIS	304	42.684	1.322	11.912	1.00	0.00	LX0	C
ATOM	2686	O	HIS	304	42.097	2.340	11.558	1.00	0.00	LX0	O
ATOM	2687	N	GLY	305	43.804	1.306	12.653	1.00	0.00	LX0	N
ATOM	2688	H	GLY	305	44.261	0.444	12.878	0.00	0.00	LX0	H
ATOM	2689	CA	GLY	305	44.390	2.576	13.073	1.00	0.00	LX0	C
ATOM	2690	C	GLY	305	44.567	2.746	14.573	1.00	0.00	LX0	C
ATOM	2691	O	GLY	305	45.022	3.775	15.061	1.00	0.00	LX0	O
ATOM	2692	N	SER	306	44.179	1.695	15.297	1.00	0.00	LX0	N
ATOM	2693	H	SER	306	43.854	0.846	14.884	0.00	0.00	LX0	H
ATOM	2694	CA	SER	306	44.220	1.804	16.750	1.00	0.00	LX0	C
ATOM	2695	CB	SER	306	43.207	0.811	17.311	1.00	0.00	LX0	C
ATOM	2696	OG	SER	306	41.999	0.892	16.539	1.00	0.00	LX0	O

ATOM	2697	HG	SER	306	41.644	0.010	16.453	0.00	0.00	LX0	H
ATOM	2698	C	SER	306	45.608	1.572	17.325	1.00	0.00	LX0	C
ATOM	2699	O	SER	306	46.316	0.664	16.908	1.00	0.00	LX0	O
ATOM	2700	N	CYS	307	45.965	2.423	18.299	1.00	0.00	LX0	N
ATOM	2701	H	CYS	307	45.360	3.162	18.597	0.00	0.00	LX0	H
ATOM	2702	CA	CYS	307	47.267	2.233	18.942	1.00	0.00	LX0	C
ATOM	2703	CB	CYS	307	47.913	3.585	19.242	1.00	0.00	LX0	C
ATOM	2704	SG	CYS	307	47.891	4.740	17.846	1.00	0.00	LX0	S
ATOM	2705	C	CYS	307	47.181	1.423	20.224	1.00	0.00	LX0	C
ATOM	2706	O	CYS	307	47.406	1.921	21.320	1.00	0.00	LX0	O
ATOM	2707	N	VAL	308	46.791	0.156	20.050	1.00	0.00	LX0	N
ATOM	2708	H	VAL	308	46.922	-0.257	19.145	0.00	0.00	LX0	H
ATOM	2709	CA	VAL	308	46.493	-0.617	21.253	1.00	0.00	LX0	C
ATOM	2710	CB	VAL	308	45.539	-1.784	20.934	1.00	0.00	LX0	C
ATOM	2711	CG1	VAL	308	44.210	-1.272	20.385	1.00	0.00	LX0	C
ATOM	2712	CG2	VAL	308	46.136	-2.794	19.960	1.00	0.00	LX0	C
ATOM	2713	C	VAL	308	47.716	-1.085	22.038	1.00	0.00	LX0	C
ATOM	2714	O	VAL	308	48.827	-1.201	21.541	1.00	0.00	LX0	O
ATOM	2715	N	ARG	309	47.453	-1.372	23.320	1.00	0.00	LX0	N
ATOM	2716	H	ARG	309	46.539	-1.203	23.672	0.00	0.00	LX0	H
ATOM	2717	CA	ARG	309	48.500	-1.989	24.139	1.00	0.00	LX0	C
ATOM	2718	CB	ARG	309	48.206	-1.855	25.630	1.00	0.00	LX0	C
ATOM	2719	CG	ARG	309	48.160	-0.469	26.266	1.00	0.00	LX0	C
ATOM	2720	CD	ARG	309	47.538	-0.646	27.650	1.00	0.00	LX0	C
ATOM	2721	NE	ARG	309	47.503	0.570	28.462	1.00	0.00	LX0	N
ATOM	2722	HE	ARG	309	48.312	1.158	28.474	0.00	0.00	LX0	H
ATOM	2723	CZ	ARG	309	46.523	0.659	29.392	1.00	0.00	LX0	C
ATOM	2724	NH1	ARG	309	45.404	-0.052	29.279	1.00	0.00	LX0	N
ATOM	2725	HH11	ARG	309	44.792	-0.096	30.081	0.00	0.00	LX0	H
ATOM	2726	HH12	ARG	309	45.144	-0.539	28.443	0.00	0.00	LX0	H
ATOM	2727	NH2	ARG	309	46.679	1.446	30.450	1.00	0.00	LX0	N
ATOM	2728	HH21	ARG	309	45.976	1.416	31.185	0.00	0.00	LX0	H
ATOM	2729	HH22	ARG	309	47.463	2.044	30.583	0.00	0.00	LX0	H
ATOM	2730	C	ARG	309	48.659	-3.479	23.897	1.00	0.00	LX0	C
ATOM	2731	O	ARG	309	49.714	-4.062	24.123	1.00	0.00	LX0	O
ATOM	2732	N	ALA	310	47.529	-4.077	23.501	1.00	0.00	LX0	N
ATOM	2733	H	ALA	310	46.762	-3.575	23.110	0.00	0.00	LX0	H
ATOM	2734	CA	ALA	310	47.413	-5.515	23.695	1.00	0.00	LX0	C
ATOM	2735	CB	ALA	310	46.297	-5.802	24.698	1.00	0.00	LX0	C
ATOM	2736	C	ALA	310	47.085	-6.235	22.412	1.00	0.00	LX0	C
ATOM	2737	O	ALA	310	46.782	-5.633	21.391	1.00	0.00	LX0	O
ATOM	2738	N	CYS	311	47.127	-7.568	22.518	1.00	0.00	LX0	N
ATOM	2739	H	CYS	311	47.367	-7.996	23.387	0.00	0.00	LX0	H
ATOM	2740	CA	CYS	311	46.545	-8.331	21.419	1.00	0.00	LX0	C
ATOM	2741	CB	CYS	311	46.976	-9.793	21.506	1.00	0.00	LX0	C
ATOM	2742	SG	CYS	311	48.773	-9.998	21.588	1.00	0.00	LX0	S
ATOM	2743	C	CYS	311	45.031	-8.251	21.464	1.00	0.00	LX0	C
ATOM	2744	O	CYS	311	44.430	-8.296	22.532	1.00	0.00	LX0	O
ATOM	2745	N	GLY	312	44.443	-8.158	20.259	1.00	0.00	LX0	N
ATOM	2746	H	GLY	312	44.993	-8.020	19.439	0.00	0.00	LX0	H
ATOM	2747	CA	GLY	312	42.994	-8.351	20.186	1.00	0.00	LX0	C
ATOM	2748	C	GLY	312	42.613	-9.754	20.631	1.00	0.00	LX0	C
ATOM	2749	O	GLY	312	43.466	-10.629	20.712	1.00	0.00	LX0	O
ATOM	2750	N	ALA	313	41.316	-9.929	20.924	1.00	0.00	LX0	N
ATOM	2751	H	ALA	313	40.710	-9.136	20.841	0.00	0.00	LX0	H
ATOM	2752	CA	ALA	313	40.854	-11.164	21.569	1.00	0.00	LX0	C
ATOM	2753	CB	ALA	313	39.324	-11.234	21.539	1.00	0.00	LX0	C
ATOM	2754	C	ALA	313	41.423	-12.481	21.049	1.00	0.00	LX0	C
ATOM	2755	O	ALA	313	42.020	-13.261	21.781	1.00	0.00	LX0	O
ATOM	2756	N	ASP	314	41.210	-12.681	19.747	1.00	0.00	LX0	N
ATOM	2757	H	ASP	314	40.768	-11.992	19.167	0.00	0.00	LX0	H

ATOM	2758	CA	ASP	314	41.675	-13.884	19.049	1.00	0.00	LX0 C
ATOM	2759	CB	ASP	314	40.613	-14.297	18.019	1.00	0.00	LX0 C
ATOM	2760	CG	ASP	314	40.285	-13.175	17.028	1.00	0.00	LX0 C
ATOM	2761	OD1	ASP	314	40.921	-12.119	17.053	1.00	0.00	LX0 O
ATOM	2762	OD2	ASP	314	39.387	-13.367	16.216	1.00	0.00	LX0 O
ATOM	2763	C	ASP	314	43.029	-13.728	18.363	1.00	0.00	LX0 C
ATOM	2764	O	ASP	314	43.508	-14.570	17.610	1.00	0.00	LX0 O
ATOM	2765	N	SER	315	43.620	-12.565	18.627	1.00	0.00	LX0 N
ATOM	2766	H	SER	315	43.318	-12.000	19.392	0.00	0.00	LX0 H
ATOM	2767	CA	SER	315	44.762	-12.165	17.828	1.00	0.00	LX0 C
ATOM	2768	CB	SER	315	44.545	-10.711	17.418	1.00	0.00	LX0 C
ATOM	2769	OG	SER	315	43.391	-10.588	16.575	1.00	0.00	LX0 O
ATOM	2770	HG	SER	315	42.702	-11.183	16.885	0.00	0.00	LX0 H
ATOM	2771	C	SER	315	46.077	-12.350	18.558	1.00	0.00	LX0 C
ATOM	2772	O	SER	315	46.123	-12.502	19.772	1.00	0.00	LX0 O
ATOM	2773	N	TYR	316	47.159	-12.311	17.771	1.00	0.00	LX0 N
ATOM	2774	H	TYR	316	47.083	-12.213	16.775	0.00	0.00	LX0 H
ATOM	2775	CA	TYR	316	48.450	-12.219	18.441	1.00	0.00	LX0 C
ATOM	2776	CB	TYR	316	49.285	-13.492	18.216	1.00	0.00	LX0 C
ATOM	2777	CG	TYR	316	50.239	-13.706	19.376	1.00	0.00	LX0 C
ATOM	2778	CD1	TYR	316	49.771	-14.379	20.523	1.00	0.00	LX0 C
ATOM	2779	CE1	TYR	316	50.625	-14.519	21.631	1.00	0.00	LX0 C
ATOM	2780	CD2	TYR	316	51.559	-13.215	19.293	1.00	0.00	LX0 C
ATOM	2781	CE2	TYR	316	52.410	-13.345	20.405	1.00	0.00	LX0 C
ATOM	2782	CZ	TYR	316	51.926	-13.980	21.568	1.00	0.00	LX0 C
ATOM	2783	OH	TYR	316	52.740	-14.077	22.680	1.00	0.00	LX0 O
ATOM	2784	HH	TYR	316	53.469	-13.468	22.588	0.00	0.00	LX0 H
ATOM	2785	C	TYR	316	49.176	-10.973	17.978	1.00	0.00	LX0 C
ATOM	2786	O	TYR	316	48.931	-10.471	16.887	1.00	0.00	LX0 O
ATOM	2787	N	GLU	317	50.066	-10.490	18.856	1.00	0.00	LX0 N
ATOM	2788	H	GLU	317	50.195	-10.934	19.741	0.00	0.00	LX0 H
ATOM	2789	CA	GLU	317	50.935	-9.390	18.451	1.00	0.00	LX0 C
ATOM	2790	CB	GLU	317	51.504	-8.686	19.683	1.00	0.00	LX0 C
ATOM	2791	CG	GLU	317	52.020	-7.286	19.351	1.00	0.00	LX0 C
ATOM	2792	CD	GLU	317	53.023	-6.803	20.384	1.00	0.00	LX0 C
ATOM	2793	OE1	GLU	317	54.072	-6.306	20.000	1.00	0.00	LX0 O
ATOM	2794	OE2	GLU	317	52.784	-6.870	21.582	1.00	0.00	LX0 O
ATOM	2795	C	GLU	317	52.066	-9.879	17.560	1.00	0.00	LX0 C
ATOM	2796	O	GLU	317	53.079	-10.398	18.025	1.00	0.00	LX0 O
ATOM	2797	N	MET	318	51.835	-9.712	16.258	1.00	0.00	LX0 N
ATOM	2798	H	MET	318	51.020	-9.215	15.949	0.00	0.00	LX0 H
ATOM	2799	CA	MET	318	52.824	-10.178	15.296	1.00	0.00	LX0 C
ATOM	2800	CB	MET	318	52.164	-11.179	14.345	1.00	0.00	LX0 C
ATOM	2801	CG	MET	318	53.144	-12.129	13.652	1.00	0.00	LX0 C
ATOM	2802	SD	MET	318	53.986	-13.215	14.817	1.00	0.00	LX0 S
ATOM	2803	CE	MET	318	52.546	-14.089	15.451	1.00	0.00	LX0 C
ATOM	2804	C	MET	318	53.421	-9.012	14.538	1.00	0.00	LX0 C
ATOM	2805	O	MET	318	52.756	-8.020	14.273	1.00	0.00	LX0 O
ATOM	2806	N	GLU	319	54.704	-9.148	14.208	1.00	0.00	LX0 N
ATOM	2807	H	GLU	319	55.184	-10.013	14.366	0.00	0.00	LX0 H
ATOM	2808	CA	GLU	319	55.277	-8.134	13.329	1.00	0.00	LX0 C
ATOM	2809	CB	GLU	319	56.813	-8.086	13.423	1.00	0.00	LX0 C
ATOM	2810	CG	GLU	319	57.580	-9.422	13.402	1.00	0.00	LX0 C
ATOM	2811	CD	GLU	319	57.509	-10.102	14.760	1.00	0.00	LX0 C
ATOM	2812	OE1	GLU	319	58.160	-9.649	15.694	1.00	0.00	LX0 O
ATOM	2813	OE2	GLU	319	56.760	-11.061	14.921	1.00	0.00	LX0 O
ATOM	2814	C	GLU	319	54.805	-8.326	11.901	1.00	0.00	LX0 C
ATOM	2815	O	GLU	319	54.775	-9.433	11.377	1.00	0.00	LX0 O
ATOM	2816	N	GLU	320	54.394	-7.198	11.314	1.00	0.00	LX0 N
ATOM	2817	H	GLU	320	54.424	-6.327	11.807	0.00	0.00	LX0 H
ATOM	2818	CA	GLU	320	53.867	-7.304	9.958	1.00	0.00	LX0 C

ATOM	2819	CB	GLU	320	52.337	-7.201	9.998	1.00	0.00	LX0	C
ATOM	2820	CG	GLU	320	51.614	-7.715	8.746	1.00	0.00	LX0	C
ATOM	2821	CD	GLU	320	50.128	-7.433	8.877	1.00	0.00	LX0	C
ATOM	2822	OE1	GLU	320	49.566	-6.751	8.024	1.00	0.00	LX0	O
ATOM	2823	OE2	GLU	320	49.518	-7.843	9.860	1.00	0.00	LX0	O
ATOM	2824	C	GLU	320	54.545	-6.304	9.034	1.00	0.00	LX0	C
ATOM	2825	O	GLU	320	55.643	-6.543	8.544	1.00	0.00	LX0	O
ATOM	2826	N	ASP	321	53.897	-5.148	8.839	1.00	0.00	LX0	N
ATOM	2827	H	ASP	321	53.032	-4.900	9.277	0.00	0.00	LX0	H
ATOM	2828	CA	ASP	321	54.538	-4.101	8.042	1.00	0.00	LX0	C
ATOM	2829	CB	ASP	321	53.515	-3.255	7.251	1.00	0.00	LX0	C
ATOM	2830	CG	ASP	321	52.155	-3.113	7.928	1.00	0.00	LX0	C
ATOM	2831	OD1	ASP	321	51.144	-3.173	7.237	1.00	0.00	LX0	O
ATOM	2832	OD2	ASP	321	52.071	-2.986	9.146	1.00	0.00	LX0	O
ATOM	2833	C	ASP	321	55.489	-3.235	8.850	1.00	0.00	LX0	C
ATOM	2834	O	ASP	321	55.346	-2.031	9.027	1.00	0.00	LX0	O
ATOM	2835	N	GLY	322	56.514	-3.930	9.366	1.00	0.00	LX0	N
ATOM	2836	H	GLY	322	56.556	-4.917	9.200	0.00	0.00	LX0	H
ATOM	2837	CA	GLY	322	57.465	-3.241	10.234	1.00	0.00	LX0	C
ATOM	2838	C	GLY	322	56.992	-3.135	11.672	1.00	0.00	LX0	C
ATOM	2839	O	GLY	322	57.605	-3.645	12.600	1.00	0.00	LX0	O
ATOM	2840	N	VAL	323	55.858	-2.440	11.817	1.00	0.00	LX0	N
ATOM	2841	H	VAL	323	55.391	-2.101	10.998	0.00	0.00	LX0	H
ATOM	2842	CA	VAL	323	55.246	-2.399	13.142	1.00	0.00	LX0	C
ATOM	2843	CB	VAL	323	54.204	-1.276	13.215	1.00	0.00	LX0	C
ATOM	2844	CG1	VAL	323	54.885	0.093	13.165	1.00	0.00	LX0	C
ATOM	2845	CG2	VAL	323	53.122	-1.422	12.138	1.00	0.00	LX0	C
ATOM	2846	C	VAL	323	54.642	-3.734	13.542	1.00	0.00	LX0	C
ATOM	2847	O	VAL	323	54.389	-4.606	12.716	1.00	0.00	LX0	O
ATOM	2848	N	ARG	324	54.427	-3.857	14.858	1.00	0.00	LX0	N
ATOM	2849	H	ARG	324	54.580	-3.101	15.492	0.00	0.00	LX0	H
ATOM	2850	CA	ARG	324	53.743	-5.073	15.277	1.00	0.00	LX0	C
ATOM	2851	CB	ARG	324	54.339	-5.651	16.566	1.00	0.00	LX0	C
ATOM	2852	CG	ARG	324	55.872	-5.631	16.594	1.00	0.00	LX0	C
ATOM	2853	CD	ARG	324	56.498	-6.500	17.692	1.00	0.00	LX0	C
ATOM	2854	NE	ARG	324	56.576	-7.902	17.278	1.00	0.00	LX0	N
ATOM	2855	HE	ARG	324	57.210	-8.164	16.540	0.00	0.00	LX0	H
ATOM	2856	CZ	ARG	324	55.823	-8.880	17.818	1.00	0.00	LX0	C
ATOM	2857	NH1	ARG	324	54.978	-8.627	18.802	1.00	0.00	LX0	N
ATOM	2858	HH11	ARG	324	54.394	-9.356	19.168	0.00	0.00	LX0	H
ATOM	2859	HH12	ARG	324	54.872	-7.703	19.191	0.00	0.00	LX0	H
ATOM	2860	NH2	ARG	324	55.915	-10.115	17.350	1.00	0.00	LX0	N
ATOM	2861	HH21	ARG	324	55.329	-10.853	17.679	0.00	0.00	LX0	H
ATOM	2862	HH22	ARG	324	56.587	-10.327	16.616	0.00	0.00	LX0	H
ATOM	2863	C	ARG	324	52.257	-4.824	15.410	1.00	0.00	LX0	C
ATOM	2864	O	ARG	324	51.805	-3.972	16.167	1.00	0.00	LX0	O
ATOM	2865	N	LYS	325	51.517	-5.568	14.592	1.00	0.00	LX0	N
ATOM	2866	H	LYS	325	51.936	-6.317	14.076	0.00	0.00	LX0	H
ATOM	2867	CA	LYS	325	50.073	-5.409	14.640	1.00	0.00	LX0	C
ATOM	2868	CB	LYS	325	49.521	-5.119	13.247	1.00	0.00	LX0	C
ATOM	2869	CG	LYS	325	50.005	-3.762	12.732	1.00	0.00	LX0	C
ATOM	2870	CD	LYS	325	49.442	-3.405	11.362	1.00	0.00	LX0	C
ATOM	2871	CE	LYS	325	49.726	-4.561	10.419	1.00	0.00	LX0	C
ATOM	2872	NZ	LYS	325	49.563	-4.185	9.015	1.00	0.00	LX0	N
ATOM	2873	HZ1	LYS	325	49.785	-5.004	8.412	0.00	0.00	LX0	H
ATOM	2874	HZ2	LYS	325	48.595	-3.872	8.804	0.00	0.00	LX0	H
ATOM	2875	HZ3	LYS	325	50.265	-3.451	8.755	0.00	0.00	LX0	H
ATOM	2876	C	LYS	325	49.413	-6.622	15.244	1.00	0.00	LX0	C
ATOM	2877	O	LYS	325	50.047	-7.638	15.499	1.00	0.00	LX0	O
ATOM	2878	N	CYS	326	48.104	-6.494	15.465	1.00	0.00	LX0	N
ATOM	2879	H	CYS	326	47.618	-5.633	15.302	0.00	0.00	LX0	H

ATOM	2880	CA	CYS	326	47.452	-7.724	15.890	1.00	0.00	LX0	C
ATOM	2881	CB	CYS	326	46.801	-7.568	17.265	1.00	0.00	LX0	C
ATOM	2882	SG	CYS	326	45.614	-6.207	17.402	1.00	0.00	LX0	S
ATOM	2883	C	CYS	326	46.495	-8.269	14.859	1.00	0.00	LX0	C
ATOM	2884	O	CYS	326	45.454	-7.695	14.569	1.00	0.00	LX0	O
ATOM	2885	N	LYS	327	46.906	-9.416	14.307	1.00	0.00	LX0	N
ATOM	2886	H	LYS	327	47.759	-9.848	14.603	0.00	0.00	LX0	H
ATOM	2887	CA	LYS	327	45.934	-10.129	13.488	1.00	0.00	LX0	C
ATOM	2888	CB	LYS	327	46.407	-10.315	12.044	1.00	0.00	LX0	C
ATOM	2889	CG	LYS	327	46.234	-9.048	11.202	1.00	0.00	LX0	C
ATOM	2890	CD	LYS	327	46.429	-9.315	9.706	1.00	0.00	LX0	C
ATOM	2891	CE	LYS	327	46.179	-8.084	8.830	1.00	0.00	LX0	C
ATOM	2892	NZ	LYS	327	47.189	-7.061	9.101	1.00	0.00	LX0	N
ATOM	2893	HZ1	LYS	327	47.840	-6.938	8.299	0.00	0.00	LX0	H
ATOM	2894	HZ2	LYS	327	46.772	-6.132	9.328	0.00	0.00	LX0	H
ATOM	2895	HZ3	LYS	327	47.824	-7.342	9.875	0.00	0.00	LX0	H
ATOM	2896	C	LYS	327	45.590	-11.453	14.123	1.00	0.00	LX0	C
ATOM	2897	O	LYS	327	46.321	-11.970	14.964	1.00	0.00	LX0	O
ATOM	2898	N	LYS	328	44.424	-11.958	13.712	1.00	0.00	LX0	N
ATOM	2899	H	LYS	328	43.907	-11.527	12.977	0.00	0.00	LX0	H
ATOM	2900	CA	LYS	328	43.929	-13.131	14.416	1.00	0.00	LX0	C
ATOM	2901	CB	LYS	328	42.406	-13.231	14.325	1.00	0.00	LX0	C
ATOM	2902	CG	LYS	328	41.833	-13.472	12.928	1.00	0.00	LX0	C
ATOM	2903	CD	LYS	328	40.309	-13.634	12.900	1.00	0.00	LX0	C
ATOM	2904	CE	LYS	328	39.751	-15.033	13.195	1.00	0.00	LX0	C
ATOM	2905	NZ	LYS	328	39.899	-15.469	14.590	1.00	0.00	LX0	N
ATOM	2906	HZ1	LYS	328	39.403	-16.385	14.711	0.00	0.00	LX0	H
ATOM	2907	HZ2	LYS	328	40.890	-15.627	14.852	0.00	0.00	LX0	H
ATOM	2908	HZ3	LYS	328	39.484	-14.783	15.254	0.00	0.00	LX0	H
ATOM	2909	C	LYS	328	44.567	-14.439	14.020	1.00	0.00	LX0	C
ATOM	2910	O	LYS	328	44.895	-14.696	12.868	1.00	0.00	LX0	O
ATOM	2911	N	CYS	329	44.684	-15.279	15.045	1.00	0.00	LX0	N
ATOM	2912	H	CYS	329	44.420	-14.981	15.965	0.00	0.00	LX0	H
ATOM	2913	CA	CYS	329	44.708	-16.696	14.723	1.00	0.00	LX0	C
ATOM	2914	CB	CYS	329	45.821	-17.388	15.514	1.00	0.00	LX0	C
ATOM	2915	SG	CYS	329	45.870	-16.958	17.273	1.00	0.00	LX0	S
ATOM	2916	C	CYS	329	43.312	-17.220	15.009	1.00	0.00	LX0	C
ATOM	2917	O	CYS	329	42.367	-16.436	15.061	1.00	0.00	LX0	O
ATOM	2918	N	GLU	330	43.187	-18.539	15.212	1.00	0.00	LX0	N
ATOM	2919	H	GLU	330	43.915	-19.213	15.128	0.00	0.00	LX0	H
ATOM	2920	CA	GLU	330	41.882	-18.889	15.753	1.00	0.00	LX0	C
ATOM	2921	CB	GLU	330	41.137	-19.898	14.870	1.00	0.00	LX0	C
ATOM	2922	CG	GLU	330	39.612	-19.834	15.067	1.00	0.00	LX0	C
ATOM	2923	CD	GLU	330	39.142	-18.395	14.919	1.00	0.00	LX0	C
ATOM	2924	OE1	GLU	330	39.027	-17.684	15.914	1.00	0.00	LX0	O
ATOM	2925	OE2	GLU	330	38.953	-17.943	13.799	1.00	0.00	LX0	O
ATOM	2926	C	GLU	330	41.936	-19.277	17.212	1.00	0.00	LX0	C
ATOM	2927	O	GLU	330	42.902	-19.886	17.676	1.00	0.00	LX0	O
ATOM	2928	N	GLY	331	40.884	-18.850	17.922	1.00	0.00	LX0	N
ATOM	2929	H	GLY	331	40.165	-18.357	17.423	0.00	0.00	LX0	H
ATOM	2930	CA	GLY	331	40.904	-18.909	19.378	1.00	0.00	LX0	C
ATOM	2931	C	GLY	331	41.735	-17.776	19.951	1.00	0.00	LX0	C
ATOM	2932	O	GLY	331	42.633	-17.260	19.297	1.00	0.00	LX0	O
ATOM	2933	N	PRO	332	41.417	-17.408	21.212	1.00	0.00	LX0	N
ATOM	2934	CD	PRO	332	40.375	-17.992	22.048	1.00	0.00	LX0	C
ATOM	2935	CA	PRO	332	42.108	-16.292	21.870	1.00	0.00	LX0	C
ATOM	2936	CB	PRO	332	41.574	-16.363	23.304	1.00	0.00	LX0	C
ATOM	2937	CG	PRO	332	40.179	-16.966	23.158	1.00	0.00	LX0	C
ATOM	2938	C	PRO	332	43.629	-16.280	21.797	1.00	0.00	LX0	C
ATOM	2939	O	PRO	332	44.291	-17.271	21.483	1.00	0.00	LX0	O
ATOM	2940	N	CYS	333	44.169	-15.099	22.134	1.00	0.00	LX0	N

ATOM	2941	H	CYS	333	43.553	-14.323	22.284	0.00	0.00	LX0	H
ATOM	2942	CA	CYS	333	45.617	-14.970	22.309	1.00	0.00	LX0	C
ATOM	2943	CB	CYS	333	45.950	-13.572	22.831	1.00	0.00	LX0	C
ATOM	2944	SG	CYS	333	47.728	-13.248	22.949	1.00	0.00	LX0	S
ATOM	2945	C	CYS	333	46.155	-16.054	23.230	1.00	0.00	LX0	C
ATOM	2946	O	CYS	333	45.471	-16.511	24.138	1.00	0.00	LX0	O
ATOM	2947	N	ARG	334	47.372	-16.512	22.904	1.00	0.00	LX0	N
ATOM	2948	H	ARG	334	47.966	-15.974	22.305	0.00	0.00	LX0	H
ATOM	2949	CA	ARG	334	47.821	-17.751	23.542	1.00	0.00	LX0	C
ATOM	2950	CB	ARG	334	49.041	-18.334	22.824	1.00	0.00	LX0	C
ATOM	2951	CG	ARG	334	48.765	-19.249	21.621	1.00	0.00	LX0	C
ATOM	2952	CD	ARG	334	48.214	-18.596	20.346	1.00	0.00	LX0	C
ATOM	2953	NE	ARG	334	46.754	-18.449	20.352	1.00	0.00	LX0	N
ATOM	2954	HE	ARG	334	46.283	-17.758	20.911	0.00	0.00	LX0	H
ATOM	2955	CZ	ARG	334	45.986	-19.195	19.527	1.00	0.00	LX0	C
ATOM	2956	NH1	ARG	334	46.506	-20.188	18.810	1.00	0.00	LX0	N
ATOM	2957	HH11	ARG	334	45.934	-20.726	18.190	0.00	0.00	LX0	H
ATOM	2958	HH12	ARG	334	47.478	-20.406	18.883	0.00	0.00	LX0	H
ATOM	2959	NH2	ARG	334	44.700	-18.920	19.411	1.00	0.00	LX0	N
ATOM	2960	HH21	ARG	334	44.076	-19.417	18.800	0.00	0.00	LX0	H
ATOM	2961	HH22	ARG	334	44.295	-18.152	19.926	0.00	0.00	LX0	H
ATOM	2962	C	ARG	334	48.104	-17.628	25.028	1.00	0.00	LX0	C
ATOM	2963	O	ARG	334	49.198	-17.274	25.444	1.00	0.00	LX0	O
ATOM	2964	N	LYS	335	47.054	-17.956	25.802	1.00	0.00	LX0	N
ATOM	2965	H	LYS	335	46.196	-18.133	25.320	0.00	0.00	LX0	H
ATOM	2966	CA	LYS	335	47.050	-17.851	27.263	1.00	0.00	LX0	C
ATOM	2967	CB	LYS	335	47.072	-19.237	27.945	1.00	0.00	LX0	C
ATOM	2968	CG	LYS	335	48.390	-19.981	28.195	1.00	0.00	LX0	C
ATOM	2969	CD	LYS	335	49.222	-20.357	26.966	1.00	0.00	LX0	C
ATOM	2970	CE	LYS	335	50.549	-21.016	27.355	1.00	0.00	LX0	C
ATOM	2971	NZ	LYS	335	51.316	-20.126	28.236	1.00	0.00	LX0	N
ATOM	2972	HZ1	LYS	335	52.087	-20.631	28.718	0.00	0.00	LX0	H
ATOM	2973	HZ2	LYS	335	51.714	-19.299	27.743	0.00	0.00	LX0	H
ATOM	2974	HZ3	LYS	335	50.715	-19.757	28.998	0.00	0.00	LX0	H
ATOM	2975	C	LYS	335	47.949	-16.785	27.879	1.00	0.00	LX0	C
ATOM	2976	O	LYS	335	49.098	-16.976	28.272	1.00	0.00	LX0	O
ATOM	2977	N	VAL	336	47.333	-15.600	27.869	1.00	0.00	LX0	N
ATOM	2978	H	VAL	336	46.375	-15.526	27.598	0.00	0.00	LX0	H
ATOM	2979	CA	VAL	336	48.014	-14.416	28.369	1.00	0.00	LX0	C
ATOM	2980	CB	VAL	336	48.253	-13.406	27.229	1.00	0.00	LX0	C
ATOM	2981	CG1	VAL	336	49.149	-14.004	26.145	1.00	0.00	LX0	C
ATOM	2982	CG2	VAL	336	46.954	-12.868	26.619	1.00	0.00	LX0	C
ATOM	2983	C	VAL	336	47.178	-13.796	29.466	1.00	0.00	LX0	C
ATOM	2984	O	VAL	336	45.957	-13.905	29.465	1.00	0.00	LX0	O
ATOM	2985	N	CYS	337	47.876	-13.133	30.386	1.00	0.00	LX0	N
ATOM	2986	H	CYS	337	48.869	-13.036	30.317	0.00	0.00	LX0	H
ATOM	2987	CA	CYS	337	47.102	-12.279	31.278	1.00	0.00	LX0	C
ATOM	2988	CB	CYS	337	47.406	-12.600	32.741	1.00	0.00	LX0	C
ATOM	2989	SG	CYS	337	46.907	-14.275	33.214	1.00	0.00	LX0	S
ATOM	2990	C	CYS	337	47.469	-10.855	30.957	1.00	0.00	LX0	C
ATOM	2991	O	CYS	337	48.463	-10.625	30.278	1.00	0.00	LX0	O
ATOM	2992	N	ASN	338	46.667	-9.907	31.465	1.00	0.00	LX0	N
ATOM	2993	H	ASN	338	45.793	-10.128	31.906	0.00	0.00	LX0	H
ATOM	2994	CA	ASN	338	47.160	-8.535	31.331	1.00	0.00	LX0	C
ATOM	2995	CB	ASN	338	46.058	-7.515	31.597	1.00	0.00	LX0	C
ATOM	2996	CG	ASN	338	45.468	-6.981	30.301	1.00	0.00	LX0	C
ATOM	2997	OD1	ASN	338	46.128	-6.415	29.446	1.00	0.00	LX0	O
ATOM	2998	ND2	ASN	338	44.143	-7.107	30.204	1.00	0.00	LX0	N
ATOM	2999	HD21	ASN	338	43.636	-7.655	30.875	0.00	0.00	LX0	H
ATOM	3000	HD22	ASN	338	43.686	-6.620	29.464	0.00	0.00	LX0	H
ATOM	3001	C	ASN	338	48.390	-8.281	32.191	1.00	0.00	LX0	C

ATOM	3002	O	ASN	338	49.501	-8.172	31.693	1.00	0.00	LX0	O
ATOM	3003	N	GLY	339	48.168	-8.266	33.513	1.00	0.00	LX0	N
ATOM	3004	H	GLY	339	47.258	-8.278	33.932	0.00	0.00	LX0	H
ATOM	3005	CA	GLY	339	49.336	-8.177	34.384	1.00	0.00	LX0	C
ATOM	3006	C	GLY	339	48.896	-7.957	35.810	1.00	0.00	LX0	C
ATOM	3007	O	GLY	339	47.811	-8.365	36.196	1.00	0.00	LX0	O
ATOM	3008	N	ILE	340	49.762	-7.281	36.570	1.00	0.00	LX0	N
ATOM	3009	H	ILE	340	50.609	-6.943	36.156	0.00	0.00	LX0	H
ATOM	3010	CA	ILE	340	49.316	-6.799	37.874	1.00	0.00	LX0	C
ATOM	3011	CB	ILE	340	50.340	-7.173	38.959	1.00	0.00	LX0	C
ATOM	3012	CG2	ILE	340	50.113	-6.449	40.291	1.00	0.00	LX0	C
ATOM	3013	CG1	ILE	340	50.346	-8.688	39.169	1.00	0.00	LX0	C
ATOM	3014	CD1	ILE	340	49.059	-9.197	39.828	1.00	0.00	LX0	C
ATOM	3015	C	ILE	340	49.117	-5.304	37.765	1.00	0.00	LX0	C
ATOM	3016	O	ILE	340	49.992	-4.586	37.297	1.00	0.00	LX0	O
ATOM	3017	N	GLY	341	47.922	-4.872	38.174	1.00	0.00	LX0	N
ATOM	3018	H	GLY	341	47.242	-5.484	38.577	0.00	0.00	LX0	H
ATOM	3019	CA	GLY	341	47.557	-3.506	37.827	1.00	0.00	LX0	C
ATOM	3020	C	GLY	341	46.781	-3.477	36.525	1.00	0.00	LX0	C
ATOM	3021	O	GLY	341	45.565	-3.338	36.493	1.00	0.00	LX0	O
ATOM	3022	N	ILE	342	47.547	-3.622	35.438	1.00	0.00	LX0	N
ATOM	3023	H	ILE	342	48.532	-3.779	35.530	0.00	0.00	LX0	H
ATOM	3024	CA	ILE	342	46.902	-3.475	34.134	1.00	0.00	LX0	C
ATOM	3025	CB	ILE	342	47.977	-3.393	33.045	1.00	0.00	LX0	C
ATOM	3026	CG2	ILE	342	48.656	-4.749	32.892	1.00	0.00	LX0	C
ATOM	3027	CG1	ILE	342	47.447	-2.802	31.732	1.00	0.00	LX0	C
ATOM	3028	CD1	ILE	342	48.489	-2.671	30.630	1.00	0.00	LX0	C
ATOM	3029	C	ILE	342	45.818	-4.511	33.817	1.00	0.00	LX0	C
ATOM	3030	O	ILE	342	45.839	-5.648	34.281	1.00	0.00	LX0	O
ATOM	3031	N	GLY	343	44.857	-4.054	32.995	1.00	0.00	LX0	N
ATOM	3032	H	GLY	343	44.909	-3.101	32.694	0.00	0.00	LX0	H
ATOM	3033	CA	GLY	343	43.763	-4.913	32.561	1.00	0.00	LX0	C
ATOM	3034	C	GLY	343	42.936	-5.463	33.691	1.00	0.00	LX0	C
ATOM	3035	O	GLY	343	42.386	-4.723	34.488	1.00	0.00	LX0	O
ATOM	3036	N	GLU	344	42.863	-6.787	33.744	1.00	0.00	LX0	N
ATOM	3037	H	GLU	344	43.447	-7.362	33.176	0.00	0.00	LX0	H
ATOM	3038	CA	GLU	344	41.945	-7.365	34.719	1.00	0.00	LX0	C
ATOM	3039	CB	GLU	344	41.438	-8.736	34.211	1.00	0.00	LX0	C
ATOM	3040	CG	GLU	344	42.428	-9.669	33.463	1.00	0.00	LX0	C
ATOM	3041	CD	GLU	344	42.585	-9.295	31.974	1.00	0.00	LX0	C
ATOM	3042	OE1	GLU	344	43.551	-9.705	31.327	1.00	0.00	LX0	O
ATOM	3043	OE2	GLU	344	41.734	-8.604	31.411	1.00	0.00	LX0	O
ATOM	3044	C	GLU	344	42.447	-7.412	36.167	1.00	0.00	LX0	C
ATOM	3045	O	GLU	344	42.300	-8.424	36.841	1.00	0.00	LX0	O
ATOM	3046	N	PHE	345	43.070	-6.295	36.614	1.00	0.00	LX0	N
ATOM	3047	H	PHE	345	43.111	-5.475	36.048	0.00	0.00	LX0	H
ATOM	3048	CA	PHE	345	43.817	-6.338	37.869	1.00	0.00	LX0	C
ATOM	3049	CB	PHE	345	45.182	-7.009	37.638	1.00	0.00	LX0	C
ATOM	3050	CG	PHE	345	45.151	-8.490	37.940	1.00	0.00	LX0	C
ATOM	3051	CD1	PHE	345	45.137	-8.910	39.286	1.00	0.00	LX0	C
ATOM	3052	CD2	PHE	345	45.154	-9.427	36.882	1.00	0.00	LX0	C
ATOM	3053	CE1	PHE	345	45.145	-10.286	39.581	1.00	0.00	LX0	C
ATOM	3054	CE2	PHE	345	45.162	-10.804	37.176	1.00	0.00	LX0	C
ATOM	3055	CZ	PHE	345	45.166	-11.219	38.524	1.00	0.00	LX0	C
ATOM	3056	C	PHE	345	44.054	-5.007	38.590	1.00	0.00	LX0	C
ATOM	3057	O	PHE	345	44.967	-4.929	39.407	1.00	0.00	LX0	O
ATOM	3058	N	LYS	346	43.248	-3.959	38.298	1.00	0.00	LX0	N
ATOM	3059	H	LYS	346	42.472	-4.067	37.674	0.00	0.00	LX0	H
ATOM	3060	CA	LYS	346	43.650	-2.643	38.847	1.00	0.00	LX0	C
ATOM	3061	CB	LYS	346	42.846	-1.483	38.223	1.00	0.00	LX0	C
ATOM	3062	CG	LYS	346	43.180	-0.031	38.626	1.00	0.00	LX0	C

ATOM	3063	CD	LYS	346	42.318	1.022	37.901	1.00	0.00	LX0	C
ATOM	3064	CE	LYS	346	42.645	2.482	38.266	1.00	0.00	LX0	C
ATOM	3065	NZ	LYS	346	42.041	3.450	37.327	1.00	0.00	LX0	N
ATOM	3066	HZ1	LYS	346	42.137	4.432	37.670	0.00	0.00	LX0	H
ATOM	3067	HZ2	LYS	346	42.459	3.450	36.380	0.00	0.00	LX0	H
ATOM	3068	HZ3	LYS	346	41.007	3.397	37.192	0.00	0.00	LX0	H
ATOM	3069	C	LYS	346	43.739	-2.522	40.368	1.00	0.00	LX0	C
ATOM	3070	O	LYS	346	44.583	-1.809	40.898	1.00	0.00	LX0	O
ATOM	3071	N	ASP	347	42.874	-3.283	41.058	1.00	0.00	LX0	N
ATOM	3072	H	ASP	347	42.204	-3.840	40.571	0.00	0.00	LX0	H
ATOM	3073	CA	ASP	347	42.937	-3.283	42.529	1.00	0.00	LX0	C
ATOM	3074	CB	ASP	347	41.562	-3.689	43.088	1.00	0.00	LX0	C
ATOM	3075	CG	ASP	347	41.043	-2.750	44.174	1.00	0.00	LX0	C
ATOM	3076	OD1	ASP	347	40.160	-3.146	44.932	1.00	0.00	LX0	O
ATOM	3077	OD2	ASP	347	41.433	-1.585	44.242	1.00	0.00	LX0	O
ATOM	3078	C	ASP	347	44.058	-4.141	43.133	1.00	0.00	LX0	C
ATOM	3079	O	ASP	347	43.896	-4.846	44.121	1.00	0.00	LX0	O
ATOM	3080	N	SER	348	45.228	-4.087	42.473	1.00	0.00	LX0	N
ATOM	3081	H	SER	348	45.368	-3.428	41.734	0.00	0.00	LX0	H
ATOM	3082	CA	SER	348	46.289	-5.003	42.886	1.00	0.00	LX0	C
ATOM	3083	CB	SER	348	46.385	-6.205	41.940	1.00	0.00	LX0	C
ATOM	3084	OG	SER	348	45.076	-6.671	41.593	1.00	0.00	LX0	O
ATOM	3085	HG	SER	348	44.737	-6.010	40.996	0.00	0.00	LX0	H
ATOM	3086	C	SER	348	47.646	-4.338	42.982	1.00	0.00	LX0	C
ATOM	3087	O	SER	348	48.355	-4.181	41.998	1.00	0.00	LX0	O
ATOM	3088	N	LEU	349	47.996	-3.963	44.223	1.00	0.00	LX0	N
ATOM	3089	H	LEU	349	47.353	-4.097	44.977	0.00	0.00	LX0	H
ATOM	3090	CA	LEU	349	49.277	-3.264	44.368	1.00	0.00	LX0	C
ATOM	3091	CB	LEU	349	49.329	-2.475	45.691	1.00	0.00	LX0	C
ATOM	3092	CG	LEU	349	49.104	-3.232	47.011	1.00	0.00	LX0	C
ATOM	3093	CD1	LEU	349	50.331	-4.007	47.499	1.00	0.00	LX0	C
ATOM	3094	CD2	LEU	349	48.612	-2.278	48.101	1.00	0.00	LX0	C
ATOM	3095	C	LEU	349	50.544	-4.076	44.106	1.00	0.00	LX0	C
ATOM	3096	O	LEU	349	51.583	-3.543	43.744	1.00	0.00	LX0	O
ATOM	3097	N	SER	350	50.416	-5.396	44.298	1.00	0.00	LX0	N
ATOM	3098	H	SER	350	49.562	-5.810	44.606	0.00	0.00	LX0	H
ATOM	3099	CA	SER	350	51.573	-6.245	44.026	1.00	0.00	LX0	C
ATOM	3100	CB	SER	350	52.500	-6.324	45.250	1.00	0.00	LX0	C
ATOM	3101	OG	SER	350	53.716	-7.030	44.948	1.00	0.00	LX0	O
ATOM	3102	HG	SER	350	53.542	-7.958	45.116	0.00	0.00	LX0	H
ATOM	3103	C	SER	350	51.109	-7.627	43.647	1.00	0.00	LX0	C
ATOM	3104	O	SER	350	50.028	-8.068	44.032	1.00	0.00	LX0	O
ATOM	3105	N	ILE	351	51.998	-8.309	42.913	1.00	0.00	LX0	N
ATOM	3106	H	ILE	351	52.846	-7.844	42.648	0.00	0.00	LX0	H
ATOM	3107	CA	ILE	351	51.852	-9.756	42.807	1.00	0.00	LX0	C
ATOM	3108	CB	ILE	351	52.794	-10.283	41.705	1.00	0.00	LX0	C
ATOM	3109	CG2	ILE	351	54.271	-10.139	42.087	1.00	0.00	LX0	C
ATOM	3110	CG1	ILE	351	52.421	-11.690	41.227	1.00	0.00	LX0	C
ATOM	3111	CD1	ILE	351	53.168	-12.092	39.953	1.00	0.00	LX0	C
ATOM	3112	C	ILE	351	52.086	-10.384	44.177	1.00	0.00	LX0	C
ATOM	3113	O	ILE	351	52.758	-9.803	45.024	1.00	0.00	LX0	O
ATOM	3114	N	ASN	352	51.438	-11.539	44.371	1.00	0.00	LX0	N
ATOM	3115	H	ASN	352	50.962	-11.988	43.614	0.00	0.00	LX0	H
ATOM	3116	CA	ASN	352	51.326	-12.151	45.695	1.00	0.00	LX0	C
ATOM	3117	CB	ASN	352	50.365	-11.353	46.598	1.00	0.00	LX0	C
ATOM	3118	CG	ASN	352	48.975	-11.269	45.985	1.00	0.00	LX0	C
ATOM	3119	OD1	ASN	352	48.246	-12.247	45.891	1.00	0.00	LX0	O
ATOM	3120	ND2	ASN	352	48.633	-10.053	45.555	1.00	0.00	LX0	N
ATOM	3121	HD21	ASN	352	49.275	-9.283	45.537	0.00	0.00	LX0	H
ATOM	3122	HD22	ASN	352	47.708	-9.889	45.221	0.00	0.00	LX0	H
ATOM	3123	C	ASN	352	50.828	-13.567	45.499	1.00	0.00	LX0	C

ATOM	3124	O	ASN	352	50.340	-13.888	44.419	1.00	0.00	LX0	O
ATOM	3125	N	ALA	353	50.958	-14.394	46.552	1.00	0.00	LX0	N
ATOM	3126	H	ALA	353	51.306	-14.079	47.438	0.00	0.00	LX0	H
ATOM	3127	CA	ALA	353	50.550	-15.790	46.364	1.00	0.00	LX0	C
ATOM	3128	CB	ALA	353	50.829	-16.618	47.618	1.00	0.00	LX0	C
ATOM	3129	C	ALA	353	49.090	-15.954	45.977	1.00	0.00	LX0	C
ATOM	3130	O	ALA	353	48.727	-16.666	45.045	1.00	0.00	LX0	O
ATOM	3131	N	THR	354	48.264	-15.192	46.709	1.00	0.00	LX0	N
ATOM	3132	H	THR	354	48.591	-14.640	47.477	0.00	0.00	LX0	H
ATOM	3133	CA	THR	354	46.823	-15.196	46.466	1.00	0.00	LX0	C
ATOM	3134	CB	THR	354	46.172	-14.140	47.360	1.00	0.00	LX0	C
ATOM	3135	OG1	THR	354	46.912	-14.027	48.580	1.00	0.00	LX0	O
ATOM	3136	HG1	THR	354	46.362	-13.522	49.170	0.00	0.00	LX0	H
ATOM	3137	CG2	THR	354	44.697	-14.433	47.646	1.00	0.00	LX0	C
ATOM	3138	C	THR	354	46.381	-15.023	45.019	1.00	0.00	LX0	C
ATOM	3139	O	THR	354	45.337	-15.523	44.603	1.00	0.00	LX0	O
ATOM	3140	N	ASN	355	47.217	-14.311	44.246	1.00	0.00	LX0	N
ATOM	3141	H	ASN	355	48.020	-13.858	44.643	0.00	0.00	LX0	H
ATOM	3142	CA	ASN	355	46.834	-14.216	42.843	1.00	0.00	LX0	C
ATOM	3143	CB	ASN	355	46.382	-12.806	42.464	1.00	0.00	LX0	C
ATOM	3144	CG	ASN	355	44.873	-12.838	42.306	1.00	0.00	LX0	C
ATOM	3145	OD1	ASN	355	44.248	-13.887	42.166	1.00	0.00	LX0	O
ATOM	3146	ND2	ASN	355	44.292	-11.637	42.366	1.00	0.00	LX0	N
ATOM	3147	HD21	ASN	355	44.819	-10.794	42.471	0.00	0.00	LX0	H
ATOM	3148	HD22	ASN	355	43.299	-11.558	42.295	0.00	0.00	LX0	H
ATOM	3149	C	ASN	355	47.737	-14.824	41.792	1.00	0.00	LX0	C
ATOM	3150	O	ASN	355	47.308	-15.035	40.662	1.00	0.00	LX0	O
ATOM	3151	N	ILE	356	48.985	-15.148	42.191	1.00	0.00	LX0	N
ATOM	3152	H	ILE	356	49.278	-14.990	43.137	0.00	0.00	LX0	H
ATOM	3153	CA	ILE	356	49.937	-15.644	41.184	1.00	0.00	LX0	C
ATOM	3154	CB	ILE	356	51.345	-15.863	41.773	1.00	0.00	LX0	C
ATOM	3155	CG2	ILE	356	51.403	-17.073	42.714	1.00	0.00	LX0	C
ATOM	3156	CG1	ILE	356	52.393	-15.915	40.648	1.00	0.00	LX0	C
ATOM	3157	CD1	ILE	356	53.847	-16.059	41.102	1.00	0.00	LX0	C
ATOM	3158	C	ILE	356	49.479	-16.851	40.361	1.00	0.00	LX0	C
ATOM	3159	O	ILE	356	49.859	-17.033	39.213	1.00	0.00	LX0	O
ATOM	3160	N	LYS	357	48.573	-17.632	40.982	1.00	0.00	LX0	N
ATOM	3161	H	LYS	357	48.487	-17.474	41.964	0.00	0.00	LX0	H
ATOM	3162	CA	LYS	357	47.905	-18.740	40.280	1.00	0.00	LX0	C
ATOM	3163	CB	LYS	357	46.604	-19.159	40.975	1.00	0.00	LX0	C
ATOM	3164	CG	LYS	357	46.547	-18.975	42.494	1.00	0.00	LX0	C
ATOM	3165	CD	LYS	357	45.190	-18.405	42.923	1.00	0.00	LX0	C
ATOM	3166	CE	LYS	357	44.844	-17.191	42.058	1.00	0.00	LX0	C
ATOM	3167	NZ	LYS	357	43.792	-16.358	42.647	1.00	0.00	LX0	N
ATOM	3168	HZ1	LYS	357	43.694	-15.494	42.067	0.00	0.00	LX0	H
ATOM	3169	HZ2	LYS	357	42.885	-16.854	42.696	0.00	0.00	LX0	H
ATOM	3170	HZ3	LYS	357	44.103	-16.041	43.590	0.00	0.00	LX0	H
ATOM	3171	C	LYS	357	47.549	-18.483	38.817	1.00	0.00	LX0	C
ATOM	3172	O	LYS	357	47.748	-19.319	37.949	1.00	0.00	LX0	O
ATOM	3173	N	HIS	358	47.005	-17.273	38.581	1.00	0.00	LX0	N
ATOM	3174	H	HIS	358	46.989	-16.578	39.302	0.00	0.00	LX0	H
ATOM	3175	CA	HIS	358	46.552	-16.972	37.216	1.00	0.00	LX0	C
ATOM	3176	CB	HIS	358	45.807	-15.628	37.143	1.00	0.00	LX0	C
ATOM	3177	CG	HIS	358	44.620	-15.541	38.084	1.00	0.00	LX0	C
ATOM	3178	ND1	HIS	358	44.026	-14.375	38.395	1.00	0.00	LX0	N
ATOM	3179	HD1	HIS	358	44.253	-13.493	38.035	0.00	0.00	LX0	H
ATOM	3180	CD2	HIS	358	43.967	-16.563	38.782	1.00	0.00	LX0	C
ATOM	3181	NE2	HIS	358	42.983	-15.994	39.525	1.00	0.00	LX0	N
ATOM	3182	CE1	HIS	358	43.016	-14.645	39.279	1.00	0.00	LX0	C
ATOM	3183	C	HIS	358	47.684	-16.968	36.198	1.00	0.00	LX0	C
ATOM	3184	O	HIS	358	47.566	-17.397	35.059	1.00	0.00	LX0	O

ATOM	3185	N	PHE	359	48.822	-16.469	36.696	1.00	0.00	LX0	N
ATOM	3186	H	PHE	359	48.898	-16.271	37.672	0.00	0.00	LX0	H
ATOM	3187	CA	PHE	359	50.002	-16.350	35.846	1.00	0.00	LX0	C
ATOM	3188	CB	PHE	359	50.968	-15.308	36.430	1.00	0.00	LX0	C
ATOM	3189	CG	PHE	359	50.256	-13.995	36.675	1.00	0.00	LX0	C
ATOM	3190	CD1	PHE	359	50.102	-13.080	35.611	1.00	0.00	LX0	C
ATOM	3191	CD2	PHE	359	49.750	-13.710	37.963	1.00	0.00	LX0	C
ATOM	3192	CE1	PHE	359	49.414	-11.871	35.831	1.00	0.00	LX0	C
ATOM	3193	CE2	PHE	359	49.058	-12.504	38.184	1.00	0.00	LX0	C
ATOM	3194	CZ	PHE	359	48.889	-11.602	37.112	1.00	0.00	LX0	C
ATOM	3195	C	PHE	359	50.740	-17.655	35.590	1.00	0.00	LX0	C
ATOM	3196	O	PHE	359	51.715	-17.696	34.850	1.00	0.00	LX0	O
ATOM	3197	N	LYS	360	50.246	-18.732	36.241	1.00	0.00	LX0	N
ATOM	3198	H	LYS	360	49.408	-18.643	36.779	0.00	0.00	LX0	H
ATOM	3199	CA	LYS	360	51.002	-19.988	36.295	1.00	0.00	LX0	C
ATOM	3200	CB	LYS	360	50.226	-21.037	37.099	1.00	0.00	LX0	C
ATOM	3201	CG	LYS	360	51.133	-22.027	37.833	1.00	0.00	LX0	C
ATOM	3202	CD	LYS	360	50.396	-22.851	38.891	1.00	0.00	LX0	C
ATOM	3203	CE	LYS	360	51.340	-23.692	39.759	1.00	0.00	LX0	C
ATOM	3204	NZ	LYS	360	52.250	-22.821	40.509	1.00	0.00	LX0	N
ATOM	3205	HZ1	LYS	360	52.932	-22.292	39.937	0.00	0.00	LX0	H
ATOM	3206	HZ2	LYS	360	51.772	-22.121	41.116	0.00	0.00	LX0	H
ATOM	3207	HZ3	LYS	360	52.838	-23.323	41.212	0.00	0.00	LX0	H
ATOM	3208	C	LYS	360	51.523	-20.551	34.981	1.00	0.00	LX0	C
ATOM	3209	O	LYS	360	52.523	-21.259	34.933	1.00	0.00	LX0	O
ATOM	3210	N	ASN	361	50.810	-20.189	33.907	1.00	0.00	LX0	N
ATOM	3211	H	ASN	361	49.978	-19.644	34.007	0.00	0.00	LX0	H
ATOM	3212	CA	ASN	361	51.431	-20.432	32.612	1.00	0.00	LX0	C
ATOM	3213	CB	ASN	361	51.064	-21.811	32.058	1.00	0.00	LX0	C
ATOM	3214	CG	ASN	361	52.139	-22.252	31.084	1.00	0.00	LX0	C
ATOM	3215	OD1	ASN	361	52.251	-21.781	29.958	1.00	0.00	LX0	O
ATOM	3216	ND2	ASN	361	52.942	-23.199	31.572	1.00	0.00	LX0	N
ATOM	3217	HD21	ASN	361	52.838	-23.513	32.515	0.00	0.00	LX0	H
ATOM	3218	HD22	ASN	361	53.658	-23.583	30.993	0.00	0.00	LX0	H
ATOM	3219	C	ASN	361	51.131	-19.342	31.607	1.00	0.00	LX0	C
ATOM	3220	O	ASN	361	50.428	-19.553	30.624	1.00	0.00	LX0	O
ATOM	3221	N	CYS	362	51.687	-18.160	31.889	1.00	0.00	LX0	N
ATOM	3222	H	CYS	362	52.293	-18.065	32.685	0.00	0.00	LX0	H
ATOM	3223	CA	CYS	362	51.514	-17.068	30.929	1.00	0.00	LX0	C
ATOM	3224	CB	CYS	362	51.939	-15.745	31.566	1.00	0.00	LX0	C
ATOM	3225	SG	CYS	362	50.749	-15.166	32.798	1.00	0.00	LX0	S
ATOM	3226	C	CYS	362	52.238	-17.302	29.609	1.00	0.00	LX0	C
ATOM	3227	O	CYS	362	52.792	-18.373	29.366	1.00	0.00	LX0	O
ATOM	3228	N	THR	363	52.204	-16.264	28.762	1.00	0.00	LX0	N
ATOM	3229	H	THR	363	51.618	-15.470	28.928	0.00	0.00	LX0	H
ATOM	3230	CA	THR	363	53.054	-16.321	27.575	1.00	0.00	LX0	C
ATOM	3231	CB	THR	363	52.297	-16.933	26.390	1.00	0.00	LX0	C
ATOM	3232	OG1	THR	363	51.377	-17.936	26.847	1.00	0.00	LX0	O
ATOM	3233	HG1	THR	363	50.514	-17.511	26.799	0.00	0.00	LX0	H
ATOM	3234	CG2	THR	363	53.247	-17.496	25.332	1.00	0.00	LX0	C
ATOM	3235	C	THR	363	53.566	-14.931	27.248	1.00	0.00	LX0	C
ATOM	3236	O	THR	363	54.721	-14.580	27.448	1.00	0.00	LX0	O
ATOM	3237	N	SER	364	52.610	-14.116	26.799	1.00	0.00	LX0	N
ATOM	3238	H	SER	364	51.676	-14.420	26.634	0.00	0.00	LX0	H
ATOM	3239	CA	SER	364	52.875	-12.697	26.944	1.00	0.00	LX0	C
ATOM	3240	CB	SER	364	52.445	-11.943	25.676	1.00	0.00	LX0	C
ATOM	3241	OG	SER	364	52.932	-10.597	25.694	1.00	0.00	LX0	O
ATOM	3242	HG	SER	364	53.412	-10.471	24.869	0.00	0.00	LX0	H
ATOM	3243	C	SER	364	52.144	-12.240	28.192	1.00	0.00	LX0	C
ATOM	3244	O	SER	364	51.263	-12.931	28.705	1.00	0.00	LX0	O
ATOM	3245	N	ILE	365	52.572	-11.070	28.649	1.00	0.00	LX0	N

ATOM	3246	H	ILE	365	53.324	-10.630	28.153	0.00	0.00	LX0	H
ATOM	3247	CA	ILE	365	51.907	-10.323	29.700	1.00	0.00	LX0	C
ATOM	3248	CB	ILE	365	52.832	-10.209	30.929	1.00	0.00	LX0	C
ATOM	3249	CG2	ILE	365	52.583	-8.991	31.827	1.00	0.00	LX0	C
ATOM	3250	CG1	ILE	365	52.726	-11.500	31.743	1.00	0.00	LX0	C
ATOM	3251	CD1	ILE	365	51.334	-11.708	32.344	1.00	0.00	LX0	C
ATOM	3252	C	ILE	365	51.587	-8.994	29.065	1.00	0.00	LX0	C
ATOM	3253	O	ILE	365	52.454	-8.261	28.597	1.00	0.00	LX0	O
ATOM	3254	N	SER	366	50.282	-8.757	29.005	1.00	0.00	LX0	N
ATOM	3255	H	SER	366	49.674	-9.327	29.559	0.00	0.00	LX0	H
ATOM	3256	CA	SER	366	49.788	-7.550	28.366	1.00	0.00	LX0	C
ATOM	3257	CB	SER	366	48.390	-7.860	27.826	1.00	0.00	LX0	C
ATOM	3258	OG	SER	366	47.757	-6.699	27.289	1.00	0.00	LX0	O
ATOM	3259	HG	SER	366	47.387	-6.256	28.058	0.00	0.00	LX0	H
ATOM	3260	C	SER	366	49.849	-6.378	29.330	1.00	0.00	LX0	C
ATOM	3261	O	SER	366	48.858	-5.918	29.882	1.00	0.00	LX0	O
ATOM	3262	N	GLY	367	51.092	-5.933	29.515	1.00	0.00	LX0	N
ATOM	3263	H	GLY	367	51.842	-6.342	28.997	0.00	0.00	LX0	H
ATOM	3264	CA	GLY	367	51.321	-4.888	30.493	1.00	0.00	LX0	C
ATOM	3265	C	GLY	367	52.369	-5.281	31.500	1.00	0.00	LX0	C
ATOM	3266	O	GLY	367	53.406	-5.840	31.154	1.00	0.00	LX0	O
ATOM	3267	N	ASP	368	52.055	-4.926	32.744	1.00	0.00	LX0	N
ATOM	3268	H	ASP	368	51.172	-4.503	32.936	0.00	0.00	LX0	H
ATOM	3269	CA	ASP	368	53.114	-4.742	33.726	1.00	0.00	LX0	C
ATOM	3270	CB	ASP	368	52.981	-3.356	34.367	1.00	0.00	LX0	C
ATOM	3271	CG	ASP	368	52.506	-2.310	33.372	1.00	0.00	LX0	C
ATOM	3272	OD1	ASP	368	53.266	-1.922	32.494	1.00	0.00	LX0	O
ATOM	3273	OD2	ASP	368	51.358	-1.892	33.459	1.00	0.00	LX0	O
ATOM	3274	C	ASP	368	53.110	-5.775	34.828	1.00	0.00	LX0	C
ATOM	3275	O	ASP	368	52.099	-6.401	35.132	1.00	0.00	LX0	O
ATOM	3276	N	LEU	369	54.286	-5.909	35.455	1.00	0.00	LX0	N
ATOM	3277	H	LEU	369	55.086	-5.416	35.106	0.00	0.00	LX0	H
ATOM	3278	CA	LEU	369	54.334	-6.709	36.676	1.00	0.00	LX0	C
ATOM	3279	CB	LEU	369	55.119	-8.007	36.463	1.00	0.00	LX0	C
ATOM	3280	CG	LEU	369	54.397	-9.024	35.570	1.00	0.00	LX0	C
ATOM	3281	CD1	LEU	369	55.295	-10.209	35.214	1.00	0.00	LX0	C
ATOM	3282	CD2	LEU	369	53.073	-9.492	36.179	1.00	0.00	LX0	C
ATOM	3283	C	LEU	369	54.885	-5.926	37.851	1.00	0.00	LX0	C
ATOM	3284	O	LEU	369	56.040	-5.515	37.884	1.00	0.00	LX0	O
ATOM	3285	N	HIS	370	53.980	-5.732	38.820	1.00	0.00	LX0	N
ATOM	3286	H	HIS	370	53.077	-6.147	38.732	0.00	0.00	LX0	H
ATOM	3287	CA	HIS	370	54.364	-5.029	40.045	1.00	0.00	LX0	C
ATOM	3288	CB	HIS	370	53.170	-4.249	40.601	1.00	0.00	LX0	C
ATOM	3289	CG	HIS	370	52.843	-3.035	39.765	1.00	0.00	LX0	C
ATOM	3290	ND1	HIS	370	51.971	-3.015	38.737	1.00	0.00	LX0	N
ATOM	3291	HD1	HIS	370	51.432	-3.750	38.371	0.00	0.00	LX0	H
ATOM	3292	CD2	HIS	370	53.363	-1.750	39.932	1.00	0.00	LX0	C
ATOM	3293	NE2	HIS	370	52.797	-0.952	38.996	1.00	0.00	LX0	N
ATOM	3294	CE1	HIS	370	51.937	-1.732	38.261	1.00	0.00	LX0	C
ATOM	3295	C	HIS	370	54.847	-5.990	41.115	1.00	0.00	LX0	C
ATOM	3296	O	HIS	370	54.076	-6.800	41.618	1.00	0.00	LX0	O
ATOM	3297	N	ILE	371	56.139	-5.873	41.449	1.00	0.00	LX0	N
ATOM	3298	H	ILE	371	56.756	-5.240	40.977	0.00	0.00	LX0	H
ATOM	3299	CA	ILE	371	56.651	-6.689	42.552	1.00	0.00	LX0	C
ATOM	3300	CB	ILE	371	57.756	-7.652	42.082	1.00	0.00	LX0	C
ATOM	3301	CG2	ILE	371	58.078	-8.682	43.172	1.00	0.00	LX0	C
ATOM	3302	CG1	ILE	371	57.397	-8.329	40.754	1.00	0.00	LX0	C
ATOM	3303	CD1	ILE	371	58.490	-9.262	40.235	1.00	0.00	LX0	C
ATOM	3304	C	ILE	371	57.140	-5.818	43.700	1.00	0.00	LX0	C
ATOM	3305	O	ILE	371	58.318	-5.495	43.830	1.00	0.00	LX0	O
ATOM	3306	N	LEU	372	56.153	-5.425	44.513	1.00	0.00	LX0	N

ATOM	3307	H	LEU	372	55.259	-5.871	44.433	0.00	0.00	LX0	H
ATOM	3308	CA	LEU	372	56.435	-4.515	45.626	1.00	0.00	LX0	C
ATOM	3309	CB	LEU	372	55.141	-3.793	46.031	1.00	0.00	LX0	C
ATOM	3310	CG	LEU	372	54.879	-2.455	45.327	1.00	0.00	LX0	C
ATOM	3311	CD1	LEU	372	54.766	-2.566	43.806	1.00	0.00	LX0	C
ATOM	3312	CD2	LEU	372	53.663	-1.754	45.930	1.00	0.00	LX0	C
ATOM	3313	C	LEU	372	57.017	-5.273	46.811	1.00	0.00	LX0	C
ATOM	3314	O	LEU	372	56.721	-6.449	46.992	1.00	0.00	LX0	O
ATOM	3315	N	PRO	373	57.853	-4.582	47.639	1.00	0.00	LX0	N
ATOM	3316	CD	PRO	373	58.232	-3.174	47.543	1.00	0.00	LX0	C
ATOM	3317	CA	PRO	373	58.527	-5.247	48.767	1.00	0.00	LX0	C
ATOM	3318	CB	PRO	373	59.086	-4.072	49.573	1.00	0.00	LX0	C
ATOM	3319	CG	PRO	373	59.396	-3.019	48.514	1.00	0.00	LX0	C
ATOM	3320	C	PRO	373	57.696	-6.224	49.586	1.00	0.00	LX0	C
ATOM	3321	O	PRO	373	58.096	-7.344	49.888	1.00	0.00	LX0	O
ATOM	3322	N	VAL	374	56.488	-5.743	49.917	1.00	0.00	LX0	N
ATOM	3323	H	VAL	374	56.217	-4.833	49.610	0.00	0.00	LX0	H
ATOM	3324	CA	VAL	374	55.619	-6.542	50.778	1.00	0.00	LX0	C
ATOM	3325	CB	VAL	374	54.373	-5.718	51.148	1.00	0.00	LX0	C
ATOM	3326	CG1	VAL	374	53.382	-5.578	49.986	1.00	0.00	LX0	C
ATOM	3327	CG2	VAL	374	53.742	-6.221	52.447	1.00	0.00	LX0	C
ATOM	3328	C	VAL	374	55.285	-7.953	50.286	1.00	0.00	LX0	C
ATOM	3329	O	VAL	374	54.981	-8.847	51.066	1.00	0.00	LX0	O
ATOM	3330	N	ALA	375	55.407	-8.145	48.957	1.00	0.00	LX0	N
ATOM	3331	H	ALA	375	55.686	-7.393	48.360	0.00	0.00	LX0	H
ATOM	3332	CA	ALA	375	55.167	-9.473	48.388	1.00	0.00	LX0	C
ATOM	3333	CB	ALA	375	55.515	-9.495	46.898	1.00	0.00	LX0	C
ATOM	3334	C	ALA	375	55.919	-10.606	49.070	1.00	0.00	LX0	C
ATOM	3335	O	ALA	375	55.385	-11.679	49.318	1.00	0.00	LX0	O
ATOM	3336	N	PHE	376	57.189	-10.319	49.397	1.00	0.00	LX0	N
ATOM	3337	H	PHE	376	57.562	-9.395	49.281	0.00	0.00	LX0	H
ATOM	3338	CA	PHE	376	57.946	-11.402	50.025	1.00	0.00	LX0	C
ATOM	3339	CB	PHE	376	59.360	-11.485	49.443	1.00	0.00	LX0	C
ATOM	3340	CG	PHE	376	59.258	-11.817	47.973	1.00	0.00	LX0	C
ATOM	3341	CD1	PHE	376	59.580	-10.831	47.020	1.00	0.00	LX0	C
ATOM	3342	CD2	PHE	376	58.826	-13.099	47.573	1.00	0.00	LX0	C
ATOM	3343	CE1	PHE	376	59.449	-11.117	45.648	1.00	0.00	LX0	C
ATOM	3344	CE2	PHE	376	58.695	-13.391	46.202	1.00	0.00	LX0	C
ATOM	3345	CZ	PHE	376	58.998	-12.392	45.254	1.00	0.00	LX0	C
ATOM	3346	C	PHE	376	57.948	-11.404	51.543	1.00	0.00	LX0	C
ATOM	3347	O	PHE	376	58.837	-11.936	52.200	1.00	0.00	LX0	O
ATOM	3348	N	ARG	377	56.888	-10.781	52.074	1.00	0.00	LX0	N
ATOM	3349	H	ARG	377	56.262	-10.276	51.482	0.00	0.00	LX0	H
ATOM	3350	CA	ARG	377	56.607	-10.904	53.502	1.00	0.00	LX0	C
ATOM	3351	CB	ARG	377	56.942	-9.612	54.253	1.00	0.00	LX0	C
ATOM	3352	CG	ARG	377	58.447	-9.413	54.452	1.00	0.00	LX0	C
ATOM	3353	CD	ARG	377	59.081	-10.630	55.134	1.00	0.00	LX0	C
ATOM	3354	NE	ARG	377	60.495	-10.420	55.430	1.00	0.00	LX0	N
ATOM	3355	HE	ARG	377	60.714	-9.868	56.233	0.00	0.00	LX0	H
ATOM	3356	CZ	ARG	377	61.475	-10.972	54.677	1.00	0.00	LX0	C
ATOM	3357	NH1	ARG	377	61.227	-11.661	53.568	1.00	0.00	LX0	N
ATOM	3358	HH11	ARG	377	62.022	-12.020	53.050	0.00	0.00	LX0	H
ATOM	3359	HH12	ARG	377	60.302	-11.831	53.210	0.00	0.00	LX0	H
ATOM	3360	NH2	ARG	377	62.733	-10.824	55.061	1.00	0.00	LX0	N
ATOM	3361	HH21	ARG	377	63.435	-11.299	54.509	0.00	0.00	LX0	H
ATOM	3362	HH22	ARG	377	63.018	-10.293	55.853	0.00	0.00	LX0	H
ATOM	3363	C	ARG	377	55.181	-11.331	53.793	1.00	0.00	LX0	C
ATOM	3364	O	ARG	377	54.899	-11.978	54.791	1.00	0.00	LX0	O
ATOM	3365	N	GLY	378	54.302	-10.963	52.852	1.00	0.00	LX0	N
ATOM	3366	H	GLY	378	54.571	-10.368	52.099	0.00	0.00	LX0	H
ATOM	3367	CA	GLY	378	52.886	-11.193	53.094	1.00	0.00	LX0	C

ATOM	3368	C	GLY	378	52.224	-9.918	53.558	1.00	0.00	LX0	C
ATOM	3369	O	GLY	378	52.617	-9.319	54.553	1.00	0.00	LX0	O
ATOM	3370	N	ASP	379	51.229	-9.490	52.771	1.00	0.00	LX0	N
ATOM	3371	H	ASP	379	50.806	-10.073	52.073	0.00	0.00	LX0	H
ATOM	3372	CA	ASP	379	50.632	-8.234	53.207	1.00	0.00	LX0	C
ATOM	3373	CB	ASP	379	50.161	-7.371	52.027	1.00	0.00	LX0	C
ATOM	3374	CG	ASP	379	49.568	-6.046	52.498	1.00	0.00	LX0	C
ATOM	3375	OD1	ASP	379	49.990	-5.519	53.525	1.00	0.00	LX0	O
ATOM	3376	OD2	ASP	379	48.636	-5.567	51.864	1.00	0.00	LX0	O
ATOM	3377	C	ASP	379	49.544	-8.444	54.231	1.00	0.00	LX0	C
ATOM	3378	O	ASP	379	48.627	-9.247	54.088	1.00	0.00	LX0	O
ATOM	3379	N	SER	380	49.703	-7.661	55.300	1.00	0.00	LX0	N
ATOM	3380	H	SER	380	50.353	-6.907	55.179	0.00	0.00	LX0	H
ATOM	3381	CA	SER	380	48.674	-7.693	56.326	1.00	0.00	LX0	C
ATOM	3382	CB	SER	380	49.209	-7.030	57.592	1.00	0.00	LX0	C
ATOM	3383	OG	SER	380	50.440	-7.659	57.975	1.00	0.00	LX0	O
ATOM	3384	HG	SER	380	50.859	-7.968	57.178	0.00	0.00	LX0	H
ATOM	3385	C	SER	380	47.364	-7.072	55.873	1.00	0.00	LX0	C
ATOM	3386	O	SER	380	46.286	-7.575	56.166	1.00	0.00	LX0	O
ATOM	3387	N	PHE	381	47.494	-5.964	55.124	1.00	0.00	LX0	N
ATOM	3388	H	PHE	381	48.381	-5.688	54.741	0.00	0.00	LX0	H
ATOM	3389	CA	PHE	381	46.279	-5.252	54.734	1.00	0.00	LX0	C
ATOM	3390	CB	PHE	381	46.643	-3.858	54.203	1.00	0.00	LX0	C
ATOM	3391	CG	PHE	381	45.406	-3.011	54.003	1.00	0.00	LX0	C
ATOM	3392	CD1	PHE	381	44.749	-2.461	55.126	1.00	0.00	LX0	C
ATOM	3393	CD2	PHE	381	44.927	-2.788	52.694	1.00	0.00	LX0	C
ATOM	3394	CE1	PHE	381	43.592	-1.680	54.937	1.00	0.00	LX0	C
ATOM	3395	CE2	PHE	381	43.770	-2.007	52.503	1.00	0.00	LX0	C
ATOM	3396	CZ	PHE	381	43.114	-1.464	53.627	1.00	0.00	LX0	C
ATOM	3397	C	PHE	381	45.408	-6.026	53.757	1.00	0.00	LX0	C
ATOM	3398	O	PHE	381	44.196	-6.114	53.893	1.00	0.00	LX0	O
ATOM	3399	N	THR	382	46.092	-6.616	52.773	1.00	0.00	LX0	N
ATOM	3400	H	THR	382	47.082	-6.485	52.695	0.00	0.00	LX0	H
ATOM	3401	CA	THR	382	45.328	-7.416	51.820	1.00	0.00	LX0	C
ATOM	3402	CB	THR	382	45.948	-7.300	50.426	1.00	0.00	LX0	C
ATOM	3403	OG1	THR	382	47.340	-7.645	50.461	1.00	0.00	LX0	O
ATOM	3404	HG1	THR	382	47.798	-6.861	50.776	0.00	0.00	LX0	H
ATOM	3405	CG2	THR	382	45.761	-5.899	49.840	1.00	0.00	LX0	C
ATOM	3406	C	THR	382	45.145	-8.876	52.209	1.00	0.00	LX0	C
ATOM	3407	O	THR	382	44.614	-9.677	51.450	1.00	0.00	LX0	O
ATOM	3408	N	HIS	383	45.642	-9.195	53.422	1.00	0.00	LX0	N
ATOM	3409	H	HIS	383	46.054	-8.481	53.984	0.00	0.00	LX0	H
ATOM	3410	CA	HIS	383	45.620	-10.574	53.925	1.00	0.00	LX0	C
ATOM	3411	CB	HIS	383	44.201	-11.052	54.289	1.00	0.00	LX0	C
ATOM	3412	CG	HIS	383	43.419	-10.068	55.136	1.00	0.00	LX0	C
ATOM	3413	ND1	HIS	383	43.947	-9.091	55.899	1.00	0.00	LX0	N
ATOM	3414	HD1	HIS	383	44.893	-8.853	56.007	0.00	0.00	LX0	H
ATOM	3415	CD2	HIS	383	42.028	-10.019	55.264	1.00	0.00	LX0	C
ATOM	3416	NE2	HIS	383	41.728	-9.003	56.109	1.00	0.00	LX0	N
ATOM	3417	CE1	HIS	383	42.910	-8.430	56.499	1.00	0.00	LX0	C
ATOM	3418	C	HIS	383	46.276	-11.581	52.988	1.00	0.00	LX0	C
ATOM	3419	O	HIS	383	45.825	-12.708	52.817	1.00	0.00	LX0	O
ATOM	3420	N	THR	384	47.354	-11.115	52.349	1.00	0.00	LX0	N
ATOM	3421	H	THR	384	47.799	-10.261	52.623	0.00	0.00	LX0	H
ATOM	3422	CA	THR	384	47.930	-11.967	51.318	1.00	0.00	LX0	C
ATOM	3423	CB	THR	384	48.295	-11.124	50.094	1.00	0.00	LX0	C
ATOM	3424	OG1	THR	384	48.972	-9.923	50.483	1.00	0.00	LX0	O
ATOM	3425	HG1	THR	384	48.319	-9.223	50.500	0.00	0.00	LX0	H
ATOM	3426	CG2	THR	384	47.063	-10.786	49.253	1.00	0.00	LX0	C
ATOM	3427	C	THR	384	49.115	-12.790	51.789	1.00	0.00	LX0	C
ATOM	3428	O	THR	384	50.139	-12.266	52.213	1.00	0.00	LX0	O

ATOM	3429	N	PRO	385	48.955	-14.133	51.690	1.00	0.00	LX0	N
ATOM	3430	CD	PRO	385	47.719	-14.855	51.409	1.00	0.00	LX0	C
ATOM	3431	CA	PRO	385	50.099	-15.032	51.877	1.00	0.00	LX0	C
ATOM	3432	CB	PRO	385	49.525	-16.392	51.458	1.00	0.00	LX0	C
ATOM	3433	CG	PRO	385	48.035	-16.299	51.768	1.00	0.00	LX0	C
ATOM	3434	C	PRO	385	51.329	-14.632	51.068	1.00	0.00	LX0	C
ATOM	3435	O	PRO	385	51.232	-14.198	49.920	1.00	0.00	LX0	O
ATOM	3436	N	PRO	386	52.502	-14.795	51.730	1.00	0.00	LX0	N
ATOM	3437	CD	PRO	386	52.674	-15.276	53.099	1.00	0.00	LX0	C
ATOM	3438	CA	PRO	386	53.777	-14.479	51.079	1.00	0.00	LX0	C
ATOM	3439	CB	PRO	386	54.817	-14.969	52.091	1.00	0.00	LX0	C
ATOM	3440	CG	PRO	386	54.115	-14.929	53.445	1.00	0.00	LX0	C
ATOM	3441	C	PRO	386	53.939	-15.149	49.731	1.00	0.00	LX0	C
ATOM	3442	O	PRO	386	53.560	-16.295	49.521	1.00	0.00	LX0	O
ATOM	3443	N	LEU	387	54.522	-14.373	48.817	1.00	0.00	LX0	N
ATOM	3444	H	LEU	387	54.860	-13.467	49.070	0.00	0.00	LX0	H
ATOM	3445	CA	LEU	387	54.832	-14.978	47.532	1.00	0.00	LX0	C
ATOM	3446	CB	LEU	387	55.071	-13.871	46.502	1.00	0.00	LX0	C
ATOM	3447	CG	LEU	387	55.231	-14.377	45.069	1.00	0.00	LX0	C
ATOM	3448	CD1	LEU	387	54.095	-15.314	44.672	1.00	0.00	LX0	C
ATOM	3449	CD2	LEU	387	55.387	-13.223	44.081	1.00	0.00	LX0	C
ATOM	3450	C	LEU	387	55.998	-15.943	47.645	1.00	0.00	LX0	C
ATOM	3451	O	LEU	387	57.127	-15.560	47.919	1.00	0.00	LX0	O
ATOM	3452	N	ASP	388	55.654	-17.219	47.442	1.00	0.00	LX0	N
ATOM	3453	H	ASP	388	54.697	-17.455	47.286	0.00	0.00	LX0	H
ATOM	3454	CA	ASP	388	56.668	-18.265	47.571	1.00	0.00	LX0	C
ATOM	3455	CB	ASP	388	55.986	-19.641	47.444	1.00	0.00	LX0	C
ATOM	3456	CG	ASP	388	56.970	-20.804	47.402	1.00	0.00	LX0	C
ATOM	3457	OD1	ASP	388	57.981	-20.789	48.096	1.00	0.00	LX0	O
ATOM	3458	OD2	ASP	388	56.758	-21.716	46.619	1.00	0.00	LX0	O
ATOM	3459	C	ASP	388	57.865	-18.105	46.635	1.00	0.00	LX0	C
ATOM	3460	O	ASP	388	57.754	-18.038	45.412	1.00	0.00	LX0	O
ATOM	3461	N	PRO	389	59.057	-18.061	47.283	1.00	0.00	LX0	N
ATOM	3462	CD	PRO	389	59.246	-17.947	48.729	1.00	0.00	LX0	C
ATOM	3463	CA	PRO	389	60.327	-18.110	46.554	1.00	0.00	LX0	C
ATOM	3464	CB	PRO	389	61.364	-18.186	47.680	1.00	0.00	LX0	C
ATOM	3465	CG	PRO	389	60.699	-17.530	48.886	1.00	0.00	LX0	C
ATOM	3466	C	PRO	389	60.511	-19.238	45.541	1.00	0.00	LX0	C
ATOM	3467	O	PRO	389	61.426	-19.185	44.722	1.00	0.00	LX0	O
ATOM	3468	N	GLN	390	59.669	-20.276	45.643	1.00	0.00	LX0	N
ATOM	3469	H	GLN	390	58.957	-20.319	46.351	0.00	0.00	LX0	H
ATOM	3470	CA	GLN	390	59.714	-21.312	44.614	1.00	0.00	LX0	C
ATOM	3471	CB	GLN	390	59.470	-22.687	45.232	1.00	0.00	LX0	C
ATOM	3472	CG	GLN	390	60.308	-22.909	46.492	1.00	0.00	LX0	C
ATOM	3473	CD	GLN	390	59.726	-24.056	47.289	1.00	0.00	LX0	C
ATOM	3474	OE1	GLN	390	60.325	-25.114	47.431	1.00	0.00	LX0	O
ATOM	3475	NE2	GLN	390	58.532	-23.794	47.821	1.00	0.00	LX0	N
ATOM	3476	HE21	GLN	390	58.083	-22.899	47.675	0.00	0.00	LX0	H
ATOM	3477	HE22	GLN	390	58.049	-24.472	48.364	0.00	0.00	LX0	H
ATOM	3478	C	GLN	390	58.734	-21.031	43.490	1.00	0.00	LX0	C
ATOM	3479	O	GLN	390	59.100	-21.007	42.320	1.00	0.00	LX0	O
ATOM	3480	N	GLU	391	57.481	-20.748	43.901	1.00	0.00	LX0	N
ATOM	3481	H	GLU	391	57.237	-20.895	44.863	0.00	0.00	LX0	H
ATOM	3482	CA	GLU	391	56.444	-20.355	42.938	1.00	0.00	LX0	C
ATOM	3483	CB	GLU	391	55.151	-19.933	43.645	1.00	0.00	LX0	C
ATOM	3484	CG	GLU	391	54.232	-21.096	44.045	1.00	0.00	LX0	C
ATOM	3485	CD	GLU	391	53.539	-21.686	42.826	1.00	0.00	LX0	C
ATOM	3486	OE1	GLU	391	52.406	-21.301	42.531	1.00	0.00	LX0	O
ATOM	3487	OE2	GLU	391	54.107	-22.552	42.162	1.00	0.00	LX0	O
ATOM	3488	C	GLU	391	56.853	-19.279	41.949	1.00	0.00	LX0	C
ATOM	3489	O	GLU	391	56.430	-19.264	40.802	1.00	0.00	LX0	O

ATOM	3490	N	LEU	392	57.756	-18.399	42.414	1.00	0.00	LX0	N
ATOM	3491	H	LEU	392	57.922	-18.391	43.402	0.00	0.00	LX0	H
ATOM	3492	CA	LEU	392	58.410	-17.457	41.497	1.00	0.00	LX0	C
ATOM	3493	CB	LEU	392	59.698	-16.923	42.110	1.00	0.00	LX0	C
ATOM	3494	CG	LEU	392	59.456	-15.796	43.104	1.00	0.00	LX0	C
ATOM	3495	CD1	LEU	392	60.770	-15.343	43.729	1.00	0.00	LX0	C
ATOM	3496	CD2	LEU	392	58.701	-14.629	42.463	1.00	0.00	LX0	C
ATOM	3497	C	LEU	392	58.715	-17.933	40.082	1.00	0.00	LX0	C
ATOM	3498	O	LEU	392	58.459	-17.235	39.107	1.00	0.00	LX0	O
ATOM	3499	N	ASP	393	59.264	-19.156	40.002	1.00	0.00	LX0	N
ATOM	3500	H	ASP	393	59.369	-19.725	40.820	0.00	0.00	LX0	H
ATOM	3501	CA	ASP	393	59.715	-19.635	38.694	1.00	0.00	LX0	C
ATOM	3502	CB	ASP	393	60.602	-20.873	38.864	1.00	0.00	LX0	C
ATOM	3503	CG	ASP	393	61.939	-20.694	38.155	1.00	0.00	LX0	C
ATOM	3504	OD1	ASP	393	62.470	-21.669	37.632	1.00	0.00	LX0	O
ATOM	3505	OD2	ASP	393	62.480	-19.589	38.113	1.00	0.00	LX0	O
ATOM	3506	C	ASP	393	58.645	-19.813	37.615	1.00	0.00	LX0	C
ATOM	3507	O	ASP	393	58.928	-19.940	36.430	1.00	0.00	LX0	O
ATOM	3508	N	ILE	394	57.371	-19.720	38.060	1.00	0.00	LX0	N
ATOM	3509	H	ILE	394	57.217	-19.717	39.049	0.00	0.00	LX0	H
ATOM	3510	CA	ILE	394	56.240	-19.481	37.145	1.00	0.00	LX0	C
ATOM	3511	CB	ILE	394	55.026	-19.009	37.975	1.00	0.00	LX0	C
ATOM	3512	CG2	ILE	394	53.977	-18.201	37.202	1.00	0.00	LX0	C
ATOM	3513	CG1	ILE	394	54.383	-20.188	38.700	1.00	0.00	LX0	C
ATOM	3514	CD1	ILE	394	53.339	-19.699	39.706	1.00	0.00	LX0	C
ATOM	3515	C	ILE	394	56.543	-18.478	36.035	1.00	0.00	LX0	C
ATOM	3516	O	ILE	394	56.191	-18.640	34.869	1.00	0.00	LX0	O
ATOM	3517	N	LEU	395	57.227	-17.411	36.468	1.00	0.00	LX0	N
ATOM	3518	H	LEU	395	57.548	-17.385	37.417	0.00	0.00	LX0	H
ATOM	3519	CA	LEU	395	57.480	-16.300	35.560	1.00	0.00	LX0	C
ATOM	3520	CB	LEU	395	57.900	-15.082	36.382	1.00	0.00	LX0	C
ATOM	3521	CG	LEU	395	56.815	-14.733	37.414	1.00	0.00	LX0	C
ATOM	3522	CD1	LEU	395	57.343	-13.899	38.581	1.00	0.00	LX0	C
ATOM	3523	CD2	LEU	395	55.579	-14.112	36.761	1.00	0.00	LX0	C
ATOM	3524	C	LEU	395	58.398	-16.603	34.384	1.00	0.00	LX0	C
ATOM	3525	O	LEU	395	58.479	-15.847	33.426	1.00	0.00	LX0	O
ATOM	3526	N	LYS	396	59.007	-17.802	34.434	1.00	0.00	LX0	N
ATOM	3527	H	LYS	396	58.985	-18.356	35.266	0.00	0.00	LX0	H
ATOM	3528	CA	LYS	396	59.678	-18.303	33.235	1.00	0.00	LX0	C
ATOM	3529	CB	LYS	396	60.460	-19.579	33.536	1.00	0.00	LX0	C
ATOM	3530	CG	LYS	396	61.799	-19.366	34.241	1.00	0.00	LX0	C
ATOM	3531	CD	LYS	396	62.524	-20.704	34.388	1.00	0.00	LX0	C
ATOM	3532	CE	LYS	396	63.982	-20.598	34.840	1.00	0.00	LX0	C
ATOM	3533	NZ	LYS	396	64.095	-20.018	36.180	1.00	0.00	LX0	N
ATOM	3534	HZ1	LYS	396	65.094	-19.872	36.431	0.00	0.00	LX0	H
ATOM	3535	HZ2	LYS	396	63.617	-19.098	36.255	0.00	0.00	LX0	H
ATOM	3536	HZ3	LYS	396	63.623	-20.603	36.906	0.00	0.00	LX0	H
ATOM	3537	C	LYS	396	58.778	-18.556	32.031	1.00	0.00	LX0	C
ATOM	3538	O	LYS	396	59.250	-18.788	30.925	1.00	0.00	LX0	O
ATOM	3539	N	THR	397	57.461	-18.510	32.273	1.00	0.00	LX0	N
ATOM	3540	H	THR	397	57.111	-18.354	33.195	0.00	0.00	LX0	H
ATOM	3541	CA	THR	397	56.563	-18.580	31.122	1.00	0.00	LX0	C
ATOM	3542	CB	THR	397	55.197	-19.119	31.558	1.00	0.00	LX0	C
ATOM	3543	OG1	THR	397	54.613	-18.316	32.597	1.00	0.00	LX0	O
ATOM	3544	HG1	THR	397	55.036	-18.564	33.419	0.00	0.00	LX0	H
ATOM	3545	CG2	THR	397	55.314	-20.571	32.021	1.00	0.00	LX0	C
ATOM	3546	C	THR	397	56.427	-17.271	30.353	1.00	0.00	LX0	C
ATOM	3547	O	THR	397	55.959	-17.217	29.221	1.00	0.00	LX0	O
ATOM	3548	N	VAL	398	56.857	-16.199	31.032	1.00	0.00	LX0	N
ATOM	3549	H	VAL	398	57.367	-16.276	31.888	0.00	0.00	LX0	H
ATOM	3550	CA	VAL	398	56.676	-14.884	30.437	1.00	0.00	LX0	C

ATOM	3551	CB	VAL	398	56.571	-13.819	31.533	1.00	0.00	LX0	C
ATOM	3552	CG1	VAL	398	56.217	-12.463	30.933	1.00	0.00	LX0	C
ATOM	3553	CG2	VAL	398	55.562	-14.221	32.613	1.00	0.00	LX0	C
ATOM	3554	C	VAL	398	57.750	-14.542	29.420	1.00	0.00	LX0	C
ATOM	3555	O	VAL	398	58.837	-14.074	29.727	1.00	0.00	LX0	O
ATOM	3556	N	LYS	399	57.361	-14.793	28.170	1.00	0.00	LX0	N
ATOM	3557	H	LYS	399	56.456	-15.202	28.040	0.00	0.00	LX0	H
ATOM	3558	CA	LYS	399	58.193	-14.385	27.042	1.00	0.00	LX0	C
ATOM	3559	CB	LYS	399	57.698	-15.074	25.765	1.00	0.00	LX0	C
ATOM	3560	CG	LYS	399	58.437	-16.380	25.459	1.00	0.00	LX0	C
ATOM	3561	CD	LYS	399	58.389	-17.412	26.589	1.00	0.00	LX0	C
ATOM	3562	CE	LYS	399	59.625	-18.310	26.607	1.00	0.00	LX0	C
ATOM	3563	NZ	LYS	399	60.816	-17.481	26.813	1.00	0.00	LX0	N
ATOM	3564	HZ1	LYS	399	60.985	-16.868	25.985	0.00	0.00	LX0	H
ATOM	3565	HZ2	LYS	399	60.724	-16.803	27.598	0.00	0.00	LX0	H
ATOM	3566	HZ3	LYS	399	61.700	-18.009	26.945	0.00	0.00	LX0	H
ATOM	3567	C	LYS	399	58.230	-12.879	26.865	1.00	0.00	LX0	C
ATOM	3568	O	LYS	399	59.274	-12.258	26.697	1.00	0.00	LX0	O
ATOM	3569	N	GLU	400	57.016	-12.313	26.920	1.00	0.00	LX0	N
ATOM	3570	H	GLU	400	56.196	-12.861	27.093	0.00	0.00	LX0	H
ATOM	3571	CA	GLU	400	56.956	-10.863	26.782	1.00	0.00	LX0	C
ATOM	3572	CB	GLU	400	56.188	-10.434	25.526	1.00	0.00	LX0	C
ATOM	3573	CG	GLU	400	56.405	-11.267	24.255	1.00	0.00	LX0	C
ATOM	3574	CD	GLU	400	55.657	-10.648	23.082	1.00	0.00	LX0	C
ATOM	3575	OE1	GLU	400	56.220	-10.548	21.994	1.00	0.00	LX0	O
ATOM	3576	OE2	GLU	400	54.520	-10.215	23.246	1.00	0.00	LX0	O
ATOM	3577	C	GLU	400	56.288	-10.222	27.977	1.00	0.00	LX0	C
ATOM	3578	O	GLU	400	55.202	-10.616	28.374	1.00	0.00	LX0	O
ATOM	3579	N	ILE	401	56.954	-9.203	28.515	1.00	0.00	LX0	N
ATOM	3580	H	ILE	401	57.881	-9.011	28.205	0.00	0.00	LX0	H
ATOM	3581	CA	ILE	401	56.246	-8.207	29.310	1.00	0.00	LX0	C
ATOM	3582	CB	ILE	401	57.060	-7.840	30.558	1.00	0.00	LX0	C
ATOM	3583	CG2	ILE	401	56.480	-6.649	31.328	1.00	0.00	LX0	C
ATOM	3584	CG1	ILE	401	57.190	-9.063	31.460	1.00	0.00	LX0	C
ATOM	3585	CD1	ILE	401	58.022	-8.806	32.711	1.00	0.00	LX0	C
ATOM	3586	C	ILE	401	56.065	-7.004	28.414	1.00	0.00	LX0	C
ATOM	3587	O	ILE	401	57.023	-6.411	27.936	1.00	0.00	LX0	O
ATOM	3588	N	THR	402	54.805	-6.669	28.155	1.00	0.00	LX0	N
ATOM	3589	H	THR	402	54.026	-7.119	28.595	0.00	0.00	LX0	H
ATOM	3590	CA	THR	402	54.694	-5.562	27.213	1.00	0.00	LX0	C
ATOM	3591	CB	THR	402	53.518	-5.784	26.267	1.00	0.00	LX0	C
ATOM	3592	OG1	THR	402	52.349	-6.154	26.996	1.00	0.00	LX0	O
ATOM	3593	HG1	THR	402	52.431	-7.094	27.157	0.00	0.00	LX0	H
ATOM	3594	CG2	THR	402	53.850	-6.879	25.249	1.00	0.00	LX0	C
ATOM	3595	C	THR	402	54.722	-4.174	27.833	1.00	0.00	LX0	C
ATOM	3596	O	THR	402	54.973	-3.175	27.169	1.00	0.00	LX0	O
ATOM	3597	N	GLY	403	54.492	-4.153	29.149	1.00	0.00	LX0	N
ATOM	3598	H	GLY	403	54.309	-4.994	29.659	0.00	0.00	LX0	H
ATOM	3599	CA	GLY	403	54.703	-2.904	29.867	1.00	0.00	LX0	C
ATOM	3600	C	GLY	403	56.044	-2.908	30.565	1.00	0.00	LX0	C
ATOM	3601	O	GLY	403	57.082	-3.059	29.927	1.00	0.00	LX0	O
ATOM	3602	N	PHE	404	55.974	-2.759	31.891	1.00	0.00	LX0	N
ATOM	3603	H	PHE	404	55.078	-2.657	32.329	0.00	0.00	LX0	H
ATOM	3604	CA	PHE	404	57.207	-2.716	32.667	1.00	0.00	LX0	C
ATOM	3605	CB	PHE	404	57.360	-1.354	33.366	1.00	0.00	LX0	C
ATOM	3606	CG	PHE	404	56.246	-1.063	34.348	1.00	0.00	LX0	C
ATOM	3607	CD1	PHE	404	56.199	-1.728	35.594	1.00	0.00	LX0	C
ATOM	3608	CD2	PHE	404	55.270	-0.107	34.000	1.00	0.00	LX0	C
ATOM	3609	CE1	PHE	404	55.162	-1.434	36.499	1.00	0.00	LX0	C
ATOM	3610	CE2	PHE	404	54.239	0.200	34.905	1.00	0.00	LX0	C
ATOM	3611	CZ	PHE	404	54.197	-0.468	36.144	1.00	0.00	LX0	C

ATOM	3612	C	PHE	404	57.370	-3.858	33.655	1.00	0.00	LX0	C
ATOM	3613	O	PHE	404	56.433	-4.555	34.027	1.00	0.00	LX0	O
ATOM	3614	N	LEU	405	58.623	-3.987	34.096	1.00	0.00	LX0	N
ATOM	3615	H	LEU	405	59.339	-3.396	33.726	0.00	0.00	LX0	H
ATOM	3616	CA	LEU	405	58.937	-4.930	35.156	1.00	0.00	LX0	C
ATOM	3617	CB	LEU	405	59.923	-5.966	34.614	1.00	0.00	LX0	C
ATOM	3618	CG	LEU	405	60.270	-7.093	35.587	1.00	0.00	LX0	C
ATOM	3619	CD1	LEU	405	59.026	-7.758	36.180	1.00	0.00	LX0	C
ATOM	3620	CD2	LEU	405	61.225	-8.100	34.946	1.00	0.00	LX0	C
ATOM	3621	C	LEU	405	59.469	-4.209	36.381	1.00	0.00	LX0	C
ATOM	3622	O	LEU	405	60.589	-3.708	36.411	1.00	0.00	LX0	O
ATOM	3623	N	LEU	406	58.593	-4.167	37.392	1.00	0.00	LX0	N
ATOM	3624	H	LEU	406	57.720	-4.655	37.328	0.00	0.00	LX0	H
ATOM	3625	CA	LEU	406	58.963	-3.482	38.626	1.00	0.00	LX0	C
ATOM	3626	CB	LEU	406	57.781	-2.621	39.095	1.00	0.00	LX0	C
ATOM	3627	CG	LEU	406	58.047	-1.748	40.326	1.00	0.00	LX0	C
ATOM	3628	CD1	LEU	406	59.112	-0.685	40.075	1.00	0.00	LX0	C
ATOM	3629	CD2	LEU	406	56.766	-1.124	40.870	1.00	0.00	LX0	C
ATOM	3630	C	LEU	406	59.407	-4.453	39.706	1.00	0.00	LX0	C
ATOM	3631	O	LEU	406	58.644	-4.823	40.591	1.00	0.00	LX0	O
ATOM	3632	N	ILE	407	60.676	-4.860	39.604	1.00	0.00	LX0	N
ATOM	3633	H	ILE	407	61.289	-4.415	38.949	0.00	0.00	LX0	H
ATOM	3634	CA	ILE	407	61.173	-5.692	40.696	1.00	0.00	LX0	C
ATOM	3635	CB	ILE	407	62.169	-6.758	40.224	1.00	0.00	LX0	C
ATOM	3636	CG2	ILE	407	62.413	-7.780	41.337	1.00	0.00	LX0	C
ATOM	3637	CG1	ILE	407	61.693	-7.461	38.956	1.00	0.00	LX0	C
ATOM	3638	CD1	ILE	407	62.665	-8.539	38.474	1.00	0.00	LX0	C
ATOM	3639	C	ILE	407	61.762	-4.868	41.827	1.00	0.00	LX0	C
ATOM	3640	O	ILE	407	62.943	-4.537	41.871	1.00	0.00	LX0	O
ATOM	3641	N	GLN	408	60.857	-4.558	42.760	1.00	0.00	LX0	N
ATOM	3642	H	GLN	408	59.900	-4.843	42.656	0.00	0.00	LX0	H
ATOM	3643	CA	GLN	408	61.351	-3.954	43.994	1.00	0.00	LX0	C
ATOM	3644	CB	GLN	408	60.352	-2.937	44.530	1.00	0.00	LX0	C
ATOM	3645	CG	GLN	408	60.042	-1.815	43.546	1.00	0.00	LX0	C
ATOM	3646	CD	GLN	408	58.956	-0.930	44.118	1.00	0.00	LX0	C
ATOM	3647	OE1	GLN	408	58.068	-1.357	44.842	1.00	0.00	LX0	O
ATOM	3648	NE2	GLN	408	59.064	0.352	43.761	1.00	0.00	LX0	N
ATOM	3649	HE21	GLN	408	59.803	0.667	43.170	0.00	0.00	LX0	H
ATOM	3650	HE22	GLN	408	58.376	0.986	44.109	0.00	0.00	LX0	H
ATOM	3651	C	GLN	408	61.665	-4.976	45.070	1.00	0.00	LX0	C
ATOM	3652	O	GLN	408	62.455	-4.747	45.976	1.00	0.00	LX0	O
ATOM	3653	N	ALA	409	60.994	-6.125	44.939	1.00	0.00	LX0	N
ATOM	3654	H	ALA	409	60.324	-6.266	44.209	0.00	0.00	LX0	H
ATOM	3655	CA	ALA	409	61.252	-7.157	45.932	1.00	0.00	LX0	C
ATOM	3656	CB	ALA	409	59.973	-7.502	46.676	1.00	0.00	LX0	C
ATOM	3657	C	ALA	409	61.773	-8.428	45.317	1.00	0.00	LX0	C
ATOM	3658	O	ALA	409	61.387	-8.810	44.221	1.00	0.00	LX0	O
ATOM	3659	N	TRP	410	62.643	-9.075	46.098	1.00	0.00	LX0	N
ATOM	3660	H	TRP	410	62.964	-8.661	46.952	0.00	0.00	LX0	H
ATOM	3661	CA	TRP	410	63.157	-10.397	45.750	1.00	0.00	LX0	C
ATOM	3662	CB	TRP	410	64.378	-10.257	44.842	1.00	0.00	LX0	C
ATOM	3663	CG	TRP	410	64.400	-11.240	43.694	1.00	0.00	LX0	C
ATOM	3664	CD2	TRP	410	63.354	-11.603	42.764	1.00	0.00	LX0	C
ATOM	3665	CE2	TRP	410	63.925	-12.532	41.828	1.00	0.00	LX0	C
ATOM	3666	CE3	TRP	410	61.999	-11.229	42.633	1.00	0.00	LX0	C
ATOM	3667	CD1	TRP	410	65.525	-11.961	43.275	1.00	0.00	LX0	C
ATOM	3668	NE1	TRP	410	65.259	-12.722	42.182	1.00	0.00	LX0	N
ATOM	3669	HE1	TRP	410	65.921	-13.281	41.719	0.00	0.00	LX0	H
ATOM	3670	CZ2	TRP	410	63.131	-13.049	40.782	1.00	0.00	LX0	C
ATOM	3671	CZ3	TRP	410	61.216	-11.756	41.586	1.00	0.00	LX0	C
ATOM	3672	CH2	TRP	410	61.781	-12.660	40.664	1.00	0.00	LX0	C

ATOM	3673	C	TRP	410	63.561	-11.030	47.061	1.00	0.00	LX0	C
ATOM	3674	O	TRP	410	64.080	-10.339	47.929	1.00	0.00	LX0	O
ATOM	3675	N	PRO	411	63.278	-12.344	47.209	1.00	0.00	LX0	N
ATOM	3676	CD	PRO	411	62.627	-13.227	46.253	1.00	0.00	LX0	C
ATOM	3677	CA	PRO	411	63.618	-13.014	48.467	1.00	0.00	LX0	C
ATOM	3678	CB	PRO	411	63.181	-14.459	48.225	1.00	0.00	LX0	C
ATOM	3679	CG	PRO	411	62.144	-14.386	47.111	1.00	0.00	LX0	C
ATOM	3680	C	PRO	411	65.093	-12.931	48.799	1.00	0.00	LX0	C
ATOM	3681	O	PRO	411	65.956	-13.026	47.934	1.00	0.00	LX0	O
ATOM	3682	N	GLU	412	65.334	-12.792	50.105	1.00	0.00	LX0	N
ATOM	3683	H	GLU	412	64.552	-12.621	50.708	0.00	0.00	LX0	H
ATOM	3684	CA	GLU	412	66.698	-12.666	50.620	1.00	0.00	LX0	C
ATOM	3685	CB	GLU	412	66.711	-12.728	52.153	1.00	0.00	LX0	C
ATOM	3686	CG	GLU	412	65.850	-11.675	52.868	1.00	0.00	LX0	C
ATOM	3687	CD	GLU	412	64.375	-12.049	52.867	1.00	0.00	LX0	C
ATOM	3688	OE1	GLU	412	63.959	-12.854	53.687	1.00	0.00	LX0	O
ATOM	3689	OE2	GLU	412	63.601	-11.489	52.102	1.00	0.00	LX0	O
ATOM	3690	C	GLU	412	67.687	-13.670	50.047	1.00	0.00	LX0	C
ATOM	3691	O	GLU	412	68.778	-13.337	49.597	1.00	0.00	LX0	O
ATOM	3692	N	ASN	413	67.235	-14.930	50.039	1.00	0.00	LX0	N
ATOM	3693	H	ASN	413	66.354	-15.130	50.467	0.00	0.00	LX0	H
ATOM	3694	CA	ASN	413	68.095	-15.958	49.452	1.00	0.00	LX0	C
ATOM	3695	CB	ASN	413	67.941	-17.306	50.166	1.00	0.00	LX0	C
ATOM	3696	CG	ASN	413	68.346	-17.182	51.619	1.00	0.00	LX0	C
ATOM	3697	OD1	ASN	413	67.631	-16.631	52.439	1.00	0.00	LX0	O
ATOM	3698	ND2	ASN	413	69.529	-17.727	51.913	1.00	0.00	LX0	N
ATOM	3699	HD21	ASN	413	70.105	-18.177	51.235	0.00	0.00	LX0	H
ATOM	3700	HD22	ASN	413	69.822	-17.665	52.866	0.00	0.00	LX0	H
ATOM	3701	C	ASN	413	67.902	-16.146	47.958	1.00	0.00	LX0	C
ATOM	3702	O	ASN	413	67.621	-17.244	47.477	1.00	0.00	LX0	O
ATOM	3703	N	ARG	414	68.066	-15.028	47.237	1.00	0.00	LX0	N
ATOM	3704	H	ARG	414	68.191	-14.142	47.693	0.00	0.00	LX0	H
ATOM	3705	CA	ARG	414	68.056	-15.093	45.779	1.00	0.00	LX0	C
ATOM	3706	CB	ARG	414	66.709	-14.672	45.189	1.00	0.00	LX0	C
ATOM	3707	CG	ARG	414	65.596	-15.704	45.355	1.00	0.00	LX0	C
ATOM	3708	CD	ARG	414	65.975	-17.047	44.730	1.00	0.00	LX0	C
ATOM	3709	NE	ARG	414	64.857	-17.988	44.761	1.00	0.00	LX0	N
ATOM	3710	HE	ARG	414	64.132	-17.876	44.071	0.00	0.00	LX0	H
ATOM	3711	CZ	ARG	414	64.829	-18.993	45.658	1.00	0.00	LX0	C
ATOM	3712	NH1	ARG	414	65.777	-19.100	46.592	1.00	0.00	LX0	N
ATOM	3713	HH11	ARG	414	65.799	-19.857	47.242	0.00	0.00	LX0	H
ATOM	3714	HH12	ARG	414	66.502	-18.402	46.675	0.00	0.00	LX0	H
ATOM	3715	NH2	ARG	414	63.837	-19.876	45.595	1.00	0.00	LX0	N
ATOM	3716	HH21	ARG	414	63.781	-20.679	46.187	0.00	0.00	LX0	H
ATOM	3717	HH22	ARG	414	63.087	-19.737	44.931	0.00	0.00	LX0	H
ATOM	3718	C	ARG	414	69.157	-14.261	45.162	1.00	0.00	LX0	C
ATOM	3719	O	ARG	414	69.205	-13.041	45.262	1.00	0.00	LX0	O
ATOM	3720	N	THR	415	70.050	-15.002	44.506	1.00	0.00	LX0	N
ATOM	3721	H	THR	415	70.035	-16.001	44.488	0.00	0.00	LX0	H
ATOM	3722	CA	THR	415	71.195	-14.343	43.893	1.00	0.00	LX0	C
ATOM	3723	CB	THR	415	72.423	-15.203	44.168	1.00	0.00	LX0	C
ATOM	3724	OG1	THR	415	72.025	-16.578	44.309	1.00	0.00	LX0	O
ATOM	3725	HG1	THR	415	72.828	-17.086	44.334	0.00	0.00	LX0	H
ATOM	3726	CG2	THR	415	73.151	-14.733	45.429	1.00	0.00	LX0	C
ATOM	3727	C	THR	415	71.058	-14.030	42.411	1.00	0.00	LX0	C
ATOM	3728	O	THR	415	71.885	-13.329	41.840	1.00	0.00	LX0	O
ATOM	3729	N	ASP	416	69.982	-14.568	41.823	1.00	0.00	LX0	N
ATOM	3730	H	ASP	416	69.305	-15.089	42.336	0.00	0.00	LX0	H
ATOM	3731	CA	ASP	416	69.769	-14.367	40.392	1.00	0.00	LX0	C
ATOM	3732	CB	ASP	416	70.234	-15.622	39.635	1.00	0.00	LX0	C
ATOM	3733	CG	ASP	416	70.244	-15.407	38.131	1.00	0.00	LX0	C

```
ATOM   3734  OD1 ASP   416      71.284 -15.063  37.580  1.00  0.00      LX0  O
ATOM   3735  OD2 ASP   416      69.209 -15.591  37.503  1.00  0.00      LX0  O
ATOM   3736  C   ASP   416      68.301 -14.054  40.163  1.00  0.00      LX0  C
ATOM   3737  O   ASP   416      67.453 -14.318  41.018  1.00  0.00      LX0  O
ATOM   3738  N   LEU   417      68.012 -13.463  38.997  1.00  0.00      LX0  N
ATOM   3739  H   LEU   417      68.718 -13.449  38.284  0.00  0.00      LX0  H
ATOM   3740  CA  LEU   417      66.607 -13.149  38.749  1.00  0.00      LX0  C
ATOM   3741  CB  LEU   417      66.422 -11.978  37.778  1.00  0.00      LX0  C
ATOM   3742  CG  LEU   417      66.645 -10.605  38.426  1.00  0.00      LX0  C
ATOM   3743  CD1 LEU   417      66.334  -9.459  37.463  1.00  0.00      LX0  C
ATOM   3744  CD2 LEU   417      65.842 -10.428  39.711  1.00  0.00      LX0  C
ATOM   3745  C   LEU   417      65.711 -14.309  38.353  1.00  0.00      LX0  C
ATOM   3746  O   LEU   417      64.519 -14.110  38.176  1.00  0.00      LX0  O
ATOM   3747  N   HIS   418      66.314 -15.514  38.269  1.00  0.00      LX0  N
ATOM   3748  H   HIS   418      67.317 -15.518  38.269  0.00  0.00      LX0  H
ATOM   3749  CA  HIS   418      65.645 -16.817  38.093  1.00  0.00      LX0  C
ATOM   3750  CB  HIS   418      65.823 -17.708  39.339  1.00  0.00      LX0  C
ATOM   3751  CG  HIS   418      64.969 -17.255  40.500  1.00  0.00      LX0  C
ATOM   3752  ND1 HIS   418      65.052 -16.032  41.052  1.00  0.00      LX0  N
ATOM   3753  HD1 HIS   418      65.702 -15.331  40.825  0.00  0.00      LX0  H
ATOM   3754  CD2 HIS   418      63.972 -17.983  41.157  1.00  0.00      LX0  C
ATOM   3755  NE2 HIS   418      63.453 -17.174  42.115  1.00  0.00      LX0  N
ATOM   3756  CE1 HIS   418      64.113 -15.976  42.043  1.00  0.00      LX0  C
ATOM   3757  C   HIS   418      64.244 -16.913  37.481  1.00  0.00      LX0  C
ATOM   3758  O   HIS   418      64.059 -17.444  36.389  1.00  0.00      LX0  O
ATOM   3759  N   ALA   419      63.252 -16.359  38.190  1.00  0.00      LX0  N
ATOM   3760  H   ALA   419      63.481 -15.876  39.034  0.00  0.00      LX0  H
ATOM   3761  CA  ALA   419      61.904 -16.297  37.628  1.00  0.00      LX0  C
ATOM   3762  CB  ALA   419      61.018 -15.463  38.545  1.00  0.00      LX0  C
ATOM   3763  C   ALA   419      61.860 -15.697  36.229  1.00  0.00      LX0  C
ATOM   3764  O   ALA   419      61.233 -16.199  35.309  1.00  0.00      LX0  O
ATOM   3765  N   PHE   420      62.603 -14.594  36.098  1.00  0.00      LX0  N
ATOM   3766  H   PHE   420      63.145 -14.259  36.870  0.00  0.00      LX0  H
ATOM   3767  CA  PHE   420      62.603 -13.928  34.801  1.00  0.00      LX0  C
ATOM   3768  CB  PHE   420      62.512 -12.415  34.995  1.00  0.00      LX0  C
ATOM   3769  CG  PHE   420      61.194 -12.032  35.622  1.00  0.00      LX0  C
ATOM   3770  CD1 PHE   420      60.028 -12.003  34.824  1.00  0.00      LX0  C
ATOM   3771  CD2 PHE   420      61.153 -11.697  36.992  1.00  0.00      LX0  C
ATOM   3772  CE1 PHE   420      58.806 -11.614  35.401  1.00  0.00      LX0  C
ATOM   3773  CE2 PHE   420      59.930 -11.310  37.570  1.00  0.00      LX0  C
ATOM   3774  CZ  PHE   420      58.772 -11.263  36.767  1.00  0.00      LX0  C
ATOM   3775  C   PHE   420      63.754 -14.284  33.873  1.00  0.00      LX0  C
ATOM   3776  O   PHE   420      64.124 -13.519  32.992  1.00  0.00      LX0  O
ATOM   3777  N   GLU   421      64.311 -15.491  34.076  1.00  0.00      LX0  N
ATOM   3778  H   GLU   421      64.008 -16.079  34.827  0.00  0.00      LX0  H
ATOM   3779  CA  GLU   421      65.384 -15.898  33.158  1.00  0.00      LX0  C
ATOM   3780  CB  GLU   421      65.955 -17.256  33.541  1.00  0.00      LX0  C
ATOM   3781  CG  GLU   421      66.754 -17.240  34.834  1.00  0.00      LX0  C
ATOM   3782  CD  GLU   421      67.240 -18.642  35.134  1.00  0.00      LX0  C
ATOM   3783  OE1 GLU   421      68.182 -19.082  34.493  1.00  0.00      LX0  O
ATOM   3784  OE2 GLU   421      66.686 -19.301  36.004  1.00  0.00      LX0  O
ATOM   3785  C   GLU   421      64.948 -15.995  31.710  1.00  0.00      LX0  C
ATOM   3786  O   GLU   421      65.613 -15.570  30.771  1.00  0.00      LX0  O
ATOM   3787  N   ASN   422      63.770 -16.621  31.587  1.00  0.00      LX0  N
ATOM   3788  H   ASN   422      63.232 -16.774  32.415  0.00  0.00      LX0  H
ATOM   3789  CA  ASN   422      63.259 -17.012  30.274  1.00  0.00      LX0  C
ATOM   3790  CB  ASN   422      62.554 -18.361  30.454  1.00  0.00      LX0  C
ATOM   3791  CG  ASN   422      62.160 -19.024  29.148  1.00  0.00      LX0  C
ATOM   3792  OD1 ASN   422      62.705 -18.794  28.077  1.00  0.00      LX0  O
ATOM   3793  ND2 ASN   422      61.183 -19.920  29.283  1.00  0.00      LX0  N
ATOM   3794 HD21 ASN   422      60.641 -19.920  30.127  0.00  0.00      LX0  H
```

ATOM	3795	HD22	ASN	422	60.963	-20.566	28.557	0.00	0.00	LX0	H
ATOM	3796	C	ASN	422	62.363	-15.950	29.654	1.00	0.00	LX0	C
ATOM	3797	O	ASN	422	61.324	-16.240	29.070	1.00	0.00	LX0	O
ATOM	3798	N	LEU	423	62.816	-14.707	29.868	1.00	0.00	LX0	N
ATOM	3799	H	LEU	423	63.737	-14.569	30.232	0.00	0.00	LX0	H
ATOM	3800	CA	LEU	423	62.075	-13.524	29.448	1.00	0.00	LX0	C
ATOM	3801	CB	LEU	423	62.066	-12.532	30.614	1.00	0.00	LX0	C
ATOM	3802	CG	LEU	423	60.873	-11.589	30.811	1.00	0.00	LX0	C
ATOM	3803	CD1	LEU	423	61.257	-10.496	31.804	1.00	0.00	LX0	C
ATOM	3804	CD2	LEU	423	60.311	-10.970	29.535	1.00	0.00	LX0	C
ATOM	3805	C	LEU	423	62.826	-12.906	28.290	1.00	0.00	LX0	C
ATOM	3806	O	LEU	423	64.009	-12.613	28.413	1.00	0.00	LX0	O
ATOM	3807	N	GLU	424	62.114	-12.718	27.181	1.00	0.00	LX0	N
ATOM	3808	H	GLU	424	61.174	-13.059	27.102	0.00	0.00	LX0	H
ATOM	3809	CA	GLU	424	62.810	-12.174	26.023	1.00	0.00	LX0	C
ATOM	3810	CB	GLU	424	62.471	-12.943	24.741	1.00	0.00	LX0	C
ATOM	3811	CG	GLU	424	63.052	-14.357	24.605	1.00	0.00	LX0	C
ATOM	3812	CD	GLU	424	62.145	-15.416	25.204	1.00	0.00	LX0	C
ATOM	3813	OE1	GLU	424	61.357	-15.124	26.098	1.00	0.00	LX0	O
ATOM	3814	OE2	GLU	424	62.207	-16.565	24.774	1.00	0.00	LX0	O
ATOM	3815	C	GLU	424	62.559	-10.704	25.778	1.00	0.00	LX0	C
ATOM	3816	O	GLU	424	63.386	-9.990	25.220	1.00	0.00	LX0	O
ATOM	3817	N	ILE	425	61.351	-10.277	26.157	1.00	0.00	LX0	N
ATOM	3818	H	ILE	425	60.718	-10.883	26.644	0.00	0.00	LX0	H
ATOM	3819	CA	ILE	425	60.891	-8.993	25.635	1.00	0.00	LX0	C
ATOM	3820	CB	ILE	425	59.868	-9.292	24.523	1.00	0.00	LX0	C
ATOM	3821	CG2	ILE	425	59.108	-8.048	24.080	1.00	0.00	LX0	C
ATOM	3822	CG1	ILE	425	60.536	-10.015	23.345	1.00	0.00	LX0	C
ATOM	3823	CD1	ILE	425	59.603	-10.762	22.398	1.00	0.00	LX0	C
ATOM	3824	C	ILE	425	60.290	-8.118	26.721	1.00	0.00	LX0	C
ATOM	3825	O	ILE	425	59.483	-8.588	27.506	1.00	0.00	LX0	O
ATOM	3826	N	ILE	426	60.682	-6.834	26.720	1.00	0.00	LX0	N
ATOM	3827	H	ILE	426	61.435	-6.542	26.131	0.00	0.00	LX0	H
ATOM	3828	CA	ILE	426	59.973	-5.832	27.518	1.00	0.00	LX0	C
ATOM	3829	CB	ILE	426	60.763	-5.450	28.787	1.00	0.00	LX0	C
ATOM	3830	CG2	ILE	426	60.041	-4.375	29.606	1.00	0.00	LX0	C
ATOM	3831	CG1	ILE	426	61.037	-6.681	29.659	1.00	0.00	LX0	C
ATOM	3832	CD1	ILE	426	61.883	-6.390	30.897	1.00	0.00	LX0	C
ATOM	3833	C	ILE	426	59.667	-4.609	26.659	1.00	0.00	LX0	C
ATOM	3834	O	ILE	426	60.560	-3.920	26.178	1.00	0.00	LX0	O
ATOM	3835	N	ARG	427	58.360	-4.381	26.449	1.00	0.00	LX0	N
ATOM	3836	H	ARG	427	57.691	-4.941	26.938	0.00	0.00	LX0	H
ATOM	3837	CA	ARG	427	58.007	-3.335	25.486	1.00	0.00	LX0	C
ATOM	3838	CB	ARG	427	56.844	-3.743	24.573	1.00	0.00	LX0	C
ATOM	3839	CG	ARG	427	57.233	-4.940	23.716	1.00	0.00	LX0	C
ATOM	3840	CD	ARG	427	56.386	-5.188	22.468	1.00	0.00	LX0	C
ATOM	3841	NE	ARG	427	57.096	-6.151	21.633	1.00	0.00	LX0	N
ATOM	3842	HE	ARG	427	58.016	-5.914	21.304	0.00	0.00	LX0	H
ATOM	3843	CZ	ARG	427	56.733	-7.445	21.584	1.00	0.00	LX0	C
ATOM	3844	NH1	ARG	427	55.511	-7.849	21.924	1.00	0.00	LX0	N
ATOM	3845	HH11	ARG	427	55.329	-8.826	22.127	0.00	0.00	LX0	H
ATOM	3846	HH12	ARG	427	54.726	-7.221	21.968	0.00	0.00	LX0	H
ATOM	3847	NH2	ARG	427	57.645	-8.327	21.202	1.00	0.00	LX0	N
ATOM	3848	HH21	ARG	427	57.404	-9.306	21.208	0.00	0.00	LX0	H
ATOM	3849	HH22	ARG	427	58.577	-8.031	20.948	0.00	0.00	LX0	H
ATOM	3850	C	ARG	427	57.778	-1.932	26.010	1.00	0.00	LX0	C
ATOM	3851	O	ARG	427	57.709	-0.985	25.234	1.00	0.00	LX0	O
ATOM	3852	N	GLY	428	57.653	-1.815	27.342	1.00	0.00	LX0	N
ATOM	3853	H	GLY	428	57.646	-2.619	27.940	0.00	0.00	LX0	H
ATOM	3854	CA	GLY	428	57.533	-0.473	27.918	1.00	0.00	LX0	C
ATOM	3855	C	GLY	428	56.382	0.375	27.396	1.00	0.00	LX0	C

ATOM	3856	O	GLY	428	56.510	1.577	27.174	1.00	0.00	LX0	O
ATOM	3857	N	ARG	429	55.243	-0.308	27.198	1.00	0.00	LX0	N
ATOM	3858	H	ARG	429	55.234	-1.289	27.392	0.00	0.00	LX0	H
ATOM	3859	CA	ARG	429	54.045	0.417	26.775	1.00	0.00	LX0	C
ATOM	3860	CB	ARG	429	52.965	-0.553	26.303	1.00	0.00	LX0	C
ATOM	3861	CG	ARG	429	53.384	-1.179	24.982	1.00	0.00	LX0	C
ATOM	3862	CD	ARG	429	52.381	-2.173	24.413	1.00	0.00	LX0	C
ATOM	3863	NE	ARG	429	52.864	-2.555	23.097	1.00	0.00	LX0	N
ATOM	3864	HE	ARG	429	53.347	-1.863	22.558	0.00	0.00	LX0	H
ATOM	3865	CZ	ARG	429	52.782	-3.791	22.585	1.00	0.00	LX0	C
ATOM	3866	NH1	ARG	429	52.088	-4.753	23.176	1.00	0.00	LX0	N
ATOM	3867	HH11	ARG	429	52.134	-5.692	22.810	0.00	0.00	LX0	H
ATOM	3868	HH12	ARG	429	51.480	-4.569	23.956	0.00	0.00	LX0	H
ATOM	3869	NH2	ARG	429	53.416	-4.028	21.449	1.00	0.00	LX0	N
ATOM	3870	HH21	ARG	429	53.414	-4.947	21.032	0.00	0.00	LX0	H
ATOM	3871	HH22	ARG	429	53.916	-3.296	20.977	0.00	0.00	LX0	H
ATOM	3872	C	ARG	429	53.510	1.327	27.855	1.00	0.00	LX0	C
ATOM	3873	O	ARG	429	53.404	2.538	27.697	1.00	0.00	LX0	O
ATOM	3874	N	THR	430	53.217	0.670	28.980	1.00	0.00	LX0	N
ATOM	3875	H	THR	430	53.369	-0.313	29.058	0.00	0.00	LX0	H
ATOM	3876	CA	THR	430	53.141	1.466	30.193	1.00	0.00	LX0	C
ATOM	3877	CB	THR	430	51.979	1.003	31.111	1.00	0.00	LX0	C
ATOM	3878	OG1	THR	430	52.379	0.809	32.469	1.00	0.00	LX0	O
ATOM	3879	HG1	THR	430	52.319	-0.141	32.652	0.00	0.00	LX0	H
ATOM	3880	CG2	THR	430	51.204	-0.195	30.557	1.00	0.00	LX0	C
ATOM	3881	C	THR	430	54.529	1.435	30.808	1.00	0.00	LX0	C
ATOM	3882	O	THR	430	55.351	0.591	30.457	1.00	0.00	LX0	O
ATOM	3883	N	LYS	431	54.790	2.456	31.633	1.00	0.00	LX0	N
ATOM	3884	H	LYS	431	54.042	3.017	31.986	0.00	0.00	LX0	H
ATOM	3885	CA	LYS	431	56.159	2.675	32.088	1.00	0.00	LX0	C
ATOM	3886	CB	LYS	431	56.875	3.738	31.241	1.00	0.00	LX0	C
ATOM	3887	CG	LYS	431	56.733	3.687	29.715	1.00	0.00	LX0	C
ATOM	3888	CD	LYS	431	57.427	4.877	29.044	1.00	0.00	LX0	C
ATOM	3889	CE	LYS	431	57.018	5.122	27.586	1.00	0.00	LX0	C
ATOM	3890	NZ	LYS	431	57.462	4.028	26.721	1.00	0.00	LX0	N
ATOM	3891	HZ1	LYS	431	57.119	4.130	25.741	0.00	0.00	LX0	H
ATOM	3892	HZ2	LYS	431	58.494	4.018	26.575	0.00	0.00	LX0	H
ATOM	3893	HZ3	LYS	431	57.153	3.095	27.054	0.00	0.00	LX0	H
ATOM	3894	C	LYS	431	56.117	3.176	33.516	1.00	0.00	LX0	C
ATOM	3895	O	LYS	431	55.366	4.095	33.825	1.00	0.00	LX0	O
ATOM	3896	N	GLN	432	56.939	2.570	34.385	1.00	0.00	LX0	N
ATOM	3897	H	GLN	432	57.573	1.871	34.051	0.00	0.00	LX0	H
ATOM	3898	CA	GLN	432	56.938	3.073	35.759	1.00	0.00	LX0	C
ATOM	3899	CB	GLN	432	57.767	2.197	36.700	1.00	0.00	LX0	C
ATOM	3900	CG	GLN	432	56.911	1.321	37.623	1.00	0.00	LX0	C
ATOM	3901	CD	GLN	432	55.966	2.160	38.467	1.00	0.00	LX0	C
ATOM	3902	OE1	GLN	432	56.201	3.334	38.732	1.00	0.00	LX0	O
ATOM	3903	NE2	GLN	432	54.866	1.514	38.866	1.00	0.00	LX0	N
ATOM	3904	HE21	GLN	432	54.661	0.564	38.621	0.00	0.00	LX0	H
ATOM	3905	HE22	GLN	432	54.190	1.985	39.430	0.00	0.00	LX0	H
ATOM	3906	C	GLN	432	57.385	4.516	35.861	1.00	0.00	LX0	C
ATOM	3907	O	GLN	432	58.322	4.942	35.197	1.00	0.00	LX0	O
ATOM	3908	N	HIS	433	56.617	5.267	36.667	1.00	0.00	LX0	N
ATOM	3909	H	HIS	433	55.951	4.790	37.243	0.00	0.00	LX0	H
ATOM	3910	CA	HIS	433	56.779	6.725	36.742	1.00	0.00	LX0	C
ATOM	3911	CB	HIS	433	58.053	7.113	37.509	1.00	0.00	LX0	C
ATOM	3912	CG	HIS	433	57.909	6.884	38.998	1.00	0.00	LX0	C
ATOM	3913	ND1	HIS	433	57.492	5.737	39.569	1.00	0.00	LX0	N
ATOM	3914	HD1	HIS	433	57.215	4.907	39.125	0.00	0.00	LX0	H
ATOM	3915	CD2	HIS	433	58.187	7.805	40.011	1.00	0.00	LX0	C
ATOM	3916	NE2	HIS	433	57.933	7.199	41.195	1.00	0.00	LX0	N

ATOM	3917	CE1	HIS	433	57.504	5.926	40.924	1.00	0.00	LX0	C
ATOM	3918	C	HIS	433	56.724	7.464	35.407	1.00	0.00	LX0	C
ATOM	3919	O	HIS	433	57.241	8.565	35.258	1.00	0.00	LX0	O
ATOM	3920	N	GLY	434	56.087	6.811	34.422	1.00	0.00	LX0	N
ATOM	3921	H	GLY	434	55.707	5.894	34.561	0.00	0.00	LX0	H
ATOM	3922	CA	GLY	434	56.061	7.414	33.090	1.00	0.00	LX0	C
ATOM	3923	C	GLY	434	57.313	7.194	32.249	1.00	0.00	LX0	C
ATOM	3924	O	GLY	434	57.375	7.575	31.087	1.00	0.00	LX0	O
ATOM	3925	N	GLN	435	58.317	6.561	32.875	1.00	0.00	LX0	N
ATOM	3926	H	GLN	435	58.209	6.236	33.812	0.00	0.00	LX0	H
ATOM	3927	CA	GLN	435	59.588	6.453	32.168	1.00	0.00	LX0	C
ATOM	3928	CB	GLN	435	60.621	7.360	32.847	1.00	0.00	LX0	C
ATOM	3929	CG	GLN	435	61.819	7.694	31.954	1.00	0.00	LX0	C
ATOM	3930	CD	GLN	435	62.835	8.517	32.722	1.00	0.00	LX0	C
ATOM	3931	OE1	GLN	435	63.659	8.018	33.486	1.00	0.00	LX0	O
ATOM	3932	NE2	GLN	435	62.748	9.829	32.479	1.00	0.00	LX0	N
ATOM	3933	HE21	GLN	435	62.065	10.196	31.847	0.00	0.00	LX0	H
ATOM	3934	HE22	GLN	435	63.378	10.454	32.935	0.00	0.00	LX0	H
ATOM	3935	C	GLN	435	60.112	5.032	31.996	1.00	0.00	LX0	C
ATOM	3936	O	GLN	435	60.468	4.598	30.907	1.00	0.00	LX0	O
ATOM	3937	N	PHE	436	60.181	4.326	33.130	1.00	0.00	LX0	N
ATOM	3938	H	PHE	436	59.725	4.642	33.961	0.00	0.00	LX0	H
ATOM	3939	CA	PHE	436	60.967	3.091	33.122	1.00	0.00	LX0	C
ATOM	3940	CB	PHE	436	61.498	2.738	34.520	1.00	0.00	LX0	C
ATOM	3941	CG	PHE	436	61.729	3.955	35.388	1.00	0.00	LX0	C
ATOM	3942	CD1	PHE	436	61.004	4.061	36.594	1.00	0.00	LX0	C
ATOM	3943	CD2	PHE	436	62.647	4.955	34.997	1.00	0.00	LX0	C
ATOM	3944	CE1	PHE	436	61.187	5.186	37.420	1.00	0.00	LX0	C
ATOM	3945	CE2	PHE	436	62.823	6.084	35.820	1.00	0.00	LX0	C
ATOM	3946	CZ	PHE	436	62.092	6.192	37.021	1.00	0.00	LX0	C
ATOM	3947	C	PHE	436	60.241	1.876	32.585	1.00	0.00	LX0	C
ATOM	3948	O	PHE	436	59.091	1.632	32.924	1.00	0.00	LX0	O
ATOM	3949	N	SER	437	60.964	1.098	31.777	1.00	0.00	LX0	N
ATOM	3950	H	SER	437	61.878	1.348	31.458	0.00	0.00	LX0	H
ATOM	3951	CA	SER	437	60.484	-0.258	31.549	1.00	0.00	LX0	C
ATOM	3952	CB	SER	437	60.665	-0.644	30.087	1.00	0.00	LX0	C
ATOM	3953	OG	SER	437	61.898	-0.109	29.598	1.00	0.00	LX0	O
ATOM	3954	HG	SER	437	61.842	-0.187	28.645	0.00	0.00	LX0	H
ATOM	3955	C	SER	437	61.137	-1.264	32.477	1.00	0.00	LX0	C
ATOM	3956	O	SER	437	60.503	-2.181	32.980	1.00	0.00	LX0	O
ATOM	3957	N	LEU	438	62.435	-1.049	32.722	1.00	0.00	LX0	N
ATOM	3958	H	LEU	438	62.924	-0.272	32.320	0.00	0.00	LX0	H
ATOM	3959	CA	LEU	438	63.027	-1.907	33.743	1.00	0.00	LX0	C
ATOM	3960	CB	LEU	438	64.350	-2.499	33.265	1.00	0.00	LX0	C
ATOM	3961	CG	LEU	438	64.207	-3.937	32.771	1.00	0.00	LX0	C
ATOM	3962	CD1	LEU	438	65.483	-4.429	32.091	1.00	0.00	LX0	C
ATOM	3963	CD2	LEU	438	63.764	-4.878	33.894	1.00	0.00	LX0	C
ATOM	3964	C	LEU	438	63.235	-1.162	35.035	1.00	0.00	LX0	C
ATOM	3965	O	LEU	438	63.777	-0.064	35.043	1.00	0.00	LX0	O
ATOM	3966	N	ALA	439	62.799	-1.800	36.123	1.00	0.00	LX0	N
ATOM	3967	H	ALA	439	62.253	-2.640	36.061	0.00	0.00	LX0	H
ATOM	3968	CA	ALA	439	63.035	-1.168	37.414	1.00	0.00	LX0	C
ATOM	3969	CB	ALA	439	61.836	-0.308	37.810	1.00	0.00	LX0	C
ATOM	3970	C	ALA	439	63.312	-2.195	38.489	1.00	0.00	LX0	C
ATOM	3971	O	ALA	439	62.410	-2.795	39.059	1.00	0.00	LX0	O
ATOM	3972	N	VAL	440	64.617	-2.400	38.701	1.00	0.00	LX0	N
ATOM	3973	H	VAL	440	65.281	-1.768	38.295	0.00	0.00	LX0	H
ATOM	3974	CA	VAL	440	65.061	-3.453	39.613	1.00	0.00	LX0	C
ATOM	3975	CB	VAL	440	65.796	-4.547	38.816	1.00	0.00	LX0	C
ATOM	3976	CG1	VAL	440	66.414	-5.623	39.707	1.00	0.00	LX0	C
ATOM	3977	CG2	VAL	440	64.892	-5.167	37.748	1.00	0.00	LX0	C

ATOM	3978	C	VAL	440	65.960	-2.838	40.671	1.00	0.00	LX0	C
ATOM	3979	O	VAL	440	67.013	-2.287	40.359	1.00	0.00	LX0	O
ATOM	3980	N	VAL	441	65.489	-2.901	41.923	1.00	0.00	LX0	N
ATOM	3981	H	VAL	441	64.669	-3.437	42.144	0.00	0.00	LX0	H
ATOM	3982	CA	VAL	441	66.225	-2.138	42.932	1.00	0.00	LX0	C
ATOM	3983	CB	VAL	441	65.482	-0.841	43.307	1.00	0.00	LX0	C
ATOM	3984	CG1	VAL	441	65.498	0.181	42.164	1.00	0.00	LX0	C
ATOM	3985	CG2	VAL	441	64.063	-1.117	43.811	1.00	0.00	LX0	C
ATOM	3986	C	VAL	441	66.596	-2.912	44.185	1.00	0.00	LX0	C
ATOM	3987	O	VAL	441	65.851	-3.754	44.663	1.00	0.00	LX0	O
ATOM	3988	N	SER	442	67.777	-2.559	44.718	1.00	0.00	LX0	N
ATOM	3989	H	SER	442	68.299	-1.887	44.192	0.00	0.00	LX0	H
ATOM	3990	CA	SER	442	68.218	-2.985	46.060	1.00	0.00	LX0	C
ATOM	3991	CB	SER	442	67.236	-2.509	47.139	1.00	0.00	LX0	C
ATOM	3992	OG	SER	442	66.629	-1.275	46.733	1.00	0.00	LX0	O
ATOM	3993	HG	SER	442	65.774	-1.515	46.395	0.00	0.00	LX0	H
ATOM	3994	C	SER	442	68.607	-4.444	46.310	1.00	0.00	LX0	C
ATOM	3995	O	SER	442	69.171	-4.807	47.346	1.00	0.00	LX0	O
ATOM	3996	N	LEU	443	68.268	-5.283	45.324	1.00	0.00	LX0	N
ATOM	3997	H	LEU	443	67.952	-4.898	44.455	0.00	0.00	LX0	H
ATOM	3998	CA	LEU	443	68.195	-6.724	45.554	1.00	0.00	LX0	C
ATOM	3999	CB	LEU	443	67.623	-7.431	44.325	1.00	0.00	LX0	C
ATOM	4000	CG	LEU	443	66.295	-6.879	43.809	1.00	0.00	LX0	C
ATOM	4001	CD1	LEU	443	65.836	-7.678	42.597	1.00	0.00	LX0	C
ATOM	4002	CD2	LEU	443	65.212	-6.795	44.887	1.00	0.00	LX0	C
ATOM	4003	C	LEU	443	69.476	-7.416	45.988	1.00	0.00	LX0	C
ATOM	4004	O	LEU	443	70.561	-6.844	46.062	1.00	0.00	LX0	O
ATOM	4005	N	ASN	444	69.288	-8.712	46.286	1.00	0.00	LX0	N
ATOM	4006	H	ASN	444	68.381	-9.129	46.209	0.00	0.00	LX0	H
ATOM	4007	CA	ASN	444	70.450	-9.527	46.645	1.00	0.00	LX0	C
ATOM	4008	CB	ASN	444	70.101	-10.553	47.723	1.00	0.00	LX0	C
ATOM	4009	CG	ASN	444	70.052	-9.870	49.073	1.00	0.00	LX0	C
ATOM	4010	OD1	ASN	444	70.229	-8.662	49.204	1.00	0.00	LX0	O
ATOM	4011	ND2	ASN	444	69.805	-10.697	50.088	1.00	0.00	LX0	N
ATOM	4012	HD21	ASN	444	69.666	-11.674	49.908	0.00	0.00	LX0	H
ATOM	4013	HD22	ASN	444	69.740	-10.361	51.025	0.00	0.00	LX0	H
ATOM	4014	C	ASN	444	71.124	-10.211	45.475	1.00	0.00	LX0	C
ATOM	4015	O	ASN	444	72.080	-10.964	45.623	1.00	0.00	LX0	O
ATOM	4016	N	ILE	445	70.586	-9.899	44.288	1.00	0.00	LX0	N
ATOM	4017	H	ILE	445	69.841	-9.244	44.218	0.00	0.00	LX0	H
ATOM	4018	CA	ILE	445	71.143	-10.501	43.085	1.00	0.00	LX0	C
ATOM	4019	CB	ILE	445	70.240	-10.209	41.877	1.00	0.00	LX0	C
ATOM	4020	CG2	ILE	445	68.898	-10.915	42.069	1.00	0.00	LX0	C
ATOM	4021	CG1	ILE	445	70.065	-8.709	41.601	1.00	0.00	LX0	C
ATOM	4022	CD1	ILE	445	69.288	-8.411	40.319	1.00	0.00	LX0	C
ATOM	4023	C	ILE	445	72.589	-10.126	42.811	1.00	0.00	LX0	C
ATOM	4024	O	ILE	445	72.963	-8.962	42.733	1.00	0.00	LX0	O
ATOM	4025	N	THR	446	73.397	-11.180	42.681	1.00	0.00	LX0	N
ATOM	4026	H	THR	446	73.014	-12.100	42.753	0.00	0.00	LX0	H
ATOM	4027	CA	THR	446	74.719	-10.926	42.133	1.00	0.00	LX0	C
ATOM	4028	CB	THR	446	75.786	-11.854	42.736	1.00	0.00	LX0	C
ATOM	4029	OG1	THR	446	77.106	-11.424	42.354	1.00	0.00	LX0	O
ATOM	4030	HG1	THR	446	77.017	-11.087	41.462	0.00	0.00	LX0	H
ATOM	4031	CG2	THR	446	75.561	-13.331	42.387	1.00	0.00	LX0	C
ATOM	4032	C	THR	446	74.736	-10.974	40.617	1.00	0.00	LX0	C
ATOM	4033	O	THR	446	75.682	-10.500	39.997	1.00	0.00	LX0	O
ATOM	4034	N	SER	447	73.665	-11.552	40.066	1.00	0.00	LX0	N
ATOM	4035	H	SER	447	72.966	-12.059	40.575	0.00	0.00	LX0	H
ATOM	4036	CA	SER	447	73.546	-11.564	38.620	1.00	0.00	LX0	C
ATOM	4037	CB	SER	447	73.769	-12.987	38.112	1.00	0.00	LX0	C
ATOM	4038	OG	SER	447	73.448	-13.937	39.135	1.00	0.00	LX0	O

ATOM	4039	HG	SER	447	72.684	-14.415	38.803	0.00	0.00	LX0	H
ATOM	4040	C	SER	447	72.205	-11.009	38.201	1.00	0.00	LX0	C
ATOM	4041	O	SER	447	71.204	-11.131	38.897	1.00	0.00	LX0	O
ATOM	4042	N	LEU	448	72.244	-10.348	37.041	1.00	0.00	LX0	N
ATOM	4043	H	LEU	448	73.077	-10.371	36.487	0.00	0.00	LX0	H
ATOM	4044	CA	LEU	448	71.013	-9.761	36.530	1.00	0.00	LX0	C
ATOM	4045	CB	LEU	448	71.336	-8.680	35.501	1.00	0.00	LX0	C
ATOM	4046	CG	LEU	448	70.934	-7.276	35.958	1.00	0.00	LX0	C
ATOM	4047	CD1	LEU	448	71.428	-6.203	34.989	1.00	0.00	LX0	C
ATOM	4048	CD2	LEU	448	69.433	-7.157	36.228	1.00	0.00	LX0	C
ATOM	4049	C	LEU	448	70.027	-10.768	35.973	1.00	0.00	LX0	C
ATOM	4050	O	LEU	448	68.830	-10.534	35.943	1.00	0.00	LX0	O
ATOM	4051	N	GLY	449	70.568	-11.909	35.525	1.00	0.00	LX0	N
ATOM	4052	H	GLY	449	71.564	-12.003	35.475	0.00	0.00	LX0	H
ATOM	4053	CA	GLY	449	69.691	-13.061	35.295	1.00	0.00	LX0	C
ATOM	4054	C	GLY	449	68.619	-12.999	34.211	1.00	0.00	LX0	C
ATOM	4055	O	GLY	449	67.924	-13.976	33.957	1.00	0.00	LX0	O
ATOM	4056	N	LEU	450	68.512	-11.837	33.547	1.00	0.00	LX0	N
ATOM	4057	H	LEU	450	69.071	-11.064	33.834	0.00	0.00	LX0	H
ATOM	4058	CA	LEU	450	67.549	-11.706	32.451	1.00	0.00	LX0	C
ATOM	4059	CB	LEU	450	67.159	-10.237	32.272	1.00	0.00	LX0	C
ATOM	4060	CG	LEU	450	66.489	-9.609	33.495	1.00	0.00	LX0	C
ATOM	4061	CD1	LEU	450	66.477	-8.083	33.425	1.00	0.00	LX0	C
ATOM	4062	CD2	LEU	450	65.086	-10.167	33.713	1.00	0.00	LX0	C
ATOM	4063	C	LEU	450	68.086	-12.263	31.144	1.00	0.00	LX0	C
ATOM	4064	O	LEU	450	68.160	-11.597	30.123	1.00	0.00	LX0	O
ATOM	4065	N	ARG	451	68.508	-13.526	31.235	1.00	0.00	LX0	N
ATOM	4066	H	ARG	451	68.264	-14.030	32.063	0.00	0.00	LX0	H
ATOM	4067	CA	ARG	451	69.370	-14.067	30.185	1.00	0.00	LX0	C
ATOM	4068	CB	ARG	451	70.067	-15.347	30.669	1.00	0.00	LX0	C
ATOM	4069	CG	ARG	451	69.210	-16.253	31.559	1.00	0.00	LX0	C
ATOM	4070	CD	ARG	451	70.008	-17.333	32.291	1.00	0.00	LX0	C
ATOM	4071	NE	ARG	451	71.090	-16.775	33.100	1.00	0.00	LX0	N
ATOM	4072	HE	ARG	451	71.926	-16.423	32.662	0.00	0.00	LX0	H
ATOM	4073	CZ	ARG	451	71.026	-16.544	34.430	1.00	0.00	LX0	C
ATOM	4074	NH1	ARG	451	69.994	-16.929	35.170	1.00	0.00	LX0	N
ATOM	4075	HH11	ARG	451	69.920	-16.618	36.128	0.00	0.00	LX0	H
ATOM	4076	HH12	ARG	451	69.269	-17.523	34.809	0.00	0.00	LX0	H
ATOM	4077	NH2	ARG	451	72.025	-15.894	35.000	1.00	0.00	LX0	N
ATOM	4078	HH21	ARG	451	72.008	-15.679	35.982	0.00	0.00	LX0	H
ATOM	4079	HH22	ARG	451	72.806	-15.624	34.416	0.00	0.00	LX0	H
ATOM	4080	C	ARG	451	68.768	-14.196	28.795	1.00	0.00	LX0	C
ATOM	4081	O	ARG	451	69.468	-14.186	27.793	1.00	0.00	LX0	O
ATOM	4082	N	SER	452	67.431	-14.277	28.760	1.00	0.00	LX0	N
ATOM	4083	H	SER	452	66.870	-14.245	29.587	0.00	0.00	LX0	H
ATOM	4084	CA	SER	452	66.853	-14.327	27.418	1.00	0.00	LX0	C
ATOM	4085	CB	SER	452	65.660	-15.286	27.402	1.00	0.00	LX0	C
ATOM	4086	OG	SER	452	65.970	-16.456	28.177	1.00	0.00	LX0	O
ATOM	4087	HG	SER	452	66.081	-16.148	29.071	0.00	0.00	LX0	H
ATOM	4088	C	SER	452	66.496	-12.979	26.795	1.00	0.00	LX0	C
ATOM	4089	O	SER	452	65.930	-12.902	25.709	1.00	0.00	LX0	O
ATOM	4090	N	LEU	453	66.829	-11.901	27.529	1.00	0.00	LX0	N
ATOM	4091	H	LEU	453	67.386	-11.983	28.356	0.00	0.00	LX0	H
ATOM	4092	CA	LEU	453	66.297	-10.601	27.127	1.00	0.00	LX0	C
ATOM	4093	CB	LEU	453	66.440	-9.592	28.272	1.00	0.00	LX0	C
ATOM	4094	CG	LEU	453	65.363	-8.504	28.304	1.00	0.00	LX0	C
ATOM	4095	CD1	LEU	453	63.968	-9.087	28.526	1.00	0.00	LX0	C
ATOM	4096	CD2	LEU	453	65.670	-7.440	29.356	1.00	0.00	LX0	C
ATOM	4097	C	LEU	453	66.839	-10.050	25.817	1.00	0.00	LX0	C
ATOM	4098	O	LEU	453	67.898	-9.443	25.738	1.00	0.00	LX0	O
ATOM	4099	N	LYS	454	66.031	-10.279	24.780	1.00	0.00	LX0	N

ATOM	4100	H	LYS	454	65.208	-10.816	24.976	0.00	0.00	LX0	H
ATOM	4101	CA	LYS	454	66.361	-9.763	23.455	1.00	0.00	LX0	C
ATOM	4102	CB	LYS	454	66.193	-10.853	22.382	1.00	0.00	LX0	C
ATOM	4103	CG	LYS	454	64.783	-11.462	22.373	1.00	0.00	LX0	C
ATOM	4104	CD	LYS	454	64.171	-11.830	21.014	1.00	0.00	LX0	C
ATOM	4105	CE	LYS	454	63.898	-10.655	20.064	1.00	0.00	LX0	C
ATOM	4106	NZ	LYS	454	63.256	-9.523	20.746	1.00	0.00	LX0	N
ATOM	4107	HZ1	LYS	454	62.673	-8.949	20.095	0.00	0.00	LX0	H
ATOM	4108	HZ2	LYS	454	62.573	-9.814	21.471	0.00	0.00	LX0	H
ATOM	4109	HZ3	LYS	454	63.980	-8.891	21.157	0.00	0.00	LX0	H
ATOM	4110	C	LYS	454	65.566	-8.535	23.027	1.00	0.00	LX0	C
ATOM	4111	O	LYS	454	65.420	-8.290	21.829	1.00	0.00	LX0	O
ATOM	4112	N	GLU	455	65.002	-7.802	24.000	1.00	0.00	LX0	N
ATOM	4113	H	GLU	455	65.084	-8.047	24.967	0.00	0.00	LX0	H
ATOM	4114	CA	GLU	455	64.172	-6.660	23.606	1.00	0.00	LX0	C
ATOM	4115	CB	GLU	455	62.838	-7.117	22.987	1.00	0.00	LX0	C
ATOM	4116	CG	GLU	455	62.270	-6.247	21.856	1.00	0.00	LX0	C
ATOM	4117	CD	GLU	455	61.069	-6.937	21.214	1.00	0.00	LX0	C
ATOM	4118	OE1	GLU	455	61.223	-8.043	20.698	1.00	0.00	LX0	O
ATOM	4119	OE2	GLU	455	59.975	-6.379	21.223	1.00	0.00	LX0	O
ATOM	4120	C	GLU	455	63.871	-5.751	24.771	1.00	0.00	LX0	C
ATOM	4121	O	GLU	455	63.208	-6.154	25.716	1.00	0.00	LX0	O
ATOM	4122	N	ILE	456	64.339	-4.504	24.655	1.00	0.00	LX0	N
ATOM	4123	H	ILE	456	65.046	-4.238	23.995	0.00	0.00	LX0	H
ATOM	4124	CA	ILE	456	63.612	-3.486	25.404	1.00	0.00	LX0	C
ATOM	4125	CB	ILE	456	64.427	-2.873	26.551	1.00	0.00	LX0	C
ATOM	4126	CG2	ILE	456	63.525	-1.995	27.424	1.00	0.00	LX0	C
ATOM	4127	CG1	ILE	456	65.154	-3.923	27.394	1.00	0.00	LX0	C
ATOM	4128	CD1	ILE	456	66.123	-3.305	28.403	1.00	0.00	LX0	C
ATOM	4129	C	ILE	456	63.131	-2.432	24.425	1.00	0.00	LX0	C
ATOM	4130	O	ILE	456	63.833	-1.505	24.030	1.00	0.00	LX0	O
ATOM	4131	N	SER	457	61.892	-2.664	23.991	1.00	0.00	LX0	N
ATOM	4132	H	SER	457	61.326	-3.388	24.397	0.00	0.00	LX0	H
ATOM	4133	CA	SER	457	61.392	-1.936	22.828	1.00	0.00	LX0	C
ATOM	4134	CB	SER	457	60.139	-2.645	22.307	1.00	0.00	LX0	C
ATOM	4135	OG	SER	457	60.109	-3.982	22.832	1.00	0.00	LX0	O
ATOM	4136	HG	SER	457	60.136	-4.602	22.101	0.00	0.00	LX0	H
ATOM	4137	C	SER	457	61.166	-0.441	23.028	1.00	0.00	LX0	C
ATOM	4138	O	SER	457	61.145	0.339	22.077	1.00	0.00	LX0	O
ATOM	4139	N	ASP	458	61.018	-0.093	24.319	1.00	0.00	LX0	N
ATOM	4140	H	ASP	458	61.014	-0.805	25.024	0.00	0.00	LX0	H
ATOM	4141	CA	ASP	458	60.781	1.277	24.776	1.00	0.00	LX0	C
ATOM	4142	CB	ASP	458	59.358	1.687	24.350	1.00	0.00	LX0	C
ATOM	4143	CG	ASP	458	58.952	3.138	24.582	1.00	0.00	LX0	C
ATOM	4144	OD1	ASP	458	59.678	3.920	25.185	1.00	0.00	LX0	O
ATOM	4145	OD2	ASP	458	57.830	3.488	24.240	1.00	0.00	LX0	O
ATOM	4146	C	ASP	458	60.983	1.275	26.288	1.00	0.00	LX0	C
ATOM	4147	O	ASP	458	60.986	0.220	26.915	1.00	0.00	LX0	O
ATOM	4148	N	GLY	459	61.179	2.474	26.845	1.00	0.00	LX0	N
ATOM	4149	H	GLY	459	61.201	3.270	26.239	0.00	0.00	LX0	H
ATOM	4150	CA	GLY	459	61.293	2.617	28.292	1.00	0.00	LX0	C
ATOM	4151	C	GLY	459	62.735	2.740	28.743	1.00	0.00	LX0	C
ATOM	4152	O	GLY	459	63.654	2.288	28.070	1.00	0.00	LX0	O
ATOM	4153	N	ASP	460	62.887	3.425	29.881	1.00	0.00	LX0	N
ATOM	4154	H	ASP	460	62.060	3.653	30.397	0.00	0.00	LX0	H
ATOM	4155	CA	ASP	460	64.220	3.683	30.433	1.00	0.00	LX0	C
ATOM	4156	CB	ASP	460	64.223	5.152	30.923	1.00	0.00	LX0	C
ATOM	4157	CG	ASP	460	65.547	5.757	31.393	1.00	0.00	LX0	C
ATOM	4158	OD1	ASP	460	65.734	6.963	31.298	1.00	0.00	LX0	O
ATOM	4159	OD2	ASP	460	66.376	5.091	31.986	1.00	0.00	LX0	O
ATOM	4160	C	ASP	460	64.496	2.633	31.503	1.00	0.00	LX0	C

ATOM	4161	O	ASP	460	63.594	1.942	31.967	1.00	0.00	LX0	O
ATOM	4162	N	VAL	461	65.771	2.504	31.855	1.00	0.00	LX0	N
ATOM	4163	H	VAL	461	66.450	3.153	31.502	0.00	0.00	LX0	H
ATOM	4164	CA	VAL	461	66.145	1.555	32.888	1.00	0.00	LX0	C
ATOM	4165	CB	VAL	461	67.280	0.695	32.316	1.00	0.00	LX0	C
ATOM	4166	CG1	VAL	461	68.099	-0.100	33.335	1.00	0.00	LX0	C
ATOM	4167	CG2	VAL	461	66.676	-0.218	31.251	1.00	0.00	LX0	C
ATOM	4168	C	VAL	461	66.514	2.253	34.185	1.00	0.00	LX0	C
ATOM	4169	O	VAL	461	67.233	3.244	34.224	1.00	0.00	LX0	O
ATOM	4170	N	ILE	462	65.999	1.659	35.263	1.00	0.00	LX0	N
ATOM	4171	H	ILE	462	65.294	0.963	35.134	0.00	0.00	LX0	H
ATOM	4172	CA	ILE	462	66.539	1.944	36.583	1.00	0.00	LX0	C
ATOM	4173	CB	ILE	462	65.695	2.990	37.338	1.00	0.00	LX0	C
ATOM	4174	CG2	ILE	462	64.232	2.581	37.509	1.00	0.00	LX0	C
ATOM	4175	CG1	ILE	462	66.368	3.396	38.653	1.00	0.00	LX0	C
ATOM	4176	CD1	ILE	462	65.695	4.599	39.315	1.00	0.00	LX0	C
ATOM	4177	C	ILE	462	66.775	0.658	37.360	1.00	0.00	LX0	C
ATOM	4178	O	ILE	462	65.949	0.129	38.096	1.00	0.00	LX0	O
ATOM	4179	N	ILE	463	67.982	0.144	37.126	1.00	0.00	LX0	N
ATOM	4180	H	ILE	463	68.647	0.651	36.576	0.00	0.00	LX0	H
ATOM	4181	CA	ILE	463	68.376	-1.003	37.931	1.00	0.00	LX0	C
ATOM	4182	CB	ILE	463	68.802	-2.176	37.036	1.00	0.00	LX0	C
ATOM	4183	CG2	ILE	463	69.270	-3.387	37.847	1.00	0.00	LX0	C
ATOM	4184	CG1	ILE	463	67.637	-2.559	36.116	1.00	0.00	LX0	C
ATOM	4185	CD1	ILE	463	67.935	-3.740	35.195	1.00	0.00	LX0	C
ATOM	4186	C	ILE	463	69.443	-0.558	38.908	1.00	0.00	LX0	C
ATOM	4187	O	ILE	463	70.635	-0.570	38.634	1.00	0.00	LX0	O
ATOM	4188	N	SER	464	68.938	-0.083	40.047	1.00	0.00	LX0	N
ATOM	4189	H	SER	464	67.983	-0.274	40.284	0.00	0.00	LX0	H
ATOM	4190	CA	SER	464	69.837	0.620	40.955	1.00	0.00	LX0	C
ATOM	4191	CB	SER	464	69.551	2.122	40.909	1.00	0.00	LX0	C
ATOM	4192	OG	SER	464	70.007	2.678	39.668	1.00	0.00	LX0	O
ATOM	4193	HG	SER	464	69.567	2.203	38.967	0.00	0.00	LX0	H
ATOM	4194	C	SER	464	69.769	0.104	42.377	1.00	0.00	LX0	C
ATOM	4195	O	SER	464	68.845	-0.595	42.772	1.00	0.00	LX0	O
ATOM	4196	N	GLY	465	70.801	0.475	43.145	1.00	0.00	LX0	N
ATOM	4197	H	GLY	465	71.574	0.946	42.720	0.00	0.00	LX0	H
ATOM	4198	CA	GLY	465	70.805	0.099	44.558	1.00	0.00	LX0	C
ATOM	4199	C	GLY	465	71.111	-1.366	44.828	1.00	0.00	LX0	C
ATOM	4200	O	GLY	465	71.079	-1.848	45.955	1.00	0.00	LX0	O
ATOM	4201	N	ASN	466	71.384	-2.090	43.737	1.00	0.00	LX0	N
ATOM	4202	H	ASN	466	71.577	-1.650	42.859	0.00	0.00	LX0	H
ATOM	4203	CA	ASN	466	71.558	-3.527	43.909	1.00	0.00	LX0	C
ATOM	4204	CB	ASN	466	71.173	-4.339	42.668	1.00	0.00	LX0	C
ATOM	4205	CG	ASN	466	69.770	-4.016	42.207	1.00	0.00	LX0	C
ATOM	4206	OD1	ASN	466	68.782	-4.547	42.695	1.00	0.00	LX0	O
ATOM	4207	ND2	ASN	466	69.722	-3.100	41.242	1.00	0.00	LX0	N
ATOM	4208	HD21	ASN	466	70.565	-2.702	40.873	0.00	0.00	LX0	H
ATOM	4209	HD22	ASN	466	68.825	-2.805	40.906	0.00	0.00	LX0	H
ATOM	4210	C	ASN	466	72.969	-3.843	44.335	1.00	0.00	LX0	C
ATOM	4211	O	ASN	466	73.875	-4.112	43.554	1.00	0.00	LX0	O
ATOM	4212	N	LYS	467	73.088	-3.807	45.665	1.00	0.00	LX0	N
ATOM	4213	H	LYS	467	72.290	-3.382	46.094	0.00	0.00	LX0	H
ATOM	4214	CA	LYS	467	74.330	-4.073	46.404	1.00	0.00	LX0	C
ATOM	4215	CB	LYS	467	73.999	-4.457	47.852	1.00	0.00	LX0	C
ATOM	4216	CG	LYS	467	72.754	-3.769	48.424	1.00	0.00	LX0	C
ATOM	4217	CD	LYS	467	72.215	-4.404	49.706	1.00	0.00	LX0	C
ATOM	4218	CE	LYS	467	71.979	-5.917	49.618	1.00	0.00	LX0	C
ATOM	4219	NZ	LYS	467	71.124	-6.295	48.483	1.00	0.00	LX0	N
ATOM	4220	HZ1	LYS	467	70.528	-7.094	48.773	0.00	0.00	LX0	H
ATOM	4221	HZ2	LYS	467	70.500	-5.530	48.149	0.00	0.00	LX0	H

ATOM	4222	HZ3	LYS	467	71.689	-6.644	47.674	0.00	0.00	LX0	H
ATOM	4223	C	LYS	467	75.222	-5.162	45.822	1.00	0.00	LX0	C
ATOM	4224	O	LYS	467	76.441	-5.086	45.782	1.00	0.00	LX0	O
ATOM	4225	N	ASN	468	74.529	-6.220	45.392	1.00	0.00	LX0	N
ATOM	4226	H	ASN	468	73.529	-6.207	45.389	0.00	0.00	LX0	H
ATOM	4227	CA	ASN	468	75.264	-7.429	45.040	1.00	0.00	LX0	C
ATOM	4228	CB	ASN	468	74.494	-8.675	45.504	1.00	0.00	LX0	C
ATOM	4229	CG	ASN	468	73.960	-8.552	46.925	1.00	0.00	LX0	C
ATOM	4230	OD1	ASN	468	73.116	-7.718	47.255	1.00	0.00	LX0	O
ATOM	4231	ND2	ASN	468	74.482	-9.442	47.771	1.00	0.00	LX0	N
ATOM	4232	HD21	ASN	468	75.141	-10.122	47.453	0.00	0.00	LX0	H
ATOM	4233	HD22	ASN	468	74.201	-9.446	48.729	0.00	0.00	LX0	H
ATOM	4234	C	ASN	468	75.616	-7.545	43.561	1.00	0.00	LX0	C
ATOM	4235	O	ASN	468	76.415	-8.384	43.155	1.00	0.00	LX0	O
ATOM	4236	N	LEU	469	74.975	-6.670	42.772	1.00	0.00	LX0	N
ATOM	4237	H	LEU	469	74.474	-5.913	43.188	0.00	0.00	LX0	H
ATOM	4238	CA	LEU	469	74.882	-6.855	41.324	1.00	0.00	LX0	C
ATOM	4239	CB	LEU	469	73.765	-5.962	40.798	1.00	0.00	LX0	C
ATOM	4240	CG	LEU	469	73.324	-6.247	39.368	1.00	0.00	LX0	C
ATOM	4241	CD1	LEU	469	72.923	-7.706	39.186	1.00	0.00	LX0	C
ATOM	4242	CD2	LEU	469	72.221	-5.288	38.937	1.00	0.00	LX0	C
ATOM	4243	C	LEU	469	76.140	-6.667	40.495	1.00	0.00	LX0	C
ATOM	4244	O	LEU	469	76.518	-5.564	40.111	1.00	0.00	LX0	O
ATOM	4245	N	CYS	470	76.752	-7.814	40.194	1.00	0.00	LX0	N
ATOM	4246	H	CYS	470	76.341	-8.674	40.496	0.00	0.00	LX0	H
ATOM	4247	CA	CYS	470	77.765	-7.787	39.144	1.00	0.00	LX0	C
ATOM	4248	CB	CYS	470	78.619	-9.047	39.222	1.00	0.00	LX0	C
ATOM	4249	SG	CYS	470	79.679	-9.066	40.682	1.00	0.00	LX0	S
ATOM	4250	C	CYS	470	77.121	-7.669	37.775	1.00	0.00	LX0	C
ATOM	4251	O	CYS	470	75.903	-7.682	37.649	1.00	0.00	LX0	O
ATOM	4252	N	TYR	471	77.987	-7.553	36.751	1.00	0.00	LX0	N
ATOM	4253	H	TYR	471	78.965	-7.470	36.944	0.00	0.00	LX0	H
ATOM	4254	CA	TYR	471	77.554	-7.646	35.349	1.00	0.00	LX0	C
ATOM	4255	CB	TYR	471	76.905	-9.003	35.032	1.00	0.00	LX0	C
ATOM	4256	CG	TYR	471	77.873	-10.134	35.293	1.00	0.00	LX0	C
ATOM	4257	CD1	TYR	471	77.653	-10.980	36.399	1.00	0.00	LX0	C
ATOM	4258	CE1	TYR	471	78.545	-12.038	36.635	1.00	0.00	LX0	C
ATOM	4259	CD2	TYR	471	78.965	-10.318	34.422	1.00	0.00	LX0	C
ATOM	4260	CE2	TYR	471	79.847	-11.385	34.651	1.00	0.00	LX0	C
ATOM	4261	CZ	TYR	471	79.626	-12.234	35.754	1.00	0.00	LX0	C
ATOM	4262	OH	TYR	471	80.490	-13.287	35.990	1.00	0.00	LX0	O
ATOM	4263	HH	TYR	471	81.115	-13.367	35.270	0.00	0.00	LX0	H
ATOM	4264	C	TYR	471	76.735	-6.513	34.742	1.00	0.00	LX0	C
ATOM	4265	O	TYR	471	76.800	-6.286	33.541	1.00	0.00	LX0	O
ATOM	4266	N	ALA	472	75.996	-5.775	35.594	1.00	0.00	LX0	N
ATOM	4267	H	ALA	472	75.907	-6.099	36.535	0.00	0.00	LX0	H
ATOM	4268	CA	ALA	472	75.125	-4.693	35.114	1.00	0.00	LX0	C
ATOM	4269	CB	ALA	472	74.607	-3.868	36.293	1.00	0.00	LX0	C
ATOM	4270	C	ALA	472	75.713	-3.726	34.093	1.00	0.00	LX0	C
ATOM	4271	O	ALA	472	75.047	-3.235	33.191	1.00	0.00	LX0	O
ATOM	4272	N	ASN	473	77.013	-3.469	34.274	1.00	0.00	LX0	N
ATOM	4273	H	ASN	473	77.522	-3.988	34.961	0.00	0.00	LX0	H
ATOM	4274	CA	ASN	473	77.718	-2.623	33.308	1.00	0.00	LX0	C
ATOM	4275	CB	ASN	473	79.096	-2.208	33.855	1.00	0.00	LX0	C
ATOM	4276	CG	ASN	473	79.986	-3.415	34.119	1.00	0.00	LX0	C
ATOM	4277	OD1	ASN	473	79.530	-4.480	34.517	1.00	0.00	LX0	O
ATOM	4278	ND2	ASN	473	81.281	-3.206	33.876	1.00	0.00	LX0	N
ATOM	4279	HD21	ASN	473	81.630	-2.332	33.542	0.00	0.00	LX0	H
ATOM	4280	HD22	ASN	473	81.928	-3.949	34.046	0.00	0.00	LX0	H
ATOM	4281	C	ASN	473	77.843	-3.222	31.911	1.00	0.00	LX0	C
ATOM	4282	O	ASN	473	77.713	-2.551	30.897	1.00	0.00	LX0	O

ATOM	4283	N	THR	474	78.099	-4.535	31.899	1.00	0.00	LX0	N
ATOM	4284	H	THR	474	78.104	-5.043	32.762	0.00	0.00	LX0	H
ATOM	4285	CA	THR	474	78.426	-5.183	30.630	1.00	0.00	LX0	C
ATOM	4286	CB	THR	474	79.102	-6.536	30.890	1.00	0.00	LX0	C
ATOM	4287	OG1	THR	474	78.182	-7.502	31.419	1.00	0.00	LX0	O
ATOM	4288	HG1	THR	474	77.736	-7.117	32.170	0.00	0.00	LX0	H
ATOM	4289	CG2	THR	474	80.330	-6.406	31.792	1.00	0.00	LX0	C
ATOM	4290	C	THR	474	77.294	-5.325	29.617	1.00	0.00	LX0	C
ATOM	4291	O	THR	474	77.494	-5.712	28.474	1.00	0.00	LX0	O
ATOM	4292	N	ILE	475	76.077	-5.000	30.083	1.00	0.00	LX0	N
ATOM	4293	H	ILE	475	75.993	-4.620	31.003	0.00	0.00	LX0	H
ATOM	4294	CA	ILE	475	74.892	-5.293	29.271	1.00	0.00	LX0	C
ATOM	4295	CB	ILE	475	73.621	-5.048	30.115	1.00	0.00	LX0	C
ATOM	4296	CG2	ILE	475	72.322	-5.448	29.404	1.00	0.00	LX0	C
ATOM	4297	CG1	ILE	475	73.715	-5.736	31.481	1.00	0.00	LX0	C
ATOM	4298	CD1	ILE	475	73.763	-7.260	31.402	1.00	0.00	LX0	C
ATOM	4299	C	ILE	475	74.819	-4.573	27.920	1.00	0.00	LX0	C
ATOM	4300	O	ILE	475	74.088	-4.966	27.022	1.00	0.00	LX0	O
ATOM	4301	N	ASN	476	75.586	-3.467	27.820	1.00	0.00	LX0	N
ATOM	4302	H	ASN	476	76.240	-3.271	28.549	0.00	0.00	LX0	H
ATOM	4303	CA	ASN	476	75.464	-2.597	26.639	1.00	0.00	LX0	C
ATOM	4304	CB	ASN	476	76.110	-3.185	25.376	1.00	0.00	LX0	C
ATOM	4305	CG	ASN	476	76.083	-2.153	24.258	1.00	0.00	LX0	C
ATOM	4306	OD1	ASN	476	75.869	-0.964	24.472	1.00	0.00	LX0	O
ATOM	4307	ND2	ASN	476	76.315	-2.660	23.048	1.00	0.00	LX0	N
ATOM	4308	HD21	ASN	476	76.365	-3.658	22.940	0.00	0.00	LX0	H
ATOM	4309	HD22	ASN	476	76.409	-2.071	22.251	0.00	0.00	LX0	H
ATOM	4310	C	ASN	476	74.035	-2.146	26.386	1.00	0.00	LX0	C
ATOM	4311	O	ASN	476	73.373	-2.403	25.387	1.00	0.00	LX0	O
ATOM	4312	N	TRP	477	73.584	-1.426	27.413	1.00	0.00	LX0	N
ATOM	4313	H	TRP	477	74.204	-1.275	28.181	0.00	0.00	LX0	H
ATOM	4314	CA	TRP	477	72.176	-1.062	27.481	1.00	0.00	LX0	C
ATOM	4315	CB	TRP	477	71.964	-0.199	28.713	1.00	0.00	LX0	C
ATOM	4316	CG	TRP	477	72.079	-1.042	29.958	1.00	0.00	LX0	C
ATOM	4317	CD2	TRP	477	71.079	-1.913	30.528	1.00	0.00	LX0	C
ATOM	4318	CE2	TRP	477	71.645	-2.480	31.717	1.00	0.00	LX0	C
ATOM	4319	CE3	TRP	477	69.767	-2.258	30.137	1.00	0.00	LX0	C
ATOM	4320	CD1	TRP	477	73.188	-1.135	30.813	1.00	0.00	LX0	C
ATOM	4321	NE1	TRP	477	72.936	-1.979	31.846	1.00	0.00	LX0	N
ATOM	4322	HE1	TRP	477	73.577	-2.226	32.553	0.00	0.00	LX0	H
ATOM	4323	CZ2	TRP	477	70.892	-3.391	32.487	1.00	0.00	LX0	C
ATOM	4324	CZ3	TRP	477	69.025	-3.171	30.915	1.00	0.00	LX0	C
ATOM	4325	CH2	TRP	477	69.584	-3.731	32.084	1.00	0.00	LX0	C
ATOM	4326	C	TRP	477	71.551	-0.432	26.245	1.00	0.00	LX0	C
ATOM	4327	O	TRP	477	70.404	-0.699	25.917	1.00	0.00	LX0	O
ATOM	4328	N	LYS	478	72.335	0.409	25.550	1.00	0.00	LX0	N
ATOM	4329	H	LYS	478	73.310	0.484	25.757	0.00	0.00	LX0	H
ATOM	4330	CA	LYS	478	71.718	1.028	24.375	1.00	0.00	LX0	C
ATOM	4331	CB	LYS	478	72.459	2.303	23.960	1.00	0.00	LX0	C
ATOM	4332	CG	LYS	478	71.679	3.608	24.210	1.00	0.00	LX0	C
ATOM	4333	CD	LYS	478	70.853	4.157	23.030	1.00	0.00	LX0	C
ATOM	4334	CE	LYS	478	69.594	3.382	22.618	1.00	0.00	LX0	C
ATOM	4335	NZ	LYS	478	68.539	3.511	23.633	1.00	0.00	LX0	N
ATOM	4336	HZ1	LYS	478	67.947	2.653	23.648	0.00	0.00	LX0	H
ATOM	4337	HZ2	LYS	478	68.918	3.628	24.587	0.00	0.00	LX0	H
ATOM	4338	HZ3	LYS	478	67.878	4.284	23.413	0.00	0.00	LX0	H
ATOM	4339	C	LYS	478	71.460	0.113	23.187	1.00	0.00	LX0	C
ATOM	4340	O	LYS	478	70.625	0.407	22.343	1.00	0.00	LX0	O
ATOM	4341	N	LYS	479	72.183	-1.017	23.179	1.00	0.00	LX0	N
ATOM	4342	H	LYS	479	72.861	-1.205	23.891	0.00	0.00	LX0	H
ATOM	4343	CA	LYS	479	71.844	-2.065	22.215	1.00	0.00	LX0	C

ATOM	4344	CB	LYS	479	73.015	-3.064	22.165	1.00	0.00	LX0	C
ATOM	4345	CG	LYS	479	72.903	-4.412	21.426	1.00	0.00	LX0	C
ATOM	4346	CD	LYS	479	72.228	-5.524	22.245	1.00	0.00	LX0	C
ATOM	4347	CE	LYS	479	72.553	-6.952	21.786	1.00	0.00	LX0	C
ATOM	4348	NZ	LYS	479	73.942	-7.286	22.123	1.00	0.00	LX0	N
ATOM	4349	HZ1	LYS	479	74.111	-8.302	22.269	0.00	0.00	LX0	H
ATOM	4350	HZ2	LYS	479	74.635	-6.927	21.428	0.00	0.00	LX0	H
ATOM	4351	HZ3	LYS	479	74.253	-6.776	22.978	0.00	0.00	LX0	H
ATOM	4352	C	LYS	479	70.508	-2.717	22.529	1.00	0.00	LX0	C
ATOM	4353	O	LYS	479	69.721	-3.047	21.652	1.00	0.00	LX0	O
ATOM	4354	N	LEU	480	70.292	-2.907	23.838	1.00	0.00	LX0	N
ATOM	4355	H	LEU	480	70.932	-2.561	24.525	0.00	0.00	LX0	H
ATOM	4356	CA	LEU	480	69.089	-3.658	24.175	1.00	0.00	LX0	C
ATOM	4357	CB	LEU	480	69.295	-4.440	25.466	1.00	0.00	LX0	C
ATOM	4358	CG	LEU	480	68.325	-5.616	25.536	1.00	0.00	LX0	C
ATOM	4359	CD1	LEU	480	68.517	-6.579	24.363	1.00	0.00	LX0	C
ATOM	4360	CD2	LEU	480	68.406	-6.318	26.881	1.00	0.00	LX0	C
ATOM	4361	C	LEU	480	67.811	-2.844	24.214	1.00	0.00	LX0	C
ATOM	4362	O	LEU	480	66.773	-3.219	23.677	1.00	0.00	LX0	O
ATOM	4363	N	PHE	481	67.932	-1.688	24.879	1.00	0.00	LX0	N
ATOM	4364	H	PHE	481	68.803	-1.414	25.283	0.00	0.00	LX0	H
ATOM	4365	CA	PHE	481	66.809	-0.777	24.748	1.00	0.00	LX0	C
ATOM	4366	CB	PHE	481	66.502	-0.019	26.054	1.00	0.00	LX0	C
ATOM	4367	CG	PHE	481	67.638	0.716	26.733	1.00	0.00	LX0	C
ATOM	4368	CD1	PHE	481	68.372	1.719	26.067	1.00	0.00	LX0	C
ATOM	4369	CD2	PHE	481	67.902	0.416	28.084	1.00	0.00	LX0	C
ATOM	4370	CE1	PHE	481	69.341	2.464	26.761	1.00	0.00	LX0	C
ATOM	4371	CE2	PHE	481	68.867	1.160	28.788	1.00	0.00	LX0	C
ATOM	4372	CZ	PHE	481	69.565	2.189	28.123	1.00	0.00	LX0	C
ATOM	4373	C	PHE	481	66.903	0.086	23.509	1.00	0.00	LX0	C
ATOM	4374	O	PHE	481	67.655	1.049	23.413	1.00	0.00	LX0	O
ATOM	4375	N	GLY	482	66.121	-0.331	22.514	1.00	0.00	LX0	N
ATOM	4376	H	GLY	482	65.442	-1.045	22.703	0.00	0.00	LX0	H
ATOM	4377	CA	GLY	482	66.387	0.225	21.189	1.00	0.00	LX0	C
ATOM	4378	C	GLY	482	65.627	1.491	20.835	1.00	0.00	LX0	C
ATOM	4379	O	GLY	482	65.007	1.587	19.781	1.00	0.00	LX0	O
ATOM	4380	N	THR	483	65.684	2.473	21.739	1.00	0.00	LX0	N
ATOM	4381	H	THR	483	66.127	2.330	22.624	0.00	0.00	LX0	H
ATOM	4382	CA	THR	483	64.861	3.657	21.497	1.00	0.00	LX0	C
ATOM	4383	CB	THR	483	63.510	3.433	22.213	1.00	0.00	LX0	C
ATOM	4384	OG1	THR	483	63.086	2.087	21.950	1.00	0.00	LX0	O
ATOM	4385	HG1	THR	483	62.257	1.952	22.409	0.00	0.00	LX0	H
ATOM	4386	CG2	THR	483	62.387	4.400	21.824	1.00	0.00	LX0	C
ATOM	4387	C	THR	483	65.579	4.947	21.890	1.00	0.00	LX0	C
ATOM	4388	O	THR	483	66.585	4.944	22.599	1.00	0.00	LX0	O
ATOM	4389	N	SER	484	65.049	6.059	21.370	1.00	0.00	LX0	N
ATOM	4390	H	SER	484	64.319	6.042	20.686	0.00	0.00	LX0	H
ATOM	4391	CA	SER	484	65.528	7.375	21.780	1.00	0.00	LX0	C
ATOM	4392	CB	SER	484	64.849	8.404	20.875	1.00	0.00	LX0	C
ATOM	4393	OG	SER	484	64.659	7.817	19.578	1.00	0.00	LX0	O
ATOM	4394	HG	SER	484	64.422	8.525	18.989	0.00	0.00	LX0	H
ATOM	4395	C	SER	484	65.271	7.661	23.255	1.00	0.00	LX0	C
ATOM	4396	O	SER	484	64.563	6.923	23.930	1.00	0.00	LX0	O
ATOM	4397	N	GLY	485	65.873	8.761	23.737	1.00	0.00	LX0	N
ATOM	4398	H	GLY	485	66.499	9.288	23.166	0.00	0.00	LX0	H
ATOM	4399	CA	GLY	485	65.589	9.151	25.120	1.00	0.00	LX0	C
ATOM	4400	C	GLY	485	66.385	8.388	26.167	1.00	0.00	LX0	C
ATOM	4401	O	GLY	485	67.351	8.897	26.731	1.00	0.00	LX0	O
ATOM	4402	N	GLN	486	65.926	7.143	26.367	1.00	0.00	LX0	N
ATOM	4403	H	GLN	486	65.119	6.920	25.815	0.00	0.00	LX0	H
ATOM	4404	CA	GLN	486	66.433	6.152	27.329	1.00	0.00	LX0	C

ATOM	4405	CB	GLN	486	66.374	4.762	26.706	1.00	0.00	LX0	C
ATOM	4406	CG	GLN	486	65.018	4.371	26.126	1.00	0.00	LX0	C
ATOM	4407	CD	GLN	486	65.217	3.157	25.250	1.00	0.00	LX0	C
ATOM	4408	OE1	GLN	486	66.171	3.069	24.482	1.00	0.00	LX0	O
ATOM	4409	NE2	GLN	486	64.265	2.232	25.361	1.00	0.00	LX0	N
ATOM	4410	HE21	GLN	486	63.637	2.301	26.139	0.00	0.00	LX0	H
ATOM	4411	HE22	GLN	486	64.192	1.447	24.749	0.00	0.00	LX0	H
ATOM	4412	C	GLN	486	67.831	6.336	27.902	1.00	0.00	LX0	C
ATOM	4413	O	GLN	486	68.795	6.672	27.210	1.00	0.00	LX0	O
ATOM	4414	N	LYS	487	67.903	6.076	29.208	1.00	0.00	LX0	N
ATOM	4415	H	LYS	487	67.090	5.851	29.755	0.00	0.00	LX0	H
ATOM	4416	CA	LYS	487	69.195	6.005	29.876	1.00	0.00	LX0	C
ATOM	4417	CB	LYS	487	69.333	7.160	30.875	1.00	0.00	LX0	C
ATOM	4418	CG	LYS	487	69.493	8.497	30.154	1.00	0.00	LX0	C
ATOM	4419	CD	LYS	487	70.805	8.541	29.366	1.00	0.00	LX0	C
ATOM	4420	CE	LYS	487	70.772	9.526	28.199	1.00	0.00	LX0	C
ATOM	4421	NZ	LYS	487	69.788	9.060	27.214	1.00	0.00	LX0	N
ATOM	4422	HZ1	LYS	487	68.811	9.240	27.533	0.00	0.00	LX0	H
ATOM	4423	HZ2	LYS	487	69.908	9.531	26.300	0.00	0.00	LX0	H
ATOM	4424	HZ3	LYS	487	69.836	8.027	27.081	0.00	0.00	LX0	H
ATOM	4425	C	LYS	487	69.351	4.664	30.556	1.00	0.00	LX0	C
ATOM	4426	O	LYS	487	68.497	3.794	30.452	1.00	0.00	LX0	O
ATOM	4427	N	THR	488	70.482	4.536	31.257	1.00	0.00	LX0	N
ATOM	4428	H	THR	488	71.201	5.228	31.290	0.00	0.00	LX0	H
ATOM	4429	CA	THR	488	70.537	3.406	32.166	1.00	0.00	LX0	C
ATOM	4430	CB	THR	488	71.350	2.231	31.602	1.00	0.00	LX0	C
ATOM	4431	OG1	THR	488	71.250	1.101	32.477	1.00	0.00	LX0	O
ATOM	4432	HG1	THR	488	71.704	0.375	32.066	0.00	0.00	LX0	H
ATOM	4433	CG2	THR	488	72.808	2.575	31.272	1.00	0.00	LX0	C
ATOM	4434	C	THR	488	70.985	3.858	33.536	1.00	0.00	LX0	C
ATOM	4435	O	THR	488	72.142	4.118	33.847	1.00	0.00	LX0	O
ATOM	4436	N	LYS	489	69.951	3.991	34.358	1.00	0.00	LX0	N
ATOM	4437	H	LYS	489	69.039	3.722	34.045	0.00	0.00	LX0	H
ATOM	4438	CA	LYS	489	70.210	4.306	35.749	1.00	0.00	LX0	C
ATOM	4439	CB	LYS	489	69.006	5.069	36.306	1.00	0.00	LX0	C
ATOM	4440	CG	LYS	489	68.726	6.347	35.500	1.00	0.00	LX0	C
ATOM	4441	CD	LYS	489	67.425	7.056	35.886	1.00	0.00	LX0	C
ATOM	4442	CE	LYS	489	66.166	6.373	35.352	1.00	0.00	LX0	C
ATOM	4443	NZ	LYS	489	65.933	6.675	33.932	1.00	0.00	LX0	N
ATOM	4444	HZ1	LYS	489	65.557	5.826	33.463	0.00	0.00	LX0	H
ATOM	4445	HZ2	LYS	489	65.240	7.432	33.739	0.00	0.00	LX0	H
ATOM	4446	HZ3	LYS	489	66.773	6.842	33.343	0.00	0.00	LX0	H
ATOM	4447	C	LYS	489	70.535	3.027	36.499	1.00	0.00	LX0	C
ATOM	4448	O	LYS	489	69.691	2.381	37.110	1.00	0.00	LX0	O
ATOM	4449	N	ILE	490	71.821	2.678	36.375	1.00	0.00	LX0	N
ATOM	4450	H	ILE	490	72.411	3.245	35.797	0.00	0.00	LX0	H
ATOM	4451	CA	ILE	490	72.329	1.493	37.066	1.00	0.00	LX0	C
ATOM	4452	CB	ILE	490	72.954	0.485	36.076	1.00	0.00	LX0	C
ATOM	4453	CG2	ILE	490	71.843	-0.385	35.486	1.00	0.00	LX0	C
ATOM	4454	CG1	ILE	490	73.790	1.110	34.945	1.00	0.00	LX0	C
ATOM	4455	CD1	ILE	490	75.132	1.731	35.340	1.00	0.00	LX0	C
ATOM	4456	C	ILE	490	73.262	1.833	38.219	1.00	0.00	LX0	C
ATOM	4457	O	ILE	490	74.331	1.262	38.413	1.00	0.00	LX0	O
ATOM	4458	N	ILE	491	72.819	2.855	38.959	1.00	0.00	LX0	N
ATOM	4459	H	ILE	491	71.842	3.076	38.945	0.00	0.00	LX0	H
ATOM	4460	CA	ILE	491	73.720	3.390	39.972	1.00	0.00	LX0	C
ATOM	4461	CB	ILE	491	73.468	4.892	40.175	1.00	0.00	LX0	C
ATOM	4462	CG2	ILE	491	73.775	5.647	38.878	1.00	0.00	LX0	C
ATOM	4463	CG1	ILE	491	72.057	5.185	40.700	1.00	0.00	LX0	C
ATOM	4464	CD1	ILE	491	71.820	6.663	41.015	1.00	0.00	LX0	C
ATOM	4465	C	ILE	491	73.644	2.622	41.277	1.00	0.00	LX0	C

ATOM	4466	O	ILE	491	72.715	1.866	41.525	1.00	0.00	LX0	O
ATOM	4467	N	SER	492	74.681	2.832	42.108	1.00	0.00	LX0	N
ATOM	4468	H	SER	492	75.395	3.476	41.841	0.00	0.00	LX0	H
ATOM	4469	CA	SER	492	74.708	2.193	43.430	1.00	0.00	LX0	C
ATOM	4470	CB	SER	492	73.719	2.891	44.380	1.00	0.00	LX0	C
ATOM	4471	OG	SER	492	73.972	2.524	45.738	1.00	0.00	LX0	O
ATOM	4472	HG	SER	492	73.909	1.572	45.771	0.00	0.00	LX0	H
ATOM	4473	C	SER	492	74.520	0.677	43.437	1.00	0.00	LX0	C
ATOM	4474	O	SER	492	73.983	0.089	44.370	1.00	0.00	LX0	O
ATOM	4475	N	ASN	493	74.983	0.072	42.337	1.00	0.00	LX0	N
ATOM	4476	H	ASN	493	75.475	0.604	41.653	0.00	0.00	LX0	H
ATOM	4477	CA	ASN	493	75.051	-1.386	42.354	1.00	0.00	LX0	C
ATOM	4478	CB	ASN	493	74.834	-1.957	40.948	1.00	0.00	LX0	C
ATOM	4479	CG	ASN	493	73.417	-1.702	40.465	1.00	0.00	LX0	C
ATOM	4480	OD1	ASN	493	72.427	-1.925	41.149	1.00	0.00	LX0	O
ATOM	4481	ND2	ASN	493	73.350	-1.233	39.225	1.00	0.00	LX0	N
ATOM	4482	HD21	ASN	493	74.150	-0.941	38.701	0.00	0.00	LX0	H
ATOM	4483	HD22	ASN	493	72.442	-1.121	38.819	0.00	0.00	LX0	H
ATOM	4484	C	ASN	493	76.417	-1.763	42.885	1.00	0.00	LX0	C
ATOM	4485	O	ASN	493	77.223	-0.876	43.133	1.00	0.00	LX0	O
ATOM	4486	N	ARG	494	76.660	-3.082	42.999	1.00	0.00	LX0	N
ATOM	4487	H	ARG	494	75.867	-3.686	42.907	0.00	0.00	LX0	H
ATOM	4488	CA	ARG	494	77.973	-3.650	43.364	1.00	0.00	LX0	C
ATOM	4489	CB	ARG	494	78.083	-5.014	42.687	1.00	0.00	LX0	C
ATOM	4490	CG	ARG	494	79.291	-5.920	42.928	1.00	0.00	LX0	C
ATOM	4491	CD	ARG	494	79.220	-6.761	44.202	1.00	0.00	LX0	C
ATOM	4492	NE	ARG	494	80.296	-7.750	44.201	1.00	0.00	LX0	N
ATOM	4493	HE	ARG	494	81.239	-7.454	44.376	0.00	0.00	LX0	H
ATOM	4494	CZ	ARG	494	80.090	-9.024	43.802	1.00	0.00	LX0	C
ATOM	4495	NH1	ARG	494	78.885	-9.482	43.467	1.00	0.00	LX0	N
ATOM	4496	HH11	ARG	494	78.766	-10.432	43.157	0.00	0.00	LX0	H
ATOM	4497	HH12	ARG	494	78.063	-8.907	43.487	0.00	0.00	LX0	H
ATOM	4498	NH2	ARG	494	81.117	-9.851	43.727	1.00	0.00	LX0	N
ATOM	4499	HH21	ARG	494	80.965	-10.774	43.363	0.00	0.00	LX0	H
ATOM	4500	HH22	ARG	494	82.055	-9.613	44.008	0.00	0.00	LX0	H
ATOM	4501	C	ARG	494	79.211	-2.819	43.031	1.00	0.00	LX0	C
ATOM	4502	O	ARG	494	80.107	-2.619	43.844	1.00	0.00	LX0	O
ATOM	4503	N	GLY	495	79.218	-2.360	41.772	1.00	0.00	LX0	N
ATOM	4504	H	GLY	495	78.480	-2.621	41.156	0.00	0.00	LX0	H
ATOM	4505	CA	GLY	495	80.376	-1.593	41.337	1.00	0.00	LX0	C
ATOM	4506	C	GLY	495	81.327	-2.472	40.562	1.00	0.00	LX0	C
ATOM	4507	O	GLY	495	81.571	-3.625	40.901	1.00	0.00	LX0	O
ATOM	4508	N	GLU	496	81.833	-1.883	39.469	1.00	0.00	LX0	N
ATOM	4509	H	GLU	496	81.626	-0.920	39.305	0.00	0.00	LX0	H
ATOM	4510	CA	GLU	496	82.713	-2.635	38.570	1.00	0.00	LX0	C
ATOM	4511	CB	GLU	496	83.044	-1.723	37.380	1.00	0.00	LX0	C
ATOM	4512	CG	GLU	496	83.832	-2.337	36.215	1.00	0.00	LX0	C
ATOM	4513	CD	GLU	496	85.270	-2.606	36.609	1.00	0.00	LX0	C
ATOM	4514	OE1	GLU	496	85.802	-3.652	36.249	1.00	0.00	LX0	O
ATOM	4515	OE2	GLU	496	85.873	-1.794	37.311	1.00	0.00	LX0	O
ATOM	4516	C	GLU	496	83.933	-3.202	39.293	1.00	0.00	LX0	C
ATOM	4517	O	GLU	496	84.275	-4.379	39.220	1.00	0.00	LX0	O
ATOM	4518	N	ASN	497	84.517	-2.303	40.097	1.00	0.00	LX0	N
ATOM	4519	H	ASN	497	84.179	-1.363	40.077	0.00	0.00	LX0	H
ATOM	4520	CA	ASN	497	85.539	-2.705	41.067	1.00	0.00	LX0	C
ATOM	4521	CB	ASN	497	85.899	-1.524	41.993	1.00	0.00	LX0	C
ATOM	4522	CG	ASN	497	84.687	-1.072	42.792	1.00	0.00	LX0	C
ATOM	4523	OD1	ASN	497	83.614	-0.848	42.247	1.00	0.00	LX0	O
ATOM	4524	ND2	ASN	497	84.870	-1.057	44.111	1.00	0.00	LX0	N
ATOM	4525	HD21	ASN	497	85.762	-1.152	44.549	0.00	0.00	LX0	H
ATOM	4526	HD22	ASN	497	84.047	-0.988	44.676	0.00	0.00	LX0	H

ATOM	4527	C	ASN	497	85.190	-3.957	41.866	1.00	0.00	LX0	C
ATOM	4528	O	ASN	497	85.930	-4.931	41.888	1.00	0.00	LX0	O
ATOM	4529	N	SER	498	84.008	-3.909	42.490	1.00	0.00	LX0	N
ATOM	4530	H	SER	498	83.406	-3.109	42.440	0.00	0.00	LX0	H
ATOM	4531	CA	SER	498	83.604	-5.019	43.347	1.00	0.00	LX0	C
ATOM	4532	CB	SER	498	82.565	-4.513	44.343	1.00	0.00	LX0	C
ATOM	4533	OG	SER	498	82.736	-3.102	44.542	1.00	0.00	LX0	O
ATOM	4534	HG	SER	498	81.853	-2.733	44.480	0.00	0.00	LX0	H
ATOM	4535	C	SER	498	83.119	-6.262	42.609	1.00	0.00	LX0	C
ATOM	4536	O	SER	498	82.976	-7.345	43.168	1.00	0.00	LX0	O
ATOM	4537	N	CYS	499	82.887	-6.053	41.306	1.00	0.00	LX0	N
ATOM	4538	H	CYS	499	82.942	-5.120	40.946	0.00	0.00	LX0	H
ATOM	4539	CA	CYS	499	82.579	-7.153	40.397	1.00	0.00	LX0	C
ATOM	4540	CB	CYS	499	81.973	-6.544	39.127	1.00	0.00	LX0	C
ATOM	4541	SG	CYS	499	81.338	-7.695	37.890	1.00	0.00	LX0	S
ATOM	4542	C	CYS	499	83.820	-7.990	40.125	1.00	0.00	LX0	C
ATOM	4543	O	CYS	499	83.886	-9.190	40.388	1.00	0.00	LX0	O
ATOM	4544	N	LYS	500	84.858	-7.281	39.643	1.00	0.00	LX0	N
ATOM	4545	H	LYS	500	84.772	-6.297	39.464	0.00	0.00	LX0	H
ATOM	4546	CA	LYS	500	86.116	-8.010	39.477	1.00	0.00	LX0	C
ATOM	4547	CB	LYS	500	87.113	-7.280	38.572	1.00	0.00	LX0	C
ATOM	4548	CG	LYS	500	87.462	-5.840	38.949	1.00	0.00	LX0	C
ATOM	4549	CD	LYS	500	88.696	-5.368	38.177	1.00	0.00	LX0	C
ATOM	4550	CE	LYS	500	88.932	-3.860	38.260	1.00	0.00	LX0	C
ATOM	4551	NZ	LYS	500	87.906	-3.184	37.466	1.00	0.00	LX0	N
ATOM	4552	HZ1	LYS	500	87.915	-2.146	37.479	0.00	0.00	LX0	H
ATOM	4553	HZ2	LYS	500	87.846	-3.489	36.474	0.00	0.00	LX0	H
ATOM	4554	HZ3	LYS	500	86.927	-3.393	37.773	0.00	0.00	LX0	H
ATOM	4555	C	LYS	500	86.771	-8.464	40.772	1.00	0.00	LX0	C
ATOM	4556	O	LYS	500	87.477	-9.462	40.814	1.00	0.00	LX0	O
ATOM	4557	N	ALA	501	86.437	-7.739	41.852	1.00	0.00	LX0	N
ATOM	4558	H	ALA	501	85.951	-6.871	41.741	0.00	0.00	LX0	H
ATOM	4559	CA	ALA	501	86.867	-8.190	43.177	1.00	0.00	LX0	C
ATOM	4560	CB	ALA	501	86.650	-7.095	44.222	1.00	0.00	LX0	C
ATOM	4561	C	ALA	501	86.232	-9.478	43.690	1.00	0.00	LX0	C
ATOM	4562	O	ALA	501	86.464	-9.910	44.811	1.00	0.00	LX0	O
ATOM	4563	N	THR	502	85.424	-10.105	42.828	1.00	0.00	LX0	N
ATOM	4564	H	THR	502	85.041	-9.658	42.021	0.00	0.00	LX0	H
ATOM	4565	CA	THR	502	85.110	-11.496	43.138	1.00	0.00	LX0	C
ATOM	4566	CB	THR	502	83.785	-11.578	43.901	1.00	0.00	LX0	C
ATOM	4567	OG1	THR	502	83.580	-10.367	44.654	1.00	0.00	LX0	O
ATOM	4568	HG1	THR	502	84.396	-10.240	45.141	0.00	0.00	LX0	H
ATOM	4569	CG2	THR	502	83.714	-12.797	44.826	1.00	0.00	LX0	C
ATOM	4570	C	THR	502	85.123	-12.375	41.898	1.00	0.00	LX0	C
ATOM	4571	O	THR	502	84.412	-13.363	41.774	1.00	0.00	LX0	O
ATOM	4572	N	GLY	503	85.961	-11.930	40.946	1.00	0.00	LX0	N
ATOM	4573	H	GLY	503	86.549	-11.140	41.121	0.00	0.00	LX0	H
ATOM	4574	CA	GLY	503	86.101	-12.667	39.691	1.00	0.00	LX0	C
ATOM	4575	C	GLY	503	84.857	-12.779	38.823	1.00	0.00	LX0	C
ATOM	4576	O	GLY	503	84.805	-13.568	37.889	1.00	0.00	LX0	O
ATOM	4577	N	GLN	504	83.850	-11.953	39.146	1.00	0.00	LX0	N
ATOM	4578	H	GLN	504	83.965	-11.205	39.800	0.00	0.00	LX0	H
ATOM	4579	CA	GLN	504	82.604	-12.112	38.403	1.00	0.00	LX0	C
ATOM	4580	CB	GLN	504	81.406	-11.822	39.307	1.00	0.00	LX0	C
ATOM	4581	CG	GLN	504	81.276	-12.839	40.441	1.00	0.00	LX0	C
ATOM	4582	CD	GLN	504	80.062	-12.522	41.284	1.00	0.00	LX0	C
ATOM	4583	OE1	GLN	504	80.141	-11.855	42.309	1.00	0.00	LX0	O
ATOM	4584	NE2	GLN	504	78.919	-13.029	40.821	1.00	0.00	LX0	N
ATOM	4585	HE21	GLN	504	78.905	-13.544	39.964	0.00	0.00	LX0	H
ATOM	4586	HE22	GLN	504	78.090	-12.862	41.358	0.00	0.00	LX0	H
ATOM	4587	C	GLN	504	82.565	-11.291	37.130	1.00	0.00	LX0	C

ATOM	4588	O	GLN	504	81.782	-10.363	36.962	1.00	0.00	LX0	O
ATOM	4589	N	VAL	505	83.486	-11.673	36.241	1.00	0.00	LX0	N
ATOM	4590	H	VAL	505	84.006	-12.510	36.431	0.00	0.00	LX0	H
ATOM	4591	CA	VAL	505	83.558	-11.009	34.947	1.00	0.00	LX0	C
ATOM	4592	CB	VAL	505	84.971	-10.461	34.708	1.00	0.00	LX0	C
ATOM	4593	CG1	VAL	505	85.247	-9.279	35.639	1.00	0.00	LX0	C
ATOM	4594	CG2	VAL	505	86.046	-11.548	34.822	1.00	0.00	LX0	C
ATOM	4595	C	VAL	505	83.118	-11.968	33.858	1.00	0.00	LX0	C
ATOM	4596	O	VAL	505	82.656	-13.065	34.145	1.00	0.00	LX0	O
ATOM	4597	N	CYS	506	83.277	-11.519	32.603	1.00	0.00	LX0	N
ATOM	4598	H	CYS	506	83.503	-10.560	32.451	0.00	0.00	LX0	H
ATOM	4599	CA	CYS	506	82.812	-12.395	31.524	1.00	0.00	LX0	C
ATOM	4600	CB	CYS	506	81.323	-12.173	31.308	1.00	0.00	LX0	C
ATOM	4601	SG	CYS	506	80.957	-10.419	31.027	1.00	0.00	LX0	S
ATOM	4602	C	CYS	506	83.530	-12.258	30.191	1.00	0.00	LX0	C
ATOM	4603	O	CYS	506	83.309	-13.026	29.258	1.00	0.00	LX0	O
ATOM	4604	N	HIS	507	84.382	-11.220	30.122	1.00	0.00	LX0	N
ATOM	4605	H	HIS	507	84.648	-10.718	30.940	0.00	0.00	LX0	H
ATOM	4606	CA	HIS	507	84.982	-10.877	28.832	1.00	0.00	LX0	C
ATOM	4607	CB	HIS	507	85.549	-9.452	28.882	1.00	0.00	LX0	C
ATOM	4608	CG	HIS	507	86.080	-9.044	27.526	1.00	0.00	LX0	C
ATOM	4609	ND1	HIS	507	87.241	-9.494	27.020	1.00	0.00	LX0	N
ATOM	4610	HD1	HIS	507	87.849	-10.131	27.459	0.00	0.00	LX0	H
ATOM	4611	CD2	HIS	507	85.488	-8.187	26.599	1.00	0.00	LX0	C
ATOM	4612	NE2	HIS	507	86.311	-8.130	25.522	1.00	0.00	LX0	N
ATOM	4613	CE1	HIS	507	87.391	-8.934	25.781	1.00	0.00	LX0	C
ATOM	4614	C	HIS	507	86.022	-11.881	28.357	1.00	0.00	LX0	C
ATOM	4615	O	HIS	507	87.210	-11.772	28.636	1.00	0.00	LX0	O
ATOM	4616	N	ALA	508	85.485	-12.855	27.618	1.00	0.00	LX0	N
ATOM	4617	H	ALA	508	84.509	-12.758	27.409	0.00	0.00	LX0	H
ATOM	4618	CA	ALA	508	86.247	-13.965	27.054	1.00	0.00	LX0	C
ATOM	4619	CB	ALA	508	86.886	-14.847	28.138	1.00	0.00	LX0	C
ATOM	4620	C	ALA	508	85.289	-14.809	26.244	1.00	0.00	LX0	C
ATOM	4621	O	ALA	508	85.382	-14.915	25.030	1.00	0.00	LX0	O
ATOM	4622	N	LEU	509	84.319	-15.366	26.990	1.00	0.00	LX0	N
ATOM	4623	H	LEU	509	84.291	-15.181	27.971	0.00	0.00	LX0	H
ATOM	4624	CA	LEU	509	83.209	-16.007	26.288	1.00	0.00	LX0	C
ATOM	4625	CB	LEU	509	82.603	-17.176	27.088	1.00	0.00	LX0	C
ATOM	4626	CG	LEU	509	81.796	-16.853	28.357	1.00	0.00	LX0	C
ATOM	4627	CD1	LEU	509	80.764	-17.941	28.641	1.00	0.00	LX0	C
ATOM	4628	CD2	LEU	509	82.665	-16.570	29.586	1.00	0.00	LX0	C
ATOM	4629	C	LEU	509	82.159	-14.991	25.879	1.00	0.00	LX0	C
ATOM	4630	O	LEU	509	81.599	-15.000	24.790	1.00	0.00	LX0	O
ATOM	4631	N	CYS	510	81.957	-14.052	26.810	1.00	0.00	LX0	N
ATOM	4632	H	CYS	510	82.430	-14.046	27.690	0.00	0.00	LX0	H
ATOM	4633	CA	CYS	510	81.157	-12.913	26.398	1.00	0.00	LX0	C
ATOM	4634	CB	CYS	510	80.453	-12.309	27.601	1.00	0.00	LX0	C
ATOM	4635	SG	CYS	510	79.663	-13.567	28.633	1.00	0.00	LX0	S
ATOM	4636	C	CYS	510	82.042	-11.900	25.716	1.00	0.00	LX0	C
ATOM	4637	O	CYS	510	83.259	-11.890	25.885	1.00	0.00	LX0	O
ATOM	4638	N	SER	511	81.380	-11.083	24.902	1.00	0.00	LX0	N
ATOM	4639	H	SER	511	80.377	-11.103	24.923	0.00	0.00	LX0	H
ATOM	4640	CA	SER	511	82.122	-10.074	24.159	1.00	0.00	LX0	C
ATOM	4641	CB	SER	511	81.314	-9.869	22.872	1.00	0.00	LX0	C
ATOM	4642	OG	SER	511	80.081	-9.209	23.161	1.00	0.00	LX0	O
ATOM	4643	HG	SER	511	79.695	-9.619	23.937	0.00	0.00	LX0	H
ATOM	4644	C	SER	511	82.302	-8.826	25.031	1.00	0.00	LX0	C
ATOM	4645	O	SER	511	81.939	-8.866	26.201	1.00	0.00	LX0	O
ATOM	4646	N	PRO	512	82.811	-7.691	24.460	1.00	0.00	LX0	N
ATOM	4647	CD	PRO	512	83.625	-7.533	23.252	1.00	0.00	LX0	C
ATOM	4648	CA	PRO	512	82.558	-6.397	25.118	1.00	0.00	LX0	C

ATOM	4649	CB	PRO	512	82.996	-5.384	24.058	1.00	0.00	LX0	C
ATOM	4650	CG	PRO	512	84.116	-6.092	23.302	1.00	0.00	LX0	C
ATOM	4651	C	PRO	512	81.123	-6.192	25.598	1.00	0.00	LX0	C
ATOM	4652	O	PRO	512	80.870	-5.668	26.676	1.00	0.00	LX0	O
ATOM	4653	N	GLU	513	80.186	-6.680	24.762	1.00	0.00	LX0	N
ATOM	4654	H	GLU	513	80.413	-7.121	23.895	0.00	0.00	LX0	H
ATOM	4655	CA	GLU	513	78.872	-6.901	25.354	1.00	0.00	LX0	C
ATOM	4656	CB	GLU	513	77.774	-7.021	24.308	1.00	0.00	LX0	C
ATOM	4657	CG	GLU	513	77.839	-6.013	23.165	1.00	0.00	LX0	C
ATOM	4658	CD	GLU	513	76.509	-6.066	22.452	1.00	0.00	LX0	C
ATOM	4659	OE1	GLU	513	75.549	-5.518	22.977	1.00	0.00	LX0	O
ATOM	4660	OE2	GLU	513	76.390	-6.689	21.401	1.00	0.00	LX0	O
ATOM	4661	C	GLU	513	78.903	-8.174	26.170	1.00	0.00	LX0	C
ATOM	4662	O	GLU	513	78.991	-9.280	25.639	1.00	0.00	LX0	O
ATOM	4663	N	GLY	514	78.887	-7.937	27.480	1.00	0.00	LX0	N
ATOM	4664	H	GLY	514	78.743	-6.992	27.775	0.00	0.00	LX0	H
ATOM	4665	CA	GLY	514	79.091	-9.026	28.421	1.00	0.00	LX0	C
ATOM	4666	C	GLY	514	77.885	-9.914	28.611	1.00	0.00	LX0	C
ATOM	4667	O	GLY	514	77.452	-10.621	27.707	1.00	0.00	LX0	O
ATOM	4668	N	CYS	515	77.372	-9.878	29.843	1.00	0.00	LX0	N
ATOM	4669	H	CYS	515	77.690	-9.236	30.547	0.00	0.00	LX0	H
ATOM	4670	CA	CYS	515	76.399	-10.911	30.179	1.00	0.00	LX0	C
ATOM	4671	CB	CYS	515	77.121	-12.216	30.505	1.00	0.00	LX0	C
ATOM	4672	SG	CYS	515	78.226	-12.020	31.915	1.00	0.00	LX0	S
ATOM	4673	C	CYS	515	75.516	-10.515	31.337	1.00	0.00	LX0	C
ATOM	4674	O	CYS	515	75.826	-9.604	32.092	1.00	0.00	LX0	O
ATOM	4675	N	TRP	516	74.408	-11.255	31.460	1.00	0.00	LX0	N
ATOM	4676	H	TRP	516	74.196	-11.977	30.798	0.00	0.00	LX0	H
ATOM	4677	CA	TRP	516	73.555	-11.040	32.626	1.00	0.00	LX0	C
ATOM	4678	CB	TRP	516	72.112	-11.464	32.343	1.00	0.00	LX0	C
ATOM	4679	CG	TRP	516	71.530	-10.753	31.147	1.00	0.00	LX0	C
ATOM	4680	CD2	TRP	516	70.807	-9.507	31.129	1.00	0.00	LX0	C
ATOM	4681	CE2	TRP	516	70.422	-9.260	29.771	1.00	0.00	LX0	C
ATOM	4682	CE3	TRP	516	70.445	-8.593	32.141	1.00	0.00	LX0	C
ATOM	4683	CD1	TRP	516	71.551	-11.187	29.812	1.00	0.00	LX0	C
ATOM	4684	NE1	TRP	516	70.901	-10.313	28.999	1.00	0.00	LX0	N
ATOM	4685	HE1	TRP	516	70.764	-10.428	28.034	0.00	0.00	LX0	H
ATOM	4686	CZ2	TRP	516	69.687	-8.101	29.456	1.00	0.00	LX0	C
ATOM	4687	CZ3	TRP	516	69.710	-7.436	31.811	1.00	0.00	LX0	C
ATOM	4688	CH2	TRP	516	69.329	-7.193	30.475	1.00	0.00	LX0	C
ATOM	4689	C	TRP	516	74.039	-11.733	33.892	1.00	0.00	LX0	C
ATOM	4690	O	TRP	516	73.531	-11.494	34.982	1.00	0.00	LX0	O
ATOM	4691	N	GLY	517	75.040	-12.601	33.719	1.00	0.00	LX0	N
ATOM	4692	H	GLY	517	75.394	-12.847	32.818	0.00	0.00	LX0	H
ATOM	4693	CA	GLY	517	75.476	-13.401	34.852	1.00	0.00	LX0	C
ATOM	4694	C	GLY	517	76.643	-14.278	34.458	1.00	0.00	LX0	C
ATOM	4695	O	GLY	517	77.130	-14.224	33.337	1.00	0.00	LX0	O
ATOM	4696	N	PRO	518	77.104	-15.079	35.443	1.00	0.00	LX0	N
ATOM	4697	CD	PRO	518	76.579	-15.196	36.796	1.00	0.00	LX0	C
ATOM	4698	CA	PRO	518	78.304	-15.894	35.225	1.00	0.00	LX0	C
ATOM	4699	CB	PRO	518	78.615	-16.427	36.632	1.00	0.00	LX0	C
ATOM	4700	CG	PRO	518	77.806	-15.575	37.612	1.00	0.00	LX0	C
ATOM	4701	C	PRO	518	78.156	-17.033	34.222	1.00	0.00	LX0	C
ATOM	4702	O	PRO	518	79.130	-17.645	33.798	1.00	0.00	LX0	O
ATOM	4703	N	GLU	519	76.895	-17.356	33.914	1.00	0.00	LX0	N
ATOM	4704	H	GLU	519	76.114	-16.737	34.024	0.00	0.00	LX0	H
ATOM	4705	CA	GLU	519	76.699	-18.620	33.219	1.00	0.00	LX0	C
ATOM	4706	CB	GLU	519	75.297	-19.147	33.532	1.00	0.00	LX0	C
ATOM	4707	CG	GLU	519	75.073	-19.364	35.030	1.00	0.00	LX0	C
ATOM	4708	CD	GLU	519	73.649	-19.819	35.257	1.00	0.00	LX0	C
ATOM	4709	OE1	GLU	519	72.812	-19.000	35.618	1.00	0.00	LX0	O

ATOM	4710	OE2	GLU	519	73.352	-20.992	35.050	1.00	0.00	LX0	O
ATOM	4711	C	GLU	519	76.936	-18.505	31.724	1.00	0.00	LX0	C
ATOM	4712	O	GLU	519	76.662	-17.489	31.099	1.00	0.00	LX0	O
ATOM	4713	N	PRO	520	77.433	-19.615	31.122	1.00	0.00	LX0	N
ATOM	4714	CD	PRO	520	77.907	-20.830	31.778	1.00	0.00	LX0	C
ATOM	4715	CA	PRO	520	77.610	-19.661	29.660	1.00	0.00	LX0	C
ATOM	4716	CB	PRO	520	78.130	-21.083	29.426	1.00	0.00	LX0	C
ATOM	4717	CG	PRO	520	78.804	-21.483	30.736	1.00	0.00	LX0	C
ATOM	4718	C	PRO	520	76.408	-19.336	28.769	1.00	0.00	LX0	C
ATOM	4719	O	PRO	520	76.535	-19.282	27.553	1.00	0.00	LX0	O
ATOM	4720	N	ARG	521	75.242	-19.157	29.408	1.00	0.00	LX0	N
ATOM	4721	H	ARG	521	75.225	-19.131	30.404	0.00	0.00	LX0	H
ATOM	4722	CA	ARG	521	74.018	-18.849	28.672	1.00	0.00	LX0	C
ATOM	4723	CB	ARG	521	72.912	-19.795	29.158	1.00	0.00	LX0	C
ATOM	4724	CG	ARG	521	72.720	-19.764	30.678	1.00	0.00	LX0	C
ATOM	4725	CD	ARG	521	71.845	-20.891	31.229	1.00	0.00	LX0	C
ATOM	4726	NE	ARG	521	71.778	-20.794	32.686	1.00	0.00	LX0	N
ATOM	4727	HE	ARG	521	72.616	-20.781	33.246	0.00	0.00	LX0	H
ATOM	4728	CZ	ARG	521	70.626	-20.576	33.344	1.00	0.00	LX0	C
ATOM	4729	NH1	ARG	521	69.462	-20.619	32.707	1.00	0.00	LX0	N
ATOM	4730	HH11	ARG	521	68.641	-20.336	33.220	0.00	0.00	LX0	H
ATOM	4731	HH12	ARG	521	69.393	-20.895	31.753	0.00	0.00	LX0	H
ATOM	4732	NH2	ARG	521	70.647	-20.287	34.635	1.00	0.00	LX0	N
ATOM	4733	HH21	ARG	521	69.806	-20.070	35.139	0.00	0.00	LX0	H
ATOM	4734	HH22	ARG	521	71.540	-20.239	35.121	0.00	0.00	LX0	H
ATOM	4735	C	ARG	521	73.589	-17.387	28.761	1.00	0.00	LX0	C
ATOM	4736	O	ARG	521	72.578	-16.979	28.210	1.00	0.00	LX0	O
ATOM	4737	N	ASP	522	74.395	-16.610	29.498	1.00	0.00	LX0	N
ATOM	4738	H	ASP	522	75.255	-16.948	29.879	0.00	0.00	LX0	H
ATOM	4739	CA	ASP	522	73.984	-15.248	29.846	1.00	0.00	LX0	C
ATOM	4740	CB	ASP	522	74.633	-14.862	31.174	1.00	0.00	LX0	C
ATOM	4741	CG	ASP	522	73.786	-15.199	32.380	1.00	0.00	LX0	C
ATOM	4742	OD1	ASP	522	74.149	-16.078	33.150	1.00	0.00	LX0	O
ATOM	4743	OD2	ASP	522	72.782	-14.541	32.608	1.00	0.00	LX0	O
ATOM	4744	C	ASP	522	74.345	-14.160	28.843	1.00	0.00	LX0	C
ATOM	4745	O	ASP	522	74.287	-12.973	29.146	1.00	0.00	LX0	O
ATOM	4746	N	CYS	523	74.816	-14.581	27.664	1.00	0.00	LX0	N
ATOM	4747	H	CYS	523	74.643	-15.507	27.336	0.00	0.00	LX0	H
ATOM	4748	CA	CYS	523	75.593	-13.593	26.913	1.00	0.00	LX0	C
ATOM	4749	CB	CYS	523	76.682	-14.287	26.103	1.00	0.00	LX0	C
ATOM	4750	SG	CYS	523	77.652	-15.426	27.126	1.00	0.00	LX0	S
ATOM	4751	C	CYS	523	74.822	-12.609	26.052	1.00	0.00	LX0	C
ATOM	4752	O	CYS	523	74.041	-12.965	25.182	1.00	0.00	LX0	O
ATOM	4753	N	VAL	524	75.116	-11.328	26.322	1.00	0.00	LX0	N
ATOM	4754	H	VAL	524	75.782	-11.144	27.044	0.00	0.00	LX0	H
ATOM	4755	CA	VAL	524	74.542	-10.246	25.518	1.00	0.00	LX0	C
ATOM	4756	CB	VAL	524	74.643	-8.907	26.274	1.00	0.00	LX0	C
ATOM	4757	CG1	VAL	524	73.874	-7.788	25.569	1.00	0.00	LX0	C
ATOM	4758	CG2	VAL	524	74.159	-9.047	27.719	1.00	0.00	LX0	C
ATOM	4759	C	VAL	524	75.147	-10.148	24.116	1.00	0.00	LX0	C
ATOM	4760	O	VAL	524	74.515	-9.691	23.162	1.00	0.00	LX0	O
ATOM	4761	N	SER	525	76.400	-10.630	24.039	1.00	0.00	LX0	N
ATOM	4762	H	SER	525	76.931	-10.810	24.871	0.00	0.00	LX0	H
ATOM	4763	CA	SER	525	76.989	-11.012	22.756	1.00	0.00	LX0	C
ATOM	4764	CB	SER	525	77.329	-9.799	21.884	1.00	0.00	LX0	C
ATOM	4765	OG	SER	525	77.581	-10.198	20.532	1.00	0.00	LX0	O
ATOM	4766	HG	SER	525	77.582	-9.379	20.045	0.00	0.00	LX0	H
ATOM	4767	C	SER	525	78.200	-11.886	23.012	1.00	0.00	LX0	C
ATOM	4768	O	SER	525	78.712	-11.952	24.127	1.00	0.00	LX0	O
ATOM	4769	N	CYS	526	78.606	-12.603	21.959	1.00	0.00	LX0	N
ATOM	4770	H	CYS	526	78.279	-12.355	21.046	0.00	0.00	LX0	H

ATOM	4771	CA	CYS	526	79.325	-13.839	22.258	1.00	0.00	LX0	C
ATOM	4772	CB	CYS	526	78.425	-15.028	21.925	1.00	0.00	LX0	C
ATOM	4773	SG	CYS	526	76.724	-14.800	22.504	1.00	0.00	LX0	S
ATOM	4774	C	CYS	526	80.674	-14.005	21.587	1.00	0.00	LX0	C
ATOM	4775	O	CYS	526	81.061	-13.280	20.670	1.00	0.00	LX0	O
ATOM	4776	N	ARG	527	81.391	-15.028	22.067	1.00	0.00	LX0	N
ATOM	4777	H	ARG	527	81.108	-15.536	22.886	0.00	0.00	LX0	H
ATOM	4778	CA	ARG	527	82.610	-15.376	21.357	1.00	0.00	LX0	C
ATOM	4779	CB	ARG	527	83.711	-15.847	22.311	1.00	0.00	LX0	C
ATOM	4780	CG	ARG	527	85.141	-15.805	21.742	1.00	0.00	LX0	C
ATOM	4781	CD	ARG	527	85.651	-14.420	21.310	1.00	0.00	LX0	C
ATOM	4782	NE	ARG	527	85.125	-14.023	20.000	1.00	0.00	LX0	N
ATOM	4783	HE	ARG	527	85.325	-14.607	19.208	0.00	0.00	LX0	H
ATOM	4784	CZ	ARG	527	84.330	-12.946	19.836	1.00	0.00	LX0	C
ATOM	4785	NH1	ARG	527	84.048	-12.148	20.865	1.00	0.00	LX0	N
ATOM	4786	HH11	ARG	527	83.426	-11.373	20.768	0.00	0.00	LX0	H
ATOM	4787	HH12	ARG	527	84.454	-12.330	21.763	0.00	0.00	LX0	H
ATOM	4788	NH2	ARG	527	83.821	-12.713	18.631	1.00	0.00	LX0	N
ATOM	4789	HH21	ARG	527	83.236	-11.928	18.428	0.00	0.00	LX0	H
ATOM	4790	HH22	ARG	527	84.026	-13.368	17.891	0.00	0.00	LX0	H
ATOM	4791	C	ARG	527	82.403	-16.323	20.195	1.00	0.00	LX0	C
ATOM	4792	O	ARG	527	82.634	-17.522	20.259	1.00	0.00	LX0	O
ATOM	4793	N	ASN	528	81.997	-15.671	19.091	1.00	0.00	LX0	N
ATOM	4794	H	ASN	528	81.657	-14.742	19.235	0.00	0.00	LX0	H
ATOM	4795	CA	ASN	528	81.832	-16.345	17.795	1.00	0.00	LX0	C
ATOM	4796	CB	ASN	528	83.084	-17.135	17.339	1.00	0.00	LX0	C
ATOM	4797	CG	ASN	528	84.349	-16.295	17.369	1.00	0.00	LX0	C
ATOM	4798	OD1	ASN	528	84.346	-15.071	17.467	1.00	0.00	LX0	O
ATOM	4799	ND2	ASN	528	85.467	-17.021	17.294	1.00	0.00	LX0	N
ATOM	4800	HD21	ASN	528	85.428	-18.020	17.272	0.00	0.00	LX0	H
ATOM	4801	HD22	ASN	528	86.362	-16.578	17.256	0.00	0.00	LX0	H
ATOM	4802	C	ASN	528	80.577	-17.204	17.785	1.00	0.00	LX0	C
ATOM	4803	O	ASN	528	79.723	-17.061	18.653	1.00	0.00	LX0	O
ATOM	4804	N	VAL	529	80.470	-18.080	16.766	1.00	0.00	LX0	N
ATOM	4805	H	VAL	529	81.193	-18.173	16.087	0.00	0.00	LX0	H
ATOM	4806	CA	VAL	529	79.255	-18.896	16.648	1.00	0.00	LX0	C
ATOM	4807	CB	VAL	529	79.307	-19.768	15.381	1.00	0.00	LX0	C
ATOM	4808	CG1	VAL	529	78.009	-20.559	15.173	1.00	0.00	LX0	C
ATOM	4809	CG2	VAL	529	79.636	-18.926	14.144	1.00	0.00	LX0	C
ATOM	4810	C	VAL	529	78.966	-19.741	17.883	1.00	0.00	LX0	C
ATOM	4811	O	VAL	529	79.672	-20.689	18.202	1.00	0.00	LX0	O
ATOM	4812	N	SER	530	77.900	-19.308	18.557	1.00	0.00	LX0	N
ATOM	4813	H	SER	530	77.325	-18.592	18.166	0.00	0.00	LX0	H
ATOM	4814	CA	SER	530	77.496	-19.938	19.806	1.00	0.00	LX0	C
ATOM	4815	CB	SER	530	77.249	-18.818	20.808	1.00	0.00	LX0	C
ATOM	4816	OG	SER	530	78.453	-18.050	20.958	1.00	0.00	LX0	O
ATOM	4817	HG	SER	530	78.763	-17.828	20.083	0.00	0.00	LX0	H
ATOM	4818	C	SER	530	76.275	-20.808	19.564	1.00	0.00	LX0	C
ATOM	4819	O	SER	530	75.555	-20.609	18.590	1.00	0.00	LX0	O
ATOM	4820	N	ARG	531	76.104	-21.827	20.415	1.00	0.00	LX0	N
ATOM	4821	H	ARG	531	76.572	-21.883	21.306	0.00	0.00	LX0	H
ATOM	4822	CA	ARG	531	75.233	-22.908	19.961	1.00	0.00	LX0	C
ATOM	4823	CB	ARG	531	76.055	-24.157	19.624	1.00	0.00	LX0	C
ATOM	4824	CG	ARG	531	77.126	-23.918	18.551	1.00	0.00	LX0	C
ATOM	4825	CD	ARG	531	78.477	-24.527	18.934	1.00	0.00	LX0	C
ATOM	4826	NE	ARG	531	78.903	-24.012	20.233	1.00	0.00	LX0	N
ATOM	4827	HE	ARG	531	78.337	-24.188	21.049	0.00	0.00	LX0	H
ATOM	4828	CZ	ARG	531	79.952	-23.186	20.406	1.00	0.00	LX0	C
ATOM	4829	NH1	ARG	531	80.743	-22.858	19.394	1.00	0.00	LX0	N
ATOM	4830	HH11	ARG	531	81.511	-22.235	19.530	0.00	0.00	LX0	H
ATOM	4831	HH12	ARG	531	80.552	-23.196	18.475	0.00	0.00	LX0	H

ATOM	4832	NH2	ARG	531	80.181	-22.693	21.609	1.00	0.00	LX0	N
ATOM	4833	HH21	ARG	531	80.962	-22.109	21.843	0.00	0.00	LX0	H
ATOM	4834	HH22	ARG	531	79.527	-22.903	22.356	0.00	0.00	LX0	H
ATOM	4835	C	ARG	531	74.127	-23.252	20.931	1.00	0.00	LX0	C
ATOM	4836	O	ARG	531	74.334	-23.656	22.065	1.00	0.00	LX0	O
ATOM	4837	N	GLY	532	72.898	-23.078	20.426	1.00	0.00	LX0	N
ATOM	4838	H	GLY	532	72.817	-22.673	19.515	0.00	0.00	LX0	H
ATOM	4839	CA	GLY	532	71.748	-23.632	21.149	1.00	0.00	LX0	C
ATOM	4840	C	GLY	532	71.453	-23.110	22.555	1.00	0.00	LX0	C
ATOM	4841	O	GLY	532	70.622	-23.682	23.258	1.00	0.00	LX0	O
ATOM	4842	N	ARG	533	72.124	-21.979	22.877	1.00	0.00	LX0	N
ATOM	4843	H	ARG	533	72.785	-21.673	22.196	0.00	0.00	LX0	H
ATOM	4844	CA	ARG	533	72.135	-21.248	24.158	1.00	0.00	LX0	C
ATOM	4845	CB	ARG	533	70.850	-21.315	24.995	1.00	0.00	LX0	C
ATOM	4846	CG	ARG	533	69.580	-20.834	24.300	1.00	0.00	LX0	C
ATOM	4847	CD	ARG	533	68.378	-21.370	25.077	1.00	0.00	LX0	C
ATOM	4848	NE	ARG	533	67.193	-21.489	24.230	1.00	0.00	LX0	N
ATOM	4849	HE	ARG	533	66.548	-20.723	24.269	0.00	0.00	LX0	H
ATOM	4850	CZ	ARG	533	67.001	-22.592	23.467	1.00	0.00	LX0	C
ATOM	4851	NH1	ARG	533	67.922	-23.557	23.393	1.00	0.00	LX0	N
ATOM	4852	HH11	ARG	533	67.772	-24.413	22.901	0.00	0.00	LX0	H
ATOM	4853	HH12	ARG	533	68.830	-23.452	23.824	0.00	0.00	LX0	H
ATOM	4854	NH2	ARG	533	65.866	-22.703	22.778	1.00	0.00	LX0	N
ATOM	4855	HH21	ARG	533	65.679	-23.496	22.197	0.00	0.00	LX0	H
ATOM	4856	HH22	ARG	533	65.172	-21.983	22.830	0.00	0.00	LX0	H
ATOM	4857	C	ARG	533	73.306	-21.540	25.078	1.00	0.00	LX0	C
ATOM	4858	O	ARG	533	73.239	-21.284	26.273	1.00	0.00	LX0	O
ATOM	4859	N	GLU	534	74.397	-22.038	24.488	1.00	0.00	LX0	N
ATOM	4860	H	GLU	534	74.451	-22.392	23.555	0.00	0.00	LX0	H
ATOM	4861	CA	GLU	534	75.636	-21.705	25.177	1.00	0.00	LX0	C
ATOM	4862	CB	GLU	534	76.417	-22.950	25.638	1.00	0.00	LX0	C
ATOM	4863	CG	GLU	534	76.880	-23.963	24.581	1.00	0.00	LX0	C
ATOM	4864	CD	GLU	534	77.954	-23.370	23.691	1.00	0.00	LX0	C
ATOM	4865	OE1	GLU	534	77.663	-23.070	22.543	1.00	0.00	LX0	O
ATOM	4866	OE2	GLU	534	79.091	-23.199	24.122	1.00	0.00	LX0	O
ATOM	4867	C	GLU	534	76.438	-20.752	24.322	1.00	0.00	LX0	C
ATOM	4868	O	GLU	534	76.171	-20.592	23.134	1.00	0.00	LX0	O
ATOM	4869	N	CYS	535	77.399	-20.107	24.989	1.00	0.00	LX0	N
ATOM	4870	H	CYS	535	77.433	-20.183	25.985	0.00	0.00	LX0	H
ATOM	4871	CA	CYS	535	78.203	-19.122	24.276	1.00	0.00	LX0	C
ATOM	4872	CB	CYS	535	77.720	-17.717	24.621	1.00	0.00	LX0	C
ATOM	4873	SG	CYS	535	75.931	-17.501	24.409	1.00	0.00	LX0	S
ATOM	4874	C	CYS	535	79.685	-19.258	24.552	1.00	0.00	LX0	C
ATOM	4875	O	CYS	535	80.440	-18.295	24.603	1.00	0.00	LX0	O
ATOM	4876	N	VAL	536	80.069	-20.520	24.774	1.00	0.00	LX0	N
ATOM	4877	H	VAL	536	79.423	-21.278	24.655	0.00	0.00	LX0	H
ATOM	4878	CA	VAL	536	81.465	-20.756	25.125	1.00	0.00	LX0	C
ATOM	4879	CB	VAL	536	81.596	-21.159	26.604	1.00	0.00	LX0	C
ATOM	4880	CG1	VAL	536	80.848	-22.457	26.923	1.00	0.00	LX0	C
ATOM	4881	CG2	VAL	536	83.064	-21.200	27.043	1.00	0.00	LX0	C
ATOM	4882	C	VAL	536	82.136	-21.761	24.208	1.00	0.00	LX0	C
ATOM	4883	O	VAL	536	82.164	-21.631	22.985	1.00	0.00	LX0	O
TER											
