# Supplementary material for: Structural analysis of hubs in human NR-RTK network
Source: Biol Direct. 2011 Oct 5;6:49. doi: 10.1186/1745-6150-6-49 (PMC3220635; doi:10.1186/1745-6150-6-49)
Supplement: Additional file 7 — ESR1-Erbb2. ESR1-Erbb2 complex structure. [file 1745-6150-6-49-S7.PDF]

```
HEADER ESR1-ERBB2
REMARK original generated coordinate pdb file
ATOM      1  N   ALA   156      10.627  12.174   8.322  1.00  0.00      RX0  N
ATOM      2  H   ALA   156      11.197  11.353   8.275  0.00  0.00      RX0  H
ATOM      3  CA  ALA   156       9.864  12.527   9.538  1.00  0.00      RX0  C
ATOM      4  CB  ALA   156      10.757  12.403  10.764  1.00  0.00      RX0  C
ATOM      5  C   ALA   156       9.377  13.991   9.496  1.00  0.00      RX0  C
ATOM      6  O   ALA   156       9.121  14.644  10.500  1.00  0.00      RX0  O
ATOM      7  N   LEU   157       9.039  14.416   8.289  1.00  0.00      RX0  N
ATOM      8  H   LEU   157       9.130  13.765   7.532  0.00  0.00      RX0  H
ATOM      9  CA  LEU   157       8.850  15.849   7.979  1.00  0.00      RX0  C
ATOM     10  CB  LEU   157       9.701  16.234   6.775  1.00  0.00      RX0  C
ATOM     11  CG  LEU   157      11.024  15.477   6.701  1.00  0.00      RX0  C
ATOM     12  CD1 LEU   157      11.606  15.544   5.293  1.00  0.00      RX0  C
ATOM     13  CD2 LEU   157      12.013  15.910   7.781  1.00  0.00      RX0  C
ATOM     14  C   LEU   157       7.387  16.184   7.642  1.00  0.00      RX0  C
ATOM     15  O   LEU   157       7.075  17.216   7.039  1.00  0.00      RX0  O
ATOM     16  N   SER   158       6.522  15.244   7.955  1.00  0.00      RX0  N
ATOM     17  H   SER   158       6.876  14.329   8.116  0.00  0.00      RX0  H
ATOM     18  CA  SER   158       5.051  15.362   7.847  1.00  0.00      RX0  C
ATOM     19  CB  SER   158       4.718  14.914   6.431  1.00  0.00      RX0  C
ATOM     20  OG  SER   158       5.881  15.171   5.634  1.00  0.00      RX0  O
ATOM     21  HG  SER   158       6.020  16.116   5.673  0.00  0.00      RX0  H
ATOM     22  C   SER   158       4.335  14.560   8.949  1.00  0.00      RX0  C
ATOM     23  O   SER   158       3.148  14.670   9.188  1.00  0.00      RX0  O
ATOM     24  N   LEU   159       5.132  13.681   9.591  1.00  0.00      RX0  N
ATOM     25  H   LEU   159       6.085  13.590   9.330  0.00  0.00      RX0  H
ATOM     26  CA  LEU   159       4.759  12.951  10.797  1.00  0.00      RX0  C
ATOM     27  CB  LEU   159       5.860  11.981  11.242  1.00  0.00      RX0  C
ATOM     28  CG  LEU   159       5.962  10.653  10.479  1.00  0.00      RX0  C
ATOM     29  CD1 LEU   159       6.508  10.794   9.056  1.00  0.00      RX0  C
ATOM     30  CD2 LEU   159       6.770   9.634  11.284  1.00  0.00      RX0  C
ATOM     31  C   LEU   159       4.518  13.965  11.920  1.00  0.00      RX0  C
ATOM     32  O   LEU   159       5.291  14.932  12.058  1.00  0.00      RX0  O
ATOM     33  N   THR   160       3.434  13.807  12.646  1.00  0.00      RX0  N
ATOM     34  H   THR   160       2.847  13.019  12.458  0.00  0.00      RX0  H
ATOM     35  CA  THR   160       3.156  14.665  13.825  1.00  0.00      RX0  C
ATOM     36  CB  THR   160       1.666  14.648  14.227  1.00  0.00      RX0  C
ATOM     37  OG1 THR   160       1.375  15.741  15.105  1.00  0.00      RX0  O
ATOM     38  HG1 THR   160       0.428  15.831  15.119  0.00  0.00      RX0  H
ATOM     39  CG2 THR   160       1.173  13.337  14.822  1.00  0.00      RX0  C
ATOM     40  C   THR   160       4.203  14.411  14.921  1.00  0.00      RX0  C
ATOM     41  O   THR   160       4.902  13.383  14.913  1.00  0.00      RX0  O
ATOM     42  N   ALA   161       4.153  15.229  15.953  1.00  0.00      RX0  N
ATOM     43  H   ALA   161       3.464  15.956  15.915  0.00  0.00      RX0  H
ATOM     44  CA  ALA   161       4.942  15.044  17.184  1.00  0.00      RX0  C
ATOM     45  CB  ALA   161       4.758  16.234  18.117  1.00  0.00      RX0  C
ATOM     46  C   ALA   161       4.543  13.746  17.920  1.00  0.00      RX0  C
ATOM     47  O   ALA   161       5.387  12.950  18.288  1.00  0.00      RX0  O
ATOM     48  N   ASP   162       3.226  13.461  17.917  1.00  0.00      RX0  N
ATOM     49  H   ASP   162       2.575  14.179  17.680  0.00  0.00      RX0  H
ATOM     50  CA  ASP   162       2.690  12.196  18.469  1.00  0.00      RX0  C
ATOM     51  CB  ASP   162       1.216  12.309  18.878  1.00  0.00      RX0  C
ATOM     52  CG  ASP   162       1.150  13.112  20.177  1.00  0.00      RX0  C
ATOM     53  OD1 ASP   162       2.149  13.712  20.566  1.00  0.00      RX0  O
ATOM     54  OD2 ASP   162       0.114  13.133  20.840  1.00  0.00      RX0  O
ATOM     55  C   ASP   162       3.088  10.948  17.668  1.00  0.00      RX0  C
ATOM     56  O   ASP   162       3.397   9.903  18.257  1.00  0.00      RX0  O
ATOM     57  N   GLN   163       3.164  11.087  16.353  1.00  0.00      RX0  N
ATOM     58  H   GLN   163       3.081  12.014  15.997  0.00  0.00      RX0  H
ATOM     59  CA  GLN   163       3.593   9.998  15.449  1.00  0.00      RX0  C
```

ATOM	60	CB	GLN	163	3.245	10.284	13.996	1.00	0.00	RX0	C
ATOM	61	CG	GLN	163	1.818	9.876	13.642	1.00	0.00	RX0	C
ATOM	62	CD	GLN	163	1.536	10.385	12.250	1.00	0.00	RX0	C
ATOM	63	OE1	GLN	163	2.075	11.407	11.838	1.00	0.00	RX0	O
ATOM	64	NE2	GLN	163	0.675	9.625	11.553	1.00	0.00	RX0	N
ATOM	65	HE21	GLN	163	0.276	8.802	11.961	0.00	0.00	RX0	H
ATOM	66	HE22	GLN	163	0.406	9.853	10.617	0.00	0.00	RX0	H
ATOM	67	C	GLN	163	5.089	9.698	15.572	1.00	0.00	RX0	C
ATOM	68	O	GLN	163	5.477	8.537	15.545	1.00	0.00	RX0	O
ATOM	69	N	MET	164	5.882	10.740	15.840	1.00	0.00	RX0	N
ATOM	70	H	MET	164	5.494	11.662	15.888	0.00	0.00	RX0	H
ATOM	71	CA	MET	164	7.331	10.587	16.060	1.00	0.00	RX0	C
ATOM	72	CB	MET	164	8.015	11.955	16.081	1.00	0.00	RX0	C
ATOM	73	CG	MET	164	9.452	11.878	16.603	1.00	0.00	RX0	C
ATOM	74	SD	MET	164	10.534	10.861	15.591	1.00	0.00	RX0	S
ATOM	75	CE	MET	164	11.030	12.132	14.425	1.00	0.00	RX0	C
ATOM	76	C	MET	164	7.610	9.825	17.366	1.00	0.00	RX0	C
ATOM	77	O	MET	164	8.404	8.887	17.381	1.00	0.00	RX0	O
ATOM	78	N	VAL	165	6.828	10.145	18.396	1.00	0.00	RX0	N
ATOM	79	H	VAL	165	6.175	10.897	18.281	0.00	0.00	RX0	H
ATOM	80	CA	VAL	165	6.992	9.563	19.744	1.00	0.00	RX0	C
ATOM	81	CB	VAL	165	6.098	10.257	20.777	1.00	0.00	RX0	C
ATOM	82	CG1	VAL	165	6.170	9.555	22.133	1.00	0.00	RX0	C
ATOM	83	CG2	VAL	165	6.451	11.729	20.925	1.00	0.00	RX0	C
ATOM	84	C	VAL	165	6.649	8.067	19.731	1.00	0.00	RX0	C
ATOM	85	O	VAL	165	7.442	7.255	20.191	1.00	0.00	RX0	O
ATOM	86	N	SER	166	5.467	7.742	19.205	1.00	0.00	RX0	N
ATOM	87	H	SER	166	4.870	8.478	18.870	0.00	0.00	RX0	H
ATOM	88	CA	SER	166	5.029	6.335	19.106	1.00	0.00	RX0	C
ATOM	89	CB	SER	166	3.566	6.115	18.703	1.00	0.00	RX0	C
ATOM	90	OG	SER	166	3.131	4.804	19.127	1.00	0.00	RX0	O
ATOM	91	HG	SER	166	3.746	4.153	18.748	0.00	0.00	RX0	H
ATOM	92	C	SER	166	5.941	5.501	18.195	1.00	0.00	RX0	C
ATOM	93	O	SER	166	6.295	4.379	18.542	1.00	0.00	RX0	O
ATOM	94	N	ALA	167	6.456	6.129	17.133	1.00	0.00	RX0	N
ATOM	95	H	ALA	167	6.160	7.058	16.916	0.00	0.00	RX0	H
ATOM	96	CA	ALA	167	7.397	5.466	16.208	1.00	0.00	RX0	C
ATOM	97	CB	ALA	167	7.730	6.358	15.013	1.00	0.00	RX0	C
ATOM	98	C	ALA	167	8.706	5.103	16.927	1.00	0.00	RX0	C
ATOM	99	O	ALA	167	9.113	3.946	16.932	1.00	0.00	RX0	O
ATOM	100	N	LEU	168	9.179	6.054	17.734	1.00	0.00	RX0	N
ATOM	101	H	LEU	168	8.725	6.947	17.747	0.00	0.00	RX0	H
ATOM	102	CA	LEU	168	10.385	5.875	18.562	1.00	0.00	RX0	C
ATOM	103	CB	LEU	168	10.907	7.213	19.074	1.00	0.00	RX0	C
ATOM	104	CG	LEU	168	11.568	8.043	17.977	1.00	0.00	RX0	C
ATOM	105	CD1	LEU	168	12.102	9.370	18.519	1.00	0.00	RX0	C
ATOM	106	CD2	LEU	168	12.647	7.241	17.249	1.00	0.00	RX0	C
ATOM	107	C	LEU	168	10.197	4.896	19.724	1.00	0.00	RX0	C
ATOM	108	O	LEU	168	11.077	4.078	19.994	1.00	0.00	RX0	O
ATOM	109	N	LEU	169	9.007	4.918	20.317	1.00	0.00	RX0	N
ATOM	110	H	LEU	169	8.336	5.594	20.009	0.00	0.00	RX0	H
ATOM	111	CA	LEU	169	8.640	3.970	21.384	1.00	0.00	RX0	C
ATOM	112	CB	LEU	169	7.360	4.383	22.113	1.00	0.00	RX0	C
ATOM	113	CG	LEU	169	7.534	5.596	23.031	1.00	0.00	RX0	C
ATOM	114	CD1	LEU	169	6.208	5.998	23.676	1.00	0.00	RX0	C
ATOM	115	CD2	LEU	169	8.625	5.381	24.083	1.00	0.00	RX0	C
ATOM	116	C	LEU	169	8.505	2.536	20.864	1.00	0.00	RX0	C
ATOM	117	O	LEU	169	9.003	1.602	21.486	1.00	0.00	RX0	O
ATOM	118	N	ASP	170	7.977	2.423	19.645	1.00	0.00	RX0	N
ATOM	119	H	ASP	170	7.529	3.214	19.236	0.00	0.00	RX0	H
ATOM	120	CA	ASP	170	7.822	1.133	18.952	1.00	0.00	RX0	C

ATOM	121	CB	ASP	170	6.723	1.241	17.864	1.00	0.00	RX0	C
ATOM	122	CG	ASP	170	5.369	1.727	18.438	1.00	0.00	RX0	C
ATOM	123	OD1	ASP	170	5.118	1.580	19.634	1.00	0.00	RX0	O
ATOM	124	OD2	ASP	170	4.562	2.293	17.694	1.00	0.00	RX0	O
ATOM	125	C	ASP	170	9.164	0.506	18.541	1.00	0.00	RX0	C
ATOM	126	O	ASP	170	9.313	-0.704	18.571	1.00	0.00	RX0	O
ATOM	127	N	ALA	171	10.119	1.387	18.228	1.00	0.00	RX0	N
ATOM	128	H	ALA	171	9.870	2.356	18.291	0.00	0.00	RX0	H
ATOM	129	CA	ALA	171	11.447	1.008	17.717	1.00	0.00	RX0	C
ATOM	130	CB	ALA	171	12.103	2.224	17.065	1.00	0.00	RX0	C
ATOM	131	C	ALA	171	12.418	0.479	18.779	1.00	0.00	RX0	C
ATOM	132	O	ALA	171	13.427	-0.136	18.431	1.00	0.00	RX0	O
ATOM	133	N	GLU	172	12.125	0.739	20.058	1.00	0.00	RX0	N
ATOM	134	H	GLU	172	11.277	1.225	20.272	0.00	0.00	RX0	H
ATOM	135	CA	GLU	172	13.017	0.374	21.170	1.00	0.00	RX0	C
ATOM	136	CB	GLU	172	12.418	0.772	22.520	1.00	0.00	RX0	C
ATOM	137	CG	GLU	172	12.443	2.293	22.737	1.00	0.00	RX0	C
ATOM	138	CD	GLU	172	13.868	2.830	22.661	1.00	0.00	RX0	C
ATOM	139	OE1	GLU	172	14.620	2.710	23.628	1.00	0.00	RX0	O
ATOM	140	OE2	GLU	172	14.255	3.367	21.624	1.00	0.00	RX0	O
ATOM	141	C	GLU	172	13.554	-1.065	21.099	1.00	0.00	RX0	C
ATOM	142	O	GLU	172	12.785	-2.004	20.837	1.00	0.00	RX0	O
ATOM	143	N	PRO	173	14.865	-1.209	21.269	1.00	0.00	RX0	N
ATOM	144	CD	PRO	173	15.802	-0.100	21.442	1.00	0.00	RX0	C
ATOM	145	CA	PRO	173	15.538	-2.517	21.328	1.00	0.00	RX0	C
ATOM	146	CB	PRO	173	17.016	-2.116	21.196	1.00	0.00	RX0	C
ATOM	147	CG	PRO	173	17.115	-0.746	21.859	1.00	0.00	RX0	C
ATOM	148	C	PRO	173	15.206	-3.249	22.640	1.00	0.00	RX0	C
ATOM	149	O	PRO	173	14.829	-2.595	23.631	1.00	0.00	RX0	O
ATOM	150	N	PRO	174	15.294	-4.574	22.646	1.00	0.00	RX0	N
ATOM	151	CD	PRO	174	15.575	-5.399	21.472	1.00	0.00	RX0	C
ATOM	152	CA	PRO	174	15.084	-5.400	23.852	1.00	0.00	RX0	C
ATOM	153	CB	PRO	174	14.955	-6.816	23.281	1.00	0.00	RX0	C
ATOM	154	CG	PRO	174	15.808	-6.802	22.019	1.00	0.00	RX0	C
ATOM	155	C	PRO	174	16.250	-5.248	24.838	1.00	0.00	RX0	C
ATOM	156	O	PRO	174	17.379	-4.922	24.444	1.00	0.00	RX0	O
ATOM	157	N	ILE	175	15.956	-5.464	26.106	1.00	0.00	RX0	N
ATOM	158	H	ILE	175	15.021	-5.746	26.316	0.00	0.00	RX0	H
ATOM	159	CA	ILE	175	16.988	-5.556	27.159	1.00	0.00	RX0	C
ATOM	160	CB	ILE	175	16.417	-5.147	28.515	1.00	0.00	RX0	C
ATOM	161	CG2	ILE	175	17.508	-5.109	29.585	1.00	0.00	RX0	C
ATOM	162	CG1	ILE	175	15.755	-3.773	28.358	1.00	0.00	RX0	C
ATOM	163	CD1	ILE	175	14.977	-3.292	29.579	1.00	0.00	RX0	C
ATOM	164	C	ILE	175	17.586	-6.969	27.112	1.00	0.00	RX0	C
ATOM	165	O	ILE	175	16.886	-7.963	27.343	1.00	0.00	RX0	O
ATOM	166	N	LEU	176	18.884	-7.017	26.884	1.00	0.00	RX0	N
ATOM	167	H	LEU	176	19.409	-6.171	26.830	0.00	0.00	RX0	H
ATOM	168	CA	LEU	176	19.617	-8.291	26.770	1.00	0.00	RX0	C
ATOM	169	CB	LEU	176	20.635	-8.241	25.630	1.00	0.00	RX0	C
ATOM	170	CG	LEU	176	20.011	-7.922	24.267	1.00	0.00	RX0	C
ATOM	171	CD1	LEU	176	21.072	-7.898	23.171	1.00	0.00	RX0	C
ATOM	172	CD2	LEU	176	18.861	-8.863	23.900	1.00	0.00	RX0	C
ATOM	173	C	LEU	176	20.277	-8.690	28.089	1.00	0.00	RX0	C
ATOM	174	O	LEU	176	20.563	-7.852	28.952	1.00	0.00	RX0	O
ATOM	175	N	TYR	177	20.459	-9.989	28.237	1.00	0.00	RX0	N
ATOM	176	H	TYR	177	20.217	-10.579	27.470	0.00	0.00	RX0	H
ATOM	177	CA	TYR	177	21.114	-10.573	29.420	1.00	0.00	RX0	C
ATOM	178	CB	TYR	177	20.481	-11.933	29.700	1.00	0.00	RX0	C
ATOM	179	CG	TYR	177	19.545	-11.948	30.882	1.00	0.00	RX0	C
ATOM	180	CD1	TYR	177	18.442	-11.071	30.933	1.00	0.00	RX0	C
ATOM	181	CE1	TYR	177	17.533	-11.194	31.997	1.00	0.00	RX0	C

ATOM	182	CD2	TYR	177	19.794	-12.896	31.894	1.00	0.00	RX0	C
ATOM	183	CE2	TYR	177	18.888	-13.015	32.958	1.00	0.00	RX0	C
ATOM	184	CZ	TYR	177	17.756	-12.179	32.980	1.00	0.00	RX0	C
ATOM	185	OH	TYR	177	16.830	-12.339	33.992	1.00	0.00	RX0	O
ATOM	186	HH	TYR	177	17.277	-12.561	34.800	0.00	0.00	RX0	H
ATOM	187	C	TYR	177	22.589	-10.858	29.163	1.00	0.00	RX0	C
ATOM	188	O	TYR	177	22.985	-11.163	28.046	1.00	0.00	RX0	O
ATOM	189	N	SER	178	23.381	-10.750	30.220	1.00	0.00	RX0	N
ATOM	190	H	SER	178	23.016	-10.526	31.126	0.00	0.00	RX0	H
ATOM	191	CA	SER	178	24.788	-11.188	30.183	1.00	0.00	RX0	C
ATOM	192	CB	SER	178	25.485	-10.623	31.404	1.00	0.00	RX0	C
ATOM	193	OG	SER	178	25.192	-9.230	31.395	1.00	0.00	RX0	O
ATOM	194	HG	SER	178	25.018	-9.025	30.483	0.00	0.00	RX0	H
ATOM	195	C	SER	178	24.834	-12.718	30.070	1.00	0.00	RX0	C
ATOM	196	O	SER	178	23.999	-13.413	30.674	1.00	0.00	RX0	O
ATOM	197	N	GLU	179	25.827	-13.218	29.362	1.00	0.00	RX0	N
ATOM	198	H	GLU	179	26.358	-12.614	28.763	0.00	0.00	RX0	H
ATOM	199	CA	GLU	179	26.033	-14.670	29.175	1.00	0.00	RX0	C
ATOM	200	CB	GLU	179	26.295	-15.027	27.709	1.00	0.00	RX0	C
ATOM	201	CG	GLU	179	25.139	-14.746	26.747	1.00	0.00	RX0	C
ATOM	202	CD	GLU	179	25.285	-13.391	26.085	1.00	0.00	RX0	C
ATOM	203	OE1	GLU	179	25.370	-13.342	24.865	1.00	0.00	RX0	O
ATOM	204	OE2	GLU	179	25.227	-12.372	26.762	1.00	0.00	RX0	O
ATOM	205	C	GLU	179	27.192	-15.208	30.012	1.00	0.00	RX0	C
ATOM	206	O	GLU	179	28.361	-15.227	29.589	1.00	0.00	RX0	O
ATOM	207	N	TYR	180	26.873	-15.475	31.254	1.00	0.00	RX0	N
ATOM	208	H	TYR	180	25.923	-15.385	31.561	0.00	0.00	RX0	H
ATOM	209	CA	TYR	180	27.735	-16.233	32.177	1.00	0.00	RX0	C
ATOM	210	CB	TYR	180	28.496	-15.323	33.154	1.00	0.00	RX0	C
ATOM	211	CG	TYR	180	27.556	-14.674	34.142	1.00	0.00	RX0	C
ATOM	212	CD1	TYR	180	26.938	-13.468	33.832	1.00	0.00	RX0	C
ATOM	213	CE1	TYR	180	26.030	-12.908	34.721	1.00	0.00	RX0	C
ATOM	214	CD2	TYR	180	27.303	-15.290	35.363	1.00	0.00	RX0	C
ATOM	215	CE2	TYR	180	26.383	-14.739	36.244	1.00	0.00	RX0	C
ATOM	216	CZ	TYR	180	25.730	-13.559	35.912	1.00	0.00	RX0	C
ATOM	217	OH	TYR	180	24.774	-13.044	36.760	1.00	0.00	RX0	O
ATOM	218	HH	TYR	180	24.680	-12.111	36.591	0.00	0.00	RX0	H
ATOM	219	C	TYR	180	26.838	-17.226	32.909	1.00	0.00	RX0	C
ATOM	220	O	TYR	180	25.642	-16.953	33.094	1.00	0.00	RX0	O
ATOM	221	N	ASP	181	27.404	-18.345	33.318	1.00	0.00	RX0	N
ATOM	222	H	ASP	181	28.387	-18.477	33.232	0.00	0.00	RX0	H
ATOM	223	CA	ASP	181	26.630	-19.347	34.059	1.00	0.00	RX0	C
ATOM	224	CB	ASP	181	27.231	-20.738	34.030	1.00	0.00	RX0	C
ATOM	225	CG	ASP	181	26.368	-21.569	34.946	1.00	0.00	RX0	C
ATOM	226	OD1	ASP	181	25.173	-21.675	34.696	1.00	0.00	RX0	O
ATOM	227	OD2	ASP	181	26.870	-22.067	35.942	1.00	0.00	RX0	O
ATOM	228	C	ASP	181	26.420	-18.851	35.504	1.00	0.00	RX0	C
ATOM	229	O	ASP	181	27.391	-18.832	36.273	1.00	0.00	RX0	O
ATOM	230	N	PRO	182	25.185	-18.489	35.856	1.00	0.00	RX0	N
ATOM	231	CD	PRO	182	24.010	-18.603	34.995	1.00	0.00	RX0	C
ATOM	232	CA	PRO	182	24.825	-17.989	37.201	1.00	0.00	RX0	C
ATOM	233	CB	PRO	182	23.394	-17.485	36.997	1.00	0.00	RX0	C
ATOM	234	CG	PRO	182	22.821	-18.404	35.924	1.00	0.00	RX0	C
ATOM	235	C	PRO	182	24.941	-19.052	38.308	1.00	0.00	RX0	C
ATOM	236	O	PRO	182	24.654	-18.763	39.474	1.00	0.00	RX0	O
ATOM	237	N	THR	183	25.345	-20.259	37.948	1.00	0.00	RX0	N
ATOM	238	H	THR	183	25.554	-20.522	37.004	0.00	0.00	RX0	H
ATOM	239	CA	THR	183	25.568	-21.363	38.913	1.00	0.00	RX0	C
ATOM	240	CB	THR	183	24.917	-22.544	38.229	1.00	0.00	RX0	C
ATOM	241	OG1	THR	183	23.986	-22.018	37.267	1.00	0.00	RX0	O
ATOM	242	HG1	THR	183	24.470	-22.003	36.436	0.00	0.00	RX0	H

ATOM	243	CG2	THR	183	24.240	-23.501	39.210	1.00	0.00	RX0	C
ATOM	244	C	THR	183	27.063	-21.532	39.218	1.00	0.00	RX0	C
ATOM	245	O	THR	183	27.455	-22.345	40.058	1.00	0.00	RX0	O
ATOM	246	N	ARG	184	27.887	-20.699	38.573	1.00	0.00	RX0	N
ATOM	247	H	ARG	184	27.515	-20.002	37.958	0.00	0.00	RX0	H
ATOM	248	CA	ARG	184	29.343	-20.701	38.681	1.00	0.00	RX0	C
ATOM	249	CB	ARG	184	29.892	-20.616	37.273	1.00	0.00	RX0	C
ATOM	250	CG	ARG	184	30.161	-21.869	36.447	1.00	0.00	RX0	C
ATOM	251	CD	ARG	184	30.592	-21.392	35.056	1.00	0.00	RX0	C
ATOM	252	NE	ARG	184	31.205	-20.071	35.177	1.00	0.00	RX0	N
ATOM	253	HE	ARG	184	30.583	-19.289	35.287	0.00	0.00	RX0	H
ATOM	254	CZ	ARG	184	32.524	-20.037	35.487	1.00	0.00	RX0	C
ATOM	255	NH1	ARG	184	33.292	-21.114	35.234	1.00	0.00	RX0	N
ATOM	256	HH11	ARG	184	34.252	-21.151	35.564	0.00	0.00	RX0	H
ATOM	257	HH12	ARG	184	32.977	-21.918	34.734	0.00	0.00	RX0	H
ATOM	258	NH2	ARG	184	33.032	-18.950	36.085	1.00	0.00	RX0	N
ATOM	259	HH21	ARG	184	33.989	-19.014	36.406	0.00	0.00	RX0	H
ATOM	260	HH22	ARG	184	32.555	-18.099	36.306	0.00	0.00	RX0	H
ATOM	261	C	ARG	184	29.836	-19.410	39.407	1.00	0.00	RX0	C
ATOM	262	O	ARG	184	29.116	-18.390	39.334	1.00	0.00	RX0	O
ATOM	263	N	PRO	185	30.940	-19.460	40.113	1.00	0.00	RX0	N
ATOM	264	CD	PRO	185	31.744	-20.665	40.286	1.00	0.00	RX0	C
ATOM	265	CA	PRO	185	31.574	-18.285	40.781	1.00	0.00	RX0	C
ATOM	266	CB	PRO	185	32.859	-18.871	41.380	1.00	0.00	RX0	C
ATOM	267	CG	PRO	185	33.142	-20.137	40.576	1.00	0.00	RX0	C
ATOM	268	C	PRO	185	31.820	-17.125	39.813	1.00	0.00	RX0	C
ATOM	269	O	PRO	185	31.836	-17.275	38.592	1.00	0.00	RX0	O
ATOM	270	N	PHE	186	32.164	-15.998	40.422	1.00	0.00	RX0	N
ATOM	271	H	PHE	186	32.239	-15.992	41.418	0.00	0.00	RX0	H
ATOM	272	CA	PHE	186	32.333	-14.726	39.697	1.00	0.00	RX0	C
ATOM	273	CB	PHE	186	31.516	-13.614	40.357	1.00	0.00	RX0	C
ATOM	274	CG	PHE	186	31.415	-12.441	39.410	1.00	0.00	RX0	C
ATOM	275	CD1	PHE	186	30.863	-12.620	38.146	1.00	0.00	RX0	C
ATOM	276	CD2	PHE	186	31.873	-11.187	39.796	1.00	0.00	RX0	C
ATOM	277	CE1	PHE	186	30.773	-11.546	37.268	1.00	0.00	RX0	C
ATOM	278	CE2	PHE	186	31.782	-10.113	38.918	1.00	0.00	RX0	C
ATOM	279	CZ	PHE	186	31.234	-10.293	37.653	1.00	0.00	RX0	C
ATOM	280	C	PHE	186	33.791	-14.305	39.507	1.00	0.00	RX0	C
ATOM	281	O	PHE	186	34.127	-13.678	38.496	1.00	0.00	RX0	O
ATOM	282	N	SER	187	34.655	-14.802	40.380	1.00	0.00	RX0	N
ATOM	283	H	SER	187	34.310	-15.347	41.139	0.00	0.00	RX0	H
ATOM	284	CA	SER	187	36.113	-14.542	40.380	1.00	0.00	RX0	C
ATOM	285	CB	SER	187	36.612	-15.272	41.614	1.00	0.00	RX0	C
ATOM	286	OG	SER	187	35.491	-15.373	42.511	1.00	0.00	RX0	O
ATOM	287	HG	SER	187	35.866	-15.444	43.382	0.00	0.00	RX0	H
ATOM	288	C	SER	187	36.764	-14.980	39.057	1.00	0.00	RX0	C
ATOM	289	O	SER	187	37.834	-14.531	38.683	1.00	0.00	RX0	O
ATOM	290	N	GLU	188	36.054	-15.878	38.369	1.00	0.00	RX0	N
ATOM	291	H	GLU	188	35.131	-16.111	38.660	0.00	0.00	RX0	H
ATOM	292	CA	GLU	188	36.561	-16.586	37.191	1.00	0.00	RX0	C
ATOM	293	CB	GLU	188	36.203	-18.042	37.395	1.00	0.00	RX0	C
ATOM	294	CG	GLU	188	36.848	-19.063	36.472	1.00	0.00	RX0	C
ATOM	295	CD	GLU	188	35.906	-20.230	36.559	1.00	0.00	RX0	C
ATOM	296	OE1	GLU	188	34.990	-20.160	37.373	1.00	0.00	RX0	O
ATOM	297	OE2	GLU	188	36.014	-21.172	35.786	1.00	0.00	RX0	O
ATOM	298	C	GLU	188	36.028	-16.018	35.856	1.00	0.00	RX0	C
ATOM	299	O	GLU	188	36.494	-16.416	34.788	1.00	0.00	RX0	O
ATOM	300	N	ALA	189	35.058	-15.107	35.914	1.00	0.00	RX0	N
ATOM	301	H	ALA	189	34.842	-14.673	36.792	0.00	0.00	RX0	H
ATOM	302	CA	ALA	189	34.543	-14.432	34.708	1.00	0.00	RX0	C
ATOM	303	CB	ALA	189	33.067	-14.083	34.892	1.00	0.00	RX0	C

ATOM	304	C	ALA	189	35.336	-13.151	34.407	1.00	0.00	RX0	C
ATOM	305	O	ALA	189	35.533	-12.292	35.270	1.00	0.00	RX0	O
ATOM	306	N	SER	190	35.819	-13.065	33.173	1.00	0.00	RX0	N
ATOM	307	H	SER	190	35.615	-13.817	32.549	0.00	0.00	RX0	H
ATOM	308	CA	SER	190	36.430	-11.825	32.646	1.00	0.00	RX0	C
ATOM	309	CB	SER	190	37.101	-12.195	31.298	1.00	0.00	RX0	C
ATOM	310	OG	SER	190	38.256	-11.386	30.967	1.00	0.00	RX0	O
ATOM	311	HG	SER	190	38.783	-11.420	31.767	0.00	0.00	RX0	H
ATOM	312	C	SER	190	35.341	-10.761	32.513	1.00	0.00	RX0	C
ATOM	313	O	SER	190	34.465	-10.869	31.639	1.00	0.00	RX0	O
ATOM	314	N	MET	191	35.401	-9.751	33.361	1.00	0.00	RX0	N
ATOM	315	H	MET	191	36.037	-9.820	34.131	0.00	0.00	RX0	H
ATOM	316	CA	MET	191	34.414	-8.652	33.337	1.00	0.00	RX0	C
ATOM	317	CB	MET	191	34.642	-7.665	34.479	1.00	0.00	RX0	C
ATOM	318	CG	MET	191	33.546	-6.599	34.499	1.00	0.00	RX0	C
ATOM	319	SD	MET	191	33.640	-5.544	35.948	1.00	0.00	RX0	S
ATOM	320	CE	MET	191	33.290	-6.808	37.183	1.00	0.00	RX0	C
ATOM	321	C	MET	191	34.384	-7.939	31.976	1.00	0.00	RX0	C
ATOM	322	O	MET	191	33.329	-7.841	31.363	1.00	0.00	RX0	O
ATOM	323	N	MET	192	35.577	-7.654	31.438	1.00	0.00	RX0	N
ATOM	324	H	MET	192	36.383	-7.740	32.020	0.00	0.00	RX0	H
ATOM	325	CA	MET	192	35.697	-7.082	30.089	1.00	0.00	RX0	C
ATOM	326	CB	MET	192	37.136	-6.648	29.804	1.00	0.00	RX0	C
ATOM	327	CG	MET	192	37.281	-5.928	28.460	1.00	0.00	RX0	C
ATOM	328	SD	MET	192	36.170	-4.520	28.285	1.00	0.00	RX0	S
ATOM	329	CE	MET	192	36.763	-3.523	29.662	1.00	0.00	RX0	C
ATOM	330	C	MET	192	35.151	-8.021	28.999	1.00	0.00	RX0	C
ATOM	331	O	MET	192	34.484	-7.587	28.093	1.00	0.00	RX0	O
ATOM	332	N	GLY	193	35.358	-9.342	29.220	1.00	0.00	RX0	N
ATOM	333	H	GLY	193	35.698	-9.610	30.117	0.00	0.00	RX0	H
ATOM	334	CA	GLY	193	34.804	-10.378	28.330	1.00	0.00	RX0	C
ATOM	335	C	GLY	193	33.267	-10.338	28.334	1.00	0.00	RX0	C
ATOM	336	O	GLY	193	32.637	-10.184	27.296	1.00	0.00	RX0	O
ATOM	337	N	LEU	194	32.696	-10.293	29.537	1.00	0.00	RX0	N
ATOM	338	H	LEU	194	33.262	-10.302	30.358	0.00	0.00	RX0	H
ATOM	339	CA	LEU	194	31.235	-10.169	29.722	1.00	0.00	RX0	C
ATOM	340	CB	LEU	194	30.848	-10.222	31.199	1.00	0.00	RX0	C
ATOM	341	CG	LEU	194	31.148	-11.558	31.871	1.00	0.00	RX0	C
ATOM	342	CD1	LEU	194	30.709	-11.542	33.335	1.00	0.00	RX0	C
ATOM	343	CD2	LEU	194	30.542	-12.731	31.100	1.00	0.00	RX0	C
ATOM	344	C	LEU	194	30.647	-8.891	29.116	1.00	0.00	RX0	C
ATOM	345	O	LEU	194	29.706	-8.959	28.317	1.00	0.00	RX0	O
ATOM	346	N	LEU	195	31.327	-7.782	29.364	1.00	0.00	RX0	N
ATOM	347	H	LEU	195	32.131	-7.841	29.951	0.00	0.00	RX0	H
ATOM	348	CA	LEU	195	30.920	-6.462	28.846	1.00	0.00	RX0	C
ATOM	349	CB	LEU	195	31.732	-5.338	29.492	1.00	0.00	RX0	C
ATOM	350	CG	LEU	195	31.537	-5.239	31.005	1.00	0.00	RX0	C
ATOM	351	CD1	LEU	195	32.374	-4.111	31.609	1.00	0.00	RX0	C
ATOM	352	CD2	LEU	195	30.062	-5.128	31.388	1.00	0.00	RX0	C
ATOM	353	C	LEU	195	31.020	-6.357	27.321	1.00	0.00	RX0	C
ATOM	354	O	LEU	195	30.051	-5.942	26.671	1.00	0.00	RX0	O
ATOM	355	N	THR	196	32.075	-6.931	26.767	1.00	0.00	RX0	N
ATOM	356	H	THR	196	32.805	-7.314	27.331	0.00	0.00	RX0	H
ATOM	357	CA	THR	196	32.335	-6.901	25.309	1.00	0.00	RX0	C
ATOM	358	CB	THR	196	33.782	-7.304	25.031	1.00	0.00	RX0	C
ATOM	359	OG1	THR	196	34.666	-6.397	25.703	1.00	0.00	RX0	O
ATOM	360	HG1	THR	196	34.398	-5.525	25.440	0.00	0.00	RX0	H
ATOM	361	CG2	THR	196	34.089	-7.346	23.533	1.00	0.00	RX0	C
ATOM	362	C	THR	196	31.317	-7.765	24.552	1.00	0.00	RX0	C
ATOM	363	O	THR	196	30.772	-7.327	23.532	1.00	0.00	RX0	O
ATOM	364	N	ASN	197	31.003	-8.928	25.107	1.00	0.00	RX0	N

ATOM	365	H	ASN	197	31.411	-9.167	25.990	0.00	0.00	RX0	H
ATOM	366	CA	ASN	197	30.010	-9.840	24.504	1.00	0.00	RX0	C
ATOM	367	CB	ASN	197	29.968	-11.202	25.197	1.00	0.00	RX0	C
ATOM	368	CG	ASN	197	30.966	-12.143	24.559	1.00	0.00	RX0	C
ATOM	369	OD1	ASN	197	30.710	-12.803	23.558	1.00	0.00	RX0	O
ATOM	370	ND2	ASN	197	32.140	-12.182	25.211	1.00	0.00	RX0	N
ATOM	371	HD21	ASN	197	32.275	-11.592	26.010	0.00	0.00	RX0	H
ATOM	372	HD22	ASN	197	32.872	-12.788	24.906	0.00	0.00	RX0	H
ATOM	373	C	ASN	197	28.594	-9.255	24.528	1.00	0.00	RX0	C
ATOM	374	O	ASN	197	27.900	-9.272	23.514	1.00	0.00	RX0	O
ATOM	375	N	LEU	198	28.277	-8.575	25.633	1.00	0.00	RX0	N
ATOM	376	H	LEU	198	28.899	-8.589	26.418	0.00	0.00	RX0	H
ATOM	377	CA	LEU	198	27.002	-7.850	25.760	1.00	0.00	RX0	C
ATOM	378	CB	LEU	198	26.852	-7.334	27.187	1.00	0.00	RX0	C
ATOM	379	CG	LEU	198	25.449	-6.823	27.502	1.00	0.00	RX0	C
ATOM	380	CD1	LEU	198	24.375	-7.884	27.253	1.00	0.00	RX0	C
ATOM	381	CD2	LEU	198	25.388	-6.279	28.925	1.00	0.00	RX0	C
ATOM	382	C	LEU	198	26.885	-6.719	24.724	1.00	0.00	RX0	C
ATOM	383	O	LEU	198	25.930	-6.676	23.947	1.00	0.00	RX0	O
ATOM	384	N	ALA	199	27.942	-5.914	24.641	1.00	0.00	RX0	N
ATOM	385	H	ALA	199	28.696	-6.063	25.281	0.00	0.00	RX0	H
ATOM	386	CA	ALA	199	28.029	-4.784	23.694	1.00	0.00	RX0	C
ATOM	387	CB	ALA	199	29.343	-4.025	23.882	1.00	0.00	RX0	C
ATOM	388	C	ALA	199	27.921	-5.230	22.227	1.00	0.00	RX0	C
ATOM	389	O	ALA	199	27.138	-4.660	21.467	1.00	0.00	RX0	O
ATOM	390	N	ASP	200	28.555	-6.360	21.908	1.00	0.00	RX0	N
ATOM	391	H	ASP	200	29.131	-6.815	22.589	0.00	0.00	RX0	H
ATOM	392	CA	ASP	200	28.494	-6.940	20.550	1.00	0.00	RX0	C
ATOM	393	CB	ASP	200	29.532	-8.049	20.367	1.00	0.00	RX0	C
ATOM	394	CG	ASP	200	30.213	-7.910	19.014	1.00	0.00	RX0	C
ATOM	395	OD1	ASP	200	30.566	-6.798	18.624	1.00	0.00	RX0	O
ATOM	396	OD2	ASP	200	30.461	-8.915	18.349	1.00	0.00	RX0	O
ATOM	397	C	ASP	200	27.084	-7.410	20.171	1.00	0.00	RX0	C
ATOM	398	O	ASP	200	26.604	-7.102	19.080	1.00	0.00	RX0	O
ATOM	399	N	ARG	201	26.390	-7.998	21.143	1.00	0.00	RX0	N
ATOM	400	H	ARG	201	26.838	-8.159	22.026	0.00	0.00	RX0	H
ATOM	401	CA	ARG	201	24.992	-8.434	20.957	1.00	0.00	RX0	C
ATOM	402	CB	ARG	201	24.532	-9.424	22.021	1.00	0.00	RX0	C
ATOM	403	CG	ARG	201	24.392	-10.810	21.394	1.00	0.00	RX0	C
ATOM	404	CD	ARG	201	23.269	-11.634	22.021	1.00	0.00	RX0	C
ATOM	405	NE	ARG	201	23.465	-11.808	23.455	1.00	0.00	RX0	N
ATOM	406	HE	ARG	201	24.406	-11.883	23.824	0.00	0.00	RX0	H
ATOM	407	CZ	ARG	201	22.397	-12.073	24.251	1.00	0.00	RX0	C
ATOM	408	NH1	ARG	201	21.170	-12.128	23.697	1.00	0.00	RX0	N
ATOM	409	HH11	ARG	201	20.315	-12.422	24.152	0.00	0.00	RX0	H
ATOM	410	HH12	ARG	201	21.005	-11.893	22.738	0.00	0.00	RX0	H
ATOM	411	NH2	ARG	201	22.598	-12.274	25.564	1.00	0.00	RX0	N
ATOM	412	HH21	ARG	201	21.880	-12.458	26.233	0.00	0.00	RX0	H
ATOM	413	HH22	ARG	201	23.544	-12.243	25.944	0.00	0.00	RX0	H
ATOM	414	C	ARG	201	23.991	-7.279	20.827	1.00	0.00	RX0	C
ATOM	415	O	ARG	201	23.123	-7.308	19.955	1.00	0.00	RX0	O
ATOM	416	N	GLU	202	24.240	-6.201	21.568	1.00	0.00	RX0	N
ATOM	417	H	GLU	202	24.995	-6.229	22.229	0.00	0.00	RX0	H
ATOM	418	CA	GLU	202	23.401	-4.988	21.493	1.00	0.00	RX0	C
ATOM	419	CB	GLU	202	23.562	-4.061	22.721	1.00	0.00	RX0	C
ATOM	420	CG	GLU	202	22.897	-4.617	24.000	1.00	0.00	RX0	C
ATOM	421	CD	GLU	202	22.833	-3.609	25.153	1.00	0.00	RX0	C
ATOM	422	OE1	GLU	202	21.786	-2.999	25.377	1.00	0.00	RX0	O
ATOM	423	OE2	GLU	202	23.802	-3.470	25.893	1.00	0.00	RX0	O
ATOM	424	C	GLU	202	23.526	-4.262	20.149	1.00	0.00	RX0	C
ATOM	425	O	GLU	202	22.539	-3.754	19.625	1.00	0.00	RX0	O

ATOM	426	N	LEU	203	24.712	-4.358	19.546	1.00	0.00	RX0	N
ATOM	427	H	LEU	203	25.461	-4.814	20.036	0.00	0.00	RX0	H
ATOM	428	CA	LEU	203	25.004	-3.680	18.270	1.00	0.00	RX0	C
ATOM	429	CB	LEU	203	26.480	-3.871	17.917	1.00	0.00	RX0	C
ATOM	430	CG	LEU	203	26.922	-3.125	16.656	1.00	0.00	RX0	C
ATOM	431	CD1	LEU	203	26.718	-1.613	16.777	1.00	0.00	RX0	C
ATOM	432	CD2	LEU	203	28.361	-3.476	16.273	1.00	0.00	RX0	C
ATOM	433	C	LEU	203	24.099	-4.160	17.127	1.00	0.00	RX0	C
ATOM	434	O	LEU	203	23.593	-3.349	16.346	1.00	0.00	RX0	O
ATOM	435	N	VAL	204	23.782	-5.447	17.151	1.00	0.00	RX0	N
ATOM	436	H	VAL	204	24.169	-6.003	17.890	0.00	0.00	RX0	H
ATOM	437	CA	VAL	204	22.925	-6.083	16.127	1.00	0.00	RX0	C
ATOM	438	CB	VAL	204	22.899	-7.597	16.335	1.00	0.00	RX0	C
ATOM	439	CG1	VAL	204	22.008	-8.281	15.297	1.00	0.00	RX0	C
ATOM	440	CG2	VAL	204	24.320	-8.163	16.352	1.00	0.00	RX0	C
ATOM	441	C	VAL	204	21.502	-5.497	16.213	1.00	0.00	RX0	C
ATOM	442	O	VAL	204	20.938	-5.041	15.221	1.00	0.00	RX0	O
ATOM	443	N	HIS	205	21.015	-5.393	17.448	1.00	0.00	RX0	N
ATOM	444	H	HIS	205	21.599	-5.683	18.209	0.00	0.00	RX0	H
ATOM	445	CA	HIS	205	19.703	-4.786	17.746	1.00	0.00	RX0	C
ATOM	446	CB	HIS	205	19.264	-5.117	19.176	1.00	0.00	RX0	C
ATOM	447	CG	HIS	205	19.016	-6.604	19.296	1.00	0.00	RX0	C
ATOM	448	ND1	HIS	205	17.816	-7.181	19.092	1.00	0.00	RX0	N
ATOM	449	HD1	HIS	205	16.977	-6.732	18.866	0.00	0.00	RX0	H
ATOM	450	CD2	HIS	205	19.943	-7.602	19.610	1.00	0.00	RX0	C
ATOM	451	NE2	HIS	205	19.284	-8.785	19.589	1.00	0.00	RX0	N
ATOM	452	CE1	HIS	205	17.977	-8.531	19.272	1.00	0.00	RX0	C
ATOM	453	C	HIS	205	19.668	-3.277	17.476	1.00	0.00	RX0	C
ATOM	454	O	HIS	205	18.642	-2.756	17.030	1.00	0.00	RX0	O
ATOM	455	N	MET	206	20.820	-2.627	17.609	1.00	0.00	RX0	N
ATOM	456	H	MET	206	21.610	-3.126	17.973	0.00	0.00	RX0	H
ATOM	457	CA	MET	206	20.969	-1.185	17.340	1.00	0.00	RX0	C
ATOM	458	CB	MET	206	22.357	-0.694	17.746	1.00	0.00	RX0	C
ATOM	459	CG	MET	206	22.542	0.805	17.510	1.00	0.00	RX0	C
ATOM	460	SD	MET	206	24.243	1.326	17.771	1.00	0.00	RX0	S
ATOM	461	CE	MET	206	24.497	0.519	19.356	1.00	0.00	RX0	C
ATOM	462	C	MET	206	20.721	-0.870	15.856	1.00	0.00	RX0	C
ATOM	463	O	MET	206	20.035	0.103	15.544	1.00	0.00	RX0	O
ATOM	464	N	ILE	207	21.183	-1.758	14.977	1.00	0.00	RX0	N
ATOM	465	H	ILE	207	21.730	-2.518	15.339	0.00	0.00	RX0	H
ATOM	466	CA	ILE	207	20.975	-1.628	13.516	1.00	0.00	RX0	C
ATOM	467	CB	ILE	207	21.671	-2.789	12.800	1.00	0.00	RX0	C
ATOM	468	CG2	ILE	207	21.419	-2.767	11.294	1.00	0.00	RX0	C
ATOM	469	CG1	ILE	207	23.164	-2.811	13.123	1.00	0.00	RX0	C
ATOM	470	CD1	ILE	207	23.908	-1.601	12.560	1.00	0.00	RX0	C
ATOM	471	C	ILE	207	19.470	-1.621	13.197	1.00	0.00	RX0	C
ATOM	472	O	ILE	207	18.988	-0.755	12.467	1.00	0.00	RX0	O
ATOM	473	N	ASN	208	18.761	-2.558	13.816	1.00	0.00	RX0	N
ATOM	474	H	ASN	208	19.235	-3.132	14.486	0.00	0.00	RX0	H
ATOM	475	CA	ASN	208	17.313	-2.734	13.586	1.00	0.00	RX0	C
ATOM	476	CB	ASN	208	16.793	-4.061	14.137	1.00	0.00	RX0	C
ATOM	477	CG	ASN	208	16.799	-5.084	13.016	1.00	0.00	RX0	C
ATOM	478	OD1	ASN	208	17.363	-4.874	11.938	1.00	0.00	RX0	O
ATOM	479	ND2	ASN	208	16.141	-6.216	13.327	1.00	0.00	RX0	N
ATOM	480	HD21	ASN	208	15.696	-6.323	14.218	0.00	0.00	RX0	H
ATOM	481	HD22	ASN	208	16.063	-6.995	12.704	0.00	0.00	RX0	H
ATOM	482	C	ASN	208	16.516	-1.532	14.103	1.00	0.00	RX0	C
ATOM	483	O	ASN	208	15.637	-1.017	13.417	1.00	0.00	RX0	O
ATOM	484	N	TRP	209	16.982	-1.008	15.238	1.00	0.00	RX0	N
ATOM	485	H	TRP	209	17.723	-1.476	15.724	0.00	0.00	RX0	H
ATOM	486	CA	TRP	209	16.425	0.201	15.864	1.00	0.00	RX0	C

ATOM	487	CB	TRP	209	17.092	0.414	17.230	1.00	0.00	RX0	C
ATOM	488	CG	TRP	209	16.719	1.753	17.830	1.00	0.00	RX0	C
ATOM	489	CD2	TRP	209	17.458	2.993	17.783	1.00	0.00	RX0	C
ATOM	490	CE2	TRP	209	16.709	3.966	18.486	1.00	0.00	RX0	C
ATOM	491	CE3	TRP	209	18.695	3.331	17.247	1.00	0.00	RX0	C
ATOM	492	CD1	TRP	209	15.553	2.061	18.541	1.00	0.00	RX0	C
ATOM	493	NE1	TRP	209	15.533	3.363	18.929	1.00	0.00	RX0	N
ATOM	494	HE1	TRP	209	14.802	3.771	19.453	0.00	0.00	RX0	H
ATOM	495	CZ2	TRP	209	17.194	5.267	18.575	1.00	0.00	RX0	C
ATOM	496	CZ3	TRP	209	19.172	4.632	17.356	1.00	0.00	RX0	C
ATOM	497	CH2	TRP	209	18.428	5.595	18.025	1.00	0.00	RX0	C
ATOM	498	C	TRP	209	16.619	1.438	14.972	1.00	0.00	RX0	C
ATOM	499	O	TRP	209	15.652	2.120	14.634	1.00	0.00	RX0	O
ATOM	500	N	ALA	210	17.853	1.624	14.503	1.00	0.00	RX0	N
ATOM	501	H	ALA	210	18.567	0.988	14.791	0.00	0.00	RX0	H
ATOM	502	CA	ALA	210	18.233	2.756	13.635	1.00	0.00	RX0	C
ATOM	503	CB	ALA	210	19.715	2.665	13.273	1.00	0.00	RX0	C
ATOM	504	C	ALA	210	17.400	2.800	12.347	1.00	0.00	RX0	C
ATOM	505	O	ALA	210	16.892	3.855	11.979	1.00	0.00	RX0	O
ATOM	506	N	LYS	211	17.095	1.613	11.820	1.00	0.00	RX0	N
ATOM	507	H	LYS	211	17.501	0.798	12.237	0.00	0.00	RX0	H
ATOM	508	CA	LYS	211	16.258	1.472	10.614	1.00	0.00	RX0	C
ATOM	509	CB	LYS	211	16.402	0.068	10.024	1.00	0.00	RX0	C
ATOM	510	CG	LYS	211	17.789	-0.100	9.392	1.00	0.00	RX0	C
ATOM	511	CD	LYS	211	18.059	-1.511	8.865	1.00	0.00	RX0	C
ATOM	512	CE	LYS	211	17.834	-2.521	9.984	1.00	0.00	RX0	C
ATOM	513	NZ	LYS	211	18.327	-3.863	9.642	1.00	0.00	RX0	N
ATOM	514	HZ1	LYS	211	18.120	-4.484	10.458	0.00	0.00	RX0	H
ATOM	515	HZ2	LYS	211	17.840	-4.227	8.802	0.00	0.00	RX0	H
ATOM	516	HZ3	LYS	211	19.353	-3.845	9.469	0.00	0.00	RX0	H
ATOM	517	C	LYS	211	14.795	1.898	10.823	1.00	0.00	RX0	C
ATOM	518	O	LYS	211	14.129	2.337	9.881	1.00	0.00	RX0	O
ATOM	519	N	ARG	212	14.357	1.870	12.071	1.00	0.00	RX0	N
ATOM	520	H	ARG	212	14.979	1.606	12.809	0.00	0.00	RX0	H
ATOM	521	CA	ARG	212	13.005	2.320	12.466	1.00	0.00	RX0	C
ATOM	522	CB	ARG	212	12.398	1.421	13.561	1.00	0.00	RX0	C
ATOM	523	CG	ARG	212	12.143	-0.046	13.180	1.00	0.00	RX0	C
ATOM	524	CD	ARG	212	11.636	-0.951	14.324	1.00	0.00	RX0	C
ATOM	525	NE	ARG	212	10.233	-0.722	14.707	1.00	0.00	RX0	N
ATOM	526	HE	ARG	212	9.728	0.029	14.278	0.00	0.00	RX0	H
ATOM	527	CZ	ARG	212	9.637	-1.495	15.658	1.00	0.00	RX0	C
ATOM	528	NH1	ARG	212	10.320	-2.452	16.287	1.00	0.00	RX0	N
ATOM	529	HH11	ARG	212	9.887	-3.090	16.944	0.00	0.00	RX0	H
ATOM	530	HH12	ARG	212	11.293	-2.602	16.132	0.00	0.00	RX0	H
ATOM	531	NH2	ARG	212	8.356	-1.311	15.976	1.00	0.00	RX0	N
ATOM	532	HH21	ARG	212	8.002	-1.746	16.808	0.00	0.00	RX0	H
ATOM	533	HH22	ARG	212	7.698	-0.759	15.452	0.00	0.00	RX0	H
ATOM	534	C	ARG	212	12.933	3.790	12.894	1.00	0.00	RX0	C
ATOM	535	O	ARG	212	11.827	4.344	12.989	1.00	0.00	RX0	O
ATOM	536	N	VAL	213	14.074	4.417	13.148	1.00	0.00	RX0	N
ATOM	537	H	VAL	213	14.933	3.927	12.994	0.00	0.00	RX0	H
ATOM	538	CA	VAL	213	14.155	5.874	13.374	1.00	0.00	RX0	C
ATOM	539	CB	VAL	213	15.581	6.281	13.749	1.00	0.00	RX0	C
ATOM	540	CG1	VAL	213	15.751	7.800	13.837	1.00	0.00	RX0	C
ATOM	541	CG2	VAL	213	15.980	5.585	15.049	1.00	0.00	RX0	C
ATOM	542	C	VAL	213	13.672	6.590	12.095	1.00	0.00	RX0	C
ATOM	543	O	VAL	213	14.288	6.431	11.023	1.00	0.00	RX0	O
ATOM	544	N	PRO	214	12.622	7.395	12.213	1.00	0.00	RX0	N
ATOM	545	CD	PRO	214	11.896	7.608	13.459	1.00	0.00	RX0	C
ATOM	546	CA	PRO	214	12.035	8.145	11.084	1.00	0.00	RX0	C
ATOM	547	CB	PRO	214	10.927	8.954	11.762	1.00	0.00	RX0	C

ATOM	548	CG	PRO	214	10.552	8.162	13.010	1.00	0.00	RX0	C
ATOM	549	C	PRO	214	13.102	9.001	10.387	1.00	0.00	RX0	C
ATOM	550	O	PRO	214	13.853	9.727	11.025	1.00	0.00	RX0	O
ATOM	551	N	GLY	215	13.244	8.730	9.080	1.00	0.00	RX0	N
ATOM	552	H	GLY	215	12.749	7.981	8.637	0.00	0.00	RX0	H
ATOM	553	CA	GLY	215	14.194	9.473	8.227	1.00	0.00	RX0	C
ATOM	554	C	GLY	215	15.511	8.732	7.950	1.00	0.00	RX0	C
ATOM	555	O	GLY	215	16.085	8.889	6.862	1.00	0.00	RX0	O
ATOM	556	N	PHE	216	15.917	7.845	8.848	1.00	0.00	RX0	N
ATOM	557	H	PHE	216	15.329	7.652	9.637	0.00	0.00	RX0	H
ATOM	558	CA	PHE	216	17.224	7.160	8.764	1.00	0.00	RX0	C
ATOM	559	CB	PHE	216	17.452	6.293	9.997	1.00	0.00	RX0	C
ATOM	560	CG	PHE	216	18.891	5.843	10.056	1.00	0.00	RX0	C
ATOM	561	CD1	PHE	216	19.896	6.772	10.296	1.00	0.00	RX0	C
ATOM	562	CD2	PHE	216	19.211	4.501	9.885	1.00	0.00	RX0	C
ATOM	563	CE1	PHE	216	21.216	6.354	10.405	1.00	0.00	RX0	C
ATOM	564	CE2	PHE	216	20.531	4.084	9.996	1.00	0.00	RX0	C
ATOM	565	CZ	PHE	216	21.531	5.007	10.277	1.00	0.00	RX0	C
ATOM	566	C	PHE	216	17.435	6.347	7.474	1.00	0.00	RX0	C
ATOM	567	O	PHE	216	18.358	6.641	6.711	1.00	0.00	RX0	O
ATOM	568	N	VAL	217	16.482	5.483	7.151	1.00	0.00	RX0	N
ATOM	569	H	VAL	217	15.674	5.447	7.737	0.00	0.00	RX0	H
ATOM	570	CA	VAL	217	16.570	4.608	5.958	1.00	0.00	RX0	C
ATOM	571	CB	VAL	217	15.527	3.495	6.007	1.00	0.00	RX0	C
ATOM	572	CG1	VAL	217	15.894	2.483	7.083	1.00	0.00	RX0	C
ATOM	573	CG2	VAL	217	14.110	4.053	6.163	1.00	0.00	RX0	C
ATOM	574	C	VAL	217	16.469	5.342	4.608	1.00	0.00	RX0	C
ATOM	575	O	VAL	217	16.660	4.747	3.556	1.00	0.00	RX0	O
ATOM	576	N	ASP	218	16.058	6.613	4.671	1.00	0.00	RX0	N
ATOM	577	H	ASP	218	15.764	7.060	5.519	0.00	0.00	RX0	H
ATOM	578	CA	ASP	218	16.006	7.470	3.472	1.00	0.00	RX0	C
ATOM	579	CB	ASP	218	15.079	8.660	3.757	1.00	0.00	RX0	C
ATOM	580	CG	ASP	218	13.736	8.206	4.327	1.00	0.00	RX0	C
ATOM	581	OD1	ASP	218	12.743	8.269	3.605	1.00	0.00	RX0	O
ATOM	582	OD2	ASP	218	13.668	7.819	5.498	1.00	0.00	RX0	O
ATOM	583	C	ASP	218	17.401	7.924	3.023	1.00	0.00	RX0	C
ATOM	584	O	ASP	218	17.595	8.369	1.896	1.00	0.00	RX0	O
ATOM	585	N	LEU	219	18.344	7.857	3.967	1.00	0.00	RX0	N
ATOM	586	H	LEU	219	18.094	7.491	4.864	0.00	0.00	RX0	H
ATOM	587	CA	LEU	219	19.767	8.083	3.697	1.00	0.00	RX0	C
ATOM	588	CB	LEU	219	20.543	8.278	4.996	1.00	0.00	RX0	C
ATOM	589	CG	LEU	219	19.991	9.474	5.773	1.00	0.00	RX0	C
ATOM	590	CD1	LEU	219	20.567	9.547	7.185	1.00	0.00	RX0	C
ATOM	591	CD2	LEU	219	20.159	10.783	4.999	1.00	0.00	RX0	C
ATOM	592	C	LEU	219	20.350	6.970	2.832	1.00	0.00	RX0	C
ATOM	593	O	LEU	219	19.843	5.836	2.789	1.00	0.00	RX0	O
ATOM	594	N	THR	220	21.442	7.293	2.191	1.00	0.00	RX0	N
ATOM	595	H	THR	220	21.838	8.186	2.407	0.00	0.00	RX0	H
ATOM	596	CA	THR	220	22.263	6.312	1.453	1.00	0.00	RX0	C
ATOM	597	CB	THR	220	23.276	7.190	0.729	1.00	0.00	RX0	C
ATOM	598	OG1	THR	220	23.171	8.517	1.254	1.00	0.00	RX0	O
ATOM	599	HG1	THR	220	22.470	9.003	0.816	0.00	0.00	RX0	H
ATOM	600	CG2	THR	220	23.063	7.220	-0.787	1.00	0.00	RX0	C
ATOM	601	C	THR	220	22.829	5.303	2.454	1.00	0.00	RX0	C
ATOM	602	O	THR	220	23.112	5.646	3.611	1.00	0.00	RX0	O
ATOM	603	N	LEU	221	23.130	4.116	1.957	1.00	0.00	RX0	N
ATOM	604	H	LEU	221	22.852	3.905	1.022	0.00	0.00	RX0	H
ATOM	605	CA	LEU	221	23.750	3.065	2.781	1.00	0.00	RX0	C
ATOM	606	CB	LEU	221	23.959	1.859	1.873	1.00	0.00	RX0	C
ATOM	607	CG	LEU	221	24.867	0.785	2.457	1.00	0.00	RX0	C
ATOM	608	CD1	LEU	221	24.216	0.049	3.626	1.00	0.00	RX0	C

ATOM	609	CD2	LEU	221	25.361	-0.151	1.357	1.00	0.00	RX0	C
ATOM	610	C	LEU	221	25.092	3.509	3.394	1.00	0.00	RX0	C
ATOM	611	O	LEU	221	25.324	3.347	4.578	1.00	0.00	RX0	O
ATOM	612	N	HIS	222	25.854	4.270	2.593	1.00	0.00	RX0	N
ATOM	613	H	HIS	222	25.542	4.455	1.665	0.00	0.00	RX0	H
ATOM	614	CA	HIS	222	27.131	4.847	3.045	1.00	0.00	RX0	C
ATOM	615	CB	HIS	222	27.829	5.618	1.925	1.00	0.00	RX0	C
ATOM	616	CG	HIS	222	29.080	6.269	2.475	1.00	0.00	RX0	C
ATOM	617	ND1	HIS	222	30.044	5.606	3.145	1.00	0.00	RX0	N
ATOM	618	HD1	HIS	222	30.069	4.648	3.377	0.00	0.00	RX0	H
ATOM	619	CD2	HIS	222	29.447	7.616	2.381	1.00	0.00	RX0	C
ATOM	620	NE2	HIS	222	30.646	7.755	3.002	1.00	0.00	RX0	N
ATOM	621	CE1	HIS	222	31.010	6.520	3.473	1.00	0.00	RX0	C
ATOM	622	C	HIS	222	26.942	5.765	4.264	1.00	0.00	RX0	C
ATOM	623	O	HIS	222	27.673	5.642	5.246	1.00	0.00	RX0	O
ATOM	624	N	ASP	223	25.958	6.658	4.167	1.00	0.00	RX0	N
ATOM	625	H	ASP	223	25.495	6.854	3.302	0.00	0.00	RX0	H
ATOM	626	CA	ASP	223	25.694	7.636	5.242	1.00	0.00	RX0	C
ATOM	627	CB	ASP	223	24.857	8.795	4.684	1.00	0.00	RX0	C
ATOM	628	CG	ASP	223	25.667	9.547	3.627	1.00	0.00	RX0	C
ATOM	629	OD1	ASP	223	26.107	8.933	2.650	1.00	0.00	RX0	O
ATOM	630	OD2	ASP	223	25.871	10.751	3.784	1.00	0.00	RX0	O
ATOM	631	C	ASP	223	25.148	6.996	6.516	1.00	0.00	RX0	C
ATOM	632	O	ASP	223	25.558	7.375	7.616	1.00	0.00	RX0	O
ATOM	633	N	GLN	224	24.393	5.921	6.332	1.00	0.00	RX0	N
ATOM	634	H	GLN	224	24.151	5.690	5.387	0.00	0.00	RX0	H
ATOM	635	CA	GLN	224	23.868	5.118	7.452	1.00	0.00	RX0	C
ATOM	636	CB	GLN	224	22.844	4.095	6.960	1.00	0.00	RX0	C
ATOM	637	CG	GLN	224	21.592	4.769	6.393	1.00	0.00	RX0	C
ATOM	638	CD	GLN	224	20.546	3.719	6.084	1.00	0.00	RX0	C
ATOM	639	OE1	GLN	224	20.387	2.738	6.806	1.00	0.00	RX0	O
ATOM	640	NE2	GLN	224	19.840	3.973	4.969	1.00	0.00	RX0	N
ATOM	641	HE21	GLN	224	20.005	4.798	4.418	0.00	0.00	RX0	H
ATOM	642	HE22	GLN	224	19.109	3.389	4.621	0.00	0.00	RX0	H
ATOM	643	C	GLN	224	25.003	4.453	8.243	1.00	0.00	RX0	C
ATOM	644	O	GLN	224	25.073	4.591	9.468	1.00	0.00	RX0	O
ATOM	645	N	VAL	225	25.993	3.956	7.505	1.00	0.00	RX0	N
ATOM	646	H	VAL	225	25.897	3.982	6.507	0.00	0.00	RX0	H
ATOM	647	CA	VAL	225	27.191	3.319	8.093	1.00	0.00	RX0	C
ATOM	648	CB	VAL	225	28.045	2.587	7.059	1.00	0.00	RX0	C
ATOM	649	CG1	VAL	225	29.210	1.891	7.758	1.00	0.00	RX0	C
ATOM	650	CG2	VAL	225	27.224	1.570	6.273	1.00	0.00	RX0	C
ATOM	651	C	VAL	225	28.021	4.368	8.852	1.00	0.00	RX0	C
ATOM	652	O	VAL	225	28.415	4.141	9.995	1.00	0.00	RX0	O
ATOM	653	N	HIS	226	28.182	5.534	8.231	1.00	0.00	RX0	N
ATOM	654	H	HIS	226	27.808	5.644	7.307	0.00	0.00	RX0	H
ATOM	655	CA	HIS	226	28.959	6.641	8.815	1.00	0.00	RX0	C
ATOM	656	CB	HIS	226	29.130	7.826	7.867	1.00	0.00	RX0	C
ATOM	657	CG	HIS	226	30.075	8.821	8.510	1.00	0.00	RX0	C
ATOM	658	ND1	HIS	226	31.345	8.531	8.847	1.00	0.00	RX0	N
ATOM	659	HD1	HIS	226	31.794	7.663	8.750	0.00	0.00	RX0	H
ATOM	660	CD2	HIS	226	29.821	10.154	8.853	1.00	0.00	RX0	C
ATOM	661	NE2	HIS	226	30.952	10.669	9.398	1.00	0.00	RX0	N
ATOM	662	CE1	HIS	226	31.889	9.666	9.393	1.00	0.00	RX0	C
ATOM	663	C	HIS	226	28.363	7.118	10.150	1.00	0.00	RX0	C
ATOM	664	O	HIS	226	29.071	7.189	11.155	1.00	0.00	RX0	O
ATOM	665	N	LEU	227	27.047	7.317	10.166	1.00	0.00	RX0	N
ATOM	666	H	LEU	227	26.531	7.184	9.316	0.00	0.00	RX0	H
ATOM	667	CA	LEU	227	26.344	7.787	11.375	1.00	0.00	RX0	C
ATOM	668	CB	LEU	227	24.875	8.083	11.077	1.00	0.00	RX0	C
ATOM	669	CG	LEU	227	24.670	9.353	10.254	1.00	0.00	RX0	C

ATOM	670	CD1	LEU	227	23.200	9.552	9.892	1.00	0.00	RX0	C
ATOM	671	CD2	LEU	227	25.238	10.585	10.960	1.00	0.00	RX0	C
ATOM	672	C	LEU	227	26.435	6.799	12.540	1.00	0.00	RX0	C
ATOM	673	O	LEU	227	26.853	7.165	13.635	1.00	0.00	RX0	O
ATOM	674	N	LEU	228	26.270	5.522	12.200	1.00	0.00	RX0	N
ATOM	675	H	LEU	228	26.042	5.297	11.249	0.00	0.00	RX0	H
ATOM	676	CA	LEU	228	26.384	4.431	13.181	1.00	0.00	RX0	C
ATOM	677	CB	LEU	228	25.763	3.155	12.619	1.00	0.00	RX0	C
ATOM	678	CG	LEU	228	24.258	3.145	12.877	1.00	0.00	RX0	C
ATOM	679	CD1	LEU	228	23.516	2.136	12.007	1.00	0.00	RX0	C
ATOM	680	CD2	LEU	228	23.963	2.937	14.363	1.00	0.00	RX0	C
ATOM	681	C	LEU	228	27.805	4.188	13.685	1.00	0.00	RX0	C
ATOM	682	O	LEU	228	28.004	4.019	14.891	1.00	0.00	RX0	O
ATOM	683	N	GLU	229	28.784	4.376	12.809	1.00	0.00	RX0	N
ATOM	684	H	GLU	229	28.582	4.583	11.850	0.00	0.00	RX0	H
ATOM	685	CA	GLU	229	30.199	4.229	13.197	1.00	0.00	RX0	C
ATOM	686	CB	GLU	229	31.135	4.105	11.990	1.00	0.00	RX0	C
ATOM	687	CG	GLU	229	32.469	3.474	12.407	1.00	0.00	RX0	C
ATOM	688	CD	GLU	229	33.256	3.028	11.192	1.00	0.00	RX0	C
ATOM	689	OE1	GLU	229	33.035	3.575	10.115	1.00	0.00	RX0	O
ATOM	690	OE2	GLU	229	34.079	2.119	11.325	1.00	0.00	RX0	O
ATOM	691	C	GLU	229	30.618	5.338	14.175	1.00	0.00	RX0	C
ATOM	692	O	GLU	229	31.393	5.088	15.099	1.00	0.00	RX0	O
ATOM	693	N	CYS	230	30.060	6.523	13.970	1.00	0.00	RX0	N
ATOM	694	H	CYS	230	29.478	6.652	13.163	0.00	0.00	RX0	H
ATOM	695	CA	CYS	230	30.321	7.692	14.829	1.00	0.00	RX0	C
ATOM	696	CB	CYS	230	30.011	8.967	14.056	1.00	0.00	RX0	C
ATOM	697	SG	CYS	230	31.049	9.131	12.584	1.00	0.00	RX0	S
ATOM	698	C	CYS	230	29.592	7.653	16.182	1.00	0.00	RX0	C
ATOM	699	O	CYS	230	30.123	8.119	17.188	1.00	0.00	RX0	O
ATOM	700	N	ALA	231	28.434	7.000	16.215	1.00	0.00	RX0	N
ATOM	701	H	ALA	231	28.115	6.531	15.389	0.00	0.00	RX0	H
ATOM	702	CA	ALA	231	27.494	7.141	17.345	1.00	0.00	RX0	C
ATOM	703	CB	ALA	231	26.145	7.670	16.853	1.00	0.00	RX0	C
ATOM	704	C	ALA	231	27.249	5.885	18.186	1.00	0.00	RX0	C
ATOM	705	O	ALA	231	26.768	6.021	19.321	1.00	0.00	RX0	O
ATOM	706	N	TRP	232	27.687	4.719	17.731	1.00	0.00	RX0	N
ATOM	707	H	TRP	232	28.111	4.685	16.823	0.00	0.00	RX0	H
ATOM	708	CA	TRP	232	27.348	3.433	18.379	1.00	0.00	RX0	C
ATOM	709	CB	TRP	232	27.967	2.238	17.642	1.00	0.00	RX0	C
ATOM	710	CG	TRP	232	29.470	2.258	17.779	1.00	0.00	RX0	C
ATOM	711	CD2	TRP	232	30.296	1.489	18.678	1.00	0.00	RX0	C
ATOM	712	CE2	TRP	232	31.639	1.876	18.460	1.00	0.00	RX0	C
ATOM	713	CE3	TRP	232	30.003	0.526	19.635	1.00	0.00	RX0	C
ATOM	714	CD1	TRP	232	30.364	3.062	17.065	1.00	0.00	RX0	C
ATOM	715	NE1	TRP	232	31.643	2.844	17.460	1.00	0.00	RX0	N
ATOM	716	HE1	TRP	232	32.424	3.298	17.072	0.00	0.00	RX0	H
ATOM	717	CZ2	TRP	232	32.653	1.285	19.205	1.00	0.00	RX0	C
ATOM	718	CZ3	TRP	232	31.026	-0.056	20.373	1.00	0.00	RX0	C
ATOM	719	CH2	TRP	232	32.347	0.323	20.160	1.00	0.00	RX0	C
ATOM	720	C	TRP	232	27.676	3.373	19.884	1.00	0.00	RX0	C
ATOM	721	O	TRP	232	26.862	2.914	20.672	1.00	0.00	RX0	O
ATOM	722	N	LEU	233	28.801	3.989	20.277	1.00	0.00	RX0	N
ATOM	723	H	LEU	233	29.365	4.445	19.590	0.00	0.00	RX0	H
ATOM	724	CA	LEU	233	29.211	3.960	21.691	1.00	0.00	RX0	C
ATOM	725	CB	LEU	233	30.716	4.175	21.838	1.00	0.00	RX0	C
ATOM	726	CG	LEU	233	31.187	3.778	23.239	1.00	0.00	RX0	C
ATOM	727	CD1	LEU	233	30.800	2.337	23.575	1.00	0.00	RX0	C
ATOM	728	CD2	LEU	233	32.681	4.022	23.440	1.00	0.00	RX0	C
ATOM	729	C	LEU	233	28.415	4.936	22.566	1.00	0.00	RX0	C
ATOM	730	O	LEU	233	27.943	4.566	23.634	1.00	0.00	RX0	O

ATOM	731	N	GLU	234	28.150	6.122	22.016	1.00	0.00	RX0	N
ATOM	732	H	GLU	234	28.478	6.313	21.093	0.00	0.00	RX0	H
ATOM	733	CA	GLU	234	27.227	7.090	22.644	1.00	0.00	RX0	C
ATOM	734	CB	GLU	234	27.079	8.302	21.716	1.00	0.00	RX0	C
ATOM	735	CG	GLU	234	27.540	9.658	22.256	1.00	0.00	RX0	C
ATOM	736	CD	GLU	234	27.233	10.754	21.251	1.00	0.00	RX0	C
ATOM	737	OE1	GLU	234	26.795	11.833	21.638	1.00	0.00	RX0	O
ATOM	738	OE2	GLU	234	27.475	10.571	20.067	1.00	0.00	RX0	O
ATOM	739	C	GLU	234	25.830	6.482	22.841	1.00	0.00	RX0	C
ATOM	740	O	GLU	234	25.253	6.598	23.926	1.00	0.00	RX0	O
ATOM	741	N	ILE	235	25.389	5.711	21.848	1.00	0.00	RX0	N
ATOM	742	H	ILE	235	25.963	5.633	21.031	0.00	0.00	RX0	H
ATOM	743	CA	ILE	235	24.069	5.045	21.863	1.00	0.00	RX0	C
ATOM	744	CB	ILE	235	23.696	4.485	20.487	1.00	0.00	RX0	C
ATOM	745	CG2	ILE	235	22.408	3.661	20.554	1.00	0.00	RX0	C
ATOM	746	CG1	ILE	235	23.560	5.612	19.464	1.00	0.00	RX0	C
ATOM	747	CD1	ILE	235	23.220	5.096	18.066	1.00	0.00	RX0	C
ATOM	748	C	ILE	235	24.018	3.945	22.939	1.00	0.00	RX0	C
ATOM	749	O	ILE	235	23.068	3.902	23.724	1.00	0.00	RX0	O
ATOM	750	N	LEU	236	25.072	3.140	23.020	1.00	0.00	RX0	N
ATOM	751	H	LEU	236	25.813	3.235	22.352	0.00	0.00	RX0	H
ATOM	752	CA	LEU	236	25.176	2.114	24.078	1.00	0.00	RX0	C
ATOM	753	CB	LEU	236	26.439	1.275	23.891	1.00	0.00	RX0	C
ATOM	754	CG	LEU	236	26.325	0.300	22.724	1.00	0.00	RX0	C
ATOM	755	CD1	LEU	236	27.642	-0.422	22.443	1.00	0.00	RX0	C
ATOM	756	CD2	LEU	236	25.172	-0.679	22.943	1.00	0.00	RX0	C
ATOM	757	C	LEU	236	25.182	2.733	25.479	1.00	0.00	RX0	C
ATOM	758	O	LEU	236	24.381	2.362	26.336	1.00	0.00	RX0	O
ATOM	759	N	MET	237	25.933	3.823	25.600	1.00	0.00	RX0	N
ATOM	760	H	MET	237	26.458	4.135	24.805	0.00	0.00	RX0	H
ATOM	761	CA	MET	237	26.132	4.523	26.881	1.00	0.00	RX0	C
ATOM	762	CB	MET	237	27.280	5.529	26.805	1.00	0.00	RX0	C
ATOM	763	CG	MET	237	28.652	4.856	26.817	1.00	0.00	RX0	C
ATOM	764	SD	MET	237	29.997	6.050	26.828	1.00	0.00	RX0	S
ATOM	765	CE	MET	237	31.348	4.901	27.128	1.00	0.00	RX0	C
ATOM	766	C	MET	237	24.875	5.215	27.409	1.00	0.00	RX0	C
ATOM	767	O	MET	237	24.517	5.003	28.572	1.00	0.00	RX0	O
ATOM	768	N	ILE	238	24.128	5.878	26.531	1.00	0.00	RX0	N
ATOM	769	H	ILE	238	24.459	5.963	25.588	0.00	0.00	RX0	H
ATOM	770	CA	ILE	238	22.871	6.546	26.925	1.00	0.00	RX0	C
ATOM	771	CB	ILE	238	22.355	7.528	25.859	1.00	0.00	RX0	C
ATOM	772	CG2	ILE	238	21.939	6.846	24.557	1.00	0.00	RX0	C
ATOM	773	CG1	ILE	238	21.227	8.386	26.435	1.00	0.00	RX0	C
ATOM	774	CD1	ILE	238	20.627	9.348	25.408	1.00	0.00	RX0	C
ATOM	775	C	ILE	238	21.800	5.514	27.357	1.00	0.00	RX0	C
ATOM	776	O	ILE	238	21.031	5.731	28.268	1.00	0.00	RX0	O
ATOM	777	N	GLY	239	21.845	4.355	26.660	1.00	0.00	RX0	N
ATOM	778	H	GLY	239	22.522	4.243	25.929	0.00	0.00	RX0	H
ATOM	779	CA	GLY	239	20.969	3.213	26.975	1.00	0.00	RX0	C
ATOM	780	C	GLY	239	21.301	2.634	28.356	1.00	0.00	RX0	C
ATOM	781	O	GLY	239	20.417	2.460	29.193	1.00	0.00	RX0	O
ATOM	782	N	LEU	240	22.605	2.583	28.639	1.00	0.00	RX0	N
ATOM	783	H	LEU	240	23.260	2.804	27.913	0.00	0.00	RX0	H
ATOM	784	CA	LEU	240	23.124	2.082	29.919	1.00	0.00	RX0	C
ATOM	785	CB	LEU	240	24.644	1.943	29.849	1.00	0.00	RX0	C
ATOM	786	CG	LEU	240	25.287	1.616	31.197	1.00	0.00	RX0	C
ATOM	787	CD1	LEU	240	24.824	0.271	31.756	1.00	0.00	RX0	C
ATOM	788	CD2	LEU	240	26.808	1.715	31.125	1.00	0.00	RX0	C
ATOM	789	C	LEU	240	22.728	2.995	31.086	1.00	0.00	RX0	C
ATOM	790	O	LEU	240	22.214	2.535	32.097	1.00	0.00	RX0	O
ATOM	791	N	VAL	241	22.901	4.295	30.880	1.00	0.00	RX0	N

ATOM	792	H	VAL	241	23.276	4.586	29.997	0.00	0.00	RX0	H
ATOM	793	CA	VAL	241	22.596	5.307	31.912	1.00	0.00	RX0	C
ATOM	794	CB	VAL	241	23.256	6.669	31.662	1.00	0.00	RX0	C
ATOM	795	CG1	VAL	241	24.774	6.500	31.610	1.00	0.00	RX0	C
ATOM	796	CG2	VAL	241	22.716	7.397	30.438	1.00	0.00	RX0	C
ATOM	797	C	VAL	241	21.084	5.392	32.193	1.00	0.00	RX0	C
ATOM	798	O	VAL	241	20.670	5.516	33.338	1.00	0.00	RX0	O
ATOM	799	N	TRP	242	20.290	5.181	31.134	1.00	0.00	RX0	N
ATOM	800	H	TRP	242	20.709	5.094	30.225	0.00	0.00	RX0	H
ATOM	801	CA	TRP	242	18.822	5.192	31.222	1.00	0.00	RX0	C
ATOM	802	CB	TRP	242	18.213	5.213	29.812	1.00	0.00	RX0	C
ATOM	803	CG	TRP	242	16.724	4.984	29.896	1.00	0.00	RX0	C
ATOM	804	CD2	TRP	242	15.692	5.895	30.330	1.00	0.00	RX0	C
ATOM	805	CE2	TRP	242	14.461	5.161	30.334	1.00	0.00	RX0	C
ATOM	806	CE3	TRP	242	15.699	7.247	30.725	1.00	0.00	RX0	C
ATOM	807	CD1	TRP	242	16.071	3.771	29.647	1.00	0.00	RX0	C
ATOM	808	NE1	TRP	242	14.746	3.874	29.906	1.00	0.00	RX0	N
ATOM	809	HE1	TRP	242	14.087	3.153	29.849	0.00	0.00	RX0	H
ATOM	810	CZ2	TRP	242	13.264	5.784	30.743	1.00	0.00	RX0	C
ATOM	811	CZ3	TRP	242	14.494	7.863	31.126	1.00	0.00	RX0	C
ATOM	812	CH2	TRP	242	13.288	7.135	31.144	1.00	0.00	RX0	C
ATOM	813	C	TRP	242	18.281	4.038	32.076	1.00	0.00	RX0	C
ATOM	814	O	TRP	242	17.477	4.269	32.979	1.00	0.00	RX0	O
ATOM	815	N	ARG	243	18.818	2.841	31.865	1.00	0.00	RX0	N
ATOM	816	H	ARG	243	19.488	2.749	31.125	0.00	0.00	RX0	H
ATOM	817	CA	ARG	243	18.359	1.661	32.627	1.00	0.00	RX0	C
ATOM	818	CB	ARG	243	18.473	0.404	31.735	1.00	0.00	RX0	C
ATOM	819	CG	ARG	243	19.871	-0.079	31.291	1.00	0.00	RX0	C
ATOM	820	CD	ARG	243	19.795	-1.126	30.158	1.00	0.00	RX0	C
ATOM	821	NE	ARG	243	21.078	-1.785	29.866	1.00	0.00	RX0	N
ATOM	822	HE	ARG	243	21.626	-2.048	30.675	0.00	0.00	RX0	H
ATOM	823	CZ	ARG	243	21.409	-2.137	28.574	1.00	0.00	RX0	C
ATOM	824	NH1	ARG	243	20.649	-1.713	27.552	1.00	0.00	RX0	N
ATOM	825	HH11	ARG	243	20.885	-2.000	26.605	0.00	0.00	RX0	H
ATOM	826	HH12	ARG	243	19.857	-1.126	27.694	0.00	0.00	RX0	H
ATOM	827	NH2	ARG	243	22.476	-2.909	28.298	1.00	0.00	RX0	N
ATOM	828	HH21	ARG	243	22.764	-3.101	27.337	0.00	0.00	RX0	H
ATOM	829	HH22	ARG	243	23.030	-3.353	29.012	0.00	0.00	RX0	H
ATOM	830	C	ARG	243	19.043	1.510	33.998	1.00	0.00	RX0	C
ATOM	831	O	ARG	243	18.610	0.722	34.836	1.00	0.00	RX0	O
ATOM	832	N	SER	244	20.027	2.366	34.245	1.00	0.00	RX0	N
ATOM	833	H	SER	244	20.300	2.985	33.510	0.00	0.00	RX0	H
ATOM	834	CA	SER	244	20.722	2.464	35.548	1.00	0.00	RX0	C
ATOM	835	CB	SER	244	22.209	2.751	35.367	1.00	0.00	RX0	C
ATOM	836	OG	SER	244	22.809	1.736	34.558	1.00	0.00	RX0	O
ATOM	837	HG	SER	244	22.408	1.812	33.697	0.00	0.00	RX0	H
ATOM	838	C	SER	244	20.112	3.547	36.442	1.00	0.00	RX0	C
ATOM	839	O	SER	244	20.448	3.642	37.630	1.00	0.00	RX0	O
ATOM	840	N	MET	245	19.184	4.322	35.895	1.00	0.00	RX0	N
ATOM	841	H	MET	245	18.851	4.105	34.975	0.00	0.00	RX0	H
ATOM	842	CA	MET	245	18.600	5.504	36.550	1.00	0.00	RX0	C
ATOM	843	CB	MET	245	17.592	6.214	35.642	1.00	0.00	RX0	C
ATOM	844	CG	MET	245	17.039	7.487	36.295	1.00	0.00	RX0	C
ATOM	845	SD	MET	245	15.567	8.153	35.516	1.00	0.00	RX0	S
ATOM	846	CE	MET	245	16.305	8.485	33.921	1.00	0.00	RX0	C
ATOM	847	C	MET	245	17.925	5.204	37.895	1.00	0.00	RX0	C
ATOM	848	O	MET	245	18.105	5.946	38.853	1.00	0.00	RX0	O
ATOM	849	N	GLU	246	17.212	4.082	37.945	1.00	0.00	RX0	N
ATOM	850	H	GLU	246	17.108	3.524	37.115	0.00	0.00	RX0	H
ATOM	851	CA	GLU	246	16.494	3.692	39.178	1.00	0.00	RX0	C
ATOM	852	CB	GLU	246	15.210	2.924	38.854	1.00	0.00	RX0	C

ATOM	853	CG	GLU	246	14.163	3.741	38.092	1.00	0.00	RX0	C
ATOM	854	CD	GLU	246	12.937	2.884	37.830	1.00	0.00	RX0	C
ATOM	855	OE1	GLU	246	12.761	2.404	36.713	1.00	0.00	RX0	O
ATOM	856	OE2	GLU	246	12.154	2.668	38.748	1.00	0.00	RX0	O
ATOM	857	C	GLU	246	17.379	2.867	40.123	1.00	0.00	RX0	C
ATOM	858	O	GLU	246	16.897	2.295	41.108	1.00	0.00	RX0	O
ATOM	859	N	HIS	247	18.674	2.844	39.836	1.00	0.00	RX0	N
ATOM	860	H	HIS	247	19.005	3.286	39.004	0.00	0.00	RX0	H
ATOM	861	CA	HIS	247	19.658	2.055	40.601	1.00	0.00	RX0	C
ATOM	862	CB	HIS	247	20.137	0.831	39.822	1.00	0.00	RX0	C
ATOM	863	CG	HIS	247	19.014	-0.164	39.645	1.00	0.00	RX0	C
ATOM	864	ND1	HIS	247	17.893	-0.219	40.394	1.00	0.00	RX0	N
ATOM	865	HD1	HIS	247	17.610	0.389	41.112	0.00	0.00	RX0	H
ATOM	866	CD2	HIS	247	18.958	-1.177	38.690	1.00	0.00	RX0	C
ATOM	867	NE2	HIS	247	17.794	-1.839	38.869	1.00	0.00	RX0	N
ATOM	868	CE1	HIS	247	17.137	-1.257	39.919	1.00	0.00	RX0	C
ATOM	869	C	HIS	247	20.841	2.947	41.015	1.00	0.00	RX0	C
ATOM	870	O	HIS	247	21.962	2.788	40.490	1.00	0.00	RX0	O
ATOM	871	N	PRO	248	20.632	3.827	41.991	1.00	0.00	RX0	N
ATOM	872	CD	PRO	248	19.384	3.972	42.733	1.00	0.00	RX0	C
ATOM	873	CA	PRO	248	21.659	4.767	42.484	1.00	0.00	RX0	C
ATOM	874	CB	PRO	248	20.980	5.449	43.675	1.00	0.00	RX0	C
ATOM	875	CG	PRO	248	19.487	5.342	43.386	1.00	0.00	RX0	C
ATOM	876	C	PRO	248	22.939	4.013	42.877	1.00	0.00	RX0	C
ATOM	877	O	PRO	248	22.892	2.963	43.503	1.00	0.00	RX0	O
ATOM	878	N	GLY	249	24.055	4.541	42.350	1.00	0.00	RX0	N
ATOM	879	H	GLY	249	23.973	5.296	41.705	0.00	0.00	RX0	H
ATOM	880	CA	GLY	249	25.407	3.996	42.610	1.00	0.00	RX0	C
ATOM	881	C	GLY	249	25.783	2.749	41.794	1.00	0.00	RX0	C
ATOM	882	O	GLY	249	26.914	2.250	41.927	1.00	0.00	RX0	O
ATOM	883	N	LYS	250	24.877	2.267	40.961	1.00	0.00	RX0	N
ATOM	884	H	LYS	250	23.981	2.694	40.814	0.00	0.00	RX0	H
ATOM	885	CA	LYS	250	25.097	1.050	40.158	1.00	0.00	RX0	C
ATOM	886	CB	LYS	250	24.372	-0.144	40.780	1.00	0.00	RX0	C
ATOM	887	CG	LYS	250	25.227	-0.856	41.838	1.00	0.00	RX0	C
ATOM	888	CD	LYS	250	24.581	-2.119	42.411	1.00	0.00	RX0	C
ATOM	889	CE	LYS	250	25.527	-2.993	43.243	1.00	0.00	RX0	C
ATOM	890	NZ	LYS	250	26.640	-3.491	42.422	1.00	0.00	RX0	N
ATOM	891	HZ1	LYS	250	27.368	-3.909	43.045	0.00	0.00	RX0	H
ATOM	892	HZ2	LYS	250	26.338	-4.185	41.701	0.00	0.00	RX0	H
ATOM	893	HZ3	LYS	250	27.131	-2.684	41.992	0.00	0.00	RX0	H
ATOM	894	C	LYS	250	24.802	1.266	38.671	1.00	0.00	RX0	C
ATOM	895	O	LYS	250	24.040	2.166	38.282	1.00	0.00	RX0	O
ATOM	896	N	LEU	251	25.472	0.476	37.863	1.00	0.00	RX0	N
ATOM	897	H	LEU	251	26.045	-0.242	38.245	0.00	0.00	RX0	H
ATOM	898	CA	LEU	251	25.292	0.430	36.401	1.00	0.00	RX0	C
ATOM	899	CB	LEU	251	26.626	0.590	35.684	1.00	0.00	RX0	C
ATOM	900	CG	LEU	251	27.164	2.014	35.765	1.00	0.00	RX0	C
ATOM	901	CD1	LEU	251	28.587	2.110	35.219	1.00	0.00	RX0	C
ATOM	902	CD2	LEU	251	26.214	3.010	35.096	1.00	0.00	RX0	C
ATOM	903	C	LEU	251	24.646	-0.892	36.017	1.00	0.00	RX0	C
ATOM	904	O	LEU	251	25.224	-1.976	36.286	1.00	0.00	RX0	O
ATOM	905	N	LEU	252	23.437	-0.808	35.533	1.00	0.00	RX0	N
ATOM	906	H	LEU	252	23.078	0.099	35.321	0.00	0.00	RX0	H
ATOM	907	CA	LEU	252	22.659	-1.981	35.114	1.00	0.00	RX0	C
ATOM	908	CB	LEU	252	21.163	-1.738	35.319	1.00	0.00	RX0	C
ATOM	909	CG	LEU	252	20.326	-3.018	35.243	1.00	0.00	RX0	C
ATOM	910	CD1	LEU	252	20.839	-4.117	36.161	1.00	0.00	RX0	C
ATOM	911	CD2	LEU	252	18.873	-2.744	35.580	1.00	0.00	RX0	C
ATOM	912	C	LEU	252	23.007	-2.330	33.663	1.00	0.00	RX0	C
ATOM	913	O	LEU	252	22.274	-2.025	32.731	1.00	0.00	RX0	O

ATOM	914	N	PHE	253	24.144	-3.005	33.506	1.00	0.00	RX0	N
ATOM	915	H	PHE	253	24.623	-3.314	34.333	0.00	0.00	RX0	H
ATOM	916	CA	PHE	253	24.599	-3.457	32.174	1.00	0.00	RX0	C
ATOM	917	CB	PHE	253	25.969	-4.124	32.266	1.00	0.00	RX0	C
ATOM	918	CG	PHE	253	27.032	-3.096	32.548	1.00	0.00	RX0	C
ATOM	919	CD1	PHE	253	27.564	-2.354	31.500	1.00	0.00	RX0	C
ATOM	920	CD2	PHE	253	27.487	-2.898	33.845	1.00	0.00	RX0	C
ATOM	921	CE1	PHE	253	28.561	-1.420	31.746	1.00	0.00	RX0	C
ATOM	922	CE2	PHE	253	28.484	-1.963	34.090	1.00	0.00	RX0	C
ATOM	923	CZ	PHE	253	29.022	-1.227	33.042	1.00	0.00	RX0	C
ATOM	924	C	PHE	253	23.603	-4.446	31.564	1.00	0.00	RX0	C
ATOM	925	O	PHE	253	23.259	-4.379	30.390	1.00	0.00	RX0	O
ATOM	926	N	ALA	254	23.094	-5.300	32.445	1.00	0.00	RX0	N
ATOM	927	H	ALA	254	23.453	-5.299	33.385	0.00	0.00	RX0	H
ATOM	928	CA	ALA	254	22.050	-6.280	32.141	1.00	0.00	RX0	C
ATOM	929	CB	ALA	254	22.698	-7.557	31.616	1.00	0.00	RX0	C
ATOM	930	C	ALA	254	21.288	-6.584	33.440	1.00	0.00	RX0	C
ATOM	931	O	ALA	254	21.887	-6.418	34.526	1.00	0.00	RX0	O
ATOM	932	N	PRO	255	20.056	-7.064	33.372	1.00	0.00	RX0	N
ATOM	933	CD	PRO	255	19.301	-7.250	32.132	1.00	0.00	RX0	C
ATOM	934	CA	PRO	255	19.236	-7.444	34.545	1.00	0.00	RX0	C
ATOM	935	CB	PRO	255	17.990	-8.083	33.930	1.00	0.00	RX0	C
ATOM	936	CG	PRO	255	17.853	-7.415	32.570	1.00	0.00	RX0	C
ATOM	937	C	PRO	255	19.972	-8.395	35.506	1.00	0.00	RX0	C
ATOM	938	O	PRO	255	19.756	-8.342	36.714	1.00	0.00	RX0	O
ATOM	939	N	ASN	256	20.900	-9.179	34.970	1.00	0.00	RX0	N
ATOM	940	H	ASN	256	21.119	-9.122	33.997	0.00	0.00	RX0	H
ATOM	941	CA	ASN	256	21.722	-10.125	35.761	1.00	0.00	RX0	C
ATOM	942	CB	ASN	256	21.718	-11.528	35.150	1.00	0.00	RX0	C
ATOM	943	CG	ASN	256	22.413	-11.525	33.797	1.00	0.00	RX0	C
ATOM	944	OD1	ASN	256	22.279	-10.594	33.003	1.00	0.00	RX0	O
ATOM	945	ND2	ASN	256	23.067	-12.669	33.525	1.00	0.00	RX0	N
ATOM	946	HD21	ASN	256	23.226	-13.337	34.255	0.00	0.00	RX0	H
ATOM	947	HD22	ASN	256	23.408	-12.915	32.611	0.00	0.00	RX0	H
ATOM	948	C	ASN	256	23.191	-9.669	35.876	1.00	0.00	RX0	C
ATOM	949	O	ASN	256	24.101	-10.505	36.011	1.00	0.00	RX0	O
ATOM	950	N	LEU	257	23.439	-8.384	35.742	1.00	0.00	RX0	N
ATOM	951	H	LEU	257	22.690	-7.724	35.649	0.00	0.00	RX0	H
ATOM	952	CA	LEU	257	24.796	-7.805	35.783	1.00	0.00	RX0	C
ATOM	953	CB	LEU	257	25.509	-7.969	34.442	1.00	0.00	RX0	C
ATOM	954	CG	LEU	257	27.020	-7.761	34.558	1.00	0.00	RX0	C
ATOM	955	CD1	LEU	257	27.653	-8.793	35.494	1.00	0.00	RX0	C
ATOM	956	CD2	LEU	257	27.706	-7.737	33.193	1.00	0.00	RX0	C
ATOM	957	C	LEU	257	24.720	-6.327	36.168	1.00	0.00	RX0	C
ATOM	958	O	LEU	257	24.738	-5.412	35.328	1.00	0.00	RX0	O
ATOM	959	N	LEU	258	24.604	-6.148	37.469	1.00	0.00	RX0	N
ATOM	960	H	LEU	258	24.656	-6.943	38.072	0.00	0.00	RX0	H
ATOM	961	CA	LEU	258	24.482	-4.834	38.118	1.00	0.00	RX0	C
ATOM	962	CB	LEU	258	23.224	-4.895	38.991	1.00	0.00	RX0	C
ATOM	963	CG	LEU	258	22.828	-3.618	39.729	1.00	0.00	RX0	C
ATOM	964	CD1	LEU	258	22.601	-2.441	38.788	1.00	0.00	RX0	C
ATOM	965	CD2	LEU	258	21.620	-3.850	40.638	1.00	0.00	RX0	C
ATOM	966	C	LEU	258	25.743	-4.564	38.938	1.00	0.00	RX0	C
ATOM	967	O	LEU	258	26.013	-5.237	39.948	1.00	0.00	RX0	O
ATOM	968	N	LEU	259	26.528	-3.622	38.460	1.00	0.00	RX0	N
ATOM	969	H	LEU	259	26.212	-3.064	37.685	0.00	0.00	RX0	H
ATOM	970	CA	LEU	259	27.862	-3.349	39.027	1.00	0.00	RX0	C
ATOM	971	CB	LEU	259	28.937	-3.491	37.947	1.00	0.00	RX0	C
ATOM	972	CG	LEU	259	28.878	-4.805	37.162	1.00	0.00	RX0	C
ATOM	973	CD1	LEU	259	29.893	-4.823	36.018	1.00	0.00	RX0	C
ATOM	974	CD2	LEU	259	29.028	-6.031	38.062	1.00	0.00	RX0	C

ATOM	975	C	LEU	259	27.958	-1.956	39.652	1.00	0.00	RX0	C
ATOM	976	O	LEU	259	27.419	-0.984	39.137	1.00	0.00	RX0	O
ATOM	977	N	ASP	260	28.645	-1.912	40.785	1.00	0.00	RX0	N
ATOM	978	H	ASP	260	29.222	-2.689	41.050	0.00	0.00	RX0	H
ATOM	979	CA	ASP	260	29.043	-0.657	41.454	1.00	0.00	RX0	C
ATOM	980	CB	ASP	260	29.076	-0.875	42.961	1.00	0.00	RX0	C
ATOM	981	CG	ASP	260	29.750	-2.205	43.205	1.00	0.00	RX0	C
ATOM	982	OD1	ASP	260	30.977	-2.254	43.233	1.00	0.00	RX0	O
ATOM	983	OD2	ASP	260	29.034	-3.202	43.315	1.00	0.00	RX0	O
ATOM	984	C	ASP	260	30.443	-0.222	40.970	1.00	0.00	RX0	C
ATOM	985	O	ASP	260	31.127	-1.008	40.295	1.00	0.00	RX0	O
ATOM	986	N	ARG	261	30.963	0.873	41.504	1.00	0.00	RX0	N
ATOM	987	H	ARG	261	30.385	1.409	42.118	0.00	0.00	RX0	H
ATOM	988	CA	ARG	261	32.263	1.408	41.044	1.00	0.00	RX0	C
ATOM	989	CB	ARG	261	32.415	2.885	41.438	1.00	0.00	RX0	C
ATOM	990	CG	ARG	261	32.596	3.166	42.932	1.00	0.00	RX0	C
ATOM	991	CD	ARG	261	32.497	4.658	43.270	1.00	0.00	RX0	C
ATOM	992	NE	ARG	261	33.371	5.463	42.420	1.00	0.00	RX0	N
ATOM	993	HE	ARG	261	33.087	5.667	41.466	0.00	0.00	RX0	H
ATOM	994	CZ	ARG	261	34.560	5.974	42.849	1.00	0.00	RX0	C
ATOM	995	NH1	ARG	261	34.931	5.805	44.137	1.00	0.00	RX0	N
ATOM	996	HH11	ARG	261	35.795	6.169	44.490	0.00	0.00	RX0	H
ATOM	997	HH12	ARG	261	34.333	5.322	44.779	0.00	0.00	RX0	H
ATOM	998	NH2	ARG	261	35.336	6.642	41.976	1.00	0.00	RX0	N
ATOM	999	HH21	ARG	261	36.244	7.025	42.137	0.00	0.00	RX0	H
ATOM	1000	HH22	ARG	261	34.941	6.791	41.047	0.00	0.00	RX0	H
ATOM	1001	C	ARG	261	33.476	0.540	41.436	1.00	0.00	RX0	C
ATOM	1002	O	ARG	261	34.378	0.347	40.637	1.00	0.00	RX0	O
ATOM	1003	N	ASN	262	33.410	-0.067	42.632	1.00	0.00	RX0	N
ATOM	1004	H	ASN	262	32.532	-0.042	43.113	0.00	0.00	RX0	H
ATOM	1005	CA	ASN	262	34.456	-1.000	43.094	1.00	0.00	RX0	C
ATOM	1006	CB	ASN	262	34.128	-1.509	44.490	1.00	0.00	RX0	C
ATOM	1007	CG	ASN	262	35.126	-2.589	44.850	1.00	0.00	RX0	C
ATOM	1008	OD1	ASN	262	36.269	-2.289	45.200	1.00	0.00	RX0	O
ATOM	1009	ND2	ASN	262	34.631	-3.839	44.798	1.00	0.00	RX0	N
ATOM	1010	HD21	ASN	262	33.677	-4.004	44.535	0.00	0.00	RX0	H
ATOM	1011	HD22	ASN	262	35.177	-4.652	45.004	0.00	0.00	RX0	H
ATOM	1012	C	ASN	262	34.635	-2.233	42.204	1.00	0.00	RX0	C
ATOM	1013	O	ASN	262	35.755	-2.665	41.964	1.00	0.00	RX0	O
ATOM	1014	N	GLN	263	33.530	-2.679	41.603	1.00	0.00	RX0	N
ATOM	1015	H	GLN	263	32.635	-2.280	41.829	0.00	0.00	RX0	H
ATOM	1016	CA	GLN	263	33.559	-3.756	40.599	1.00	0.00	RX0	C
ATOM	1017	CB	GLN	263	32.198	-4.424	40.443	1.00	0.00	RX0	C
ATOM	1018	CG	GLN	263	31.928	-5.195	41.733	1.00	0.00	RX0	C
ATOM	1019	CD	GLN	263	30.707	-6.069	41.588	1.00	0.00	RX0	C
ATOM	1020	OE1	GLN	263	30.630	-6.947	40.739	1.00	0.00	RX0	O
ATOM	1021	NE2	GLN	263	29.766	-5.816	42.509	1.00	0.00	RX0	N
ATOM	1022	HE21	GLN	263	29.879	-5.008	43.102	0.00	0.00	RX0	H
ATOM	1023	HE22	GLN	263	28.979	-6.418	42.621	0.00	0.00	RX0	H
ATOM	1024	C	GLN	263	34.189	-3.308	39.265	1.00	0.00	RX0	C
ATOM	1025	O	GLN	263	34.644	-4.116	38.479	1.00	0.00	RX0	O
ATOM	1026	N	GLY	264	34.180	-1.978	39.039	1.00	0.00	RX0	N
ATOM	1027	H	GLY	264	33.764	-1.376	39.720	0.00	0.00	RX0	H
ATOM	1028	CA	GLY	264	34.831	-1.337	37.881	1.00	0.00	RX0	C
ATOM	1029	C	GLY	264	36.364	-1.310	37.978	1.00	0.00	RX0	C
ATOM	1030	O	GLY	264	37.050	-1.510	36.977	1.00	0.00	RX0	O
ATOM	1031	N	LYS	265	36.881	-1.155	39.202	1.00	0.00	RX0	N
ATOM	1032	H	LYS	265	36.232	-1.008	39.949	0.00	0.00	RX0	H
ATOM	1033	CA	LYS	265	38.336	-1.184	39.469	1.00	0.00	RX0	C
ATOM	1034	CB	LYS	265	38.661	-1.122	40.953	1.00	0.00	RX0	C
ATOM	1035	CG	LYS	265	38.094	-0.025	41.842	1.00	0.00	RX0	C

ATOM	1036	CD	LYS	265	38.464	-0.474	43.254	1.00	0.00	RX0	C
ATOM	1037	CE	LYS	265	37.918	0.346	44.415	1.00	0.00	RX0	C
ATOM	1038	NZ	LYS	265	38.104	-0.472	45.622	1.00	0.00	RX0	N
ATOM	1039	HZ1	LYS	265	37.678	-0.019	46.453	0.00	0.00	RX0	H
ATOM	1040	HZ2	LYS	265	37.620	-1.386	45.469	0.00	0.00	RX0	H
ATOM	1041	HZ3	LYS	265	39.112	-0.657	45.784	0.00	0.00	RX0	H
ATOM	1042	C	LYS	265	38.994	-2.500	39.030	1.00	0.00	RX0	C
ATOM	1043	O	LYS	265	40.184	-2.535	38.765	1.00	0.00	RX0	O
ATOM	1044	N	CYS	266	38.153	-3.548	38.917	1.00	0.00	RX0	N
ATOM	1045	H	CYS	266	37.198	-3.455	39.194	0.00	0.00	RX0	H
ATOM	1046	CA	CYS	266	38.538	-4.854	38.355	1.00	0.00	RX0	C
ATOM	1047	CB	CYS	266	37.315	-5.759	38.264	1.00	0.00	RX0	C
ATOM	1048	SG	CYS	266	36.555	-5.944	39.899	1.00	0.00	RX0	S
ATOM	1049	C	CYS	266	39.318	-4.732	37.033	1.00	0.00	RX0	C
ATOM	1050	O	CYS	266	40.108	-5.603	36.695	1.00	0.00	RX0	O
ATOM	1051	N	VAL	267	39.075	-3.636	36.304	1.00	0.00	RX0	N
ATOM	1052	H	VAL	267	38.470	-2.902	36.620	0.00	0.00	RX0	H
ATOM	1053	CA	VAL	267	39.804	-3.345	35.058	1.00	0.00	RX0	C
ATOM	1054	CB	VAL	267	38.857	-3.352	33.851	1.00	0.00	RX0	C
ATOM	1055	CG1	VAL	267	39.586	-3.007	32.550	1.00	0.00	RX0	C
ATOM	1056	CG2	VAL	267	38.128	-4.693	33.738	1.00	0.00	RX0	C
ATOM	1057	C	VAL	267	40.557	-2.013	35.191	1.00	0.00	RX0	C
ATOM	1058	O	VAL	267	39.969	-0.956	35.468	1.00	0.00	RX0	O
ATOM	1059	N	GLU	268	41.829	-2.078	34.821	1.00	0.00	RX0	N
ATOM	1060	H	GLU	268	42.209	-2.964	34.565	0.00	0.00	RX0	H
ATOM	1061	CA	GLU	268	42.726	-0.908	34.727	1.00	0.00	RX0	C
ATOM	1062	CB	GLU	268	44.097	-1.333	34.209	1.00	0.00	RX0	C
ATOM	1063	CG	GLU	268	45.141	-0.224	34.335	1.00	0.00	RX0	C
ATOM	1064	CD	GLU	268	46.504	-0.806	34.039	1.00	0.00	RX0	C
ATOM	1065	OE1	GLU	268	46.593	-2.020	33.866	1.00	0.00	RX0	O
ATOM	1066	OE2	GLU	268	47.471	-0.048	33.993	1.00	0.00	RX0	O
ATOM	1067	C	GLU	268	42.079	0.194	33.866	1.00	0.00	RX0	C
ATOM	1068	O	GLU	268	41.697	-0.027	32.727	1.00	0.00	RX0	O
ATOM	1069	N	GLY	269	41.924	1.355	34.524	1.00	0.00	RX0	N
ATOM	1070	H	GLY	269	42.193	1.398	35.484	0.00	0.00	RX0	H
ATOM	1071	CA	GLY	269	41.377	2.576	33.902	1.00	0.00	RX0	C
ATOM	1072	C	GLY	269	39.898	2.490	33.494	1.00	0.00	RX0	C
ATOM	1073	O	GLY	269	39.424	3.343	32.745	1.00	0.00	RX0	O
ATOM	1074	N	MET	270	39.146	1.598	34.134	1.00	0.00	RX0	N
ATOM	1075	H	MET	270	39.580	0.956	34.768	0.00	0.00	RX0	H
ATOM	1076	CA	MET	270	37.704	1.465	33.841	1.00	0.00	RX0	C
ATOM	1077	CB	MET	270	37.280	0.008	33.653	1.00	0.00	RX0	C
ATOM	1078	CG	MET	270	35.924	-0.097	32.949	1.00	0.00	RX0	C
ATOM	1079	SD	MET	270	35.416	-1.787	32.584	1.00	0.00	RX0	S
ATOM	1080	CE	MET	270	35.156	-2.354	34.270	1.00	0.00	RX0	C
ATOM	1081	C	MET	270	36.825	2.181	34.877	1.00	0.00	RX0	C
ATOM	1082	O	MET	270	35.781	2.734	34.514	1.00	0.00	RX0	O
ATOM	1083	N	VAL	271	37.310	2.290	36.107	1.00	0.00	RX0	N
ATOM	1084	H	VAL	271	38.235	1.949	36.273	0.00	0.00	RX0	H
ATOM	1085	CA	VAL	271	36.591	3.043	37.171	1.00	0.00	RX0	C
ATOM	1086	CB	VAL	271	37.248	2.855	38.550	1.00	0.00	RX0	C
ATOM	1087	CG1	VAL	271	38.735	3.196	38.560	1.00	0.00	RX0	C
ATOM	1088	CG2	VAL	271	36.456	3.559	39.653	1.00	0.00	RX0	C
ATOM	1089	C	VAL	271	36.343	4.502	36.749	1.00	0.00	RX0	C
ATOM	1090	O	VAL	271	35.261	5.071	37.095	1.00	0.00	RX0	O
ATOM	1091	N	GLU	272	37.228	5.074	36.011	1.00	0.00	RX0	N
ATOM	1092	H	GLU	272	38.100	4.599	35.878	0.00	0.00	RX0	H
ATOM	1093	CA	GLU	272	37.141	6.453	35.460	1.00	0.00	RX0	C
ATOM	1094	CB	GLU	272	38.430	6.788	34.700	1.00	0.00	RX0	C
ATOM	1095	CG	GLU	272	39.677	7.024	35.568	1.00	0.00	RX0	C
ATOM	1096	CD	GLU	272	40.048	5.793	36.376	1.00	0.00	RX0	C

ATOM	1097	OE1	GLU	272	40.058	4.694	35.824	1.00	0.00	RX0	O
ATOM	1098	OE2	GLU	272	40.293	5.931	37.570	1.00	0.00	RX0	O
ATOM	1099	C	GLU	272	35.927	6.588	34.526	1.00	0.00	RX0	C
ATOM	1100	O	GLU	272	35.142	7.521	34.681	1.00	0.00	RX0	O
ATOM	1101	N	ILE	273	35.702	5.552	33.729	1.00	0.00	RX0	N
ATOM	1102	H	ILE	273	36.304	4.758	33.824	0.00	0.00	RX0	H
ATOM	1103	CA	ILE	273	34.533	5.484	32.820	1.00	0.00	RX0	C
ATOM	1104	CB	ILE	273	34.734	4.376	31.780	1.00	0.00	RX0	C
ATOM	1105	CG2	ILE	273	33.623	4.408	30.729	1.00	0.00	RX0	C
ATOM	1106	CG1	ILE	273	36.127	4.432	31.146	1.00	0.00	RX0	C
ATOM	1107	CD1	ILE	273	36.339	5.656	30.254	1.00	0.00	RX0	C
ATOM	1108	C	ILE	273	33.249	5.235	33.628	1.00	0.00	RX0	C
ATOM	1109	O	ILE	273	32.257	5.951	33.452	1.00	0.00	RX0	O
ATOM	1110	N	PHE	274	33.320	4.302	34.575	1.00	0.00	RX0	N
ATOM	1111	H	PHE	274	34.190	3.821	34.693	0.00	0.00	RX0	H
ATOM	1112	CA	PHE	274	32.191	3.976	35.472	1.00	0.00	RX0	C
ATOM	1113	CB	PHE	274	32.611	2.939	36.516	1.00	0.00	RX0	C
ATOM	1114	CG	PHE	274	32.271	1.538	36.073	1.00	0.00	RX0	C
ATOM	1115	CD1	PHE	274	32.533	1.121	34.774	1.00	0.00	RX0	C
ATOM	1116	CD2	PHE	274	31.690	0.662	36.983	1.00	0.00	RX0	C
ATOM	1117	CE1	PHE	274	32.221	-0.178	34.390	1.00	0.00	RX0	C
ATOM	1118	CE2	PHE	274	31.379	-0.637	36.600	1.00	0.00	RX0	C
ATOM	1119	CZ	PHE	274	31.653	-1.058	35.304	1.00	0.00	RX0	C
ATOM	1120	C	PHE	274	31.669	5.203	36.222	1.00	0.00	RX0	C
ATOM	1121	O	PHE	274	30.484	5.521	36.143	1.00	0.00	RX0	O
ATOM	1122	N	ASP	275	32.607	5.983	36.757	1.00	0.00	RX0	N
ATOM	1123	H	ASP	275	33.560	5.685	36.801	0.00	0.00	RX0	H
ATOM	1124	CA	ASP	275	32.273	7.222	37.483	1.00	0.00	RX0	C
ATOM	1125	CB	ASP	275	33.488	7.800	38.200	1.00	0.00	RX0	C
ATOM	1126	CG	ASP	275	33.578	7.163	39.571	1.00	0.00	RX0	C
ATOM	1127	OD1	ASP	275	33.907	5.985	39.678	1.00	0.00	RX0	O
ATOM	1128	OD2	ASP	275	33.348	7.841	40.566	1.00	0.00	RX0	O
ATOM	1129	C	ASP	275	31.555	8.270	36.629	1.00	0.00	RX0	C
ATOM	1130	O	ASP	275	30.604	8.890	37.102	1.00	0.00	RX0	O
ATOM	1131	N	MET	276	31.921	8.336	35.353	1.00	0.00	RX0	N
ATOM	1132	H	MET	276	32.664	7.739	35.041	0.00	0.00	RX0	H
ATOM	1133	CA	MET	276	31.257	9.254	34.408	1.00	0.00	RX0	C
ATOM	1134	CB	MET	276	32.089	9.446	33.142	1.00	0.00	RX0	C
ATOM	1135	CG	MET	276	33.423	10.146	33.403	1.00	0.00	RX0	C
ATOM	1136	SD	MET	276	34.295	10.536	31.877	1.00	0.00	RX0	S
ATOM	1137	CE	MET	276	34.322	8.878	31.182	1.00	0.00	RX0	C
ATOM	1138	C	MET	276	29.833	8.798	34.061	1.00	0.00	RX0	C
ATOM	1139	O	MET	276	28.893	9.589	34.158	1.00	0.00	RX0	O
ATOM	1140	N	LEU	277	29.673	7.488	33.891	1.00	0.00	RX0	N
ATOM	1141	H	LEU	277	30.490	6.907	33.947	0.00	0.00	RX0	H
ATOM	1142	CA	LEU	277	28.362	6.866	33.606	1.00	0.00	RX0	C
ATOM	1143	CB	LEU	277	28.562	5.401	33.227	1.00	0.00	RX0	C
ATOM	1144	CG	LEU	277	29.434	5.218	31.986	1.00	0.00	RX0	C
ATOM	1145	CD1	LEU	277	29.930	3.779	31.845	1.00	0.00	RX0	C
ATOM	1146	CD2	LEU	277	28.731	5.710	30.723	1.00	0.00	RX0	C
ATOM	1147	C	LEU	277	27.393	6.992	34.787	1.00	0.00	RX0	C
ATOM	1148	O	LEU	277	26.257	7.447	34.627	1.00	0.00	RX0	O
ATOM	1149	N	LEU	278	27.939	6.795	35.983	1.00	0.00	RX0	N
ATOM	1150	H	LEU	278	28.900	6.516	36.020	0.00	0.00	RX0	H
ATOM	1151	CA	LEU	278	27.196	6.926	37.250	1.00	0.00	RX0	C
ATOM	1152	CB	LEU	278	28.067	6.470	38.419	1.00	0.00	RX0	C
ATOM	1153	CG	LEU	278	28.220	4.953	38.467	1.00	0.00	RX0	C
ATOM	1154	CD1	LEU	278	29.273	4.502	39.479	1.00	0.00	RX0	C
ATOM	1155	CD2	LEU	278	26.867	4.291	38.711	1.00	0.00	RX0	C
ATOM	1156	C	LEU	278	26.716	8.359	37.508	1.00	0.00	RX0	C
ATOM	1157	O	LEU	278	25.554	8.575	37.840	1.00	0.00	RX0	O

ATOM	1158	N	ALA	279	27.582	9.317	37.175	1.00	0.00	RX0	N
ATOM	1159	H	ALA	279	28.513	9.059	36.898	0.00	0.00	RX0	H
ATOM	1160	CA	ALA	279	27.275	10.754	37.296	1.00	0.00	RX0	C
ATOM	1161	CB	ALA	279	28.528	11.595	37.048	1.00	0.00	RX0	C
ATOM	1162	C	ALA	279	26.182	11.196	36.310	1.00	0.00	RX0	C
ATOM	1163	O	ALA	279	25.263	11.922	36.684	1.00	0.00	RX0	O
ATOM	1164	N	THR	280	26.210	10.618	35.109	1.00	0.00	RX0	N
ATOM	1165	H	THR	280	26.970	10.007	34.881	0.00	0.00	RX0	H
ATOM	1166	CA	THR	280	25.203	10.899	34.059	1.00	0.00	RX0	C
ATOM	1167	CB	THR	280	25.748	10.367	32.740	1.00	0.00	RX0	C
ATOM	1168	OG1	THR	280	27.059	10.907	32.532	1.00	0.00	RX0	O
ATOM	1169	HG1	THR	280	27.667	10.445	33.101	0.00	0.00	RX0	H
ATOM	1170	CG2	THR	280	24.830	10.714	31.566	1.00	0.00	RX0	C
ATOM	1171	C	THR	280	23.835	10.327	34.462	1.00	0.00	RX0	C
ATOM	1172	O	THR	280	22.822	11.023	34.397	1.00	0.00	RX0	O
ATOM	1173	N	SER	281	23.868	9.112	35.003	1.00	0.00	RX0	N
ATOM	1174	H	SER	281	24.739	8.618	35.047	0.00	0.00	RX0	H
ATOM	1175	CA	SER	281	22.669	8.413	35.507	1.00	0.00	RX0	C
ATOM	1176	CB	SER	281	23.145	6.984	35.865	1.00	0.00	RX0	C
ATOM	1177	OG	SER	281	22.391	6.341	36.913	1.00	0.00	RX0	O
ATOM	1178	HG	SER	281	22.583	5.411	36.814	0.00	0.00	RX0	H
ATOM	1179	C	SER	281	22.019	9.180	36.675	1.00	0.00	RX0	C
ATOM	1180	O	SER	281	20.814	9.399	36.693	1.00	0.00	RX0	O
ATOM	1181	N	SER	282	22.889	9.788	37.491	1.00	0.00	RX0	N
ATOM	1182	H	SER	282	23.868	9.616	37.376	0.00	0.00	RX0	H
ATOM	1183	CA	SER	282	22.489	10.660	38.613	1.00	0.00	RX0	C
ATOM	1184	CB	SER	282	23.670	10.725	39.560	1.00	0.00	RX0	C
ATOM	1185	OG	SER	282	23.946	9.347	39.873	1.00	0.00	RX0	O
ATOM	1186	HG	SER	282	24.724	9.121	39.362	0.00	0.00	RX0	H
ATOM	1187	C	SER	282	21.828	11.963	38.130	1.00	0.00	RX0	C
ATOM	1188	O	SER	282	20.788	12.371	38.639	1.00	0.00	RX0	O
ATOM	1189	N	ARG	283	22.365	12.516	37.039	1.00	0.00	RX0	N
ATOM	1190	H	ARG	283	23.190	12.111	36.638	0.00	0.00	RX0	H
ATOM	1191	CA	ARG	283	21.822	13.735	36.412	1.00	0.00	RX0	C
ATOM	1192	CB	ARG	283	22.813	14.208	35.349	1.00	0.00	RX0	C
ATOM	1193	CG	ARG	283	22.352	15.403	34.517	1.00	0.00	RX0	C
ATOM	1194	CD	ARG	283	22.162	16.688	35.321	1.00	0.00	RX0	C
ATOM	1195	NE	ARG	283	21.785	17.772	34.418	1.00	0.00	RX0	N
ATOM	1196	HE	ARG	283	22.220	17.743	33.511	0.00	0.00	RX0	H
ATOM	1197	CZ	ARG	283	20.894	18.723	34.823	1.00	0.00	RX0	C
ATOM	1198	NH1	ARG	283	20.396	18.686	36.080	1.00	0.00	RX0	N
ATOM	1199	HH11	ARG	283	19.732	19.365	36.408	0.00	0.00	RX0	H
ATOM	1200	HH12	ARG	283	20.669	17.977	36.738	0.00	0.00	RX0	H
ATOM	1201	NH2	ARG	283	20.518	19.680	33.952	1.00	0.00	RX0	N
ATOM	1202	HH21	ARG	283	19.822	20.378	34.181	0.00	0.00	RX0	H
ATOM	1203	HH22	ARG	283	20.900	19.734	33.026	0.00	0.00	RX0	H
ATOM	1204	C	ARG	283	20.432	13.477	35.812	1.00	0.00	RX0	C
ATOM	1205	O	ARG	283	19.498	14.246	36.035	1.00	0.00	RX0	O
ATOM	1206	N	PHE	284	20.293	12.325	35.169	1.00	0.00	RX0	N
ATOM	1207	H	PHE	284	21.097	11.740	35.042	0.00	0.00	RX0	H
ATOM	1208	CA	PHE	284	19.003	11.899	34.606	1.00	0.00	RX0	C
ATOM	1209	CB	PHE	284	19.209	10.636	33.777	1.00	0.00	RX0	C
ATOM	1210	CG	PHE	284	19.682	10.996	32.392	1.00	0.00	RX0	C
ATOM	1211	CD1	PHE	284	19.376	12.241	31.855	1.00	0.00	RX0	C
ATOM	1212	CD2	PHE	284	20.398	10.072	31.642	1.00	0.00	RX0	C
ATOM	1213	CE1	PHE	284	19.752	12.543	30.552	1.00	0.00	RX0	C
ATOM	1214	CE2	PHE	284	20.777	10.379	30.341	1.00	0.00	RX0	C
ATOM	1215	CZ	PHE	284	20.440	11.608	29.789	1.00	0.00	RX0	C
ATOM	1216	C	PHE	284	17.921	11.680	35.654	1.00	0.00	RX0	C
ATOM	1217	O	PHE	284	16.817	12.216	35.524	1.00	0.00	RX0	O
ATOM	1218	N	ARG	285	18.348	11.087	36.760	1.00	0.00	RX0	N

ATOM	1219	H	ARG	285	19.279	10.716	36.763	0.00	0.00	RX0	H
ATOM	1220	CA	ARG	285	17.485	10.841	37.923	1.00	0.00	RX0	C
ATOM	1221	CB	ARG	285	18.336	10.058	38.925	1.00	0.00	RX0	C
ATOM	1222	CG	ARG	285	17.771	9.787	40.320	1.00	0.00	RX0	C
ATOM	1223	CD	ARG	285	18.801	9.030	41.172	1.00	0.00	RX0	C
ATOM	1224	NE	ARG	285	19.213	7.816	40.470	1.00	0.00	RX0	N
ATOM	1225	HE	ARG	285	18.456	7.216	40.178	0.00	0.00	RX0	H
ATOM	1226	CZ	ARG	285	20.502	7.635	40.052	1.00	0.00	RX0	C
ATOM	1227	NH1	ARG	285	21.474	8.412	40.575	1.00	0.00	RX0	N
ATOM	1228	HH11	ARG	285	22.435	8.403	40.258	0.00	0.00	RX0	H
ATOM	1229	HH12	ARG	285	21.272	9.064	41.310	0.00	0.00	RX0	H
ATOM	1230	NH2	ARG	285	20.759	6.698	39.114	1.00	0.00	RX0	N
ATOM	1231	HH21	ARG	285	21.644	6.564	38.645	0.00	0.00	RX0	H
ATOM	1232	HH22	ARG	285	20.015	6.089	38.814	0.00	0.00	RX0	H
ATOM	1233	C	ARG	285	17.003	12.164	38.534	1.00	0.00	RX0	C
ATOM	1234	O	ARG	285	15.822	12.321	38.816	1.00	0.00	RX0	O
ATOM	1235	N	MET	286	17.922	13.130	38.591	1.00	0.00	RX0	N
ATOM	1236	H	MET	286	18.856	12.922	38.296	0.00	0.00	RX0	H
ATOM	1237	CA	MET	286	17.643	14.467	39.144	1.00	0.00	RX0	C
ATOM	1238	CB	MET	286	18.961	15.233	39.261	1.00	0.00	RX0	C
ATOM	1239	CG	MET	286	18.793	16.699	39.662	1.00	0.00	RX0	C
ATOM	1240	SD	MET	286	20.309	17.644	39.439	1.00	0.00	RX0	S
ATOM	1241	CE	MET	286	19.712	19.217	40.080	1.00	0.00	RX0	C
ATOM	1242	C	MET	286	16.681	15.257	38.243	1.00	0.00	RX0	C
ATOM	1243	O	MET	286	15.799	15.962	38.735	1.00	0.00	RX0	O
ATOM	1244	N	MET	287	16.911	15.161	36.942	1.00	0.00	RX0	N
ATOM	1245	H	MET	287	17.630	14.536	36.630	0.00	0.00	RX0	H
ATOM	1246	CA	MET	287	16.049	15.814	35.941	1.00	0.00	RX0	C
ATOM	1247	CB	MET	287	16.684	15.859	34.555	1.00	0.00	RX0	C
ATOM	1248	CG	MET	287	17.873	16.810	34.515	1.00	0.00	RX0	C
ATOM	1249	SD	MET	287	18.232	17.350	32.841	1.00	0.00	RX0	S
ATOM	1250	CE	MET	287	16.608	18.046	32.496	1.00	0.00	RX0	C
ATOM	1251	C	MET	287	14.674	15.156	35.849	1.00	0.00	RX0	C
ATOM	1252	O	MET	287	13.755	15.729	35.264	1.00	0.00	RX0	O
ATOM	1253	N	ASN	288	14.583	13.920	36.346	1.00	0.00	RX0	N
ATOM	1254	H	ASN	288	15.393	13.490	36.746	0.00	0.00	RX0	H
ATOM	1255	CA	ASN	288	13.391	13.066	36.242	1.00	0.00	RX0	C
ATOM	1256	CB	ASN	288	12.174	13.628	36.977	1.00	0.00	RX0	C
ATOM	1257	CG	ASN	288	11.001	12.691	36.752	1.00	0.00	RX0	C
ATOM	1258	OD1	ASN	288	11.147	11.482	36.613	1.00	0.00	RX0	O
ATOM	1259	ND2	ASN	288	9.815	13.325	36.709	1.00	0.00	RX0	N
ATOM	1260	HD21	ASN	288	9.763	14.316	36.837	0.00	0.00	RX0	H
ATOM	1261	HD22	ASN	288	8.966	12.823	36.543	0.00	0.00	RX0	H
ATOM	1262	C	ASN	288	13.076	12.836	34.753	1.00	0.00	RX0	C
ATOM	1263	O	ASN	288	11.986	13.121	34.256	1.00	0.00	RX0	O
ATOM	1264	N	LEU	289	14.125	12.439	34.028	1.00	0.00	RX0	N
ATOM	1265	H	LEU	289	14.954	12.170	34.522	0.00	0.00	RX0	H
ATOM	1266	CA	LEU	289	14.030	12.183	32.583	1.00	0.00	RX0	C
ATOM	1267	CB	LEU	289	15.381	11.672	32.071	1.00	0.00	RX0	C
ATOM	1268	CG	LEU	289	15.461	11.452	30.557	1.00	0.00	RX0	C
ATOM	1269	CD1	LEU	289	15.495	12.773	29.803	1.00	0.00	RX0	C
ATOM	1270	CD2	LEU	289	16.646	10.580	30.151	1.00	0.00	RX0	C
ATOM	1271	C	LEU	289	12.926	11.152	32.321	1.00	0.00	RX0	C
ATOM	1272	O	LEU	289	12.814	10.137	33.014	1.00	0.00	RX0	O
ATOM	1273	N	GLN	290	12.165	11.420	31.281	1.00	0.00	RX0	N
ATOM	1274	H	GLN	290	12.392	12.206	30.698	0.00	0.00	RX0	H
ATOM	1275	CA	GLN	290	11.011	10.584	30.916	1.00	0.00	RX0	C
ATOM	1276	CB	GLN	290	9.766	11.433	30.661	1.00	0.00	RX0	C
ATOM	1277	CG	GLN	290	9.296	12.160	31.927	1.00	0.00	RX0	C
ATOM	1278	CD	GLN	290	8.965	11.151	33.015	1.00	0.00	RX0	C
ATOM	1279	OE1	GLN	290	8.015	10.385	32.934	1.00	0.00	RX0	O

ATOM	1280	NE2	GLN	290	9.812	11.173	34.045	1.00	0.00	RX0	N
ATOM	1281	HE21	GLN	290	10.584	11.809	34.079	0.00	0.00	RX0	H
ATOM	1282	HE22	GLN	290	9.737	10.540	34.815	0.00	0.00	RX0	H
ATOM	1283	C	GLN	290	11.379	9.701	29.727	1.00	0.00	RX0	C
ATOM	1284	O	GLN	290	12.115	10.141	28.832	1.00	0.00	RX0	O
ATOM	1285	N	GLY	291	10.739	8.533	29.672	1.00	0.00	RX0	N
ATOM	1286	H	GLY	291	10.121	8.322	30.428	0.00	0.00	RX0	H
ATOM	1287	CA	GLY	291	10.952	7.542	28.589	1.00	0.00	RX0	C
ATOM	1288	C	GLY	291	10.769	8.134	27.181	1.00	0.00	RX0	C
ATOM	1289	O	GLY	291	11.559	7.865	26.269	1.00	0.00	RX0	O
ATOM	1290	N	GLU	292	9.854	9.086	27.078	1.00	0.00	RX0	N
ATOM	1291	H	GLU	292	9.205	9.179	27.836	0.00	0.00	RX0	H
ATOM	1292	CA	GLU	292	9.541	9.789	25.813	1.00	0.00	RX0	C
ATOM	1293	CB	GLU	292	8.224	10.557	25.961	1.00	0.00	RX0	C
ATOM	1294	CG	GLU	292	6.957	9.692	26.068	1.00	0.00	RX0	C
ATOM	1295	CD	GLU	292	7.023	8.741	27.249	1.00	0.00	RX0	C
ATOM	1296	OE1	GLU	292	7.186	9.181	28.384	1.00	0.00	RX0	O
ATOM	1297	OE2	GLU	292	6.949	7.538	27.045	1.00	0.00	RX0	O
ATOM	1298	C	GLU	292	10.671	10.744	25.400	1.00	0.00	RX0	C
ATOM	1299	O	GLU	292	11.065	10.802	24.241	1.00	0.00	RX0	O
ATOM	1300	N	GLU	293	11.241	11.417	26.395	1.00	0.00	RX0	N
ATOM	1301	H	GLU	293	10.969	11.202	27.333	0.00	0.00	RX0	H
ATOM	1302	CA	GLU	293	12.389	12.320	26.184	1.00	0.00	RX0	C
ATOM	1303	CB	GLU	293	12.613	13.166	27.433	1.00	0.00	RX0	C
ATOM	1304	CG	GLU	293	11.374	13.935	27.889	1.00	0.00	RX0	C
ATOM	1305	CD	GLU	293	11.645	14.516	29.261	1.00	0.00	RX0	C
ATOM	1306	OE1	GLU	293	12.533	14.028	29.949	1.00	0.00	RX0	O
ATOM	1307	OE2	GLU	293	10.970	15.453	29.668	1.00	0.00	RX0	O
ATOM	1308	C	GLU	293	13.669	11.544	25.843	1.00	0.00	RX0	C
ATOM	1309	O	GLU	293	14.364	11.888	24.886	1.00	0.00	RX0	O
ATOM	1310	N	PHE	294	13.855	10.409	26.517	1.00	0.00	RX0	N
ATOM	1311	H	PHE	294	13.198	10.198	27.243	0.00	0.00	RX0	H
ATOM	1312	CA	PHE	294	14.999	9.503	26.300	1.00	0.00	RX0	C
ATOM	1313	CB	PHE	294	14.900	8.308	27.249	1.00	0.00	RX0	C
ATOM	1314	CG	PHE	294	15.864	7.230	26.813	1.00	0.00	RX0	C
ATOM	1315	CD1	PHE	294	17.254	7.475	26.852	1.00	0.00	RX0	C
ATOM	1316	CD2	PHE	294	15.350	5.997	26.358	1.00	0.00	RX0	C
ATOM	1317	CE1	PHE	294	18.142	6.477	26.405	1.00	0.00	RX0	C
ATOM	1318	CE2	PHE	294	16.238	5.001	25.911	1.00	0.00	RX0	C
ATOM	1319	CZ	PHE	294	17.625	5.253	25.934	1.00	0.00	RX0	C
ATOM	1320	C	PHE	294	15.115	9.012	24.847	1.00	0.00	RX0	C
ATOM	1321	O	PHE	294	16.186	9.132	24.238	1.00	0.00	RX0	O
ATOM	1322	N	VAL	295	14.000	8.581	24.280	1.00	0.00	RX0	N
ATOM	1323	H	VAL	295	13.159	8.562	24.829	0.00	0.00	RX0	H
ATOM	1324	CA	VAL	295	13.976	8.026	22.907	1.00	0.00	RX0	C
ATOM	1325	CB	VAL	295	12.683	7.252	22.620	1.00	0.00	RX0	C
ATOM	1326	CG1	VAL	295	12.575	6.075	23.587	1.00	0.00	RX0	C
ATOM	1327	CG2	VAL	295	11.428	8.122	22.657	1.00	0.00	RX0	C
ATOM	1328	C	VAL	295	14.286	9.097	21.847	1.00	0.00	RX0	C
ATOM	1329	O	VAL	295	14.999	8.845	20.884	1.00	0.00	RX0	O
ATOM	1330	N	CYS	296	13.866	10.330	22.156	1.00	0.00	RX0	N
ATOM	1331	H	CYS	296	13.305	10.479	22.974	0.00	0.00	RX0	H
ATOM	1332	CA	CYS	296	14.167	11.498	21.314	1.00	0.00	RX0	C
ATOM	1333	CB	CYS	296	13.265	12.653	21.731	1.00	0.00	RX0	C
ATOM	1334	SG	CYS	296	11.518	12.260	21.469	1.00	0.00	RX0	S
ATOM	1335	C	CYS	296	15.661	11.843	21.352	1.00	0.00	RX0	C
ATOM	1336	O	CYS	296	16.290	11.969	20.305	1.00	0.00	RX0	O
ATOM	1337	N	LEU	297	16.247	11.750	22.547	1.00	0.00	RX0	N
ATOM	1338	H	LEU	297	15.681	11.545	23.350	0.00	0.00	RX0	H
ATOM	1339	CA	LEU	297	17.681	12.031	22.752	1.00	0.00	RX0	C
ATOM	1340	CB	LEU	297	18.015	12.112	24.240	1.00	0.00	RX0	C

ATOM	1341	CG	LEU	297	17.338	13.284	24.948	1.00	0.00	RX0	C
ATOM	1342	CD1	LEU	297	17.611	13.260	26.452	1.00	0.00	RX0	C
ATOM	1343	CD2	LEU	297	17.711	14.626	24.316	1.00	0.00	RX0	C
ATOM	1344	C	LEU	297	18.589	11.009	22.068	1.00	0.00	RX0	C
ATOM	1345	O	LEU	297	19.526	11.385	21.359	1.00	0.00	RX0	O
ATOM	1346	N	LYS	298	18.173	9.750	22.123	1.00	0.00	RX0	N
ATOM	1347	H	LYS	298	17.359	9.543	22.671	0.00	0.00	RX0	H
ATOM	1348	CA	LYS	298	18.945	8.654	21.515	1.00	0.00	RX0	C
ATOM	1349	CB	LYS	298	18.415	7.325	22.049	1.00	0.00	RX0	C
ATOM	1350	CG	LYS	298	19.299	6.121	21.734	1.00	0.00	RX0	C
ATOM	1351	CD	LYS	298	18.720	4.850	22.351	1.00	0.00	RX0	C
ATOM	1352	CE	LYS	298	17.252	4.703	21.958	1.00	0.00	RX0	C
ATOM	1353	NZ	LYS	298	16.704	3.430	22.424	1.00	0.00	RX0	N
ATOM	1354	HZ1	LYS	298	15.948	3.128	21.770	0.00	0.00	RX0	H
ATOM	1355	HZ2	LYS	298	17.386	2.664	22.536	0.00	0.00	RX0	H
ATOM	1356	HZ3	LYS	298	16.138	3.540	23.295	0.00	0.00	RX0	H
ATOM	1357	C	LYS	298	18.925	8.733	19.978	1.00	0.00	RX0	C
ATOM	1358	O	LYS	298	19.964	8.557	19.332	1.00	0.00	RX0	O
ATOM	1359	N	SER	299	17.791	9.152	19.431	1.00	0.00	RX0	N
ATOM	1360	H	SER	299	16.964	9.263	19.983	0.00	0.00	RX0	H
ATOM	1361	CA	SER	299	17.645	9.383	17.977	1.00	0.00	RX0	C
ATOM	1362	CB	SER	299	16.155	9.360	17.703	1.00	0.00	RX0	C
ATOM	1363	OG	SER	299	15.660	8.195	18.370	1.00	0.00	RX0	O
ATOM	1364	HG	SER	299	15.146	8.490	19.115	0.00	0.00	RX0	H
ATOM	1365	C	SER	299	18.416	10.621	17.504	1.00	0.00	RX0	C
ATOM	1366	O	SER	299	19.051	10.583	16.444	1.00	0.00	RX0	O
ATOM	1367	N	ILE	300	18.478	11.644	18.354	1.00	0.00	RX0	N
ATOM	1368	H	ILE	300	17.952	11.595	19.206	0.00	0.00	RX0	H
ATOM	1369	CA	ILE	300	19.283	12.860	18.096	1.00	0.00	RX0	C
ATOM	1370	CB	ILE	300	19.080	13.934	19.168	1.00	0.00	RX0	C
ATOM	1371	CG2	ILE	300	20.123	15.048	19.044	1.00	0.00	RX0	C
ATOM	1372	CG1	ILE	300	17.675	14.518	19.095	1.00	0.00	RX0	C
ATOM	1373	CD1	ILE	300	17.416	15.534	20.205	1.00	0.00	RX0	C
ATOM	1374	C	ILE	300	20.773	12.490	18.013	1.00	0.00	RX0	C
ATOM	1375	O	ILE	300	21.456	12.924	17.087	1.00	0.00	RX0	O
ATOM	1376	N	ILE	301	21.227	11.630	18.921	1.00	0.00	RX0	N
ATOM	1377	H	ILE	301	20.604	11.321	19.644	0.00	0.00	RX0	H
ATOM	1378	CA	ILE	301	22.629	11.156	18.925	1.00	0.00	RX0	C
ATOM	1379	CB	ILE	301	22.865	10.197	20.088	1.00	0.00	RX0	C
ATOM	1380	CG2	ILE	301	24.137	9.384	19.874	1.00	0.00	RX0	C
ATOM	1381	CG1	ILE	301	22.900	10.957	21.412	1.00	0.00	RX0	C
ATOM	1382	CD1	ILE	301	23.257	10.041	22.582	1.00	0.00	RX0	C
ATOM	1383	C	ILE	301	22.948	10.474	17.584	1.00	0.00	RX0	C
ATOM	1384	O	ILE	301	23.927	10.823	16.926	1.00	0.00	RX0	O
ATOM	1385	N	LEU	302	22.047	9.582	17.175	1.00	0.00	RX0	N
ATOM	1386	H	LEU	302	21.274	9.366	17.776	0.00	0.00	RX0	H
ATOM	1387	CA	LEU	302	22.205	8.836	15.917	1.00	0.00	RX0	C
ATOM	1388	CB	LEU	302	20.992	7.930	15.709	1.00	0.00	RX0	C
ATOM	1389	CG	LEU	302	21.000	7.210	14.359	1.00	0.00	RX0	C
ATOM	1390	CD1	LEU	302	22.181	6.251	14.224	1.00	0.00	RX0	C
ATOM	1391	CD2	LEU	302	19.665	6.526	14.079	1.00	0.00	RX0	C
ATOM	1392	C	LEU	302	22.391	9.757	14.699	1.00	0.00	RX0	C
ATOM	1393	O	LEU	302	23.285	9.544	13.882	1.00	0.00	RX0	O
ATOM	1394	N	LEU	303	21.581	10.806	14.658	1.00	0.00	RX0	N
ATOM	1395	H	LEU	303	20.929	10.942	15.408	0.00	0.00	RX0	H
ATOM	1396	CA	LEU	303	21.546	11.719	13.502	1.00	0.00	RX0	C
ATOM	1397	CB	LEU	303	20.124	12.232	13.311	1.00	0.00	RX0	C
ATOM	1398	CG	LEU	303	19.163	11.066	13.082	1.00	0.00	RX0	C
ATOM	1399	CD1	LEU	303	17.707	11.488	13.251	1.00	0.00	RX0	C
ATOM	1400	CD2	LEU	303	19.418	10.369	11.745	1.00	0.00	RX0	C
ATOM	1401	C	LEU	303	22.576	12.849	13.543	1.00	0.00	RX0	C

ATOM	1402	O	LEU	303	23.073	13.268	12.494	1.00	0.00	RX0	O
ATOM	1403	N	ASN	304	22.971	13.240	14.745	1.00	0.00	RX0	N
ATOM	1404	H	ASN	304	22.642	12.724	15.537	0.00	0.00	RX0	H
ATOM	1405	CA	ASN	304	23.832	14.419	14.940	1.00	0.00	RX0	C
ATOM	1406	CB	ASN	304	23.520	15.282	16.178	1.00	0.00	RX0	C
ATOM	1407	CG	ASN	304	24.445	14.903	17.332	1.00	0.00	RX0	C
ATOM	1408	OD1	ASN	304	25.502	15.475	17.601	1.00	0.00	RX0	O
ATOM	1409	ND2	ASN	304	24.002	13.821	17.978	1.00	0.00	RX0	N
ATOM	1410	HD21	ASN	304	23.083	13.499	17.749	0.00	0.00	RX0	H
ATOM	1411	HD22	ASN	304	24.526	13.241	18.608	0.00	0.00	RX0	H
ATOM	1412	C	ASN	304	25.330	14.097	14.987	1.00	0.00	RX0	C
ATOM	1413	O	ASN	304	26.132	14.783	14.344	1.00	0.00	RX0	O
ATOM	1414	N	SER	305	25.693	13.015	15.656	1.00	0.00	RX0	N
ATOM	1415	H	SER	305	25.044	12.412	16.130	0.00	0.00	RX0	H
ATOM	1416	CA	SER	305	27.104	12.765	16.012	1.00	0.00	RX0	C
ATOM	1417	CB	SER	305	27.087	11.570	16.950	1.00	0.00	RX0	C
ATOM	1418	OG	SER	305	26.111	11.864	17.962	1.00	0.00	RX0	O
ATOM	1419	HG	SER	305	26.310	11.245	18.672	0.00	0.00	RX0	H
ATOM	1420	C	SER	305	28.103	12.695	14.846	1.00	0.00	RX0	C
ATOM	1421	O	SER	305	29.198	13.228	14.946	1.00	0.00	RX0	O
ATOM	1422	N	GLY	306	27.634	12.177	13.693	1.00	0.00	RX0	N
ATOM	1423	H	GLY	306	26.686	11.863	13.636	0.00	0.00	RX0	H
ATOM	1424	CA	GLY	306	28.500	12.079	12.501	1.00	0.00	RX0	C
ATOM	1425	C	GLY	306	28.091	12.961	11.315	1.00	0.00	RX0	C
ATOM	1426	O	GLY	306	28.756	12.897	10.274	1.00	0.00	RX0	O
ATOM	1427	N	VAL	307	27.242	13.954	11.536	1.00	0.00	RX0	N
ATOM	1428	H	VAL	307	26.873	14.100	12.457	0.00	0.00	RX0	H
ATOM	1429	CA	VAL	307	26.760	14.815	10.435	1.00	0.00	RX0	C
ATOM	1430	CB	VAL	307	25.402	15.462	10.752	1.00	0.00	RX0	C
ATOM	1431	CG1	VAL	307	25.497	16.579	11.788	1.00	0.00	RX0	C
ATOM	1432	CG2	VAL	307	24.721	15.937	9.467	1.00	0.00	RX0	C
ATOM	1433	C	VAL	307	27.809	15.841	9.948	1.00	0.00	RX0	C
ATOM	1434	O	VAL	307	27.790	16.280	8.811	1.00	0.00	RX0	O
ATOM	1435	N	TYR	308	28.719	16.199	10.859	1.00	0.00	RX0	N
ATOM	1436	H	TYR	308	28.744	15.711	11.730	0.00	0.00	RX0	H
ATOM	1437	CA	TYR	308	29.761	17.211	10.584	1.00	0.00	RX0	C
ATOM	1438	CB	TYR	308	30.102	17.968	11.866	1.00	0.00	RX0	C
ATOM	1439	CG	TYR	308	28.897	18.757	12.312	1.00	0.00	RX0	C
ATOM	1440	CD1	TYR	308	28.439	19.809	11.528	1.00	0.00	RX0	C
ATOM	1441	CE1	TYR	308	27.335	20.549	11.933	1.00	0.00	RX0	C
ATOM	1442	CD2	TYR	308	28.247	18.438	13.499	1.00	0.00	RX0	C
ATOM	1443	CE2	TYR	308	27.139	19.175	13.902	1.00	0.00	RX0	C
ATOM	1444	CZ	TYR	308	26.686	20.233	13.120	1.00	0.00	RX0	C
ATOM	1445	OH	TYR	308	25.595	20.975	13.522	1.00	0.00	RX0	O
ATOM	1446	HH	TYR	308	24.975	20.415	13.972	0.00	0.00	RX0	H
ATOM	1447	C	TYR	308	31.032	16.660	9.940	1.00	0.00	RX0	C
ATOM	1448	O	TYR	308	31.912	17.411	9.537	1.00	0.00	RX0	O
ATOM	1449	N	THR	309	31.091	15.334	9.861	1.00	0.00	RX0	N
ATOM	1450	H	THR	309	30.342	14.756	10.179	0.00	0.00	RX0	H
ATOM	1451	CA	THR	309	32.303	14.643	9.386	1.00	0.00	RX0	C
ATOM	1452	CB	THR	309	32.578	13.750	10.573	1.00	0.00	RX0	C
ATOM	1453	OG1	THR	309	31.323	13.589	11.261	1.00	0.00	RX0	O
ATOM	1454	HG1	THR	309	30.870	12.876	10.808	0.00	0.00	RX0	H
ATOM	1455	CG2	THR	309	33.613	14.357	11.522	1.00	0.00	RX0	C
ATOM	1456	C	THR	309	32.183	13.866	8.071	1.00	0.00	RX0	C
ATOM	1457	O	THR	309	33.137	13.195	7.681	1.00	0.00	RX0	O
ATOM	1458	N	PHE	310	31.041	13.953	7.382	1.00	0.00	RX0	N
ATOM	1459	H	PHE	310	30.300	14.509	7.752	0.00	0.00	RX0	H
ATOM	1460	CA	PHE	310	30.972	13.467	5.988	1.00	0.00	RX0	C
ATOM	1461	CB	PHE	310	29.596	13.721	5.372	1.00	0.00	RX0	C
ATOM	1462	CG	PHE	310	28.515	12.939	6.078	1.00	0.00	RX0	C

ATOM	1463	CD1	PHE	310	28.442	11.560	5.926	1.00	0.00	RX0	C
ATOM	1464	CD2	PHE	310	27.581	13.601	6.865	1.00	0.00	RX0	C
ATOM	1465	CE1	PHE	310	27.428	10.847	6.555	1.00	0.00	RX0	C
ATOM	1466	CE2	PHE	310	26.567	12.886	7.492	1.00	0.00	RX0	C
ATOM	1467	CZ	PHE	310	26.488	11.509	7.335	1.00	0.00	RX0	C
ATOM	1468	C	PHE	310	32.019	14.245	5.180	1.00	0.00	RX0	C
ATOM	1469	O	PHE	310	32.102	15.468	5.301	1.00	0.00	RX0	O
ATOM	1470	N	LEU	311	32.854	13.508	4.462	1.00	0.00	RX0	N
ATOM	1471	H	LEU	311	32.719	12.519	4.489	0.00	0.00	RX0	H
ATOM	1472	CA	LEU	311	33.988	14.087	3.701	1.00	0.00	RX0	C
ATOM	1473	CB	LEU	311	34.758	12.991	2.963	1.00	0.00	RX0	C
ATOM	1474	CG	LEU	311	35.315	11.930	3.914	1.00	0.00	RX0	C
ATOM	1475	CD1	LEU	311	35.976	10.779	3.154	1.00	0.00	RX0	C
ATOM	1476	CD2	LEU	311	36.246	12.536	4.966	1.00	0.00	RX0	C
ATOM	1477	C	LEU	311	33.468	15.166	2.739	1.00	0.00	RX0	C
ATOM	1478	O	LEU	311	33.554	16.351	2.969	1.00	0.00	RX0	O
ATOM	1479	N	SER	312	32.784	14.619	1.713	1.00	0.00	RX0	N
ATOM	1480	H	SER	312	32.577	13.642	1.741	0.00	0.00	RX0	H
ATOM	1481	CA	SER	312	32.345	15.333	0.526	1.00	0.00	RX0	C
ATOM	1482	CB	SER	312	31.786	14.208	-0.318	1.00	0.00	RX0	C
ATOM	1483	OG	SER	312	32.586	13.060	-0.012	1.00	0.00	RX0	O
ATOM	1484	HG	SER	312	33.400	13.154	-0.493	0.00	0.00	RX0	H
ATOM	1485	C	SER	312	31.423	16.517	0.816	1.00	0.00	RX0	C
ATOM	1486	O	SER	312	31.042	16.847	1.948	1.00	0.00	RX0	O
ATOM	1487	N	SER	313	30.837	16.923	-0.276	1.00	0.00	RX0	N
ATOM	1488	H	SER	313	31.194	16.607	-1.156	0.00	0.00	RX0	H
ATOM	1489	CA	SER	313	29.838	17.983	-0.455	1.00	0.00	RX0	C
ATOM	1490	CB	SER	313	30.335	19.323	0.093	1.00	0.00	RX0	C
ATOM	1491	OG	SER	313	30.311	19.223	1.533	1.00	0.00	RX0	O
ATOM	1492	HG	SER	313	31.130	18.789	1.769	0.00	0.00	RX0	H
ATOM	1493	C	SER	313	29.353	17.880	-1.909	1.00	0.00	RX0	C
ATOM	1494	O	SER	313	29.168	18.836	-2.628	1.00	0.00	RX0	O
ATOM	1495	N	THR	314	29.278	16.602	-2.358	1.00	0.00	RX0	N
ATOM	1496	H	THR	314	29.439	15.836	-1.741	0.00	0.00	RX0	H
ATOM	1497	CA	THR	314	28.609	16.250	-3.614	1.00	0.00	RX0	C
ATOM	1498	CB	THR	314	28.748	14.743	-3.668	1.00	0.00	RX0	C
ATOM	1499	OG1	THR	314	29.854	14.390	-2.823	1.00	0.00	RX0	O
ATOM	1500	HG1	THR	314	30.034	13.469	-2.992	0.00	0.00	RX0	H
ATOM	1501	CG2	THR	314	28.926	14.207	-5.090	1.00	0.00	RX0	C
ATOM	1502	C	THR	314	27.167	16.747	-3.490	1.00	0.00	RX0	C
ATOM	1503	O	THR	314	26.675	16.978	-2.368	1.00	0.00	RX0	O
ATOM	1504	N	LEU	315	26.451	16.801	-4.589	1.00	0.00	RX0	N
ATOM	1505	H	LEU	315	26.878	16.639	-5.477	0.00	0.00	RX0	H
ATOM	1506	CA	LEU	315	25.050	17.256	-4.537	1.00	0.00	RX0	C
ATOM	1507	CB	LEU	315	24.457	17.331	-5.943	1.00	0.00	RX0	C
ATOM	1508	CG	LEU	315	23.032	17.887	-5.943	1.00	0.00	RX0	C
ATOM	1509	CD1	LEU	315	22.968	19.296	-5.347	1.00	0.00	RX0	C
ATOM	1510	CD2	LEU	315	22.396	17.820	-7.332	1.00	0.00	RX0	C
ATOM	1511	C	LEU	315	24.190	16.343	-3.639	1.00	0.00	RX0	C
ATOM	1512	O	LEU	315	23.484	16.800	-2.757	1.00	0.00	RX0	O
ATOM	1513	N	LYS	316	24.524	15.049	-3.730	1.00	0.00	RX0	N
ATOM	1514	H	LYS	316	25.102	14.782	-4.495	0.00	0.00	RX0	H
ATOM	1515	CA	LYS	316	23.912	13.990	-2.918	1.00	0.00	RX0	C
ATOM	1516	CB	LYS	316	24.332	12.617	-3.442	1.00	0.00	RX0	C
ATOM	1517	CG	LYS	316	23.144	11.704	-3.762	1.00	0.00	RX0	C
ATOM	1518	CD	LYS	316	22.483	11.039	-2.554	1.00	0.00	RX0	C
ATOM	1519	CE	LYS	316	20.956	11.078	-2.638	1.00	0.00	RX0	C
ATOM	1520	NZ	LYS	316	20.497	12.359	-2.093	1.00	0.00	RX0	N
ATOM	1521	HZ1	LYS	316	19.523	12.604	-2.366	0.00	0.00	RX0	H
ATOM	1522	HZ2	LYS	316	20.538	12.320	-1.054	0.00	0.00	RX0	H
ATOM	1523	HZ3	LYS	316	21.019	13.200	-2.425	0.00	0.00	RX0	H

ATOM	1524	C	LYS	316	24.213	14.187	-1.422	1.00	0.00	RX0	C
ATOM	1525	O	LYS	316	23.297	14.194	-0.611	1.00	0.00	RX0	O
ATOM	1526	N	SER	317	25.467	14.540	-1.122	1.00	0.00	RX0	N
ATOM	1527	H	SER	317	26.099	14.717	-1.869	0.00	0.00	RX0	H
ATOM	1528	CA	SER	317	25.934	14.787	0.260	1.00	0.00	RX0	C
ATOM	1529	CB	SER	317	27.466	14.861	0.211	1.00	0.00	RX0	C
ATOM	1530	OG	SER	317	28.077	14.577	1.476	1.00	0.00	RX0	O
ATOM	1531	HG	SER	317	27.713	13.737	1.762	0.00	0.00	RX0	H
ATOM	1532	C	SER	317	25.242	16.001	0.898	1.00	0.00	RX0	C
ATOM	1533	O	SER	317	24.716	15.915	2.007	1.00	0.00	RX0	O
ATOM	1534	N	LEU	318	25.067	17.043	0.088	1.00	0.00	RX0	N
ATOM	1535	H	LEU	318	25.352	16.977	-0.870	0.00	0.00	RX0	H
ATOM	1536	CA	LEU	318	24.405	18.288	0.525	1.00	0.00	RX0	C
ATOM	1537	CB	LEU	318	24.580	19.375	-0.532	1.00	0.00	RX0	C
ATOM	1538	CG	LEU	318	26.045	19.771	-0.691	1.00	0.00	RX0	C
ATOM	1539	CD1	LEU	318	26.256	20.719	-1.872	1.00	0.00	RX0	C
ATOM	1540	CD2	LEU	318	26.617	20.325	0.615	1.00	0.00	RX0	C
ATOM	1541	C	LEU	318	22.918	18.061	0.823	1.00	0.00	RX0	C
ATOM	1542	O	LEU	318	22.412	18.453	1.877	1.00	0.00	RX0	O
ATOM	1543	N	GLU	319	22.299	17.244	-0.024	1.00	0.00	RX0	N
ATOM	1544	H	GLU	319	22.768	16.913	-0.846	0.00	0.00	RX0	H
ATOM	1545	CA	GLU	319	20.900	16.808	0.157	1.00	0.00	RX0	C
ATOM	1546	CB	GLU	319	20.408	15.846	-0.925	1.00	0.00	RX0	C
ATOM	1547	CG	GLU	319	20.243	16.240	-2.389	1.00	0.00	RX0	C
ATOM	1548	CD	GLU	319	20.058	14.932	-3.140	1.00	0.00	RX0	C
ATOM	1549	OE1	GLU	319	20.992	14.479	-3.797	1.00	0.00	RX0	O
ATOM	1550	OE2	GLU	319	19.034	14.273	-2.977	1.00	0.00	RX0	O
ATOM	1551	C	GLU	319	20.725	15.924	1.402	1.00	0.00	RX0	C
ATOM	1552	O	GLU	319	19.808	16.153	2.196	1.00	0.00	RX0	O
ATOM	1553	N	GLU	320	21.706	15.058	1.645	1.00	0.00	RX0	N
ATOM	1554	H	GLU	320	22.382	14.893	0.928	0.00	0.00	RX0	H
ATOM	1555	CA	GLU	320	21.731	14.149	2.812	1.00	0.00	RX0	C
ATOM	1556	CB	GLU	320	22.733	12.983	2.716	1.00	0.00	RX0	C
ATOM	1557	CG	GLU	320	22.756	12.137	1.422	1.00	0.00	RX0	C
ATOM	1558	CD	GLU	320	21.398	11.655	0.903	1.00	0.00	RX0	C
ATOM	1559	OE1	GLU	320	21.239	10.465	0.651	1.00	0.00	RX0	O
ATOM	1560	OE2	GLU	320	20.520	12.464	0.619	1.00	0.00	RX0	O
ATOM	1561	C	GLU	320	21.765	14.931	4.131	1.00	0.00	RX0	C
ATOM	1562	O	GLU	320	20.881	14.754	4.973	1.00	0.00	RX0	O
ATOM	1563	N	LYS	321	22.647	15.926	4.188	1.00	0.00	RX0	N
ATOM	1564	H	LYS	321	23.234	16.045	3.384	0.00	0.00	RX0	H
ATOM	1565	CA	LYS	321	22.801	16.771	5.391	1.00	0.00	RX0	C
ATOM	1566	CB	LYS	321	24.080	17.633	5.232	1.00	0.00	RX0	C
ATOM	1567	CG	LYS	321	25.361	16.834	4.881	1.00	0.00	RX0	C
ATOM	1568	CD	LYS	321	26.626	17.639	4.482	1.00	0.00	RX0	C
ATOM	1569	CE	LYS	321	27.750	16.752	3.887	1.00	0.00	RX0	C
ATOM	1570	NZ	LYS	321	29.005	17.472	3.559	1.00	0.00	RX0	N
ATOM	1571	HZ1	LYS	321	29.681	16.829	3.085	0.00	0.00	RX0	H
ATOM	1572	HZ2	LYS	321	28.869	18.274	2.909	0.00	0.00	RX0	H
ATOM	1573	HZ3	LYS	321	29.469	17.807	4.426	0.00	0.00	RX0	H
ATOM	1574	C	LYS	321	21.549	17.612	5.656	1.00	0.00	RX0	C
ATOM	1575	O	LYS	321	21.102	17.724	6.798	1.00	0.00	RX0	O
ATOM	1576	N	ASP	322	20.935	18.099	4.575	1.00	0.00	RX0	N
ATOM	1577	H	ASP	322	21.312	17.934	3.660	0.00	0.00	RX0	H
ATOM	1578	CA	ASP	322	19.715	18.915	4.684	1.00	0.00	RX0	C
ATOM	1579	CB	ASP	322	19.386	19.459	3.291	1.00	0.00	RX0	C
ATOM	1580	CG	ASP	322	18.039	20.144	3.270	1.00	0.00	RX0	C
ATOM	1581	OD1	ASP	322	17.648	20.736	4.265	1.00	0.00	RX0	O
ATOM	1582	OD2	ASP	322	17.349	20.049	2.260	1.00	0.00	RX0	O
ATOM	1583	C	ASP	322	18.561	18.097	5.282	1.00	0.00	RX0	C
ATOM	1584	O	ASP	322	17.955	18.512	6.263	1.00	0.00	RX0	O

ATOM	1585	N	HIS	323	18.423	16.872	4.772	1.00	0.00	RX0	N
ATOM	1586	H	HIS	323	19.038	16.602	4.025	0.00	0.00	RX0	H
ATOM	1587	CA	HIS	323	17.423	15.916	5.274	1.00	0.00	RX0	C
ATOM	1588	CB	HIS	323	17.459	14.618	4.464	1.00	0.00	RX0	C
ATOM	1589	CG	HIS	323	16.296	13.748	4.877	1.00	0.00	RX0	C
ATOM	1590	ND1	HIS	323	16.324	12.403	4.928	1.00	0.00	RX0	N
ATOM	1591	HD1	HIS	323	17.076	11.810	4.707	0.00	0.00	RX0	H
ATOM	1592	CD2	HIS	323	15.022	14.181	5.248	1.00	0.00	RX0	C
ATOM	1593	NE2	HIS	323	14.277	13.089	5.525	1.00	0.00	RX0	N
ATOM	1594	CE1	HIS	323	15.077	11.988	5.328	1.00	0.00	RX0	C
ATOM	1595	C	HIS	323	17.630	15.607	6.766	1.00	0.00	RX0	C
ATOM	1596	O	HIS	323	16.677	15.663	7.540	1.00	0.00	RX0	O
ATOM	1597	N	ILE	324	18.888	15.420	7.164	1.00	0.00	RX0	N
ATOM	1598	H	ILE	324	19.613	15.432	6.470	0.00	0.00	RX0	H
ATOM	1599	CA	ILE	324	19.235	15.107	8.570	1.00	0.00	RX0	C
ATOM	1600	CB	ILE	324	20.719	14.765	8.707	1.00	0.00	RX0	C
ATOM	1601	CG2	ILE	324	21.116	14.614	10.175	1.00	0.00	RX0	C
ATOM	1602	CG1	ILE	324	21.045	13.500	7.913	1.00	0.00	RX0	C
ATOM	1603	CD1	ILE	324	22.533	13.149	7.930	1.00	0.00	RX0	C
ATOM	1604	C	ILE	324	18.844	16.271	9.496	1.00	0.00	RX0	C
ATOM	1605	O	ILE	324	18.200	16.053	10.527	1.00	0.00	RX0	O
ATOM	1606	N	HIS	325	19.159	17.485	9.071	1.00	0.00	RX0	N
ATOM	1607	H	HIS	325	19.601	17.591	8.178	0.00	0.00	RX0	H
ATOM	1608	CA	HIS	325	18.840	18.694	9.856	1.00	0.00	RX0	C
ATOM	1609	CB	HIS	325	19.592	19.915	9.334	1.00	0.00	RX0	C
ATOM	1610	CG	HIS	325	21.048	19.764	9.699	1.00	0.00	RX0	C
ATOM	1611	ND1	HIS	325	21.994	19.371	8.831	1.00	0.00	RX0	N
ATOM	1612	HD1	HIS	325	21.837	19.125	7.893	0.00	0.00	RX0	H
ATOM	1613	CD2	HIS	325	21.642	19.975	10.947	1.00	0.00	RX0	C
ATOM	1614	NE2	HIS	325	22.965	19.702	10.817	1.00	0.00	RX0	N
ATOM	1615	CE1	HIS	325	23.180	19.332	9.514	1.00	0.00	RX0	C
ATOM	1616	C	HIS	325	17.335	18.955	9.948	1.00	0.00	RX0	C
ATOM	1617	O	HIS	325	16.820	19.290	11.021	1.00	0.00	RX0	O
ATOM	1618	N	ARG	326	16.631	18.584	8.887	1.00	0.00	RX0	N
ATOM	1619	H	ARG	326	17.127	18.354	8.048	0.00	0.00	RX0	H
ATOM	1620	CA	ARG	326	15.160	18.594	8.873	1.00	0.00	RX0	C
ATOM	1621	CB	ARG	326	14.655	18.404	7.441	1.00	0.00	RX0	C
ATOM	1622	CG	ARG	326	14.680	19.703	6.633	1.00	0.00	RX0	C
ATOM	1623	CD	ARG	326	14.139	19.551	5.207	1.00	0.00	RX0	C
ATOM	1624	NE	ARG	326	15.179	19.162	4.254	1.00	0.00	RX0	N
ATOM	1625	HE	ARG	326	15.919	19.841	4.101	0.00	0.00	RX0	H
ATOM	1626	CZ	ARG	326	15.138	17.981	3.577	1.00	0.00	RX0	C
ATOM	1627	NH1	ARG	326	14.165	17.094	3.866	1.00	0.00	RX0	N
ATOM	1628	HH11	ARG	326	14.077	16.212	3.395	0.00	0.00	RX0	H
ATOM	1629	HH12	ARG	326	13.492	17.295	4.582	0.00	0.00	RX0	H
ATOM	1630	NH2	ARG	326	16.064	17.715	2.633	1.00	0.00	RX0	N
ATOM	1631	HH21	ARG	326	16.147	16.864	2.112	0.00	0.00	RX0	H
ATOM	1632	HH22	ARG	326	16.744	18.436	2.416	0.00	0.00	RX0	H
ATOM	1633	C	ARG	326	14.537	17.576	9.843	1.00	0.00	RX0	C
ATOM	1634	O	ARG	326	13.617	17.928	10.589	1.00	0.00	RX0	O
ATOM	1635	N	VAL	327	15.171	16.419	9.987	1.00	0.00	RX0	N
ATOM	1636	H	VAL	327	15.982	16.249	9.423	0.00	0.00	RX0	H
ATOM	1637	CA	VAL	327	14.709	15.376	10.935	1.00	0.00	RX0	C
ATOM	1638	CB	VAL	327	15.276	13.993	10.606	1.00	0.00	RX0	C
ATOM	1639	CG1	VAL	327	14.753	12.958	11.601	1.00	0.00	RX0	C
ATOM	1640	CG2	VAL	327	14.932	13.578	9.176	1.00	0.00	RX0	C
ATOM	1641	C	VAL	327	15.041	15.781	12.381	1.00	0.00	RX0	C
ATOM	1642	O	VAL	327	14.187	15.661	13.270	1.00	0.00	RX0	O
ATOM	1643	N	LEU	328	16.212	16.371	12.574	1.00	0.00	RX0	N
ATOM	1644	H	LEU	328	16.818	16.509	11.789	0.00	0.00	RX0	H
ATOM	1645	CA	LEU	328	16.631	16.899	13.887	1.00	0.00	RX0	C

ATOM	1646	CB	LEU	328	18.071	17.406	13.828	1.00	0.00	RX0	C
ATOM	1647	CG	LEU	328	19.077	16.259	13.764	1.00	0.00	RX0	C
ATOM	1648	CD1	LEU	328	20.500	16.754	13.500	1.00	0.00	RX0	C
ATOM	1649	CD2	LEU	328	18.994	15.385	15.016	1.00	0.00	RX0	C
ATOM	1650	C	LEU	328	15.692	18.005	14.386	1.00	0.00	RX0	C
ATOM	1651	O	LEU	328	15.231	17.955	15.519	1.00	0.00	RX0	O
ATOM	1652	N	ASP	329	15.222	18.821	13.436	1.00	0.00	RX0	N
ATOM	1653	H	ASP	329	15.682	18.904	12.550	0.00	0.00	RX0	H
ATOM	1654	CA	ASP	329	14.223	19.870	13.722	1.00	0.00	RX0	C
ATOM	1655	CB	ASP	329	14.069	20.831	12.541	1.00	0.00	RX0	C
ATOM	1656	CG	ASP	329	15.289	21.728	12.442	1.00	0.00	RX0	C
ATOM	1657	OD1	ASP	329	16.078	21.761	13.388	1.00	0.00	RX0	O
ATOM	1658	OD2	ASP	329	15.445	22.398	11.421	1.00	0.00	RX0	O
ATOM	1659	C	ASP	329	12.864	19.312	14.154	1.00	0.00	RX0	C
ATOM	1660	O	ASP	329	12.272	19.788	15.128	1.00	0.00	RX0	O
ATOM	1661	N	LYS	330	12.463	18.218	13.512	1.00	0.00	RX0	N
ATOM	1662	H	LYS	330	13.022	17.921	12.736	0.00	0.00	RX0	H
ATOM	1663	CA	LYS	330	11.217	17.525	13.871	1.00	0.00	RX0	C
ATOM	1664	CB	LYS	330	10.800	16.458	12.861	1.00	0.00	RX0	C
ATOM	1665	CG	LYS	330	9.628	15.599	13.368	1.00	0.00	RX0	C
ATOM	1666	CD	LYS	330	8.371	16.369	13.802	1.00	0.00	RX0	C
ATOM	1667	CE	LYS	330	7.825	17.300	12.728	1.00	0.00	RX0	C
ATOM	1668	NZ	LYS	330	7.441	16.476	11.582	1.00	0.00	RX0	N
ATOM	1669	HZ1	LYS	330	7.131	17.100	10.816	0.00	0.00	RX0	H
ATOM	1670	HZ2	LYS	330	8.253	15.893	11.283	0.00	0.00	RX0	H
ATOM	1671	HZ3	LYS	330	6.659	15.855	11.878	0.00	0.00	RX0	H
ATOM	1672	C	LYS	330	11.283	16.915	15.278	1.00	0.00	RX0	C
ATOM	1673	O	LYS	330	10.354	17.095	16.067	1.00	0.00	RX0	O
ATOM	1674	N	ILE	331	12.441	16.375	15.625	1.00	0.00	RX0	N
ATOM	1675	H	ILE	331	13.185	16.378	14.951	0.00	0.00	RX0	H
ATOM	1676	CA	ILE	331	12.656	15.802	16.972	1.00	0.00	RX0	C
ATOM	1677	CB	ILE	331	13.953	14.997	17.053	1.00	0.00	RX0	C
ATOM	1678	CG2	ILE	331	14.062	14.329	18.421	1.00	0.00	RX0	C
ATOM	1679	CG1	ILE	331	14.043	13.952	15.942	1.00	0.00	RX0	C
ATOM	1680	CD1	ILE	331	15.358	13.173	15.984	1.00	0.00	RX0	C
ATOM	1681	C	ILE	331	12.642	16.922	18.027	1.00	0.00	RX0	C
ATOM	1682	O	ILE	331	12.078	16.732	19.120	1.00	0.00	RX0	O
ATOM	1683	N	THR	332	13.158	18.084	17.675	1.00	0.00	RX0	N
ATOM	1684	H	THR	332	13.577	18.203	16.774	0.00	0.00	RX0	H
ATOM	1685	CA	THR	332	13.155	19.265	18.570	1.00	0.00	RX0	C
ATOM	1686	CB	THR	332	14.034	20.315	17.913	1.00	0.00	RX0	C
ATOM	1687	OG1	THR	332	15.301	19.707	17.625	1.00	0.00	RX0	O
ATOM	1688	HG1	THR	332	15.251	19.375	16.732	0.00	0.00	RX0	H
ATOM	1689	CG2	THR	332	14.211	21.553	18.794	1.00	0.00	RX0	C
ATOM	1690	C	THR	332	11.706	19.696	18.836	1.00	0.00	RX0	C
ATOM	1691	O	THR	332	11.302	19.832	19.995	1.00	0.00	RX0	O
ATOM	1692	N	ASP	333	10.912	19.714	17.772	1.00	0.00	RX0	N
ATOM	1693	H	ASP	333	11.280	19.672	16.837	0.00	0.00	RX0	H
ATOM	1694	CA	ASP	333	9.466	20.017	17.863	1.00	0.00	RX0	C
ATOM	1695	CB	ASP	333	8.670	19.790	16.568	1.00	0.00	RX0	C
ATOM	1696	CG	ASP	333	9.095	20.573	15.353	1.00	0.00	RX0	C
ATOM	1697	OD1	ASP	333	9.499	21.722	15.501	1.00	0.00	RX0	O
ATOM	1698	OD2	ASP	333	8.967	20.030	14.251	1.00	0.00	RX0	O
ATOM	1699	C	ASP	333	8.732	19.000	18.747	1.00	0.00	RX0	C
ATOM	1700	O	ASP	333	7.880	19.374	19.559	1.00	0.00	RX0	O
ATOM	1701	N	THR	334	9.187	17.759	18.682	1.00	0.00	RX0	N
ATOM	1702	H	THR	334	9.941	17.582	18.049	0.00	0.00	RX0	H
ATOM	1703	CA	THR	334	8.631	16.638	19.462	1.00	0.00	RX0	C
ATOM	1704	CB	THR	334	9.175	15.372	18.821	1.00	0.00	RX0	C
ATOM	1705	OG1	THR	334	8.792	15.339	17.438	1.00	0.00	RX0	O
ATOM	1706	HG1	THR	334	9.148	16.125	17.032	0.00	0.00	RX0	H

ATOM	1707	CG2	THR	334	8.721	14.119	19.557	1.00	0.00	RX0	C
ATOM	1708	C	THR	334	8.961	16.782	20.953	1.00	0.00	RX0	C
ATOM	1709	O	THR	334	8.059	16.689	21.789	1.00	0.00	RX0	O
ATOM	1710	N	LEU	335	10.217	17.081	21.263	1.00	0.00	RX0	N
ATOM	1711	H	LEU	335	10.880	17.204	20.523	0.00	0.00	RX0	H
ATOM	1712	CA	LEU	335	10.648	17.349	22.650	1.00	0.00	RX0	C
ATOM	1713	CB	LEU	335	12.150	17.611	22.695	1.00	0.00	RX0	C
ATOM	1714	CG	LEU	335	12.959	16.321	22.777	1.00	0.00	RX0	C
ATOM	1715	CD1	LEU	335	14.457	16.569	22.603	1.00	0.00	RX0	C
ATOM	1716	CD2	LEU	335	12.654	15.571	24.075	1.00	0.00	RX0	C
ATOM	1717	C	LEU	335	9.903	18.522	23.297	1.00	0.00	RX0	C
ATOM	1718	O	LEU	335	9.384	18.379	24.399	1.00	0.00	RX0	O
ATOM	1719	N	ILE	336	9.668	19.567	22.501	1.00	0.00	RX0	N
ATOM	1720	H	ILE	336	10.071	19.579	21.582	0.00	0.00	RX0	H
ATOM	1721	CA	ILE	336	8.897	20.743	22.962	1.00	0.00	RX0	C
ATOM	1722	CB	ILE	336	9.048	21.912	21.989	1.00	0.00	RX0	C
ATOM	1723	CG2	ILE	336	8.089	23.056	22.324	1.00	0.00	RX0	C
ATOM	1724	CG1	ILE	336	10.500	22.389	21.997	1.00	0.00	RX0	C
ATOM	1725	CD1	ILE	336	10.926	22.872	23.385	1.00	0.00	RX0	C
ATOM	1726	C	ILE	336	7.427	20.365	23.180	1.00	0.00	RX0	C
ATOM	1727	O	ILE	336	6.836	20.730	24.203	1.00	0.00	RX0	O
ATOM	1728	N	HIS	337	6.891	19.571	22.265	1.00	0.00	RX0	N
ATOM	1729	H	HIS	337	7.437	19.322	21.462	0.00	0.00	RX0	H
ATOM	1730	CA	HIS	337	5.501	19.096	22.359	1.00	0.00	RX0	C
ATOM	1731	CB	HIS	337	5.071	18.338	21.112	1.00	0.00	RX0	C
ATOM	1732	CG	HIS	337	3.600	18.033	21.239	1.00	0.00	RX0	C
ATOM	1733	ND1	HIS	337	2.626	18.944	21.065	1.00	0.00	RX0	N
ATOM	1734	HD1	HIS	337	2.747	19.888	20.823	0.00	0.00	RX0	H
ATOM	1735	CD2	HIS	337	3.017	16.809	21.570	1.00	0.00	RX0	C
ATOM	1736	NE2	HIS	337	1.672	16.991	21.600	1.00	0.00	RX0	N
ATOM	1737	CE1	HIS	337	1.433	18.307	21.287	1.00	0.00	RX0	C
ATOM	1738	C	HIS	337	5.301	18.263	23.631	1.00	0.00	RX0	C
ATOM	1739	O	HIS	337	4.339	18.490	24.365	1.00	0.00	RX0	O
ATOM	1740	N	LEU	338	6.274	17.405	23.914	1.00	0.00	RX0	N
ATOM	1741	H	LEU	338	7.051	17.336	23.285	0.00	0.00	RX0	H
ATOM	1742	CA	LEU	338	6.246	16.526	25.097	1.00	0.00	RX0	C
ATOM	1743	CB	LEU	338	7.440	15.574	25.089	1.00	0.00	RX0	C
ATOM	1744	CG	LEU	338	7.338	14.530	23.981	1.00	0.00	RX0	C
ATOM	1745	CD1	LEU	338	8.639	13.743	23.814	1.00	0.00	RX0	C
ATOM	1746	CD2	LEU	338	6.117	13.632	24.180	1.00	0.00	RX0	C
ATOM	1747	C	LEU	338	6.240	17.331	26.400	1.00	0.00	RX0	C
ATOM	1748	O	LEU	338	5.410	17.098	27.277	1.00	0.00	RX0	O
ATOM	1749	N	MET	339	7.027	18.402	26.394	1.00	0.00	RX0	N
ATOM	1750	H	MET	339	7.615	18.549	25.594	0.00	0.00	RX0	H
ATOM	1751	CA	MET	339	7.168	19.308	27.550	1.00	0.00	RX0	C
ATOM	1752	CB	MET	339	8.407	20.188	27.400	1.00	0.00	RX0	C
ATOM	1753	CG	MET	339	9.705	19.383	27.373	1.00	0.00	RX0	C
ATOM	1754	SD	MET	339	11.142	20.415	27.054	1.00	0.00	RX0	S
ATOM	1755	CE	MET	339	12.273	19.097	26.587	1.00	0.00	RX0	C
ATOM	1756	C	MET	339	5.924	20.183	27.753	1.00	0.00	RX0	C
ATOM	1757	O	MET	339	5.433	20.322	28.878	1.00	0.00	RX0	O
ATOM	1758	N	ALA	340	5.370	20.672	26.646	1.00	0.00	RX0	N
ATOM	1759	H	ALA	340	5.815	20.473	25.772	0.00	0.00	RX0	H
ATOM	1760	CA	ALA	340	4.120	21.456	26.636	1.00	0.00	RX0	C
ATOM	1761	CB	ALA	340	3.835	21.992	25.232	1.00	0.00	RX0	C
ATOM	1762	C	ALA	340	2.921	20.619	27.100	1.00	0.00	RX0	C
ATOM	1763	O	ALA	340	2.176	21.064	27.967	1.00	0.00	RX0	O
ATOM	1764	N	LYS	341	2.865	19.360	26.655	1.00	0.00	RX0	N
ATOM	1765	H	LYS	341	3.552	19.058	25.993	0.00	0.00	RX0	H
ATOM	1766	CA	LYS	341	1.846	18.397	27.117	1.00	0.00	RX0	C
ATOM	1767	CB	LYS	341	1.977	17.109	26.283	1.00	0.00	RX0	C

ATOM	1768	CG	LYS	341	0.691	16.282	26.160	1.00	0.00	RX0	C
ATOM	1769	CD	LYS	341	0.413	15.770	24.733	1.00	0.00	RX0	C
ATOM	1770	CE	LYS	341	1.384	14.716	24.180	1.00	0.00	RX0	C
ATOM	1771	NZ	LYS	341	1.099	14.472	22.757	1.00	0.00	RX0	N
ATOM	1772	HZ1	LYS	341	1.826	13.892	22.285	0.00	0.00	RX0	H
ATOM	1773	HZ2	LYS	341	0.232	13.938	22.536	0.00	0.00	RX0	H
ATOM	1774	HZ3	LYS	341	1.072	15.341	22.187	0.00	0.00	RX0	H
ATOM	1775	C	LYS	341	1.927	18.179	28.637	1.00	0.00	RX0	C
ATOM	1776	O	LYS	341	0.908	18.032	29.304	1.00	0.00	RX0	O
ATOM	1777	N	ALA	342	3.166	18.103	29.120	1.00	0.00	RX0	N
ATOM	1778	H	ALA	342	3.940	18.176	28.489	0.00	0.00	RX0	H
ATOM	1779	CA	ALA	342	3.459	17.942	30.556	1.00	0.00	RX0	C
ATOM	1780	CB	ALA	342	4.953	17.706	30.786	1.00	0.00	RX0	C
ATOM	1781	C	ALA	342	3.007	19.166	31.371	1.00	0.00	RX0	C
ATOM	1782	O	ALA	342	2.879	19.099	32.588	1.00	0.00	RX0	O
ATOM	1783	N	GLY	343	2.900	20.305	30.669	1.00	0.00	RX0	N
ATOM	1784	H	GLY	343	3.153	20.318	29.702	0.00	0.00	RX0	H
ATOM	1785	CA	GLY	343	2.393	21.560	31.244	1.00	0.00	RX0	C
ATOM	1786	C	GLY	343	3.505	22.433	31.829	1.00	0.00	RX0	C
ATOM	1787	O	GLY	343	3.244	23.282	32.678	1.00	0.00	RX0	O
ATOM	1788	N	LEU	344	4.736	22.226	31.356	1.00	0.00	RX0	N
ATOM	1789	H	LEU	344	4.854	21.555	30.622	0.00	0.00	RX0	H
ATOM	1790	CA	LEU	344	5.823	23.174	31.634	1.00	0.00	RX0	C
ATOM	1791	CB	LEU	344	7.150	22.610	31.139	1.00	0.00	RX0	C
ATOM	1792	CG	LEU	344	7.551	21.337	31.880	1.00	0.00	RX0	C
ATOM	1793	CD1	LEU	344	8.831	20.738	31.302	1.00	0.00	RX0	C
ATOM	1794	CD2	LEU	344	7.657	21.566	33.389	1.00	0.00	RX0	C
ATOM	1795	C	LEU	344	5.508	24.488	30.929	1.00	0.00	RX0	C
ATOM	1796	O	LEU	344	4.977	24.500	29.792	1.00	0.00	RX0	O
ATOM	1797	N	THR	345	5.822	25.577	31.575	1.00	0.00	RX0	N
ATOM	1798	H	THR	345	6.284	25.494	32.458	0.00	0.00	RX0	H
ATOM	1799	CA	THR	345	5.705	26.912	30.948	1.00	0.00	RX0	C
ATOM	1800	CB	THR	345	5.996	27.967	32.007	1.00	0.00	RX0	C
ATOM	1801	OG1	THR	345	7.175	27.610	32.724	1.00	0.00	RX0	O
ATOM	1802	HG1	THR	345	6.900	26.982	33.388	0.00	0.00	RX0	H
ATOM	1803	CG2	THR	345	4.821	28.110	32.977	1.00	0.00	RX0	C
ATOM	1804	C	THR	345	6.639	26.971	29.731	1.00	0.00	RX0	C
ATOM	1805	O	THR	345	7.615	26.233	29.623	1.00	0.00	RX0	O
ATOM	1806	N	LEU	346	6.390	27.971	28.898	1.00	0.00	RX0	N
ATOM	1807	H	LEU	346	5.564	28.512	29.045	0.00	0.00	RX0	H
ATOM	1808	CA	LEU	346	7.214	28.242	27.708	1.00	0.00	RX0	C
ATOM	1809	CB	LEU	346	6.672	29.451	26.948	1.00	0.00	RX0	C
ATOM	1810	CG	LEU	346	7.380	29.659	25.608	1.00	0.00	RX0	C
ATOM	1811	CD1	LEU	346	7.250	28.433	24.700	1.00	0.00	RX0	C
ATOM	1812	CD2	LEU	346	6.916	30.940	24.914	1.00	0.00	RX0	C
ATOM	1813	C	LEU	346	8.705	28.433	28.051	1.00	0.00	RX0	C
ATOM	1814	O	LEU	346	9.594	27.857	27.432	1.00	0.00	RX0	O
ATOM	1815	N	GLN	347	8.927	29.083	29.198	1.00	0.00	RX0	N
ATOM	1816	H	GLN	347	8.145	29.439	29.706	0.00	0.00	RX0	H
ATOM	1817	CA	GLN	347	10.275	29.275	29.754	1.00	0.00	RX0	C
ATOM	1818	CB	GLN	347	10.217	30.281	30.899	1.00	0.00	RX0	C
ATOM	1819	CG	GLN	347	11.595	30.615	31.467	1.00	0.00	RX0	C
ATOM	1820	CD	GLN	347	11.421	31.578	32.618	1.00	0.00	RX0	C
ATOM	1821	OE1	GLN	347	10.440	31.529	33.348	1.00	0.00	RX0	O
ATOM	1822	NE2	GLN	347	12.424	32.467	32.730	1.00	0.00	RX0	N
ATOM	1823	HE21	GLN	347	13.192	32.461	32.090	0.00	0.00	RX0	H
ATOM	1824	HE22	GLN	347	12.413	33.158	33.453	0.00	0.00	RX0	H
ATOM	1825	C	GLN	347	10.911	27.956	30.229	1.00	0.00	RX0	C
ATOM	1826	O	GLN	347	12.052	27.652	29.875	1.00	0.00	RX0	O
ATOM	1827	N	GLN	348	10.120	27.140	30.905	1.00	0.00	RX0	N
ATOM	1828	H	GLN	348	9.171	27.399	31.084	0.00	0.00	RX0	H

ATOM	1829	CA	GLN	348	10.582	25.823	31.394	1.00	0.00	RX0	C
ATOM	1830	CB	GLN	348	9.593	25.216	32.375	1.00	0.00	RX0	C
ATOM	1831	CG	GLN	348	9.653	25.880	33.746	1.00	0.00	RX0	C
ATOM	1832	CD	GLN	348	8.480	25.382	34.557	1.00	0.00	RX0	C
ATOM	1833	OE1	GLN	348	7.355	25.304	34.068	1.00	0.00	RX0	O
ATOM	1834	NE2	GLN	348	8.796	25.052	35.820	1.00	0.00	RX0	N
ATOM	1835	HE21	GLN	348	9.737	25.163	36.141	0.00	0.00	RX0	H
ATOM	1836	HE22	GLN	348	8.115	24.705	36.467	0.00	0.00	RX0	H
ATOM	1837	C	GLN	348	10.871	24.847	30.248	1.00	0.00	RX0	C
ATOM	1838	O	GLN	348	11.861	24.115	30.296	1.00	0.00	RX0	O
ATOM	1839	N	GLN	349	10.121	24.983	29.160	1.00	0.00	RX0	N
ATOM	1840	H	GLN	349	9.375	25.648	29.186	0.00	0.00	RX0	H
ATOM	1841	CA	GLN	349	10.299	24.172	27.940	1.00	0.00	RX0	C
ATOM	1842	CB	GLN	349	9.205	24.481	26.921	1.00	0.00	RX0	C
ATOM	1843	CG	GLN	349	7.821	24.031	27.384	1.00	0.00	RX0	C
ATOM	1844	CD	GLN	349	6.787	24.590	26.434	1.00	0.00	RX0	C
ATOM	1845	OE1	GLN	349	7.076	24.918	25.290	1.00	0.00	RX0	O
ATOM	1846	NE2	GLN	349	5.564	24.698	26.978	1.00	0.00	RX0	N
ATOM	1847	HE21	GLN	349	5.399	24.433	27.934	0.00	0.00	RX0	H
ATOM	1848	HE22	GLN	349	4.781	25.036	26.459	0.00	0.00	RX0	H
ATOM	1849	C	GLN	349	11.682	24.372	27.306	1.00	0.00	RX0	C
ATOM	1850	O	GLN	349	12.447	23.422	27.181	1.00	0.00	RX0	O
ATOM	1851	N	HIS	350	12.056	25.641	27.113	1.00	0.00	RX0	N
ATOM	1852	H	HIS	350	11.422	26.375	27.365	0.00	0.00	RX0	H
ATOM	1853	CA	HIS	350	13.344	25.953	26.463	1.00	0.00	RX0	C
ATOM	1854	CB	HIS	350	13.379	27.327	25.781	1.00	0.00	RX0	C
ATOM	1855	CG	HIS	350	13.577	28.469	26.746	1.00	0.00	RX0	C
ATOM	1856	ND1	HIS	350	12.561	29.183	27.258	1.00	0.00	RX0	N
ATOM	1857	HD1	HIS	350	11.603	29.024	27.101	0.00	0.00	RX0	H
ATOM	1858	CD2	HIS	350	14.785	28.992	27.219	1.00	0.00	RX0	C
ATOM	1859	NE2	HIS	350	14.487	30.038	28.025	1.00	0.00	RX0	N
ATOM	1860	CE1	HIS	350	13.122	30.156	28.046	1.00	0.00	RX0	C
ATOM	1861	C	HIS	350	14.540	25.670	27.386	1.00	0.00	RX0	C
ATOM	1862	O	HIS	350	15.573	25.182	26.934	1.00	0.00	RX0	O
ATOM	1863	N	GLN	351	14.320	25.854	28.691	1.00	0.00	RX0	N
ATOM	1864	H	GLN	351	13.441	26.232	28.990	0.00	0.00	RX0	H
ATOM	1865	CA	GLN	351	15.341	25.538	29.706	1.00	0.00	RX0	C
ATOM	1866	CB	GLN	351	14.917	26.062	31.072	1.00	0.00	RX0	C
ATOM	1867	CG	GLN	351	14.894	27.585	31.147	1.00	0.00	RX0	C
ATOM	1868	CD	GLN	351	14.210	27.984	32.435	1.00	0.00	RX0	C
ATOM	1869	OE1	GLN	351	13.354	27.278	32.958	1.00	0.00	RX0	O
ATOM	1870	NE2	GLN	351	14.652	29.152	32.933	1.00	0.00	RX0	N
ATOM	1871	HE21	GLN	351	15.356	29.668	32.443	0.00	0.00	RX0	H
ATOM	1872	HE22	GLN	351	14.297	29.517	33.793	0.00	0.00	RX0	H
ATOM	1873	C	GLN	351	15.597	24.030	29.805	1.00	0.00	RX0	C
ATOM	1874	O	GLN	351	16.752	23.608	29.740	1.00	0.00	RX0	O
ATOM	1875	N	ARG	352	14.527	23.242	29.744	1.00	0.00	RX0	N
ATOM	1876	H	ARG	352	13.617	23.653	29.656	0.00	0.00	RX0	H
ATOM	1877	CA	ARG	352	14.627	21.772	29.816	1.00	0.00	RX0	C
ATOM	1878	CB	ARG	352	13.310	21.109	30.246	1.00	0.00	RX0	C
ATOM	1879	CG	ARG	352	13.414	19.586	30.434	1.00	0.00	RX0	C
ATOM	1880	CD	ARG	352	12.214	18.996	31.187	1.00	0.00	RX0	C
ATOM	1881	NE	ARG	352	12.179	17.530	31.162	1.00	0.00	RX0	N
ATOM	1882	HE	ARG	352	11.951	17.068	30.290	0.00	0.00	RX0	H
ATOM	1883	CZ	ARG	352	12.321	16.775	32.295	1.00	0.00	RX0	C
ATOM	1884	NH1	ARG	352	12.647	17.374	33.458	1.00	0.00	RX0	N
ATOM	1885	HH11	ARG	352	12.832	16.827	34.288	0.00	0.00	RX0	H
ATOM	1886	HH12	ARG	352	12.730	18.368	33.535	0.00	0.00	RX0	H
ATOM	1887	NH2	ARG	352	12.129	15.442	32.236	1.00	0.00	RX0	N
ATOM	1888	HH21	ARG	352	12.178	14.805	33.014	0.00	0.00	RX0	H
ATOM	1889	HH22	ARG	352	11.911	15.026	31.331	0.00	0.00	RX0	H

ATOM	1890	C	ARG	352	15.192	21.188	28.514	1.00	0.00	RX0	C
ATOM	1891	O	ARG	352	16.048	20.305	28.551	1.00	0.00	RX0	O
ATOM	1892	N	LEU	353	14.843	21.816	27.391	1.00	0.00	RX0	N
ATOM	1893	H	LEU	353	14.131	22.519	27.429	0.00	0.00	RX0	H
ATOM	1894	CA	LEU	353	15.400	21.446	26.079	1.00	0.00	RX0	C
ATOM	1895	CB	LEU	353	14.765	22.302	24.985	1.00	0.00	RX0	C
ATOM	1896	CG	LEU	353	15.233	21.922	23.581	1.00	0.00	RX0	C
ATOM	1897	CD1	LEU	353	14.794	20.508	23.199	1.00	0.00	RX0	C
ATOM	1898	CD2	LEU	353	14.808	22.959	22.542	1.00	0.00	RX0	C
ATOM	1899	C	LEU	353	16.930	21.610	26.063	1.00	0.00	RX0	C
ATOM	1900	O	LEU	353	17.658	20.692	25.694	1.00	0.00	RX0	O
ATOM	1901	N	ALA	354	17.375	22.739	26.614	1.00	0.00	RX0	N
ATOM	1902	H	ALA	354	16.705	23.421	26.916	0.00	0.00	RX0	H
ATOM	1903	CA	ALA	354	18.808	23.073	26.716	1.00	0.00	RX0	C
ATOM	1904	CB	ALA	354	18.993	24.518	27.181	1.00	0.00	RX0	C
ATOM	1905	C	ALA	354	19.540	22.141	27.690	1.00	0.00	RX0	C
ATOM	1906	O	ALA	354	20.574	21.567	27.334	1.00	0.00	RX0	O
ATOM	1907	N	GLN	355	18.893	21.844	28.811	1.00	0.00	RX0	N
ATOM	1908	H	GLN	355	18.018	22.305	28.967	0.00	0.00	RX0	H
ATOM	1909	CA	GLN	355	19.427	20.920	29.833	1.00	0.00	RX0	C
ATOM	1910	CB	GLN	355	18.563	20.902	31.084	1.00	0.00	RX0	C
ATOM	1911	CG	GLN	355	18.680	22.209	31.861	1.00	0.00	RX0	C
ATOM	1912	CD	GLN	355	17.957	22.050	33.175	1.00	0.00	RX0	C
ATOM	1913	OE1	GLN	355	18.399	21.314	34.058	1.00	0.00	RX0	O
ATOM	1914	NE2	GLN	355	16.832	22.777	33.251	1.00	0.00	RX0	N
ATOM	1915	HE21	GLN	355	16.564	23.336	32.464	0.00	0.00	RX0	H
ATOM	1916	HE22	GLN	355	16.242	22.802	34.059	0.00	0.00	RX0	H
ATOM	1917	C	GLN	355	19.631	19.504	29.273	1.00	0.00	RX0	C
ATOM	1918	O	GLN	355	20.705	18.919	29.430	1.00	0.00	RX0	O
ATOM	1919	N	LEU	356	18.681	19.086	28.443	1.00	0.00	RX0	N
ATOM	1920	H	LEU	356	17.883	19.669	28.276	0.00	0.00	RX0	H
ATOM	1921	CA	LEU	356	18.716	17.770	27.778	1.00	0.00	RX0	C
ATOM	1922	CB	LEU	356	17.350	17.404	27.202	1.00	0.00	RX0	C
ATOM	1923	CG	LEU	356	16.352	17.084	28.311	1.00	0.00	RX0	C
ATOM	1924	CD1	LEU	356	15.008	16.608	27.760	1.00	0.00	RX0	C
ATOM	1925	CD2	LEU	356	16.948	16.094	29.309	1.00	0.00	RX0	C
ATOM	1926	C	LEU	356	19.795	17.661	26.699	1.00	0.00	RX0	C
ATOM	1927	O	LEU	356	20.593	16.720	26.700	1.00	0.00	RX0	O
ATOM	1928	N	LEU	357	19.916	18.722	25.913	1.00	0.00	RX0	N
ATOM	1929	H	LEU	357	19.280	19.489	26.028	0.00	0.00	RX0	H
ATOM	1930	CA	LEU	357	20.885	18.765	24.803	1.00	0.00	RX0	C
ATOM	1931	CB	LEU	357	20.531	19.867	23.805	1.00	0.00	RX0	C
ATOM	1932	CG	LEU	357	19.159	19.682	23.152	1.00	0.00	RX0	C
ATOM	1933	CD1	LEU	357	18.827	20.838	22.209	1.00	0.00	RX0	C
ATOM	1934	CD2	LEU	357	19.018	18.323	22.468	1.00	0.00	RX0	C
ATOM	1935	C	LEU	357	22.335	18.932	25.264	1.00	0.00	RX0	C
ATOM	1936	O	LEU	357	23.247	18.345	24.683	1.00	0.00	RX0	O
ATOM	1937	N	LEU	358	22.501	19.595	26.404	1.00	0.00	RX0	N
ATOM	1938	H	LEU	358	21.707	20.033	26.830	0.00	0.00	RX0	H
ATOM	1939	CA	LEU	358	23.826	19.754	27.029	1.00	0.00	RX0	C
ATOM	1940	CB	LEU	358	23.803	20.838	28.104	1.00	0.00	RX0	C
ATOM	1941	CG	LEU	358	23.626	22.235	27.510	1.00	0.00	RX0	C
ATOM	1942	CD1	LEU	358	23.473	23.301	28.596	1.00	0.00	RX0	C
ATOM	1943	CD2	LEU	358	24.737	22.571	26.514	1.00	0.00	RX0	C
ATOM	1944	C	LEU	358	24.390	18.455	27.609	1.00	0.00	RX0	C
ATOM	1945	O	LEU	358	25.603	18.227	27.557	1.00	0.00	RX0	O
ATOM	1946	N	ILE	359	23.510	17.559	28.043	1.00	0.00	RX0	N
ATOM	1947	H	ILE	359	22.534	17.782	28.000	0.00	0.00	RX0	H
ATOM	1948	CA	ILE	359	23.928	16.212	28.495	1.00	0.00	RX0	C
ATOM	1949	CB	ILE	359	22.752	15.476	29.134	1.00	0.00	RX0	C
ATOM	1950	CG2	ILE	359	23.131	14.060	29.569	1.00	0.00	RX0	C

ATOM	1951	CG1	ILE	359	22.238	16.301	30.314	1.00	0.00	RX0	C
ATOM	1952	CD1	ILE	359	20.906	15.804	30.869	1.00	0.00	RX0	C
ATOM	1953	C	ILE	359	24.559	15.428	27.334	1.00	0.00	RX0	C
ATOM	1954	O	ILE	359	25.552	14.723	27.543	1.00	0.00	RX0	O
ATOM	1955	N	LEU	360	24.038	15.620	26.133	1.00	0.00	RX0	N
ATOM	1956	H	LEU	360	23.268	16.251	26.029	0.00	0.00	RX0	H
ATOM	1957	CA	LEU	360	24.601	14.974	24.928	1.00	0.00	RX0	C
ATOM	1958	CB	LEU	360	23.756	15.278	23.691	1.00	0.00	RX0	C
ATOM	1959	CG	LEU	360	22.270	14.958	23.885	1.00	0.00	RX0	C
ATOM	1960	CD1	LEU	360	21.458	15.316	22.643	1.00	0.00	RX0	C
ATOM	1961	CD2	LEU	360	22.024	13.510	24.312	1.00	0.00	RX0	C
ATOM	1962	C	LEU	360	26.079	15.308	24.694	1.00	0.00	RX0	C
ATOM	1963	O	LEU	360	26.843	14.455	24.246	1.00	0.00	RX0	O
ATOM	1964	N	SER	361	26.491	16.472	25.202	1.00	0.00	RX0	N
ATOM	1965	H	SER	361	25.853	17.142	25.590	0.00	0.00	RX0	H
ATOM	1966	CA	SER	361	27.906	16.894	25.202	1.00	0.00	RX0	C
ATOM	1967	CB	SER	361	27.866	18.374	25.507	1.00	0.00	RX0	C
ATOM	1968	OG	SER	361	26.609	18.818	24.975	1.00	0.00	RX0	O
ATOM	1969	HG	SER	361	26.623	18.642	24.043	0.00	0.00	RX0	H
ATOM	1970	C	SER	361	28.769	15.985	26.099	1.00	0.00	RX0	C
ATOM	1971	O	SER	361	29.797	15.456	25.681	1.00	0.00	RX0	O
ATOM	1972	N	HIS	362	28.237	15.704	27.286	1.00	0.00	RX0	N
ATOM	1973	H	HIS	362	27.305	16.024	27.457	0.00	0.00	RX0	H
ATOM	1974	CA	HIS	362	28.854	14.793	28.272	1.00	0.00	RX0	C
ATOM	1975	CB	HIS	362	28.100	14.897	29.599	1.00	0.00	RX0	C
ATOM	1976	CG	HIS	362	28.139	16.320	30.110	1.00	0.00	RX0	C
ATOM	1977	ND1	HIS	362	27.354	17.323	29.659	1.00	0.00	RX0	N
ATOM	1978	HD1	HIS	362	26.671	17.296	28.952	0.00	0.00	RX0	H
ATOM	1979	CD2	HIS	362	28.977	16.826	31.107	1.00	0.00	RX0	C
ATOM	1980	NE2	HIS	362	28.691	18.143	31.253	1.00	0.00	RX0	N
ATOM	1981	CE1	HIS	362	27.694	18.448	30.363	1.00	0.00	RX0	C
ATOM	1982	C	HIS	362	28.890	13.339	27.777	1.00	0.00	RX0	C
ATOM	1983	O	HIS	362	29.902	12.656	27.936	1.00	0.00	RX0	O
ATOM	1984	N	ILE	363	27.856	12.940	27.039	1.00	0.00	RX0	N
ATOM	1985	H	ILE	363	27.097	13.579	26.907	0.00	0.00	RX0	H
ATOM	1986	CA	ILE	363	27.773	11.584	26.446	1.00	0.00	RX0	C
ATOM	1987	CB	ILE	363	26.349	11.258	25.990	1.00	0.00	RX0	C
ATOM	1988	CG2	ILE	363	26.216	9.795	25.568	1.00	0.00	RX0	C
ATOM	1989	CG1	ILE	363	25.374	11.544	27.132	1.00	0.00	RX0	C
ATOM	1990	CD1	ILE	363	23.926	11.232	26.763	1.00	0.00	RX0	C
ATOM	1991	C	ILE	363	28.830	11.416	25.343	1.00	0.00	RX0	C
ATOM	1992	O	ILE	363	29.487	10.370	25.263	1.00	0.00	RX0	O
ATOM	1993	N	ARG	364	29.035	12.469	24.564	1.00	0.00	RX0	N
ATOM	1994	H	ARG	364	28.398	13.240	24.623	0.00	0.00	RX0	H
ATOM	1995	CA	ARG	364	30.102	12.498	23.547	1.00	0.00	RX0	C
ATOM	1996	CB	ARG	364	30.041	13.833	22.802	1.00	0.00	RX0	C
ATOM	1997	CG	ARG	364	30.972	14.005	21.595	1.00	0.00	RX0	C
ATOM	1998	CD	ARG	364	30.628	13.072	20.431	1.00	0.00	RX0	C
ATOM	1999	NE	ARG	364	29.179	12.983	20.280	1.00	0.00	RX0	N
ATOM	2000	HE	ARG	364	28.708	12.278	20.831	0.00	0.00	RX0	H
ATOM	2001	CZ	ARG	364	28.418	13.804	19.504	1.00	0.00	RX0	C
ATOM	2002	NH1	ARG	364	28.998	14.638	18.614	1.00	0.00	RX0	N
ATOM	2003	HH11	ARG	364	28.449	15.245	18.031	0.00	0.00	RX0	H
ATOM	2004	HH12	ARG	364	29.993	14.659	18.495	0.00	0.00	RX0	H
ATOM	2005	NH2	ARG	364	27.082	13.755	19.644	1.00	0.00	RX0	N
ATOM	2006	HH21	ARG	364	26.437	14.321	19.117	0.00	0.00	RX0	H
ATOM	2007	HH22	ARG	364	26.700	13.081	20.302	0.00	0.00	RX0	H
ATOM	2008	C	ARG	364	31.469	12.319	24.223	1.00	0.00	RX0	C
ATOM	2009	O	ARG	364	32.264	11.465	23.831	1.00	0.00	RX0	O
ATOM	2010	N	HIS	365	31.645	13.058	25.316	1.00	0.00	RX0	N
ATOM	2011	H	HIS	365	30.908	13.685	25.580	0.00	0.00	RX0	H

ATOM	2012	CA	HIS	365	32.879	13.027	26.115	1.00	0.00	RX0	C
ATOM	2013	CB	HIS	365	32.813	14.039	27.260	1.00	0.00	RX0	C
ATOM	2014	CG	HIS	365	34.145	14.101	27.969	1.00	0.00	RX0	C
ATOM	2015	ND1	HIS	365	35.171	14.865	27.550	1.00	0.00	RX0	N
ATOM	2016	HD1	HIS	365	35.175	15.461	26.771	0.00	0.00	RX0	H
ATOM	2017	CD2	HIS	365	34.536	13.421	29.128	1.00	0.00	RX0	C
ATOM	2018	NE2	HIS	365	35.814	13.785	29.399	1.00	0.00	RX0	N
ATOM	2019	CE1	HIS	365	36.204	14.673	28.430	1.00	0.00	RX0	C
ATOM	2020	C	HIS	365	33.175	11.617	26.649	1.00	0.00	RX0	C
ATOM	2021	O	HIS	365	34.274	11.105	26.425	1.00	0.00	RX0	O
ATOM	2022	N	MET	366	32.138	10.946	27.137	1.00	0.00	RX0	N
ATOM	2023	H	MET	366	31.251	11.408	27.194	0.00	0.00	RX0	H
ATOM	2024	CA	MET	366	32.267	9.572	27.662	1.00	0.00	RX0	C
ATOM	2025	CB	MET	366	31.015	9.142	28.427	1.00	0.00	RX0	C
ATOM	2026	CG	MET	366	30.716	10.016	29.644	1.00	0.00	RX0	C
ATOM	2027	SD	MET	366	29.366	9.372	30.646	1.00	0.00	RX0	S
ATOM	2028	CE	MET	366	28.102	9.320	29.369	1.00	0.00	RX0	C
ATOM	2029	C	MET	366	32.567	8.557	26.555	1.00	0.00	RX0	C
ATOM	2030	O	MET	366	33.398	7.674	26.741	1.00	0.00	RX0	O
ATOM	2031	N	SER	367	31.985	8.782	25.378	1.00	0.00	RX0	N
ATOM	2032	H	SER	367	31.290	9.497	25.274	0.00	0.00	RX0	H
ATOM	2033	CA	SER	367	32.231	7.934	24.196	1.00	0.00	RX0	C
ATOM	2034	CB	SER	367	31.294	8.290	23.069	1.00	0.00	RX0	C
ATOM	2035	OG	SER	367	30.027	7.725	23.386	1.00	0.00	RX0	O
ATOM	2036	HG	SER	367	29.456	8.068	22.712	0.00	0.00	RX0	H
ATOM	2037	C	SER	367	33.681	8.046	23.711	1.00	0.00	RX0	C
ATOM	2038	O	SER	367	34.361	7.038	23.567	1.00	0.00	RX0	O
ATOM	2039	N	ASN	368	34.193	9.278	23.688	1.00	0.00	RX0	N
ATOM	2040	H	ASN	368	33.582	10.045	23.894	0.00	0.00	RX0	H
ATOM	2041	CA	ASN	368	35.583	9.549	23.266	1.00	0.00	RX0	C
ATOM	2042	CB	ASN	368	35.869	11.050	23.184	1.00	0.00	RX0	C
ATOM	2043	CG	ASN	368	35.134	11.694	22.030	1.00	0.00	RX0	C
ATOM	2044	OD1	ASN	368	34.613	11.040	21.134	1.00	0.00	RX0	O
ATOM	2045	ND2	ASN	368	35.117	13.037	22.104	1.00	0.00	RX0	N
ATOM	2046	HD21	ASN	368	35.570	13.503	22.863	0.00	0.00	RX0	H
ATOM	2047	HD22	ASN	368	34.657	13.582	21.403	0.00	0.00	RX0	H
ATOM	2048	C	ASN	368	36.615	8.948	24.226	1.00	0.00	RX0	C
ATOM	2049	O	ASN	368	37.514	8.215	23.806	1.00	0.00	RX0	O
ATOM	2050	N	LYS	369	36.353	9.119	25.515	1.00	0.00	RX0	N
ATOM	2051	H	LYS	369	35.549	9.662	25.770	0.00	0.00	RX0	H
ATOM	2052	CA	LYS	369	37.205	8.554	26.580	1.00	0.00	RX0	C
ATOM	2053	CB	LYS	369	36.874	9.130	27.960	1.00	0.00	RX0	C
ATOM	2054	CG	LYS	369	37.010	10.650	28.069	1.00	0.00	RX0	C
ATOM	2055	CD	LYS	369	38.354	11.179	27.569	1.00	0.00	RX0	C
ATOM	2056	CE	LYS	369	39.568	10.607	28.304	1.00	0.00	RX0	C
ATOM	2057	NZ	LYS	369	40.760	10.904	27.505	1.00	0.00	RX0	N
ATOM	2058	HZ1	LYS	369	41.293	11.738	27.795	0.00	0.00	RX0	H
ATOM	2059	HZ2	LYS	369	41.387	10.073	27.427	0.00	0.00	RX0	H
ATOM	2060	HZ3	LYS	369	40.485	11.008	26.503	0.00	0.00	RX0	H
ATOM	2061	C	LYS	369	37.117	7.021	26.620	1.00	0.00	RX0	C
ATOM	2062	O	LYS	369	38.123	6.337	26.771	1.00	0.00	RX0	O
ATOM	2063	N	GLY	370	35.908	6.527	26.299	1.00	0.00	RX0	N
ATOM	2064	H	GLY	370	35.156	7.164	26.127	0.00	0.00	RX0	H
ATOM	2065	CA	GLY	370	35.597	5.090	26.225	1.00	0.00	RX0	C
ATOM	2066	C	GLY	370	36.324	4.429	25.048	1.00	0.00	RX0	C
ATOM	2067	O	GLY	370	36.946	3.386	25.214	1.00	0.00	RX0	O
ATOM	2068	N	MET	371	36.380	5.143	23.928	1.00	0.00	RX0	N
ATOM	2069	H	MET	371	35.916	6.029	23.909	0.00	0.00	RX0	H
ATOM	2070	CA	MET	371	37.103	4.709	22.719	1.00	0.00	RX0	C
ATOM	2071	CB	MET	371	36.782	5.587	21.506	1.00	0.00	RX0	C
ATOM	2072	CG	MET	371	35.326	5.491	21.048	1.00	0.00	RX0	C

ATOM	2073	SD	MET	371	34.845	3.843	20.509	1.00	0.00	RX0	S
ATOM	2074	CE	MET	371	35.437	3.956	18.815	1.00	0.00	RX0	C
ATOM	2075	C	MET	371	38.619	4.671	22.937	1.00	0.00	RX0	C
ATOM	2076	O	MET	371	39.258	3.669	22.631	1.00	0.00	RX0	O
ATOM	2077	N	GLU	372	39.117	5.692	23.643	1.00	0.00	RX0	N
ATOM	2078	H	GLU	372	38.530	6.478	23.848	0.00	0.00	RX0	H
ATOM	2079	CA	GLU	372	40.525	5.740	24.082	1.00	0.00	RX0	C
ATOM	2080	CB	GLU	372	40.823	7.025	24.858	1.00	0.00	RX0	C
ATOM	2081	CG	GLU	372	40.720	8.316	24.047	1.00	0.00	RX0	C
ATOM	2082	CD	GLU	372	40.970	9.477	24.986	1.00	0.00	RX0	C
ATOM	2083	OE1	GLU	372	40.279	10.489	24.899	1.00	0.00	RX0	O
ATOM	2084	OE2	GLU	372	41.828	9.365	25.858	1.00	0.00	RX0	O
ATOM	2085	C	GLU	372	40.884	4.541	24.968	1.00	0.00	RX0	C
ATOM	2086	O	GLU	372	41.865	3.853	24.723	1.00	0.00	RX0	O
ATOM	2087	N	HIS	373	40.006	4.291	25.945	1.00	0.00	RX0	N
ATOM	2088	H	HIS	373	39.222	4.905	26.041	0.00	0.00	RX0	H
ATOM	2089	CA	HIS	373	40.194	3.212	26.919	1.00	0.00	RX0	C
ATOM	2090	CB	HIS	373	39.267	3.437	28.123	1.00	0.00	RX0	C
ATOM	2091	CG	HIS	373	38.389	2.247	28.437	1.00	0.00	RX0	C
ATOM	2092	ND1	HIS	373	37.275	1.940	27.746	1.00	0.00	RX0	N
ATOM	2093	HD1	HIS	373	36.937	2.408	26.949	0.00	0.00	RX0	H
ATOM	2094	CD2	HIS	373	38.541	1.327	29.479	1.00	0.00	RX0	C
ATOM	2095	NE2	HIS	373	37.499	0.461	29.409	1.00	0.00	RX0	N
ATOM	2096	CE1	HIS	373	36.722	0.839	28.346	1.00	0.00	RX0	C
ATOM	2097	C	HIS	373	40.078	1.832	26.262	1.00	0.00	RX0	C
ATOM	2098	O	HIS	373	40.934	1.001	26.470	1.00	0.00	RX0	O
ATOM	2099	N	LEU	374	39.111	1.679	25.351	1.00	0.00	RX0	N
ATOM	2100	H	LEU	374	38.558	2.474	25.113	0.00	0.00	RX0	H
ATOM	2101	CA	LEU	374	38.919	0.408	24.629	1.00	0.00	RX0	C
ATOM	2102	CB	LEU	374	37.662	0.451	23.760	1.00	0.00	RX0	C
ATOM	2103	CG	LEU	374	36.363	0.305	24.551	1.00	0.00	RX0	C
ATOM	2104	CD1	LEU	374	35.137	0.608	23.688	1.00	0.00	RX0	C
ATOM	2105	CD2	LEU	374	36.269	-1.064	25.227	1.00	0.00	RX0	C
ATOM	2106	C	LEU	374	40.113	0.051	23.744	1.00	0.00	RX0	C
ATOM	2107	O	LEU	374	40.555	-1.109	23.754	1.00	0.00	RX0	O
ATOM	2108	N	TYR	375	40.664	1.068	23.103	1.00	0.00	RX0	N
ATOM	2109	H	TYR	375	40.300	1.988	23.267	0.00	0.00	RX0	H
ATOM	2110	CA	TYR	375	41.854	0.983	22.222	1.00	0.00	RX0	C
ATOM	2111	CB	TYR	375	41.976	2.221	21.333	1.00	0.00	RX0	C
ATOM	2112	CG	TYR	375	40.871	2.220	20.305	1.00	0.00	RX0	C
ATOM	2113	CD1	TYR	375	40.278	1.023	19.923	1.00	0.00	RX0	C
ATOM	2114	CE1	TYR	375	39.273	1.019	18.964	1.00	0.00	RX0	C
ATOM	2115	CD2	TYR	375	40.456	3.416	19.731	1.00	0.00	RX0	C
ATOM	2116	CE2	TYR	375	39.452	3.413	18.770	1.00	0.00	RX0	C
ATOM	2117	CZ	TYR	375	38.865	2.214	18.383	1.00	0.00	RX0	C
ATOM	2118	OH	TYR	375	37.877	2.207	17.421	1.00	0.00	RX0	O
ATOM	2119	HH	TYR	375	37.767	3.080	17.066	0.00	0.00	RX0	H
ATOM	2120	C	TYR	375	43.165	0.750	22.977	1.00	0.00	RX0	C
ATOM	2121	O	TYR	375	44.176	1.452	22.753	1.00	0.00	RX0	O
ATOM	2122	N	SER	376	43.162	-0.226	23.845	1.00	0.00	RX0	N
ATOM	2123	H	SER	376	42.296	-0.614	24.145	0.00	0.00	RX0	H
ATOM	2124	CA	SER	376	44.345	-0.646	24.634	1.00	0.00	RX0	C
ATOM	2125	CB	SER	376	44.677	0.418	25.692	1.00	0.00	RX0	C
ATOM	2126	OG	SER	376	44.088	1.682	25.370	1.00	0.00	RX0	O
ATOM	2127	HG	SER	376	44.418	1.942	24.516	0.00	0.00	RX0	H
ATOM	2128	C	SER	376	44.183	-1.992	25.350	1.00	0.00	RX0	C
ATOM	2129	O	SER	376	44.985	-2.369	26.199	1.00	0.00	RX0	O
ATOM	2130	N	MET	377	43.156	-2.746	24.938	1.00	0.00	RX0	N
ATOM	2131	H	MET	377	42.557	-2.429	24.202	0.00	0.00	RX0	H
ATOM	2132	CA	MET	377	42.915	-4.112	25.422	1.00	0.00	RX0	C
ATOM	2133	CB	MET	377	41.735	-4.071	26.399	1.00	0.00	RX0	C

ATOM	2134	CG	MET	377	42.038	-3.409	27.744	1.00	0.00	RX0	C
ATOM	2135	SD	MET	377	40.549	-3.245	28.736	1.00	0.00	RX0	S
ATOM	2136	CE	MET	377	39.726	-2.022	27.703	1.00	0.00	RX0	C
ATOM	2137	C	MET	377	42.647	-5.080	24.257	1.00	0.00	RX0	C
ATOM	2138	O	MET	377	43.092	-4.854	23.130	1.00	0.00	RX0	O
ATOM	2139	N	LYS	378	41.856	-6.107	24.527	1.00	0.00	RX0	N
ATOM	2140	H	LYS	378	41.482	-6.208	25.443	0.00	0.00	RX0	H
ATOM	2141	CA	LYS	378	41.570	-7.191	23.576	1.00	0.00	RX0	C
ATOM	2142	CB	LYS	378	41.441	-8.520	24.363	1.00	0.00	RX0	C
ATOM	2143	CG	LYS	378	40.449	-8.516	25.552	1.00	0.00	RX0	C
ATOM	2144	CD	LYS	378	40.403	-9.806	26.399	1.00	0.00	RX0	C
ATOM	2145	CE	LYS	378	39.448	-9.726	27.610	1.00	0.00	RX0	C
ATOM	2146	NZ	LYS	378	39.481	-10.968	28.411	1.00	0.00	RX0	N
ATOM	2147	HZ1	LYS	378	38.891	-10.895	29.275	0.00	0.00	RX0	H
ATOM	2148	HZ2	LYS	378	39.130	-11.761	27.838	0.00	0.00	RX0	H
ATOM	2149	HZ3	LYS	378	40.458	-11.169	28.700	0.00	0.00	RX0	H
ATOM	2150	C	LYS	378	40.316	-6.920	22.720	1.00	0.00	RX0	C
ATOM	2151	O	LYS	378	40.245	-7.352	21.611	1.00	0.00	RX0	O
ATOM	2152	N	CYS	379	39.320	-6.266	23.410	1.00	0.00	RX0	N
ATOM	2153	H	CYS	379	39.564	-5.889	24.298	0.00	0.00	RX0	H
ATOM	2154	CA	CYS	379	37.939	-6.053	22.985	1.00	0.00	RX0	C
ATOM	2155	CB	CYS	379	37.612	-4.559	23.098	1.00	0.00	RX0	C
ATOM	2156	SG	CYS	379	35.839	-4.200	23.183	1.00	0.00	RX0	S
ATOM	2157	C	CYS	379	37.599	-6.654	21.614	1.00	0.00	RX0	C
ATOM	2158	O	CYS	379	37.115	-7.773	21.559	1.00	0.00	RX0	O
ATOM	2159	N	LYS	380	38.012	-5.930	20.563	1.00	0.00	RX0	N
ATOM	2160	H	LYS	380	38.380	-5.003	20.639	0.00	0.00	RX0	H
ATOM	2161	CA	LYS	380	37.949	-6.383	19.165	1.00	0.00	RX0	C
ATOM	2162	CB	LYS	380	36.478	-6.764	18.914	1.00	0.00	RX0	C
ATOM	2163	CG	LYS	380	35.894	-7.096	17.539	1.00	0.00	RX0	C
ATOM	2164	CD	LYS	380	34.384	-7.299	17.739	1.00	0.00	RX0	C
ATOM	2165	CE	LYS	380	33.563	-7.592	16.481	1.00	0.00	RX0	C
ATOM	2166	NZ	LYS	380	32.147	-7.714	16.853	1.00	0.00	RX0	N
ATOM	2167	HZ1	LYS	380	31.498	-7.913	16.072	0.00	0.00	RX0	H
ATOM	2168	HZ2	LYS	380	31.970	-8.458	17.565	0.00	0.00	RX0	H
ATOM	2169	HZ3	LYS	380	31.780	-6.877	17.356	0.00	0.00	RX0	H
ATOM	2170	C	LYS	380	38.311	-5.215	18.248	1.00	0.00	RX0	C
ATOM	2171	O	LYS	380	37.971	-4.057	18.518	1.00	0.00	RX0	O
ATOM	2172	N	ASN	381	38.881	-5.592	17.123	1.00	0.00	RX0	N
ATOM	2173	H	ASN	381	39.187	-6.541	17.050	0.00	0.00	RX0	H
ATOM	2174	CA	ASN	381	39.208	-4.700	15.996	1.00	0.00	RX0	C
ATOM	2175	CB	ASN	381	40.304	-3.692	16.354	1.00	0.00	RX0	C
ATOM	2176	CG	ASN	381	40.511	-2.736	15.201	1.00	0.00	RX0	C
ATOM	2177	OD1	ASN	381	39.578	-2.371	14.466	1.00	0.00	RX0	O
ATOM	2178	ND2	ASN	381	41.776	-2.344	15.040	1.00	0.00	RX0	N
ATOM	2179	HD21	ASN	381	42.471	-2.584	15.724	0.00	0.00	RX0	H
ATOM	2180	HD22	ASN	381	42.068	-1.819	14.245	0.00	0.00	RX0	H
ATOM	2181	C	ASN	381	39.620	-5.523	14.759	1.00	0.00	RX0	C
ATOM	2182	O	ASN	381	40.286	-5.062	13.834	1.00	0.00	RX0	O
ATOM	2183	N	VAL	382	39.176	-6.776	14.737	1.00	0.00	RX0	N
ATOM	2184	H	VAL	382	38.429	-7.014	15.352	0.00	0.00	RX0	H
ATOM	2185	CA	VAL	382	39.338	-7.659	13.572	1.00	0.00	RX0	C
ATOM	2186	CB	VAL	382	39.226	-9.119	14.010	1.00	0.00	RX0	C
ATOM	2187	CG1	VAL	382	39.384	-10.078	12.828	1.00	0.00	RX0	C
ATOM	2188	CG2	VAL	382	40.220	-9.411	15.135	1.00	0.00	RX0	C
ATOM	2189	C	VAL	382	38.195	-7.277	12.635	1.00	0.00	RX0	C
ATOM	2190	O	VAL	382	37.049	-7.276	13.059	1.00	0.00	RX0	O
ATOM	2191	N	VAL	383	38.551	-7.063	11.363	1.00	0.00	RX0	N
ATOM	2192	H	VAL	383	39.526	-7.039	11.156	0.00	0.00	RX0	H
ATOM	2193	CA	VAL	383	37.600	-6.539	10.366	1.00	0.00	RX0	C
ATOM	2194	CB	VAL	383	36.434	-7.503	10.109	1.00	0.00	RX0	C

ATOM	2195	CG1	VAL	383	35.391	-6.887	9.174	1.00	0.00	RX0	C
ATOM	2196	CG2	VAL	383	36.950	-8.847	9.591	1.00	0.00	RX0	C
ATOM	2197	C	VAL	383	37.102	-5.173	10.877	1.00	0.00	RX0	C
ATOM	2198	O	VAL	383	36.355	-5.097	11.860	1.00	0.00	RX0	O
ATOM	2199	N	PRO	384	37.530	-4.088	10.236	1.00	0.00	RX0	N
ATOM	2200	CD	PRO	384	38.349	-4.086	9.033	1.00	0.00	RX0	C
ATOM	2201	CA	PRO	384	37.142	-2.731	10.663	1.00	0.00	RX0	C
ATOM	2202	CB	PRO	384	37.738	-1.832	9.572	1.00	0.00	RX0	C
ATOM	2203	CG	PRO	384	38.030	-2.746	8.383	1.00	0.00	RX0	C
ATOM	2204	C	PRO	384	35.624	-2.628	10.828	1.00	0.00	RX0	C
ATOM	2205	O	PRO	384	34.847	-3.282	10.112	1.00	0.00	RX0	O
ATOM	2206	N	LEU	385	35.228	-1.769	11.750	1.00	0.00	RX0	N
ATOM	2207	H	LEU	385	35.910	-1.200	12.206	0.00	0.00	RX0	H
ATOM	2208	CA	LEU	385	33.812	-1.621	12.127	1.00	0.00	RX0	C
ATOM	2209	CB	LEU	385	33.675	-0.670	13.315	1.00	0.00	RX0	C
ATOM	2210	CG	LEU	385	32.278	-0.710	13.936	1.00	0.00	RX0	C
ATOM	2211	CD1	LEU	385	31.900	-2.115	14.409	1.00	0.00	RX0	C
ATOM	2212	CD2	LEU	385	32.123	0.327	15.045	1.00	0.00	RX0	C
ATOM	2213	C	LEU	385	32.896	-1.213	10.964	1.00	0.00	RX0	C
ATOM	2214	O	LEU	385	31.809	-1.761	10.815	1.00	0.00	RX0	O
ATOM	2215	N	TYR	386	33.449	-0.406	10.055	1.00	0.00	RX0	N
ATOM	2216	H	TYR	386	34.229	0.150	10.348	0.00	0.00	RX0	H
ATOM	2217	CA	TYR	386	32.718	0.069	8.867	1.00	0.00	RX0	C
ATOM	2218	CB	TYR	386	33.663	0.897	7.981	1.00	0.00	RX0	C
ATOM	2219	CG	TYR	386	32.950	1.450	6.764	1.00	0.00	RX0	C
ATOM	2220	CD1	TYR	386	32.462	2.753	6.775	1.00	0.00	RX0	C
ATOM	2221	CE1	TYR	386	31.766	3.248	5.676	1.00	0.00	RX0	C
ATOM	2222	CD2	TYR	386	32.778	0.663	5.629	1.00	0.00	RX0	C
ATOM	2223	CE2	TYR	386	32.073	1.151	4.537	1.00	0.00	RX0	C
ATOM	2224	CZ	TYR	386	31.552	2.437	4.566	1.00	0.00	RX0	C
ATOM	2225	OH	TYR	386	30.818	2.898	3.489	1.00	0.00	RX0	O
ATOM	2226	HH	TYR	386	30.768	2.195	2.852	0.00	0.00	RX0	H
ATOM	2227	C	TYR	386	32.144	-1.103	8.049	1.00	0.00	RX0	C
ATOM	2228	O	TYR	386	30.957	-1.141	7.757	1.00	0.00	RX0	O
ATOM	2229	N	ASP	387	32.969	-2.141	7.903	1.00	0.00	RX0	N
ATOM	2230	H	ASP	387	33.927	-2.075	8.184	0.00	0.00	RX0	H
ATOM	2231	CA	ASP	387	32.619	-3.323	7.093	1.00	0.00	RX0	C
ATOM	2232	CB	ASP	387	33.860	-4.171	6.793	1.00	0.00	RX0	C
ATOM	2233	CG	ASP	387	34.960	-3.382	6.101	1.00	0.00	RX0	C
ATOM	2234	OD1	ASP	387	34.736	-2.236	5.712	1.00	0.00	RX0	O
ATOM	2235	OD2	ASP	387	36.055	-3.923	5.957	1.00	0.00	RX0	O
ATOM	2236	C	ASP	387	31.563	-4.207	7.765	1.00	0.00	RX0	C
ATOM	2237	O	ASP	387	30.619	-4.649	7.114	1.00	0.00	RX0	O
ATOM	2238	N	LEU	388	31.677	-4.332	9.088	1.00	0.00	RX0	N
ATOM	2239	H	LEU	388	32.449	-3.862	9.522	0.00	0.00	RX0	H
ATOM	2240	CA	LEU	388	30.691	-5.072	9.891	1.00	0.00	RX0	C
ATOM	2241	CB	LEU	388	31.205	-5.218	11.324	1.00	0.00	RX0	C
ATOM	2242	CG	LEU	388	30.279	-6.049	12.214	1.00	0.00	RX0	C
ATOM	2243	CD1	LEU	388	30.091	-7.468	11.676	1.00	0.00	RX0	C
ATOM	2244	CD2	LEU	388	30.737	-6.041	13.673	1.00	0.00	RX0	C
ATOM	2245	C	LEU	388	29.315	-4.386	9.871	1.00	0.00	RX0	C
ATOM	2246	O	LEU	388	28.299	-5.023	9.588	1.00	0.00	RX0	O
ATOM	2247	N	LEU	389	29.339	-3.070	10.049	1.00	0.00	RX0	N
ATOM	2248	H	LEU	389	30.229	-2.626	10.175	0.00	0.00	RX0	H
ATOM	2249	CA	LEU	389	28.129	-2.229	9.970	1.00	0.00	RX0	C
ATOM	2250	CB	LEU	389	28.426	-0.786	10.358	1.00	0.00	RX0	C
ATOM	2251	CG	LEU	389	28.866	-0.628	11.809	1.00	0.00	RX0	C
ATOM	2252	CD1	LEU	389	29.259	0.815	12.100	1.00	0.00	RX0	C
ATOM	2253	CD2	LEU	389	27.814	-1.128	12.797	1.00	0.00	RX0	C
ATOM	2254	C	LEU	389	27.489	-2.268	8.583	1.00	0.00	RX0	C
ATOM	2255	O	LEU	389	26.284	-2.473	8.458	1.00	0.00	RX0	O

ATOM	2256	N	LEU	390	28.350	-2.246	7.565	1.00	0.00	RX0	N
ATOM	2257	H	LEU	390	29.326	-2.133	7.756	0.00	0.00	RX0	H
ATOM	2258	CA	LEU	390	27.929	-2.335	6.162	1.00	0.00	RX0	C
ATOM	2259	CB	LEU	390	29.173	-2.218	5.282	1.00	0.00	RX0	C
ATOM	2260	CG	LEU	390	28.913	-2.075	3.787	1.00	0.00	RX0	C
ATOM	2261	CD1	LEU	390	28.400	-0.679	3.444	1.00	0.00	RX0	C
ATOM	2262	CD2	LEU	390	30.155	-2.428	2.968	1.00	0.00	RX0	C
ATOM	2263	C	LEU	390	27.202	-3.653	5.862	1.00	0.00	RX0	C
ATOM	2264	O	LEU	390	26.123	-3.627	5.302	1.00	0.00	RX0	O
ATOM	2265	N	GLU	391	27.733	-4.754	6.404	1.00	0.00	RX0	N
ATOM	2266	H	GLU	391	28.630	-4.700	6.849	0.00	0.00	RX0	H
ATOM	2267	CA	GLU	391	27.124	-6.090	6.264	1.00	0.00	RX0	C
ATOM	2268	CB	GLU	391	27.986	-7.213	6.841	1.00	0.00	RX0	C
ATOM	2269	CG	GLU	391	29.037	-7.706	5.848	1.00	0.00	RX0	C
ATOM	2270	CD	GLU	391	29.242	-9.194	6.060	1.00	0.00	RX0	C
ATOM	2271	OE1	GLU	391	28.772	-9.711	7.074	1.00	0.00	RX0	O
ATOM	2272	OE2	GLU	391	29.843	-9.836	5.199	1.00	0.00	RX0	O
ATOM	2273	C	GLU	391	25.713	-6.159	6.867	1.00	0.00	RX0	C
ATOM	2274	O	GLU	391	24.778	-6.600	6.214	1.00	0.00	RX0	O
ATOM	2275	N	MET	392	25.589	-5.588	8.070	1.00	0.00	RX0	N
ATOM	2276	H	MET	392	26.423	-5.203	8.473	0.00	0.00	RX0	H
ATOM	2277	CA	MET	392	24.313	-5.555	8.807	1.00	0.00	RX0	C
ATOM	2278	CB	MET	392	24.544	-5.227	10.281	1.00	0.00	RX0	C
ATOM	2279	CG	MET	392	25.239	-6.381	11.007	1.00	0.00	RX0	C
ATOM	2280	SD	MET	392	25.400	-6.112	12.780	1.00	0.00	RX0	S
ATOM	2281	CE	MET	392	26.595	-4.772	12.719	1.00	0.00	RX0	C
ATOM	2282	C	MET	392	23.266	-4.630	8.173	1.00	0.00	RX0	C
ATOM	2283	O	MET	392	22.107	-5.006	8.028	1.00	0.00	RX0	O
ATOM	2284	N	LEU	393	23.721	-3.452	7.754	1.00	0.00	RX0	N
ATOM	2285	H	LEU	393	24.707	-3.284	7.786	0.00	0.00	RX0	H
ATOM	2286	CA	LEU	393	22.852	-2.457	7.099	1.00	0.00	RX0	C
ATOM	2287	CB	LEU	393	23.510	-1.081	7.147	1.00	0.00	RX0	C
ATOM	2288	CG	LEU	393	23.446	-0.450	8.536	1.00	0.00	RX0	C
ATOM	2289	CD1	LEU	393	24.507	0.631	8.716	1.00	0.00	RX0	C
ATOM	2290	CD2	LEU	393	22.042	0.071	8.848	1.00	0.00	RX0	C
ATOM	2291	C	LEU	393	22.462	-2.815	5.663	1.00	0.00	RX0	C
ATOM	2292	O	LEU	393	21.313	-2.601	5.257	1.00	0.00	RX0	O
ATOM	2293	N	ASP	394	23.400	-3.384	4.923	1.00	0.00	RX0	N
ATOM	2294	H	ASP	394	24.269	-3.668	5.322	0.00	0.00	RX0	H
ATOM	2295	CA	ASP	394	23.187	-3.768	3.516	1.00	0.00	RX0	C
ATOM	2296	CB	ASP	394	24.414	-3.706	2.611	1.00	0.00	RX0	C
ATOM	2297	CG	ASP	394	23.913	-3.347	1.222	1.00	0.00	RX0	C
ATOM	2298	OD1	ASP	394	22.754	-2.942	1.105	1.00	0.00	RX0	O
ATOM	2299	OD2	ASP	394	24.683	-3.435	0.265	1.00	0.00	RX0	O
ATOM	2300	C	ASP	394	22.492	-5.132	3.432	1.00	0.00	RX0	C
ATOM	2301	O	ASP	394	23.034	-6.108	2.888	1.00	0.00	RX0	O
ATOM	2302	N	ALA	395	21.256	-5.134	3.874	1.00	0.00	RX0	N
ATOM	2303	H	ALA	395	20.886	-4.231	4.093	0.00	0.00	RX0	H
ATOM	2304	CA	ALA	395	20.412	-6.338	3.880	1.00	0.00	RX0	C
ATOM	2305	CB	ALA	395	19.422	-6.296	5.045	1.00	0.00	RX0	C
ATOM	2306	C	ALA	395	19.639	-6.454	2.565	1.00	0.00	RX0	C
ATOM	2307	O	ALA	395	19.303	-5.463	1.911	1.00	0.00	RX0	O
ATOM	2308	N	HIS	396	19.441	-7.696	2.156	1.00	0.00	RX0	N
ATOM	2309	H	HIS	396	19.740	-8.406	2.789	0.00	0.00	RX0	H
ATOM	2310	CA	HIS	396	18.536	-7.999	1.035	1.00	0.00	RX0	C
ATOM	2311	CB	HIS	396	18.856	-9.399	0.508	1.00	0.00	RX0	C
ATOM	2312	CG	HIS	396	19.033	-10.343	1.678	1.00	0.00	RX0	C
ATOM	2313	ND1	HIS	396	20.236	-10.729	2.144	1.00	0.00	RX0	N
ATOM	2314	HD1	HIS	396	21.117	-10.467	1.793	0.00	0.00	RX0	H
ATOM	2315	CD2	HIS	396	18.042	-10.935	2.466	1.00	0.00	RX0	C
ATOM	2316	NE2	HIS	396	18.657	-11.680	3.412	1.00	0.00	RX0	N

```
ATOM   2317  CE1 HIS   396      20.011 -11.555   3.216  1.00  0.00      RX0  C
ATOM   2318  C   HIS   396      17.077  -7.909   1.523  1.00  0.00      RX0  C
ATOM   2319  O   HIS   396      16.821  -7.755   2.721  1.00  0.00      RX0  O
ATOM   2320  N   ARG   397      16.142  -8.174   0.629  1.00  0.00      RX0  N
ATOM   2321  H   ARG   397      16.392  -8.364  -0.320  0.00  0.00      RX0  H
ATOM   2322  CA  ARG   397      14.704  -8.151   0.982  1.00  0.00      RX0  C
ATOM   2323  CB  ARG   397      14.040  -7.001   0.213  1.00  0.00      RX0  C
ATOM   2324  CG  ARG   397      14.837  -5.735   0.560  1.00  0.00      RX0  C
ATOM   2325  CD  ARG   397      14.595  -4.454  -0.236  1.00  0.00      RX0  C
ATOM   2326  NE  ARG   397      15.662  -3.503   0.090  1.00  0.00      RX0  N
ATOM   2327  HE  ARG   397      16.571  -3.913   0.240  0.00  0.00      RX0  H
ATOM   2328  CZ  ARG   397      15.421  -2.160   0.164  1.00  0.00      RX0  C
ATOM   2329  NH1 ARG   397      14.170  -1.699  -0.067  1.00  0.00      RX0  N
ATOM   2330 HH11 ARG   397      13.932  -0.724  -0.023  0.00  0.00      RX0  H
ATOM   2331 HH12 ARG   397      13.419  -2.326  -0.293  0.00  0.00      RX0  H
ATOM   2332  NH2 ARG   397      16.440  -1.321   0.469  1.00  0.00      RX0  N
ATOM   2333 HH21 ARG   397      16.329  -0.325   0.543  0.00  0.00      RX0  H
ATOM   2334 HH22 ARG   397      17.370  -1.668   0.641  0.00  0.00      RX0  H
ATOM   2335  C   ARG   397      14.082  -9.540   0.774  1.00  0.00      RX0  C
ATOM   2336  O   ARG   397      12.875  -9.706   0.613  1.00  0.00      RX0  O
ATOM   2337  N   LEU   398      14.938 -10.556   0.835  1.00  0.00      RX0  N
ATOM   2338  H   LEU   398      15.872 -10.362   1.123  0.00  0.00      RX0  H
ATOM   2339  CA  LEU   398      14.565 -11.951   0.542  1.00  0.00      RX0  C
ATOM   2340  CB  LEU   398      15.798 -12.796   0.229  1.00  0.00      RX0  C
ATOM   2341  CG  LEU   398      16.579 -12.273  -0.977  1.00  0.00      RX0  C
ATOM   2342  CD1 LEU   398      17.867 -13.067  -1.202  1.00  0.00      RX0  C
ATOM   2343  CD2 LEU   398      15.713 -12.211  -2.237  1.00  0.00      RX0  C
ATOM   2344  C   LEU   398      13.757 -12.584   1.680  1.00  0.00      RX0  C
ATOM   2345  O   LEU   398      12.772 -13.259   1.439  1.00  0.00      RX0  O
ATOM   2346  N   HIS   399      14.100 -12.169   2.904  1.00  0.00      RX0  N
ATOM   2347  H   HIS   399      14.876 -11.548   3.000  0.00  0.00      RX0  H
ATOM   2348  CA  HIS   399      13.392 -12.616   4.118  1.00  0.00      RX0  C
ATOM   2349  CB  HIS   399      14.361 -12.626   5.301  1.00  0.00      RX0  C
ATOM   2350  CG  HIS   399      15.418 -13.673   5.045  1.00  0.00      RX0  C
ATOM   2351  ND1 HIS   399      16.720 -13.405   4.829  1.00  0.00      RX0  N
ATOM   2352  HD1 HIS   399      17.162 -12.528   4.814  0.00  0.00      RX0  H
ATOM   2353  CD2 HIS   399      15.227 -15.055   4.970  1.00  0.00      RX0  C
ATOM   2354  NE2 HIS   399      16.430 -15.618   4.705  1.00  0.00      RX0  N
ATOM   2355  CE1 HIS   399      17.348 -14.605   4.620  1.00  0.00      RX0  C
ATOM   2356  C   HIS   399      12.131 -11.786   4.401  1.00  0.00      RX0  C
ATOM   2357  O   HIS   399      11.630 -11.743   5.524  1.00  0.00      RX0  O
ATOM   2358  N   ALA   400      11.619 -11.143   3.351  1.00  0.00      RX0  N
ATOM   2359  H   ALA   400      12.033 -11.299   2.457  0.00  0.00      RX0  H
ATOM   2360  CA  ALA   400      10.356 -10.385   3.402  1.00  0.00      RX0  C
ATOM   2361  CB  ALA   400      10.143  -9.554   2.137  1.00  0.00      RX0  C
ATOM   2362  C   ALA   400       9.147 -11.323   3.579  1.00  0.00      RX0  C
ATOM   2363  O   ALA   400       8.508 -11.220   4.647  1.00  0.00      RX0  O
TER
HEADER lig.000.00.pdb
ATOM      1  N   SER    22     -16.808  -6.482  42.503  1.00  0.00      LX0  N
ATOM      2  H   SER    22     -16.562  -6.368  41.538  0.00  0.00      LX0  H
ATOM      3  CA  SER    22     -16.954  -7.844  43.019  1.00  0.00      LX0  C
ATOM      4  CB  SER    22     -18.344  -8.387  42.654  1.00  0.00      LX0  C
ATOM      5  OG  SER    22     -18.600  -9.636  43.308  1.00  0.00      LX0  O
ATOM      6  HG  SER    22     -17.886 -10.214  43.043  0.00  0.00      LX0  H
ATOM      7  C   SER    22     -15.852  -8.761  42.510  1.00  0.00      LX0  C
ATOM      8  O   SER    22     -16.031  -9.964  42.353  1.00  0.00      LX0  O
ATOM      9  N   THR    23     -14.716  -8.119  42.226  1.00  0.00      LX0  N
ATOM     10  H   THR    23     -14.605  -7.140  42.392  0.00  0.00      LX0  H
ATOM     11  CA  THR    23     -13.675  -8.798  41.466  1.00  0.00      LX0  C
ATOM     12  CB  THR    23     -13.924  -8.542  39.971  1.00  0.00      LX0  C
```

ATOM	13	OG1	THR	23	-14.617	-7.294	39.792	1.00	0.00	LX0	O
ATOM	14	HG1	THR	23	-14.622	-7.120	38.859	0.00	0.00	LX0	H
ATOM	15	CG2	THR	23	-14.714	-9.667	39.299	1.00	0.00	LX0	C
ATOM	16	C	THR	23	-12.316	-8.272	41.883	1.00	0.00	LX0	C
ATOM	17	O	THR	23	-12.198	-7.174	42.414	1.00	0.00	LX0	O
ATOM	18	N	GLN	24	-11.287	-9.085	41.603	1.00	0.00	LX0	N
ATOM	19	H	GLN	24	-11.468	-9.998	41.235	0.00	0.00	LX0	H
ATOM	20	CA	GLN	24	-9.930	-8.550	41.711	1.00	0.00	LX0	C
ATOM	21	CB	GLN	24	-8.927	-9.717	41.719	1.00	0.00	LX0	C
ATOM	22	CG	GLN	24	-7.441	-9.413	41.460	1.00	0.00	LX0	C
ATOM	23	CD	GLN	24	-6.801	-8.546	42.526	1.00	0.00	LX0	C
ATOM	24	OE1	GLN	24	-7.438	-7.771	43.226	1.00	0.00	LX0	O
ATOM	25	NE2	GLN	24	-5.478	-8.696	42.597	1.00	0.00	LX0	N
ATOM	26	HE21	GLN	24	-4.986	-9.352	42.018	0.00	0.00	LX0	H
ATOM	27	HE22	GLN	24	-4.954	-8.138	43.236	0.00	0.00	LX0	H
ATOM	28	C	GLN	24	-9.660	-7.544	40.603	1.00	0.00	LX0	C
ATOM	29	O	GLN	24	-9.600	-7.883	39.425	1.00	0.00	LX0	O
ATOM	30	N	VAL	25	-9.546	-6.286	41.035	1.00	0.00	LX0	N
ATOM	31	H	VAL	25	-9.546	-6.093	42.017	0.00	0.00	LX0	H
ATOM	32	CA	VAL	25	-9.387	-5.206	40.066	1.00	0.00	LX0	C
ATOM	33	CB	VAL	25	-10.622	-4.282	40.094	1.00	0.00	LX0	C
ATOM	34	CG1	VAL	25	-10.488	-3.084	39.152	1.00	0.00	LX0	C
ATOM	35	CG2	VAL	25	-11.909	-5.053	39.795	1.00	0.00	LX0	C
ATOM	36	C	VAL	25	-8.127	-4.427	40.384	1.00	0.00	LX0	C
ATOM	37	O	VAL	25	-7.909	-4.012	41.515	1.00	0.00	LX0	O
ATOM	38	N	CYS	26	-7.309	-4.236	39.347	1.00	0.00	LX0	N
ATOM	39	H	CYS	26	-7.535	-4.570	38.428	0.00	0.00	LX0	H
ATOM	40	CA	CYS	26	-6.153	-3.376	39.574	1.00	0.00	LX0	C
ATOM	41	CB	CYS	26	-4.866	-4.196	39.715	1.00	0.00	LX0	C
ATOM	42	SG	CYS	26	-4.380	-5.070	38.206	1.00	0.00	LX0	S
ATOM	43	C	CYS	26	-6.051	-2.369	38.456	1.00	0.00	LX0	C
ATOM	44	O	CYS	26	-6.747	-2.476	37.455	1.00	0.00	LX0	O
ATOM	45	N	THR	27	-5.174	-1.380	38.644	1.00	0.00	LX0	N
ATOM	46	H	THR	27	-4.592	-1.288	39.451	0.00	0.00	LX0	H
ATOM	47	CA	THR	27	-4.887	-0.550	37.480	1.00	0.00	LX0	C
ATOM	48	CB	THR	27	-4.299	0.768	37.970	1.00	0.00	LX0	C
ATOM	49	OG1	THR	27	-3.411	0.519	39.070	1.00	0.00	LX0	O
ATOM	50	HG1	THR	27	-2.645	0.093	38.698	0.00	0.00	LX0	H
ATOM	51	CG2	THR	27	-5.407	1.731	38.401	1.00	0.00	LX0	C
ATOM	52	C	THR	27	-3.927	-1.271	36.558	1.00	0.00	LX0	C
ATOM	53	O	THR	27	-3.163	-2.119	36.999	1.00	0.00	LX0	O
ATOM	54	N	GLY	28	-3.996	-0.912	35.278	1.00	0.00	LX0	N
ATOM	55	H	GLY	28	-4.588	-0.155	35.000	0.00	0.00	LX0	H
ATOM	56	CA	GLY	28	-2.938	-1.409	34.410	1.00	0.00	LX0	C
ATOM	57	C	GLY	28	-1.810	-0.416	34.395	1.00	0.00	LX0	C
ATOM	58	O	GLY	28	-1.886	0.626	35.041	1.00	0.00	LX0	O
ATOM	59	N	THR	29	-0.774	-0.750	33.630	1.00	0.00	LX0	N
ATOM	60	H	THR	29	-0.728	-1.605	33.106	0.00	0.00	LX0	H
ATOM	61	CA	THR	29	0.125	0.359	33.360	1.00	0.00	LX0	C
ATOM	62	CB	THR	29	1.578	-0.128	33.321	1.00	0.00	LX0	C
ATOM	63	OG1	THR	29	1.710	-1.321	32.543	1.00	0.00	LX0	O
ATOM	64	HG1	THR	29	1.686	-1.029	31.628	0.00	0.00	LX0	H
ATOM	65	CG2	THR	29	2.101	-0.377	34.735	1.00	0.00	LX0	C
ATOM	66	C	THR	29	-0.301	1.092	32.103	1.00	0.00	LX0	C
ATOM	67	O	THR	29	-1.182	0.640	31.375	1.00	0.00	LX0	O
ATOM	68	N	ASP	30	0.353	2.242	31.908	1.00	0.00	LX0	N
ATOM	69	H	ASP	30	0.965	2.629	32.592	0.00	0.00	LX0	H
ATOM	70	CA	ASP	30	0.286	2.872	30.598	1.00	0.00	LX0	C
ATOM	71	CB	ASP	30	-0.793	3.960	30.542	1.00	0.00	LX0	C
ATOM	72	CG	ASP	30	-1.154	4.323	29.106	1.00	0.00	LX0	C
ATOM	73	OD1	ASP	30	-0.561	3.792	28.167	1.00	0.00	LX0	O

ATOM	74	OD2	ASP	30	-2.056	5.131	28.924	1.00	0.00	LX0	O
ATOM	75	C	ASP	30	1.654	3.421	30.266	1.00	0.00	LX0	C
ATOM	76	O	ASP	30	2.172	4.320	30.933	1.00	0.00	LX0	O
ATOM	77	N	MET	31	2.260	2.796	29.255	1.00	0.00	LX0	N
ATOM	78	H	MET	31	1.809	1.974	28.902	0.00	0.00	LX0	H
ATOM	79	CA	MET	31	3.618	3.182	28.900	1.00	0.00	LX0	C
ATOM	80	CB	MET	31	4.656	2.517	29.822	1.00	0.00	LX0	C
ATOM	81	CG	MET	31	4.887	1.014	29.654	1.00	0.00	LX0	C
ATOM	82	SD	MET	31	6.125	0.395	30.804	1.00	0.00	LX0	S
ATOM	83	CE	MET	31	5.236	0.734	32.330	1.00	0.00	LX0	C
ATOM	84	C	MET	31	4.021	3.034	27.448	1.00	0.00	LX0	C
ATOM	85	O	MET	31	5.061	3.541	27.049	1.00	0.00	LX0	O
ATOM	86	N	LYS	32	3.201	2.306	26.673	1.00	0.00	LX0	N
ATOM	87	H	LYS	32	2.378	1.904	27.076	0.00	0.00	LX0	H
ATOM	88	CA	LYS	32	3.691	1.847	25.368	1.00	0.00	LX0	C
ATOM	89	CB	LYS	32	3.727	2.973	24.317	1.00	0.00	LX0	C
ATOM	90	CG	LYS	32	2.329	3.356	23.831	1.00	0.00	LX0	C
ATOM	91	CD	LYS	32	2.324	3.883	22.395	1.00	0.00	LX0	C
ATOM	92	CE	LYS	32	0.916	3.836	21.797	1.00	0.00	LX0	C
ATOM	93	NZ	LYS	32	0.951	4.166	20.371	1.00	0.00	LX0	N
ATOM	94	HZ1	LYS	32	0.221	3.624	19.857	0.00	0.00	LX0	H
ATOM	95	HZ2	LYS	32	0.680	5.140	20.142	0.00	0.00	LX0	H
ATOM	96	HZ3	LYS	32	1.859	3.968	19.901	0.00	0.00	LX0	H
ATOM	97	C	LYS	32	5.011	1.095	25.508	1.00	0.00	LX0	C
ATOM	98	O	LYS	32	5.265	0.486	26.540	1.00	0.00	LX0	O
ATOM	99	N	LEU	33	5.879	1.161	24.491	1.00	0.00	LX0	N
ATOM	100	H	LEU	33	5.726	1.646	23.632	0.00	0.00	LX0	H
ATOM	101	CA	LEU	33	7.134	0.454	24.746	1.00	0.00	LX0	C
ATOM	102	CB	LEU	33	7.685	-0.143	23.456	1.00	0.00	LX0	C
ATOM	103	CG	LEU	33	6.671	-1.007	22.715	1.00	0.00	LX0	C
ATOM	104	CD1	LEU	33	7.067	-1.329	21.279	1.00	0.00	LX0	C
ATOM	105	CD2	LEU	33	6.381	-2.272	23.497	1.00	0.00	LX0	C
ATOM	106	C	LEU	33	8.201	1.251	25.483	1.00	0.00	LX0	C
ATOM	107	O	LEU	33	9.393	1.061	25.280	1.00	0.00	LX0	O
ATOM	108	N	ARG	34	7.737	2.154	26.368	1.00	0.00	LX0	N
ATOM	109	H	ARG	34	6.755	2.248	26.535	0.00	0.00	LX0	H
ATOM	110	CA	ARG	34	8.680	2.932	27.172	1.00	0.00	LX0	C
ATOM	111	CB	ARG	34	7.898	3.907	28.066	1.00	0.00	LX0	C
ATOM	112	CG	ARG	34	8.668	4.684	29.137	1.00	0.00	LX0	C
ATOM	113	CD	ARG	34	7.864	5.842	29.744	1.00	0.00	LX0	C
ATOM	114	NE	ARG	34	6.587	5.422	30.323	1.00	0.00	LX0	N
ATOM	115	HE	ARG	34	6.590	4.599	30.891	0.00	0.00	LX0	H
ATOM	116	CZ	ARG	34	5.474	6.166	30.111	1.00	0.00	LX0	C
ATOM	117	NH1	ARG	34	5.497	7.229	29.321	1.00	0.00	LX0	N
ATOM	118	HH11	ARG	34	4.713	7.822	29.162	0.00	0.00	LX0	H
ATOM	119	HH12	ARG	34	6.330	7.497	28.805	0.00	0.00	LX0	H
ATOM	120	NH2	ARG	34	4.328	5.826	30.691	1.00	0.00	LX0	N
ATOM	121	HH21	ARG	34	3.482	6.340	30.541	0.00	0.00	LX0	H
ATOM	122	HH22	ARG	34	4.235	5.016	31.273	0.00	0.00	LX0	H
ATOM	123	C	ARG	34	9.667	2.080	27.952	1.00	0.00	LX0	C
ATOM	124	O	ARG	34	9.364	1.492	28.984	1.00	0.00	LX0	O
ATOM	125	N	LEU	35	10.885	2.067	27.387	1.00	0.00	LX0	N
ATOM	126	H	LEU	35	10.949	2.420	26.452	0.00	0.00	LX0	H
ATOM	127	CA	LEU	35	12.027	1.416	28.024	1.00	0.00	LX0	C
ATOM	128	CB	LEU	35	13.290	1.826	27.250	1.00	0.00	LX0	C
ATOM	129	CG	LEU	35	14.530	0.953	27.469	1.00	0.00	LX0	C
ATOM	130	CD1	LEU	35	14.318	-0.470	26.966	1.00	0.00	LX0	C
ATOM	131	CD2	LEU	35	15.776	1.553	26.819	1.00	0.00	LX0	C
ATOM	132	C	LEU	35	12.131	1.760	29.506	1.00	0.00	LX0	C
ATOM	133	O	LEU	35	12.194	2.925	29.874	1.00	0.00	LX0	O
ATOM	134	N	PRO	36	12.116	0.713	30.359	1.00	0.00	LX0	N

ATOM	135	CD	PRO	36	11.967	-0.695	30.014	1.00	0.00	LX0	C
ATOM	136	CA	PRO	36	12.204	0.950	31.805	1.00	0.00	LX0	C
ATOM	137	CB	PRO	36	12.140	-0.468	32.373	1.00	0.00	LX0	C
ATOM	138	CG	PRO	36	11.469	-1.325	31.301	1.00	0.00	LX0	C
ATOM	139	C	PRO	36	13.476	1.673	32.222	1.00	0.00	LX0	C
ATOM	140	O	PRO	36	14.541	1.430	31.667	1.00	0.00	LX0	O
ATOM	141	N	ALA	37	13.318	2.546	33.233	1.00	0.00	LX0	N
ATOM	142	H	ALA	37	12.439	2.664	33.695	0.00	0.00	LX0	H
ATOM	143	CA	ALA	37	14.518	3.175	33.787	1.00	0.00	LX0	C
ATOM	144	CB	ALA	37	14.177	4.524	34.419	1.00	0.00	LX0	C
ATOM	145	C	ALA	37	15.273	2.290	34.771	1.00	0.00	LX0	C
ATOM	146	O	ALA	37	16.403	2.564	35.173	1.00	0.00	LX0	O
ATOM	147	N	SER	38	14.601	1.162	35.062	1.00	0.00	LX0	N
ATOM	148	H	SER	38	13.613	1.237	35.223	0.00	0.00	LX0	H
ATOM	149	CA	SER	38	15.295	-0.086	35.334	1.00	0.00	LX0	C
ATOM	150	CB	SER	38	15.882	-0.120	36.751	1.00	0.00	LX0	C
ATOM	151	OG	SER	38	14.877	-0.394	37.734	1.00	0.00	LX0	O
ATOM	152	HG	SER	38	14.123	0.174	37.555	0.00	0.00	LX0	H
ATOM	153	C	SER	38	14.394	-1.287	35.077	1.00	0.00	LX0	C
ATOM	154	O	SER	38	13.246	-1.355	35.500	1.00	0.00	LX0	O
ATOM	155	N	PRO	39	14.954	-2.271	34.339	1.00	0.00	LX0	N
ATOM	156	CD	PRO	39	16.171	-2.159	33.550	1.00	0.00	LX0	C
ATOM	157	CA	PRO	39	14.342	-3.605	34.262	1.00	0.00	LX0	C
ATOM	158	CB	PRO	39	15.480	-4.458	33.697	1.00	0.00	LX0	C
ATOM	159	CG	PRO	39	16.306	-3.501	32.845	1.00	0.00	LX0	C
ATOM	160	C	PRO	39	13.804	-4.158	35.577	1.00	0.00	LX0	C
ATOM	161	O	PRO	39	12.689	-4.662	35.654	1.00	0.00	LX0	O
ATOM	162	N	GLU	40	14.663	-4.050	36.608	1.00	0.00	LX0	N
ATOM	163	H	GLU	40	15.545	-3.588	36.519	0.00	0.00	LX0	H
ATOM	164	CA	GLU	40	14.301	-4.646	37.894	1.00	0.00	LX0	C
ATOM	165	CB	GLU	40	15.482	-4.570	38.863	1.00	0.00	LX0	C
ATOM	166	CG	GLU	40	16.687	-5.381	38.369	1.00	0.00	LX0	C
ATOM	167	CD	GLU	40	17.863	-5.255	39.323	1.00	0.00	LX0	C
ATOM	168	OE1	GLU	40	17.670	-5.367	40.534	1.00	0.00	LX0	O
ATOM	169	OE2	GLU	40	18.979	-5.058	38.845	1.00	0.00	LX0	O
ATOM	170	C	GLU	40	13.031	-4.090	38.514	1.00	0.00	LX0	C
ATOM	171	O	GLU	40	12.109	-4.828	38.853	1.00	0.00	LX0	O
ATOM	172	N	THR	41	12.988	-2.746	38.607	1.00	0.00	LX0	N
ATOM	173	H	THR	41	13.721	-2.147	38.272	0.00	0.00	LX0	H
ATOM	174	CA	THR	41	11.770	-2.156	39.166	1.00	0.00	LX0	C
ATOM	175	CB	THR	41	11.905	-0.636	39.258	1.00	0.00	LX0	C
ATOM	176	OG1	THR	41	12.280	-0.107	37.980	1.00	0.00	LX0	O
ATOM	177	HG1	THR	41	12.183	0.854	38.035	0.00	0.00	LX0	H
ATOM	178	CG2	THR	41	12.914	-0.232	40.336	1.00	0.00	LX0	C
ATOM	179	C	THR	41	10.523	-2.534	38.386	1.00	0.00	LX0	C
ATOM	180	O	THR	41	9.531	-3.009	38.928	1.00	0.00	LX0	O
ATOM	181	N	HIS	42	10.658	-2.353	37.061	1.00	0.00	LX0	N
ATOM	182	H	HIS	42	11.488	-1.871	36.758	0.00	0.00	LX0	H
ATOM	183	CA	HIS	42	9.589	-2.729	36.136	1.00	0.00	LX0	C
ATOM	184	CB	HIS	42	10.107	-2.606	34.701	1.00	0.00	LX0	C
ATOM	185	CG	HIS	42	9.014	-2.830	33.680	1.00	0.00	LX0	C
ATOM	186	ND1	HIS	42	7.965	-2.006	33.506	1.00	0.00	LX0	N
ATOM	187	HD1	HIS	42	7.772	-1.187	34.003	0.00	0.00	LX0	H
ATOM	188	CD2	HIS	42	8.920	-3.880	32.763	1.00	0.00	LX0	C
ATOM	189	NE2	HIS	42	7.798	-3.673	32.035	1.00	0.00	LX0	N
ATOM	190	CE1	HIS	42	7.209	-2.524	32.487	1.00	0.00	LX0	C
ATOM	191	C	HIS	42	8.979	-4.101	36.392	1.00	0.00	LX0	C
ATOM	192	O	HIS	42	7.784	-4.238	36.619	1.00	0.00	LX0	O
ATOM	193	N	LEU	43	9.868	-5.108	36.364	1.00	0.00	LX0	N
ATOM	194	H	LEU	43	10.839	-4.897	36.236	0.00	0.00	LX0	H
ATOM	195	CA	LEU	43	9.415	-6.490	36.533	1.00	0.00	LX0	C

ATOM	196	CB	LEU	43	10.631	-7.427	36.523	1.00	0.00	LX0 C
ATOM	197	CG	LEU	43	10.329	-8.897	36.844	1.00	0.00	LX0 C
ATOM	198	CD1	LEU	43	9.377	-9.545	35.838	1.00	0.00	LX0 C
ATOM	199	CD2	LEU	43	11.612	-9.697	37.061	1.00	0.00	LX0 C
ATOM	200	C	LEU	43	8.559	-6.720	37.769	1.00	0.00	LX0 C
ATOM	201	O	LEU	43	7.419	-7.179	37.712	1.00	0.00	LX0 O
ATOM	202	N	ASP	44	9.169	-6.387	38.909	1.00	0.00	LX0 N
ATOM	203	H	ASP	44	10.077	-5.960	38.936	0.00	0.00	LX0 H
ATOM	204	CA	ASP	44	8.444	-6.790	40.106	1.00	0.00	LX0 C
ATOM	205	CB	ASP	44	9.415	-7.063	41.254	1.00	0.00	LX0 C
ATOM	206	CG	ASP	44	9.721	-8.555	41.294	1.00	0.00	LX0 C
ATOM	207	OD1	ASP	44	9.744	-9.123	42.380	1.00	0.00	LX0 O
ATOM	208	OD2	ASP	44	9.853	-9.199	40.249	1.00	0.00	LX0 O
ATOM	209	C	ASP	44	7.263	-5.910	40.456	1.00	0.00	LX0 C
ATOM	210	O	ASP	44	6.269	-6.348	41.028	1.00	0.00	LX0 O
ATOM	211	N	MET	45	7.362	-4.653	39.987	1.00	0.00	LX0 N
ATOM	212	H	MET	45	8.214	-4.335	39.564	0.00	0.00	LX0 H
ATOM	213	CA	MET	45	6.167	-3.809	40.000	1.00	0.00	LX0 C
ATOM	214	CB	MET	45	6.530	-2.387	39.548	1.00	0.00	LX0 C
ATOM	215	CG	MET	45	5.392	-1.362	39.486	1.00	0.00	LX0 C
ATOM	216	SD	MET	45	4.367	-1.518	38.013	1.00	0.00	LX0 S
ATOM	217	CE	MET	45	5.642	-1.164	36.791	1.00	0.00	LX0 C
ATOM	218	C	MET	45	5.007	-4.406	39.215	1.00	0.00	LX0 C
ATOM	219	O	MET	45	3.873	-4.430	39.678	1.00	0.00	LX0 O
ATOM	220	N	LEU	46	5.342	-4.947	38.028	1.00	0.00	LX0 N
ATOM	221	H	LEU	46	6.279	-4.879	37.678	0.00	0.00	LX0 H
ATOM	222	CA	LEU	46	4.293	-5.629	37.267	1.00	0.00	LX0 C
ATOM	223	CB	LEU	46	4.789	-6.172	35.927	1.00	0.00	LX0 C
ATOM	224	CG	LEU	46	5.238	-5.125	34.910	1.00	0.00	LX0 C
ATOM	225	CD1	LEU	46	5.628	-5.806	33.602	1.00	0.00	LX0 C
ATOM	226	CD2	LEU	46	4.206	-4.021	34.679	1.00	0.00	LX0 C
ATOM	227	C	LEU	46	3.617	-6.757	38.019	1.00	0.00	LX0 C
ATOM	228	O	LEU	46	2.404	-6.909	37.985	1.00	0.00	LX0 O
ATOM	229	N	ARG	47	4.445	-7.530	38.742	1.00	0.00	LX0 N
ATOM	230	H	ARG	47	5.434	-7.364	38.710	0.00	0.00	LX0 H
ATOM	231	CA	ARG	47	3.822	-8.567	39.568	1.00	0.00	LX0 C
ATOM	232	CB	ARG	47	4.877	-9.476	40.200	1.00	0.00	LX0 C
ATOM	233	CG	ARG	47	4.308	-10.811	40.708	1.00	0.00	LX0 C
ATOM	234	CD	ARG	47	5.361	-11.843	41.136	1.00	0.00	LX0 C
ATOM	235	NE	ARG	47	6.376	-12.017	40.094	1.00	0.00	LX0 N
ATOM	236	HE	ARG	47	6.137	-12.457	39.224	0.00	0.00	LX0 H
ATOM	237	CZ	ARG	47	7.561	-11.389	40.241	1.00	0.00	LX0 C
ATOM	238	NH1	ARG	47	7.917	-10.894	41.418	1.00	0.00	LX0 N
ATOM	239	HH11	ARG	47	8.768	-10.347	41.501	0.00	0.00	LX0 H
ATOM	240	HH12	ARG	47	7.376	-11.009	42.247	0.00	0.00	LX0 H
ATOM	241	NH2	ARG	47	8.355	-11.233	39.193	1.00	0.00	LX0 N
ATOM	242	HH21	ARG	47	9.178	-10.654	39.306	0.00	0.00	LX0 H
ATOM	243	HH22	ARG	47	8.129	-11.649	38.310	0.00	0.00	LX0 H
ATOM	244	C	ARG	47	2.873	-8.024	40.621	1.00	0.00	LX0 C
ATOM	245	O	ARG	47	1.710	-8.400	40.708	1.00	0.00	LX0 O
ATOM	246	N	HIS	48	3.429	-7.082	41.400	1.00	0.00	LX0 N
ATOM	247	H	HIS	48	4.379	-6.810	41.236	0.00	0.00	LX0 H
ATOM	248	CA	HIS	48	2.652	-6.478	42.483	1.00	0.00	LX0 C
ATOM	249	CB	HIS	48	3.511	-5.433	43.206	1.00	0.00	LX0 C
ATOM	250	CG	HIS	48	2.995	-5.117	44.596	1.00	0.00	LX0 C
ATOM	251	ND1	HIS	48	1.696	-5.033	44.948	1.00	0.00	LX0 N
ATOM	252	HD1	HIS	48	0.911	-5.180	44.374	0.00	0.00	LX0 H
ATOM	253	CD2	HIS	48	3.774	-4.850	45.726	1.00	0.00	LX0 C
ATOM	254	NE2	HIS	48	2.928	-4.606	46.756	1.00	0.00	LX0 N
ATOM	255	CE1	HIS	48	1.649	-4.718	46.279	1.00	0.00	LX0 C
ATOM	256	C	HIS	48	1.328	-5.873	42.038	1.00	0.00	LX0 C

ATOM	257	O	HIS	48	0.316	-5.948	42.723	1.00	0.00	LX0	O
ATOM	258	N	LEU	49	1.393	-5.260	40.852	1.00	0.00	LX0	N
ATOM	259	H	LEU	49	2.257	-5.240	40.347	0.00	0.00	LX0	H
ATOM	260	CA	LEU	49	0.189	-4.637	40.320	1.00	0.00	LX0	C
ATOM	261	CB	LEU	49	0.605	-3.592	39.280	1.00	0.00	LX0	C
ATOM	262	CG	LEU	49	-0.512	-2.662	38.808	1.00	0.00	LX0	C
ATOM	263	CD1	LEU	49	-1.174	-1.913	39.966	1.00	0.00	LX0	C
ATOM	264	CD2	LEU	49	-0.009	-1.708	37.725	1.00	0.00	LX0	C
ATOM	265	C	LEU	49	-0.809	-5.637	39.756	1.00	0.00	LX0	C
ATOM	266	O	LEU	49	-1.977	-5.675	40.112	1.00	0.00	LX0	O
ATOM	267	N	TYR	50	-0.282	-6.446	38.832	1.00	0.00	LX0	N
ATOM	268	H	TYR	50	0.706	-6.465	38.681	0.00	0.00	LX0	H
ATOM	269	CA	TYR	50	-1.184	-7.259	38.021	1.00	0.00	LX0	C
ATOM	270	CB	TYR	50	-0.533	-7.607	36.681	1.00	0.00	LX0	C
ATOM	271	CG	TYR	50	-0.288	-6.421	35.773	1.00	0.00	LX0	C
ATOM	272	CD1	TYR	50	-1.217	-5.361	35.714	1.00	0.00	LX0	C
ATOM	273	CE1	TYR	50	-1.018	-4.334	34.779	1.00	0.00	LX0	C
ATOM	274	CD2	TYR	50	0.865	-6.441	34.962	1.00	0.00	LX0	C
ATOM	275	CE2	TYR	50	1.061	-5.415	34.024	1.00	0.00	LX0	C
ATOM	276	CZ	TYR	50	0.102	-4.388	33.927	1.00	0.00	LX0	C
ATOM	277	OH	TYR	50	0.243	-3.415	32.960	1.00	0.00	LX0	O
ATOM	278	HH	TYR	50	1.107	-3.005	33.023	0.00	0.00	LX0	H
ATOM	279	C	TYR	50	-1.697	-8.543	38.647	1.00	0.00	LX0	C
ATOM	280	O	TYR	50	-2.575	-9.199	38.098	1.00	0.00	LX0	O
ATOM	281	N	GLN	51	-1.095	-8.913	39.788	1.00	0.00	LX0	N
ATOM	282	H	GLN	51	-0.380	-8.327	40.175	0.00	0.00	LX0	H
ATOM	283	CA	GLN	51	-1.322	-10.252	40.339	1.00	0.00	LX0	C
ATOM	284	CB	GLN	51	-0.639	-10.344	41.704	1.00	0.00	LX0	C
ATOM	285	CG	GLN	51	-0.303	-11.774	42.128	1.00	0.00	LX0	C
ATOM	286	CD	GLN	51	0.752	-11.732	43.214	1.00	0.00	LX0	C
ATOM	287	OE1	GLN	51	1.923	-12.005	42.992	1.00	0.00	LX0	O
ATOM	288	NE2	GLN	51	0.290	-11.380	44.417	1.00	0.00	LX0	N
ATOM	289	HE21	GLN	51	-0.672	-11.158	44.565	0.00	0.00	LX0	H
ATOM	290	HE22	GLN	51	0.947	-11.342	45.167	0.00	0.00	LX0	H
ATOM	291	C	GLN	51	-2.752	-10.787	40.369	1.00	0.00	LX0	C
ATOM	292	O	GLN	51	-3.580	-10.402	41.188	1.00	0.00	LX0	O
ATOM	293	N	GLY	52	-2.991	-11.719	39.429	1.00	0.00	LX0	N
ATOM	294	H	GLY	52	-2.295	-11.914	38.734	0.00	0.00	LX0	H
ATOM	295	CA	GLY	52	-4.292	-12.391	39.375	1.00	0.00	LX0	C
ATOM	296	C	GLY	52	-5.473	-11.506	39.000	1.00	0.00	LX0	C
ATOM	297	O	GLY	52	-6.616	-11.759	39.360	1.00	0.00	LX0	O
ATOM	298	N	CYS	53	-5.152	-10.432	38.269	1.00	0.00	LX0	N
ATOM	299	H	CYS	53	-4.209	-10.274	37.973	0.00	0.00	LX0	H
ATOM	300	CA	CYS	53	-6.206	-9.450	38.036	1.00	0.00	LX0	C
ATOM	301	CB	CYS	53	-5.627	-8.135	37.532	1.00	0.00	LX0	C
ATOM	302	SG	CYS	53	-6.846	-6.807	37.655	1.00	0.00	LX0	S
ATOM	303	C	CYS	53	-7.343	-9.893	37.139	1.00	0.00	LX0	C
ATOM	304	O	CYS	53	-7.176	-10.250	35.978	1.00	0.00	LX0	O
ATOM	305	N	GLN	54	-8.543	-9.825	37.717	1.00	0.00	LX0	N
ATOM	306	H	GLN	54	-8.637	-9.457	38.643	0.00	0.00	LX0	H
ATOM	307	CA	GLN	54	-9.693	-10.110	36.870	1.00	0.00	LX0	C
ATOM	308	CB	GLN	54	-10.915	-10.466	37.706	1.00	0.00	LX0	C
ATOM	309	CG	GLN	54	-10.652	-11.657	38.621	1.00	0.00	LX0	C
ATOM	310	CD	GLN	54	-11.935	-12.009	39.328	1.00	0.00	LX0	C
ATOM	311	OE1	GLN	54	-12.189	-11.589	40.447	1.00	0.00	LX0	O
ATOM	312	NE2	GLN	54	-12.754	-12.789	38.617	1.00	0.00	LX0	N
ATOM	313	HE21	GLN	54	-12.532	-13.080	37.682	0.00	0.00	LX0	H
ATOM	314	HE22	GLN	54	-13.614	-13.072	39.036	0.00	0.00	LX0	H
ATOM	315	C	GLN	54	-10.018	-8.984	35.912	1.00	0.00	LX0	C
ATOM	316	O	GLN	54	-10.239	-9.199	34.727	1.00	0.00	LX0	O
ATOM	317	N	VAL	55	-10.037	-7.773	36.485	1.00	0.00	LX0	N

ATOM	318	H	VAL	55	-9.832	-7.662	37.460	0.00	0.00	LX0	H
ATOM	319	CA	VAL	55	-10.273	-6.608	35.637	1.00	0.00	LX0	C
ATOM	320	CB	VAL	55	-11.587	-5.896	36.008	1.00	0.00	LX0	C
ATOM	321	CG1	VAL	55	-11.895	-4.750	35.036	1.00	0.00	LX0	C
ATOM	322	CG2	VAL	55	-12.765	-6.870	36.105	1.00	0.00	LX0	C
ATOM	323	C	VAL	55	-9.105	-5.648	35.729	1.00	0.00	LX0	C
ATOM	324	O	VAL	55	-8.958	-4.902	36.692	1.00	0.00	LX0	O
ATOM	325	N	VAL	56	-8.267	-5.706	34.690	1.00	0.00	LX0	N
ATOM	326	H	VAL	56	-8.446	-6.284	33.893	0.00	0.00	LX0	H
ATOM	327	CA	VAL	56	-7.177	-4.745	34.711	1.00	0.00	LX0	C
ATOM	328	CB	VAL	56	-5.835	-5.400	34.330	1.00	0.00	LX0	C
ATOM	329	CG1	VAL	56	-5.697	-5.789	32.861	1.00	0.00	LX0	C
ATOM	330	CG2	VAL	56	-4.675	-4.536	34.800	1.00	0.00	LX0	C
ATOM	331	C	VAL	56	-7.504	-3.459	33.963	1.00	0.00	LX0	C
ATOM	332	O	VAL	56	-7.633	-3.371	32.744	1.00	0.00	LX0	O
ATOM	333	N	GLN	57	-7.692	-2.446	34.809	1.00	0.00	LX0	N
ATOM	334	H	GLN	57	-7.530	-2.610	35.782	0.00	0.00	LX0	H
ATOM	335	CA	GLN	57	-8.078	-1.136	34.304	1.00	0.00	LX0	C
ATOM	336	CB	GLN	57	-8.906	-0.380	35.343	1.00	0.00	LX0	C
ATOM	337	CG	GLN	57	-10.050	-1.251	35.861	1.00	0.00	LX0	C
ATOM	338	CD	GLN	57	-11.170	-0.383	36.386	1.00	0.00	LX0	C
ATOM	339	OE1	GLN	57	-12.231	-0.269	35.788	1.00	0.00	LX0	O
ATOM	340	NE2	GLN	57	-10.900	0.224	37.545	1.00	0.00	LX0	N
ATOM	341	HE21	GLN	57	-10.022	0.097	38.002	0.00	0.00	LX0	H
ATOM	342	HE22	GLN	57	-11.607	0.808	37.937	0.00	0.00	LX0	H
ATOM	343	C	GLN	57	-6.903	-0.319	33.822	1.00	0.00	LX0	C
ATOM	344	O	GLN	57	-6.334	0.505	34.530	1.00	0.00	LX0	O
ATOM	345	N	GLY	58	-6.548	-0.629	32.577	1.00	0.00	LX0	N
ATOM	346	H	GLY	58	-7.023	-1.374	32.103	0.00	0.00	LX0	H
ATOM	347	CA	GLY	58	-5.407	0.011	31.938	1.00	0.00	LX0	C
ATOM	348	C	GLY	58	-4.877	-0.965	30.918	1.00	0.00	LX0	C
ATOM	349	O	GLY	58	-5.584	-1.895	30.537	1.00	0.00	LX0	O
ATOM	350	N	ASN	59	-3.631	-0.739	30.493	1.00	0.00	LX0	N
ATOM	351	H	ASN	59	-3.034	-0.036	30.888	0.00	0.00	LX0	H
ATOM	352	CA	ASN	59	-3.087	-1.745	29.584	1.00	0.00	LX0	C
ATOM	353	CB	ASN	59	-1.938	-1.216	28.730	1.00	0.00	LX0	C
ATOM	354	CG	ASN	59	-2.286	0.044	27.979	1.00	0.00	LX0	C
ATOM	355	OD1	ASN	59	-3.265	0.126	27.242	1.00	0.00	LX0	O
ATOM	356	ND2	ASN	59	-1.418	1.026	28.218	1.00	0.00	LX0	N
ATOM	357	HD21	ASN	59	-0.591	0.822	28.752	0.00	0.00	LX0	H
ATOM	358	HD22	ASN	59	-1.472	1.987	27.932	0.00	0.00	LX0	H
ATOM	359	C	ASN	59	-2.569	-2.931	30.364	1.00	0.00	LX0	C
ATOM	360	O	ASN	59	-2.456	-2.890	31.583	1.00	0.00	LX0	O
ATOM	361	N	LEU	60	-2.244	-3.983	29.608	1.00	0.00	LX0	N
ATOM	362	H	LEU	60	-2.409	-3.959	28.621	0.00	0.00	LX0	H
ATOM	363	CA	LEU	60	-1.500	-5.071	30.227	1.00	0.00	LX0	C
ATOM	364	CB	LEU	60	-2.227	-6.400	30.032	1.00	0.00	LX0	C
ATOM	365	CG	LEU	60	-1.711	-7.529	30.923	1.00	0.00	LX0	C
ATOM	366	CD1	LEU	60	-1.883	-7.206	32.405	1.00	0.00	LX0	C
ATOM	367	CD2	LEU	60	-2.357	-8.864	30.566	1.00	0.00	LX0	C
ATOM	368	C	LEU	60	-0.113	-5.117	29.631	1.00	0.00	LX0	C
ATOM	369	O	LEU	60	0.160	-5.827	28.667	1.00	0.00	LX0	O
ATOM	370	N	GLU	61	0.737	-4.271	30.210	1.00	0.00	LX0	N
ATOM	371	H	GLU	61	0.490	-3.798	31.059	0.00	0.00	LX0	H
ATOM	372	CA	GLU	61	2.055	-4.145	29.601	1.00	0.00	LX0	C
ATOM	373	CB	GLU	61	2.512	-2.693	29.647	1.00	0.00	LX0	C
ATOM	374	CG	GLU	61	1.512	-1.918	28.790	1.00	0.00	LX0	C
ATOM	375	CD	GLU	61	1.605	-0.420	28.956	1.00	0.00	LX0	C
ATOM	376	OE1	GLU	61	1.765	0.054	30.074	1.00	0.00	LX0	O
ATOM	377	OE2	GLU	61	1.462	0.284	27.964	1.00	0.00	LX0	O
ATOM	378	C	GLU	61	3.054	-5.124	30.159	1.00	0.00	LX0	C

ATOM	379	O	GLU	61	3.360	-5.168	31.342	1.00	0.00	LX0	O
ATOM	380	N	LEU	62	3.484	-5.960	29.215	1.00	0.00	LX0	N
ATOM	381	H	LEU	62	3.202	-5.796	28.271	0.00	0.00	LX0	H
ATOM	382	CA	LEU	62	4.346	-7.095	29.512	1.00	0.00	LX0	C
ATOM	383	CB	LEU	62	3.599	-8.402	29.254	1.00	0.00	LX0	C
ATOM	384	CG	LEU	62	2.296	-8.496	30.043	1.00	0.00	LX0	C
ATOM	385	CD1	LEU	62	1.458	-9.688	29.592	1.00	0.00	LX0	C
ATOM	386	CD2	LEU	62	2.540	-8.461	31.554	1.00	0.00	LX0	C
ATOM	387	C	LEU	62	5.574	-7.009	28.642	1.00	0.00	LX0	C
ATOM	388	O	LEU	62	5.712	-7.659	27.607	1.00	0.00	LX0	O
ATOM	389	N	THR	63	6.441	-6.112	29.097	1.00	0.00	LX0	N
ATOM	390	H	THR	63	6.311	-5.585	29.939	0.00	0.00	LX0	H
ATOM	391	CA	THR	63	7.494	-5.694	28.196	1.00	0.00	LX0	C
ATOM	392	CB	THR	63	7.210	-4.244	27.806	1.00	0.00	LX0	C
ATOM	393	OG1	THR	63	6.445	-3.587	28.829	1.00	0.00	LX0	O
ATOM	394	HG1	THR	63	6.355	-2.677	28.570	0.00	0.00	LX0	H
ATOM	395	CG2	THR	63	6.430	-4.203	26.499	1.00	0.00	LX0	C
ATOM	396	C	THR	63	8.874	-5.876	28.782	1.00	0.00	LX0	C
ATOM	397	O	THR	63	9.097	-5.716	29.974	1.00	0.00	LX0	O
ATOM	398	N	TYR	64	9.804	-6.234	27.878	1.00	0.00	LX0	N
ATOM	399	H	TYR	64	9.493	-6.454	26.952	0.00	0.00	LX0	H
ATOM	400	CA	TYR	64	11.221	-6.335	28.250	1.00	0.00	LX0	C
ATOM	401	CB	TYR	64	11.813	-4.968	28.624	1.00	0.00	LX0	C
ATOM	402	CG	TYR	64	11.606	-3.932	27.545	1.00	0.00	LX0	C
ATOM	403	CD1	TYR	64	12.490	-3.907	26.449	1.00	0.00	LX0	C
ATOM	404	CE1	TYR	64	12.322	-2.918	25.469	1.00	0.00	LX0	C
ATOM	405	CD2	TYR	64	10.549	-3.008	27.675	1.00	0.00	LX0	C
ATOM	406	CE2	TYR	64	10.383	-2.015	26.696	1.00	0.00	LX0	C
ATOM	407	CZ	TYR	64	11.281	-1.981	25.612	1.00	0.00	LX0	C
ATOM	408	OH	TYR	64	11.154	-1.007	24.652	1.00	0.00	LX0	O
ATOM	409	HH	TYR	64	10.472	-0.377	24.864	0.00	0.00	LX0	H
ATOM	410	C	TYR	64	11.545	-7.319	29.366	1.00	0.00	LX0	C
ATOM	411	O	TYR	64	12.585	-7.244	30.011	1.00	0.00	LX0	O
ATOM	412	N	LEU	65	10.597	-8.236	29.592	1.00	0.00	LX0	N
ATOM	413	H	LEU	65	9.808	-8.329	28.984	0.00	0.00	LX0	H
ATOM	414	CA	LEU	65	10.710	-9.034	30.807	1.00	0.00	LX0	C
ATOM	415	CB	LEU	65	9.369	-9.702	31.123	1.00	0.00	LX0	C
ATOM	416	CG	LEU	65	8.275	-8.660	31.372	1.00	0.00	LX0	C
ATOM	417	CD1	LEU	65	6.883	-9.278	31.455	1.00	0.00	LX0	C
ATOM	418	CD2	LEU	65	8.594	-7.790	32.587	1.00	0.00	LX0	C
ATOM	419	C	LEU	65	11.859	-10.020	30.768	1.00	0.00	LX0	C
ATOM	420	O	LEU	65	12.048	-10.746	29.796	1.00	0.00	LX0	O
ATOM	421	N	PRO	66	12.649	-9.980	31.871	1.00	0.00	LX0	N
ATOM	422	CD	PRO	66	12.489	-9.066	33.000	1.00	0.00	LX0	C
ATOM	423	CA	PRO	66	13.821	-10.850	32.020	1.00	0.00	LX0	C
ATOM	424	CB	PRO	66	14.232	-10.587	33.472	1.00	0.00	LX0	C
ATOM	425	CG	PRO	66	13.815	-9.146	33.740	1.00	0.00	LX0	C
ATOM	426	C	PRO	66	13.596	-12.323	31.712	1.00	0.00	LX0	C
ATOM	427	O	PRO	66	12.548	-12.897	31.975	1.00	0.00	LX0	O
ATOM	428	N	THR	67	14.659	-12.908	31.142	1.00	0.00	LX0	N
ATOM	429	H	THR	67	15.499	-12.387	30.989	0.00	0.00	LX0	H
ATOM	430	CA	THR	67	14.615	-14.249	30.556	1.00	0.00	LX0	C
ATOM	431	CB	THR	67	16.057	-14.676	30.290	1.00	0.00	LX0	C
ATOM	432	OG1	THR	67	16.855	-13.526	29.977	1.00	0.00	LX0	O
ATOM	433	HG1	THR	67	16.858	-13.432	29.022	0.00	0.00	LX0	H
ATOM	434	CG2	THR	67	16.172	-15.742	29.207	1.00	0.00	LX0	C
ATOM	435	C	THR	67	13.851	-15.325	31.317	1.00	0.00	LX0	C
ATOM	436	O	THR	67	13.001	-16.040	30.798	1.00	0.00	LX0	O
ATOM	437	N	ASN	68	14.213	-15.399	32.604	1.00	0.00	LX0	N
ATOM	438	H	ASN	68	14.828	-14.713	32.989	0.00	0.00	LX0	H
ATOM	439	CA	ASN	68	13.602	-16.458	33.406	1.00	0.00	LX0	C

ATOM	440	CB	ASN	68	14.661	-17.391	34.004	1.00	0.00	LX0	C
ATOM	441	CG	ASN	68	15.552	-17.984	32.932	1.00	0.00	LX0	C
ATOM	442	OD1	ASN	68	15.125	-18.614	31.974	1.00	0.00	LX0	O
ATOM	443	ND2	ASN	68	16.849	-17.753	33.142	1.00	0.00	LX0	N
ATOM	444	HD21	ASN	68	17.184	-17.232	33.924	0.00	0.00	LX0	H
ATOM	445	HD22	ASN	68	17.492	-18.146	32.481	0.00	0.00	LX0	H
ATOM	446	C	ASN	68	12.759	-15.878	34.519	1.00	0.00	LX0	C
ATOM	447	O	ASN	68	12.752	-16.346	35.651	1.00	0.00	LX0	O
ATOM	448	N	ALA	69	12.090	-14.774	34.170	1.00	0.00	LX0	N
ATOM	449	H	ALA	69	12.037	-14.468	33.216	0.00	0.00	LX0	H
ATOM	450	CA	ALA	69	11.389	-14.070	35.235	1.00	0.00	LX0	C
ATOM	451	CB	ALA	69	11.048	-12.654	34.791	1.00	0.00	LX0	C
ATOM	452	C	ALA	69	10.116	-14.757	35.681	1.00	0.00	LX0	C
ATOM	453	O	ALA	69	9.397	-15.376	34.907	1.00	0.00	LX0	O
ATOM	454	N	SER	70	9.840	-14.594	36.984	1.00	0.00	LX0	N
ATOM	455	H	SER	70	10.469	-14.092	37.573	0.00	0.00	LX0	H
ATOM	456	CA	SER	70	8.545	-15.101	37.424	1.00	0.00	LX0	C
ATOM	457	CB	SER	70	8.504	-15.316	38.941	1.00	0.00	LX0	C
ATOM	458	OG	SER	70	7.483	-16.269	39.276	1.00	0.00	LX0	O
ATOM	459	HG	SER	70	7.943	-17.083	39.462	0.00	0.00	LX0	H
ATOM	460	C	SER	70	7.399	-14.225	36.949	1.00	0.00	LX0	C
ATOM	461	O	SER	70	7.089	-13.178	37.515	1.00	0.00	LX0	O
ATOM	462	N	LEU	71	6.799	-14.735	35.865	1.00	0.00	LX0	N
ATOM	463	H	LEU	71	7.232	-15.521	35.418	0.00	0.00	LX0	H
ATOM	464	CA	LEU	71	5.601	-14.116	35.301	1.00	0.00	LX0	C
ATOM	465	CB	LEU	71	5.639	-14.200	33.778	1.00	0.00	LX0	C
ATOM	466	CG	LEU	71	6.070	-12.901	33.100	1.00	0.00	LX0	C
ATOM	467	CD1	LEU	71	7.464	-12.432	33.517	1.00	0.00	LX0	C
ATOM	468	CD2	LEU	71	5.937	-13.023	31.585	1.00	0.00	LX0	C
ATOM	469	C	LEU	71	4.293	-14.687	35.820	1.00	0.00	LX0	C
ATOM	470	O	LEU	71	3.218	-14.421	35.303	1.00	0.00	LX0	O
ATOM	471	N	SER	72	4.443	-15.484	36.888	1.00	0.00	LX0	N
ATOM	472	H	SER	72	5.343	-15.581	37.305	0.00	0.00	LX0	H
ATOM	473	CA	SER	72	3.346	-16.164	37.582	1.00	0.00	LX0	C
ATOM	474	CB	SER	72	3.873	-16.485	38.983	1.00	0.00	LX0	C
ATOM	475	OG	SER	72	4.832	-15.473	39.348	1.00	0.00	LX0	O
ATOM	476	HG	SER	72	5.627	-15.926	39.628	0.00	0.00	LX0	H
ATOM	477	C	SER	72	2.016	-15.418	37.628	1.00	0.00	LX0	C
ATOM	478	O	SER	72	0.966	-15.909	37.239	1.00	0.00	LX0	O
ATOM	479	N	PHE	73	2.139	-14.162	38.085	1.00	0.00	LX0	N
ATOM	480	H	PHE	73	3.041	-13.907	38.431	0.00	0.00	LX0	H
ATOM	481	CA	PHE	73	0.998	-13.247	38.198	1.00	0.00	LX0	C
ATOM	482	CB	PHE	73	1.499	-11.837	38.543	1.00	0.00	LX0	C
ATOM	483	CG	PHE	73	2.364	-11.214	37.463	1.00	0.00	LX0	C
ATOM	484	CD1	PHE	73	1.804	-10.226	36.626	1.00	0.00	LX0	C
ATOM	485	CD2	PHE	73	3.717	-11.596	37.324	1.00	0.00	LX0	C
ATOM	486	CE1	PHE	73	2.608	-9.597	35.656	1.00	0.00	LX0	C
ATOM	487	CE2	PHE	73	4.524	-10.968	36.356	1.00	0.00	LX0	C
ATOM	488	CZ	PHE	73	3.962	-9.970	35.534	1.00	0.00	LX0	C
ATOM	489	C	PHE	73	-0.025	-13.190	37.065	1.00	0.00	LX0	C
ATOM	490	O	PHE	73	-1.191	-12.871	37.277	1.00	0.00	LX0	O
ATOM	491	N	LEU	74	0.471	-13.493	35.858	1.00	0.00	LX0	N
ATOM	492	H	LEU	74	1.407	-13.836	35.771	0.00	0.00	LX0	H
ATOM	493	CA	LEU	74	-0.376	-13.411	34.672	1.00	0.00	LX0	C
ATOM	494	CB	LEU	74	0.488	-13.475	33.421	1.00	0.00	LX0	C
ATOM	495	CG	LEU	74	1.333	-12.224	33.246	1.00	0.00	LX0	C
ATOM	496	CD1	LEU	74	2.353	-12.396	32.130	1.00	0.00	LX0	C
ATOM	497	CD2	LEU	74	0.453	-10.998	33.029	1.00	0.00	LX0	C
ATOM	498	C	LEU	74	-1.500	-14.421	34.571	1.00	0.00	LX0	C
ATOM	499	O	LEU	74	-2.499	-14.175	33.905	1.00	0.00	LX0	O
ATOM	500	N	GLN	75	-1.310	-15.553	35.274	1.00	0.00	LX0	N

```
ATOM    501  H   GLN    75      -0.449 -15.668  35.771  0.00  0.00      LX0  H
ATOM    502  CA  GLN    75      -2.229 -16.691  35.146  1.00  0.00      LX0  C
ATOM    503  CB  GLN    75      -2.012 -17.679  36.300  1.00  0.00      LX0  C
ATOM    504  CG  GLN    75      -2.113 -17.045  37.694  1.00  0.00      LX0  C
ATOM    505  CD  GLN    75      -2.151 -18.123  38.754  1.00  0.00      LX0  C
ATOM    506  OE1 GLN    75      -1.143 -18.674  39.175  1.00  0.00      LX0  O
ATOM    507  NE2 GLN    75      -3.381 -18.389  39.197  1.00  0.00      LX0  N
ATOM    508 HE21 GLN    75      -4.186 -17.922  38.811  0.00  0.00      LX0  H
ATOM    509 HE22 GLN    75      -3.508 -19.054  39.925  0.00  0.00      LX0  H
ATOM    510  C   GLN    75      -3.717 -16.390  34.974  1.00  0.00      LX0  C
ATOM    511  O   GLN    75      -4.410 -16.992  34.161  1.00  0.00      LX0  O
ATOM    512  N   ASP    76      -4.172 -15.417  35.774  1.00  0.00      LX0  N
ATOM    513  H   ASP    76      -3.534 -14.896  36.335  0.00  0.00      LX0  H
ATOM    514  CA  ASP    76      -5.616 -15.242  35.865  1.00  0.00      LX0  C
ATOM    515  CB  ASP    76      -6.072 -15.319  37.325  1.00  0.00      LX0  C
ATOM    516  CG  ASP    76      -5.889 -16.704  37.921  1.00  0.00      LX0  C
ATOM    517  OD1 ASP    76      -5.655 -17.672  37.206  1.00  0.00      LX0  O
ATOM    518  OD2 ASP    76      -6.052 -16.863  39.123  1.00  0.00      LX0  O
ATOM    519  C   ASP    76      -6.156 -13.973  35.251  1.00  0.00      LX0  C
ATOM    520  O   ASP    76      -7.290 -13.588  35.518  1.00  0.00      LX0  O
ATOM    521  N   ILE    77      -5.310 -13.305  34.453  1.00  0.00      LX0  N
ATOM    522  H   ILE    77      -4.441 -13.701  34.146  0.00  0.00      LX0  H
ATOM    523  CA  ILE    77      -5.753 -11.970  34.056  1.00  0.00      LX0  C
ATOM    524  CB  ILE    77      -4.570 -11.044  33.769  1.00  0.00      LX0  C
ATOM    525  CG2 ILE    77      -5.016  -9.621  33.404  1.00  0.00      LX0  C
ATOM    526  CG1 ILE    77      -3.669 -11.047  35.006  1.00  0.00      LX0  C
ATOM    527  CD1 ILE    77      -2.484 -10.100  34.892  1.00  0.00      LX0  C
ATOM    528  C   ILE    77      -6.824 -11.931  32.983  1.00  0.00      LX0  C
ATOM    529  O   ILE    77      -6.597 -12.117  31.796  1.00  0.00      LX0  O
ATOM    530  N   GLN    78      -8.034 -11.699  33.497  1.00  0.00      LX0  N
ATOM    531  H   GLN    78      -8.066 -11.462  34.470  0.00  0.00      LX0  H
ATOM    532  CA  GLN    78      -9.208 -11.953  32.671  1.00  0.00      LX0  C
ATOM    533  CB  GLN    78     -10.391 -12.290  33.571  1.00  0.00      LX0  C
ATOM    534  CG  GLN    78     -10.231 -13.689  34.158  1.00  0.00      LX0  C
ATOM    535  CD  GLN    78     -10.808 -13.734  35.552  1.00  0.00      LX0  C
ATOM    536  OE1 GLN    78     -11.969 -13.428  35.801  1.00  0.00      LX0  O
ATOM    537  NE2 GLN    78      -9.928 -14.138  36.468  1.00  0.00      LX0  N
ATOM    538 HE21 GLN    78      -8.970 -14.258  36.188  0.00  0.00      LX0  H
ATOM    539 HE22 GLN    78     -10.165 -14.291  37.423  0.00  0.00      LX0  H
ATOM    540  C   GLN    78      -9.556 -10.909  31.631  1.00  0.00      LX0  C
ATOM    541  O   GLN    78      -9.885 -11.237  30.497  1.00  0.00      LX0  O
ATOM    542  N   GLU    79      -9.494  -9.640  32.034  1.00  0.00      LX0  N
ATOM    543  H   GLU    79      -9.293  -9.372  32.978  0.00  0.00      LX0  H
ATOM    544  CA  GLU    79      -9.784  -8.656  30.997  1.00  0.00      LX0  C
ATOM    545  CB  GLU    79     -11.260  -8.265  30.958  1.00  0.00      LX0  C
ATOM    546  CG  GLU    79     -11.868  -7.745  32.256  1.00  0.00      LX0  C
ATOM    547  CD  GLU    79     -13.315  -7.380  32.002  1.00  0.00      LX0  C
ATOM    548  OE1 GLU    79     -13.655  -6.219  32.166  1.00  0.00      LX0  O
ATOM    549  OE2 GLU    79     -14.111  -8.233  31.607  1.00  0.00      LX0  O
ATOM    550  C   GLU    79      -8.918  -7.434  31.060  1.00  0.00      LX0  C
ATOM    551  O   GLU    79      -8.597  -6.921  32.123  1.00  0.00      LX0  O
ATOM    552  N   VAL    80      -8.544  -7.008  29.853  1.00  0.00      LX0  N
ATOM    553  H   VAL    80      -8.905  -7.462  29.035  0.00  0.00      LX0  H
ATOM    554  CA  VAL    80      -7.676  -5.842  29.758  1.00  0.00      LX0  C
ATOM    555  CB  VAL    80      -6.459  -6.183  28.894  1.00  0.00      LX0  C
ATOM    556  CG1 VAL    80      -5.495  -5.001  28.769  1.00  0.00      LX0  C
ATOM    557  CG2 VAL    80      -5.773  -7.447  29.412  1.00  0.00      LX0  C
ATOM    558  C   VAL    80      -8.443  -4.699  29.146  1.00  0.00      LX0  C
ATOM    559  O   VAL    80      -8.992  -4.845  28.064  1.00  0.00      LX0  O
ATOM    560  N   GLN    81      -8.479  -3.565  29.855  1.00  0.00      LX0  N
ATOM    561  H   GLN    81      -7.991  -3.486  30.729  0.00  0.00      LX0  H
```

ATOM	562	CA	GLN	81	-9.224	-2.469	29.234	1.00	0.00	LX0	C
ATOM	563	CB	GLN	81	-9.654	-1.449	30.283	1.00	0.00	LX0	C
ATOM	564	CG	GLN	81	-10.638	-2.057	31.287	1.00	0.00	LX0	C
ATOM	565	CD	GLN	81	-11.039	-1.011	32.303	1.00	0.00	LX0	C
ATOM	566	OE1	GLN	81	-10.455	0.058	32.399	1.00	0.00	LX0	O
ATOM	567	NE2	GLN	81	-12.055	-1.379	33.088	1.00	0.00	LX0	N
ATOM	568	HE21	GLN	81	-12.534	-2.248	32.983	0.00	0.00	LX0	H
ATOM	569	HE22	GLN	81	-12.326	-0.762	33.833	0.00	0.00	LX0	H
ATOM	570	C	GLN	81	-8.524	-1.810	28.054	1.00	0.00	LX0	C
ATOM	571	O	GLN	81	-9.115	-1.514	27.018	1.00	0.00	LX0	O
ATOM	572	N	GLY	82	-7.221	-1.599	28.256	1.00	0.00	LX0	N
ATOM	573	H	GLY	82	-6.787	-1.938	29.090	0.00	0.00	LX0	H
ATOM	574	CA	GLY	82	-6.430	-1.050	27.161	1.00	0.00	LX0	C
ATOM	575	C	GLY	82	-5.999	-2.132	26.196	1.00	0.00	LX0	C
ATOM	576	O	GLY	82	-6.748	-3.054	25.884	1.00	0.00	LX0	O
ATOM	577	N	TYR	83	-4.754	-1.975	25.743	1.00	0.00	LX0	N
ATOM	578	H	TYR	83	-4.176	-1.240	26.108	0.00	0.00	LX0	H
ATOM	579	CA	TYR	83	-4.212	-3.022	24.884	1.00	0.00	LX0	C
ATOM	580	CB	TYR	83	-3.412	-2.407	23.719	1.00	0.00	LX0	C
ATOM	581	CG	TYR	83	-2.366	-1.416	24.181	1.00	0.00	LX0	C
ATOM	582	CD1	TYR	83	-1.217	-1.877	24.853	1.00	0.00	LX0	C
ATOM	583	CE1	TYR	83	-0.272	-0.944	25.303	1.00	0.00	LX0	C
ATOM	584	CD2	TYR	83	-2.574	-0.047	23.918	1.00	0.00	LX0	C
ATOM	585	CE2	TYR	83	-1.624	0.887	24.357	1.00	0.00	LX0	C
ATOM	586	CZ	TYR	83	-0.492	0.424	25.055	1.00	0.00	LX0	C
ATOM	587	OH	TYR	83	0.429	1.335	25.516	1.00	0.00	LX0	O
ATOM	588	HH	TYR	83	0.728	1.039	26.375	0.00	0.00	LX0	H
ATOM	589	C	TYR	83	-3.401	-4.034	25.675	1.00	0.00	LX0	C
ATOM	590	O	TYR	83	-2.990	-3.779	26.803	1.00	0.00	LX0	O
ATOM	591	N	VAL	84	-3.183	-5.195	25.048	1.00	0.00	LX0	N
ATOM	592	H	VAL	84	-3.478	-5.316	24.098	0.00	0.00	LX0	H
ATOM	593	CA	VAL	84	-2.225	-6.111	25.662	1.00	0.00	LX0	C
ATOM	594	CB	VAL	84	-2.706	-7.566	25.584	1.00	0.00	LX0	C
ATOM	595	CG1	VAL	84	-1.787	-8.519	26.352	1.00	0.00	LX0	C
ATOM	596	CG2	VAL	84	-4.136	-7.694	26.094	1.00	0.00	LX0	C
ATOM	597	C	VAL	84	-0.901	-5.950	24.952	1.00	0.00	LX0	C
ATOM	598	O	VAL	84	-0.842	-5.949	23.729	1.00	0.00	LX0	O
ATOM	599	N	LEU	85	0.141	-5.777	25.765	1.00	0.00	LX0	N
ATOM	600	H	LEU	85	0.045	-5.846	26.760	0.00	0.00	LX0	H
ATOM	601	CA	LEU	85	1.411	-5.400	25.166	1.00	0.00	LX0	C
ATOM	602	CB	LEU	85	1.733	-3.968	25.578	1.00	0.00	LX0	C
ATOM	603	CG	LEU	85	2.981	-3.386	24.925	1.00	0.00	LX0	C
ATOM	604	CD1	LEU	85	2.923	-3.461	23.402	1.00	0.00	LX0	C
ATOM	605	CD2	LEU	85	3.257	-1.982	25.447	1.00	0.00	LX0	C
ATOM	606	C	LEU	85	2.528	-6.353	25.527	1.00	0.00	LX0	C
ATOM	607	O	LEU	85	3.317	-6.120	26.432	1.00	0.00	LX0	O
ATOM	608	N	ILE	86	2.557	-7.460	24.790	1.00	0.00	LX0	N
ATOM	609	H	ILE	86	2.000	-7.514	23.958	0.00	0.00	LX0	H
ATOM	610	CA	ILE	86	3.612	-8.411	25.113	1.00	0.00	LX0	C
ATOM	611	CB	ILE	86	3.073	-9.846	25.102	1.00	0.00	LX0	C
ATOM	612	CG2	ILE	86	4.057	-10.781	25.801	1.00	0.00	LX0	C
ATOM	613	CG1	ILE	86	1.683	-9.938	25.739	1.00	0.00	LX0	C
ATOM	614	CD1	ILE	86	1.066	-11.336	25.670	1.00	0.00	LX0	C
ATOM	615	C	ILE	86	4.791	-8.250	24.171	1.00	0.00	LX0	C
ATOM	616	O	ILE	86	4.795	-8.762	23.057	1.00	0.00	LX0	O
ATOM	617	N	ALA	87	5.786	-7.489	24.638	1.00	0.00	LX0	N
ATOM	618	H	ALA	87	5.782	-7.130	25.575	0.00	0.00	LX0	H
ATOM	619	CA	ALA	87	6.825	-7.168	23.665	1.00	0.00	LX0	C
ATOM	620	CB	ALA	87	6.516	-5.834	22.992	1.00	0.00	LX0	C
ATOM	621	C	ALA	87	8.239	-7.145	24.208	1.00	0.00	LX0	C
ATOM	622	O	ALA	87	8.474	-6.934	25.392	1.00	0.00	LX0	O

ATOM	623	N	HIS	88	9.177	-7.374	23.269	1.00	0.00	LX0	N
ATOM	624	H	HIS	88	8.853	-7.518	22.331	0.00	0.00	LX0	H
ATOM	625	CA	HIS	88	10.621	-7.379	23.552	1.00	0.00	LX0	C
ATOM	626	CB	HIS	88	11.195	-5.991	23.847	1.00	0.00	LX0	C
ATOM	627	CG	HIS	88	10.613	-4.920	22.965	1.00	0.00	LX0	C
ATOM	628	ND1	HIS	88	10.950	-4.709	21.682	1.00	0.00	LX0	N
ATOM	629	HD1	HIS	88	11.591	-5.209	21.137	0.00	0.00	LX0	H
ATOM	630	CD2	HIS	88	9.653	-3.983	23.342	1.00	0.00	LX0	C
ATOM	631	NE2	HIS	88	9.417	-3.204	22.268	1.00	0.00	LX0	N
ATOM	632	CE1	HIS	88	10.212	-3.640	21.243	1.00	0.00	LX0	C
ATOM	633	C	HIS	88	11.055	-8.304	24.671	1.00	0.00	LX0	C
ATOM	634	O	HIS	88	11.956	-8.019	25.453	1.00	0.00	LX0	O
ATOM	635	N	ASN	89	10.334	-9.421	24.745	1.00	0.00	LX0	N
ATOM	636	H	ASN	89	9.674	-9.669	24.037	0.00	0.00	LX0	H
ATOM	637	CA	ASN	89	10.531	-10.233	25.935	1.00	0.00	LX0	C
ATOM	638	CB	ASN	89	9.253	-10.958	26.325	1.00	0.00	LX0	C
ATOM	639	CG	ASN	89	8.310	-9.947	26.923	1.00	0.00	LX0	C
ATOM	640	OD1	ASN	89	8.538	-9.396	27.990	1.00	0.00	LX0	O
ATOM	641	ND2	ASN	89	7.237	-9.712	26.177	1.00	0.00	LX0	N
ATOM	642	HD21	ASN	89	7.131	-10.142	25.278	0.00	0.00	LX0	H
ATOM	643	HD22	ASN	89	6.571	-9.063	26.550	0.00	0.00	LX0	H
ATOM	644	C	ASN	89	11.661	-11.210	25.827	1.00	0.00	LX0	C
ATOM	645	O	ASN	89	11.777	-11.974	24.879	1.00	0.00	LX0	O
ATOM	646	N	GLN	90	12.475	-11.147	26.884	1.00	0.00	LX0	N
ATOM	647	H	GLN	90	12.297	-10.480	27.610	0.00	0.00	LX0	H
ATOM	648	CA	GLN	90	13.459	-12.201	27.090	1.00	0.00	LX0	C
ATOM	649	CB	GLN	90	14.575	-11.659	27.977	1.00	0.00	LX0	C
ATOM	650	CG	GLN	90	15.638	-10.845	27.249	1.00	0.00	LX0	C
ATOM	651	CD	GLN	90	16.676	-11.785	26.677	1.00	0.00	LX0	C
ATOM	652	OE1	GLN	90	17.134	-12.714	27.335	1.00	0.00	LX0	O
ATOM	653	NE2	GLN	90	17.027	-11.498	25.424	1.00	0.00	LX0	N
ATOM	654	HE21	GLN	90	16.652	-10.715	24.937	0.00	0.00	LX0	H
ATOM	655	HE22	GLN	90	17.649	-12.098	24.914	0.00	0.00	LX0	H
ATOM	656	C	GLN	90	12.835	-13.439	27.714	1.00	0.00	LX0	C
ATOM	657	O	GLN	90	13.316	-14.558	27.570	1.00	0.00	LX0	O
ATOM	658	N	VAL	91	11.736	-13.175	28.442	1.00	0.00	LX0	N
ATOM	659	H	VAL	91	11.457	-12.228	28.592	0.00	0.00	LX0	H
ATOM	660	CA	VAL	91	11.042	-14.268	29.111	1.00	0.00	LX0	C
ATOM	661	CB	VAL	91	9.981	-13.725	30.084	1.00	0.00	LX0	C
ATOM	662	CG1	VAL	91	8.824	-13.035	29.361	1.00	0.00	LX0	C
ATOM	663	CG2	VAL	91	9.516	-14.797	31.075	1.00	0.00	LX0	C
ATOM	664	C	VAL	91	10.488	-15.319	28.165	1.00	0.00	LX0	C
ATOM	665	O	VAL	91	9.826	-15.044	27.171	1.00	0.00	LX0	O
ATOM	666	N	ARG	92	10.823	-16.555	28.541	1.00	0.00	LX0	N
ATOM	667	H	ARG	92	11.383	-16.637	29.368	0.00	0.00	LX0	H
ATOM	668	CA	ARG	92	10.551	-17.663	27.630	1.00	0.00	LX0	C
ATOM	669	CB	ARG	92	11.312	-18.905	28.105	1.00	0.00	LX0	C
ATOM	670	CG	ARG	92	12.772	-18.583	28.452	1.00	0.00	LX0	C
ATOM	671	CD	ARG	92	13.601	-19.807	28.850	1.00	0.00	LX0	C
ATOM	672	NE	ARG	92	14.971	-19.466	29.248	1.00	0.00	LX0	N
ATOM	673	HE	ARG	92	15.159	-19.445	30.236	0.00	0.00	LX0	H
ATOM	674	CZ	ARG	92	15.945	-19.158	28.362	1.00	0.00	LX0	C
ATOM	675	NH1	ARG	92	15.669	-19.014	27.068	1.00	0.00	LX0	N
ATOM	676	HH11	ARG	92	16.368	-18.734	26.399	0.00	0.00	LX0	H
ATOM	677	HH12	ARG	92	14.741	-19.152	26.728	0.00	0.00	LX0	H
ATOM	678	NH2	ARG	92	17.190	-18.982	28.788	1.00	0.00	LX0	N
ATOM	679	HH21	ARG	92	17.925	-18.735	28.148	0.00	0.00	LX0	H
ATOM	680	HH22	ARG	92	17.434	-19.111	29.754	0.00	0.00	LX0	H
ATOM	681	C	ARG	92	9.076	-17.938	27.343	1.00	0.00	LX0	C
ATOM	682	O	ARG	92	8.675	-18.196	26.213	1.00	0.00	LX0	O
ATOM	683	N	GLN	93	8.266	-17.850	28.409	1.00	0.00	LX0	N

ATOM	684	H	GLN	93	8.620	-17.613	29.311	0.00	0.00	LX0	H
ATOM	685	CA	GLN	93	6.834	-18.067	28.204	1.00	0.00	LX0	C
ATOM	686	CB	GLN	93	6.381	-19.427	28.749	1.00	0.00	LX0	C
ATOM	687	CG	GLN	93	6.336	-20.582	27.742	1.00	0.00	LX0	C
ATOM	688	CD	GLN	93	7.711	-21.138	27.424	1.00	0.00	LX0	C
ATOM	689	OE1	GLN	93	8.708	-20.869	28.081	1.00	0.00	LX0	O
ATOM	690	NE2	GLN	93	7.717	-21.981	26.391	1.00	0.00	LX0	N
ATOM	691	HE21	GLN	93	6.897	-22.167	25.835	0.00	0.00	LX0	H
ATOM	692	HE22	GLN	93	8.556	-22.454	26.147	0.00	0.00	LX0	H
ATOM	693	C	GLN	93	6.006	-16.989	28.871	1.00	0.00	LX0	C
ATOM	694	O	GLN	93	6.401	-16.421	29.882	1.00	0.00	LX0	O
ATOM	695	N	VAL	94	4.823	-16.757	28.278	1.00	0.00	LX0	N
ATOM	696	H	VAL	94	4.593	-17.200	27.412	0.00	0.00	LX0	H
ATOM	697	CA	VAL	94	3.831	-15.939	28.981	1.00	0.00	LX0	C
ATOM	698	CB	VAL	94	3.453	-14.700	28.145	1.00	0.00	LX0	C
ATOM	699	CG1	VAL	94	2.259	-13.917	28.702	1.00	0.00	LX0	C
ATOM	700	CG2	VAL	94	4.658	-13.771	28.020	1.00	0.00	LX0	C
ATOM	701	C	VAL	94	2.607	-16.766	29.354	1.00	0.00	LX0	C
ATOM	702	O	VAL	94	1.767	-17.080	28.516	1.00	0.00	LX0	O
ATOM	703	N	PRO	95	2.550	-17.117	30.660	1.00	0.00	LX0	N
ATOM	704	CD	PRO	95	3.563	-16.848	31.679	1.00	0.00	LX0	C
ATOM	705	CA	PRO	95	1.408	-17.876	31.179	1.00	0.00	LX0	C
ATOM	706	CB	PRO	95	2.020	-18.510	32.433	1.00	0.00	LX0	C
ATOM	707	CG	PRO	95	3.015	-17.472	32.957	1.00	0.00	LX0	C
ATOM	708	C	PRO	95	0.212	-16.976	31.471	1.00	0.00	LX0	C
ATOM	709	O	PRO	95	0.010	-16.485	32.576	1.00	0.00	LX0	O
ATOM	710	N	LEU	96	-0.571	-16.749	30.418	1.00	0.00	LX0	N
ATOM	711	H	LEU	96	-0.422	-17.233	29.552	0.00	0.00	LX0	H
ATOM	712	CA	LEU	96	-1.721	-15.868	30.598	1.00	0.00	LX0	C
ATOM	713	CB	LEU	96	-1.600	-14.684	29.644	1.00	0.00	LX0	C
ATOM	714	CG	LEU	96	-1.724	-13.292	30.262	1.00	0.00	LX0	C
ATOM	715	CD1	LEU	96	-1.444	-12.222	29.209	1.00	0.00	LX0	C
ATOM	716	CD2	LEU	96	-3.064	-13.053	30.955	1.00	0.00	LX0	C
ATOM	717	C	LEU	96	-3.024	-16.612	30.377	1.00	0.00	LX0	C
ATOM	718	O	LEU	96	-3.922	-16.184	29.660	1.00	0.00	LX0	O
ATOM	719	N	GLN	97	-3.073	-17.787	31.017	1.00	0.00	LX0	N
ATOM	720	H	GLN	97	-2.302	-18.036	31.607	0.00	0.00	LX0	H
ATOM	721	CA	GLN	97	-4.119	-18.759	30.697	1.00	0.00	LX0	C
ATOM	722	CB	GLN	97	-4.011	-19.972	31.627	1.00	0.00	LX0	C
ATOM	723	CG	GLN	97	-2.965	-21.022	31.224	1.00	0.00	LX0	C
ATOM	724	CD	GLN	97	-1.548	-20.482	31.254	1.00	0.00	LX0	C
ATOM	725	OE1	GLN	97	-1.190	-19.624	32.051	1.00	0.00	LX0	O
ATOM	726	NE2	GLN	97	-0.745	-21.048	30.355	1.00	0.00	LX0	N
ATOM	727	HE21	GLN	97	-1.121	-21.673	29.661	0.00	0.00	LX0	H
ATOM	728	HE22	GLN	97	0.239	-20.872	30.302	0.00	0.00	LX0	H
ATOM	729	C	GLN	97	-5.540	-18.217	30.692	1.00	0.00	LX0	C
ATOM	730	O	GLN	97	-6.321	-18.422	29.766	1.00	0.00	LX0	O
ATOM	731	N	ARG	98	-5.852	-17.499	31.773	1.00	0.00	LX0	N
ATOM	732	H	ARG	98	-5.160	-17.301	32.470	0.00	0.00	LX0	H
ATOM	733	CA	ARG	98	-7.214	-16.991	31.855	1.00	0.00	LX0	C
ATOM	734	CB	ARG	98	-7.780	-17.147	33.270	1.00	0.00	LX0	C
ATOM	735	CG	ARG	98	-7.982	-18.627	33.604	1.00	0.00	LX0	C
ATOM	736	CD	ARG	98	-8.826	-18.890	34.853	1.00	0.00	LX0	C
ATOM	737	NE	ARG	98	-8.115	-18.606	36.099	1.00	0.00	LX0	N
ATOM	738	HE	ARG	98	-7.203	-18.173	36.078	0.00	0.00	LX0	H
ATOM	739	CZ	ARG	98	-8.645	-18.950	37.293	1.00	0.00	LX0	C
ATOM	740	NH1	ARG	98	-9.849	-19.520	37.356	1.00	0.00	LX0	N
ATOM	741	HH11	ARG	98	-10.257	-19.778	38.231	0.00	0.00	LX0	H
ATOM	742	HH12	ARG	98	-10.360	-19.695	36.516	0.00	0.00	LX0	H
ATOM	743	NH2	ARG	98	-7.967	-18.720	38.410	1.00	0.00	LX0	N
ATOM	744	HH21	ARG	98	-8.278	-18.945	39.328	0.00	0.00	LX0	H

ATOM	745	HH22	ARG	98	-7.062	-18.263	38.344	0.00	0.00	LX0	H
ATOM	746	C	ARG	98	-7.455	-15.603	31.293	1.00	0.00	LX0	C
ATOM	747	O	ARG	98	-8.392	-14.918	31.687	1.00	0.00	LX0	O
ATOM	748	N	LEU	99	-6.611	-15.230	30.314	1.00	0.00	LX0	N
ATOM	749	H	LEU	99	-5.830	-15.795	30.044	0.00	0.00	LX0	H
ATOM	750	CA	LEU	99	-6.992	-14.068	29.513	1.00	0.00	LX0	C
ATOM	751	CB	LEU	99	-5.864	-13.655	28.560	1.00	0.00	LX0	C
ATOM	752	CG	LEU	99	-6.135	-12.384	27.739	1.00	0.00	LX0	C
ATOM	753	CD1	LEU	99	-6.515	-11.180	28.605	1.00	0.00	LX0	C
ATOM	754	CD2	LEU	99	-4.966	-12.058	26.810	1.00	0.00	LX0	C
ATOM	755	C	LEU	99	-8.267	-14.348	28.751	1.00	0.00	LX0	C
ATOM	756	O	LEU	99	-8.335	-15.247	27.930	1.00	0.00	LX0	O
ATOM	757	N	ARG	100	-9.284	-13.569	29.103	1.00	0.00	LX0	N
ATOM	758	H	ARG	100	-9.111	-12.844	29.769	0.00	0.00	LX0	H
ATOM	759	CA	ARG	100	-10.598	-13.797	28.517	1.00	0.00	LX0	C
ATOM	760	CB	ARG	100	-11.625	-13.780	29.662	1.00	0.00	LX0	C
ATOM	761	CG	ARG	100	-13.100	-13.571	29.307	1.00	0.00	LX0	C
ATOM	762	CD	ARG	100	-14.021	-13.598	30.537	1.00	0.00	LX0	C
ATOM	763	NE	ARG	100	-13.540	-12.748	31.634	1.00	0.00	LX0	N
ATOM	764	HE	ARG	100	-12.950	-13.187	32.314	0.00	0.00	LX0	H
ATOM	765	CZ	ARG	100	-13.826	-11.428	31.717	1.00	0.00	LX0	C
ATOM	766	NH1	ARG	100	-14.579	-10.830	30.802	1.00	0.00	LX0	N
ATOM	767	HH11	ARG	100	-14.718	-9.833	30.874	0.00	0.00	LX0	H
ATOM	768	HH12	ARG	100	-14.991	-11.337	30.046	0.00	0.00	LX0	H
ATOM	769	NH2	ARG	100	-13.354	-10.697	32.720	1.00	0.00	LX0	N
ATOM	770	HH21	ARG	100	-13.531	-9.701	32.718	0.00	0.00	LX0	H
ATOM	771	HH22	ARG	100	-12.822	-11.084	33.472	0.00	0.00	LX0	H
ATOM	772	C	ARG	100	-10.893	-12.820	27.392	1.00	0.00	LX0	C
ATOM	773	O	ARG	100	-11.257	-13.198	26.286	1.00	0.00	LX0	O
ATOM	774	N	ILE	101	-10.718	-11.531	27.713	1.00	0.00	LX0	N
ATOM	775	H	ILE	101	-10.380	-11.249	28.613	0.00	0.00	LX0	H
ATOM	776	CA	ILE	101	-11.106	-10.553	26.703	1.00	0.00	LX0	C
ATOM	777	CB	ILE	101	-12.610	-10.227	26.850	1.00	0.00	LX0	C
ATOM	778	CG2	ILE	101	-12.944	-9.630	28.211	1.00	0.00	LX0	C
ATOM	779	CG1	ILE	101	-13.178	-9.371	25.721	1.00	0.00	LX0	C
ATOM	780	CD1	ILE	101	-14.700	-9.277	25.828	1.00	0.00	LX0	C
ATOM	781	C	ILE	101	-10.216	-9.324	26.701	1.00	0.00	LX0	C
ATOM	782	O	ILE	101	-9.916	-8.720	27.728	1.00	0.00	LX0	O
ATOM	783	N	VAL	102	-9.788	-8.987	25.481	1.00	0.00	LX0	N
ATOM	784	H	VAL	102	-10.135	-9.490	24.684	0.00	0.00	LX0	H
ATOM	785	CA	VAL	102	-9.120	-7.697	25.345	1.00	0.00	LX0	C
ATOM	786	CB	VAL	102	-8.050	-7.754	24.245	1.00	0.00	LX0	C
ATOM	787	CG1	VAL	102	-7.231	-6.461	24.167	1.00	0.00	LX0	C
ATOM	788	CG2	VAL	102	-7.151	-8.980	24.414	1.00	0.00	LX0	C
ATOM	789	C	VAL	102	-10.185	-6.678	25.003	1.00	0.00	LX0	C
ATOM	790	O	VAL	102	-11.075	-6.956	24.212	1.00	0.00	LX0	O
ATOM	791	N	ARG	103	-10.093	-5.507	25.634	1.00	0.00	LX0	N
ATOM	792	H	ARG	103	-9.354	-5.320	26.281	0.00	0.00	LX0	H
ATOM	793	CA	ARG	103	-11.084	-4.505	25.264	1.00	0.00	LX0	C
ATOM	794	CB	ARG	103	-11.615	-3.771	26.499	1.00	0.00	LX0	C
ATOM	795	CG	ARG	103	-12.264	-4.740	27.497	1.00	0.00	LX0	C
ATOM	796	CD	ARG	103	-12.898	-4.038	28.700	1.00	0.00	LX0	C
ATOM	797	NE	ARG	103	-13.616	-4.977	29.566	1.00	0.00	LX0	N
ATOM	798	HE	ARG	103	-13.133	-5.451	30.315	0.00	0.00	LX0	H
ATOM	799	CZ	ARG	103	-14.934	-5.227	29.419	1.00	0.00	LX0	C
ATOM	800	NH1	ARG	103	-15.639	-4.603	28.475	1.00	0.00	LX0	N
ATOM	801	HH11	ARG	103	-16.616	-4.767	28.350	0.00	0.00	LX0	H
ATOM	802	HH12	ARG	103	-15.182	-3.948	27.872	0.00	0.00	LX0	H
ATOM	803	NH2	ARG	103	-15.526	-6.098	30.229	1.00	0.00	LX0	N
ATOM	804	HH21	ARG	103	-16.492	-6.338	30.189	0.00	0.00	LX0	H
ATOM	805	HH22	ARG	103	-14.960	-6.541	30.948	0.00	0.00	LX0	H

ATOM	806	C	ARG	103	-10.606	-3.572	24.169	1.00	0.00	LX0	C
ATOM	807	O	ARG	103	-11.326	-3.286	23.220	1.00	0.00	LX0	O
ATOM	808	N	GLY	104	-9.338	-3.156	24.311	1.00	0.00	LX0	N
ATOM	809	H	GLY	104	-8.807	-3.329	25.144	0.00	0.00	LX0	H
ATOM	810	CA	GLY	104	-8.759	-2.404	23.199	1.00	0.00	LX0	C
ATOM	811	C	GLY	104	-9.223	-0.965	23.106	1.00	0.00	LX0	C
ATOM	812	O	GLY	104	-9.523	-0.427	22.049	1.00	0.00	LX0	O
ATOM	813	N	THR	105	-9.249	-0.345	24.294	1.00	0.00	LX0	N
ATOM	814	H	THR	105	-9.021	-0.848	25.127	0.00	0.00	LX0	H
ATOM	815	CA	THR	105	-9.631	1.069	24.299	1.00	0.00	LX0	C
ATOM	816	CB	THR	105	-10.021	1.484	25.725	1.00	0.00	LX0	C
ATOM	817	OG1	THR	105	-10.884	0.492	26.303	1.00	0.00	LX0	O
ATOM	818	HG1	THR	105	-10.341	-0.265	26.492	0.00	0.00	LX0	H
ATOM	819	CG2	THR	105	-10.694	2.859	25.786	1.00	0.00	LX0	C
ATOM	820	C	THR	105	-8.583	2.002	23.687	1.00	0.00	LX0	C
ATOM	821	O	THR	105	-8.880	3.054	23.135	1.00	0.00	LX0	O
ATOM	822	N	GLN	106	-7.325	1.552	23.800	1.00	0.00	LX0	N
ATOM	823	H	GLN	106	-7.119	0.649	24.169	0.00	0.00	LX0	H
ATOM	824	CA	GLN	106	-6.247	2.329	23.196	1.00	0.00	LX0	C
ATOM	825	CB	GLN	106	-5.504	3.130	24.270	1.00	0.00	LX0	C
ATOM	826	CG	GLN	106	-5.063	2.328	25.496	1.00	0.00	LX0	C
ATOM	827	CD	GLN	106	-4.745	3.305	26.608	1.00	0.00	LX0	C
ATOM	828	OE1	GLN	106	-5.592	4.089	27.017	1.00	0.00	LX0	O
ATOM	829	NE2	GLN	106	-3.488	3.246	27.044	1.00	0.00	LX0	N
ATOM	830	HE21	GLN	106	-2.861	2.530	26.744	0.00	0.00	LX0	H
ATOM	831	HE22	GLN	106	-3.115	3.922	27.692	0.00	0.00	LX0	H
ATOM	832	C	GLN	106	-5.357	1.414	22.385	1.00	0.00	LX0	C
ATOM	833	O	GLN	106	-5.426	0.200	22.537	1.00	0.00	LX0	O
ATOM	834	N	LEU	107	-4.583	2.037	21.482	1.00	0.00	LX0	N
ATOM	835	H	LEU	107	-4.430	3.023	21.513	0.00	0.00	LX0	H
ATOM	836	CA	LEU	107	-4.007	1.228	20.410	1.00	0.00	LX0	C
ATOM	837	CB	LEU	107	-4.548	1.686	19.049	1.00	0.00	LX0	C
ATOM	838	CG	LEU	107	-6.072	1.629	18.904	1.00	0.00	LX0	C
ATOM	839	CD1	LEU	107	-6.523	2.254	17.585	1.00	0.00	LX0	C
ATOM	840	CD2	LEU	107	-6.634	0.217	19.086	1.00	0.00	LX0	C
ATOM	841	C	LEU	107	-2.490	1.191	20.374	1.00	0.00	LX0	C
ATOM	842	O	LEU	107	-1.798	1.929	21.067	1.00	0.00	LX0	O
ATOM	843	N	PHE	108	-2.024	0.284	19.506	1.00	0.00	LX0	N
ATOM	844	H	PHE	108	-2.672	-0.331	19.059	0.00	0.00	LX0	H
ATOM	845	CA	PHE	108	-0.598	0.080	19.293	1.00	0.00	LX0	C
ATOM	846	CB	PHE	108	-0.174	-1.152	20.070	1.00	0.00	LX0	C
ATOM	847	CG	PHE	108	1.118	-0.838	20.764	1.00	0.00	LX0	C
ATOM	848	CD1	PHE	108	1.069	-0.277	22.054	1.00	0.00	LX0	C
ATOM	849	CD2	PHE	108	2.339	-1.091	20.109	1.00	0.00	LX0	C
ATOM	850	CE1	PHE	108	2.270	0.071	22.691	1.00	0.00	LX0	C
ATOM	851	CE2	PHE	108	3.540	-0.742	20.749	1.00	0.00	LX0	C
ATOM	852	CZ	PHE	108	3.491	-0.146	22.025	1.00	0.00	LX0	C
ATOM	853	C	PHE	108	-0.304	-0.083	17.815	1.00	0.00	LX0	C
ATOM	854	O	PHE	108	-1.198	-0.506	17.082	1.00	0.00	LX0	O
ATOM	855	N	GLU	109	0.931	0.309	17.406	1.00	0.00	LX0	N
ATOM	856	H	GLU	109	1.614	0.558	18.094	0.00	0.00	LX0	H
ATOM	857	CA	GLU	109	1.266	0.582	15.993	1.00	0.00	LX0	C
ATOM	858	CB	GLU	109	2.128	-0.538	15.356	1.00	0.00	LX0	C
ATOM	859	CG	GLU	109	3.506	-0.672	16.050	1.00	0.00	LX0	C
ATOM	860	CD	GLU	109	4.445	-1.713	15.426	1.00	0.00	LX0	C
ATOM	861	OE1	GLU	109	4.084	-2.880	15.310	1.00	0.00	LX0	O
ATOM	862	OE2	GLU	109	5.593	-1.395	15.110	1.00	0.00	LX0	O
ATOM	863	C	GLU	109	0.067	1.061	15.168	1.00	0.00	LX0	C
ATOM	864	O	GLU	109	-0.538	0.383	14.340	1.00	0.00	LX0	O
ATOM	865	N	ASP	110	-0.309	2.284	15.580	1.00	0.00	LX0	N
ATOM	866	H	ASP	110	0.398	2.828	16.032	0.00	0.00	LX0	H

ATOM	867	CA	ASP	110	-1.647	2.490	16.150	1.00	0.00	LX0	C
ATOM	868	CB	ASP	110	-1.737	3.881	16.797	1.00	0.00	LX0	C
ATOM	869	CG	ASP	110	-0.802	3.971	18.006	1.00	0.00	LX0	C
ATOM	870	OD1	ASP	110	-0.836	4.957	18.736	1.00	0.00	LX0	O
ATOM	871	OD2	ASP	110	-0.021	3.057	18.253	1.00	0.00	LX0	O
ATOM	872	C	ASP	110	-2.900	2.123	15.385	1.00	0.00	LX0	C
ATOM	873	O	ASP	110	-3.661	2.951	14.905	1.00	0.00	LX0	O
ATOM	874	N	ASN	111	-3.110	0.802	15.357	1.00	0.00	LX0	N
ATOM	875	H	ASN	111	-2.335	0.216	15.607	0.00	0.00	LX0	H
ATOM	876	CA	ASN	111	-4.384	0.263	14.873	1.00	0.00	LX0	C
ATOM	877	CB	ASN	111	-4.293	-0.242	13.429	1.00	0.00	LX0	C
ATOM	878	CG	ASN	111	-3.965	0.849	12.431	1.00	0.00	LX0	C
ATOM	879	OD1	ASN	111	-4.823	1.376	11.739	1.00	0.00	LX0	O
ATOM	880	ND2	ASN	111	-2.660	1.130	12.336	1.00	0.00	LX0	N
ATOM	881	HD21	ASN	111	-1.969	0.702	12.926	0.00	0.00	LX0	H
ATOM	882	HD22	ASN	111	-2.386	1.814	11.665	0.00	0.00	LX0	H
ATOM	883	C	ASN	111	-4.862	-0.907	15.718	1.00	0.00	LX0	C
ATOM	884	O	ASN	111	-5.905	-1.513	15.492	1.00	0.00	LX0	O
ATOM	885	N	TYR	112	-4.007	-1.269	16.684	1.00	0.00	LX0	N
ATOM	886	H	TYR	112	-3.197	-0.741	16.938	0.00	0.00	LX0	H
ATOM	887	CA	TYR	112	-4.176	-2.619	17.202	1.00	0.00	LX0	C
ATOM	888	CB	TYR	112	-3.013	-3.490	16.748	1.00	0.00	LX0	C
ATOM	889	CG	TYR	112	-2.835	-3.480	15.245	1.00	0.00	LX0	C
ATOM	890	CD1	TYR	112	-3.649	-4.316	14.458	1.00	0.00	LX0	C
ATOM	891	CE1	TYR	112	-3.482	-4.319	13.067	1.00	0.00	LX0	C
ATOM	892	CD2	TYR	112	-1.853	-2.647	14.671	1.00	0.00	LX0	C
ATOM	893	CE2	TYR	112	-1.687	-2.646	13.276	1.00	0.00	LX0	C
ATOM	894	CZ	TYR	112	-2.514	-3.476	12.492	1.00	0.00	LX0	C
ATOM	895	OH	TYR	112	-2.385	-3.465	11.118	1.00	0.00	LX0	O
ATOM	896	HH	TYR	112	-1.680	-2.880	10.869	0.00	0.00	LX0	H
ATOM	897	C	TYR	112	-4.296	-2.696	18.704	1.00	0.00	LX0	C
ATOM	898	O	TYR	112	-3.825	-1.829	19.425	1.00	0.00	LX0	O
ATOM	899	N	ALA	113	-4.940	-3.784	19.135	1.00	0.00	LX0	N
ATOM	900	H	ALA	113	-5.291	-4.446	18.473	0.00	0.00	LX0	H
ATOM	901	CA	ALA	113	-5.056	-4.019	20.569	1.00	0.00	LX0	C
ATOM	902	CB	ALA	113	-6.505	-4.315	20.939	1.00	0.00	LX0	C
ATOM	903	C	ALA	113	-4.193	-5.162	21.070	1.00	0.00	LX0	C
ATOM	904	O	ALA	113	-3.689	-5.161	22.187	1.00	0.00	LX0	O
ATOM	905	N	LEU	114	-4.036	-6.168	20.201	1.00	0.00	LX0	N
ATOM	906	H	LEU	114	-4.415	-6.131	19.276	0.00	0.00	LX0	H
ATOM	907	CA	LEU	114	-3.199	-7.274	20.654	1.00	0.00	LX0	C
ATOM	908	CB	LEU	114	-3.809	-8.609	20.227	1.00	0.00	LX0	C
ATOM	909	CG	LEU	114	-3.165	-9.809	20.926	1.00	0.00	LX0	C
ATOM	910	CD1	LEU	114	-3.427	-9.815	22.434	1.00	0.00	LX0	C
ATOM	911	CD2	LEU	114	-3.579	-11.119	20.269	1.00	0.00	LX0	C
ATOM	912	C	LEU	114	-1.767	-7.137	20.177	1.00	0.00	LX0	C
ATOM	913	O	LEU	114	-1.362	-7.715	19.175	1.00	0.00	LX0	O
ATOM	914	N	ALA	115	-1.030	-6.313	20.922	1.00	0.00	LX0	N
ATOM	915	H	ALA	115	-1.363	-5.974	21.807	0.00	0.00	LX0	H
ATOM	916	CA	ALA	115	0.322	-6.014	20.472	1.00	0.00	LX0	C
ATOM	917	CB	ALA	115	0.667	-4.566	20.777	1.00	0.00	LX0	C
ATOM	918	C	ALA	115	1.380	-6.910	21.076	1.00	0.00	LX0	C
ATOM	919	O	ALA	115	1.876	-6.707	22.178	1.00	0.00	LX0	O
ATOM	920	N	VAL	116	1.710	-7.930	20.288	1.00	0.00	LX0	N
ATOM	921	H	VAL	116	1.299	-8.009	19.378	0.00	0.00	LX0	H
ATOM	922	CA	VAL	116	2.817	-8.782	20.700	1.00	0.00	LX0	C
ATOM	923	CB	VAL	116	2.349	-10.241	20.781	1.00	0.00	LX0	C
ATOM	924	CG1	VAL	116	3.450	-11.163	21.303	1.00	0.00	LX0	C
ATOM	925	CG2	VAL	116	1.073	-10.373	21.617	1.00	0.00	LX0	C
ATOM	926	C	VAL	116	3.973	-8.596	19.732	1.00	0.00	LX0	C
ATOM	927	O	VAL	116	3.904	-8.997	18.577	1.00	0.00	LX0	O

ATOM	928	N	LEU	117	5.010	-7.910	20.230	1.00	0.00	LX0	N
ATOM	929	H	LEU	117	5.078	-7.749	21.216	0.00	0.00	LX0	H
ATOM	930	CA	LEU	117	5.980	-7.352	19.283	1.00	0.00	LX0	C
ATOM	931	CB	LEU	117	5.858	-5.828	19.230	1.00	0.00	LX0	C
ATOM	932	CG	LEU	117	4.493	-5.306	18.803	1.00	0.00	LX0	C
ATOM	933	CD1	LEU	117	4.402	-3.791	18.958	1.00	0.00	LX0	C
ATOM	934	CD2	LEU	117	4.151	-5.750	17.388	1.00	0.00	LX0	C
ATOM	935	C	LEU	117	7.423	-7.676	19.595	1.00	0.00	LX0	C
ATOM	936	O	LEU	117	7.800	-7.834	20.751	1.00	0.00	LX0	O
ATOM	937	N	ASP	118	8.221	-7.715	18.512	1.00	0.00	LX0	N
ATOM	938	H	ASP	118	7.786	-7.686	17.611	0.00	0.00	LX0	H
ATOM	939	CA	ASP	118	9.689	-7.642	18.621	1.00	0.00	LX0	C
ATOM	940	CB	ASP	118	10.158	-6.186	18.803	1.00	0.00	LX0	C
ATOM	941	CG	ASP	118	9.804	-5.266	17.641	1.00	0.00	LX0	C
ATOM	942	OD1	ASP	118	10.534	-5.232	16.658	1.00	0.00	LX0	O
ATOM	943	OD2	ASP	118	8.820	-4.530	17.712	1.00	0.00	LX0	O
ATOM	944	C	ASP	118	10.314	-8.530	19.698	1.00	0.00	LX0	C
ATOM	945	O	ASP	118	11.147	-8.113	20.499	1.00	0.00	LX0	O
ATOM	946	N	ASN	119	9.832	-9.782	19.731	1.00	0.00	LX0	N
ATOM	947	H	ASN	119	9.254	-10.093	18.973	0.00	0.00	LX0	H
ATOM	948	CA	ASN	119	10.020	-10.531	20.980	1.00	0.00	LX0	C
ATOM	949	CB	ASN	119	8.820	-11.427	21.281	1.00	0.00	LX0	C
ATOM	950	CG	ASN	119	7.824	-10.688	22.150	1.00	0.00	LX0	C
ATOM	951	OD1	ASN	119	8.051	-10.386	23.317	1.00	0.00	LX0	O
ATOM	952	ND2	ASN	119	6.680	-10.416	21.533	1.00	0.00	LX0	N
ATOM	953	HD21	ASN	119	6.563	-10.667	20.570	0.00	0.00	LX0	H
ATOM	954	HD22	ASN	119	5.952	-9.936	22.022	0.00	0.00	LX0	H
ATOM	955	C	ASN	119	11.305	-11.312	21.206	1.00	0.00	LX0	C
ATOM	956	O	ASN	119	11.319	-12.539	21.210	1.00	0.00	LX0	O
ATOM	957	N	GLY	120	12.362	-10.532	21.479	1.00	0.00	LX0	N
ATOM	958	H	GLY	120	12.301	-9.560	21.253	0.00	0.00	LX0	H
ATOM	959	CA	GLY	120	13.552	-11.119	22.103	1.00	0.00	LX0	C
ATOM	960	C	GLY	120	14.513	-11.850	21.179	1.00	0.00	LX0	C
ATOM	961	O	GLY	120	14.439	-11.758	19.958	1.00	0.00	LX0	O
ATOM	962	N	ASP	121	15.441	-12.567	21.837	1.00	0.00	LX0	N
ATOM	963	H	ASP	121	15.400	-12.631	22.836	0.00	0.00	LX0	H
ATOM	964	CA	ASP	121	16.464	-13.316	21.099	1.00	0.00	LX0	C
ATOM	965	CB	ASP	121	17.487	-13.984	22.032	1.00	0.00	LX0	C
ATOM	966	CG	ASP	121	18.368	-13.057	22.857	1.00	0.00	LX0	C
ATOM	967	OD1	ASP	121	18.719	-11.973	22.409	1.00	0.00	LX0	O
ATOM	968	OD2	ASP	121	18.758	-13.443	23.959	1.00	0.00	LX0	O
ATOM	969	C	ASP	121	15.872	-14.425	20.234	1.00	0.00	LX0	C
ATOM	970	O	ASP	121	15.037	-15.211	20.670	1.00	0.00	LX0	O
ATOM	971	N	PRO	122	16.335	-14.474	18.965	1.00	0.00	LX0	N
ATOM	972	CD	PRO	122	17.237	-13.517	18.336	1.00	0.00	LX0	C
ATOM	973	CA	PRO	122	15.862	-15.506	18.031	1.00	0.00	LX0	C
ATOM	974	CB	PRO	122	16.406	-15.011	16.681	1.00	0.00	LX0	C
ATOM	975	CG	PRO	122	16.806	-13.549	16.878	1.00	0.00	LX0	C
ATOM	976	C	PRO	122	16.308	-16.942	18.317	1.00	0.00	LX0	C
ATOM	977	O	PRO	122	17.149	-17.494	17.616	1.00	0.00	LX0	O
ATOM	978	N	LEU	123	15.708	-17.566	19.342	1.00	0.00	LX0	N
ATOM	979	H	LEU	123	15.051	-17.081	19.925	0.00	0.00	LX0	H
ATOM	980	CA	LEU	123	15.967	-19.003	19.449	1.00	0.00	LX0	C
ATOM	981	CB	LEU	123	15.802	-19.461	20.906	1.00	0.00	LX0	C
ATOM	982	CG	LEU	123	15.984	-20.948	21.244	1.00	0.00	LX0	C
ATOM	983	CD1	LEU	123	17.411	-21.447	21.014	1.00	0.00	LX0	C
ATOM	984	CD2	LEU	123	15.494	-21.255	22.659	1.00	0.00	LX0	C
ATOM	985	C	LEU	123	15.108	-19.801	18.476	1.00	0.00	LX0	C
ATOM	986	O	LEU	123	13.993	-19.428	18.124	1.00	0.00	LX0	O
ATOM	987	N	ASN	124	15.710	-20.909	18.028	1.00	0.00	LX0	N
ATOM	988	H	ASN	124	16.600	-21.147	18.411	0.00	0.00	LX0	H

```
ATOM    989  CA  ASN   124      14.968 -21.846  17.189  1.00  0.00      LX0  C
ATOM    990  CB  ASN   124      15.904 -22.469  16.133  1.00  0.00      LX0  C
ATOM    991  CG  ASN   124      16.980 -23.333  16.762  1.00  0.00      LX0  C
ATOM    992  OD1 ASN   124      16.799 -23.946  17.806  1.00  0.00      LX0  O
ATOM    993  ND2 ASN   124      18.129 -23.349  16.084  1.00  0.00      LX0  N
ATOM    994 HD21 ASN   124      18.236 -22.832  15.236  0.00  0.00      LX0  H
ATOM    995 HD22 ASN   124      18.895 -23.890  16.430  0.00  0.00      LX0  H
ATOM    996  C   ASN   124      14.199 -22.873  18.019  1.00  0.00      LX0  C
ATOM    997  O   ASN   124      13.952 -22.673  19.202  1.00  0.00      LX0  O
ATOM    998  N   ASN   125      13.831 -23.995  17.368  1.00  0.00      LX0  N
ATOM    999  H   ASN   125      13.967 -24.080  16.381  0.00  0.00      LX0  H
ATOM   1000  CA  ASN   125      13.040 -25.005  18.081  1.00  0.00      LX0  C
ATOM   1001  CB  ASN   125      12.666 -26.193  17.198  1.00  0.00      LX0  C
ATOM   1002  CG  ASN   125      11.507 -25.782  16.331  1.00  0.00      LX0  C
ATOM   1003  OD1 ASN   125      11.679 -25.086  15.342  1.00  0.00      LX0  O
ATOM   1004  ND2 ASN   125      10.313 -26.185  16.772  1.00  0.00      LX0  N
ATOM   1005 HD21 ASN   125      10.204 -26.809  17.548  0.00  0.00      LX0  H
ATOM   1006 HD22 ASN   125       9.483 -25.853  16.321  0.00  0.00      LX0  H
ATOM   1007  C   ASN   125      13.569 -25.555  19.386  1.00  0.00      LX0  C
ATOM   1008  O   ASN   125      12.780 -25.992  20.219  1.00  0.00      LX0  O
ATOM   1009  N   THR   126      14.905 -25.527  19.537  1.00  0.00      LX0  N
ATOM   1010  H   THR   126      15.482 -25.146  18.815  0.00  0.00      LX0  H
ATOM   1011  CA  THR   126      15.537 -26.054  20.753  1.00  0.00      LX0  C
ATOM   1012  CB  THR   126      16.998 -25.581  20.784  1.00  0.00      LX0  C
ATOM   1013  OG1 THR   126      17.611 -25.894  19.528  1.00  0.00      LX0  O
ATOM   1014  HG1 THR   126      17.513 -25.123  18.978  0.00  0.00      LX0  H
ATOM   1015  CG2 THR   126      17.829 -26.173  21.928  1.00  0.00      LX0  C
ATOM   1016  C   THR   126      14.799 -25.717  22.046  1.00  0.00      LX0  C
ATOM   1017  O   THR   126      14.252 -24.631  22.215  1.00  0.00      LX0  O
ATOM   1018  N   THR   127      14.757 -26.724  22.934  1.00  0.00      LX0  N
ATOM   1019  H   THR   127      15.263 -27.565  22.756  0.00  0.00      LX0  H
ATOM   1020  CA  THR   127      14.120 -26.468  24.225  1.00  0.00      LX0  C
ATOM   1021  CB  THR   127      14.050 -27.772  25.045  1.00  0.00      LX0  C
ATOM   1022  OG1 THR   127      13.178 -27.615  26.166  1.00  0.00      LX0  O
ATOM   1023  HG1 THR   127      13.148 -28.452  26.614  0.00  0.00      LX0  H
ATOM   1024  CG2 THR   127      15.417 -28.317  25.474  1.00  0.00      LX0  C
ATOM   1025  C   THR   127      14.820 -25.328  24.959  1.00  0.00      LX0  C
ATOM   1026  O   THR   127      16.042 -25.249  24.982  1.00  0.00      LX0  O
ATOM   1027  N   PRO   128      13.999 -24.394  25.487  1.00  0.00      LX0  N
ATOM   1028  CD  PRO   128      12.539 -24.400  25.513  1.00  0.00      LX0  C
ATOM   1029  CA  PRO   128      14.587 -23.187  26.066  1.00  0.00      LX0  C
ATOM   1030  CB  PRO   128      13.378 -22.244  26.079  1.00  0.00      LX0  C
ATOM   1031  CG  PRO   128      12.166 -23.149  26.298  1.00  0.00      LX0  C
ATOM   1032  C   PRO   128      15.219 -23.438  27.427  1.00  0.00      LX0  C
ATOM   1033  O   PRO   128      14.631 -23.201  28.474  1.00  0.00      LX0  O
ATOM   1034  N   VAL   129      16.470 -23.914  27.367  1.00  0.00      LX0  N
ATOM   1035  H   VAL   129      16.898 -24.040  26.466  0.00  0.00      LX0  H
ATOM   1036  CA  VAL   129      17.211 -24.148  28.605  1.00  0.00      LX0  C
ATOM   1037  CB  VAL   129      18.593 -24.749  28.294  1.00  0.00      LX0  C
ATOM   1038  CG1 VAL   129      19.419 -25.023  29.556  1.00  0.00      LX0  C
ATOM   1039  CG2 VAL   129      18.458 -26.007  27.433  1.00  0.00      LX0  C
ATOM   1040  C   VAL   129      17.341 -22.876  29.426  1.00  0.00      LX0  C
ATOM   1041  O   VAL   129      17.864 -21.863  28.975  1.00  0.00      LX0  O
ATOM   1042  N   THR   130      16.835 -22.990  30.663  1.00  0.00      LX0  N
ATOM   1043  H   THR   130      16.353 -23.825  30.931  0.00  0.00      LX0  H
ATOM   1044  CA  THR   130      16.752 -21.863  31.591  1.00  0.00      LX0  C
ATOM   1045  CB  THR   130      16.509 -22.430  32.992  1.00  0.00      LX0  C
ATOM   1046  OG1 THR   130      15.825 -23.687  32.887  1.00  0.00      LX0  O
ATOM   1047  HG1 THR   130      15.516 -23.907  33.758  0.00  0.00      LX0  H
ATOM   1048  CG2 THR   130      15.747 -21.460  33.896  1.00  0.00      LX0  C
ATOM   1049  C   THR   130      17.935 -20.903  31.569  1.00  0.00      LX0  C
```

ATOM	1050	O	THR	130	17.804	-19.703	31.348	1.00	0.00	LX0	O
ATOM	1051	N	GLY	131	19.116	-21.506	31.762	1.00	0.00	LX0	N
ATOM	1052	H	GLY	131	19.154	-22.496	31.881	0.00	0.00	LX0	H
ATOM	1053	CA	GLY	131	20.312	-20.697	31.559	1.00	0.00	LX0	C
ATOM	1054	C	GLY	131	20.659	-20.555	30.089	1.00	0.00	LX0	C
ATOM	1055	O	GLY	131	20.280	-19.607	29.411	1.00	0.00	LX0	O
ATOM	1056	N	ALA	132	21.415	-21.565	29.634	1.00	0.00	LX0	N
ATOM	1057	H	ALA	132	21.614	-22.341	30.228	0.00	0.00	LX0	H
ATOM	1058	CA	ALA	132	21.991	-21.494	28.293	1.00	0.00	LX0	C
ATOM	1059	CB	ALA	132	23.193	-22.436	28.186	1.00	0.00	LX0	C
ATOM	1060	C	ALA	132	21.036	-21.766	27.141	1.00	0.00	LX0	C
ATOM	1061	O	ALA	132	21.041	-22.818	26.516	1.00	0.00	LX0	O
ATOM	1062	N	SER	133	20.240	-20.734	26.864	1.00	0.00	LX0	N
ATOM	1063	H	SER	133	20.200	-19.982	27.525	0.00	0.00	LX0	H
ATOM	1064	CA	SER	133	19.441	-20.645	25.646	1.00	0.00	LX0	C
ATOM	1065	CB	SER	133	18.179	-21.508	25.708	1.00	0.00	LX0	C
ATOM	1066	OG	SER	133	18.473	-22.897	25.532	1.00	0.00	LX0	O
ATOM	1067	HG	SER	133	19.377	-23.039	25.807	0.00	0.00	LX0	H
ATOM	1068	C	SER	133	19.001	-19.207	25.511	1.00	0.00	LX0	C
ATOM	1069	O	SER	133	18.540	-18.618	26.487	1.00	0.00	LX0	O
ATOM	1070	N	PRO	134	19.152	-18.661	24.280	1.00	0.00	LX0	N
ATOM	1071	CD	PRO	134	19.791	-19.294	23.129	1.00	0.00	LX0	C
ATOM	1072	CA	PRO	134	18.647	-17.314	23.975	1.00	0.00	LX0	C
ATOM	1073	CB	PRO	134	18.689	-17.307	22.445	1.00	0.00	LX0	C
ATOM	1074	CG	PRO	134	19.883	-18.185	22.089	1.00	0.00	LX0	C
ATOM	1075	C	PRO	134	17.272	-17.020	24.560	1.00	0.00	LX0	C
ATOM	1076	O	PRO	134	16.465	-17.923	24.776	1.00	0.00	LX0	O
ATOM	1077	N	GLY	135	17.072	-15.740	24.874	1.00	0.00	LX0	N
ATOM	1078	H	GLY	135	17.723	-15.020	24.618	0.00	0.00	LX0	H
ATOM	1079	CA	GLY	135	15.822	-15.384	25.526	1.00	0.00	LX0	C
ATOM	1080	C	GLY	135	14.902	-14.585	24.637	1.00	0.00	LX0	C
ATOM	1081	O	GLY	135	14.923	-13.360	24.597	1.00	0.00	LX0	O
ATOM	1082	N	GLY	136	14.079	-15.342	23.919	1.00	0.00	LX0	N
ATOM	1083	H	GLY	136	14.154	-16.339	23.869	0.00	0.00	LX0	H
ATOM	1084	CA	GLY	136	12.951	-14.671	23.297	1.00	0.00	LX0	C
ATOM	1085	C	GLY	136	11.668	-15.239	23.846	1.00	0.00	LX0	C
ATOM	1086	O	GLY	136	11.667	-16.158	24.661	1.00	0.00	LX0	O
ATOM	1087	N	LEU	137	10.572	-14.682	23.331	1.00	0.00	LX0	N
ATOM	1088	H	LEU	137	10.669	-13.961	22.642	0.00	0.00	LX0	H
ATOM	1089	CA	LEU	137	9.293	-15.265	23.717	1.00	0.00	LX0	C
ATOM	1090	CB	LEU	137	8.236	-14.164	23.738	1.00	0.00	LX0	C
ATOM	1091	CG	LEU	137	6.852	-14.607	24.202	1.00	0.00	LX0	C
ATOM	1092	CD1	LEU	137	6.879	-15.252	25.585	1.00	0.00	LX0	C
ATOM	1093	CD2	LEU	137	5.859	-13.455	24.114	1.00	0.00	LX0	C
ATOM	1094	C	LEU	137	8.906	-16.420	22.810	1.00	0.00	LX0	C
ATOM	1095	O	LEU	137	8.940	-16.318	21.592	1.00	0.00	LX0	O
ATOM	1096	N	ARG	138	8.561	-17.540	23.461	1.00	0.00	LX0	N
ATOM	1097	H	ARG	138	8.529	-17.522	24.462	0.00	0.00	LX0	H
ATOM	1098	CA	ARG	138	8.310	-18.761	22.701	1.00	0.00	LX0	C
ATOM	1099	CB	ARG	138	8.776	-19.977	23.508	1.00	0.00	LX0	C
ATOM	1100	CG	ARG	138	9.074	-21.184	22.626	1.00	0.00	LX0	C
ATOM	1101	CD	ARG	138	9.566	-22.412	23.385	1.00	0.00	LX0	C
ATOM	1102	NE	ARG	138	9.490	-23.603	22.541	1.00	0.00	LX0	N
ATOM	1103	HE	ARG	138	8.581	-24.014	22.391	0.00	0.00	LX0	H
ATOM	1104	CZ	ARG	138	10.574	-24.170	21.973	1.00	0.00	LX0	C
ATOM	1105	NH1	ARG	138	11.783	-23.634	22.126	1.00	0.00	LX0	N
ATOM	1106	HH11	ARG	138	12.614	-24.076	21.764	0.00	0.00	LX0	H
ATOM	1107	HH12	ARG	138	11.902	-22.775	22.618	0.00	0.00	LX0	H
ATOM	1108	NH2	ARG	138	10.417	-25.277	21.261	1.00	0.00	LX0	N
ATOM	1109	HH21	ARG	138	11.195	-25.760	20.850	0.00	0.00	LX0	H
ATOM	1110	HH22	ARG	138	9.486	-25.644	21.123	0.00	0.00	LX0	H

ATOM	1111	C	ARG	138	6.893	-18.961	22.190	1.00	0.00	LX0	C
ATOM	1112	O	ARG	138	6.663	-19.483	21.099	1.00	0.00	LX0	O
ATOM	1113	N	GLU	139	5.947	-18.546	23.046	1.00	0.00	LX0	N
ATOM	1114	H	GLU	139	6.182	-18.107	23.913	0.00	0.00	LX0	H
ATOM	1115	CA	GLU	139	4.542	-18.840	22.761	1.00	0.00	LX0	C
ATOM	1116	CB	GLU	139	4.260	-20.314	23.028	1.00	0.00	LX0	C
ATOM	1117	CG	GLU	139	4.560	-20.690	24.476	1.00	0.00	LX0	C
ATOM	1118	CD	GLU	139	5.042	-22.116	24.518	1.00	0.00	LX0	C
ATOM	1119	OE1	GLU	139	6.085	-22.390	23.932	1.00	0.00	LX0	O
ATOM	1120	OE2	GLU	139	4.398	-22.942	25.154	1.00	0.00	LX0	O
ATOM	1121	C	GLU	139	3.621	-17.952	23.569	1.00	0.00	LX0	C
ATOM	1122	O	GLU	139	4.046	-17.275	24.501	1.00	0.00	LX0	O
ATOM	1123	N	LEU	140	2.350	-17.968	23.155	1.00	0.00	LX0	N
ATOM	1124	H	LEU	140	2.029	-18.640	22.482	0.00	0.00	LX0	H
ATOM	1125	CA	LEU	140	1.402	-17.072	23.804	1.00	0.00	LX0	C
ATOM	1126	CB	LEU	140	0.740	-16.156	22.776	1.00	0.00	LX0	C
ATOM	1127	CG	LEU	140	1.668	-15.100	22.190	1.00	0.00	LX0	C
ATOM	1128	CD1	LEU	140	0.986	-14.337	21.056	1.00	0.00	LX0	C
ATOM	1129	CD2	LEU	140	2.208	-14.166	23.274	1.00	0.00	LX0	C
ATOM	1130	C	LEU	140	0.330	-17.852	24.518	1.00	0.00	LX0	C
ATOM	1131	O	LEU	140	-0.710	-18.147	23.953	1.00	0.00	LX0	O
ATOM	1132	N	GLN	141	0.610	-18.192	25.778	1.00	0.00	LX0	N
ATOM	1133	H	GLN	141	1.389	-17.803	26.272	0.00	0.00	LX0	H
ATOM	1134	CA	GLN	141	-0.350	-19.065	26.453	1.00	0.00	LX0	C
ATOM	1135	CB	GLN	141	0.372	-19.867	27.527	1.00	0.00	LX0	C
ATOM	1136	CG	GLN	141	1.536	-20.695	26.987	1.00	0.00	LX0	C
ATOM	1137	CD	GLN	141	2.277	-21.331	28.144	1.00	0.00	LX0	C
ATOM	1138	OE1	GLN	141	2.065	-21.028	29.313	1.00	0.00	LX0	O
ATOM	1139	NE2	GLN	141	3.182	-22.235	27.764	1.00	0.00	LX0	N
ATOM	1140	HE21	GLN	141	3.376	-22.433	26.797	0.00	0.00	LX0	H
ATOM	1141	HE22	GLN	141	3.700	-22.733	28.453	0.00	0.00	LX0	H
ATOM	1142	C	GLN	141	-1.553	-18.332	27.027	1.00	0.00	LX0	C
ATOM	1143	O	GLN	141	-1.681	-18.132	28.227	1.00	0.00	LX0	O
ATOM	1144	N	LEU	142	-2.424	-17.901	26.105	1.00	0.00	LX0	N
ATOM	1145	H	LEU	142	-2.274	-18.132	25.142	0.00	0.00	LX0	H
ATOM	1146	CA	LEU	142	-3.535	-17.052	26.537	1.00	0.00	LX0	C
ATOM	1147	CB	LEU	142	-3.602	-15.740	25.732	1.00	0.00	LX0	C
ATOM	1148	CG	LEU	142	-2.318	-15.174	25.102	1.00	0.00	LX0	C
ATOM	1149	CD1	LEU	142	-2.639	-14.013	24.159	1.00	0.00	LX0	C
ATOM	1150	CD2	LEU	142	-1.247	-14.764	26.109	1.00	0.00	LX0	C
ATOM	1151	C	LEU	142	-4.875	-17.759	26.421	1.00	0.00	LX0	C
ATOM	1152	O	LEU	142	-5.820	-17.242	25.838	1.00	0.00	LX0	O
ATOM	1153	N	ARG	143	-4.912	-19.001	26.942	1.00	0.00	LX0	N
ATOM	1154	H	ARG	143	-4.126	-19.330	27.468	0.00	0.00	LX0	H
ATOM	1155	CA	ARG	143	-5.944	-19.926	26.454	1.00	0.00	LX0	C
ATOM	1156	CB	ARG	143	-5.852	-21.299	27.127	1.00	0.00	LX0	C
ATOM	1157	CG	ARG	143	-6.404	-21.385	28.549	1.00	0.00	LX0	C
ATOM	1158	CD	ARG	143	-6.310	-22.795	29.119	1.00	0.00	LX0	C
ATOM	1159	NE	ARG	143	-4.918	-23.208	29.262	1.00	0.00	LX0	N
ATOM	1160	HE	ARG	143	-4.171	-22.635	28.901	0.00	0.00	LX0	H
ATOM	1161	CZ	ARG	143	-4.612	-24.376	29.852	1.00	0.00	LX0	C
ATOM	1162	NH1	ARG	143	-5.566	-25.195	30.278	1.00	0.00	LX0	N
ATOM	1163	HH11	ARG	143	-5.306	-26.090	30.683	0.00	0.00	LX0	H
ATOM	1164	HH12	ARG	143	-6.535	-24.992	30.188	0.00	0.00	LX0	H
ATOM	1165	NH2	ARG	143	-3.343	-24.717	30.006	1.00	0.00	LX0	N
ATOM	1166	HH21	ARG	143	-3.126	-25.570	30.497	0.00	0.00	LX0	H
ATOM	1167	HH22	ARG	143	-2.597	-24.151	29.634	0.00	0.00	LX0	H
ATOM	1168	C	ARG	143	-7.397	-19.481	26.327	1.00	0.00	LX0	C
ATOM	1169	O	ARG	143	-8.106	-19.864	25.397	1.00	0.00	LX0	O
ATOM	1170	N	SER	144	-7.816	-18.667	27.303	1.00	0.00	LX0	N
ATOM	1171	H	SER	144	-7.151	-18.266	27.935	0.00	0.00	LX0	H

```
ATOM   1172  CA  SER   144      -9.236 -18.342  27.356  1.00  0.00      LX0  C
ATOM   1173  CB  SER   144      -9.633 -18.009  28.795  1.00  0.00      LX0  C
ATOM   1174  OG  SER   144      -9.078 -18.988  29.686  1.00  0.00      LX0  O
ATOM   1175  HG  SER   144      -8.131 -18.878  29.645  0.00  0.00      LX0  H
ATOM   1176  C   SER   144      -9.722 -17.275  26.382  1.00  0.00      LX0  C
ATOM   1177  O   SER   144     -10.916 -16.993  26.294  1.00  0.00      LX0  O
ATOM   1178  N   LEU   145      -8.751 -16.702  25.647  1.00  0.00      LX0  N
ATOM   1179  H   LEU   145      -7.794 -16.959  25.779  0.00  0.00      LX0  H
ATOM   1180  CA  LEU   145      -9.076 -15.544  24.822  1.00  0.00      LX0  C
ATOM   1181  CB  LEU   145      -7.802 -14.995  24.175  1.00  0.00      LX0  C
ATOM   1182  CG  LEU   145      -7.924 -13.594  23.570  1.00  0.00      LX0  C
ATOM   1183  CD1 LEU   145      -8.456 -12.568  24.569  1.00  0.00      LX0  C
ATOM   1184  CD2 LEU   145      -6.604 -13.136  22.948  1.00  0.00      LX0  C
ATOM   1185  C   LEU   145     -10.187 -15.802  23.826  1.00  0.00      LX0  C
ATOM   1186  O   LEU   145     -10.126 -16.673  22.965  1.00  0.00      LX0  O
ATOM   1187  N   THR   146     -11.240 -15.017  24.043  1.00  0.00      LX0  N
ATOM   1188  H   THR   146     -11.237 -14.357  24.795  0.00  0.00      LX0  H
ATOM   1189  CA  THR   146     -12.446 -15.259  23.273  1.00  0.00      LX0  C
ATOM   1190  CB  THR   146     -13.580 -15.670  24.215  1.00  0.00      LX0  C
ATOM   1191  OG1 THR   146     -13.231 -15.406  25.585  1.00  0.00      LX0  O
ATOM   1192  HG1 THR   146     -12.458 -15.936  25.782  0.00  0.00      LX0  H
ATOM   1193  CG2 THR   146     -13.920 -17.151  24.036  1.00  0.00      LX0  C
ATOM   1194  C   THR   146     -12.831 -14.108  22.370  1.00  0.00      LX0  C
ATOM   1195  O   THR   146     -13.160 -14.311  21.210  1.00  0.00      LX0  O
ATOM   1196  N   GLU   147     -12.733 -12.894  22.943  1.00  0.00      LX0  N
ATOM   1197  H   GLU   147     -12.453 -12.796  23.899  0.00  0.00      LX0  H
ATOM   1198  CA  GLU   147     -12.969 -11.711  22.116  1.00  0.00      LX0  C
ATOM   1199  CB  GLU   147     -14.355 -11.093  22.373  1.00  0.00      LX0  C
ATOM   1200  CG  GLU   147     -15.588 -11.860  21.862  1.00  0.00      LX0  C
ATOM   1201  CD  GLU   147     -15.756 -11.744  20.353  1.00  0.00      LX0  C
ATOM   1202  OE1 GLU   147     -16.853 -11.460  19.875  1.00  0.00      LX0  O
ATOM   1203  OE2 GLU   147     -14.807 -11.958  19.613  1.00  0.00      LX0  O
ATOM   1204  C   GLU   147     -11.903 -10.650  22.320  1.00  0.00      LX0  C
ATOM   1205  O   GLU   147     -11.306 -10.515  23.385  1.00  0.00      LX0  O
ATOM   1206  N   ILE   148     -11.714  -9.880  21.246  1.00  0.00      LX0  N
ATOM   1207  H   ILE   148     -12.150 -10.183  20.396  0.00  0.00      LX0  H
ATOM   1208  CA  ILE   148     -10.937  -8.643  21.294  1.00  0.00      LX0  C
ATOM   1209  CB  ILE   148      -9.659  -8.743  20.441  1.00  0.00      LX0  C
ATOM   1210  CG2 ILE   148      -8.877  -7.422  20.428  1.00  0.00      LX0  C
ATOM   1211  CG1 ILE   148      -8.772  -9.906  20.891  1.00  0.00      LX0  C
ATOM   1212  CD1 ILE   148      -7.586 -10.151  19.958  1.00  0.00      LX0  C
ATOM   1213  C   ILE   148     -11.831  -7.542  20.767  1.00  0.00      LX0  C
ATOM   1214  O   ILE   148     -12.053  -7.434  19.564  1.00  0.00      LX0  O
ATOM   1215  N   LEU   149     -12.370  -6.771  21.728  1.00  0.00      LX0  N
ATOM   1216  H   LEU   149     -12.079  -6.930  22.669  0.00  0.00      LX0  H
ATOM   1217  CA  LEU   149     -13.449  -5.832  21.420  1.00  0.00      LX0  C
ATOM   1218  CB  LEU   149     -13.925  -5.050  22.648  1.00  0.00      LX0  C
ATOM   1219  CG  LEU   149     -14.529  -5.891  23.773  1.00  0.00      LX0  C
ATOM   1220  CD1 LEU   149     -15.023  -5.007  24.916  1.00  0.00      LX0  C
ATOM   1221  CD2 LEU   149     -15.651  -6.805  23.288  1.00  0.00      LX0  C
ATOM   1222  C   LEU   149     -13.198  -4.884  20.270  1.00  0.00      LX0  C
ATOM   1223  O   LEU   149     -13.901  -4.914  19.265  1.00  0.00      LX0  O
ATOM   1224  N   LYS   150     -12.184  -4.029  20.457  1.00  0.00      LX0  N
ATOM   1225  H   LYS   150     -11.657  -3.985  21.310  0.00  0.00      LX0  H
ATOM   1226  CA  LYS   150     -11.810  -3.161  19.347  1.00  0.00      LX0  C
ATOM   1227  CB  LYS   150     -12.431  -1.760  19.497  1.00  0.00      LX0  C
ATOM   1228  CG  LYS   150     -13.959  -1.838  19.365  1.00  0.00      LX0  C
ATOM   1229  CD  LYS   150     -14.766  -0.553  19.523  1.00  0.00      LX0  C
ATOM   1230  CE  LYS   150     -16.278  -0.833  19.474  1.00  0.00      LX0  C
ATOM   1231  NZ  LYS   150     -16.663  -1.474  18.207  1.00  0.00      LX0  N
ATOM   1232  HZ1 LYS   150     -17.697  -1.542  18.106  0.00  0.00      LX0  H
```

ATOM	1233	HZ2	LYS	150	-16.286	-0.952	17.392	0.00	0.00	LX0	H
ATOM	1234	HZ3	LYS	150	-16.308	-2.451	18.130	0.00	0.00	LX0	H
ATOM	1235	C	LYS	150	-10.307	-3.135	19.182	1.00	0.00	LX0	C
ATOM	1236	O	LYS	150	-9.560	-3.320	20.133	1.00	0.00	LX0	O
ATOM	1237	N	GLY	151	-9.900	-2.918	17.925	1.00	0.00	LX0	N
ATOM	1238	H	GLY	151	-10.568	-2.972	17.182	0.00	0.00	LX0	H
ATOM	1239	CA	GLY	151	-8.463	-2.940	17.668	1.00	0.00	LX0	C
ATOM	1240	C	GLY	151	-7.997	-4.309	17.219	1.00	0.00	LX0	C
ATOM	1241	O	GLY	151	-8.504	-5.337	17.651	1.00	0.00	LX0	O
ATOM	1242	N	GLY	152	-7.025	-4.280	16.301	1.00	0.00	LX0	N
ATOM	1243	H	GLY	152	-6.653	-3.405	15.982	0.00	0.00	LX0	H
ATOM	1244	CA	GLY	152	-6.680	-5.559	15.685	1.00	0.00	LX0	C
ATOM	1245	C	GLY	152	-5.574	-6.342	16.360	1.00	0.00	LX0	C
ATOM	1246	O	GLY	152	-5.078	-6.010	17.433	1.00	0.00	LX0	O
ATOM	1247	N	VAL	153	-5.197	-7.409	15.652	1.00	0.00	LX0	N
ATOM	1248	H	VAL	153	-5.592	-7.539	14.741	0.00	0.00	LX0	H
ATOM	1249	CA	VAL	153	-4.064	-8.219	16.079	1.00	0.00	LX0	C
ATOM	1250	CB	VAL	153	-4.337	-9.673	15.659	1.00	0.00	LX0	C
ATOM	1251	CG1	VAL	153	-3.116	-10.593	15.665	1.00	0.00	LX0	C
ATOM	1252	CG2	VAL	153	-5.449	-10.233	16.546	1.00	0.00	LX0	C
ATOM	1253	C	VAL	153	-2.774	-7.664	15.500	1.00	0.00	LX0	C
ATOM	1254	O	VAL	153	-2.697	-7.306	14.331	1.00	0.00	LX0	O
ATOM	1255	N	LEU	154	-1.781	-7.592	16.391	1.00	0.00	LX0	N
ATOM	1256	H	LEU	154	-1.919	-7.905	17.331	0.00	0.00	LX0	H
ATOM	1257	CA	LEU	154	-0.482	-7.030	16.038	1.00	0.00	LX0	C
ATOM	1258	CB	LEU	154	-0.365	-5.622	16.605	1.00	0.00	LX0	C
ATOM	1259	CG	LEU	154	0.901	-4.832	16.283	1.00	0.00	LX0	C
ATOM	1260	CD1	LEU	154	1.137	-4.643	14.788	1.00	0.00	LX0	C
ATOM	1261	CD2	LEU	154	0.917	-3.521	17.060	1.00	0.00	LX0	C
ATOM	1262	C	LEU	154	0.612	-7.900	16.602	1.00	0.00	LX0	C
ATOM	1263	O	LEU	154	1.350	-7.544	17.512	1.00	0.00	LX0	O
ATOM	1264	N	ILE	155	0.675	-9.099	16.040	1.00	0.00	LX0	N
ATOM	1265	H	ILE	155	0.146	-9.317	15.219	0.00	0.00	LX0	H
ATOM	1266	CA	ILE	155	1.744	-9.936	16.552	1.00	0.00	LX0	C
ATOM	1267	CB	ILE	155	1.233	-11.344	16.862	1.00	0.00	LX0	C
ATOM	1268	CG2	ILE	155	2.263	-12.100	17.691	1.00	0.00	LX0	C
ATOM	1269	CG1	ILE	155	-0.089	-11.295	17.630	1.00	0.00	LX0	C
ATOM	1270	CD1	ILE	155	-0.668	-12.684	17.888	1.00	0.00	LX0	C
ATOM	1271	C	ILE	155	2.937	-9.913	15.616	1.00	0.00	LX0	C
ATOM	1272	O	ILE	155	3.291	-10.895	14.971	1.00	0.00	LX0	O
ATOM	1273	N	GLN	156	3.521	-8.713	15.539	1.00	0.00	LX0	N
ATOM	1274	H	GLN	156	3.287	-8.040	16.243	0.00	0.00	LX0	H
ATOM	1275	CA	GLN	156	4.536	-8.456	14.521	1.00	0.00	LX0	C
ATOM	1276	CB	GLN	156	4.211	-7.123	13.831	1.00	0.00	LX0	C
ATOM	1277	CG	GLN	156	4.198	-7.142	12.295	1.00	0.00	LX0	C
ATOM	1278	CD	GLN	156	5.553	-6.759	11.736	1.00	0.00	LX0	C
ATOM	1279	OE1	GLN	156	5.998	-5.624	11.856	1.00	0.00	LX0	O
ATOM	1280	NE2	GLN	156	6.207	-7.744	11.118	1.00	0.00	LX0	N
ATOM	1281	HE21	GLN	156	5.868	-8.684	11.007	0.00	0.00	LX0	H
ATOM	1282	HE22	GLN	156	7.110	-7.569	10.731	0.00	0.00	LX0	H
ATOM	1283	C	GLN	156	5.967	-8.527	15.037	1.00	0.00	LX0	C
ATOM	1284	O	GLN	156	6.262	-8.146	16.165	1.00	0.00	LX0	O
ATOM	1285	N	ARG	157	6.839	-9.031	14.143	1.00	0.00	LX0	N
ATOM	1286	H	ARG	157	6.479	-9.469	13.317	0.00	0.00	LX0	H
ATOM	1287	CA	ARG	157	8.281	-9.155	14.390	1.00	0.00	LX0	C
ATOM	1288	CB	ARG	157	8.929	-7.775	14.589	1.00	0.00	LX0	C
ATOM	1289	CG	ARG	157	8.589	-6.860	13.408	1.00	0.00	LX0	C
ATOM	1290	CD	ARG	157	8.797	-5.363	13.641	1.00	0.00	LX0	C
ATOM	1291	NE	ARG	157	8.185	-4.934	14.896	1.00	0.00	LX0	N
ATOM	1292	HE	ARG	157	8.705	-5.136	15.735	0.00	0.00	LX0	H
ATOM	1293	CZ	ARG	157	7.025	-4.252	15.000	1.00	0.00	LX0	C

ATOM	1294	NH1	ARG	157	6.262	-3.969	13.954	1.00	0.00	LX0	N
ATOM	1295	HH11	ARG	157	5.476	-3.347	14.110	0.00	0.00	LX0	H
ATOM	1296	HH12	ARG	157	6.419	-4.352	13.039	0.00	0.00	LX0	H
ATOM	1297	NH2	ARG	157	6.631	-3.837	16.191	1.00	0.00	LX0	N
ATOM	1298	HH21	ARG	157	5.785	-3.281	16.248	0.00	0.00	LX0	H
ATOM	1299	HH22	ARG	157	7.161	-4.068	17.017	0.00	0.00	LX0	H
ATOM	1300	C	ARG	157	8.623	-10.139	15.495	1.00	0.00	LX0	C
ATOM	1301	O	ARG	157	9.182	-9.809	16.534	1.00	0.00	LX0	O
ATOM	1302	N	ASN	158	8.230	-11.394	15.238	1.00	0.00	LX0	N
ATOM	1303	H	ASN	158	7.802	-11.628	14.358	0.00	0.00	LX0	H
ATOM	1304	CA	ASN	158	8.333	-12.330	16.360	1.00	0.00	LX0	C
ATOM	1305	CB	ASN	158	6.951	-12.661	16.903	1.00	0.00	LX0	C
ATOM	1306	CG	ASN	158	6.424	-11.485	17.682	1.00	0.00	LX0	C
ATOM	1307	OD1	ASN	158	6.908	-11.121	18.745	1.00	0.00	LX0	O
ATOM	1308	ND2	ASN	158	5.400	-10.893	17.085	1.00	0.00	LX0	N
ATOM	1309	HD21	ASN	158	4.994	-11.240	16.237	0.00	0.00	LX0	H
ATOM	1310	HD22	ASN	158	5.031	-10.056	17.495	0.00	0.00	LX0	H
ATOM	1311	C	ASN	158	9.095	-13.616	16.114	1.00	0.00	LX0	C
ATOM	1312	O	ASN	158	8.526	-14.634	15.736	1.00	0.00	LX0	O
ATOM	1313	N	PRO	159	10.419	-13.547	16.400	1.00	0.00	LX0	N
ATOM	1314	CD	PRO	159	11.123	-12.413	16.993	1.00	0.00	LX0	C
ATOM	1315	CA	PRO	159	11.307	-14.667	16.067	1.00	0.00	LX0	C
ATOM	1316	CB	PRO	159	12.696	-14.073	16.331	1.00	0.00	LX0	C
ATOM	1317	CG	PRO	159	12.495	-12.959	17.358	1.00	0.00	LX0	C
ATOM	1318	C	PRO	159	11.010	-15.979	16.785	1.00	0.00	LX0	C
ATOM	1319	O	PRO	159	10.621	-16.977	16.189	1.00	0.00	LX0	O
ATOM	1320	N	GLN	160	11.193	-15.963	18.113	1.00	0.00	LX0	N
ATOM	1321	H	GLN	160	11.437	-15.124	18.598	0.00	0.00	LX0	H
ATOM	1322	CA	GLN	160	11.016	-17.228	18.827	1.00	0.00	LX0	C
ATOM	1323	CB	GLN	160	11.764	-17.130	20.160	1.00	0.00	LX0	C
ATOM	1324	CG	GLN	160	12.122	-18.464	20.816	1.00	0.00	LX0	C
ATOM	1325	CD	GLN	160	12.661	-18.191	22.203	1.00	0.00	LX0	C
ATOM	1326	OE1	GLN	160	13.787	-17.773	22.419	1.00	0.00	LX0	O
ATOM	1327	NE2	GLN	160	11.768	-18.401	23.159	1.00	0.00	LX0	N
ATOM	1328	HE21	GLN	160	10.886	-18.807	22.952	0.00	0.00	LX0	H
ATOM	1329	HE22	GLN	160	11.960	-18.069	24.085	0.00	0.00	LX0	H
ATOM	1330	C	GLN	160	9.557	-17.663	19.006	1.00	0.00	LX0	C
ATOM	1331	O	GLN	160	9.238	-18.687	19.606	1.00	0.00	LX0	O
ATOM	1332	N	LEU	161	8.670	-16.813	18.471	1.00	0.00	LX0	N
ATOM	1333	H	LEU	161	8.933	-16.162	17.763	0.00	0.00	LX0	H
ATOM	1334	CA	LEU	161	7.268	-16.960	18.814	1.00	0.00	LX0	C
ATOM	1335	CB	LEU	161	6.659	-15.572	18.966	1.00	0.00	LX0	C
ATOM	1336	CG	LEU	161	5.268	-15.553	19.588	1.00	0.00	LX0	C
ATOM	1337	CD1	LEU	161	5.324	-15.855	21.074	1.00	0.00	LX0	C
ATOM	1338	CD2	LEU	161	4.565	-14.228	19.351	1.00	0.00	LX0	C
ATOM	1339	C	LEU	161	6.505	-17.768	17.793	1.00	0.00	LX0	C
ATOM	1340	O	LEU	161	6.465	-17.457	16.608	1.00	0.00	LX0	O
ATOM	1341	N	CYS	162	5.880	-18.820	18.316	1.00	0.00	LX0	N
ATOM	1342	H	CYS	162	5.943	-18.995	19.300	0.00	0.00	LX0	H
ATOM	1343	CA	CYS	162	4.928	-19.495	17.448	1.00	0.00	LX0	C
ATOM	1344	CB	CYS	162	5.196	-20.992	17.435	1.00	0.00	LX0	C
ATOM	1345	SG	CYS	162	6.709	-21.383	16.532	1.00	0.00	LX0	S
ATOM	1346	C	CYS	162	3.499	-19.165	17.825	1.00	0.00	LX0	C
ATOM	1347	O	CYS	162	3.256	-18.362	18.717	1.00	0.00	LX0	O
ATOM	1348	N	TYR	163	2.572	-19.825	17.102	1.00	0.00	LX0	N
ATOM	1349	H	TYR	163	2.838	-20.392	16.322	0.00	0.00	LX0	H
ATOM	1350	CA	TYR	163	1.142	-19.809	17.435	1.00	0.00	LX0	C
ATOM	1351	CB	TYR	163	0.881	-20.279	18.878	1.00	0.00	LX0	C
ATOM	1352	CG	TYR	163	1.523	-21.639	19.060	1.00	0.00	LX0	C
ATOM	1353	CD1	TYR	163	0.985	-22.750	18.378	1.00	0.00	LX0	C
ATOM	1354	CE1	TYR	163	1.628	-23.992	18.470	1.00	0.00	LX0	C

ATOM	1355	CD2	TYR	163	2.663	-21.755	19.878	1.00	0.00	LX0	C
ATOM	1356	CE2	TYR	163	3.312	-22.996	19.966	1.00	0.00	LX0	C
ATOM	1357	CZ	TYR	163	2.794	-24.095	19.249	1.00	0.00	LX0	C
ATOM	1358	OH	TYR	163	3.449	-25.307	19.296	1.00	0.00	LX0	O
ATOM	1359	HH	TYR	163	4.077	-25.286	20.017	0.00	0.00	LX0	H
ATOM	1360	C	TYR	163	0.323	-18.591	17.037	1.00	0.00	LX0	C
ATOM	1361	O	TYR	163	-0.896	-18.556	17.142	1.00	0.00	LX0	O
ATOM	1362	N	GLN	164	1.051	-17.610	16.471	1.00	0.00	LX0	N
ATOM	1363	H	GLN	164	2.035	-17.620	16.638	0.00	0.00	LX0	H
ATOM	1364	CA	GLN	164	0.393	-16.495	15.776	1.00	0.00	LX0	C
ATOM	1365	CB	GLN	164	1.396	-15.606	15.026	1.00	0.00	LX0	C
ATOM	1366	CG	GLN	164	2.583	-15.041	15.809	1.00	0.00	LX0	C
ATOM	1367	CD	GLN	164	3.802	-15.925	15.663	1.00	0.00	LX0	C
ATOM	1368	OE1	GLN	164	3.749	-17.129	15.872	1.00	0.00	LX0	O
ATOM	1369	NE2	GLN	164	4.921	-15.281	15.336	1.00	0.00	LX0	N
ATOM	1370	HE21	GLN	164	4.847	-14.362	14.940	0.00	0.00	LX0	H
ATOM	1371	HE22	GLN	164	5.821	-15.707	15.462	0.00	0.00	LX0	H
ATOM	1372	C	GLN	164	-0.668	-16.906	14.759	1.00	0.00	LX0	C
ATOM	1373	O	GLN	164	-1.673	-16.242	14.558	1.00	0.00	LX0	O
ATOM	1374	N	ASP	165	-0.339	-18.023	14.106	1.00	0.00	LX0	N
ATOM	1375	H	ASP	165	0.448	-18.557	14.407	0.00	0.00	LX0	H
ATOM	1376	CA	ASP	165	-1.145	-18.599	13.032	1.00	0.00	LX0	C
ATOM	1377	CB	ASP	165	-0.242	-19.289	11.981	1.00	0.00	LX0	C
ATOM	1378	CG	ASP	165	1.002	-19.981	12.553	1.00	0.00	LX0	C
ATOM	1379	OD1	ASP	165	1.035	-20.371	13.720	1.00	0.00	LX0	O
ATOM	1380	OD2	ASP	165	2.009	-20.056	11.857	1.00	0.00	LX0	O
ATOM	1381	C	ASP	165	-2.230	-19.546	13.513	1.00	0.00	LX0	C
ATOM	1382	O	ASP	165	-3.397	-19.436	13.155	1.00	0.00	LX0	O
ATOM	1383	N	THR	166	-1.793	-20.488	14.359	1.00	0.00	LX0	N
ATOM	1384	H	THR	166	-0.821	-20.528	14.596	0.00	0.00	LX0	H
ATOM	1385	CA	THR	166	-2.692	-21.524	14.868	1.00	0.00	LX0	C
ATOM	1386	CB	THR	166	-1.895	-22.408	15.816	1.00	0.00	LX0	C
ATOM	1387	OG1	THR	166	-1.163	-21.578	16.721	1.00	0.00	LX0	O
ATOM	1388	HG1	THR	166	-1.437	-21.803	17.610	0.00	0.00	LX0	H
ATOM	1389	CG2	THR	166	-0.930	-23.325	15.061	1.00	0.00	LX0	C
ATOM	1390	C	THR	166	-3.933	-20.983	15.559	1.00	0.00	LX0	C
ATOM	1391	O	THR	166	-5.050	-21.477	15.426	1.00	0.00	LX0	O
ATOM	1392	N	ILE	167	-3.687	-19.905	16.313	1.00	0.00	LX0	N
ATOM	1393	H	ILE	167	-2.758	-19.533	16.394	0.00	0.00	LX0	H
ATOM	1394	CA	ILE	167	-4.855	-19.234	16.863	1.00	0.00	LX0	C
ATOM	1395	CB	ILE	167	-4.491	-18.443	18.120	1.00	0.00	LX0	C
ATOM	1396	CG2	ILE	167	-5.727	-17.785	18.736	1.00	0.00	LX0	C
ATOM	1397	CG1	ILE	167	-3.808	-19.359	19.135	1.00	0.00	LX0	C
ATOM	1398	CD1	ILE	167	-4.745	-20.462	19.630	1.00	0.00	LX0	C
ATOM	1399	C	ILE	167	-5.556	-18.377	15.829	1.00	0.00	LX0	C
ATOM	1400	O	ILE	167	-5.259	-17.212	15.609	1.00	0.00	LX0	O
ATOM	1401	N	LEU	168	-6.542	-19.024	15.198	1.00	0.00	LX0	N
ATOM	1402	H	LEU	168	-6.598	-20.013	15.346	0.00	0.00	LX0	H
ATOM	1403	CA	LEU	168	-7.302	-18.305	14.182	1.00	0.00	LX0	C
ATOM	1404	CB	LEU	168	-8.184	-19.293	13.414	1.00	0.00	LX0	C
ATOM	1405	CG	LEU	168	-8.796	-18.737	12.127	1.00	0.00	LX0	C
ATOM	1406	CD1	LEU	168	-7.730	-18.281	11.129	1.00	0.00	LX0	C
ATOM	1407	CD2	LEU	168	-9.784	-19.726	11.507	1.00	0.00	LX0	C
ATOM	1408	C	LEU	168	-8.085	-17.101	14.704	1.00	0.00	LX0	C
ATOM	1409	O	LEU	168	-9.225	-17.187	15.154	1.00	0.00	LX0	O
ATOM	1410	N	TRP	169	-7.407	-15.948	14.576	1.00	0.00	LX0	N
ATOM	1411	H	TRP	169	-6.427	-16.058	14.389	0.00	0.00	LX0	H
ATOM	1412	CA	TRP	169	-7.945	-14.671	15.057	1.00	0.00	LX0	C
ATOM	1413	CB	TRP	169	-6.936	-13.540	14.847	1.00	0.00	LX0	C
ATOM	1414	CG	TRP	169	-5.650	-13.903	15.547	1.00	0.00	LX0	C
ATOM	1415	CD2	TRP	169	-5.407	-14.022	16.964	1.00	0.00	LX0	C

ATOM	1416	CE2	TRP	169	-4.047	-14.449	17.125	1.00	0.00	LX0	C
ATOM	1417	CE3	TRP	169	-6.211	-13.787	18.100	1.00	0.00	LX0	C
ATOM	1418	CD1	TRP	169	-4.446	-14.272	14.933	1.00	0.00	LX0	C
ATOM	1419	NE1	TRP	169	-3.505	-14.601	15.853	1.00	0.00	LX0	N
ATOM	1420	HE1	TRP	169	-2.615	-14.956	15.632	0.00	0.00	LX0	H
ATOM	1421	CZ2	TRP	169	-3.531	-14.674	18.418	1.00	0.00	LX0	C
ATOM	1422	CZ3	TRP	169	-5.681	-14.007	19.388	1.00	0.00	LX0	C
ATOM	1423	CH2	TRP	169	-4.347	-14.443	19.545	1.00	0.00	LX0	C
ATOM	1424	C	TRP	169	-9.304	-14.278	14.515	1.00	0.00	LX0	C
ATOM	1425	O	TRP	169	-10.076	-13.578	15.154	1.00	0.00	LX0	O
ATOM	1426	N	LYS	170	-9.601	-14.839	13.330	1.00	0.00	LX0	N
ATOM	1427	H	LYS	170	-8.861	-15.324	12.867	0.00	0.00	LX0	H
ATOM	1428	CA	LYS	170	-10.940	-14.772	12.731	1.00	0.00	LX0	C
ATOM	1429	CB	LYS	170	-10.968	-15.727	11.531	1.00	0.00	LX0	C
ATOM	1430	CG	LYS	170	-12.185	-15.660	10.598	1.00	0.00	LX0	C
ATOM	1431	CD	LYS	170	-12.739	-17.053	10.265	1.00	0.00	LX0	C
ATOM	1432	CE	LYS	170	-14.001	-17.472	11.041	1.00	0.00	LX0	C
ATOM	1433	NZ	LYS	170	-13.818	-17.357	12.493	1.00	0.00	LX0	N
ATOM	1434	HZ1	LYS	170	-14.480	-17.947	13.042	0.00	0.00	LX0	H
ATOM	1435	HZ2	LYS	170	-12.891	-17.721	12.809	0.00	0.00	LX0	H
ATOM	1436	HZ3	LYS	170	-13.912	-16.371	12.817	0.00	0.00	LX0	H
ATOM	1437	C	LYS	170	-12.119	-15.088	13.659	1.00	0.00	LX0	C
ATOM	1438	O	LYS	170	-13.281	-14.863	13.336	1.00	0.00	LX0	O
ATOM	1439	N	ASP	171	-11.803	-15.693	14.801	1.00	0.00	LX0	N
ATOM	1440	H	ASP	171	-10.875	-16.021	14.982	0.00	0.00	LX0	H
ATOM	1441	CA	ASP	171	-12.892	-15.994	15.722	1.00	0.00	LX0	C
ATOM	1442	CB	ASP	171	-12.823	-17.475	16.124	1.00	0.00	LX0	C
ATOM	1443	CG	ASP	171	-12.846	-18.345	14.872	1.00	0.00	LX0	C
ATOM	1444	OD1	ASP	171	-13.868	-18.925	14.540	1.00	0.00	LX0	O
ATOM	1445	OD2	ASP	171	-11.860	-18.412	14.147	1.00	0.00	LX0	O
ATOM	1446	C	ASP	171	-12.917	-15.050	16.909	1.00	0.00	LX0	C
ATOM	1447	O	ASP	171	-13.969	-14.658	17.406	1.00	0.00	LX0	O
ATOM	1448	N	ILE	172	-11.686	-14.684	17.305	1.00	0.00	LX0	N
ATOM	1449	H	ILE	172	-10.897	-14.931	16.746	0.00	0.00	LX0	H
ATOM	1450	CA	ILE	172	-11.481	-13.832	18.477	1.00	0.00	LX0	C
ATOM	1451	CB	ILE	172	-10.122	-14.178	19.113	1.00	0.00	LX0	C
ATOM	1452	CG2	ILE	172	-9.870	-13.458	20.440	1.00	0.00	LX0	C
ATOM	1453	CG1	ILE	172	-10.005	-15.690	19.313	1.00	0.00	LX0	C
ATOM	1454	CD1	ILE	172	-8.692	-16.084	19.987	1.00	0.00	LX0	C
ATOM	1455	C	ILE	172	-11.616	-12.338	18.186	1.00	0.00	LX0	C
ATOM	1456	O	ILE	172	-11.702	-11.491	19.065	1.00	0.00	LX0	O
ATOM	1457	N	PHE	173	-11.660	-12.016	16.885	1.00	0.00	LX0	N
ATOM	1458	H	PHE	173	-11.494	-12.692	16.170	0.00	0.00	LX0	H
ATOM	1459	CA	PHE	173	-12.087	-10.650	16.593	1.00	0.00	LX0	C
ATOM	1460	CB	PHE	173	-11.941	-10.313	15.111	1.00	0.00	LX0	C
ATOM	1461	CG	PHE	173	-10.505	-10.009	14.760	1.00	0.00	LX0	C
ATOM	1462	CD1	PHE	173	-9.818	-8.978	15.439	1.00	0.00	LX0	C
ATOM	1463	CD2	PHE	173	-9.883	-10.752	13.735	1.00	0.00	LX0	C
ATOM	1464	CE1	PHE	173	-8.493	-8.676	15.075	1.00	0.00	LX0	C
ATOM	1465	CE2	PHE	173	-8.558	-10.451	13.372	1.00	0.00	LX0	C
ATOM	1466	CZ	PHE	173	-7.880	-9.410	14.039	1.00	0.00	LX0	C
ATOM	1467	C	PHE	173	-13.523	-10.436	17.006	1.00	0.00	LX0	C
ATOM	1468	O	PHE	173	-14.418	-11.174	16.596	1.00	0.00	LX0	O
ATOM	1469	N	HIS	174	-13.699	-9.401	17.841	1.00	0.00	LX0	N
ATOM	1470	H	HIS	174	-12.921	-8.881	18.192	0.00	0.00	LX0	H
ATOM	1471	CA	HIS	174	-15.055	-9.147	18.312	1.00	0.00	LX0	C
ATOM	1472	CB	HIS	174	-15.063	-8.002	19.319	1.00	0.00	LX0	C
ATOM	1473	CG	HIS	174	-16.424	-7.729	19.911	1.00	0.00	LX0	C
ATOM	1474	ND1	HIS	174	-17.225	-8.669	20.443	1.00	0.00	LX0	N
ATOM	1475	HD1	HIS	174	-17.021	-9.629	20.548	0.00	0.00	LX0	H
ATOM	1476	CD2	HIS	174	-17.068	-6.493	19.990	1.00	0.00	LX0	C

ATOM	1477	NE2	HIS	174	-18.272	-6.706	20.570	1.00	0.00	LX0	N
ATOM	1478	CE1	HIS	174	-18.372	-8.045	20.853	1.00	0.00	LX0	C
ATOM	1479	C	HIS	174	-16.029	-8.912	17.188	1.00	0.00	LX0	C
ATOM	1480	O	HIS	174	-15.714	-8.360	16.142	1.00	0.00	LX0	O
ATOM	1481	N	LYS	175	-17.252	-9.365	17.453	1.00	0.00	LX0	N
ATOM	1482	H	LYS	175	-17.414	-9.804	18.341	0.00	0.00	LX0	H
ATOM	1483	CA	LYS	175	-18.237	-9.275	16.380	1.00	0.00	LX0	C
ATOM	1484	CB	LYS	175	-19.380	-10.259	16.668	1.00	0.00	LX0	C
ATOM	1485	CG	LYS	175	-18.863	-11.620	17.181	1.00	0.00	LX0	C
ATOM	1486	CD	LYS	175	-17.919	-12.368	16.224	1.00	0.00	LX0	C
ATOM	1487	CE	LYS	175	-17.128	-13.520	16.871	1.00	0.00	LX0	C
ATOM	1488	NZ	LYS	175	-16.014	-13.020	17.687	1.00	0.00	LX0	N
ATOM	1489	HZ1	LYS	175	-16.302	-12.557	18.578	0.00	0.00	LX0	H
ATOM	1490	HZ2	LYS	175	-15.336	-13.761	17.966	0.00	0.00	LX0	H
ATOM	1491	HZ3	LYS	175	-15.448	-12.309	17.189	0.00	0.00	LX0	H
ATOM	1492	C	LYS	175	-18.681	-7.847	16.040	1.00	0.00	LX0	C
ATOM	1493	O	LYS	175	-19.304	-7.573	15.025	1.00	0.00	LX0	O
ATOM	1494	N	ASN	176	-18.271	-6.924	16.928	1.00	0.00	LX0	N
ATOM	1495	H	ASN	176	-17.887	-7.231	17.795	0.00	0.00	LX0	H
ATOM	1496	CA	ASN	176	-18.310	-5.502	16.578	1.00	0.00	LX0	C
ATOM	1497	CB	ASN	176	-19.293	-4.758	17.497	1.00	0.00	LX0	C
ATOM	1498	CG	ASN	176	-19.554	-3.343	17.004	1.00	0.00	LX0	C
ATOM	1499	OD1	ASN	176	-19.023	-2.358	17.510	1.00	0.00	LX0	O
ATOM	1500	ND2	ASN	176	-20.414	-3.280	15.983	1.00	0.00	LX0	N
ATOM	1501	HD21	ASN	176	-20.820	-4.107	15.595	0.00	0.00	LX0	H
ATOM	1502	HD22	ASN	176	-20.665	-2.391	15.604	0.00	0.00	LX0	H
ATOM	1503	C	ASN	176	-16.923	-4.855	16.584	1.00	0.00	LX0	C
ATOM	1504	O	ASN	176	-16.712	-3.741	17.059	1.00	0.00	LX0	O
ATOM	1505	N	ASN	177	-15.955	-5.602	16.045	1.00	0.00	LX0	N
ATOM	1506	H	ASN	177	-16.118	-6.507	15.646	0.00	0.00	LX0	H
ATOM	1507	CA	ASN	177	-14.636	-4.977	15.965	1.00	0.00	LX0	C
ATOM	1508	CB	ASN	177	-13.504	-5.951	16.303	1.00	0.00	LX0	C
ATOM	1509	CG	ASN	177	-12.224	-5.182	16.583	1.00	0.00	LX0	C
ATOM	1510	OD1	ASN	177	-12.044	-4.026	16.205	1.00	0.00	LX0	O
ATOM	1511	ND2	ASN	177	-11.319	-5.892	17.258	1.00	0.00	LX0	N
ATOM	1512	HD21	ASN	177	-11.575	-6.740	17.724	0.00	0.00	LX0	H
ATOM	1513	HD22	ASN	177	-10.368	-5.596	17.358	0.00	0.00	LX0	H
ATOM	1514	C	ASN	177	-14.389	-4.340	14.618	1.00	0.00	LX0	C
ATOM	1515	O	ASN	177	-13.951	-4.969	13.666	1.00	0.00	LX0	O
ATOM	1516	N	GLN	178	-14.681	-3.033	14.584	1.00	0.00	LX0	N
ATOM	1517	H	GLN	178	-15.041	-2.589	15.404	0.00	0.00	LX0	H
ATOM	1518	CA	GLN	178	-14.436	-2.315	13.332	1.00	0.00	LX0	C
ATOM	1519	CB	GLN	178	-15.052	-0.904	13.375	1.00	0.00	LX0	C
ATOM	1520	CG	GLN	178	-14.309	0.191	14.160	1.00	0.00	LX0	C
ATOM	1521	CD	GLN	178	-14.197	-0.154	15.633	1.00	0.00	LX0	C
ATOM	1522	OE1	GLN	178	-15.096	-0.714	16.252	1.00	0.00	LX0	O
ATOM	1523	NE2	GLN	178	-13.027	0.190	16.174	1.00	0.00	LX0	N
ATOM	1524	HE21	GLN	178	-12.333	0.656	15.624	0.00	0.00	LX0	H
ATOM	1525	HE22	GLN	178	-12.820	-0.002	17.132	0.00	0.00	LX0	H
ATOM	1526	C	GLN	178	-12.981	-2.293	12.875	1.00	0.00	LX0	C
ATOM	1527	O	GLN	178	-12.666	-2.199	11.699	1.00	0.00	LX0	O
ATOM	1528	N	LEU	179	-12.095	-2.394	13.876	1.00	0.00	LX0	N
ATOM	1529	H	LEU	179	-12.380	-2.607	14.810	0.00	0.00	LX0	H
ATOM	1530	CA	LEU	179	-10.691	-2.512	13.510	1.00	0.00	LX0	C
ATOM	1531	CB	LEU	179	-9.822	-1.570	14.345	1.00	0.00	LX0	C
ATOM	1532	CG	LEU	179	-10.104	-0.085	14.121	1.00	0.00	LX0	C
ATOM	1533	CD1	LEU	179	-9.312	0.780	15.101	1.00	0.00	LX0	C
ATOM	1534	CD2	LEU	179	-9.876	0.339	12.668	1.00	0.00	LX0	C
ATOM	1535	C	LEU	179	-10.214	-3.938	13.665	1.00	0.00	LX0	C
ATOM	1536	O	LEU	179	-9.296	-4.232	14.418	1.00	0.00	LX0	O
ATOM	1537	N	ALA	180	-10.874	-4.826	12.912	1.00	0.00	LX0	N

ATOM	1538	H	ALA	180	-11.619	-4.523	12.317	0.00	0.00	LX0	H
ATOM	1539	CA	ALA	180	-10.442	-6.224	12.944	1.00	0.00	LX0	C
ATOM	1540	CB	ALA	180	-11.602	-7.141	12.552	1.00	0.00	LX0	C
ATOM	1541	C	ALA	180	-9.244	-6.503	12.044	1.00	0.00	LX0	C
ATOM	1542	O	ALA	180	-9.267	-7.339	11.151	1.00	0.00	LX0	O
ATOM	1543	N	LEU	181	-8.191	-5.718	12.298	1.00	0.00	LX0	N
ATOM	1544	H	LEU	181	-8.168	-5.186	13.145	0.00	0.00	LX0	H
ATOM	1545	CA	LEU	181	-7.066	-5.753	11.372	1.00	0.00	LX0	C
ATOM	1546	CB	LEU	181	-6.435	-4.365	11.273	1.00	0.00	LX0	C
ATOM	1547	CG	LEU	181	-7.437	-3.264	10.906	1.00	0.00	LX0	C
ATOM	1548	CD1	LEU	181	-6.808	-1.876	11.004	1.00	0.00	LX0	C
ATOM	1549	CD2	LEU	181	-8.093	-3.499	9.543	1.00	0.00	LX0	C
ATOM	1550	C	LEU	181	-6.053	-6.816	11.740	1.00	0.00	LX0	C
ATOM	1551	O	LEU	181	-5.922	-7.213	12.893	1.00	0.00	LX0	O
ATOM	1552	N	THR	182	-5.364	-7.283	10.700	1.00	0.00	LX0	N
ATOM	1553	H	THR	182	-5.506	-6.944	9.771	0.00	0.00	LX0	H
ATOM	1554	CA	THR	182	-4.695	-8.568	10.848	1.00	0.00	LX0	C
ATOM	1555	CB	THR	182	-5.351	-9.532	9.859	1.00	0.00	LX0	C
ATOM	1556	OG1	THR	182	-5.894	-8.791	8.752	1.00	0.00	LX0	O
ATOM	1557	HG1	THR	182	-6.232	-9.434	8.141	0.00	0.00	LX0	H
ATOM	1558	CG2	THR	182	-6.448	-10.367	10.519	1.00	0.00	LX0	C
ATOM	1559	C	THR	182	-3.188	-8.563	10.672	1.00	0.00	LX0	C
ATOM	1560	O	THR	182	-2.649	-9.223	9.791	1.00	0.00	LX0	O
ATOM	1561	N	LEU	183	-2.498	-7.824	11.554	1.00	0.00	LX0	N
ATOM	1562	H	LEU	183	-2.936	-7.362	12.328	0.00	0.00	LX0	H
ATOM	1563	CA	LEU	183	-1.046	-7.988	11.484	1.00	0.00	LX0	C
ATOM	1564	CB	LEU	183	-0.295	-6.716	11.882	1.00	0.00	LX0	C
ATOM	1565	CG	LEU	183	0.070	-5.870	10.657	1.00	0.00	LX0	C
ATOM	1566	CD1	LEU	183	0.815	-4.591	11.039	1.00	0.00	LX0	C
ATOM	1567	CD2	LEU	183	0.858	-6.675	9.620	1.00	0.00	LX0	C
ATOM	1568	C	LEU	183	-0.528	-9.206	12.225	1.00	0.00	LX0	C
ATOM	1569	O	LEU	183	0.039	-9.151	13.311	1.00	0.00	LX0	O
ATOM	1570	N	ILE	184	-0.769	-10.336	11.558	1.00	0.00	LX0	N
ATOM	1571	H	ILE	184	-1.181	-10.272	10.647	0.00	0.00	LX0	H
ATOM	1572	CA	ILE	184	-0.245	-11.604	12.048	1.00	0.00	LX0	C
ATOM	1573	CB	ILE	184	-1.217	-12.732	11.675	1.00	0.00	LX0	C
ATOM	1574	CG2	ILE	184	-0.754	-14.086	12.218	1.00	0.00	LX0	C
ATOM	1575	CG1	ILE	184	-2.651	-12.394	12.093	1.00	0.00	LX0	C
ATOM	1576	CD1	ILE	184	-3.682	-13.333	11.464	1.00	0.00	LX0	C
ATOM	1577	C	ILE	184	1.110	-11.842	11.414	1.00	0.00	LX0	C
ATOM	1578	O	ILE	184	1.212	-12.046	10.212	1.00	0.00	LX0	O
ATOM	1579	N	ASP	185	2.144	-11.779	12.254	1.00	0.00	LX0	N
ATOM	1580	H	ASP	185	2.064	-11.648	13.243	0.00	0.00	LX0	H
ATOM	1581	CA	ASP	185	3.458	-11.989	11.663	1.00	0.00	LX0	C
ATOM	1582	CB	ASP	185	4.344	-10.793	12.013	1.00	0.00	LX0	C
ATOM	1583	CG	ASP	185	5.830	-11.023	11.821	1.00	0.00	LX0	C
ATOM	1584	OD1	ASP	185	6.358	-10.668	10.776	1.00	0.00	LX0	O
ATOM	1585	OD2	ASP	185	6.468	-11.519	12.744	1.00	0.00	LX0	O
ATOM	1586	C	ASP	185	4.042	-13.321	12.067	1.00	0.00	LX0	C
ATOM	1587	O	ASP	185	3.821	-13.844	13.155	1.00	0.00	LX0	O
ATOM	1588	N	THR	186	4.788	-13.854	11.104	1.00	0.00	LX0	N
ATOM	1589	H	THR	186	4.882	-13.389	10.224	0.00	0.00	LX0	H
ATOM	1590	CA	THR	186	5.407	-15.151	11.311	1.00	0.00	LX0	C
ATOM	1591	CB	THR	186	4.673	-16.172	10.440	1.00	0.00	LX0	C
ATOM	1592	OG1	THR	186	4.302	-15.569	9.191	1.00	0.00	LX0	O
ATOM	1593	HG1	THR	186	3.801	-16.219	8.715	0.00	0.00	LX0	H
ATOM	1594	CG2	THR	186	3.422	-16.708	11.142	1.00	0.00	LX0	C
ATOM	1595	C	THR	186	6.900	-15.124	11.032	1.00	0.00	LX0	C
ATOM	1596	O	THR	186	7.482	-16.086	10.542	1.00	0.00	LX0	O
ATOM	1597	N	ASN	187	7.501	-13.969	11.374	1.00	0.00	LX0	N
ATOM	1598	H	ASN	187	6.960	-13.220	11.766	0.00	0.00	LX0	H

```
ATOM   1599  CA  ASN   187       8.951 -13.803  11.229  1.00  0.00      LX0  C
ATOM   1600  CB  ASN   187       9.338 -12.331  11.337  1.00  0.00      LX0  C
ATOM   1601  CG  ASN   187       9.850 -11.859  10.002  1.00  0.00      LX0  C
ATOM   1602  OD1 ASN   187      11.001 -12.056   9.642  1.00  0.00      LX0  O
ATOM   1603  ND2 ASN   187       8.930 -11.228   9.269  1.00  0.00      LX0  N
ATOM   1604 HD21 ASN   187       8.016 -11.061   9.652  0.00  0.00      LX0  H
ATOM   1605 HD22 ASN   187       9.162 -10.939   8.342  0.00  0.00      LX0  H
ATOM   1606  C   ASN   187       9.751 -14.597  12.239  1.00  0.00      LX0  C
ATOM   1607  O   ASN   187      10.260 -14.078  13.226  1.00  0.00      LX0  O
ATOM   1608  N   ARG   188       9.794 -15.901  11.964  1.00  0.00      LX0  N
ATOM   1609  H   ARG   188       9.433 -16.232  11.092  0.00  0.00      LX0  H
ATOM   1610  CA  ARG   188      10.239 -16.818  13.002  1.00  0.00      LX0  C
ATOM   1611  CB  ARG   188       9.266 -17.982  13.117  1.00  0.00      LX0  C
ATOM   1612  CG  ARG   188       7.866 -17.626  13.597  1.00  0.00      LX0  C
ATOM   1613  CD  ARG   188       6.991 -18.873  13.540  1.00  0.00      LX0  C
ATOM   1614  NE  ARG   188       5.662 -18.615  14.074  1.00  0.00      LX0  N
ATOM   1615  HE  ARG   188       5.596 -18.032  14.894  0.00  0.00      LX0  H
ATOM   1616  CZ  ARG   188       4.587 -19.208  13.525  1.00  0.00      LX0  C
ATOM   1617  NH1 ARG   188       4.679 -20.002  12.465  1.00  0.00      LX0  N
ATOM   1618 HH11 ARG   188       3.819 -20.360  12.071  0.00  0.00      LX0  H
ATOM   1619 HH12 ARG   188       5.556 -20.236  12.051  0.00  0.00      LX0  H
ATOM   1620  NH2 ARG   188       3.400 -18.990  14.047  1.00  0.00      LX0  N
ATOM   1621 HH21 ARG   188       2.594 -19.463  13.656  0.00  0.00      LX0  H
ATOM   1622 HH22 ARG   188       3.282 -18.356  14.811  0.00  0.00      LX0  H
ATOM   1623  C   ARG   188      11.620 -17.392  12.805  1.00  0.00      LX0  C
ATOM   1624  O   ARG   188      12.076 -17.665  11.704  1.00  0.00      LX0  O
ATOM   1625  N   SER   189      12.233 -17.633  13.965  1.00  0.00      LX0  N
ATOM   1626  H   SER   189      11.859 -17.287  14.823  0.00  0.00      LX0  H
ATOM   1627  CA  SER   189      13.373 -18.532  14.025  1.00  0.00      LX0  C
ATOM   1628  CB  SER   189      14.343 -18.004  15.082  1.00  0.00      LX0  C
ATOM   1629  OG  SER   189      13.609 -17.464  16.195  1.00  0.00      LX0  O
ATOM   1630  HG  SER   189      13.560 -18.161  16.848  0.00  0.00      LX0  H
ATOM   1631  C   SER   189      12.973 -19.978  14.298  1.00  0.00      LX0  C
ATOM   1632  O   SER   189      13.771 -20.900  14.174  1.00  0.00      LX0  O
ATOM   1633  N   ARG   190      11.698 -20.151  14.691  1.00  0.00      LX0  N
ATOM   1634  H   ARG   190      11.057 -19.390  14.792  0.00  0.00      LX0  H
ATOM   1635  CA  ARG   190      11.280 -21.521  14.965  1.00  0.00      LX0  C
ATOM   1636  CB  ARG   190      11.197 -21.784  16.471  1.00  0.00      LX0  C
ATOM   1637  CG  ARG   190      10.022 -21.138  17.204  1.00  0.00      LX0  C
ATOM   1638  CD  ARG   190       9.823 -21.843  18.542  1.00  0.00      LX0  C
ATOM   1639  NE  ARG   190       8.595 -21.439  19.223  1.00  0.00      LX0  N
ATOM   1640  HE  ARG   190       8.525 -20.491  19.556  0.00  0.00      LX0  H
ATOM   1641  CZ  ARG   190       7.703 -22.397  19.559  1.00  0.00      LX0  C
ATOM   1642  NH1 ARG   190       7.748 -23.597  18.989  1.00  0.00      LX0  N
ATOM   1643 HH11 ARG   190       7.236 -24.350  19.436  0.00  0.00      LX0  H
ATOM   1644 HH12 ARG   190       8.259 -23.783  18.144  0.00  0.00      LX0  H
ATOM   1645  NH2 ARG   190       6.777 -22.156  20.480  1.00  0.00      LX0  N
ATOM   1646 HH21 ARG   190       6.184 -22.912  20.794  0.00  0.00      LX0  H
ATOM   1647 HH22 ARG   190       6.659 -21.249  20.896  0.00  0.00      LX0  H
ATOM   1648  C   ARG   190       9.986 -21.936  14.294  1.00  0.00      LX0  C
ATOM   1649  O   ARG   190       9.186 -21.121  13.857  1.00  0.00      LX0  O
ATOM   1650  N   ALA   191       9.803 -23.257  14.265  1.00  0.00      LX0  N
ATOM   1651  H   ALA   191      10.523 -23.859  14.604  0.00  0.00      LX0  H
ATOM   1652  CA  ALA   191       8.490 -23.780  13.921  1.00  0.00      LX0  C
ATOM   1653  CB  ALA   191       8.631 -25.045  13.072  1.00  0.00      LX0  C
ATOM   1654  C   ALA   191       7.686 -24.106  15.167  1.00  0.00      LX0  C
ATOM   1655  O   ALA   191       8.204 -24.209  16.280  1.00  0.00      LX0  O
ATOM   1656  N   CYS   192       6.380 -24.270  14.917  1.00  0.00      LX0  N
ATOM   1657  H   CYS   192       6.048 -24.213  13.978  0.00  0.00      LX0  H
ATOM   1658  CA  CYS   192       5.502 -24.737  15.988  1.00  0.00      LX0  C
ATOM   1659  CB  CYS   192       4.041 -24.503  15.599  1.00  0.00      LX0  C
```

ATOM	1660	SG	CYS	192	3.742	-22.804	15.040	1.00	0.00	LX0	S
ATOM	1661	C	CYS	192	5.740	-26.203	16.285	1.00	0.00	LX0	C
ATOM	1662	O	CYS	192	6.375	-26.908	15.509	1.00	0.00	LX0	O
ATOM	1663	N	HIS	193	5.221	-26.629	17.436	1.00	0.00	LX0	N
ATOM	1664	H	HIS	193	4.641	-26.044	18.006	0.00	0.00	LX0	H
ATOM	1665	CA	HIS	193	5.238	-28.064	17.690	1.00	0.00	LX0	C
ATOM	1666	CB	HIS	193	5.503	-28.302	19.186	1.00	0.00	LX0	C
ATOM	1667	CG	HIS	193	6.986	-28.198	19.494	1.00	0.00	LX0	C
ATOM	1668	ND1	HIS	193	7.529	-28.641	20.642	1.00	0.00	LX0	N
ATOM	1669	HD1	HIS	193	7.044	-29.030	21.398	0.00	0.00	LX0	H
ATOM	1670	CD2	HIS	193	8.013	-27.663	18.705	1.00	0.00	LX0	C
ATOM	1671	NE2	HIS	193	9.179	-27.788	19.389	1.00	0.00	LX0	N
ATOM	1672	CE1	HIS	193	8.875	-28.393	20.584	1.00	0.00	LX0	C
ATOM	1673	C	HIS	193	3.921	-28.647	17.200	1.00	0.00	LX0	C
ATOM	1674	O	HIS	193	2.940	-27.925	17.077	1.00	0.00	LX0	O
ATOM	1675	N	PRO	194	3.920	-29.962	16.863	1.00	0.00	LX0	N
ATOM	1676	CD	PRO	194	5.049	-30.890	16.863	1.00	0.00	LX0	C
ATOM	1677	CA	PRO	194	2.680	-30.589	16.382	1.00	0.00	LX0	C
ATOM	1678	CB	PRO	194	3.059	-32.072	16.307	1.00	0.00	LX0	C
ATOM	1679	CG	PRO	194	4.562	-32.072	16.036	1.00	0.00	LX0	C
ATOM	1680	C	PRO	194	1.440	-30.327	17.228	1.00	0.00	LX0	C
ATOM	1681	O	PRO	194	1.343	-30.720	18.384	1.00	0.00	LX0	O
ATOM	1682	N	CYS	195	0.487	-29.646	16.574	1.00	0.00	LX0	N
ATOM	1683	H	CYS	195	0.684	-29.282	15.666	0.00	0.00	LX0	H
ATOM	1684	CA	CYS	195	-0.775	-29.328	17.242	1.00	0.00	LX0	C
ATOM	1685	CB	CYS	195	-1.688	-28.557	16.290	1.00	0.00	LX0	C
ATOM	1686	SG	CYS	195	-0.817	-27.246	15.393	1.00	0.00	LX0	S
ATOM	1687	C	CYS	195	-1.512	-30.547	17.767	1.00	0.00	LX0	C
ATOM	1688	O	CYS	195	-1.735	-31.521	17.057	1.00	0.00	LX0	O
ATOM	1689	N	SER	196	-1.882	-30.461	19.048	1.00	0.00	LX0	N
ATOM	1690	H	SER	196	-1.715	-29.637	19.588	0.00	0.00	LX0	H
ATOM	1691	CA	SER	196	-2.615	-31.597	19.595	1.00	0.00	LX0	C
ATOM	1692	CB	SER	196	-2.492	-31.547	21.121	1.00	0.00	LX0	C
ATOM	1693	OG	SER	196	-3.157	-30.387	21.629	1.00	0.00	LX0	O
ATOM	1694	HG	SER	196	-2.478	-29.740	21.834	0.00	0.00	LX0	H
ATOM	1695	C	SER	196	-4.071	-31.606	19.133	1.00	0.00	LX0	C
ATOM	1696	O	SER	196	-4.614	-30.569	18.769	1.00	0.00	LX0	O
ATOM	1697	N	PRO	197	-4.730	-32.793	19.183	1.00	0.00	LX0	N
ATOM	1698	CD	PRO	197	-4.194	-34.123	19.466	1.00	0.00	LX0	C
ATOM	1699	CA	PRO	197	-6.176	-32.800	18.915	1.00	0.00	LX0	C
ATOM	1700	CB	PRO	197	-6.518	-34.295	18.954	1.00	0.00	LX0	C
ATOM	1701	CG	PRO	197	-5.427	-34.953	19.799	1.00	0.00	LX0	C
ATOM	1702	C	PRO	197	-6.998	-31.934	19.872	1.00	0.00	LX0	C
ATOM	1703	O	PRO	197	-8.033	-31.383	19.518	1.00	0.00	LX0	O
ATOM	1704	N	MET	198	-6.475	-31.796	21.108	1.00	0.00	LX0	N
ATOM	1705	H	MET	198	-5.621	-32.253	21.346	0.00	0.00	LX0	H
ATOM	1706	CA	MET	198	-7.136	-30.887	22.053	1.00	0.00	LX0	C
ATOM	1707	CB	MET	198	-6.523	-31.003	23.447	1.00	0.00	LX0	C
ATOM	1708	CG	MET	198	-6.437	-32.440	23.967	1.00	0.00	LX0	C
ATOM	1709	SD	MET	198	-8.030	-33.277	24.011	1.00	0.00	LX0	S
ATOM	1710	CE	MET	198	-7.440	-34.895	24.534	1.00	0.00	LX0	C
ATOM	1711	C	MET	198	-7.138	-29.436	21.593	1.00	0.00	LX0	C
ATOM	1712	O	MET	198	-8.051	-28.648	21.831	1.00	0.00	LX0	O
ATOM	1713	N	CYS	199	-6.089	-29.141	20.817	1.00	0.00	LX0	N
ATOM	1714	H	CYS	199	-5.328	-29.781	20.706	0.00	0.00	LX0	H
ATOM	1715	CA	CYS	199	-6.109	-27.929	20.011	1.00	0.00	LX0	C
ATOM	1716	CB	CYS	199	-4.682	-27.535	19.653	1.00	0.00	LX0	C
ATOM	1717	SG	CYS	199	-3.584	-27.537	21.091	1.00	0.00	LX0	S
ATOM	1718	C	CYS	199	-6.945	-28.043	18.746	1.00	0.00	LX0	C
ATOM	1719	O	CYS	199	-6.498	-27.726	17.654	1.00	0.00	LX0	O
ATOM	1720	N	LYS	200	-8.204	-28.482	18.936	1.00	0.00	LX0	N

ATOM	1721	H	LYS	200	-8.435	-28.899	19.814	0.00	0.00	LX0	H
ATOM	1722	CA	LYS	200	-9.173	-28.532	17.838	1.00	0.00	LX0	C
ATOM	1723	CB	LYS	200	-10.610	-28.753	18.359	1.00	0.00	LX0	C
ATOM	1724	CG	LYS	200	-11.306	-27.601	19.109	1.00	0.00	LX0	C
ATOM	1725	CD	LYS	200	-10.683	-27.269	20.466	1.00	0.00	LX0	C
ATOM	1726	CE	LYS	200	-11.279	-26.046	21.156	1.00	0.00	LX0	C
ATOM	1727	NZ	LYS	200	-10.424	-25.711	22.300	1.00	0.00	LX0	N
ATOM	1728	HZ1	LYS	200	-10.703	-24.783	22.687	0.00	0.00	LX0	H
ATOM	1729	HZ2	LYS	200	-10.475	-26.441	23.039	0.00	0.00	LX0	H
ATOM	1730	HZ3	LYS	200	-9.445	-25.617	21.967	0.00	0.00	LX0	H
ATOM	1731	C	LYS	200	-9.114	-27.367	16.865	1.00	0.00	LX0	C
ATOM	1732	O	LYS	200	-9.074	-26.197	17.235	1.00	0.00	LX0	O
ATOM	1733	N	GLY	201	-9.061	-27.763	15.591	1.00	0.00	LX0	N
ATOM	1734	H	GLY	201	-9.011	-28.738	15.381	0.00	0.00	LX0	H
ATOM	1735	CA	GLY	201	-8.913	-26.752	14.549	1.00	0.00	LX0	C
ATOM	1736	C	GLY	201	-7.538	-26.106	14.494	1.00	0.00	LX0	C
ATOM	1737	O	GLY	201	-7.380	-25.004	13.987	1.00	0.00	LX0	O
ATOM	1738	N	SER	202	-6.561	-26.858	15.035	1.00	0.00	LX0	N
ATOM	1739	H	SER	202	-6.788	-27.710	15.502	0.00	0.00	LX0	H
ATOM	1740	CA	SER	202	-5.156	-26.438	15.107	1.00	0.00	LX0	C
ATOM	1741	CB	SER	202	-4.575	-26.093	13.727	1.00	0.00	LX0	C
ATOM	1742	OG	SER	202	-5.351	-26.738	12.707	1.00	0.00	LX0	O
ATOM	1743	HG	SER	202	-6.013	-26.097	12.471	0.00	0.00	LX0	H
ATOM	1744	C	SER	202	-4.847	-25.344	16.120	1.00	0.00	LX0	C
ATOM	1745	O	SER	202	-3.765	-24.777	16.163	1.00	0.00	LX0	O
ATOM	1746	N	ARG	203	-5.857	-25.048	16.947	1.00	0.00	LX0	N
ATOM	1747	H	ARG	203	-6.644	-25.662	17.014	0.00	0.00	LX0	H
ATOM	1748	CA	ARG	203	-5.732	-23.838	17.752	1.00	0.00	LX0	C
ATOM	1749	CB	ARG	203	-7.115	-23.234	17.988	1.00	0.00	LX0	C
ATOM	1750	CG	ARG	203	-7.931	-22.986	16.717	1.00	0.00	LX0	C
ATOM	1751	CD	ARG	203	-9.305	-22.414	17.064	1.00	0.00	LX0	C
ATOM	1752	NE	ARG	203	-10.192	-22.332	15.906	1.00	0.00	LX0	N
ATOM	1753	HE	ARG	203	-10.451	-23.201	15.483	0.00	0.00	LX0	H
ATOM	1754	CZ	ARG	203	-10.688	-21.146	15.486	1.00	0.00	LX0	C
ATOM	1755	NH1	ARG	203	-10.250	-19.990	15.981	1.00	0.00	LX0	N
ATOM	1756	HH11	ARG	203	-10.587	-19.121	15.597	0.00	0.00	LX0	H
ATOM	1757	HH12	ARG	203	-9.573	-19.945	16.728	0.00	0.00	LX0	H
ATOM	1758	NH2	ARG	203	-11.640	-21.135	14.558	1.00	0.00	LX0	N
ATOM	1759	HH21	ARG	203	-12.051	-20.250	14.292	0.00	0.00	LX0	H
ATOM	1760	HH22	ARG	203	-11.980	-21.965	14.124	0.00	0.00	LX0	H
ATOM	1761	C	ARG	203	-4.979	-24.018	19.063	1.00	0.00	LX0	C
ATOM	1762	O	ARG	203	-5.566	-24.076	20.141	1.00	0.00	LX0	O
ATOM	1763	N	CYS	204	-3.650	-24.104	18.922	1.00	0.00	LX0	N
ATOM	1764	H	CYS	204	-3.251	-24.082	18.002	0.00	0.00	LX0	H
ATOM	1765	CA	CYS	204	-2.796	-24.118	20.111	1.00	0.00	LX0	C
ATOM	1766	CB	CYS	204	-1.701	-25.180	19.996	1.00	0.00	LX0	C
ATOM	1767	SG	CYS	204	-1.267	-25.575	18.284	1.00	0.00	LX0	S
ATOM	1768	C	CYS	204	-2.160	-22.774	20.371	1.00	0.00	LX0	C
ATOM	1769	O	CYS	204	-1.808	-22.054	19.446	1.00	0.00	LX0	O
ATOM	1770	N	TRP	205	-2.051	-22.481	21.673	1.00	0.00	LX0	N
ATOM	1771	H	TRP	205	-2.390	-23.146	22.338	0.00	0.00	LX0	H
ATOM	1772	CA	TRP	205	-1.409	-21.250	22.137	1.00	0.00	LX0	C
ATOM	1773	CB	TRP	205	-2.058	-20.790	23.435	1.00	0.00	LX0	C
ATOM	1774	CG	TRP	205	-3.461	-20.277	23.249	1.00	0.00	LX0	C
ATOM	1775	CD2	TRP	205	-3.894	-18.966	22.827	1.00	0.00	LX0	C
ATOM	1776	CE2	TRP	205	-5.327	-18.955	22.890	1.00	0.00	LX0	C
ATOM	1777	CE3	TRP	205	-3.204	-17.815	22.392	1.00	0.00	LX0	C
ATOM	1778	CD1	TRP	205	-4.636	-20.975	23.544	1.00	0.00	LX0	C
ATOM	1779	NE1	TRP	205	-5.735	-20.201	23.345	1.00	0.00	LX0	N
ATOM	1780	HE1	TRP	205	-6.660	-20.445	23.558	0.00	0.00	LX0	H
ATOM	1781	CZ2	TRP	205	-6.040	-17.789	22.547	1.00	0.00	LX0	C

ATOM	1782	CZ3	TRP	205	-3.927	-16.657	22.046	1.00	0.00	LX0	C
ATOM	1783	CH2	TRP	205	-5.336	-16.644	22.120	1.00	0.00	LX0	C
ATOM	1784	C	TRP	205	0.074	-21.424	22.426	1.00	0.00	LX0	C
ATOM	1785	O	TRP	205	0.902	-20.535	22.254	1.00	0.00	LX0	O
ATOM	1786	N	GLY	206	0.355	-22.638	22.913	1.00	0.00	LX0	N
ATOM	1787	H	GLY	206	-0.354	-23.335	22.998	0.00	0.00	LX0	H
ATOM	1788	CA	GLY	206	1.727	-22.972	23.267	1.00	0.00	LX0	C
ATOM	1789	C	GLY	206	2.063	-24.333	22.713	1.00	0.00	LX0	C
ATOM	1790	O	GLY	206	1.248	-24.909	21.998	1.00	0.00	LX0	O
ATOM	1791	N	GLU	207	3.250	-24.840	23.084	1.00	0.00	LX0	N
ATOM	1792	H	GLU	207	3.849	-24.291	23.679	0.00	0.00	LX0	H
ATOM	1793	CA	GLU	207	3.585	-26.224	22.717	1.00	0.00	LX0	C
ATOM	1794	CB	GLU	207	5.066	-26.547	22.980	1.00	0.00	LX0	C
ATOM	1795	CG	GLU	207	6.104	-25.428	22.828	1.00	0.00	LX0	C
ATOM	1796	CD	GLU	207	6.542	-25.181	21.396	1.00	0.00	LX0	C
ATOM	1797	OE1	GLU	207	5.762	-24.720	20.577	1.00	0.00	LX0	O
ATOM	1798	OE2	GLU	207	7.711	-25.373	21.095	1.00	0.00	LX0	O
ATOM	1799	C	GLU	207	2.739	-27.216	23.512	1.00	0.00	LX0	C
ATOM	1800	O	GLU	207	3.176	-27.779	24.508	1.00	0.00	LX0	O
ATOM	1801	N	SER	208	1.478	-27.365	23.087	1.00	0.00	LX0	N
ATOM	1802	H	SER	208	1.163	-26.919	22.248	0.00	0.00	LX0	H
ATOM	1803	CA	SER	208	0.561	-27.818	24.126	1.00	0.00	LX0	C
ATOM	1804	CB	SER	208	0.050	-26.590	24.893	1.00	0.00	LX0	C
ATOM	1805	OG	SER	208	-0.336	-26.945	26.227	1.00	0.00	LX0	O
ATOM	1806	HG	SER	208	0.421	-26.741	26.774	0.00	0.00	LX0	H
ATOM	1807	C	SER	208	-0.591	-28.705	23.692	1.00	0.00	LX0	C
ATOM	1808	O	SER	208	-1.069	-28.703	22.561	1.00	0.00	LX0	O
ATOM	1809	N	SER	209	-1.038	-29.448	24.707	1.00	0.00	LX0	N
ATOM	1810	H	SER	209	-0.498	-29.559	25.541	0.00	0.00	LX0	H
ATOM	1811	CA	SER	209	-2.352	-30.081	24.679	1.00	0.00	LX0	C
ATOM	1812	CB	SER	209	-2.177	-31.542	25.086	1.00	0.00	LX0	C
ATOM	1813	OG	SER	209	-1.072	-31.643	25.998	1.00	0.00	LX0	O
ATOM	1814	HG	SER	209	-1.145	-32.492	26.417	0.00	0.00	LX0	H
ATOM	1815	C	SER	209	-3.362	-29.391	25.589	1.00	0.00	LX0	C
ATOM	1816	O	SER	209	-4.533	-29.739	25.636	1.00	0.00	LX0	O
ATOM	1817	N	GLU	210	-2.826	-28.418	26.339	1.00	0.00	LX0	N
ATOM	1818	H	GLU	210	-1.887	-28.115	26.190	0.00	0.00	LX0	H
ATOM	1819	CA	GLU	210	-3.585	-27.801	27.419	1.00	0.00	LX0	C
ATOM	1820	CB	GLU	210	-2.694	-27.863	28.662	1.00	0.00	LX0	C
ATOM	1821	CG	GLU	210	-3.246	-28.689	29.827	1.00	0.00	LX0	C
ATOM	1822	CD	GLU	210	-4.182	-27.828	30.644	1.00	0.00	LX0	C
ATOM	1823	OE1	GLU	210	-3.705	-27.008	31.423	1.00	0.00	LX0	O
ATOM	1824	OE2	GLU	210	-5.391	-27.916	30.473	1.00	0.00	LX0	O
ATOM	1825	C	GLU	210	-3.995	-26.382	27.061	1.00	0.00	LX0	C
ATOM	1826	O	GLU	210	-5.156	-25.985	27.085	1.00	0.00	LX0	O
ATOM	1827	N	ASP	211	-2.966	-25.613	26.672	1.00	0.00	LX0	N
ATOM	1828	H	ASP	211	-2.032	-25.968	26.672	0.00	0.00	LX0	H
ATOM	1829	CA	ASP	211	-3.270	-24.274	26.169	1.00	0.00	LX0	C
ATOM	1830	CB	ASP	211	-2.072	-23.334	26.305	1.00	0.00	LX0	C
ATOM	1831	CG	ASP	211	-2.042	-22.772	27.710	1.00	0.00	LX0	C
ATOM	1832	OD1	ASP	211	-2.669	-21.748	27.957	1.00	0.00	LX0	O
ATOM	1833	OD2	ASP	211	-1.411	-23.366	28.576	1.00	0.00	LX0	O
ATOM	1834	C	ASP	211	-3.807	-24.278	24.757	1.00	0.00	LX0	C
ATOM	1835	O	ASP	211	-3.093	-24.153	23.765	1.00	0.00	LX0	O
ATOM	1836	N	CYS	212	-5.131	-24.439	24.725	1.00	0.00	LX0	N
ATOM	1837	H	CYS	212	-5.615	-24.577	25.593	0.00	0.00	LX0	H
ATOM	1838	CA	CYS	212	-5.829	-24.511	23.448	1.00	0.00	LX0	C
ATOM	1839	CB	CYS	212	-6.387	-25.919	23.268	1.00	0.00	LX0	C
ATOM	1840	SG	CYS	212	-5.185	-27.209	23.672	1.00	0.00	LX0	S
ATOM	1841	C	CYS	212	-6.952	-23.495	23.434	1.00	0.00	LX0	C
ATOM	1842	O	CYS	212	-7.514	-23.187	24.476	1.00	0.00	LX0	O

```
ATOM   1843  N   GLN   213      -7.274 -22.972  22.238  1.00  0.00      LX0  N
ATOM   1844  H   GLN   213      -6.752 -23.233  21.423  0.00  0.00      LX0  H
ATOM   1845  CA  GLN   213      -8.230 -21.858  22.212  1.00  0.00      LX0  C
ATOM   1846  CB  GLN   213      -8.438 -21.343  20.785  1.00  0.00      LX0  C
ATOM   1847  CG  GLN   213      -8.865 -19.873  20.723  1.00  0.00      LX0  C
ATOM   1848  CD  GLN   213      -9.509 -19.558  19.386  1.00  0.00      LX0  C
ATOM   1849  OE1 GLN   213      -8.900 -19.565  18.321  1.00  0.00      LX0  O
ATOM   1850  NE2 GLN   213     -10.804 -19.256  19.485  1.00  0.00      LX0  N
ATOM   1851 HE21 GLN   213     -11.271 -19.381  20.364  0.00  0.00      LX0  H
ATOM   1852 HE22 GLN   213     -11.320 -18.897  18.712  0.00  0.00      LX0  H
ATOM   1853  C   GLN   213      -9.578 -22.140  22.858  1.00  0.00      LX0  C
ATOM   1854  O   GLN   213     -10.185 -23.191  22.642  1.00  0.00      LX0  O
ATOM   1855  N   SER   214     -10.019 -21.148  23.640  1.00  0.00      LX0  N
ATOM   1856  H   SER   214      -9.414 -20.382  23.870  0.00  0.00      LX0  H
ATOM   1857  CA  SER   214     -11.420 -21.140  24.048  1.00  0.00      LX0  C
ATOM   1858  CB  SER   214     -11.607 -20.277  25.292  1.00  0.00      LX0  C
ATOM   1859  OG  SER   214     -12.932 -20.449  25.801  1.00  0.00      LX0  O
ATOM   1860  HG  SER   214     -13.009 -19.873  26.555  0.00  0.00      LX0  H
ATOM   1861  C   SER   214     -12.329 -20.659  22.931  1.00  0.00      LX0  C
ATOM   1862  O   SER   214     -11.932 -20.593  21.769  1.00  0.00      LX0  O
TER
```
