# Supplementary material for: Structural analysis of hubs in human NR-RTK network
Source: Biol Direct. 2011 Oct 5;6:49. doi: 10.1186/1745-6150-6-49 (PMC3220635; doi:10.1186/1745-6150-6-49)
Supplement: Additional file 8 — ESR1-PGR. ESR1-PGR complex structure. [file 1745-6150-6-49-S8.PDF]

```
HEADER ESR1-PGR
REMARK original generated coordinate pdb file
ATOM      1  N   ALA   156      10.627  12.174   8.322  1.00  0.00      RX0  N
ATOM      2  H   ALA   156      11.188  11.347   8.270  0.00  0.00      RX0  H
ATOM      3  CA  ALA   156       9.864  12.527   9.538  1.00  0.00      RX0  C
ATOM      4  CB  ALA   156      10.757  12.402  10.765  1.00  0.00      RX0  C
ATOM      5  C   ALA   156       9.377  13.991   9.496  1.00  0.00      RX0  C
ATOM      6  O   ALA   156       9.121  14.644  10.500  1.00  0.00      RX0  O
ATOM      7  N   LEU   157       9.039  14.416   8.289  1.00  0.00      RX0  N
ATOM      8  H   LEU   157       9.127  13.765   7.532  0.00  0.00      RX0  H
ATOM      9  CA  LEU   157       8.850  15.849   7.979  1.00  0.00      RX0  C
ATOM     10  CB  LEU   157       9.703  16.234   6.776  1.00  0.00      RX0  C
ATOM     11  CG  LEU   157      11.025  15.473   6.705  1.00  0.00      RX0  C
ATOM     12  CD1 LEU   157      11.611  15.541   5.299  1.00  0.00      RX0  C
ATOM     13  CD2 LEU   157      12.015  15.904   7.788  1.00  0.00      RX0  C
ATOM     14  C   LEU   157       7.387  16.184   7.642  1.00  0.00      RX0  C
ATOM     15  O   LEU   157       7.075  17.216   7.039  1.00  0.00      RX0  O
ATOM     16  N   SER   158       6.522  15.244   7.955  1.00  0.00      RX0  N
ATOM     17  H   SER   158       6.874  14.326   8.099  0.00  0.00      RX0  H
ATOM     18  CA  SER   158       5.051  15.362   7.847  1.00  0.00      RX0  C
ATOM     19  CB  SER   158       4.728  14.894   6.437  1.00  0.00      RX0  C
ATOM     20  OG  SER   158       5.937  15.035   5.681  1.00  0.00      RX0  O
ATOM     21  HG  SER   158       6.093  15.975   5.623  0.00  0.00      RX0  H
ATOM     22  C   SER   158       4.335  14.560   8.949  1.00  0.00      RX0  C
ATOM     23  O   SER   158       3.148  14.670   9.188  1.00  0.00      RX0  O
ATOM     24  N   LEU   159       5.132  13.681   9.591  1.00  0.00      RX0  N
ATOM     25  H   LEU   159       6.084  13.587   9.326  0.00  0.00      RX0  H
ATOM     26  CA  LEU   159       4.759  12.951  10.797  1.00  0.00      RX0  C
ATOM     27  CB  LEU   159       5.860  11.985  11.247  1.00  0.00      RX0  C
ATOM     28  CG  LEU   159       5.923  10.637  10.518  1.00  0.00      RX0  C
ATOM     29  CD1 LEU   159       6.416  10.739   9.072  1.00  0.00      RX0  C
ATOM     30  CD2 LEU   159       6.753   9.632  11.320  1.00  0.00      RX0  C
ATOM     31  C   LEU   159       4.518  13.965  11.920  1.00  0.00      RX0  C
ATOM     32  O   LEU   159       5.291  14.932  12.058  1.00  0.00      RX0  O
ATOM     33  N   THR   160       3.434  13.807  12.646  1.00  0.00      RX0  N
ATOM     34  H   THR   160       2.848  13.018  12.460  0.00  0.00      RX0  H
ATOM     35  CA  THR   160       3.156  14.665  13.825  1.00  0.00      RX0  C
ATOM     36  CB  THR   160       1.666  14.646  14.226  1.00  0.00      RX0  C
ATOM     37  OG1 THR   160       1.373  15.741  15.101  1.00  0.00      RX0  O
ATOM     38  HG1 THR   160       0.426  15.817  15.130  0.00  0.00      RX0  H
ATOM     39  CG2 THR   160       1.177  13.336  14.825  1.00  0.00      RX0  C
ATOM     40  C   THR   160       4.203  14.411  14.921  1.00  0.00      RX0  C
ATOM     41  O   THR   160       4.902  13.383  14.913  1.00  0.00      RX0  O
ATOM     42  N   ALA   161       4.153  15.229  15.953  1.00  0.00      RX0  N
ATOM     43  H   ALA   161       3.464  15.955  15.914  0.00  0.00      RX0  H
ATOM     44  CA  ALA   161       4.942  15.044  17.184  1.00  0.00      RX0  C
ATOM     45  CB  ALA   161       4.756  16.236  18.115  1.00  0.00      RX0  C
ATOM     46  C   ALA   161       4.543  13.746  17.920  1.00  0.00      RX0  C
ATOM     47  O   ALA   161       5.387  12.950  18.288  1.00  0.00      RX0  O
ATOM     48  N   ASP   162       3.226  13.461  17.917  1.00  0.00      RX0  N
ATOM     49  H   ASP   162       2.572  14.176  17.682  0.00  0.00      RX0  H
ATOM     50  CA  ASP   162       2.690  12.196  18.469  1.00  0.00      RX0  C
ATOM     51  CB  ASP   162       1.215  12.328  18.866  1.00  0.00      RX0  C
ATOM     52  CG  ASP   162       1.144  13.135  20.162  1.00  0.00      RX0  C
ATOM     53  OD1 ASP   162       2.124  13.783  20.525  1.00  0.00      RX0  O
ATOM     54  OD2 ASP   162       0.124  13.115  20.849  1.00  0.00      RX0  O
ATOM     55  C   ASP   162       3.088  10.948  17.668  1.00  0.00      RX0  C
ATOM     56  O   ASP   162       3.397   9.903  18.257  1.00  0.00      RX0  O
ATOM     57  N   GLN   163       3.164  11.087  16.353  1.00  0.00      RX0  N
ATOM     58  H   GLN   163       3.080  12.014  16.000  0.00  0.00      RX0  H
ATOM     59  CA  GLN   163       3.593   9.998  15.449  1.00  0.00      RX0  C
```

ATOM	60	CB	GLN	163	3.250	10.286	13.995	1.00	0.00	RX0	C
ATOM	61	CG	GLN	163	1.826	9.877	13.632	1.00	0.00	RX0	C
ATOM	62	CD	GLN	163	1.555	10.379	12.235	1.00	0.00	RX0	C
ATOM	63	OE1	GLN	163	2.081	11.410	11.829	1.00	0.00	RX0	O
ATOM	64	NE2	GLN	163	0.719	9.603	11.526	1.00	0.00	RX0	N
ATOM	65	HE21	GLN	163	0.331	8.772	11.926	0.00	0.00	RX0	H
ATOM	66	HE22	GLN	163	0.461	9.830	10.586	0.00	0.00	RX0	H
ATOM	67	C	GLN	163	5.089	9.698	15.572	1.00	0.00	RX0	C
ATOM	68	O	GLN	163	5.477	8.537	15.545	1.00	0.00	RX0	O
ATOM	69	N	MET	164	5.882	10.740	15.840	1.00	0.00	RX0	N
ATOM	70	H	MET	164	5.492	11.661	15.892	0.00	0.00	RX0	H
ATOM	71	CA	MET	164	7.331	10.587	16.060	1.00	0.00	RX0	C
ATOM	72	CB	MET	164	8.015	11.955	16.081	1.00	0.00	RX0	C
ATOM	73	CG	MET	164	9.451	11.879	16.606	1.00	0.00	RX0	C
ATOM	74	SD	MET	164	10.536	10.861	15.596	1.00	0.00	RX0	S
ATOM	75	CE	MET	164	11.025	12.129	14.424	1.00	0.00	RX0	C
ATOM	76	C	MET	164	7.610	9.825	17.366	1.00	0.00	RX0	C
ATOM	77	O	MET	164	8.404	8.887	17.381	1.00	0.00	RX0	O
ATOM	78	N	VAL	165	6.828	10.145	18.396	1.00	0.00	RX0	N
ATOM	79	H	VAL	165	6.166	10.889	18.279	0.00	0.00	RX0	H
ATOM	80	CA	VAL	165	6.992	9.563	19.744	1.00	0.00	RX0	C
ATOM	81	CB	VAL	165	6.100	10.259	20.778	1.00	0.00	RX0	C
ATOM	82	CG1	VAL	165	6.181	9.567	22.138	1.00	0.00	RX0	C
ATOM	83	CG2	VAL	165	6.449	11.732	20.918	1.00	0.00	RX0	C
ATOM	84	C	VAL	165	6.649	8.067	19.731	1.00	0.00	RX0	C
ATOM	85	O	VAL	165	7.442	7.255	20.191	1.00	0.00	RX0	O
ATOM	86	N	SER	166	5.467	7.742	19.205	1.00	0.00	RX0	N
ATOM	87	H	SER	166	4.815	8.431	18.873	0.00	0.00	RX0	H
ATOM	88	CA	SER	166	5.029	6.335	19.106	1.00	0.00	RX0	C
ATOM	89	CB	SER	166	3.570	6.382	18.709	1.00	0.00	RX0	C
ATOM	90	OG	SER	166	3.003	7.438	19.488	1.00	0.00	RX0	O
ATOM	91	HG	SER	166	3.380	7.369	20.355	0.00	0.00	RX0	H
ATOM	92	C	SER	166	5.941	5.501	18.195	1.00	0.00	RX0	C
ATOM	93	O	SER	166	6.295	4.379	18.542	1.00	0.00	RX0	O
ATOM	94	N	ALA	167	6.456	6.129	17.133	1.00	0.00	RX0	N
ATOM	95	H	ALA	167	6.170	7.064	16.912	0.00	0.00	RX0	H
ATOM	96	CA	ALA	167	7.397	5.466	16.208	1.00	0.00	RX0	C
ATOM	97	CB	ALA	167	7.731	6.357	15.013	1.00	0.00	RX0	C
ATOM	98	C	ALA	167	8.706	5.103	16.927	1.00	0.00	RX0	C
ATOM	99	O	ALA	167	9.113	3.946	16.932	1.00	0.00	RX0	O
ATOM	100	N	LEU	168	9.179	6.054	17.734	1.00	0.00	RX0	N
ATOM	101	H	LEU	168	8.734	6.952	17.737	0.00	0.00	RX0	H
ATOM	102	CA	LEU	168	10.385	5.875	18.562	1.00	0.00	RX0	C
ATOM	103	CB	LEU	168	10.907	7.213	19.074	1.00	0.00	RX0	C
ATOM	104	CG	LEU	168	11.571	8.041	17.978	1.00	0.00	RX0	C
ATOM	105	CD1	LEU	168	12.103	9.369	18.519	1.00	0.00	RX0	C
ATOM	106	CD2	LEU	168	12.652	7.238	17.253	1.00	0.00	RX0	C
ATOM	107	C	LEU	168	10.197	4.896	19.724	1.00	0.00	RX0	C
ATOM	108	O	LEU	168	11.077	4.078	19.994	1.00	0.00	RX0	O
ATOM	109	N	LEU	169	9.007	4.918	20.317	1.00	0.00	RX0	N
ATOM	110	H	LEU	169	8.333	5.589	20.010	0.00	0.00	RX0	H
ATOM	111	CA	LEU	169	8.640	3.970	21.384	1.00	0.00	RX0	C
ATOM	112	CB	LEU	169	7.358	4.393	22.101	1.00	0.00	RX0	C
ATOM	113	CG	LEU	169	7.538	5.639	22.970	1.00	0.00	RX0	C
ATOM	114	CD1	LEU	169	6.226	6.046	23.640	1.00	0.00	RX0	C
ATOM	115	CD2	LEU	169	8.667	5.470	23.989	1.00	0.00	RX0	C
ATOM	116	C	LEU	169	8.505	2.536	20.864	1.00	0.00	RX0	C
ATOM	117	O	LEU	169	9.003	1.602	21.486	1.00	0.00	RX0	O
ATOM	118	N	ASP	170	7.977	2.423	19.645	1.00	0.00	RX0	N
ATOM	119	H	ASP	170	7.671	3.242	19.161	0.00	0.00	RX0	H
ATOM	120	CA	ASP	170	7.822	1.133	18.952	1.00	0.00	RX0	C

ATOM	121	CB	ASP	170	6.876	1.395	17.764	1.00	0.00	RX0	C
ATOM	122	CG	ASP	170	6.640	0.249	16.790	1.00	0.00	RX0	C
ATOM	123	OD1	ASP	170	7.085	-0.874	16.994	1.00	0.00	RX0	O
ATOM	124	OD2	ASP	170	6.019	0.487	15.757	1.00	0.00	RX0	O
ATOM	125	C	ASP	170	9.164	0.506	18.541	1.00	0.00	RX0	C
ATOM	126	O	ASP	170	9.313	-0.704	18.571	1.00	0.00	RX0	O
ATOM	127	N	ALA	171	10.119	1.387	18.228	1.00	0.00	RX0	N
ATOM	128	H	ALA	171	9.895	2.361	18.289	0.00	0.00	RX0	H
ATOM	129	CA	ALA	171	11.447	1.008	17.717	1.00	0.00	RX0	C
ATOM	130	CB	ALA	171	12.102	2.217	17.051	1.00	0.00	RX0	C
ATOM	131	C	ALA	171	12.418	0.479	18.779	1.00	0.00	RX0	C
ATOM	132	O	ALA	171	13.427	-0.136	18.431	1.00	0.00	RX0	O
ATOM	133	N	GLU	172	12.125	0.739	20.058	1.00	0.00	RX0	N
ATOM	134	H	GLU	172	11.281	1.230	20.277	0.00	0.00	RX0	H
ATOM	135	CA	GLU	172	13.017	0.374	21.170	1.00	0.00	RX0	C
ATOM	136	CB	GLU	172	12.364	0.708	22.510	1.00	0.00	RX0	C
ATOM	137	CG	GLU	172	12.323	2.226	22.680	1.00	0.00	RX0	C
ATOM	138	CD	GLU	172	13.729	2.777	22.517	1.00	0.00	RX0	C
ATOM	139	OE1	GLU	172	14.552	2.616	23.418	1.00	0.00	RX0	O
ATOM	140	OE2	GLU	172	14.036	3.383	21.490	1.00	0.00	RX0	O
ATOM	141	C	GLU	172	13.554	-1.065	21.099	1.00	0.00	RX0	C
ATOM	142	O	GLU	172	12.785	-2.004	20.837	1.00	0.00	RX0	O
ATOM	143	N	PRO	173	14.865	-1.209	21.269	1.00	0.00	RX0	N
ATOM	144	CD	PRO	173	15.789	-0.095	21.453	1.00	0.00	RX0	C
ATOM	145	CA	PRO	173	15.538	-2.517	21.328	1.00	0.00	RX0	C
ATOM	146	CB	PRO	173	17.014	-2.108	21.199	1.00	0.00	RX0	C
ATOM	147	CG	PRO	173	17.103	-0.741	21.869	1.00	0.00	RX0	C
ATOM	148	C	PRO	173	15.206	-3.249	22.640	1.00	0.00	RX0	C
ATOM	149	O	PRO	173	14.829	-2.595	23.631	1.00	0.00	RX0	O
ATOM	150	N	PRO	174	15.294	-4.574	22.646	1.00	0.00	RX0	N
ATOM	151	CD	PRO	174	15.605	-5.388	21.475	1.00	0.00	RX0	C
ATOM	152	CA	PRO	174	15.084	-5.400	23.852	1.00	0.00	RX0	C
ATOM	153	CB	PRO	174	14.968	-6.812	23.273	1.00	0.00	RX0	C
ATOM	154	CG	PRO	174	15.853	-6.784	22.032	1.00	0.00	RX0	C
ATOM	155	C	PRO	174	16.250	-5.248	24.838	1.00	0.00	RX0	C
ATOM	156	O	PRO	174	17.379	-4.922	24.444	1.00	0.00	RX0	O
ATOM	157	N	ILE	175	15.956	-5.464	26.106	1.00	0.00	RX0	N
ATOM	158	H	ILE	175	15.039	-5.788	26.327	0.00	0.00	RX0	H
ATOM	159	CA	ILE	175	16.988	-5.556	27.159	1.00	0.00	RX0	C
ATOM	160	CB	ILE	175	16.447	-5.212	28.551	1.00	0.00	RX0	C
ATOM	161	CG2	ILE	175	17.624	-5.097	29.519	1.00	0.00	RX0	C
ATOM	162	CG1	ILE	175	15.624	-3.917	28.563	1.00	0.00	RX0	C
ATOM	163	CD1	ILE	175	14.111	-4.135	28.454	1.00	0.00	RX0	C
ATOM	164	C	ILE	175	17.586	-6.969	27.112	1.00	0.00	RX0	C
ATOM	165	O	ILE	175	16.886	-7.963	27.343	1.00	0.00	RX0	O
ATOM	166	N	LEU	176	18.884	-7.017	26.884	1.00	0.00	RX0	N
ATOM	167	H	LEU	176	19.406	-6.165	26.835	0.00	0.00	RX0	H
ATOM	168	CA	LEU	176	19.617	-8.291	26.770	1.00	0.00	RX0	C
ATOM	169	CB	LEU	176	20.648	-8.225	25.645	1.00	0.00	RX0	C
ATOM	170	CG	LEU	176	20.005	-8.000	24.276	1.00	0.00	RX0	C
ATOM	171	CD1	LEU	176	21.058	-7.946	23.172	1.00	0.00	RX0	C
ATOM	172	CD2	LEU	176	18.923	-9.038	23.970	1.00	0.00	RX0	C
ATOM	173	C	LEU	176	20.277	-8.690	28.089	1.00	0.00	RX0	C
ATOM	174	O	LEU	176	20.563	-7.852	28.952	1.00	0.00	RX0	O
ATOM	175	N	TYR	177	20.459	-9.989	28.237	1.00	0.00	RX0	N
ATOM	176	H	TYR	177	20.209	-10.605	27.493	0.00	0.00	RX0	H
ATOM	177	CA	TYR	177	21.114	-10.573	29.420	1.00	0.00	RX0	C
ATOM	178	CB	TYR	177	20.420	-11.864	29.855	1.00	0.00	RX0	C
ATOM	179	CG	TYR	177	19.155	-11.562	30.620	1.00	0.00	RX0	C
ATOM	180	CD1	TYR	177	18.031	-11.068	29.968	1.00	0.00	RX0	C
ATOM	181	CE1	TYR	177	16.868	-10.811	30.685	1.00	0.00	RX0	C

ATOM	182	CD2	TYR	177	19.118	-11.793	31.990	1.00	0.00	RX0	C
ATOM	183	CE2	TYR	177	17.954	-11.543	32.706	1.00	0.00	RX0	C
ATOM	184	CZ	TYR	177	16.830	-11.051	32.054	1.00	0.00	RX0	C
ATOM	185	OH	TYR	177	15.677	-10.795	32.767	1.00	0.00	RX0	O
ATOM	186	HH	TYR	177	15.786	-11.080	33.665	0.00	0.00	RX0	H
ATOM	187	C	TYR	177	22.589	-10.858	29.163	1.00	0.00	RX0	C
ATOM	188	O	TYR	177	22.985	-11.163	28.046	1.00	0.00	RX0	O
ATOM	189	N	SER	178	23.381	-10.750	30.220	1.00	0.00	RX0	N
ATOM	190	H	SER	178	23.027	-10.490	31.122	0.00	0.00	RX0	H
ATOM	191	CA	SER	178	24.788	-11.188	30.183	1.00	0.00	RX0	C
ATOM	192	CB	SER	178	25.488	-10.617	31.402	1.00	0.00	RX0	C
ATOM	193	OG	SER	178	25.214	-9.216	31.413	1.00	0.00	RX0	O
ATOM	194	HG	SER	178	25.715	-8.836	30.702	0.00	0.00	RX0	H
ATOM	195	C	SER	178	24.834	-12.718	30.070	1.00	0.00	RX0	C
ATOM	196	O	SER	178	23.999	-13.413	30.674	1.00	0.00	RX0	O
ATOM	197	N	GLU	179	25.827	-13.218	29.362	1.00	0.00	RX0	N
ATOM	198	H	GLU	179	26.520	-12.638	28.926	0.00	0.00	RX0	H
ATOM	199	CA	GLU	179	26.033	-14.670	29.175	1.00	0.00	RX0	C
ATOM	200	CB	GLU	179	26.341	-15.051	27.722	1.00	0.00	RX0	C
ATOM	201	CG	GLU	179	25.833	-14.100	26.641	1.00	0.00	RX0	C
ATOM	202	CD	GLU	179	26.872	-13.039	26.296	1.00	0.00	RX0	C
ATOM	203	OE1	GLU	179	27.281	-12.977	25.146	1.00	0.00	RX0	O
ATOM	204	OE2	GLU	179	27.207	-12.200	27.130	1.00	0.00	RX0	O
ATOM	205	C	GLU	179	27.192	-15.208	30.012	1.00	0.00	RX0	C
ATOM	206	O	GLU	179	28.361	-15.227	29.589	1.00	0.00	RX0	O
ATOM	207	N	TYR	180	26.873	-15.475	31.254	1.00	0.00	RX0	N
ATOM	208	H	TYR	180	25.923	-15.394	31.565	0.00	0.00	RX0	H
ATOM	209	CA	TYR	180	27.735	-16.233	32.177	1.00	0.00	RX0	C
ATOM	210	CB	TYR	180	28.496	-15.323	33.154	1.00	0.00	RX0	C
ATOM	211	CG	TYR	180	27.556	-14.674	34.143	1.00	0.00	RX0	C
ATOM	212	CD1	TYR	180	26.931	-13.473	33.831	1.00	0.00	RX0	C
ATOM	213	CE1	TYR	180	26.028	-12.911	34.725	1.00	0.00	RX0	C
ATOM	214	CD2	TYR	180	27.312	-15.286	35.368	1.00	0.00	RX0	C
ATOM	215	CE2	TYR	180	26.398	-14.733	36.253	1.00	0.00	RX0	C
ATOM	216	CZ	TYR	180	25.740	-13.556	35.921	1.00	0.00	RX0	C
ATOM	217	OH	TYR	180	24.793	-13.036	36.776	1.00	0.00	RX0	O
ATOM	218	HH	TYR	180	24.692	-12.106	36.595	0.00	0.00	RX0	H
ATOM	219	C	TYR	180	26.838	-17.226	32.909	1.00	0.00	RX0	C
ATOM	220	O	TYR	180	25.642	-16.953	33.094	1.00	0.00	RX0	O
ATOM	221	N	ASP	181	27.404	-18.345	33.318	1.00	0.00	RX0	N
ATOM	222	H	ASP	181	28.388	-18.474	33.236	0.00	0.00	RX0	H
ATOM	223	CA	ASP	181	26.630	-19.347	34.059	1.00	0.00	RX0	C
ATOM	224	CB	ASP	181	27.274	-20.721	34.067	1.00	0.00	RX0	C
ATOM	225	CG	ASP	181	26.440	-21.553	35.010	1.00	0.00	RX0	C
ATOM	226	OD1	ASP	181	25.233	-21.626	34.821	1.00	0.00	RX0	O
ATOM	227	OD2	ASP	181	26.977	-22.083	35.971	1.00	0.00	RX0	O
ATOM	228	C	ASP	181	26.420	-18.851	35.504	1.00	0.00	RX0	C
ATOM	229	O	ASP	181	27.391	-18.832	36.273	1.00	0.00	RX0	O
ATOM	230	N	PRO	182	25.185	-18.489	35.856	1.00	0.00	RX0	N
ATOM	231	CD	PRO	182	24.009	-18.603	34.996	1.00	0.00	RX0	C
ATOM	232	CA	PRO	182	24.825	-17.989	37.201	1.00	0.00	RX0	C
ATOM	233	CB	PRO	182	23.394	-17.487	36.998	1.00	0.00	RX0	C
ATOM	234	CG	PRO	182	22.821	-18.410	35.929	1.00	0.00	RX0	C
ATOM	235	C	PRO	182	24.941	-19.052	38.308	1.00	0.00	RX0	C
ATOM	236	O	PRO	182	24.654	-18.763	39.474	1.00	0.00	RX0	O
ATOM	237	N	THR	183	25.345	-20.259	37.948	1.00	0.00	RX0	N
ATOM	238	H	THR	183	25.549	-20.526	37.004	0.00	0.00	RX0	H
ATOM	239	CA	THR	183	25.568	-21.363	38.913	1.00	0.00	RX0	C
ATOM	240	CB	THR	183	24.906	-22.560	38.262	1.00	0.00	RX0	C
ATOM	241	OG1	THR	183	23.953	-22.054	37.318	1.00	0.00	RX0	O
ATOM	242	HG1	THR	183	24.430	-22.033	36.485	0.00	0.00	RX0	H

ATOM	243	CG2	THR	183	24.242	-23.492	39.276	1.00	0.00	RX0	C
ATOM	244	C	THR	183	27.063	-21.532	39.218	1.00	0.00	RX0	C
ATOM	245	O	THR	183	27.455	-22.345	40.058	1.00	0.00	RX0	O
ATOM	246	N	ARG	184	27.887	-20.699	38.573	1.00	0.00	RX0	N
ATOM	247	H	ARG	184	27.514	-20.005	37.955	0.00	0.00	RX0	H
ATOM	248	CA	ARG	184	29.343	-20.701	38.681	1.00	0.00	RX0	C
ATOM	249	CB	ARG	184	29.887	-20.668	37.266	1.00	0.00	RX0	C
ATOM	250	CG	ARG	184	30.195	-21.989	36.567	1.00	0.00	RX0	C
ATOM	251	CD	ARG	184	30.731	-21.642	35.179	1.00	0.00	RX0	C
ATOM	252	NE	ARG	184	31.402	-20.346	35.281	1.00	0.00	RX0	N
ATOM	253	HE	ARG	184	30.801	-19.541	35.260	0.00	0.00	RX0	H
ATOM	254	CZ	ARG	184	32.702	-20.331	35.691	1.00	0.00	RX0	C
ATOM	255	NH1	ARG	184	33.462	-21.428	35.510	1.00	0.00	RX0	N
ATOM	256	HH11	ARG	184	34.437	-21.421	35.813	0.00	0.00	RX0	H
ATOM	257	HH12	ARG	184	33.131	-22.264	35.075	0.00	0.00	RX0	H
ATOM	258	NH2	ARG	184	33.197	-19.235	36.299	1.00	0.00	RX0	N
ATOM	259	HH21	ARG	184	34.162	-19.267	36.609	0.00	0.00	RX0	H
ATOM	260	HH22	ARG	184	32.669	-18.404	36.501	0.00	0.00	RX0	H
ATOM	261	C	ARG	184	29.836	-19.410	39.407	1.00	0.00	RX0	C
ATOM	262	O	ARG	184	29.116	-18.390	39.334	1.00	0.00	RX0	O
ATOM	263	N	PRO	185	30.940	-19.460	40.113	1.00	0.00	RX0	N
ATOM	264	CD	PRO	185	31.743	-20.666	40.282	1.00	0.00	RX0	C
ATOM	265	CA	PRO	185	31.574	-18.285	40.781	1.00	0.00	RX0	C
ATOM	266	CB	PRO	185	32.858	-18.875	41.378	1.00	0.00	RX0	C
ATOM	267	CG	PRO	185	33.142	-20.138	40.568	1.00	0.00	RX0	C
ATOM	268	C	PRO	185	31.820	-17.125	39.813	1.00	0.00	RX0	C
ATOM	269	O	PRO	185	31.836	-17.275	38.592	1.00	0.00	RX0	O
ATOM	270	N	PHE	186	32.164	-15.998	40.422	1.00	0.00	RX0	N
ATOM	271	H	PHE	186	32.237	-15.990	41.418	0.00	0.00	RX0	H
ATOM	272	CA	PHE	186	32.333	-14.726	39.697	1.00	0.00	RX0	C
ATOM	273	CB	PHE	186	31.516	-13.614	40.356	1.00	0.00	RX0	C
ATOM	274	CG	PHE	186	31.409	-12.444	39.405	1.00	0.00	RX0	C
ATOM	275	CD1	PHE	186	30.853	-12.628	38.144	1.00	0.00	RX0	C
ATOM	276	CD2	PHE	186	31.868	-11.188	39.785	1.00	0.00	RX0	C
ATOM	277	CE1	PHE	186	30.758	-11.558	37.262	1.00	0.00	RX0	C
ATOM	278	CE2	PHE	186	31.773	-10.118	38.903	1.00	0.00	RX0	C
ATOM	279	CZ	PHE	186	31.219	-10.303	37.641	1.00	0.00	RX0	C
ATOM	280	C	PHE	186	33.791	-14.305	39.507	1.00	0.00	RX0	C
ATOM	281	O	PHE	186	34.127	-13.678	38.496	1.00	0.00	RX0	O
ATOM	282	N	SER	187	34.655	-14.802	40.380	1.00	0.00	RX0	N
ATOM	283	H	SER	187	34.309	-15.342	41.142	0.00	0.00	RX0	H
ATOM	284	CA	SER	187	36.113	-14.542	40.380	1.00	0.00	RX0	C
ATOM	285	CB	SER	187	36.613	-15.272	41.614	1.00	0.00	RX0	C
ATOM	286	OG	SER	187	35.493	-15.373	42.511	1.00	0.00	RX0	O
ATOM	287	HG	SER	187	35.867	-15.423	43.384	0.00	0.00	RX0	H
ATOM	288	C	SER	187	36.764	-14.980	39.057	1.00	0.00	RX0	C
ATOM	289	O	SER	187	37.834	-14.531	38.683	1.00	0.00	RX0	O
ATOM	290	N	GLU	188	36.054	-15.878	38.369	1.00	0.00	RX0	N
ATOM	291	H	GLU	188	35.127	-16.099	38.656	0.00	0.00	RX0	H
ATOM	292	CA	GLU	188	36.561	-16.586	37.191	1.00	0.00	RX0	C
ATOM	293	CB	GLU	188	36.254	-18.063	37.365	1.00	0.00	RX0	C
ATOM	294	CG	GLU	188	37.000	-19.017	36.441	1.00	0.00	RX0	C
ATOM	295	CD	GLU	188	36.147	-20.257	36.415	1.00	0.00	RX0	C
ATOM	296	OE1	GLU	188	35.378	-20.453	37.351	1.00	0.00	RX0	O
ATOM	297	OE2	GLU	188	36.148	-20.975	35.424	1.00	0.00	RX0	O
ATOM	298	C	GLU	188	36.028	-16.018	35.856	1.00	0.00	RX0	C
ATOM	299	O	GLU	188	36.494	-16.416	34.788	1.00	0.00	RX0	O
ATOM	300	N	ALA	189	35.058	-15.107	35.914	1.00	0.00	RX0	N
ATOM	301	H	ALA	189	34.839	-14.673	36.791	0.00	0.00	RX0	H
ATOM	302	CA	ALA	189	34.543	-14.432	34.708	1.00	0.00	RX0	C
ATOM	303	CB	ALA	189	33.067	-14.082	34.892	1.00	0.00	RX0	C

ATOM	304	C	ALA	189	35.336	-13.151	34.407	1.00	0.00	RX0	C
ATOM	305	O	ALA	189	35.533	-12.292	35.270	1.00	0.00	RX0	O
ATOM	306	N	SER	190	35.819	-13.065	33.173	1.00	0.00	RX0	N
ATOM	307	H	SER	190	35.612	-13.815	32.547	0.00	0.00	RX0	H
ATOM	308	CA	SER	190	36.430	-11.825	32.646	1.00	0.00	RX0	C
ATOM	309	CB	SER	190	37.099	-12.193	31.297	1.00	0.00	RX0	C
ATOM	310	OG	SER	190	38.282	-11.415	30.990	1.00	0.00	RX0	O
ATOM	311	HG	SER	190	38.800	-11.481	31.793	0.00	0.00	RX0	H
ATOM	312	C	SER	190	35.341	-10.761	32.513	1.00	0.00	RX0	C
ATOM	313	O	SER	190	34.465	-10.869	31.639	1.00	0.00	RX0	O
ATOM	314	N	MET	191	35.401	-9.751	33.361	1.00	0.00	RX0	N
ATOM	315	H	MET	191	36.037	-9.822	34.131	0.00	0.00	RX0	H
ATOM	316	CA	MET	191	34.414	-8.652	33.337	1.00	0.00	RX0	C
ATOM	317	CB	MET	191	34.641	-7.665	34.479	1.00	0.00	RX0	C
ATOM	318	CG	MET	191	33.544	-6.601	34.500	1.00	0.00	RX0	C
ATOM	319	SD	MET	191	33.634	-5.545	35.948	1.00	0.00	RX0	S
ATOM	320	CE	MET	191	33.275	-6.806	37.183	1.00	0.00	RX0	C
ATOM	321	C	MET	191	34.384	-7.939	31.976	1.00	0.00	RX0	C
ATOM	322	O	MET	191	33.329	-7.841	31.363	1.00	0.00	RX0	O
ATOM	323	N	MET	192	35.577	-7.654	31.438	1.00	0.00	RX0	N
ATOM	324	H	MET	192	36.383	-7.743	32.020	0.00	0.00	RX0	H
ATOM	325	CA	MET	192	35.697	-7.082	30.089	1.00	0.00	RX0	C
ATOM	326	CB	MET	192	37.135	-6.645	29.803	1.00	0.00	RX0	C
ATOM	327	CG	MET	192	37.270	-5.931	28.455	1.00	0.00	RX0	C
ATOM	328	SD	MET	192	36.116	-4.559	28.272	1.00	0.00	RX0	S
ATOM	329	CE	MET	192	36.710	-3.514	29.613	1.00	0.00	RX0	C
ATOM	330	C	MET	192	35.151	-8.021	28.999	1.00	0.00	RX0	C
ATOM	331	O	MET	192	34.484	-7.587	28.093	1.00	0.00	RX0	O
ATOM	332	N	GLY	193	35.358	-9.342	29.220	1.00	0.00	RX0	N
ATOM	333	H	GLY	193	35.697	-9.615	30.117	0.00	0.00	RX0	H
ATOM	334	CA	GLY	193	34.804	-10.378	28.330	1.00	0.00	RX0	C
ATOM	335	C	GLY	193	33.267	-10.338	28.334	1.00	0.00	RX0	C
ATOM	336	O	GLY	193	32.637	-10.184	27.296	1.00	0.00	RX0	O
ATOM	337	N	LEU	194	32.696	-10.293	29.537	1.00	0.00	RX0	N
ATOM	338	H	LEU	194	33.259	-10.321	30.358	0.00	0.00	RX0	H
ATOM	339	CA	LEU	194	31.235	-10.169	29.722	1.00	0.00	RX0	C
ATOM	340	CB	LEU	194	30.847	-10.224	31.198	1.00	0.00	RX0	C
ATOM	341	CG	LEU	194	31.124	-11.571	31.858	1.00	0.00	RX0	C
ATOM	342	CD1	LEU	194	30.684	-11.563	33.322	1.00	0.00	RX0	C
ATOM	343	CD2	LEU	194	30.499	-12.725	31.073	1.00	0.00	RX0	C
ATOM	344	C	LEU	194	30.647	-8.891	29.116	1.00	0.00	RX0	C
ATOM	345	O	LEU	194	29.706	-8.959	28.317	1.00	0.00	RX0	O
ATOM	346	N	LEU	195	31.327	-7.782	29.364	1.00	0.00	RX0	N
ATOM	347	H	LEU	195	32.122	-7.844	29.963	0.00	0.00	RX0	H
ATOM	348	CA	LEU	195	30.920	-6.462	28.846	1.00	0.00	RX0	C
ATOM	349	CB	LEU	195	31.733	-5.338	29.490	1.00	0.00	RX0	C
ATOM	350	CG	LEU	195	31.536	-5.238	31.004	1.00	0.00	RX0	C
ATOM	351	CD1	LEU	195	32.374	-4.110	31.608	1.00	0.00	RX0	C
ATOM	352	CD2	LEU	195	30.061	-5.125	31.383	1.00	0.00	RX0	C
ATOM	353	C	LEU	195	31.020	-6.357	27.321	1.00	0.00	RX0	C
ATOM	354	O	LEU	195	30.051	-5.942	26.671	1.00	0.00	RX0	O
ATOM	355	N	THR	196	32.075	-6.931	26.767	1.00	0.00	RX0	N
ATOM	356	H	THR	196	32.735	-7.387	27.363	0.00	0.00	RX0	H
ATOM	357	CA	THR	196	32.335	-6.901	25.309	1.00	0.00	RX0	C
ATOM	358	CB	THR	196	33.776	-7.306	25.015	1.00	0.00	RX0	C
ATOM	359	OG1	THR	196	34.657	-6.432	25.724	1.00	0.00	RX0	O
ATOM	360	HG1	THR	196	34.408	-6.481	26.639	0.00	0.00	RX0	H
ATOM	361	CG2	THR	196	34.122	-7.299	23.522	1.00	0.00	RX0	C
ATOM	362	C	THR	196	31.317	-7.765	24.552	1.00	0.00	RX0	C
ATOM	363	O	THR	196	30.772	-7.327	23.532	1.00	0.00	RX0	O
ATOM	364	N	ASN	197	31.003	-8.928	25.107	1.00	0.00	RX0	N

ATOM	365	H	ASN	197	31.448	-9.188	25.967	0.00	0.00	RX0	H
ATOM	366	CA	ASN	197	30.010	-9.840	24.504	1.00	0.00	RX0	C
ATOM	367	CB	ASN	197	30.095	-11.230	25.118	1.00	0.00	RX0	C
ATOM	368	CG	ASN	197	30.519	-12.188	24.027	1.00	0.00	RX0	C
ATOM	369	OD1	ASN	197	31.376	-11.896	23.193	1.00	0.00	RX0	O
ATOM	370	ND2	ASN	197	29.888	-13.358	24.062	1.00	0.00	RX0	N
ATOM	371	HD21	ASN	197	29.143	-13.469	24.731	0.00	0.00	RX0	H
ATOM	372	HD22	ASN	197	30.096	-14.110	23.445	0.00	0.00	RX0	H
ATOM	373	C	ASN	197	28.594	-9.255	24.528	1.00	0.00	RX0	C
ATOM	374	O	ASN	197	27.900	-9.272	23.514	1.00	0.00	RX0	O
ATOM	375	N	LEU	198	28.277	-8.575	25.633	1.00	0.00	RX0	N
ATOM	376	H	LEU	198	28.914	-8.586	26.405	0.00	0.00	RX0	H
ATOM	377	CA	LEU	198	27.002	-7.850	25.760	1.00	0.00	RX0	C
ATOM	378	CB	LEU	198	26.864	-7.329	27.188	1.00	0.00	RX0	C
ATOM	379	CG	LEU	198	25.549	-6.606	27.469	1.00	0.00	RX0	C
ATOM	380	CD1	LEU	198	24.323	-7.503	27.285	1.00	0.00	RX0	C
ATOM	381	CD2	LEU	198	25.584	-5.971	28.854	1.00	0.00	RX0	C
ATOM	382	C	LEU	198	26.885	-6.719	24.724	1.00	0.00	RX0	C
ATOM	383	O	LEU	198	25.930	-6.676	23.947	1.00	0.00	RX0	O
ATOM	384	N	ALA	199	27.942	-5.914	24.641	1.00	0.00	RX0	N
ATOM	385	H	ALA	199	28.689	-6.056	25.292	0.00	0.00	RX0	H
ATOM	386	CA	ALA	199	28.029	-4.784	23.694	1.00	0.00	RX0	C
ATOM	387	CB	ALA	199	29.338	-4.020	23.891	1.00	0.00	RX0	C
ATOM	388	C	ALA	199	27.921	-5.230	22.227	1.00	0.00	RX0	C
ATOM	389	O	ALA	199	27.138	-4.660	21.467	1.00	0.00	RX0	O
ATOM	390	N	ASP	200	28.555	-6.360	21.908	1.00	0.00	RX0	N
ATOM	391	H	ASP	200	29.232	-6.726	22.549	0.00	0.00	RX0	H
ATOM	392	CA	ASP	200	28.494	-6.940	20.550	1.00	0.00	RX0	C
ATOM	393	CB	ASP	200	29.502	-8.082	20.447	1.00	0.00	RX0	C
ATOM	394	CG	ASP	200	30.910	-7.526	20.560	1.00	0.00	RX0	C
ATOM	395	OD1	ASP	200	31.170	-6.458	20.027	1.00	0.00	RX0	O
ATOM	396	OD2	ASP	200	31.771	-8.180	21.134	1.00	0.00	RX0	O
ATOM	397	C	ASP	200	27.084	-7.410	20.171	1.00	0.00	RX0	C
ATOM	398	O	ASP	200	26.604	-7.102	19.080	1.00	0.00	RX0	O
ATOM	399	N	ARG	201	26.390	-7.998	21.143	1.00	0.00	RX0	N
ATOM	400	H	ARG	201	26.854	-8.133	22.022	0.00	0.00	RX0	H
ATOM	401	CA	ARG	201	24.992	-8.434	20.957	1.00	0.00	RX0	C
ATOM	402	CB	ARG	201	24.560	-9.385	22.059	1.00	0.00	RX0	C
ATOM	403	CG	ARG	201	25.023	-10.809	21.778	1.00	0.00	RX0	C
ATOM	404	CD	ARG	201	24.543	-11.756	22.870	1.00	0.00	RX0	C
ATOM	405	NE	ARG	201	25.164	-11.412	24.143	1.00	0.00	RX0	N
ATOM	406	HE	ARG	201	26.157	-11.587	24.232	0.00	0.00	RX0	H
ATOM	407	CZ	ARG	201	24.415	-11.031	25.212	1.00	0.00	RX0	C
ATOM	408	NH1	ARG	201	23.079	-10.897	25.093	1.00	0.00	RX0	N
ATOM	409	HH11	ARG	201	22.524	-10.709	25.908	0.00	0.00	RX0	H
ATOM	410	HH12	ARG	201	22.615	-10.992	24.209	0.00	0.00	RX0	H
ATOM	411	NH2	ARG	201	25.011	-10.814	26.392	1.00	0.00	RX0	N
ATOM	412	HH21	ARG	201	24.495	-10.552	27.213	0.00	0.00	RX0	H
ATOM	413	HH22	ARG	201	26.007	-10.979	26.504	0.00	0.00	RX0	H
ATOM	414	C	ARG	201	23.991	-7.279	20.827	1.00	0.00	RX0	C
ATOM	415	O	ARG	201	23.123	-7.308	19.955	1.00	0.00	RX0	O
ATOM	416	N	GLU	202	24.240	-6.201	21.568	1.00	0.00	RX0	N
ATOM	417	H	GLU	202	25.003	-6.207	22.220	0.00	0.00	RX0	H
ATOM	418	CA	GLU	202	23.401	-4.988	21.493	1.00	0.00	RX0	C
ATOM	419	CB	GLU	202	23.556	-4.080	22.734	1.00	0.00	RX0	C
ATOM	420	CG	GLU	202	22.899	-4.685	23.994	1.00	0.00	RX0	C
ATOM	421	CD	GLU	202	22.851	-3.727	25.188	1.00	0.00	RX0	C
ATOM	422	OE1	GLU	202	21.825	-3.086	25.419	1.00	0.00	RX0	O
ATOM	423	OE2	GLU	202	23.809	-3.665	25.953	1.00	0.00	RX0	O
ATOM	424	C	GLU	202	23.526	-4.262	20.149	1.00	0.00	RX0	C
ATOM	425	O	GLU	202	22.539	-3.754	19.625	1.00	0.00	RX0	O

ATOM	426	N	LEU	203	24.712	-4.358	19.546	1.00	0.00	RX0	N
ATOM	427	H	LEU	203	25.459	-4.782	20.065	0.00	0.00	RX0	H
ATOM	428	CA	LEU	203	25.004	-3.680	18.270	1.00	0.00	RX0	C
ATOM	429	CB	LEU	203	26.494	-3.846	17.947	1.00	0.00	RX0	C
ATOM	430	CG	LEU	203	27.018	-3.038	16.754	1.00	0.00	RX0	C
ATOM	431	CD1	LEU	203	26.714	-1.544	16.872	1.00	0.00	RX0	C
ATOM	432	CD2	LEU	203	28.511	-3.288	16.536	1.00	0.00	RX0	C
ATOM	433	C	LEU	203	24.099	-4.160	17.127	1.00	0.00	RX0	C
ATOM	434	O	LEU	203	23.593	-3.349	16.346	1.00	0.00	RX0	O
ATOM	435	N	VAL	204	23.782	-5.447	17.151	1.00	0.00	RX0	N
ATOM	436	H	VAL	204	24.159	-6.006	17.893	0.00	0.00	RX0	H
ATOM	437	CA	VAL	204	22.925	-6.083	16.127	1.00	0.00	RX0	C
ATOM	438	CB	VAL	204	22.899	-7.597	16.333	1.00	0.00	RX0	C
ATOM	439	CG1	VAL	204	22.012	-8.278	15.289	1.00	0.00	RX0	C
ATOM	440	CG2	VAL	204	24.320	-8.163	16.351	1.00	0.00	RX0	C
ATOM	441	C	VAL	204	21.502	-5.497	16.213	1.00	0.00	RX0	C
ATOM	442	O	VAL	204	20.938	-5.041	15.221	1.00	0.00	RX0	O
ATOM	443	N	HIS	205	21.015	-5.393	17.448	1.00	0.00	RX0	N
ATOM	444	H	HIS	205	21.602	-5.675	18.210	0.00	0.00	RX0	H
ATOM	445	CA	HIS	205	19.703	-4.786	17.746	1.00	0.00	RX0	C
ATOM	446	CB	HIS	205	19.274	-5.100	19.184	1.00	0.00	RX0	C
ATOM	447	CG	HIS	205	19.000	-6.579	19.311	1.00	0.00	RX0	C
ATOM	448	ND1	HIS	205	17.801	-7.139	19.068	1.00	0.00	RX0	N
ATOM	449	HD1	HIS	205	16.982	-6.673	18.805	0.00	0.00	RX0	H
ATOM	450	CD2	HIS	205	19.898	-7.584	19.679	1.00	0.00	RX0	C
ATOM	451	NE2	HIS	205	19.224	-8.760	19.656	1.00	0.00	RX0	N
ATOM	452	CE1	HIS	205	17.936	-8.486	19.280	1.00	0.00	RX0	C
ATOM	453	C	HIS	205	19.668	-3.277	17.476	1.00	0.00	RX0	C
ATOM	454	O	HIS	205	18.642	-2.756	17.030	1.00	0.00	RX0	O
ATOM	455	N	MET	206	20.820	-2.627	17.609	1.00	0.00	RX0	N
ATOM	456	H	MET	206	21.609	-3.124	17.976	0.00	0.00	RX0	H
ATOM	457	CA	MET	206	20.969	-1.185	17.340	1.00	0.00	RX0	C
ATOM	458	CB	MET	206	22.353	-0.693	17.755	1.00	0.00	RX0	C
ATOM	459	CG	MET	206	22.523	0.813	17.554	1.00	0.00	RX0	C
ATOM	460	SD	MET	206	24.209	1.345	17.875	1.00	0.00	RX0	S
ATOM	461	CE	MET	206	24.431	0.498	19.446	1.00	0.00	RX0	C
ATOM	462	C	MET	206	20.721	-0.870	15.856	1.00	0.00	RX0	C
ATOM	463	O	MET	206	20.035	0.103	15.544	1.00	0.00	RX0	O
ATOM	464	N	ILE	207	21.183	-1.758	14.977	1.00	0.00	RX0	N
ATOM	465	H	ILE	207	21.731	-2.518	15.338	0.00	0.00	RX0	H
ATOM	466	CA	ILE	207	20.975	-1.628	13.516	1.00	0.00	RX0	C
ATOM	467	CB	ILE	207	21.678	-2.781	12.801	1.00	0.00	RX0	C
ATOM	468	CG2	ILE	207	21.410	-2.786	11.298	1.00	0.00	RX0	C
ATOM	469	CG1	ILE	207	23.171	-2.744	13.107	1.00	0.00	RX0	C
ATOM	470	CD1	ILE	207	23.863	-1.515	12.521	1.00	0.00	RX0	C
ATOM	471	C	ILE	207	19.470	-1.621	13.197	1.00	0.00	RX0	C
ATOM	472	O	ILE	207	18.988	-0.755	12.467	1.00	0.00	RX0	O
ATOM	473	N	ASN	208	18.761	-2.558	13.816	1.00	0.00	RX0	N
ATOM	474	H	ASN	208	19.237	-3.126	14.490	0.00	0.00	RX0	H
ATOM	475	CA	ASN	208	17.313	-2.734	13.586	1.00	0.00	RX0	C
ATOM	476	CB	ASN	208	16.808	-4.070	14.127	1.00	0.00	RX0	C
ATOM	477	CG	ASN	208	17.155	-5.154	13.122	1.00	0.00	RX0	C
ATOM	478	OD1	ASN	208	17.524	-4.888	11.973	1.00	0.00	RX0	O
ATOM	479	ND2	ASN	208	17.037	-6.401	13.616	1.00	0.00	RX0	N
ATOM	480	HD21	ASN	208	16.730	-6.562	14.556	0.00	0.00	RX0	H
ATOM	481	HD22	ASN	208	17.257	-7.216	13.079	0.00	0.00	RX0	H
ATOM	482	C	ASN	208	16.516	-1.532	14.103	1.00	0.00	RX0	C
ATOM	483	O	ASN	208	15.637	-1.017	13.417	1.00	0.00	RX0	O
ATOM	484	N	TRP	209	16.982	-1.008	15.238	1.00	0.00	RX0	N
ATOM	485	H	TRP	209	17.725	-1.473	15.722	0.00	0.00	RX0	H
ATOM	486	CA	TRP	209	16.425	0.201	15.864	1.00	0.00	RX0	C

ATOM	487	CB	TRP	209	17.093	0.419	17.231	1.00	0.00	RX0	C
ATOM	488	CG	TRP	209	16.695	1.748	17.837	1.00	0.00	RX0	C
ATOM	489	CD2	TRP	209	17.414	3.000	17.806	1.00	0.00	RX0	C
ATOM	490	CE2	TRP	209	16.634	3.960	18.496	1.00	0.00	RX0	C
ATOM	491	CE3	TRP	209	18.633	3.371	17.253	1.00	0.00	RX0	C
ATOM	492	CD1	TRP	209	15.527	2.029	18.555	1.00	0.00	RX0	C
ATOM	493	NE1	TRP	209	15.480	3.326	18.951	1.00	0.00	RX0	N
ATOM	494	HE1	TRP	209	14.724	3.718	19.455	0.00	0.00	RX0	H
ATOM	495	CZ2	TRP	209	17.098	5.266	18.599	1.00	0.00	RX0	C
ATOM	496	CZ3	TRP	209	19.086	4.679	17.370	1.00	0.00	RX0	C
ATOM	497	CH2	TRP	209	18.317	5.625	18.036	1.00	0.00	RX0	C
ATOM	498	C	TRP	209	16.619	1.438	14.972	1.00	0.00	RX0	C
ATOM	499	O	TRP	209	15.652	2.120	14.634	1.00	0.00	RX0	O
ATOM	500	N	ALA	210	17.853	1.624	14.503	1.00	0.00	RX0	N
ATOM	501	H	ALA	210	18.568	0.988	14.792	0.00	0.00	RX0	H
ATOM	502	CA	ALA	210	18.233	2.756	13.635	1.00	0.00	RX0	C
ATOM	503	CB	ALA	210	19.715	2.665	13.273	1.00	0.00	RX0	C
ATOM	504	C	ALA	210	17.400	2.800	12.347	1.00	0.00	RX0	C
ATOM	505	O	ALA	210	16.892	3.855	11.979	1.00	0.00	RX0	O
ATOM	506	N	LYS	211	17.095	1.613	11.820	1.00	0.00	RX0	N
ATOM	507	H	LYS	211	17.503	0.799	12.236	0.00	0.00	RX0	H
ATOM	508	CA	LYS	211	16.258	1.472	10.614	1.00	0.00	RX0	C
ATOM	509	CB	LYS	211	16.405	0.071	10.022	1.00	0.00	RX0	C
ATOM	510	CG	LYS	211	17.792	-0.083	9.389	1.00	0.00	RX0	C
ATOM	511	CD	LYS	211	18.069	-1.490	8.860	1.00	0.00	RX0	C
ATOM	512	CE	LYS	211	17.852	-2.503	9.977	1.00	0.00	RX0	C
ATOM	513	NZ	LYS	211	18.357	-3.837	9.626	1.00	0.00	RX0	N
ATOM	514	HZ1	LYS	211	18.194	-4.458	10.453	0.00	0.00	RX0	H
ATOM	515	HZ2	LYS	211	17.844	-4.212	8.806	0.00	0.00	RX0	H
ATOM	516	HZ3	LYS	211	19.374	-3.797	9.412	0.00	0.00	RX0	H
ATOM	517	C	LYS	211	14.795	1.898	10.823	1.00	0.00	RX0	C
ATOM	518	O	LYS	211	14.129	2.337	9.881	1.00	0.00	RX0	O
ATOM	519	N	ARG	212	14.357	1.870	12.071	1.00	0.00	RX0	N
ATOM	520	H	ARG	212	14.980	1.610	12.810	0.00	0.00	RX0	H
ATOM	521	CA	ARG	212	13.005	2.320	12.466	1.00	0.00	RX0	C
ATOM	522	CB	ARG	212	12.388	1.396	13.516	1.00	0.00	RX0	C
ATOM	523	CG	ARG	212	12.542	-0.088	13.182	1.00	0.00	RX0	C
ATOM	524	CD	ARG	212	11.665	-0.987	14.056	1.00	0.00	RX0	C
ATOM	525	NE	ARG	212	10.259	-0.783	13.718	1.00	0.00	RX0	N
ATOM	526	HE	ARG	212	10.062	-0.550	12.761	0.00	0.00	RX0	H
ATOM	527	CZ	ARG	212	9.288	-0.890	14.673	1.00	0.00	RX0	C
ATOM	528	NH1	ARG	212	9.605	-1.229	15.938	1.00	0.00	RX0	N
ATOM	529	HH11	ARG	212	8.886	-1.236	16.662	0.00	0.00	RX0	H
ATOM	530	HH12	ARG	212	10.526	-1.462	16.252	0.00	0.00	RX0	H
ATOM	531	NH2	ARG	212	8.011	-0.638	14.339	1.00	0.00	RX0	N
ATOM	532	HH21	ARG	212	7.295	-0.630	15.066	0.00	0.00	RX0	H
ATOM	533	HH22	ARG	212	7.684	-0.412	13.425	0.00	0.00	RX0	H
ATOM	534	C	ARG	212	12.933	3.790	12.894	1.00	0.00	RX0	C
ATOM	535	O	ARG	212	11.827	4.344	12.989	1.00	0.00	RX0	O
ATOM	536	N	VAL	213	14.074	4.417	13.148	1.00	0.00	RX0	N
ATOM	537	H	VAL	213	14.933	3.927	12.994	0.00	0.00	RX0	H
ATOM	538	CA	VAL	213	14.155	5.874	13.374	1.00	0.00	RX0	C
ATOM	539	CB	VAL	213	15.581	6.281	13.748	1.00	0.00	RX0	C
ATOM	540	CG1	VAL	213	15.751	7.800	13.838	1.00	0.00	RX0	C
ATOM	541	CG2	VAL	213	15.980	5.583	15.046	1.00	0.00	RX0	C
ATOM	542	C	VAL	213	13.672	6.590	12.095	1.00	0.00	RX0	C
ATOM	543	O	VAL	213	14.288	6.431	11.023	1.00	0.00	RX0	O
ATOM	544	N	PRO	214	12.622	7.395	12.213	1.00	0.00	RX0	N
ATOM	545	CD	PRO	214	11.895	7.609	13.458	1.00	0.00	RX0	C
ATOM	546	CA	PRO	214	12.035	8.145	11.084	1.00	0.00	RX0	C
ATOM	547	CB	PRO	214	10.927	8.954	11.761	1.00	0.00	RX0	C

ATOM	548	CG	PRO	214	10.552	8.163	13.010	1.00	0.00	RX0	C
ATOM	549	C	PRO	214	13.102	9.001	10.387	1.00	0.00	RX0	C
ATOM	550	O	PRO	214	13.853	9.727	11.025	1.00	0.00	RX0	O
ATOM	551	N	GLY	215	13.244	8.730	9.080	1.00	0.00	RX0	N
ATOM	552	H	GLY	215	12.749	7.982	8.637	0.00	0.00	RX0	H
ATOM	553	CA	GLY	215	14.194	9.473	8.227	1.00	0.00	RX0	C
ATOM	554	C	GLY	215	15.511	8.732	7.950	1.00	0.00	RX0	C
ATOM	555	O	GLY	215	16.085	8.889	6.862	1.00	0.00	RX0	O
ATOM	556	N	PHE	216	15.917	7.845	8.848	1.00	0.00	RX0	N
ATOM	557	H	PHE	216	15.330	7.653	9.637	0.00	0.00	RX0	H
ATOM	558	CA	PHE	216	17.224	7.160	8.764	1.00	0.00	RX0	C
ATOM	559	CB	PHE	216	17.451	6.293	9.997	1.00	0.00	RX0	C
ATOM	560	CG	PHE	216	18.892	5.848	10.057	1.00	0.00	RX0	C
ATOM	561	CD1	PHE	216	19.893	6.780	10.299	1.00	0.00	RX0	C
ATOM	562	CD2	PHE	216	19.217	4.508	9.885	1.00	0.00	RX0	C
ATOM	563	CE1	PHE	216	21.215	6.368	10.406	1.00	0.00	RX0	C
ATOM	564	CE2	PHE	216	20.539	4.095	9.994	1.00	0.00	RX0	C
ATOM	565	CZ	PHE	216	21.535	5.023	10.275	1.00	0.00	RX0	C
ATOM	566	C	PHE	216	17.435	6.347	7.474	1.00	0.00	RX0	C
ATOM	567	O	PHE	216	18.358	6.641	6.711	1.00	0.00	RX0	O
ATOM	568	N	VAL	217	16.482	5.483	7.151	1.00	0.00	RX0	N
ATOM	569	H	VAL	217	15.675	5.447	7.738	0.00	0.00	RX0	H
ATOM	570	CA	VAL	217	16.570	4.608	5.958	1.00	0.00	RX0	C
ATOM	571	CB	VAL	217	15.526	3.497	6.007	1.00	0.00	RX0	C
ATOM	572	CG1	VAL	217	15.896	2.479	7.076	1.00	0.00	RX0	C
ATOM	573	CG2	VAL	217	14.111	4.056	6.173	1.00	0.00	RX0	C
ATOM	574	C	VAL	217	16.469	5.342	4.608	1.00	0.00	RX0	C
ATOM	575	O	VAL	217	16.660	4.747	3.556	1.00	0.00	RX0	O
ATOM	576	N	ASP	218	16.058	6.613	4.671	1.00	0.00	RX0	N
ATOM	577	H	ASP	218	15.764	7.060	5.519	0.00	0.00	RX0	H
ATOM	578	CA	ASP	218	16.006	7.470	3.472	1.00	0.00	RX0	C
ATOM	579	CB	ASP	218	15.084	8.664	3.757	1.00	0.00	RX0	C
ATOM	580	CG	ASP	218	13.739	8.218	4.327	1.00	0.00	RX0	C
ATOM	581	OD1	ASP	218	12.745	8.296	3.607	1.00	0.00	RX0	O
ATOM	582	OD2	ASP	218	13.669	7.822	5.495	1.00	0.00	RX0	O
ATOM	583	C	ASP	218	17.401	7.924	3.023	1.00	0.00	RX0	C
ATOM	584	O	ASP	218	17.595	8.369	1.896	1.00	0.00	RX0	O
ATOM	585	N	LEU	219	18.344	7.857	3.967	1.00	0.00	RX0	N
ATOM	586	H	LEU	219	18.094	7.489	4.863	0.00	0.00	RX0	H
ATOM	587	CA	LEU	219	19.767	8.083	3.697	1.00	0.00	RX0	C
ATOM	588	CB	LEU	219	20.540	8.282	4.997	1.00	0.00	RX0	C
ATOM	589	CG	LEU	219	19.972	9.467	5.781	1.00	0.00	RX0	C
ATOM	590	CD1	LEU	219	20.525	9.527	7.203	1.00	0.00	RX0	C
ATOM	591	CD2	LEU	219	20.144	10.786	5.024	1.00	0.00	RX0	C
ATOM	592	C	LEU	219	20.350	6.970	2.832	1.00	0.00	RX0	C
ATOM	593	O	LEU	219	19.843	5.836	2.789	1.00	0.00	RX0	O
ATOM	594	N	THR	220	21.442	7.293	2.191	1.00	0.00	RX0	N
ATOM	595	H	THR	220	21.808	8.211	2.346	0.00	0.00	RX0	H
ATOM	596	CA	THR	220	22.263	6.312	1.453	1.00	0.00	RX0	C
ATOM	597	CB	THR	220	23.292	7.150	0.726	1.00	0.00	RX0	C
ATOM	598	OG1	THR	220	23.311	8.444	1.325	1.00	0.00	RX0	O
ATOM	599	HG1	THR	220	22.687	9.013	0.873	0.00	0.00	RX0	H
ATOM	600	CG2	THR	220	22.972	7.266	-0.764	1.00	0.00	RX0	C
ATOM	601	C	THR	220	22.829	5.303	2.454	1.00	0.00	RX0	C
ATOM	602	O	THR	220	23.112	5.646	3.611	1.00	0.00	RX0	O
ATOM	603	N	LEU	221	23.130	4.116	1.957	1.00	0.00	RX0	N
ATOM	604	H	LEU	221	22.846	3.902	1.025	0.00	0.00	RX0	H
ATOM	605	CA	LEU	221	23.750	3.065	2.781	1.00	0.00	RX0	C
ATOM	606	CB	LEU	221	23.957	1.860	1.872	1.00	0.00	RX0	C
ATOM	607	CG	LEU	221	24.852	0.776	2.457	1.00	0.00	RX0	C
ATOM	608	CD1	LEU	221	24.193	0.052	3.629	1.00	0.00	RX0	C

ATOM	609	CD2	LEU	221	25.328	-0.171	1.358	1.00	0.00	RX0	C
ATOM	610	C	LEU	221	25.092	3.509	3.394	1.00	0.00	RX0	C
ATOM	611	O	LEU	221	25.324	3.347	4.578	1.00	0.00	RX0	O
ATOM	612	N	HIS	222	25.854	4.270	2.593	1.00	0.00	RX0	N
ATOM	613	H	HIS	222	25.546	4.445	1.662	0.00	0.00	RX0	H
ATOM	614	CA	HIS	222	27.131	4.847	3.045	1.00	0.00	RX0	C
ATOM	615	CB	HIS	222	27.830	5.627	1.931	1.00	0.00	RX0	C
ATOM	616	CG	HIS	222	29.068	6.299	2.489	1.00	0.00	RX0	C
ATOM	617	ND1	HIS	222	30.063	5.645	3.122	1.00	0.00	RX0	N
ATOM	618	HD1	HIS	222	30.125	4.682	3.317	0.00	0.00	RX0	H
ATOM	619	CD2	HIS	222	29.393	7.660	2.441	1.00	0.00	RX0	C
ATOM	620	NE2	HIS	222	30.596	7.816	3.051	1.00	0.00	RX0	N
ATOM	621	CE1	HIS	222	31.005	6.577	3.470	1.00	0.00	RX0	C
ATOM	622	C	HIS	222	26.942	5.765	4.264	1.00	0.00	RX0	C
ATOM	623	O	HIS	222	27.673	5.642	5.246	1.00	0.00	RX0	O
ATOM	624	N	ASP	223	25.958	6.658	4.167	1.00	0.00	RX0	N
ATOM	625	H	ASP	223	25.463	6.827	3.315	0.00	0.00	RX0	H
ATOM	626	CA	ASP	223	25.694	7.636	5.242	1.00	0.00	RX0	C
ATOM	627	CB	ASP	223	24.873	8.819	4.713	1.00	0.00	RX0	C
ATOM	628	CG	ASP	223	25.749	9.655	3.783	1.00	0.00	RX0	C
ATOM	629	OD1	ASP	223	26.258	9.114	2.799	1.00	0.00	RX0	O
ATOM	630	OD2	ASP	223	25.934	10.844	4.051	1.00	0.00	RX0	O
ATOM	631	C	ASP	223	25.148	6.996	6.516	1.00	0.00	RX0	C
ATOM	632	O	ASP	223	25.558	7.375	7.616	1.00	0.00	RX0	O
ATOM	633	N	GLN	224	24.393	5.921	6.332	1.00	0.00	RX0	N
ATOM	634	H	GLN	224	24.149	5.692	5.387	0.00	0.00	RX0	H
ATOM	635	CA	GLN	224	23.868	5.118	7.452	1.00	0.00	RX0	C
ATOM	636	CB	GLN	224	22.845	4.094	6.959	1.00	0.00	RX0	C
ATOM	637	CG	GLN	224	21.593	4.765	6.390	1.00	0.00	RX0	C
ATOM	638	CD	GLN	224	20.547	3.715	6.084	1.00	0.00	RX0	C
ATOM	639	OE1	GLN	224	20.390	2.736	6.808	1.00	0.00	RX0	O
ATOM	640	NE2	GLN	224	19.839	3.966	4.970	1.00	0.00	RX0	N
ATOM	641	HE21	GLN	224	19.999	4.788	4.415	0.00	0.00	RX0	H
ATOM	642	HE22	GLN	224	19.110	3.376	4.627	0.00	0.00	RX0	H
ATOM	643	C	GLN	224	25.003	4.453	8.243	1.00	0.00	RX0	C
ATOM	644	O	GLN	224	25.073	4.591	9.468	1.00	0.00	RX0	O
ATOM	645	N	VAL	225	25.993	3.956	7.505	1.00	0.00	RX0	N
ATOM	646	H	VAL	225	25.892	3.979	6.507	0.00	0.00	RX0	H
ATOM	647	CA	VAL	225	27.191	3.319	8.093	1.00	0.00	RX0	C
ATOM	648	CB	VAL	225	28.045	2.593	7.053	1.00	0.00	RX0	C
ATOM	649	CG1	VAL	225	29.235	1.920	7.733	1.00	0.00	RX0	C
ATOM	650	CG2	VAL	225	27.223	1.561	6.285	1.00	0.00	RX0	C
ATOM	651	C	VAL	225	28.021	4.368	8.852	1.00	0.00	RX0	C
ATOM	652	O	VAL	225	28.415	4.141	9.995	1.00	0.00	RX0	O
ATOM	653	N	HIS	226	28.182	5.534	8.231	1.00	0.00	RX0	N
ATOM	654	H	HIS	226	27.811	5.620	7.303	0.00	0.00	RX0	H
ATOM	655	CA	HIS	226	28.959	6.641	8.815	1.00	0.00	RX0	C
ATOM	656	CB	HIS	226	29.084	7.807	7.818	1.00	0.00	RX0	C
ATOM	657	CG	HIS	226	30.104	8.844	8.256	1.00	0.00	RX0	C
ATOM	658	ND1	HIS	226	31.306	8.983	7.672	1.00	0.00	RX0	N
ATOM	659	HD1	HIS	226	31.653	8.452	6.927	0.00	0.00	RX0	H
ATOM	660	CD2	HIS	226	29.991	9.813	9.262	1.00	0.00	RX0	C
ATOM	661	NE2	HIS	226	31.140	10.535	9.273	1.00	0.00	RX0	N
ATOM	662	CE1	HIS	226	31.949	10.022	8.292	1.00	0.00	RX0	C
ATOM	663	C	HIS	226	28.363	7.118	10.150	1.00	0.00	RX0	C
ATOM	664	O	HIS	226	29.071	7.189	11.155	1.00	0.00	RX0	O
ATOM	665	N	LEU	227	27.047	7.317	10.166	1.00	0.00	RX0	N
ATOM	666	H	LEU	227	26.532	7.187	9.317	0.00	0.00	RX0	H
ATOM	667	CA	LEU	227	26.344	7.787	11.375	1.00	0.00	RX0	C
ATOM	668	CB	LEU	227	24.876	8.086	11.076	1.00	0.00	RX0	C
ATOM	669	CG	LEU	227	24.677	9.371	10.273	1.00	0.00	RX0	C

ATOM	670	CD1	LEU	227	23.209	9.582	9.909	1.00	0.00	RX0	C
ATOM	671	CD2	LEU	227	25.248	10.587	11.001	1.00	0.00	RX0	C
ATOM	672	C	LEU	227	26.435	6.799	12.540	1.00	0.00	RX0	C
ATOM	673	O	LEU	227	26.853	7.165	13.635	1.00	0.00	RX0	O
ATOM	674	N	LEU	228	26.270	5.522	12.200	1.00	0.00	RX0	N
ATOM	675	H	LEU	228	26.028	5.299	11.252	0.00	0.00	RX0	H
ATOM	676	CA	LEU	228	26.384	4.431	13.181	1.00	0.00	RX0	C
ATOM	677	CB	LEU	228	25.754	3.160	12.621	1.00	0.00	RX0	C
ATOM	678	CG	LEU	228	24.250	3.162	12.888	1.00	0.00	RX0	C
ATOM	679	CD1	LEU	228	23.488	2.194	11.988	1.00	0.00	RX0	C
ATOM	680	CD2	LEU	228	23.960	2.913	14.369	1.00	0.00	RX0	C
ATOM	681	C	LEU	228	27.805	4.188	13.685	1.00	0.00	RX0	C
ATOM	682	O	LEU	228	28.004	4.019	14.891	1.00	0.00	RX0	O
ATOM	683	N	GLU	229	28.784	4.376	12.809	1.00	0.00	RX0	N
ATOM	684	H	GLU	229	28.552	4.544	11.850	0.00	0.00	RX0	H
ATOM	685	CA	GLU	229	30.199	4.229	13.197	1.00	0.00	RX0	C
ATOM	686	CB	GLU	229	31.098	4.216	11.962	1.00	0.00	RX0	C
ATOM	687	CG	GLU	229	31.775	2.876	11.645	1.00	0.00	RX0	C
ATOM	688	CD	GLU	229	32.976	2.630	12.538	1.00	0.00	RX0	C
ATOM	689	OE1	GLU	229	34.094	2.554	12.033	1.00	0.00	RX0	O
ATOM	690	OE2	GLU	229	32.834	2.530	13.753	1.00	0.00	RX0	O
ATOM	691	C	GLU	229	30.618	5.338	14.175	1.00	0.00	RX0	C
ATOM	692	O	GLU	229	31.393	5.088	15.099	1.00	0.00	RX0	O
ATOM	693	N	CYS	230	30.060	6.523	13.970	1.00	0.00	RX0	N
ATOM	694	H	CYS	230	29.470	6.618	13.163	0.00	0.00	RX0	H
ATOM	695	CA	CYS	230	30.321	7.692	14.829	1.00	0.00	RX0	C
ATOM	696	CB	CYS	230	29.975	8.989	14.104	1.00	0.00	RX0	C
ATOM	697	SG	CYS	230	31.442	9.837	13.472	1.00	0.00	RX0	S
ATOM	698	C	CYS	230	29.592	7.653	16.182	1.00	0.00	RX0	C
ATOM	699	O	CYS	230	30.123	8.119	17.188	1.00	0.00	RX0	O
ATOM	700	N	ALA	231	28.434	7.000	16.215	1.00	0.00	RX0	N
ATOM	701	H	ALA	231	28.101	6.540	15.391	0.00	0.00	RX0	H
ATOM	702	CA	ALA	231	27.494	7.141	17.345	1.00	0.00	RX0	C
ATOM	703	CB	ALA	231	26.144	7.665	16.849	1.00	0.00	RX0	C
ATOM	704	C	ALA	231	27.249	5.885	18.186	1.00	0.00	RX0	C
ATOM	705	O	ALA	231	26.768	6.021	19.321	1.00	0.00	RX0	O
ATOM	706	N	TRP	232	27.687	4.719	17.731	1.00	0.00	RX0	N
ATOM	707	H	TRP	232	28.089	4.679	16.813	0.00	0.00	RX0	H
ATOM	708	CA	TRP	232	27.348	3.433	18.379	1.00	0.00	RX0	C
ATOM	709	CB	TRP	232	27.716	2.195	17.560	1.00	0.00	RX0	C
ATOM	710	CG	TRP	232	29.179	1.888	17.640	1.00	0.00	RX0	C
ATOM	711	CD2	TRP	232	29.859	1.126	18.653	1.00	0.00	RX0	C
ATOM	712	CE2	TRP	232	31.245	1.109	18.300	1.00	0.00	RX0	C
ATOM	713	CE3	TRP	232	29.415	0.469	19.821	1.00	0.00	RX0	C
ATOM	714	CD1	TRP	232	30.162	2.289	16.732	1.00	0.00	RX0	C
ATOM	715	NE1	TRP	232	31.381	1.833	17.113	1.00	0.00	RX0	N
ATOM	716	HE1	TRP	232	32.193	1.996	16.578	0.00	0.00	RX0	H
ATOM	717	CZ2	TRP	232	32.159	0.432	19.131	1.00	0.00	RX0	C
ATOM	718	CZ3	TRP	232	30.345	-0.202	20.641	1.00	0.00	RX0	C
ATOM	719	CH2	TRP	232	31.712	-0.218	20.299	1.00	0.00	RX0	C
ATOM	720	C	TRP	232	27.676	3.373	19.884	1.00	0.00	RX0	C
ATOM	721	O	TRP	232	26.862	2.914	20.672	1.00	0.00	RX0	O
ATOM	722	N	LEU	233	28.801	3.989	20.277	1.00	0.00	RX0	N
ATOM	723	H	LEU	233	29.346	4.436	19.571	0.00	0.00	RX0	H
ATOM	724	CA	LEU	233	29.211	3.960	21.691	1.00	0.00	RX0	C
ATOM	725	CB	LEU	233	30.724	4.154	21.805	1.00	0.00	RX0	C
ATOM	726	CG	LEU	233	31.298	3.744	23.164	1.00	0.00	RX0	C
ATOM	727	CD1	LEU	233	30.964	2.293	23.523	1.00	0.00	RX0	C
ATOM	728	CD2	LEU	233	32.800	4.007	23.238	1.00	0.00	RX0	C
ATOM	729	C	LEU	233	28.415	4.936	22.566	1.00	0.00	RX0	C
ATOM	730	O	LEU	233	27.943	4.566	23.634	1.00	0.00	RX0	O

ATOM	731	N	GLU	234	28.150	6.122	22.016	1.00	0.00	RX0	N
ATOM	732	H	GLU	234	28.421	6.295	21.071	0.00	0.00	RX0	H
ATOM	733	CA	GLU	234	27.227	7.090	22.644	1.00	0.00	RX0	C
ATOM	734	CB	GLU	234	27.014	8.298	21.745	1.00	0.00	RX0	C
ATOM	735	CG	GLU	234	28.078	9.381	21.735	1.00	0.00	RX0	C
ATOM	736	CD	GLU	234	27.899	10.182	20.465	1.00	0.00	RX0	C
ATOM	737	OE1	GLU	234	28.014	11.400	20.479	1.00	0.00	RX0	O
ATOM	738	OE2	GLU	234	27.724	9.581	19.415	1.00	0.00	RX0	O
ATOM	739	C	GLU	234	25.830	6.482	22.841	1.00	0.00	RX0	C
ATOM	740	O	GLU	234	25.253	6.598	23.926	1.00	0.00	RX0	O
ATOM	741	N	ILE	235	25.389	5.711	21.848	1.00	0.00	RX0	N
ATOM	742	H	ILE	235	25.953	5.645	21.024	0.00	0.00	RX0	H
ATOM	743	CA	ILE	235	24.069	5.045	21.863	1.00	0.00	RX0	C
ATOM	744	CB	ILE	235	23.693	4.489	20.486	1.00	0.00	RX0	C
ATOM	745	CG2	ILE	235	22.400	3.674	20.555	1.00	0.00	RX0	C
ATOM	746	CG1	ILE	235	23.563	5.619	19.466	1.00	0.00	RX0	C
ATOM	747	CD1	ILE	235	23.218	5.105	18.068	1.00	0.00	RX0	C
ATOM	748	C	ILE	235	24.018	3.945	22.939	1.00	0.00	RX0	C
ATOM	749	O	ILE	235	23.068	3.902	23.724	1.00	0.00	RX0	O
ATOM	750	N	LEU	236	25.072	3.140	23.020	1.00	0.00	RX0	N
ATOM	751	H	LEU	236	25.808	3.229	22.346	0.00	0.00	RX0	H
ATOM	752	CA	LEU	236	25.176	2.114	24.078	1.00	0.00	RX0	C
ATOM	753	CB	LEU	236	26.404	1.225	23.886	1.00	0.00	RX0	C
ATOM	754	CG	LEU	236	26.175	0.148	22.827	1.00	0.00	RX0	C
ATOM	755	CD1	LEU	236	27.398	-0.750	22.656	1.00	0.00	RX0	C
ATOM	756	CD2	LEU	236	24.921	-0.675	23.124	1.00	0.00	RX0	C
ATOM	757	C	LEU	236	25.182	2.733	25.479	1.00	0.00	RX0	C
ATOM	758	O	LEU	236	24.381	2.362	26.336	1.00	0.00	RX0	O
ATOM	759	N	MET	237	25.933	3.823	25.600	1.00	0.00	RX0	N
ATOM	760	H	MET	237	26.453	4.134	24.802	0.00	0.00	RX0	H
ATOM	761	CA	MET	237	26.132	4.523	26.881	1.00	0.00	RX0	C
ATOM	762	CB	MET	237	27.281	5.528	26.799	1.00	0.00	RX0	C
ATOM	763	CG	MET	237	28.651	4.851	26.797	1.00	0.00	RX0	C
ATOM	764	SD	MET	237	30.003	6.035	26.723	1.00	0.00	RX0	S
ATOM	765	CE	MET	237	31.355	4.886	27.022	1.00	0.00	RX0	C
ATOM	766	C	MET	237	24.875	5.215	27.409	1.00	0.00	RX0	C
ATOM	767	O	MET	237	24.517	5.003	28.572	1.00	0.00	RX0	O
ATOM	768	N	ILE	238	24.128	5.878	26.531	1.00	0.00	RX0	N
ATOM	769	H	ILE	238	24.453	5.957	25.586	0.00	0.00	RX0	H
ATOM	770	CA	ILE	238	22.871	6.546	26.925	1.00	0.00	RX0	C
ATOM	771	CB	ILE	238	22.348	7.524	25.857	1.00	0.00	RX0	C
ATOM	772	CG2	ILE	238	21.924	6.837	24.559	1.00	0.00	RX0	C
ATOM	773	CG1	ILE	238	21.219	8.378	26.440	1.00	0.00	RX0	C
ATOM	774	CD1	ILE	238	20.604	9.334	25.418	1.00	0.00	RX0	C
ATOM	775	C	ILE	238	21.800	5.514	27.357	1.00	0.00	RX0	C
ATOM	776	O	ILE	238	21.031	5.731	28.268	1.00	0.00	RX0	O
ATOM	777	N	GLY	239	21.845	4.355	26.660	1.00	0.00	RX0	N
ATOM	778	H	GLY	239	22.519	4.243	25.926	0.00	0.00	RX0	H
ATOM	779	CA	GLY	239	20.969	3.213	26.975	1.00	0.00	RX0	C
ATOM	780	C	GLY	239	21.301	2.634	28.356	1.00	0.00	RX0	C
ATOM	781	O	GLY	239	20.417	2.460	29.193	1.00	0.00	RX0	O
ATOM	782	N	LEU	240	22.605	2.583	28.639	1.00	0.00	RX0	N
ATOM	783	H	LEU	240	23.260	2.803	27.913	0.00	0.00	RX0	H
ATOM	784	CA	LEU	240	23.124	2.082	29.919	1.00	0.00	RX0	C
ATOM	785	CB	LEU	240	24.644	1.943	29.849	1.00	0.00	RX0	C
ATOM	786	CG	LEU	240	25.286	1.614	31.198	1.00	0.00	RX0	C
ATOM	787	CD1	LEU	240	24.824	0.267	31.752	1.00	0.00	RX0	C
ATOM	788	CD2	LEU	240	26.807	1.715	31.128	1.00	0.00	RX0	C
ATOM	789	C	LEU	240	22.728	2.995	31.086	1.00	0.00	RX0	C
ATOM	790	O	LEU	240	22.214	2.535	32.097	1.00	0.00	RX0	O
ATOM	791	N	VAL	241	22.901	4.295	30.880	1.00	0.00	RX0	N

ATOM	792	H	VAL	241	23.278	4.586	29.998	0.00	0.00	RX0	H
ATOM	793	CA	VAL	241	22.596	5.307	31.912	1.00	0.00	RX0	C
ATOM	794	CB	VAL	241	23.252	6.671	31.660	1.00	0.00	RX0	C
ATOM	795	CG1	VAL	241	24.770	6.506	31.594	1.00	0.00	RX0	C
ATOM	796	CG2	VAL	241	22.696	7.406	30.447	1.00	0.00	RX0	C
ATOM	797	C	VAL	241	21.084	5.392	32.193	1.00	0.00	RX0	C
ATOM	798	O	VAL	241	20.670	5.516	33.338	1.00	0.00	RX0	O
ATOM	799	N	TRP	242	20.290	5.181	31.134	1.00	0.00	RX0	N
ATOM	800	H	TRP	242	20.699	5.072	30.225	0.00	0.00	RX0	H
ATOM	801	CA	TRP	242	18.822	5.192	31.222	1.00	0.00	RX0	C
ATOM	802	CB	TRP	242	18.252	5.149	29.801	1.00	0.00	RX0	C
ATOM	803	CG	TRP	242	16.824	4.661	29.780	1.00	0.00	RX0	C
ATOM	804	CD2	TRP	242	15.637	5.341	30.238	1.00	0.00	RX0	C
ATOM	805	CE2	TRP	242	14.541	4.475	30.022	1.00	0.00	RX0	C
ATOM	806	CE3	TRP	242	15.425	6.590	30.803	1.00	0.00	RX0	C
ATOM	807	CD1	TRP	242	16.374	3.420	29.305	1.00	0.00	RX0	C
ATOM	808	NE1	TRP	242	15.027	3.306	29.446	1.00	0.00	RX0	N
ATOM	809	HE1	TRP	242	14.483	2.533	29.188	0.00	0.00	RX0	H
ATOM	810	CZ2	TRP	242	13.264	4.881	30.389	1.00	0.00	RX0	C
ATOM	811	CZ3	TRP	242	14.143	6.988	31.163	1.00	0.00	RX0	C
ATOM	812	CH2	TRP	242	13.068	6.132	30.960	1.00	0.00	RX0	C
ATOM	813	C	TRP	242	18.281	4.038	32.076	1.00	0.00	RX0	C
ATOM	814	O	TRP	242	17.477	4.269	32.979	1.00	0.00	RX0	O
ATOM	815	N	ARG	243	18.818	2.841	31.865	1.00	0.00	RX0	N
ATOM	816	H	ARG	243	19.515	2.737	31.152	0.00	0.00	RX0	H
ATOM	817	CA	ARG	243	18.359	1.661	32.627	1.00	0.00	RX0	C
ATOM	818	CB	ARG	243	18.486	0.403	31.737	1.00	0.00	RX0	C
ATOM	819	CG	ARG	243	19.890	-0.065	31.297	1.00	0.00	RX0	C
ATOM	820	CD	ARG	243	19.828	-1.102	30.153	1.00	0.00	RX0	C
ATOM	821	NE	ARG	243	21.110	-1.762	29.863	1.00	0.00	RX0	N
ATOM	822	HE	ARG	243	21.660	-2.019	30.672	0.00	0.00	RX0	H
ATOM	823	CZ	ARG	243	21.427	-2.132	28.569	1.00	0.00	RX0	C
ATOM	824	NH1	ARG	243	20.661	-1.724	27.537	1.00	0.00	RX0	N
ATOM	825	HH11	ARG	243	20.905	-2.037	26.601	0.00	0.00	RX0	H
ATOM	826	HH12	ARG	243	19.862	-1.133	27.641	0.00	0.00	RX0	H
ATOM	827	NH2	ARG	243	22.492	-2.914	28.303	1.00	0.00	RX0	N
ATOM	828	HH21	ARG	243	22.771	-3.132	27.346	0.00	0.00	RX0	H
ATOM	829	HH22	ARG	243	23.050	-3.344	29.022	0.00	0.00	RX0	H
ATOM	830	C	ARG	243	19.043	1.510	33.998	1.00	0.00	RX0	C
ATOM	831	O	ARG	243	18.610	0.722	34.836	1.00	0.00	RX0	O
ATOM	832	N	SER	244	20.027	2.366	34.245	1.00	0.00	RX0	N
ATOM	833	H	SER	244	20.316	3.010	33.538	0.00	0.00	RX0	H
ATOM	834	CA	SER	244	20.722	2.464	35.548	1.00	0.00	RX0	C
ATOM	835	CB	SER	244	22.206	2.696	35.311	1.00	0.00	RX0	C
ATOM	836	OG	SER	244	22.700	1.647	34.476	1.00	0.00	RX0	O
ATOM	837	HG	SER	244	22.225	1.717	33.654	0.00	0.00	RX0	H
ATOM	838	C	SER	244	20.112	3.547	36.442	1.00	0.00	RX0	C
ATOM	839	O	SER	244	20.448	3.642	37.630	1.00	0.00	RX0	O
ATOM	840	N	MET	245	19.184	4.322	35.895	1.00	0.00	RX0	N
ATOM	841	H	MET	245	18.884	4.151	34.954	0.00	0.00	RX0	H
ATOM	842	CA	MET	245	18.600	5.504	36.550	1.00	0.00	RX0	C
ATOM	843	CB	MET	245	17.595	6.196	35.632	1.00	0.00	RX0	C
ATOM	844	CG	MET	245	17.035	7.466	36.272	1.00	0.00	RX0	C
ATOM	845	SD	MET	245	15.728	8.226	35.305	1.00	0.00	RX0	S
ATOM	846	CE	MET	245	16.546	8.111	33.713	1.00	0.00	RX0	C
ATOM	847	C	MET	245	17.925	5.204	37.895	1.00	0.00	RX0	C
ATOM	848	O	MET	245	18.105	5.946	38.853	1.00	0.00	RX0	O
ATOM	849	N	GLU	246	17.212	4.082	37.945	1.00	0.00	RX0	N
ATOM	850	H	GLU	246	17.124	3.482	37.148	0.00	0.00	RX0	H
ATOM	851	CA	GLU	246	16.494	3.692	39.178	1.00	0.00	RX0	C
ATOM	852	CB	GLU	246	15.245	2.901	38.818	1.00	0.00	RX0	C

ATOM	853	CG	GLU	246	14.334	3.663	37.859	1.00	0.00	RX0	C
ATOM	854	CD	GLU	246	13.173	2.765	37.501	1.00	0.00	RX0	C
ATOM	855	OE1	GLU	246	13.302	1.555	37.675	1.00	0.00	RX0	O
ATOM	856	OE2	GLU	246	12.147	3.276	37.058	1.00	0.00	RX0	O
ATOM	857	C	GLU	246	17.379	2.867	40.123	1.00	0.00	RX0	C
ATOM	858	O	GLU	246	16.897	2.295	41.108	1.00	0.00	RX0	O
ATOM	859	N	HIS	247	18.674	2.844	39.836	1.00	0.00	RX0	N
ATOM	860	H	HIS	247	19.044	3.322	39.040	0.00	0.00	RX0	H
ATOM	861	CA	HIS	247	19.658	2.055	40.601	1.00	0.00	RX0	C
ATOM	862	CB	HIS	247	20.175	0.869	39.789	1.00	0.00	RX0	C
ATOM	863	CG	HIS	247	19.089	-0.143	39.495	1.00	0.00	RX0	C
ATOM	864	ND1	HIS	247	17.873	-0.174	40.078	1.00	0.00	RX0	N
ATOM	865	HD1	HIS	247	17.503	0.454	40.742	0.00	0.00	RX0	H
ATOM	866	CD2	HIS	247	19.170	-1.205	38.590	1.00	0.00	RX0	C
ATOM	867	NE2	HIS	247	17.995	-1.876	38.633	1.00	0.00	RX0	N
ATOM	868	CE1	HIS	247	17.194	-1.241	39.547	1.00	0.00	RX0	C
ATOM	869	C	HIS	247	20.841	2.947	41.015	1.00	0.00	RX0	C
ATOM	870	O	HIS	247	21.962	2.788	40.490	1.00	0.00	RX0	O
ATOM	871	N	PRO	248	20.632	3.827	41.991	1.00	0.00	RX0	N
ATOM	872	CD	PRO	248	19.383	3.973	42.733	1.00	0.00	RX0	C
ATOM	873	CA	PRO	248	21.659	4.767	42.484	1.00	0.00	RX0	C
ATOM	874	CB	PRO	248	20.980	5.450	43.675	1.00	0.00	RX0	C
ATOM	875	CG	PRO	248	19.487	5.344	43.385	1.00	0.00	RX0	C
ATOM	876	C	PRO	248	22.939	4.013	42.877	1.00	0.00	RX0	C
ATOM	877	O	PRO	248	22.892	2.963	43.503	1.00	0.00	RX0	O
ATOM	878	N	GLY	249	24.055	4.541	42.350	1.00	0.00	RX0	N
ATOM	879	H	GLY	249	23.974	5.299	41.708	0.00	0.00	RX0	H
ATOM	880	CA	GLY	249	25.407	3.996	42.610	1.00	0.00	RX0	C
ATOM	881	C	GLY	249	25.783	2.749	41.794	1.00	0.00	RX0	C
ATOM	882	O	GLY	249	26.914	2.250	41.927	1.00	0.00	RX0	O
ATOM	883	N	LYS	250	24.877	2.267	40.961	1.00	0.00	RX0	N
ATOM	884	H	LYS	250	23.984	2.698	40.812	0.00	0.00	RX0	H
ATOM	885	CA	LYS	250	25.097	1.050	40.158	1.00	0.00	RX0	C
ATOM	886	CB	LYS	250	24.369	-0.146	40.773	1.00	0.00	RX0	C
ATOM	887	CG	LYS	250	25.211	-0.824	41.862	1.00	0.00	RX0	C
ATOM	888	CD	LYS	250	24.590	-2.100	42.434	1.00	0.00	RX0	C
ATOM	889	CE	LYS	250	25.532	-2.896	43.344	1.00	0.00	RX0	C
ATOM	890	NZ	LYS	250	26.695	-3.379	42.585	1.00	0.00	RX0	N
ATOM	891	HZ1	LYS	250	27.400	-3.792	43.237	0.00	0.00	RX0	H
ATOM	892	HZ2	LYS	250	26.442	-4.073	41.849	0.00	0.00	RX0	H
ATOM	893	HZ3	LYS	250	27.211	-2.577	42.174	0.00	0.00	RX0	H
ATOM	894	C	LYS	250	24.802	1.266	38.671	1.00	0.00	RX0	C
ATOM	895	O	LYS	250	24.040	2.166	38.282	1.00	0.00	RX0	O
ATOM	896	N	LEU	251	25.472	0.476	37.863	1.00	0.00	RX0	N
ATOM	897	H	LEU	251	26.053	-0.236	38.243	0.00	0.00	RX0	H
ATOM	898	CA	LEU	251	25.292	0.430	36.401	1.00	0.00	RX0	C
ATOM	899	CB	LEU	251	26.626	0.591	35.684	1.00	0.00	RX0	C
ATOM	900	CG	LEU	251	27.161	2.016	35.763	1.00	0.00	RX0	C
ATOM	901	CD1	LEU	251	28.584	2.114	35.218	1.00	0.00	RX0	C
ATOM	902	CD2	LEU	251	26.208	3.008	35.093	1.00	0.00	RX0	C
ATOM	903	C	LEU	251	24.646	-0.892	36.017	1.00	0.00	RX0	C
ATOM	904	O	LEU	251	25.224	-1.976	36.286	1.00	0.00	RX0	O
ATOM	905	N	LEU	252	23.437	-0.808	35.533	1.00	0.00	RX0	N
ATOM	906	H	LEU	252	23.084	0.097	35.312	0.00	0.00	RX0	H
ATOM	907	CA	LEU	252	22.659	-1.981	35.114	1.00	0.00	RX0	C
ATOM	908	CB	LEU	252	21.157	-1.712	35.230	1.00	0.00	RX0	C
ATOM	909	CG	LEU	252	20.300	-2.958	34.979	1.00	0.00	RX0	C
ATOM	910	CD1	LEU	252	20.457	-3.993	36.091	1.00	0.00	RX0	C
ATOM	911	CD2	LEU	252	18.831	-2.612	34.736	1.00	0.00	RX0	C
ATOM	912	C	LEU	252	23.007	-2.330	33.663	1.00	0.00	RX0	C
ATOM	913	O	LEU	252	22.274	-2.025	32.731	1.00	0.00	RX0	O

ATOM	914	N	PHE	253	24.144	-3.005	33.506	1.00	0.00	RX0	N
ATOM	915	H	PHE	253	24.631	-3.308	34.330	0.00	0.00	RX0	H
ATOM	916	CA	PHE	253	24.599	-3.457	32.174	1.00	0.00	RX0	C
ATOM	917	CB	PHE	253	25.968	-4.123	32.271	1.00	0.00	RX0	C
ATOM	918	CG	PHE	253	27.033	-3.092	32.539	1.00	0.00	RX0	C
ATOM	919	CD1	PHE	253	27.566	-2.366	31.482	1.00	0.00	RX0	C
ATOM	920	CD2	PHE	253	27.488	-2.878	33.834	1.00	0.00	RX0	C
ATOM	921	CE1	PHE	253	28.565	-1.430	31.716	1.00	0.00	RX0	C
ATOM	922	CE2	PHE	253	28.487	-1.942	34.066	1.00	0.00	RX0	C
ATOM	923	CZ	PHE	253	29.027	-1.220	33.008	1.00	0.00	RX0	C
ATOM	924	C	PHE	253	23.603	-4.446	31.564	1.00	0.00	RX0	C
ATOM	925	O	PHE	253	23.259	-4.379	30.390	1.00	0.00	RX0	O
ATOM	926	N	ALA	254	23.094	-5.300	32.445	1.00	0.00	RX0	N
ATOM	927	H	ALA	254	23.408	-5.312	33.400	0.00	0.00	RX0	H
ATOM	928	CA	ALA	254	22.050	-6.280	32.141	1.00	0.00	RX0	C
ATOM	929	CB	ALA	254	22.697	-7.549	31.594	1.00	0.00	RX0	C
ATOM	930	C	ALA	254	21.288	-6.584	33.440	1.00	0.00	RX0	C
ATOM	931	O	ALA	254	21.887	-6.418	34.526	1.00	0.00	RX0	O
ATOM	932	N	PRO	255	20.056	-7.064	33.372	1.00	0.00	RX0	N
ATOM	933	CD	PRO	255	19.307	-7.243	32.131	1.00	0.00	RX0	C
ATOM	934	CA	PRO	255	19.236	-7.444	34.545	1.00	0.00	RX0	C
ATOM	935	CB	PRO	255	17.987	-8.069	33.924	1.00	0.00	RX0	C
ATOM	936	CG	PRO	255	17.856	-7.386	32.569	1.00	0.00	RX0	C
ATOM	937	C	PRO	255	19.972	-8.395	35.506	1.00	0.00	RX0	C
ATOM	938	O	PRO	255	19.756	-8.342	36.714	1.00	0.00	RX0	O
ATOM	939	N	ASN	256	20.900	-9.179	34.970	1.00	0.00	RX0	N
ATOM	940	H	ASN	256	21.114	-9.138	33.993	0.00	0.00	RX0	H
ATOM	941	CA	ASN	256	21.722	-10.125	35.761	1.00	0.00	RX0	C
ATOM	942	CB	ASN	256	21.709	-11.529	35.151	1.00	0.00	RX0	C
ATOM	943	CG	ASN	256	22.406	-11.538	33.800	1.00	0.00	RX0	C
ATOM	944	OD1	ASN	256	22.286	-10.607	33.003	1.00	0.00	RX0	O
ATOM	945	ND2	ASN	256	23.051	-12.689	33.536	1.00	0.00	RX0	N
ATOM	946	HD21	ASN	256	23.182	-13.360	34.270	0.00	0.00	RX0	H
ATOM	947	HD22	ASN	256	23.411	-12.942	32.632	0.00	0.00	RX0	H
ATOM	948	C	ASN	256	23.191	-9.669	35.876	1.00	0.00	RX0	C
ATOM	949	O	ASN	256	24.101	-10.505	36.011	1.00	0.00	RX0	O
ATOM	950	N	LEU	257	23.439	-8.384	35.742	1.00	0.00	RX0	N
ATOM	951	H	LEU	257	22.692	-7.726	35.629	0.00	0.00	RX0	H
ATOM	952	CA	LEU	257	24.796	-7.805	35.783	1.00	0.00	RX0	C
ATOM	953	CB	LEU	257	25.515	-7.965	34.445	1.00	0.00	RX0	C
ATOM	954	CG	LEU	257	27.025	-7.762	34.574	1.00	0.00	RX0	C
ATOM	955	CD1	LEU	257	27.653	-8.815	35.489	1.00	0.00	RX0	C
ATOM	956	CD2	LEU	257	27.719	-7.705	33.213	1.00	0.00	RX0	C
ATOM	957	C	LEU	257	24.720	-6.327	36.168	1.00	0.00	RX0	C
ATOM	958	O	LEU	257	24.738	-5.412	35.328	1.00	0.00	RX0	O
ATOM	959	N	LEU	258	24.604	-6.148	37.469	1.00	0.00	RX0	N
ATOM	960	H	LEU	258	24.690	-6.942	38.070	0.00	0.00	RX0	H
ATOM	961	CA	LEU	258	24.482	-4.834	38.118	1.00	0.00	RX0	C
ATOM	962	CB	LEU	258	23.225	-4.903	38.985	1.00	0.00	RX0	C
ATOM	963	CG	LEU	258	22.828	-3.611	39.686	1.00	0.00	RX0	C
ATOM	964	CD1	LEU	258	22.659	-2.464	38.700	1.00	0.00	RX0	C
ATOM	965	CD2	LEU	258	21.579	-3.800	40.548	1.00	0.00	RX0	C
ATOM	966	C	LEU	258	25.743	-4.564	38.938	1.00	0.00	RX0	C
ATOM	967	O	LEU	258	26.013	-5.237	39.948	1.00	0.00	RX0	O
ATOM	968	N	LEU	259	26.528	-3.622	38.460	1.00	0.00	RX0	N
ATOM	969	H	LEU	259	26.218	-3.062	37.684	0.00	0.00	RX0	H
ATOM	970	CA	LEU	259	27.862	-3.349	39.027	1.00	0.00	RX0	C
ATOM	971	CB	LEU	259	28.937	-3.490	37.946	1.00	0.00	RX0	C
ATOM	972	CG	LEU	259	28.868	-4.794	37.145	1.00	0.00	RX0	C
ATOM	973	CD1	LEU	259	29.885	-4.808	36.004	1.00	0.00	RX0	C
ATOM	974	CD2	LEU	259	29.004	-6.033	38.029	1.00	0.00	RX0	C

ATOM	975	C	LEU	259	27.958	-1.956	39.652	1.00	0.00	RX0	C
ATOM	976	O	LEU	259	27.419	-0.984	39.137	1.00	0.00	RX0	O
ATOM	977	N	ASP	260	28.645	-1.912	40.785	1.00	0.00	RX0	N
ATOM	978	H	ASP	260	29.231	-2.685	41.040	0.00	0.00	RX0	H
ATOM	979	CA	ASP	260	29.043	-0.657	41.454	1.00	0.00	RX0	C
ATOM	980	CB	ASP	260	29.086	-0.860	42.963	1.00	0.00	RX0	C
ATOM	981	CG	ASP	260	29.796	-2.169	43.220	1.00	0.00	RX0	C
ATOM	982	OD1	ASP	260	31.023	-2.188	43.249	1.00	0.00	RX0	O
ATOM	983	OD2	ASP	260	29.106	-3.180	43.349	1.00	0.00	RX0	O
ATOM	984	C	ASP	260	30.443	-0.222	40.970	1.00	0.00	RX0	C
ATOM	985	O	ASP	260	31.127	-1.008	40.295	1.00	0.00	RX0	O
ATOM	986	N	ARG	261	30.963	0.873	41.504	1.00	0.00	RX0	N
ATOM	987	H	ARG	261	30.390	1.411	42.121	0.00	0.00	RX0	H
ATOM	988	CA	ARG	261	32.263	1.408	41.044	1.00	0.00	RX0	C
ATOM	989	CB	ARG	261	32.418	2.885	41.437	1.00	0.00	RX0	C
ATOM	990	CG	ARG	261	32.612	3.166	42.930	1.00	0.00	RX0	C
ATOM	991	CD	ARG	261	32.522	4.659	43.271	1.00	0.00	RX0	C
ATOM	992	NE	ARG	261	33.393	5.461	42.414	1.00	0.00	RX0	N
ATOM	993	HE	ARG	261	33.102	5.665	41.462	0.00	0.00	RX0	H
ATOM	994	CZ	ARG	261	34.587	5.968	42.833	1.00	0.00	RX0	C
ATOM	995	NH1	ARG	261	34.967	5.803	44.120	1.00	0.00	RX0	N
ATOM	996	HH11	ARG	261	35.835	6.164	44.465	0.00	0.00	RX0	H
ATOM	997	HH12	ARG	261	34.369	5.326	44.767	0.00	0.00	RX0	H
ATOM	998	NH2	ARG	261	35.361	6.628	41.952	1.00	0.00	RX0	N
ATOM	999	HH21	ARG	261	36.272	7.008	42.105	0.00	0.00	RX0	H
ATOM	1000	HH22	ARG	261	34.960	6.777	41.025	0.00	0.00	RX0	H
ATOM	1001	C	ARG	261	33.476	0.540	41.436	1.00	0.00	RX0	C
ATOM	1002	O	ARG	261	34.378	0.347	40.637	1.00	0.00	RX0	O
ATOM	1003	N	ASN	262	33.410	-0.067	42.632	1.00	0.00	RX0	N
ATOM	1004	H	ASN	262	32.528	-0.056	43.105	0.00	0.00	RX0	H
ATOM	1005	CA	ASN	262	34.456	-1.000	43.094	1.00	0.00	RX0	C
ATOM	1006	CB	ASN	262	34.144	-1.494	44.500	1.00	0.00	RX0	C
ATOM	1007	CG	ASN	262	35.166	-2.543	44.872	1.00	0.00	RX0	C
ATOM	1008	OD1	ASN	262	36.300	-2.210	45.220	1.00	0.00	RX0	O
ATOM	1009	ND2	ASN	262	34.701	-3.804	44.830	1.00	0.00	RX0	N
ATOM	1010	HD21	ASN	262	33.750	-3.985	44.567	0.00	0.00	RX0	H
ATOM	1011	HD22	ASN	262	35.262	-4.605	45.042	0.00	0.00	RX0	H
ATOM	1012	C	ASN	262	34.635	-2.233	42.204	1.00	0.00	RX0	C
ATOM	1013	O	ASN	262	35.755	-2.665	41.964	1.00	0.00	RX0	O
ATOM	1014	N	GLN	263	33.530	-2.679	41.603	1.00	0.00	RX0	N
ATOM	1015	H	GLN	263	32.638	-2.270	41.822	0.00	0.00	RX0	H
ATOM	1016	CA	GLN	263	33.559	-3.756	40.599	1.00	0.00	RX0	C
ATOM	1017	CB	GLN	263	32.196	-4.414	40.430	1.00	0.00	RX0	C
ATOM	1018	CG	GLN	263	31.889	-5.190	41.708	1.00	0.00	RX0	C
ATOM	1019	CD	GLN	263	30.680	-6.070	41.512	1.00	0.00	RX0	C
ATOM	1020	OE1	GLN	263	30.642	-6.951	40.662	1.00	0.00	RX0	O
ATOM	1021	NE2	GLN	263	29.698	-5.810	42.386	1.00	0.00	RX0	N
ATOM	1022	HE21	GLN	263	29.813	-5.019	42.998	0.00	0.00	RX0	H
ATOM	1023	HE22	GLN	263	28.888	-6.391	42.444	0.00	0.00	RX0	H
ATOM	1024	C	GLN	263	34.189	-3.308	39.265	1.00	0.00	RX0	C
ATOM	1025	O	GLN	263	34.644	-4.116	38.479	1.00	0.00	RX0	O
ATOM	1026	N	GLY	264	34.180	-1.978	39.039	1.00	0.00	RX0	N
ATOM	1027	H	GLY	264	33.764	-1.376	39.720	0.00	0.00	RX0	H
ATOM	1028	CA	GLY	264	34.831	-1.337	37.881	1.00	0.00	RX0	C
ATOM	1029	C	GLY	264	36.364	-1.310	37.978	1.00	0.00	RX0	C
ATOM	1030	O	GLY	264	37.050	-1.510	36.977	1.00	0.00	RX0	O
ATOM	1031	N	LYS	265	36.881	-1.155	39.202	1.00	0.00	RX0	N
ATOM	1032	H	LYS	265	36.232	-1.009	39.950	0.00	0.00	RX0	H
ATOM	1033	CA	LYS	265	38.336	-1.184	39.469	1.00	0.00	RX0	C
ATOM	1034	CB	LYS	265	38.659	-1.113	40.953	1.00	0.00	RX0	C
ATOM	1035	CG	LYS	265	38.094	0.001	41.822	1.00	0.00	RX0	C

ATOM	1036	CD	LYS	265	38.466	-0.414	43.244	1.00	0.00	RX0	C
ATOM	1037	CE	LYS	265	37.917	0.437	44.381	1.00	0.00	RX0	C
ATOM	1038	NZ	LYS	265	38.099	-0.346	45.611	1.00	0.00	RX0	N
ATOM	1039	HZ1	LYS	265	37.669	0.130	46.426	0.00	0.00	RX0	H
ATOM	1040	HZ2	LYS	265	37.620	-1.265	45.480	0.00	0.00	RX0	H
ATOM	1041	HZ3	LYS	265	39.107	-0.524	45.783	0.00	0.00	RX0	H
ATOM	1042	C	LYS	265	38.994	-2.500	39.030	1.00	0.00	RX0	C
ATOM	1043	O	LYS	265	40.184	-2.535	38.765	1.00	0.00	RX0	O
ATOM	1044	N	CYS	266	38.153	-3.548	38.917	1.00	0.00	RX0	N
ATOM	1045	H	CYS	266	37.198	-3.455	39.194	0.00	0.00	RX0	H
ATOM	1046	CA	CYS	266	38.538	-4.854	38.355	1.00	0.00	RX0	C
ATOM	1047	CB	CYS	266	37.315	-5.759	38.262	1.00	0.00	RX0	C
ATOM	1048	SG	CYS	266	36.555	-5.950	39.896	1.00	0.00	RX0	S
ATOM	1049	C	CYS	266	39.318	-4.732	37.033	1.00	0.00	RX0	C
ATOM	1050	O	CYS	266	40.108	-5.603	36.695	1.00	0.00	RX0	O
ATOM	1051	N	VAL	267	39.075	-3.636	36.304	1.00	0.00	RX0	N
ATOM	1052	H	VAL	267	38.470	-2.902	36.621	0.00	0.00	RX0	H
ATOM	1053	CA	VAL	267	39.804	-3.345	35.058	1.00	0.00	RX0	C
ATOM	1054	CB	VAL	267	38.856	-3.352	33.851	1.00	0.00	RX0	C
ATOM	1055	CG1	VAL	267	39.583	-3.008	32.549	1.00	0.00	RX0	C
ATOM	1056	CG2	VAL	267	38.126	-4.693	33.740	1.00	0.00	RX0	C
ATOM	1057	C	VAL	267	40.557	-2.013	35.191	1.00	0.00	RX0	C
ATOM	1058	O	VAL	267	39.969	-0.956	35.468	1.00	0.00	RX0	O
ATOM	1059	N	GLU	268	41.829	-2.078	34.821	1.00	0.00	RX0	N
ATOM	1060	H	GLU	268	42.209	-2.964	34.566	0.00	0.00	RX0	H
ATOM	1061	CA	GLU	268	42.726	-0.908	34.727	1.00	0.00	RX0	C
ATOM	1062	CB	GLU	268	44.094	-1.333	34.201	1.00	0.00	RX0	C
ATOM	1063	CG	GLU	268	45.133	-0.216	34.298	1.00	0.00	RX0	C
ATOM	1064	CD	GLU	268	46.490	-0.788	33.959	1.00	0.00	RX0	C
ATOM	1065	OE1	GLU	268	46.580	-2.000	33.771	1.00	0.00	RX0	O
ATOM	1066	OE2	GLU	268	47.452	-0.025	33.895	1.00	0.00	RX0	O
ATOM	1067	C	GLU	268	42.079	0.194	33.866	1.00	0.00	RX0	C
ATOM	1068	O	GLU	268	41.697	-0.027	32.727	1.00	0.00	RX0	O
ATOM	1069	N	GLY	269	41.924	1.355	34.524	1.00	0.00	RX0	N
ATOM	1070	H	GLY	269	42.192	1.398	35.484	0.00	0.00	RX0	H
ATOM	1071	CA	GLY	269	41.377	2.576	33.902	1.00	0.00	RX0	C
ATOM	1072	C	GLY	269	39.898	2.490	33.494	1.00	0.00	RX0	C
ATOM	1073	O	GLY	269	39.424	3.343	32.745	1.00	0.00	RX0	O
ATOM	1074	N	MET	270	39.146	1.598	34.134	1.00	0.00	RX0	N
ATOM	1075	H	MET	270	39.580	0.956	34.770	0.00	0.00	RX0	H
ATOM	1076	CA	MET	270	37.704	1.465	33.841	1.00	0.00	RX0	C
ATOM	1077	CB	MET	270	37.280	0.008	33.653	1.00	0.00	RX0	C
ATOM	1078	CG	MET	270	35.925	-0.099	32.948	1.00	0.00	RX0	C
ATOM	1079	SD	MET	270	35.420	-1.789	32.583	1.00	0.00	RX0	S
ATOM	1080	CE	MET	270	35.152	-2.354	34.268	1.00	0.00	RX0	C
ATOM	1081	C	MET	270	36.825	2.181	34.877	1.00	0.00	RX0	C
ATOM	1082	O	MET	270	35.781	2.734	34.514	1.00	0.00	RX0	O
ATOM	1083	N	VAL	271	37.310	2.290	36.107	1.00	0.00	RX0	N
ATOM	1084	H	VAL	271	38.234	1.949	36.273	0.00	0.00	RX0	H
ATOM	1085	CA	VAL	271	36.591	3.043	37.171	1.00	0.00	RX0	C
ATOM	1086	CB	VAL	271	37.247	2.853	38.551	1.00	0.00	RX0	C
ATOM	1087	CG1	VAL	271	38.735	3.184	38.561	1.00	0.00	RX0	C
ATOM	1088	CG2	VAL	271	36.459	3.564	39.652	1.00	0.00	RX0	C
ATOM	1089	C	VAL	271	36.343	4.502	36.749	1.00	0.00	RX0	C
ATOM	1090	O	VAL	271	35.261	5.071	37.095	1.00	0.00	RX0	O
ATOM	1091	N	GLU	272	37.228	5.074	36.011	1.00	0.00	RX0	N
ATOM	1092	H	GLU	272	38.100	4.600	35.878	0.00	0.00	RX0	H
ATOM	1093	CA	GLU	272	37.141	6.453	35.460	1.00	0.00	RX0	C
ATOM	1094	CB	GLU	272	38.432	6.787	34.703	1.00	0.00	RX0	C
ATOM	1095	CG	GLU	272	39.677	7.019	35.574	1.00	0.00	RX0	C
ATOM	1096	CD	GLU	272	40.043	5.788	36.384	1.00	0.00	RX0	C

ATOM	1097	OE1	GLU	272	40.060	4.689	35.832	1.00	0.00	RX0	O
ATOM	1098	OE2	GLU	272	40.278	5.926	37.581	1.00	0.00	RX0	O
ATOM	1099	C	GLU	272	35.927	6.588	34.526	1.00	0.00	RX0	C
ATOM	1100	O	GLU	272	35.142	7.521	34.681	1.00	0.00	RX0	O
ATOM	1101	N	ILE	273	35.702	5.552	33.729	1.00	0.00	RX0	N
ATOM	1102	H	ILE	273	36.304	4.758	33.825	0.00	0.00	RX0	H
ATOM	1103	CA	ILE	273	34.533	5.484	32.820	1.00	0.00	RX0	C
ATOM	1104	CB	ILE	273	34.734	4.377	31.779	1.00	0.00	RX0	C
ATOM	1105	CG2	ILE	273	33.622	4.407	30.730	1.00	0.00	RX0	C
ATOM	1106	CG1	ILE	273	36.126	4.436	31.143	1.00	0.00	RX0	C
ATOM	1107	CD1	ILE	273	36.334	5.661	30.250	1.00	0.00	RX0	C
ATOM	1108	C	ILE	273	33.249	5.235	33.628	1.00	0.00	RX0	C
ATOM	1109	O	ILE	273	32.257	5.951	33.452	1.00	0.00	RX0	O
ATOM	1110	N	PHE	274	33.320	4.302	34.575	1.00	0.00	RX0	N
ATOM	1111	H	PHE	274	34.190	3.821	34.693	0.00	0.00	RX0	H
ATOM	1112	CA	PHE	274	32.191	3.976	35.472	1.00	0.00	RX0	C
ATOM	1113	CB	PHE	274	32.610	2.938	36.515	1.00	0.00	RX0	C
ATOM	1114	CG	PHE	274	32.269	1.538	36.070	1.00	0.00	RX0	C
ATOM	1115	CD1	PHE	274	32.531	1.123	34.770	1.00	0.00	RX0	C
ATOM	1116	CD2	PHE	274	31.688	0.660	36.979	1.00	0.00	RX0	C
ATOM	1117	CE1	PHE	274	32.217	-0.175	34.384	1.00	0.00	RX0	C
ATOM	1118	CE2	PHE	274	31.375	-0.637	36.593	1.00	0.00	RX0	C
ATOM	1119	CZ	PHE	274	31.648	-1.056	35.297	1.00	0.00	RX0	C
ATOM	1120	C	PHE	274	31.669	5.203	36.222	1.00	0.00	RX0	C
ATOM	1121	O	PHE	274	30.484	5.521	36.143	1.00	0.00	RX0	O
ATOM	1122	N	ASP	275	32.607	5.983	36.757	1.00	0.00	RX0	N
ATOM	1123	H	ASP	275	33.560	5.686	36.802	0.00	0.00	RX0	H
ATOM	1124	CA	ASP	275	32.273	7.222	37.483	1.00	0.00	RX0	C
ATOM	1125	CB	ASP	275	33.488	7.801	38.198	1.00	0.00	RX0	C
ATOM	1126	CG	ASP	275	33.583	7.159	39.567	1.00	0.00	RX0	C
ATOM	1127	OD1	ASP	275	33.907	5.981	39.671	1.00	0.00	RX0	O
ATOM	1128	OD2	ASP	275	33.360	7.835	40.565	1.00	0.00	RX0	O
ATOM	1129	C	ASP	275	31.555	8.270	36.629	1.00	0.00	RX0	C
ATOM	1130	O	ASP	275	30.604	8.890	37.102	1.00	0.00	RX0	O
ATOM	1131	N	MET	276	31.921	8.336	35.353	1.00	0.00	RX0	N
ATOM	1132	H	MET	276	32.664	7.739	35.041	0.00	0.00	RX0	H
ATOM	1133	CA	MET	276	31.257	9.254	34.408	1.00	0.00	RX0	C
ATOM	1134	CB	MET	276	32.089	9.446	33.141	1.00	0.00	RX0	C
ATOM	1135	CG	MET	276	33.421	10.150	33.399	1.00	0.00	RX0	C
ATOM	1136	SD	MET	276	34.286	10.549	31.871	1.00	0.00	RX0	S
ATOM	1137	CE	MET	276	34.341	8.891	31.179	1.00	0.00	RX0	C
ATOM	1138	C	MET	276	29.833	8.798	34.061	1.00	0.00	RX0	C
ATOM	1139	O	MET	276	28.893	9.589	34.158	1.00	0.00	RX0	O
ATOM	1140	N	LEU	277	29.673	7.488	33.891	1.00	0.00	RX0	N
ATOM	1141	H	LEU	277	30.490	6.907	33.950	0.00	0.00	RX0	H
ATOM	1142	CA	LEU	277	28.362	6.866	33.606	1.00	0.00	RX0	C
ATOM	1143	CB	LEU	277	28.561	5.401	33.227	1.00	0.00	RX0	C
ATOM	1144	CG	LEU	277	29.434	5.217	31.986	1.00	0.00	RX0	C
ATOM	1145	CD1	LEU	277	29.932	3.778	31.848	1.00	0.00	RX0	C
ATOM	1146	CD2	LEU	277	28.732	5.706	30.721	1.00	0.00	RX0	C
ATOM	1147	C	LEU	277	27.393	6.992	34.787	1.00	0.00	RX0	C
ATOM	1148	O	LEU	277	26.257	7.447	34.627	1.00	0.00	RX0	O
ATOM	1149	N	LEU	278	27.939	6.795	35.983	1.00	0.00	RX0	N
ATOM	1150	H	LEU	278	28.900	6.516	36.019	0.00	0.00	RX0	H
ATOM	1151	CA	LEU	278	27.196	6.926	37.250	1.00	0.00	RX0	C
ATOM	1152	CB	LEU	278	28.067	6.470	38.419	1.00	0.00	RX0	C
ATOM	1153	CG	LEU	278	28.220	4.953	38.467	1.00	0.00	RX0	C
ATOM	1154	CD1	LEU	278	29.272	4.502	39.480	1.00	0.00	RX0	C
ATOM	1155	CD2	LEU	278	26.867	4.291	38.710	1.00	0.00	RX0	C
ATOM	1156	C	LEU	278	26.716	8.359	37.508	1.00	0.00	RX0	C
ATOM	1157	O	LEU	278	25.554	8.575	37.840	1.00	0.00	RX0	O

ATOM	1158	N	ALA	279	27.582	9.317	37.175	1.00	0.00	RX0	N
ATOM	1159	H	ALA	279	28.513	9.059	36.898	0.00	0.00	RX0	H
ATOM	1160	CA	ALA	279	27.275	10.754	37.296	1.00	0.00	RX0	C
ATOM	1161	CB	ALA	279	28.528	11.595	37.048	1.00	0.00	RX0	C
ATOM	1162	C	ALA	279	26.182	11.196	36.310	1.00	0.00	RX0	C
ATOM	1163	O	ALA	279	25.263	11.922	36.684	1.00	0.00	RX0	O
ATOM	1164	N	THR	280	26.210	10.618	35.109	1.00	0.00	RX0	N
ATOM	1165	H	THR	280	26.969	10.006	34.881	0.00	0.00	RX0	H
ATOM	1166	CA	THR	280	25.203	10.899	34.059	1.00	0.00	RX0	C
ATOM	1167	CB	THR	280	25.747	10.367	32.739	1.00	0.00	RX0	C
ATOM	1168	OG1	THR	280	27.060	10.904	32.529	1.00	0.00	RX0	O
ATOM	1169	HG1	THR	280	27.665	10.447	33.105	0.00	0.00	RX0	H
ATOM	1170	CG2	THR	280	24.828	10.715	31.566	1.00	0.00	RX0	C
ATOM	1171	C	THR	280	23.835	10.327	34.462	1.00	0.00	RX0	C
ATOM	1172	O	THR	280	22.822	11.023	34.397	1.00	0.00	RX0	O
ATOM	1173	N	SER	281	23.868	9.112	35.003	1.00	0.00	RX0	N
ATOM	1174	H	SER	281	24.740	8.620	35.048	0.00	0.00	RX0	H
ATOM	1175	CA	SER	281	22.669	8.413	35.507	1.00	0.00	RX0	C
ATOM	1176	CB	SER	281	23.148	6.985	35.865	1.00	0.00	RX0	C
ATOM	1177	OG	SER	281	22.387	6.335	36.902	1.00	0.00	RX0	O
ATOM	1178	HG	SER	281	22.602	5.409	36.818	0.00	0.00	RX0	H
ATOM	1179	C	SER	281	22.019	9.180	36.675	1.00	0.00	RX0	C
ATOM	1180	O	SER	281	20.814	9.399	36.693	1.00	0.00	RX0	O
ATOM	1181	N	SER	282	22.889	9.788	37.491	1.00	0.00	RX0	N
ATOM	1182	H	SER	282	23.868	9.611	37.382	0.00	0.00	RX0	H
ATOM	1183	CA	SER	282	22.489	10.660	38.613	1.00	0.00	RX0	C
ATOM	1184	CB	SER	282	23.674	10.724	39.558	1.00	0.00	RX0	C
ATOM	1185	OG	SER	282	23.948	9.346	39.873	1.00	0.00	RX0	O
ATOM	1186	HG	SER	282	24.737	9.128	39.377	0.00	0.00	RX0	H
ATOM	1187	C	SER	282	21.828	11.963	38.130	1.00	0.00	RX0	C
ATOM	1188	O	SER	282	20.788	12.371	38.639	1.00	0.00	RX0	O
ATOM	1189	N	ARG	283	22.365	12.516	37.039	1.00	0.00	RX0	N
ATOM	1190	H	ARG	283	23.190	12.111	36.638	0.00	0.00	RX0	H
ATOM	1191	CA	ARG	283	21.822	13.735	36.412	1.00	0.00	RX0	C
ATOM	1192	CB	ARG	283	22.812	14.209	35.348	1.00	0.00	RX0	C
ATOM	1193	CG	ARG	283	22.351	15.406	34.521	1.00	0.00	RX0	C
ATOM	1194	CD	ARG	283	22.160	16.689	35.330	1.00	0.00	RX0	C
ATOM	1195	NE	ARG	283	21.780	17.775	34.431	1.00	0.00	RX0	N
ATOM	1196	HE	ARG	283	22.217	17.751	33.525	0.00	0.00	RX0	H
ATOM	1197	CZ	ARG	283	20.884	18.720	34.838	1.00	0.00	RX0	C
ATOM	1198	NH1	ARG	283	20.387	18.678	36.095	1.00	0.00	RX0	N
ATOM	1199	HH11	ARG	283	19.719	19.353	36.426	0.00	0.00	RX0	H
ATOM	1200	HH12	ARG	283	20.664	17.970	36.752	0.00	0.00	RX0	H
ATOM	1201	NH2	ARG	283	20.502	19.677	33.968	1.00	0.00	RX0	N
ATOM	1202	HH21	ARG	283	19.804	20.372	34.200	0.00	0.00	RX0	H
ATOM	1203	HH22	ARG	283	20.883	19.734	33.042	0.00	0.00	RX0	H
ATOM	1204	C	ARG	283	20.432	13.477	35.812	1.00	0.00	RX0	C
ATOM	1205	O	ARG	283	19.498	14.246	36.035	1.00	0.00	RX0	O
ATOM	1206	N	PHE	284	20.293	12.325	35.169	1.00	0.00	RX0	N
ATOM	1207	H	PHE	284	21.098	11.741	35.041	0.00	0.00	RX0	H
ATOM	1208	CA	PHE	284	19.003	11.899	34.606	1.00	0.00	RX0	C
ATOM	1209	CB	PHE	284	19.202	10.642	33.766	1.00	0.00	RX0	C
ATOM	1210	CG	PHE	284	19.645	11.021	32.375	1.00	0.00	RX0	C
ATOM	1211	CD1	PHE	284	19.181	12.197	31.798	1.00	0.00	RX0	C
ATOM	1212	CD2	PHE	284	20.491	10.182	31.662	1.00	0.00	RX0	C
ATOM	1213	CE1	PHE	284	19.531	12.512	30.491	1.00	0.00	RX0	C
ATOM	1214	CE2	PHE	284	20.843	10.502	30.356	1.00	0.00	RX0	C
ATOM	1215	CZ	PHE	284	20.349	11.657	29.763	1.00	0.00	RX0	C
ATOM	1216	C	PHE	284	17.921	11.680	35.654	1.00	0.00	RX0	C
ATOM	1217	O	PHE	284	16.817	12.216	35.524	1.00	0.00	RX0	O
ATOM	1218	N	ARG	285	18.348	11.087	36.760	1.00	0.00	RX0	N

ATOM	1219	H	ARG	285	19.279	10.718	36.763	0.00	0.00	RX0	H
ATOM	1220	CA	ARG	285	17.485	10.841	37.923	1.00	0.00	RX0	C
ATOM	1221	CB	ARG	285	18.332	10.059	38.927	1.00	0.00	RX0	C
ATOM	1222	CG	ARG	285	17.761	9.782	40.318	1.00	0.00	RX0	C
ATOM	1223	CD	ARG	285	18.788	9.023	41.171	1.00	0.00	RX0	C
ATOM	1224	NE	ARG	285	19.211	7.819	40.461	1.00	0.00	RX0	N
ATOM	1225	HE	ARG	285	18.456	7.220	40.162	0.00	0.00	RX0	H
ATOM	1226	CZ	ARG	285	20.503	7.648	40.048	1.00	0.00	RX0	C
ATOM	1227	NH1	ARG	285	21.466	8.430	40.579	1.00	0.00	RX0	N
ATOM	1228	HH11	ARG	285	22.431	8.428	40.270	0.00	0.00	RX0	H
ATOM	1229	HH12	ARG	285	21.255	9.080	41.313	0.00	0.00	RX0	H
ATOM	1230	NH2	ARG	285	20.769	6.716	39.107	1.00	0.00	RX0	N
ATOM	1231	HH21	ARG	285	21.657	6.588	38.643	0.00	0.00	RX0	H
ATOM	1232	HH22	ARG	285	20.029	6.105	38.801	0.00	0.00	RX0	H
ATOM	1233	C	ARG	285	17.003	12.164	38.534	1.00	0.00	RX0	C
ATOM	1234	O	ARG	285	15.822	12.321	38.816	1.00	0.00	RX0	O
ATOM	1235	N	MET	286	17.922	13.130	38.591	1.00	0.00	RX0	N
ATOM	1236	H	MET	286	18.856	12.922	38.295	0.00	0.00	RX0	H
ATOM	1237	CA	MET	286	17.643	14.467	39.144	1.00	0.00	RX0	C
ATOM	1238	CB	MET	286	18.961	15.233	39.262	1.00	0.00	RX0	C
ATOM	1239	CG	MET	286	18.792	16.699	39.665	1.00	0.00	RX0	C
ATOM	1240	SD	MET	286	20.309	17.644	39.445	1.00	0.00	RX0	S
ATOM	1241	CE	MET	286	19.711	19.216	40.087	1.00	0.00	RX0	C
ATOM	1242	C	MET	286	16.681	15.257	38.243	1.00	0.00	RX0	C
ATOM	1243	O	MET	286	15.799	15.962	38.735	1.00	0.00	RX0	O
ATOM	1244	N	MET	287	16.911	15.161	36.942	1.00	0.00	RX0	N
ATOM	1245	H	MET	287	17.629	14.536	36.630	0.00	0.00	RX0	H
ATOM	1246	CA	MET	287	16.049	15.814	35.941	1.00	0.00	RX0	C
ATOM	1247	CB	MET	287	16.684	15.856	34.555	1.00	0.00	RX0	C
ATOM	1248	CG	MET	287	17.866	16.816	34.509	1.00	0.00	RX0	C
ATOM	1249	SD	MET	287	18.271	17.295	32.827	1.00	0.00	RX0	S
ATOM	1250	CE	MET	287	16.651	17.953	32.400	1.00	0.00	RX0	C
ATOM	1251	C	MET	287	14.674	15.156	35.849	1.00	0.00	RX0	C
ATOM	1252	O	MET	287	13.755	15.729	35.264	1.00	0.00	RX0	O
ATOM	1253	N	ASN	288	14.583	13.920	36.346	1.00	0.00	RX0	N
ATOM	1254	H	ASN	288	15.392	13.490	36.747	0.00	0.00	RX0	H
ATOM	1255	CA	ASN	288	13.391	13.066	36.242	1.00	0.00	RX0	C
ATOM	1256	CB	ASN	288	12.161	13.613	36.968	1.00	0.00	RX0	C
ATOM	1257	CG	ASN	288	11.009	12.654	36.724	1.00	0.00	RX0	C
ATOM	1258	OD1	ASN	288	11.177	11.442	36.648	1.00	0.00	RX0	O
ATOM	1259	ND2	ASN	288	9.819	13.269	36.581	1.00	0.00	RX0	N
ATOM	1260	HD21	ASN	288	9.740	14.261	36.671	0.00	0.00	RX0	H
ATOM	1261	HD22	ASN	288	8.993	12.747	36.362	0.00	0.00	RX0	H
ATOM	1262	C	ASN	288	13.076	12.836	34.753	1.00	0.00	RX0	C
ATOM	1263	O	ASN	288	11.986	13.121	34.256	1.00	0.00	RX0	O
ATOM	1264	N	LEU	289	14.125	12.439	34.028	1.00	0.00	RX0	N
ATOM	1265	H	LEU	289	14.956	12.174	34.521	0.00	0.00	RX0	H
ATOM	1266	CA	LEU	289	14.030	12.183	32.583	1.00	0.00	RX0	C
ATOM	1267	CB	LEU	289	15.372	11.628	32.098	1.00	0.00	RX0	C
ATOM	1268	CG	LEU	289	15.410	11.218	30.622	1.00	0.00	RX0	C
ATOM	1269	CD1	LEU	289	15.551	12.426	29.703	1.00	0.00	RX0	C
ATOM	1270	CD2	LEU	289	16.489	10.175	30.337	1.00	0.00	RX0	C
ATOM	1271	C	LEU	289	12.926	11.152	32.321	1.00	0.00	RX0	C
ATOM	1272	O	LEU	289	12.814	10.137	33.014	1.00	0.00	RX0	O
ATOM	1273	N	GLN	290	12.165	11.420	31.281	1.00	0.00	RX0	N
ATOM	1274	H	GLN	290	12.394	12.178	30.664	0.00	0.00	RX0	H
ATOM	1275	CA	GLN	290	11.011	10.584	30.916	1.00	0.00	RX0	C
ATOM	1276	CB	GLN	290	9.778	11.452	30.673	1.00	0.00	RX0	C
ATOM	1277	CG	GLN	290	9.412	12.303	31.899	1.00	0.00	RX0	C
ATOM	1278	CD	GLN	290	8.986	11.431	33.072	1.00	0.00	RX0	C
ATOM	1279	OE1	GLN	290	7.826	11.061	33.214	1.00	0.00	RX0	O

ATOM	1280	NE2	GLN	290	9.973	11.157	33.941	1.00	0.00	RX0	N
ATOM	1281	HE21	GLN	290	10.899	11.513	33.799	0.00	0.00	RX0	H
ATOM	1282	HE22	GLN	290	9.878	10.609	34.774	0.00	0.00	RX0	H
ATOM	1283	C	GLN	290	11.379	9.701	29.727	1.00	0.00	RX0	C
ATOM	1284	O	GLN	290	12.115	10.141	28.832	1.00	0.00	RX0	O
ATOM	1285	N	GLY	291	10.739	8.533	29.672	1.00	0.00	RX0	N
ATOM	1286	H	GLY	291	10.026	8.353	30.350	0.00	0.00	RX0	H
ATOM	1287	CA	GLY	291	10.952	7.542	28.589	1.00	0.00	RX0	C
ATOM	1288	C	GLY	291	10.769	8.134	27.181	1.00	0.00	RX0	C
ATOM	1289	O	GLY	291	11.559	7.865	26.269	1.00	0.00	RX0	O
ATOM	1290	N	GLU	292	9.854	9.086	27.078	1.00	0.00	RX0	N
ATOM	1291	H	GLU	292	9.255	9.248	27.866	0.00	0.00	RX0	H
ATOM	1292	CA	GLU	292	9.541	9.789	25.813	1.00	0.00	RX0	C
ATOM	1293	CB	GLU	292	8.215	10.552	25.943	1.00	0.00	RX0	C
ATOM	1294	CG	GLU	292	6.976	9.707	26.281	1.00	0.00	RX0	C
ATOM	1295	CD	GLU	292	7.097	9.120	27.675	1.00	0.00	RX0	C
ATOM	1296	OE1	GLU	292	7.476	9.849	28.591	1.00	0.00	RX0	O
ATOM	1297	OE2	GLU	292	6.911	7.917	27.829	1.00	0.00	RX0	O
ATOM	1298	C	GLU	292	10.671	10.744	25.400	1.00	0.00	RX0	C
ATOM	1299	O	GLU	292	11.065	10.802	24.241	1.00	0.00	RX0	O
ATOM	1300	N	GLU	293	11.241	11.417	26.395	1.00	0.00	RX0	N
ATOM	1301	H	GLU	293	10.951	11.211	27.330	0.00	0.00	RX0	H
ATOM	1302	CA	GLU	293	12.389	12.320	26.184	1.00	0.00	RX0	C
ATOM	1303	CB	GLU	293	12.636	13.156	27.435	1.00	0.00	RX0	C
ATOM	1304	CG	GLU	293	11.406	13.909	27.931	1.00	0.00	RX0	C
ATOM	1305	CD	GLU	293	11.723	14.505	29.286	1.00	0.00	RX0	C
ATOM	1306	OE1	GLU	293	12.638	14.032	29.953	1.00	0.00	RX0	O
ATOM	1307	OE2	GLU	293	11.054	15.443	29.700	1.00	0.00	RX0	O
ATOM	1308	C	GLU	293	13.669	11.544	25.843	1.00	0.00	RX0	C
ATOM	1309	O	GLU	293	14.364	11.888	24.886	1.00	0.00	RX0	O
ATOM	1310	N	PHE	294	13.855	10.409	26.517	1.00	0.00	RX0	N
ATOM	1311	H	PHE	294	13.194	10.199	27.241	0.00	0.00	RX0	H
ATOM	1312	CA	PHE	294	14.999	9.503	26.300	1.00	0.00	RX0	C
ATOM	1313	CB	PHE	294	14.905	8.308	27.252	1.00	0.00	RX0	C
ATOM	1314	CG	PHE	294	15.867	7.227	26.816	1.00	0.00	RX0	C
ATOM	1315	CD1	PHE	294	17.239	7.452	26.841	1.00	0.00	RX0	C
ATOM	1316	CD2	PHE	294	15.373	6.005	26.374	1.00	0.00	RX0	C
ATOM	1317	CE1	PHE	294	18.110	6.465	26.397	1.00	0.00	RX0	C
ATOM	1318	CE2	PHE	294	16.245	5.018	25.932	1.00	0.00	RX0	C
ATOM	1319	CZ	PHE	294	17.615	5.252	25.935	1.00	0.00	RX0	C
ATOM	1320	C	PHE	294	15.115	9.012	24.847	1.00	0.00	RX0	C
ATOM	1321	O	PHE	294	16.186	9.132	24.238	1.00	0.00	RX0	O
ATOM	1322	N	VAL	295	14.000	8.581	24.280	1.00	0.00	RX0	N
ATOM	1323	H	VAL	295	13.158	8.569	24.828	0.00	0.00	RX0	H
ATOM	1324	CA	VAL	295	13.976	8.026	22.907	1.00	0.00	RX0	C
ATOM	1325	CB	VAL	295	12.686	7.251	22.616	1.00	0.00	RX0	C
ATOM	1326	CG1	VAL	295	12.586	6.064	23.571	1.00	0.00	RX0	C
ATOM	1327	CG2	VAL	295	11.430	8.119	22.660	1.00	0.00	RX0	C
ATOM	1328	C	VAL	295	14.286	9.097	21.847	1.00	0.00	RX0	C
ATOM	1329	O	VAL	295	14.999	8.845	20.884	1.00	0.00	RX0	O
ATOM	1330	N	CYS	296	13.866	10.330	22.156	1.00	0.00	RX0	N
ATOM	1331	H	CYS	296	13.306	10.481	22.974	0.00	0.00	RX0	H
ATOM	1332	CA	CYS	296	14.167	11.498	21.314	1.00	0.00	RX0	C
ATOM	1333	CB	CYS	296	13.264	12.653	21.730	1.00	0.00	RX0	C
ATOM	1334	SG	CYS	296	11.518	12.259	21.464	1.00	0.00	RX0	S
ATOM	1335	C	CYS	296	15.661	11.843	21.352	1.00	0.00	RX0	C
ATOM	1336	O	CYS	296	16.290	11.969	20.305	1.00	0.00	RX0	O
ATOM	1337	N	LEU	297	16.247	11.750	22.547	1.00	0.00	RX0	N
ATOM	1338	H	LEU	297	15.681	11.549	23.351	0.00	0.00	RX0	H
ATOM	1339	CA	LEU	297	17.681	12.031	22.752	1.00	0.00	RX0	C
ATOM	1340	CB	LEU	297	18.019	12.108	24.239	1.00	0.00	RX0	C

ATOM	1341	CG	LEU	297	17.346	13.279	24.953	1.00	0.00	RX0	C
ATOM	1342	CD1	LEU	297	17.641	13.253	26.452	1.00	0.00	RX0	C
ATOM	1343	CD2	LEU	297	17.709	14.622	24.317	1.00	0.00	RX0	C
ATOM	1344	C	LEU	297	18.589	11.009	22.068	1.00	0.00	RX0	C
ATOM	1345	O	LEU	297	19.526	11.385	21.359	1.00	0.00	RX0	O
ATOM	1346	N	LYS	298	18.173	9.750	22.123	1.00	0.00	RX0	N
ATOM	1347	H	LYS	298	17.359	9.545	22.671	0.00	0.00	RX0	H
ATOM	1348	CA	LYS	298	18.945	8.654	21.515	1.00	0.00	RX0	C
ATOM	1349	CB	LYS	298	18.431	7.321	22.046	1.00	0.00	RX0	C
ATOM	1350	CG	LYS	298	19.253	6.122	21.582	1.00	0.00	RX0	C
ATOM	1351	CD	LYS	298	18.614	4.836	22.091	1.00	0.00	RX0	C
ATOM	1352	CE	LYS	298	17.097	5.003	22.051	1.00	0.00	RX0	C
ATOM	1353	NZ	LYS	298	16.429	3.710	22.169	1.00	0.00	RX0	N
ATOM	1354	HZ1	LYS	298	15.436	3.867	22.466	0.00	0.00	RX0	H
ATOM	1355	HZ2	LYS	298	16.293	3.223	21.262	0.00	0.00	RX0	H
ATOM	1356	HZ3	LYS	298	16.789	3.064	22.892	0.00	0.00	RX0	H
ATOM	1357	C	LYS	298	18.925	8.733	19.978	1.00	0.00	RX0	C
ATOM	1358	O	LYS	298	19.964	8.557	19.332	1.00	0.00	RX0	O
ATOM	1359	N	SER	299	17.791	9.152	19.431	1.00	0.00	RX0	N
ATOM	1360	H	SER	299	16.964	9.267	19.982	0.00	0.00	RX0	H
ATOM	1361	CA	SER	299	17.645	9.383	17.977	1.00	0.00	RX0	C
ATOM	1362	CB	SER	299	16.155	9.360	17.703	1.00	0.00	RX0	C
ATOM	1363	OG	SER	299	15.660	8.202	18.384	1.00	0.00	RX0	O
ATOM	1364	HG	SER	299	15.127	8.510	19.109	0.00	0.00	RX0	H
ATOM	1365	C	SER	299	18.416	10.621	17.504	1.00	0.00	RX0	C
ATOM	1366	O	SER	299	19.051	10.583	16.444	1.00	0.00	RX0	O
ATOM	1367	N	ILE	300	18.478	11.644	18.354	1.00	0.00	RX0	N
ATOM	1368	H	ILE	300	17.956	11.596	19.208	0.00	0.00	RX0	H
ATOM	1369	CA	ILE	300	19.283	12.860	18.096	1.00	0.00	RX0	C
ATOM	1370	CB	ILE	300	19.078	13.935	19.167	1.00	0.00	RX0	C
ATOM	1371	CG2	ILE	300	20.117	15.051	19.043	1.00	0.00	RX0	C
ATOM	1372	CG1	ILE	300	17.672	14.515	19.094	1.00	0.00	RX0	C
ATOM	1373	CD1	ILE	300	17.411	15.532	20.204	1.00	0.00	RX0	C
ATOM	1374	C	ILE	300	20.773	12.490	18.013	1.00	0.00	RX0	C
ATOM	1375	O	ILE	300	21.456	12.924	17.087	1.00	0.00	RX0	O
ATOM	1376	N	ILE	301	21.227	11.630	18.921	1.00	0.00	RX0	N
ATOM	1377	H	ILE	301	20.606	11.326	19.646	0.00	0.00	RX0	H
ATOM	1378	CA	ILE	301	22.629	11.156	18.925	1.00	0.00	RX0	C
ATOM	1379	CB	ILE	301	22.873	10.195	20.082	1.00	0.00	RX0	C
ATOM	1380	CG2	ILE	301	24.192	9.458	19.889	1.00	0.00	RX0	C
ATOM	1381	CG1	ILE	301	22.834	10.931	21.419	1.00	0.00	RX0	C
ATOM	1382	CD1	ILE	301	23.180	10.001	22.579	1.00	0.00	RX0	C
ATOM	1383	C	ILE	301	22.948	10.474	17.584	1.00	0.00	RX0	C
ATOM	1384	O	ILE	301	23.927	10.823	16.926	1.00	0.00	RX0	O
ATOM	1385	N	LEU	302	22.047	9.582	17.175	1.00	0.00	RX0	N
ATOM	1386	H	LEU	302	21.272	9.375	17.776	0.00	0.00	RX0	H
ATOM	1387	CA	LEU	302	22.205	8.836	15.917	1.00	0.00	RX0	C
ATOM	1388	CB	LEU	302	20.992	7.929	15.709	1.00	0.00	RX0	C
ATOM	1389	CG	LEU	302	21.002	7.206	14.362	1.00	0.00	RX0	C
ATOM	1390	CD1	LEU	302	22.184	6.246	14.231	1.00	0.00	RX0	C
ATOM	1391	CD2	LEU	302	19.667	6.521	14.082	1.00	0.00	RX0	C
ATOM	1392	C	LEU	302	22.391	9.757	14.699	1.00	0.00	RX0	C
ATOM	1393	O	LEU	302	23.285	9.544	13.882	1.00	0.00	RX0	O
ATOM	1394	N	LEU	303	21.581	10.806	14.658	1.00	0.00	RX0	N
ATOM	1395	H	LEU	303	20.947	10.957	15.419	0.00	0.00	RX0	H
ATOM	1396	CA	LEU	303	21.546	11.719	13.502	1.00	0.00	RX0	C
ATOM	1397	CB	LEU	303	20.125	12.231	13.311	1.00	0.00	RX0	C
ATOM	1398	CG	LEU	303	19.164	11.064	13.081	1.00	0.00	RX0	C
ATOM	1399	CD1	LEU	303	17.707	11.485	13.249	1.00	0.00	RX0	C
ATOM	1400	CD2	LEU	303	19.421	10.367	11.744	1.00	0.00	RX0	C
ATOM	1401	C	LEU	303	22.576	12.849	13.543	1.00	0.00	RX0	C

ATOM	1402	O	LEU	303	23.073	13.268	12.494	1.00	0.00	RX0	O
ATOM	1403	N	ASN	304	22.971	13.240	14.745	1.00	0.00	RX0	N
ATOM	1404	H	ASN	304	22.641	12.745	15.550	0.00	0.00	RX0	H
ATOM	1405	CA	ASN	304	23.832	14.419	14.940	1.00	0.00	RX0	C
ATOM	1406	CB	ASN	304	23.503	15.139	16.243	1.00	0.00	RX0	C
ATOM	1407	CG	ASN	304	24.734	15.884	16.731	1.00	0.00	RX0	C
ATOM	1408	OD1	ASN	304	25.042	16.984	16.282	1.00	0.00	RX0	O
ATOM	1409	ND2	ASN	304	25.384	15.261	17.738	1.00	0.00	RX0	N
ATOM	1410	HD21	ASN	304	25.146	14.333	18.041	0.00	0.00	RX0	H
ATOM	1411	HD22	ASN	304	26.157	15.688	18.207	0.00	0.00	RX0	H
ATOM	1412	C	ASN	304	25.330	14.097	14.987	1.00	0.00	RX0	C
ATOM	1413	O	ASN	304	26.132	14.783	14.344	1.00	0.00	RX0	O
ATOM	1414	N	SER	305	25.693	13.015	15.656	1.00	0.00	RX0	N
ATOM	1415	H	SER	305	25.041	12.407	16.119	0.00	0.00	RX0	H
ATOM	1416	CA	SER	305	27.104	12.765	16.012	1.00	0.00	RX0	C
ATOM	1417	CB	SER	305	27.126	11.615	17.002	1.00	0.00	RX0	C
ATOM	1418	OG	SER	305	26.251	11.968	18.082	1.00	0.00	RX0	O
ATOM	1419	HG	SER	305	26.560	11.438	18.826	0.00	0.00	RX0	H
ATOM	1420	C	SER	305	28.103	12.695	14.846	1.00	0.00	RX0	C
ATOM	1421	O	SER	305	29.198	13.228	14.946	1.00	0.00	RX0	O
ATOM	1422	N	GLY	306	27.634	12.177	13.693	1.00	0.00	RX0	N
ATOM	1423	H	GLY	306	26.681	11.878	13.636	0.00	0.00	RX0	H
ATOM	1424	CA	GLY	306	28.500	12.079	12.501	1.00	0.00	RX0	C
ATOM	1425	C	GLY	306	28.091	12.961	11.315	1.00	0.00	RX0	C
ATOM	1426	O	GLY	306	28.756	12.897	10.274	1.00	0.00	RX0	O
ATOM	1427	N	VAL	307	27.242	13.954	11.536	1.00	0.00	RX0	N
ATOM	1428	H	VAL	307	26.877	14.106	12.458	0.00	0.00	RX0	H
ATOM	1429	CA	VAL	307	26.760	14.815	10.435	1.00	0.00	RX0	C
ATOM	1430	CB	VAL	307	25.400	15.460	10.751	1.00	0.00	RX0	C
ATOM	1431	CG1	VAL	307	25.493	16.573	11.793	1.00	0.00	RX0	C
ATOM	1432	CG2	VAL	307	24.720	15.942	9.470	1.00	0.00	RX0	C
ATOM	1433	C	VAL	307	27.809	15.841	9.948	1.00	0.00	RX0	C
ATOM	1434	O	VAL	307	27.790	16.280	8.811	1.00	0.00	RX0	O
ATOM	1435	N	TYR	308	28.719	16.199	10.859	1.00	0.00	RX0	N
ATOM	1436	H	TYR	308	28.724	15.728	11.740	0.00	0.00	RX0	H
ATOM	1437	CA	TYR	308	29.761	17.211	10.584	1.00	0.00	RX0	C
ATOM	1438	CB	TYR	308	30.116	17.961	11.867	1.00	0.00	RX0	C
ATOM	1439	CG	TYR	308	28.883	18.626	12.428	1.00	0.00	RX0	C
ATOM	1440	CD1	TYR	308	28.267	19.653	11.722	1.00	0.00	RX0	C
ATOM	1441	CE1	TYR	308	27.140	20.277	12.246	1.00	0.00	RX0	C
ATOM	1442	CD2	TYR	308	28.367	18.215	13.652	1.00	0.00	RX0	C
ATOM	1443	CE2	TYR	308	27.239	18.836	14.174	1.00	0.00	RX0	C
ATOM	1444	CZ	TYR	308	26.628	19.870	13.473	1.00	0.00	RX0	C
ATOM	1445	OH	TYR	308	25.515	20.492	13.997	1.00	0.00	RX0	O
ATOM	1446	HH	TYR	308	25.117	19.916	14.640	0.00	0.00	RX0	H
ATOM	1447	C	TYR	308	31.032	16.660	9.940	1.00	0.00	RX0	C
ATOM	1448	O	TYR	308	31.912	17.411	9.537	1.00	0.00	RX0	O
ATOM	1449	N	THR	309	31.091	15.334	9.861	1.00	0.00	RX0	N
ATOM	1450	H	THR	309	30.367	14.745	10.228	0.00	0.00	RX0	H
ATOM	1451	CA	THR	309	32.303	14.643	9.386	1.00	0.00	RX0	C
ATOM	1452	CB	THR	309	32.722	13.696	10.509	1.00	0.00	RX0	C
ATOM	1453	OG1	THR	309	31.552	13.086	11.081	1.00	0.00	RX0	O
ATOM	1454	HG1	THR	309	31.294	12.364	10.509	0.00	0.00	RX0	H
ATOM	1455	CG2	THR	309	33.497	14.435	11.602	1.00	0.00	RX0	C
ATOM	1456	C	THR	309	32.183	13.866	8.071	1.00	0.00	RX0	C
ATOM	1457	O	THR	309	33.137	13.195	7.681	1.00	0.00	RX0	O
ATOM	1458	N	PHE	310	31.041	13.953	7.382	1.00	0.00	RX0	N
ATOM	1459	H	PHE	310	30.302	14.505	7.759	0.00	0.00	RX0	H
ATOM	1460	CA	PHE	310	30.972	13.467	5.988	1.00	0.00	RX0	C
ATOM	1461	CB	PHE	310	29.596	13.717	5.371	1.00	0.00	RX0	C
ATOM	1462	CG	PHE	310	28.499	13.027	6.145	1.00	0.00	RX0	C

ATOM	1463	CD1	PHE	310	28.549	11.657	6.370	1.00	0.00	RX0	C
ATOM	1464	CD2	PHE	310	27.422	13.768	6.616	1.00	0.00	RX0	C
ATOM	1465	CE1	PHE	310	27.515	11.030	7.055	1.00	0.00	RX0	C
ATOM	1466	CE2	PHE	310	26.389	13.140	7.299	1.00	0.00	RX0	C
ATOM	1467	CZ	PHE	310	26.433	11.769	7.517	1.00	0.00	RX0	C
ATOM	1468	C	PHE	310	32.019	14.245	5.180	1.00	0.00	RX0	C
ATOM	1469	O	PHE	310	32.102	15.468	5.301	1.00	0.00	RX0	O
ATOM	1470	N	LEU	311	32.854	13.508	4.462	1.00	0.00	RX0	N
ATOM	1471	H	LEU	311	32.727	12.522	4.539	0.00	0.00	RX0	H
ATOM	1472	CA	LEU	311	33.988	14.087	3.701	1.00	0.00	RX0	C
ATOM	1473	CB	LEU	311	34.898	13.020	3.049	1.00	0.00	RX0	C
ATOM	1474	CG	LEU	311	34.296	11.824	2.289	1.00	0.00	RX0	C
ATOM	1475	CD1	LEU	311	35.249	11.340	1.195	1.00	0.00	RX0	C
ATOM	1476	CD2	LEU	311	33.878	10.662	3.196	1.00	0.00	RX0	C
ATOM	1477	C	LEU	311	33.468	15.166	2.739	1.00	0.00	RX0	C
ATOM	1478	O	LEU	311	33.554	16.351	2.969	1.00	0.00	RX0	O
ATOM	1479	N	SER	312	32.784	14.619	1.713	1.00	0.00	RX0	N
ATOM	1480	H	SER	312	32.670	13.631	1.632	0.00	0.00	RX0	H
ATOM	1481	CA	SER	312	32.345	15.333	0.526	1.00	0.00	RX0	C
ATOM	1482	CB	SER	312	31.712	14.248	-0.345	1.00	0.00	RX0	C
ATOM	1483	OG	SER	312	32.514	13.061	-0.273	1.00	0.00	RX0	O
ATOM	1484	HG	SER	312	32.020	12.355	-0.674	0.00	0.00	RX0	H
ATOM	1485	C	SER	312	31.423	16.517	0.816	1.00	0.00	RX0	C
ATOM	1486	O	SER	312	31.042	16.847	1.948	1.00	0.00	RX0	O
ATOM	1487	N	SER	313	30.837	16.923	-0.276	1.00	0.00	RX0	N
ATOM	1488	H	SER	313	31.208	16.620	-1.155	0.00	0.00	RX0	H
ATOM	1489	CA	SER	313	29.838	17.983	-0.455	1.00	0.00	RX0	C
ATOM	1490	CB	SER	313	30.335	19.323	0.093	1.00	0.00	RX0	C
ATOM	1491	OG	SER	313	30.310	19.223	1.533	1.00	0.00	RX0	O
ATOM	1492	HG	SER	313	31.130	18.789	1.768	0.00	0.00	RX0	H
ATOM	1493	C	SER	313	29.353	17.880	-1.909	1.00	0.00	RX0	C
ATOM	1494	O	SER	313	29.168	18.836	-2.628	1.00	0.00	RX0	O
ATOM	1495	N	THR	314	29.278	16.602	-2.358	1.00	0.00	RX0	N
ATOM	1496	H	THR	314	29.453	15.829	-1.754	0.00	0.00	RX0	H
ATOM	1497	CA	THR	314	28.609	16.250	-3.614	1.00	0.00	RX0	C
ATOM	1498	CB	THR	314	28.742	14.745	-3.677	1.00	0.00	RX0	C
ATOM	1499	OG1	THR	314	29.879	14.387	-2.879	1.00	0.00	RX0	O
ATOM	1500	HG1	THR	314	30.172	13.543	-3.209	0.00	0.00	RX0	H
ATOM	1501	CG2	THR	314	28.860	14.210	-5.105	1.00	0.00	RX0	C
ATOM	1502	C	THR	314	27.167	16.747	-3.490	1.00	0.00	RX0	C
ATOM	1503	O	THR	314	26.675	16.978	-2.368	1.00	0.00	RX0	O
ATOM	1504	N	LEU	315	26.451	16.801	-4.589	1.00	0.00	RX0	N
ATOM	1505	H	LEU	315	26.877	16.640	-5.478	0.00	0.00	RX0	H
ATOM	1506	CA	LEU	315	25.050	17.256	-4.537	1.00	0.00	RX0	C
ATOM	1507	CB	LEU	315	24.459	17.329	-5.944	1.00	0.00	RX0	C
ATOM	1508	CG	LEU	315	23.032	17.883	-5.947	1.00	0.00	RX0	C
ATOM	1509	CD1	LEU	315	22.966	19.293	-5.354	1.00	0.00	RX0	C
ATOM	1510	CD2	LEU	315	22.399	17.812	-7.337	1.00	0.00	RX0	C
ATOM	1511	C	LEU	315	24.190	16.343	-3.639	1.00	0.00	RX0	C
ATOM	1512	O	LEU	315	23.484	16.800	-2.757	1.00	0.00	RX0	O
ATOM	1513	N	LYS	316	24.524	15.049	-3.730	1.00	0.00	RX0	N
ATOM	1514	H	LYS	316	25.092	14.779	-4.502	0.00	0.00	RX0	H
ATOM	1515	CA	LYS	316	23.912	13.990	-2.918	1.00	0.00	RX0	C
ATOM	1516	CB	LYS	316	24.338	12.603	-3.417	1.00	0.00	RX0	C
ATOM	1517	CG	LYS	316	23.131	11.696	-3.689	1.00	0.00	RX0	C
ATOM	1518	CD	LYS	316	22.244	11.662	-2.449	1.00	0.00	RX0	C
ATOM	1519	CE	LYS	316	20.785	11.256	-2.626	1.00	0.00	RX0	C
ATOM	1520	NZ	LYS	316	20.050	11.948	-1.567	1.00	0.00	RX0	N
ATOM	1521	HZ1	LYS	316	19.027	11.844	-1.627	0.00	0.00	RX0	H
ATOM	1522	HZ2	LYS	316	20.369	11.641	-0.620	0.00	0.00	RX0	H
ATOM	1523	HZ3	LYS	316	20.218	12.973	-1.672	0.00	0.00	RX0	H

ATOM	1524	C	LYS	316	24.213	14.187	-1.422	1.00	0.00	RX0	C
ATOM	1525	O	LYS	316	23.297	14.194	-0.611	1.00	0.00	RX0	O
ATOM	1526	N	SER	317	25.467	14.540	-1.122	1.00	0.00	RX0	N
ATOM	1527	H	SER	317	26.098	14.714	-1.871	0.00	0.00	RX0	H
ATOM	1528	CA	SER	317	25.934	14.787	0.260	1.00	0.00	RX0	C
ATOM	1529	CB	SER	317	27.466	14.862	0.209	1.00	0.00	RX0	C
ATOM	1530	OG	SER	317	28.080	14.563	1.469	1.00	0.00	RX0	O
ATOM	1531	HG	SER	317	27.680	13.746	1.772	0.00	0.00	RX0	H
ATOM	1532	C	SER	317	25.242	16.001	0.898	1.00	0.00	RX0	C
ATOM	1533	O	SER	317	24.716	15.915	2.007	1.00	0.00	RX0	O
ATOM	1534	N	LEU	318	25.067	17.043	0.088	1.00	0.00	RX0	N
ATOM	1535	H	LEU	318	25.355	16.979	-0.869	0.00	0.00	RX0	H
ATOM	1536	CA	LEU	318	24.405	18.288	0.525	1.00	0.00	RX0	C
ATOM	1537	CB	LEU	318	24.580	19.375	-0.531	1.00	0.00	RX0	C
ATOM	1538	CG	LEU	318	26.046	19.772	-0.690	1.00	0.00	RX0	C
ATOM	1539	CD1	LEU	318	26.256	20.719	-1.872	1.00	0.00	RX0	C
ATOM	1540	CD2	LEU	318	26.616	20.328	0.616	1.00	0.00	RX0	C
ATOM	1541	C	LEU	318	22.918	18.061	0.823	1.00	0.00	RX0	C
ATOM	1542	O	LEU	318	22.412	18.453	1.877	1.00	0.00	RX0	O
ATOM	1543	N	GLU	319	22.299	17.244	-0.024	1.00	0.00	RX0	N
ATOM	1544	H	GLU	319	22.765	16.927	-0.853	0.00	0.00	RX0	H
ATOM	1545	CA	GLU	319	20.900	16.808	0.157	1.00	0.00	RX0	C
ATOM	1546	CB	GLU	319	20.455	15.801	-0.901	1.00	0.00	RX0	C
ATOM	1547	CG	GLU	319	20.359	16.115	-2.385	1.00	0.00	RX0	C
ATOM	1548	CD	GLU	319	20.019	14.780	-3.037	1.00	0.00	RX0	C
ATOM	1549	OE1	GLU	319	20.737	14.344	-3.932	1.00	0.00	RX0	O
ATOM	1550	OE2	GLU	319	19.076	14.123	-2.594	1.00	0.00	RX0	O
ATOM	1551	C	GLU	319	20.725	15.924	1.402	1.00	0.00	RX0	C
ATOM	1552	O	GLU	319	19.808	16.153	2.196	1.00	0.00	RX0	O
ATOM	1553	N	GLU	320	21.706	15.058	1.645	1.00	0.00	RX0	N
ATOM	1554	H	GLU	320	22.402	14.916	0.942	0.00	0.00	RX0	H
ATOM	1555	CA	GLU	320	21.731	14.149	2.812	1.00	0.00	RX0	C
ATOM	1556	CB	GLU	320	22.753	13.009	2.765	1.00	0.00	RX0	C
ATOM	1557	CG	GLU	320	22.777	12.092	1.528	1.00	0.00	RX0	C
ATOM	1558	CD	GLU	320	21.424	11.542	1.069	1.00	0.00	RX0	C
ATOM	1559	OE1	GLU	320	21.368	10.413	0.590	1.00	0.00	RX0	O
ATOM	1560	OE2	GLU	320	20.429	12.258	1.016	1.00	0.00	RX0	O
ATOM	1561	C	GLU	320	21.765	14.931	4.131	1.00	0.00	RX0	C
ATOM	1562	O	GLU	320	20.881	14.754	4.973	1.00	0.00	RX0	O
ATOM	1563	N	LYS	321	22.647	15.926	4.188	1.00	0.00	RX0	N
ATOM	1564	H	LYS	321	23.237	16.045	3.387	0.00	0.00	RX0	H
ATOM	1565	CA	LYS	321	22.801	16.771	5.391	1.00	0.00	RX0	C
ATOM	1566	CB	LYS	321	24.079	17.634	5.231	1.00	0.00	RX0	C
ATOM	1567	CG	LYS	321	25.361	16.838	4.881	1.00	0.00	RX0	C
ATOM	1568	CD	LYS	321	26.623	17.647	4.478	1.00	0.00	RX0	C
ATOM	1569	CE	LYS	321	27.751	16.764	3.885	1.00	0.00	RX0	C
ATOM	1570	NZ	LYS	321	29.001	17.492	3.554	1.00	0.00	RX0	N
ATOM	1571	HZ1	LYS	321	29.683	16.853	3.082	0.00	0.00	RX0	H
ATOM	1572	HZ2	LYS	321	28.858	18.289	2.899	0.00	0.00	RX0	H
ATOM	1573	HZ3	LYS	321	29.463	17.835	4.418	0.00	0.00	RX0	H
ATOM	1574	C	LYS	321	21.549	17.612	5.656	1.00	0.00	RX0	C
ATOM	1575	O	LYS	321	21.102	17.724	6.798	1.00	0.00	RX0	O
ATOM	1576	N	ASP	322	20.935	18.099	4.575	1.00	0.00	RX0	N
ATOM	1577	H	ASP	322	21.314	17.936	3.660	0.00	0.00	RX0	H
ATOM	1578	CA	ASP	322	19.715	18.915	4.684	1.00	0.00	RX0	C
ATOM	1579	CB	ASP	322	19.383	19.461	3.293	1.00	0.00	RX0	C
ATOM	1580	CG	ASP	322	18.040	20.155	3.284	1.00	0.00	RX0	C
ATOM	1581	OD1	ASP	322	17.660	20.747	4.283	1.00	0.00	RX0	O
ATOM	1582	OD2	ASP	322	17.340	20.070	2.280	1.00	0.00	RX0	O
ATOM	1583	C	ASP	322	18.561	18.097	5.282	1.00	0.00	RX0	C
ATOM	1584	O	ASP	322	17.955	18.512	6.263	1.00	0.00	RX0	O

ATOM	1585	N	HIS	323	18.423	16.872	4.772	1.00	0.00	RX0	N
ATOM	1586	H	HIS	323	19.041	16.602	4.028	0.00	0.00	RX0	H
ATOM	1587	CA	HIS	323	17.423	15.916	5.274	1.00	0.00	RX0	C
ATOM	1588	CB	HIS	323	17.458	14.617	4.468	1.00	0.00	RX0	C
ATOM	1589	CG	HIS	323	16.297	13.748	4.888	1.00	0.00	RX0	C
ATOM	1590	ND1	HIS	323	16.333	12.406	4.975	1.00	0.00	RX0	N
ATOM	1591	HD1	HIS	323	17.092	11.812	4.781	0.00	0.00	RX0	H
ATOM	1592	CD2	HIS	323	15.017	14.182	5.233	1.00	0.00	RX0	C
ATOM	1593	NE2	HIS	323	14.275	13.093	5.530	1.00	0.00	RX0	N
ATOM	1594	CE1	HIS	323	15.085	11.993	5.372	1.00	0.00	RX0	C
ATOM	1595	C	HIS	323	17.630	15.607	6.766	1.00	0.00	RX0	C
ATOM	1596	O	HIS	323	16.677	15.663	7.540	1.00	0.00	RX0	O
ATOM	1597	N	ILE	324	18.888	15.420	7.164	1.00	0.00	RX0	N
ATOM	1598	H	ILE	324	19.613	15.432	6.471	0.00	0.00	RX0	H
ATOM	1599	CA	ILE	324	19.235	15.107	8.570	1.00	0.00	RX0	C
ATOM	1600	CB	ILE	324	20.717	14.760	8.706	1.00	0.00	RX0	C
ATOM	1601	CG2	ILE	324	21.116	14.611	10.174	1.00	0.00	RX0	C
ATOM	1602	CG1	ILE	324	21.034	13.492	7.914	1.00	0.00	RX0	C
ATOM	1603	CD1	ILE	324	22.520	13.133	7.928	1.00	0.00	RX0	C
ATOM	1604	C	ILE	324	18.844	16.271	9.496	1.00	0.00	RX0	C
ATOM	1605	O	ILE	324	18.200	16.053	10.527	1.00	0.00	RX0	O
ATOM	1606	N	HIS	325	19.159	17.485	9.071	1.00	0.00	RX0	N
ATOM	1607	H	HIS	325	19.605	17.591	8.180	0.00	0.00	RX0	H
ATOM	1608	CA	HIS	325	18.840	18.694	9.856	1.00	0.00	RX0	C
ATOM	1609	CB	HIS	325	19.581	19.920	9.324	1.00	0.00	RX0	C
ATOM	1610	CG	HIS	325	21.041	19.789	9.678	1.00	0.00	RX0	C
ATOM	1611	ND1	HIS	325	21.982	19.390	8.809	1.00	0.00	RX0	N
ATOM	1612	HD1	HIS	325	21.819	19.126	7.878	0.00	0.00	RX0	H
ATOM	1613	CD2	HIS	325	21.645	20.033	10.914	1.00	0.00	RX0	C
ATOM	1614	NE2	HIS	325	22.969	19.774	10.774	1.00	0.00	RX0	N
ATOM	1615	CE1	HIS	325	23.175	19.380	9.478	1.00	0.00	RX0	C
ATOM	1616	C	HIS	325	17.335	18.955	9.948	1.00	0.00	RX0	C
ATOM	1617	O	HIS	325	16.820	19.290	11.021	1.00	0.00	RX0	O
ATOM	1618	N	ARG	326	16.631	18.584	8.887	1.00	0.00	RX0	N
ATOM	1619	H	ARG	326	17.127	18.354	8.048	0.00	0.00	RX0	H
ATOM	1620	CA	ARG	326	15.160	18.594	8.873	1.00	0.00	RX0	C
ATOM	1621	CB	ARG	326	14.653	18.412	7.441	1.00	0.00	RX0	C
ATOM	1622	CG	ARG	326	14.680	19.720	6.648	1.00	0.00	RX0	C
ATOM	1623	CD	ARG	326	14.142	19.585	5.219	1.00	0.00	RX0	C
ATOM	1624	NE	ARG	326	15.179	19.192	4.266	1.00	0.00	RX0	N
ATOM	1625	HE	ARG	326	15.923	19.867	4.111	0.00	0.00	RX0	H
ATOM	1626	CZ	ARG	326	15.133	18.007	3.594	1.00	0.00	RX0	C
ATOM	1627	NH1	ARG	326	14.158	17.123	3.890	1.00	0.00	RX0	N
ATOM	1628	HH11	ARG	326	14.067	16.240	3.424	0.00	0.00	RX0	H
ATOM	1629	HH12	ARG	326	13.488	17.329	4.607	0.00	0.00	RX0	H
ATOM	1630	NH2	ARG	326	16.055	17.735	2.649	1.00	0.00	RX0	N
ATOM	1631	HH21	ARG	326	16.135	16.881	2.132	0.00	0.00	RX0	H
ATOM	1632	HH22	ARG	326	16.736	18.454	2.427	0.00	0.00	RX0	H
ATOM	1633	C	ARG	326	14.537	17.576	9.843	1.00	0.00	RX0	C
ATOM	1634	O	ARG	326	13.617	17.928	10.589	1.00	0.00	RX0	O
ATOM	1635	N	VAL	327	15.171	16.419	9.987	1.00	0.00	RX0	N
ATOM	1636	H	VAL	327	15.983	16.249	9.424	0.00	0.00	RX0	H
ATOM	1637	CA	VAL	327	14.709	15.376	10.935	1.00	0.00	RX0	C
ATOM	1638	CB	VAL	327	15.278	13.994	10.608	1.00	0.00	RX0	C
ATOM	1639	CG1	VAL	327	14.753	12.958	11.601	1.00	0.00	RX0	C
ATOM	1640	CG2	VAL	327	14.942	13.578	9.177	1.00	0.00	RX0	C
ATOM	1641	C	VAL	327	15.041	15.781	12.381	1.00	0.00	RX0	C
ATOM	1642	O	VAL	327	14.187	15.661	13.270	1.00	0.00	RX0	O
ATOM	1643	N	LEU	328	16.212	16.371	12.574	1.00	0.00	RX0	N
ATOM	1644	H	LEU	328	16.819	16.508	11.790	0.00	0.00	RX0	H
ATOM	1645	CA	LEU	328	16.631	16.899	13.887	1.00	0.00	RX0	C

ATOM	1646	CB	LEU	328	18.070	17.407	13.829	1.00	0.00	RX0	C
ATOM	1647	CG	LEU	328	19.079	16.261	13.765	1.00	0.00	RX0	C
ATOM	1648	CD1	LEU	328	20.502	16.758	13.506	1.00	0.00	RX0	C
ATOM	1649	CD2	LEU	328	18.994	15.385	15.016	1.00	0.00	RX0	C
ATOM	1650	C	LEU	328	15.692	18.005	14.386	1.00	0.00	RX0	C
ATOM	1651	O	LEU	328	15.231	17.955	15.519	1.00	0.00	RX0	O
ATOM	1652	N	ASP	329	15.222	18.821	13.436	1.00	0.00	RX0	N
ATOM	1653	H	ASP	329	15.683	18.903	12.550	0.00	0.00	RX0	H
ATOM	1654	CA	ASP	329	14.223	19.870	13.722	1.00	0.00	RX0	C
ATOM	1655	CB	ASP	329	14.063	20.827	12.538	1.00	0.00	RX0	C
ATOM	1656	CG	ASP	329	15.277	21.731	12.434	1.00	0.00	RX0	C
ATOM	1657	OD1	ASP	329	16.057	21.786	13.386	1.00	0.00	RX0	O
ATOM	1658	OD2	ASP	329	15.436	22.386	11.403	1.00	0.00	RX0	O
ATOM	1659	C	ASP	329	12.864	19.312	14.154	1.00	0.00	RX0	C
ATOM	1660	O	ASP	329	12.272	19.788	15.128	1.00	0.00	RX0	O
ATOM	1661	N	LYS	330	12.463	18.218	13.512	1.00	0.00	RX0	N
ATOM	1662	H	LYS	330	13.022	17.921	12.736	0.00	0.00	RX0	H
ATOM	1663	CA	LYS	330	11.217	17.525	13.871	1.00	0.00	RX0	C
ATOM	1664	CB	LYS	330	10.799	16.459	12.861	1.00	0.00	RX0	C
ATOM	1665	CG	LYS	330	9.625	15.603	13.369	1.00	0.00	RX0	C
ATOM	1666	CD	LYS	330	8.370	16.378	13.800	1.00	0.00	RX0	C
ATOM	1667	CE	LYS	330	7.826	17.305	12.721	1.00	0.00	RX0	C
ATOM	1668	NZ	LYS	330	7.439	16.475	11.581	1.00	0.00	RX0	N
ATOM	1669	HZ1	LYS	330	7.126	17.094	10.812	0.00	0.00	RX0	H
ATOM	1670	HZ2	LYS	330	8.249	15.890	11.282	0.00	0.00	RX0	H
ATOM	1671	HZ3	LYS	330	6.659	15.855	11.882	0.00	0.00	RX0	H
ATOM	1672	C	LYS	330	11.283	16.915	15.278	1.00	0.00	RX0	C
ATOM	1673	O	LYS	330	10.354	17.095	16.067	1.00	0.00	RX0	O
ATOM	1674	N	ILE	331	12.441	16.375	15.625	1.00	0.00	RX0	N
ATOM	1675	H	ILE	331	13.185	16.378	14.951	0.00	0.00	RX0	H
ATOM	1676	CA	ILE	331	12.656	15.802	16.972	1.00	0.00	RX0	C
ATOM	1677	CB	ILE	331	13.952	14.996	17.053	1.00	0.00	RX0	C
ATOM	1678	CG2	ILE	331	14.060	14.327	18.420	1.00	0.00	RX0	C
ATOM	1679	CG1	ILE	331	14.042	13.952	15.942	1.00	0.00	RX0	C
ATOM	1680	CD1	ILE	331	15.356	13.172	15.982	1.00	0.00	RX0	C
ATOM	1681	C	ILE	331	12.642	16.922	18.027	1.00	0.00	RX0	C
ATOM	1682	O	ILE	331	12.078	16.732	19.120	1.00	0.00	RX0	O
ATOM	1683	N	THR	332	13.158	18.084	17.675	1.00	0.00	RX0	N
ATOM	1684	H	THR	332	13.577	18.203	16.774	0.00	0.00	RX0	H
ATOM	1685	CA	THR	332	13.155	19.265	18.570	1.00	0.00	RX0	C
ATOM	1686	CB	THR	332	14.034	20.315	17.913	1.00	0.00	RX0	C
ATOM	1687	OG1	THR	332	15.301	19.707	17.627	1.00	0.00	RX0	O
ATOM	1688	HG1	THR	332	15.254	19.373	16.735	0.00	0.00	RX0	H
ATOM	1689	CG2	THR	332	14.208	21.554	18.792	1.00	0.00	RX0	C
ATOM	1690	C	THR	332	11.706	19.696	18.836	1.00	0.00	RX0	C
ATOM	1691	O	THR	332	11.302	19.832	19.995	1.00	0.00	RX0	O
ATOM	1692	N	ASP	333	10.912	19.714	17.772	1.00	0.00	RX0	N
ATOM	1693	H	ASP	333	11.281	19.672	16.838	0.00	0.00	RX0	H
ATOM	1694	CA	ASP	333	9.466	20.017	17.863	1.00	0.00	RX0	C
ATOM	1695	CB	ASP	333	8.670	19.790	16.568	1.00	0.00	RX0	C
ATOM	1696	CG	ASP	333	9.094	20.575	15.354	1.00	0.00	RX0	C
ATOM	1697	OD1	ASP	333	9.497	21.724	15.503	1.00	0.00	RX0	O
ATOM	1698	OD2	ASP	333	8.967	20.033	14.252	1.00	0.00	RX0	O
ATOM	1699	C	ASP	333	8.732	19.000	18.747	1.00	0.00	RX0	C
ATOM	1700	O	ASP	333	7.880	19.374	19.559	1.00	0.00	RX0	O
ATOM	1701	N	THR	334	9.187	17.759	18.682	1.00	0.00	RX0	N
ATOM	1702	H	THR	334	9.941	17.583	18.048	0.00	0.00	RX0	H
ATOM	1703	CA	THR	334	8.631	16.638	19.462	1.00	0.00	RX0	C
ATOM	1704	CB	THR	334	9.176	15.373	18.821	1.00	0.00	RX0	C
ATOM	1705	OG1	THR	334	8.793	15.340	17.438	1.00	0.00	RX0	O
ATOM	1706	HG1	THR	334	9.149	16.126	17.032	0.00	0.00	RX0	H

ATOM	1707	CG2	THR	334	8.724	14.119	19.557	1.00	0.00	RX0	C
ATOM	1708	C	THR	334	8.961	16.782	20.953	1.00	0.00	RX0	C
ATOM	1709	O	THR	334	8.059	16.689	21.789	1.00	0.00	RX0	O
ATOM	1710	N	LEU	335	10.217	17.081	21.263	1.00	0.00	RX0	N
ATOM	1711	H	LEU	335	10.880	17.204	20.523	0.00	0.00	RX0	H
ATOM	1712	CA	LEU	335	10.648	17.349	22.650	1.00	0.00	RX0	C
ATOM	1713	CB	LEU	335	12.150	17.610	22.696	1.00	0.00	RX0	C
ATOM	1714	CG	LEU	335	12.958	16.320	22.781	1.00	0.00	RX0	C
ATOM	1715	CD1	LEU	335	14.457	16.566	22.610	1.00	0.00	RX0	C
ATOM	1716	CD2	LEU	335	12.649	15.571	24.078	1.00	0.00	RX0	C
ATOM	1717	C	LEU	335	9.903	18.522	23.297	1.00	0.00	RX0	C
ATOM	1718	O	LEU	335	9.384	18.379	24.399	1.00	0.00	RX0	O
ATOM	1719	N	ILE	336	9.668	19.567	22.501	1.00	0.00	RX0	N
ATOM	1720	H	ILE	336	10.072	19.579	21.582	0.00	0.00	RX0	H
ATOM	1721	CA	ILE	336	8.897	20.743	22.962	1.00	0.00	RX0	C
ATOM	1722	CB	ILE	336	9.048	21.912	21.989	1.00	0.00	RX0	C
ATOM	1723	CG2	ILE	336	8.088	23.056	22.324	1.00	0.00	RX0	C
ATOM	1724	CG1	ILE	336	10.501	22.389	21.997	1.00	0.00	RX0	C
ATOM	1725	CD1	ILE	336	10.928	22.872	23.385	1.00	0.00	RX0	C
ATOM	1726	C	ILE	336	7.427	20.365	23.180	1.00	0.00	RX0	C
ATOM	1727	O	ILE	336	6.836	20.730	24.203	1.00	0.00	RX0	O
ATOM	1728	N	HIS	337	6.891	19.571	22.265	1.00	0.00	RX0	N
ATOM	1729	H	HIS	337	7.438	19.319	21.464	0.00	0.00	RX0	H
ATOM	1730	CA	HIS	337	5.501	19.096	22.359	1.00	0.00	RX0	C
ATOM	1731	CB	HIS	337	5.075	18.324	21.120	1.00	0.00	RX0	C
ATOM	1732	CG	HIS	337	3.604	18.022	21.252	1.00	0.00	RX0	C
ATOM	1733	ND1	HIS	337	2.634	18.933	21.055	1.00	0.00	RX0	N
ATOM	1734	HD1	HIS	337	2.760	19.870	20.792	0.00	0.00	RX0	H
ATOM	1735	CD2	HIS	337	3.017	16.810	21.614	1.00	0.00	RX0	C
ATOM	1736	NE2	HIS	337	1.674	16.998	21.641	1.00	0.00	RX0	N
ATOM	1737	CE1	HIS	337	1.439	18.307	21.295	1.00	0.00	RX0	C
ATOM	1738	C	HIS	337	5.301	18.263	23.631	1.00	0.00	RX0	C
ATOM	1739	O	HIS	337	4.339	18.490	24.365	1.00	0.00	RX0	O
ATOM	1740	N	LEU	338	6.274	17.405	23.914	1.00	0.00	RX0	N
ATOM	1741	H	LEU	338	7.051	17.336	23.286	0.00	0.00	RX0	H
ATOM	1742	CA	LEU	338	6.246	16.526	25.097	1.00	0.00	RX0	C
ATOM	1743	CB	LEU	338	7.440	15.574	25.088	1.00	0.00	RX0	C
ATOM	1744	CG	LEU	338	7.338	14.531	23.980	1.00	0.00	RX0	C
ATOM	1745	CD1	LEU	338	8.640	13.746	23.812	1.00	0.00	RX0	C
ATOM	1746	CD2	LEU	338	6.118	13.630	24.180	1.00	0.00	RX0	C
ATOM	1747	C	LEU	338	6.240	17.331	26.400	1.00	0.00	RX0	C
ATOM	1748	O	LEU	338	5.410	17.098	27.277	1.00	0.00	RX0	O
ATOM	1749	N	MET	339	7.027	18.402	26.394	1.00	0.00	RX0	N
ATOM	1750	H	MET	339	7.615	18.548	25.595	0.00	0.00	RX0	H
ATOM	1751	CA	MET	339	7.168	19.308	27.550	1.00	0.00	RX0	C
ATOM	1752	CB	MET	339	8.406	20.190	27.400	1.00	0.00	RX0	C
ATOM	1753	CG	MET	339	9.705	19.389	27.372	1.00	0.00	RX0	C
ATOM	1754	SD	MET	339	11.138	20.426	27.054	1.00	0.00	RX0	S
ATOM	1755	CE	MET	339	12.272	19.113	26.580	1.00	0.00	RX0	C
ATOM	1756	C	MET	339	5.924	20.183	27.753	1.00	0.00	RX0	C
ATOM	1757	O	MET	339	5.433	20.322	28.878	1.00	0.00	RX0	O
ATOM	1758	N	ALA	340	5.370	20.672	26.646	1.00	0.00	RX0	N
ATOM	1759	H	ALA	340	5.814	20.473	25.772	0.00	0.00	RX0	H
ATOM	1760	CA	ALA	340	4.120	21.456	26.636	1.00	0.00	RX0	C
ATOM	1761	CB	ALA	340	3.835	21.992	25.232	1.00	0.00	RX0	C
ATOM	1762	C	ALA	340	2.921	20.619	27.100	1.00	0.00	RX0	C
ATOM	1763	O	ALA	340	2.176	21.064	27.967	1.00	0.00	RX0	O
ATOM	1764	N	LYS	341	2.865	19.360	26.655	1.00	0.00	RX0	N
ATOM	1765	H	LYS	341	3.553	19.058	25.994	0.00	0.00	RX0	H
ATOM	1766	CA	LYS	341	1.846	18.397	27.117	1.00	0.00	RX0	C
ATOM	1767	CB	LYS	341	1.956	17.112	26.275	1.00	0.00	RX0	C

ATOM	1768	CG	LYS	341	0.664	16.290	26.159	1.00	0.00	RX0	C
ATOM	1769	CD	LYS	341	0.390	15.751	24.740	1.00	0.00	RX0	C
ATOM	1770	CE	LYS	341	1.381	14.712	24.190	1.00	0.00	RX0	C
ATOM	1771	NZ	LYS	341	1.108	14.469	22.763	1.00	0.00	RX0	N
ATOM	1772	HZ1	LYS	341	1.841	13.904	22.279	0.00	0.00	RX0	H
ATOM	1773	HZ2	LYS	341	0.242	13.937	22.532	0.00	0.00	RX0	H
ATOM	1774	HZ3	LYS	341	1.077	15.342	22.203	0.00	0.00	RX0	H
ATOM	1775	C	LYS	341	1.927	18.179	28.637	1.00	0.00	RX0	C
ATOM	1776	O	LYS	341	0.908	18.032	29.304	1.00	0.00	RX0	O
ATOM	1777	N	ALA	342	3.166	18.103	29.120	1.00	0.00	RX0	N
ATOM	1778	H	ALA	342	3.940	18.171	28.489	0.00	0.00	RX0	H
ATOM	1779	CA	ALA	342	3.459	17.942	30.556	1.00	0.00	RX0	C
ATOM	1780	CB	ALA	342	4.954	17.706	30.786	1.00	0.00	RX0	C
ATOM	1781	C	ALA	342	3.007	19.166	31.371	1.00	0.00	RX0	C
ATOM	1782	O	ALA	342	2.879	19.099	32.588	1.00	0.00	RX0	O
ATOM	1783	N	GLY	343	2.900	20.305	30.669	1.00	0.00	RX0	N
ATOM	1784	H	GLY	343	3.153	20.317	29.702	0.00	0.00	RX0	H
ATOM	1785	CA	GLY	343	2.393	21.560	31.244	1.00	0.00	RX0	C
ATOM	1786	C	GLY	343	3.505	22.433	31.829	1.00	0.00	RX0	C
ATOM	1787	O	GLY	343	3.244	23.282	32.678	1.00	0.00	RX0	O
ATOM	1788	N	LEU	344	4.736	22.226	31.356	1.00	0.00	RX0	N
ATOM	1789	H	LEU	344	4.853	21.555	30.623	0.00	0.00	RX0	H
ATOM	1790	CA	LEU	344	5.823	23.174	31.634	1.00	0.00	RX0	C
ATOM	1791	CB	LEU	344	7.151	22.611	31.138	1.00	0.00	RX0	C
ATOM	1792	CG	LEU	344	7.555	21.338	31.877	1.00	0.00	RX0	C
ATOM	1793	CD1	LEU	344	8.838	20.743	31.302	1.00	0.00	RX0	C
ATOM	1794	CD2	LEU	344	7.657	21.566	33.386	1.00	0.00	RX0	C
ATOM	1795	C	LEU	344	5.508	24.488	30.929	1.00	0.00	RX0	C
ATOM	1796	O	LEU	344	4.977	24.500	29.792	1.00	0.00	RX0	O
ATOM	1797	N	THR	345	5.822	25.577	31.575	1.00	0.00	RX0	N
ATOM	1798	H	THR	345	6.293	25.481	32.451	0.00	0.00	RX0	H
ATOM	1799	CA	THR	345	5.705	26.912	30.948	1.00	0.00	RX0	C
ATOM	1800	CB	THR	345	5.997	27.965	32.011	1.00	0.00	RX0	C
ATOM	1801	OG1	THR	345	7.170	27.605	32.740	1.00	0.00	RX0	O
ATOM	1802	HG1	THR	345	6.901	26.963	33.394	0.00	0.00	RX0	H
ATOM	1803	CG2	THR	345	4.819	28.124	32.973	1.00	0.00	RX0	C
ATOM	1804	C	THR	345	6.639	26.971	29.731	1.00	0.00	RX0	C
ATOM	1805	O	THR	345	7.615	26.233	29.623	1.00	0.00	RX0	O
ATOM	1806	N	LEU	346	6.390	27.971	28.898	1.00	0.00	RX0	N
ATOM	1807	H	LEU	346	5.563	28.512	29.043	0.00	0.00	RX0	H
ATOM	1808	CA	LEU	346	7.214	28.242	27.708	1.00	0.00	RX0	C
ATOM	1809	CB	LEU	346	6.672	29.451	26.948	1.00	0.00	RX0	C
ATOM	1810	CG	LEU	346	7.379	29.658	25.607	1.00	0.00	RX0	C
ATOM	1811	CD1	LEU	346	7.248	28.432	24.700	1.00	0.00	RX0	C
ATOM	1812	CD2	LEU	346	6.916	30.939	24.913	1.00	0.00	RX0	C
ATOM	1813	C	LEU	346	8.705	28.433	28.051	1.00	0.00	RX0	C
ATOM	1814	O	LEU	346	9.594	27.857	27.432	1.00	0.00	RX0	O
ATOM	1815	N	GLN	347	8.927	29.083	29.198	1.00	0.00	RX0	N
ATOM	1816	H	GLN	347	8.145	29.438	29.706	0.00	0.00	RX0	H
ATOM	1817	CA	GLN	347	10.275	29.275	29.754	1.00	0.00	RX0	C
ATOM	1818	CB	GLN	347	10.215	30.281	30.899	1.00	0.00	RX0	C
ATOM	1819	CG	GLN	347	11.593	30.612	31.471	1.00	0.00	RX0	C
ATOM	1820	CD	GLN	347	11.418	31.568	32.627	1.00	0.00	RX0	C
ATOM	1821	OE1	GLN	347	10.437	31.515	33.357	1.00	0.00	RX0	O
ATOM	1822	NE2	GLN	347	12.420	32.458	32.744	1.00	0.00	RX0	N
ATOM	1823	HE21	GLN	347	13.188	32.458	32.105	0.00	0.00	RX0	H
ATOM	1824	HE22	GLN	347	12.408	33.145	33.472	0.00	0.00	RX0	H
ATOM	1825	C	GLN	347	10.911	27.956	30.229	1.00	0.00	RX0	C
ATOM	1826	O	GLN	347	12.052	27.652	29.875	1.00	0.00	RX0	O
ATOM	1827	N	GLN	348	10.120	27.140	30.905	1.00	0.00	RX0	N
ATOM	1828	H	GLN	348	9.171	27.398	31.089	0.00	0.00	RX0	H

ATOM	1829	CA	GLN	348	10.582	25.823	31.394	1.00	0.00	RX0	C
ATOM	1830	CB	GLN	348	9.592	25.220	32.378	1.00	0.00	RX0	C
ATOM	1831	CG	GLN	348	9.645	25.896	33.744	1.00	0.00	RX0	C
ATOM	1832	CD	GLN	348	8.477	25.389	34.557	1.00	0.00	RX0	C
ATOM	1833	OE1	GLN	348	7.356	25.284	34.064	1.00	0.00	RX0	O
ATOM	1834	NE2	GLN	348	8.794	25.078	35.825	1.00	0.00	RX0	N
ATOM	1835	HE21	GLN	348	9.731	25.203	36.154	0.00	0.00	RX0	H
ATOM	1836	HE22	GLN	348	8.114	24.723	36.468	0.00	0.00	RX0	H
ATOM	1837	C	GLN	348	10.871	24.847	30.248	1.00	0.00	RX0	C
ATOM	1838	O	GLN	348	11.861	24.115	30.296	1.00	0.00	RX0	O
ATOM	1839	N	GLN	349	10.121	24.983	29.160	1.00	0.00	RX0	N
ATOM	1840	H	GLN	349	9.375	25.648	29.187	0.00	0.00	RX0	H
ATOM	1841	CA	GLN	349	10.299	24.172	27.940	1.00	0.00	RX0	C
ATOM	1842	CB	GLN	349	9.205	24.481	26.921	1.00	0.00	RX0	C
ATOM	1843	CG	GLN	349	7.821	24.032	27.384	1.00	0.00	RX0	C
ATOM	1844	CD	GLN	349	6.787	24.590	26.434	1.00	0.00	RX0	C
ATOM	1845	OE1	GLN	349	7.076	24.919	25.290	1.00	0.00	RX0	O
ATOM	1846	NE2	GLN	349	5.564	24.698	26.977	1.00	0.00	RX0	N
ATOM	1847	HE21	GLN	349	5.398	24.432	27.933	0.00	0.00	RX0	H
ATOM	1848	HE22	GLN	349	4.780	25.036	26.459	0.00	0.00	RX0	H
ATOM	1849	C	GLN	349	11.682	24.372	27.306	1.00	0.00	RX0	C
ATOM	1850	O	GLN	349	12.447	23.422	27.181	1.00	0.00	RX0	O
ATOM	1851	N	HIS	350	12.056	25.641	27.113	1.00	0.00	RX0	N
ATOM	1852	H	HIS	350	11.422	26.375	27.364	0.00	0.00	RX0	H
ATOM	1853	CA	HIS	350	13.344	25.953	26.463	1.00	0.00	RX0	C
ATOM	1854	CB	HIS	350	13.380	27.329	25.781	1.00	0.00	RX0	C
ATOM	1855	CG	HIS	350	13.578	28.468	26.748	1.00	0.00	RX0	C
ATOM	1856	ND1	HIS	350	12.564	29.182	27.263	1.00	0.00	RX0	N
ATOM	1857	HD1	HIS	350	11.606	29.023	27.107	0.00	0.00	RX0	H
ATOM	1858	CD2	HIS	350	14.788	28.991	27.219	1.00	0.00	RX0	C
ATOM	1859	NE2	HIS	350	14.491	30.036	28.027	1.00	0.00	RX0	N
ATOM	1860	CE1	HIS	350	13.127	30.153	28.050	1.00	0.00	RX0	C
ATOM	1861	C	HIS	350	14.540	25.670	27.386	1.00	0.00	RX0	C
ATOM	1862	O	HIS	350	15.573	25.182	26.934	1.00	0.00	RX0	O
ATOM	1863	N	GLN	351	14.320	25.854	28.691	1.00	0.00	RX0	N
ATOM	1864	H	GLN	351	13.441	26.233	28.990	0.00	0.00	RX0	H
ATOM	1865	CA	GLN	351	15.341	25.538	29.706	1.00	0.00	RX0	C
ATOM	1866	CB	GLN	351	14.917	26.062	31.072	1.00	0.00	RX0	C
ATOM	1867	CG	GLN	351	14.894	27.585	31.147	1.00	0.00	RX0	C
ATOM	1868	CD	GLN	351	14.211	27.984	32.435	1.00	0.00	RX0	C
ATOM	1869	OE1	GLN	351	13.352	27.281	32.957	1.00	0.00	RX0	O
ATOM	1870	NE2	GLN	351	14.654	29.152	32.932	1.00	0.00	RX0	N
ATOM	1871	HE21	GLN	351	15.360	29.667	32.445	0.00	0.00	RX0	H
ATOM	1872	HE22	GLN	351	14.298	29.517	33.793	0.00	0.00	RX0	H
ATOM	1873	C	GLN	351	15.597	24.030	29.805	1.00	0.00	RX0	C
ATOM	1874	O	GLN	351	16.752	23.608	29.740	1.00	0.00	RX0	O
ATOM	1875	N	ARG	352	14.527	23.242	29.744	1.00	0.00	RX0	N
ATOM	1876	H	ARG	352	13.617	23.653	29.656	0.00	0.00	RX0	H
ATOM	1877	CA	ARG	352	14.627	21.772	29.816	1.00	0.00	RX0	C
ATOM	1878	CB	ARG	352	13.311	21.110	30.250	1.00	0.00	RX0	C
ATOM	1879	CG	ARG	352	13.424	19.588	30.444	1.00	0.00	RX0	C
ATOM	1880	CD	ARG	352	12.235	18.993	31.208	1.00	0.00	RX0	C
ATOM	1881	NE	ARG	352	12.215	17.528	31.184	1.00	0.00	RX0	N
ATOM	1882	HE	ARG	352	12.011	17.063	30.308	0.00	0.00	RX0	H
ATOM	1883	CZ	ARG	352	12.338	16.772	32.319	1.00	0.00	RX0	C
ATOM	1884	NH1	ARG	352	12.625	17.376	33.490	1.00	0.00	RX0	N
ATOM	1885	HH11	ARG	352	12.802	16.830	34.322	0.00	0.00	RX0	H
ATOM	1886	HH12	ARG	352	12.685	18.371	33.570	0.00	0.00	RX0	H
ATOM	1887	NH2	ARG	352	12.169	15.436	32.256	1.00	0.00	RX0	N
ATOM	1888	HH21	ARG	352	12.204	14.800	33.035	0.00	0.00	RX0	H
ATOM	1889	HH22	ARG	352	11.981	15.017	31.344	0.00	0.00	RX0	H

ATOM	1890	C	ARG	352	15.192	21.188	28.514	1.00	0.00	RX0	C
ATOM	1891	O	ARG	352	16.048	20.305	28.551	1.00	0.00	RX0	O
ATOM	1892	N	LEU	353	14.843	21.816	27.391	1.00	0.00	RX0	N
ATOM	1893	H	LEU	353	14.131	22.519	27.429	0.00	0.00	RX0	H
ATOM	1894	CA	LEU	353	15.400	21.446	26.079	1.00	0.00	RX0	C
ATOM	1895	CB	LEU	353	14.765	22.302	24.985	1.00	0.00	RX0	C
ATOM	1896	CG	LEU	353	15.233	21.922	23.581	1.00	0.00	RX0	C
ATOM	1897	CD1	LEU	353	14.794	20.508	23.199	1.00	0.00	RX0	C
ATOM	1898	CD2	LEU	353	14.808	22.959	22.542	1.00	0.00	RX0	C
ATOM	1899	C	LEU	353	16.930	21.610	26.063	1.00	0.00	RX0	C
ATOM	1900	O	LEU	353	17.658	20.692	25.694	1.00	0.00	RX0	O
ATOM	1901	N	ALA	354	17.375	22.739	26.614	1.00	0.00	RX0	N
ATOM	1902	H	ALA	354	16.705	23.421	26.916	0.00	0.00	RX0	H
ATOM	1903	CA	ALA	354	18.808	23.073	26.716	1.00	0.00	RX0	C
ATOM	1904	CB	ALA	354	18.993	24.518	27.181	1.00	0.00	RX0	C
ATOM	1905	C	ALA	354	19.540	22.141	27.690	1.00	0.00	RX0	C
ATOM	1906	O	ALA	354	20.574	21.567	27.334	1.00	0.00	RX0	O
ATOM	1907	N	GLN	355	18.893	21.844	28.811	1.00	0.00	RX0	N
ATOM	1908	H	GLN	355	18.018	22.305	28.967	0.00	0.00	RX0	H
ATOM	1909	CA	GLN	355	19.427	20.920	29.833	1.00	0.00	RX0	C
ATOM	1910	CB	GLN	355	18.563	20.902	31.084	1.00	0.00	RX0	C
ATOM	1911	CG	GLN	355	18.677	22.209	31.861	1.00	0.00	RX0	C
ATOM	1912	CD	GLN	355	17.950	22.051	33.172	1.00	0.00	RX0	C
ATOM	1913	OE1	GLN	355	18.392	21.320	34.058	1.00	0.00	RX0	O
ATOM	1914	NE2	GLN	355	16.822	22.775	33.245	1.00	0.00	RX0	N
ATOM	1915	HE21	GLN	355	16.552	23.328	32.454	0.00	0.00	RX0	H
ATOM	1916	HE22	GLN	355	16.232	22.800	34.052	0.00	0.00	RX0	H
ATOM	1917	C	GLN	355	19.631	19.504	29.273	1.00	0.00	RX0	C
ATOM	1918	O	GLN	355	20.705	18.919	29.430	1.00	0.00	RX0	O
ATOM	1919	N	LEU	356	18.681	19.086	28.443	1.00	0.00	RX0	N
ATOM	1920	H	LEU	356	17.883	19.669	28.276	0.00	0.00	RX0	H
ATOM	1921	CA	LEU	356	18.716	17.770	27.778	1.00	0.00	RX0	C
ATOM	1922	CB	LEU	356	17.349	17.402	27.204	1.00	0.00	RX0	C
ATOM	1923	CG	LEU	356	16.354	17.031	28.303	1.00	0.00	RX0	C
ATOM	1924	CD1	LEU	356	14.993	16.629	27.734	1.00	0.00	RX0	C
ATOM	1925	CD2	LEU	356	16.924	15.954	29.225	1.00	0.00	RX0	C
ATOM	1926	C	LEU	356	19.795	17.661	26.699	1.00	0.00	RX0	C
ATOM	1927	O	LEU	356	20.593	16.720	26.700	1.00	0.00	RX0	O
ATOM	1928	N	LEU	357	19.916	18.722	25.913	1.00	0.00	RX0	N
ATOM	1929	H	LEU	357	19.280	19.489	26.028	0.00	0.00	RX0	H
ATOM	1930	CA	LEU	357	20.885	18.765	24.803	1.00	0.00	RX0	C
ATOM	1931	CB	LEU	357	20.531	19.867	23.805	1.00	0.00	RX0	C
ATOM	1932	CG	LEU	357	19.159	19.684	23.153	1.00	0.00	RX0	C
ATOM	1933	CD1	LEU	357	18.827	20.841	22.211	1.00	0.00	RX0	C
ATOM	1934	CD2	LEU	357	19.015	18.326	22.468	1.00	0.00	RX0	C
ATOM	1935	C	LEU	357	22.335	18.932	25.264	1.00	0.00	RX0	C
ATOM	1936	O	LEU	357	23.247	18.345	24.683	1.00	0.00	RX0	O
ATOM	1937	N	LEU	358	22.501	19.595	26.404	1.00	0.00	RX0	N
ATOM	1938	H	LEU	358	21.707	20.033	26.830	0.00	0.00	RX0	H
ATOM	1939	CA	LEU	358	23.826	19.754	27.029	1.00	0.00	RX0	C
ATOM	1940	CB	LEU	358	23.803	20.839	28.104	1.00	0.00	RX0	C
ATOM	1941	CG	LEU	358	23.626	22.235	27.509	1.00	0.00	RX0	C
ATOM	1942	CD1	LEU	358	23.473	23.301	28.595	1.00	0.00	RX0	C
ATOM	1943	CD2	LEU	358	24.737	22.571	26.513	1.00	0.00	RX0	C
ATOM	1944	C	LEU	358	24.390	18.455	27.609	1.00	0.00	RX0	C
ATOM	1945	O	LEU	358	25.603	18.227	27.557	1.00	0.00	RX0	O
ATOM	1946	N	ILE	359	23.510	17.559	28.043	1.00	0.00	RX0	N
ATOM	1947	H	ILE	359	22.534	17.783	28.003	0.00	0.00	RX0	H
ATOM	1948	CA	ILE	359	23.928	16.212	28.495	1.00	0.00	RX0	C
ATOM	1949	CB	ILE	359	22.747	15.469	29.118	1.00	0.00	RX0	C
ATOM	1950	CG2	ILE	359	23.121	14.045	29.534	1.00	0.00	RX0	C

ATOM	1951	CG1	ILE	359	22.217	16.274	30.304	1.00	0.00	RX0	C
ATOM	1952	CD1	ILE	359	20.898	15.734	30.854	1.00	0.00	RX0	C
ATOM	1953	C	ILE	359	24.559	15.428	27.334	1.00	0.00	RX0	C
ATOM	1954	O	ILE	359	25.552	14.723	27.543	1.00	0.00	RX0	O
ATOM	1955	N	LEU	360	24.038	15.620	26.133	1.00	0.00	RX0	N
ATOM	1956	H	LEU	360	23.269	16.253	26.027	0.00	0.00	RX0	H
ATOM	1957	CA	LEU	360	24.601	14.974	24.928	1.00	0.00	RX0	C
ATOM	1958	CB	LEU	360	23.754	15.278	23.692	1.00	0.00	RX0	C
ATOM	1959	CG	LEU	360	22.267	14.962	23.889	1.00	0.00	RX0	C
ATOM	1960	CD1	LEU	360	21.453	15.324	22.650	1.00	0.00	RX0	C
ATOM	1961	CD2	LEU	360	22.018	13.514	24.315	1.00	0.00	RX0	C
ATOM	1962	C	LEU	360	26.079	15.308	24.694	1.00	0.00	RX0	C
ATOM	1963	O	LEU	360	26.843	14.455	24.246	1.00	0.00	RX0	O
ATOM	1964	N	SER	361	26.491	16.472	25.202	1.00	0.00	RX0	N
ATOM	1965	H	SER	361	25.850	17.145	25.581	0.00	0.00	RX0	H
ATOM	1966	CA	SER	361	27.906	16.894	25.202	1.00	0.00	RX0	C
ATOM	1967	CB	SER	361	27.870	18.375	25.504	1.00	0.00	RX0	C
ATOM	1968	OG	SER	361	26.631	18.829	24.940	1.00	0.00	RX0	O
ATOM	1969	HG	SER	361	26.656	18.618	24.015	0.00	0.00	RX0	H
ATOM	1970	C	SER	361	28.769	15.985	26.099	1.00	0.00	RX0	C
ATOM	1971	O	SER	361	29.797	15.456	25.681	1.00	0.00	RX0	O
ATOM	1972	N	HIS	362	28.237	15.704	27.286	1.00	0.00	RX0	N
ATOM	1973	H	HIS	362	27.307	16.026	27.460	0.00	0.00	RX0	H
ATOM	1974	CA	HIS	362	28.854	14.793	28.272	1.00	0.00	RX0	C
ATOM	1975	CB	HIS	362	28.100	14.898	29.601	1.00	0.00	RX0	C
ATOM	1976	CG	HIS	362	28.142	16.322	30.109	1.00	0.00	RX0	C
ATOM	1977	ND1	HIS	362	27.358	17.326	29.659	1.00	0.00	RX0	N
ATOM	1978	HD1	HIS	362	26.675	17.300	28.953	0.00	0.00	RX0	H
ATOM	1979	CD2	HIS	362	28.982	16.826	31.106	1.00	0.00	RX0	C
ATOM	1980	NE2	HIS	362	28.696	18.143	31.252	1.00	0.00	RX0	N
ATOM	1981	CE1	HIS	362	27.699	18.450	30.363	1.00	0.00	RX0	C
ATOM	1982	C	HIS	362	28.890	13.339	27.777	1.00	0.00	RX0	C
ATOM	1983	O	HIS	362	29.902	12.656	27.936	1.00	0.00	RX0	O
ATOM	1984	N	ILE	363	27.856	12.940	27.039	1.00	0.00	RX0	N
ATOM	1985	H	ILE	363	27.092	13.576	26.919	0.00	0.00	RX0	H
ATOM	1986	CA	ILE	363	27.773	11.584	26.446	1.00	0.00	RX0	C
ATOM	1987	CB	ILE	363	26.356	11.305	25.945	1.00	0.00	RX0	C
ATOM	1988	CG2	ILE	363	26.244	9.928	25.289	1.00	0.00	RX0	C
ATOM	1989	CG1	ILE	363	25.378	11.442	27.112	1.00	0.00	RX0	C
ATOM	1990	CD1	ILE	363	23.927	11.207	26.702	1.00	0.00	RX0	C
ATOM	1991	C	ILE	363	28.830	11.416	25.343	1.00	0.00	RX0	C
ATOM	1992	O	ILE	363	29.487	10.370	25.263	1.00	0.00	RX0	O
ATOM	1993	N	ARG	364	29.035	12.469	24.564	1.00	0.00	RX0	N
ATOM	1994	H	ARG	364	28.424	13.259	24.645	0.00	0.00	RX0	H
ATOM	1995	CA	ARG	364	30.102	12.498	23.547	1.00	0.00	RX0	C
ATOM	1996	CB	ARG	364	30.000	13.748	22.666	1.00	0.00	RX0	C
ATOM	1997	CG	ARG	364	31.195	14.023	21.738	1.00	0.00	RX0	C
ATOM	1998	CD	ARG	364	31.776	12.821	20.978	1.00	0.00	RX0	C
ATOM	1999	NE	ARG	364	30.775	12.051	20.247	1.00	0.00	RX0	N
ATOM	2000	HE	ARG	364	29.794	12.262	20.380	0.00	0.00	RX0	H
ATOM	2001	CZ	ARG	364	31.182	11.019	19.454	1.00	0.00	RX0	C
ATOM	2002	NH1	ARG	364	32.492	10.700	19.377	1.00	0.00	RX0	N
ATOM	2003	HH11	ARG	364	32.840	9.996	18.758	0.00	0.00	RX0	H
ATOM	2004	HH12	ARG	364	33.186	11.145	19.962	0.00	0.00	RX0	H
ATOM	2005	NH2	ARG	364	30.263	10.319	18.766	1.00	0.00	RX0	N
ATOM	2006	HH21	ARG	364	30.453	9.582	18.108	0.00	0.00	RX0	H
ATOM	2007	HH22	ARG	364	29.275	10.491	18.928	0.00	0.00	RX0	H
ATOM	2008	C	ARG	364	31.469	12.319	24.223	1.00	0.00	RX0	C
ATOM	2009	O	ARG	364	32.264	11.465	23.831	1.00	0.00	RX0	O
ATOM	2010	N	HIS	365	31.645	13.058	25.316	1.00	0.00	RX0	N
ATOM	2011	H	HIS	365	30.915	13.696	25.573	0.00	0.00	RX0	H

ATOM	2012	CA	HIS	365	32.879	13.027	26.115	1.00	0.00	RX0	C
ATOM	2013	CB	HIS	365	32.827	14.044	27.259	1.00	0.00	RX0	C
ATOM	2014	CG	HIS	365	34.194	14.153	27.892	1.00	0.00	RX0	C
ATOM	2015	ND1	HIS	365	35.166	14.949	27.413	1.00	0.00	RX0	N
ATOM	2016	HD1	HIS	365	35.106	15.541	26.635	0.00	0.00	RX0	H
ATOM	2017	CD2	HIS	365	34.673	13.488	29.024	1.00	0.00	RX0	C
ATOM	2018	NE2	HIS	365	35.952	13.894	29.220	1.00	0.00	RX0	N
ATOM	2019	CE1	HIS	365	36.253	14.793	28.231	1.00	0.00	RX0	C
ATOM	2020	C	HIS	365	33.175	11.617	26.649	1.00	0.00	RX0	C
ATOM	2021	O	HIS	365	34.274	11.105	26.425	1.00	0.00	RX0	O
ATOM	2022	N	MET	366	32.138	10.946	27.137	1.00	0.00	RX0	N
ATOM	2023	H	MET	366	31.256	11.417	27.208	0.00	0.00	RX0	H
ATOM	2024	CA	MET	366	32.267	9.572	27.662	1.00	0.00	RX0	C
ATOM	2025	CB	MET	366	31.014	9.140	28.426	1.00	0.00	RX0	C
ATOM	2026	CG	MET	366	30.714	10.011	29.644	1.00	0.00	RX0	C
ATOM	2027	SD	MET	366	29.363	9.365	30.641	1.00	0.00	RX0	S
ATOM	2028	CE	MET	366	28.099	9.318	29.363	1.00	0.00	RX0	C
ATOM	2029	C	MET	366	32.567	8.557	26.555	1.00	0.00	RX0	C
ATOM	2030	O	MET	366	33.398	7.674	26.741	1.00	0.00	RX0	O
ATOM	2031	N	SER	367	31.985	8.782	25.378	1.00	0.00	RX0	N
ATOM	2032	H	SER	367	31.327	9.532	25.282	0.00	0.00	RX0	H
ATOM	2033	CA	SER	367	32.231	7.934	24.196	1.00	0.00	RX0	C
ATOM	2034	CB	SER	367	31.218	8.364	23.137	1.00	0.00	RX0	C
ATOM	2035	OG	SER	367	31.147	7.461	22.027	1.00	0.00	RX0	O
ATOM	2036	HG	SER	367	30.433	7.796	21.496	0.00	0.00	RX0	H
ATOM	2037	C	SER	367	33.681	8.046	23.711	1.00	0.00	RX0	C
ATOM	2038	O	SER	367	34.361	7.038	23.567	1.00	0.00	RX0	O
ATOM	2039	N	ASN	368	34.193	9.278	23.688	1.00	0.00	RX0	N
ATOM	2040	H	ASN	368	33.584	10.039	23.911	0.00	0.00	RX0	H
ATOM	2041	CA	ASN	368	35.583	9.549	23.266	1.00	0.00	RX0	C
ATOM	2042	CB	ASN	368	35.840	11.047	23.054	1.00	0.00	RX0	C
ATOM	2043	CG	ASN	368	35.521	11.428	21.619	1.00	0.00	RX0	C
ATOM	2044	OD1	ASN	368	34.556	10.974	21.016	1.00	0.00	RX0	O
ATOM	2045	ND2	ASN	368	36.386	12.278	21.062	1.00	0.00	RX0	N
ATOM	2046	HD21	ASN	368	37.187	12.678	21.514	0.00	0.00	RX0	H
ATOM	2047	HD22	ASN	368	36.278	12.564	20.112	0.00	0.00	RX0	H
ATOM	2048	C	ASN	368	36.615	8.948	24.226	1.00	0.00	RX0	C
ATOM	2049	O	ASN	368	37.514	8.215	23.806	1.00	0.00	RX0	O
ATOM	2050	N	LYS	369	36.353	9.119	25.515	1.00	0.00	RX0	N
ATOM	2051	H	LYS	369	35.543	9.653	25.762	0.00	0.00	RX0	H
ATOM	2052	CA	LYS	369	37.205	8.554	26.580	1.00	0.00	RX0	C
ATOM	2053	CB	LYS	369	36.975	9.198	27.950	1.00	0.00	RX0	C
ATOM	2054	CG	LYS	369	37.156	10.722	27.947	1.00	0.00	RX0	C
ATOM	2055	CD	LYS	369	38.373	11.229	27.160	1.00	0.00	RX0	C
ATOM	2056	CE	LYS	369	39.743	10.739	27.640	1.00	0.00	RX0	C
ATOM	2057	NZ	LYS	369	40.741	11.132	26.640	1.00	0.00	RX0	N
ATOM	2058	HZ1	LYS	369	41.558	10.482	26.615	0.00	0.00	RX0	H
ATOM	2059	HZ2	LYS	369	40.357	10.991	25.680	0.00	0.00	RX0	H
ATOM	2060	HZ3	LYS	369	41.065	12.111	26.723	0.00	0.00	RX0	H
ATOM	2061	C	LYS	369	37.117	7.021	26.620	1.00	0.00	RX0	C
ATOM	2062	O	LYS	369	38.123	6.337	26.771	1.00	0.00	RX0	O
ATOM	2063	N	GLY	370	35.908	6.527	26.299	1.00	0.00	RX0	N
ATOM	2064	H	GLY	370	35.153	7.174	26.172	0.00	0.00	RX0	H
ATOM	2065	CA	GLY	370	35.597	5.090	26.225	1.00	0.00	RX0	C
ATOM	2066	C	GLY	370	36.324	4.429	25.048	1.00	0.00	RX0	C
ATOM	2067	O	GLY	370	36.946	3.386	25.214	1.00	0.00	RX0	O
ATOM	2068	N	MET	371	36.380	5.143	23.928	1.00	0.00	RX0	N
ATOM	2069	H	MET	371	35.881	6.009	23.924	0.00	0.00	RX0	H
ATOM	2070	CA	MET	371	37.103	4.709	22.719	1.00	0.00	RX0	C
ATOM	2071	CB	MET	371	36.896	5.653	21.527	1.00	0.00	RX0	C
ATOM	2072	CG	MET	371	35.477	5.798	20.983	1.00	0.00	RX0	C

ATOM	2073	SD	MET	371	35.448	6.625	19.383	1.00	0.00	RX0	S
ATOM	2074	CE	MET	371	36.325	8.133	19.823	1.00	0.00	RX0	C
ATOM	2075	C	MET	371	38.619	4.671	22.937	1.00	0.00	RX0	C
ATOM	2076	O	MET	371	39.258	3.669	22.631	1.00	0.00	RX0	O
ATOM	2077	N	GLU	372	39.117	5.692	23.643	1.00	0.00	RX0	N
ATOM	2078	H	GLU	372	38.513	6.466	23.846	0.00	0.00	RX0	H
ATOM	2079	CA	GLU	372	40.525	5.740	24.082	1.00	0.00	RX0	C
ATOM	2080	CB	GLU	372	40.840	7.085	24.741	1.00	0.00	RX0	C
ATOM	2081	CG	GLU	372	40.855	8.187	23.677	1.00	0.00	RX0	C
ATOM	2082	CD	GLU	372	41.106	9.558	24.278	1.00	0.00	RX0	C
ATOM	2083	OE1	GLU	372	40.341	10.484	24.016	1.00	0.00	RX0	O
ATOM	2084	OE2	GLU	372	42.058	9.731	25.028	1.00	0.00	RX0	O
ATOM	2085	C	GLU	372	40.884	4.541	24.968	1.00	0.00	RX0	C
ATOM	2086	O	GLU	372	41.865	3.853	24.723	1.00	0.00	RX0	O
ATOM	2087	N	HIS	373	40.006	4.291	25.945	1.00	0.00	RX0	N
ATOM	2088	H	HIS	373	39.215	4.895	26.053	0.00	0.00	RX0	H
ATOM	2089	CA	HIS	373	40.194	3.212	26.919	1.00	0.00	RX0	C
ATOM	2090	CB	HIS	373	39.269	3.445	28.123	1.00	0.00	RX0	C
ATOM	2091	CG	HIS	373	38.394	2.258	28.451	1.00	0.00	RX0	C
ATOM	2092	ND1	HIS	373	37.265	1.959	27.783	1.00	0.00	RX0	N
ATOM	2093	HD1	HIS	373	36.906	2.432	26.999	0.00	0.00	RX0	H
ATOM	2094	CD2	HIS	373	38.566	1.335	29.488	1.00	0.00	RX0	C
ATOM	2095	NE2	HIS	373	37.518	0.475	29.438	1.00	0.00	RX0	N
ATOM	2096	CE1	HIS	373	36.720	0.860	28.394	1.00	0.00	RX0	C
ATOM	2097	C	HIS	373	40.078	1.832	26.262	1.00	0.00	RX0	C
ATOM	2098	O	HIS	373	40.934	1.001	26.470	1.00	0.00	RX0	O
ATOM	2099	N	LEU	374	39.111	1.679	25.351	1.00	0.00	RX0	N
ATOM	2100	H	LEU	374	38.527	2.465	25.171	0.00	0.00	RX0	H
ATOM	2101	CA	LEU	374	38.919	0.408	24.629	1.00	0.00	RX0	C
ATOM	2102	CB	LEU	374	37.608	0.408	23.847	1.00	0.00	RX0	C
ATOM	2103	CG	LEU	374	36.341	0.308	24.695	1.00	0.00	RX0	C
ATOM	2104	CD1	LEU	374	35.097	0.673	23.883	1.00	0.00	RX0	C
ATOM	2105	CD2	LEU	374	36.208	-1.060	25.366	1.00	0.00	RX0	C
ATOM	2106	C	LEU	374	40.113	0.051	23.744	1.00	0.00	RX0	C
ATOM	2107	O	LEU	374	40.555	-1.109	23.754	1.00	0.00	RX0	O
ATOM	2108	N	TYR	375	40.664	1.068	23.103	1.00	0.00	RX0	N
ATOM	2109	H	TYR	375	40.264	1.973	23.273	0.00	0.00	RX0	H
ATOM	2110	CA	TYR	375	41.854	0.983	22.222	1.00	0.00	RX0	C
ATOM	2111	CB	TYR	375	42.033	2.324	21.501	1.00	0.00	RX0	C
ATOM	2112	CG	TYR	375	41.656	2.370	20.036	1.00	0.00	RX0	C
ATOM	2113	CD1	TYR	375	40.950	3.509	19.604	1.00	0.00	RX0	C
ATOM	2114	CE1	TYR	375	40.639	3.645	18.244	1.00	0.00	RX0	C
ATOM	2115	CD2	TYR	375	42.037	1.348	19.137	1.00	0.00	RX0	C
ATOM	2116	CE2	TYR	375	41.722	1.485	17.770	1.00	0.00	RX0	C
ATOM	2117	CZ	TYR	375	41.036	2.641	17.339	1.00	0.00	RX0	C
ATOM	2118	OH	TYR	375	40.740	2.814	15.998	1.00	0.00	RX0	O
ATOM	2119	HH	TYR	375	40.308	3.655	15.905	0.00	0.00	RX0	H
ATOM	2120	C	TYR	375	43.165	0.750	22.977	1.00	0.00	RX0	C
ATOM	2121	O	TYR	375	44.176	1.452	22.753	1.00	0.00	RX0	O
ATOM	2122	N	SER	376	43.162	-0.226	23.845	1.00	0.00	RX0	N
ATOM	2123	H	SER	376	42.302	-0.619	24.151	0.00	0.00	RX0	H
ATOM	2124	CA	SER	376	44.345	-0.646	24.634	1.00	0.00	RX0	C
ATOM	2125	CB	SER	376	44.579	0.462	25.664	1.00	0.00	RX0	C
ATOM	2126	OG	SER	376	43.558	1.459	25.502	1.00	0.00	RX0	O
ATOM	2127	HG	SER	376	42.828	1.225	26.073	0.00	0.00	RX0	H
ATOM	2128	C	SER	376	44.183	-1.992	25.350	1.00	0.00	RX0	C
ATOM	2129	O	SER	376	44.985	-2.369	26.199	1.00	0.00	RX0	O
ATOM	2130	N	MET	377	43.156	-2.746	24.938	1.00	0.00	RX0	N
ATOM	2131	H	MET	377	42.543	-2.438	24.210	0.00	0.00	RX0	H
ATOM	2132	CA	MET	377	42.915	-4.112	25.422	1.00	0.00	RX0	C
ATOM	2133	CB	MET	377	41.735	-4.067	26.400	1.00	0.00	RX0	C

ATOM	2134	CG	MET	377	42.038	-3.400	27.742	1.00	0.00	RX0	C
ATOM	2135	SD	MET	377	40.553	-3.238	28.741	1.00	0.00	RX0	S
ATOM	2136	CE	MET	377	39.715	-2.029	27.705	1.00	0.00	RX0	C
ATOM	2137	C	MET	377	42.647	-5.080	24.257	1.00	0.00	RX0	C
ATOM	2138	O	MET	377	43.092	-4.854	23.130	1.00	0.00	RX0	O
ATOM	2139	N	LYS	378	41.856	-6.107	24.527	1.00	0.00	RX0	N
ATOM	2140	H	LYS	378	41.525	-6.206	25.459	0.00	0.00	RX0	H
ATOM	2141	CA	LYS	378	41.570	-7.191	23.576	1.00	0.00	RX0	C
ATOM	2142	CB	LYS	378	41.446	-8.520	24.365	1.00	0.00	RX0	C
ATOM	2143	CG	LYS	378	40.476	-8.516	25.573	1.00	0.00	RX0	C
ATOM	2144	CD	LYS	378	40.444	-9.808	26.418	1.00	0.00	RX0	C
ATOM	2145	CE	LYS	378	39.504	-9.735	27.642	1.00	0.00	RX0	C
ATOM	2146	NZ	LYS	378	39.554	-10.977	28.443	1.00	0.00	RX0	N
ATOM	2147	HZ1	LYS	378	38.958	-10.914	29.303	0.00	0.00	RX0	H
ATOM	2148	HZ2	LYS	378	39.222	-11.776	27.868	0.00	0.00	RX0	H
ATOM	2149	HZ3	LYS	378	40.534	-11.160	28.738	0.00	0.00	RX0	H
ATOM	2150	C	LYS	378	40.316	-6.920	22.720	1.00	0.00	RX0	C
ATOM	2151	O	LYS	378	40.245	-7.352	21.611	1.00	0.00	RX0	O
ATOM	2152	N	CYS	379	39.320	-6.266	23.410	1.00	0.00	RX0	N
ATOM	2153	H	CYS	379	39.571	-5.896	24.301	0.00	0.00	RX0	H
ATOM	2154	CA	CYS	379	37.939	-6.053	22.985	1.00	0.00	RX0	C
ATOM	2155	CB	CYS	379	37.587	-4.570	23.105	1.00	0.00	RX0	C
ATOM	2156	SG	CYS	379	38.259	-3.859	24.630	1.00	0.00	RX0	S
ATOM	2157	C	CYS	379	37.599	-6.654	21.614	1.00	0.00	RX0	C
ATOM	2158	O	CYS	379	37.115	-7.773	21.559	1.00	0.00	RX0	O
ATOM	2159	N	LYS	380	38.012	-5.930	20.563	1.00	0.00	RX0	N
ATOM	2160	H	LYS	380	38.441	-5.048	20.736	0.00	0.00	RX0	H
ATOM	2161	CA	LYS	380	37.949	-6.383	19.165	1.00	0.00	RX0	C
ATOM	2162	CB	LYS	380	36.537	-6.910	18.822	1.00	0.00	RX0	C
ATOM	2163	CG	LYS	380	36.316	-7.761	17.564	1.00	0.00	RX0	C
ATOM	2164	CD	LYS	380	36.040	-6.914	16.325	1.00	0.00	RX0	C
ATOM	2165	CE	LYS	380	35.290	-7.650	15.218	1.00	0.00	RX0	C
ATOM	2166	NZ	LYS	380	34.844	-6.662	14.214	1.00	0.00	RX0	N
ATOM	2167	HZ1	LYS	380	33.985	-7.011	13.737	0.00	0.00	RX0	H
ATOM	2168	HZ2	LYS	380	34.564	-5.773	14.682	0.00	0.00	RX0	H
ATOM	2169	HZ3	LYS	380	35.583	-6.482	13.496	0.00	0.00	RX0	H
ATOM	2170	C	LYS	380	38.311	-5.215	18.248	1.00	0.00	RX0	C
ATOM	2171	O	LYS	380	37.971	-4.057	18.518	1.00	0.00	RX0	O
ATOM	2172	N	ASN	381	38.881	-5.592	17.123	1.00	0.00	RX0	N
ATOM	2173	H	ASN	381	39.215	-6.535	17.124	0.00	0.00	RX0	H
ATOM	2174	CA	ASN	381	39.208	-4.700	15.996	1.00	0.00	RX0	C
ATOM	2175	CB	ASN	381	40.365	-3.778	16.369	1.00	0.00	RX0	C
ATOM	2176	CG	ASN	381	40.096	-2.379	15.867	1.00	0.00	RX0	C
ATOM	2177	OD1	ASN	381	39.893	-1.451	16.638	1.00	0.00	RX0	O
ATOM	2178	ND2	ASN	381	40.153	-2.255	14.546	1.00	0.00	RX0	N
ATOM	2179	HD21	ASN	381	40.245	-3.052	13.946	0.00	0.00	RX0	H
ATOM	2180	HD22	ASN	381	40.168	-1.328	14.174	0.00	0.00	RX0	H
ATOM	2181	C	ASN	381	39.620	-5.523	14.759	1.00	0.00	RX0	C
ATOM	2182	O	ASN	381	40.286	-5.062	13.834	1.00	0.00	RX0	O
ATOM	2183	N	VAL	382	39.176	-6.776	14.737	1.00	0.00	RX0	N
ATOM	2184	H	VAL	382	38.565	-7.084	15.459	0.00	0.00	RX0	H
ATOM	2185	CA	VAL	382	39.338	-7.659	13.572	1.00	0.00	RX0	C
ATOM	2186	CB	VAL	382	39.241	-9.128	14.005	1.00	0.00	RX0	C
ATOM	2187	CG1	VAL	382	39.427	-10.090	12.827	1.00	0.00	RX0	C
ATOM	2188	CG2	VAL	382	40.228	-9.426	15.136	1.00	0.00	RX0	C
ATOM	2189	C	VAL	382	38.195	-7.277	12.635	1.00	0.00	RX0	C
ATOM	2190	O	VAL	382	37.049	-7.276	13.059	1.00	0.00	RX0	O
ATOM	2191	N	VAL	383	38.551	-7.063	11.363	1.00	0.00	RX0	N
ATOM	2192	H	VAL	383	39.520	-7.125	11.129	0.00	0.00	RX0	H
ATOM	2193	CA	VAL	383	37.600	-6.539	10.366	1.00	0.00	RX0	C
ATOM	2194	CB	VAL	383	36.438	-7.513	10.084	1.00	0.00	RX0	C

ATOM	2195	CG1	VAL	383	35.417	-6.938	9.097	1.00	0.00	RX0	C
ATOM	2196	CG2	VAL	383	36.972	-8.866	9.609	1.00	0.00	RX0	C
ATOM	2197	C	VAL	383	37.102	-5.173	10.877	1.00	0.00	RX0	C
ATOM	2198	O	VAL	383	36.355	-5.097	11.860	1.00	0.00	RX0	O
ATOM	2199	N	PRO	384	37.530	-4.088	10.236	1.00	0.00	RX0	N
ATOM	2200	CD	PRO	384	38.340	-4.087	9.022	1.00	0.00	RX0	C
ATOM	2201	CA	PRO	384	37.142	-2.731	10.663	1.00	0.00	RX0	C
ATOM	2202	CB	PRO	384	37.762	-1.840	9.591	1.00	0.00	RX0	C
ATOM	2203	CG	PRO	384	38.061	-2.732	8.391	1.00	0.00	RX0	C
ATOM	2204	C	PRO	384	35.624	-2.628	10.828	1.00	0.00	RX0	C
ATOM	2205	O	PRO	384	34.847	-3.282	10.112	1.00	0.00	RX0	O
ATOM	2206	N	LEU	385	35.228	-1.769	11.750	1.00	0.00	RX0	N
ATOM	2207	H	LEU	385	35.917	-1.200	12.191	0.00	0.00	RX0	H
ATOM	2208	CA	LEU	385	33.812	-1.621	12.127	1.00	0.00	RX0	C
ATOM	2209	CB	LEU	385	33.506	-0.818	13.377	1.00	0.00	RX0	C
ATOM	2210	CG	LEU	385	32.142	-1.288	13.900	1.00	0.00	RX0	C
ATOM	2211	CD1	LEU	385	32.141	-2.775	14.237	1.00	0.00	RX0	C
ATOM	2212	CD2	LEU	385	31.627	-0.472	15.066	1.00	0.00	RX0	C
ATOM	2213	C	LEU	385	32.896	-1.213	10.964	1.00	0.00	RX0	C
ATOM	2214	O	LEU	385	31.809	-1.761	10.815	1.00	0.00	RX0	O
ATOM	2215	N	TYR	386	33.449	-0.406	10.055	1.00	0.00	RX0	N
ATOM	2216	H	TYR	386	34.329	0.012	10.269	0.00	0.00	RX0	H
ATOM	2217	CA	TYR	386	32.718	0.069	8.867	1.00	0.00	RX0	C
ATOM	2218	CB	TYR	386	33.681	0.935	8.031	1.00	0.00	RX0	C
ATOM	2219	CG	TYR	386	33.027	1.521	6.795	1.00	0.00	RX0	C
ATOM	2220	CD1	TYR	386	32.536	2.842	6.837	1.00	0.00	RX0	C
ATOM	2221	CE1	TYR	386	31.897	3.369	5.700	1.00	0.00	RX0	C
ATOM	2222	CD2	TYR	386	32.927	0.728	5.632	1.00	0.00	RX0	C
ATOM	2223	CE2	TYR	386	32.284	1.251	4.500	1.00	0.00	RX0	C
ATOM	2224	CZ	TYR	386	31.766	2.560	4.553	1.00	0.00	RX0	C
ATOM	2225	OH	TYR	386	31.105	3.058	3.444	1.00	0.00	RX0	O
ATOM	2226	HH	TYR	386	31.208	2.437	2.732	0.00	0.00	RX0	H
ATOM	2227	C	TYR	386	32.144	-1.103	8.049	1.00	0.00	RX0	C
ATOM	2228	O	TYR	386	30.957	-1.141	7.757	1.00	0.00	RX0	O
ATOM	2229	N	ASP	387	32.969	-2.141	7.903	1.00	0.00	RX0	N
ATOM	2230	H	ASP	387	33.945	-2.037	8.104	0.00	0.00	RX0	H
ATOM	2231	CA	ASP	387	32.619	-3.323	7.093	1.00	0.00	RX0	C
ATOM	2232	CB	ASP	387	33.847	-4.212	6.830	1.00	0.00	RX0	C
ATOM	2233	CG	ASP	387	35.101	-3.490	6.344	1.00	0.00	RX0	C
ATOM	2234	OD1	ASP	387	35.097	-2.268	6.188	1.00	0.00	RX0	O
ATOM	2235	OD2	ASP	387	36.105	-4.170	6.143	1.00	0.00	RX0	O
ATOM	2236	C	ASP	387	31.563	-4.207	7.765	1.00	0.00	RX0	C
ATOM	2237	O	ASP	387	30.619	-4.649	7.114	1.00	0.00	RX0	O
ATOM	2238	N	LEU	388	31.677	-4.332	9.088	1.00	0.00	RX0	N
ATOM	2239	H	LEU	388	32.453	-3.864	9.514	0.00	0.00	RX0	H
ATOM	2240	CA	LEU	388	30.691	-5.072	9.891	1.00	0.00	RX0	C
ATOM	2241	CB	LEU	388	31.106	-5.243	11.344	1.00	0.00	RX0	C
ATOM	2242	CG	LEU	388	29.947	-5.946	12.055	1.00	0.00	RX0	C
ATOM	2243	CD1	LEU	388	30.078	-7.470	12.018	1.00	0.00	RX0	C
ATOM	2244	CD2	LEU	388	29.663	-5.365	13.436	1.00	0.00	RX0	C
ATOM	2245	C	LEU	388	29.315	-4.386	9.871	1.00	0.00	RX0	C
ATOM	2246	O	LEU	388	28.299	-5.023	9.588	1.00	0.00	RX0	O
ATOM	2247	N	LEU	389	29.339	-3.070	10.049	1.00	0.00	RX0	N
ATOM	2248	H	LEU	389	30.230	-2.628	10.169	0.00	0.00	RX0	H
ATOM	2249	CA	LEU	389	28.129	-2.229	9.970	1.00	0.00	RX0	C
ATOM	2250	CB	LEU	389	28.398	-0.800	10.435	1.00	0.00	RX0	C
ATOM	2251	CG	LEU	389	28.775	-0.754	11.916	1.00	0.00	RX0	C
ATOM	2252	CD1	LEU	389	28.919	0.678	12.417	1.00	0.00	RX0	C
ATOM	2253	CD2	LEU	389	27.795	-1.539	12.785	1.00	0.00	RX0	C
ATOM	2254	C	LEU	389	27.489	-2.268	8.583	1.00	0.00	RX0	C
ATOM	2255	O	LEU	389	26.284	-2.473	8.458	1.00	0.00	RX0	O

ATOM	2256	N	LEU	390	28.350	-2.246	7.565	1.00	0.00	RX0	N
ATOM	2257	H	LEU	390	29.327	-2.144	7.757	0.00	0.00	RX0	H
ATOM	2258	CA	LEU	390	27.929	-2.335	6.162	1.00	0.00	RX0	C
ATOM	2259	CB	LEU	390	29.172	-2.218	5.281	1.00	0.00	RX0	C
ATOM	2260	CG	LEU	390	28.906	-2.086	3.785	1.00	0.00	RX0	C
ATOM	2261	CD1	LEU	390	28.372	-0.699	3.437	1.00	0.00	RX0	C
ATOM	2262	CD2	LEU	390	30.151	-2.426	2.964	1.00	0.00	RX0	C
ATOM	2263	C	LEU	390	27.202	-3.653	5.862	1.00	0.00	RX0	C
ATOM	2264	O	LEU	390	26.123	-3.627	5.302	1.00	0.00	RX0	O
ATOM	2265	N	GLU	391	27.733	-4.754	6.404	1.00	0.00	RX0	N
ATOM	2266	H	GLU	391	28.628	-4.704	6.854	0.00	0.00	RX0	H
ATOM	2267	CA	GLU	391	27.124	-6.090	6.264	1.00	0.00	RX0	C
ATOM	2268	CB	GLU	391	27.965	-7.200	6.902	1.00	0.00	RX0	C
ATOM	2269	CG	GLU	391	29.159	-7.671	6.072	1.00	0.00	RX0	C
ATOM	2270	CD	GLU	391	29.688	-8.960	6.677	1.00	0.00	RX0	C
ATOM	2271	OE1	GLU	391	29.496	-9.170	7.875	1.00	0.00	RX0	O
ATOM	2272	OE2	GLU	391	30.267	-9.763	5.944	1.00	0.00	RX0	O
ATOM	2273	C	GLU	391	25.713	-6.159	6.867	1.00	0.00	RX0	C
ATOM	2274	O	GLU	391	24.778	-6.600	6.214	1.00	0.00	RX0	O
ATOM	2275	N	MET	392	25.589	-5.588	8.070	1.00	0.00	RX0	N
ATOM	2276	H	MET	392	26.424	-5.209	8.476	0.00	0.00	RX0	H
ATOM	2277	CA	MET	392	24.313	-5.555	8.807	1.00	0.00	RX0	C
ATOM	2278	CB	MET	392	24.520	-5.280	10.295	1.00	0.00	RX0	C
ATOM	2279	CG	MET	392	25.158	-6.495	10.973	1.00	0.00	RX0	C
ATOM	2280	SD	MET	392	25.086	-6.428	12.769	1.00	0.00	RX0	S
ATOM	2281	CE	MET	392	26.079	-4.951	12.997	1.00	0.00	RX0	C
ATOM	2282	C	MET	392	23.266	-4.630	8.173	1.00	0.00	RX0	C
ATOM	2283	O	MET	392	22.107	-5.006	8.028	1.00	0.00	RX0	O
ATOM	2284	N	LEU	393	23.721	-3.452	7.754	1.00	0.00	RX0	N
ATOM	2285	H	LEU	393	24.708	-3.284	7.787	0.00	0.00	RX0	H
ATOM	2286	CA	LEU	393	22.852	-2.457	7.099	1.00	0.00	RX0	C
ATOM	2287	CB	LEU	393	23.508	-1.080	7.151	1.00	0.00	RX0	C
ATOM	2288	CG	LEU	393	23.439	-0.454	8.542	1.00	0.00	RX0	C
ATOM	2289	CD1	LEU	393	24.496	0.630	8.730	1.00	0.00	RX0	C
ATOM	2290	CD2	LEU	393	22.032	0.061	8.853	1.00	0.00	RX0	C
ATOM	2291	C	LEU	393	22.462	-2.815	5.663	1.00	0.00	RX0	C
ATOM	2292	O	LEU	393	21.313	-2.601	5.257	1.00	0.00	RX0	O
ATOM	2293	N	ASP	394	23.400	-3.384	4.923	1.00	0.00	RX0	N
ATOM	2294	H	ASP	394	24.270	-3.667	5.323	0.00	0.00	RX0	H
ATOM	2295	CA	ASP	394	23.187	-3.768	3.516	1.00	0.00	RX0	C
ATOM	2296	CB	ASP	394	24.427	-3.720	2.626	1.00	0.00	RX0	C
ATOM	2297	CG	ASP	394	23.952	-3.396	1.219	1.00	0.00	RX0	C
ATOM	2298	OD1	ASP	394	22.795	-2.999	1.068	1.00	0.00	RX0	O
ATOM	2299	OD2	ASP	394	24.744	-3.503	0.282	1.00	0.00	RX0	O
ATOM	2300	C	ASP	394	22.492	-5.132	3.432	1.00	0.00	RX0	C
ATOM	2301	O	ASP	394	23.034	-6.108	2.888	1.00	0.00	RX0	O
ATOM	2302	N	ALA	395	21.256	-5.134	3.874	1.00	0.00	RX0	N
ATOM	2303	H	ALA	395	20.884	-4.231	4.090	0.00	0.00	RX0	H
ATOM	2304	CA	ALA	395	20.412	-6.338	3.880	1.00	0.00	RX0	C
ATOM	2305	CB	ALA	395	19.422	-6.296	5.045	1.00	0.00	RX0	C
ATOM	2306	C	ALA	395	19.639	-6.454	2.565	1.00	0.00	RX0	C
ATOM	2307	O	ALA	395	19.303	-5.463	1.911	1.00	0.00	RX0	O
ATOM	2308	N	HIS	396	19.441	-7.696	2.156	1.00	0.00	RX0	N
ATOM	2309	H	HIS	396	19.739	-8.406	2.789	0.00	0.00	RX0	H
ATOM	2310	CA	HIS	396	18.536	-7.999	1.035	1.00	0.00	RX0	C
ATOM	2311	CB	HIS	396	18.854	-9.404	0.512	1.00	0.00	RX0	C
ATOM	2312	CG	HIS	396	19.023	-10.345	1.684	1.00	0.00	RX0	C
ATOM	2313	ND1	HIS	396	20.222	-10.752	2.139	1.00	0.00	RX0	N
ATOM	2314	HD1	HIS	396	21.102	-10.508	1.778	0.00	0.00	RX0	H
ATOM	2315	CD2	HIS	396	18.029	-10.915	2.484	1.00	0.00	RX0	C
ATOM	2316	NE2	HIS	396	18.639	-11.669	3.426	1.00	0.00	RX0	N

```
ATOM   2317  CE1 HIS   396      19.992 -11.570   3.215  1.00  0.00      RX0  C
ATOM   2318  C   HIS   396      17.077  -7.909   1.523  1.00  0.00      RX0  C
ATOM   2319  O   HIS   396      16.821  -7.755   2.721  1.00  0.00      RX0  O
ATOM   2320  N   ARG   397      16.142  -8.174   0.629  1.00  0.00      RX0  N
ATOM   2321  H   ARG   397      16.393  -8.364  -0.320  0.00  0.00      RX0  H
ATOM   2322  CA  ARG   397      14.704  -8.151   0.982  1.00  0.00      RX0  C
ATOM   2323  CB  ARG   397      14.031  -7.015   0.202  1.00  0.00      RX0  C
ATOM   2324  CG  ARG   397      14.817  -5.733   0.508  1.00  0.00      RX0  C
ATOM   2325  CD  ARG   397      14.494  -4.472  -0.290  1.00  0.00      RX0  C
ATOM   2326  NE  ARG   397      15.506  -3.457   0.012  1.00  0.00      RX0  N
ATOM   2327  HE  ARG   397      16.430  -3.814   0.198  0.00  0.00      RX0  H
ATOM   2328  CZ  ARG   397      15.191  -2.127   0.014  1.00  0.00      RX0  C
ATOM   2329  NH1 ARG   397      13.921  -1.748  -0.265  1.00  0.00      RX0  N
ATOM   2330 HH11 ARG   397      13.630  -0.787  -0.275  0.00  0.00      RX0  H
ATOM   2331 HH12 ARG   397      13.211  -2.427  -0.475  0.00  0.00      RX0  H
ATOM   2332  NH2 ARG   397      16.154  -1.218   0.297  1.00  0.00      RX0  N
ATOM   2333 HH21 ARG   397      15.986  -0.228   0.319  0.00  0.00      RX0  H
ATOM   2334 HH22 ARG   397      17.098  -1.505   0.502  0.00  0.00      RX0  H
ATOM   2335  C   ARG   397      14.082  -9.540   0.774  1.00  0.00      RX0  C
ATOM   2336  O   ARG   397      12.875  -9.706   0.613  1.00  0.00      RX0  O
ATOM   2337  N   LEU   398      14.938 -10.556   0.835  1.00  0.00      RX0  N
ATOM   2338  H   LEU   398      15.873 -10.362   1.120  0.00  0.00      RX0  H
ATOM   2339  CA  LEU   398      14.565 -11.951   0.542  1.00  0.00      RX0  C
ATOM   2340  CB  LEU   398      15.799 -12.795   0.229  1.00  0.00      RX0  C
ATOM   2341  CG  LEU   398      16.576 -12.272  -0.980  1.00  0.00      RX0  C
ATOM   2342  CD1 LEU   398      17.865 -13.063  -1.208  1.00  0.00      RX0  C
ATOM   2343  CD2 LEU   398      15.706 -12.212  -2.237  1.00  0.00      RX0  C
ATOM   2344  C   LEU   398      13.757 -12.584   1.680  1.00  0.00      RX0  C
ATOM   2345  O   LEU   398      12.772 -13.259   1.439  1.00  0.00      RX0  O
ATOM   2346  N   HIS   399      14.100 -12.169   2.904  1.00  0.00      RX0  N
ATOM   2347  H   HIS   399      14.843 -11.513   3.010  0.00  0.00      RX0  H
ATOM   2348  CA  HIS   399      13.392 -12.616   4.118  1.00  0.00      RX0  C
ATOM   2349  CB  HIS   399      14.353 -12.613   5.307  1.00  0.00      RX0  C
ATOM   2350  CG  HIS   399      15.407 -13.668   5.069  1.00  0.00      RX0  C
ATOM   2351  ND1 HIS   399      16.709 -13.406   4.834  1.00  0.00      RX0  N
ATOM   2352  HD1 HIS   399      17.150 -12.529   4.788  0.00  0.00      RX0  H
ATOM   2353  CD2 HIS   399      15.214 -15.052   5.036  1.00  0.00      RX0  C
ATOM   2354  NE2 HIS   399      16.416 -15.622   4.778  1.00  0.00      RX0  N
ATOM   2355  CE1 HIS   399      17.335 -14.613   4.654  1.00  0.00      RX0  C
ATOM   2356  C   HIS   399      12.131 -11.786   4.401  1.00  0.00      RX0  C
ATOM   2357  O   HIS   399      11.630 -11.743   5.524  1.00  0.00      RX0  O
ATOM   2358  N   ALA   400      11.619 -11.143   3.351  1.00  0.00      RX0  N
ATOM   2359  H   ALA   400      11.998 -11.314   2.443  0.00  0.00      RX0  H
ATOM   2360  CA  ALA   400      10.356 -10.385   3.402  1.00  0.00      RX0  C
ATOM   2361  CB  ALA   400      10.144  -9.552   2.138  1.00  0.00      RX0  C
ATOM   2362  C   ALA   400       9.147 -11.323   3.579  1.00  0.00      RX0  C
ATOM   2363  O   ALA   400       8.508 -11.220   4.647  1.00  0.00      RX0  O
TER
HEADER lig.000.00.pdb
ATOM      1  N   PRO   563      51.469 -12.423   1.587  1.00  0.00      LX0  N
ATOM      2  CD  PRO   563      52.361 -13.447   2.120  1.00  0.00      LX0  C
ATOM      3  CA  PRO   563      50.272 -12.201   2.407  1.00  0.00      LX0  C
ATOM      4  CB  PRO   563      50.323 -13.403   3.358  1.00  0.00      LX0  C
ATOM      5  CG  PRO   563      51.811 -13.719   3.516  1.00  0.00      LX0  C
ATOM      6  C   PRO   563      50.291 -10.860   3.129  1.00  0.00      LX0  C
ATOM      7  O   PRO   563      49.563 -10.620   4.087  1.00  0.00      LX0  O
ATOM      8  N   GLN   564      51.174  -9.980   2.635  1.00  0.00      LX0  N
ATOM      9  H   GLN   564      51.699 -10.184   1.811  0.00  0.00      LX0  H
ATOM     10  CA  GLN   564      51.275  -8.715   3.348  1.00  0.00      LX0  C
ATOM     11  CB  GLN   564      52.631  -8.042   3.123  1.00  0.00      LX0  C
ATOM     12  CG  GLN   564      53.017  -7.125   4.291  1.00  0.00      LX0  C
```

ATOM	13	CD	GLN	564	53.181	-7.964	5.544	1.00	0.00	LX0	C
ATOM	14	OE1	GLN	564	53.903	-8.950	5.575	1.00	0.00	LX0	O
ATOM	15	NE2	GLN	564	52.453	-7.552	6.582	1.00	0.00	LX0	N
ATOM	16	HE21	GLN	564	51.865	-6.738	6.575	0.00	0.00	LX0	H
ATOM	17	HE22	GLN	564	52.522	-8.060	7.437	0.00	0.00	LX0	H
ATOM	18	C	GLN	564	50.134	-7.759	3.080	1.00	0.00	LX0	C
ATOM	19	O	GLN	564	50.081	-7.060	2.078	1.00	0.00	LX0	O
ATOM	20	N	LYS	565	49.226	-7.756	4.060	1.00	0.00	LX0	N
ATOM	21	H	LYS	565	49.288	-8.512	4.712	0.00	0.00	LX0	H
ATOM	22	CA	LYS	565	48.169	-6.744	4.095	1.00	0.00	LX0	C
ATOM	23	CB	LYS	565	47.327	-6.946	5.366	1.00	0.00	LX0	C
ATOM	24	CG	LYS	565	48.167	-7.130	6.636	1.00	0.00	LX0	C
ATOM	25	CD	LYS	565	47.365	-7.478	7.892	1.00	0.00	LX0	C
ATOM	26	CE	LYS	565	48.245	-7.664	9.137	1.00	0.00	LX0	C
ATOM	27	NZ	LYS	565	48.888	-6.396	9.487	1.00	0.00	LX0	N
ATOM	28	HZ1	LYS	565	48.902	-6.215	10.511	0.00	0.00	LX0	H
ATOM	29	HZ2	LYS	565	49.868	-6.306	9.127	0.00	0.00	LX0	H
ATOM	30	HZ3	LYS	565	48.369	-5.618	9.045	0.00	0.00	LX0	H
ATOM	31	C	LYS	565	48.682	-5.313	3.948	1.00	0.00	LX0	C
ATOM	32	O	LYS	565	49.418	-4.790	4.775	1.00	0.00	LX0	O
ATOM	33	N	ILE	566	48.277	-4.706	2.826	1.00	0.00	LX0	N
ATOM	34	H	ILE	566	47.654	-5.163	2.194	0.00	0.00	LX0	H
ATOM	35	CA	ILE	566	48.775	-3.356	2.584	1.00	0.00	LX0	C
ATOM	36	CB	ILE	566	49.035	-3.121	1.084	1.00	0.00	LX0	C
ATOM	37	CG2	ILE	566	49.643	-1.738	0.809	1.00	0.00	LX0	C
ATOM	38	CG1	ILE	566	49.909	-4.233	0.497	1.00	0.00	LX0	C
ATOM	39	CD1	ILE	566	50.044	-4.152	-1.025	1.00	0.00	LX0	C
ATOM	40	C	ILE	566	47.838	-2.299	3.132	1.00	0.00	LX0	C
ATOM	41	O	ILE	566	46.654	-2.248	2.824	1.00	0.00	LX0	O
ATOM	42	N	CYS	567	48.437	-1.416	3.937	1.00	0.00	LX0	N
ATOM	43	H	CYS	567	49.392	-1.578	4.187	0.00	0.00	LX0	H
ATOM	44	CA	CYS	567	47.734	-0.190	4.302	1.00	0.00	LX0	C
ATOM	45	CB	CYS	567	48.461	0.542	5.432	1.00	0.00	LX0	C
ATOM	46	SG	CYS	567	47.715	2.136	5.871	1.00	0.00	LX0	S
ATOM	47	C	CYS	567	47.594	0.731	3.113	1.00	0.00	LX0	C
ATOM	48	O	CYS	567	48.422	1.603	2.867	1.00	0.00	LX0	O
ATOM	49	N	LEU	568	46.484	0.515	2.392	1.00	0.00	LX0	N
ATOM	50	H	LEU	568	45.955	-0.306	2.625	0.00	0.00	LX0	H
ATOM	51	CA	LEU	568	46.209	1.300	1.185	1.00	0.00	LX0	C
ATOM	52	CB	LEU	568	44.821	0.962	0.639	1.00	0.00	LX0	C
ATOM	53	CG	LEU	568	44.639	-0.524	0.309	1.00	0.00	LX0	C
ATOM	54	CD1	LEU	568	43.193	-0.842	-0.070	1.00	0.00	LX0	C
ATOM	55	CD2	LEU	568	45.632	-1.013	-0.749	1.00	0.00	LX0	C
ATOM	56	C	LEU	568	46.357	2.805	1.355	1.00	0.00	LX0	C
ATOM	57	O	LEU	568	46.813	3.531	0.474	1.00	0.00	LX0	O
ATOM	58	N	ILE	569	45.986	3.244	2.570	1.00	0.00	LX0	N
ATOM	59	H	ILE	569	45.598	2.589	3.218	0.00	0.00	LX0	H
ATOM	60	CA	ILE	569	46.182	4.653	2.911	1.00	0.00	LX0	C
ATOM	61	CB	ILE	569	45.633	4.966	4.315	1.00	0.00	LX0	C
ATOM	62	CG2	ILE	569	45.782	6.449	4.680	1.00	0.00	LX0	C
ATOM	63	CG1	ILE	569	44.178	4.510	4.438	1.00	0.00	LX0	C
ATOM	64	CD1	ILE	569	43.249	5.218	3.452	1.00	0.00	LX0	C
ATOM	65	C	ILE	569	47.623	5.122	2.767	1.00	0.00	LX0	C
ATOM	66	O	ILE	569	47.914	6.066	2.040	1.00	0.00	LX0	O
ATOM	67	N	CYS	570	48.520	4.424	3.466	1.00	0.00	LX0	N
ATOM	68	H	CYS	570	48.275	3.593	3.969	0.00	0.00	LX0	H
ATOM	69	CA	CYS	570	49.848	5.024	3.529	1.00	0.00	LX0	C
ATOM	70	CB	CYS	570	50.054	5.647	4.908	1.00	0.00	LX0	C
ATOM	71	SG	CYS	570	49.586	4.560	6.273	1.00	0.00	LX0	S
ATOM	72	C	CYS	570	51.018	4.158	3.103	1.00	0.00	LX0	C
ATOM	73	O	CYS	570	52.166	4.419	3.445	1.00	0.00	LX0	O

ATOM	74	N	GLY	571	50.683	3.127	2.314	1.00	0.00	LX0	N
ATOM	75	H	GLY	571	49.719	2.884	2.196	0.00	0.00	LX0	H
ATOM	76	CA	GLY	571	51.715	2.381	1.592	1.00	0.00	LX0	C
ATOM	77	C	GLY	571	52.738	1.629	2.429	1.00	0.00	LX0	C
ATOM	78	O	GLY	571	53.915	1.562	2.087	1.00	0.00	LX0	O
ATOM	79	N	ASP	572	52.242	1.059	3.532	1.00	0.00	LX0	N
ATOM	80	H	ASP	572	51.264	1.096	3.737	0.00	0.00	LX0	H
ATOM	81	CA	ASP	572	53.104	0.205	4.351	1.00	0.00	LX0	C
ATOM	82	CB	ASP	572	53.894	1.011	5.389	1.00	0.00	LX0	C
ATOM	83	CG	ASP	572	55.344	1.034	4.948	1.00	0.00	LX0	C
ATOM	84	OD1	ASP	572	55.925	-0.024	4.751	1.00	0.00	LX0	O
ATOM	85	OD2	ASP	572	55.894	2.101	4.690	1.00	0.00	LX0	O
ATOM	86	C	ASP	572	52.275	-0.879	4.992	1.00	0.00	LX0	C
ATOM	87	O	ASP	572	51.123	-1.037	4.608	1.00	0.00	LX0	O
ATOM	88	N	GLU	573	52.866	-1.590	5.970	1.00	0.00	LX0	N
ATOM	89	H	GLU	573	53.834	-1.439	6.172	0.00	0.00	LX0	H
ATOM	90	CA	GLU	573	52.099	-2.608	6.701	1.00	0.00	LX0	C
ATOM	91	CB	GLU	573	52.918	-3.129	7.888	1.00	0.00	LX0	C
ATOM	92	CG	GLU	573	52.874	-4.643	8.155	1.00	0.00	LX0	C
ATOM	93	CD	GLU	573	51.501	-5.153	8.571	1.00	0.00	LX0	C
ATOM	94	OE1	GLU	573	50.675	-5.449	7.717	1.00	0.00	LX0	O
ATOM	95	OE2	GLU	573	51.250	-5.332	9.755	1.00	0.00	LX0	O
ATOM	96	C	GLU	573	50.754	-2.108	7.203	1.00	0.00	LX0	C
ATOM	97	O	GLU	573	50.647	-1.062	7.836	1.00	0.00	LX0	O
ATOM	98	N	ALA	574	49.720	-2.885	6.883	1.00	0.00	LX0	N
ATOM	99	H	ALA	574	49.901	-3.775	6.453	0.00	0.00	LX0	H
ATOM	100	CA	ALA	574	48.433	-2.567	7.485	1.00	0.00	LX0	C
ATOM	101	CB	ALA	574	47.290	-3.081	6.618	1.00	0.00	LX0	C
ATOM	102	C	ALA	574	48.322	-3.218	8.839	1.00	0.00	LX0	C
ATOM	103	O	ALA	574	47.580	-4.180	9.015	1.00	0.00	LX0	O
ATOM	104	N	SER	575	49.121	-2.684	9.776	1.00	0.00	LX0	N
ATOM	105	H	SER	575	49.694	-1.902	9.521	0.00	0.00	LX0	H
ATOM	106	CA	SER	575	49.305	-3.312	11.087	1.00	0.00	LX0	C
ATOM	107	CB	SER	575	49.903	-2.301	12.066	1.00	0.00	LX0	C
ATOM	108	OG	SER	575	49.772	-0.977	11.528	1.00	0.00	LX0	O
ATOM	109	HG	SER	575	48.870	-0.896	11.217	0.00	0.00	LX0	H
ATOM	110	C	SER	575	48.088	-4.017	11.659	1.00	0.00	LX0	C
ATOM	111	O	SER	575	48.043	-5.242	11.755	1.00	0.00	LX0	O
ATOM	112	N	GLY	576	47.091	-3.174	11.949	1.00	0.00	LX0	N
ATOM	113	H	GLY	576	47.171	-2.198	11.740	0.00	0.00	LX0	H
ATOM	114	CA	GLY	576	45.774	-3.681	12.299	1.00	0.00	LX0	C
ATOM	115	C	GLY	576	44.774	-2.772	11.628	1.00	0.00	LX0	C
ATOM	116	O	GLY	576	45.118	-2.012	10.732	1.00	0.00	LX0	O
ATOM	117	N	CYS	577	43.530	-2.844	12.098	1.00	0.00	LX0	N
ATOM	118	H	CYS	577	43.274	-3.446	12.851	0.00	0.00	LX0	H
ATOM	119	CA	CYS	577	42.618	-1.851	11.543	1.00	0.00	LX0	C
ATOM	120	CB	CYS	577	41.242	-2.465	11.312	1.00	0.00	LX0	C
ATOM	121	SG	CYS	577	41.317	-4.156	10.668	1.00	0.00	LX0	S
ATOM	122	C	CYS	577	42.515	-0.654	12.460	1.00	0.00	LX0	C
ATOM	123	O	CYS	577	42.306	-0.800	13.659	1.00	0.00	LX0	O
ATOM	124	N	HIS	578	42.673	0.536	11.884	1.00	0.00	LX0	N
ATOM	125	H	HIS	578	42.893	0.648	10.912	0.00	0.00	LX0	H
ATOM	126	CA	HIS	578	42.477	1.703	12.735	1.00	0.00	LX0	C
ATOM	127	CB	HIS	578	43.775	2.492	12.952	1.00	0.00	LX0	C
ATOM	128	CG	HIS	578	44.907	1.586	13.383	1.00	0.00	LX0	C
ATOM	129	ND1	HIS	578	44.769	0.537	14.217	1.00	0.00	LX0	N
ATOM	130	HD1	HIS	578	43.928	0.208	14.606	0.00	0.00	LX0	H
ATOM	131	CD2	HIS	578	46.247	1.675	12.998	1.00	0.00	LX0	C
ATOM	132	NE2	HIS	578	46.913	0.666	13.611	1.00	0.00	LX0	N
ATOM	133	CE1	HIS	578	46.005	-0.034	14.361	1.00	0.00	LX0	C
ATOM	134	C	HIS	578	41.413	2.593	12.148	1.00	0.00	LX0	C

ATOM	135	O	HIS	578	41.390	2.866	10.955	1.00	0.00	LX0	O
ATOM	136	N	TYR	579	40.499	2.992	13.045	1.00	0.00	LX0	N
ATOM	137	H	TYR	579	40.641	2.667	13.983	0.00	0.00	LX0	H
ATOM	138	CA	TYR	579	39.438	3.966	12.746	1.00	0.00	LX0	C
ATOM	139	CB	TYR	579	39.966	5.406	12.736	1.00	0.00	LX0	C
ATOM	140	CG	TYR	579	40.578	5.774	14.065	1.00	0.00	LX0	C
ATOM	141	CD1	TYR	579	39.741	5.987	15.179	1.00	0.00	LX0	C
ATOM	142	CE1	TYR	579	40.324	6.326	16.412	1.00	0.00	LX0	C
ATOM	143	CD2	TYR	579	41.977	5.901	14.146	1.00	0.00	LX0	C
ATOM	144	CE2	TYR	579	42.554	6.242	15.376	1.00	0.00	LX0	C
ATOM	145	CZ	TYR	579	41.726	6.439	16.497	1.00	0.00	LX0	C
ATOM	146	OH	TYR	579	42.304	6.738	17.714	1.00	0.00	LX0	O
ATOM	147	HH	TYR	579	43.195	7.053	17.569	0.00	0.00	LX0	H
ATOM	148	C	TYR	579	38.503	3.790	11.553	1.00	0.00	LX0	C
ATOM	149	O	TYR	579	37.592	4.586	11.387	1.00	0.00	LX0	O
ATOM	150	N	GLY	580	38.731	2.752	10.744	1.00	0.00	LX0	N
ATOM	151	H	GLY	580	39.519	2.154	10.866	0.00	0.00	LX0	H
ATOM	152	CA	GLY	580	37.900	2.653	9.551	1.00	0.00	LX0	C
ATOM	153	C	GLY	580	38.378	1.584	8.599	1.00	0.00	LX0	C
ATOM	154	O	GLY	580	37.594	0.795	8.103	1.00	0.00	LX0	O
ATOM	155	N	VAL	581	39.708	1.562	8.394	1.00	0.00	LX0	N
ATOM	156	H	VAL	581	40.311	2.235	8.825	0.00	0.00	LX0	H
ATOM	157	CA	VAL	581	40.263	0.512	7.533	1.00	0.00	LX0	C
ATOM	158	CB	VAL	581	40.478	0.980	6.082	1.00	0.00	LX0	C
ATOM	159	CG1	VAL	581	39.178	0.993	5.279	1.00	0.00	LX0	C
ATOM	160	CG2	VAL	581	41.263	2.292	6.016	1.00	0.00	LX0	C
ATOM	161	C	VAL	581	41.564	-0.045	8.073	1.00	0.00	LX0	C
ATOM	162	O	VAL	581	42.099	0.416	9.077	1.00	0.00	LX0	O
ATOM	163	N	LEU	582	42.072	-1.050	7.343	1.00	0.00	LX0	N
ATOM	164	H	LEU	582	41.562	-1.376	6.548	0.00	0.00	LX0	H
ATOM	165	CA	LEU	582	43.428	-1.538	7.606	1.00	0.00	LX0	C
ATOM	166	CB	LEU	582	43.716	-2.728	6.695	1.00	0.00	LX0	C
ATOM	167	CG	LEU	582	43.172	-4.061	7.208	1.00	0.00	LX0	C
ATOM	168	CD1	LEU	582	43.179	-5.128	6.113	1.00	0.00	LX0	C
ATOM	169	CD2	LEU	582	43.929	-4.537	8.450	1.00	0.00	LX0	C
ATOM	170	C	LEU	582	44.486	-0.463	7.413	1.00	0.00	LX0	C
ATOM	171	O	LEU	582	44.632	0.117	6.342	1.00	0.00	LX0	O
ATOM	172	N	THR	583	45.200	-0.201	8.509	1.00	0.00	LX0	N
ATOM	173	H	THR	583	45.077	-0.710	9.362	0.00	0.00	LX0	H
ATOM	174	CA	THR	583	46.107	0.939	8.500	1.00	0.00	LX0	C
ATOM	175	CB	THR	583	45.386	2.178	9.043	1.00	0.00	LX0	C
ATOM	176	OG1	THR	583	44.310	1.794	9.905	1.00	0.00	LX0	O
ATOM	177	HG1	THR	583	43.580	1.554	9.339	0.00	0.00	LX0	H
ATOM	178	CG2	THR	583	44.860	3.079	7.927	1.00	0.00	LX0	C
ATOM	179	C	THR	583	47.399	0.679	9.256	1.00	0.00	LX0	C
ATOM	180	O	THR	583	47.533	-0.269	10.025	1.00	0.00	LX0	O
ATOM	181	N	CYS	584	48.373	1.564	8.990	1.00	0.00	LX0	N
ATOM	182	H	CYS	584	48.224	2.343	8.384	0.00	0.00	LX0	H
ATOM	183	CA	CYS	584	49.584	1.438	9.794	1.00	0.00	LX0	C
ATOM	184	CB	CYS	584	50.827	1.915	9.037	1.00	0.00	LX0	C
ATOM	185	SG	CYS	584	50.945	3.717	8.859	1.00	0.00	LX0	S
ATOM	186	C	CYS	584	49.440	2.166	11.112	1.00	0.00	LX0	C
ATOM	187	O	CYS	584	48.721	3.156	11.195	1.00	0.00	LX0	O
ATOM	188	N	GLY	585	50.174	1.658	12.119	1.00	0.00	LX0	N
ATOM	189	H	GLY	585	50.592	0.764	11.940	0.00	0.00	LX0	H
ATOM	190	CA	GLY	585	50.098	2.149	13.501	1.00	0.00	LX0	C
ATOM	191	C	GLY	585	49.867	3.639	13.707	1.00	0.00	LX0	C
ATOM	192	O	GLY	585	49.057	4.068	14.520	1.00	0.00	LX0	O
ATOM	193	N	SER	586	50.607	4.425	12.898	1.00	0.00	LX0	N
ATOM	194	H	SER	586	51.272	3.975	12.307	0.00	0.00	LX0	H
ATOM	195	CA	SER	586	50.473	5.883	12.983	1.00	0.00	LX0	C

ATOM	196	CB	SER	586	51.265	6.571	11.859	1.00	0.00	LX0	C
ATOM	197	OG	SER	586	50.741	6.241	10.558	1.00	0.00	LX0	O
ATOM	198	HG	SER	586	50.974	5.328	10.399	0.00	0.00	LX0	H
ATOM	199	C	SER	586	49.046	6.419	13.044	1.00	0.00	LX0	C
ATOM	200	O	SER	586	48.730	7.361	13.759	1.00	0.00	LX0	O
ATOM	201	N	CYS	587	48.181	5.756	12.264	1.00	0.00	LX0	N
ATOM	202	H	CYS	587	48.482	4.942	11.766	0.00	0.00	LX0	H
ATOM	203	CA	CYS	587	46.793	6.195	12.195	1.00	0.00	LX0	C
ATOM	204	CB	CYS	587	46.053	5.432	11.099	1.00	0.00	LX0	C
ATOM	205	SG	CYS	587	46.874	5.664	9.496	1.00	0.00	LX0	S
ATOM	206	C	CYS	587	46.016	6.222	13.500	1.00	0.00	LX0	C
ATOM	207	O	CYS	587	45.104	7.027	13.644	1.00	0.00	LX0	O
ATOM	208	N	LYS	588	46.437	5.373	14.467	1.00	0.00	LX0	N
ATOM	209	H	LYS	588	47.204	4.746	14.311	0.00	0.00	LX0	H
ATOM	210	CA	LYS	588	45.800	5.478	15.786	1.00	0.00	LX0	C
ATOM	211	CB	LYS	588	46.323	4.438	16.797	1.00	0.00	LX0	C
ATOM	212	CG	LYS	588	45.515	4.474	18.109	1.00	0.00	LX0	C
ATOM	213	CD	LYS	588	46.031	3.634	19.285	1.00	0.00	LX0	C
ATOM	214	CE	LYS	588	45.210	3.928	20.551	1.00	0.00	LX0	C
ATOM	215	NZ	LYS	588	45.626	3.093	21.690	1.00	0.00	LX0	N
ATOM	216	HZ1	LYS	588	44.912	3.130	22.455	0.00	0.00	LX0	H
ATOM	217	HZ2	LYS	588	45.633	2.085	21.436	0.00	0.00	LX0	H
ATOM	218	HZ3	LYS	588	46.548	3.371	22.071	0.00	0.00	LX0	H
ATOM	219	C	LYS	588	45.877	6.876	16.387	1.00	0.00	LX0	C
ATOM	220	O	LYS	588	44.920	7.400	16.947	1.00	0.00	LX0	O
ATOM	221	N	VAL	589	47.066	7.475	16.227	1.00	0.00	LX0	N
ATOM	222	H	VAL	589	47.796	7.047	15.691	0.00	0.00	LX0	H
ATOM	223	CA	VAL	589	47.098	8.862	16.671	1.00	0.00	LX0	C
ATOM	224	CB	VAL	589	48.453	9.251	17.290	1.00	0.00	LX0	C
ATOM	225	CG1	VAL	589	48.668	8.465	18.583	1.00	0.00	LX0	C
ATOM	226	CG2	VAL	589	49.644	9.113	16.337	1.00	0.00	LX0	C
ATOM	227	C	VAL	589	46.626	9.849	15.619	1.00	0.00	LX0	C
ATOM	228	O	VAL	589	45.901	10.786	15.918	1.00	0.00	LX0	O
ATOM	229	N	PHE	590	47.043	9.585	14.368	1.00	0.00	LX0	N
ATOM	230	H	PHE	590	47.626	8.789	14.219	0.00	0.00	LX0	H
ATOM	231	CA	PHE	590	46.734	10.509	13.276	1.00	0.00	LX0	C
ATOM	232	CB	PHE	590	47.294	9.983	11.938	1.00	0.00	LX0	C
ATOM	233	CG	PHE	590	46.920	10.894	10.783	1.00	0.00	LX0	C
ATOM	234	CD1	PHE	590	47.569	12.139	10.630	1.00	0.00	LX0	C
ATOM	235	CD2	PHE	590	45.902	10.489	9.890	1.00	0.00	LX0	C
ATOM	236	CE1	PHE	590	47.169	13.006	9.594	1.00	0.00	LX0	C
ATOM	237	CE2	PHE	590	45.499	11.356	8.856	1.00	0.00	LX0	C
ATOM	238	CZ	PHE	590	46.127	12.613	8.728	1.00	0.00	LX0	C
ATOM	239	C	PHE	590	45.263	10.862	13.156	1.00	0.00	LX0	C
ATOM	240	O	PHE	590	44.879	12.018	13.247	1.00	0.00	LX0	O
ATOM	241	N	PHE	591	44.455	9.816	12.930	1.00	0.00	LX0	N
ATOM	242	H	PHE	591	44.779	8.873	13.010	0.00	0.00	LX0	H
ATOM	243	CA	PHE	591	43.078	10.142	12.570	1.00	0.00	LX0	C
ATOM	244	CB	PHE	591	42.364	8.907	12.014	1.00	0.00	LX0	C
ATOM	245	CG	PHE	591	41.180	9.330	11.175	1.00	0.00	LX0	C
ATOM	246	CD1	PHE	591	41.404	9.940	9.920	1.00	0.00	LX0	C
ATOM	247	CD2	PHE	591	39.873	9.115	11.660	1.00	0.00	LX0	C
ATOM	248	CE1	PHE	591	40.304	10.359	9.148	1.00	0.00	LX0	C
ATOM	249	CE2	PHE	591	38.773	9.535	10.887	1.00	0.00	LX0	C
ATOM	250	CZ	PHE	591	39.000	10.161	9.644	1.00	0.00	LX0	C
ATOM	251	C	PHE	591	42.297	10.809	13.687	1.00	0.00	LX0	C
ATOM	252	O	PHE	591	41.523	11.734	13.486	1.00	0.00	LX0	O
ATOM	253	N	LYS	592	42.583	10.313	14.902	1.00	0.00	LX0	N
ATOM	254	H	LYS	592	43.260	9.587	15.008	0.00	0.00	LX0	H
ATOM	255	CA	LYS	592	41.940	10.956	16.043	1.00	0.00	LX0	C
ATOM	256	CB	LYS	592	42.155	10.128	17.312	1.00	0.00	LX0	C

ATOM	257	CG	LYS	592	41.159	10.513	18.405	1.00	0.00	LX0	C
ATOM	258	CD	LYS	592	41.147	9.561	19.599	1.00	0.00	LX0	C
ATOM	259	CE	LYS	592	40.081	9.950	20.627	1.00	0.00	LX0	C
ATOM	260	NZ	LYS	592	40.494	11.131	21.393	1.00	0.00	LX0	N
ATOM	261	HZ1	LYS	592	40.832	10.890	22.349	0.00	0.00	LX0	H
ATOM	262	HZ2	LYS	592	41.184	11.718	20.878	0.00	0.00	LX0	H
ATOM	263	HZ3	LYS	592	39.696	11.791	21.488	0.00	0.00	LX0	H
ATOM	264	C	LYS	592	42.345	12.409	16.222	1.00	0.00	LX0	C
ATOM	265	O	LYS	592	41.526	13.317	16.202	1.00	0.00	LX0	O
ATOM	266	N	ARG	593	43.669	12.595	16.345	1.00	0.00	LX0	N
ATOM	267	H	ARG	593	44.296	11.821	16.256	0.00	0.00	LX0	H
ATOM	268	CA	ARG	593	44.163	13.957	16.558	1.00	0.00	LX0	C
ATOM	269	CB	ARG	593	45.677	13.971	16.755	1.00	0.00	LX0	C
ATOM	270	CG	ARG	593	46.129	13.246	18.021	1.00	0.00	LX0	C
ATOM	271	CD	ARG	593	47.652	13.239	18.138	1.00	0.00	LX0	C
ATOM	272	NE	ARG	593	48.086	12.552	19.354	1.00	0.00	LX0	N
ATOM	273	HE	ARG	593	47.363	12.301	20.002	0.00	0.00	LX0	H
ATOM	274	CZ	ARG	593	49.397	12.320	19.581	1.00	0.00	LX0	C
ATOM	275	NH1	ARG	593	50.314	12.694	18.686	1.00	0.00	LX0	N
ATOM	276	HH11	ARG	593	51.292	12.536	18.824	0.00	0.00	LX0	H
ATOM	277	HH12	ARG	593	50.030	13.153	17.842	0.00	0.00	LX0	H
ATOM	278	NH2	ARG	593	49.768	11.715	20.709	1.00	0.00	LX0	N
ATOM	279	HH21	ARG	593	50.728	11.525	20.916	0.00	0.00	LX0	H
ATOM	280	HH22	ARG	593	49.082	11.439	21.384	0.00	0.00	LX0	H
ATOM	281	C	ARG	593	43.783	14.950	15.477	1.00	0.00	LX0	C
ATOM	282	O	ARG	593	43.514	16.111	15.746	1.00	0.00	LX0	O
ATOM	283	N	ALA	594	43.750	14.435	14.240	1.00	0.00	LX0	N
ATOM	284	H	ALA	594	43.984	13.477	14.077	0.00	0.00	LX0	H
ATOM	285	CA	ALA	594	43.355	15.311	13.142	1.00	0.00	LX0	C
ATOM	286	CB	ALA	594	43.563	14.622	11.792	1.00	0.00	LX0	C
ATOM	287	C	ALA	594	41.921	15.798	13.228	1.00	0.00	LX0	C
ATOM	288	O	ALA	594	41.608	16.937	12.906	1.00	0.00	LX0	O
ATOM	289	N	MET	595	41.054	14.883	13.685	1.00	0.00	LX0	N
ATOM	290	H	MET	595	41.347	13.972	13.983	0.00	0.00	LX0	H
ATOM	291	CA	MET	595	39.659	15.308	13.747	1.00	0.00	LX0	C
ATOM	292	CB	MET	595	38.714	14.139	13.463	1.00	0.00	LX0	C
ATOM	293	CG	MET	595	39.026	13.414	12.148	1.00	0.00	LX0	C
ATOM	294	SD	MET	595	39.045	14.489	10.702	1.00	0.00	LX0	S
ATOM	295	CE	MET	595	37.308	14.959	10.690	1.00	0.00	LX0	C
ATOM	296	C	MET	595	39.265	16.045	15.015	1.00	0.00	LX0	C
ATOM	297	O	MET	595	38.370	16.880	15.020	1.00	0.00	LX0	O
ATOM	298	N	GLU	596	39.981	15.717	16.102	1.00	0.00	LX0	N
ATOM	299	H	GLU	596	40.680	15.005	16.052	0.00	0.00	LX0	H
ATOM	300	CA	GLU	596	39.733	16.468	17.333	1.00	0.00	LX0	C
ATOM	301	CB	GLU	596	40.192	15.675	18.566	1.00	0.00	LX0	C
ATOM	302	CG	GLU	596	39.433	14.352	18.751	1.00	0.00	LX0	C
ATOM	303	CD	GLU	596	39.885	13.615	20.004	1.00	0.00	LX0	C
ATOM	304	OE1	GLU	596	41.071	13.326	20.152	1.00	0.00	LX0	O
ATOM	305	OE2	GLU	596	39.041	13.263	20.829	1.00	0.00	LX0	O
ATOM	306	C	GLU	596	40.357	17.857	17.305	1.00	0.00	LX0	C
ATOM	307	O	GLU	596	39.753	18.864	17.659	1.00	0.00	LX0	O
ATOM	308	N	GLY	597	41.609	17.873	16.835	1.00	0.00	LX0	N
ATOM	309	H	GLY	597	42.024	17.041	16.469	0.00	0.00	LX0	H
ATOM	310	CA	GLY	597	42.287	19.156	16.696	1.00	0.00	LX0	C
ATOM	311	C	GLY	597	41.861	19.893	15.444	1.00	0.00	LX0	C
ATOM	312	O	GLY	597	42.129	19.487	14.320	1.00	0.00	LX0	O
ATOM	313	N	GLN	598	41.168	21.010	15.697	1.00	0.00	LX0	N
ATOM	314	H	GLN	598	41.030	21.282	16.648	0.00	0.00	LX0	H
ATOM	315	CA	GLN	598	40.623	21.781	14.580	1.00	0.00	LX0	C
ATOM	316	CB	GLN	598	39.458	22.656	15.060	1.00	0.00	LX0	C
ATOM	317	CG	GLN	598	38.377	21.905	15.848	1.00	0.00	LX0	C

ATOM	318	CD	GLN	598	37.773	20.811	14.992	1.00	0.00	LX0	C
ATOM	319	OE1	GLN	598	37.195	21.048	13.942	1.00	0.00	LX0	O
ATOM	320	NE2	GLN	598	37.967	19.589	15.486	1.00	0.00	LX0	N
ATOM	321	HE21	GLN	598	38.413	19.442	16.370	0.00	0.00	LX0	H
ATOM	322	HE22	GLN	598	37.695	18.769	14.978	0.00	0.00	LX0	H
ATOM	323	C	GLN	598	41.644	22.620	13.828	1.00	0.00	LX0	C
ATOM	324	O	GLN	598	41.712	23.836	13.959	1.00	0.00	LX0	O
ATOM	325	N	HIS	599	42.451	21.916	13.032	1.00	0.00	LX0	N
ATOM	326	H	HIS	599	42.326	20.926	12.941	0.00	0.00	LX0	H
ATOM	327	CA	HIS	599	43.412	22.665	12.230	1.00	0.00	LX0	C
ATOM	328	CB	HIS	599	44.792	22.002	12.250	1.00	0.00	LX0	C
ATOM	329	CG	HIS	599	45.221	21.690	13.663	1.00	0.00	LX0	C
ATOM	330	ND1	HIS	599	45.119	20.466	14.212	1.00	0.00	LX0	N
ATOM	331	HD1	HIS	599	44.750	19.660	13.784	0.00	0.00	LX0	H
ATOM	332	CD2	HIS	599	45.773	22.562	14.605	1.00	0.00	LX0	C
ATOM	333	NE2	HIS	599	46.002	21.841	15.730	1.00	0.00	LX0	N
ATOM	334	CE1	HIS	599	45.602	20.553	15.489	1.00	0.00	LX0	C
ATOM	335	C	HIS	599	42.933	22.779	10.803	1.00	0.00	LX0	C
ATOM	336	O	HIS	599	42.199	21.934	10.307	1.00	0.00	LX0	O
ATOM	337	N	ASN	600	43.390	23.847	10.137	1.00	0.00	LX0	N
ATOM	338	H	ASN	600	44.027	24.487	10.561	0.00	0.00	LX0	H
ATOM	339	CA	ASN	600	43.105	23.834	8.703	1.00	0.00	LX0	C
ATOM	340	CB	ASN	600	43.038	25.249	8.119	1.00	0.00	LX0	C
ATOM	341	CG	ASN	600	42.420	25.198	6.731	1.00	0.00	LX0	C
ATOM	342	OD1	ASN	600	42.508	24.218	6.007	1.00	0.00	LX0	O
ATOM	343	ND2	ASN	600	41.757	26.305	6.389	1.00	0.00	LX0	N
ATOM	344	HD21	ASN	600	41.691	27.096	6.994	0.00	0.00	LX0	H
ATOM	345	HD22	ASN	600	41.317	26.313	5.492	0.00	0.00	LX0	H
ATOM	346	C	ASN	600	44.112	22.974	7.966	1.00	0.00	LX0	C
ATOM	347	O	ASN	600	45.273	23.331	7.798	1.00	0.00	LX0	O
ATOM	348	N	TYR	601	43.618	21.797	7.572	1.00	0.00	LX0	N
ATOM	349	H	TYR	601	42.646	21.609	7.708	0.00	0.00	LX0	H
ATOM	350	CA	TYR	601	44.506	20.871	6.879	1.00	0.00	LX0	C
ATOM	351	CB	TYR	601	44.018	19.426	7.060	1.00	0.00	LX0	C
ATOM	352	CG	TYR	601	43.978	19.089	8.536	1.00	0.00	LX0	C
ATOM	353	CD1	TYR	601	45.190	18.871	9.222	1.00	0.00	LX0	C
ATOM	354	CE1	TYR	601	45.157	18.626	10.604	1.00	0.00	LX0	C
ATOM	355	CD2	TYR	601	42.731	19.023	9.192	1.00	0.00	LX0	C
ATOM	356	CE2	TYR	601	42.698	18.779	10.575	1.00	0.00	LX0	C
ATOM	357	CZ	TYR	601	43.914	18.605	11.266	1.00	0.00	LX0	C
ATOM	358	OH	TYR	601	43.906	18.421	12.634	1.00	0.00	LX0	O
ATOM	359	HH	TYR	601	42.997	18.327	12.941	0.00	0.00	LX0	H
ATOM	360	C	TYR	601	44.714	21.230	5.421	1.00	0.00	LX0	C
ATOM	361	O	TYR	601	44.080	20.704	4.517	1.00	0.00	LX0	O
ATOM	362	N	LEU	602	45.652	22.168	5.244	1.00	0.00	LX0	N
ATOM	363	H	LEU	602	46.066	22.577	6.058	0.00	0.00	LX0	H
ATOM	364	CA	LEU	602	45.967	22.624	3.892	1.00	0.00	LX0	C
ATOM	365	CB	LEU	602	46.772	23.923	3.960	1.00	0.00	LX0	C
ATOM	366	CG	LEU	602	45.976	25.082	4.567	1.00	0.00	LX0	C
ATOM	367	CD1	LEU	602	46.866	26.290	4.858	1.00	0.00	LX0	C
ATOM	368	CD2	LEU	602	44.767	25.459	3.708	1.00	0.00	LX0	C
ATOM	369	C	LEU	602	46.671	21.587	3.032	1.00	0.00	LX0	C
ATOM	370	O	LEU	602	47.117	20.539	3.493	1.00	0.00	LX0	O
ATOM	371	N	CYS	603	46.732	21.928	1.739	1.00	0.00	LX0	N
ATOM	372	H	CYS	603	46.452	22.835	1.428	0.00	0.00	LX0	H
ATOM	373	CA	CYS	603	47.413	21.020	0.825	1.00	0.00	LX0	C
ATOM	374	CB	CYS	603	46.551	20.768	-0.415	1.00	0.00	LX0	C
ATOM	375	SG	CYS	603	47.114	19.339	-1.376	1.00	0.00	LX0	S
ATOM	376	C	CYS	603	48.774	21.556	0.439	1.00	0.00	LX0	C
ATOM	377	O	CYS	603	49.041	22.744	0.557	1.00	0.00	LX0	O
ATOM	378	N	ALA	604	49.617	20.617	-0.011	1.00	0.00	LX0	N

ATOM	379	H	ALA	604	49.303	19.671	-0.039	0.00	0.00	LX0	H
ATOM	380	CA	ALA	604	50.891	21.028	-0.597	1.00	0.00	LX0	C
ATOM	381	CB	ALA	604	52.015	20.095	-0.141	1.00	0.00	LX0	C
ATOM	382	C	ALA	604	50.835	21.035	-2.115	1.00	0.00	LX0	C
ATOM	383	O	ALA	604	51.450	21.844	-2.795	1.00	0.00	LX0	O
ATOM	384	N	GLY	605	50.041	20.076	-2.620	1.00	0.00	LX0	N
ATOM	385	H	GLY	605	49.540	19.457	-2.023	0.00	0.00	LX0	H
ATOM	386	CA	GLY	605	49.664	20.194	-4.022	1.00	0.00	LX0	C
ATOM	387	C	GLY	605	48.419	21.047	-4.122	1.00	0.00	LX0	C
ATOM	388	O	GLY	605	48.075	21.784	-3.208	1.00	0.00	LX0	O
ATOM	389	N	ARG	606	47.736	20.896	-5.263	1.00	0.00	LX0	N
ATOM	390	H	ARG	606	47.980	20.141	-5.875	0.00	0.00	LX0	H
ATOM	391	CA	ARG	606	46.480	21.640	-5.398	1.00	0.00	LX0	C
ATOM	392	CB	ARG	606	46.078	21.703	-6.875	1.00	0.00	LX0	C
ATOM	393	CG	ARG	606	47.274	22.044	-7.768	1.00	0.00	LX0	C
ATOM	394	CD	ARG	606	47.209	21.329	-9.121	1.00	0.00	LX0	C
ATOM	395	NE	ARG	606	48.529	21.285	-9.754	1.00	0.00	LX0	N
ATOM	396	HE	ARG	606	48.772	22.056	-10.345	0.00	0.00	LX0	H
ATOM	397	CZ	ARG	606	49.386	20.271	-9.478	1.00	0.00	LX0	C
ATOM	398	NH1	ARG	606	49.029	19.281	-8.669	1.00	0.00	LX0	N
ATOM	399	HH11	ARG	606	49.605	18.509	-8.382	0.00	0.00	LX0	H
ATOM	400	HH12	ARG	606	48.112	19.233	-8.249	0.00	0.00	LX0	H
ATOM	401	NH2	ARG	606	50.601	20.269	-10.021	1.00	0.00	LX0	N
ATOM	402	HH21	ARG	606	51.244	19.528	-9.823	0.00	0.00	LX0	H
ATOM	403	HH22	ARG	606	50.893	21.004	-10.633	0.00	0.00	LX0	H
ATOM	404	C	ARG	606	45.391	20.984	-4.563	1.00	0.00	LX0	C
ATOM	405	O	ARG	606	44.620	21.599	-3.838	1.00	0.00	LX0	O
ATOM	406	N	ASN	607	45.423	19.652	-4.691	1.00	0.00	LX0	N
ATOM	407	H	ASN	607	46.063	19.273	-5.363	0.00	0.00	LX0	H
ATOM	408	CA	ASN	607	44.719	18.761	-3.773	1.00	0.00	LX0	C
ATOM	409	CB	ASN	607	43.199	18.768	-3.977	1.00	0.00	LX0	C
ATOM	410	CG	ASN	607	42.537	18.063	-2.808	1.00	0.00	LX0	C
ATOM	411	OD1	ASN	607	42.515	16.842	-2.701	1.00	0.00	LX0	O
ATOM	412	ND2	ASN	607	41.994	18.898	-1.919	1.00	0.00	LX0	N
ATOM	413	HD21	ASN	607	42.059	19.887	-2.051	0.00	0.00	LX0	H
ATOM	414	HD22	ASN	607	41.523	18.530	-1.120	0.00	0.00	LX0	H
ATOM	415	C	ASN	607	45.261	17.362	-3.950	1.00	0.00	LX0	C
ATOM	416	O	ASN	607	45.602	16.663	-3.007	1.00	0.00	LX0	O
ATOM	417	N	ASP	608	45.345	17.014	-5.244	1.00	0.00	LX0	N
ATOM	418	H	ASP	608	45.046	17.682	-5.924	0.00	0.00	LX0	H
ATOM	419	CA	ASP	608	45.995	15.808	-5.764	1.00	0.00	LX0	C
ATOM	420	CB	ASP	608	46.739	16.165	-7.068	1.00	0.00	LX0	C
ATOM	421	CG	ASP	608	47.607	17.409	-6.912	1.00	0.00	LX0	C
ATOM	422	OD1	ASP	608	47.077	18.518	-6.925	1.00	0.00	LX0	O
ATOM	423	OD2	ASP	608	48.823	17.286	-6.816	1.00	0.00	LX0	O
ATOM	424	C	ASP	608	46.805	14.942	-4.796	1.00	0.00	LX0	C
ATOM	425	O	ASP	608	46.216	14.148	-4.069	1.00	0.00	LX0	O
ATOM	426	N	CYS	609	48.142	15.104	-4.819	1.00	0.00	LX0	N
ATOM	427	H	CYS	609	48.516	15.854	-5.372	0.00	0.00	LX0	H
ATOM	428	CA	CYS	609	49.066	14.324	-3.986	1.00	0.00	LX0	C
ATOM	429	CB	CYS	609	49.520	15.128	-2.770	1.00	0.00	LX0	C
ATOM	430	SG	CYS	609	50.304	16.701	-3.205	1.00	0.00	LX0	S
ATOM	431	C	CYS	609	48.687	12.908	-3.568	1.00	0.00	LX0	C
ATOM	432	O	CYS	609	47.859	12.643	-2.697	1.00	0.00	LX0	O
ATOM	433	N	ILE	610	49.393	11.985	-4.240	1.00	0.00	LX0	N
ATOM	434	H	ILE	610	50.070	12.284	-4.908	0.00	0.00	LX0	H
ATOM	435	CA	ILE	610	49.309	10.584	-3.825	1.00	0.00	LX0	C
ATOM	436	CB	ILE	610	50.059	9.696	-4.833	1.00	0.00	LX0	C
ATOM	437	CG2	ILE	610	49.964	8.202	-4.494	1.00	0.00	LX0	C
ATOM	438	CG1	ILE	610	49.577	9.977	-6.260	1.00	0.00	LX0	C
ATOM	439	CD1	ILE	610	50.420	9.276	-7.327	1.00	0.00	LX0	C

ATOM	440	C	ILE	610	49.868	10.421	-2.420	1.00	0.00	LX0	C
ATOM	441	O	ILE	610	50.872	11.022	-2.064	1.00	0.00	LX0	O
ATOM	442	N	VAL	611	49.142	9.624	-1.628	1.00	0.00	LX0	N
ATOM	443	H	VAL	611	48.344	9.159	-1.998	0.00	0.00	LX0	H
ATOM	444	CA	VAL	611	49.671	9.352	-0.297	1.00	0.00	LX0	C
ATOM	445	CB	VAL	611	48.603	9.610	0.772	1.00	0.00	LX0	C
ATOM	446	CG1	VAL	611	49.093	9.346	2.198	1.00	0.00	LX0	C
ATOM	447	CG2	VAL	611	48.069	11.031	0.625	1.00	0.00	LX0	C
ATOM	448	C	VAL	611	50.201	7.938	-0.233	1.00	0.00	LX0	C
ATOM	449	O	VAL	611	49.529	6.983	-0.624	1.00	0.00	LX0	O
ATOM	450	N	ASP	612	51.436	7.891	0.264	1.00	0.00	LX0	N
ATOM	451	H	ASP	612	51.876	8.747	0.530	0.00	0.00	LX0	H
ATOM	452	CA	ASP	612	52.312	6.727	0.178	1.00	0.00	LX0	C
ATOM	453	CB	ASP	612	52.965	6.675	-1.211	1.00	0.00	LX0	C
ATOM	454	CG	ASP	612	53.517	8.040	-1.586	1.00	0.00	LX0	C
ATOM	455	OD1	ASP	612	54.494	8.498	-0.996	1.00	0.00	LX0	O
ATOM	456	OD2	ASP	612	52.951	8.665	-2.470	1.00	0.00	LX0	O
ATOM	457	C	ASP	612	53.365	6.869	1.260	1.00	0.00	LX0	C
ATOM	458	O	ASP	612	53.226	7.712	2.141	1.00	0.00	LX0	O
ATOM	459	N	LYS	613	54.426	6.044	1.153	1.00	0.00	LX0	N
ATOM	460	H	LYS	613	54.478	5.422	0.374	0.00	0.00	LX0	H
ATOM	461	CA	LYS	613	55.490	6.075	2.161	1.00	0.00	LX0	C
ATOM	462	CB	LYS	613	56.664	5.168	1.785	1.00	0.00	LX0	C
ATOM	463	CG	LYS	613	56.319	3.688	1.617	1.00	0.00	LX0	C
ATOM	464	CD	LYS	613	57.584	2.823	1.551	1.00	0.00	LX0	C
ATOM	465	CE	LYS	613	57.334	1.322	1.356	1.00	0.00	LX0	C
ATOM	466	NZ	LYS	613	56.464	0.802	2.414	1.00	0.00	LX0	N
ATOM	467	HZ1	LYS	613	56.419	-0.232	2.466	0.00	0.00	LX0	H
ATOM	468	HZ2	LYS	613	56.738	1.099	3.380	0.00	0.00	LX0	H
ATOM	469	HZ3	LYS	613	55.493	1.155	2.313	0.00	0.00	LX0	H
ATOM	470	C	LYS	613	56.043	7.442	2.537	1.00	0.00	LX0	C
ATOM	471	O	LYS	613	56.381	7.697	3.687	1.00	0.00	LX0	O
ATOM	472	N	ILE	614	56.130	8.299	1.511	1.00	0.00	LX0	N
ATOM	473	H	ILE	614	55.738	8.064	0.618	0.00	0.00	LX0	H
ATOM	474	CA	ILE	614	56.646	9.635	1.790	1.00	0.00	LX0	C
ATOM	475	CB	ILE	614	57.385	10.187	0.561	1.00	0.00	LX0	C
ATOM	476	CG2	ILE	614	58.062	11.530	0.863	1.00	0.00	LX0	C
ATOM	477	CG1	ILE	614	58.367	9.159	-0.009	1.00	0.00	LX0	C
ATOM	478	CD1	ILE	614	58.926	9.569	-1.372	1.00	0.00	LX0	C
ATOM	479	C	ILE	614	55.534	10.579	2.208	1.00	0.00	LX0	C
ATOM	480	O	ILE	614	55.550	11.230	3.250	1.00	0.00	LX0	O
ATOM	481	N	ARG	615	54.537	10.642	1.314	1.00	0.00	LX0	N
ATOM	482	H	ARG	615	54.502	10.002	0.539	0.00	0.00	LX0	H
ATOM	483	CA	ARG	615	53.565	11.713	1.502	1.00	0.00	LX0	C
ATOM	484	CB	ARG	615	52.875	12.022	0.180	1.00	0.00	LX0	C
ATOM	485	CG	ARG	615	53.819	12.861	-0.690	1.00	0.00	LX0	C
ATOM	486	CD	ARG	615	53.561	12.742	-2.192	1.00	0.00	LX0	C
ATOM	487	NE	ARG	615	53.840	11.374	-2.622	1.00	0.00	LX0	N
ATOM	488	HE	ARG	615	53.209	10.641	-2.328	0.00	0.00	LX0	H
ATOM	489	CZ	ARG	615	54.957	11.039	-3.297	1.00	0.00	LX0	C
ATOM	490	NH1	ARG	615	55.763	11.980	-3.785	1.00	0.00	LX0	N
ATOM	491	HH11	ARG	615	56.603	11.747	-4.273	0.00	0.00	LX0	H
ATOM	492	HH12	ARG	615	55.525	12.944	-3.667	0.00	0.00	LX0	H
ATOM	493	NH2	ARG	615	55.245	9.757	-3.465	1.00	0.00	LX0	N
ATOM	494	HH21	ARG	615	56.014	9.403	-3.989	0.00	0.00	LX0	H
ATOM	495	HH22	ARG	615	54.631	9.094	-3.006	0.00	0.00	LX0	H
ATOM	496	C	ARG	615	52.621	11.584	2.685	1.00	0.00	LX0	C
ATOM	497	O	ARG	615	51.993	12.551	3.107	1.00	0.00	LX0	O
ATOM	498	N	ARG	616	52.625	10.371	3.266	1.00	0.00	LX0	N
ATOM	499	H	ARG	616	53.103	9.608	2.823	0.00	0.00	LX0	H
ATOM	500	CA	ARG	616	51.963	10.176	4.559	1.00	0.00	LX0	C

ATOM	501	CB	ARG	616	52.070	8.719	5.008	1.00	0.00	LX0	C
ATOM	502	CG	ARG	616	53.504	8.264	5.295	1.00	0.00	LX0	C
ATOM	503	CD	ARG	616	53.566	6.810	5.745	1.00	0.00	LX0	C
ATOM	504	NE	ARG	616	54.945	6.350	5.881	1.00	0.00	LX0	N
ATOM	505	HE	ARG	616	55.637	6.986	6.229	0.00	0.00	LX0	H
ATOM	506	CZ	ARG	616	55.253	5.084	5.536	1.00	0.00	LX0	C
ATOM	507	NH1	ARG	616	54.359	4.314	4.931	1.00	0.00	LX0	N
ATOM	508	HH11	ARG	616	54.596	3.356	4.736	0.00	0.00	LX0	H
ATOM	509	HH12	ARG	616	53.458	4.640	4.632	0.00	0.00	LX0	H
ATOM	510	NH2	ARG	616	56.458	4.584	5.786	1.00	0.00	LX0	N
ATOM	511	HH21	ARG	616	56.637	3.621	5.543	0.00	0.00	LX0	H
ATOM	512	HH22	ARG	616	57.185	5.126	6.204	0.00	0.00	LX0	H
ATOM	513	C	ARG	616	52.420	11.074	5.700	1.00	0.00	LX0	C
ATOM	514	O	ARG	616	51.722	11.238	6.697	1.00	0.00	LX0	O
ATOM	515	N	LYS	617	53.626	11.629	5.522	1.00	0.00	LX0	N
ATOM	516	H	LYS	617	54.194	11.389	4.731	0.00	0.00	LX0	H
ATOM	517	CA	LYS	617	54.078	12.618	6.491	1.00	0.00	LX0	C
ATOM	518	CB	LYS	617	55.249	12.075	7.318	1.00	0.00	LX0	C
ATOM	519	CG	LYS	617	56.481	11.671	6.498	1.00	0.00	LX0	C
ATOM	520	CD	LYS	617	57.768	12.102	7.205	1.00	0.00	LX0	C
ATOM	521	CE	LYS	617	58.611	13.106	6.406	1.00	0.00	LX0	C
ATOM	522	NZ	LYS	617	57.802	14.256	5.977	1.00	0.00	LX0	N
ATOM	523	HZ1	LYS	617	58.384	15.077	5.733	0.00	0.00	LX0	H
ATOM	524	HZ2	LYS	617	57.265	13.999	5.122	0.00	0.00	LX0	H
ATOM	525	HZ3	LYS	617	57.075	14.533	6.672	0.00	0.00	LX0	H
ATOM	526	C	LYS	617	54.450	13.931	5.826	1.00	0.00	LX0	C
ATOM	527	O	LYS	617	55.354	14.642	6.258	1.00	0.00	LX0	O
ATOM	528	N	ASN	618	53.749	14.188	4.715	1.00	0.00	LX0	N
ATOM	529	H	ASN	618	53.002	13.581	4.443	0.00	0.00	LX0	H
ATOM	530	CA	ASN	618	54.086	15.382	3.944	1.00	0.00	LX0	C
ATOM	531	CB	ASN	618	54.670	15.043	2.570	1.00	0.00	LX0	C
ATOM	532	CG	ASN	618	56.172	14.846	2.630	1.00	0.00	LX0	C
ATOM	533	OD1	ASN	618	56.760	14.464	3.635	1.00	0.00	LX0	O
ATOM	534	ND2	ASN	618	56.789	15.128	1.480	1.00	0.00	LX0	N
ATOM	535	HD21	ASN	618	56.283	15.468	0.689	0.00	0.00	LX0	H
ATOM	536	HD22	ASN	618	57.778	15.008	1.411	0.00	0.00	LX0	H
ATOM	537	C	ASN	618	52.907	16.303	3.750	1.00	0.00	LX0	C
ATOM	538	O	ASN	618	53.010	17.509	3.904	1.00	0.00	LX0	O
ATOM	539	N	CYS	619	51.770	15.691	3.390	1.00	0.00	LX0	N
ATOM	540	H	CYS	619	51.680	14.698	3.289	0.00	0.00	LX0	H
ATOM	541	CA	CYS	619	50.656	16.596	3.118	1.00	0.00	LX0	C
ATOM	542	CB	CYS	619	50.345	16.640	1.623	1.00	0.00	LX0	C
ATOM	543	SG	CYS	619	49.071	17.865	1.236	1.00	0.00	LX0	S
ATOM	544	C	CYS	619	49.406	16.286	3.912	1.00	0.00	LX0	C
ATOM	545	O	CYS	619	48.740	15.280	3.687	1.00	0.00	LX0	O
ATOM	546	N	PRO	620	49.096	17.211	4.855	1.00	0.00	LX0	N
ATOM	547	CD	PRO	620	49.820	18.448	5.136	1.00	0.00	LX0	C
ATOM	548	CA	PRO	620	47.935	17.016	5.730	1.00	0.00	LX0	C
ATOM	549	CB	PRO	620	47.957	18.260	6.631	1.00	0.00	LX0	C
ATOM	550	CG	PRO	620	48.829	19.296	5.922	1.00	0.00	LX0	C
ATOM	551	C	PRO	620	46.628	16.761	4.994	1.00	0.00	LX0	C
ATOM	552	O	PRO	620	46.023	15.707	5.157	1.00	0.00	LX0	O
ATOM	553	N	ALA	621	46.234	17.732	4.141	1.00	0.00	LX0	N
ATOM	554	H	ALA	621	46.725	18.605	4.074	0.00	0.00	LX0	H
ATOM	555	CA	ALA	621	44.985	17.540	3.394	1.00	0.00	LX0	C
ATOM	556	CB	ALA	621	44.774	18.621	2.337	1.00	0.00	LX0	C
ATOM	557	C	ALA	621	44.898	16.221	2.663	1.00	0.00	LX0	C
ATOM	558	O	ALA	621	43.926	15.487	2.750	1.00	0.00	LX0	O
ATOM	559	N	CYS	622	45.986	15.936	1.939	1.00	0.00	LX0	N
ATOM	560	H	CYS	622	46.754	16.573	1.911	0.00	0.00	LX0	H
ATOM	561	CA	CYS	622	45.952	14.722	1.130	1.00	0.00	LX0	C

ATOM	562	CB	CYS	622	47.155	14.687	0.201	1.00	0.00	LX0 C
ATOM	563	SG	CYS	622	47.350	16.272	-0.640	1.00	0.00	LX0 S
ATOM	564	C	CYS	622	45.828	13.428	1.908	1.00	0.00	LX0 C
ATOM	565	O	CYS	622	45.119	12.510	1.509	1.00	0.00	LX0 O
ATOM	566	N	ARG	623	46.540	13.391	3.050	1.00	0.00	LX0 N
ATOM	567	H	ARG	623	47.064	14.184	3.369	0.00	0.00	LX0 H
ATOM	568	CA	ARG	623	46.416	12.180	3.857	1.00	0.00	LX0 C
ATOM	569	CB	ARG	623	47.523	12.083	4.914	1.00	0.00	LX0 C
ATOM	570	CG	ARG	623	47.519	10.715	5.606	1.00	0.00	LX0 C
ATOM	571	CD	ARG	623	48.553	10.572	6.720	1.00	0.00	LX0 C
ATOM	572	NE	ARG	623	48.385	9.296	7.415	1.00	0.00	LX0 N
ATOM	573	HE	ARG	623	47.497	8.843	7.311	0.00	0.00	LX0 H
ATOM	574	CZ	ARG	623	49.352	8.810	8.224	1.00	0.00	LX0 C
ATOM	575	NH1	ARG	623	50.509	9.457	8.365	1.00	0.00	LX0 N
ATOM	576	HH11	ARG	623	51.217	9.134	8.990	0.00	0.00	LX0 H
ATOM	577	HH12	ARG	623	50.705	10.295	7.836	0.00	0.00	LX0 H
ATOM	578	NH2	ARG	623	49.137	7.671	8.881	1.00	0.00	LX0 N
ATOM	579	HH21	ARG	623	49.829	7.263	9.491	0.00	0.00	LX0 H
ATOM	580	HH22	ARG	623	48.272	7.175	8.786	0.00	0.00	LX0 H
ATOM	581	C	ARG	623	45.042	12.027	4.481	1.00	0.00	LX0 C
ATOM	582	O	ARG	623	44.414	10.979	4.394	1.00	0.00	LX0 O
ATOM	583	N	LEU	624	44.588	13.139	5.082	1.00	0.00	LX0 N
ATOM	584	H	LEU	624	45.143	13.973	5.090	0.00	0.00	LX0 H
ATOM	585	CA	LEU	624	43.258	13.121	5.693	1.00	0.00	LX0 C
ATOM	586	CB	LEU	624	42.961	14.479	6.335	1.00	0.00	LX0 C
ATOM	587	CG	LEU	624	41.750	14.463	7.271	1.00	0.00	LX0 C
ATOM	588	CD1	LEU	624	41.949	13.501	8.442	1.00	0.00	LX0 C
ATOM	589	CD2	LEU	624	41.383	15.868	7.748	1.00	0.00	LX0 C
ATOM	590	C	LEU	624	42.169	12.712	4.712	1.00	0.00	LX0 C
ATOM	591	O	LEU	624	41.379	11.807	4.948	1.00	0.00	LX0 O
ATOM	592	N	ARG	625	42.236	13.383	3.549	1.00	0.00	LX0 N
ATOM	593	H	ARG	625	42.869	14.152	3.485	0.00	0.00	LX0 H
ATOM	594	CA	ARG	625	41.394	13.015	2.412	1.00	0.00	LX0 C
ATOM	595	CB	ARG	625	41.831	13.760	1.149	1.00	0.00	LX0 C
ATOM	596	CG	ARG	625	40.925	13.521	-0.062	1.00	0.00	LX0 C
ATOM	597	CD	ARG	625	41.643	13.806	-1.380	1.00	0.00	LX0 C
ATOM	598	NE	ARG	625	42.789	12.909	-1.548	1.00	0.00	LX0 N
ATOM	599	HE	ARG	625	42.645	11.940	-1.321	0.00	0.00	LX0 H
ATOM	600	CZ	ARG	625	43.948	13.383	-2.056	1.00	0.00	LX0 C
ATOM	601	NH1	ARG	625	44.051	14.654	-2.413	1.00	0.00	LX0 N
ATOM	602	HH11	ARG	625	44.915	15.021	-2.781	0.00	0.00	LX0 H
ATOM	603	HH12	ARG	625	43.288	15.306	-2.342	0.00	0.00	LX0 H
ATOM	604	NH2	ARG	625	44.999	12.583	-2.205	1.00	0.00	LX0 N
ATOM	605	HH21	ARG	625	45.841	12.930	-2.649	0.00	0.00	LX0 H
ATOM	606	HH22	ARG	625	44.977	11.637	-1.893	0.00	0.00	LX0 H
ATOM	607	C	ARG	625	41.396	11.525	2.136	1.00	0.00	LX0 C
ATOM	608	O	ARG	625	40.355	10.888	2.111	1.00	0.00	LX0 O
ATOM	609	N	LYS	626	42.614	10.989	1.951	1.00	0.00	LX0 N
ATOM	610	H	LYS	626	43.434	11.557	2.041	0.00	0.00	LX0 H
ATOM	611	CA	LYS	626	42.698	9.567	1.622	1.00	0.00	LX0 C
ATOM	612	CB	LYS	626	44.145	9.176	1.319	1.00	0.00	LX0 C
ATOM	613	CG	LYS	626	44.267	7.980	0.369	1.00	0.00	LX0 C
ATOM	614	CD	LYS	626	45.725	7.561	0.193	1.00	0.00	LX0 C
ATOM	615	CE	LYS	626	45.945	6.375	-0.748	1.00	0.00	LX0 C
ATOM	616	NZ	LYS	626	47.302	5.848	-0.538	1.00	0.00	LX0 N
ATOM	617	HZ1	LYS	626	47.291	4.806	-0.563	0.00	0.00	LX0 H
ATOM	618	HZ2	LYS	626	48.021	6.219	-1.193	0.00	0.00	LX0 H
ATOM	619	HZ3	LYS	626	47.638	6.119	0.408	0.00	0.00	LX0 H
ATOM	620	C	LYS	626	42.066	8.649	2.660	1.00	0.00	LX0 C
ATOM	621	O	LYS	626	41.353	7.703	2.345	1.00	0.00	LX0 O
ATOM	622	N	CYS	627	42.317	9.009	3.926	1.00	0.00	LX0 N

ATOM	623	H	CYS	627	42.909	9.796	4.114	0.00	0.00	LX0 H
ATOM	624	CA	CYS	627	41.655	8.296	5.020	1.00	0.00	LX0 C
ATOM	625	CB	CYS	627	42.122	8.860	6.362	1.00	0.00	LX0 C
ATOM	626	SG	CYS	627	43.915	8.740	6.613	1.00	0.00	LX0 S
ATOM	627	C	CYS	627	40.135	8.309	4.926	1.00	0.00	LX0 C
ATOM	628	O	CYS	627	39.452	7.298	5.049	1.00	0.00	LX0 O
ATOM	629	N	CYS	628	39.627	9.514	4.645	1.00	0.00	LX0 N
ATOM	630	H	CYS	628	40.222	10.315	4.547	0.00	0.00	LX0 H
ATOM	631	CA	CYS	628	38.184	9.633	4.453	1.00	0.00	LX0 C
ATOM	632	CB	CYS	628	37.791	11.105	4.441	1.00	0.00	LX0 C
ATOM	633	SG	CYS	628	38.339	11.990	5.923	1.00	0.00	LX0 S
ATOM	634	C	CYS	628	37.650	8.908	3.225	1.00	0.00	LX0 C
ATOM	635	O	CYS	628	36.547	8.379	3.215	1.00	0.00	LX0 O
ATOM	636	N	GLN	629	38.507	8.860	2.189	1.00	0.00	LX0 N
ATOM	637	H	GLN	629	39.402	9.294	2.280	0.00	0.00	LX0 H
ATOM	638	CA	GLN	629	38.165	8.098	0.986	1.00	0.00	LX0 C
ATOM	639	CB	GLN	629	39.231	8.273	-0.104	1.00	0.00	LX0 C
ATOM	640	CG	GLN	629	39.366	9.705	-0.630	1.00	0.00	LX0 C
ATOM	641	CD	GLN	629	40.507	9.790	-1.629	1.00	0.00	LX0 C
ATOM	642	OE1	GLN	629	41.600	10.280	-1.361	1.00	0.00	LX0 O
ATOM	643	NE2	GLN	629	40.197	9.299	-2.832	1.00	0.00	LX0 N
ATOM	644	HE21	GLN	629	39.295	8.907	-3.008	0.00	0.00	LX0 H
ATOM	645	HE22	GLN	629	40.875	9.328	-3.563	0.00	0.00	LX0 H
ATOM	646	C	GLN	629	37.958	6.623	1.279	1.00	0.00	LX0 C
ATOM	647	O	GLN	629	37.087	5.967	0.726	1.00	0.00	LX0 O
ATOM	648	N	ALA	630	38.782	6.140	2.218	1.00	0.00	LX0 N
ATOM	649	H	ALA	630	39.497	6.710	2.627	0.00	0.00	LX0 H
ATOM	650	CA	ALA	630	38.521	4.789	2.696	1.00	0.00	LX0 C
ATOM	651	CB	ALA	630	39.826	4.004	2.809	1.00	0.00	LX0 C
ATOM	652	C	ALA	630	37.765	4.746	4.015	1.00	0.00	LX0 C
ATOM	653	O	ALA	630	38.078	3.988	4.922	1.00	0.00	LX0 O
ATOM	654	N	GLY	631	36.733	5.603	4.074	1.00	0.00	LX0 N
ATOM	655	H	GLY	631	36.590	6.251	3.325	0.00	0.00	LX0 H
ATOM	656	CA	GLY	631	35.724	5.494	5.130	1.00	0.00	LX0 C
ATOM	657	C	GLY	631	36.193	5.408	6.575	1.00	0.00	LX0 C
ATOM	658	O	GLY	631	35.569	4.764	7.409	1.00	0.00	LX0 O
ATOM	659	N	MET	632	37.299	6.109	6.864	1.00	0.00	LX0 N
ATOM	660	H	MET	632	37.806	6.595	6.150	0.00	0.00	LX0 H
ATOM	661	CA	MET	632	37.672	6.143	8.277	1.00	0.00	LX0 C
ATOM	662	CB	MET	632	39.157	6.483	8.432	1.00	0.00	LX0 C
ATOM	663	CG	MET	632	40.034	5.405	7.790	1.00	0.00	LX0 C
ATOM	664	SD	MET	632	41.801	5.756	7.804	1.00	0.00	LX0 S
ATOM	665	CE	MET	632	42.062	5.773	9.581	1.00	0.00	LX0 C
ATOM	666	C	MET	632	36.778	7.060	9.091	1.00	0.00	LX0 C
ATOM	667	O	MET	632	36.707	8.261	8.864	1.00	0.00	LX0 O
ATOM	668	N	VAL	633	36.066	6.434	10.034	1.00	0.00	LX0 N
ATOM	669	H	VAL	633	36.205	5.458	10.209	0.00	0.00	LX0 H
ATOM	670	CA	VAL	633	35.126	7.220	10.822	1.00	0.00	LX0 C
ATOM	671	CB	VAL	633	33.704	6.648	10.712	1.00	0.00	LX0 C
ATOM	672	CG1	VAL	633	32.686	7.582	11.368	1.00	0.00	LX0 C
ATOM	673	CG2	VAL	633	33.305	6.360	9.261	1.00	0.00	LX0 C
ATOM	674	C	VAL	633	35.554	7.357	12.275	1.00	0.00	LX0 C
ATOM	675	O	VAL	633	35.603	6.398	13.050	1.00	0.00	LX0 O
ATOM	676	N	LEU	634	35.847	8.629	12.607	1.00	0.00	LX0 N
ATOM	677	H	LEU	634	35.732	9.327	11.900	0.00	0.00	LX0 H
ATOM	678	CA	LEU	634	36.222	9.007	13.972	1.00	0.00	LX0 C
ATOM	679	CB	LEU	634	37.019	10.325	13.955	1.00	0.00	LX0 C
ATOM	680	CG	LEU	634	37.909	10.638	15.175	1.00	0.00	LX0 C
ATOM	681	CD1	LEU	634	37.240	11.520	16.232	1.00	0.00	LX0 C
ATOM	682	CD2	LEU	634	38.531	9.380	15.773	1.00	0.00	LX0 C
ATOM	683	C	LEU	634	35.047	9.045	14.936	1.00	0.00	LX0 C

ATOM	684	O	LEU	634	34.580	10.070	15.415	1.00	0.00	LX0	O
ATOM	685	N	GLY	635	34.595	7.834	15.218	1.00	0.00	LX0	N
ATOM	686	H	GLY	635	34.991	7.026	14.781	0.00	0.00	LX0	H
ATOM	687	CA	GLY	635	33.543	7.666	16.198	1.00	0.00	LX0	C
ATOM	688	C	GLY	635	33.640	6.248	16.677	1.00	0.00	LX0	C
ATOM	689	O	GLY	635	34.539	5.530	16.245	1.00	0.00	LX0	O
ATOM	690	N	GLY	636	32.707	5.917	17.588	1.00	0.00	LX0	N
ATOM	691	H	GLY	636	31.999	6.602	17.756	0.00	0.00	LX0	H
ATOM	692	CA	GLY	636	32.602	4.606	18.238	1.00	0.00	LX0	C
ATOM	693	C	GLY	636	33.635	3.536	17.900	1.00	0.00	LX0	C
ATOM	694	O	GLY	636	33.849	3.130	16.762	1.00	0.00	LX0	O
ATOM	695	N	ARG	637	34.294	3.078	18.965	1.00	0.00	LX0	N
ATOM	696	H	ARG	637	34.015	3.316	19.894	0.00	0.00	LX0	H
ATOM	697	CA	ARG	637	35.435	2.222	18.672	1.00	0.00	LX0	C
ATOM	698	CB	ARG	637	36.535	2.464	19.697	1.00	0.00	LX0	C
ATOM	699	CG	ARG	637	37.846	1.775	19.353	1.00	0.00	LX0	C
ATOM	700	CD	ARG	637	38.198	0.857	20.510	1.00	0.00	LX0	C
ATOM	701	NE	ARG	637	39.204	-0.133	20.153	1.00	0.00	LX0	N
ATOM	702	HE	ARG	637	40.165	0.106	20.276	0.00	0.00	LX0	H
ATOM	703	CZ	ARG	637	38.824	-1.313	19.627	1.00	0.00	LX0	C
ATOM	704	NH1	ARG	637	37.546	-1.598	19.405	1.00	0.00	LX0	N
ATOM	705	HH11	ARG	637	37.272	-2.444	18.922	0.00	0.00	LX0	H
ATOM	706	HH12	ARG	637	36.797	-0.985	19.671	0.00	0.00	LX0	H
ATOM	707	NH2	ARG	637	39.745	-2.208	19.318	1.00	0.00	LX0	N
ATOM	708	HH21	ARG	637	39.426	-3.057	18.890	0.00	0.00	LX0	H
ATOM	709	HH22	ARG	637	40.720	-2.058	19.466	0.00	0.00	LX0	H
ATOM	710	C	ARG	637	35.156	0.739	18.447	1.00	0.00	LX0	C
ATOM	711	O	ARG	637	35.360	-0.114	19.301	1.00	0.00	LX0	O
ATOM	712	N	LYS	638	34.758	0.486	17.197	1.00	0.00	LX0	N
ATOM	713	H	LYS	638	34.388	1.262	16.684	0.00	0.00	LX0	H
ATOM	714	CA	LYS	638	35.102	-0.740	16.469	1.00	0.00	LX0	C
ATOM	715	CB	LYS	638	36.488	-0.596	15.788	1.00	0.00	LX0	C
ATOM	716	CG	LYS	638	36.964	0.833	15.434	1.00	0.00	LX0	C
ATOM	717	CD	LYS	638	36.001	1.662	14.572	1.00	0.00	LX0	C
ATOM	718	CE	LYS	638	36.274	3.173	14.577	1.00	0.00	LX0	C
ATOM	719	NZ	LYS	638	35.040	3.897	14.250	1.00	0.00	LX0	N
ATOM	720	HZ1	LYS	638	35.224	4.802	13.770	0.00	0.00	LX0	H
ATOM	721	HZ2	LYS	638	34.458	3.309	13.613	0.00	0.00	LX0	H
ATOM	722	HZ3	LYS	638	34.462	4.056	15.107	0.00	0.00	LX0	H
ATOM	723	C	LYS	638	34.962	-2.085	17.183	1.00	0.00	LX0	C
ATOM	724	O	LYS	638	35.945	-2.714	17.551	1.00	0.00	LX0	O
ATOM	725	N	PHE	639	33.701	-2.507	17.359	1.00	0.00	LX0	N
ATOM	726	H	PHE	639	32.937	-1.972	17.003	0.00	0.00	LX0	H
ATOM	727	CA	PHE	639	33.476	-3.790	18.036	1.00	0.00	LX0	C
ATOM	728	CB	PHE	639	32.382	-3.604	19.092	1.00	0.00	LX0	C
ATOM	729	CG	PHE	639	32.985	-3.415	20.472	1.00	0.00	LX0	C
ATOM	730	CD1	PHE	639	34.176	-2.670	20.651	1.00	0.00	LX0	C
ATOM	731	CD2	PHE	639	32.327	-3.996	21.578	1.00	0.00	LX0	C
ATOM	732	CE1	PHE	639	34.714	-2.508	21.940	1.00	0.00	LX0	C
ATOM	733	CE2	PHE	639	32.862	-3.836	22.871	1.00	0.00	LX0	C
ATOM	734	CZ	PHE	639	34.050	-3.095	23.037	1.00	0.00	LX0	C
ATOM	735	C	PHE	639	33.251	-4.977	17.097	1.00	0.00	LX0	C
ATOM	736	O	PHE	639	33.692	-4.940	15.948	1.00	0.00	LX0	O
ATOM	737	N	LYS	640	32.590	-6.050	17.591	1.00	0.00	LX0	N
ATOM	738	H	LYS	640	32.249	-6.060	18.533	0.00	0.00	LX0	H
ATOM	739	CA	LYS	640	32.389	-7.224	16.730	1.00	0.00	LX0	C
ATOM	740	CB	LYS	640	31.772	-8.461	17.391	1.00	0.00	LX0	C
ATOM	741	CG	LYS	640	32.710	-9.289	18.279	1.00	0.00	LX0	C
ATOM	742	CD	LYS	640	32.037	-10.600	18.714	1.00	0.00	LX0	C
ATOM	743	CE	LYS	640	32.637	-11.288	19.949	1.00	0.00	LX0	C
ATOM	744	NZ	LYS	640	32.286	-10.549	21.167	1.00	0.00	LX0	N

```
ATOM    745  HZ1 LYS   640       32.304 -11.131  22.030  0.00  0.00      LX0  H
ATOM    746  HZ2 LYS   640       32.861  -9.687  21.290  0.00  0.00      LX0  H
ATOM    747  HZ3 LYS   640       31.338 -10.124  21.090  0.00  0.00      LX0  H
ATOM    748  C   LYS   640       31.686  -6.985  15.418  1.00  0.00      LX0  C
ATOM    749  O   LYS   640       32.338  -7.033  14.377  1.00  0.00      LX0  O
TER
```
