# Supplementary material for: Structural analysis of hubs in human NR-RTK network
Source: Biol Direct. 2011 Oct 5;6:49. doi: 10.1186/1745-6150-6-49 (PMC3220635; doi:10.1186/1745-6150-6-49)
Supplement: Additional file 9 — ESR1-IGF1-R. ESR1-IGF1-R complex structure. [file 1745-6150-6-49-S9.PDF]

```
HEADER ESR1-IGF1R
REMARK original generated coordinate pdb file
ATOM      1  N   ALA   156      10.627  12.174   8.322  1.00  0.00      RX0  N
ATOM      2  H   ALA   156      11.190  11.348   8.271  0.00  0.00      RX0  H
ATOM      3  CA  ALA   156       9.864  12.527   9.538  1.00  0.00      RX0  C
ATOM      4  CB  ALA   156      10.756  12.402  10.764  1.00  0.00      RX0  C
ATOM      5  C   ALA   156       9.377  13.991   9.496  1.00  0.00      RX0  C
ATOM      6  O   ALA   156       9.121  14.644  10.500  1.00  0.00      RX0  O
ATOM      7  N   LEU   157       9.039  14.416   8.289  1.00  0.00      RX0  N
ATOM      8  H   LEU   157       9.126  13.765   7.532  0.00  0.00      RX0  H
ATOM      9  CA  LEU   157       8.850  15.849   7.979  1.00  0.00      RX0  C
ATOM     10  CB  LEU   157       9.703  16.230   6.775  1.00  0.00      RX0  C
ATOM     11  CG  LEU   157      11.024  15.469   6.703  1.00  0.00      RX0  C
ATOM     12  CD1 LEU   157      11.606  15.527   5.295  1.00  0.00      RX0  C
ATOM     13  CD2 LEU   157      12.017  15.904   7.780  1.00  0.00      RX0  C
ATOM     14  C   LEU   157       7.387  16.184   7.642  1.00  0.00      RX0  C
ATOM     15  O   LEU   157       7.075  17.216   7.039  1.00  0.00      RX0  O
ATOM     16  N   SER   158       6.522  15.244   7.955  1.00  0.00      RX0  N
ATOM     17  H   SER   158       6.875  14.327   8.104  0.00  0.00      RX0  H
ATOM     18  CA  SER   158       5.051  15.362   7.847  1.00  0.00      RX0  C
ATOM     19  CB  SER   158       4.730  14.896   6.436  1.00  0.00      RX0  C
ATOM     20  OG  SER   158       5.929  15.067   5.669  1.00  0.00      RX0  O
ATOM     21  HG  SER   158       6.079  16.010   5.634  0.00  0.00      RX0  H
ATOM     22  C   SER   158       4.335  14.560   8.949  1.00  0.00      RX0  C
ATOM     23  O   SER   158       3.148  14.670   9.188  1.00  0.00      RX0  O
ATOM     24  N   LEU   159       5.132  13.681   9.591  1.00  0.00      RX0  N
ATOM     25  H   LEU   159       6.083  13.585   9.325  0.00  0.00      RX0  H
ATOM     26  CA  LEU   159       4.759  12.951  10.797  1.00  0.00      RX0  C
ATOM     27  CB  LEU   159       5.859  11.984  11.246  1.00  0.00      RX0  C
ATOM     28  CG  LEU   159       5.897  10.622  10.542  1.00  0.00      RX0  C
ATOM     29  CD1 LEU   159       6.344  10.696   9.080  1.00  0.00      RX0  C
ATOM     30  CD2 LEU   159       6.746   9.629  11.338  1.00  0.00      RX0  C
ATOM     31  C   LEU   159       4.518  13.965  11.920  1.00  0.00      RX0  C
ATOM     32  O   LEU   159       5.291  14.932  12.058  1.00  0.00      RX0  O
ATOM     33  N   THR   160       3.434  13.807  12.646  1.00  0.00      RX0  N
ATOM     34  H   THR   160       2.848  13.019  12.459  0.00  0.00      RX0  H
ATOM     35  CA  THR   160       3.156  14.665  13.825  1.00  0.00      RX0  C
ATOM     36  CB  THR   160       1.666  14.646  14.226  1.00  0.00      RX0  C
ATOM     37  OG1 THR   160       1.372  15.742  15.099  1.00  0.00      RX0  O
ATOM     38  HG1 THR   160       0.424  15.807  15.140  0.00  0.00      RX0  H
ATOM     39  CG2 THR   160       1.177  13.336  14.828  1.00  0.00      RX0  C
ATOM     40  C   THR   160       4.203  14.411  14.921  1.00  0.00      RX0  C
ATOM     41  O   THR   160       4.902  13.383  14.913  1.00  0.00      RX0  O
ATOM     42  N   ALA   161       4.153  15.229  15.953  1.00  0.00      RX0  N
ATOM     43  H   ALA   161       3.461  15.953  15.917  0.00  0.00      RX0  H
ATOM     44  CA  ALA   161       4.942  15.044  17.184  1.00  0.00      RX0  C
ATOM     45  CB  ALA   161       4.758  16.234  18.117  1.00  0.00      RX0  C
ATOM     46  C   ALA   161       4.543  13.746  17.920  1.00  0.00      RX0  C
ATOM     47  O   ALA   161       5.387  12.950  18.288  1.00  0.00      RX0  O
ATOM     48  N   ASP   162       3.226  13.461  17.917  1.00  0.00      RX0  N
ATOM     49  H   ASP   162       2.570  14.184  17.711  0.00  0.00      RX0  H
ATOM     50  CA  ASP   162       2.690  12.196  18.469  1.00  0.00      RX0  C
ATOM     51  CB  ASP   162       1.205  12.331  18.825  1.00  0.00      RX0  C
ATOM     52  CG  ASP   162       1.076  13.179  20.093  1.00  0.00      RX0  C
ATOM     53  OD1 ASP   162       1.918  14.036  20.341  1.00  0.00      RX0  O
ATOM     54  OD2 ASP   162       0.150  12.985  20.879  1.00  0.00      RX0  O
ATOM     55  C   ASP   162       3.088  10.948  17.668  1.00  0.00      RX0  C
ATOM     56  O   ASP   162       3.397   9.903  18.257  1.00  0.00      RX0  O
ATOM     57  N   GLN   163       3.164  11.087  16.353  1.00  0.00      RX0  N
ATOM     58  H   GLN   163       3.080  12.015  16.001  0.00  0.00      RX0  H
ATOM     59  CA  GLN   163       3.593   9.998  15.449  1.00  0.00      RX0  C
```

ATOM	60	CB	GLN	163	3.252	10.287	13.995	1.00	0.00	RX0	C
ATOM	61	CG	GLN	163	1.829	9.878	13.626	1.00	0.00	RX0	C
ATOM	62	CD	GLN	163	1.567	10.374	12.226	1.00	0.00	RX0	C
ATOM	63	OE1	GLN	163	2.084	11.411	11.823	1.00	0.00	RX0	O
ATOM	64	NE2	GLN	163	0.752	9.584	11.507	1.00	0.00	RX0	N
ATOM	65	HE21	GLN	163	0.371	8.748	11.904	0.00	0.00	RX0	H
ATOM	66	HE22	GLN	163	0.503	9.805	10.564	0.00	0.00	RX0	H
ATOM	67	C	GLN	163	5.089	9.698	15.572	1.00	0.00	RX0	C
ATOM	68	O	GLN	163	5.477	8.537	15.545	1.00	0.00	RX0	O
ATOM	69	N	MET	164	5.882	10.740	15.840	1.00	0.00	RX0	N
ATOM	70	H	MET	164	5.492	11.661	15.892	0.00	0.00	RX0	H
ATOM	71	CA	MET	164	7.331	10.587	16.060	1.00	0.00	RX0	C
ATOM	72	CB	MET	164	8.014	11.955	16.082	1.00	0.00	RX0	C
ATOM	73	CG	MET	164	9.451	11.879	16.606	1.00	0.00	RX0	C
ATOM	74	SD	MET	164	10.536	10.864	15.594	1.00	0.00	RX0	S
ATOM	75	CE	MET	164	11.024	12.134	14.424	1.00	0.00	RX0	C
ATOM	76	C	MET	164	7.610	9.825	17.366	1.00	0.00	RX0	C
ATOM	77	O	MET	164	8.404	8.887	17.381	1.00	0.00	RX0	O
ATOM	78	N	VAL	165	6.828	10.145	18.396	1.00	0.00	RX0	N
ATOM	79	H	VAL	165	6.165	10.888	18.278	0.00	0.00	RX0	H
ATOM	80	CA	VAL	165	6.992	9.563	19.744	1.00	0.00	RX0	C
ATOM	81	CB	VAL	165	6.100	10.259	20.777	1.00	0.00	RX0	C
ATOM	82	CG1	VAL	165	6.180	9.566	22.138	1.00	0.00	RX0	C
ATOM	83	CG2	VAL	165	6.451	11.732	20.917	1.00	0.00	RX0	C
ATOM	84	C	VAL	165	6.649	8.067	19.731	1.00	0.00	RX0	C
ATOM	85	O	VAL	165	7.442	7.255	20.191	1.00	0.00	RX0	O
ATOM	86	N	SER	166	5.467	7.742	19.205	1.00	0.00	RX0	N
ATOM	87	H	SER	166	4.817	8.431	18.869	0.00	0.00	RX0	H
ATOM	88	CA	SER	166	5.029	6.335	19.106	1.00	0.00	RX0	C
ATOM	89	CB	SER	166	3.572	6.381	18.707	1.00	0.00	RX0	C
ATOM	90	OG	SER	166	3.001	7.448	19.466	1.00	0.00	RX0	O
ATOM	91	HG	SER	166	3.358	7.384	20.342	0.00	0.00	RX0	H
ATOM	92	C	SER	166	5.941	5.501	18.195	1.00	0.00	RX0	C
ATOM	93	O	SER	166	6.295	4.379	18.542	1.00	0.00	RX0	O
ATOM	94	N	ALA	167	6.456	6.129	17.133	1.00	0.00	RX0	N
ATOM	95	H	ALA	167	6.171	7.064	16.912	0.00	0.00	RX0	H
ATOM	96	CA	ALA	167	7.397	5.466	16.208	1.00	0.00	RX0	C
ATOM	97	CB	ALA	167	7.731	6.357	15.012	1.00	0.00	RX0	C
ATOM	98	C	ALA	167	8.706	5.103	16.927	1.00	0.00	RX0	C
ATOM	99	O	ALA	167	9.113	3.946	16.932	1.00	0.00	RX0	O
ATOM	100	N	LEU	168	9.179	6.054	17.734	1.00	0.00	RX0	N
ATOM	101	H	LEU	168	8.734	6.952	17.737	0.00	0.00	RX0	H
ATOM	102	CA	LEU	168	10.385	5.875	18.562	1.00	0.00	RX0	C
ATOM	103	CB	LEU	168	10.907	7.214	19.074	1.00	0.00	RX0	C
ATOM	104	CG	LEU	168	11.571	8.041	17.977	1.00	0.00	RX0	C
ATOM	105	CD1	LEU	168	12.103	9.369	18.518	1.00	0.00	RX0	C
ATOM	106	CD2	LEU	168	12.652	7.238	17.253	1.00	0.00	RX0	C
ATOM	107	C	LEU	168	10.197	4.896	19.724	1.00	0.00	RX0	C
ATOM	108	O	LEU	168	11.077	4.078	19.994	1.00	0.00	RX0	O
ATOM	109	N	LEU	169	9.007	4.918	20.317	1.00	0.00	RX0	N
ATOM	110	H	LEU	169	8.333	5.589	20.010	0.00	0.00	RX0	H
ATOM	111	CA	LEU	169	8.640	3.970	21.384	1.00	0.00	RX0	C
ATOM	112	CB	LEU	169	7.358	4.393	22.101	1.00	0.00	RX0	C
ATOM	113	CG	LEU	169	7.538	5.638	22.970	1.00	0.00	RX0	C
ATOM	114	CD1	LEU	169	6.226	6.046	23.641	1.00	0.00	RX0	C
ATOM	115	CD2	LEU	169	8.667	5.470	23.988	1.00	0.00	RX0	C
ATOM	116	C	LEU	169	8.505	2.536	20.864	1.00	0.00	RX0	C
ATOM	117	O	LEU	169	9.003	1.602	21.486	1.00	0.00	RX0	O
ATOM	118	N	ASP	170	7.977	2.423	19.645	1.00	0.00	RX0	N
ATOM	119	H	ASP	170	7.673	3.242	19.160	0.00	0.00	RX0	H
ATOM	120	CA	ASP	170	7.822	1.133	18.952	1.00	0.00	RX0	C

ATOM	121	CB	ASP	170	6.887	1.398	17.755	1.00	0.00	RX0	C
ATOM	122	CG	ASP	170	6.640	0.254	16.783	1.00	0.00	RX0	C
ATOM	123	OD1	ASP	170	7.096	-0.865	16.981	1.00	0.00	RX0	O
ATOM	124	OD2	ASP	170	6.021	0.495	15.750	1.00	0.00	RX0	O
ATOM	125	C	ASP	170	9.164	0.506	18.541	1.00	0.00	RX0	C
ATOM	126	O	ASP	170	9.313	-0.704	18.571	1.00	0.00	RX0	O
ATOM	127	N	ALA	171	10.119	1.387	18.228	1.00	0.00	RX0	N
ATOM	128	H	ALA	171	9.895	2.361	18.290	0.00	0.00	RX0	H
ATOM	129	CA	ALA	171	11.447	1.008	17.717	1.00	0.00	RX0	C
ATOM	130	CB	ALA	171	12.102	2.214	17.045	1.00	0.00	RX0	C
ATOM	131	C	ALA	171	12.418	0.479	18.779	1.00	0.00	RX0	C
ATOM	132	O	ALA	171	13.427	-0.136	18.431	1.00	0.00	RX0	O
ATOM	133	N	GLU	172	12.125	0.739	20.058	1.00	0.00	RX0	N
ATOM	134	H	GLU	172	11.282	1.229	20.278	0.00	0.00	RX0	H
ATOM	135	CA	GLU	172	13.017	0.374	21.170	1.00	0.00	RX0	C
ATOM	136	CB	GLU	172	12.362	0.712	22.510	1.00	0.00	RX0	C
ATOM	137	CG	GLU	172	12.323	2.231	22.679	1.00	0.00	RX0	C
ATOM	138	CD	GLU	172	13.729	2.779	22.511	1.00	0.00	RX0	C
ATOM	139	OE1	GLU	172	14.557	2.617	23.407	1.00	0.00	RX0	O
ATOM	140	OE2	GLU	172	14.031	3.386	21.483	1.00	0.00	RX0	O
ATOM	141	C	GLU	172	13.554	-1.065	21.099	1.00	0.00	RX0	C
ATOM	142	O	GLU	172	12.785	-2.004	20.837	1.00	0.00	RX0	O
ATOM	143	N	PRO	173	14.865	-1.209	21.269	1.00	0.00	RX0	N
ATOM	144	CD	PRO	173	15.790	-0.096	21.451	1.00	0.00	RX0	C
ATOM	145	CA	PRO	173	15.538	-2.517	21.328	1.00	0.00	RX0	C
ATOM	146	CB	PRO	173	17.014	-2.109	21.199	1.00	0.00	RX0	C
ATOM	147	CG	PRO	173	17.103	-0.741	21.869	1.00	0.00	RX0	C
ATOM	148	C	PRO	173	15.206	-3.249	22.640	1.00	0.00	RX0	C
ATOM	149	O	PRO	173	14.829	-2.595	23.631	1.00	0.00	RX0	O
ATOM	150	N	PRO	174	15.294	-4.574	22.646	1.00	0.00	RX0	N
ATOM	151	CD	PRO	174	15.604	-5.387	21.475	1.00	0.00	RX0	C
ATOM	152	CA	PRO	174	15.084	-5.400	23.852	1.00	0.00	RX0	C
ATOM	153	CB	PRO	174	14.968	-6.812	23.273	1.00	0.00	RX0	C
ATOM	154	CG	PRO	174	15.853	-6.784	22.031	1.00	0.00	RX0	C
ATOM	155	C	PRO	174	16.250	-5.248	24.838	1.00	0.00	RX0	C
ATOM	156	O	PRO	174	17.379	-4.922	24.444	1.00	0.00	RX0	O
ATOM	157	N	ILE	175	15.956	-5.464	26.106	1.00	0.00	RX0	N
ATOM	158	H	ILE	175	15.039	-5.788	26.328	0.00	0.00	RX0	H
ATOM	159	CA	ILE	175	16.988	-5.556	27.159	1.00	0.00	RX0	C
ATOM	160	CB	ILE	175	16.446	-5.212	28.551	1.00	0.00	RX0	C
ATOM	161	CG2	ILE	175	17.623	-5.099	29.520	1.00	0.00	RX0	C
ATOM	162	CG1	ILE	175	15.623	-3.918	28.562	1.00	0.00	RX0	C
ATOM	163	CD1	ILE	175	14.110	-4.136	28.453	1.00	0.00	RX0	C
ATOM	164	C	ILE	175	17.586	-6.969	27.112	1.00	0.00	RX0	C
ATOM	165	O	ILE	175	16.886	-7.963	27.343	1.00	0.00	RX0	O
ATOM	166	N	LEU	176	18.884	-7.017	26.884	1.00	0.00	RX0	N
ATOM	167	H	LEU	176	19.405	-6.165	26.840	0.00	0.00	RX0	H
ATOM	168	CA	LEU	176	19.617	-8.291	26.770	1.00	0.00	RX0	C
ATOM	169	CB	LEU	176	20.636	-8.205	25.638	1.00	0.00	RX0	C
ATOM	170	CG	LEU	176	19.969	-7.976	24.281	1.00	0.00	RX0	C
ATOM	171	CD1	LEU	176	20.999	-7.856	23.161	1.00	0.00	RX0	C
ATOM	172	CD2	LEU	176	18.923	-9.048	23.969	1.00	0.00	RX0	C
ATOM	173	C	LEU	176	20.277	-8.690	28.089	1.00	0.00	RX0	C
ATOM	174	O	LEU	176	20.563	-7.852	28.952	1.00	0.00	RX0	O
ATOM	175	N	TYR	177	20.459	-9.989	28.237	1.00	0.00	RX0	N
ATOM	176	H	TYR	177	20.230	-10.608	27.489	0.00	0.00	RX0	H
ATOM	177	CA	TYR	177	21.114	-10.573	29.420	1.00	0.00	RX0	C
ATOM	178	CB	TYR	177	20.421	-11.864	29.854	1.00	0.00	RX0	C
ATOM	179	CG	TYR	177	19.152	-11.562	30.615	1.00	0.00	RX0	C
ATOM	180	CD1	TYR	177	18.030	-11.070	29.958	1.00	0.00	RX0	C
ATOM	181	CE1	TYR	177	16.866	-10.812	30.672	1.00	0.00	RX0	C

ATOM	182	CD2	TYR	177	19.111	-11.791	31.985	1.00	0.00	RX0	C
ATOM	183	CE2	TYR	177	17.945	-11.540	32.697	1.00	0.00	RX0	C
ATOM	184	CZ	TYR	177	16.823	-11.049	32.040	1.00	0.00	RX0	C
ATOM	185	OH	TYR	177	15.669	-10.789	32.750	1.00	0.00	RX0	O
ATOM	186	HH	TYR	177	15.778	-11.068	33.650	0.00	0.00	RX0	H
ATOM	187	C	TYR	177	22.589	-10.858	29.163	1.00	0.00	RX0	C
ATOM	188	O	TYR	177	22.985	-11.163	28.046	1.00	0.00	RX0	O
ATOM	189	N	SER	178	23.381	-10.750	30.220	1.00	0.00	RX0	N
ATOM	190	H	SER	178	23.019	-10.516	31.126	0.00	0.00	RX0	H
ATOM	191	CA	SER	178	24.788	-11.188	30.183	1.00	0.00	RX0	C
ATOM	192	CB	SER	178	25.480	-10.623	31.408	1.00	0.00	RX0	C
ATOM	193	OG	SER	178	25.175	-9.230	31.438	1.00	0.00	RX0	O
ATOM	194	HG	SER	178	25.380	-8.887	30.578	0.00	0.00	RX0	H
ATOM	195	C	SER	178	24.834	-12.718	30.070	1.00	0.00	RX0	C
ATOM	196	O	SER	178	23.999	-13.413	30.674	1.00	0.00	RX0	O
ATOM	197	N	GLU	179	25.827	-13.218	29.362	1.00	0.00	RX0	N
ATOM	198	H	GLU	179	26.520	-12.638	28.936	0.00	0.00	RX0	H
ATOM	199	CA	GLU	179	26.033	-14.670	29.175	1.00	0.00	RX0	C
ATOM	200	CB	GLU	179	26.338	-14.994	27.723	1.00	0.00	RX0	C
ATOM	201	CG	GLU	179	25.133	-14.822	26.816	1.00	0.00	RX0	C
ATOM	202	CD	GLU	179	25.638	-14.231	25.524	1.00	0.00	RX0	C
ATOM	203	OE1	GLU	179	25.127	-14.582	24.469	1.00	0.00	RX0	O
ATOM	204	OE2	GLU	179	26.494	-13.350	25.584	1.00	0.00	RX0	O
ATOM	205	C	GLU	179	27.192	-15.208	30.012	1.00	0.00	RX0	C
ATOM	206	O	GLU	179	28.361	-15.227	29.589	1.00	0.00	RX0	O
ATOM	207	N	TYR	180	26.873	-15.475	31.254	1.00	0.00	RX0	N
ATOM	208	H	TYR	180	25.922	-15.405	31.564	0.00	0.00	RX0	H
ATOM	209	CA	TYR	180	27.735	-16.233	32.177	1.00	0.00	RX0	C
ATOM	210	CB	TYR	180	28.495	-15.323	33.155	1.00	0.00	RX0	C
ATOM	211	CG	TYR	180	27.554	-14.675	34.144	1.00	0.00	RX0	C
ATOM	212	CD1	TYR	180	26.926	-13.476	33.829	1.00	0.00	RX0	C
ATOM	213	CE1	TYR	180	26.018	-12.917	34.718	1.00	0.00	RX0	C
ATOM	214	CD2	TYR	180	27.311	-15.286	35.369	1.00	0.00	RX0	C
ATOM	215	CE2	TYR	180	26.391	-14.735	36.251	1.00	0.00	RX0	C
ATOM	216	CZ	TYR	180	25.728	-13.563	35.914	1.00	0.00	RX0	C
ATOM	217	OH	TYR	180	24.772	-13.047	36.762	1.00	0.00	RX0	O
ATOM	218	HH	TYR	180	24.677	-12.114	36.590	0.00	0.00	RX0	H
ATOM	219	C	TYR	180	26.838	-17.226	32.909	1.00	0.00	RX0	C
ATOM	220	O	TYR	180	25.642	-16.953	33.094	1.00	0.00	RX0	O
ATOM	221	N	ASP	181	27.404	-18.345	33.318	1.00	0.00	RX0	N
ATOM	222	H	ASP	181	28.389	-18.473	33.247	0.00	0.00	RX0	H
ATOM	223	CA	ASP	181	26.630	-19.347	34.059	1.00	0.00	RX0	C
ATOM	224	CB	ASP	181	27.317	-20.701	34.094	1.00	0.00	RX0	C
ATOM	225	CG	ASP	181	26.491	-21.562	35.015	1.00	0.00	RX0	C
ATOM	226	OD1	ASP	181	25.291	-21.657	34.802	1.00	0.00	RX0	O
ATOM	227	OD2	ASP	181	27.024	-22.088	35.981	1.00	0.00	RX0	O
ATOM	228	C	ASP	181	26.420	-18.851	35.504	1.00	0.00	RX0	C
ATOM	229	O	ASP	181	27.391	-18.832	36.273	1.00	0.00	RX0	O
ATOM	230	N	PRO	182	25.185	-18.489	35.856	1.00	0.00	RX0	N
ATOM	231	CD	PRO	182	24.010	-18.612	34.996	1.00	0.00	RX0	C
ATOM	232	CA	PRO	182	24.825	-17.989	37.201	1.00	0.00	RX0	C
ATOM	233	CB	PRO	182	23.393	-17.490	36.996	1.00	0.00	RX0	C
ATOM	234	CG	PRO	182	22.822	-18.417	35.928	1.00	0.00	RX0	C
ATOM	235	C	PRO	182	24.941	-19.052	38.308	1.00	0.00	RX0	C
ATOM	236	O	PRO	182	24.654	-18.763	39.474	1.00	0.00	RX0	O
ATOM	237	N	THR	183	25.345	-20.259	37.948	1.00	0.00	RX0	N
ATOM	238	H	THR	183	25.539	-20.528	37.003	0.00	0.00	RX0	H
ATOM	239	CA	THR	183	25.568	-21.363	38.913	1.00	0.00	RX0	C
ATOM	240	CB	THR	183	24.912	-22.556	38.255	1.00	0.00	RX0	C
ATOM	241	OG1	THR	183	23.979	-22.056	37.287	1.00	0.00	RX0	O
ATOM	242	HG1	THR	183	24.469	-22.059	36.460	0.00	0.00	RX0	H

ATOM	243	CG2	THR	183	24.228	-23.475	39.266	1.00	0.00	RX0	C
ATOM	244	C	THR	183	27.063	-21.532	39.218	1.00	0.00	RX0	C
ATOM	245	O	THR	183	27.455	-22.345	40.058	1.00	0.00	RX0	O
ATOM	246	N	ARG	184	27.887	-20.699	38.573	1.00	0.00	RX0	N
ATOM	247	H	ARG	184	27.516	-20.004	37.954	0.00	0.00	RX0	H
ATOM	248	CA	ARG	184	29.343	-20.701	38.681	1.00	0.00	RX0	C
ATOM	249	CB	ARG	184	29.911	-20.761	37.268	1.00	0.00	RX0	C
ATOM	250	CG	ARG	184	30.303	-22.149	36.755	1.00	0.00	RX0	C
ATOM	251	CD	ARG	184	30.858	-21.998	35.338	1.00	0.00	RX0	C
ATOM	252	NE	ARG	184	31.572	-20.725	35.273	1.00	0.00	RX0	N
ATOM	253	HE	ARG	184	31.002	-19.913	35.112	0.00	0.00	RX0	H
ATOM	254	CZ	ARG	184	32.861	-20.665	35.714	1.00	0.00	RX0	C
ATOM	255	NH1	ARG	184	33.605	-21.784	35.817	1.00	0.00	RX0	N
ATOM	256	HH11	ARG	184	34.576	-21.696	36.125	0.00	0.00	RX0	H
ATOM	257	HH12	ARG	184	33.273	-22.702	35.600	0.00	0.00	RX0	H
ATOM	258	NH2	ARG	184	33.376	-19.477	36.076	1.00	0.00	RX0	N
ATOM	259	HH21	ARG	184	34.329	-19.484	36.419	0.00	0.00	RX0	H
ATOM	260	HH22	ARG	184	32.886	-18.602	36.064	0.00	0.00	RX0	H
ATOM	261	C	ARG	184	29.836	-19.410	39.407	1.00	0.00	RX0	C
ATOM	262	O	ARG	184	29.116	-18.390	39.334	1.00	0.00	RX0	O
ATOM	263	N	PRO	185	30.940	-19.460	40.113	1.00	0.00	RX0	N
ATOM	264	CD	PRO	185	31.744	-20.665	40.290	1.00	0.00	RX0	C
ATOM	265	CA	PRO	185	31.574	-18.285	40.781	1.00	0.00	RX0	C
ATOM	266	CB	PRO	185	32.859	-18.871	41.379	1.00	0.00	RX0	C
ATOM	267	CG	PRO	185	33.143	-20.136	40.574	1.00	0.00	RX0	C
ATOM	268	C	PRO	185	31.820	-17.125	39.813	1.00	0.00	RX0	C
ATOM	269	O	PRO	185	31.836	-17.275	38.592	1.00	0.00	RX0	O
ATOM	270	N	PHE	186	32.164	-15.998	40.422	1.00	0.00	RX0	N
ATOM	271	H	PHE	186	32.239	-15.991	41.418	0.00	0.00	RX0	H
ATOM	272	CA	PHE	186	32.333	-14.726	39.697	1.00	0.00	RX0	C
ATOM	273	CB	PHE	186	31.516	-13.614	40.357	1.00	0.00	RX0	C
ATOM	274	CG	PHE	186	31.418	-12.439	39.413	1.00	0.00	RX0	C
ATOM	275	CD1	PHE	186	30.868	-12.613	38.148	1.00	0.00	RX0	C
ATOM	276	CD2	PHE	186	31.879	-11.187	39.803	1.00	0.00	RX0	C
ATOM	277	CE1	PHE	186	30.781	-11.538	37.272	1.00	0.00	RX0	C
ATOM	278	CE2	PHE	186	31.792	-10.111	38.927	1.00	0.00	RX0	C
ATOM	279	CZ	PHE	186	31.245	-10.286	37.661	1.00	0.00	RX0	C
ATOM	280	C	PHE	186	33.791	-14.305	39.507	1.00	0.00	RX0	C
ATOM	281	O	PHE	186	34.127	-13.678	38.496	1.00	0.00	RX0	O
ATOM	282	N	SER	187	34.655	-14.802	40.380	1.00	0.00	RX0	N
ATOM	283	H	SER	187	34.310	-15.346	41.140	0.00	0.00	RX0	H
ATOM	284	CA	SER	187	36.113	-14.542	40.380	1.00	0.00	RX0	C
ATOM	285	CB	SER	187	36.611	-15.271	41.614	1.00	0.00	RX0	C
ATOM	286	OG	SER	187	35.492	-15.364	42.514	1.00	0.00	RX0	O
ATOM	287	HG	SER	187	35.867	-15.410	43.386	0.00	0.00	RX0	H
ATOM	288	C	SER	187	36.764	-14.980	39.057	1.00	0.00	RX0	C
ATOM	289	O	SER	187	37.834	-14.531	38.683	1.00	0.00	RX0	O
ATOM	290	N	GLU	188	36.054	-15.878	38.369	1.00	0.00	RX0	N
ATOM	291	H	GLU	188	35.128	-16.106	38.658	0.00	0.00	RX0	H
ATOM	292	CA	GLU	188	36.561	-16.586	37.191	1.00	0.00	RX0	C
ATOM	293	CB	GLU	188	36.219	-18.056	37.353	1.00	0.00	RX0	C
ATOM	294	CG	GLU	188	36.975	-19.026	36.449	1.00	0.00	RX0	C
ATOM	295	CD	GLU	188	36.220	-20.326	36.549	1.00	0.00	RX0	C
ATOM	296	OE1	GLU	188	35.468	-20.487	37.504	1.00	0.00	RX0	O
ATOM	297	OE2	GLU	188	36.255	-21.130	35.628	1.00	0.00	RX0	O
ATOM	298	C	GLU	188	36.028	-16.018	35.856	1.00	0.00	RX0	C
ATOM	299	O	GLU	188	36.494	-16.416	34.788	1.00	0.00	RX0	O
ATOM	300	N	ALA	189	35.058	-15.107	35.914	1.00	0.00	RX0	N
ATOM	301	H	ALA	189	34.838	-14.675	36.792	0.00	0.00	RX0	H
ATOM	302	CA	ALA	189	34.543	-14.432	34.708	1.00	0.00	RX0	C
ATOM	303	CB	ALA	189	33.067	-14.082	34.892	1.00	0.00	RX0	C

ATOM	304	C	ALA	189	35.336	-13.151	34.407	1.00	0.00	RX0	C
ATOM	305	O	ALA	189	35.533	-12.292	35.270	1.00	0.00	RX0	O
ATOM	306	N	SER	190	35.819	-13.065	33.173	1.00	0.00	RX0	N
ATOM	307	H	SER	190	35.615	-13.816	32.549	0.00	0.00	RX0	H
ATOM	308	CA	SER	190	36.430	-11.825	32.646	1.00	0.00	RX0	C
ATOM	309	CB	SER	190	37.099	-12.195	31.297	1.00	0.00	RX0	C
ATOM	310	OG	SER	190	38.258	-11.391	30.967	1.00	0.00	RX0	O
ATOM	311	HG	SER	190	38.785	-11.429	31.766	0.00	0.00	RX0	H
ATOM	312	C	SER	190	35.341	-10.761	32.513	1.00	0.00	RX0	C
ATOM	313	O	SER	190	34.465	-10.869	31.639	1.00	0.00	RX0	O
ATOM	314	N	MET	191	35.401	-9.751	33.361	1.00	0.00	RX0	N
ATOM	315	H	MET	191	36.038	-9.820	34.130	0.00	0.00	RX0	H
ATOM	316	CA	MET	191	34.414	-8.652	33.337	1.00	0.00	RX0	C
ATOM	317	CB	MET	191	34.643	-7.665	34.478	1.00	0.00	RX0	C
ATOM	318	CG	MET	191	33.549	-6.597	34.497	1.00	0.00	RX0	C
ATOM	319	SD	MET	191	33.642	-5.541	35.945	1.00	0.00	RX0	S
ATOM	320	CE	MET	191	33.286	-6.802	37.180	1.00	0.00	RX0	C
ATOM	321	C	MET	191	34.384	-7.939	31.976	1.00	0.00	RX0	C
ATOM	322	O	MET	191	33.329	-7.841	31.363	1.00	0.00	RX0	O
ATOM	323	N	MET	192	35.577	-7.654	31.438	1.00	0.00	RX0	N
ATOM	324	H	MET	192	36.383	-7.740	32.020	0.00	0.00	RX0	H
ATOM	325	CA	MET	192	35.697	-7.082	30.089	1.00	0.00	RX0	C
ATOM	326	CB	MET	192	37.136	-6.648	29.804	1.00	0.00	RX0	C
ATOM	327	CG	MET	192	37.281	-5.928	28.460	1.00	0.00	RX0	C
ATOM	328	SD	MET	192	36.171	-4.519	28.287	1.00	0.00	RX0	S
ATOM	329	CE	MET	192	36.763	-3.524	29.666	1.00	0.00	RX0	C
ATOM	330	C	MET	192	35.151	-8.021	28.999	1.00	0.00	RX0	C
ATOM	331	O	MET	192	34.484	-7.587	28.093	1.00	0.00	RX0	O
ATOM	332	N	GLY	193	35.358	-9.342	29.220	1.00	0.00	RX0	N
ATOM	333	H	GLY	193	35.699	-9.610	30.117	0.00	0.00	RX0	H
ATOM	334	CA	GLY	193	34.804	-10.378	28.330	1.00	0.00	RX0	C
ATOM	335	C	GLY	193	33.267	-10.338	28.334	1.00	0.00	RX0	C
ATOM	336	O	GLY	193	32.637	-10.184	27.296	1.00	0.00	RX0	O
ATOM	337	N	LEU	194	32.696	-10.293	29.537	1.00	0.00	RX0	N
ATOM	338	H	LEU	194	33.263	-10.301	30.357	0.00	0.00	RX0	H
ATOM	339	CA	LEU	194	31.235	-10.169	29.722	1.00	0.00	RX0	C
ATOM	340	CB	LEU	194	30.848	-10.222	31.199	1.00	0.00	RX0	C
ATOM	341	CG	LEU	194	31.144	-11.559	31.870	1.00	0.00	RX0	C
ATOM	342	CD1	LEU	194	30.704	-11.543	33.334	1.00	0.00	RX0	C
ATOM	343	CD2	LEU	194	30.537	-12.730	31.097	1.00	0.00	RX0	C
ATOM	344	C	LEU	194	30.647	-8.891	29.116	1.00	0.00	RX0	C
ATOM	345	O	LEU	194	29.706	-8.959	28.317	1.00	0.00	RX0	O
ATOM	346	N	LEU	195	31.327	-7.782	29.364	1.00	0.00	RX0	N
ATOM	347	H	LEU	195	32.135	-7.840	29.946	0.00	0.00	RX0	H
ATOM	348	CA	LEU	195	30.920	-6.462	28.846	1.00	0.00	RX0	C
ATOM	349	CB	LEU	195	31.730	-5.338	29.494	1.00	0.00	RX0	C
ATOM	350	CG	LEU	195	31.538	-5.245	31.008	1.00	0.00	RX0	C
ATOM	351	CD1	LEU	195	32.371	-4.114	31.613	1.00	0.00	RX0	C
ATOM	352	CD2	LEU	195	30.064	-5.145	31.396	1.00	0.00	RX0	C
ATOM	353	C	LEU	195	31.020	-6.357	27.321	1.00	0.00	RX0	C
ATOM	354	O	LEU	195	30.051	-5.942	26.671	1.00	0.00	RX0	O
ATOM	355	N	THR	196	32.075	-6.931	26.767	1.00	0.00	RX0	N
ATOM	356	H	THR	196	32.808	-7.311	27.329	0.00	0.00	RX0	H
ATOM	357	CA	THR	196	32.335	-6.901	25.309	1.00	0.00	RX0	C
ATOM	358	CB	THR	196	33.782	-7.302	25.031	1.00	0.00	RX0	C
ATOM	359	OG1	THR	196	34.664	-6.395	25.707	1.00	0.00	RX0	O
ATOM	360	HG1	THR	196	34.381	-5.523	25.461	0.00	0.00	RX0	H
ATOM	361	CG2	THR	196	34.092	-7.339	23.534	1.00	0.00	RX0	C
ATOM	362	C	THR	196	31.317	-7.765	24.552	1.00	0.00	RX0	C
ATOM	363	O	THR	196	30.772	-7.327	23.532	1.00	0.00	RX0	O
ATOM	364	N	ASN	197	31.003	-8.928	25.107	1.00	0.00	RX0	N

ATOM	365	H	ASN	197	31.431	-9.177	25.978	0.00	0.00	RX0	H
ATOM	366	CA	ASN	197	30.010	-9.840	24.504	1.00	0.00	RX0	C
ATOM	367	CB	ASN	197	29.980	-11.209	25.183	1.00	0.00	RX0	C
ATOM	368	CG	ASN	197	31.011	-12.118	24.552	1.00	0.00	RX0	C
ATOM	369	OD1	ASN	197	30.835	-12.672	23.474	1.00	0.00	RX0	O
ATOM	370	ND2	ASN	197	32.120	-12.248	25.300	1.00	0.00	RX0	N
ATOM	371	HD21	ASN	197	32.207	-11.717	26.145	0.00	0.00	RX0	H
ATOM	372	HD22	ASN	197	32.854	-12.863	25.015	0.00	0.00	RX0	H
ATOM	373	C	ASN	197	28.594	-9.255	24.528	1.00	0.00	RX0	C
ATOM	374	O	ASN	197	27.900	-9.272	23.514	1.00	0.00	RX0	O
ATOM	375	N	LEU	198	28.277	-8.575	25.633	1.00	0.00	RX0	N
ATOM	376	H	LEU	198	28.923	-8.558	26.399	0.00	0.00	RX0	H
ATOM	377	CA	LEU	198	27.002	-7.850	25.760	1.00	0.00	RX0	C
ATOM	378	CB	LEU	198	26.850	-7.339	27.189	1.00	0.00	RX0	C
ATOM	379	CG	LEU	198	25.449	-6.823	27.500	1.00	0.00	RX0	C
ATOM	380	CD1	LEU	198	24.368	-7.872	27.230	1.00	0.00	RX0	C
ATOM	381	CD2	LEU	198	25.383	-6.296	28.930	1.00	0.00	RX0	C
ATOM	382	C	LEU	198	26.885	-6.719	24.724	1.00	0.00	RX0	C
ATOM	383	O	LEU	198	25.930	-6.676	23.947	1.00	0.00	RX0	O
ATOM	384	N	ALA	199	27.942	-5.914	24.641	1.00	0.00	RX0	N
ATOM	385	H	ALA	199	28.700	-6.057	25.279	0.00	0.00	RX0	H
ATOM	386	CA	ALA	199	28.029	-4.784	23.694	1.00	0.00	RX0	C
ATOM	387	CB	ALA	199	29.343	-4.024	23.882	1.00	0.00	RX0	C
ATOM	388	C	ALA	199	27.921	-5.230	22.227	1.00	0.00	RX0	C
ATOM	389	O	ALA	199	27.138	-4.660	21.467	1.00	0.00	RX0	O
ATOM	390	N	ASP	200	28.555	-6.360	21.908	1.00	0.00	RX0	N
ATOM	391	H	ASP	200	29.144	-6.807	22.583	0.00	0.00	RX0	H
ATOM	392	CA	ASP	200	28.494	-6.940	20.550	1.00	0.00	RX0	C
ATOM	393	CB	ASP	200	29.534	-8.051	20.372	1.00	0.00	RX0	C
ATOM	394	CG	ASP	200	30.225	-7.920	19.023	1.00	0.00	RX0	C
ATOM	395	OD1	ASP	200	30.631	-6.819	18.653	1.00	0.00	RX0	O
ATOM	396	OD2	ASP	200	30.418	-8.926	18.341	1.00	0.00	RX0	O
ATOM	397	C	ASP	200	27.084	-7.410	20.171	1.00	0.00	RX0	C
ATOM	398	O	ASP	200	26.604	-7.102	19.080	1.00	0.00	RX0	O
ATOM	399	N	ARG	201	26.390	-7.998	21.143	1.00	0.00	RX0	N
ATOM	400	H	ARG	201	26.849	-8.157	22.021	0.00	0.00	RX0	H
ATOM	401	CA	ARG	201	24.992	-8.434	20.957	1.00	0.00	RX0	C
ATOM	402	CB	ARG	201	24.551	-9.435	22.006	1.00	0.00	RX0	C
ATOM	403	CG	ARG	201	24.627	-10.848	21.432	1.00	0.00	RX0	C
ATOM	404	CD	ARG	201	23.864	-11.869	22.275	1.00	0.00	RX0	C
ATOM	405	NE	ARG	201	24.441	-12.013	23.608	1.00	0.00	RX0	N
ATOM	406	HE	ARG	201	25.207	-12.671	23.721	0.00	0.00	RX0	H
ATOM	407	CZ	ARG	201	23.892	-11.406	24.695	1.00	0.00	RX0	C
ATOM	408	NH1	ARG	201	22.776	-10.661	24.559	1.00	0.00	RX0	N
ATOM	409	HH11	ARG	201	22.358	-10.201	25.344	0.00	0.00	RX0	H
ATOM	410	HH12	ARG	201	22.341	-10.549	23.662	0.00	0.00	RX0	H
ATOM	411	NH2	ARG	201	24.467	-11.562	25.895	1.00	0.00	RX0	N
ATOM	412	HH21	ARG	201	24.104	-11.194	26.756	0.00	0.00	RX0	H
ATOM	413	HH22	ARG	201	25.306	-12.126	25.950	0.00	0.00	RX0	H
ATOM	414	C	ARG	201	23.991	-7.279	20.827	1.00	0.00	RX0	C
ATOM	415	O	ARG	201	23.123	-7.308	19.955	1.00	0.00	RX0	O
ATOM	416	N	GLU	202	24.240	-6.201	21.568	1.00	0.00	RX0	N
ATOM	417	H	GLU	202	25.010	-6.214	22.211	0.00	0.00	RX0	H
ATOM	418	CA	GLU	202	23.401	-4.988	21.493	1.00	0.00	RX0	C
ATOM	419	CB	GLU	202	23.558	-4.079	22.733	1.00	0.00	RX0	C
ATOM	420	CG	GLU	202	22.916	-4.694	23.995	1.00	0.00	RX0	C
ATOM	421	CD	GLU	202	22.872	-3.749	25.198	1.00	0.00	RX0	C
ATOM	422	OE1	GLU	202	21.856	-3.087	25.422	1.00	0.00	RX0	O
ATOM	423	OE2	GLU	202	23.820	-3.717	25.976	1.00	0.00	RX0	O
ATOM	424	C	GLU	202	23.526	-4.262	20.149	1.00	0.00	RX0	C
ATOM	425	O	GLU	202	22.539	-3.754	19.625	1.00	0.00	RX0	O

ATOM	426	N	LEU	203	24.712	-4.358	19.546	1.00	0.00	RX0	N
ATOM	427	H	LEU	203	25.464	-4.809	20.035	0.00	0.00	RX0	H
ATOM	428	CA	LEU	203	25.004	-3.680	18.270	1.00	0.00	RX0	C
ATOM	429	CB	LEU	203	26.480	-3.871	17.917	1.00	0.00	RX0	C
ATOM	430	CG	LEU	203	26.921	-3.124	16.656	1.00	0.00	RX0	C
ATOM	431	CD1	LEU	203	26.718	-1.613	16.780	1.00	0.00	RX0	C
ATOM	432	CD2	LEU	203	28.358	-3.476	16.271	1.00	0.00	RX0	C
ATOM	433	C	LEU	203	24.099	-4.160	17.127	1.00	0.00	RX0	C
ATOM	434	O	LEU	203	23.593	-3.349	16.346	1.00	0.00	RX0	O
ATOM	435	N	VAL	204	23.782	-5.447	17.151	1.00	0.00	RX0	N
ATOM	436	H	VAL	204	24.176	-6.003	17.886	0.00	0.00	RX0	H
ATOM	437	CA	VAL	204	22.925	-6.083	16.127	1.00	0.00	RX0	C
ATOM	438	CB	VAL	204	22.898	-7.597	16.334	1.00	0.00	RX0	C
ATOM	439	CG1	VAL	204	22.009	-8.279	15.293	1.00	0.00	RX0	C
ATOM	440	CG2	VAL	204	24.320	-8.163	16.351	1.00	0.00	RX0	C
ATOM	441	C	VAL	204	21.502	-5.497	16.213	1.00	0.00	RX0	C
ATOM	442	O	VAL	204	20.938	-5.041	15.221	1.00	0.00	RX0	O
ATOM	443	N	HIS	205	21.015	-5.393	17.448	1.00	0.00	RX0	N
ATOM	444	H	HIS	205	21.605	-5.673	18.208	0.00	0.00	RX0	H
ATOM	445	CA	HIS	205	19.703	-4.786	17.746	1.00	0.00	RX0	C
ATOM	446	CB	HIS	205	19.274	-5.101	19.182	1.00	0.00	RX0	C
ATOM	447	CG	HIS	205	19.002	-6.581	19.309	1.00	0.00	RX0	C
ATOM	448	ND1	HIS	205	17.799	-7.140	19.083	1.00	0.00	RX0	N
ATOM	449	HD1	HIS	205	16.975	-6.673	18.832	0.00	0.00	RX0	H
ATOM	450	CD2	HIS	205	19.904	-7.588	19.661	1.00	0.00	RX0	C
ATOM	451	NE2	HIS	205	19.230	-8.763	19.645	1.00	0.00	RX0	N
ATOM	452	CE1	HIS	205	17.936	-8.489	19.289	1.00	0.00	RX0	C
ATOM	453	C	HIS	205	19.668	-3.277	17.476	1.00	0.00	RX0	C
ATOM	454	O	HIS	205	18.642	-2.756	17.030	1.00	0.00	RX0	O
ATOM	455	N	MET	206	20.820	-2.627	17.609	1.00	0.00	RX0	N
ATOM	456	H	MET	206	21.611	-3.124	17.973	0.00	0.00	RX0	H
ATOM	457	CA	MET	206	20.969	-1.185	17.340	1.00	0.00	RX0	C
ATOM	458	CB	MET	206	22.357	-0.694	17.746	1.00	0.00	RX0	C
ATOM	459	CG	MET	206	22.540	0.806	17.512	1.00	0.00	RX0	C
ATOM	460	SD	MET	206	24.240	1.328	17.775	1.00	0.00	RX0	S
ATOM	461	CE	MET	206	24.495	0.519	19.359	1.00	0.00	RX0	C
ATOM	462	C	MET	206	20.721	-0.870	15.856	1.00	0.00	RX0	C
ATOM	463	O	MET	206	20.035	0.103	15.544	1.00	0.00	RX0	O
ATOM	464	N	ILE	207	21.183	-1.758	14.977	1.00	0.00	RX0	N
ATOM	465	H	ILE	207	21.732	-2.517	15.339	0.00	0.00	RX0	H
ATOM	466	CA	ILE	207	20.975	-1.628	13.516	1.00	0.00	RX0	C
ATOM	467	CB	ILE	207	21.670	-2.792	12.803	1.00	0.00	RX0	C
ATOM	468	CG2	ILE	207	21.417	-2.776	11.297	1.00	0.00	RX0	C
ATOM	469	CG1	ILE	207	23.164	-2.811	13.124	1.00	0.00	RX0	C
ATOM	470	CD1	ILE	207	23.906	-1.605	12.551	1.00	0.00	RX0	C
ATOM	471	C	ILE	207	19.470	-1.621	13.197	1.00	0.00	RX0	C
ATOM	472	O	ILE	207	18.988	-0.755	12.467	1.00	0.00	RX0	O
ATOM	473	N	ASN	208	18.761	-2.558	13.816	1.00	0.00	RX0	N
ATOM	474	H	ASN	208	19.238	-3.125	14.491	0.00	0.00	RX0	H
ATOM	475	CA	ASN	208	17.313	-2.734	13.586	1.00	0.00	RX0	C
ATOM	476	CB	ASN	208	16.802	-4.071	14.120	1.00	0.00	RX0	C
ATOM	477	CG	ASN	208	17.153	-5.161	13.121	1.00	0.00	RX0	C
ATOM	478	OD1	ASN	208	17.541	-4.906	11.976	1.00	0.00	RX0	O
ATOM	479	ND2	ASN	208	17.014	-6.401	13.621	1.00	0.00	RX0	N
ATOM	480	HD21	ASN	208	16.687	-6.537	14.558	0.00	0.00	RX0	H
ATOM	481	HD22	ASN	208	17.232	-7.229	13.103	0.00	0.00	RX0	H
ATOM	482	C	ASN	208	16.516	-1.532	14.103	1.00	0.00	RX0	C
ATOM	483	O	ASN	208	15.637	-1.017	13.417	1.00	0.00	RX0	O
ATOM	484	N	TRP	209	16.982	-1.008	15.238	1.00	0.00	RX0	N
ATOM	485	H	TRP	209	17.725	-1.474	15.722	0.00	0.00	RX0	H
ATOM	486	CA	TRP	209	16.425	0.201	15.864	1.00	0.00	RX0	C

ATOM	487	CB	TRP	209	17.093	0.420	17.231	1.00	0.00	RX0	C
ATOM	488	CG	TRP	209	16.695	1.749	17.836	1.00	0.00	RX0	C
ATOM	489	CD2	TRP	209	17.414	3.001	17.807	1.00	0.00	RX0	C
ATOM	490	CE2	TRP	209	16.632	3.962	18.493	1.00	0.00	RX0	C
ATOM	491	CE3	TRP	209	18.634	3.371	17.254	1.00	0.00	RX0	C
ATOM	492	CD1	TRP	209	15.525	2.030	18.551	1.00	0.00	RX0	C
ATOM	493	NE1	TRP	209	15.477	3.328	18.947	1.00	0.00	RX0	N
ATOM	494	HE1	TRP	209	14.720	3.720	19.449	0.00	0.00	RX0	H
ATOM	495	CZ2	TRP	209	17.096	5.268	18.596	1.00	0.00	RX0	C
ATOM	496	CZ3	TRP	209	19.087	4.679	17.371	1.00	0.00	RX0	C
ATOM	497	CH2	TRP	209	18.316	5.626	18.034	1.00	0.00	RX0	C
ATOM	498	C	TRP	209	16.619	1.438	14.972	1.00	0.00	RX0	C
ATOM	499	O	TRP	209	15.652	2.120	14.634	1.00	0.00	RX0	O
ATOM	500	N	ALA	210	17.853	1.624	14.503	1.00	0.00	RX0	N
ATOM	501	H	ALA	210	18.567	0.988	14.791	0.00	0.00	RX0	H
ATOM	502	CA	ALA	210	18.233	2.756	13.635	1.00	0.00	RX0	C
ATOM	503	CB	ALA	210	19.715	2.665	13.272	1.00	0.00	RX0	C
ATOM	504	C	ALA	210	17.400	2.800	12.347	1.00	0.00	RX0	C
ATOM	505	O	ALA	210	16.892	3.855	11.979	1.00	0.00	RX0	O
ATOM	506	N	LYS	211	17.095	1.613	11.820	1.00	0.00	RX0	N
ATOM	507	H	LYS	211	17.503	0.799	12.236	0.00	0.00	RX0	H
ATOM	508	CA	LYS	211	16.258	1.472	10.614	1.00	0.00	RX0	C
ATOM	509	CB	LYS	211	16.400	0.065	10.029	1.00	0.00	RX0	C
ATOM	510	CG	LYS	211	17.792	-0.113	9.409	1.00	0.00	RX0	C
ATOM	511	CD	LYS	211	18.062	-1.522	8.875	1.00	0.00	RX0	C
ATOM	512	CE	LYS	211	17.850	-2.547	9.983	1.00	0.00	RX0	C
ATOM	513	NZ	LYS	211	18.351	-3.881	9.621	1.00	0.00	RX0	N
ATOM	514	HZ1	LYS	211	18.173	-4.511	10.437	0.00	0.00	RX0	H
ATOM	515	HZ2	LYS	211	17.848	-4.244	8.790	0.00	0.00	RX0	H
ATOM	516	HZ3	LYS	211	19.372	-3.851	9.425	0.00	0.00	RX0	H
ATOM	517	C	LYS	211	14.795	1.898	10.823	1.00	0.00	RX0	C
ATOM	518	O	LYS	211	14.129	2.337	9.881	1.00	0.00	RX0	O
ATOM	519	N	ARG	212	14.357	1.870	12.071	1.00	0.00	RX0	N
ATOM	520	H	ARG	212	14.980	1.608	12.810	0.00	0.00	RX0	H
ATOM	521	CA	ARG	212	13.005	2.320	12.466	1.00	0.00	RX0	C
ATOM	522	CB	ARG	212	12.377	1.397	13.507	1.00	0.00	RX0	C
ATOM	523	CG	ARG	212	12.523	-0.080	13.139	1.00	0.00	RX0	C
ATOM	524	CD	ARG	212	11.655	-1.003	13.998	1.00	0.00	RX0	C
ATOM	525	NE	ARG	212	10.244	-0.799	13.674	1.00	0.00	RX0	N
ATOM	526	HE	ARG	212	10.037	-0.578	12.717	0.00	0.00	RX0	H
ATOM	527	CZ	ARG	212	9.285	-0.879	14.645	1.00	0.00	RX0	C
ATOM	528	NH1	ARG	212	9.622	-1.190	15.911	1.00	0.00	RX0	N
ATOM	529	HH11	ARG	212	8.911	-1.200	16.643	0.00	0.00	RX0	H
ATOM	530	HH12	ARG	212	10.552	-1.401	16.213	0.00	0.00	RX0	H
ATOM	531	NH2	ARG	212	8.004	-0.630	14.326	1.00	0.00	RX0	N
ATOM	532	HH21	ARG	212	7.299	-0.612	15.064	0.00	0.00	RX0	H
ATOM	533	HH22	ARG	212	7.659	-0.419	13.413	0.00	0.00	RX0	H
ATOM	534	C	ARG	212	12.933	3.790	12.894	1.00	0.00	RX0	C
ATOM	535	O	ARG	212	11.827	4.344	12.989	1.00	0.00	RX0	O
ATOM	536	N	VAL	213	14.074	4.417	13.148	1.00	0.00	RX0	N
ATOM	537	H	VAL	213	14.933	3.928	12.995	0.00	0.00	RX0	H
ATOM	538	CA	VAL	213	14.155	5.874	13.374	1.00	0.00	RX0	C
ATOM	539	CB	VAL	213	15.581	6.281	13.748	1.00	0.00	RX0	C
ATOM	540	CG1	VAL	213	15.751	7.800	13.839	1.00	0.00	RX0	C
ATOM	541	CG2	VAL	213	15.979	5.583	15.046	1.00	0.00	RX0	C
ATOM	542	C	VAL	213	13.672	6.590	12.095	1.00	0.00	RX0	C
ATOM	543	O	VAL	213	14.288	6.431	11.023	1.00	0.00	RX0	O
ATOM	544	N	PRO	214	12.622	7.395	12.213	1.00	0.00	RX0	N
ATOM	545	CD	PRO	214	11.896	7.609	13.458	1.00	0.00	RX0	C
ATOM	546	CA	PRO	214	12.035	8.145	11.084	1.00	0.00	RX0	C
ATOM	547	CB	PRO	214	10.927	8.954	11.761	1.00	0.00	RX0	C

ATOM	548	CG	PRO	214	10.552	8.163	13.010	1.00	0.00	RX0	C
ATOM	549	C	PRO	214	13.102	9.001	10.387	1.00	0.00	RX0	C
ATOM	550	O	PRO	214	13.853	9.727	11.025	1.00	0.00	RX0	O
ATOM	551	N	GLY	215	13.244	8.730	9.080	1.00	0.00	RX0	N
ATOM	552	H	GLY	215	12.750	7.982	8.636	0.00	0.00	RX0	H
ATOM	553	CA	GLY	215	14.194	9.473	8.227	1.00	0.00	RX0	C
ATOM	554	C	GLY	215	15.511	8.732	7.950	1.00	0.00	RX0	C
ATOM	555	O	GLY	215	16.085	8.889	6.862	1.00	0.00	RX0	O
ATOM	556	N	PHE	216	15.917	7.845	8.848	1.00	0.00	RX0	N
ATOM	557	H	PHE	216	15.329	7.653	9.637	0.00	0.00	RX0	H
ATOM	558	CA	PHE	216	17.224	7.160	8.764	1.00	0.00	RX0	C
ATOM	559	CB	PHE	216	17.451	6.293	9.997	1.00	0.00	RX0	C
ATOM	560	CG	PHE	216	18.888	5.835	10.053	1.00	0.00	RX0	C
ATOM	561	CD1	PHE	216	19.898	6.755	10.305	1.00	0.00	RX0	C
ATOM	562	CD2	PHE	216	19.201	4.493	9.866	1.00	0.00	RX0	C
ATOM	563	CE1	PHE	216	21.216	6.330	10.410	1.00	0.00	RX0	C
ATOM	564	CE2	PHE	216	20.519	4.067	9.973	1.00	0.00	RX0	C
ATOM	565	CZ	PHE	216	21.523	4.983	10.266	1.00	0.00	RX0	C
ATOM	566	C	PHE	216	17.435	6.347	7.474	1.00	0.00	RX0	C
ATOM	567	O	PHE	216	18.358	6.641	6.711	1.00	0.00	RX0	O
ATOM	568	N	VAL	217	16.482	5.483	7.151	1.00	0.00	RX0	N
ATOM	569	H	VAL	217	15.673	5.450	7.736	0.00	0.00	RX0	H
ATOM	570	CA	VAL	217	16.570	4.608	5.958	1.00	0.00	RX0	C
ATOM	571	CB	VAL	217	15.528	3.494	6.008	1.00	0.00	RX0	C
ATOM	572	CG1	VAL	217	15.900	2.479	7.079	1.00	0.00	RX0	C
ATOM	573	CG2	VAL	217	14.111	4.050	6.172	1.00	0.00	RX0	C
ATOM	574	C	VAL	217	16.469	5.342	4.608	1.00	0.00	RX0	C
ATOM	575	O	VAL	217	16.660	4.747	3.556	1.00	0.00	RX0	O
ATOM	576	N	ASP	218	16.058	6.613	4.671	1.00	0.00	RX0	N
ATOM	577	H	ASP	218	15.770	7.063	5.519	0.00	0.00	RX0	H
ATOM	578	CA	ASP	218	16.006	7.470	3.472	1.00	0.00	RX0	C
ATOM	579	CB	ASP	218	15.079	8.660	3.758	1.00	0.00	RX0	C
ATOM	580	CG	ASP	218	13.736	8.210	4.334	1.00	0.00	RX0	C
ATOM	581	OD1	ASP	218	12.738	8.287	3.620	1.00	0.00	RX0	O
ATOM	582	OD2	ASP	218	13.671	7.813	5.503	1.00	0.00	RX0	O
ATOM	583	C	ASP	218	17.401	7.924	3.023	1.00	0.00	RX0	C
ATOM	584	O	ASP	218	17.595	8.369	1.896	1.00	0.00	RX0	O
ATOM	585	N	LEU	219	18.344	7.857	3.967	1.00	0.00	RX0	N
ATOM	586	H	LEU	219	18.096	7.493	4.865	0.00	0.00	RX0	H
ATOM	587	CA	LEU	219	19.767	8.083	3.697	1.00	0.00	RX0	C
ATOM	588	CB	LEU	219	20.550	8.256	4.997	1.00	0.00	RX0	C
ATOM	589	CG	LEU	219	19.934	9.332	5.894	1.00	0.00	RX0	C
ATOM	590	CD1	LEU	219	20.554	9.323	7.289	1.00	0.00	RX0	C
ATOM	591	CD2	LEU	219	19.964	10.719	5.250	1.00	0.00	RX0	C
ATOM	592	C	LEU	219	20.350	6.970	2.832	1.00	0.00	RX0	C
ATOM	593	O	LEU	219	19.843	5.836	2.789	1.00	0.00	RX0	O
ATOM	594	N	THR	220	21.442	7.293	2.191	1.00	0.00	RX0	N
ATOM	595	H	THR	220	21.726	8.256	2.262	0.00	0.00	RX0	H
ATOM	596	CA	THR	220	22.263	6.312	1.453	1.00	0.00	RX0	C
ATOM	597	CB	THR	220	23.375	6.911	0.580	1.00	0.00	RX0	C
ATOM	598	OG1	THR	220	24.431	7.497	1.347	1.00	0.00	RX0	O
ATOM	599	HG1	THR	220	24.081	8.295	1.770	0.00	0.00	RX0	H
ATOM	600	CG2	THR	220	22.822	7.871	-0.466	1.00	0.00	RX0	C
ATOM	601	C	THR	220	22.829	5.303	2.454	1.00	0.00	RX0	C
ATOM	602	O	THR	220	23.112	5.646	3.611	1.00	0.00	RX0	O
ATOM	603	N	LEU	221	23.130	4.116	1.957	1.00	0.00	RX0	N
ATOM	604	H	LEU	221	22.804	3.912	1.036	0.00	0.00	RX0	H
ATOM	605	CA	LEU	221	23.750	3.065	2.781	1.00	0.00	RX0	C
ATOM	606	CB	LEU	221	23.936	1.845	1.884	1.00	0.00	RX0	C
ATOM	607	CG	LEU	221	24.599	0.639	2.539	1.00	0.00	RX0	C
ATOM	608	CD1	LEU	221	23.730	0.049	3.645	1.00	0.00	RX0	C

ATOM	609	CD2	LEU	221	25.006	-0.391	1.490	1.00	0.00	RX0	C
ATOM	610	C	LEU	221	25.092	3.509	3.394	1.00	0.00	RX0	C
ATOM	611	O	LEU	221	25.324	3.347	4.578	1.00	0.00	RX0	O
ATOM	612	N	HIS	222	25.854	4.270	2.593	1.00	0.00	RX0	N
ATOM	613	H	HIS	222	25.503	4.469	1.682	0.00	0.00	RX0	H
ATOM	614	CA	HIS	222	27.131	4.847	3.045	1.00	0.00	RX0	C
ATOM	615	CB	HIS	222	27.624	5.677	1.848	1.00	0.00	RX0	C
ATOM	616	CG	HIS	222	28.827	6.564	2.104	1.00	0.00	RX0	C
ATOM	617	ND1	HIS	222	30.089	6.196	1.832	1.00	0.00	RX0	N
ATOM	618	HD1	HIS	222	30.376	5.324	1.483	0.00	0.00	RX0	H
ATOM	619	CD2	HIS	222	28.843	7.884	2.571	1.00	0.00	RX0	C
ATOM	620	NE2	HIS	222	30.131	8.302	2.567	1.00	0.00	RX0	N
ATOM	621	CE1	HIS	222	30.897	7.265	2.111	1.00	0.00	RX0	C
ATOM	622	C	HIS	222	26.942	5.765	4.264	1.00	0.00	RX0	C
ATOM	623	O	HIS	222	27.673	5.642	5.246	1.00	0.00	RX0	O
ATOM	624	N	ASP	223	25.958	6.658	4.167	1.00	0.00	RX0	N
ATOM	625	H	ASP	223	25.442	6.783	3.317	0.00	0.00	RX0	H
ATOM	626	CA	ASP	223	25.694	7.636	5.242	1.00	0.00	RX0	C
ATOM	627	CB	ASP	223	24.827	8.807	4.780	1.00	0.00	RX0	C
ATOM	628	CG	ASP	223	25.663	9.686	3.873	1.00	0.00	RX0	C
ATOM	629	OD1	ASP	223	26.021	10.797	4.252	1.00	0.00	RX0	O
ATOM	630	OD2	ASP	223	25.990	9.253	2.777	1.00	0.00	RX0	O
ATOM	631	C	ASP	223	25.148	6.996	6.516	1.00	0.00	RX0	C
ATOM	632	O	ASP	223	25.558	7.375	7.616	1.00	0.00	RX0	O
ATOM	633	N	GLN	224	24.393	5.921	6.332	1.00	0.00	RX0	N
ATOM	634	H	GLN	224	24.133	5.692	5.391	0.00	0.00	RX0	H
ATOM	635	CA	GLN	224	23.868	5.118	7.452	1.00	0.00	RX0	C
ATOM	636	CB	GLN	224	22.853	4.089	6.956	1.00	0.00	RX0	C
ATOM	637	CG	GLN	224	21.597	4.752	6.385	1.00	0.00	RX0	C
ATOM	638	CD	GLN	224	20.574	3.691	6.044	1.00	0.00	RX0	C
ATOM	639	OE1	GLN	224	20.456	2.671	6.717	1.00	0.00	RX0	O
ATOM	640	NE2	GLN	224	19.840	3.981	4.956	1.00	0.00	RX0	N
ATOM	641	HE21	GLN	224	19.976	4.835	4.443	0.00	0.00	RX0	H
ATOM	642	HE22	GLN	224	19.116	3.399	4.589	0.00	0.00	RX0	H
ATOM	643	C	GLN	224	25.003	4.453	8.243	1.00	0.00	RX0	C
ATOM	644	O	GLN	224	25.073	4.591	9.468	1.00	0.00	RX0	O
ATOM	645	N	VAL	225	25.993	3.956	7.505	1.00	0.00	RX0	N
ATOM	646	H	VAL	225	25.892	3.982	6.508	0.00	0.00	RX0	H
ATOM	647	CA	VAL	225	27.191	3.319	8.093	1.00	0.00	RX0	C
ATOM	648	CB	VAL	225	28.039	2.566	7.062	1.00	0.00	RX0	C
ATOM	649	CG1	VAL	225	29.172	1.810	7.754	1.00	0.00	RX0	C
ATOM	650	CG2	VAL	225	27.194	1.581	6.258	1.00	0.00	RX0	C
ATOM	651	C	VAL	225	28.021	4.368	8.852	1.00	0.00	RX0	C
ATOM	652	O	VAL	225	28.415	4.141	9.995	1.00	0.00	RX0	O
ATOM	653	N	HIS	226	28.182	5.534	8.231	1.00	0.00	RX0	N
ATOM	654	H	HIS	226	27.798	5.635	7.313	0.00	0.00	RX0	H
ATOM	655	CA	HIS	226	28.959	6.641	8.815	1.00	0.00	RX0	C
ATOM	656	CB	HIS	226	29.007	7.785	7.791	1.00	0.00	RX0	C
ATOM	657	CG	HIS	226	30.014	8.873	8.115	1.00	0.00	RX0	C
ATOM	658	ND1	HIS	226	31.019	9.194	7.280	1.00	0.00	RX0	N
ATOM	659	HD1	HIS	226	31.208	8.776	6.415	0.00	0.00	RX0	H
ATOM	660	CD2	HIS	226	30.069	9.730	9.222	1.00	0.00	RX0	C
ATOM	661	NE2	HIS	226	31.119	10.571	9.039	1.00	0.00	RX0	N
ATOM	662	CE1	HIS	226	31.702	10.238	7.844	1.00	0.00	RX0	C
ATOM	663	C	HIS	226	28.363	7.118	10.150	1.00	0.00	RX0	C
ATOM	664	O	HIS	226	29.071	7.189	11.155	1.00	0.00	RX0	O
ATOM	665	N	LEU	227	27.047	7.317	10.166	1.00	0.00	RX0	N
ATOM	666	H	LEU	227	26.522	7.185	9.321	0.00	0.00	RX0	H
ATOM	667	CA	LEU	227	26.344	7.787	11.375	1.00	0.00	RX0	C
ATOM	668	CB	LEU	227	24.877	8.088	11.076	1.00	0.00	RX0	C
ATOM	669	CG	LEU	227	24.682	9.371	10.271	1.00	0.00	RX0	C

ATOM	670	CD1	LEU	227	23.213	9.596	9.926	1.00	0.00	RX0	C
ATOM	671	CD2	LEU	227	25.272	10.584	10.988	1.00	0.00	RX0	C
ATOM	672	C	LEU	227	26.435	6.799	12.540	1.00	0.00	RX0	C
ATOM	673	O	LEU	227	26.853	7.165	13.635	1.00	0.00	RX0	O
ATOM	674	N	LEU	228	26.270	5.522	12.200	1.00	0.00	RX0	N
ATOM	675	H	LEU	228	26.043	5.298	11.248	0.00	0.00	RX0	H
ATOM	676	CA	LEU	228	26.384	4.431	13.181	1.00	0.00	RX0	C
ATOM	677	CB	LEU	228	25.763	3.155	12.620	1.00	0.00	RX0	C
ATOM	678	CG	LEU	228	24.257	3.145	12.879	1.00	0.00	RX0	C
ATOM	679	CD1	LEU	228	23.514	2.133	12.013	1.00	0.00	RX0	C
ATOM	680	CD2	LEU	228	23.964	2.944	14.365	1.00	0.00	RX0	C
ATOM	681	C	LEU	228	27.805	4.188	13.685	1.00	0.00	RX0	C
ATOM	682	O	LEU	228	28.004	4.019	14.891	1.00	0.00	RX0	O
ATOM	683	N	GLU	229	28.784	4.376	12.809	1.00	0.00	RX0	N
ATOM	684	H	GLU	229	28.568	4.555	11.846	0.00	0.00	RX0	H
ATOM	685	CA	GLU	229	30.199	4.229	13.197	1.00	0.00	RX0	C
ATOM	686	CB	GLU	229	31.147	4.063	12.019	1.00	0.00	RX0	C
ATOM	687	CG	GLU	229	32.341	3.224	12.498	1.00	0.00	RX0	C
ATOM	688	CD	GLU	229	33.210	2.736	11.356	1.00	0.00	RX0	C
ATOM	689	OE1	GLU	229	33.016	3.177	10.228	1.00	0.00	RX0	O
ATOM	690	OE2	GLU	229	34.094	1.914	11.603	1.00	0.00	RX0	O
ATOM	691	C	GLU	229	30.618	5.338	14.175	1.00	0.00	RX0	C
ATOM	692	O	GLU	229	31.393	5.088	15.099	1.00	0.00	RX0	O
ATOM	693	N	CYS	230	30.060	6.523	13.970	1.00	0.00	RX0	N
ATOM	694	H	CYS	230	29.478	6.646	13.165	0.00	0.00	RX0	H
ATOM	695	CA	CYS	230	30.321	7.692	14.829	1.00	0.00	RX0	C
ATOM	696	CB	CYS	230	30.017	8.977	14.063	1.00	0.00	RX0	C
ATOM	697	SG	CYS	230	31.102	9.206	12.632	1.00	0.00	RX0	S
ATOM	698	C	CYS	230	29.592	7.653	16.182	1.00	0.00	RX0	C
ATOM	699	O	CYS	230	30.123	8.119	17.188	1.00	0.00	RX0	O
ATOM	700	N	ALA	231	28.434	7.000	16.215	1.00	0.00	RX0	N
ATOM	701	H	ALA	231	28.113	6.530	15.390	0.00	0.00	RX0	H
ATOM	702	CA	ALA	231	27.494	7.141	17.345	1.00	0.00	RX0	C
ATOM	703	CB	ALA	231	26.144	7.665	16.852	1.00	0.00	RX0	C
ATOM	704	C	ALA	231	27.249	5.885	18.186	1.00	0.00	RX0	C
ATOM	705	O	ALA	231	26.768	6.021	19.321	1.00	0.00	RX0	O
ATOM	706	N	TRP	232	27.687	4.719	17.731	1.00	0.00	RX0	N
ATOM	707	H	TRP	232	28.108	4.685	16.821	0.00	0.00	RX0	H
ATOM	708	CA	TRP	232	27.348	3.433	18.379	1.00	0.00	RX0	C
ATOM	709	CB	TRP	232	27.971	2.235	17.648	1.00	0.00	RX0	C
ATOM	710	CG	TRP	232	29.476	2.276	17.752	1.00	0.00	RX0	C
ATOM	711	CD2	TRP	232	30.335	1.559	18.666	1.00	0.00	RX0	C
ATOM	712	CE2	TRP	232	31.666	1.962	18.386	1.00	0.00	RX0	C
ATOM	713	CE3	TRP	232	30.072	0.620	19.668	1.00	0.00	RX0	C
ATOM	714	CD1	TRP	232	30.338	3.066	16.984	1.00	0.00	RX0	C
ATOM	715	NE1	TRP	232	31.631	2.892	17.352	1.00	0.00	RX0	N
ATOM	716	HE1	TRP	232	32.389	3.349	16.926	0.00	0.00	RX0	H
ATOM	717	CZ2	TRP	232	32.687	1.403	19.154	1.00	0.00	RX0	C
ATOM	718	CZ3	TRP	232	31.098	0.062	20.432	1.00	0.00	RX0	C
ATOM	719	CH2	TRP	232	32.405	0.467	20.155	1.00	0.00	RX0	C
ATOM	720	C	TRP	232	27.676	3.373	19.884	1.00	0.00	RX0	C
ATOM	721	O	TRP	232	26.862	2.914	20.672	1.00	0.00	RX0	O
ATOM	722	N	LEU	233	28.801	3.989	20.277	1.00	0.00	RX0	N
ATOM	723	H	LEU	233	29.371	4.441	19.592	0.00	0.00	RX0	H
ATOM	724	CA	LEU	233	29.211	3.960	21.691	1.00	0.00	RX0	C
ATOM	725	CB	LEU	233	30.722	4.141	21.827	1.00	0.00	RX0	C
ATOM	726	CG	LEU	233	31.212	3.776	23.229	1.00	0.00	RX0	C
ATOM	727	CD1	LEU	233	30.803	2.355	23.620	1.00	0.00	RX0	C
ATOM	728	CD2	LEU	233	32.715	3.995	23.387	1.00	0.00	RX0	C
ATOM	729	C	LEU	233	28.415	4.936	22.566	1.00	0.00	RX0	C
ATOM	730	O	LEU	233	27.943	4.566	23.634	1.00	0.00	RX0	O

ATOM	731	N	GLU	234	28.150	6.122	22.016	1.00	0.00	RX0	N
ATOM	732	H	GLU	234	28.478	6.311	21.093	0.00	0.00	RX0	H
ATOM	733	CA	GLU	234	27.227	7.090	22.644	1.00	0.00	RX0	C
ATOM	734	CB	GLU	234	27.039	8.337	21.783	1.00	0.00	RX0	C
ATOM	735	CG	GLU	234	28.186	9.337	21.786	1.00	0.00	RX0	C
ATOM	736	CD	GLU	234	27.844	10.524	20.916	1.00	0.00	RX0	C
ATOM	737	OE1	GLU	234	27.723	11.630	21.427	1.00	0.00	RX0	O
ATOM	738	OE2	GLU	234	27.744	10.373	19.707	1.00	0.00	RX0	O
ATOM	739	C	GLU	234	25.830	6.482	22.841	1.00	0.00	RX0	C
ATOM	740	O	GLU	234	25.253	6.598	23.926	1.00	0.00	RX0	O
ATOM	741	N	ILE	235	25.389	5.711	21.848	1.00	0.00	RX0	N
ATOM	742	H	ILE	235	25.963	5.633	21.032	0.00	0.00	RX0	H
ATOM	743	CA	ILE	235	24.069	5.045	21.863	1.00	0.00	RX0	C
ATOM	744	CB	ILE	235	23.697	4.487	20.486	1.00	0.00	RX0	C
ATOM	745	CG2	ILE	235	22.411	3.660	20.553	1.00	0.00	RX0	C
ATOM	746	CG1	ILE	235	23.558	5.617	19.466	1.00	0.00	RX0	C
ATOM	747	CD1	ILE	235	23.218	5.101	18.068	1.00	0.00	RX0	C
ATOM	748	C	ILE	235	24.018	3.945	22.939	1.00	0.00	RX0	C
ATOM	749	O	ILE	235	23.068	3.902	23.724	1.00	0.00	RX0	O
ATOM	750	N	LEU	236	25.072	3.140	23.020	1.00	0.00	RX0	N
ATOM	751	H	LEU	236	25.811	3.233	22.349	0.00	0.00	RX0	H
ATOM	752	CA	LEU	236	25.176	2.114	24.078	1.00	0.00	RX0	C
ATOM	753	CB	LEU	236	26.441	1.277	23.892	1.00	0.00	RX0	C
ATOM	754	CG	LEU	236	26.335	0.305	22.721	1.00	0.00	RX0	C
ATOM	755	CD1	LEU	236	27.662	-0.396	22.430	1.00	0.00	RX0	C
ATOM	756	CD2	LEU	236	25.197	-0.691	22.938	1.00	0.00	RX0	C
ATOM	757	C	LEU	236	25.182	2.733	25.479	1.00	0.00	RX0	C
ATOM	758	O	LEU	236	24.381	2.362	26.336	1.00	0.00	RX0	O
ATOM	759	N	MET	237	25.933	3.823	25.600	1.00	0.00	RX0	N
ATOM	760	H	MET	237	26.455	4.134	24.803	0.00	0.00	RX0	H
ATOM	761	CA	MET	237	26.132	4.523	26.881	1.00	0.00	RX0	C
ATOM	762	CB	MET	237	27.280	5.529	26.802	1.00	0.00	RX0	C
ATOM	763	CG	MET	237	28.652	4.855	26.814	1.00	0.00	RX0	C
ATOM	764	SD	MET	237	29.998	6.048	26.789	1.00	0.00	RX0	S
ATOM	765	CE	MET	237	31.350	4.902	27.104	1.00	0.00	RX0	C
ATOM	766	C	MET	237	24.875	5.215	27.409	1.00	0.00	RX0	C
ATOM	767	O	MET	237	24.517	5.003	28.572	1.00	0.00	RX0	O
ATOM	768	N	ILE	238	24.128	5.878	26.531	1.00	0.00	RX0	N
ATOM	769	H	ILE	238	24.456	5.960	25.587	0.00	0.00	RX0	H
ATOM	770	CA	ILE	238	22.871	6.546	26.925	1.00	0.00	RX0	C
ATOM	771	CB	ILE	238	22.348	7.524	25.857	1.00	0.00	RX0	C
ATOM	772	CG2	ILE	238	21.925	6.838	24.559	1.00	0.00	RX0	C
ATOM	773	CG1	ILE	238	21.220	8.379	26.438	1.00	0.00	RX0	C
ATOM	774	CD1	ILE	238	20.608	9.338	25.416	1.00	0.00	RX0	C
ATOM	775	C	ILE	238	21.800	5.514	27.357	1.00	0.00	RX0	C
ATOM	776	O	ILE	238	21.031	5.731	28.268	1.00	0.00	RX0	O
ATOM	777	N	GLY	239	21.845	4.355	26.660	1.00	0.00	RX0	N
ATOM	778	H	GLY	239	22.520	4.244	25.926	0.00	0.00	RX0	H
ATOM	779	CA	GLY	239	20.969	3.213	26.975	1.00	0.00	RX0	C
ATOM	780	C	GLY	239	21.301	2.634	28.356	1.00	0.00	RX0	C
ATOM	781	O	GLY	239	20.417	2.460	29.193	1.00	0.00	RX0	O
ATOM	782	N	LEU	240	22.605	2.583	28.639	1.00	0.00	RX0	N
ATOM	783	H	LEU	240	23.260	2.804	27.913	0.00	0.00	RX0	H
ATOM	784	CA	LEU	240	23.124	2.082	29.919	1.00	0.00	RX0	C
ATOM	785	CB	LEU	240	24.644	1.943	29.850	1.00	0.00	RX0	C
ATOM	786	CG	LEU	240	25.285	1.619	31.201	1.00	0.00	RX0	C
ATOM	787	CD1	LEU	240	24.826	0.272	31.758	1.00	0.00	RX0	C
ATOM	788	CD2	LEU	240	26.806	1.724	31.133	1.00	0.00	RX0	C
ATOM	789	C	LEU	240	22.728	2.995	31.086	1.00	0.00	RX0	C
ATOM	790	O	LEU	240	22.214	2.535	32.097	1.00	0.00	RX0	O
ATOM	791	N	VAL	241	22.901	4.295	30.880	1.00	0.00	RX0	N

ATOM	792	H	VAL	241	23.278	4.586	29.998	0.00	0.00	RX0	H
ATOM	793	CA	VAL	241	22.596	5.307	31.912	1.00	0.00	RX0	C
ATOM	794	CB	VAL	241	23.252	6.672	31.661	1.00	0.00	RX0	C
ATOM	795	CG1	VAL	241	24.770	6.506	31.594	1.00	0.00	RX0	C
ATOM	796	CG2	VAL	241	22.696	7.407	30.447	1.00	0.00	RX0	C
ATOM	797	C	VAL	241	21.084	5.392	32.193	1.00	0.00	RX0	C
ATOM	798	O	VAL	241	20.670	5.516	33.338	1.00	0.00	RX0	O
ATOM	799	N	TRP	242	20.290	5.181	31.134	1.00	0.00	RX0	N
ATOM	800	H	TRP	242	20.699	5.072	30.225	0.00	0.00	RX0	H
ATOM	801	CA	TRP	242	18.822	5.192	31.222	1.00	0.00	RX0	C
ATOM	802	CB	TRP	242	18.253	5.150	29.801	1.00	0.00	RX0	C
ATOM	803	CG	TRP	242	16.827	4.657	29.777	1.00	0.00	RX0	C
ATOM	804	CD2	TRP	242	15.636	5.332	30.235	1.00	0.00	RX0	C
ATOM	805	CE2	TRP	242	14.544	4.462	30.016	1.00	0.00	RX0	C
ATOM	806	CE3	TRP	242	15.419	6.581	30.800	1.00	0.00	RX0	C
ATOM	807	CD1	TRP	242	16.383	3.414	29.300	1.00	0.00	RX0	C
ATOM	808	NE1	TRP	242	15.036	3.295	29.439	1.00	0.00	RX0	N
ATOM	809	HE1	TRP	242	14.494	2.519	29.180	0.00	0.00	RX0	H
ATOM	810	CZ2	TRP	242	13.264	4.862	30.381	1.00	0.00	RX0	C
ATOM	811	CZ3	TRP	242	14.135	6.972	31.158	1.00	0.00	RX0	C
ATOM	812	CH2	TRP	242	13.063	6.112	30.953	1.00	0.00	RX0	C
ATOM	813	C	TRP	242	18.281	4.038	32.076	1.00	0.00	RX0	C
ATOM	814	O	TRP	242	17.477	4.269	32.979	1.00	0.00	RX0	O
ATOM	815	N	ARG	243	18.818	2.841	31.865	1.00	0.00	RX0	N
ATOM	816	H	ARG	243	19.516	2.737	31.152	0.00	0.00	RX0	H
ATOM	817	CA	ARG	243	18.359	1.661	32.627	1.00	0.00	RX0	C
ATOM	818	CB	ARG	243	18.485	0.403	31.737	1.00	0.00	RX0	C
ATOM	819	CG	ARG	243	19.890	-0.063	31.297	1.00	0.00	RX0	C
ATOM	820	CD	ARG	243	19.830	-1.094	30.146	1.00	0.00	RX0	C
ATOM	821	NE	ARG	243	21.113	-1.757	29.860	1.00	0.00	RX0	N
ATOM	822	HE	ARG	243	21.661	-2.013	30.670	0.00	0.00	RX0	H
ATOM	823	CZ	ARG	243	21.433	-2.129	28.568	1.00	0.00	RX0	C
ATOM	824	NH1	ARG	243	20.671	-1.725	27.532	1.00	0.00	RX0	N
ATOM	825	HH11	ARG	243	20.920	-2.039	26.598	0.00	0.00	RX0	H
ATOM	826	HH12	ARG	243	19.870	-1.135	27.632	0.00	0.00	RX0	H
ATOM	827	NH2	ARG	243	22.499	-2.911	28.306	1.00	0.00	RX0	N
ATOM	828	HH21	ARG	243	22.781	-3.131	27.350	0.00	0.00	RX0	H
ATOM	829	HH22	ARG	243	23.057	-3.339	29.027	0.00	0.00	RX0	H
ATOM	830	C	ARG	243	19.043	1.510	33.998	1.00	0.00	RX0	C
ATOM	831	O	ARG	243	18.610	0.722	34.836	1.00	0.00	RX0	O
ATOM	832	N	SER	244	20.027	2.366	34.245	1.00	0.00	RX0	N
ATOM	833	H	SER	244	20.316	3.011	33.538	0.00	0.00	RX0	H
ATOM	834	CA	SER	244	20.722	2.464	35.548	1.00	0.00	RX0	C
ATOM	835	CB	SER	244	22.206	2.697	35.311	1.00	0.00	RX0	C
ATOM	836	OG	SER	244	22.700	1.648	34.476	1.00	0.00	RX0	O
ATOM	837	HG	SER	244	22.226	1.717	33.654	0.00	0.00	RX0	H
ATOM	838	C	SER	244	20.112	3.547	36.442	1.00	0.00	RX0	C
ATOM	839	O	SER	244	20.448	3.642	37.630	1.00	0.00	RX0	O
ATOM	840	N	MET	245	19.184	4.322	35.895	1.00	0.00	RX0	N
ATOM	841	H	MET	245	18.885	4.151	34.954	0.00	0.00	RX0	H
ATOM	842	CA	MET	245	18.600	5.504	36.550	1.00	0.00	RX0	C
ATOM	843	CB	MET	245	17.597	6.196	35.631	1.00	0.00	RX0	C
ATOM	844	CG	MET	245	17.041	7.470	36.267	1.00	0.00	RX0	C
ATOM	845	SD	MET	245	15.739	8.232	35.295	1.00	0.00	RX0	S
ATOM	846	CE	MET	245	16.556	8.100	33.703	1.00	0.00	RX0	C
ATOM	847	C	MET	245	17.925	5.204	37.895	1.00	0.00	RX0	C
ATOM	848	O	MET	245	18.105	5.946	38.853	1.00	0.00	RX0	O
ATOM	849	N	GLU	246	17.212	4.082	37.945	1.00	0.00	RX0	N
ATOM	850	H	GLU	246	17.124	3.482	37.149	0.00	0.00	RX0	H
ATOM	851	CA	GLU	246	16.494	3.692	39.178	1.00	0.00	RX0	C
ATOM	852	CB	GLU	246	15.245	2.899	38.818	1.00	0.00	RX0	C

ATOM	853	CG	GLU	246	14.333	3.659	37.858	1.00	0.00	RX0	C
ATOM	854	CD	GLU	246	13.171	2.761	37.507	1.00	0.00	RX0	C
ATOM	855	OE1	GLU	246	13.269	1.562	37.758	1.00	0.00	RX0	O
ATOM	856	OE2	GLU	246	12.175	3.262	36.990	1.00	0.00	RX0	O
ATOM	857	C	GLU	246	17.379	2.867	40.123	1.00	0.00	RX0	C
ATOM	858	O	GLU	246	16.897	2.295	41.108	1.00	0.00	RX0	O
ATOM	859	N	HIS	247	18.674	2.844	39.836	1.00	0.00	RX0	N
ATOM	860	H	HIS	247	19.044	3.322	39.040	0.00	0.00	RX0	H
ATOM	861	CA	HIS	247	19.658	2.055	40.601	1.00	0.00	RX0	C
ATOM	862	CB	HIS	247	20.176	0.866	39.787	1.00	0.00	RX0	C
ATOM	863	CG	HIS	247	19.085	-0.137	39.486	1.00	0.00	RX0	C
ATOM	864	ND1	HIS	247	17.869	-0.172	40.069	1.00	0.00	RX0	N
ATOM	865	HD1	HIS	247	17.500	0.449	40.739	0.00	0.00	RX0	H
ATOM	866	CD2	HIS	247	19.164	-1.188	38.569	1.00	0.00	RX0	C
ATOM	867	NE2	HIS	247	17.989	-1.859	38.605	1.00	0.00	RX0	N
ATOM	868	CE1	HIS	247	17.189	-1.232	39.526	1.00	0.00	RX0	C
ATOM	869	C	HIS	247	20.841	2.947	41.015	1.00	0.00	RX0	C
ATOM	870	O	HIS	247	21.962	2.788	40.490	1.00	0.00	RX0	O
ATOM	871	N	PRO	248	20.632	3.827	41.991	1.00	0.00	RX0	N
ATOM	872	CD	PRO	248	19.384	3.973	42.733	1.00	0.00	RX0	C
ATOM	873	CA	PRO	248	21.659	4.767	42.484	1.00	0.00	RX0	C
ATOM	874	CB	PRO	248	20.980	5.450	43.675	1.00	0.00	RX0	C
ATOM	875	CG	PRO	248	19.487	5.344	43.385	1.00	0.00	RX0	C
ATOM	876	C	PRO	248	22.939	4.013	42.877	1.00	0.00	RX0	C
ATOM	877	O	PRO	248	22.892	2.963	43.503	1.00	0.00	RX0	O
ATOM	878	N	GLY	249	24.055	4.541	42.350	1.00	0.00	RX0	N
ATOM	879	H	GLY	249	23.974	5.299	41.708	0.00	0.00	RX0	H
ATOM	880	CA	GLY	249	25.407	3.996	42.610	1.00	0.00	RX0	C
ATOM	881	C	GLY	249	25.783	2.749	41.794	1.00	0.00	RX0	C
ATOM	882	O	GLY	249	26.914	2.250	41.927	1.00	0.00	RX0	O
ATOM	883	N	LYS	250	24.877	2.267	40.961	1.00	0.00	RX0	N
ATOM	884	H	LYS	250	23.983	2.698	40.813	0.00	0.00	RX0	H
ATOM	885	CA	LYS	250	25.097	1.050	40.158	1.00	0.00	RX0	C
ATOM	886	CB	LYS	250	24.370	-0.146	40.775	1.00	0.00	RX0	C
ATOM	887	CG	LYS	250	25.221	-0.841	41.846	1.00	0.00	RX0	C
ATOM	888	CD	LYS	250	24.602	-2.127	42.400	1.00	0.00	RX0	C
ATOM	889	CE	LYS	250	25.545	-2.952	43.285	1.00	0.00	RX0	C
ATOM	890	NZ	LYS	250	26.688	-3.454	42.508	1.00	0.00	RX0	N
ATOM	891	HZ1	LYS	250	27.408	-3.848	43.155	0.00	0.00	RX0	H
ATOM	892	HZ2	LYS	250	26.419	-4.168	41.796	0.00	0.00	RX0	H
ATOM	893	HZ3	LYS	250	27.184	-2.657	42.065	0.00	0.00	RX0	H
ATOM	894	C	LYS	250	24.802	1.266	38.671	1.00	0.00	RX0	C
ATOM	895	O	LYS	250	24.040	2.166	38.282	1.00	0.00	RX0	O
ATOM	896	N	LEU	251	25.472	0.476	37.863	1.00	0.00	RX0	N
ATOM	897	H	LEU	251	26.054	-0.236	38.243	0.00	0.00	RX0	H
ATOM	898	CA	LEU	251	25.292	0.430	36.401	1.00	0.00	RX0	C
ATOM	899	CB	LEU	251	26.626	0.592	35.684	1.00	0.00	RX0	C
ATOM	900	CG	LEU	251	27.159	2.018	35.763	1.00	0.00	RX0	C
ATOM	901	CD1	LEU	251	28.582	2.118	35.218	1.00	0.00	RX0	C
ATOM	902	CD2	LEU	251	26.205	3.009	35.094	1.00	0.00	RX0	C
ATOM	903	C	LEU	251	24.646	-0.892	36.017	1.00	0.00	RX0	C
ATOM	904	O	LEU	251	25.224	-1.976	36.286	1.00	0.00	RX0	O
ATOM	905	N	LEU	252	23.437	-0.808	35.533	1.00	0.00	RX0	N
ATOM	906	H	LEU	252	23.083	0.097	35.313	0.00	0.00	RX0	H
ATOM	907	CA	LEU	252	22.659	-1.981	35.114	1.00	0.00	RX0	C
ATOM	908	CB	LEU	252	21.157	-1.712	35.230	1.00	0.00	RX0	C
ATOM	909	CG	LEU	252	20.300	-2.958	34.976	1.00	0.00	RX0	C
ATOM	910	CD1	LEU	252	20.450	-3.993	36.089	1.00	0.00	RX0	C
ATOM	911	CD2	LEU	252	18.834	-2.611	34.723	1.00	0.00	RX0	C
ATOM	912	C	LEU	252	23.007	-2.330	33.663	1.00	0.00	RX0	C
ATOM	913	O	LEU	252	22.274	-2.025	32.731	1.00	0.00	RX0	O

ATOM	914	N	PHE	253	24.144	-3.005	33.506	1.00	0.00	RX0	N
ATOM	915	H	PHE	253	24.630	-3.308	34.330	0.00	0.00	RX0	H
ATOM	916	CA	PHE	253	24.599	-3.457	32.174	1.00	0.00	RX0	C
ATOM	917	CB	PHE	253	25.968	-4.125	32.266	1.00	0.00	RX0	C
ATOM	918	CG	PHE	253	27.032	-3.097	32.548	1.00	0.00	RX0	C
ATOM	919	CD1	PHE	253	27.568	-2.360	31.500	1.00	0.00	RX0	C
ATOM	920	CD2	PHE	253	27.483	-2.896	33.846	1.00	0.00	RX0	C
ATOM	921	CE1	PHE	253	28.566	-1.426	31.747	1.00	0.00	RX0	C
ATOM	922	CE2	PHE	253	28.480	-1.961	34.092	1.00	0.00	RX0	C
ATOM	923	CZ	PHE	253	29.022	-1.228	33.043	1.00	0.00	RX0	C
ATOM	924	C	PHE	253	23.603	-4.446	31.564	1.00	0.00	RX0	C
ATOM	925	O	PHE	253	23.259	-4.379	30.390	1.00	0.00	RX0	O
ATOM	926	N	ALA	254	23.094	-5.300	32.445	1.00	0.00	RX0	N
ATOM	927	H	ALA	254	23.408	-5.311	33.400	0.00	0.00	RX0	H
ATOM	928	CA	ALA	254	22.050	-6.280	32.141	1.00	0.00	RX0	C
ATOM	929	CB	ALA	254	22.690	-7.550	31.589	1.00	0.00	RX0	C
ATOM	930	C	ALA	254	21.288	-6.584	33.440	1.00	0.00	RX0	C
ATOM	931	O	ALA	254	21.887	-6.418	34.526	1.00	0.00	RX0	O
ATOM	932	N	PRO	255	20.056	-7.064	33.372	1.00	0.00	RX0	N
ATOM	933	CD	PRO	255	19.306	-7.244	32.131	1.00	0.00	RX0	C
ATOM	934	CA	PRO	255	19.236	-7.444	34.545	1.00	0.00	RX0	C
ATOM	935	CB	PRO	255	17.988	-8.070	33.923	1.00	0.00	RX0	C
ATOM	936	CG	PRO	255	17.856	-7.383	32.571	1.00	0.00	RX0	C
ATOM	937	C	PRO	255	19.972	-8.395	35.506	1.00	0.00	RX0	C
ATOM	938	O	PRO	255	19.756	-8.342	36.714	1.00	0.00	RX0	O
ATOM	939	N	ASN	256	20.900	-9.179	34.970	1.00	0.00	RX0	N
ATOM	940	H	ASN	256	21.114	-9.136	33.993	0.00	0.00	RX0	H
ATOM	941	CA	ASN	256	21.722	-10.125	35.761	1.00	0.00	RX0	C
ATOM	942	CB	ASN	256	21.706	-11.530	35.154	1.00	0.00	RX0	C
ATOM	943	CG	ASN	256	22.403	-11.539	33.803	1.00	0.00	RX0	C
ATOM	944	OD1	ASN	256	22.283	-10.610	33.004	1.00	0.00	RX0	O
ATOM	945	ND2	ASN	256	23.048	-12.690	33.541	1.00	0.00	RX0	N
ATOM	946	HD21	ASN	256	23.187	-13.356	34.279	0.00	0.00	RX0	H
ATOM	947	HD22	ASN	256	23.399	-12.949	32.636	0.00	0.00	RX0	H
ATOM	948	C	ASN	256	23.191	-9.669	35.876	1.00	0.00	RX0	C
ATOM	949	O	ASN	256	24.101	-10.505	36.011	1.00	0.00	RX0	O
ATOM	950	N	LEU	257	23.439	-8.384	35.742	1.00	0.00	RX0	N
ATOM	951	H	LEU	257	22.692	-7.727	35.627	0.00	0.00	RX0	H
ATOM	952	CA	LEU	257	24.796	-7.805	35.783	1.00	0.00	RX0	C
ATOM	953	CB	LEU	257	25.511	-7.966	34.443	1.00	0.00	RX0	C
ATOM	954	CG	LEU	257	27.022	-7.762	34.564	1.00	0.00	RX0	C
ATOM	955	CD1	LEU	257	27.650	-8.800	35.496	1.00	0.00	RX0	C
ATOM	956	CD2	LEU	257	27.712	-7.732	33.201	1.00	0.00	RX0	C
ATOM	957	C	LEU	257	24.720	-6.327	36.168	1.00	0.00	RX0	C
ATOM	958	O	LEU	257	24.738	-5.412	35.328	1.00	0.00	RX0	O
ATOM	959	N	LEU	258	24.604	-6.148	37.469	1.00	0.00	RX0	N
ATOM	960	H	LEU	258	24.691	-6.942	38.070	0.00	0.00	RX0	H
ATOM	961	CA	LEU	258	24.482	-4.834	38.118	1.00	0.00	RX0	C
ATOM	962	CB	LEU	258	23.222	-4.898	38.982	1.00	0.00	RX0	C
ATOM	963	CG	LEU	258	22.824	-3.605	39.682	1.00	0.00	RX0	C
ATOM	964	CD1	LEU	258	22.657	-2.455	38.699	1.00	0.00	RX0	C
ATOM	965	CD2	LEU	258	21.574	-3.796	40.542	1.00	0.00	RX0	C
ATOM	966	C	LEU	258	25.743	-4.564	38.938	1.00	0.00	RX0	C
ATOM	967	O	LEU	258	26.013	-5.237	39.948	1.00	0.00	RX0	O
ATOM	968	N	LEU	259	26.528	-3.622	38.460	1.00	0.00	RX0	N
ATOM	969	H	LEU	259	26.218	-3.063	37.684	0.00	0.00	RX0	H
ATOM	970	CA	LEU	259	27.862	-3.349	39.027	1.00	0.00	RX0	C
ATOM	971	CB	LEU	259	28.937	-3.494	37.946	1.00	0.00	RX0	C
ATOM	972	CG	LEU	259	28.870	-4.805	37.158	1.00	0.00	RX0	C
ATOM	973	CD1	LEU	259	29.882	-4.827	36.013	1.00	0.00	RX0	C
ATOM	974	CD2	LEU	259	29.013	-6.035	38.054	1.00	0.00	RX0	C

ATOM	975	C	LEU	259	27.958	-1.956	39.652	1.00	0.00	RX0	C
ATOM	976	O	LEU	259	27.419	-0.984	39.137	1.00	0.00	RX0	O
ATOM	977	N	ASP	260	28.645	-1.912	40.785	1.00	0.00	RX0	N
ATOM	978	H	ASP	260	29.222	-2.690	41.046	0.00	0.00	RX0	H
ATOM	979	CA	ASP	260	29.043	-0.657	41.454	1.00	0.00	RX0	C
ATOM	980	CB	ASP	260	29.082	-0.866	42.964	1.00	0.00	RX0	C
ATOM	981	CG	ASP	260	29.782	-2.177	43.220	1.00	0.00	RX0	C
ATOM	982	OD1	ASP	260	31.010	-2.204	43.239	1.00	0.00	RX0	O
ATOM	983	OD2	ASP	260	29.082	-3.181	43.349	1.00	0.00	RX0	O
ATOM	984	C	ASP	260	30.443	-0.222	40.970	1.00	0.00	RX0	C
ATOM	985	O	ASP	260	31.127	-1.008	40.295	1.00	0.00	RX0	O
ATOM	986	N	ARG	261	30.963	0.873	41.504	1.00	0.00	RX0	N
ATOM	987	H	ARG	261	30.389	1.408	42.122	0.00	0.00	RX0	H
ATOM	988	CA	ARG	261	32.263	1.408	41.044	1.00	0.00	RX0	C
ATOM	989	CB	ARG	261	32.415	2.885	41.438	1.00	0.00	RX0	C
ATOM	990	CG	ARG	261	32.594	3.165	42.932	1.00	0.00	RX0	C
ATOM	991	CD	ARG	261	32.495	4.658	43.271	1.00	0.00	RX0	C
ATOM	992	NE	ARG	261	33.370	5.462	42.420	1.00	0.00	RX0	N
ATOM	993	HE	ARG	261	33.082	5.672	41.468	0.00	0.00	RX0	H
ATOM	994	CZ	ARG	261	34.565	5.960	42.845	1.00	0.00	RX0	C
ATOM	995	NH1	ARG	261	34.942	5.783	44.130	1.00	0.00	RX0	N
ATOM	996	HH11	ARG	261	35.810	6.138	44.481	0.00	0.00	RX0	H
ATOM	997	HH12	ARG	261	34.343	5.300	44.773	0.00	0.00	RX0	H
ATOM	998	NH2	ARG	261	35.341	6.626	41.970	1.00	0.00	RX0	N
ATOM	999	HH21	ARG	261	36.253	7.001	42.128	0.00	0.00	RX0	H
ATOM	1000	HH22	ARG	261	34.942	6.782	41.044	0.00	0.00	RX0	H
ATOM	1001	C	ARG	261	33.476	0.540	41.436	1.00	0.00	RX0	C
ATOM	1002	O	ARG	261	34.378	0.347	40.637	1.00	0.00	RX0	O
ATOM	1003	N	ASN	262	33.410	-0.067	42.632	1.00	0.00	RX0	N
ATOM	1004	H	ASN	262	32.529	-0.053	43.107	0.00	0.00	RX0	H
ATOM	1005	CA	ASN	262	34.456	-1.000	43.094	1.00	0.00	RX0	C
ATOM	1006	CB	ASN	262	34.124	-1.503	44.493	1.00	0.00	RX0	C
ATOM	1007	CG	ASN	262	35.089	-2.613	44.844	1.00	0.00	RX0	C
ATOM	1008	OD1	ASN	262	36.242	-2.352	45.190	1.00	0.00	RX0	O
ATOM	1009	ND2	ASN	262	34.553	-3.845	44.780	1.00	0.00	RX0	N
ATOM	1010	HD21	ASN	262	33.589	-3.968	44.526	0.00	0.00	RX0	H
ATOM	1011	HD22	ASN	262	35.071	-4.682	44.961	0.00	0.00	RX0	H
ATOM	1012	C	ASN	262	34.635	-2.233	42.204	1.00	0.00	RX0	C
ATOM	1013	O	ASN	262	35.755	-2.665	41.964	1.00	0.00	RX0	O
ATOM	1014	N	GLN	263	33.530	-2.679	41.603	1.00	0.00	RX0	N
ATOM	1015	H	GLN	263	32.638	-2.271	41.822	0.00	0.00	RX0	H
ATOM	1016	CA	GLN	263	33.559	-3.756	40.599	1.00	0.00	RX0	C
ATOM	1017	CB	GLN	263	32.195	-4.415	40.443	1.00	0.00	RX0	C
ATOM	1018	CG	GLN	263	31.907	-5.155	41.748	1.00	0.00	RX0	C
ATOM	1019	CD	GLN	263	30.684	-6.030	41.616	1.00	0.00	RX0	C
ATOM	1020	OE1	GLN	263	30.585	-6.891	40.751	1.00	0.00	RX0	O
ATOM	1021	NE2	GLN	263	29.771	-5.800	42.570	1.00	0.00	RX0	N
ATOM	1022	HE21	GLN	263	29.902	-4.999	43.167	0.00	0.00	RX0	H
ATOM	1023	HE22	GLN	263	28.990	-6.405	42.707	0.00	0.00	RX0	H
ATOM	1024	C	GLN	263	34.189	-3.308	39.265	1.00	0.00	RX0	C
ATOM	1025	O	GLN	263	34.644	-4.116	38.479	1.00	0.00	RX0	O
ATOM	1026	N	GLY	264	34.180	-1.978	39.039	1.00	0.00	RX0	N
ATOM	1027	H	GLY	264	33.763	-1.376	39.720	0.00	0.00	RX0	H
ATOM	1028	CA	GLY	264	34.831	-1.337	37.881	1.00	0.00	RX0	C
ATOM	1029	C	GLY	264	36.364	-1.310	37.978	1.00	0.00	RX0	C
ATOM	1030	O	GLY	264	37.050	-1.510	36.977	1.00	0.00	RX0	O
ATOM	1031	N	LYS	265	36.881	-1.155	39.202	1.00	0.00	RX0	N
ATOM	1032	H	LYS	265	36.232	-1.005	39.949	0.00	0.00	RX0	H
ATOM	1033	CA	LYS	265	38.336	-1.184	39.469	1.00	0.00	RX0	C
ATOM	1034	CB	LYS	265	38.664	-1.128	40.952	1.00	0.00	RX0	C
ATOM	1035	CG	LYS	265	38.095	-0.043	41.854	1.00	0.00	RX0	C

ATOM	1036	CD	LYS	265	38.475	-0.513	43.256	1.00	0.00	RX0	C
ATOM	1037	CE	LYS	265	37.932	0.281	44.435	1.00	0.00	RX0	C
ATOM	1038	NZ	LYS	265	38.103	-0.573	45.620	1.00	0.00	RX0	N
ATOM	1039	HZ1	LYS	265	37.668	-0.144	46.458	0.00	0.00	RX0	H
ATOM	1040	HZ2	LYS	265	37.618	-1.480	45.433	0.00	0.00	RX0	H
ATOM	1041	HZ3	LYS	265	39.109	-0.765	45.790	0.00	0.00	RX0	H
ATOM	1042	C	LYS	265	38.994	-2.500	39.030	1.00	0.00	RX0	C
ATOM	1043	O	LYS	265	40.184	-2.535	38.765	1.00	0.00	RX0	O
ATOM	1044	N	CYS	266	38.153	-3.548	38.917	1.00	0.00	RX0	N
ATOM	1045	H	CYS	266	37.198	-3.454	39.193	0.00	0.00	RX0	H
ATOM	1046	CA	CYS	266	38.538	-4.854	38.355	1.00	0.00	RX0	C
ATOM	1047	CB	CYS	266	37.315	-5.759	38.262	1.00	0.00	RX0	C
ATOM	1048	SG	CYS	266	36.554	-5.948	39.896	1.00	0.00	RX0	S
ATOM	1049	C	CYS	266	39.318	-4.732	37.033	1.00	0.00	RX0	C
ATOM	1050	O	CYS	266	40.108	-5.603	36.695	1.00	0.00	RX0	O
ATOM	1051	N	VAL	267	39.075	-3.636	36.304	1.00	0.00	RX0	N
ATOM	1052	H	VAL	267	38.470	-2.902	36.620	0.00	0.00	RX0	H
ATOM	1053	CA	VAL	267	39.804	-3.345	35.058	1.00	0.00	RX0	C
ATOM	1054	CB	VAL	267	38.857	-3.352	33.851	1.00	0.00	RX0	C
ATOM	1055	CG1	VAL	267	39.587	-3.009	32.550	1.00	0.00	RX0	C
ATOM	1056	CG2	VAL	267	38.126	-4.693	33.740	1.00	0.00	RX0	C
ATOM	1057	C	VAL	267	40.557	-2.013	35.191	1.00	0.00	RX0	C
ATOM	1058	O	VAL	267	39.969	-0.956	35.468	1.00	0.00	RX0	O
ATOM	1059	N	GLU	268	41.829	-2.078	34.821	1.00	0.00	RX0	N
ATOM	1060	H	GLU	268	42.209	-2.964	34.565	0.00	0.00	RX0	H
ATOM	1061	CA	GLU	268	42.726	-0.908	34.727	1.00	0.00	RX0	C
ATOM	1062	CB	GLU	268	44.097	-1.333	34.209	1.00	0.00	RX0	C
ATOM	1063	CG	GLU	268	45.140	-0.223	34.334	1.00	0.00	RX0	C
ATOM	1064	CD	GLU	268	46.504	-0.805	34.040	1.00	0.00	RX0	C
ATOM	1065	OE1	GLU	268	46.594	-2.020	33.870	1.00	0.00	RX0	O
ATOM	1066	OE2	GLU	268	47.471	-0.047	33.996	1.00	0.00	RX0	O
ATOM	1067	C	GLU	268	42.079	0.194	33.866	1.00	0.00	RX0	C
ATOM	1068	O	GLU	268	41.697	-0.027	32.727	1.00	0.00	RX0	O
ATOM	1069	N	GLY	269	41.924	1.355	34.524	1.00	0.00	RX0	N
ATOM	1070	H	GLY	269	42.193	1.398	35.484	0.00	0.00	RX0	H
ATOM	1071	CA	GLY	269	41.377	2.576	33.902	1.00	0.00	RX0	C
ATOM	1072	C	GLY	269	39.898	2.490	33.494	1.00	0.00	RX0	C
ATOM	1073	O	GLY	269	39.424	3.343	32.745	1.00	0.00	RX0	O
ATOM	1074	N	MET	270	39.146	1.598	34.134	1.00	0.00	RX0	N
ATOM	1075	H	MET	270	39.580	0.956	34.769	0.00	0.00	RX0	H
ATOM	1076	CA	MET	270	37.704	1.465	33.841	1.00	0.00	RX0	C
ATOM	1077	CB	MET	270	37.280	0.008	33.652	1.00	0.00	RX0	C
ATOM	1078	CG	MET	270	35.925	-0.098	32.947	1.00	0.00	RX0	C
ATOM	1079	SD	MET	270	35.419	-1.787	32.583	1.00	0.00	RX0	S
ATOM	1080	CE	MET	270	35.154	-2.353	34.268	1.00	0.00	RX0	C
ATOM	1081	C	MET	270	36.825	2.181	34.877	1.00	0.00	RX0	C
ATOM	1082	O	MET	270	35.781	2.734	34.514	1.00	0.00	RX0	O
ATOM	1083	N	VAL	271	37.310	2.290	36.107	1.00	0.00	RX0	N
ATOM	1084	H	VAL	271	38.235	1.950	36.273	0.00	0.00	RX0	H
ATOM	1085	CA	VAL	271	36.591	3.043	37.171	1.00	0.00	RX0	C
ATOM	1086	CB	VAL	271	37.245	2.853	38.552	1.00	0.00	RX0	C
ATOM	1087	CG1	VAL	271	38.734	3.183	38.564	1.00	0.00	RX0	C
ATOM	1088	CG2	VAL	271	36.456	3.563	39.652	1.00	0.00	RX0	C
ATOM	1089	C	VAL	271	36.343	4.502	36.749	1.00	0.00	RX0	C
ATOM	1090	O	VAL	271	35.261	5.071	37.095	1.00	0.00	RX0	O
ATOM	1091	N	GLU	272	37.228	5.074	36.011	1.00	0.00	RX0	N
ATOM	1092	H	GLU	272	38.100	4.599	35.878	0.00	0.00	RX0	H
ATOM	1093	CA	GLU	272	37.141	6.453	35.460	1.00	0.00	RX0	C
ATOM	1094	CB	GLU	272	38.433	6.785	34.704	1.00	0.00	RX0	C
ATOM	1095	CG	GLU	272	39.679	7.017	35.574	1.00	0.00	RX0	C
ATOM	1096	CD	GLU	272	40.044	5.788	36.387	1.00	0.00	RX0	C

ATOM	1097	OE1	GLU	272	40.058	4.687	35.838	1.00	0.00	RX0	O
ATOM	1098	OE2	GLU	272	40.279	5.927	37.583	1.00	0.00	RX0	O
ATOM	1099	C	GLU	272	35.927	6.588	34.526	1.00	0.00	RX0	C
ATOM	1100	O	GLU	272	35.142	7.521	34.681	1.00	0.00	RX0	O
ATOM	1101	N	ILE	273	35.702	5.552	33.729	1.00	0.00	RX0	N
ATOM	1102	H	ILE	273	36.306	4.759	33.821	0.00	0.00	RX0	H
ATOM	1103	CA	ILE	273	34.533	5.484	32.820	1.00	0.00	RX0	C
ATOM	1104	CB	ILE	273	34.734	4.376	31.780	1.00	0.00	RX0	C
ATOM	1105	CG2	ILE	273	33.622	4.406	30.730	1.00	0.00	RX0	C
ATOM	1106	CG1	ILE	273	36.126	4.434	31.144	1.00	0.00	RX0	C
ATOM	1107	CD1	ILE	273	36.335	5.657	30.250	1.00	0.00	RX0	C
ATOM	1108	C	ILE	273	33.249	5.235	33.628	1.00	0.00	RX0	C
ATOM	1109	O	ILE	273	32.257	5.951	33.452	1.00	0.00	RX0	O
ATOM	1110	N	PHE	274	33.320	4.302	34.575	1.00	0.00	RX0	N
ATOM	1111	H	PHE	274	34.190	3.821	34.693	0.00	0.00	RX0	H
ATOM	1112	CA	PHE	274	32.191	3.976	35.472	1.00	0.00	RX0	C
ATOM	1113	CB	PHE	274	32.611	2.939	36.515	1.00	0.00	RX0	C
ATOM	1114	CG	PHE	274	32.269	1.538	36.073	1.00	0.00	RX0	C
ATOM	1115	CD1	PHE	274	32.532	1.120	34.775	1.00	0.00	RX0	C
ATOM	1116	CD2	PHE	274	31.686	0.663	36.983	1.00	0.00	RX0	C
ATOM	1117	CE1	PHE	274	32.219	-0.178	34.391	1.00	0.00	RX0	C
ATOM	1118	CE2	PHE	274	31.374	-0.635	36.600	1.00	0.00	RX0	C
ATOM	1119	CZ	PHE	274	31.649	-1.057	35.306	1.00	0.00	RX0	C
ATOM	1120	C	PHE	274	31.669	5.203	36.222	1.00	0.00	RX0	C
ATOM	1121	O	PHE	274	30.484	5.521	36.143	1.00	0.00	RX0	O
ATOM	1122	N	ASP	275	32.607	5.983	36.757	1.00	0.00	RX0	N
ATOM	1123	H	ASP	275	33.560	5.685	36.802	0.00	0.00	RX0	H
ATOM	1124	CA	ASP	275	32.273	7.222	37.483	1.00	0.00	RX0	C
ATOM	1125	CB	ASP	275	33.489	7.800	38.200	1.00	0.00	RX0	C
ATOM	1126	CG	ASP	275	33.579	7.161	39.571	1.00	0.00	RX0	C
ATOM	1127	OD1	ASP	275	33.901	5.982	39.678	1.00	0.00	RX0	O
ATOM	1128	OD2	ASP	275	33.357	7.841	40.567	1.00	0.00	RX0	O
ATOM	1129	C	ASP	275	31.555	8.270	36.629	1.00	0.00	RX0	C
ATOM	1130	O	ASP	275	30.604	8.890	37.102	1.00	0.00	RX0	O
ATOM	1131	N	MET	276	31.921	8.336	35.353	1.00	0.00	RX0	N
ATOM	1132	H	MET	276	32.663	7.739	35.041	0.00	0.00	RX0	H
ATOM	1133	CA	MET	276	31.257	9.254	34.408	1.00	0.00	RX0	C
ATOM	1134	CB	MET	276	32.088	9.445	33.141	1.00	0.00	RX0	C
ATOM	1135	CG	MET	276	33.422	10.145	33.399	1.00	0.00	RX0	C
ATOM	1136	SD	MET	276	34.289	10.540	31.871	1.00	0.00	RX0	S
ATOM	1137	CE	MET	276	34.332	8.882	31.179	1.00	0.00	RX0	C
ATOM	1138	C	MET	276	29.833	8.798	34.061	1.00	0.00	RX0	C
ATOM	1139	O	MET	276	28.893	9.589	34.158	1.00	0.00	RX0	O
ATOM	1140	N	LEU	277	29.673	7.488	33.891	1.00	0.00	RX0	N
ATOM	1141	H	LEU	277	30.490	6.906	33.949	0.00	0.00	RX0	H
ATOM	1142	CA	LEU	277	28.362	6.866	33.606	1.00	0.00	RX0	C
ATOM	1143	CB	LEU	277	28.562	5.402	33.227	1.00	0.00	RX0	C
ATOM	1144	CG	LEU	277	29.434	5.219	31.985	1.00	0.00	RX0	C
ATOM	1145	CD1	LEU	277	29.934	3.782	31.845	1.00	0.00	RX0	C
ATOM	1146	CD2	LEU	277	28.730	5.708	30.721	1.00	0.00	RX0	C
ATOM	1147	C	LEU	277	27.393	6.992	34.787	1.00	0.00	RX0	C
ATOM	1148	O	LEU	277	26.257	7.447	34.627	1.00	0.00	RX0	O
ATOM	1149	N	LEU	278	27.939	6.795	35.983	1.00	0.00	RX0	N
ATOM	1150	H	LEU	278	28.900	6.516	36.019	0.00	0.00	RX0	H
ATOM	1151	CA	LEU	278	27.196	6.926	37.250	1.00	0.00	RX0	C
ATOM	1152	CB	LEU	278	28.067	6.470	38.419	1.00	0.00	RX0	C
ATOM	1153	CG	LEU	278	28.220	4.953	38.467	1.00	0.00	RX0	C
ATOM	1154	CD1	LEU	278	29.273	4.502	39.479	1.00	0.00	RX0	C
ATOM	1155	CD2	LEU	278	26.868	4.291	38.710	1.00	0.00	RX0	C
ATOM	1156	C	LEU	278	26.716	8.359	37.508	1.00	0.00	RX0	C
ATOM	1157	O	LEU	278	25.554	8.575	37.840	1.00	0.00	RX0	O

ATOM	1158	N	ALA	279	27.582	9.317	37.175	1.00	0.00	RX0	N
ATOM	1159	H	ALA	279	28.513	9.059	36.898	0.00	0.00	RX0	H
ATOM	1160	CA	ALA	279	27.275	10.754	37.296	1.00	0.00	RX0	C
ATOM	1161	CB	ALA	279	28.527	11.595	37.048	1.00	0.00	RX0	C
ATOM	1162	C	ALA	279	26.182	11.196	36.310	1.00	0.00	RX0	C
ATOM	1163	O	ALA	279	25.263	11.922	36.684	1.00	0.00	RX0	O
ATOM	1164	N	THR	280	26.210	10.618	35.109	1.00	0.00	RX0	N
ATOM	1165	H	THR	280	26.969	10.006	34.882	0.00	0.00	RX0	H
ATOM	1166	CA	THR	280	25.203	10.899	34.059	1.00	0.00	RX0	C
ATOM	1167	CB	THR	280	25.748	10.367	32.740	1.00	0.00	RX0	C
ATOM	1168	OG1	THR	280	27.060	10.904	32.531	1.00	0.00	RX0	O
ATOM	1169	HG1	THR	280	27.666	10.445	33.104	0.00	0.00	RX0	H
ATOM	1170	CG2	THR	280	24.830	10.715	31.566	1.00	0.00	RX0	C
ATOM	1171	C	THR	280	23.835	10.327	34.462	1.00	0.00	RX0	C
ATOM	1172	O	THR	280	22.822	11.023	34.397	1.00	0.00	RX0	O
ATOM	1173	N	SER	281	23.868	9.112	35.003	1.00	0.00	RX0	N
ATOM	1174	H	SER	281	24.740	8.620	35.048	0.00	0.00	RX0	H
ATOM	1175	CA	SER	281	22.669	8.413	35.507	1.00	0.00	RX0	C
ATOM	1176	CB	SER	281	23.149	6.986	35.867	1.00	0.00	RX0	C
ATOM	1177	OG	SER	281	22.387	6.335	36.904	1.00	0.00	RX0	O
ATOM	1178	HG	SER	281	22.607	5.409	36.822	0.00	0.00	RX0	H
ATOM	1179	C	SER	281	22.019	9.180	36.675	1.00	0.00	RX0	C
ATOM	1180	O	SER	281	20.814	9.399	36.693	1.00	0.00	RX0	O
ATOM	1181	N	SER	282	22.889	9.788	37.491	1.00	0.00	RX0	N
ATOM	1182	H	SER	282	23.869	9.616	37.379	0.00	0.00	RX0	H
ATOM	1183	CA	SER	282	22.489	10.660	38.613	1.00	0.00	RX0	C
ATOM	1184	CB	SER	282	23.679	10.722	39.554	1.00	0.00	RX0	C
ATOM	1185	OG	SER	282	23.950	9.344	39.870	1.00	0.00	RX0	O
ATOM	1186	HG	SER	282	24.737	9.124	39.370	0.00	0.00	RX0	H
ATOM	1187	C	SER	282	21.828	11.963	38.130	1.00	0.00	RX0	C
ATOM	1188	O	SER	282	20.788	12.371	38.639	1.00	0.00	RX0	O
ATOM	1189	N	ARG	283	22.365	12.516	37.039	1.00	0.00	RX0	N
ATOM	1190	H	ARG	283	23.191	12.111	36.639	0.00	0.00	RX0	H
ATOM	1191	CA	ARG	283	21.822	13.735	36.412	1.00	0.00	RX0	C
ATOM	1192	CB	ARG	283	22.812	14.215	35.349	1.00	0.00	RX0	C
ATOM	1193	CG	ARG	283	22.346	15.417	34.531	1.00	0.00	RX0	C
ATOM	1194	CD	ARG	283	22.157	16.694	35.349	1.00	0.00	RX0	C
ATOM	1195	NE	ARG	283	21.763	17.784	34.460	1.00	0.00	RX0	N
ATOM	1196	HE	ARG	283	22.192	17.769	33.549	0.00	0.00	RX0	H
ATOM	1197	CZ	ARG	283	20.865	18.721	34.881	1.00	0.00	RX0	C
ATOM	1198	NH1	ARG	283	20.376	18.666	36.141	1.00	0.00	RX0	N
ATOM	1199	HH11	ARG	283	19.708	19.335	36.483	0.00	0.00	RX0	H
ATOM	1200	HH12	ARG	283	20.662	17.953	36.790	0.00	0.00	RX0	H
ATOM	1201	NH2	ARG	283	20.471	19.683	34.021	1.00	0.00	RX0	N
ATOM	1202	HH21	ARG	283	19.770	20.372	34.260	0.00	0.00	RX0	H
ATOM	1203	HH22	ARG	283	20.845	19.748	33.093	0.00	0.00	RX0	H
ATOM	1204	C	ARG	283	20.432	13.477	35.812	1.00	0.00	RX0	C
ATOM	1205	O	ARG	283	19.498	14.246	36.035	1.00	0.00	RX0	O
ATOM	1206	N	PHE	284	20.293	12.325	35.169	1.00	0.00	RX0	N
ATOM	1207	H	PHE	284	21.098	11.741	35.041	0.00	0.00	RX0	H
ATOM	1208	CA	PHE	284	19.003	11.899	34.606	1.00	0.00	RX0	C
ATOM	1209	CB	PHE	284	19.202	10.642	33.766	1.00	0.00	RX0	C
ATOM	1210	CG	PHE	284	19.644	11.021	32.375	1.00	0.00	RX0	C
ATOM	1211	CD1	PHE	284	19.180	12.197	31.797	1.00	0.00	RX0	C
ATOM	1212	CD2	PHE	284	20.490	10.182	31.661	1.00	0.00	RX0	C
ATOM	1213	CE1	PHE	284	19.529	12.512	30.490	1.00	0.00	RX0	C
ATOM	1214	CE2	PHE	284	20.842	10.502	30.355	1.00	0.00	RX0	C
ATOM	1215	CZ	PHE	284	20.348	11.657	29.763	1.00	0.00	RX0	C
ATOM	1216	C	PHE	284	17.921	11.680	35.654	1.00	0.00	RX0	C
ATOM	1217	O	PHE	284	16.817	12.216	35.524	1.00	0.00	RX0	O
ATOM	1218	N	ARG	285	18.348	11.087	36.760	1.00	0.00	RX0	N

ATOM	1219	H	ARG	285	19.279	10.718	36.763	0.00	0.00	RX0	H
ATOM	1220	CA	ARG	285	17.485	10.841	37.923	1.00	0.00	RX0	C
ATOM	1221	CB	ARG	285	18.333	10.060	38.926	1.00	0.00	RX0	C
ATOM	1222	CG	ARG	285	17.763	9.787	40.319	1.00	0.00	RX0	C
ATOM	1223	CD	ARG	285	18.791	9.029	41.171	1.00	0.00	RX0	C
ATOM	1224	NE	ARG	285	19.207	7.819	40.465	1.00	0.00	RX0	N
ATOM	1225	HE	ARG	285	18.452	7.220	40.169	0.00	0.00	RX0	H
ATOM	1226	CZ	ARG	285	20.498	7.646	40.050	1.00	0.00	RX0	C
ATOM	1227	NH1	ARG	285	21.465	8.424	40.580	1.00	0.00	RX0	N
ATOM	1228	HH11	ARG	285	22.429	8.416	40.268	0.00	0.00	RX0	H
ATOM	1229	HH12	ARG	285	21.258	9.074	41.315	0.00	0.00	RX0	H
ATOM	1230	NH2	ARG	285	20.761	6.715	39.108	1.00	0.00	RX0	N
ATOM	1231	HH21	ARG	285	21.649	6.586	38.645	0.00	0.00	RX0	H
ATOM	1232	HH22	ARG	285	20.019	6.108	38.800	0.00	0.00	RX0	H
ATOM	1233	C	ARG	285	17.003	12.164	38.534	1.00	0.00	RX0	C
ATOM	1234	O	ARG	285	15.822	12.321	38.816	1.00	0.00	RX0	O
ATOM	1235	N	MET	286	17.922	13.130	38.591	1.00	0.00	RX0	N
ATOM	1236	H	MET	286	18.856	12.922	38.295	0.00	0.00	RX0	H
ATOM	1237	CA	MET	286	17.643	14.467	39.144	1.00	0.00	RX0	C
ATOM	1238	CB	MET	286	18.961	15.234	39.262	1.00	0.00	RX0	C
ATOM	1239	CG	MET	286	18.791	16.697	39.672	1.00	0.00	RX0	C
ATOM	1240	SD	MET	286	20.307	17.645	39.454	1.00	0.00	RX0	S
ATOM	1241	CE	MET	286	19.712	19.211	40.112	1.00	0.00	RX0	C
ATOM	1242	C	MET	286	16.681	15.257	38.243	1.00	0.00	RX0	C
ATOM	1243	O	MET	286	15.799	15.962	38.735	1.00	0.00	RX0	O
ATOM	1244	N	MET	287	16.911	15.161	36.942	1.00	0.00	RX0	N
ATOM	1245	H	MET	287	17.629	14.536	36.630	0.00	0.00	RX0	H
ATOM	1246	CA	MET	287	16.049	15.814	35.941	1.00	0.00	RX0	C
ATOM	1247	CB	MET	287	16.685	15.857	34.555	1.00	0.00	RX0	C
ATOM	1248	CG	MET	287	17.865	16.818	34.511	1.00	0.00	RX0	C
ATOM	1249	SD	MET	287	18.274	17.297	32.830	1.00	0.00	RX0	S
ATOM	1250	CE	MET	287	16.654	17.952	32.399	1.00	0.00	RX0	C
ATOM	1251	C	MET	287	14.674	15.156	35.849	1.00	0.00	RX0	C
ATOM	1252	O	MET	287	13.755	15.729	35.264	1.00	0.00	RX0	O
ATOM	1253	N	ASN	288	14.583	13.920	36.346	1.00	0.00	RX0	N
ATOM	1254	H	ASN	288	15.392	13.490	36.747	0.00	0.00	RX0	H
ATOM	1255	CA	ASN	288	13.391	13.066	36.242	1.00	0.00	RX0	C
ATOM	1256	CB	ASN	288	12.161	13.612	36.968	1.00	0.00	RX0	C
ATOM	1257	CG	ASN	288	11.009	12.654	36.724	1.00	0.00	RX0	C
ATOM	1258	OD1	ASN	288	11.178	11.442	36.647	1.00	0.00	RX0	O
ATOM	1259	ND2	ASN	288	9.819	13.267	36.582	1.00	0.00	RX0	N
ATOM	1260	HD21	ASN	288	9.739	14.259	36.673	0.00	0.00	RX0	H
ATOM	1261	HD22	ASN	288	8.993	12.745	36.363	0.00	0.00	RX0	H
ATOM	1262	C	ASN	288	13.076	12.836	34.753	1.00	0.00	RX0	C
ATOM	1263	O	ASN	288	11.986	13.121	34.256	1.00	0.00	RX0	O
ATOM	1264	N	LEU	289	14.125	12.439	34.028	1.00	0.00	RX0	N
ATOM	1265	H	LEU	289	14.956	12.173	34.521	0.00	0.00	RX0	H
ATOM	1266	CA	LEU	289	14.030	12.183	32.583	1.00	0.00	RX0	C
ATOM	1267	CB	LEU	289	15.372	11.628	32.098	1.00	0.00	RX0	C
ATOM	1268	CG	LEU	289	15.410	11.217	30.623	1.00	0.00	RX0	C
ATOM	1269	CD1	LEU	289	15.551	12.424	29.703	1.00	0.00	RX0	C
ATOM	1270	CD2	LEU	289	16.487	10.172	30.338	1.00	0.00	RX0	C
ATOM	1271	C	LEU	289	12.926	11.152	32.321	1.00	0.00	RX0	C
ATOM	1272	O	LEU	289	12.814	10.137	33.014	1.00	0.00	RX0	O
ATOM	1273	N	GLN	290	12.165	11.420	31.281	1.00	0.00	RX0	N
ATOM	1274	H	GLN	290	12.394	12.178	30.664	0.00	0.00	RX0	H
ATOM	1275	CA	GLN	290	11.011	10.584	30.916	1.00	0.00	RX0	C
ATOM	1276	CB	GLN	290	9.778	11.452	30.673	1.00	0.00	RX0	C
ATOM	1277	CG	GLN	290	9.411	12.301	31.898	1.00	0.00	RX0	C
ATOM	1278	CD	GLN	290	8.986	11.428	33.070	1.00	0.00	RX0	C
ATOM	1279	OE1	GLN	290	7.827	11.056	33.212	1.00	0.00	RX0	O

ATOM	1280	NE2	GLN	290	9.973	11.156	33.940	1.00	0.00	RX0	N
ATOM	1281	HE21	GLN	290	10.898	11.515	33.799	0.00	0.00	RX0	H
ATOM	1282	HE22	GLN	290	9.878	10.607	34.772	0.00	0.00	RX0	H
ATOM	1283	C	GLN	290	11.379	9.701	29.727	1.00	0.00	RX0	C
ATOM	1284	O	GLN	290	12.115	10.141	28.832	1.00	0.00	RX0	O
ATOM	1285	N	GLY	291	10.739	8.533	29.672	1.00	0.00	RX0	N
ATOM	1286	H	GLY	291	10.026	8.353	30.350	0.00	0.00	RX0	H
ATOM	1287	CA	GLY	291	10.952	7.542	28.589	1.00	0.00	RX0	C
ATOM	1288	C	GLY	291	10.769	8.134	27.181	1.00	0.00	RX0	C
ATOM	1289	O	GLY	291	11.559	7.865	26.269	1.00	0.00	RX0	O
ATOM	1290	N	GLU	292	9.854	9.086	27.078	1.00	0.00	RX0	N
ATOM	1291	H	GLU	292	9.255	9.248	27.866	0.00	0.00	RX0	H
ATOM	1292	CA	GLU	292	9.541	9.789	25.813	1.00	0.00	RX0	C
ATOM	1293	CB	GLU	292	8.215	10.551	25.942	1.00	0.00	RX0	C
ATOM	1294	CG	GLU	292	6.976	9.705	26.282	1.00	0.00	RX0	C
ATOM	1295	CD	GLU	292	7.097	9.118	27.675	1.00	0.00	RX0	C
ATOM	1296	OE1	GLU	292	7.474	9.847	28.590	1.00	0.00	RX0	O
ATOM	1297	OE2	GLU	292	6.917	7.914	27.828	1.00	0.00	RX0	O
ATOM	1298	C	GLU	292	10.671	10.744	25.400	1.00	0.00	RX0	C
ATOM	1299	O	GLU	292	11.065	10.802	24.241	1.00	0.00	RX0	O
ATOM	1300	N	GLU	293	11.241	11.417	26.395	1.00	0.00	RX0	N
ATOM	1301	H	GLU	293	10.950	11.211	27.330	0.00	0.00	RX0	H
ATOM	1302	CA	GLU	293	12.389	12.320	26.184	1.00	0.00	RX0	C
ATOM	1303	CB	GLU	293	12.636	13.158	27.433	1.00	0.00	RX0	C
ATOM	1304	CG	GLU	293	11.405	13.910	27.930	1.00	0.00	RX0	C
ATOM	1305	CD	GLU	293	11.723	14.506	29.285	1.00	0.00	RX0	C
ATOM	1306	OE1	GLU	293	12.638	14.031	29.951	1.00	0.00	RX0	O
ATOM	1307	OE2	GLU	293	11.057	15.445	29.699	1.00	0.00	RX0	O
ATOM	1308	C	GLU	293	13.669	11.544	25.843	1.00	0.00	RX0	C
ATOM	1309	O	GLU	293	14.364	11.888	24.886	1.00	0.00	RX0	O
ATOM	1310	N	PHE	294	13.855	10.409	26.517	1.00	0.00	RX0	N
ATOM	1311	H	PHE	294	13.194	10.199	27.240	0.00	0.00	RX0	H
ATOM	1312	CA	PHE	294	14.999	9.503	26.300	1.00	0.00	RX0	C
ATOM	1313	CB	PHE	294	14.905	8.308	27.252	1.00	0.00	RX0	C
ATOM	1314	CG	PHE	294	15.866	7.227	26.816	1.00	0.00	RX0	C
ATOM	1315	CD1	PHE	294	17.238	7.451	26.840	1.00	0.00	RX0	C
ATOM	1316	CD2	PHE	294	15.372	6.004	26.374	1.00	0.00	RX0	C
ATOM	1317	CE1	PHE	294	18.110	6.464	26.396	1.00	0.00	RX0	C
ATOM	1318	CE2	PHE	294	16.244	5.017	25.932	1.00	0.00	RX0	C
ATOM	1319	CZ	PHE	294	17.613	5.251	25.934	1.00	0.00	RX0	C
ATOM	1320	C	PHE	294	15.115	9.012	24.847	1.00	0.00	RX0	C
ATOM	1321	O	PHE	294	16.186	9.132	24.238	1.00	0.00	RX0	O
ATOM	1322	N	VAL	295	14.000	8.581	24.280	1.00	0.00	RX0	N
ATOM	1323	H	VAL	295	13.158	8.569	24.828	0.00	0.00	RX0	H
ATOM	1324	CA	VAL	295	13.976	8.026	22.907	1.00	0.00	RX0	C
ATOM	1325	CB	VAL	295	12.685	7.251	22.616	1.00	0.00	RX0	C
ATOM	1326	CG1	VAL	295	12.585	6.064	23.571	1.00	0.00	RX0	C
ATOM	1327	CG2	VAL	295	11.429	8.119	22.661	1.00	0.00	RX0	C
ATOM	1328	C	VAL	295	14.286	9.097	21.847	1.00	0.00	RX0	C
ATOM	1329	O	VAL	295	14.999	8.845	20.884	1.00	0.00	RX0	O
ATOM	1330	N	CYS	296	13.866	10.330	22.156	1.00	0.00	RX0	N
ATOM	1331	H	CYS	296	13.306	10.480	22.974	0.00	0.00	RX0	H
ATOM	1332	CA	CYS	296	14.167	11.498	21.314	1.00	0.00	RX0	C
ATOM	1333	CB	CYS	296	13.264	12.652	21.730	1.00	0.00	RX0	C
ATOM	1334	SG	CYS	296	11.518	12.259	21.464	1.00	0.00	RX0	S
ATOM	1335	C	CYS	296	15.661	11.843	21.352	1.00	0.00	RX0	C
ATOM	1336	O	CYS	296	16.290	11.969	20.305	1.00	0.00	RX0	O
ATOM	1337	N	LEU	297	16.247	11.750	22.547	1.00	0.00	RX0	N
ATOM	1338	H	LEU	297	15.681	11.549	23.351	0.00	0.00	RX0	H
ATOM	1339	CA	LEU	297	17.681	12.031	22.752	1.00	0.00	RX0	C
ATOM	1340	CB	LEU	297	18.019	12.108	24.239	1.00	0.00	RX0	C

ATOM	1341	CG	LEU	297	17.347	13.280	24.952	1.00	0.00	RX0	C
ATOM	1342	CD1	LEU	297	17.641	13.254	26.451	1.00	0.00	RX0	C
ATOM	1343	CD2	LEU	297	17.710	14.622	24.317	1.00	0.00	RX0	C
ATOM	1344	C	LEU	297	18.589	11.009	22.068	1.00	0.00	RX0	C
ATOM	1345	O	LEU	297	19.526	11.385	21.359	1.00	0.00	RX0	O
ATOM	1346	N	LYS	298	18.173	9.750	22.123	1.00	0.00	RX0	N
ATOM	1347	H	LYS	298	17.359	9.546	22.671	0.00	0.00	RX0	H
ATOM	1348	CA	LYS	298	18.945	8.654	21.515	1.00	0.00	RX0	C
ATOM	1349	CB	LYS	298	18.433	7.319	22.046	1.00	0.00	RX0	C
ATOM	1350	CG	LYS	298	19.255	6.120	21.581	1.00	0.00	RX0	C
ATOM	1351	CD	LYS	298	18.612	4.834	22.086	1.00	0.00	RX0	C
ATOM	1352	CE	LYS	298	17.096	5.006	22.045	1.00	0.00	RX0	C
ATOM	1353	NZ	LYS	298	16.426	3.714	22.148	1.00	0.00	RX0	N
ATOM	1354	HZ1	LYS	298	15.432	3.870	22.444	0.00	0.00	RX0	H
ATOM	1355	HZ2	LYS	298	16.294	3.234	21.237	0.00	0.00	RX0	H
ATOM	1356	HZ3	LYS	298	16.781	3.060	22.867	0.00	0.00	RX0	H
ATOM	1357	C	LYS	298	18.925	8.733	19.978	1.00	0.00	RX0	C
ATOM	1358	O	LYS	298	19.964	8.557	19.332	1.00	0.00	RX0	O
ATOM	1359	N	SER	299	17.791	9.152	19.431	1.00	0.00	RX0	N
ATOM	1360	H	SER	299	16.964	9.266	19.981	0.00	0.00	RX0	H
ATOM	1361	CA	SER	299	17.645	9.383	17.977	1.00	0.00	RX0	C
ATOM	1362	CB	SER	299	16.155	9.360	17.703	1.00	0.00	RX0	C
ATOM	1363	OG	SER	299	15.661	8.203	18.385	1.00	0.00	RX0	O
ATOM	1364	HG	SER	299	15.126	8.511	19.109	0.00	0.00	RX0	H
ATOM	1365	C	SER	299	18.416	10.621	17.504	1.00	0.00	RX0	C
ATOM	1366	O	SER	299	19.051	10.583	16.444	1.00	0.00	RX0	O
ATOM	1367	N	ILE	300	18.478	11.644	18.354	1.00	0.00	RX0	N
ATOM	1368	H	ILE	300	17.955	11.596	19.207	0.00	0.00	RX0	H
ATOM	1369	CA	ILE	300	19.283	12.860	18.096	1.00	0.00	RX0	C
ATOM	1370	CB	ILE	300	19.078	13.935	19.167	1.00	0.00	RX0	C
ATOM	1371	CG2	ILE	300	20.117	15.052	19.042	1.00	0.00	RX0	C
ATOM	1372	CG1	ILE	300	17.672	14.515	19.094	1.00	0.00	RX0	C
ATOM	1373	CD1	ILE	300	17.411	15.533	20.203	1.00	0.00	RX0	C
ATOM	1374	C	ILE	300	20.773	12.490	18.013	1.00	0.00	RX0	C
ATOM	1375	O	ILE	300	21.456	12.924	17.087	1.00	0.00	RX0	O
ATOM	1376	N	ILE	301	21.227	11.630	18.921	1.00	0.00	RX0	N
ATOM	1377	H	ILE	301	20.605	11.327	19.646	0.00	0.00	RX0	H
ATOM	1378	CA	ILE	301	22.629	11.156	18.925	1.00	0.00	RX0	C
ATOM	1379	CB	ILE	301	22.882	10.194	20.082	1.00	0.00	RX0	C
ATOM	1380	CG2	ILE	301	24.203	9.455	19.892	1.00	0.00	RX0	C
ATOM	1381	CG1	ILE	301	22.845	10.932	21.417	1.00	0.00	RX0	C
ATOM	1382	CD1	ILE	301	23.191	10.007	22.583	1.00	0.00	RX0	C
ATOM	1383	C	ILE	301	22.948	10.474	17.584	1.00	0.00	RX0	C
ATOM	1384	O	ILE	301	23.927	10.823	16.926	1.00	0.00	RX0	O
ATOM	1385	N	LEU	302	22.047	9.582	17.175	1.00	0.00	RX0	N
ATOM	1386	H	LEU	302	21.270	9.376	17.773	0.00	0.00	RX0	H
ATOM	1387	CA	LEU	302	22.205	8.836	15.917	1.00	0.00	RX0	C
ATOM	1388	CB	LEU	302	20.992	7.929	15.709	1.00	0.00	RX0	C
ATOM	1389	CG	LEU	302	20.999	7.210	14.360	1.00	0.00	RX0	C
ATOM	1390	CD1	LEU	302	22.180	6.252	14.222	1.00	0.00	RX0	C
ATOM	1391	CD2	LEU	302	19.664	6.525	14.081	1.00	0.00	RX0	C
ATOM	1392	C	LEU	302	22.391	9.757	14.699	1.00	0.00	RX0	C
ATOM	1393	O	LEU	302	23.285	9.544	13.882	1.00	0.00	RX0	O
ATOM	1394	N	LEU	303	21.581	10.806	14.658	1.00	0.00	RX0	N
ATOM	1395	H	LEU	303	20.943	10.954	15.417	0.00	0.00	RX0	H
ATOM	1396	CA	LEU	303	21.546	11.719	13.502	1.00	0.00	RX0	C
ATOM	1397	CB	LEU	303	20.124	12.231	13.311	1.00	0.00	RX0	C
ATOM	1398	CG	LEU	303	19.164	11.064	13.082	1.00	0.00	RX0	C
ATOM	1399	CD1	LEU	303	17.707	11.485	13.248	1.00	0.00	RX0	C
ATOM	1400	CD2	LEU	303	19.421	10.366	11.745	1.00	0.00	RX0	C
ATOM	1401	C	LEU	303	22.576	12.849	13.543	1.00	0.00	RX0	C

ATOM	1402	O	LEU	303	23.073	13.268	12.494	1.00	0.00	RX0	O
ATOM	1403	N	ASN	304	22.971	13.240	14.745	1.00	0.00	RX0	N
ATOM	1404	H	ASN	304	22.634	12.747	15.549	0.00	0.00	RX0	H
ATOM	1405	CA	ASN	304	23.832	14.419	14.940	1.00	0.00	RX0	C
ATOM	1406	CB	ASN	304	23.488	15.175	16.213	1.00	0.00	RX0	C
ATOM	1407	CG	ASN	304	24.639	16.107	16.524	1.00	0.00	RX0	C
ATOM	1408	OD1	ASN	304	24.916	17.068	15.822	1.00	0.00	RX0	O
ATOM	1409	ND2	ASN	304	25.281	15.813	17.660	1.00	0.00	RX0	N
ATOM	1410	HD21	ASN	304	25.045	15.010	18.206	0.00	0.00	RX0	H
ATOM	1411	HD22	ASN	304	25.992	16.454	17.944	0.00	0.00	RX0	H
ATOM	1412	C	ASN	304	25.330	14.097	14.987	1.00	0.00	RX0	C
ATOM	1413	O	ASN	304	26.132	14.783	14.344	1.00	0.00	RX0	O
ATOM	1414	N	SER	305	25.693	13.015	15.656	1.00	0.00	RX0	N
ATOM	1415	H	SER	305	25.035	12.407	16.113	0.00	0.00	RX0	H
ATOM	1416	CA	SER	305	27.104	12.765	16.012	1.00	0.00	RX0	C
ATOM	1417	CB	SER	305	27.024	11.514	16.849	1.00	0.00	RX0	C
ATOM	1418	OG	SER	305	26.187	11.884	17.952	1.00	0.00	RX0	O
ATOM	1419	HG	SER	305	26.305	11.179	18.597	0.00	0.00	RX0	H
ATOM	1420	C	SER	305	28.103	12.695	14.846	1.00	0.00	RX0	C
ATOM	1421	O	SER	305	29.198	13.228	14.946	1.00	0.00	RX0	O
ATOM	1422	N	GLY	306	27.634	12.177	13.693	1.00	0.00	RX0	N
ATOM	1423	H	GLY	306	26.685	11.867	13.629	0.00	0.00	RX0	H
ATOM	1424	CA	GLY	306	28.500	12.079	12.501	1.00	0.00	RX0	C
ATOM	1425	C	GLY	306	28.091	12.961	11.315	1.00	0.00	RX0	C
ATOM	1426	O	GLY	306	28.756	12.897	10.274	1.00	0.00	RX0	O
ATOM	1427	N	VAL	307	27.242	13.954	11.536	1.00	0.00	RX0	N
ATOM	1428	H	VAL	307	26.876	14.106	12.457	0.00	0.00	RX0	H
ATOM	1429	CA	VAL	307	26.760	14.815	10.435	1.00	0.00	RX0	C
ATOM	1430	CB	VAL	307	25.396	15.437	10.773	1.00	0.00	RX0	C
ATOM	1431	CG1	VAL	307	25.480	16.561	11.799	1.00	0.00	RX0	C
ATOM	1432	CG2	VAL	307	24.673	15.878	9.505	1.00	0.00	RX0	C
ATOM	1433	C	VAL	307	27.809	15.841	9.948	1.00	0.00	RX0	C
ATOM	1434	O	VAL	307	27.790	16.280	8.811	1.00	0.00	RX0	O
ATOM	1435	N	TYR	308	28.719	16.199	10.859	1.00	0.00	RX0	N
ATOM	1436	H	TYR	308	28.697	15.740	11.745	0.00	0.00	RX0	H
ATOM	1437	CA	TYR	308	29.761	17.211	10.584	1.00	0.00	RX0	C
ATOM	1438	CB	TYR	308	29.982	17.993	11.876	1.00	0.00	RX0	C
ATOM	1439	CG	TYR	308	28.715	18.762	12.209	1.00	0.00	RX0	C
ATOM	1440	CD1	TYR	308	28.214	19.680	11.262	1.00	0.00	RX0	C
ATOM	1441	CE1	TYR	308	27.034	20.381	11.544	1.00	0.00	RX0	C
ATOM	1442	CD2	TYR	308	28.067	18.554	13.447	1.00	0.00	RX0	C
ATOM	1443	CE2	TYR	308	26.883	19.265	13.729	1.00	0.00	RX0	C
ATOM	1444	CZ	TYR	308	26.372	20.160	12.763	1.00	0.00	RX0	C
ATOM	1445	OH	TYR	308	25.187	20.840	12.990	1.00	0.00	RX0	O
ATOM	1446	HH	TYR	308	24.791	20.499	13.790	0.00	0.00	RX0	H
ATOM	1447	C	TYR	308	31.032	16.660	9.940	1.00	0.00	RX0	C
ATOM	1448	O	TYR	308	31.912	17.411	9.537	1.00	0.00	RX0	O
ATOM	1449	N	THR	309	31.091	15.334	9.861	1.00	0.00	RX0	N
ATOM	1450	H	THR	309	30.357	14.753	10.213	0.00	0.00	RX0	H
ATOM	1451	CA	THR	309	32.303	14.643	9.386	1.00	0.00	RX0	C
ATOM	1452	CB	THR	309	32.721	13.696	10.511	1.00	0.00	RX0	C
ATOM	1453	OG1	THR	309	31.554	13.061	11.064	1.00	0.00	RX0	O
ATOM	1454	HG1	THR	309	31.286	12.371	10.456	0.00	0.00	RX0	H
ATOM	1455	CG2	THR	309	33.467	14.439	11.623	1.00	0.00	RX0	C
ATOM	1456	C	THR	309	32.183	13.866	8.071	1.00	0.00	RX0	C
ATOM	1457	O	THR	309	33.137	13.195	7.681	1.00	0.00	RX0	O
ATOM	1458	N	PHE	310	31.041	13.953	7.382	1.00	0.00	RX0	N
ATOM	1459	H	PHE	310	30.315	14.529	7.753	0.00	0.00	RX0	H
ATOM	1460	CA	PHE	310	30.972	13.467	5.988	1.00	0.00	RX0	C
ATOM	1461	CB	PHE	310	29.592	13.704	5.357	1.00	0.00	RX0	C
ATOM	1462	CG	PHE	310	28.462	13.077	6.145	1.00	0.00	RX0	C

ATOM	1463	CD1	PHE	310	28.492	11.705	6.480	1.00	0.00	RX0	C
ATOM	1464	CD2	PHE	310	27.371	13.892	6.515	1.00	0.00	RX0	C
ATOM	1465	CE1	PHE	310	27.416	11.142	7.194	1.00	0.00	RX0	C
ATOM	1466	CE2	PHE	310	26.293	13.329	7.224	1.00	0.00	RX0	C
ATOM	1467	CZ	PHE	310	26.326	11.959	7.558	1.00	0.00	RX0	C
ATOM	1468	C	PHE	310	32.019	14.245	5.180	1.00	0.00	RX0	C
ATOM	1469	O	PHE	310	32.102	15.468	5.301	1.00	0.00	RX0	O
ATOM	1470	N	LEU	311	32.854	13.508	4.462	1.00	0.00	RX0	N
ATOM	1471	H	LEU	311	32.727	12.519	4.506	0.00	0.00	RX0	H
ATOM	1472	CA	LEU	311	33.988	14.087	3.701	1.00	0.00	RX0	C
ATOM	1473	CB	LEU	311	34.898	13.011	3.095	1.00	0.00	RX0	C
ATOM	1474	CG	LEU	311	35.449	12.014	4.137	1.00	0.00	RX0	C
ATOM	1475	CD1	LEU	311	36.682	11.277	3.628	1.00	0.00	RX0	C
ATOM	1476	CD2	LEU	311	35.782	12.636	5.492	1.00	0.00	RX0	C
ATOM	1477	C	LEU	311	33.468	15.166	2.739	1.00	0.00	RX0	C
ATOM	1478	O	LEU	311	33.554	16.351	2.969	1.00	0.00	RX0	O
ATOM	1479	N	SER	312	32.784	14.619	1.713	1.00	0.00	RX0	N
ATOM	1480	H	SER	312	32.575	13.644	1.766	0.00	0.00	RX0	H
ATOM	1481	CA	SER	312	32.345	15.333	0.526	1.00	0.00	RX0	C
ATOM	1482	CB	SER	312	31.760	14.274	-0.433	1.00	0.00	RX0	C
ATOM	1483	OG	SER	312	31.409	13.067	0.273	1.00	0.00	RX0	O
ATOM	1484	HG	SER	312	32.103	12.430	0.093	0.00	0.00	RX0	H
ATOM	1485	C	SER	312	31.423	16.517	0.816	1.00	0.00	RX0	C
ATOM	1486	O	SER	312	31.042	16.847	1.948	1.00	0.00	RX0	O
ATOM	1487	N	SER	313	30.837	16.923	-0.276	1.00	0.00	RX0	N
ATOM	1488	H	SER	313	31.306	16.657	-1.118	0.00	0.00	RX0	H
ATOM	1489	CA	SER	313	29.838	17.983	-0.455	1.00	0.00	RX0	C
ATOM	1490	CB	SER	313	30.472	19.366	-0.222	1.00	0.00	RX0	C
ATOM	1491	OG	SER	313	31.441	19.341	0.844	1.00	0.00	RX0	O
ATOM	1492	HG	SER	313	31.095	18.769	1.531	0.00	0.00	RX0	H
ATOM	1493	C	SER	313	29.353	17.880	-1.909	1.00	0.00	RX0	C
ATOM	1494	O	SER	313	29.168	18.836	-2.628	1.00	0.00	RX0	O
ATOM	1495	N	THR	314	29.278	16.602	-2.358	1.00	0.00	RX0	N
ATOM	1496	H	THR	314	29.398	15.850	-1.714	0.00	0.00	RX0	H
ATOM	1497	CA	THR	314	28.609	16.250	-3.614	1.00	0.00	RX0	C
ATOM	1498	CB	THR	314	28.686	14.736	-3.782	1.00	0.00	RX0	C
ATOM	1499	OG1	THR	314	28.286	14.078	-2.573	1.00	0.00	RX0	O
ATOM	1500	HG1	THR	314	28.615	13.187	-2.653	0.00	0.00	RX0	H
ATOM	1501	CG2	THR	314	30.100	14.299	-4.148	1.00	0.00	RX0	C
ATOM	1502	C	THR	314	27.167	16.747	-3.490	1.00	0.00	RX0	C
ATOM	1503	O	THR	314	26.675	16.978	-2.368	1.00	0.00	RX0	O
ATOM	1504	N	LEU	315	26.451	16.801	-4.589	1.00	0.00	RX0	N
ATOM	1505	H	LEU	315	26.885	16.524	-5.446	0.00	0.00	RX0	H
ATOM	1506	CA	LEU	315	25.050	17.256	-4.537	1.00	0.00	RX0	C
ATOM	1507	CB	LEU	315	24.323	17.655	-5.803	1.00	0.00	RX0	C
ATOM	1508	CG	LEU	315	23.197	18.641	-5.499	1.00	0.00	RX0	C
ATOM	1509	CD1	LEU	315	23.608	20.085	-5.786	1.00	0.00	RX0	C
ATOM	1510	CD2	LEU	315	21.895	18.241	-6.178	1.00	0.00	RX0	C
ATOM	1511	C	LEU	315	24.190	16.343	-3.639	1.00	0.00	RX0	C
ATOM	1512	O	LEU	315	23.484	16.800	-2.757	1.00	0.00	RX0	O
ATOM	1513	N	LYS	316	24.524	15.049	-3.730	1.00	0.00	RX0	N
ATOM	1514	H	LYS	316	25.169	14.810	-4.450	0.00	0.00	RX0	H
ATOM	1515	CA	LYS	316	23.912	13.990	-2.918	1.00	0.00	RX0	C
ATOM	1516	CB	LYS	316	24.314	12.597	-3.410	1.00	0.00	RX0	C
ATOM	1517	CG	LYS	316	23.162	11.599	-3.247	1.00	0.00	RX0	C
ATOM	1518	CD	LYS	316	23.465	10.206	-3.805	1.00	0.00	RX0	C
ATOM	1519	CE	LYS	316	22.254	9.266	-3.736	1.00	0.00	RX0	C
ATOM	1520	NZ	LYS	316	22.663	7.923	-4.164	1.00	0.00	RX0	N
ATOM	1521	HZ1	LYS	316	21.916	7.200	-4.055	0.00	0.00	RX0	H
ATOM	1522	HZ2	LYS	316	22.925	7.928	-5.169	0.00	0.00	RX0	H
ATOM	1523	HZ3	LYS	316	23.477	7.589	-3.609	0.00	0.00	RX0	H

ATOM	1524	C	LYS	316	24.213	14.187	-1.422	1.00	0.00	RX0	C
ATOM	1525	O	LYS	316	23.297	14.194	-0.611	1.00	0.00	RX0	O
ATOM	1526	N	SER	317	25.467	14.540	-1.122	1.00	0.00	RX0	N
ATOM	1527	H	SER	317	26.122	14.632	-1.871	0.00	0.00	RX0	H
ATOM	1528	CA	SER	317	25.934	14.787	0.260	1.00	0.00	RX0	C
ATOM	1529	CB	SER	317	27.459	14.931	0.326	1.00	0.00	RX0	C
ATOM	1530	OG	SER	317	28.016	14.180	1.420	1.00	0.00	RX0	O
ATOM	1531	HG	SER	317	28.195	14.816	2.108	0.00	0.00	RX0	H
ATOM	1532	C	SER	317	25.242	16.001	0.898	1.00	0.00	RX0	C
ATOM	1533	O	SER	317	24.716	15.915	2.007	1.00	0.00	RX0	O
ATOM	1534	N	LEU	318	25.067	17.043	0.088	1.00	0.00	RX0	N
ATOM	1535	H	LEU	318	25.361	16.971	-0.866	0.00	0.00	RX0	H
ATOM	1536	CA	LEU	318	24.405	18.288	0.525	1.00	0.00	RX0	C
ATOM	1537	CB	LEU	318	24.568	19.373	-0.539	1.00	0.00	RX0	C
ATOM	1538	CG	LEU	318	26.028	19.758	-0.782	1.00	0.00	RX0	C
ATOM	1539	CD1	LEU	318	26.183	20.625	-2.032	1.00	0.00	RX0	C
ATOM	1540	CD2	LEU	318	26.666	20.402	0.451	1.00	0.00	RX0	C
ATOM	1541	C	LEU	318	22.918	18.061	0.823	1.00	0.00	RX0	C
ATOM	1542	O	LEU	318	22.412	18.453	1.877	1.00	0.00	RX0	O
ATOM	1543	N	GLU	319	22.299	17.244	-0.024	1.00	0.00	RX0	N
ATOM	1544	H	GLU	319	22.773	16.916	-0.845	0.00	0.00	RX0	H
ATOM	1545	CA	GLU	319	20.900	16.808	0.157	1.00	0.00	RX0	C
ATOM	1546	CB	GLU	319	20.436	16.073	-1.133	1.00	0.00	RX0	C
ATOM	1547	CG	GLU	319	20.422	16.932	-2.423	1.00	0.00	RX0	C
ATOM	1548	CD	GLU	319	20.273	16.135	-3.735	1.00	0.00	RX0	C
ATOM	1549	OE1	GLU	319	21.114	15.289	-4.047	1.00	0.00	RX0	O
ATOM	1550	OE2	GLU	319	19.348	16.413	-4.502	1.00	0.00	RX0	O
ATOM	1551	C	GLU	319	20.725	15.924	1.402	1.00	0.00	RX0	C
ATOM	1552	O	GLU	319	19.808	16.153	2.196	1.00	0.00	RX0	O
ATOM	1553	N	GLU	320	21.706	15.058	1.645	1.00	0.00	RX0	N
ATOM	1554	H	GLU	320	22.404	14.930	0.939	0.00	0.00	RX0	H
ATOM	1555	CA	GLU	320	21.731	14.149	2.812	1.00	0.00	RX0	C
ATOM	1556	CB	GLU	320	22.927	13.177	2.790	1.00	0.00	RX0	C
ATOM	1557	CG	GLU	320	23.020	12.155	1.645	1.00	0.00	RX0	C
ATOM	1558	CD	GLU	320	22.256	10.869	1.912	1.00	0.00	RX0	C
ATOM	1559	OE1	GLU	320	21.030	10.880	1.937	1.00	0.00	RX0	O
ATOM	1560	OE2	GLU	320	22.894	9.829	2.040	1.00	0.00	RX0	O
ATOM	1561	C	GLU	320	21.765	14.931	4.131	1.00	0.00	RX0	C
ATOM	1562	O	GLU	320	20.881	14.754	4.973	1.00	0.00	RX0	O
ATOM	1563	N	LYS	321	22.647	15.926	4.188	1.00	0.00	RX0	N
ATOM	1564	H	LYS	321	23.257	16.041	3.400	0.00	0.00	RX0	H
ATOM	1565	CA	LYS	321	22.801	16.771	5.391	1.00	0.00	RX0	C
ATOM	1566	CB	LYS	321	24.036	17.662	5.225	1.00	0.00	RX0	C
ATOM	1567	CG	LYS	321	24.675	18.054	6.558	1.00	0.00	RX0	C
ATOM	1568	CD	LYS	321	25.867	19.010	6.444	1.00	0.00	RX0	C
ATOM	1569	CE	LYS	321	26.645	19.079	7.762	1.00	0.00	RX0	C
ATOM	1570	NZ	LYS	321	27.642	20.151	7.733	1.00	0.00	RX0	N
ATOM	1571	HZ1	LYS	321	28.316	20.085	8.525	0.00	0.00	RX0	H
ATOM	1572	HZ2	LYS	321	28.281	20.127	6.914	0.00	0.00	RX0	H
ATOM	1573	HZ3	LYS	321	27.230	21.103	7.802	0.00	0.00	RX0	H
ATOM	1574	C	LYS	321	21.549	17.612	5.656	1.00	0.00	RX0	C
ATOM	1575	O	LYS	321	21.102	17.724	6.798	1.00	0.00	RX0	O
ATOM	1576	N	ASP	322	20.935	18.099	4.575	1.00	0.00	RX0	N
ATOM	1577	H	ASP	322	21.321	17.944	3.662	0.00	0.00	RX0	H
ATOM	1578	CA	ASP	322	19.715	18.915	4.684	1.00	0.00	RX0	C
ATOM	1579	CB	ASP	322	19.383	19.459	3.293	1.00	0.00	RX0	C
ATOM	1580	CG	ASP	322	18.038	20.149	3.280	1.00	0.00	RX0	C
ATOM	1581	OD1	ASP	322	17.652	20.737	4.280	1.00	0.00	RX0	O
ATOM	1582	OD2	ASP	322	17.342	20.066	2.274	1.00	0.00	RX0	O
ATOM	1583	C	ASP	322	18.561	18.097	5.282	1.00	0.00	RX0	C
ATOM	1584	O	ASP	322	17.955	18.512	6.263	1.00	0.00	RX0	O

ATOM	1585	N	HIS	323	18.423	16.872	4.772	1.00	0.00	RX0	N
ATOM	1586	H	HIS	323	19.040	16.603	4.026	0.00	0.00	RX0	H
ATOM	1587	CA	HIS	323	17.423	15.916	5.274	1.00	0.00	RX0	C
ATOM	1588	CB	HIS	323	17.463	14.618	4.467	1.00	0.00	RX0	C
ATOM	1589	CG	HIS	323	16.306	13.744	4.885	1.00	0.00	RX0	C
ATOM	1590	ND1	HIS	323	16.351	12.401	4.972	1.00	0.00	RX0	N
ATOM	1591	HD1	HIS	323	17.115	11.813	4.779	0.00	0.00	RX0	H
ATOM	1592	CD2	HIS	323	15.022	14.170	5.227	1.00	0.00	RX0	C
ATOM	1593	NE2	HIS	323	14.287	13.076	5.522	1.00	0.00	RX0	N
ATOM	1594	CE1	HIS	323	15.104	11.981	5.366	1.00	0.00	RX0	C
ATOM	1595	C	HIS	323	17.630	15.607	6.766	1.00	0.00	RX0	C
ATOM	1596	O	HIS	323	16.677	15.663	7.540	1.00	0.00	RX0	O
ATOM	1597	N	ILE	324	18.888	15.420	7.164	1.00	0.00	RX0	N
ATOM	1598	H	ILE	324	19.611	15.424	6.468	0.00	0.00	RX0	H
ATOM	1599	CA	ILE	324	19.235	15.107	8.570	1.00	0.00	RX0	C
ATOM	1600	CB	ILE	324	20.715	14.752	8.706	1.00	0.00	RX0	C
ATOM	1601	CG2	ILE	324	21.109	14.592	10.174	1.00	0.00	RX0	C
ATOM	1602	CG1	ILE	324	21.020	13.483	7.910	1.00	0.00	RX0	C
ATOM	1603	CD1	ILE	324	22.499	13.101	7.930	1.00	0.00	RX0	C
ATOM	1604	C	ILE	324	18.844	16.271	9.496	1.00	0.00	RX0	C
ATOM	1605	O	ILE	324	18.200	16.053	10.527	1.00	0.00	RX0	O
ATOM	1606	N	HIS	325	19.159	17.485	9.071	1.00	0.00	RX0	N
ATOM	1607	H	HIS	325	19.581	17.591	8.168	0.00	0.00	RX0	H
ATOM	1608	CA	HIS	325	18.840	18.694	9.856	1.00	0.00	RX0	C
ATOM	1609	CB	HIS	325	19.567	19.924	9.311	1.00	0.00	RX0	C
ATOM	1610	CG	HIS	325	21.010	19.883	9.749	1.00	0.00	RX0	C
ATOM	1611	ND1	HIS	325	21.976	19.236	9.075	1.00	0.00	RX0	N
ATOM	1612	HD1	HIS	325	21.851	18.730	8.243	0.00	0.00	RX0	H
ATOM	1613	CD2	HIS	325	21.573	20.476	10.884	1.00	0.00	RX0	C
ATOM	1614	NE2	HIS	325	22.896	20.174	10.881	1.00	0.00	RX0	N
ATOM	1615	CE1	HIS	325	23.142	19.412	9.769	1.00	0.00	RX0	C
ATOM	1616	C	HIS	325	17.335	18.955	9.948	1.00	0.00	RX0	C
ATOM	1617	O	HIS	325	16.820	19.290	11.021	1.00	0.00	RX0	O
ATOM	1618	N	ARG	326	16.631	18.584	8.887	1.00	0.00	RX0	N
ATOM	1619	H	ARG	326	17.125	18.357	8.047	0.00	0.00	RX0	H
ATOM	1620	CA	ARG	326	15.160	18.594	8.873	1.00	0.00	RX0	C
ATOM	1621	CB	ARG	326	14.653	18.414	7.441	1.00	0.00	RX0	C
ATOM	1622	CG	ARG	326	14.672	19.726	6.653	1.00	0.00	RX0	C
ATOM	1623	CD	ARG	326	14.132	19.596	5.225	1.00	0.00	RX0	C
ATOM	1624	NE	ARG	326	15.166	19.202	4.268	1.00	0.00	RX0	N
ATOM	1625	HE	ARG	326	15.909	19.877	4.109	0.00	0.00	RX0	H
ATOM	1626	CZ	ARG	326	15.123	18.014	3.601	1.00	0.00	RX0	C
ATOM	1627	NH1	ARG	326	14.149	17.130	3.899	1.00	0.00	RX0	N
ATOM	1628	HH11	ARG	326	14.060	16.244	3.437	0.00	0.00	RX0	H
ATOM	1629	HH12	ARG	326	13.476	17.340	4.613	0.00	0.00	RX0	H
ATOM	1630	NH2	ARG	326	16.047	17.738	2.658	1.00	0.00	RX0	N
ATOM	1631	HH21	ARG	326	16.127	16.883	2.143	0.00	0.00	RX0	H
ATOM	1632	HH22	ARG	326	16.729	18.455	2.435	0.00	0.00	RX0	H
ATOM	1633	C	ARG	326	14.537	17.576	9.843	1.00	0.00	RX0	C
ATOM	1634	O	ARG	326	13.617	17.928	10.589	1.00	0.00	RX0	O
ATOM	1635	N	VAL	327	15.171	16.419	9.987	1.00	0.00	RX0	N
ATOM	1636	H	VAL	327	15.982	16.248	9.423	0.00	0.00	RX0	H
ATOM	1637	CA	VAL	327	14.709	15.376	10.935	1.00	0.00	RX0	C
ATOM	1638	CB	VAL	327	15.277	13.993	10.606	1.00	0.00	RX0	C
ATOM	1639	CG1	VAL	327	14.752	12.958	11.601	1.00	0.00	RX0	C
ATOM	1640	CG2	VAL	327	14.937	13.578	9.176	1.00	0.00	RX0	C
ATOM	1641	C	VAL	327	15.041	15.781	12.381	1.00	0.00	RX0	C
ATOM	1642	O	VAL	327	14.187	15.661	13.270	1.00	0.00	RX0	O
ATOM	1643	N	LEU	328	16.212	16.371	12.574	1.00	0.00	RX0	N
ATOM	1644	H	LEU	328	16.818	16.508	11.789	0.00	0.00	RX0	H
ATOM	1645	CA	LEU	328	16.631	16.899	13.887	1.00	0.00	RX0	C

ATOM	1646	CB	LEU	328	18.068	17.414	13.827	1.00	0.00	RX0	C
ATOM	1647	CG	LEU	328	19.088	16.279	13.758	1.00	0.00	RX0	C
ATOM	1648	CD1	LEU	328	20.505	16.793	13.495	1.00	0.00	RX0	C
ATOM	1649	CD2	LEU	328	19.017	15.400	15.007	1.00	0.00	RX0	C
ATOM	1650	C	LEU	328	15.692	18.005	14.386	1.00	0.00	RX0	C
ATOM	1651	O	LEU	328	15.231	17.955	15.519	1.00	0.00	RX0	O
ATOM	1652	N	ASP	329	15.222	18.821	13.436	1.00	0.00	RX0	N
ATOM	1653	H	ASP	329	15.683	18.907	12.550	0.00	0.00	RX0	H
ATOM	1654	CA	ASP	329	14.223	19.870	13.722	1.00	0.00	RX0	C
ATOM	1655	CB	ASP	329	14.040	20.807	12.527	1.00	0.00	RX0	C
ATOM	1656	CG	ASP	329	15.256	21.695	12.358	1.00	0.00	RX0	C
ATOM	1657	OD1	ASP	329	16.058	21.778	13.288	1.00	0.00	RX0	O
ATOM	1658	OD2	ASP	329	15.396	22.307	11.299	1.00	0.00	RX0	O
ATOM	1659	C	ASP	329	12.864	19.312	14.154	1.00	0.00	RX0	C
ATOM	1660	O	ASP	329	12.272	19.788	15.128	1.00	0.00	RX0	O
ATOM	1661	N	LYS	330	12.463	18.218	13.512	1.00	0.00	RX0	N
ATOM	1662	H	LYS	330	13.022	17.922	12.735	0.00	0.00	RX0	H
ATOM	1663	CA	LYS	330	11.217	17.525	13.871	1.00	0.00	RX0	C
ATOM	1664	CB	LYS	330	10.799	16.459	12.860	1.00	0.00	RX0	C
ATOM	1665	CG	LYS	330	9.621	15.608	13.368	1.00	0.00	RX0	C
ATOM	1666	CD	LYS	330	8.369	16.388	13.797	1.00	0.00	RX0	C
ATOM	1667	CE	LYS	330	7.827	17.311	12.714	1.00	0.00	RX0	C
ATOM	1668	NZ	LYS	330	7.436	16.478	11.578	1.00	0.00	RX0	N
ATOM	1669	HZ1	LYS	330	7.122	17.094	10.808	0.00	0.00	RX0	H
ATOM	1670	HZ2	LYS	330	8.246	15.891	11.280	0.00	0.00	RX0	H
ATOM	1671	HZ3	LYS	330	6.656	15.859	11.884	0.00	0.00	RX0	H
ATOM	1672	C	LYS	330	11.283	16.915	15.278	1.00	0.00	RX0	C
ATOM	1673	O	LYS	330	10.354	17.095	16.067	1.00	0.00	RX0	O
ATOM	1674	N	ILE	331	12.441	16.375	15.625	1.00	0.00	RX0	N
ATOM	1675	H	ILE	331	13.185	16.378	14.951	0.00	0.00	RX0	H
ATOM	1676	CA	ILE	331	12.656	15.802	16.972	1.00	0.00	RX0	C
ATOM	1677	CB	ILE	331	13.953	14.996	17.053	1.00	0.00	RX0	C
ATOM	1678	CG2	ILE	331	14.060	14.327	18.421	1.00	0.00	RX0	C
ATOM	1679	CG1	ILE	331	14.043	13.952	15.942	1.00	0.00	RX0	C
ATOM	1680	CD1	ILE	331	15.358	13.173	15.983	1.00	0.00	RX0	C
ATOM	1681	C	ILE	331	12.642	16.922	18.027	1.00	0.00	RX0	C
ATOM	1682	O	ILE	331	12.078	16.732	19.120	1.00	0.00	RX0	O
ATOM	1683	N	THR	332	13.158	18.084	17.675	1.00	0.00	RX0	N
ATOM	1684	H	THR	332	13.578	18.202	16.774	0.00	0.00	RX0	H
ATOM	1685	CA	THR	332	13.155	19.265	18.570	1.00	0.00	RX0	C
ATOM	1686	CB	THR	332	14.035	20.314	17.913	1.00	0.00	RX0	C
ATOM	1687	OG1	THR	332	15.300	19.703	17.627	1.00	0.00	RX0	O
ATOM	1688	HG1	THR	332	15.254	19.373	16.733	0.00	0.00	RX0	H
ATOM	1689	CG2	THR	332	14.210	21.553	18.791	1.00	0.00	RX0	C
ATOM	1690	C	THR	332	11.706	19.696	18.836	1.00	0.00	RX0	C
ATOM	1691	O	THR	332	11.302	19.832	19.995	1.00	0.00	RX0	O
ATOM	1692	N	ASP	333	10.912	19.714	17.772	1.00	0.00	RX0	N
ATOM	1693	H	ASP	333	11.280	19.673	16.837	0.00	0.00	RX0	H
ATOM	1694	CA	ASP	333	9.466	20.017	17.863	1.00	0.00	RX0	C
ATOM	1695	CB	ASP	333	8.671	19.792	16.567	1.00	0.00	RX0	C
ATOM	1696	CG	ASP	333	9.097	20.581	15.356	1.00	0.00	RX0	C
ATOM	1697	OD1	ASP	333	9.504	21.728	15.510	1.00	0.00	RX0	O
ATOM	1698	OD2	ASP	333	8.968	20.044	14.251	1.00	0.00	RX0	O
ATOM	1699	C	ASP	333	8.732	19.000	18.747	1.00	0.00	RX0	C
ATOM	1700	O	ASP	333	7.880	19.374	19.559	1.00	0.00	RX0	O
ATOM	1701	N	THR	334	9.187	17.759	18.682	1.00	0.00	RX0	N
ATOM	1702	H	THR	334	9.941	17.583	18.048	0.00	0.00	RX0	H
ATOM	1703	CA	THR	334	8.631	16.638	19.462	1.00	0.00	RX0	C
ATOM	1704	CB	THR	334	9.177	15.373	18.822	1.00	0.00	RX0	C
ATOM	1705	OG1	THR	334	8.793	15.339	17.438	1.00	0.00	RX0	O
ATOM	1706	HG1	THR	334	9.149	16.125	17.032	0.00	0.00	RX0	H

ATOM	1707	CG2	THR	334	8.725	14.119	19.558	1.00	0.00	RX0	C
ATOM	1708	C	THR	334	8.961	16.782	20.953	1.00	0.00	RX0	C
ATOM	1709	O	THR	334	8.059	16.689	21.789	1.00	0.00	RX0	O
ATOM	1710	N	LEU	335	10.217	17.081	21.263	1.00	0.00	RX0	N
ATOM	1711	H	LEU	335	10.880	17.204	20.523	0.00	0.00	RX0	H
ATOM	1712	CA	LEU	335	10.648	17.349	22.650	1.00	0.00	RX0	C
ATOM	1713	CB	LEU	335	12.150	17.610	22.696	1.00	0.00	RX0	C
ATOM	1714	CG	LEU	335	12.958	16.320	22.781	1.00	0.00	RX0	C
ATOM	1715	CD1	LEU	335	14.457	16.566	22.610	1.00	0.00	RX0	C
ATOM	1716	CD2	LEU	335	12.649	15.571	24.077	1.00	0.00	RX0	C
ATOM	1717	C	LEU	335	9.903	18.522	23.297	1.00	0.00	RX0	C
ATOM	1718	O	LEU	335	9.384	18.379	24.399	1.00	0.00	RX0	O
ATOM	1719	N	ILE	336	9.668	19.567	22.501	1.00	0.00	RX0	N
ATOM	1720	H	ILE	336	10.072	19.579	21.582	0.00	0.00	RX0	H
ATOM	1721	CA	ILE	336	8.897	20.743	22.962	1.00	0.00	RX0	C
ATOM	1722	CB	ILE	336	9.048	21.912	21.989	1.00	0.00	RX0	C
ATOM	1723	CG2	ILE	336	8.089	23.056	22.324	1.00	0.00	RX0	C
ATOM	1724	CG1	ILE	336	10.500	22.388	21.997	1.00	0.00	RX0	C
ATOM	1725	CD1	ILE	336	10.926	22.872	23.385	1.00	0.00	RX0	C
ATOM	1726	C	ILE	336	7.427	20.365	23.180	1.00	0.00	RX0	C
ATOM	1727	O	ILE	336	6.836	20.730	24.203	1.00	0.00	RX0	O
ATOM	1728	N	HIS	337	6.891	19.571	22.265	1.00	0.00	RX0	N
ATOM	1729	H	HIS	337	7.438	19.319	21.464	0.00	0.00	RX0	H
ATOM	1730	CA	HIS	337	5.501	19.096	22.359	1.00	0.00	RX0	C
ATOM	1731	CB	HIS	337	5.079	18.326	21.119	1.00	0.00	RX0	C
ATOM	1732	CG	HIS	337	3.599	18.067	21.226	1.00	0.00	RX0	C
ATOM	1733	ND1	HIS	337	2.659	18.997	20.978	1.00	0.00	RX0	N
ATOM	1734	HD1	HIS	337	2.820	19.918	20.677	0.00	0.00	RX0	H
ATOM	1735	CD2	HIS	337	2.972	16.881	21.605	1.00	0.00	RX0	C
ATOM	1736	NE2	HIS	337	1.633	17.103	21.589	1.00	0.00	RX0	N
ATOM	1737	CE1	HIS	337	1.441	18.407	21.201	1.00	0.00	RX0	C
ATOM	1738	C	HIS	337	5.301	18.263	23.631	1.00	0.00	RX0	C
ATOM	1739	O	HIS	337	4.339	18.490	24.365	1.00	0.00	RX0	O
ATOM	1740	N	LEU	338	6.274	17.405	23.914	1.00	0.00	RX0	N
ATOM	1741	H	LEU	338	7.051	17.336	23.286	0.00	0.00	RX0	H
ATOM	1742	CA	LEU	338	6.246	16.526	25.097	1.00	0.00	RX0	C
ATOM	1743	CB	LEU	338	7.441	15.575	25.089	1.00	0.00	RX0	C
ATOM	1744	CG	LEU	338	7.340	14.531	23.981	1.00	0.00	RX0	C
ATOM	1745	CD1	LEU	338	8.642	13.746	23.814	1.00	0.00	RX0	C
ATOM	1746	CD2	LEU	338	6.121	13.630	24.182	1.00	0.00	RX0	C
ATOM	1747	C	LEU	338	6.240	17.331	26.400	1.00	0.00	RX0	C
ATOM	1748	O	LEU	338	5.410	17.098	27.277	1.00	0.00	RX0	O
ATOM	1749	N	MET	339	7.027	18.402	26.394	1.00	0.00	RX0	N
ATOM	1750	H	MET	339	7.615	18.548	25.595	0.00	0.00	RX0	H
ATOM	1751	CA	MET	339	7.168	19.308	27.550	1.00	0.00	RX0	C
ATOM	1752	CB	MET	339	8.406	20.190	27.400	1.00	0.00	RX0	C
ATOM	1753	CG	MET	339	9.705	19.389	27.371	1.00	0.00	RX0	C
ATOM	1754	SD	MET	339	11.138	20.427	27.053	1.00	0.00	RX0	S
ATOM	1755	CE	MET	339	12.272	19.114	26.582	1.00	0.00	RX0	C
ATOM	1756	C	MET	339	5.924	20.183	27.753	1.00	0.00	RX0	C
ATOM	1757	O	MET	339	5.433	20.322	28.878	1.00	0.00	RX0	O
ATOM	1758	N	ALA	340	5.370	20.672	26.646	1.00	0.00	RX0	N
ATOM	1759	H	ALA	340	5.815	20.473	25.772	0.00	0.00	RX0	H
ATOM	1760	CA	ALA	340	4.120	21.456	26.636	1.00	0.00	RX0	C
ATOM	1761	CB	ALA	340	3.834	21.992	25.232	1.00	0.00	RX0	C
ATOM	1762	C	ALA	340	2.921	20.619	27.100	1.00	0.00	RX0	C
ATOM	1763	O	ALA	340	2.176	21.064	27.967	1.00	0.00	RX0	O
ATOM	1764	N	LYS	341	2.865	19.360	26.655	1.00	0.00	RX0	N
ATOM	1765	H	LYS	341	3.553	19.058	25.994	0.00	0.00	RX0	H
ATOM	1766	CA	LYS	341	1.846	18.397	27.117	1.00	0.00	RX0	C
ATOM	1767	CB	LYS	341	1.971	17.104	26.283	1.00	0.00	RX0	C

ATOM	1768	CG	LYS	341	0.693	16.261	26.157	1.00	0.00	RX0	C
ATOM	1769	CD	LYS	341	0.387	15.789	24.720	1.00	0.00	RX0	C
ATOM	1770	CE	LYS	341	1.368	14.781	24.096	1.00	0.00	RX0	C
ATOM	1771	NZ	LYS	341	1.037	14.570	22.676	1.00	0.00	RX0	N
ATOM	1772	HZ1	LYS	341	1.758	14.034	22.142	0.00	0.00	RX0	H
ATOM	1773	HZ2	LYS	341	0.179	14.012	22.479	0.00	0.00	RX0	H
ATOM	1774	HZ3	LYS	341	0.967	15.460	22.144	0.00	0.00	RX0	H
ATOM	1775	C	LYS	341	1.927	18.179	28.637	1.00	0.00	RX0	C
ATOM	1776	O	LYS	341	0.908	18.032	29.304	1.00	0.00	RX0	O
ATOM	1777	N	ALA	342	3.166	18.103	29.120	1.00	0.00	RX0	N
ATOM	1778	H	ALA	342	3.940	18.169	28.488	0.00	0.00	RX0	H
ATOM	1779	CA	ALA	342	3.459	17.942	30.556	1.00	0.00	RX0	C
ATOM	1780	CB	ALA	342	4.953	17.707	30.786	1.00	0.00	RX0	C
ATOM	1781	C	ALA	342	3.007	19.166	31.371	1.00	0.00	RX0	C
ATOM	1782	O	ALA	342	2.879	19.099	32.588	1.00	0.00	RX0	O
ATOM	1783	N	GLY	343	2.900	20.305	30.669	1.00	0.00	RX0	N
ATOM	1784	H	GLY	343	3.153	20.317	29.702	0.00	0.00	RX0	H
ATOM	1785	CA	GLY	343	2.393	21.560	31.244	1.00	0.00	RX0	C
ATOM	1786	C	GLY	343	3.505	22.433	31.829	1.00	0.00	RX0	C
ATOM	1787	O	GLY	343	3.244	23.282	32.678	1.00	0.00	RX0	O
ATOM	1788	N	LEU	344	4.736	22.226	31.356	1.00	0.00	RX0	N
ATOM	1789	H	LEU	344	4.853	21.555	30.623	0.00	0.00	RX0	H
ATOM	1790	CA	LEU	344	5.823	23.174	31.634	1.00	0.00	RX0	C
ATOM	1791	CB	LEU	344	7.151	22.611	31.138	1.00	0.00	RX0	C
ATOM	1792	CG	LEU	344	7.557	21.338	31.877	1.00	0.00	RX0	C
ATOM	1793	CD1	LEU	344	8.841	20.746	31.302	1.00	0.00	RX0	C
ATOM	1794	CD2	LEU	344	7.658	21.566	33.387	1.00	0.00	RX0	C
ATOM	1795	C	LEU	344	5.508	24.488	30.929	1.00	0.00	RX0	C
ATOM	1796	O	LEU	344	4.977	24.500	29.792	1.00	0.00	RX0	O
ATOM	1797	N	THR	345	5.822	25.577	31.575	1.00	0.00	RX0	N
ATOM	1798	H	THR	345	6.277	25.488	32.460	0.00	0.00	RX0	H
ATOM	1799	CA	THR	345	5.705	26.912	30.948	1.00	0.00	RX0	C
ATOM	1800	CB	THR	345	6.002	27.965	32.009	1.00	0.00	RX0	C
ATOM	1801	OG1	THR	345	7.181	27.611	32.728	1.00	0.00	RX0	O
ATOM	1802	HG1	THR	345	6.913	26.964	33.377	0.00	0.00	RX0	H
ATOM	1803	CG2	THR	345	4.833	28.111	32.982	1.00	0.00	RX0	C
ATOM	1804	C	THR	345	6.639	26.971	29.731	1.00	0.00	RX0	C
ATOM	1805	O	THR	345	7.615	26.233	29.623	1.00	0.00	RX0	O
ATOM	1806	N	LEU	346	6.390	27.971	28.898	1.00	0.00	RX0	N
ATOM	1807	H	LEU	346	5.564	28.513	29.045	0.00	0.00	RX0	H
ATOM	1808	CA	LEU	346	7.214	28.242	27.708	1.00	0.00	RX0	C
ATOM	1809	CB	LEU	346	6.672	29.451	26.948	1.00	0.00	RX0	C
ATOM	1810	CG	LEU	346	7.379	29.659	25.608	1.00	0.00	RX0	C
ATOM	1811	CD1	LEU	346	7.250	28.434	24.700	1.00	0.00	RX0	C
ATOM	1812	CD2	LEU	346	6.916	30.941	24.914	1.00	0.00	RX0	C
ATOM	1813	C	LEU	346	8.705	28.433	28.051	1.00	0.00	RX0	C
ATOM	1814	O	LEU	346	9.594	27.857	27.432	1.00	0.00	RX0	O
ATOM	1815	N	GLN	347	8.927	29.083	29.198	1.00	0.00	RX0	N
ATOM	1816	H	GLN	347	8.146	29.440	29.705	0.00	0.00	RX0	H
ATOM	1817	CA	GLN	347	10.275	29.275	29.754	1.00	0.00	RX0	C
ATOM	1818	CB	GLN	347	10.217	30.283	30.897	1.00	0.00	RX0	C
ATOM	1819	CG	GLN	347	11.596	30.616	31.463	1.00	0.00	RX0	C
ATOM	1820	CD	GLN	347	11.426	31.582	32.612	1.00	0.00	RX0	C
ATOM	1821	OE1	GLN	347	10.448	31.536	33.347	1.00	0.00	RX0	O
ATOM	1822	NE2	GLN	347	12.429	32.472	32.718	1.00	0.00	RX0	N
ATOM	1823	HE21	GLN	347	13.195	32.466	32.076	0.00	0.00	RX0	H
ATOM	1824	HE22	GLN	347	12.419	33.165	33.440	0.00	0.00	RX0	H
ATOM	1825	C	GLN	347	10.911	27.956	30.229	1.00	0.00	RX0	C
ATOM	1826	O	GLN	347	12.052	27.652	29.875	1.00	0.00	RX0	O
ATOM	1827	N	GLN	348	10.120	27.140	30.905	1.00	0.00	RX0	N
ATOM	1828	H	GLN	348	9.171	27.399	31.086	0.00	0.00	RX0	H

ATOM	1829	CA	GLN	348	10.582	25.823	31.394	1.00	0.00	RX0	C
ATOM	1830	CB	GLN	348	9.593	25.217	32.378	1.00	0.00	RX0	C
ATOM	1831	CG	GLN	348	9.650	25.878	33.751	1.00	0.00	RX0	C
ATOM	1832	CD	GLN	348	8.480	25.366	34.558	1.00	0.00	RX0	C
ATOM	1833	OE1	GLN	348	7.357	25.281	34.066	1.00	0.00	RX0	O
ATOM	1834	NE2	GLN	348	8.799	25.028	35.818	1.00	0.00	RX0	N
ATOM	1835	HE21	GLN	348	9.739	25.134	36.146	0.00	0.00	RX0	H
ATOM	1836	HE22	GLN	348	8.116	24.671	36.457	0.00	0.00	RX0	H
ATOM	1837	C	GLN	348	10.871	24.847	30.248	1.00	0.00	RX0	C
ATOM	1838	O	GLN	348	11.861	24.115	30.296	1.00	0.00	RX0	O
ATOM	1839	N	GLN	349	10.121	24.983	29.160	1.00	0.00	RX0	N
ATOM	1840	H	GLN	349	9.375	25.648	29.186	0.00	0.00	RX0	H
ATOM	1841	CA	GLN	349	10.299	24.172	27.940	1.00	0.00	RX0	C
ATOM	1842	CB	GLN	349	9.205	24.481	26.921	1.00	0.00	RX0	C
ATOM	1843	CG	GLN	349	7.821	24.032	27.384	1.00	0.00	RX0	C
ATOM	1844	CD	GLN	349	6.787	24.590	26.434	1.00	0.00	RX0	C
ATOM	1845	OE1	GLN	349	7.076	24.918	25.290	1.00	0.00	RX0	O
ATOM	1846	NE2	GLN	349	5.564	24.698	26.977	1.00	0.00	RX0	N
ATOM	1847	HE21	GLN	349	5.398	24.433	27.933	0.00	0.00	RX0	H
ATOM	1848	HE22	GLN	349	4.781	25.035	26.457	0.00	0.00	RX0	H
ATOM	1849	C	GLN	349	11.682	24.372	27.306	1.00	0.00	RX0	C
ATOM	1850	O	GLN	349	12.447	23.422	27.181	1.00	0.00	RX0	O
ATOM	1851	N	HIS	350	12.056	25.641	27.113	1.00	0.00	RX0	N
ATOM	1852	H	HIS	350	11.422	26.375	27.364	0.00	0.00	RX0	H
ATOM	1853	CA	HIS	350	13.344	25.953	26.463	1.00	0.00	RX0	C
ATOM	1854	CB	HIS	350	13.379	27.327	25.781	1.00	0.00	RX0	C
ATOM	1855	CG	HIS	350	13.575	28.469	26.746	1.00	0.00	RX0	C
ATOM	1856	ND1	HIS	350	12.559	29.184	27.256	1.00	0.00	RX0	N
ATOM	1857	HD1	HIS	350	11.601	29.024	27.098	0.00	0.00	RX0	H
ATOM	1858	CD2	HIS	350	14.783	28.992	27.219	1.00	0.00	RX0	C
ATOM	1859	NE2	HIS	350	14.484	30.040	28.025	1.00	0.00	RX0	N
ATOM	1860	CE1	HIS	350	13.119	30.158	28.043	1.00	0.00	RX0	C
ATOM	1861	C	HIS	350	14.540	25.670	27.386	1.00	0.00	RX0	C
ATOM	1862	O	HIS	350	15.573	25.182	26.934	1.00	0.00	RX0	O
ATOM	1863	N	GLN	351	14.320	25.854	28.691	1.00	0.00	RX0	N
ATOM	1864	H	GLN	351	13.441	26.232	28.990	0.00	0.00	RX0	H
ATOM	1865	CA	GLN	351	15.341	25.538	29.706	1.00	0.00	RX0	C
ATOM	1866	CB	GLN	351	14.917	26.060	31.073	1.00	0.00	RX0	C
ATOM	1867	CG	GLN	351	14.892	27.583	31.150	1.00	0.00	RX0	C
ATOM	1868	CD	GLN	351	14.207	27.979	32.438	1.00	0.00	RX0	C
ATOM	1869	OE1	GLN	351	13.351	27.272	32.959	1.00	0.00	RX0	O
ATOM	1870	NE2	GLN	351	14.647	29.148	32.937	1.00	0.00	RX0	N
ATOM	1871	HE21	GLN	351	15.351	29.666	32.450	0.00	0.00	RX0	H
ATOM	1872	HE22	GLN	351	14.288	29.511	33.798	0.00	0.00	RX0	H
ATOM	1873	C	GLN	351	15.597	24.030	29.805	1.00	0.00	RX0	C
ATOM	1874	O	GLN	351	16.752	23.608	29.740	1.00	0.00	RX0	O
ATOM	1875	N	ARG	352	14.527	23.242	29.744	1.00	0.00	RX0	N
ATOM	1876	H	ARG	352	13.617	23.654	29.656	0.00	0.00	RX0	H
ATOM	1877	CA	ARG	352	14.627	21.772	29.816	1.00	0.00	RX0	C
ATOM	1878	CB	ARG	352	13.311	21.110	30.249	1.00	0.00	RX0	C
ATOM	1879	CG	ARG	352	13.426	19.589	30.445	1.00	0.00	RX0	C
ATOM	1880	CD	ARG	352	12.237	18.993	31.209	1.00	0.00	RX0	C
ATOM	1881	NE	ARG	352	12.218	17.528	31.185	1.00	0.00	RX0	N
ATOM	1882	HE	ARG	352	12.015	17.063	30.308	0.00	0.00	RX0	H
ATOM	1883	CZ	ARG	352	12.340	16.772	32.319	1.00	0.00	RX0	C
ATOM	1884	NH1	ARG	352	12.627	17.376	33.490	1.00	0.00	RX0	N
ATOM	1885	HH11	ARG	352	12.802	16.830	34.322	0.00	0.00	RX0	H
ATOM	1886	HH12	ARG	352	12.689	18.371	33.570	0.00	0.00	RX0	H
ATOM	1887	NH2	ARG	352	12.170	15.436	32.255	1.00	0.00	RX0	N
ATOM	1888	HH21	ARG	352	12.205	14.800	33.034	0.00	0.00	RX0	H
ATOM	1889	HH22	ARG	352	11.983	15.017	31.344	0.00	0.00	RX0	H

ATOM	1890	C	ARG	352	15.192	21.188	28.514	1.00	0.00	RX0	C
ATOM	1891	O	ARG	352	16.048	20.305	28.551	1.00	0.00	RX0	O
ATOM	1892	N	LEU	353	14.843	21.816	27.391	1.00	0.00	RX0	N
ATOM	1893	H	LEU	353	14.131	22.519	27.429	0.00	0.00	RX0	H
ATOM	1894	CA	LEU	353	15.400	21.446	26.079	1.00	0.00	RX0	C
ATOM	1895	CB	LEU	353	14.766	22.302	24.985	1.00	0.00	RX0	C
ATOM	1896	CG	LEU	353	15.235	21.922	23.581	1.00	0.00	RX0	C
ATOM	1897	CD1	LEU	353	14.798	20.508	23.199	1.00	0.00	RX0	C
ATOM	1898	CD2	LEU	353	14.809	22.959	22.541	1.00	0.00	RX0	C
ATOM	1899	C	LEU	353	16.930	21.610	26.063	1.00	0.00	RX0	C
ATOM	1900	O	LEU	353	17.658	20.692	25.694	1.00	0.00	RX0	O
ATOM	1901	N	ALA	354	17.375	22.739	26.614	1.00	0.00	RX0	N
ATOM	1902	H	ALA	354	16.705	23.421	26.916	0.00	0.00	RX0	H
ATOM	1903	CA	ALA	354	18.808	23.073	26.716	1.00	0.00	RX0	C
ATOM	1904	CB	ALA	354	18.993	24.518	27.181	1.00	0.00	RX0	C
ATOM	1905	C	ALA	354	19.540	22.141	27.690	1.00	0.00	RX0	C
ATOM	1906	O	ALA	354	20.574	21.567	27.334	1.00	0.00	RX0	O
ATOM	1907	N	GLN	355	18.893	21.844	28.811	1.00	0.00	RX0	N
ATOM	1908	H	GLN	355	18.017	22.305	28.967	0.00	0.00	RX0	H
ATOM	1909	CA	GLN	355	19.427	20.920	29.833	1.00	0.00	RX0	C
ATOM	1910	CB	GLN	355	18.563	20.901	31.084	1.00	0.00	RX0	C
ATOM	1911	CG	GLN	355	18.675	22.209	31.860	1.00	0.00	RX0	C
ATOM	1912	CD	GLN	355	17.934	22.056	33.165	1.00	0.00	RX0	C
ATOM	1913	OE1	GLN	355	18.366	21.328	34.059	1.00	0.00	RX0	O
ATOM	1914	NE2	GLN	355	16.805	22.778	33.221	1.00	0.00	RX0	N
ATOM	1915	HE21	GLN	355	16.547	23.328	32.423	0.00	0.00	RX0	H
ATOM	1916	HE22	GLN	355	16.206	22.808	34.022	0.00	0.00	RX0	H
ATOM	1917	C	GLN	355	19.631	19.504	29.273	1.00	0.00	RX0	C
ATOM	1918	O	GLN	355	20.705	18.919	29.430	1.00	0.00	RX0	O
ATOM	1919	N	LEU	356	18.681	19.086	28.443	1.00	0.00	RX0	N
ATOM	1920	H	LEU	356	17.882	19.668	28.278	0.00	0.00	RX0	H
ATOM	1921	CA	LEU	356	18.716	17.770	27.778	1.00	0.00	RX0	C
ATOM	1922	CB	LEU	356	17.348	17.401	27.205	1.00	0.00	RX0	C
ATOM	1923	CG	LEU	356	16.354	17.031	28.303	1.00	0.00	RX0	C
ATOM	1924	CD1	LEU	356	14.993	16.629	27.734	1.00	0.00	RX0	C
ATOM	1925	CD2	LEU	356	16.924	15.953	29.225	1.00	0.00	RX0	C
ATOM	1926	C	LEU	356	19.795	17.661	26.699	1.00	0.00	RX0	C
ATOM	1927	O	LEU	356	20.593	16.720	26.700	1.00	0.00	RX0	O
ATOM	1928	N	LEU	357	19.916	18.722	25.913	1.00	0.00	RX0	N
ATOM	1929	H	LEU	357	19.277	19.487	26.027	0.00	0.00	RX0	H
ATOM	1930	CA	LEU	357	20.885	18.765	24.803	1.00	0.00	RX0	C
ATOM	1931	CB	LEU	357	20.532	19.868	23.805	1.00	0.00	RX0	C
ATOM	1932	CG	LEU	357	19.161	19.687	23.151	1.00	0.00	RX0	C
ATOM	1933	CD1	LEU	357	18.832	20.845	22.209	1.00	0.00	RX0	C
ATOM	1934	CD2	LEU	357	19.018	18.330	22.463	1.00	0.00	RX0	C
ATOM	1935	C	LEU	357	22.335	18.932	25.264	1.00	0.00	RX0	C
ATOM	1936	O	LEU	357	23.247	18.345	24.683	1.00	0.00	RX0	O
ATOM	1937	N	LEU	358	22.501	19.595	26.404	1.00	0.00	RX0	N
ATOM	1938	H	LEU	358	21.705	20.031	26.829	0.00	0.00	RX0	H
ATOM	1939	CA	LEU	358	23.826	19.754	27.029	1.00	0.00	RX0	C
ATOM	1940	CB	LEU	358	23.804	20.841	28.102	1.00	0.00	RX0	C
ATOM	1941	CG	LEU	358	23.625	22.237	27.505	1.00	0.00	RX0	C
ATOM	1942	CD1	LEU	358	23.474	23.304	28.590	1.00	0.00	RX0	C
ATOM	1943	CD2	LEU	358	24.733	22.572	26.505	1.00	0.00	RX0	C
ATOM	1944	C	LEU	358	24.390	18.455	27.609	1.00	0.00	RX0	C
ATOM	1945	O	LEU	358	25.603	18.227	27.557	1.00	0.00	RX0	O
ATOM	1946	N	ILE	359	23.510	17.559	28.043	1.00	0.00	RX0	N
ATOM	1947	H	ILE	359	22.533	17.781	28.008	0.00	0.00	RX0	H
ATOM	1948	CA	ILE	359	23.928	16.212	28.495	1.00	0.00	RX0	C
ATOM	1949	CB	ILE	359	22.747	15.468	29.119	1.00	0.00	RX0	C
ATOM	1950	CG2	ILE	359	23.123	14.046	29.538	1.00	0.00	RX0	C

ATOM	1951	CG1	ILE	359	22.216	16.275	30.303	1.00	0.00	RX0	C
ATOM	1952	CD1	ILE	359	20.898	15.734	30.854	1.00	0.00	RX0	C
ATOM	1953	C	ILE	359	24.559	15.428	27.334	1.00	0.00	RX0	C
ATOM	1954	O	ILE	359	25.552	14.723	27.543	1.00	0.00	RX0	O
ATOM	1955	N	LEU	360	24.038	15.620	26.133	1.00	0.00	RX0	N
ATOM	1956	H	LEU	360	23.250	16.230	26.031	0.00	0.00	RX0	H
ATOM	1957	CA	LEU	360	24.601	14.974	24.928	1.00	0.00	RX0	C
ATOM	1958	CB	LEU	360	23.754	15.278	23.691	1.00	0.00	RX0	C
ATOM	1959	CG	LEU	360	22.267	14.962	23.886	1.00	0.00	RX0	C
ATOM	1960	CD1	LEU	360	21.455	15.325	22.647	1.00	0.00	RX0	C
ATOM	1961	CD2	LEU	360	22.018	13.515	24.311	1.00	0.00	RX0	C
ATOM	1962	C	LEU	360	26.079	15.308	24.694	1.00	0.00	RX0	C
ATOM	1963	O	LEU	360	26.843	14.455	24.246	1.00	0.00	RX0	O
ATOM	1964	N	SER	361	26.491	16.472	25.202	1.00	0.00	RX0	N
ATOM	1965	H	SER	361	25.812	17.093	25.593	0.00	0.00	RX0	H
ATOM	1966	CA	SER	361	27.906	16.894	25.202	1.00	0.00	RX0	C
ATOM	1967	CB	SER	361	28.025	18.375	25.580	1.00	0.00	RX0	C
ATOM	1968	OG	SER	361	29.212	18.945	25.023	1.00	0.00	RX0	O
ATOM	1969	HG	SER	361	29.256	19.843	25.323	0.00	0.00	RX0	H
ATOM	1970	C	SER	361	28.769	15.985	26.099	1.00	0.00	RX0	C
ATOM	1971	O	SER	361	29.797	15.456	25.681	1.00	0.00	RX0	O
ATOM	1972	N	HIS	362	28.237	15.704	27.286	1.00	0.00	RX0	N
ATOM	1973	H	HIS	362	27.307	16.021	27.461	0.00	0.00	RX0	H
ATOM	1974	CA	HIS	362	28.854	14.793	28.272	1.00	0.00	RX0	C
ATOM	1975	CB	HIS	362	28.103	14.893	29.602	1.00	0.00	RX0	C
ATOM	1976	CG	HIS	362	28.138	16.316	30.112	1.00	0.00	RX0	C
ATOM	1977	ND1	HIS	362	27.327	17.306	29.683	1.00	0.00	RX0	N
ATOM	1978	HD1	HIS	362	26.627	17.263	28.995	0.00	0.00	RX0	H
ATOM	1979	CD2	HIS	362	28.996	16.834	31.086	1.00	0.00	RX0	C
ATOM	1980	NE2	HIS	362	28.692	18.146	31.240	1.00	0.00	RX0	N
ATOM	1981	CE1	HIS	362	27.668	18.437	30.378	1.00	0.00	RX0	C
ATOM	1982	C	HIS	362	28.890	13.339	27.777	1.00	0.00	RX0	C
ATOM	1983	O	HIS	362	29.902	12.656	27.936	1.00	0.00	RX0	O
ATOM	1984	N	ILE	363	27.856	12.940	27.039	1.00	0.00	RX0	N
ATOM	1985	H	ILE	363	27.096	13.579	26.907	0.00	0.00	RX0	H
ATOM	1986	CA	ILE	363	27.773	11.584	26.446	1.00	0.00	RX0	C
ATOM	1987	CB	ILE	363	26.356	11.299	25.950	1.00	0.00	RX0	C
ATOM	1988	CG2	ILE	363	26.241	9.910	25.320	1.00	0.00	RX0	C
ATOM	1989	CG1	ILE	363	25.378	11.453	27.115	1.00	0.00	RX0	C
ATOM	1990	CD1	ILE	363	23.929	11.210	26.706	1.00	0.00	RX0	C
ATOM	1991	C	ILE	363	28.830	11.416	25.343	1.00	0.00	RX0	C
ATOM	1992	O	ILE	363	29.487	10.370	25.263	1.00	0.00	RX0	O
ATOM	1993	N	ARG	364	29.035	12.469	24.564	1.00	0.00	RX0	N
ATOM	1994	H	ARG	364	28.404	13.246	24.618	0.00	0.00	RX0	H
ATOM	1995	CA	ARG	364	30.102	12.498	23.547	1.00	0.00	RX0	C
ATOM	1996	CB	ARG	364	30.091	13.831	22.795	1.00	0.00	RX0	C
ATOM	1997	CG	ARG	364	31.316	14.087	21.899	1.00	0.00	RX0	C
ATOM	1998	CD	ARG	364	31.559	12.989	20.858	1.00	0.00	RX0	C
ATOM	1999	NE	ARG	364	30.276	12.577	20.310	1.00	0.00	RX0	N
ATOM	2000	HE	ARG	364	29.725	11.909	20.833	0.00	0.00	RX0	H
ATOM	2001	CZ	ARG	364	29.683	13.253	19.295	1.00	0.00	RX0	C
ATOM	2002	NH1	ARG	364	30.401	14.075	18.493	1.00	0.00	RX0	N
ATOM	2003	HH11	ARG	364	30.000	14.557	17.707	0.00	0.00	RX0	H
ATOM	2004	HH12	ARG	364	31.381	14.220	18.655	0.00	0.00	RX0	H
ATOM	2005	NH2	ARG	364	28.367	13.068	19.141	1.00	0.00	RX0	N
ATOM	2006	HH21	ARG	364	27.782	13.500	18.455	0.00	0.00	RX0	H
ATOM	2007	HH22	ARG	364	27.899	12.398	19.752	0.00	0.00	RX0	H
ATOM	2008	C	ARG	364	31.469	12.319	24.223	1.00	0.00	RX0	C
ATOM	2009	O	ARG	364	32.264	11.465	23.831	1.00	0.00	RX0	O
ATOM	2010	N	HIS	365	31.645	13.058	25.316	1.00	0.00	RX0	N
ATOM	2011	H	HIS	365	30.921	13.706	25.567	0.00	0.00	RX0	H

ATOM	2012	CA	HIS	365	32.879	13.027	26.115	1.00	0.00	RX0	C
ATOM	2013	CB	HIS	365	32.892	14.081	27.219	1.00	0.00	RX0	C
ATOM	2014	CG	HIS	365	34.328	14.313	27.629	1.00	0.00	RX0	C
ATOM	2015	ND1	HIS	365	35.234	14.946	26.856	1.00	0.00	RX0	N
ATOM	2016	HD1	HIS	365	35.103	15.331	25.962	0.00	0.00	RX0	H
ATOM	2017	CD2	HIS	365	34.950	13.927	28.820	1.00	0.00	RX0	C
ATOM	2018	NE2	HIS	365	36.242	14.336	28.751	1.00	0.00	RX0	N
ATOM	2019	CE1	HIS	365	36.417	14.964	27.543	1.00	0.00	RX0	C
ATOM	2020	C	HIS	365	33.175	11.617	26.649	1.00	0.00	RX0	C
ATOM	2021	O	HIS	365	34.274	11.105	26.425	1.00	0.00	RX0	O
ATOM	2022	N	MET	366	32.138	10.946	27.137	1.00	0.00	RX0	N
ATOM	2023	H	MET	366	31.256	11.417	27.199	0.00	0.00	RX0	H
ATOM	2024	CA	MET	366	32.267	9.572	27.662	1.00	0.00	RX0	C
ATOM	2025	CB	MET	366	31.014	9.141	28.426	1.00	0.00	RX0	C
ATOM	2026	CG	MET	366	30.713	10.013	29.643	1.00	0.00	RX0	C
ATOM	2027	SD	MET	366	29.363	9.367	30.641	1.00	0.00	RX0	S
ATOM	2028	CE	MET	366	28.099	9.318	29.363	1.00	0.00	RX0	C
ATOM	2029	C	MET	366	32.567	8.557	26.555	1.00	0.00	RX0	C
ATOM	2030	O	MET	366	33.398	7.674	26.741	1.00	0.00	RX0	O
ATOM	2031	N	SER	367	31.985	8.782	25.378	1.00	0.00	RX0	N
ATOM	2032	H	SER	367	31.325	9.529	25.277	0.00	0.00	RX0	H
ATOM	2033	CA	SER	367	32.231	7.934	24.196	1.00	0.00	RX0	C
ATOM	2034	CB	SER	367	31.237	8.388	23.134	1.00	0.00	RX0	C
ATOM	2035	OG	SER	367	30.806	7.316	22.290	1.00	0.00	RX0	O
ATOM	2036	HG	SER	367	30.353	7.742	21.571	0.00	0.00	RX0	H
ATOM	2037	C	SER	367	33.681	8.046	23.711	1.00	0.00	RX0	C
ATOM	2038	O	SER	367	34.361	7.038	23.567	1.00	0.00	RX0	O
ATOM	2039	N	ASN	368	34.193	9.278	23.688	1.00	0.00	RX0	N
ATOM	2040	H	ASN	368	33.603	10.051	23.929	0.00	0.00	RX0	H
ATOM	2041	CA	ASN	368	35.583	9.549	23.266	1.00	0.00	RX0	C
ATOM	2042	CB	ASN	368	35.864	11.045	23.130	1.00	0.00	RX0	C
ATOM	2043	CG	ASN	368	37.241	11.233	22.517	1.00	0.00	RX0	C
ATOM	2044	OD1	ASN	368	37.768	10.336	21.869	1.00	0.00	RX0	O
ATOM	2045	ND2	ASN	368	37.773	12.460	22.706	1.00	0.00	RX0	N
ATOM	2046	HD21	ASN	368	37.328	13.260	23.119	0.00	0.00	RX0	H
ATOM	2047	HD22	ASN	368	38.694	12.695	22.385	0.00	0.00	RX0	H
ATOM	2048	C	ASN	368	36.615	8.948	24.226	1.00	0.00	RX0	C
ATOM	2049	O	ASN	368	37.514	8.215	23.806	1.00	0.00	RX0	O
ATOM	2050	N	LYS	369	36.353	9.119	25.515	1.00	0.00	RX0	N
ATOM	2051	H	LYS	369	35.554	9.671	25.764	0.00	0.00	RX0	H
ATOM	2052	CA	LYS	369	37.205	8.554	26.580	1.00	0.00	RX0	C
ATOM	2053	CB	LYS	369	36.939	9.159	27.963	1.00	0.00	RX0	C
ATOM	2054	CG	LYS	369	36.882	10.691	28.021	1.00	0.00	RX0	C
ATOM	2055	CD	LYS	369	37.990	11.420	27.257	1.00	0.00	RX0	C
ATOM	2056	CE	LYS	369	39.400	11.222	27.803	1.00	0.00	RX0	C
ATOM	2057	NZ	LYS	369	40.330	11.671	26.765	1.00	0.00	RX0	N
ATOM	2058	HZ1	LYS	369	41.137	11.011	26.697	0.00	0.00	RX0	H
ATOM	2059	HZ2	LYS	369	39.881	11.527	25.834	0.00	0.00	RX0	H
ATOM	2060	HZ3	LYS	369	40.638	12.653	26.841	0.00	0.00	RX0	H
ATOM	2061	C	LYS	369	37.117	7.021	26.620	1.00	0.00	RX0	C
ATOM	2062	O	LYS	369	38.123	6.337	26.771	1.00	0.00	RX0	O
ATOM	2063	N	GLY	370	35.908	6.527	26.299	1.00	0.00	RX0	N
ATOM	2064	H	GLY	370	35.160	7.167	26.118	0.00	0.00	RX0	H
ATOM	2065	CA	GLY	370	35.597	5.090	26.225	1.00	0.00	RX0	C
ATOM	2066	C	GLY	370	36.324	4.429	25.048	1.00	0.00	RX0	C
ATOM	2067	O	GLY	370	36.946	3.386	25.214	1.00	0.00	RX0	O
ATOM	2068	N	MET	371	36.380	5.143	23.928	1.00	0.00	RX0	N
ATOM	2069	H	MET	371	35.927	6.035	23.907	0.00	0.00	RX0	H
ATOM	2070	CA	MET	371	37.103	4.709	22.719	1.00	0.00	RX0	C
ATOM	2071	CB	MET	371	36.781	5.592	21.513	1.00	0.00	RX0	C
ATOM	2072	CG	MET	371	35.392	5.330	20.928	1.00	0.00	RX0	C

ATOM	2073	SD	MET	371	35.179	3.629	20.373	1.00	0.00	RX0	S
ATOM	2074	CE	MET	371	36.391	3.627	19.040	1.00	0.00	RX0	C
ATOM	2075	C	MET	371	38.619	4.671	22.937	1.00	0.00	RX0	C
ATOM	2076	O	MET	371	39.258	3.669	22.631	1.00	0.00	RX0	O
ATOM	2077	N	GLU	372	39.117	5.692	23.643	1.00	0.00	RX0	N
ATOM	2078	H	GLU	372	38.541	6.494	23.824	0.00	0.00	RX0	H
ATOM	2079	CA	GLU	372	40.525	5.740	24.082	1.00	0.00	RX0	C
ATOM	2080	CB	GLU	372	40.810	7.039	24.838	1.00	0.00	RX0	C
ATOM	2081	CG	GLU	372	40.755	8.286	23.955	1.00	0.00	RX0	C
ATOM	2082	CD	GLU	372	40.796	9.531	24.818	1.00	0.00	RX0	C
ATOM	2083	OE1	GLU	372	40.087	10.486	24.514	1.00	0.00	RX0	O
ATOM	2084	OE2	GLU	372	41.514	9.563	25.817	1.00	0.00	RX0	O
ATOM	2085	C	GLU	372	40.884	4.541	24.968	1.00	0.00	RX0	C
ATOM	2086	O	GLU	372	41.865	3.853	24.723	1.00	0.00	RX0	O
ATOM	2087	N	HIS	373	40.006	4.291	25.945	1.00	0.00	RX0	N
ATOM	2088	H	HIS	373	39.228	4.912	26.044	0.00	0.00	RX0	H
ATOM	2089	CA	HIS	373	40.194	3.212	26.919	1.00	0.00	RX0	C
ATOM	2090	CB	HIS	373	39.268	3.432	28.124	1.00	0.00	RX0	C
ATOM	2091	CG	HIS	373	38.377	2.250	28.427	1.00	0.00	RX0	C
ATOM	2092	ND1	HIS	373	37.255	1.964	27.738	1.00	0.00	RX0	N
ATOM	2093	HD1	HIS	373	36.922	2.445	26.947	0.00	0.00	RX0	H
ATOM	2094	CD2	HIS	373	38.521	1.314	29.458	1.00	0.00	RX0	C
ATOM	2095	NE2	HIS	373	37.467	0.462	29.382	1.00	0.00	RX0	N
ATOM	2096	CE1	HIS	373	36.690	0.863	28.326	1.00	0.00	RX0	C
ATOM	2097	C	HIS	373	40.078	1.832	26.262	1.00	0.00	RX0	C
ATOM	2098	O	HIS	373	40.934	1.001	26.470	1.00	0.00	RX0	O
ATOM	2099	N	LEU	374	39.111	1.679	25.351	1.00	0.00	RX0	N
ATOM	2100	H	LEU	374	38.559	2.475	25.113	0.00	0.00	RX0	H
ATOM	2101	CA	LEU	374	38.919	0.408	24.629	1.00	0.00	RX0	C
ATOM	2102	CB	LEU	374	37.665	0.452	23.755	1.00	0.00	RX0	C
ATOM	2103	CG	LEU	374	36.361	0.300	24.537	1.00	0.00	RX0	C
ATOM	2104	CD1	LEU	374	35.141	0.595	23.663	1.00	0.00	RX0	C
ATOM	2105	CD2	LEU	374	36.269	-1.069	25.212	1.00	0.00	RX0	C
ATOM	2106	C	LEU	374	40.113	0.051	23.744	1.00	0.00	RX0	C
ATOM	2107	O	LEU	374	40.555	-1.109	23.754	1.00	0.00	RX0	O
ATOM	2108	N	TYR	375	40.664	1.068	23.103	1.00	0.00	RX0	N
ATOM	2109	H	TYR	375	40.301	1.989	23.264	0.00	0.00	RX0	H
ATOM	2110	CA	TYR	375	41.854	0.983	22.222	1.00	0.00	RX0	C
ATOM	2111	CB	TYR	375	41.975	2.235	21.354	1.00	0.00	RX0	C
ATOM	2112	CG	TYR	375	41.318	1.994	20.017	1.00	0.00	RX0	C
ATOM	2113	CD1	TYR	375	40.237	1.094	19.930	1.00	0.00	RX0	C
ATOM	2114	CE1	TYR	375	39.672	0.828	18.673	1.00	0.00	RX0	C
ATOM	2115	CD2	TYR	375	41.821	2.669	18.887	1.00	0.00	RX0	C
ATOM	2116	CE2	TYR	375	41.256	2.404	17.630	1.00	0.00	RX0	C
ATOM	2117	CZ	TYR	375	40.206	1.468	17.536	1.00	0.00	RX0	C
ATOM	2118	OH	TYR	375	39.693	1.156	16.293	1.00	0.00	RX0	O
ATOM	2119	HH	TYR	375	40.326	1.444	15.640	0.00	0.00	RX0	H
ATOM	2120	C	TYR	375	43.165	0.750	22.977	1.00	0.00	RX0	C
ATOM	2121	O	TYR	375	44.176	1.452	22.753	1.00	0.00	RX0	O
ATOM	2122	N	SER	376	43.162	-0.226	23.845	1.00	0.00	RX0	N
ATOM	2123	H	SER	376	42.297	-0.617	24.142	0.00	0.00	RX0	H
ATOM	2124	CA	SER	376	44.345	-0.646	24.634	1.00	0.00	RX0	C
ATOM	2125	CB	SER	376	44.681	0.415	25.693	1.00	0.00	RX0	C
ATOM	2126	OG	SER	376	44.113	1.686	25.364	1.00	0.00	RX0	O
ATOM	2127	HG	SER	376	44.449	1.934	24.508	0.00	0.00	RX0	H
ATOM	2128	C	SER	376	44.183	-1.992	25.350	1.00	0.00	RX0	C
ATOM	2129	O	SER	376	44.985	-2.369	26.199	1.00	0.00	RX0	O
ATOM	2130	N	MET	377	43.156	-2.746	24.938	1.00	0.00	RX0	N
ATOM	2131	H	MET	377	42.565	-2.431	24.194	0.00	0.00	RX0	H
ATOM	2132	CA	MET	377	42.915	-4.112	25.422	1.00	0.00	RX0	C
ATOM	2133	CB	MET	377	41.735	-4.071	26.399	1.00	0.00	RX0	C

ATOM	2134	CG	MET	377	42.038	-3.410	27.744	1.00	0.00	RX0	C
ATOM	2135	SD	MET	377	40.550	-3.249	28.739	1.00	0.00	RX0	S
ATOM	2136	CE	MET	377	39.727	-2.020	27.712	1.00	0.00	RX0	C
ATOM	2137	C	MET	377	42.647	-5.080	24.257	1.00	0.00	RX0	C
ATOM	2138	O	MET	377	43.092	-4.854	23.130	1.00	0.00	RX0	O
ATOM	2139	N	LYS	378	41.856	-6.107	24.527	1.00	0.00	RX0	N
ATOM	2140	H	LYS	378	41.483	-6.208	25.443	0.00	0.00	RX0	H
ATOM	2141	CA	LYS	378	41.570	-7.191	23.576	1.00	0.00	RX0	C
ATOM	2142	CB	LYS	378	41.440	-8.519	24.363	1.00	0.00	RX0	C
ATOM	2143	CG	LYS	378	40.448	-8.516	25.552	1.00	0.00	RX0	C
ATOM	2144	CD	LYS	378	40.402	-9.805	26.398	1.00	0.00	RX0	C
ATOM	2145	CE	LYS	378	39.446	-9.726	27.609	1.00	0.00	RX0	C
ATOM	2146	NZ	LYS	378	39.479	-10.968	28.410	1.00	0.00	RX0	N
ATOM	2147	HZ1	LYS	378	38.889	-10.896	29.273	0.00	0.00	RX0	H
ATOM	2148	HZ2	LYS	378	39.130	-11.762	27.837	0.00	0.00	RX0	H
ATOM	2149	HZ3	LYS	378	40.457	-11.168	28.700	0.00	0.00	RX0	H
ATOM	2150	C	LYS	378	40.316	-6.920	22.720	1.00	0.00	RX0	C
ATOM	2151	O	LYS	378	40.245	-7.352	21.611	1.00	0.00	RX0	O
ATOM	2152	N	CYS	379	39.320	-6.266	23.410	1.00	0.00	RX0	N
ATOM	2153	H	CYS	379	39.564	-5.891	24.298	0.00	0.00	RX0	H
ATOM	2154	CA	CYS	379	37.939	-6.053	22.985	1.00	0.00	RX0	C
ATOM	2155	CB	CYS	379	37.616	-4.558	23.097	1.00	0.00	RX0	C
ATOM	2156	SG	CYS	379	35.844	-4.185	23.105	1.00	0.00	RX0	S
ATOM	2157	C	CYS	379	37.599	-6.654	21.614	1.00	0.00	RX0	C
ATOM	2158	O	CYS	379	37.115	-7.773	21.559	1.00	0.00	RX0	O
ATOM	2159	N	LYS	380	38.012	-5.930	20.563	1.00	0.00	RX0	N
ATOM	2160	H	LYS	380	38.389	-5.007	20.647	0.00	0.00	RX0	H
ATOM	2161	CA	LYS	380	37.949	-6.383	19.165	1.00	0.00	RX0	C
ATOM	2162	CB	LYS	380	36.479	-6.760	18.913	1.00	0.00	RX0	C
ATOM	2163	CG	LYS	380	35.908	-7.144	17.546	1.00	0.00	RX0	C
ATOM	2164	CD	LYS	380	34.397	-7.338	17.742	1.00	0.00	RX0	C
ATOM	2165	CE	LYS	380	33.576	-7.686	16.497	1.00	0.00	RX0	C
ATOM	2166	NZ	LYS	380	32.160	-7.794	16.876	1.00	0.00	RX0	N
ATOM	2167	HZ1	LYS	380	31.514	-8.033	16.105	0.00	0.00	RX0	H
ATOM	2168	HZ2	LYS	380	31.978	-8.503	17.622	0.00	0.00	RX0	H
ATOM	2169	HZ3	LYS	380	31.786	-6.936	17.339	0.00	0.00	RX0	H
ATOM	2170	C	LYS	380	38.311	-5.215	18.248	1.00	0.00	RX0	C
ATOM	2171	O	LYS	380	37.971	-4.057	18.518	1.00	0.00	RX0	O
ATOM	2172	N	ASN	381	38.881	-5.592	17.123	1.00	0.00	RX0	N
ATOM	2173	H	ASN	381	39.199	-6.541	17.104	0.00	0.00	RX0	H
ATOM	2174	CA	ASN	381	39.208	-4.700	15.996	1.00	0.00	RX0	C
ATOM	2175	CB	ASN	381	40.353	-3.771	16.397	1.00	0.00	RX0	C
ATOM	2176	CG	ASN	381	40.116	-2.390	15.836	1.00	0.00	RX0	C
ATOM	2177	OD1	ASN	381	40.768	-1.943	14.902	1.00	0.00	RX0	O
ATOM	2178	ND2	ASN	381	39.185	-1.700	16.498	1.00	0.00	RX0	N
ATOM	2179	HD21	ASN	381	38.629	-2.110	17.222	0.00	0.00	RX0	H
ATOM	2180	HD22	ASN	381	39.068	-0.728	16.272	0.00	0.00	RX0	H
ATOM	2181	C	ASN	381	39.620	-5.523	14.759	1.00	0.00	RX0	C
ATOM	2182	O	ASN	381	40.286	-5.062	13.834	1.00	0.00	RX0	O
ATOM	2183	N	VAL	382	39.176	-6.776	14.737	1.00	0.00	RX0	N
ATOM	2184	H	VAL	382	38.481	-7.033	15.402	0.00	0.00	RX0	H
ATOM	2185	CA	VAL	382	39.338	-7.659	13.572	1.00	0.00	RX0	C
ATOM	2186	CB	VAL	382	39.247	-9.129	14.003	1.00	0.00	RX0	C
ATOM	2187	CG1	VAL	382	39.431	-10.089	12.824	1.00	0.00	RX0	C
ATOM	2188	CG2	VAL	382	40.244	-9.424	15.127	1.00	0.00	RX0	C
ATOM	2189	C	VAL	382	38.195	-7.277	12.635	1.00	0.00	RX0	C
ATOM	2190	O	VAL	382	37.049	-7.276	13.059	1.00	0.00	RX0	O
ATOM	2191	N	VAL	383	38.551	-7.063	11.363	1.00	0.00	RX0	N
ATOM	2192	H	VAL	383	39.525	-7.106	11.151	0.00	0.00	RX0	H
ATOM	2193	CA	VAL	383	37.600	-6.539	10.366	1.00	0.00	RX0	C
ATOM	2194	CB	VAL	383	36.435	-7.507	10.076	1.00	0.00	RX0	C

ATOM	2195	CG1	VAL	383	35.426	-6.924	9.079	1.00	0.00	RX0	C
ATOM	2196	CG2	VAL	383	36.965	-8.863	9.604	1.00	0.00	RX0	C
ATOM	2197	C	VAL	383	37.102	-5.173	10.877	1.00	0.00	RX0	C
ATOM	2198	O	VAL	383	36.355	-5.097	11.860	1.00	0.00	RX0	O
ATOM	2199	N	PRO	384	37.530	-4.088	10.236	1.00	0.00	RX0	N
ATOM	2200	CD	PRO	384	38.353	-4.077	9.033	1.00	0.00	RX0	C
ATOM	2201	CA	PRO	384	37.142	-2.731	10.663	1.00	0.00	RX0	C
ATOM	2202	CB	PRO	384	37.698	-1.843	9.546	1.00	0.00	RX0	C
ATOM	2203	CG	PRO	384	37.986	-2.763	8.361	1.00	0.00	RX0	C
ATOM	2204	C	PRO	384	35.624	-2.628	10.828	1.00	0.00	RX0	C
ATOM	2205	O	PRO	384	34.847	-3.282	10.112	1.00	0.00	RX0	O
ATOM	2206	N	LEU	385	35.228	-1.769	11.750	1.00	0.00	RX0	N
ATOM	2207	H	LEU	385	35.913	-1.180	12.174	0.00	0.00	RX0	H
ATOM	2208	CA	LEU	385	33.812	-1.621	12.127	1.00	0.00	RX0	C
ATOM	2209	CB	LEU	385	33.662	-0.731	13.364	1.00	0.00	RX0	C
ATOM	2210	CG	LEU	385	32.280	-0.815	14.020	1.00	0.00	RX0	C
ATOM	2211	CD1	LEU	385	31.932	-2.235	14.472	1.00	0.00	RX0	C
ATOM	2212	CD2	LEU	385	32.140	0.190	15.160	1.00	0.00	RX0	C
ATOM	2213	C	LEU	385	32.896	-1.213	10.964	1.00	0.00	RX0	C
ATOM	2214	O	LEU	385	31.809	-1.761	10.815	1.00	0.00	RX0	O
ATOM	2215	N	TYR	386	33.449	-0.406	10.055	1.00	0.00	RX0	N
ATOM	2216	H	TYR	386	34.206	0.165	10.379	0.00	0.00	RX0	H
ATOM	2217	CA	TYR	386	32.718	0.069	8.867	1.00	0.00	RX0	C
ATOM	2218	CB	TYR	386	33.693	0.888	8.001	1.00	0.00	RX0	C
ATOM	2219	CG	TYR	386	33.092	1.253	6.661	1.00	0.00	RX0	C
ATOM	2220	CD1	TYR	386	32.322	2.426	6.541	1.00	0.00	RX0	C
ATOM	2221	CE1	TYR	386	31.684	2.697	5.319	1.00	0.00	RX0	C
ATOM	2222	CD2	TYR	386	33.299	0.383	5.571	1.00	0.00	RX0	C
ATOM	2223	CE2	TYR	386	32.657	0.653	4.355	1.00	0.00	RX0	C
ATOM	2224	CZ	TYR	386	31.855	1.803	4.245	1.00	0.00	RX0	C
ATOM	2225	OH	TYR	386	31.232	2.053	3.037	1.00	0.00	RX0	O
ATOM	2226	HH	TYR	386	30.654	2.804	3.131	0.00	0.00	RX0	H
ATOM	2227	C	TYR	386	32.144	-1.103	8.049	1.00	0.00	RX0	C
ATOM	2228	O	TYR	386	30.957	-1.141	7.757	1.00	0.00	RX0	O
ATOM	2229	N	ASP	387	32.969	-2.141	7.903	1.00	0.00	RX0	N
ATOM	2230	H	ASP	387	33.927	-2.042	8.176	0.00	0.00	RX0	H
ATOM	2231	CA	ASP	387	32.619	-3.323	7.093	1.00	0.00	RX0	C
ATOM	2232	CB	ASP	387	33.891	-4.032	6.621	1.00	0.00	RX0	C
ATOM	2233	CG	ASP	387	34.663	-3.082	5.709	1.00	0.00	RX0	C
ATOM	2234	OD1	ASP	387	34.497	-3.156	4.492	1.00	0.00	RX0	O
ATOM	2235	OD2	ASP	387	35.411	-2.248	6.219	1.00	0.00	RX0	O
ATOM	2236	C	ASP	387	31.563	-4.207	7.765	1.00	0.00	RX0	C
ATOM	2237	O	ASP	387	30.619	-4.649	7.114	1.00	0.00	RX0	O
ATOM	2238	N	LEU	388	31.677	-4.332	9.088	1.00	0.00	RX0	N
ATOM	2239	H	LEU	388	32.433	-3.839	9.524	0.00	0.00	RX0	H
ATOM	2240	CA	LEU	388	30.691	-5.072	9.891	1.00	0.00	RX0	C
ATOM	2241	CB	LEU	388	31.204	-5.222	11.324	1.00	0.00	RX0	C
ATOM	2242	CG	LEU	388	30.278	-6.056	12.211	1.00	0.00	RX0	C
ATOM	2243	CD1	LEU	388	30.083	-7.471	11.662	1.00	0.00	RX0	C
ATOM	2244	CD2	LEU	388	30.741	-6.064	13.668	1.00	0.00	RX0	C
ATOM	2245	C	LEU	388	29.315	-4.386	9.871	1.00	0.00	RX0	C
ATOM	2246	O	LEU	388	28.299	-5.023	9.588	1.00	0.00	RX0	O
ATOM	2247	N	LEU	389	29.339	-3.070	10.049	1.00	0.00	RX0	N
ATOM	2248	H	LEU	389	30.227	-2.622	10.175	0.00	0.00	RX0	H
ATOM	2249	CA	LEU	389	28.129	-2.229	9.970	1.00	0.00	RX0	C
ATOM	2250	CB	LEU	389	28.425	-0.790	10.375	1.00	0.00	RX0	C
ATOM	2251	CG	LEU	389	28.857	-0.655	11.832	1.00	0.00	RX0	C
ATOM	2252	CD1	LEU	389	29.235	0.783	12.157	1.00	0.00	RX0	C
ATOM	2253	CD2	LEU	389	27.804	-1.183	12.803	1.00	0.00	RX0	C
ATOM	2254	C	LEU	389	27.489	-2.268	8.583	1.00	0.00	RX0	C
ATOM	2255	O	LEU	389	26.284	-2.473	8.458	1.00	0.00	RX0	O

ATOM	2256	N	LEU	390	28.350	-2.246	7.565	1.00	0.00	RX0	N
ATOM	2257	H	LEU	390	29.327	-2.132	7.754	0.00	0.00	RX0	H
ATOM	2258	CA	LEU	390	27.929	-2.335	6.162	1.00	0.00	RX0	C
ATOM	2259	CB	LEU	390	29.177	-2.195	5.286	1.00	0.00	RX0	C
ATOM	2260	CG	LEU	390	28.962	-2.090	3.778	1.00	0.00	RX0	C
ATOM	2261	CD1	LEU	390	28.323	-0.760	3.382	1.00	0.00	RX0	C
ATOM	2262	CD2	LEU	390	30.268	-2.338	3.024	1.00	0.00	RX0	C
ATOM	2263	C	LEU	390	27.202	-3.653	5.862	1.00	0.00	RX0	C
ATOM	2264	O	LEU	390	26.123	-3.627	5.302	1.00	0.00	RX0	O
ATOM	2265	N	GLU	391	27.733	-4.754	6.404	1.00	0.00	RX0	N
ATOM	2266	H	GLU	391	28.654	-4.695	6.795	0.00	0.00	RX0	H
ATOM	2267	CA	GLU	391	27.124	-6.090	6.264	1.00	0.00	RX0	C
ATOM	2268	CB	GLU	391	28.074	-7.236	6.596	1.00	0.00	RX0	C
ATOM	2269	CG	GLU	391	29.241	-7.216	5.596	1.00	0.00	RX0	C
ATOM	2270	CD	GLU	391	28.757	-6.812	4.203	1.00	0.00	RX0	C
ATOM	2271	OE1	GLU	391	28.129	-7.610	3.507	1.00	0.00	RX0	O
ATOM	2272	OE2	GLU	391	29.008	-5.681	3.793	1.00	0.00	RX0	O
ATOM	2273	C	GLU	391	25.713	-6.159	6.867	1.00	0.00	RX0	C
ATOM	2274	O	GLU	391	24.778	-6.600	6.214	1.00	0.00	RX0	O
ATOM	2275	N	MET	392	25.589	-5.588	8.070	1.00	0.00	RX0	N
ATOM	2276	H	MET	392	26.414	-5.173	8.461	0.00	0.00	RX0	H
ATOM	2277	CA	MET	392	24.313	-5.555	8.807	1.00	0.00	RX0	C
ATOM	2278	CB	MET	392	24.544	-5.229	10.282	1.00	0.00	RX0	C
ATOM	2279	CG	MET	392	25.233	-6.388	11.004	1.00	0.00	RX0	C
ATOM	2280	SD	MET	392	25.394	-6.130	12.778	1.00	0.00	RX0	S
ATOM	2281	CE	MET	392	26.592	-4.792	12.725	1.00	0.00	RX0	C
ATOM	2282	C	MET	392	23.266	-4.630	8.173	1.00	0.00	RX0	C
ATOM	2283	O	MET	392	22.107	-5.006	8.028	1.00	0.00	RX0	O
ATOM	2284	N	LEU	393	23.721	-3.452	7.754	1.00	0.00	RX0	N
ATOM	2285	H	LEU	393	24.706	-3.278	7.794	0.00	0.00	RX0	H
ATOM	2286	CA	LEU	393	22.852	-2.457	7.099	1.00	0.00	RX0	C
ATOM	2287	CB	LEU	393	23.512	-1.082	7.155	1.00	0.00	RX0	C
ATOM	2288	CG	LEU	393	23.445	-0.455	8.545	1.00	0.00	RX0	C
ATOM	2289	CD1	LEU	393	24.506	0.625	8.730	1.00	0.00	RX0	C
ATOM	2290	CD2	LEU	393	22.041	0.065	8.855	1.00	0.00	RX0	C
ATOM	2291	C	LEU	393	22.462	-2.815	5.663	1.00	0.00	RX0	C
ATOM	2292	O	LEU	393	21.313	-2.601	5.257	1.00	0.00	RX0	O
ATOM	2293	N	ASP	394	23.400	-3.384	4.923	1.00	0.00	RX0	N
ATOM	2294	H	ASP	394	24.264	-3.670	5.333	0.00	0.00	RX0	H
ATOM	2295	CA	ASP	394	23.187	-3.768	3.516	1.00	0.00	RX0	C
ATOM	2296	CB	ASP	394	24.516	-3.813	2.761	1.00	0.00	RX0	C
ATOM	2297	CG	ASP	394	24.314	-4.014	1.273	1.00	0.00	RX0	C
ATOM	2298	OD1	ASP	394	23.340	-3.498	0.732	1.00	0.00	RX0	O
ATOM	2299	OD2	ASP	394	25.153	-4.668	0.653	1.00	0.00	RX0	O
ATOM	2300	C	ASP	394	22.492	-5.132	3.432	1.00	0.00	RX0	C
ATOM	2301	O	ASP	394	23.034	-6.108	2.888	1.00	0.00	RX0	O
ATOM	2302	N	ALA	395	21.256	-5.134	3.874	1.00	0.00	RX0	N
ATOM	2303	H	ALA	395	20.881	-4.239	4.116	0.00	0.00	RX0	H
ATOM	2304	CA	ALA	395	20.412	-6.338	3.880	1.00	0.00	RX0	C
ATOM	2305	CB	ALA	395	19.423	-6.296	5.046	1.00	0.00	RX0	C
ATOM	2306	C	ALA	395	19.639	-6.454	2.565	1.00	0.00	RX0	C
ATOM	2307	O	ALA	395	19.303	-5.463	1.911	1.00	0.00	RX0	O
ATOM	2308	N	HIS	396	19.441	-7.696	2.156	1.00	0.00	RX0	N
ATOM	2309	H	HIS	396	19.739	-8.409	2.787	0.00	0.00	RX0	H
ATOM	2310	CA	HIS	396	18.536	-7.999	1.035	1.00	0.00	RX0	C
ATOM	2311	CB	HIS	396	18.855	-9.400	0.503	1.00	0.00	RX0	C
ATOM	2312	CG	HIS	396	19.026	-10.351	1.668	1.00	0.00	RX0	C
ATOM	2313	ND1	HIS	396	20.225	-10.784	2.098	1.00	0.00	RX0	N
ATOM	2314	HD1	HIS	396	21.103	-10.556	1.721	0.00	0.00	RX0	H
ATOM	2315	CD2	HIS	396	18.035	-10.906	2.482	1.00	0.00	RX0	C
ATOM	2316	NE2	HIS	396	18.647	-11.678	3.408	1.00	0.00	RX0	N

```
ATOM   2317  CE1 HIS   396      19.998 -11.605   3.173  1.00  0.00      RX0  C
ATOM   2318  C   HIS   396      17.077  -7.909   1.523  1.00  0.00      RX0  C
ATOM   2319  O   HIS   396      16.821  -7.755   2.721  1.00  0.00      RX0  O
ATOM   2320  N   ARG   397      16.142  -8.174   0.629  1.00  0.00      RX0  N
ATOM   2321  H   ARG   397      16.392  -8.365  -0.320  0.00  0.00      RX0  H
ATOM   2322  CA  ARG   397      14.704  -8.151   0.982  1.00  0.00      RX0  C
ATOM   2323  CB  ARG   397      14.033  -7.010   0.208  1.00  0.00      RX0  C
ATOM   2324  CG  ARG   397      14.826  -5.734   0.519  1.00  0.00      RX0  C
ATOM   2325  CD  ARG   397      14.516  -4.464  -0.270  1.00  0.00      RX0  C
ATOM   2326  NE  ARG   397      15.554  -3.471   0.024  1.00  0.00      RX0  N
ATOM   2327  HE  ARG   397      16.467  -3.852   0.219  0.00  0.00      RX0  H
ATOM   2328  CZ  ARG   397      15.280  -2.132  -0.003  1.00  0.00      RX0  C
ATOM   2329  NH1 ARG   397      14.022  -1.720  -0.287  1.00  0.00      RX0  N
ATOM   2330 HH11 ARG   397      13.762  -0.750  -0.319  0.00  0.00      RX0  H
ATOM   2331 HH12 ARG   397      13.290  -2.380  -0.479  0.00  0.00      RX0  H
ATOM   2332  NH2 ARG   397      16.272  -1.247   0.257  1.00  0.00      RX0  N
ATOM   2333 HH21 ARG   397      16.134  -0.251   0.257  0.00  0.00      RX0  H
ATOM   2334 HH22 ARG   397      17.207  -1.556   0.467  0.00  0.00      RX0  H
ATOM   2335  C   ARG   397      14.082  -9.540   0.774  1.00  0.00      RX0  C
ATOM   2336  O   ARG   397      12.875  -9.706   0.613  1.00  0.00      RX0  O
ATOM   2337  N   LEU   398      14.938 -10.556   0.835  1.00  0.00      RX0  N
ATOM   2338  H   LEU   398      15.873 -10.363   1.120  0.00  0.00      RX0  H
ATOM   2339  CA  LEU   398      14.565 -11.951   0.542  1.00  0.00      RX0  C
ATOM   2340  CB  LEU   398      15.800 -12.796   0.232  1.00  0.00      RX0  C
ATOM   2341  CG  LEU   398      16.583 -12.273  -0.973  1.00  0.00      RX0  C
ATOM   2342  CD1 LEU   398      17.872 -13.067  -1.194  1.00  0.00      RX0  C
ATOM   2343  CD2 LEU   398      15.720 -12.211  -2.234  1.00  0.00      RX0  C
ATOM   2344  C   LEU   398      13.757 -12.584   1.680  1.00  0.00      RX0  C
ATOM   2345  O   LEU   398      12.772 -13.259   1.439  1.00  0.00      RX0  O
ATOM   2346  N   HIS   399      14.100 -12.169   2.904  1.00  0.00      RX0  N
ATOM   2347  H   HIS   399      14.842 -11.513   3.010  0.00  0.00      RX0  H
ATOM   2348  CA  HIS   399      13.392 -12.616   4.118  1.00  0.00      RX0  C
ATOM   2349  CB  HIS   399      14.354 -12.610   5.307  1.00  0.00      RX0  C
ATOM   2350  CG  HIS   399      15.409 -13.665   5.068  1.00  0.00      RX0  C
ATOM   2351  ND1 HIS   399      16.707 -13.401   4.816  1.00  0.00      RX0  N
ATOM   2352  HD1 HIS   399      17.146 -12.524   4.758  0.00  0.00      RX0  H
ATOM   2353  CD2 HIS   399      15.221 -15.051   5.052  1.00  0.00      RX0  C
ATOM   2354  NE2 HIS   399      16.423 -15.619   4.787  1.00  0.00      RX0  N
ATOM   2355  CE1 HIS   399      17.337 -14.607   4.642  1.00  0.00      RX0  C
ATOM   2356  C   HIS   399      12.131 -11.786   4.401  1.00  0.00      RX0  C
ATOM   2357  O   HIS   399      11.630 -11.743   5.524  1.00  0.00      RX0  O
ATOM   2358  N   ALA   400      11.619 -11.143   3.351  1.00  0.00      RX0  N
ATOM   2359  H   ALA   400      11.998 -11.314   2.443  0.00  0.00      RX0  H
ATOM   2360  CA  ALA   400      10.356 -10.385   3.402  1.00  0.00      RX0  C
ATOM   2361  CB  ALA   400      10.144  -9.553   2.138  1.00  0.00      RX0  C
ATOM   2362  C   ALA   400       9.147 -11.323   3.579  1.00  0.00      RX0  C
ATOM   2363  O   ALA   400       8.508 -11.220   4.647  1.00  0.00      RX0  O
TER
HEADER lig.000.00.pdb
ATOM      1  N   ASP   985      23.924  20.918  15.697  1.00  0.00      LX0  N
ATOM      2  H   ASP   985      24.274  20.277  16.387  0.00  0.00      LX0  H
ATOM      3  CA  ASP   985      24.313  22.312  15.909  1.00  0.00      LX0  C
ATOM      4  CB  ASP   985      23.077  23.206  16.100  1.00  0.00      LX0  C
ATOM      5  CG  ASP   985      23.233  24.495  15.304  1.00  0.00      LX0  C
ATOM      6  OD1 ASP   985      22.277  24.910  14.657  1.00  0.00      LX0  O
ATOM      7  OD2 ASP   985      24.324  25.051  15.253  1.00  0.00      LX0  O
ATOM      8  C   ASP   985      25.281  22.422  17.075  1.00  0.00      LX0  C
ATOM      9  O   ASP   985      25.662  21.407  17.645  1.00  0.00      LX0  O
ATOM     10  N   VAL   986      25.652  23.674  17.401  1.00  0.00      LX0  N
ATOM     11  H   VAL   986      25.265  24.406  16.835  0.00  0.00      LX0  H
ATOM     12  CA  VAL   986      26.584  23.979  18.488  1.00  0.00      LX0  C
```

ATOM	13	CB	VAL	986	26.004	23.616	19.873	1.00	0.00	LX0	C
ATOM	14	CG1	VAL	986	26.927	24.040	21.022	1.00	0.00	LX0	C
ATOM	15	CG2	VAL	986	24.605	24.214	20.053	1.00	0.00	LX0	C
ATOM	16	C	VAL	986	27.987	23.419	18.298	1.00	0.00	LX0	C
ATOM	17	O	VAL	986	28.295	22.257	18.531	1.00	0.00	LX0	O
ATOM	18	N	TYR	987	28.878	24.339	17.896	1.00	0.00	LX0	N
ATOM	19	H	TYR	987	28.612	25.287	17.735	0.00	0.00	LX0	H
ATOM	20	CA	TYR	987	30.264	23.924	18.080	1.00	0.00	LX0	C
ATOM	21	CB	TYR	987	31.246	24.629	17.127	1.00	0.00	LX0	C
ATOM	22	CG	TYR	987	32.612	23.986	17.271	1.00	0.00	LX0	C
ATOM	23	CD1	TYR	987	32.851	22.739	16.656	1.00	0.00	LX0	C
ATOM	24	CE1	TYR	987	34.078	22.093	16.878	1.00	0.00	LX0	C
ATOM	25	CD2	TYR	987	33.597	24.630	18.050	1.00	0.00	LX0	C
ATOM	26	CE2	TYR	987	34.822	23.981	18.277	1.00	0.00	LX0	C
ATOM	27	CZ	TYR	987	35.036	22.712	17.705	1.00	0.00	LX0	C
ATOM	28	OH	TYR	987	36.218	22.047	17.963	1.00	0.00	LX0	O
ATOM	29	HH	TYR	987	36.108	21.582	18.801	0.00	0.00	LX0	H
ATOM	30	C	TYR	987	30.676	24.088	19.528	1.00	0.00	LX0	C
ATOM	31	O	TYR	987	31.042	25.161	19.989	1.00	0.00	LX0	O
ATOM	32	N	VAL	988	30.588	22.955	20.225	1.00	0.00	LX0	N
ATOM	33	H	VAL	988	30.237	22.138	19.764	0.00	0.00	LX0	H
ATOM	34	CA	VAL	988	31.201	22.929	21.548	1.00	0.00	LX0	C
ATOM	35	CB	VAL	988	30.658	21.719	22.346	1.00	0.00	LX0	C
ATOM	36	CG1	VAL	988	30.890	20.381	21.634	1.00	0.00	LX0	C
ATOM	37	CG2	VAL	988	31.157	21.698	23.794	1.00	0.00	LX0	C
ATOM	38	C	VAL	988	32.722	22.933	21.419	1.00	0.00	LX0	C
ATOM	39	O	VAL	988	33.287	22.182	20.635	1.00	0.00	LX0	O
ATOM	40	N	PRO	989	33.375	23.836	22.186	1.00	0.00	LX0	N
ATOM	41	CD	PRO	989	32.804	24.959	22.919	1.00	0.00	LX0	C
ATOM	42	CA	PRO	989	34.834	23.757	22.311	1.00	0.00	LX0	C
ATOM	43	CB	PRO	989	35.168	24.979	23.177	1.00	0.00	LX0	C
ATOM	44	CG	PRO	989	33.974	25.924	23.042	1.00	0.00	LX0	C
ATOM	45	C	PRO	989	35.285	22.446	22.943	1.00	0.00	LX0	C
ATOM	46	O	PRO	989	35.376	22.309	24.157	1.00	0.00	LX0	O
ATOM	47	N	ASP	990	35.532	21.486	22.048	1.00	0.00	LX0	N
ATOM	48	H	ASP	990	35.360	21.657	21.078	0.00	0.00	LX0	H
ATOM	49	CA	ASP	990	36.098	20.206	22.463	1.00	0.00	LX0	C
ATOM	50	CB	ASP	990	35.857	19.143	21.375	1.00	0.00	LX0	C
ATOM	51	CG	ASP	990	36.264	19.630	19.992	1.00	0.00	LX0	C
ATOM	52	OD1	ASP	990	37.407	20.018	19.774	1.00	0.00	LX0	O
ATOM	53	OD2	ASP	990	35.432	19.632	19.095	1.00	0.00	LX0	O
ATOM	54	C	ASP	990	37.567	20.328	22.823	1.00	0.00	LX0	C
ATOM	55	O	ASP	990	38.133	21.419	22.845	1.00	0.00	LX0	O
ATOM	56	N	GLU	991	38.185	19.168	23.097	1.00	0.00	LX0	N
ATOM	57	H	GLU	991	37.730	18.275	23.013	0.00	0.00	LX0	H
ATOM	58	CA	GLU	991	39.567	19.216	23.567	1.00	0.00	LX0	C
ATOM	59	CB	GLU	991	39.949	17.896	24.265	1.00	0.00	LX0	C
ATOM	60	CG	GLU	991	40.026	16.601	23.429	1.00	0.00	LX0	C
ATOM	61	CD	GLU	991	38.667	16.010	23.060	1.00	0.00	LX0	C
ATOM	62	OE1	GLU	991	37.686	16.236	23.770	1.00	0.00	LX0	O
ATOM	63	OE2	GLU	991	38.601	15.292	22.064	1.00	0.00	LX0	O
ATOM	64	C	GLU	991	40.642	19.680	22.581	1.00	0.00	LX0	C
ATOM	65	O	GLU	991	41.836	19.586	22.849	1.00	0.00	LX0	O
ATOM	66	N	TRP	992	40.182	20.197	21.429	1.00	0.00	LX0	N
ATOM	67	H	TRP	992	39.201	20.247	21.236	0.00	0.00	LX0	H
ATOM	68	CA	TRP	992	41.141	20.676	20.440	1.00	0.00	LX0	C
ATOM	69	CB	TRP	992	40.957	19.957	19.093	1.00	0.00	LX0	C
ATOM	70	CG	TRP	992	40.952	18.444	19.215	1.00	0.00	LX0	C
ATOM	71	CD2	TRP	992	41.889	17.503	18.648	1.00	0.00	LX0	C
ATOM	72	CE2	TRP	992	41.459	16.185	19.032	1.00	0.00	LX0	C
ATOM	73	CE3	TRP	992	43.055	17.664	17.868	1.00	0.00	LX0	C

ATOM	74	CD1	TRP	992	40.008	17.647	19.882	1.00	0.00	LX0	C
ATOM	75	NE1	TRP	992	40.294	16.325	19.783	1.00	0.00	LX0	N
ATOM	76	HE1	TRP	992	39.769	15.625	20.243	0.00	0.00	LX0	H
ATOM	77	CZ2	TRP	992	42.192	15.057	18.604	1.00	0.00	LX0	C
ATOM	78	CZ3	TRP	992	43.779	16.527	17.450	1.00	0.00	LX0	C
ATOM	79	CH2	TRP	992	43.354	15.232	17.822	1.00	0.00	LX0	C
ATOM	80	C	TRP	992	41.078	22.182	20.214	1.00	0.00	LX0	C
ATOM	81	O	TRP	992	41.677	22.703	19.280	1.00	0.00	LX0	O
ATOM	82	N	GLU	993	40.307	22.864	21.078	1.00	0.00	LX0	N
ATOM	83	H	GLU	993	39.842	22.404	21.835	0.00	0.00	LX0	H
ATOM	84	CA	GLU	993	40.224	24.320	20.925	1.00	0.00	LX0	C
ATOM	85	CB	GLU	993	38.973	24.820	21.659	1.00	0.00	LX0	C
ATOM	86	CG	GLU	993	38.678	26.330	21.628	1.00	0.00	LX0	C
ATOM	87	CD	GLU	993	38.393	26.880	20.236	1.00	0.00	LX0	C
ATOM	88	OE1	GLU	993	37.976	26.154	19.341	1.00	0.00	LX0	O
ATOM	89	OE2	GLU	993	38.537	28.081	20.049	1.00	0.00	LX0	O
ATOM	90	C	GLU	993	41.505	25.029	21.360	1.00	0.00	LX0	C
ATOM	91	O	GLU	993	42.182	24.629	22.301	1.00	0.00	LX0	O
ATOM	92	N	VAL	994	41.836	26.073	20.593	1.00	0.00	LX0	N
ATOM	93	H	VAL	994	41.171	26.399	19.916	0.00	0.00	LX0	H
ATOM	94	CA	VAL	994	43.160	26.683	20.663	1.00	0.00	LX0	C
ATOM	95	CB	VAL	994	43.939	26.320	19.379	1.00	0.00	LX0	C
ATOM	96	CG1	VAL	994	45.114	27.238	19.034	1.00	0.00	LX0	C
ATOM	97	CG2	VAL	994	44.403	24.866	19.459	1.00	0.00	LX0	C
ATOM	98	C	VAL	994	43.070	28.182	20.892	1.00	0.00	LX0	C
ATOM	99	O	VAL	994	42.292	28.907	20.284	1.00	0.00	LX0	O
ATOM	100	N	ALA	995	43.921	28.628	21.828	1.00	0.00	LX0	N
ATOM	101	H	ALA	995	44.587	28.006	22.233	0.00	0.00	LX0	H
ATOM	102	CA	ALA	995	43.919	30.060	22.101	1.00	0.00	LX0	C
ATOM	103	CB	ALA	995	44.627	30.360	23.422	1.00	0.00	LX0	C
ATOM	104	C	ALA	995	44.534	30.894	20.990	1.00	0.00	LX0	C
ATOM	105	O	ALA	995	45.402	30.477	20.232	1.00	0.00	LX0	O
ATOM	106	N	ARG	996	44.022	32.130	20.924	1.00	0.00	LX0	N
ATOM	107	H	ARG	996	43.342	32.409	21.599	0.00	0.00	LX0	H
ATOM	108	CA	ARG	996	44.387	32.973	19.787	1.00	0.00	LX0	C
ATOM	109	CB	ARG	996	43.332	34.078	19.638	1.00	0.00	LX0	C
ATOM	110	CG	ARG	996	43.342	34.822	18.298	1.00	0.00	LX0	C
ATOM	111	CD	ARG	996	42.046	35.593	18.037	1.00	0.00	LX0	C
ATOM	112	NE	ARG	996	40.939	34.660	17.833	1.00	0.00	LX0	N
ATOM	113	HE	ARG	996	40.574	34.130	18.606	0.00	0.00	LX0	H
ATOM	114	CZ	ARG	996	40.418	34.447	16.609	1.00	0.00	LX0	C
ATOM	115	NH1	ARG	996	40.773	35.194	15.572	1.00	0.00	LX0	N
ATOM	116	HH11	ARG	996	40.384	34.983	14.669	0.00	0.00	LX0	H
ATOM	117	HH12	ARG	996	41.399	35.967	15.671	0.00	0.00	LX0	H
ATOM	118	NH2	ARG	996	39.529	33.482	16.438	1.00	0.00	LX0	N
ATOM	119	HH21	ARG	996	39.094	33.328	15.545	0.00	0.00	LX0	H
ATOM	120	HH22	ARG	996	39.245	32.913	17.216	0.00	0.00	LX0	H
ATOM	121	C	ARG	996	45.829	33.480	19.750	1.00	0.00	LX0	C
ATOM	122	O	ARG	996	46.307	33.985	18.741	1.00	0.00	LX0	O
ATOM	123	N	GLU	997	46.535	33.292	20.880	1.00	0.00	LX0	N
ATOM	124	H	GLU	997	46.162	32.770	21.644	0.00	0.00	LX0	H
ATOM	125	CA	GLU	997	47.951	33.675	20.851	1.00	0.00	LX0	C
ATOM	126	CB	GLU	997	48.568	33.717	22.259	1.00	0.00	LX0	C
ATOM	127	CG	GLU	997	48.220	32.593	23.250	1.00	0.00	LX0	C
ATOM	128	CD	GLU	997	48.682	31.222	22.788	1.00	0.00	LX0	C
ATOM	129	OE1	GLU	997	49.826	31.056	22.376	1.00	0.00	LX0	O
ATOM	130	OE2	GLU	997	47.899	30.283	22.853	1.00	0.00	LX0	O
ATOM	131	C	GLU	997	48.823	32.894	19.874	1.00	0.00	LX0	C
ATOM	132	O	GLU	997	49.912	33.306	19.495	1.00	0.00	LX0	O
ATOM	133	N	LYS	998	48.260	31.763	19.425	1.00	0.00	LX0	N
ATOM	134	H	LYS	998	47.383	31.449	19.790	0.00	0.00	LX0	H

ATOM	135	CA	LYS	998	49.021	30.984	18.455	1.00	0.00	LX0	C
ATOM	136	CB	LYS	998	48.582	29.517	18.458	1.00	0.00	LX0	C
ATOM	137	CG	LYS	998	48.403	29.066	19.898	1.00	0.00	LX0	C
ATOM	138	CD	LYS	998	48.650	27.598	20.204	1.00	0.00	LX0	C
ATOM	139	CE	LYS	998	48.850	27.412	21.710	1.00	0.00	LX0	C
ATOM	140	NZ	LYS	998	49.797	28.432	22.184	1.00	0.00	LX0	N
ATOM	141	HZ1	LYS	998	50.147	28.245	23.136	0.00	0.00	LX0	H
ATOM	142	HZ2	LYS	998	50.591	28.544	21.521	0.00	0.00	LX0	H
ATOM	143	HZ3	LYS	998	49.311	29.361	22.228	0.00	0.00	LX0	H
ATOM	144	C	LYS	998	49.052	31.503	17.033	1.00	0.00	LX0	C
ATOM	145	O	LYS	998	49.749	30.955	16.190	1.00	0.00	LX0	O
ATOM	146	N	ILE	999	48.229	32.523	16.764	1.00	0.00	LX0	N
ATOM	147	H	ILE	999	47.809	33.087	17.477	0.00	0.00	LX0	H
ATOM	148	CA	ILE	999	47.925	32.689	15.345	1.00	0.00	LX0	C
ATOM	149	CB	ILE	999	46.426	32.957	15.125	1.00	0.00	LX0	C
ATOM	150	CG2	ILE	999	46.044	32.658	13.673	1.00	0.00	LX0	C
ATOM	151	CG1	ILE	999	45.531	32.188	16.106	1.00	0.00	LX0	C
ATOM	152	CD1	ILE	999	45.621	30.667	15.995	1.00	0.00	LX0	C
ATOM	153	C	ILE	999	48.787	33.697	14.602	1.00	0.00	LX0	C
ATOM	154	O	ILE	999	48.359	34.789	14.242	1.00	0.00	LX0	O
ATOM	155	N	THR	1000	50.026	33.272	14.349	1.00	0.00	LX0	N
ATOM	156	H	THR	1000	50.366	32.373	14.640	0.00	0.00	LX0	H
ATOM	157	CA	THR	1000	50.905	34.154	13.590	1.00	0.00	LX0	C
ATOM	158	CB	THR	1000	52.364	33.837	13.943	1.00	0.00	LX0	C
ATOM	159	OG1	THR	1000	52.478	33.312	15.275	1.00	0.00	LX0	O
ATOM	160	HG1	THR	1000	53.277	32.782	15.255	0.00	0.00	LX0	H
ATOM	161	CG2	THR	1000	53.274	35.053	13.755	1.00	0.00	LX0	C
ATOM	162	C	THR	1000	50.668	34.085	12.079	1.00	0.00	LX0	C
ATOM	163	O	THR	1000	51.441	33.523	11.311	1.00	0.00	LX0	O
ATOM	164	N	MET	1001	49.534	34.670	11.654	1.00	0.00	LX0	N
ATOM	165	H	MET	1001	48.957	35.131	12.331	0.00	0.00	LX0	H
ATOM	166	CA	MET	1001	49.240	34.621	10.215	1.00	0.00	LX0	C
ATOM	167	CB	MET	1001	47.885	35.255	9.900	1.00	0.00	LX0	C
ATOM	168	CG	MET	1001	46.720	34.396	10.390	1.00	0.00	LX0	C
ATOM	169	SD	MET	1001	45.103	35.099	10.031	1.00	0.00	LX0	S
ATOM	170	CE	MET	1001	45.205	36.533	11.114	1.00	0.00	LX0	C
ATOM	171	C	MET	1001	50.326	35.230	9.342	1.00	0.00	LX0	C
ATOM	172	O	MET	1001	50.883	36.277	9.642	1.00	0.00	LX0	O
ATOM	173	N	SER	1002	50.632	34.497	8.264	1.00	0.00	LX0	N
ATOM	174	H	SER	1002	50.142	33.657	8.026	0.00	0.00	LX0	H
ATOM	175	CA	SER	1002	51.846	34.884	7.557	1.00	0.00	LX0	C
ATOM	176	CB	SER	1002	52.906	33.794	7.780	1.00	0.00	LX0	C
ATOM	177	OG	SER	1002	54.221	34.232	7.386	1.00	0.00	LX0	O
ATOM	178	HG	SER	1002	54.487	34.828	8.084	0.00	0.00	LX0	H
ATOM	179	C	SER	1002	51.647	35.255	6.092	1.00	0.00	LX0	C
ATOM	180	O	SER	1002	52.358	36.103	5.564	1.00	0.00	LX0	O
ATOM	181	N	ARG	1003	50.662	34.597	5.454	1.00	0.00	LX0	N
ATOM	182	H	ARG	1003	50.181	33.832	5.892	0.00	0.00	LX0	H
ATOM	183	CA	ARG	1003	50.238	34.985	4.103	1.00	0.00	LX0	C
ATOM	184	CB	ARG	1003	51.316	34.696	3.043	1.00	0.00	LX0	C
ATOM	185	CG	ARG	1003	51.615	33.217	2.776	1.00	0.00	LX0	C
ATOM	186	CD	ARG	1003	53.070	32.991	2.355	1.00	0.00	LX0	C
ATOM	187	NE	ARG	1003	53.802	32.292	3.412	1.00	0.00	LX0	N
ATOM	188	HE	ARG	1003	53.814	31.283	3.380	0.00	0.00	LX0	H
ATOM	189	CZ	ARG	1003	54.273	32.918	4.511	1.00	0.00	LX0	C
ATOM	190	NH1	ARG	1003	54.258	34.242	4.620	1.00	0.00	LX0	N
ATOM	191	HH11	ARG	1003	54.509	34.675	5.491	0.00	0.00	LX0	H
ATOM	192	HH12	ARG	1003	53.964	34.829	3.866	0.00	0.00	LX0	H
ATOM	193	NH2	ARG	1003	54.732	32.202	5.523	1.00	0.00	LX0	N
ATOM	194	HH21	ARG	1003	55.024	32.639	6.377	0.00	0.00	LX0	H
ATOM	195	HH22	ARG	1003	54.758	31.196	5.459	0.00	0.00	LX0	H

ATOM	196	C	ARG	1003	48.950	34.276	3.756	1.00	0.00	LX0	C
ATOM	197	O	ARG	1003	48.531	33.379	4.477	1.00	0.00	LX0	O
ATOM	198	N	GLU	1004	48.350	34.693	2.637	1.00	0.00	LX0	N
ATOM	199	H	GLU	1004	48.738	35.400	2.048	0.00	0.00	LX0	H
ATOM	200	CA	GLU	1004	47.207	33.909	2.185	1.00	0.00	LX0	C
ATOM	201	CB	GLU	1004	46.124	34.808	1.593	1.00	0.00	LX0	C
ATOM	202	CG	GLU	1004	45.814	35.998	2.504	1.00	0.00	LX0	C
ATOM	203	CD	GLU	1004	44.497	36.631	2.117	1.00	0.00	LX0	C
ATOM	204	OE1	GLU	1004	44.371	37.092	0.986	1.00	0.00	LX0	O
ATOM	205	OE2	GLU	1004	43.598	36.656	2.957	1.00	0.00	LX0	O
ATOM	206	C	GLU	1004	47.638	32.852	1.192	1.00	0.00	LX0	C
ATOM	207	O	GLU	1004	48.749	32.888	0.678	1.00	0.00	LX0	O
ATOM	208	N	LEU	1005	46.720	31.908	0.967	1.00	0.00	LX0	N
ATOM	209	H	LEU	1005	45.854	31.956	1.464	0.00	0.00	LX0	H
ATOM	210	CA	LEU	1005	46.977	30.860	-0.018	1.00	0.00	LX0	C
ATOM	211	CB	LEU	1005	47.057	29.474	0.634	1.00	0.00	LX0	C
ATOM	212	CG	LEU	1005	48.213	29.271	1.617	1.00	0.00	LX0	C
ATOM	213	CD1	LEU	1005	48.123	27.906	2.301	1.00	0.00	LX0	C
ATOM	214	CD2	LEU	1005	49.583	29.487	0.970	1.00	0.00	LX0	C
ATOM	215	C	LEU	1005	45.927	30.839	-1.111	1.00	0.00	LX0	C
ATOM	216	O	LEU	1005	46.231	30.820	-2.296	1.00	0.00	LX0	O
ATOM	217	N	GLY	1006	44.664	30.840	-0.662	1.00	0.00	LX0	N
ATOM	218	H	GLY	1006	44.434	30.854	0.314	0.00	0.00	LX0	H
ATOM	219	CA	GLY	1006	43.609	30.790	-1.670	1.00	0.00	LX0	C
ATOM	220	C	GLY	1006	42.235	30.691	-1.051	1.00	0.00	LX0	C
ATOM	221	O	GLY	1006	42.093	30.499	0.150	1.00	0.00	LX0	O
ATOM	222	N	GLN	1007	41.230	30.850	-1.922	1.00	0.00	LX0	N
ATOM	223	H	GLN	1007	41.434	30.897	-2.897	0.00	0.00	LX0	H
ATOM	224	CA	GLN	1007	39.846	30.787	-1.453	1.00	0.00	LX0	C
ATOM	225	CB	GLN	1007	38.936	31.293	-2.586	1.00	0.00	LX0	C
ATOM	226	CG	GLN	1007	37.427	31.375	-2.314	1.00	0.00	LX0	C
ATOM	227	CD	GLN	1007	37.148	32.322	-1.166	1.00	0.00	LX0	C
ATOM	228	OE1	GLN	1007	37.511	33.488	-1.171	1.00	0.00	LX0	O
ATOM	229	NE2	GLN	1007	36.493	31.760	-0.150	1.00	0.00	LX0	N
ATOM	230	HE21	GLN	1007	36.214	30.797	-0.170	0.00	0.00	LX0	H
ATOM	231	HE22	GLN	1007	36.293	32.321	0.649	0.00	0.00	LX0	H
ATOM	232	C	GLN	1007	39.468	29.387	-0.993	1.00	0.00	LX0	C
ATOM	233	O	GLN	1007	39.973	28.399	-1.509	1.00	0.00	LX0	O
ATOM	234	N	GLY	1008	38.564	29.352	-0.008	1.00	0.00	LX0	N
ATOM	235	H	GLY	1008	38.289	30.174	0.493	0.00	0.00	LX0	H
ATOM	236	CA	GLY	1008	38.017	28.060	0.373	1.00	0.00	LX0	C
ATOM	237	C	GLY	1008	36.647	28.180	1.008	1.00	0.00	LX0	C
ATOM	238	O	GLY	1008	35.998	29.227	1.028	1.00	0.00	LX0	O
ATOM	239	N	SER	1009	36.258	27.033	1.571	1.00	0.00	LX0	N
ATOM	240	H	SER	1009	36.865	26.243	1.489	0.00	0.00	LX0	H
ATOM	241	CA	SER	1009	34.923	26.791	2.118	1.00	0.00	LX0	C
ATOM	242	CB	SER	1009	35.037	25.607	3.076	1.00	0.00	LX0	C
ATOM	243	OG	SER	1009	36.093	24.750	2.614	1.00	0.00	LX0	O
ATOM	244	HG	SER	1009	35.776	24.429	1.770	0.00	0.00	LX0	H
ATOM	245	C	SER	1009	34.174	27.969	2.735	1.00	0.00	LX0	C
ATOM	246	O	SER	1009	33.150	28.426	2.233	1.00	0.00	LX0	O
ATOM	247	N	PHE	1010	34.736	28.474	3.840	1.00	0.00	LX0	N
ATOM	248	H	PHE	1010	35.580	28.111	4.233	0.00	0.00	LX0	H
ATOM	249	CA	PHE	1010	34.038	29.597	4.463	1.00	0.00	LX0	C
ATOM	250	CB	PHE	1010	33.384	29.157	5.775	1.00	0.00	LX0	C
ATOM	251	CG	PHE	1010	32.087	28.424	5.514	1.00	0.00	LX0	C
ATOM	252	CD1	PHE	1010	30.880	29.155	5.490	1.00	0.00	LX0	C
ATOM	253	CD2	PHE	1010	32.096	27.027	5.303	1.00	0.00	LX0	C
ATOM	254	CE1	PHE	1010	29.666	28.481	5.257	1.00	0.00	LX0	C
ATOM	255	CE2	PHE	1010	30.884	26.350	5.071	1.00	0.00	LX0	C
ATOM	256	CZ	PHE	1010	29.680	27.085	5.052	1.00	0.00	LX0	C

ATOM	257	C	PHE	1010	34.905	30.825	4.664	1.00	0.00	LX0	C
ATOM	258	O	PHE	1010	34.678	31.653	5.538	1.00	0.00	LX0	O
ATOM	259	N	GLY	1011	35.926	30.905	3.804	1.00	0.00	LX0	N
ATOM	260	H	GLY	1011	36.067	30.259	3.051	0.00	0.00	LX0	H
ATOM	261	CA	GLY	1011	36.860	32.008	3.976	1.00	0.00	LX0	C
ATOM	262	C	GLY	1011	38.138	31.697	3.242	1.00	0.00	LX0	C
ATOM	263	O	GLY	1011	38.172	30.828	2.379	1.00	0.00	LX0	O
ATOM	264	N	MET	1012	39.179	32.441	3.614	1.00	0.00	LX0	N
ATOM	265	H	MET	1012	39.107	33.016	4.431	0.00	0.00	LX0	H
ATOM	266	CA	MET	1012	40.461	32.181	2.971	1.00	0.00	LX0	C
ATOM	267	CB	MET	1012	41.279	33.477	2.965	1.00	0.00	LX0	C
ATOM	268	CG	MET	1012	42.405	33.547	1.929	1.00	0.00	LX0	C
ATOM	269	SD	MET	1012	41.796	33.644	0.239	1.00	0.00	LX0	S
ATOM	270	CE	MET	1012	41.110	35.306	0.321	1.00	0.00	LX0	C
ATOM	271	C	MET	1012	41.189	31.050	3.682	1.00	0.00	LX0	C
ATOM	272	O	MET	1012	40.929	30.754	4.845	1.00	0.00	LX0	O
ATOM	273	N	VAL	1013	42.107	30.432	2.943	1.00	0.00	LX0	N
ATOM	274	H	VAL	1013	42.231	30.693	1.987	0.00	0.00	LX0	H
ATOM	275	CA	VAL	1013	43.084	29.573	3.595	1.00	0.00	LX0	C
ATOM	276	CB	VAL	1013	43.234	28.247	2.834	1.00	0.00	LX0	C
ATOM	277	CG1	VAL	1013	44.163	27.272	3.565	1.00	0.00	LX0	C
ATOM	278	CG2	VAL	1013	41.868	27.613	2.556	1.00	0.00	LX0	C
ATOM	279	C	VAL	1013	44.392	30.338	3.633	1.00	0.00	LX0	C
ATOM	280	O	VAL	1013	44.784	30.943	2.643	1.00	0.00	LX0	O
ATOM	281	N	TYR	1014	45.019	30.321	4.810	1.00	0.00	LX0	N
ATOM	282	H	TYR	1014	44.637	29.764	5.545	0.00	0.00	LX0	H
ATOM	283	CA	TYR	1014	46.257	31.066	5.011	1.00	0.00	LX0	C
ATOM	284	CB	TYR	1014	46.165	31.906	6.292	1.00	0.00	LX0	C
ATOM	285	CG	TYR	1014	45.199	33.058	6.151	1.00	0.00	LX0	C
ATOM	286	CD1	TYR	1014	45.722	34.343	5.912	1.00	0.00	LX0	C
ATOM	287	CE1	TYR	1014	44.836	35.419	5.750	1.00	0.00	LX0	C
ATOM	288	CD2	TYR	1014	43.813	32.826	6.268	1.00	0.00	LX0	C
ATOM	289	CE2	TYR	1014	42.927	33.902	6.100	1.00	0.00	LX0	C
ATOM	290	CZ	TYR	1014	43.450	35.179	5.817	1.00	0.00	LX0	C
ATOM	291	OH	TYR	1014	42.577	36.219	5.585	1.00	0.00	LX0	O
ATOM	292	HH	TYR	1014	42.813	36.603	4.734	0.00	0.00	LX0	H
ATOM	293	C	TYR	1014	47.416	30.112	5.180	1.00	0.00	LX0	C
ATOM	294	O	TYR	1014	47.233	29.003	5.664	1.00	0.00	LX0	O
ATOM	295	N	GLU	1015	48.614	30.600	4.832	1.00	0.00	LX0	N
ATOM	296	H	GLU	1015	48.679	31.494	4.391	0.00	0.00	LX0	H
ATOM	297	CA	GLU	1015	49.744	30.043	5.566	1.00	0.00	LX0	C
ATOM	298	CB	GLU	1015	51.053	30.008	4.767	1.00	0.00	LX0	C
ATOM	299	CG	GLU	1015	52.140	29.251	5.552	1.00	0.00	LX0	C
ATOM	300	CD	GLU	1015	53.526	29.374	4.942	1.00	0.00	LX0	C
ATOM	301	OE1	GLU	1015	53.686	29.350	3.729	1.00	0.00	LX0	O
ATOM	302	OE2	GLU	1015	54.493	29.485	5.684	1.00	0.00	LX0	O
ATOM	303	C	GLU	1015	49.939	30.872	6.815	1.00	0.00	LX0	C
ATOM	304	O	GLU	1015	50.322	32.040	6.782	1.00	0.00	LX0	O
ATOM	305	N	GLY	1016	49.620	30.226	7.927	1.00	0.00	LX0	N
ATOM	306	H	GLY	1016	49.381	29.254	7.883	0.00	0.00	LX0	H
ATOM	307	CA	GLY	1016	50.032	30.844	9.172	1.00	0.00	LX0	C
ATOM	308	C	GLY	1016	51.260	30.132	9.668	1.00	0.00	LX0	C
ATOM	309	O	GLY	1016	51.602	29.049	9.214	1.00	0.00	LX0	O
ATOM	310	N	VAL	1017	51.895	30.783	10.627	1.00	0.00	LX0	N
ATOM	311	H	VAL	1017	51.597	31.682	10.947	0.00	0.00	LX0	H
ATOM	312	CA	VAL	1017	52.782	30.020	11.485	1.00	0.00	LX0	C
ATOM	313	CB	VAL	1017	54.212	30.565	11.391	1.00	0.00	LX0	C
ATOM	314	CG1	VAL	1017	54.883	30.053	10.119	1.00	0.00	LX0	C
ATOM	315	CG2	VAL	1017	54.284	32.092	11.435	1.00	0.00	LX0	C
ATOM	316	C	VAL	1017	52.165	30.093	12.868	1.00	0.00	LX0	C
ATOM	317	O	VAL	1017	51.324	30.955	13.110	1.00	0.00	LX0	O

ATOM	318	N	ALA	1018	52.525	29.133	13.721	1.00	0.00	LX0	N
ATOM	319	H	ALA	1018	53.185	28.432	13.453	0.00	0.00	LX0	H
ATOM	320	CA	ALA	1018	51.694	29.012	14.910	1.00	0.00	LX0	C
ATOM	321	CB	ALA	1018	50.627	27.936	14.716	1.00	0.00	LX0	C
ATOM	322	C	ALA	1018	52.441	28.734	16.191	1.00	0.00	LX0	C
ATOM	323	O	ALA	1018	53.189	27.775	16.328	1.00	0.00	LX0	O
ATOM	324	N	LYS	1019	52.171	29.628	17.148	1.00	0.00	LX0	N
ATOM	325	H	LYS	1019	51.526	30.350	16.891	0.00	0.00	LX0	H
ATOM	326	CA	LYS	1019	52.836	29.586	18.448	1.00	0.00	LX0	C
ATOM	327	CB	LYS	1019	52.568	30.921	19.154	1.00	0.00	LX0	C
ATOM	328	CG	LYS	1019	53.391	31.224	20.410	1.00	0.00	LX0	C
ATOM	329	CD	LYS	1019	54.908	31.317	20.210	1.00	0.00	LX0	C
ATOM	330	CE	LYS	1019	55.375	32.360	19.186	1.00	0.00	LX0	C
ATOM	331	NZ	LYS	1019	55.372	31.791	17.836	1.00	0.00	LX0	N
ATOM	332	HZ1	LYS	1019	54.446	31.747	17.358	0.00	0.00	LX0	H
ATOM	333	HZ2	LYS	1019	55.933	32.360	17.168	0.00	0.00	LX0	H
ATOM	334	HZ3	LYS	1019	55.749	30.825	17.795	0.00	0.00	LX0	H
ATOM	335	C	LYS	1019	52.504	28.390	19.334	1.00	0.00	LX0	C
ATOM	336	O	LYS	1019	51.700	28.469	20.260	1.00	0.00	LX0	O
ATOM	337	N	GLY	1020	53.182	27.277	19.036	1.00	0.00	LX0	N
ATOM	338	H	GLY	1020	53.756	27.220	18.213	0.00	0.00	LX0	H
ATOM	339	CA	GLY	1020	52.991	26.107	19.886	1.00	0.00	LX0	C
ATOM	340	C	GLY	1020	51.726	25.336	19.578	1.00	0.00	LX0	C
ATOM	341	O	GLY	1020	50.836	25.206	20.411	1.00	0.00	LX0	O
ATOM	342	N	VAL	1021	51.665	24.833	18.333	1.00	0.00	LX0	N
ATOM	343	H	VAL	1021	52.410	24.997	17.683	0.00	0.00	LX0	H
ATOM	344	CA	VAL	1021	50.560	23.896	18.122	1.00	0.00	LX0	C
ATOM	345	CB	VAL	1021	49.673	24.227	16.909	1.00	0.00	LX0	C
ATOM	346	CG1	VAL	1021	48.896	25.522	17.139	1.00	0.00	LX0	C
ATOM	347	CG2	VAL	1021	50.434	24.241	15.590	1.00	0.00	LX0	C
ATOM	348	C	VAL	1021	50.976	22.438	18.126	1.00	0.00	LX0	C
ATOM	349	O	VAL	1021	50.283	21.577	18.658	1.00	0.00	LX0	O
ATOM	350	N	VAL	1022	52.169	22.174	17.564	1.00	0.00	LX0	N
ATOM	351	H	VAL	1022	52.731	22.882	17.130	0.00	0.00	LX0	H
ATOM	352	CA	VAL	1022	52.687	20.828	17.807	1.00	0.00	LX0	C
ATOM	353	CB	VAL	1022	53.586	20.345	16.658	1.00	0.00	LX0	C
ATOM	354	CG1	VAL	1022	54.074	18.906	16.862	1.00	0.00	LX0	C
ATOM	355	CG2	VAL	1022	52.838	20.457	15.330	1.00	0.00	LX0	C
ATOM	356	C	VAL	1022	53.370	20.768	19.163	1.00	0.00	LX0	C
ATOM	357	O	VAL	1022	54.581	20.835	19.316	1.00	0.00	LX0	O
ATOM	358	N	LYS	1023	52.483	20.686	20.167	1.00	0.00	LX0	N
ATOM	359	H	LYS	1023	51.517	20.697	19.906	0.00	0.00	LX0	H
ATOM	360	CA	LYS	1023	52.900	20.874	21.556	1.00	0.00	LX0	C
ATOM	361	CB	LYS	1023	53.584	19.613	22.117	1.00	0.00	LX0	C
ATOM	362	CG	LYS	1023	52.691	18.371	22.047	1.00	0.00	LX0	C
ATOM	363	CD	LYS	1023	51.387	18.544	22.829	1.00	0.00	LX0	C
ATOM	364	CE	LYS	1023	50.458	17.336	22.707	1.00	0.00	LX0	C
ATOM	365	NZ	LYS	1023	49.213	17.612	23.436	1.00	0.00	LX0	N
ATOM	366	HZ1	LYS	1023	48.579	16.790	23.372	0.00	0.00	LX0	H
ATOM	367	HZ2	LYS	1023	49.432	17.807	24.434	0.00	0.00	LX0	H
ATOM	368	HZ3	LYS	1023	48.744	18.441	23.016	0.00	0.00	LX0	H
ATOM	369	C	LYS	1023	53.669	22.173	21.775	1.00	0.00	LX0	C
ATOM	370	O	LYS	1023	53.085	23.246	21.695	1.00	0.00	LX0	O
ATOM	371	N	ASP	1024	54.968	22.044	22.044	1.00	0.00	LX0	N
ATOM	372	H	ASP	1024	55.485	21.184	22.007	0.00	0.00	LX0	H
ATOM	373	CA	ASP	1024	55.772	23.238	22.284	1.00	0.00	LX0	C
ATOM	374	CB	ASP	1024	56.993	22.871	23.138	1.00	0.00	LX0	C
ATOM	375	CG	ASP	1024	57.727	21.691	22.524	1.00	0.00	LX0	C
ATOM	376	OD1	ASP	1024	58.667	21.916	21.768	1.00	0.00	LX0	O
ATOM	377	OD2	ASP	1024	57.325	20.555	22.777	1.00	0.00	LX0	O
ATOM	378	C	ASP	1024	56.179	23.995	21.030	1.00	0.00	LX0	C

ATOM	379	O	ASP	1024	56.381	25.205	21.071	1.00	0.00	LX0	O
ATOM	380	N	GLU	1025	56.259	23.236	19.914	1.00	0.00	LX0	N
ATOM	381	H	GLU	1025	56.116	22.251	20.024	0.00	0.00	LX0	H
ATOM	382	CA	GLU	1025	56.796	23.754	18.647	1.00	0.00	LX0	C
ATOM	383	CB	GLU	1025	56.424	22.810	17.498	1.00	0.00	LX0	C
ATOM	384	CG	GLU	1025	57.437	22.758	16.346	1.00	0.00	LX0	C
ATOM	385	CD	GLU	1025	57.206	23.875	15.347	1.00	0.00	LX0	C
ATOM	386	OE1	GLU	1025	57.610	25.007	15.595	1.00	0.00	LX0	O
ATOM	387	OE2	GLU	1025	56.612	23.606	14.310	1.00	0.00	LX0	O
ATOM	388	C	GLU	1025	56.501	25.217	18.331	1.00	0.00	LX0	C
ATOM	389	O	GLU	1025	55.384	25.622	18.015	1.00	0.00	LX0	O
ATOM	390	N	PRO	1026	57.582	26.018	18.507	1.00	0.00	LX0	N
ATOM	391	CD	PRO	1026	58.952	25.565	18.740	1.00	0.00	LX0	C
ATOM	392	CA	PRO	1026	57.460	27.474	18.579	1.00	0.00	LX0	C
ATOM	393	CB	PRO	1026	58.920	27.937	18.588	1.00	0.00	LX0	C
ATOM	394	CG	PRO	1026	59.673	26.798	19.268	1.00	0.00	LX0	C
ATOM	395	C	PRO	1026	56.611	28.196	17.549	1.00	0.00	LX0	C
ATOM	396	O	PRO	1026	55.973	29.196	17.878	1.00	0.00	LX0	O
ATOM	397	N	GLU	1027	56.671	27.712	16.303	1.00	0.00	LX0	N
ATOM	398	H	GLU	1027	57.120	26.835	16.104	0.00	0.00	LX0	H
ATOM	399	CA	GLU	1027	56.064	28.493	15.230	1.00	0.00	LX0	C
ATOM	400	CB	GLU	1027	57.007	29.637	14.826	1.00	0.00	LX0	C
ATOM	401	CG	GLU	1027	56.347	30.845	14.148	1.00	0.00	LX0	C
ATOM	402	CD	GLU	1027	55.481	31.654	15.100	1.00	0.00	LX0	C
ATOM	403	OE1	GLU	1027	54.419	31.203	15.521	1.00	0.00	LX0	O
ATOM	404	OE2	GLU	1027	55.881	32.748	15.481	1.00	0.00	LX0	O
ATOM	405	C	GLU	1027	55.631	27.643	14.044	1.00	0.00	LX0	C
ATOM	406	O	GLU	1027	55.972	27.888	12.892	1.00	0.00	LX0	O
ATOM	407	N	THR	1028	54.843	26.614	14.381	1.00	0.00	LX0	N
ATOM	408	H	THR	1028	54.595	26.464	15.341	0.00	0.00	LX0	H
ATOM	409	CA	THR	1028	54.461	25.632	13.368	1.00	0.00	LX0	C
ATOM	410	CB	THR	1028	53.549	24.584	14.004	1.00	0.00	LX0	C
ATOM	411	OG1	THR	1028	53.993	24.262	15.331	1.00	0.00	LX0	O
ATOM	412	HG1	THR	1028	54.939	24.099	15.233	0.00	0.00	LX0	H
ATOM	413	CG2	THR	1028	53.435	23.326	13.137	1.00	0.00	LX0	C
ATOM	414	C	THR	1028	53.820	26.186	12.104	1.00	0.00	LX0	C
ATOM	415	O	THR	1028	52.833	26.910	12.144	1.00	0.00	LX0	O
ATOM	416	N	ARG	1029	54.417	25.802	10.968	1.00	0.00	LX0	N
ATOM	417	H	ARG	1029	55.203	25.189	11.026	0.00	0.00	LX0	H
ATOM	418	CA	ARG	1029	53.793	26.197	9.706	1.00	0.00	LX0	C
ATOM	419	CB	ARG	1029	54.785	26.037	8.555	1.00	0.00	LX0	C
ATOM	420	CG	ARG	1029	56.073	26.844	8.744	1.00	0.00	LX0	C
ATOM	421	CD	ARG	1029	56.981	26.825	7.512	1.00	0.00	LX0	C
ATOM	422	NE	ARG	1029	56.289	27.383	6.351	1.00	0.00	LX0	N
ATOM	423	HE	ARG	1029	55.732	28.214	6.458	0.00	0.00	LX0	H
ATOM	424	CZ	ARG	1029	56.318	26.779	5.148	1.00	0.00	LX0	C
ATOM	425	NH1	ARG	1029	57.034	25.676	4.972	1.00	0.00	LX0	N
ATOM	426	HH11	ARG	1029	56.975	25.210	4.079	0.00	0.00	LX0	H
ATOM	427	HH12	ARG	1029	57.606	25.292	5.692	0.00	0.00	LX0	H
ATOM	428	NH2	ARG	1029	55.613	27.264	4.133	1.00	0.00	LX0	N
ATOM	429	HH21	ARG	1029	55.600	26.777	3.253	0.00	0.00	LX0	H
ATOM	430	HH22	ARG	1029	55.056	28.106	4.228	0.00	0.00	LX0	H
ATOM	431	C	ARG	1029	52.509	25.430	9.431	1.00	0.00	LX0	C
ATOM	432	O	ARG	1029	52.497	24.206	9.369	1.00	0.00	LX0	O
ATOM	433	N	VAL	1030	51.425	26.204	9.309	1.00	0.00	LX0	N
ATOM	434	H	VAL	1030	51.529	27.198	9.310	0.00	0.00	LX0	H
ATOM	435	CA	VAL	1030	50.089	25.611	9.274	1.00	0.00	LX0	C
ATOM	436	CB	VAL	1030	49.407	25.740	10.647	1.00	0.00	LX0	C
ATOM	437	CG1	VAL	1030	50.065	24.829	11.681	1.00	0.00	LX0	C
ATOM	438	CG2	VAL	1030	49.332	27.193	11.128	1.00	0.00	LX0	C
ATOM	439	C	VAL	1030	49.201	26.214	8.200	1.00	0.00	LX0	C

ATOM	440	O	VAL	1030	49.334	27.373	7.826	1.00	0.00	LX0	O
ATOM	441	N	ALA	1031	48.273	25.379	7.721	1.00	0.00	LX0	N
ATOM	442	H	ALA	1031	48.172	24.469	8.123	0.00	0.00	LX0	H
ATOM	443	CA	ALA	1031	47.251	25.920	6.832	1.00	0.00	LX0	C
ATOM	444	CB	ALA	1031	46.874	24.917	5.742	1.00	0.00	LX0	C
ATOM	445	C	ALA	1031	46.006	26.276	7.612	1.00	0.00	LX0	C
ATOM	446	O	ALA	1031	45.340	25.434	8.198	1.00	0.00	LX0	O
ATOM	447	N	ILE	1032	45.725	27.576	7.623	1.00	0.00	LX0	N
ATOM	448	H	ILE	1032	46.277	28.199	7.070	0.00	0.00	LX0	H
ATOM	449	CA	ILE	1032	44.555	28.000	8.384	1.00	0.00	LX0	C
ATOM	450	CB	ILE	1032	44.856	29.308	9.127	1.00	0.00	LX0	C
ATOM	451	CG2	ILE	1032	43.711	29.702	10.064	1.00	0.00	LX0	C
ATOM	452	CG1	ILE	1032	46.197	29.224	9.859	1.00	0.00	LX0	C
ATOM	453	CD1	ILE	1032	46.585	30.541	10.527	1.00	0.00	LX0	C
ATOM	454	C	ILE	1032	43.345	28.171	7.491	1.00	0.00	LX0	C
ATOM	455	O	ILE	1032	43.309	29.077	6.669	1.00	0.00	LX0	O
ATOM	456	N	LYS	1033	42.349	27.293	7.668	1.00	0.00	LX0	N
ATOM	457	H	LYS	1033	42.405	26.644	8.430	0.00	0.00	LX0	H
ATOM	458	CA	LYS	1033	41.115	27.654	6.969	1.00	0.00	LX0	C
ATOM	459	CB	LYS	1033	40.282	26.433	6.543	1.00	0.00	LX0	C
ATOM	460	CG	LYS	1033	41.042	25.307	5.835	1.00	0.00	LX0	C
ATOM	461	CD	LYS	1033	40.185	24.300	5.041	1.00	0.00	LX0	C
ATOM	462	CE	LYS	1033	39.095	23.502	5.784	1.00	0.00	LX0	C
ATOM	463	NZ	LYS	1033	38.780	22.280	5.025	1.00	0.00	LX0	N
ATOM	464	HZ1	LYS	1033	37.907	21.763	5.257	0.00	0.00	LX0	H
ATOM	465	HZ2	LYS	1033	38.856	22.419	4.000	0.00	0.00	LX0	H
ATOM	466	HZ3	LYS	1033	39.565	21.613	5.193	0.00	0.00	LX0	H
ATOM	467	C	LYS	1033	40.295	28.597	7.834	1.00	0.00	LX0	C
ATOM	468	O	LYS	1033	40.355	28.530	9.058	1.00	0.00	LX0	O
ATOM	469	N	THR	1034	39.552	29.495	7.178	1.00	0.00	LX0	N
ATOM	470	H	THR	1034	39.553	29.576	6.181	0.00	0.00	LX0	H
ATOM	471	CA	THR	1034	38.802	30.423	8.020	1.00	0.00	LX0	C
ATOM	472	CB	THR	1034	39.290	31.859	7.795	1.00	0.00	LX0	C
ATOM	473	OG1	THR	1034	39.259	32.192	6.404	1.00	0.00	LX0	O
ATOM	474	HG1	THR	1034	40.020	31.753	6.030	0.00	0.00	LX0	H
ATOM	475	CG2	THR	1034	40.702	32.063	8.336	1.00	0.00	LX0	C
ATOM	476	C	THR	1034	37.297	30.343	7.859	1.00	0.00	LX0	C
ATOM	477	O	THR	1034	36.773	29.784	6.900	1.00	0.00	LX0	O
ATOM	478	N	VAL	1035	36.638	30.962	8.851	1.00	0.00	LX0	N
ATOM	479	H	VAL	1035	37.154	31.287	9.645	0.00	0.00	LX0	H
ATOM	480	CA	VAL	1035	35.270	31.425	8.648	1.00	0.00	LX0	C
ATOM	481	CB	VAL	1035	34.275	30.869	9.672	1.00	0.00	LX0	C
ATOM	482	CG1	VAL	1035	32.897	30.717	9.035	1.00	0.00	LX0	C
ATOM	483	CG2	VAL	1035	34.749	29.601	10.371	1.00	0.00	LX0	C
ATOM	484	C	VAL	1035	35.298	32.931	8.784	1.00	0.00	LX0	C
ATOM	485	O	VAL	1035	35.628	33.450	9.851	1.00	0.00	LX0	O
ATOM	486	N	ASN	1036	34.995	33.579	7.654	1.00	0.00	LX0	N
ATOM	487	H	ASN	1036	34.637	33.031	6.894	0.00	0.00	LX0	H
ATOM	488	CA	ASN	1036	35.267	35.006	7.463	1.00	0.00	LX0	C
ATOM	489	CB	ASN	1036	34.983	35.387	5.995	1.00	0.00	LX0	C
ATOM	490	CG	ASN	1036	33.516	35.718	5.757	1.00	0.00	LX0	C
ATOM	491	OD1	ASN	1036	32.614	35.046	6.236	1.00	0.00	LX0	O
ATOM	492	ND2	ASN	1036	33.307	36.798	5.010	1.00	0.00	LX0	N
ATOM	493	HD21	ASN	1036	34.059	37.442	4.812	0.00	0.00	LX0	H
ATOM	494	HD22	ASN	1036	32.406	37.056	4.675	0.00	0.00	LX0	H
ATOM	495	C	ASN	1036	34.619	35.975	8.450	1.00	0.00	LX0	C
ATOM	496	O	ASN	1036	33.901	35.601	9.373	1.00	0.00	LX0	O
ATOM	497	N	GLU	1037	34.885	37.267	8.201	1.00	0.00	LX0	N
ATOM	498	H	GLU	1037	35.437	37.516	7.401	0.00	0.00	LX0	H
ATOM	499	CA	GLU	1037	34.194	38.301	8.978	1.00	0.00	LX0	C
ATOM	500	CB	GLU	1037	34.600	39.745	8.614	1.00	0.00	LX0	C

ATOM	501	CG	GLU	1037	35.486	40.002	7.383	1.00	0.00	LX0	C
ATOM	502	CD	GLU	1037	34.905	39.349	6.143	1.00	0.00	LX0	C
ATOM	503	OE1	GLU	1037	35.579	38.499	5.573	1.00	0.00	LX0	O
ATOM	504	OE2	GLU	1037	33.761	39.618	5.791	1.00	0.00	LX0	O
ATOM	505	C	GLU	1037	32.678	38.192	8.960	1.00	0.00	LX0	C
ATOM	506	O	GLU	1037	32.055	38.070	10.008	1.00	0.00	LX0	O
ATOM	507	N	ALA	1038	32.118	38.193	7.743	1.00	0.00	LX0	N
ATOM	508	H	ALA	1038	32.716	38.362	6.953	0.00	0.00	LX0	H
ATOM	509	CA	ALA	1038	30.660	38.236	7.606	1.00	0.00	LX0	C
ATOM	510	CB	ALA	1038	30.270	38.389	6.135	1.00	0.00	LX0	C
ATOM	511	C	ALA	1038	29.860	37.082	8.199	1.00	0.00	LX0	C
ATOM	512	O	ALA	1038	28.863	37.291	8.875	1.00	0.00	LX0	O
ATOM	513	N	ALA	1039	30.341	35.859	7.907	1.00	0.00	LX0	N
ATOM	514	H	ALA	1039	31.163	35.792	7.340	0.00	0.00	LX0	H
ATOM	515	CA	ALA	1039	29.577	34.627	8.160	1.00	0.00	LX0	C
ATOM	516	CB	ALA	1039	30.528	33.432	8.172	1.00	0.00	LX0	C
ATOM	517	C	ALA	1039	28.716	34.545	9.418	1.00	0.00	LX0	C
ATOM	518	O	ALA	1039	29.099	34.995	10.499	1.00	0.00	LX0	O
ATOM	519	N	SER	1040	27.534	33.937	9.244	1.00	0.00	LX0	N
ATOM	520	H	SER	1040	27.308	33.526	8.353	0.00	0.00	LX0	H
ATOM	521	CA	SER	1040	26.654	33.846	10.408	1.00	0.00	LX0	C
ATOM	522	CB	SER	1040	25.245	33.385	10.015	1.00	0.00	LX0	C
ATOM	523	OG	SER	1040	25.233	31.980	9.729	1.00	0.00	LX0	O
ATOM	524	HG	SER	1040	25.319	31.939	8.770	0.00	0.00	LX0	H
ATOM	525	C	SER	1040	27.195	32.966	11.521	1.00	0.00	LX0	C
ATOM	526	O	SER	1040	28.171	32.236	11.374	1.00	0.00	LX0	O
ATOM	527	N	MET	1041	26.480	33.032	12.656	1.00	0.00	LX0	N
ATOM	528	H	MET	1041	25.705	33.657	12.727	0.00	0.00	LX0	H
ATOM	529	CA	MET	1041	26.834	32.112	13.735	1.00	0.00	LX0	C
ATOM	530	CB	MET	1041	26.031	32.414	15.014	1.00	0.00	LX0	C
ATOM	531	CG	MET	1041	24.538	32.052	15.008	1.00	0.00	LX0	C
ATOM	532	SD	MET	1041	23.593	32.870	13.711	1.00	0.00	LX0	S
ATOM	533	CE	MET	1041	22.073	31.921	13.878	1.00	0.00	LX0	C
ATOM	534	C	MET	1041	26.784	30.640	13.345	1.00	0.00	LX0	C
ATOM	535	O	MET	1041	27.666	29.864	13.692	1.00	0.00	LX0	O
ATOM	536	N	ARG	1042	25.735	30.287	12.572	1.00	0.00	LX0	N
ATOM	537	H	ARG	1042	25.102	30.959	12.189	0.00	0.00	LX0	H
ATOM	538	CA	ARG	1042	25.718	28.877	12.197	1.00	0.00	LX0	C
ATOM	539	CB	ARG	1042	24.336	28.326	11.816	1.00	0.00	LX0	C
ATOM	540	CG	ARG	1042	24.453	26.794	11.796	1.00	0.00	LX0	C
ATOM	541	CD	ARG	1042	23.316	25.982	11.182	1.00	0.00	LX0	C
ATOM	542	NE	ARG	1042	23.874	24.732	10.664	1.00	0.00	LX0	N
ATOM	543	HE	ARG	1042	24.331	24.724	9.767	0.00	0.00	LX0	H
ATOM	544	CZ	ARG	1042	23.959	23.605	11.398	1.00	0.00	LX0	C
ATOM	545	NH1	ARG	1042	23.407	23.510	12.594	1.00	0.00	LX0	N
ATOM	546	HH11	ARG	1042	23.444	22.657	13.112	0.00	0.00	LX0	H
ATOM	547	HH12	ARG	1042	22.954	24.289	13.055	0.00	0.00	LX0	H
ATOM	548	NH2	ARG	1042	24.626	22.571	10.921	1.00	0.00	LX0	N
ATOM	549	HH21	ARG	1042	24.693	21.710	11.437	0.00	0.00	LX0	H
ATOM	550	HH22	ARG	1042	25.105	22.642	10.032	0.00	0.00	LX0	H
ATOM	551	C	ARG	1042	26.761	28.531	11.154	1.00	0.00	LX0	C
ATOM	552	O	ARG	1042	27.396	27.494	11.236	1.00	0.00	LX0	O
ATOM	553	N	GLU	1043	26.983	29.456	10.209	1.00	0.00	LX0	N
ATOM	554	H	GLU	1043	26.426	30.287	10.141	0.00	0.00	LX0	H
ATOM	555	CA	GLU	1043	28.082	29.198	9.267	1.00	0.00	LX0	C
ATOM	556	CB	GLU	1043	28.215	30.370	8.309	1.00	0.00	LX0	C
ATOM	557	CG	GLU	1043	27.041	30.436	7.332	1.00	0.00	LX0	C
ATOM	558	CD	GLU	1043	26.833	31.869	6.891	1.00	0.00	LX0	C
ATOM	559	OE1	GLU	1043	25.710	32.353	6.986	1.00	0.00	LX0	O
ATOM	560	OE2	GLU	1043	27.793	32.531	6.516	1.00	0.00	LX0	O
ATOM	561	C	GLU	1043	29.421	28.875	9.925	1.00	0.00	LX0	C

ATOM	562	O	GLU	1043	30.179	28.014	9.493	1.00	0.00	LX0	O
ATOM	563	N	ARG	1044	29.648	29.573	11.049	1.00	0.00	LX0	N
ATOM	564	H	ARG	1044	29.000	30.294	11.302	0.00	0.00	LX0	H
ATOM	565	CA	ARG	1044	30.806	29.210	11.868	1.00	0.00	LX0	C
ATOM	566	CB	ARG	1044	31.074	30.274	12.930	1.00	0.00	LX0	C
ATOM	567	CG	ARG	1044	31.161	31.623	12.236	1.00	0.00	LX0	C
ATOM	568	CD	ARG	1044	31.336	32.830	13.144	1.00	0.00	LX0	C
ATOM	569	NE	ARG	1044	31.055	34.026	12.360	1.00	0.00	LX0	N
ATOM	570	HE	ARG	1044	30.101	34.316	12.232	0.00	0.00	LX0	H
ATOM	571	CZ	ARG	1044	31.992	34.606	11.590	1.00	0.00	LX0	C
ATOM	572	NH1	ARG	1044	33.255	34.208	11.579	1.00	0.00	LX0	N
ATOM	573	HH11	ARG	1044	33.910	34.655	10.959	0.00	0.00	LX0	H
ATOM	574	HH12	ARG	1044	33.597	33.464	12.161	0.00	0.00	LX0	H
ATOM	575	NH2	ARG	1044	31.625	35.596	10.804	1.00	0.00	LX0	N
ATOM	576	HH21	ARG	1044	32.296	36.074	10.231	0.00	0.00	LX0	H
ATOM	577	HH22	ARG	1044	30.658	35.871	10.747	0.00	0.00	LX0	H
ATOM	578	C	ARG	1044	30.718	27.831	12.490	1.00	0.00	LX0	C
ATOM	579	O	ARG	1044	31.624	27.017	12.376	1.00	0.00	LX0	O
ATOM	580	N	ILE	1045	29.571	27.585	13.140	1.00	0.00	LX0	N
ATOM	581	H	ILE	1045	28.860	28.290	13.168	0.00	0.00	LX0	H
ATOM	582	CA	ILE	1045	29.369	26.263	13.743	1.00	0.00	LX0	C
ATOM	583	CB	ILE	1045	27.979	26.206	14.391	1.00	0.00	LX0	C
ATOM	584	CG2	ILE	1045	27.570	24.782	14.779	1.00	0.00	LX0	C
ATOM	585	CG1	ILE	1045	27.924	27.180	15.575	1.00	0.00	LX0	C
ATOM	586	CD1	ILE	1045	26.524	27.384	16.157	1.00	0.00	LX0	C
ATOM	587	C	ILE	1045	29.604	25.097	12.788	1.00	0.00	LX0	C
ATOM	588	O	ILE	1045	30.360	24.178	13.080	1.00	0.00	LX0	O
ATOM	589	N	GLU	1046	28.947	25.218	11.626	1.00	0.00	LX0	N
ATOM	590	H	GLU	1046	28.301	25.968	11.530	0.00	0.00	LX0	H
ATOM	591	CA	GLU	1046	29.137	24.322	10.492	1.00	0.00	LX0	C
ATOM	592	CB	GLU	1046	28.393	24.892	9.277	1.00	0.00	LX0	C
ATOM	593	CG	GLU	1046	27.928	23.869	8.233	1.00	0.00	LX0	C
ATOM	594	CD	GLU	1046	26.628	23.206	8.663	1.00	0.00	LX0	C
ATOM	595	OE1	GLU	1046	25.569	23.804	8.535	1.00	0.00	LX0	O
ATOM	596	OE2	GLU	1046	26.648	22.074	9.124	1.00	0.00	LX0	O
ATOM	597	C	GLU	1046	30.613	24.142	10.187	1.00	0.00	LX0	C
ATOM	598	O	GLU	1046	31.181	23.075	10.347	1.00	0.00	LX0	O
ATOM	599	N	PHE	1047	31.243	25.263	9.803	1.00	0.00	LX0	N
ATOM	600	H	PHE	1047	30.745	26.130	9.729	0.00	0.00	LX0	H
ATOM	601	CA	PHE	1047	32.641	25.156	9.390	1.00	0.00	LX0	C
ATOM	602	CB	PHE	1047	33.156	26.500	8.874	1.00	0.00	LX0	C
ATOM	603	CG	PHE	1047	34.509	26.317	8.225	1.00	0.00	LX0	C
ATOM	604	CD1	PHE	1047	34.602	25.650	6.986	1.00	0.00	LX0	C
ATOM	605	CD2	PHE	1047	35.659	26.806	8.880	1.00	0.00	LX0	C
ATOM	606	CE1	PHE	1047	35.863	25.483	6.388	1.00	0.00	LX0	C
ATOM	607	CE2	PHE	1047	36.921	26.645	8.284	1.00	0.00	LX0	C
ATOM	608	CZ	PHE	1047	37.003	25.992	7.039	1.00	0.00	LX0	C
ATOM	609	C	PHE	1047	33.617	24.553	10.392	1.00	0.00	LX0	C
ATOM	610	O	PHE	1047	34.467	23.749	10.033	1.00	0.00	LX0	O
ATOM	611	N	LEU	1048	33.488	24.962	11.662	1.00	0.00	LX0	N
ATOM	612	H	LEU	1048	32.747	25.584	11.917	0.00	0.00	LX0	H
ATOM	613	CA	LEU	1048	34.412	24.360	12.629	1.00	0.00	LX0	C
ATOM	614	CB	LEU	1048	34.530	25.183	13.919	1.00	0.00	LX0	C
ATOM	615	CG	LEU	1048	35.299	26.514	13.817	1.00	0.00	LX0	C
ATOM	616	CD1	LEU	1048	36.410	26.496	12.762	1.00	0.00	LX0	C
ATOM	617	CD2	LEU	1048	34.386	27.730	13.688	1.00	0.00	LX0	C
ATOM	618	C	LEU	1048	34.094	22.906	12.947	1.00	0.00	LX0	C
ATOM	619	O	LEU	1048	34.946	22.090	13.294	1.00	0.00	LX0	O
ATOM	620	N	ASN	1049	32.805	22.604	12.775	1.00	0.00	LX0	N
ATOM	621	H	ASN	1049	32.138	23.283	12.457	0.00	0.00	LX0	H
ATOM	622	CA	ASN	1049	32.403	21.213	12.926	1.00	0.00	LX0	C

ATOM	623	CB	ASN	1049	30.898	21.150	13.105	1.00	0.00	LX0	C
ATOM	624	CG	ASN	1049	30.561	20.806	14.537	1.00	0.00	LX0	C
ATOM	625	OD1	ASN	1049	30.964	19.789	15.082	1.00	0.00	LX0	O
ATOM	626	ND2	ASN	1049	29.756	21.695	15.124	1.00	0.00	LX0	N
ATOM	627	HD21	ASN	1049	29.512	22.526	14.624	0.00	0.00	LX0	H
ATOM	628	HD22	ASN	1049	29.411	21.506	16.045	0.00	0.00	LX0	H
ATOM	629	C	ASN	1049	32.910	20.313	11.809	1.00	0.00	LX0	C
ATOM	630	O	ASN	1049	33.589	19.328	12.061	1.00	0.00	LX0	O
ATOM	631	N	GLU	1050	32.657	20.744	10.563	1.00	0.00	LX0	N
ATOM	632	H	GLU	1050	31.932	21.407	10.387	0.00	0.00	LX0	H
ATOM	633	CA	GLU	1050	33.310	20.120	9.409	1.00	0.00	LX0	C
ATOM	634	CB	GLU	1050	32.943	20.844	8.113	1.00	0.00	LX0	C
ATOM	635	CG	GLU	1050	31.993	20.039	7.222	1.00	0.00	LX0	C
ATOM	636	CD	GLU	1050	30.538	20.351	7.517	1.00	0.00	LX0	C
ATOM	637	OE1	GLU	1050	29.847	20.830	6.619	1.00	0.00	LX0	O
ATOM	638	OE2	GLU	1050	30.063	20.090	8.621	1.00	0.00	LX0	O
ATOM	639	C	GLU	1050	34.821	20.023	9.519	1.00	0.00	LX0	C
ATOM	640	O	GLU	1050	35.461	19.030	9.208	1.00	0.00	LX0	O
ATOM	641	N	ALA	1051	35.401	21.107	10.045	1.00	0.00	LX0	N
ATOM	642	H	ALA	1051	34.844	21.919	10.218	0.00	0.00	LX0	H
ATOM	643	CA	ALA	1051	36.845	21.062	10.278	1.00	0.00	LX0	C
ATOM	644	CB	ALA	1051	37.325	22.383	10.864	1.00	0.00	LX0	C
ATOM	645	C	ALA	1051	37.286	19.928	11.193	1.00	0.00	LX0	C
ATOM	646	O	ALA	1051	38.347	19.327	11.049	1.00	0.00	LX0	O
ATOM	647	N	SER	1052	36.383	19.633	12.138	1.00	0.00	LX0	N
ATOM	648	H	SER	1052	35.503	20.110	12.153	0.00	0.00	LX0	H
ATOM	649	CA	SER	1052	36.638	18.536	13.060	1.00	0.00	LX0	C
ATOM	650	CB	SER	1052	35.582	18.532	14.167	1.00	0.00	LX0	C
ATOM	651	OG	SER	1052	35.677	19.769	14.898	1.00	0.00	LX0	O
ATOM	652	HG	SER	1052	35.305	20.444	14.323	0.00	0.00	LX0	H
ATOM	653	C	SER	1052	36.860	17.153	12.463	1.00	0.00	LX0	C
ATOM	654	O	SER	1052	37.408	16.289	13.137	1.00	0.00	LX0	O
ATOM	655	N	VAL	1053	36.507	16.984	11.167	1.00	0.00	LX0	N
ATOM	656	H	VAL	1053	35.990	17.694	10.682	0.00	0.00	LX0	H
ATOM	657	CA	VAL	1053	36.930	15.761	10.461	1.00	0.00	LX0	C
ATOM	658	CB	VAL	1053	36.696	15.872	8.940	1.00	0.00	LX0	C
ATOM	659	CG1	VAL	1053	37.128	14.607	8.207	1.00	0.00	LX0	C
ATOM	660	CG2	VAL	1053	35.246	16.155	8.573	1.00	0.00	LX0	C
ATOM	661	C	VAL	1053	38.393	15.402	10.713	1.00	0.00	LX0	C
ATOM	662	O	VAL	1053	38.763	14.286	11.057	1.00	0.00	LX0	O
ATOM	663	N	MET	1054	39.230	16.438	10.549	1.00	0.00	LX0	N
ATOM	664	H	MET	1054	38.882	17.369	10.413	0.00	0.00	LX0	H
ATOM	665	CA	MET	1054	40.651	16.109	10.583	1.00	0.00	LX0	C
ATOM	666	CB	MET	1054	41.474	17.103	9.753	1.00	0.00	LX0	C
ATOM	667	CG	MET	1054	40.851	17.494	8.408	1.00	0.00	LX0	C
ATOM	668	SD	MET	1054	40.361	16.090	7.396	1.00	0.00	LX0	S
ATOM	669	CE	MET	1054	41.977	15.334	7.169	1.00	0.00	LX0	C
ATOM	670	C	MET	1054	41.256	15.903	11.964	1.00	0.00	LX0	C
ATOM	671	O	MET	1054	42.459	15.707	12.115	1.00	0.00	LX0	O
ATOM	672	N	LYS	1055	40.388	15.955	12.991	1.00	0.00	LX0	N
ATOM	673	H	LYS	1055	39.403	16.032	12.824	0.00	0.00	LX0	H
ATOM	674	CA	LYS	1055	40.904	15.694	14.335	1.00	0.00	LX0	C
ATOM	675	CB	LYS	1055	39.846	15.983	15.399	1.00	0.00	LX0	C
ATOM	676	CG	LYS	1055	39.649	17.475	15.606	1.00	0.00	LX0	C
ATOM	677	CD	LYS	1055	38.438	17.834	16.459	1.00	0.00	LX0	C
ATOM	678	CE	LYS	1055	38.372	19.351	16.596	1.00	0.00	LX0	C
ATOM	679	NZ	LYS	1055	37.087	19.786	17.135	1.00	0.00	LX0	N
ATOM	680	HZ1	LYS	1055	37.090	20.809	17.311	0.00	0.00	LX0	H
ATOM	681	HZ2	LYS	1055	36.282	19.588	16.507	0.00	0.00	LX0	H
ATOM	682	HZ3	LYS	1055	36.896	19.379	18.080	0.00	0.00	LX0	H
ATOM	683	C	LYS	1055	41.396	14.276	14.498	1.00	0.00	LX0	C

ATOM	684	O	LYS	1055	42.567	14.022	14.755	1.00	0.00	LX0	O
ATOM	685	N	GLU	1056	40.429	13.369	14.317	1.00	0.00	LX0	N
ATOM	686	H	GLU	1056	39.530	13.664	13.992	0.00	0.00	LX0	H
ATOM	687	CA	GLU	1056	40.645	11.981	14.713	1.00	0.00	LX0	C
ATOM	688	CB	GLU	1056	39.311	11.244	14.570	1.00	0.00	LX0	C
ATOM	689	CG	GLU	1056	39.286	9.799	15.075	1.00	0.00	LX0	C
ATOM	690	CD	GLU	1056	39.637	8.835	13.957	1.00	0.00	LX0	C
ATOM	691	OE1	GLU	1056	39.036	8.910	12.890	1.00	0.00	LX0	O
ATOM	692	OE2	GLU	1056	40.433	7.926	14.172	1.00	0.00	LX0	O
ATOM	693	C	GLU	1056	41.815	11.282	14.038	1.00	0.00	LX0	C
ATOM	694	O	GLU	1056	42.574	10.560	14.667	1.00	0.00	LX0	O
ATOM	695	N	PHE	1057	41.949	11.577	12.734	1.00	0.00	LX0	N
ATOM	696	H	PHE	1057	41.250	12.157	12.321	0.00	0.00	LX0	H
ATOM	697	CA	PHE	1057	42.939	10.873	11.911	1.00	0.00	LX0	C
ATOM	698	CB	PHE	1057	42.804	11.242	10.431	1.00	0.00	LX0	C
ATOM	699	CG	PHE	1057	41.379	11.248	9.933	1.00	0.00	LX0	C
ATOM	700	CD1	PHE	1057	40.483	10.210	10.272	1.00	0.00	LX0	C
ATOM	701	CD2	PHE	1057	40.978	12.319	9.109	1.00	0.00	LX0	C
ATOM	702	CE1	PHE	1057	39.163	10.259	9.793	1.00	0.00	LX0	C
ATOM	703	CE2	PHE	1057	39.661	12.361	8.621	1.00	0.00	LX0	C
ATOM	704	CZ	PHE	1057	38.761	11.335	8.975	1.00	0.00	LX0	C
ATOM	705	C	PHE	1057	44.405	11.040	12.279	1.00	0.00	LX0	C
ATOM	706	O	PHE	1057	45.142	11.765	11.619	1.00	0.00	LX0	O
ATOM	707	N	ASN	1058	44.837	10.354	13.338	1.00	0.00	LX0	N
ATOM	708	H	ASN	1058	44.223	9.728	13.833	0.00	0.00	LX0	H
ATOM	709	CA	ASN	1058	46.194	10.676	13.764	1.00	0.00	LX0	C
ATOM	710	CB	ASN	1058	46.294	10.794	15.286	1.00	0.00	LX0	C
ATOM	711	CG	ASN	1058	47.434	11.738	15.622	1.00	0.00	LX0	C
ATOM	712	OD1	ASN	1058	47.285	12.957	15.629	1.00	0.00	LX0	O
ATOM	713	ND2	ASN	1058	48.588	11.132	15.901	1.00	0.00	LX0	N
ATOM	714	HD21	ASN	1058	48.656	10.141	15.756	0.00	0.00	LX0	H
ATOM	715	HD22	ASN	1058	49.378	11.650	16.221	0.00	0.00	LX0	H
ATOM	716	C	ASN	1058	47.283	9.793	13.195	1.00	0.00	LX0	C
ATOM	717	O	ASN	1058	48.097	9.222	13.911	1.00	0.00	LX0	O
ATOM	718	N	CYS	1059	47.275	9.716	11.862	1.00	0.00	LX0	N
ATOM	719	H	CYS	1059	46.647	10.275	11.316	0.00	0.00	LX0	H
ATOM	720	CA	CYS	1059	48.365	8.968	11.249	1.00	0.00	LX0	C
ATOM	721	CB	CYS	1059	47.882	7.645	10.663	1.00	0.00	LX0	C
ATOM	722	SG	CYS	1059	49.258	6.510	10.372	1.00	0.00	LX0	S
ATOM	723	C	CYS	1059	49.063	9.806	10.209	1.00	0.00	LX0	C
ATOM	724	O	CYS	1059	48.537	10.817	9.758	1.00	0.00	LX0	O
ATOM	725	N	HIS	1060	50.284	9.364	9.866	1.00	0.00	LX0	N
ATOM	726	H	HIS	1060	50.612	8.480	10.204	0.00	0.00	LX0	H
ATOM	727	CA	HIS	1060	51.164	10.230	9.082	1.00	0.00	LX0	C
ATOM	728	CB	HIS	1060	52.482	9.515	8.787	1.00	0.00	LX0	C
ATOM	729	CG	HIS	1060	53.606	10.516	8.659	1.00	0.00	LX0	C
ATOM	730	ND1	HIS	1060	54.518	10.718	9.627	1.00	0.00	LX0	N
ATOM	731	HD1	HIS	1060	54.576	10.244	10.480	0.00	0.00	LX0	H
ATOM	732	CD2	HIS	1060	53.890	11.369	7.587	1.00	0.00	LX0	C
ATOM	733	NE2	HIS	1060	54.985	12.087	7.932	1.00	0.00	LX0	N
ATOM	734	CE1	HIS	1060	55.375	11.690	9.182	1.00	0.00	LX0	C
ATOM	735	C	HIS	1060	50.564	10.793	7.807	1.00	0.00	LX0	C
ATOM	736	O	HIS	1060	50.542	11.998	7.584	1.00	0.00	LX0	O
ATOM	737	N	HIS	1061	50.062	9.866	6.980	1.00	0.00	LX0	N
ATOM	738	H	HIS	1061	50.031	8.895	7.223	0.00	0.00	LX0	H
ATOM	739	CA	HIS	1061	49.657	10.316	5.650	1.00	0.00	LX0	C
ATOM	740	CB	HIS	1061	49.956	9.234	4.607	1.00	0.00	LX0	C
ATOM	741	CG	HIS	1061	51.392	8.786	4.760	1.00	0.00	LX0	C
ATOM	742	ND1	HIS	1061	51.747	7.595	5.271	1.00	0.00	LX0	N
ATOM	743	HD1	HIS	1061	51.137	6.875	5.549	0.00	0.00	LX0	H
ATOM	744	CD2	HIS	1061	52.553	9.502	4.458	1.00	0.00	LX0	C

ATOM	745	NE2	HIS	1061	53.608	8.723	4.802	1.00	0.00	LX0	N
ATOM	746	CE1	HIS	1061	53.112	7.548	5.303	1.00	0.00	LX0	C
ATOM	747	C	HIS	1061	48.239	10.851	5.530	1.00	0.00	LX0	C
ATOM	748	O	HIS	1061	47.611	10.806	4.479	1.00	0.00	LX0	O
ATOM	749	N	VAL	1062	47.752	11.387	6.657	1.00	0.00	LX0	N
ATOM	750	H	VAL	1062	48.280	11.433	7.507	0.00	0.00	LX0	H
ATOM	751	CA	VAL	1062	46.513	12.151	6.559	1.00	0.00	LX0	C
ATOM	752	CB	VAL	1062	45.411	11.554	7.454	1.00	0.00	LX0	C
ATOM	753	CG1	VAL	1062	44.026	11.895	6.896	1.00	0.00	LX0	C
ATOM	754	CG2	VAL	1062	45.550	10.042	7.662	1.00	0.00	LX0	C
ATOM	755	C	VAL	1062	46.829	13.582	6.965	1.00	0.00	LX0	C
ATOM	756	O	VAL	1062	47.846	13.827	7.611	1.00	0.00	LX0	O
ATOM	757	N	VAL	1063	45.944	14.512	6.574	1.00	0.00	LX0	N
ATOM	758	H	VAL	1063	45.201	14.273	5.946	0.00	0.00	LX0	H
ATOM	759	CA	VAL	1063	46.082	15.858	7.133	1.00	0.00	LX0	C
ATOM	760	CB	VAL	1063	45.190	16.851	6.363	1.00	0.00	LX0	C
ATOM	761	CG1	VAL	1063	45.219	18.287	6.903	1.00	0.00	LX0	C
ATOM	762	CG2	VAL	1063	45.573	16.837	4.887	1.00	0.00	LX0	C
ATOM	763	C	VAL	1063	45.806	15.889	8.632	1.00	0.00	LX0	C
ATOM	764	O	VAL	1063	44.889	15.262	9.158	1.00	0.00	LX0	O
ATOM	765	N	ARG	1064	46.659	16.646	9.322	1.00	0.00	LX0	N
ATOM	766	H	ARG	1064	47.445	17.068	8.860	0.00	0.00	LX0	H
ATOM	767	CA	ARG	1064	46.358	16.832	10.731	1.00	0.00	LX0	C
ATOM	768	CB	ARG	1064	47.627	16.720	11.581	1.00	0.00	LX0	C
ATOM	769	CG	ARG	1064	48.361	15.383	11.406	1.00	0.00	LX0	C
ATOM	770	CD	ARG	1064	47.492	14.145	11.666	1.00	0.00	LX0	C
ATOM	771	NE	ARG	1064	46.955	14.125	13.028	1.00	0.00	LX0	N
ATOM	772	HE	ARG	1064	47.577	13.985	13.804	0.00	0.00	LX0	H
ATOM	773	CZ	ARG	1064	45.635	14.274	13.267	1.00	0.00	LX0	C
ATOM	774	NH1	ARG	1064	44.770	14.440	12.269	1.00	0.00	LX0	N
ATOM	775	HH11	ARG	1064	43.803	14.648	12.441	0.00	0.00	LX0	H
ATOM	776	HH12	ARG	1064	45.058	14.350	11.311	0.00	0.00	LX0	H
ATOM	777	NH2	ARG	1064	45.213	14.245	14.524	1.00	0.00	LX0	N
ATOM	778	HH21	ARG	1064	44.242	14.356	14.762	0.00	0.00	LX0	H
ATOM	779	HH22	ARG	1064	45.873	14.079	15.264	0.00	0.00	LX0	H
ATOM	780	C	ARG	1064	45.606	18.110	11.031	1.00	0.00	LX0	C
ATOM	781	O	ARG	1064	46.015	19.207	10.674	1.00	0.00	LX0	O
ATOM	782	N	LEU	1065	44.484	17.930	11.741	1.00	0.00	LX0	N
ATOM	783	H	LEU	1065	44.126	17.021	11.952	0.00	0.00	LX0	H
ATOM	784	CA	LEU	1065	44.000	19.115	12.440	1.00	0.00	LX0	C
ATOM	785	CB	LEU	1065	42.490	19.049	12.685	1.00	0.00	LX0	C
ATOM	786	CG	LEU	1065	41.873	20.307	13.298	1.00	0.00	LX0	C
ATOM	787	CD1	LEU	1065	42.242	21.552	12.513	1.00	0.00	LX0	C
ATOM	788	CD2	LEU	1065	40.358	20.211	13.409	1.00	0.00	LX0	C
ATOM	789	C	LEU	1065	44.754	19.258	13.740	1.00	0.00	LX0	C
ATOM	790	O	LEU	1065	44.764	18.363	14.575	1.00	0.00	LX0	O
ATOM	791	N	LEU	1066	45.410	20.413	13.837	1.00	0.00	LX0	N
ATOM	792	H	LEU	1066	45.339	21.095	13.107	0.00	0.00	LX0	H
ATOM	793	CA	LEU	1066	46.162	20.687	15.053	1.00	0.00	LX0	C
ATOM	794	CB	LEU	1066	47.457	21.416	14.695	1.00	0.00	LX0	C
ATOM	795	CG	LEU	1066	48.331	20.569	13.763	1.00	0.00	LX0	C
ATOM	796	CD1	LEU	1066	49.447	21.381	13.110	1.00	0.00	LX0	C
ATOM	797	CD2	LEU	1066	48.867	19.318	14.464	1.00	0.00	LX0	C
ATOM	798	C	LEU	1066	45.349	21.423	16.099	1.00	0.00	LX0	C
ATOM	799	O	LEU	1066	45.555	21.265	17.294	1.00	0.00	LX0	O
ATOM	800	N	GLY	1067	44.383	22.209	15.598	1.00	0.00	LX0	N
ATOM	801	H	GLY	1067	44.303	22.410	14.619	0.00	0.00	LX0	H
ATOM	802	CA	GLY	1067	43.464	22.805	16.562	1.00	0.00	LX0	C
ATOM	803	C	GLY	1067	42.385	23.637	15.910	1.00	0.00	LX0	C
ATOM	804	O	GLY	1067	42.463	23.974	14.735	1.00	0.00	LX0	O
ATOM	805	N	VAL	1068	41.365	23.943	16.711	1.00	0.00	LX0	N

ATOM	806	H	VAL	1068	41.412	23.708	17.685	0.00	0.00	LX0	H
ATOM	807	CA	VAL	1068	40.307	24.834	16.240	1.00	0.00	LX0	C
ATOM	808	CB	VAL	1068	38.942	24.206	16.564	1.00	0.00	LX0	C
ATOM	809	CG1	VAL	1068	37.750	25.072	16.160	1.00	0.00	LX0	C
ATOM	810	CG2	VAL	1068	38.832	22.837	15.902	1.00	0.00	LX0	C
ATOM	811	C	VAL	1068	40.498	26.168	16.932	1.00	0.00	LX0	C
ATOM	812	O	VAL	1068	41.028	26.205	18.028	1.00	0.00	LX0	O
ATOM	813	N	VAL	1069	40.105	27.254	16.252	1.00	0.00	LX0	N
ATOM	814	H	VAL	1069	39.736	27.188	15.325	0.00	0.00	LX0	H
ATOM	815	CA	VAL	1069	40.094	28.527	16.969	1.00	0.00	LX0	C
ATOM	816	CB	VAL	1069	41.240	29.465	16.550	1.00	0.00	LX0	C
ATOM	817	CG1	VAL	1069	41.399	30.623	17.544	1.00	0.00	LX0	C
ATOM	818	CG2	VAL	1069	42.562	28.723	16.375	1.00	0.00	LX0	C
ATOM	819	C	VAL	1069	38.752	29.210	16.786	1.00	0.00	LX0	C
ATOM	820	O	VAL	1069	38.603	30.221	16.097	1.00	0.00	LX0	O
ATOM	821	N	SER	1070	37.759	28.589	17.428	1.00	0.00	LX0	N
ATOM	822	H	SER	1070	37.995	27.852	18.073	0.00	0.00	LX0	H
ATOM	823	CA	SER	1070	36.433	29.197	17.400	1.00	0.00	LX0	C
ATOM	824	CB	SER	1070	35.372	28.183	17.844	1.00	0.00	LX0	C
ATOM	825	OG	SER	1070	35.607	27.738	19.185	1.00	0.00	LX0	O
ATOM	826	HG	SER	1070	36.419	27.226	19.183	0.00	0.00	LX0	H
ATOM	827	C	SER	1070	36.338	30.503	18.177	1.00	0.00	LX0	C
ATOM	828	O	SER	1070	35.527	31.378	17.892	1.00	0.00	LX0	O
ATOM	829	N	GLN	1071	37.241	30.627	19.165	1.00	0.00	LX0	N
ATOM	830	H	GLN	1071	37.839	29.851	19.383	0.00	0.00	LX0	H
ATOM	831	CA	GLN	1071	37.205	31.848	19.965	1.00	0.00	LX0	C
ATOM	832	CB	GLN	1071	38.074	31.749	21.228	1.00	0.00	LX0	C
ATOM	833	CG	GLN	1071	37.947	30.463	22.056	1.00	0.00	LX0	C
ATOM	834	CD	GLN	1071	36.494	30.122	22.316	1.00	0.00	LX0	C
ATOM	835	OE1	GLN	1071	35.764	30.833	22.993	1.00	0.00	LX0	O
ATOM	836	NE2	GLN	1071	36.100	29.001	21.711	1.00	0.00	LX0	N
ATOM	837	HE21	GLN	1071	36.749	28.466	21.159	0.00	0.00	LX0	H
ATOM	838	HE22	GLN	1071	35.159	28.676	21.754	0.00	0.00	LX0	H
ATOM	839	C	GLN	1071	37.576	33.105	19.201	1.00	0.00	LX0	C
ATOM	840	O	GLN	1071	38.746	33.411	18.988	1.00	0.00	LX0	O
ATOM	841	N	GLY	1072	36.523	33.837	18.825	1.00	0.00	LX0	N
ATOM	842	H	GLY	1072	35.641	33.364	18.772	0.00	0.00	LX0	H
ATOM	843	CA	GLY	1072	36.736	35.201	18.349	1.00	0.00	LX0	C
ATOM	844	C	GLY	1072	36.854	35.358	16.845	1.00	0.00	LX0	C
ATOM	845	O	GLY	1072	37.017	34.414	16.082	1.00	0.00	LX0	O
ATOM	846	N	GLN	1073	36.761	36.633	16.452	1.00	0.00	LX0	N
ATOM	847	H	GLN	1073	36.741	37.360	17.135	0.00	0.00	LX0	H
ATOM	848	CA	GLN	1073	36.885	36.951	15.032	1.00	0.00	LX0	C
ATOM	849	CB	GLN	1073	36.028	38.179	14.712	1.00	0.00	LX0	C
ATOM	850	CG	GLN	1073	34.528	37.882	14.638	1.00	0.00	LX0	C
ATOM	851	CD	GLN	1073	34.178	37.281	13.288	1.00	0.00	LX0	C
ATOM	852	OE1	GLN	1073	34.719	36.277	12.842	1.00	0.00	LX0	O
ATOM	853	NE2	GLN	1073	33.238	37.976	12.643	1.00	0.00	LX0	N
ATOM	854	HE21	GLN	1073	32.815	38.783	13.051	0.00	0.00	LX0	H
ATOM	855	HE22	GLN	1073	32.930	37.725	11.725	0.00	0.00	LX0	H
ATOM	856	C	GLN	1073	38.331	37.183	14.620	1.00	0.00	LX0	C
ATOM	857	O	GLN	1073	39.123	37.708	15.393	1.00	0.00	LX0	O
ATOM	858	N	PRO	1074	38.671	36.767	13.375	1.00	0.00	LX0	N
ATOM	859	CD	PRO	1074	39.888	37.165	12.674	1.00	0.00	LX0	C
ATOM	860	CA	PRO	1074	37.851	35.824	12.606	1.00	0.00	LX0	C
ATOM	861	CB	PRO	1074	38.428	35.978	11.196	1.00	0.00	LX0	C
ATOM	862	CG	PRO	1074	39.906	36.306	11.414	1.00	0.00	LX0	C
ATOM	863	C	PRO	1074	38.015	34.426	13.176	1.00	0.00	LX0	C
ATOM	864	O	PRO	1074	39.009	34.111	13.830	1.00	0.00	LX0	O
ATOM	865	N	THR	1075	36.993	33.615	12.927	1.00	0.00	LX0	N
ATOM	866	H	THR	1075	36.204	33.938	12.403	0.00	0.00	LX0	H

ATOM	867	CA	THR	1075	37.084	32.226	13.369	1.00	0.00	LX0	C
ATOM	868	CB	THR	1075	35.676	31.647	13.341	1.00	0.00	LX0	C
ATOM	869	OG1	THR	1075	34.904	32.318	12.330	1.00	0.00	LX0	O
ATOM	870	HG1	THR	1075	35.436	32.302	11.532	0.00	0.00	LX0	H
ATOM	871	CG2	THR	1075	34.983	31.806	14.692	1.00	0.00	LX0	C
ATOM	872	C	THR	1075	38.050	31.435	12.501	1.00	0.00	LX0	C
ATOM	873	O	THR	1075	38.058	31.576	11.283	1.00	0.00	LX0	O
ATOM	874	N	LEU	1076	38.896	30.653	13.178	1.00	0.00	LX0	N
ATOM	875	H	LEU	1076	38.779	30.464	14.155	0.00	0.00	LX0	H
ATOM	876	CA	LEU	1076	40.015	30.056	12.451	1.00	0.00	LX0	C
ATOM	877	CB	LEU	1076	41.338	30.629	12.963	1.00	0.00	LX0	C
ATOM	878	CG	LEU	1076	41.409	32.153	13.046	1.00	0.00	LX0	C
ATOM	879	CD1	LEU	1076	42.383	32.598	14.127	1.00	0.00	LX0	C
ATOM	880	CD2	LEU	1076	41.722	32.812	11.708	1.00	0.00	LX0	C
ATOM	881	C	LEU	1076	40.050	28.561	12.660	1.00	0.00	LX0	C
ATOM	882	O	LEU	1076	39.478	28.052	13.617	1.00	0.00	LX0	O
ATOM	883	N	VAL	1077	40.788	27.886	11.771	1.00	0.00	LX0	N
ATOM	884	H	VAL	1077	41.068	28.291	10.897	0.00	0.00	LX0	H
ATOM	885	CA	VAL	1077	41.118	26.503	12.085	1.00	0.00	LX0	C
ATOM	886	CB	VAL	1077	40.000	25.560	11.605	1.00	0.00	LX0	C
ATOM	887	CG1	VAL	1077	39.998	25.286	10.101	1.00	0.00	LX0	C
ATOM	888	CG2	VAL	1077	39.978	24.298	12.453	1.00	0.00	LX0	C
ATOM	889	C	VAL	1077	42.512	26.118	11.604	1.00	0.00	LX0	C
ATOM	890	O	VAL	1077	42.916	26.398	10.482	1.00	0.00	LX0	O
ATOM	891	N	ILE	1078	43.248	25.526	12.549	1.00	0.00	LX0	N
ATOM	892	H	ILE	1078	42.796	25.206	13.381	0.00	0.00	LX0	H
ATOM	893	CA	ILE	1078	44.677	25.275	12.367	1.00	0.00	LX0	C
ATOM	894	CB	ILE	1078	45.404	25.498	13.707	1.00	0.00	LX0	C
ATOM	895	CG2	ILE	1078	46.915	25.275	13.614	1.00	0.00	LX0	C
ATOM	896	CG1	ILE	1078	45.090	26.879	14.284	1.00	0.00	LX0	C
ATOM	897	CD1	ILE	1078	45.617	28.021	13.415	1.00	0.00	LX0	C
ATOM	898	C	ILE	1078	44.947	23.879	11.835	1.00	0.00	LX0	C
ATOM	899	O	ILE	1078	45.036	22.909	12.582	1.00	0.00	LX0	O
ATOM	900	N	MET	1079	45.062	23.817	10.508	1.00	0.00	LX0	N
ATOM	901	H	MET	1079	45.025	24.643	9.941	0.00	0.00	LX0	H
ATOM	902	CA	MET	1079	45.384	22.530	9.899	1.00	0.00	LX0	C
ATOM	903	CB	MET	1079	44.488	22.285	8.684	1.00	0.00	LX0	C
ATOM	904	CG	MET	1079	43.022	22.244	9.098	1.00	0.00	LX0	C
ATOM	905	SD	MET	1079	41.873	21.979	7.750	1.00	0.00	LX0	S
ATOM	906	CE	MET	1079	40.471	21.480	8.760	1.00	0.00	LX0	C
ATOM	907	C	MET	1079	46.845	22.425	9.518	1.00	0.00	LX0	C
ATOM	908	O	MET	1079	47.602	23.386	9.571	1.00	0.00	LX0	O
ATOM	909	N	GLU	1080	47.210	21.204	9.122	1.00	0.00	LX0	N
ATOM	910	H	GLU	1080	46.549	20.456	9.197	0.00	0.00	LX0	H
ATOM	911	CA	GLU	1080	48.559	20.946	8.626	1.00	0.00	LX0	C
ATOM	912	CB	GLU	1080	48.696	19.433	8.525	1.00	0.00	LX0	C
ATOM	913	CG	GLU	1080	50.089	18.839	8.326	1.00	0.00	LX0	C
ATOM	914	CD	GLU	1080	49.957	17.329	8.296	1.00	0.00	LX0	C
ATOM	915	OE1	GLU	1080	49.066	16.817	7.624	1.00	0.00	LX0	O
ATOM	916	OE2	GLU	1080	50.743	16.642	8.940	1.00	0.00	LX0	O
ATOM	917	C	GLU	1080	48.835	21.653	7.305	1.00	0.00	LX0	C
ATOM	918	O	GLU	1080	47.939	21.904	6.507	1.00	0.00	LX0	O
ATOM	919	N	LEU	1081	50.111	22.009	7.116	1.00	0.00	LX0	N
ATOM	920	H	LEU	1081	50.827	21.718	7.749	0.00	0.00	LX0	H
ATOM	921	CA	LEU	1081	50.404	22.797	5.923	1.00	0.00	LX0	C
ATOM	922	CB	LEU	1081	51.587	23.721	6.183	1.00	0.00	LX0	C
ATOM	923	CG	LEU	1081	51.786	24.780	5.100	1.00	0.00	LX0	C
ATOM	924	CD1	LEU	1081	50.563	25.668	4.870	1.00	0.00	LX0	C
ATOM	925	CD2	LEU	1081	53.031	25.599	5.385	1.00	0.00	LX0	C
ATOM	926	C	LEU	1081	50.578	22.001	4.640	1.00	0.00	LX0	C
ATOM	927	O	LEU	1081	51.634	21.472	4.310	1.00	0.00	LX0	O

ATOM	928	N	MET	1082	49.466	21.973	3.902	1.00	0.00	LX0	N
ATOM	929	H	MET	1082	48.638	22.400	4.267	0.00	0.00	LX0	H
ATOM	930	CA	MET	1082	49.504	21.265	2.625	1.00	0.00	LX0	C
ATOM	931	CB	MET	1082	48.155	20.599	2.347	1.00	0.00	LX0	C
ATOM	932	CG	MET	1082	47.544	19.924	3.582	1.00	0.00	LX0	C
ATOM	933	SD	MET	1082	48.659	18.782	4.419	1.00	0.00	LX0	S
ATOM	934	CE	MET	1082	48.836	17.573	3.102	1.00	0.00	LX0	C
ATOM	935	C	MET	1082	49.957	22.126	1.457	1.00	0.00	LX0	C
ATOM	936	O	MET	1082	49.222	22.425	0.523	1.00	0.00	LX0	O
ATOM	937	N	THR	1083	51.221	22.547	1.563	1.00	0.00	LX0	N
ATOM	938	H	THR	1083	51.793	22.277	2.342	0.00	0.00	LX0	H
ATOM	939	CA	THR	1083	51.696	23.619	0.689	1.00	0.00	LX0	C
ATOM	940	CB	THR	1083	52.922	24.274	1.312	1.00	0.00	LX0	C
ATOM	941	OG1	THR	1083	53.251	23.637	2.553	1.00	0.00	LX0	O
ATOM	942	HG1	THR	1083	54.088	24.022	2.812	0.00	0.00	LX0	H
ATOM	943	CG2	THR	1083	52.736	25.785	1.479	1.00	0.00	LX0	C
ATOM	944	C	THR	1083	51.976	23.327	-0.778	1.00	0.00	LX0	C
ATOM	945	O	THR	1083	52.719	24.055	-1.431	1.00	0.00	LX0	O
ATOM	946	N	ARG	1084	51.383	22.244	-1.290	1.00	0.00	LX0	N
ATOM	947	H	ARG	1084	50.869	21.592	-0.726	0.00	0.00	LX0	H
ATOM	948	CA	ARG	1084	51.410	22.131	-2.746	1.00	0.00	LX0	C
ATOM	949	CB	ARG	1084	52.141	20.872	-3.215	1.00	0.00	LX0	C
ATOM	950	CG	ARG	1084	53.539	20.705	-2.621	1.00	0.00	LX0	C
ATOM	951	CD	ARG	1084	54.489	21.843	-2.981	1.00	0.00	LX0	C
ATOM	952	NE	ARG	1084	55.731	21.709	-2.229	1.00	0.00	LX0	N
ATOM	953	HE	ARG	1084	56.254	20.860	-2.331	0.00	0.00	LX0	H
ATOM	954	CZ	ARG	1084	56.143	22.671	-1.381	1.00	0.00	LX0	C
ATOM	955	NH1	ARG	1084	55.420	23.762	-1.143	1.00	0.00	LX0	N
ATOM	956	HH11	ARG	1084	55.764	24.425	-0.463	0.00	0.00	LX0	H
ATOM	957	HH12	ARG	1084	54.534	23.937	-1.587	0.00	0.00	LX0	H
ATOM	958	NH2	ARG	1084	57.290	22.518	-0.742	1.00	0.00	LX0	N
ATOM	959	HH21	ARG	1084	57.623	23.259	-0.146	0.00	0.00	LX0	H
ATOM	960	HH22	ARG	1084	57.814	21.661	-0.776	0.00	0.00	LX0	H
ATOM	961	C	ARG	1084	50.036	22.190	-3.380	1.00	0.00	LX0	C
ATOM	962	O	ARG	1084	49.895	22.154	-4.594	1.00	0.00	LX0	O
ATOM	963	N	GLY	1085	49.019	22.280	-2.507	1.00	0.00	LX0	N
ATOM	964	H	GLY	1085	49.153	22.236	-1.516	0.00	0.00	LX0	H
ATOM	965	CA	GLY	1085	47.679	22.153	-3.066	1.00	0.00	LX0	C
ATOM	966	C	GLY	1085	47.336	20.697	-3.287	1.00	0.00	LX0	C
ATOM	967	O	GLY	1085	47.876	19.807	-2.636	1.00	0.00	LX0	O
ATOM	968	N	ASP	1086	46.414	20.495	-4.223	1.00	0.00	LX0	N
ATOM	969	H	ASP	1086	46.058	21.217	-4.820	0.00	0.00	LX0	H
ATOM	970	CA	ASP	1086	46.002	19.130	-4.512	1.00	0.00	LX0	C
ATOM	971	CB	ASP	1086	44.549	19.123	-5.006	1.00	0.00	LX0	C
ATOM	972	CG	ASP	1086	44.386	19.865	-6.324	1.00	0.00	LX0	C
ATOM	973	OD1	ASP	1086	44.670	21.055	-6.390	1.00	0.00	LX0	O
ATOM	974	OD2	ASP	1086	43.992	19.243	-7.299	1.00	0.00	LX0	O
ATOM	975	C	ASP	1086	46.929	18.441	-5.491	1.00	0.00	LX0	C
ATOM	976	O	ASP	1086	47.610	19.062	-6.302	1.00	0.00	LX0	O
ATOM	977	N	LEU	1087	46.920	17.103	-5.398	1.00	0.00	LX0	N
ATOM	978	H	LEU	1087	46.354	16.660	-4.700	0.00	0.00	LX0	H
ATOM	979	CA	LEU	1087	47.740	16.329	-6.327	1.00	0.00	LX0	C
ATOM	980	CB	LEU	1087	47.666	14.831	-6.014	1.00	0.00	LX0	C
ATOM	981	CG	LEU	1087	48.542	13.952	-6.919	1.00	0.00	LX0	C
ATOM	982	CD1	LEU	1087	50.028	14.303	-6.822	1.00	0.00	LX0	C
ATOM	983	CD2	LEU	1087	48.278	12.463	-6.701	1.00	0.00	LX0	C
ATOM	984	C	LEU	1087	47.364	16.576	-7.770	1.00	0.00	LX0	C
ATOM	985	O	LEU	1087	48.201	16.661	-8.654	1.00	0.00	LX0	O
ATOM	986	N	LYS	1088	46.049	16.698	-7.969	1.00	0.00	LX0	N
ATOM	987	H	LYS	1088	45.430	16.736	-7.183	0.00	0.00	LX0	H
ATOM	988	CA	LYS	1088	45.561	16.888	-9.329	1.00	0.00	LX0	C

ATOM	989	CB	LYS	1088	44.039	16.845	-9.253	1.00	0.00	LX0 C
ATOM	990	CG	LYS	1088	43.276	17.108	-10.533	1.00	0.00	LX0 C
ATOM	991	CD	LYS	1088	41.859	16.576	-10.414	1.00	0.00	LX0 C
ATOM	992	CE	LYS	1088	41.172	16.764	-11.749	1.00	0.00	LX0 C
ATOM	993	NZ	LYS	1088	40.111	15.781	-11.926	1.00	0.00	LX0 N
ATOM	994	HZ1	LYS	1088	39.765	15.830	-12.904	0.00	0.00	LX0 H
ATOM	995	HZ2	LYS	1088	40.477	14.822	-11.728	0.00	0.00	LX0 H
ATOM	996	HZ3	LYS	1088	39.305	16.003	-11.301	0.00	0.00	LX0 H
ATOM	997	C	LYS	1088	46.134	18.116	-10.028	1.00	0.00	LX0 C
ATOM	998	O	LYS	1088	46.662	18.053	-11.135	1.00	0.00	LX0 O
ATOM	999	N	SER	1089	46.062	19.237	-9.310	1.00	0.00	LX0 N
ATOM	1000	H	SER	1089	45.602	19.271	-8.419	0.00	0.00	LX0 H
ATOM	1001	CA	SER	1089	46.660	20.429	-9.892	1.00	0.00	LX0 C
ATOM	1002	CB	SER	1089	46.085	21.665	-9.215	1.00	0.00	LX0 C
ATOM	1003	OG	SER	1089	44.658	21.524	-9.198	1.00	0.00	LX0 O
ATOM	1004	HG	SER	1089	44.450	21.191	-8.313	0.00	0.00	LX0 H
ATOM	1005	C	SER	1089	48.177	20.404	-9.942	1.00	0.00	LX0 C
ATOM	1006	O	SER	1089	48.784	20.838	-10.912	1.00	0.00	LX0 O
ATOM	1007	N	TYR	1090	48.766	19.790	-8.900	1.00	0.00	LX0 N
ATOM	1008	H	TYR	1090	48.219	19.525	-8.102	0.00	0.00	LX0 H
ATOM	1009	CA	TYR	1090	50.213	19.548	-8.928	1.00	0.00	LX0 C
ATOM	1010	CB	TYR	1090	50.629	18.815	-7.643	1.00	0.00	LX0 C
ATOM	1011	CG	TYR	1090	52.128	18.631	-7.556	1.00	0.00	LX0 C
ATOM	1012	CD1	TYR	1090	52.944	19.739	-7.249	1.00	0.00	LX0 C
ATOM	1013	CE1	TYR	1090	54.335	19.560	-7.178	1.00	0.00	LX0 C
ATOM	1014	CD2	TYR	1090	52.665	17.349	-7.787	1.00	0.00	LX0 C
ATOM	1015	CE2	TYR	1090	54.056	17.170	-7.716	1.00	0.00	LX0 C
ATOM	1016	CZ	TYR	1090	54.872	18.279	-7.414	1.00	0.00	LX0 C
ATOM	1017	OH	TYR	1090	56.241	18.109	-7.346	1.00	0.00	LX0 O
ATOM	1018	HH	TYR	1090	56.462	17.207	-7.536	0.00	0.00	LX0 H
ATOM	1019	C	TYR	1090	50.695	18.820	-10.183	1.00	0.00	LX0 C
ATOM	1020	O	TYR	1090	51.688	19.172	-10.812	1.00	0.00	LX0 O
ATOM	1021	N	LEU	1091	49.907	17.799	-10.547	1.00	0.00	LX0 N
ATOM	1022	H	LEU	1091	49.116	17.580	-9.977	0.00	0.00	LX0 H
ATOM	1023	CA	LEU	1091	50.166	17.069	-11.785	1.00	0.00	LX0 C
ATOM	1024	CB	LEU	1091	49.198	15.897	-11.915	1.00	0.00	LX0 C
ATOM	1025	CG	LEU	1091	49.362	14.806	-10.859	1.00	0.00	LX0 C
ATOM	1026	CD1	LEU	1091	48.125	13.911	-10.802	1.00	0.00	LX0 C
ATOM	1027	CD2	LEU	1091	50.658	14.012	-11.030	1.00	0.00	LX0 C
ATOM	1028	C	LEU	1091	50.066	17.952	-13.014	1.00	0.00	LX0 C
ATOM	1029	O	LEU	1091	50.868	17.889	-13.936	1.00	0.00	LX0 O
ATOM	1030	N	ARG	1092	49.048	18.820	-12.970	1.00	0.00	LX0 N
ATOM	1031	H	ARG	1092	48.422	18.819	-12.187	0.00	0.00	LX0 H
ATOM	1032	CA	ARG	1092	48.918	19.787	-14.058	1.00	0.00	LX0 C
ATOM	1033	CB	ARG	1092	47.572	20.504	-13.956	1.00	0.00	LX0 C
ATOM	1034	CG	ARG	1092	46.477	19.470	-14.208	1.00	0.00	LX0 C
ATOM	1035	CD	ARG	1092	45.031	19.926	-14.024	1.00	0.00	LX0 C
ATOM	1036	NE	ARG	1092	44.182	18.848	-14.518	1.00	0.00	LX0 N
ATOM	1037	HE	ARG	1092	44.626	18.154	-15.090	0.00	0.00	LX0 H
ATOM	1038	CZ	ARG	1092	42.866	18.721	-14.276	1.00	0.00	LX0 C
ATOM	1039	NH1	ARG	1092	42.230	19.580	-13.490	1.00	0.00	LX0 N
ATOM	1040	HH11	ARG	1092	41.237	19.564	-13.390	0.00	0.00	LX0 H
ATOM	1041	HH12	ARG	1092	42.736	20.259	-12.937	0.00	0.00	LX0 H
ATOM	1042	NH2	ARG	1092	42.221	17.697	-14.827	1.00	0.00	LX0 N
ATOM	1043	HH21	ARG	1092	41.239	17.511	-14.713	0.00	0.00	LX0 H
ATOM	1044	HH22	ARG	1092	42.739	17.031	-15.376	0.00	0.00	LX0 H
ATOM	1045	C	ARG	1092	50.095	20.738	-14.221	1.00	0.00	LX0 C
ATOM	1046	O	ARG	1092	50.508	21.047	-15.330	1.00	0.00	LX0 O
ATOM	1047	N	SER	1093	50.676	21.136	-13.080	1.00	0.00	LX0 N
ATOM	1048	H	SER	1093	50.283	20.947	-12.177	0.00	0.00	LX0 H
ATOM	1049	CA	SER	1093	51.913	21.913	-13.193	1.00	0.00	LX0 C

ATOM	1050	CB	SER	1093	52.228	22.602	-11.864	1.00	0.00	LX0	C
ATOM	1051	OG	SER	1093	51.663	21.864	-10.774	1.00	0.00	LX0	O
ATOM	1052	HG	SER	1093	52.043	20.990	-10.804	0.00	0.00	LX0	H
ATOM	1053	C	SER	1093	53.133	21.170	-13.729	1.00	0.00	LX0	C
ATOM	1054	O	SER	1093	54.150	21.762	-14.061	1.00	0.00	LX0	O
ATOM	1055	N	LEU	1094	52.992	19.838	-13.811	1.00	0.00	LX0	N
ATOM	1056	H	LEU	1094	52.149	19.377	-13.539	0.00	0.00	LX0	H
ATOM	1057	CA	LEU	1094	54.073	19.076	-14.433	1.00	0.00	LX0	C
ATOM	1058	CB	LEU	1094	54.223	17.714	-13.750	1.00	0.00	LX0	C
ATOM	1059	CG	LEU	1094	54.396	17.786	-12.230	1.00	0.00	LX0	C
ATOM	1060	CD1	LEU	1094	54.273	16.406	-11.585	1.00	0.00	LX0	C
ATOM	1061	CD2	LEU	1094	55.687	18.499	-11.821	1.00	0.00	LX0	C
ATOM	1062	C	LEU	1094	53.919	18.894	-15.937	1.00	0.00	LX0	C
ATOM	1063	O	LEU	1094	54.706	18.219	-16.587	1.00	0.00	LX0	O
ATOM	1064	N	ARG	1095	52.853	19.506	-16.480	1.00	0.00	LX0	N
ATOM	1065	H	ARG	1095	52.266	20.118	-15.950	0.00	0.00	LX0	H
ATOM	1066	CA	ARG	1095	52.692	19.387	-17.927	1.00	0.00	LX0	C
ATOM	1067	CB	ARG	1095	51.283	19.802	-18.353	1.00	0.00	LX0	C
ATOM	1068	CG	ARG	1095	50.237	18.822	-17.840	1.00	0.00	LX0	C
ATOM	1069	CD	ARG	1095	48.841	19.106	-18.381	1.00	0.00	LX0	C
ATOM	1070	NE	ARG	1095	47.896	18.175	-17.781	1.00	0.00	LX0	N
ATOM	1071	HE	ARG	1095	47.998	17.932	-16.811	0.00	0.00	LX0	H
ATOM	1072	CZ	ARG	1095	46.883	17.620	-18.470	1.00	0.00	LX0	C
ATOM	1073	NH1	ARG	1095	46.708	17.817	-19.772	1.00	0.00	LX0	N
ATOM	1074	HH11	ARG	1095	45.951	17.328	-20.242	0.00	0.00	LX0	H
ATOM	1075	HH12	ARG	1095	47.300	18.410	-20.309	0.00	0.00	LX0	H
ATOM	1076	NH2	ARG	1095	46.039	16.856	-17.815	1.00	0.00	LX0	N
ATOM	1077	HH21	ARG	1095	45.234	16.481	-18.293	0.00	0.00	LX0	H
ATOM	1078	HH22	ARG	1095	46.181	16.639	-16.846	0.00	0.00	LX0	H
ATOM	1079	C	ARG	1095	53.722	20.181	-18.707	1.00	0.00	LX0	C
ATOM	1080	O	ARG	1095	54.136	21.262	-18.308	1.00	0.00	LX0	O
ATOM	1081	N	PRO	1096	54.125	19.611	-19.867	1.00	0.00	LX0	N
ATOM	1082	CD	PRO	1096	53.797	18.283	-20.379	1.00	0.00	LX0	C
ATOM	1083	CA	PRO	1096	54.993	20.375	-20.764	1.00	0.00	LX0	C
ATOM	1084	CB	PRO	1096	55.287	19.368	-21.886	1.00	0.00	LX0	C
ATOM	1085	CG	PRO	1096	54.134	18.365	-21.861	1.00	0.00	LX0	C
ATOM	1086	C	PRO	1096	54.358	21.665	-21.254	1.00	0.00	LX0	C
ATOM	1087	O	PRO	1096	53.557	21.678	-22.180	1.00	0.00	LX0	O
ATOM	1088	N	GLU	1097	54.800	22.759	-20.611	1.00	0.00	LX0	N
ATOM	1089	H	GLU	1097	55.295	22.646	-19.749	0.00	0.00	LX0	H
ATOM	1090	CA	GLU	1097	54.495	24.090	-21.147	1.00	0.00	LX0	C
ATOM	1091	CB	GLU	1097	55.048	25.156	-20.204	1.00	0.00	LX0	C
ATOM	1092	CG	GLU	1097	54.565	26.569	-20.536	1.00	0.00	LX0	C
ATOM	1093	CD	GLU	1097	55.441	27.569	-19.820	1.00	0.00	LX0	C
ATOM	1094	OE1	GLU	1097	56.461	27.953	-20.387	1.00	0.00	LX0	O
ATOM	1095	OE2	GLU	1097	55.106	27.958	-18.704	1.00	0.00	LX0	O
ATOM	1096	C	GLU	1097	55.042	24.273	-22.560	1.00	0.00	LX0	C
ATOM	1097	O	GLU	1097	54.461	24.912	-23.429	1.00	0.00	LX0	O
ATOM	1098	N	MET	1098	56.185	23.588	-22.749	1.00	0.00	LX0	N
ATOM	1099	H	MET	1098	56.741	23.409	-21.934	0.00	0.00	LX0	H
ATOM	1100	CA	MET	1098	56.707	23.216	-24.063	1.00	0.00	LX0	C
ATOM	1101	CB	MET	1098	55.641	22.561	-24.962	1.00	0.00	LX0	C
ATOM	1102	CG	MET	1098	56.200	21.845	-26.194	1.00	0.00	LX0	C
ATOM	1103	SD	MET	1098	57.261	20.454	-25.767	1.00	0.00	LX0	S
ATOM	1104	CE	MET	1098	57.665	19.934	-27.442	1.00	0.00	LX0	C
ATOM	1105	C	MET	1098	57.480	24.290	-24.797	1.00	0.00	LX0	C
ATOM	1106	O	MET	1098	58.582	24.033	-25.266	1.00	0.00	LX0	O
ATOM	1107	N	GLU	1099	56.885	25.494	-24.877	1.00	0.00	LX0	N
ATOM	1108	H	GLU	1099	55.992	25.643	-24.449	0.00	0.00	LX0	H
ATOM	1109	CA	GLU	1099	57.546	26.548	-25.651	1.00	0.00	LX0	C
ATOM	1110	CB	GLU	1099	56.687	27.818	-25.711	1.00	0.00	LX0	C

ATOM	1111	CG	GLU	1099	57.270	28.934	-26.596	1.00	0.00	LX0	C
ATOM	1112	CD	GLU	1099	57.584	28.410	-27.988	1.00	0.00	LX0	C
ATOM	1113	OE1	GLU	1099	56.696	28.403	-28.834	1.00	0.00	LX0	O
ATOM	1114	OE2	GLU	1099	58.722	28.004	-28.221	1.00	0.00	LX0	O
ATOM	1115	C	GLU	1099	58.973	26.842	-25.214	1.00	0.00	LX0	C
ATOM	1116	O	GLU	1099	59.248	27.366	-24.140	1.00	0.00	LX0	O
ATOM	1117	N	ASN	1100	59.874	26.370	-26.091	1.00	0.00	LX0	N
ATOM	1118	H	ASN	1100	59.489	26.176	-26.996	0.00	0.00	LX0	H
ATOM	1119	CA	ASN	1100	61.312	26.297	-25.814	1.00	0.00	LX0	C
ATOM	1120	CB	ASN	1100	62.015	27.585	-26.251	1.00	0.00	LX0	C
ATOM	1121	CG	ASN	1100	62.489	27.433	-27.681	1.00	0.00	LX0	C
ATOM	1122	OD1	ASN	1100	63.609	27.026	-27.957	1.00	0.00	LX0	O
ATOM	1123	ND2	ASN	1100	61.586	27.782	-28.599	1.00	0.00	LX0	N
ATOM	1124	HD21	ASN	1100	60.659	28.082	-28.349	0.00	0.00	LX0	H
ATOM	1125	HD22	ASN	1100	61.809	27.735	-29.568	0.00	0.00	LX0	H
ATOM	1126	C	ASN	1100	61.735	25.899	-24.405	1.00	0.00	LX0	C
ATOM	1127	O	ASN	1100	62.734	26.368	-23.872	1.00	0.00	LX0	O
ATOM	1128	N	ASN	1101	60.935	25.000	-23.807	1.00	0.00	LX0	N
ATOM	1129	H	ASN	1101	60.150	24.607	-24.287	0.00	0.00	LX0	H
ATOM	1130	CA	ASN	1101	61.237	24.704	-22.409	1.00	0.00	LX0	C
ATOM	1131	CB	ASN	1101	60.367	25.501	-21.418	1.00	0.00	LX0	C
ATOM	1132	CG	ASN	1101	58.888	25.160	-21.454	1.00	0.00	LX0	C
ATOM	1133	OD1	ASN	1101	58.429	24.092	-21.066	1.00	0.00	LX0	O
ATOM	1134	ND2	ASN	1101	58.146	26.200	-21.829	1.00	0.00	LX0	N
ATOM	1135	HD21	ASN	1101	58.536	26.968	-22.341	0.00	0.00	LX0	H
ATOM	1136	HD22	ASN	1101	57.198	26.350	-21.538	0.00	0.00	LX0	H
ATOM	1137	C	ASN	1101	61.328	23.240	-22.037	1.00	0.00	LX0	C
ATOM	1138	O	ASN	1101	60.425	22.432	-22.224	1.00	0.00	LX0	O
ATOM	1139	N	PRO	1102	62.521	22.909	-21.492	1.00	0.00	LX0	N
ATOM	1140	CD	PRO	1102	63.716	23.741	-21.419	1.00	0.00	LX0	C
ATOM	1141	CA	PRO	1102	62.728	21.576	-20.923	1.00	0.00	LX0	C
ATOM	1142	CB	PRO	1102	64.238	21.546	-20.649	1.00	0.00	LX0	C
ATOM	1143	CG	PRO	1102	64.845	22.722	-21.417	1.00	0.00	LX0	C
ATOM	1144	C	PRO	1102	61.920	21.366	-19.652	1.00	0.00	LX0	C
ATOM	1145	O	PRO	1102	62.378	21.633	-18.547	1.00	0.00	LX0	O
ATOM	1146	N	VAL	1103	60.696	20.863	-19.853	1.00	0.00	LX0	N
ATOM	1147	H	VAL	1103	60.346	20.804	-20.789	0.00	0.00	LX0	H
ATOM	1148	CA	VAL	1103	59.914	20.466	-18.682	1.00	0.00	LX0	C
ATOM	1149	CB	VAL	1103	58.488	20.088	-19.128	1.00	0.00	LX0	C
ATOM	1150	CG1	VAL	1103	58.470	18.789	-19.942	1.00	0.00	LX0	C
ATOM	1151	CG2	VAL	1103	57.485	20.081	-17.970	1.00	0.00	LX0	C
ATOM	1152	C	VAL	1103	60.596	19.350	-17.890	1.00	0.00	LX0	C
ATOM	1153	O	VAL	1103	61.367	18.564	-18.430	1.00	0.00	LX0	O
ATOM	1154	N	LEU	1104	60.292	19.318	-16.584	1.00	0.00	LX0	N
ATOM	1155	H	LEU	1104	59.611	19.947	-16.217	0.00	0.00	LX0	H
ATOM	1156	CA	LEU	1104	60.787	18.175	-15.822	1.00	0.00	LX0	C
ATOM	1157	CB	LEU	1104	60.644	18.419	-14.317	1.00	0.00	LX0	C
ATOM	1158	CG	LEU	1104	61.927	18.906	-13.631	1.00	0.00	LX0	C
ATOM	1159	CD1	LEU	1104	63.042	17.860	-13.714	1.00	0.00	LX0	C
ATOM	1160	CD2	LEU	1104	62.387	20.282	-14.119	1.00	0.00	LX0	C
ATOM	1161	C	LEU	1104	60.083	16.893	-16.219	1.00	0.00	LX0	C
ATOM	1162	O	LEU	1104	58.957	16.895	-16.700	1.00	0.00	LX0	O
ATOM	1163	N	ALA	1105	60.812	15.794	-16.001	1.00	0.00	LX0	N
ATOM	1164	H	ALA	1105	61.690	15.862	-15.535	0.00	0.00	LX0	H
ATOM	1165	CA	ALA	1105	60.193	14.506	-16.294	1.00	0.00	LX0	C
ATOM	1166	CB	ALA	1105	61.263	13.409	-16.324	1.00	0.00	LX0	C
ATOM	1167	C	ALA	1105	59.140	14.150	-15.259	1.00	0.00	LX0	C
ATOM	1168	O	ALA	1105	59.327	14.371	-14.067	1.00	0.00	LX0	O
ATOM	1169	N	PRO	1106	58.014	13.580	-15.752	1.00	0.00	LX0	N
ATOM	1170	CD	PRO	1106	57.654	13.389	-17.154	1.00	0.00	LX0	C
ATOM	1171	CA	PRO	1106	57.027	13.028	-14.817	1.00	0.00	LX0	C

ATOM	1172	CB	PRO	1106	55.914	12.553	-15.767	1.00	0.00	LX0	C
ATOM	1173	CG	PRO	1106	56.591	12.301	-17.113	1.00	0.00	LX0	C
ATOM	1174	C	PRO	1106	57.653	11.926	-13.967	1.00	0.00	LX0	C
ATOM	1175	O	PRO	1106	58.570	11.236	-14.401	1.00	0.00	LX0	O
ATOM	1176	N	PRO	1107	57.154	11.813	-12.710	1.00	0.00	LX0	N
ATOM	1177	CD	PRO	1107	56.083	12.605	-12.112	1.00	0.00	LX0	C
ATOM	1178	CA	PRO	1107	57.725	10.838	-11.772	1.00	0.00	LX0	C
ATOM	1179	CB	PRO	1107	56.759	10.908	-10.586	1.00	0.00	LX0	C
ATOM	1180	CG	PRO	1107	56.206	12.331	-10.619	1.00	0.00	LX0	C
ATOM	1181	C	PRO	1107	57.889	9.432	-12.323	1.00	0.00	LX0	C
ATOM	1182	O	PRO	1107	57.035	8.900	-13.022	1.00	0.00	LX0	O
ATOM	1183	N	SER	1108	59.041	8.851	-11.961	1.00	0.00	LX0	N
ATOM	1184	H	SER	1108	59.699	9.288	-11.350	0.00	0.00	LX0	H
ATOM	1185	CA	SER	1108	59.272	7.456	-12.318	1.00	0.00	LX0	C
ATOM	1186	CB	SER	1108	60.708	7.102	-11.927	1.00	0.00	LX0	C
ATOM	1187	OG	SER	1108	61.043	7.770	-10.701	1.00	0.00	LX0	O
ATOM	1188	HG	SER	1108	61.671	7.207	-10.258	0.00	0.00	LX0	H
ATOM	1189	C	SER	1108	58.277	6.539	-11.631	1.00	0.00	LX0	C
ATOM	1190	O	SER	1108	57.609	6.939	-10.680	1.00	0.00	LX0	O
ATOM	1191	N	LEU	1109	58.225	5.288	-12.138	1.00	0.00	LX0	N
ATOM	1192	H	LEU	1109	58.788	5.037	-12.922	0.00	0.00	LX0	H
ATOM	1193	CA	LEU	1109	57.299	4.318	-11.545	1.00	0.00	LX0	C
ATOM	1194	CB	LEU	1109	57.465	2.934	-12.183	1.00	0.00	LX0	C
ATOM	1195	CG	LEU	1109	56.316	1.963	-11.870	1.00	0.00	LX0	C
ATOM	1196	CD1	LEU	1109	54.939	2.524	-12.235	1.00	0.00	LX0	C
ATOM	1197	CD2	LEU	1109	56.556	0.588	-12.493	1.00	0.00	LX0	C
ATOM	1198	C	LEU	1109	57.342	4.291	-10.027	1.00	0.00	LX0	C
ATOM	1199	O	LEU	1109	56.327	4.494	-9.382	1.00	0.00	LX0	O
ATOM	1200	N	SER	1110	58.576	4.211	-9.514	1.00	0.00	LX0	N
ATOM	1201	H	SER	1110	59.287	3.746	-10.042	0.00	0.00	LX0	H
ATOM	1202	CA	SER	1110	58.845	4.454	-8.095	1.00	0.00	LX0	C
ATOM	1203	CB	SER	1110	60.332	4.767	-7.922	1.00	0.00	LX0	C
ATOM	1204	OG	SER	1110	61.067	4.239	-9.038	1.00	0.00	LX0	O
ATOM	1205	HG	SER	1110	60.905	3.290	-9.018	0.00	0.00	LX0	H
ATOM	1206	C	SER	1110	57.980	5.481	-7.371	1.00	0.00	LX0	C
ATOM	1207	O	SER	1110	57.247	5.163	-6.447	1.00	0.00	LX0	O
ATOM	1208	N	LYS	1111	58.060	6.746	-7.828	1.00	0.00	LX0	N
ATOM	1209	H	LYS	1111	58.500	6.946	-8.704	0.00	0.00	LX0	H
ATOM	1210	CA	LYS	1111	57.240	7.712	-7.091	1.00	0.00	LX0	C
ATOM	1211	CB	LYS	1111	57.823	9.137	-7.124	1.00	0.00	LX0	C
ATOM	1212	CG	LYS	1111	57.872	9.927	-5.789	1.00	0.00	LX0	C
ATOM	1213	CD	LYS	1111	56.544	10.397	-5.158	1.00	0.00	LX0	C
ATOM	1214	CE	LYS	1111	56.632	11.386	-3.968	1.00	0.00	LX0	C
ATOM	1215	NZ	LYS	1111	56.850	10.757	-2.654	1.00	0.00	LX0	N
ATOM	1216	HZ1	LYS	1111	56.971	11.474	-1.916	0.00	0.00	LX0	H
ATOM	1217	HZ2	LYS	1111	56.017	10.213	-2.336	0.00	0.00	LX0	H
ATOM	1218	HZ3	LYS	1111	57.709	10.158	-2.611	0.00	0.00	LX0	H
ATOM	1219	C	LYS	1111	55.765	7.692	-7.459	1.00	0.00	LX0	C
ATOM	1220	O	LYS	1111	54.931	8.197	-6.727	1.00	0.00	LX0	O
ATOM	1221	N	MET	1112	55.441	7.068	-8.602	1.00	0.00	LX0	N
ATOM	1222	H	MET	1112	56.136	6.632	-9.179	0.00	0.00	LX0	H
ATOM	1223	CA	MET	1112	54.006	6.893	-8.844	1.00	0.00	LX0	C
ATOM	1224	CB	MET	1112	53.721	6.591	-10.316	1.00	0.00	LX0	C
ATOM	1225	CG	MET	1112	54.116	7.762	-11.220	1.00	0.00	LX0	C
ATOM	1226	SD	MET	1112	53.536	7.584	-12.915	1.00	0.00	LX0	S
ATOM	1227	CE	MET	1112	54.644	6.266	-13.430	1.00	0.00	LX0	C
ATOM	1228	C	MET	1112	53.355	5.866	-7.926	1.00	0.00	LX0	C
ATOM	1229	O	MET	1112	52.277	6.063	-7.379	1.00	0.00	LX0	O
ATOM	1230	N	ILE	1113	54.105	4.773	-7.744	1.00	0.00	LX0	N
ATOM	1231	H	ILE	1113	54.979	4.715	-8.217	0.00	0.00	LX0	H
ATOM	1232	CA	ILE	1113	53.767	3.731	-6.777	1.00	0.00	LX0	C

ATOM	1233	CB	ILE	1113	54.785	2.587	-6.930	1.00	0.00	LX0	C
ATOM	1234	CG2	ILE	1113	54.779	1.593	-5.770	1.00	0.00	LX0	C
ATOM	1235	CG1	ILE	1113	54.591	1.883	-8.275	1.00	0.00	LX0	C
ATOM	1236	CD1	ILE	1113	53.224	1.209	-8.418	1.00	0.00	LX0	C
ATOM	1237	C	ILE	1113	53.734	4.290	-5.366	1.00	0.00	LX0	C
ATOM	1238	O	ILE	1113	52.800	4.090	-4.602	1.00	0.00	LX0	O
ATOM	1239	N	GLN	1114	54.784	5.073	-5.087	1.00	0.00	LX0	N
ATOM	1240	H	GLN	1114	55.562	5.072	-5.712	0.00	0.00	LX0	H
ATOM	1241	CA	GLN	1114	54.828	5.802	-3.827	1.00	0.00	LX0	C
ATOM	1242	CB	GLN	1114	56.113	6.622	-3.737	1.00	0.00	LX0	C
ATOM	1243	CG	GLN	1114	56.206	7.542	-2.522	1.00	0.00	LX0	C
ATOM	1244	CD	GLN	1114	56.383	6.792	-1.219	1.00	0.00	LX0	C
ATOM	1245	OE1	GLN	1114	56.689	5.609	-1.173	1.00	0.00	LX0	O
ATOM	1246	NE2	GLN	1114	56.195	7.549	-0.142	1.00	0.00	LX0	N
ATOM	1247	HE21	GLN	1114	55.946	8.516	-0.251	0.00	0.00	LX0	H
ATOM	1248	HE22	GLN	1114	56.313	7.187	0.776	0.00	0.00	LX0	H
ATOM	1249	C	GLN	1114	53.590	6.640	-3.576	1.00	0.00	LX0	C
ATOM	1250	O	GLN	1114	52.920	6.450	-2.578	1.00	0.00	LX0	O
ATOM	1251	N	MET	1115	53.276	7.521	-4.542	1.00	0.00	LX0	N
ATOM	1252	H	MET	1115	53.885	7.628	-5.323	0.00	0.00	LX0	H
ATOM	1253	CA	MET	1115	52.069	8.343	-4.407	1.00	0.00	LX0	C
ATOM	1254	CB	MET	1115	51.872	9.268	-5.609	1.00	0.00	LX0	C
ATOM	1255	CG	MET	1115	52.832	10.455	-5.586	1.00	0.00	LX0	C
ATOM	1256	SD	MET	1115	52.575	11.630	-6.925	1.00	0.00	LX0	S
ATOM	1257	CE	MET	1115	53.286	10.657	-8.256	1.00	0.00	LX0	C
ATOM	1258	C	MET	1115	50.812	7.537	-4.172	1.00	0.00	LX0	C
ATOM	1259	O	MET	1115	49.998	7.828	-3.306	1.00	0.00	LX0	O
ATOM	1260	N	ALA	1116	50.719	6.450	-4.956	1.00	0.00	LX0	N
ATOM	1261	H	ALA	1116	51.403	6.295	-5.670	0.00	0.00	LX0	H
ATOM	1262	CA	ALA	1116	49.640	5.493	-4.720	1.00	0.00	LX0	C
ATOM	1263	CB	ALA	1116	49.813	4.260	-5.607	1.00	0.00	LX0	C
ATOM	1264	C	ALA	1116	49.544	5.045	-3.271	1.00	0.00	LX0	C
ATOM	1265	O	ALA	1116	48.483	5.041	-2.665	1.00	0.00	LX0	O
ATOM	1266	N	GLY	1117	50.715	4.702	-2.727	1.00	0.00	LX0	N
ATOM	1267	H	GLY	1117	51.561	4.776	-3.259	0.00	0.00	LX0	H
ATOM	1268	CA	GLY	1117	50.748	4.316	-1.323	1.00	0.00	LX0	C
ATOM	1269	C	GLY	1117	50.488	5.440	-0.338	1.00	0.00	LX0	C
ATOM	1270	O	GLY	1117	49.853	5.225	0.679	1.00	0.00	LX0	O
ATOM	1271	N	GLU	1118	50.978	6.644	-0.668	1.00	0.00	LX0	N
ATOM	1272	H	GLU	1118	51.441	6.761	-1.543	0.00	0.00	LX0	H
ATOM	1273	CA	GLU	1118	50.787	7.801	0.214	1.00	0.00	LX0	C
ATOM	1274	CB	GLU	1118	51.553	9.008	-0.360	1.00	0.00	LX0	C
ATOM	1275	CG	GLU	1118	53.075	8.799	-0.415	1.00	0.00	LX0	C
ATOM	1276	CD	GLU	1118	53.823	9.873	-1.209	1.00	0.00	LX0	C
ATOM	1277	OE1	GLU	1118	53.302	10.406	-2.179	1.00	0.00	LX0	O
ATOM	1278	OE2	GLU	1118	54.970	10.163	-0.886	1.00	0.00	LX0	O
ATOM	1279	C	GLU	1118	49.305	8.087	0.430	1.00	0.00	LX0	C
ATOM	1280	O	GLU	1118	48.782	8.152	1.539	1.00	0.00	LX0	O
ATOM	1281	N	ILE	1119	48.627	8.143	-0.725	1.00	0.00	LX0	N
ATOM	1282	H	ILE	1119	49.157	8.119	-1.575	0.00	0.00	LX0	H
ATOM	1283	CA	ILE	1119	47.166	8.223	-0.724	1.00	0.00	LX0	C
ATOM	1284	CB	ILE	1119	46.674	8.350	-2.174	1.00	0.00	LX0	C
ATOM	1285	CG2	ILE	1119	45.146	8.386	-2.301	1.00	0.00	LX0	C
ATOM	1286	CG1	ILE	1119	47.324	9.563	-2.840	1.00	0.00	LX0	C
ATOM	1287	CD1	ILE	1119	47.241	9.510	-4.364	1.00	0.00	LX0	C
ATOM	1288	C	ILE	1119	46.515	7.038	-0.017	1.00	0.00	LX0	C
ATOM	1289	O	ILE	1119	45.658	7.172	0.849	1.00	0.00	LX0	O
ATOM	1290	N	ALA	1120	46.977	5.846	-0.419	1.00	0.00	LX0	N
ATOM	1291	H	ALA	1120	47.699	5.795	-1.110	0.00	0.00	LX0	H
ATOM	1292	CA	ALA	1120	46.372	4.641	0.143	1.00	0.00	LX0	C
ATOM	1293	CB	ALA	1120	46.858	3.375	-0.563	1.00	0.00	LX0	C

ATOM	1294	C	ALA	1120	46.557	4.457	1.634	1.00	0.00	LX0	C
ATOM	1295	O	ALA	1120	45.743	3.814	2.277	1.00	0.00	LX0	O
ATOM	1296	N	ASP	1121	47.628	5.060	2.168	1.00	0.00	LX0	N
ATOM	1297	H	ASP	1121	48.259	5.579	1.593	0.00	0.00	LX0	H
ATOM	1298	CA	ASP	1121	47.861	4.974	3.608	1.00	0.00	LX0	C
ATOM	1299	CB	ASP	1121	49.289	5.397	3.949	1.00	0.00	LX0	C
ATOM	1300	CG	ASP	1121	49.607	5.052	5.390	1.00	0.00	LX0	C
ATOM	1301	OD1	ASP	1121	49.868	3.888	5.668	1.00	0.00	LX0	O
ATOM	1302	OD2	ASP	1121	49.604	5.945	6.235	1.00	0.00	LX0	O
ATOM	1303	C	ASP	1121	46.851	5.798	4.372	1.00	0.00	LX0	C
ATOM	1304	O	ASP	1121	46.174	5.334	5.282	1.00	0.00	LX0	O
ATOM	1305	N	GLY	1122	46.697	7.042	3.883	1.00	0.00	LX0	N
ATOM	1306	H	GLY	1122	47.321	7.375	3.172	0.00	0.00	LX0	H
ATOM	1307	CA	GLY	1122	45.610	7.862	4.420	1.00	0.00	LX0	C
ATOM	1308	C	GLY	1122	44.255	7.168	4.367	1.00	0.00	LX0	C
ATOM	1309	O	GLY	1122	43.528	7.064	5.348	1.00	0.00	LX0	O
ATOM	1310	N	MET	1123	43.973	6.642	3.165	1.00	0.00	LX0	N
ATOM	1311	H	MET	1123	44.618	6.785	2.412	0.00	0.00	LX0	H
ATOM	1312	CA	MET	1123	42.743	5.865	2.998	1.00	0.00	LX0	C
ATOM	1313	CB	MET	1123	42.507	5.507	1.532	1.00	0.00	LX0	C
ATOM	1314	CG	MET	1123	42.255	6.694	0.603	1.00	0.00	LX0	C
ATOM	1315	SD	MET	1123	40.830	7.697	1.064	1.00	0.00	LX0	S
ATOM	1316	CE	MET	1123	39.539	6.453	0.891	1.00	0.00	LX0	C
ATOM	1317	C	MET	1123	42.646	4.603	3.842	1.00	0.00	LX0	C
ATOM	1318	O	MET	1123	41.569	4.174	4.235	1.00	0.00	LX0	O
ATOM	1319	N	ALA	1124	43.820	4.025	4.123	1.00	0.00	LX0	N
ATOM	1320	H	ALA	1124	44.676	4.413	3.781	0.00	0.00	LX0	H
ATOM	1321	CA	ALA	1124	43.848	2.835	4.965	1.00	0.00	LX0	C
ATOM	1322	CB	ALA	1124	45.228	2.177	4.950	1.00	0.00	LX0	C
ATOM	1323	C	ALA	1124	43.465	3.153	6.389	1.00	0.00	LX0	C
ATOM	1324	O	ALA	1124	42.630	2.489	6.985	1.00	0.00	LX0	O
ATOM	1325	N	TYR	1125	44.060	4.249	6.892	1.00	0.00	LX0	N
ATOM	1326	H	TYR	1125	44.769	4.721	6.366	0.00	0.00	LX0	H
ATOM	1327	CA	TYR	1125	43.596	4.750	8.184	1.00	0.00	LX0	C
ATOM	1328	CB	TYR	1125	44.395	5.984	8.624	1.00	0.00	LX0	C
ATOM	1329	CG	TYR	1125	44.046	6.392	10.043	1.00	0.00	LX0	C
ATOM	1330	CD1	TYR	1125	44.865	5.953	11.103	1.00	0.00	LX0	C
ATOM	1331	CE1	TYR	1125	44.591	6.393	12.409	1.00	0.00	LX0	C
ATOM	1332	CD2	TYR	1125	42.919	7.208	10.272	1.00	0.00	LX0	C
ATOM	1333	CE2	TYR	1125	42.625	7.620	11.581	1.00	0.00	LX0	C
ATOM	1334	CZ	TYR	1125	43.494	7.250	12.625	1.00	0.00	LX0	C
ATOM	1335	OH	TYR	1125	43.295	7.768	13.889	1.00	0.00	LX0	O
ATOM	1336	HH	TYR	1125	42.364	7.980	14.014	0.00	0.00	LX0	H
ATOM	1337	C	TYR	1125	42.107	5.037	8.181	1.00	0.00	LX0	C
ATOM	1338	O	TYR	1125	41.402	4.701	9.118	1.00	0.00	LX0	O
ATOM	1339	N	LEU	1126	41.654	5.638	7.068	1.00	0.00	LX0	N
ATOM	1340	H	LEU	1126	42.300	5.944	6.368	0.00	0.00	LX0	H
ATOM	1341	CA	LEU	1126	40.214	5.864	6.961	1.00	0.00	LX0	C
ATOM	1342	CB	LEU	1126	39.852	6.634	5.683	1.00	0.00	LX0	C
ATOM	1343	CG	LEU	1126	40.520	8.001	5.502	1.00	0.00	LX0	C
ATOM	1344	CD1	LEU	1126	39.938	8.738	4.299	1.00	0.00	LX0	C
ATOM	1345	CD2	LEU	1126	40.475	8.870	6.756	1.00	0.00	LX0	C
ATOM	1346	C	LEU	1126	39.387	4.591	7.107	1.00	0.00	LX0	C
ATOM	1347	O	LEU	1126	38.788	4.354	8.143	1.00	0.00	LX0	O
ATOM	1348	N	ASN	1127	39.413	3.749	6.064	1.00	0.00	LX0	N
ATOM	1349	H	ASN	1127	39.958	3.982	5.258	0.00	0.00	LX0	H
ATOM	1350	CA	ASN	1127	38.576	2.543	6.108	1.00	0.00	LX0	C
ATOM	1351	CB	ASN	1127	38.728	1.788	4.782	1.00	0.00	LX0	C
ATOM	1352	CG	ASN	1127	37.998	0.446	4.659	1.00	0.00	LX0	C
ATOM	1353	OD1	ASN	1127	38.321	-0.309	3.752	1.00	0.00	LX0	O
ATOM	1354	ND2	ASN	1127	37.013	0.167	5.524	1.00	0.00	LX0	N

ATOM	1355	HD21	ASN	1127	36.716	0.735	6.294	0.00	0.00	LX0	H
ATOM	1356	HD22	ASN	1127	36.520	-0.710	5.450	0.00	0.00	LX0	H
ATOM	1357	C	ASN	1127	38.832	1.643	7.305	1.00	0.00	LX0	C
ATOM	1358	O	ASN	1127	37.926	1.253	8.029	1.00	0.00	LX0	O
ATOM	1359	N	ALA	1128	40.120	1.317	7.484	1.00	0.00	LX0	N
ATOM	1360	H	ALA	1128	40.844	1.762	6.956	0.00	0.00	LX0	H
ATOM	1361	CA	ALA	1128	40.416	0.398	8.579	1.00	0.00	LX0	C
ATOM	1362	CB	ALA	1128	41.847	-0.133	8.493	1.00	0.00	LX0	C
ATOM	1363	C	ALA	1128	40.162	0.975	9.960	1.00	0.00	LX0	C
ATOM	1364	O	ALA	1128	39.953	0.259	10.931	1.00	0.00	LX0	O
ATOM	1365	N	ASN	1129	40.160	2.310	10.002	1.00	0.00	LX0	N
ATOM	1366	H	ASN	1129	40.388	2.894	9.220	0.00	0.00	LX0	H
ATOM	1367	CA	ASN	1129	39.730	2.945	11.236	1.00	0.00	LX0	C
ATOM	1368	CB	ASN	1129	40.949	3.513	11.978	1.00	0.00	LX0	C
ATOM	1369	CG	ASN	1129	40.612	3.844	13.416	1.00	0.00	LX0	C
ATOM	1370	OD1	ASN	1129	39.523	4.288	13.754	1.00	0.00	LX0	O
ATOM	1371	ND2	ASN	1129	41.611	3.623	14.268	1.00	0.00	LX0	N
ATOM	1372	HD21	ASN	1129	42.460	3.172	13.973	0.00	0.00	LX0	H
ATOM	1373	HD22	ASN	1129	41.551	3.892	15.226	0.00	0.00	LX0	H
ATOM	1374	C	ASN	1129	38.639	3.972	10.970	1.00	0.00	LX0	C
ATOM	1375	O	ASN	1129	38.788	5.165	11.234	1.00	0.00	LX0	O
ATOM	1376	N	LYS	1130	37.512	3.415	10.470	1.00	0.00	LX0	N
ATOM	1377	H	LYS	1130	37.584	2.483	10.113	0.00	0.00	LX0	H
ATOM	1378	CA	LYS	1130	36.217	4.085	10.247	1.00	0.00	LX0	C
ATOM	1379	CB	LYS	1130	35.862	5.142	11.316	1.00	0.00	LX0	C
ATOM	1380	CG	LYS	1130	35.893	4.700	12.782	1.00	0.00	LX0	C
ATOM	1381	CD	LYS	1130	35.857	5.913	13.716	1.00	0.00	LX0	C
ATOM	1382	CE	LYS	1130	37.195	6.245	14.393	1.00	0.00	LX0	C
ATOM	1383	NZ	LYS	1130	38.289	6.468	13.437	1.00	0.00	LX0	N
ATOM	1384	HZ1	LYS	1130	39.169	6.687	13.952	0.00	0.00	LX0	H
ATOM	1385	HZ2	LYS	1130	38.504	5.631	12.856	0.00	0.00	LX0	H
ATOM	1386	HZ3	LYS	1130	38.170	7.312	12.838	0.00	0.00	LX0	H
ATOM	1387	C	LYS	1130	35.969	4.681	8.861	1.00	0.00	LX0	C
ATOM	1388	O	LYS	1130	35.254	4.156	8.015	1.00	0.00	LX0	O
ATOM	1389	N	PHE	1131	36.548	5.879	8.688	1.00	0.00	LX0	N
ATOM	1390	H	PHE	1131	37.368	6.061	9.229	0.00	0.00	LX0	H
ATOM	1391	CA	PHE	1131	36.010	6.789	7.674	1.00	0.00	LX0	C
ATOM	1392	CB	PHE	1131	36.575	8.198	7.888	1.00	0.00	LX0	C
ATOM	1393	CG	PHE	1131	35.999	8.820	9.144	1.00	0.00	LX0	C
ATOM	1394	CD1	PHE	1131	36.522	8.478	10.412	1.00	0.00	LX0	C
ATOM	1395	CD2	PHE	1131	34.941	9.746	9.024	1.00	0.00	LX0	C
ATOM	1396	CE1	PHE	1131	35.978	9.062	11.572	1.00	0.00	LX0	C
ATOM	1397	CE2	PHE	1131	34.395	10.333	10.182	1.00	0.00	LX0	C
ATOM	1398	CZ	PHE	1131	34.918	9.984	11.445	1.00	0.00	LX0	C
ATOM	1399	C	PHE	1131	36.112	6.363	6.214	1.00	0.00	LX0	C
ATOM	1400	O	PHE	1131	36.924	5.544	5.805	1.00	0.00	LX0	O
ATOM	1401	N	VAL	1132	35.219	6.991	5.434	1.00	0.00	LX0	N
ATOM	1402	H	VAL	1132	34.635	7.708	5.811	0.00	0.00	LX0	H
ATOM	1403	CA	VAL	1132	35.025	6.604	4.037	1.00	0.00	LX0	C
ATOM	1404	CB	VAL	1132	33.935	5.514	4.006	1.00	0.00	LX0	C
ATOM	1405	CG1	VAL	1132	32.672	5.968	4.747	1.00	0.00	LX0	C
ATOM	1406	CG2	VAL	1132	33.673	4.941	2.611	1.00	0.00	LX0	C
ATOM	1407	C	VAL	1132	34.680	7.866	3.249	1.00	0.00	LX0	C
ATOM	1408	O	VAL	1132	34.233	8.843	3.840	1.00	0.00	LX0	O
ATOM	1409	N	HIS	1133	34.969	7.855	1.937	1.00	0.00	LX0	N
ATOM	1410	H	HIS	1133	35.110	7.011	1.411	0.00	0.00	LX0	H
ATOM	1411	CA	HIS	1133	35.142	9.153	1.286	1.00	0.00	LX0	C
ATOM	1412	CB	HIS	1133	36.443	9.117	0.473	1.00	0.00	LX0	C
ATOM	1413	CG	HIS	1133	36.993	10.484	0.136	1.00	0.00	LX0	C
ATOM	1414	ND1	HIS	1133	36.265	11.605	-0.033	1.00	0.00	LX0	N
ATOM	1415	HD1	HIS	1133	35.293	11.706	0.049	0.00	0.00	LX0	H

ATOM	1416	CD2	HIS	1133	38.338	10.793	-0.071	1.00	0.00	LX0	C
ATOM	1417	NE2	HIS	1133	38.407	12.111	-0.368	1.00	0.00	LX0	N
ATOM	1418	CE1	HIS	1133	37.134	12.613	-0.343	1.00	0.00	LX0	C
ATOM	1419	C	HIS	1133	33.969	9.703	0.483	1.00	0.00	LX0	C
ATOM	1420	O	HIS	1133	33.647	10.891	0.554	1.00	0.00	LX0	O
ATOM	1421	N	ARG	1134	33.365	8.795	-0.303	1.00	0.00	LX0	N
ATOM	1422	H	ARG	1134	33.817	7.901	-0.394	0.00	0.00	LX0	H
ATOM	1423	CA	ARG	1134	32.281	9.136	-1.234	1.00	0.00	LX0	C
ATOM	1424	CB	ARG	1134	31.094	9.822	-0.521	1.00	0.00	LX0	C
ATOM	1425	CG	ARG	1134	29.727	9.632	-1.180	1.00	0.00	LX0	C
ATOM	1426	CD	ARG	1134	28.872	10.901	-1.284	1.00	0.00	LX0	C
ATOM	1427	NE	ARG	1134	28.555	11.494	0.015	1.00	0.00	LX0	N
ATOM	1428	HE	ARG	1134	29.136	12.235	0.369	0.00	0.00	LX0	H
ATOM	1429	CZ	ARG	1134	27.543	11.025	0.783	1.00	0.00	LX0	C
ATOM	1430	NH1	ARG	1134	26.793	10.003	0.386	1.00	0.00	LX0	N
ATOM	1431	HH11	ARG	1134	26.100	9.654	1.038	0.00	0.00	LX0	H
ATOM	1432	HH12	ARG	1134	26.897	9.559	-0.497	0.00	0.00	LX0	H
ATOM	1433	NH2	ARG	1134	27.295	11.591	1.952	1.00	0.00	LX0	N
ATOM	1434	HH21	ARG	1134	26.661	11.141	2.610	0.00	0.00	LX0	H
ATOM	1435	HH22	ARG	1134	27.716	12.459	2.214	0.00	0.00	LX0	H
ATOM	1436	C	ARG	1134	32.715	9.956	-2.450	1.00	0.00	LX0	C
ATOM	1437	O	ARG	1134	31.888	10.477	-3.190	1.00	0.00	LX0	O
ATOM	1438	N	ASP	1135	34.046	10.104	-2.585	1.00	0.00	LX0	N
ATOM	1439	H	ASP	1135	34.652	9.460	-2.112	0.00	0.00	LX0	H
ATOM	1440	CA	ASP	1135	34.533	11.233	-3.387	1.00	0.00	LX0	C
ATOM	1441	CB	ASP	1135	34.217	12.545	-2.636	1.00	0.00	LX0	C
ATOM	1442	CG	ASP	1135	34.173	13.766	-3.538	1.00	0.00	LX0	C
ATOM	1443	OD1	ASP	1135	33.396	13.807	-4.481	1.00	0.00	LX0	O
ATOM	1444	OD2	ASP	1135	34.936	14.698	-3.324	1.00	0.00	LX0	O
ATOM	1445	C	ASP	1135	36.011	11.166	-3.770	1.00	0.00	LX0	C
ATOM	1446	O	ASP	1135	36.621	12.149	-4.179	1.00	0.00	LX0	O
ATOM	1447	N	LEU	1136	36.607	9.972	-3.605	1.00	0.00	LX0	N
ATOM	1448	H	LEU	1136	36.100	9.143	-3.352	0.00	0.00	LX0	H
ATOM	1449	CA	LEU	1136	38.056	9.935	-3.836	1.00	0.00	LX0	C
ATOM	1450	CB	LEU	1136	38.625	8.586	-3.375	1.00	0.00	LX0	C
ATOM	1451	CG	LEU	1136	40.156	8.483	-3.361	1.00	0.00	LX0	C
ATOM	1452	CD1	LEU	1136	40.823	9.565	-2.509	1.00	0.00	LX0	C
ATOM	1453	CD2	LEU	1136	40.616	7.091	-2.941	1.00	0.00	LX0	C
ATOM	1454	C	LEU	1136	38.500	10.294	-5.254	1.00	0.00	LX0	C
ATOM	1455	O	LEU	1136	37.976	9.808	-6.248	1.00	0.00	LX0	O
ATOM	1456	N	ALA	1137	39.500	11.188	-5.284	1.00	0.00	LX0	N
ATOM	1457	H	ALA	1137	39.868	11.554	-4.428	0.00	0.00	LX0	H
ATOM	1458	CA	ALA	1137	40.121	11.619	-6.535	1.00	0.00	LX0	C
ATOM	1459	CB	ALA	1137	39.240	12.615	-7.278	1.00	0.00	LX0	C
ATOM	1460	C	ALA	1137	41.408	12.327	-6.188	1.00	0.00	LX0	C
ATOM	1461	O	ALA	1137	41.628	12.641	-5.024	1.00	0.00	LX0	O
ATOM	1462	N	ALA	1138	42.234	12.602	-7.211	1.00	0.00	LX0	N
ATOM	1463	H	ALA	1138	41.999	12.384	-8.164	0.00	0.00	LX0	H
ATOM	1464	CA	ALA	1138	43.501	13.268	-6.895	1.00	0.00	LX0	C
ATOM	1465	CB	ALA	1138	44.430	13.287	-8.109	1.00	0.00	LX0	C
ATOM	1466	C	ALA	1138	43.364	14.677	-6.332	1.00	0.00	LX0	C
ATOM	1467	O	ALA	1138	44.227	15.175	-5.619	1.00	0.00	LX0	O
ATOM	1468	N	ARG	1139	42.200	15.284	-6.636	1.00	0.00	LX0	N
ATOM	1469	H	ARG	1139	41.601	14.866	-7.316	0.00	0.00	LX0	H
ATOM	1470	CA	ARG	1139	41.871	16.559	-5.984	1.00	0.00	LX0	C
ATOM	1471	CB	ARG	1139	40.554	17.130	-6.537	1.00	0.00	LX0	C
ATOM	1472	CG	ARG	1139	39.312	16.314	-6.157	1.00	0.00	LX0	C
ATOM	1473	CD	ARG	1139	37.976	16.895	-6.623	1.00	0.00	LX0	C
ATOM	1474	NE	ARG	1139	36.868	16.103	-6.094	1.00	0.00	LX0	N
ATOM	1475	HE	ARG	1139	36.432	16.363	-5.225	0.00	0.00	LX0	H
ATOM	1476	CZ	ARG	1139	36.472	14.968	-6.696	1.00	0.00	LX0	C

ATOM	1477	NH1	ARG	1139	36.857	14.678	-7.935	1.00	0.00	LX0	N
ATOM	1478	HH11	ARG	1139	36.624	13.817	-8.380	0.00	0.00	LX0	H
ATOM	1479	HH12	ARG	1139	37.399	15.340	-8.473	0.00	0.00	LX0	H
ATOM	1480	NH2	ARG	1139	35.694	14.134	-6.032	1.00	0.00	LX0	N
ATOM	1481	HH21	ARG	1139	35.410	13.238	-6.362	0.00	0.00	LX0	H
ATOM	1482	HH22	ARG	1139	35.340	14.385	-5.117	0.00	0.00	LX0	H
ATOM	1483	C	ARG	1139	41.836	16.494	-4.456	1.00	0.00	LX0	C
ATOM	1484	O	ARG	1139	42.133	17.435	-3.738	1.00	0.00	LX0	O
ATOM	1485	N	ASN	1140	41.468	15.296	-3.983	1.00	0.00	LX0	N
ATOM	1486	H	ASN	1140	41.347	14.512	-4.588	0.00	0.00	LX0	H
ATOM	1487	CA	ASN	1140	41.339	15.142	-2.538	1.00	0.00	LX0	C
ATOM	1488	CB	ASN	1140	40.090	14.325	-2.202	1.00	0.00	LX0	C
ATOM	1489	CG	ASN	1140	38.845	15.134	-2.511	1.00	0.00	LX0	C
ATOM	1490	OD1	ASN	1140	38.802	16.347	-2.376	1.00	0.00	LX0	O
ATOM	1491	ND2	ASN	1140	37.811	14.409	-2.941	1.00	0.00	LX0	N
ATOM	1492	HD21	ASN	1140	37.825	13.426	-3.124	0.00	0.00	LX0	H
ATOM	1493	HD22	ASN	1140	36.919	14.850	-3.067	0.00	0.00	LX0	H
ATOM	1494	C	ASN	1140	42.573	14.543	-1.899	1.00	0.00	LX0	C
ATOM	1495	O	ASN	1140	42.557	14.033	-0.785	1.00	0.00	LX0	O
ATOM	1496	N	CYS	1141	43.669	14.621	-2.661	1.00	0.00	LX0	N
ATOM	1497	H	CYS	1141	43.672	15.058	-3.562	0.00	0.00	LX0	H
ATOM	1498	CA	CYS	1141	44.924	14.140	-2.107	1.00	0.00	LX0	C
ATOM	1499	CB	CYS	1141	45.470	13.013	-2.977	1.00	0.00	LX0	C
ATOM	1500	SG	CYS	1141	44.249	11.709	-3.280	1.00	0.00	LX0	S
ATOM	1501	C	CYS	1141	45.894	15.294	-2.029	1.00	0.00	LX0	C
ATOM	1502	O	CYS	1141	46.446	15.733	-3.028	1.00	0.00	LX0	O
ATOM	1503	N	MET	1142	46.023	15.830	-0.814	1.00	0.00	LX0	N
ATOM	1504	H	MET	1142	45.818	15.261	-0.017	0.00	0.00	LX0	H
ATOM	1505	CA	MET	1142	46.792	17.068	-0.739	1.00	0.00	LX0	C
ATOM	1506	CB	MET	1142	46.244	17.985	0.352	1.00	0.00	LX0	C
ATOM	1507	CG	MET	1142	44.822	18.494	0.104	1.00	0.00	LX0	C
ATOM	1508	SD	MET	1142	44.686	19.608	-1.297	1.00	0.00	LX0	S
ATOM	1509	CE	MET	1142	42.895	19.791	-1.290	1.00	0.00	LX0	C
ATOM	1510	C	MET	1142	48.264	16.802	-0.520	1.00	0.00	LX0	C
ATOM	1511	O	MET	1142	48.656	15.906	0.217	1.00	0.00	LX0	O
ATOM	1512	N	VAL	1143	49.068	17.616	-1.202	1.00	0.00	LX0	N
ATOM	1513	H	VAL	1143	48.690	18.381	-1.726	0.00	0.00	LX0	H
ATOM	1514	CA	VAL	1143	50.505	17.432	-1.056	1.00	0.00	LX0	C
ATOM	1515	CB	VAL	1143	51.189	17.528	-2.428	1.00	0.00	LX0	C
ATOM	1516	CG1	VAL	1143	52.609	16.967	-2.385	1.00	0.00	LX0	C
ATOM	1517	CG2	VAL	1143	50.363	16.876	-3.539	1.00	0.00	LX0	C
ATOM	1518	C	VAL	1143	51.058	18.466	-0.092	1.00	0.00	LX0	C
ATOM	1519	O	VAL	1143	50.752	19.653	-0.187	1.00	0.00	LX0	O
ATOM	1520	N	ALA	1144	51.854	17.982	0.867	1.00	0.00	LX0	N
ATOM	1521	H	ALA	1144	52.155	17.024	0.864	0.00	0.00	LX0	H
ATOM	1522	CA	ALA	1144	52.403	18.973	1.787	1.00	0.00	LX0	C
ATOM	1523	CB	ALA	1144	52.522	18.396	3.202	1.00	0.00	LX0	C
ATOM	1524	C	ALA	1144	53.732	19.530	1.303	1.00	0.00	LX0	C
ATOM	1525	O	ALA	1144	54.216	19.191	0.228	1.00	0.00	LX0	O
ATOM	1526	N	GLU	1145	54.309	20.417	2.137	1.00	0.00	LX0	N
ATOM	1527	H	GLU	1145	53.813	20.691	2.963	0.00	0.00	LX0	H
ATOM	1528	CA	GLU	1145	55.606	21.023	1.802	1.00	0.00	LX0	C
ATOM	1529	CB	GLU	1145	56.083	21.873	2.988	1.00	0.00	LX0	C
ATOM	1530	CG	GLU	1145	57.186	22.906	2.697	1.00	0.00	LX0	C
ATOM	1531	CD	GLU	1145	56.620	24.178	2.080	1.00	0.00	LX0	C
ATOM	1532	OE1	GLU	1145	55.811	24.840	2.715	1.00	0.00	LX0	O
ATOM	1533	OE2	GLU	1145	57.014	24.546	0.982	1.00	0.00	LX0	O
ATOM	1534	C	GLU	1145	56.696	20.027	1.388	1.00	0.00	LX0	C
ATOM	1535	O	GLU	1145	57.504	20.249	0.491	1.00	0.00	LX0	O
ATOM	1536	N	ASP	1146	56.651	18.906	2.110	1.00	0.00	LX0	N
ATOM	1537	H	ASP	1146	55.868	18.727	2.707	0.00	0.00	LX0	H

ATOM	1538	CA	ASP	1146	57.565	17.778	1.936	1.00	0.00	LX0	C
ATOM	1539	CB	ASP	1146	57.304	16.764	3.062	1.00	0.00	LX0	C
ATOM	1540	CG	ASP	1146	55.845	16.306	3.131	1.00	0.00	LX0	C
ATOM	1541	OD1	ASP	1146	55.022	16.724	2.318	1.00	0.00	LX0	O
ATOM	1542	OD2	ASP	1146	55.526	15.513	4.006	1.00	0.00	LX0	O
ATOM	1543	C	ASP	1146	57.562	17.076	0.581	1.00	0.00	LX0	C
ATOM	1544	O	ASP	1146	58.550	16.458	0.205	1.00	0.00	LX0	O
ATOM	1545	N	PHE	1147	56.418	17.216	-0.124	1.00	0.00	LX0	N
ATOM	1546	H	PHE	1147	55.677	17.673	0.374	0.00	0.00	LX0	H
ATOM	1547	CA	PHE	1147	56.104	16.491	-1.365	1.00	0.00	LX0	C
ATOM	1548	CB	PHE	1147	57.214	16.499	-2.435	1.00	0.00	LX0	C
ATOM	1549	CG	PHE	1147	57.514	17.889	-2.948	1.00	0.00	LX0	C
ATOM	1550	CD1	PHE	1147	56.794	18.384	-4.058	1.00	0.00	LX0	C
ATOM	1551	CD2	PHE	1147	58.527	18.657	-2.334	1.00	0.00	LX0	C
ATOM	1552	CE1	PHE	1147	57.117	19.650	-4.583	1.00	0.00	LX0	C
ATOM	1553	CE2	PHE	1147	58.849	19.925	-2.856	1.00	0.00	LX0	C
ATOM	1554	CZ	PHE	1147	58.151	20.401	-3.986	1.00	0.00	LX0	C
ATOM	1555	C	PHE	1147	55.589	15.071	-1.165	1.00	0.00	LX0	C
ATOM	1556	O	PHE	1147	55.638	14.233	-2.062	1.00	0.00	LX0	O
ATOM	1557	N	THR	1148	55.053	14.853	0.038	1.00	0.00	LX0	N
ATOM	1558	H	THR	1148	55.088	15.533	0.770	0.00	0.00	LX0	H
ATOM	1559	CA	THR	1148	54.293	13.631	0.272	1.00	0.00	LX0	C
ATOM	1560	CB	THR	1148	54.470	13.221	1.735	1.00	0.00	LX0	C
ATOM	1561	OG1	THR	1148	55.842	13.351	2.119	1.00	0.00	LX0	O
ATOM	1562	HG1	THR	1148	55.854	14.080	2.742	0.00	0.00	LX0	H
ATOM	1563	CG2	THR	1148	53.953	11.814	2.033	1.00	0.00	LX0	C
ATOM	1564	C	THR	1148	52.827	13.862	-0.059	1.00	0.00	LX0	C
ATOM	1565	O	THR	1148	52.245	14.891	0.283	1.00	0.00	LX0	O
ATOM	1566	N	VAL	1149	52.246	12.888	-0.770	1.00	0.00	LX0	N
ATOM	1567	H	VAL	1149	52.739	12.046	-1.020	0.00	0.00	LX0	H
ATOM	1568	CA	VAL	1149	50.853	13.101	-1.150	1.00	0.00	LX0	C
ATOM	1569	CB	VAL	1149	50.611	12.665	-2.596	1.00	0.00	LX0	C
ATOM	1570	CG1	VAL	1149	49.208	13.058	-3.052	1.00	0.00	LX0	C
ATOM	1571	CG2	VAL	1149	51.675	13.259	-3.520	1.00	0.00	LX0	C
ATOM	1572	C	VAL	1149	49.861	12.466	-0.193	1.00	0.00	LX0	C
ATOM	1573	O	VAL	1149	49.461	11.314	-0.299	1.00	0.00	LX0	O
ATOM	1574	N	LYS	1150	49.486	13.293	0.781	1.00	0.00	LX0	N
ATOM	1575	H	LYS	1150	49.705	14.268	0.709	0.00	0.00	LX0	H
ATOM	1576	CA	LYS	1150	48.570	12.770	1.785	1.00	0.00	LX0	C
ATOM	1577	CB	LYS	1150	48.630	13.623	3.044	1.00	0.00	LX0	C
ATOM	1578	CG	LYS	1150	50.006	13.779	3.682	1.00	0.00	LX0	C
ATOM	1579	CD	LYS	1150	49.768	14.386	5.056	1.00	0.00	LX0	C
ATOM	1580	CE	LYS	1150	50.988	14.596	5.941	1.00	0.00	LX0	C
ATOM	1581	NZ	LYS	1150	50.475	14.643	7.313	1.00	0.00	LX0	N
ATOM	1582	HZ1	LYS	1150	51.074	15.223	7.937	0.00	0.00	LX0	H
ATOM	1583	HZ2	LYS	1150	49.560	15.145	7.343	0.00	0.00	LX0	H
ATOM	1584	HZ3	LYS	1150	50.329	13.692	7.704	0.00	0.00	LX0	H
ATOM	1585	C	LYS	1150	47.126	12.705	1.328	1.00	0.00	LX0	C
ATOM	1586	O	LYS	1150	46.706	13.354	0.375	1.00	0.00	LX0	O
ATOM	1587	N	ILE	1151	46.353	11.934	2.107	1.00	0.00	LX0	N
ATOM	1588	H	ILE	1151	46.768	11.414	2.855	0.00	0.00	LX0	H
ATOM	1589	CA	ILE	1151	44.912	12.153	2.024	1.00	0.00	LX0	C
ATOM	1590	CB	ILE	1151	44.151	10.968	2.637	1.00	0.00	LX0	C
ATOM	1591	CG2	ILE	1151	42.756	11.278	3.194	1.00	0.00	LX0	C
ATOM	1592	CG1	ILE	1151	44.065	9.909	1.543	1.00	0.00	LX0	C
ATOM	1593	CD1	ILE	1151	43.295	10.406	0.316	1.00	0.00	LX0	C
ATOM	1594	C	ILE	1151	44.534	13.495	2.611	1.00	0.00	LX0	C
ATOM	1595	O	ILE	1151	45.002	13.902	3.671	1.00	0.00	LX0	O
ATOM	1596	N	GLY	1152	43.740	14.179	1.783	1.00	0.00	LX0	N
ATOM	1597	H	GLY	1152	43.287	13.721	1.016	0.00	0.00	LX0	H
ATOM	1598	CA	GLY	1152	43.567	15.606	1.969	1.00	0.00	LX0	C

ATOM	1599	C	GLY	1152	42.590	16.032	3.032	1.00	0.00	LX0	C
ATOM	1600	O	GLY	1152	42.175	15.289	3.911	1.00	0.00	LX0	O
ATOM	1601	N	ASP	1153	42.285	17.320	2.892	1.00	0.00	LX0	N
ATOM	1602	H	ASP	1153	42.526	17.794	2.049	0.00	0.00	LX0	H
ATOM	1603	CA	ASP	1153	41.426	18.000	3.846	1.00	0.00	LX0	C
ATOM	1604	CB	ASP	1153	42.042	19.393	4.036	1.00	0.00	LX0	C
ATOM	1605	CG	ASP	1153	41.046	20.406	4.542	1.00	0.00	LX0	C
ATOM	1606	OD1	ASP	1153	40.486	20.229	5.620	1.00	0.00	LX0	O
ATOM	1607	OD2	ASP	1153	40.771	21.355	3.821	1.00	0.00	LX0	O
ATOM	1608	C	ASP	1153	39.992	17.971	3.336	1.00	0.00	LX0	C
ATOM	1609	O	ASP	1153	39.741	17.757	2.155	1.00	0.00	LX0	O
ATOM	1610	N	PHE	1154	39.062	18.133	4.287	1.00	0.00	LX0	N
ATOM	1611	H	PHE	1154	39.360	18.493	5.174	0.00	0.00	LX0	H
ATOM	1612	CA	PHE	1154	37.668	17.876	3.949	1.00	0.00	LX0	C
ATOM	1613	CB	PHE	1154	37.216	16.537	4.561	1.00	0.00	LX0	C
ATOM	1614	CG	PHE	1154	38.066	15.399	4.032	1.00	0.00	LX0	C
ATOM	1615	CD1	PHE	1154	38.108	15.135	2.644	1.00	0.00	LX0	C
ATOM	1616	CD2	PHE	1154	38.817	14.622	4.939	1.00	0.00	LX0	C
ATOM	1617	CE1	PHE	1154	38.935	14.105	2.159	1.00	0.00	LX0	C
ATOM	1618	CE2	PHE	1154	39.637	13.584	4.457	1.00	0.00	LX0	C
ATOM	1619	CZ	PHE	1154	39.697	13.344	3.069	1.00	0.00	LX0	C
ATOM	1620	C	PHE	1154	36.725	19.024	4.278	1.00	0.00	LX0	C
ATOM	1621	O	PHE	1154	37.129	20.122	4.672	1.00	0.00	LX0	O
ATOM	1622	N	GLY	1155	35.429	18.723	4.050	1.00	0.00	LX0	N
ATOM	1623	H	GLY	1155	35.131	17.811	3.756	0.00	0.00	LX0	H
ATOM	1624	CA	GLY	1155	34.395	19.742	4.232	1.00	0.00	LX0	C
ATOM	1625	C	GLY	1155	34.474	20.885	3.236	1.00	0.00	LX0	C
ATOM	1626	O	GLY	1155	33.981	21.984	3.455	1.00	0.00	LX0	O
ATOM	1627	N	MET	1156	35.153	20.587	2.114	1.00	0.00	LX0	N
ATOM	1628	H	MET	1156	35.419	19.644	1.927	0.00	0.00	LX0	H
ATOM	1629	CA	MET	1156	35.606	21.704	1.286	1.00	0.00	LX0	C
ATOM	1630	CB	MET	1156	36.642	21.261	0.253	1.00	0.00	LX0	C
ATOM	1631	CG	MET	1156	38.006	21.020	0.907	1.00	0.00	LX0	C
ATOM	1632	SD	MET	1156	38.591	22.462	1.822	1.00	0.00	LX0	S
ATOM	1633	CE	MET	1156	38.764	23.616	0.451	1.00	0.00	LX0	C
ATOM	1634	C	MET	1156	34.554	22.595	0.659	1.00	0.00	LX0	C
ATOM	1635	O	MET	1156	34.830	23.742	0.339	1.00	0.00	LX0	O
ATOM	1636	N	THR	1157	33.339	22.035	0.547	1.00	0.00	LX0	N
ATOM	1637	H	THR	1157	33.213	21.069	0.773	0.00	0.00	LX0	H
ATOM	1638	CA	THR	1157	32.184	22.793	0.066	1.00	0.00	LX0	C
ATOM	1639	CB	THR	1157	31.886	24.053	0.908	1.00	0.00	LX0	C
ATOM	1640	OG1	THR	1157	32.440	23.983	2.232	1.00	0.00	LX0	O
ATOM	1641	HG1	THR	1157	32.553	23.067	2.482	0.00	0.00	LX0	H
ATOM	1642	CG2	THR	1157	30.383	24.332	0.967	1.00	0.00	LX0	C
ATOM	1643	C	THR	1157	32.156	23.118	-1.425	1.00	0.00	LX0	C
ATOM	1644	O	THR	1157	31.309	22.611	-2.149	1.00	0.00	LX0	O
ATOM	1645	N	ARG	1158	33.080	23.999	-1.842	1.00	0.00	LX0	N
ATOM	1646	H	ARG	1158	33.825	24.317	-1.249	0.00	0.00	LX0	H
ATOM	1647	CA	ARG	1158	33.114	24.460	-3.231	1.00	0.00	LX0	C
ATOM	1648	CB	ARG	1158	32.324	25.768	-3.421	1.00	0.00	LX0	C
ATOM	1649	CG	ARG	1158	31.104	26.056	-2.546	1.00	0.00	LX0	C
ATOM	1650	CD	ARG	1158	30.867	27.566	-2.458	1.00	0.00	LX0	C
ATOM	1651	NE	ARG	1158	32.088	28.233	-2.005	1.00	0.00	LX0	N
ATOM	1652	HE	ARG	1158	32.885	28.320	-2.622	0.00	0.00	LX0	H
ATOM	1653	CZ	ARG	1158	32.322	28.547	-0.718	1.00	0.00	LX0	C
ATOM	1654	NH1	ARG	1158	31.390	28.395	0.217	1.00	0.00	LX0	N
ATOM	1655	HH11	ARG	1158	31.647	28.548	1.178	0.00	0.00	LX0	H
ATOM	1656	HH12	ARG	1158	30.462	28.113	-0.015	0.00	0.00	LX0	H
ATOM	1657	NH2	ARG	1158	33.518	28.993	-0.383	1.00	0.00	LX0	N
ATOM	1658	HH21	ARG	1158	33.765	29.296	0.537	0.00	0.00	LX0	H
ATOM	1659	HH22	ARG	1158	34.242	28.954	-1.093	0.00	0.00	LX0	H

ATOM	1660	C	ARG	1158	34.570	24.737	-3.594	1.00	0.00	LX0	C
ATOM	1661	O	ARG	1158	35.480	24.215	-2.956	1.00	0.00	LX0	O
ATOM	1662	N	ASP	1159	34.724	25.647	-4.578	1.00	0.00	LX0	N
ATOM	1663	H	ASP	1159	34.071	25.581	-5.336	0.00	0.00	LX0	H
ATOM	1664	CA	ASP	1159	35.892	26.523	-4.746	1.00	0.00	LX0	C
ATOM	1665	CB	ASP	1159	36.434	27.160	-3.443	1.00	0.00	LX0	C
ATOM	1666	CG	ASP	1159	35.364	27.766	-2.548	1.00	0.00	LX0	C
ATOM	1667	OD1	ASP	1159	34.868	28.859	-2.818	1.00	0.00	LX0	O
ATOM	1668	OD2	ASP	1159	35.039	27.162	-1.533	1.00	0.00	LX0	O
ATOM	1669	C	ASP	1159	37.060	25.901	-5.482	1.00	0.00	LX0	C
ATOM	1670	O	ASP	1159	37.975	26.588	-5.924	1.00	0.00	LX0	O
ATOM	1671	N	ILE	1160	37.026	24.565	-5.564	1.00	0.00	LX0	N
ATOM	1672	H	ILE	1160	36.176	24.074	-5.360	0.00	0.00	LX0	H
ATOM	1673	CA	ILE	1160	38.260	23.908	-5.980	1.00	0.00	LX0	C
ATOM	1674	CB	ILE	1160	38.781	22.966	-4.871	1.00	0.00	LX0	C
ATOM	1675	CG2	ILE	1160	40.147	22.363	-5.224	1.00	0.00	LX0	C
ATOM	1676	CG1	ILE	1160	38.827	23.644	-3.494	1.00	0.00	LX0	C
ATOM	1677	CD1	ILE	1160	39.858	24.773	-3.380	1.00	0.00	LX0	C
ATOM	1678	C	ILE	1160	38.128	23.177	-7.303	1.00	0.00	LX0	C
ATOM	1679	O	ILE	1160	38.878	23.394	-8.248	1.00	0.00	LX0	O
ATOM	1680	N	TYR	1161	37.158	22.253	-7.321	1.00	0.00	LX0	N
ATOM	1681	H	TYR	1161	36.451	22.248	-6.607	0.00	0.00	LX0	H
ATOM	1682	CA	TYR	1161	37.169	21.295	-8.424	1.00	0.00	LX0	C
ATOM	1683	CB	TYR	1161	38.121	20.132	-8.143	1.00	0.00	LX0	C
ATOM	1684	CG	TYR	1161	39.382	20.298	-8.953	1.00	0.00	LX0	C
ATOM	1685	CD1	TYR	1161	39.303	20.234	-10.358	1.00	0.00	LX0	C
ATOM	1686	CE1	TYR	1161	40.472	20.416	-11.109	1.00	0.00	LX0	C
ATOM	1687	CD2	TYR	1161	40.600	20.521	-8.281	1.00	0.00	LX0	C
ATOM	1688	CE2	TYR	1161	41.769	20.698	-9.036	1.00	0.00	LX0	C
ATOM	1689	CZ	TYR	1161	41.693	20.641	-10.441	1.00	0.00	LX0	C
ATOM	1690	OH	TYR	1161	42.842	20.797	-11.199	1.00	0.00	LX0	O
ATOM	1691	HH	TYR	1161	43.533	21.075	-10.586	0.00	0.00	LX0	H
ATOM	1692	C	TYR	1161	35.818	20.764	-8.832	1.00	0.00	LX0	C
ATOM	1693	O	TYR	1161	35.638	19.603	-9.178	1.00	0.00	LX0	O
ATOM	1694	N	GLU	1162	34.858	21.688	-8.819	1.00	0.00	LX0	N
ATOM	1695	H	GLU	1162	35.044	22.581	-8.398	0.00	0.00	LX0	H
ATOM	1696	CA	GLU	1162	33.493	21.299	-9.165	1.00	0.00	LX0	C
ATOM	1697	CB	GLU	1162	32.533	22.460	-8.868	1.00	0.00	LX0	C
ATOM	1698	CG	GLU	1162	32.457	22.889	-7.386	1.00	0.00	LX0	C
ATOM	1699	CD	GLU	1162	33.721	23.603	-6.921	1.00	0.00	LX0	C
ATOM	1700	OE1	GLU	1162	34.538	22.996	-6.232	1.00	0.00	LX0	O
ATOM	1701	OE2	GLU	1162	33.905	24.766	-7.260	1.00	0.00	LX0	O
ATOM	1702	C	GLU	1162	33.329	20.741	-10.582	1.00	0.00	LX0	C
ATOM	1703	O	GLU	1162	32.409	19.998	-10.903	1.00	0.00	LX0	O
ATOM	1704	N	THR	1163	34.314	21.094	-11.420	1.00	0.00	LX0	N
ATOM	1705	H	THR	1163	35.065	21.674	-11.104	0.00	0.00	LX0	H
ATOM	1706	CA	THR	1163	34.407	20.555	-12.778	1.00	0.00	LX0	C
ATOM	1707	CB	THR	1163	35.528	21.310	-13.487	1.00	0.00	LX0	C
ATOM	1708	OG1	THR	1163	36.528	21.695	-12.528	1.00	0.00	LX0	O
ATOM	1709	HG1	THR	1163	37.146	22.255	-12.983	0.00	0.00	LX0	H
ATOM	1710	CG2	THR	1163	34.987	22.554	-14.194	1.00	0.00	LX0	C
ATOM	1711	C	THR	1163	34.584	19.041	-12.909	1.00	0.00	LX0	C
ATOM	1712	O	THR	1163	34.346	18.445	-13.956	1.00	0.00	LX0	O
ATOM	1713	N	ASP	1164	34.984	18.429	-11.786	1.00	0.00	LX0	N
ATOM	1714	H	ASP	1164	35.235	18.955	-10.972	0.00	0.00	LX0	H
ATOM	1715	CA	ASP	1164	35.056	16.968	-11.740	1.00	0.00	LX0	C
ATOM	1716	CB	ASP	1164	35.888	16.538	-10.526	1.00	0.00	LX0	C
ATOM	1717	CG	ASP	1164	37.390	16.557	-10.781	1.00	0.00	LX0	C
ATOM	1718	OD1	ASP	1164	37.850	17.090	-11.786	1.00	0.00	LX0	O
ATOM	1719	OD2	ASP	1164	38.132	15.975	-9.993	1.00	0.00	LX0	O
ATOM	1720	C	ASP	1164	33.703	16.264	-11.696	1.00	0.00	LX0	C

```
ATOM   1721  O   ASP  1164      33.587  15.045 -11.797  1.00  0.00      LX0  O
ATOM   1722  N   TYR  1165      32.666  17.080 -11.491  1.00  0.00      LX0  N
ATOM   1723  H   TYR  1165      32.749  18.077 -11.518  0.00  0.00      LX0  H
ATOM   1724  CA  TYR  1165      31.385  16.480 -11.152  1.00  0.00      LX0  C
ATOM   1725  CB  TYR  1165      30.841  17.172  -9.896  1.00  0.00      LX0  C
ATOM   1726  CG  TYR  1165      31.726  17.010  -8.671  1.00  0.00      LX0  C
ATOM   1727  CD1 TYR  1165      32.908  17.770  -8.540  1.00  0.00      LX0  C
ATOM   1728  CE1 TYR  1165      33.720  17.599  -7.406  1.00  0.00      LX0  C
ATOM   1729  CD2 TYR  1165      31.327  16.105  -7.667  1.00  0.00      LX0  C
ATOM   1730  CE2 TYR  1165      32.135  15.938  -6.529  1.00  0.00      LX0  C
ATOM   1731  CZ  TYR  1165      33.330  16.674  -6.417  1.00  0.00      LX0  C
ATOM   1732  OH  TYR  1165      34.153  16.485  -5.323  1.00  0.00      LX0  O
ATOM   1733  HH  TYR  1165      33.826  15.771  -4.764  0.00  0.00      LX0  H
ATOM   1734  C   TYR  1165      30.384  16.537 -12.302  1.00  0.00      LX0  C
ATOM   1735  O   TYR  1165      30.610  17.161 -13.333  1.00  0.00      LX0  O
ATOM   1736  N   TYR  1166      29.244  15.867 -12.079  1.00  0.00      LX0  N
ATOM   1737  H   TYR  1166      29.185  15.250 -11.291  0.00  0.00      LX0  H
ATOM   1738  CA  TYR  1166      28.089  16.037 -12.963  1.00  0.00      LX0  C
ATOM   1739  CB  TYR  1166      28.122  14.999 -14.109  1.00  0.00      LX0  C
ATOM   1740  CG  TYR  1166      26.750  14.586 -14.618  1.00  0.00      LX0  C
ATOM   1741  CD1 TYR  1166      25.938  15.487 -15.341  1.00  0.00      LX0  C
ATOM   1742  CE1 TYR  1166      24.636  15.092 -15.704  1.00  0.00      LX0  C
ATOM   1743  CD2 TYR  1166      26.314  13.282 -14.318  1.00  0.00      LX0  C
ATOM   1744  CE2 TYR  1166      25.027  12.879 -14.702  1.00  0.00      LX0  C
ATOM   1745  CZ  TYR  1166      24.189  13.800 -15.357  1.00  0.00      LX0  C
ATOM   1746  OH  TYR  1166      22.891  13.420 -15.639  1.00  0.00      LX0  O
ATOM   1747  HH  TYR  1166      22.919  12.716 -16.298  0.00  0.00      LX0  H
ATOM   1748  C   TYR  1166      26.833  15.942 -12.125  1.00  0.00      LX0  C
ATOM   1749  O   TYR  1166      26.731  15.091 -11.251  1.00  0.00      LX0  O
ATOM   1750  N   ARG  1167      25.892  16.856 -12.418  1.00  0.00      LX0  N
ATOM   1751  H   ARG  1167      26.019  17.467 -13.197  0.00  0.00      LX0  H
ATOM   1752  CA  ARG  1167      24.652  16.860 -11.647  1.00  0.00      LX0  C
ATOM   1753  CB  ARG  1167      24.072  18.279 -11.571  1.00  0.00      LX0  C
ATOM   1754  CG  ARG  1167      23.058  18.393 -10.433  1.00  0.00      LX0  C
ATOM   1755  CD  ARG  1167      22.460  19.777 -10.191  1.00  0.00      LX0  C
ATOM   1756  NE  ARG  1167      21.360  19.617  -9.246  1.00  0.00      LX0  N
ATOM   1757  HE  ARG  1167      21.020  18.684  -9.103  0.00  0.00      LX0  H
ATOM   1758  CZ  ARG  1167      20.747  20.605  -8.571  1.00  0.00      LX0  C
ATOM   1759  NH1 ARG  1167      21.179  21.861  -8.664  1.00  0.00      LX0  N
ATOM   1760 HH11 ARG  1167      20.741  22.610  -8.167  0.00  0.00      LX0  H
ATOM   1761 HH12 ARG  1167      21.968  22.071  -9.244  0.00  0.00      LX0  H
ATOM   1762  NH2 ARG  1167      19.705  20.281  -7.808  1.00  0.00      LX0  N
ATOM   1763 HH21 ARG  1167      19.217  20.934  -7.230  0.00  0.00      LX0  H
ATOM   1764 HH22 ARG  1167      19.393  19.321  -7.807  0.00  0.00      LX0  H
ATOM   1765  C   ARG  1167      23.623  15.826 -12.082  1.00  0.00      LX0  C
ATOM   1766  O   ARG  1167      22.641  16.119 -12.753  1.00  0.00      LX0  O
ATOM   1767  N   LYS  1168      23.921  14.591 -11.655  1.00  0.00      LX0  N
ATOM   1768  H   LYS  1168      24.755  14.490 -11.109  0.00  0.00      LX0  H
ATOM   1769  CA  LYS  1168      23.133  13.404 -11.994  1.00  0.00      LX0  C
ATOM   1770  CB  LYS  1168      23.624  12.268 -11.096  1.00  0.00      LX0  C
ATOM   1771  CG  LYS  1168      22.969  10.901 -11.268  1.00  0.00      LX0  C
ATOM   1772  CD  LYS  1168      23.500   9.944 -10.202  1.00  0.00      LX0  C
ATOM   1773  CE  LYS  1168      22.695   8.652 -10.100  1.00  0.00      LX0  C
ATOM   1774  NZ  LYS  1168      22.816   7.863 -11.331  1.00  0.00      LX0  N
ATOM   1775  HZ1 LYS  1168      22.298   6.968 -11.203  0.00  0.00      LX0  H
ATOM   1776  HZ2 LYS  1168      22.454   8.366 -12.160  0.00  0.00      LX0  H
ATOM   1777  HZ3 LYS  1168      23.812   7.600 -11.485  0.00  0.00      LX0  H
ATOM   1778  C   LYS  1168      21.617  13.559 -11.963  1.00  0.00      LX0  C
ATOM   1779  O   LYS  1168      20.968  13.486 -10.924  1.00  0.00      LX0  O
ATOM   1780  N   GLY  1169      21.067  13.784 -13.168  1.00  0.00      LX0  N
ATOM   1781  H   GLY  1169      21.665  13.832 -13.976  0.00  0.00      LX0  H
```

ATOM	1782	CA	GLY	1169	19.613	13.949	-13.279	1.00	0.00	LX0	C
ATOM	1783	C	GLY	1169	19.010	14.991	-12.340	1.00	0.00	LX0	C
ATOM	1784	O	GLY	1169	17.937	14.831	-11.754	1.00	0.00	LX0	O
ATOM	1785	N	GLY	1170	19.776	16.080	-12.183	1.00	0.00	LX0	N
ATOM	1786	H	GLY	1170	20.678	16.111	-12.623	0.00	0.00	LX0	H
ATOM	1787	CA	GLY	1170	19.313	17.145	-11.297	1.00	0.00	LX0	C
ATOM	1788	C	GLY	1170	19.458	16.889	-9.800	1.00	0.00	LX0	C
ATOM	1789	O	GLY	1170	19.308	17.797	-8.987	1.00	0.00	LX0	O
ATOM	1790	N	LYS	1171	19.733	15.624	-9.464	1.00	0.00	LX0	N
ATOM	1791	H	LYS	1171	20.006	14.957	-10.156	0.00	0.00	LX0	H
ATOM	1792	CA	LYS	1171	20.036	15.301	-8.072	1.00	0.00	LX0	C
ATOM	1793	CB	LYS	1171	19.445	13.938	-7.680	1.00	0.00	LX0	C
ATOM	1794	CG	LYS	1171	18.050	13.526	-8.173	1.00	0.00	LX0	C
ATOM	1795	CD	LYS	1171	16.852	14.330	-7.650	1.00	0.00	LX0	C
ATOM	1796	CE	LYS	1171	16.424	15.486	-8.558	1.00	0.00	LX0	C
ATOM	1797	NZ	LYS	1171	16.139	14.970	-9.907	1.00	0.00	LX0	N
ATOM	1798	HZ1	LYS	1171	15.759	15.721	-10.514	0.00	0.00	LX0	H
ATOM	1799	HZ2	LYS	1171	17.017	14.630	-10.357	0.00	0.00	LX0	H
ATOM	1800	HZ3	LYS	1171	15.464	14.184	-9.857	0.00	0.00	LX0	H
ATOM	1801	C	LYS	1171	21.547	15.214	-7.975	1.00	0.00	LX0	C
ATOM	1802	O	LYS	1171	22.240	15.559	-8.927	1.00	0.00	LX0	O
ATOM	1803	N	GLY	1172	22.018	14.682	-6.839	1.00	0.00	LX0	N
ATOM	1804	H	GLY	1172	21.446	14.752	-6.010	0.00	0.00	LX0	H
ATOM	1805	CA	GLY	1172	23.210	13.827	-6.821	1.00	0.00	LX0	C
ATOM	1806	C	GLY	1172	24.368	14.055	-7.785	1.00	0.00	LX0	C
ATOM	1807	O	GLY	1172	24.924	13.124	-8.348	1.00	0.00	LX0	O
ATOM	1808	N	LEU	1173	24.760	15.328	-7.897	1.00	0.00	LX0	N
ATOM	1809	H	LEU	1173	24.073	16.012	-7.664	0.00	0.00	LX0	H
ATOM	1810	CA	LEU	1173	26.069	15.655	-8.464	1.00	0.00	LX0	C
ATOM	1811	CB	LEU	1173	26.238	17.183	-8.487	1.00	0.00	LX0	C
ATOM	1812	CG	LEU	1173	27.608	17.821	-8.695	1.00	0.00	LX0	C
ATOM	1813	CD1	LEU	1173	27.524	19.015	-9.647	1.00	0.00	LX0	C
ATOM	1814	CD2	LEU	1173	28.250	18.227	-7.364	1.00	0.00	LX0	C
ATOM	1815	C	LEU	1173	27.201	14.914	-7.780	1.00	0.00	LX0	C
ATOM	1816	O	LEU	1173	27.407	15.015	-6.576	1.00	0.00	LX0	O
ATOM	1817	N	LEU	1174	27.881	14.134	-8.620	1.00	0.00	LX0	N
ATOM	1818	H	LEU	1174	27.628	14.149	-9.590	0.00	0.00	LX0	H
ATOM	1819	CA	LEU	1174	28.885	13.189	-8.143	1.00	0.00	LX0	C
ATOM	1820	CB	LEU	1174	28.254	11.789	-8.148	1.00	0.00	LX0	C
ATOM	1821	CG	LEU	1174	27.276	11.516	-6.997	1.00	0.00	LX0	C
ATOM	1822	CD1	LEU	1174	26.400	10.289	-7.258	1.00	0.00	LX0	C
ATOM	1823	CD2	LEU	1174	27.983	11.422	-5.645	1.00	0.00	LX0	C
ATOM	1824	C	LEU	1174	30.086	13.284	-9.072	1.00	0.00	LX0	C
ATOM	1825	O	LEU	1174	29.935	13.738	-10.204	1.00	0.00	LX0	O
ATOM	1826	N	PRO	1175	31.291	12.888	-8.582	1.00	0.00	LX0	N
ATOM	1827	CD	PRO	1175	31.603	12.411	-7.234	1.00	0.00	LX0	C
ATOM	1828	CA	PRO	1175	32.481	12.960	-9.443	1.00	0.00	LX0	C
ATOM	1829	CB	PRO	1175	33.611	12.897	-8.417	1.00	0.00	LX0	C
ATOM	1830	CG	PRO	1175	33.070	12.005	-7.300	1.00	0.00	LX0	C
ATOM	1831	C	PRO	1175	32.538	11.819	-10.449	1.00	0.00	LX0	C
ATOM	1832	O	PRO	1175	33.321	10.885	-10.338	1.00	0.00	LX0	O
ATOM	1833	N	VAL	1176	31.646	11.922	-11.439	1.00	0.00	LX0	N
ATOM	1834	H	VAL	1176	31.102	12.761	-11.479	0.00	0.00	LX0	H
ATOM	1835	CA	VAL	1176	31.294	10.735	-12.221	1.00	0.00	LX0	C
ATOM	1836	CB	VAL	1176	30.206	11.090	-13.239	1.00	0.00	LX0	C
ATOM	1837	CG1	VAL	1176	28.936	11.504	-12.491	1.00	0.00	LX0	C
ATOM	1838	CG2	VAL	1176	30.660	12.154	-14.242	1.00	0.00	LX0	C
ATOM	1839	C	VAL	1176	32.422	9.927	-12.852	1.00	0.00	LX0	C
ATOM	1840	O	VAL	1176	32.378	8.707	-12.926	1.00	0.00	LX0	O
ATOM	1841	N	ARG	1177	33.462	10.659	-13.280	1.00	0.00	LX0	N
ATOM	1842	H	ARG	1177	33.494	11.634	-13.069	0.00	0.00	LX0	H

ATOM	1843	CA	ARG	1177	34.557	9.967	-13.964	1.00	0.00	LX0	C
ATOM	1844	CB	ARG	1177	35.413	10.980	-14.728	1.00	0.00	LX0	C
ATOM	1845	CG	ARG	1177	34.615	11.918	-15.639	1.00	0.00	LX0	C
ATOM	1846	CD	ARG	1177	35.501	12.948	-16.341	1.00	0.00	LX0	C
ATOM	1847	NE	ARG	1177	34.751	13.711	-17.336	1.00	0.00	LX0	N
ATOM	1848	HE	ARG	1177	34.014	14.318	-17.028	0.00	0.00	LX0	H
ATOM	1849	CZ	ARG	1177	34.972	13.601	-18.666	1.00	0.00	LX0	C
ATOM	1850	NH1	ARG	1177	35.908	12.810	-19.176	1.00	0.00	LX0	N
ATOM	1851	HH11	ARG	1177	36.092	12.773	-20.165	0.00	0.00	LX0	H
ATOM	1852	HH12	ARG	1177	36.476	12.215	-18.594	0.00	0.00	LX0	H
ATOM	1853	NH2	ARG	1177	34.212	14.317	-19.477	1.00	0.00	LX0	N
ATOM	1854	HH21	ARG	1177	34.280	14.219	-20.478	0.00	0.00	LX0	H
ATOM	1855	HH22	ARG	1177	33.537	14.950	-19.089	0.00	0.00	LX0	H
ATOM	1856	C	ARG	1177	35.425	9.070	-13.085	1.00	0.00	LX0	C
ATOM	1857	O	ARG	1177	36.275	8.330	-13.556	1.00	0.00	LX0	O
ATOM	1858	N	TRP	1178	35.177	9.185	-11.773	1.00	0.00	LX0	N
ATOM	1859	H	TRP	1178	34.440	9.771	-11.438	0.00	0.00	LX0	H
ATOM	1860	CA	TRP	1178	35.961	8.426	-10.805	1.00	0.00	LX0	C
ATOM	1861	CB	TRP	1178	36.382	9.343	-9.646	1.00	0.00	LX0	C
ATOM	1862	CG	TRP	1178	37.367	10.375	-10.139	1.00	0.00	LX0	C
ATOM	1863	CD2	TRP	1178	37.117	11.557	-10.932	1.00	0.00	LX0	C
ATOM	1864	CE2	TRP	1178	38.387	12.154	-11.212	1.00	0.00	LX0	C
ATOM	1865	CE3	TRP	1178	35.942	12.152	-11.437	1.00	0.00	LX0	C
ATOM	1866	CD1	TRP	1178	38.758	10.337	-9.968	1.00	0.00	LX0	C
ATOM	1867	NE1	TRP	1178	39.360	11.379	-10.597	1.00	0.00	LX0	N
ATOM	1868	HE1	TRP	1178	40.327	11.571	-10.619	0.00	0.00	LX0	H
ATOM	1869	CZ2	TRP	1178	38.459	13.312	-12.009	1.00	0.00	LX0	C
ATOM	1870	CZ3	TRP	1178	36.026	13.314	-12.229	1.00	0.00	LX0	C
ATOM	1871	CH2	TRP	1178	37.280	13.886	-12.524	1.00	0.00	LX0	C
ATOM	1872	C	TRP	1178	35.218	7.227	-10.250	1.00	0.00	LX0	C
ATOM	1873	O	TRP	1178	35.702	6.516	-9.379	1.00	0.00	LX0	O
ATOM	1874	N	MET	1179	33.977	7.072	-10.725	1.00	0.00	LX0	N
ATOM	1875	H	MET	1179	33.644	7.495	-11.570	0.00	0.00	LX0	H
ATOM	1876	CA	MET	1179	33.106	6.263	-9.888	1.00	0.00	LX0	C
ATOM	1877	CB	MET	1179	31.815	7.023	-9.622	1.00	0.00	LX0	C
ATOM	1878	CG	MET	1179	32.060	8.286	-8.798	1.00	0.00	LX0	C
ATOM	1879	SD	MET	1179	30.693	9.443	-8.914	1.00	0.00	LX0	S
ATOM	1880	CE	MET	1179	29.376	8.303	-8.497	1.00	0.00	LX0	C
ATOM	1881	C	MET	1179	32.827	4.867	-10.385	1.00	0.00	LX0	C
ATOM	1882	O	MET	1179	32.801	4.563	-11.569	1.00	0.00	LX0	O
ATOM	1883	N	SER	1180	32.608	4.028	-9.375	1.00	0.00	LX0	N
ATOM	1884	H	SER	1180	32.656	4.338	-8.424	0.00	0.00	LX0	H
ATOM	1885	CA	SER	1180	32.222	2.644	-9.605	1.00	0.00	LX0	C
ATOM	1886	CB	SER	1180	32.341	1.938	-8.258	1.00	0.00	LX0	C
ATOM	1887	OG	SER	1180	32.191	2.880	-7.189	1.00	0.00	LX0	O
ATOM	1888	HG	SER	1180	31.300	2.775	-6.844	0.00	0.00	LX0	H
ATOM	1889	C	SER	1180	30.826	2.510	-10.193	1.00	0.00	LX0	C
ATOM	1890	O	SER	1180	29.970	3.359	-9.970	1.00	0.00	LX0	O
ATOM	1891	N	PRO	1181	30.607	1.402	-10.950	1.00	0.00	LX0	N
ATOM	1892	CD	PRO	1181	31.583	0.373	-11.299	1.00	0.00	LX0	C
ATOM	1893	CA	PRO	1181	29.287	1.117	-11.526	1.00	0.00	LX0	C
ATOM	1894	CB	PRO	1181	29.411	-0.364	-11.893	1.00	0.00	LX0	C
ATOM	1895	CG	PRO	1181	30.870	-0.526	-12.299	1.00	0.00	LX0	C
ATOM	1896	C	PRO	1181	28.093	1.431	-10.636	1.00	0.00	LX0	C
ATOM	1897	O	PRO	1181	27.253	2.263	-10.953	1.00	0.00	LX0	O
ATOM	1898	N	GLU	1182	28.061	0.737	-9.492	1.00	0.00	LX0	N
ATOM	1899	H	GLU	1182	28.798	0.090	-9.298	0.00	0.00	LX0	H
ATOM	1900	CA	GLU	1182	27.016	0.940	-8.488	1.00	0.00	LX0	C
ATOM	1901	CB	GLU	1182	27.256	0.015	-7.273	1.00	0.00	LX0	C
ATOM	1902	CG	GLU	1182	28.408	0.297	-6.276	1.00	0.00	LX0	C
ATOM	1903	CD	GLU	1182	29.821	0.005	-6.786	1.00	0.00	LX0	C

ATOM	1904	OE1	GLU	1182	30.012	-0.418	-7.920	1.00	0.00	LX0	O
ATOM	1905	OE2	GLU	1182	30.768	0.187	-6.030	1.00	0.00	LX0	O
ATOM	1906	C	GLU	1182	26.794	2.397	-8.092	1.00	0.00	LX0	C
ATOM	1907	O	GLU	1182	25.688	2.931	-8.071	1.00	0.00	LX0	O
ATOM	1908	N	SER	1183	27.935	3.044	-7.848	1.00	0.00	LX0	N
ATOM	1909	H	SER	1183	28.812	2.563	-7.846	0.00	0.00	LX0	H
ATOM	1910	CA	SER	1183	27.900	4.457	-7.508	1.00	0.00	LX0	C
ATOM	1911	CB	SER	1183	29.276	4.828	-6.967	1.00	0.00	LX0	C
ATOM	1912	OG	SER	1183	29.760	3.739	-6.164	1.00	0.00	LX0	O
ATOM	1913	HG	SER	1183	29.184	3.683	-5.398	0.00	0.00	LX0	H
ATOM	1914	C	SER	1183	27.415	5.368	-8.634	1.00	0.00	LX0	C
ATOM	1915	O	SER	1183	26.813	6.412	-8.426	1.00	0.00	LX0	O
ATOM	1916	N	LEU	1184	27.651	4.910	-9.865	1.00	0.00	LX0	N
ATOM	1917	H	LEU	1184	28.163	4.063	-10.018	0.00	0.00	LX0	H
ATOM	1918	CA	LEU	1184	27.041	5.652	-10.961	1.00	0.00	LX0	C
ATOM	1919	CB	LEU	1184	27.797	5.402	-12.260	1.00	0.00	LX0	C
ATOM	1920	CG	LEU	1184	29.175	6.053	-12.246	1.00	0.00	LX0	C
ATOM	1921	CD1	LEU	1184	30.006	5.644	-13.451	1.00	0.00	LX0	C
ATOM	1922	CD2	LEU	1184	29.077	7.572	-12.142	1.00	0.00	LX0	C
ATOM	1923	C	LEU	1184	25.555	5.398	-11.125	1.00	0.00	LX0	C
ATOM	1924	O	LEU	1184	24.800	6.272	-11.547	1.00	0.00	LX0	O
ATOM	1925	N	LYS	1185	25.145	4.176	-10.744	1.00	0.00	LX0	N
ATOM	1926	H	LYS	1185	25.815	3.519	-10.395	0.00	0.00	LX0	H
ATOM	1927	CA	LYS	1185	23.719	3.863	-10.832	1.00	0.00	LX0	C
ATOM	1928	CB	LYS	1185	23.464	2.349	-10.722	1.00	0.00	LX0	C
ATOM	1929	CG	LYS	1185	24.319	1.655	-11.784	1.00	0.00	LX0	C
ATOM	1930	CD	LYS	1185	24.236	0.140	-11.988	1.00	0.00	LX0	C
ATOM	1931	CE	LYS	1185	25.520	-0.228	-12.733	1.00	0.00	LX0	C
ATOM	1932	NZ	LYS	1185	25.466	-1.462	-13.524	1.00	0.00	LX0	N
ATOM	1933	HZ1	LYS	1185	26.300	-1.493	-14.154	0.00	0.00	LX0	H
ATOM	1934	HZ2	LYS	1185	25.492	-2.359	-13.001	0.00	0.00	LX0	H
ATOM	1935	HZ3	LYS	1185	24.711	-1.475	-14.246	0.00	0.00	LX0	H
ATOM	1936	C	LYS	1185	22.872	4.707	-9.897	1.00	0.00	LX0	C
ATOM	1937	O	LYS	1185	22.100	5.546	-10.356	1.00	0.00	LX0	O
ATOM	1938	N	ASP	1186	23.095	4.535	-8.588	1.00	0.00	LX0	N
ATOM	1939	H	ASP	1186	23.704	3.819	-8.236	0.00	0.00	LX0	H
ATOM	1940	CA	ASP	1186	22.467	5.529	-7.713	1.00	0.00	LX0	C
ATOM	1941	CB	ASP	1186	21.489	4.887	-6.701	1.00	0.00	LX0	C
ATOM	1942	CG	ASP	1186	22.153	4.665	-5.352	1.00	0.00	LX0	C
ATOM	1943	OD1	ASP	1186	21.933	5.470	-4.447	1.00	0.00	LX0	O
ATOM	1944	OD2	ASP	1186	23.002	3.787	-5.264	1.00	0.00	LX0	O
ATOM	1945	C	ASP	1186	23.457	6.461	-7.027	1.00	0.00	LX0	C
ATOM	1946	O	ASP	1186	23.216	7.647	-6.809	1.00	0.00	LX0	O
ATOM	1947	N	GLY	1187	24.572	5.842	-6.637	1.00	0.00	LX0	N
ATOM	1948	H	GLY	1187	24.622	4.849	-6.757	0.00	0.00	LX0	H
ATOM	1949	CA	GLY	1187	25.454	6.558	-5.728	1.00	0.00	LX0	C
ATOM	1950	C	GLY	1187	25.278	6.142	-4.286	1.00	0.00	LX0	C
ATOM	1951	O	GLY	1187	25.066	6.974	-3.407	1.00	0.00	LX0	O
ATOM	1952	N	VAL	1188	25.402	4.829	-4.087	1.00	0.00	LX0	N
ATOM	1953	H	VAL	1188	25.243	4.192	-4.841	0.00	0.00	LX0	H
ATOM	1954	CA	VAL	1188	25.835	4.324	-2.792	1.00	0.00	LX0	C
ATOM	1955	CB	VAL	1188	25.098	3.006	-2.476	1.00	0.00	LX0	C
ATOM	1956	CG1	VAL	1188	25.777	2.117	-1.427	1.00	0.00	LX0	C
ATOM	1957	CG2	VAL	1188	23.661	3.325	-2.058	1.00	0.00	LX0	C
ATOM	1958	C	VAL	1188	27.342	4.161	-2.847	1.00	0.00	LX0	C
ATOM	1959	O	VAL	1188	27.919	3.818	-3.875	1.00	0.00	LX0	O
ATOM	1960	N	PHE	1189	27.953	4.471	-1.698	1.00	0.00	LX0	N
ATOM	1961	H	PHE	1189	27.437	4.773	-0.899	0.00	0.00	LX0	H
ATOM	1962	CA	PHE	1189	29.397	4.304	-1.635	1.00	0.00	LX0	C
ATOM	1963	CB	PHE	1189	30.110	5.654	-1.512	1.00	0.00	LX0	C
ATOM	1964	CG	PHE	1189	30.094	6.436	-2.809	1.00	0.00	LX0	C

ATOM	1965	CD1	PHE	1189	28.898	7.028	-3.275	1.00	0.00	LX0	C
ATOM	1966	CD2	PHE	1189	31.304	6.588	-3.520	1.00	0.00	LX0	C
ATOM	1967	CE1	PHE	1189	28.919	7.810	-4.444	1.00	0.00	LX0	C
ATOM	1968	CE2	PHE	1189	31.329	7.370	-4.691	1.00	0.00	LX0	C
ATOM	1969	CZ	PHE	1189	30.140	7.988	-5.127	1.00	0.00	LX0	C
ATOM	1970	C	PHE	1189	29.808	3.390	-0.501	1.00	0.00	LX0	C
ATOM	1971	O	PHE	1189	29.711	3.717	0.675	1.00	0.00	LX0	O
ATOM	1972	N	THR	1190	30.283	2.217	-0.907	1.00	0.00	LX0	N
ATOM	1973	H	THR	1190	30.423	2.097	-1.889	0.00	0.00	LX0	H
ATOM	1974	CA	THR	1190	31.034	1.432	0.066	1.00	0.00	LX0	C
ATOM	1975	CB	THR	1190	31.073	-0.016	-0.423	1.00	0.00	LX0	C
ATOM	1976	OG1	THR	1190	31.650	-0.080	-1.737	1.00	0.00	LX0	O
ATOM	1977	HG1	THR	1190	30.968	0.163	-2.358	0.00	0.00	LX0	H
ATOM	1978	CG2	THR	1190	29.683	-0.653	-0.431	1.00	0.00	LX0	C
ATOM	1979	C	THR	1190	32.443	2.011	0.180	1.00	0.00	LX0	C
ATOM	1980	O	THR	1190	32.809	2.879	-0.605	1.00	0.00	LX0	O
ATOM	1981	N	THR	1191	33.256	1.456	1.095	1.00	0.00	LX0	N
ATOM	1982	H	THR	1191	32.853	0.962	1.867	0.00	0.00	LX0	H
ATOM	1983	CA	THR	1191	34.699	1.716	0.958	1.00	0.00	LX0	C
ATOM	1984	CB	THR	1191	35.499	1.017	2.081	1.00	0.00	LX0	C
ATOM	1985	OG1	THR	1191	36.901	1.248	1.924	1.00	0.00	LX0	O
ATOM	1986	HG1	THR	1191	37.345	0.554	2.410	0.00	0.00	LX0	H
ATOM	1987	CG2	THR	1191	35.222	-0.485	2.202	1.00	0.00	LX0	C
ATOM	1988	C	THR	1191	35.219	1.338	-0.430	1.00	0.00	LX0	C
ATOM	1989	O	THR	1191	36.022	2.001	-1.076	1.00	0.00	LX0	O
ATOM	1990	N	TYR	1192	34.637	0.229	-0.903	1.00	0.00	LX0	N
ATOM	1991	H	TYR	1192	33.937	-0.233	-0.364	0.00	0.00	LX0	H
ATOM	1992	CA	TYR	1192	35.046	-0.312	-2.189	1.00	0.00	LX0	C
ATOM	1993	CB	TYR	1192	34.420	-1.690	-2.384	1.00	0.00	LX0	C
ATOM	1994	CG	TYR	1192	34.608	-2.563	-1.159	1.00	0.00	LX0	C
ATOM	1995	CD1	TYR	1192	35.886	-3.080	-0.862	1.00	0.00	LX0	C
ATOM	1996	CE1	TYR	1192	36.054	-3.867	0.290	1.00	0.00	LX0	C
ATOM	1997	CD2	TYR	1192	33.491	-2.833	-0.340	1.00	0.00	LX0	C
ATOM	1998	CE2	TYR	1192	33.659	-3.616	0.814	1.00	0.00	LX0	C
ATOM	1999	CZ	TYR	1192	34.941	-4.120	1.117	1.00	0.00	LX0	C
ATOM	2000	OH	TYR	1192	35.112	-4.884	2.251	1.00	0.00	LX0	O
ATOM	2001	HH	TYR	1192	34.893	-4.362	3.029	0.00	0.00	LX0	H
ATOM	2002	C	TYR	1192	34.783	0.592	-3.381	1.00	0.00	LX0	C
ATOM	2003	O	TYR	1192	35.432	0.495	-4.417	1.00	0.00	LX0	O
ATOM	2004	N	SER	1193	33.816	1.504	-3.221	1.00	0.00	LX0	N
ATOM	2005	H	SER	1193	33.236	1.540	-2.406	0.00	0.00	LX0	H
ATOM	2006	CA	SER	1193	33.715	2.516	-4.273	1.00	0.00	LX0	C
ATOM	2007	CB	SER	1193	32.419	3.306	-4.171	1.00	0.00	LX0	C
ATOM	2008	OG	SER	1193	31.764	2.968	-2.952	1.00	0.00	LX0	O
ATOM	2009	HG	SER	1193	32.267	3.411	-2.268	0.00	0.00	LX0	H
ATOM	2010	C	SER	1193	34.912	3.441	-4.329	1.00	0.00	LX0	C
ATOM	2011	O	SER	1193	35.539	3.603	-5.366	1.00	0.00	LX0	O
ATOM	2012	N	ASP	1194	35.261	3.968	-3.144	1.00	0.00	LX0	N
ATOM	2013	H	ASP	1194	34.750	3.831	-2.295	0.00	0.00	LX0	H
ATOM	2014	CA	ASP	1194	36.474	4.784	-3.100	1.00	0.00	LX0	C
ATOM	2015	CB	ASP	1194	36.628	5.471	-1.735	1.00	0.00	LX0	C
ATOM	2016	CG	ASP	1194	35.470	6.423	-1.449	1.00	0.00	LX0	C
ATOM	2017	OD1	ASP	1194	35.367	7.471	-2.090	1.00	0.00	LX0	O
ATOM	2018	OD2	ASP	1194	34.684	6.138	-0.547	1.00	0.00	LX0	O
ATOM	2019	C	ASP	1194	37.740	4.033	-3.507	1.00	0.00	LX0	C
ATOM	2020	O	ASP	1194	38.683	4.607	-4.034	1.00	0.00	LX0	O
ATOM	2021	N	VAL	1195	37.707	2.697	-3.321	1.00	0.00	LX0	N
ATOM	2022	H	VAL	1195	36.983	2.329	-2.738	0.00	0.00	LX0	H
ATOM	2023	CA	VAL	1195	38.751	1.859	-3.930	1.00	0.00	LX0	C
ATOM	2024	CB	VAL	1195	38.642	0.393	-3.462	1.00	0.00	LX0	C
ATOM	2025	CG1	VAL	1195	39.721	-0.501	-4.080	1.00	0.00	LX0	C

```
ATOM   2026  CG2 VAL  1195      38.692   0.285  -1.937  1.00  0.00      LX0  C
ATOM   2027  C   VAL  1195      38.805   1.944  -5.458  1.00  0.00      LX0  C
ATOM   2028  O   VAL  1195      39.856   2.140  -6.059  1.00  0.00      LX0  O
ATOM   2029  N   TRP  1196      37.617   1.828  -6.080  1.00  0.00      LX0  N
ATOM   2030  H   TRP  1196      36.787   1.707  -5.532  0.00  0.00      LX0  H
ATOM   2031  CA  TRP  1196      37.548   2.046  -7.533  1.00  0.00      LX0  C
ATOM   2032  CB  TRP  1196      36.088   1.885  -7.995  1.00  0.00      LX0  C
ATOM   2033  CG  TRP  1196      35.890   2.008  -9.495  1.00  0.00      LX0  C
ATOM   2034  CD2 TRP  1196      35.500   0.972 -10.423  1.00  0.00      LX0  C
ATOM   2035  CE2 TRP  1196      35.430   1.569 -11.728  1.00  0.00      LX0  C
ATOM   2036  CE3 TRP  1196      35.199  -0.397 -10.263  1.00  0.00      LX0  C
ATOM   2037  CD1 TRP  1196      36.024   3.164 -10.284  1.00  0.00      LX0  C
ATOM   2038  NE1 TRP  1196      35.756   2.913 -11.593  1.00  0.00      LX0  N
ATOM   2039  HE1 TRP  1196      35.773   3.572 -12.322  0.00  0.00      LX0  H
ATOM   2040  CZ2 TRP  1196      35.073   0.784 -12.844  1.00  0.00      LX0  C
ATOM   2041  CZ3 TRP  1196      34.837  -1.168 -11.389  1.00  0.00      LX0  C
ATOM   2042  CH2 TRP  1196      34.780  -0.584 -12.672  1.00  0.00      LX0  C
ATOM   2043  C   TRP  1196      38.135   3.401  -7.921  1.00  0.00      LX0  C
ATOM   2044  O   TRP  1196      39.021   3.531  -8.764  1.00  0.00      LX0  O
ATOM   2045  N   SER  1197      37.627   4.406  -7.198  1.00  0.00      LX0  N
ATOM   2046  H   SER  1197      36.854   4.266  -6.576  0.00  0.00      LX0  H
ATOM   2047  CA  SER  1197      38.137   5.760  -7.363  1.00  0.00      LX0  C
ATOM   2048  CB  SER  1197      37.331   6.685  -6.460  1.00  0.00      LX0  C
ATOM   2049  OG  SER  1197      35.944   6.360  -6.610  1.00  0.00      LX0  O
ATOM   2050  HG  SER  1197      35.781   6.367  -7.550  0.00  0.00      LX0  H
ATOM   2051  C   SER  1197      39.640   5.913  -7.191  1.00  0.00      LX0  C
ATOM   2052  O   SER  1197      40.282   6.658  -7.913  1.00  0.00      LX0  O
ATOM   2053  N   PHE  1198      40.198   5.122  -6.260  1.00  0.00      LX0  N
ATOM   2054  H   PHE  1198      39.606   4.562  -5.682  0.00  0.00      LX0  H
ATOM   2055  CA  PHE  1198      41.652   5.071  -6.096  1.00  0.00      LX0  C
ATOM   2056  CB  PHE  1198      42.011   4.138  -4.930  1.00  0.00      LX0  C
ATOM   2057  CG  PHE  1198      43.505   4.047  -4.729  1.00  0.00      LX0  C
ATOM   2058  CD1 PHE  1198      44.182   5.073  -4.037  1.00  0.00      LX0  C
ATOM   2059  CD2 PHE  1198      44.196   2.932  -5.251  1.00  0.00      LX0  C
ATOM   2060  CE1 PHE  1198      45.577   4.987  -3.873  1.00  0.00      LX0  C
ATOM   2061  CE2 PHE  1198      45.590   2.846  -5.089  1.00  0.00      LX0  C
ATOM   2062  CZ  PHE  1198      46.265   3.875  -4.403  1.00  0.00      LX0  C
ATOM   2063  C   PHE  1198      42.382   4.686  -7.373  1.00  0.00      LX0  C
ATOM   2064  O   PHE  1198      43.339   5.327  -7.794  1.00  0.00      LX0  O
ATOM   2065  N   GLY  1199      41.851   3.631  -8.011  1.00  0.00      LX0  N
ATOM   2066  H   GLY  1199      41.073   3.148  -7.600  0.00  0.00      LX0  H
ATOM   2067  CA  GLY  1199      42.402   3.269  -9.320  1.00  0.00      LX0  C
ATOM   2068  C   GLY  1199      42.362   4.418 -10.320  1.00  0.00      LX0  C
ATOM   2069  O   GLY  1199      43.289   4.683 -11.077  1.00  0.00      LX0  O
ATOM   2070  N   VAL  1200      41.229   5.129 -10.246  1.00  0.00      LX0  N
ATOM   2071  H   VAL  1200      40.533   4.889  -9.567  0.00  0.00      LX0  H
ATOM   2072  CA  VAL  1200      41.100   6.309 -11.098  1.00  0.00      LX0  C
ATOM   2073  CB  VAL  1200      39.641   6.782 -11.131  1.00  0.00      LX0  C
ATOM   2074  CG1 VAL  1200      39.431   7.813 -12.233  1.00  0.00      LX0  C
ATOM   2075  CG2 VAL  1200      38.669   5.616 -11.325  1.00  0.00      LX0  C
ATOM   2076  C   VAL  1200      42.071   7.443 -10.755  1.00  0.00      LX0  C
ATOM   2077  O   VAL  1200      42.578   8.141 -11.622  1.00  0.00      LX0  O
ATOM   2078  N   VAL  1201      42.380   7.561  -9.452  1.00  0.00      LX0  N
ATOM   2079  H   VAL  1201      41.918   6.974  -8.788  0.00  0.00      LX0  H
ATOM   2080  CA  VAL  1201      43.430   8.501  -9.040  1.00  0.00      LX0  C
ATOM   2081  CB  VAL  1201      43.608   8.518  -7.510  1.00  0.00      LX0  C
ATOM   2082  CG1 VAL  1201      44.672   9.523  -7.064  1.00  0.00      LX0  C
ATOM   2083  CG2 VAL  1201      42.293   8.784  -6.786  1.00  0.00      LX0  C
ATOM   2084  C   VAL  1201      44.758   8.187  -9.710  1.00  0.00      LX0  C
ATOM   2085  O   VAL  1201      45.479   9.055 -10.184  1.00  0.00      LX0  O
ATOM   2086  N   LEU  1202      45.033   6.876  -9.768  1.00  0.00      LX0  N
```

ATOM	2087	H	LEU	1202	44.400	6.227	-9.340	0.00	0.00	LX0	H
ATOM	2088	CA	LEU	1202	46.260	6.450	-10.445	1.00	0.00	LX0	C
ATOM	2089	CB	LEU	1202	46.465	4.943	-10.304	1.00	0.00	LX0	C
ATOM	2090	CG	LEU	1202	46.267	4.426	-8.880	1.00	0.00	LX0	C
ATOM	2091	CD1	LEU	1202	46.336	2.903	-8.822	1.00	0.00	LX0	C
ATOM	2092	CD2	LEU	1202	47.204	5.103	-7.886	1.00	0.00	LX0	C
ATOM	2093	C	LEU	1202	46.304	6.850	-11.910	1.00	0.00	LX0	C
ATOM	2094	O	LEU	1202	47.309	7.308	-12.442	1.00	0.00	LX0	O
ATOM	2095	N	TRP	1203	45.122	6.703	-12.528	1.00	0.00	LX0	N
ATOM	2096	H	TRP	1203	44.372	6.253	-12.039	0.00	0.00	LX0	H
ATOM	2097	CA	TRP	1203	44.939	7.223	-13.883	1.00	0.00	LX0	C
ATOM	2098	CB	TRP	1203	43.515	6.909	-14.338	1.00	0.00	LX0	C
ATOM	2099	CG	TRP	1203	43.307	7.188	-15.806	1.00	0.00	LX0	C
ATOM	2100	CD2	TRP	1203	43.000	8.440	-16.459	1.00	0.00	LX0	C
ATOM	2101	CE2	TRP	1203	42.852	8.160	-17.857	1.00	0.00	LX0	C
ATOM	2102	CE3	TRP	1203	42.844	9.762	-15.992	1.00	0.00	LX0	C
ATOM	2103	CD1	TRP	1203	43.329	6.233	-16.827	1.00	0.00	LX0	C
ATOM	2104	NE1	TRP	1203	43.057	6.799	-18.030	1.00	0.00	LX0	N
ATOM	2105	HE1	TRP	1203	42.975	6.308	-18.877	0.00	0.00	LX0	H
ATOM	2106	CZ2	TRP	1203	42.531	9.198	-18.757	1.00	0.00	LX0	C
ATOM	2107	CZ3	TRP	1203	42.529	10.793	-16.901	1.00	0.00	LX0	C
ATOM	2108	CH2	TRP	1203	42.371	10.513	-18.276	1.00	0.00	LX0	C
ATOM	2109	C	TRP	1203	45.248	8.710	-14.005	1.00	0.00	LX0	C
ATOM	2110	O	TRP	1203	45.933	9.161	-14.920	1.00	0.00	LX0	O
ATOM	2111	N	GLU	1204	44.733	9.463	-13.016	1.00	0.00	LX0	N
ATOM	2112	H	GLU	1204	44.148	9.053	-12.314	0.00	0.00	LX0	H
ATOM	2113	CA	GLU	1204	45.065	10.886	-13.005	1.00	0.00	LX0	C
ATOM	2114	CB	GLU	1204	44.311	11.671	-11.924	1.00	0.00	LX0	C
ATOM	2115	CG	GLU	1204	42.790	11.559	-12.063	1.00	0.00	LX0	C
ATOM	2116	CD	GLU	1204	42.098	12.735	-11.398	1.00	0.00	LX0	C
ATOM	2117	OE1	GLU	1204	41.705	13.652	-12.109	1.00	0.00	LX0	O
ATOM	2118	OE2	GLU	1204	41.898	12.727	-10.185	1.00	0.00	LX0	O
ATOM	2119	C	GLU	1204	46.557	11.143	-12.932	1.00	0.00	LX0	C
ATOM	2120	O	GLU	1204	47.106	11.906	-13.711	1.00	0.00	LX0	O
ATOM	2121	N	ILE	1205	47.225	10.417	-12.023	1.00	0.00	LX0	N
ATOM	2122	H	ILE	1205	46.708	9.817	-11.412	0.00	0.00	LX0	H
ATOM	2123	CA	ILE	1205	48.690	10.517	-11.984	1.00	0.00	LX0	C
ATOM	2124	CB	ILE	1205	49.274	9.540	-10.949	1.00	0.00	LX0	C
ATOM	2125	CG2	ILE	1205	50.800	9.638	-10.879	1.00	0.00	LX0	C
ATOM	2126	CG1	ILE	1205	48.645	9.752	-9.569	1.00	0.00	LX0	C
ATOM	2127	CD1	ILE	1205	49.066	8.695	-8.545	1.00	0.00	LX0	C
ATOM	2128	C	ILE	1205	49.340	10.333	-13.355	1.00	0.00	LX0	C
ATOM	2129	O	ILE	1205	50.097	11.163	-13.846	1.00	0.00	LX0	O
ATOM	2130	N	ALA	1206	48.941	9.216	-13.976	1.00	0.00	LX0	N
ATOM	2131	H	ALA	1206	48.297	8.606	-13.512	0.00	0.00	LX0	H
ATOM	2132	CA	ALA	1206	49.470	8.890	-15.298	1.00	0.00	LX0	C
ATOM	2133	CB	ALA	1206	48.975	7.509	-15.722	1.00	0.00	LX0	C
ATOM	2134	C	ALA	1206	49.170	9.888	-16.411	1.00	0.00	LX0	C
ATOM	2135	O	ALA	1206	49.859	9.940	-17.421	1.00	0.00	LX0	O
ATOM	2136	N	THR	1207	48.113	10.682	-16.202	1.00	0.00	LX0	N
ATOM	2137	H	THR	1207	47.580	10.641	-15.356	0.00	0.00	LX0	H
ATOM	2138	CA	THR	1207	47.799	11.651	-17.249	1.00	0.00	LX0	C
ATOM	2139	CB	THR	1207	46.307	11.636	-17.551	1.00	0.00	LX0	C
ATOM	2140	OG1	THR	1207	45.540	11.540	-16.339	1.00	0.00	LX0	O
ATOM	2141	HG1	THR	1207	45.638	10.638	-16.036	0.00	0.00	LX0	H
ATOM	2142	CG2	THR	1207	45.940	10.530	-18.529	1.00	0.00	LX0	C
ATOM	2143	C	THR	1207	48.213	13.079	-16.967	1.00	0.00	LX0	C
ATOM	2144	O	THR	1207	47.828	14.000	-17.679	1.00	0.00	LX0	O
ATOM	2145	N	LEU	1208	48.945	13.257	-15.848	1.00	0.00	LX0	N
ATOM	2146	H	LEU	1208	49.298	12.468	-15.339	0.00	0.00	LX0	H
ATOM	2147	CA	LEU	1208	49.084	14.618	-15.312	1.00	0.00	LX0	C

```
ATOM   2148  CB  LEU  1208      50.110  15.436 -16.108  1.00  0.00      LX0  C
ATOM   2149  CG  LEU  1208      51.488  14.767 -16.157  1.00  0.00      LX0  C
ATOM   2150  CD1 LEU  1208      52.447  15.506 -17.089  1.00  0.00      LX0  C
ATOM   2151  CD2 LEU  1208      52.090  14.563 -14.764  1.00  0.00      LX0  C
ATOM   2152  C   LEU  1208      47.733  15.318 -15.153  1.00  0.00      LX0  C
ATOM   2153  O   LEU  1208      47.476  16.459 -15.531  1.00  0.00      LX0  O
ATOM   2154  N   ALA  1209      46.860  14.476 -14.585  1.00  0.00      LX0  N
ATOM   2155  H   ALA  1209      47.250  13.597 -14.325  0.00  0.00      LX0  H
ATOM   2156  CA  ALA  1209      45.444  14.707 -14.332  1.00  0.00      LX0  C
ATOM   2157  CB  ALA  1209      45.257  15.658 -13.154  1.00  0.00      LX0  C
ATOM   2158  C   ALA  1209      44.646  15.194 -15.518  1.00  0.00      LX0  C
ATOM   2159  O   ALA  1209      44.413  16.387 -15.685  1.00  0.00      LX0  O
ATOM   2160  N   GLU  1210      44.217  14.242 -16.350  1.00  0.00      LX0  N
ATOM   2161  H   GLU  1210      44.447  13.276 -16.203  0.00  0.00      LX0  H
ATOM   2162  CA  GLU  1210      43.207  14.687 -17.310  1.00  0.00      LX0  C
ATOM   2163  CB  GLU  1210      43.301  13.902 -18.625  1.00  0.00      LX0  C
ATOM   2164  CG  GLU  1210      44.518  14.241 -19.503  1.00  0.00      LX0  C
ATOM   2165  CD  GLU  1210      44.484  15.659 -20.075  1.00  0.00      LX0  C
ATOM   2166  OE1 GLU  1210      45.144  15.911 -21.073  1.00  0.00      LX0  O
ATOM   2167  OE2 GLU  1210      43.858  16.551 -19.509  1.00  0.00      LX0  O
ATOM   2168  C   GLU  1210      41.819  14.721 -16.689  1.00  0.00      LX0  C
ATOM   2169  O   GLU  1210      41.699  14.957 -15.492  1.00  0.00      LX0  O
ATOM   2170  N   GLN  1211      40.781  14.490 -17.502  1.00  0.00      LX0  N
ATOM   2171  H   GLN  1211      40.859  14.418 -18.500  0.00  0.00      LX0  H
ATOM   2172  CA  GLN  1211      39.497  14.174 -16.885  1.00  0.00      LX0  C
ATOM   2173  CB  GLN  1211      38.443  15.243 -17.210  1.00  0.00      LX0  C
ATOM   2174  CG  GLN  1211      38.728  16.661 -16.691  1.00  0.00      LX0  C
ATOM   2175  CD  GLN  1211      38.662  16.739 -15.173  1.00  0.00      LX0  C
ATOM   2176  OE1 GLN  1211      39.670  16.769 -14.472  1.00  0.00      LX0  O
ATOM   2177  NE2 GLN  1211      37.426  16.832 -14.682  1.00  0.00      LX0  N
ATOM   2178 HE21 GLN  1211      36.599  16.829 -15.242  0.00  0.00      LX0  H
ATOM   2179 HE22 GLN  1211      37.312  16.939 -13.688  0.00  0.00      LX0  H
ATOM   2180  C   GLN  1211      39.053  12.807 -17.379  1.00  0.00      LX0  C
ATOM   2181  O   GLN  1211      38.604  12.660 -18.507  1.00  0.00      LX0  O
ATOM   2182  N   PRO  1212      39.209  11.800 -16.486  1.00  0.00      LX0  N
ATOM   2183  CD  PRO  1212      39.563  11.978 -15.081  1.00  0.00      LX0  C
ATOM   2184  CA  PRO  1212      39.045  10.380 -16.853  1.00  0.00      LX0  C
ATOM   2185  CB  PRO  1212      38.871   9.725 -15.485  1.00  0.00      LX0  C
ATOM   2186  CG  PRO  1212      39.730  10.566 -14.544  1.00  0.00      LX0  C
ATOM   2187  C   PRO  1212      37.932  10.057 -17.839  1.00  0.00      LX0  C
ATOM   2188  O   PRO  1212      36.881  10.687 -17.815  1.00  0.00      LX0  O
ATOM   2189  N   TYR  1213      38.214   9.076 -18.723  1.00  0.00      LX0  N
ATOM   2190  H   TYR  1213      39.111   8.625 -18.739  0.00  0.00      LX0  H
ATOM   2191  CA  TYR  1213      37.323   8.787 -19.855  1.00  0.00      LX0  C
ATOM   2192  CB  TYR  1213      35.910   8.336 -19.431  1.00  0.00      LX0  C
ATOM   2193  CG  TYR  1213      35.959   7.145 -18.503  1.00  0.00      LX0  C
ATOM   2194  CD1 TYR  1213      36.002   5.852 -19.059  1.00  0.00      LX0  C
ATOM   2195  CE1 TYR  1213      36.054   4.745 -18.199  1.00  0.00      LX0  C
ATOM   2196  CD2 TYR  1213      35.958   7.357 -17.108  1.00  0.00      LX0  C
ATOM   2197  CE2 TYR  1213      36.023   6.249 -16.247  1.00  0.00      LX0  C
ATOM   2198  CZ  TYR  1213      36.074   4.955 -16.806  1.00  0.00      LX0  C
ATOM   2199  OH  TYR  1213      36.143   3.862 -15.965  1.00  0.00      LX0  O
ATOM   2200  HH  TYR  1213      35.641   3.150 -16.357  0.00  0.00      LX0  H
ATOM   2201  C   TYR  1213      37.257   9.994 -20.767  1.00  0.00      LX0  C
ATOM   2202  O   TYR  1213      36.205  10.575 -21.023  1.00  0.00      LX0  O
ATOM   2203  N   GLN  1214      38.477  10.404 -21.163  1.00  0.00      LX0  N
ATOM   2204  H   GLN  1214      39.213   9.719 -21.090  0.00  0.00      LX0  H
ATOM   2205  CA  GLN  1214      38.719  11.812 -21.506  1.00  0.00      LX0  C
ATOM   2206  CB  GLN  1214      40.102  11.968 -22.166  1.00  0.00      LX0  C
ATOM   2207  CG  GLN  1214      40.532  13.388 -22.588  1.00  0.00      LX0  C
ATOM   2208  CD  GLN  1214      40.901  14.279 -21.412  1.00  0.00      LX0  C
```

ATOM	2209	OE1	GLN	1214	40.312	14.259	-20.340	1.00	0.00	LX0	O
ATOM	2210	NE2	GLN	1214	41.939	15.078	-21.666	1.00	0.00	LX0	N
ATOM	2211	HE21	GLN	1214	42.427	15.090	-22.536	0.00	0.00	LX0	H
ATOM	2212	HE22	GLN	1214	42.320	15.680	-20.958	0.00	0.00	LX0	H
ATOM	2213	C	GLN	1214	37.612	12.490	-22.306	1.00	0.00	LX0	C
ATOM	2214	O	GLN	1214	36.875	13.339	-21.811	1.00	0.00	LX0	O
ATOM	2215	N	GLY	1215	37.512	12.039	-23.566	1.00	0.00	LX0	N
ATOM	2216	H	GLY	1215	38.038	11.232	-23.831	0.00	0.00	LX0	H
ATOM	2217	CA	GLY	1215	36.645	12.749	-24.505	1.00	0.00	LX0	C
ATOM	2218	C	GLY	1215	35.146	12.531	-24.373	1.00	0.00	LX0	C
ATOM	2219	O	GLY	1215	34.360	13.089	-25.129	1.00	0.00	LX0	O
ATOM	2220	N	LEU	1216	34.758	11.692	-23.401	1.00	0.00	LX0	N
ATOM	2221	H	LEU	1216	35.399	11.347	-22.714	0.00	0.00	LX0	H
ATOM	2222	CA	LEU	1216	33.314	11.558	-23.230	1.00	0.00	LX0	C
ATOM	2223	CB	LEU	1216	32.977	10.307	-22.417	1.00	0.00	LX0	C
ATOM	2224	CG	LEU	1216	33.344	8.990	-23.101	1.00	0.00	LX0	C
ATOM	2225	CD1	LEU	1216	33.085	7.796	-22.181	1.00	0.00	LX0	C
ATOM	2226	CD2	LEU	1216	32.641	8.825	-24.451	1.00	0.00	LX0	C
ATOM	2227	C	LEU	1216	32.727	12.779	-22.554	1.00	0.00	LX0	C
ATOM	2228	O	LEU	1216	33.404	13.491	-21.817	1.00	0.00	LX0	O
ATOM	2229	N	SER	1217	31.430	12.994	-22.801	1.00	0.00	LX0	N
ATOM	2230	H	SER	1217	30.916	12.400	-23.421	0.00	0.00	LX0	H
ATOM	2231	CA	SER	1217	30.785	13.970	-21.930	1.00	0.00	LX0	C
ATOM	2232	CB	SER	1217	29.452	14.430	-22.525	1.00	0.00	LX0	C
ATOM	2233	OG	SER	1217	28.595	13.303	-22.731	1.00	0.00	LX0	O
ATOM	2234	HG	SER	1217	28.821	12.972	-23.599	0.00	0.00	LX0	H
ATOM	2235	C	SER	1217	30.596	13.380	-20.546	1.00	0.00	LX0	C
ATOM	2236	O	SER	1217	30.596	12.166	-20.372	1.00	0.00	LX0	O
ATOM	2237	N	ASN	1218	30.431	14.277	-19.560	1.00	0.00	LX0	N
ATOM	2238	H	ASN	1218	30.483	15.254	-19.759	0.00	0.00	LX0	H
ATOM	2239	CA	ASN	1218	30.300	13.767	-18.189	1.00	0.00	LX0	C
ATOM	2240	CB	ASN	1218	30.192	14.910	-17.169	1.00	0.00	LX0	C
ATOM	2241	CG	ASN	1218	31.553	15.543	-16.932	1.00	0.00	LX0	C
ATOM	2242	OD1	ASN	1218	32.487	15.382	-17.712	1.00	0.00	LX0	O
ATOM	2243	ND2	ASN	1218	31.643	16.280	-15.821	1.00	0.00	LX0	N
ATOM	2244	HD21	ASN	1218	30.898	16.420	-15.164	0.00	0.00	LX0	H
ATOM	2245	HD22	ASN	1218	32.486	16.761	-15.579	0.00	0.00	LX0	H
ATOM	2246	C	ASN	1218	29.176	12.759	-18.002	1.00	0.00	LX0	C
ATOM	2247	O	ASN	1218	29.305	11.779	-17.283	1.00	0.00	LX0	O
ATOM	2248	N	GLU	1219	28.084	13.027	-18.739	1.00	0.00	LX0	N
ATOM	2249	H	GLU	1219	28.055	13.843	-19.310	0.00	0.00	LX0	H
ATOM	2250	CA	GLU	1219	26.979	12.067	-18.783	1.00	0.00	LX0	C
ATOM	2251	CB	GLU	1219	25.815	12.696	-19.554	1.00	0.00	LX0	C
ATOM	2252	CG	GLU	1219	24.487	12.757	-18.785	1.00	0.00	LX0	C
ATOM	2253	CD	GLU	1219	23.911	11.377	-18.508	1.00	0.00	LX0	C
ATOM	2254	OE1	GLU	1219	24.274	10.429	-19.195	1.00	0.00	LX0	O
ATOM	2255	OE2	GLU	1219	23.077	11.250	-17.614	1.00	0.00	LX0	O
ATOM	2256	C	GLU	1219	27.371	10.711	-19.368	1.00	0.00	LX0	C
ATOM	2257	O	GLU	1219	27.252	9.654	-18.757	1.00	0.00	LX0	O
ATOM	2258	N	GLN	1220	27.933	10.794	-20.592	1.00	0.00	LX0	N
ATOM	2259	H	GLN	1220	28.021	11.683	-21.042	0.00	0.00	LX0	H
ATOM	2260	CA	GLN	1220	28.408	9.564	-21.235	1.00	0.00	LX0	C
ATOM	2261	CB	GLN	1220	29.106	9.864	-22.554	1.00	0.00	LX0	C
ATOM	2262	CG	GLN	1220	28.152	10.188	-23.699	1.00	0.00	LX0	C
ATOM	2263	CD	GLN	1220	28.984	10.715	-24.845	1.00	0.00	LX0	C
ATOM	2264	OE1	GLN	1220	29.624	11.753	-24.743	1.00	0.00	LX0	O
ATOM	2265	NE2	GLN	1220	28.963	9.956	-25.942	1.00	0.00	LX0	N
ATOM	2266	HE21	GLN	1220	28.433	9.112	-25.990	0.00	0.00	LX0	H
ATOM	2267	HE22	GLN	1220	29.501	10.256	-26.730	0.00	0.00	LX0	H
ATOM	2268	C	GLN	1220	29.313	8.685	-20.389	1.00	0.00	LX0	C
ATOM	2269	O	GLN	1220	29.262	7.464	-20.465	1.00	0.00	LX0	O

ATOM	2270	N	VAL	1221	30.114	9.360	-19.546	1.00	0.00	LX0	N
ATOM	2271	H	VAL	1221	30.132	10.361	-19.583	0.00	0.00	LX0	H
ATOM	2272	CA	VAL	1221	30.918	8.603	-18.587	1.00	0.00	LX0	C
ATOM	2273	CB	VAL	1221	31.729	9.540	-17.682	1.00	0.00	LX0	C
ATOM	2274	CG1	VAL	1221	32.565	8.759	-16.668	1.00	0.00	LX0	C
ATOM	2275	CG2	VAL	1221	32.617	10.466	-18.515	1.00	0.00	LX0	C
ATOM	2276	C	VAL	1221	30.115	7.598	-17.770	1.00	0.00	LX0	C
ATOM	2277	O	VAL	1221	30.456	6.425	-17.713	1.00	0.00	LX0	O
ATOM	2278	N	LEU	1222	28.998	8.078	-17.187	1.00	0.00	LX0	N
ATOM	2279	H	LEU	1222	28.713	9.023	-17.359	0.00	0.00	LX0	H
ATOM	2280	CA	LEU	1222	28.147	7.125	-16.459	1.00	0.00	LX0	C
ATOM	2281	CB	LEU	1222	26.838	7.721	-15.924	1.00	0.00	LX0	C
ATOM	2282	CG	LEU	1222	26.932	8.818	-14.869	1.00	0.00	LX0	C
ATOM	2283	CD1	LEU	1222	27.163	10.183	-15.498	1.00	0.00	LX0	C
ATOM	2284	CD2	LEU	1222	25.698	8.836	-13.965	1.00	0.00	LX0	C
ATOM	2285	C	LEU	1222	27.744	5.948	-17.320	1.00	0.00	LX0	C
ATOM	2286	O	LEU	1222	27.881	4.786	-16.963	1.00	0.00	LX0	O
ATOM	2287	N	ARG	1223	27.252	6.332	-18.505	1.00	0.00	LX0	N
ATOM	2288	H	ARG	1223	27.293	7.309	-18.722	0.00	0.00	LX0	H
ATOM	2289	CA	ARG	1223	26.703	5.327	-19.416	1.00	0.00	LX0	C
ATOM	2290	CB	ARG	1223	26.056	6.005	-20.631	1.00	0.00	LX0	C
ATOM	2291	CG	ARG	1223	25.284	7.264	-20.220	1.00	0.00	LX0	C
ATOM	2292	CD	ARG	1223	24.543	7.985	-21.347	1.00	0.00	LX0	C
ATOM	2293	NE	ARG	1223	23.222	7.401	-21.577	1.00	0.00	LX0	N
ATOM	2294	HE	ARG	1223	23.179	6.623	-22.204	0.00	0.00	LX0	H
ATOM	2295	CZ	ARG	1223	22.137	7.893	-20.929	1.00	0.00	LX0	C
ATOM	2296	NH1	ARG	1223	22.244	8.897	-20.060	1.00	0.00	LX0	N
ATOM	2297	HH11	ARG	1223	21.486	9.309	-19.557	0.00	0.00	LX0	H
ATOM	2298	HH12	ARG	1223	23.142	9.311	-19.852	0.00	0.00	LX0	H
ATOM	2299	NH2	ARG	1223	20.944	7.355	-21.171	1.00	0.00	LX0	N
ATOM	2300	HH21	ARG	1223	20.124	7.696	-20.712	0.00	0.00	LX0	H
ATOM	2301	HH22	ARG	1223	20.849	6.600	-21.820	0.00	0.00	LX0	H
ATOM	2302	C	ARG	1223	27.683	4.240	-19.831	1.00	0.00	LX0	C
ATOM	2303	O	ARG	1223	27.315	3.104	-20.093	1.00	0.00	LX0	O
ATOM	2304	N	PHE	1224	28.960	4.645	-19.850	1.00	0.00	LX0	N
ATOM	2305	H	PHE	1224	29.182	5.600	-19.643	0.00	0.00	LX0	H
ATOM	2306	CA	PHE	1224	30.009	3.667	-20.109	1.00	0.00	LX0	C
ATOM	2307	CB	PHE	1224	31.223	4.420	-20.672	1.00	0.00	LX0	C
ATOM	2308	CG	PHE	1224	32.316	3.502	-21.175	1.00	0.00	LX0	C
ATOM	2309	CD1	PHE	1224	32.016	2.459	-22.080	1.00	0.00	LX0	C
ATOM	2310	CD2	PHE	1224	33.639	3.729	-20.737	1.00	0.00	LX0	C
ATOM	2311	CE1	PHE	1224	33.055	1.637	-22.559	1.00	0.00	LX0	C
ATOM	2312	CE2	PHE	1224	34.679	2.911	-21.217	1.00	0.00	LX0	C
ATOM	2313	CZ	PHE	1224	34.377	1.875	-22.126	1.00	0.00	LX0	C
ATOM	2314	C	PHE	1224	30.357	2.823	-18.888	1.00	0.00	LX0	C
ATOM	2315	O	PHE	1224	30.330	1.597	-18.895	1.00	0.00	LX0	O
ATOM	2316	N	VAL	1225	30.707	3.542	-17.813	1.00	0.00	LX0	N
ATOM	2317	H	VAL	1225	30.589	4.536	-17.818	0.00	0.00	LX0	H
ATOM	2318	CA	VAL	1225	31.278	2.825	-16.671	1.00	0.00	LX0	C
ATOM	2319	CB	VAL	1225	31.937	3.781	-15.670	1.00	0.00	LX0	C
ATOM	2320	CG1	VAL	1225	32.788	3.045	-14.629	1.00	0.00	LX0	C
ATOM	2321	CG2	VAL	1225	32.765	4.840	-16.386	1.00	0.00	LX0	C
ATOM	2322	C	VAL	1225	30.307	1.891	-15.970	1.00	0.00	LX0	C
ATOM	2323	O	VAL	1225	30.684	0.839	-15.466	1.00	0.00	LX0	O
ATOM	2324	N	MET	1226	29.024	2.298	-15.994	1.00	0.00	LX0	N
ATOM	2325	H	MET	1226	28.798	3.170	-16.432	0.00	0.00	LX0	H
ATOM	2326	CA	MET	1226	27.992	1.502	-15.323	1.00	0.00	LX0	C
ATOM	2327	CB	MET	1226	26.584	2.021	-15.620	1.00	0.00	LX0	C
ATOM	2328	CG	MET	1226	26.225	3.257	-14.797	1.00	0.00	LX0	C
ATOM	2329	SD	MET	1226	24.498	3.749	-14.938	1.00	0.00	LX0	S
ATOM	2330	CE	MET	1226	24.423	3.937	-16.725	1.00	0.00	LX0	C

ATOM	2331	C	MET	1226	28.036	0.002	-15.550	1.00	0.00	LX0	C
ATOM	2332	O	MET	1226	27.841	-0.780	-14.623	1.00	0.00	LX0	O
ATOM	2333	N	GLU	1227	28.325	-0.376	-16.802	1.00	0.00	LX0	N
ATOM	2334	H	GLU	1227	28.487	0.276	-17.543	0.00	0.00	LX0	H
ATOM	2335	CA	GLU	1227	28.416	-1.819	-16.982	1.00	0.00	LX0	C
ATOM	2336	CB	GLU	1227	27.376	-2.335	-17.989	1.00	0.00	LX0	C
ATOM	2337	CG	GLU	1227	25.946	-1.777	-17.825	1.00	0.00	LX0	C
ATOM	2338	CD	GLU	1227	25.422	-1.858	-16.394	1.00	0.00	LX0	C
ATOM	2339	OE1	GLU	1227	25.642	-2.848	-15.700	1.00	0.00	LX0	O
ATOM	2340	OE2	GLU	1227	24.811	-0.898	-15.934	1.00	0.00	LX0	O
ATOM	2341	C	GLU	1227	29.820	-2.325	-17.260	1.00	0.00	LX0	C
ATOM	2342	O	GLU	1227	30.078	-3.131	-18.143	1.00	0.00	LX0	O
ATOM	2343	N	GLY	1228	30.737	-1.810	-16.427	1.00	0.00	LX0	N
ATOM	2344	H	GLY	1228	30.478	-1.073	-15.801	0.00	0.00	LX0	H
ATOM	2345	CA	GLY	1228	32.112	-2.298	-16.507	1.00	0.00	LX0	C
ATOM	2346	C	GLY	1228	33.011	-1.569	-17.494	1.00	0.00	LX0	C
ATOM	2347	O	GLY	1228	34.042	-2.073	-17.923	1.00	0.00	LX0	O
ATOM	2348	N	GLY	1229	32.592	-0.342	-17.832	1.00	0.00	LX0	N
ATOM	2349	H	GLY	1229	31.756	0.045	-17.445	0.00	0.00	LX0	H
ATOM	2350	CA	GLY	1229	33.444	0.426	-18.738	1.00	0.00	LX0	C
ATOM	2351	C	GLY	1229	34.720	0.928	-18.086	1.00	0.00	LX0	C
ATOM	2352	O	GLY	1229	34.726	1.887	-17.323	1.00	0.00	LX0	O
ATOM	2353	N	LEU	1230	35.804	0.214	-18.412	1.00	0.00	LX0	N
ATOM	2354	H	LEU	1230	35.697	-0.569	-19.023	0.00	0.00	LX0	H
ATOM	2355	CA	LEU	1230	37.093	0.604	-17.844	1.00	0.00	LX0	C
ATOM	2356	CB	LEU	1230	38.052	-0.590	-17.815	1.00	0.00	LX0	C
ATOM	2357	CG	LEU	1230	37.521	-1.783	-17.014	1.00	0.00	LX0	C
ATOM	2358	CD1	LEU	1230	38.491	-2.964	-17.057	1.00	0.00	LX0	C
ATOM	2359	CD2	LEU	1230	37.149	-1.406	-15.579	1.00	0.00	LX0	C
ATOM	2360	C	LEU	1230	37.730	1.799	-18.530	1.00	0.00	LX0	C
ATOM	2361	O	LEU	1230	37.306	2.236	-19.592	1.00	0.00	LX0	O
ATOM	2362	N	LEU	1231	38.760	2.310	-17.843	1.00	0.00	LX0	N
ATOM	2363	H	LEU	1231	39.072	1.855	-17.013	0.00	0.00	LX0	H
ATOM	2364	CA	LEU	1231	39.420	3.532	-18.296	1.00	0.00	LX0	C
ATOM	2365	CB	LEU	1231	40.245	4.094	-17.142	1.00	0.00	LX0	C
ATOM	2366	CG	LEU	1231	39.487	5.173	-16.379	1.00	0.00	LX0	C
ATOM	2367	CD1	LEU	1231	40.018	5.419	-14.978	1.00	0.00	LX0	C
ATOM	2368	CD2	LEU	1231	39.490	6.470	-17.163	1.00	0.00	LX0	C
ATOM	2369	C	LEU	1231	40.278	3.410	-19.540	1.00	0.00	LX0	C
ATOM	2370	O	LEU	1231	40.726	2.335	-19.925	1.00	0.00	LX0	O
ATOM	2371	N	ASP	1232	40.514	4.600	-20.117	1.00	0.00	LX0	N
ATOM	2372	H	ASP	1232	40.083	5.432	-19.764	0.00	0.00	LX0	H
ATOM	2373	CA	ASP	1232	41.520	4.766	-21.168	1.00	0.00	LX0	C
ATOM	2374	CB	ASP	1232	41.625	6.238	-21.657	1.00	0.00	LX0	C
ATOM	2375	CG	ASP	1232	40.480	7.180	-21.251	1.00	0.00	LX0	C
ATOM	2376	OD1	ASP	1232	39.765	7.676	-22.119	1.00	0.00	LX0	O
ATOM	2377	OD2	ASP	1232	40.323	7.463	-20.067	1.00	0.00	LX0	O
ATOM	2378	C	ASP	1232	42.888	4.342	-20.643	1.00	0.00	LX0	C
ATOM	2379	O	ASP	1232	43.164	4.487	-19.459	1.00	0.00	LX0	O
ATOM	2380	N	LYS	1233	43.759	3.828	-21.527	1.00	0.00	LX0	N
ATOM	2381	H	LYS	1233	43.543	3.722	-22.496	0.00	0.00	LX0	H
ATOM	2382	CA	LYS	1233	45.119	3.728	-20.995	1.00	0.00	LX0	C
ATOM	2383	CB	LYS	1233	45.784	2.352	-21.164	1.00	0.00	LX0	C
ATOM	2384	CG	LYS	1233	46.835	2.194	-20.053	1.00	0.00	LX0	C
ATOM	2385	CD	LYS	1233	47.958	1.169	-20.228	1.00	0.00	LX0	C
ATOM	2386	CE	LYS	1233	49.061	1.602	-21.197	1.00	0.00	LX0	C
ATOM	2387	NZ	LYS	1233	50.270	0.810	-20.934	1.00	0.00	LX0	N
ATOM	2388	HZ1	LYS	1233	51.007	0.910	-21.650	0.00	0.00	LX0	H
ATOM	2389	HZ2	LYS	1233	50.702	1.039	-20.014	0.00	0.00	LX0	H
ATOM	2390	HZ3	LYS	1233	50.071	-0.214	-20.860	0.00	0.00	LX0	H
ATOM	2391	C	LYS	1233	46.030	4.780	-21.586	1.00	0.00	LX0	C

ATOM	2392	O	LYS	1233	46.252	4.804	-22.788	1.00	0.00	LX0	O
ATOM	2393	N	PRO	1234	46.564	5.649	-20.699	1.00	0.00	LX0	N
ATOM	2394	CD	PRO	1234	46.293	5.723	-19.268	1.00	0.00	LX0	C
ATOM	2395	CA	PRO	1234	47.529	6.652	-21.159	1.00	0.00	LX0	C
ATOM	2396	CB	PRO	1234	47.911	7.377	-19.864	1.00	0.00	LX0	C
ATOM	2397	CG	PRO	1234	46.776	7.105	-18.878	1.00	0.00	LX0	C
ATOM	2398	C	PRO	1234	48.748	6.034	-21.825	1.00	0.00	LX0	C
ATOM	2399	O	PRO	1234	49.323	5.061	-21.335	1.00	0.00	LX0	O
ATOM	2400	N	ASP	1235	49.145	6.668	-22.937	1.00	0.00	LX0	N
ATOM	2401	H	ASP	1235	48.565	7.323	-23.429	0.00	0.00	LX0	H
ATOM	2402	CA	ASP	1235	50.418	6.316	-23.565	1.00	0.00	LX0	C
ATOM	2403	CB	ASP	1235	50.745	7.271	-24.711	1.00	0.00	LX0	C
ATOM	2404	CG	ASP	1235	49.651	7.201	-25.747	1.00	0.00	LX0	C
ATOM	2405	OD1	ASP	1235	49.806	6.450	-26.705	1.00	0.00	LX0	O
ATOM	2406	OD2	ASP	1235	48.646	7.889	-25.576	1.00	0.00	LX0	O
ATOM	2407	C	ASP	1235	51.566	6.335	-22.587	1.00	0.00	LX0	C
ATOM	2408	O	ASP	1235	51.606	7.127	-21.652	1.00	0.00	LX0	O
ATOM	2409	N	ASN	1236	52.486	5.387	-22.814	1.00	0.00	LX0	N
ATOM	2410	H	ASN	1236	52.348	4.820	-23.626	0.00	0.00	LX0	H
ATOM	2411	CA	ASN	1236	53.660	5.205	-21.945	1.00	0.00	LX0	C
ATOM	2412	CB	ASN	1236	54.557	6.451	-21.826	1.00	0.00	LX0	C
ATOM	2413	CG	ASN	1236	55.001	6.927	-23.191	1.00	0.00	LX0	C
ATOM	2414	OD1	ASN	1236	55.896	6.382	-23.817	1.00	0.00	LX0	O
ATOM	2415	ND2	ASN	1236	54.316	7.984	-23.637	1.00	0.00	LX0	N
ATOM	2416	HD21	ASN	1236	53.587	8.397	-23.091	0.00	0.00	LX0	H
ATOM	2417	HD22	ASN	1236	54.540	8.347	-24.540	0.00	0.00	LX0	H
ATOM	2418	C	ASN	1236	53.409	4.655	-20.550	1.00	0.00	LX0	C
ATOM	2419	O	ASN	1236	54.281	4.018	-19.976	1.00	0.00	LX0	O
ATOM	2420	N	CYS	1237	52.194	4.892	-20.016	1.00	0.00	LX0	N
ATOM	2421	H	CYS	1237	51.529	5.455	-20.508	0.00	0.00	LX0	H
ATOM	2422	CA	CYS	1237	51.886	4.439	-18.655	1.00	0.00	LX0	C
ATOM	2423	CB	CYS	1237	50.419	4.714	-18.318	1.00	0.00	LX0	C
ATOM	2424	SG	CYS	1237	49.953	4.234	-16.633	1.00	0.00	LX0	S
ATOM	2425	C	CYS	1237	52.212	2.977	-18.395	1.00	0.00	LX0	C
ATOM	2426	O	CYS	1237	51.646	2.074	-19.012	1.00	0.00	LX0	O
ATOM	2427	N	PRO	1238	53.174	2.788	-17.456	1.00	0.00	LX0	N
ATOM	2428	CD	PRO	1238	53.880	3.824	-16.706	1.00	0.00	LX0	C
ATOM	2429	CA	PRO	1238	53.645	1.442	-17.127	1.00	0.00	LX0	C
ATOM	2430	CB	PRO	1238	54.707	1.694	-16.053	1.00	0.00	LX0	C
ATOM	2431	CG	PRO	1238	55.163	3.136	-16.263	1.00	0.00	LX0	C
ATOM	2432	C	PRO	1238	52.534	0.538	-16.641	1.00	0.00	LX0	C
ATOM	2433	O	PRO	1238	51.881	0.782	-15.631	1.00	0.00	LX0	O
ATOM	2434	N	ASP	1239	52.359	-0.537	-17.411	1.00	0.00	LX0	N
ATOM	2435	H	ASP	1239	52.835	-0.629	-18.288	0.00	0.00	LX0	H
ATOM	2436	CA	ASP	1239	51.201	-1.398	-17.187	1.00	0.00	LX0	C
ATOM	2437	CB	ASP	1239	51.111	-2.444	-18.299	1.00	0.00	LX0	C
ATOM	2438	CG	ASP	1239	50.841	-1.711	-19.601	1.00	0.00	LX0	C
ATOM	2439	OD1	ASP	1239	51.753	-1.101	-20.156	1.00	0.00	LX0	O
ATOM	2440	OD2	ASP	1239	49.697	-1.675	-20.039	1.00	0.00	LX0	O
ATOM	2441	C	ASP	1239	51.042	-1.992	-15.802	1.00	0.00	LX0	C
ATOM	2442	O	ASP	1239	49.939	-2.274	-15.366	1.00	0.00	LX0	O
ATOM	2443	N	MET	1240	52.173	-2.089	-15.076	1.00	0.00	LX0	N
ATOM	2444	H	MET	1240	53.044	-1.918	-15.530	0.00	0.00	LX0	H
ATOM	2445	CA	MET	1240	52.083	-2.422	-13.647	1.00	0.00	LX0	C
ATOM	2446	CB	MET	1240	53.485	-2.361	-13.024	1.00	0.00	LX0	C
ATOM	2447	CG	MET	1240	53.616	-2.906	-11.594	1.00	0.00	LX0	C
ATOM	2448	SD	MET	1240	52.827	-1.886	-10.336	1.00	0.00	LX0	S
ATOM	2449	CE	MET	1240	53.219	-2.897	-8.899	1.00	0.00	LX0	C
ATOM	2450	C	MET	1240	51.076	-1.572	-12.870	1.00	0.00	LX0	C
ATOM	2451	O	MET	1240	50.202	-2.060	-12.163	1.00	0.00	LX0	O
ATOM	2452	N	LEU	1241	51.205	-0.251	-13.080	1.00	0.00	LX0	N

```
ATOM   2453  H   LEU  1241      51.872   0.093 -13.742  0.00  0.00      LX0  H
ATOM   2454  CA  LEU  1241      50.241   0.644 -12.441  1.00  0.00      LX0  C
ATOM   2455  CB  LEU  1241      50.682   2.100 -12.636  1.00  0.00      LX0  C
ATOM   2456  CG  LEU  1241      49.954   3.120 -11.751  1.00  0.00      LX0  C
ATOM   2457  CD1 LEU  1241      50.150   2.839 -10.260  1.00  0.00      LX0  C
ATOM   2458  CD2 LEU  1241      50.341   4.556 -12.112  1.00  0.00      LX0  C
ATOM   2459  C   LEU  1241      48.814   0.408 -12.919  1.00  0.00      LX0  C
ATOM   2460  O   LEU  1241      47.858   0.389 -12.155  1.00  0.00      LX0  O
ATOM   2461  N   PHE  1242      48.722   0.173 -14.237  1.00  0.00      LX0  N
ATOM   2462  H   PHE  1242      49.556   0.160 -14.789  0.00  0.00      LX0  H
ATOM   2463  CA  PHE  1242      47.420  -0.168 -14.816  1.00  0.00      LX0  C
ATOM   2464  CB  PHE  1242      47.503  -0.048 -16.343  1.00  0.00      LX0  C
ATOM   2465  CG  PHE  1242      46.138   0.113 -16.979  1.00  0.00      LX0  C
ATOM   2466  CD1 PHE  1242      45.352   1.248 -16.679  1.00  0.00      LX0  C
ATOM   2467  CD2 PHE  1242      45.680  -0.869 -17.885  1.00  0.00      LX0  C
ATOM   2468  CE1 PHE  1242      44.098   1.412 -17.298  1.00  0.00      LX0  C
ATOM   2469  CE2 PHE  1242      44.428  -0.704 -18.510  1.00  0.00      LX0  C
ATOM   2470  CZ  PHE  1242      43.650   0.435 -18.212  1.00  0.00      LX0  C
ATOM   2471  C   PHE  1242      46.845  -1.516 -14.375  1.00  0.00      LX0  C
ATOM   2472  O   PHE  1242      45.653  -1.793 -14.489  1.00  0.00      LX0  O
ATOM   2473  N   GLU  1243      47.744  -2.350 -13.837  1.00  0.00      LX0  N
ATOM   2474  H   GLU  1243      48.719  -2.131 -13.840  0.00  0.00      LX0  H
ATOM   2475  CA  GLU  1243      47.276  -3.582 -13.221  1.00  0.00      LX0  C
ATOM   2476  CB  GLU  1243      48.400  -4.623 -13.157  1.00  0.00      LX0  C
ATOM   2477  CG  GLU  1243      47.907  -6.069 -13.003  1.00  0.00      LX0  C
ATOM   2478  CD  GLU  1243      46.977  -6.437 -14.149  1.00  0.00      LX0  C
ATOM   2479  OE1 GLU  1243      47.346  -6.295 -15.312  1.00  0.00      LX0  O
ATOM   2480  OE2 GLU  1243      45.841  -6.811 -13.895  1.00  0.00      LX0  O
ATOM   2481  C   GLU  1243      46.640  -3.308 -11.881  1.00  0.00      LX0  C
ATOM   2482  O   GLU  1243      45.495  -3.654 -11.632  1.00  0.00      LX0  O
ATOM   2483  N   LEU  1244      47.401  -2.565 -11.057  1.00  0.00      LX0  N
ATOM   2484  H   LEU  1244      48.345  -2.357 -11.326  0.00  0.00      LX0  H
ATOM   2485  CA  LEU  1244      46.840  -2.102  -9.783  1.00  0.00      LX0  C
ATOM   2486  CB  LEU  1244      47.837  -1.176  -9.080  1.00  0.00      LX0  C
ATOM   2487  CG  LEU  1244      47.458  -0.841  -7.634  1.00  0.00      LX0  C
ATOM   2488  CD1 LEU  1244      47.466  -2.078  -6.733  1.00  0.00      LX0  C
ATOM   2489  CD2 LEU  1244      48.320   0.290  -7.078  1.00  0.00      LX0  C
ATOM   2490  C   LEU  1244      45.469  -1.441  -9.912  1.00  0.00      LX0  C
ATOM   2491  O   LEU  1244      44.519  -1.746  -9.200  1.00  0.00      LX0  O
ATOM   2492  N   MET  1245      45.395  -0.556 -10.921  1.00  0.00      LX0  N
ATOM   2493  H   MET  1245      46.241  -0.308 -11.395  0.00  0.00      LX0  H
ATOM   2494  CA  MET  1245      44.103   0.047 -11.260  1.00  0.00      LX0  C
ATOM   2495  CB  MET  1245      44.210   0.897 -12.526  1.00  0.00      LX0  C
ATOM   2496  CG  MET  1245      45.182   2.074 -12.476  1.00  0.00      LX0  C
ATOM   2497  SD  MET  1245      45.108   3.054 -13.984  1.00  0.00      LX0  S
ATOM   2498  CE  MET  1245      46.779   3.717 -13.959  1.00  0.00      LX0  C
ATOM   2499  C   MET  1245      42.981  -0.965 -11.448  1.00  0.00      LX0  C
ATOM   2500  O   MET  1245      41.934  -0.915 -10.814  1.00  0.00      LX0  O
ATOM   2501  N   ARG  1246      43.258  -1.918 -12.348  1.00  0.00      LX0  N
ATOM   2502  H   ARG  1246      44.166  -1.974 -12.769  0.00  0.00      LX0  H
ATOM   2503  CA  ARG  1246      42.240  -2.935 -12.606  1.00  0.00      LX0  C
ATOM   2504  CB  ARG  1246      42.591  -3.699 -13.873  1.00  0.00      LX0  C
ATOM   2505  CG  ARG  1246      42.148  -2.947 -15.132  1.00  0.00      LX0  C
ATOM   2506  CD  ARG  1246      43.029  -3.187 -16.364  1.00  0.00      LX0  C
ATOM   2507  NE  ARG  1246      43.298  -4.605 -16.603  1.00  0.00      LX0  N
ATOM   2508  HE  ARG  1246      42.565  -5.182 -16.963  0.00  0.00      LX0  H
ATOM   2509  CZ  ARG  1246      44.491  -5.120 -16.226  1.00  0.00      LX0  C
ATOM   2510  NH1 ARG  1246      45.438  -4.343 -15.717  1.00  0.00      LX0  N
ATOM   2511 HH11 ARG  1246      46.300  -4.794 -15.444  0.00  0.00      LX0  H
ATOM   2512 HH12 ARG  1246      45.338  -3.357 -15.563  0.00  0.00      LX0  H
ATOM   2513  NH2 ARG  1246      44.728  -6.419 -16.327  1.00  0.00      LX0  N
```

ATOM	2514	HH21	ARG	1246	45.570	-6.757	-15.866	0.00	0.00	LX0	H
ATOM	2515	HH22	ARG	1246	44.130	-7.073	-16.778	0.00	0.00	LX0	H
ATOM	2516	C	ARG	1246	41.930	-3.867	-11.447	1.00	0.00	LX0	C
ATOM	2517	O	ARG	1246	40.821	-4.367	-11.318	1.00	0.00	LX0	O
ATOM	2518	N	MET	1247	42.927	-4.033	-10.564	1.00	0.00	LX0	N
ATOM	2519	H	MET	1247	43.828	-3.643	-10.751	0.00	0.00	LX0	H
ATOM	2520	CA	MET	1247	42.643	-4.719	-9.301	1.00	0.00	LX0	C
ATOM	2521	CB	MET	1247	43.903	-4.832	-8.442	1.00	0.00	LX0	C
ATOM	2522	CG	MET	1247	44.985	-5.735	-9.037	1.00	0.00	LX0	C
ATOM	2523	SD	MET	1247	46.521	-5.683	-8.096	1.00	0.00	LX0	S
ATOM	2524	CE	MET	1247	45.863	-6.117	-6.476	1.00	0.00	LX0	C
ATOM	2525	C	MET	1247	41.544	-4.019	-8.523	1.00	0.00	LX0	C
ATOM	2526	O	MET	1247	40.554	-4.613	-8.114	1.00	0.00	LX0	O
ATOM	2527	N	CYS	1248	41.735	-2.698	-8.401	1.00	0.00	LX0	N
ATOM	2528	H	CYS	1248	42.580	-2.277	-8.742	0.00	0.00	LX0	H
ATOM	2529	CA	CYS	1248	40.678	-1.883	-7.800	1.00	0.00	LX0	C
ATOM	2530	CB	CYS	1248	41.151	-0.437	-7.663	1.00	0.00	LX0	C
ATOM	2531	SG	CYS	1248	42.724	-0.288	-6.777	1.00	0.00	LX0	S
ATOM	2532	C	CYS	1248	39.346	-1.926	-8.538	1.00	0.00	LX0	C
ATOM	2533	O	CYS	1248	38.270	-1.841	-7.956	1.00	0.00	LX0	O
ATOM	2534	N	TRP	1249	39.459	-2.063	-9.864	1.00	0.00	LX0	N
ATOM	2535	H	TRP	1249	40.363	-2.156	-10.282	0.00	0.00	LX0	H
ATOM	2536	CA	TRP	1249	38.260	-1.973	-10.692	1.00	0.00	LX0	C
ATOM	2537	CB	TRP	1249	38.592	-1.396	-12.069	1.00	0.00	LX0	C
ATOM	2538	CG	TRP	1249	39.163	-0.001	-11.965	1.00	0.00	LX0	C
ATOM	2539	CD2	TRP	1249	40.023	0.659	-12.913	1.00	0.00	LX0	C
ATOM	2540	CE2	TRP	1249	40.314	1.962	-12.389	1.00	0.00	LX0	C
ATOM	2541	CE3	TRP	1249	40.566	0.255	-14.151	1.00	0.00	LX0	C
ATOM	2542	CD1	TRP	1249	38.974	0.932	-10.934	1.00	0.00	LX0	C
ATOM	2543	NE1	TRP	1249	39.644	2.088	-11.174	1.00	0.00	LX0	N
ATOM	2544	HE1	TRP	1249	39.621	2.871	-10.579	0.00	0.00	LX0	H
ATOM	2545	CZ2	TRP	1249	41.149	2.833	-13.117	1.00	0.00	LX0	C
ATOM	2546	CZ3	TRP	1249	41.397	1.137	-14.871	1.00	0.00	LX0	C
ATOM	2547	CH2	TRP	1249	41.689	2.417	-14.354	1.00	0.00	LX0	C
ATOM	2548	C	TRP	1249	37.439	-3.241	-10.850	1.00	0.00	LX0	C
ATOM	2549	O	TRP	1249	37.155	-3.701	-11.950	1.00	0.00	LX0	O
ATOM	2550	N	GLN	1250	37.022	-3.784	-9.701	1.00	0.00	LX0	N
ATOM	2551	H	GLN	1250	37.225	-3.326	-8.835	0.00	0.00	LX0	H
ATOM	2552	CA	GLN	1250	36.089	-4.899	-9.845	1.00	0.00	LX0	C
ATOM	2553	CB	GLN	1250	36.184	-5.850	-8.649	1.00	0.00	LX0	C
ATOM	2554	CG	GLN	1250	37.608	-6.344	-8.374	1.00	0.00	LX0	C
ATOM	2555	CD	GLN	1250	38.157	-7.025	-9.610	1.00	0.00	LX0	C
ATOM	2556	OE1	GLN	1250	37.557	-7.924	-10.184	1.00	0.00	LX0	O
ATOM	2557	NE2	GLN	1250	39.309	-6.504	-10.030	1.00	0.00	LX0	N
ATOM	2558	HE21	GLN	1250	39.757	-5.781	-9.499	0.00	0.00	LX0	H
ATOM	2559	HE22	GLN	1250	39.730	-6.794	-10.886	0.00	0.00	LX0	H
ATOM	2560	C	GLN	1250	34.666	-4.418	-10.026	1.00	0.00	LX0	C
ATOM	2561	O	GLN	1250	34.243	-3.455	-9.401	1.00	0.00	LX0	O
ATOM	2562	N	TYR	1251	33.916	-5.129	-10.886	1.00	0.00	LX0	N
ATOM	2563	H	TYR	1251	34.354	-5.846	-11.428	0.00	0.00	LX0	H
ATOM	2564	CA	TYR	1251	32.512	-4.727	-11.038	1.00	0.00	LX0	C
ATOM	2565	CB	TYR	1251	31.808	-5.599	-12.094	1.00	0.00	LX0	C
ATOM	2566	CG	TYR	1251	30.416	-5.086	-12.418	1.00	0.00	LX0	C
ATOM	2567	CD1	TYR	1251	30.240	-4.227	-13.523	1.00	0.00	LX0	C
ATOM	2568	CE1	TYR	1251	28.949	-3.759	-13.827	1.00	0.00	LX0	C
ATOM	2569	CD2	TYR	1251	29.329	-5.484	-11.611	1.00	0.00	LX0	C
ATOM	2570	CE2	TYR	1251	28.042	-5.006	-11.906	1.00	0.00	LX0	C
ATOM	2571	CZ	TYR	1251	27.867	-4.152	-13.013	1.00	0.00	LX0	C
ATOM	2572	OH	TYR	1251	26.596	-3.693	-13.301	1.00	0.00	LX0	O
ATOM	2573	HH	TYR	1251	26.467	-3.769	-14.254	0.00	0.00	LX0	H
ATOM	2574	C	TYR	1251	31.761	-4.725	-9.709	1.00	0.00	LX0	C

ATOM	2575	O	TYR	1251	31.289	-3.704	-9.229	1.00	0.00	LX0	O
ATOM	2576	N	ASN	1252	31.719	-5.931	-9.119	1.00	0.00	LX0	N
ATOM	2577	H	ASN	1252	32.198	-6.690	-9.554	0.00	0.00	LX0	H
ATOM	2578	CA	ASN	1252	31.109	-6.079	-7.793	1.00	0.00	LX0	C
ATOM	2579	CB	ASN	1252	31.135	-7.576	-7.415	1.00	0.00	LX0	C
ATOM	2580	CG	ASN	1252	30.727	-7.842	-5.971	1.00	0.00	LX0	C
ATOM	2581	OD1	ASN	1252	30.079	-7.045	-5.309	1.00	0.00	LX0	O
ATOM	2582	ND2	ASN	1252	31.209	-8.984	-5.480	1.00	0.00	LX0	N
ATOM	2583	HD21	ASN	1252	31.661	-9.668	-6.047	0.00	0.00	LX0	H
ATOM	2584	HD22	ASN	1252	31.157	-9.151	-4.492	0.00	0.00	LX0	H
ATOM	2585	C	ASN	1252	31.797	-5.210	-6.745	1.00	0.00	LX0	C
ATOM	2586	O	ASN	1252	33.003	-5.307	-6.552	1.00	0.00	LX0	O
ATOM	2587	N	PRO	1253	30.985	-4.360	-6.066	1.00	0.00	LX0	N
ATOM	2588	CD	PRO	1253	29.576	-4.082	-6.329	1.00	0.00	LX0	C
ATOM	2589	CA	PRO	1253	31.506	-3.615	-4.916	1.00	0.00	LX0	C
ATOM	2590	CB	PRO	1253	30.276	-2.852	-4.404	1.00	0.00	LX0	C
ATOM	2591	CG	PRO	1253	29.055	-3.553	-5.000	1.00	0.00	LX0	C
ATOM	2592	C	PRO	1253	32.185	-4.488	-3.873	1.00	0.00	LX0	C
ATOM	2593	O	PRO	1253	33.331	-4.271	-3.508	1.00	0.00	LX0	O
ATOM	2594	N	LYS	1254	31.450	-5.507	-3.408	1.00	0.00	LX0	N
ATOM	2595	H	LYS	1254	30.587	-5.757	-3.852	0.00	0.00	LX0	H
ATOM	2596	CA	LYS	1254	32.066	-6.376	-2.408	1.00	0.00	LX0	C
ATOM	2597	CB	LYS	1254	30.994	-6.959	-1.476	1.00	0.00	LX0	C
ATOM	2598	CG	LYS	1254	30.132	-5.874	-0.815	1.00	0.00	LX0	C
ATOM	2599	CD	LYS	1254	29.106	-6.407	0.194	1.00	0.00	LX0	C
ATOM	2600	CE	LYS	1254	28.221	-5.284	0.746	1.00	0.00	LX0	C
ATOM	2601	NZ	LYS	1254	27.335	-5.766	1.815	1.00	0.00	LX0	N
ATOM	2602	HZ1	LYS	1254	27.626	-5.331	2.717	0.00	0.00	LX0	H
ATOM	2603	HZ2	LYS	1254	26.332	-5.544	1.620	0.00	0.00	LX0	H
ATOM	2604	HZ3	LYS	1254	27.473	-6.776	2.030	0.00	0.00	LX0	H
ATOM	2605	C	LYS	1254	32.947	-7.454	-3.026	1.00	0.00	LX0	C
ATOM	2606	O	LYS	1254	32.635	-8.639	-3.009	1.00	0.00	LX0	O
ATOM	2607	N	MET	1255	34.062	-6.959	-3.592	1.00	0.00	LX0	N
ATOM	2608	H	MET	1255	34.145	-5.964	-3.635	0.00	0.00	LX0	H
ATOM	2609	CA	MET	1255	35.101	-7.792	-4.204	1.00	0.00	LX0	C
ATOM	2610	CB	MET	1255	34.615	-8.409	-5.521	1.00	0.00	LX0	C
ATOM	2611	CG	MET	1255	34.701	-9.939	-5.554	1.00	0.00	LX0	C
ATOM	2612	SD	MET	1255	36.378	-10.593	-5.519	1.00	0.00	LX0	S
ATOM	2613	CE	MET	1255	36.875	-10.119	-7.184	1.00	0.00	LX0	C
ATOM	2614	C	MET	1255	36.385	-7.018	-4.475	1.00	0.00	LX0	C
ATOM	2615	O	MET	1255	37.243	-7.438	-5.238	1.00	0.00	LX0	O
ATOM	2616	N	ARG	1256	36.466	-5.829	-3.862	1.00	0.00	LX0	N
ATOM	2617	H	ARG	1256	35.868	-5.563	-3.108	0.00	0.00	LX0	H
ATOM	2618	CA	ARG	1256	37.621	-5.003	-4.201	1.00	0.00	LX0	C
ATOM	2619	CB	ARG	1256	37.170	-3.572	-4.512	1.00	0.00	LX0	C
ATOM	2620	CG	ARG	1256	36.111	-3.564	-5.611	1.00	0.00	LX0	C
ATOM	2621	CD	ARG	1256	35.650	-2.176	-6.039	1.00	0.00	LX0	C
ATOM	2622	NE	ARG	1256	34.524	-2.306	-6.955	1.00	0.00	LX0	N
ATOM	2623	HE	ARG	1256	34.538	-3.057	-7.617	0.00	0.00	LX0	H
ATOM	2624	CZ	ARG	1256	33.454	-1.496	-6.909	1.00	0.00	LX0	C
ATOM	2625	NH1	ARG	1256	33.427	-0.460	-6.085	1.00	0.00	LX0	N
ATOM	2626	HH11	ARG	1256	32.573	0.070	-5.990	0.00	0.00	LX0	H
ATOM	2627	HH12	ARG	1256	34.238	-0.190	-5.558	0.00	0.00	LX0	H
ATOM	2628	NH2	ARG	1256	32.408	-1.742	-7.682	1.00	0.00	LX0	N
ATOM	2629	HH21	ARG	1256	31.572	-1.180	-7.596	0.00	0.00	LX0	H
ATOM	2630	HH22	ARG	1256	32.393	-2.485	-8.359	0.00	0.00	LX0	H
ATOM	2631	C	ARG	1256	38.651	-5.043	-3.092	1.00	0.00	LX0	C
ATOM	2632	O	ARG	1256	38.292	-5.019	-1.922	1.00	0.00	LX0	O
ATOM	2633	N	PRO	1257	39.940	-5.129	-3.499	1.00	0.00	LX0	N
ATOM	2634	CD	PRO	1257	40.433	-5.154	-4.872	1.00	0.00	LX0	C
ATOM	2635	CA	PRO	1257	41.017	-5.204	-2.505	1.00	0.00	LX0	C

ATOM	2636	CB	PRO	1257	42.264	-5.377	-3.383	1.00	0.00	LX0	C
ATOM	2637	CG	PRO	1257	41.902	-4.780	-4.740	1.00	0.00	LX0	C
ATOM	2638	C	PRO	1257	41.072	-3.973	-1.619	1.00	0.00	LX0	C
ATOM	2639	O	PRO	1257	40.791	-2.857	-2.049	1.00	0.00	LX0	O
ATOM	2640	N	SER	1258	41.436	-4.210	-0.355	1.00	0.00	LX0	N
ATOM	2641	H	SER	1258	41.687	-5.141	-0.066	0.00	0.00	LX0	H
ATOM	2642	CA	SER	1258	41.531	-3.039	0.510	1.00	0.00	LX0	C
ATOM	2643	CB	SER	1258	41.552	-3.443	1.988	1.00	0.00	LX0	C
ATOM	2644	OG	SER	1258	42.812	-4.046	2.320	1.00	0.00	LX0	O
ATOM	2645	HG	SER	1258	42.685	-4.981	2.123	0.00	0.00	LX0	H
ATOM	2646	C	SER	1258	42.737	-2.176	0.189	1.00	0.00	LX0	C
ATOM	2647	O	SER	1258	43.691	-2.591	-0.460	1.00	0.00	LX0	O
ATOM	2648	N	PHE	1259	42.696	-0.955	0.745	1.00	0.00	LX0	N
ATOM	2649	H	PHE	1259	41.857	-0.645	1.189	0.00	0.00	LX0	H
ATOM	2650	CA	PHE	1259	43.894	-0.116	0.651	1.00	0.00	LX0	C
ATOM	2651	CB	PHE	1259	43.638	1.253	1.284	1.00	0.00	LX0	C
ATOM	2652	CG	PHE	1259	42.470	1.933	0.606	1.00	0.00	LX0	C
ATOM	2653	CD1	PHE	1259	42.638	2.488	-0.683	1.00	0.00	LX0	C
ATOM	2654	CD2	PHE	1259	41.227	2.002	1.274	1.00	0.00	LX0	C
ATOM	2655	CE1	PHE	1259	41.549	3.123	-1.311	1.00	0.00	LX0	C
ATOM	2656	CE2	PHE	1259	40.137	2.636	0.647	1.00	0.00	LX0	C
ATOM	2657	CZ	PHE	1259	40.311	3.192	-0.638	1.00	0.00	LX0	C
ATOM	2658	C	PHE	1259	45.154	-0.760	1.222	1.00	0.00	LX0	C
ATOM	2659	O	PHE	1259	46.257	-0.603	0.715	1.00	0.00	LX0	O
ATOM	2660	N	LEU	1260	44.928	-1.547	2.289	1.00	0.00	LX0	N
ATOM	2661	H	LEU	1260	43.995	-1.714	2.604	0.00	0.00	LX0	H
ATOM	2662	CA	LEU	1260	46.050	-2.293	2.860	1.00	0.00	LX0	C
ATOM	2663	CB	LEU	1260	45.622	-2.974	4.159	1.00	0.00	LX0	C
ATOM	2664	CG	LEU	1260	45.282	-1.974	5.263	1.00	0.00	LX0	C
ATOM	2665	CD1	LEU	1260	44.526	-2.637	6.415	1.00	0.00	LX0	C
ATOM	2666	CD2	LEU	1260	46.524	-1.220	5.742	1.00	0.00	LX0	C
ATOM	2667	C	LEU	1260	46.654	-3.307	1.905	1.00	0.00	LX0	C
ATOM	2668	O	LEU	1260	47.867	-3.447	1.781	1.00	0.00	LX0	O
ATOM	2669	N	GLU	1261	45.749	-3.997	1.199	1.00	0.00	LX0	N
ATOM	2670	H	GLU	1261	44.765	-3.862	1.347	0.00	0.00	LX0	H
ATOM	2671	CA	GLU	1261	46.223	-4.928	0.174	1.00	0.00	LX0	C
ATOM	2672	CB	GLU	1261	45.051	-5.736	-0.360	1.00	0.00	LX0	C
ATOM	2673	CG	GLU	1261	44.491	-6.660	0.720	1.00	0.00	LX0	C
ATOM	2674	CD	GLU	1261	43.020	-6.889	0.463	1.00	0.00	LX0	C
ATOM	2675	OE1	GLU	1261	42.226	-6.624	1.357	1.00	0.00	LX0	O
ATOM	2676	OE2	GLU	1261	42.658	-7.275	-0.642	1.00	0.00	LX0	O
ATOM	2677	C	GLU	1261	46.983	-4.255	-0.953	1.00	0.00	LX0	C
ATOM	2678	O	GLU	1261	48.024	-4.726	-1.401	1.00	0.00	LX0	O
ATOM	2679	N	ILE	1262	46.452	-3.085	-1.343	1.00	0.00	LX0	N
ATOM	2680	H	ILE	1262	45.553	-2.828	-0.981	0.00	0.00	LX0	H
ATOM	2681	CA	ILE	1262	47.163	-2.238	-2.305	1.00	0.00	LX0	C
ATOM	2682	CB	ILE	1262	46.342	-0.964	-2.569	1.00	0.00	LX0	C
ATOM	2683	CG2	ILE	1262	47.092	0.079	-3.405	1.00	0.00	LX0	C
ATOM	2684	CG1	ILE	1262	44.999	-1.342	-3.204	1.00	0.00	LX0	C
ATOM	2685	CD1	ILE	1262	43.995	-0.189	-3.247	1.00	0.00	LX0	C
ATOM	2686	C	ILE	1262	48.599	-1.931	-1.887	1.00	0.00	LX0	C
ATOM	2687	O	ILE	1262	49.556	-2.197	-2.603	1.00	0.00	LX0	O
ATOM	2688	N	ILE	1263	48.735	-1.415	-0.652	1.00	0.00	LX0	N
ATOM	2689	H	ILE	1263	47.920	-1.201	-0.109	0.00	0.00	LX0	H
ATOM	2690	CA	ILE	1263	50.105	-1.165	-0.187	1.00	0.00	LX0	C
ATOM	2691	CB	ILE	1263	50.121	-0.455	1.176	1.00	0.00	LX0	C
ATOM	2692	CG2	ILE	1263	51.544	-0.043	1.572	1.00	0.00	LX0	C
ATOM	2693	CG1	ILE	1263	49.189	0.761	1.174	1.00	0.00	LX0	C
ATOM	2694	CD1	ILE	1263	48.973	1.364	2.563	1.00	0.00	LX0	C
ATOM	2695	C	ILE	1263	50.969	-2.425	-0.179	1.00	0.00	LX0	C
ATOM	2696	O	ILE	1263	52.116	-2.448	-0.608	1.00	0.00	LX0	O

ATOM	2697	N	SER	1264	50.326	-3.512	0.263	1.00	0.00	LX0	N
ATOM	2698	H	SER	1264	49.388	-3.423	0.598	0.00	0.00	LX0	H
ATOM	2699	CA	SER	1264	51.004	-4.806	0.207	1.00	0.00	LX0	C
ATOM	2700	CB	SER	1264	50.133	-5.877	0.861	1.00	0.00	LX0	C
ATOM	2701	OG	SER	1264	49.779	-5.448	2.184	1.00	0.00	LX0	O
ATOM	2702	HG	SER	1264	49.042	-4.848	2.086	0.00	0.00	LX0	H
ATOM	2703	C	SER	1264	51.485	-5.255	-1.170	1.00	0.00	LX0	C
ATOM	2704	O	SER	1264	52.485	-5.956	-1.301	1.00	0.00	LX0	O
ATOM	2705	N	SER	1265	50.753	-4.812	-2.198	1.00	0.00	LX0	N
ATOM	2706	H	SER	1265	49.968	-4.197	-2.093	0.00	0.00	LX0	H
ATOM	2707	CA	SER	1265	51.207	-5.164	-3.538	1.00	0.00	LX0	C
ATOM	2708	CB	SER	1265	50.000	-5.416	-4.450	1.00	0.00	LX0	C
ATOM	2709	OG	SER	1265	48.863	-4.663	-4.007	1.00	0.00	LX0	O
ATOM	2710	HG	SER	1265	48.575	-5.036	-3.179	0.00	0.00	LX0	H
ATOM	2711	C	SER	1265	52.203	-4.197	-4.163	1.00	0.00	LX0	C
ATOM	2712	O	SER	1265	52.836	-4.501	-5.163	1.00	0.00	LX0	O
ATOM	2713	N	ILE	1266	52.321	-3.016	-3.530	1.00	0.00	LX0	N
ATOM	2714	H	ILE	1266	51.731	-2.785	-2.756	0.00	0.00	LX0	H
ATOM	2715	CA	ILE	1266	53.208	-2.026	-4.142	1.00	0.00	LX0	C
ATOM	2716	CB	ILE	1266	52.416	-0.775	-4.540	1.00	0.00	LX0	C
ATOM	2717	CG2	ILE	1266	51.344	-1.130	-5.570	1.00	0.00	LX0	C
ATOM	2718	CG1	ILE	1266	51.861	-0.032	-3.319	1.00	0.00	LX0	C
ATOM	2719	CD1	ILE	1266	51.065	1.219	-3.682	1.00	0.00	LX0	C
ATOM	2720	C	ILE	1266	54.451	-1.640	-3.353	1.00	0.00	LX0	C
ATOM	2721	O	ILE	1266	55.320	-0.925	-3.839	1.00	0.00	LX0	O
ATOM	2722	N	LYS	1267	54.510	-2.137	-2.104	1.00	0.00	LX0	N
ATOM	2723	H	LYS	1267	53.727	-2.659	-1.769	0.00	0.00	LX0	H
ATOM	2724	CA	LYS	1267	55.573	-1.715	-1.181	1.00	0.00	LX0	C
ATOM	2725	CB	LYS	1267	55.423	-2.458	0.154	1.00	0.00	LX0	C
ATOM	2726	CG	LYS	1267	55.792	-3.934	0.035	1.00	0.00	LX0	C
ATOM	2727	CD	LYS	1267	54.891	-4.893	0.807	1.00	0.00	LX0	C
ATOM	2728	CE	LYS	1267	55.197	-6.345	0.426	1.00	0.00	LX0	C
ATOM	2729	NZ	LYS	1267	55.070	-6.512	-1.030	1.00	0.00	LX0	N
ATOM	2730	HZ1	LYS	1267	55.480	-7.403	-1.365	0.00	0.00	LX0	H
ATOM	2731	HZ2	LYS	1267	54.101	-6.386	-1.383	0.00	0.00	LX0	H
ATOM	2732	HZ3	LYS	1267	55.676	-5.819	-1.528	0.00	0.00	LX0	H
ATOM	2733	C	LYS	1267	57.013	-1.740	-1.702	1.00	0.00	LX0	C
ATOM	2734	O	LYS	1267	57.880	-0.998	-1.258	1.00	0.00	LX0	O
ATOM	2735	N	GLU	1268	57.203	-2.618	-2.691	1.00	0.00	LX0	N
ATOM	2736	H	GLU	1268	56.445	-3.216	-2.951	0.00	0.00	LX0	H
ATOM	2737	CA	GLU	1268	58.473	-2.810	-3.378	1.00	0.00	LX0	C
ATOM	2738	CB	GLU	1268	58.242	-3.829	-4.501	1.00	0.00	LX0	C
ATOM	2739	CG	GLU	1268	58.146	-5.313	-4.081	1.00	0.00	LX0	C
ATOM	2740	CD	GLU	1268	57.066	-5.627	-3.045	1.00	0.00	LX0	C
ATOM	2741	OE1	GLU	1268	55.932	-5.157	-3.143	1.00	0.00	LX0	O
ATOM	2742	OE2	GLU	1268	57.347	-6.366	-2.107	1.00	0.00	LX0	O
ATOM	2743	C	GLU	1268	59.161	-1.543	-3.883	1.00	0.00	LX0	C
ATOM	2744	O	GLU	1268	60.368	-1.392	-3.750	1.00	0.00	LX0	O
ATOM	2745	N	GLU	1269	58.358	-0.629	-4.459	1.00	0.00	LX0	N
ATOM	2746	H	GLU	1269	57.364	-0.754	-4.487	0.00	0.00	LX0	H
ATOM	2747	CA	GLU	1269	59.014	0.595	-4.937	1.00	0.00	LX0	C
ATOM	2748	CB	GLU	1269	58.595	0.979	-6.361	1.00	0.00	LX0	C
ATOM	2749	CG	GLU	1269	59.381	0.300	-7.489	1.00	0.00	LX0	C
ATOM	2750	CD	GLU	1269	59.162	1.057	-8.793	1.00	0.00	LX0	C
ATOM	2751	OE1	GLU	1269	58.027	1.142	-9.251	1.00	0.00	LX0	O
ATOM	2752	OE2	GLU	1269	60.126	1.587	-9.345	1.00	0.00	LX0	O
ATOM	2753	C	GLU	1269	58.857	1.818	-4.047	1.00	0.00	LX0	C
ATOM	2754	O	GLU	1269	59.058	2.951	-4.470	1.00	0.00	LX0	O
ATOM	2755	N	MET	1270	58.457	1.567	-2.792	1.00	0.00	LX0	N
ATOM	2756	H	MET	1270	58.457	0.639	-2.417	0.00	0.00	LX0	H
ATOM	2757	CA	MET	1270	58.197	2.750	-1.971	1.00	0.00	LX0	C

ATOM	2758	CB	MET	1270	57.302	2.411	-0.778	1.00	0.00	LX0	C
ATOM	2759	CG	MET	1270	56.005	1.706	-1.173	1.00	0.00	LX0	C
ATOM	2760	SD	MET	1270	54.901	2.660	-2.216	1.00	0.00	LX0	S
ATOM	2761	CE	MET	1270	54.215	3.677	-0.910	1.00	0.00	LX0	C
ATOM	2762	C	MET	1270	59.457	3.435	-1.477	1.00	0.00	LX0	C
ATOM	2763	O	MET	1270	60.451	2.799	-1.143	1.00	0.00	LX0	O
ATOM	2764	N	GLU	1271	59.359	4.771	-1.401	1.00	0.00	LX0	N
ATOM	2765	H	GLU	1271	58.492	5.190	-1.673	0.00	0.00	LX0	H
ATOM	2766	CA	GLU	1271	60.388	5.540	-0.696	1.00	0.00	LX0	C
ATOM	2767	CB	GLU	1271	60.035	7.030	-0.705	1.00	0.00	LX0	C
ATOM	2768	CG	GLU	1271	59.999	7.655	-2.107	1.00	0.00	LX0	C
ATOM	2769	CD	GLU	1271	59.307	9.015	-2.101	1.00	0.00	LX0	C
ATOM	2770	OE1	GLU	1271	58.548	9.327	-1.189	1.00	0.00	LX0	O
ATOM	2771	OE2	GLU	1271	59.451	9.766	-3.056	1.00	0.00	LX0	O
ATOM	2772	C	GLU	1271	60.508	5.046	0.737	1.00	0.00	LX0	C
ATOM	2773	O	GLU	1271	59.554	5.086	1.503	1.00	0.00	LX0	O
ATOM	2774	N	PRO	1272	61.715	4.531	1.081	1.00	0.00	LX0	N
ATOM	2775	CD	PRO	1272	62.950	4.630	0.309	1.00	0.00	LX0	C
ATOM	2776	CA	PRO	1272	61.876	3.722	2.296	1.00	0.00	LX0	C
ATOM	2777	CB	PRO	1272	63.397	3.707	2.473	1.00	0.00	LX0	C
ATOM	2778	CG	PRO	1272	63.925	3.710	1.038	1.00	0.00	LX0	C
ATOM	2779	C	PRO	1272	61.083	4.135	3.530	1.00	0.00	LX0	C
ATOM	2780	O	PRO	1272	60.289	3.362	4.059	1.00	0.00	LX0	O
ATOM	2781	N	GLY	1273	61.331	5.395	3.936	1.00	0.00	LX0	N
ATOM	2782	H	GLY	1273	61.953	5.955	3.393	0.00	0.00	LX0	H
ATOM	2783	CA	GLY	1273	60.759	5.924	5.178	1.00	0.00	LX0	C
ATOM	2784	C	GLY	1273	59.242	5.899	5.305	1.00	0.00	LX0	C
ATOM	2785	O	GLY	1273	58.692	5.892	6.401	1.00	0.00	LX0	O
ATOM	2786	N	PHE	1274	58.578	5.864	4.128	1.00	0.00	LX0	N
ATOM	2787	H	PHE	1274	59.084	5.872	3.265	0.00	0.00	LX0	H
ATOM	2788	CA	PHE	1274	57.117	5.721	4.097	1.00	0.00	LX0	C
ATOM	2789	CB	PHE	1274	56.660	5.398	2.661	1.00	0.00	LX0	C
ATOM	2790	CG	PHE	1274	55.184	5.067	2.564	1.00	0.00	LX0	C
ATOM	2791	CD1	PHE	1274	54.247	6.101	2.357	1.00	0.00	LX0	C
ATOM	2792	CD2	PHE	1274	54.769	3.720	2.687	1.00	0.00	LX0	C
ATOM	2793	CE1	PHE	1274	52.876	5.787	2.294	1.00	0.00	LX0	C
ATOM	2794	CE2	PHE	1274	53.398	3.405	2.629	1.00	0.00	LX0	C
ATOM	2795	CZ	PHE	1274	52.465	4.445	2.440	1.00	0.00	LX0	C
ATOM	2796	C	PHE	1274	56.624	4.658	5.059	1.00	0.00	LX0	C
ATOM	2797	O	PHE	1274	55.723	4.856	5.863	1.00	0.00	LX0	O
ATOM	2798	N	ARG	1275	57.281	3.497	4.931	1.00	0.00	LX0	N
ATOM	2799	H	ARG	1275	58.110	3.451	4.370	0.00	0.00	LX0	H
ATOM	2800	CA	ARG	1275	56.783	2.386	5.728	1.00	0.00	LX0	C
ATOM	2801	CB	ARG	1275	57.463	1.088	5.346	1.00	0.00	LX0	C
ATOM	2802	CG	ARG	1275	57.514	0.708	3.861	1.00	0.00	LX0	C
ATOM	2803	CD	ARG	1275	58.905	0.193	3.445	1.00	0.00	LX0	C
ATOM	2804	NE	ARG	1275	59.478	-0.709	4.451	1.00	0.00	LX0	N
ATOM	2805	HE	ARG	1275	59.103	-1.631	4.570	0.00	0.00	LX0	H
ATOM	2806	CZ	ARG	1275	60.321	-0.221	5.389	1.00	0.00	LX0	C
ATOM	2807	NH1	ARG	1275	60.795	1.013	5.300	1.00	0.00	LX0	N
ATOM	2808	HH11	ARG	1275	61.289	1.420	6.089	0.00	0.00	LX0	H
ATOM	2809	HH12	ARG	1275	60.636	1.627	4.524	0.00	0.00	LX0	H
ATOM	2810	NH2	ARG	1275	60.610	-0.952	6.454	1.00	0.00	LX0	N
ATOM	2811	HH21	ARG	1275	61.180	-0.537	7.178	0.00	0.00	LX0	H
ATOM	2812	HH22	ARG	1275	60.232	-1.874	6.572	0.00	0.00	LX0	H
ATOM	2813	C	ARG	1275	56.901	2.617	7.222	1.00	0.00	LX0	C
ATOM	2814	O	ARG	1275	55.937	2.438	7.951	1.00	0.00	LX0	O
ATOM	2815	N	GLU	1276	58.090	3.071	7.651	1.00	0.00	LX0	N
ATOM	2816	H	GLU	1276	58.884	3.187	7.048	0.00	0.00	LX0	H
ATOM	2817	CA	GLU	1276	58.252	3.360	9.080	1.00	0.00	LX0	C
ATOM	2818	CB	GLU	1276	59.650	3.913	9.382	1.00	0.00	LX0	C

ATOM	2819	CG	GLU	1276	60.796	2.887	9.420	1.00	0.00	LX0	C
ATOM	2820	CD	GLU	1276	61.112	2.300	8.054	1.00	0.00	LX0	C
ATOM	2821	OE1	GLU	1276	61.031	3.007	7.053	1.00	0.00	LX0	O
ATOM	2822	OE2	GLU	1276	61.443	1.119	7.976	1.00	0.00	LX0	O
ATOM	2823	C	GLU	1276	57.204	4.289	9.681	1.00	0.00	LX0	C
ATOM	2824	O	GLU	1276	56.809	4.159	10.833	1.00	0.00	LX0	O
ATOM	2825	N	VAL	1277	56.755	5.237	8.844	1.00	0.00	LX0	N
ATOM	2826	H	VAL	1277	57.111	5.305	7.909	0.00	0.00	LX0	H
ATOM	2827	CA	VAL	1277	55.731	6.139	9.369	1.00	0.00	LX0	C
ATOM	2828	CB	VAL	1277	56.064	7.592	9.002	1.00	0.00	LX0	C
ATOM	2829	CG1	VAL	1277	57.366	8.031	9.674	1.00	0.00	LX0	C
ATOM	2830	CG2	VAL	1277	56.101	7.821	7.489	1.00	0.00	LX0	C
ATOM	2831	C	VAL	1277	54.281	5.801	9.026	1.00	0.00	LX0	C
ATOM	2832	O	VAL	1277	53.354	6.502	9.416	1.00	0.00	LX0	O
ATOM	2833	N	SER	1278	54.120	4.718	8.258	1.00	0.00	LX0	N
ATOM	2834	H	SER	1278	54.912	4.164	8.008	0.00	0.00	LX0	H
ATOM	2835	CA	SER	1278	52.788	4.442	7.723	1.00	0.00	LX0	C
ATOM	2836	CB	SER	1278	52.917	3.538	6.493	1.00	0.00	LX0	C
ATOM	2837	OG	SER	1278	53.444	2.252	6.863	1.00	0.00	LX0	O
ATOM	2838	HG	SER	1278	54.231	2.426	7.371	0.00	0.00	LX0	H
ATOM	2839	C	SER	1278	51.799	3.848	8.713	1.00	0.00	LX0	C
ATOM	2840	O	SER	1278	52.162	3.142	9.652	1.00	0.00	LX0	O
ATOM	2841	N	PHE	1279	50.513	4.094	8.416	1.00	0.00	LX0	N
ATOM	2842	H	PHE	1279	50.286	4.645	7.606	0.00	0.00	LX0	H
ATOM	2843	CA	PHE	1279	49.471	3.289	9.050	1.00	0.00	LX0	C
ATOM	2844	CB	PHE	1279	48.075	3.753	8.604	1.00	0.00	LX0	C
ATOM	2845	CG	PHE	1279	46.993	2.915	9.249	1.00	0.00	LX0	C
ATOM	2846	CD1	PHE	1279	46.718	3.066	10.625	1.00	0.00	LX0	C
ATOM	2847	CD2	PHE	1279	46.285	1.984	8.459	1.00	0.00	LX0	C
ATOM	2848	CE1	PHE	1279	45.722	2.269	11.219	1.00	0.00	LX0	C
ATOM	2849	CE2	PHE	1279	45.288	1.188	9.053	1.00	0.00	LX0	C
ATOM	2850	CZ	PHE	1279	45.017	1.338	10.429	1.00	0.00	LX0	C
ATOM	2851	C	PHE	1279	49.671	1.808	8.777	1.00	0.00	LX0	C
ATOM	2852	O	PHE	1279	49.541	0.974	9.663	1.00	0.00	LX0	O
ATOM	2853	N	TYR	1280	50.066	1.529	7.526	1.00	0.00	LX0	N
ATOM	2854	H	TYR	1280	50.061	2.282	6.861	0.00	0.00	LX0	H
ATOM	2855	CA	TYR	1280	50.438	0.170	7.129	1.00	0.00	LX0	C
ATOM	2856	CB	TYR	1280	51.043	0.234	5.721	1.00	0.00	LX0	C
ATOM	2857	CG	TYR	1280	51.327	-1.131	5.137	1.00	0.00	LX0	C
ATOM	2858	CD1	TYR	1280	52.671	-1.515	4.955	1.00	0.00	LX0	C
ATOM	2859	CE1	TYR	1280	52.952	-2.762	4.374	1.00	0.00	LX0	C
ATOM	2860	CD2	TYR	1280	50.254	-1.971	4.774	1.00	0.00	LX0	C
ATOM	2861	CE2	TYR	1280	50.536	-3.218	4.194	1.00	0.00	LX0	C
ATOM	2862	CZ	TYR	1280	51.881	-3.594	3.993	1.00	0.00	LX0	C
ATOM	2863	OH	TYR	1280	52.163	-4.810	3.403	1.00	0.00	LX0	O
ATOM	2864	HH	TYR	1280	51.336	-5.234	3.176	0.00	0.00	LX0	H
ATOM	2865	C	TYR	1280	51.336	-0.585	8.112	1.00	0.00	LX0	C
ATOM	2866	O	TYR	1280	51.095	-1.743	8.433	1.00	0.00	LX0	O
ATOM	2867	N	TYR	1281	52.369	0.129	8.586	1.00	0.00	LX0	N
ATOM	2868	H	TYR	1281	52.509	1.068	8.266	0.00	0.00	LX0	H
ATOM	2869	CA	TYR	1281	53.241	-0.482	9.593	1.00	0.00	LX0	C
ATOM	2870	CB	TYR	1281	54.656	0.095	9.504	1.00	0.00	LX0	C
ATOM	2871	CG	TYR	1281	55.622	-0.704	8.649	1.00	0.00	LX0	C
ATOM	2872	CD1	TYR	1281	55.188	-1.449	7.530	1.00	0.00	LX0	C
ATOM	2873	CE1	TYR	1281	56.138	-2.140	6.756	1.00	0.00	LX0	C
ATOM	2874	CD2	TYR	1281	56.983	-0.652	9.013	1.00	0.00	LX0	C
ATOM	2875	CE2	TYR	1281	57.933	-1.336	8.239	1.00	0.00	LX0	C
ATOM	2876	CZ	TYR	1281	57.502	-2.064	7.112	1.00	0.00	LX0	C
ATOM	2877	OH	TYR	1281	58.449	-2.711	6.332	1.00	0.00	LX0	O
ATOM	2878	HH	TYR	1281	58.059	-3.522	6.020	0.00	0.00	LX0	H
ATOM	2879	C	TYR	1281	52.786	-0.341	11.039	1.00	0.00	LX0	C

```
ATOM   2880  O    TYR  1281      53.292  -1.011  11.930  1.00  0.00      LX0  O
ATOM   2881  N    SER  1282      51.855   0.597  11.255  1.00  0.00      LX0  N
ATOM   2882  H    SER  1282      51.450   1.095  10.489  0.00  0.00      LX0  H
ATOM   2883  CA   SER  1282      51.564   0.951  12.642  1.00  0.00      LX0  C
ATOM   2884  CB   SER  1282      50.715   2.225  12.684  1.00  0.00      LX0  C
ATOM   2885  OG   SER  1282      49.375   1.953  12.253  1.00  0.00      LX0  O
ATOM   2886  HG   SER  1282      49.439   1.464  11.435  0.00  0.00      LX0  H
ATOM   2887  C    SER  1282      50.932  -0.148  13.483  1.00  0.00      LX0  C
ATOM   2888  O    SER  1282      50.254  -1.041  12.993  1.00  0.00      LX0  O
ATOM   2889  N    GLU  1283      51.137   0.007  14.800  1.00  0.00      LX0  N
ATOM   2890  H    GLU  1283      51.780   0.698  15.122  0.00  0.00      LX0  H
ATOM   2891  CA   GLU  1283      50.492  -0.887  15.766  1.00  0.00      LX0  C
ATOM   2892  CB   GLU  1283      50.939  -0.452  17.160  1.00  0.00      LX0  C
ATOM   2893  CG   GLU  1283      50.613  -1.417  18.299  1.00  0.00      LX0  C
ATOM   2894  CD   GLU  1283      51.220  -0.864  19.571  1.00  0.00      LX0  C
ATOM   2895  OE1  GLU  1283      52.173  -1.458  20.072  1.00  0.00      LX0  O
ATOM   2896  OE2  GLU  1283      50.750   0.169  20.046  1.00  0.00      LX0  O
ATOM   2897  C    GLU  1283      48.970  -0.970  15.639  1.00  0.00      LX0  C
ATOM   2898  O    GLU  1283      48.351  -2.020  15.757  1.00  0.00      LX0  O
ATOM   2899  N    GLU  1284      48.386   0.204  15.323  1.00  0.00      LX0  N
ATOM   2900  H    GLU  1284      48.946   1.024  15.230  0.00  0.00      LX0  H
ATOM   2901  CA   GLU  1284      46.936   0.249  15.103  1.00  0.00      LX0  C
ATOM   2902  CB   GLU  1284      46.480   1.701  14.895  1.00  0.00      LX0  C
ATOM   2903  CG   GLU  1284      45.131   2.112  15.521  1.00  0.00      LX0  C
ATOM   2904  CD   GLU  1284      43.916   1.551  14.792  1.00  0.00      LX0  C
ATOM   2905  OE1  GLU  1284      43.805   1.690  13.579  1.00  0.00      LX0  O
ATOM   2906  OE2  GLU  1284      43.030   0.987  15.425  1.00  0.00      LX0  O
ATOM   2907  C    GLU  1284      46.436  -0.655  13.981  1.00  0.00      LX0  C
ATOM   2908  O    GLU  1284      45.279  -1.060  13.944  1.00  0.00      LX0  O
ATOM   2909  N    ASN  1285      47.359  -0.991  13.065  1.00  0.00      LX0  N
ATOM   2910  H    ASN  1285      48.319  -0.739  13.194  0.00  0.00      LX0  H
ATOM   2911  CA   ASN  1285      46.982  -1.784  11.895  1.00  0.00      LX0  C
ATOM   2912  CB   ASN  1285      47.954  -1.509  10.746  1.00  0.00      LX0  C
ATOM   2913  CG   ASN  1285      47.366  -1.994   9.437  1.00  0.00      LX0  C
ATOM   2914  OD1  ASN  1285      46.159  -2.002   9.230  1.00  0.00      LX0  O
ATOM   2915  ND2  ASN  1285      48.277  -2.422   8.562  1.00  0.00      LX0  N
ATOM   2916 HD21 ASN  1285      49.256  -2.370   8.778  0.00  0.00      LX0  H
ATOM   2917 HD22 ASN  1285      47.973  -2.799   7.693  0.00  0.00      LX0  H
ATOM   2918  C    ASN  1285      46.776  -3.285  12.099  1.00  0.00      LX0  C
ATOM   2919  O    ASN  1285      47.290  -4.128  11.375  1.00  0.00      LX0  O
ATOM   2920  N    LYS  1286      45.940  -3.592  13.105  1.00  0.00      LX0  N
ATOM   2921  H    LYS  1286      45.704  -2.870  13.750  0.00  0.00      LX0  H
ATOM   2922  CA   LYS  1286      45.234  -4.874  13.174  1.00  0.00      LX0  C
ATOM   2923  CB   LYS  1286      43.905  -4.824  12.395  1.00  0.00      LX0  C
ATOM   2924  CG   LYS  1286      42.981  -3.628  12.689  1.00  0.00      LX0  C
ATOM   2925  CD   LYS  1286      43.234  -2.397  11.808  1.00  0.00      LX0  C
ATOM   2926  CE   LYS  1286      42.400  -1.164  12.172  1.00  0.00      LX0  C
ATOM   2927  NZ   LYS  1286      42.679  -0.717  13.538  1.00  0.00      LX0  N
ATOM   2928  HZ1  LYS  1286      42.029  -1.121  14.237  0.00  0.00      LX0  H
ATOM   2929  HZ2  LYS  1286      43.653  -0.912  13.831  0.00  0.00      LX0  H
ATOM   2930  HZ3  LYS  1286      42.630   0.322  13.640  0.00  0.00      LX0  H
ATOM   2931  C    LYS  1286      46.022  -6.121  12.795  1.00  0.00      LX0  C
ATOM   2932  O    LYS  1286      45.677  -7.239  13.163  1.00  0.00      LX0  O
TER
```
