# Supplementary material for: Structural analysis of hubs in human NR-RTK network
Source: Biol Direct. 2011 Oct 5;6:49. doi: 10.1186/1745-6150-6-49 (PMC3220635; doi:10.1186/1745-6150-6-49)
Supplement: Additional file 10 — ESR1-IGF1R-PGR. ESR1-IGF1-R-PGR complex structure. [file 1745-6150-6-49-S10.PDF]

```
HEADER ESR1-IGF1R-PGR
REMARK original generated coordinate pdb file
ATOM      1  N   ALA   156      10.627  12.174   8.322  1.00  0.00      RX0  N
ATOM      2  H   ALA   156      11.188  11.347   8.269  1.00  0.00      RX0  H
ATOM      3  CA  ALA   156       9.864  12.527   9.538  1.00  0.00      RX0  C
ATOM      4  CB  ALA   156      10.757  12.402  10.765  1.00  0.00      RX0  C
ATOM      5  C   ALA   156       9.377  13.991   9.496  1.00  0.00      RX0  C
ATOM      6  O   ALA   156       9.121  14.644  10.500  1.00  0.00      RX0  O
ATOM      7  N   LEU   157       9.039  14.416   8.289  1.00  0.00      RX0  N
ATOM      8  H   LEU   157       9.128  13.766   7.531  1.00  0.00      RX0  H
ATOM      9  CA  LEU   157       8.850  15.849   7.979  1.00  0.00      RX0  C
ATOM     10  CB  LEU   157       9.706  16.234   6.779  1.00  0.00      RX0  C
ATOM     11  CG  LEU   157      11.042  15.499   6.731  1.00  0.00      RX0  C
ATOM     12  CD1 LEU   157      11.660  15.593   5.341  1.00  0.00      RX0  C
ATOM     13  CD2 LEU   157      11.996  15.936   7.841  1.00  0.00      RX0  C
ATOM     14  C   LEU   157       7.387  16.184   7.642  1.00  0.00      RX0  C
ATOM     15  O   LEU   157       7.075  17.216   7.039  1.00  0.00      RX0  O
ATOM     16  N   SER   158       6.522  15.244   7.955  1.00  0.00      RX0  N
ATOM     17  H   SER   158       6.875  14.326   8.100  1.00  0.00      RX0  H
ATOM     18  CA  SER   158       5.051  15.362   7.847  1.00  0.00      RX0  C
ATOM     19  CB  SER   158       4.729  14.894   6.436  1.00  0.00      RX0  C
ATOM     20  OG  SER   158       5.938  15.038   5.679  1.00  0.00      RX0  O
ATOM     21  HG  SER   158       6.094  15.978   5.623  1.00  0.00      RX0  H
ATOM     22  C   SER   158       4.335  14.560   8.949  1.00  0.00      RX0  C
ATOM     23  O   SER   158       3.148  14.670   9.188  1.00  0.00      RX0  O
ATOM     24  N   LEU   159       5.132  13.681   9.591  1.00  0.00      RX0  N
ATOM     25  H   LEU   159       6.083  13.586   9.325  1.00  0.00      RX0  H
ATOM     26  CA  LEU   159       4.759  12.951  10.797  1.00  0.00      RX0  C
ATOM     27  CB  LEU   159       5.860  11.985  11.247  1.00  0.00      RX0  C
ATOM     28  CG  LEU   159       5.904  10.626  10.539  1.00  0.00      RX0  C
ATOM     29  CD1 LEU   159       6.366  10.705   9.082  1.00  0.00      RX0  C
ATOM     30  CD2 LEU   159       6.746   9.630  11.339  1.00  0.00      RX0  C
ATOM     31  C   LEU   159       4.518  13.965  11.920  1.00  0.00      RX0  C
ATOM     32  O   LEU   159       5.291  14.932  12.058  1.00  0.00      RX0  O
ATOM     33  N   THR   160       3.434  13.807  12.646  1.00  0.00      RX0  N
ATOM     34  H   THR   160       2.847  13.019  12.460  1.00  0.00      RX0  H
ATOM     35  CA  THR   160       3.156  14.665  13.825  1.00  0.00      RX0  C
ATOM     36  CB  THR   160       1.666  14.648  14.226  1.00  0.00      RX0  C
ATOM     37  OG1 THR   160       1.372  15.747  15.093  1.00  0.00      RX0  O
ATOM     38  HG1 THR   160       0.424  15.812  15.136  1.00  0.00      RX0  H
ATOM     39  CG2 THR   160       1.177  13.340  14.832  1.00  0.00      RX0  C
ATOM     40  C   THR   160       4.203  14.411  14.921  1.00  0.00      RX0  C
ATOM     41  O   THR   160       4.902  13.383  14.913  1.00  0.00      RX0  O
ATOM     42  N   ALA   161       4.153  15.229  15.953  1.00  0.00      RX0  N
ATOM     43  H   ALA   161       3.461  15.953  15.917  1.00  0.00      RX0  H
ATOM     44  CA  ALA   161       4.942  15.044  17.184  1.00  0.00      RX0  C
ATOM     45  CB  ALA   161       4.755  16.234  18.117  1.00  0.00      RX0  C
ATOM     46  C   ALA   161       4.543  13.746  17.920  1.00  0.00      RX0  C
ATOM     47  O   ALA   161       5.387  12.950  18.288  1.00  0.00      RX0  O
ATOM     48  N   ASP   162       3.226  13.461  17.917  1.00  0.00      RX0  N
ATOM     49  H   ASP   162       2.570  14.188  17.722  1.00  0.00      RX0  H
ATOM     50  CA  ASP   162       2.690  12.196  18.469  1.00  0.00      RX0  C
ATOM     51  CB  ASP   162       1.202  12.325  18.828  1.00  0.00      RX0  C
ATOM     52  CG  ASP   162       1.055  13.198  20.077  1.00  0.00      RX0  C
ATOM     53  OD1 ASP   162       1.807  14.156  20.247  1.00  0.00      RX0  O
ATOM     54  OD2 ASP   162       0.203  12.927  20.922  1.00  0.00      RX0  O
ATOM     55  C   ASP   162       3.088  10.948  17.668  1.00  0.00      RX0  C
ATOM     56  O   ASP   162       3.397   9.903  18.257  1.00  0.00      RX0  O
ATOM     57  N   GLN   163       3.164  11.087  16.353  1.00  0.00      RX0  N
ATOM     58  H   GLN   163       3.079  12.014  15.999  1.00  0.00      RX0  H
ATOM     59  CA  GLN   163       3.593   9.998  15.449  1.00  0.00      RX0  C
```

ATOM	60	CB	GLN	163	3.250	10.287	13.995	1.00	0.00	RX0	C
ATOM	61	CG	GLN	163	1.824	9.881	13.632	1.00	0.00	RX0	C
ATOM	62	CD	GLN	163	1.555	10.381	12.235	1.00	0.00	RX0	C
ATOM	63	OE1	GLN	163	2.081	11.412	11.828	1.00	0.00	RX0	O
ATOM	64	NE2	GLN	163	0.721	9.603	11.524	1.00	0.00	RX0	N
ATOM	65	HE21	GLN	163	0.334	8.771	11.924	1.00	0.00	RX0	H
ATOM	66	HE22	GLN	163	0.465	9.829	10.584	1.00	0.00	RX0	H
ATOM	67	C	GLN	163	5.089	9.698	15.572	1.00	0.00	RX0	C
ATOM	68	O	GLN	163	5.477	8.537	15.545	1.00	0.00	RX0	O
ATOM	69	N	MET	164	5.882	10.740	15.840	1.00	0.00	RX0	N
ATOM	70	H	MET	164	5.492	11.661	15.891	1.00	0.00	RX0	H
ATOM	71	CA	MET	164	7.331	10.587	16.060	1.00	0.00	RX0	C
ATOM	72	CB	MET	164	8.015	11.955	16.082	1.00	0.00	RX0	C
ATOM	73	CG	MET	164	9.451	11.879	16.606	1.00	0.00	RX0	C
ATOM	74	SD	MET	164	10.536	10.864	15.595	1.00	0.00	RX0	S
ATOM	75	CE	MET	164	11.024	12.134	14.424	1.00	0.00	RX0	C
ATOM	76	C	MET	164	7.610	9.825	17.366	1.00	0.00	RX0	C
ATOM	77	O	MET	164	8.404	8.887	17.381	1.00	0.00	RX0	O
ATOM	78	N	VAL	165	6.828	10.145	18.396	1.00	0.00	RX0	N
ATOM	79	H	VAL	165	6.165	10.888	18.278	1.00	0.00	RX0	H
ATOM	80	CA	VAL	165	6.992	9.563	19.744	1.00	0.00	RX0	C
ATOM	81	CB	VAL	165	6.101	10.259	20.778	1.00	0.00	RX0	C
ATOM	82	CG1	VAL	165	6.181	9.567	22.138	1.00	0.00	RX0	C
ATOM	83	CG2	VAL	165	6.451	11.732	20.918	1.00	0.00	RX0	C
ATOM	84	C	VAL	165	6.649	8.067	19.731	1.00	0.00	RX0	C
ATOM	85	O	VAL	165	7.442	7.255	20.191	1.00	0.00	RX0	O
ATOM	86	N	SER	166	5.467	7.742	19.205	1.00	0.00	RX0	N
ATOM	87	H	SER	166	4.814	8.431	18.872	1.00	0.00	RX0	H
ATOM	88	CA	SER	166	5.029	6.335	19.106	1.00	0.00	RX0	C
ATOM	89	CB	SER	166	3.569	6.385	18.712	1.00	0.00	RX0	C
ATOM	90	OG	SER	166	3.007	7.453	19.477	1.00	0.00	RX0	O
ATOM	91	HG	SER	166	3.347	7.366	20.358	1.00	0.00	RX0	H
ATOM	92	C	SER	166	5.941	5.501	18.195	1.00	0.00	RX0	C
ATOM	93	O	SER	166	6.295	4.379	18.542	1.00	0.00	RX0	O
ATOM	94	N	ALA	167	6.456	6.129	17.133	1.00	0.00	RX0	N
ATOM	95	H	ALA	167	6.171	7.064	16.912	1.00	0.00	RX0	H
ATOM	96	CA	ALA	167	7.397	5.466	16.208	1.00	0.00	RX0	C
ATOM	97	CB	ALA	167	7.731	6.357	15.012	1.00	0.00	RX0	C
ATOM	98	C	ALA	167	8.706	5.103	16.927	1.00	0.00	RX0	C
ATOM	99	O	ALA	167	9.113	3.946	16.932	1.00	0.00	RX0	O
ATOM	100	N	LEU	168	9.179	6.054	17.734	1.00	0.00	RX0	N
ATOM	101	H	LEU	168	8.734	6.951	17.738	1.00	0.00	RX0	H
ATOM	102	CA	LEU	168	10.385	5.875	18.562	1.00	0.00	RX0	C
ATOM	103	CB	LEU	168	10.907	7.213	19.074	1.00	0.00	RX0	C
ATOM	104	CG	LEU	168	11.571	8.041	17.977	1.00	0.00	RX0	C
ATOM	105	CD1	LEU	168	12.103	9.369	18.519	1.00	0.00	RX0	C
ATOM	106	CD2	LEU	168	12.651	7.238	17.253	1.00	0.00	RX0	C
ATOM	107	C	LEU	168	10.197	4.896	19.724	1.00	0.00	RX0	C
ATOM	108	O	LEU	168	11.077	4.078	19.994	1.00	0.00	RX0	O
ATOM	109	N	LEU	169	9.007	4.918	20.317	1.00	0.00	RX0	N
ATOM	110	H	LEU	169	8.333	5.589	20.011	1.00	0.00	RX0	H
ATOM	111	CA	LEU	169	8.640	3.970	21.384	1.00	0.00	RX0	C
ATOM	112	CB	LEU	169	7.358	4.393	22.101	1.00	0.00	RX0	C
ATOM	113	CG	LEU	169	7.538	5.639	22.970	1.00	0.00	RX0	C
ATOM	114	CD1	LEU	169	6.226	6.046	23.641	1.00	0.00	RX0	C
ATOM	115	CD2	LEU	169	8.667	5.470	23.988	1.00	0.00	RX0	C
ATOM	116	C	LEU	169	8.505	2.536	20.864	1.00	0.00	RX0	C
ATOM	117	O	LEU	169	9.003	1.602	21.486	1.00	0.00	RX0	O
ATOM	118	N	ASP	170	7.977	2.423	19.645	1.00	0.00	RX0	N
ATOM	119	H	ASP	170	7.672	3.241	19.159	1.00	0.00	RX0	H
ATOM	120	CA	ASP	170	7.822	1.133	18.952	1.00	0.00	RX0	C

ATOM	121	CB	ASP	170	6.882	1.395	17.759	1.00	0.00	RX0	C
ATOM	122	CG	ASP	170	6.638	0.242	16.794	1.00	0.00	RX0	C
ATOM	123	OD1	ASP	170	7.092	-0.877	16.999	1.00	0.00	RX0	O
ATOM	124	OD2	ASP	170	6.018	0.475	15.760	1.00	0.00	RX0	O
ATOM	125	C	ASP	170	9.164	0.506	18.541	1.00	0.00	RX0	C
ATOM	126	O	ASP	170	9.313	-0.704	18.571	1.00	0.00	RX0	O
ATOM	127	N	ALA	171	10.119	1.387	18.228	1.00	0.00	RX0	N
ATOM	128	H	ALA	171	9.894	2.361	18.290	1.00	0.00	RX0	H
ATOM	129	CA	ALA	171	11.447	1.008	17.717	1.00	0.00	RX0	C
ATOM	130	CB	ALA	171	12.102	2.218	17.052	1.00	0.00	RX0	C
ATOM	131	C	ALA	171	12.418	0.479	18.779	1.00	0.00	RX0	C
ATOM	132	O	ALA	171	13.427	-0.136	18.431	1.00	0.00	RX0	O
ATOM	133	N	GLU	172	12.125	0.739	20.058	1.00	0.00	RX0	N
ATOM	134	H	GLU	172	11.281	1.230	20.277	1.00	0.00	RX0	H
ATOM	135	CA	GLU	172	13.017	0.374	21.170	1.00	0.00	RX0	C
ATOM	136	CB	GLU	172	12.365	0.709	22.510	1.00	0.00	RX0	C
ATOM	137	CG	GLU	172	12.323	2.228	22.680	1.00	0.00	RX0	C
ATOM	138	CD	GLU	172	13.728	2.778	22.513	1.00	0.00	RX0	C
ATOM	139	OE1	GLU	172	14.555	2.616	23.411	1.00	0.00	RX0	O
ATOM	140	OE2	GLU	172	14.033	3.385	21.485	1.00	0.00	RX0	O
ATOM	141	C	GLU	172	13.554	-1.065	21.099	1.00	0.00	RX0	C
ATOM	142	O	GLU	172	12.785	-2.004	20.837	1.00	0.00	RX0	O
ATOM	143	N	PRO	173	14.865	-1.209	21.269	1.00	0.00	RX0	N
ATOM	144	CD	PRO	173	15.789	-0.095	21.453	1.00	0.00	RX0	C
ATOM	145	CA	PRO	173	15.538	-2.517	21.328	1.00	0.00	RX0	C
ATOM	146	CB	PRO	173	17.014	-2.108	21.199	1.00	0.00	RX0	C
ATOM	147	CG	PRO	173	17.103	-0.741	21.869	1.00	0.00	RX0	C
ATOM	148	C	PRO	173	15.206	-3.249	22.640	1.00	0.00	RX0	C
ATOM	149	O	PRO	173	14.829	-2.595	23.631	1.00	0.00	RX0	O
ATOM	150	N	PRO	174	15.294	-4.574	22.646	1.00	0.00	RX0	N
ATOM	151	CD	PRO	174	15.605	-5.388	21.475	1.00	0.00	RX0	C
ATOM	152	CA	PRO	174	15.084	-5.400	23.852	1.00	0.00	RX0	C
ATOM	153	CB	PRO	174	14.968	-6.812	23.273	1.00	0.00	RX0	C
ATOM	154	CG	PRO	174	15.853	-6.784	22.031	1.00	0.00	RX0	C
ATOM	155	C	PRO	174	16.250	-5.248	24.838	1.00	0.00	RX0	C
ATOM	156	O	PRO	174	17.379	-4.922	24.444	1.00	0.00	RX0	O
ATOM	157	N	ILE	175	15.956	-5.464	26.106	1.00	0.00	RX0	N
ATOM	158	H	ILE	175	15.039	-5.788	26.327	1.00	0.00	RX0	H
ATOM	159	CA	ILE	175	16.988	-5.556	27.159	1.00	0.00	RX0	C
ATOM	160	CB	ILE	175	16.446	-5.212	28.551	1.00	0.00	RX0	C
ATOM	161	CG2	ILE	175	17.623	-5.099	29.520	1.00	0.00	RX0	C
ATOM	162	CG1	ILE	175	15.624	-3.918	28.563	1.00	0.00	RX0	C
ATOM	163	CD1	ILE	175	14.111	-4.134	28.454	1.00	0.00	RX0	C
ATOM	164	C	ILE	175	17.586	-6.969	27.112	1.00	0.00	RX0	C
ATOM	165	O	ILE	175	16.886	-7.963	27.343	1.00	0.00	RX0	O
ATOM	166	N	LEU	176	18.884	-7.017	26.884	1.00	0.00	RX0	N
ATOM	167	H	LEU	176	19.406	-6.165	26.837	1.00	0.00	RX0	H
ATOM	168	CA	LEU	176	19.617	-8.291	26.770	1.00	0.00	RX0	C
ATOM	169	CB	LEU	176	20.651	-8.225	25.648	1.00	0.00	RX0	C
ATOM	170	CG	LEU	176	20.016	-7.992	24.277	1.00	0.00	RX0	C
ATOM	171	CD1	LEU	176	21.074	-7.935	23.179	1.00	0.00	RX0	C
ATOM	172	CD2	LEU	176	18.932	-9.024	23.961	1.00	0.00	RX0	C
ATOM	173	C	LEU	176	20.277	-8.690	28.089	1.00	0.00	RX0	C
ATOM	174	O	LEU	176	20.563	-7.852	28.952	1.00	0.00	RX0	O
ATOM	175	N	TYR	177	20.459	-9.989	28.237	1.00	0.00	RX0	N
ATOM	176	H	TYR	177	20.229	-10.605	27.487	1.00	0.00	RX0	H
ATOM	177	CA	TYR	177	21.114	-10.573	29.420	1.00	0.00	RX0	C
ATOM	178	CB	TYR	177	20.420	-11.864	29.855	1.00	0.00	RX0	C
ATOM	179	CG	TYR	177	19.153	-11.561	30.618	1.00	0.00	RX0	C
ATOM	180	CD1	TYR	177	18.029	-11.069	29.963	1.00	0.00	RX0	C
ATOM	181	CE1	TYR	177	16.866	-10.812	30.677	1.00	0.00	RX0	C

ATOM	182	CD2	TYR	177	19.113	-11.790	31.988	1.00	0.00	RX0	C
ATOM	183	CE2	TYR	177	17.948	-11.540	32.701	1.00	0.00	RX0	C
ATOM	184	CZ	TYR	177	16.825	-11.049	32.046	1.00	0.00	RX0	C
ATOM	185	OH	TYR	177	15.671	-10.793	32.757	1.00	0.00	RX0	O
ATOM	186	HH	TYR	177	15.779	-11.075	33.655	1.00	0.00	RX0	H
ATOM	187	C	TYR	177	22.589	-10.858	29.163	1.00	0.00	RX0	C
ATOM	188	O	TYR	177	22.985	-11.163	28.046	1.00	0.00	RX0	O
ATOM	189	N	SER	178	23.381	-10.750	30.220	1.00	0.00	RX0	N
ATOM	190	H	SER	178	23.026	-10.499	31.124	1.00	0.00	RX0	H
ATOM	191	CA	SER	178	24.788	-11.188	30.183	1.00	0.00	RX0	C
ATOM	192	CB	SER	178	25.490	-10.624	31.403	1.00	0.00	RX0	C
ATOM	193	OG	SER	178	25.205	-9.226	31.425	1.00	0.00	RX0	O
ATOM	194	HG	SER	178	25.574	-8.863	30.630	1.00	0.00	RX0	H
ATOM	195	C	SER	178	24.834	-12.718	30.070	1.00	0.00	RX0	C
ATOM	196	O	SER	178	23.999	-13.413	30.674	1.00	0.00	RX0	O
ATOM	197	N	GLU	179	25.827	-13.218	29.362	1.00	0.00	RX0	N
ATOM	198	H	GLU	179	26.532	-12.639	28.946	1.00	0.00	RX0	H
ATOM	199	CA	GLU	179	26.033	-14.670	29.175	1.00	0.00	RX0	C
ATOM	200	CB	GLU	179	26.345	-15.031	27.725	1.00	0.00	RX0	C
ATOM	201	CG	GLU	179	25.805	-14.057	26.688	1.00	0.00	RX0	C
ATOM	202	CD	GLU	179	26.834	-12.989	26.353	1.00	0.00	RX0	C
ATOM	203	OE1	GLU	179	27.323	-12.996	25.233	1.00	0.00	RX0	O
ATOM	204	OE2	GLU	179	27.068	-12.079	27.149	1.00	0.00	RX0	O
ATOM	205	C	GLU	179	27.192	-15.208	30.012	1.00	0.00	RX0	C
ATOM	206	O	GLU	179	28.361	-15.227	29.589	1.00	0.00	RX0	O
ATOM	207	N	TYR	180	26.873	-15.475	31.254	1.00	0.00	RX0	N
ATOM	208	H	TYR	180	25.923	-15.393	31.565	1.00	0.00	RX0	H
ATOM	209	CA	TYR	180	27.735	-16.233	32.177	1.00	0.00	RX0	C
ATOM	210	CB	TYR	180	28.494	-15.322	33.155	1.00	0.00	RX0	C
ATOM	211	CG	TYR	180	27.553	-14.674	34.143	1.00	0.00	RX0	C
ATOM	212	CD1	TYR	180	26.927	-13.474	33.830	1.00	0.00	RX0	C
ATOM	213	CE1	TYR	180	26.023	-12.913	34.723	1.00	0.00	RX0	C
ATOM	214	CD2	TYR	180	27.309	-15.286	35.368	1.00	0.00	RX0	C
ATOM	215	CE2	TYR	180	26.393	-14.734	36.253	1.00	0.00	RX0	C
ATOM	216	CZ	TYR	180	25.735	-13.558	35.920	1.00	0.00	RX0	C
ATOM	217	OH	TYR	180	24.786	-13.038	36.773	1.00	0.00	RX0	O
ATOM	218	HH	TYR	180	24.687	-12.107	36.594	1.00	0.00	RX0	H
ATOM	219	C	TYR	180	26.838	-17.226	32.909	1.00	0.00	RX0	C
ATOM	220	O	TYR	180	25.642	-16.953	33.094	1.00	0.00	RX0	O
ATOM	221	N	ASP	181	27.404	-18.345	33.318	1.00	0.00	RX0	N
ATOM	222	H	ASP	181	28.392	-18.460	33.264	1.00	0.00	RX0	H
ATOM	223	CA	ASP	181	26.630	-19.347	34.059	1.00	0.00	RX0	C
ATOM	224	CB	ASP	181	27.318	-20.705	34.095	1.00	0.00	RX0	C
ATOM	225	CG	ASP	181	26.527	-21.560	35.060	1.00	0.00	RX0	C
ATOM	226	OD1	ASP	181	25.306	-21.577	34.967	1.00	0.00	RX0	O
ATOM	227	OD2	ASP	181	27.118	-22.172	35.939	1.00	0.00	RX0	O
ATOM	228	C	ASP	181	26.420	-18.851	35.504	1.00	0.00	RX0	C
ATOM	229	O	ASP	181	27.391	-18.832	36.273	1.00	0.00	RX0	O
ATOM	230	N	PRO	182	25.185	-18.489	35.856	1.00	0.00	RX0	N
ATOM	231	CD	PRO	182	24.009	-18.601	34.995	1.00	0.00	RX0	C
ATOM	232	CA	PRO	182	24.825	-17.989	37.201	1.00	0.00	RX0	C
ATOM	233	CB	PRO	182	23.394	-17.487	36.998	1.00	0.00	RX0	C
ATOM	234	CG	PRO	182	22.821	-18.409	35.928	1.00	0.00	RX0	C
ATOM	235	C	PRO	182	24.941	-19.052	38.308	1.00	0.00	RX0	C
ATOM	236	O	PRO	182	24.654	-18.763	39.474	1.00	0.00	RX0	O
ATOM	237	N	THR	183	25.345	-20.259	37.948	1.00	0.00	RX0	N
ATOM	238	H	THR	183	25.556	-20.521	37.004	1.00	0.00	RX0	H
ATOM	239	CA	THR	183	25.568	-21.363	38.913	1.00	0.00	RX0	C
ATOM	240	CB	THR	183	24.915	-22.567	38.265	1.00	0.00	RX0	C
ATOM	241	OG1	THR	183	23.896	-22.082	37.378	1.00	0.00	RX0	O
ATOM	242	HG1	THR	183	24.336	-22.014	36.528	1.00	0.00	RX0	H

ATOM	243	CG2	THR	183	24.347	-23.553	39.287	1.00	0.00	RX0	C
ATOM	244	C	THR	183	27.063	-21.532	39.218	1.00	0.00	RX0	C
ATOM	245	O	THR	183	27.455	-22.345	40.058	1.00	0.00	RX0	O
ATOM	246	N	ARG	184	27.887	-20.699	38.573	1.00	0.00	RX0	N
ATOM	247	H	ARG	184	27.521	-20.000	37.955	1.00	0.00	RX0	H
ATOM	248	CA	ARG	184	29.343	-20.701	38.681	1.00	0.00	RX0	C
ATOM	249	CB	ARG	184	29.931	-20.838	37.278	1.00	0.00	RX0	C
ATOM	250	CG	ARG	184	30.366	-22.276	36.994	1.00	0.00	RX0	C
ATOM	251	CD	ARG	184	30.803	-22.466	35.542	1.00	0.00	RX0	C
ATOM	252	NE	ARG	184	31.547	-21.300	35.069	1.00	0.00	RX0	N
ATOM	253	HE	ARG	184	30.980	-20.605	34.618	1.00	0.00	RX0	H
ATOM	254	CZ	ARG	184	32.881	-21.158	35.338	1.00	0.00	RX0	C
ATOM	255	NH1	ARG	184	33.554	-22.119	36.000	1.00	0.00	RX0	N
ATOM	256	HH11	ARG	184	34.526	-21.930	36.252	1.00	0.00	RX0	H
ATOM	257	HH12	ARG	184	33.162	-22.994	36.280	1.00	0.00	RX0	H
ATOM	258	NH2	ARG	184	33.530	-20.046	34.945	1.00	0.00	RX0	N
ATOM	259	HH21	ARG	184	34.524	-19.991	35.144	1.00	0.00	RX0	H
ATOM	260	HH22	ARG	184	33.105	-19.271	34.478	1.00	0.00	RX0	H
ATOM	261	C	ARG	184	29.836	-19.410	39.407	1.00	0.00	RX0	C
ATOM	262	O	ARG	184	29.116	-18.390	39.334	1.00	0.00	RX0	O
ATOM	263	N	PRO	185	30.940	-19.460	40.113	1.00	0.00	RX0	N
ATOM	264	CD	PRO	185	31.743	-20.664	40.296	1.00	0.00	RX0	C
ATOM	265	CA	PRO	185	31.574	-18.285	40.781	1.00	0.00	RX0	C
ATOM	266	CB	PRO	185	32.858	-18.870	41.383	1.00	0.00	RX0	C
ATOM	267	CG	PRO	185	33.142	-20.137	40.582	1.00	0.00	RX0	C
ATOM	268	C	PRO	185	31.820	-17.125	39.813	1.00	0.00	RX0	C
ATOM	269	O	PRO	185	31.836	-17.275	38.592	1.00	0.00	RX0	O
ATOM	270	N	PHE	186	32.164	-15.998	40.422	1.00	0.00	RX0	N
ATOM	271	H	PHE	186	32.240	-15.993	41.417	1.00	0.00	RX0	H
ATOM	272	CA	PHE	186	32.333	-14.726	39.697	1.00	0.00	RX0	C
ATOM	273	CB	PHE	186	31.517	-13.614	40.357	1.00	0.00	RX0	C
ATOM	274	CG	PHE	186	31.420	-12.439	39.412	1.00	0.00	RX0	C
ATOM	275	CD1	PHE	186	30.869	-12.612	38.148	1.00	0.00	RX0	C
ATOM	276	CD2	PHE	186	31.883	-11.187	39.802	1.00	0.00	RX0	C
ATOM	277	CE1	PHE	186	30.784	-11.536	37.272	1.00	0.00	RX0	C
ATOM	278	CE2	PHE	186	31.797	-10.111	38.926	1.00	0.00	RX0	C
ATOM	279	CZ	PHE	186	31.249	-10.286	37.660	1.00	0.00	RX0	C
ATOM	280	C	PHE	186	33.791	-14.305	39.507	1.00	0.00	RX0	C
ATOM	281	O	PHE	186	34.127	-13.678	38.496	1.00	0.00	RX0	O
ATOM	282	N	SER	187	34.655	-14.802	40.380	1.00	0.00	RX0	N
ATOM	283	H	SER	187	34.311	-15.351	41.137	1.00	0.00	RX0	H
ATOM	284	CA	SER	187	36.113	-14.542	40.380	1.00	0.00	RX0	C
ATOM	285	CB	SER	187	36.611	-15.271	41.614	1.00	0.00	RX0	C
ATOM	286	OG	SER	187	35.492	-15.361	42.515	1.00	0.00	RX0	O
ATOM	287	HG	SER	187	35.869	-15.438	43.385	1.00	0.00	RX0	H
ATOM	288	C	SER	187	36.764	-14.980	39.057	1.00	0.00	RX0	C
ATOM	289	O	SER	187	37.834	-14.531	38.683	1.00	0.00	RX0	O
ATOM	290	N	GLU	188	36.054	-15.878	38.369	1.00	0.00	RX0	N
ATOM	291	H	GLU	188	35.126	-16.109	38.649	1.00	0.00	RX0	H
ATOM	292	CA	GLU	188	36.561	-16.586	37.191	1.00	0.00	RX0	C
ATOM	293	CB	GLU	188	36.157	-18.051	37.352	1.00	0.00	RX0	C
ATOM	294	CG	GLU	188	36.900	-19.068	36.488	1.00	0.00	RX0	C
ATOM	295	CD	GLU	188	36.075	-20.335	36.476	1.00	0.00	RX0	C
ATOM	296	OE1	GLU	188	35.330	-20.574	37.418	1.00	0.00	RX0	O
ATOM	297	OE2	GLU	188	36.063	-21.037	35.472	1.00	0.00	RX0	O
ATOM	298	C	GLU	188	36.028	-16.018	35.856	1.00	0.00	RX0	C
ATOM	299	O	GLU	188	36.494	-16.416	34.788	1.00	0.00	RX0	O
ATOM	300	N	ALA	189	35.058	-15.107	35.914	1.00	0.00	RX0	N
ATOM	301	H	ALA	189	34.827	-14.686	36.794	1.00	0.00	RX0	H
ATOM	302	CA	ALA	189	34.543	-14.432	34.708	1.00	0.00	RX0	C
ATOM	303	CB	ALA	189	33.067	-14.081	34.891	1.00	0.00	RX0	C

ATOM	304	C	ALA	189	35.336	-13.151	34.407	1.00	0.00	RX0	C
ATOM	305	O	ALA	189	35.533	-12.292	35.270	1.00	0.00	RX0	O
ATOM	306	N	SER	190	35.819	-13.065	33.173	1.00	0.00	RX0	N
ATOM	307	H	SER	190	35.612	-13.815	32.548	1.00	0.00	RX0	H
ATOM	308	CA	SER	190	36.430	-11.825	32.646	1.00	0.00	RX0	C
ATOM	309	CB	SER	190	37.099	-12.195	31.297	1.00	0.00	RX0	C
ATOM	310	OG	SER	190	38.255	-11.389	30.965	1.00	0.00	RX0	O
ATOM	311	HG	SER	190	38.783	-11.424	31.764	1.00	0.00	RX0	H
ATOM	312	C	SER	190	35.341	-10.761	32.513	1.00	0.00	RX0	C
ATOM	313	O	SER	190	34.465	-10.869	31.639	1.00	0.00	RX0	O
ATOM	314	N	MET	191	35.401	-9.751	33.361	1.00	0.00	RX0	N
ATOM	315	H	MET	191	36.036	-9.822	34.132	1.00	0.00	RX0	H
ATOM	316	CA	MET	191	34.414	-8.652	33.337	1.00	0.00	RX0	C
ATOM	317	CB	MET	191	34.642	-7.665	34.478	1.00	0.00	RX0	C
ATOM	318	CG	MET	191	33.545	-6.599	34.497	1.00	0.00	RX0	C
ATOM	319	SD	MET	191	33.636	-5.542	35.945	1.00	0.00	RX0	S
ATOM	320	CE	MET	191	33.284	-6.804	37.181	1.00	0.00	RX0	C
ATOM	321	C	MET	191	34.384	-7.939	31.976	1.00	0.00	RX0	C
ATOM	322	O	MET	191	33.329	-7.841	31.363	1.00	0.00	RX0	O
ATOM	323	N	MET	192	35.577	-7.654	31.438	1.00	0.00	RX0	N
ATOM	324	H	MET	192	36.383	-7.741	32.020	1.00	0.00	RX0	H
ATOM	325	CA	MET	192	35.697	-7.082	30.089	1.00	0.00	RX0	C
ATOM	326	CB	MET	192	37.136	-6.648	29.804	1.00	0.00	RX0	C
ATOM	327	CG	MET	192	37.280	-5.929	28.459	1.00	0.00	RX0	C
ATOM	328	SD	MET	192	36.169	-4.521	28.284	1.00	0.00	RX0	S
ATOM	329	CE	MET	192	36.763	-3.523	29.660	1.00	0.00	RX0	C
ATOM	330	C	MET	192	35.151	-8.021	28.999	1.00	0.00	RX0	C
ATOM	331	O	MET	192	34.484	-7.587	28.093	1.00	0.00	RX0	O
ATOM	332	N	GLY	193	35.358	-9.342	29.220	1.00	0.00	RX0	N
ATOM	333	H	GLY	193	35.695	-9.611	30.118	1.00	0.00	RX0	H
ATOM	334	CA	GLY	193	34.804	-10.378	28.330	1.00	0.00	RX0	C
ATOM	335	C	GLY	193	33.267	-10.338	28.334	1.00	0.00	RX0	C
ATOM	336	O	GLY	193	32.637	-10.184	27.296	1.00	0.00	RX0	O
ATOM	337	N	LEU	194	32.696	-10.293	29.537	1.00	0.00	RX0	N
ATOM	338	H	LEU	194	33.261	-10.304	30.358	1.00	0.00	RX0	H
ATOM	339	CA	LEU	194	31.235	-10.169	29.722	1.00	0.00	RX0	C
ATOM	340	CB	LEU	194	30.847	-10.225	31.198	1.00	0.00	RX0	C
ATOM	341	CG	LEU	194	31.125	-11.572	31.856	1.00	0.00	RX0	C
ATOM	342	CD1	LEU	194	30.683	-11.567	33.320	1.00	0.00	RX0	C
ATOM	343	CD2	LEU	194	30.503	-12.726	31.069	1.00	0.00	RX0	C
ATOM	344	C	LEU	194	30.647	-8.891	29.116	1.00	0.00	RX0	C
ATOM	345	O	LEU	194	29.706	-8.959	28.317	1.00	0.00	RX0	O
ATOM	346	N	LEU	195	31.327	-7.782	29.364	1.00	0.00	RX0	N
ATOM	347	H	LEU	195	32.135	-7.843	29.946	1.00	0.00	RX0	H
ATOM	348	CA	LEU	195	30.920	-6.462	28.846	1.00	0.00	RX0	C
ATOM	349	CB	LEU	195	31.731	-5.338	29.492	1.00	0.00	RX0	C
ATOM	350	CG	LEU	195	31.535	-5.240	31.006	1.00	0.00	RX0	C
ATOM	351	CD1	LEU	195	32.369	-4.109	31.610	1.00	0.00	RX0	C
ATOM	352	CD2	LEU	195	30.060	-5.133	31.388	1.00	0.00	RX0	C
ATOM	353	C	LEU	195	31.020	-6.357	27.321	1.00	0.00	RX0	C
ATOM	354	O	LEU	195	30.051	-5.942	26.671	1.00	0.00	RX0	O
ATOM	355	N	THR	196	32.075	-6.931	26.767	1.00	0.00	RX0	N
ATOM	356	H	THR	196	32.801	-7.325	27.328	1.00	0.00	RX0	H
ATOM	357	CA	THR	196	32.335	-6.901	25.309	1.00	0.00	RX0	C
ATOM	358	CB	THR	196	33.782	-7.302	25.032	1.00	0.00	RX0	C
ATOM	359	OG1	THR	196	34.664	-6.393	25.705	1.00	0.00	RX0	O
ATOM	360	HG1	THR	196	34.364	-5.523	25.476	1.00	0.00	RX0	H
ATOM	361	CG2	THR	196	34.091	-7.342	23.534	1.00	0.00	RX0	C
ATOM	362	C	THR	196	31.317	-7.765	24.552	1.00	0.00	RX0	C
ATOM	363	O	THR	196	30.772	-7.327	23.532	1.00	0.00	RX0	O
ATOM	364	N	ASN	197	31.003	-8.928	25.107	1.00	0.00	RX0	N

ATOM	365	H	ASN	197	31.427	-9.187	25.977	1.00	0.00	RX0	H
ATOM	366	CA	ASN	197	30.010	-9.840	24.504	1.00	0.00	RX0	C
ATOM	367	CB	ASN	197	30.011	-11.206	25.182	1.00	0.00	RX0	C
ATOM	368	CG	ASN	197	31.098	-12.076	24.594	1.00	0.00	RX0	C
ATOM	369	OD1	ASN	197	31.107	-12.436	23.422	1.00	0.00	RX0	O
ATOM	370	ND2	ASN	197	32.047	-12.396	25.491	1.00	0.00	RX0	N
ATOM	371	HD21	ASN	197	31.988	-11.989	26.405	1.00	0.00	RX0	H
ATOM	372	HD22	ASN	197	32.791	-13.017	25.254	1.00	0.00	RX0	H
ATOM	373	C	ASN	197	28.594	-9.255	24.528	1.00	0.00	RX0	C
ATOM	374	O	ASN	197	27.900	-9.272	23.514	1.00	0.00	RX0	O
ATOM	375	N	LEU	198	28.277	-8.575	25.633	1.00	0.00	RX0	N
ATOM	376	H	LEU	198	28.907	-8.599	26.412	1.00	0.00	RX0	H
ATOM	377	CA	LEU	198	27.002	-7.850	25.760	1.00	0.00	RX0	C
ATOM	378	CB	LEU	198	26.853	-7.308	27.177	1.00	0.00	RX0	C
ATOM	379	CG	LEU	198	25.503	-6.636	27.418	1.00	0.00	RX0	C
ATOM	380	CD1	LEU	198	24.338	-7.627	27.381	1.00	0.00	RX0	C
ATOM	381	CD2	LEU	198	25.531	-5.825	28.708	1.00	0.00	RX0	C
ATOM	382	C	LEU	198	26.885	-6.719	24.724	1.00	0.00	RX0	C
ATOM	383	O	LEU	198	25.930	-6.676	23.947	1.00	0.00	RX0	O
ATOM	384	N	ALA	199	27.942	-5.914	24.641	1.00	0.00	RX0	N
ATOM	385	H	ALA	199	28.700	-6.063	25.277	1.00	0.00	RX0	H
ATOM	386	CA	ALA	199	28.029	-4.784	23.694	1.00	0.00	RX0	C
ATOM	387	CB	ALA	199	29.343	-4.024	23.882	1.00	0.00	RX0	C
ATOM	388	C	ALA	199	27.921	-5.230	22.227	1.00	0.00	RX0	C
ATOM	389	O	ALA	199	27.138	-4.660	21.467	1.00	0.00	RX0	O
ATOM	390	N	ASP	200	28.555	-6.360	21.908	1.00	0.00	RX0	N
ATOM	391	H	ASP	200	29.141	-6.810	22.584	1.00	0.00	RX0	H
ATOM	392	CA	ASP	200	28.494	-6.940	20.550	1.00	0.00	RX0	C
ATOM	393	CB	ASP	200	29.537	-8.048	20.365	1.00	0.00	RX0	C
ATOM	394	CG	ASP	200	30.201	-7.923	19.001	1.00	0.00	RX0	C
ATOM	395	OD1	ASP	200	30.596	-6.823	18.616	1.00	0.00	RX0	O
ATOM	396	OD2	ASP	200	30.385	-8.930	18.318	1.00	0.00	RX0	O
ATOM	397	C	ASP	200	27.084	-7.410	20.171	1.00	0.00	RX0	C
ATOM	398	O	ASP	200	26.604	-7.102	19.080	1.00	0.00	RX0	O
ATOM	399	N	ARG	201	26.390	-7.998	21.143	1.00	0.00	RX0	N
ATOM	400	H	ARG	201	26.848	-8.151	22.022	1.00	0.00	RX0	H
ATOM	401	CA	ARG	201	24.992	-8.434	20.957	1.00	0.00	RX0	C
ATOM	402	CB	ARG	201	24.576	-9.442	22.007	1.00	0.00	RX0	C
ATOM	403	CG	ARG	201	25.164	-10.806	21.660	1.00	0.00	RX0	C
ATOM	404	CD	ARG	201	24.688	-11.887	22.622	1.00	0.00	RX0	C
ATOM	405	NE	ARG	201	25.173	-11.613	23.968	1.00	0.00	RX0	N
ATOM	406	HE	ARG	201	26.148	-11.822	24.154	1.00	0.00	RX0	H
ATOM	407	CZ	ARG	201	24.335	-11.161	24.941	1.00	0.00	RX0	C
ATOM	408	NH1	ARG	201	23.026	-10.968	24.673	1.00	0.00	RX0	N
ATOM	409	HH11	ARG	201	22.396	-10.670	25.392	1.00	0.00	RX0	H
ATOM	410	HH12	ARG	201	22.656	-11.121	23.753	1.00	0.00	RX0	H
ATOM	411	NH2	ARG	201	24.822	-10.928	26.166	1.00	0.00	RX0	N
ATOM	412	HH21	ARG	201	24.262	-10.643	26.950	1.00	0.00	RX0	H
ATOM	413	HH22	ARG	201	25.805	-11.103	26.352	1.00	0.00	RX0	H
ATOM	414	C	ARG	201	23.991	-7.279	20.827	1.00	0.00	RX0	C
ATOM	415	O	ARG	201	23.123	-7.308	19.955	1.00	0.00	RX0	O
ATOM	416	N	GLU	202	24.240	-6.201	21.568	1.00	0.00	RX0	N
ATOM	417	H	GLU	202	25.008	-6.215	22.213	1.00	0.00	RX0	H
ATOM	418	CA	GLU	202	23.401	-4.988	21.493	1.00	0.00	RX0	C
ATOM	419	CB	GLU	202	23.559	-4.075	22.731	1.00	0.00	RX0	C
ATOM	420	CG	GLU	202	22.918	-4.682	23.998	1.00	0.00	RX0	C
ATOM	421	CD	GLU	202	22.866	-3.721	25.189	1.00	0.00	RX0	C
ATOM	422	OE1	GLU	202	21.835	-3.088	25.421	1.00	0.00	RX0	O
ATOM	423	OE2	GLU	202	23.825	-3.648	25.950	1.00	0.00	RX0	O
ATOM	424	C	GLU	202	23.526	-4.262	20.149	1.00	0.00	RX0	C
ATOM	425	O	GLU	202	22.539	-3.754	19.625	1.00	0.00	RX0	O

ATOM	426	N	LEU	203	24.712	-4.358	19.546	1.00	0.00	RX0	N
ATOM	427	H	LEU	203	25.464	-4.809	20.035	1.00	0.00	RX0	H
ATOM	428	CA	LEU	203	25.004	-3.680	18.270	1.00	0.00	RX0	C
ATOM	429	CB	LEU	203	26.480	-3.871	17.917	1.00	0.00	RX0	C
ATOM	430	CG	LEU	203	26.922	-3.125	16.656	1.00	0.00	RX0	C
ATOM	431	CD1	LEU	203	26.718	-1.614	16.777	1.00	0.00	RX0	C
ATOM	432	CD2	LEU	203	28.360	-3.477	16.273	1.00	0.00	RX0	C
ATOM	433	C	LEU	203	24.099	-4.160	17.127	1.00	0.00	RX0	C
ATOM	434	O	LEU	203	23.593	-3.349	16.346	1.00	0.00	RX0	O
ATOM	435	N	VAL	204	23.782	-5.447	17.151	1.00	0.00	RX0	N
ATOM	436	H	VAL	204	24.176	-6.003	17.886	1.00	0.00	RX0	H
ATOM	437	CA	VAL	204	22.925	-6.083	16.127	1.00	0.00	RX0	C
ATOM	438	CB	VAL	204	22.899	-7.597	16.333	1.00	0.00	RX0	C
ATOM	439	CG1	VAL	204	22.010	-8.279	15.292	1.00	0.00	RX0	C
ATOM	440	CG2	VAL	204	24.320	-8.163	16.350	1.00	0.00	RX0	C
ATOM	441	C	VAL	204	21.502	-5.497	16.213	1.00	0.00	RX0	C
ATOM	442	O	VAL	204	20.938	-5.041	15.221	1.00	0.00	RX0	O
ATOM	443	N	HIS	205	21.015	-5.393	17.448	1.00	0.00	RX0	N
ATOM	444	H	HIS	205	21.604	-5.675	18.208	1.00	0.00	RX0	H
ATOM	445	CA	HIS	205	19.703	-4.786	17.746	1.00	0.00	RX0	C
ATOM	446	CB	HIS	205	19.276	-5.096	19.182	1.00	0.00	RX0	C
ATOM	447	CG	HIS	205	19.006	-6.576	19.317	1.00	0.00	RX0	C
ATOM	448	ND1	HIS	205	17.811	-7.143	19.064	1.00	0.00	RX0	N
ATOM	449	HD1	HIS	205	16.993	-6.681	18.785	1.00	0.00	RX0	H
ATOM	450	CD2	HIS	205	19.903	-7.576	19.704	1.00	0.00	RX0	C
ATOM	451	NE2	HIS	205	19.233	-8.755	19.681	1.00	0.00	RX0	N
ATOM	452	CE1	HIS	205	17.948	-8.489	19.287	1.00	0.00	RX0	C
ATOM	453	C	HIS	205	19.668	-3.277	17.476	1.00	0.00	RX0	C
ATOM	454	O	HIS	205	18.642	-2.756	17.030	1.00	0.00	RX0	O
ATOM	455	N	MET	206	20.820	-2.627	17.609	1.00	0.00	RX0	N
ATOM	456	H	MET	206	21.610	-3.124	17.974	1.00	0.00	RX0	H
ATOM	457	CA	MET	206	20.969	-1.185	17.340	1.00	0.00	RX0	C
ATOM	458	CB	MET	206	22.357	-0.694	17.746	1.00	0.00	RX0	C
ATOM	459	CG	MET	206	22.542	0.805	17.511	1.00	0.00	RX0	C
ATOM	460	SD	MET	206	24.243	1.325	17.774	1.00	0.00	RX0	S
ATOM	461	CE	MET	206	24.495	0.518	19.360	1.00	0.00	RX0	C
ATOM	462	C	MET	206	20.721	-0.870	15.856	1.00	0.00	RX0	C
ATOM	463	O	MET	206	20.035	0.103	15.544	1.00	0.00	RX0	O
ATOM	464	N	ILE	207	21.183	-1.758	14.977	1.00	0.00	RX0	N
ATOM	465	H	ILE	207	21.732	-2.517	15.339	1.00	0.00	RX0	H
ATOM	466	CA	ILE	207	20.975	-1.628	13.516	1.00	0.00	RX0	C
ATOM	467	CB	ILE	207	21.670	-2.791	12.803	1.00	0.00	RX0	C
ATOM	468	CG2	ILE	207	21.418	-2.774	11.297	1.00	0.00	RX0	C
ATOM	469	CG1	ILE	207	23.164	-2.812	13.124	1.00	0.00	RX0	C
ATOM	470	CD1	ILE	207	23.906	-1.605	12.554	1.00	0.00	RX0	C
ATOM	471	C	ILE	207	19.470	-1.621	13.197	1.00	0.00	RX0	C
ATOM	472	O	ILE	207	18.988	-0.755	12.467	1.00	0.00	RX0	O
ATOM	473	N	ASN	208	18.761	-2.558	13.816	1.00	0.00	RX0	N
ATOM	474	H	ASN	208	19.238	-3.128	14.488	1.00	0.00	RX0	H
ATOM	475	CA	ASN	208	17.313	-2.734	13.586	1.00	0.00	RX0	C
ATOM	476	CB	ASN	208	16.798	-4.064	14.134	1.00	0.00	RX0	C
ATOM	477	CG	ASN	208	17.042	-5.148	13.099	1.00	0.00	RX0	C
ATOM	478	OD1	ASN	208	17.482	-4.898	11.973	1.00	0.00	RX0	O
ATOM	479	ND2	ASN	208	16.740	-6.381	13.543	1.00	0.00	RX0	N
ATOM	480	HD21	ASN	208	16.385	-6.512	14.471	1.00	0.00	RX0	H
ATOM	481	HD22	ASN	208	16.856	-7.208	12.991	1.00	0.00	RX0	H
ATOM	482	C	ASN	208	16.516	-1.532	14.103	1.00	0.00	RX0	C
ATOM	483	O	ASN	208	15.637	-1.017	13.417	1.00	0.00	RX0	O
ATOM	484	N	TRP	209	16.982	-1.008	15.238	1.00	0.00	RX0	N
ATOM	485	H	TRP	209	17.725	-1.473	15.722	1.00	0.00	RX0	H
ATOM	486	CA	TRP	209	16.425	0.201	15.864	1.00	0.00	RX0	C

ATOM	487	CB	TRP	209	17.092	0.419	17.231	1.00	0.00	RX0	C
ATOM	488	CG	TRP	209	16.696	1.748	17.837	1.00	0.00	RX0	C
ATOM	489	CD2	TRP	209	17.414	3.000	17.805	1.00	0.00	RX0	C
ATOM	490	CE2	TRP	209	16.635	3.960	18.495	1.00	0.00	RX0	C
ATOM	491	CE3	TRP	209	18.634	3.370	17.253	1.00	0.00	RX0	C
ATOM	492	CD1	TRP	209	15.527	2.029	18.554	1.00	0.00	RX0	C
ATOM	493	NE1	TRP	209	15.480	3.327	18.949	1.00	0.00	RX0	N
ATOM	494	HE1	TRP	209	14.723	3.719	19.453	1.00	0.00	RX0	H
ATOM	495	CZ2	TRP	209	17.098	5.267	18.597	1.00	0.00	RX0	C
ATOM	496	CZ3	TRP	209	19.087	4.679	17.369	1.00	0.00	RX0	C
ATOM	497	CH2	TRP	209	18.318	5.625	18.035	1.00	0.00	RX0	C
ATOM	498	C	TRP	209	16.619	1.438	14.972	1.00	0.00	RX0	C
ATOM	499	O	TRP	209	15.652	2.120	14.634	1.00	0.00	RX0	O
ATOM	500	N	ALA	210	17.853	1.624	14.503	1.00	0.00	RX0	N
ATOM	501	H	ALA	210	18.567	0.988	14.791	1.00	0.00	RX0	H
ATOM	502	CA	ALA	210	18.233	2.756	13.635	1.00	0.00	RX0	C
ATOM	503	CB	ALA	210	19.715	2.666	13.272	1.00	0.00	RX0	C
ATOM	504	C	ALA	210	17.400	2.800	12.347	1.00	0.00	RX0	C
ATOM	505	O	ALA	210	16.892	3.855	11.979	1.00	0.00	RX0	O
ATOM	506	N	LYS	211	17.095	1.613	11.820	1.00	0.00	RX0	N
ATOM	507	H	LYS	211	17.503	0.799	12.236	1.00	0.00	RX0	H
ATOM	508	CA	LYS	211	16.258	1.472	10.614	1.00	0.00	RX0	C
ATOM	509	CB	LYS	211	16.401	0.067	10.027	1.00	0.00	RX0	C
ATOM	510	CG	LYS	211	17.792	-0.105	9.403	1.00	0.00	RX0	C
ATOM	511	CD	LYS	211	18.064	-1.517	8.880	1.00	0.00	RX0	C
ATOM	512	CE	LYS	211	17.842	-2.528	9.999	1.00	0.00	RX0	C
ATOM	513	NZ	LYS	211	18.344	-3.866	9.655	1.00	0.00	RX0	N
ATOM	514	HZ1	LYS	211	18.163	-4.486	10.478	1.00	0.00	RX0	H
ATOM	515	HZ2	LYS	211	17.842	-4.241	8.829	1.00	0.00	RX0	H
ATOM	516	HZ3	LYS	211	19.366	-3.838	9.464	1.00	0.00	RX0	H
ATOM	517	C	LYS	211	14.795	1.898	10.823	1.00	0.00	RX0	C
ATOM	518	O	LYS	211	14.129	2.337	9.881	1.00	0.00	RX0	O
ATOM	519	N	ARG	212	14.357	1.870	12.071	1.00	0.00	RX0	N
ATOM	520	H	ARG	212	14.980	1.608	12.810	1.00	0.00	RX0	H
ATOM	521	CA	ARG	212	13.005	2.320	12.466	1.00	0.00	RX0	C
ATOM	522	CB	ARG	212	12.392	1.398	13.519	1.00	0.00	RX0	C
ATOM	523	CG	ARG	212	12.551	-0.087	13.192	1.00	0.00	RX0	C
ATOM	524	CD	ARG	212	11.672	-0.984	14.067	1.00	0.00	RX0	C
ATOM	525	NE	ARG	212	10.267	-0.778	13.723	1.00	0.00	RX0	N
ATOM	526	HE	ARG	212	10.079	-0.540	12.767	1.00	0.00	RX0	H
ATOM	527	CZ	ARG	212	9.291	-0.885	14.674	1.00	0.00	RX0	C
ATOM	528	NH1	ARG	212	9.607	-1.219	15.941	1.00	0.00	RX0	N
ATOM	529	HH11	ARG	212	8.884	-1.240	16.661	1.00	0.00	RX0	H
ATOM	530	HH12	ARG	212	10.530	-1.434	16.260	1.00	0.00	RX0	H
ATOM	531	NH2	ARG	212	8.013	-0.643	14.335	1.00	0.00	RX0	N
ATOM	532	HH21	ARG	212	7.293	-0.635	15.059	1.00	0.00	RX0	H
ATOM	533	HH22	ARG	212	7.690	-0.424	13.415	1.00	0.00	RX0	H
ATOM	534	C	ARG	212	12.933	3.790	12.894	1.00	0.00	RX0	C
ATOM	535	O	ARG	212	11.827	4.344	12.989	1.00	0.00	RX0	O
ATOM	536	N	VAL	213	14.074	4.417	13.148	1.00	0.00	RX0	N
ATOM	537	H	VAL	213	14.933	3.928	12.994	1.00	0.00	RX0	H
ATOM	538	CA	VAL	213	14.155	5.874	13.374	1.00	0.00	RX0	C
ATOM	539	CB	VAL	213	15.581	6.281	13.748	1.00	0.00	RX0	C
ATOM	540	CG1	VAL	213	15.751	7.800	13.839	1.00	0.00	RX0	C
ATOM	541	CG2	VAL	213	15.979	5.583	15.046	1.00	0.00	RX0	C
ATOM	542	C	VAL	213	13.672	6.590	12.095	1.00	0.00	RX0	C
ATOM	543	O	VAL	213	14.288	6.431	11.023	1.00	0.00	RX0	O
ATOM	544	N	PRO	214	12.622	7.395	12.213	1.00	0.00	RX0	N
ATOM	545	CD	PRO	214	11.895	7.609	13.458	1.00	0.00	RX0	C
ATOM	546	CA	PRO	214	12.035	8.145	11.084	1.00	0.00	RX0	C
ATOM	547	CB	PRO	214	10.927	8.954	11.761	1.00	0.00	RX0	C

ATOM	548	CG	PRO	214	10.552	8.163	13.010	1.00	0.00	RX0	C
ATOM	549	C	PRO	214	13.102	9.001	10.387	1.00	0.00	RX0	C
ATOM	550	O	PRO	214	13.853	9.727	11.025	1.00	0.00	RX0	O
ATOM	551	N	GLY	215	13.244	8.730	9.080	1.00	0.00	RX0	N
ATOM	552	H	GLY	215	12.749	7.982	8.636	1.00	0.00	RX0	H
ATOM	553	CA	GLY	215	14.194	9.473	8.227	1.00	0.00	RX0	C
ATOM	554	C	GLY	215	15.511	8.732	7.950	1.00	0.00	RX0	C
ATOM	555	O	GLY	215	16.085	8.889	6.862	1.00	0.00	RX0	O
ATOM	556	N	PHE	216	15.917	7.845	8.848	1.00	0.00	RX0	N
ATOM	557	H	PHE	216	15.330	7.654	9.637	1.00	0.00	RX0	H
ATOM	558	CA	PHE	216	17.224	7.160	8.764	1.00	0.00	RX0	C
ATOM	559	CB	PHE	216	17.451	6.293	9.997	1.00	0.00	RX0	C
ATOM	560	CG	PHE	216	18.887	5.831	10.050	1.00	0.00	RX0	C
ATOM	561	CD1	PHE	216	19.899	6.749	10.302	1.00	0.00	RX0	C
ATOM	562	CD2	PHE	216	19.196	4.489	9.861	1.00	0.00	RX0	C
ATOM	563	CE1	PHE	216	21.217	6.319	10.405	1.00	0.00	RX0	C
ATOM	564	CE2	PHE	216	20.513	4.059	9.965	1.00	0.00	RX0	C
ATOM	565	CZ	PHE	216	21.520	4.972	10.258	1.00	0.00	RX0	C
ATOM	566	C	PHE	216	17.435	6.347	7.474	1.00	0.00	RX0	C
ATOM	567	O	PHE	216	18.358	6.641	6.711	1.00	0.00	RX0	O
ATOM	568	N	VAL	217	16.482	5.483	7.151	1.00	0.00	RX0	N
ATOM	569	H	VAL	217	15.672	5.451	7.734	1.00	0.00	RX0	H
ATOM	570	CA	VAL	217	16.570	4.608	5.958	1.00	0.00	RX0	C
ATOM	571	CB	VAL	217	15.527	3.495	6.007	1.00	0.00	RX0	C
ATOM	572	CG1	VAL	217	15.896	2.481	7.081	1.00	0.00	RX0	C
ATOM	573	CG2	VAL	217	14.111	4.052	6.167	1.00	0.00	RX0	C
ATOM	574	C	VAL	217	16.469	5.342	4.608	1.00	0.00	RX0	C
ATOM	575	O	VAL	217	16.660	4.747	3.556	1.00	0.00	RX0	O
ATOM	576	N	ASP	218	16.058	6.613	4.671	1.00	0.00	RX0	N
ATOM	577	H	ASP	218	15.767	7.062	5.518	1.00	0.00	RX0	H
ATOM	578	CA	ASP	218	16.006	7.470	3.472	1.00	0.00	RX0	C
ATOM	579	CB	ASP	218	15.089	8.669	3.759	1.00	0.00	RX0	C
ATOM	580	CG	ASP	218	13.740	8.244	4.345	1.00	0.00	RX0	C
ATOM	581	OD1	ASP	218	12.734	8.388	3.654	1.00	0.00	RX0	O
ATOM	582	OD2	ASP	218	13.681	7.807	5.499	1.00	0.00	RX0	O
ATOM	583	C	ASP	218	17.401	7.924	3.023	1.00	0.00	RX0	C
ATOM	584	O	ASP	218	17.595	8.369	1.896	1.00	0.00	RX0	O
ATOM	585	N	LEU	219	18.344	7.857	3.967	1.00	0.00	RX0	N
ATOM	586	H	LEU	219	18.095	7.494	4.865	1.00	0.00	RX0	H
ATOM	587	CA	LEU	219	19.767	8.083	3.697	1.00	0.00	RX0	C
ATOM	588	CB	LEU	219	20.546	8.267	4.996	1.00	0.00	RX0	C
ATOM	589	CG	LEU	219	19.962	9.402	5.840	1.00	0.00	RX0	C
ATOM	590	CD1	LEU	219	20.550	9.418	7.249	1.00	0.00	RX0	C
ATOM	591	CD2	LEU	219	20.077	10.759	5.145	1.00	0.00	RX0	C
ATOM	592	C	LEU	219	20.350	6.970	2.832	1.00	0.00	RX0	C
ATOM	593	O	LEU	219	19.843	5.836	2.789	1.00	0.00	RX0	O
ATOM	594	N	THR	220	21.442	7.293	2.191	1.00	0.00	RX0	N
ATOM	595	H	THR	220	21.760	8.241	2.276	1.00	0.00	RX0	H
ATOM	596	CA	THR	220	22.263	6.312	1.453	1.00	0.00	RX0	C
ATOM	597	CB	THR	220	23.317	6.974	0.559	1.00	0.00	RX0	C
ATOM	598	OG1	THR	220	24.323	7.667	1.298	1.00	0.00	RX0	O
ATOM	599	HG1	THR	220	23.913	8.434	1.719	1.00	0.00	RX0	H
ATOM	600	CG2	THR	220	22.656	7.881	-0.470	1.00	0.00	RX0	C
ATOM	601	C	THR	220	22.829	5.303	2.454	1.00	0.00	RX0	C
ATOM	602	O	THR	220	23.112	5.646	3.611	1.00	0.00	RX0	O
ATOM	603	N	LEU	221	23.130	4.116	1.957	1.00	0.00	RX0	N
ATOM	604	H	LEU	221	22.841	3.910	1.024	1.00	0.00	RX0	H
ATOM	605	CA	LEU	221	23.750	3.065	2.781	1.00	0.00	RX0	C
ATOM	606	CB	LEU	221	23.942	1.851	1.880	1.00	0.00	RX0	C
ATOM	607	CG	LEU	221	24.693	0.691	2.517	1.00	0.00	RX0	C
ATOM	608	CD1	LEU	221	23.929	0.103	3.699	1.00	0.00	RX0	C

ATOM	609	CD2	LEU	221	25.051	-0.360	1.468	1.00	0.00	RX0	C
ATOM	610	C	LEU	221	25.092	3.509	3.394	1.00	0.00	RX0	C
ATOM	611	O	LEU	221	25.324	3.347	4.578	1.00	0.00	RX0	O
ATOM	612	N	HIS	222	25.854	4.270	2.593	1.00	0.00	RX0	N
ATOM	613	H	HIS	222	25.507	4.497	1.687	1.00	0.00	RX0	H
ATOM	614	CA	HIS	222	27.131	4.847	3.045	1.00	0.00	RX0	C
ATOM	615	CB	HIS	222	27.618	5.687	1.861	1.00	0.00	RX0	C
ATOM	616	CG	HIS	222	28.808	6.576	2.149	1.00	0.00	RX0	C
ATOM	617	ND1	HIS	222	30.078	6.213	1.902	1.00	0.00	RX0	N
ATOM	618	HD1	HIS	222	30.371	5.339	1.557	1.00	0.00	RX0	H
ATOM	619	CD2	HIS	222	28.809	7.898	2.609	1.00	0.00	RX0	C
ATOM	620	NE2	HIS	222	30.092	8.330	2.629	1.00	0.00	RX0	N
ATOM	621	CE1	HIS	222	30.871	7.292	2.193	1.00	0.00	RX0	C
ATOM	622	C	HIS	222	26.942	5.765	4.264	1.00	0.00	RX0	C
ATOM	623	O	HIS	222	27.673	5.642	5.246	1.00	0.00	RX0	O
ATOM	624	N	ASP	223	25.958	6.658	4.167	1.00	0.00	RX0	N
ATOM	625	H	ASP	223	25.408	6.776	3.338	1.00	0.00	RX0	H
ATOM	626	CA	ASP	223	25.694	7.636	5.242	1.00	0.00	RX0	C
ATOM	627	CB	ASP	223	24.792	8.782	4.785	1.00	0.00	RX0	C
ATOM	628	CG	ASP	223	25.604	9.700	3.899	1.00	0.00	RX0	C
ATOM	629	OD1	ASP	223	25.881	10.834	4.281	1.00	0.00	RX0	O
ATOM	630	OD2	ASP	223	26.005	9.274	2.825	1.00	0.00	RX0	O
ATOM	631	C	ASP	223	25.148	6.996	6.516	1.00	0.00	RX0	C
ATOM	632	O	ASP	223	25.558	7.375	7.616	1.00	0.00	RX0	O
ATOM	633	N	GLN	224	24.393	5.921	6.332	1.00	0.00	RX0	N
ATOM	634	H	GLN	224	24.136	5.697	5.389	1.00	0.00	RX0	H
ATOM	635	CA	GLN	224	23.868	5.118	7.452	1.00	0.00	RX0	C
ATOM	636	CB	GLN	224	22.857	4.085	6.953	1.00	0.00	RX0	C
ATOM	637	CG	GLN	224	21.601	4.739	6.373	1.00	0.00	RX0	C
ATOM	638	CD	GLN	224	20.594	3.669	6.012	1.00	0.00	RX0	C
ATOM	639	OE1	GLN	224	20.508	2.624	6.652	1.00	0.00	RX0	O
ATOM	640	NE2	GLN	224	19.835	3.980	4.948	1.00	0.00	RX0	N
ATOM	641	HE21	GLN	224	19.950	4.850	4.457	1.00	0.00	RX0	H
ATOM	642	HE22	GLN	224	19.112	3.401	4.575	1.00	0.00	RX0	H
ATOM	643	C	GLN	224	25.003	4.453	8.243	1.00	0.00	RX0	C
ATOM	644	O	GLN	224	25.073	4.591	9.468	1.00	0.00	RX0	O
ATOM	645	N	VAL	225	25.993	3.956	7.505	1.00	0.00	RX0	N
ATOM	646	H	VAL	225	25.891	3.978	6.508	1.00	0.00	RX0	H
ATOM	647	CA	VAL	225	27.191	3.319	8.093	1.00	0.00	RX0	C
ATOM	648	CB	VAL	225	28.048	2.587	7.062	1.00	0.00	RX0	C
ATOM	649	CG1	VAL	225	29.205	1.893	7.776	1.00	0.00	RX0	C
ATOM	650	CG2	VAL	225	27.236	1.568	6.267	1.00	0.00	RX0	C
ATOM	651	C	VAL	225	28.021	4.368	8.852	1.00	0.00	RX0	C
ATOM	652	O	VAL	225	28.415	4.141	9.995	1.00	0.00	RX0	O
ATOM	653	N	HIS	226	28.182	5.534	8.231	1.00	0.00	RX0	N
ATOM	654	H	HIS	226	27.800	5.644	7.311	1.00	0.00	RX0	H
ATOM	655	CA	HIS	226	28.959	6.641	8.815	1.00	0.00	RX0	C
ATOM	656	CB	HIS	226	29.041	7.797	7.813	1.00	0.00	RX0	C
ATOM	657	CG	HIS	226	30.011	8.878	8.249	1.00	0.00	RX0	C
ATOM	658	ND1	HIS	226	31.202	9.071	7.654	1.00	0.00	RX0	N
ATOM	659	HD1	HIS	226	31.571	8.542	6.915	1.00	0.00	RX0	H
ATOM	660	CD2	HIS	226	29.854	9.848	9.247	1.00	0.00	RX0	C
ATOM	661	NE2	HIS	226	30.963	10.629	9.245	1.00	0.00	RX0	N
ATOM	662	CE1	HIS	226	31.791	10.148	8.263	1.00	0.00	RX0	C
ATOM	663	C	HIS	226	28.363	7.118	10.150	1.00	0.00	RX0	C
ATOM	664	O	HIS	226	29.071	7.189	11.155	1.00	0.00	RX0	O
ATOM	665	N	LEU	227	27.047	7.317	10.166	1.00	0.00	RX0	N
ATOM	666	H	LEU	227	26.527	7.181	9.319	1.00	0.00	RX0	H
ATOM	667	CA	LEU	227	26.344	7.787	11.375	1.00	0.00	RX0	C
ATOM	668	CB	LEU	227	24.877	8.087	11.076	1.00	0.00	RX0	C
ATOM	669	CG	LEU	227	24.680	9.365	10.264	1.00	0.00	RX0	C

ATOM	670	CD1	LEU	227	23.212	9.580	9.908	1.00	0.00	RX0	C
ATOM	671	CD2	LEU	227	25.260	10.585	10.979	1.00	0.00	RX0	C
ATOM	672	C	LEU	227	26.435	6.799	12.540	1.00	0.00	RX0	C
ATOM	673	O	LEU	227	26.853	7.165	13.635	1.00	0.00	RX0	O
ATOM	674	N	LEU	228	26.270	5.522	12.200	1.00	0.00	RX0	N
ATOM	675	H	LEU	228	26.043	5.297	11.248	1.00	0.00	RX0	H
ATOM	676	CA	LEU	228	26.384	4.431	13.181	1.00	0.00	RX0	C
ATOM	677	CB	LEU	228	25.763	3.155	12.619	1.00	0.00	RX0	C
ATOM	678	CG	LEU	228	24.258	3.145	12.879	1.00	0.00	RX0	C
ATOM	679	CD1	LEU	228	23.515	2.133	12.012	1.00	0.00	RX0	C
ATOM	680	CD2	LEU	228	23.964	2.941	14.365	1.00	0.00	RX0	C
ATOM	681	C	LEU	228	27.805	4.188	13.685	1.00	0.00	RX0	C
ATOM	682	O	LEU	228	28.004	4.019	14.891	1.00	0.00	RX0	O
ATOM	683	N	GLU	229	28.784	4.376	12.809	1.00	0.00	RX0	N
ATOM	684	H	GLU	229	28.582	4.580	11.850	1.00	0.00	RX0	H
ATOM	685	CA	GLU	229	30.199	4.229	13.197	1.00	0.00	RX0	C
ATOM	686	CB	GLU	229	31.156	4.053	12.023	1.00	0.00	RX0	C
ATOM	687	CG	GLU	229	32.337	3.221	12.530	1.00	0.00	RX0	C
ATOM	688	CD	GLU	229	33.180	2.749	11.373	1.00	0.00	RX0	C
ATOM	689	OE1	GLU	229	32.930	3.195	10.260	1.00	0.00	RX0	O
ATOM	690	OE2	GLU	229	34.085	1.940	11.586	1.00	0.00	RX0	O
ATOM	691	C	GLU	229	30.618	5.338	14.175	1.00	0.00	RX0	C
ATOM	692	O	GLU	229	31.393	5.088	15.099	1.00	0.00	RX0	O
ATOM	693	N	CYS	230	30.060	6.523	13.970	1.00	0.00	RX0	N
ATOM	694	H	CYS	230	29.476	6.650	13.164	1.00	0.00	RX0	H
ATOM	695	CA	CYS	230	30.321	7.692	14.829	1.00	0.00	RX0	C
ATOM	696	CB	CYS	230	30.024	8.966	14.047	1.00	0.00	RX0	C
ATOM	697	SG	CYS	230	31.067	9.109	12.575	1.00	0.00	RX0	S
ATOM	698	C	CYS	230	29.592	7.653	16.182	1.00	0.00	RX0	C
ATOM	699	O	CYS	230	30.123	8.119	17.188	1.00	0.00	RX0	O
ATOM	700	N	ALA	231	28.434	7.000	16.215	1.00	0.00	RX0	N
ATOM	701	H	ALA	231	28.113	6.531	15.390	1.00	0.00	RX0	H
ATOM	702	CA	ALA	231	27.494	7.141	17.345	1.00	0.00	RX0	C
ATOM	703	CB	ALA	231	26.145	7.668	16.853	1.00	0.00	RX0	C
ATOM	704	C	ALA	231	27.249	5.885	18.186	1.00	0.00	RX0	C
ATOM	705	O	ALA	231	26.768	6.021	19.321	1.00	0.00	RX0	O
ATOM	706	N	TRP	232	27.687	4.719	17.731	1.00	0.00	RX0	N
ATOM	707	H	TRP	232	28.107	4.685	16.821	1.00	0.00	RX0	H
ATOM	708	CA	TRP	232	27.348	3.433	18.379	1.00	0.00	RX0	C
ATOM	709	CB	TRP	232	27.965	2.232	17.648	1.00	0.00	RX0	C
ATOM	710	CG	TRP	232	29.470	2.273	17.749	1.00	0.00	RX0	C
ATOM	711	CD2	TRP	232	30.333	1.555	18.657	1.00	0.00	RX0	C
ATOM	712	CE2	TRP	232	31.663	1.952	18.385	1.00	0.00	RX0	C
ATOM	713	CE3	TRP	232	30.082	0.625	19.658	1.00	0.00	RX0	C
ATOM	714	CD1	TRP	232	30.332	3.059	16.981	1.00	0.00	RX0	C
ATOM	715	NE1	TRP	232	31.624	2.879	17.348	1.00	0.00	RX0	N
ATOM	716	HE1	TRP	232	32.382	3.336	16.920	1.00	0.00	RX0	H
ATOM	717	CZ2	TRP	232	32.706	1.411	19.127	1.00	0.00	RX0	C
ATOM	718	CZ3	TRP	232	31.135	0.092	20.392	1.00	0.00	RX0	C
ATOM	719	CH2	TRP	232	32.442	0.483	20.128	1.00	0.00	RX0	C
ATOM	720	C	TRP	232	27.676	3.373	19.884	1.00	0.00	RX0	C
ATOM	721	O	TRP	232	26.862	2.914	20.672	1.00	0.00	RX0	O
ATOM	722	N	LEU	233	28.801	3.989	20.277	1.00	0.00	RX0	N
ATOM	723	H	LEU	233	29.361	4.449	19.590	1.00	0.00	RX0	H
ATOM	724	CA	LEU	233	29.211	3.960	21.691	1.00	0.00	RX0	C
ATOM	725	CB	LEU	233	30.717	4.176	21.830	1.00	0.00	RX0	C
ATOM	726	CG	LEU	233	31.209	3.798	23.228	1.00	0.00	RX0	C
ATOM	727	CD1	LEU	233	30.812	2.368	23.601	1.00	0.00	RX0	C
ATOM	728	CD2	LEU	233	32.711	4.025	23.390	1.00	0.00	RX0	C
ATOM	729	C	LEU	233	28.415	4.936	22.566	1.00	0.00	RX0	C
ATOM	730	O	LEU	233	27.943	4.566	23.634	1.00	0.00	RX0	O

ATOM	731	N	GLU	234	28.150	6.122	22.016	1.00	0.00	RX0	N
ATOM	732	H	GLU	234	28.483	6.333	21.099	1.00	0.00	RX0	H
ATOM	733	CA	GLU	234	27.227	7.090	22.644	1.00	0.00	RX0	C
ATOM	734	CB	GLU	234	27.008	8.334	21.789	1.00	0.00	RX0	C
ATOM	735	CG	GLU	234	28.097	9.394	21.814	1.00	0.00	RX0	C
ATOM	736	CD	GLU	234	27.595	10.620	21.094	1.00	0.00	RX0	C
ATOM	737	OE1	GLU	234	27.461	11.669	21.711	1.00	0.00	RX0	O
ATOM	738	OE2	GLU	234	27.375	10.560	19.895	1.00	0.00	RX0	O
ATOM	739	C	GLU	234	25.830	6.482	22.841	1.00	0.00	RX0	C
ATOM	740	O	GLU	234	25.253	6.598	23.926	1.00	0.00	RX0	O
ATOM	741	N	ILE	235	25.389	5.711	21.848	1.00	0.00	RX0	N
ATOM	742	H	ILE	235	25.965	5.636	21.033	1.00	0.00	RX0	H
ATOM	743	CA	ILE	235	24.069	5.045	21.863	1.00	0.00	RX0	C
ATOM	744	CB	ILE	235	23.697	4.484	20.487	1.00	0.00	RX0	C
ATOM	745	CG2	ILE	235	22.411	3.658	20.554	1.00	0.00	RX0	C
ATOM	746	CG1	ILE	235	23.560	5.612	19.464	1.00	0.00	RX0	C
ATOM	747	CD1	ILE	235	23.222	5.094	18.066	1.00	0.00	RX0	C
ATOM	748	C	ILE	235	24.018	3.945	22.939	1.00	0.00	RX0	C
ATOM	749	O	ILE	235	23.068	3.902	23.724	1.00	0.00	RX0	O
ATOM	750	N	LEU	236	25.072	3.140	23.020	1.00	0.00	RX0	N
ATOM	751	H	LEU	236	25.813	3.237	22.352	1.00	0.00	RX0	H
ATOM	752	CA	LEU	236	25.176	2.114	24.078	1.00	0.00	RX0	C
ATOM	753	CB	LEU	236	26.442	1.278	23.892	1.00	0.00	RX0	C
ATOM	754	CG	LEU	236	26.338	0.307	22.720	1.00	0.00	RX0	C
ATOM	755	CD1	LEU	236	27.668	-0.387	22.426	1.00	0.00	RX0	C
ATOM	756	CD2	LEU	236	25.205	-0.695	22.939	1.00	0.00	RX0	C
ATOM	757	C	LEU	236	25.182	2.733	25.479	1.00	0.00	RX0	C
ATOM	758	O	LEU	236	24.381	2.362	26.336	1.00	0.00	RX0	O
ATOM	759	N	MET	237	25.933	3.823	25.600	1.00	0.00	RX0	N
ATOM	760	H	MET	237	26.456	4.136	24.805	1.00	0.00	RX0	H
ATOM	761	CA	MET	237	26.132	4.523	26.881	1.00	0.00	RX0	C
ATOM	762	CB	MET	237	27.281	5.529	26.803	1.00	0.00	RX0	C
ATOM	763	CG	MET	237	28.653	4.854	26.811	1.00	0.00	RX0	C
ATOM	764	SD	MET	237	30.001	6.045	26.814	1.00	0.00	RX0	S
ATOM	765	CE	MET	237	31.349	4.895	27.123	1.00	0.00	RX0	C
ATOM	766	C	MET	237	24.875	5.215	27.409	1.00	0.00	RX0	C
ATOM	767	O	MET	237	24.517	5.003	28.572	1.00	0.00	RX0	O
ATOM	768	N	ILE	238	24.128	5.878	26.531	1.00	0.00	RX0	N
ATOM	769	H	ILE	238	24.458	5.962	25.587	1.00	0.00	RX0	H
ATOM	770	CA	ILE	238	22.871	6.546	26.925	1.00	0.00	RX0	C
ATOM	771	CB	ILE	238	22.349	7.523	25.856	1.00	0.00	RX0	C
ATOM	772	CG2	ILE	238	21.931	6.836	24.556	1.00	0.00	RX0	C
ATOM	773	CG1	ILE	238	21.216	8.375	26.433	1.00	0.00	RX0	C
ATOM	774	CD1	ILE	238	20.604	9.329	25.407	1.00	0.00	RX0	C
ATOM	775	C	ILE	238	21.800	5.514	27.357	1.00	0.00	RX0	C
ATOM	776	O	ILE	238	21.031	5.731	28.268	1.00	0.00	RX0	O
ATOM	777	N	GLY	239	21.845	4.355	26.660	1.00	0.00	RX0	N
ATOM	778	H	GLY	239	22.520	4.244	25.927	1.00	0.00	RX0	H
ATOM	779	CA	GLY	239	20.969	3.213	26.975	1.00	0.00	RX0	C
ATOM	780	C	GLY	239	21.301	2.634	28.356	1.00	0.00	RX0	C
ATOM	781	O	GLY	239	20.417	2.460	29.193	1.00	0.00	RX0	O
ATOM	782	N	LEU	240	22.605	2.583	28.639	1.00	0.00	RX0	N
ATOM	783	H	LEU	240	23.260	2.804	27.913	1.00	0.00	RX0	H
ATOM	784	CA	LEU	240	23.124	2.082	29.919	1.00	0.00	RX0	C
ATOM	785	CB	LEU	240	24.644	1.942	29.848	1.00	0.00	RX0	C
ATOM	786	CG	LEU	240	25.285	1.607	31.196	1.00	0.00	RX0	C
ATOM	787	CD1	LEU	240	24.817	0.261	31.747	1.00	0.00	RX0	C
ATOM	788	CD2	LEU	240	26.806	1.702	31.124	1.00	0.00	RX0	C
ATOM	789	C	LEU	240	22.728	2.995	31.086	1.00	0.00	RX0	C
ATOM	790	O	LEU	240	22.214	2.535	32.097	1.00	0.00	RX0	O
ATOM	791	N	VAL	241	22.901	4.295	30.880	1.00	0.00	RX0	N

ATOM	792	H	VAL	241	23.279	4.586	29.999	1.00	0.00	RX0	H
ATOM	793	CA	VAL	241	22.596	5.307	31.912	1.00	0.00	RX0	C
ATOM	794	CB	VAL	241	23.251	6.672	31.660	1.00	0.00	RX0	C
ATOM	795	CG1	VAL	241	24.769	6.507	31.594	1.00	0.00	RX0	C
ATOM	796	CG2	VAL	241	22.696	7.406	30.446	1.00	0.00	RX0	C
ATOM	797	C	VAL	241	21.084	5.392	32.193	1.00	0.00	RX0	C
ATOM	798	O	VAL	241	20.670	5.516	33.338	1.00	0.00	RX0	O
ATOM	799	N	TRP	242	20.290	5.181	31.134	1.00	0.00	RX0	N
ATOM	800	H	TRP	242	20.699	5.072	30.225	1.00	0.00	RX0	H
ATOM	801	CA	TRP	242	18.822	5.192	31.222	1.00	0.00	RX0	C
ATOM	802	CB	TRP	242	18.253	5.150	29.801	1.00	0.00	RX0	C
ATOM	803	CG	TRP	242	16.826	4.657	29.777	1.00	0.00	RX0	C
ATOM	804	CD2	TRP	242	15.636	5.331	30.234	1.00	0.00	RX0	C
ATOM	805	CE2	TRP	242	14.544	4.461	30.016	1.00	0.00	RX0	C
ATOM	806	CE3	TRP	242	15.419	6.579	30.801	1.00	0.00	RX0	C
ATOM	807	CD1	TRP	242	16.382	3.414	29.300	1.00	0.00	RX0	C
ATOM	808	NE1	TRP	242	15.035	3.295	29.439	1.00	0.00	RX0	N
ATOM	809	HE1	TRP	242	14.494	2.520	29.180	1.00	0.00	RX0	H
ATOM	810	CZ2	TRP	242	13.265	4.862	30.380	1.00	0.00	RX0	C
ATOM	811	CZ3	TRP	242	14.135	6.971	31.159	1.00	0.00	RX0	C
ATOM	812	CH2	TRP	242	13.063	6.112	30.953	1.00	0.00	RX0	C
ATOM	813	C	TRP	242	18.281	4.038	32.076	1.00	0.00	RX0	C
ATOM	814	O	TRP	242	17.477	4.269	32.979	1.00	0.00	RX0	O
ATOM	815	N	ARG	243	18.818	2.841	31.865	1.00	0.00	RX0	N
ATOM	816	H	ARG	243	19.515	2.737	31.151	1.00	0.00	RX0	H
ATOM	817	CA	ARG	243	18.359	1.661	32.627	1.00	0.00	RX0	C
ATOM	818	CB	ARG	243	18.485	0.403	31.737	1.00	0.00	RX0	C
ATOM	819	CG	ARG	243	19.890	-0.065	31.297	1.00	0.00	RX0	C
ATOM	820	CD	ARG	243	19.829	-1.103	30.153	1.00	0.00	RX0	C
ATOM	821	NE	ARG	243	21.113	-1.762	29.863	1.00	0.00	RX0	N
ATOM	822	HE	ARG	243	21.663	-2.017	30.672	1.00	0.00	RX0	H
ATOM	823	CZ	ARG	243	21.430	-2.133	28.569	1.00	0.00	RX0	C
ATOM	824	NH1	ARG	243	20.664	-1.729	27.537	1.00	0.00	RX0	N
ATOM	825	HH11	ARG	243	20.909	-2.042	26.601	1.00	0.00	RX0	H
ATOM	826	HH12	ARG	243	19.863	-1.140	27.639	1.00	0.00	RX0	H
ATOM	827	NH2	ARG	243	22.498	-2.912	28.304	1.00	0.00	RX0	N
ATOM	828	HH21	ARG	243	22.780	-3.128	27.347	1.00	0.00	RX0	H
ATOM	829	HH22	ARG	243	23.055	-3.342	29.023	1.00	0.00	RX0	H
ATOM	830	C	ARG	243	19.043	1.510	33.998	1.00	0.00	RX0	C
ATOM	831	O	ARG	243	18.610	0.722	34.836	1.00	0.00	RX0	O
ATOM	832	N	SER	244	20.027	2.366	34.245	1.00	0.00	RX0	N
ATOM	833	H	SER	244	20.316	3.011	33.538	1.00	0.00	RX0	H
ATOM	834	CA	SER	244	20.722	2.464	35.548	1.00	0.00	RX0	C
ATOM	835	CB	SER	244	22.206	2.697	35.311	1.00	0.00	RX0	C
ATOM	836	OG	SER	244	22.700	1.647	34.476	1.00	0.00	RX0	O
ATOM	837	HG	SER	244	22.226	1.717	33.654	1.00	0.00	RX0	H
ATOM	838	C	SER	244	20.112	3.547	36.442	1.00	0.00	RX0	C
ATOM	839	O	SER	244	20.448	3.642	37.630	1.00	0.00	RX0	O
ATOM	840	N	MET	245	19.184	4.322	35.895	1.00	0.00	RX0	N
ATOM	841	H	MET	245	18.885	4.152	34.954	1.00	0.00	RX0	H
ATOM	842	CA	MET	245	18.600	5.504	36.550	1.00	0.00	RX0	C
ATOM	843	CB	MET	245	17.596	6.196	35.632	1.00	0.00	RX0	C
ATOM	844	CG	MET	245	17.039	7.469	36.269	1.00	0.00	RX0	C
ATOM	845	SD	MET	245	15.735	8.230	35.299	1.00	0.00	RX0	S
ATOM	846	CE	MET	245	16.550	8.101	33.706	1.00	0.00	RX0	C
ATOM	847	C	MET	245	17.925	5.204	37.895	1.00	0.00	RX0	C
ATOM	848	O	MET	245	18.105	5.946	38.853	1.00	0.00	RX0	O
ATOM	849	N	GLU	246	17.212	4.082	37.945	1.00	0.00	RX0	N
ATOM	850	H	GLU	246	17.124	3.484	37.147	1.00	0.00	RX0	H
ATOM	851	CA	GLU	246	16.494	3.692	39.178	1.00	0.00	RX0	C
ATOM	852	CB	GLU	246	15.245	2.899	38.818	1.00	0.00	RX0	C

ATOM	853	CG	GLU	246	14.342	3.651	37.845	1.00	0.00	RX0 C
ATOM	854	CD	GLU	246	13.169	2.761	37.505	1.00	0.00	RX0 C
ATOM	855	OE1	GLU	246	13.042	1.708	38.125	1.00	0.00	RX0 O
ATOM	856	OE2	GLU	246	12.395	3.120	36.621	1.00	0.00	RX0 O
ATOM	857	C	GLU	246	17.379	2.867	40.123	1.00	0.00	RX0 C
ATOM	858	O	GLU	246	16.897	2.295	41.108	1.00	0.00	RX0 O
ATOM	859	N	HIS	247	18.674	2.844	39.836	1.00	0.00	RX0 N
ATOM	860	H	HIS	247	19.044	3.323	39.041	1.00	0.00	RX0 H
ATOM	861	CA	HIS	247	19.658	2.055	40.601	1.00	0.00	RX0 C
ATOM	862	CB	HIS	247	20.174	0.865	39.788	1.00	0.00	RX0 C
ATOM	863	CG	HIS	247	19.081	-0.138	39.488	1.00	0.00	RX0 C
ATOM	864	ND1	HIS	247	17.866	-0.170	40.073	1.00	0.00	RX0 N
ATOM	865	HD1	HIS	247	17.499	0.453	40.743	1.00	0.00	RX0 H
ATOM	866	CD2	HIS	247	19.157	-1.190	38.573	1.00	0.00	RX0 C
ATOM	867	NE2	HIS	247	17.980	-1.858	38.611	1.00	0.00	RX0 N
ATOM	868	CE1	HIS	247	17.183	-1.229	39.533	1.00	0.00	RX0 C
ATOM	869	C	HIS	247	20.841	2.947	41.015	1.00	0.00	RX0 C
ATOM	870	O	HIS	247	21.962	2.788	40.490	1.00	0.00	RX0 O
ATOM	871	N	PRO	248	20.632	3.827	41.991	1.00	0.00	RX0 N
ATOM	872	CD	PRO	248	19.384	3.973	42.733	1.00	0.00	RX0 C
ATOM	873	CA	PRO	248	21.659	4.767	42.484	1.00	0.00	RX0 C
ATOM	874	CB	PRO	248	20.980	5.449	43.675	1.00	0.00	RX0 C
ATOM	875	CG	PRO	248	19.487	5.344	43.385	1.00	0.00	RX0 C
ATOM	876	C	PRO	248	22.939	4.013	42.877	1.00	0.00	RX0 C
ATOM	877	O	PRO	248	22.892	2.963	43.503	1.00	0.00	RX0 O
ATOM	878	N	GLY	249	24.055	4.541	42.350	1.00	0.00	RX0 N
ATOM	879	H	GLY	249	23.972	5.298	41.707	1.00	0.00	RX0 H
ATOM	880	CA	GLY	249	25.407	3.996	42.610	1.00	0.00	RX0 C
ATOM	881	C	GLY	249	25.783	2.749	41.794	1.00	0.00	RX0 C
ATOM	882	O	GLY	249	26.914	2.250	41.927	1.00	0.00	RX0 O
ATOM	883	N	LYS	250	24.877	2.267	40.961	1.00	0.00	RX0 N
ATOM	884	H	LYS	250	23.983	2.697	40.812	1.00	0.00	RX0 H
ATOM	885	CA	LYS	250	25.097	1.050	40.158	1.00	0.00	RX0 C
ATOM	886	CB	LYS	250	24.371	-0.147	40.774	1.00	0.00	RX0 C
ATOM	887	CG	LYS	250	25.229	-0.851	41.835	1.00	0.00	RX0 C
ATOM	888	CD	LYS	250	24.610	-2.140	42.383	1.00	0.00	RX0 C
ATOM	889	CE	LYS	250	25.549	-2.971	43.267	1.00	0.00	RX0 C
ATOM	890	NZ	LYS	250	26.698	-3.462	42.491	1.00	0.00	RX0 N
ATOM	891	HZ1	LYS	250	27.411	-3.874	43.135	1.00	0.00	RX0 H
ATOM	892	HZ2	LYS	250	26.434	-4.157	41.759	1.00	0.00	RX0 H
ATOM	893	HZ3	LYS	250	27.206	-2.657	42.076	1.00	0.00	RX0 H
ATOM	894	C	LYS	250	24.802	1.266	38.671	1.00	0.00	RX0 C
ATOM	895	O	LYS	250	24.040	2.166	38.282	1.00	0.00	RX0 O
ATOM	896	N	LEU	251	25.472	0.476	37.863	1.00	0.00	RX0 N
ATOM	897	H	LEU	251	26.053	-0.236	38.243	1.00	0.00	RX0 H
ATOM	898	CA	LEU	251	25.292	0.430	36.401	1.00	0.00	RX0 C
ATOM	899	CB	LEU	251	26.626	0.590	35.684	1.00	0.00	RX0 C
ATOM	900	CG	LEU	251	27.160	2.016	35.762	1.00	0.00	RX0 C
ATOM	901	CD1	LEU	251	28.584	2.114	35.220	1.00	0.00	RX0 C
ATOM	902	CD2	LEU	251	26.208	3.006	35.088	1.00	0.00	RX0 C
ATOM	903	C	LEU	251	24.646	-0.892	36.017	1.00	0.00	RX0 C
ATOM	904	O	LEU	251	25.224	-1.976	36.286	1.00	0.00	RX0 O
ATOM	905	N	LEU	252	23.437	-0.808	35.533	1.00	0.00	RX0 N
ATOM	906	H	LEU	252	23.084	0.097	35.312	1.00	0.00	RX0 H
ATOM	907	CA	LEU	252	22.659	-1.981	35.114	1.00	0.00	RX0 C
ATOM	908	CB	LEU	252	21.157	-1.712	35.230	1.00	0.00	RX0 C
ATOM	909	CG	LEU	252	20.298	-2.957	34.974	1.00	0.00	RX0 C
ATOM	910	CD1	LEU	252	20.449	-3.993	36.086	1.00	0.00	RX0 C
ATOM	911	CD2	LEU	252	18.831	-2.608	34.727	1.00	0.00	RX0 C
ATOM	912	C	LEU	252	23.007	-2.330	33.663	1.00	0.00	RX0 C
ATOM	913	O	LEU	252	22.274	-2.025	32.731	1.00	0.00	RX0 O

ATOM	914	N	PHE	253	24.144	-3.005	33.506	1.00	0.00	RX0	N
ATOM	915	H	PHE	253	24.631	-3.308	34.330	1.00	0.00	RX0	H
ATOM	916	CA	PHE	253	24.599	-3.457	32.174	1.00	0.00	RX0	C
ATOM	917	CB	PHE	253	25.968	-4.125	32.268	1.00	0.00	RX0	C
ATOM	918	CG	PHE	253	27.032	-3.095	32.540	1.00	0.00	RX0	C
ATOM	919	CD1	PHE	253	27.563	-2.362	31.485	1.00	0.00	RX0	C
ATOM	920	CD2	PHE	253	27.488	-2.886	33.835	1.00	0.00	RX0	C
ATOM	921	CE1	PHE	253	28.562	-1.427	31.723	1.00	0.00	RX0	C
ATOM	922	CE2	PHE	253	28.485	-1.950	34.071	1.00	0.00	RX0	C
ATOM	923	CZ	PHE	253	29.023	-1.222	33.017	1.00	0.00	RX0	C
ATOM	924	C	PHE	253	23.603	-4.446	31.564	1.00	0.00	RX0	C
ATOM	925	O	PHE	253	23.259	-4.379	30.390	1.00	0.00	RX0	O
ATOM	926	N	ALA	254	23.094	-5.300	32.445	1.00	0.00	RX0	N
ATOM	927	H	ALA	254	23.408	-5.312	33.400	1.00	0.00	RX0	H
ATOM	928	CA	ALA	254	22.050	-6.280	32.141	1.00	0.00	RX0	C
ATOM	929	CB	ALA	254	22.697	-7.548	31.590	1.00	0.00	RX0	C
ATOM	930	C	ALA	254	21.288	-6.584	33.440	1.00	0.00	RX0	C
ATOM	931	O	ALA	254	21.887	-6.418	34.526	1.00	0.00	RX0	O
ATOM	932	N	PRO	255	20.056	-7.064	33.372	1.00	0.00	RX0	N
ATOM	933	CD	PRO	255	19.307	-7.244	32.131	1.00	0.00	RX0	C
ATOM	934	CA	PRO	255	19.236	-7.444	34.545	1.00	0.00	RX0	C
ATOM	935	CB	PRO	255	17.987	-8.068	33.924	1.00	0.00	RX0	C
ATOM	936	CG	PRO	255	17.856	-7.385	32.569	1.00	0.00	RX0	C
ATOM	937	C	PRO	255	19.972	-8.395	35.506	1.00	0.00	RX0	C
ATOM	938	O	PRO	255	19.756	-8.342	36.714	1.00	0.00	RX0	O
ATOM	939	N	ASN	256	20.900	-9.179	34.970	1.00	0.00	RX0	N
ATOM	940	H	ASN	256	21.113	-9.138	33.993	1.00	0.00	RX0	H
ATOM	941	CA	ASN	256	21.722	-10.125	35.761	1.00	0.00	RX0	C
ATOM	942	CB	ASN	256	21.709	-11.530	35.152	1.00	0.00	RX0	C
ATOM	943	CG	ASN	256	22.401	-11.537	33.797	1.00	0.00	RX0	C
ATOM	944	OD1	ASN	256	22.275	-10.607	33.000	1.00	0.00	RX0	O
ATOM	945	ND2	ASN	256	23.049	-12.686	33.532	1.00	0.00	RX0	N
ATOM	946	HD21	ASN	256	23.189	-13.356	34.265	1.00	0.00	RX0	H
ATOM	947	HD22	ASN	256	23.404	-12.938	32.626	1.00	0.00	RX0	H
ATOM	948	C	ASN	256	23.191	-9.669	35.876	1.00	0.00	RX0	C
ATOM	949	O	ASN	256	24.101	-10.505	36.011	1.00	0.00	RX0	O
ATOM	950	N	LEU	257	23.439	-8.384	35.742	1.00	0.00	RX0	N
ATOM	951	H	LEU	257	22.692	-7.727	35.628	1.00	0.00	RX0	H
ATOM	952	CA	LEU	257	24.796	-7.805	35.783	1.00	0.00	RX0	C
ATOM	953	CB	LEU	257	25.513	-7.966	34.444	1.00	0.00	RX0	C
ATOM	954	CG	LEU	257	27.025	-7.763	34.567	1.00	0.00	RX0	C
ATOM	955	CD1	LEU	257	27.654	-8.806	35.493	1.00	0.00	RX0	C
ATOM	956	CD2	LEU	257	27.715	-7.724	33.204	1.00	0.00	RX0	C
ATOM	957	C	LEU	257	24.720	-6.327	36.168	1.00	0.00	RX0	C
ATOM	958	O	LEU	257	24.738	-5.412	35.328	1.00	0.00	RX0	O
ATOM	959	N	LEU	258	24.604	-6.148	37.469	1.00	0.00	RX0	N
ATOM	960	H	LEU	258	24.689	-6.942	38.070	1.00	0.00	RX0	H
ATOM	961	CA	LEU	258	24.482	-4.834	38.118	1.00	0.00	RX0	C
ATOM	962	CB	LEU	258	23.222	-4.903	38.981	1.00	0.00	RX0	C
ATOM	963	CG	LEU	258	22.813	-3.612	39.677	1.00	0.00	RX0	C
ATOM	964	CD1	LEU	258	22.656	-2.462	38.693	1.00	0.00	RX0	C
ATOM	965	CD2	LEU	258	21.554	-3.805	40.524	1.00	0.00	RX0	C
ATOM	966	C	LEU	258	25.743	-4.564	38.938	1.00	0.00	RX0	C
ATOM	967	O	LEU	258	26.013	-5.237	39.948	1.00	0.00	RX0	O
ATOM	968	N	LEU	259	26.528	-3.622	38.460	1.00	0.00	RX0	N
ATOM	969	H	LEU	259	26.218	-3.063	37.684	1.00	0.00	RX0	H
ATOM	970	CA	LEU	259	27.862	-3.349	39.027	1.00	0.00	RX0	C
ATOM	971	CB	LEU	259	28.937	-3.489	37.946	1.00	0.00	RX0	C
ATOM	972	CG	LEU	259	28.873	-4.798	37.152	1.00	0.00	RX0	C
ATOM	973	CD1	LEU	259	29.889	-4.812	36.009	1.00	0.00	RX0	C
ATOM	974	CD2	LEU	259	29.017	-6.031	38.042	1.00	0.00	RX0	C

ATOM	975	C	LEU	259	27.958	-1.956	39.652	1.00	0.00	RX0	C
ATOM	976	O	LEU	259	27.419	-0.984	39.137	1.00	0.00	RX0	O
ATOM	977	N	ASP	260	28.645	-1.912	40.785	1.00	0.00	RX0	N
ATOM	978	H	ASP	260	29.225	-2.689	41.044	1.00	0.00	RX0	H
ATOM	979	CA	ASP	260	29.043	-0.657	41.454	1.00	0.00	RX0	C
ATOM	980	CB	ASP	260	29.078	-0.868	42.963	1.00	0.00	RX0	C
ATOM	981	CG	ASP	260	29.781	-2.181	43.220	1.00	0.00	RX0	C
ATOM	982	OD1	ASP	260	31.008	-2.205	43.259	1.00	0.00	RX0	O
ATOM	983	OD2	ASP	260	29.087	-3.191	43.338	1.00	0.00	RX0	O
ATOM	984	C	ASP	260	30.443	-0.222	40.970	1.00	0.00	RX0	C
ATOM	985	O	ASP	260	31.127	-1.008	40.295	1.00	0.00	RX0	O
ATOM	986	N	ARG	261	30.963	0.873	41.504	1.00	0.00	RX0	N
ATOM	987	H	ARG	261	30.386	1.405	42.121	1.00	0.00	RX0	H
ATOM	988	CA	ARG	261	32.263	1.408	41.044	1.00	0.00	RX0	C
ATOM	989	CB	ARG	261	32.404	2.878	41.449	1.00	0.00	RX0	C
ATOM	990	CG	ARG	261	32.499	3.139	42.954	1.00	0.00	RX0	C
ATOM	991	CD	ARG	261	32.337	4.622	43.293	1.00	0.00	RX0	C
ATOM	992	NE	ARG	261	33.211	5.453	42.470	1.00	0.00	RX0	N
ATOM	993	HE	ARG	261	33.210	5.365	41.464	1.00	0.00	RX0	H
ATOM	994	CZ	ARG	261	33.952	6.448	42.991	1.00	0.00	RX0	C
ATOM	995	NH1	ARG	261	33.954	6.690	44.304	1.00	0.00	RX0	N
ATOM	996	HH11	ARG	261	34.511	7.430	44.678	1.00	0.00	RX0	H
ATOM	997	HH12	ARG	261	33.398	6.149	44.936	1.00	0.00	RX0	H
ATOM	998	NH2	ARG	261	34.683	7.190	42.175	1.00	0.00	RX0	N
ATOM	999	HH21	ARG	261	35.277	7.942	42.443	1.00	0.00	RX0	H
ATOM	1000	HH22	ARG	261	34.638	7.002	41.175	1.00	0.00	RX0	H
ATOM	1001	C	ARG	261	33.476	0.540	41.436	1.00	0.00	RX0	C
ATOM	1002	O	ARG	261	34.378	0.347	40.637	1.00	0.00	RX0	O
ATOM	1003	N	ASN	262	33.410	-0.067	42.632	1.00	0.00	RX0	N
ATOM	1004	H	ASN	262	32.532	-0.049	43.112	1.00	0.00	RX0	H
ATOM	1005	CA	ASN	262	34.456	-1.000	43.094	1.00	0.00	RX0	C
ATOM	1006	CB	ASN	262	34.119	-1.490	44.495	1.00	0.00	RX0	C
ATOM	1007	CG	ASN	262	35.056	-2.624	44.844	1.00	0.00	RX0	C
ATOM	1008	OD1	ASN	262	36.214	-2.391	45.197	1.00	0.00	RX0	O
ATOM	1009	ND2	ASN	262	34.488	-3.842	44.780	1.00	0.00	RX0	N
ATOM	1010	HD21	ASN	262	33.524	-3.933	44.513	1.00	0.00	RX0	H
ATOM	1011	HD22	ASN	262	34.977	-4.692	44.979	1.00	0.00	RX0	H
ATOM	1012	C	ASN	262	34.635	-2.233	42.204	1.00	0.00	RX0	C
ATOM	1013	O	ASN	262	35.755	-2.665	41.964	1.00	0.00	RX0	O
ATOM	1014	N	GLN	263	33.530	-2.679	41.603	1.00	0.00	RX0	N
ATOM	1015	H	GLN	263	32.639	-2.268	41.820	1.00	0.00	RX0	H
ATOM	1016	CA	GLN	263	33.559	-3.756	40.599	1.00	0.00	RX0	C
ATOM	1017	CB	GLN	263	32.196	-4.419	40.447	1.00	0.00	RX0	C
ATOM	1018	CG	GLN	263	31.905	-5.155	41.752	1.00	0.00	RX0	C
ATOM	1019	CD	GLN	263	30.689	-6.037	41.613	1.00	0.00	RX0	C
ATOM	1020	OE1	GLN	263	30.593	-6.887	40.737	1.00	0.00	RX0	O
ATOM	1021	NE2	GLN	263	29.777	-5.827	42.574	1.00	0.00	RX0	N
ATOM	1022	HE21	GLN	263	29.901	-5.028	43.176	1.00	0.00	RX0	H
ATOM	1023	HE22	GLN	263	29.005	-6.445	42.707	1.00	0.00	RX0	H
ATOM	1024	C	GLN	263	34.189	-3.308	39.265	1.00	0.00	RX0	C
ATOM	1025	O	GLN	263	34.644	-4.116	38.479	1.00	0.00	RX0	O
ATOM	1026	N	GLY	264	34.180	-1.978	39.039	1.00	0.00	RX0	N
ATOM	1027	H	GLY	264	33.761	-1.378	39.720	1.00	0.00	RX0	H
ATOM	1028	CA	GLY	264	34.831	-1.337	37.881	1.00	0.00	RX0	C
ATOM	1029	C	GLY	264	36.364	-1.310	37.978	1.00	0.00	RX0	C
ATOM	1030	O	GLY	264	37.050	-1.510	36.977	1.00	0.00	RX0	O
ATOM	1031	N	LYS	265	36.881	-1.155	39.202	1.00	0.00	RX0	N
ATOM	1032	H	LYS	265	36.233	-1.021	39.953	1.00	0.00	RX0	H
ATOM	1033	CA	LYS	265	38.336	-1.184	39.469	1.00	0.00	RX0	C
ATOM	1034	CB	LYS	265	38.665	-1.127	40.954	1.00	0.00	RX0	C
ATOM	1035	CG	LYS	265	38.091	-0.048	41.861	1.00	0.00	RX0	C

ATOM	1036	CD	LYS	265	38.450	-0.522	43.268	1.00	0.00	RX0	C
ATOM	1037	CE	LYS	265	37.883	0.274	44.436	1.00	0.00	RX0	C
ATOM	1038	NZ	LYS	265	38.035	-0.568	45.631	1.00	0.00	RX0	N
ATOM	1039	HZ1	LYS	265	37.581	-0.131	46.455	1.00	0.00	RX0	H
ATOM	1040	HZ2	LYS	265	37.560	-1.480	45.446	1.00	0.00	RX0	H
ATOM	1041	HZ3	LYS	265	39.038	-0.754	45.823	1.00	0.00	RX0	H
ATOM	1042	C	LYS	265	38.994	-2.500	39.030	1.00	0.00	RX0	C
ATOM	1043	O	LYS	265	40.184	-2.535	38.765	1.00	0.00	RX0	O
ATOM	1044	N	CYS	266	38.153	-3.548	38.917	1.00	0.00	RX0	N
ATOM	1045	H	CYS	266	37.197	-3.465	39.197	1.00	0.00	RX0	H
ATOM	1046	CA	CYS	266	38.538	-4.854	38.355	1.00	0.00	RX0	C
ATOM	1047	CB	CYS	266	37.315	-5.759	38.263	1.00	0.00	RX0	C
ATOM	1048	SG	CYS	266	36.553	-5.947	39.897	1.00	0.00	RX0	S
ATOM	1049	C	CYS	266	39.318	-4.732	37.033	1.00	0.00	RX0	C
ATOM	1050	O	CYS	266	40.108	-5.603	36.695	1.00	0.00	RX0	O
ATOM	1051	N	VAL	267	39.075	-3.636	36.304	1.00	0.00	RX0	N
ATOM	1052	H	VAL	267	38.464	-2.907	36.621	1.00	0.00	RX0	H
ATOM	1053	CA	VAL	267	39.804	-3.345	35.058	1.00	0.00	RX0	C
ATOM	1054	CB	VAL	267	38.857	-3.352	33.851	1.00	0.00	RX0	C
ATOM	1055	CG1	VAL	267	39.587	-3.008	32.550	1.00	0.00	RX0	C
ATOM	1056	CG2	VAL	267	38.128	-4.694	33.738	1.00	0.00	RX0	C
ATOM	1057	C	VAL	267	40.557	-2.013	35.191	1.00	0.00	RX0	C
ATOM	1058	O	VAL	267	39.969	-0.956	35.468	1.00	0.00	RX0	O
ATOM	1059	N	GLU	268	41.829	-2.078	34.821	1.00	0.00	RX0	N
ATOM	1060	H	GLU	268	42.197	-2.986	34.625	1.00	0.00	RX0	H
ATOM	1061	CA	GLU	268	42.726	-0.908	34.727	1.00	0.00	RX0	C
ATOM	1062	CB	GLU	268	44.066	-1.342	34.148	1.00	0.00	RX0	C
ATOM	1063	CG	GLU	268	45.045	-1.757	35.243	1.00	0.00	RX0	C
ATOM	1064	CD	GLU	268	45.849	-0.542	35.661	1.00	0.00	RX0	C
ATOM	1065	OE1	GLU	268	45.307	0.560	35.757	1.00	0.00	RX0	O
ATOM	1066	OE2	GLU	268	47.066	-0.653	35.772	1.00	0.00	RX0	O
ATOM	1067	C	GLU	268	42.079	0.194	33.866	1.00	0.00	RX0	C
ATOM	1068	O	GLU	268	41.697	-0.027	32.727	1.00	0.00	RX0	O
ATOM	1069	N	GLY	269	41.924	1.355	34.524	1.00	0.00	RX0	N
ATOM	1070	H	GLY	269	42.290	1.363	35.454	1.00	0.00	RX0	H
ATOM	1071	CA	GLY	269	41.377	2.576	33.902	1.00	0.00	RX0	C
ATOM	1072	C	GLY	269	39.898	2.490	33.494	1.00	0.00	RX0	C
ATOM	1073	O	GLY	269	39.424	3.343	32.745	1.00	0.00	RX0	O
ATOM	1074	N	MET	270	39.146	1.598	34.134	1.00	0.00	RX0	N
ATOM	1075	H	MET	270	39.600	0.908	34.703	1.00	0.00	RX0	H
ATOM	1076	CA	MET	270	37.704	1.465	33.841	1.00	0.00	RX0	C
ATOM	1077	CB	MET	270	37.282	0.008	33.653	1.00	0.00	RX0	C
ATOM	1078	CG	MET	270	35.928	-0.098	32.947	1.00	0.00	RX0	C
ATOM	1079	SD	MET	270	35.423	-1.788	32.582	1.00	0.00	RX0	S
ATOM	1080	CE	MET	270	35.156	-2.355	34.267	1.00	0.00	RX0	C
ATOM	1081	C	MET	270	36.825	2.181	34.877	1.00	0.00	RX0	C
ATOM	1082	O	MET	270	35.781	2.734	34.514	1.00	0.00	RX0	O
ATOM	1083	N	VAL	271	37.310	2.290	36.107	1.00	0.00	RX0	N
ATOM	1084	H	VAL	271	38.135	1.764	36.307	1.00	0.00	RX0	H
ATOM	1085	CA	VAL	271	36.591	3.043	37.171	1.00	0.00	RX0	C
ATOM	1086	CB	VAL	271	37.304	2.885	38.527	1.00	0.00	RX0	C
ATOM	1087	CG1	VAL	271	38.768	3.322	38.474	1.00	0.00	RX0	C
ATOM	1088	CG2	VAL	271	36.515	3.528	39.672	1.00	0.00	RX0	C
ATOM	1089	C	VAL	271	36.343	4.502	36.749	1.00	0.00	RX0	C
ATOM	1090	O	VAL	271	35.261	5.071	37.095	1.00	0.00	RX0	O
ATOM	1091	N	GLU	272	37.228	5.074	36.011	1.00	0.00	RX0	N
ATOM	1092	H	GLU	272	38.064	4.560	35.837	1.00	0.00	RX0	H
ATOM	1093	CA	GLU	272	37.141	6.453	35.460	1.00	0.00	RX0	C
ATOM	1094	CB	GLU	272	38.425	6.719	34.675	1.00	0.00	RX0	C
ATOM	1095	CG	GLU	272	38.822	8.192	34.594	1.00	0.00	RX0	C
ATOM	1096	CD	GLU	272	39.224	8.710	35.962	1.00	0.00	RX0	C

ATOM	1097	OE1	GLU	272	39.802	7.970	36.753	1.00	0.00	RX0	O
ATOM	1098	OE2	GLU	272	38.990	9.879	36.233	1.00	0.00	RX0	O
ATOM	1099	C	GLU	272	35.927	6.588	34.526	1.00	0.00	RX0	C
ATOM	1100	O	GLU	272	35.142	7.521	34.681	1.00	0.00	RX0	O
ATOM	1101	N	ILE	273	35.702	5.552	33.729	1.00	0.00	RX0	N
ATOM	1102	H	ILE	273	36.324	4.773	33.795	1.00	0.00	RX0	H
ATOM	1103	CA	ILE	273	34.533	5.484	32.820	1.00	0.00	RX0	C
ATOM	1104	CB	ILE	273	34.724	4.374	31.776	1.00	0.00	RX0	C
ATOM	1105	CG2	ILE	273	33.599	4.413	30.742	1.00	0.00	RX0	C
ATOM	1106	CG1	ILE	273	36.107	4.407	31.116	1.00	0.00	RX0	C
ATOM	1107	CD1	ILE	273	36.332	5.632	30.228	1.00	0.00	RX0	C
ATOM	1108	C	ILE	273	33.249	5.235	33.628	1.00	0.00	RX0	C
ATOM	1109	O	ILE	273	32.257	5.951	33.452	1.00	0.00	RX0	O
ATOM	1110	N	PHE	274	33.320	4.302	34.575	1.00	0.00	RX0	N
ATOM	1111	H	PHE	274	34.185	3.812	34.684	1.00	0.00	RX0	H
ATOM	1112	CA	PHE	274	32.191	3.976	35.472	1.00	0.00	RX0	C
ATOM	1113	CB	PHE	274	32.608	2.931	36.511	1.00	0.00	RX0	C
ATOM	1114	CG	PHE	274	32.271	1.533	36.056	1.00	0.00	RX0	C
ATOM	1115	CD1	PHE	274	32.534	1.127	34.754	1.00	0.00	RX0	C
ATOM	1116	CD2	PHE	274	31.693	0.648	36.960	1.00	0.00	RX0	C
ATOM	1117	CE1	PHE	274	32.225	-0.169	34.360	1.00	0.00	RX0	C
ATOM	1118	CE2	PHE	274	31.385	-0.648	36.566	1.00	0.00	RX0	C
ATOM	1119	CZ	PHE	274	31.659	-1.058	35.268	1.00	0.00	RX0	C
ATOM	1120	C	PHE	274	31.669	5.203	36.222	1.00	0.00	RX0	C
ATOM	1121	O	PHE	274	30.484	5.521	36.143	1.00	0.00	RX0	O
ATOM	1122	N	ASP	275	32.607	5.983	36.757	1.00	0.00	RX0	N
ATOM	1123	H	ASP	275	33.552	5.665	36.811	1.00	0.00	RX0	H
ATOM	1124	CA	ASP	275	32.273	7.222	37.483	1.00	0.00	RX0	C
ATOM	1125	CB	ASP	275	33.529	7.899	38.038	1.00	0.00	RX0	C
ATOM	1126	CG	ASP	275	34.183	7.162	39.194	1.00	0.00	RX0	C
ATOM	1127	OD1	ASP	275	33.577	6.275	39.789	1.00	0.00	RX0	O
ATOM	1128	OD2	ASP	275	35.302	7.517	39.560	1.00	0.00	RX0	O
ATOM	1129	C	ASP	275	31.555	8.270	36.629	1.00	0.00	RX0	C
ATOM	1130	O	ASP	275	30.604	8.890	37.102	1.00	0.00	RX0	O
ATOM	1131	N	MET	276	31.921	8.336	35.353	1.00	0.00	RX0	N
ATOM	1132	H	MET	276	32.685	7.755	35.064	1.00	0.00	RX0	H
ATOM	1133	CA	MET	276	31.257	9.254	34.408	1.00	0.00	RX0	C
ATOM	1134	CB	MET	276	32.066	9.396	33.119	1.00	0.00	RX0	C
ATOM	1135	CG	MET	276	33.430	10.056	33.305	1.00	0.00	RX0	C
ATOM	1136	SD	MET	276	34.230	10.480	31.749	1.00	0.00	RX0	S
ATOM	1137	CE	MET	276	34.344	8.825	31.059	1.00	0.00	RX0	C
ATOM	1138	C	MET	276	29.833	8.798	34.061	1.00	0.00	RX0	C
ATOM	1139	O	MET	276	28.893	9.589	34.158	1.00	0.00	RX0	O
ATOM	1140	N	LEU	277	29.673	7.488	33.891	1.00	0.00	RX0	N
ATOM	1141	H	LEU	277	30.501	6.921	33.924	1.00	0.00	RX0	H
ATOM	1142	CA	LEU	277	28.362	6.866	33.606	1.00	0.00	RX0	C
ATOM	1143	CB	LEU	277	28.561	5.401	33.226	1.00	0.00	RX0	C
ATOM	1144	CG	LEU	277	29.417	5.209	31.974	1.00	0.00	RX0	C
ATOM	1145	CD1	LEU	277	29.911	3.769	31.835	1.00	0.00	RX0	C
ATOM	1146	CD2	LEU	277	28.701	5.697	30.717	1.00	0.00	RX0	C
ATOM	1147	C	LEU	277	27.393	6.992	34.787	1.00	0.00	RX0	C
ATOM	1148	O	LEU	277	26.257	7.447	34.627	1.00	0.00	RX0	O
ATOM	1149	N	LEU	278	27.939	6.795	35.983	1.00	0.00	RX0	N
ATOM	1150	H	LEU	278	28.900	6.514	36.017	1.00	0.00	RX0	H
ATOM	1151	CA	LEU	278	27.196	6.926	37.250	1.00	0.00	RX0	C
ATOM	1152	CB	LEU	278	28.066	6.468	38.419	1.00	0.00	RX0	C
ATOM	1153	CG	LEU	278	28.211	4.951	38.467	1.00	0.00	RX0	C
ATOM	1154	CD1	LEU	278	29.258	4.494	39.483	1.00	0.00	RX0	C
ATOM	1155	CD2	LEU	278	26.855	4.296	38.705	1.00	0.00	RX0	C
ATOM	1156	C	LEU	278	26.716	8.359	37.508	1.00	0.00	RX0	C
ATOM	1157	O	LEU	278	25.554	8.575	37.840	1.00	0.00	RX0	O

ATOM	1158	N	ALA	279	27.582	9.317	37.175	1.00	0.00	RX0	N
ATOM	1159	H	ALA	279	28.516	9.044	36.925	1.00	0.00	RX0	H
ATOM	1160	CA	ALA	279	27.275	10.754	37.296	1.00	0.00	RX0	C
ATOM	1161	CB	ALA	279	28.528	11.596	37.050	1.00	0.00	RX0	C
ATOM	1162	C	ALA	279	26.182	11.196	36.310	1.00	0.00	RX0	C
ATOM	1163	O	ALA	279	25.263	11.922	36.684	1.00	0.00	RX0	O
ATOM	1164	N	THR	280	26.210	10.618	35.109	1.00	0.00	RX0	N
ATOM	1165	H	THR	280	26.997	10.033	34.902	1.00	0.00	RX0	H
ATOM	1166	CA	THR	280	25.203	10.899	34.059	1.00	0.00	RX0	C
ATOM	1167	CB	THR	280	25.709	10.366	32.715	1.00	0.00	RX0	C
ATOM	1168	OG1	THR	280	27.025	10.872	32.452	1.00	0.00	RX0	O
ATOM	1169	HG1	THR	280	27.642	10.400	33.007	1.00	0.00	RX0	H
ATOM	1170	CG2	THR	280	24.758	10.733	31.573	1.00	0.00	RX0	C
ATOM	1171	C	THR	280	23.835	10.327	34.462	1.00	0.00	RX0	C
ATOM	1172	O	THR	280	22.822	11.023	34.397	1.00	0.00	RX0	O
ATOM	1173	N	SER	281	23.868	9.112	35.003	1.00	0.00	RX0	N
ATOM	1174	H	SER	281	24.740	8.620	35.047	1.00	0.00	RX0	H
ATOM	1175	CA	SER	281	22.669	8.413	35.507	1.00	0.00	RX0	C
ATOM	1176	CB	SER	281	23.147	6.985	35.866	1.00	0.00	RX0	C
ATOM	1177	OG	SER	281	22.390	6.339	36.909	1.00	0.00	RX0	O
ATOM	1178	HG	SER	281	22.606	5.412	36.828	1.00	0.00	RX0	H
ATOM	1179	C	SER	281	22.019	9.180	36.675	1.00	0.00	RX0	C
ATOM	1180	O	SER	281	20.814	9.399	36.693	1.00	0.00	RX0	O
ATOM	1181	N	SER	282	22.889	9.788	37.491	1.00	0.00	RX0	N
ATOM	1182	H	SER	282	23.868	9.616	37.378	1.00	0.00	RX0	H
ATOM	1183	CA	SER	282	22.489	10.660	38.613	1.00	0.00	RX0	C
ATOM	1184	CB	SER	282	23.672	10.721	39.559	1.00	0.00	RX0	C
ATOM	1185	OG	SER	282	23.939	9.341	39.872	1.00	0.00	RX0	O
ATOM	1186	HG	SER	282	24.721	9.118	39.367	1.00	0.00	RX0	H
ATOM	1187	C	SER	282	21.828	11.963	38.130	1.00	0.00	RX0	C
ATOM	1188	O	SER	282	20.788	12.371	38.639	1.00	0.00	RX0	O
ATOM	1189	N	ARG	283	22.365	12.516	37.039	1.00	0.00	RX0	N
ATOM	1190	H	ARG	283	23.192	12.114	36.639	1.00	0.00	RX0	H
ATOM	1191	CA	ARG	283	21.822	13.735	36.412	1.00	0.00	RX0	C
ATOM	1192	CB	ARG	283	22.798	14.310	35.377	1.00	0.00	RX0	C
ATOM	1193	CG	ARG	283	22.325	15.621	34.735	1.00	0.00	RX0	C
ATOM	1194	CD	ARG	283	22.157	16.794	35.708	1.00	0.00	RX0	C
ATOM	1195	NE	ARG	283	21.377	17.853	35.068	1.00	0.00	RX0	N
ATOM	1196	HE	ARG	283	21.028	17.665	34.148	1.00	0.00	RX0	H
ATOM	1197	CZ	ARG	283	21.035	18.988	35.715	1.00	0.00	RX0	C
ATOM	1198	NH1	ARG	283	21.522	19.287	36.917	1.00	0.00	RX0	N
ATOM	1199	HH11	ARG	283	21.203	20.118	37.387	1.00	0.00	RX0	H
ATOM	1200	HH12	ARG	283	22.202	18.717	37.382	1.00	0.00	RX0	H
ATOM	1201	NH2	ARG	283	20.180	19.827	35.142	1.00	0.00	RX0	N
ATOM	1202	HH21	ARG	283	19.889	20.650	35.644	1.00	0.00	RX0	H
ATOM	1203	HH22	ARG	283	19.782	19.689	34.235	1.00	0.00	RX0	H
ATOM	1204	C	ARG	283	20.432	13.477	35.812	1.00	0.00	RX0	C
ATOM	1205	O	ARG	283	19.498	14.246	36.035	1.00	0.00	RX0	O
ATOM	1206	N	PHE	284	20.293	12.325	35.169	1.00	0.00	RX0	N
ATOM	1207	H	PHE	284	21.100	11.744	35.040	1.00	0.00	RX0	H
ATOM	1208	CA	PHE	284	19.003	11.899	34.606	1.00	0.00	RX0	C
ATOM	1209	CB	PHE	284	19.201	10.641	33.768	1.00	0.00	RX0	C
ATOM	1210	CG	PHE	284	19.637	11.017	32.374	1.00	0.00	RX0	C
ATOM	1211	CD1	PHE	284	19.175	12.194	31.798	1.00	0.00	RX0	C
ATOM	1212	CD2	PHE	284	20.474	10.173	31.655	1.00	0.00	RX0	C
ATOM	1213	CE1	PHE	284	19.519	12.507	30.488	1.00	0.00	RX0	C
ATOM	1214	CE2	PHE	284	20.821	10.490	30.347	1.00	0.00	RX0	C
ATOM	1215	CZ	PHE	284	20.329	11.647	29.757	1.00	0.00	RX0	C
ATOM	1216	C	PHE	284	17.921	11.680	35.654	1.00	0.00	RX0	C
ATOM	1217	O	PHE	284	16.817	12.216	35.524	1.00	0.00	RX0	O
ATOM	1218	N	ARG	285	18.348	11.087	36.760	1.00	0.00	RX0	N

ATOM	1219	H	ARG	285	19.282	10.726	36.767	1.00	0.00	RX0	H
ATOM	1220	CA	ARG	285	17.485	10.841	37.923	1.00	0.00	RX0	C
ATOM	1221	CB	ARG	285	18.332	10.060	38.927	1.00	0.00	RX0	C
ATOM	1222	CG	ARG	285	17.761	9.791	40.320	1.00	0.00	RX0	C
ATOM	1223	CD	ARG	285	18.784	9.030	41.175	1.00	0.00	RX0	C
ATOM	1224	NE	ARG	285	19.205	7.823	40.467	1.00	0.00	RX0	N
ATOM	1225	HE	ARG	285	18.451	7.224	40.167	1.00	0.00	RX0	H
ATOM	1226	CZ	ARG	285	20.498	7.653	40.055	1.00	0.00	RX0	C
ATOM	1227	NH1	ARG	285	21.460	8.436	40.587	1.00	0.00	RX0	N
ATOM	1228	HH11	ARG	285	22.425	8.432	40.280	1.00	0.00	RX0	H
ATOM	1229	HH12	ARG	285	21.248	9.087	41.320	1.00	0.00	RX0	H
ATOM	1230	NH2	ARG	285	20.766	6.721	39.115	1.00	0.00	RX0	N
ATOM	1231	HH21	ARG	285	21.655	6.595	38.651	1.00	0.00	RX0	H
ATOM	1232	HH22	ARG	285	20.027	6.110	38.808	1.00	0.00	RX0	H
ATOM	1233	C	ARG	285	17.003	12.164	38.534	1.00	0.00	RX0	C
ATOM	1234	O	ARG	285	15.822	12.321	38.816	1.00	0.00	RX0	O
ATOM	1235	N	MET	286	17.922	13.130	38.591	1.00	0.00	RX0	N
ATOM	1236	H	MET	286	18.858	12.930	38.296	1.00	0.00	RX0	H
ATOM	1237	CA	MET	286	17.643	14.467	39.144	1.00	0.00	RX0	C
ATOM	1238	CB	MET	286	18.975	15.206	39.274	1.00	0.00	RX0	C
ATOM	1239	CG	MET	286	18.871	16.685	39.642	1.00	0.00	RX0	C
ATOM	1240	SD	MET	286	20.486	17.477	39.577	1.00	0.00	RX0	S
ATOM	1241	CE	MET	286	19.979	19.120	40.102	1.00	0.00	RX0	C
ATOM	1242	C	MET	286	16.681	15.257	38.243	1.00	0.00	RX0	C
ATOM	1243	O	MET	286	15.799	15.962	38.735	1.00	0.00	RX0	O
ATOM	1244	N	MET	287	16.911	15.161	36.942	1.00	0.00	RX0	N
ATOM	1245	H	MET	287	17.645	14.553	36.634	1.00	0.00	RX0	H
ATOM	1246	CA	MET	287	16.049	15.814	35.941	1.00	0.00	RX0	C
ATOM	1247	CB	MET	287	16.720	15.894	34.574	1.00	0.00	RX0	C
ATOM	1248	CG	MET	287	17.875	16.893	34.607	1.00	0.00	RX0	C
ATOM	1249	SD	MET	287	18.402	17.390	32.962	1.00	0.00	RX0	S
ATOM	1250	CE	MET	287	16.809	18.017	32.401	1.00	0.00	RX0	C
ATOM	1251	C	MET	287	14.674	15.156	35.849	1.00	0.00	RX0	C
ATOM	1252	O	MET	287	13.755	15.729	35.264	1.00	0.00	RX0	O
ATOM	1253	N	ASN	288	14.583	13.920	36.346	1.00	0.00	RX0	N
ATOM	1254	H	ASN	288	15.392	13.495	36.753	1.00	0.00	RX0	H
ATOM	1255	CA	ASN	288	13.391	13.066	36.242	1.00	0.00	RX0	C
ATOM	1256	CB	ASN	288	12.162	13.613	36.970	1.00	0.00	RX0	C
ATOM	1257	CG	ASN	288	11.009	12.656	36.726	1.00	0.00	RX0	C
ATOM	1258	OD1	ASN	288	11.176	11.443	36.647	1.00	0.00	RX0	O
ATOM	1259	ND2	ASN	288	9.820	13.272	36.586	1.00	0.00	RX0	N
ATOM	1260	HD21	ASN	288	9.743	14.264	36.677	1.00	0.00	RX0	H
ATOM	1261	HD22	ASN	288	8.992	12.752	36.367	1.00	0.00	RX0	H
ATOM	1262	C	ASN	288	13.076	12.836	34.753	1.00	0.00	RX0	C
ATOM	1263	O	ASN	288	11.986	13.121	34.256	1.00	0.00	RX0	O
ATOM	1264	N	LEU	289	14.125	12.439	34.028	1.00	0.00	RX0	N
ATOM	1265	H	LEU	289	14.957	12.177	34.522	1.00	0.00	RX0	H
ATOM	1266	CA	LEU	289	14.030	12.183	32.583	1.00	0.00	RX0	C
ATOM	1267	CB	LEU	289	15.372	11.627	32.097	1.00	0.00	RX0	C
ATOM	1268	CG	LEU	289	15.408	11.218	30.622	1.00	0.00	RX0	C
ATOM	1269	CD1	LEU	289	15.555	12.426	29.703	1.00	0.00	RX0	C
ATOM	1270	CD2	LEU	289	16.482	10.169	30.337	1.00	0.00	RX0	C
ATOM	1271	C	LEU	289	12.926	11.152	32.321	1.00	0.00	RX0	C
ATOM	1272	O	LEU	289	12.814	10.137	33.014	1.00	0.00	RX0	O
ATOM	1273	N	GLN	290	12.165	11.420	31.281	1.00	0.00	RX0	N
ATOM	1274	H	GLN	290	12.394	12.178	30.664	1.00	0.00	RX0	H
ATOM	1275	CA	GLN	290	11.011	10.584	30.916	1.00	0.00	RX0	C
ATOM	1276	CB	GLN	290	9.778	11.452	30.673	1.00	0.00	RX0	C
ATOM	1277	CG	GLN	290	9.409	12.301	31.898	1.00	0.00	RX0	C
ATOM	1278	CD	GLN	290	8.985	11.427	33.070	1.00	0.00	RX0	C
ATOM	1279	OE1	GLN	290	7.827	11.051	33.210	1.00	0.00	RX0	O

ATOM	1280	NE2	GLN	290	9.973	11.158	33.941	1.00	0.00	RX0	N
ATOM	1281	HE21	GLN	290	10.897	11.518	33.800	1.00	0.00	RX0	H
ATOM	1282	HE22	GLN	290	9.879	10.609	34.773	1.00	0.00	RX0	H
ATOM	1283	C	GLN	290	11.379	9.701	29.727	1.00	0.00	RX0	C
ATOM	1284	O	GLN	290	12.115	10.141	28.832	1.00	0.00	RX0	O
ATOM	1285	N	GLY	291	10.739	8.533	29.672	1.00	0.00	RX0	N
ATOM	1286	H	GLY	291	10.027	8.353	30.351	1.00	0.00	RX0	H
ATOM	1287	CA	GLY	291	10.952	7.542	28.589	1.00	0.00	RX0	C
ATOM	1288	C	GLY	291	10.769	8.134	27.181	1.00	0.00	RX0	C
ATOM	1289	O	GLY	291	11.559	7.865	26.269	1.00	0.00	RX0	O
ATOM	1290	N	GLU	292	9.854	9.086	27.078	1.00	0.00	RX0	N
ATOM	1291	H	GLU	292	9.255	9.248	27.866	1.00	0.00	RX0	H
ATOM	1292	CA	GLU	292	9.541	9.789	25.813	1.00	0.00	RX0	C
ATOM	1293	CB	GLU	292	8.215	10.552	25.942	1.00	0.00	RX0	C
ATOM	1294	CG	GLU	292	6.976	9.706	26.279	1.00	0.00	RX0	C
ATOM	1295	CD	GLU	292	7.096	9.116	27.672	1.00	0.00	RX0	C
ATOM	1296	OE1	GLU	292	7.473	9.844	28.589	1.00	0.00	RX0	O
ATOM	1297	OE2	GLU	292	6.914	7.912	27.823	1.00	0.00	RX0	O
ATOM	1298	C	GLU	292	10.671	10.744	25.400	1.00	0.00	RX0	C
ATOM	1299	O	GLU	292	11.065	10.802	24.241	1.00	0.00	RX0	O
ATOM	1300	N	GLU	293	11.241	11.417	26.395	1.00	0.00	RX0	N
ATOM	1301	H	GLU	293	10.951	11.211	27.330	1.00	0.00	RX0	H
ATOM	1302	CA	GLU	293	12.389	12.320	26.184	1.00	0.00	RX0	C
ATOM	1303	CB	GLU	293	12.636	13.156	27.436	1.00	0.00	RX0	C
ATOM	1304	CG	GLU	293	11.407	13.909	27.935	1.00	0.00	RX0	C
ATOM	1305	CD	GLU	293	11.726	14.503	29.291	1.00	0.00	RX0	C
ATOM	1306	OE1	GLU	293	12.642	14.031	29.956	1.00	0.00	RX0	O
ATOM	1307	OE2	GLU	293	11.057	15.440	29.707	1.00	0.00	RX0	O
ATOM	1308	C	GLU	293	13.669	11.544	25.843	1.00	0.00	RX0	C
ATOM	1309	O	GLU	293	14.364	11.888	24.886	1.00	0.00	RX0	O
ATOM	1310	N	PHE	294	13.855	10.409	26.517	1.00	0.00	RX0	N
ATOM	1311	H	PHE	294	13.194	10.199	27.241	1.00	0.00	RX0	H
ATOM	1312	CA	PHE	294	14.999	9.503	26.300	1.00	0.00	RX0	C
ATOM	1313	CB	PHE	294	14.905	8.307	27.252	1.00	0.00	RX0	C
ATOM	1314	CG	PHE	294	15.866	7.226	26.816	1.00	0.00	RX0	C
ATOM	1315	CD1	PHE	294	17.238	7.450	26.840	1.00	0.00	RX0	C
ATOM	1316	CD2	PHE	294	15.370	6.004	26.373	1.00	0.00	RX0	C
ATOM	1317	CE1	PHE	294	18.108	6.462	26.396	1.00	0.00	RX0	C
ATOM	1318	CE2	PHE	294	16.242	5.017	25.931	1.00	0.00	RX0	C
ATOM	1319	CZ	PHE	294	17.612	5.250	25.933	1.00	0.00	RX0	C
ATOM	1320	C	PHE	294	15.115	9.012	24.847	1.00	0.00	RX0	C
ATOM	1321	O	PHE	294	16.186	9.132	24.238	1.00	0.00	RX0	O
ATOM	1322	N	VAL	295	14.000	8.581	24.280	1.00	0.00	RX0	N
ATOM	1323	H	VAL	295	13.158	8.569	24.828	1.00	0.00	RX0	H
ATOM	1324	CA	VAL	295	13.976	8.026	22.907	1.00	0.00	RX0	C
ATOM	1325	CB	VAL	295	12.686	7.251	22.616	1.00	0.00	RX0	C
ATOM	1326	CG1	VAL	295	12.586	6.064	23.571	1.00	0.00	RX0	C
ATOM	1327	CG2	VAL	295	11.430	8.119	22.660	1.00	0.00	RX0	C
ATOM	1328	C	VAL	295	14.286	9.097	21.847	1.00	0.00	RX0	C
ATOM	1329	O	VAL	295	14.999	8.845	20.884	1.00	0.00	RX0	O
ATOM	1330	N	CYS	296	13.866	10.330	22.156	1.00	0.00	RX0	N
ATOM	1331	H	CYS	296	13.306	10.481	22.974	1.00	0.00	RX0	H
ATOM	1332	CA	CYS	296	14.167	11.498	21.314	1.00	0.00	RX0	C
ATOM	1333	CB	CYS	296	13.264	12.653	21.730	1.00	0.00	RX0	C
ATOM	1334	SG	CYS	296	11.518	12.259	21.464	1.00	0.00	RX0	S
ATOM	1335	C	CYS	296	15.661	11.843	21.352	1.00	0.00	RX0	C
ATOM	1336	O	CYS	296	16.290	11.969	20.305	1.00	0.00	RX0	O
ATOM	1337	N	LEU	297	16.247	11.750	22.547	1.00	0.00	RX0	N
ATOM	1338	H	LEU	297	15.681	11.549	23.351	1.00	0.00	RX0	H
ATOM	1339	CA	LEU	297	17.681	12.031	22.752	1.00	0.00	RX0	C
ATOM	1340	CB	LEU	297	18.018	12.110	24.239	1.00	0.00	RX0	C

ATOM	1341	CG	LEU	297	17.347	13.283	24.950	1.00	0.00	RX0	C
ATOM	1342	CD1	LEU	297	17.640	13.261	26.449	1.00	0.00	RX0	C
ATOM	1343	CD2	LEU	297	17.713	14.625	24.311	1.00	0.00	RX0	C
ATOM	1344	C	LEU	297	18.589	11.009	22.068	1.00	0.00	RX0	C
ATOM	1345	O	LEU	297	19.526	11.385	21.359	1.00	0.00	RX0	O
ATOM	1346	N	LYS	298	18.173	9.750	22.123	1.00	0.00	RX0	N
ATOM	1347	H	LYS	298	17.359	9.545	22.671	1.00	0.00	RX0	H
ATOM	1348	CA	LYS	298	18.945	8.654	21.515	1.00	0.00	RX0	C
ATOM	1349	CB	LYS	298	18.432	7.321	22.046	1.00	0.00	RX0	C
ATOM	1350	CG	LYS	298	19.254	6.122	21.582	1.00	0.00	RX0	C
ATOM	1351	CD	LYS	298	18.614	4.835	22.087	1.00	0.00	RX0	C
ATOM	1352	CE	LYS	298	17.097	5.003	22.047	1.00	0.00	RX0	C
ATOM	1353	NZ	LYS	298	16.429	3.710	22.157	1.00	0.00	RX0	N
ATOM	1354	HZ1	LYS	298	15.436	3.866	22.454	1.00	0.00	RX0	H
ATOM	1355	HZ2	LYS	298	16.294	3.227	21.248	1.00	0.00	RX0	H
ATOM	1356	HZ3	LYS	298	16.787	3.060	22.878	1.00	0.00	RX0	H
ATOM	1357	C	LYS	298	18.925	8.733	19.978	1.00	0.00	RX0	C
ATOM	1358	O	LYS	298	19.964	8.557	19.332	1.00	0.00	RX0	O
ATOM	1359	N	SER	299	17.791	9.152	19.431	1.00	0.00	RX0	N
ATOM	1360	H	SER	299	16.964	9.266	19.982	1.00	0.00	RX0	H
ATOM	1361	CA	SER	299	17.645	9.383	17.977	1.00	0.00	RX0	C
ATOM	1362	CB	SER	299	16.155	9.360	17.703	1.00	0.00	RX0	C
ATOM	1363	OG	SER	299	15.661	8.202	18.384	1.00	0.00	RX0	O
ATOM	1364	HG	SER	299	15.126	8.510	19.109	1.00	0.00	RX0	H
ATOM	1365	C	SER	299	18.416	10.621	17.504	1.00	0.00	RX0	C
ATOM	1366	O	SER	299	19.051	10.583	16.444	1.00	0.00	RX0	O
ATOM	1367	N	ILE	300	18.478	11.644	18.354	1.00	0.00	RX0	N
ATOM	1368	H	ILE	300	17.954	11.596	19.207	1.00	0.00	RX0	H
ATOM	1369	CA	ILE	300	19.283	12.860	18.096	1.00	0.00	RX0	C
ATOM	1370	CB	ILE	300	19.078	13.935	19.167	1.00	0.00	RX0	C
ATOM	1371	CG2	ILE	300	20.118	15.051	19.044	1.00	0.00	RX0	C
ATOM	1372	CG1	ILE	300	17.672	14.515	19.093	1.00	0.00	RX0	C
ATOM	1373	CD1	ILE	300	17.410	15.531	20.203	1.00	0.00	RX0	C
ATOM	1374	C	ILE	300	20.773	12.490	18.013	1.00	0.00	RX0	C
ATOM	1375	O	ILE	300	21.456	12.924	17.087	1.00	0.00	RX0	O
ATOM	1376	N	ILE	301	21.227	11.630	18.921	1.00	0.00	RX0	N
ATOM	1377	H	ILE	301	20.605	11.325	19.646	1.00	0.00	RX0	H
ATOM	1378	CA	ILE	301	22.629	11.156	18.925	1.00	0.00	RX0	C
ATOM	1379	CB	ILE	301	22.862	10.193	20.085	1.00	0.00	RX0	C
ATOM	1380	CG2	ILE	301	24.145	9.396	19.882	1.00	0.00	RX0	C
ATOM	1381	CG1	ILE	301	22.872	10.943	21.414	1.00	0.00	RX0	C
ATOM	1382	CD1	ILE	301	23.211	10.018	22.581	1.00	0.00	RX0	C
ATOM	1383	C	ILE	301	22.948	10.474	17.584	1.00	0.00	RX0	C
ATOM	1384	O	ILE	301	23.927	10.823	16.926	1.00	0.00	RX0	O
ATOM	1385	N	LEU	302	22.047	9.582	17.175	1.00	0.00	RX0	N
ATOM	1386	H	LEU	302	21.271	9.374	17.774	1.00	0.00	RX0	H
ATOM	1387	CA	LEU	302	22.205	8.836	15.917	1.00	0.00	RX0	C
ATOM	1388	CB	LEU	302	20.992	7.929	15.709	1.00	0.00	RX0	C
ATOM	1389	CG	LEU	302	20.999	7.210	14.360	1.00	0.00	RX0	C
ATOM	1390	CD1	LEU	302	22.180	6.251	14.223	1.00	0.00	RX0	C
ATOM	1391	CD2	LEU	302	19.664	6.526	14.081	1.00	0.00	RX0	C
ATOM	1392	C	LEU	302	22.391	9.757	14.699	1.00	0.00	RX0	C
ATOM	1393	O	LEU	302	23.285	9.544	13.882	1.00	0.00	RX0	O
ATOM	1394	N	LEU	303	21.581	10.806	14.658	1.00	0.00	RX0	N
ATOM	1395	H	LEU	303	20.942	10.954	15.416	1.00	0.00	RX0	H
ATOM	1396	CA	LEU	303	21.546	11.719	13.502	1.00	0.00	RX0	C
ATOM	1397	CB	LEU	303	20.124	12.231	13.311	1.00	0.00	RX0	C
ATOM	1398	CG	LEU	303	19.163	11.064	13.084	1.00	0.00	RX0	C
ATOM	1399	CD1	LEU	303	17.707	11.486	13.251	1.00	0.00	RX0	C
ATOM	1400	CD2	LEU	303	19.420	10.363	11.748	1.00	0.00	RX0	C
ATOM	1401	C	LEU	303	22.576	12.849	13.543	1.00	0.00	RX0	C

ATOM	1402	O	LEU	303	23.073	13.268	12.494	1.00	0.00	RX0	O
ATOM	1403	N	ASN	304	22.971	13.240	14.745	1.00	0.00	RX0	N
ATOM	1404	H	ASN	304	22.638	12.744	15.549	1.00	0.00	RX0	H
ATOM	1405	CA	ASN	304	23.832	14.419	14.940	1.00	0.00	RX0	C
ATOM	1406	CB	ASN	304	23.463	15.185	16.202	1.00	0.00	RX0	C
ATOM	1407	CG	ASN	304	24.573	16.164	16.521	1.00	0.00	RX0	C
ATOM	1408	OD1	ASN	304	24.934	17.032	15.735	1.00	0.00	RX0	O
ATOM	1409	ND2	ASN	304	25.057	16.013	17.768	1.00	0.00	RX0	N
ATOM	1410	HD21	ASN	304	24.715	15.277	18.355	1.00	0.00	RX0	H
ATOM	1411	HD22	ASN	304	25.729	16.661	18.129	1.00	0.00	RX0	H
ATOM	1412	C	ASN	304	25.330	14.097	14.987	1.00	0.00	RX0	C
ATOM	1413	O	ASN	304	26.132	14.783	14.344	1.00	0.00	RX0	O
ATOM	1414	N	SER	305	25.693	13.015	15.656	1.00	0.00	RX0	N
ATOM	1415	H	SER	305	25.039	12.406	16.117	1.00	0.00	RX0	H
ATOM	1416	CA	SER	305	27.104	12.765	16.012	1.00	0.00	RX0	C
ATOM	1417	CB	SER	305	27.035	11.557	16.902	1.00	0.00	RX0	C
ATOM	1418	OG	SER	305	26.070	11.931	17.895	1.00	0.00	RX0	O
ATOM	1419	HG	SER	305	26.124	11.230	18.550	1.00	0.00	RX0	H
ATOM	1420	C	SER	305	28.103	12.695	14.846	1.00	0.00	RX0	C
ATOM	1421	O	SER	305	29.198	13.228	14.946	1.00	0.00	RX0	O
ATOM	1422	N	GLY	306	27.634	12.177	13.693	1.00	0.00	RX0	N
ATOM	1423	H	GLY	306	26.684	11.869	13.632	1.00	0.00	RX0	H
ATOM	1424	CA	GLY	306	28.500	12.079	12.501	1.00	0.00	RX0	C
ATOM	1425	C	GLY	306	28.091	12.961	11.315	1.00	0.00	RX0	C
ATOM	1426	O	GLY	306	28.756	12.897	10.274	1.00	0.00	RX0	O
ATOM	1427	N	VAL	307	27.242	13.954	11.536	1.00	0.00	RX0	N
ATOM	1428	H	VAL	307	26.882	14.112	12.459	1.00	0.00	RX0	H
ATOM	1429	CA	VAL	307	26.760	14.815	10.435	1.00	0.00	RX0	C
ATOM	1430	CB	VAL	307	25.397	15.438	10.774	1.00	0.00	RX0	C
ATOM	1431	CG1	VAL	307	25.484	16.559	11.804	1.00	0.00	RX0	C
ATOM	1432	CG2	VAL	307	24.676	15.883	9.508	1.00	0.00	RX0	C
ATOM	1433	C	VAL	307	27.809	15.841	9.948	1.00	0.00	RX0	C
ATOM	1434	O	VAL	307	27.790	16.280	8.811	1.00	0.00	RX0	O
ATOM	1435	N	TYR	308	28.719	16.199	10.859	1.00	0.00	RX0	N
ATOM	1436	H	TYR	308	28.746	15.721	11.735	1.00	0.00	RX0	H
ATOM	1437	CA	TYR	308	29.761	17.211	10.584	1.00	0.00	RX0	C
ATOM	1438	CB	TYR	308	30.003	17.935	11.896	1.00	0.00	RX0	C
ATOM	1439	CG	TYR	308	28.722	18.666	12.230	1.00	0.00	RX0	C
ATOM	1440	CD1	TYR	308	28.138	19.460	11.255	1.00	0.00	RX0	C
ATOM	1441	CE1	TYR	308	26.963	20.144	11.527	1.00	0.00	RX0	C
ATOM	1442	CD2	TYR	308	28.127	18.562	13.485	1.00	0.00	RX0	C
ATOM	1443	CE2	TYR	308	26.950	19.257	13.758	1.00	0.00	RX0	C
ATOM	1444	CZ	TYR	308	26.363	20.045	12.773	1.00	0.00	RX0	C
ATOM	1445	OH	TYR	308	25.185	20.731	13.007	1.00	0.00	RX0	O
ATOM	1446	HH	TYR	308	24.812	20.412	13.827	1.00	0.00	RX0	H
ATOM	1447	C	TYR	308	31.032	16.660	9.940	1.00	0.00	RX0	C
ATOM	1448	O	TYR	308	31.912	17.411	9.537	1.00	0.00	RX0	O
ATOM	1449	N	THR	309	31.091	15.334	9.861	1.00	0.00	RX0	N
ATOM	1450	H	THR	309	30.336	14.763	10.177	1.00	0.00	RX0	H
ATOM	1451	CA	THR	309	32.303	14.643	9.386	1.00	0.00	RX0	C
ATOM	1452	CB	THR	309	32.567	13.747	10.578	1.00	0.00	RX0	C
ATOM	1453	OG1	THR	309	31.299	13.549	11.233	1.00	0.00	RX0	O
ATOM	1454	HG1	THR	309	30.846	12.872	10.730	1.00	0.00	RX0	H
ATOM	1455	CG2	THR	309	33.552	14.371	11.569	1.00	0.00	RX0	C
ATOM	1456	C	THR	309	32.183	13.866	8.071	1.00	0.00	RX0	C
ATOM	1457	O	THR	309	33.137	13.195	7.681	1.00	0.00	RX0	O
ATOM	1458	N	PHE	310	31.041	13.953	7.382	1.00	0.00	RX0	N
ATOM	1459	H	PHE	310	30.335	14.575	7.717	1.00	0.00	RX0	H
ATOM	1460	CA	PHE	310	30.972	13.467	5.988	1.00	0.00	RX0	C
ATOM	1461	CB	PHE	310	29.596	13.724	5.371	1.00	0.00	RX0	C
ATOM	1462	CG	PHE	310	28.504	13.019	6.137	1.00	0.00	RX0	C

ATOM	1463	CD1	PHE	310	28.540	11.640	6.303	1.00	0.00	RX0	C
ATOM	1464	CD2	PHE	310	27.448	13.754	6.664	1.00	0.00	RX0	C
ATOM	1465	CE1	PHE	310	27.515	11.000	6.989	1.00	0.00	RX0	C
ATOM	1466	CE2	PHE	310	26.425	13.110	7.348	1.00	0.00	RX0	C
ATOM	1467	CZ	PHE	310	26.456	11.732	7.511	1.00	0.00	RX0	C
ATOM	1468	C	PHE	310	32.019	14.245	5.180	1.00	0.00	RX0	C
ATOM	1469	O	PHE	310	32.102	15.468	5.301	1.00	0.00	RX0	O
ATOM	1470	N	LEU	311	32.854	13.508	4.462	1.00	0.00	RX0	N
ATOM	1471	H	LEU	311	32.728	12.518	4.482	1.00	0.00	RX0	H
ATOM	1472	CA	LEU	311	33.988	14.087	3.701	1.00	0.00	RX0	C
ATOM	1473	CB	LEU	311	34.881	13.007	3.081	1.00	0.00	RX0	C
ATOM	1474	CG	LEU	311	35.441	12.050	4.149	1.00	0.00	RX0	C
ATOM	1475	CD1	LEU	311	36.663	11.281	3.659	1.00	0.00	RX0	C
ATOM	1476	CD2	LEU	311	35.782	12.744	5.466	1.00	0.00	RX0	C
ATOM	1477	C	LEU	311	33.468	15.166	2.739	1.00	0.00	RX0	C
ATOM	1478	O	LEU	311	33.554	16.351	2.969	1.00	0.00	RX0	O
ATOM	1479	N	SER	312	32.784	14.619	1.713	1.00	0.00	RX0	N
ATOM	1480	H	SER	312	32.591	13.648	1.802	1.00	0.00	RX0	H
ATOM	1481	CA	SER	312	32.345	15.333	0.526	1.00	0.00	RX0	C
ATOM	1482	CB	SER	312	31.762	14.220	-0.345	1.00	0.00	RX0	C
ATOM	1483	OG	SER	312	31.621	13.013	0.434	1.00	0.00	RX0	O
ATOM	1484	HG	SER	312	32.351	12.439	0.191	1.00	0.00	RX0	H
ATOM	1485	C	SER	312	31.423	16.517	0.816	1.00	0.00	RX0	C
ATOM	1486	O	SER	312	31.042	16.847	1.948	1.00	0.00	RX0	O
ATOM	1487	N	SER	313	30.837	16.923	-0.276	1.00	0.00	RX0	N
ATOM	1488	H	SER	313	31.244	16.662	-1.151	1.00	0.00	RX0	H
ATOM	1489	CA	SER	313	29.838	17.983	-0.455	1.00	0.00	RX0	C
ATOM	1490	CB	SER	313	30.513	19.339	-0.188	1.00	0.00	RX0	C
ATOM	1491	OG	SER	313	31.567	19.217	0.785	1.00	0.00	RX0	O
ATOM	1492	HG	SER	313	31.205	18.725	1.524	1.00	0.00	RX0	H
ATOM	1493	C	SER	313	29.353	17.880	-1.909	1.00	0.00	RX0	C
ATOM	1494	O	SER	313	29.168	18.836	-2.628	1.00	0.00	RX0	O
ATOM	1495	N	THR	314	29.278	16.602	-2.358	1.00	0.00	RX0	N
ATOM	1496	H	THR	314	29.348	15.891	-1.668	1.00	0.00	RX0	H
ATOM	1497	CA	THR	314	28.609	16.250	-3.614	1.00	0.00	RX0	C
ATOM	1498	CB	THR	314	28.890	14.758	-3.704	1.00	0.00	RX0	C
ATOM	1499	OG1	THR	314	29.680	14.378	-2.565	1.00	0.00	RX0	O
ATOM	1500	HG1	THR	314	30.471	13.997	-2.952	1.00	0.00	RX0	H
ATOM	1501	CG2	THR	314	29.773	14.461	-4.900	1.00	0.00	RX0	C
ATOM	1502	C	THR	314	27.167	16.747	-3.490	1.00	0.00	RX0	C
ATOM	1503	O	THR	314	26.675	16.978	-2.368	1.00	0.00	RX0	O
ATOM	1504	N	LEU	315	26.451	16.801	-4.589	1.00	0.00	RX0	N
ATOM	1505	H	LEU	315	26.868	16.503	-5.448	1.00	0.00	RX0	H
ATOM	1506	CA	LEU	315	25.050	17.256	-4.537	1.00	0.00	RX0	C
ATOM	1507	CB	LEU	315	24.298	17.644	-5.790	1.00	0.00	RX0	C
ATOM	1508	CG	LEU	315	23.039	18.410	-5.392	1.00	0.00	RX0	C
ATOM	1509	CD1	LEU	315	23.358	19.834	-4.939	1.00	0.00	RX0	C
ATOM	1510	CD2	LEU	315	21.966	18.355	-6.466	1.00	0.00	RX0	C
ATOM	1511	C	LEU	315	24.190	16.343	-3.639	1.00	0.00	RX0	C
ATOM	1512	O	LEU	315	23.484	16.800	-2.757	1.00	0.00	RX0	O
ATOM	1513	N	LYS	316	24.524	15.049	-3.730	1.00	0.00	RX0	N
ATOM	1514	H	LYS	316	25.191	14.796	-4.425	1.00	0.00	RX0	H
ATOM	1515	CA	LYS	316	23.912	13.990	-2.918	1.00	0.00	RX0	C
ATOM	1516	CB	LYS	316	24.333	12.607	-3.405	1.00	0.00	RX0	C
ATOM	1517	CG	LYS	316	23.181	11.617	-3.234	1.00	0.00	RX0	C
ATOM	1518	CD	LYS	316	23.490	10.233	-3.800	1.00	0.00	RX0	C
ATOM	1519	CE	LYS	316	22.274	9.305	-3.742	1.00	0.00	RX0	C
ATOM	1520	NZ	LYS	316	22.687	7.965	-4.165	1.00	0.00	RX0	N
ATOM	1521	HZ1	LYS	316	21.941	7.244	-4.054	1.00	0.00	RX0	H
ATOM	1522	HZ2	LYS	316	22.951	7.961	-5.170	1.00	0.00	RX0	H
ATOM	1523	HZ3	LYS	316	23.501	7.631	-3.608	1.00	0.00	RX0	H

ATOM	1524	C	LYS	316	24.213	14.187	-1.422	1.00	0.00	RX0	C
ATOM	1525	O	LYS	316	23.297	14.194	-0.611	1.00	0.00	RX0	O
ATOM	1526	N	SER	317	25.467	14.540	-1.122	1.00	0.00	RX0	N
ATOM	1527	H	SER	317	26.150	14.652	-1.839	1.00	0.00	RX0	H
ATOM	1528	CA	SER	317	25.934	14.787	0.260	1.00	0.00	RX0	C
ATOM	1529	CB	SER	317	27.431	14.813	0.119	1.00	0.00	RX0	C
ATOM	1530	OG	SER	317	27.658	13.931	-0.988	1.00	0.00	RX0	O
ATOM	1531	HG	SER	317	28.597	13.902	-1.152	1.00	0.00	RX0	H
ATOM	1532	C	SER	317	25.242	16.001	0.898	1.00	0.00	RX0	C
ATOM	1533	O	SER	317	24.716	15.915	2.007	1.00	0.00	RX0	O
ATOM	1534	N	LEU	318	25.067	17.043	0.088	1.00	0.00	RX0	N
ATOM	1535	H	LEU	318	25.365	16.971	-0.866	1.00	0.00	RX0	H
ATOM	1536	CA	LEU	318	24.405	18.288	0.525	1.00	0.00	RX0	C
ATOM	1537	CB	LEU	318	24.576	19.374	-0.535	1.00	0.00	RX0	C
ATOM	1538	CG	LEU	318	26.043	19.750	-0.744	1.00	0.00	RX0	C
ATOM	1539	CD1	LEU	318	26.229	20.680	-1.942	1.00	0.00	RX0	C
ATOM	1540	CD2	LEU	318	26.668	20.313	0.533	1.00	0.00	RX0	C
ATOM	1541	C	LEU	318	22.918	18.061	0.823	1.00	0.00	RX0	C
ATOM	1542	O	LEU	318	22.412	18.453	1.877	1.00	0.00	RX0	O
ATOM	1543	N	GLU	319	22.299	17.244	-0.024	1.00	0.00	RX0	N
ATOM	1544	H	GLU	319	22.775	16.912	-0.843	1.00	0.00	RX0	H
ATOM	1545	CA	GLU	319	20.900	16.808	0.157	1.00	0.00	RX0	C
ATOM	1546	CB	GLU	319	20.443	16.083	-1.143	1.00	0.00	RX0	C
ATOM	1547	CG	GLU	319	20.434	16.962	-2.420	1.00	0.00	RX0	C
ATOM	1548	CD	GLU	319	20.311	16.188	-3.749	1.00	0.00	RX0	C
ATOM	1549	OE1	GLU	319	21.094	15.273	-4.012	1.00	0.00	RX0	O
ATOM	1550	OE2	GLU	319	19.465	16.545	-4.573	1.00	0.00	RX0	O
ATOM	1551	C	GLU	319	20.725	15.924	1.402	1.00	0.00	RX0	C
ATOM	1552	O	GLU	319	19.808	16.153	2.196	1.00	0.00	RX0	O
ATOM	1553	N	GLU	320	21.706	15.058	1.645	1.00	0.00	RX0	N
ATOM	1554	H	GLU	320	22.399	14.923	0.936	1.00	0.00	RX0	H
ATOM	1555	CA	GLU	320	21.731	14.149	2.812	1.00	0.00	RX0	C
ATOM	1556	CB	GLU	320	22.945	13.206	2.801	1.00	0.00	RX0	C
ATOM	1557	CG	GLU	320	23.060	12.193	1.655	1.00	0.00	RX0	C
ATOM	1558	CD	GLU	320	22.188	10.971	1.862	1.00	0.00	RX0	C
ATOM	1559	OE1	GLU	320	20.970	11.070	1.761	1.00	0.00	RX0	O
ATOM	1560	OE2	GLU	320	22.739	9.899	2.085	1.00	0.00	RX0	O
ATOM	1561	C	GLU	320	21.765	14.931	4.131	1.00	0.00	RX0	C
ATOM	1562	O	GLU	320	20.881	14.754	4.973	1.00	0.00	RX0	O
ATOM	1563	N	LYS	321	22.647	15.926	4.188	1.00	0.00	RX0	N
ATOM	1564	H	LYS	321	23.257	16.042	3.400	1.00	0.00	RX0	H
ATOM	1565	CA	LYS	321	22.801	16.771	5.391	1.00	0.00	RX0	C
ATOM	1566	CB	LYS	321	24.036	17.671	5.231	1.00	0.00	RX0	C
ATOM	1567	CG	LYS	321	24.696	18.054	6.560	1.00	0.00	RX0	C
ATOM	1568	CD	LYS	321	25.876	19.032	6.451	1.00	0.00	RX0	C
ATOM	1569	CE	LYS	321	26.655	19.130	7.771	1.00	0.00	RX0	C
ATOM	1570	NZ	LYS	321	27.622	20.232	7.755	1.00	0.00	RX0	N
ATOM	1571	HZ1	LYS	321	28.316	20.152	8.529	1.00	0.00	RX0	H
ATOM	1572	HZ2	LYS	321	28.244	20.264	6.922	1.00	0.00	RX0	H
ATOM	1573	HZ3	LYS	321	27.186	21.166	7.893	1.00	0.00	RX0	H
ATOM	1574	C	LYS	321	21.549	17.612	5.656	1.00	0.00	RX0	C
ATOM	1575	O	LYS	321	21.102	17.724	6.798	1.00	0.00	RX0	O
ATOM	1576	N	ASP	322	20.935	18.099	4.575	1.00	0.00	RX0	N
ATOM	1577	H	ASP	322	21.320	17.942	3.662	1.00	0.00	RX0	H
ATOM	1578	CA	ASP	322	19.715	18.915	4.684	1.00	0.00	RX0	C
ATOM	1579	CB	ASP	322	19.384	19.462	3.293	1.00	0.00	RX0	C
ATOM	1580	CG	ASP	322	18.048	20.168	3.285	1.00	0.00	RX0	C
ATOM	1581	OD1	ASP	322	17.664	20.745	4.292	1.00	0.00	RX0	O
ATOM	1582	OD2	ASP	322	17.354	20.107	2.275	1.00	0.00	RX0	O
ATOM	1583	C	ASP	322	18.561	18.097	5.282	1.00	0.00	RX0	C
ATOM	1584	O	ASP	322	17.955	18.512	6.263	1.00	0.00	RX0	O

ATOM	1585	N	HIS	323	18.423	16.872	4.772	1.00	0.00	RX0	N
ATOM	1586	H	HIS	323	19.042	16.602	4.028	1.00	0.00	RX0	H
ATOM	1587	CA	HIS	323	17.423	15.916	5.274	1.00	0.00	RX0	C
ATOM	1588	CB	HIS	323	17.464	14.618	4.466	1.00	0.00	RX0	C
ATOM	1589	CG	HIS	323	16.311	13.740	4.889	1.00	0.00	RX0	C
ATOM	1590	ND1	HIS	323	16.359	12.398	4.973	1.00	0.00	RX0	N
ATOM	1591	HD1	HIS	323	17.124	11.811	4.774	1.00	0.00	RX0	H
ATOM	1592	CD2	HIS	323	15.028	14.163	5.240	1.00	0.00	RX0	C
ATOM	1593	NE2	HIS	323	14.297	13.066	5.537	1.00	0.00	RX0	N
ATOM	1594	CE1	HIS	323	15.116	11.973	5.374	1.00	0.00	RX0	C
ATOM	1595	C	HIS	323	17.630	15.607	6.766	1.00	0.00	RX0	C
ATOM	1596	O	HIS	323	16.677	15.663	7.540	1.00	0.00	RX0	O
ATOM	1597	N	ILE	324	18.888	15.420	7.164	1.00	0.00	RX0	N
ATOM	1598	H	ILE	324	19.611	15.423	6.468	1.00	0.00	RX0	H
ATOM	1599	CA	ILE	324	19.235	15.107	8.570	1.00	0.00	RX0	C
ATOM	1600	CB	ILE	324	20.716	14.755	8.707	1.00	0.00	RX0	C
ATOM	1601	CG2	ILE	324	21.109	14.598	10.175	1.00	0.00	RX0	C
ATOM	1602	CG1	ILE	324	21.027	13.487	7.912	1.00	0.00	RX0	C
ATOM	1603	CD1	ILE	324	22.512	13.122	7.916	1.00	0.00	RX0	C
ATOM	1604	C	ILE	324	18.844	16.271	9.496	1.00	0.00	RX0	C
ATOM	1605	O	ILE	324	18.200	16.053	10.527	1.00	0.00	RX0	O
ATOM	1606	N	HIS	325	19.159	17.485	9.071	1.00	0.00	RX0	N
ATOM	1607	H	HIS	325	19.583	17.591	8.169	1.00	0.00	RX0	H
ATOM	1608	CA	HIS	325	18.840	18.694	9.856	1.00	0.00	RX0	C
ATOM	1609	CB	HIS	325	19.570	19.920	9.312	1.00	0.00	RX0	C
ATOM	1610	CG	HIS	325	21.011	19.868	9.755	1.00	0.00	RX0	C
ATOM	1611	ND1	HIS	325	21.980	19.238	9.069	1.00	0.00	RX0	N
ATOM	1612	HD1	HIS	325	21.857	18.753	8.223	1.00	0.00	RX0	H
ATOM	1613	CD2	HIS	325	21.566	20.429	10.909	1.00	0.00	RX0	C
ATOM	1614	NE2	HIS	325	22.889	20.128	10.909	1.00	0.00	RX0	N
ATOM	1615	CE1	HIS	325	23.141	19.396	9.778	1.00	0.00	RX0	C
ATOM	1616	C	HIS	325	17.335	18.955	9.948	1.00	0.00	RX0	C
ATOM	1617	O	HIS	325	16.820	19.290	11.021	1.00	0.00	RX0	O
ATOM	1618	N	ARG	326	16.631	18.584	8.887	1.00	0.00	RX0	N
ATOM	1619	H	ARG	326	17.125	18.357	8.047	1.00	0.00	RX0	H
ATOM	1620	CA	ARG	326	15.160	18.594	8.873	1.00	0.00	RX0	C
ATOM	1621	CB	ARG	326	14.655	18.406	7.441	1.00	0.00	RX0	C
ATOM	1622	CG	ARG	326	14.682	19.706	6.634	1.00	0.00	RX0	C
ATOM	1623	CD	ARG	326	14.146	19.555	5.206	1.00	0.00	RX0	C
ATOM	1624	NE	ARG	326	15.192	19.184	4.252	1.00	0.00	RX0	N
ATOM	1625	HE	ARG	326	15.928	19.869	4.107	1.00	0.00	RX0	H
ATOM	1626	CZ	ARG	326	15.164	18.006	3.567	1.00	0.00	RX0	C
ATOM	1627	NH1	ARG	326	14.197	17.108	3.846	1.00	0.00	RX0	N
ATOM	1628	HH11	ARG	326	14.121	16.228	3.370	1.00	0.00	RX0	H
ATOM	1629	HH12	ARG	326	13.518	17.300	4.558	1.00	0.00	RX0	H
ATOM	1630	NH2	ARG	326	16.096	17.753	2.625	1.00	0.00	RX0	N
ATOM	1631	HH21	ARG	326	16.191	16.905	2.102	1.00	0.00	RX0	H
ATOM	1632	HH22	ARG	326	16.769	18.482	2.414	1.00	0.00	RX0	H
ATOM	1633	C	ARG	326	14.537	17.576	9.843	1.00	0.00	RX0	C
ATOM	1634	O	ARG	326	13.617	17.928	10.589	1.00	0.00	RX0	O
ATOM	1635	N	VAL	327	15.171	16.419	9.987	1.00	0.00	RX0	N
ATOM	1636	H	VAL	327	15.982	16.248	9.422	1.00	0.00	RX0	H
ATOM	1637	CA	VAL	327	14.709	15.376	10.935	1.00	0.00	RX0	C
ATOM	1638	CB	VAL	327	15.278	13.994	10.607	1.00	0.00	RX0	C
ATOM	1639	CG1	VAL	327	14.753	12.958	11.601	1.00	0.00	RX0	C
ATOM	1640	CG2	VAL	327	14.939	13.578	9.176	1.00	0.00	RX0	C
ATOM	1641	C	VAL	327	15.041	15.781	12.381	1.00	0.00	RX0	C
ATOM	1642	O	VAL	327	14.187	15.661	13.270	1.00	0.00	RX0	O
ATOM	1643	N	LEU	328	16.212	16.371	12.574	1.00	0.00	RX0	N
ATOM	1644	H	LEU	328	16.818	16.508	11.789	1.00	0.00	RX0	H
ATOM	1645	CA	LEU	328	16.631	16.899	13.887	1.00	0.00	RX0	C

ATOM	1646	CB	LEU	328	18.068	17.414	13.827	1.00	0.00	RX0	C
ATOM	1647	CG	LEU	328	19.088	16.278	13.756	1.00	0.00	RX0	C
ATOM	1648	CD1	LEU	328	20.504	16.792	13.489	1.00	0.00	RX0	C
ATOM	1649	CD2	LEU	328	19.020	15.401	15.007	1.00	0.00	RX0	C
ATOM	1650	C	LEU	328	15.692	18.005	14.386	1.00	0.00	RX0	C
ATOM	1651	O	LEU	328	15.231	17.955	15.519	1.00	0.00	RX0	O
ATOM	1652	N	ASP	329	15.222	18.821	13.436	1.00	0.00	RX0	N
ATOM	1653	H	ASP	329	15.683	18.906	12.550	1.00	0.00	RX0	H
ATOM	1654	CA	ASP	329	14.223	19.870	13.722	1.00	0.00	RX0	C
ATOM	1655	CB	ASP	329	14.041	20.809	12.527	1.00	0.00	RX0	C
ATOM	1656	CG	ASP	329	15.257	21.699	12.365	1.00	0.00	RX0	C
ATOM	1657	OD1	ASP	329	16.057	21.781	13.297	1.00	0.00	RX0	O
ATOM	1658	OD2	ASP	329	15.398	22.315	11.308	1.00	0.00	RX0	O
ATOM	1659	C	ASP	329	12.864	19.312	14.154	1.00	0.00	RX0	C
ATOM	1660	O	ASP	329	12.272	19.788	15.128	1.00	0.00	RX0	O
ATOM	1661	N	LYS	330	12.463	18.218	13.512	1.00	0.00	RX0	N
ATOM	1662	H	LYS	330	13.021	17.922	12.735	1.00	0.00	RX0	H
ATOM	1663	CA	LYS	330	11.217	17.525	13.871	1.00	0.00	RX0	C
ATOM	1664	CB	LYS	330	10.799	16.459	12.860	1.00	0.00	RX0	C
ATOM	1665	CG	LYS	330	9.623	15.606	13.368	1.00	0.00	RX0	C
ATOM	1666	CD	LYS	330	8.369	16.384	13.797	1.00	0.00	RX0	C
ATOM	1667	CE	LYS	330	7.826	17.309	12.716	1.00	0.00	RX0	C
ATOM	1668	NZ	LYS	330	7.437	16.477	11.578	1.00	0.00	RX0	N
ATOM	1669	HZ1	LYS	330	7.123	17.094	10.809	1.00	0.00	RX0	H
ATOM	1670	HZ2	LYS	330	8.247	15.891	11.280	1.00	0.00	RX0	H
ATOM	1671	HZ3	LYS	330	6.657	15.858	11.882	1.00	0.00	RX0	H
ATOM	1672	C	LYS	330	11.283	16.915	15.278	1.00	0.00	RX0	C
ATOM	1673	O	LYS	330	10.354	17.095	16.067	1.00	0.00	RX0	O
ATOM	1674	N	ILE	331	12.441	16.375	15.625	1.00	0.00	RX0	N
ATOM	1675	H	ILE	331	13.185	16.378	14.951	1.00	0.00	RX0	H
ATOM	1676	CA	ILE	331	12.656	15.802	16.972	1.00	0.00	RX0	C
ATOM	1677	CB	ILE	331	13.952	14.996	17.053	1.00	0.00	RX0	C
ATOM	1678	CG2	ILE	331	14.060	14.327	18.421	1.00	0.00	RX0	C
ATOM	1679	CG1	ILE	331	14.043	13.952	15.942	1.00	0.00	RX0	C
ATOM	1680	CD1	ILE	331	15.358	13.174	15.983	1.00	0.00	RX0	C
ATOM	1681	C	ILE	331	12.642	16.922	18.027	1.00	0.00	RX0	C
ATOM	1682	O	ILE	331	12.078	16.732	19.120	1.00	0.00	RX0	O
ATOM	1683	N	THR	332	13.158	18.084	17.675	1.00	0.00	RX0	N
ATOM	1684	H	THR	332	13.578	18.202	16.774	1.00	0.00	RX0	H
ATOM	1685	CA	THR	332	13.155	19.265	18.570	1.00	0.00	RX0	C
ATOM	1686	CB	THR	332	14.035	20.314	17.913	1.00	0.00	RX0	C
ATOM	1687	OG1	THR	332	15.301	19.703	17.627	1.00	0.00	RX0	O
ATOM	1688	HG1	THR	332	15.254	19.373	16.734	1.00	0.00	RX0	H
ATOM	1689	CG2	THR	332	14.211	21.553	18.792	1.00	0.00	RX0	C
ATOM	1690	C	THR	332	11.706	19.696	18.836	1.00	0.00	RX0	C
ATOM	1691	O	THR	332	11.302	19.832	19.995	1.00	0.00	RX0	O
ATOM	1692	N	ASP	333	10.912	19.714	17.772	1.00	0.00	RX0	N
ATOM	1693	H	ASP	333	11.280	19.672	16.837	1.00	0.00	RX0	H
ATOM	1694	CA	ASP	333	9.466	20.017	17.863	1.00	0.00	RX0	C
ATOM	1695	CB	ASP	333	8.671	19.792	16.567	1.00	0.00	RX0	C
ATOM	1696	CG	ASP	333	9.096	20.580	15.355	1.00	0.00	RX0	C
ATOM	1697	OD1	ASP	333	9.501	21.728	15.508	1.00	0.00	RX0	O
ATOM	1698	OD2	ASP	333	8.968	20.041	14.252	1.00	0.00	RX0	O
ATOM	1699	C	ASP	333	8.732	19.000	18.747	1.00	0.00	RX0	C
ATOM	1700	O	ASP	333	7.880	19.374	19.559	1.00	0.00	RX0	O
ATOM	1701	N	THR	334	9.187	17.759	18.682	1.00	0.00	RX0	N
ATOM	1702	H	THR	334	9.941	17.583	18.048	1.00	0.00	RX0	H
ATOM	1703	CA	THR	334	8.631	16.638	19.462	1.00	0.00	RX0	C
ATOM	1704	CB	THR	334	9.177	15.373	18.821	1.00	0.00	RX0	C
ATOM	1705	OG1	THR	334	8.793	15.339	17.438	1.00	0.00	RX0	O
ATOM	1706	HG1	THR	334	9.149	16.125	17.032	1.00	0.00	RX0	H

ATOM	1707	CG2	THR	334	8.726	14.119	19.558	1.00	0.00	RX0	C
ATOM	1708	C	THR	334	8.961	16.782	20.953	1.00	0.00	RX0	C
ATOM	1709	O	THR	334	8.059	16.689	21.789	1.00	0.00	RX0	O
ATOM	1710	N	LEU	335	10.217	17.081	21.263	1.00	0.00	RX0	N
ATOM	1711	H	LEU	335	10.880	17.204	20.523	1.00	0.00	RX0	H
ATOM	1712	CA	LEU	335	10.648	17.349	22.650	1.00	0.00	RX0	C
ATOM	1713	CB	LEU	335	12.150	17.610	22.696	1.00	0.00	RX0	C
ATOM	1714	CG	LEU	335	12.958	16.320	22.781	1.00	0.00	RX0	C
ATOM	1715	CD1	LEU	335	14.457	16.567	22.609	1.00	0.00	RX0	C
ATOM	1716	CD2	LEU	335	12.650	15.571	24.078	1.00	0.00	RX0	C
ATOM	1717	C	LEU	335	9.903	18.522	23.297	1.00	0.00	RX0	C
ATOM	1718	O	LEU	335	9.384	18.379	24.399	1.00	0.00	RX0	O
ATOM	1719	N	ILE	336	9.668	19.567	22.501	1.00	0.00	RX0	N
ATOM	1720	H	ILE	336	10.071	19.579	21.582	1.00	0.00	RX0	H
ATOM	1721	CA	ILE	336	8.897	20.743	22.962	1.00	0.00	RX0	C
ATOM	1722	CB	ILE	336	9.048	21.912	21.989	1.00	0.00	RX0	C
ATOM	1723	CG2	ILE	336	8.088	23.056	22.324	1.00	0.00	RX0	C
ATOM	1724	CG1	ILE	336	10.500	22.389	21.997	1.00	0.00	RX0	C
ATOM	1725	CD1	ILE	336	10.927	22.872	23.385	1.00	0.00	RX0	C
ATOM	1726	C	ILE	336	7.427	20.365	23.180	1.00	0.00	RX0	C
ATOM	1727	O	ILE	336	6.836	20.730	24.203	1.00	0.00	RX0	O
ATOM	1728	N	HIS	337	6.891	19.571	22.265	1.00	0.00	RX0	N
ATOM	1729	H	HIS	337	7.438	19.318	21.465	1.00	0.00	RX0	H
ATOM	1730	CA	HIS	337	5.501	19.096	22.359	1.00	0.00	RX0	C
ATOM	1731	CB	HIS	337	5.081	18.332	21.113	1.00	0.00	RX0	C
ATOM	1732	CG	HIS	337	3.596	18.091	21.195	1.00	0.00	RX0	C
ATOM	1733	ND1	HIS	337	2.668	19.012	20.880	1.00	0.00	RX0	N
ATOM	1734	HD1	HIS	337	2.840	19.920	20.547	1.00	0.00	RX0	H
ATOM	1735	CD2	HIS	337	2.950	16.932	21.621	1.00	0.00	RX0	C
ATOM	1736	NE2	HIS	337	1.613	17.161	21.566	1.00	0.00	RX0	N
ATOM	1737	CE1	HIS	337	1.441	18.444	21.106	1.00	0.00	RX0	C
ATOM	1738	C	HIS	337	5.301	18.263	23.631	1.00	0.00	RX0	C
ATOM	1739	O	HIS	337	4.339	18.490	24.365	1.00	0.00	RX0	O
ATOM	1740	N	LEU	338	6.274	17.405	23.914	1.00	0.00	RX0	N
ATOM	1741	H	LEU	338	7.051	17.336	23.286	1.00	0.00	RX0	H
ATOM	1742	CA	LEU	338	6.246	16.526	25.097	1.00	0.00	RX0	C
ATOM	1743	CB	LEU	338	7.441	15.575	25.089	1.00	0.00	RX0	C
ATOM	1744	CG	LEU	338	7.342	14.530	23.982	1.00	0.00	RX0	C
ATOM	1745	CD1	LEU	338	8.646	13.749	23.814	1.00	0.00	RX0	C
ATOM	1746	CD2	LEU	338	6.125	13.627	24.183	1.00	0.00	RX0	C
ATOM	1747	C	LEU	338	6.240	17.331	26.400	1.00	0.00	RX0	C
ATOM	1748	O	LEU	338	5.410	17.098	27.277	1.00	0.00	RX0	O
ATOM	1749	N	MET	339	7.027	18.402	26.394	1.00	0.00	RX0	N
ATOM	1750	H	MET	339	7.615	18.549	25.595	1.00	0.00	RX0	H
ATOM	1751	CA	MET	339	7.168	19.308	27.550	1.00	0.00	RX0	C
ATOM	1752	CB	MET	339	8.406	20.190	27.400	1.00	0.00	RX0	C
ATOM	1753	CG	MET	339	9.705	19.389	27.372	1.00	0.00	RX0	C
ATOM	1754	SD	MET	339	11.139	20.426	27.055	1.00	0.00	RX0	S
ATOM	1755	CE	MET	339	12.272	19.113	26.583	1.00	0.00	RX0	C
ATOM	1756	C	MET	339	5.924	20.183	27.753	1.00	0.00	RX0	C
ATOM	1757	O	MET	339	5.433	20.322	28.878	1.00	0.00	RX0	O
ATOM	1758	N	ALA	340	5.370	20.672	26.646	1.00	0.00	RX0	N
ATOM	1759	H	ALA	340	5.814	20.472	25.772	1.00	0.00	RX0	H
ATOM	1760	CA	ALA	340	4.120	21.456	26.636	1.00	0.00	RX0	C
ATOM	1761	CB	ALA	340	3.835	21.992	25.232	1.00	0.00	RX0	C
ATOM	1762	C	ALA	340	2.921	20.619	27.100	1.00	0.00	RX0	C
ATOM	1763	O	ALA	340	2.176	21.064	27.967	1.00	0.00	RX0	O
ATOM	1764	N	LYS	341	2.865	19.360	26.655	1.00	0.00	RX0	N
ATOM	1765	H	LYS	341	3.552	19.058	25.994	1.00	0.00	RX0	H
ATOM	1766	CA	LYS	341	1.846	18.397	27.117	1.00	0.00	RX0	C
ATOM	1767	CB	LYS	341	1.979	17.108	26.280	1.00	0.00	RX0	C

ATOM	1768	CG	LYS	341	0.705	16.260	26.146	1.00	0.00	RX0	C
ATOM	1769	CD	LYS	341	0.384	15.817	24.703	1.00	0.00	RX0	C
ATOM	1770	CE	LYS	341	1.352	14.815	24.047	1.00	0.00	RX0	C
ATOM	1771	NZ	LYS	341	1.001	14.626	22.627	1.00	0.00	RX0	N
ATOM	1772	HZ1	LYS	341	1.714	14.096	22.075	1.00	0.00	RX0	H
ATOM	1773	HZ2	LYS	341	0.149	14.058	22.435	1.00	0.00	RX0	H
ATOM	1774	HZ3	LYS	341	0.917	15.518	22.101	1.00	0.00	RX0	H
ATOM	1775	C	LYS	341	1.927	18.179	28.637	1.00	0.00	RX0	C
ATOM	1776	O	LYS	341	0.908	18.032	29.304	1.00	0.00	RX0	O
ATOM	1777	N	ALA	342	3.166	18.103	29.120	1.00	0.00	RX0	N
ATOM	1778	H	ALA	342	3.940	18.170	28.488	1.00	0.00	RX0	H
ATOM	1779	CA	ALA	342	3.459	17.942	30.556	1.00	0.00	RX0	C
ATOM	1780	CB	ALA	342	4.954	17.706	30.786	1.00	0.00	RX0	C
ATOM	1781	C	ALA	342	3.007	19.166	31.371	1.00	0.00	RX0	C
ATOM	1782	O	ALA	342	2.879	19.099	32.588	1.00	0.00	RX0	O
ATOM	1783	N	GLY	343	2.900	20.305	30.669	1.00	0.00	RX0	N
ATOM	1784	H	GLY	343	3.153	20.317	29.702	1.00	0.00	RX0	H
ATOM	1785	CA	GLY	343	2.393	21.560	31.244	1.00	0.00	RX0	C
ATOM	1786	C	GLY	343	3.505	22.433	31.829	1.00	0.00	RX0	C
ATOM	1787	O	GLY	343	3.244	23.282	32.678	1.00	0.00	RX0	O
ATOM	1788	N	LEU	344	4.736	22.226	31.356	1.00	0.00	RX0	N
ATOM	1789	H	LEU	344	4.853	21.555	30.623	1.00	0.00	RX0	H
ATOM	1790	CA	LEU	344	5.823	23.174	31.634	1.00	0.00	RX0	C
ATOM	1791	CB	LEU	344	7.151	22.611	31.138	1.00	0.00	RX0	C
ATOM	1792	CG	LEU	344	7.554	21.337	31.877	1.00	0.00	RX0	C
ATOM	1793	CD1	LEU	344	8.837	20.743	31.301	1.00	0.00	RX0	C
ATOM	1794	CD2	LEU	344	7.655	21.562	33.386	1.00	0.00	RX0	C
ATOM	1795	C	LEU	344	5.508	24.488	30.929	1.00	0.00	RX0	C
ATOM	1796	O	LEU	344	4.977	24.500	29.792	1.00	0.00	RX0	O
ATOM	1797	N	THR	345	5.822	25.577	31.575	1.00	0.00	RX0	N
ATOM	1798	H	THR	345	6.293	25.483	32.451	1.00	0.00	RX0	H
ATOM	1799	CA	THR	345	5.705	26.912	30.948	1.00	0.00	RX0	C
ATOM	1800	CB	THR	345	5.999	27.963	32.012	1.00	0.00	RX0	C
ATOM	1801	OG1	THR	345	7.167	27.596	32.747	1.00	0.00	RX0	O
ATOM	1802	HG1	THR	345	6.888	26.953	33.396	1.00	0.00	RX0	H
ATOM	1803	CG2	THR	345	4.817	28.130	32.967	1.00	0.00	RX0	C
ATOM	1804	C	THR	345	6.639	26.971	29.731	1.00	0.00	RX0	C
ATOM	1805	O	THR	345	7.615	26.233	29.623	1.00	0.00	RX0	O
ATOM	1806	N	LEU	346	6.390	27.971	28.898	1.00	0.00	RX0	N
ATOM	1807	H	LEU	346	5.564	28.513	29.044	1.00	0.00	RX0	H
ATOM	1808	CA	LEU	346	7.214	28.242	27.708	1.00	0.00	RX0	C
ATOM	1809	CB	LEU	346	6.671	29.451	26.948	1.00	0.00	RX0	C
ATOM	1810	CG	LEU	346	7.373	29.654	25.604	1.00	0.00	RX0	C
ATOM	1811	CD1	LEU	346	7.238	28.426	24.700	1.00	0.00	RX0	C
ATOM	1812	CD2	LEU	346	6.909	30.934	24.908	1.00	0.00	RX0	C
ATOM	1813	C	LEU	346	8.705	28.433	28.051	1.00	0.00	RX0	C
ATOM	1814	O	LEU	346	9.594	27.857	27.432	1.00	0.00	RX0	O
ATOM	1815	N	GLN	347	8.927	29.083	29.198	1.00	0.00	RX0	N
ATOM	1816	H	GLN	347	8.140	29.477	29.672	1.00	0.00	RX0	H
ATOM	1817	CA	GLN	347	10.275	29.275	29.754	1.00	0.00	RX0	C
ATOM	1818	CB	GLN	347	10.271	30.321	30.870	1.00	0.00	RX0	C
ATOM	1819	CG	GLN	347	11.682	30.791	31.249	1.00	0.00	RX0	C
ATOM	1820	CD	GLN	347	11.617	31.786	32.389	1.00	0.00	RX0	C
ATOM	1821	OE1	GLN	347	10.568	32.064	32.949	1.00	0.00	RX0	O
ATOM	1822	NE2	GLN	347	12.796	32.324	32.716	1.00	0.00	RX0	N
ATOM	1823	HE21	GLN	347	13.668	32.088	32.282	1.00	0.00	RX0	H
ATOM	1824	HE22	GLN	347	12.792	33.012	33.438	1.00	0.00	RX0	H
ATOM	1825	C	GLN	347	10.911	27.956	30.229	1.00	0.00	RX0	C
ATOM	1826	O	GLN	347	12.052	27.652	29.875	1.00	0.00	RX0	O
ATOM	1827	N	GLN	348	10.120	27.140	30.905	1.00	0.00	RX0	N
ATOM	1828	H	GLN	348	9.171	27.401	31.070	1.00	0.00	RX0	H

ATOM	1829	CA	GLN	348	10.582	25.823	31.394	1.00	0.00	RX0	C
ATOM	1830	CB	GLN	348	9.593	25.215	32.375	1.00	0.00	RX0	C
ATOM	1831	CG	GLN	348	9.649	25.870	33.751	1.00	0.00	RX0	C
ATOM	1832	CD	GLN	348	8.483	25.348	34.557	1.00	0.00	RX0	C
ATOM	1833	OE1	GLN	348	7.354	25.283	34.074	1.00	0.00	RX0	O
ATOM	1834	NE2	GLN	348	8.810	24.978	35.806	1.00	0.00	RX0	N
ATOM	1835	HE21	GLN	348	9.752	25.073	36.130	1.00	0.00	RX0	H
ATOM	1836	HE22	GLN	348	8.131	24.606	36.440	1.00	0.00	RX0	H
ATOM	1837	C	GLN	348	10.871	24.847	30.248	1.00	0.00	RX0	C
ATOM	1838	O	GLN	348	11.861	24.115	30.296	1.00	0.00	RX0	O
ATOM	1839	N	GLN	349	10.121	24.983	29.160	1.00	0.00	RX0	N
ATOM	1840	H	GLN	349	9.373	25.646	29.186	1.00	0.00	RX0	H
ATOM	1841	CA	GLN	349	10.299	24.172	27.940	1.00	0.00	RX0	C
ATOM	1842	CB	GLN	349	9.204	24.479	26.922	1.00	0.00	RX0	C
ATOM	1843	CG	GLN	349	7.821	24.030	27.386	1.00	0.00	RX0	C
ATOM	1844	CD	GLN	349	6.787	24.585	26.434	1.00	0.00	RX0	C
ATOM	1845	OE1	GLN	349	7.076	24.911	25.289	1.00	0.00	RX0	O
ATOM	1846	NE2	GLN	349	5.563	24.694	26.976	1.00	0.00	RX0	N
ATOM	1847	HE21	GLN	349	5.396	24.431	27.932	1.00	0.00	RX0	H
ATOM	1848	HE22	GLN	349	4.780	25.031	26.455	1.00	0.00	RX0	H
ATOM	1849	C	GLN	349	11.682	24.372	27.306	1.00	0.00	RX0	C
ATOM	1850	O	GLN	349	12.447	23.422	27.181	1.00	0.00	RX0	O
ATOM	1851	N	HIS	350	12.056	25.641	27.113	1.00	0.00	RX0	N
ATOM	1852	H	HIS	350	11.386	26.361	27.313	1.00	0.00	RX0	H
ATOM	1853	CA	HIS	350	13.344	25.953	26.463	1.00	0.00	RX0	C
ATOM	1854	CB	HIS	350	13.357	27.350	25.816	1.00	0.00	RX0	C
ATOM	1855	CG	HIS	350	13.861	28.423	26.753	1.00	0.00	RX0	C
ATOM	1856	ND1	HIS	350	13.098	29.033	27.672	1.00	0.00	RX0	N
ATOM	1857	HD1	HIS	350	12.153	28.853	27.855	1.00	0.00	RX0	H
ATOM	1858	CD2	HIS	350	15.160	28.930	26.833	1.00	0.00	RX0	C
ATOM	1859	NE2	HIS	350	15.166	29.855	27.822	1.00	0.00	RX0	N
ATOM	1860	CE1	HIS	350	13.900	29.922	28.338	1.00	0.00	RX0	C
ATOM	1861	C	HIS	350	14.540	25.670	27.386	1.00	0.00	RX0	C
ATOM	1862	O	HIS	350	15.573	25.182	26.934	1.00	0.00	RX0	O
ATOM	1863	N	GLN	351	14.320	25.854	28.691	1.00	0.00	RX0	N
ATOM	1864	H	GLN	351	13.445	26.253	28.973	1.00	0.00	RX0	H
ATOM	1865	CA	GLN	351	15.341	25.538	29.706	1.00	0.00	RX0	C
ATOM	1866	CB	GLN	351	14.919	26.043	31.081	1.00	0.00	RX0	C
ATOM	1867	CG	GLN	351	14.879	27.562	31.228	1.00	0.00	RX0	C
ATOM	1868	CD	GLN	351	14.123	27.899	32.497	1.00	0.00	RX0	C
ATOM	1869	OE1	GLN	351	13.457	27.073	33.105	1.00	0.00	RX0	O
ATOM	1870	NE2	GLN	351	14.264	29.162	32.897	1.00	0.00	RX0	N
ATOM	1871	HE21	GLN	351	14.793	29.848	32.393	1.00	0.00	RX0	H
ATOM	1872	HE22	GLN	351	13.801	29.445	33.735	1.00	0.00	RX0	H
ATOM	1873	C	GLN	351	15.597	24.030	29.805	1.00	0.00	RX0	C
ATOM	1874	O	GLN	351	16.752	23.608	29.740	1.00	0.00	RX0	O
ATOM	1875	N	ARG	352	14.527	23.242	29.744	1.00	0.00	RX0	N
ATOM	1876	H	ARG	352	13.620	23.655	29.658	1.00	0.00	RX0	H
ATOM	1877	CA	ARG	352	14.627	21.772	29.816	1.00	0.00	RX0	C
ATOM	1878	CB	ARG	352	13.312	21.110	30.250	1.00	0.00	RX0	C
ATOM	1879	CG	ARG	352	13.430	19.590	30.448	1.00	0.00	RX0	C
ATOM	1880	CD	ARG	352	12.237	18.994	31.205	1.00	0.00	RX0	C
ATOM	1881	NE	ARG	352	12.220	17.530	31.183	1.00	0.00	RX0	N
ATOM	1882	HE	ARG	352	12.011	17.063	30.309	1.00	0.00	RX0	H
ATOM	1883	CZ	ARG	352	12.354	16.778	32.318	1.00	0.00	RX0	C
ATOM	1884	NH1	ARG	352	12.653	17.385	33.484	1.00	0.00	RX0	N
ATOM	1885	HH11	ARG	352	12.830	16.842	34.318	1.00	0.00	RX0	H
ATOM	1886	HH12	ARG	352	12.722	18.380	33.561	1.00	0.00	RX0	H
ATOM	1887	NH2	ARG	352	12.183	15.441	32.259	1.00	0.00	RX0	N
ATOM	1888	HH21	ARG	352	12.222	14.808	33.040	1.00	0.00	RX0	H
ATOM	1889	HH22	ARG	352	11.990	15.020	31.350	1.00	0.00	RX0	H

ATOM	1890	C	ARG	352	15.192	21.188	28.514	1.00	0.00	RX0	C
ATOM	1891	O	ARG	352	16.048	20.305	28.551	1.00	0.00	RX0	O
ATOM	1892	N	LEU	353	14.843	21.816	27.391	1.00	0.00	RX0	N
ATOM	1893	H	LEU	353	14.130	22.518	27.423	1.00	0.00	RX0	H
ATOM	1894	CA	LEU	353	15.400	21.446	26.079	1.00	0.00	RX0	C
ATOM	1895	CB	LEU	353	14.765	22.303	24.985	1.00	0.00	RX0	C
ATOM	1896	CG	LEU	353	15.233	21.924	23.581	1.00	0.00	RX0	C
ATOM	1897	CD1	LEU	353	14.793	20.511	23.198	1.00	0.00	RX0	C
ATOM	1898	CD2	LEU	353	14.808	22.962	22.543	1.00	0.00	RX0	C
ATOM	1899	C	LEU	353	16.930	21.610	26.063	1.00	0.00	RX0	C
ATOM	1900	O	LEU	353	17.658	20.692	25.694	1.00	0.00	RX0	O
ATOM	1901	N	ALA	354	17.375	22.739	26.614	1.00	0.00	RX0	N
ATOM	1902	H	ALA	354	16.704	23.417	26.923	1.00	0.00	RX0	H
ATOM	1903	CA	ALA	354	18.808	23.073	26.716	1.00	0.00	RX0	C
ATOM	1904	CB	ALA	354	18.997	24.519	27.176	1.00	0.00	RX0	C
ATOM	1905	C	ALA	354	19.540	22.141	27.690	1.00	0.00	RX0	C
ATOM	1906	O	ALA	354	20.574	21.567	27.334	1.00	0.00	RX0	O
ATOM	1907	N	GLN	355	18.893	21.844	28.811	1.00	0.00	RX0	N
ATOM	1908	H	GLN	355	18.023	22.311	28.964	1.00	0.00	RX0	H
ATOM	1909	CA	GLN	355	19.427	20.920	29.833	1.00	0.00	RX0	C
ATOM	1910	CB	GLN	355	18.588	20.942	31.094	1.00	0.00	RX0	C
ATOM	1911	CG	GLN	355	18.840	22.238	31.853	1.00	0.00	RX0	C
ATOM	1912	CD	GLN	355	18.005	22.217	33.103	1.00	0.00	RX0	C
ATOM	1913	OE1	GLN	355	18.272	21.486	34.049	1.00	0.00	RX0	O
ATOM	1914	NE2	GLN	355	16.968	23.054	33.060	1.00	0.00	RX0	N
ATOM	1915	HE21	GLN	355	16.849	23.615	32.241	1.00	0.00	RX0	H
ATOM	1916	HE22	GLN	355	16.325	23.127	33.820	1.00	0.00	RX0	H
ATOM	1917	C	GLN	355	19.631	19.504	29.273	1.00	0.00	RX0	C
ATOM	1918	O	GLN	355	20.705	18.919	29.430	1.00	0.00	RX0	O
ATOM	1919	N	LEU	356	18.681	19.086	28.443	1.00	0.00	RX0	N
ATOM	1920	H	LEU	356	17.883	19.671	28.283	1.00	0.00	RX0	H
ATOM	1921	CA	LEU	356	18.716	17.770	27.778	1.00	0.00	RX0	C
ATOM	1922	CB	LEU	356	17.348	17.400	27.206	1.00	0.00	RX0	C
ATOM	1923	CG	LEU	356	16.351	17.037	28.306	1.00	0.00	RX0	C
ATOM	1924	CD1	LEU	356	14.993	16.630	27.736	1.00	0.00	RX0	C
ATOM	1925	CD2	LEU	356	16.919	15.967	29.238	1.00	0.00	RX0	C
ATOM	1926	C	LEU	356	19.795	17.661	26.699	1.00	0.00	RX0	C
ATOM	1927	O	LEU	356	20.593	16.720	26.700	1.00	0.00	RX0	O
ATOM	1928	N	LEU	357	19.916	18.722	25.913	1.00	0.00	RX0	N
ATOM	1929	H	LEU	357	19.258	19.473	26.026	1.00	0.00	RX0	H
ATOM	1930	CA	LEU	357	20.885	18.765	24.803	1.00	0.00	RX0	C
ATOM	1931	CB	LEU	357	20.533	19.867	23.803	1.00	0.00	RX0	C
ATOM	1932	CG	LEU	357	19.162	19.700	23.142	1.00	0.00	RX0	C
ATOM	1933	CD1	LEU	357	18.831	20.892	22.245	1.00	0.00	RX0	C
ATOM	1934	CD2	LEU	357	19.019	18.372	22.399	1.00	0.00	RX0	C
ATOM	1935	C	LEU	357	22.335	18.932	25.264	1.00	0.00	RX0	C
ATOM	1936	O	LEU	357	23.247	18.345	24.683	1.00	0.00	RX0	O
ATOM	1937	N	LEU	358	22.501	19.595	26.404	1.00	0.00	RX0	N
ATOM	1938	H	LEU	358	21.701	20.042	26.810	1.00	0.00	RX0	H
ATOM	1939	CA	LEU	358	23.826	19.754	27.029	1.00	0.00	RX0	C
ATOM	1940	CB	LEU	358	23.756	20.795	28.143	1.00	0.00	RX0	C
ATOM	1941	CG	LEU	358	23.453	22.214	27.667	1.00	0.00	RX0	C
ATOM	1942	CD1	LEU	358	23.256	23.166	28.846	1.00	0.00	RX0	C
ATOM	1943	CD2	LEU	358	24.472	22.718	26.652	1.00	0.00	RX0	C
ATOM	1944	C	LEU	358	24.390	18.455	27.609	1.00	0.00	RX0	C
ATOM	1945	O	LEU	358	25.603	18.227	27.557	1.00	0.00	RX0	O
ATOM	1946	N	ILE	359	23.510	17.559	28.043	1.00	0.00	RX0	N
ATOM	1947	H	ILE	359	22.537	17.791	28.023	1.00	0.00	RX0	H
ATOM	1948	CA	ILE	359	23.928	16.212	28.495	1.00	0.00	RX0	C
ATOM	1949	CB	ILE	359	22.749	15.469	29.127	1.00	0.00	RX0	C
ATOM	1950	CG2	ILE	359	23.117	14.034	29.508	1.00	0.00	RX0	C

ATOM	1951	CG1	ILE	359	22.244	16.252	30.341	1.00	0.00	RX0	C
ATOM	1952	CD1	ILE	359	20.944	15.697	30.920	1.00	0.00	RX0	C
ATOM	1953	C	ILE	359	24.559	15.428	27.334	1.00	0.00	RX0	C
ATOM	1954	O	ILE	359	25.552	14.723	27.543	1.00	0.00	RX0	O
ATOM	1955	N	LEU	360	24.038	15.620	26.133	1.00	0.00	RX0	N
ATOM	1956	H	LEU	360	23.266	16.249	26.024	1.00	0.00	RX0	H
ATOM	1957	CA	LEU	360	24.601	14.974	24.928	1.00	0.00	RX0	C
ATOM	1958	CB	LEU	360	23.755	15.280	23.691	1.00	0.00	RX0	C
ATOM	1959	CG	LEU	360	22.267	14.969	23.887	1.00	0.00	RX0	C
ATOM	1960	CD1	LEU	360	21.453	15.345	22.652	1.00	0.00	RX0	C
ATOM	1961	CD2	LEU	360	22.011	13.521	24.303	1.00	0.00	RX0	C
ATOM	1962	C	LEU	360	26.079	15.308	24.694	1.00	0.00	RX0	C
ATOM	1963	O	LEU	360	26.843	14.455	24.246	1.00	0.00	RX0	O
ATOM	1964	N	SER	361	26.491	16.472	25.202	1.00	0.00	RX0	N
ATOM	1965	H	SER	361	25.812	17.134	25.522	1.00	0.00	RX0	H
ATOM	1966	CA	SER	361	27.906	16.894	25.202	1.00	0.00	RX0	C
ATOM	1967	CB	SER	361	28.050	18.355	25.596	1.00	0.00	RX0	C
ATOM	1968	OG	SER	361	27.578	19.199	24.542	1.00	0.00	RX0	O
ATOM	1969	HG	SER	361	28.368	19.618	24.213	1.00	0.00	RX0	H
ATOM	1970	C	SER	361	28.769	15.985	26.099	1.00	0.00	RX0	C
ATOM	1971	O	SER	361	29.797	15.456	25.681	1.00	0.00	RX0	O
ATOM	1972	N	HIS	362	28.237	15.704	27.286	1.00	0.00	RX0	N
ATOM	1973	H	HIS	362	27.329	16.081	27.465	1.00	0.00	RX0	H
ATOM	1974	CA	HIS	362	28.854	14.793	28.272	1.00	0.00	RX0	C
ATOM	1975	CB	HIS	362	28.093	14.822	29.594	1.00	0.00	RX0	C
ATOM	1976	CG	HIS	362	28.349	16.135	30.274	1.00	0.00	RX0	C
ATOM	1977	ND1	HIS	362	27.676	17.265	30.002	1.00	0.00	RX0	N
ATOM	1978	HD1	HIS	362	26.963	17.401	29.341	1.00	0.00	RX0	H
ATOM	1979	CD2	HIS	362	29.316	16.394	31.245	1.00	0.00	RX0	C
ATOM	1980	NE2	HIS	362	29.223	17.705	31.557	1.00	0.00	RX0	N
ATOM	1981	CE1	HIS	362	28.216	18.233	30.797	1.00	0.00	RX0	C
ATOM	1982	C	HIS	362	28.890	13.339	27.777	1.00	0.00	RX0	C
ATOM	1983	O	HIS	362	29.902	12.656	27.936	1.00	0.00	RX0	O
ATOM	1984	N	ILE	363	27.856	12.940	27.039	1.00	0.00	RX0	N
ATOM	1985	H	ILE	363	27.092	13.580	26.942	1.00	0.00	RX0	H
ATOM	1986	CA	ILE	363	27.773	11.584	26.446	1.00	0.00	RX0	C
ATOM	1987	CB	ILE	363	26.355	11.298	25.946	1.00	0.00	RX0	C
ATOM	1988	CG2	ILE	363	26.255	9.919	25.294	1.00	0.00	RX0	C
ATOM	1989	CG1	ILE	363	25.365	11.425	27.105	1.00	0.00	RX0	C
ATOM	1990	CD1	ILE	363	23.919	11.196	26.676	1.00	0.00	RX0	C
ATOM	1991	C	ILE	363	28.830	11.416	25.343	1.00	0.00	RX0	C
ATOM	1992	O	ILE	363	29.487	10.370	25.263	1.00	0.00	RX0	O
ATOM	1993	N	ARG	364	29.035	12.469	24.564	1.00	0.00	RX0	N
ATOM	1994	H	ARG	364	28.404	13.246	24.621	1.00	0.00	RX0	H
ATOM	1995	CA	ARG	364	30.102	12.498	23.547	1.00	0.00	RX0	C
ATOM	1996	CB	ARG	364	30.070	13.842	22.816	1.00	0.00	RX0	C
ATOM	1997	CG	ARG	364	31.185	14.074	21.787	1.00	0.00	RX0	C
ATOM	1998	CD	ARG	364	31.167	13.042	20.657	1.00	0.00	RX0	C
ATOM	1999	NE	ARG	364	29.784	12.739	20.316	1.00	0.00	RX0	N
ATOM	2000	HE	ARG	364	29.317	12.013	20.843	1.00	0.00	RX0	H
ATOM	2001	CZ	ARG	364	29.038	13.501	19.476	1.00	0.00	RX0	C
ATOM	2002	NH1	ARG	364	29.628	14.419	18.676	1.00	0.00	RX0	N
ATOM	2003	HH11	ARG	364	29.115	14.988	18.028	1.00	0.00	RX0	H
ATOM	2004	HH12	ARG	364	30.623	14.550	18.702	1.00	0.00	RX0	H
ATOM	2005	NH2	ARG	364	27.712	13.294	19.481	1.00	0.00	RX0	N
ATOM	2006	HH21	ARG	364	27.032	13.731	18.891	1.00	0.00	RX0	H
ATOM	2007	HH22	ARG	364	27.347	12.579	20.110	1.00	0.00	RX0	H
ATOM	2008	C	ARG	364	31.469	12.319	24.223	1.00	0.00	RX0	C
ATOM	2009	O	ARG	364	32.264	11.465	23.831	1.00	0.00	RX0	O
ATOM	2010	N	HIS	365	31.645	13.058	25.316	1.00	0.00	RX0	N
ATOM	2011	H	HIS	365	30.931	13.726	25.540	1.00	0.00	RX0	H

ATOM	2012	CA	HIS	365	32.879	13.027	26.115	1.00	0.00	RX0	C
ATOM	2013	CB	HIS	365	32.892	14.107	27.201	1.00	0.00	RX0	C
ATOM	2014	CG	HIS	365	34.324	14.399	27.591	1.00	0.00	RX0	C
ATOM	2015	ND1	HIS	365	35.112	15.268	26.932	1.00	0.00	RX0	N
ATOM	2016	HD1	HIS	365	34.862	15.846	26.178	1.00	0.00	RX0	H
ATOM	2017	CD2	HIS	365	35.065	13.828	28.631	1.00	0.00	RX0	C
ATOM	2018	NE2	HIS	365	36.313	14.359	28.584	1.00	0.00	RX0	N
ATOM	2019	CE1	HIS	365	36.339	15.245	27.538	1.00	0.00	RX0	C
ATOM	2020	C	HIS	365	33.175	11.617	26.649	1.00	0.00	RX0	C
ATOM	2021	O	HIS	365	34.274	11.105	26.425	1.00	0.00	RX0	O
ATOM	2022	N	MET	366	32.138	10.946	27.137	1.00	0.00	RX0	N
ATOM	2023	H	MET	366	31.272	11.443	27.219	1.00	0.00	RX0	H
ATOM	2024	CA	MET	366	32.267	9.572	27.662	1.00	0.00	RX0	C
ATOM	2025	CB	MET	366	31.014	9.137	28.424	1.00	0.00	RX0	C
ATOM	2026	CG	MET	366	30.700	10.007	29.641	1.00	0.00	RX0	C
ATOM	2027	SD	MET	366	29.360	9.350	30.649	1.00	0.00	RX0	S
ATOM	2028	CE	MET	366	28.059	9.363	29.408	1.00	0.00	RX0	C
ATOM	2029	C	MET	366	32.567	8.557	26.555	1.00	0.00	RX0	C
ATOM	2030	O	MET	366	33.398	7.674	26.741	1.00	0.00	RX0	O
ATOM	2031	N	SER	367	31.985	8.782	25.378	1.00	0.00	RX0	N
ATOM	2032	H	SER	367	31.305	9.509	25.284	1.00	0.00	RX0	H
ATOM	2033	CA	SER	367	32.231	7.934	24.196	1.00	0.00	RX0	C
ATOM	2034	CB	SER	367	31.111	8.195	23.224	1.00	0.00	RX0	C
ATOM	2035	OG	SER	367	29.915	7.949	23.973	1.00	0.00	RX0	O
ATOM	2036	HG	SER	367	29.665	8.768	24.391	1.00	0.00	RX0	H
ATOM	2037	C	SER	367	33.681	8.046	23.711	1.00	0.00	RX0	C
ATOM	2038	O	SER	367	34.361	7.038	23.567	1.00	0.00	RX0	O
ATOM	2039	N	ASN	368	34.193	9.278	23.688	1.00	0.00	RX0	N
ATOM	2040	H	ASN	368	33.601	10.050	23.931	1.00	0.00	RX0	H
ATOM	2041	CA	ASN	368	35.583	9.549	23.266	1.00	0.00	RX0	C
ATOM	2042	CB	ASN	368	35.802	11.064	23.175	1.00	0.00	RX0	C
ATOM	2043	CG	ASN	368	37.196	11.366	22.658	1.00	0.00	RX0	C
ATOM	2044	OD1	ASN	368	37.858	10.513	22.085	1.00	0.00	RX0	O
ATOM	2045	ND2	ASN	368	37.573	12.651	22.838	1.00	0.00	RX0	N
ATOM	2046	HD21	ASN	368	37.041	13.369	23.293	1.00	0.00	RX0	H
ATOM	2047	HD22	ASN	368	38.415	13.062	22.469	1.00	0.00	RX0	H
ATOM	2048	C	ASN	368	36.615	8.948	24.226	1.00	0.00	RX0	C
ATOM	2049	O	ASN	368	37.514	8.215	23.806	1.00	0.00	RX0	O
ATOM	2050	N	LYS	369	36.353	9.119	25.515	1.00	0.00	RX0	N
ATOM	2051	H	LYS	369	35.547	9.660	25.767	1.00	0.00	RX0	H
ATOM	2052	CA	LYS	369	37.205	8.554	26.580	1.00	0.00	RX0	C
ATOM	2053	CB	LYS	369	36.978	9.197	27.954	1.00	0.00	RX0	C
ATOM	2054	CG	LYS	369	36.936	10.733	27.956	1.00	0.00	RX0	C
ATOM	2055	CD	LYS	369	37.980	11.439	27.081	1.00	0.00	RX0	C
ATOM	2056	CE	LYS	369	39.428	11.256	27.525	1.00	0.00	RX0	C
ATOM	2057	NZ	LYS	369	40.305	11.605	26.404	1.00	0.00	RX0	N
ATOM	2058	HZ1	LYS	369	41.195	11.056	26.447	1.00	0.00	RX0	H
ATOM	2059	HZ2	LYS	369	39.946	11.211	25.504	1.00	0.00	RX0	H
ATOM	2060	HZ3	LYS	369	40.495	12.611	26.265	1.00	0.00	RX0	H
ATOM	2061	C	LYS	369	37.117	7.021	26.620	1.00	0.00	RX0	C
ATOM	2062	O	LYS	369	38.123	6.337	26.771	1.00	0.00	RX0	O
ATOM	2063	N	GLY	370	35.908	6.527	26.299	1.00	0.00	RX0	N
ATOM	2064	H	GLY	370	35.157	7.166	26.129	1.00	0.00	RX0	H
ATOM	2065	CA	GLY	370	35.597	5.090	26.225	1.00	0.00	RX0	C
ATOM	2066	C	GLY	370	36.324	4.429	25.048	1.00	0.00	RX0	C
ATOM	2067	O	GLY	370	36.946	3.386	25.214	1.00	0.00	RX0	O
ATOM	2068	N	MET	371	36.380	5.143	23.928	1.00	0.00	RX0	N
ATOM	2069	H	MET	371	35.926	6.034	23.910	1.00	0.00	RX0	H
ATOM	2070	CA	MET	371	37.103	4.709	22.719	1.00	0.00	RX0	C
ATOM	2071	CB	MET	371	36.782	5.592	21.512	1.00	0.00	RX0	C
ATOM	2072	CG	MET	371	35.390	5.343	20.934	1.00	0.00	RX0	C

ATOM	2073	SD	MET	371	35.164	3.646	20.376	1.00	0.00	RX0	S
ATOM	2074	CE	MET	371	36.411	3.629	19.077	1.00	0.00	RX0	C
ATOM	2075	C	MET	371	38.619	4.671	22.937	1.00	0.00	RX0	C
ATOM	2076	O	MET	371	39.258	3.669	22.631	1.00	0.00	RX0	O
ATOM	2077	N	GLU	372	39.117	5.692	23.643	1.00	0.00	RX0	N
ATOM	2078	H	GLU	372	38.539	6.490	23.830	1.00	0.00	RX0	H
ATOM	2079	CA	GLU	372	40.525	5.740	24.082	1.00	0.00	RX0	C
ATOM	2080	CB	GLU	372	40.840	7.035	24.829	1.00	0.00	RX0	C
ATOM	2081	CG	GLU	372	40.888	8.266	23.927	1.00	0.00	RX0	C
ATOM	2082	CD	GLU	372	41.199	9.483	24.773	1.00	0.00	RX0	C
ATOM	2083	OE1	GLU	372	41.117	10.602	24.274	1.00	0.00	RX0	O
ATOM	2084	OE2	GLU	372	41.477	9.344	25.961	1.00	0.00	RX0	O
ATOM	2085	C	GLU	372	40.884	4.541	24.968	1.00	0.00	RX0	C
ATOM	2086	O	GLU	372	41.865	3.853	24.723	1.00	0.00	RX0	O
ATOM	2087	N	HIS	373	40.006	4.291	25.945	1.00	0.00	RX0	N
ATOM	2088	H	HIS	373	39.230	4.914	26.046	1.00	0.00	RX0	H
ATOM	2089	CA	HIS	373	40.194	3.212	26.919	1.00	0.00	RX0	C
ATOM	2090	CB	HIS	373	39.262	3.439	28.118	1.00	0.00	RX0	C
ATOM	2091	CG	HIS	373	38.391	2.246	28.438	1.00	0.00	RX0	C
ATOM	2092	ND1	HIS	373	37.286	1.920	27.740	1.00	0.00	RX0	N
ATOM	2093	HD1	HIS	373	36.953	2.374	26.933	1.00	0.00	RX0	H
ATOM	2094	CD2	HIS	373	38.541	1.342	29.494	1.00	0.00	RX0	C
ATOM	2095	NE2	HIS	373	37.508	0.465	29.426	1.00	0.00	RX0	N
ATOM	2096	CE1	HIS	373	36.738	0.822	28.350	1.00	0.00	RX0	C
ATOM	2097	C	HIS	373	40.078	1.832	26.262	1.00	0.00	RX0	C
ATOM	2098	O	HIS	373	40.934	1.001	26.470	1.00	0.00	RX0	O
ATOM	2099	N	LEU	374	39.111	1.679	25.351	1.00	0.00	RX0	N
ATOM	2100	H	LEU	374	38.561	2.476	25.112	1.00	0.00	RX0	H
ATOM	2101	CA	LEU	374	38.919	0.408	24.629	1.00	0.00	RX0	C
ATOM	2102	CB	LEU	374	37.659	0.450	23.764	1.00	0.00	RX0	C
ATOM	2103	CG	LEU	374	36.365	0.292	24.560	1.00	0.00	RX0	C
ATOM	2104	CD1	LEU	374	35.133	0.601	23.707	1.00	0.00	RX0	C
ATOM	2105	CD2	LEU	374	36.278	-1.084	25.221	1.00	0.00	RX0	C
ATOM	2106	C	LEU	374	40.113	0.051	23.744	1.00	0.00	RX0	C
ATOM	2107	O	LEU	374	40.555	-1.109	23.754	1.00	0.00	RX0	O
ATOM	2108	N	TYR	375	40.664	1.068	23.103	1.00	0.00	RX0	N
ATOM	2109	H	TYR	375	40.302	1.989	23.265	1.00	0.00	RX0	H
ATOM	2110	CA	TYR	375	41.854	0.983	22.222	1.00	0.00	RX0	C
ATOM	2111	CB	TYR	375	41.970	2.253	21.383	1.00	0.00	RX0	C
ATOM	2112	CG	TYR	375	41.487	1.976	19.983	1.00	0.00	RX0	C
ATOM	2113	CD1	TYR	375	40.437	1.092	19.760	1.00	0.00	RX0	C
ATOM	2114	CE1	TYR	375	40.045	0.798	18.460	1.00	0.00	RX0	C
ATOM	2115	CD2	TYR	375	42.112	2.604	18.915	1.00	0.00	RX0	C
ATOM	2116	CE2	TYR	375	41.719	2.310	17.617	1.00	0.00	RX0	C
ATOM	2117	CZ	TYR	375	40.705	1.387	17.388	1.00	0.00	RX0	C
ATOM	2118	OH	TYR	375	40.373	1.049	16.093	1.00	0.00	RX0	O
ATOM	2119	HH	TYR	375	41.173	1.167	15.577	1.00	0.00	RX0	H
ATOM	2120	C	TYR	375	43.165	0.750	22.977	1.00	0.00	RX0	C
ATOM	2121	O	TYR	375	44.176	1.452	22.753	1.00	0.00	RX0	O
ATOM	2122	N	SER	376	43.162	-0.226	23.845	1.00	0.00	RX0	N
ATOM	2123	H	SER	376	42.297	-0.621	24.137	1.00	0.00	RX0	H
ATOM	2124	CA	SER	376	44.345	-0.646	24.634	1.00	0.00	RX0	C
ATOM	2125	CB	SER	376	44.689	0.415	25.690	1.00	0.00	RX0	C
ATOM	2126	OG	SER	376	44.114	1.687	25.375	1.00	0.00	RX0	O
ATOM	2127	HG	SER	376	44.430	1.936	24.511	1.00	0.00	RX0	H
ATOM	2128	C	SER	376	44.183	-1.992	25.350	1.00	0.00	RX0	C
ATOM	2129	O	SER	376	44.985	-2.369	26.199	1.00	0.00	RX0	O
ATOM	2130	N	MET	377	43.156	-2.746	24.938	1.00	0.00	RX0	N
ATOM	2131	H	MET	377	42.566	-2.433	24.192	1.00	0.00	RX0	H
ATOM	2132	CA	MET	377	42.915	-4.112	25.422	1.00	0.00	RX0	C
ATOM	2133	CB	MET	377	41.737	-4.070	26.401	1.00	0.00	RX0	C

ATOM	2134	CG	MET	377	42.045	-3.403	27.742	1.00	0.00	RX0	C
ATOM	2135	SD	MET	377	40.564	-3.233	28.745	1.00	0.00	RX0	S
ATOM	2136	CE	MET	377	39.733	-2.015	27.713	1.00	0.00	RX0	C
ATOM	2137	C	MET	377	42.647	-5.080	24.257	1.00	0.00	RX0	C
ATOM	2138	O	MET	377	43.092	-4.854	23.130	1.00	0.00	RX0	O
ATOM	2139	N	LYS	378	41.856	-6.107	24.527	1.00	0.00	RX0	N
ATOM	2140	H	LYS	378	41.481	-6.207	25.443	1.00	0.00	RX0	H
ATOM	2141	CA	LYS	378	41.570	-7.191	23.576	1.00	0.00	RX0	C
ATOM	2142	CB	LYS	378	41.440	-8.519	24.363	1.00	0.00	RX0	C
ATOM	2143	CG	LYS	378	40.447	-8.515	25.551	1.00	0.00	RX0	C
ATOM	2144	CD	LYS	378	40.399	-9.805	26.396	1.00	0.00	RX0	C
ATOM	2145	CE	LYS	378	39.442	-9.725	27.606	1.00	0.00	RX0	C
ATOM	2146	NZ	LYS	378	39.472	-10.969	28.405	1.00	0.00	RX0	N
ATOM	2147	HZ1	LYS	378	38.884	-10.895	29.269	1.00	0.00	RX0	H
ATOM	2148	HZ2	LYS	378	39.118	-11.759	27.830	1.00	0.00	RX0	H
ATOM	2149	HZ3	LYS	378	40.449	-11.174	28.693	1.00	0.00	RX0	H
ATOM	2150	C	LYS	378	40.316	-6.920	22.720	1.00	0.00	RX0	C
ATOM	2151	O	LYS	378	40.245	-7.352	21.611	1.00	0.00	RX0	O
ATOM	2152	N	CYS	379	39.320	-6.266	23.410	1.00	0.00	RX0	N
ATOM	2153	H	CYS	379	39.564	-5.891	24.299	1.00	0.00	RX0	H
ATOM	2154	CA	CYS	379	37.939	-6.053	22.985	1.00	0.00	RX0	C
ATOM	2155	CB	CYS	379	37.615	-4.558	23.096	1.00	0.00	RX0	C
ATOM	2156	SG	CYS	379	35.844	-4.184	23.102	1.00	0.00	RX0	S
ATOM	2157	C	CYS	379	37.599	-6.654	21.614	1.00	0.00	RX0	C
ATOM	2158	O	CYS	379	37.115	-7.773	21.559	1.00	0.00	RX0	O
ATOM	2159	N	LYS	380	38.012	-5.930	20.563	1.00	0.00	RX0	N
ATOM	2160	H	LYS	380	38.389	-5.007	20.645	1.00	0.00	RX0	H
ATOM	2161	CA	LYS	380	37.949	-6.383	19.165	1.00	0.00	RX0	C
ATOM	2162	CB	LYS	380	36.478	-6.758	18.911	1.00	0.00	RX0	C
ATOM	2163	CG	LYS	380	35.911	-7.167	17.550	1.00	0.00	RX0	C
ATOM	2164	CD	LYS	380	34.399	-7.356	17.747	1.00	0.00	RX0	C
ATOM	2165	CE	LYS	380	33.579	-7.732	16.510	1.00	0.00	RX0	C
ATOM	2166	NZ	LYS	380	32.163	-7.822	16.890	1.00	0.00	RX0	N
ATOM	2167	HZ1	LYS	380	31.514	-8.074	16.126	1.00	0.00	RX0	H
ATOM	2168	HZ2	LYS	380	31.973	-8.513	17.652	1.00	0.00	RX0	H
ATOM	2169	HZ3	LYS	380	31.793	-6.951	17.332	1.00	0.00	RX0	H
ATOM	2170	C	LYS	380	38.311	-5.215	18.248	1.00	0.00	RX0	C
ATOM	2171	O	LYS	380	37.971	-4.057	18.518	1.00	0.00	RX0	O
ATOM	2172	N	ASN	381	38.881	-5.592	17.123	1.00	0.00	RX0	N
ATOM	2173	H	ASN	381	39.200	-6.537	17.056	1.00	0.00	RX0	H
ATOM	2174	CA	ASN	381	39.208	-4.700	15.996	1.00	0.00	RX0	C
ATOM	2175	CB	ASN	381	40.351	-3.768	16.389	1.00	0.00	RX0	C
ATOM	2176	CG	ASN	381	40.272	-2.479	15.603	1.00	0.00	RX0	C
ATOM	2177	OD1	ASN	381	41.028	-2.226	14.670	1.00	0.00	RX0	O
ATOM	2178	ND2	ASN	381	39.374	-1.619	16.111	1.00	0.00	RX0	N
ATOM	2179	HD21	ASN	381	38.730	-1.899	16.826	1.00	0.00	RX0	H
ATOM	2180	HD22	ASN	381	39.351	-0.651	15.835	1.00	0.00	RX0	H
ATOM	2181	C	ASN	381	39.620	-5.523	14.759	1.00	0.00	RX0	C
ATOM	2182	O	ASN	381	40.286	-5.062	13.834	1.00	0.00	RX0	O
ATOM	2183	N	VAL	382	39.176	-6.776	14.737	1.00	0.00	RX0	N
ATOM	2184	H	VAL	382	38.442	-7.029	15.362	1.00	0.00	RX0	H
ATOM	2185	CA	VAL	382	39.338	-7.659	13.572	1.00	0.00	RX0	C
ATOM	2186	CB	VAL	382	39.229	-9.119	14.010	1.00	0.00	RX0	C
ATOM	2187	CG1	VAL	382	39.386	-10.077	12.827	1.00	0.00	RX0	C
ATOM	2188	CG2	VAL	382	40.229	-9.410	15.131	1.00	0.00	RX0	C
ATOM	2189	C	VAL	382	38.195	-7.277	12.635	1.00	0.00	RX0	C
ATOM	2190	O	VAL	382	37.049	-7.276	13.059	1.00	0.00	RX0	O
ATOM	2191	N	VAL	383	38.551	-7.063	11.363	1.00	0.00	RX0	N
ATOM	2192	H	VAL	383	39.526	-7.055	11.153	1.00	0.00	RX0	H
ATOM	2193	CA	VAL	383	37.600	-6.539	10.366	1.00	0.00	RX0	C
ATOM	2194	CB	VAL	383	36.432	-7.500	10.106	1.00	0.00	RX0	C

ATOM	2195	CG1	VAL	383	35.394	-6.877	9.167	1.00	0.00	RX0	C
ATOM	2196	CG2	VAL	383	36.944	-8.845	9.588	1.00	0.00	RX0	C
ATOM	2197	C	VAL	383	37.102	-5.173	10.877	1.00	0.00	RX0	C
ATOM	2198	O	VAL	383	36.355	-5.097	11.860	1.00	0.00	RX0	O
ATOM	2199	N	PRO	384	37.530	-4.088	10.236	1.00	0.00	RX0	N
ATOM	2200	CD	PRO	384	38.352	-4.089	9.035	1.00	0.00	RX0	C
ATOM	2201	CA	PRO	384	37.142	-2.731	10.663	1.00	0.00	RX0	C
ATOM	2202	CB	PRO	384	37.704	-1.847	9.546	1.00	0.00	RX0	C
ATOM	2203	CG	PRO	384	37.999	-2.770	8.364	1.00	0.00	RX0	C
ATOM	2204	C	PRO	384	35.624	-2.628	10.828	1.00	0.00	RX0	C
ATOM	2205	O	PRO	384	34.847	-3.282	10.112	1.00	0.00	RX0	O
ATOM	2206	N	LEU	385	35.228	-1.769	11.750	1.00	0.00	RX0	N
ATOM	2207	H	LEU	385	35.899	-1.154	12.161	1.00	0.00	RX0	H
ATOM	2208	CA	LEU	385	33.812	-1.621	12.127	1.00	0.00	RX0	C
ATOM	2209	CB	LEU	385	33.661	-0.722	13.354	1.00	0.00	RX0	C
ATOM	2210	CG	LEU	385	32.265	-0.812	13.972	1.00	0.00	RX0	C
ATOM	2211	CD1	LEU	385	31.907	-2.245	14.369	1.00	0.00	RX0	C
ATOM	2212	CD2	LEU	385	32.098	0.159	15.138	1.00	0.00	RX0	C
ATOM	2213	C	LEU	385	32.896	-1.213	10.964	1.00	0.00	RX0	C
ATOM	2214	O	LEU	385	31.809	-1.761	10.815	1.00	0.00	RX0	O
ATOM	2215	N	TYR	386	33.449	-0.406	10.055	1.00	0.00	RX0	N
ATOM	2216	H	TYR	386	34.219	0.159	10.356	1.00	0.00	RX0	H
ATOM	2217	CA	TYR	386	32.718	0.069	8.867	1.00	0.00	RX0	C
ATOM	2218	CB	TYR	386	33.702	0.864	7.996	1.00	0.00	RX0	C
ATOM	2219	CG	TYR	386	33.087	1.197	6.658	1.00	0.00	RX0	C
ATOM	2220	CD1	TYR	386	32.341	2.358	6.505	1.00	0.00	RX0	C
ATOM	2221	CE1	TYR	386	31.715	2.624	5.294	1.00	0.00	RX0	C
ATOM	2222	CD2	TYR	386	33.254	0.334	5.581	1.00	0.00	RX0	C
ATOM	2223	CE2	TYR	386	32.620	0.595	4.376	1.00	0.00	RX0	C
ATOM	2224	CZ	TYR	386	31.846	1.737	4.234	1.00	0.00	RX0	C
ATOM	2225	OH	TYR	386	31.215	1.988	3.031	1.00	0.00	RX0	O
ATOM	2226	HH	TYR	386	30.652	2.747	3.134	1.00	0.00	RX0	H
ATOM	2227	C	TYR	386	32.144	-1.103	8.049	1.00	0.00	RX0	C
ATOM	2228	O	TYR	386	30.957	-1.141	7.757	1.00	0.00	RX0	O
ATOM	2229	N	ASP	387	32.969	-2.141	7.903	1.00	0.00	RX0	N
ATOM	2230	H	ASP	387	33.927	-2.068	8.186	1.00	0.00	RX0	H
ATOM	2231	CA	ASP	387	32.619	-3.323	7.093	1.00	0.00	RX0	C
ATOM	2232	CB	ASP	387	33.890	-4.035	6.626	1.00	0.00	RX0	C
ATOM	2233	CG	ASP	387	34.674	-3.065	5.751	1.00	0.00	RX0	C
ATOM	2234	OD1	ASP	387	34.537	-3.115	4.529	1.00	0.00	RX0	O
ATOM	2235	OD2	ASP	387	35.412	-2.243	6.292	1.00	0.00	RX0	O
ATOM	2236	C	ASP	387	31.563	-4.207	7.765	1.00	0.00	RX0	C
ATOM	2237	O	ASP	387	30.619	-4.649	7.114	1.00	0.00	RX0	O
ATOM	2238	N	LEU	388	31.677	-4.332	9.088	1.00	0.00	RX0	N
ATOM	2239	H	LEU	388	32.433	-3.840	9.525	1.00	0.00	RX0	H
ATOM	2240	CA	LEU	388	30.691	-5.072	9.891	1.00	0.00	RX0	C
ATOM	2241	CB	LEU	388	31.205	-5.224	11.323	1.00	0.00	RX0	C
ATOM	2242	CG	LEU	388	30.284	-6.066	12.208	1.00	0.00	RX0	C
ATOM	2243	CD1	LEU	388	30.097	-7.480	11.653	1.00	0.00	RX0	C
ATOM	2244	CD2	LEU	388	30.749	-6.078	13.665	1.00	0.00	RX0	C
ATOM	2245	C	LEU	388	29.315	-4.386	9.871	1.00	0.00	RX0	C
ATOM	2246	O	LEU	388	28.299	-5.023	9.588	1.00	0.00	RX0	O
ATOM	2247	N	LEU	389	29.339	-3.070	10.049	1.00	0.00	RX0	N
ATOM	2248	H	LEU	389	30.227	-2.621	10.175	1.00	0.00	RX0	H
ATOM	2249	CA	LEU	389	28.129	-2.229	9.970	1.00	0.00	RX0	C
ATOM	2250	CB	LEU	389	28.425	-0.788	10.369	1.00	0.00	RX0	C
ATOM	2251	CG	LEU	389	28.856	-0.648	11.825	1.00	0.00	RX0	C
ATOM	2252	CD1	LEU	389	29.230	0.793	12.144	1.00	0.00	RX0	C
ATOM	2253	CD2	LEU	389	27.803	-1.175	12.798	1.00	0.00	RX0	C
ATOM	2254	C	LEU	389	27.489	-2.268	8.583	1.00	0.00	RX0	C
ATOM	2255	O	LEU	389	26.284	-2.473	8.458	1.00	0.00	RX0	O

ATOM	2256	N	LEU	390	28.350	-2.246	7.565	1.00	0.00	RX0	N
ATOM	2257	H	LEU	390	29.327	-2.132	7.754	1.00	0.00	RX0	H
ATOM	2258	CA	LEU	390	27.929	-2.335	6.162	1.00	0.00	RX0	C
ATOM	2259	CB	LEU	390	29.177	-2.205	5.287	1.00	0.00	RX0	C
ATOM	2260	CG	LEU	390	28.940	-2.123	3.783	1.00	0.00	RX0	C
ATOM	2261	CD1	LEU	390	28.276	-0.806	3.389	1.00	0.00	RX0	C
ATOM	2262	CD2	LEU	390	30.238	-2.356	3.009	1.00	0.00	RX0	C
ATOM	2263	C	LEU	390	27.202	-3.653	5.862	1.00	0.00	RX0	C
ATOM	2264	O	LEU	390	26.123	-3.627	5.302	1.00	0.00	RX0	O
ATOM	2265	N	GLU	391	27.733	-4.754	6.404	1.00	0.00	RX0	N
ATOM	2266	H	GLU	391	28.655	-4.694	6.792	1.00	0.00	RX0	H
ATOM	2267	CA	GLU	391	27.124	-6.090	6.264	1.00	0.00	RX0	C
ATOM	2268	CB	GLU	391	28.081	-7.240	6.550	1.00	0.00	RX0	C
ATOM	2269	CG	GLU	391	29.206	-7.202	5.501	1.00	0.00	RX0	C
ATOM	2270	CD	GLU	391	28.674	-6.780	4.130	1.00	0.00	RX0	C
ATOM	2271	OE1	GLU	391	28.021	-7.566	3.444	1.00	0.00	RX0	O
ATOM	2272	OE2	GLU	391	28.917	-5.645	3.722	1.00	0.00	RX0	O
ATOM	2273	C	GLU	391	25.713	-6.159	6.867	1.00	0.00	RX0	C
ATOM	2274	O	GLU	391	24.778	-6.600	6.214	1.00	0.00	RX0	O
ATOM	2275	N	MET	392	25.589	-5.588	8.070	1.00	0.00	RX0	N
ATOM	2276	H	MET	392	26.414	-5.173	8.461	1.00	0.00	RX0	H
ATOM	2277	CA	MET	392	24.313	-5.555	8.807	1.00	0.00	RX0	C
ATOM	2278	CB	MET	392	24.543	-5.230	10.282	1.00	0.00	RX0	C
ATOM	2279	CG	MET	392	25.230	-6.391	11.004	1.00	0.00	RX0	C
ATOM	2280	SD	MET	392	25.390	-6.135	12.778	1.00	0.00	RX0	S
ATOM	2281	CE	MET	392	26.590	-4.799	12.728	1.00	0.00	RX0	C
ATOM	2282	C	MET	392	23.266	-4.630	8.173	1.00	0.00	RX0	C
ATOM	2283	O	MET	392	22.107	-5.006	8.028	1.00	0.00	RX0	O
ATOM	2284	N	LEU	393	23.721	-3.452	7.754	1.00	0.00	RX0	N
ATOM	2285	H	LEU	393	24.706	-3.278	7.797	1.00	0.00	RX0	H
ATOM	2286	CA	LEU	393	22.852	-2.457	7.099	1.00	0.00	RX0	C
ATOM	2287	CB	LEU	393	23.511	-1.082	7.155	1.00	0.00	RX0	C
ATOM	2288	CG	LEU	393	23.448	-0.454	8.544	1.00	0.00	RX0	C
ATOM	2289	CD1	LEU	393	24.510	0.627	8.724	1.00	0.00	RX0	C
ATOM	2290	CD2	LEU	393	22.045	0.067	8.859	1.00	0.00	RX0	C
ATOM	2291	C	LEU	393	22.462	-2.815	5.663	1.00	0.00	RX0	C
ATOM	2292	O	LEU	393	21.313	-2.601	5.257	1.00	0.00	RX0	O
ATOM	2293	N	ASP	394	23.400	-3.384	4.923	1.00	0.00	RX0	N
ATOM	2294	H	ASP	394	24.265	-3.668	5.334	1.00	0.00	RX0	H
ATOM	2295	CA	ASP	394	23.187	-3.768	3.516	1.00	0.00	RX0	C
ATOM	2296	CB	ASP	394	24.494	-3.795	2.730	1.00	0.00	RX0	C
ATOM	2297	CG	ASP	394	24.210	-3.997	1.257	1.00	0.00	RX0	C
ATOM	2298	OD1	ASP	394	23.158	-3.559	0.792	1.00	0.00	RX0	O
ATOM	2299	OD2	ASP	394	25.053	-4.589	0.582	1.00	0.00	RX0	O
ATOM	2300	C	ASP	394	22.492	-5.132	3.432	1.00	0.00	RX0	C
ATOM	2301	O	ASP	394	23.034	-6.108	2.888	1.00	0.00	RX0	O
ATOM	2302	N	ALA	395	21.256	-5.134	3.874	1.00	0.00	RX0	N
ATOM	2303	H	ALA	395	20.903	-4.248	4.174	1.00	0.00	RX0	H
ATOM	2304	CA	ALA	395	20.412	-6.338	3.880	1.00	0.00	RX0	C
ATOM	2305	CB	ALA	395	19.421	-6.294	5.044	1.00	0.00	RX0	C
ATOM	2306	C	ALA	395	19.639	-6.454	2.565	1.00	0.00	RX0	C
ATOM	2307	O	ALA	395	19.303	-5.463	1.911	1.00	0.00	RX0	O
ATOM	2308	N	HIS	396	19.441	-7.696	2.156	1.00	0.00	RX0	N
ATOM	2309	H	HIS	396	19.745	-8.413	2.781	1.00	0.00	RX0	H
ATOM	2310	CA	HIS	396	18.536	-7.999	1.035	1.00	0.00	RX0	C
ATOM	2311	CB	HIS	396	18.852	-9.401	0.508	1.00	0.00	RX0	C
ATOM	2312	CG	HIS	396	19.007	-10.356	1.672	1.00	0.00	RX0	C
ATOM	2313	ND1	HIS	396	20.197	-10.820	2.097	1.00	0.00	RX0	N
ATOM	2314	HD1	HIS	396	21.080	-10.620	1.713	1.00	0.00	RX0	H
ATOM	2315	CD2	HIS	396	18.008	-10.886	2.494	1.00	0.00	RX0	C
ATOM	2316	NE2	HIS	396	18.605	-11.671	3.418	1.00	0.00	RX0	N

ATOM	2317	CE1	HIS	396	19.956	-11.634	3.175	1.00	0.00	RX0	C
ATOM	2318	C	HIS	396	17.077	-7.909	1.523	1.00	0.00	RX0	C
ATOM	2319	O	HIS	396	16.821	-7.755	2.721	1.00	0.00	RX0	O
ATOM	2320	N	ARG	397	16.142	-8.174	0.629	1.00	0.00	RX0	N
ATOM	2321	H	ARG	397	16.382	-8.379	-0.320	1.00	0.00	RX0	H
ATOM	2322	CA	ARG	397	14.704	-8.151	0.982	1.00	0.00	RX0	C
ATOM	2323	CB	ARG	397	14.000	-6.986	0.268	1.00	0.00	RX0	C
ATOM	2324	CG	ARG	397	14.503	-5.597	0.727	1.00	0.00	RX0	C
ATOM	2325	CD	ARG	397	15.561	-4.923	-0.170	1.00	0.00	RX0	C
ATOM	2326	NE	ARG	397	16.802	-4.582	0.543	1.00	0.00	RX0	N
ATOM	2327	HE	ARG	397	17.338	-5.317	0.975	1.00	0.00	RX0	H
ATOM	2328	CZ	ARG	397	17.301	-3.305	0.535	1.00	0.00	RX0	C
ATOM	2329	NH1	ARG	397	16.544	-2.298	0.038	1.00	0.00	RX0	N
ATOM	2330	HH11	ARG	397	16.870	-1.348	-0.000	1.00	0.00	RX0	H
ATOM	2331	HH12	ARG	397	15.617	-2.462	-0.312	1.00	0.00	RX0	H
ATOM	2332	NH2	ARG	397	18.545	-3.079	1.022	1.00	0.00	RX0	N
ATOM	2333	HH21	ARG	397	18.992	-2.181	1.064	1.00	0.00	RX0	H
ATOM	2334	HH22	ARG	397	19.104	-3.850	1.370	1.00	0.00	RX0	H
ATOM	2335	C	ARG	397	14.082	-9.540	0.774	1.00	0.00	RX0	C
ATOM	2336	O	ARG	397	12.875	-9.706	0.613	1.00	0.00	RX0	O
ATOM	2337	N	LEU	398	14.938	-10.556	0.835	1.00	0.00	RX0	N
ATOM	2338	H	LEU	398	15.872	-10.366	1.123	1.00	0.00	RX0	H
ATOM	2339	CA	LEU	398	14.565	-11.951	0.542	1.00	0.00	RX0	C
ATOM	2340	CB	LEU	398	15.800	-12.794	0.230	1.00	0.00	RX0	C
ATOM	2341	CG	LEU	398	16.578	-12.270	-0.977	1.00	0.00	RX0	C
ATOM	2342	CD1	LEU	398	17.869	-13.060	-1.203	1.00	0.00	RX0	C
ATOM	2343	CD2	LEU	398	15.711	-12.212	-2.236	1.00	0.00	RX0	C
ATOM	2344	C	LEU	398	13.757	-12.584	1.680	1.00	0.00	RX0	C
ATOM	2345	O	LEU	398	12.772	-13.259	1.439	1.00	0.00	RX0	O
ATOM	2346	N	HIS	399	14.100	-12.169	2.904	1.00	0.00	RX0	N
ATOM	2347	H	HIS	399	14.844	-11.515	3.011	1.00	0.00	RX0	H
ATOM	2348	CA	HIS	399	13.392	-12.616	4.118	1.00	0.00	RX0	C
ATOM	2349	CB	HIS	399	14.353	-12.615	5.307	1.00	0.00	RX0	C
ATOM	2350	CG	HIS	399	15.403	-13.674	5.069	1.00	0.00	RX0	C
ATOM	2351	ND1	HIS	399	16.706	-13.418	4.842	1.00	0.00	RX0	N
ATOM	2352	HD1	HIS	399	17.152	-12.543	4.802	1.00	0.00	RX0	H
ATOM	2353	CD2	HIS	399	15.203	-15.057	5.030	1.00	0.00	RX0	C
ATOM	2354	NE2	HIS	399	16.403	-15.633	4.774	1.00	0.00	RX0	N
ATOM	2355	CE1	HIS	399	17.327	-14.627	4.659	1.00	0.00	RX0	C
ATOM	2356	C	HIS	399	12.131	-11.786	4.401	1.00	0.00	RX0	C
ATOM	2357	O	HIS	399	11.630	-11.743	5.524	1.00	0.00	RX0	O
ATOM	2358	N	ALA	400	11.619	-11.143	3.351	1.00	0.00	RX0	N
ATOM	2359	H	ALA	400	11.997	-11.315	2.443	1.00	0.00	RX0	H
ATOM	2360	CA	ALA	400	10.356	-10.385	3.402	1.00	0.00	RX0	C
ATOM	2361	CB	ALA	400	10.144	-9.552	2.138	1.00	0.00	RX0	C
ATOM	2362	C	ALA	400	9.147	-11.323	3.579	1.00	0.00	RX0	C
ATOM	2363	O	ALA	400	8.508	-11.220	4.647	1.00	0.00	RX0	O
ATOM	2364	N	ASP	985	23.924	20.918	15.697	1.00	0.00	RX1	N
ATOM	2365	H	ASP	985	24.341	20.350	16.413	1.00	0.00	RX1	H
ATOM	2366	CA	ASP	985	24.313	22.312	15.909	1.00	0.00	RX1	C
ATOM	2367	CB	ASP	985	23.083	23.205	16.115	1.00	0.00	RX1	C
ATOM	2368	CG	ASP	985	23.265	24.491	15.327	1.00	0.00	RX1	C
ATOM	2369	OD1	ASP	985	22.298	24.988	14.759	1.00	0.00	RX1	O
ATOM	2370	OD2	ASP	985	24.391	24.947	15.180	1.00	0.00	RX1	O
ATOM	2371	C	ASP	985	25.281	22.422	17.075	1.00	0.00	RX1	C
ATOM	2372	O	ASP	985	25.662	21.407	17.645	1.00	0.00	RX1	O
ATOM	2373	N	VAL	986	25.652	23.674	17.401	1.00	0.00	RX1	N
ATOM	2374	H	VAL	986	25.299	24.409	16.818	1.00	0.00	RX1	H
ATOM	2375	CA	VAL	986	26.584	23.979	18.488	1.00	0.00	RX1	C
ATOM	2376	CB	VAL	986	26.008	23.646	19.874	1.00	0.00	RX1	C
ATOM	2377	CG1	VAL	986	26.904	24.227	20.964	1.00	0.00	RX1	C

ATOM	2378	CG2	VAL	986	24.574	24.161	20.035	1.00	0.00	RX1	C
ATOM	2379	C	VAL	986	27.987	23.419	18.298	1.00	0.00	RX1	C
ATOM	2380	O	VAL	986	28.295	22.257	18.531	1.00	0.00	RX1	O
ATOM	2381	N	TYR	987	28.878	24.339	17.896	1.00	0.00	RX1	N
ATOM	2382	H	TYR	987	28.630	25.297	17.767	1.00	0.00	RX1	H
ATOM	2383	CA	TYR	987	30.264	23.924	18.080	1.00	0.00	RX1	C
ATOM	2384	CB	TYR	987	31.243	24.651	17.156	1.00	0.00	RX1	C
ATOM	2385	CG	TYR	987	32.592	23.991	17.332	1.00	0.00	RX1	C
ATOM	2386	CD1	TYR	987	32.788	22.699	16.858	1.00	0.00	RX1	C
ATOM	2387	CE1	TYR	987	33.998	22.052	17.079	1.00	0.00	RX1	C
ATOM	2388	CD2	TYR	987	33.623	24.652	17.991	1.00	0.00	RX1	C
ATOM	2389	CE2	TYR	987	34.832	24.003	18.217	1.00	0.00	RX1	C
ATOM	2390	CZ	TYR	987	35.013	22.699	17.773	1.00	0.00	RX1	C
ATOM	2391	OH	TYR	987	36.201	22.039	18.020	1.00	0.00	RX1	O
ATOM	2392	HH	TYR	987	36.076	21.520	18.824	1.00	0.00	RX1	H
ATOM	2393	C	TYR	987	30.676	24.088	19.528	1.00	0.00	RX1	C
ATOM	2394	O	TYR	987	31.042	25.161	19.989	1.00	0.00	RX1	O
ATOM	2395	N	VAL	988	30.588	22.955	20.225	1.00	0.00	RX1	N
ATOM	2396	H	VAL	988	30.248	22.132	19.766	1.00	0.00	RX1	H
ATOM	2397	CA	VAL	988	31.201	22.929	21.548	1.00	0.00	RX1	C
ATOM	2398	CB	VAL	988	30.683	21.690	22.297	1.00	0.00	RX1	C
ATOM	2399	CG1	VAL	988	29.156	21.696	22.326	1.00	0.00	RX1	C
ATOM	2400	CG2	VAL	988	31.166	20.345	21.743	1.00	0.00	RX1	C
ATOM	2401	C	VAL	988	32.722	22.933	21.419	1.00	0.00	RX1	C
ATOM	2402	O	VAL	988	33.287	22.182	20.635	1.00	0.00	RX1	O
ATOM	2403	N	PRO	989	33.375	23.836	22.186	1.00	0.00	RX1	N
ATOM	2404	CD	PRO	989	32.800	24.936	22.943	1.00	0.00	RX1	C
ATOM	2405	CA	PRO	989	34.834	23.757	22.311	1.00	0.00	RX1	C
ATOM	2406	CB	PRO	989	35.169	24.976	23.178	1.00	0.00	RX1	C
ATOM	2407	CG	PRO	989	33.967	25.907	23.052	1.00	0.00	RX1	C
ATOM	2408	C	PRO	989	35.285	22.446	22.943	1.00	0.00	RX1	C
ATOM	2409	O	PRO	989	35.376	22.309	24.157	1.00	0.00	RX1	O
ATOM	2410	N	ASP	990	35.532	21.486	22.048	1.00	0.00	RX1	N
ATOM	2411	H	ASP	990	35.351	21.650	21.078	1.00	0.00	RX1	H
ATOM	2412	CA	ASP	990	36.098	20.206	22.463	1.00	0.00	RX1	C
ATOM	2413	CB	ASP	990	35.854	19.136	21.384	1.00	0.00	RX1	C
ATOM	2414	CG	ASP	990	36.266	19.605	19.996	1.00	0.00	RX1	C
ATOM	2415	OD1	ASP	990	37.408	19.997	19.778	1.00	0.00	RX1	O
ATOM	2416	OD2	ASP	990	35.437	19.593	19.095	1.00	0.00	RX1	O
ATOM	2417	C	ASP	990	37.567	20.328	22.823	1.00	0.00	RX1	C
ATOM	2418	O	ASP	990	38.133	21.419	22.845	1.00	0.00	RX1	O
ATOM	2419	N	GLU	991	38.185	19.168	23.097	1.00	0.00	RX1	N
ATOM	2420	H	GLU	991	37.733	18.274	23.014	1.00	0.00	RX1	H
ATOM	2421	CA	GLU	991	39.567	19.216	23.567	1.00	0.00	RX1	C
ATOM	2422	CB	GLU	991	39.957	17.912	24.290	1.00	0.00	RX1	C
ATOM	2423	CG	GLU	991	40.105	16.616	23.468	1.00	0.00	RX1	C
ATOM	2424	CD	GLU	991	38.780	15.981	23.059	1.00	0.00	RX1	C
ATOM	2425	OE1	GLU	991	37.765	16.199	23.721	1.00	0.00	RX1	O
ATOM	2426	OE2	GLU	991	38.774	15.235	22.081	1.00	0.00	RX1	O
ATOM	2427	C	GLU	991	40.642	19.680	22.581	1.00	0.00	RX1	C
ATOM	2428	O	GLU	991	41.836	19.586	22.849	1.00	0.00	RX1	O
ATOM	2429	N	TRP	992	40.182	20.197	21.429	1.00	0.00	RX1	N
ATOM	2430	H	TRP	992	39.200	20.239	21.232	1.00	0.00	RX1	H
ATOM	2431	CA	TRP	992	41.141	20.676	20.440	1.00	0.00	RX1	C
ATOM	2432	CB	TRP	992	40.966	19.961	19.091	1.00	0.00	RX1	C
ATOM	2433	CG	TRP	992	40.985	18.448	19.210	1.00	0.00	RX1	C
ATOM	2434	CD2	TRP	992	41.922	17.523	18.620	1.00	0.00	RX1	C
ATOM	2435	CE2	TRP	992	41.532	16.214	18.997	1.00	0.00	RX1	C
ATOM	2436	CE3	TRP	992	43.038	17.703	17.811	1.00	0.00	RX1	C
ATOM	2437	CD1	TRP	992	40.079	17.635	19.905	1.00	0.00	RX1	C
ATOM	2438	NE1	TRP	992	40.393	16.322	19.792	1.00	0.00	RX1	N

ATOM	2439	HE1	TRP	992	39.898	15.607	20.262	1.00	0.00	RX1	H
ATOM	2440	CZ2	TRP	992	42.272	15.124	18.554	1.00	0.00	RX1	C
ATOM	2441	CZ3	TRP	992	43.767	16.603	17.373	1.00	0.00	RX1	C
ATOM	2442	CH2	TRP	992	43.386	15.319	17.745	1.00	0.00	RX1	C
ATOM	2443	C	TRP	992	41.078	22.182	20.214	1.00	0.00	RX1	C
ATOM	2444	O	TRP	992	41.677	22.703	19.280	1.00	0.00	RX1	O
ATOM	2445	N	GLU	993	40.307	22.864	21.078	1.00	0.00	RX1	N
ATOM	2446	H	GLU	993	39.834	22.401	21.829	1.00	0.00	RX1	H
ATOM	2447	CA	GLU	993	40.224	24.320	20.925	1.00	0.00	RX1	C
ATOM	2448	CB	GLU	993	38.954	24.833	21.620	1.00	0.00	RX1	C
ATOM	2449	CG	GLU	993	38.682	26.348	21.605	1.00	0.00	RX1	C
ATOM	2450	CD	GLU	993	38.467	26.926	20.212	1.00	0.00	RX1	C
ATOM	2451	OE1	GLU	993	38.144	26.203	19.275	1.00	0.00	RX1	O
ATOM	2452	OE2	GLU	993	38.576	28.136	20.062	1.00	0.00	RX1	O
ATOM	2453	C	GLU	993	41.505	25.029	21.360	1.00	0.00	RX1	C
ATOM	2454	O	GLU	993	42.182	24.629	22.301	1.00	0.00	RX1	O
ATOM	2455	N	VAL	994	41.836	26.073	20.593	1.00	0.00	RX1	N
ATOM	2456	H	VAL	994	41.171	26.398	19.915	1.00	0.00	RX1	H
ATOM	2457	CA	VAL	994	43.160	26.683	20.663	1.00	0.00	RX1	C
ATOM	2458	CB	VAL	994	43.940	26.317	19.381	1.00	0.00	RX1	C
ATOM	2459	CG1	VAL	994	45.108	27.241	19.029	1.00	0.00	RX1	C
ATOM	2460	CG2	VAL	994	44.413	24.866	19.471	1.00	0.00	RX1	C
ATOM	2461	C	VAL	994	43.070	28.182	20.892	1.00	0.00	RX1	C
ATOM	2462	O	VAL	994	42.292	28.907	20.284	1.00	0.00	RX1	O
ATOM	2463	N	ALA	995	43.921	28.628	21.828	1.00	0.00	RX1	N
ATOM	2464	H	ALA	995	44.620	28.018	22.192	1.00	0.00	RX1	H
ATOM	2465	CA	ALA	995	43.919	30.060	22.101	1.00	0.00	RX1	C
ATOM	2466	CB	ALA	995	44.622	30.362	23.426	1.00	0.00	RX1	C
ATOM	2467	C	ALA	995	44.534	30.894	20.990	1.00	0.00	RX1	C
ATOM	2468	O	ALA	995	45.402	30.477	20.232	1.00	0.00	RX1	O
ATOM	2469	N	ARG	996	44.022	32.130	20.924	1.00	0.00	RX1	N
ATOM	2470	H	ARG	996	43.346	32.412	21.601	1.00	0.00	RX1	H
ATOM	2471	CA	ARG	996	44.387	32.973	19.787	1.00	0.00	RX1	C
ATOM	2472	CB	ARG	996	43.332	34.076	19.630	1.00	0.00	RX1	C
ATOM	2473	CG	ARG	996	43.356	34.805	18.283	1.00	0.00	RX1	C
ATOM	2474	CD	ARG	996	42.062	35.571	18.006	1.00	0.00	RX1	C
ATOM	2475	NE	ARG	996	40.954	34.633	17.839	1.00	0.00	RX1	N
ATOM	2476	HE	ARG	996	40.618	34.123	18.643	1.00	0.00	RX1	H
ATOM	2477	CZ	ARG	996	40.422	34.424	16.602	1.00	0.00	RX1	C
ATOM	2478	NH1	ARG	996	40.796	35.201	15.567	1.00	0.00	RX1	N
ATOM	2479	HH11	ARG	996	40.419	35.019	14.649	1.00	0.00	RX1	H
ATOM	2480	HH12	ARG	996	41.427	35.974	15.667	1.00	0.00	RX1	H
ATOM	2481	NH2	ARG	996	39.517	33.447	16.423	1.00	0.00	RX1	N
ATOM	2482	HH21	ARG	996	39.070	33.292	15.531	1.00	0.00	RX1	H
ATOM	2483	HH22	ARG	996	39.225	32.860	17.187	1.00	0.00	RX1	H
ATOM	2484	C	ARG	996	45.829	33.480	19.750	1.00	0.00	RX1	C
ATOM	2485	O	ARG	996	46.307	33.985	18.741	1.00	0.00	RX1	O
ATOM	2486	N	GLU	997	46.535	33.292	20.880	1.00	0.00	RX1	N
ATOM	2487	H	GLU	997	46.176	32.739	21.629	1.00	0.00	RX1	H
ATOM	2488	CA	GLU	997	47.951	33.675	20.851	1.00	0.00	RX1	C
ATOM	2489	CB	GLU	997	48.587	33.703	22.254	1.00	0.00	RX1	C
ATOM	2490	CG	GLU	997	48.201	32.631	23.288	1.00	0.00	RX1	C
ATOM	2491	CD	GLU	997	48.605	31.219	22.896	1.00	0.00	RX1	C
ATOM	2492	OE1	GLU	997	49.781	30.868	22.947	1.00	0.00	RX1	O
ATOM	2493	OE2	GLU	997	47.729	30.425	22.586	1.00	0.00	RX1	O
ATOM	2494	C	GLU	997	48.823	32.894	19.874	1.00	0.00	RX1	C
ATOM	2495	O	GLU	997	49.912	33.306	19.495	1.00	0.00	RX1	O
ATOM	2496	N	LYS	998	48.260	31.763	19.425	1.00	0.00	RX1	N
ATOM	2497	H	LYS	998	47.371	31.448	19.763	1.00	0.00	RX1	H
ATOM	2498	CA	LYS	998	49.021	30.984	18.455	1.00	0.00	RX1	C
ATOM	2499	CB	LYS	998	48.587	29.526	18.434	1.00	0.00	RX1	C

ATOM	2500	CG	LYS	998	48.460	29.093	19.874	1.00	0.00	RX1	C
ATOM	2501	CD	LYS	998	48.637	27.611	20.150	1.00	0.00	RX1	C
ATOM	2502	CE	LYS	998	48.646	27.365	21.654	1.00	0.00	RX1	C
ATOM	2503	NZ	LYS	998	49.539	28.329	22.312	1.00	0.00	RX1	N
ATOM	2504	HZ1	LYS	998	49.602	28.104	23.324	1.00	0.00	RX1	H
ATOM	2505	HZ2	LYS	998	50.479	28.364	21.862	1.00	0.00	RX1	H
ATOM	2506	HZ3	LYS	998	49.129	29.294	22.282	1.00	0.00	RX1	H
ATOM	2507	C	LYS	998	49.052	31.503	17.033	1.00	0.00	RX1	C
ATOM	2508	O	LYS	998	49.749	30.955	16.190	1.00	0.00	RX1	O
ATOM	2509	N	ILE	999	48.229	32.523	16.764	1.00	0.00	RX1	N
ATOM	2510	H	ILE	999	47.822	33.115	17.462	1.00	0.00	RX1	H
ATOM	2511	CA	ILE	999	47.925	32.689	15.345	1.00	0.00	RX1	C
ATOM	2512	CB	ILE	999	46.437	32.977	15.143	1.00	0.00	RX1	C
ATOM	2513	CG2	ILE	999	46.043	32.711	13.690	1.00	0.00	RX1	C
ATOM	2514	CG1	ILE	999	45.573	32.169	16.113	1.00	0.00	RX1	C
ATOM	2515	CD1	ILE	999	45.649	30.661	15.882	1.00	0.00	RX1	C
ATOM	2516	C	ILE	999	48.787	33.697	14.602	1.00	0.00	RX1	C
ATOM	2517	O	ILE	999	48.359	34.789	14.242	1.00	0.00	RX1	O
ATOM	2518	N	THR	1000	50.026	33.272	14.349	1.00	0.00	RX1	N
ATOM	2519	H	THR	1000	50.400	32.383	14.629	1.00	0.00	RX1	H
ATOM	2520	CA	THR	1000	50.905	34.154	13.590	1.00	0.00	RX1	C
ATOM	2521	CB	THR	1000	52.297	33.749	14.030	1.00	0.00	RX1	C
ATOM	2522	OG1	THR	1000	52.191	33.038	15.273	1.00	0.00	RX1	O
ATOM	2523	HG1	THR	1000	52.962	32.469	15.276	1.00	0.00	RX1	H
ATOM	2524	CG2	THR	1000	53.258	34.934	14.134	1.00	0.00	RX1	C
ATOM	2525	C	THR	1000	50.668	34.085	12.079	1.00	0.00	RX1	C
ATOM	2526	O	THR	1000	51.441	33.523	11.311	1.00	0.00	RX1	O
ATOM	2527	N	MET	1001	49.534	34.670	11.654	1.00	0.00	RX1	N
ATOM	2528	H	MET	1001	48.961	35.153	12.319	1.00	0.00	RX1	H
ATOM	2529	CA	MET	1001	49.240	34.621	10.215	1.00	0.00	RX1	C
ATOM	2530	CB	MET	1001	47.885	35.252	9.895	1.00	0.00	RX1	C
ATOM	2531	CG	MET	1001	46.722	34.391	10.382	1.00	0.00	RX1	C
ATOM	2532	SD	MET	1001	45.108	35.089	10.000	1.00	0.00	RX1	S
ATOM	2533	CE	MET	1001	45.249	36.587	10.986	1.00	0.00	RX1	C
ATOM	2534	C	MET	1001	50.326	35.230	9.342	1.00	0.00	RX1	C
ATOM	2535	O	MET	1001	50.883	36.277	9.642	1.00	0.00	RX1	O
ATOM	2536	N	SER	1002	50.632	34.497	8.264	1.00	0.00	RX1	N
ATOM	2537	H	SER	1002	50.161	33.657	7.993	1.00	0.00	RX1	H
ATOM	2538	CA	SER	1002	51.846	34.884	7.557	1.00	0.00	RX1	C
ATOM	2539	CB	SER	1002	52.861	33.763	7.841	1.00	0.00	RX1	C
ATOM	2540	OG	SER	1002	54.220	34.172	7.598	1.00	0.00	RX1	O
ATOM	2541	HG	SER	1002	54.425	34.743	8.337	1.00	0.00	RX1	H
ATOM	2542	C	SER	1002	51.647	35.255	6.092	1.00	0.00	RX1	C
ATOM	2543	O	SER	1002	52.358	36.103	5.564	1.00	0.00	RX1	O
ATOM	2544	N	ARG	1003	50.662	34.597	5.454	1.00	0.00	RX1	N
ATOM	2545	H	ARG	1003	50.140	33.839	5.857	1.00	0.00	RX1	H
ATOM	2546	CA	ARG	1003	50.238	34.985	4.103	1.00	0.00	RX1	C
ATOM	2547	CB	ARG	1003	51.315	34.691	3.054	1.00	0.00	RX1	C
ATOM	2548	CG	ARG	1003	51.669	33.210	2.931	1.00	0.00	RX1	C
ATOM	2549	CD	ARG	1003	53.126	33.015	2.506	1.00	0.00	RX1	C
ATOM	2550	NE	ARG	1003	53.853	32.275	3.536	1.00	0.00	RX1	N
ATOM	2551	HE	ARG	1003	53.844	31.266	3.454	1.00	0.00	RX1	H
ATOM	2552	CZ	ARG	1003	54.322	32.908	4.653	1.00	0.00	RX1	C
ATOM	2553	NH1	ARG	1003	54.293	34.254	4.742	1.00	0.00	RX1	N
ATOM	2554	HH11	ARG	1003	54.551	34.722	5.597	1.00	0.00	RX1	H
ATOM	2555	HH12	ARG	1003	53.982	34.842	3.990	1.00	0.00	RX1	H
ATOM	2556	NH2	ARG	1003	54.789	32.184	5.684	1.00	0.00	RX1	N
ATOM	2557	HH21	ARG	1003	55.077	32.607	6.550	1.00	0.00	RX1	H
ATOM	2558	HH22	ARG	1003	54.828	31.175	5.629	1.00	0.00	RX1	H
ATOM	2559	C	ARG	1003	48.950	34.276	3.756	1.00	0.00	RX1	C
ATOM	2560	O	ARG	1003	48.531	33.379	4.477	1.00	0.00	RX1	O

ATOM	2561	N	GLU	1004	48.350	34.693	2.637	1.00	0.00	RX1	N
ATOM	2562	H	GLU	1004	48.749	35.359	2.010	1.00	0.00	RX1	H
ATOM	2563	CA	GLU	1004	47.207	33.909	2.185	1.00	0.00	RX1	C
ATOM	2564	CB	GLU	1004	46.127	34.806	1.595	1.00	0.00	RX1	C
ATOM	2565	CG	GLU	1004	45.806	35.958	2.541	1.00	0.00	RX1	C
ATOM	2566	CD	GLU	1004	44.468	36.547	2.167	1.00	0.00	RX1	C
ATOM	2567	OE1	GLU	1004	44.217	36.711	0.978	1.00	0.00	RX1	O
ATOM	2568	OE2	GLU	1004	43.674	36.812	3.068	1.00	0.00	RX1	O
ATOM	2569	C	GLU	1004	47.638	32.852	1.192	1.00	0.00	RX1	C
ATOM	2570	O	GLU	1004	48.749	32.888	0.678	1.00	0.00	RX1	O
ATOM	2571	N	LEU	1005	46.720	31.908	0.967	1.00	0.00	RX1	N
ATOM	2572	H	LEU	1005	45.830	31.953	1.422	1.00	0.00	RX1	H
ATOM	2573	CA	LEU	1005	46.977	30.860	-0.018	1.00	0.00	RX1	C
ATOM	2574	CB	LEU	1005	47.044	29.476	0.635	1.00	0.00	RX1	C
ATOM	2575	CG	LEU	1005	48.200	29.281	1.617	1.00	0.00	RX1	C
ATOM	2576	CD1	LEU	1005	48.132	27.910	2.292	1.00	0.00	RX1	C
ATOM	2577	CD2	LEU	1005	49.562	29.524	0.965	1.00	0.00	RX1	C
ATOM	2578	C	LEU	1005	45.927	30.839	-1.111	1.00	0.00	RX1	C
ATOM	2579	O	LEU	1005	46.231	30.820	-2.296	1.00	0.00	RX1	O
ATOM	2580	N	GLY	1006	44.664	30.840	-0.662	1.00	0.00	RX1	N
ATOM	2581	H	GLY	1006	44.388	30.848	0.302	1.00	0.00	RX1	H
ATOM	2582	CA	GLY	1006	43.609	30.790	-1.670	1.00	0.00	RX1	C
ATOM	2583	C	GLY	1006	42.235	30.691	-1.051	1.00	0.00	RX1	C
ATOM	2584	O	GLY	1006	42.093	30.499	0.150	1.00	0.00	RX1	O
ATOM	2585	N	GLN	1007	41.230	30.850	-1.922	1.00	0.00	RX1	N
ATOM	2586	H	GLN	1007	41.417	30.858	-2.901	1.00	0.00	RX1	H
ATOM	2587	CA	GLN	1007	39.846	30.787	-1.453	1.00	0.00	RX1	C
ATOM	2588	CB	GLN	1007	38.948	31.271	-2.600	1.00	0.00	RX1	C
ATOM	2589	CG	GLN	1007	37.448	31.349	-2.307	1.00	0.00	RX1	C
ATOM	2590	CD	GLN	1007	37.213	32.291	-1.149	1.00	0.00	RX1	C
ATOM	2591	OE1	GLN	1007	37.641	33.439	-1.149	1.00	0.00	RX1	O
ATOM	2592	NE2	GLN	1007	36.518	31.735	-0.145	1.00	0.00	RX1	N
ATOM	2593	HE21	GLN	1007	36.204	30.782	-0.206	1.00	0.00	RX1	H
ATOM	2594	HE22	GLN	1007	36.307	32.233	0.694	1.00	0.00	RX1	H
ATOM	2595	C	GLN	1007	39.468	29.387	-0.993	1.00	0.00	RX1	C
ATOM	2596	O	GLN	1007	39.973	28.399	-1.509	1.00	0.00	RX1	O
ATOM	2597	N	GLY	1008	38.564	29.352	-0.008	1.00	0.00	RX1	N
ATOM	2598	H	GLY	1008	38.240	30.155	0.496	1.00	0.00	RX1	H
ATOM	2599	CA	GLY	1008	38.017	28.060	0.373	1.00	0.00	RX1	C
ATOM	2600	C	GLY	1008	36.647	28.180	1.008	1.00	0.00	RX1	C
ATOM	2601	O	GLY	1008	35.998	29.227	1.028	1.00	0.00	RX1	O
ATOM	2602	N	SER	1009	36.258	27.033	1.571	1.00	0.00	RX1	N
ATOM	2603	H	SER	1009	36.893	26.267	1.494	1.00	0.00	RX1	H
ATOM	2604	CA	SER	1009	34.923	26.791	2.118	1.00	0.00	RX1	C
ATOM	2605	CB	SER	1009	35.114	25.589	3.021	1.00	0.00	RX1	C
ATOM	2606	OG	SER	1009	36.215	24.860	2.462	1.00	0.00	RX1	O
ATOM	2607	HG	SER	1009	35.897	24.570	1.605	1.00	0.00	RX1	H
ATOM	2608	C	SER	1009	34.174	27.969	2.735	1.00	0.00	RX1	C
ATOM	2609	O	SER	1009	33.150	28.426	2.233	1.00	0.00	RX1	O
ATOM	2610	N	PHE	1010	34.736	28.474	3.840	1.00	0.00	RX1	N
ATOM	2611	H	PHE	1010	35.598	28.156	4.235	1.00	0.00	RX1	H
ATOM	2612	CA	PHE	1010	34.038	29.597	4.463	1.00	0.00	RX1	C
ATOM	2613	CB	PHE	1010	33.411	29.168	5.789	1.00	0.00	RX1	C
ATOM	2614	CG	PHE	1010	32.158	28.353	5.563	1.00	0.00	RX1	C
ATOM	2615	CD1	PHE	1010	30.917	28.973	5.624	1.00	0.00	RX1	C
ATOM	2616	CD2	PHE	1010	32.238	26.990	5.304	1.00	0.00	RX1	C
ATOM	2617	CE1	PHE	1010	29.759	28.228	5.435	1.00	0.00	RX1	C
ATOM	2618	CE2	PHE	1010	31.081	26.245	5.114	1.00	0.00	RX1	C
ATOM	2619	CZ	PHE	1010	29.839	26.863	5.185	1.00	0.00	RX1	C
ATOM	2620	C	PHE	1010	34.905	30.825	4.664	1.00	0.00	RX1	C
ATOM	2621	O	PHE	1010	34.678	31.653	5.538	1.00	0.00	RX1	O

ATOM	2622	N	GLY	1011	35.926	30.905	3.804	1.00	0.00	RX1	N
ATOM	2623	H	GLY	1011	36.123	30.268	3.055	1.00	0.00	RX1	H
ATOM	2624	CA	GLY	1011	36.860	32.008	3.976	1.00	0.00	RX1	C
ATOM	2625	C	GLY	1011	38.138	31.697	3.242	1.00	0.00	RX1	C
ATOM	2626	O	GLY	1011	38.172	30.828	2.379	1.00	0.00	RX1	O
ATOM	2627	N	MET	1012	39.179	32.441	3.614	1.00	0.00	RX1	N
ATOM	2628	H	MET	1012	39.145	32.969	4.464	1.00	0.00	RX1	H
ATOM	2629	CA	MET	1012	40.461	32.181	2.971	1.00	0.00	RX1	C
ATOM	2630	CB	MET	1012	41.280	33.475	2.966	1.00	0.00	RX1	C
ATOM	2631	CG	MET	1012	42.392	33.542	1.918	1.00	0.00	RX1	C
ATOM	2632	SD	MET	1012	41.746	33.612	0.242	1.00	0.00	RX1	S
ATOM	2633	CE	MET	1012	40.929	35.211	0.364	1.00	0.00	RX1	C
ATOM	2634	C	MET	1012	41.189	31.050	3.682	1.00	0.00	RX1	C
ATOM	2635	O	MET	1012	40.929	30.754	4.845	1.00	0.00	RX1	O
ATOM	2636	N	VAL	1013	42.107	30.432	2.943	1.00	0.00	RX1	N
ATOM	2637	H	VAL	1013	42.279	30.711	1.998	1.00	0.00	RX1	H
ATOM	2638	CA	VAL	1013	43.084	29.573	3.595	1.00	0.00	RX1	C
ATOM	2639	CB	VAL	1013	43.236	28.262	2.822	1.00	0.00	RX1	C
ATOM	2640	CG1	VAL	1013	44.186	27.298	3.537	1.00	0.00	RX1	C
ATOM	2641	CG2	VAL	1013	41.866	27.635	2.557	1.00	0.00	RX1	C
ATOM	2642	C	VAL	1013	44.392	30.338	3.633	1.00	0.00	RX1	C
ATOM	2643	O	VAL	1013	44.784	30.943	2.643	1.00	0.00	RX1	O
ATOM	2644	N	TYR	1014	45.019	30.321	4.810	1.00	0.00	RX1	N
ATOM	2645	H	TYR	1014	44.677	29.741	5.549	1.00	0.00	RX1	H
ATOM	2646	CA	TYR	1014	46.257	31.066	5.011	1.00	0.00	RX1	C
ATOM	2647	CB	TYR	1014	46.170	31.899	6.294	1.00	0.00	RX1	C
ATOM	2648	CG	TYR	1014	45.170	33.019	6.159	1.00	0.00	RX1	C
ATOM	2649	CD1	TYR	1014	45.625	34.306	5.905	1.00	0.00	RX1	C
ATOM	2650	CE1	TYR	1014	44.715	35.340	5.738	1.00	0.00	RX1	C
ATOM	2651	CD2	TYR	1014	43.808	32.775	6.291	1.00	0.00	RX1	C
ATOM	2652	CE2	TYR	1014	42.899	33.810	6.117	1.00	0.00	RX1	C
ATOM	2653	CZ	TYR	1014	43.352	35.087	5.810	1.00	0.00	RX1	C
ATOM	2654	OH	TYR	1014	42.450	36.097	5.560	1.00	0.00	RX1	O
ATOM	2655	HH	TYR	1014	42.739	36.543	4.757	1.00	0.00	RX1	H
ATOM	2656	C	TYR	1014	47.416	30.112	5.180	1.00	0.00	RX1	C
ATOM	2657	O	TYR	1014	47.233	29.003	5.664	1.00	0.00	RX1	O
ATOM	2658	N	GLU	1015	48.614	30.600	4.832	1.00	0.00	RX1	N
ATOM	2659	H	GLU	1015	48.696	31.511	4.432	1.00	0.00	RX1	H
ATOM	2660	CA	GLU	1015	49.744	30.043	5.566	1.00	0.00	RX1	C
ATOM	2661	CB	GLU	1015	51.054	29.994	4.775	1.00	0.00	RX1	C
ATOM	2662	CG	GLU	1015	52.147	29.375	5.656	1.00	0.00	RX1	C
ATOM	2663	CD	GLU	1015	53.509	29.389	4.987	1.00	0.00	RX1	C
ATOM	2664	OE1	GLU	1015	53.603	29.329	3.770	1.00	0.00	RX1	O
ATOM	2665	OE2	GLU	1015	54.513	29.464	5.683	1.00	0.00	RX1	O
ATOM	2666	C	GLU	1015	49.939	30.872	6.815	1.00	0.00	RX1	C
ATOM	2667	O	GLU	1015	50.322	32.040	6.782	1.00	0.00	RX1	O
ATOM	2668	N	GLY	1016	49.620	30.226	7.927	1.00	0.00	RX1	N
ATOM	2669	H	GLY	1016	49.426	29.243	7.906	1.00	0.00	RX1	H
ATOM	2670	CA	GLY	1016	50.032	30.844	9.172	1.00	0.00	RX1	C
ATOM	2671	C	GLY	1016	51.260	30.132	9.668	1.00	0.00	RX1	C
ATOM	2672	O	GLY	1016	51.602	29.049	9.214	1.00	0.00	RX1	O
ATOM	2673	N	VAL	1017	51.895	30.783	10.627	1.00	0.00	RX1	N
ATOM	2674	H	VAL	1017	51.578	31.652	11.010	1.00	0.00	RX1	H
ATOM	2675	CA	VAL	1017	52.782	30.020	11.485	1.00	0.00	RX1	C
ATOM	2676	CB	VAL	1017	54.204	30.573	11.390	1.00	0.00	RX1	C
ATOM	2677	CG1	VAL	1017	54.877	30.077	10.112	1.00	0.00	RX1	C
ATOM	2678	CG2	VAL	1017	54.235	32.098	11.444	1.00	0.00	RX1	C
ATOM	2679	C	VAL	1017	52.165	30.093	12.868	1.00	0.00	RX1	C
ATOM	2680	O	VAL	1017	51.324	30.955	13.110	1.00	0.00	RX1	O
ATOM	2681	N	ALA	1018	52.525	29.133	13.721	1.00	0.00	RX1	N
ATOM	2682	H	ALA	1018	53.199	28.431	13.494	1.00	0.00	RX1	H

ATOM	2683	CA	ALA	1018	51.694	29.012	14.910	1.00	0.00	RX1	C
ATOM	2684	CB	ALA	1018	50.629	27.937	14.708	1.00	0.00	RX1	C
ATOM	2685	C	ALA	1018	52.441	28.734	16.191	1.00	0.00	RX1	C
ATOM	2686	O	ALA	1018	53.189	27.775	16.328	1.00	0.00	RX1	O
ATOM	2687	N	LYS	1019	52.171	29.628	17.148	1.00	0.00	RX1	N
ATOM	2688	H	LYS	1019	51.516	30.346	16.896	1.00	0.00	RX1	H
ATOM	2689	CA	LYS	1019	52.836	29.586	18.448	1.00	0.00	RX1	C
ATOM	2690	CB	LYS	1019	52.564	30.933	19.128	1.00	0.00	RX1	C
ATOM	2691	CG	LYS	1019	53.380	31.286	20.372	1.00	0.00	RX1	C
ATOM	2692	CD	LYS	1019	54.900	31.368	20.190	1.00	0.00	RX1	C
ATOM	2693	CE	LYS	1019	55.383	32.388	19.153	1.00	0.00	RX1	C
ATOM	2694	NZ	LYS	1019	55.385	31.794	17.814	1.00	0.00	RX1	N
ATOM	2695	HZ1	LYS	1019	54.470	31.780	17.315	1.00	0.00	RX1	H
ATOM	2696	HZ2	LYS	1019	55.983	32.327	17.150	1.00	0.00	RX1	H
ATOM	2697	HZ3	LYS	1019	55.725	30.814	17.798	1.00	0.00	RX1	H
ATOM	2698	C	LYS	1019	52.504	28.390	19.334	1.00	0.00	RX1	C
ATOM	2699	O	LYS	1019	51.700	28.469	20.260	1.00	0.00	RX1	O
ATOM	2700	N	GLY	1020	53.182	27.277	19.036	1.00	0.00	RX1	N
ATOM	2701	H	GLY	1020	53.733	27.204	18.201	1.00	0.00	RX1	H
ATOM	2702	CA	GLY	1020	52.991	26.107	19.886	1.00	0.00	RX1	C
ATOM	2703	C	GLY	1020	51.726	25.336	19.578	1.00	0.00	RX1	C
ATOM	2704	O	GLY	1020	50.836	25.206	20.411	1.00	0.00	RX1	O
ATOM	2705	N	VAL	1021	51.665	24.833	18.333	1.00	0.00	RX1	N
ATOM	2706	H	VAL	1021	52.416	24.968	17.681	1.00	0.00	RX1	H
ATOM	2707	CA	VAL	1021	50.560	23.896	18.122	1.00	0.00	RX1	C
ATOM	2708	CB	VAL	1021	49.674	24.225	16.912	1.00	0.00	RX1	C
ATOM	2709	CG1	VAL	1021	48.931	25.542	17.117	1.00	0.00	RX1	C
ATOM	2710	CG2	VAL	1021	50.434	24.192	15.595	1.00	0.00	RX1	C
ATOM	2711	C	VAL	1021	50.976	22.438	18.126	1.00	0.00	RX1	C
ATOM	2712	O	VAL	1021	50.283	21.577	18.658	1.00	0.00	RX1	O
ATOM	2713	N	VAL	1022	52.169	22.174	17.564	1.00	0.00	RX1	N
ATOM	2714	H	VAL	1022	52.766	22.880	17.175	1.00	0.00	RX1	H
ATOM	2715	CA	VAL	1022	52.687	20.828	17.807	1.00	0.00	RX1	C
ATOM	2716	CB	VAL	1022	53.587	20.344	16.669	1.00	0.00	RX1	C
ATOM	2717	CG1	VAL	1022	54.096	18.920	16.912	1.00	0.00	RX1	C
ATOM	2718	CG2	VAL	1022	52.818	20.415	15.351	1.00	0.00	RX1	C
ATOM	2719	C	VAL	1022	53.370	20.768	19.163	1.00	0.00	RX1	C
ATOM	2720	O	VAL	1022	54.581	20.835	19.316	1.00	0.00	RX1	O
ATOM	2721	N	LYS	1023	52.483	20.686	20.167	1.00	0.00	RX1	N
ATOM	2722	H	LYS	1023	51.528	20.764	19.896	1.00	0.00	RX1	H
ATOM	2723	CA	LYS	1023	52.900	20.874	21.556	1.00	0.00	RX1	C
ATOM	2724	CB	LYS	1023	53.421	19.576	22.176	1.00	0.00	RX1	C
ATOM	2725	CG	LYS	1023	52.327	18.517	21.952	1.00	0.00	RX1	C
ATOM	2726	CD	LYS	1023	50.921	19.043	22.302	1.00	0.00	RX1	C
ATOM	2727	CE	LYS	1023	49.821	18.447	21.410	1.00	0.00	RX1	C
ATOM	2728	NZ	LYS	1023	48.735	19.427	21.212	1.00	0.00	RX1	N
ATOM	2729	HZ1	LYS	1023	47.940	18.976	20.714	1.00	0.00	RX1	H
ATOM	2730	HZ2	LYS	1023	48.401	19.785	22.129	1.00	0.00	RX1	H
ATOM	2731	HZ3	LYS	1023	49.073	20.225	20.631	1.00	0.00	RX1	H
ATOM	2732	C	LYS	1023	53.669	22.173	21.775	1.00	0.00	RX1	C
ATOM	2733	O	LYS	1023	53.085	23.246	21.695	1.00	0.00	RX1	O
ATOM	2734	N	ASP	1024	54.968	22.044	22.044	1.00	0.00	RX1	N
ATOM	2735	H	ASP	1024	55.487	21.189	21.959	1.00	0.00	RX1	H
ATOM	2736	CA	ASP	1024	55.772	23.238	22.284	1.00	0.00	RX1	C
ATOM	2737	CB	ASP	1024	57.011	22.849	23.087	1.00	0.00	RX1	C
ATOM	2738	CG	ASP	1024	57.766	21.786	22.317	1.00	0.00	RX1	C
ATOM	2739	OD1	ASP	1024	58.704	22.132	21.604	1.00	0.00	RX1	O
ATOM	2740	OD2	ASP	1024	57.399	20.615	22.417	1.00	0.00	RX1	O
ATOM	2741	C	ASP	1024	56.179	23.995	21.030	1.00	0.00	RX1	C
ATOM	2742	O	ASP	1024	56.381	25.205	21.071	1.00	0.00	RX1	O
ATOM	2743	N	GLU	1025	56.259	23.236	19.914	1.00	0.00	RX1	N

ATOM	2744	H	GLU	1025	56.142	22.248	20.022	1.00	0.00	RX1	H
ATOM	2745	CA	GLU	1025	56.796	23.754	18.647	1.00	0.00	RX1	C
ATOM	2746	CB	GLU	1025	56.423	22.811	17.499	1.00	0.00	RX1	C
ATOM	2747	CG	GLU	1025	57.441	22.758	16.354	1.00	0.00	RX1	C
ATOM	2748	CD	GLU	1025	57.204	23.871	15.354	1.00	0.00	RX1	C
ATOM	2749	OE1	GLU	1025	57.601	25.004	15.599	1.00	0.00	RX1	O
ATOM	2750	OE2	GLU	1025	56.606	23.599	14.320	1.00	0.00	RX1	O
ATOM	2751	C	GLU	1025	56.501	25.217	18.331	1.00	0.00	RX1	C
ATOM	2752	O	GLU	1025	55.384	25.622	18.015	1.00	0.00	RX1	O
ATOM	2753	N	PRO	1026	57.582	26.018	18.507	1.00	0.00	RX1	N
ATOM	2754	CD	PRO	1026	58.941	25.549	18.748	1.00	0.00	RX1	C
ATOM	2755	CA	PRO	1026	57.460	27.474	18.579	1.00	0.00	RX1	C
ATOM	2756	CB	PRO	1026	58.923	27.926	18.586	1.00	0.00	RX1	C
ATOM	2757	CG	PRO	1026	59.672	26.781	19.265	1.00	0.00	RX1	C
ATOM	2758	C	PRO	1026	56.611	28.196	17.549	1.00	0.00	RX1	C
ATOM	2759	O	PRO	1026	55.973	29.196	17.878	1.00	0.00	RX1	O
ATOM	2760	N	GLU	1027	56.671	27.712	16.303	1.00	0.00	RX1	N
ATOM	2761	H	GLU	1027	57.093	26.830	16.071	1.00	0.00	RX1	H
ATOM	2762	CA	GLU	1027	56.064	28.493	15.230	1.00	0.00	RX1	C
ATOM	2763	CB	GLU	1027	57.015	29.623	14.820	1.00	0.00	RX1	C
ATOM	2764	CG	GLU	1027	56.352	30.816	14.125	1.00	0.00	RX1	C
ATOM	2765	CD	GLU	1027	55.504	31.637	15.080	1.00	0.00	RX1	C
ATOM	2766	OE1	GLU	1027	54.428	31.208	15.495	1.00	0.00	RX1	O
ATOM	2767	OE2	GLU	1027	55.935	32.717	15.468	1.00	0.00	RX1	O
ATOM	2768	C	GLU	1027	55.631	27.643	14.044	1.00	0.00	RX1	C
ATOM	2769	O	GLU	1027	55.972	27.888	12.892	1.00	0.00	RX1	O
ATOM	2770	N	THR	1028	54.843	26.614	14.381	1.00	0.00	RX1	N
ATOM	2771	H	THR	1028	54.553	26.452	15.327	1.00	0.00	RX1	H
ATOM	2772	CA	THR	1028	54.461	25.632	13.368	1.00	0.00	RX1	C
ATOM	2773	CB	THR	1028	53.564	24.646	14.078	1.00	0.00	RX1	C
ATOM	2774	OG1	THR	1028	54.056	24.444	15.411	1.00	0.00	RX1	O
ATOM	2775	HG1	THR	1028	54.999	24.280	15.291	1.00	0.00	RX1	H
ATOM	2776	CG2	THR	1028	53.436	23.339	13.295	1.00	0.00	RX1	C
ATOM	2777	C	THR	1028	53.820	26.186	12.104	1.00	0.00	RX1	C
ATOM	2778	O	THR	1028	52.833	26.910	12.144	1.00	0.00	RX1	O
ATOM	2779	N	ARG	1029	54.417	25.802	10.968	1.00	0.00	RX1	N
ATOM	2780	H	ARG	1029	55.175	25.153	11.011	1.00	0.00	RX1	H
ATOM	2781	CA	ARG	1029	53.793	26.197	9.706	1.00	0.00	RX1	C
ATOM	2782	CB	ARG	1029	54.781	26.029	8.555	1.00	0.00	RX1	C
ATOM	2783	CG	ARG	1029	56.086	26.798	8.756	1.00	0.00	RX1	C
ATOM	2784	CD	ARG	1029	56.968	26.772	7.507	1.00	0.00	RX1	C
ATOM	2785	NE	ARG	1029	56.254	27.350	6.370	1.00	0.00	RX1	N
ATOM	2786	HE	ARG	1029	55.731	28.200	6.517	1.00	0.00	RX1	H
ATOM	2787	CZ	ARG	1029	56.247	26.718	5.162	1.00	0.00	RX1	C
ATOM	2788	NH1	ARG	1029	56.950	25.579	5.017	1.00	0.00	RX1	N
ATOM	2789	HH11	ARG	1029	56.883	25.076	4.141	1.00	0.00	RX1	H
ATOM	2790	HH12	ARG	1029	57.525	25.190	5.737	1.00	0.00	RX1	H
ATOM	2791	NH2	ARG	1029	55.530	27.212	4.132	1.00	0.00	RX1	N
ATOM	2792	HH21	ARG	1029	55.488	26.727	3.248	1.00	0.00	RX1	H
ATOM	2793	HH22	ARG	1029	54.983	28.067	4.215	1.00	0.00	RX1	H
ATOM	2794	C	ARG	1029	52.509	25.430	9.431	1.00	0.00	RX1	C
ATOM	2795	O	ARG	1029	52.497	24.206	9.369	1.00	0.00	RX1	O
ATOM	2796	N	VAL	1030	51.425	26.204	9.309	1.00	0.00	RX1	N
ATOM	2797	H	VAL	1030	51.497	27.201	9.269	1.00	0.00	RX1	H
ATOM	2798	CA	VAL	1030	50.089	25.611	9.274	1.00	0.00	RX1	C
ATOM	2799	CB	VAL	1030	49.411	25.746	10.641	1.00	0.00	RX1	C
ATOM	2800	CG1	VAL	1030	50.086	24.869	11.694	1.00	0.00	RX1	C
ATOM	2801	CG2	VAL	1030	49.326	27.208	11.079	1.00	0.00	RX1	C
ATOM	2802	C	VAL	1030	49.201	26.214	8.200	1.00	0.00	RX1	C
ATOM	2803	O	VAL	1030	49.334	27.373	7.826	1.00	0.00	RX1	O
ATOM	2804	N	ALA	1031	48.273	25.379	7.721	1.00	0.00	RX1	N

ATOM	2805	H	ALA	1031	48.135	24.479	8.134	1.00	0.00	RX1	H
ATOM	2806	CA	ALA	1031	47.251	25.920	6.832	1.00	0.00	RX1	C
ATOM	2807	CB	ALA	1031	46.863	24.912	5.751	1.00	0.00	RX1	C
ATOM	2808	C	ALA	1031	46.006	26.276	7.612	1.00	0.00	RX1	C
ATOM	2809	O	ALA	1031	45.340	25.434	8.198	1.00	0.00	RX1	O
ATOM	2810	N	ILE	1032	45.725	27.576	7.623	1.00	0.00	RX1	N
ATOM	2811	H	ILE	1032	46.242	28.208	7.046	1.00	0.00	RX1	H
ATOM	2812	CA	ILE	1032	44.555	28.000	8.384	1.00	0.00	RX1	C
ATOM	2813	CB	ILE	1032	44.857	29.313	9.097	1.00	0.00	RX1	C
ATOM	2814	CG2	ILE	1032	43.713	29.715	10.028	1.00	0.00	RX1	C
ATOM	2815	CG1	ILE	1032	46.199	29.231	9.817	1.00	0.00	RX1	C
ATOM	2816	CD1	ILE	1032	46.539	30.537	10.530	1.00	0.00	RX1	C
ATOM	2817	C	ILE	1032	43.345	28.171	7.491	1.00	0.00	RX1	C
ATOM	2818	O	ILE	1032	43.309	29.077	6.669	1.00	0.00	RX1	O
ATOM	2819	N	LYS	1033	42.349	27.293	7.668	1.00	0.00	RX1	N
ATOM	2820	H	LYS	1033	42.375	26.667	8.451	1.00	0.00	RX1	H
ATOM	2821	CA	LYS	1033	41.115	27.654	6.969	1.00	0.00	RX1	C
ATOM	2822	CB	LYS	1033	40.275	26.432	6.548	1.00	0.00	RX1	C
ATOM	2823	CG	LYS	1033	41.061	25.296	5.879	1.00	0.00	RX1	C
ATOM	2824	CD	LYS	1033	40.236	24.262	5.082	1.00	0.00	RX1	C
ATOM	2825	CE	LYS	1033	39.133	23.477	5.817	1.00	0.00	RX1	C
ATOM	2826	NZ	LYS	1033	38.749	22.294	5.028	1.00	0.00	RX1	N
ATOM	2827	HZ1	LYS	1033	37.873	21.793	5.279	1.00	0.00	RX1	H
ATOM	2828	HZ2	LYS	1033	38.793	22.455	4.004	1.00	0.00	RX1	H
ATOM	2829	HZ3	LYS	1033	39.513	21.594	5.148	1.00	0.00	RX1	H
ATOM	2830	C	LYS	1033	40.295	28.597	7.834	1.00	0.00	RX1	C
ATOM	2831	O	LYS	1033	40.355	28.530	9.058	1.00	0.00	RX1	O
ATOM	2832	N	THR	1034	39.552	29.495	7.178	1.00	0.00	RX1	N
ATOM	2833	H	THR	1034	39.513	29.602	6.184	1.00	0.00	RX1	H
ATOM	2834	CA	THR	1034	38.802	30.423	8.020	1.00	0.00	RX1	C
ATOM	2835	CB	THR	1034	39.333	31.843	7.815	1.00	0.00	RX1	C
ATOM	2836	OG1	THR	1034	39.410	32.150	6.424	1.00	0.00	RX1	O
ATOM	2837	HG1	THR	1034	40.207	31.723	6.127	1.00	0.00	RX1	H
ATOM	2838	CG2	THR	1034	40.711	32.019	8.443	1.00	0.00	RX1	C
ATOM	2839	C	THR	1034	37.297	30.343	7.859	1.00	0.00	RX1	C
ATOM	2840	O	THR	1034	36.773	29.784	6.900	1.00	0.00	RX1	O
ATOM	2841	N	VAL	1035	36.638	30.962	8.851	1.00	0.00	RX1	N
ATOM	2842	H	VAL	1035	37.135	31.346	9.631	1.00	0.00	RX1	H
ATOM	2843	CA	VAL	1035	35.270	31.425	8.648	1.00	0.00	RX1	C
ATOM	2844	CB	VAL	1035	34.320	30.857	9.696	1.00	0.00	RX1	C
ATOM	2845	CG1	VAL	1035	32.924	30.693	9.104	1.00	0.00	RX1	C
ATOM	2846	CG2	VAL	1035	34.860	29.585	10.340	1.00	0.00	RX1	C
ATOM	2847	C	VAL	1035	35.298	32.931	8.784	1.00	0.00	RX1	C
ATOM	2848	O	VAL	1035	35.628	33.450	9.851	1.00	0.00	RX1	O
ATOM	2849	N	ASN	1036	34.995	33.579	7.654	1.00	0.00	RX1	N
ATOM	2850	H	ASN	1036	34.620	33.046	6.892	1.00	0.00	RX1	H
ATOM	2851	CA	ASN	1036	35.267	35.006	7.463	1.00	0.00	RX1	C
ATOM	2852	CB	ASN	1036	34.987	35.423	6.019	1.00	0.00	RX1	C
ATOM	2853	CG	ASN	1036	33.534	35.814	5.875	1.00	0.00	RX1	C
ATOM	2854	OD1	ASN	1036	32.643	35.147	6.385	1.00	0.00	RX1	O
ATOM	2855	ND2	ASN	1036	33.330	36.900	5.118	1.00	0.00	RX1	N
ATOM	2856	HD21	ASN	1036	34.099	37.521	4.903	1.00	0.00	RX1	H
ATOM	2857	HD22	ASN	1036	32.452	37.219	4.769	1.00	0.00	RX1	H
ATOM	2858	C	ASN	1036	34.619	35.975	8.450	1.00	0.00	RX1	C
ATOM	2859	O	ASN	1036	33.901	35.601	9.373	1.00	0.00	RX1	O
ATOM	2860	N	GLU	1037	34.885	37.267	8.201	1.00	0.00	RX1	N
ATOM	2861	H	GLU	1037	35.428	37.526	7.398	1.00	0.00	RX1	H
ATOM	2862	CA	GLU	1037	34.194	38.301	8.978	1.00	0.00	RX1	C
ATOM	2863	CB	GLU	1037	34.598	39.747	8.632	1.00	0.00	RX1	C
ATOM	2864	CG	GLU	1037	35.559	39.984	7.463	1.00	0.00	RX1	C
ATOM	2865	CD	GLU	1037	34.980	39.379	6.203	1.00	0.00	RX1	C

ATOM	2866	OE1	GLU	1037	35.653	38.554	5.596	1.00	0.00	RX1 O
ATOM	2867	OE2	GLU	1037	33.829	39.651	5.880	1.00	0.00	RX1 O
ATOM	2868	C	GLU	1037	32.678	38.192	8.960	1.00	0.00	RX1 C
ATOM	2869	O	GLU	1037	32.055	38.070	10.008	1.00	0.00	RX1 O
ATOM	2870	N	ALA	1038	32.118	38.193	7.743	1.00	0.00	RX1 N
ATOM	2871	H	ALA	1038	32.712	38.351	6.948	1.00	0.00	RX1 H
ATOM	2872	CA	ALA	1038	30.660	38.236	7.606	1.00	0.00	RX1 C
ATOM	2873	CB	ALA	1038	30.268	38.385	6.135	1.00	0.00	RX1 C
ATOM	2874	C	ALA	1038	29.860	37.082	8.199	1.00	0.00	RX1 C
ATOM	2875	O	ALA	1038	28.863	37.291	8.875	1.00	0.00	RX1 O
ATOM	2876	N	ALA	1039	30.341	35.859	7.907	1.00	0.00	RX1 N
ATOM	2877	H	ALA	1039	31.171	35.786	7.355	1.00	0.00	RX1 H
ATOM	2878	CA	ALA	1039	29.577	34.627	8.160	1.00	0.00	RX1 C
ATOM	2879	CB	ALA	1039	30.534	33.436	8.183	1.00	0.00	RX1 C
ATOM	2880	C	ALA	1039	28.716	34.545	9.418	1.00	0.00	RX1 C
ATOM	2881	O	ALA	1039	29.099	34.995	10.499	1.00	0.00	RX1 O
ATOM	2882	N	SER	1040	27.534	33.937	9.244	1.00	0.00	RX1 N
ATOM	2883	H	SER	1040	27.305	33.487	8.373	1.00	0.00	RX1 H
ATOM	2884	CA	SER	1040	26.654	33.846	10.408	1.00	0.00	RX1 C
ATOM	2885	CB	SER	1040	25.249	33.427	9.964	1.00	0.00	RX1 C
ATOM	2886	OG	SER	1040	25.241	32.049	9.582	1.00	0.00	RX1 O
ATOM	2887	HG	SER	1040	25.343	32.062	8.625	1.00	0.00	RX1 H
ATOM	2888	C	SER	1040	27.195	32.966	11.521	1.00	0.00	RX1 C
ATOM	2889	O	SER	1040	28.171	32.236	11.374	1.00	0.00	RX1 O
ATOM	2890	N	MET	1041	26.480	33.032	12.656	1.00	0.00	RX1 N
ATOM	2891	H	MET	1041	25.680	33.626	12.726	1.00	0.00	RX1 H
ATOM	2892	CA	MET	1041	26.834	32.112	13.735	1.00	0.00	RX1 C
ATOM	2893	CB	MET	1041	26.041	32.409	15.019	1.00	0.00	RX1 C
ATOM	2894	CG	MET	1041	24.570	31.978	15.028	1.00	0.00	RX1 C
ATOM	2895	SD	MET	1041	23.590	32.744	13.725	1.00	0.00	RX1 S
ATOM	2896	CE	MET	1041	22.078	31.798	13.967	1.00	0.00	RX1 C
ATOM	2897	C	MET	1041	26.784	30.640	13.345	1.00	0.00	RX1 C
ATOM	2898	O	MET	1041	27.666	29.864	13.692	1.00	0.00	RX1 O
ATOM	2899	N	ARG	1042	25.735	30.287	12.572	1.00	0.00	RX1 N
ATOM	2900	H	ARG	1042	25.109	30.949	12.161	1.00	0.00	RX1 H
ATOM	2901	CA	ARG	1042	25.718	28.877	12.197	1.00	0.00	RX1 C
ATOM	2902	CB	ARG	1042	24.340	28.334	11.800	1.00	0.00	RX1 C
ATOM	2903	CG	ARG	1042	24.509	26.816	11.659	1.00	0.00	RX1 C
ATOM	2904	CD	ARG	1042	23.348	25.980	11.130	1.00	0.00	RX1 C
ATOM	2905	NE	ARG	1042	23.894	24.715	10.641	1.00	0.00	RX1 N
ATOM	2906	HE	ARG	1042	24.351	24.708	9.740	1.00	0.00	RX1 H
ATOM	2907	CZ	ARG	1042	23.961	23.604	11.431	1.00	0.00	RX1 C
ATOM	2908	NH1	ARG	1042	23.367	23.582	12.635	1.00	0.00	RX1 N
ATOM	2909	HH11	ARG	1042	23.355	22.761	13.209	1.00	0.00	RX1 H
ATOM	2910	HH12	ARG	1042	22.930	24.385	13.075	1.00	0.00	RX1 H
ATOM	2911	NH2	ARG	1042	24.644	22.531	11.002	1.00	0.00	RX1 N
ATOM	2912	HH21	ARG	1042	24.704	21.675	11.535	1.00	0.00	RX1 H
ATOM	2913	HH22	ARG	1042	25.157	22.559	10.125	1.00	0.00	RX1 H
ATOM	2914	C	ARG	1042	26.761	28.531	11.154	1.00	0.00	RX1 C
ATOM	2915	O	ARG	1042	27.396	27.494	11.236	1.00	0.00	RX1 O
ATOM	2916	N	GLU	1043	26.983	29.456	10.209	1.00	0.00	RX1 N
ATOM	2917	H	GLU	1043	26.441	30.296	10.128	1.00	0.00	RX1 H
ATOM	2918	CA	GLU	1043	28.082	29.198	9.267	1.00	0.00	RX1 C
ATOM	2919	CB	GLU	1043	28.203	30.359	8.293	1.00	0.00	RX1 C
ATOM	2920	CG	GLU	1043	26.996	30.410	7.358	1.00	0.00	RX1 C
ATOM	2921	CD	GLU	1043	26.841	31.828	6.864	1.00	0.00	RX1 C
ATOM	2922	OE1	GLU	1043	25.719	32.320	6.818	1.00	0.00	RX1 O
ATOM	2923	OE2	GLU	1043	27.846	32.473	6.605	1.00	0.00	RX1 O
ATOM	2924	C	GLU	1043	29.421	28.875	9.925	1.00	0.00	RX1 C
ATOM	2925	O	GLU	1043	30.179	28.014	9.493	1.00	0.00	RX1 O
ATOM	2926	N	ARG	1044	29.648	29.573	11.049	1.00	0.00	RX1 N

ATOM	2927	H	ARG	1044	28.993	30.276	11.334	1.00	0.00	RX1	H
ATOM	2928	CA	ARG	1044	30.806	29.210	11.868	1.00	0.00	RX1	C
ATOM	2929	CB	ARG	1044	31.075	30.274	12.926	1.00	0.00	RX1	C
ATOM	2930	CG	ARG	1044	31.127	31.622	12.225	1.00	0.00	RX1	C
ATOM	2931	CD	ARG	1044	31.336	32.829	13.127	1.00	0.00	RX1	C
ATOM	2932	NE	ARG	1044	31.008	34.025	12.361	1.00	0.00	RX1	N
ATOM	2933	HE	ARG	1044	30.034	34.267	12.265	1.00	0.00	RX1	H
ATOM	2934	CZ	ARG	1044	31.947	34.645	11.595	1.00	0.00	RX1	C
ATOM	2935	NH1	ARG	1044	33.230	34.259	11.610	1.00	0.00	RX1	N
ATOM	2936	HH11	ARG	1044	33.905	34.694	10.997	1.00	0.00	RX1	H
ATOM	2937	HH12	ARG	1044	33.589	33.532	12.208	1.00	0.00	RX1	H
ATOM	2938	NH2	ARG	1044	31.571	35.651	10.802	1.00	0.00	RX1	N
ATOM	2939	HH21	ARG	1044	32.229	36.160	10.233	1.00	0.00	RX1	H
ATOM	2940	HH22	ARG	1044	30.601	35.922	10.723	1.00	0.00	RX1	H
ATOM	2941	C	ARG	1044	30.718	27.831	12.490	1.00	0.00	RX1	C
ATOM	2942	O	ARG	1044	31.624	27.017	12.376	1.00	0.00	RX1	O
ATOM	2943	N	ILE	1045	29.571	27.585	13.140	1.00	0.00	RX1	N
ATOM	2944	H	ILE	1045	28.842	28.273	13.155	1.00	0.00	RX1	H
ATOM	2945	CA	ILE	1045	29.369	26.263	13.743	1.00	0.00	RX1	C
ATOM	2946	CB	ILE	1045	27.981	26.190	14.370	1.00	0.00	RX1	C
ATOM	2947	CG2	ILE	1045	27.614	24.754	14.737	1.00	0.00	RX1	C
ATOM	2948	CG1	ILE	1045	27.911	27.152	15.557	1.00	0.00	RX1	C
ATOM	2949	CD1	ILE	1045	26.506	27.308	16.137	1.00	0.00	RX1	C
ATOM	2950	C	ILE	1045	29.604	25.097	12.788	1.00	0.00	RX1	C
ATOM	2951	O	ILE	1045	30.360	24.178	13.080	1.00	0.00	RX1	O
ATOM	2952	N	GLU	1046	28.947	25.218	11.626	1.00	0.00	RX1	N
ATOM	2953	H	GLU	1046	28.317	25.979	11.512	1.00	0.00	RX1	H
ATOM	2954	CA	GLU	1046	29.137	24.322	10.492	1.00	0.00	RX1	C
ATOM	2955	CB	GLU	1046	28.401	24.899	9.279	1.00	0.00	RX1	C
ATOM	2956	CG	GLU	1046	27.926	23.882	8.239	1.00	0.00	RX1	C
ATOM	2957	CD	GLU	1046	26.677	23.180	8.738	1.00	0.00	RX1	C
ATOM	2958	OE1	GLU	1046	25.578	23.681	8.544	1.00	0.00	RX1	O
ATOM	2959	OE2	GLU	1046	26.776	22.109	9.316	1.00	0.00	RX1	O
ATOM	2960	C	GLU	1046	30.613	24.142	10.187	1.00	0.00	RX1	C
ATOM	2961	O	GLU	1046	31.181	23.075	10.347	1.00	0.00	RX1	O
ATOM	2962	N	PHE	1047	31.243	25.263	9.803	1.00	0.00	RX1	N
ATOM	2963	H	PHE	1047	30.761	26.140	9.747	1.00	0.00	RX1	H
ATOM	2964	CA	PHE	1047	32.641	25.156	9.390	1.00	0.00	RX1	C
ATOM	2965	CB	PHE	1047	33.167	26.490	8.882	1.00	0.00	RX1	C
ATOM	2966	CG	PHE	1047	34.415	26.213	8.086	1.00	0.00	RX1	C
ATOM	2967	CD1	PHE	1047	34.421	25.172	7.167	1.00	0.00	RX1	C
ATOM	2968	CD2	PHE	1047	35.551	26.987	8.271	1.00	0.00	RX1	C
ATOM	2969	CE1	PHE	1047	35.562	24.917	6.421	1.00	0.00	RX1	C
ATOM	2970	CE2	PHE	1047	36.691	26.735	7.523	1.00	0.00	RX1	C
ATOM	2971	CZ	PHE	1047	36.688	25.711	6.588	1.00	0.00	RX1	C
ATOM	2972	C	PHE	1047	33.617	24.553	10.392	1.00	0.00	RX1	C
ATOM	2973	O	PHE	1047	34.467	23.749	10.033	1.00	0.00	RX1	O
ATOM	2974	N	LEU	1048	33.488	24.962	11.662	1.00	0.00	RX1	N
ATOM	2975	H	LEU	1048	32.730	25.554	11.939	1.00	0.00	RX1	H
ATOM	2976	CA	LEU	1048	34.412	24.360	12.629	1.00	0.00	RX1	C
ATOM	2977	CB	LEU	1048	34.509	25.179	13.920	1.00	0.00	RX1	C
ATOM	2978	CG	LEU	1048	35.299	26.490	13.799	1.00	0.00	RX1	C
ATOM	2979	CD1	LEU	1048	36.373	26.426	12.710	1.00	0.00	RX1	C
ATOM	2980	CD2	LEU	1048	34.409	27.724	13.685	1.00	0.00	RX1	C
ATOM	2981	C	LEU	1048	34.094	22.906	12.947	1.00	0.00	RX1	C
ATOM	2982	O	LEU	1048	34.946	22.090	13.294	1.00	0.00	RX1	O
ATOM	2983	N	ASN	1049	32.805	22.604	12.775	1.00	0.00	RX1	N
ATOM	2984	H	ASN	1049	32.136	23.259	12.416	1.00	0.00	RX1	H
ATOM	2985	CA	ASN	1049	32.403	21.213	12.926	1.00	0.00	RX1	C
ATOM	2986	CB	ASN	1049	30.894	21.130	13.050	1.00	0.00	RX1	C
ATOM	2987	CG	ASN	1049	30.510	20.849	14.481	1.00	0.00	RX1	C

ATOM	2988	OD1	ASN	1049	30.793	19.796	15.043	1.00	0.00	RX1	O
ATOM	2989	ND2	ASN	1049	29.812	21.856	15.035	1.00	0.00	RX1	N
ATOM	2990	HD21	ASN	1049	29.700	22.694	14.492	1.00	0.00	RX1	H
ATOM	2991	HD22	ASN	1049	29.408	21.795	15.949	1.00	0.00	RX1	H
ATOM	2992	C	ASN	1049	32.910	20.313	11.809	1.00	0.00	RX1	C
ATOM	2993	O	ASN	1049	33.589	19.328	12.061	1.00	0.00	RX1	O
ATOM	2994	N	GLU	1050	32.657	20.744	10.563	1.00	0.00	RX1	N
ATOM	2995	H	GLU	1050	31.942	21.415	10.373	1.00	0.00	RX1	H
ATOM	2996	CA	GLU	1050	33.310	20.120	9.409	1.00	0.00	RX1	C
ATOM	2997	CB	GLU	1050	32.950	20.846	8.113	1.00	0.00	RX1	C
ATOM	2998	CG	GLU	1050	31.958	20.083	7.232	1.00	0.00	RX1	C
ATOM	2999	CD	GLU	1050	30.521	20.429	7.567	1.00	0.00	RX1	C
ATOM	3000	OE1	GLU	1050	29.829	20.949	6.695	1.00	0.00	RX1	O
ATOM	3001	OE2	GLU	1050	30.060	20.152	8.673	1.00	0.00	RX1	O
ATOM	3002	C	GLU	1050	34.821	20.023	9.519	1.00	0.00	RX1	C
ATOM	3003	O	GLU	1050	35.461	19.030	9.208	1.00	0.00	RX1	O
ATOM	3004	N	ALA	1051	35.401	21.107	10.045	1.00	0.00	RX1	N
ATOM	3005	H	ALA	1051	34.850	21.917	10.241	1.00	0.00	RX1	H
ATOM	3006	CA	ALA	1051	36.845	21.062	10.278	1.00	0.00	RX1	C
ATOM	3007	CB	ALA	1051	37.326	22.383	10.867	1.00	0.00	RX1	C
ATOM	3008	C	ALA	1051	37.286	19.928	11.193	1.00	0.00	RX1	C
ATOM	3009	O	ALA	1051	38.347	19.327	11.049	1.00	0.00	RX1	O
ATOM	3010	N	SER	1052	36.383	19.633	12.138	1.00	0.00	RX1	N
ATOM	3011	H	SER	1052	35.486	20.075	12.160	1.00	0.00	RX1	H
ATOM	3012	CA	SER	1052	36.638	18.536	13.060	1.00	0.00	RX1	C
ATOM	3013	CB	SER	1052	35.591	18.598	14.170	1.00	0.00	RX1	C
ATOM	3014	OG	SER	1052	35.730	19.865	14.842	1.00	0.00	RX1	O
ATOM	3015	HG	SER	1052	35.375	20.522	14.240	1.00	0.00	RX1	H
ATOM	3016	C	SER	1052	36.860	17.153	12.463	1.00	0.00	RX1	C
ATOM	3017	O	SER	1052	37.408	16.289	13.137	1.00	0.00	RX1	O
ATOM	3018	N	VAL	1053	36.507	16.984	11.167	1.00	0.00	RX1	N
ATOM	3019	H	VAL	1053	36.010	17.696	10.664	1.00	0.00	RX1	H
ATOM	3020	CA	VAL	1053	36.930	15.761	10.461	1.00	0.00	RX1	C
ATOM	3021	CB	VAL	1053	36.722	15.881	8.945	1.00	0.00	RX1	C
ATOM	3022	CG1	VAL	1053	37.224	14.626	8.247	1.00	0.00	RX1	C
ATOM	3023	CG2	VAL	1053	35.274	16.132	8.548	1.00	0.00	RX1	C
ATOM	3024	C	VAL	1053	38.393	15.402	10.713	1.00	0.00	RX1	C
ATOM	3025	O	VAL	1053	38.763	14.286	11.057	1.00	0.00	RX1	O
ATOM	3026	N	MET	1054	39.230	16.438	10.549	1.00	0.00	RX1	N
ATOM	3027	H	MET	1054	38.897	17.378	10.437	1.00	0.00	RX1	H
ATOM	3028	CA	MET	1054	40.651	16.109	10.583	1.00	0.00	RX1	C
ATOM	3029	CB	MET	1054	41.473	17.100	9.753	1.00	0.00	RX1	C
ATOM	3030	CG	MET	1054	40.830	17.473	8.415	1.00	0.00	RX1	C
ATOM	3031	SD	MET	1054	40.358	16.050	7.422	1.00	0.00	RX1	S
ATOM	3032	CE	MET	1054	41.980	15.304	7.215	1.00	0.00	RX1	C
ATOM	3033	C	MET	1054	41.256	15.903	11.964	1.00	0.00	RX1	C
ATOM	3034	O	MET	1054	42.459	15.707	12.115	1.00	0.00	RX1	O
ATOM	3035	N	LYS	1055	40.388	15.955	12.991	1.00	0.00	RX1	N
ATOM	3036	H	LYS	1055	39.400	16.006	12.836	1.00	0.00	RX1	H
ATOM	3037	CA	LYS	1055	40.904	15.694	14.335	1.00	0.00	RX1	C
ATOM	3038	CB	LYS	1055	39.838	15.971	15.394	1.00	0.00	RX1	C
ATOM	3039	CG	LYS	1055	39.636	17.464	15.602	1.00	0.00	RX1	C
ATOM	3040	CD	LYS	1055	38.430	17.834	16.462	1.00	0.00	RX1	C
ATOM	3041	CE	LYS	1055	38.380	19.353	16.607	1.00	0.00	RX1	C
ATOM	3042	NZ	LYS	1055	37.095	19.813	17.130	1.00	0.00	RX1	N
ATOM	3043	HZ1	LYS	1055	37.121	20.832	17.336	1.00	0.00	RX1	H
ATOM	3044	HZ2	LYS	1055	36.301	19.656	16.475	1.00	0.00	RX1	H
ATOM	3045	HZ3	LYS	1055	36.873	19.385	18.058	1.00	0.00	RX1	H
ATOM	3046	C	LYS	1055	41.396	14.276	14.498	1.00	0.00	RX1	C
ATOM	3047	O	LYS	1055	42.567	14.022	14.755	1.00	0.00	RX1	O
ATOM	3048	N	GLU	1056	40.429	13.369	14.317	1.00	0.00	RX1	N

ATOM	3049	H	GLU	1056	39.532	13.643	13.968	1.00	0.00	RX1	H
ATOM	3050	CA	GLU	1056	40.645	11.981	14.713	1.00	0.00	RX1	C
ATOM	3051	CB	GLU	1056	39.319	11.234	14.573	1.00	0.00	RX1	C
ATOM	3052	CG	GLU	1056	39.359	9.800	15.087	1.00	0.00	RX1	C
ATOM	3053	CD	GLU	1056	39.591	8.864	13.925	1.00	0.00	RX1	C
ATOM	3054	OE1	GLU	1056	38.896	8.983	12.923	1.00	0.00	RX1	O
ATOM	3055	OE2	GLU	1056	40.395	7.946	14.039	1.00	0.00	RX1	O
ATOM	3056	C	GLU	1056	41.815	11.282	14.038	1.00	0.00	RX1	C
ATOM	3057	O	GLU	1056	42.574	10.560	14.667	1.00	0.00	RX1	O
ATOM	3058	N	PHE	1057	41.949	11.577	12.734	1.00	0.00	RX1	N
ATOM	3059	H	PHE	1057	41.265	12.169	12.313	1.00	0.00	RX1	H
ATOM	3060	CA	PHE	1057	42.939	10.873	11.911	1.00	0.00	RX1	C
ATOM	3061	CB	PHE	1057	42.813	11.275	10.446	1.00	0.00	RX1	C
ATOM	3062	CG	PHE	1057	41.383	11.235	9.977	1.00	0.00	RX1	C
ATOM	3063	CD1	PHE	1057	40.532	10.211	10.373	1.00	0.00	RX1	C
ATOM	3064	CD2	PHE	1057	40.925	12.237	9.133	1.00	0.00	RX1	C
ATOM	3065	CE1	PHE	1057	39.213	10.207	9.940	1.00	0.00	RX1	C
ATOM	3066	CE2	PHE	1057	39.610	12.224	8.694	1.00	0.00	RX1	C
ATOM	3067	CZ	PHE	1057	38.750	11.217	9.108	1.00	0.00	RX1	C
ATOM	3068	C	PHE	1057	44.405	11.040	12.279	1.00	0.00	RX1	C
ATOM	3069	O	PHE	1057	45.142	11.765	11.619	1.00	0.00	RX1	O
ATOM	3070	N	ASN	1058	44.837	10.354	13.338	1.00	0.00	RX1	N
ATOM	3071	H	ASN	1058	44.243	9.703	13.826	1.00	0.00	RX1	H
ATOM	3072	CA	ASN	1058	46.194	10.676	13.764	1.00	0.00	RX1	C
ATOM	3073	CB	ASN	1058	46.341	10.758	15.276	1.00	0.00	RX1	C
ATOM	3074	CG	ASN	1058	47.529	11.656	15.552	1.00	0.00	RX1	C
ATOM	3075	OD1	ASN	1058	47.428	12.880	15.449	1.00	0.00	RX1	O
ATOM	3076	ND2	ASN	1058	48.639	11.007	15.938	1.00	0.00	RX1	N
ATOM	3077	HD21	ASN	1058	48.644	10.005	15.848	1.00	0.00	RX1	H
ATOM	3078	HD22	ASN	1058	49.468	11.450	16.278	1.00	0.00	RX1	H
ATOM	3079	C	ASN	1058	47.283	9.793	13.195	1.00	0.00	RX1	C
ATOM	3080	O	ASN	1058	48.097	9.222	13.911	1.00	0.00	RX1	O
ATOM	3081	N	CYS	1059	47.275	9.716	11.862	1.00	0.00	RX1	N
ATOM	3082	H	CYS	1059	46.684	10.282	11.284	1.00	0.00	RX1	H
ATOM	3083	CA	CYS	1059	48.365	8.968	11.249	1.00	0.00	RX1	C
ATOM	3084	CB	CYS	1059	47.863	7.635	10.702	1.00	0.00	RX1	C
ATOM	3085	SG	CYS	1059	49.222	6.467	10.459	1.00	0.00	RX1	S
ATOM	3086	C	CYS	1059	49.063	9.806	10.209	1.00	0.00	RX1	C
ATOM	3087	O	CYS	1059	48.537	10.817	9.758	1.00	0.00	RX1	O
ATOM	3088	N	HIS	1060	50.284	9.364	9.866	1.00	0.00	RX1	N
ATOM	3089	H	HIS	1060	50.614	8.472	10.181	1.00	0.00	RX1	H
ATOM	3090	CA	HIS	1060	51.164	10.230	9.082	1.00	0.00	RX1	C
ATOM	3091	CB	HIS	1060	52.483	9.524	8.777	1.00	0.00	RX1	C
ATOM	3092	CG	HIS	1060	53.576	10.542	8.548	1.00	0.00	RX1	C
ATOM	3093	ND1	HIS	1060	54.529	10.809	9.460	1.00	0.00	RX1	N
ATOM	3094	HD1	HIS	1060	54.636	10.375	10.333	1.00	0.00	RX1	H
ATOM	3095	CD2	HIS	1060	53.789	11.350	7.427	1.00	0.00	RX1	C
ATOM	3096	NE2	HIS	1060	54.883	12.108	7.680	1.00	0.00	RX1	N
ATOM	3097	CE1	HIS	1060	55.340	11.777	8.928	1.00	0.00	RX1	C
ATOM	3098	C	HIS	1060	50.564	10.793	7.807	1.00	0.00	RX1	C
ATOM	3099	O	HIS	1060	50.542	11.998	7.584	1.00	0.00	RX1	O
ATOM	3100	N	HIS	1061	50.062	9.866	6.980	1.00	0.00	RX1	N
ATOM	3101	H	HIS	1061	49.991	8.896	7.219	1.00	0.00	RX1	H
ATOM	3102	CA	HIS	1061	49.657	10.316	5.650	1.00	0.00	RX1	C
ATOM	3103	CB	HIS	1061	49.930	9.225	4.616	1.00	0.00	RX1	C
ATOM	3104	CG	HIS	1061	51.362	8.775	4.773	1.00	0.00	RX1	C
ATOM	3105	ND1	HIS	1061	51.719	7.593	5.302	1.00	0.00	RX1	N
ATOM	3106	HD1	HIS	1061	51.107	6.878	5.595	1.00	0.00	RX1	H
ATOM	3107	CD2	HIS	1061	52.520	9.487	4.449	1.00	0.00	RX1	C
ATOM	3108	NE2	HIS	1061	53.580	8.716	4.797	1.00	0.00	RX1	N
ATOM	3109	CE1	HIS	1061	53.086	7.549	5.322	1.00	0.00	RX1	C

ATOM	3110	C	HIS	1061	48.239	10.851	5.530	1.00	0.00	RX1	C
ATOM	3111	O	HIS	1061	47.611	10.806	4.479	1.00	0.00	RX1	O
ATOM	3112	N	VAL	1062	47.752	11.387	6.657	1.00	0.00	RX1	N
ATOM	3113	H	VAL	1062	48.279	11.493	7.503	1.00	0.00	RX1	H
ATOM	3114	CA	VAL	1062	46.513	12.151	6.559	1.00	0.00	RX1	C
ATOM	3115	CB	VAL	1062	45.433	11.553	7.467	1.00	0.00	RX1	C
ATOM	3116	CG1	VAL	1062	44.038	11.835	6.903	1.00	0.00	RX1	C
ATOM	3117	CG2	VAL	1062	45.651	10.056	7.705	1.00	0.00	RX1	C
ATOM	3118	C	VAL	1062	46.829	13.582	6.965	1.00	0.00	RX1	C
ATOM	3119	O	VAL	1062	47.846	13.827	7.611	1.00	0.00	RX1	O
ATOM	3120	N	VAL	1063	45.944	14.512	6.574	1.00	0.00	RX1	N
ATOM	3121	H	VAL	1063	45.169	14.278	5.984	1.00	0.00	RX1	H
ATOM	3122	CA	VAL	1063	46.082	15.858	7.133	1.00	0.00	RX1	C
ATOM	3123	CB	VAL	1063	45.180	16.839	6.373	1.00	0.00	RX1	C
ATOM	3124	CG1	VAL	1063	45.180	18.257	6.953	1.00	0.00	RX1	C
ATOM	3125	CG2	VAL	1063	45.575	16.847	4.902	1.00	0.00	RX1	C
ATOM	3126	C	VAL	1063	45.806	15.889	8.632	1.00	0.00	RX1	C
ATOM	3127	O	VAL	1063	44.889	15.262	9.158	1.00	0.00	RX1	O
ATOM	3128	N	ARG	1064	46.659	16.646	9.322	1.00	0.00	RX1	N
ATOM	3129	H	ARG	1064	47.427	17.112	8.873	1.00	0.00	RX1	H
ATOM	3130	CA	ARG	1064	46.358	16.832	10.731	1.00	0.00	RX1	C
ATOM	3131	CB	ARG	1064	47.633	16.717	11.566	1.00	0.00	RX1	C
ATOM	3132	CG	ARG	1064	48.367	15.396	11.312	1.00	0.00	RX1	C
ATOM	3133	CD	ARG	1064	47.485	14.160	11.521	1.00	0.00	RX1	C
ATOM	3134	NE	ARG	1064	46.988	14.078	12.894	1.00	0.00	RX1	N
ATOM	3135	HE	ARG	1064	47.654	13.891	13.626	1.00	0.00	RX1	H
ATOM	3136	CZ	ARG	1064	45.654	14.221	13.150	1.00	0.00	RX1	C
ATOM	3137	NH1	ARG	1064	44.790	14.444	12.135	1.00	0.00	RX1	N
ATOM	3138	HH11	ARG	1064	43.814	14.643	12.292	1.00	0.00	RX1	H
ATOM	3139	HH12	ARG	1064	45.071	14.410	11.167	1.00	0.00	RX1	H
ATOM	3140	NH2	ARG	1064	45.232	14.128	14.427	1.00	0.00	RX1	N
ATOM	3141	HH21	ARG	1064	44.262	14.225	14.692	1.00	0.00	RX1	H
ATOM	3142	HH22	ARG	1064	45.892	13.928	15.164	1.00	0.00	RX1	H
ATOM	3143	C	ARG	1064	45.606	18.110	11.031	1.00	0.00	RX1	C
ATOM	3144	O	ARG	1064	46.015	19.207	10.674	1.00	0.00	RX1	O
ATOM	3145	N	LEU	1065	44.484	17.930	11.741	1.00	0.00	RX1	N
ATOM	3146	H	LEU	1065	44.149	17.028	12.011	1.00	0.00	RX1	H
ATOM	3147	CA	LEU	1065	44.000	19.115	12.440	1.00	0.00	RX1	C
ATOM	3148	CB	LEU	1065	42.496	19.032	12.699	1.00	0.00	RX1	C
ATOM	3149	CG	LEU	1065	41.898	20.293	13.314	1.00	0.00	RX1	C
ATOM	3150	CD1	LEU	1065	42.278	21.522	12.511	1.00	0.00	RX1	C
ATOM	3151	CD2	LEU	1065	40.382	20.218	13.435	1.00	0.00	RX1	C
ATOM	3152	C	LEU	1065	44.754	19.258	13.740	1.00	0.00	RX1	C
ATOM	3153	O	LEU	1065	44.764	18.363	14.575	1.00	0.00	RX1	O
ATOM	3154	N	LEU	1066	45.410	20.413	13.837	1.00	0.00	RX1	N
ATOM	3155	H	LEU	1066	45.323	21.116	13.130	1.00	0.00	RX1	H
ATOM	3156	CA	LEU	1066	46.162	20.687	15.053	1.00	0.00	RX1	C
ATOM	3157	CB	LEU	1066	47.447	21.425	14.686	1.00	0.00	RX1	C
ATOM	3158	CG	LEU	1066	48.281	20.585	13.716	1.00	0.00	RX1	C
ATOM	3159	CD1	LEU	1066	49.407	21.384	13.064	1.00	0.00	RX1	C
ATOM	3160	CD2	LEU	1066	48.794	19.306	14.382	1.00	0.00	RX1	C
ATOM	3161	C	LEU	1066	45.349	21.423	16.099	1.00	0.00	RX1	C
ATOM	3162	O	LEU	1066	45.555	21.265	17.294	1.00	0.00	RX1	O
ATOM	3163	N	GLY	1067	44.383	22.209	15.598	1.00	0.00	RX1	N
ATOM	3164	H	GLY	1067	44.257	22.426	14.627	1.00	0.00	RX1	H
ATOM	3165	CA	GLY	1067	43.464	22.805	16.562	1.00	0.00	RX1	C
ATOM	3166	C	GLY	1067	42.385	23.637	15.910	1.00	0.00	RX1	C
ATOM	3167	O	GLY	1067	42.463	23.974	14.735	1.00	0.00	RX1	O
ATOM	3168	N	VAL	1068	41.365	23.943	16.711	1.00	0.00	RX1	N
ATOM	3169	H	VAL	1068	41.407	23.759	17.696	1.00	0.00	RX1	H
ATOM	3170	CA	VAL	1068	40.307	24.834	16.240	1.00	0.00	RX1	C

ATOM	3171	CB	VAL	1068	38.948	24.210	16.569	1.00	0.00	RX1	C
ATOM	3172	CG1	VAL	1068	37.765	25.074	16.142	1.00	0.00	RX1	C
ATOM	3173	CG2	VAL	1068	38.858	22.831	15.930	1.00	0.00	RX1	C
ATOM	3174	C	VAL	1068	40.498	26.168	16.932	1.00	0.00	RX1	C
ATOM	3175	O	VAL	1068	41.028	26.205	18.028	1.00	0.00	RX1	O
ATOM	3176	N	VAL	1069	40.105	27.254	16.252	1.00	0.00	RX1	N
ATOM	3177	H	VAL	1069	39.689	27.215	15.342	1.00	0.00	RX1	H
ATOM	3178	CA	VAL	1069	40.094	28.527	16.969	1.00	0.00	RX1	C
ATOM	3179	CB	VAL	1069	41.228	29.465	16.542	1.00	0.00	RX1	C
ATOM	3180	CG1	VAL	1069	41.379	30.616	17.542	1.00	0.00	RX1	C
ATOM	3181	CG2	VAL	1069	42.546	28.720	16.356	1.00	0.00	RX1	C
ATOM	3182	C	VAL	1069	38.752	29.210	16.786	1.00	0.00	RX1	C
ATOM	3183	O	VAL	1069	38.603	30.221	16.097	1.00	0.00	RX1	O
ATOM	3184	N	SER	1070	37.759	28.589	17.428	1.00	0.00	RX1	N
ATOM	3185	H	SER	1070	37.986	27.867	18.090	1.00	0.00	RX1	H
ATOM	3186	CA	SER	1070	36.433	29.197	17.400	1.00	0.00	RX1	C
ATOM	3187	CB	SER	1070	35.394	28.137	17.788	1.00	0.00	RX1	C
ATOM	3188	OG	SER	1070	35.689	27.553	19.061	1.00	0.00	RX1	O
ATOM	3189	HG	SER	1070	36.507	27.060	18.979	1.00	0.00	RX1	H
ATOM	3190	C	SER	1070	36.338	30.503	18.177	1.00	0.00	RX1	C
ATOM	3191	O	SER	1070	35.527	31.378	17.892	1.00	0.00	RX1	O
ATOM	3192	N	GLN	1071	37.241	30.627	19.165	1.00	0.00	RX1	N
ATOM	3193	H	GLN	1071	37.905	29.895	19.344	1.00	0.00	RX1	H
ATOM	3194	CA	GLN	1071	37.205	31.848	19.965	1.00	0.00	RX1	C
ATOM	3195	CB	GLN	1071	37.909	31.717	21.332	1.00	0.00	RX1	C
ATOM	3196	CG	GLN	1071	37.786	30.408	22.154	1.00	0.00	RX1	C
ATOM	3197	CD	GLN	1071	36.365	29.870	22.357	1.00	0.00	RX1	C
ATOM	3198	OE1	GLN	1071	35.616	30.211	23.277	1.00	0.00	RX1	O
ATOM	3199	NE2	GLN	1071	36.038	28.938	21.462	1.00	0.00	RX1	N
ATOM	3200	HE21	GLN	1071	36.736	28.608	20.813	1.00	0.00	RX1	H
ATOM	3201	HE22	GLN	1071	35.155	28.489	21.347	1.00	0.00	RX1	H
ATOM	3202	C	GLN	1071	37.576	33.105	19.201	1.00	0.00	RX1	C
ATOM	3203	O	GLN	1071	38.746	33.411	18.988	1.00	0.00	RX1	O
ATOM	3204	N	GLY	1072	36.523	33.837	18.825	1.00	0.00	RX1	N
ATOM	3205	H	GLY	1072	35.641	33.372	18.720	1.00	0.00	RX1	H
ATOM	3206	CA	GLY	1072	36.736	35.201	18.349	1.00	0.00	RX1	C
ATOM	3207	C	GLY	1072	36.854	35.358	16.845	1.00	0.00	RX1	C
ATOM	3208	O	GLY	1072	37.017	34.414	16.082	1.00	0.00	RX1	O
ATOM	3209	N	GLN	1073	36.761	36.633	16.452	1.00	0.00	RX1	N
ATOM	3210	H	GLN	1073	36.785	37.374	17.121	1.00	0.00	RX1	H
ATOM	3211	CA	GLN	1073	36.885	36.951	15.032	1.00	0.00	RX1	C
ATOM	3212	CB	GLN	1073	36.034	38.183	14.719	1.00	0.00	RX1	C
ATOM	3213	CG	GLN	1073	34.537	37.880	14.650	1.00	0.00	RX1	C
ATOM	3214	CD	GLN	1073	34.200	37.259	13.308	1.00	0.00	RX1	C
ATOM	3215	OE1	GLN	1073	34.756	36.248	12.887	1.00	0.00	RX1	O
ATOM	3216	NE2	GLN	1073	33.256	37.954	12.650	1.00	0.00	RX1	N
ATOM	3217	HE21	GLN	1073	32.838	38.763	13.065	1.00	0.00	RX1	H
ATOM	3218	HE22	GLN	1073	32.918	37.739	11.731	1.00	0.00	RX1	H
ATOM	3219	C	GLN	1073	38.331	37.183	14.620	1.00	0.00	RX1	C
ATOM	3220	O	GLN	1073	39.123	37.708	15.393	1.00	0.00	RX1	O
ATOM	3221	N	PRO	1074	38.671	36.767	13.375	1.00	0.00	RX1	N
ATOM	3222	CD	PRO	1074	39.891	37.151	12.673	1.00	0.00	RX1	C
ATOM	3223	CA	PRO	1074	37.851	35.824	12.606	1.00	0.00	RX1	C
ATOM	3224	CB	PRO	1074	38.425	35.973	11.196	1.00	0.00	RX1	C
ATOM	3225	CG	PRO	1074	39.904	36.281	11.420	1.00	0.00	RX1	C
ATOM	3226	C	PRO	1074	38.015	34.426	13.176	1.00	0.00	RX1	C
ATOM	3227	O	PRO	1074	39.009	34.111	13.830	1.00	0.00	RX1	O
ATOM	3228	N	THR	1075	36.993	33.615	12.927	1.00	0.00	RX1	N
ATOM	3229	H	THR	1075	36.205	33.939	12.405	1.00	0.00	RX1	H
ATOM	3230	CA	THR	1075	37.084	32.226	13.369	1.00	0.00	RX1	C
ATOM	3231	CB	THR	1075	35.664	31.716	13.320	1.00	0.00	RX1	C

ATOM	3232	OG1	THR	1075	34.962	32.520	12.365	1.00	0.00	RX1	O
ATOM	3233	HG1	THR	1075	35.465	32.461	11.550	1.00	0.00	RX1	H
ATOM	3234	CG2	THR	1075	34.971	31.816	14.677	1.00	0.00	RX1	C
ATOM	3235	C	THR	1075	38.050	31.435	12.501	1.00	0.00	RX1	C
ATOM	3236	O	THR	1075	38.058	31.576	11.283	1.00	0.00	RX1	O
ATOM	3237	N	LEU	1076	38.896	30.653	13.178	1.00	0.00	RX1	N
ATOM	3238	H	LEU	1076	38.786	30.417	14.147	1.00	0.00	RX1	H
ATOM	3239	CA	LEU	1076	40.015	30.056	12.451	1.00	0.00	RX1	C
ATOM	3240	CB	LEU	1076	41.340	30.614	12.966	1.00	0.00	RX1	C
ATOM	3241	CG	LEU	1076	41.397	32.133	13.105	1.00	0.00	RX1	C
ATOM	3242	CD1	LEU	1076	42.383	32.547	14.187	1.00	0.00	RX1	C
ATOM	3243	CD2	LEU	1076	41.694	32.840	11.788	1.00	0.00	RX1	C
ATOM	3244	C	LEU	1076	40.050	28.561	12.660	1.00	0.00	RX1	C
ATOM	3245	O	LEU	1076	39.478	28.052	13.617	1.00	0.00	RX1	O
ATOM	3246	N	VAL	1077	40.788	27.886	11.771	1.00	0.00	RX1	N
ATOM	3247	H	VAL	1077	41.119	28.270	10.905	1.00	0.00	RX1	H
ATOM	3248	CA	VAL	1077	41.118	26.503	12.085	1.00	0.00	RX1	C
ATOM	3249	CB	VAL	1077	40.007	25.560	11.605	1.00	0.00	RX1	C
ATOM	3250	CG1	VAL	1077	40.019	25.310	10.099	1.00	0.00	RX1	C
ATOM	3251	CG2	VAL	1077	40.014	24.281	12.428	1.00	0.00	RX1	C
ATOM	3252	C	VAL	1077	42.512	26.118	11.604	1.00	0.00	RX1	C
ATOM	3253	O	VAL	1077	42.916	26.398	10.482	1.00	0.00	RX1	O
ATOM	3254	N	ILE	1078	43.248	25.526	12.549	1.00	0.00	RX1	N
ATOM	3255	H	ILE	1078	42.807	25.175	13.374	1.00	0.00	RX1	H
ATOM	3256	CA	ILE	1078	44.677	25.275	12.367	1.00	0.00	RX1	C
ATOM	3257	CB	ILE	1078	45.395	25.476	13.704	1.00	0.00	RX1	C
ATOM	3258	CG2	ILE	1078	46.899	25.242	13.590	1.00	0.00	RX1	C
ATOM	3259	CG1	ILE	1078	45.099	26.862	14.262	1.00	0.00	RX1	C
ATOM	3260	CD1	ILE	1078	45.686	27.953	13.371	1.00	0.00	RX1	C
ATOM	3261	C	ILE	1078	44.947	23.879	11.835	1.00	0.00	RX1	C
ATOM	3262	O	ILE	1078	45.036	22.909	12.582	1.00	0.00	RX1	O
ATOM	3263	N	MET	1079	45.062	23.817	10.508	1.00	0.00	RX1	N
ATOM	3264	H	MET	1079	45.065	24.635	9.929	1.00	0.00	RX1	H
ATOM	3265	CA	MET	1079	45.384	22.530	9.899	1.00	0.00	RX1	C
ATOM	3266	CB	MET	1079	44.498	22.276	8.682	1.00	0.00	RX1	C
ATOM	3267	CG	MET	1079	43.031	22.262	9.090	1.00	0.00	RX1	C
ATOM	3268	SD	MET	1079	41.893	21.918	7.751	1.00	0.00	RX1	S
ATOM	3269	CE	MET	1079	40.492	21.444	8.775	1.00	0.00	RX1	C
ATOM	3270	C	MET	1079	46.845	22.425	9.518	1.00	0.00	RX1	C
ATOM	3271	O	MET	1079	47.602	23.386	9.571	1.00	0.00	RX1	O
ATOM	3272	N	GLU	1080	47.210	21.204	9.122	1.00	0.00	RX1	N
ATOM	3273	H	GLU	1080	46.551	20.452	9.162	1.00	0.00	RX1	H
ATOM	3274	CA	GLU	1080	48.559	20.946	8.626	1.00	0.00	RX1	C
ATOM	3275	CB	GLU	1080	48.713	19.433	8.506	1.00	0.00	RX1	C
ATOM	3276	CG	GLU	1080	50.130	18.878	8.411	1.00	0.00	RX1	C
ATOM	3277	CD	GLU	1080	50.014	17.372	8.329	1.00	0.00	RX1	C
ATOM	3278	OE1	GLU	1080	49.082	16.886	7.698	1.00	0.00	RX1	O
ATOM	3279	OE2	GLU	1080	50.843	16.667	8.896	1.00	0.00	RX1	O
ATOM	3280	C	GLU	1080	48.835	21.653	7.305	1.00	0.00	RX1	C
ATOM	3281	O	GLU	1080	47.939	21.904	6.507	1.00	0.00	RX1	O
ATOM	3282	N	LEU	1081	50.111	22.009	7.116	1.00	0.00	RX1	N
ATOM	3283	H	LEU	1081	50.844	21.693	7.718	1.00	0.00	RX1	H
ATOM	3284	CA	LEU	1081	50.404	22.797	5.923	1.00	0.00	RX1	C
ATOM	3285	CB	LEU	1081	51.584	23.719	6.186	1.00	0.00	RX1	C
ATOM	3286	CG	LEU	1081	51.746	24.785	5.109	1.00	0.00	RX1	C
ATOM	3287	CD1	LEU	1081	50.475	25.585	4.835	1.00	0.00	RX1	C
ATOM	3288	CD2	LEU	1081	52.912	25.697	5.441	1.00	0.00	RX1	C
ATOM	3289	C	LEU	1081	50.578	22.001	4.640	1.00	0.00	RX1	C
ATOM	3290	O	LEU	1081	51.634	21.472	4.310	1.00	0.00	RX1	O
ATOM	3291	N	MET	1082	49.466	21.973	3.902	1.00	0.00	RX1	N
ATOM	3292	H	MET	1082	48.643	22.436	4.232	1.00	0.00	RX1	H

```
ATOM   3293  CA  MET  1082      49.504  21.265   2.625  1.00  0.00      RX1  C
ATOM   3294  CB  MET  1082      48.152  20.615   2.345  1.00  0.00      RX1  C
ATOM   3295  CG  MET  1082      47.556  19.970   3.598  1.00  0.00      RX1  C
ATOM   3296  SD  MET  1082      48.679  18.828   4.421  1.00  0.00      RX1  S
ATOM   3297  CE  MET  1082      48.826  17.613   3.105  1.00  0.00      RX1  C
ATOM   3298  C   MET  1082      49.957  22.126   1.457  1.00  0.00      RX1  C
ATOM   3299  O   MET  1082      49.222  22.425   0.523  1.00  0.00      RX1  O
ATOM   3300  N   THR  1083      51.221  22.547   1.563  1.00  0.00      RX1  N
ATOM   3301  H   THR  1083      51.802  22.253   2.324  1.00  0.00      RX1  H
ATOM   3302  CA  THR  1083      51.696  23.619   0.689  1.00  0.00      RX1  C
ATOM   3303  CB  THR  1083      52.901  24.217   1.369  1.00  0.00      RX1  C
ATOM   3304  OG1 THR  1083      53.085  23.559   2.626  1.00  0.00      RX1  O
ATOM   3305  HG1 THR  1083      53.929  23.884   2.940  1.00  0.00      RX1  H
ATOM   3306  CG2 THR  1083      52.780  25.736   1.523  1.00  0.00      RX1  C
ATOM   3307  C   THR  1083      51.976  23.327  -0.778  1.00  0.00      RX1  C
ATOM   3308  O   THR  1083      52.719  24.055  -1.431  1.00  0.00      RX1  O
ATOM   3309  N   ARG  1084      51.383  22.244  -1.290  1.00  0.00      RX1  N
ATOM   3310  H   ARG  1084      50.834  21.600  -0.750  1.00  0.00      RX1  H
ATOM   3311  CA  ARG  1084      51.410  22.131  -2.746  1.00  0.00      RX1  C
ATOM   3312  CB  ARG  1084      52.144  20.875  -3.211  1.00  0.00      RX1  C
ATOM   3313  CG  ARG  1084      53.531  20.714  -2.593  1.00  0.00      RX1  C
ATOM   3314  CD  ARG  1084      54.482  21.859  -2.931  1.00  0.00      RX1  C
ATOM   3315  NE  ARG  1084      55.731  21.703  -2.194  1.00  0.00      RX1  N
ATOM   3316  HE  ARG  1084      56.224  20.837  -2.325  1.00  0.00      RX1  H
ATOM   3317  CZ  ARG  1084      56.148  22.685  -1.344  1.00  0.00      RX1  C
ATOM   3318  NH1 ARG  1084      55.394  23.781  -1.125  1.00  0.00      RX1  N
ATOM   3319 HH11 ARG  1084      55.704  24.465  -0.446  1.00  0.00      RX1  H
ATOM   3320 HH12 ARG  1084      54.506  23.953  -1.577  1.00  0.00      RX1  H
ATOM   3321  NH2 ARG  1084      57.315  22.544  -0.694  1.00  0.00      RX1  N
ATOM   3322 HH21 ARG  1084      57.651  23.284  -0.091  1.00  0.00      RX1  H
ATOM   3323 HH22 ARG  1084      57.858  21.694  -0.719  1.00  0.00      RX1  H
ATOM   3324  C   ARG  1084      50.036  22.190  -3.380  1.00  0.00      RX1  C
ATOM   3325  O   ARG  1084      49.895  22.154  -4.594  1.00  0.00      RX1  O
ATOM   3326  N   GLY  1085      49.019  22.280  -2.507  1.00  0.00      RX1  N
ATOM   3327  H   GLY  1085      49.135  22.198  -1.517  1.00  0.00      RX1  H
ATOM   3328  CA  GLY  1085      47.679  22.153  -3.066  1.00  0.00      RX1  C
ATOM   3329  C   GLY  1085      47.336  20.697  -3.287  1.00  0.00      RX1  C
ATOM   3330  O   GLY  1085      47.876  19.807  -2.636  1.00  0.00      RX1  O
ATOM   3331  N   ASP  1086      46.414  20.495  -4.223  1.00  0.00      RX1  N
ATOM   3332  H   ASP  1086      46.079  21.198  -4.854  1.00  0.00      RX1  H
ATOM   3333  CA  ASP  1086      46.002  19.130  -4.512  1.00  0.00      RX1  C
ATOM   3334  CB  ASP  1086      44.555  19.095  -4.987  1.00  0.00      RX1  C
ATOM   3335  CG  ASP  1086      44.426  19.795  -6.317  1.00  0.00      RX1  C
ATOM   3336  OD1 ASP  1086      44.730  20.979  -6.407  1.00  0.00      RX1  O
ATOM   3337  OD2 ASP  1086      44.020  19.160  -7.277  1.00  0.00      RX1  O
ATOM   3338  C   ASP  1086      46.929  18.441  -5.491  1.00  0.00      RX1  C
ATOM   3339  O   ASP  1086      47.610  19.062  -6.302  1.00  0.00      RX1  O
ATOM   3340  N   LEU  1087      46.920  17.103  -5.398  1.00  0.00      RX1  N
ATOM   3341  H   LEU  1087      46.338  16.647  -4.723  1.00  0.00      RX1  H
ATOM   3342  CA  LEU  1087      47.740  16.329  -6.327  1.00  0.00      RX1  C
ATOM   3343  CB  LEU  1087      47.650  14.835  -6.017  1.00  0.00      RX1  C
ATOM   3344  CG  LEU  1087      48.527  13.980  -6.936  1.00  0.00      RX1  C
ATOM   3345  CD1 LEU  1087      50.008  14.347  -6.824  1.00  0.00      RX1  C
ATOM   3346  CD2 LEU  1087      48.282  12.486  -6.733  1.00  0.00      RX1  C
ATOM   3347  C   LEU  1087      47.364  16.576  -7.770  1.00  0.00      RX1  C
ATOM   3348  O   LEU  1087      48.201  16.661  -8.654  1.00  0.00      RX1  O
ATOM   3349  N   LYS  1088      46.049  16.698  -7.969  1.00  0.00      RX1  N
ATOM   3350  H   LYS  1088      45.422  16.781  -7.192  1.00  0.00      RX1  H
ATOM   3351  CA  LYS  1088      45.561  16.888  -9.329  1.00  0.00      RX1  C
ATOM   3352  CB  LYS  1088      44.040  16.834  -9.268  1.00  0.00      RX1  C
ATOM   3353  CG  LYS  1088      43.289  17.141 -10.546  1.00  0.00      RX1  C
```

ATOM	3354	CD	LYS	1088	41.868	16.605	-10.439	1.00	0.00	RX1	C
ATOM	3355	CE	LYS	1088	41.157	16.816	-11.761	1.00	0.00	RX1	C
ATOM	3356	NZ	LYS	1088	40.107	15.821	-11.949	1.00	0.00	RX1	N
ATOM	3357	HZ1	LYS	1088	39.762	15.873	-12.927	1.00	0.00	RX1	H
ATOM	3358	HZ2	LYS	1088	40.481	14.864	-11.758	1.00	0.00	RX1	H
ATOM	3359	HZ3	LYS	1088	39.295	16.029	-11.326	1.00	0.00	RX1	H
ATOM	3360	C	LYS	1088	46.134	18.116	-10.028	1.00	0.00	RX1	C
ATOM	3361	O	LYS	1088	46.662	18.053	-11.135	1.00	0.00	RX1	O
ATOM	3362	N	SER	1089	46.062	19.237	-9.310	1.00	0.00	RX1	N
ATOM	3363	H	SER	1089	45.625	19.298	-8.410	1.00	0.00	RX1	H
ATOM	3364	CA	SER	1089	46.660	20.429	-9.892	1.00	0.00	RX1	C
ATOM	3365	CB	SER	1089	45.998	21.613	-9.223	1.00	0.00	RX1	C
ATOM	3366	OG	SER	1089	44.602	21.288	-9.200	1.00	0.00	RX1	O
ATOM	3367	HG	SER	1089	44.431	20.965	-8.304	1.00	0.00	RX1	H
ATOM	3368	C	SER	1089	48.177	20.404	-9.942	1.00	0.00	RX1	C
ATOM	3369	O	SER	1089	48.784	20.838	-10.912	1.00	0.00	RX1	O
ATOM	3370	N	TYR	1090	48.766	19.790	-8.900	1.00	0.00	RX1	N
ATOM	3371	H	TYR	1090	48.224	19.497	-8.108	1.00	0.00	RX1	H
ATOM	3372	CA	TYR	1090	50.213	19.548	-8.928	1.00	0.00	RX1	C
ATOM	3373	CB	TYR	1090	50.632	18.798	-7.662	1.00	0.00	RX1	C
ATOM	3374	CG	TYR	1090	52.128	18.609	-7.636	1.00	0.00	RX1	C
ATOM	3375	CD1	TYR	1090	52.966	19.716	-7.578	1.00	0.00	RX1	C
ATOM	3376	CE1	TYR	1090	54.344	19.540	-7.545	1.00	0.00	RX1	C
ATOM	3377	CD2	TYR	1090	52.664	17.328	-7.664	1.00	0.00	RX1	C
ATOM	3378	CE2	TYR	1090	54.042	17.152	-7.631	1.00	0.00	RX1	C
ATOM	3379	CZ	TYR	1090	54.881	18.259	-7.569	1.00	0.00	RX1	C
ATOM	3380	OH	TYR	1090	56.249	18.085	-7.528	1.00	0.00	RX1	O
ATOM	3381	HH	TYR	1090	56.448	17.159	-7.566	1.00	0.00	RX1	H
ATOM	3382	C	TYR	1090	50.695	18.820	-10.183	1.00	0.00	RX1	C
ATOM	3383	O	TYR	1090	51.688	19.172	-10.812	1.00	0.00	RX1	O
ATOM	3384	N	LEU	1091	49.907	17.799	-10.547	1.00	0.00	RX1	N
ATOM	3385	H	LEU	1091	49.096	17.586	-10.002	1.00	0.00	RX1	H
ATOM	3386	CA	LEU	1091	50.166	17.069	-11.785	1.00	0.00	RX1	C
ATOM	3387	CB	LEU	1091	49.191	15.905	-11.917	1.00	0.00	RX1	C
ATOM	3388	CG	LEU	1091	49.352	14.821	-10.855	1.00	0.00	RX1	C
ATOM	3389	CD1	LEU	1091	48.125	13.912	-10.811	1.00	0.00	RX1	C
ATOM	3390	CD2	LEU	1091	50.660	14.045	-11.007	1.00	0.00	RX1	C
ATOM	3391	C	LEU	1091	50.066	17.952	-13.014	1.00	0.00	RX1	C
ATOM	3392	O	LEU	1091	50.868	17.889	-13.936	1.00	0.00	RX1	O
ATOM	3393	N	ARG	1092	49.048	18.820	-12.970	1.00	0.00	RX1	N
ATOM	3394	H	ARG	1092	48.438	18.853	-12.175	1.00	0.00	RX1	H
ATOM	3395	CA	ARG	1092	48.918	19.787	-14.058	1.00	0.00	RX1	C
ATOM	3396	CB	ARG	1092	47.576	20.508	-13.946	1.00	0.00	RX1	C
ATOM	3397	CG	ARG	1092	46.487	19.462	-14.159	1.00	0.00	RX1	C
ATOM	3398	CD	ARG	1092	45.038	19.913	-13.987	1.00	0.00	RX1	C
ATOM	3399	NE	ARG	1092	44.203	18.825	-14.481	1.00	0.00	RX1	N
ATOM	3400	HE	ARG	1092	44.699	18.126	-15.009	1.00	0.00	RX1	H
ATOM	3401	CZ	ARG	1092	42.861	18.729	-14.284	1.00	0.00	RX1	C
ATOM	3402	NH1	ARG	1092	42.222	19.644	-13.530	1.00	0.00	RX1	N
ATOM	3403	HH11	ARG	1092	41.224	19.682	-13.451	1.00	0.00	RX1	H
ATOM	3404	HH12	ARG	1092	42.722	20.325	-12.969	1.00	0.00	RX1	H
ATOM	3405	NH2	ARG	1092	42.204	17.690	-14.842	1.00	0.00	RX1	N
ATOM	3406	HH21	ARG	1092	41.214	17.513	-14.761	1.00	0.00	RX1	H
ATOM	3407	HH22	ARG	1092	42.714	16.992	-15.366	1.00	0.00	RX1	H
ATOM	3408	C	ARG	1092	50.095	20.738	-14.221	1.00	0.00	RX1	C
ATOM	3409	O	ARG	1092	50.508	21.047	-15.330	1.00	0.00	RX1	O
ATOM	3410	N	SER	1093	50.676	21.136	-13.080	1.00	0.00	RX1	N
ATOM	3411	H	SER	1093	50.273	20.966	-12.179	1.00	0.00	RX1	H
ATOM	3412	CA	SER	1093	51.913	21.913	-13.193	1.00	0.00	RX1	C
ATOM	3413	CB	SER	1093	52.154	22.565	-11.842	1.00	0.00	RX1	C
ATOM	3414	OG	SER	1093	51.311	21.909	-10.894	1.00	0.00	RX1	O

ATOM	3415	HG	SER	1093	51.682	21.038	-10.785	1.00	0.00	RX1	H
ATOM	3416	C	SER	1093	53.133	21.170	-13.729	1.00	0.00	RX1	C
ATOM	3417	O	SER	1093	54.150	21.762	-14.061	1.00	0.00	RX1	O
ATOM	3418	N	LEU	1094	52.992	19.838	-13.811	1.00	0.00	RX1	N
ATOM	3419	H	LEU	1094	52.142	19.366	-13.581	1.00	0.00	RX1	H
ATOM	3420	CA	LEU	1094	54.073	19.076	-14.433	1.00	0.00	RX1	C
ATOM	3421	CB	LEU	1094	54.218	17.715	-13.752	1.00	0.00	RX1	C
ATOM	3422	CG	LEU	1094	54.365	17.812	-12.232	1.00	0.00	RX1	C
ATOM	3423	CD1	LEU	1094	54.261	16.440	-11.565	1.00	0.00	RX1	C
ATOM	3424	CD2	LEU	1094	55.635	18.559	-11.821	1.00	0.00	RX1	C
ATOM	3425	C	LEU	1094	53.919	18.894	-15.937	1.00	0.00	RX1	C
ATOM	3426	O	LEU	1094	54.706	18.219	-16.587	1.00	0.00	RX1	O
ATOM	3427	N	ARG	1095	52.853	19.506	-16.480	1.00	0.00	RX1	N
ATOM	3428	H	ARG	1095	52.283	20.149	-15.969	1.00	0.00	RX1	H
ATOM	3429	CA	ARG	1095	52.692	19.387	-17.927	1.00	0.00	RX1	C
ATOM	3430	CB	ARG	1095	51.283	19.799	-18.351	1.00	0.00	RX1	C
ATOM	3431	CG	ARG	1095	50.243	18.825	-17.817	1.00	0.00	RX1	C
ATOM	3432	CD	ARG	1095	48.849	19.092	-18.372	1.00	0.00	RX1	C
ATOM	3433	NE	ARG	1095	47.900	18.177	-17.752	1.00	0.00	RX1	N
ATOM	3434	HE	ARG	1095	48.008	17.986	-16.767	1.00	0.00	RX1	H
ATOM	3435	CZ	ARG	1095	46.902	17.597	-18.474	1.00	0.00	RX1	C
ATOM	3436	NH1	ARG	1095	46.805	17.770	-19.808	1.00	0.00	RX1	N
ATOM	3437	HH11	ARG	1095	46.080	17.274	-20.322	1.00	0.00	RX1	H
ATOM	3438	HH12	ARG	1095	47.425	18.348	-20.337	1.00	0.00	RX1	H
ATOM	3439	NH2	ARG	1095	46.006	16.847	-17.827	1.00	0.00	RX1	N
ATOM	3440	HH21	ARG	1095	45.212	16.457	-18.318	1.00	0.00	RX1	H
ATOM	3441	HH22	ARG	1095	46.091	16.646	-16.844	1.00	0.00	RX1	H
ATOM	3442	C	ARG	1095	53.722	20.181	-18.707	1.00	0.00	RX1	C
ATOM	3443	O	ARG	1095	54.136	21.262	-18.308	1.00	0.00	RX1	O
ATOM	3444	N	PRO	1096	54.125	19.611	-19.867	1.00	0.00	RX1	N
ATOM	3445	CD	PRO	1096	53.784	18.289	-20.377	1.00	0.00	RX1	C
ATOM	3446	CA	PRO	1096	54.993	20.375	-20.764	1.00	0.00	RX1	C
ATOM	3447	CB	PRO	1096	55.284	19.368	-21.885	1.00	0.00	RX1	C
ATOM	3448	CG	PRO	1096	54.123	18.373	-21.859	1.00	0.00	RX1	C
ATOM	3449	C	PRO	1096	54.358	21.665	-21.254	1.00	0.00	RX1	C
ATOM	3450	O	PRO	1096	53.557	21.678	-22.180	1.00	0.00	RX1	O
ATOM	3451	N	GLU	1097	54.800	22.759	-20.611	1.00	0.00	RX1	N
ATOM	3452	H	GLU	1097	55.340	22.661	-19.774	1.00	0.00	RX1	H
ATOM	3453	CA	GLU	1097	54.495	24.090	-21.147	1.00	0.00	RX1	C
ATOM	3454	CB	GLU	1097	55.038	25.166	-20.215	1.00	0.00	RX1	C
ATOM	3455	CG	GLU	1097	54.567	26.562	-20.618	1.00	0.00	RX1	C
ATOM	3456	CD	GLU	1097	55.383	27.584	-19.865	1.00	0.00	RX1	C
ATOM	3457	OE1	GLU	1097	56.448	27.948	-20.358	1.00	0.00	RX1	O
ATOM	3458	OE2	GLU	1097	54.958	28.009	-18.795	1.00	0.00	RX1	O
ATOM	3459	C	GLU	1097	55.042	24.273	-22.560	1.00	0.00	RX1	C
ATOM	3460	O	GLU	1097	54.461	24.912	-23.429	1.00	0.00	RX1	O
ATOM	3461	N	MET	1098	56.185	23.588	-22.749	1.00	0.00	RX1	N
ATOM	3462	H	MET	1098	56.761	23.408	-21.948	1.00	0.00	RX1	H
ATOM	3463	CA	MET	1098	56.707	23.216	-24.063	1.00	0.00	RX1	C
ATOM	3464	CB	MET	1098	55.632	22.586	-24.959	1.00	0.00	RX1	C
ATOM	3465	CG	MET	1098	56.205	22.039	-26.266	1.00	0.00	RX1	C
ATOM	3466	SD	MET	1098	57.417	20.738	-25.988	1.00	0.00	RX1	S
ATOM	3467	CE	MET	1098	57.976	20.558	-27.689	1.00	0.00	RX1	C
ATOM	3468	C	MET	1098	57.480	24.290	-24.797	1.00	0.00	RX1	C
ATOM	3469	O	MET	1098	58.582	24.033	-25.266	1.00	0.00	RX1	O
ATOM	3470	N	GLU	1099	56.885	25.494	-24.877	1.00	0.00	RX1	N
ATOM	3471	H	GLU	1099	55.999	25.659	-24.441	1.00	0.00	RX1	H
ATOM	3472	CA	GLU	1099	57.546	26.548	-25.651	1.00	0.00	RX1	C
ATOM	3473	CB	GLU	1099	56.705	27.819	-25.752	1.00	0.00	RX1	C
ATOM	3474	CG	GLU	1099	57.370	28.826	-26.696	1.00	0.00	RX1	C
ATOM	3475	CD	GLU	1099	57.806	28.118	-27.968	1.00	0.00	RX1	C

ATOM	3476	OE1	GLU	1099	56.956	27.586	-28.676	1.00	0.00	RX1	O
ATOM	3477	OE2	GLU	1099	59.006	28.078	-28.238	1.00	0.00	RX1	O
ATOM	3478	C	GLU	1099	58.973	26.842	-25.214	1.00	0.00	RX1	C
ATOM	3479	O	GLU	1099	59.248	27.366	-24.140	1.00	0.00	RX1	O
ATOM	3480	N	ASN	1100	59.874	26.370	-26.091	1.00	0.00	RX1	N
ATOM	3481	H	ASN	1100	59.497	26.183	-27.001	1.00	0.00	RX1	H
ATOM	3482	CA	ASN	1100	61.312	26.297	-25.814	1.00	0.00	RX1	C
ATOM	3483	CB	ASN	1100	62.012	27.603	-26.184	1.00	0.00	RX1	C
ATOM	3484	CG	ASN	1100	62.717	27.432	-27.510	1.00	0.00	RX1	C
ATOM	3485	OD1	ASN	1100	63.898	27.109	-27.590	1.00	0.00	RX1	O
ATOM	3486	ND2	ASN	1100	61.919	27.681	-28.564	1.00	0.00	RX1	N
ATOM	3487	HD21	ASN	1100	60.950	27.924	-28.417	1.00	0.00	RX1	H
ATOM	3488	HD22	ASN	1100	62.217	27.634	-29.514	1.00	0.00	RX1	H
ATOM	3489	C	ASN	1100	61.735	25.899	-24.405	1.00	0.00	RX1	C
ATOM	3490	O	ASN	1100	62.734	26.368	-23.872	1.00	0.00	RX1	O
ATOM	3491	N	ASN	1101	60.935	25.000	-23.807	1.00	0.00	RX1	N
ATOM	3492	H	ASN	1101	60.158	24.572	-24.274	1.00	0.00	RX1	H
ATOM	3493	CA	ASN	1101	61.237	24.704	-22.409	1.00	0.00	RX1	C
ATOM	3494	CB	ASN	1101	60.375	25.486	-21.406	1.00	0.00	RX1	C
ATOM	3495	CG	ASN	1101	58.908	25.108	-21.414	1.00	0.00	RX1	C
ATOM	3496	OD1	ASN	1101	58.474	24.028	-21.014	1.00	0.00	RX1	O
ATOM	3497	ND2	ASN	1101	58.153	26.155	-21.773	1.00	0.00	RX1	N
ATOM	3498	HD21	ASN	1101	58.538	26.913	-22.311	1.00	0.00	RX1	H
ATOM	3499	HD22	ASN	1101	57.217	26.357	-21.461	1.00	0.00	RX1	H
ATOM	3500	C	ASN	1101	61.328	23.240	-22.037	1.00	0.00	RX1	C
ATOM	3501	O	ASN	1101	60.425	22.432	-22.224	1.00	0.00	RX1	O
ATOM	3502	N	PRO	1102	62.521	22.909	-21.492	1.00	0.00	RX1	N
ATOM	3503	CD	PRO	1102	63.703	23.754	-21.415	1.00	0.00	RX1	C
ATOM	3504	CA	PRO	1102	62.728	21.576	-20.923	1.00	0.00	RX1	C
ATOM	3505	CB	PRO	1102	64.236	21.559	-20.635	1.00	0.00	RX1	C
ATOM	3506	CG	PRO	1102	64.840	22.743	-21.394	1.00	0.00	RX1	C
ATOM	3507	C	PRO	1102	61.920	21.366	-19.652	1.00	0.00	RX1	C
ATOM	3508	O	PRO	1102	62.378	21.633	-18.547	1.00	0.00	RX1	O
ATOM	3509	N	VAL	1103	60.696	20.863	-19.853	1.00	0.00	RX1	N
ATOM	3510	H	VAL	1103	60.348	20.763	-20.786	1.00	0.00	RX1	H
ATOM	3511	CA	VAL	1103	59.914	20.466	-18.682	1.00	0.00	RX1	C
ATOM	3512	CB	VAL	1103	58.496	20.074	-19.124	1.00	0.00	RX1	C
ATOM	3513	CG1	VAL	1103	58.515	18.753	-19.897	1.00	0.00	RX1	C
ATOM	3514	CG2	VAL	1103	57.491	20.075	-17.969	1.00	0.00	RX1	C
ATOM	3515	C	VAL	1103	60.596	19.350	-17.890	1.00	0.00	RX1	C
ATOM	3516	O	VAL	1103	61.367	18.564	-18.430	1.00	0.00	RX1	O
ATOM	3517	N	LEU	1104	60.292	19.318	-16.584	1.00	0.00	RX1	N
ATOM	3518	H	LEU	1104	59.579	19.911	-16.217	1.00	0.00	RX1	H
ATOM	3519	CA	LEU	1104	60.787	18.175	-15.822	1.00	0.00	RX1	C
ATOM	3520	CB	LEU	1104	60.652	18.398	-14.311	1.00	0.00	RX1	C
ATOM	3521	CG	LEU	1104	61.606	19.441	-13.713	1.00	0.00	RX1	C
ATOM	3522	CD1	LEU	1104	63.036	19.242	-14.219	1.00	0.00	RX1	C
ATOM	3523	CD2	LEU	1104	61.118	20.885	-13.868	1.00	0.00	RX1	C
ATOM	3524	C	LEU	1104	60.083	16.893	-16.219	1.00	0.00	RX1	C
ATOM	3525	O	LEU	1104	58.957	16.895	-16.700	1.00	0.00	RX1	O
ATOM	3526	N	ALA	1105	60.812	15.794	-16.001	1.00	0.00	RX1	N
ATOM	3527	H	ALA	1105	61.669	15.845	-15.495	1.00	0.00	RX1	H
ATOM	3528	CA	ALA	1105	60.193	14.506	-16.294	1.00	0.00	RX1	C
ATOM	3529	CB	ALA	1105	61.262	13.409	-16.323	1.00	0.00	RX1	C
ATOM	3530	C	ALA	1105	59.140	14.150	-15.259	1.00	0.00	RX1	C
ATOM	3531	O	ALA	1105	59.327	14.371	-14.067	1.00	0.00	RX1	O
ATOM	3532	N	PRO	1106	58.014	13.580	-15.752	1.00	0.00	RX1	N
ATOM	3533	CD	PRO	1106	57.669	13.383	-17.154	1.00	0.00	RX1	C
ATOM	3534	CA	PRO	1106	57.027	13.028	-14.817	1.00	0.00	RX1	C
ATOM	3535	CB	PRO	1106	55.919	12.551	-15.772	1.00	0.00	RX1	C
ATOM	3536	CG	PRO	1106	56.606	12.294	-17.112	1.00	0.00	RX1	C

ATOM	3537	C	PRO	1106	57.653	11.926	-13.967	1.00	0.00	RX1	C
ATOM	3538	O	PRO	1106	58.570	11.236	-14.401	1.00	0.00	RX1	O
ATOM	3539	N	PRO	1107	57.154	11.813	-12.710	1.00	0.00	RX1	N
ATOM	3540	CD	PRO	1107	56.082	12.608	-12.124	1.00	0.00	RX1	C
ATOM	3541	CA	PRO	1107	57.725	10.838	-11.772	1.00	0.00	RX1	C
ATOM	3542	CB	PRO	1107	56.755	10.912	-10.588	1.00	0.00	RX1	C
ATOM	3543	CG	PRO	1107	56.196	12.333	-10.629	1.00	0.00	RX1	C
ATOM	3544	C	PRO	1107	57.889	9.432	-12.323	1.00	0.00	RX1	C
ATOM	3545	O	PRO	1107	57.035	8.900	-13.022	1.00	0.00	RX1	O
ATOM	3546	N	SER	1108	59.041	8.851	-11.961	1.00	0.00	RX1	N
ATOM	3547	H	SER	1108	59.693	9.300	-11.352	1.00	0.00	RX1	H
ATOM	3548	CA	SER	1108	59.272	7.456	-12.318	1.00	0.00	RX1	C
ATOM	3549	CB	SER	1108	60.728	7.289	-11.953	1.00	0.00	RX1	C
ATOM	3550	OG	SER	1108	61.279	8.609	-12.090	1.00	0.00	RX1	O
ATOM	3551	HG	SER	1108	61.149	8.858	-12.999	1.00	0.00	RX1	H
ATOM	3552	C	SER	1108	58.277	6.539	-11.631	1.00	0.00	RX1	C
ATOM	3553	O	SER	1108	57.609	6.939	-10.680	1.00	0.00	RX1	O
ATOM	3554	N	LEU	1109	58.225	5.288	-12.138	1.00	0.00	RX1	N
ATOM	3555	H	LEU	1109	58.817	5.009	-12.891	1.00	0.00	RX1	H
ATOM	3556	CA	LEU	1109	57.299	4.318	-11.545	1.00	0.00	RX1	C
ATOM	3557	CB	LEU	1109	57.478	2.931	-12.166	1.00	0.00	RX1	C
ATOM	3558	CG	LEU	1109	56.331	1.977	-11.815	1.00	0.00	RX1	C
ATOM	3559	CD1	LEU	1109	54.958	2.551	-12.172	1.00	0.00	RX1	C
ATOM	3560	CD2	LEU	1109	56.545	0.588	-12.418	1.00	0.00	RX1	C
ATOM	3561	C	LEU	1109	57.342	4.291	-10.027	1.00	0.00	RX1	C
ATOM	3562	O	LEU	1109	56.327	4.494	-9.382	1.00	0.00	RX1	O
ATOM	3563	N	SER	1110	58.576	4.211	-9.514	1.00	0.00	RX1	N
ATOM	3564	H	SER	1110	59.277	3.764	-10.066	1.00	0.00	RX1	H
ATOM	3565	CA	SER	1110	58.845	4.454	-8.095	1.00	0.00	RX1	C
ATOM	3566	CB	SER	1110	60.335	4.727	-8.045	1.00	0.00	RX1	C
ATOM	3567	OG	SER	1110	60.876	4.200	-9.265	1.00	0.00	RX1	O
ATOM	3568	HG	SER	1110	60.756	3.247	-9.194	1.00	0.00	RX1	H
ATOM	3569	C	SER	1110	57.980	5.481	-7.371	1.00	0.00	RX1	C
ATOM	3570	O	SER	1110	57.247	5.163	-6.447	1.00	0.00	RX1	O
ATOM	3571	N	LYS	1111	58.060	6.746	-7.828	1.00	0.00	RX1	N
ATOM	3572	H	LYS	1111	58.458	6.946	-8.723	1.00	0.00	RX1	H
ATOM	3573	CA	LYS	1111	57.240	7.712	-7.091	1.00	0.00	RX1	C
ATOM	3574	CB	LYS	1111	57.819	9.139	-7.123	1.00	0.00	RX1	C
ATOM	3575	CG	LYS	1111	57.869	9.927	-5.787	1.00	0.00	RX1	C
ATOM	3576	CD	LYS	1111	56.542	10.388	-5.146	1.00	0.00	RX1	C
ATOM	3577	CE	LYS	1111	56.633	11.371	-3.950	1.00	0.00	RX1	C
ATOM	3578	NZ	LYS	1111	56.852	10.737	-2.638	1.00	0.00	RX1	N
ATOM	3579	HZ1	LYS	1111	56.979	11.453	-1.900	1.00	0.00	RX1	H
ATOM	3580	HZ2	LYS	1111	56.018	10.197	-2.320	1.00	0.00	RX1	H
ATOM	3581	HZ3	LYS	1111	57.707	10.131	-2.599	1.00	0.00	RX1	H
ATOM	3582	C	LYS	1111	55.765	7.692	-7.459	1.00	0.00	RX1	C
ATOM	3583	O	LYS	1111	54.931	8.197	-6.727	1.00	0.00	RX1	O
ATOM	3584	N	MET	1112	55.441	7.068	-8.602	1.00	0.00	RX1	N
ATOM	3585	H	MET	1112	56.121	6.593	-9.166	1.00	0.00	RX1	H
ATOM	3586	CA	MET	1112	54.006	6.893	-8.844	1.00	0.00	RX1	C
ATOM	3587	CB	MET	1112	53.724	6.595	-10.317	1.00	0.00	RX1	C
ATOM	3588	CG	MET	1112	54.141	7.766	-11.207	1.00	0.00	RX1	C
ATOM	3589	SD	MET	1112	53.565	7.615	-12.906	1.00	0.00	RX1	S
ATOM	3590	CE	MET	1112	54.611	6.245	-13.408	1.00	0.00	RX1	C
ATOM	3591	C	MET	1112	53.355	5.866	-7.926	1.00	0.00	RX1	C
ATOM	3592	O	MET	1112	52.277	6.063	-7.379	1.00	0.00	RX1	O
ATOM	3593	N	ILE	1113	54.105	4.773	-7.744	1.00	0.00	RX1	N
ATOM	3594	H	ILE	1113	54.996	4.713	-8.185	1.00	0.00	RX1	H
ATOM	3595	CA	ILE	1113	53.767	3.731	-6.777	1.00	0.00	RX1	C
ATOM	3596	CB	ILE	1113	54.789	2.603	-6.905	1.00	0.00	RX1	C
ATOM	3597	CG2	ILE	1113	54.776	1.671	-5.700	1.00	0.00	RX1	C

ATOM	3598	CG1	ILE	1113	54.564	1.851	-8.215	1.00	0.00	RX1	C
ATOM	3599	CD1	ILE	1113	53.164	1.242	-8.287	1.00	0.00	RX1	C
ATOM	3600	C	ILE	1113	53.734	4.290	-5.366	1.00	0.00	RX1	C
ATOM	3601	O	ILE	1113	52.800	4.090	-4.602	1.00	0.00	RX1	O
ATOM	3602	N	GLN	1114	54.784	5.073	-5.087	1.00	0.00	RX1	N
ATOM	3603	H	GLN	1114	55.559	5.101	-5.715	1.00	0.00	RX1	H
ATOM	3604	CA	GLN	1114	54.828	5.802	-3.827	1.00	0.00	RX1	C
ATOM	3605	CB	GLN	1114	56.095	6.639	-3.747	1.00	0.00	RX1	C
ATOM	3606	CG	GLN	1114	56.120	7.544	-2.525	1.00	0.00	RX1	C
ATOM	3607	CD	GLN	1114	56.334	6.768	-1.246	1.00	0.00	RX1	C
ATOM	3608	OE1	GLN	1114	56.665	5.587	-1.248	1.00	0.00	RX1	O
ATOM	3609	NE2	GLN	1114	56.151	7.509	-0.146	1.00	0.00	RX1	N
ATOM	3610	HE21	GLN	1114	55.884	8.474	-0.251	1.00	0.00	RX1	H
ATOM	3611	HE22	GLN	1114	56.280	7.187	0.788	1.00	0.00	RX1	H
ATOM	3612	C	GLN	1114	53.590	6.640	-3.576	1.00	0.00	RX1	C
ATOM	3613	O	GLN	1114	52.920	6.450	-2.578	1.00	0.00	RX1	O
ATOM	3614	N	MET	1115	53.276	7.521	-4.542	1.00	0.00	RX1	N
ATOM	3615	H	MET	1115	53.857	7.614	-5.346	1.00	0.00	RX1	H
ATOM	3616	CA	MET	1115	52.069	8.343	-4.407	1.00	0.00	RX1	C
ATOM	3617	CB	MET	1115	51.870	9.266	-5.610	1.00	0.00	RX1	C
ATOM	3618	CG	MET	1115	52.826	10.456	-5.583	1.00	0.00	RX1	C
ATOM	3619	SD	MET	1115	52.563	11.632	-6.921	1.00	0.00	RX1	S
ATOM	3620	CE	MET	1115	53.205	10.628	-8.265	1.00	0.00	RX1	C
ATOM	3621	C	MET	1115	50.812	7.537	-4.172	1.00	0.00	RX1	C
ATOM	3622	O	MET	1115	49.998	7.828	-3.306	1.00	0.00	RX1	O
ATOM	3623	N	ALA	1116	50.719	6.450	-4.956	1.00	0.00	RX1	N
ATOM	3624	H	ALA	1116	51.413	6.269	-5.655	1.00	0.00	RX1	H
ATOM	3625	CA	ALA	1116	49.640	5.493	-4.720	1.00	0.00	RX1	C
ATOM	3626	CB	ALA	1116	49.818	4.255	-5.599	1.00	0.00	RX1	C
ATOM	3627	C	ALA	1116	49.544	5.045	-3.271	1.00	0.00	RX1	C
ATOM	3628	O	ALA	1116	48.483	5.041	-2.665	1.00	0.00	RX1	O
ATOM	3629	N	GLY	1117	50.715	4.702	-2.727	1.00	0.00	RX1	N
ATOM	3630	H	GLY	1117	51.572	4.798	-3.238	1.00	0.00	RX1	H
ATOM	3631	CA	GLY	1117	50.748	4.316	-1.323	1.00	0.00	RX1	C
ATOM	3632	C	GLY	1117	50.488	5.440	-0.338	1.00	0.00	RX1	C
ATOM	3633	O	GLY	1117	49.853	5.225	0.679	1.00	0.00	RX1	O
ATOM	3634	N	GLU	1118	50.978	6.644	-0.668	1.00	0.00	RX1	N
ATOM	3635	H	GLU	1118	51.426	6.781	-1.549	1.00	0.00	RX1	H
ATOM	3636	CA	GLU	1118	50.787	7.801	0.214	1.00	0.00	RX1	C
ATOM	3637	CB	GLU	1118	51.548	9.004	-0.366	1.00	0.00	RX1	C
ATOM	3638	CG	GLU	1118	53.068	8.805	-0.357	1.00	0.00	RX1	C
ATOM	3639	CD	GLU	1118	53.803	9.863	-1.175	1.00	0.00	RX1	C
ATOM	3640	OE1	GLU	1118	53.251	10.385	-2.132	1.00	0.00	RX1	O
ATOM	3641	OE2	GLU	1118	54.958	10.154	-0.881	1.00	0.00	RX1	O
ATOM	3642	C	GLU	1118	49.305	8.087	0.430	1.00	0.00	RX1	C
ATOM	3643	O	GLU	1118	48.782	8.152	1.539	1.00	0.00	RX1	O
ATOM	3644	N	ILE	1119	48.627	8.143	-0.725	1.00	0.00	RX1	N
ATOM	3645	H	ILE	1119	49.144	8.088	-1.581	1.00	0.00	RX1	H
ATOM	3646	CA	ILE	1119	47.166	8.223	-0.724	1.00	0.00	RX1	C
ATOM	3647	CB	ILE	1119	46.676	8.334	-2.166	1.00	0.00	RX1	C
ATOM	3648	CG2	ILE	1119	45.150	8.351	-2.268	1.00	0.00	RX1	C
ATOM	3649	CG1	ILE	1119	47.318	9.550	-2.830	1.00	0.00	RX1	C
ATOM	3650	CD1	ILE	1119	47.191	9.516	-4.350	1.00	0.00	RX1	C
ATOM	3651	C	ILE	1119	46.515	7.038	-0.017	1.00	0.00	RX1	C
ATOM	3652	O	ILE	1119	45.658	7.172	0.849	1.00	0.00	RX1	O
ATOM	3653	N	ALA	1120	46.977	5.846	-0.419	1.00	0.00	RX1	N
ATOM	3654	H	ALA	1120	47.718	5.780	-1.088	1.00	0.00	RX1	H
ATOM	3655	CA	ALA	1120	46.372	4.641	0.143	1.00	0.00	RX1	C
ATOM	3656	CB	ALA	1120	46.856	3.375	-0.566	1.00	0.00	RX1	C
ATOM	3657	C	ALA	1120	46.557	4.457	1.634	1.00	0.00	RX1	C
ATOM	3658	O	ALA	1120	45.743	3.814	2.277	1.00	0.00	RX1	O

ATOM	3659	N	ASP	1121	47.628	5.060	2.168	1.00	0.00	RX1	N
ATOM	3660	H	ASP	1121	48.257	5.602	1.611	1.00	0.00	RX1	H
ATOM	3661	CA	ASP	1121	47.861	4.974	3.608	1.00	0.00	RX1	C
ATOM	3662	CB	ASP	1121	49.273	5.420	3.960	1.00	0.00	RX1	C
ATOM	3663	CG	ASP	1121	49.551	5.081	5.407	1.00	0.00	RX1	C
ATOM	3664	OD1	ASP	1121	49.816	3.918	5.692	1.00	0.00	RX1	O
ATOM	3665	OD2	ASP	1121	49.537	5.976	6.252	1.00	0.00	RX1	O
ATOM	3666	C	ASP	1121	46.851	5.798	4.372	1.00	0.00	RX1	C
ATOM	3667	O	ASP	1121	46.174	5.334	5.282	1.00	0.00	RX1	O
ATOM	3668	N	GLY	1122	46.697	7.042	3.883	1.00	0.00	RX1	N
ATOM	3669	H	GLY	1122	47.296	7.376	3.152	1.00	0.00	RX1	H
ATOM	3670	CA	GLY	1122	45.610	7.862	4.420	1.00	0.00	RX1	C
ATOM	3671	C	GLY	1122	44.255	7.168	4.367	1.00	0.00	RX1	C
ATOM	3672	O	GLY	1122	43.528	7.064	5.348	1.00	0.00	RX1	O
ATOM	3673	N	MET	1123	43.973	6.642	3.165	1.00	0.00	RX1	N
ATOM	3674	H	MET	1123	44.625	6.753	2.412	1.00	0.00	RX1	H
ATOM	3675	CA	MET	1123	42.743	5.865	2.998	1.00	0.00	RX1	C
ATOM	3676	CB	MET	1123	42.507	5.507	1.532	1.00	0.00	RX1	C
ATOM	3677	CG	MET	1123	42.269	6.701	0.610	1.00	0.00	RX1	C
ATOM	3678	SD	MET	1123	40.848	7.702	1.081	1.00	0.00	RX1	S
ATOM	3679	CE	MET	1123	39.561	6.456	0.902	1.00	0.00	RX1	C
ATOM	3680	C	MET	1123	42.646	4.603	3.842	1.00	0.00	RX1	C
ATOM	3681	O	MET	1123	41.569	4.174	4.235	1.00	0.00	RX1	O
ATOM	3682	N	ALA	1124	43.820	4.025	4.123	1.00	0.00	RX1	N
ATOM	3683	H	ALA	1124	44.687	4.417	3.812	1.00	0.00	RX1	H
ATOM	3684	CA	ALA	1124	43.848	2.835	4.965	1.00	0.00	RX1	C
ATOM	3685	CB	ALA	1124	45.228	2.180	4.954	1.00	0.00	RX1	C
ATOM	3686	C	ALA	1124	43.465	3.153	6.389	1.00	0.00	RX1	C
ATOM	3687	O	ALA	1124	42.630	2.489	6.985	1.00	0.00	RX1	O
ATOM	3688	N	TYR	1125	44.060	4.249	6.892	1.00	0.00	RX1	N
ATOM	3689	H	TYR	1125	44.744	4.751	6.361	1.00	0.00	RX1	H
ATOM	3690	CA	TYR	1125	43.596	4.750	8.184	1.00	0.00	RX1	C
ATOM	3691	CB	TYR	1125	44.384	5.992	8.600	1.00	0.00	RX1	C
ATOM	3692	CG	TYR	1125	44.024	6.388	10.015	1.00	0.00	RX1	C
ATOM	3693	CD1	TYR	1125	44.797	5.936	11.077	1.00	0.00	RX1	C
ATOM	3694	CE1	TYR	1125	44.527	6.368	12.370	1.00	0.00	RX1	C
ATOM	3695	CD2	TYR	1125	42.930	7.211	10.261	1.00	0.00	RX1	C
ATOM	3696	CE2	TYR	1125	42.640	7.613	11.557	1.00	0.00	RX1	C
ATOM	3697	CZ	TYR	1125	43.469	7.237	12.605	1.00	0.00	RX1	C
ATOM	3698	OH	TYR	1125	43.268	7.754	13.867	1.00	0.00	RX1	O
ATOM	3699	HH	TYR	1125	42.340	7.981	13.978	1.00	0.00	RX1	H
ATOM	3700	C	TYR	1125	42.107	5.037	8.181	1.00	0.00	RX1	C
ATOM	3701	O	TYR	1125	41.402	4.701	9.118	1.00	0.00	RX1	O
ATOM	3702	N	LEU	1126	41.654	5.638	7.068	1.00	0.00	RX1	N
ATOM	3703	H	LEU	1126	42.286	5.923	6.347	1.00	0.00	RX1	H
ATOM	3704	CA	LEU	1126	40.214	5.864	6.961	1.00	0.00	RX1	C
ATOM	3705	CB	LEU	1126	39.860	6.621	5.677	1.00	0.00	RX1	C
ATOM	3706	CG	LEU	1126	40.525	7.991	5.536	1.00	0.00	RX1	C
ATOM	3707	CD1	LEU	1126	39.971	8.758	4.337	1.00	0.00	RX1	C
ATOM	3708	CD2	LEU	1126	40.448	8.819	6.816	1.00	0.00	RX1	C
ATOM	3709	C	LEU	1126	39.387	4.591	7.107	1.00	0.00	RX1	C
ATOM	3710	O	LEU	1126	38.788	4.354	8.143	1.00	0.00	RX1	O
ATOM	3711	N	ASN	1127	39.413	3.749	6.064	1.00	0.00	RX1	N
ATOM	3712	H	ASN	1127	39.970	3.960	5.261	1.00	0.00	RX1	H
ATOM	3713	CA	ASN	1127	38.576	2.543	6.108	1.00	0.00	RX1	C
ATOM	3714	CB	ASN	1127	38.742	1.765	4.810	1.00	0.00	RX1	C
ATOM	3715	CG	ASN	1127	38.029	0.415	4.750	1.00	0.00	RX1	C
ATOM	3716	OD1	ASN	1127	38.376	-0.361	3.863	1.00	0.00	RX1	O
ATOM	3717	ND2	ASN	1127	37.013	0.171	5.602	1.00	0.00	RX1	N
ATOM	3718	HD21	ASN	1127	36.715	0.767	6.352	1.00	0.00	RX1	H
ATOM	3719	HD22	ASN	1127	36.481	-0.689	5.571	1.00	0.00	RX1	H

ATOM	3720	C	ASN	1127	38.832	1.643	7.305	1.00	0.00	RX1	C
ATOM	3721	O	ASN	1127	37.926	1.253	8.029	1.00	0.00	RX1	O
ATOM	3722	N	ALA	1128	40.120	1.317	7.484	1.00	0.00	RX1	N
ATOM	3723	H	ALA	1128	40.854	1.772	6.979	1.00	0.00	RX1	H
ATOM	3724	CA	ALA	1128	40.416	0.398	8.579	1.00	0.00	RX1	C
ATOM	3725	CB	ALA	1128	41.846	-0.131	8.476	1.00	0.00	RX1	C
ATOM	3726	C	ALA	1128	40.162	0.975	9.960	1.00	0.00	RX1	C
ATOM	3727	O	ALA	1128	39.953	0.259	10.931	1.00	0.00	RX1	O
ATOM	3728	N	ASN	1129	40.160	2.310	10.002	1.00	0.00	RX1	N
ATOM	3729	H	ASN	1129	40.333	2.921	9.226	1.00	0.00	RX1	H
ATOM	3730	CA	ASN	1129	39.730	2.945	11.236	1.00	0.00	RX1	C
ATOM	3731	CB	ASN	1129	40.915	3.555	11.982	1.00	0.00	RX1	C
ATOM	3732	CG	ASN	1129	40.519	3.859	13.410	1.00	0.00	RX1	C
ATOM	3733	OD1	ASN	1129	39.427	4.331	13.715	1.00	0.00	RX1	O
ATOM	3734	ND2	ASN	1129	41.487	3.550	14.288	1.00	0.00	RX1	N
ATOM	3735	HD21	ASN	1129	42.347	3.124	13.976	1.00	0.00	RX1	H
ATOM	3736	HD22	ASN	1129	41.419	3.707	15.272	1.00	0.00	RX1	H
ATOM	3737	C	ASN	1129	38.639	3.972	10.970	1.00	0.00	RX1	C
ATOM	3738	O	ASN	1129	38.788	5.165	11.234	1.00	0.00	RX1	O
ATOM	3739	N	LYS	1130	37.512	3.415	10.470	1.00	0.00	RX1	N
ATOM	3740	H	LYS	1130	37.572	2.492	10.087	1.00	0.00	RX1	H
ATOM	3741	CA	LYS	1130	36.217	4.085	10.247	1.00	0.00	RX1	C
ATOM	3742	CB	LYS	1130	35.883	5.166	11.289	1.00	0.00	RX1	C
ATOM	3743	CG	LYS	1130	35.861	4.742	12.759	1.00	0.00	RX1	C
ATOM	3744	CD	LYS	1130	35.784	5.963	13.677	1.00	0.00	RX1	C
ATOM	3745	CE	LYS	1130	37.114	6.319	14.354	1.00	0.00	RX1	C
ATOM	3746	NZ	LYS	1130	38.205	6.519	13.390	1.00	0.00	RX1	N
ATOM	3747	HZ1	LYS	1130	39.087	6.744	13.897	1.00	0.00	RX1	H
ATOM	3748	HZ2	LYS	1130	38.420	5.670	12.827	1.00	0.00	RX1	H
ATOM	3749	HZ3	LYS	1130	38.083	7.353	12.778	1.00	0.00	RX1	H
ATOM	3750	C	LYS	1130	35.969	4.681	8.861	1.00	0.00	RX1	C
ATOM	3751	O	LYS	1130	35.254	4.156	8.015	1.00	0.00	RX1	O
ATOM	3752	N	PHE	1131	36.548	5.879	8.688	1.00	0.00	RX1	N
ATOM	3753	H	PHE	1131	37.381	6.066	9.208	1.00	0.00	RX1	H
ATOM	3754	CA	PHE	1131	36.010	6.789	7.674	1.00	0.00	RX1	C
ATOM	3755	CB	PHE	1131	36.635	8.180	7.805	1.00	0.00	RX1	C
ATOM	3756	CG	PHE	1131	36.064	8.965	8.965	1.00	0.00	RX1	C
ATOM	3757	CD1	PHE	1131	36.239	8.538	10.276	1.00	0.00	RX1	C
ATOM	3758	CD2	PHE	1131	35.377	10.146	8.711	1.00	0.00	RX1	C
ATOM	3759	CE1	PHE	1131	35.740	9.293	11.330	1.00	0.00	RX1	C
ATOM	3760	CE2	PHE	1131	34.882	10.904	9.765	1.00	0.00	RX1	C
ATOM	3761	CZ	PHE	1131	35.067	10.481	11.075	1.00	0.00	RX1	C
ATOM	3762	C	PHE	1131	36.112	6.363	6.214	1.00	0.00	RX1	C
ATOM	3763	O	PHE	1131	36.924	5.544	5.805	1.00	0.00	RX1	O
ATOM	3764	N	VAL	1132	35.219	6.991	5.434	1.00	0.00	RX1	N
ATOM	3765	H	VAL	1132	34.651	7.751	5.751	1.00	0.00	RX1	H
ATOM	3766	CA	VAL	1132	35.025	6.604	4.037	1.00	0.00	RX1	C
ATOM	3767	CB	VAL	1132	33.935	5.523	4.020	1.00	0.00	RX1	C
ATOM	3768	CG1	VAL	1132	32.713	5.996	4.810	1.00	0.00	RX1	C
ATOM	3769	CG2	VAL	1132	33.617	4.981	2.625	1.00	0.00	RX1	C
ATOM	3770	C	VAL	1132	34.680	7.866	3.249	1.00	0.00	RX1	C
ATOM	3771	O	VAL	1132	34.233	8.843	3.840	1.00	0.00	RX1	O
ATOM	3772	N	HIS	1133	34.969	7.855	1.937	1.00	0.00	RX1	N
ATOM	3773	H	HIS	1133	35.097	7.024	1.385	1.00	0.00	RX1	H
ATOM	3774	CA	HIS	1133	35.142	9.153	1.286	1.00	0.00	RX1	C
ATOM	3775	CB	HIS	1133	36.432	9.118	0.462	1.00	0.00	RX1	C
ATOM	3776	CG	HIS	1133	36.978	10.490	0.140	1.00	0.00	RX1	C
ATOM	3777	ND1	HIS	1133	36.253	11.616	-0.003	1.00	0.00	RX1	N
ATOM	3778	HD1	HIS	1133	35.281	11.718	0.089	1.00	0.00	RX1	H
ATOM	3779	CD2	HIS	1133	38.322	10.802	-0.071	1.00	0.00	RX1	C
ATOM	3780	NE2	HIS	1133	38.398	12.124	-0.345	1.00	0.00	RX1	N

ATOM	3781	CE1	HIS	1133	37.124	12.626	-0.301	1.00	0.00	RX1	C
ATOM	3782	C	HIS	1133	33.969	9.703	0.483	1.00	0.00	RX1	C
ATOM	3783	O	HIS	1133	33.647	10.891	0.554	1.00	0.00	RX1	O
ATOM	3784	N	ARG	1134	33.365	8.795	-0.303	1.00	0.00	RX1	N
ATOM	3785	H	ARG	1134	33.822	7.908	-0.432	1.00	0.00	RX1	H
ATOM	3786	CA	ARG	1134	32.281	9.136	-1.234	1.00	0.00	RX1	C
ATOM	3787	CB	ARG	1134	31.104	9.815	-0.518	1.00	0.00	RX1	C
ATOM	3788	CG	ARG	1134	29.740	9.666	-1.191	1.00	0.00	RX1	C
ATOM	3789	CD	ARG	1134	28.913	10.945	-1.080	1.00	0.00	RX1	C
ATOM	3790	NE	ARG	1134	28.871	11.420	0.300	1.00	0.00	RX1	N
ATOM	3791	HE	ARG	1134	29.682	11.925	0.621	1.00	0.00	RX1	H
ATOM	3792	CZ	ARG	1134	27.810	11.052	1.082	1.00	0.00	RX1	C
ATOM	3793	NH1	ARG	1134	26.776	10.372	0.544	1.00	0.00	RX1	N
ATOM	3794	HH11	ARG	1134	26.055	10.019	1.170	1.00	0.00	RX1	H
ATOM	3795	HH12	ARG	1134	26.672	10.172	-0.429	1.00	0.00	RX1	H
ATOM	3796	NH2	ARG	1134	27.802	11.357	2.392	1.00	0.00	RX1	N
ATOM	3797	HH21	ARG	1134	27.038	11.008	2.972	1.00	0.00	RX1	H
ATOM	3798	HH22	ARG	1134	28.501	11.888	2.865	1.00	0.00	RX1	H
ATOM	3799	C	ARG	1134	32.715	9.956	-2.450	1.00	0.00	RX1	C
ATOM	3800	O	ARG	1134	31.888	10.477	-3.190	1.00	0.00	RX1	O
ATOM	3801	N	ASP	1135	34.046	10.104	-2.585	1.00	0.00	RX1	N
ATOM	3802	H	ASP	1135	34.681	9.475	-2.128	1.00	0.00	RX1	H
ATOM	3803	CA	ASP	1135	34.533	11.233	-3.387	1.00	0.00	RX1	C
ATOM	3804	CB	ASP	1135	34.201	12.537	-2.637	1.00	0.00	RX1	C
ATOM	3805	CG	ASP	1135	34.145	13.759	-3.539	1.00	0.00	RX1	C
ATOM	3806	OD1	ASP	1135	33.305	13.831	-4.427	1.00	0.00	RX1	O
ATOM	3807	OD2	ASP	1135	34.944	14.670	-3.366	1.00	0.00	RX1	O
ATOM	3808	C	ASP	1135	36.011	11.166	-3.770	1.00	0.00	RX1	C
ATOM	3809	O	ASP	1135	36.621	12.149	-4.179	1.00	0.00	RX1	O
ATOM	3810	N	LEU	1136	36.607	9.972	-3.605	1.00	0.00	RX1	N
ATOM	3811	H	LEU	1136	36.108	9.131	-3.377	1.00	0.00	RX1	H
ATOM	3812	CA	LEU	1136	38.056	9.935	-3.836	1.00	0.00	RX1	C
ATOM	3813	CB	LEU	1136	38.621	8.583	-3.389	1.00	0.00	RX1	C
ATOM	3814	CG	LEU	1136	40.151	8.501	-3.382	1.00	0.00	RX1	C
ATOM	3815	CD1	LEU	1136	40.797	9.587	-2.519	1.00	0.00	RX1	C
ATOM	3816	CD2	LEU	1136	40.632	7.113	-2.971	1.00	0.00	RX1	C
ATOM	3817	C	LEU	1136	38.500	10.294	-5.254	1.00	0.00	RX1	C
ATOM	3818	O	LEU	1136	37.976	9.808	-6.248	1.00	0.00	RX1	O
ATOM	3819	N	ALA	1137	39.500	11.188	-5.284	1.00	0.00	RX1	N
ATOM	3820	H	ALA	1137	39.918	11.565	-4.455	1.00	0.00	RX1	H
ATOM	3821	CA	ALA	1137	40.121	11.619	-6.535	1.00	0.00	RX1	C
ATOM	3822	CB	ALA	1137	39.240	12.614	-7.276	1.00	0.00	RX1	C
ATOM	3823	C	ALA	1137	41.408	12.327	-6.188	1.00	0.00	RX1	C
ATOM	3824	O	ALA	1137	41.628	12.641	-5.024	1.00	0.00	RX1	O
ATOM	3825	N	ALA	1138	42.234	12.602	-7.211	1.00	0.00	RX1	N
ATOM	3826	H	ALA	1138	42.002	12.409	-8.170	1.00	0.00	RX1	H
ATOM	3827	CA	ALA	1138	43.501	13.268	-6.895	1.00	0.00	RX1	C
ATOM	3828	CB	ALA	1138	44.431	13.284	-8.109	1.00	0.00	RX1	C
ATOM	3829	C	ALA	1138	43.364	14.677	-6.332	1.00	0.00	RX1	C
ATOM	3830	O	ALA	1138	44.227	15.175	-5.619	1.00	0.00	RX1	O
ATOM	3831	N	ARG	1139	42.200	15.284	-6.636	1.00	0.00	RX1	N
ATOM	3832	H	ARG	1139	41.574	14.858	-7.286	1.00	0.00	RX1	H
ATOM	3833	CA	ARG	1139	41.871	16.559	-5.984	1.00	0.00	RX1	C
ATOM	3834	CB	ARG	1139	40.555	17.127	-6.532	1.00	0.00	RX1	C
ATOM	3835	CG	ARG	1139	39.323	16.323	-6.110	1.00	0.00	RX1	C
ATOM	3836	CD	ARG	1139	37.986	16.888	-6.590	1.00	0.00	RX1	C
ATOM	3837	NE	ARG	1139	36.883	16.100	-6.048	1.00	0.00	RX1	N
ATOM	3838	HE	ARG	1139	36.466	16.383	-5.173	1.00	0.00	RX1	H
ATOM	3839	CZ	ARG	1139	36.490	14.965	-6.686	1.00	0.00	RX1	C
ATOM	3840	NH1	ARG	1139	36.906	14.723	-7.947	1.00	0.00	RX1	N
ATOM	3841	HH11	ARG	1139	36.700	13.878	-8.442	1.00	0.00	RX1	H

ATOM	3842	HH12	ARG	1139	37.447	15.402	-8.469	1.00	0.00	RX1	H
ATOM	3843	NH2	ARG	1139	35.693	14.098	-6.041	1.00	0.00	RX1	N
ATOM	3844	HH21	ARG	1139	35.403	13.207	-6.390	1.00	0.00	RX1	H
ATOM	3845	HH22	ARG	1139	35.332	14.313	-5.117	1.00	0.00	RX1	H
ATOM	3846	C	ARG	1139	41.836	16.494	-4.456	1.00	0.00	RX1	C
ATOM	3847	O	ARG	1139	42.133	17.435	-3.738	1.00	0.00	RX1	O
ATOM	3848	N	ASN	1140	41.468	15.296	-3.983	1.00	0.00	RX1	N
ATOM	3849	H	ASN	1140	41.388	14.490	-4.566	1.00	0.00	RX1	H
ATOM	3850	CA	ASN	1140	41.339	15.142	-2.538	1.00	0.00	RX1	C
ATOM	3851	CB	ASN	1140	40.114	14.296	-2.190	1.00	0.00	RX1	C
ATOM	3852	CG	ASN	1140	38.850	15.109	-2.378	1.00	0.00	RX1	C
ATOM	3853	OD1	ASN	1140	38.804	16.301	-2.099	1.00	0.00	RX1	O
ATOM	3854	ND2	ASN	1140	37.819	14.407	-2.883	1.00	0.00	RX1	N
ATOM	3855	HD21	ASN	1140	37.860	13.441	-3.146	1.00	0.00	RX1	H
ATOM	3856	HD22	ASN	1140	36.902	14.801	-2.997	1.00	0.00	RX1	H
ATOM	3857	C	ASN	1140	42.573	14.543	-1.899	1.00	0.00	RX1	C
ATOM	3858	O	ASN	1140	42.557	14.033	-0.785	1.00	0.00	RX1	O
ATOM	3859	N	CYS	1141	43.669	14.621	-2.661	1.00	0.00	RX1	N
ATOM	3860	H	CYS	1141	43.714	15.078	-3.550	1.00	0.00	RX1	H
ATOM	3861	CA	CYS	1141	44.924	14.140	-2.107	1.00	0.00	RX1	C
ATOM	3862	CB	CYS	1141	45.427	12.996	-2.977	1.00	0.00	RX1	C
ATOM	3863	SG	CYS	1141	44.137	11.760	-3.280	1.00	0.00	RX1	S
ATOM	3864	C	CYS	1141	45.894	15.294	-2.029	1.00	0.00	RX1	C
ATOM	3865	O	CYS	1141	46.446	15.733	-3.028	1.00	0.00	RX1	O
ATOM	3866	N	MET	1142	46.023	15.830	-0.814	1.00	0.00	RX1	N
ATOM	3867	H	MET	1142	45.855	15.260	-0.009	1.00	0.00	RX1	H
ATOM	3868	CA	MET	1142	46.792	17.068	-0.739	1.00	0.00	RX1	C
ATOM	3869	CB	MET	1142	46.247	17.982	0.356	1.00	0.00	RX1	C
ATOM	3870	CG	MET	1142	44.825	18.486	0.107	1.00	0.00	RX1	C
ATOM	3871	SD	MET	1142	44.687	19.595	-1.297	1.00	0.00	RX1	S
ATOM	3872	CE	MET	1142	42.897	19.783	-1.287	1.00	0.00	RX1	C
ATOM	3873	C	MET	1142	48.264	16.802	-0.520	1.00	0.00	RX1	C
ATOM	3874	O	MET	1142	48.656	15.906	0.217	1.00	0.00	RX1	O
ATOM	3875	N	VAL	1143	49.068	17.616	-1.202	1.00	0.00	RX1	N
ATOM	3876	H	VAL	1143	48.713	18.410	-1.699	1.00	0.00	RX1	H
ATOM	3877	CA	VAL	1143	50.505	17.432	-1.056	1.00	0.00	RX1	C
ATOM	3878	CB	VAL	1143	51.172	17.534	-2.426	1.00	0.00	RX1	C
ATOM	3879	CG1	VAL	1143	52.591	16.972	-2.401	1.00	0.00	RX1	C
ATOM	3880	CG2	VAL	1143	50.308	16.886	-3.506	1.00	0.00	RX1	C
ATOM	3881	C	VAL	1143	51.058	18.466	-0.092	1.00	0.00	RX1	C
ATOM	3882	O	VAL	1143	50.752	19.653	-0.187	1.00	0.00	RX1	O
ATOM	3883	N	ALA	1144	51.854	17.982	0.867	1.00	0.00	RX1	N
ATOM	3884	H	ALA	1144	52.192	17.036	0.859	1.00	0.00	RX1	H
ATOM	3885	CA	ALA	1144	52.403	18.973	1.787	1.00	0.00	RX1	C
ATOM	3886	CB	ALA	1144	52.533	18.403	3.202	1.00	0.00	RX1	C
ATOM	3887	C	ALA	1144	53.732	19.530	1.303	1.00	0.00	RX1	C
ATOM	3888	O	ALA	1144	54.216	19.191	0.228	1.00	0.00	RX1	O
ATOM	3889	N	GLU	1145	54.309	20.417	2.137	1.00	0.00	RX1	N
ATOM	3890	H	GLU	1145	53.833	20.680	2.978	1.00	0.00	RX1	H
ATOM	3891	CA	GLU	1145	55.606	21.023	1.802	1.00	0.00	RX1	C
ATOM	3892	CB	GLU	1145	56.092	21.840	3.003	1.00	0.00	RX1	C
ATOM	3893	CG	GLU	1145	57.186	22.866	2.694	1.00	0.00	RX1	C
ATOM	3894	CD	GLU	1145	56.565	24.127	2.124	1.00	0.00	RX1	C
ATOM	3895	OE1	GLU	1145	55.708	24.704	2.778	1.00	0.00	RX1	O
ATOM	3896	OE2	GLU	1145	56.949	24.561	1.046	1.00	0.00	RX1	O
ATOM	3897	C	GLU	1145	56.696	20.027	1.388	1.00	0.00	RX1	C
ATOM	3898	O	GLU	1145	57.504	20.249	0.491	1.00	0.00	RX1	O
ATOM	3899	N	ASP	1146	56.651	18.906	2.110	1.00	0.00	RX1	N
ATOM	3900	H	ASP	1146	55.861	18.705	2.691	1.00	0.00	RX1	H
ATOM	3901	CA	ASP	1146	57.565	17.778	1.936	1.00	0.00	RX1	C
ATOM	3902	CB	ASP	1146	57.314	16.775	3.065	1.00	0.00	RX1	C

ATOM	3903	CG	ASP	1146	55.852	16.349	3.126	1.00	0.00	RX1	C
ATOM	3904	OD1	ASP	1146	55.049	16.760	2.293	1.00	0.00	RX1	O
ATOM	3905	OD2	ASP	1146	55.506	15.580	4.013	1.00	0.00	RX1	O
ATOM	3906	C	ASP	1146	57.562	17.076	0.581	1.00	0.00	RX1	C
ATOM	3907	O	ASP	1146	58.550	16.458	0.205	1.00	0.00	RX1	O
ATOM	3908	N	PHE	1147	56.418	17.216	-0.124	1.00	0.00	RX1	N
ATOM	3909	H	PHE	1147	55.660	17.640	0.377	1.00	0.00	RX1	H
ATOM	3910	CA	PHE	1147	56.104	16.491	-1.365	1.00	0.00	RX1	C
ATOM	3911	CB	PHE	1147	57.221	16.486	-2.419	1.00	0.00	RX1	C
ATOM	3912	CG	PHE	1147	57.475	17.868	-2.971	1.00	0.00	RX1	C
ATOM	3913	CD1	PHE	1147	56.742	18.319	-4.062	1.00	0.00	RX1	C
ATOM	3914	CD2	PHE	1147	58.454	18.679	-2.409	1.00	0.00	RX1	C
ATOM	3915	CE1	PHE	1147	57.018	19.562	-4.617	1.00	0.00	RX1	C
ATOM	3916	CE2	PHE	1147	58.727	19.923	-2.963	1.00	0.00	RX1	C
ATOM	3917	CZ	PHE	1147	58.024	20.356	-4.080	1.00	0.00	RX1	C
ATOM	3918	C	PHE	1147	55.589	15.071	-1.165	1.00	0.00	RX1	C
ATOM	3919	O	PHE	1147	55.638	14.233	-2.062	1.00	0.00	RX1	O
ATOM	3920	N	THR	1148	55.053	14.853	0.038	1.00	0.00	RX1	N
ATOM	3921	H	THR	1148	55.064	15.509	0.792	1.00	0.00	RX1	H
ATOM	3922	CA	THR	1148	54.293	13.631	0.272	1.00	0.00	RX1	C
ATOM	3923	CB	THR	1148	54.552	13.278	1.722	1.00	0.00	RX1	C
ATOM	3924	OG1	THR	1148	55.930	13.546	1.999	1.00	0.00	RX1	O
ATOM	3925	HG1	THR	1148	55.909	14.205	2.695	1.00	0.00	RX1	H
ATOM	3926	CG2	THR	1148	54.170	11.843	2.080	1.00	0.00	RX1	C
ATOM	3927	C	THR	1148	52.827	13.862	-0.059	1.00	0.00	RX1	C
ATOM	3928	O	THR	1148	52.245	14.891	0.283	1.00	0.00	RX1	O
ATOM	3929	N	VAL	1149	52.246	12.888	-0.770	1.00	0.00	RX1	N
ATOM	3930	H	VAL	1149	52.711	12.025	-0.999	1.00	0.00	RX1	H
ATOM	3931	CA	VAL	1149	50.853	13.101	-1.150	1.00	0.00	RX1	C
ATOM	3932	CB	VAL	1149	50.610	12.653	-2.583	1.00	0.00	RX1	C
ATOM	3933	CG1	VAL	1149	49.197	13.028	-3.019	1.00	0.00	RX1	C
ATOM	3934	CG2	VAL	1149	51.670	13.255	-3.503	1.00	0.00	RX1	C
ATOM	3935	C	VAL	1149	49.861	12.466	-0.193	1.00	0.00	RX1	C
ATOM	3936	O	VAL	1149	49.461	11.314	-0.299	1.00	0.00	RX1	O
ATOM	3937	N	LYS	1150	49.486	13.293	0.781	1.00	0.00	RX1	N
ATOM	3938	H	LYS	1150	49.675	14.274	0.707	1.00	0.00	RX1	H
ATOM	3939	CA	LYS	1150	48.570	12.770	1.785	1.00	0.00	RX1	C
ATOM	3940	CB	LYS	1150	48.620	13.623	3.042	1.00	0.00	RX1	C
ATOM	3941	CG	LYS	1150	49.995	13.796	3.676	1.00	0.00	RX1	C
ATOM	3942	CD	LYS	1150	49.756	14.402	5.052	1.00	0.00	RX1	C
ATOM	3943	CE	LYS	1150	50.975	14.614	5.939	1.00	0.00	RX1	C
ATOM	3944	NZ	LYS	1150	50.466	14.667	7.313	1.00	0.00	RX1	N
ATOM	3945	HZ1	LYS	1150	51.105	15.197	7.943	1.00	0.00	RX1	H
ATOM	3946	HZ2	LYS	1150	49.587	15.228	7.357	1.00	0.00	RX1	H
ATOM	3947	HZ3	LYS	1150	50.267	13.718	7.686	1.00	0.00	RX1	H
ATOM	3948	C	LYS	1150	47.126	12.705	1.328	1.00	0.00	RX1	C
ATOM	3949	O	LYS	1150	46.706	13.354	0.375	1.00	0.00	RX1	O
ATOM	3950	N	ILE	1151	46.353	11.934	2.107	1.00	0.00	RX1	N
ATOM	3951	H	ILE	1151	46.753	11.443	2.882	1.00	0.00	RX1	H
ATOM	3952	CA	ILE	1151	44.912	12.153	2.024	1.00	0.00	RX1	C
ATOM	3953	CB	ILE	1151	44.152	10.994	2.662	1.00	0.00	RX1	C
ATOM	3954	CG2	ILE	1151	42.769	11.374	3.194	1.00	0.00	RX1	C
ATOM	3955	CG1	ILE	1151	44.039	9.921	1.590	1.00	0.00	RX1	C
ATOM	3956	CD1	ILE	1151	43.320	10.473	0.358	1.00	0.00	RX1	C
ATOM	3957	C	ILE	1151	44.534	13.495	2.611	1.00	0.00	RX1	C
ATOM	3958	O	ILE	1151	45.002	13.902	3.671	1.00	0.00	RX1	O
ATOM	3959	N	GLY	1152	43.740	14.179	1.783	1.00	0.00	RX1	N
ATOM	3960	H	GLY	1152	43.259	13.731	1.027	1.00	0.00	RX1	H
ATOM	3961	CA	GLY	1152	43.567	15.606	1.969	1.00	0.00	RX1	C
ATOM	3962	C	GLY	1152	42.590	16.032	3.032	1.00	0.00	RX1	C
ATOM	3963	O	GLY	1152	42.175	15.289	3.911	1.00	0.00	RX1	O

ATOM	3964	N	ASP	1153	42.285	17.320	2.892	1.00	0.00	RX1	N
ATOM	3965	H	ASP	1153	42.471	17.797	2.036	1.00	0.00	RX1	H
ATOM	3966	CA	ASP	1153	41.426	18.000	3.846	1.00	0.00	RX1	C
ATOM	3967	CB	ASP	1153	42.008	19.400	4.018	1.00	0.00	RX1	C
ATOM	3968	CG	ASP	1153	40.944	20.358	4.471	1.00	0.00	RX1	C
ATOM	3969	OD1	ASP	1153	40.449	20.233	5.588	1.00	0.00	RX1	O
ATOM	3970	OD2	ASP	1153	40.576	21.231	3.696	1.00	0.00	RX1	O
ATOM	3971	C	ASP	1153	39.992	17.971	3.336	1.00	0.00	RX1	C
ATOM	3972	O	ASP	1153	39.741	17.757	2.155	1.00	0.00	RX1	O
ATOM	3973	N	PHE	1154	39.062	18.133	4.287	1.00	0.00	RX1	N
ATOM	3974	H	PHE	1154	39.325	18.522	5.172	1.00	0.00	RX1	H
ATOM	3975	CA	PHE	1154	37.668	17.876	3.949	1.00	0.00	RX1	C
ATOM	3976	CB	PHE	1154	37.211	16.562	4.588	1.00	0.00	RX1	C
ATOM	3977	CG	PHE	1154	38.057	15.431	4.052	1.00	0.00	RX1	C
ATOM	3978	CD1	PHE	1154	38.099	15.186	2.686	1.00	0.00	RX1	C
ATOM	3979	CD2	PHE	1154	38.804	14.645	4.919	1.00	0.00	RX1	C
ATOM	3980	CE1	PHE	1154	38.910	14.174	2.189	1.00	0.00	RX1	C
ATOM	3981	CE2	PHE	1154	39.607	13.626	4.423	1.00	0.00	RX1	C
ATOM	3982	CZ	PHE	1154	39.668	13.396	3.054	1.00	0.00	RX1	C
ATOM	3983	C	PHE	1154	36.725	19.024	4.278	1.00	0.00	RX1	C
ATOM	3984	O	PHE	1154	37.129	20.122	4.672	1.00	0.00	RX1	O
ATOM	3985	N	GLY	1155	35.429	18.723	4.050	1.00	0.00	RX1	N
ATOM	3986	H	GLY	1155	35.122	17.825	3.726	1.00	0.00	RX1	H
ATOM	3987	CA	GLY	1155	34.395	19.742	4.232	1.00	0.00	RX1	C
ATOM	3988	C	GLY	1155	34.474	20.885	3.236	1.00	0.00	RX1	C
ATOM	3989	O	GLY	1155	33.981	21.984	3.455	1.00	0.00	RX1	O
ATOM	3990	N	MET	1156	35.153	20.587	2.114	1.00	0.00	RX1	N
ATOM	3991	H	MET	1156	35.407	19.645	1.903	1.00	0.00	RX1	H
ATOM	3992	CA	MET	1156	35.606	21.704	1.286	1.00	0.00	RX1	C
ATOM	3993	CB	MET	1156	36.635	21.256	0.250	1.00	0.00	RX1	C
ATOM	3994	CG	MET	1156	37.994	21.048	0.919	1.00	0.00	RX1	C
ATOM	3995	SD	MET	1156	38.513	22.529	1.807	1.00	0.00	RX1	S
ATOM	3996	CE	MET	1156	38.728	23.629	0.399	1.00	0.00	RX1	C
ATOM	3997	C	MET	1156	34.554	22.595	0.659	1.00	0.00	RX1	C
ATOM	3998	O	MET	1156	34.830	23.742	0.339	1.00	0.00	RX1	O
ATOM	3999	N	THR	1157	33.339	22.035	0.547	1.00	0.00	RX1	N
ATOM	4000	H	THR	1157	33.189	21.068	0.753	1.00	0.00	RX1	H
ATOM	4001	CA	THR	1157	32.184	22.793	0.066	1.00	0.00	RX1	C
ATOM	4002	CB	THR	1157	31.983	24.023	0.958	1.00	0.00	RX1	C
ATOM	4003	OG1	THR	1157	32.124	23.681	2.342	1.00	0.00	RX1	O
ATOM	4004	HG1	THR	1157	32.816	23.035	2.439	1.00	0.00	RX1	H
ATOM	4005	CG2	THR	1157	30.622	24.680	0.728	1.00	0.00	RX1	C
ATOM	4006	C	THR	1157	32.156	23.118	-1.425	1.00	0.00	RX1	C
ATOM	4007	O	THR	1157	31.309	22.611	-2.149	1.00	0.00	RX1	O
ATOM	4008	N	ARG	1158	33.080	23.999	-1.842	1.00	0.00	RX1	N
ATOM	4009	H	ARG	1158	33.869	24.308	-1.301	1.00	0.00	RX1	H
ATOM	4010	CA	ARG	1158	33.114	24.460	-3.231	1.00	0.00	RX1	C
ATOM	4011	CB	ARG	1158	32.390	25.795	-3.428	1.00	0.00	RX1	C
ATOM	4012	CG	ARG	1158	31.111	26.063	-2.640	1.00	0.00	RX1	C
ATOM	4013	CD	ARG	1158	30.891	27.571	-2.521	1.00	0.00	RX1	C
ATOM	4014	NE	ARG	1158	32.123	28.207	-2.056	1.00	0.00	RX1	N
ATOM	4015	HE	ARG	1158	32.904	28.294	-2.698	1.00	0.00	RX1	H
ATOM	4016	CZ	ARG	1158	32.340	28.518	-0.746	1.00	0.00	RX1	C
ATOM	4017	NH1	ARG	1158	31.370	28.350	0.176	1.00	0.00	RX1	N
ATOM	4018	HH11	ARG	1158	31.584	28.502	1.151	1.00	0.00	RX1	H
ATOM	4019	HH12	ARG	1158	30.444	28.057	-0.068	1.00	0.00	RX1	H
ATOM	4020	NH2	ARG	1158	33.546	28.977	-0.385	1.00	0.00	RX1	N
ATOM	4021	HH21	ARG	1158	33.793	29.287	0.537	1.00	0.00	RX1	H
ATOM	4022	HH22	ARG	1158	34.294	28.942	-1.075	1.00	0.00	RX1	H
ATOM	4023	C	ARG	1158	34.570	24.737	-3.594	1.00	0.00	RX1	C
ATOM	4024	O	ARG	1158	35.480	24.215	-2.956	1.00	0.00	RX1	O

ATOM	4025	N	ASP	1159	34.724	25.647	-4.578	1.00	0.00	RX1	N
ATOM	4026	H	ASP	1159	34.100	25.574	-5.361	1.00	0.00	RX1	H
ATOM	4027	CA	ASP	1159	35.892	26.523	-4.746	1.00	0.00	RX1	C
ATOM	4028	CB	ASP	1159	36.450	27.117	-3.434	1.00	0.00	RX1	C
ATOM	4029	CG	ASP	1159	35.398	27.734	-2.528	1.00	0.00	RX1	C
ATOM	4030	OD1	ASP	1159	34.899	28.824	-2.804	1.00	0.00	RX1	O
ATOM	4031	OD2	ASP	1159	35.095	27.144	-1.496	1.00	0.00	RX1	O
ATOM	4032	C	ASP	1159	37.060	25.901	-5.482	1.00	0.00	RX1	C
ATOM	4033	O	ASP	1159	37.975	26.588	-5.924	1.00	0.00	RX1	O
ATOM	4034	N	ILE	1160	37.026	24.565	-5.564	1.00	0.00	RX1	N
ATOM	4035	H	ILE	1160	36.178	24.055	-5.395	1.00	0.00	RX1	H
ATOM	4036	CA	ILE	1160	38.260	23.908	-5.980	1.00	0.00	RX1	C
ATOM	4037	CB	ILE	1160	38.750	22.943	-4.890	1.00	0.00	RX1	C
ATOM	4038	CG2	ILE	1160	40.086	22.304	-5.276	1.00	0.00	RX1	C
ATOM	4039	CG1	ILE	1160	38.840	23.622	-3.521	1.00	0.00	RX1	C
ATOM	4040	CD1	ILE	1160	39.936	24.689	-3.456	1.00	0.00	RX1	C
ATOM	4041	C	ILE	1160	38.128	23.177	-7.303	1.00	0.00	RX1	C
ATOM	4042	O	ILE	1160	38.878	23.394	-8.248	1.00	0.00	RX1	O
ATOM	4043	N	TYR	1161	37.158	22.253	-7.321	1.00	0.00	RX1	N
ATOM	4044	H	TYR	1161	36.429	22.226	-6.630	1.00	0.00	RX1	H
ATOM	4045	CA	TYR	1161	37.169	21.295	-8.424	1.00	0.00	RX1	C
ATOM	4046	CB	TYR	1161	38.107	20.131	-8.136	1.00	0.00	RX1	C
ATOM	4047	CG	TYR	1161	39.356	20.307	-8.957	1.00	0.00	RX1	C
ATOM	4048	CD1	TYR	1161	39.257	20.425	-10.337	1.00	0.00	RX1	C
ATOM	4049	CE1	TYR	1161	40.403	20.609	-11.096	1.00	0.00	RX1	C
ATOM	4050	CD2	TYR	1161	40.596	20.363	-8.336	1.00	0.00	RX1	C
ATOM	4051	CE2	TYR	1161	41.740	20.547	-9.098	1.00	0.00	RX1	C
ATOM	4052	CZ	TYR	1161	41.647	20.667	-10.478	1.00	0.00	RX1	C
ATOM	4053	OH	TYR	1161	42.788	20.832	-11.242	1.00	0.00	RX1	O
ATOM	4054	HH	TYR	1161	43.492	21.028	-10.614	1.00	0.00	RX1	H
ATOM	4055	C	TYR	1161	35.818	20.764	-8.832	1.00	0.00	RX1	C
ATOM	4056	O	TYR	1161	35.638	19.603	-9.178	1.00	0.00	RX1	O
ATOM	4057	N	GLU	1162	34.858	21.688	-8.819	1.00	0.00	RX1	N
ATOM	4058	H	GLU	1162	35.038	22.601	-8.442	1.00	0.00	RX1	H
ATOM	4059	CA	GLU	1162	33.493	21.299	-9.165	1.00	0.00	RX1	C
ATOM	4060	CB	GLU	1162	32.538	22.460	-8.855	1.00	0.00	RX1	C
ATOM	4061	CG	GLU	1162	32.476	22.864	-7.366	1.00	0.00	RX1	C
ATOM	4062	CD	GLU	1162	33.756	23.544	-6.896	1.00	0.00	RX1	C
ATOM	4063	OE1	GLU	1162	34.536	22.930	-6.172	1.00	0.00	RX1	O
ATOM	4064	OE2	GLU	1162	33.996	24.684	-7.273	1.00	0.00	RX1	O
ATOM	4065	C	GLU	1162	33.329	20.741	-10.582	1.00	0.00	RX1	C
ATOM	4066	O	GLU	1162	32.409	19.998	-10.903	1.00	0.00	RX1	O
ATOM	4067	N	THR	1163	34.314	21.094	-11.420	1.00	0.00	RX1	N
ATOM	4068	H	THR	1163	35.002	21.759	-11.135	1.00	0.00	RX1	H
ATOM	4069	CA	THR	1163	34.407	20.555	-12.778	1.00	0.00	RX1	C
ATOM	4070	CB	THR	1163	35.532	21.365	-13.385	1.00	0.00	RX1	C
ATOM	4071	OG1	THR	1163	35.566	22.619	-12.685	1.00	0.00	RX1	O
ATOM	4072	HG1	THR	1163	36.124	23.200	-13.187	1.00	0.00	RX1	H
ATOM	4073	CG2	THR	1163	35.386	21.564	-14.895	1.00	0.00	RX1	C
ATOM	4074	C	THR	1163	34.584	19.041	-12.909	1.00	0.00	RX1	C
ATOM	4075	O	THR	1163	34.346	18.445	-13.956	1.00	0.00	RX1	O
ATOM	4076	N	ASP	1164	34.984	18.429	-11.786	1.00	0.00	RX1	N
ATOM	4077	H	ASP	1164	35.174	18.933	-10.941	1.00	0.00	RX1	H
ATOM	4078	CA	ASP	1164	35.056	16.968	-11.740	1.00	0.00	RX1	C
ATOM	4079	CB	ASP	1164	35.881	16.535	-10.526	1.00	0.00	RX1	C
ATOM	4080	CG	ASP	1164	37.374	16.570	-10.804	1.00	0.00	RX1	C
ATOM	4081	OD1	ASP	1164	37.793	17.054	-11.849	1.00	0.00	RX1	O
ATOM	4082	OD2	ASP	1164	38.145	16.051	-10.000	1.00	0.00	RX1	O
ATOM	4083	C	ASP	1164	33.703	16.264	-11.696	1.00	0.00	RX1	C
ATOM	4084	O	ASP	1164	33.587	15.045	-11.797	1.00	0.00	RX1	O
ATOM	4085	N	TYR	1165	32.666	17.080	-11.491	1.00	0.00	RX1	N

ATOM	4086	H	TYR	1165	32.717	18.079	-11.548	1.00	0.00	RX1	H
ATOM	4087	CA	TYR	1165	31.385	16.480	-11.152	1.00	0.00	RX1	C
ATOM	4088	CB	TYR	1165	30.847	17.182	-9.907	1.00	0.00	RX1	C
ATOM	4089	CG	TYR	1165	31.754	17.027	-8.701	1.00	0.00	RX1	C
ATOM	4090	CD1	TYR	1165	32.870	17.837	-8.519	1.00	0.00	RX1	C
ATOM	4091	CE1	TYR	1165	33.686	17.679	-7.402	1.00	0.00	RX1	C
ATOM	4092	CD2	TYR	1165	31.454	16.071	-7.744	1.00	0.00	RX1	C
ATOM	4093	CE2	TYR	1165	32.267	15.913	-6.629	1.00	0.00	RX1	C
ATOM	4094	CZ	TYR	1165	33.388	16.707	-6.454	1.00	0.00	RX1	C
ATOM	4095	OH	TYR	1165	34.203	16.523	-5.353	1.00	0.00	RX1	O
ATOM	4096	HH	TYR	1165	33.872	15.806	-4.801	1.00	0.00	RX1	H
ATOM	4097	C	TYR	1165	30.384	16.537	-12.302	1.00	0.00	RX1	C
ATOM	4098	O	TYR	1165	30.610	17.161	-13.333	1.00	0.00	RX1	O
ATOM	4099	N	TYR	1166	29.244	15.867	-12.079	1.00	0.00	RX1	N
ATOM	4100	H	TYR	1166	29.130	15.272	-11.279	1.00	0.00	RX1	H
ATOM	4101	CA	TYR	1166	28.089	16.037	-12.963	1.00	0.00	RX1	C
ATOM	4102	CB	TYR	1166	28.124	14.989	-14.084	1.00	0.00	RX1	C
ATOM	4103	CG	TYR	1166	26.748	14.548	-14.538	1.00	0.00	RX1	C
ATOM	4104	CD1	TYR	1166	25.905	15.388	-15.260	1.00	0.00	RX1	C
ATOM	4105	CE1	TYR	1166	24.637	14.948	-15.634	1.00	0.00	RX1	C
ATOM	4106	CD2	TYR	1166	26.328	13.262	-14.231	1.00	0.00	RX1	C
ATOM	4107	CE2	TYR	1166	25.076	12.814	-14.628	1.00	0.00	RX1	C
ATOM	4108	CZ	TYR	1166	24.216	13.662	-15.308	1.00	0.00	RX1	C
ATOM	4109	OH	TYR	1166	22.948	13.222	-15.634	1.00	0.00	RX1	O
ATOM	4110	HH	TYR	1166	23.026	12.561	-16.331	1.00	0.00	RX1	H
ATOM	4111	C	TYR	1166	26.833	15.942	-12.125	1.00	0.00	RX1	C
ATOM	4112	O	TYR	1166	26.731	15.091	-11.251	1.00	0.00	RX1	O
ATOM	4113	N	ARG	1167	25.892	16.856	-12.418	1.00	0.00	RX1	N
ATOM	4114	H	ARG	1167	25.989	17.450	-13.216	1.00	0.00	RX1	H
ATOM	4115	CA	ARG	1167	24.652	16.860	-11.647	1.00	0.00	RX1	C
ATOM	4116	CB	ARG	1167	24.069	18.276	-11.616	1.00	0.00	RX1	C
ATOM	4117	CG	ARG	1167	23.017	18.406	-10.520	1.00	0.00	RX1	C
ATOM	4118	CD	ARG	1167	22.394	19.792	-10.358	1.00	0.00	RX1	C
ATOM	4119	NE	ARG	1167	21.292	19.657	-9.413	1.00	0.00	RX1	N
ATOM	4120	HE	ARG	1167	21.017	18.710	-9.214	1.00	0.00	RX1	H
ATOM	4121	CZ	ARG	1167	20.643	20.694	-8.817	1.00	0.00	RX1	C
ATOM	4122	NH1	ARG	1167	21.047	21.962	-9.053	1.00	0.00	RX1	N
ATOM	4123	HH11	ARG	1167	20.604	22.760	-8.635	1.00	0.00	RX1	H
ATOM	4124	HH12	ARG	1167	21.821	22.148	-9.666	1.00	0.00	RX1	H
ATOM	4125	NH2	ARG	1167	19.608	20.404	-7.998	1.00	0.00	RX1	N
ATOM	4126	HH21	ARG	1167	19.091	21.085	-7.473	1.00	0.00	RX1	H
ATOM	4127	HH22	ARG	1167	19.321	19.438	-7.889	1.00	0.00	RX1	H
ATOM	4128	C	ARG	1167	23.623	15.826	-12.082	1.00	0.00	RX1	C
ATOM	4129	O	ARG	1167	22.641	16.119	-12.753	1.00	0.00	RX1	O
ATOM	4130	N	LYS	1168	23.921	14.591	-11.655	1.00	0.00	RX1	N
ATOM	4131	H	LYS	1168	24.744	14.481	-11.094	1.00	0.00	RX1	H
ATOM	4132	CA	LYS	1168	23.133	13.404	-11.994	1.00	0.00	RX1	C
ATOM	4133	CB	LYS	1168	23.610	12.278	-11.082	1.00	0.00	RX1	C
ATOM	4134	CG	LYS	1168	22.925	10.923	-11.222	1.00	0.00	RX1	C
ATOM	4135	CD	LYS	1168	23.430	9.996	-10.117	1.00	0.00	RX1	C
ATOM	4136	CE	LYS	1168	22.649	8.691	-10.020	1.00	0.00	RX1	C
ATOM	4137	NZ	LYS	1168	22.843	7.887	-11.232	1.00	0.00	RX1	N
ATOM	4138	HZ1	LYS	1168	22.308	6.998	-11.132	1.00	0.00	RX1	H
ATOM	4139	HZ2	LYS	1168	22.541	8.386	-12.087	1.00	0.00	RX1	H
ATOM	4140	HZ3	LYS	1168	23.844	7.612	-11.323	1.00	0.00	RX1	H
ATOM	4141	C	LYS	1168	21.617	13.559	-11.963	1.00	0.00	RX1	C
ATOM	4142	O	LYS	1168	20.968	13.486	-10.924	1.00	0.00	RX1	O
ATOM	4143	N	GLY	1169	21.067	13.784	-13.168	1.00	0.00	RX1	N
ATOM	4144	H	GLY	1169	21.658	13.844	-13.979	1.00	0.00	RX1	H
ATOM	4145	CA	GLY	1169	19.613	13.949	-13.279	1.00	0.00	RX1	C
ATOM	4146	C	GLY	1169	19.010	14.991	-12.340	1.00	0.00	RX1	C

ATOM	4147	O	GLY	1169	17.937	14.831	-11.754	1.00	0.00	RX1	O
ATOM	4148	N	GLY	1170	19.776	16.080	-12.183	1.00	0.00	RX1	N
ATOM	4149	H	GLY	1170	20.691	16.114	-12.595	1.00	0.00	RX1	H
ATOM	4150	CA	GLY	1170	19.313	17.145	-11.297	1.00	0.00	RX1	C
ATOM	4151	C	GLY	1170	19.458	16.889	-9.800	1.00	0.00	RX1	C
ATOM	4152	O	GLY	1170	19.308	17.797	-8.987	1.00	0.00	RX1	O
ATOM	4153	N	LYS	1171	19.733	15.624	-9.464	1.00	0.00	RX1	N
ATOM	4154	H	LYS	1171	20.060	14.960	-10.137	1.00	0.00	RX1	H
ATOM	4155	CA	LYS	1171	20.036	15.301	-8.072	1.00	0.00	RX1	C
ATOM	4156	CB	LYS	1171	19.463	13.936	-7.679	1.00	0.00	RX1	C
ATOM	4157	CG	LYS	1171	18.060	13.547	-8.163	1.00	0.00	RX1	C
ATOM	4158	CD	LYS	1171	16.884	14.354	-7.605	1.00	0.00	RX1	C
ATOM	4159	CE	LYS	1171	16.438	15.494	-8.522	1.00	0.00	RX1	C
ATOM	4160	NZ	LYS	1171	16.184	14.952	-9.866	1.00	0.00	RX1	N
ATOM	4161	HZ1	LYS	1171	15.774	15.679	-10.482	1.00	0.00	RX1	H
ATOM	4162	HZ2	LYS	1171	17.077	14.645	-10.312	1.00	0.00	RX1	H
ATOM	4163	HZ3	LYS	1171	15.543	14.138	-9.811	1.00	0.00	RX1	H
ATOM	4164	C	LYS	1171	21.547	15.214	-7.975	1.00	0.00	RX1	C
ATOM	4165	O	LYS	1171	22.240	15.559	-8.927	1.00	0.00	RX1	O
ATOM	4166	N	GLY	1172	22.018	14.682	-6.839	1.00	0.00	RX1	N
ATOM	4167	H	GLY	1172	21.458	14.750	-6.001	1.00	0.00	RX1	H
ATOM	4168	CA	GLY	1172	23.210	13.827	-6.821	1.00	0.00	RX1	C
ATOM	4169	C	GLY	1172	24.368	14.055	-7.785	1.00	0.00	RX1	C
ATOM	4170	O	GLY	1172	24.924	13.124	-8.348	1.00	0.00	RX1	O
ATOM	4171	N	LEU	1173	24.760	15.328	-7.897	1.00	0.00	RX1	N
ATOM	4172	H	LEU	1173	24.097	16.021	-7.627	1.00	0.00	RX1	H
ATOM	4173	CA	LEU	1173	26.069	15.655	-8.464	1.00	0.00	RX1	C
ATOM	4174	CB	LEU	1173	26.241	17.180	-8.473	1.00	0.00	RX1	C
ATOM	4175	CG	LEU	1173	27.621	17.791	-8.682	1.00	0.00	RX1	C
ATOM	4176	CD1	LEU	1173	27.551	18.990	-9.630	1.00	0.00	RX1	C
ATOM	4177	CD2	LEU	1173	28.277	18.187	-7.354	1.00	0.00	RX1	C
ATOM	4178	C	LEU	1173	27.201	14.914	-7.780	1.00	0.00	RX1	C
ATOM	4179	O	LEU	1173	27.407	15.015	-6.576	1.00	0.00	RX1	O
ATOM	4180	N	LEU	1174	27.881	14.134	-8.620	1.00	0.00	RX1	N
ATOM	4181	H	LEU	1174	27.682	14.149	-9.603	1.00	0.00	RX1	H
ATOM	4182	CA	LEU	1174	28.885	13.189	-8.143	1.00	0.00	RX1	C
ATOM	4183	CB	LEU	1174	28.256	11.788	-8.173	1.00	0.00	RX1	C
ATOM	4184	CG	LEU	1174	27.281	11.477	-7.029	1.00	0.00	RX1	C
ATOM	4185	CD1	LEU	1174	26.383	10.281	-7.351	1.00	0.00	RX1	C
ATOM	4186	CD2	LEU	1174	27.996	11.288	-5.692	1.00	0.00	RX1	C
ATOM	4187	C	LEU	1174	30.086	13.284	-9.072	1.00	0.00	RX1	C
ATOM	4188	O	LEU	1174	29.935	13.738	-10.204	1.00	0.00	RX1	O
ATOM	4189	N	PRO	1175	31.291	12.888	-8.582	1.00	0.00	RX1	N
ATOM	4190	CD	PRO	1175	31.603	12.378	-7.248	1.00	0.00	RX1	C
ATOM	4191	CA	PRO	1175	32.481	12.960	-9.443	1.00	0.00	RX1	C
ATOM	4192	CB	PRO	1175	33.615	12.878	-8.424	1.00	0.00	RX1	C
ATOM	4193	CG	PRO	1175	33.070	11.973	-7.319	1.00	0.00	RX1	C
ATOM	4194	C	PRO	1175	32.538	11.819	-10.449	1.00	0.00	RX1	C
ATOM	4195	O	PRO	1175	33.321	10.885	-10.338	1.00	0.00	RX1	O
ATOM	4196	N	VAL	1176	31.646	11.922	-11.439	1.00	0.00	RX1	N
ATOM	4197	H	VAL	1176	31.103	12.761	-11.492	1.00	0.00	RX1	H
ATOM	4198	CA	VAL	1176	31.294	10.735	-12.221	1.00	0.00	RX1	C
ATOM	4199	CB	VAL	1176	30.213	11.096	-13.235	1.00	0.00	RX1	C
ATOM	4200	CG1	VAL	1176	28.955	11.526	-12.479	1.00	0.00	RX1	C
ATOM	4201	CG2	VAL	1176	30.686	12.159	-14.228	1.00	0.00	RX1	C
ATOM	4202	C	VAL	1176	32.422	9.927	-12.852	1.00	0.00	RX1	C
ATOM	4203	O	VAL	1176	32.378	8.707	-12.926	1.00	0.00	RX1	O
ATOM	4204	N	ARG	1177	33.462	10.659	-13.280	1.00	0.00	RX1	N
ATOM	4205	H	ARG	1177	33.524	11.627	-13.043	1.00	0.00	RX1	H
ATOM	4206	CA	ARG	1177	34.557	9.967	-13.964	1.00	0.00	RX1	C
ATOM	4207	CB	ARG	1177	35.413	10.978	-14.728	1.00	0.00	RX1	C

ATOM	4208	CG	ARG	1177	34.605	11.927	-15.615	1.00	0.00	RX1	C
ATOM	4209	CD	ARG	1177	35.495	12.936	-16.344	1.00	0.00	RX1	C
ATOM	4210	NE	ARG	1177	34.735	13.720	-17.314	1.00	0.00	RX1	N
ATOM	4211	HE	ARG	1177	34.007	14.318	-16.959	1.00	0.00	RX1	H
ATOM	4212	CZ	ARG	1177	34.961	13.618	-18.662	1.00	0.00	RX1	C
ATOM	4213	NH1	ARG	1177	35.913	12.808	-19.161	1.00	0.00	RX1	N
ATOM	4214	HH11	ARG	1177	36.122	12.757	-20.149	1.00	0.00	RX1	H
ATOM	4215	HH12	ARG	1177	36.481	12.205	-18.582	1.00	0.00	RX1	H
ATOM	4216	NH2	ARG	1177	34.197	14.354	-19.486	1.00	0.00	RX1	N
ATOM	4217	HH21	ARG	1177	34.258	14.269	-20.492	1.00	0.00	RX1	H
ATOM	4218	HH22	ARG	1177	33.517	14.994	-19.108	1.00	0.00	RX1	H
ATOM	4219	C	ARG	1177	35.425	9.070	-13.085	1.00	0.00	RX1	C
ATOM	4220	O	ARG	1177	36.275	8.330	-13.556	1.00	0.00	RX1	O
ATOM	4221	N	TRP	1178	35.177	9.185	-11.773	1.00	0.00	RX1	N
ATOM	4222	H	TRP	1178	34.420	9.733	-11.419	1.00	0.00	RX1	H
ATOM	4223	CA	TRP	1178	35.961	8.426	-10.805	1.00	0.00	RX1	C
ATOM	4224	CB	TRP	1178	36.379	9.339	-9.645	1.00	0.00	RX1	C
ATOM	4225	CG	TRP	1178	37.371	10.365	-10.134	1.00	0.00	RX1	C
ATOM	4226	CD2	TRP	1178	37.130	11.550	-10.922	1.00	0.00	RX1	C
ATOM	4227	CE2	TRP	1178	38.381	12.144	-11.191	1.00	0.00	RX1	C
ATOM	4228	CE3	TRP	1178	35.977	12.140	-11.423	1.00	0.00	RX1	C
ATOM	4229	CD1	TRP	1178	38.760	10.324	-9.956	1.00	0.00	RX1	C
ATOM	4230	NE1	TRP	1178	39.357	11.372	-10.578	1.00	0.00	RX1	N
ATOM	4231	HE1	TRP	1178	40.324	11.569	-10.601	1.00	0.00	RX1	H
ATOM	4232	CZ2	TRP	1178	38.445	13.290	-11.967	1.00	0.00	RX1	C
ATOM	4233	CZ3	TRP	1178	36.054	13.289	-12.195	1.00	0.00	RX1	C
ATOM	4234	CH2	TRP	1178	37.287	13.857	-12.477	1.00	0.00	RX1	C
ATOM	4235	C	TRP	1178	35.218	7.227	-10.250	1.00	0.00	RX1	C
ATOM	4236	O	TRP	1178	35.702	6.516	-9.379	1.00	0.00	RX1	O
ATOM	4237	N	MET	1179	33.977	7.072	-10.725	1.00	0.00	RX1	N
ATOM	4238	H	MET	1179	33.633	7.462	-11.582	1.00	0.00	RX1	H
ATOM	4239	CA	MET	1179	33.106	6.263	-9.888	1.00	0.00	RX1	C
ATOM	4240	CB	MET	1179	31.815	7.019	-9.611	1.00	0.00	RX1	C
ATOM	4241	CG	MET	1179	32.088	8.313	-8.852	1.00	0.00	RX1	C
ATOM	4242	SD	MET	1179	30.679	9.421	-8.905	1.00	0.00	RX1	S
ATOM	4243	CE	MET	1179	29.453	8.241	-8.350	1.00	0.00	RX1	C
ATOM	4244	C	MET	1179	32.827	4.867	-10.385	1.00	0.00	RX1	C
ATOM	4245	O	MET	1179	32.801	4.563	-11.569	1.00	0.00	RX1	O
ATOM	4246	N	SER	1180	32.608	4.028	-9.375	1.00	0.00	RX1	N
ATOM	4247	H	SER	1180	32.657	4.362	-8.436	1.00	0.00	RX1	H
ATOM	4248	CA	SER	1180	32.222	2.644	-9.605	1.00	0.00	RX1	C
ATOM	4249	CB	SER	1180	32.378	2.002	-8.240	1.00	0.00	RX1	C
ATOM	4250	OG	SER	1180	32.318	3.036	-7.252	1.00	0.00	RX1	O
ATOM	4251	HG	SER	1180	31.434	2.974	-6.882	1.00	0.00	RX1	H
ATOM	4252	C	SER	1180	30.826	2.510	-10.193	1.00	0.00	RX1	C
ATOM	4253	O	SER	1180	29.970	3.359	-9.970	1.00	0.00	RX1	O
ATOM	4254	N	PRO	1181	30.607	1.402	-10.950	1.00	0.00	RX1	N
ATOM	4255	CD	PRO	1181	31.586	0.374	-11.277	1.00	0.00	RX1	C
ATOM	4256	CA	PRO	1181	29.287	1.117	-11.526	1.00	0.00	RX1	C
ATOM	4257	CB	PRO	1181	29.413	-0.367	-11.878	1.00	0.00	RX1	C
ATOM	4258	CG	PRO	1181	30.876	-0.537	-12.270	1.00	0.00	RX1	C
ATOM	4259	C	PRO	1181	28.093	1.431	-10.636	1.00	0.00	RX1	C
ATOM	4260	O	PRO	1181	27.253	2.263	-10.953	1.00	0.00	RX1	O
ATOM	4261	N	GLU	1182	28.061	0.737	-9.492	1.00	0.00	RX1	N
ATOM	4262	H	GLU	1182	28.800	0.098	-9.283	1.00	0.00	RX1	H
ATOM	4263	CA	GLU	1182	27.016	0.940	-8.488	1.00	0.00	RX1	C
ATOM	4264	CB	GLU	1182	27.256	0.016	-7.272	1.00	0.00	RX1	C
ATOM	4265	CG	GLU	1182	28.406	0.303	-6.280	1.00	0.00	RX1	C
ATOM	4266	CD	GLU	1182	29.796	-0.027	-6.813	1.00	0.00	RX1	C
ATOM	4267	OE1	GLU	1182	29.932	-0.547	-7.913	1.00	0.00	RX1	O
ATOM	4268	OE2	GLU	1182	30.774	0.211	-6.113	1.00	0.00	RX1	O

ATOM	4269	C	GLU	1182	26.794	2.397	-8.092	1.00	0.00	RX1	C
ATOM	4270	O	GLU	1182	25.688	2.931	-8.071	1.00	0.00	RX1	O
ATOM	4271	N	SER	1183	27.935	3.044	-7.848	1.00	0.00	RX1	N
ATOM	4272	H	SER	1183	28.831	2.597	-7.876	1.00	0.00	RX1	H
ATOM	4273	CA	SER	1183	27.900	4.457	-7.508	1.00	0.00	RX1	C
ATOM	4274	CB	SER	1183	29.290	4.720	-6.971	1.00	0.00	RX1	C
ATOM	4275	OG	SER	1183	29.705	3.492	-6.357	1.00	0.00	RX1	O
ATOM	4276	HG	SER	1183	29.145	3.383	-5.586	1.00	0.00	RX1	H
ATOM	4277	C	SER	1183	27.415	5.368	-8.634	1.00	0.00	RX1	C
ATOM	4278	O	SER	1183	26.813	6.412	-8.426	1.00	0.00	RX1	O
ATOM	4279	N	LEU	1184	27.651	4.910	-9.865	1.00	0.00	RX1	N
ATOM	4280	H	LEU	1184	28.134	4.048	-10.031	1.00	0.00	RX1	H
ATOM	4281	CA	LEU	1184	27.041	5.652	-10.961	1.00	0.00	RX1	C
ATOM	4282	CB	LEU	1184	27.801	5.401	-12.257	1.00	0.00	RX1	C
ATOM	4283	CG	LEU	1184	29.162	6.085	-12.214	1.00	0.00	RX1	C
ATOM	4284	CD1	LEU	1184	30.009	5.765	-13.438	1.00	0.00	RX1	C
ATOM	4285	CD2	LEU	1184	29.005	7.590	-12.026	1.00	0.00	RX1	C
ATOM	4286	C	LEU	1184	25.555	5.398	-11.125	1.00	0.00	RX1	C
ATOM	4287	O	LEU	1184	24.800	6.272	-11.547	1.00	0.00	RX1	O
ATOM	4288	N	LYS	1185	25.145	4.176	-10.744	1.00	0.00	RX1	N
ATOM	4289	H	LYS	1185	25.798	3.521	-10.359	1.00	0.00	RX1	H
ATOM	4290	CA	LYS	1185	23.719	3.863	-10.832	1.00	0.00	RX1	C
ATOM	4291	CB	LYS	1185	23.464	2.353	-10.718	1.00	0.00	RX1	C
ATOM	4292	CG	LYS	1185	24.341	1.670	-11.768	1.00	0.00	RX1	C
ATOM	4293	CD	LYS	1185	24.244	0.159	-11.996	1.00	0.00	RX1	C
ATOM	4294	CE	LYS	1185	25.524	-0.218	-12.745	1.00	0.00	RX1	C
ATOM	4295	NZ	LYS	1185	25.461	-1.451	-13.537	1.00	0.00	RX1	N
ATOM	4296	HZ1	LYS	1185	26.294	-1.487	-14.169	1.00	0.00	RX1	H
ATOM	4297	HZ2	LYS	1185	25.487	-2.348	-13.013	1.00	0.00	RX1	H
ATOM	4298	HZ3	LYS	1185	24.705	-1.461	-14.258	1.00	0.00	RX1	H
ATOM	4299	C	LYS	1185	22.872	4.707	-9.897	1.00	0.00	RX1	C
ATOM	4300	O	LYS	1185	22.100	5.546	-10.356	1.00	0.00	RX1	O
ATOM	4301	N	ASP	1186	23.095	4.535	-8.588	1.00	0.00	RX1	N
ATOM	4302	H	ASP	1186	23.727	3.850	-8.215	1.00	0.00	RX1	H
ATOM	4303	CA	ASP	1186	22.467	5.529	-7.713	1.00	0.00	RX1	C
ATOM	4304	CB	ASP	1186	21.502	4.909	-6.700	1.00	0.00	RX1	C
ATOM	4305	CG	ASP	1186	22.233	4.734	-5.393	1.00	0.00	RX1	C
ATOM	4306	OD1	ASP	1186	22.024	5.533	-4.481	1.00	0.00	RX1	O
ATOM	4307	OD2	ASP	1186	23.073	3.845	-5.319	1.00	0.00	RX1	O
ATOM	4308	C	ASP	1186	23.457	6.461	-7.027	1.00	0.00	RX1	C
ATOM	4309	O	ASP	1186	23.216	7.647	-6.809	1.00	0.00	RX1	O
ATOM	4310	N	GLY	1187	24.572	5.842	-6.637	1.00	0.00	RX1	N
ATOM	4311	H	GLY	1187	24.598	4.842	-6.692	1.00	0.00	RX1	H
ATOM	4312	CA	GLY	1187	25.454	6.558	-5.728	1.00	0.00	RX1	C
ATOM	4313	C	GLY	1187	25.278	6.142	-4.286	1.00	0.00	RX1	C
ATOM	4314	O	GLY	1187	25.066	6.974	-3.407	1.00	0.00	RX1	O
ATOM	4315	N	VAL	1188	25.402	4.829	-4.087	1.00	0.00	RX1	N
ATOM	4316	H	VAL	1188	25.325	4.183	-4.847	1.00	0.00	RX1	H
ATOM	4317	CA	VAL	1188	25.835	4.324	-2.792	1.00	0.00	RX1	C
ATOM	4318	CB	VAL	1188	25.117	3.000	-2.495	1.00	0.00	RX1	C
ATOM	4319	CG1	VAL	1188	25.837	2.122	-1.469	1.00	0.00	RX1	C
ATOM	4320	CG2	VAL	1188	23.675	3.288	-2.071	1.00	0.00	RX1	C
ATOM	4321	C	VAL	1188	27.342	4.161	-2.847	1.00	0.00	RX1	C
ATOM	4322	O	VAL	1188	27.919	3.818	-3.875	1.00	0.00	RX1	O
ATOM	4323	N	PHE	1189	27.953	4.471	-1.698	1.00	0.00	RX1	N
ATOM	4324	H	PHE	1189	27.462	4.737	-0.871	1.00	0.00	RX1	H
ATOM	4325	CA	PHE	1189	29.397	4.304	-1.635	1.00	0.00	RX1	C
ATOM	4326	CB	PHE	1189	30.112	5.643	-1.460	1.00	0.00	RX1	C
ATOM	4327	CG	PHE	1189	30.322	6.353	-2.775	1.00	0.00	RX1	C
ATOM	4328	CD1	PHE	1189	29.256	6.936	-3.448	1.00	0.00	RX1	C
ATOM	4329	CD2	PHE	1189	31.606	6.441	-3.299	1.00	0.00	RX1	C

ATOM	4330	CE1	PHE	1189	29.483	7.640	-4.624	1.00	0.00	RX1	C
ATOM	4331	CE2	PHE	1189	31.834	7.141	-4.477	1.00	0.00	RX1	C
ATOM	4332	CZ	PHE	1189	30.773	7.757	-5.126	1.00	0.00	RX1	C
ATOM	4333	C	PHE	1189	29.808	3.390	-0.501	1.00	0.00	RX1	C
ATOM	4334	O	PHE	1189	29.711	3.717	0.675	1.00	0.00	RX1	O
ATOM	4335	N	THR	1190	30.283	2.217	-0.907	1.00	0.00	RX1	N
ATOM	4336	H	THR	1190	30.390	2.037	-1.882	1.00	0.00	RX1	H
ATOM	4337	CA	THR	1190	31.034	1.432	0.066	1.00	0.00	RX1	C
ATOM	4338	CB	THR	1190	31.008	0.001	-0.462	1.00	0.00	RX1	C
ATOM	4339	OG1	THR	1190	31.513	-0.039	-1.806	1.00	0.00	RX1	O
ATOM	4340	HG1	THR	1190	30.873	0.369	-2.381	1.00	0.00	RX1	H
ATOM	4341	CG2	THR	1190	29.593	-0.576	-0.426	1.00	0.00	RX1	C
ATOM	4342	C	THR	1190	32.443	2.011	0.180	1.00	0.00	RX1	C
ATOM	4343	O	THR	1190	32.809	2.879	-0.605	1.00	0.00	RX1	O
ATOM	4344	N	THR	1191	33.256	1.456	1.095	1.00	0.00	RX1	N
ATOM	4345	H	THR	1191	32.887	0.917	1.853	1.00	0.00	RX1	H
ATOM	4346	CA	THR	1191	34.699	1.716	0.958	1.00	0.00	RX1	C
ATOM	4347	CB	THR	1191	35.436	1.027	2.133	1.00	0.00	RX1	C
ATOM	4348	OG1	THR	1191	36.831	1.330	2.140	1.00	0.00	RX1	O
ATOM	4349	HG1	THR	1191	37.262	0.598	2.580	1.00	0.00	RX1	H
ATOM	4350	CG2	THR	1191	35.222	-0.486	2.185	1.00	0.00	RX1	C
ATOM	4351	C	THR	1191	35.219	1.338	-0.430	1.00	0.00	RX1	C
ATOM	4352	O	THR	1191	36.022	2.001	-1.076	1.00	0.00	RX1	O
ATOM	4353	N	TYR	1192	34.637	0.229	-0.903	1.00	0.00	RX1	N
ATOM	4354	H	TYR	1192	33.922	-0.232	-0.382	1.00	0.00	RX1	H
ATOM	4355	CA	TYR	1192	35.046	-0.312	-2.189	1.00	0.00	RX1	C
ATOM	4356	CB	TYR	1192	34.389	-1.670	-2.380	1.00	0.00	RX1	C
ATOM	4357	CG	TYR	1192	34.592	-2.525	-1.150	1.00	0.00	RX1	C
ATOM	4358	CD1	TYR	1192	35.860	-2.996	-0.827	1.00	0.00	RX1	C
ATOM	4359	CE1	TYR	1192	36.043	-3.768	0.316	1.00	0.00	RX1	C
ATOM	4360	CD2	TYR	1192	33.508	-2.840	-0.337	1.00	0.00	RX1	C
ATOM	4361	CE2	TYR	1192	33.694	-3.605	0.809	1.00	0.00	RX1	C
ATOM	4362	CZ	TYR	1192	34.963	-4.066	1.140	1.00	0.00	RX1	C
ATOM	4363	OH	TYR	1192	35.151	-4.821	2.278	1.00	0.00	RX1	O
ATOM	4364	HH	TYR	1192	34.923	-4.299	3.054	1.00	0.00	RX1	H
ATOM	4365	C	TYR	1192	34.783	0.592	-3.381	1.00	0.00	RX1	C
ATOM	4366	O	TYR	1192	35.432	0.495	-4.417	1.00	0.00	RX1	O
ATOM	4367	N	SER	1193	33.816	1.504	-3.221	1.00	0.00	RX1	N
ATOM	4368	H	SER	1193	33.249	1.546	-2.401	1.00	0.00	RX1	H
ATOM	4369	CA	SER	1193	33.715	2.516	-4.273	1.00	0.00	RX1	C
ATOM	4370	CB	SER	1193	32.379	3.195	-4.134	1.00	0.00	RX1	C
ATOM	4371	OG	SER	1193	31.771	2.586	-3.004	1.00	0.00	RX1	O
ATOM	4372	HG	SER	1193	32.162	3.059	-2.271	1.00	0.00	RX1	H
ATOM	4373	C	SER	1193	34.912	3.441	-4.329	1.00	0.00	RX1	C
ATOM	4374	O	SER	1193	35.539	3.603	-5.366	1.00	0.00	RX1	O
ATOM	4375	N	ASP	1194	35.261	3.968	-3.144	1.00	0.00	RX1	N
ATOM	4376	H	ASP	1194	34.771	3.822	-2.284	1.00	0.00	RX1	H
ATOM	4377	CA	ASP	1194	36.474	4.784	-3.100	1.00	0.00	RX1	C
ATOM	4378	CB	ASP	1194	36.625	5.473	-1.737	1.00	0.00	RX1	C
ATOM	4379	CG	ASP	1194	35.463	6.421	-1.457	1.00	0.00	RX1	C
ATOM	4380	OD1	ASP	1194	35.354	7.464	-2.107	1.00	0.00	RX1	O
ATOM	4381	OD2	ASP	1194	34.683	6.144	-0.546	1.00	0.00	RX1	O
ATOM	4382	C	ASP	1194	37.740	4.033	-3.507	1.00	0.00	RX1	C
ATOM	4383	O	ASP	1194	38.683	4.607	-4.034	1.00	0.00	RX1	O
ATOM	4384	N	VAL	1195	37.707	2.697	-3.321	1.00	0.00	RX1	N
ATOM	4385	H	VAL	1195	36.965	2.311	-2.773	1.00	0.00	RX1	H
ATOM	4386	CA	VAL	1195	38.751	1.859	-3.930	1.00	0.00	RX1	C
ATOM	4387	CB	VAL	1195	38.625	0.397	-3.481	1.00	0.00	RX1	C
ATOM	4388	CG1	VAL	1195	39.678	-0.483	-4.153	1.00	0.00	RX1	C
ATOM	4389	CG2	VAL	1195	38.701	0.267	-1.960	1.00	0.00	RX1	C
ATOM	4390	C	VAL	1195	38.805	1.944	-5.458	1.00	0.00	RX1	C

ATOM	4391	O	VAL	1195	39.856	2.140	-6.059	1.00	0.00	RX1	O
ATOM	4392	N	TRP	1196	37.617	1.828	-6.080	1.00	0.00	RX1	N
ATOM	4393	H	TRP	1196	36.776	1.746	-5.543	1.00	0.00	RX1	H
ATOM	4394	CA	TRP	1196	37.548	2.046	-7.533	1.00	0.00	RX1	C
ATOM	4395	CB	TRP	1196	36.089	1.898	-7.990	1.00	0.00	RX1	C
ATOM	4396	CG	TRP	1196	35.900	2.027	-9.488	1.00	0.00	RX1	C
ATOM	4397	CD2	TRP	1196	35.510	0.992	-10.415	1.00	0.00	RX1	C
ATOM	4398	CE2	TRP	1196	35.439	1.570	-11.704	1.00	0.00	RX1	C
ATOM	4399	CE3	TRP	1196	35.212	-0.354	-10.252	1.00	0.00	RX1	C
ATOM	4400	CD1	TRP	1196	36.035	3.179	-10.279	1.00	0.00	RX1	C
ATOM	4401	NE1	TRP	1196	35.766	2.916	-11.585	1.00	0.00	RX1	N
ATOM	4402	HE1	TRP	1196	35.782	3.566	-12.321	1.00	0.00	RX1	H
ATOM	4403	CZ2	TRP	1196	35.086	0.786	-12.795	1.00	0.00	RX1	C
ATOM	4404	CZ3	TRP	1196	34.855	-1.125	-11.352	1.00	0.00	RX1	C
ATOM	4405	CH2	TRP	1196	34.796	-0.561	-12.620	1.00	0.00	RX1	C
ATOM	4406	C	TRP	1196	38.135	3.401	-7.921	1.00	0.00	RX1	C
ATOM	4407	O	TRP	1196	39.021	3.531	-8.764	1.00	0.00	RX1	O
ATOM	4408	N	SER	1197	37.627	4.406	-7.198	1.00	0.00	RX1	N
ATOM	4409	H	SER	1197	36.868	4.295	-6.552	1.00	0.00	RX1	H
ATOM	4410	CA	SER	1197	38.137	5.760	-7.363	1.00	0.00	RX1	C
ATOM	4411	CB	SER	1197	37.254	6.603	-6.469	1.00	0.00	RX1	C
ATOM	4412	OG	SER	1197	35.941	6.061	-6.640	1.00	0.00	RX1	O
ATOM	4413	HG	SER	1197	35.783	6.092	-7.581	1.00	0.00	RX1	H
ATOM	4414	C	SER	1197	39.640	5.913	-7.191	1.00	0.00	RX1	C
ATOM	4415	O	SER	1197	40.282	6.658	-7.913	1.00	0.00	RX1	O
ATOM	4416	N	PHE	1198	40.198	5.122	-6.260	1.00	0.00	RX1	N
ATOM	4417	H	PHE	1198	39.619	4.539	-5.690	1.00	0.00	RX1	H
ATOM	4418	CA	PHE	1198	41.652	5.071	-6.096	1.00	0.00	RX1	C
ATOM	4419	CB	PHE	1198	42.022	4.131	-4.949	1.00	0.00	RX1	C
ATOM	4420	CG	PHE	1198	43.519	4.065	-4.800	1.00	0.00	RX1	C
ATOM	4421	CD1	PHE	1198	44.211	5.137	-4.251	1.00	0.00	RX1	C
ATOM	4422	CD2	PHE	1198	44.202	2.929	-5.217	1.00	0.00	RX1	C
ATOM	4423	CE1	PHE	1198	45.590	5.068	-4.113	1.00	0.00	RX1	C
ATOM	4424	CE2	PHE	1198	45.581	2.862	-5.079	1.00	0.00	RX1	C
ATOM	4425	CZ	PHE	1198	46.273	3.930	-4.523	1.00	0.00	RX1	C
ATOM	4426	C	PHE	1198	42.382	4.686	-7.373	1.00	0.00	RX1	C
ATOM	4427	O	PHE	1198	43.339	5.327	-7.794	1.00	0.00	RX1	O
ATOM	4428	N	GLY	1199	41.851	3.631	-8.011	1.00	0.00	RX1	N
ATOM	4429	H	GLY	1199	41.050	3.163	-7.627	1.00	0.00	RX1	H
ATOM	4430	CA	GLY	1199	42.402	3.269	-9.320	1.00	0.00	RX1	C
ATOM	4431	C	GLY	1199	42.362	4.418	-10.320	1.00	0.00	RX1	C
ATOM	4432	O	GLY	1199	43.289	4.683	-11.077	1.00	0.00	RX1	O
ATOM	4433	N	VAL	1200	41.229	5.129	-10.246	1.00	0.00	RX1	N
ATOM	4434	H	VAL	1200	40.540	4.918	-9.551	1.00	0.00	RX1	H
ATOM	4435	CA	VAL	1200	41.100	6.309	-11.098	1.00	0.00	RX1	C
ATOM	4436	CB	VAL	1200	39.648	6.779	-11.124	1.00	0.00	RX1	C
ATOM	4437	CG1	VAL	1200	39.449	7.814	-12.221	1.00	0.00	RX1	C
ATOM	4438	CG2	VAL	1200	38.693	5.602	-11.317	1.00	0.00	RX1	C
ATOM	4439	C	VAL	1200	42.071	7.443	-10.755	1.00	0.00	RX1	C
ATOM	4440	O	VAL	1200	42.578	8.141	-11.622	1.00	0.00	RX1	O
ATOM	4441	N	VAL	1201	42.380	7.561	-9.452	1.00	0.00	RX1	N
ATOM	4442	H	VAL	1201	41.943	6.962	-8.781	1.00	0.00	RX1	H
ATOM	4443	CA	VAL	1201	43.430	8.501	-9.040	1.00	0.00	RX1	C
ATOM	4444	CB	VAL	1201	43.618	8.500	-7.520	1.00	0.00	RX1	C
ATOM	4445	CG1	VAL	1201	44.722	9.472	-7.108	1.00	0.00	RX1	C
ATOM	4446	CG2	VAL	1201	42.316	8.794	-6.785	1.00	0.00	RX1	C
ATOM	4447	C	VAL	1201	44.758	8.187	-9.710	1.00	0.00	RX1	C
ATOM	4448	O	VAL	1201	45.479	9.055	-10.184	1.00	0.00	RX1	O
ATOM	4449	N	LEU	1202	45.033	6.876	-9.768	1.00	0.00	RX1	N
ATOM	4450	H	LEU	1202	44.391	6.215	-9.374	1.00	0.00	RX1	H
ATOM	4451	CA	LEU	1202	46.260	6.450	-10.445	1.00	0.00	RX1	C

ATOM	4452	CB	LEU	1202	46.448	4.945	-10.310	1.00	0.00	RX1	C
ATOM	4453	CG	LEU	1202	46.273	4.472	-8.872	1.00	0.00	RX1	C
ATOM	4454	CD1	LEU	1202	46.311	2.951	-8.770	1.00	0.00	RX1	C
ATOM	4455	CD2	LEU	1202	47.256	5.162	-7.933	1.00	0.00	RX1	C
ATOM	4456	C	LEU	1202	46.304	6.850	-11.910	1.00	0.00	RX1	C
ATOM	4457	O	LEU	1202	47.309	7.308	-12.442	1.00	0.00	RX1	O
ATOM	4458	N	TRP	1203	45.122	6.703	-12.528	1.00	0.00	RX1	N
ATOM	4459	H	TRP	1203	44.354	6.285	-12.039	1.00	0.00	RX1	H
ATOM	4460	CA	TRP	1203	44.939	7.223	-13.883	1.00	0.00	RX1	C
ATOM	4461	CB	TRP	1203	43.514	6.918	-14.331	1.00	0.00	RX1	C
ATOM	4462	CG	TRP	1203	43.314	7.199	-15.797	1.00	0.00	RX1	C
ATOM	4463	CD2	TRP	1203	43.012	8.450	-16.450	1.00	0.00	RX1	C
ATOM	4464	CE2	TRP	1203	42.867	8.184	-17.829	1.00	0.00	RX1	C
ATOM	4465	CE3	TRP	1203	42.850	9.748	-15.985	1.00	0.00	RX1	C
ATOM	4466	CD1	TRP	1203	43.342	6.248	-16.821	1.00	0.00	RX1	C
ATOM	4467	NE1	TRP	1203	43.076	6.825	-18.019	1.00	0.00	RX1	N
ATOM	4468	HE1	TRP	1203	42.992	6.341	-18.870	1.00	0.00	RX1	H
ATOM	4469	CZ2	TRP	1203	42.559	9.217	-18.704	1.00	0.00	RX1	C
ATOM	4470	CZ3	TRP	1203	42.545	10.774	-16.870	1.00	0.00	RX1	C
ATOM	4471	CH2	TRP	1203	42.395	10.509	-18.225	1.00	0.00	RX1	C
ATOM	4472	C	TRP	1203	45.248	8.710	-14.005	1.00	0.00	RX1	C
ATOM	4473	O	TRP	1203	45.933	9.161	-14.920	1.00	0.00	RX1	O
ATOM	4474	N	GLU	1204	44.733	9.463	-13.016	1.00	0.00	RX1	N
ATOM	4475	H	GLU	1204	44.169	9.061	-12.292	1.00	0.00	RX1	H
ATOM	4476	CA	GLU	1204	45.065	10.886	-13.005	1.00	0.00	RX1	C
ATOM	4477	CB	GLU	1204	44.307	11.672	-11.927	1.00	0.00	RX1	C
ATOM	4478	CG	GLU	1204	42.789	11.580	-12.096	1.00	0.00	RX1	C
ATOM	4479	CD	GLU	1204	42.098	12.743	-11.404	1.00	0.00	RX1	C
ATOM	4480	OE1	GLU	1204	41.700	13.675	-12.093	1.00	0.00	RX1	O
ATOM	4481	OE2	GLU	1204	41.909	12.709	-10.190	1.00	0.00	RX1	O
ATOM	4482	C	GLU	1204	46.557	11.143	-12.932	1.00	0.00	RX1	C
ATOM	4483	O	GLU	1204	47.106	11.906	-13.711	1.00	0.00	RX1	O
ATOM	4484	N	ILE	1205	47.225	10.417	-12.023	1.00	0.00	RX1	N
ATOM	4485	H	ILE	1205	46.720	9.796	-11.422	1.00	0.00	RX1	H
ATOM	4486	CA	ILE	1205	48.690	10.517	-11.984	1.00	0.00	RX1	C
ATOM	4487	CB	ILE	1205	49.266	9.520	-10.978	1.00	0.00	RX1	C
ATOM	4488	CG2	ILE	1205	50.791	9.584	-10.970	1.00	0.00	RX1	C
ATOM	4489	CG1	ILE	1205	48.682	9.741	-9.583	1.00	0.00	RX1	C
ATOM	4490	CD1	ILE	1205	49.171	8.701	-8.574	1.00	0.00	RX1	C
ATOM	4491	C	ILE	1205	49.340	10.333	-13.355	1.00	0.00	RX1	C
ATOM	4492	O	ILE	1205	50.097	11.163	-13.846	1.00	0.00	RX1	O
ATOM	4493	N	ALA	1206	48.941	9.216	-13.976	1.00	0.00	RX1	N
ATOM	4494	H	ALA	1206	48.275	8.611	-13.535	1.00	0.00	RX1	H
ATOM	4495	CA	ALA	1206	49.470	8.890	-15.298	1.00	0.00	RX1	C
ATOM	4496	CB	ALA	1206	48.974	7.509	-15.722	1.00	0.00	RX1	C
ATOM	4497	C	ALA	1206	49.170	9.888	-16.411	1.00	0.00	RX1	C
ATOM	4498	O	ALA	1206	49.859	9.940	-17.421	1.00	0.00	RX1	O
ATOM	4499	N	THR	1207	48.113	10.682	-16.202	1.00	0.00	RX1	N
ATOM	4500	H	THR	1207	47.563	10.684	-15.365	1.00	0.00	RX1	H
ATOM	4501	CA	THR	1207	47.799	11.651	-17.249	1.00	0.00	RX1	C
ATOM	4502	CB	THR	1207	46.312	11.599	-17.544	1.00	0.00	RX1	C
ATOM	4503	OG1	THR	1207	45.576	11.394	-16.332	1.00	0.00	RX1	O
ATOM	4504	HG1	THR	1207	45.688	10.471	-16.117	1.00	0.00	RX1	H
ATOM	4505	CG2	THR	1207	45.973	10.543	-18.585	1.00	0.00	RX1	C
ATOM	4506	C	THR	1207	48.213	13.079	-16.967	1.00	0.00	RX1	C
ATOM	4507	O	THR	1207	47.828	14.000	-17.679	1.00	0.00	RX1	O
ATOM	4508	N	LEU	1208	48.945	13.257	-15.848	1.00	0.00	RX1	N
ATOM	4509	H	LEU	1208	49.275	12.474	-15.315	1.00	0.00	RX1	H
ATOM	4510	CA	LEU	1208	49.084	14.618	-15.312	1.00	0.00	RX1	C
ATOM	4511	CB	LEU	1208	50.094	15.432	-16.124	1.00	0.00	RX1	C
ATOM	4512	CG	LEU	1208	51.466	14.755	-16.164	1.00	0.00	RX1	C

ATOM	4513	CD1	LEU	1208	52.443	15.495	-17.077	1.00	0.00	RX1	C
ATOM	4514	CD2	LEU	1208	52.041	14.540	-14.763	1.00	0.00	RX1	C
ATOM	4515	C	LEU	1208	47.733	15.318	-15.153	1.00	0.00	RX1	C
ATOM	4516	O	LEU	1208	47.476	16.459	-15.531	1.00	0.00	RX1	O
ATOM	4517	N	ALA	1209	46.860	14.476	-14.585	1.00	0.00	RX1	N
ATOM	4518	H	ALA	1209	47.237	13.586	-14.345	1.00	0.00	RX1	H
ATOM	4519	CA	ALA	1209	45.444	14.707	-14.332	1.00	0.00	RX1	C
ATOM	4520	CB	ALA	1209	45.264	15.676	-13.172	1.00	0.00	RX1	C
ATOM	4521	C	ALA	1209	44.646	15.194	-15.518	1.00	0.00	RX1	C
ATOM	4522	O	ALA	1209	44.413	16.387	-15.685	1.00	0.00	RX1	O
ATOM	4523	N	GLU	1210	44.217	14.242	-16.350	1.00	0.00	RX1	N
ATOM	4524	H	GLU	1210	44.405	13.269	-16.188	1.00	0.00	RX1	H
ATOM	4525	CA	GLU	1210	43.207	14.687	-17.310	1.00	0.00	RX1	C
ATOM	4526	CB	GLU	1210	43.274	13.896	-18.619	1.00	0.00	RX1	C
ATOM	4527	CG	GLU	1210	44.509	14.167	-19.493	1.00	0.00	RX1	C
ATOM	4528	CD	GLU	1210	44.516	15.577	-20.072	1.00	0.00	RX1	C
ATOM	4529	OE1	GLU	1210	45.244	15.827	-21.021	1.00	0.00	RX1	O
ATOM	4530	OE2	GLU	1210	43.838	16.461	-19.559	1.00	0.00	RX1	O
ATOM	4531	C	GLU	1210	41.819	14.721	-16.689	1.00	0.00	RX1	C
ATOM	4532	O	GLU	1210	41.699	14.957	-15.492	1.00	0.00	RX1	O
ATOM	4533	N	GLN	1211	40.781	14.490	-17.502	1.00	0.00	RX1	N
ATOM	4534	H	GLN	1211	40.832	14.362	-18.497	1.00	0.00	RX1	H
ATOM	4535	CA	GLN	1211	39.497	14.174	-16.885	1.00	0.00	RX1	C
ATOM	4536	CB	GLN	1211	38.445	15.228	-17.243	1.00	0.00	RX1	C
ATOM	4537	CG	GLN	1211	38.728	16.637	-16.714	1.00	0.00	RX1	C
ATOM	4538	CD	GLN	1211	38.655	16.673	-15.198	1.00	0.00	RX1	C
ATOM	4539	OE1	GLN	1211	39.665	16.765	-14.500	1.00	0.00	RX1	O
ATOM	4540	NE2	GLN	1211	37.401	16.654	-14.719	1.00	0.00	RX1	N
ATOM	4541	HE21	GLN	1211	36.594	16.591	-15.307	1.00	0.00	RX1	H
ATOM	4542	HE22	GLN	1211	37.216	16.737	-13.733	1.00	0.00	RX1	H
ATOM	4543	C	GLN	1211	39.053	12.807	-17.379	1.00	0.00	RX1	C
ATOM	4544	O	GLN	1211	38.604	12.660	-18.507	1.00	0.00	RX1	O
ATOM	4545	N	PRO	1212	39.209	11.800	-16.486	1.00	0.00	RX1	N
ATOM	4546	CD	PRO	1212	39.563	11.994	-15.087	1.00	0.00	RX1	C
ATOM	4547	CA	PRO	1212	39.045	10.380	-16.853	1.00	0.00	RX1	C
ATOM	4548	CB	PRO	1212	38.875	9.731	-15.480	1.00	0.00	RX1	C
ATOM	4549	CG	PRO	1212	39.726	10.584	-14.540	1.00	0.00	RX1	C
ATOM	4550	C	PRO	1212	37.932	10.057	-17.839	1.00	0.00	RX1	C
ATOM	4551	O	PRO	1212	36.881	10.687	-17.815	1.00	0.00	RX1	O
ATOM	4552	N	TYR	1213	38.214	9.076	-18.723	1.00	0.00	RX1	N
ATOM	4553	H	TYR	1213	39.116	8.636	-18.765	1.00	0.00	RX1	H
ATOM	4554	CA	TYR	1213	37.323	8.787	-19.855	1.00	0.00	RX1	C
ATOM	4555	CB	TYR	1213	35.910	8.362	-19.423	1.00	0.00	RX1	C
ATOM	4556	CG	TYR	1213	35.956	7.173	-18.494	1.00	0.00	RX1	C
ATOM	4557	CD1	TYR	1213	36.012	5.889	-19.021	1.00	0.00	RX1	C
ATOM	4558	CE1	TYR	1213	36.055	4.794	-18.169	1.00	0.00	RX1	C
ATOM	4559	CD2	TYR	1213	35.938	7.358	-17.115	1.00	0.00	RX1	C
ATOM	4560	CE2	TYR	1213	35.997	6.262	-16.263	1.00	0.00	RX1	C
ATOM	4561	CZ	TYR	1213	36.059	4.977	-16.791	1.00	0.00	RX1	C
ATOM	4562	OH	TYR	1213	36.118	3.882	-15.953	1.00	0.00	RX1	O
ATOM	4563	HH	TYR	1213	35.639	3.165	-16.365	1.00	0.00	RX1	H
ATOM	4564	C	TYR	1213	37.257	9.994	-20.767	1.00	0.00	RX1	C
ATOM	4565	O	TYR	1213	36.205	10.575	-21.023	1.00	0.00	RX1	O
ATOM	4566	N	GLN	1214	38.477	10.404	-21.163	1.00	0.00	RX1	N
ATOM	4567	H	GLN	1214	39.218	9.725	-21.090	1.00	0.00	RX1	H
ATOM	4568	CA	GLN	1214	38.719	11.812	-21.506	1.00	0.00	RX1	C
ATOM	4569	CB	GLN	1214	40.082	11.963	-22.196	1.00	0.00	RX1	C
ATOM	4570	CG	GLN	1214	40.474	13.395	-22.602	1.00	0.00	RX1	C
ATOM	4571	CD	GLN	1214	40.875	14.251	-21.412	1.00	0.00	RX1	C
ATOM	4572	OE1	GLN	1214	40.281	14.223	-20.340	1.00	0.00	RX1	O
ATOM	4573	NE2	GLN	1214	41.945	15.022	-21.670	1.00	0.00	RX1	N

ATOM	4574	HE21	GLN	1214	42.417	15.016	-22.551	1.00	0.00	RX1	H
ATOM	4575	HE22	GLN	1214	42.385	15.623	-20.992	1.00	0.00	RX1	H
ATOM	4576	C	GLN	1214	37.612	12.490	-22.306	1.00	0.00	RX1	C
ATOM	4577	O	GLN	1214	36.875	13.339	-21.811	1.00	0.00	RX1	O
ATOM	4578	N	GLY	1215	37.512	12.039	-23.566	1.00	0.00	RX1	N
ATOM	4579	H	GLY	1215	38.006	11.214	-23.837	1.00	0.00	RX1	H
ATOM	4580	CA	GLY	1215	36.645	12.749	-24.505	1.00	0.00	RX1	C
ATOM	4581	C	GLY	1215	35.146	12.531	-24.373	1.00	0.00	RX1	C
ATOM	4582	O	GLY	1215	34.360	13.089	-25.129	1.00	0.00	RX1	O
ATOM	4583	N	LEU	1216	34.758	11.692	-23.401	1.00	0.00	RX1	N
ATOM	4584	H	LEU	1216	35.382	11.369	-22.688	1.00	0.00	RX1	H
ATOM	4585	CA	LEU	1216	33.314	11.558	-23.230	1.00	0.00	RX1	C
ATOM	4586	CB	LEU	1216	32.980	10.314	-22.409	1.00	0.00	RX1	C
ATOM	4587	CG	LEU	1216	33.387	9.005	-23.083	1.00	0.00	RX1	C
ATOM	4588	CD1	LEU	1216	33.101	7.801	-22.183	1.00	0.00	RX1	C
ATOM	4589	CD2	LEU	1216	32.742	8.853	-24.461	1.00	0.00	RX1	C
ATOM	4590	C	LEU	1216	32.727	12.779	-22.554	1.00	0.00	RX1	C
ATOM	4591	O	LEU	1216	33.404	13.491	-21.817	1.00	0.00	RX1	O
ATOM	4592	N	SER	1217	31.430	12.994	-22.801	1.00	0.00	RX1	N
ATOM	4593	H	SER	1217	30.889	12.329	-23.315	1.00	0.00	RX1	H
ATOM	4594	CA	SER	1217	30.785	13.970	-21.930	1.00	0.00	RX1	C
ATOM	4595	CB	SER	1217	29.513	14.430	-22.632	1.00	0.00	RX1	C
ATOM	4596	OG	SER	1217	28.960	13.326	-23.348	1.00	0.00	RX1	O
ATOM	4597	HG	SER	1217	29.522	13.156	-24.102	1.00	0.00	RX1	H
ATOM	4598	C	SER	1217	30.596	13.380	-20.546	1.00	0.00	RX1	C
ATOM	4599	O	SER	1217	30.596	12.166	-20.372	1.00	0.00	RX1	O
ATOM	4600	N	ASN	1218	30.431	14.277	-19.560	1.00	0.00	RX1	N
ATOM	4601	H	ASN	1218	30.464	15.257	-19.746	1.00	0.00	RX1	H
ATOM	4602	CA	ASN	1218	30.300	13.767	-18.189	1.00	0.00	RX1	C
ATOM	4603	CB	ASN	1218	30.181	14.903	-17.163	1.00	0.00	RX1	C
ATOM	4604	CG	ASN	1218	31.542	15.525	-16.903	1.00	0.00	RX1	C
ATOM	4605	OD1	ASN	1218	32.474	15.361	-17.689	1.00	0.00	RX1	O
ATOM	4606	ND2	ASN	1218	31.618	16.257	-15.778	1.00	0.00	RX1	N
ATOM	4607	HD21	ASN	1218	30.855	16.385	-15.136	1.00	0.00	RX1	H
ATOM	4608	HD22	ASN	1218	32.431	16.762	-15.476	1.00	0.00	RX1	H
ATOM	4609	C	ASN	1218	29.176	12.759	-18.002	1.00	0.00	RX1	C
ATOM	4610	O	ASN	1218	29.305	11.779	-17.283	1.00	0.00	RX1	O
ATOM	4611	N	GLU	1219	28.084	13.027	-18.739	1.00	0.00	RX1	N
ATOM	4612	H	GLU	1219	28.048	13.833	-19.323	1.00	0.00	RX1	H
ATOM	4613	CA	GLU	1219	26.979	12.067	-18.783	1.00	0.00	RX1	C
ATOM	4614	CB	GLU	1219	25.834	12.737	-19.544	1.00	0.00	RX1	C
ATOM	4615	CG	GLU	1219	24.527	12.786	-18.755	1.00	0.00	RX1	C
ATOM	4616	CD	GLU	1219	23.985	11.384	-18.670	1.00	0.00	RX1	C
ATOM	4617	OE1	GLU	1219	24.296	10.610	-19.567	1.00	0.00	RX1	O
ATOM	4618	OE2	GLU	1219	23.255	11.061	-17.731	1.00	0.00	RX1	O
ATOM	4619	C	GLU	1219	27.371	10.711	-19.368	1.00	0.00	RX1	C
ATOM	4620	O	GLU	1219	27.252	9.654	-18.757	1.00	0.00	RX1	O
ATOM	4621	N	GLN	1220	27.933	10.794	-20.592	1.00	0.00	RX1	N
ATOM	4622	H	GLN	1220	28.030	11.674	-21.052	1.00	0.00	RX1	H
ATOM	4623	CA	GLN	1220	28.408	9.564	-21.235	1.00	0.00	RX1	C
ATOM	4624	CB	GLN	1220	29.116	9.863	-22.550	1.00	0.00	RX1	C
ATOM	4625	CG	GLN	1220	28.181	10.271	-23.684	1.00	0.00	RX1	C
ATOM	4626	CD	GLN	1220	29.034	10.655	-24.874	1.00	0.00	RX1	C
ATOM	4627	OE1	GLN	1220	30.019	11.380	-24.755	1.00	0.00	RX1	O
ATOM	4628	NE2	GLN	1220	28.611	10.122	-26.033	1.00	0.00	RX1	N
ATOM	4629	HE21	GLN	1220	27.796	9.542	-26.058	1.00	0.00	RX1	H
ATOM	4630	HE22	GLN	1220	29.094	10.288	-26.893	1.00	0.00	RX1	H
ATOM	4631	C	GLN	1220	29.313	8.685	-20.389	1.00	0.00	RX1	C
ATOM	4632	O	GLN	1220	29.262	7.464	-20.465	1.00	0.00	RX1	O
ATOM	4633	N	VAL	1221	30.114	9.360	-19.546	1.00	0.00	RX1	N
ATOM	4634	H	VAL	1221	30.127	10.361	-19.561	1.00	0.00	RX1	H

ATOM	4635	CA	VAL	1221	30.918	8.603	-18.587	1.00	0.00	RX1	C
ATOM	4636	CB	VAL	1221	31.697	9.541	-17.670	1.00	0.00	RX1	C
ATOM	4637	CG1	VAL	1221	32.456	8.744	-16.613	1.00	0.00	RX1	C
ATOM	4638	CG2	VAL	1221	32.624	10.446	-18.481	1.00	0.00	RX1	C
ATOM	4639	C	VAL	1221	30.115	7.598	-17.770	1.00	0.00	RX1	C
ATOM	4640	O	VAL	1221	30.456	6.425	-17.713	1.00	0.00	RX1	O
ATOM	4641	N	LEU	1222	28.998	8.078	-17.187	1.00	0.00	RX1	N
ATOM	4642	H	LEU	1222	28.692	9.014	-17.375	1.00	0.00	RX1	H
ATOM	4643	CA	LEU	1222	28.147	7.125	-16.459	1.00	0.00	RX1	C
ATOM	4644	CB	LEU	1222	26.835	7.721	-15.934	1.00	0.00	RX1	C
ATOM	4645	CG	LEU	1222	26.932	8.805	-14.866	1.00	0.00	RX1	C
ATOM	4646	CD1	LEU	1222	27.202	10.171	-15.476	1.00	0.00	RX1	C
ATOM	4647	CD2	LEU	1222	25.683	8.838	-13.983	1.00	0.00	RX1	C
ATOM	4648	C	LEU	1222	27.744	5.948	-17.320	1.00	0.00	RX1	C
ATOM	4649	O	LEU	1222	27.881	4.786	-16.963	1.00	0.00	RX1	O
ATOM	4650	N	ARG	1223	27.252	6.332	-18.505	1.00	0.00	RX1	N
ATOM	4651	H	ARG	1223	27.342	7.298	-18.755	1.00	0.00	RX1	H
ATOM	4652	CA	ARG	1223	26.703	5.327	-19.416	1.00	0.00	RX1	C
ATOM	4653	CB	ARG	1223	25.991	5.976	-20.604	1.00	0.00	RX1	C
ATOM	4654	CG	ARG	1223	25.303	7.269	-20.168	1.00	0.00	RX1	C
ATOM	4655	CD	ARG	1223	24.114	7.699	-21.027	1.00	0.00	RX1	C
ATOM	4656	NE	ARG	1223	22.896	6.993	-20.628	1.00	0.00	RX1	N
ATOM	4657	HE	ARG	1223	22.737	6.114	-21.086	1.00	0.00	RX1	H
ATOM	4658	CZ	ARG	1223	22.106	7.552	-19.655	1.00	0.00	RX1	C
ATOM	4659	NH1	ARG	1223	22.458	8.726	-19.094	1.00	0.00	RX1	N
ATOM	4660	HH11	ARG	1223	21.973	9.234	-18.376	1.00	0.00	RX1	H
ATOM	4661	HH12	ARG	1223	23.293	9.229	-19.368	1.00	0.00	RX1	H
ATOM	4662	NH2	ARG	1223	20.977	6.920	-19.269	1.00	0.00	RX1	N
ATOM	4663	HH21	ARG	1223	20.372	7.308	-18.568	1.00	0.00	RX1	H
ATOM	4664	HH22	ARG	1223	20.703	6.043	-19.671	1.00	0.00	RX1	H
ATOM	4665	C	ARG	1223	27.683	4.240	-19.831	1.00	0.00	RX1	C
ATOM	4666	O	ARG	1223	27.315	3.104	-20.093	1.00	0.00	RX1	O
ATOM	4667	N	PHE	1224	28.960	4.645	-19.850	1.00	0.00	RX1	N
ATOM	4668	H	PHE	1224	29.201	5.589	-19.618	1.00	0.00	RX1	H
ATOM	4669	CA	PHE	1224	30.009	3.667	-20.109	1.00	0.00	RX1	C
ATOM	4670	CB	PHE	1224	31.230	4.427	-20.628	1.00	0.00	RX1	C
ATOM	4671	CG	PHE	1224	32.307	3.491	-21.116	1.00	0.00	RX1	C
ATOM	4672	CD1	PHE	1224	31.982	2.398	-21.910	1.00	0.00	RX1	C
ATOM	4673	CD2	PHE	1224	33.633	3.741	-20.785	1.00	0.00	RX1	C
ATOM	4674	CE1	PHE	1224	32.987	1.565	-22.385	1.00	0.00	RX1	C
ATOM	4675	CE2	PHE	1224	34.637	2.909	-21.262	1.00	0.00	RX1	C
ATOM	4676	CZ	PHE	1224	34.315	1.824	-22.069	1.00	0.00	RX1	C
ATOM	4677	C	PHE	1224	30.357	2.823	-18.888	1.00	0.00	RX1	C
ATOM	4678	O	PHE	1224	30.330	1.597	-18.895	1.00	0.00	RX1	O
ATOM	4679	N	VAL	1225	30.707	3.542	-17.813	1.00	0.00	RX1	N
ATOM	4680	H	VAL	1225	30.571	4.533	-17.796	1.00	0.00	RX1	H
ATOM	4681	CA	VAL	1225	31.278	2.825	-16.671	1.00	0.00	RX1	C
ATOM	4682	CB	VAL	1225	31.908	3.789	-15.669	1.00	0.00	RX1	C
ATOM	4683	CG1	VAL	1225	32.723	3.056	-14.602	1.00	0.00	RX1	C
ATOM	4684	CG2	VAL	1225	32.749	4.826	-16.396	1.00	0.00	RX1	C
ATOM	4685	C	VAL	1225	30.307	1.891	-15.970	1.00	0.00	RX1	C
ATOM	4686	O	VAL	1225	30.684	0.839	-15.466	1.00	0.00	RX1	O
ATOM	4687	N	MET	1226	29.024	2.298	-15.994	1.00	0.00	RX1	N
ATOM	4688	H	MET	1226	28.784	3.159	-16.446	1.00	0.00	RX1	H
ATOM	4689	CA	MET	1226	27.992	1.502	-15.323	1.00	0.00	RX1	C
ATOM	4690	CB	MET	1226	26.585	2.015	-15.632	1.00	0.00	RX1	C
ATOM	4691	CG	MET	1226	26.209	3.254	-14.822	1.00	0.00	RX1	C
ATOM	4692	SD	MET	1226	24.492	3.742	-15.047	1.00	0.00	RX1	S
ATOM	4693	CE	MET	1226	24.459	3.683	-16.844	1.00	0.00	RX1	C
ATOM	4694	C	MET	1226	28.036	0.002	-15.550	1.00	0.00	RX1	C
ATOM	4695	O	MET	1226	27.841	-0.780	-14.623	1.00	0.00	RX1	O

ATOM	4696	N	GLU	1227	28.325	-0.376	-16.802	1.00	0.00	RX1	N
ATOM	4697	H	GLU	1227	28.533	0.256	-17.550	1.00	0.00	RX1	H
ATOM	4698	CA	GLU	1227	28.416	-1.819	-16.982	1.00	0.00	RX1	C
ATOM	4699	CB	GLU	1227	27.387	-2.330	-17.996	1.00	0.00	RX1	C
ATOM	4700	CG	GLU	1227	25.978	-1.731	-17.841	1.00	0.00	RX1	C
ATOM	4701	CD	GLU	1227	25.457	-1.832	-16.414	1.00	0.00	RX1	C
ATOM	4702	OE1	GLU	1227	25.715	-2.816	-15.725	1.00	0.00	RX1	O
ATOM	4703	OE2	GLU	1227	24.809	-0.897	-15.951	1.00	0.00	RX1	O
ATOM	4704	C	GLU	1227	29.820	-2.325	-17.260	1.00	0.00	RX1	C
ATOM	4705	O	GLU	1227	30.078	-3.131	-18.143	1.00	0.00	RX1	O
ATOM	4706	N	GLY	1228	30.737	-1.810	-16.427	1.00	0.00	RX1	N
ATOM	4707	H	GLY	1228	30.499	-1.059	-15.810	1.00	0.00	RX1	H
ATOM	4708	CA	GLY	1228	32.112	-2.298	-16.507	1.00	0.00	RX1	C
ATOM	4709	C	GLY	1228	33.011	-1.569	-17.494	1.00	0.00	RX1	C
ATOM	4710	O	GLY	1228	34.042	-2.073	-17.923	1.00	0.00	RX1	O
ATOM	4711	N	GLY	1229	32.592	-0.342	-17.832	1.00	0.00	RX1	N
ATOM	4712	H	GLY	1229	31.782	0.072	-17.418	1.00	0.00	RX1	H
ATOM	4713	CA	GLY	1229	33.444	0.426	-18.738	1.00	0.00	RX1	C
ATOM	4714	C	GLY	1229	34.720	0.928	-18.086	1.00	0.00	RX1	C
ATOM	4715	O	GLY	1229	34.726	1.887	-17.323	1.00	0.00	RX1	O
ATOM	4716	N	LEU	1230	35.804	0.214	-18.412	1.00	0.00	RX1	N
ATOM	4717	H	LEU	1230	35.723	-0.540	-19.062	1.00	0.00	RX1	H
ATOM	4718	CA	LEU	1230	37.093	0.604	-17.844	1.00	0.00	RX1	C
ATOM	4719	CB	LEU	1230	38.053	-0.585	-17.814	1.00	0.00	RX1	C
ATOM	4720	CG	LEU	1230	37.505	-1.749	-16.989	1.00	0.00	RX1	C
ATOM	4721	CD1	LEU	1230	38.474	-2.932	-16.961	1.00	0.00	RX1	C
ATOM	4722	CD2	LEU	1230	37.101	-1.308	-15.581	1.00	0.00	RX1	C
ATOM	4723	C	LEU	1230	37.730	1.799	-18.530	1.00	0.00	RX1	C
ATOM	4724	O	LEU	1230	37.306	2.236	-19.592	1.00	0.00	RX1	O
ATOM	4725	N	LEU	1231	38.760	2.310	-17.843	1.00	0.00	RX1	N
ATOM	4726	H	LEU	1231	39.110	1.844	-17.034	1.00	0.00	RX1	H
ATOM	4727	CA	LEU	1231	39.420	3.532	-18.296	1.00	0.00	RX1	C
ATOM	4728	CB	LEU	1231	40.252	4.090	-17.146	1.00	0.00	RX1	C
ATOM	4729	CG	LEU	1231	39.493	5.153	-16.363	1.00	0.00	RX1	C
ATOM	4730	CD1	LEU	1231	40.052	5.410	-14.974	1.00	0.00	RX1	C
ATOM	4731	CD2	LEU	1231	39.457	6.449	-17.148	1.00	0.00	RX1	C
ATOM	4732	C	LEU	1231	40.278	3.410	-19.540	1.00	0.00	RX1	C
ATOM	4733	O	LEU	1231	40.726	2.335	-19.925	1.00	0.00	RX1	O
ATOM	4734	N	ASP	1232	40.514	4.600	-20.117	1.00	0.00	RX1	N
ATOM	4735	H	ASP	1232	40.118	5.440	-19.741	1.00	0.00	RX1	H
ATOM	4736	CA	ASP	1232	41.520	4.766	-21.168	1.00	0.00	RX1	C
ATOM	4737	CB	ASP	1232	41.611	6.236	-21.654	1.00	0.00	RX1	C
ATOM	4738	CG	ASP	1232	40.474	7.158	-21.196	1.00	0.00	RX1	C
ATOM	4739	OD1	ASP	1232	39.705	7.633	-22.030	1.00	0.00	RX1	O
ATOM	4740	OD2	ASP	1232	40.379	7.460	-20.010	1.00	0.00	RX1	O
ATOM	4741	C	ASP	1232	42.888	4.342	-20.643	1.00	0.00	RX1	C
ATOM	4742	O	ASP	1232	43.164	4.487	-19.459	1.00	0.00	RX1	O
ATOM	4743	N	LYS	1233	43.759	3.828	-21.527	1.00	0.00	RX1	N
ATOM	4744	H	LYS	1233	43.581	3.762	-22.507	1.00	0.00	RX1	H
ATOM	4745	CA	LYS	1233	45.119	3.728	-20.995	1.00	0.00	RX1	C
ATOM	4746	CB	LYS	1233	45.784	2.355	-21.166	1.00	0.00	RX1	C
ATOM	4747	CG	LYS	1233	46.832	2.214	-20.052	1.00	0.00	RX1	C
ATOM	4748	CD	LYS	1233	47.956	1.189	-20.215	1.00	0.00	RX1	C
ATOM	4749	CE	LYS	1233	49.056	1.612	-21.194	1.00	0.00	RX1	C
ATOM	4750	NZ	LYS	1233	50.264	0.819	-20.933	1.00	0.00	RX1	N
ATOM	4751	HZ1	LYS	1233	51.002	0.922	-21.648	1.00	0.00	RX1	H
ATOM	4752	HZ2	LYS	1233	50.696	1.043	-20.012	1.00	0.00	RX1	H
ATOM	4753	HZ3	LYS	1233	50.066	-0.205	-20.863	1.00	0.00	RX1	H
ATOM	4754	C	LYS	1233	46.030	4.780	-21.586	1.00	0.00	RX1	C
ATOM	4755	O	LYS	1233	46.252	4.804	-22.788	1.00	0.00	RX1	O
ATOM	4756	N	PRO	1234	46.564	5.649	-20.699	1.00	0.00	RX1	N

ATOM	4757	CD	PRO	1234	46.288	5.715	-19.271	1.00	0.00	RX1	C
ATOM	4758	CA	PRO	1234	47.529	6.652	-21.159	1.00	0.00	RX1	C
ATOM	4759	CB	PRO	1234	47.907	7.374	-19.861	1.00	0.00	RX1	C
ATOM	4760	CG	PRO	1234	46.772	7.097	-18.876	1.00	0.00	RX1	C
ATOM	4761	C	PRO	1234	48.748	6.034	-21.825	1.00	0.00	RX1	C
ATOM	4762	O	PRO	1234	49.323	5.061	-21.335	1.00	0.00	RX1	O
ATOM	4763	N	ASP	1235	49.145	6.668	-22.937	1.00	0.00	RX1	N
ATOM	4764	H	ASP	1235	48.593	7.358	-23.415	1.00	0.00	RX1	H
ATOM	4765	CA	ASP	1235	50.418	6.316	-23.565	1.00	0.00	RX1	C
ATOM	4766	CB	ASP	1235	50.739	7.273	-24.707	1.00	0.00	RX1	C
ATOM	4767	CG	ASP	1235	49.587	7.283	-25.678	1.00	0.00	RX1	C
ATOM	4768	OD1	ASP	1235	49.632	6.522	-26.641	1.00	0.00	RX1	O
ATOM	4769	OD2	ASP	1235	48.647	8.045	-25.458	1.00	0.00	RX1	O
ATOM	4770	C	ASP	1235	51.566	6.335	-22.587	1.00	0.00	RX1	C
ATOM	4771	O	ASP	1235	51.606	7.127	-21.652	1.00	0.00	RX1	O
ATOM	4772	N	ASN	1236	52.486	5.387	-22.814	1.00	0.00	RX1	N
ATOM	4773	H	ASN	1236	52.340	4.786	-23.599	1.00	0.00	RX1	H
ATOM	4774	CA	ASN	1236	53.660	5.205	-21.945	1.00	0.00	RX1	C
ATOM	4775	CB	ASN	1236	54.533	6.462	-21.786	1.00	0.00	RX1	C
ATOM	4776	CG	ASN	1236	54.985	6.999	-23.123	1.00	0.00	RX1	C
ATOM	4777	OD1	ASN	1236	55.916	6.501	-23.745	1.00	0.00	RX1	O
ATOM	4778	ND2	ASN	1236	54.262	8.058	-23.534	1.00	0.00	RX1	N
ATOM	4779	HD21	ASN	1236	53.510	8.403	-22.966	1.00	0.00	RX1	H
ATOM	4780	HD22	ASN	1236	54.437	8.516	-24.405	1.00	0.00	RX1	H
ATOM	4781	C	ASN	1236	53.409	4.655	-20.550	1.00	0.00	RX1	C
ATOM	4782	O	ASN	1236	54.281	4.018	-19.976	1.00	0.00	RX1	O
ATOM	4783	N	CYS	1237	52.194	4.892	-20.016	1.00	0.00	RX1	N
ATOM	4784	H	CYS	1237	51.501	5.427	-20.502	1.00	0.00	RX1	H
ATOM	4785	CA	CYS	1237	51.886	4.439	-18.655	1.00	0.00	RX1	C
ATOM	4786	CB	CYS	1237	50.422	4.744	-18.334	1.00	0.00	RX1	C
ATOM	4787	SG	CYS	1237	49.930	4.256	-16.661	1.00	0.00	RX1	S
ATOM	4788	C	CYS	1237	52.212	2.977	-18.395	1.00	0.00	RX1	C
ATOM	4789	O	CYS	1237	51.646	2.074	-19.012	1.00	0.00	RX1	O
ATOM	4790	N	PRO	1238	53.174	2.788	-17.456	1.00	0.00	RX1	N
ATOM	4791	CD	PRO	1238	53.864	3.832	-16.711	1.00	0.00	RX1	C
ATOM	4792	CA	PRO	1238	53.645	1.442	-17.127	1.00	0.00	RX1	C
ATOM	4793	CB	PRO	1238	54.698	1.704	-16.045	1.00	0.00	RX1	C
ATOM	4794	CG	PRO	1238	55.145	3.151	-16.249	1.00	0.00	RX1	C
ATOM	4795	C	PRO	1238	52.534	0.538	-16.641	1.00	0.00	RX1	C
ATOM	4796	O	PRO	1238	51.881	0.782	-15.631	1.00	0.00	RX1	O
ATOM	4797	N	ASP	1239	52.359	-0.537	-17.411	1.00	0.00	RX1	N
ATOM	4798	H	ASP	1239	52.838	-0.649	-18.284	1.00	0.00	RX1	H
ATOM	4799	CA	ASP	1239	51.201	-1.398	-17.187	1.00	0.00	RX1	C
ATOM	4800	CB	ASP	1239	51.113	-2.440	-18.299	1.00	0.00	RX1	C
ATOM	4801	CG	ASP	1239	50.845	-1.696	-19.592	1.00	0.00	RX1	C
ATOM	4802	OD1	ASP	1239	51.756	-1.079	-20.142	1.00	0.00	RX1	O
ATOM	4803	OD2	ASP	1239	49.703	-1.663	-20.037	1.00	0.00	RX1	O
ATOM	4804	C	ASP	1239	51.042	-1.992	-15.802	1.00	0.00	RX1	C
ATOM	4805	O	ASP	1239	49.939	-2.274	-15.366	1.00	0.00	RX1	O
ATOM	4806	N	MET	1240	52.173	-2.089	-15.076	1.00	0.00	RX1	N
ATOM	4807	H	MET	1240	53.048	-1.883	-15.508	1.00	0.00	RX1	H
ATOM	4808	CA	MET	1240	52.083	-2.422	-13.647	1.00	0.00	RX1	C
ATOM	4809	CB	MET	1240	53.478	-2.353	-13.015	1.00	0.00	RX1	C
ATOM	4810	CG	MET	1240	53.570	-2.853	-11.567	1.00	0.00	RX1	C
ATOM	4811	SD	MET	1240	52.817	-1.764	-10.346	1.00	0.00	RX1	S
ATOM	4812	CE	MET	1240	53.126	-2.766	-8.882	1.00	0.00	RX1	C
ATOM	4813	C	MET	1240	51.076	-1.572	-12.870	1.00	0.00	RX1	C
ATOM	4814	O	MET	1240	50.202	-2.060	-12.163	1.00	0.00	RX1	O
ATOM	4815	N	LEU	1241	51.205	-0.251	-13.080	1.00	0.00	RX1	N
ATOM	4816	H	LEU	1241	51.849	0.104	-13.758	1.00	0.00	RX1	H
ATOM	4817	CA	LEU	1241	50.241	0.644	-12.441	1.00	0.00	RX1	C

ATOM	4818	CB	LEU	1241	50.672	2.099	-12.642	1.00	0.00	RX1	C
ATOM	4819	CG	LEU	1241	49.916	3.090	-11.755	1.00	0.00	RX1	C
ATOM	4820	CD1	LEU	1241	50.078	2.763	-10.270	1.00	0.00	RX1	C
ATOM	4821	CD2	LEU	1241	50.301	4.539	-12.064	1.00	0.00	RX1	C
ATOM	4822	C	LEU	1241	48.814	0.408	-12.919	1.00	0.00	RX1	C
ATOM	4823	O	LEU	1241	47.858	0.389	-12.155	1.00	0.00	RX1	O
ATOM	4824	N	PHE	1242	48.722	0.173	-14.237	1.00	0.00	RX1	N
ATOM	4825	H	PHE	1242	49.551	0.122	-14.794	1.00	0.00	RX1	H
ATOM	4826	CA	PHE	1242	47.420	-0.168	-14.816	1.00	0.00	RX1	C
ATOM	4827	CB	PHE	1242	47.514	-0.075	-16.340	1.00	0.00	RX1	C
ATOM	4828	CG	PHE	1242	46.145	0.032	-16.965	1.00	0.00	RX1	C
ATOM	4829	CD1	PHE	1242	45.290	1.061	-16.592	1.00	0.00	RX1	C
ATOM	4830	CD2	PHE	1242	45.745	-0.886	-17.929	1.00	0.00	RX1	C
ATOM	4831	CE1	PHE	1242	44.044	1.187	-17.193	1.00	0.00	RX1	C
ATOM	4832	CE2	PHE	1242	44.500	-0.758	-18.532	1.00	0.00	RX1	C
ATOM	4833	CZ	PHE	1242	43.651	0.282	-18.171	1.00	0.00	RX1	C
ATOM	4834	C	PHE	1242	46.845	-1.516	-14.375	1.00	0.00	RX1	C
ATOM	4835	O	PHE	1242	45.653	-1.793	-14.489	1.00	0.00	RX1	O
ATOM	4836	N	GLU	1243	47.744	-2.350	-13.837	1.00	0.00	RX1	N
ATOM	4837	H	GLU	1243	48.719	-2.132	-13.803	1.00	0.00	RX1	H
ATOM	4838	CA	GLU	1243	47.276	-3.582	-13.221	1.00	0.00	RX1	C
ATOM	4839	CB	GLU	1243	48.407	-4.608	-13.131	1.00	0.00	RX1	C
ATOM	4840	CG	GLU	1243	47.892	-6.028	-12.900	1.00	0.00	RX1	C
ATOM	4841	CD	GLU	1243	46.959	-6.397	-14.036	1.00	0.00	RX1	C
ATOM	4842	OE1	GLU	1243	47.325	-6.244	-15.198	1.00	0.00	RX1	O
ATOM	4843	OE2	GLU	1243	45.830	-6.794	-13.778	1.00	0.00	RX1	O
ATOM	4844	C	GLU	1243	46.640	-3.308	-11.881	1.00	0.00	RX1	C
ATOM	4845	O	GLU	1243	45.495	-3.654	-11.632	1.00	0.00	RX1	O
ATOM	4846	N	LEU	1244	47.401	-2.565	-11.057	1.00	0.00	RX1	N
ATOM	4847	H	LEU	1244	48.339	-2.332	-11.326	1.00	0.00	RX1	H
ATOM	4848	CA	LEU	1244	46.840	-2.102	-9.783	1.00	0.00	RX1	C
ATOM	4849	CB	LEU	1244	47.828	-1.166	-9.089	1.00	0.00	RX1	C
ATOM	4850	CG	LEU	1244	47.397	-0.802	-7.668	1.00	0.00	RX1	C
ATOM	4851	CD1	LEU	1244	47.324	-2.033	-6.761	1.00	0.00	RX1	C
ATOM	4852	CD2	LEU	1244	48.276	0.299	-7.078	1.00	0.00	RX1	C
ATOM	4853	C	LEU	1244	45.469	-1.441	-9.912	1.00	0.00	RX1	C
ATOM	4854	O	LEU	1244	44.519	-1.746	-9.200	1.00	0.00	RX1	O
ATOM	4855	N	MET	1245	45.395	-0.556	-10.921	1.00	0.00	RX1	N
ATOM	4856	H	MET	1245	46.230	-0.321	-11.420	1.00	0.00	RX1	H
ATOM	4857	CA	MET	1245	44.103	0.047	-11.260	1.00	0.00	RX1	C
ATOM	4858	CB	MET	1245	44.206	0.885	-12.532	1.00	0.00	RX1	C
ATOM	4859	CG	MET	1245	45.173	2.066	-12.488	1.00	0.00	RX1	C
ATOM	4860	SD	MET	1245	45.096	3.036	-14.003	1.00	0.00	RX1	S
ATOM	4861	CE	MET	1245	46.760	3.718	-13.975	1.00	0.00	RX1	C
ATOM	4862	C	MET	1245	42.981	-0.965	-11.448	1.00	0.00	RX1	C
ATOM	4863	O	MET	1245	41.934	-0.915	-10.814	1.00	0.00	RX1	O
ATOM	4864	N	ARG	1246	43.258	-1.918	-12.348	1.00	0.00	RX1	N
ATOM	4865	H	ARG	1246	44.172	-2.006	-12.750	1.00	0.00	RX1	H
ATOM	4866	CA	ARG	1246	42.240	-2.935	-12.606	1.00	0.00	RX1	C
ATOM	4867	CB	ARG	1246	42.589	-3.697	-13.874	1.00	0.00	RX1	C
ATOM	4868	CG	ARG	1246	42.194	-2.913	-15.129	1.00	0.00	RX1	C
ATOM	4869	CD	ARG	1246	43.055	-3.222	-16.358	1.00	0.00	RX1	C
ATOM	4870	NE	ARG	1246	43.261	-4.655	-16.552	1.00	0.00	RX1	N
ATOM	4871	HE	ARG	1246	42.486	-5.199	-16.887	1.00	0.00	RX1	H
ATOM	4872	CZ	ARG	1246	44.470	-5.165	-16.165	1.00	0.00	RX1	C
ATOM	4873	NH1	ARG	1246	45.421	-4.334	-15.699	1.00	0.00	RX1	N
ATOM	4874	HH11	ARG	1246	46.303	-4.744	-15.414	1.00	0.00	RX1	H
ATOM	4875	HH12	ARG	1246	45.321	-3.340	-15.578	1.00	0.00	RX1	H
ATOM	4876	NH2	ARG	1246	44.709	-6.488	-16.212	1.00	0.00	RX1	N
ATOM	4877	HH21	ARG	1246	45.553	-6.821	-15.743	1.00	0.00	RX1	H
ATOM	4878	HH22	ARG	1246	44.114	-7.173	-16.629	1.00	0.00	RX1	H

ATOM	4879	C	ARG	1246	41.930	-3.867	-11.447	1.00	0.00	RX1	C
ATOM	4880	O	ARG	1246	40.821	-4.367	-11.318	1.00	0.00	RX1	O
ATOM	4881	N	MET	1247	42.927	-4.033	-10.564	1.00	0.00	RX1	N
ATOM	4882	H	MET	1247	43.825	-3.624	-10.725	1.00	0.00	RX1	H
ATOM	4883	CA	MET	1247	42.643	-4.719	-9.301	1.00	0.00	RX1	C
ATOM	4884	CB	MET	1247	43.900	-4.826	-8.438	1.00	0.00	RX1	C
ATOM	4885	CG	MET	1247	44.988	-5.715	-9.038	1.00	0.00	RX1	C
ATOM	4886	SD	MET	1247	46.507	-5.659	-8.073	1.00	0.00	RX1	S
ATOM	4887	CE	MET	1247	45.789	-5.969	-6.451	1.00	0.00	RX1	C
ATOM	4888	C	MET	1247	41.544	-4.019	-8.523	1.00	0.00	RX1	C
ATOM	4889	O	MET	1247	40.554	-4.613	-8.114	1.00	0.00	RX1	O
ATOM	4890	N	CYS	1248	41.735	-2.698	-8.401	1.00	0.00	RX1	N
ATOM	4891	H	CYS	1248	42.563	-2.250	-8.750	1.00	0.00	RX1	H
ATOM	4892	CA	CYS	1248	40.678	-1.883	-7.800	1.00	0.00	RX1	C
ATOM	4893	CB	CYS	1248	41.201	-0.456	-7.657	1.00	0.00	RX1	C
ATOM	4894	SG	CYS	1248	42.823	-0.410	-6.855	1.00	0.00	RX1	S
ATOM	4895	C	CYS	1248	39.346	-1.926	-8.538	1.00	0.00	RX1	C
ATOM	4896	O	CYS	1248	38.270	-1.841	-7.956	1.00	0.00	RX1	O
ATOM	4897	N	TRP	1249	39.459	-2.063	-9.864	1.00	0.00	RX1	N
ATOM	4898	H	TRP	1249	40.352	-2.189	-10.295	1.00	0.00	RX1	H
ATOM	4899	CA	TRP	1249	38.260	-1.973	-10.692	1.00	0.00	RX1	C
ATOM	4900	CB	TRP	1249	38.596	-1.427	-12.075	1.00	0.00	RX1	C
ATOM	4901	CG	TRP	1249	39.156	-0.034	-11.958	1.00	0.00	RX1	C
ATOM	4902	CD2	TRP	1249	40.009	0.643	-12.897	1.00	0.00	RX1	C
ATOM	4903	CE2	TRP	1249	40.290	1.924	-12.370	1.00	0.00	RX1	C
ATOM	4904	CE3	TRP	1249	40.540	0.261	-14.122	1.00	0.00	RX1	C
ATOM	4905	CD1	TRP	1249	38.964	0.880	-10.914	1.00	0.00	RX1	C
ATOM	4906	NE1	TRP	1249	39.628	2.039	-11.149	1.00	0.00	RX1	N
ATOM	4907	HE1	TRP	1249	39.606	2.820	-10.551	1.00	0.00	RX1	H
ATOM	4908	CZ2	TRP	1249	41.107	2.785	-13.087	1.00	0.00	RX1	C
ATOM	4909	CZ3	TRP	1249	41.352	1.135	-14.830	1.00	0.00	RX1	C
ATOM	4910	CH2	TRP	1249	41.636	2.391	-14.311	1.00	0.00	RX1	C
ATOM	4911	C	TRP	1249	37.439	-3.241	-10.850	1.00	0.00	RX1	C
ATOM	4912	O	TRP	1249	37.155	-3.701	-11.950	1.00	0.00	RX1	O
ATOM	4913	N	GLN	1250	37.022	-3.784	-9.701	1.00	0.00	RX1	N
ATOM	4914	H	GLN	1250	37.183	-3.311	-8.833	1.00	0.00	RX1	H
ATOM	4915	CA	GLN	1250	36.089	-4.899	-9.845	1.00	0.00	RX1	C
ATOM	4916	CB	GLN	1250	36.166	-5.828	-8.636	1.00	0.00	RX1	C
ATOM	4917	CG	GLN	1250	37.591	-6.319	-8.408	1.00	0.00	RX1	C
ATOM	4918	CD	GLN	1250	38.084	-6.923	-9.701	1.00	0.00	RX1	C
ATOM	4919	OE1	GLN	1250	37.433	-7.769	-10.308	1.00	0.00	RX1	O
ATOM	4920	NE2	GLN	1250	39.246	-6.394	-10.108	1.00	0.00	RX1	N
ATOM	4921	HE21	GLN	1250	39.716	-5.728	-9.521	1.00	0.00	RX1	H
ATOM	4922	HE22	GLN	1250	39.687	-6.575	-10.987	1.00	0.00	RX1	H
ATOM	4923	C	GLN	1250	34.666	-4.418	-10.026	1.00	0.00	RX1	C
ATOM	4924	O	GLN	1250	34.243	-3.455	-9.401	1.00	0.00	RX1	O
ATOM	4925	N	TYR	1251	33.916	-5.129	-10.886	1.00	0.00	RX1	N
ATOM	4926	H	TYR	1251	34.332	-5.876	-11.404	1.00	0.00	RX1	H
ATOM	4927	CA	TYR	1251	32.512	-4.727	-11.038	1.00	0.00	RX1	C
ATOM	4928	CB	TYR	1251	31.804	-5.613	-12.071	1.00	0.00	RX1	C
ATOM	4929	CG	TYR	1251	30.419	-5.083	-12.387	1.00	0.00	RX1	C
ATOM	4930	CD1	TYR	1251	30.228	-4.249	-13.483	1.00	0.00	RX1	C
ATOM	4931	CE1	TYR	1251	28.955	-3.781	-13.791	1.00	0.00	RX1	C
ATOM	4932	CD2	TYR	1251	29.331	-5.434	-11.594	1.00	0.00	RX1	C
ATOM	4933	CE2	TYR	1251	28.062	-4.954	-11.891	1.00	0.00	RX1	C
ATOM	4934	CZ	TYR	1251	27.870	-4.133	-12.995	1.00	0.00	RX1	C
ATOM	4935	OH	TYR	1251	26.603	-3.675	-13.300	1.00	0.00	RX1	O
ATOM	4936	HH	TYR	1251	26.497	-3.738	-14.256	1.00	0.00	RX1	H
ATOM	4937	C	TYR	1251	31.761	-4.725	-9.709	1.00	0.00	RX1	C
ATOM	4938	O	TYR	1251	31.289	-3.704	-9.229	1.00	0.00	RX1	O
ATOM	4939	N	ASN	1252	31.719	-5.931	-9.119	1.00	0.00	RX1	N

ATOM	4940	H	ASN	1252	32.222	-6.689	-9.528	1.00	0.00	RX1	H
ATOM	4941	CA	ASN	1252	31.109	-6.079	-7.793	1.00	0.00	RX1	C
ATOM	4942	CB	ASN	1252	31.151	-7.558	-7.400	1.00	0.00	RX1	C
ATOM	4943	CG	ASN	1252	30.770	-7.761	-5.945	1.00	0.00	RX1	C
ATOM	4944	OD1	ASN	1252	30.078	-6.969	-5.318	1.00	0.00	RX1	O
ATOM	4945	ND2	ASN	1252	31.286	-8.888	-5.432	1.00	0.00	RX1	N
ATOM	4946	HD21	ASN	1252	31.781	-9.547	-5.996	1.00	0.00	RX1	H
ATOM	4947	HD22	ASN	1252	31.243	-9.108	-4.451	1.00	0.00	RX1	H
ATOM	4948	C	ASN	1252	31.797	-5.210	-6.745	1.00	0.00	RX1	C
ATOM	4949	O	ASN	1252	33.003	-5.307	-6.552	1.00	0.00	RX1	O
ATOM	4950	N	PRO	1253	30.985	-4.360	-6.066	1.00	0.00	RX1	N
ATOM	4951	CD	PRO	1253	29.579	-4.080	-6.338	1.00	0.00	RX1	C
ATOM	4952	CA	PRO	1253	31.506	-3.615	-4.916	1.00	0.00	RX1	C
ATOM	4953	CB	PRO	1253	30.275	-2.856	-4.403	1.00	0.00	RX1	C
ATOM	4954	CG	PRO	1253	29.055	-3.552	-5.009	1.00	0.00	RX1	C
ATOM	4955	C	PRO	1253	32.185	-4.488	-3.873	1.00	0.00	RX1	C
ATOM	4956	O	PRO	1253	33.331	-4.271	-3.508	1.00	0.00	RX1	O
ATOM	4957	N	LYS	1254	31.450	-5.507	-3.408	1.00	0.00	RX1	N
ATOM	4958	H	LYS	1254	30.593	-5.776	-3.852	1.00	0.00	RX1	H
ATOM	4959	CA	LYS	1254	32.066	-6.376	-2.408	1.00	0.00	RX1	C
ATOM	4960	CB	LYS	1254	30.994	-6.964	-1.482	1.00	0.00	RX1	C
ATOM	4961	CG	LYS	1254	30.083	-5.882	-0.884	1.00	0.00	RX1	C
ATOM	4962	CD	LYS	1254	29.073	-6.399	0.150	1.00	0.00	RX1	C
ATOM	4963	CE	LYS	1254	28.128	-5.290	0.633	1.00	0.00	RX1	C
ATOM	4964	NZ	LYS	1254	27.250	-5.743	1.724	1.00	0.00	RX1	N
ATOM	4965	HZ1	LYS	1254	27.573	-5.318	2.619	1.00	0.00	RX1	H
ATOM	4966	HZ2	LYS	1254	26.253	-5.484	1.547	1.00	0.00	RX1	H
ATOM	4967	HZ3	LYS	1254	27.354	-6.757	1.940	1.00	0.00	RX1	H
ATOM	4968	C	LYS	1254	32.947	-7.454	-3.026	1.00	0.00	RX1	C
ATOM	4969	O	LYS	1254	32.635	-8.639	-3.009	1.00	0.00	RX1	O
ATOM	4970	N	MET	1255	34.062	-6.959	-3.592	1.00	0.00	RX1	N
ATOM	4971	H	MET	1255	34.186	-5.967	-3.638	1.00	0.00	RX1	H
ATOM	4972	CA	MET	1255	35.101	-7.792	-4.204	1.00	0.00	RX1	C
ATOM	4973	CB	MET	1255	34.623	-8.411	-5.519	1.00	0.00	RX1	C
ATOM	4974	CG	MET	1255	34.740	-9.938	-5.555	1.00	0.00	RX1	C
ATOM	4975	SD	MET	1255	36.430	-10.557	-5.524	1.00	0.00	RX1	S
ATOM	4976	CE	MET	1255	36.906	-10.082	-7.195	1.00	0.00	RX1	C
ATOM	4977	C	MET	1255	36.385	-7.018	-4.475	1.00	0.00	RX1	C
ATOM	4978	O	MET	1255	37.243	-7.438	-5.238	1.00	0.00	RX1	O
ATOM	4979	N	ARG	1256	36.466	-5.829	-3.862	1.00	0.00	RX1	N
ATOM	4980	H	ARG	1256	35.911	-5.565	-3.075	1.00	0.00	RX1	H
ATOM	4981	CA	ARG	1256	37.621	-5.003	-4.201	1.00	0.00	RX1	C
ATOM	4982	CB	ARG	1256	37.165	-3.573	-4.501	1.00	0.00	RX1	C
ATOM	4983	CG	ARG	1256	36.088	-3.579	-5.581	1.00	0.00	RX1	C
ATOM	4984	CD	ARG	1256	35.627	-2.193	-6.019	1.00	0.00	RX1	C
ATOM	4985	NE	ARG	1256	34.521	-2.335	-6.956	1.00	0.00	RX1	N
ATOM	4986	HE	ARG	1256	34.577	-3.098	-7.607	1.00	0.00	RX1	H
ATOM	4987	CZ	ARG	1256	33.441	-1.510	-6.912	1.00	0.00	RX1	C
ATOM	4988	NH1	ARG	1256	33.433	-0.467	-6.066	1.00	0.00	RX1	N
ATOM	4989	HH11	ARG	1256	32.586	0.077	-5.949	1.00	0.00	RX1	H
ATOM	4990	HH12	ARG	1256	34.245	-0.194	-5.538	1.00	0.00	RX1	H
ATOM	4991	NH2	ARG	1256	32.381	-1.750	-7.701	1.00	0.00	RX1	N
ATOM	4992	HH21	ARG	1256	31.537	-1.194	-7.622	1.00	0.00	RX1	H
ATOM	4993	HH22	ARG	1256	32.350	-2.488	-8.389	1.00	0.00	RX1	H
ATOM	4994	C	ARG	1256	38.651	-5.043	-3.092	1.00	0.00	RX1	C
ATOM	4995	O	ARG	1256	38.292	-5.019	-1.922	1.00	0.00	RX1	O
ATOM	4996	N	PRO	1257	39.940	-5.129	-3.499	1.00	0.00	RX1	N
ATOM	4997	CD	PRO	1257	40.423	-5.143	-4.873	1.00	0.00	RX1	C
ATOM	4998	CA	PRO	1257	41.017	-5.204	-2.505	1.00	0.00	RX1	C
ATOM	4999	CB	PRO	1257	42.261	-5.367	-3.390	1.00	0.00	RX1	C
ATOM	5000	CG	PRO	1257	41.890	-4.762	-4.741	1.00	0.00	RX1	C

ATOM	5001	C	PRO	1257	41.072	-3.973	-1.619	1.00	0.00	RX1	C
ATOM	5002	O	PRO	1257	40.791	-2.857	-2.049	1.00	0.00	RX1	O
ATOM	5003	N	SER	1258	41.436	-4.210	-0.355	1.00	0.00	RX1	N
ATOM	5004	H	SER	1258	41.721	-5.126	-0.058	1.00	0.00	RX1	H
ATOM	5005	CA	SER	1258	41.531	-3.039	0.510	1.00	0.00	RX1	C
ATOM	5006	CB	SER	1258	41.495	-3.495	1.970	1.00	0.00	RX1	C
ATOM	5007	OG	SER	1258	42.665	-4.263	2.270	1.00	0.00	RX1	O
ATOM	5008	HG	SER	1258	42.458	-5.160	1.993	1.00	0.00	RX1	H
ATOM	5009	C	SER	1258	42.737	-2.176	0.189	1.00	0.00	RX1	C
ATOM	5010	O	SER	1258	43.691	-2.591	-0.460	1.00	0.00	RX1	O
ATOM	5011	N	PHE	1259	42.696	-0.955	0.745	1.00	0.00	RX1	N
ATOM	5012	H	PHE	1259	41.874	-0.646	1.221	1.00	0.00	RX1	H
ATOM	5013	CA	PHE	1259	43.894	-0.116	0.651	1.00	0.00	RX1	C
ATOM	5014	CB	PHE	1259	43.634	1.239	1.302	1.00	0.00	RX1	C
ATOM	5015	CG	PHE	1259	42.460	1.903	0.625	1.00	0.00	RX1	C
ATOM	5016	CD1	PHE	1259	42.599	2.407	-0.663	1.00	0.00	RX1	C
ATOM	5017	CD2	PHE	1259	41.243	2.012	1.288	1.00	0.00	RX1	C
ATOM	5018	CE1	PHE	1259	41.524	3.030	-1.284	1.00	0.00	RX1	C
ATOM	5019	CE2	PHE	1259	40.168	2.634	0.666	1.00	0.00	RX1	C
ATOM	5020	CZ	PHE	1259	40.311	3.151	-0.617	1.00	0.00	RX1	C
ATOM	5021	C	PHE	1259	45.154	-0.760	1.222	1.00	0.00	RX1	C
ATOM	5022	O	PHE	1259	46.257	-0.603	0.715	1.00	0.00	RX1	O
ATOM	5023	N	LEU	1260	44.928	-1.547	2.289	1.00	0.00	RX1	N
ATOM	5024	H	LEU	1260	43.999	-1.724	2.609	1.00	0.00	RX1	H
ATOM	5025	CA	LEU	1260	46.050	-2.293	2.860	1.00	0.00	RX1	C
ATOM	5026	CB	LEU	1260	45.613	-2.976	4.153	1.00	0.00	RX1	C
ATOM	5027	CG	LEU	1260	45.302	-1.966	5.256	1.00	0.00	RX1	C
ATOM	5028	CD1	LEU	1260	44.546	-2.605	6.420	1.00	0.00	RX1	C
ATOM	5029	CD2	LEU	1260	46.567	-1.239	5.715	1.00	0.00	RX1	C
ATOM	5030	C	LEU	1260	46.654	-3.307	1.905	1.00	0.00	RX1	C
ATOM	5031	O	LEU	1260	47.867	-3.447	1.781	1.00	0.00	RX1	O
ATOM	5032	N	GLU	1261	45.749	-3.997	1.199	1.00	0.00	RX1	N
ATOM	5033	H	GLU	1261	44.762	-3.856	1.312	1.00	0.00	RX1	H
ATOM	5034	CA	GLU	1261	46.223	-4.928	0.174	1.00	0.00	RX1	C
ATOM	5035	CB	GLU	1261	45.054	-5.736	-0.361	1.00	0.00	RX1	C
ATOM	5036	CG	GLU	1261	44.441	-6.583	0.748	1.00	0.00	RX1	C
ATOM	5037	CD	GLU	1261	43.043	-6.952	0.325	1.00	0.00	RX1	C
ATOM	5038	OE1	GLU	1261	42.123	-6.777	1.117	1.00	0.00	RX1	O
ATOM	5039	OE2	GLU	1261	42.864	-7.349	-0.818	1.00	0.00	RX1	O
ATOM	5040	C	GLU	1261	46.983	-4.255	-0.953	1.00	0.00	RX1	C
ATOM	5041	O	GLU	1261	48.024	-4.726	-1.401	1.00	0.00	RX1	O
ATOM	5042	N	ILE	1262	46.452	-3.085	-1.343	1.00	0.00	RX1	N
ATOM	5043	H	ILE	1262	45.564	-2.806	-0.972	1.00	0.00	RX1	H
ATOM	5044	CA	ILE	1262	47.163	-2.238	-2.305	1.00	0.00	RX1	C
ATOM	5045	CB	ILE	1262	46.358	-0.962	-2.550	1.00	0.00	RX1	C
ATOM	5046	CG2	ILE	1262	47.145	0.069	-3.359	1.00	0.00	RX1	C
ATOM	5047	CG1	ILE	1262	45.018	-1.321	-3.197	1.00	0.00	RX1	C
ATOM	5048	CD1	ILE	1262	44.043	-0.146	-3.257	1.00	0.00	RX1	C
ATOM	5049	C	ILE	1262	48.599	-1.931	-1.887	1.00	0.00	RX1	C
ATOM	5050	O	ILE	1262	49.556	-2.197	-2.603	1.00	0.00	RX1	O
ATOM	5051	N	ILE	1263	48.735	-1.415	-0.652	1.00	0.00	RX1	N
ATOM	5052	H	ILE	1263	47.929	-1.223	-0.088	1.00	0.00	RX1	H
ATOM	5053	CA	ILE	1263	50.105	-1.165	-0.187	1.00	0.00	RX1	C
ATOM	5054	CB	ILE	1263	50.112	-0.480	1.180	1.00	0.00	RX1	C
ATOM	5055	CG2	ILE	1263	51.539	-0.116	1.595	1.00	0.00	RX1	C
ATOM	5056	CG1	ILE	1263	49.209	0.754	1.171	1.00	0.00	RX1	C
ATOM	5057	CD1	ILE	1263	49.067	1.393	2.552	1.00	0.00	RX1	C
ATOM	5058	C	ILE	1263	50.969	-2.425	-0.179	1.00	0.00	RX1	C
ATOM	5059	O	ILE	1263	52.116	-2.448	-0.608	1.00	0.00	RX1	O
ATOM	5060	N	SER	1264	50.326	-3.512	0.263	1.00	0.00	RX1	N
ATOM	5061	H	SER	1264	49.376	-3.461	0.567	1.00	0.00	RX1	H

ATOM	5062	CA	SER	1264	51.004	-4.806	0.207	1.00	0.00	RX1	C
ATOM	5063	CB	SER	1264	50.128	-5.826	0.924	1.00	0.00	RX1	C
ATOM	5064	OG	SER	1264	49.834	-5.305	2.226	1.00	0.00	RX1	O
ATOM	5065	HG	SER	1264	49.083	-4.728	2.121	1.00	0.00	RX1	H
ATOM	5066	C	SER	1264	51.485	-5.255	-1.170	1.00	0.00	RX1	C
ATOM	5067	O	SER	1264	52.485	-5.956	-1.301	1.00	0.00	RX1	O
ATOM	5068	N	SER	1265	50.753	-4.812	-2.198	1.00	0.00	RX1	N
ATOM	5069	H	SER	1265	49.967	-4.191	-2.139	1.00	0.00	RX1	H
ATOM	5070	CA	SER	1265	51.207	-5.164	-3.538	1.00	0.00	RX1	C
ATOM	5071	CB	SER	1265	49.982	-5.405	-4.412	1.00	0.00	RX1	C
ATOM	5072	OG	SER	1265	48.854	-4.775	-3.804	1.00	0.00	RX1	O
ATOM	5073	HG	SER	1265	48.623	-5.284	-3.034	1.00	0.00	RX1	H
ATOM	5074	C	SER	1265	52.203	-4.197	-4.163	1.00	0.00	RX1	C
ATOM	5075	O	SER	1265	52.836	-4.501	-5.163	1.00	0.00	RX1	O
ATOM	5076	N	ILE	1266	52.321	-3.016	-3.530	1.00	0.00	RX1	N
ATOM	5077	H	ILE	1266	51.769	-2.776	-2.730	1.00	0.00	RX1	H
ATOM	5078	CA	ILE	1266	53.208	-2.026	-4.142	1.00	0.00	RX1	C
ATOM	5079	CB	ILE	1266	52.417	-0.775	-4.517	1.00	0.00	RX1	C
ATOM	5080	CG2	ILE	1266	51.261	-1.125	-5.451	1.00	0.00	RX1	C
ATOM	5081	CG1	ILE	1266	51.956	-0.017	-3.273	1.00	0.00	RX1	C
ATOM	5082	CD1	ILE	1266	51.085	1.186	-3.622	1.00	0.00	RX1	C
ATOM	5083	C	ILE	1266	54.451	-1.640	-3.353	1.00	0.00	RX1	C
ATOM	5084	O	ILE	1266	55.320	-0.925	-3.839	1.00	0.00	RX1	O
ATOM	5085	N	LYS	1267	54.510	-2.137	-2.104	1.00	0.00	RX1	N
ATOM	5086	H	LYS	1267	53.738	-2.671	-1.761	1.00	0.00	RX1	H
ATOM	5087	CA	LYS	1267	55.573	-1.715	-1.181	1.00	0.00	RX1	C
ATOM	5088	CB	LYS	1267	55.419	-2.471	0.141	1.00	0.00	RX1	C
ATOM	5089	CG	LYS	1267	55.736	-3.948	-0.052	1.00	0.00	RX1	C
ATOM	5090	CD	LYS	1267	54.946	-4.912	0.825	1.00	0.00	RX1	C
ATOM	5091	CE	LYS	1267	55.252	-6.351	0.403	1.00	0.00	RX1	C
ATOM	5092	NZ	LYS	1267	55.090	-6.477	-1.052	1.00	0.00	RX1	N
ATOM	5093	HZ1	LYS	1267	55.460	-7.376	-1.410	1.00	0.00	RX1	H
ATOM	5094	HZ2	LYS	1267	54.118	-6.318	-1.383	1.00	0.00	RX1	H
ATOM	5095	HZ3	LYS	1267	55.711	-5.800	-1.555	1.00	0.00	RX1	H
ATOM	5096	C	LYS	1267	57.013	-1.740	-1.702	1.00	0.00	RX1	C
ATOM	5097	O	LYS	1267	57.880	-0.998	-1.258	1.00	0.00	RX1	O
ATOM	5098	N	GLU	1268	57.203	-2.618	-2.691	1.00	0.00	RX1	N
ATOM	5099	H	GLU	1268	56.447	-3.205	-2.982	1.00	0.00	RX1	H
ATOM	5100	CA	GLU	1268	58.473	-2.810	-3.378	1.00	0.00	RX1	C
ATOM	5101	CB	GLU	1268	58.243	-3.818	-4.510	1.00	0.00	RX1	C
ATOM	5102	CG	GLU	1268	58.177	-5.301	-4.099	1.00	0.00	RX1	C
ATOM	5103	CD	GLU	1268	57.096	-5.619	-3.072	1.00	0.00	RX1	C
ATOM	5104	OE1	GLU	1268	55.963	-5.153	-3.181	1.00	0.00	RX1	O
ATOM	5105	OE2	GLU	1268	57.373	-6.356	-2.130	1.00	0.00	RX1	O
ATOM	5106	C	GLU	1268	59.161	-1.543	-3.883	1.00	0.00	RX1	C
ATOM	5107	O	GLU	1268	60.368	-1.392	-3.750	1.00	0.00	RX1	O
ATOM	5108	N	GLU	1269	58.358	-0.629	-4.459	1.00	0.00	RX1	N
ATOM	5109	H	GLU	1269	57.359	-0.720	-4.470	1.00	0.00	RX1	H
ATOM	5110	CA	GLU	1269	59.014	0.595	-4.937	1.00	0.00	RX1	C
ATOM	5111	CB	GLU	1269	58.590	0.978	-6.359	1.00	0.00	RX1	C
ATOM	5112	CG	GLU	1269	59.417	0.333	-7.476	1.00	0.00	RX1	C
ATOM	5113	CD	GLU	1269	59.130	1.055	-8.782	1.00	0.00	RX1	C
ATOM	5114	OE1	GLU	1269	57.964	1.210	-9.128	1.00	0.00	RX1	O
ATOM	5115	OE2	GLU	1269	60.070	1.498	-9.442	1.00	0.00	RX1	O
ATOM	5116	C	GLU	1269	58.857	1.818	-4.047	1.00	0.00	RX1	C
ATOM	5117	O	GLU	1269	59.058	2.951	-4.470	1.00	0.00	RX1	O
ATOM	5118	N	MET	1270	58.457	1.567	-2.792	1.00	0.00	RX1	N
ATOM	5119	H	MET	1270	58.483	0.648	-2.397	1.00	0.00	RX1	H
ATOM	5120	CA	MET	1270	58.197	2.750	-1.971	1.00	0.00	RX1	C
ATOM	5121	CB	MET	1270	57.305	2.408	-0.778	1.00	0.00	RX1	C
ATOM	5122	CG	MET	1270	56.016	1.703	-1.190	1.00	0.00	RX1	C

ATOM	5123	SD	MET	1270	54.909	2.673	-2.212	1.00	0.00	RX1	S
ATOM	5124	CE	MET	1270	54.212	3.654	-0.884	1.00	0.00	RX1	C
ATOM	5125	C	MET	1270	59.457	3.435	-1.477	1.00	0.00	RX1	C
ATOM	5126	O	MET	1270	60.451	2.799	-1.143	1.00	0.00	RX1	O
ATOM	5127	N	GLU	1271	59.359	4.771	-1.401	1.00	0.00	RX1	N
ATOM	5128	H	GLU	1271	58.486	5.195	-1.644	1.00	0.00	RX1	H
ATOM	5129	CA	GLU	1271	60.388	5.540	-0.696	1.00	0.00	RX1	C
ATOM	5130	CB	GLU	1271	60.020	7.027	-0.719	1.00	0.00	RX1	C
ATOM	5131	CG	GLU	1271	59.992	7.616	-2.131	1.00	0.00	RX1	C
ATOM	5132	CD	GLU	1271	59.290	8.963	-2.113	1.00	0.00	RX1	C
ATOM	5133	OE1	GLU	1271	58.536	9.243	-1.191	1.00	0.00	RX1	O
ATOM	5134	OE2	GLU	1271	59.431	9.734	-3.053	1.00	0.00	RX1	O
ATOM	5135	C	GLU	1271	60.508	5.046	0.737	1.00	0.00	RX1	C
ATOM	5136	O	GLU	1271	59.554	5.086	1.503	1.00	0.00	RX1	O
ATOM	5137	N	PRO	1272	61.715	4.531	1.081	1.00	0.00	RX1	N
ATOM	5138	CD	PRO	1272	62.943	4.633	0.301	1.00	0.00	RX1	C
ATOM	5139	CA	PRO	1272	61.876	3.722	2.296	1.00	0.00	RX1	C
ATOM	5140	CB	PRO	1272	63.396	3.720	2.472	1.00	0.00	RX1	C
ATOM	5141	CG	PRO	1272	63.926	3.726	1.037	1.00	0.00	RX1	C
ATOM	5142	C	PRO	1272	61.083	4.135	3.530	1.00	0.00	RX1	C
ATOM	5143	O	PRO	1272	60.289	3.362	4.059	1.00	0.00	RX1	O
ATOM	5144	N	GLY	1273	61.331	5.395	3.936	1.00	0.00	RX1	N
ATOM	5145	H	GLY	1273	61.929	5.975	3.387	1.00	0.00	RX1	H
ATOM	5146	CA	GLY	1273	60.759	5.924	5.178	1.00	0.00	RX1	C
ATOM	5147	C	GLY	1273	59.242	5.899	5.305	1.00	0.00	RX1	C
ATOM	5148	O	GLY	1273	58.692	5.892	6.401	1.00	0.00	RX1	O
ATOM	5149	N	PHE	1274	58.578	5.864	4.128	1.00	0.00	RX1	N
ATOM	5150	H	PHE	1274	59.074	5.848	3.259	1.00	0.00	RX1	H
ATOM	5151	CA	PHE	1274	57.117	5.721	4.097	1.00	0.00	RX1	C
ATOM	5152	CB	PHE	1274	56.664	5.368	2.675	1.00	0.00	RX1	C
ATOM	5153	CG	PHE	1274	55.193	5.019	2.612	1.00	0.00	RX1	C
ATOM	5154	CD1	PHE	1274	54.247	6.020	2.437	1.00	0.00	RX1	C
ATOM	5155	CD2	PHE	1274	54.782	3.693	2.718	1.00	0.00	RX1	C
ATOM	5156	CE1	PHE	1274	52.896	5.697	2.383	1.00	0.00	RX1	C
ATOM	5157	CE2	PHE	1274	53.432	3.370	2.669	1.00	0.00	RX1	C
ATOM	5158	CZ	PHE	1274	52.488	4.375	2.506	1.00	0.00	RX1	C
ATOM	5159	C	PHE	1274	56.624	4.658	5.059	1.00	0.00	RX1	C
ATOM	5160	O	PHE	1274	55.723	4.856	5.863	1.00	0.00	RX1	O
ATOM	5161	N	ARG	1275	57.281	3.497	4.931	1.00	0.00	RX1	N
ATOM	5162	H	ARG	1275	58.130	3.449	4.401	1.00	0.00	RX1	H
ATOM	5163	CA	ARG	1275	56.783	2.386	5.728	1.00	0.00	RX1	C
ATOM	5164	CB	ARG	1275	57.449	1.087	5.331	1.00	0.00	RX1	C
ATOM	5165	CG	ARG	1275	57.520	0.768	3.836	1.00	0.00	RX1	C
ATOM	5166	CD	ARG	1275	58.909	0.239	3.442	1.00	0.00	RX1	C
ATOM	5167	NE	ARG	1275	59.424	-0.707	4.435	1.00	0.00	RX1	N
ATOM	5168	HE	ARG	1275	59.010	-1.622	4.491	1.00	0.00	RX1	H
ATOM	5169	CZ	ARG	1275	60.278	-0.237	5.395	1.00	0.00	RX1	C
ATOM	5170	NH1	ARG	1275	60.759	1.015	5.297	1.00	0.00	RX1	N
ATOM	5171	HH11	ARG	1275	61.255	1.436	6.082	1.00	0.00	RX1	H
ATOM	5172	HH12	ARG	1275	60.611	1.640	4.524	1.00	0.00	RX1	H
ATOM	5173	NH2	ARG	1275	60.567	-0.995	6.466	1.00	0.00	RX1	N
ATOM	5174	HH21	ARG	1275	61.138	-0.602	7.207	1.00	0.00	RX1	H
ATOM	5175	HH22	ARG	1275	60.193	-1.922	6.583	1.00	0.00	RX1	H
ATOM	5176	C	ARG	1275	56.901	2.617	7.222	1.00	0.00	RX1	C
ATOM	5177	O	ARG	1275	55.937	2.438	7.951	1.00	0.00	RX1	O
ATOM	5178	N	GLU	1276	58.090	3.071	7.651	1.00	0.00	RX1	N
ATOM	5179	H	GLU	1276	58.884	3.214	7.055	1.00	0.00	RX1	H
ATOM	5180	CA	GLU	1276	58.252	3.360	9.080	1.00	0.00	RX1	C
ATOM	5181	CB	GLU	1276	59.655	3.902	9.377	1.00	0.00	RX1	C
ATOM	5182	CG	GLU	1276	60.773	2.851	9.435	1.00	0.00	RX1	C
ATOM	5183	CD	GLU	1276	61.077	2.275	8.066	1.00	0.00	RX1	C

ATOM	5184	OE1	GLU	1276	60.983	2.996	7.078	1.00	0.00	RX1	O
ATOM	5185	OE2	GLU	1276	61.408	1.094	7.972	1.00	0.00	RX1	O
ATOM	5186	C	GLU	1276	57.204	4.289	9.681	1.00	0.00	RX1	C
ATOM	5187	O	GLU	1276	56.809	4.159	10.833	1.00	0.00	RX1	O
ATOM	5188	N	VAL	1277	56.755	5.237	8.844	1.00	0.00	RX1	N
ATOM	5189	H	VAL	1277	57.072	5.298	7.895	1.00	0.00	RX1	H
ATOM	5190	CA	VAL	1277	55.731	6.139	9.369	1.00	0.00	RX1	C
ATOM	5191	CB	VAL	1277	56.063	7.584	8.992	1.00	0.00	RX1	C
ATOM	5192	CG1	VAL	1277	57.401	8.003	9.602	1.00	0.00	RX1	C
ATOM	5193	CG2	VAL	1277	56.042	7.791	7.477	1.00	0.00	RX1	C
ATOM	5194	C	VAL	1277	54.281	5.801	9.026	1.00	0.00	RX1	C
ATOM	5195	O	VAL	1277	53.354	6.502	9.416	1.00	0.00	RX1	O
ATOM	5196	N	SER	1278	54.120	4.718	8.258	1.00	0.00	RX1	N
ATOM	5197	H	SER	1278	54.896	4.146	8.001	1.00	0.00	RX1	H
ATOM	5198	CA	SER	1278	52.788	4.442	7.723	1.00	0.00	RX1	C
ATOM	5199	CB	SER	1278	52.934	3.591	6.457	1.00	0.00	RX1	C
ATOM	5200	OG	SER	1278	53.554	2.333	6.762	1.00	0.00	RX1	O
ATOM	5201	HG	SER	1278	54.251	2.527	7.378	1.00	0.00	RX1	H
ATOM	5202	C	SER	1278	51.799	3.848	8.713	1.00	0.00	RX1	C
ATOM	5203	O	SER	1278	52.162	3.142	9.652	1.00	0.00	RX1	O
ATOM	5204	N	PHE	1279	50.513	4.094	8.416	1.00	0.00	RX1	N
ATOM	5205	H	PHE	1279	50.269	4.629	7.600	1.00	0.00	RX1	H
ATOM	5206	CA	PHE	1279	49.471	3.289	9.050	1.00	0.00	RX1	C
ATOM	5207	CB	PHE	1279	48.082	3.735	8.585	1.00	0.00	RX1	C
ATOM	5208	CG	PHE	1279	47.029	2.845	9.197	1.00	0.00	RX1	C
ATOM	5209	CD1	PHE	1279	46.747	2.936	10.553	1.00	0.00	RX1	C
ATOM	5210	CD2	PHE	1279	46.345	1.933	8.403	1.00	0.00	RX1	C
ATOM	5211	CE1	PHE	1279	45.779	2.113	11.112	1.00	0.00	RX1	C
ATOM	5212	CE2	PHE	1279	45.377	1.110	8.963	1.00	0.00	RX1	C
ATOM	5213	CZ	PHE	1279	45.092	1.202	10.320	1.00	0.00	RX1	C
ATOM	5214	C	PHE	1279	49.671	1.808	8.777	1.00	0.00	RX1	C
ATOM	5215	O	PHE	1279	49.541	0.974	9.663	1.00	0.00	RX1	O
ATOM	5216	N	TYR	1280	50.066	1.529	7.526	1.00	0.00	RX1	N
ATOM	5217	H	TYR	1280	50.073	2.279	6.856	1.00	0.00	RX1	H
ATOM	5218	CA	TYR	1280	50.438	0.170	7.129	1.00	0.00	RX1	C
ATOM	5219	CB	TYR	1280	51.081	0.253	5.746	1.00	0.00	RX1	C
ATOM	5220	CG	TYR	1280	51.388	-1.113	5.191	1.00	0.00	RX1	C
ATOM	5221	CD1	TYR	1280	52.707	-1.479	4.955	1.00	0.00	RX1	C
ATOM	5222	CE1	TYR	1280	52.995	-2.713	4.385	1.00	0.00	RX1	C
ATOM	5223	CD2	TYR	1280	50.354	-1.991	4.891	1.00	0.00	RX1	C
ATOM	5224	CE2	TYR	1280	50.643	-3.224	4.320	1.00	0.00	RX1	C
ATOM	5225	CZ	TYR	1280	51.961	-3.579	4.053	1.00	0.00	RX1	C
ATOM	5226	OH	TYR	1280	52.240	-4.789	3.452	1.00	0.00	RX1	O
ATOM	5227	HH	TYR	1280	51.406	-5.190	3.210	1.00	0.00	RX1	H
ATOM	5228	C	TYR	1280	51.336	-0.585	8.112	1.00	0.00	RX1	C
ATOM	5229	O	TYR	1280	51.095	-1.743	8.433	1.00	0.00	RX1	O
ATOM	5230	N	TYR	1281	52.369	0.129	8.586	1.00	0.00	RX1	N
ATOM	5231	H	TYR	1281	52.502	1.084	8.313	1.00	0.00	RX1	H
ATOM	5232	CA	TYR	1281	53.241	-0.482	9.593	1.00	0.00	RX1	C
ATOM	5233	CB	TYR	1281	54.645	0.110	9.532	1.00	0.00	RX1	C
ATOM	5234	CG	TYR	1281	55.595	-0.690	8.672	1.00	0.00	RX1	C
ATOM	5235	CD1	TYR	1281	55.144	-1.480	7.621	1.00	0.00	RX1	C
ATOM	5236	CE1	TYR	1281	56.060	-2.173	6.833	1.00	0.00	RX1	C
ATOM	5237	CD2	TYR	1281	56.955	-0.612	8.944	1.00	0.00	RX1	C
ATOM	5238	CE2	TYR	1281	57.868	-1.300	8.157	1.00	0.00	RX1	C
ATOM	5239	CZ	TYR	1281	57.424	-2.072	7.091	1.00	0.00	RX1	C
ATOM	5240	OH	TYR	1281	58.343	-2.727	6.285	1.00	0.00	RX1	O
ATOM	5241	HH	TYR	1281	57.914	-3.505	5.940	1.00	0.00	RX1	H
ATOM	5242	C	TYR	1281	52.786	-0.341	11.039	1.00	0.00	RX1	C
ATOM	5243	O	TYR	1281	53.292	-1.011	11.930	1.00	0.00	RX1	O
ATOM	5244	N	SER	1282	51.855	0.597	11.255	1.00	0.00	RX1	N

```
ATOM   5245  H    SER  1282      51.428   1.108  10.510  1.00  0.00      RX1  H
ATOM   5246  CA   SER  1282      51.564   0.951  12.642  1.00  0.00      RX1  C
ATOM   5247  CB   SER  1282      50.800   2.279  12.669  1.00  0.00      RX1  C
ATOM   5248  OG   SER  1282      49.504   2.133  12.086  1.00  0.00      RX1  O
ATOM   5249  HG   SER  1282      49.581   1.480  11.395  1.00  0.00      RX1  H
ATOM   5250  C    SER  1282      50.932  -0.148  13.483  1.00  0.00      RX1  C
ATOM   5251  O    SER  1282      50.254  -1.041  12.993  1.00  0.00      RX1  O
ATOM   5252  N    GLU  1283      51.137   0.007  14.800  1.00  0.00      RX1  N
ATOM   5253  H    GLU  1283      51.744   0.720  15.145  1.00  0.00      RX1  H
ATOM   5254  CA   GLU  1283      50.492  -0.887  15.766  1.00  0.00      RX1  C
ATOM   5255  CB   GLU  1283      50.913  -0.425  17.155  1.00  0.00      RX1  C
ATOM   5256  CG   GLU  1283      50.486  -1.321  18.311  1.00  0.00      RX1  C
ATOM   5257  CD   GLU  1283      51.021  -0.683  19.571  1.00  0.00      RX1  C
ATOM   5258  OE1 GLU  1283      51.149  -1.375  20.579  1.00  0.00      RX1  O
ATOM   5259  OE2 GLU  1283      51.326   0.508  19.526  1.00  0.00      RX1  O
ATOM   5260  C    GLU  1283      48.970  -0.970  15.639  1.00  0.00      RX1  C
ATOM   5261  O    GLU  1283      48.351  -2.020  15.757  1.00  0.00      RX1  O
ATOM   5262  N    GLU  1284      48.386   0.204  15.323  1.00  0.00      RX1  N
ATOM   5263  H    GLU  1284      48.938   1.028  15.225  1.00  0.00      RX1  H
ATOM   5264  CA   GLU  1284      46.936   0.249  15.103  1.00  0.00      RX1  C
ATOM   5265  CB   GLU  1284      46.473   1.698  14.876  1.00  0.00      RX1  C
ATOM   5266  CG   GLU  1284      45.094   2.097  15.442  1.00  0.00      RX1  C
ATOM   5267  CD   GLU  1284      43.917   1.506  14.676  1.00  0.00      RX1  C
ATOM   5268  OE1 GLU  1284      43.790   1.717  13.476  1.00  0.00      RX1  O
ATOM   5269  OE2 GLU  1284      43.072   0.849  15.270  1.00  0.00      RX1  O
ATOM   5270  C    GLU  1284      46.436  -0.655  13.981  1.00  0.00      RX1  C
ATOM   5271  O    GLU  1284      45.279  -1.060  13.944  1.00  0.00      RX1  O
ATOM   5272  N    ASN  1285      47.359  -0.991  13.065  1.00  0.00      RX1  N
ATOM   5273  H    ASN  1285      48.328  -0.786  13.202  1.00  0.00      RX1  H
ATOM   5274  CA   ASN  1285      46.982  -1.784  11.895  1.00  0.00      RX1  C
ATOM   5275  CB   ASN  1285      47.945  -1.551  10.739  1.00  0.00      RX1  C
ATOM   5276  CG   ASN  1285      47.345  -2.151   9.488  1.00  0.00      RX1  C
ATOM   5277  OD1 ASN  1285      46.128  -2.227   9.329  1.00  0.00      RX1  O
ATOM   5278  ND2 ASN  1285      48.269  -2.565   8.602  1.00  0.00      RX1  N
ATOM   5279 HD21 ASN  1285      49.247  -2.439   8.805  1.00  0.00      RX1  H
ATOM   5280 HD22 ASN  1285      48.028  -3.006   7.741  1.00  0.00      RX1  H
ATOM   5281  C    ASN  1285      46.776  -3.285  12.099  1.00  0.00      RX1  C
ATOM   5282  O    ASN  1285      47.290  -4.128  11.375  1.00  0.00      RX1  O
ATOM   5283  N    LYS  1286      45.940  -3.592  13.105  1.00  0.00      RX1  N
ATOM   5284  H    LYS  1286      45.698  -2.880  13.760  1.00  0.00      RX1  H
ATOM   5285  CA   LYS  1286      45.234  -4.874  13.174  1.00  0.00      RX1  C
ATOM   5286  CB   LYS  1286      43.929  -4.841  12.359  1.00  0.00      RX1  C
ATOM   5287  CG   LYS  1286      43.020  -3.622  12.567  1.00  0.00      RX1  C
ATOM   5288  CD   LYS  1286      43.318  -2.447  11.631  1.00  0.00      RX1  C
ATOM   5289  CE   LYS  1286      42.503  -1.187  11.928  1.00  0.00      RX1  C
ATOM   5290  NZ   LYS  1286      42.726  -0.733  13.300  1.00  0.00      RX1  N
ATOM   5291  HZ1 LYS  1286      42.069  -1.167  13.973  1.00  0.00      RX1  H
ATOM   5292  HZ2 LYS  1286      43.689  -0.917  13.631  1.00  0.00      RX1  H
ATOM   5293  HZ3 LYS  1286      42.656   0.303  13.407  1.00  0.00      RX1  H
ATOM   5294  C    LYS  1286      46.022  -6.121  12.795  1.00  0.00      RX1  C
ATOM   5295  O    LYS  1286      45.677  -7.239  13.163  1.00  0.00      RX1  O
TER
HEADER lig.000.00.pdb
ATOM      1  N    PRO   563      52.222  27.997  23.768  1.00  0.00      LX0  N
ATOM      2  CD   PRO   563      53.181  28.929  23.192  1.00  0.00      LX0  C
ATOM      3  CA   PRO   563      52.482  27.701  25.179  1.00  0.00      LX0  C
ATOM      4  CB   PRO   563      53.740  28.531  25.474  1.00  0.00      LX0  C
ATOM      5  CG   PRO   563      53.789  29.622  24.403  1.00  0.00      LX0  C
ATOM      6  C    PRO   563      51.295  28.010  26.076  1.00  0.00      LX0  C
ATOM      7  O    PRO   563      50.963  27.260  26.989  1.00  0.00      LX0  O
ATOM      8  N    GLN   564      50.629  29.132  25.755  1.00  0.00      LX0  N
```

ATOM	9	H	GLN	564	50.876	29.713	24.977	0.00	0.00	LX0	H
ATOM	10	CA	GLN	564	49.355	29.288	26.441	1.00	0.00	LX0	C
ATOM	11	CB	GLN	564	48.935	30.758	26.506	1.00	0.00	LX0	C
ATOM	12	CG	GLN	564	48.421	31.173	27.890	1.00	0.00	LX0	C
ATOM	13	CD	GLN	564	49.537	31.034	28.909	1.00	0.00	LX0	C
ATOM	14	OE1	GLN	564	50.587	31.652	28.818	1.00	0.00	LX0	O
ATOM	15	NE2	GLN	564	49.273	30.172	29.892	1.00	0.00	LX0	N
ATOM	16	HE21	GLN	564	48.405	29.674	29.970	0.00	0.00	LX0	H
ATOM	17	HE22	GLN	564	49.970	30.031	30.589	0.00	0.00	LX0	H
ATOM	18	C	GLN	564	48.312	28.394	25.805	1.00	0.00	LX0	C
ATOM	19	O	GLN	564	48.518	27.856	24.719	1.00	0.00	LX0	O
ATOM	20	N	LYS	565	47.227	28.208	26.563	1.00	0.00	LX0	N
ATOM	21	H	LYS	565	47.101	28.711	27.420	0.00	0.00	LX0	H
ATOM	22	CA	LYS	565	46.244	27.183	26.234	1.00	0.00	LX0	C
ATOM	23	CB	LYS	565	46.686	25.837	26.834	1.00	0.00	LX0	C
ATOM	24	CG	LYS	565	47.328	26.025	28.212	1.00	0.00	LX0	C
ATOM	25	CD	LYS	565	47.978	24.802	28.856	1.00	0.00	LX0	C
ATOM	26	CE	LYS	565	48.745	25.204	30.123	1.00	0.00	LX0	C
ATOM	27	NZ	LYS	565	47.819	25.809	31.086	1.00	0.00	LX0	N
ATOM	28	HZ1	LYS	565	48.257	26.277	31.905	0.00	0.00	LX0	H
ATOM	29	HZ2	LYS	565	47.284	26.602	30.671	0.00	0.00	LX0	H
ATOM	30	HZ3	LYS	565	47.111	25.138	31.438	0.00	0.00	LX0	H
ATOM	31	C	LYS	565	44.915	27.647	26.783	1.00	0.00	LX0	C
ATOM	32	O	LYS	565	44.848	28.249	27.848	1.00	0.00	LX0	O
ATOM	33	N	ILE	566	43.869	27.407	25.988	1.00	0.00	LX0	N
ATOM	34	H	ILE	566	43.911	26.771	25.219	0.00	0.00	LX0	H
ATOM	35	CA	ILE	566	42.625	28.039	26.402	1.00	0.00	LX0	C
ATOM	36	CB	ILE	566	41.842	28.539	25.168	1.00	0.00	LX0	C
ATOM	37	CG2	ILE	566	41.334	27.396	24.287	1.00	0.00	LX0	C
ATOM	38	CG1	ILE	566	40.739	29.538	25.534	1.00	0.00	LX0	C
ATOM	39	CD1	ILE	566	41.288	30.801	26.200	1.00	0.00	LX0	C
ATOM	40	C	ILE	566	41.789	27.167	27.321	1.00	0.00	LX0	C
ATOM	41	O	ILE	566	41.581	25.983	27.095	1.00	0.00	LX0	O
ATOM	42	N	CYS	567	41.290	27.821	28.375	1.00	0.00	LX0	N
ATOM	43	H	CYS	567	41.597	28.756	28.549	0.00	0.00	LX0	H
ATOM	44	CA	CYS	567	40.164	27.217	29.078	1.00	0.00	LX0	C
ATOM	45	CB	CYS	567	39.844	27.970	30.370	1.00	0.00	LX0	C
ATOM	46	SG	CYS	567	38.317	27.419	31.186	1.00	0.00	LX0	S
ATOM	47	C	CYS	567	38.959	27.223	28.174	1.00	0.00	LX0	C
ATOM	48	O	CYS	567	38.313	28.250	27.973	1.00	0.00	LX0	O
ATOM	49	N	LEU	568	38.695	26.017	27.646	1.00	0.00	LX0	N
ATOM	50	H	LEU	568	39.341	25.282	27.876	0.00	0.00	LX0	H
ATOM	51	CA	LEU	568	37.654	25.818	26.631	1.00	0.00	LX0	C
ATOM	52	CB	LEU	568	37.392	24.324	26.409	1.00	0.00	LX0	C
ATOM	53	CG	LEU	568	38.640	23.449	26.236	1.00	0.00	LX0	C
ATOM	54	CD1	LEU	568	38.264	21.977	26.108	1.00	0.00	LX0	C
ATOM	55	CD2	LEU	568	39.551	23.892	25.093	1.00	0.00	LX0	C
ATOM	56	C	LEU	568	36.344	26.520	26.955	1.00	0.00	LX0	C
ATOM	57	O	LEU	568	35.646	27.085	26.117	1.00	0.00	LX0	O
ATOM	58	N	ILE	569	36.067	26.493	28.268	1.00	0.00	LX0	N
ATOM	59	H	ILE	569	36.701	26.015	28.873	0.00	0.00	LX0	H
ATOM	60	CA	ILE	569	34.929	27.268	28.744	1.00	0.00	LX0	C
ATOM	61	CB	ILE	569	34.476	26.820	30.149	1.00	0.00	LX0	C
ATOM	62	CG2	ILE	569	33.089	27.374	30.489	1.00	0.00	LX0	C
ATOM	63	CG1	ILE	569	34.540	25.304	30.342	1.00	0.00	LX0	C
ATOM	64	CD1	ILE	569	33.775	24.480	29.310	1.00	0.00	LX0	C
ATOM	65	C	ILE	569	35.182	28.772	28.694	1.00	0.00	LX0	C
ATOM	66	O	ILE	569	34.791	29.461	27.756	1.00	0.00	LX0	O
ATOM	67	N	CYS	570	35.817	29.269	29.760	1.00	0.00	LX0	N
ATOM	68	H	CYS	570	36.317	28.695	30.407	0.00	0.00	LX0	H
ATOM	69	CA	CYS	570	35.575	30.685	30.007	1.00	0.00	LX0	C

ATOM	70	CB	CYS	570	34.917	30.856	31.372	1.00	0.00	LX0	C
ATOM	71	SG	CYS	570	35.875	30.141	32.734	1.00	0.00	LX0	S
ATOM	72	C	CYS	570	36.748	31.619	29.826	1.00	0.00	LX0	C
ATOM	73	O	CYS	570	37.021	32.480	30.657	1.00	0.00	LX0	O
ATOM	74	N	GLY	571	37.420	31.415	28.681	1.00	0.00	LX0	N
ATOM	75	H	GLY	571	37.189	30.612	28.130	0.00	0.00	LX0	H
ATOM	76	CA	GLY	571	38.323	32.447	28.164	1.00	0.00	LX0	C
ATOM	77	C	GLY	571	39.402	32.954	29.108	1.00	0.00	LX0	C
ATOM	78	O	GLY	571	39.603	34.154	29.282	1.00	0.00	LX0	O
ATOM	79	N	ASP	572	40.091	31.972	29.697	1.00	0.00	LX0	N
ATOM	80	H	ASP	572	39.894	31.016	29.481	0.00	0.00	LX0	H
ATOM	81	CA	ASP	572	41.254	32.267	30.530	1.00	0.00	LX0	C
ATOM	82	CB	ASP	572	40.857	32.604	31.974	1.00	0.00	LX0	C
ATOM	83	CG	ASP	572	41.064	34.091	32.181	1.00	0.00	LX0	C
ATOM	84	OD1	ASP	572	42.134	34.599	31.874	1.00	0.00	LX0	O
ATOM	85	OD2	ASP	572	40.133	34.795	32.556	1.00	0.00	LX0	O
ATOM	86	C	ASP	572	42.203	31.097	30.468	1.00	0.00	LX0	C
ATOM	87	O	ASP	572	41.992	30.210	29.650	1.00	0.00	LX0	O
ATOM	88	N	GLU	573	43.218	31.109	31.344	1.00	0.00	LX0	N
ATOM	89	H	GLU	573	43.341	31.898	31.946	0.00	0.00	LX0	H
ATOM	90	CA	GLU	573	44.166	29.992	31.370	1.00	0.00	LX0	C
ATOM	91	CB	GLU	573	45.187	30.222	32.488	1.00	0.00	LX0	C
ATOM	92	CG	GLU	573	46.652	30.002	32.095	1.00	0.00	LX0	C
ATOM	93	CD	GLU	573	46.909	28.570	31.664	1.00	0.00	LX0	C
ATOM	94	OE1	GLU	573	46.975	28.306	30.467	1.00	0.00	LX0	O
ATOM	95	OE2	GLU	573	47.065	27.702	32.515	1.00	0.00	LX0	O
ATOM	96	C	GLU	573	43.525	28.618	31.531	1.00	0.00	LX0	C
ATOM	97	O	GLU	573	42.623	28.414	32.340	1.00	0.00	LX0	O
ATOM	98	N	ALA	574	44.019	27.677	30.719	1.00	0.00	LX0	N
ATOM	99	H	ALA	574	44.788	27.916	30.121	0.00	0.00	LX0	H
ATOM	100	CA	ALA	574	43.588	26.305	30.967	1.00	0.00	LX0	C
ATOM	101	CB	ALA	574	43.608	25.468	29.689	1.00	0.00	LX0	C
ATOM	102	C	ALA	574	44.485	25.621	31.969	1.00	0.00	LX0	C
ATOM	103	O	ALA	574	45.473	24.989	31.605	1.00	0.00	LX0	O
ATOM	104	N	SER	575	44.102	25.752	33.245	1.00	0.00	LX0	N
ATOM	105	H	SER	575	43.287	26.278	33.487	0.00	0.00	LX0	H
ATOM	106	CA	SER	575	44.921	25.128	34.286	1.00	0.00	LX0	C
ATOM	107	CB	SER	575	44.338	25.440	35.667	1.00	0.00	LX0	C
ATOM	108	OG	SER	575	42.915	25.616	35.585	1.00	0.00	LX0	O
ATOM	109	HG	SER	575	42.565	24.794	35.237	0.00	0.00	LX0	H
ATOM	110	C	SER	575	45.200	23.641	34.098	1.00	0.00	LX0	C
ATOM	111	O	SER	575	46.282	23.150	34.392	1.00	0.00	LX0	O
ATOM	112	N	GLY	576	44.195	22.948	33.543	1.00	0.00	LX0	N
ATOM	113	H	GLY	576	43.296	23.352	33.352	0.00	0.00	LX0	H
ATOM	114	CA	GLY	576	44.514	21.593	33.112	1.00	0.00	LX0	C
ATOM	115	C	GLY	576	43.270	20.781	32.859	1.00	0.00	LX0	C
ATOM	116	O	GLY	576	42.194	21.316	32.635	1.00	0.00	LX0	O
ATOM	117	N	CYS	577	43.462	19.457	32.926	1.00	0.00	LX0	N
ATOM	118	H	CYS	577	44.367	19.103	33.155	0.00	0.00	LX0	H
ATOM	119	CA	CYS	577	42.294	18.580	32.846	1.00	0.00	LX0	C
ATOM	120	CB	CYS	577	42.741	17.126	32.649	1.00	0.00	LX0	C
ATOM	121	SG	CYS	577	41.443	15.875	32.881	1.00	0.00	LX0	S
ATOM	122	C	CYS	577	41.434	18.704	34.086	1.00	0.00	LX0	C
ATOM	123	O	CYS	577	41.766	18.207	35.160	1.00	0.00	LX0	O
ATOM	124	N	HIS	578	40.311	19.399	33.916	1.00	0.00	LX0	N
ATOM	125	H	HIS	578	40.078	19.845	33.047	0.00	0.00	LX0	H
ATOM	126	CA	HIS	578	39.376	19.351	35.029	1.00	0.00	LX0	C
ATOM	127	CB	HIS	578	39.160	20.711	35.698	1.00	0.00	LX0	C
ATOM	128	CG	HIS	578	40.465	21.395	36.046	1.00	0.00	LX0	C
ATOM	129	ND1	HIS	578	41.610	20.762	36.372	1.00	0.00	LX0	N
ATOM	130	HD1	HIS	578	41.762	19.790	36.368	0.00	0.00	LX0	H

ATOM	131	CD2	HIS	578	40.694	22.772	36.090	1.00	0.00	LX0	C
ATOM	132	NE2	HIS	578	41.988	22.960	36.446	1.00	0.00	LX0	N
ATOM	133	CE1	HIS	578	42.553	21.724	36.619	1.00	0.00	LX0	C
ATOM	134	C	HIS	578	38.081	18.763	34.546	1.00	0.00	LX0	C
ATOM	135	O	HIS	578	37.445	19.254	33.626	1.00	0.00	LX0	O
ATOM	136	N	TYR	579	37.770	17.620	35.180	1.00	0.00	LX0	N
ATOM	137	H	TYR	579	38.323	17.358	35.967	0.00	0.00	LX0	H
ATOM	138	CA	TYR	579	36.656	16.782	34.723	1.00	0.00	LX0	C
ATOM	139	CB	TYR	579	35.288	17.344	35.142	1.00	0.00	LX0	C
ATOM	140	CG	TYR	579	35.240	17.864	36.563	1.00	0.00	LX0	C
ATOM	141	CD1	TYR	579	35.785	17.122	37.634	1.00	0.00	LX0	C
ATOM	142	CE1	TYR	579	35.736	17.663	38.931	1.00	0.00	LX0	C
ATOM	143	CD2	TYR	579	34.620	19.112	36.765	1.00	0.00	LX0	C
ATOM	144	CE2	TYR	579	34.564	19.646	38.059	1.00	0.00	LX0	C
ATOM	145	CZ	TYR	579	35.141	18.926	39.124	1.00	0.00	LX0	C
ATOM	146	OH	TYR	579	35.136	19.485	40.387	1.00	0.00	LX0	O
ATOM	147	HH	TYR	579	34.755	20.360	40.329	0.00	0.00	LX0	H
ATOM	148	C	TYR	579	36.690	16.490	33.225	1.00	0.00	LX0	C
ATOM	149	O	TYR	579	35.731	16.640	32.482	1.00	0.00	LX0	O
ATOM	150	N	GLY	580	37.900	16.080	32.817	1.00	0.00	LX0	N
ATOM	151	H	GLY	580	38.679	16.072	33.438	0.00	0.00	LX0	H
ATOM	152	CA	GLY	580	38.093	15.881	31.387	1.00	0.00	LX0	C
ATOM	153	C	GLY	580	38.713	17.094	30.725	1.00	0.00	LX0	C
ATOM	154	O	GLY	580	39.924	17.209	30.582	1.00	0.00	LX0	O
ATOM	155	N	VAL	581	37.817	17.991	30.310	1.00	0.00	LX0	N
ATOM	156	H	VAL	581	36.861	17.876	30.585	0.00	0.00	LX0	H
ATOM	157	CA	VAL	581	38.284	19.070	29.439	1.00	0.00	LX0	C
ATOM	158	CB	VAL	581	37.098	19.751	28.753	1.00	0.00	LX0	C
ATOM	159	CG1	VAL	581	36.442	18.809	27.747	1.00	0.00	LX0	C
ATOM	160	CG2	VAL	581	36.096	20.302	29.765	1.00	0.00	LX0	C
ATOM	161	C	VAL	581	39.228	20.101	30.049	1.00	0.00	LX0	C
ATOM	162	O	VAL	581	39.295	20.328	31.255	1.00	0.00	LX0	O
ATOM	163	N	LEU	582	39.966	20.730	29.117	1.00	0.00	LX0	N
ATOM	164	H	LEU	582	39.804	20.518	28.155	0.00	0.00	LX0	H
ATOM	165	CA	LEU	582	40.933	21.759	29.500	1.00	0.00	LX0	C
ATOM	166	CB	LEU	582	41.841	22.109	28.321	1.00	0.00	LX0	C
ATOM	167	CG	LEU	582	42.673	20.928	27.820	1.00	0.00	LX0	C
ATOM	168	CD1	LEU	582	43.390	21.265	26.512	1.00	0.00	LX0	C
ATOM	169	CD2	LEU	582	43.636	20.408	28.890	1.00	0.00	LX0	C
ATOM	170	C	LEU	582	40.286	23.014	30.047	1.00	0.00	LX0	C
ATOM	171	O	LEU	582	39.730	23.846	29.337	1.00	0.00	LX0	O
ATOM	172	N	THR	583	40.359	23.101	31.371	1.00	0.00	LX0	N
ATOM	173	H	THR	583	40.814	22.400	31.923	0.00	0.00	LX0	H
ATOM	174	CA	THR	583	39.600	24.156	32.015	1.00	0.00	LX0	C
ATOM	175	CB	THR	583	38.324	23.552	32.599	1.00	0.00	LX0	C
ATOM	176	OG1	THR	583	38.605	22.292	33.208	1.00	0.00	LX0	O
ATOM	177	HG1	THR	583	38.661	21.638	32.510	0.00	0.00	LX0	H
ATOM	178	CG2	THR	583	37.240	23.376	31.543	1.00	0.00	LX0	C
ATOM	179	C	THR	583	40.397	24.905	33.062	1.00	0.00	LX0	C
ATOM	180	O	THR	583	41.506	24.536	33.443	1.00	0.00	LX0	O
ATOM	181	N	CYS	584	39.771	26.003	33.503	1.00	0.00	LX0	N
ATOM	182	H	CYS	584	38.866	26.256	33.167	0.00	0.00	LX0	H
ATOM	183	CA	CYS	584	40.370	26.712	34.623	1.00	0.00	LX0	C
ATOM	184	CB	CYS	584	40.200	28.225	34.470	1.00	0.00	LX0	C
ATOM	185	SG	CYS	584	38.471	28.769	34.564	1.00	0.00	LX0	S
ATOM	186	C	CYS	584	39.783	26.237	35.933	1.00	0.00	LX0	C
ATOM	187	O	CYS	584	38.656	25.750	35.986	1.00	0.00	LX0	O
ATOM	188	N	GLY	585	40.567	26.447	37.002	1.00	0.00	LX0	N
ATOM	189	H	GLY	585	41.536	26.623	36.806	0.00	0.00	LX0	H
ATOM	190	CA	GLY	585	40.104	26.120	38.356	1.00	0.00	LX0	C
ATOM	191	C	GLY	585	38.663	26.495	38.693	1.00	0.00	LX0	C

ATOM	192	O	GLY	585	37.912	25.732	39.291	1.00	0.00	LX0	O
ATOM	193	N	SER	586	38.281	27.708	38.255	1.00	0.00	LX0	N
ATOM	194	H	SER	586	38.920	28.283	37.749	0.00	0.00	LX0	H
ATOM	195	CA	SER	586	36.904	28.097	38.561	1.00	0.00	LX0	C
ATOM	196	CB	SER	586	36.684	29.597	38.326	1.00	0.00	LX0	C
ATOM	197	OG	SER	586	36.835	29.933	36.936	1.00	0.00	LX0	O
ATOM	198	HG	SER	586	37.730	30.250	36.835	0.00	0.00	LX0	H
ATOM	199	C	SER	586	35.824	27.244	37.901	1.00	0.00	LX0	C
ATOM	200	O	SER	586	34.755	27.009	38.447	1.00	0.00	LX0	O
ATOM	201	N	CYS	587	36.155	26.754	36.694	1.00	0.00	LX0	N
ATOM	202	H	CYS	587	37.073	26.892	36.321	0.00	0.00	LX0	H
ATOM	203	CA	CYS	587	35.227	25.805	36.079	1.00	0.00	LX0	C
ATOM	204	CB	CYS	587	35.587	25.547	34.618	1.00	0.00	LX0	C
ATOM	205	SG	CYS	587	35.598	27.081	33.656	1.00	0.00	LX0	S
ATOM	206	C	CYS	587	35.117	24.505	36.850	1.00	0.00	LX0	C
ATOM	207	O	CYS	587	34.033	23.988	37.073	1.00	0.00	LX0	O
ATOM	208	N	LYS	588	36.287	24.041	37.322	1.00	0.00	LX0	N
ATOM	209	H	LYS	588	37.142	24.497	37.067	0.00	0.00	LX0	H
ATOM	210	CA	LYS	588	36.286	22.880	38.218	1.00	0.00	LX0	C
ATOM	211	CB	LYS	588	37.720	22.600	38.679	1.00	0.00	LX0	C
ATOM	212	CG	LYS	588	37.891	21.364	39.565	1.00	0.00	LX0	C
ATOM	213	CD	LYS	588	39.307	21.242	40.125	1.00	0.00	LX0	C
ATOM	214	CE	LYS	588	39.453	20.048	41.071	1.00	0.00	LX0	C
ATOM	215	NZ	LYS	588	40.806	20.042	41.644	1.00	0.00	LX0	N
ATOM	216	HZ1	LYS	588	40.909	19.230	42.286	0.00	0.00	LX0	H
ATOM	217	HZ2	LYS	588	41.510	19.973	40.881	0.00	0.00	LX0	H
ATOM	218	HZ3	LYS	588	40.961	20.922	42.177	0.00	0.00	LX0	H
ATOM	219	C	LYS	588	35.349	23.023	39.417	1.00	0.00	LX0	C
ATOM	220	O	LYS	588	34.550	22.151	39.744	1.00	0.00	LX0	O
ATOM	221	N	VAL	589	35.479	24.191	40.063	1.00	0.00	LX0	N
ATOM	222	H	VAL	589	36.142	24.875	39.753	0.00	0.00	LX0	H
ATOM	223	CA	VAL	589	34.625	24.378	41.234	1.00	0.00	LX0	C
ATOM	224	CB	VAL	589	35.256	25.352	42.242	1.00	0.00	LX0	C
ATOM	225	CG1	VAL	589	36.629	24.839	42.682	1.00	0.00	LX0	C
ATOM	226	CG2	VAL	589	35.315	26.793	41.730	1.00	0.00	LX0	C
ATOM	227	C	VAL	589	33.170	24.728	40.949	1.00	0.00	LX0	C
ATOM	228	O	VAL	589	32.320	24.665	41.835	1.00	0.00	LX0	O
ATOM	229	N	PHE	590	32.915	25.090	39.683	1.00	0.00	LX0	N
ATOM	230	H	PHE	590	33.652	25.171	39.012	0.00	0.00	LX0	H
ATOM	231	CA	PHE	590	31.525	25.302	39.294	1.00	0.00	LX0	C
ATOM	232	CB	PHE	590	31.441	26.320	38.145	1.00	0.00	LX0	C
ATOM	233	CG	PHE	590	30.001	26.640	37.804	1.00	0.00	LX0	C
ATOM	234	CD1	PHE	590	29.288	27.573	38.590	1.00	0.00	LX0	C
ATOM	235	CD2	PHE	590	29.390	25.993	36.707	1.00	0.00	LX0	C
ATOM	236	CE1	PHE	590	27.942	27.853	38.282	1.00	0.00	LX0	C
ATOM	237	CE2	PHE	590	28.045	26.270	36.401	1.00	0.00	LX0	C
ATOM	238	CZ	PHE	590	27.332	27.193	37.194	1.00	0.00	LX0	C
ATOM	239	C	PHE	590	30.814	24.007	38.944	1.00	0.00	LX0	C
ATOM	240	O	PHE	590	29.775	23.671	39.497	1.00	0.00	LX0	O
ATOM	241	N	PHE	591	31.412	23.284	37.985	1.00	0.00	LX0	N
ATOM	242	H	PHE	591	32.296	23.564	37.612	0.00	0.00	LX0	H
ATOM	243	CA	PHE	591	30.650	22.189	37.388	1.00	0.00	LX0	C
ATOM	244	CB	PHE	591	31.366	21.597	36.171	1.00	0.00	LX0	C
ATOM	245	CG	PHE	591	30.346	20.836	35.358	1.00	0.00	LX0	C
ATOM	246	CD1	PHE	591	29.416	21.553	34.576	1.00	0.00	LX0	C
ATOM	247	CD2	PHE	591	30.314	19.426	35.424	1.00	0.00	LX0	C
ATOM	248	CE1	PHE	591	28.408	20.856	33.887	1.00	0.00	LX0	C
ATOM	249	CE2	PHE	591	29.308	18.727	34.733	1.00	0.00	LX0	C
ATOM	250	CZ	PHE	591	28.350	19.452	33.993	1.00	0.00	LX0	C
ATOM	251	C	PHE	591	30.189	21.102	38.342	1.00	0.00	LX0	C
ATOM	252	O	PHE	591	29.089	20.575	38.238	1.00	0.00	LX0	O

ATOM	253	N	LYS	592	31.053	20.835	39.334	1.00	0.00	LX0	N
ATOM	254	H	LYS	592	31.957	21.259	39.336	0.00	0.00	LX0	H
ATOM	255	CA	LYS	592	30.598	19.910	40.374	1.00	0.00	LX0	C
ATOM	256	CB	LYS	592	31.706	19.716	41.409	1.00	0.00	LX0	C
ATOM	257	CG	LYS	592	31.636	18.355	42.101	1.00	0.00	LX0	C
ATOM	258	CD	LYS	592	32.688	18.211	43.203	1.00	0.00	LX0	C
ATOM	259	CE	LYS	592	32.673	16.846	43.899	1.00	0.00	LX0	C
ATOM	260	NZ	LYS	592	31.355	16.587	44.486	1.00	0.00	LX0	N
ATOM	261	HZ1	LYS	592	31.307	15.821	45.179	0.00	0.00	LX0	H
ATOM	262	HZ2	LYS	592	30.880	17.426	44.884	0.00	0.00	LX0	H
ATOM	263	HZ3	LYS	592	30.644	16.324	43.765	0.00	0.00	LX0	H
ATOM	264	C	LYS	592	29.278	20.332	41.022	1.00	0.00	LX0	C
ATOM	265	O	LYS	592	28.331	19.570	41.154	1.00	0.00	LX0	O
ATOM	266	N	ARG	593	29.239	21.633	41.351	1.00	0.00	LX0	N
ATOM	267	H	ARG	593	29.990	22.234	41.080	0.00	0.00	LX0	H
ATOM	268	CA	ARG	593	27.991	22.202	41.861	1.00	0.00	LX0	C
ATOM	269	CB	ARG	593	28.221	23.625	42.362	1.00	0.00	LX0	C
ATOM	270	CG	ARG	593	29.094	23.694	43.611	1.00	0.00	LX0	C
ATOM	271	CD	ARG	593	29.401	25.145	43.982	1.00	0.00	LX0	C
ATOM	272	NE	ARG	593	30.011	25.238	45.308	1.00	0.00	LX0	N
ATOM	273	HE	ARG	593	29.374	25.220	46.080	0.00	0.00	LX0	H
ATOM	274	CZ	ARG	593	31.348	25.339	45.465	1.00	0.00	LX0	C
ATOM	275	NH1	ARG	593	32.167	25.306	44.415	1.00	0.00	LX0	N
ATOM	276	HH11	ARG	593	33.154	25.420	44.501	0.00	0.00	LX0	H
ATOM	277	HH12	ARG	593	31.812	25.155	43.481	0.00	0.00	LX0	H
ATOM	278	NH2	ARG	593	31.845	25.472	46.694	1.00	0.00	LX0	N
ATOM	279	HH21	ARG	593	32.828	25.549	46.859	0.00	0.00	LX0	H
ATOM	280	HH22	ARG	593	31.233	25.500	47.487	0.00	0.00	LX0	H
ATOM	281	C	ARG	593	26.838	22.197	40.871	1.00	0.00	LX0	C
ATOM	282	O	ARG	593	25.676	22.109	41.243	1.00	0.00	LX0	O
ATOM	283	N	ALA	594	27.192	22.274	39.584	1.00	0.00	LX0	N
ATOM	284	H	ALA	594	28.155	22.379	39.336	0.00	0.00	LX0	H
ATOM	285	CA	ALA	594	26.141	22.162	38.571	1.00	0.00	LX0	C
ATOM	286	CB	ALA	594	26.700	22.512	37.195	1.00	0.00	LX0	C
ATOM	287	C	ALA	594	25.455	20.801	38.517	1.00	0.00	LX0	C
ATOM	288	O	ALA	594	24.291	20.662	38.154	1.00	0.00	LX0	O
ATOM	289	N	MET	595	26.236	19.791	38.922	1.00	0.00	LX0	N
ATOM	290	H	MET	595	27.194	19.960	39.163	0.00	0.00	LX0	H
ATOM	291	CA	MET	595	25.638	18.464	39.032	1.00	0.00	LX0	C
ATOM	292	CB	MET	595	26.715	17.397	38.835	1.00	0.00	LX0	C
ATOM	293	CG	MET	595	27.395	17.496	37.470	1.00	0.00	LX0	C
ATOM	294	SD	MET	595	26.320	17.012	36.111	1.00	0.00	LX0	S
ATOM	295	CE	MET	595	26.389	15.234	36.383	1.00	0.00	LX0	C
ATOM	296	C	MET	595	24.903	18.243	40.343	1.00	0.00	LX0	C
ATOM	297	O	MET	595	23.737	17.869	40.391	1.00	0.00	LX0	O
ATOM	298	N	GLU	596	25.662	18.484	41.419	1.00	0.00	LX0	N
ATOM	299	H	GLU	596	26.590	18.839	41.304	0.00	0.00	LX0	H
ATOM	300	CA	GLU	596	25.168	18.124	42.747	1.00	0.00	LX0	C
ATOM	301	CB	GLU	596	26.352	17.918	43.691	1.00	0.00	LX0	C
ATOM	302	CG	GLU	596	27.340	16.896	43.123	1.00	0.00	LX0	C
ATOM	303	CD	GLU	596	28.587	16.796	43.979	1.00	0.00	LX0	C
ATOM	304	OE1	GLU	596	29.169	17.817	44.349	1.00	0.00	LX0	O
ATOM	305	OE2	GLU	596	29.041	15.683	44.234	1.00	0.00	LX0	O
ATOM	306	C	GLU	596	24.171	19.098	43.346	1.00	0.00	LX0	C
ATOM	307	O	GLU	596	23.328	18.754	44.169	1.00	0.00	LX0	O
ATOM	308	N	GLY	597	24.300	20.352	42.899	1.00	0.00	LX0	N
ATOM	309	H	GLY	597	24.933	20.561	42.154	0.00	0.00	LX0	H
ATOM	310	CA	GLY	597	23.443	21.396	43.448	1.00	0.00	LX0	C
ATOM	311	C	GLY	597	22.000	21.289	43.005	1.00	0.00	LX0	C
ATOM	312	O	GLY	597	21.580	21.816	41.984	1.00	0.00	LX0	O
ATOM	313	N	GLN	598	21.242	20.596	43.870	1.00	0.00	LX0	N

ATOM	314	H	GLN	598	21.736	20.114	44.595	0.00	0.00	LX0	H
ATOM	315	CA	GLN	598	19.822	20.362	43.587	1.00	0.00	LX0	C
ATOM	316	CB	GLN	598	19.205	19.448	44.652	1.00	0.00	LX0	C
ATOM	317	CG	GLN	598	19.945	18.113	44.837	1.00	0.00	LX0	C
ATOM	318	CD	GLN	598	19.966	17.326	43.538	1.00	0.00	LX0	C
ATOM	319	OE1	GLN	598	18.950	16.886	43.023	1.00	0.00	LX0	O
ATOM	320	NE2	GLN	598	21.185	17.185	43.013	1.00	0.00	LX0	N
ATOM	321	HE21	GLN	598	21.992	17.534	43.492	0.00	0.00	LX0	H
ATOM	322	HE22	GLN	598	21.292	16.739	42.126	0.00	0.00	LX0	H
ATOM	323	C	GLN	598	18.968	21.602	43.350	1.00	0.00	LX0	C
ATOM	324	O	GLN	598	17.904	21.556	42.745	1.00	0.00	LX0	O
ATOM	325	N	HIS	599	19.509	22.742	43.813	1.00	0.00	LX0	N
ATOM	326	H	HIS	599	20.374	22.690	44.305	0.00	0.00	LX0	H
ATOM	327	CA	HIS	599	19.015	24.018	43.291	1.00	0.00	LX0	C
ATOM	328	CB	HIS	599	19.457	25.183	44.192	1.00	0.00	LX0	C
ATOM	329	CG	HIS	599	20.925	25.095	44.557	1.00	0.00	LX0	C
ATOM	330	ND1	HIS	599	21.934	25.152	43.671	1.00	0.00	LX0	N
ATOM	331	HD1	HIS	599	21.849	25.249	42.696	0.00	0.00	LX0	H
ATOM	332	CD2	HIS	599	21.466	24.939	45.836	1.00	0.00	LX0	C
ATOM	333	NE2	HIS	599	22.814	24.903	45.703	1.00	0.00	LX0	N
ATOM	334	CE1	HIS	599	23.105	25.036	44.371	1.00	0.00	LX0	C
ATOM	335	C	HIS	599	19.402	24.240	41.832	1.00	0.00	LX0	C
ATOM	336	O	HIS	599	20.329	24.964	41.480	1.00	0.00	LX0	O
ATOM	337	N	ASN	600	18.635	23.546	40.983	1.00	0.00	LX0	N
ATOM	338	H	ASN	600	17.901	22.978	41.362	0.00	0.00	LX0	H
ATOM	339	CA	ASN	600	18.990	23.493	39.570	1.00	0.00	LX0	C
ATOM	340	CB	ASN	600	18.236	22.355	38.880	1.00	0.00	LX0	C
ATOM	341	CG	ASN	600	18.786	22.136	37.484	1.00	0.00	LX0	C
ATOM	342	OD1	ASN	600	19.695	21.347	37.249	1.00	0.00	LX0	O
ATOM	343	ND2	ASN	600	18.165	22.854	36.548	1.00	0.00	LX0	N
ATOM	344	HD21	ASN	600	17.519	23.573	36.817	0.00	0.00	LX0	H
ATOM	345	HD22	ASN	600	18.350	22.697	35.579	0.00	0.00	LX0	H
ATOM	346	C	ASN	600	18.815	24.799	38.817	1.00	0.00	LX0	C
ATOM	347	O	ASN	600	17.755	25.106	38.287	1.00	0.00	LX0	O
ATOM	348	N	TYR	601	19.947	25.509	38.767	1.00	0.00	LX0	N
ATOM	349	H	TYR	601	20.637	25.192	39.419	0.00	0.00	LX0	H
ATOM	350	CA	TYR	601	20.137	26.743	37.994	1.00	0.00	LX0	C
ATOM	351	CB	TYR	601	21.625	26.948	37.646	1.00	0.00	LX0	C
ATOM	352	CG	TYR	601	22.535	26.389	38.719	1.00	0.00	LX0	C
ATOM	353	CD1	TYR	601	22.801	27.147	39.877	1.00	0.00	LX0	C
ATOM	354	CE1	TYR	601	23.591	26.582	40.893	1.00	0.00	LX0	C
ATOM	355	CD2	TYR	601	23.073	25.098	38.533	1.00	0.00	LX0	C
ATOM	356	CE2	TYR	601	23.851	24.531	39.553	1.00	0.00	LX0	C
ATOM	357	CZ	TYR	601	24.091	25.275	40.725	1.00	0.00	LX0	C
ATOM	358	OH	TYR	601	24.833	24.705	41.739	1.00	0.00	LX0	O
ATOM	359	HH	TYR	601	24.903	23.766	41.583	0.00	0.00	LX0	H
ATOM	360	C	TYR	601	19.314	26.876	36.719	1.00	0.00	LX0	C
ATOM	361	O	TYR	601	19.626	26.302	35.683	1.00	0.00	LX0	O
ATOM	362	N	LEU	602	18.249	27.674	36.846	1.00	0.00	LX0	N
ATOM	363	H	LEU	602	18.058	28.119	37.718	0.00	0.00	LX0	H
ATOM	364	CA	LEU	602	17.511	28.011	35.634	1.00	0.00	LX0	C
ATOM	365	CB	LEU	602	16.004	28.008	35.900	1.00	0.00	LX0	C
ATOM	366	CG	LEU	602	15.454	26.638	36.308	1.00	0.00	LX0	C
ATOM	367	CD1	LEU	602	13.965	26.710	36.648	1.00	0.00	LX0	C
ATOM	368	CD2	LEU	602	15.746	25.557	35.264	1.00	0.00	LX0	C
ATOM	369	C	LEU	602	17.969	29.353	35.103	1.00	0.00	LX0	C
ATOM	370	O	LEU	602	18.751	30.050	35.741	1.00	0.00	LX0	O
ATOM	371	N	CYS	603	17.460	29.697	33.915	1.00	0.00	LX0	N
ATOM	372	H	CYS	603	16.822	29.112	33.417	0.00	0.00	LX0	H
ATOM	373	CA	CYS	603	17.898	30.985	33.390	1.00	0.00	LX0	C
ATOM	374	CB	CYS	603	18.735	30.793	32.127	1.00	0.00	LX0	C

ATOM	375	SG	CYS	603	19.720	32.254	31.703	1.00	0.00	LX0	S
ATOM	376	C	CYS	603	16.761	31.950	33.144	1.00	0.00	LX0	C
ATOM	377	O	CYS	603	15.651	31.570	32.786	1.00	0.00	LX0	O
ATOM	378	N	ALA	604	17.097	33.228	33.376	1.00	0.00	LX0	N
ATOM	379	H	ALA	604	18.023	33.438	33.684	0.00	0.00	LX0	H
ATOM	380	CA	ALA	604	16.148	34.276	33.003	1.00	0.00	LX0	C
ATOM	381	CB	ALA	604	16.372	35.531	33.849	1.00	0.00	LX0	C
ATOM	382	C	ALA	604	16.255	34.637	31.532	1.00	0.00	LX0	C
ATOM	383	O	ALA	604	15.271	34.836	30.834	1.00	0.00	LX0	O
ATOM	384	N	GLY	605	17.524	34.680	31.090	1.00	0.00	LX0	N
ATOM	385	H	GLY	605	18.283	34.469	31.700	0.00	0.00	LX0	H
ATOM	386	CA	GLY	605	17.733	34.701	29.647	1.00	0.00	LX0	C
ATOM	387	C	GLY	605	17.547	33.302	29.098	1.00	0.00	LX0	C
ATOM	388	O	GLY	605	17.160	32.384	29.813	1.00	0.00	LX0	O
ATOM	389	N	ARG	606	17.849	33.159	27.808	1.00	0.00	LX0	N
ATOM	390	H	ARG	606	18.381	33.880	27.351	0.00	0.00	LX0	H
ATOM	391	CA	ARG	606	17.535	31.892	27.144	1.00	0.00	LX0	C
ATOM	392	CB	ARG	606	17.282	32.149	25.659	1.00	0.00	LX0	C
ATOM	393	CG	ARG	606	16.307	33.302	25.425	1.00	0.00	LX0	C
ATOM	394	CD	ARG	606	16.495	33.917	24.039	1.00	0.00	LX0	C
ATOM	395	NE	ARG	606	15.723	35.152	23.898	1.00	0.00	LX0	N
ATOM	396	HE	ARG	606	14.787	35.051	23.558	0.00	0.00	LX0	H
ATOM	397	CZ	ARG	606	16.251	36.347	24.254	1.00	0.00	LX0	C
ATOM	398	NH1	ARG	606	17.482	36.440	24.750	1.00	0.00	LX0	N
ATOM	399	HH11	ARG	606	17.921	37.299	25.005	0.00	0.00	LX0	H
ATOM	400	HH12	ARG	606	18.046	35.617	24.915	0.00	0.00	LX0	H
ATOM	401	NH2	ARG	606	15.517	37.448	24.106	1.00	0.00	LX0	N
ATOM	402	HH21	ARG	606	15.875	38.346	24.365	0.00	0.00	LX0	H
ATOM	403	HH22	ARG	606	14.590	37.395	23.734	0.00	0.00	LX0	H
ATOM	404	C	ARG	606	18.596	30.815	27.312	1.00	0.00	LX0	C
ATOM	405	O	ARG	606	18.859	30.018	26.419	1.00	0.00	LX0	O
ATOM	406	N	ASN	607	19.244	30.856	28.494	1.00	0.00	LX0	N
ATOM	407	H	ASN	607	18.833	31.413	29.214	0.00	0.00	LX0	H
ATOM	408	CA	ASN	607	20.517	30.149	28.720	1.00	0.00	LX0	C
ATOM	409	CB	ASN	607	20.410	28.610	28.662	1.00	0.00	LX0	C
ATOM	410	CG	ASN	607	19.138	28.100	29.313	1.00	0.00	LX0	C
ATOM	411	OD1	ASN	607	19.024	27.931	30.520	1.00	0.00	LX0	O
ATOM	412	ND2	ASN	607	18.163	27.853	28.432	1.00	0.00	LX0	N
ATOM	413	HD21	ASN	607	18.307	28.076	27.465	0.00	0.00	LX0	H
ATOM	414	HD22	ASN	607	17.291	27.475	28.730	0.00	0.00	LX0	H
ATOM	415	C	ASN	607	21.602	30.625	27.763	1.00	0.00	LX0	C
ATOM	416	O	ASN	607	22.510	29.905	27.361	1.00	0.00	LX0	O
ATOM	417	N	ASP	608	21.423	31.895	27.381	1.00	0.00	LX0	N
ATOM	418	H	ASP	608	20.793	32.499	27.872	0.00	0.00	LX0	H
ATOM	419	CA	ASP	608	22.066	32.450	26.193	1.00	0.00	LX0	C
ATOM	420	CB	ASP	608	21.123	33.413	25.446	1.00	0.00	LX0	C
ATOM	421	CG	ASP	608	20.354	34.378	26.348	1.00	0.00	LX0	C
ATOM	422	OD1	ASP	608	19.375	34.946	25.880	1.00	0.00	LX0	O
ATOM	423	OD2	ASP	608	20.670	34.528	27.526	1.00	0.00	LX0	O
ATOM	424	C	ASP	608	23.431	33.072	26.394	1.00	0.00	LX0	C
ATOM	425	O	ASP	608	24.360	32.777	25.651	1.00	0.00	LX0	O
ATOM	426	N	CYS	609	23.548	33.897	27.445	1.00	0.00	LX0	N
ATOM	427	H	CYS	609	22.687	34.140	27.902	0.00	0.00	LX0	H
ATOM	428	CA	CYS	609	24.792	34.643	27.688	1.00	0.00	LX0	C
ATOM	429	CB	CYS	609	24.840	35.162	29.126	1.00	0.00	LX0	C
ATOM	430	SG	CYS	609	23.435	36.215	29.561	1.00	0.00	LX0	S
ATOM	431	C	CYS	609	26.136	33.993	27.349	1.00	0.00	LX0	C
ATOM	432	O	CYS	609	26.364	32.784	27.445	1.00	0.00	LX0	O
ATOM	433	N	ILE	610	27.035	34.897	26.932	1.00	0.00	LX0	N
ATOM	434	H	ILE	610	26.786	35.864	26.931	0.00	0.00	LX0	H
ATOM	435	CA	ILE	610	28.415	34.490	26.671	1.00	0.00	LX0	C

ATOM	436	CB	ILE	610	29.144	35.599	25.888	1.00	0.00	LX0	C
ATOM	437	CG2	ILE	610	30.543	35.175	25.422	1.00	0.00	LX0	C
ATOM	438	CG1	ILE	610	28.295	36.084	24.708	1.00	0.00	LX0	C
ATOM	439	CD1	ILE	610	28.872	37.331	24.033	1.00	0.00	LX0	C
ATOM	440	C	ILE	610	29.109	34.180	27.988	1.00	0.00	LX0	C
ATOM	441	O	ILE	610	28.804	34.767	29.016	1.00	0.00	LX0	O
ATOM	442	N	VAL	611	30.014	33.194	27.930	1.00	0.00	LX0	N
ATOM	443	H	VAL	611	30.295	32.818	27.050	0.00	0.00	LX0	H
ATOM	444	CA	VAL	611	30.697	32.838	29.172	1.00	0.00	LX0	C
ATOM	445	CB	VAL	611	30.609	31.323	29.409	1.00	0.00	LX0	C
ATOM	446	CG1	VAL	611	31.108	30.904	30.793	1.00	0.00	LX0	C
ATOM	447	CG2	VAL	611	29.163	30.862	29.235	1.00	0.00	LX0	C
ATOM	448	C	VAL	611	32.121	33.370	29.171	1.00	0.00	LX0	C
ATOM	449	O	VAL	611	33.119	32.663	29.248	1.00	0.00	LX0	O
ATOM	450	N	ASP	612	32.142	34.694	29.024	1.00	0.00	LX0	N
ATOM	451	H	ASP	612	31.294	35.227	29.003	0.00	0.00	LX0	H
ATOM	452	CA	ASP	612	33.407	35.418	29.054	1.00	0.00	LX0	C
ATOM	453	CB	ASP	612	33.269	36.733	28.281	1.00	0.00	LX0	C
ATOM	454	CG	ASP	612	32.057	37.495	28.780	1.00	0.00	LX0	C
ATOM	455	OD1	ASP	612	32.128	38.175	29.800	1.00	0.00	LX0	O
ATOM	456	OD2	ASP	612	31.018	37.398	28.146	1.00	0.00	LX0	O
ATOM	457	C	ASP	612	33.853	35.685	30.475	1.00	0.00	LX0	C
ATOM	458	O	ASP	612	33.219	35.269	31.440	1.00	0.00	LX0	O
ATOM	459	N	LYS	613	34.973	36.427	30.561	1.00	0.00	LX0	N
ATOM	460	H	LYS	613	35.392	36.749	29.715	0.00	0.00	LX0	H
ATOM	461	CA	LYS	613	35.560	36.734	31.865	1.00	0.00	LX0	C
ATOM	462	CB	LYS	613	36.785	37.645	31.727	1.00	0.00	LX0	C
ATOM	463	CG	LYS	613	37.926	37.080	30.874	1.00	0.00	LX0	C
ATOM	464	CD	LYS	613	39.172	37.971	30.963	1.00	0.00	LX0	C
ATOM	465	CE	LYS	613	40.350	37.553	30.072	1.00	0.00	LX0	C
ATOM	466	NZ	LYS	613	40.802	36.198	30.400	1.00	0.00	LX0	N
ATOM	467	HZ1	LYS	613	41.701	35.932	29.961	0.00	0.00	LX0	H
ATOM	468	HZ2	LYS	613	40.956	36.032	31.423	0.00	0.00	LX0	H
ATOM	469	HZ3	LYS	613	40.103	35.479	30.129	0.00	0.00	LX0	H
ATOM	470	C	LYS	613	34.619	37.296	32.923	1.00	0.00	LX0	C
ATOM	471	O	LYS	613	34.792	37.051	34.114	1.00	0.00	LX0	O
ATOM	472	N	ILE	614	33.626	38.056	32.436	1.00	0.00	LX0	N
ATOM	473	H	ILE	614	33.475	38.134	31.447	0.00	0.00	LX0	H
ATOM	474	CA	ILE	614	32.665	38.617	33.381	1.00	0.00	LX0	C
ATOM	475	CB	ILE	614	32.147	39.973	32.876	1.00	0.00	LX0	C
ATOM	476	CG2	ILE	614	31.242	40.655	33.910	1.00	0.00	LX0	C
ATOM	477	CG1	ILE	614	33.301	40.876	32.432	1.00	0.00	LX0	C
ATOM	478	CD1	ILE	614	32.822	42.127	31.693	1.00	0.00	LX0	C
ATOM	479	C	ILE	614	31.505	37.671	33.642	1.00	0.00	LX0	C
ATOM	480	O	ILE	614	31.173	37.319	34.771	1.00	0.00	LX0	O
ATOM	481	N	ARG	615	30.872	37.275	32.529	1.00	0.00	LX0	N
ATOM	482	H	ARG	615	31.247	37.484	31.620	0.00	0.00	LX0	H
ATOM	483	CA	ARG	615	29.612	36.560	32.718	1.00	0.00	LX0	C
ATOM	484	CB	ARG	615	28.739	36.686	31.476	1.00	0.00	LX0	C
ATOM	485	CG	ARG	615	28.008	38.032	31.476	1.00	0.00	LX0	C
ATOM	486	CD	ARG	615	27.644	38.516	30.074	1.00	0.00	LX0	C
ATOM	487	NE	ARG	615	28.876	38.762	29.334	1.00	0.00	LX0	N
ATOM	488	HE	ARG	615	29.513	37.994	29.191	0.00	0.00	LX0	H
ATOM	489	CZ	ARG	615	29.273	39.977	28.916	1.00	0.00	LX0	C
ATOM	490	NH1	ARG	615	28.461	41.029	29.011	1.00	0.00	LX0	N
ATOM	491	HH11	ARG	615	28.747	41.940	28.716	0.00	0.00	LX0	H
ATOM	492	HH12	ARG	615	27.540	40.909	29.380	0.00	0.00	LX0	H
ATOM	493	NH2	ARG	615	30.493	40.102	28.413	1.00	0.00	LX0	N
ATOM	494	HH21	ARG	615	30.881	40.943	28.046	0.00	0.00	LX0	H
ATOM	495	HH22	ARG	615	31.069	39.267	28.416	0.00	0.00	LX0	H
ATOM	496	C	ARG	615	29.700	35.145	33.262	1.00	0.00	LX0	C

ATOM	497	O	ARG	615	28.735	34.608	33.800	1.00	0.00	LX0	O
ATOM	498	N	ARG	616	30.931	34.606	33.204	1.00	0.00	LX0	N
ATOM	499	H	ARG	616	31.640	35.058	32.656	0.00	0.00	LX0	H
ATOM	500	CA	ARG	616	31.225	33.371	33.940	1.00	0.00	LX0	C
ATOM	501	CB	ARG	616	32.661	32.921	33.669	1.00	0.00	LX0	C
ATOM	502	CG	ARG	616	33.702	33.876	34.256	1.00	0.00	LX0	C
ATOM	503	CD	ARG	616	35.133	33.467	33.939	1.00	0.00	LX0	C
ATOM	504	NE	ARG	616	36.072	34.477	34.413	1.00	0.00	LX0	N
ATOM	505	HE	ARG	616	35.742	35.166	35.065	0.00	0.00	LX0	H
ATOM	506	CZ	ARG	616	37.324	34.514	33.917	1.00	0.00	LX0	C
ATOM	507	NH1	ARG	616	37.682	33.732	32.905	1.00	0.00	LX0	N
ATOM	508	HH11	ARG	616	38.633	33.768	32.578	0.00	0.00	LX0	H
ATOM	509	HH12	ARG	616	37.058	33.120	32.410	0.00	0.00	LX0	H
ATOM	510	NH2	ARG	616	38.216	35.355	34.426	1.00	0.00	LX0	N
ATOM	511	HH21	ARG	616	39.147	35.361	34.035	0.00	0.00	LX0	H
ATOM	512	HH22	ARG	616	37.998	35.978	35.176	0.00	0.00	LX0	H
ATOM	513	C	ARG	616	30.996	33.413	35.449	1.00	0.00	LX0	C
ATOM	514	O	ARG	616	30.953	32.388	36.123	1.00	0.00	LX0	O
ATOM	515	N	LYS	617	30.878	34.645	35.959	1.00	0.00	LX0	N
ATOM	516	H	LYS	617	31.029	35.455	35.388	0.00	0.00	LX0	H
ATOM	517	CA	LYS	617	30.477	34.791	37.351	1.00	0.00	LX0	C
ATOM	518	CB	LYS	617	31.655	35.273	38.208	1.00	0.00	LX0	C
ATOM	519	CG	LYS	617	32.280	36.595	37.748	1.00	0.00	LX0	C
ATOM	520	CD	LYS	617	32.521	37.537	38.929	1.00	0.00	LX0	C
ATOM	521	CE	LYS	617	31.798	38.883	38.796	1.00	0.00	LX0	C
ATOM	522	NZ	LYS	617	30.359	38.681	38.585	1.00	0.00	LX0	N
ATOM	523	HZ1	LYS	617	29.802	39.519	38.828	0.00	0.00	LX0	H
ATOM	524	HZ2	LYS	617	30.168	38.480	37.580	0.00	0.00	LX0	H
ATOM	525	HZ3	LYS	617	29.984	37.856	39.102	0.00	0.00	LX0	H
ATOM	526	C	LYS	617	29.277	35.713	37.484	1.00	0.00	LX0	C
ATOM	527	O	LYS	617	29.152	36.487	38.430	1.00	0.00	LX0	O
ATOM	528	N	ASN	618	28.426	35.629	36.455	1.00	0.00	LX0	N
ATOM	529	H	ASN	618	28.596	34.968	35.723	0.00	0.00	LX0	H
ATOM	530	CA	ASN	618	27.227	36.465	36.453	1.00	0.00	LX0	C
ATOM	531	CB	ASN	618	27.246	37.517	35.339	1.00	0.00	LX0	C
ATOM	532	CG	ASN	618	27.959	38.785	35.762	1.00	0.00	LX0	C
ATOM	533	OD1	ASN	618	28.907	38.799	36.539	1.00	0.00	LX0	O
ATOM	534	ND2	ASN	618	27.458	39.886	35.197	1.00	0.00	LX0	N
ATOM	535	HD21	ASN	618	26.667	39.842	34.588	0.00	0.00	LX0	H
ATOM	536	HD22	ASN	618	27.871	40.774	35.391	0.00	0.00	LX0	H
ATOM	537	C	ASN	618	25.978	35.631	36.301	1.00	0.00	LX0	C
ATOM	538	O	ASN	618	25.043	35.728	37.079	1.00	0.00	LX0	O
ATOM	539	N	CYS	619	25.994	34.791	35.255	1.00	0.00	LX0	N
ATOM	540	H	CYS	619	26.788	34.682	34.652	0.00	0.00	LX0	H
ATOM	541	CA	CYS	619	24.787	33.985	35.089	1.00	0.00	LX0	C
ATOM	542	CB	CYS	619	24.096	34.289	33.758	1.00	0.00	LX0	C
ATOM	543	SG	CYS	619	22.540	33.377	33.568	1.00	0.00	LX0	S
ATOM	544	C	CYS	619	25.056	32.503	35.227	1.00	0.00	LX0	C
ATOM	545	O	CYS	619	25.661	31.878	34.362	1.00	0.00	LX0	O
ATOM	546	N	PRO	620	24.566	31.942	36.360	1.00	0.00	LX0	N
ATOM	547	CD	PRO	620	23.741	32.590	37.376	1.00	0.00	LX0	C
ATOM	548	CA	PRO	620	24.855	30.538	36.672	1.00	0.00	LX0	C
ATOM	549	CB	PRO	620	24.104	30.312	37.993	1.00	0.00	LX0	C
ATOM	550	CG	PRO	620	23.064	31.428	38.091	1.00	0.00	LX0	C
ATOM	551	C	PRO	620	24.505	29.568	35.555	1.00	0.00	LX0	C
ATOM	552	O	PRO	620	25.348	28.820	35.084	1.00	0.00	LX0	O
ATOM	553	N	ALA	621	23.239	29.639	35.109	1.00	0.00	LX0	N
ATOM	554	H	ALA	621	22.577	30.260	35.526	0.00	0.00	LX0	H
ATOM	555	CA	ALA	621	22.843	28.713	34.046	1.00	0.00	LX0	C
ATOM	556	CB	ALA	621	21.343	28.789	33.787	1.00	0.00	LX0	C
ATOM	557	C	ALA	621	23.582	28.875	32.725	1.00	0.00	LX0	C

ATOM	558	O	ALA	621	23.791	27.928	31.982	1.00	0.00	LX0	O
ATOM	559	N	CYS	622	24.014	30.116	32.462	1.00	0.00	LX0	N
ATOM	560	H	CYS	622	23.871	30.871	33.099	0.00	0.00	LX0	H
ATOM	561	CA	CYS	622	24.815	30.256	31.246	1.00	0.00	LX0	C
ATOM	562	CB	CYS	622	24.853	31.702	30.772	1.00	0.00	LX0	C
ATOM	563	SG	CYS	622	23.191	32.332	30.433	1.00	0.00	LX0	S
ATOM	564	C	CYS	622	26.215	29.682	31.352	1.00	0.00	LX0	C
ATOM	565	O	CYS	622	26.760	29.129	30.402	1.00	0.00	LX0	O
ATOM	566	N	ARG	623	26.774	29.791	32.572	1.00	0.00	LX0	N
ATOM	567	H	ARG	623	26.300	30.244	33.330	0.00	0.00	LX0	H
ATOM	568	CA	ARG	623	28.047	29.099	32.760	1.00	0.00	LX0	C
ATOM	569	CB	ARG	623	28.735	29.506	34.062	1.00	0.00	LX0	C
ATOM	570	CG	ARG	623	30.194	29.054	34.022	1.00	0.00	LX0	C
ATOM	571	CD	ARG	623	30.975	29.325	35.298	1.00	0.00	LX0	C
ATOM	572	NE	ARG	623	32.381	28.987	35.093	1.00	0.00	LX0	N
ATOM	573	HE	ARG	623	32.572	28.196	34.507	0.00	0.00	LX0	H
ATOM	574	CZ	ARG	623	33.351	29.687	35.715	1.00	0.00	LX0	C
ATOM	575	NH1	ARG	623	33.046	30.703	36.520	1.00	0.00	LX0	N
ATOM	576	HH11	ARG	623	33.739	31.180	37.057	0.00	0.00	LX0	H
ATOM	577	HH12	ARG	623	32.092	31.027	36.595	0.00	0.00	LX0	H
ATOM	578	NH2	ARG	623	34.620	29.354	35.503	1.00	0.00	LX0	N
ATOM	579	HH21	ARG	623	35.385	29.801	35.981	0.00	0.00	LX0	H
ATOM	580	HH22	ARG	623	34.857	28.635	34.847	0.00	0.00	LX0	H
ATOM	581	C	ARG	623	27.905	27.590	32.667	1.00	0.00	LX0	C
ATOM	582	O	ARG	623	28.645	26.915	31.961	1.00	0.00	LX0	O
ATOM	583	N	LEU	624	26.854	27.115	33.361	1.00	0.00	LX0	N
ATOM	584	H	LEU	624	26.377	27.741	33.976	0.00	0.00	LX0	H
ATOM	585	CA	LEU	624	26.378	25.736	33.228	1.00	0.00	LX0	C
ATOM	586	CB	LEU	624	25.005	25.623	33.912	1.00	0.00	LX0	C
ATOM	587	CG	LEU	624	24.155	24.371	33.648	1.00	0.00	LX0	C
ATOM	588	CD1	LEU	624	24.812	23.083	34.138	1.00	0.00	LX0	C
ATOM	589	CD2	LEU	624	22.745	24.535	34.217	1.00	0.00	LX0	C
ATOM	590	C	LEU	624	26.352	25.280	31.781	1.00	0.00	LX0	C
ATOM	591	O	LEU	624	26.902	24.247	31.434	1.00	0.00	LX0	O
ATOM	592	N	ARG	625	25.758	26.143	30.942	1.00	0.00	LX0	N
ATOM	593	H	ARG	625	25.254	26.922	31.313	0.00	0.00	LX0	H
ATOM	594	CA	ARG	625	25.777	25.860	29.513	1.00	0.00	LX0	C
ATOM	595	CB	ARG	625	25.085	26.964	28.712	1.00	0.00	LX0	C
ATOM	596	CG	ARG	625	24.793	26.521	27.277	1.00	0.00	LX0	C
ATOM	597	CD	ARG	625	24.765	27.684	26.292	1.00	0.00	LX0	C
ATOM	598	NE	ARG	625	26.066	28.355	26.262	1.00	0.00	LX0	N
ATOM	599	HE	ARG	625	26.880	27.789	26.090	0.00	0.00	LX0	H
ATOM	600	CZ	ARG	625	26.119	29.690	26.453	1.00	0.00	LX0	C
ATOM	601	NH1	ARG	625	25.020	30.392	26.659	1.00	0.00	LX0	N
ATOM	602	HH11	ARG	625	25.064	31.397	26.694	0.00	0.00	LX0	H
ATOM	603	HH12	ARG	625	24.118	29.960	26.769	0.00	0.00	LX0	H
ATOM	604	NH2	ARG	625	27.277	30.332	26.437	1.00	0.00	LX0	N
ATOM	605	HH21	ARG	625	27.269	31.320	26.635	0.00	0.00	LX0	H
ATOM	606	HH22	ARG	625	28.133	29.857	26.247	0.00	0.00	LX0	H
ATOM	607	C	ARG	625	27.153	25.565	28.936	1.00	0.00	LX0	C
ATOM	608	O	ARG	625	27.387	24.468	28.453	1.00	0.00	LX0	O
ATOM	609	N	LYS	626	28.064	26.559	28.994	1.00	0.00	LX0	N
ATOM	610	H	LYS	626	27.875	27.384	29.532	0.00	0.00	LX0	H
ATOM	611	CA	LYS	626	29.332	26.270	28.307	1.00	0.00	LX0	C
ATOM	612	CB	LYS	626	30.194	27.519	28.056	1.00	0.00	LX0	C
ATOM	613	CG	LYS	626	30.975	27.470	26.726	1.00	0.00	LX0	C
ATOM	614	CD	LYS	626	32.070	28.542	26.578	1.00	0.00	LX0	C
ATOM	615	CE	LYS	626	32.752	28.594	25.194	1.00	0.00	LX0	C
ATOM	616	NZ	LYS	626	34.138	29.097	25.278	1.00	0.00	LX0	N
ATOM	617	HZ1	LYS	626	34.784	28.288	25.387	0.00	0.00	LX0	H
ATOM	618	HZ2	LYS	626	34.450	29.620	24.428	0.00	0.00	LX0	H

ATOM	619	HZ3	LYS	626	34.278	29.682	26.127	0.00	0.00	LX0	H
ATOM	620	C	LYS	626	30.154	25.144	28.921	1.00	0.00	LX0	C
ATOM	621	O	LYS	626	30.777	24.348	28.229	1.00	0.00	LX0	O
ATOM	622	N	CYS	627	30.087	25.082	30.261	1.00	0.00	LX0	N
ATOM	623	H	CYS	627	29.545	25.753	30.768	0.00	0.00	LX0	H
ATOM	624	CA	CYS	627	30.731	23.963	30.955	1.00	0.00	LX0	C
ATOM	625	CB	CYS	627	30.606	24.130	32.470	1.00	0.00	LX0	C
ATOM	626	SG	CYS	627	31.413	25.631	33.094	1.00	0.00	LX0	S
ATOM	627	C	CYS	627	30.222	22.603	30.504	1.00	0.00	LX0	C
ATOM	628	O	CYS	627	30.964	21.670	30.212	1.00	0.00	LX0	O
ATOM	629	N	CYS	628	28.890	22.563	30.393	1.00	0.00	LX0	N
ATOM	630	H	CYS	628	28.324	23.350	30.634	0.00	0.00	LX0	H
ATOM	631	CA	CYS	628	28.278	21.373	29.822	1.00	0.00	LX0	C
ATOM	632	CB	CYS	628	26.759	21.434	29.915	1.00	0.00	LX0	C
ATOM	633	SG	CYS	628	26.122	21.194	31.590	1.00	0.00	LX0	S
ATOM	634	C	CYS	628	28.690	21.092	28.396	1.00	0.00	LX0	C
ATOM	635	O	CYS	628	28.962	19.958	28.032	1.00	0.00	LX0	O
ATOM	636	N	GLN	629	28.757	22.184	27.619	1.00	0.00	LX0	N
ATOM	637	H	GLN	629	28.553	23.072	28.027	0.00	0.00	LX0	H
ATOM	638	CA	GLN	629	29.090	22.082	26.201	1.00	0.00	LX0	C
ATOM	639	CB	GLN	629	29.082	23.477	25.553	1.00	0.00	LX0	C
ATOM	640	CG	GLN	629	27.637	23.967	25.427	1.00	0.00	LX0	C
ATOM	641	CD	GLN	629	27.508	25.398	24.939	1.00	0.00	LX0	C
ATOM	642	OE1	GLN	629	28.081	26.359	25.440	1.00	0.00	LX0	O
ATOM	643	NE2	GLN	629	26.644	25.519	23.933	1.00	0.00	LX0	N
ATOM	644	HE21	GLN	629	26.178	24.710	23.575	0.00	0.00	LX0	H
ATOM	645	HE22	GLN	629	26.457	26.410	23.527	0.00	0.00	LX0	H
ATOM	646	C	GLN	629	30.365	21.312	25.915	1.00	0.00	LX0	C
ATOM	647	O	GLN	629	30.387	20.425	25.074	1.00	0.00	LX0	O
ATOM	648	N	ALA	630	31.414	21.664	26.672	1.00	0.00	LX0	N
ATOM	649	H	ALA	630	31.324	22.383	27.364	0.00	0.00	LX0	H
ATOM	650	CA	ALA	630	32.640	20.892	26.467	1.00	0.00	LX0	C
ATOM	651	CB	ALA	630	33.845	21.645	27.019	1.00	0.00	LX0	C
ATOM	652	C	ALA	630	32.616	19.480	27.044	1.00	0.00	LX0	C
ATOM	653	O	ALA	630	33.280	18.573	26.560	1.00	0.00	LX0	O
ATOM	654	N	GLY	631	31.801	19.328	28.099	1.00	0.00	LX0	N
ATOM	655	H	GLY	631	31.313	20.104	28.499	0.00	0.00	LX0	H
ATOM	656	CA	GLY	631	31.671	17.978	28.638	1.00	0.00	LX0	C
ATOM	657	C	GLY	631	32.563	17.725	29.833	1.00	0.00	LX0	C
ATOM	658	O	GLY	631	33.485	16.919	29.809	1.00	0.00	LX0	O
ATOM	659	N	MET	632	32.240	18.462	30.904	1.00	0.00	LX0	N
ATOM	660	H	MET	632	31.410	19.019	30.896	0.00	0.00	LX0	H
ATOM	661	CA	MET	632	33.028	18.273	32.122	1.00	0.00	LX0	C
ATOM	662	CB	MET	632	33.033	19.548	32.970	1.00	0.00	LX0	C
ATOM	663	CG	MET	632	33.489	20.801	32.223	1.00	0.00	LX0	C
ATOM	664	SD	MET	632	33.669	22.242	33.287	1.00	0.00	LX0	S
ATOM	665	CE	MET	632	35.042	21.637	34.279	1.00	0.00	LX0	C
ATOM	666	C	MET	632	32.579	17.083	32.960	1.00	0.00	LX0	C
ATOM	667	O	MET	632	32.008	17.224	34.035	1.00	0.00	LX0	O
ATOM	668	N	VAL	633	32.832	15.883	32.419	1.00	0.00	LX0	N
ATOM	669	H	VAL	633	33.470	15.840	31.646	0.00	0.00	LX0	H
ATOM	670	CA	VAL	633	32.282	14.737	33.144	1.00	0.00	LX0	C
ATOM	671	CB	VAL	633	31.978	13.532	32.237	1.00	0.00	LX0	C
ATOM	672	CG1	VAL	633	30.668	12.871	32.677	1.00	0.00	LX0	C
ATOM	673	CG2	VAL	633	31.923	13.880	30.751	1.00	0.00	LX0	C
ATOM	674	C	VAL	633	33.099	14.311	34.355	1.00	0.00	LX0	C
ATOM	675	O	VAL	633	34.326	14.304	34.357	1.00	0.00	LX0	O
ATOM	676	N	LEU	634	32.348	13.958	35.408	1.00	0.00	LX0	N
ATOM	677	H	LEU	634	31.357	13.926	35.301	0.00	0.00	LX0	H
ATOM	678	CA	LEU	634	32.980	13.689	36.699	1.00	0.00	LX0	C
ATOM	679	CB	LEU	634	31.985	13.917	37.844	1.00	0.00	LX0	C

ATOM	680	CG	LEU	634	31.176	15.219	37.814	1.00	0.00	LX0	C
ATOM	681	CD1	LEU	634	30.144	15.235	38.943	1.00	0.00	LX0	C
ATOM	682	CD2	LEU	634	32.048	16.475	37.848	1.00	0.00	LX0	C
ATOM	683	C	LEU	634	33.620	12.315	36.859	1.00	0.00	LX0	C
ATOM	684	O	LEU	634	33.229	11.529	37.708	1.00	0.00	LX0	O
ATOM	685	N	GLY	635	34.638	12.057	36.026	1.00	0.00	LX0	N
ATOM	686	H	GLY	635	34.921	12.759	35.374	0.00	0.00	LX0	H
ATOM	687	CA	GLY	635	35.421	10.846	36.278	1.00	0.00	LX0	C
ATOM	688	C	GLY	635	36.434	11.095	37.375	1.00	0.00	LX0	C
ATOM	689	O	GLY	635	37.027	12.174	37.430	1.00	0.00	LX0	O
ATOM	690	N	GLY	636	36.587	10.080	38.239	1.00	0.00	LX0	N
ATOM	691	H	GLY	636	36.084	9.221	38.106	0.00	0.00	LX0	H
ATOM	692	CA	GLY	636	37.412	10.230	39.437	1.00	0.00	LX0	C
ATOM	693	C	GLY	636	38.889	10.539	39.242	1.00	0.00	LX0	C
ATOM	694	O	GLY	636	39.295	11.601	38.770	1.00	0.00	LX0	O
ATOM	695	N	ARG	637	39.706	9.585	39.705	1.00	0.00	LX0	N
ATOM	696	H	ARG	637	39.361	8.657	39.843	0.00	0.00	LX0	H
ATOM	697	CA	ARG	637	41.118	9.944	39.784	1.00	0.00	LX0	C
ATOM	698	CB	ARG	637	41.686	9.712	41.200	1.00	0.00	LX0	C
ATOM	699	CG	ARG	637	43.058	10.366	41.460	1.00	0.00	LX0	C
ATOM	700	CD	ARG	637	44.225	9.365	41.469	1.00	0.00	LX0	C
ATOM	701	NE	ARG	637	45.498	10.000	41.116	1.00	0.00	LX0	N
ATOM	702	HE	ARG	637	45.925	10.568	41.822	0.00	0.00	LX0	H
ATOM	703	CZ	ARG	637	46.034	9.833	39.880	1.00	0.00	LX0	C
ATOM	704	NH1	ARG	637	45.420	9.086	38.966	1.00	0.00	LX0	N
ATOM	705	HH11	ARG	637	45.730	8.950	38.019	0.00	0.00	LX0	H
ATOM	706	HH12	ARG	637	44.555	8.623	39.193	0.00	0.00	LX0	H
ATOM	707	NH2	ARG	637	47.185	10.433	39.579	1.00	0.00	LX0	N
ATOM	708	HH21	ARG	637	47.596	10.334	38.672	0.00	0.00	LX0	H
ATOM	709	HH22	ARG	637	47.662	10.996	40.256	0.00	0.00	LX0	H
ATOM	710	C	ARG	637	41.974	9.352	38.683	1.00	0.00	LX0	C
ATOM	711	O	ARG	637	42.873	8.552	38.910	1.00	0.00	LX0	O
ATOM	712	N	LYS	638	41.690	9.858	37.478	1.00	0.00	LX0	N
ATOM	713	H	LYS	638	40.730	10.122	37.360	0.00	0.00	LX0	H
ATOM	714	CA	LYS	638	42.562	9.735	36.307	1.00	0.00	LX0	C
ATOM	715	CB	LYS	638	43.317	11.057	36.108	1.00	0.00	LX0	C
ATOM	716	CG	LYS	638	42.756	11.925	34.966	1.00	0.00	LX0	C
ATOM	717	CD	LYS	638	41.230	12.118	34.962	1.00	0.00	LX0	C
ATOM	718	CE	LYS	638	40.645	12.859	36.171	1.00	0.00	LX0	C
ATOM	719	NZ	LYS	638	39.233	12.479	36.299	1.00	0.00	LX0	N
ATOM	720	HZ1	LYS	638	38.938	12.370	37.297	0.00	0.00	LX0	H
ATOM	721	HZ2	LYS	638	38.560	13.118	35.844	0.00	0.00	LX0	H
ATOM	722	HZ3	LYS	638	39.098	11.528	35.902	0.00	0.00	LX0	H
ATOM	723	C	LYS	638	43.442	8.498	36.259	1.00	0.00	LX0	C
ATOM	724	O	LYS	638	44.666	8.542	36.391	1.00	0.00	LX0	O
ATOM	725	N	PHE	639	42.724	7.380	36.114	1.00	0.00	LX0	N
ATOM	726	H	PHE	639	41.722	7.448	36.052	0.00	0.00	LX0	H
ATOM	727	CA	PHE	639	43.424	6.105	36.061	1.00	0.00	LX0	C
ATOM	728	CB	PHE	639	42.611	5.012	36.762	1.00	0.00	LX0	C
ATOM	729	CG	PHE	639	42.466	5.330	38.233	1.00	0.00	LX0	C
ATOM	730	CD1	PHE	639	43.572	5.160	39.095	1.00	0.00	LX0	C
ATOM	731	CD2	PHE	639	41.225	5.790	38.724	1.00	0.00	LX0	C
ATOM	732	CE1	PHE	639	43.434	5.450	40.466	1.00	0.00	LX0	C
ATOM	733	CE2	PHE	639	41.083	6.077	40.095	1.00	0.00	LX0	C
ATOM	734	CZ	PHE	639	42.190	5.904	40.953	1.00	0.00	LX0	C
ATOM	735	C	PHE	639	43.770	5.705	34.644	1.00	0.00	LX0	C
ATOM	736	O	PHE	639	42.931	5.604	33.761	1.00	0.00	LX0	O
ATOM	737	N	LYS	640	45.076	5.488	34.454	1.00	0.00	LX0	N
ATOM	738	H	LYS	640	45.702	5.575	35.225	0.00	0.00	LX0	H
ATOM	739	CA	LYS	640	45.522	5.172	33.097	1.00	0.00	LX0	C
ATOM	740	CB	LYS	640	47.057	5.286	33.033	1.00	0.00	LX0	C

ATOM	741	CG	LYS	640	47.817	4.365	33.998	1.00	0.00	LX0	C
ATOM	742	CD	LYS	640	48.509	3.210	33.269	1.00	0.00	LX0	C
ATOM	743	CE	LYS	640	48.572	1.922	34.094	1.00	0.00	LX0	C
ATOM	744	NZ	LYS	640	47.223	1.366	34.254	1.00	0.00	LX0	N
ATOM	745	HZ1	LYS	640	46.800	1.515	35.199	0.00	0.00	LX0	H
ATOM	746	HZ2	LYS	640	46.519	1.666	33.550	0.00	0.00	LX0	H
ATOM	747	HZ3	LYS	640	47.258	0.323	34.269	0.00	0.00	LX0	H
ATOM	748	C	LYS	640	44.980	3.857	32.537	1.00	0.00	LX0	C
ATOM	749	O	LYS	640	45.388	2.766	32.937	1.00	0.00	LX0	O
TER											
