# Supplementary material for: Structural analysis of hubs in human NR-RTK network
Source: Biol Direct. 2011 Oct 5;6:49. doi: 10.1186/1745-6150-6-49 (PMC3220635; doi:10.1186/1745-6150-6-49)
Supplement: Additional file 11 — ESR1-Erbb2-PGR. ESR1-Erbb2-PGR complex structure. [file 1745-6150-6-49-S11.PDF]

```
HEADER ESR1-ERBB2-PGR
REMARK original generated coordinate pdb file
ATOM      1  N   ALA   156      10.627  12.174   8.322  1.00  0.00      RX0  N
ATOM      2  H   ALA   156      11.184  11.345   8.267  1.00  0.00      RX0  H
ATOM      3  CA  ALA   156       9.864  12.527   9.538  1.00  0.00      RX0  C
ATOM      4  CB  ALA   156      10.757  12.402  10.765  1.00  0.00      RX0  C
ATOM      5  C   ALA   156       9.377  13.991   9.496  1.00  0.00      RX0  C
ATOM      6  O   ALA   156       9.121  14.644  10.500  1.00  0.00      RX0  O
ATOM      7  N   LEU   157       9.039  14.416   8.289  1.00  0.00      RX0  N
ATOM      8  H   LEU   157       9.125  13.764   7.532  1.00  0.00      RX0  H
ATOM      9  CA  LEU   157       8.850  15.849   7.979  1.00  0.00      RX0  C
ATOM     10  CB  LEU   157       9.701  16.233   6.774  1.00  0.00      RX0  C
ATOM     11  CG  LEU   157      11.010  15.454   6.683  1.00  0.00      RX0  C
ATOM     12  CD1 LEU   157      11.566  15.497   5.264  1.00  0.00      RX0  C
ATOM     13  CD2 LEU   157      12.028  15.879   7.740  1.00  0.00      RX0  C
ATOM     14  C   LEU   157       7.387  16.184   7.642  1.00  0.00      RX0  C
ATOM     15  O   LEU   157       7.075  17.216   7.039  1.00  0.00      RX0  O
ATOM     16  N   SER   158       6.522  15.244   7.955  1.00  0.00      RX0  N
ATOM     17  H   SER   158       6.874  14.326   8.100  1.00  0.00      RX0  H
ATOM     18  CA  SER   158       5.051  15.362   7.847  1.00  0.00      RX0  C
ATOM     19  CB  SER   158       4.729  14.894   6.436  1.00  0.00      RX0  C
ATOM     20  OG  SER   158       5.938  15.038   5.679  1.00  0.00      RX0  O
ATOM     21  HG  SER   158       6.094  15.978   5.623  1.00  0.00      RX0  H
ATOM     22  C   SER   158       4.335  14.560   8.949  1.00  0.00      RX0  C
ATOM     23  O   SER   158       3.148  14.670   9.188  1.00  0.00      RX0  O
ATOM     24  N   LEU   159       5.132  13.681   9.591  1.00  0.00      RX0  N
ATOM     25  H   LEU   159       6.083  13.585   9.324  1.00  0.00      RX0  H
ATOM     26  CA  LEU   159       4.759  12.951  10.797  1.00  0.00      RX0  C
ATOM     27  CB  LEU   159       5.860  11.985  11.247  1.00  0.00      RX0  C
ATOM     28  CG  LEU   159       5.904  10.626  10.539  1.00  0.00      RX0  C
ATOM     29  CD1 LEU   159       6.367  10.706   9.082  1.00  0.00      RX0  C
ATOM     30  CD2 LEU   159       6.746   9.631  11.339  1.00  0.00      RX0  C
ATOM     31  C   LEU   159       4.518  13.965  11.920  1.00  0.00      RX0  C
ATOM     32  O   LEU   159       5.291  14.932  12.058  1.00  0.00      RX0  O
ATOM     33  N   THR   160       3.434  13.807  12.646  1.00  0.00      RX0  N
ATOM     34  H   THR   160       2.847  13.019  12.458  1.00  0.00      RX0  H
ATOM     35  CA  THR   160       3.156  14.665  13.825  1.00  0.00      RX0  C
ATOM     36  CB  THR   160       1.666  14.649  14.226  1.00  0.00      RX0  C
ATOM     37  OG1 THR   160       1.374  15.747  15.097  1.00  0.00      RX0  O
ATOM     38  HG1 THR   160       0.426  15.831  15.117  1.00  0.00      RX0  H
ATOM     39  CG2 THR   160       1.175  13.341  14.831  1.00  0.00      RX0  C
ATOM     40  C   THR   160       4.203  14.411  14.921  1.00  0.00      RX0  C
ATOM     41  O   THR   160       4.902  13.383  14.913  1.00  0.00      RX0  O
ATOM     42  N   ALA   161       4.153  15.229  15.953  1.00  0.00      RX0  N
ATOM     43  H   ALA   161       3.464  15.956  15.915  1.00  0.00      RX0  H
ATOM     44  CA  ALA   161       4.942  15.044  17.184  1.00  0.00      RX0  C
ATOM     45  CB  ALA   161       4.756  16.235  18.116  1.00  0.00      RX0  C
ATOM     46  C   ALA   161       4.543  13.746  17.920  1.00  0.00      RX0  C
ATOM     47  O   ALA   161       5.387  12.950  18.288  1.00  0.00      RX0  O
ATOM     48  N   ASP   162       3.226  13.461  17.917  1.00  0.00      RX0  N
ATOM     49  H   ASP   162       2.574  14.184  17.699  1.00  0.00      RX0  H
ATOM     50  CA  ASP   162       2.690  12.196  18.469  1.00  0.00      RX0  C
ATOM     51  CB  ASP   162       1.209  12.322  18.853  1.00  0.00      RX0  C
ATOM     52  CG  ASP   162       1.108  13.150  20.135  1.00  0.00      RX0  C
ATOM     53  OD1 ASP   162       2.017  13.924  20.429  1.00  0.00      RX0  O
ATOM     54  OD2 ASP   162       0.137  13.024  20.880  1.00  0.00      RX0  O
ATOM     55  C   ASP   162       3.088  10.948  17.668  1.00  0.00      RX0  C
ATOM     56  O   ASP   162       3.397   9.903  18.257  1.00  0.00      RX0  O
ATOM     57  N   GLN   163       3.164  11.087  16.353  1.00  0.00      RX0  N
ATOM     58  H   GLN   163       3.079  12.014  15.997  1.00  0.00      RX0  H
ATOM     59  CA  GLN   163       3.593   9.998  15.449  1.00  0.00      RX0  C
```

ATOM	60	CB	GLN	163	3.243	10.284	13.996	1.00	0.00	RX0	C
ATOM	61	CG	GLN	163	1.812	9.882	13.650	1.00	0.00	RX0	C
ATOM	62	CD	GLN	163	1.525	10.392	12.259	1.00	0.00	RX0	C
ATOM	63	OE1	GLN	163	2.074	11.405	11.840	1.00	0.00	RX0	O
ATOM	64	NE2	GLN	163	0.647	9.642	11.571	1.00	0.00	RX0	N
ATOM	65	HE21	GLN	163	0.240	8.826	11.983	1.00	0.00	RX0	H
ATOM	66	HE22	GLN	163	0.374	9.873	10.636	1.00	0.00	RX0	H
ATOM	67	C	GLN	163	5.089	9.698	15.572	1.00	0.00	RX0	C
ATOM	68	O	GLN	163	5.477	8.537	15.545	1.00	0.00	RX0	O
ATOM	69	N	MET	164	5.882	10.740	15.840	1.00	0.00	RX0	N
ATOM	70	H	MET	164	5.494	11.662	15.887	1.00	0.00	RX0	H
ATOM	71	CA	MET	164	7.331	10.587	16.060	1.00	0.00	RX0	C
ATOM	72	CB	MET	164	8.015	11.955	16.082	1.00	0.00	RX0	C
ATOM	73	CG	MET	164	9.452	11.879	16.605	1.00	0.00	RX0	C
ATOM	74	SD	MET	164	10.535	10.863	15.592	1.00	0.00	RX0	S
ATOM	75	CE	MET	164	11.026	12.135	14.424	1.00	0.00	RX0	C
ATOM	76	C	MET	164	7.610	9.825	17.366	1.00	0.00	RX0	C
ATOM	77	O	MET	164	8.404	8.887	17.381	1.00	0.00	RX0	O
ATOM	78	N	VAL	165	6.828	10.145	18.396	1.00	0.00	RX0	N
ATOM	79	H	VAL	165	6.176	10.898	18.281	1.00	0.00	RX0	H
ATOM	80	CA	VAL	165	6.992	9.563	19.744	1.00	0.00	RX0	C
ATOM	81	CB	VAL	165	6.099	10.256	20.778	1.00	0.00	RX0	C
ATOM	82	CG1	VAL	165	6.170	9.551	22.133	1.00	0.00	RX0	C
ATOM	83	CG2	VAL	165	6.451	11.727	20.927	1.00	0.00	RX0	C
ATOM	84	C	VAL	165	6.649	8.067	19.731	1.00	0.00	RX0	C
ATOM	85	O	VAL	165	7.442	7.255	20.191	1.00	0.00	RX0	O
ATOM	86	N	SER	166	5.467	7.742	19.205	1.00	0.00	RX0	N
ATOM	87	H	SER	166	4.863	8.457	18.845	1.00	0.00	RX0	H
ATOM	88	CA	SER	166	5.029	6.335	19.106	1.00	0.00	RX0	C
ATOM	89	CB	SER	166	3.545	6.211	18.738	1.00	0.00	RX0	C
ATOM	90	OG	SER	166	3.007	4.980	19.260	1.00	0.00	RX0	O
ATOM	91	HG	SER	166	3.591	4.277	18.936	1.00	0.00	RX0	H
ATOM	92	C	SER	166	5.941	5.501	18.195	1.00	0.00	RX0	C
ATOM	93	O	SER	166	6.295	4.379	18.542	1.00	0.00	RX0	O
ATOM	94	N	ALA	167	6.456	6.129	17.133	1.00	0.00	RX0	N
ATOM	95	H	ALA	167	6.168	7.061	16.908	1.00	0.00	RX0	H
ATOM	96	CA	ALA	167	7.397	5.466	16.208	1.00	0.00	RX0	C
ATOM	97	CB	ALA	167	7.731	6.357	15.013	1.00	0.00	RX0	C
ATOM	98	C	ALA	167	8.706	5.103	16.927	1.00	0.00	RX0	C
ATOM	99	O	ALA	167	9.113	3.946	16.932	1.00	0.00	RX0	O
ATOM	100	N	LEU	168	9.179	6.054	17.734	1.00	0.00	RX0	N
ATOM	101	H	LEU	168	8.726	6.947	17.747	1.00	0.00	RX0	H
ATOM	102	CA	LEU	168	10.385	5.875	18.562	1.00	0.00	RX0	C
ATOM	103	CB	LEU	168	10.907	7.213	19.075	1.00	0.00	RX0	C
ATOM	104	CG	LEU	168	11.568	8.043	17.978	1.00	0.00	RX0	C
ATOM	105	CD1	LEU	168	12.100	9.370	18.520	1.00	0.00	RX0	C
ATOM	106	CD2	LEU	168	12.648	7.242	17.250	1.00	0.00	RX0	C
ATOM	107	C	LEU	168	10.197	4.896	19.724	1.00	0.00	RX0	C
ATOM	108	O	LEU	168	11.077	4.078	19.994	1.00	0.00	RX0	O
ATOM	109	N	LEU	169	9.007	4.918	20.317	1.00	0.00	RX0	N
ATOM	110	H	LEU	169	8.319	5.569	19.998	1.00	0.00	RX0	H
ATOM	111	CA	LEU	169	8.640	3.970	21.384	1.00	0.00	RX0	C
ATOM	112	CB	LEU	169	7.359	4.385	22.108	1.00	0.00	RX0	C
ATOM	113	CG	LEU	169	7.565	5.582	23.037	1.00	0.00	RX0	C
ATOM	114	CD1	LEU	169	6.266	5.983	23.736	1.00	0.00	RX0	C
ATOM	115	CD2	LEU	169	8.691	5.333	24.042	1.00	0.00	RX0	C
ATOM	116	C	LEU	169	8.505	2.536	20.864	1.00	0.00	RX0	C
ATOM	117	O	LEU	169	9.003	1.602	21.486	1.00	0.00	RX0	O
ATOM	118	N	ASP	170	7.977	2.423	19.645	1.00	0.00	RX0	N
ATOM	119	H	ASP	170	7.549	3.210	19.207	1.00	0.00	RX0	H
ATOM	120	CA	ASP	170	7.822	1.133	18.952	1.00	0.00	RX0	C

ATOM	121	CB	ASP	170	6.725	1.243	17.869	1.00	0.00	RX0	C
ATOM	122	CG	ASP	170	5.382	1.685	18.486	1.00	0.00	RX0	C
ATOM	123	OD1	ASP	170	5.193	1.541	19.694	1.00	0.00	RX0	O
ATOM	124	OD2	ASP	170	4.521	2.205	17.769	1.00	0.00	RX0	O
ATOM	125	C	ASP	170	9.164	0.506	18.541	1.00	0.00	RX0	C
ATOM	126	O	ASP	170	9.313	-0.704	18.571	1.00	0.00	RX0	O
ATOM	127	N	ALA	171	10.119	1.387	18.228	1.00	0.00	RX0	N
ATOM	128	H	ALA	171	9.898	2.360	18.318	1.00	0.00	RX0	H
ATOM	129	CA	ALA	171	11.447	1.008	17.717	1.00	0.00	RX0	C
ATOM	130	CB	ALA	171	12.104	2.224	17.066	1.00	0.00	RX0	C
ATOM	131	C	ALA	171	12.418	0.479	18.779	1.00	0.00	RX0	C
ATOM	132	O	ALA	171	13.427	-0.136	18.431	1.00	0.00	RX0	O
ATOM	133	N	GLU	172	12.125	0.739	20.058	1.00	0.00	RX0	N
ATOM	134	H	GLU	172	11.270	1.205	20.288	1.00	0.00	RX0	H
ATOM	135	CA	GLU	172	13.017	0.374	21.170	1.00	0.00	RX0	C
ATOM	136	CB	GLU	172	12.396	0.762	22.509	1.00	0.00	RX0	C
ATOM	137	CG	GLU	172	12.417	2.281	22.697	1.00	0.00	RX0	C
ATOM	138	CD	GLU	172	13.847	2.788	22.599	1.00	0.00	RX0	C
ATOM	139	OE1	GLU	172	14.617	2.620	23.545	1.00	0.00	RX0	O
ATOM	140	OE2	GLU	172	14.222	3.361	21.575	1.00	0.00	RX0	O
ATOM	141	C	GLU	172	13.554	-1.065	21.099	1.00	0.00	RX0	C
ATOM	142	O	GLU	172	12.785	-2.004	20.837	1.00	0.00	RX0	O
ATOM	143	N	PRO	173	14.865	-1.209	21.269	1.00	0.00	RX0	N
ATOM	144	CD	PRO	173	15.788	-0.096	21.453	1.00	0.00	RX0	C
ATOM	145	CA	PRO	173	15.538	-2.517	21.328	1.00	0.00	RX0	C
ATOM	146	CB	PRO	173	17.014	-2.107	21.203	1.00	0.00	RX0	C
ATOM	147	CG	PRO	173	17.100	-0.741	21.875	1.00	0.00	RX0	C
ATOM	148	C	PRO	173	15.206	-3.249	22.640	1.00	0.00	RX0	C
ATOM	149	O	PRO	173	14.829	-2.595	23.631	1.00	0.00	RX0	O
ATOM	150	N	PRO	174	15.294	-4.574	22.646	1.00	0.00	RX0	N
ATOM	151	CD	PRO	174	15.590	-5.390	21.473	1.00	0.00	RX0	C
ATOM	152	CA	PRO	174	15.084	-5.400	23.852	1.00	0.00	RX0	C
ATOM	153	CB	PRO	174	14.962	-6.814	23.277	1.00	0.00	RX0	C
ATOM	154	CG	PRO	174	15.830	-6.791	22.023	1.00	0.00	RX0	C
ATOM	155	C	PRO	174	16.250	-5.248	24.838	1.00	0.00	RX0	C
ATOM	156	O	PRO	174	17.379	-4.922	24.444	1.00	0.00	RX0	O
ATOM	157	N	ILE	175	15.956	-5.464	26.106	1.00	0.00	RX0	N
ATOM	158	H	ILE	175	15.040	-5.796	26.324	1.00	0.00	RX0	H
ATOM	159	CA	ILE	175	16.988	-5.556	27.159	1.00	0.00	RX0	C
ATOM	160	CB	ILE	175	16.403	-5.167	28.506	1.00	0.00	RX0	C
ATOM	161	CG2	ILE	175	17.489	-5.104	29.577	1.00	0.00	RX0	C
ATOM	162	CG1	ILE	175	15.706	-3.817	28.333	1.00	0.00	RX0	C
ATOM	163	CD1	ILE	175	14.987	-3.318	29.582	1.00	0.00	RX0	C
ATOM	164	C	ILE	175	17.586	-6.969	27.112	1.00	0.00	RX0	C
ATOM	165	O	ILE	175	16.886	-7.963	27.343	1.00	0.00	RX0	O
ATOM	166	N	LEU	176	18.884	-7.017	26.884	1.00	0.00	RX0	N
ATOM	167	H	LEU	176	19.412	-6.170	26.826	1.00	0.00	RX0	H
ATOM	168	CA	LEU	176	19.617	-8.291	26.770	1.00	0.00	RX0	C
ATOM	169	CB	LEU	176	20.640	-8.239	25.636	1.00	0.00	RX0	C
ATOM	170	CG	LEU	176	19.996	-7.960	24.276	1.00	0.00	RX0	C
ATOM	171	CD1	LEU	176	21.037	-7.949	23.159	1.00	0.00	RX0	C
ATOM	172	CD2	LEU	176	18.854	-8.928	23.966	1.00	0.00	RX0	C
ATOM	173	C	LEU	176	20.277	-8.690	28.089	1.00	0.00	RX0	C
ATOM	174	O	LEU	176	20.563	-7.852	28.952	1.00	0.00	RX0	O
ATOM	175	N	TYR	177	20.459	-9.989	28.237	1.00	0.00	RX0	N
ATOM	176	H	TYR	177	20.270	-10.589	27.463	1.00	0.00	RX0	H
ATOM	177	CA	TYR	177	21.114	-10.573	29.420	1.00	0.00	RX0	C
ATOM	178	CB	TYR	177	20.496	-11.936	29.705	1.00	0.00	RX0	C
ATOM	179	CG	TYR	177	19.572	-11.933	30.893	1.00	0.00	RX0	C
ATOM	180	CD1	TYR	177	18.528	-11.021	30.985	1.00	0.00	RX0	C
ATOM	181	CE1	TYR	177	17.635	-11.114	32.046	1.00	0.00	RX0	C

ATOM	182	CD2	TYR	177	19.760	-12.889	31.884	1.00	0.00	RX0	C
ATOM	183	CE2	TYR	177	18.873	-12.974	32.948	1.00	0.00	RX0	C
ATOM	184	CZ	TYR	177	17.797	-12.099	33.015	1.00	0.00	RX0	C
ATOM	185	OH	TYR	177	16.885	-12.219	34.045	1.00	0.00	RX0	O
ATOM	186	HH	TYR	177	17.328	-12.546	34.818	1.00	0.00	RX0	H
ATOM	187	C	TYR	177	22.589	-10.858	29.163	1.00	0.00	RX0	C
ATOM	188	O	TYR	177	22.985	-11.163	28.046	1.00	0.00	RX0	O
ATOM	189	N	SER	178	23.381	-10.750	30.220	1.00	0.00	RX0	N
ATOM	190	H	SER	178	23.009	-10.523	31.122	1.00	0.00	RX0	H
ATOM	191	CA	SER	178	24.788	-11.188	30.183	1.00	0.00	RX0	C
ATOM	192	CB	SER	178	25.494	-10.612	31.393	1.00	0.00	RX0	C
ATOM	193	OG	SER	178	25.203	-9.216	31.395	1.00	0.00	RX0	O
ATOM	194	HG	SER	178	25.045	-8.983	30.487	1.00	0.00	RX0	H
ATOM	195	C	SER	178	24.834	-12.718	30.070	1.00	0.00	RX0	C
ATOM	196	O	SER	178	23.999	-13.413	30.674	1.00	0.00	RX0	O
ATOM	197	N	GLU	179	25.827	-13.218	29.362	1.00	0.00	RX0	N
ATOM	198	H	GLU	179	26.399	-12.609	28.811	1.00	0.00	RX0	H
ATOM	199	CA	GLU	179	26.033	-14.670	29.175	1.00	0.00	RX0	C
ATOM	200	CB	GLU	179	26.280	-15.014	27.698	1.00	0.00	RX0	C
ATOM	201	CG	GLU	179	25.187	-14.598	26.703	1.00	0.00	RX0	C
ATOM	202	CD	GLU	179	25.519	-13.263	26.046	1.00	0.00	RX0	C
ATOM	203	OE1	GLU	179	26.112	-13.255	24.970	1.00	0.00	RX0	O
ATOM	204	OE2	GLU	179	25.128	-12.225	26.569	1.00	0.00	RX0	O
ATOM	205	C	GLU	179	27.192	-15.208	30.012	1.00	0.00	RX0	C
ATOM	206	O	GLU	179	28.361	-15.227	29.589	1.00	0.00	RX0	O
ATOM	207	N	TYR	180	26.873	-15.475	31.254	1.00	0.00	RX0	N
ATOM	208	H	TYR	180	25.924	-15.387	31.565	1.00	0.00	RX0	H
ATOM	209	CA	TYR	180	27.735	-16.233	32.177	1.00	0.00	RX0	C
ATOM	210	CB	TYR	180	28.493	-15.322	33.156	1.00	0.00	RX0	C
ATOM	211	CG	TYR	180	27.547	-14.673	34.139	1.00	0.00	RX0	C
ATOM	212	CD1	TYR	180	26.931	-13.467	33.826	1.00	0.00	RX0	C
ATOM	213	CE1	TYR	180	26.012	-12.912	34.707	1.00	0.00	RX0	C
ATOM	214	CD2	TYR	180	27.286	-15.290	35.358	1.00	0.00	RX0	C
ATOM	215	CE2	TYR	180	26.355	-14.744	36.230	1.00	0.00	RX0	C
ATOM	216	CZ	TYR	180	25.699	-13.568	35.891	1.00	0.00	RX0	C
ATOM	217	OH	TYR	180	24.726	-13.062	36.726	1.00	0.00	RX0	O
ATOM	218	HH	TYR	180	24.652	-12.123	36.577	1.00	0.00	RX0	H
ATOM	219	C	TYR	180	26.838	-17.226	32.909	1.00	0.00	RX0	C
ATOM	220	O	TYR	180	25.642	-16.953	33.094	1.00	0.00	RX0	O
ATOM	221	N	ASP	181	27.404	-18.345	33.318	1.00	0.00	RX0	N
ATOM	222	H	ASP	181	28.392	-18.459	33.263	1.00	0.00	RX0	H
ATOM	223	CA	ASP	181	26.630	-19.347	34.059	1.00	0.00	RX0	C
ATOM	224	CB	ASP	181	27.307	-20.707	34.082	1.00	0.00	RX0	C
ATOM	225	CG	ASP	181	26.452	-21.570	34.977	1.00	0.00	RX0	C
ATOM	226	OD1	ASP	181	25.254	-21.659	34.734	1.00	0.00	RX0	O
ATOM	227	OD2	ASP	181	26.962	-22.109	35.947	1.00	0.00	RX0	O
ATOM	228	C	ASP	181	26.420	-18.851	35.504	1.00	0.00	RX0	C
ATOM	229	O	ASP	181	27.391	-18.832	36.273	1.00	0.00	RX0	O
ATOM	230	N	PRO	182	25.185	-18.489	35.856	1.00	0.00	RX0	N
ATOM	231	CD	PRO	182	24.010	-18.609	34.996	1.00	0.00	RX0	C
ATOM	232	CA	PRO	182	24.825	-17.989	37.201	1.00	0.00	RX0	C
ATOM	233	CB	PRO	182	23.393	-17.490	36.996	1.00	0.00	RX0	C
ATOM	234	CG	PRO	182	22.822	-18.416	35.928	1.00	0.00	RX0	C
ATOM	235	C	PRO	182	24.941	-19.052	38.308	1.00	0.00	RX0	C
ATOM	236	O	PRO	182	24.654	-18.763	39.474	1.00	0.00	RX0	O
ATOM	237	N	THR	183	25.345	-20.259	37.948	1.00	0.00	RX0	N
ATOM	238	H	THR	183	25.556	-20.524	37.006	1.00	0.00	RX0	H
ATOM	239	CA	THR	183	25.568	-21.363	38.913	1.00	0.00	RX0	C
ATOM	240	CB	THR	183	24.913	-22.558	38.256	1.00	0.00	RX0	C
ATOM	241	OG1	THR	183	24.015	-22.056	37.256	1.00	0.00	RX0	O
ATOM	242	HG1	THR	183	24.532	-22.062	36.445	1.00	0.00	RX0	H

ATOM	243	CG2	THR	183	24.189	-23.459	39.259	1.00	0.00	RX0	C
ATOM	244	C	THR	183	27.063	-21.532	39.218	1.00	0.00	RX0	C
ATOM	245	O	THR	183	27.455	-22.345	40.058	1.00	0.00	RX0	O
ATOM	246	N	ARG	184	27.887	-20.699	38.573	1.00	0.00	RX0	N
ATOM	247	H	ARG	184	27.521	-19.996	37.961	1.00	0.00	RX0	H
ATOM	248	CA	ARG	184	29.343	-20.701	38.681	1.00	0.00	RX0	C
ATOM	249	CB	ARG	184	29.922	-20.817	37.272	1.00	0.00	RX0	C
ATOM	250	CG	ARG	184	30.321	-22.252	36.930	1.00	0.00	RX0	C
ATOM	251	CD	ARG	184	30.753	-22.386	35.470	1.00	0.00	RX0	C
ATOM	252	NE	ARG	184	31.491	-21.200	35.036	1.00	0.00	RX0	N
ATOM	253	HE	ARG	184	30.919	-20.484	34.626	1.00	0.00	RX0	H
ATOM	254	CZ	ARG	184	32.830	-21.073	35.286	1.00	0.00	RX0	C
ATOM	255	NH1	ARG	184	33.511	-22.063	35.894	1.00	0.00	RX0	N
ATOM	256	HH11	ARG	184	34.488	-21.888	36.137	1.00	0.00	RX0	H
ATOM	257	HH12	ARG	184	33.120	-22.948	36.142	1.00	0.00	RX0	H
ATOM	258	NH2	ARG	184	33.477	-19.947	34.929	1.00	0.00	RX0	N
ATOM	259	HH21	ARG	184	34.474	-19.903	35.116	1.00	0.00	RX0	H
ATOM	260	HH22	ARG	184	33.049	-19.152	34.501	1.00	0.00	RX0	H
ATOM	261	C	ARG	184	29.836	-19.410	39.407	1.00	0.00	RX0	C
ATOM	262	O	ARG	184	29.116	-18.390	39.334	1.00	0.00	RX0	O
ATOM	263	N	PRO	185	30.940	-19.460	40.113	1.00	0.00	RX0	N
ATOM	264	CD	PRO	185	31.743	-20.664	40.293	1.00	0.00	RX0	C
ATOM	265	CA	PRO	185	31.574	-18.285	40.781	1.00	0.00	RX0	C
ATOM	266	CB	PRO	185	32.858	-18.870	41.382	1.00	0.00	RX0	C
ATOM	267	CG	PRO	185	33.142	-20.137	40.579	1.00	0.00	RX0	C
ATOM	268	C	PRO	185	31.820	-17.125	39.813	1.00	0.00	RX0	C
ATOM	269	O	PRO	185	31.836	-17.275	38.592	1.00	0.00	RX0	O
ATOM	270	N	PHE	186	32.164	-15.998	40.422	1.00	0.00	RX0	N
ATOM	271	H	PHE	186	32.240	-15.993	41.417	1.00	0.00	RX0	H
ATOM	272	CA	PHE	186	32.333	-14.726	39.697	1.00	0.00	RX0	C
ATOM	273	CB	PHE	186	31.517	-13.614	40.358	1.00	0.00	RX0	C
ATOM	274	CG	PHE	186	31.422	-12.437	39.416	1.00	0.00	RX0	C
ATOM	275	CD1	PHE	186	30.871	-12.607	38.151	1.00	0.00	RX0	C
ATOM	276	CD2	PHE	186	31.885	-11.186	39.809	1.00	0.00	RX0	C
ATOM	277	CE1	PHE	186	30.787	-11.529	37.277	1.00	0.00	RX0	C
ATOM	278	CE2	PHE	186	31.801	-10.108	38.935	1.00	0.00	RX0	C
ATOM	279	CZ	PHE	186	31.253	-10.280	37.669	1.00	0.00	RX0	C
ATOM	280	C	PHE	186	33.791	-14.305	39.507	1.00	0.00	RX0	C
ATOM	281	O	PHE	186	34.127	-13.678	38.496	1.00	0.00	RX0	O
ATOM	282	N	SER	187	34.655	-14.802	40.380	1.00	0.00	RX0	N
ATOM	283	H	SER	187	34.311	-15.350	41.137	1.00	0.00	RX0	H
ATOM	284	CA	SER	187	36.113	-14.542	40.380	1.00	0.00	RX0	C
ATOM	285	CB	SER	187	36.611	-15.271	41.615	1.00	0.00	RX0	C
ATOM	286	OG	SER	187	35.492	-15.360	42.516	1.00	0.00	RX0	O
ATOM	287	HG	SER	187	35.869	-15.431	43.386	1.00	0.00	RX0	H
ATOM	288	C	SER	187	36.764	-14.980	39.057	1.00	0.00	RX0	C
ATOM	289	O	SER	187	37.834	-14.531	38.683	1.00	0.00	RX0	O
ATOM	290	N	GLU	188	36.054	-15.878	38.369	1.00	0.00	RX0	N
ATOM	291	H	GLU	188	35.126	-16.108	38.649	1.00	0.00	RX0	H
ATOM	292	CA	GLU	188	36.561	-16.586	37.191	1.00	0.00	RX0	C
ATOM	293	CB	GLU	188	36.157	-18.052	37.348	1.00	0.00	RX0	C
ATOM	294	CG	GLU	188	36.897	-19.061	36.473	1.00	0.00	RX0	C
ATOM	295	CD	GLU	188	36.048	-20.311	36.412	1.00	0.00	RX0	C
ATOM	296	OE1	GLU	188	35.311	-20.584	37.352	1.00	0.00	RX0	O
ATOM	297	OE2	GLU	188	36.013	-20.965	35.376	1.00	0.00	RX0	O
ATOM	298	C	GLU	188	36.028	-16.018	35.856	1.00	0.00	RX0	C
ATOM	299	O	GLU	188	36.494	-16.416	34.788	1.00	0.00	RX0	O
ATOM	300	N	ALA	189	35.058	-15.107	35.914	1.00	0.00	RX0	N
ATOM	301	H	ALA	189	34.828	-14.685	36.794	1.00	0.00	RX0	H
ATOM	302	CA	ALA	189	34.543	-14.432	34.708	1.00	0.00	RX0	C
ATOM	303	CB	ALA	189	33.067	-14.082	34.891	1.00	0.00	RX0	C

ATOM	304	C	ALA	189	35.336	-13.151	34.407	1.00	0.00	RX0	C
ATOM	305	O	ALA	189	35.533	-12.292	35.270	1.00	0.00	RX0	O
ATOM	306	N	SER	190	35.819	-13.065	33.173	1.00	0.00	RX0	N
ATOM	307	H	SER	190	35.614	-13.816	32.549	1.00	0.00	RX0	H
ATOM	308	CA	SER	190	36.430	-11.825	32.646	1.00	0.00	RX0	C
ATOM	309	CB	SER	190	37.096	-12.196	31.296	1.00	0.00	RX0	C
ATOM	310	OG	SER	190	38.271	-11.411	30.976	1.00	0.00	RX0	O
ATOM	311	HG	SER	190	38.794	-11.465	31.776	1.00	0.00	RX0	H
ATOM	312	C	SER	190	35.341	-10.761	32.513	1.00	0.00	RX0	C
ATOM	313	O	SER	190	34.465	-10.869	31.639	1.00	0.00	RX0	O
ATOM	314	N	MET	191	35.401	-9.751	33.361	1.00	0.00	RX0	N
ATOM	315	H	MET	191	36.035	-9.823	34.132	1.00	0.00	RX0	H
ATOM	316	CA	MET	191	34.414	-8.652	33.337	1.00	0.00	RX0	C
ATOM	317	CB	MET	191	34.642	-7.665	34.478	1.00	0.00	RX0	C
ATOM	318	CG	MET	191	33.544	-6.601	34.500	1.00	0.00	RX0	C
ATOM	319	SD	MET	191	33.638	-5.545	35.948	1.00	0.00	RX0	S
ATOM	320	CE	MET	191	33.293	-6.808	37.185	1.00	0.00	RX0	C
ATOM	321	C	MET	191	34.384	-7.939	31.976	1.00	0.00	RX0	C
ATOM	322	O	MET	191	33.329	-7.841	31.363	1.00	0.00	RX0	O
ATOM	323	N	MET	192	35.577	-7.654	31.438	1.00	0.00	RX0	N
ATOM	324	H	MET	192	36.382	-7.744	32.021	1.00	0.00	RX0	H
ATOM	325	CA	MET	192	35.697	-7.082	30.089	1.00	0.00	RX0	C
ATOM	326	CB	MET	192	37.137	-6.649	29.805	1.00	0.00	RX0	C
ATOM	327	CG	MET	192	37.287	-5.957	28.446	1.00	0.00	RX0	C
ATOM	328	SD	MET	192	36.147	-4.579	28.226	1.00	0.00	RX0	S
ATOM	329	CE	MET	192	36.715	-3.527	29.574	1.00	0.00	RX0	C
ATOM	330	C	MET	192	35.151	-8.021	28.999	1.00	0.00	RX0	C
ATOM	331	O	MET	192	34.484	-7.587	28.093	1.00	0.00	RX0	O
ATOM	332	N	GLY	193	35.358	-9.342	29.220	1.00	0.00	RX0	N
ATOM	333	H	GLY	193	35.698	-9.615	30.116	1.00	0.00	RX0	H
ATOM	334	CA	GLY	193	34.804	-10.378	28.330	1.00	0.00	RX0	C
ATOM	335	C	GLY	193	33.267	-10.338	28.334	1.00	0.00	RX0	C
ATOM	336	O	GLY	193	32.637	-10.184	27.296	1.00	0.00	RX0	O
ATOM	337	N	LEU	194	32.696	-10.293	29.537	1.00	0.00	RX0	N
ATOM	338	H	LEU	194	33.259	-10.319	30.359	1.00	0.00	RX0	H
ATOM	339	CA	LEU	194	31.235	-10.169	29.722	1.00	0.00	RX0	C
ATOM	340	CB	LEU	194	30.848	-10.220	31.199	1.00	0.00	RX0	C
ATOM	341	CG	LEU	194	31.149	-11.553	31.875	1.00	0.00	RX0	C
ATOM	342	CD1	LEU	194	30.712	-11.533	33.340	1.00	0.00	RX0	C
ATOM	343	CD2	LEU	194	30.541	-12.729	31.109	1.00	0.00	RX0	C
ATOM	344	C	LEU	194	30.647	-8.891	29.116	1.00	0.00	RX0	C
ATOM	345	O	LEU	194	29.706	-8.959	28.317	1.00	0.00	RX0	O
ATOM	346	N	LEU	195	31.327	-7.782	29.364	1.00	0.00	RX0	N
ATOM	347	H	LEU	195	32.115	-7.843	29.972	1.00	0.00	RX0	H
ATOM	348	CA	LEU	195	30.920	-6.462	28.846	1.00	0.00	RX0	C
ATOM	349	CB	LEU	195	31.733	-5.338	29.491	1.00	0.00	RX0	C
ATOM	350	CG	LEU	195	31.540	-5.239	31.004	1.00	0.00	RX0	C
ATOM	351	CD1	LEU	195	32.379	-4.111	31.607	1.00	0.00	RX0	C
ATOM	352	CD2	LEU	195	30.066	-5.127	31.389	1.00	0.00	RX0	C
ATOM	353	C	LEU	195	31.020	-6.357	27.321	1.00	0.00	RX0	C
ATOM	354	O	LEU	195	30.051	-5.942	26.671	1.00	0.00	RX0	O
ATOM	355	N	THR	196	32.075	-6.931	26.767	1.00	0.00	RX0	N
ATOM	356	H	THR	196	32.732	-7.386	27.369	1.00	0.00	RX0	H
ATOM	357	CA	THR	196	32.335	-6.901	25.309	1.00	0.00	RX0	C
ATOM	358	CB	THR	196	33.768	-7.351	25.054	1.00	0.00	RX0	C
ATOM	359	OG1	THR	196	34.644	-6.455	25.737	1.00	0.00	RX0	O
ATOM	360	HG1	THR	196	34.385	-6.488	26.651	1.00	0.00	RX0	H
ATOM	361	CG2	THR	196	34.153	-7.455	23.574	1.00	0.00	RX0	C
ATOM	362	C	THR	196	31.317	-7.765	24.552	1.00	0.00	RX0	C
ATOM	363	O	THR	196	30.772	-7.327	23.532	1.00	0.00	RX0	O
ATOM	364	N	ASN	197	31.003	-8.928	25.107	1.00	0.00	RX0	N

ATOM	365	H	ASN	197	31.422	-9.187	25.978	1.00	0.00	RX0	H
ATOM	366	CA	ASN	197	30.010	-9.840	24.504	1.00	0.00	RX0	C
ATOM	367	CB	ASN	197	30.063	-11.314	24.846	1.00	0.00	RX0	C
ATOM	368	CG	ASN	197	29.692	-11.942	23.513	1.00	0.00	RX0	C
ATOM	369	OD1	ASN	197	30.415	-11.786	22.516	1.00	0.00	RX0	O
ATOM	370	ND2	ASN	197	28.527	-12.615	23.533	1.00	0.00	RX0	N
ATOM	371	HD21	ASN	197	27.976	-12.742	24.373	1.00	0.00	RX0	H
ATOM	372	HD22	ASN	197	28.095	-13.011	22.725	1.00	0.00	RX0	H
ATOM	373	C	ASN	197	28.594	-9.255	24.528	1.00	0.00	RX0	C
ATOM	374	O	ASN	197	27.900	-9.272	23.514	1.00	0.00	RX0	O
ATOM	375	N	LEU	198	28.277	-8.575	25.633	1.00	0.00	RX0	N
ATOM	376	H	LEU	198	28.901	-8.597	26.414	1.00	0.00	RX0	H
ATOM	377	CA	LEU	198	27.002	-7.850	25.760	1.00	0.00	RX0	C
ATOM	378	CB	LEU	198	26.847	-7.342	27.190	1.00	0.00	RX0	C
ATOM	379	CG	LEU	198	25.440	-6.842	27.502	1.00	0.00	RX0	C
ATOM	380	CD1	LEU	198	24.373	-7.906	27.236	1.00	0.00	RX0	C
ATOM	381	CD2	LEU	198	25.369	-6.313	28.931	1.00	0.00	RX0	C
ATOM	382	C	LEU	198	26.885	-6.719	24.724	1.00	0.00	RX0	C
ATOM	383	O	LEU	198	25.930	-6.676	23.947	1.00	0.00	RX0	O
ATOM	384	N	ALA	199	27.942	-5.914	24.641	1.00	0.00	RX0	N
ATOM	385	H	ALA	199	28.674	-6.050	25.309	1.00	0.00	RX0	H
ATOM	386	CA	ALA	199	28.029	-4.784	23.694	1.00	0.00	RX0	C
ATOM	387	CB	ALA	199	29.327	-4.006	23.902	1.00	0.00	RX0	C
ATOM	388	C	ALA	199	27.921	-5.230	22.227	1.00	0.00	RX0	C
ATOM	389	O	ALA	199	27.138	-4.660	21.467	1.00	0.00	RX0	O
ATOM	390	N	ASP	200	28.555	-6.360	21.908	1.00	0.00	RX0	N
ATOM	391	H	ASP	200	29.165	-6.771	22.587	1.00	0.00	RX0	H
ATOM	392	CA	ASP	200	28.494	-6.940	20.550	1.00	0.00	RX0	C
ATOM	393	CB	ASP	200	29.280	-8.209	20.750	1.00	0.00	RX0	C
ATOM	394	CG	ASP	200	30.259	-8.475	19.675	1.00	0.00	RX0	C
ATOM	395	OD1	ASP	200	31.385	-8.004	19.783	1.00	0.00	RX0	O
ATOM	396	OD2	ASP	200	29.974	-9.307	18.823	1.00	0.00	RX0	O
ATOM	397	C	ASP	200	27.084	-7.410	20.171	1.00	0.00	RX0	C
ATOM	398	O	ASP	200	26.604	-7.102	19.080	1.00	0.00	RX0	O
ATOM	399	N	ARG	201	26.390	-7.998	21.143	1.00	0.00	RX0	N
ATOM	400	H	ARG	201	26.843	-8.153	22.024	1.00	0.00	RX0	H
ATOM	401	CA	ARG	201	24.992	-8.434	20.957	1.00	0.00	RX0	C
ATOM	402	CB	ARG	201	24.523	-9.406	22.045	1.00	0.00	RX0	C
ATOM	403	CG	ARG	201	24.951	-10.847	21.768	1.00	0.00	RX0	C
ATOM	404	CD	ARG	201	23.910	-11.875	22.232	1.00	0.00	RX0	C
ATOM	405	NE	ARG	201	23.766	-11.922	23.685	1.00	0.00	RX0	N
ATOM	406	HE	ARG	201	24.612	-11.955	24.247	1.00	0.00	RX0	H
ATOM	407	CZ	ARG	201	22.539	-12.142	24.246	1.00	0.00	RX0	C
ATOM	408	NH1	ARG	201	21.429	-12.193	23.462	1.00	0.00	RX0	N
ATOM	409	HH11	ARG	201	20.494	-12.439	23.777	1.00	0.00	RX0	H
ATOM	410	HH12	ARG	201	21.451	-11.989	22.481	1.00	0.00	RX0	H
ATOM	411	NH2	ARG	201	22.472	-12.309	25.583	1.00	0.00	RX0	N
ATOM	412	HH21	ARG	201	21.629	-12.524	26.073	1.00	0.00	RX0	H
ATOM	413	HH22	ARG	201	23.314	-12.214	26.151	1.00	0.00	RX0	H
ATOM	414	C	ARG	201	23.991	-7.279	20.827	1.00	0.00	RX0	C
ATOM	415	O	ARG	201	23.123	-7.308	19.955	1.00	0.00	RX0	O
ATOM	416	N	GLU	202	24.240	-6.201	21.568	1.00	0.00	RX0	N
ATOM	417	H	GLU	202	24.992	-6.221	22.232	1.00	0.00	RX0	H
ATOM	418	CA	GLU	202	23.401	-4.988	21.493	1.00	0.00	RX0	C
ATOM	419	CB	GLU	202	23.560	-4.066	22.724	1.00	0.00	RX0	C
ATOM	420	CG	GLU	202	22.892	-4.629	23.999	1.00	0.00	RX0	C
ATOM	421	CD	GLU	202	22.831	-3.625	25.155	1.00	0.00	RX0	C
ATOM	422	OE1	GLU	202	21.789	-3.005	25.375	1.00	0.00	RX0	O
ATOM	423	OE2	GLU	202	23.797	-3.498	25.901	1.00	0.00	RX0	O
ATOM	424	C	GLU	202	23.526	-4.262	20.149	1.00	0.00	RX0	C
ATOM	425	O	GLU	202	22.539	-3.754	19.625	1.00	0.00	RX0	O

ATOM	426	N	LEU	203	24.712	-4.358	19.546	1.00	0.00	RX0	N
ATOM	427	H	LEU	203	25.457	-4.795	20.057	1.00	0.00	RX0	H
ATOM	428	CA	LEU	203	25.004	-3.680	18.270	1.00	0.00	RX0	C
ATOM	429	CB	LEU	203	26.491	-3.830	17.926	1.00	0.00	RX0	C
ATOM	430	CG	LEU	203	26.965	-2.999	16.725	1.00	0.00	RX0	C
ATOM	431	CD1	LEU	203	26.681	-1.506	16.894	1.00	0.00	RX0	C
ATOM	432	CD2	LEU	203	28.440	-3.251	16.416	1.00	0.00	RX0	C
ATOM	433	C	LEU	203	24.099	-4.160	17.127	1.00	0.00	RX0	C
ATOM	434	O	LEU	203	23.593	-3.349	16.346	1.00	0.00	RX0	O
ATOM	435	N	VAL	204	23.782	-5.447	17.151	1.00	0.00	RX0	N
ATOM	436	H	VAL	204	24.171	-6.011	17.883	1.00	0.00	RX0	H
ATOM	437	CA	VAL	204	22.925	-6.083	16.127	1.00	0.00	RX0	C
ATOM	438	CB	VAL	204	22.895	-7.600	16.328	1.00	0.00	RX0	C
ATOM	439	CG1	VAL	204	21.968	-8.268	15.311	1.00	0.00	RX0	C
ATOM	440	CG2	VAL	204	24.308	-8.190	16.298	1.00	0.00	RX0	C
ATOM	441	C	VAL	204	21.502	-5.497	16.213	1.00	0.00	RX0	C
ATOM	442	O	VAL	204	20.938	-5.041	15.221	1.00	0.00	RX0	O
ATOM	443	N	HIS	205	21.015	-5.393	17.448	1.00	0.00	RX0	N
ATOM	444	H	HIS	205	21.600	-5.682	18.209	1.00	0.00	RX0	H
ATOM	445	CA	HIS	205	19.703	-4.786	17.746	1.00	0.00	RX0	C
ATOM	446	CB	HIS	205	19.262	-5.118	19.174	1.00	0.00	RX0	C
ATOM	447	CG	HIS	205	19.025	-6.606	19.293	1.00	0.00	RX0	C
ATOM	448	ND1	HIS	205	17.825	-7.193	19.113	1.00	0.00	RX0	N
ATOM	449	HD1	HIS	205	16.978	-6.748	18.904	1.00	0.00	RX0	H
ATOM	450	CD2	HIS	205	19.966	-7.598	19.582	1.00	0.00	RX0	C
ATOM	451	NE2	HIS	205	19.321	-8.788	19.572	1.00	0.00	RX0	N
ATOM	452	CE1	HIS	205	18.004	-8.543	19.285	1.00	0.00	RX0	C
ATOM	453	C	HIS	205	19.668	-3.277	17.476	1.00	0.00	RX0	C
ATOM	454	O	HIS	205	18.642	-2.756	17.030	1.00	0.00	RX0	O
ATOM	455	N	MET	206	20.820	-2.627	17.609	1.00	0.00	RX0	N
ATOM	456	H	MET	206	21.610	-3.126	17.971	1.00	0.00	RX0	H
ATOM	457	CA	MET	206	20.969	-1.185	17.340	1.00	0.00	RX0	C
ATOM	458	CB	MET	206	22.354	-0.693	17.753	1.00	0.00	RX0	C
ATOM	459	CG	MET	206	22.532	0.809	17.535	1.00	0.00	RX0	C
ATOM	460	SD	MET	206	24.221	1.340	17.845	1.00	0.00	RX0	S
ATOM	461	CE	MET	206	24.438	0.528	19.434	1.00	0.00	RX0	C
ATOM	462	C	MET	206	20.721	-0.870	15.856	1.00	0.00	RX0	C
ATOM	463	O	MET	206	20.035	0.103	15.544	1.00	0.00	RX0	O
ATOM	464	N	ILE	207	21.183	-1.758	14.977	1.00	0.00	RX0	N
ATOM	465	H	ILE	207	21.732	-2.518	15.337	1.00	0.00	RX0	H
ATOM	466	CA	ILE	207	20.975	-1.628	13.516	1.00	0.00	RX0	C
ATOM	467	CB	ILE	207	21.665	-2.793	12.800	1.00	0.00	RX0	C
ATOM	468	CG2	ILE	207	21.388	-2.787	11.298	1.00	0.00	RX0	C
ATOM	469	CG1	ILE	207	23.162	-2.809	13.104	1.00	0.00	RX0	C
ATOM	470	CD1	ILE	207	23.895	-1.604	12.516	1.00	0.00	RX0	C
ATOM	471	C	ILE	207	19.470	-1.621	13.197	1.00	0.00	RX0	C
ATOM	472	O	ILE	207	18.988	-0.755	12.467	1.00	0.00	RX0	O
ATOM	473	N	ASN	208	18.761	-2.558	13.816	1.00	0.00	RX0	N
ATOM	474	H	ASN	208	19.235	-3.132	14.487	1.00	0.00	RX0	H
ATOM	475	CA	ASN	208	17.313	-2.734	13.586	1.00	0.00	RX0	C
ATOM	476	CB	ASN	208	16.788	-4.061	14.123	1.00	0.00	RX0	C
ATOM	477	CG	ASN	208	16.197	-4.818	12.949	1.00	0.00	RX0	C
ATOM	478	OD1	ASN	208	16.732	-4.797	11.836	1.00	0.00	RX0	O
ATOM	479	ND2	ASN	208	15.081	-5.503	13.254	1.00	0.00	RX0	N
ATOM	480	HD21	ASN	208	14.691	-5.471	14.177	1.00	0.00	RX0	H
ATOM	481	HD22	ASN	208	14.591	-6.078	12.596	1.00	0.00	RX0	H
ATOM	482	C	ASN	208	16.516	-1.532	14.103	1.00	0.00	RX0	C
ATOM	483	O	ASN	208	15.637	-1.017	13.417	1.00	0.00	RX0	O
ATOM	484	N	TRP	209	16.982	-1.008	15.238	1.00	0.00	RX0	N
ATOM	485	H	TRP	209	17.724	-1.475	15.723	1.00	0.00	RX0	H
ATOM	486	CA	TRP	209	16.425	0.201	15.864	1.00	0.00	RX0	C

ATOM	487	CB	TRP	209	17.093	0.416	17.230	1.00	0.00	RX0	C
ATOM	488	CG	TRP	209	16.717	1.756	17.824	1.00	0.00	RX0	C
ATOM	489	CD2	TRP	209	17.462	2.992	17.789	1.00	0.00	RX0	C
ATOM	490	CE2	TRP	209	16.701	3.972	18.468	1.00	0.00	RX0	C
ATOM	491	CE3	TRP	209	18.694	3.334	17.243	1.00	0.00	RX0	C
ATOM	492	CD1	TRP	209	15.551	2.065	18.533	1.00	0.00	RX0	C
ATOM	493	NE1	TRP	209	15.531	3.366	18.920	1.00	0.00	RX0	N
ATOM	494	HE1	TRP	209	14.794	3.775	19.435	1.00	0.00	RX0	H
ATOM	495	CZ2	TRP	209	17.192	5.268	18.568	1.00	0.00	RX0	C
ATOM	496	CZ3	TRP	209	19.175	4.632	17.357	1.00	0.00	RX0	C
ATOM	497	CH2	TRP	209	18.424	5.598	18.015	1.00	0.00	RX0	C
ATOM	498	C	TRP	209	16.619	1.438	14.972	1.00	0.00	RX0	C
ATOM	499	O	TRP	209	15.652	2.120	14.634	1.00	0.00	RX0	O
ATOM	500	N	ALA	210	17.853	1.624	14.503	1.00	0.00	RX0	N
ATOM	501	H	ALA	210	18.568	0.988	14.791	1.00	0.00	RX0	H
ATOM	502	CA	ALA	210	18.233	2.756	13.635	1.00	0.00	RX0	C
ATOM	503	CB	ALA	210	19.715	2.665	13.273	1.00	0.00	RX0	C
ATOM	504	C	ALA	210	17.400	2.800	12.347	1.00	0.00	RX0	C
ATOM	505	O	ALA	210	16.892	3.855	11.979	1.00	0.00	RX0	O
ATOM	506	N	LYS	211	17.095	1.613	11.820	1.00	0.00	RX0	N
ATOM	507	H	LYS	211	17.505	0.799	12.235	1.00	0.00	RX0	H
ATOM	508	CA	LYS	211	16.258	1.472	10.614	1.00	0.00	RX0	C
ATOM	509	CB	LYS	211	16.392	0.067	10.028	1.00	0.00	RX0	C
ATOM	510	CG	LYS	211	17.773	-0.125	9.390	1.00	0.00	RX0	C
ATOM	511	CD	LYS	211	17.985	-1.548	8.874	1.00	0.00	RX0	C
ATOM	512	CE	LYS	211	17.729	-2.523	10.016	1.00	0.00	RX0	C
ATOM	513	NZ	LYS	211	18.000	-3.917	9.643	1.00	0.00	RX0	N
ATOM	514	HZ1	LYS	211	17.675	-4.518	10.437	1.00	0.00	RX0	H
ATOM	515	HZ2	LYS	211	17.468	-4.169	8.789	1.00	0.00	RX0	H
ATOM	516	HZ3	LYS	211	19.017	-4.062	9.483	1.00	0.00	RX0	H
ATOM	517	C	LYS	211	14.795	1.898	10.823	1.00	0.00	RX0	C
ATOM	518	O	LYS	211	14.129	2.337	9.881	1.00	0.00	RX0	O
ATOM	519	N	ARG	212	14.357	1.870	12.071	1.00	0.00	RX0	N
ATOM	520	H	ARG	212	14.980	1.608	12.809	1.00	0.00	RX0	H
ATOM	521	CA	ARG	212	13.005	2.320	12.466	1.00	0.00	RX0	C
ATOM	522	CB	ARG	212	12.399	1.423	13.563	1.00	0.00	RX0	C
ATOM	523	CG	ARG	212	12.167	-0.053	13.200	1.00	0.00	RX0	C
ATOM	524	CD	ARG	212	11.637	-0.941	14.348	1.00	0.00	RX0	C
ATOM	525	NE	ARG	212	10.229	-0.699	14.698	1.00	0.00	RX0	N
ATOM	526	HE	ARG	212	9.758	0.056	14.231	1.00	0.00	RX0	H
ATOM	527	CZ	ARG	212	9.624	-1.480	15.661	1.00	0.00	RX0	C
ATOM	528	NH1	ARG	212	10.340	-2.436	16.297	1.00	0.00	RX0	N
ATOM	529	HH11	ARG	212	9.927	-3.093	16.955	1.00	0.00	RX0	H
ATOM	530	HH12	ARG	212	11.320	-2.576	16.160	1.00	0.00	RX0	H
ATOM	531	NH2	ARG	212	8.320	-1.300	15.975	1.00	0.00	RX0	N
ATOM	532	HH21	ARG	212	7.945	-1.718	16.809	1.00	0.00	RX0	H
ATOM	533	HH22	ARG	212	7.648	-0.763	15.445	1.00	0.00	RX0	H
ATOM	534	C	ARG	212	12.933	3.790	12.894	1.00	0.00	RX0	C
ATOM	535	O	ARG	212	11.827	4.344	12.989	1.00	0.00	RX0	O
ATOM	536	N	VAL	213	14.074	4.417	13.148	1.00	0.00	RX0	N
ATOM	537	H	VAL	213	14.933	3.928	12.993	1.00	0.00	RX0	H
ATOM	538	CA	VAL	213	14.155	5.874	13.374	1.00	0.00	RX0	C
ATOM	539	CB	VAL	213	15.581	6.281	13.749	1.00	0.00	RX0	C
ATOM	540	CG1	VAL	213	15.751	7.800	13.838	1.00	0.00	RX0	C
ATOM	541	CG2	VAL	213	15.980	5.584	15.048	1.00	0.00	RX0	C
ATOM	542	C	VAL	213	13.672	6.590	12.095	1.00	0.00	RX0	C
ATOM	543	O	VAL	213	14.288	6.431	11.023	1.00	0.00	RX0	O
ATOM	544	N	PRO	214	12.622	7.395	12.213	1.00	0.00	RX0	N
ATOM	545	CD	PRO	214	11.895	7.609	13.458	1.00	0.00	RX0	C
ATOM	546	CA	PRO	214	12.035	8.145	11.084	1.00	0.00	RX0	C
ATOM	547	CB	PRO	214	10.927	8.954	11.761	1.00	0.00	RX0	C

ATOM	548	CG	PRO	214	10.552	8.163	13.010	1.00	0.00	RX0 C
ATOM	549	C	PRO	214	13.102	9.001	10.387	1.00	0.00	RX0 C
ATOM	550	O	PRO	214	13.853	9.727	11.025	1.00	0.00	RX0 O
ATOM	551	N	GLY	215	13.244	8.730	9.080	1.00	0.00	RX0 N
ATOM	552	H	GLY	215	12.749	7.982	8.637	1.00	0.00	RX0 H
ATOM	553	CA	GLY	215	14.194	9.473	8.227	1.00	0.00	RX0 C
ATOM	554	C	GLY	215	15.511	8.732	7.950	1.00	0.00	RX0 C
ATOM	555	O	GLY	215	16.085	8.889	6.862	1.00	0.00	RX0 O
ATOM	556	N	PHE	216	15.917	7.845	8.848	1.00	0.00	RX0 N
ATOM	557	H	PHE	216	15.329	7.653	9.636	1.00	0.00	RX0 H
ATOM	558	CA	PHE	216	17.224	7.160	8.764	1.00	0.00	RX0 C
ATOM	559	CB	PHE	216	17.452	6.293	9.997	1.00	0.00	RX0 C
ATOM	560	CG	PHE	216	18.890	5.840	10.054	1.00	0.00	RX0 C
ATOM	561	CD1	PHE	216	19.897	6.765	10.297	1.00	0.00	RX0 C
ATOM	562	CD2	PHE	216	19.207	4.498	9.877	1.00	0.00	RX0 C
ATOM	563	CE1	PHE	216	21.217	6.344	10.404	1.00	0.00	RX0 C
ATOM	564	CE2	PHE	216	20.526	4.077	9.985	1.00	0.00	RX0 C
ATOM	565	CZ	PHE	216	21.528	4.997	10.269	1.00	0.00	RX0 C
ATOM	566	C	PHE	216	17.435	6.347	7.474	1.00	0.00	RX0 C
ATOM	567	O	PHE	216	18.358	6.641	6.711	1.00	0.00	RX0 O
ATOM	568	N	VAL	217	16.482	5.483	7.151	1.00	0.00	RX0 N
ATOM	569	H	VAL	217	15.673	5.447	7.736	1.00	0.00	RX0 H
ATOM	570	CA	VAL	217	16.570	4.608	5.958	1.00	0.00	RX0 C
ATOM	571	CB	VAL	217	15.528	3.495	6.008	1.00	0.00	RX0 C
ATOM	572	CG1	VAL	217	15.897	2.481	7.081	1.00	0.00	RX0 C
ATOM	573	CG2	VAL	217	14.111	4.052	6.168	1.00	0.00	RX0 C
ATOM	574	C	VAL	217	16.469	5.342	4.608	1.00	0.00	RX0 C
ATOM	575	O	VAL	217	16.660	4.747	3.556	1.00	0.00	RX0 O
ATOM	576	N	ASP	218	16.058	6.613	4.671	1.00	0.00	RX0 N
ATOM	577	H	ASP	218	15.763	7.060	5.518	1.00	0.00	RX0 H
ATOM	578	CA	ASP	218	16.006	7.470	3.472	1.00	0.00	RX0 C
ATOM	579	CB	ASP	218	15.098	8.676	3.760	1.00	0.00	RX0 C
ATOM	580	CG	ASP	218	13.746	8.270	4.349	1.00	0.00	RX0 C
ATOM	581	OD1	ASP	218	12.737	8.455	3.672	1.00	0.00	RX0 O
ATOM	582	OD2	ASP	218	13.687	7.810	5.494	1.00	0.00	RX0 O
ATOM	583	C	ASP	218	17.401	7.924	3.023	1.00	0.00	RX0 C
ATOM	584	O	ASP	218	17.595	8.369	1.896	1.00	0.00	RX0 O
ATOM	585	N	LEU	219	18.344	7.857	3.967	1.00	0.00	RX0 N
ATOM	586	H	LEU	219	18.095	7.487	4.862	1.00	0.00	RX0 H
ATOM	587	CA	LEU	219	19.767	8.083	3.697	1.00	0.00	RX0 C
ATOM	588	CB	LEU	219	20.544	8.274	4.996	1.00	0.00	RX0 C
ATOM	589	CG	LEU	219	19.979	9.447	5.798	1.00	0.00	RX0 C
ATOM	590	CD1	LEU	219	20.554	9.496	7.211	1.00	0.00	RX0 C
ATOM	591	CD2	LEU	219	20.131	10.775	5.053	1.00	0.00	RX0 C
ATOM	592	C	LEU	219	20.350	6.970	2.832	1.00	0.00	RX0 C
ATOM	593	O	LEU	219	19.843	5.836	2.789	1.00	0.00	RX0 O
ATOM	594	N	THR	220	21.442	7.293	2.191	1.00	0.00	RX0 N
ATOM	595	H	THR	220	21.800	8.221	2.307	1.00	0.00	RX0 H
ATOM	596	CA	THR	220	22.263	6.312	1.453	1.00	0.00	RX0 C
ATOM	597	CB	THR	220	23.300	7.147	0.726	1.00	0.00	RX0 C
ATOM	598	OG1	THR	220	23.470	8.371	1.439	1.00	0.00	RX0 O
ATOM	599	HG1	THR	220	22.858	9.027	1.101	1.00	0.00	RX0 H
ATOM	600	CG2	THR	220	22.893	7.435	-0.720	1.00	0.00	RX0 C
ATOM	601	C	THR	220	22.829	5.303	2.454	1.00	0.00	RX0 C
ATOM	602	O	THR	220	23.112	5.646	3.611	1.00	0.00	RX0 O
ATOM	603	N	LEU	221	23.130	4.116	1.957	1.00	0.00	RX0 N
ATOM	604	H	LEU	221	22.843	3.904	1.025	1.00	0.00	RX0 H
ATOM	605	CA	LEU	221	23.750	3.065	2.781	1.00	0.00	RX0 C
ATOM	606	CB	LEU	221	23.960	1.859	1.873	1.00	0.00	RX0 C
ATOM	607	CG	LEU	221	24.874	0.788	2.454	1.00	0.00	RX0 C
ATOM	608	CD1	LEU	221	24.242	0.072	3.645	1.00	0.00	RX0 C

ATOM	609	CD2	LEU	221	25.347	-0.164	1.358	1.00	0.00	RX0	C
ATOM	610	C	LEU	221	25.092	3.509	3.394	1.00	0.00	RX0	C
ATOM	611	O	LEU	221	25.324	3.347	4.578	1.00	0.00	RX0	O
ATOM	612	N	HIS	222	25.854	4.270	2.593	1.00	0.00	RX0	N
ATOM	613	H	HIS	222	25.541	4.455	1.665	1.00	0.00	RX0	H
ATOM	614	CA	HIS	222	27.131	4.847	3.045	1.00	0.00	RX0	C
ATOM	615	CB	HIS	222	27.881	5.606	1.951	1.00	0.00	RX0	C
ATOM	616	CG	HIS	222	29.163	6.155	2.546	1.00	0.00	RX0	C
ATOM	617	ND1	HIS	222	30.159	5.387	3.035	1.00	0.00	RX0	N
ATOM	618	HD1	HIS	222	30.205	4.405	3.083	1.00	0.00	RX0	H
ATOM	619	CD2	HIS	222	29.526	7.499	2.700	1.00	0.00	RX0	C
ATOM	620	NE2	HIS	222	30.751	7.525	3.286	1.00	0.00	RX0	N
ATOM	621	CE1	HIS	222	31.137	6.228	3.491	1.00	0.00	RX0	C
ATOM	622	C	HIS	222	26.942	5.765	4.264	1.00	0.00	RX0	C
ATOM	623	O	HIS	222	27.673	5.642	5.246	1.00	0.00	RX0	O
ATOM	624	N	ASP	223	25.958	6.658	4.167	1.00	0.00	RX0	N
ATOM	625	H	ASP	223	25.454	6.820	3.319	1.00	0.00	RX0	H
ATOM	626	CA	ASP	223	25.694	7.636	5.242	1.00	0.00	RX0	C
ATOM	627	CB	ASP	223	24.892	8.835	4.720	1.00	0.00	RX0	C
ATOM	628	CG	ASP	223	25.805	9.713	3.866	1.00	0.00	RX0	C
ATOM	629	OD1	ASP	223	26.443	9.195	2.946	1.00	0.00	RX0	O
ATOM	630	OD2	ASP	223	25.895	10.913	4.131	1.00	0.00	RX0	O
ATOM	631	C	ASP	223	25.148	6.996	6.516	1.00	0.00	RX0	C
ATOM	632	O	ASP	223	25.558	7.375	7.616	1.00	0.00	RX0	O
ATOM	633	N	GLN	224	24.393	5.921	6.332	1.00	0.00	RX0	N
ATOM	634	H	GLN	224	24.147	5.694	5.387	1.00	0.00	RX0	H
ATOM	635	CA	GLN	224	23.868	5.118	7.452	1.00	0.00	RX0	C
ATOM	636	CB	GLN	224	22.846	4.093	6.958	1.00	0.00	RX0	C
ATOM	637	CG	GLN	224	21.595	4.762	6.382	1.00	0.00	RX0	C
ATOM	638	CD	GLN	224	20.554	3.707	6.075	1.00	0.00	RX0	C
ATOM	639	OE1	GLN	224	20.409	2.721	6.792	1.00	0.00	RX0	O
ATOM	640	NE2	GLN	224	19.837	3.962	4.966	1.00	0.00	RX0	N
ATOM	641	HE21	GLN	224	19.987	4.790	4.416	1.00	0.00	RX0	H
ATOM	642	HE22	GLN	224	19.109	3.372	4.621	1.00	0.00	RX0	H
ATOM	643	C	GLN	224	25.003	4.453	8.243	1.00	0.00	RX0	C
ATOM	644	O	GLN	224	25.073	4.591	9.468	1.00	0.00	RX0	O
ATOM	645	N	VAL	225	25.993	3.956	7.505	1.00	0.00	RX0	N
ATOM	646	H	VAL	225	25.871	3.966	6.507	1.00	0.00	RX0	H
ATOM	647	CA	VAL	225	27.191	3.319	8.093	1.00	0.00	RX0	C
ATOM	648	CB	VAL	225	28.035	2.560	7.064	1.00	0.00	RX0	C
ATOM	649	CG1	VAL	225	29.163	1.795	7.754	1.00	0.00	RX0	C
ATOM	650	CG2	VAL	225	27.180	1.580	6.268	1.00	0.00	RX0	C
ATOM	651	C	VAL	225	28.021	4.368	8.852	1.00	0.00	RX0	C
ATOM	652	O	VAL	225	28.415	4.141	9.995	1.00	0.00	RX0	O
ATOM	653	N	HIS	226	28.182	5.534	8.231	1.00	0.00	RX0	N
ATOM	654	H	HIS	226	27.815	5.627	7.302	1.00	0.00	RX0	H
ATOM	655	CA	HIS	226	28.959	6.641	8.815	1.00	0.00	RX0	C
ATOM	656	CB	HIS	226	29.042	7.795	7.802	1.00	0.00	RX0	C
ATOM	657	CG	HIS	226	30.076	8.848	8.162	1.00	0.00	RX0	C
ATOM	658	ND1	HIS	226	31.165	9.084	7.409	1.00	0.00	RX0	N
ATOM	659	HD1	HIS	226	31.399	8.620	6.577	1.00	0.00	RX0	H
ATOM	660	CD2	HIS	226	30.087	9.744	9.240	1.00	0.00	RX0	C
ATOM	661	NE2	HIS	226	31.195	10.519	9.123	1.00	0.00	RX0	N
ATOM	662	CE1	HIS	226	31.859	10.111	7.994	1.00	0.00	RX0	C
ATOM	663	C	HIS	226	28.363	7.118	10.150	1.00	0.00	RX0	C
ATOM	664	O	HIS	226	29.071	7.189	11.155	1.00	0.00	RX0	O
ATOM	665	N	LEU	227	27.047	7.317	10.166	1.00	0.00	RX0	N
ATOM	666	H	LEU	227	26.533	7.190	9.316	1.00	0.00	RX0	H
ATOM	667	CA	LEU	227	26.344	7.787	11.375	1.00	0.00	RX0	C
ATOM	668	CB	LEU	227	24.876	8.087	11.076	1.00	0.00	RX0	C
ATOM	669	CG	LEU	227	24.674	9.373	10.275	1.00	0.00	RX0	C

ATOM	670	CD1	LEU	227	23.205	9.581	9.914	1.00	0.00	RX0	C
ATOM	671	CD2	LEU	227	25.243	10.589	11.005	1.00	0.00	RX0	C
ATOM	672	C	LEU	227	26.435	6.799	12.540	1.00	0.00	RX0	C
ATOM	673	O	LEU	227	26.853	7.165	13.635	1.00	0.00	RX0	O
ATOM	674	N	LEU	228	26.270	5.522	12.200	1.00	0.00	RX0	N
ATOM	675	H	LEU	228	26.031	5.300	11.251	1.00	0.00	RX0	H
ATOM	676	CA	LEU	228	26.384	4.431	13.181	1.00	0.00	RX0	C
ATOM	677	CB	LEU	228	25.755	3.158	12.622	1.00	0.00	RX0	C
ATOM	678	CG	LEU	228	24.252	3.147	12.895	1.00	0.00	RX0	C
ATOM	679	CD1	LEU	228	23.502	2.143	12.025	1.00	0.00	RX0	C
ATOM	680	CD2	LEU	228	23.971	2.930	14.382	1.00	0.00	RX0	C
ATOM	681	C	LEU	228	27.805	4.188	13.685	1.00	0.00	RX0	C
ATOM	682	O	LEU	228	28.004	4.019	14.891	1.00	0.00	RX0	O
ATOM	683	N	GLU	229	28.784	4.376	12.809	1.00	0.00	RX0	N
ATOM	684	H	GLU	229	28.553	4.551	11.851	1.00	0.00	RX0	H
ATOM	685	CA	GLU	229	30.199	4.229	13.197	1.00	0.00	RX0	C
ATOM	686	CB	GLU	229	31.151	4.197	11.987	1.00	0.00	RX0	C
ATOM	687	CG	GLU	229	31.931	2.884	11.754	1.00	0.00	RX0	C
ATOM	688	CD	GLU	229	33.269	2.767	12.486	1.00	0.00	RX0	C
ATOM	689	OE1	GLU	229	33.387	3.133	13.653	1.00	0.00	RX0	O
ATOM	690	OE2	GLU	229	34.226	2.276	11.887	1.00	0.00	RX0	O
ATOM	691	C	GLU	229	30.618	5.338	14.175	1.00	0.00	RX0	C
ATOM	692	O	GLU	229	31.393	5.088	15.099	1.00	0.00	RX0	O
ATOM	693	N	CYS	230	30.060	6.523	13.970	1.00	0.00	RX0	N
ATOM	694	H	CYS	230	29.483	6.631	13.156	1.00	0.00	RX0	H
ATOM	695	CA	CYS	230	30.321	7.692	14.829	1.00	0.00	RX0	C
ATOM	696	CB	CYS	230	30.020	8.987	14.083	1.00	0.00	RX0	C
ATOM	697	SG	CYS	230	31.073	9.196	12.627	1.00	0.00	RX0	S
ATOM	698	C	CYS	230	29.592	7.653	16.182	1.00	0.00	RX0	C
ATOM	699	O	CYS	230	30.123	8.119	17.188	1.00	0.00	RX0	O
ATOM	700	N	ALA	231	28.434	7.000	16.215	1.00	0.00	RX0	N
ATOM	701	H	ALA	231	28.110	6.541	15.387	1.00	0.00	RX0	H
ATOM	702	CA	ALA	231	27.494	7.141	17.345	1.00	0.00	RX0	C
ATOM	703	CB	ALA	231	26.145	7.670	16.853	1.00	0.00	RX0	C
ATOM	704	C	ALA	231	27.249	5.885	18.186	1.00	0.00	RX0	C
ATOM	705	O	ALA	231	26.768	6.021	19.321	1.00	0.00	RX0	O
ATOM	706	N	TRP	232	27.687	4.719	17.731	1.00	0.00	RX0	N
ATOM	707	H	TRP	232	28.084	4.696	16.811	1.00	0.00	RX0	H
ATOM	708	CA	TRP	232	27.348	3.433	18.379	1.00	0.00	RX0	C
ATOM	709	CB	TRP	232	27.838	2.221	17.582	1.00	0.00	RX0	C
ATOM	710	CG	TRP	232	29.325	2.055	17.720	1.00	0.00	RX0	C
ATOM	711	CD2	TRP	232	30.025	1.250	18.686	1.00	0.00	RX0	C
ATOM	712	CE2	TRP	232	31.426	1.413	18.435	1.00	0.00	RX0	C
ATOM	713	CE3	TRP	232	29.584	0.416	19.734	1.00	0.00	RX0	C
ATOM	714	CD1	TRP	232	30.315	2.661	16.940	1.00	0.00	RX0	C
ATOM	715	NE1	TRP	232	31.553	2.286	17.353	1.00	0.00	RX0	N
ATOM	716	HE1	TRP	232	32.365	2.574	16.889	1.00	0.00	RX0	H
ATOM	717	CZ2	TRP	232	32.356	0.732	19.247	1.00	0.00	RX0	C
ATOM	718	CZ3	TRP	232	30.530	-0.256	20.536	1.00	0.00	RX0	C
ATOM	719	CH2	TRP	232	31.910	-0.098	20.294	1.00	0.00	RX0	C
ATOM	720	C	TRP	232	27.676	3.373	19.884	1.00	0.00	RX0	C
ATOM	721	O	TRP	232	26.862	2.914	20.672	1.00	0.00	RX0	O
ATOM	722	N	LEU	233	28.801	3.989	20.277	1.00	0.00	RX0	N
ATOM	723	H	LEU	233	29.346	4.443	19.575	1.00	0.00	RX0	H
ATOM	724	CA	LEU	233	29.211	3.960	21.691	1.00	0.00	RX0	C
ATOM	725	CB	LEU	233	30.723	4.178	21.787	1.00	0.00	RX0	C
ATOM	726	CG	LEU	233	31.336	3.775	23.132	1.00	0.00	RX0	C
ATOM	727	CD1	LEU	233	30.998	2.333	23.518	1.00	0.00	RX0	C
ATOM	728	CD2	LEU	233	32.844	4.020	23.150	1.00	0.00	RX0	C
ATOM	729	C	LEU	233	28.415	4.936	22.566	1.00	0.00	RX0	C
ATOM	730	O	LEU	233	27.943	4.566	23.634	1.00	0.00	RX0	O

ATOM	731	N	GLU	234	28.150	6.122	22.016	1.00	0.00	RX0	N
ATOM	732	H	GLU	234	28.484	6.339	21.101	1.00	0.00	RX0	H
ATOM	733	CA	GLU	234	27.227	7.090	22.644	1.00	0.00	RX0	C
ATOM	734	CB	GLU	234	27.212	8.352	21.767	1.00	0.00	RX0	C
ATOM	735	CG	GLU	234	26.695	9.602	22.475	1.00	0.00	RX0	C
ATOM	736	CD	GLU	234	26.710	10.816	21.565	1.00	0.00	RX0	C
ATOM	737	OE1	GLU	234	26.379	11.906	22.023	1.00	0.00	RX0	O
ATOM	738	OE2	GLU	234	27.058	10.698	20.396	1.00	0.00	RX0	O
ATOM	739	C	GLU	234	25.830	6.482	22.841	1.00	0.00	RX0	C
ATOM	740	O	GLU	234	25.253	6.598	23.926	1.00	0.00	RX0	O
ATOM	741	N	ILE	235	25.389	5.711	21.848	1.00	0.00	RX0	N
ATOM	742	H	ILE	235	25.962	5.642	21.030	1.00	0.00	RX0	H
ATOM	743	CA	ILE	235	24.069	5.045	21.863	1.00	0.00	RX0	C
ATOM	744	CB	ILE	235	23.694	4.475	20.490	1.00	0.00	RX0	C
ATOM	745	CG2	ILE	235	22.395	3.671	20.565	1.00	0.00	RX0	C
ATOM	746	CG1	ILE	235	23.575	5.588	19.450	1.00	0.00	RX0	C
ATOM	747	CD1	ILE	235	23.236	5.055	18.058	1.00	0.00	RX0	C
ATOM	748	C	ILE	235	24.018	3.945	22.939	1.00	0.00	RX0	C
ATOM	749	O	ILE	235	23.068	3.902	23.724	1.00	0.00	RX0	O
ATOM	750	N	LEU	236	25.072	3.140	23.020	1.00	0.00	RX0	N
ATOM	751	H	LEU	236	25.813	3.236	22.352	1.00	0.00	RX0	H
ATOM	752	CA	LEU	236	25.176	2.114	24.078	1.00	0.00	RX0	C
ATOM	753	CB	LEU	236	26.418	1.244	23.890	1.00	0.00	RX0	C
ATOM	754	CG	LEU	236	26.244	0.200	22.790	1.00	0.00	RX0	C
ATOM	755	CD1	LEU	236	27.513	-0.623	22.581	1.00	0.00	RX0	C
ATOM	756	CD2	LEU	236	25.036	-0.698	23.057	1.00	0.00	RX0	C
ATOM	757	C	LEU	236	25.182	2.733	25.479	1.00	0.00	RX0	C
ATOM	758	O	LEU	236	24.381	2.362	26.336	1.00	0.00	RX0	O
ATOM	759	N	MET	237	25.933	3.823	25.600	1.00	0.00	RX0	N
ATOM	760	H	MET	237	26.452	4.139	24.803	1.00	0.00	RX0	H
ATOM	761	CA	MET	237	26.132	4.523	26.881	1.00	0.00	RX0	C
ATOM	762	CB	MET	237	27.283	5.527	26.807	1.00	0.00	RX0	C
ATOM	763	CG	MET	237	28.653	4.849	26.798	1.00	0.00	RX0	C
ATOM	764	SD	MET	237	30.005	6.036	26.805	1.00	0.00	RX0	S
ATOM	765	CE	MET	237	31.360	4.869	27.003	1.00	0.00	RX0	C
ATOM	766	C	MET	237	24.875	5.215	27.409	1.00	0.00	RX0	C
ATOM	767	O	MET	237	24.517	5.003	28.572	1.00	0.00	RX0	O
ATOM	768	N	ILE	238	24.128	5.878	26.531	1.00	0.00	RX0	N
ATOM	769	H	ILE	238	24.459	5.965	25.588	1.00	0.00	RX0	H
ATOM	770	CA	ILE	238	22.871	6.546	26.925	1.00	0.00	RX0	C
ATOM	771	CB	ILE	238	22.342	7.528	25.866	1.00	0.00	RX0	C
ATOM	772	CG2	ILE	238	21.915	6.843	24.568	1.00	0.00	RX0	C
ATOM	773	CG1	ILE	238	21.214	8.372	26.465	1.00	0.00	RX0	C
ATOM	774	CD1	ILE	238	20.568	9.329	25.463	1.00	0.00	RX0	C
ATOM	775	C	ILE	238	21.800	5.514	27.357	1.00	0.00	RX0	C
ATOM	776	O	ILE	238	21.031	5.731	28.268	1.00	0.00	RX0	O
ATOM	777	N	GLY	239	21.845	4.355	26.660	1.00	0.00	RX0	N
ATOM	778	H	GLY	239	22.522	4.244	25.928	1.00	0.00	RX0	H
ATOM	779	CA	GLY	239	20.969	3.213	26.975	1.00	0.00	RX0	C
ATOM	780	C	GLY	239	21.301	2.634	28.356	1.00	0.00	RX0	C
ATOM	781	O	GLY	239	20.417	2.460	29.193	1.00	0.00	RX0	O
ATOM	782	N	LEU	240	22.605	2.583	28.639	1.00	0.00	RX0	N
ATOM	783	H	LEU	240	23.260	2.804	27.913	1.00	0.00	RX0	H
ATOM	784	CA	LEU	240	23.124	2.082	29.919	1.00	0.00	RX0	C
ATOM	785	CB	LEU	240	24.644	1.943	29.848	1.00	0.00	RX0	C
ATOM	786	CG	LEU	240	25.286	1.611	31.196	1.00	0.00	RX0	C
ATOM	787	CD1	LEU	240	24.821	0.265	31.750	1.00	0.00	RX0	C
ATOM	788	CD2	LEU	240	26.807	1.710	31.125	1.00	0.00	RX0	C
ATOM	789	C	LEU	240	22.728	2.995	31.086	1.00	0.00	RX0	C
ATOM	790	O	LEU	240	22.214	2.535	32.097	1.00	0.00	RX0	O
ATOM	791	N	VAL	241	22.901	4.295	30.880	1.00	0.00	RX0	N

ATOM	792	H	VAL	241	23.276	4.586	29.997	1.00	0.00	RX0	H
ATOM	793	CA	VAL	241	22.596	5.307	31.912	1.00	0.00	RX0	C
ATOM	794	CB	VAL	241	23.256	6.669	31.662	1.00	0.00	RX0	C
ATOM	795	CG1	VAL	241	24.774	6.500	31.611	1.00	0.00	RX0	C
ATOM	796	CG2	VAL	241	22.716	7.396	30.437	1.00	0.00	RX0	C
ATOM	797	C	VAL	241	21.084	5.392	32.193	1.00	0.00	RX0	C
ATOM	798	O	VAL	241	20.670	5.516	33.338	1.00	0.00	RX0	O
ATOM	799	N	TRP	242	20.290	5.181	31.134	1.00	0.00	RX0	N
ATOM	800	H	TRP	242	20.698	5.068	30.225	1.00	0.00	RX0	H
ATOM	801	CA	TRP	242	18.822	5.192	31.222	1.00	0.00	RX0	C
ATOM	802	CB	TRP	242	18.214	5.181	29.823	1.00	0.00	RX0	C
ATOM	803	CG	TRP	242	16.734	4.951	29.949	1.00	0.00	RX0	C
ATOM	804	CD2	TRP	242	15.715	5.872	30.390	1.00	0.00	RX0	C
ATOM	805	CE2	TRP	242	14.491	5.173	30.400	1.00	0.00	RX0	C
ATOM	806	CE3	TRP	242	15.742	7.204	30.773	1.00	0.00	RX0	C
ATOM	807	CD1	TRP	242	16.065	3.747	29.709	1.00	0.00	RX0	C
ATOM	808	NE1	TRP	242	14.746	3.878	29.977	1.00	0.00	RX0	N
ATOM	809	HE1	TRP	242	14.073	3.170	29.924	1.00	0.00	RX0	H
ATOM	810	CZ2	TRP	242	13.327	5.813	30.802	1.00	0.00	RX0	C
ATOM	811	CZ3	TRP	242	14.569	7.837	31.168	1.00	0.00	RX0	C
ATOM	812	CH2	TRP	242	13.367	7.145	31.189	1.00	0.00	RX0	C
ATOM	813	C	TRP	242	18.281	4.038	32.076	1.00	0.00	RX0	C
ATOM	814	O	TRP	242	17.477	4.269	32.979	1.00	0.00	RX0	O
ATOM	815	N	ARG	243	18.818	2.841	31.865	1.00	0.00	RX0	N
ATOM	816	H	ARG	243	19.513	2.737	31.150	1.00	0.00	RX0	H
ATOM	817	CA	ARG	243	18.359	1.661	32.627	1.00	0.00	RX0	C
ATOM	818	CB	ARG	243	18.475	0.404	31.736	1.00	0.00	RX0	C
ATOM	819	CG	ARG	243	19.876	-0.076	31.302	1.00	0.00	RX0	C
ATOM	820	CD	ARG	243	19.807	-1.118	30.163	1.00	0.00	RX0	C
ATOM	821	NE	ARG	243	21.089	-1.778	29.870	1.00	0.00	RX0	N
ATOM	822	HE	ARG	243	21.639	-2.041	30.676	1.00	0.00	RX0	H
ATOM	823	CZ	ARG	243	21.410	-2.132	28.573	1.00	0.00	RX0	C
ATOM	824	NH1	ARG	243	20.646	-1.712	27.544	1.00	0.00	RX0	N
ATOM	825	HH11	ARG	243	20.892	-2.010	26.604	1.00	0.00	RX0	H
ATOM	826	HH12	ARG	243	19.845	-1.123	27.653	1.00	0.00	RX0	H
ATOM	827	NH2	ARG	243	22.477	-2.909	28.301	1.00	0.00	RX0	N
ATOM	828	HH21	ARG	243	22.765	-3.106	27.341	1.00	0.00	RX0	H
ATOM	829	HH22	ARG	243	23.030	-3.352	29.016	1.00	0.00	RX0	H
ATOM	830	C	ARG	243	19.043	1.510	33.998	1.00	0.00	RX0	C
ATOM	831	O	ARG	243	18.610	0.722	34.836	1.00	0.00	RX0	O
ATOM	832	N	SER	244	20.027	2.366	34.245	1.00	0.00	RX0	N
ATOM	833	H	SER	244	20.298	3.026	33.545	1.00	0.00	RX0	H
ATOM	834	CA	SER	244	20.722	2.464	35.548	1.00	0.00	RX0	C
ATOM	835	CB	SER	244	22.206	2.695	35.312	1.00	0.00	RX0	C
ATOM	836	OG	SER	244	22.701	1.650	34.473	1.00	0.00	RX0	O
ATOM	837	HG	SER	244	22.213	1.715	33.657	1.00	0.00	RX0	H
ATOM	838	C	SER	244	20.112	3.547	36.442	1.00	0.00	RX0	C
ATOM	839	O	SER	244	20.448	3.642	37.630	1.00	0.00	RX0	O
ATOM	840	N	MET	245	19.184	4.322	35.895	1.00	0.00	RX0	N
ATOM	841	H	MET	245	18.819	4.099	34.988	1.00	0.00	RX0	H
ATOM	842	CA	MET	245	18.600	5.504	36.550	1.00	0.00	RX0	C
ATOM	843	CB	MET	245	17.574	6.191	35.650	1.00	0.00	RX0	C
ATOM	844	CG	MET	245	16.863	7.319	36.403	1.00	0.00	RX0	C
ATOM	845	SD	MET	245	15.431	7.990	35.556	1.00	0.00	RX0	S
ATOM	846	CE	MET	245	16.282	8.494	34.064	1.00	0.00	RX0	C
ATOM	847	C	MET	245	17.925	5.204	37.895	1.00	0.00	RX0	C
ATOM	848	O	MET	245	18.105	5.946	38.853	1.00	0.00	RX0	O
ATOM	849	N	GLU	246	17.212	4.082	37.945	1.00	0.00	RX0	N
ATOM	850	H	GLU	246	17.123	3.488	37.138	1.00	0.00	RX0	H
ATOM	851	CA	GLU	246	16.494	3.692	39.178	1.00	0.00	RX0	C
ATOM	852	CB	GLU	246	15.180	2.970	38.900	1.00	0.00	RX0	C

ATOM	853	CG	GLU	246	14.149	3.743	38.077	1.00	0.00	RX0	C
ATOM	854	CD	GLU	246	12.955	2.839	37.828	1.00	0.00	RX0	C
ATOM	855	OE1	GLU	246	12.795	2.336	36.720	1.00	0.00	RX0	O
ATOM	856	OE2	GLU	246	12.191	2.591	38.753	1.00	0.00	RX0	O
ATOM	857	C	GLU	246	17.379	2.867	40.123	1.00	0.00	RX0	C
ATOM	858	O	GLU	246	16.897	2.295	41.108	1.00	0.00	RX0	O
ATOM	859	N	HIS	247	18.674	2.844	39.836	1.00	0.00	RX0	N
ATOM	860	H	HIS	247	19.039	3.308	39.030	1.00	0.00	RX0	H
ATOM	861	CA	HIS	247	19.658	2.055	40.601	1.00	0.00	RX0	C
ATOM	862	CB	HIS	247	20.165	0.855	39.809	1.00	0.00	RX0	C
ATOM	863	CG	HIS	247	19.053	-0.142	39.599	1.00	0.00	RX0	C
ATOM	864	ND1	HIS	247	17.925	-0.210	40.334	1.00	0.00	RX0	N
ATOM	865	HD1	HIS	247	17.633	0.388	41.058	1.00	0.00	RX0	H
ATOM	866	CD2	HIS	247	19.013	-1.136	38.624	1.00	0.00	RX0	C
ATOM	867	NE2	HIS	247	17.848	-1.806	38.775	1.00	0.00	RX0	N
ATOM	868	CE1	HIS	247	17.178	-1.243	39.829	1.00	0.00	RX0	C
ATOM	869	C	HIS	247	20.841	2.947	41.015	1.00	0.00	RX0	C
ATOM	870	O	HIS	247	21.962	2.788	40.490	1.00	0.00	RX0	O
ATOM	871	N	PRO	248	20.632	3.827	41.991	1.00	0.00	RX0	N
ATOM	872	CD	PRO	248	19.383	3.972	42.733	1.00	0.00	RX0	C
ATOM	873	CA	PRO	248	21.659	4.767	42.484	1.00	0.00	RX0	C
ATOM	874	CB	PRO	248	20.980	5.449	43.675	1.00	0.00	RX0	C
ATOM	875	CG	PRO	248	19.487	5.343	43.386	1.00	0.00	RX0	C
ATOM	876	C	PRO	248	22.939	4.013	42.877	1.00	0.00	RX0	C
ATOM	877	O	PRO	248	22.892	2.963	43.503	1.00	0.00	RX0	O
ATOM	878	N	GLY	249	24.055	4.541	42.350	1.00	0.00	RX0	N
ATOM	879	H	GLY	249	23.973	5.297	41.706	1.00	0.00	RX0	H
ATOM	880	CA	GLY	249	25.407	3.996	42.610	1.00	0.00	RX0	C
ATOM	881	C	GLY	249	25.783	2.749	41.794	1.00	0.00	RX0	C
ATOM	882	O	GLY	249	26.914	2.250	41.927	1.00	0.00	RX0	O
ATOM	883	N	LYS	250	24.877	2.267	40.961	1.00	0.00	RX0	N
ATOM	884	H	LYS	250	23.982	2.694	40.813	1.00	0.00	RX0	H
ATOM	885	CA	LYS	250	25.097	1.050	40.158	1.00	0.00	RX0	C
ATOM	886	CB	LYS	250	24.369	-0.138	40.786	1.00	0.00	RX0	C
ATOM	887	CG	LYS	250	25.229	-0.846	41.841	1.00	0.00	RX0	C
ATOM	888	CD	LYS	250	24.587	-2.110	42.416	1.00	0.00	RX0	C
ATOM	889	CE	LYS	250	25.528	-2.977	43.261	1.00	0.00	RX0	C
ATOM	890	NZ	LYS	250	26.641	-3.486	42.446	1.00	0.00	RX0	N
ATOM	891	HZ1	LYS	250	27.366	-3.908	43.068	1.00	0.00	RX0	H
ATOM	892	HZ2	LYS	250	26.339	-4.177	41.723	1.00	0.00	RX0	H
ATOM	893	HZ3	LYS	250	27.142	-2.687	42.013	1.00	0.00	RX0	H
ATOM	894	C	LYS	250	24.802	1.266	38.671	1.00	0.00	RX0	C
ATOM	895	O	LYS	250	24.040	2.166	38.282	1.00	0.00	RX0	O
ATOM	896	N	LEU	251	25.472	0.476	37.863	1.00	0.00	RX0	N
ATOM	897	H	LEU	251	26.046	-0.241	38.244	1.00	0.00	RX0	H
ATOM	898	CA	LEU	251	25.292	0.430	36.401	1.00	0.00	RX0	C
ATOM	899	CB	LEU	251	26.626	0.591	35.684	1.00	0.00	RX0	C
ATOM	900	CG	LEU	251	27.161	2.016	35.762	1.00	0.00	RX0	C
ATOM	901	CD1	LEU	251	28.584	2.114	35.219	1.00	0.00	RX0	C
ATOM	902	CD2	LEU	251	26.208	3.007	35.091	1.00	0.00	RX0	C
ATOM	903	C	LEU	251	24.646	-0.892	36.017	1.00	0.00	RX0	C
ATOM	904	O	LEU	251	25.224	-1.976	36.286	1.00	0.00	RX0	O
ATOM	905	N	LEU	252	23.437	-0.808	35.533	1.00	0.00	RX0	N
ATOM	906	H	LEU	252	23.078	0.097	35.319	1.00	0.00	RX0	H
ATOM	907	CA	LEU	252	22.659	-1.981	35.114	1.00	0.00	RX0	C
ATOM	908	CB	LEU	252	21.165	-1.732	35.294	1.00	0.00	RX0	C
ATOM	909	CG	LEU	252	20.360	-3.028	35.220	1.00	0.00	RX0	C
ATOM	910	CD1	LEU	252	20.904	-4.096	36.154	1.00	0.00	RX0	C
ATOM	911	CD2	LEU	252	18.897	-2.796	35.546	1.00	0.00	RX0	C
ATOM	912	C	LEU	252	23.007	-2.330	33.663	1.00	0.00	RX0	C
ATOM	913	O	LEU	252	22.274	-2.025	32.731	1.00	0.00	RX0	O

ATOM	914	N	PHE	253	24.144	-3.005	33.506	1.00	0.00	RX0	N
ATOM	915	H	PHE	253	24.622	-3.315	34.333	1.00	0.00	RX0	H
ATOM	916	CA	PHE	253	24.599	-3.457	32.174	1.00	0.00	RX0	C
ATOM	917	CB	PHE	253	25.968	-4.125	32.266	1.00	0.00	RX0	C
ATOM	918	CG	PHE	253	27.032	-3.098	32.554	1.00	0.00	RX0	C
ATOM	919	CD1	PHE	253	27.565	-2.351	31.510	1.00	0.00	RX0	C
ATOM	920	CD2	PHE	253	27.484	-2.906	33.853	1.00	0.00	RX0	C
ATOM	921	CE1	PHE	253	28.562	-1.418	31.763	1.00	0.00	RX0	C
ATOM	922	CE2	PHE	253	28.481	-1.973	34.104	1.00	0.00	RX0	C
ATOM	923	CZ	PHE	253	29.021	-1.231	33.060	1.00	0.00	RX0	C
ATOM	924	C	PHE	253	23.603	-4.446	31.564	1.00	0.00	RX0	C
ATOM	925	O	PHE	253	23.259	-4.379	30.390	1.00	0.00	RX0	O
ATOM	926	N	ALA	254	23.094	-5.300	32.445	1.00	0.00	RX0	N
ATOM	927	H	ALA	254	23.402	-5.311	33.402	1.00	0.00	RX0	H
ATOM	928	CA	ALA	254	22.050	-6.280	32.141	1.00	0.00	RX0	C
ATOM	929	CB	ALA	254	22.693	-7.552	31.598	1.00	0.00	RX0	C
ATOM	930	C	ALA	254	21.288	-6.584	33.440	1.00	0.00	RX0	C
ATOM	931	O	ALA	254	21.887	-6.418	34.526	1.00	0.00	RX0	O
ATOM	932	N	PRO	255	20.056	-7.064	33.372	1.00	0.00	RX0	N
ATOM	933	CD	PRO	255	19.307	-7.244	32.131	1.00	0.00	RX0	C
ATOM	934	CA	PRO	255	19.236	-7.444	34.545	1.00	0.00	RX0	C
ATOM	935	CB	PRO	255	17.989	-8.076	33.927	1.00	0.00	RX0	C
ATOM	936	CG	PRO	255	17.857	-7.403	32.568	1.00	0.00	RX0	C
ATOM	937	C	PRO	255	19.972	-8.395	35.506	1.00	0.00	RX0	C
ATOM	938	O	PRO	255	19.756	-8.342	36.714	1.00	0.00	RX0	O
ATOM	939	N	ASN	256	20.900	-9.179	34.970	1.00	0.00	RX0	N
ATOM	940	H	ASN	256	21.117	-9.128	33.994	1.00	0.00	RX0	H
ATOM	941	CA	ASN	256	21.722	-10.125	35.761	1.00	0.00	RX0	C
ATOM	942	CB	ASN	256	21.714	-11.529	35.152	1.00	0.00	RX0	C
ATOM	943	CG	ASN	256	22.407	-11.531	33.799	1.00	0.00	RX0	C
ATOM	944	OD1	ASN	256	22.272	-10.605	33.000	1.00	0.00	RX0	O
ATOM	945	ND2	ASN	256	23.063	-12.676	33.533	1.00	0.00	RX0	N
ATOM	946	HD21	ASN	256	23.220	-13.338	34.269	1.00	0.00	RX0	H
ATOM	947	HD22	ASN	256	23.408	-12.927	32.622	1.00	0.00	RX0	H
ATOM	948	C	ASN	256	23.191	-9.669	35.876	1.00	0.00	RX0	C
ATOM	949	O	ASN	256	24.101	-10.505	36.011	1.00	0.00	RX0	O
ATOM	950	N	LEU	257	23.439	-8.384	35.742	1.00	0.00	RX0	N
ATOM	951	H	LEU	257	22.690	-7.724	35.650	1.00	0.00	RX0	H
ATOM	952	CA	LEU	257	24.796	-7.805	35.783	1.00	0.00	RX0	C
ATOM	953	CB	LEU	257	25.513	-7.967	34.444	1.00	0.00	RX0	C
ATOM	954	CG	LEU	257	27.023	-7.755	34.565	1.00	0.00	RX0	C
ATOM	955	CD1	LEU	257	27.658	-8.790	35.494	1.00	0.00	RX0	C
ATOM	956	CD2	LEU	257	27.712	-7.720	33.201	1.00	0.00	RX0	C
ATOM	957	C	LEU	257	24.720	-6.327	36.168	1.00	0.00	RX0	C
ATOM	958	O	LEU	257	24.738	-5.412	35.328	1.00	0.00	RX0	O
ATOM	959	N	LEU	258	24.604	-6.148	37.469	1.00	0.00	RX0	N
ATOM	960	H	LEU	258	24.657	-6.942	38.073	1.00	0.00	RX0	H
ATOM	961	CA	LEU	258	24.482	-4.834	38.118	1.00	0.00	RX0	C
ATOM	962	CB	LEU	258	23.233	-4.901	38.998	1.00	0.00	RX0	C
ATOM	963	CG	LEU	258	22.854	-3.619	39.730	1.00	0.00	RX0	C
ATOM	964	CD1	LEU	258	22.662	-2.446	38.778	1.00	0.00	RX0	C
ATOM	965	CD2	LEU	258	21.629	-3.822	40.623	1.00	0.00	RX0	C
ATOM	966	C	LEU	258	25.743	-4.564	38.938	1.00	0.00	RX0	C
ATOM	967	O	LEU	258	26.013	-5.237	39.948	1.00	0.00	RX0	O
ATOM	968	N	LEU	259	26.528	-3.622	38.460	1.00	0.00	RX0	N
ATOM	969	H	LEU	259	26.212	-3.064	37.686	1.00	0.00	RX0	H
ATOM	970	CA	LEU	259	27.862	-3.349	39.027	1.00	0.00	RX0	C
ATOM	971	CB	LEU	259	28.938	-3.493	37.947	1.00	0.00	RX0	C
ATOM	972	CG	LEU	259	28.880	-4.809	37.167	1.00	0.00	RX0	C
ATOM	973	CD1	LEU	259	29.895	-4.832	36.024	1.00	0.00	RX0	C
ATOM	974	CD2	LEU	259	29.029	-6.032	38.071	1.00	0.00	RX0	C

ATOM	975	C	LEU	259	27.958	-1.956	39.652	1.00	0.00	RX0	C
ATOM	976	O	LEU	259	27.419	-0.984	39.137	1.00	0.00	RX0	O
ATOM	977	N	ASP	260	28.645	-1.912	40.785	1.00	0.00	RX0	N
ATOM	978	H	ASP	260	29.220	-2.691	41.048	1.00	0.00	RX0	H
ATOM	979	CA	ASP	260	29.043	-0.657	41.454	1.00	0.00	RX0	C
ATOM	980	CB	ASP	260	29.077	-0.877	42.962	1.00	0.00	RX0	C
ATOM	981	CG	ASP	260	29.748	-2.210	43.211	1.00	0.00	RX0	C
ATOM	982	OD1	ASP	260	30.974	-2.264	43.249	1.00	0.00	RX0	O
ATOM	983	OD2	ASP	260	29.029	-3.204	43.321	1.00	0.00	RX0	O
ATOM	984	C	ASP	260	30.443	-0.222	40.970	1.00	0.00	RX0	C
ATOM	985	O	ASP	260	31.127	-1.008	40.295	1.00	0.00	RX0	O
ATOM	986	N	ARG	261	30.963	0.873	41.504	1.00	0.00	RX0	N
ATOM	987	H	ARG	261	30.385	1.411	42.117	1.00	0.00	RX0	H
ATOM	988	CA	ARG	261	32.263	1.408	41.044	1.00	0.00	RX0	C
ATOM	989	CB	ARG	261	32.422	2.884	41.442	1.00	0.00	RX0	C
ATOM	990	CG	ARG	261	32.657	3.157	42.930	1.00	0.00	RX0	C
ATOM	991	CD	ARG	261	32.610	4.651	43.278	1.00	0.00	RX0	C
ATOM	992	NE	ARG	261	33.470	5.435	42.394	1.00	0.00	RX0	N
ATOM	993	HE	ARG	261	33.133	5.681	41.468	1.00	0.00	RX0	H
ATOM	994	CZ	ARG	261	34.718	5.855	42.746	1.00	0.00	RX0	C
ATOM	995	NH1	ARG	261	35.170	5.629	43.999	1.00	0.00	RX0	N
ATOM	996	HH11	ARG	261	36.081	5.925	44.293	1.00	0.00	RX0	H
ATOM	997	HH12	ARG	261	34.587	5.172	44.673	1.00	0.00	RX0	H
ATOM	998	NH2	ARG	261	35.473	6.491	41.831	1.00	0.00	RX0	N
ATOM	999	HH21	ARG	261	36.414	6.812	41.933	1.00	0.00	RX0	H
ATOM	1000	HH22	ARG	261	35.025	6.685	40.935	1.00	0.00	RX0	H
ATOM	1001	C	ARG	261	33.476	0.540	41.436	1.00	0.00	RX0	C
ATOM	1002	O	ARG	261	34.378	0.347	40.637	1.00	0.00	RX0	O
ATOM	1003	N	ASN	262	33.410	-0.067	42.632	1.00	0.00	RX0	N
ATOM	1004	H	ASN	262	32.533	-0.040	43.114	1.00	0.00	RX0	H
ATOM	1005	CA	ASN	262	34.456	-1.000	43.094	1.00	0.00	RX0	C
ATOM	1006	CB	ASN	262	34.112	-1.492	44.493	1.00	0.00	RX0	C
ATOM	1007	CG	ASN	262	35.023	-2.651	44.833	1.00	0.00	RX0	C
ATOM	1008	OD1	ASN	262	36.186	-2.449	45.185	1.00	0.00	RX0	O
ATOM	1009	ND2	ASN	262	34.424	-3.853	44.762	1.00	0.00	RX0	N
ATOM	1010	HD21	ASN	262	33.457	-3.920	44.496	1.00	0.00	RX0	H
ATOM	1011	HD22	ASN	262	34.892	-4.717	44.956	1.00	0.00	RX0	H
ATOM	1012	C	ASN	262	34.635	-2.233	42.204	1.00	0.00	RX0	C
ATOM	1013	O	ASN	262	35.755	-2.665	41.964	1.00	0.00	RX0	O
ATOM	1014	N	GLN	263	33.530	-2.679	41.603	1.00	0.00	RX0	N
ATOM	1015	H	GLN	263	32.637	-2.270	41.818	1.00	0.00	RX0	H
ATOM	1016	CA	GLN	263	33.559	-3.756	40.599	1.00	0.00	RX0	C
ATOM	1017	CB	GLN	263	32.198	-4.423	40.447	1.00	0.00	RX0	C
ATOM	1018	CG	GLN	263	31.925	-5.186	41.741	1.00	0.00	RX0	C
ATOM	1019	CD	GLN	263	30.707	-6.065	41.599	1.00	0.00	RX0	C
ATOM	1020	OE1	GLN	263	30.630	-6.944	40.749	1.00	0.00	RX0	O
ATOM	1021	NE2	GLN	263	29.772	-5.817	42.526	1.00	0.00	RX0	N
ATOM	1022	HE21	GLN	263	29.888	-5.006	43.113	1.00	0.00	RX0	H
ATOM	1023	HE22	GLN	263	28.986	-6.419	42.651	1.00	0.00	RX0	H
ATOM	1024	C	GLN	263	34.189	-3.308	39.265	1.00	0.00	RX0	C
ATOM	1025	O	GLN	263	34.644	-4.116	38.479	1.00	0.00	RX0	O
ATOM	1026	N	GLY	264	34.180	-1.978	39.039	1.00	0.00	RX0	N
ATOM	1027	H	GLY	264	33.763	-1.376	39.719	1.00	0.00	RX0	H
ATOM	1028	CA	GLY	264	34.831	-1.337	37.881	1.00	0.00	RX0	C
ATOM	1029	C	GLY	264	36.364	-1.310	37.978	1.00	0.00	RX0	C
ATOM	1030	O	GLY	264	37.050	-1.510	36.977	1.00	0.00	RX0	O
ATOM	1031	N	LYS	265	36.881	-1.155	39.202	1.00	0.00	RX0	N
ATOM	1032	H	LYS	265	36.232	-1.005	39.949	1.00	0.00	RX0	H
ATOM	1033	CA	LYS	265	38.336	-1.184	39.469	1.00	0.00	RX0	C
ATOM	1034	CB	LYS	265	38.670	-1.145	40.952	1.00	0.00	RX0	C
ATOM	1035	CG	LYS	265	38.098	-0.088	41.885	1.00	0.00	RX0	C

ATOM	1036	CD	LYS	265	38.474	-0.603	43.274	1.00	0.00	RX0	C
ATOM	1037	CE	LYS	265	37.930	0.156	44.477	1.00	0.00	RX0	C
ATOM	1038	NZ	LYS	265	38.090	-0.732	45.638	1.00	0.00	RX0	N
ATOM	1039	HZ1	LYS	265	37.665	-0.318	46.488	1.00	0.00	RX0	H
ATOM	1040	HZ2	LYS	265	37.589	-1.625	45.430	1.00	0.00	RX0	H
ATOM	1041	HZ3	LYS	265	39.093	-0.947	45.799	1.00	0.00	RX0	H
ATOM	1042	C	LYS	265	38.994	-2.500	39.030	1.00	0.00	RX0	C
ATOM	1043	O	LYS	265	40.184	-2.535	38.765	1.00	0.00	RX0	O
ATOM	1044	N	CYS	266	38.153	-3.548	38.917	1.00	0.00	RX0	N
ATOM	1045	H	CYS	266	37.197	-3.455	39.192	1.00	0.00	RX0	H
ATOM	1046	CA	CYS	266	38.538	-4.854	38.355	1.00	0.00	RX0	C
ATOM	1047	CB	CYS	266	37.315	-5.759	38.263	1.00	0.00	RX0	C
ATOM	1048	SG	CYS	266	36.554	-5.947	39.897	1.00	0.00	RX0	S
ATOM	1049	C	CYS	266	39.318	-4.732	37.033	1.00	0.00	RX0	C
ATOM	1050	O	CYS	266	40.108	-5.603	36.695	1.00	0.00	RX0	O
ATOM	1051	N	VAL	267	39.075	-3.636	36.304	1.00	0.00	RX0	N
ATOM	1052	H	VAL	267	38.470	-2.902	36.621	1.00	0.00	RX0	H
ATOM	1053	CA	VAL	267	39.804	-3.345	35.058	1.00	0.00	RX0	C
ATOM	1054	CB	VAL	267	38.856	-3.352	33.851	1.00	0.00	RX0	C
ATOM	1055	CG1	VAL	267	39.583	-3.008	32.548	1.00	0.00	RX0	C
ATOM	1056	CG2	VAL	267	38.126	-4.694	33.740	1.00	0.00	RX0	C
ATOM	1057	C	VAL	267	40.557	-2.013	35.191	1.00	0.00	RX0	C
ATOM	1058	O	VAL	267	39.969	-0.956	35.468	1.00	0.00	RX0	O
ATOM	1059	N	GLU	268	41.829	-2.078	34.821	1.00	0.00	RX0	N
ATOM	1060	H	GLU	268	42.209	-2.964	34.565	1.00	0.00	RX0	H
ATOM	1061	CA	GLU	268	42.726	-0.908	34.727	1.00	0.00	RX0	C
ATOM	1062	CB	GLU	268	44.097	-1.333	34.209	1.00	0.00	RX0	C
ATOM	1063	CG	GLU	268	45.139	-0.222	34.330	1.00	0.00	RX0	C
ATOM	1064	CD	GLU	268	46.503	-0.802	34.033	1.00	0.00	RX0	C
ATOM	1065	OE1	GLU	268	46.592	-2.016	33.854	1.00	0.00	RX0	O
ATOM	1066	OE2	GLU	268	47.470	-0.044	33.993	1.00	0.00	RX0	O
ATOM	1067	C	GLU	268	42.079	0.194	33.866	1.00	0.00	RX0	C
ATOM	1068	O	GLU	268	41.697	-0.027	32.727	1.00	0.00	RX0	O
ATOM	1069	N	GLY	269	41.924	1.355	34.524	1.00	0.00	RX0	N
ATOM	1070	H	GLY	269	42.193	1.397	35.484	1.00	0.00	RX0	H
ATOM	1071	CA	GLY	269	41.377	2.576	33.902	1.00	0.00	RX0	C
ATOM	1072	C	GLY	269	39.898	2.490	33.494	1.00	0.00	RX0	C
ATOM	1073	O	GLY	269	39.424	3.343	32.745	1.00	0.00	RX0	O
ATOM	1074	N	MET	270	39.146	1.598	34.134	1.00	0.00	RX0	N
ATOM	1075	H	MET	270	39.580	0.955	34.768	1.00	0.00	RX0	H
ATOM	1076	CA	MET	270	37.704	1.465	33.841	1.00	0.00	RX0	C
ATOM	1077	CB	MET	270	37.281	0.008	33.653	1.00	0.00	RX0	C
ATOM	1078	CG	MET	270	35.929	-0.101	32.942	1.00	0.00	RX0	C
ATOM	1079	SD	MET	270	35.428	-1.792	32.577	1.00	0.00	RX0	S
ATOM	1080	CE	MET	270	35.155	-2.355	34.262	1.00	0.00	RX0	C
ATOM	1081	C	MET	270	36.825	2.181	34.877	1.00	0.00	RX0	C
ATOM	1082	O	MET	270	35.781	2.734	34.514	1.00	0.00	RX0	O
ATOM	1083	N	VAL	271	37.310	2.290	36.107	1.00	0.00	RX0	N
ATOM	1084	H	VAL	271	38.234	1.949	36.273	1.00	0.00	RX0	H
ATOM	1085	CA	VAL	271	36.591	3.043	37.171	1.00	0.00	RX0	C
ATOM	1086	CB	VAL	271	37.245	2.851	38.551	1.00	0.00	RX0	C
ATOM	1087	CG1	VAL	271	38.734	3.178	38.565	1.00	0.00	RX0	C
ATOM	1088	CG2	VAL	271	36.455	3.560	39.652	1.00	0.00	RX0	C
ATOM	1089	C	VAL	271	36.343	4.502	36.749	1.00	0.00	RX0	C
ATOM	1090	O	VAL	271	35.261	5.071	37.095	1.00	0.00	RX0	O
ATOM	1091	N	GLU	272	37.228	5.074	36.011	1.00	0.00	RX0	N
ATOM	1092	H	GLU	272	38.101	4.600	35.879	1.00	0.00	RX0	H
ATOM	1093	CA	GLU	272	37.141	6.453	35.460	1.00	0.00	RX0	C
ATOM	1094	CB	GLU	272	38.433	6.786	34.705	1.00	0.00	RX0	C
ATOM	1095	CG	GLU	272	39.677	7.020	35.578	1.00	0.00	RX0	C
ATOM	1096	CD	GLU	272	40.034	5.793	36.399	1.00	0.00	RX0	C

ATOM	1097	OE1	GLU	272	40.064	4.691	35.851	1.00	0.00	RX0	O
ATOM	1098	OE2	GLU	272	40.247	5.935	37.599	1.00	0.00	RX0	O
ATOM	1099	C	GLU	272	35.927	6.588	34.526	1.00	0.00	RX0	C
ATOM	1100	O	GLU	272	35.142	7.521	34.681	1.00	0.00	RX0	O
ATOM	1101	N	ILE	273	35.702	5.552	33.729	1.00	0.00	RX0	N
ATOM	1102	H	ILE	273	36.305	4.759	33.822	1.00	0.00	RX0	H
ATOM	1103	CA	ILE	273	34.533	5.484	32.820	1.00	0.00	RX0	C
ATOM	1104	CB	ILE	273	34.734	4.378	31.778	1.00	0.00	RX0	C
ATOM	1105	CG2	ILE	273	33.621	4.407	30.730	1.00	0.00	RX0	C
ATOM	1106	CG1	ILE	273	36.125	4.438	31.141	1.00	0.00	RX0	C
ATOM	1107	CD1	ILE	273	36.332	5.663	30.249	1.00	0.00	RX0	C
ATOM	1108	C	ILE	273	33.249	5.235	33.628	1.00	0.00	RX0	C
ATOM	1109	O	ILE	273	32.257	5.951	33.452	1.00	0.00	RX0	O
ATOM	1110	N	PHE	274	33.320	4.302	34.575	1.00	0.00	RX0	N
ATOM	1111	H	PHE	274	34.190	3.822	34.693	1.00	0.00	RX0	H
ATOM	1112	CA	PHE	274	32.191	3.976	35.472	1.00	0.00	RX0	C
ATOM	1113	CB	PHE	274	32.611	2.940	36.516	1.00	0.00	RX0	C
ATOM	1114	CG	PHE	274	32.271	1.538	36.075	1.00	0.00	RX0	C
ATOM	1115	CD1	PHE	274	32.536	1.119	34.777	1.00	0.00	RX0	C
ATOM	1116	CD2	PHE	274	31.687	0.664	36.985	1.00	0.00	RX0	C
ATOM	1117	CE1	PHE	274	32.225	-0.180	34.396	1.00	0.00	RX0	C
ATOM	1118	CE2	PHE	274	31.376	-0.635	36.603	1.00	0.00	RX0	C
ATOM	1119	CZ	PHE	274	31.653	-1.058	35.310	1.00	0.00	RX0	C
ATOM	1120	C	PHE	274	31.669	5.203	36.222	1.00	0.00	RX0	C
ATOM	1121	O	PHE	274	30.484	5.521	36.143	1.00	0.00	RX0	O
ATOM	1122	N	ASP	275	32.607	5.983	36.757	1.00	0.00	RX0	N
ATOM	1123	H	ASP	275	33.558	5.683	36.808	1.00	0.00	RX0	H
ATOM	1124	CA	ASP	275	32.273	7.222	37.483	1.00	0.00	RX0	C
ATOM	1125	CB	ASP	275	33.487	7.803	38.196	1.00	0.00	RX0	C
ATOM	1126	CG	ASP	275	33.599	7.142	39.555	1.00	0.00	RX0	C
ATOM	1127	OD1	ASP	275	33.846	5.943	39.634	1.00	0.00	RX0	O
ATOM	1128	OD2	ASP	275	33.468	7.820	40.567	1.00	0.00	RX0	O
ATOM	1129	C	ASP	275	31.555	8.270	36.629	1.00	0.00	RX0	C
ATOM	1130	O	ASP	275	30.604	8.890	37.102	1.00	0.00	RX0	O
ATOM	1131	N	MET	276	31.921	8.336	35.353	1.00	0.00	RX0	N
ATOM	1132	H	MET	276	32.664	7.739	35.041	1.00	0.00	RX0	H
ATOM	1133	CA	MET	276	31.257	9.254	34.408	1.00	0.00	RX0	C
ATOM	1134	CB	MET	276	32.091	9.446	33.143	1.00	0.00	RX0	C
ATOM	1135	CG	MET	276	33.422	10.151	33.402	1.00	0.00	RX0	C
ATOM	1136	SD	MET	276	34.286	10.559	31.876	1.00	0.00	RX0	S
ATOM	1137	CE	MET	276	34.363	8.902	31.183	1.00	0.00	RX0	C
ATOM	1138	C	MET	276	29.833	8.798	34.061	1.00	0.00	RX0	C
ATOM	1139	O	MET	276	28.893	9.589	34.158	1.00	0.00	RX0	O
ATOM	1140	N	LEU	277	29.673	7.488	33.891	1.00	0.00	RX0	N
ATOM	1141	H	LEU	277	30.490	6.907	33.949	1.00	0.00	RX0	H
ATOM	1142	CA	LEU	277	28.362	6.866	33.606	1.00	0.00	RX0	C
ATOM	1143	CB	LEU	277	28.561	5.402	33.226	1.00	0.00	RX0	C
ATOM	1144	CG	LEU	277	29.433	5.220	31.984	1.00	0.00	RX0	C
ATOM	1145	CD1	LEU	277	29.926	3.780	31.840	1.00	0.00	RX0	C
ATOM	1146	CD2	LEU	277	28.733	5.717	30.721	1.00	0.00	RX0	C
ATOM	1147	C	LEU	277	27.393	6.992	34.787	1.00	0.00	RX0	C
ATOM	1148	O	LEU	277	26.257	7.447	34.627	1.00	0.00	RX0	O
ATOM	1149	N	LEU	278	27.939	6.795	35.983	1.00	0.00	RX0	N
ATOM	1150	H	LEU	278	28.900	6.516	36.019	1.00	0.00	RX0	H
ATOM	1151	CA	LEU	278	27.196	6.926	37.250	1.00	0.00	RX0	C
ATOM	1152	CB	LEU	278	28.067	6.470	38.419	1.00	0.00	RX0	C
ATOM	1153	CG	LEU	278	28.221	4.953	38.466	1.00	0.00	RX0	C
ATOM	1154	CD1	LEU	278	29.274	4.502	39.479	1.00	0.00	RX0	C
ATOM	1155	CD2	LEU	278	26.869	4.290	38.709	1.00	0.00	RX0	C
ATOM	1156	C	LEU	278	26.716	8.359	37.508	1.00	0.00	RX0	C
ATOM	1157	O	LEU	278	25.554	8.575	37.840	1.00	0.00	RX0	O

ATOM	1158	N	ALA	279	27.582	9.317	37.175	1.00	0.00	RX0	N
ATOM	1159	H	ALA	279	28.513	9.059	36.898	1.00	0.00	RX0	H
ATOM	1160	CA	ALA	279	27.275	10.754	37.296	1.00	0.00	RX0	C
ATOM	1161	CB	ALA	279	28.528	11.595	37.048	1.00	0.00	RX0	C
ATOM	1162	C	ALA	279	26.182	11.196	36.310	1.00	0.00	RX0	C
ATOM	1163	O	ALA	279	25.263	11.922	36.684	1.00	0.00	RX0	O
ATOM	1164	N	THR	280	26.210	10.618	35.109	1.00	0.00	RX0	N
ATOM	1165	H	THR	280	26.970	10.007	34.881	1.00	0.00	RX0	H
ATOM	1166	CA	THR	280	25.203	10.899	34.059	1.00	0.00	RX0	C
ATOM	1167	CB	THR	280	25.747	10.368	32.739	1.00	0.00	RX0	C
ATOM	1168	OG1	THR	280	27.060	10.906	32.531	1.00	0.00	RX0	O
ATOM	1169	HG1	THR	280	27.666	10.444	33.101	1.00	0.00	RX0	H
ATOM	1170	CG2	THR	280	24.829	10.717	31.566	1.00	0.00	RX0	C
ATOM	1171	C	THR	280	23.835	10.327	34.462	1.00	0.00	RX0	C
ATOM	1172	O	THR	280	22.822	11.023	34.397	1.00	0.00	RX0	O
ATOM	1173	N	SER	281	23.868	9.112	35.003	1.00	0.00	RX0	N
ATOM	1174	H	SER	281	24.739	8.619	35.048	1.00	0.00	RX0	H
ATOM	1175	CA	SER	281	22.669	8.413	35.507	1.00	0.00	RX0	C
ATOM	1176	CB	SER	281	23.145	6.984	35.866	1.00	0.00	RX0	C
ATOM	1177	OG	SER	281	22.381	6.337	36.903	1.00	0.00	RX0	O
ATOM	1178	HG	SER	281	22.559	5.405	36.792	1.00	0.00	RX0	H
ATOM	1179	C	SER	281	22.019	9.180	36.675	1.00	0.00	RX0	C
ATOM	1180	O	SER	281	20.814	9.399	36.693	1.00	0.00	RX0	O
ATOM	1181	N	SER	282	22.889	9.788	37.491	1.00	0.00	RX0	N
ATOM	1182	H	SER	282	23.868	9.614	37.378	1.00	0.00	RX0	H
ATOM	1183	CA	SER	282	22.489	10.660	38.613	1.00	0.00	RX0	C
ATOM	1184	CB	SER	282	23.674	10.723	39.557	1.00	0.00	RX0	C
ATOM	1185	OG	SER	282	23.944	9.344	39.873	1.00	0.00	RX0	O
ATOM	1186	HG	SER	282	24.725	9.120	39.367	1.00	0.00	RX0	H
ATOM	1187	C	SER	282	21.828	11.963	38.130	1.00	0.00	RX0	C
ATOM	1188	O	SER	282	20.788	12.371	38.639	1.00	0.00	RX0	O
ATOM	1189	N	ARG	283	22.365	12.516	37.039	1.00	0.00	RX0	N
ATOM	1190	H	ARG	283	23.190	12.111	36.638	1.00	0.00	RX0	H
ATOM	1191	CA	ARG	283	21.822	13.735	36.412	1.00	0.00	RX0	C
ATOM	1192	CB	ARG	283	22.814	14.204	35.348	1.00	0.00	RX0	C
ATOM	1193	CG	ARG	283	22.356	15.390	34.504	1.00	0.00	RX0	C
ATOM	1194	CD	ARG	283	22.168	16.685	35.292	1.00	0.00	RX0	C
ATOM	1195	NE	ARG	283	21.808	17.760	34.371	1.00	0.00	RX0	N
ATOM	1196	HE	ARG	283	22.251	17.716	33.469	1.00	0.00	RX0	H
ATOM	1197	CZ	ARG	283	20.921	18.723	34.756	1.00	0.00	RX0	C
ATOM	1198	NH1	ARG	283	20.411	18.709	36.008	1.00	0.00	RX0	N
ATOM	1199	HH11	ARG	283	19.749	19.398	36.319	1.00	0.00	RX0	H
ATOM	1200	HH12	ARG	283	20.673	18.008	36.680	1.00	0.00	RX0	H
ATOM	1201	NH2	ARG	283	20.561	19.671	33.868	1.00	0.00	RX0	N
ATOM	1202	HH21	ARG	283	19.871	20.378	34.088	1.00	0.00	RX0	H
ATOM	1203	HH22	ARG	283	20.949	19.709	32.943	1.00	0.00	RX0	H
ATOM	1204	C	ARG	283	20.432	13.477	35.812	1.00	0.00	RX0	C
ATOM	1205	O	ARG	283	19.498	14.246	36.035	1.00	0.00	RX0	O
ATOM	1206	N	PHE	284	20.293	12.325	35.169	1.00	0.00	RX0	N
ATOM	1207	H	PHE	284	21.097	11.740	35.042	1.00	0.00	RX0	H
ATOM	1208	CA	PHE	284	19.003	11.899	34.606	1.00	0.00	RX0	C
ATOM	1209	CB	PHE	284	19.206	10.636	33.776	1.00	0.00	RX0	C
ATOM	1210	CG	PHE	284	19.681	10.996	32.391	1.00	0.00	RX0	C
ATOM	1211	CD1	PHE	284	19.371	12.239	31.853	1.00	0.00	RX0	C
ATOM	1212	CD2	PHE	284	20.401	10.073	31.644	1.00	0.00	RX0	C
ATOM	1213	CE1	PHE	284	19.750	12.542	30.550	1.00	0.00	RX0	C
ATOM	1214	CE2	PHE	284	20.781	10.380	30.343	1.00	0.00	RX0	C
ATOM	1215	CZ	PHE	284	20.441	11.608	29.789	1.00	0.00	RX0	C
ATOM	1216	C	PHE	284	17.921	11.680	35.654	1.00	0.00	RX0	C
ATOM	1217	O	PHE	284	16.817	12.216	35.524	1.00	0.00	RX0	O
ATOM	1218	N	ARG	285	18.348	11.087	36.760	1.00	0.00	RX0	N

ATOM	1219	H	ARG	285	19.279	10.717	36.763	1.00	0.00	RX0	H
ATOM	1220	CA	ARG	285	17.485	10.841	37.923	1.00	0.00	RX0	C
ATOM	1221	CB	ARG	285	18.334	10.055	38.923	1.00	0.00	RX0	C
ATOM	1222	CG	ARG	285	17.771	9.784	40.319	1.00	0.00	RX0	C
ATOM	1223	CD	ARG	285	18.800	9.024	41.168	1.00	0.00	RX0	C
ATOM	1224	NE	ARG	285	19.214	7.813	40.462	1.00	0.00	RX0	N
ATOM	1225	HE	ARG	285	18.456	7.214	40.168	1.00	0.00	RX0	H
ATOM	1226	CZ	ARG	285	20.503	7.633	40.045	1.00	0.00	RX0	C
ATOM	1227	NH1	ARG	285	21.472	8.414	40.568	1.00	0.00	RX0	N
ATOM	1228	HH11	ARG	285	22.435	8.407	40.255	1.00	0.00	RX0	H
ATOM	1229	HH12	ARG	285	21.266	9.069	41.299	1.00	0.00	RX0	H
ATOM	1230	NH2	ARG	285	20.764	6.694	39.110	1.00	0.00	RX0	N
ATOM	1231	HH21	ARG	285	21.649	6.562	38.642	1.00	0.00	RX0	H
ATOM	1232	HH22	ARG	285	20.022	6.081	38.811	1.00	0.00	RX0	H
ATOM	1233	C	ARG	285	17.003	12.164	38.534	1.00	0.00	RX0	C
ATOM	1234	O	ARG	285	15.822	12.321	38.816	1.00	0.00	RX0	O
ATOM	1235	N	MET	286	17.922	13.130	38.591	1.00	0.00	RX0	N
ATOM	1236	H	MET	286	18.856	12.922	38.296	1.00	0.00	RX0	H
ATOM	1237	CA	MET	286	17.643	14.467	39.144	1.00	0.00	RX0	C
ATOM	1238	CB	MET	286	18.961	15.233	39.261	1.00	0.00	RX0	C
ATOM	1239	CG	MET	286	18.793	16.701	39.652	1.00	0.00	RX0	C
ATOM	1240	SD	MET	286	20.311	17.644	39.426	1.00	0.00	RX0	S
ATOM	1241	CE	MET	286	19.711	19.224	40.045	1.00	0.00	RX0	C
ATOM	1242	C	MET	286	16.681	15.257	38.243	1.00	0.00	RX0	C
ATOM	1243	O	MET	286	15.799	15.962	38.735	1.00	0.00	RX0	O
ATOM	1244	N	MET	287	16.911	15.161	36.942	1.00	0.00	RX0	N
ATOM	1245	H	MET	287	17.631	14.537	36.630	1.00	0.00	RX0	H
ATOM	1246	CA	MET	287	16.049	15.814	35.941	1.00	0.00	RX0	C
ATOM	1247	CB	MET	287	16.684	15.859	34.555	1.00	0.00	RX0	C
ATOM	1248	CG	MET	287	17.872	16.812	34.515	1.00	0.00	RX0	C
ATOM	1249	SD	MET	287	18.234	17.349	32.841	1.00	0.00	RX0	S
ATOM	1250	CE	MET	287	16.609	18.040	32.489	1.00	0.00	RX0	C
ATOM	1251	C	MET	287	14.674	15.156	35.849	1.00	0.00	RX0	C
ATOM	1252	O	MET	287	13.755	15.729	35.264	1.00	0.00	RX0	O
ATOM	1253	N	ASN	288	14.583	13.920	36.346	1.00	0.00	RX0	N
ATOM	1254	H	ASN	288	15.393	13.490	36.746	1.00	0.00	RX0	H
ATOM	1255	CA	ASN	288	13.391	13.066	36.242	1.00	0.00	RX0	C
ATOM	1256	CB	ASN	288	12.173	13.625	36.977	1.00	0.00	RX0	C
ATOM	1257	CG	ASN	288	11.006	12.682	36.757	1.00	0.00	RX0	C
ATOM	1258	OD1	ASN	288	11.159	11.475	36.605	1.00	0.00	RX0	O
ATOM	1259	ND2	ASN	288	9.813	13.306	36.738	1.00	0.00	RX0	N
ATOM	1260	HD21	ASN	288	9.755	14.295	36.872	1.00	0.00	RX0	H
ATOM	1261	HD22	ASN	288	8.966	12.797	36.586	1.00	0.00	RX0	H
ATOM	1262	C	ASN	288	13.076	12.836	34.753	1.00	0.00	RX0	C
ATOM	1263	O	ASN	288	11.986	13.121	34.256	1.00	0.00	RX0	O
ATOM	1264	N	LEU	289	14.125	12.439	34.028	1.00	0.00	RX0	N
ATOM	1265	H	LEU	289	14.954	12.171	34.521	1.00	0.00	RX0	H
ATOM	1266	CA	LEU	289	14.030	12.183	32.583	1.00	0.00	RX0	C
ATOM	1267	CB	LEU	289	15.381	11.667	32.074	1.00	0.00	RX0	C
ATOM	1268	CG	LEU	289	15.461	11.443	30.561	1.00	0.00	RX0	C
ATOM	1269	CD1	LEU	289	15.500	12.762	29.803	1.00	0.00	RX0	C
ATOM	1270	CD2	LEU	289	16.645	10.567	30.158	1.00	0.00	RX0	C
ATOM	1271	C	LEU	289	12.926	11.152	32.321	1.00	0.00	RX0	C
ATOM	1272	O	LEU	289	12.814	10.137	33.014	1.00	0.00	RX0	O
ATOM	1273	N	GLN	290	12.165	11.420	31.281	1.00	0.00	RX0	N
ATOM	1274	H	GLN	290	12.397	12.178	30.666	1.00	0.00	RX0	H
ATOM	1275	CA	GLN	290	11.011	10.584	30.916	1.00	0.00	RX0	C
ATOM	1276	CB	GLN	290	9.773	11.433	30.644	1.00	0.00	RX0	C
ATOM	1277	CG	GLN	290	9.291	12.061	31.950	1.00	0.00	RX0	C
ATOM	1278	CD	GLN	290	9.105	10.951	32.966	1.00	0.00	RX0	C
ATOM	1279	OE1	GLN	290	8.313	10.031	32.783	1.00	0.00	RX0	O

ATOM	1280	NE2	GLN	290	9.892	11.085	34.046	1.00	0.00	RX0	N
ATOM	1281	HE21	GLN	290	10.537	11.849	34.122	1.00	0.00	RX0	H
ATOM	1282	HE22	GLN	290	9.943	10.447	34.818	1.00	0.00	RX0	H
ATOM	1283	C	GLN	290	11.379	9.701	29.727	1.00	0.00	RX0	C
ATOM	1284	O	GLN	290	12.115	10.141	28.832	1.00	0.00	RX0	O
ATOM	1285	N	GLY	291	10.739	8.533	29.672	1.00	0.00	RX0	N
ATOM	1286	H	GLY	291	10.097	8.326	30.409	1.00	0.00	RX0	H
ATOM	1287	CA	GLY	291	10.952	7.542	28.589	1.00	0.00	RX0	C
ATOM	1288	C	GLY	291	10.769	8.134	27.181	1.00	0.00	RX0	C
ATOM	1289	O	GLY	291	11.559	7.865	26.269	1.00	0.00	RX0	O
ATOM	1290	N	GLU	292	9.854	9.086	27.078	1.00	0.00	RX0	N
ATOM	1291	H	GLU	292	9.225	9.228	27.846	1.00	0.00	RX0	H
ATOM	1292	CA	GLU	292	9.541	9.789	25.813	1.00	0.00	RX0	C
ATOM	1293	CB	GLU	292	8.231	10.572	25.945	1.00	0.00	RX0	C
ATOM	1294	CG	GLU	292	6.968	9.717	26.090	1.00	0.00	RX0	C
ATOM	1295	CD	GLU	292	7.055	8.867	27.338	1.00	0.00	RX0	C
ATOM	1296	OE1	GLU	292	7.221	9.407	28.429	1.00	0.00	RX0	O
ATOM	1297	OE2	GLU	292	7.019	7.649	27.233	1.00	0.00	RX0	O
ATOM	1298	C	GLU	292	10.671	10.744	25.400	1.00	0.00	RX0	C
ATOM	1299	O	GLU	292	11.065	10.802	24.241	1.00	0.00	RX0	O
ATOM	1300	N	GLU	293	11.241	11.417	26.395	1.00	0.00	RX0	N
ATOM	1301	H	GLU	293	10.968	11.204	27.333	1.00	0.00	RX0	H
ATOM	1302	CA	GLU	293	12.389	12.320	26.184	1.00	0.00	RX0	C
ATOM	1303	CB	GLU	293	12.614	13.166	27.433	1.00	0.00	RX0	C
ATOM	1304	CG	GLU	293	11.375	13.936	27.888	1.00	0.00	RX0	C
ATOM	1305	CD	GLU	293	11.648	14.513	29.261	1.00	0.00	RX0	C
ATOM	1306	OE1	GLU	293	12.536	14.022	29.948	1.00	0.00	RX0	O
ATOM	1307	OE2	GLU	293	10.976	15.451	29.670	1.00	0.00	RX0	O
ATOM	1308	C	GLU	293	13.669	11.544	25.843	1.00	0.00	RX0	C
ATOM	1309	O	GLU	293	14.364	11.888	24.886	1.00	0.00	RX0	O
ATOM	1310	N	PHE	294	13.855	10.409	26.517	1.00	0.00	RX0	N
ATOM	1311	H	PHE	294	13.198	10.198	27.243	1.00	0.00	RX0	H
ATOM	1312	CA	PHE	294	14.999	9.503	26.300	1.00	0.00	RX0	C
ATOM	1313	CB	PHE	294	14.901	8.311	27.253	1.00	0.00	RX0	C
ATOM	1314	CG	PHE	294	15.864	7.231	26.822	1.00	0.00	RX0	C
ATOM	1315	CD1	PHE	294	17.235	7.454	26.865	1.00	0.00	RX0	C
ATOM	1316	CD2	PHE	294	15.371	6.014	26.366	1.00	0.00	RX0	C
ATOM	1317	CE1	PHE	294	18.110	6.467	26.425	1.00	0.00	RX0	C
ATOM	1318	CE2	PHE	294	16.247	5.030	25.927	1.00	0.00	RX0	C
ATOM	1319	CZ	PHE	294	17.617	5.259	25.950	1.00	0.00	RX0	C
ATOM	1320	C	PHE	294	15.115	9.012	24.847	1.00	0.00	RX0	C
ATOM	1321	O	PHE	294	16.186	9.132	24.238	1.00	0.00	RX0	O
ATOM	1322	N	VAL	295	14.000	8.581	24.280	1.00	0.00	RX0	N
ATOM	1323	H	VAL	295	13.159	8.564	24.829	1.00	0.00	RX0	H
ATOM	1324	CA	VAL	295	13.976	8.026	22.907	1.00	0.00	RX0	C
ATOM	1325	CB	VAL	295	12.685	7.249	22.619	1.00	0.00	RX0	C
ATOM	1326	CG1	VAL	295	12.586	6.065	23.579	1.00	0.00	RX0	C
ATOM	1327	CG2	VAL	295	11.428	8.115	22.664	1.00	0.00	RX0	C
ATOM	1328	C	VAL	295	14.286	9.097	21.847	1.00	0.00	RX0	C
ATOM	1329	O	VAL	295	14.999	8.845	20.884	1.00	0.00	RX0	O
ATOM	1330	N	CYS	296	13.866	10.330	22.156	1.00	0.00	RX0	N
ATOM	1331	H	CYS	296	13.306	10.479	22.974	1.00	0.00	RX0	H
ATOM	1332	CA	CYS	296	14.167	11.498	21.314	1.00	0.00	RX0	C
ATOM	1333	CB	CYS	296	13.264	12.653	21.730	1.00	0.00	RX0	C
ATOM	1334	SG	CYS	296	11.518	12.259	21.468	1.00	0.00	RX0	S
ATOM	1335	C	CYS	296	15.661	11.843	21.352	1.00	0.00	RX0	C
ATOM	1336	O	CYS	296	16.290	11.969	20.305	1.00	0.00	RX0	O
ATOM	1337	N	LEU	297	16.247	11.750	22.547	1.00	0.00	RX0	N
ATOM	1338	H	LEU	297	15.681	11.545	23.350	1.00	0.00	RX0	H
ATOM	1339	CA	LEU	297	17.681	12.031	22.752	1.00	0.00	RX0	C
ATOM	1340	CB	LEU	297	18.016	12.112	24.240	1.00	0.00	RX0	C

ATOM	1341	CG	LEU	297	17.338	13.284	24.949	1.00	0.00	RX0	C
ATOM	1342	CD1	LEU	297	17.612	13.259	26.452	1.00	0.00	RX0	C
ATOM	1343	CD2	LEU	297	17.711	14.626	24.317	1.00	0.00	RX0	C
ATOM	1344	C	LEU	297	18.589	11.009	22.068	1.00	0.00	RX0	C
ATOM	1345	O	LEU	297	19.526	11.385	21.359	1.00	0.00	RX0	O
ATOM	1346	N	LYS	298	18.173	9.750	22.123	1.00	0.00	RX0	N
ATOM	1347	H	LYS	298	17.359	9.543	22.671	1.00	0.00	RX0	H
ATOM	1348	CA	LYS	298	18.945	8.654	21.515	1.00	0.00	RX0	C
ATOM	1349	CB	LYS	298	18.411	7.327	22.050	1.00	0.00	RX0	C
ATOM	1350	CG	LYS	298	19.286	6.119	21.722	1.00	0.00	RX0	C
ATOM	1351	CD	LYS	298	18.696	4.846	22.326	1.00	0.00	RX0	C
ATOM	1352	CE	LYS	298	17.223	4.724	21.945	1.00	0.00	RX0	C
ATOM	1353	NZ	LYS	298	16.675	3.434	22.361	1.00	0.00	RX0	N
ATOM	1354	HZ1	LYS	298	15.924	3.150	21.693	1.00	0.00	RX0	H
ATOM	1355	HZ2	LYS	298	17.357	2.664	22.448	1.00	0.00	RX0	H
ATOM	1356	HZ3	LYS	298	16.104	3.509	23.233	1.00	0.00	RX0	H
ATOM	1357	C	LYS	298	18.925	8.733	19.978	1.00	0.00	RX0	C
ATOM	1358	O	LYS	298	19.964	8.557	19.332	1.00	0.00	RX0	O
ATOM	1359	N	SER	299	17.791	9.152	19.431	1.00	0.00	RX0	N
ATOM	1360	H	SER	299	16.964	9.263	19.983	1.00	0.00	RX0	H
ATOM	1361	CA	SER	299	17.645	9.383	17.977	1.00	0.00	RX0	C
ATOM	1362	CB	SER	299	16.155	9.360	17.703	1.00	0.00	RX0	C
ATOM	1363	OG	SER	299	15.662	8.195	18.374	1.00	0.00	RX0	O
ATOM	1364	HG	SER	299	15.142	8.493	19.114	1.00	0.00	RX0	H
ATOM	1365	C	SER	299	18.416	10.621	17.504	1.00	0.00	RX0	C
ATOM	1366	O	SER	299	19.051	10.583	16.444	1.00	0.00	RX0	O
ATOM	1367	N	ILE	300	18.478	11.644	18.354	1.00	0.00	RX0	N
ATOM	1368	H	ILE	300	17.952	11.595	19.206	1.00	0.00	RX0	H
ATOM	1369	CA	ILE	300	19.283	12.860	18.096	1.00	0.00	RX0	C
ATOM	1370	CB	ILE	300	19.081	13.935	19.167	1.00	0.00	RX0	C
ATOM	1371	CG2	ILE	300	20.123	15.049	19.042	1.00	0.00	RX0	C
ATOM	1372	CG1	ILE	300	17.675	14.517	19.095	1.00	0.00	RX0	C
ATOM	1373	CD1	ILE	300	17.416	15.533	20.205	1.00	0.00	RX0	C
ATOM	1374	C	ILE	300	20.773	12.490	18.013	1.00	0.00	RX0	C
ATOM	1375	O	ILE	300	21.456	12.924	17.087	1.00	0.00	RX0	O
ATOM	1376	N	ILE	301	21.227	11.630	18.921	1.00	0.00	RX0	N
ATOM	1377	H	ILE	301	20.604	11.318	19.642	1.00	0.00	RX0	H
ATOM	1378	CA	ILE	301	22.629	11.156	18.925	1.00	0.00	RX0	C
ATOM	1379	CB	ILE	301	22.812	10.180	20.079	1.00	0.00	RX0	C
ATOM	1380	CG2	ILE	301	24.031	9.288	19.874	1.00	0.00	RX0	C
ATOM	1381	CG1	ILE	301	22.840	10.937	21.403	1.00	0.00	RX0	C
ATOM	1382	CD1	ILE	301	22.939	9.991	22.595	1.00	0.00	RX0	C
ATOM	1383	C	ILE	301	22.948	10.474	17.584	1.00	0.00	RX0	C
ATOM	1384	O	ILE	301	23.927	10.823	16.926	1.00	0.00	RX0	O
ATOM	1385	N	LEU	302	22.047	9.582	17.175	1.00	0.00	RX0	N
ATOM	1386	H	LEU	302	21.275	9.365	17.777	1.00	0.00	RX0	H
ATOM	1387	CA	LEU	302	22.205	8.836	15.917	1.00	0.00	RX0	C
ATOM	1388	CB	LEU	302	20.992	7.930	15.709	1.00	0.00	RX0	C
ATOM	1389	CG	LEU	302	21.002	7.207	14.361	1.00	0.00	RX0	C
ATOM	1390	CD1	LEU	302	22.182	6.246	14.230	1.00	0.00	RX0	C
ATOM	1391	CD2	LEU	302	19.666	6.525	14.079	1.00	0.00	RX0	C
ATOM	1392	C	LEU	302	22.391	9.757	14.699	1.00	0.00	RX0	C
ATOM	1393	O	LEU	302	23.285	9.544	13.882	1.00	0.00	RX0	O
ATOM	1394	N	LEU	303	21.581	10.806	14.658	1.00	0.00	RX0	N
ATOM	1395	H	LEU	303	20.929	10.941	15.407	1.00	0.00	RX0	H
ATOM	1396	CA	LEU	303	21.546	11.719	13.502	1.00	0.00	RX0	C
ATOM	1397	CB	LEU	303	20.124	12.231	13.311	1.00	0.00	RX0	C
ATOM	1398	CG	LEU	303	19.163	11.065	13.084	1.00	0.00	RX0	C
ATOM	1399	CD1	LEU	303	17.707	11.487	13.252	1.00	0.00	RX0	C
ATOM	1400	CD2	LEU	303	19.418	10.365	11.748	1.00	0.00	RX0	C
ATOM	1401	C	LEU	303	22.576	12.849	13.543	1.00	0.00	RX0	C

ATOM	1402	O	LEU	303	23.073	13.268	12.494	1.00	0.00	RX0	O
ATOM	1403	N	ASN	304	22.971	13.240	14.745	1.00	0.00	RX0	N
ATOM	1404	H	ASN	304	22.641	12.728	15.539	1.00	0.00	RX0	H
ATOM	1405	CA	ASN	304	23.832	14.419	14.940	1.00	0.00	RX0	C
ATOM	1406	CB	ASN	304	23.533	15.282	16.186	1.00	0.00	RX0	C
ATOM	1407	CG	ASN	304	24.464	14.899	17.337	1.00	0.00	RX0	C
ATOM	1408	OD1	ASN	304	25.519	15.472	17.617	1.00	0.00	RX0	O
ATOM	1409	ND2	ASN	304	24.044	13.790	17.952	1.00	0.00	RX0	N
ATOM	1410	HD21	ASN	304	23.089	13.513	17.831	1.00	0.00	RX0	H
ATOM	1411	HD22	ASN	304	24.632	13.132	18.436	1.00	0.00	RX0	H
ATOM	1412	C	ASN	304	25.330	14.097	14.987	1.00	0.00	RX0	C
ATOM	1413	O	ASN	304	26.132	14.783	14.344	1.00	0.00	RX0	O
ATOM	1414	N	SER	305	25.693	13.015	15.656	1.00	0.00	RX0	N
ATOM	1415	H	SER	305	25.042	12.400	16.111	1.00	0.00	RX0	H
ATOM	1416	CA	SER	305	27.104	12.765	16.012	1.00	0.00	RX0	C
ATOM	1417	CB	SER	305	27.109	11.591	16.979	1.00	0.00	RX0	C
ATOM	1418	OG	SER	305	26.151	11.907	17.999	1.00	0.00	RX0	O
ATOM	1419	HG	SER	305	26.345	11.300	18.721	1.00	0.00	RX0	H
ATOM	1420	C	SER	305	28.103	12.695	14.846	1.00	0.00	RX0	C
ATOM	1421	O	SER	305	29.198	13.228	14.946	1.00	0.00	RX0	O
ATOM	1422	N	GLY	306	27.634	12.177	13.693	1.00	0.00	RX0	N
ATOM	1423	H	GLY	306	26.686	11.864	13.633	1.00	0.00	RX0	H
ATOM	1424	CA	GLY	306	28.500	12.079	12.501	1.00	0.00	RX0	C
ATOM	1425	C	GLY	306	28.091	12.961	11.315	1.00	0.00	RX0	C
ATOM	1426	O	GLY	306	28.756	12.897	10.274	1.00	0.00	RX0	O
ATOM	1427	N	VAL	307	27.242	13.954	11.536	1.00	0.00	RX0	N
ATOM	1428	H	VAL	307	26.875	14.098	12.458	1.00	0.00	RX0	H
ATOM	1429	CA	VAL	307	26.760	14.815	10.435	1.00	0.00	RX0	C
ATOM	1430	CB	VAL	307	25.378	15.425	10.728	1.00	0.00	RX0	C
ATOM	1431	CG1	VAL	307	25.403	16.459	11.846	1.00	0.00	RX0	C
ATOM	1432	CG2	VAL	307	24.738	15.976	9.453	1.00	0.00	RX0	C
ATOM	1433	C	VAL	307	27.809	15.841	9.948	1.00	0.00	RX0	C
ATOM	1434	O	VAL	307	27.790	16.280	8.811	1.00	0.00	RX0	O
ATOM	1435	N	TYR	308	28.719	16.199	10.859	1.00	0.00	RX0	N
ATOM	1436	H	TYR	308	28.732	15.718	11.734	1.00	0.00	RX0	H
ATOM	1437	CA	TYR	308	29.761	17.211	10.584	1.00	0.00	RX0	C
ATOM	1438	CB	TYR	308	30.021	18.004	11.858	1.00	0.00	RX0	C
ATOM	1439	CG	TYR	308	28.703	18.611	12.281	1.00	0.00	RX0	C
ATOM	1440	CD1	TYR	308	27.867	19.186	11.328	1.00	0.00	RX0	C
ATOM	1441	CE1	TYR	308	26.667	19.773	11.712	1.00	0.00	RX0	C
ATOM	1442	CD2	TYR	308	28.324	18.601	13.617	1.00	0.00	RX0	C
ATOM	1443	CE2	TYR	308	27.127	19.195	14.003	1.00	0.00	RX0	C
ATOM	1444	CZ	TYR	308	26.307	19.791	13.053	1.00	0.00	RX0	C
ATOM	1445	OH	TYR	308	25.136	20.408	13.443	1.00	0.00	RX0	O
ATOM	1446	HH	TYR	308	24.605	20.598	12.677	1.00	0.00	RX0	H
ATOM	1447	C	TYR	308	31.032	16.660	9.940	1.00	0.00	RX0	C
ATOM	1448	O	TYR	308	31.912	17.411	9.537	1.00	0.00	RX0	O
ATOM	1449	N	THR	309	31.091	15.334	9.861	1.00	0.00	RX0	N
ATOM	1450	H	THR	309	30.367	14.741	10.223	1.00	0.00	RX0	H
ATOM	1451	CA	THR	309	32.303	14.643	9.386	1.00	0.00	RX0	C
ATOM	1452	CB	THR	309	32.710	13.693	10.512	1.00	0.00	RX0	C
ATOM	1453	OG1	THR	309	31.534	13.065	11.053	1.00	0.00	RX0	O
ATOM	1454	HG1	THR	309	31.302	12.340	10.472	1.00	0.00	RX0	H
ATOM	1455	CG2	THR	309	33.441	14.436	11.631	1.00	0.00	RX0	C
ATOM	1456	C	THR	309	32.183	13.866	8.071	1.00	0.00	RX0	C
ATOM	1457	O	THR	309	33.137	13.195	7.681	1.00	0.00	RX0	O
ATOM	1458	N	PHE	310	31.041	13.953	7.382	1.00	0.00	RX0	N
ATOM	1459	H	PHE	310	30.301	14.503	7.758	1.00	0.00	RX0	H
ATOM	1460	CA	PHE	310	30.972	13.467	5.988	1.00	0.00	RX0	C
ATOM	1461	CB	PHE	310	29.595	13.717	5.371	1.00	0.00	RX0	C
ATOM	1462	CG	PHE	310	28.497	13.041	6.155	1.00	0.00	RX0	C

ATOM	1463	CD1	PHE	310	28.553	11.678	6.419	1.00	0.00	RX0	C
ATOM	1464	CD2	PHE	310	27.411	13.788	6.595	1.00	0.00	RX0	C
ATOM	1465	CE1	PHE	310	27.518	11.064	7.115	1.00	0.00	RX0	C
ATOM	1466	CE2	PHE	310	26.376	13.173	7.288	1.00	0.00	RX0	C
ATOM	1467	CZ	PHE	310	26.428	11.809	7.546	1.00	0.00	RX0	C
ATOM	1468	C	PHE	310	32.019	14.245	5.180	1.00	0.00	RX0	C
ATOM	1469	O	PHE	310	32.102	15.468	5.301	1.00	0.00	RX0	O
ATOM	1470	N	LEU	311	32.854	13.508	4.462	1.00	0.00	RX0	N
ATOM	1471	H	LEU	311	32.713	12.522	4.520	1.00	0.00	RX0	H
ATOM	1472	CA	LEU	311	33.988	14.087	3.701	1.00	0.00	RX0	C
ATOM	1473	CB	LEU	311	34.741	12.994	2.942	1.00	0.00	RX0	C
ATOM	1474	CG	LEU	311	35.045	11.779	3.810	1.00	0.00	RX0	C
ATOM	1475	CD1	LEU	311	35.478	10.594	2.953	1.00	0.00	RX0	C
ATOM	1476	CD2	LEU	311	36.003	12.119	4.954	1.00	0.00	RX0	C
ATOM	1477	C	LEU	311	33.468	15.166	2.739	1.00	0.00	RX0	C
ATOM	1478	O	LEU	311	33.554	16.351	2.969	1.00	0.00	RX0	O
ATOM	1479	N	SER	312	32.784	14.619	1.713	1.00	0.00	RX0	N
ATOM	1480	H	SER	312	32.654	13.627	1.687	1.00	0.00	RX0	H
ATOM	1481	CA	SER	312	32.345	15.333	0.526	1.00	0.00	RX0	C
ATOM	1482	CB	SER	312	31.734	14.265	-0.379	1.00	0.00	RX0	C
ATOM	1483	OG	SER	312	32.554	13.089	-0.325	1.00	0.00	RX0	O
ATOM	1484	HG	SER	312	33.281	13.223	-0.921	1.00	0.00	RX0	H
ATOM	1485	C	SER	312	31.423	16.517	0.816	1.00	0.00	RX0	C
ATOM	1486	O	SER	312	31.042	16.847	1.948	1.00	0.00	RX0	O
ATOM	1487	N	SER	313	30.837	16.923	-0.276	1.00	0.00	RX0	N
ATOM	1488	H	SER	313	31.197	16.622	-1.159	1.00	0.00	RX0	H
ATOM	1489	CA	SER	313	29.838	17.983	-0.455	1.00	0.00	RX0	C
ATOM	1490	CB	SER	313	30.333	19.323	0.095	1.00	0.00	RX0	C
ATOM	1491	OG	SER	313	30.303	19.221	1.535	1.00	0.00	RX0	O
ATOM	1492	HG	SER	313	31.121	18.787	1.774	1.00	0.00	RX0	H
ATOM	1493	C	SER	313	29.353	17.880	-1.909	1.00	0.00	RX0	C
ATOM	1494	O	SER	313	29.168	18.836	-2.628	1.00	0.00	RX0	O
ATOM	1495	N	THR	314	29.278	16.602	-2.358	1.00	0.00	RX0	N
ATOM	1496	H	THR	314	29.441	15.834	-1.744	1.00	0.00	RX0	H
ATOM	1497	CA	THR	314	28.609	16.250	-3.614	1.00	0.00	RX0	C
ATOM	1498	CB	THR	314	28.743	14.744	-3.675	1.00	0.00	RX0	C
ATOM	1499	OG1	THR	314	29.850	14.385	-2.837	1.00	0.00	RX0	O
ATOM	1500	HG1	THR	314	30.025	13.463	-3.004	1.00	0.00	RX0	H
ATOM	1501	CG2	THR	314	28.915	14.214	-5.100	1.00	0.00	RX0	C
ATOM	1502	C	THR	314	27.167	16.747	-3.490	1.00	0.00	RX0	C
ATOM	1503	O	THR	314	26.675	16.978	-2.368	1.00	0.00	RX0	O
ATOM	1504	N	LEU	315	26.451	16.801	-4.589	1.00	0.00	RX0	N
ATOM	1505	H	LEU	315	26.878	16.641	-5.477	1.00	0.00	RX0	H
ATOM	1506	CA	LEU	315	25.050	17.256	-4.537	1.00	0.00	RX0	C
ATOM	1507	CB	LEU	315	24.456	17.329	-5.942	1.00	0.00	RX0	C
ATOM	1508	CG	LEU	315	23.026	17.873	-5.940	1.00	0.00	RX0	C
ATOM	1509	CD1	LEU	315	22.951	19.282	-5.346	1.00	0.00	RX0	C
ATOM	1510	CD2	LEU	315	22.387	17.798	-7.327	1.00	0.00	RX0	C
ATOM	1511	C	LEU	315	24.190	16.343	-3.639	1.00	0.00	RX0	C
ATOM	1512	O	LEU	315	23.484	16.800	-2.757	1.00	0.00	RX0	O
ATOM	1513	N	LYS	316	24.524	15.049	-3.730	1.00	0.00	RX0	N
ATOM	1514	H	LYS	316	25.093	14.784	-4.503	1.00	0.00	RX0	H
ATOM	1515	CA	LYS	316	23.912	13.990	-2.918	1.00	0.00	RX0	C
ATOM	1516	CB	LYS	316	24.342	12.615	-3.430	1.00	0.00	RX0	C
ATOM	1517	CG	LYS	316	23.142	11.699	-3.693	1.00	0.00	RX0	C
ATOM	1518	CD	LYS	316	22.534	11.070	-2.438	1.00	0.00	RX0	C
ATOM	1519	CE	LYS	316	21.008	11.194	-2.371	1.00	0.00	RX0	C
ATOM	1520	NZ	LYS	316	20.668	12.542	-1.913	1.00	0.00	RX0	N
ATOM	1521	HZ1	LYS	316	19.654	12.759	-1.999	1.00	0.00	RX0	H
ATOM	1522	HZ2	LYS	316	20.937	12.659	-0.912	1.00	0.00	RX0	H
ATOM	1523	HZ3	LYS	316	21.106	13.284	-2.498	1.00	0.00	RX0	H

ATOM	1524	C	LYS	316	24.213	14.187	-1.422	1.00	0.00	RX0	C
ATOM	1525	O	LYS	316	23.297	14.194	-0.611	1.00	0.00	RX0	O
ATOM	1526	N	SER	317	25.467	14.540	-1.122	1.00	0.00	RX0	N
ATOM	1527	H	SER	317	26.102	14.717	-1.867	1.00	0.00	RX0	H
ATOM	1528	CA	SER	317	25.934	14.787	0.260	1.00	0.00	RX0	C
ATOM	1529	CB	SER	317	27.467	14.863	0.209	1.00	0.00	RX0	C
ATOM	1530	OG	SER	317	28.081	14.610	1.478	1.00	0.00	RX0	O
ATOM	1531	HG	SER	317	27.723	13.775	1.784	1.00	0.00	RX0	H
ATOM	1532	C	SER	317	25.242	16.001	0.898	1.00	0.00	RX0	C
ATOM	1533	O	SER	317	24.716	15.915	2.007	1.00	0.00	RX0	O
ATOM	1534	N	LEU	318	25.067	17.043	0.088	1.00	0.00	RX0	N
ATOM	1535	H	LEU	318	25.359	16.982	-0.868	1.00	0.00	RX0	H
ATOM	1536	CA	LEU	318	24.405	18.288	0.525	1.00	0.00	RX0	C
ATOM	1537	CB	LEU	318	24.579	19.375	-0.532	1.00	0.00	RX0	C
ATOM	1538	CG	LEU	318	26.045	19.771	-0.691	1.00	0.00	RX0	C
ATOM	1539	CD1	LEU	318	26.256	20.718	-1.873	1.00	0.00	RX0	C
ATOM	1540	CD2	LEU	318	26.616	20.328	0.614	1.00	0.00	RX0	C
ATOM	1541	C	LEU	318	22.918	18.061	0.823	1.00	0.00	RX0	C
ATOM	1542	O	LEU	318	22.412	18.453	1.877	1.00	0.00	RX0	O
ATOM	1543	N	GLU	319	22.299	17.244	-0.024	1.00	0.00	RX0	N
ATOM	1544	H	GLU	319	22.772	16.931	-0.851	1.00	0.00	RX0	H
ATOM	1545	CA	GLU	319	20.900	16.808	0.157	1.00	0.00	RX0	C
ATOM	1546	CB	GLU	319	20.421	15.874	-0.946	1.00	0.00	RX0	C
ATOM	1547	CG	GLU	319	20.368	16.291	-2.408	1.00	0.00	RX0	C
ATOM	1548	CD	GLU	319	20.128	14.989	-3.140	1.00	0.00	RX0	C
ATOM	1549	OE1	GLU	319	21.024	14.506	-3.826	1.00	0.00	RX0	O
ATOM	1550	OE2	GLU	319	19.110	14.346	-2.897	1.00	0.00	RX0	O
ATOM	1551	C	GLU	319	20.725	15.924	1.402	1.00	0.00	RX0	C
ATOM	1552	O	GLU	319	19.808	16.153	2.196	1.00	0.00	RX0	O
ATOM	1553	N	GLU	320	21.706	15.058	1.645	1.00	0.00	RX0	N
ATOM	1554	H	GLU	320	22.408	14.922	0.947	1.00	0.00	RX0	H
ATOM	1555	CA	GLU	320	21.731	14.149	2.812	1.00	0.00	RX0	C
ATOM	1556	CB	GLU	320	22.814	13.059	2.780	1.00	0.00	RX0	C
ATOM	1557	CG	GLU	320	22.895	12.154	1.538	1.00	0.00	RX0	C
ATOM	1558	CD	GLU	320	21.566	11.544	1.106	1.00	0.00	RX0	C
ATOM	1559	OE1	GLU	320	21.473	10.321	1.032	1.00	0.00	RX0	O
ATOM	1560	OE2	GLU	320	20.652	12.277	0.735	1.00	0.00	RX0	O
ATOM	1561	C	GLU	320	21.765	14.931	4.131	1.00	0.00	RX0	C
ATOM	1562	O	GLU	320	20.881	14.754	4.973	1.00	0.00	RX0	O
ATOM	1563	N	LYS	321	22.647	15.926	4.188	1.00	0.00	RX0	N
ATOM	1564	H	LYS	321	23.239	16.043	3.388	1.00	0.00	RX0	H
ATOM	1565	CA	LYS	321	22.801	16.771	5.391	1.00	0.00	RX0	C
ATOM	1566	CB	LYS	321	24.078	17.636	5.231	1.00	0.00	RX0	C
ATOM	1567	CG	LYS	321	25.360	16.839	4.883	1.00	0.00	RX0	C
ATOM	1568	CD	LYS	321	26.621	17.647	4.478	1.00	0.00	RX0	C
ATOM	1569	CE	LYS	321	27.747	16.760	3.889	1.00	0.00	RX0	C
ATOM	1570	NZ	LYS	321	28.995	17.484	3.547	1.00	0.00	RX0	N
ATOM	1571	HZ1	LYS	321	29.670	16.842	3.070	1.00	0.00	RX0	H
ATOM	1572	HZ2	LYS	321	28.850	18.282	2.894	1.00	0.00	RX0	H
ATOM	1573	HZ3	LYS	321	29.466	17.826	4.407	1.00	0.00	RX0	H
ATOM	1574	C	LYS	321	21.549	17.612	5.656	1.00	0.00	RX0	C
ATOM	1575	O	LYS	321	21.102	17.724	6.798	1.00	0.00	RX0	O
ATOM	1576	N	ASP	322	20.935	18.099	4.575	1.00	0.00	RX0	N
ATOM	1577	H	ASP	322	21.314	17.938	3.660	1.00	0.00	RX0	H
ATOM	1578	CA	ASP	322	19.715	18.915	4.684	1.00	0.00	RX0	C
ATOM	1579	CB	ASP	322	19.385	19.454	3.290	1.00	0.00	RX0	C
ATOM	1580	CG	ASP	322	18.031	20.127	3.269	1.00	0.00	RX0	C
ATOM	1581	OD1	ASP	322	17.641	20.729	4.258	1.00	0.00	RX0	O
ATOM	1582	OD2	ASP	322	17.336	20.018	2.264	1.00	0.00	RX0	O
ATOM	1583	C	ASP	322	18.561	18.097	5.282	1.00	0.00	RX0	C
ATOM	1584	O	ASP	322	17.955	18.512	6.263	1.00	0.00	RX0	O

ATOM	1585	N	HIS	323	18.423	16.872	4.772	1.00	0.00	RX0	N
ATOM	1586	H	HIS	323	19.038	16.603	4.025	1.00	0.00	RX0	H
ATOM	1587	CA	HIS	323	17.423	15.916	5.274	1.00	0.00	RX0	C
ATOM	1588	CB	HIS	323	17.456	14.616	4.468	1.00	0.00	RX0	C
ATOM	1589	CG	HIS	323	16.284	13.757	4.882	1.00	0.00	RX0	C
ATOM	1590	ND1	HIS	323	16.302	12.413	4.953	1.00	0.00	RX0	N
ATOM	1591	HD1	HIS	323	17.053	11.811	4.750	1.00	0.00	RX0	H
ATOM	1592	CD2	HIS	323	15.010	14.204	5.235	1.00	0.00	RX0	C
ATOM	1593	NE2	HIS	323	14.255	13.121	5.521	1.00	0.00	RX0	N
ATOM	1594	CE1	HIS	323	15.049	12.012	5.348	1.00	0.00	RX0	C
ATOM	1595	C	HIS	323	17.630	15.607	6.766	1.00	0.00	RX0	C
ATOM	1596	O	HIS	323	16.677	15.663	7.540	1.00	0.00	RX0	O
ATOM	1597	N	ILE	324	18.888	15.420	7.164	1.00	0.00	RX0	N
ATOM	1598	H	ILE	324	19.612	15.429	6.469	1.00	0.00	RX0	H
ATOM	1599	CA	ILE	324	19.235	15.107	8.570	1.00	0.00	RX0	C
ATOM	1600	CB	ILE	324	20.718	14.763	8.705	1.00	0.00	RX0	C
ATOM	1601	CG2	ILE	324	21.120	14.616	10.172	1.00	0.00	RX0	C
ATOM	1602	CG1	ILE	324	21.038	13.495	7.914	1.00	0.00	RX0	C
ATOM	1603	CD1	ILE	324	22.526	13.144	7.922	1.00	0.00	RX0	C
ATOM	1604	C	ILE	324	18.844	16.271	9.496	1.00	0.00	RX0	C
ATOM	1605	O	ILE	324	18.200	16.053	10.527	1.00	0.00	RX0	O
ATOM	1606	N	HIS	325	19.159	17.485	9.071	1.00	0.00	RX0	N
ATOM	1607	H	HIS	325	19.589	17.591	8.172	1.00	0.00	RX0	H
ATOM	1608	CA	HIS	325	18.840	18.694	9.856	1.00	0.00	RX0	C
ATOM	1609	CB	HIS	325	19.577	19.925	9.325	1.00	0.00	RX0	C
ATOM	1610	CG	HIS	325	21.039	19.861	9.710	1.00	0.00	RX0	C
ATOM	1611	ND1	HIS	325	21.987	19.300	8.941	1.00	0.00	RX0	N
ATOM	1612	HD1	HIS	325	21.833	18.872	8.071	1.00	0.00	RX0	H
ATOM	1613	CD2	HIS	325	21.643	20.349	10.874	1.00	0.00	RX0	C
ATOM	1614	NE2	HIS	325	22.972	20.075	10.794	1.00	0.00	RX0	N
ATOM	1615	CE1	HIS	325	23.180	19.429	9.602	1.00	0.00	RX0	C
ATOM	1616	C	HIS	325	17.335	18.955	9.948	1.00	0.00	RX0	C
ATOM	1617	O	HIS	325	16.820	19.290	11.021	1.00	0.00	RX0	O
ATOM	1618	N	ARG	326	16.631	18.584	8.887	1.00	0.00	RX0	N
ATOM	1619	H	ARG	326	17.126	18.354	8.048	1.00	0.00	RX0	H
ATOM	1620	CA	ARG	326	15.160	18.594	8.873	1.00	0.00	RX0	C
ATOM	1621	CB	ARG	326	14.652	18.414	7.441	1.00	0.00	RX0	C
ATOM	1622	CG	ARG	326	14.667	19.727	6.655	1.00	0.00	RX0	C
ATOM	1623	CD	ARG	326	14.126	19.598	5.227	1.00	0.00	RX0	C
ATOM	1624	NE	ARG	326	15.156	19.187	4.274	1.00	0.00	RX0	N
ATOM	1625	HE	ARG	326	15.908	19.852	4.114	1.00	0.00	RX0	H
ATOM	1626	CZ	ARG	326	15.096	17.999	3.609	1.00	0.00	RX0	C
ATOM	1627	NH1	ARG	326	14.107	17.131	3.907	1.00	0.00	RX0	N
ATOM	1628	HH11	ARG	326	14.006	16.245	3.447	1.00	0.00	RX0	H
ATOM	1629	HH12	ARG	326	13.435	17.353	4.617	1.00	0.00	RX0	H
ATOM	1630	NH2	ARG	326	16.017	17.708	2.669	1.00	0.00	RX0	N
ATOM	1631	HH21	ARG	326	16.086	16.850	2.156	1.00	0.00	RX0	H
ATOM	1632	HH22	ARG	326	16.709	18.415	2.446	1.00	0.00	RX0	H
ATOM	1633	C	ARG	326	14.537	17.576	9.843	1.00	0.00	RX0	C
ATOM	1634	O	ARG	326	13.617	17.928	10.589	1.00	0.00	RX0	O
ATOM	1635	N	VAL	327	15.171	16.419	9.987	1.00	0.00	RX0	N
ATOM	1636	H	VAL	327	15.982	16.248	9.422	1.00	0.00	RX0	H
ATOM	1637	CA	VAL	327	14.709	15.376	10.935	1.00	0.00	RX0	C
ATOM	1638	CB	VAL	327	15.279	13.994	10.608	1.00	0.00	RX0	C
ATOM	1639	CG1	VAL	327	14.752	12.958	11.602	1.00	0.00	RX0	C
ATOM	1640	CG2	VAL	327	14.942	13.577	9.177	1.00	0.00	RX0	C
ATOM	1641	C	VAL	327	15.041	15.781	12.381	1.00	0.00	RX0	C
ATOM	1642	O	VAL	327	14.187	15.661	13.270	1.00	0.00	RX0	O
ATOM	1643	N	LEU	328	16.212	16.371	12.574	1.00	0.00	RX0	N
ATOM	1644	H	LEU	328	16.818	16.509	11.789	1.00	0.00	RX0	H
ATOM	1645	CA	LEU	328	16.631	16.899	13.887	1.00	0.00	RX0	C

ATOM	1646	CB	LEU	328	18.070	17.409	13.828	1.00	0.00	RX0	C
ATOM	1647	CG	LEU	328	19.081	16.266	13.760	1.00	0.00	RX0	C
ATOM	1648	CD1	LEU	328	20.501	16.768	13.492	1.00	0.00	RX0	C
ATOM	1649	CD2	LEU	328	19.006	15.392	15.012	1.00	0.00	RX0	C
ATOM	1650	C	LEU	328	15.692	18.005	14.386	1.00	0.00	RX0	C
ATOM	1651	O	LEU	328	15.231	17.955	15.519	1.00	0.00	RX0	O
ATOM	1652	N	ASP	329	15.222	18.821	13.436	1.00	0.00	RX0	N
ATOM	1653	H	ASP	329	15.682	18.904	12.549	1.00	0.00	RX0	H
ATOM	1654	CA	ASP	329	14.223	19.870	13.722	1.00	0.00	RX0	C
ATOM	1655	CB	ASP	329	14.065	20.828	12.539	1.00	0.00	RX0	C
ATOM	1656	CG	ASP	329	15.285	21.725	12.430	1.00	0.00	RX0	C
ATOM	1657	OD1	ASP	329	16.071	21.772	13.377	1.00	0.00	RX0	O
ATOM	1658	OD2	ASP	329	15.442	22.382	11.400	1.00	0.00	RX0	O
ATOM	1659	C	ASP	329	12.864	19.312	14.154	1.00	0.00	RX0	C
ATOM	1660	O	ASP	329	12.272	19.788	15.128	1.00	0.00	RX0	O
ATOM	1661	N	LYS	330	12.463	18.218	13.512	1.00	0.00	RX0	N
ATOM	1662	H	LYS	330	13.022	17.921	12.736	1.00	0.00	RX0	H
ATOM	1663	CA	LYS	330	11.217	17.525	13.871	1.00	0.00	RX0	C
ATOM	1664	CB	LYS	330	10.799	16.459	12.860	1.00	0.00	RX0	C
ATOM	1665	CG	LYS	330	9.623	15.605	13.368	1.00	0.00	RX0	C
ATOM	1666	CD	LYS	330	8.369	16.382	13.798	1.00	0.00	RX0	C
ATOM	1667	CE	LYS	330	7.826	17.307	12.718	1.00	0.00	RX0	C
ATOM	1668	NZ	LYS	330	7.438	16.477	11.579	1.00	0.00	RX0	N
ATOM	1669	HZ1	LYS	330	7.123	17.095	10.810	1.00	0.00	RX0	H
ATOM	1670	HZ2	LYS	330	8.248	15.891	11.280	1.00	0.00	RX0	H
ATOM	1671	HZ3	LYS	330	6.658	15.856	11.882	1.00	0.00	RX0	H
ATOM	1672	C	LYS	330	11.283	16.915	15.278	1.00	0.00	RX0	C
ATOM	1673	O	LYS	330	10.354	17.095	16.067	1.00	0.00	RX0	O
ATOM	1674	N	ILE	331	12.441	16.375	15.625	1.00	0.00	RX0	N
ATOM	1675	H	ILE	331	13.185	16.378	14.951	1.00	0.00	RX0	H
ATOM	1676	CA	ILE	331	12.656	15.802	16.972	1.00	0.00	RX0	C
ATOM	1677	CB	ILE	331	13.953	14.997	17.053	1.00	0.00	RX0	C
ATOM	1678	CG2	ILE	331	14.062	14.330	18.421	1.00	0.00	RX0	C
ATOM	1679	CG1	ILE	331	14.043	13.952	15.943	1.00	0.00	RX0	C
ATOM	1680	CD1	ILE	331	15.358	13.173	15.985	1.00	0.00	RX0	C
ATOM	1681	C	ILE	331	12.642	16.922	18.027	1.00	0.00	RX0	C
ATOM	1682	O	ILE	331	12.078	16.732	19.120	1.00	0.00	RX0	O
ATOM	1683	N	THR	332	13.158	18.084	17.675	1.00	0.00	RX0	N
ATOM	1684	H	THR	332	13.577	18.203	16.774	1.00	0.00	RX0	H
ATOM	1685	CA	THR	332	13.155	19.265	18.570	1.00	0.00	RX0	C
ATOM	1686	CB	THR	332	14.034	20.315	17.913	1.00	0.00	RX0	C
ATOM	1687	OG1	THR	332	15.301	19.706	17.626	1.00	0.00	RX0	O
ATOM	1688	HG1	THR	332	15.252	19.374	16.733	1.00	0.00	RX0	H
ATOM	1689	CG2	THR	332	14.209	21.554	18.792	1.00	0.00	RX0	C
ATOM	1690	C	THR	332	11.706	19.696	18.836	1.00	0.00	RX0	C
ATOM	1691	O	THR	332	11.302	19.832	19.995	1.00	0.00	RX0	O
ATOM	1692	N	ASP	333	10.912	19.714	17.772	1.00	0.00	RX0	N
ATOM	1693	H	ASP	333	11.281	19.673	16.838	1.00	0.00	RX0	H
ATOM	1694	CA	ASP	333	9.466	20.017	17.863	1.00	0.00	RX0	C
ATOM	1695	CB	ASP	333	8.671	19.791	16.568	1.00	0.00	RX0	C
ATOM	1696	CG	ASP	333	9.096	20.578	15.354	1.00	0.00	RX0	C
ATOM	1697	OD1	ASP	333	9.499	21.726	15.506	1.00	0.00	RX0	O
ATOM	1698	OD2	ASP	333	8.968	20.038	14.251	1.00	0.00	RX0	O
ATOM	1699	C	ASP	333	8.732	19.000	18.747	1.00	0.00	RX0	C
ATOM	1700	O	ASP	333	7.880	19.374	19.559	1.00	0.00	RX0	O
ATOM	1701	N	THR	334	9.187	17.759	18.682	1.00	0.00	RX0	N
ATOM	1702	H	THR	334	9.941	17.583	18.048	1.00	0.00	RX0	H
ATOM	1703	CA	THR	334	8.631	16.638	19.462	1.00	0.00	RX0	C
ATOM	1704	CB	THR	334	9.175	15.372	18.822	1.00	0.00	RX0	C
ATOM	1705	OG1	THR	334	8.792	15.339	17.438	1.00	0.00	RX0	O
ATOM	1706	HG1	THR	334	9.149	16.124	17.032	1.00	0.00	RX0	H

ATOM	1707	CG2	THR	334	8.722	14.119	19.558	1.00	0.00	RX0	C
ATOM	1708	C	THR	334	8.961	16.782	20.953	1.00	0.00	RX0	C
ATOM	1709	O	THR	334	8.059	16.689	21.789	1.00	0.00	RX0	O
ATOM	1710	N	LEU	335	10.217	17.081	21.263	1.00	0.00	RX0	N
ATOM	1711	H	LEU	335	10.880	17.204	20.523	1.00	0.00	RX0	H
ATOM	1712	CA	LEU	335	10.648	17.349	22.650	1.00	0.00	RX0	C
ATOM	1713	CB	LEU	335	12.150	17.611	22.696	1.00	0.00	RX0	C
ATOM	1714	CG	LEU	335	12.959	16.321	22.777	1.00	0.00	RX0	C
ATOM	1715	CD1	LEU	335	14.457	16.569	22.602	1.00	0.00	RX0	C
ATOM	1716	CD2	LEU	335	12.654	15.571	24.074	1.00	0.00	RX0	C
ATOM	1717	C	LEU	335	9.903	18.522	23.297	1.00	0.00	RX0	C
ATOM	1718	O	LEU	335	9.384	18.379	24.399	1.00	0.00	RX0	O
ATOM	1719	N	ILE	336	9.668	19.567	22.501	1.00	0.00	RX0	N
ATOM	1720	H	ILE	336	10.071	19.579	21.582	1.00	0.00	RX0	H
ATOM	1721	CA	ILE	336	8.897	20.743	22.962	1.00	0.00	RX0	C
ATOM	1722	CB	ILE	336	9.048	21.912	21.989	1.00	0.00	RX0	C
ATOM	1723	CG2	ILE	336	8.089	23.056	22.324	1.00	0.00	RX0	C
ATOM	1724	CG1	ILE	336	10.500	22.389	21.997	1.00	0.00	RX0	C
ATOM	1725	CD1	ILE	336	10.927	22.872	23.385	1.00	0.00	RX0	C
ATOM	1726	C	ILE	336	7.427	20.365	23.180	1.00	0.00	RX0	C
ATOM	1727	O	ILE	336	6.836	20.730	24.203	1.00	0.00	RX0	O
ATOM	1728	N	HIS	337	6.891	19.571	22.265	1.00	0.00	RX0	N
ATOM	1729	H	HIS	337	7.438	19.318	21.465	1.00	0.00	RX0	H
ATOM	1730	CA	HIS	337	5.501	19.096	22.359	1.00	0.00	RX0	C
ATOM	1731	CB	HIS	337	5.080	18.324	21.118	1.00	0.00	RX0	C
ATOM	1732	CG	HIS	337	3.602	18.050	21.223	1.00	0.00	RX0	C
ATOM	1733	ND1	HIS	337	2.651	18.971	20.983	1.00	0.00	RX0	N
ATOM	1734	HD1	HIS	337	2.800	19.899	20.700	1.00	0.00	RX0	H
ATOM	1735	CD2	HIS	337	2.986	16.856	21.597	1.00	0.00	RX0	C
ATOM	1736	NE2	HIS	337	1.645	17.065	21.587	1.00	0.00	RX0	N
ATOM	1737	CE1	HIS	337	1.440	18.369	21.206	1.00	0.00	RX0	C
ATOM	1738	C	HIS	337	5.301	18.263	23.631	1.00	0.00	RX0	C
ATOM	1739	O	HIS	337	4.339	18.490	24.365	1.00	0.00	RX0	O
ATOM	1740	N	LEU	338	6.274	17.405	23.914	1.00	0.00	RX0	N
ATOM	1741	H	LEU	338	7.051	17.336	23.285	1.00	0.00	RX0	H
ATOM	1742	CA	LEU	338	6.246	16.526	25.097	1.00	0.00	RX0	C
ATOM	1743	CB	LEU	338	7.441	15.575	25.089	1.00	0.00	RX0	C
ATOM	1744	CG	LEU	338	7.340	14.533	23.979	1.00	0.00	RX0	C
ATOM	1745	CD1	LEU	338	8.644	13.751	23.809	1.00	0.00	RX0	C
ATOM	1746	CD2	LEU	338	6.122	13.631	24.179	1.00	0.00	RX0	C
ATOM	1747	C	LEU	338	6.240	17.331	26.400	1.00	0.00	RX0	C
ATOM	1748	O	LEU	338	5.410	17.098	27.277	1.00	0.00	RX0	O
ATOM	1749	N	MET	339	7.027	18.402	26.394	1.00	0.00	RX0	N
ATOM	1750	H	MET	339	7.614	18.550	25.594	1.00	0.00	RX0	H
ATOM	1751	CA	MET	339	7.168	19.308	27.550	1.00	0.00	RX0	C
ATOM	1752	CB	MET	339	8.407	20.188	27.400	1.00	0.00	RX0	C
ATOM	1753	CG	MET	339	9.705	19.384	27.373	1.00	0.00	RX0	C
ATOM	1754	SD	MET	339	11.142	20.416	27.053	1.00	0.00	RX0	S
ATOM	1755	CE	MET	339	12.273	19.098	26.588	1.00	0.00	RX0	C
ATOM	1756	C	MET	339	5.924	20.183	27.753	1.00	0.00	RX0	C
ATOM	1757	O	MET	339	5.433	20.322	28.878	1.00	0.00	RX0	O
ATOM	1758	N	ALA	340	5.370	20.672	26.646	1.00	0.00	RX0	N
ATOM	1759	H	ALA	340	5.814	20.472	25.772	1.00	0.00	RX0	H
ATOM	1760	CA	ALA	340	4.120	21.456	26.636	1.00	0.00	RX0	C
ATOM	1761	CB	ALA	340	3.835	21.992	25.232	1.00	0.00	RX0	C
ATOM	1762	C	ALA	340	2.921	20.619	27.100	1.00	0.00	RX0	C
ATOM	1763	O	ALA	340	2.176	21.064	27.967	1.00	0.00	RX0	O
ATOM	1764	N	LYS	341	2.865	19.360	26.655	1.00	0.00	RX0	N
ATOM	1765	H	LYS	341	3.552	19.058	25.993	1.00	0.00	RX0	H
ATOM	1766	CA	LYS	341	1.846	18.397	27.117	1.00	0.00	RX0	C
ATOM	1767	CB	LYS	341	1.969	17.108	26.280	1.00	0.00	RX0	C

ATOM	1768	CG	LYS	341	0.686	16.273	26.166	1.00	0.00	RX0	C
ATOM	1769	CD	LYS	341	0.377	15.789	24.734	1.00	0.00	RX0	C
ATOM	1770	CE	LYS	341	1.357	14.772	24.127	1.00	0.00	RX0	C
ATOM	1771	NZ	LYS	341	1.045	14.552	22.703	1.00	0.00	RX0	N
ATOM	1772	HZ1	LYS	341	1.771	13.999	22.194	1.00	0.00	RX0	H
ATOM	1773	HZ2	LYS	341	0.184	14.005	22.490	1.00	0.00	RX0	H
ATOM	1774	HZ3	LYS	341	0.996	15.432	22.154	1.00	0.00	RX0	H
ATOM	1775	C	LYS	341	1.927	18.179	28.637	1.00	0.00	RX0	C
ATOM	1776	O	LYS	341	0.908	18.032	29.304	1.00	0.00	RX0	O
ATOM	1777	N	ALA	342	3.166	18.103	29.120	1.00	0.00	RX0	N
ATOM	1778	H	ALA	342	3.940	18.176	28.488	1.00	0.00	RX0	H
ATOM	1779	CA	ALA	342	3.459	17.942	30.556	1.00	0.00	RX0	C
ATOM	1780	CB	ALA	342	4.953	17.706	30.786	1.00	0.00	RX0	C
ATOM	1781	C	ALA	342	3.007	19.166	31.371	1.00	0.00	RX0	C
ATOM	1782	O	ALA	342	2.879	19.099	32.588	1.00	0.00	RX0	O
ATOM	1783	N	GLY	343	2.900	20.305	30.669	1.00	0.00	RX0	N
ATOM	1784	H	GLY	343	3.153	20.317	29.702	1.00	0.00	RX0	H
ATOM	1785	CA	GLY	343	2.393	21.560	31.244	1.00	0.00	RX0	C
ATOM	1786	C	GLY	343	3.505	22.433	31.829	1.00	0.00	RX0	C
ATOM	1787	O	GLY	343	3.244	23.282	32.678	1.00	0.00	RX0	O
ATOM	1788	N	LEU	344	4.736	22.226	31.356	1.00	0.00	RX0	N
ATOM	1789	H	LEU	344	4.854	21.555	30.622	1.00	0.00	RX0	H
ATOM	1790	CA	LEU	344	5.823	23.174	31.634	1.00	0.00	RX0	C
ATOM	1791	CB	LEU	344	7.150	22.610	31.139	1.00	0.00	RX0	C
ATOM	1792	CG	LEU	344	7.551	21.336	31.878	1.00	0.00	RX0	C
ATOM	1793	CD1	LEU	344	8.830	20.737	31.301	1.00	0.00	RX0	C
ATOM	1794	CD2	LEU	344	7.656	21.564	33.387	1.00	0.00	RX0	C
ATOM	1795	C	LEU	344	5.508	24.488	30.929	1.00	0.00	RX0	C
ATOM	1796	O	LEU	344	4.977	24.500	29.792	1.00	0.00	RX0	O
ATOM	1797	N	THR	345	5.822	25.577	31.575	1.00	0.00	RX0	N
ATOM	1798	H	THR	345	6.293	25.483	32.451	1.00	0.00	RX0	H
ATOM	1799	CA	THR	345	5.705	26.912	30.948	1.00	0.00	RX0	C
ATOM	1800	CB	THR	345	5.997	27.964	32.011	1.00	0.00	RX0	C
ATOM	1801	OG1	THR	345	7.172	27.606	32.737	1.00	0.00	RX0	O
ATOM	1802	HG1	THR	345	6.902	26.963	33.390	1.00	0.00	RX0	H
ATOM	1803	CG2	THR	345	4.820	28.123	32.973	1.00	0.00	RX0	C
ATOM	1804	C	THR	345	6.639	26.971	29.731	1.00	0.00	RX0	C
ATOM	1805	O	THR	345	7.615	26.233	29.623	1.00	0.00	RX0	O
ATOM	1806	N	LEU	346	6.390	27.971	28.898	1.00	0.00	RX0	N
ATOM	1807	H	LEU	346	5.564	28.512	29.044	1.00	0.00	RX0	H
ATOM	1808	CA	LEU	346	7.214	28.242	27.708	1.00	0.00	RX0	C
ATOM	1809	CB	LEU	346	6.672	29.451	26.948	1.00	0.00	RX0	C
ATOM	1810	CG	LEU	346	7.379	29.658	25.607	1.00	0.00	RX0	C
ATOM	1811	CD1	LEU	346	7.248	28.432	24.700	1.00	0.00	RX0	C
ATOM	1812	CD2	LEU	346	6.916	30.939	24.913	1.00	0.00	RX0	C
ATOM	1813	C	LEU	346	8.705	28.433	28.051	1.00	0.00	RX0	C
ATOM	1814	O	LEU	346	9.594	27.857	27.432	1.00	0.00	RX0	O
ATOM	1815	N	GLN	347	8.927	29.083	29.198	1.00	0.00	RX0	N
ATOM	1816	H	GLN	347	8.145	29.438	29.706	1.00	0.00	RX0	H
ATOM	1817	CA	GLN	347	10.275	29.275	29.754	1.00	0.00	RX0	C
ATOM	1818	CB	GLN	347	10.218	30.283	30.897	1.00	0.00	RX0	C
ATOM	1819	CG	GLN	347	11.597	30.619	31.460	1.00	0.00	RX0	C
ATOM	1820	CD	GLN	347	11.430	31.584	32.609	1.00	0.00	RX0	C
ATOM	1821	OE1	GLN	347	10.467	31.521	33.362	1.00	0.00	RX0	O
ATOM	1822	NE2	GLN	347	12.418	32.493	32.696	1.00	0.00	RX0	N
ATOM	1823	HE21	GLN	347	13.173	32.499	32.041	1.00	0.00	RX0	H
ATOM	1824	HE22	GLN	347	12.408	33.187	33.417	1.00	0.00	RX0	H
ATOM	1825	C	GLN	347	10.911	27.956	30.229	1.00	0.00	RX0	C
ATOM	1826	O	GLN	347	12.052	27.652	29.875	1.00	0.00	RX0	O
ATOM	1827	N	GLN	348	10.120	27.140	30.905	1.00	0.00	RX0	N
ATOM	1828	H	GLN	348	9.171	27.398	31.088	1.00	0.00	RX0	H

ATOM	1829	CA	GLN	348	10.582	25.823	31.394	1.00	0.00	RX0	C
ATOM	1830	CB	GLN	348	9.593	25.218	32.378	1.00	0.00	RX0	C
ATOM	1831	CG	GLN	348	9.648	25.886	33.748	1.00	0.00	RX0	C
ATOM	1832	CD	GLN	348	8.479	25.377	34.558	1.00	0.00	RX0	C
ATOM	1833	OE1	GLN	348	7.355	25.288	34.068	1.00	0.00	RX0	O
ATOM	1834	NE2	GLN	348	8.798	25.044	35.819	1.00	0.00	RX0	N
ATOM	1835	HE21	GLN	348	9.737	25.160	36.148	1.00	0.00	RX0	H
ATOM	1836	HE22	GLN	348	8.119	24.682	36.459	1.00	0.00	RX0	H
ATOM	1837	C	GLN	348	10.871	24.847	30.248	1.00	0.00	RX0	C
ATOM	1838	O	GLN	348	11.861	24.115	30.296	1.00	0.00	RX0	O
ATOM	1839	N	GLN	349	10.121	24.983	29.160	1.00	0.00	RX0	N
ATOM	1840	H	GLN	349	9.375	25.648	29.187	1.00	0.00	RX0	H
ATOM	1841	CA	GLN	349	10.299	24.172	27.940	1.00	0.00	RX0	C
ATOM	1842	CB	GLN	349	9.205	24.481	26.921	1.00	0.00	RX0	C
ATOM	1843	CG	GLN	349	7.821	24.032	27.384	1.00	0.00	RX0	C
ATOM	1844	CD	GLN	349	6.787	24.591	26.435	1.00	0.00	RX0	C
ATOM	1845	OE1	GLN	349	7.076	24.921	25.290	1.00	0.00	RX0	O
ATOM	1846	NE2	GLN	349	5.564	24.698	26.977	1.00	0.00	RX0	N
ATOM	1847	HE21	GLN	349	5.398	24.432	27.933	1.00	0.00	RX0	H
ATOM	1848	HE22	GLN	349	4.780	25.036	26.458	1.00	0.00	RX0	H
ATOM	1849	C	GLN	349	11.682	24.372	27.306	1.00	0.00	RX0	C
ATOM	1850	O	GLN	349	12.447	23.422	27.181	1.00	0.00	RX0	O
ATOM	1851	N	HIS	350	12.056	25.641	27.113	1.00	0.00	RX0	N
ATOM	1852	H	HIS	350	11.422	26.375	27.365	1.00	0.00	RX0	H
ATOM	1853	CA	HIS	350	13.344	25.953	26.463	1.00	0.00	RX0	C
ATOM	1854	CB	HIS	350	13.379	27.327	25.781	1.00	0.00	RX0	C
ATOM	1855	CG	HIS	350	13.575	28.469	26.745	1.00	0.00	RX0	C
ATOM	1856	ND1	HIS	350	12.559	29.184	27.256	1.00	0.00	RX0	N
ATOM	1857	HD1	HIS	350	11.602	29.024	27.097	1.00	0.00	RX0	H
ATOM	1858	CD2	HIS	350	14.783	28.993	27.219	1.00	0.00	RX0	C
ATOM	1859	NE2	HIS	350	14.484	30.040	28.024	1.00	0.00	RX0	N
ATOM	1860	CE1	HIS	350	13.119	30.157	28.043	1.00	0.00	RX0	C
ATOM	1861	C	HIS	350	14.540	25.670	27.386	1.00	0.00	RX0	C
ATOM	1862	O	HIS	350	15.573	25.182	26.934	1.00	0.00	RX0	O
ATOM	1863	N	GLN	351	14.320	25.854	28.691	1.00	0.00	RX0	N
ATOM	1864	H	GLN	351	13.441	26.232	28.990	1.00	0.00	RX0	H
ATOM	1865	CA	GLN	351	15.341	25.538	29.706	1.00	0.00	RX0	C
ATOM	1866	CB	GLN	351	14.916	26.060	31.073	1.00	0.00	RX0	C
ATOM	1867	CG	GLN	351	14.892	27.583	31.151	1.00	0.00	RX0	C
ATOM	1868	CD	GLN	351	14.205	27.978	32.438	1.00	0.00	RX0	C
ATOM	1869	OE1	GLN	351	13.356	27.264	32.963	1.00	0.00	RX0	O
ATOM	1870	NE2	GLN	351	14.633	29.153	32.931	1.00	0.00	RX0	N
ATOM	1871	HE21	GLN	351	15.332	29.676	32.442	1.00	0.00	RX0	H
ATOM	1872	HE22	GLN	351	14.272	29.516	33.791	1.00	0.00	RX0	H
ATOM	1873	C	GLN	351	15.597	24.030	29.805	1.00	0.00	RX0	C
ATOM	1874	O	GLN	351	16.752	23.608	29.740	1.00	0.00	RX0	O
ATOM	1875	N	ARG	352	14.527	23.242	29.744	1.00	0.00	RX0	N
ATOM	1876	H	ARG	352	13.617	23.654	29.656	1.00	0.00	RX0	H
ATOM	1877	CA	ARG	352	14.627	21.772	29.816	1.00	0.00	RX0	C
ATOM	1878	CB	ARG	352	13.310	21.110	30.247	1.00	0.00	RX0	C
ATOM	1879	CG	ARG	352	13.414	19.586	30.434	1.00	0.00	RX0	C
ATOM	1880	CD	ARG	352	12.214	18.995	31.185	1.00	0.00	RX0	C
ATOM	1881	NE	ARG	352	12.181	17.530	31.160	1.00	0.00	RX0	N
ATOM	1882	HE	ARG	352	11.954	17.067	30.288	1.00	0.00	RX0	H
ATOM	1883	CZ	ARG	352	12.325	16.774	32.292	1.00	0.00	RX0	C
ATOM	1884	NH1	ARG	352	12.649	17.373	33.456	1.00	0.00	RX0	N
ATOM	1885	HH11	ARG	352	12.834	16.826	34.286	1.00	0.00	RX0	H
ATOM	1886	HH12	ARG	352	12.729	18.367	33.533	1.00	0.00	RX0	H
ATOM	1887	NH2	ARG	352	12.137	15.440	32.233	1.00	0.00	RX0	N
ATOM	1888	HH21	ARG	352	12.188	14.803	33.010	1.00	0.00	RX0	H
ATOM	1889	HH22	ARG	352	11.921	15.024	31.327	1.00	0.00	RX0	H

ATOM	1890	C	ARG	352	15.192	21.188	28.514	1.00	0.00	RX0	C
ATOM	1891	O	ARG	352	16.048	20.305	28.551	1.00	0.00	RX0	O
ATOM	1892	N	LEU	353	14.843	21.816	27.391	1.00	0.00	RX0	N
ATOM	1893	H	LEU	353	14.132	22.520	27.429	1.00	0.00	RX0	H
ATOM	1894	CA	LEU	353	15.400	21.446	26.079	1.00	0.00	RX0	C
ATOM	1895	CB	LEU	353	14.765	22.302	24.985	1.00	0.00	RX0	C
ATOM	1896	CG	LEU	353	15.233	21.922	23.581	1.00	0.00	RX0	C
ATOM	1897	CD1	LEU	353	14.794	20.508	23.199	1.00	0.00	RX0	C
ATOM	1898	CD2	LEU	353	14.809	22.959	22.542	1.00	0.00	RX0	C
ATOM	1899	C	LEU	353	16.930	21.610	26.063	1.00	0.00	RX0	C
ATOM	1900	O	LEU	353	17.658	20.692	25.694	1.00	0.00	RX0	O
ATOM	1901	N	ALA	354	17.375	22.739	26.614	1.00	0.00	RX0	N
ATOM	1902	H	ALA	354	16.705	23.421	26.916	1.00	0.00	RX0	H
ATOM	1903	CA	ALA	354	18.808	23.073	26.716	1.00	0.00	RX0	C
ATOM	1904	CB	ALA	354	18.993	24.518	27.181	1.00	0.00	RX0	C
ATOM	1905	C	ALA	354	19.540	22.141	27.690	1.00	0.00	RX0	C
ATOM	1906	O	ALA	354	20.574	21.567	27.334	1.00	0.00	RX0	O
ATOM	1907	N	GLN	355	18.893	21.844	28.811	1.00	0.00	RX0	N
ATOM	1908	H	GLN	355	18.018	22.306	28.967	1.00	0.00	RX0	H
ATOM	1909	CA	GLN	355	19.427	20.920	29.833	1.00	0.00	RX0	C
ATOM	1910	CB	GLN	355	18.564	20.902	31.085	1.00	0.00	RX0	C
ATOM	1911	CG	GLN	355	18.674	22.212	31.858	1.00	0.00	RX0	C
ATOM	1912	CD	GLN	355	17.977	22.044	33.186	1.00	0.00	RX0	C
ATOM	1913	OE1	GLN	355	18.438	21.301	34.052	1.00	0.00	RX0	O
ATOM	1914	NE2	GLN	355	16.854	22.770	33.293	1.00	0.00	RX0	N
ATOM	1915	HE21	GLN	355	16.566	23.336	32.518	1.00	0.00	RX0	H
ATOM	1916	HE22	GLN	355	16.284	22.788	34.116	1.00	0.00	RX0	H
ATOM	1917	C	GLN	355	19.631	19.504	29.273	1.00	0.00	RX0	C
ATOM	1918	O	GLN	355	20.705	18.919	29.430	1.00	0.00	RX0	O
ATOM	1919	N	LEU	356	18.681	19.086	28.443	1.00	0.00	RX0	N
ATOM	1920	H	LEU	356	17.883	19.669	28.276	1.00	0.00	RX0	H
ATOM	1921	CA	LEU	356	18.716	17.770	27.778	1.00	0.00	RX0	C
ATOM	1922	CB	LEU	356	17.350	17.404	27.202	1.00	0.00	RX0	C
ATOM	1923	CG	LEU	356	16.352	17.081	28.310	1.00	0.00	RX0	C
ATOM	1924	CD1	LEU	356	15.007	16.608	27.758	1.00	0.00	RX0	C
ATOM	1925	CD2	LEU	356	16.947	16.088	29.305	1.00	0.00	RX0	C
ATOM	1926	C	LEU	356	19.795	17.661	26.699	1.00	0.00	RX0	C
ATOM	1927	O	LEU	356	20.593	16.720	26.700	1.00	0.00	RX0	O
ATOM	1928	N	LEU	357	19.916	18.722	25.913	1.00	0.00	RX0	N
ATOM	1929	H	LEU	357	19.280	19.489	26.028	1.00	0.00	RX0	H
ATOM	1930	CA	LEU	357	20.885	18.765	24.803	1.00	0.00	RX0	C
ATOM	1931	CB	LEU	357	20.530	19.866	23.804	1.00	0.00	RX0	C
ATOM	1932	CG	LEU	357	19.160	19.676	23.150	1.00	0.00	RX0	C
ATOM	1933	CD1	LEU	357	18.823	20.831	22.207	1.00	0.00	RX0	C
ATOM	1934	CD2	LEU	357	19.025	18.317	22.465	1.00	0.00	RX0	C
ATOM	1935	C	LEU	357	22.335	18.932	25.264	1.00	0.00	RX0	C
ATOM	1936	O	LEU	357	23.247	18.345	24.683	1.00	0.00	RX0	O
ATOM	1937	N	LEU	358	22.501	19.595	26.404	1.00	0.00	RX0	N
ATOM	1938	H	LEU	358	21.707	20.033	26.830	1.00	0.00	RX0	H
ATOM	1939	CA	LEU	358	23.826	19.754	27.029	1.00	0.00	RX0	C
ATOM	1940	CB	LEU	358	23.803	20.838	28.104	1.00	0.00	RX0	C
ATOM	1941	CG	LEU	358	23.626	22.235	27.510	1.00	0.00	RX0	C
ATOM	1942	CD1	LEU	358	23.473	23.301	28.596	1.00	0.00	RX0	C
ATOM	1943	CD2	LEU	358	24.737	22.571	26.513	1.00	0.00	RX0	C
ATOM	1944	C	LEU	358	24.390	18.455	27.609	1.00	0.00	RX0	C
ATOM	1945	O	LEU	358	25.603	18.227	27.557	1.00	0.00	RX0	O
ATOM	1946	N	ILE	359	23.510	17.559	28.043	1.00	0.00	RX0	N
ATOM	1947	H	ILE	359	22.534	17.781	27.999	1.00	0.00	RX0	H
ATOM	1948	CA	ILE	359	23.928	16.212	28.495	1.00	0.00	RX0	C
ATOM	1949	CB	ILE	359	22.752	15.476	29.133	1.00	0.00	RX0	C
ATOM	1950	CG2	ILE	359	23.132	14.060	29.569	1.00	0.00	RX0	C

ATOM	1951	CG1	ILE	359	22.236	16.302	30.312	1.00	0.00	RX0	C
ATOM	1952	CD1	ILE	359	20.904	15.804	30.867	1.00	0.00	RX0	C
ATOM	1953	C	ILE	359	24.559	15.428	27.334	1.00	0.00	RX0	C
ATOM	1954	O	ILE	359	25.552	14.723	27.543	1.00	0.00	RX0	O
ATOM	1955	N	LEU	360	24.038	15.620	26.133	1.00	0.00	RX0	N
ATOM	1956	H	LEU	360	23.266	16.249	26.029	1.00	0.00	RX0	H
ATOM	1957	CA	LEU	360	24.601	14.974	24.928	1.00	0.00	RX0	C
ATOM	1958	CB	LEU	360	23.756	15.275	23.690	1.00	0.00	RX0	C
ATOM	1959	CG	LEU	360	22.272	14.948	23.884	1.00	0.00	RX0	C
ATOM	1960	CD1	LEU	360	21.459	15.294	22.640	1.00	0.00	RX0	C
ATOM	1961	CD2	LEU	360	22.035	13.502	24.321	1.00	0.00	RX0	C
ATOM	1962	C	LEU	360	26.079	15.308	24.694	1.00	0.00	RX0	C
ATOM	1963	O	LEU	360	26.843	14.455	24.246	1.00	0.00	RX0	O
ATOM	1964	N	SER	361	26.491	16.472	25.202	1.00	0.00	RX0	N
ATOM	1965	H	SER	361	25.850	17.141	25.586	1.00	0.00	RX0	H
ATOM	1966	CA	SER	361	27.906	16.894	25.202	1.00	0.00	RX0	C
ATOM	1967	CB	SER	361	27.872	18.374	25.513	1.00	0.00	RX0	C
ATOM	1968	OG	SER	361	26.623	18.831	24.974	1.00	0.00	RX0	O
ATOM	1969	HG	SER	361	26.644	18.659	24.041	1.00	0.00	RX0	H
ATOM	1970	C	SER	361	28.769	15.985	26.099	1.00	0.00	RX0	C
ATOM	1971	O	SER	361	29.797	15.456	25.681	1.00	0.00	RX0	O
ATOM	1972	N	HIS	362	28.237	15.704	27.286	1.00	0.00	RX0	N
ATOM	1973	H	HIS	362	27.305	16.023	27.458	1.00	0.00	RX0	H
ATOM	1974	CA	HIS	362	28.854	14.793	28.272	1.00	0.00	RX0	C
ATOM	1975	CB	HIS	362	28.100	14.896	29.599	1.00	0.00	RX0	C
ATOM	1976	CG	HIS	362	28.138	16.320	30.110	1.00	0.00	RX0	C
ATOM	1977	ND1	HIS	362	27.354	17.323	29.658	1.00	0.00	RX0	N
ATOM	1978	HD1	HIS	362	26.672	17.296	28.950	1.00	0.00	RX0	H
ATOM	1979	CD2	HIS	362	28.975	16.825	31.108	1.00	0.00	RX0	C
ATOM	1980	NE2	HIS	362	28.688	18.142	31.254	1.00	0.00	RX0	N
ATOM	1981	CE1	HIS	362	27.693	18.448	30.363	1.00	0.00	RX0	C
ATOM	1982	C	HIS	362	28.890	13.339	27.777	1.00	0.00	RX0	C
ATOM	1983	O	HIS	362	29.902	12.656	27.936	1.00	0.00	RX0	O
ATOM	1984	N	ILE	363	27.856	12.940	27.039	1.00	0.00	RX0	N
ATOM	1985	H	ILE	363	27.101	13.583	26.901	1.00	0.00	RX0	H
ATOM	1986	CA	ILE	363	27.773	11.584	26.446	1.00	0.00	RX0	C
ATOM	1987	CB	ILE	363	26.354	11.178	26.043	1.00	0.00	RX0	C
ATOM	1988	CG2	ILE	363	26.263	9.655	25.919	1.00	0.00	RX0	C
ATOM	1989	CG1	ILE	363	25.353	11.625	27.105	1.00	0.00	RX0	C
ATOM	1990	CD1	ILE	363	23.924	11.199	26.782	1.00	0.00	RX0	C
ATOM	1991	C	ILE	363	28.830	11.416	25.343	1.00	0.00	RX0	C
ATOM	1992	O	ILE	363	29.487	10.370	25.263	1.00	0.00	RX0	O
ATOM	1993	N	ARG	364	29.035	12.469	24.564	1.00	0.00	RX0	N
ATOM	1994	H	ARG	364	28.393	13.236	24.621	1.00	0.00	RX0	H
ATOM	1995	CA	ARG	364	30.102	12.498	23.547	1.00	0.00	RX0	C
ATOM	1996	CB	ARG	364	29.999	13.836	22.804	1.00	0.00	RX0	C
ATOM	1997	CG	ARG	364	30.653	13.986	21.423	1.00	0.00	RX0	C
ATOM	1998	CD	ARG	364	29.985	13.167	20.312	1.00	0.00	RX0	C
ATOM	1999	NE	ARG	364	28.532	13.160	20.464	1.00	0.00	RX0	N
ATOM	2000	HE	ARG	364	28.149	12.486	21.116	1.00	0.00	RX0	H
ATOM	2001	CZ	ARG	364	27.645	13.921	19.756	1.00	0.00	RX0	C
ATOM	2002	NH1	ARG	364	28.051	14.729	18.752	1.00	0.00	RX0	N
ATOM	2003	HH11	ARG	364	27.374	15.250	18.210	1.00	0.00	RX0	H
ATOM	2004	HH12	ARG	364	29.018	14.817	18.502	1.00	0.00	RX0	H
ATOM	2005	NH2	ARG	364	26.343	13.840	20.077	1.00	0.00	RX0	N
ATOM	2006	HH21	ARG	364	25.635	14.388	19.624	1.00	0.00	RX0	H
ATOM	2007	HH22	ARG	364	26.066	13.177	20.795	1.00	0.00	RX0	H
ATOM	2008	C	ARG	364	31.469	12.319	24.223	1.00	0.00	RX0	C
ATOM	2009	O	ARG	364	32.264	11.465	23.831	1.00	0.00	RX0	O
ATOM	2010	N	HIS	365	31.645	13.058	25.316	1.00	0.00	RX0	N
ATOM	2011	H	HIS	365	30.912	13.691	25.576	1.00	0.00	RX0	H

ATOM	2012	CA	HIS	365	32.879	13.027	26.115	1.00	0.00	RX0	C
ATOM	2013	CB	HIS	365	32.818	14.041	27.258	1.00	0.00	RX0	C
ATOM	2014	CG	HIS	365	34.177	14.159	27.906	1.00	0.00	RX0	C
ATOM	2015	ND1	HIS	365	35.131	15.003	27.471	1.00	0.00	RX0	N
ATOM	2016	HD1	HIS	365	35.055	15.631	26.722	1.00	0.00	RX0	H
ATOM	2017	CD2	HIS	365	34.669	13.456	29.012	1.00	0.00	RX0	C
ATOM	2018	NE2	HIS	365	35.935	13.889	29.234	1.00	0.00	RX0	N
ATOM	2019	CE1	HIS	365	36.219	14.840	28.289	1.00	0.00	RX0	C
ATOM	2020	C	HIS	365	33.175	11.617	26.649	1.00	0.00	RX0	C
ATOM	2021	O	HIS	365	34.274	11.105	26.425	1.00	0.00	RX0	O
ATOM	2022	N	MET	366	32.138	10.946	27.137	1.00	0.00	RX0	N
ATOM	2023	H	MET	366	31.256	11.416	27.209	1.00	0.00	RX0	H
ATOM	2024	CA	MET	366	32.267	9.572	27.662	1.00	0.00	RX0	C
ATOM	2025	CB	MET	366	31.017	9.144	28.432	1.00	0.00	RX0	C
ATOM	2026	CG	MET	366	30.736	10.018	29.653	1.00	0.00	RX0	C
ATOM	2027	SD	MET	366	29.402	9.378	30.676	1.00	0.00	RX0	S
ATOM	2028	CE	MET	366	28.108	9.360	29.429	1.00	0.00	RX0	C
ATOM	2029	C	MET	366	32.567	8.557	26.555	1.00	0.00	RX0	C
ATOM	2030	O	MET	366	33.398	7.674	26.741	1.00	0.00	RX0	O
ATOM	2031	N	SER	367	31.985	8.782	25.378	1.00	0.00	RX0	N
ATOM	2032	H	SER	367	31.329	9.531	25.280	1.00	0.00	RX0	H
ATOM	2033	CA	SER	367	32.231	7.934	24.196	1.00	0.00	RX0	C
ATOM	2034	CB	SER	367	31.162	8.274	23.171	1.00	0.00	RX0	C
ATOM	2035	OG	SER	367	29.908	8.068	23.823	1.00	0.00	RX0	O
ATOM	2036	HG	SER	367	29.672	8.890	24.240	1.00	0.00	RX0	H
ATOM	2037	C	SER	367	33.681	8.046	23.711	1.00	0.00	RX0	C
ATOM	2038	O	SER	367	34.361	7.038	23.567	1.00	0.00	RX0	O
ATOM	2039	N	ASN	368	34.193	9.278	23.688	1.00	0.00	RX0	N
ATOM	2040	H	ASN	368	33.595	10.046	23.928	1.00	0.00	RX0	H
ATOM	2041	CA	ASN	368	35.583	9.549	23.266	1.00	0.00	RX0	C
ATOM	2042	CB	ASN	368	35.821	11.037	23.148	1.00	0.00	RX0	C
ATOM	2043	CG	ASN	368	37.153	11.292	22.472	1.00	0.00	RX0	C
ATOM	2044	OD1	ASN	368	37.838	10.413	21.972	1.00	0.00	RX0	O
ATOM	2045	ND2	ASN	368	37.467	12.586	22.411	1.00	0.00	RX0	N
ATOM	2046	HD21	ASN	368	36.956	13.281	22.905	1.00	0.00	RX0	H
ATOM	2047	HD22	ASN	368	38.231	12.849	21.811	1.00	0.00	RX0	H
ATOM	2048	C	ASN	368	36.615	8.948	24.226	1.00	0.00	RX0	C
ATOM	2049	O	ASN	368	37.514	8.215	23.806	1.00	0.00	RX0	O
ATOM	2050	N	LYS	369	36.353	9.119	25.515	1.00	0.00	RX0	N
ATOM	2051	H	LYS	369	35.538	9.644	25.774	1.00	0.00	RX0	H
ATOM	2052	CA	LYS	369	37.205	8.554	26.580	1.00	0.00	RX0	C
ATOM	2053	CB	LYS	369	37.081	9.274	27.926	1.00	0.00	RX0	C
ATOM	2054	CG	LYS	369	37.279	10.798	27.810	1.00	0.00	RX0	C
ATOM	2055	CD	LYS	369	38.391	11.291	26.861	1.00	0.00	RX0	C
ATOM	2056	CE	LYS	369	39.833	10.906	27.204	1.00	0.00	RX0	C
ATOM	2057	NZ	LYS	369	40.701	11.260	26.074	1.00	0.00	RX0	N
ATOM	2058	HZ1	LYS	369	40.838	12.273	25.925	1.00	0.00	RX0	H
ATOM	2059	HZ2	LYS	369	41.628	10.783	26.148	1.00	0.00	RX0	H
ATOM	2060	HZ3	LYS	369	40.359	10.844	25.178	1.00	0.00	RX0	H
ATOM	2061	C	LYS	369	37.117	7.021	26.620	1.00	0.00	RX0	C
ATOM	2062	O	LYS	369	38.123	6.337	26.771	1.00	0.00	RX0	O
ATOM	2063	N	GLY	370	35.908	6.527	26.299	1.00	0.00	RX0	N
ATOM	2064	H	GLY	370	35.154	7.174	26.170	1.00	0.00	RX0	H
ATOM	2065	CA	GLY	370	35.597	5.090	26.225	1.00	0.00	RX0	C
ATOM	2066	C	GLY	370	36.324	4.429	25.048	1.00	0.00	RX0	C
ATOM	2067	O	GLY	370	36.946	3.386	25.214	1.00	0.00	RX0	O
ATOM	2068	N	MET	371	36.380	5.143	23.928	1.00	0.00	RX0	N
ATOM	2069	H	MET	371	35.894	6.017	23.921	1.00	0.00	RX0	H
ATOM	2070	CA	MET	371	37.103	4.709	22.719	1.00	0.00	RX0	C
ATOM	2071	CB	MET	371	36.727	5.708	21.618	1.00	0.00	RX0	C
ATOM	2072	CG	MET	371	36.889	5.284	20.159	1.00	0.00	RX0	C

ATOM	2073	SD	MET	371	38.577	4.984	19.625	1.00	0.00	RX0	S
ATOM	2074	CE	MET	371	39.135	6.688	19.685	1.00	0.00	RX0	C
ATOM	2075	C	MET	371	38.619	4.671	22.937	1.00	0.00	RX0	C
ATOM	2076	O	MET	371	39.258	3.669	22.631	1.00	0.00	RX0	O
ATOM	2077	N	GLU	372	39.117	5.692	23.643	1.00	0.00	RX0	N
ATOM	2078	H	GLU	372	38.525	6.472	23.849	1.00	0.00	RX0	H
ATOM	2079	CA	GLU	372	40.525	5.740	24.082	1.00	0.00	RX0	C
ATOM	2080	CB	GLU	372	40.898	7.089	24.687	1.00	0.00	RX0	C
ATOM	2081	CG	GLU	372	41.034	8.092	23.541	1.00	0.00	RX0	C
ATOM	2082	CD	GLU	372	41.460	9.445	24.070	1.00	0.00	RX0	C
ATOM	2083	OE1	GLU	372	41.002	10.464	23.556	1.00	0.00	RX0	O
ATOM	2084	OE2	GLU	372	42.191	9.508	25.053	1.00	0.00	RX0	O
ATOM	2085	C	GLU	372	40.884	4.541	24.968	1.00	0.00	RX0	C
ATOM	2086	O	GLU	372	41.865	3.853	24.723	1.00	0.00	RX0	O
ATOM	2087	N	HIS	373	40.006	4.291	25.945	1.00	0.00	RX0	N
ATOM	2088	H	HIS	373	39.224	4.906	26.050	1.00	0.00	RX0	H
ATOM	2089	CA	HIS	373	40.194	3.212	26.919	1.00	0.00	RX0	C
ATOM	2090	CB	HIS	373	39.267	3.437	28.122	1.00	0.00	RX0	C
ATOM	2091	CG	HIS	373	38.388	2.249	28.438	1.00	0.00	RX0	C
ATOM	2092	ND1	HIS	373	37.264	1.953	27.759	1.00	0.00	RX0	N
ATOM	2093	HD1	HIS	373	36.917	2.431	26.973	1.00	0.00	RX0	H
ATOM	2094	CD2	HIS	373	38.550	1.320	29.471	1.00	0.00	RX0	C
ATOM	2095	NE2	HIS	373	37.502	0.459	29.407	1.00	0.00	RX0	N
ATOM	2096	CE1	HIS	373	36.714	0.850	28.357	1.00	0.00	RX0	C
ATOM	2097	C	HIS	373	40.078	1.832	26.262	1.00	0.00	RX0	C
ATOM	2098	O	HIS	373	40.934	1.001	26.470	1.00	0.00	RX0	O
ATOM	2099	N	LEU	374	39.111	1.679	25.351	1.00	0.00	RX0	N
ATOM	2100	H	LEU	374	38.541	2.475	25.157	1.00	0.00	RX0	H
ATOM	2101	CA	LEU	374	38.919	0.408	24.629	1.00	0.00	RX0	C
ATOM	2102	CB	LEU	374	37.602	0.396	23.856	1.00	0.00	RX0	C
ATOM	2103	CG	LEU	374	36.372	0.155	24.731	1.00	0.00	RX0	C
ATOM	2104	CD1	LEU	374	35.076	0.478	23.988	1.00	0.00	RX0	C
ATOM	2105	CD2	LEU	374	36.357	-1.261	25.311	1.00	0.00	RX0	C
ATOM	2106	C	LEU	374	40.113	0.051	23.744	1.00	0.00	RX0	C
ATOM	2107	O	LEU	374	40.555	-1.109	23.754	1.00	0.00	RX0	O
ATOM	2108	N	TYR	375	40.664	1.068	23.103	1.00	0.00	RX0	N
ATOM	2109	H	TYR	375	40.295	1.982	23.290	1.00	0.00	RX0	H
ATOM	2110	CA	TYR	375	41.854	0.983	22.222	1.00	0.00	RX0	C
ATOM	2111	CB	TYR	375	42.024	2.322	21.486	1.00	0.00	RX0	C
ATOM	2112	CG	TYR	375	41.971	2.260	19.969	1.00	0.00	RX0	C
ATOM	2113	CD1	TYR	375	42.269	1.075	19.262	1.00	0.00	RX0	C
ATOM	2114	CE1	TYR	375	42.250	1.099	17.856	1.00	0.00	RX0	C
ATOM	2115	CD2	TYR	375	41.631	3.451	19.293	1.00	0.00	RX0	C
ATOM	2116	CE2	TYR	375	41.617	3.479	17.889	1.00	0.00	RX0	C
ATOM	2117	CZ	TYR	375	41.945	2.304	17.187	1.00	0.00	RX0	C
ATOM	2118	OH	TYR	375	41.986	2.319	15.805	1.00	0.00	RX0	O
ATOM	2119	HH	TYR	375	41.885	3.218	15.482	1.00	0.00	RX0	H
ATOM	2120	C	TYR	375	43.165	0.750	22.977	1.00	0.00	RX0	C
ATOM	2121	O	TYR	375	44.176	1.452	22.753	1.00	0.00	RX0	O
ATOM	2122	N	SER	376	43.162	-0.226	23.845	1.00	0.00	RX0	N
ATOM	2123	H	SER	376	42.298	-0.601	24.167	1.00	0.00	RX0	H
ATOM	2124	CA	SER	376	44.345	-0.646	24.634	1.00	0.00	RX0	C
ATOM	2125	CB	SER	376	44.671	0.427	25.683	1.00	0.00	RX0	C
ATOM	2126	OG	SER	376	44.007	1.658	25.381	1.00	0.00	RX0	O
ATOM	2127	HG	SER	376	44.350	1.969	24.550	1.00	0.00	RX0	H
ATOM	2128	C	SER	376	44.183	-1.992	25.350	1.00	0.00	RX0	C
ATOM	2129	O	SER	376	44.985	-2.369	26.199	1.00	0.00	RX0	O
ATOM	2130	N	MET	377	43.156	-2.746	24.938	1.00	0.00	RX0	N
ATOM	2131	H	MET	377	42.551	-2.426	24.208	1.00	0.00	RX0	H
ATOM	2132	CA	MET	377	42.915	-4.112	25.422	1.00	0.00	RX0	C
ATOM	2133	CB	MET	377	41.736	-4.071	26.400	1.00	0.00	RX0	C

ATOM	2134	CG	MET	377	42.050	-3.432	27.753	1.00	0.00	RX0	C
ATOM	2135	SD	MET	377	40.566	-3.267	28.754	1.00	0.00	RX0	S
ATOM	2136	CE	MET	377	39.764	-2.002	27.755	1.00	0.00	RX0	C
ATOM	2137	C	MET	377	42.647	-5.080	24.257	1.00	0.00	RX0	C
ATOM	2138	O	MET	377	43.092	-4.854	23.130	1.00	0.00	RX0	O
ATOM	2139	N	LYS	378	41.856	-6.107	24.527	1.00	0.00	RX0	N
ATOM	2140	H	LYS	378	41.490	-6.210	25.446	1.00	0.00	RX0	H
ATOM	2141	CA	LYS	378	41.570	-7.191	23.576	1.00	0.00	RX0	C
ATOM	2142	CB	LYS	378	41.445	-8.522	24.360	1.00	0.00	RX0	C
ATOM	2143	CG	LYS	378	40.460	-8.527	25.556	1.00	0.00	RX0	C
ATOM	2144	CD	LYS	378	40.427	-9.822	26.395	1.00	0.00	RX0	C
ATOM	2145	CE	LYS	378	39.480	-9.757	27.615	1.00	0.00	RX0	C
ATOM	2146	NZ	LYS	378	39.522	-11.004	28.407	1.00	0.00	RX0	N
ATOM	2147	HZ1	LYS	378	38.936	-10.939	29.273	1.00	0.00	RX0	H
ATOM	2148	HZ2	LYS	378	39.174	-11.796	27.831	1.00	0.00	RX0	H
ATOM	2149	HZ3	LYS	378	40.502	-11.203	28.691	1.00	0.00	RX0	H
ATOM	2150	C	LYS	378	40.316	-6.920	22.720	1.00	0.00	RX0	C
ATOM	2151	O	LYS	378	40.245	-7.352	21.611	1.00	0.00	RX0	O
ATOM	2152	N	CYS	379	39.320	-6.266	23.410	1.00	0.00	RX0	N
ATOM	2153	H	CYS	379	39.589	-5.920	24.304	1.00	0.00	RX0	H
ATOM	2154	CA	CYS	379	37.939	-6.053	22.985	1.00	0.00	RX0	C
ATOM	2155	CB	CYS	379	37.355	-4.660	23.283	1.00	0.00	RX0	C
ATOM	2156	SG	CYS	379	38.055	-3.275	22.357	1.00	0.00	RX0	S
ATOM	2157	C	CYS	379	37.599	-6.654	21.614	1.00	0.00	RX0	C
ATOM	2158	O	CYS	379	37.115	-7.773	21.559	1.00	0.00	RX0	O
ATOM	2159	N	LYS	380	38.012	-5.930	20.563	1.00	0.00	RX0	N
ATOM	2160	H	LYS	380	38.453	-5.057	20.757	1.00	0.00	RX0	H
ATOM	2161	CA	LYS	380	37.949	-6.383	19.165	1.00	0.00	RX0	C
ATOM	2162	CB	LYS	380	36.537	-6.893	18.792	1.00	0.00	RX0	C
ATOM	2163	CG	LYS	380	36.287	-7.690	17.497	1.00	0.00	RX0	C
ATOM	2164	CD	LYS	380	35.979	-6.831	16.266	1.00	0.00	RX0	C
ATOM	2165	CE	LYS	380	35.177	-7.528	15.162	1.00	0.00	RX0	C
ATOM	2166	NZ	LYS	380	34.832	-6.563	14.097	1.00	0.00	RX0	N
ATOM	2167	HZ1	LYS	380	34.177	-6.995	13.419	1.00	0.00	RX0	H
ATOM	2168	HZ2	LYS	380	34.371	-5.724	14.513	1.00	0.00	RX0	H
ATOM	2169	HZ3	LYS	380	35.684	-6.284	13.554	1.00	0.00	RX0	H
ATOM	2170	C	LYS	380	38.311	-5.215	18.248	1.00	0.00	RX0	C
ATOM	2171	O	LYS	380	37.971	-4.057	18.518	1.00	0.00	RX0	O
ATOM	2172	N	ASN	381	38.881	-5.592	17.123	1.00	0.00	RX0	N
ATOM	2173	H	ASN	381	39.211	-6.536	17.128	1.00	0.00	RX0	H
ATOM	2174	CA	ASN	381	39.208	-4.700	15.996	1.00	0.00	RX0	C
ATOM	2175	CB	ASN	381	40.416	-3.801	16.344	1.00	0.00	RX0	C
ATOM	2176	CG	ASN	381	40.559	-2.563	15.452	1.00	0.00	RX0	C
ATOM	2177	OD1	ASN	381	40.089	-1.483	15.778	1.00	0.00	RX0	O
ATOM	2178	ND2	ASN	381	41.305	-2.707	14.361	1.00	0.00	RX0	N
ATOM	2179	HD21	ASN	381	41.570	-3.624	14.054	1.00	0.00	RX0	H
ATOM	2180	HD22	ASN	381	41.555	-1.902	13.827	1.00	0.00	RX0	H
ATOM	2181	C	ASN	381	39.620	-5.523	14.759	1.00	0.00	RX0	C
ATOM	2182	O	ASN	381	40.286	-5.062	13.834	1.00	0.00	RX0	O
ATOM	2183	N	VAL	382	39.176	-6.776	14.737	1.00	0.00	RX0	N
ATOM	2184	H	VAL	382	38.558	-7.079	15.455	1.00	0.00	RX0	H
ATOM	2185	CA	VAL	382	39.338	-7.659	13.572	1.00	0.00	RX0	C
ATOM	2186	CB	VAL	382	39.229	-9.124	14.005	1.00	0.00	RX0	C
ATOM	2187	CG1	VAL	382	39.324	-10.072	12.808	1.00	0.00	RX0	C
ATOM	2188	CG2	VAL	382	40.265	-9.453	15.082	1.00	0.00	RX0	C
ATOM	2189	C	VAL	382	38.195	-7.277	12.635	1.00	0.00	RX0	C
ATOM	2190	O	VAL	382	37.049	-7.276	13.059	1.00	0.00	RX0	O
ATOM	2191	N	VAL	383	38.551	-7.063	11.363	1.00	0.00	RX0	N
ATOM	2192	H	VAL	383	39.518	-7.137	11.127	1.00	0.00	RX0	H
ATOM	2193	CA	VAL	383	37.600	-6.539	10.366	1.00	0.00	RX0	C
ATOM	2194	CB	VAL	383	36.439	-7.517	10.102	1.00	0.00	RX0	C

ATOM	2195	CG1	VAL	383	35.397	-6.945	9.135	1.00	0.00	RX0	C
ATOM	2196	CG2	VAL	383	36.972	-8.865	9.611	1.00	0.00	RX0	C
ATOM	2197	C	VAL	383	37.102	-5.173	10.877	1.00	0.00	RX0	C
ATOM	2198	O	VAL	383	36.355	-5.097	11.860	1.00	0.00	RX0	O
ATOM	2199	N	PRO	384	37.530	-4.088	10.236	1.00	0.00	RX0	N
ATOM	2200	CD	PRO	384	38.366	-4.077	9.044	1.00	0.00	RX0	C
ATOM	2201	CA	PRO	384	37.142	-2.731	10.663	1.00	0.00	RX0	C
ATOM	2202	CB	PRO	384	37.752	-1.813	9.604	1.00	0.00	RX0	C
ATOM	2203	CG	PRO	384	38.149	-2.700	8.430	1.00	0.00	RX0	C
ATOM	2204	C	PRO	384	35.624	-2.628	10.828	1.00	0.00	RX0	C
ATOM	2205	O	PRO	384	34.847	-3.282	10.112	1.00	0.00	RX0	O
ATOM	2206	N	LEU	385	35.228	-1.769	11.750	1.00	0.00	RX0	N
ATOM	2207	H	LEU	385	35.925	-1.219	12.198	1.00	0.00	RX0	H
ATOM	2208	CA	LEU	385	33.812	-1.621	12.127	1.00	0.00	RX0	C
ATOM	2209	CB	LEU	385	33.516	-0.706	13.303	1.00	0.00	RX0	C
ATOM	2210	CG	LEU	385	32.101	-0.870	13.859	1.00	0.00	RX0	C
ATOM	2211	CD1	LEU	385	31.805	-2.296	14.321	1.00	0.00	RX0	C
ATOM	2212	CD2	LEU	385	31.817	0.145	14.953	1.00	0.00	RX0	C
ATOM	2213	C	LEU	385	32.896	-1.213	10.964	1.00	0.00	RX0	C
ATOM	2214	O	LEU	385	31.809	-1.761	10.815	1.00	0.00	RX0	O
ATOM	2215	N	TYR	386	33.449	-0.406	10.055	1.00	0.00	RX0	N
ATOM	2216	H	TYR	386	34.313	0.034	10.287	1.00	0.00	RX0	H
ATOM	2217	CA	TYR	386	32.718	0.069	8.867	1.00	0.00	RX0	C
ATOM	2218	CB	TYR	386	33.675	0.932	8.025	1.00	0.00	RX0	C
ATOM	2219	CG	TYR	386	33.042	1.432	6.743	1.00	0.00	RX0	C
ATOM	2220	CD1	TYR	386	32.331	2.648	6.756	1.00	0.00	RX0	C
ATOM	2221	CE1	TYR	386	31.723	3.100	5.572	1.00	0.00	RX0	C
ATOM	2222	CD2	TYR	386	33.182	0.668	5.565	1.00	0.00	RX0	C
ATOM	2223	CE2	TYR	386	32.571	1.116	4.382	1.00	0.00	RX0	C
ATOM	2224	CZ	TYR	386	31.841	2.322	4.403	1.00	0.00	RX0	C
ATOM	2225	OH	TYR	386	31.218	2.751	3.243	1.00	0.00	RX0	O
ATOM	2226	HH	TYR	386	31.432	2.139	2.549	1.00	0.00	RX0	H
ATOM	2227	C	TYR	386	32.144	-1.103	8.049	1.00	0.00	RX0	C
ATOM	2228	O	TYR	386	30.957	-1.141	7.757	1.00	0.00	RX0	O
ATOM	2229	N	ASP	387	32.969	-2.141	7.903	1.00	0.00	RX0	N
ATOM	2230	H	ASP	387	33.935	-2.064	8.159	1.00	0.00	RX0	H
ATOM	2231	CA	ASP	387	32.619	-3.323	7.093	1.00	0.00	RX0	C
ATOM	2232	CB	ASP	387	33.840	-4.233	6.836	1.00	0.00	RX0	C
ATOM	2233	CG	ASP	387	35.204	-3.565	6.626	1.00	0.00	RX0	C
ATOM	2234	OD1	ASP	387	35.345	-2.343	6.720	1.00	0.00	RX0	O
ATOM	2235	OD2	ASP	387	36.158	-4.306	6.400	1.00	0.00	RX0	O
ATOM	2236	C	ASP	387	31.563	-4.207	7.765	1.00	0.00	RX0	C
ATOM	2237	O	ASP	387	30.619	-4.649	7.114	1.00	0.00	RX0	O
ATOM	2238	N	LEU	388	31.677	-4.332	9.088	1.00	0.00	RX0	N
ATOM	2239	H	LEU	388	32.463	-3.887	9.519	1.00	0.00	RX0	H
ATOM	2240	CA	LEU	388	30.691	-5.072	9.891	1.00	0.00	RX0	C
ATOM	2241	CB	LEU	388	31.189	-5.213	11.324	1.00	0.00	RX0	C
ATOM	2242	CG	LEU	388	30.295	-6.129	12.156	1.00	0.00	RX0	C
ATOM	2243	CD1	LEU	388	30.581	-7.611	11.911	1.00	0.00	RX0	C
ATOM	2244	CD2	LEU	388	30.354	-5.747	13.626	1.00	0.00	RX0	C
ATOM	2245	C	LEU	388	29.315	-4.386	9.871	1.00	0.00	RX0	C
ATOM	2246	O	LEU	388	28.299	-5.023	9.588	1.00	0.00	RX0	O
ATOM	2247	N	LEU	389	29.339	-3.070	10.049	1.00	0.00	RX0	N
ATOM	2248	H	LEU	389	30.232	-2.633	10.173	1.00	0.00	RX0	H
ATOM	2249	CA	LEU	389	28.129	-2.229	9.970	1.00	0.00	RX0	C
ATOM	2250	CB	LEU	389	28.397	-0.791	10.397	1.00	0.00	RX0	C
ATOM	2251	CG	LEU	389	28.765	-0.669	11.873	1.00	0.00	RX0	C
ATOM	2252	CD1	LEU	389	28.910	0.792	12.274	1.00	0.00	RX0	C
ATOM	2253	CD2	LEU	389	27.775	-1.388	12.787	1.00	0.00	RX0	C
ATOM	2254	C	LEU	389	27.489	-2.268	8.583	1.00	0.00	RX0	C
ATOM	2255	O	LEU	389	26.284	-2.473	8.458	1.00	0.00	RX0	O

ATOM	2256	N	LEU	390	28.350	-2.246	7.565	1.00	0.00	RX0	N
ATOM	2257	H	LEU	390	29.328	-2.153	7.759	1.00	0.00	RX0	H
ATOM	2258	CA	LEU	390	27.929	-2.335	6.162	1.00	0.00	RX0	C
ATOM	2259	CB	LEU	390	29.173	-2.216	5.282	1.00	0.00	RX0	C
ATOM	2260	CG	LEU	390	28.915	-2.053	3.788	1.00	0.00	RX0	C
ATOM	2261	CD1	LEU	390	28.416	-0.649	3.459	1.00	0.00	RX0	C
ATOM	2262	CD2	LEU	390	30.155	-2.408	2.967	1.00	0.00	RX0	C
ATOM	2263	C	LEU	390	27.202	-3.653	5.862	1.00	0.00	RX0	C
ATOM	2264	O	LEU	390	26.123	-3.627	5.302	1.00	0.00	RX0	O
ATOM	2265	N	GLU	391	27.733	-4.754	6.404	1.00	0.00	RX0	N
ATOM	2266	H	GLU	391	28.624	-4.704	6.864	1.00	0.00	RX0	H
ATOM	2267	CA	GLU	391	27.124	-6.090	6.264	1.00	0.00	RX0	C
ATOM	2268	CB	GLU	391	27.937	-7.167	6.988	1.00	0.00	RX0	C
ATOM	2269	CG	GLU	391	29.341	-7.465	6.466	1.00	0.00	RX0	C
ATOM	2270	CD	GLU	391	29.964	-8.526	7.356	1.00	0.00	RX0	C
ATOM	2271	OE1	GLU	391	30.544	-8.175	8.384	1.00	0.00	RX0	O
ATOM	2272	OE2	GLU	391	29.860	-9.706	7.022	1.00	0.00	RX0	O
ATOM	2273	C	GLU	391	25.713	-6.159	6.867	1.00	0.00	RX0	C
ATOM	2274	O	GLU	391	24.778	-6.600	6.214	1.00	0.00	RX0	O
ATOM	2275	N	MET	392	25.589	-5.588	8.070	1.00	0.00	RX0	N
ATOM	2276	H	MET	392	26.424	-5.212	8.481	1.00	0.00	RX0	H
ATOM	2277	CA	MET	392	24.313	-5.555	8.807	1.00	0.00	RX0	C
ATOM	2278	CB	MET	392	24.537	-5.243	10.286	1.00	0.00	RX0	C
ATOM	2279	CG	MET	392	25.170	-6.434	11.009	1.00	0.00	RX0	C
ATOM	2280	SD	MET	392	25.255	-6.225	12.795	1.00	0.00	RX0	S
ATOM	2281	CE	MET	392	26.526	-4.956	12.838	1.00	0.00	RX0	C
ATOM	2282	C	MET	392	23.266	-4.630	8.173	1.00	0.00	RX0	C
ATOM	2283	O	MET	392	22.107	-5.006	8.028	1.00	0.00	RX0	O
ATOM	2284	N	LEU	393	23.721	-3.452	7.754	1.00	0.00	RX0	N
ATOM	2285	H	LEU	393	24.706	-3.281	7.798	1.00	0.00	RX0	H
ATOM	2286	CA	LEU	393	22.852	-2.457	7.099	1.00	0.00	RX0	C
ATOM	2287	CB	LEU	393	23.508	-1.080	7.151	1.00	0.00	RX0	C
ATOM	2288	CG	LEU	393	23.437	-0.453	8.542	1.00	0.00	RX0	C
ATOM	2289	CD1	LEU	393	24.489	0.636	8.728	1.00	0.00	RX0	C
ATOM	2290	CD2	LEU	393	22.029	0.055	8.855	1.00	0.00	RX0	C
ATOM	2291	C	LEU	393	22.462	-2.815	5.663	1.00	0.00	RX0	C
ATOM	2292	O	LEU	393	21.313	-2.601	5.257	1.00	0.00	RX0	O
ATOM	2293	N	ASP	394	23.400	-3.384	4.923	1.00	0.00	RX0	N
ATOM	2294	H	ASP	394	24.268	-3.661	5.331	1.00	0.00	RX0	H
ATOM	2295	CA	ASP	394	23.187	-3.768	3.516	1.00	0.00	RX0	C
ATOM	2296	CB	ASP	394	24.187	-3.532	2.396	1.00	0.00	RX0	C
ATOM	2297	CG	ASP	394	23.308	-3.292	1.152	1.00	0.00	RX0	C
ATOM	2298	OD1	ASP	394	22.072	-3.259	1.265	1.00	0.00	RX0	O
ATOM	2299	OD2	ASP	394	23.852	-3.106	0.065	1.00	0.00	RX0	O
ATOM	2300	C	ASP	394	22.492	-5.132	3.432	1.00	0.00	RX0	C
ATOM	2301	O	ASP	394	23.034	-6.108	2.888	1.00	0.00	RX0	O
ATOM	2302	N	ALA	395	21.256	-5.134	3.874	1.00	0.00	RX0	N
ATOM	2303	H	ALA	395	20.895	-4.229	4.104	1.00	0.00	RX0	H
ATOM	2304	CA	ALA	395	20.412	-6.338	3.880	1.00	0.00	RX0	C
ATOM	2305	CB	ALA	395	19.421	-6.294	5.044	1.00	0.00	RX0	C
ATOM	2306	C	ALA	395	19.639	-6.454	2.565	1.00	0.00	RX0	C
ATOM	2307	O	ALA	395	19.303	-5.463	1.911	1.00	0.00	RX0	O
ATOM	2308	N	HIS	396	19.441	-7.696	2.156	1.00	0.00	RX0	N
ATOM	2309	H	HIS	396	19.740	-8.411	2.785	1.00	0.00	RX0	H
ATOM	2310	CA	HIS	396	18.536	-7.999	1.035	1.00	0.00	RX0	C
ATOM	2311	CB	HIS	396	18.852	-9.400	0.508	1.00	0.00	RX0	C
ATOM	2312	CG	HIS	396	19.012	-10.353	1.673	1.00	0.00	RX0	C
ATOM	2313	ND1	HIS	396	20.205	-10.808	2.100	1.00	0.00	RX0	N
ATOM	2314	HD1	HIS	396	21.087	-10.602	1.717	1.00	0.00	RX0	H
ATOM	2315	CD2	HIS	396	18.015	-10.887	2.496	1.00	0.00	RX0	C
ATOM	2316	NE2	HIS	396	18.618	-11.667	3.422	1.00	0.00	RX0	N

ATOM	2317	CE1	HIS	396	19.969	-11.621	3.180	1.00	0.00	RX0	C
ATOM	2318	C	HIS	396	17.077	-7.909	1.523	1.00	0.00	RX0	C
ATOM	2319	O	HIS	396	16.821	-7.755	2.721	1.00	0.00	RX0	O
ATOM	2320	N	ARG	397	16.142	-8.174	0.629	1.00	0.00	RX0	N
ATOM	2321	H	ARG	397	16.384	-8.366	-0.322	1.00	0.00	RX0	H
ATOM	2322	CA	ARG	397	14.704	-8.151	0.982	1.00	0.00	RX0	C
ATOM	2323	CB	ARG	397	14.090	-6.922	0.280	1.00	0.00	RX0	C
ATOM	2324	CG	ARG	397	14.781	-5.601	0.728	1.00	0.00	RX0	C
ATOM	2325	CD	ARG	397	15.824	-4.973	-0.230	1.00	0.00	RX0	C
ATOM	2326	NE	ARG	397	17.073	-4.548	0.434	1.00	0.00	RX0	N
ATOM	2327	HE	ARG	397	17.413	-5.069	1.228	1.00	0.00	RX0	H
ATOM	2328	CZ	ARG	397	17.883	-3.581	-0.122	1.00	0.00	RX0	C
ATOM	2329	NH1	ARG	397	17.386	-2.791	-1.104	1.00	0.00	RX0	N
ATOM	2330	HH11	ARG	397	17.948	-2.080	-1.535	1.00	0.00	RX0	H
ATOM	2331	HH12	ARG	397	16.442	-2.881	-1.432	1.00	0.00	RX0	H
ATOM	2332	NH2	ARG	397	19.165	-3.427	0.301	1.00	0.00	RX0	N
ATOM	2333	HH21	ARG	397	19.822	-2.740	-0.028	1.00	0.00	RX0	H
ATOM	2334	HH22	ARG	397	19.575	-4.038	0.999	1.00	0.00	RX0	H
ATOM	2335	C	ARG	397	14.082	-9.540	0.774	1.00	0.00	RX0	C
ATOM	2336	O	ARG	397	12.875	-9.706	0.613	1.00	0.00	RX0	O
ATOM	2337	N	LEU	398	14.938	-10.556	0.835	1.00	0.00	RX0	N
ATOM	2338	H	LEU	398	15.871	-10.365	1.127	1.00	0.00	RX0	H
ATOM	2339	CA	LEU	398	14.565	-11.951	0.542	1.00	0.00	RX0	C
ATOM	2340	CB	LEU	398	15.800	-12.794	0.231	1.00	0.00	RX0	C
ATOM	2341	CG	LEU	398	16.580	-12.270	-0.976	1.00	0.00	RX0	C
ATOM	2342	CD1	LEU	398	17.870	-13.060	-1.200	1.00	0.00	RX0	C
ATOM	2343	CD2	LEU	398	15.714	-12.212	-2.235	1.00	0.00	RX0	C
ATOM	2344	C	LEU	398	13.757	-12.584	1.680	1.00	0.00	RX0	C
ATOM	2345	O	LEU	398	12.772	-13.259	1.439	1.00	0.00	RX0	O
ATOM	2346	N	HIS	399	14.100	-12.169	2.904	1.00	0.00	RX0	N
ATOM	2347	H	HIS	399	14.845	-11.517	3.013	1.00	0.00	RX0	H
ATOM	2348	CA	HIS	399	13.392	-12.616	4.118	1.00	0.00	RX0	C
ATOM	2349	CB	HIS	399	14.353	-12.620	5.306	1.00	0.00	RX0	C
ATOM	2350	CG	HIS	399	15.412	-13.666	5.053	1.00	0.00	RX0	C
ATOM	2351	ND1	HIS	399	16.717	-13.396	4.850	1.00	0.00	RX0	N
ATOM	2352	HD1	HIS	399	17.157	-12.518	4.843	1.00	0.00	RX0	H
ATOM	2353	CD2	HIS	399	15.222	-15.048	4.968	1.00	0.00	RX0	C
ATOM	2354	NE2	HIS	399	16.428	-15.609	4.711	1.00	0.00	RX0	N
ATOM	2355	CE1	HIS	399	17.347	-14.595	4.639	1.00	0.00	RX0	C
ATOM	2356	C	HIS	399	12.131	-11.786	4.401	1.00	0.00	RX0	C
ATOM	2357	O	HIS	399	11.630	-11.743	5.524	1.00	0.00	RX0	O
ATOM	2358	N	ALA	400	11.619	-11.143	3.351	1.00	0.00	RX0	N
ATOM	2359	H	ALA	400	11.998	-11.316	2.444	1.00	0.00	RX0	H
ATOM	2360	CA	ALA	400	10.356	-10.385	3.402	1.00	0.00	RX0	C
ATOM	2361	CB	ALA	400	10.144	-9.552	2.138	1.00	0.00	RX0	C
ATOM	2362	C	ALA	400	9.147	-11.323	3.579	1.00	0.00	RX0	C
ATOM	2363	O	ALA	400	8.508	-11.220	4.647	1.00	0.00	RX0	O
ATOM	2364	N	SER	22	-16.808	-6.482	42.503	1.00	0.00	RX1	N
ATOM	2365	H	SER	22	-16.383	-6.486	41.595	1.00	0.00	RX1	H
ATOM	2366	CA	SER	22	-16.954	-7.844	43.019	1.00	0.00	RX1	C
ATOM	2367	CB	SER	22	-18.370	-8.333	42.665	1.00	0.00	RX1	C
ATOM	2368	OG	SER	22	-18.747	-9.459	43.464	1.00	0.00	RX1	O
ATOM	2369	HG	SER	22	-18.071	-10.111	43.298	1.00	0.00	RX1	H
ATOM	2370	C	SER	22	-15.852	-8.761	42.510	1.00	0.00	RX1	C
ATOM	2371	O	SER	22	-16.031	-9.964	42.353	1.00	0.00	RX1	O
ATOM	2372	N	THR	23	-14.716	-8.119	42.226	1.00	0.00	RX1	N
ATOM	2373	H	THR	23	-14.566	-7.142	42.363	1.00	0.00	RX1	H
ATOM	2374	CA	THR	23	-13.675	-8.798	41.466	1.00	0.00	RX1	C
ATOM	2375	CB	THR	23	-14.066	-8.419	40.049	1.00	0.00	RX1	C
ATOM	2376	OG1	THR	23	-15.111	-7.430	40.144	1.00	0.00	RX1	O
ATOM	2377	HG1	THR	23	-15.288	-7.157	39.252	1.00	0.00	RX1	H

ATOM	2378	CG2	THR	23	-14.547	-9.607	39.214	1.00	0.00	RX1	C
ATOM	2379	C	THR	23	-12.316	-8.272	41.883	1.00	0.00	RX1	C
ATOM	2380	O	THR	23	-12.198	-7.174	42.414	1.00	0.00	RX1	O
ATOM	2381	N	GLN	24	-11.287	-9.085	41.603	1.00	0.00	RX1	N
ATOM	2382	H	GLN	24	-11.453	-9.984	41.197	1.00	0.00	RX1	H
ATOM	2383	CA	GLN	24	-9.930	-8.550	41.711	1.00	0.00	RX1	C
ATOM	2384	CB	GLN	24	-8.913	-9.698	41.721	1.00	0.00	RX1	C
ATOM	2385	CG	GLN	24	-7.451	-9.337	41.413	1.00	0.00	RX1	C
ATOM	2386	CD	GLN	24	-6.830	-8.404	42.432	1.00	0.00	RX1	C
ATOM	2387	OE1	GLN	24	-7.486	-7.572	43.049	1.00	0.00	RX1	O
ATOM	2388	NE2	GLN	24	-5.501	-8.574	42.548	1.00	0.00	RX1	N
ATOM	2389	HE21	GLN	24	-5.009	-9.282	42.028	1.00	0.00	RX1	H
ATOM	2390	HE22	GLN	24	-4.945	-7.998	43.145	1.00	0.00	RX1	H
ATOM	2391	C	GLN	24	-9.660	-7.544	40.603	1.00	0.00	RX1	C
ATOM	2392	O	GLN	24	-9.600	-7.883	39.425	1.00	0.00	RX1	O
ATOM	2393	N	VAL	25	-9.546	-6.286	41.035	1.00	0.00	RX1	N
ATOM	2394	H	VAL	25	-9.470	-6.071	42.011	1.00	0.00	RX1	H
ATOM	2395	CA	VAL	25	-9.387	-5.206	40.066	1.00	0.00	RX1	C
ATOM	2396	CB	VAL	25	-10.612	-4.283	40.115	1.00	0.00	RX1	C
ATOM	2397	CG1	VAL	25	-10.431	-3.053	39.228	1.00	0.00	RX1	C
ATOM	2398	CG2	VAL	25	-11.898	-5.037	39.773	1.00	0.00	RX1	C
ATOM	2399	C	VAL	25	-8.127	-4.427	40.384	1.00	0.00	RX1	C
ATOM	2400	O	VAL	25	-7.909	-4.012	41.515	1.00	0.00	RX1	O
ATOM	2401	N	CYS	26	-7.309	-4.236	39.347	1.00	0.00	RX1	N
ATOM	2402	H	CYS	26	-7.514	-4.523	38.407	1.00	0.00	RX1	H
ATOM	2403	CA	CYS	26	-6.153	-3.376	39.574	1.00	0.00	RX1	C
ATOM	2404	CB	CYS	26	-4.884	-4.215	39.735	1.00	0.00	RX1	C
ATOM	2405	SG	CYS	26	-4.416	-5.108	38.235	1.00	0.00	RX1	S
ATOM	2406	C	CYS	26	-6.051	-2.369	38.456	1.00	0.00	RX1	C
ATOM	2407	O	CYS	26	-6.747	-2.476	37.455	1.00	0.00	RX1	O
ATOM	2408	N	THR	27	-5.174	-1.380	38.644	1.00	0.00	RX1	N
ATOM	2409	H	THR	27	-4.569	-1.312	39.433	1.00	0.00	RX1	H
ATOM	2410	CA	THR	27	-4.887	-0.550	37.480	1.00	0.00	RX1	C
ATOM	2411	CB	THR	27	-4.290	0.700	38.084	1.00	0.00	RX1	C
ATOM	2412	OG1	THR	27	-4.251	0.517	39.511	1.00	0.00	RX1	O
ATOM	2413	HG1	THR	27	-3.456	0.945	39.804	1.00	0.00	RX1	H
ATOM	2414	CG2	THR	27	-5.078	1.958	37.712	1.00	0.00	RX1	C
ATOM	2415	C	THR	27	-3.927	-1.271	36.558	1.00	0.00	RX1	C
ATOM	2416	O	THR	27	-3.163	-2.119	36.999	1.00	0.00	RX1	O
ATOM	2417	N	GLY	28	-3.996	-0.912	35.278	1.00	0.00	RX1	N
ATOM	2418	H	GLY	28	-4.555	-0.133	34.993	1.00	0.00	RX1	H
ATOM	2419	CA	GLY	28	-2.938	-1.409	34.410	1.00	0.00	RX1	C
ATOM	2420	C	GLY	28	-1.810	-0.416	34.395	1.00	0.00	RX1	C
ATOM	2421	O	GLY	28	-1.886	0.626	35.041	1.00	0.00	RX1	O
ATOM	2422	N	THR	29	-0.774	-0.750	33.630	1.00	0.00	RX1	N
ATOM	2423	H	THR	29	-0.715	-1.584	33.075	1.00	0.00	RX1	H
ATOM	2424	CA	THR	29	0.125	0.359	33.360	1.00	0.00	RX1	C
ATOM	2425	CB	THR	29	1.554	-0.177	33.337	1.00	0.00	RX1	C
ATOM	2426	OG1	THR	29	1.615	-1.404	32.607	1.00	0.00	RX1	O
ATOM	2427	HG1	THR	29	1.604	-1.129	31.685	1.00	0.00	RX1	H
ATOM	2428	CG2	THR	29	2.078	-0.400	34.755	1.00	0.00	RX1	C
ATOM	2429	C	THR	29	-0.301	1.092	32.103	1.00	0.00	RX1	C
ATOM	2430	O	THR	29	-1.182	0.640	31.375	1.00	0.00	RX1	O
ATOM	2431	N	ASP	30	0.353	2.242	31.908	1.00	0.00	RX1	N
ATOM	2432	H	ASP	30	1.026	2.626	32.536	1.00	0.00	RX1	H
ATOM	2433	CA	ASP	30	0.286	2.872	30.598	1.00	0.00	RX1	C
ATOM	2434	CB	ASP	30	-0.788	3.957	30.511	1.00	0.00	RX1	C
ATOM	2435	CG	ASP	30	-1.038	4.302	29.051	1.00	0.00	RX1	C
ATOM	2436	OD1	ASP	30	-0.425	3.699	28.174	1.00	0.00	RX1	O
ATOM	2437	OD2	ASP	30	-1.874	5.158	28.782	1.00	0.00	RX1	O
ATOM	2438	C	ASP	30	1.654	3.421	30.266	1.00	0.00	RX1	C

ATOM	2439	O	ASP	30	2.172	4.320	30.933	1.00	0.00	RX1	O
ATOM	2440	N	MET	31	2.260	2.796	29.255	1.00	0.00	RX1	N
ATOM	2441	H	MET	31	1.803	2.004	28.845	1.00	0.00	RX1	H
ATOM	2442	CA	MET	31	3.618	3.182	28.900	1.00	0.00	RX1	C
ATOM	2443	CB	MET	31	4.638	2.505	29.825	1.00	0.00	RX1	C
ATOM	2444	CG	MET	31	4.890	1.018	29.590	1.00	0.00	RX1	C
ATOM	2445	SD	MET	31	6.085	0.362	30.763	1.00	0.00	RX1	S
ATOM	2446	CE	MET	31	5.174	0.759	32.263	1.00	0.00	RX1	C
ATOM	2447	C	MET	31	4.021	3.034	27.448	1.00	0.00	RX1	C
ATOM	2448	O	MET	31	5.061	3.541	27.049	1.00	0.00	RX1	O
ATOM	2449	N	LYS	32	3.201	2.306	26.673	1.00	0.00	RX1	N
ATOM	2450	H	LYS	32	2.393	1.872	27.074	1.00	0.00	RX1	H
ATOM	2451	CA	LYS	32	3.691	1.847	25.368	1.00	0.00	RX1	C
ATOM	2452	CB	LYS	32	3.734	2.962	24.310	1.00	0.00	RX1	C
ATOM	2453	CG	LYS	32	2.333	3.380	23.855	1.00	0.00	RX1	C
ATOM	2454	CD	LYS	32	2.264	3.681	22.355	1.00	0.00	RX1	C
ATOM	2455	CE	LYS	32	0.827	3.903	21.871	1.00	0.00	RX1	C
ATOM	2456	NZ	LYS	32	0.803	4.085	20.417	1.00	0.00	RX1	N
ATOM	2457	HZ1	LYS	32	0.780	3.192	19.884	1.00	0.00	RX1	H
ATOM	2458	HZ2	LYS	32	-0.077	4.538	20.095	1.00	0.00	RX1	H
ATOM	2459	HZ3	LYS	32	1.598	4.641	20.049	1.00	0.00	RX1	H
ATOM	2460	C	LYS	32	5.011	1.095	25.508	1.00	0.00	RX1	C
ATOM	2461	O	LYS	32	5.265	0.486	26.540	1.00	0.00	RX1	O
ATOM	2462	N	LEU	33	5.879	1.161	24.491	1.00	0.00	RX1	N
ATOM	2463	H	LEU	33	5.760	1.673	23.641	1.00	0.00	RX1	H
ATOM	2464	CA	LEU	33	7.134	0.454	24.746	1.00	0.00	RX1	C
ATOM	2465	CB	LEU	33	7.694	-0.138	23.460	1.00	0.00	RX1	C
ATOM	2466	CG	LEU	33	6.662	-0.964	22.702	1.00	0.00	RX1	C
ATOM	2467	CD1	LEU	33	7.073	-1.309	21.275	1.00	0.00	RX1	C
ATOM	2468	CD2	LEU	33	6.322	-2.221	23.474	1.00	0.00	RX1	C
ATOM	2469	C	LEU	33	8.201	1.251	25.483	1.00	0.00	RX1	C
ATOM	2470	O	LEU	33	9.393	1.061	25.280	1.00	0.00	RX1	O
ATOM	2471	N	ARG	34	7.737	2.154	26.368	1.00	0.00	RX1	N
ATOM	2472	H	ARG	34	6.759	2.232	26.565	1.00	0.00	RX1	H
ATOM	2473	CA	ARG	34	8.680	2.932	27.172	1.00	0.00	RX1	C
ATOM	2474	CB	ARG	34	7.886	3.904	28.057	1.00	0.00	RX1	C
ATOM	2475	CG	ARG	34	8.605	4.615	29.204	1.00	0.00	RX1	C
ATOM	2476	CD	ARG	34	7.775	5.766	29.794	1.00	0.00	RX1	C
ATOM	2477	NE	ARG	34	6.423	5.364	30.193	1.00	0.00	RX1	N
ATOM	2478	HE	ARG	34	6.327	4.433	30.554	1.00	0.00	RX1	H
ATOM	2479	CZ	ARG	34	5.415	6.294	30.104	1.00	0.00	RX1	C
ATOM	2480	NH1	ARG	34	5.672	7.501	29.574	1.00	0.00	RX1	N
ATOM	2481	HH11	ARG	34	5.048	8.282	29.578	1.00	0.00	RX1	H
ATOM	2482	HH12	ARG	34	6.530	7.712	29.066	1.00	0.00	RX1	H
ATOM	2483	NH2	ARG	34	4.164	6.011	30.534	1.00	0.00	RX1	N
ATOM	2484	HH21	ARG	34	3.417	6.678	30.484	1.00	0.00	RX1	H
ATOM	2485	HH22	ARG	34	3.876	5.115	30.900	1.00	0.00	RX1	H
ATOM	2486	C	ARG	34	9.667	2.080	27.952	1.00	0.00	RX1	C
ATOM	2487	O	ARG	34	9.364	1.492	28.984	1.00	0.00	RX1	O
ATOM	2488	N	LEU	35	10.885	2.067	27.387	1.00	0.00	RX1	N
ATOM	2489	H	LEU	35	10.971	2.444	26.465	1.00	0.00	RX1	H
ATOM	2490	CA	LEU	35	12.027	1.416	28.024	1.00	0.00	RX1	C
ATOM	2491	CB	LEU	35	13.287	1.838	27.256	1.00	0.00	RX1	C
ATOM	2492	CG	LEU	35	14.535	0.985	27.500	1.00	0.00	RX1	C
ATOM	2493	CD1	LEU	35	14.335	-0.451	27.033	1.00	0.00	RX1	C
ATOM	2494	CD2	LEU	35	15.775	1.580	26.835	1.00	0.00	RX1	C
ATOM	2495	C	LEU	35	12.131	1.760	29.506	1.00	0.00	RX1	C
ATOM	2496	O	LEU	35	12.194	2.925	29.874	1.00	0.00	RX1	O
ATOM	2497	N	PRO	36	12.116	0.713	30.359	1.00	0.00	RX1	N
ATOM	2498	CD	PRO	36	11.972	-0.690	30.009	1.00	0.00	RX1	C
ATOM	2499	CA	PRO	36	12.204	0.950	31.805	1.00	0.00	RX1	C

ATOM	2500	CB	PRO	36	12.139	-0.470	32.371	1.00	0.00	RX1	C
ATOM	2501	CG	PRO	36	11.475	-1.326	31.295	1.00	0.00	RX1	C
ATOM	2502	C	PRO	36	13.476	1.673	32.222	1.00	0.00	RX1	C
ATOM	2503	O	PRO	36	14.541	1.430	31.667	1.00	0.00	RX1	O
ATOM	2504	N	ALA	37	13.318	2.546	33.233	1.00	0.00	RX1	N
ATOM	2505	H	ALA	37	12.455	2.653	33.725	1.00	0.00	RX1	H
ATOM	2506	CA	ALA	37	14.518	3.175	33.787	1.00	0.00	RX1	C
ATOM	2507	CB	ALA	37	14.175	4.520	34.427	1.00	0.00	RX1	C
ATOM	2508	C	ALA	37	15.273	2.290	34.771	1.00	0.00	RX1	C
ATOM	2509	O	ALA	37	16.403	2.564	35.173	1.00	0.00	RX1	O
ATOM	2510	N	SER	38	14.601	1.162	35.062	1.00	0.00	RX1	N
ATOM	2511	H	SER	38	13.604	1.164	35.159	1.00	0.00	RX1	H
ATOM	2512	CA	SER	38	15.295	-0.086	35.334	1.00	0.00	RX1	C
ATOM	2513	CB	SER	38	15.983	-0.047	36.696	1.00	0.00	RX1	C
ATOM	2514	OG	SER	38	15.059	0.229	37.742	1.00	0.00	RX1	O
ATOM	2515	HG	SER	38	14.311	0.696	37.366	1.00	0.00	RX1	H
ATOM	2516	C	SER	38	14.394	-1.287	35.077	1.00	0.00	RX1	C
ATOM	2517	O	SER	38	13.246	-1.355	35.500	1.00	0.00	RX1	O
ATOM	2518	N	PRO	39	14.954	-2.271	34.339	1.00	0.00	RX1	N
ATOM	2519	CD	PRO	39	16.176	-2.147	33.562	1.00	0.00	RX1	C
ATOM	2520	CA	PRO	39	14.342	-3.605	34.262	1.00	0.00	RX1	C
ATOM	2521	CB	PRO	39	15.487	-4.450	33.704	1.00	0.00	RX1	C
ATOM	2522	CG	PRO	39	16.315	-3.488	32.855	1.00	0.00	RX1	C
ATOM	2523	C	PRO	39	13.804	-4.158	35.577	1.00	0.00	RX1	C
ATOM	2524	O	PRO	39	12.689	-4.662	35.654	1.00	0.00	RX1	O
ATOM	2525	N	GLU	40	14.663	-4.050	36.608	1.00	0.00	RX1	N
ATOM	2526	H	GLU	40	15.541	-3.578	36.534	1.00	0.00	RX1	H
ATOM	2527	CA	GLU	40	14.301	-4.646	37.894	1.00	0.00	RX1	C
ATOM	2528	CB	GLU	40	15.480	-4.562	38.862	1.00	0.00	RX1	C
ATOM	2529	CG	GLU	40	16.728	-5.244	38.294	1.00	0.00	RX1	C
ATOM	2530	CD	GLU	40	17.865	-5.133	39.289	1.00	0.00	RX1	C
ATOM	2531	OE1	GLU	40	17.591	-4.979	40.478	1.00	0.00	RX1	O
ATOM	2532	OE2	GLU	40	19.019	-5.200	38.871	1.00	0.00	RX1	O
ATOM	2533	C	GLU	40	13.031	-4.090	38.514	1.00	0.00	RX1	C
ATOM	2534	O	GLU	40	12.109	-4.828	38.853	1.00	0.00	RX1	O
ATOM	2535	N	THR	41	12.988	-2.746	38.607	1.00	0.00	RX1	N
ATOM	2536	H	THR	41	13.667	-2.103	38.245	1.00	0.00	RX1	H
ATOM	2537	CA	THR	41	11.770	-2.156	39.166	1.00	0.00	RX1	C
ATOM	2538	CB	THR	41	11.998	-0.659	39.237	1.00	0.00	RX1	C
ATOM	2539	OG1	THR	41	12.533	-0.223	37.988	1.00	0.00	RX1	O
ATOM	2540	HG1	THR	41	12.440	0.738	37.996	1.00	0.00	RX1	H
ATOM	2541	CG2	THR	41	12.959	-0.303	40.375	1.00	0.00	RX1	C
ATOM	2542	C	THR	41	10.523	-2.534	38.386	1.00	0.00	RX1	C
ATOM	2543	O	THR	41	9.531	-3.009	38.928	1.00	0.00	RX1	O
ATOM	2544	N	HIS	42	10.658	-2.353	37.061	1.00	0.00	RX1	N
ATOM	2545	H	HIS	42	11.499	-1.902	36.744	1.00	0.00	RX1	H
ATOM	2546	CA	HIS	42	9.589	-2.729	36.136	1.00	0.00	RX1	C
ATOM	2547	CB	HIS	42	10.097	-2.639	34.701	1.00	0.00	RX1	C
ATOM	2548	CG	HIS	42	8.971	-2.835	33.714	1.00	0.00	RX1	C
ATOM	2549	ND1	HIS	42	7.879	-2.049	33.654	1.00	0.00	RX1	N
ATOM	2550	HD1	HIS	42	7.671	-1.288	34.234	1.00	0.00	RX1	H
ATOM	2551	CD2	HIS	42	8.886	-3.801	32.707	1.00	0.00	RX1	C
ATOM	2552	NE2	HIS	42	7.729	-3.584	32.035	1.00	0.00	RX1	N
ATOM	2553	CE1	HIS	42	7.109	-2.509	32.616	1.00	0.00	RX1	C
ATOM	2554	C	HIS	42	8.979	-4.101	36.392	1.00	0.00	RX1	C
ATOM	2555	O	HIS	42	7.784	-4.238	36.619	1.00	0.00	RX1	O
ATOM	2556	N	LEU	43	9.868	-5.108	36.364	1.00	0.00	RX1	N
ATOM	2557	H	LEU	43	10.845	-4.908	36.259	1.00	0.00	RX1	H
ATOM	2558	CA	LEU	43	9.415	-6.490	36.533	1.00	0.00	RX1	C
ATOM	2559	CB	LEU	43	10.624	-7.426	36.551	1.00	0.00	RX1	C
ATOM	2560	CG	LEU	43	10.266	-8.877	36.887	1.00	0.00	RX1	C

ATOM	2561	CD1	LEU	43	9.331	-9.511	35.857	1.00	0.00	RX1	C
ATOM	2562	CD2	LEU	43	11.518	-9.714	37.147	1.00	0.00	RX1	C
ATOM	2563	C	LEU	43	8.559	-6.720	37.769	1.00	0.00	RX1	C
ATOM	2564	O	LEU	43	7.419	-7.179	37.712	1.00	0.00	RX1	O
ATOM	2565	N	ASP	44	9.169	-6.387	38.909	1.00	0.00	RX1	N
ATOM	2566	H	ASP	44	10.066	-5.939	38.954	1.00	0.00	RX1	H
ATOM	2567	CA	ASP	44	8.444	-6.790	40.106	1.00	0.00	RX1	C
ATOM	2568	CB	ASP	44	9.403	-7.002	41.274	1.00	0.00	RX1	C
ATOM	2569	CG	ASP	44	9.679	-8.490	41.386	1.00	0.00	RX1	C
ATOM	2570	OD1	ASP	44	9.635	-9.023	42.491	1.00	0.00	RX1	O
ATOM	2571	OD2	ASP	44	9.856	-9.157	40.367	1.00	0.00	RX1	O
ATOM	2572	C	ASP	44	7.263	-5.910	40.456	1.00	0.00	RX1	C
ATOM	2573	O	ASP	44	6.269	-6.348	41.028	1.00	0.00	RX1	O
ATOM	2574	N	MET	45	7.362	-4.653	39.987	1.00	0.00	RX1	N
ATOM	2575	H	MET	45	8.198	-4.335	39.534	1.00	0.00	RX1	H
ATOM	2576	CA	MET	45	6.167	-3.809	40.000	1.00	0.00	RX1	C
ATOM	2577	CB	MET	45	6.512	-2.390	39.536	1.00	0.00	RX1	C
ATOM	2578	CG	MET	45	5.334	-1.415	39.438	1.00	0.00	RX1	C
ATOM	2579	SD	MET	45	4.384	-1.589	37.918	1.00	0.00	RX1	S
ATOM	2580	CE	MET	45	5.719	-1.225	36.765	1.00	0.00	RX1	C
ATOM	2581	C	MET	45	5.007	-4.406	39.215	1.00	0.00	RX1	C
ATOM	2582	O	MET	45	3.873	-4.430	39.678	1.00	0.00	RX1	O
ATOM	2583	N	LEU	46	5.342	-4.947	38.028	1.00	0.00	RX1	N
ATOM	2584	H	LEU	46	6.282	-4.913	37.681	1.00	0.00	RX1	H
ATOM	2585	CA	LEU	46	4.293	-5.629	37.267	1.00	0.00	RX1	C
ATOM	2586	CB	LEU	46	4.799	-6.189	35.940	1.00	0.00	RX1	C
ATOM	2587	CG	LEU	46	5.225	-5.142	34.918	1.00	0.00	RX1	C
ATOM	2588	CD1	LEU	46	5.634	-5.817	33.613	1.00	0.00	RX1	C
ATOM	2589	CD2	LEU	46	4.160	-4.069	34.693	1.00	0.00	RX1	C
ATOM	2590	C	LEU	46	3.617	-6.757	38.019	1.00	0.00	RX1	C
ATOM	2591	O	LEU	46	2.404	-6.909	37.985	1.00	0.00	RX1	O
ATOM	2592	N	ARG	47	4.445	-7.530	38.742	1.00	0.00	RX1	N
ATOM	2593	H	ARG	47	5.432	-7.351	38.745	1.00	0.00	RX1	H
ATOM	2594	CA	ARG	47	3.822	-8.567	39.568	1.00	0.00	RX1	C
ATOM	2595	CB	ARG	47	4.885	-9.466	40.194	1.00	0.00	RX1	C
ATOM	2596	CG	ARG	47	4.326	-10.801	40.700	1.00	0.00	RX1	C
ATOM	2597	CD	ARG	47	5.403	-11.813	41.106	1.00	0.00	RX1	C
ATOM	2598	NE	ARG	47	6.408	-11.959	40.052	1.00	0.00	RX1	N
ATOM	2599	HE	ARG	47	6.159	-12.378	39.170	1.00	0.00	RX1	H
ATOM	2600	CZ	ARG	47	7.610	-11.347	40.252	1.00	0.00	RX1	C
ATOM	2601	NH1	ARG	47	7.902	-10.857	41.470	1.00	0.00	RX1	N
ATOM	2602	HH11	ARG	47	8.748	-10.305	41.610	1.00	0.00	RX1	H
ATOM	2603	HH12	ARG	47	7.333	-10.965	42.285	1.00	0.00	RX1	H
ATOM	2604	NH2	ARG	47	8.472	-11.209	39.229	1.00	0.00	RX1	N
ATOM	2605	HH21	ARG	47	9.298	-10.635	39.364	1.00	0.00	RX1	H
ATOM	2606	HH22	ARG	47	8.313	-11.633	38.333	1.00	0.00	RX1	H
ATOM	2607	C	ARG	47	2.873	-8.024	40.621	1.00	0.00	RX1	C
ATOM	2608	O	ARG	47	1.710	-8.400	40.708	1.00	0.00	RX1	O
ATOM	2609	N	HIS	48	3.429	-7.082	41.400	1.00	0.00	RX1	N
ATOM	2610	H	HIS	48	4.372	-6.784	41.233	1.00	0.00	RX1	H
ATOM	2611	CA	HIS	48	2.652	-6.478	42.483	1.00	0.00	RX1	C
ATOM	2612	CB	HIS	48	3.498	-5.429	43.208	1.00	0.00	RX1	C
ATOM	2613	CG	HIS	48	2.974	-5.151	44.603	1.00	0.00	RX1	C
ATOM	2614	ND1	HIS	48	1.674	-5.064	44.950	1.00	0.00	RX1	N
ATOM	2615	HD1	HIS	48	0.891	-5.183	44.366	1.00	0.00	RX1	H
ATOM	2616	CD2	HIS	48	3.750	-4.932	45.746	1.00	0.00	RX1	C
ATOM	2617	NE2	HIS	48	2.904	-4.714	46.782	1.00	0.00	RX1	N
ATOM	2618	CE1	HIS	48	1.627	-4.795	46.293	1.00	0.00	RX1	C
ATOM	2619	C	HIS	48	1.328	-5.873	42.038	1.00	0.00	RX1	C
ATOM	2620	O	HIS	48	0.316	-5.948	42.723	1.00	0.00	RX1	O
ATOM	2621	N	LEU	49	1.393	-5.260	40.852	1.00	0.00	RX1	N

ATOM	2622	H	LEU	49	2.241	-5.256	40.321	1.00	0.00	RX1	H
ATOM	2623	CA	LEU	49	0.189	-4.637	40.320	1.00	0.00	RX1	C
ATOM	2624	CB	LEU	49	0.607	-3.608	39.271	1.00	0.00	RX1	C
ATOM	2625	CG	LEU	49	-0.527	-2.719	38.767	1.00	0.00	RX1	C
ATOM	2626	CD1	LEU	49	-1.240	-1.986	39.905	1.00	0.00	RX1	C
ATOM	2627	CD2	LEU	49	-0.024	-1.754	37.693	1.00	0.00	RX1	C
ATOM	2628	C	LEU	49	-0.809	-5.637	39.756	1.00	0.00	RX1	C
ATOM	2629	O	LEU	49	-1.977	-5.675	40.112	1.00	0.00	RX1	O
ATOM	2630	N	TYR	50	-0.282	-6.446	38.832	1.00	0.00	RX1	N
ATOM	2631	H	TYR	50	0.706	-6.492	38.685	1.00	0.00	RX1	H
ATOM	2632	CA	TYR	50	-1.184	-7.259	38.021	1.00	0.00	RX1	C
ATOM	2633	CB	TYR	50	-0.510	-7.648	36.712	1.00	0.00	RX1	C
ATOM	2634	CG	TYR	50	-0.285	-6.471	35.800	1.00	0.00	RX1	C
ATOM	2635	CD1	TYR	50	-1.197	-5.424	35.760	1.00	0.00	RX1	C
ATOM	2636	CE1	TYR	50	-1.032	-4.407	34.828	1.00	0.00	RX1	C
ATOM	2637	CD2	TYR	50	0.825	-6.466	34.963	1.00	0.00	RX1	C
ATOM	2638	CE2	TYR	50	0.987	-5.450	34.031	1.00	0.00	RX1	C
ATOM	2639	CZ	TYR	50	0.039	-4.439	33.945	1.00	0.00	RX1	C
ATOM	2640	OH	TYR	50	0.151	-3.474	32.969	1.00	0.00	RX1	O
ATOM	2641	HH	TYR	50	1.007	-3.050	33.037	1.00	0.00	RX1	H
ATOM	2642	C	TYR	50	-1.697	-8.543	38.647	1.00	0.00	RX1	C
ATOM	2643	O	TYR	50	-2.575	-9.199	38.098	1.00	0.00	RX1	O
ATOM	2644	N	GLN	51	-1.095	-8.913	39.788	1.00	0.00	RX1	N
ATOM	2645	H	GLN	51	-0.386	-8.330	40.190	1.00	0.00	RX1	H
ATOM	2646	CA	GLN	51	-1.322	-10.252	40.339	1.00	0.00	RX1	C
ATOM	2647	CB	GLN	51	-0.633	-10.378	41.694	1.00	0.00	RX1	C
ATOM	2648	CG	GLN	51	-0.362	-11.836	42.058	1.00	0.00	RX1	C
ATOM	2649	CD	GLN	51	0.755	-11.884	43.076	1.00	0.00	RX1	C
ATOM	2650	OE1	GLN	51	1.871	-12.303	42.785	1.00	0.00	RX1	O
ATOM	2651	NE2	GLN	51	0.394	-11.427	44.288	1.00	0.00	RX1	N
ATOM	2652	HE21	GLN	51	-0.537	-11.097	44.451	1.00	0.00	RX1	H
ATOM	2653	HE22	GLN	51	1.043	-11.400	45.048	1.00	0.00	RX1	H
ATOM	2654	C	GLN	51	-2.752	-10.787	40.369	1.00	0.00	RX1	C
ATOM	2655	O	GLN	51	-3.580	-10.402	41.188	1.00	0.00	RX1	O
ATOM	2656	N	GLY	52	-2.991	-11.719	39.429	1.00	0.00	RX1	N
ATOM	2657	H	GLY	52	-2.319	-11.891	38.705	1.00	0.00	RX1	H
ATOM	2658	CA	GLY	52	-4.292	-12.391	39.375	1.00	0.00	RX1	C
ATOM	2659	C	GLY	52	-5.473	-11.506	39.000	1.00	0.00	RX1	C
ATOM	2660	O	GLY	52	-6.616	-11.759	39.360	1.00	0.00	RX1	O
ATOM	2661	N	CYS	53	-5.152	-10.432	38.269	1.00	0.00	RX1	N
ATOM	2662	H	CYS	53	-4.222	-10.260	37.941	1.00	0.00	RX1	H
ATOM	2663	CA	CYS	53	-6.206	-9.450	38.036	1.00	0.00	RX1	C
ATOM	2664	CB	CYS	53	-5.609	-8.138	37.551	1.00	0.00	RX1	C
ATOM	2665	SG	CYS	53	-6.837	-6.816	37.641	1.00	0.00	RX1	S
ATOM	2666	C	CYS	53	-7.343	-9.893	37.139	1.00	0.00	RX1	C
ATOM	2667	O	CYS	53	-7.176	-10.250	35.978	1.00	0.00	RX1	O
ATOM	2668	N	GLN	54	-8.543	-9.825	37.717	1.00	0.00	RX1	N
ATOM	2669	H	GLN	54	-8.652	-9.424	38.627	1.00	0.00	RX1	H
ATOM	2670	CA	GLN	54	-9.693	-10.110	36.870	1.00	0.00	RX1	C
ATOM	2671	CB	GLN	54	-10.907	-10.468	37.710	1.00	0.00	RX1	C
ATOM	2672	CG	GLN	54	-10.598	-11.635	38.639	1.00	0.00	RX1	C
ATOM	2673	CD	GLN	54	-11.870	-12.024	39.346	1.00	0.00	RX1	C
ATOM	2674	OE1	GLN	54	-12.120	-11.619	40.475	1.00	0.00	RX1	O
ATOM	2675	NE2	GLN	54	-12.670	-12.815	38.610	1.00	0.00	RX1	N
ATOM	2676	HE21	GLN	54	-12.425	-13.072	37.669	1.00	0.00	RX1	H
ATOM	2677	HE22	GLN	54	-13.538	-13.160	38.967	1.00	0.00	RX1	H
ATOM	2678	C	GLN	54	-10.018	-8.984	35.912	1.00	0.00	RX1	C
ATOM	2679	O	GLN	54	-10.239	-9.199	34.727	1.00	0.00	RX1	O
ATOM	2680	N	VAL	55	-10.037	-7.773	36.485	1.00	0.00	RX1	N
ATOM	2681	H	VAL	55	-9.795	-7.629	37.448	1.00	0.00	RX1	H
ATOM	2682	CA	VAL	55	-10.273	-6.608	35.637	1.00	0.00	RX1	C

ATOM	2683	CB	VAL	55	-11.571	-5.894	36.027	1.00	0.00	RX1	C
ATOM	2684	CG1	VAL	55	-11.841	-4.715	35.089	1.00	0.00	RX1	C
ATOM	2685	CG2	VAL	55	-12.755	-6.861	36.087	1.00	0.00	RX1	C
ATOM	2686	C	VAL	55	-9.105	-5.648	35.729	1.00	0.00	RX1	C
ATOM	2687	O	VAL	55	-8.958	-4.902	36.692	1.00	0.00	RX1	O
ATOM	2688	N	VAL	56	-8.267	-5.706	34.690	1.00	0.00	RX1	N
ATOM	2689	H	VAL	56	-8.452	-6.248	33.870	1.00	0.00	RX1	H
ATOM	2690	CA	VAL	56	-7.177	-4.745	34.711	1.00	0.00	RX1	C
ATOM	2691	CB	VAL	56	-5.837	-5.393	34.326	1.00	0.00	RX1	C
ATOM	2692	CG1	VAL	56	-5.707	-5.775	32.856	1.00	0.00	RX1	C
ATOM	2693	CG2	VAL	56	-4.689	-4.511	34.788	1.00	0.00	RX1	C
ATOM	2694	C	VAL	56	-7.504	-3.459	33.963	1.00	0.00	RX1	C
ATOM	2695	O	VAL	56	-7.633	-3.371	32.744	1.00	0.00	RX1	O
ATOM	2696	N	GLN	57	-7.692	-2.446	34.809	1.00	0.00	RX1	N
ATOM	2697	H	GLN	57	-7.494	-2.585	35.779	1.00	0.00	RX1	H
ATOM	2698	CA	GLN	57	-8.078	-1.136	34.304	1.00	0.00	RX1	C
ATOM	2699	CB	GLN	57	-8.891	-0.388	35.351	1.00	0.00	RX1	C
ATOM	2700	CG	GLN	57	-9.972	-1.314	35.897	1.00	0.00	RX1	C
ATOM	2701	CD	GLN	57	-11.123	-0.497	36.432	1.00	0.00	RX1	C
ATOM	2702	OE1	GLN	57	-12.219	-0.499	35.879	1.00	0.00	RX1	O
ATOM	2703	NE2	GLN	57	-10.817	0.203	37.539	1.00	0.00	RX1	N
ATOM	2704	HE21	GLN	57	-9.899	0.142	37.933	1.00	0.00	RX1	H
ATOM	2705	HE22	GLN	57	-11.491	0.790	37.986	1.00	0.00	RX1	H
ATOM	2706	C	GLN	57	-6.903	-0.319	33.822	1.00	0.00	RX1	C
ATOM	2707	O	GLN	57	-6.334	0.505	34.530	1.00	0.00	RX1	O
ATOM	2708	N	GLY	58	-6.548	-0.629	32.577	1.00	0.00	RX1	N
ATOM	2709	H	GLY	58	-6.973	-1.386	32.075	1.00	0.00	RX1	H
ATOM	2710	CA	GLY	58	-5.407	0.011	31.938	1.00	0.00	RX1	C
ATOM	2711	C	GLY	58	-4.877	-0.965	30.918	1.00	0.00	RX1	C
ATOM	2712	O	GLY	58	-5.584	-1.895	30.537	1.00	0.00	RX1	O
ATOM	2713	N	ASN	59	-3.631	-0.739	30.493	1.00	0.00	RX1	N
ATOM	2714	H	ASN	59	-3.013	-0.063	30.902	1.00	0.00	RX1	H
ATOM	2715	CA	ASN	59	-3.087	-1.745	29.584	1.00	0.00	RX1	C
ATOM	2716	CB	ASN	59	-1.926	-1.226	28.747	1.00	0.00	RX1	C
ATOM	2717	CG	ASN	59	-2.289	-0.001	27.953	1.00	0.00	RX1	C
ATOM	2718	OD1	ASN	59	-3.262	0.021	27.200	1.00	0.00	RX1	O
ATOM	2719	ND2	ASN	59	-1.445	1.014	28.178	1.00	0.00	RX1	N
ATOM	2720	HD21	ASN	59	-0.618	0.854	28.730	1.00	0.00	RX1	H
ATOM	2721	HD22	ASN	59	-1.507	1.971	27.874	1.00	0.00	RX1	H
ATOM	2722	C	ASN	59	-2.569	-2.931	30.364	1.00	0.00	RX1	C
ATOM	2723	O	ASN	59	-2.456	-2.890	31.583	1.00	0.00	RX1	O
ATOM	2724	N	LEU	60	-2.244	-3.983	29.608	1.00	0.00	RX1	N
ATOM	2725	H	LEU	60	-2.355	-3.963	28.613	1.00	0.00	RX1	H
ATOM	2726	CA	LEU	60	-1.500	-5.071	30.227	1.00	0.00	RX1	C
ATOM	2727	CB	LEU	60	-2.219	-6.395	29.997	1.00	0.00	RX1	C
ATOM	2728	CG	LEU	60	-1.690	-7.527	30.873	1.00	0.00	RX1	C
ATOM	2729	CD1	LEU	60	-1.843	-7.198	32.353	1.00	0.00	RX1	C
ATOM	2730	CD2	LEU	60	-2.346	-8.861	30.527	1.00	0.00	RX1	C
ATOM	2731	C	LEU	60	-0.113	-5.117	29.631	1.00	0.00	RX1	C
ATOM	2732	O	LEU	60	0.160	-5.827	28.667	1.00	0.00	RX1	O
ATOM	2733	N	GLU	61	0.737	-4.271	30.210	1.00	0.00	RX1	N
ATOM	2734	H	GLU	61	0.513	-3.818	31.076	1.00	0.00	RX1	H
ATOM	2735	CA	GLU	61	2.055	-4.145	29.601	1.00	0.00	RX1	C
ATOM	2736	CB	GLU	61	2.510	-2.696	29.667	1.00	0.00	RX1	C
ATOM	2737	CG	GLU	61	1.502	-1.935	28.818	1.00	0.00	RX1	C
ATOM	2738	CD	GLU	61	1.608	-0.446	29.005	1.00	0.00	RX1	C
ATOM	2739	OE1	GLU	61	1.809	-0.004	30.130	1.00	0.00	RX1	O
ATOM	2740	OE2	GLU	61	1.451	0.277	28.027	1.00	0.00	RX1	O
ATOM	2741	C	GLU	61	3.054	-5.124	30.159	1.00	0.00	RX1	C
ATOM	2742	O	GLU	61	3.360	-5.168	31.342	1.00	0.00	RX1	O
ATOM	2743	N	LEU	62	3.484	-5.960	29.215	1.00	0.00	RX1	N

ATOM	2744	H	LEU	62	3.297	-5.770	28.252	1.00	0.00	RX1	H
ATOM	2745	CA	LEU	62	4.346	-7.095	29.512	1.00	0.00	RX1	C
ATOM	2746	CB	LEU	62	3.606	-8.398	29.233	1.00	0.00	RX1	C
ATOM	2747	CG	LEU	62	2.306	-8.491	30.023	1.00	0.00	RX1	C
ATOM	2748	CD1	LEU	62	1.463	-9.682	29.577	1.00	0.00	RX1	C
ATOM	2749	CD2	LEU	62	2.561	-8.462	31.531	1.00	0.00	RX1	C
ATOM	2750	C	LEU	62	5.574	-7.009	28.642	1.00	0.00	RX1	C
ATOM	2751	O	LEU	62	5.712	-7.659	27.607	1.00	0.00	RX1	O
ATOM	2752	N	THR	63	6.441	-6.112	29.097	1.00	0.00	RX1	N
ATOM	2753	H	THR	63	6.312	-5.571	29.926	1.00	0.00	RX1	H
ATOM	2754	CA	THR	63	7.494	-5.694	28.196	1.00	0.00	RX1	C
ATOM	2755	CB	THR	63	7.103	-4.259	27.942	1.00	0.00	RX1	C
ATOM	2756	OG1	THR	63	5.729	-4.111	28.339	1.00	0.00	RX1	O
ATOM	2757	HG1	THR	63	5.516	-3.193	28.216	1.00	0.00	RX1	H
ATOM	2758	CG2	THR	63	7.300	-3.861	26.486	1.00	0.00	RX1	C
ATOM	2759	C	THR	63	8.874	-5.876	28.782	1.00	0.00	RX1	C
ATOM	2760	O	THR	63	9.097	-5.716	29.974	1.00	0.00	RX1	O
ATOM	2761	N	TYR	64	9.804	-6.234	27.878	1.00	0.00	RX1	N
ATOM	2762	H	TYR	64	9.502	-6.473	26.954	1.00	0.00	RX1	H
ATOM	2763	CA	TYR	64	11.221	-6.335	28.250	1.00	0.00	RX1	C
ATOM	2764	CB	TYR	64	11.794	-4.978	28.658	1.00	0.00	RX1	C
ATOM	2765	CG	TYR	64	11.608	-3.938	27.583	1.00	0.00	RX1	C
ATOM	2766	CD1	TYR	64	12.455	-3.921	26.484	1.00	0.00	RX1	C
ATOM	2767	CE1	TYR	64	12.318	-2.925	25.528	1.00	0.00	RX1	C
ATOM	2768	CD2	TYR	64	10.606	-2.982	27.708	1.00	0.00	RX1	C
ATOM	2769	CE2	TYR	64	10.469	-1.983	26.753	1.00	0.00	RX1	C
ATOM	2770	CZ	TYR	64	11.338	-1.950	25.670	1.00	0.00	RX1	C
ATOM	2771	OH	TYR	64	11.245	-0.952	24.732	1.00	0.00	RX1	O
ATOM	2772	HH	TYR	64	10.526	-0.354	24.919	1.00	0.00	RX1	H
ATOM	2773	C	TYR	64	11.545	-7.319	29.366	1.00	0.00	RX1	C
ATOM	2774	O	TYR	64	12.585	-7.244	30.011	1.00	0.00	RX1	O
ATOM	2775	N	LEU	65	10.597	-8.236	29.592	1.00	0.00	RX1	N
ATOM	2776	H	LEU	65	9.819	-8.366	28.976	1.00	0.00	RX1	H
ATOM	2777	CA	LEU	65	10.710	-9.034	30.807	1.00	0.00	RX1	C
ATOM	2778	CB	LEU	65	9.372	-9.701	31.128	1.00	0.00	RX1	C
ATOM	2779	CG	LEU	65	8.299	-8.641	31.378	1.00	0.00	RX1	C
ATOM	2780	CD1	LEU	65	6.900	-9.236	31.515	1.00	0.00	RX1	C
ATOM	2781	CD2	LEU	65	8.668	-7.749	32.562	1.00	0.00	RX1	C
ATOM	2782	C	LEU	65	11.859	-10.020	30.768	1.00	0.00	RX1	C
ATOM	2783	O	LEU	65	12.048	-10.746	29.796	1.00	0.00	RX1	O
ATOM	2784	N	PRO	66	12.649	-9.980	31.871	1.00	0.00	RX1	N
ATOM	2785	CD	PRO	66	12.473	-9.067	32.994	1.00	0.00	RX1	C
ATOM	2786	CA	PRO	66	13.821	-10.850	32.020	1.00	0.00	RX1	C
ATOM	2787	CB	PRO	66	14.226	-10.584	33.473	1.00	0.00	RX1	C
ATOM	2788	CG	PRO	66	13.793	-9.147	33.747	1.00	0.00	RX1	C
ATOM	2789	C	PRO	66	13.596	-12.323	31.712	1.00	0.00	RX1	C
ATOM	2790	O	PRO	66	12.548	-12.897	31.975	1.00	0.00	RX1	O
ATOM	2791	N	THR	67	14.659	-12.908	31.142	1.00	0.00	RX1	N
ATOM	2792	H	THR	67	15.507	-12.394	31.017	1.00	0.00	RX1	H
ATOM	2793	CA	THR	67	14.615	-14.249	30.556	1.00	0.00	RX1	C
ATOM	2794	CB	THR	67	16.073	-14.595	30.345	1.00	0.00	RX1	C
ATOM	2795	OG1	THR	67	16.781	-13.375	30.095	1.00	0.00	RX1	O
ATOM	2796	HG1	THR	67	16.830	-13.282	29.142	1.00	0.00	RX1	H
ATOM	2797	CG2	THR	67	16.299	-15.623	29.242	1.00	0.00	RX1	C
ATOM	2798	C	THR	67	13.851	-15.325	31.317	1.00	0.00	RX1	C
ATOM	2799	O	THR	67	13.001	-16.040	30.798	1.00	0.00	RX1	O
ATOM	2800	N	ASN	68	14.213	-15.399	32.604	1.00	0.00	RX1	N
ATOM	2801	H	ASN	68	14.788	-14.698	33.024	1.00	0.00	RX1	H
ATOM	2802	CA	ASN	68	13.602	-16.458	33.406	1.00	0.00	RX1	C
ATOM	2803	CB	ASN	68	14.641	-17.378	34.052	1.00	0.00	RX1	C
ATOM	2804	CG	ASN	68	15.514	-18.058	33.022	1.00	0.00	RX1	C

ATOM	2805	OD1	ASN	68	15.060	-18.689	32.071	1.00	0.00	RX1	O
ATOM	2806	ND2	ASN	68	16.825	-17.887	33.264	1.00	0.00	RX1	N
ATOM	2807	HD21	ASN	68	17.148	-17.377	34.061	1.00	0.00	RX1	H
ATOM	2808	HD22	ASN	68	17.509	-18.282	32.645	1.00	0.00	RX1	H
ATOM	2809	C	ASN	68	12.759	-15.878	34.519	1.00	0.00	RX1	C
ATOM	2810	O	ASN	68	12.752	-16.346	35.651	1.00	0.00	RX1	O
ATOM	2811	N	ALA	69	12.090	-14.774	34.170	1.00	0.00	RX1	N
ATOM	2812	H	ALA	69	12.003	-14.473	33.218	1.00	0.00	RX1	H
ATOM	2813	CA	ALA	69	11.389	-14.070	35.235	1.00	0.00	RX1	C
ATOM	2814	CB	ALA	69	11.048	-12.650	34.798	1.00	0.00	RX1	C
ATOM	2815	C	ALA	69	10.116	-14.757	35.681	1.00	0.00	RX1	C
ATOM	2816	O	ALA	69	9.397	-15.376	34.907	1.00	0.00	RX1	O
ATOM	2817	N	SER	70	9.840	-14.594	36.984	1.00	0.00	RX1	N
ATOM	2818	H	SER	70	10.433	-14.114	37.628	1.00	0.00	RX1	H
ATOM	2819	CA	SER	70	8.545	-15.101	37.424	1.00	0.00	RX1	C
ATOM	2820	CB	SER	70	8.693	-15.179	38.927	1.00	0.00	RX1	C
ATOM	2821	OG	SER	70	10.096	-15.021	39.181	1.00	0.00	RX1	O
ATOM	2822	HG	SER	70	10.423	-15.875	39.436	1.00	0.00	RX1	H
ATOM	2823	C	SER	70	7.399	-14.225	36.949	1.00	0.00	RX1	C
ATOM	2824	O	SER	70	7.089	-13.178	37.515	1.00	0.00	RX1	O
ATOM	2825	N	LEU	71	6.799	-14.735	35.865	1.00	0.00	RX1	N
ATOM	2826	H	LEU	71	7.204	-15.539	35.422	1.00	0.00	RX1	H
ATOM	2827	CA	LEU	71	5.601	-14.116	35.301	1.00	0.00	RX1	C
ATOM	2828	CB	LEU	71	5.630	-14.196	33.778	1.00	0.00	RX1	C
ATOM	2829	CG	LEU	71	6.062	-12.893	33.108	1.00	0.00	RX1	C
ATOM	2830	CD1	LEU	71	7.447	-12.423	33.550	1.00	0.00	RX1	C
ATOM	2831	CD2	LEU	71	5.947	-13.003	31.591	1.00	0.00	RX1	C
ATOM	2832	C	LEU	71	4.293	-14.687	35.820	1.00	0.00	RX1	C
ATOM	2833	O	LEU	71	3.218	-14.421	35.303	1.00	0.00	RX1	O
ATOM	2834	N	SER	72	4.443	-15.484	36.888	1.00	0.00	RX1	N
ATOM	2835	H	SER	72	5.359	-15.672	37.227	1.00	0.00	RX1	H
ATOM	2836	CA	SER	72	3.346	-16.164	37.582	1.00	0.00	RX1	C
ATOM	2837	CB	SER	72	3.930	-16.526	38.938	1.00	0.00	RX1	C
ATOM	2838	OG	SER	72	5.355	-16.631	38.766	1.00	0.00	RX1	O
ATOM	2839	HG	SER	72	5.603	-17.420	39.237	1.00	0.00	RX1	H
ATOM	2840	C	SER	72	2.016	-15.418	37.628	1.00	0.00	RX1	C
ATOM	2841	O	SER	72	0.966	-15.909	37.239	1.00	0.00	RX1	O
ATOM	2842	N	PHE	73	2.139	-14.162	38.085	1.00	0.00	RX1	N
ATOM	2843	H	PHE	73	3.043	-13.846	38.362	1.00	0.00	RX1	H
ATOM	2844	CA	PHE	73	0.998	-13.247	38.198	1.00	0.00	RX1	C
ATOM	2845	CB	PHE	73	1.494	-11.837	38.538	1.00	0.00	RX1	C
ATOM	2846	CG	PHE	73	2.338	-11.223	37.440	1.00	0.00	RX1	C
ATOM	2847	CD1	PHE	73	1.788	-10.262	36.602	1.00	0.00	RX1	C
ATOM	2848	CD2	PHE	73	3.672	-11.582	37.281	1.00	0.00	RX1	C
ATOM	2849	CE1	PHE	73	2.569	-9.645	35.633	1.00	0.00	RX1	C
ATOM	2850	CE2	PHE	73	4.456	-10.966	36.313	1.00	0.00	RX1	C
ATOM	2851	CZ	PHE	73	3.907	-9.991	35.491	1.00	0.00	RX1	C
ATOM	2852	C	PHE	73	-0.025	-13.190	37.065	1.00	0.00	RX1	C
ATOM	2853	O	PHE	73	-1.191	-12.871	37.277	1.00	0.00	RX1	O
ATOM	2854	N	LEU	74	0.471	-13.493	35.858	1.00	0.00	RX1	N
ATOM	2855	H	LEU	74	1.395	-13.864	35.756	1.00	0.00	RX1	H
ATOM	2856	CA	LEU	74	-0.376	-13.411	34.672	1.00	0.00	RX1	C
ATOM	2857	CB	LEU	74	0.489	-13.484	33.424	1.00	0.00	RX1	C
ATOM	2858	CG	LEU	74	1.315	-12.224	33.246	1.00	0.00	RX1	C
ATOM	2859	CD1	LEU	74	2.340	-12.380	32.131	1.00	0.00	RX1	C
ATOM	2860	CD2	LEU	74	0.410	-11.017	33.029	1.00	0.00	RX1	C
ATOM	2861	C	LEU	74	-1.500	-14.421	34.571	1.00	0.00	RX1	C
ATOM	2862	O	LEU	74	-2.499	-14.175	33.905	1.00	0.00	RX1	O
ATOM	2863	N	GLN	75	-1.310	-15.553	35.274	1.00	0.00	RX1	N
ATOM	2864	H	GLN	75	-0.455	-15.677	35.780	1.00	0.00	RX1	H
ATOM	2865	CA	GLN	75	-2.229	-16.691	35.146	1.00	0.00	RX1	C

ATOM	2866	CB	GLN	75	-2.011	-17.666	36.306	1.00	0.00	RX1	C
ATOM	2867	CG	GLN	75	-2.080	-16.984	37.676	1.00	0.00	RX1	C
ATOM	2868	CD	GLN	75	-2.086	-18.024	38.772	1.00	0.00	RX1	C
ATOM	2869	OE1	GLN	75	-1.053	-18.523	39.207	1.00	0.00	RX1	O
ATOM	2870	NE2	GLN	75	-3.322	-18.304	39.217	1.00	0.00	RX1	N
ATOM	2871	HE21	GLN	75	-4.124	-17.859	38.796	1.00	0.00	RX1	H
ATOM	2872	HE22	GLN	75	-3.501	-18.936	39.967	1.00	0.00	RX1	H
ATOM	2873	C	GLN	75	-3.717	-16.390	34.974	1.00	0.00	RX1	C
ATOM	2874	O	GLN	75	-4.410	-16.992	34.161	1.00	0.00	RX1	O
ATOM	2875	N	ASP	76	-4.172	-15.417	35.774	1.00	0.00	RX1	N
ATOM	2876	H	ASP	76	-3.550	-14.861	36.322	1.00	0.00	RX1	H
ATOM	2877	CA	ASP	76	-5.616	-15.242	35.865	1.00	0.00	RX1	C
ATOM	2878	CB	ASP	76	-6.080	-15.317	37.322	1.00	0.00	RX1	C
ATOM	2879	CG	ASP	76	-5.871	-16.704	37.893	1.00	0.00	RX1	C
ATOM	2880	OD1	ASP	76	-5.566	-17.635	37.158	1.00	0.00	RX1	O
ATOM	2881	OD2	ASP	76	-6.058	-16.904	39.086	1.00	0.00	RX1	O
ATOM	2882	C	ASP	76	-6.156	-13.973	35.251	1.00	0.00	RX1	C
ATOM	2883	O	ASP	76	-7.290	-13.588	35.518	1.00	0.00	RX1	O
ATOM	2884	N	ILE	77	-5.310	-13.305	34.453	1.00	0.00	RX1	N
ATOM	2885	H	ILE	77	-4.449	-13.697	34.121	1.00	0.00	RX1	H
ATOM	2886	CA	ILE	77	-5.753	-11.970	34.056	1.00	0.00	RX1	C
ATOM	2887	CB	ILE	77	-4.574	-11.061	33.742	1.00	0.00	RX1	C
ATOM	2888	CG2	ILE	77	-5.037	-9.649	33.373	1.00	0.00	RX1	C
ATOM	2889	CG1	ILE	77	-3.658	-11.051	34.962	1.00	0.00	RX1	C
ATOM	2890	CD1	ILE	77	-2.564	-10.002	34.840	1.00	0.00	RX1	C
ATOM	2891	C	ILE	77	-6.824	-11.931	32.983	1.00	0.00	RX1	C
ATOM	2892	O	ILE	77	-6.597	-12.117	31.796	1.00	0.00	RX1	O
ATOM	2893	N	GLN	78	-8.034	-11.699	33.497	1.00	0.00	RX1	N
ATOM	2894	H	GLN	78	-8.078	-11.431	34.462	1.00	0.00	RX1	H
ATOM	2895	CA	GLN	78	-9.208	-11.953	32.671	1.00	0.00	RX1	C
ATOM	2896	CB	GLN	78	-10.382	-12.295	33.576	1.00	0.00	RX1	C
ATOM	2897	CG	GLN	78	-10.168	-13.674	34.190	1.00	0.00	RX1	C
ATOM	2898	CD	GLN	78	-10.773	-13.716	35.570	1.00	0.00	RX1	C
ATOM	2899	OE1	GLN	78	-11.934	-13.374	35.786	1.00	0.00	RX1	O
ATOM	2900	NE2	GLN	78	-9.910	-14.157	36.499	1.00	0.00	RX1	N
ATOM	2901	HE21	GLN	78	-8.952	-14.290	36.217	1.00	0.00	RX1	H
ATOM	2902	HE22	GLN	78	-10.112	-14.336	37.460	1.00	0.00	RX1	H
ATOM	2903	C	GLN	78	-9.556	-10.909	31.631	1.00	0.00	RX1	C
ATOM	2904	O	GLN	78	-9.885	-11.237	30.497	1.00	0.00	RX1	O
ATOM	2905	N	GLU	79	-9.494	-9.640	32.034	1.00	0.00	RX1	N
ATOM	2906	H	GLU	79	-9.263	-9.337	32.962	1.00	0.00	RX1	H
ATOM	2907	CA	GLU	79	-9.784	-8.656	30.997	1.00	0.00	RX1	C
ATOM	2908	CB	GLU	79	-11.257	-8.262	30.972	1.00	0.00	RX1	C
ATOM	2909	CG	GLU	79	-11.780	-7.587	32.230	1.00	0.00	RX1	C
ATOM	2910	CD	GLU	79	-13.239	-7.257	32.014	1.00	0.00	RX1	C
ATOM	2911	OE1	GLU	79	-13.615	-6.113	32.220	1.00	0.00	RX1	O
ATOM	2912	OE2	GLU	79	-14.009	-8.122	31.598	1.00	0.00	RX1	O
ATOM	2913	C	GLU	79	-8.918	-7.434	31.060	1.00	0.00	RX1	C
ATOM	2914	O	GLU	79	-8.597	-6.921	32.123	1.00	0.00	RX1	O
ATOM	2915	N	VAL	80	-8.544	-7.008	29.853	1.00	0.00	RX1	N
ATOM	2916	H	VAL	80	-8.923	-7.421	29.020	1.00	0.00	RX1	H
ATOM	2917	CA	VAL	80	-7.676	-5.842	29.758	1.00	0.00	RX1	C
ATOM	2918	CB	VAL	80	-6.478	-6.183	28.883	1.00	0.00	RX1	C
ATOM	2919	CG1	VAL	80	-5.534	-4.990	28.747	1.00	0.00	RX1	C
ATOM	2920	CG2	VAL	80	-5.793	-7.447	29.399	1.00	0.00	RX1	C
ATOM	2921	C	VAL	80	-8.443	-4.699	29.146	1.00	0.00	RX1	C
ATOM	2922	O	VAL	80	-8.992	-4.845	28.064	1.00	0.00	RX1	O
ATOM	2923	N	GLN	81	-8.479	-3.565	29.855	1.00	0.00	RX1	N
ATOM	2924	H	GLN	81	-7.967	-3.461	30.713	1.00	0.00	RX1	H
ATOM	2925	CA	GLN	81	-9.224	-2.469	29.234	1.00	0.00	RX1	C
ATOM	2926	CB	GLN	81	-9.657	-1.461	30.291	1.00	0.00	RX1	C

ATOM	2927	CG	GLN	81	-10.601	-2.119	31.297	1.00	0.00	RX1	C
ATOM	2928	CD	GLN	81	-11.017	-1.091	32.321	1.00	0.00	RX1	C
ATOM	2929	OE1	GLN	81	-10.469	0.004	32.376	1.00	0.00	RX1	O
ATOM	2930	NE2	GLN	81	-11.994	-1.513	33.143	1.00	0.00	RX1	N
ATOM	2931	HE21	GLN	81	-12.422	-2.411	33.039	1.00	0.00	RX1	H
ATOM	2932	HE22	GLN	81	-12.313	-0.953	33.916	1.00	0.00	RX1	H
ATOM	2933	C	GLN	81	-8.524	-1.810	28.054	1.00	0.00	RX1	C
ATOM	2934	O	GLN	81	-9.115	-1.514	27.018	1.00	0.00	RX1	O
ATOM	2935	N	GLY	82	-7.221	-1.599	28.256	1.00	0.00	RX1	N
ATOM	2936	H	GLY	82	-6.764	-1.958	29.069	1.00	0.00	RX1	H
ATOM	2937	CA	GLY	82	-6.430	-1.050	27.161	1.00	0.00	RX1	C
ATOM	2938	C	GLY	82	-5.999	-2.132	26.196	1.00	0.00	RX1	C
ATOM	2939	O	GLY	82	-6.748	-3.054	25.884	1.00	0.00	RX1	O
ATOM	2940	N	TYR	83	-4.754	-1.975	25.743	1.00	0.00	RX1	N
ATOM	2941	H	TYR	83	-4.152	-1.268	26.120	1.00	0.00	RX1	H
ATOM	2942	CA	TYR	83	-4.212	-3.022	24.884	1.00	0.00	RX1	C
ATOM	2943	CB	TYR	83	-3.418	-2.419	23.715	1.00	0.00	RX1	C
ATOM	2944	CG	TYR	83	-2.357	-1.449	24.177	1.00	0.00	RX1	C
ATOM	2945	CD1	TYR	83	-1.258	-1.890	24.905	1.00	0.00	RX1	C
ATOM	2946	CE1	TYR	83	-0.310	-0.980	25.350	1.00	0.00	RX1	C
ATOM	2947	CD2	TYR	83	-2.481	-0.102	23.861	1.00	0.00	RX1	C
ATOM	2948	CE2	TYR	83	-1.528	0.809	24.297	1.00	0.00	RX1	C
ATOM	2949	CZ	TYR	83	-0.452	0.369	25.056	1.00	0.00	RX1	C
ATOM	2950	OH	TYR	83	0.476	1.269	25.524	1.00	0.00	RX1	O
ATOM	2951	HH	TYR	83	0.742	0.980	26.397	1.00	0.00	RX1	H
ATOM	2952	C	TYR	83	-3.401	-4.034	25.675	1.00	0.00	RX1	C
ATOM	2953	O	TYR	83	-2.990	-3.779	26.803	1.00	0.00	RX1	O
ATOM	2954	N	VAL	84	-3.183	-5.195	25.048	1.00	0.00	RX1	N
ATOM	2955	H	VAL	84	-3.428	-5.317	24.085	1.00	0.00	RX1	H
ATOM	2956	CA	VAL	84	-2.225	-6.111	25.662	1.00	0.00	RX1	C
ATOM	2957	CB	VAL	84	-2.708	-7.557	25.563	1.00	0.00	RX1	C
ATOM	2958	CG1	VAL	84	-1.777	-8.514	26.307	1.00	0.00	RX1	C
ATOM	2959	CG2	VAL	84	-4.138	-7.675	26.071	1.00	0.00	RX1	C
ATOM	2960	C	VAL	84	-0.901	-5.950	24.952	1.00	0.00	RX1	C
ATOM	2961	O	VAL	84	-0.842	-5.949	23.729	1.00	0.00	RX1	O
ATOM	2962	N	LEU	85	0.141	-5.777	25.765	1.00	0.00	RX1	N
ATOM	2963	H	LEU	85	0.084	-5.874	26.761	1.00	0.00	RX1	H
ATOM	2964	CA	LEU	85	1.411	-5.400	25.166	1.00	0.00	RX1	C
ATOM	2965	CB	LEU	85	1.741	-3.985	25.611	1.00	0.00	RX1	C
ATOM	2966	CG	LEU	85	3.003	-3.432	24.969	1.00	0.00	RX1	C
ATOM	2967	CD1	LEU	85	2.932	-3.505	23.447	1.00	0.00	RX1	C
ATOM	2968	CD2	LEU	85	3.308	-2.035	25.497	1.00	0.00	RX1	C
ATOM	2969	C	LEU	85	2.528	-6.353	25.527	1.00	0.00	RX1	C
ATOM	2970	O	LEU	85	3.317	-6.120	26.432	1.00	0.00	RX1	O
ATOM	2971	N	ILE	86	2.557	-7.460	24.790	1.00	0.00	RX1	N
ATOM	2972	H	ILE	86	2.033	-7.516	23.938	1.00	0.00	RX1	H
ATOM	2973	CA	ILE	86	3.612	-8.411	25.113	1.00	0.00	RX1	C
ATOM	2974	CB	ILE	86	3.066	-9.834	25.062	1.00	0.00	RX1	C
ATOM	2975	CG2	ILE	86	4.053	-10.803	25.702	1.00	0.00	RX1	C
ATOM	2976	CG1	ILE	86	1.692	-9.915	25.722	1.00	0.00	RX1	C
ATOM	2977	CD1	ILE	86	1.102	-11.325	25.687	1.00	0.00	RX1	C
ATOM	2978	C	ILE	86	4.791	-8.250	24.171	1.00	0.00	RX1	C
ATOM	2979	O	ILE	86	4.795	-8.762	23.057	1.00	0.00	RX1	O
ATOM	2980	N	ALA	87	5.786	-7.489	24.638	1.00	0.00	RX1	N
ATOM	2981	H	ALA	87	5.820	-7.130	25.576	1.00	0.00	RX1	H
ATOM	2982	CA	ALA	87	6.825	-7.168	23.665	1.00	0.00	RX1	C
ATOM	2983	CB	ALA	87	6.514	-5.826	23.010	1.00	0.00	RX1	C
ATOM	2984	C	ALA	87	8.239	-7.145	24.208	1.00	0.00	RX1	C
ATOM	2985	O	ALA	87	8.474	-6.934	25.392	1.00	0.00	RX1	O
ATOM	2986	N	HIS	88	9.177	-7.374	23.269	1.00	0.00	RX1	N
ATOM	2987	H	HIS	88	8.864	-7.541	22.332	1.00	0.00	RX1	H

ATOM	2988	CA	HIS	88	10.621	-7.379	23.552	1.00	0.00	RX1	C
ATOM	2989	CB	HIS	88	11.189	-6.001	23.880	1.00	0.00	RX1	C
ATOM	2990	CG	HIS	88	10.603	-4.917	23.019	1.00	0.00	RX1	C
ATOM	2991	ND1	HIS	88	10.933	-4.685	21.736	1.00	0.00	RX1	N
ATOM	2992	HD1	HIS	88	11.575	-5.176	21.182	1.00	0.00	RX1	H
ATOM	2993	CD2	HIS	88	9.647	-3.985	23.417	1.00	0.00	RX1	C
ATOM	2994	NE2	HIS	88	9.403	-3.185	22.360	1.00	0.00	RX1	N
ATOM	2995	CE1	HIS	88	10.192	-3.607	21.323	1.00	0.00	RX1	C
ATOM	2996	C	HIS	88	11.055	-8.304	24.671	1.00	0.00	RX1	C
ATOM	2997	O	HIS	88	11.956	-8.019	25.453	1.00	0.00	RX1	O
ATOM	2998	N	ASN	89	10.334	-9.421	24.745	1.00	0.00	RX1	N
ATOM	2999	H	ASN	89	9.700	-9.706	24.027	1.00	0.00	RX1	H
ATOM	3000	CA	ASN	89	10.531	-10.233	25.935	1.00	0.00	RX1	C
ATOM	3001	CB	ASN	89	9.262	-10.978	26.319	1.00	0.00	RX1	C
ATOM	3002	CG	ASN	89	8.318	-9.999	26.962	1.00	0.00	RX1	C
ATOM	3003	OD1	ASN	89	8.558	-9.499	28.055	1.00	0.00	RX1	O
ATOM	3004	ND2	ASN	89	7.239	-9.736	26.215	1.00	0.00	RX1	N
ATOM	3005	HD21	ASN	89	7.149	-10.151	25.304	1.00	0.00	RX1	H
ATOM	3006	HD22	ASN	89	6.540	-9.100	26.554	1.00	0.00	RX1	H
ATOM	3007	C	ASN	89	11.661	-11.210	25.827	1.00	0.00	RX1	C
ATOM	3008	O	ASN	89	11.777	-11.974	24.879	1.00	0.00	RX1	O
ATOM	3009	N	GLN	90	12.475	-11.147	26.884	1.00	0.00	RX1	N
ATOM	3010	H	GLN	90	12.284	-10.515	27.636	1.00	0.00	RX1	H
ATOM	3011	CA	GLN	90	13.459	-12.201	27.090	1.00	0.00	RX1	C
ATOM	3012	CB	GLN	90	14.596	-11.648	27.944	1.00	0.00	RX1	C
ATOM	3013	CG	GLN	90	15.653	-10.888	27.150	1.00	0.00	RX1	C
ATOM	3014	CD	GLN	90	16.705	-11.865	26.674	1.00	0.00	RX1	C
ATOM	3015	OE1	GLN	90	17.124	-12.754	27.415	1.00	0.00	RX1	O
ATOM	3016	NE2	GLN	90	17.115	-11.647	25.415	1.00	0.00	RX1	N
ATOM	3017	HE21	GLN	90	16.734	-10.903	24.869	1.00	0.00	RX1	H
ATOM	3018	HE22	GLN	90	17.774	-12.223	24.920	1.00	0.00	RX1	H
ATOM	3019	C	GLN	90	12.835	-13.439	27.714	1.00	0.00	RX1	C
ATOM	3020	O	GLN	90	13.316	-14.558	27.570	1.00	0.00	RX1	O
ATOM	3021	N	VAL	91	11.736	-13.175	28.442	1.00	0.00	RX1	N
ATOM	3022	H	VAL	91	11.432	-12.234	28.581	1.00	0.00	RX1	H
ATOM	3023	CA	VAL	91	11.042	-14.268	29.111	1.00	0.00	RX1	C
ATOM	3024	CB	VAL	91	9.976	-13.732	30.076	1.00	0.00	RX1	C
ATOM	3025	CG1	VAL	91	8.827	-13.056	29.331	1.00	0.00	RX1	C
ATOM	3026	CG2	VAL	91	9.500	-14.817	31.045	1.00	0.00	RX1	C
ATOM	3027	C	VAL	91	10.488	-15.319	28.165	1.00	0.00	RX1	C
ATOM	3028	O	VAL	91	9.826	-15.044	27.171	1.00	0.00	RX1	O
ATOM	3029	N	ARG	92	10.823	-16.555	28.541	1.00	0.00	RX1	N
ATOM	3030	H	ARG	92	11.350	-16.649	29.388	1.00	0.00	RX1	H
ATOM	3031	CA	ARG	92	10.551	-17.663	27.630	1.00	0.00	RX1	C
ATOM	3032	CB	ARG	92	11.311	-18.900	28.110	1.00	0.00	RX1	C
ATOM	3033	CG	ARG	92	12.753	-18.546	28.492	1.00	0.00	RX1	C
ATOM	3034	CD	ARG	92	13.601	-19.766	28.865	1.00	0.00	RX1	C
ATOM	3035	NE	ARG	92	14.958	-19.413	29.292	1.00	0.00	RX1	N
ATOM	3036	HE	ARG	92	15.107	-19.409	30.291	1.00	0.00	RX1	H
ATOM	3037	CZ	ARG	92	15.938	-19.107	28.387	1.00	0.00	RX1	C
ATOM	3038	NH1	ARG	92	15.626	-18.969	27.080	1.00	0.00	RX1	N
ATOM	3039	HH11	ARG	92	16.306	-18.702	26.380	1.00	0.00	RX1	H
ATOM	3040	HH12	ARG	92	14.692	-19.097	26.743	1.00	0.00	RX1	H
ATOM	3041	NH2	ARG	92	17.208	-18.936	28.805	1.00	0.00	RX1	N
ATOM	3042	HH21	ARG	92	17.951	-18.697	28.167	1.00	0.00	RX1	H
ATOM	3043	HH22	ARG	92	17.475	-19.063	29.769	1.00	0.00	RX1	H
ATOM	3044	C	ARG	92	9.076	-17.938	27.343	1.00	0.00	RX1	C
ATOM	3045	O	ARG	92	8.675	-18.196	26.213	1.00	0.00	RX1	O
ATOM	3046	N	GLN	93	8.266	-17.850	28.409	1.00	0.00	RX1	N
ATOM	3047	H	GLN	93	8.577	-17.568	29.315	1.00	0.00	RX1	H
ATOM	3048	CA	GLN	93	6.834	-18.067	28.204	1.00	0.00	RX1	C

ATOM	3049	CB	GLN	93	6.380	-19.422	28.756	1.00	0.00	RX1	C
ATOM	3050	CG	GLN	93	6.307	-20.570	27.744	1.00	0.00	RX1	C
ATOM	3051	CD	GLN	93	7.676	-21.125	27.410	1.00	0.00	RX1	C
ATOM	3052	OE1	GLN	93	8.674	-20.852	28.069	1.00	0.00	RX1	O
ATOM	3053	NE2	GLN	93	7.659	-21.968	26.364	1.00	0.00	RX1	N
ATOM	3054	HE21	GLN	93	6.819	-22.140	25.830	1.00	0.00	RX1	H
ATOM	3055	HE22	GLN	93	8.460	-22.472	26.053	1.00	0.00	RX1	H
ATOM	3056	C	GLN	93	6.006	-16.989	28.871	1.00	0.00	RX1	C
ATOM	3057	O	GLN	93	6.401	-16.421	29.882	1.00	0.00	RX1	O
ATOM	3058	N	VAL	94	4.823	-16.757	28.278	1.00	0.00	RX1	N
ATOM	3059	H	VAL	94	4.550	-17.226	27.437	1.00	0.00	RX1	H
ATOM	3060	CA	VAL	94	3.831	-15.939	28.981	1.00	0.00	RX1	C
ATOM	3061	CB	VAL	94	3.436	-14.718	28.141	1.00	0.00	RX1	C
ATOM	3062	CG1	VAL	94	2.234	-13.975	28.728	1.00	0.00	RX1	C
ATOM	3063	CG2	VAL	94	4.627	-13.774	27.991	1.00	0.00	RX1	C
ATOM	3064	C	VAL	94	2.607	-16.766	29.354	1.00	0.00	RX1	C
ATOM	3065	O	VAL	94	1.767	-17.080	28.516	1.00	0.00	RX1	O
ATOM	3066	N	PRO	95	2.550	-17.117	30.660	1.00	0.00	RX1	N
ATOM	3067	CD	PRO	95	3.559	-16.829	31.672	1.00	0.00	RX1	C
ATOM	3068	CA	PRO	95	1.408	-17.876	31.179	1.00	0.00	RX1	C
ATOM	3069	CB	PRO	95	2.019	-18.492	32.441	1.00	0.00	RX1	C
ATOM	3070	CG	PRO	95	3.004	-17.438	32.955	1.00	0.00	RX1	C
ATOM	3071	C	PRO	95	0.212	-16.976	31.471	1.00	0.00	RX1	C
ATOM	3072	O	PRO	95	0.010	-16.485	32.576	1.00	0.00	RX1	O
ATOM	3073	N	LEU	96	-0.571	-16.749	30.418	1.00	0.00	RX1	N
ATOM	3074	H	LEU	96	-0.464	-17.249	29.555	1.00	0.00	RX1	H
ATOM	3075	CA	LEU	96	-1.721	-15.868	30.598	1.00	0.00	RX1	C
ATOM	3076	CB	LEU	96	-1.613	-14.696	29.632	1.00	0.00	RX1	C
ATOM	3077	CG	LEU	96	-1.735	-13.301	30.241	1.00	0.00	RX1	C
ATOM	3078	CD1	LEU	96	-1.478	-12.233	29.179	1.00	0.00	RX1	C
ATOM	3079	CD2	LEU	96	-3.066	-13.073	30.953	1.00	0.00	RX1	C
ATOM	3080	C	LEU	96	-3.024	-16.612	30.377	1.00	0.00	RX1	C
ATOM	3081	O	LEU	96	-3.922	-16.184	29.660	1.00	0.00	RX1	O
ATOM	3082	N	GLN	97	-3.073	-17.787	31.017	1.00	0.00	RX1	N
ATOM	3083	H	GLN	97	-2.319	-18.036	31.628	1.00	0.00	RX1	H
ATOM	3084	CA	GLN	97	-4.119	-18.759	30.697	1.00	0.00	RX1	C
ATOM	3085	CB	GLN	97	-4.023	-19.968	31.630	1.00	0.00	RX1	C
ATOM	3086	CG	GLN	97	-2.977	-21.014	31.223	1.00	0.00	RX1	C
ATOM	3087	CD	GLN	97	-1.569	-20.457	31.268	1.00	0.00	RX1	C
ATOM	3088	OE1	GLN	97	-1.232	-19.614	32.095	1.00	0.00	RX1	O
ATOM	3089	NE2	GLN	97	-0.762	-21.001	30.348	1.00	0.00	RX1	N
ATOM	3090	HE21	GLN	97	-1.144	-21.612	29.642	1.00	0.00	RX1	H
ATOM	3091	HE22	GLN	97	0.227	-20.850	30.259	1.00	0.00	RX1	H
ATOM	3092	C	GLN	97	-5.540	-18.217	30.692	1.00	0.00	RX1	C
ATOM	3093	O	GLN	97	-6.321	-18.422	29.766	1.00	0.00	RX1	O
ATOM	3094	N	ARG	98	-5.852	-17.499	31.773	1.00	0.00	RX1	N
ATOM	3095	H	ARG	98	-5.166	-17.267	32.464	1.00	0.00	RX1	H
ATOM	3096	CA	ARG	98	-7.214	-16.991	31.855	1.00	0.00	RX1	C
ATOM	3097	CB	ARG	98	-7.778	-17.163	33.266	1.00	0.00	RX1	C
ATOM	3098	CG	ARG	98	-7.949	-18.656	33.557	1.00	0.00	RX1	C
ATOM	3099	CD	ARG	98	-8.722	-18.973	34.838	1.00	0.00	RX1	C
ATOM	3100	NE	ARG	98	-7.941	-18.724	36.047	1.00	0.00	RX1	N
ATOM	3101	HE	ARG	98	-7.107	-18.152	35.992	1.00	0.00	RX1	H
ATOM	3102	CZ	ARG	98	-8.351	-19.272	37.230	1.00	0.00	RX1	C
ATOM	3103	NH1	ARG	98	-9.460	-20.047	37.247	1.00	0.00	RX1	N
ATOM	3104	HH11	ARG	98	-9.805	-20.465	38.091	1.00	0.00	RX1	H
ATOM	3105	HH12	ARG	98	-9.979	-20.229	36.408	1.00	0.00	RX1	H
ATOM	3106	NH2	ARG	98	-7.654	-19.036	38.358	1.00	0.00	RX1	N
ATOM	3107	HH21	ARG	98	-7.864	-19.394	39.267	1.00	0.00	RX1	H
ATOM	3108	HH22	ARG	98	-6.839	-18.424	38.319	1.00	0.00	RX1	H
ATOM	3109	C	ARG	98	-7.455	-15.603	31.293	1.00	0.00	RX1	C

ATOM	3110	O	ARG	98	-8.392	-14.918	31.687	1.00	0.00	RX1	O
ATOM	3111	N	LEU	99	-6.611	-15.230	30.314	1.00	0.00	RX1	N
ATOM	3112	H	LEU	99	-5.855	-15.809	30.007	1.00	0.00	RX1	H
ATOM	3113	CA	LEU	99	-6.992	-14.068	29.513	1.00	0.00	RX1	C
ATOM	3114	CB	LEU	99	-5.874	-13.665	28.550	1.00	0.00	RX1	C
ATOM	3115	CG	LEU	99	-6.191	-12.412	27.726	1.00	0.00	RX1	C
ATOM	3116	CD1	LEU	99	-6.563	-11.212	28.599	1.00	0.00	RX1	C
ATOM	3117	CD2	LEU	99	-5.055	-12.073	26.761	1.00	0.00	RX1	C
ATOM	3118	C	LEU	99	-8.267	-14.348	28.751	1.00	0.00	RX1	C
ATOM	3119	O	LEU	99	-8.335	-15.247	27.930	1.00	0.00	RX1	O
ATOM	3120	N	ARG	100	-9.284	-13.569	29.103	1.00	0.00	RX1	N
ATOM	3121	H	ARG	100	-9.123	-12.818	29.742	1.00	0.00	RX1	H
ATOM	3122	CA	ARG	100	-10.598	-13.797	28.517	1.00	0.00	RX1	C
ATOM	3123	CB	ARG	100	-11.622	-13.766	29.660	1.00	0.00	RX1	C
ATOM	3124	CG	ARG	100	-13.072	-13.453	29.294	1.00	0.00	RX1	C
ATOM	3125	CD	ARG	100	-13.987	-13.443	30.528	1.00	0.00	RX1	C
ATOM	3126	NE	ARG	100	-13.438	-12.642	31.628	1.00	0.00	RX1	N
ATOM	3127	HE	ARG	100	-12.826	-13.135	32.256	1.00	0.00	RX1	H
ATOM	3128	CZ	ARG	100	-13.728	-11.307	31.740	1.00	0.00	RX1	C
ATOM	3129	NH1	ARG	100	-14.533	-10.718	30.837	1.00	0.00	RX1	N
ATOM	3130	HH11	ARG	100	-14.685	-9.718	30.893	1.00	0.00	RX1	H
ATOM	3131	HH12	ARG	100	-14.980	-11.221	30.094	1.00	0.00	RX1	H
ATOM	3132	NH2	ARG	100	-13.213	-10.569	32.746	1.00	0.00	RX1	N
ATOM	3133	HH21	ARG	100	-13.381	-9.569	32.767	1.00	0.00	RX1	H
ATOM	3134	HH22	ARG	100	-12.649	-10.947	33.486	1.00	0.00	RX1	H
ATOM	3135	C	ARG	100	-10.893	-12.820	27.392	1.00	0.00	RX1	C
ATOM	3136	O	ARG	100	-11.257	-13.198	26.286	1.00	0.00	RX1	O
ATOM	3137	N	ILE	101	-10.718	-11.531	27.713	1.00	0.00	RX1	N
ATOM	3138	H	ILE	101	-10.358	-11.215	28.594	1.00	0.00	RX1	H
ATOM	3139	CA	ILE	101	-11.106	-10.553	26.703	1.00	0.00	RX1	C
ATOM	3140	CB	ILE	101	-12.601	-10.226	26.851	1.00	0.00	RX1	C
ATOM	3141	CG2	ILE	101	-12.917	-9.620	28.207	1.00	0.00	RX1	C
ATOM	3142	CG1	ILE	101	-13.156	-9.346	25.741	1.00	0.00	RX1	C
ATOM	3143	CD1	ILE	101	-14.657	-9.156	25.947	1.00	0.00	RX1	C
ATOM	3144	C	ILE	101	-10.216	-9.324	26.701	1.00	0.00	RX1	C
ATOM	3145	O	ILE	101	-9.916	-8.720	27.728	1.00	0.00	RX1	O
ATOM	3146	N	VAL	102	-9.788	-8.987	25.481	1.00	0.00	RX1	N
ATOM	3147	H	VAL	102	-10.161	-9.452	24.673	1.00	0.00	RX1	H
ATOM	3148	CA	VAL	102	-9.120	-7.697	25.345	1.00	0.00	RX1	C
ATOM	3149	CB	VAL	102	-8.067	-7.764	24.238	1.00	0.00	RX1	C
ATOM	3150	CG1	VAL	102	-7.258	-6.468	24.144	1.00	0.00	RX1	C
ATOM	3151	CG2	VAL	102	-7.181	-8.995	24.417	1.00	0.00	RX1	C
ATOM	3152	C	VAL	102	-10.185	-6.678	25.003	1.00	0.00	RX1	C
ATOM	3153	O	VAL	102	-11.075	-6.956	24.212	1.00	0.00	RX1	O
ATOM	3154	N	ARG	103	-10.093	-5.507	25.634	1.00	0.00	RX1	N
ATOM	3155	H	ARG	103	-9.340	-5.288	26.257	1.00	0.00	RX1	H
ATOM	3156	CA	ARG	103	-11.084	-4.505	25.264	1.00	0.00	RX1	C
ATOM	3157	CB	ARG	103	-11.606	-3.760	26.493	1.00	0.00	RX1	C
ATOM	3158	CG	ARG	103	-12.243	-4.720	27.505	1.00	0.00	RX1	C
ATOM	3159	CD	ARG	103	-12.870	-3.991	28.695	1.00	0.00	RX1	C
ATOM	3160	NE	ARG	103	-13.561	-4.913	29.597	1.00	0.00	RX1	N
ATOM	3161	HE	ARG	103	-13.041	-5.362	30.340	1.00	0.00	RX1	H
ATOM	3162	CZ	ARG	103	-14.898	-5.152	29.455	1.00	0.00	RX1	C
ATOM	3163	NH1	ARG	103	-15.587	-4.514	28.481	1.00	0.00	RX1	N
ATOM	3164	HH11	ARG	103	-16.570	-4.650	28.338	1.00	0.00	RX1	H
ATOM	3165	HH12	ARG	103	-15.127	-3.872	27.862	1.00	0.00	RX1	H
ATOM	3166	NH2	ARG	103	-15.507	-6.015	30.294	1.00	0.00	RX1	N
ATOM	3167	HH21	ARG	103	-16.478	-6.252	30.277	1.00	0.00	RX1	H
ATOM	3168	HH22	ARG	103	-14.948	-6.462	31.022	1.00	0.00	RX1	H
ATOM	3169	C	ARG	103	-10.606	-3.572	24.169	1.00	0.00	RX1	C
ATOM	3170	O	ARG	103	-11.326	-3.286	23.220	1.00	0.00	RX1	O

ATOM	3171	N	GLY	104	-9.338	-3.156	24.311	1.00	0.00	RX1	N
ATOM	3172	H	GLY	104	-8.805	-3.291	25.149	1.00	0.00	RX1	H
ATOM	3173	CA	GLY	104	-8.759	-2.404	23.199	1.00	0.00	RX1	C
ATOM	3174	C	GLY	104	-9.223	-0.965	23.106	1.00	0.00	RX1	C
ATOM	3175	O	GLY	104	-9.523	-0.427	22.049	1.00	0.00	RX1	O
ATOM	3176	N	THR	105	-9.249	-0.345	24.294	1.00	0.00	RX1	N
ATOM	3177	H	THR	105	-8.984	-0.809	25.138	1.00	0.00	RX1	H
ATOM	3178	CA	THR	105	-9.631	1.069	24.299	1.00	0.00	RX1	C
ATOM	3179	CB	THR	105	-10.040	1.413	25.726	1.00	0.00	RX1	C
ATOM	3180	OG1	THR	105	-10.910	0.379	26.211	1.00	0.00	RX1	O
ATOM	3181	HG1	THR	105	-10.361	-0.374	26.390	1.00	0.00	RX1	H
ATOM	3182	CG2	THR	105	-10.713	2.783	25.835	1.00	0.00	RX1	C
ATOM	3183	C	THR	105	-8.583	2.002	23.687	1.00	0.00	RX1	C
ATOM	3184	O	THR	105	-8.880	3.054	23.135	1.00	0.00	RX1	O
ATOM	3185	N	GLN	106	-7.325	1.552	23.800	1.00	0.00	RX1	N
ATOM	3186	H	GLN	106	-7.085	0.638	24.122	1.00	0.00	RX1	H
ATOM	3187	CA	GLN	106	-6.247	2.329	23.196	1.00	0.00	RX1	C
ATOM	3188	CB	GLN	106	-5.487	3.093	24.287	1.00	0.00	RX1	C
ATOM	3189	CG	GLN	106	-4.830	2.192	25.334	1.00	0.00	RX1	C
ATOM	3190	CD	GLN	106	-4.624	2.974	26.618	1.00	0.00	RX1	C
ATOM	3191	OE1	GLN	106	-5.581	3.214	27.348	1.00	0.00	RX1	O
ATOM	3192	NE2	GLN	106	-3.353	3.366	26.836	1.00	0.00	RX1	N
ATOM	3193	HE21	GLN	106	-2.583	3.114	26.249	1.00	0.00	RX1	H
ATOM	3194	HE22	GLN	106	-3.053	3.936	27.614	1.00	0.00	RX1	H
ATOM	3195	C	GLN	106	-5.357	1.414	22.385	1.00	0.00	RX1	C
ATOM	3196	O	GLN	106	-5.426	0.200	22.537	1.00	0.00	RX1	O
ATOM	3197	N	LEU	107	-4.583	2.037	21.482	1.00	0.00	RX1	N
ATOM	3198	H	LEU	107	-4.388	3.016	21.503	1.00	0.00	RX1	H
ATOM	3199	CA	LEU	107	-4.007	1.228	20.410	1.00	0.00	RX1	C
ATOM	3200	CB	LEU	107	-4.536	1.684	19.048	1.00	0.00	RX1	C
ATOM	3201	CG	LEU	107	-6.059	1.632	18.914	1.00	0.00	RX1	C
ATOM	3202	CD1	LEU	107	-6.519	2.239	17.589	1.00	0.00	RX1	C
ATOM	3203	CD2	LEU	107	-6.617	0.222	19.118	1.00	0.00	RX1	C
ATOM	3204	C	LEU	107	-2.490	1.191	20.374	1.00	0.00	RX1	C
ATOM	3205	O	LEU	107	-1.798	1.929	21.067	1.00	0.00	RX1	O
ATOM	3206	N	PHE	108	-2.024	0.284	19.506	1.00	0.00	RX1	N
ATOM	3207	H	PHE	108	-2.648	-0.326	19.018	1.00	0.00	RX1	H
ATOM	3208	CA	PHE	108	-0.598	0.080	19.293	1.00	0.00	RX1	C
ATOM	3209	CB	PHE	108	-0.180	-1.155	20.063	1.00	0.00	RX1	C
ATOM	3210	CG	PHE	108	1.103	-0.841	20.769	1.00	0.00	RX1	C
ATOM	3211	CD1	PHE	108	1.056	-0.320	22.052	1.00	0.00	RX1	C
ATOM	3212	CD2	PHE	108	2.318	-1.056	20.136	1.00	0.00	RX1	C
ATOM	3213	CE1	PHE	108	2.234	0.014	22.698	1.00	0.00	RX1	C
ATOM	3214	CE2	PHE	108	3.494	-0.720	20.785	1.00	0.00	RX1	C
ATOM	3215	CZ	PHE	108	3.450	-0.167	22.057	1.00	0.00	RX1	C
ATOM	3216	C	PHE	108	-0.304	-0.083	17.815	1.00	0.00	RX1	C
ATOM	3217	O	PHE	108	-1.198	-0.506	17.082	1.00	0.00	RX1	O
ATOM	3218	N	GLU	109	0.931	0.309	17.406	1.00	0.00	RX1	N
ATOM	3219	H	GLU	109	1.625	0.562	18.081	1.00	0.00	RX1	H
ATOM	3220	CA	GLU	109	1.266	0.582	15.993	1.00	0.00	RX1	C
ATOM	3221	CB	GLU	109	2.124	-0.542	15.357	1.00	0.00	RX1	C
ATOM	3222	CG	GLU	109	3.501	-0.680	16.052	1.00	0.00	RX1	C
ATOM	3223	CD	GLU	109	4.443	-1.714	15.419	1.00	0.00	RX1	C
ATOM	3224	OE1	GLU	109	4.075	-2.875	15.272	1.00	0.00	RX1	O
ATOM	3225	OE2	GLU	109	5.597	-1.395	15.125	1.00	0.00	RX1	O
ATOM	3226	C	GLU	109	0.067	1.061	15.168	1.00	0.00	RX1	C
ATOM	3227	O	GLU	109	-0.538	0.383	14.340	1.00	0.00	RX1	O
ATOM	3228	N	ASP	110	-0.309	2.284	15.580	1.00	0.00	RX1	N
ATOM	3229	H	ASP	110	0.385	2.842	16.033	1.00	0.00	RX1	H
ATOM	3230	CA	ASP	110	-1.647	2.490	16.150	1.00	0.00	RX1	C
ATOM	3231	CB	ASP	110	-1.768	3.892	16.750	1.00	0.00	RX1	C

ATOM	3232	CG	ASP	110	-0.961	3.967	18.034	1.00	0.00	RX1	C
ATOM	3233	OD1	ASP	110	-1.142	4.909	18.801	1.00	0.00	RX1	O
ATOM	3234	OD2	ASP	110	-0.146	3.084	18.291	1.00	0.00	RX1	O
ATOM	3235	C	ASP	110	-2.900	2.123	15.385	1.00	0.00	RX1	C
ATOM	3236	O	ASP	110	-3.661	2.951	14.905	1.00	0.00	RX1	O
ATOM	3237	N	ASN	111	-3.110	0.802	15.357	1.00	0.00	RX1	N
ATOM	3238	H	ASN	111	-2.365	0.192	15.639	1.00	0.00	RX1	H
ATOM	3239	CA	ASN	111	-4.384	0.263	14.873	1.00	0.00	RX1	C
ATOM	3240	CB	ASN	111	-4.299	-0.263	13.436	1.00	0.00	RX1	C
ATOM	3241	CG	ASN	111	-3.992	0.806	12.411	1.00	0.00	RX1	C
ATOM	3242	OD1	ASN	111	-4.867	1.285	11.698	1.00	0.00	RX1	O
ATOM	3243	ND2	ASN	111	-2.681	1.113	12.329	1.00	0.00	RX1	N
ATOM	3244	HD21	ASN	111	-2.002	0.705	12.949	1.00	0.00	RX1	H
ATOM	3245	HD22	ASN	111	-2.347	1.780	11.664	1.00	0.00	RX1	H
ATOM	3246	C	ASN	111	-4.862	-0.907	15.718	1.00	0.00	RX1	C
ATOM	3247	O	ASN	111	-5.905	-1.513	15.492	1.00	0.00	RX1	O
ATOM	3248	N	TYR	112	-4.007	-1.269	16.684	1.00	0.00	RX1	N
ATOM	3249	H	TYR	112	-3.207	-0.745	16.978	1.00	0.00	RX1	H
ATOM	3250	CA	TYR	112	-4.176	-2.619	17.202	1.00	0.00	RX1	C
ATOM	3251	CB	TYR	112	-3.008	-3.482	16.764	1.00	0.00	RX1	C
ATOM	3252	CG	TYR	112	-2.836	-3.464	15.264	1.00	0.00	RX1	C
ATOM	3253	CD1	TYR	112	-3.650	-4.259	14.470	1.00	0.00	RX1	C
ATOM	3254	CE1	TYR	112	-3.494	-4.254	13.092	1.00	0.00	RX1	C
ATOM	3255	CD2	TYR	112	-1.860	-2.666	14.678	1.00	0.00	RX1	C
ATOM	3256	CE2	TYR	112	-1.705	-2.657	13.296	1.00	0.00	RX1	C
ATOM	3257	CZ	TYR	112	-2.534	-3.443	12.504	1.00	0.00	RX1	C
ATOM	3258	OH	TYR	112	-2.414	-3.416	11.130	1.00	0.00	RX1	O
ATOM	3259	HH	TYR	112	-1.702	-2.836	10.892	1.00	0.00	RX1	H
ATOM	3260	C	TYR	112	-4.296	-2.696	18.704	1.00	0.00	RX1	C
ATOM	3261	O	TYR	112	-3.825	-1.829	19.425	1.00	0.00	RX1	O
ATOM	3262	N	ALA	113	-4.940	-3.784	19.135	1.00	0.00	RX1	N
ATOM	3263	H	ALA	113	-5.274	-4.474	18.494	1.00	0.00	RX1	H
ATOM	3264	CA	ALA	113	-5.056	-4.019	20.569	1.00	0.00	RX1	C
ATOM	3265	CB	ALA	113	-6.504	-4.321	20.937	1.00	0.00	RX1	C
ATOM	3266	C	ALA	113	-4.193	-5.162	21.070	1.00	0.00	RX1	C
ATOM	3267	O	ALA	113	-3.689	-5.161	22.187	1.00	0.00	RX1	O
ATOM	3268	N	LEU	114	-4.036	-6.168	20.201	1.00	0.00	RX1	N
ATOM	3269	H	LEU	114	-4.374	-6.134	19.259	1.00	0.00	RX1	H
ATOM	3270	CA	LEU	114	-3.199	-7.274	20.654	1.00	0.00	RX1	C
ATOM	3271	CB	LEU	114	-3.786	-8.612	20.215	1.00	0.00	RX1	C
ATOM	3272	CG	LEU	114	-3.084	-9.787	20.895	1.00	0.00	RX1	C
ATOM	3273	CD1	LEU	114	-3.283	-9.774	22.411	1.00	0.00	RX1	C
ATOM	3274	CD2	LEU	114	-3.489	-11.119	20.274	1.00	0.00	RX1	C
ATOM	3275	C	LEU	114	-1.767	-7.137	20.177	1.00	0.00	RX1	C
ATOM	3276	O	LEU	114	-1.362	-7.715	19.175	1.00	0.00	RX1	O
ATOM	3277	N	ALA	115	-1.030	-6.313	20.922	1.00	0.00	RX1	N
ATOM	3278	H	ALA	115	-1.334	-5.991	21.823	1.00	0.00	RX1	H
ATOM	3279	CA	ALA	115	0.322	-6.014	20.472	1.00	0.00	RX1	C
ATOM	3280	CB	ALA	115	0.674	-4.571	20.790	1.00	0.00	RX1	C
ATOM	3281	C	ALA	115	1.380	-6.910	21.076	1.00	0.00	RX1	C
ATOM	3282	O	ALA	115	1.876	-6.707	22.178	1.00	0.00	RX1	O
ATOM	3283	N	VAL	116	1.710	-7.930	20.288	1.00	0.00	RX1	N
ATOM	3284	H	VAL	116	1.342	-8.009	19.360	1.00	0.00	RX1	H
ATOM	3285	CA	VAL	116	2.817	-8.782	20.700	1.00	0.00	RX1	C
ATOM	3286	CB	VAL	116	2.349	-10.235	20.754	1.00	0.00	RX1	C
ATOM	3287	CG1	VAL	116	3.453	-11.158	21.262	1.00	0.00	RX1	C
ATOM	3288	CG2	VAL	116	1.064	-10.365	21.574	1.00	0.00	RX1	C
ATOM	3289	C	VAL	116	3.973	-8.596	19.732	1.00	0.00	RX1	C
ATOM	3290	O	VAL	116	3.904	-8.997	18.577	1.00	0.00	RX1	O
ATOM	3291	N	LEU	117	5.010	-7.910	20.230	1.00	0.00	RX1	N
ATOM	3292	H	LEU	117	5.119	-7.757	21.214	1.00	0.00	RX1	H

ATOM	3293	CA	LEU	117	5.980	-7.352	19.283	1.00	0.00	RX1	C
ATOM	3294	CB	LEU	117	5.874	-5.829	19.251	1.00	0.00	RX1	C
ATOM	3295	CG	LEU	117	4.506	-5.298	18.852	1.00	0.00	RX1	C
ATOM	3296	CD1	LEU	117	4.424	-3.782	19.013	1.00	0.00	RX1	C
ATOM	3297	CD2	LEU	117	4.146	-5.741	17.441	1.00	0.00	RX1	C
ATOM	3298	C	LEU	117	7.423	-7.676	19.595	1.00	0.00	RX1	C
ATOM	3299	O	LEU	117	7.800	-7.834	20.751	1.00	0.00	RX1	O
ATOM	3300	N	ASP	118	8.221	-7.715	18.512	1.00	0.00	RX1	N
ATOM	3301	H	ASP	118	7.797	-7.692	17.604	1.00	0.00	RX1	H
ATOM	3302	CA	ASP	118	9.689	-7.642	18.621	1.00	0.00	RX1	C
ATOM	3303	CB	ASP	118	10.152	-6.187	18.823	1.00	0.00	RX1	C
ATOM	3304	CG	ASP	118	9.819	-5.262	17.657	1.00	0.00	RX1	C
ATOM	3305	OD1	ASP	118	10.565	-5.224	16.684	1.00	0.00	RX1	O
ATOM	3306	OD2	ASP	118	8.836	-4.523	17.713	1.00	0.00	RX1	O
ATOM	3307	C	ASP	118	10.314	-8.530	19.698	1.00	0.00	RX1	C
ATOM	3308	O	ASP	118	11.147	-8.113	20.499	1.00	0.00	RX1	O
ATOM	3309	N	ASN	119	9.832	-9.782	19.731	1.00	0.00	RX1	N
ATOM	3310	H	ASN	119	9.276	-10.120	18.968	1.00	0.00	RX1	H
ATOM	3311	CA	ASN	119	10.020	-10.531	20.980	1.00	0.00	RX1	C
ATOM	3312	CB	ASN	119	8.838	-11.448	21.285	1.00	0.00	RX1	C
ATOM	3313	CG	ASN	119	7.847	-10.745	22.192	1.00	0.00	RX1	C
ATOM	3314	OD1	ASN	119	8.097	-10.469	23.363	1.00	0.00	RX1	O
ATOM	3315	ND2	ASN	119	6.688	-10.467	21.584	1.00	0.00	RX1	N
ATOM	3316	HD21	ASN	119	6.570	-10.727	20.620	1.00	0.00	RX1	H
ATOM	3317	HD22	ASN	119	5.933	-9.986	22.034	1.00	0.00	RX1	H
ATOM	3318	C	ASN	119	11.305	-11.312	21.206	1.00	0.00	RX1	C
ATOM	3319	O	ASN	119	11.319	-12.539	21.210	1.00	0.00	RX1	O
ATOM	3320	N	GLY	120	12.362	-10.532	21.479	1.00	0.00	RX1	N
ATOM	3321	H	GLY	120	12.326	-9.561	21.238	1.00	0.00	RX1	H
ATOM	3322	CA	GLY	120	13.552	-11.119	22.103	1.00	0.00	RX1	C
ATOM	3323	C	GLY	120	14.513	-11.850	21.179	1.00	0.00	RX1	C
ATOM	3324	O	GLY	120	14.439	-11.758	19.958	1.00	0.00	RX1	O
ATOM	3325	N	ASP	121	15.441	-12.567	21.837	1.00	0.00	RX1	N
ATOM	3326	H	ASP	121	15.385	-12.666	22.831	1.00	0.00	RX1	H
ATOM	3327	CA	ASP	121	16.464	-13.316	21.099	1.00	0.00	RX1	C
ATOM	3328	CB	ASP	121	17.483	-13.994	22.038	1.00	0.00	RX1	C
ATOM	3329	CG	ASP	121	18.438	-13.096	22.823	1.00	0.00	RX1	C
ATOM	3330	OD1	ASP	121	18.749	-11.995	22.394	1.00	0.00	RX1	O
ATOM	3331	OD2	ASP	121	18.923	-13.518	23.874	1.00	0.00	RX1	O
ATOM	3332	C	ASP	121	15.872	-14.425	20.234	1.00	0.00	RX1	C
ATOM	3333	O	ASP	121	15.037	-15.211	20.670	1.00	0.00	RX1	O
ATOM	3334	N	PRO	122	16.335	-14.474	18.965	1.00	0.00	RX1	N
ATOM	3335	CD	PRO	122	17.239	-13.515	18.350	1.00	0.00	RX1	C
ATOM	3336	CA	PRO	122	15.862	-15.506	18.031	1.00	0.00	RX1	C
ATOM	3337	CB	PRO	122	16.425	-15.018	16.688	1.00	0.00	RX1	C
ATOM	3338	CG	PRO	122	16.829	-13.556	16.885	1.00	0.00	RX1	C
ATOM	3339	C	PRO	122	16.308	-16.942	18.317	1.00	0.00	RX1	C
ATOM	3340	O	PRO	122	17.149	-17.494	17.616	1.00	0.00	RX1	O
ATOM	3341	N	LEU	123	15.708	-17.566	19.342	1.00	0.00	RX1	N
ATOM	3342	H	LEU	123	15.025	-17.103	19.913	1.00	0.00	RX1	H
ATOM	3343	CA	LEU	123	15.967	-19.003	19.449	1.00	0.00	RX1	C
ATOM	3344	CB	LEU	123	15.796	-19.464	20.902	1.00	0.00	RX1	C
ATOM	3345	CG	LEU	123	16.004	-20.948	21.223	1.00	0.00	RX1	C
ATOM	3346	CD1	LEU	123	17.448	-21.404	21.018	1.00	0.00	RX1	C
ATOM	3347	CD2	LEU	123	15.496	-21.288	22.625	1.00	0.00	RX1	C
ATOM	3348	C	LEU	123	15.108	-19.801	18.476	1.00	0.00	RX1	C
ATOM	3349	O	LEU	123	13.993	-19.428	18.124	1.00	0.00	RX1	O
ATOM	3350	N	ASN	124	15.710	-20.909	18.028	1.00	0.00	RX1	N
ATOM	3351	H	ASN	124	16.582	-21.208	18.417	1.00	0.00	RX1	H
ATOM	3352	CA	ASN	124	14.968	-21.846	17.189	1.00	0.00	RX1	C
ATOM	3353	CB	ASN	124	15.930	-22.575	16.248	1.00	0.00	RX1	C

ATOM	3354	CG	ASN	124	16.744	-23.554	17.069	1.00	0.00	RX1 C
ATOM	3355	OD1	ASN	124	17.243	-23.226	18.142	1.00	0.00	RX1 O
ATOM	3356	ND2	ASN	124	16.820	-24.789	16.541	1.00	0.00	RX1 N
ATOM	3357	HD21	ASN	124	16.471	-24.997	15.626	1.00	0.00	RX1 H
ATOM	3358	HD22	ASN	124	17.249	-25.537	17.058	1.00	0.00	RX1 H
ATOM	3359	C	ASN	124	14.199	-22.873	18.019	1.00	0.00	RX1 C
ATOM	3360	O	ASN	124	13.952	-22.673	19.202	1.00	0.00	RX1 O
ATOM	3361	N	ASN	125	13.831	-23.995	17.368	1.00	0.00	RX1 N
ATOM	3362	H	ASN	125	13.929	-24.079	16.377	1.00	0.00	RX1 H
ATOM	3363	CA	ASN	125	13.040	-25.005	18.081	1.00	0.00	RX1 C
ATOM	3364	CB	ASN	125	12.660	-26.202	17.212	1.00	0.00	RX1 C
ATOM	3365	CG	ASN	125	11.485	-25.812	16.358	1.00	0.00	RX1 C
ATOM	3366	OD1	ASN	125	11.654	-25.111	15.367	1.00	0.00	RX1 O
ATOM	3367	ND2	ASN	125	10.295	-26.241	16.812	1.00	0.00	RX1 N
ATOM	3368	HD21	ASN	125	10.213	-26.869	17.592	1.00	0.00	RX1 H
ATOM	3369	HD22	ASN	125	9.426	-25.955	16.400	1.00	0.00	RX1 H
ATOM	3370	C	ASN	125	13.569	-25.555	19.386	1.00	0.00	RX1 C
ATOM	3371	O	ASN	125	12.780	-25.992	20.219	1.00	0.00	RX1 O
ATOM	3372	N	THR	126	14.905	-25.527	19.537	1.00	0.00	RX1 N
ATOM	3373	H	THR	126	15.522	-25.129	18.861	1.00	0.00	RX1 H
ATOM	3374	CA	THR	126	15.537	-26.054	20.753	1.00	0.00	RX1 C
ATOM	3375	CB	THR	126	16.948	-25.511	20.675	1.00	0.00	RX1 C
ATOM	3376	OG1	THR	126	17.333	-25.549	19.293	1.00	0.00	RX1 O
ATOM	3377	HG1	THR	126	17.785	-24.719	19.142	1.00	0.00	RX1 H
ATOM	3378	CG2	THR	126	17.948	-26.250	21.566	1.00	0.00	RX1 C
ATOM	3379	C	THR	126	14.799	-25.717	22.046	1.00	0.00	RX1 C
ATOM	3380	O	THR	126	14.252	-24.631	22.215	1.00	0.00	RX1 O
ATOM	3381	N	THR	127	14.757	-26.724	22.934	1.00	0.00	RX1 N
ATOM	3382	H	THR	127	15.165	-27.620	22.764	1.00	0.00	RX1 H
ATOM	3383	CA	THR	127	14.120	-26.468	24.225	1.00	0.00	RX1 C
ATOM	3384	CB	THR	127	14.250	-27.814	24.918	1.00	0.00	RX1 C
ATOM	3385	OG1	THR	127	14.190	-28.833	23.904	1.00	0.00	RX1 O
ATOM	3386	HG1	THR	127	14.245	-29.661	24.368	1.00	0.00	RX1 H
ATOM	3387	CG2	THR	127	13.215	-28.032	26.028	1.00	0.00	RX1 C
ATOM	3388	C	THR	127	14.820	-25.328	24.959	1.00	0.00	RX1 C
ATOM	3389	O	THR	127	16.042	-25.249	24.982	1.00	0.00	RX1 O
ATOM	3390	N	PRO	128	13.999	-24.394	25.487	1.00	0.00	RX1 N
ATOM	3391	CD	PRO	128	12.541	-24.397	25.500	1.00	0.00	RX1 C
ATOM	3392	CA	PRO	128	14.587	-23.187	26.066	1.00	0.00	RX1 C
ATOM	3393	CB	PRO	128	13.379	-22.246	26.092	1.00	0.00	RX1 C
ATOM	3394	CG	PRO	128	12.171	-23.157	26.306	1.00	0.00	RX1 C
ATOM	3395	C	PRO	128	15.219	-23.438	27.427	1.00	0.00	RX1 C
ATOM	3396	O	PRO	128	14.631	-23.201	28.474	1.00	0.00	RX1 O
ATOM	3397	N	VAL	129	16.470	-23.914	27.367	1.00	0.00	RX1 N
ATOM	3398	H	VAL	129	16.928	-24.008	26.477	1.00	0.00	RX1 H
ATOM	3399	CA	VAL	129	17.211	-24.148	28.605	1.00	0.00	RX1 C
ATOM	3400	CB	VAL	129	18.591	-24.735	28.298	1.00	0.00	RX1 C
ATOM	3401	CG1	VAL	129	19.420	-24.938	29.568	1.00	0.00	RX1 C
ATOM	3402	CG2	VAL	129	18.455	-26.019	27.478	1.00	0.00	RX1 C
ATOM	3403	C	VAL	129	17.341	-22.876	29.426	1.00	0.00	RX1 C
ATOM	3404	O	VAL	129	17.864	-21.863	28.975	1.00	0.00	RX1 O
ATOM	3405	N	THR	130	16.835	-22.990	30.663	1.00	0.00	RX1 N
ATOM	3406	H	THR	130	16.368	-23.835	30.919	1.00	0.00	RX1 H
ATOM	3407	CA	THR	130	16.752	-21.863	31.591	1.00	0.00	RX1 C
ATOM	3408	CB	THR	130	16.501	-22.508	32.941	1.00	0.00	RX1 C
ATOM	3409	OG1	THR	130	15.947	-23.811	32.702	1.00	0.00	RX1 O
ATOM	3410	HG1	THR	130	15.595	-24.112	33.532	1.00	0.00	RX1 H
ATOM	3411	CG2	THR	130	15.596	-21.668	33.842	1.00	0.00	RX1 C
ATOM	3412	C	THR	130	17.935	-20.903	31.569	1.00	0.00	RX1 C
ATOM	3413	O	THR	130	17.804	-19.703	31.348	1.00	0.00	RX1 O
ATOM	3414	N	GLY	131	19.116	-21.506	31.762	1.00	0.00	RX1 N

ATOM	3415	H	GLY	131	19.169	-22.500	31.835	1.00	0.00	RX1	H
ATOM	3416	CA	GLY	131	20.312	-20.697	31.559	1.00	0.00	RX1	C
ATOM	3417	C	GLY	131	20.659	-20.555	30.089	1.00	0.00	RX1	C
ATOM	3418	O	GLY	131	20.280	-19.607	29.411	1.00	0.00	RX1	O
ATOM	3419	N	ALA	132	21.415	-21.565	29.634	1.00	0.00	RX1	N
ATOM	3420	H	ALA	132	21.595	-22.365	30.203	1.00	0.00	RX1	H
ATOM	3421	CA	ALA	132	21.991	-21.494	28.293	1.00	0.00	RX1	C
ATOM	3422	CB	ALA	132	23.178	-22.451	28.176	1.00	0.00	RX1	C
ATOM	3423	C	ALA	132	21.036	-21.766	27.141	1.00	0.00	RX1	C
ATOM	3424	O	ALA	132	21.041	-22.818	26.516	1.00	0.00	RX1	O
ATOM	3425	N	SER	133	20.240	-20.734	26.864	1.00	0.00	RX1	N
ATOM	3426	H	SER	133	20.167	-19.955	27.491	1.00	0.00	RX1	H
ATOM	3427	CA	SER	133	19.441	-20.645	25.646	1.00	0.00	RX1	C
ATOM	3428	CB	SER	133	18.193	-21.527	25.724	1.00	0.00	RX1	C
ATOM	3429	OG	SER	133	18.502	-22.920	25.625	1.00	0.00	RX1	O
ATOM	3430	HG	SER	133	19.440	-23.015	25.761	1.00	0.00	RX1	H
ATOM	3431	C	SER	133	19.001	-19.207	25.511	1.00	0.00	RX1	C
ATOM	3432	O	SER	133	18.540	-18.618	26.487	1.00	0.00	RX1	O
ATOM	3433	N	PRO	134	19.152	-18.661	24.280	1.00	0.00	RX1	N
ATOM	3434	CD	PRO	134	19.791	-19.304	23.139	1.00	0.00	RX1	C
ATOM	3435	CA	PRO	134	18.647	-17.314	23.975	1.00	0.00	RX1	C
ATOM	3436	CB	PRO	134	18.694	-17.314	22.445	1.00	0.00	RX1	C
ATOM	3437	CG	PRO	134	19.886	-18.200	22.094	1.00	0.00	RX1	C
ATOM	3438	C	PRO	134	17.272	-17.020	24.560	1.00	0.00	RX1	C
ATOM	3439	O	PRO	134	16.465	-17.923	24.776	1.00	0.00	RX1	O
ATOM	3440	N	GLY	135	17.072	-15.740	24.874	1.00	0.00	RX1	N
ATOM	3441	H	GLY	135	17.697	-15.003	24.597	1.00	0.00	RX1	H
ATOM	3442	CA	GLY	135	15.822	-15.384	25.526	1.00	0.00	RX1	C
ATOM	3443	C	GLY	135	14.902	-14.585	24.637	1.00	0.00	RX1	C
ATOM	3444	O	GLY	135	14.923	-13.360	24.597	1.00	0.00	RX1	O
ATOM	3445	N	GLY	136	14.079	-15.342	23.919	1.00	0.00	RX1	N
ATOM	3446	H	GLY	136	14.096	-16.343	23.894	1.00	0.00	RX1	H
ATOM	3447	CA	GLY	136	12.951	-14.671	23.297	1.00	0.00	RX1	C
ATOM	3448	C	GLY	136	11.668	-15.239	23.846	1.00	0.00	RX1	C
ATOM	3449	O	GLY	136	11.667	-16.158	24.661	1.00	0.00	RX1	O
ATOM	3450	N	LEU	137	10.572	-14.682	23.331	1.00	0.00	RX1	N
ATOM	3451	H	LEU	137	10.653	-13.989	22.612	1.00	0.00	RX1	H
ATOM	3452	CA	LEU	137	9.293	-15.265	23.717	1.00	0.00	RX1	C
ATOM	3453	CB	LEU	137	8.229	-14.175	23.724	1.00	0.00	RX1	C
ATOM	3454	CG	LEU	137	6.851	-14.664	24.149	1.00	0.00	RX1	C
ATOM	3455	CD1	LEU	137	6.869	-15.290	25.540	1.00	0.00	RX1	C
ATOM	3456	CD2	LEU	137	5.817	-13.551	24.028	1.00	0.00	RX1	C
ATOM	3457	C	LEU	137	8.906	-16.420	22.810	1.00	0.00	RX1	C
ATOM	3458	O	LEU	137	8.940	-16.318	21.592	1.00	0.00	RX1	O
ATOM	3459	N	ARG	138	8.561	-17.540	23.461	1.00	0.00	RX1	N
ATOM	3460	H	ARG	138	8.491	-17.533	24.460	1.00	0.00	RX1	H
ATOM	3461	CA	ARG	138	8.310	-18.761	22.701	1.00	0.00	RX1	C
ATOM	3462	CB	ARG	138	8.776	-19.974	23.509	1.00	0.00	RX1	C
ATOM	3463	CG	ARG	138	9.055	-21.178	22.618	1.00	0.00	RX1	C
ATOM	3464	CD	ARG	138	9.574	-22.401	23.366	1.00	0.00	RX1	C
ATOM	3465	NE	ARG	138	9.465	-23.596	22.533	1.00	0.00	RX1	N
ATOM	3466	HE	ARG	138	8.533	-23.975	22.408	1.00	0.00	RX1	H
ATOM	3467	CZ	ARG	138	10.563	-24.176	21.967	1.00	0.00	RX1	C
ATOM	3468	NH1	ARG	138	11.780	-23.612	22.128	1.00	0.00	RX1	N
ATOM	3469	HH11	ARG	138	12.631	-24.043	21.785	1.00	0.00	RX1	H
ATOM	3470	HH12	ARG	138	11.903	-22.739	22.602	1.00	0.00	RX1	H
ATOM	3471	NH2	ARG	138	10.407	-25.311	21.260	1.00	0.00	RX1	N
ATOM	3472	HH21	ARG	138	11.182	-25.812	20.853	1.00	0.00	RX1	H
ATOM	3473	HH22	ARG	138	9.479	-25.692	21.115	1.00	0.00	RX1	H
ATOM	3474	C	ARG	138	6.893	-18.961	22.190	1.00	0.00	RX1	C
ATOM	3475	O	ARG	138	6.663	-19.483	21.099	1.00	0.00	RX1	O

ATOM	3476	N	GLU	139	5.947	-18.546	23.046	1.00	0.00	RX1	N
ATOM	3477	H	GLU	139	6.134	-18.085	23.915	1.00	0.00	RX1	H
ATOM	3478	CA	GLU	139	4.542	-18.840	22.761	1.00	0.00	RX1	C
ATOM	3479	CB	GLU	139	4.263	-20.310	23.008	1.00	0.00	RX1	C
ATOM	3480	CG	GLU	139	4.519	-20.670	24.461	1.00	0.00	RX1	C
ATOM	3481	CD	GLU	139	5.039	-22.080	24.485	1.00	0.00	RX1	C
ATOM	3482	OE1	GLU	139	6.045	-22.328	23.830	1.00	0.00	RX1	O
ATOM	3483	OE2	GLU	139	4.459	-22.920	25.165	1.00	0.00	RX1	O
ATOM	3484	C	GLU	139	3.621	-17.952	23.569	1.00	0.00	RX1	C
ATOM	3485	O	GLU	139	4.046	-17.275	24.501	1.00	0.00	RX1	O
ATOM	3486	N	LEU	140	2.350	-17.968	23.155	1.00	0.00	RX1	N
ATOM	3487	H	LEU	140	1.996	-18.652	22.510	1.00	0.00	RX1	H
ATOM	3488	CA	LEU	140	1.402	-17.072	23.804	1.00	0.00	RX1	C
ATOM	3489	CB	LEU	140	0.717	-16.187	22.768	1.00	0.00	RX1	C
ATOM	3490	CG	LEU	140	1.629	-15.121	22.177	1.00	0.00	RX1	C
ATOM	3491	CD1	LEU	140	0.937	-14.360	21.047	1.00	0.00	RX1	C
ATOM	3492	CD2	LEU	140	2.158	-14.190	23.265	1.00	0.00	RX1	C
ATOM	3493	C	LEU	140	0.330	-17.852	24.518	1.00	0.00	RX1	C
ATOM	3494	O	LEU	140	-0.710	-18.147	23.953	1.00	0.00	RX1	O
ATOM	3495	N	GLN	141	0.610	-18.192	25.778	1.00	0.00	RX1	N
ATOM	3496	H	GLN	141	1.363	-17.787	26.298	1.00	0.00	RX1	H
ATOM	3497	CA	GLN	141	-0.350	-19.065	26.453	1.00	0.00	RX1	C
ATOM	3498	CB	GLN	141	0.372	-19.856	27.529	1.00	0.00	RX1	C
ATOM	3499	CG	GLN	141	1.545	-20.659	26.977	1.00	0.00	RX1	C
ATOM	3500	CD	GLN	141	2.274	-21.290	28.139	1.00	0.00	RX1	C
ATOM	3501	OE1	GLN	141	2.043	-20.958	29.299	1.00	0.00	RX1	O
ATOM	3502	NE2	GLN	141	3.176	-22.215	27.766	1.00	0.00	RX1	N
ATOM	3503	HE21	GLN	141	3.370	-22.411	26.796	1.00	0.00	RX1	H
ATOM	3504	HE22	GLN	141	3.705	-22.749	28.423	1.00	0.00	RX1	H
ATOM	3505	C	GLN	141	-1.553	-18.332	27.027	1.00	0.00	RX1	C
ATOM	3506	O	GLN	141	-1.681	-18.132	28.227	1.00	0.00	RX1	O
ATOM	3507	N	LEU	142	-2.424	-17.901	26.105	1.00	0.00	RX1	N
ATOM	3508	H	LEU	142	-2.306	-18.148	25.141	1.00	0.00	RX1	H
ATOM	3509	CA	LEU	142	-3.535	-17.052	26.537	1.00	0.00	RX1	C
ATOM	3510	CB	LEU	142	-3.619	-15.756	25.717	1.00	0.00	RX1	C
ATOM	3511	CG	LEU	142	-2.330	-15.203	25.088	1.00	0.00	RX1	C
ATOM	3512	CD1	LEU	142	-2.641	-14.057	24.125	1.00	0.00	RX1	C
ATOM	3513	CD2	LEU	142	-1.269	-14.775	26.099	1.00	0.00	RX1	C
ATOM	3514	C	LEU	142	-4.875	-17.759	26.421	1.00	0.00	RX1	C
ATOM	3515	O	LEU	142	-5.820	-17.242	25.838	1.00	0.00	RX1	O
ATOM	3516	N	ARG	143	-4.912	-19.001	26.942	1.00	0.00	RX1	N
ATOM	3517	H	ARG	143	-4.134	-19.342	27.472	1.00	0.00	RX1	H
ATOM	3518	CA	ARG	143	-5.944	-19.926	26.454	1.00	0.00	RX1	C
ATOM	3519	CB	ARG	143	-5.855	-21.301	27.118	1.00	0.00	RX1	C
ATOM	3520	CG	ARG	143	-6.456	-21.392	28.516	1.00	0.00	RX1	C
ATOM	3521	CD	ARG	143	-6.317	-22.796	29.086	1.00	0.00	RX1	C
ATOM	3522	NE	ARG	143	-4.910	-23.160	29.212	1.00	0.00	RX1	N
ATOM	3523	HE	ARG	143	-4.195	-22.547	28.843	1.00	0.00	RX1	H
ATOM	3524	CZ	ARG	143	-4.597	-24.344	29.804	1.00	0.00	RX1	C
ATOM	3525	NH1	ARG	143	-5.584	-25.166	30.210	1.00	0.00	RX1	N
ATOM	3526	HH11	ARG	143	-5.350	-26.071	30.617	1.00	0.00	RX1	H
ATOM	3527	HH12	ARG	143	-6.557	-24.971	30.111	1.00	0.00	RX1	H
ATOM	3528	NH2	ARG	143	-3.309	-24.685	29.974	1.00	0.00	RX1	N
ATOM	3529	HH21	ARG	143	-3.078	-25.538	30.466	1.00	0.00	RX1	H
ATOM	3530	HH22	ARG	143	-2.550	-24.123	29.614	1.00	0.00	RX1	H
ATOM	3531	C	ARG	143	-7.397	-19.481	26.327	1.00	0.00	RX1	C
ATOM	3532	O	ARG	143	-8.106	-19.864	25.397	1.00	0.00	RX1	O
ATOM	3533	N	SER	144	-7.816	-18.667	27.303	1.00	0.00	RX1	N
ATOM	3534	H	SER	144	-7.176	-18.239	27.941	1.00	0.00	RX1	H
ATOM	3535	CA	SER	144	-9.236	-18.342	27.356	1.00	0.00	RX1	C
ATOM	3536	CB	SER	144	-9.591	-18.086	28.818	1.00	0.00	RX1	C

ATOM	3537	OG	SER	144	-8.962	-19.094	29.626	1.00	0.00	RX1	O
ATOM	3538	HG	SER	144	-8.025	-18.973	29.526	1.00	0.00	RX1	H
ATOM	3539	C	SER	144	-9.722	-17.275	26.382	1.00	0.00	RX1	C
ATOM	3540	O	SER	144	-10.916	-16.993	26.294	1.00	0.00	RX1	O
ATOM	3541	N	LEU	145	-8.751	-16.702	25.647	1.00	0.00	RX1	N
ATOM	3542	H	LEU	145	-7.792	-16.964	25.755	1.00	0.00	RX1	H
ATOM	3543	CA	LEU	145	-9.076	-15.544	24.822	1.00	0.00	RX1	C
ATOM	3544	CB	LEU	145	-7.803	-15.009	24.165	1.00	0.00	RX1	C
ATOM	3545	CG	LEU	145	-7.950	-13.624	23.537	1.00	0.00	RX1	C
ATOM	3546	CD1	LEU	145	-8.507	-12.600	24.524	1.00	0.00	RX1	C
ATOM	3547	CD2	LEU	145	-6.639	-13.146	22.911	1.00	0.00	RX1	C
ATOM	3548	C	LEU	145	-10.187	-15.802	23.826	1.00	0.00	RX1	C
ATOM	3549	O	LEU	145	-10.126	-16.673	22.965	1.00	0.00	RX1	O
ATOM	3550	N	THR	146	-11.240	-15.017	24.043	1.00	0.00	RX1	N
ATOM	3551	H	THR	146	-11.285	-14.352	24.790	1.00	0.00	RX1	H
ATOM	3552	CA	THR	146	-12.446	-15.259	23.273	1.00	0.00	RX1	C
ATOM	3553	CB	THR	146	-13.509	-15.612	24.294	1.00	0.00	RX1	C
ATOM	3554	OG1	THR	146	-12.994	-15.316	25.598	1.00	0.00	RX1	O
ATOM	3555	HG1	THR	146	-12.293	-15.939	25.771	1.00	0.00	RX1	H
ATOM	3556	CG2	THR	146	-13.917	-17.084	24.203	1.00	0.00	RX1	C
ATOM	3557	C	THR	146	-12.831	-14.108	22.370	1.00	0.00	RX1	C
ATOM	3558	O	THR	146	-13.160	-14.311	21.210	1.00	0.00	RX1	O
ATOM	3559	N	GLU	147	-12.733	-12.894	22.943	1.00	0.00	RX1	N
ATOM	3560	H	GLU	147	-12.411	-12.759	23.882	1.00	0.00	RX1	H
ATOM	3561	CA	GLU	147	-12.969	-11.711	22.116	1.00	0.00	RX1	C
ATOM	3562	CB	GLU	147	-14.350	-11.088	22.381	1.00	0.00	RX1	C
ATOM	3563	CG	GLU	147	-15.588	-11.856	21.892	1.00	0.00	RX1	C
ATOM	3564	CD	GLU	147	-15.759	-11.740	20.386	1.00	0.00	RX1	C
ATOM	3565	OE1	GLU	147	-16.861	-11.476	19.908	1.00	0.00	RX1	O
ATOM	3566	OE2	GLU	147	-14.804	-11.926	19.648	1.00	0.00	RX1	O
ATOM	3567	C	GLU	147	-11.903	-10.650	22.320	1.00	0.00	RX1	C
ATOM	3568	O	GLU	147	-11.306	-10.515	23.385	1.00	0.00	RX1	O
ATOM	3569	N	ILE	148	-11.714	-9.880	21.246	1.00	0.00	RX1	N
ATOM	3570	H	ILE	148	-12.170	-10.139	20.391	1.00	0.00	RX1	H
ATOM	3571	CA	ILE	148	-10.937	-8.643	21.294	1.00	0.00	RX1	C
ATOM	3572	CB	ILE	148	-9.681	-8.739	20.423	1.00	0.00	RX1	C
ATOM	3573	CG2	ILE	148	-8.927	-7.407	20.402	1.00	0.00	RX1	C
ATOM	3574	CG1	ILE	148	-8.778	-9.892	20.859	1.00	0.00	RX1	C
ATOM	3575	CD1	ILE	148	-7.577	-10.073	19.932	1.00	0.00	RX1	C
ATOM	3576	C	ILE	148	-11.831	-7.542	20.767	1.00	0.00	RX1	C
ATOM	3577	O	ILE	148	-12.053	-7.434	19.564	1.00	0.00	RX1	O
ATOM	3578	N	LEU	149	-12.370	-6.771	21.728	1.00	0.00	RX1	N
ATOM	3579	H	LEU	149	-12.075	-6.909	22.671	1.00	0.00	RX1	H
ATOM	3580	CA	LEU	149	-13.449	-5.832	21.420	1.00	0.00	RX1	C
ATOM	3581	CB	LEU	149	-13.911	-5.044	22.646	1.00	0.00	RX1	C
ATOM	3582	CG	LEU	149	-14.500	-5.907	23.760	1.00	0.00	RX1	C
ATOM	3583	CD1	LEU	149	-15.027	-5.050	24.909	1.00	0.00	RX1	C
ATOM	3584	CD2	LEU	149	-15.585	-6.848	23.244	1.00	0.00	RX1	C
ATOM	3585	C	LEU	149	-13.198	-4.884	20.270	1.00	0.00	RX1	C
ATOM	3586	O	LEU	149	-13.901	-4.914	19.265	1.00	0.00	RX1	O
ATOM	3587	N	LYS	150	-12.184	-4.029	20.457	1.00	0.00	RX1	N
ATOM	3588	H	LYS	150	-11.610	-3.987	21.281	1.00	0.00	RX1	H
ATOM	3589	CA	LYS	150	-11.810	-3.161	19.347	1.00	0.00	RX1	C
ATOM	3590	CB	LYS	150	-12.420	-1.759	19.501	1.00	0.00	RX1	C
ATOM	3591	CG	LYS	150	-13.947	-1.828	19.366	1.00	0.00	RX1	C
ATOM	3592	CD	LYS	150	-14.731	-0.531	19.527	1.00	0.00	RX1	C
ATOM	3593	CE	LYS	150	-16.240	-0.806	19.465	1.00	0.00	RX1	C
ATOM	3594	NZ	LYS	150	-16.595	-1.470	18.203	1.00	0.00	RX1	N
ATOM	3595	HZ1	LYS	150	-17.628	-1.519	18.081	1.00	0.00	RX1	H
ATOM	3596	HZ2	LYS	150	-16.195	-0.967	17.387	1.00	0.00	RX1	H
ATOM	3597	HZ3	LYS	150	-16.260	-2.455	18.150	1.00	0.00	RX1	H

ATOM	3598	C	LYS	150	-10.307	-3.135	19.182	1.00	0.00	RX1	C
ATOM	3599	O	LYS	150	-9.560	-3.320	20.133	1.00	0.00	RX1	O
ATOM	3600	N	GLY	151	-9.900	-2.918	17.925	1.00	0.00	RX1	N
ATOM	3601	H	GLY	151	-10.549	-3.002	17.168	1.00	0.00	RX1	H
ATOM	3602	CA	GLY	151	-8.463	-2.940	17.668	1.00	0.00	RX1	C
ATOM	3603	C	GLY	151	-7.997	-4.309	17.219	1.00	0.00	RX1	C
ATOM	3604	O	GLY	151	-8.504	-5.337	17.651	1.00	0.00	RX1	O
ATOM	3605	N	GLY	152	-7.025	-4.280	16.301	1.00	0.00	RX1	N
ATOM	3606	H	GLY	152	-6.620	-3.416	15.992	1.00	0.00	RX1	H
ATOM	3607	CA	GLY	152	-6.680	-5.559	15.685	1.00	0.00	RX1	C
ATOM	3608	C	GLY	152	-5.574	-6.342	16.360	1.00	0.00	RX1	C
ATOM	3609	O	GLY	152	-5.078	-6.010	17.433	1.00	0.00	RX1	O
ATOM	3610	N	VAL	153	-5.197	-7.409	15.652	1.00	0.00	RX1	N
ATOM	3611	H	VAL	153	-5.555	-7.534	14.726	1.00	0.00	RX1	H
ATOM	3612	CA	VAL	153	-4.064	-8.219	16.079	1.00	0.00	RX1	C
ATOM	3613	CB	VAL	153	-4.323	-9.661	15.638	1.00	0.00	RX1	C
ATOM	3614	CG1	VAL	153	-3.062	-10.515	15.544	1.00	0.00	RX1	C
ATOM	3615	CG2	VAL	153	-5.370	-10.280	16.562	1.00	0.00	RX1	C
ATOM	3616	C	VAL	153	-2.774	-7.664	15.500	1.00	0.00	RX1	C
ATOM	3617	O	VAL	153	-2.697	-7.306	14.331	1.00	0.00	RX1	O
ATOM	3618	N	LEU	154	-1.781	-7.592	16.391	1.00	0.00	RX1	N
ATOM	3619	H	LEU	154	-1.875	-7.923	17.332	1.00	0.00	RX1	H
ATOM	3620	CA	LEU	154	-0.482	-7.030	16.038	1.00	0.00	RX1	C
ATOM	3621	CB	LEU	154	-0.359	-5.634	16.624	1.00	0.00	RX1	C
ATOM	3622	CG	LEU	154	0.907	-4.853	16.290	1.00	0.00	RX1	C
ATOM	3623	CD1	LEU	154	1.122	-4.669	14.792	1.00	0.00	RX1	C
ATOM	3624	CD2	LEU	154	0.938	-3.539	17.062	1.00	0.00	RX1	C
ATOM	3625	C	LEU	154	0.612	-7.900	16.602	1.00	0.00	RX1	C
ATOM	3626	O	LEU	154	1.350	-7.544	17.512	1.00	0.00	RX1	O
ATOM	3627	N	ILE	155	0.675	-9.099	16.040	1.00	0.00	RX1	N
ATOM	3628	H	ILE	155	0.180	-9.323	15.200	1.00	0.00	RX1	H
ATOM	3629	CA	ILE	155	1.744	-9.936	16.552	1.00	0.00	RX1	C
ATOM	3630	CB	ILE	155	1.242	-11.348	16.816	1.00	0.00	RX1	C
ATOM	3631	CG2	ILE	155	2.251	-12.095	17.671	1.00	0.00	RX1	C
ATOM	3632	CG1	ILE	155	-0.107	-11.332	17.526	1.00	0.00	RX1	C
ATOM	3633	CD1	ILE	155	-0.614	-12.748	17.787	1.00	0.00	RX1	C
ATOM	3634	C	ILE	155	2.937	-9.913	15.616	1.00	0.00	RX1	C
ATOM	3635	O	ILE	155	3.291	-10.895	14.971	1.00	0.00	RX1	O
ATOM	3636	N	GLN	156	3.521	-8.713	15.539	1.00	0.00	RX1	N
ATOM	3637	H	GLN	156	3.336	-8.045	16.263	1.00	0.00	RX1	H
ATOM	3638	CA	GLN	156	4.536	-8.456	14.521	1.00	0.00	RX1	C
ATOM	3639	CB	GLN	156	4.198	-7.120	13.851	1.00	0.00	RX1	C
ATOM	3640	CG	GLN	156	4.132	-7.148	12.320	1.00	0.00	RX1	C
ATOM	3641	CD	GLN	156	5.492	-6.837	11.732	1.00	0.00	RX1	C
ATOM	3642	OE1	GLN	156	5.976	-5.711	11.835	1.00	0.00	RX1	O
ATOM	3643	NE2	GLN	156	6.089	-7.874	11.118	1.00	0.00	RX1	N
ATOM	3644	HE21	GLN	156	5.703	-8.802	11.048	1.00	0.00	RX1	H
ATOM	3645	HE22	GLN	156	6.986	-7.806	10.681	1.00	0.00	RX1	H
ATOM	3646	C	GLN	156	5.967	-8.527	15.037	1.00	0.00	RX1	C
ATOM	3647	O	GLN	156	6.262	-8.146	16.165	1.00	0.00	RX1	O
ATOM	3648	N	ARG	157	6.839	-9.031	14.143	1.00	0.00	RX1	N
ATOM	3649	H	ARG	157	6.486	-9.513	13.338	1.00	0.00	RX1	H
ATOM	3650	CA	ARG	157	8.281	-9.155	14.390	1.00	0.00	RX1	C
ATOM	3651	CB	ARG	157	8.909	-7.774	14.596	1.00	0.00	RX1	C
ATOM	3652	CG	ARG	157	8.544	-6.879	13.410	1.00	0.00	RX1	C
ATOM	3653	CD	ARG	157	8.772	-5.386	13.631	1.00	0.00	RX1	C
ATOM	3654	NE	ARG	157	8.199	-4.955	14.902	1.00	0.00	RX1	N
ATOM	3655	HE	ARG	157	8.765	-5.156	15.717	1.00	0.00	RX1	H
ATOM	3656	CZ	ARG	157	7.017	-4.276	15.010	1.00	0.00	RX1	C
ATOM	3657	NH1	ARG	157	6.241	-4.032	13.938	1.00	0.00	RX1	N
ATOM	3658	HH11	ARG	157	5.439	-3.418	14.060	1.00	0.00	RX1	H

ATOM	3659	HH12	ARG	157	6.393	-4.434	13.025	1.00	0.00	RX1	H
ATOM	3660	NH2	ARG	157	6.628	-3.830	16.215	1.00	0.00	RX1	N
ATOM	3661	HH21	ARG	157	5.783	-3.269	16.286	1.00	0.00	RX1	H
ATOM	3662	HH22	ARG	157	7.160	-4.039	17.050	1.00	0.00	RX1	H
ATOM	3663	C	ARG	157	8.623	-10.139	15.495	1.00	0.00	RX1	C
ATOM	3664	O	ARG	157	9.182	-9.809	16.534	1.00	0.00	RX1	O
ATOM	3665	N	ASN	158	8.230	-11.394	15.238	1.00	0.00	RX1	N
ATOM	3666	H	ASN	158	7.825	-11.665	14.359	1.00	0.00	RX1	H
ATOM	3667	CA	ASN	158	8.333	-12.330	16.360	1.00	0.00	RX1	C
ATOM	3668	CB	ASN	158	6.949	-12.687	16.877	1.00	0.00	RX1	C
ATOM	3669	CG	ASN	158	6.416	-11.556	17.713	1.00	0.00	RX1	C
ATOM	3670	OD1	ASN	158	6.865	-11.289	18.824	1.00	0.00	RX1	O
ATOM	3671	ND2	ASN	158	5.433	-10.890	17.099	1.00	0.00	RX1	N
ATOM	3672	HD21	ASN	158	5.072	-11.190	16.211	1.00	0.00	RX1	H
ATOM	3673	HD22	ASN	158	5.032	-10.058	17.492	1.00	0.00	RX1	H
ATOM	3674	C	ASN	158	9.095	-13.616	16.114	1.00	0.00	RX1	C
ATOM	3675	O	ASN	158	8.526	-14.634	15.736	1.00	0.00	RX1	O
ATOM	3676	N	PRO	159	10.419	-13.547	16.400	1.00	0.00	RX1	N
ATOM	3677	CD	PRO	159	11.110	-12.410	16.997	1.00	0.00	RX1	C
ATOM	3678	CA	PRO	159	11.307	-14.667	16.067	1.00	0.00	RX1	C
ATOM	3679	CB	PRO	159	12.692	-14.071	16.348	1.00	0.00	RX1	C
ATOM	3680	CG	PRO	159	12.479	-12.956	17.373	1.00	0.00	RX1	C
ATOM	3681	C	PRO	159	11.010	-15.979	16.785	1.00	0.00	RX1	C
ATOM	3682	O	PRO	159	10.621	-16.977	16.189	1.00	0.00	RX1	O
ATOM	3683	N	GLN	160	11.193	-15.963	18.113	1.00	0.00	RX1	N
ATOM	3684	H	GLN	160	11.402	-15.126	18.617	1.00	0.00	RX1	H
ATOM	3685	CA	GLN	160	11.016	-17.228	18.827	1.00	0.00	RX1	C
ATOM	3686	CB	GLN	160	11.762	-17.127	20.159	1.00	0.00	RX1	C
ATOM	3687	CG	GLN	160	12.116	-18.462	20.814	1.00	0.00	RX1	C
ATOM	3688	CD	GLN	160	12.653	-18.185	22.201	1.00	0.00	RX1	C
ATOM	3689	OE1	GLN	160	13.788	-17.779	22.411	1.00	0.00	RX1	O
ATOM	3690	NE2	GLN	160	11.738	-18.385	23.150	1.00	0.00	RX1	N
ATOM	3691	HE21	GLN	160	10.854	-18.772	22.908	1.00	0.00	RX1	H
ATOM	3692	HE22	GLN	160	11.868	-18.077	24.098	1.00	0.00	RX1	H
ATOM	3693	C	GLN	160	9.557	-17.663	19.006	1.00	0.00	RX1	C
ATOM	3694	O	GLN	160	9.238	-18.687	19.606	1.00	0.00	RX1	O
ATOM	3695	N	LEU	161	8.670	-16.813	18.471	1.00	0.00	RX1	N
ATOM	3696	H	LEU	161	8.908	-16.182	17.736	1.00	0.00	RX1	H
ATOM	3697	CA	LEU	161	7.268	-16.960	18.814	1.00	0.00	RX1	C
ATOM	3698	CB	LEU	161	6.653	-15.576	18.956	1.00	0.00	RX1	C
ATOM	3699	CG	LEU	161	5.240	-15.592	19.519	1.00	0.00	RX1	C
ATOM	3700	CD1	LEU	161	5.249	-15.932	20.996	1.00	0.00	RX1	C
ATOM	3701	CD2	LEU	161	4.525	-14.270	19.298	1.00	0.00	RX1	C
ATOM	3702	C	LEU	161	6.505	-17.768	17.793	1.00	0.00	RX1	C
ATOM	3703	O	LEU	161	6.465	-17.457	16.608	1.00	0.00	RX1	O
ATOM	3704	N	CYS	162	5.880	-18.820	18.316	1.00	0.00	RX1	N
ATOM	3705	H	CYS	162	5.900	-18.998	19.301	1.00	0.00	RX1	H
ATOM	3706	CA	CYS	162	4.928	-19.495	17.448	1.00	0.00	RX1	C
ATOM	3707	CB	CYS	162	5.209	-20.987	17.431	1.00	0.00	RX1	C
ATOM	3708	SG	CYS	162	6.732	-21.352	16.535	1.00	0.00	RX1	S
ATOM	3709	C	CYS	162	3.499	-19.165	17.825	1.00	0.00	RX1	C
ATOM	3710	O	CYS	162	3.256	-18.362	18.717	1.00	0.00	RX1	O
ATOM	3711	N	TYR	163	2.572	-19.825	17.102	1.00	0.00	RX1	N
ATOM	3712	H	TYR	163	2.818	-20.373	16.302	1.00	0.00	RX1	H
ATOM	3713	CA	TYR	163	1.142	-19.809	17.435	1.00	0.00	RX1	C
ATOM	3714	CB	TYR	163	0.902	-20.247	18.884	1.00	0.00	RX1	C
ATOM	3715	CG	TYR	163	1.535	-21.609	19.053	1.00	0.00	RX1	C
ATOM	3716	CD1	TYR	163	1.015	-22.700	18.368	1.00	0.00	RX1	C
ATOM	3717	CE1	TYR	163	1.630	-23.941	18.451	1.00	0.00	RX1	C
ATOM	3718	CD2	TYR	163	2.652	-21.768	19.862	1.00	0.00	RX1	C
ATOM	3719	CE2	TYR	163	3.275	-23.007	19.939	1.00	0.00	RX1	C

ATOM	3720	CZ	TYR	163	2.774	-24.089	19.223	1.00	0.00	RX1	C
ATOM	3721	OH	TYR	163	3.418	-25.307	19.269	1.00	0.00	RX1	O
ATOM	3722	HH	TYR	163	4.057	-25.278	19.981	1.00	0.00	RX1	H
ATOM	3723	C	TYR	163	0.323	-18.591	17.037	1.00	0.00	RX1	C
ATOM	3724	O	TYR	163	-0.896	-18.556	17.142	1.00	0.00	RX1	O
ATOM	3725	N	GLN	164	1.051	-17.610	16.471	1.00	0.00	RX1	N
ATOM	3726	H	GLN	164	2.038	-17.622	16.613	1.00	0.00	RX1	H
ATOM	3727	CA	GLN	164	0.393	-16.495	15.776	1.00	0.00	RX1	C
ATOM	3728	CB	GLN	164	1.396	-15.615	15.017	1.00	0.00	RX1	C
ATOM	3729	CG	GLN	164	2.576	-15.038	15.798	1.00	0.00	RX1	C
ATOM	3730	CD	GLN	164	3.799	-15.916	15.662	1.00	0.00	RX1	C
ATOM	3731	OE1	GLN	164	3.739	-17.119	15.895	1.00	0.00	RX1	O
ATOM	3732	NE2	GLN	164	4.917	-15.262	15.317	1.00	0.00	RX1	N
ATOM	3733	HE21	GLN	164	4.823	-14.348	14.906	1.00	0.00	RX1	H
ATOM	3734	HE22	GLN	164	5.844	-15.634	15.426	1.00	0.00	RX1	H
ATOM	3735	C	GLN	164	-0.668	-16.906	14.759	1.00	0.00	RX1	C
ATOM	3736	O	GLN	164	-1.673	-16.242	14.558	1.00	0.00	RX1	O
ATOM	3737	N	ASP	165	-0.339	-18.023	14.106	1.00	0.00	RX1	N
ATOM	3738	H	ASP	165	0.421	-18.584	14.423	1.00	0.00	RX1	H
ATOM	3739	CA	ASP	165	-1.145	-18.599	13.032	1.00	0.00	RX1	C
ATOM	3740	CB	ASP	165	-0.240	-19.293	11.992	1.00	0.00	RX1	C
ATOM	3741	CG	ASP	165	0.994	-19.978	12.587	1.00	0.00	RX1	C
ATOM	3742	OD1	ASP	165	1.010	-20.367	13.754	1.00	0.00	RX1	O
ATOM	3743	OD2	ASP	165	2.013	-20.060	11.906	1.00	0.00	RX1	O
ATOM	3744	C	ASP	165	-2.230	-19.546	13.513	1.00	0.00	RX1	C
ATOM	3745	O	ASP	165	-3.397	-19.436	13.155	1.00	0.00	RX1	O
ATOM	3746	N	THR	166	-1.793	-20.488	14.359	1.00	0.00	RX1	N
ATOM	3747	H	THR	166	-0.834	-20.550	14.642	1.00	0.00	RX1	H
ATOM	3748	CA	THR	166	-2.692	-21.524	14.868	1.00	0.00	RX1	C
ATOM	3749	CB	THR	166	-1.829	-22.402	15.749	1.00	0.00	RX1	C
ATOM	3750	OG1	THR	166	-0.949	-21.552	16.481	1.00	0.00	RX1	O
ATOM	3751	HG1	THR	166	-1.285	-21.529	17.377	1.00	0.00	RX1	H
ATOM	3752	CG2	THR	166	-1.002	-23.389	14.922	1.00	0.00	RX1	C
ATOM	3753	C	THR	166	-3.933	-20.983	15.559	1.00	0.00	RX1	C
ATOM	3754	O	THR	166	-5.050	-21.477	15.426	1.00	0.00	RX1	O
ATOM	3755	N	ILE	167	-3.687	-19.905	16.313	1.00	0.00	RX1	N
ATOM	3756	H	ILE	167	-2.780	-19.477	16.345	1.00	0.00	RX1	H
ATOM	3757	CA	ILE	167	-4.855	-19.234	16.863	1.00	0.00	RX1	C
ATOM	3758	CB	ILE	167	-4.493	-18.428	18.100	1.00	0.00	RX1	C
ATOM	3759	CG2	ILE	167	-5.748	-17.804	18.703	1.00	0.00	RX1	C
ATOM	3760	CG1	ILE	167	-3.806	-19.337	19.110	1.00	0.00	RX1	C
ATOM	3761	CD1	ILE	167	-4.770	-20.421	19.588	1.00	0.00	RX1	C
ATOM	3762	C	ILE	167	-5.556	-18.377	15.829	1.00	0.00	RX1	C
ATOM	3763	O	ILE	167	-5.259	-17.212	15.609	1.00	0.00	RX1	O
ATOM	3764	N	LEU	168	-6.542	-19.024	15.198	1.00	0.00	RX1	N
ATOM	3765	H	LEU	168	-6.627	-20.009	15.357	1.00	0.00	RX1	H
ATOM	3766	CA	LEU	168	-7.302	-18.305	14.182	1.00	0.00	RX1	C
ATOM	3767	CB	LEU	168	-8.187	-19.285	13.415	1.00	0.00	RX1	C
ATOM	3768	CG	LEU	168	-8.784	-18.690	12.142	1.00	0.00	RX1	C
ATOM	3769	CD1	LEU	168	-7.700	-18.179	11.192	1.00	0.00	RX1	C
ATOM	3770	CD2	LEU	168	-9.740	-19.669	11.459	1.00	0.00	RX1	C
ATOM	3771	C	LEU	168	-8.085	-17.101	14.704	1.00	0.00	RX1	C
ATOM	3772	O	LEU	168	-9.225	-17.187	15.154	1.00	0.00	RX1	O
ATOM	3773	N	TRP	169	-7.407	-15.948	14.576	1.00	0.00	RX1	N
ATOM	3774	H	TRP	169	-6.430	-16.043	14.368	1.00	0.00	RX1	H
ATOM	3775	CA	TRP	169	-7.945	-14.671	15.057	1.00	0.00	RX1	C
ATOM	3776	CB	TRP	169	-6.938	-13.543	14.841	1.00	0.00	RX1	C
ATOM	3777	CG	TRP	169	-5.656	-13.917	15.538	1.00	0.00	RX1	C
ATOM	3778	CD2	TRP	169	-5.413	-14.024	16.956	1.00	0.00	RX1	C
ATOM	3779	CE2	TRP	169	-4.077	-14.452	17.126	1.00	0.00	RX1	C
ATOM	3780	CE3	TRP	169	-6.213	-13.798	18.069	1.00	0.00	RX1	C

ATOM	3781	CD1	TRP	169	-4.449	-14.282	14.932	1.00	0.00	RX1	C
ATOM	3782	NE1	TRP	169	-3.516	-14.602	15.862	1.00	0.00	RX1	N
ATOM	3783	HE1	TRP	169	-2.623	-14.955	15.652	1.00	0.00	RX1	H
ATOM	3784	CZ2	TRP	169	-3.578	-14.652	18.407	1.00	0.00	RX1	C
ATOM	3785	CZ3	TRP	169	-5.701	-13.998	19.344	1.00	0.00	RX1	C
ATOM	3786	CH2	TRP	169	-4.389	-14.426	19.511	1.00	0.00	RX1	C
ATOM	3787	C	TRP	169	-9.304	-14.278	14.515	1.00	0.00	RX1	C
ATOM	3788	O	TRP	169	-10.076	-13.578	15.154	1.00	0.00	RX1	O
ATOM	3789	N	LYS	170	-9.601	-14.839	13.330	1.00	0.00	RX1	N
ATOM	3790	H	LYS	170	-8.880	-15.357	12.873	1.00	0.00	RX1	H
ATOM	3791	CA	LYS	170	-10.940	-14.772	12.731	1.00	0.00	RX1	C
ATOM	3792	CB	LYS	170	-10.972	-15.726	11.532	1.00	0.00	RX1	C
ATOM	3793	CG	LYS	170	-12.206	-15.660	10.623	1.00	0.00	RX1	C
ATOM	3794	CD	LYS	170	-12.729	-17.053	10.252	1.00	0.00	RX1	C
ATOM	3795	CE	LYS	170	-13.977	-17.502	11.030	1.00	0.00	RX1	C
ATOM	3796	NZ	LYS	170	-13.788	-17.360	12.478	1.00	0.00	RX1	N
ATOM	3797	HZ1	LYS	170	-14.438	-17.955	13.038	1.00	0.00	RX1	H
ATOM	3798	HZ2	LYS	170	-12.855	-17.705	12.795	1.00	0.00	RX1	H
ATOM	3799	HZ3	LYS	170	-13.898	-16.373	12.790	1.00	0.00	RX1	H
ATOM	3800	C	LYS	170	-12.119	-15.088	13.659	1.00	0.00	RX1	C
ATOM	3801	O	LYS	170	-13.281	-14.863	13.336	1.00	0.00	RX1	O
ATOM	3802	N	ASP	171	-11.803	-15.693	14.801	1.00	0.00	RX1	N
ATOM	3803	H	ASP	171	-10.874	-15.996	15.018	1.00	0.00	RX1	H
ATOM	3804	CA	ASP	171	-12.892	-15.994	15.722	1.00	0.00	RX1	C
ATOM	3805	CB	ASP	171	-12.809	-17.472	16.120	1.00	0.00	RX1	C
ATOM	3806	CG	ASP	171	-12.812	-18.329	14.860	1.00	0.00	RX1	C
ATOM	3807	OD1	ASP	171	-13.821	-18.930	14.523	1.00	0.00	RX1	O
ATOM	3808	OD2	ASP	171	-11.822	-18.382	14.139	1.00	0.00	RX1	O
ATOM	3809	C	ASP	171	-12.917	-15.050	16.909	1.00	0.00	RX1	C
ATOM	3810	O	ASP	171	-13.969	-14.658	17.406	1.00	0.00	RX1	O
ATOM	3811	N	ILE	172	-11.686	-14.684	17.305	1.00	0.00	RX1	N
ATOM	3812	H	ILE	172	-10.893	-14.884	16.733	1.00	0.00	RX1	H
ATOM	3813	CA	ILE	172	-11.481	-13.832	18.477	1.00	0.00	RX1	C
ATOM	3814	CB	ILE	172	-10.115	-14.171	19.079	1.00	0.00	RX1	C
ATOM	3815	CG2	ILE	172	-9.859	-13.483	20.416	1.00	0.00	RX1	C
ATOM	3816	CG1	ILE	172	-9.986	-15.682	19.238	1.00	0.00	RX1	C
ATOM	3817	CD1	ILE	172	-8.714	-16.036	20.002	1.00	0.00	RX1	C
ATOM	3818	C	ILE	172	-11.616	-12.338	18.186	1.00	0.00	RX1	C
ATOM	3819	O	ILE	172	-11.702	-11.491	19.065	1.00	0.00	RX1	O
ATOM	3820	N	PHE	173	-11.660	-12.016	16.885	1.00	0.00	RX1	N
ATOM	3821	H	PHE	173	-11.536	-12.690	16.160	1.00	0.00	RX1	H
ATOM	3822	CA	PHE	173	-12.087	-10.650	16.593	1.00	0.00	RX1	C
ATOM	3823	CB	PHE	173	-11.948	-10.340	15.105	1.00	0.00	RX1	C
ATOM	3824	CG	PHE	173	-10.511	-10.035	14.766	1.00	0.00	RX1	C
ATOM	3825	CD1	PHE	173	-9.791	-9.141	15.550	1.00	0.00	RX1	C
ATOM	3826	CD2	PHE	173	-9.918	-10.628	13.658	1.00	0.00	RX1	C
ATOM	3827	CE1	PHE	173	-8.484	-8.821	15.208	1.00	0.00	RX1	C
ATOM	3828	CE2	PHE	173	-8.610	-10.308	13.317	1.00	0.00	RX1	C
ATOM	3829	CZ	PHE	173	-7.899	-9.395	14.086	1.00	0.00	RX1	C
ATOM	3830	C	PHE	173	-13.523	-10.436	17.006	1.00	0.00	RX1	C
ATOM	3831	O	PHE	173	-14.418	-11.174	16.596	1.00	0.00	RX1	O
ATOM	3832	N	HIS	174	-13.699	-9.401	17.841	1.00	0.00	RX1	N
ATOM	3833	H	HIS	174	-12.933	-8.853	18.177	1.00	0.00	RX1	H
ATOM	3834	CA	HIS	174	-15.055	-9.147	18.312	1.00	0.00	RX1	C
ATOM	3835	CB	HIS	174	-15.061	-7.995	19.305	1.00	0.00	RX1	C
ATOM	3836	CG	HIS	174	-16.427	-7.725	19.880	1.00	0.00	RX1	C
ATOM	3837	ND1	HIS	174	-17.232	-8.666	20.405	1.00	0.00	RX1	N
ATOM	3838	HD1	HIS	174	-17.024	-9.626	20.514	1.00	0.00	RX1	H
ATOM	3839	CD2	HIS	174	-17.067	-6.487	19.963	1.00	0.00	RX1	C
ATOM	3840	NE2	HIS	174	-18.276	-6.696	20.537	1.00	0.00	RX1	N
ATOM	3841	CE1	HIS	174	-18.378	-8.038	20.813	1.00	0.00	RX1	C

ATOM	3842	C	HIS	174	-16.029	-8.912	17.188	1.00	0.00	RX1	C
ATOM	3843	O	HIS	174	-15.714	-8.360	16.142	1.00	0.00	RX1	O
ATOM	3844	N	LYS	175	-17.252	-9.365	17.453	1.00	0.00	RX1	N
ATOM	3845	H	LYS	175	-17.438	-9.766	18.353	1.00	0.00	RX1	H
ATOM	3846	CA	LYS	175	-18.237	-9.275	16.380	1.00	0.00	RX1	C
ATOM	3847	CB	LYS	175	-19.374	-10.254	16.683	1.00	0.00	RX1	C
ATOM	3848	CG	LYS	175	-18.819	-11.590	17.213	1.00	0.00	RX1	C
ATOM	3849	CD	LYS	175	-17.891	-12.344	16.246	1.00	0.00	RX1	C
ATOM	3850	CE	LYS	175	-17.107	-13.503	16.886	1.00	0.00	RX1	C
ATOM	3851	NZ	LYS	175	-16.001	-13.014	17.721	1.00	0.00	RX1	N
ATOM	3852	HZ1	LYS	175	-16.298	-12.565	18.617	1.00	0.00	RX1	H
ATOM	3853	HZ2	LYS	175	-15.325	-13.759	17.991	1.00	0.00	RX1	H
ATOM	3854	HZ3	LYS	175	-15.432	-12.294	17.239	1.00	0.00	RX1	H
ATOM	3855	C	LYS	175	-18.681	-7.847	16.040	1.00	0.00	RX1	C
ATOM	3856	O	LYS	175	-19.304	-7.573	15.025	1.00	0.00	RX1	O
ATOM	3857	N	ASN	176	-18.271	-6.924	16.928	1.00	0.00	RX1	N
ATOM	3858	H	ASN	176	-17.832	-7.206	17.777	1.00	0.00	RX1	H
ATOM	3859	CA	ASN	176	-18.310	-5.502	16.578	1.00	0.00	RX1	C
ATOM	3860	CB	ASN	176	-19.269	-4.712	17.471	1.00	0.00	RX1	C
ATOM	3861	CG	ASN	176	-19.417	-3.303	16.921	1.00	0.00	RX1	C
ATOM	3862	OD1	ASN	176	-18.958	-2.319	17.503	1.00	0.00	RX1	O
ATOM	3863	ND2	ASN	176	-20.102	-3.256	15.764	1.00	0.00	RX1	N
ATOM	3864	HD21	ASN	176	-20.455	-4.096	15.344	1.00	0.00	RX1	H
ATOM	3865	HD22	ASN	176	-20.301	-2.400	15.285	1.00	0.00	RX1	H
ATOM	3866	C	ASN	176	-16.923	-4.855	16.584	1.00	0.00	RX1	C
ATOM	3867	O	ASN	176	-16.712	-3.741	17.059	1.00	0.00	RX1	O
ATOM	3868	N	ASN	177	-15.955	-5.602	16.045	1.00	0.00	RX1	N
ATOM	3869	H	ASN	177	-16.105	-6.495	15.613	1.00	0.00	RX1	H
ATOM	3870	CA	ASN	177	-14.636	-4.977	15.965	1.00	0.00	RX1	C
ATOM	3871	CB	ASN	177	-13.504	-5.949	16.271	1.00	0.00	RX1	C
ATOM	3872	CG	ASN	177	-12.235	-5.162	16.528	1.00	0.00	RX1	C
ATOM	3873	OD1	ASN	177	-12.082	-3.994	16.166	1.00	0.00	RX1	O
ATOM	3874	ND2	ASN	177	-11.315	-5.880	17.194	1.00	0.00	RX1	N
ATOM	3875	HD21	ASN	177	-11.573	-6.738	17.648	1.00	0.00	RX1	H
ATOM	3876	HD22	ASN	177	-10.354	-5.623	17.322	1.00	0.00	RX1	H
ATOM	3877	C	ASN	177	-14.389	-4.340	14.618	1.00	0.00	RX1	C
ATOM	3878	O	ASN	177	-13.951	-4.969	13.666	1.00	0.00	RX1	O
ATOM	3879	N	GLN	178	-14.681	-3.033	14.584	1.00	0.00	RX1	N
ATOM	3880	H	GLN	178	-15.005	-2.572	15.409	1.00	0.00	RX1	H
ATOM	3881	CA	GLN	178	-14.436	-2.315	13.332	1.00	0.00	RX1	C
ATOM	3882	CB	GLN	178	-15.041	-0.903	13.373	1.00	0.00	RX1	C
ATOM	3883	CG	GLN	178	-14.249	0.170	14.132	1.00	0.00	RX1	C
ATOM	3884	CD	GLN	178	-14.126	-0.178	15.602	1.00	0.00	RX1	C
ATOM	3885	OE1	GLN	178	-15.038	-0.723	16.223	1.00	0.00	RX1	O
ATOM	3886	NE2	GLN	178	-12.935	0.154	16.126	1.00	0.00	RX1	N
ATOM	3887	HE21	GLN	178	-12.250	0.609	15.551	1.00	0.00	RX1	H
ATOM	3888	HE22	GLN	178	-12.665	-0.012	17.075	1.00	0.00	RX1	H
ATOM	3889	C	GLN	178	-12.981	-2.293	12.875	1.00	0.00	RX1	C
ATOM	3890	O	GLN	178	-12.666	-2.199	11.699	1.00	0.00	RX1	O
ATOM	3891	N	LEU	179	-12.095	-2.394	13.876	1.00	0.00	RX1	N
ATOM	3892	H	LEU	179	-12.360	-2.635	14.809	1.00	0.00	RX1	H
ATOM	3893	CA	LEU	179	-10.691	-2.512	13.510	1.00	0.00	RX1	C
ATOM	3894	CB	LEU	179	-9.811	-1.591	14.351	1.00	0.00	RX1	C
ATOM	3895	CG	LEU	179	-10.062	-0.107	14.097	1.00	0.00	RX1	C
ATOM	3896	CD1	LEU	179	-9.215	0.766	15.022	1.00	0.00	RX1	C
ATOM	3897	CD2	LEU	179	-9.874	0.260	12.624	1.00	0.00	RX1	C
ATOM	3898	C	LEU	179	-10.214	-3.938	13.665	1.00	0.00	RX1	C
ATOM	3899	O	LEU	179	-9.296	-4.232	14.418	1.00	0.00	RX1	O
ATOM	3900	N	ALA	180	-10.874	-4.826	12.912	1.00	0.00	RX1	N
ATOM	3901	H	ALA	180	-11.592	-4.529	12.281	1.00	0.00	RX1	H
ATOM	3902	CA	ALA	180	-10.442	-6.224	12.944	1.00	0.00	RX1	C

ATOM	3903	CB	ALA	180	-11.591	-7.146	12.536	1.00	0.00	RX1	C
ATOM	3904	C	ALA	180	-9.244	-6.503	12.044	1.00	0.00	RX1	C
ATOM	3905	O	ALA	180	-9.267	-7.339	11.151	1.00	0.00	RX1	O
ATOM	3906	N	LEU	181	-8.191	-5.718	12.298	1.00	0.00	RX1	N
ATOM	3907	H	LEU	181	-8.145	-5.196	13.150	1.00	0.00	RX1	H
ATOM	3908	CA	LEU	181	-7.066	-5.753	11.372	1.00	0.00	RX1	C
ATOM	3909	CB	LEU	181	-6.428	-4.370	11.281	1.00	0.00	RX1	C
ATOM	3910	CG	LEU	181	-7.441	-3.280	10.925	1.00	0.00	RX1	C
ATOM	3911	CD1	LEU	181	-6.816	-1.888	10.987	1.00	0.00	RX1	C
ATOM	3912	CD2	LEU	181	-8.122	-3.543	9.581	1.00	0.00	RX1	C
ATOM	3913	C	LEU	181	-6.053	-6.816	11.740	1.00	0.00	RX1	C
ATOM	3914	O	LEU	181	-5.922	-7.213	12.893	1.00	0.00	RX1	O
ATOM	3915	N	THR	182	-5.364	-7.283	10.700	1.00	0.00	RX1	N
ATOM	3916	H	THR	182	-5.487	-6.949	9.766	1.00	0.00	RX1	H
ATOM	3917	CA	THR	182	-4.695	-8.568	10.848	1.00	0.00	RX1	C
ATOM	3918	CB	THR	182	-5.469	-9.381	9.846	1.00	0.00	RX1	C
ATOM	3919	OG1	THR	182	-6.570	-8.547	9.440	1.00	0.00	RX1	O
ATOM	3920	HG1	THR	182	-7.255	-8.640	10.096	1.00	0.00	RX1	H
ATOM	3921	CG2	THR	182	-5.970	-10.711	10.409	1.00	0.00	RX1	C
ATOM	3922	C	THR	182	-3.188	-8.563	10.672	1.00	0.00	RX1	C
ATOM	3923	O	THR	182	-2.649	-9.223	9.791	1.00	0.00	RX1	O
ATOM	3924	N	LEU	183	-2.498	-7.824	11.554	1.00	0.00	RX1	N
ATOM	3925	H	LEU	183	-2.917	-7.386	12.352	1.00	0.00	RX1	H
ATOM	3926	CA	LEU	183	-1.046	-7.988	11.484	1.00	0.00	RX1	C
ATOM	3927	CB	LEU	183	-0.288	-6.727	11.891	1.00	0.00	RX1	C
ATOM	3928	CG	LEU	183	-0.120	-5.791	10.694	1.00	0.00	RX1	C
ATOM	3929	CD1	LEU	183	0.788	-4.602	11.011	1.00	0.00	RX1	C
ATOM	3930	CD2	LEU	183	0.365	-6.551	9.459	1.00	0.00	RX1	C
ATOM	3931	C	LEU	183	-0.528	-9.206	12.225	1.00	0.00	RX1	C
ATOM	3932	O	LEU	183	0.039	-9.151	13.311	1.00	0.00	RX1	O
ATOM	3933	N	ILE	184	-0.769	-10.336	11.558	1.00	0.00	RX1	N
ATOM	3934	H	ILE	184	-1.145	-10.286	10.630	1.00	0.00	RX1	H
ATOM	3935	CA	ILE	184	-0.245	-11.604	12.048	1.00	0.00	RX1	C
ATOM	3936	CB	ILE	184	-1.184	-12.739	11.649	1.00	0.00	RX1	C
ATOM	3937	CG2	ILE	184	-0.674	-14.084	12.165	1.00	0.00	RX1	C
ATOM	3938	CG1	ILE	184	-2.623	-12.455	12.070	1.00	0.00	RX1	C
ATOM	3939	CD1	ILE	184	-3.578	-13.512	11.514	1.00	0.00	RX1	C
ATOM	3940	C	ILE	184	1.110	-11.842	11.414	1.00	0.00	RX1	C
ATOM	3941	O	ILE	184	1.212	-12.046	10.212	1.00	0.00	RX1	O
ATOM	3942	N	ASP	185	2.144	-11.779	12.254	1.00	0.00	RX1	N
ATOM	3943	H	ASP	185	2.092	-11.696	13.250	1.00	0.00	RX1	H
ATOM	3944	CA	ASP	185	3.458	-11.989	11.663	1.00	0.00	RX1	C
ATOM	3945	CB	ASP	185	4.356	-10.820	12.028	1.00	0.00	RX1	C
ATOM	3946	CG	ASP	185	5.828	-11.135	11.900	1.00	0.00	RX1	C
ATOM	3947	OD1	ASP	185	6.409	-10.847	10.860	1.00	0.00	RX1	O
ATOM	3948	OD2	ASP	185	6.417	-11.604	12.869	1.00	0.00	RX1	O
ATOM	3949	C	ASP	185	4.042	-13.321	12.067	1.00	0.00	RX1	C
ATOM	3950	O	ASP	185	3.821	-13.844	13.155	1.00	0.00	RX1	O
ATOM	3951	N	THR	186	4.788	-13.854	11.104	1.00	0.00	RX1	N
ATOM	3952	H	THR	186	4.873	-13.427	10.206	1.00	0.00	RX1	H
ATOM	3953	CA	THR	186	5.407	-15.151	11.311	1.00	0.00	RX1	C
ATOM	3954	CB	THR	186	4.582	-15.957	10.343	1.00	0.00	RX1	C
ATOM	3955	OG1	THR	186	3.807	-14.992	9.610	1.00	0.00	RX1	O
ATOM	3956	HG1	THR	186	3.153	-15.480	9.125	1.00	0.00	RX1	H
ATOM	3957	CG2	THR	186	3.637	-16.931	11.048	1.00	0.00	RX1	C
ATOM	3958	C	THR	186	6.900	-15.124	11.032	1.00	0.00	RX1	C
ATOM	3959	O	THR	186	7.482	-16.086	10.542	1.00	0.00	RX1	O
ATOM	3960	N	ASN	187	7.501	-13.969	11.374	1.00	0.00	RX1	N
ATOM	3961	H	ASN	187	6.975	-13.223	11.792	1.00	0.00	RX1	H
ATOM	3962	CA	ASN	187	8.951	-13.803	11.229	1.00	0.00	RX1	C
ATOM	3963	CB	ASN	187	9.350	-12.346	11.423	1.00	0.00	RX1	C

ATOM	3964	CG	ASN	187	9.999	-11.835	10.166	1.00	0.00	RX1	C
ATOM	3965	OD1	ASN	187	11.199	-11.966	9.947	1.00	0.00	RX1	O
ATOM	3966	ND2	ASN	187	9.114	-11.245	9.342	1.00	0.00	RX1	N
ATOM	3967	HD21	ASN	187	8.163	-11.139	9.660	1.00	0.00	RX1	H
ATOM	3968	HD22	ASN	187	9.357	-10.920	8.428	1.00	0.00	RX1	H
ATOM	3969	C	ASN	187	9.751	-14.597	12.239	1.00	0.00	RX1	C
ATOM	3970	O	ASN	187	10.260	-14.078	13.226	1.00	0.00	RX1	O
ATOM	3971	N	ARG	188	9.794	-15.901	11.964	1.00	0.00	RX1	N
ATOM	3972	H	ARG	188	9.462	-16.252	11.087	1.00	0.00	RX1	H
ATOM	3973	CA	ARG	188	10.239	-16.818	13.002	1.00	0.00	RX1	C
ATOM	3974	CB	ARG	188	9.270	-17.984	13.113	1.00	0.00	RX1	C
ATOM	3975	CG	ARG	188	7.872	-17.626	13.593	1.00	0.00	RX1	C
ATOM	3976	CD	ARG	188	6.989	-18.869	13.535	1.00	0.00	RX1	C
ATOM	3977	NE	ARG	188	5.676	-18.606	14.103	1.00	0.00	RX1	N
ATOM	3978	HE	ARG	188	5.655	-18.006	14.918	1.00	0.00	RX1	H
ATOM	3979	CZ	ARG	188	4.589	-19.217	13.561	1.00	0.00	RX1	C
ATOM	3980	NH1	ARG	188	4.707	-20.032	12.495	1.00	0.00	RX1	N
ATOM	3981	HH11	ARG	188	3.859	-20.407	12.080	1.00	0.00	RX1	H
ATOM	3982	HH12	ARG	188	5.587	-20.276	12.084	1.00	0.00	RX1	H
ATOM	3983	NH2	ARG	188	3.384	-18.993	14.091	1.00	0.00	RX1	N
ATOM	3984	HH21	ARG	188	2.567	-19.465	13.714	1.00	0.00	RX1	H
ATOM	3985	HH22	ARG	188	3.252	-18.350	14.849	1.00	0.00	RX1	H
ATOM	3986	C	ARG	188	11.620	-17.392	12.805	1.00	0.00	RX1	C
ATOM	3987	O	ARG	188	12.076	-17.665	11.704	1.00	0.00	RX1	O
ATOM	3988	N	SER	189	12.233	-17.633	13.965	1.00	0.00	RX1	N
ATOM	3989	H	SER	189	11.880	-17.319	14.844	1.00	0.00	RX1	H
ATOM	3990	CA	SER	189	13.373	-18.532	14.025	1.00	0.00	RX1	C
ATOM	3991	CB	SER	189	14.299	-17.944	15.081	1.00	0.00	RX1	C
ATOM	3992	OG	SER	189	13.494	-17.370	16.121	1.00	0.00	RX1	O
ATOM	3993	HG	SER	189	13.473	-18.027	16.817	1.00	0.00	RX1	H
ATOM	3994	C	SER	189	12.973	-19.978	14.298	1.00	0.00	RX1	C
ATOM	3995	O	SER	189	13.771	-20.900	14.174	1.00	0.00	RX1	O
ATOM	3996	N	ARG	190	11.698	-20.151	14.691	1.00	0.00	RX1	N
ATOM	3997	H	ARG	190	11.024	-19.415	14.762	1.00	0.00	RX1	H
ATOM	3998	CA	ARG	190	11.280	-21.521	14.965	1.00	0.00	RX1	C
ATOM	3999	CB	ARG	190	11.191	-21.794	16.465	1.00	0.00	RX1	C
ATOM	4000	CG	ARG	190	9.993	-21.169	17.175	1.00	0.00	RX1	C
ATOM	4001	CD	ARG	190	9.772	-21.880	18.508	1.00	0.00	RX1	C
ATOM	4002	NE	ARG	190	8.566	-21.437	19.206	1.00	0.00	RX1	N
ATOM	4003	HE	ARG	190	8.536	-20.474	19.510	1.00	0.00	RX1	H
ATOM	4004	CZ	ARG	190	7.678	-22.409	19.573	1.00	0.00	RX1	C
ATOM	4005	NH1	ARG	190	7.751	-23.624	18.994	1.00	0.00	RX1	N
ATOM	4006	HH11	ARG	190	7.260	-24.399	19.436	1.00	0.00	RX1	H
ATOM	4007	HH12	ARG	190	8.258	-23.816	18.144	1.00	0.00	RX1	H
ATOM	4008	NH2	ARG	190	6.745	-22.164	20.517	1.00	0.00	RX1	N
ATOM	4009	HH21	ARG	190	6.152	-22.914	20.857	1.00	0.00	RX1	H
ATOM	4010	HH22	ARG	190	6.614	-21.256	20.937	1.00	0.00	RX1	H
ATOM	4011	C	ARG	190	9.986	-21.936	14.294	1.00	0.00	RX1	C
ATOM	4012	O	ARG	190	9.186	-21.121	13.857	1.00	0.00	RX1	O
ATOM	4013	N	ALA	191	9.803	-23.257	14.265	1.00	0.00	RX1	N
ATOM	4014	H	ALA	191	10.486	-23.873	14.652	1.00	0.00	RX1	H
ATOM	4015	CA	ALA	191	8.490	-23.780	13.921	1.00	0.00	RX1	C
ATOM	4016	CB	ALA	191	8.623	-25.046	13.074	1.00	0.00	RX1	C
ATOM	4017	C	ALA	191	7.686	-24.106	15.167	1.00	0.00	RX1	C
ATOM	4018	O	ALA	191	8.204	-24.209	16.280	1.00	0.00	RX1	O
ATOM	4019	N	CYS	192	6.380	-24.270	14.917	1.00	0.00	RX1	N
ATOM	4020	H	CYS	192	6.027	-24.247	13.985	1.00	0.00	RX1	H
ATOM	4021	CA	CYS	192	5.502	-24.737	15.988	1.00	0.00	RX1	C
ATOM	4022	CB	CYS	192	4.061	-24.456	15.570	1.00	0.00	RX1	C
ATOM	4023	SG	CYS	192	3.920	-22.792	14.866	1.00	0.00	RX1	S
ATOM	4024	C	CYS	192	5.740	-26.203	16.285	1.00	0.00	RX1	C

ATOM	4025	O	CYS	192	6.375	-26.908	15.509	1.00	0.00	RX1	O
ATOM	4026	N	HIS	193	5.221	-26.629	17.436	1.00	0.00	RX1	N
ATOM	4027	H	HIS	193	4.596	-26.074	17.988	1.00	0.00	RX1	H
ATOM	4028	CA	HIS	193	5.238	-28.064	17.690	1.00	0.00	RX1	C
ATOM	4029	CB	HIS	193	5.475	-28.305	19.188	1.00	0.00	RX1	C
ATOM	4030	CG	HIS	193	6.956	-28.221	19.488	1.00	0.00	RX1	C
ATOM	4031	ND1	HIS	193	7.503	-28.596	20.658	1.00	0.00	RX1	N
ATOM	4032	HD1	HIS	193	7.017	-28.918	21.446	1.00	0.00	RX1	H
ATOM	4033	CD2	HIS	193	7.978	-27.766	18.648	1.00	0.00	RX1	C
ATOM	4034	NE2	HIS	193	9.152	-27.868	19.321	1.00	0.00	RX1	N
ATOM	4035	CE1	HIS	193	8.854	-28.382	20.560	1.00	0.00	RX1	C
ATOM	4036	C	HIS	193	3.921	-28.647	17.200	1.00	0.00	RX1	C
ATOM	4037	O	HIS	193	2.940	-27.925	17.077	1.00	0.00	RX1	O
ATOM	4038	N	PRO	194	3.920	-29.962	16.863	1.00	0.00	RX1	N
ATOM	4039	CD	PRO	194	5.051	-30.884	16.869	1.00	0.00	RX1	C
ATOM	4040	CA	PRO	194	2.680	-30.589	16.382	1.00	0.00	RX1	C
ATOM	4041	CB	PRO	194	3.061	-32.071	16.312	1.00	0.00	RX1	C
ATOM	4042	CG	PRO	194	4.565	-32.072	16.047	1.00	0.00	RX1	C
ATOM	4043	C	PRO	194	1.440	-30.327	17.228	1.00	0.00	RX1	C
ATOM	4044	O	PRO	194	1.343	-30.720	18.384	1.00	0.00	RX1	O
ATOM	4045	N	CYS	195	0.487	-29.646	16.574	1.00	0.00	RX1	N
ATOM	4046	H	CYS	195	0.640	-29.311	15.647	1.00	0.00	RX1	H
ATOM	4047	CA	CYS	195	-0.775	-29.328	17.242	1.00	0.00	RX1	C
ATOM	4048	CB	CYS	195	-1.634	-28.560	16.244	1.00	0.00	RX1	C
ATOM	4049	SG	CYS	195	-0.631	-27.397	15.283	1.00	0.00	RX1	S
ATOM	4050	C	CYS	195	-1.512	-30.547	17.767	1.00	0.00	RX1	C
ATOM	4051	O	CYS	195	-1.735	-31.521	17.057	1.00	0.00	RX1	O
ATOM	4052	N	SER	196	-1.882	-30.461	19.048	1.00	0.00	RX1	N
ATOM	4053	H	SER	196	-1.783	-29.634	19.598	1.00	0.00	RX1	H
ATOM	4054	CA	SER	196	-2.615	-31.597	19.595	1.00	0.00	RX1	C
ATOM	4055	CB	SER	196	-2.453	-31.517	21.110	1.00	0.00	RX1	C
ATOM	4056	OG	SER	196	-3.059	-30.310	21.571	1.00	0.00	RX1	O
ATOM	4057	HG	SER	196	-2.352	-29.696	21.771	1.00	0.00	RX1	H
ATOM	4058	C	SER	196	-4.071	-31.606	19.133	1.00	0.00	RX1	C
ATOM	4059	O	SER	196	-4.614	-30.569	18.769	1.00	0.00	RX1	O
ATOM	4060	N	PRO	197	-4.730	-32.793	19.183	1.00	0.00	RX1	N
ATOM	4061	CD	PRO	197	-4.192	-34.115	19.485	1.00	0.00	RX1	C
ATOM	4062	CA	PRO	197	-6.176	-32.800	18.915	1.00	0.00	RX1	C
ATOM	4063	CB	PRO	197	-6.516	-34.295	18.976	1.00	0.00	RX1	C
ATOM	4064	CG	PRO	197	-5.427	-34.934	19.840	1.00	0.00	RX1	C
ATOM	4065	C	PRO	197	-6.998	-31.934	19.872	1.00	0.00	RX1	C
ATOM	4066	O	PRO	197	-8.033	-31.383	19.518	1.00	0.00	RX1	O
ATOM	4067	N	MET	198	-6.475	-31.796	21.108	1.00	0.00	RX1	N
ATOM	4068	H	MET	198	-5.602	-32.215	21.345	1.00	0.00	RX1	H
ATOM	4069	CA	MET	198	-7.136	-30.887	22.053	1.00	0.00	RX1	C
ATOM	4070	CB	MET	198	-6.505	-31.002	23.437	1.00	0.00	RX1	C
ATOM	4071	CG	MET	198	-6.472	-32.440	23.952	1.00	0.00	RX1	C
ATOM	4072	SD	MET	198	-8.106	-33.192	23.993	1.00	0.00	RX1	S
ATOM	4073	CE	MET	198	-7.615	-34.817	24.589	1.00	0.00	RX1	C
ATOM	4074	C	MET	198	-7.138	-29.436	21.593	1.00	0.00	RX1	C
ATOM	4075	O	MET	198	-8.051	-28.648	21.831	1.00	0.00	RX1	O
ATOM	4076	N	CYS	199	-6.089	-29.141	20.817	1.00	0.00	RX1	N
ATOM	4077	H	CYS	199	-5.330	-29.774	20.659	1.00	0.00	RX1	H
ATOM	4078	CA	CYS	199	-6.109	-27.929	20.011	1.00	0.00	RX1	C
ATOM	4079	CB	CYS	199	-4.673	-27.555	19.691	1.00	0.00	RX1	C
ATOM	4080	SG	CYS	199	-3.652	-27.524	21.180	1.00	0.00	RX1	S
ATOM	4081	C	CYS	199	-6.945	-28.043	18.746	1.00	0.00	RX1	C
ATOM	4082	O	CYS	199	-6.498	-27.726	17.654	1.00	0.00	RX1	O
ATOM	4083	N	LYS	200	-8.204	-28.482	18.936	1.00	0.00	RX1	N
ATOM	4084	H	LYS	200	-8.465	-28.846	19.828	1.00	0.00	RX1	H
ATOM	4085	CA	LYS	200	-9.173	-28.532	17.838	1.00	0.00	RX1	C

ATOM	4086	CB	LYS	200	-10.608	-28.724	18.367	1.00	0.00	RX1	C
ATOM	4087	CG	LYS	200	-11.224	-27.536	19.127	1.00	0.00	RX1	C
ATOM	4088	CD	LYS	200	-10.669	-27.330	20.538	1.00	0.00	RX1	C
ATOM	4089	CE	LYS	200	-11.015	-25.978	21.157	1.00	0.00	RX1	C
ATOM	4090	NZ	LYS	200	-10.333	-25.861	22.453	1.00	0.00	RX1	N
ATOM	4091	HZ1	LYS	200	-10.275	-24.851	22.711	1.00	0.00	RX1	H
ATOM	4092	HZ2	LYS	200	-10.860	-26.371	23.188	1.00	0.00	RX1	H
ATOM	4093	HZ3	LYS	200	-9.377	-26.263	22.379	1.00	0.00	RX1	H
ATOM	4094	C	LYS	200	-9.114	-27.367	16.865	1.00	0.00	RX1	C
ATOM	4095	O	LYS	200	-9.074	-26.197	17.235	1.00	0.00	RX1	O
ATOM	4096	N	GLY	201	-9.061	-27.763	15.591	1.00	0.00	RX1	N
ATOM	4097	H	GLY	201	-9.010	-28.735	15.368	1.00	0.00	RX1	H
ATOM	4098	CA	GLY	201	-8.913	-26.752	14.549	1.00	0.00	RX1	C
ATOM	4099	C	GLY	201	-7.538	-26.106	14.494	1.00	0.00	RX1	C
ATOM	4100	O	GLY	201	-7.380	-25.004	13.987	1.00	0.00	RX1	O
ATOM	4101	N	SER	202	-6.561	-26.858	15.035	1.00	0.00	RX1	N
ATOM	4102	H	SER	202	-6.771	-27.703	15.521	1.00	0.00	RX1	H
ATOM	4103	CA	SER	202	-5.156	-26.438	15.107	1.00	0.00	RX1	C
ATOM	4104	CB	SER	202	-4.655	-26.167	13.690	1.00	0.00	RX1	C
ATOM	4105	OG	SER	202	-5.498	-26.899	12.789	1.00	0.00	RX1	O
ATOM	4106	HG	SER	202	-6.119	-26.250	12.478	1.00	0.00	RX1	H
ATOM	4107	C	SER	202	-4.847	-25.344	16.120	1.00	0.00	RX1	C
ATOM	4108	O	SER	202	-3.765	-24.777	16.163	1.00	0.00	RX1	O
ATOM	4109	N	ARG	203	-5.857	-25.048	16.947	1.00	0.00	RX1	N
ATOM	4110	H	ARG	203	-6.642	-25.662	17.036	1.00	0.00	RX1	H
ATOM	4111	CA	ARG	203	-5.732	-23.838	17.752	1.00	0.00	RX1	C
ATOM	4112	CB	ARG	203	-7.108	-23.235	18.001	1.00	0.00	RX1	C
ATOM	4113	CG	ARG	203	-7.921	-23.009	16.728	1.00	0.00	RX1	C
ATOM	4114	CD	ARG	203	-9.272	-22.379	17.058	1.00	0.00	RX1	C
ATOM	4115	NE	ARG	203	-10.160	-22.328	15.901	1.00	0.00	RX1	N
ATOM	4116	HE	ARG	203	-10.388	-23.222	15.503	1.00	0.00	RX1	H
ATOM	4117	CZ	ARG	203	-10.665	-21.126	15.484	1.00	0.00	RX1	C
ATOM	4118	NH1	ARG	203	-10.214	-19.968	16.018	1.00	0.00	RX1	N
ATOM	4119	HH11	ARG	203	-10.524	-19.077	15.651	1.00	0.00	RX1	H
ATOM	4120	HH12	ARG	203	-9.551	-19.925	16.780	1.00	0.00	RX1	H
ATOM	4121	NH2	ARG	203	-11.622	-21.109	14.535	1.00	0.00	RX1	N
ATOM	4122	HH21	ARG	203	-12.047	-20.228	14.264	1.00	0.00	RX1	H
ATOM	4123	HH22	ARG	203	-11.963	-21.933	14.080	1.00	0.00	RX1	H
ATOM	4124	C	ARG	203	-4.979	-24.018	19.063	1.00	0.00	RX1	C
ATOM	4125	O	ARG	203	-5.566	-24.076	20.141	1.00	0.00	RX1	O
ATOM	4126	N	CYS	204	-3.650	-24.104	18.922	1.00	0.00	RX1	N
ATOM	4127	H	CYS	204	-3.226	-24.063	18.014	1.00	0.00	RX1	H
ATOM	4128	CA	CYS	204	-2.796	-24.118	20.111	1.00	0.00	RX1	C
ATOM	4129	CB	CYS	204	-1.699	-25.159	19.929	1.00	0.00	RX1	C
ATOM	4130	SG	CYS	204	-1.965	-26.098	18.410	1.00	0.00	RX1	S
ATOM	4131	C	CYS	204	-2.160	-22.774	20.371	1.00	0.00	RX1	C
ATOM	4132	O	CYS	204	-1.808	-22.054	19.446	1.00	0.00	RX1	O
ATOM	4133	N	TRP	205	-2.051	-22.481	21.673	1.00	0.00	RX1	N
ATOM	4134	H	TRP	205	-2.356	-23.148	22.353	1.00	0.00	RX1	H
ATOM	4135	CA	TRP	205	-1.409	-21.250	22.137	1.00	0.00	RX1	C
ATOM	4136	CB	TRP	205	-2.052	-20.793	23.436	1.00	0.00	RX1	C
ATOM	4137	CG	TRP	205	-3.454	-20.280	23.250	1.00	0.00	RX1	C
ATOM	4138	CD2	TRP	205	-3.883	-18.974	22.811	1.00	0.00	RX1	C
ATOM	4139	CE2	TRP	205	-5.295	-18.946	22.885	1.00	0.00	RX1	C
ATOM	4140	CE3	TRP	205	-3.198	-17.846	22.379	1.00	0.00	RX1	C
ATOM	4141	CD1	TRP	205	-4.631	-20.970	23.551	1.00	0.00	RX1	C
ATOM	4142	NE1	TRP	205	-5.719	-20.185	23.343	1.00	0.00	RX1	N
ATOM	4143	HE1	TRP	205	-6.648	-20.415	23.557	1.00	0.00	RX1	H
ATOM	4144	CZ2	TRP	205	-5.984	-17.795	22.529	1.00	0.00	RX1	C
ATOM	4145	CZ3	TRP	205	-3.898	-16.702	22.023	1.00	0.00	RX1	C
ATOM	4146	CH2	TRP	205	-5.285	-16.674	22.101	1.00	0.00	RX1	C

ATOM	4147	C	TRP	205	0.074	-21.424	22.426	1.00	0.00	RX1	C
ATOM	4148	O	TRP	205	0.902	-20.535	22.254	1.00	0.00	RX1	O
ATOM	4149	N	GLY	206	0.355	-22.638	22.913	1.00	0.00	RX1	N
ATOM	4150	H	GLY	206	-0.318	-23.374	22.969	1.00	0.00	RX1	H
ATOM	4151	CA	GLY	206	1.727	-22.972	23.267	1.00	0.00	RX1	C
ATOM	4152	C	GLY	206	2.063	-24.333	22.713	1.00	0.00	RX1	C
ATOM	4153	O	GLY	206	1.248	-24.909	21.998	1.00	0.00	RX1	O
ATOM	4154	N	GLU	207	3.250	-24.840	23.084	1.00	0.00	RX1	N
ATOM	4155	H	GLU	207	3.843	-24.309	23.701	1.00	0.00	RX1	H
ATOM	4156	CA	GLU	207	3.585	-26.224	22.717	1.00	0.00	RX1	C
ATOM	4157	CB	GLU	207	5.059	-26.546	22.998	1.00	0.00	RX1	C
ATOM	4158	CG	GLU	207	6.084	-25.417	22.834	1.00	0.00	RX1	C
ATOM	4159	CD	GLU	207	6.525	-25.196	21.400	1.00	0.00	RX1	C
ATOM	4160	OE1	GLU	207	5.746	-24.744	20.574	1.00	0.00	RX1	O
ATOM	4161	OE2	GLU	207	7.695	-25.397	21.103	1.00	0.00	RX1	O
ATOM	4162	C	GLU	207	2.739	-27.216	23.512	1.00	0.00	RX1	C
ATOM	4163	O	GLU	207	3.176	-27.779	24.508	1.00	0.00	RX1	O
ATOM	4164	N	SER	208	1.478	-27.365	23.087	1.00	0.00	RX1	N
ATOM	4165	H	SER	208	1.124	-26.933	22.256	1.00	0.00	RX1	H
ATOM	4166	CA	SER	208	0.561	-27.818	24.126	1.00	0.00	RX1	C
ATOM	4167	CB	SER	208	0.101	-26.562	24.881	1.00	0.00	RX1	C
ATOM	4168	OG	SER	208	-0.267	-26.865	26.235	1.00	0.00	RX1	O
ATOM	4169	HG	SER	208	0.528	-26.732	26.747	1.00	0.00	RX1	H
ATOM	4170	C	SER	208	-0.591	-28.705	23.692	1.00	0.00	RX1	C
ATOM	4171	O	SER	208	-1.069	-28.703	22.561	1.00	0.00	RX1	O
ATOM	4172	N	SER	209	-1.038	-29.448	24.707	1.00	0.00	RX1	N
ATOM	4173	H	SER	209	-0.499	-29.579	25.537	1.00	0.00	RX1	H
ATOM	4174	CA	SER	209	-2.352	-30.081	24.679	1.00	0.00	RX1	C
ATOM	4175	CB	SER	209	-1.987	-31.488	25.077	1.00	0.00	RX1	C
ATOM	4176	OG	SER	209	-0.549	-31.484	25.089	1.00	0.00	RX1	O
ATOM	4177	HG	SER	209	-0.281	-32.385	25.219	1.00	0.00	RX1	H
ATOM	4178	C	SER	209	-3.362	-29.391	25.589	1.00	0.00	RX1	C
ATOM	4179	O	SER	209	-4.533	-29.739	25.636	1.00	0.00	RX1	O
ATOM	4180	N	GLU	210	-2.826	-28.418	26.339	1.00	0.00	RX1	N
ATOM	4181	H	GLU	210	-1.908	-28.060	26.173	1.00	0.00	RX1	H
ATOM	4182	CA	GLU	210	-3.585	-27.801	27.419	1.00	0.00	RX1	C
ATOM	4183	CB	GLU	210	-2.686	-27.855	28.655	1.00	0.00	RX1	C
ATOM	4184	CG	GLU	210	-3.242	-28.672	29.821	1.00	0.00	RX1	C
ATOM	4185	CD	GLU	210	-4.190	-27.799	30.606	1.00	0.00	RX1	C
ATOM	4186	OE1	GLU	210	-3.723	-26.968	31.378	1.00	0.00	RX1	O
ATOM	4187	OE2	GLU	210	-5.397	-27.891	30.417	1.00	0.00	RX1	O
ATOM	4188	C	GLU	210	-3.995	-26.382	27.061	1.00	0.00	RX1	C
ATOM	4189	O	GLU	210	-5.156	-25.985	27.085	1.00	0.00	RX1	O
ATOM	4190	N	ASP	211	-2.966	-25.613	26.672	1.00	0.00	RX1	N
ATOM	4191	H	ASP	211	-2.028	-25.960	26.638	1.00	0.00	RX1	H
ATOM	4192	CA	ASP	211	-3.270	-24.274	26.169	1.00	0.00	RX1	C
ATOM	4193	CB	ASP	211	-2.070	-23.332	26.288	1.00	0.00	RX1	C
ATOM	4194	CG	ASP	211	-2.038	-22.729	27.676	1.00	0.00	RX1	C
ATOM	4195	OD1	ASP	211	-2.666	-21.697	27.897	1.00	0.00	RX1	O
ATOM	4196	OD2	ASP	211	-1.399	-23.293	28.556	1.00	0.00	RX1	O
ATOM	4197	C	ASP	211	-3.807	-24.278	24.757	1.00	0.00	RX1	C
ATOM	4198	O	ASP	211	-3.093	-24.153	23.765	1.00	0.00	RX1	O
ATOM	4199	N	CYS	212	-5.131	-24.439	24.725	1.00	0.00	RX1	N
ATOM	4200	H	CYS	212	-5.650	-24.553	25.575	1.00	0.00	RX1	H
ATOM	4201	CA	CYS	212	-5.829	-24.511	23.448	1.00	0.00	RX1	C
ATOM	4202	CB	CYS	212	-6.337	-25.936	23.283	1.00	0.00	RX1	C
ATOM	4203	SG	CYS	212	-5.102	-27.152	23.795	1.00	0.00	RX1	S
ATOM	4204	C	CYS	212	-6.952	-23.495	23.434	1.00	0.00	RX1	C
ATOM	4205	O	CYS	212	-7.514	-23.187	24.476	1.00	0.00	RX1	O
ATOM	4206	N	GLN	213	-7.274	-22.972	22.238	1.00	0.00	RX1	N
ATOM	4207	H	GLN	213	-6.766	-23.245	21.418	1.00	0.00	RX1	H

```
ATOM   4208  CA  GLN   213      -8.230 -21.858  22.212  1.00  0.00      RX1  C
ATOM   4209  CB  GLN   213      -8.440 -21.338  20.790  1.00  0.00      RX1  C
ATOM   4210  CG  GLN   213      -8.870 -19.870  20.743  1.00  0.00      RX1  C
ATOM   4211  CD  GLN   213      -9.496 -19.550  19.399  1.00  0.00      RX1  C
ATOM   4212  OE1 GLN   213      -8.860 -19.549  18.347  1.00  0.00      RX1  O
ATOM   4213  NE2 GLN   213     -10.806 -19.276  19.493  1.00  0.00      RX1  N
ATOM   4214 HE21 GLN   213     -11.250 -19.379  20.389  1.00  0.00      RX1  H
ATOM   4215 HE22 GLN   213     -11.389 -18.967  18.743  1.00  0.00      RX1  H
ATOM   4216  C   GLN   213      -9.578 -22.140  22.858  1.00  0.00      RX1  C
ATOM   4217  O   GLN   213     -10.185 -23.191  22.642  1.00  0.00      RX1  O
ATOM   4218  N   SER   214     -10.019 -21.148  23.640  1.00  0.00      RX1  N
ATOM   4219  H   SER   214      -9.465 -20.332  23.815  1.00  0.00      RX1  H
ATOM   4220  CA  SER   214     -11.420 -21.140  24.048  1.00  0.00      RX1  C
ATOM   4221  CB  SER   214     -11.496 -20.370  25.347  1.00  0.00      RX1  C
ATOM   4222  OG  SER   214     -10.529 -20.970  26.216  1.00  0.00      RX1  O
ATOM   4223  HG  SER   214      -9.665 -20.814  25.842  1.00  0.00      RX1  H
ATOM   4224  C   SER   214     -12.329 -20.659  22.931  1.00  0.00      RX1  C
ATOM   4225  O   SER   214     -11.932 -20.593  21.769  1.00  0.00      RX1  O
TER
HEADER lig.000.00.pdb
ATOM      1  N   PRO   563      50.099 -12.288  -0.759  1.00  0.00      LX0  N
ATOM      2  CD  PRO   563      50.957 -13.446  -0.537  1.00  0.00      LX0  C
ATOM      3  CA  PRO   563      49.162 -12.031   0.342  1.00  0.00      LX0  C
ATOM      4  CB  PRO   563      49.289 -13.320   1.162  1.00  0.00      LX0  C
ATOM      5  CG  PRO   563      50.722 -13.799   0.927  1.00  0.00      LX0  C
ATOM      6  C   PRO   563      49.506 -10.774   1.131  1.00  0.00      LX0  C
ATOM      7  O   PRO   563      49.098 -10.586   2.271  1.00  0.00      LX0  O
ATOM      8  N   GLN   564      50.295  -9.912   0.472  1.00  0.00      LX0  N
ATOM      9  H   GLN   564      50.532 -10.081  -0.482  0.00  0.00      LX0  H
ATOM     10  CA  GLN   564      50.632  -8.675   1.165  1.00  0.00      LX0  C
ATOM     11  CB  GLN   564      51.847  -8.006   0.517  1.00  0.00      LX0  C
ATOM     12  CG  GLN   564      52.704  -7.217   1.513  1.00  0.00      LX0  C
ATOM     13  CD  GLN   564      53.339  -8.181   2.498  1.00  0.00      LX0  C
ATOM     14  OE1 GLN   564      54.044  -9.110   2.135  1.00  0.00      LX0  O
ATOM     15  NE2 GLN   564      53.041  -7.938   3.775  1.00  0.00      LX0  N
ATOM     16 HE21 GLN   564      52.465  -7.165   4.054  0.00  0.00      LX0  H
ATOM     17 HE22 GLN   564      53.418  -8.546   4.468  0.00  0.00      LX0  H
ATOM     18  C   GLN   564      49.450  -7.729   1.223  1.00  0.00      LX0  C
ATOM     19  O   GLN   564      48.696  -7.579   0.268  1.00  0.00      LX0  O
ATOM     20  N   LYS   565      49.302  -7.109   2.395  1.00  0.00      LX0  N
ATOM     21  H   LYS   565      50.009  -7.135   3.105  0.00  0.00      LX0  H
ATOM     22  CA  LYS   565      48.162  -6.213   2.514  1.00  0.00      LX0  C
ATOM     23  CB  LYS   565      47.776  -6.096   3.987  1.00  0.00      LX0  C
ATOM     24  CG  LYS   565      47.390  -7.454   4.579  1.00  0.00      LX0  C
ATOM     25  CD  LYS   565      47.390  -7.542   6.111  1.00  0.00      LX0  C
ATOM     26  CE  LYS   565      48.788  -7.560   6.747  1.00  0.00      LX0  C
ATOM     27  NZ  LYS   565      49.339  -6.207   6.853  1.00  0.00      LX0  N
ATOM     28  HZ1 LYS   565      49.332  -5.863   7.834  0.00  0.00      LX0  H
ATOM     29  HZ2 LYS   565      50.305  -6.117   6.458  0.00  0.00      LX0  H
ATOM     30  HZ3 LYS   565      48.787  -5.528   6.296  0.00  0.00      LX0  H
ATOM     31  C   LYS   565      48.481  -4.872   1.891  1.00  0.00      LX0  C
ATOM     32  O   LYS   565      49.093  -4.004   2.493  1.00  0.00      LX0  O
ATOM     33  N   ILE   566      48.068  -4.737   0.631  1.00  0.00      LX0  N
ATOM     34  H   ILE   566      47.605  -5.496   0.171  0.00  0.00      LX0  H
ATOM     35  CA  ILE   566      48.416  -3.475  -0.015  1.00  0.00      LX0  C
ATOM     36  CB  ILE   566      48.409  -3.614  -1.545  1.00  0.00      LX0  C
ATOM     37  CG2 ILE   566      48.947  -2.347  -2.222  1.00  0.00      LX0  C
ATOM     38  CG1 ILE   566      49.191  -4.858  -1.983  1.00  0.00      LX0  C
ATOM     39  CD1 ILE   566      49.069  -5.148  -3.480  1.00  0.00      LX0  C
ATOM     40  C   ILE   566      47.550  -2.311   0.440  1.00  0.00      LX0  C
ATOM     41  O   ILE   566      46.375  -2.199   0.108  1.00  0.00      LX0  O
```

ATOM	42	N	CYS	567	48.194	-1.419	1.202	1.00	0.00	LX0	N
ATOM	43	H	CYS	567	49.144	-1.598	1.470	0.00	0.00	LX0	H
ATOM	44	CA	CYS	567	47.521	-0.164	1.531	1.00	0.00	LX0	C
ATOM	45	CB	CYS	567	48.203	0.546	2.705	1.00	0.00	LX0	C
ATOM	46	SG	CYS	567	47.352	2.032	3.302	1.00	0.00	LX0	S
ATOM	47	C	CYS	567	47.460	0.771	0.346	1.00	0.00	LX0	C
ATOM	48	O	CYS	567	48.260	1.690	0.206	1.00	0.00	LX0	O
ATOM	49	N	LEU	568	46.437	0.526	-0.492	1.00	0.00	LX0	N
ATOM	50	H	LEU	568	45.918	-0.319	-0.334	0.00	0.00	LX0	H
ATOM	51	CA	LEU	568	46.172	1.394	-1.647	1.00	0.00	LX0	C
ATOM	52	CB	LEU	568	44.857	0.986	-2.314	1.00	0.00	LX0	C
ATOM	53	CG	LEU	568	44.846	-0.457	-2.825	1.00	0.00	LX0	C
ATOM	54	CD1	LEU	568	43.437	-0.907	-3.213	1.00	0.00	LX0	C
ATOM	55	CD2	LEU	568	45.847	-0.673	-3.962	1.00	0.00	LX0	C
ATOM	56	C	LEU	568	46.138	2.876	-1.302	1.00	0.00	LX0	C
ATOM	57	O	LEU	568	46.556	3.758	-2.050	1.00	0.00	LX0	O
ATOM	58	N	ILE	569	45.653	3.096	-0.071	1.00	0.00	LX0	N
ATOM	59	H	ILE	569	45.271	2.315	0.422	0.00	0.00	LX0	H
ATOM	60	CA	ILE	569	45.698	4.418	0.549	1.00	0.00	LX0	C
ATOM	61	CB	ILE	569	45.178	4.310	1.992	1.00	0.00	LX0	C
ATOM	62	CG2	ILE	569	45.273	5.629	2.759	1.00	0.00	LX0	C
ATOM	63	CG1	ILE	569	43.758	3.736	2.012	1.00	0.00	LX0	C
ATOM	64	CD1	ILE	569	42.748	4.568	1.219	1.00	0.00	LX0	C
ATOM	65	C	ILE	569	47.069	5.083	0.485	1.00	0.00	LX0	C
ATOM	66	O	ILE	569	47.240	6.129	-0.131	1.00	0.00	LX0	O
ATOM	67	N	CYS	570	48.046	4.435	1.128	1.00	0.00	LX0	N
ATOM	68	H	CYS	570	47.906	3.521	1.513	0.00	0.00	LX0	H
ATOM	69	CA	CYS	570	49.307	5.159	1.267	1.00	0.00	LX0	C
ATOM	70	CB	CYS	570	49.654	5.317	2.744	1.00	0.00	LX0	C
ATOM	71	SG	CYS	570	50.069	3.738	3.522	1.00	0.00	LX0	S
ATOM	72	C	CYS	570	50.489	4.595	0.500	1.00	0.00	LX0	C
ATOM	73	O	CYS	570	51.623	5.027	0.677	1.00	0.00	LX0	O
ATOM	74	N	GLY	571	50.181	3.603	-0.348	1.00	0.00	LX0	N
ATOM	75	H	GLY	571	49.252	3.235	-0.377	0.00	0.00	LX0	H
ATOM	76	CA	GLY	571	51.229	2.987	-1.160	1.00	0.00	LX0	C
ATOM	77	C	GLY	571	52.261	2.184	-0.383	1.00	0.00	LX0	C
ATOM	78	O	GLY	571	53.443	2.182	-0.715	1.00	0.00	LX0	O
ATOM	79	N	ASP	572	51.773	1.512	0.670	1.00	0.00	LX0	N
ATOM	80	H	ASP	572	50.792	1.473	0.858	0.00	0.00	LX0	H
ATOM	81	CA	ASP	572	52.696	0.724	1.491	1.00	0.00	LX0	C
ATOM	82	CB	ASP	572	53.391	1.587	2.550	1.00	0.00	LX0	C
ATOM	83	CG	ASP	572	54.852	1.699	2.164	1.00	0.00	LX0	C
ATOM	84	OD1	ASP	572	55.503	0.681	1.972	1.00	0.00	LX0	O
ATOM	85	OD2	ASP	572	55.343	2.800	1.942	1.00	0.00	LX0	O
ATOM	86	C	ASP	572	52.015	-0.466	2.123	1.00	0.00	LX0	C
ATOM	87	O	ASP	572	50.872	-0.755	1.788	1.00	0.00	LX0	O
ATOM	88	N	GLU	573	52.732	-1.131	3.045	1.00	0.00	LX0	N
ATOM	89	H	GLU	573	53.670	-0.855	3.258	0.00	0.00	LX0	H
ATOM	90	CA	GLU	573	52.062	-2.200	3.788	1.00	0.00	LX0	C
ATOM	91	CB	GLU	573	53.066	-3.010	4.612	1.00	0.00	LX0	C
ATOM	92	CG	GLU	573	52.884	-4.538	4.568	1.00	0.00	LX0	C
ATOM	93	CD	GLU	573	51.610	-5.039	5.240	1.00	0.00	LX0	C
ATOM	94	OE1	GLU	573	50.997	-5.979	4.741	1.00	0.00	LX0	O
ATOM	95	OE2	GLU	573	51.230	-4.540	6.293	1.00	0.00	LX0	O
ATOM	96	C	GLU	573	50.940	-1.683	4.668	1.00	0.00	LX0	C
ATOM	97	O	GLU	573	51.039	-0.677	5.368	1.00	0.00	LX0	O
ATOM	98	N	ALA	574	49.833	-2.404	4.556	1.00	0.00	LX0	N
ATOM	99	H	ALA	574	49.883	-3.267	4.051	0.00	0.00	LX0	H
ATOM	100	CA	ALA	574	48.683	-2.060	5.365	1.00	0.00	LX0	C
ATOM	101	CB	ALA	574	47.395	-2.344	4.618	1.00	0.00	LX0	C
ATOM	102	C	ALA	574	48.661	-2.919	6.586	1.00	0.00	LX0	C

ATOM	103	O	ALA	574	48.104	-4.013	6.573	1.00	0.00	LX0	O
ATOM	104	N	SER	575	49.280	-2.372	7.643	1.00	0.00	LX0	N
ATOM	105	H	SER	575	49.794	-1.529	7.485	0.00	0.00	LX0	H
ATOM	106	CA	SER	575	49.344	-3.038	8.948	1.00	0.00	LX0	C
ATOM	107	CB	SER	575	49.438	-1.975	10.045	1.00	0.00	LX0	C
ATOM	108	OG	SER	575	49.122	-0.682	9.502	1.00	0.00	LX0	O
ATOM	109	HG	SER	575	48.309	-0.781	9.003	0.00	0.00	LX0	H
ATOM	110	C	SER	575	48.226	-4.038	9.218	1.00	0.00	LX0	C
ATOM	111	O	SER	575	48.439	-5.247	9.246	1.00	0.00	LX0	O
ATOM	112	N	GLY	576	47.020	-3.461	9.305	1.00	0.00	LX0	N
ATOM	113	H	GLY	576	46.896	-2.476	9.170	0.00	0.00	LX0	H
ATOM	114	CA	GLY	576	45.826	-4.289	9.267	1.00	0.00	LX0	C
ATOM	115	C	GLY	576	44.796	-3.566	8.428	1.00	0.00	LX0	C
ATOM	116	O	GLY	576	45.064	-2.523	7.845	1.00	0.00	LX0	O
ATOM	117	N	CYS	577	43.595	-4.143	8.408	1.00	0.00	LX0	N
ATOM	118	H	CYS	577	43.400	-4.968	8.934	0.00	0.00	LX0	H
ATOM	119	CA	CYS	577	42.531	-3.362	7.789	1.00	0.00	LX0	C
ATOM	120	CB	CYS	577	41.551	-4.308	7.097	1.00	0.00	LX0	C
ATOM	121	SG	CYS	577	40.512	-3.506	5.857	1.00	0.00	LX0	S
ATOM	122	C	CYS	577	41.865	-2.513	8.852	1.00	0.00	LX0	C
ATOM	123	O	CYS	577	41.362	-3.032	9.844	1.00	0.00	LX0	O
ATOM	124	N	HIS	578	41.941	-1.193	8.663	1.00	0.00	LX0	N
ATOM	125	H	HIS	578	42.333	-0.799	7.827	0.00	0.00	LX0	H
ATOM	126	CA	HIS	578	41.389	-0.302	9.679	1.00	0.00	LX0	C
ATOM	127	CB	HIS	578	42.487	0.384	10.507	1.00	0.00	LX0	C
ATOM	128	CG	HIS	578	43.301	-0.559	11.373	1.00	0.00	LX0	C
ATOM	129	ND1	HIS	578	42.971	-1.823	11.703	1.00	0.00	LX0	N
ATOM	130	HD1	HIS	578	42.186	-2.324	11.385	0.00	0.00	LX0	H
ATOM	131	CD2	HIS	578	44.510	-0.254	12.000	1.00	0.00	LX0	C
ATOM	132	NE2	HIS	578	44.906	-1.336	12.718	1.00	0.00	LX0	N
ATOM	133	CE1	HIS	578	43.955	-2.306	12.526	1.00	0.00	LX0	C
ATOM	134	C	HIS	578	40.502	0.757	9.052	1.00	0.00	LX0	C
ATOM	135	O	HIS	578	40.665	1.152	7.905	1.00	0.00	LX0	O
ATOM	136	N	TYR	579	39.524	1.179	9.873	1.00	0.00	LX0	N
ATOM	137	H	TYR	579	39.483	0.754	10.774	0.00	0.00	LX0	H
ATOM	138	CA	TYR	579	38.566	2.244	9.536	1.00	0.00	LX0	C
ATOM	139	CB	TYR	579	39.213	3.640	9.522	1.00	0.00	LX0	C
ATOM	140	CG	TYR	579	39.970	3.943	10.795	1.00	0.00	LX0	C
ATOM	141	CD1	TYR	579	39.271	4.088	12.012	1.00	0.00	LX0	C
ATOM	142	CE1	TYR	579	39.994	4.348	13.189	1.00	0.00	LX0	C
ATOM	143	CD2	TYR	579	41.369	4.079	10.723	1.00	0.00	LX0	C
ATOM	144	CE2	TYR	579	42.087	4.351	11.897	1.00	0.00	LX0	C
ATOM	145	CZ	TYR	579	41.396	4.470	13.119	1.00	0.00	LX0	C
ATOM	146	OH	TYR	579	42.115	4.704	14.277	1.00	0.00	LX0	O
ATOM	147	HH	TYR	579	42.810	5.335	14.077	0.00	0.00	LX0	H
ATOM	148	C	TYR	579	37.672	2.109	8.305	1.00	0.00	LX0	C
ATOM	149	O	TYR	579	36.771	2.915	8.130	1.00	0.00	LX0	O
ATOM	150	N	GLY	580	37.948	1.094	7.475	1.00	0.00	LX0	N
ATOM	151	H	GLY	580	38.674	0.437	7.659	0.00	0.00	LX0	H
ATOM	152	CA	GLY	580	37.179	0.959	6.240	1.00	0.00	LX0	C
ATOM	153	C	GLY	580	37.937	0.225	5.147	1.00	0.00	LX0	C
ATOM	154	O	GLY	580	37.360	-0.404	4.273	1.00	0.00	LX0	O
ATOM	155	N	VAL	581	39.279	0.332	5.232	1.00	0.00	LX0	N
ATOM	156	H	VAL	581	39.714	0.878	5.950	0.00	0.00	LX0	H
ATOM	157	CA	VAL	581	40.115	-0.372	4.255	1.00	0.00	LX0	C
ATOM	158	CB	VAL	581	40.359	0.433	2.966	1.00	0.00	LX0	C
ATOM	159	CG1	VAL	581	39.257	0.248	1.922	1.00	0.00	LX0	C
ATOM	160	CG2	VAL	581	40.702	1.888	3.281	1.00	0.00	LX0	C
ATOM	161	C	VAL	581	41.466	-0.752	4.823	1.00	0.00	LX0	C
ATOM	162	O	VAL	581	41.846	-0.394	5.932	1.00	0.00	LX0	O
ATOM	163	N	LEU	582	42.209	-1.476	3.971	1.00	0.00	LX0	N

ATOM	164	H	LEU	582	41.792	-1.769	3.113	0.00	0.00	LX0	H
ATOM	165	CA	LEU	582	43.613	-1.763	4.263	1.00	0.00	LX0	C
ATOM	166	CB	LEU	582	44.213	-2.538	3.089	1.00	0.00	LX0	C
ATOM	167	CG	LEU	582	43.699	-3.971	2.942	1.00	0.00	LX0	C
ATOM	168	CD1	LEU	582	44.218	-4.617	1.657	1.00	0.00	LX0	C
ATOM	169	CD2	LEU	582	44.023	-4.825	4.169	1.00	0.00	LX0	C
ATOM	170	C	LEU	582	44.438	-0.512	4.535	1.00	0.00	LX0	C
ATOM	171	O	LEU	582	44.680	0.299	3.647	1.00	0.00	LX0	O
ATOM	172	N	THR	583	44.853	-0.385	5.802	1.00	0.00	LX0	N
ATOM	173	H	THR	583	44.665	-1.064	6.515	0.00	0.00	LX0	H
ATOM	174	CA	THR	583	45.515	0.862	6.163	1.00	0.00	LX0	C
ATOM	175	CB	THR	583	44.526	1.781	6.884	1.00	0.00	LX0	C
ATOM	176	OG1	THR	583	43.534	1.015	7.577	1.00	0.00	LX0	O
ATOM	177	HG1	THR	583	42.800	0.899	6.971	0.00	0.00	LX0	H
ATOM	178	CG2	THR	583	43.861	2.757	5.916	1.00	0.00	LX0	C
ATOM	179	C	THR	583	46.801	0.700	6.957	1.00	0.00	LX0	C
ATOM	180	O	THR	583	47.027	-0.250	7.705	1.00	0.00	LX0	O
ATOM	181	N	CYS	584	47.675	1.693	6.746	1.00	0.00	LX0	N
ATOM	182	H	CYS	584	47.458	2.452	6.134	0.00	0.00	LX0	H
ATOM	183	CA	CYS	584	48.895	1.663	7.544	1.00	0.00	LX0	C
ATOM	184	CB	CYS	584	50.081	2.238	6.765	1.00	0.00	LX0	C
ATOM	185	SG	CYS	584	50.017	4.044	6.599	1.00	0.00	LX0	S
ATOM	186	C	CYS	584	48.730	2.383	8.870	1.00	0.00	LX0	C
ATOM	187	O	CYS	584	47.908	3.284	9.010	1.00	0.00	LX0	O
ATOM	188	N	GLY	585	49.587	1.986	9.825	1.00	0.00	LX0	N
ATOM	189	H	GLY	585	50.047	1.108	9.670	0.00	0.00	LX0	H
ATOM	190	CA	GLY	585	49.594	2.606	11.154	1.00	0.00	LX0	C
ATOM	191	C	GLY	585	49.550	4.127	11.177	1.00	0.00	LX0	C
ATOM	192	O	GLY	585	48.843	4.750	11.961	1.00	0.00	LX0	O
ATOM	193	N	SER	586	50.322	4.715	10.245	1.00	0.00	LX0	N
ATOM	194	H	SER	586	50.877	4.162	9.629	0.00	0.00	LX0	H
ATOM	195	CA	SER	586	50.281	6.176	10.220	1.00	0.00	LX0	C
ATOM	196	CB	SER	586	51.380	6.759	9.320	1.00	0.00	LX0	C
ATOM	197	OG	SER	586	51.147	6.440	7.937	1.00	0.00	LX0	O
ATOM	198	HG	SER	586	51.625	5.631	7.765	0.00	0.00	LX0	H
ATOM	199	C	SER	586	48.911	6.764	9.909	1.00	0.00	LX0	C
ATOM	200	O	SER	586	48.504	7.765	10.478	1.00	0.00	LX0	O
ATOM	201	N	CYS	587	48.193	6.080	8.998	1.00	0.00	LX0	N
ATOM	202	H	CYS	587	48.529	5.206	8.646	0.00	0.00	LX0	H
ATOM	203	CA	CYS	587	46.815	6.509	8.745	1.00	0.00	LX0	C
ATOM	204	CB	CYS	587	46.227	5.792	7.528	1.00	0.00	LX0	C
ATOM	205	SG	CYS	587	47.264	5.983	6.054	1.00	0.00	LX0	S
ATOM	206	C	CYS	587	45.909	6.326	9.948	1.00	0.00	LX0	C
ATOM	207	O	CYS	587	45.113	7.188	10.295	1.00	0.00	LX0	O
ATOM	208	N	LYS	588	46.120	5.172	10.607	1.00	0.00	LX0	N
ATOM	209	H	LYS	588	46.766	4.505	10.232	0.00	0.00	LX0	H
ATOM	210	CA	LYS	588	45.382	4.880	11.837	1.00	0.00	LX0	C
ATOM	211	CB	LYS	588	45.920	3.588	12.457	1.00	0.00	LX0	C
ATOM	212	CG	LYS	588	45.216	3.131	13.736	1.00	0.00	LX0	C
ATOM	213	CD	LYS	588	45.899	1.901	14.323	1.00	0.00	LX0	C
ATOM	214	CE	LYS	588	45.120	1.290	15.484	1.00	0.00	LX0	C
ATOM	215	NZ	LYS	588	45.691	-0.028	15.784	1.00	0.00	LX0	N
ATOM	216	HZ1	LYS	588	45.151	-0.468	16.555	0.00	0.00	LX0	H
ATOM	217	HZ2	LYS	588	45.633	-0.620	14.928	0.00	0.00	LX0	H
ATOM	218	HZ3	LYS	588	46.684	0.080	16.071	0.00	0.00	LX0	H
ATOM	219	C	LYS	588	45.369	6.005	12.863	1.00	0.00	LX0	C
ATOM	220	O	LYS	588	44.332	6.383	13.401	1.00	0.00	LX0	O
ATOM	221	N	VAL	589	46.584	6.520	13.106	1.00	0.00	LX0	N
ATOM	222	H	VAL	589	47.387	6.153	12.630	0.00	0.00	LX0	H
ATOM	223	CA	VAL	589	46.647	7.624	14.060	1.00	0.00	LX0	C
ATOM	224	CB	VAL	589	47.990	7.647	14.806	1.00	0.00	LX0	C

ATOM	225	CG1	VAL	589	48.126	6.382	15.657	1.00	0.00	LX0	C
ATOM	226	CG2	VAL	589	49.200	7.847	13.886	1.00	0.00	LX0	C
ATOM	227	C	VAL	589	46.291	8.984	13.481	1.00	0.00	LX0	C
ATOM	228	O	VAL	589	45.701	9.827	14.148	1.00	0.00	LX0	O
ATOM	229	N	PHE	590	46.647	9.149	12.192	1.00	0.00	LX0	N
ATOM	230	H	PHE	590	47.146	8.425	11.715	0.00	0.00	LX0	H
ATOM	231	CA	PHE	590	46.317	10.387	11.484	1.00	0.00	LX0	C
ATOM	232	CB	PHE	590	46.789	10.309	10.021	1.00	0.00	LX0	C
ATOM	233	CG	PHE	590	46.516	11.579	9.241	1.00	0.00	LX0	C
ATOM	234	CD1	PHE	590	47.254	12.750	9.520	1.00	0.00	LX0	C
ATOM	235	CD2	PHE	590	45.526	11.562	8.235	1.00	0.00	LX0	C
ATOM	236	CE1	PHE	590	47.000	13.922	8.779	1.00	0.00	LX0	C
ATOM	237	CE2	PHE	590	45.272	12.732	7.493	1.00	0.00	LX0	C
ATOM	238	CZ	PHE	590	46.013	13.900	7.771	1.00	0.00	LX0	C
ATOM	239	C	PHE	590	44.845	10.730	11.564	1.00	0.00	LX0	C
ATOM	240	O	PHE	590	44.469	11.868	11.789	1.00	0.00	LX0	O
ATOM	241	N	PHE	591	44.030	9.680	11.401	1.00	0.00	LX0	N
ATOM	242	H	PHE	591	44.408	8.773	11.210	0.00	0.00	LX0	H
ATOM	243	CA	PHE	591	42.594	9.927	11.400	1.00	0.00	LX0	C
ATOM	244	CB	PHE	591	41.859	8.637	11.019	1.00	0.00	LX0	C
ATOM	245	CG	PHE	591	40.604	8.881	10.199	1.00	0.00	LX0	C
ATOM	246	CD1	PHE	591	40.145	10.188	9.901	1.00	0.00	LX0	C
ATOM	247	CD2	PHE	591	39.900	7.749	9.736	1.00	0.00	LX0	C
ATOM	248	CE1	PHE	591	38.964	10.362	9.155	1.00	0.00	LX0	C
ATOM	249	CE2	PHE	591	38.723	7.920	8.985	1.00	0.00	LX0	C
ATOM	250	CZ	PHE	591	38.259	9.224	8.713	1.00	0.00	LX0	C
ATOM	251	C	PHE	591	42.043	10.578	12.666	1.00	0.00	LX0	C
ATOM	252	O	PHE	591	41.366	11.597	12.612	1.00	0.00	LX0	O
ATOM	253	N	LYS	592	42.402	9.980	13.823	1.00	0.00	LX0	N
ATOM	254	H	LYS	592	42.983	9.168	13.811	0.00	0.00	LX0	H
ATOM	255	CA	LYS	592	41.964	10.620	15.073	1.00	0.00	LX0	C
ATOM	256	CB	LYS	592	42.385	9.819	16.316	1.00	0.00	LX0	C
ATOM	257	CG	LYS	592	41.594	10.159	17.596	1.00	0.00	LX0	C
ATOM	258	CD	LYS	592	42.250	9.596	18.868	1.00	0.00	LX0	C
ATOM	259	CE	LYS	592	41.375	9.491	20.136	1.00	0.00	LX0	C
ATOM	260	NZ	LYS	592	41.022	10.767	20.771	1.00	0.00	LX0	N
ATOM	261	HZ1	LYS	592	41.040	10.701	21.810	0.00	0.00	LX0	H
ATOM	262	HZ2	LYS	592	41.628	11.558	20.468	0.00	0.00	LX0	H
ATOM	263	HZ3	LYS	592	40.072	11.106	20.505	0.00	0.00	LX0	H
ATOM	264	C	LYS	592	42.469	12.048	15.194	1.00	0.00	LX0	C
ATOM	265	O	LYS	592	41.726	12.995	15.405	1.00	0.00	LX0	O
ATOM	266	N	ARG	593	43.793	12.155	14.998	1.00	0.00	LX0	N
ATOM	267	H	ARG	593	44.322	11.345	14.735	0.00	0.00	LX0	H
ATOM	268	CA	ARG	593	44.421	13.466	15.162	1.00	0.00	LX0	C
ATOM	269	CB	ARG	593	45.930	13.348	14.965	1.00	0.00	LX0	C
ATOM	270	CG	ARG	593	46.564	12.428	16.007	1.00	0.00	LX0	C
ATOM	271	CD	ARG	593	48.052	12.203	15.759	1.00	0.00	LX0	C
ATOM	272	NE	ARG	593	48.617	11.325	16.783	1.00	0.00	LX0	N
ATOM	273	HE	ARG	593	47.960	10.795	17.323	0.00	0.00	LX0	H
ATOM	274	CZ	ARG	593	49.953	11.236	16.957	1.00	0.00	LX0	C
ATOM	275	NH1	ARG	593	50.781	11.953	16.195	1.00	0.00	LX0	N
ATOM	276	HH11	ARG	593	51.775	11.910	16.297	0.00	0.00	LX0	H
ATOM	277	HH12	ARG	593	50.411	12.564	15.493	0.00	0.00	LX0	H
ATOM	278	NH2	ARG	593	50.435	10.427	17.899	1.00	0.00	LX0	N
ATOM	279	HH21	ARG	593	51.418	10.328	18.059	0.00	0.00	LX0	H
ATOM	280	HH22	ARG	593	49.814	9.895	18.477	0.00	0.00	LX0	H
ATOM	281	C	ARG	593	43.855	14.569	14.285	1.00	0.00	LX0	C
ATOM	282	O	ARG	593	43.691	15.706	14.705	1.00	0.00	LX0	O
ATOM	283	N	ALA	594	43.551	14.171	13.043	1.00	0.00	LX0	N
ATOM	284	H	ALA	594	43.641	13.211	12.784	0.00	0.00	LX0	H
ATOM	285	CA	ALA	594	43.029	15.151	12.099	1.00	0.00	LX0	C

ATOM	286	CB	ALA	594	43.036	14.597	10.673	1.00	0.00	LX0	C
ATOM	287	C	ALA	594	41.629	15.620	12.431	1.00	0.00	LX0	C
ATOM	288	O	ALA	594	41.279	16.777	12.242	1.00	0.00	LX0	O
ATOM	289	N	MET	595	40.835	14.666	12.943	1.00	0.00	LX0	N
ATOM	290	H	MET	595	41.162	13.734	13.113	0.00	0.00	LX0	H
ATOM	291	CA	MET	595	39.468	15.083	13.241	1.00	0.00	LX0	C
ATOM	292	CB	MET	595	38.472	13.935	13.051	1.00	0.00	LX0	C
ATOM	293	CG	MET	595	38.647	13.184	11.726	1.00	0.00	LX0	C
ATOM	294	SD	MET	595	38.708	14.232	10.259	1.00	0.00	LX0	S
ATOM	295	CE	MET	595	37.009	14.822	10.270	1.00	0.00	LX0	C
ATOM	296	C	MET	595	39.283	15.764	14.584	1.00	0.00	LX0	C
ATOM	297	O	MET	595	38.408	16.602	14.760	1.00	0.00	LX0	O
ATOM	298	N	GLU	596	40.158	15.391	15.531	1.00	0.00	LX0	N
ATOM	299	H	GLU	596	40.835	14.677	15.353	0.00	0.00	LX0	H
ATOM	300	CA	GLU	596	40.113	16.112	16.803	1.00	0.00	LX0	C
ATOM	301	CB	GLU	596	40.710	15.279	17.945	1.00	0.00	LX0	C
ATOM	302	CG	GLU	596	39.907	14.002	18.228	1.00	0.00	LX0	C
ATOM	303	CD	GLU	596	40.450	13.262	19.441	1.00	0.00	LX0	C
ATOM	304	OE1	GLU	596	41.651	13.004	19.516	1.00	0.00	LX0	O
ATOM	305	OE2	GLU	596	39.664	12.885	20.310	1.00	0.00	LX0	O
ATOM	306	C	GLU	596	40.758	17.487	16.722	1.00	0.00	LX0	C
ATOM	307	O	GLU	596	40.228	18.492	17.187	1.00	0.00	LX0	O
ATOM	308	N	GLY	597	41.931	17.505	16.076	1.00	0.00	LX0	N
ATOM	309	H	GLY	597	42.296	16.683	15.640	0.00	0.00	LX0	H
ATOM	310	CA	GLY	597	42.578	18.797	15.875	1.00	0.00	LX0	C
ATOM	311	C	GLY	597	41.936	19.599	14.760	1.00	0.00	LX0	C
ATOM	312	O	GLY	597	42.011	19.260	13.587	1.00	0.00	LX0	O
ATOM	313	N	GLN	598	41.290	20.693	15.186	1.00	0.00	LX0	N
ATOM	314	H	GLN	598	41.296	20.906	16.161	0.00	0.00	LX0	H
ATOM	315	CA	GLN	598	40.587	21.524	14.208	1.00	0.00	LX0	C
ATOM	316	CB	GLN	598	39.512	22.371	14.901	1.00	0.00	LX0	C
ATOM	317	CG	GLN	598	38.552	21.584	15.801	1.00	0.00	LX0	C
ATOM	318	CD	GLN	598	37.813	20.539	14.992	1.00	0.00	LX0	C
ATOM	319	OE1	GLN	598	37.037	20.836	14.095	1.00	0.00	LX0	O
ATOM	320	NE2	GLN	598	38.114	19.291	15.345	1.00	0.00	LX0	N
ATOM	321	HE21	GLN	598	38.731	19.096	16.109	0.00	0.00	LX0	H
ATOM	322	HE22	GLN	598	37.753	18.500	14.849	0.00	0.00	LX0	H
ATOM	323	C	GLN	598	41.490	22.408	13.361	1.00	0.00	LX0	C
ATOM	324	O	GLN	598	41.585	23.614	13.550	1.00	0.00	LX0	O
ATOM	325	N	HIS	599	42.166	21.754	12.414	1.00	0.00	LX0	N
ATOM	326	H	HIS	599	42.022	20.771	12.283	0.00	0.00	LX0	H
ATOM	327	CA	HIS	599	43.010	22.550	11.531	1.00	0.00	LX0	C
ATOM	328	CB	HIS	599	44.377	21.893	11.321	1.00	0.00	LX0	C
ATOM	329	CG	HIS	599	45.002	21.506	12.639	1.00	0.00	LX0	C
ATOM	330	ND1	HIS	599	45.002	20.248	13.114	1.00	0.00	LX0	N
ATOM	331	HD1	HIS	599	44.592	19.459	12.689	0.00	0.00	LX0	H
ATOM	332	CD2	HIS	599	45.660	22.334	13.553	1.00	0.00	LX0	C
ATOM	333	NE2	HIS	599	46.058	21.551	14.586	1.00	0.00	LX0	N
ATOM	334	CE1	HIS	599	45.655	20.269	14.316	1.00	0.00	LX0	C
ATOM	335	C	HIS	599	42.345	22.741	10.190	1.00	0.00	LX0	C
ATOM	336	O	HIS	599	41.554	21.919	9.747	1.00	0.00	LX0	O
ATOM	337	N	ASN	600	42.713	23.848	9.531	1.00	0.00	LX0	N
ATOM	338	H	ASN	600	43.393	24.471	9.910	0.00	0.00	LX0	H
ATOM	339	CA	ASN	600	42.289	23.885	8.133	1.00	0.00	LX0	C
ATOM	340	CB	ASN	600	42.170	25.320	7.607	1.00	0.00	LX0	C
ATOM	341	CG	ASN	600	41.437	25.317	6.274	1.00	0.00	LX0	C
ATOM	342	OD1	ASN	600	41.493	24.377	5.496	1.00	0.00	LX0	O
ATOM	343	ND2	ASN	600	40.717	26.417	6.046	1.00	0.00	LX0	N
ATOM	344	HD21	ASN	600	40.679	27.174	6.695	0.00	0.00	LX0	H
ATOM	345	HD22	ASN	600	40.204	26.457	5.190	0.00	0.00	LX0	H
ATOM	346	C	ASN	600	43.230	23.057	7.281	1.00	0.00	LX0	C

ATOM	347	O	ASN	600	44.384	23.408	7.064	1.00	0.00	LX0	O
ATOM	348	N	TYR	601	42.693	21.913	6.850	1.00	0.00	LX0	N
ATOM	349	H	TYR	601	41.726	21.732	7.024	0.00	0.00	LX0	H
ATOM	350	CA	TYR	601	43.525	21.023	6.047	1.00	0.00	LX0	C
ATOM	351	CB	TYR	601	43.041	19.572	6.183	1.00	0.00	LX0	C
ATOM	352	CG	TYR	601	43.157	19.143	7.631	1.00	0.00	LX0	C
ATOM	353	CD1	TYR	601	44.436	18.880	8.163	1.00	0.00	LX0	C
ATOM	354	CE1	TYR	601	44.557	18.561	9.524	1.00	0.00	LX0	C
ATOM	355	CD2	TYR	601	41.990	19.040	8.417	1.00	0.00	LX0	C
ATOM	356	CE2	TYR	601	42.111	18.721	9.781	1.00	0.00	LX0	C
ATOM	357	CZ	TYR	601	43.397	18.509	10.321	1.00	0.00	LX0	C
ATOM	358	OH	TYR	601	43.546	18.256	11.669	1.00	0.00	LX0	O
ATOM	359	HH	TYR	601	42.679	18.165	12.081	0.00	0.00	LX0	H
ATOM	360	C	TYR	601	43.643	21.470	4.604	1.00	0.00	LX0	C
ATOM	361	O	TYR	601	42.949	21.006	3.710	1.00	0.00	LX0	O
ATOM	362	N	LEU	602	44.576	22.412	4.430	1.00	0.00	LX0	N
ATOM	363	H	LEU	602	45.047	22.761	5.242	0.00	0.00	LX0	H
ATOM	364	CA	LEU	602	44.808	22.963	3.098	1.00	0.00	LX0	C
ATOM	365	CB	LEU	602	45.632	24.247	3.208	1.00	0.00	LX0	C
ATOM	366	CG	LEU	602	44.897	25.363	3.955	1.00	0.00	LX0	C
ATOM	367	CD1	LEU	602	45.825	26.530	4.291	1.00	0.00	LX0	C
ATOM	368	CD2	LEU	602	43.646	25.826	3.204	1.00	0.00	LX0	C
ATOM	369	C	LEU	602	45.447	21.987	2.125	1.00	0.00	LX0	C
ATOM	370	O	LEU	602	45.933	20.917	2.486	1.00	0.00	LX0	O
ATOM	371	N	CYS	603	45.410	22.410	0.857	1.00	0.00	LX0	N
ATOM	372	H	CYS	603	45.096	23.329	0.625	0.00	0.00	LX0	H
ATOM	373	CA	CYS	603	46.035	21.575	-0.159	1.00	0.00	LX0	C
ATOM	374	CB	CYS	603	45.068	21.341	-1.321	1.00	0.00	LX0	C
ATOM	375	SG	CYS	603	45.603	19.997	-2.410	1.00	0.00	LX0	S
ATOM	376	C	CYS	603	47.330	22.191	-0.643	1.00	0.00	LX0	C
ATOM	377	O	CYS	603	47.552	23.385	-0.495	1.00	0.00	LX0	O
ATOM	378	N	ALA	604	48.170	21.315	-1.208	1.00	0.00	LX0	N
ATOM	379	H	ALA	604	47.899	20.356	-1.248	0.00	0.00	LX0	H
ATOM	380	CA	ALA	604	49.369	21.812	-1.881	1.00	0.00	LX0	C
ATOM	381	CB	ALA	604	50.573	20.927	-1.555	1.00	0.00	LX0	C
ATOM	382	C	ALA	604	49.184	21.866	-3.387	1.00	0.00	LX0	C
ATOM	383	O	ALA	604	49.696	22.731	-4.086	1.00	0.00	LX0	O
ATOM	384	N	GLY	605	48.401	20.883	-3.860	1.00	0.00	LX0	N
ATOM	385	H	GLY	605	47.991	20.215	-3.247	0.00	0.00	LX0	H
ATOM	386	CA	GLY	605	47.885	21.039	-5.213	1.00	0.00	LX0	C
ATOM	387	C	GLY	605	46.597	21.830	-5.150	1.00	0.00	LX0	C
ATOM	388	O	GLY	605	46.321	22.524	-4.180	1.00	0.00	LX0	O
ATOM	389	N	ARG	606	45.801	21.675	-6.214	1.00	0.00	LX0	N
ATOM	390	H	ARG	606	46.013	20.957	-6.879	0.00	0.00	LX0	H
ATOM	391	CA	ARG	606	44.499	22.349	-6.176	1.00	0.00	LX0	C
ATOM	392	CB	ARG	606	43.940	22.463	-7.597	1.00	0.00	LX0	C
ATOM	393	CG	ARG	606	45.016	22.927	-8.583	1.00	0.00	LX0	C
ATOM	394	CD	ARG	606	44.863	22.276	-9.960	1.00	0.00	LX0	C
ATOM	395	NE	ARG	606	46.118	22.345	-10.714	1.00	0.00	LX0	N
ATOM	396	HE	ARG	606	46.264	23.161	-11.275	0.00	0.00	LX0	H
ATOM	397	CZ	ARG	606	47.050	21.369	-10.578	1.00	0.00	LX0	C
ATOM	398	NH1	ARG	606	46.822	20.316	-9.802	1.00	0.00	LX0	N
ATOM	399	HH11	ARG	606	47.461	19.564	-9.616	0.00	0.00	LX0	H
ATOM	400	HH12	ARG	606	45.953	20.192	-9.305	0.00	0.00	LX0	H
ATOM	401	NH2	ARG	606	48.208	21.468	-11.225	1.00	0.00	LX0	N
ATOM	402	HH21	ARG	606	48.905	20.756	-11.131	0.00	0.00	LX0	H
ATOM	403	HH22	ARG	606	48.404	22.251	-11.815	0.00	0.00	LX0	H
ATOM	404	C	ARG	606	43.547	21.586	-5.267	1.00	0.00	LX0	C
ATOM	405	O	ARG	606	42.834	22.115	-4.424	1.00	0.00	LX0	O
ATOM	406	N	ASN	607	43.635	20.267	-5.476	1.00	0.00	LX0	N
ATOM	407	H	ASN	607	44.210	19.962	-6.238	0.00	0.00	LX0	H

ATOM	408	CA	ASN	607	43.098	19.289	-4.534	1.00	0.00	LX0	C
ATOM	409	CB	ASN	607	41.567	19.218	-4.547	1.00	0.00	LX0	C
ATOM	410	CG	ASN	607	41.099	18.438	-3.333	1.00	0.00	LX0	C
ATOM	411	OD1	ASN	607	41.128	17.214	-3.286	1.00	0.00	LX0	O
ATOM	412	ND2	ASN	607	40.665	19.212	-2.336	1.00	0.00	LX0	N
ATOM	413	HD21	ASN	607	40.683	20.208	-2.427	0.00	0.00	LX0	H
ATOM	414	HD22	ASN	607	40.322	18.794	-1.497	0.00	0.00	LX0	H
ATOM	415	C	ASN	607	43.687	17.934	-4.853	1.00	0.00	LX0	C
ATOM	416	O	ASN	607	44.181	17.211	-4.000	1.00	0.00	LX0	O
ATOM	417	N	ASP	608	43.624	17.659	-6.166	1.00	0.00	LX0	N
ATOM	418	H	ASP	608	43.205	18.342	-6.762	0.00	0.00	LX0	H
ATOM	419	CA	ASP	608	44.270	16.526	-6.835	1.00	0.00	LX0	C
ATOM	420	CB	ASP	608	44.847	17.000	-8.184	1.00	0.00	LX0	C
ATOM	421	CG	ASP	608	45.667	18.277	-8.040	1.00	0.00	LX0	C
ATOM	422	OD1	ASP	608	45.088	19.354	-7.932	1.00	0.00	LX0	O
ATOM	423	OD2	ASP	608	46.891	18.213	-8.071	1.00	0.00	LX0	O
ATOM	424	C	ASP	608	45.221	15.648	-6.021	1.00	0.00	LX0	C
ATOM	425	O	ASP	608	44.754	14.775	-5.297	1.00	0.00	LX0	O
ATOM	426	N	CYS	609	46.539	15.890	-6.164	1.00	0.00	LX0	N
ATOM	427	H	CYS	609	46.814	16.691	-6.703	0.00	0.00	LX0	H
ATOM	428	CA	CYS	609	47.583	15.122	-5.474	1.00	0.00	LX0	C
ATOM	429	CB	CYS	609	48.139	15.905	-4.287	1.00	0.00	LX0	C
ATOM	430	SG	CYS	609	48.784	17.533	-4.744	1.00	0.00	LX0	S
ATOM	431	C	CYS	609	47.310	13.678	-5.068	1.00	0.00	LX0	C
ATOM	432	O	CYS	609	46.602	13.354	-4.115	1.00	0.00	LX0	O
ATOM	433	N	ILE	610	47.958	12.805	-5.852	1.00	0.00	LX0	N
ATOM	434	H	ILE	610	48.545	13.145	-6.584	0.00	0.00	LX0	H
ATOM	435	CA	ILE	610	47.965	11.393	-5.472	1.00	0.00	LX0	C
ATOM	436	CB	ILE	610	48.576	10.546	-6.603	1.00	0.00	LX0	C
ATOM	437	CG2	ILE	610	48.572	9.044	-6.286	1.00	0.00	LX0	C
ATOM	438	CG1	ILE	610	47.871	10.842	-7.931	1.00	0.00	LX0	C
ATOM	439	CD1	ILE	610	48.550	10.178	-9.131	1.00	0.00	LX0	C
ATOM	440	C	ILE	610	48.725	11.204	-4.169	1.00	0.00	LX0	C
ATOM	441	O	ILE	610	49.775	11.793	-3.954	1.00	0.00	LX0	O
ATOM	442	N	VAL	611	48.114	10.396	-3.297	1.00	0.00	LX0	N
ATOM	443	H	VAL	611	47.258	9.957	-3.552	0.00	0.00	LX0	H
ATOM	444	CA	VAL	611	48.819	10.064	-2.066	1.00	0.00	LX0	C
ATOM	445	CB	VAL	611	47.880	10.275	-0.870	1.00	0.00	LX0	C
ATOM	446	CG1	VAL	611	48.378	9.707	0.461	1.00	0.00	LX0	C
ATOM	447	CG2	VAL	611	47.583	11.768	-0.761	1.00	0.00	LX0	C
ATOM	448	C	VAL	611	49.373	8.658	-2.156	1.00	0.00	LX0	C
ATOM	449	O	VAL	611	48.719	7.732	-2.635	1.00	0.00	LX0	O
ATOM	450	N	ASP	612	50.616	8.580	-1.686	1.00	0.00	LX0	N
ATOM	451	H	ASP	612	51.048	9.407	-1.329	0.00	0.00	LX0	H
ATOM	452	CA	ASP	612	51.500	7.440	-1.898	1.00	0.00	LX0	C
ATOM	453	CB	ASP	612	52.041	7.447	-3.335	1.00	0.00	LX0	C
ATOM	454	CG	ASP	612	52.522	8.838	-3.711	1.00	0.00	LX0	C
ATOM	455	OD1	ASP	612	53.542	9.300	-3.201	1.00	0.00	LX0	O
ATOM	456	OD2	ASP	612	51.858	9.476	-4.513	1.00	0.00	LX0	O
ATOM	457	C	ASP	612	52.633	7.583	-0.904	1.00	0.00	LX0	C
ATOM	458	O	ASP	612	52.547	8.411	-0.002	1.00	0.00	LX0	O
ATOM	459	N	LYS	613	53.700	6.786	-1.112	1.00	0.00	LX0	N
ATOM	460	H	LYS	613	53.686	6.159	-1.889	0.00	0.00	LX0	H
ATOM	461	CA	LYS	613	54.843	6.832	-0.196	1.00	0.00	LX0	C
ATOM	462	CB	LYS	613	56.027	6.009	-0.711	1.00	0.00	LX0	C
ATOM	463	CG	LYS	613	55.738	4.526	-0.949	1.00	0.00	LX0	C
ATOM	464	CD	LYS	613	57.036	3.724	-1.110	1.00	0.00	LX0	C
ATOM	465	CE	LYS	613	56.849	2.227	-1.387	1.00	0.00	LX0	C
ATOM	466	NZ	LYS	613	56.028	1.603	-0.345	1.00	0.00	LX0	N
ATOM	467	HZ1	LYS	613	56.044	0.567	-0.346	0.00	0.00	LX0	H
ATOM	468	HZ2	LYS	613	56.289	1.874	0.633	0.00	0.00	LX0	H

ATOM	469	HZ3	LYS	613	55.036	1.901	-0.421	0.00	0.00	LX0	H
ATOM	470	C	LYS	613	55.345	8.212	0.200	1.00	0.00	LX0	C
ATOM	471	O	LYS	613	55.692	8.460	1.349	1.00	0.00	LX0	O
ATOM	472	N	ILE	614	55.370	9.089	-0.812	1.00	0.00	LX0	N
ATOM	473	H	ILE	614	54.946	8.856	-1.692	0.00	0.00	LX0	H
ATOM	474	CA	ILE	614	55.863	10.433	-0.536	1.00	0.00	LX0	C
ATOM	475	CB	ILE	614	56.512	11.029	-1.795	1.00	0.00	LX0	C
ATOM	476	CG2	ILE	614	57.182	12.377	-1.502	1.00	0.00	LX0	C
ATOM	477	CG1	ILE	614	57.476	10.035	-2.450	1.00	0.00	LX0	C
ATOM	478	CD1	ILE	614	57.938	10.486	-3.837	1.00	0.00	LX0	C
ATOM	479	C	ILE	614	54.758	11.341	-0.029	1.00	0.00	LX0	C
ATOM	480	O	ILE	614	54.834	11.979	1.017	1.00	0.00	LX0	O
ATOM	481	N	ARG	615	53.698	11.392	-0.847	1.00	0.00	LX0	N
ATOM	482	H	ARG	615	53.618	10.761	-1.626	0.00	0.00	LX0	H
ATOM	483	CA	ARG	615	52.722	12.441	-0.569	1.00	0.00	LX0	C
ATOM	484	CB	ARG	615	51.938	12.764	-1.834	1.00	0.00	LX0	C
ATOM	485	CG	ARG	615	52.801	13.639	-2.750	1.00	0.00	LX0	C
ATOM	486	CD	ARG	615	52.423	13.557	-4.229	1.00	0.00	LX0	C
ATOM	487	NE	ARG	615	52.676	12.203	-4.715	1.00	0.00	LX0	N
ATOM	488	HE	ARG	615	52.081	11.456	-4.380	0.00	0.00	LX0	H
ATOM	489	CZ	ARG	615	53.730	11.898	-5.496	1.00	0.00	LX0	C
ATOM	490	NH1	ARG	615	54.472	12.861	-6.041	1.00	0.00	LX0	N
ATOM	491	HH11	ARG	615	55.267	12.649	-6.607	0.00	0.00	LX0	H
ATOM	492	HH12	ARG	615	54.228	13.817	-5.887	0.00	0.00	LX0	H
ATOM	493	NH2	ARG	615	54.023	10.624	-5.710	1.00	0.00	LX0	N
ATOM	494	HH21	ARG	615	54.745	10.291	-6.310	0.00	0.00	LX0	H
ATOM	495	HH22	ARG	615	53.466	9.943	-5.204	0.00	0.00	LX0	H
ATOM	496	C	ARG	615	51.859	12.265	0.668	1.00	0.00	LX0	C
ATOM	497	O	ARG	615	51.253	13.211	1.163	1.00	0.00	LX0	O
ATOM	498	N	ARG	616	51.905	11.033	1.208	1.00	0.00	LX0	N
ATOM	499	H	ARG	616	52.376	10.289	0.728	0.00	0.00	LX0	H
ATOM	500	CA	ARG	616	51.316	10.817	2.531	1.00	0.00	LX0	C
ATOM	501	CB	ARG	616	51.384	9.335	2.917	1.00	0.00	LX0	C
ATOM	502	CG	ARG	616	52.809	8.823	3.141	1.00	0.00	LX0	C
ATOM	503	CD	ARG	616	52.850	7.324	3.412	1.00	0.00	LX0	C
ATOM	504	NE	ARG	616	54.224	6.852	3.557	1.00	0.00	LX0	N
ATOM	505	HE	ARG	616	54.888	7.436	4.026	0.00	0.00	LX0	H
ATOM	506	CZ	ARG	616	54.566	5.639	3.079	1.00	0.00	LX0	C
ATOM	507	NH1	ARG	616	53.723	4.939	2.332	1.00	0.00	LX0	N
ATOM	508	HH11	ARG	616	53.989	4.011	2.045	0.00	0.00	LX0	H
ATOM	509	HH12	ARG	616	52.838	5.287	2.009	0.00	0.00	LX0	H
ATOM	510	NH2	ARG	616	55.763	5.125	3.344	1.00	0.00	LX0	N
ATOM	511	HH21	ARG	616	55.975	4.200	2.998	0.00	0.00	LX0	H
ATOM	512	HH22	ARG	616	56.456	5.617	3.868	0.00	0.00	LX0	H
ATOM	513	C	ARG	616	51.879	11.677	3.657	1.00	0.00	LX0	C
ATOM	514	O	ARG	616	51.245	11.885	4.685	1.00	0.00	LX0	O
ATOM	515	N	LYS	617	53.105	12.167	3.428	1.00	0.00	LX0	N
ATOM	516	H	LYS	617	53.619	11.937	2.598	0.00	0.00	LX0	H
ATOM	517	CA	LYS	617	53.628	13.115	4.402	1.00	0.00	LX0	C
ATOM	518	CB	LYS	617	54.904	12.575	5.061	1.00	0.00	LX0	C
ATOM	519	CG	LYS	617	56.061	12.299	4.093	1.00	0.00	LX0	C
ATOM	520	CD	LYS	617	57.390	12.775	4.685	1.00	0.00	LX0	C
ATOM	521	CE	LYS	617	58.089	13.856	3.851	1.00	0.00	LX0	C
ATOM	522	NZ	LYS	617	57.181	14.978	3.572	1.00	0.00	LX0	N
ATOM	523	HZ1	LYS	617	57.689	15.839	3.301	0.00	0.00	LX0	H
ATOM	524	HZ2	LYS	617	56.557	14.728	2.777	0.00	0.00	LX0	H
ATOM	525	HZ3	LYS	617	56.532	15.187	4.361	0.00	0.00	LX0	H
ATOM	526	C	LYS	617	53.861	14.490	3.804	1.00	0.00	LX0	C
ATOM	527	O	LYS	617	54.776	15.213	4.190	1.00	0.00	LX0	O
ATOM	528	N	ASN	618	53.026	14.794	2.802	1.00	0.00	LX0	N
ATOM	529	H	ASN	618	52.280	14.174	2.555	0.00	0.00	LX0	H

ATOM	530	CA	ASN	618	53.236	16.059	2.102	1.00	0.00	LX0	C
ATOM	531	CB	ASN	618	53.618	15.855	0.632	1.00	0.00	LX0	C
ATOM	532	CG	ASN	618	55.094	15.544	0.468	1.00	0.00	LX0	C
ATOM	533	OD1	ASN	618	55.825	15.229	1.400	1.00	0.00	LX0	O
ATOM	534	ND2	ASN	618	55.519	15.650	-0.793	1.00	0.00	LX0	N
ATOM	535	HD21	ASN	618	54.900	15.932	-1.525	0.00	0.00	LX0	H
ATOM	536	HD22	ASN	618	56.475	15.455	-1.004	0.00	0.00	LX0	H
ATOM	537	C	ASN	618	52.038	16.976	2.167	1.00	0.00	LX0	C
ATOM	538	O	ASN	618	52.154	18.156	2.455	1.00	0.00	LX0	O
ATOM	539	N	CYS	619	50.868	16.393	1.870	1.00	0.00	LX0	N
ATOM	540	H	CYS	619	50.775	15.415	1.671	0.00	0.00	LX0	H
ATOM	541	CA	CYS	619	49.730	17.306	1.802	1.00	0.00	LX0	C
ATOM	542	CB	CYS	619	49.294	17.509	0.353	1.00	0.00	LX0	C
ATOM	543	SG	CYS	619	47.894	18.648	0.211	1.00	0.00	LX0	S
ATOM	544	C	CYS	619	48.552	16.877	2.649	1.00	0.00	LX0	C
ATOM	545	O	CYS	619	47.957	15.830	2.417	1.00	0.00	LX0	O
ATOM	546	N	PRO	620	48.219	17.739	3.641	1.00	0.00	LX0	N
ATOM	547	CD	PRO	620	48.866	19.013	3.943	1.00	0.00	LX0	C
ATOM	548	CA	PRO	620	47.116	17.425	4.557	1.00	0.00	LX0	C
ATOM	549	CB	PRO	620	47.088	18.636	5.503	1.00	0.00	LX0	C
ATOM	550	CG	PRO	620	47.852	19.758	4.800	1.00	0.00	LX0	C
ATOM	551	C	PRO	620	45.793	17.089	3.880	1.00	0.00	LX0	C
ATOM	552	O	PRO	620	45.294	15.978	4.020	1.00	0.00	LX0	O
ATOM	553	N	ALA	621	45.257	18.063	3.111	1.00	0.00	LX0	N
ATOM	554	H	ALA	621	45.675	18.973	3.045	0.00	0.00	LX0	H
ATOM	555	CA	ALA	621	43.973	17.794	2.450	1.00	0.00	LX0	C
ATOM	556	CB	ALA	621	43.566	18.926	1.508	1.00	0.00	LX0	C
ATOM	557	C	ALA	621	43.965	16.530	1.620	1.00	0.00	LX0	C
ATOM	558	O	ALA	621	43.109	15.664	1.740	1.00	0.00	LX0	O
ATOM	559	N	CYS	622	45.001	16.450	0.773	1.00	0.00	LX0	N
ATOM	560	H	CYS	622	45.659	17.199	0.714	0.00	0.00	LX0	H
ATOM	561	CA	CYS	622	45.071	15.291	-0.113	1.00	0.00	LX0	C
ATOM	562	CB	CYS	622	46.243	15.425	-1.073	1.00	0.00	LX0	C
ATOM	563	SG	CYS	622	46.302	17.072	-1.810	1.00	0.00	LX0	S
ATOM	564	C	CYS	622	45.133	13.952	0.593	1.00	0.00	LX0	C
ATOM	565	O	CYS	622	44.537	12.974	0.156	1.00	0.00	LX0	O
ATOM	566	N	ARG	623	45.875	13.954	1.716	1.00	0.00	LX0	N
ATOM	567	H	ARG	623	46.302	14.790	2.068	0.00	0.00	LX0	H
ATOM	568	CA	ARG	623	45.944	12.707	2.468	1.00	0.00	LX0	C
ATOM	569	CB	ARG	623	47.060	12.720	3.516	1.00	0.00	LX0	C
ATOM	570	CG	ARG	623	47.269	11.305	4.058	1.00	0.00	LX0	C
ATOM	571	CD	ARG	623	48.183	11.201	5.272	1.00	0.00	LX0	C
ATOM	572	NE	ARG	623	48.260	9.805	5.695	1.00	0.00	LX0	N
ATOM	573	HE	ARG	623	47.429	9.259	5.564	0.00	0.00	LX0	H
ATOM	574	CZ	ARG	623	49.373	9.310	6.274	1.00	0.00	LX0	C
ATOM	575	NH1	ARG	623	50.448	10.073	6.454	1.00	0.00	LX0	N
ATOM	576	HH11	ARG	623	51.248	9.757	6.960	0.00	0.00	LX0	H
ATOM	577	HH12	ARG	623	50.483	11.001	6.059	0.00	0.00	LX0	H
ATOM	578	NH2	ARG	623	49.387	8.037	6.653	1.00	0.00	LX0	N
ATOM	579	HH21	ARG	623	50.187	7.608	7.089	0.00	0.00	LX0	H
ATOM	580	HH22	ARG	623	48.585	7.459	6.507	0.00	0.00	LX0	H
ATOM	581	C	ARG	623	44.625	12.330	3.107	1.00	0.00	LX0	C
ATOM	582	O	ARG	623	44.116	11.236	2.905	1.00	0.00	LX0	O
ATOM	583	N	LEU	624	44.080	13.293	3.870	1.00	0.00	LX0	N
ATOM	584	H	LEU	624	44.531	14.185	3.950	0.00	0.00	LX0	H
ATOM	585	CA	LEU	624	42.811	13.024	4.548	1.00	0.00	LX0	C
ATOM	586	CB	LEU	624	42.379	14.260	5.345	1.00	0.00	LX0	C
ATOM	587	CG	LEU	624	41.205	14.031	6.304	1.00	0.00	LX0	C
ATOM	588	CD1	LEU	624	41.492	12.927	7.325	1.00	0.00	LX0	C
ATOM	589	CD2	LEU	624	40.780	15.332	6.985	1.00	0.00	LX0	C
ATOM	590	C	LEU	624	41.728	12.553	3.591	1.00	0.00	LX0	C

ATOM	591	O	LEU	624	41.061	11.546	3.793	1.00	0.00	LX0	O
ATOM	592	N	ARG	625	41.658	13.304	2.481	1.00	0.00	LX0	N
ATOM	593	H	ARG	625	42.209	14.137	2.424	0.00	0.00	LX0	H
ATOM	594	CA	ARG	625	40.799	12.891	1.377	1.00	0.00	LX0	C
ATOM	595	CB	ARG	625	40.963	13.847	0.196	1.00	0.00	LX0	C
ATOM	596	CG	ARG	625	39.930	13.640	-0.913	1.00	0.00	LX0	C
ATOM	597	CD	ARG	625	40.478	14.052	-2.277	1.00	0.00	LX0	C
ATOM	598	NE	ARG	625	41.635	13.226	-2.627	1.00	0.00	LX0	N
ATOM	599	HE	ARG	625	41.533	12.233	-2.501	0.00	0.00	LX0	H
ATOM	600	CZ	ARG	625	42.743	13.792	-3.151	1.00	0.00	LX0	C
ATOM	601	NH1	ARG	625	42.783	15.096	-3.375	1.00	0.00	LX0	N
ATOM	602	HH11	ARG	625	43.600	15.530	-3.775	0.00	0.00	LX0	H
ATOM	603	HH12	ARG	625	42.009	15.707	-3.172	0.00	0.00	LX0	H
ATOM	604	NH2	ARG	625	43.803	13.048	-3.451	1.00	0.00	LX0	N
ATOM	605	HH21	ARG	625	44.602	13.468	-3.910	0.00	0.00	LX0	H
ATOM	606	HH22	ARG	625	43.828	12.074	-3.241	0.00	0.00	LX0	H
ATOM	607	C	ARG	625	41.036	11.457	0.932	1.00	0.00	LX0	C
ATOM	608	O	ARG	625	40.170	10.608	1.077	1.00	0.00	LX0	O
ATOM	609	N	LYS	626	42.243	11.211	0.386	1.00	0.00	LX0	N
ATOM	610	H	LYS	626	42.962	11.906	0.423	0.00	0.00	LX0	H
ATOM	611	CA	LYS	626	42.457	9.897	-0.228	1.00	0.00	LX0	C
ATOM	612	CB	LYS	626	43.819	9.798	-0.923	1.00	0.00	LX0	C
ATOM	613	CG	LYS	626	43.887	8.583	-1.858	1.00	0.00	LX0	C
ATOM	614	CD	LYS	626	45.312	8.109	-2.122	1.00	0.00	LX0	C
ATOM	615	CE	LYS	626	45.382	6.781	-2.877	1.00	0.00	LX0	C
ATOM	616	NZ	LYS	626	46.738	6.235	-2.741	1.00	0.00	LX0	N
ATOM	617	HZ1	LYS	626	46.760	5.212	-2.935	0.00	0.00	LX0	H
ATOM	618	HZ2	LYS	626	47.446	6.741	-3.313	0.00	0.00	LX0	H
ATOM	619	HZ3	LYS	626	47.051	6.364	-1.759	0.00	0.00	LX0	H
ATOM	620	C	LYS	626	42.264	8.717	0.709	1.00	0.00	LX0	C
ATOM	621	O	LYS	626	41.718	7.685	0.342	1.00	0.00	LX0	O
ATOM	622	N	CYS	627	42.723	8.933	1.951	1.00	0.00	LX0	N
ATOM	623	H	CYS	627	43.147	9.809	2.179	0.00	0.00	LX0	H
ATOM	624	CA	CYS	627	42.478	7.926	2.980	1.00	0.00	LX0	C
ATOM	625	CB	CYS	627	43.057	8.361	4.333	1.00	0.00	LX0	C
ATOM	626	SG	CYS	627	44.857	8.598	4.339	1.00	0.00	LX0	S
ATOM	627	C	CYS	627	41.005	7.616	3.136	1.00	0.00	LX0	C
ATOM	628	O	CYS	627	40.528	6.504	2.944	1.00	0.00	LX0	O
ATOM	629	N	CYS	628	40.286	8.682	3.485	1.00	0.00	LX0	N
ATOM	630	H	CYS	628	40.657	9.615	3.511	0.00	0.00	LX0	H
ATOM	631	CA	CYS	628	38.914	8.410	3.876	1.00	0.00	LX0	C
ATOM	632	CB	CYS	628	38.446	9.520	4.803	1.00	0.00	LX0	C
ATOM	633	SG	CYS	628	39.726	9.924	6.021	1.00	0.00	LX0	S
ATOM	634	C	CYS	628	37.959	8.098	2.737	1.00	0.00	LX0	C
ATOM	635	O	CYS	628	36.920	7.478	2.925	1.00	0.00	LX0	O
ATOM	636	N	GLN	629	38.394	8.498	1.522	1.00	0.00	LX0	N
ATOM	637	H	GLN	629	39.233	9.039	1.477	0.00	0.00	LX0	H
ATOM	638	CA	GLN	629	37.672	8.156	0.291	1.00	0.00	LX0	C
ATOM	639	CB	GLN	629	38.500	8.474	-0.958	1.00	0.00	LX0	C
ATOM	640	CG	GLN	629	38.462	9.936	-1.407	1.00	0.00	LX0	C
ATOM	641	CD	GLN	629	39.345	10.110	-2.627	1.00	0.00	LX0	C
ATOM	642	OE1	GLN	629	40.455	10.631	-2.582	1.00	0.00	LX0	O
ATOM	643	NE2	GLN	629	38.790	9.660	-3.755	1.00	0.00	LX0	N
ATOM	644	HE21	GLN	629	37.885	9.236	-3.746	0.00	0.00	LX0	H
ATOM	645	HE22	GLN	629	39.293	9.749	-4.612	0.00	0.00	LX0	H
ATOM	646	C	GLN	629	37.242	6.706	0.204	1.00	0.00	LX0	C
ATOM	647	O	GLN	629	36.148	6.374	-0.235	1.00	0.00	LX0	O
ATOM	648	N	ALA	630	38.157	5.843	0.661	1.00	0.00	LX0	N
ATOM	649	H	ALA	630	39.007	6.155	1.091	0.00	0.00	LX0	H
ATOM	650	CA	ALA	630	37.777	4.439	0.663	1.00	0.00	LX0	C
ATOM	651	CB	ALA	630	39.006	3.573	0.402	1.00	0.00	LX0	C

ATOM	652	C	ALA	630	37.057	4.023	1.938	1.00	0.00	LX0	C
ATOM	653	O	ALA	630	37.505	3.187	2.710	1.00	0.00	LX0	O
ATOM	654	N	GLY	631	35.894	4.671	2.117	1.00	0.00	LX0	N
ATOM	655	H	GLY	631	35.642	5.375	1.453	0.00	0.00	LX0	H
ATOM	656	CA	GLY	631	34.983	4.303	3.201	1.00	0.00	LX0	C
ATOM	657	C	GLY	631	35.507	4.412	4.625	1.00	0.00	LX0	C
ATOM	658	O	GLY	631	34.976	3.799	5.541	1.00	0.00	LX0	O
ATOM	659	N	MET	632	36.563	5.218	4.788	1.00	0.00	LX0	N
ATOM	660	H	MET	632	36.854	5.816	4.042	0.00	0.00	LX0	H
ATOM	661	CA	MET	632	37.114	5.290	6.138	1.00	0.00	LX0	C
ATOM	662	CB	MET	632	38.599	5.644	6.114	1.00	0.00	LX0	C
ATOM	663	CG	MET	632	39.494	4.522	5.596	1.00	0.00	LX0	C
ATOM	664	SD	MET	632	41.225	5.012	5.470	1.00	0.00	LX0	S
ATOM	665	CE	MET	632	41.473	5.619	7.145	1.00	0.00	LX0	C
ATOM	666	C	MET	632	36.368	6.247	7.038	1.00	0.00	LX0	C
ATOM	667	O	MET	632	36.334	7.451	6.812	1.00	0.00	LX0	O
ATOM	668	N	VAL	633	35.780	5.660	8.085	1.00	0.00	LX0	N
ATOM	669	H	VAL	633	35.832	4.662	8.186	0.00	0.00	LX0	H
ATOM	670	CA	VAL	633	35.111	6.522	9.054	1.00	0.00	LX0	C
ATOM	671	CB	VAL	633	33.650	6.078	9.250	1.00	0.00	LX0	C
ATOM	672	CG1	VAL	633	32.873	7.013	10.183	1.00	0.00	LX0	C
ATOM	673	CG2	VAL	633	32.930	5.960	7.905	1.00	0.00	LX0	C
ATOM	674	C	VAL	633	35.866	6.584	10.374	1.00	0.00	LX0	C
ATOM	675	O	VAL	633	36.296	5.581	10.937	1.00	0.00	LX0	O
ATOM	676	N	LEU	634	36.003	7.824	10.871	1.00	0.00	LX0	N
ATOM	677	H	LEU	634	35.601	8.600	10.387	0.00	0.00	LX0	H
ATOM	678	CA	LEU	634	36.589	7.964	12.205	1.00	0.00	LX0	C
ATOM	679	CB	LEU	634	37.373	9.281	12.324	1.00	0.00	LX0	C
ATOM	680	CG	LEU	634	38.497	9.331	13.378	1.00	0.00	LX0	C
ATOM	681	CD1	LEU	634	38.084	10.030	14.674	1.00	0.00	LX0	C
ATOM	682	CD2	LEU	634	39.143	7.965	13.625	1.00	0.00	LX0	C
ATOM	683	C	LEU	634	35.561	7.791	13.308	1.00	0.00	LX0	C
ATOM	684	O	LEU	634	35.303	8.657	14.130	1.00	0.00	LX0	O
ATOM	685	N	GLY	635	34.962	6.603	13.268	1.00	0.00	LX0	N
ATOM	686	H	GLY	635	35.212	5.927	12.576	0.00	0.00	LX0	H
ATOM	687	CA	GLY	635	34.027	6.320	14.339	1.00	0.00	LX0	C
ATOM	688	C	GLY	635	34.662	5.442	15.391	1.00	0.00	LX0	C
ATOM	689	O	GLY	635	35.841	5.101	15.304	1.00	0.00	LX0	O
ATOM	690	N	GLY	636	33.830	5.088	16.378	1.00	0.00	LX0	N
ATOM	691	H	GLY	636	32.862	5.306	16.240	0.00	0.00	LX0	H
ATOM	692	CA	GLY	636	34.305	4.386	17.563	1.00	0.00	LX0	C
ATOM	693	C	GLY	636	34.816	2.969	17.359	1.00	0.00	LX0	C
ATOM	694	O	GLY	636	35.175	2.517	16.280	1.00	0.00	LX0	O
ATOM	695	N	ARG	637	34.862	2.288	18.506	1.00	0.00	LX0	N
ATOM	696	H	ARG	637	34.353	2.654	19.284	0.00	0.00	LX0	H
ATOM	697	CA	ARG	637	35.738	1.136	18.703	1.00	0.00	LX0	C
ATOM	698	CB	ARG	637	36.071	1.060	20.187	1.00	0.00	LX0	C
ATOM	699	CG	ARG	637	37.360	1.821	20.471	1.00	0.00	LX0	C
ATOM	700	CD	ARG	637	38.658	1.017	20.405	1.00	0.00	LX0	C
ATOM	701	NE	ARG	637	38.872	0.348	19.128	1.00	0.00	LX0	N
ATOM	702	HE	ARG	637	39.217	0.891	18.358	0.00	0.00	LX0	H
ATOM	703	CZ	ARG	637	38.540	-0.957	19.024	1.00	0.00	LX0	C
ATOM	704	NH1	ARG	637	38.112	-1.662	20.060	1.00	0.00	LX0	N
ATOM	705	HH11	ARG	637	37.781	-2.599	19.910	0.00	0.00	LX0	H
ATOM	706	HH12	ARG	637	38.085	-1.292	20.987	0.00	0.00	LX0	H
ATOM	707	NH2	ARG	637	38.594	-1.565	17.864	1.00	0.00	LX0	N
ATOM	708	HH21	ARG	637	38.233	-2.502	17.789	0.00	0.00	LX0	H
ATOM	709	HH22	ARG	637	38.984	-1.136	17.043	0.00	0.00	LX0	H
ATOM	710	C	ARG	637	35.421	-0.251	18.151	1.00	0.00	LX0	C
ATOM	711	O	ARG	637	35.595	-1.249	18.832	1.00	0.00	LX0	O
ATOM	712	N	LYS	638	35.052	-0.286	16.867	1.00	0.00	LX0	N

```
ATOM    713  H   LYS   638      34.831   0.584  16.427  0.00  0.00      LX0  H
ATOM    714  CA  LYS   638      35.215  -1.499  16.050  1.00  0.00      LX0  C
ATOM    715  CB  LYS   638      36.586  -1.512  15.333  1.00  0.00      LX0  C
ATOM    716  CG  LYS   638      37.257  -0.159  15.019  1.00  0.00      LX0  C
ATOM    717  CD  LYS   638      36.518   0.735  14.026  1.00  0.00      LX0  C
ATOM    718  CE  LYS   638      37.061   2.158  13.928  1.00  0.00      LX0  C
ATOM    719  NZ  LYS   638      35.936   3.080  13.737  1.00  0.00      LX0  N
ATOM    720  HZ1 LYS   638      36.179   4.063  13.962  0.00  0.00      LX0  H
ATOM    721  HZ2 LYS   638      35.502   3.023  12.790  0.00  0.00      LX0  H
ATOM    722  HZ3 LYS   638      35.137   2.846  14.369  0.00  0.00      LX0  H
ATOM    723  C   LYS   638      34.916  -2.884  16.641  1.00  0.00      LX0  C
ATOM    724  O   LYS   638      35.744  -3.785  16.567  1.00  0.00      LX0  O
ATOM    725  N   PHE   639      33.705  -3.018  17.209  1.00  0.00      LX0  N
ATOM    726  H   PHE   639      33.064  -2.256  17.160  0.00  0.00      LX0  H
ATOM    727  CA  PHE   639      33.287  -4.293  17.810  1.00  0.00      LX0  C
ATOM    728  CB  PHE   639      32.124  -4.046  18.778  1.00  0.00      LX0  C
ATOM    729  CG  PHE   639      32.636  -3.880  20.191  1.00  0.00      LX0  C
ATOM    730  CD1 PHE   639      32.082  -4.688  21.204  1.00  0.00      LX0  C
ATOM    731  CD2 PHE   639      33.642  -2.930  20.485  1.00  0.00      LX0  C
ATOM    732  CE1 PHE   639      32.533  -4.531  22.528  1.00  0.00      LX0  C
ATOM    733  CE2 PHE   639      34.096  -2.775  21.808  1.00  0.00      LX0  C
ATOM    734  CZ  PHE   639      33.529  -3.576  22.820  1.00  0.00      LX0  C
ATOM    735  C   PHE   639      32.951  -5.393  16.808  1.00  0.00      LX0  C
ATOM    736  O   PHE   639      33.119  -5.217  15.601  1.00  0.00      LX0  O
ATOM    737  N   LYS   640      32.527  -6.560  17.342  1.00  0.00      LX0  N
ATOM    738  H   LYS   640      32.387  -6.684  18.328  0.00  0.00      LX0  H
ATOM    739  CA  LYS   640      31.792  -7.483  16.473  1.00  0.00      LX0  C
ATOM    740  CB  LYS   640      32.246  -8.968  16.518  1.00  0.00      LX0  C
ATOM    741  CG  LYS   640      33.001  -9.484  17.754  1.00  0.00      LX0  C
ATOM    742  CD  LYS   640      32.746 -10.968  18.058  1.00  0.00      LX0  C
ATOM    743  CE  LYS   640      32.782 -11.327  19.558  1.00  0.00      LX0  C
ATOM    744  NZ  LYS   640      31.663 -10.703  20.279  1.00  0.00      LX0  N
ATOM    745  HZ1 LYS   640      31.408 -11.164  21.181  0.00  0.00      LX0  H
ATOM    746  HZ2 LYS   640      31.842  -9.683  20.430  0.00  0.00      LX0  H
ATOM    747  HZ3 LYS   640      30.818 -10.671  19.667  0.00  0.00      LX0  H
ATOM    748  C   LYS   640      30.306  -7.343  16.744  1.00  0.00      LX0  C
ATOM    749  O   LYS   640      29.831  -6.217  16.856  1.00  0.00      LX0  O
TER
```
