# Supplementary material for: Structural analysis of hubs in human NR-RTK network
Source: Biol Direct. 2011 Oct 5;6:49. doi: 10.1186/1745-6150-6-49 (PMC3220635; doi:10.1186/1745-6150-6-49)
Supplement: Additional file 12 — ESR1-Erbb2-IGF1R. ESR1-Erbb2-IGF1R complex structure. [file 1745-6150-6-49-S12.PDF]

```
HEADER ESR1-ERBB2-IGF1R
REMARK original generated coordinate pdb file
ATOM      1  N   ALA   156      10.627  12.174   8.322  1.00  0.00       RX0  N
ATOM      2  H   ALA   156      11.176  11.340   8.264  1.00  0.00       RX0  H
ATOM      3  CA  ALA   156       9.864  12.527   9.538  1.00  0.00       RX0  C
ATOM      4  CB  ALA   156      10.757  12.402  10.765  1.00  0.00       RX0  C
ATOM      5  C   ALA   156       9.377  13.991   9.496  1.00  0.00       RX0  C
ATOM      6  O   ALA   156       9.121  14.644  10.500  1.00  0.00       RX0  O
ATOM      7  N   LEU   157       9.039  14.416   8.289  1.00  0.00       RX0  N
ATOM      8  H   LEU   157       9.119  13.761   7.534  1.00  0.00       RX0  H
ATOM      9  CA  LEU   157       8.850  15.849   7.979  1.00  0.00       RX0  C
ATOM     10  CB  LEU   157       9.698  16.229   6.770  1.00  0.00       RX0  C
ATOM     11  CG  LEU   157      10.961  15.382   6.628  1.00  0.00       RX0  C
ATOM     12  CD1 LEU   157      11.411  15.315   5.172  1.00  0.00       RX0  C
ATOM     13  CD2 LEU   157      12.071  15.810   7.587  1.00  0.00       RX0  C
ATOM     14  C   LEU   157       7.387  16.184   7.642  1.00  0.00       RX0  C
ATOM     15  O   LEU   157       7.075  17.216   7.039  1.00  0.00       RX0  O
ATOM     16  N   SER   158       6.522  15.244   7.955  1.00  0.00       RX0  N
ATOM     17  H   SER   158       6.874  14.326   8.101  1.00  0.00       RX0  H
ATOM     18  CA  SER   158       5.051  15.362   7.847  1.00  0.00       RX0  C
ATOM     19  CB  SER   158       4.729  14.894   6.436  1.00  0.00       RX0  C
ATOM     20  OG  SER   158       5.937  15.040   5.678  1.00  0.00       RX0  O
ATOM     21  HG  SER   158       6.092  15.981   5.623  1.00  0.00       RX0  H
ATOM     22  C   SER   158       4.335  14.560   8.949  1.00  0.00       RX0  C
ATOM     23  O   SER   158       3.148  14.670   9.188  1.00  0.00       RX0  O
ATOM     24  N   LEU   159       5.132  13.681   9.591  1.00  0.00       RX0  N
ATOM     25  H   LEU   159       6.083  13.585   9.324  1.00  0.00       RX0  H
ATOM     26  CA  LEU   159       4.759  12.951  10.797  1.00  0.00       RX0  C
ATOM     27  CB  LEU   159       5.860  11.985  11.247  1.00  0.00       RX0  C
ATOM     28  CG  LEU   159       5.905  10.626  10.539  1.00  0.00       RX0  C
ATOM     29  CD1 LEU   159       6.368  10.707   9.082  1.00  0.00       RX0  C
ATOM     30  CD2 LEU   159       6.747   9.631  11.339  1.00  0.00       RX0  C
ATOM     31  C   LEU   159       4.518  13.965  11.920  1.00  0.00       RX0  C
ATOM     32  O   LEU   159       5.291  14.932  12.058  1.00  0.00       RX0  O
ATOM     33  N   THR   160       3.434  13.807  12.646  1.00  0.00       RX0  N
ATOM     34  H   THR   160       2.847  13.019  12.458  1.00  0.00       RX0  H
ATOM     35  CA  THR   160       3.156  14.665  13.825  1.00  0.00       RX0  C
ATOM     36  CB  THR   160       1.666  14.649  14.226  1.00  0.00       RX0  C
ATOM     37  OG1 THR   160       1.373  15.749  15.095  1.00  0.00       RX0  O
ATOM     38  HG1 THR   160       0.426  15.831  15.118  1.00  0.00       RX0  H
ATOM     39  CG2 THR   160       1.175  13.343  14.833  1.00  0.00       RX0  C
ATOM     40  C   THR   160       4.203  14.411  14.921  1.00  0.00       RX0  C
ATOM     41  O   THR   160       4.902  13.383  14.913  1.00  0.00       RX0  O
ATOM     42  N   ALA   161       4.153  15.229  15.953  1.00  0.00       RX0  N
ATOM     43  H   ALA   161       3.464  15.956  15.915  1.00  0.00       RX0  H
ATOM     44  CA  ALA   161       4.942  15.044  17.184  1.00  0.00       RX0  C
ATOM     45  CB  ALA   161       4.756  16.235  18.117  1.00  0.00       RX0  C
ATOM     46  C   ALA   161       4.543  13.746  17.920  1.00  0.00       RX0  C
ATOM     47  O   ALA   161       5.387  12.950  18.288  1.00  0.00       RX0  O
ATOM     48  N   ASP   162       3.226  13.461  17.917  1.00  0.00       RX0  N
ATOM     49  H   ASP   162       2.573  14.186  17.707  1.00  0.00       RX0  H
ATOM     50  CA  ASP   162       2.690  12.196  18.469  1.00  0.00       RX0  C
ATOM     51  CB  ASP   162       1.207  12.322  18.846  1.00  0.00       RX0  C
ATOM     52  CG  ASP   162       1.093  13.163  20.120  1.00  0.00       RX0  C
ATOM     53  OD1 ASP   162       1.953  14.004  20.372  1.00  0.00       RX0  O
ATOM     54  OD2 ASP   162       0.161  12.978  20.901  1.00  0.00       RX0  O
ATOM     55  C   ASP   162       3.088  10.948  17.668  1.00  0.00       RX0  C
ATOM     56  O   ASP   162       3.397   9.903  18.257  1.00  0.00       RX0  O
ATOM     57  N   GLN   163       3.164  11.087  16.353  1.00  0.00       RX0  N
ATOM     58  H   GLN   163       3.079  12.014  15.997  1.00  0.00       RX0  H
ATOM     59  CA  GLN   163       3.593   9.998  15.449  1.00  0.00       RX0  C
```

ATOM	60	CB	GLN	163	3.242	10.284	13.996	1.00	0.00	RX0	C
ATOM	61	CG	GLN	163	1.811	9.884	13.651	1.00	0.00	RX0	C
ATOM	62	CD	GLN	163	1.523	10.393	12.260	1.00	0.00	RX0	C
ATOM	63	OE1	GLN	163	2.074	11.405	11.840	1.00	0.00	RX0	O
ATOM	64	NE2	GLN	163	0.644	9.645	11.573	1.00	0.00	RX0	N
ATOM	65	HE21	GLN	163	0.236	8.829	11.986	1.00	0.00	RX0	H
ATOM	66	HE22	GLN	163	0.370	9.876	10.639	1.00	0.00	RX0	H
ATOM	67	C	GLN	163	5.089	9.698	15.572	1.00	0.00	RX0	C
ATOM	68	O	GLN	163	5.477	8.537	15.545	1.00	0.00	RX0	O
ATOM	69	N	MET	164	5.882	10.740	15.840	1.00	0.00	RX0	N
ATOM	70	H	MET	164	5.494	11.662	15.886	1.00	0.00	RX0	H
ATOM	71	CA	MET	164	7.331	10.587	16.060	1.00	0.00	RX0	C
ATOM	72	CB	MET	164	8.015	11.955	16.082	1.00	0.00	RX0	C
ATOM	73	CG	MET	164	9.452	11.879	16.605	1.00	0.00	RX0	C
ATOM	74	SD	MET	164	10.535	10.863	15.592	1.00	0.00	RX0	S
ATOM	75	CE	MET	164	11.027	12.134	14.424	1.00	0.00	RX0	C
ATOM	76	C	MET	164	7.610	9.825	17.366	1.00	0.00	RX0	C
ATOM	77	O	MET	164	8.404	8.887	17.381	1.00	0.00	RX0	O
ATOM	78	N	VAL	165	6.828	10.145	18.396	1.00	0.00	RX0	N
ATOM	79	H	VAL	165	6.176	10.898	18.281	1.00	0.00	RX0	H
ATOM	80	CA	VAL	165	6.992	9.563	19.744	1.00	0.00	RX0	C
ATOM	81	CB	VAL	165	6.099	10.256	20.778	1.00	0.00	RX0	C
ATOM	82	CG1	VAL	165	6.170	9.551	22.133	1.00	0.00	RX0	C
ATOM	83	CG2	VAL	165	6.452	11.727	20.927	1.00	0.00	RX0	C
ATOM	84	C	VAL	165	6.649	8.067	19.731	1.00	0.00	RX0	C
ATOM	85	O	VAL	165	7.442	7.255	20.191	1.00	0.00	RX0	O
ATOM	86	N	SER	166	5.467	7.742	19.205	1.00	0.00	RX0	N
ATOM	87	H	SER	166	4.863	8.456	18.845	1.00	0.00	RX0	H
ATOM	88	CA	SER	166	5.029	6.335	19.106	1.00	0.00	RX0	C
ATOM	89	CB	SER	166	3.546	6.214	18.736	1.00	0.00	RX0	C
ATOM	90	OG	SER	166	3.007	4.983	19.257	1.00	0.00	RX0	O
ATOM	91	HG	SER	166	3.594	4.280	18.938	1.00	0.00	RX0	H
ATOM	92	C	SER	166	5.941	5.501	18.195	1.00	0.00	RX0	C
ATOM	93	O	SER	166	6.295	4.379	18.542	1.00	0.00	RX0	O
ATOM	94	N	ALA	167	6.456	6.129	17.133	1.00	0.00	RX0	N
ATOM	95	H	ALA	167	6.168	7.061	16.908	1.00	0.00	RX0	H
ATOM	96	CA	ALA	167	7.397	5.466	16.208	1.00	0.00	RX0	C
ATOM	97	CB	ALA	167	7.731	6.357	15.013	1.00	0.00	RX0	C
ATOM	98	C	ALA	167	8.706	5.103	16.927	1.00	0.00	RX0	C
ATOM	99	O	ALA	167	9.113	3.946	16.932	1.00	0.00	RX0	O
ATOM	100	N	LEU	168	9.179	6.054	17.734	1.00	0.00	RX0	N
ATOM	101	H	LEU	168	8.726	6.947	17.747	1.00	0.00	RX0	H
ATOM	102	CA	LEU	168	10.385	5.875	18.562	1.00	0.00	RX0	C
ATOM	103	CB	LEU	168	10.907	7.213	19.075	1.00	0.00	RX0	C
ATOM	104	CG	LEU	168	11.568	8.043	17.978	1.00	0.00	RX0	C
ATOM	105	CD1	LEU	168	12.100	9.370	18.520	1.00	0.00	RX0	C
ATOM	106	CD2	LEU	168	12.648	7.242	17.251	1.00	0.00	RX0	C
ATOM	107	C	LEU	168	10.197	4.896	19.724	1.00	0.00	RX0	C
ATOM	108	O	LEU	168	11.077	4.078	19.994	1.00	0.00	RX0	O
ATOM	109	N	LEU	169	9.007	4.918	20.317	1.00	0.00	RX0	N
ATOM	110	H	LEU	169	8.319	5.569	19.998	1.00	0.00	RX0	H
ATOM	111	CA	LEU	169	8.640	3.970	21.384	1.00	0.00	RX0	C
ATOM	112	CB	LEU	169	7.359	4.385	22.108	1.00	0.00	RX0	C
ATOM	113	CG	LEU	169	7.565	5.582	23.037	1.00	0.00	RX0	C
ATOM	114	CD1	LEU	169	6.266	5.983	23.736	1.00	0.00	RX0	C
ATOM	115	CD2	LEU	169	8.690	5.333	24.042	1.00	0.00	RX0	C
ATOM	116	C	LEU	169	8.505	2.536	20.864	1.00	0.00	RX0	C
ATOM	117	O	LEU	169	9.003	1.602	21.486	1.00	0.00	RX0	O
ATOM	118	N	ASP	170	7.977	2.423	19.645	1.00	0.00	RX0	N
ATOM	119	H	ASP	170	7.550	3.210	19.207	1.00	0.00	RX0	H
ATOM	120	CA	ASP	170	7.822	1.133	18.952	1.00	0.00	RX0	C

ATOM	121	CB	ASP	170	6.724	1.243	17.869	1.00	0.00	RX0	C
ATOM	122	CG	ASP	170	5.382	1.686	18.488	1.00	0.00	RX0	C
ATOM	123	OD1	ASP	170	5.195	1.544	19.696	1.00	0.00	RX0	O
ATOM	124	OD2	ASP	170	4.519	2.204	17.772	1.00	0.00	RX0	O
ATOM	125	C	ASP	170	9.164	0.506	18.541	1.00	0.00	RX0	C
ATOM	126	O	ASP	170	9.313	-0.704	18.571	1.00	0.00	RX0	O
ATOM	127	N	ALA	171	10.119	1.387	18.228	1.00	0.00	RX0	N
ATOM	128	H	ALA	171	9.898	2.360	18.319	1.00	0.00	RX0	H
ATOM	129	CA	ALA	171	11.447	1.008	17.717	1.00	0.00	RX0	C
ATOM	130	CB	ALA	171	12.104	2.224	17.066	1.00	0.00	RX0	C
ATOM	131	C	ALA	171	12.418	0.479	18.779	1.00	0.00	RX0	C
ATOM	132	O	ALA	171	13.427	-0.136	18.431	1.00	0.00	RX0	O
ATOM	133	N	GLU	172	12.125	0.739	20.058	1.00	0.00	RX0	N
ATOM	134	H	GLU	172	11.270	1.205	20.288	1.00	0.00	RX0	H
ATOM	135	CA	GLU	172	13.017	0.374	21.170	1.00	0.00	RX0	C
ATOM	136	CB	GLU	172	12.396	0.762	22.509	1.00	0.00	RX0	C
ATOM	137	CG	GLU	172	12.417	2.281	22.696	1.00	0.00	RX0	C
ATOM	138	CD	GLU	172	13.848	2.788	22.599	1.00	0.00	RX0	C
ATOM	139	OE1	GLU	172	14.616	2.621	23.545	1.00	0.00	RX0	O
ATOM	140	OE2	GLU	172	14.224	3.359	21.574	1.00	0.00	RX0	O
ATOM	141	C	GLU	172	13.554	-1.065	21.099	1.00	0.00	RX0	C
ATOM	142	O	GLU	172	12.785	-2.004	20.837	1.00	0.00	RX0	O
ATOM	143	N	PRO	173	14.865	-1.209	21.269	1.00	0.00	RX0	N
ATOM	144	CD	PRO	173	15.789	-0.096	21.453	1.00	0.00	RX0	C
ATOM	145	CA	PRO	173	15.538	-2.517	21.328	1.00	0.00	RX0	C
ATOM	146	CB	PRO	173	17.014	-2.108	21.203	1.00	0.00	RX0	C
ATOM	147	CG	PRO	173	17.100	-0.742	21.876	1.00	0.00	RX0	C
ATOM	148	C	PRO	173	15.206	-3.249	22.640	1.00	0.00	RX0	C
ATOM	149	O	PRO	173	14.829	-2.595	23.631	1.00	0.00	RX0	O
ATOM	150	N	PRO	174	15.294	-4.574	22.646	1.00	0.00	RX0	N
ATOM	151	CD	PRO	174	15.590	-5.390	21.472	1.00	0.00	RX0	C
ATOM	152	CA	PRO	174	15.084	-5.400	23.852	1.00	0.00	RX0	C
ATOM	153	CB	PRO	174	14.962	-6.814	23.277	1.00	0.00	RX0	C
ATOM	154	CG	PRO	174	15.829	-6.791	22.023	1.00	0.00	RX0	C
ATOM	155	C	PRO	174	16.250	-5.248	24.838	1.00	0.00	RX0	C
ATOM	156	O	PRO	174	17.379	-4.922	24.444	1.00	0.00	RX0	O
ATOM	157	N	ILE	175	15.956	-5.464	26.106	1.00	0.00	RX0	N
ATOM	158	H	ILE	175	15.039	-5.794	26.324	1.00	0.00	RX0	H
ATOM	159	CA	ILE	175	16.988	-5.556	27.159	1.00	0.00	RX0	C
ATOM	160	CB	ILE	175	16.403	-5.168	28.506	1.00	0.00	RX0	C
ATOM	161	CG2	ILE	175	17.489	-5.106	29.577	1.00	0.00	RX0	C
ATOM	162	CG1	ILE	175	15.708	-3.816	28.333	1.00	0.00	RX0	C
ATOM	163	CD1	ILE	175	14.988	-3.317	29.581	1.00	0.00	RX0	C
ATOM	164	C	ILE	175	17.586	-6.969	27.112	1.00	0.00	RX0	C
ATOM	165	O	ILE	175	16.886	-7.963	27.343	1.00	0.00	RX0	O
ATOM	166	N	LEU	176	18.884	-7.017	26.884	1.00	0.00	RX0	N
ATOM	167	H	LEU	176	19.412	-6.170	26.827	1.00	0.00	RX0	H
ATOM	168	CA	LEU	176	19.617	-8.291	26.770	1.00	0.00	RX0	C
ATOM	169	CB	LEU	176	20.641	-8.236	25.637	1.00	0.00	RX0	C
ATOM	170	CG	LEU	176	20.003	-7.929	24.281	1.00	0.00	RX0	C
ATOM	171	CD1	LEU	176	21.047	-7.903	23.168	1.00	0.00	RX0	C
ATOM	172	CD2	LEU	176	18.855	-8.883	23.947	1.00	0.00	RX0	C
ATOM	173	C	LEU	176	20.277	-8.690	28.089	1.00	0.00	RX0	C
ATOM	174	O	LEU	176	20.563	-7.852	28.952	1.00	0.00	RX0	O
ATOM	175	N	TYR	177	20.459	-9.989	28.237	1.00	0.00	RX0	N
ATOM	176	H	TYR	177	20.256	-10.592	27.469	1.00	0.00	RX0	H
ATOM	177	CA	TYR	177	21.114	-10.573	29.420	1.00	0.00	RX0	C
ATOM	178	CB	TYR	177	20.498	-11.938	29.703	1.00	0.00	RX0	C
ATOM	179	CG	TYR	177	19.571	-11.938	30.888	1.00	0.00	RX0	C
ATOM	180	CD1	TYR	177	18.526	-11.028	30.982	1.00	0.00	RX0	C
ATOM	181	CE1	TYR	177	17.631	-11.126	32.041	1.00	0.00	RX0	C

ATOM	182	CD2	TYR	177	19.756	-12.896	31.876	1.00	0.00	RX0	C
ATOM	183	CE2	TYR	177	18.867	-12.986	32.937	1.00	0.00	RX0	C
ATOM	184	CZ	TYR	177	17.790	-12.114	33.006	1.00	0.00	RX0	C
ATOM	185	OH	TYR	177	16.875	-12.238	34.032	1.00	0.00	RX0	O
ATOM	186	HH	TYR	177	17.322	-12.543	34.812	1.00	0.00	RX0	H
ATOM	187	C	TYR	177	22.589	-10.858	29.163	1.00	0.00	RX0	C
ATOM	188	O	TYR	177	22.985	-11.163	28.046	1.00	0.00	RX0	O
ATOM	189	N	SER	178	23.381	-10.750	30.220	1.00	0.00	RX0	N
ATOM	190	H	SER	178	23.011	-10.520	31.122	1.00	0.00	RX0	H
ATOM	191	CA	SER	178	24.788	-11.188	30.183	1.00	0.00	RX0	C
ATOM	192	CB	SER	178	25.496	-10.611	31.391	1.00	0.00	RX0	C
ATOM	193	OG	SER	178	25.206	-9.214	31.392	1.00	0.00	RX0	O
ATOM	194	HG	SER	178	25.089	-8.977	30.479	1.00	0.00	RX0	H
ATOM	195	C	SER	178	24.834	-12.718	30.070	1.00	0.00	RX0	C
ATOM	196	O	SER	178	23.999	-13.413	30.674	1.00	0.00	RX0	O
ATOM	197	N	GLU	179	25.827	-13.218	29.362	1.00	0.00	RX0	N
ATOM	198	H	GLU	179	26.363	-12.613	28.768	1.00	0.00	RX0	H
ATOM	199	CA	GLU	179	26.033	-14.670	29.175	1.00	0.00	RX0	C
ATOM	200	CB	GLU	179	26.308	-15.004	27.704	1.00	0.00	RX0	C
ATOM	201	CG	GLU	179	25.197	-14.654	26.710	1.00	0.00	RX0	C
ATOM	202	CD	GLU	179	25.379	-13.276	26.094	1.00	0.00	RX0	C
ATOM	203	OE1	GLU	179	25.315	-13.164	24.875	1.00	0.00	RX0	O
ATOM	204	OE2	GLU	179	25.469	-12.284	26.809	1.00	0.00	RX0	O
ATOM	205	C	GLU	179	27.192	-15.208	30.012	1.00	0.00	RX0	C
ATOM	206	O	GLU	179	28.361	-15.227	29.589	1.00	0.00	RX0	O
ATOM	207	N	TYR	180	26.873	-15.475	31.254	1.00	0.00	RX0	N
ATOM	208	H	TYR	180	25.923	-15.385	31.563	1.00	0.00	RX0	H
ATOM	209	CA	TYR	180	27.735	-16.233	32.177	1.00	0.00	RX0	C
ATOM	210	CB	TYR	180	28.493	-15.322	33.156	1.00	0.00	RX0	C
ATOM	211	CG	TYR	180	27.546	-14.673	34.139	1.00	0.00	RX0	C
ATOM	212	CD1	TYR	180	26.930	-13.467	33.824	1.00	0.00	RX0	C
ATOM	213	CE1	TYR	180	26.010	-12.913	34.704	1.00	0.00	RX0	C
ATOM	214	CD2	TYR	180	27.284	-15.290	35.357	1.00	0.00	RX0	C
ATOM	215	CE2	TYR	180	26.351	-14.745	36.228	1.00	0.00	RX0	C
ATOM	216	CZ	TYR	180	25.695	-13.570	35.888	1.00	0.00	RX0	C
ATOM	217	OH	TYR	180	24.720	-13.064	36.720	1.00	0.00	RX0	O
ATOM	218	HH	TYR	180	24.647	-12.125	36.574	1.00	0.00	RX0	H
ATOM	219	C	TYR	180	26.838	-17.226	32.909	1.00	0.00	RX0	C
ATOM	220	O	TYR	180	25.642	-16.953	33.094	1.00	0.00	RX0	O
ATOM	221	N	ASP	181	27.404	-18.345	33.318	1.00	0.00	RX0	N
ATOM	222	H	ASP	181	28.392	-18.458	33.265	1.00	0.00	RX0	H
ATOM	223	CA	ASP	181	26.630	-19.347	34.059	1.00	0.00	RX0	C
ATOM	224	CB	ASP	181	27.302	-20.710	34.083	1.00	0.00	RX0	C
ATOM	225	CG	ASP	181	26.447	-21.567	34.985	1.00	0.00	RX0	C
ATOM	226	OD1	ASP	181	25.244	-21.639	34.759	1.00	0.00	RX0	O
ATOM	227	OD2	ASP	181	26.963	-22.121	35.945	1.00	0.00	RX0	O
ATOM	228	C	ASP	181	26.420	-18.851	35.504	1.00	0.00	RX0	C
ATOM	229	O	ASP	181	27.391	-18.832	36.273	1.00	0.00	RX0	O
ATOM	230	N	PRO	182	25.185	-18.489	35.856	1.00	0.00	RX0	N
ATOM	231	CD	PRO	182	24.010	-18.606	34.996	1.00	0.00	RX0	C
ATOM	232	CA	PRO	182	24.825	-17.989	37.201	1.00	0.00	RX0	C
ATOM	233	CB	PRO	182	23.393	-17.489	36.997	1.00	0.00	RX0	C
ATOM	234	CG	PRO	182	22.821	-18.413	35.927	1.00	0.00	RX0	C
ATOM	235	C	PRO	182	24.941	-19.052	38.308	1.00	0.00	RX0	C
ATOM	236	O	PRO	182	24.654	-18.763	39.474	1.00	0.00	RX0	O
ATOM	237	N	THR	183	25.345	-20.259	37.948	1.00	0.00	RX0	N
ATOM	238	H	THR	183	25.557	-20.523	37.006	1.00	0.00	RX0	H
ATOM	239	CA	THR	183	25.568	-21.363	38.913	1.00	0.00	RX0	C
ATOM	240	CB	THR	183	24.913	-22.561	38.259	1.00	0.00	RX0	C
ATOM	241	OG1	THR	183	23.993	-22.062	37.276	1.00	0.00	RX0	O
ATOM	242	HG1	THR	183	24.498	-22.053	36.458	1.00	0.00	RX0	H

ATOM	243	CG2	THR	183	24.218	-23.478	39.267	1.00	0.00	RX0	C
ATOM	244	C	THR	183	27.063	-21.532	39.218	1.00	0.00	RX0	C
ATOM	245	O	THR	183	27.455	-22.345	40.058	1.00	0.00	RX0	O
ATOM	246	N	ARG	184	27.887	-20.699	38.573	1.00	0.00	RX0	N
ATOM	247	H	ARG	184	27.521	-19.996	37.961	1.00	0.00	RX0	H
ATOM	248	CA	ARG	184	29.343	-20.701	38.681	1.00	0.00	RX0	C
ATOM	249	CB	ARG	184	29.923	-20.843	37.277	1.00	0.00	RX0	C
ATOM	250	CG	ARG	184	30.269	-22.300	36.967	1.00	0.00	RX0	C
ATOM	251	CD	ARG	184	30.711	-22.485	35.517	1.00	0.00	RX0	C
ATOM	252	NE	ARG	184	31.472	-21.324	35.057	1.00	0.00	RX0	N
ATOM	253	HE	ARG	184	30.914	-20.621	34.607	1.00	0.00	RX0	H
ATOM	254	CZ	ARG	184	32.807	-21.193	35.331	1.00	0.00	RX0	C
ATOM	255	NH1	ARG	184	33.472	-22.155	36.000	1.00	0.00	RX0	N
ATOM	256	HH11	ARG	184	34.444	-21.970	36.253	1.00	0.00	RX0	H
ATOM	257	HH12	ARG	184	33.071	-23.025	36.281	1.00	0.00	RX0	H
ATOM	258	NH2	ARG	184	33.464	-20.087	34.937	1.00	0.00	RX0	N
ATOM	259	HH21	ARG	184	34.458	-20.039	35.139	1.00	0.00	RX0	H
ATOM	260	HH22	ARG	184	33.047	-19.309	34.470	1.00	0.00	RX0	H
ATOM	261	C	ARG	184	29.836	-19.410	39.407	1.00	0.00	RX0	C
ATOM	262	O	ARG	184	29.116	-18.390	39.334	1.00	0.00	RX0	O
ATOM	263	N	PRO	185	30.940	-19.460	40.113	1.00	0.00	RX0	N
ATOM	264	CD	PRO	185	31.744	-20.664	40.293	1.00	0.00	RX0	C
ATOM	265	CA	PRO	185	31.574	-18.285	40.781	1.00	0.00	RX0	C
ATOM	266	CB	PRO	185	32.858	-18.870	41.382	1.00	0.00	RX0	C
ATOM	267	CG	PRO	185	33.142	-20.137	40.580	1.00	0.00	RX0	C
ATOM	268	C	PRO	185	31.820	-17.125	39.813	1.00	0.00	RX0	C
ATOM	269	O	PRO	185	31.836	-17.275	38.592	1.00	0.00	RX0	O
ATOM	270	N	PHE	186	32.164	-15.998	40.422	1.00	0.00	RX0	N
ATOM	271	H	PHE	186	32.241	-15.993	41.417	1.00	0.00	RX0	H
ATOM	272	CA	PHE	186	32.333	-14.726	39.697	1.00	0.00	RX0	C
ATOM	273	CB	PHE	186	31.517	-13.614	40.358	1.00	0.00	RX0	C
ATOM	274	CG	PHE	186	31.423	-12.436	39.416	1.00	0.00	RX0	C
ATOM	275	CD1	PHE	186	30.873	-12.606	38.150	1.00	0.00	RX0	C
ATOM	276	CD2	PHE	186	31.885	-11.186	39.810	1.00	0.00	RX0	C
ATOM	277	CE1	PHE	186	30.790	-11.528	37.277	1.00	0.00	RX0	C
ATOM	278	CE2	PHE	186	31.801	-10.108	38.937	1.00	0.00	RX0	C
ATOM	279	CZ	PHE	186	31.255	-10.279	37.670	1.00	0.00	RX0	C
ATOM	280	C	PHE	186	33.791	-14.305	39.507	1.00	0.00	RX0	C
ATOM	281	O	PHE	186	34.127	-13.678	38.496	1.00	0.00	RX0	O
ATOM	282	N	SER	187	34.655	-14.802	40.380	1.00	0.00	RX0	N
ATOM	283	H	SER	187	34.311	-15.351	41.137	1.00	0.00	RX0	H
ATOM	284	CA	SER	187	36.113	-14.542	40.380	1.00	0.00	RX0	C
ATOM	285	CB	SER	187	36.611	-15.271	41.615	1.00	0.00	RX0	C
ATOM	286	OG	SER	187	35.493	-15.359	42.516	1.00	0.00	RX0	O
ATOM	287	HG	SER	187	35.869	-15.431	43.386	1.00	0.00	RX0	H
ATOM	288	C	SER	187	36.764	-14.980	39.057	1.00	0.00	RX0	C
ATOM	289	O	SER	187	37.834	-14.531	38.683	1.00	0.00	RX0	O
ATOM	290	N	GLU	188	36.054	-15.878	38.369	1.00	0.00	RX0	N
ATOM	291	H	GLU	188	35.126	-16.109	38.649	1.00	0.00	RX0	H
ATOM	292	CA	GLU	188	36.561	-16.586	37.191	1.00	0.00	RX0	C
ATOM	293	CB	GLU	188	36.150	-18.052	37.351	1.00	0.00	RX0	C
ATOM	294	CG	GLU	188	36.846	-19.079	36.458	1.00	0.00	RX0	C
ATOM	295	CD	GLU	188	36.002	-20.336	36.466	1.00	0.00	RX0	C
ATOM	296	OE1	GLU	188	35.231	-20.535	37.397	1.00	0.00	RX0	O
ATOM	297	OE2	GLU	188	36.002	-21.071	35.485	1.00	0.00	RX0	O
ATOM	298	C	GLU	188	36.028	-16.018	35.856	1.00	0.00	RX0	C
ATOM	299	O	GLU	188	36.494	-16.416	34.788	1.00	0.00	RX0	O
ATOM	300	N	ALA	189	35.058	-15.107	35.914	1.00	0.00	RX0	N
ATOM	301	H	ALA	189	34.826	-14.687	36.794	1.00	0.00	RX0	H
ATOM	302	CA	ALA	189	34.543	-14.432	34.708	1.00	0.00	RX0	C
ATOM	303	CB	ALA	189	33.067	-14.082	34.892	1.00	0.00	RX0	C

ATOM	304	C	ALA	189	35.336	-13.151	34.407	1.00	0.00	RX0	C
ATOM	305	O	ALA	189	35.533	-12.292	35.270	1.00	0.00	RX0	O
ATOM	306	N	SER	190	35.819	-13.065	33.173	1.00	0.00	RX0	N
ATOM	307	H	SER	190	35.614	-13.816	32.549	1.00	0.00	RX0	H
ATOM	308	CA	SER	190	36.430	-11.825	32.646	1.00	0.00	RX0	C
ATOM	309	CB	SER	190	37.100	-12.195	31.298	1.00	0.00	RX0	C
ATOM	310	OG	SER	190	38.260	-11.391	30.970	1.00	0.00	RX0	O
ATOM	311	HG	SER	190	38.785	-11.429	31.771	1.00	0.00	RX0	H
ATOM	312	C	SER	190	35.341	-10.761	32.513	1.00	0.00	RX0	C
ATOM	313	O	SER	190	34.465	-10.869	31.639	1.00	0.00	RX0	O
ATOM	314	N	MET	191	35.401	-9.751	33.361	1.00	0.00	RX0	N
ATOM	315	H	MET	191	36.035	-9.822	34.132	1.00	0.00	RX0	H
ATOM	316	CA	MET	191	34.414	-8.652	33.337	1.00	0.00	RX0	C
ATOM	317	CB	MET	191	34.642	-7.665	34.478	1.00	0.00	RX0	C
ATOM	318	CG	MET	191	33.545	-6.600	34.500	1.00	0.00	RX0	C
ATOM	319	SD	MET	191	33.638	-5.545	35.949	1.00	0.00	RX0	S
ATOM	320	CE	MET	191	33.293	-6.809	37.184	1.00	0.00	RX0	C
ATOM	321	C	MET	191	34.384	-7.939	31.976	1.00	0.00	RX0	C
ATOM	322	O	MET	191	33.329	-7.841	31.363	1.00	0.00	RX0	O
ATOM	323	N	MET	192	35.577	-7.654	31.438	1.00	0.00	RX0	N
ATOM	324	H	MET	192	36.383	-7.741	32.020	1.00	0.00	RX0	H
ATOM	325	CA	MET	192	35.697	-7.082	30.089	1.00	0.00	RX0	C
ATOM	326	CB	MET	192	37.136	-6.648	29.804	1.00	0.00	RX0	C
ATOM	327	CG	MET	192	37.280	-5.928	28.460	1.00	0.00	RX0	C
ATOM	328	SD	MET	192	36.169	-4.520	28.285	1.00	0.00	RX0	S
ATOM	329	CE	MET	192	36.763	-3.522	29.661	1.00	0.00	RX0	C
ATOM	330	C	MET	192	35.151	-8.021	28.999	1.00	0.00	RX0	C
ATOM	331	O	MET	192	34.484	-7.587	28.093	1.00	0.00	RX0	O
ATOM	332	N	GLY	193	35.358	-9.342	29.220	1.00	0.00	RX0	N
ATOM	333	H	GLY	193	35.698	-9.610	30.117	1.00	0.00	RX0	H
ATOM	334	CA	GLY	193	34.804	-10.378	28.330	1.00	0.00	RX0	C
ATOM	335	C	GLY	193	33.267	-10.338	28.334	1.00	0.00	RX0	C
ATOM	336	O	GLY	193	32.637	-10.184	27.296	1.00	0.00	RX0	O
ATOM	337	N	LEU	194	32.696	-10.293	29.537	1.00	0.00	RX0	N
ATOM	338	H	LEU	194	33.261	-10.303	30.358	1.00	0.00	RX0	H
ATOM	339	CA	LEU	194	31.235	-10.169	29.722	1.00	0.00	RX0	C
ATOM	340	CB	LEU	194	30.848	-10.221	31.199	1.00	0.00	RX0	C
ATOM	341	CG	LEU	194	31.146	-11.557	31.871	1.00	0.00	RX0	C
ATOM	342	CD1	LEU	194	30.708	-11.541	33.336	1.00	0.00	RX0	C
ATOM	343	CD2	LEU	194	30.539	-12.730	31.100	1.00	0.00	RX0	C
ATOM	344	C	LEU	194	30.647	-8.891	29.116	1.00	0.00	RX0	C
ATOM	345	O	LEU	194	29.706	-8.959	28.317	1.00	0.00	RX0	O
ATOM	346	N	LEU	195	31.327	-7.782	29.364	1.00	0.00	RX0	N
ATOM	347	H	LEU	195	32.131	-7.841	29.951	1.00	0.00	RX0	H
ATOM	348	CA	LEU	195	30.920	-6.462	28.846	1.00	0.00	RX0	C
ATOM	349	CB	LEU	195	31.732	-5.338	29.492	1.00	0.00	RX0	C
ATOM	350	CG	LEU	195	31.538	-5.240	31.006	1.00	0.00	RX0	C
ATOM	351	CD1	LEU	195	32.376	-4.112	31.609	1.00	0.00	RX0	C
ATOM	352	CD2	LEU	195	30.063	-5.129	31.389	1.00	0.00	RX0	C
ATOM	353	C	LEU	195	31.020	-6.357	27.321	1.00	0.00	RX0	C
ATOM	354	O	LEU	195	30.051	-5.942	26.671	1.00	0.00	RX0	O
ATOM	355	N	THR	196	32.075	-6.931	26.767	1.00	0.00	RX0	N
ATOM	356	H	THR	196	32.805	-7.315	27.330	1.00	0.00	RX0	H
ATOM	357	CA	THR	196	32.335	-6.901	25.309	1.00	0.00	RX0	C
ATOM	358	CB	THR	196	33.781	-7.304	25.031	1.00	0.00	RX0	C
ATOM	359	OG1	THR	196	34.666	-6.399	25.705	1.00	0.00	RX0	O
ATOM	360	HG1	THR	196	34.391	-5.526	25.452	1.00	0.00	RX0	H
ATOM	361	CG2	THR	196	34.090	-7.344	23.533	1.00	0.00	RX0	C
ATOM	362	C	THR	196	31.317	-7.765	24.552	1.00	0.00	RX0	C
ATOM	363	O	THR	196	30.772	-7.327	23.532	1.00	0.00	RX0	O
ATOM	364	N	ASN	197	31.003	-8.928	25.107	1.00	0.00	RX0	N

ATOM	365	H	ASN	197	31.409	-9.166	25.991	1.00	0.00	RX0	H
ATOM	366	CA	ASN	197	30.010	-9.840	24.504	1.00	0.00	RX0	C
ATOM	367	CB	ASN	197	29.971	-11.204	25.194	1.00	0.00	RX0	C
ATOM	368	CG	ASN	197	30.969	-12.141	24.551	1.00	0.00	RX0	C
ATOM	369	OD1	ASN	197	30.705	-12.814	23.562	1.00	0.00	RX0	O
ATOM	370	ND2	ASN	197	32.154	-12.159	25.185	1.00	0.00	RX0	N
ATOM	371	HD21	ASN	197	32.292	-11.562	25.978	1.00	0.00	RX0	H
ATOM	372	HD22	ASN	197	32.891	-12.754	24.871	1.00	0.00	RX0	H
ATOM	373	C	ASN	197	28.594	-9.255	24.528	1.00	0.00	RX0	C
ATOM	374	O	ASN	197	27.900	-9.272	23.514	1.00	0.00	RX0	O
ATOM	375	N	LEU	198	28.277	-8.575	25.633	1.00	0.00	RX0	N
ATOM	376	H	LEU	198	28.898	-8.592	26.419	1.00	0.00	RX0	H
ATOM	377	CA	LEU	198	27.002	-7.850	25.760	1.00	0.00	RX0	C
ATOM	378	CB	LEU	198	26.851	-7.337	27.188	1.00	0.00	RX0	C
ATOM	379	CG	LEU	198	25.447	-6.832	27.504	1.00	0.00	RX0	C
ATOM	380	CD1	LEU	198	24.376	-7.895	27.248	1.00	0.00	RX0	C
ATOM	381	CD2	LEU	198	25.384	-6.295	28.929	1.00	0.00	RX0	C
ATOM	382	C	LEU	198	26.885	-6.719	24.724	1.00	0.00	RX0	C
ATOM	383	O	LEU	198	25.930	-6.676	23.947	1.00	0.00	RX0	O
ATOM	384	N	ALA	199	27.942	-5.914	24.641	1.00	0.00	RX0	N
ATOM	385	H	ALA	199	28.696	-6.063	25.281	1.00	0.00	RX0	H
ATOM	386	CA	ALA	199	28.029	-4.784	23.694	1.00	0.00	RX0	C
ATOM	387	CB	ALA	199	29.343	-4.025	23.882	1.00	0.00	RX0	C
ATOM	388	C	ALA	199	27.921	-5.230	22.227	1.00	0.00	RX0	C
ATOM	389	O	ALA	199	27.138	-4.660	21.467	1.00	0.00	RX0	O
ATOM	390	N	ASP	200	28.555	-6.360	21.908	1.00	0.00	RX0	N
ATOM	391	H	ASP	200	29.131	-6.815	22.589	1.00	0.00	RX0	H
ATOM	392	CA	ASP	200	28.494	-6.940	20.550	1.00	0.00	RX0	C
ATOM	393	CB	ASP	200	29.540	-8.045	20.362	1.00	0.00	RX0	C
ATOM	394	CG	ASP	200	30.224	-7.901	19.010	1.00	0.00	RX0	C
ATOM	395	OD1	ASP	200	30.582	-6.788	18.625	1.00	0.00	RX0	O
ATOM	396	OD2	ASP	200	30.460	-8.903	18.335	1.00	0.00	RX0	O
ATOM	397	C	ASP	200	27.084	-7.410	20.171	1.00	0.00	RX0	C
ATOM	398	O	ASP	200	26.604	-7.102	19.080	1.00	0.00	RX0	O
ATOM	399	N	ARG	201	26.390	-7.998	21.143	1.00	0.00	RX0	N
ATOM	400	H	ARG	201	26.838	-8.158	22.027	1.00	0.00	RX0	H
ATOM	401	CA	ARG	201	24.992	-8.434	20.957	1.00	0.00	RX0	C
ATOM	402	CB	ARG	201	24.519	-9.456	21.985	1.00	0.00	RX0	C
ATOM	403	CG	ARG	201	24.011	-10.686	21.229	1.00	0.00	RX0	C
ATOM	404	CD	ARG	201	22.930	-11.467	21.971	1.00	0.00	RX0	C
ATOM	405	NE	ARG	201	23.334	-11.743	23.342	1.00	0.00	RX0	N
ATOM	406	HE	ARG	201	24.314	-11.844	23.578	1.00	0.00	RX0	H
ATOM	407	CZ	ARG	201	22.378	-11.959	24.284	1.00	0.00	RX0	C
ATOM	408	NH1	ARG	201	21.078	-12.035	23.913	1.00	0.00	RX0	N
ATOM	409	HH11	ARG	201	20.319	-12.334	24.506	1.00	0.00	RX0	H
ATOM	410	HH12	ARG	201	20.734	-11.847	22.982	1.00	0.00	RX0	H
ATOM	411	NH2	ARG	201	22.776	-12.092	25.562	1.00	0.00	RX0	N
ATOM	412	HH21	ARG	201	22.190	-12.173	26.369	1.00	0.00	RX0	H
ATOM	413	HH22	ARG	201	23.771	-12.113	25.776	1.00	0.00	RX0	H
ATOM	414	C	ARG	201	23.991	-7.279	20.827	1.00	0.00	RX0	C
ATOM	415	O	ARG	201	23.123	-7.308	19.955	1.00	0.00	RX0	O
ATOM	416	N	GLU	202	24.240	-6.201	21.568	1.00	0.00	RX0	N
ATOM	417	H	GLU	202	24.995	-6.229	22.228	1.00	0.00	RX0	H
ATOM	418	CA	GLU	202	23.401	-4.988	21.493	1.00	0.00	RX0	C
ATOM	419	CB	GLU	202	23.563	-4.061	22.721	1.00	0.00	RX0	C
ATOM	420	CG	GLU	202	22.902	-4.621	24.000	1.00	0.00	RX0	C
ATOM	421	CD	GLU	202	22.838	-3.613	25.153	1.00	0.00	RX0	C
ATOM	422	OE1	GLU	202	21.792	-3.000	25.374	1.00	0.00	RX0	O
ATOM	423	OE2	GLU	202	23.805	-3.480	25.897	1.00	0.00	RX0	O
ATOM	424	C	GLU	202	23.526	-4.262	20.149	1.00	0.00	RX0	C
ATOM	425	O	GLU	202	22.539	-3.754	19.625	1.00	0.00	RX0	O

ATOM	426	N	LEU	203	24.712	-4.358	19.546	1.00	0.00	RX0	N
ATOM	427	H	LEU	203	25.461	-4.814	20.036	1.00	0.00	RX0	H
ATOM	428	CA	LEU	203	25.004	-3.680	18.270	1.00	0.00	RX0	C
ATOM	429	CB	LEU	203	26.480	-3.870	17.917	1.00	0.00	RX0	C
ATOM	430	CG	LEU	203	26.922	-3.125	16.655	1.00	0.00	RX0	C
ATOM	431	CD1	LEU	203	26.718	-1.614	16.776	1.00	0.00	RX0	C
ATOM	432	CD2	LEU	203	28.360	-3.477	16.272	1.00	0.00	RX0	C
ATOM	433	C	LEU	203	24.099	-4.160	17.127	1.00	0.00	RX0	C
ATOM	434	O	LEU	203	23.593	-3.349	16.346	1.00	0.00	RX0	O
ATOM	435	N	VAL	204	23.782	-5.447	17.151	1.00	0.00	RX0	N
ATOM	436	H	VAL	204	24.168	-6.003	17.891	1.00	0.00	RX0	H
ATOM	437	CA	VAL	204	22.925	-6.083	16.127	1.00	0.00	RX0	C
ATOM	438	CB	VAL	204	22.899	-7.596	16.336	1.00	0.00	RX0	C
ATOM	439	CG1	VAL	204	22.010	-8.281	15.297	1.00	0.00	RX0	C
ATOM	440	CG2	VAL	204	24.320	-8.163	16.355	1.00	0.00	RX0	C
ATOM	441	C	VAL	204	21.502	-5.497	16.213	1.00	0.00	RX0	C
ATOM	442	O	VAL	204	20.938	-5.041	15.221	1.00	0.00	RX0	O
ATOM	443	N	HIS	205	21.015	-5.393	17.448	1.00	0.00	RX0	N
ATOM	444	H	HIS	205	21.599	-5.684	18.209	1.00	0.00	RX0	H
ATOM	445	CA	HIS	205	19.703	-4.786	17.746	1.00	0.00	RX0	C
ATOM	446	CB	HIS	205	19.262	-5.115	19.175	1.00	0.00	RX0	C
ATOM	447	CG	HIS	205	19.031	-6.605	19.299	1.00	0.00	RX0	C
ATOM	448	ND1	HIS	205	17.835	-7.197	19.114	1.00	0.00	RX0	N
ATOM	449	HD1	HIS	205	16.987	-6.757	18.895	1.00	0.00	RX0	H
ATOM	450	CD2	HIS	205	19.974	-7.590	19.601	1.00	0.00	RX0	C
ATOM	451	NE2	HIS	205	19.334	-8.783	19.594	1.00	0.00	RX0	N
ATOM	452	CE1	HIS	205	18.018	-8.546	19.295	1.00	0.00	RX0	C
ATOM	453	C	HIS	205	19.668	-3.277	17.476	1.00	0.00	RX0	C
ATOM	454	O	HIS	205	18.642	-2.756	17.030	1.00	0.00	RX0	O
ATOM	455	N	MET	206	20.820	-2.627	17.609	1.00	0.00	RX0	N
ATOM	456	H	MET	206	21.610	-3.126	17.972	1.00	0.00	RX0	H
ATOM	457	CA	MET	206	20.969	-1.185	17.340	1.00	0.00	RX0	C
ATOM	458	CB	MET	206	22.357	-0.695	17.746	1.00	0.00	RX0	C
ATOM	459	CG	MET	206	22.545	0.803	17.508	1.00	0.00	RX0	C
ATOM	460	SD	MET	206	24.247	1.320	17.768	1.00	0.00	RX0	S
ATOM	461	CE	MET	206	24.500	0.514	19.354	1.00	0.00	RX0	C
ATOM	462	C	MET	206	20.721	-0.870	15.856	1.00	0.00	RX0	C
ATOM	463	O	MET	206	20.035	0.103	15.544	1.00	0.00	RX0	O
ATOM	464	N	ILE	207	21.183	-1.758	14.977	1.00	0.00	RX0	N
ATOM	465	H	ILE	207	21.731	-2.518	15.337	1.00	0.00	RX0	H
ATOM	466	CA	ILE	207	20.975	-1.628	13.516	1.00	0.00	RX0	C
ATOM	467	CB	ILE	207	21.663	-2.793	12.799	1.00	0.00	RX0	C
ATOM	468	CG2	ILE	207	21.381	-2.786	11.297	1.00	0.00	RX0	C
ATOM	469	CG1	ILE	207	23.160	-2.810	13.102	1.00	0.00	RX0	C
ATOM	470	CD1	ILE	207	23.895	-1.603	12.522	1.00	0.00	RX0	C
ATOM	471	C	ILE	207	19.470	-1.621	13.197	1.00	0.00	RX0	C
ATOM	472	O	ILE	207	18.988	-0.755	12.467	1.00	0.00	RX0	O
ATOM	473	N	ASN	208	18.761	-2.558	13.816	1.00	0.00	RX0	N
ATOM	474	H	ASN	208	19.235	-3.131	14.488	1.00	0.00	RX0	H
ATOM	475	CA	ASN	208	17.313	-2.734	13.586	1.00	0.00	RX0	C
ATOM	476	CB	ASN	208	16.769	-4.060	14.102	1.00	0.00	RX0	C
ATOM	477	CG	ASN	208	15.942	-4.648	12.975	1.00	0.00	RX0	C
ATOM	478	OD1	ASN	208	16.394	-4.710	11.826	1.00	0.00	RX0	O
ATOM	479	ND2	ASN	208	14.729	-5.090	13.353	1.00	0.00	RX0	N
ATOM	480	HD21	ASN	208	14.416	-5.006	14.302	1.00	0.00	RX0	H
ATOM	481	HD22	ASN	208	14.086	-5.531	12.724	1.00	0.00	RX0	H
ATOM	482	C	ASN	208	16.516	-1.532	14.103	1.00	0.00	RX0	C
ATOM	483	O	ASN	208	15.637	-1.017	13.417	1.00	0.00	RX0	O
ATOM	484	N	TRP	209	16.982	-1.008	15.238	1.00	0.00	RX0	N
ATOM	485	H	TRP	209	17.725	-1.474	15.723	1.00	0.00	RX0	H
ATOM	486	CA	TRP	209	16.425	0.201	15.864	1.00	0.00	RX0	C

ATOM	487	CB	TRP	209	17.093	0.415	17.230	1.00	0.00	RX0	C
ATOM	488	CG	TRP	209	16.717	1.755	17.824	1.00	0.00	RX0	C
ATOM	489	CD2	TRP	209	17.461	2.992	17.788	1.00	0.00	RX0	C
ATOM	490	CE2	TRP	209	16.701	3.972	18.469	1.00	0.00	RX0	C
ATOM	491	CE3	TRP	209	18.694	3.334	17.244	1.00	0.00	RX0	C
ATOM	492	CD1	TRP	209	15.550	2.064	18.533	1.00	0.00	RX0	C
ATOM	493	NE1	TRP	209	15.531	3.365	18.919	1.00	0.00	RX0	N
ATOM	494	HE1	TRP	209	14.794	3.774	19.436	1.00	0.00	RX0	H
ATOM	495	CZ2	TRP	209	17.192	5.270	18.569	1.00	0.00	RX0	C
ATOM	496	CZ3	TRP	209	19.174	4.632	17.357	1.00	0.00	RX0	C
ATOM	497	CH2	TRP	209	18.424	5.599	18.015	1.00	0.00	RX0	C
ATOM	498	C	TRP	209	16.619	1.438	14.972	1.00	0.00	RX0	C
ATOM	499	O	TRP	209	15.652	2.120	14.634	1.00	0.00	RX0	O
ATOM	500	N	ALA	210	17.853	1.624	14.503	1.00	0.00	RX0	N
ATOM	501	H	ALA	210	18.568	0.989	14.792	1.00	0.00	RX0	H
ATOM	502	CA	ALA	210	18.233	2.756	13.635	1.00	0.00	RX0	C
ATOM	503	CB	ALA	210	19.715	2.665	13.273	1.00	0.00	RX0	C
ATOM	504	C	ALA	210	17.400	2.800	12.347	1.00	0.00	RX0	C
ATOM	505	O	ALA	210	16.892	3.855	11.979	1.00	0.00	RX0	O
ATOM	506	N	LYS	211	17.095	1.613	11.820	1.00	0.00	RX0	N
ATOM	507	H	LYS	211	17.507	0.800	12.234	1.00	0.00	RX0	H
ATOM	508	CA	LYS	211	16.258	1.472	10.614	1.00	0.00	RX0	C
ATOM	509	CB	LYS	211	16.383	0.065	10.033	1.00	0.00	RX0	C
ATOM	510	CG	LYS	211	17.756	-0.151	9.387	1.00	0.00	RX0	C
ATOM	511	CD	LYS	211	17.925	-1.582	8.882	1.00	0.00	RX0	C
ATOM	512	CE	LYS	211	17.648	-2.531	10.040	1.00	0.00	RX0	C
ATOM	513	NZ	LYS	211	17.787	-3.944	9.663	1.00	0.00	RX0	N
ATOM	514	HZ1	LYS	211	17.407	-4.513	10.455	1.00	0.00	RX0	H
ATOM	515	HZ2	LYS	211	17.230	-4.140	8.811	1.00	0.00	RX0	H
ATOM	516	HZ3	LYS	211	18.786	-4.180	9.499	1.00	0.00	RX0	H
ATOM	517	C	LYS	211	14.795	1.898	10.823	1.00	0.00	RX0	C
ATOM	518	O	LYS	211	14.129	2.337	9.881	1.00	0.00	RX0	O
ATOM	519	N	ARG	212	14.357	1.870	12.071	1.00	0.00	RX0	N
ATOM	520	H	ARG	212	14.980	1.609	12.810	1.00	0.00	RX0	H
ATOM	521	CA	ARG	212	13.005	2.320	12.466	1.00	0.00	RX0	C
ATOM	522	CB	ARG	212	12.398	1.423	13.564	1.00	0.00	RX0	C
ATOM	523	CG	ARG	212	12.168	-0.054	13.204	1.00	0.00	RX0	C
ATOM	524	CD	ARG	212	11.634	-0.940	14.352	1.00	0.00	RX0	C
ATOM	525	NE	ARG	212	10.226	-0.697	14.698	1.00	0.00	RX0	N
ATOM	526	HE	ARG	212	9.755	0.056	14.227	1.00	0.00	RX0	H
ATOM	527	CZ	ARG	212	9.618	-1.474	15.663	1.00	0.00	RX0	C
ATOM	528	NH1	ARG	212	10.332	-2.426	16.305	1.00	0.00	RX0	N
ATOM	529	HH11	ARG	212	9.917	-3.082	16.963	1.00	0.00	RX0	H
ATOM	530	HH12	ARG	212	11.314	-2.565	16.174	1.00	0.00	RX0	H
ATOM	531	NH2	ARG	212	8.313	-1.295	15.971	1.00	0.00	RX0	N
ATOM	532	HH21	ARG	212	7.935	-1.712	16.804	1.00	0.00	RX0	H
ATOM	533	HH22	ARG	212	7.643	-0.759	15.436	1.00	0.00	RX0	H
ATOM	534	C	ARG	212	12.933	3.790	12.894	1.00	0.00	RX0	C
ATOM	535	O	ARG	212	11.827	4.344	12.989	1.00	0.00	RX0	O
ATOM	536	N	VAL	213	14.074	4.417	13.148	1.00	0.00	RX0	N
ATOM	537	H	VAL	213	14.933	3.928	12.993	1.00	0.00	RX0	H
ATOM	538	CA	VAL	213	14.155	5.874	13.374	1.00	0.00	RX0	C
ATOM	539	CB	VAL	213	15.581	6.281	13.749	1.00	0.00	RX0	C
ATOM	540	CG1	VAL	213	15.751	7.800	13.838	1.00	0.00	RX0	C
ATOM	541	CG2	VAL	213	15.980	5.584	15.048	1.00	0.00	RX0	C
ATOM	542	C	VAL	213	13.672	6.590	12.095	1.00	0.00	RX0	C
ATOM	543	O	VAL	213	14.288	6.431	11.023	1.00	0.00	RX0	O
ATOM	544	N	PRO	214	12.622	7.395	12.213	1.00	0.00	RX0	N
ATOM	545	CD	PRO	214	11.895	7.609	13.458	1.00	0.00	RX0	C
ATOM	546	CA	PRO	214	12.035	8.145	11.084	1.00	0.00	RX0	C
ATOM	547	CB	PRO	214	10.927	8.954	11.761	1.00	0.00	RX0	C

ATOM	548	CG	PRO	214	10.552	8.163	13.010	1.00	0.00	RX0	C
ATOM	549	C	PRO	214	13.102	9.001	10.387	1.00	0.00	RX0	C
ATOM	550	O	PRO	214	13.853	9.727	11.025	1.00	0.00	RX0	O
ATOM	551	N	GLY	215	13.244	8.730	9.080	1.00	0.00	RX0	N
ATOM	552	H	GLY	215	12.749	7.981	8.637	1.00	0.00	RX0	H
ATOM	553	CA	GLY	215	14.194	9.473	8.227	1.00	0.00	RX0	C
ATOM	554	C	GLY	215	15.511	8.732	7.950	1.00	0.00	RX0	C
ATOM	555	O	GLY	215	16.085	8.889	6.862	1.00	0.00	RX0	O
ATOM	556	N	PHE	216	15.917	7.845	8.848	1.00	0.00	RX0	N
ATOM	557	H	PHE	216	15.329	7.652	9.637	1.00	0.00	RX0	H
ATOM	558	CA	PHE	216	17.224	7.160	8.764	1.00	0.00	RX0	C
ATOM	559	CB	PHE	216	17.452	6.293	9.997	1.00	0.00	RX0	C
ATOM	560	CG	PHE	216	18.892	5.842	10.054	1.00	0.00	RX0	C
ATOM	561	CD1	PHE	216	19.897	6.771	10.295	1.00	0.00	RX0	C
ATOM	562	CD2	PHE	216	19.211	4.501	9.881	1.00	0.00	RX0	C
ATOM	563	CE1	PHE	216	21.218	6.353	10.402	1.00	0.00	RX0	C
ATOM	564	CE2	PHE	216	20.531	4.083	9.989	1.00	0.00	RX0	C
ATOM	565	CZ	PHE	216	21.531	5.006	10.271	1.00	0.00	RX0	C
ATOM	566	C	PHE	216	17.435	6.347	7.474	1.00	0.00	RX0	C
ATOM	567	O	PHE	216	18.358	6.641	6.711	1.00	0.00	RX0	O
ATOM	568	N	VAL	217	16.482	5.483	7.151	1.00	0.00	RX0	N
ATOM	569	H	VAL	217	15.674	5.446	7.737	1.00	0.00	RX0	H
ATOM	570	CA	VAL	217	16.570	4.608	5.958	1.00	0.00	RX0	C
ATOM	571	CB	VAL	217	15.528	3.495	6.008	1.00	0.00	RX0	C
ATOM	572	CG1	VAL	217	15.898	2.481	7.081	1.00	0.00	RX0	C
ATOM	573	CG2	VAL	217	14.111	4.051	6.169	1.00	0.00	RX0	C
ATOM	574	C	VAL	217	16.469	5.342	4.608	1.00	0.00	RX0	C
ATOM	575	O	VAL	217	16.660	4.747	3.556	1.00	0.00	RX0	O
ATOM	576	N	ASP	218	16.058	6.613	4.671	1.00	0.00	RX0	N
ATOM	577	H	ASP	218	15.762	7.059	5.518	1.00	0.00	RX0	H
ATOM	578	CA	ASP	218	16.006	7.470	3.472	1.00	0.00	RX0	C
ATOM	579	CB	ASP	218	15.107	8.683	3.761	1.00	0.00	RX0	C
ATOM	580	CG	ASP	218	13.751	8.292	4.351	1.00	0.00	RX0	C
ATOM	581	OD1	ASP	218	12.742	8.508	3.684	1.00	0.00	RX0	O
ATOM	582	OD2	ASP	218	13.690	7.813	5.489	1.00	0.00	RX0	O
ATOM	583	C	ASP	218	17.401	7.924	3.023	1.00	0.00	RX0	C
ATOM	584	O	ASP	218	17.595	8.369	1.896	1.00	0.00	RX0	O
ATOM	585	N	LEU	219	18.344	7.857	3.967	1.00	0.00	RX0	N
ATOM	586	H	LEU	219	18.094	7.484	4.861	1.00	0.00	RX0	H
ATOM	587	CA	LEU	219	19.767	8.083	3.697	1.00	0.00	RX0	C
ATOM	588	CB	LEU	219	20.544	8.273	4.996	1.00	0.00	RX0	C
ATOM	589	CG	LEU	219	19.987	9.454	5.791	1.00	0.00	RX0	C
ATOM	590	CD1	LEU	219	20.553	9.501	7.208	1.00	0.00	RX0	C
ATOM	591	CD2	LEU	219	20.160	10.778	5.043	1.00	0.00	RX0	C
ATOM	592	C	LEU	219	20.350	6.970	2.832	1.00	0.00	RX0	C
ATOM	593	O	LEU	219	19.843	5.836	2.789	1.00	0.00	RX0	O
ATOM	594	N	THR	220	21.442	7.293	2.191	1.00	0.00	RX0	N
ATOM	595	H	THR	220	21.753	8.246	2.252	1.00	0.00	RX0	H
ATOM	596	CA	THR	220	22.263	6.312	1.453	1.00	0.00	RX0	C
ATOM	597	CB	THR	220	23.333	6.997	0.596	1.00	0.00	RX0	C
ATOM	598	OG1	THR	220	24.015	8.023	1.317	1.00	0.00	RX0	O
ATOM	599	HG1	THR	220	23.421	8.775	1.398	1.00	0.00	RX0	H
ATOM	600	CG2	THR	220	22.732	7.556	-0.694	1.00	0.00	RX0	C
ATOM	601	C	THR	220	22.829	5.303	2.454	1.00	0.00	RX0	C
ATOM	602	O	THR	220	23.112	5.646	3.611	1.00	0.00	RX0	O
ATOM	603	N	LEU	221	23.130	4.116	1.957	1.00	0.00	RX0	N
ATOM	604	H	LEU	221	22.808	3.907	1.036	1.00	0.00	RX0	H
ATOM	605	CA	LEU	221	23.750	3.065	2.781	1.00	0.00	RX0	C
ATOM	606	CB	LEU	221	23.967	1.848	1.883	1.00	0.00	RX0	C
ATOM	607	CG	LEU	221	24.753	0.708	2.526	1.00	0.00	RX0	C
ATOM	608	CD1	LEU	221	23.971	0.037	3.652	1.00	0.00	RX0	C

ATOM	609	CD2	LEU	221	25.259	-0.272	1.473	1.00	0.00	RX0	C
ATOM	610	C	LEU	221	25.092	3.509	3.394	1.00	0.00	RX0	C
ATOM	611	O	LEU	221	25.324	3.347	4.578	1.00	0.00	RX0	O
ATOM	612	N	HIS	222	25.854	4.270	2.593	1.00	0.00	RX0	N
ATOM	613	H	HIS	222	25.535	4.427	1.662	1.00	0.00	RX0	H
ATOM	614	CA	HIS	222	27.131	4.847	3.045	1.00	0.00	RX0	C
ATOM	615	CB	HIS	222	27.812	5.608	1.884	1.00	0.00	RX0	C
ATOM	616	CG	HIS	222	28.869	6.589	2.370	1.00	0.00	RX0	C
ATOM	617	ND1	HIS	222	29.700	6.392	3.417	1.00	0.00	RX0	N
ATOM	618	HD1	HIS	222	29.770	5.584	3.981	1.00	0.00	RX0	H
ATOM	619	CD2	HIS	222	29.114	7.861	1.851	1.00	0.00	RX0	C
ATOM	620	NE2	HIS	222	30.090	8.435	2.594	1.00	0.00	RX0	N
ATOM	621	CE1	HIS	222	30.449	7.531	3.562	1.00	0.00	RX0	C
ATOM	622	C	HIS	222	26.942	5.765	4.264	1.00	0.00	RX0	C
ATOM	623	O	HIS	222	27.673	5.642	5.246	1.00	0.00	RX0	O
ATOM	624	N	ASP	223	25.958	6.658	4.167	1.00	0.00	RX0	N
ATOM	625	H	ASP	223	25.470	6.804	3.306	1.00	0.00	RX0	H
ATOM	626	CA	ASP	223	25.694	7.636	5.242	1.00	0.00	RX0	C
ATOM	627	CB	ASP	223	24.854	8.818	4.726	1.00	0.00	RX0	C
ATOM	628	CG	ASP	223	25.680	9.641	3.739	1.00	0.00	RX0	C
ATOM	629	OD1	ASP	223	26.322	9.068	2.869	1.00	0.00	RX0	O
ATOM	630	OD2	ASP	223	25.731	10.863	3.838	1.00	0.00	RX0	O
ATOM	631	C	ASP	223	25.148	6.996	6.516	1.00	0.00	RX0	C
ATOM	632	O	ASP	223	25.558	7.375	7.616	1.00	0.00	RX0	O
ATOM	633	N	GLN	224	24.393	5.921	6.332	1.00	0.00	RX0	N
ATOM	634	H	GLN	224	24.148	5.686	5.388	1.00	0.00	RX0	H
ATOM	635	CA	GLN	224	23.868	5.118	7.452	1.00	0.00	RX0	C
ATOM	636	CB	GLN	224	22.846	4.093	6.958	1.00	0.00	RX0	C
ATOM	637	CG	GLN	224	21.595	4.762	6.380	1.00	0.00	RX0	C
ATOM	638	CD	GLN	224	20.552	3.707	6.079	1.00	0.00	RX0	C
ATOM	639	OE1	GLN	224	20.404	2.728	6.804	1.00	0.00	RX0	O
ATOM	640	NE2	GLN	224	19.838	3.953	4.966	1.00	0.00	RX0	N
ATOM	641	HE21	GLN	224	19.985	4.777	4.410	1.00	0.00	RX0	H
ATOM	642	HE22	GLN	224	19.112	3.357	4.625	1.00	0.00	RX0	H
ATOM	643	C	GLN	224	25.003	4.453	8.243	1.00	0.00	RX0	C
ATOM	644	O	GLN	224	25.073	4.591	9.468	1.00	0.00	RX0	O
ATOM	645	N	VAL	225	25.993	3.956	7.505	1.00	0.00	RX0	N
ATOM	646	H	VAL	225	25.892	3.976	6.507	1.00	0.00	RX0	H
ATOM	647	CA	VAL	225	27.191	3.319	8.093	1.00	0.00	RX0	C
ATOM	648	CB	VAL	225	28.046	2.592	7.055	1.00	0.00	RX0	C
ATOM	649	CG1	VAL	225	29.223	1.910	7.749	1.00	0.00	RX0	C
ATOM	650	CG2	VAL	225	27.232	1.566	6.271	1.00	0.00	RX0	C
ATOM	651	C	VAL	225	28.021	4.368	8.852	1.00	0.00	RX0	C
ATOM	652	O	VAL	225	28.415	4.141	9.995	1.00	0.00	RX0	O
ATOM	653	N	HIS	226	28.182	5.534	8.231	1.00	0.00	RX0	N
ATOM	654	H	HIS	226	27.801	5.644	7.310	1.00	0.00	RX0	H
ATOM	655	CA	HIS	226	28.959	6.641	8.815	1.00	0.00	RX0	C
ATOM	656	CB	HIS	226	29.153	7.813	7.846	1.00	0.00	RX0	C
ATOM	657	CG	HIS	226	30.071	8.841	8.481	1.00	0.00	RX0	C
ATOM	658	ND1	HIS	226	31.346	8.596	8.834	1.00	0.00	RX0	N
ATOM	659	HD1	HIS	226	31.856	7.759	8.738	1.00	0.00	RX0	H
ATOM	660	CD2	HIS	226	29.767	10.161	8.827	1.00	0.00	RX0	C
ATOM	661	NE2	HIS	226	30.871	10.710	9.392	1.00	0.00	RX0	N
ATOM	662	CE1	HIS	226	31.840	9.741	9.396	1.00	0.00	RX0	C
ATOM	663	C	HIS	226	28.363	7.118	10.150	1.00	0.00	RX0	C
ATOM	664	O	HIS	226	29.071	7.189	11.155	1.00	0.00	RX0	O
ATOM	665	N	LEU	227	27.047	7.317	10.166	1.00	0.00	RX0	N
ATOM	666	H	LEU	227	26.528	7.178	9.319	1.00	0.00	RX0	H
ATOM	667	CA	LEU	227	26.344	7.787	11.375	1.00	0.00	RX0	C
ATOM	668	CB	LEU	227	24.877	8.089	11.075	1.00	0.00	RX0	C
ATOM	669	CG	LEU	227	24.683	9.359	10.249	1.00	0.00	RX0	C

ATOM	670	CD1	LEU	227	23.216	9.574	9.890	1.00	0.00	RX0	C
ATOM	671	CD2	LEU	227	25.267	10.585	10.950	1.00	0.00	RX0	C
ATOM	672	C	LEU	227	26.435	6.799	12.540	1.00	0.00	RX0	C
ATOM	673	O	LEU	227	26.853	7.165	13.635	1.00	0.00	RX0	O
ATOM	674	N	LEU	228	26.270	5.522	12.200	1.00	0.00	RX0	N
ATOM	675	H	LEU	228	26.041	5.297	11.249	1.00	0.00	RX0	H
ATOM	676	CA	LEU	228	26.384	4.431	13.181	1.00	0.00	RX0	C
ATOM	677	CB	LEU	228	25.762	3.155	12.619	1.00	0.00	RX0	C
ATOM	678	CG	LEU	228	24.257	3.147	12.879	1.00	0.00	RX0	C
ATOM	679	CD1	LEU	228	23.513	2.139	12.009	1.00	0.00	RX0	C
ATOM	680	CD2	LEU	228	23.963	2.939	14.364	1.00	0.00	RX0	C
ATOM	681	C	LEU	228	27.805	4.188	13.685	1.00	0.00	RX0	C
ATOM	682	O	LEU	228	28.004	4.019	14.891	1.00	0.00	RX0	O
ATOM	683	N	GLU	229	28.784	4.376	12.809	1.00	0.00	RX0	N
ATOM	684	H	GLU	229	28.566	4.560	11.847	1.00	0.00	RX0	H
ATOM	685	CA	GLU	229	30.199	4.229	13.197	1.00	0.00	RX0	C
ATOM	686	CB	GLU	229	31.147	4.082	11.995	1.00	0.00	RX0	C
ATOM	687	CG	GLU	229	32.448	3.366	12.409	1.00	0.00	RX0	C
ATOM	688	CD	GLU	229	33.328	2.963	11.231	1.00	0.00	RX0	C
ATOM	689	OE1	GLU	229	33.204	3.538	10.152	1.00	0.00	RX0	O
ATOM	690	OE2	GLU	229	34.152	2.061	11.397	1.00	0.00	RX0	O
ATOM	691	C	GLU	229	30.618	5.338	14.175	1.00	0.00	RX0	C
ATOM	692	O	GLU	229	31.393	5.088	15.099	1.00	0.00	RX0	O
ATOM	693	N	CYS	230	30.060	6.523	13.970	1.00	0.00	RX0	N
ATOM	694	H	CYS	230	29.481	6.649	13.163	1.00	0.00	RX0	H
ATOM	695	CA	CYS	230	30.321	7.692	14.829	1.00	0.00	RX0	C
ATOM	696	CB	CYS	230	30.031	8.972	14.052	1.00	0.00	RX0	C
ATOM	697	SG	CYS	230	31.095	9.113	12.596	1.00	0.00	RX0	S
ATOM	698	C	CYS	230	29.592	7.653	16.182	1.00	0.00	RX0	C
ATOM	699	O	CYS	230	30.123	8.119	17.188	1.00	0.00	RX0	O
ATOM	700	N	ALA	231	28.434	7.000	16.215	1.00	0.00	RX0	N
ATOM	701	H	ALA	231	28.113	6.531	15.389	1.00	0.00	RX0	H
ATOM	702	CA	ALA	231	27.494	7.141	17.345	1.00	0.00	RX0	C
ATOM	703	CB	ALA	231	26.145	7.670	16.853	1.00	0.00	RX0	C
ATOM	704	C	ALA	231	27.249	5.885	18.186	1.00	0.00	RX0	C
ATOM	705	O	ALA	231	26.768	6.021	19.321	1.00	0.00	RX0	O
ATOM	706	N	TRP	232	27.687	4.719	17.731	1.00	0.00	RX0	N
ATOM	707	H	TRP	232	28.108	4.684	16.821	1.00	0.00	RX0	H
ATOM	708	CA	TRP	232	27.348	3.433	18.379	1.00	0.00	RX0	C
ATOM	709	CB	TRP	232	27.967	2.236	17.645	1.00	0.00	RX0	C
ATOM	710	CG	TRP	232	29.470	2.266	17.766	1.00	0.00	RX0	C
ATOM	711	CD2	TRP	232	30.313	1.520	18.670	1.00	0.00	RX0	C
ATOM	712	CE2	TRP	232	31.650	1.912	18.425	1.00	0.00	RX0	C
ATOM	713	CE3	TRP	232	30.040	0.568	19.644	1.00	0.00	RX0	C
ATOM	714	CD1	TRP	232	30.349	3.065	17.028	1.00	0.00	RX0	C
ATOM	715	NE1	TRP	232	31.635	2.865	17.411	1.00	0.00	RX0	N
ATOM	716	HE1	TRP	232	32.405	3.321	17.006	1.00	0.00	RX0	H
ATOM	717	CZ2	TRP	232	32.677	1.345	19.170	1.00	0.00	RX0	C
ATOM	718	CZ3	TRP	232	31.077	0.009	20.381	1.00	0.00	RX0	C
ATOM	719	CH2	TRP	232	32.391	0.395	20.144	1.00	0.00	RX0	C
ATOM	720	C	TRP	232	27.676	3.373	19.884	1.00	0.00	RX0	C
ATOM	721	O	TRP	232	26.862	2.914	20.672	1.00	0.00	RX0	O
ATOM	722	N	LEU	233	28.801	3.989	20.277	1.00	0.00	RX0	N
ATOM	723	H	LEU	233	29.362	4.449	19.591	1.00	0.00	RX0	H
ATOM	724	CA	LEU	233	29.211	3.960	21.691	1.00	0.00	RX0	C
ATOM	725	CB	LEU	233	30.717	4.175	21.837	1.00	0.00	RX0	C
ATOM	726	CG	LEU	233	31.192	3.789	23.239	1.00	0.00	RX0	C
ATOM	727	CD1	LEU	233	30.807	2.350	23.589	1.00	0.00	RX0	C
ATOM	728	CD2	LEU	233	32.686	4.034	23.433	1.00	0.00	RX0	C
ATOM	729	C	LEU	233	28.415	4.936	22.566	1.00	0.00	RX0	C
ATOM	730	O	LEU	233	27.943	4.566	23.634	1.00	0.00	RX0	O

ATOM	731	N	GLU	234	28.150	6.122	22.016	1.00	0.00	RX0	N
ATOM	732	H	GLU	234	28.494	6.338	21.105	1.00	0.00	RX0	H
ATOM	733	CA	GLU	234	27.227	7.090	22.644	1.00	0.00	RX0	C
ATOM	734	CB	GLU	234	27.208	8.370	21.797	1.00	0.00	RX0	C
ATOM	735	CG	GLU	234	26.572	9.538	22.540	1.00	0.00	RX0	C
ATOM	736	CD	GLU	234	26.614	10.827	21.745	1.00	0.00	RX0	C
ATOM	737	OE1	GLU	234	26.456	11.889	22.338	1.00	0.00	RX0	O
ATOM	738	OE2	GLU	234	26.804	10.797	20.533	1.00	0.00	RX0	O
ATOM	739	C	GLU	234	25.830	6.482	22.841	1.00	0.00	RX0	C
ATOM	740	O	GLU	234	25.253	6.598	23.926	1.00	0.00	RX0	O
ATOM	741	N	ILE	235	25.389	5.711	21.848	1.00	0.00	RX0	N
ATOM	742	H	ILE	235	25.964	5.639	21.032	1.00	0.00	RX0	H
ATOM	743	CA	ILE	235	24.069	5.045	21.863	1.00	0.00	RX0	C
ATOM	744	CB	ILE	235	23.701	4.469	20.491	1.00	0.00	RX0	C
ATOM	745	CG2	ILE	235	22.411	3.648	20.565	1.00	0.00	RX0	C
ATOM	746	CG1	ILE	235	23.572	5.576	19.447	1.00	0.00	RX0	C
ATOM	747	CD1	ILE	235	23.241	5.035	18.056	1.00	0.00	RX0	C
ATOM	748	C	ILE	235	24.018	3.945	22.939	1.00	0.00	RX0	C
ATOM	749	O	ILE	235	23.068	3.902	23.724	1.00	0.00	RX0	O
ATOM	750	N	LEU	236	25.072	3.140	23.020	1.00	0.00	RX0	N
ATOM	751	H	LEU	236	25.813	3.236	22.352	1.00	0.00	RX0	H
ATOM	752	CA	LEU	236	25.176	2.114	24.078	1.00	0.00	RX0	C
ATOM	753	CB	LEU	236	26.440	1.277	23.892	1.00	0.00	RX0	C
ATOM	754	CG	LEU	236	26.331	0.305	22.721	1.00	0.00	RX0	C
ATOM	755	CD1	LEU	236	27.654	-0.405	22.435	1.00	0.00	RX0	C
ATOM	756	CD2	LEU	236	25.186	-0.683	22.937	1.00	0.00	RX0	C
ATOM	757	C	LEU	236	25.182	2.733	25.479	1.00	0.00	RX0	C
ATOM	758	O	LEU	236	24.381	2.362	26.336	1.00	0.00	RX0	O
ATOM	759	N	MET	237	25.933	3.823	25.600	1.00	0.00	RX0	N
ATOM	760	H	MET	237	26.454	4.138	24.804	1.00	0.00	RX0	H
ATOM	761	CA	MET	237	26.132	4.523	26.881	1.00	0.00	RX0	C
ATOM	762	CB	MET	237	27.281	5.529	26.806	1.00	0.00	RX0	C
ATOM	763	CG	MET	237	28.653	4.855	26.821	1.00	0.00	RX0	C
ATOM	764	SD	MET	237	29.999	6.048	26.841	1.00	0.00	RX0	S
ATOM	765	CE	MET	237	31.348	4.896	27.139	1.00	0.00	RX0	C
ATOM	766	C	MET	237	24.875	5.215	27.409	1.00	0.00	RX0	C
ATOM	767	O	MET	237	24.517	5.003	28.572	1.00	0.00	RX0	O
ATOM	768	N	ILE	238	24.128	5.878	26.531	1.00	0.00	RX0	N
ATOM	769	H	ILE	238	24.459	5.965	25.588	1.00	0.00	RX0	H
ATOM	770	CA	ILE	238	22.871	6.546	26.925	1.00	0.00	RX0	C
ATOM	771	CB	ILE	238	22.340	7.528	25.867	1.00	0.00	RX0	C
ATOM	772	CG2	ILE	238	21.912	6.845	24.570	1.00	0.00	RX0	C
ATOM	773	CG1	ILE	238	21.212	8.369	26.472	1.00	0.00	RX0	C
ATOM	774	CD1	ILE	238	20.556	9.326	25.477	1.00	0.00	RX0	C
ATOM	775	C	ILE	238	21.800	5.514	27.357	1.00	0.00	RX0	C
ATOM	776	O	ILE	238	21.031	5.731	28.268	1.00	0.00	RX0	O
ATOM	777	N	GLY	239	21.845	4.355	26.660	1.00	0.00	RX0	N
ATOM	778	H	GLY	239	22.522	4.244	25.928	1.00	0.00	RX0	H
ATOM	779	CA	GLY	239	20.969	3.213	26.975	1.00	0.00	RX0	C
ATOM	780	C	GLY	239	21.301	2.634	28.356	1.00	0.00	RX0	C
ATOM	781	O	GLY	239	20.417	2.460	29.193	1.00	0.00	RX0	O
ATOM	782	N	LEU	240	22.605	2.583	28.639	1.00	0.00	RX0	N
ATOM	783	H	LEU	240	23.260	2.804	27.913	1.00	0.00	RX0	H
ATOM	784	CA	LEU	240	23.124	2.082	29.919	1.00	0.00	RX0	C
ATOM	785	CB	LEU	240	24.644	1.943	29.849	1.00	0.00	RX0	C
ATOM	786	CG	LEU	240	25.286	1.611	31.196	1.00	0.00	RX0	C
ATOM	787	CD1	LEU	240	24.821	0.265	31.750	1.00	0.00	RX0	C
ATOM	788	CD2	LEU	240	26.807	1.710	31.125	1.00	0.00	RX0	C
ATOM	789	C	LEU	240	22.728	2.995	31.086	1.00	0.00	RX0	C
ATOM	790	O	LEU	240	22.214	2.535	32.097	1.00	0.00	RX0	O
ATOM	791	N	VAL	241	22.901	4.295	30.880	1.00	0.00	RX0	N

ATOM	792	H	VAL	241	23.276	4.586	29.997	1.00	0.00	RX0	H
ATOM	793	CA	VAL	241	22.596	5.307	31.912	1.00	0.00	RX0	C
ATOM	794	CB	VAL	241	23.256	6.669	31.662	1.00	0.00	RX0	C
ATOM	795	CG1	VAL	241	24.774	6.500	31.611	1.00	0.00	RX0	C
ATOM	796	CG2	VAL	241	22.716	7.396	30.437	1.00	0.00	RX0	C
ATOM	797	C	VAL	241	21.084	5.392	32.193	1.00	0.00	RX0	C
ATOM	798	O	VAL	241	20.670	5.516	33.338	1.00	0.00	RX0	O
ATOM	799	N	TRP	242	20.290	5.181	31.134	1.00	0.00	RX0	N
ATOM	800	H	TRP	242	20.698	5.068	30.225	1.00	0.00	RX0	H
ATOM	801	CA	TRP	242	18.822	5.192	31.222	1.00	0.00	RX0	C
ATOM	802	CB	TRP	242	18.214	5.181	29.823	1.00	0.00	RX0	C
ATOM	803	CG	TRP	242	16.734	4.951	29.949	1.00	0.00	RX0	C
ATOM	804	CD2	TRP	242	15.715	5.873	30.389	1.00	0.00	RX0	C
ATOM	805	CE2	TRP	242	14.491	5.172	30.400	1.00	0.00	RX0	C
ATOM	806	CE3	TRP	242	15.742	7.205	30.773	1.00	0.00	RX0	C
ATOM	807	CD1	TRP	242	16.065	3.747	29.709	1.00	0.00	RX0	C
ATOM	808	NE1	TRP	242	14.746	3.878	29.977	1.00	0.00	RX0	N
ATOM	809	HE1	TRP	242	14.071	3.170	29.924	1.00	0.00	RX0	H
ATOM	810	CZ2	TRP	242	13.327	5.812	30.802	1.00	0.00	RX0	C
ATOM	811	CZ3	TRP	242	14.569	7.837	31.168	1.00	0.00	RX0	C
ATOM	812	CH2	TRP	242	13.368	7.144	31.189	1.00	0.00	RX0	C
ATOM	813	C	TRP	242	18.281	4.038	32.076	1.00	0.00	RX0	C
ATOM	814	O	TRP	242	17.477	4.269	32.979	1.00	0.00	RX0	O
ATOM	815	N	ARG	243	18.818	2.841	31.865	1.00	0.00	RX0	N
ATOM	816	H	ARG	243	19.513	2.737	31.150	1.00	0.00	RX0	H
ATOM	817	CA	ARG	243	18.359	1.661	32.627	1.00	0.00	RX0	C
ATOM	818	CB	ARG	243	18.476	0.404	31.736	1.00	0.00	RX0	C
ATOM	819	CG	ARG	243	19.878	-0.075	31.303	1.00	0.00	RX0	C
ATOM	820	CD	ARG	243	19.810	-1.116	30.163	1.00	0.00	RX0	C
ATOM	821	NE	ARG	243	21.093	-1.776	29.871	1.00	0.00	RX0	N
ATOM	822	HE	ARG	243	21.642	-2.039	30.678	1.00	0.00	RX0	H
ATOM	823	CZ	ARG	243	21.415	-2.127	28.573	1.00	0.00	RX0	C
ATOM	824	NH1	ARG	243	20.653	-1.705	27.545	1.00	0.00	RX0	N
ATOM	825	HH11	ARG	243	20.901	-2.003	26.604	1.00	0.00	RX0	H
ATOM	826	HH12	ARG	243	19.852	-1.116	27.653	1.00	0.00	RX0	H
ATOM	827	NH2	ARG	243	22.483	-2.904	28.301	1.00	0.00	RX0	N
ATOM	828	HH21	ARG	243	22.772	-3.099	27.341	1.00	0.00	RX0	H
ATOM	829	HH22	ARG	243	23.035	-3.349	29.016	1.00	0.00	RX0	H
ATOM	830	C	ARG	243	19.043	1.510	33.998	1.00	0.00	RX0	C
ATOM	831	O	ARG	243	18.610	0.722	34.836	1.00	0.00	RX0	O
ATOM	832	N	SER	244	20.027	2.366	34.245	1.00	0.00	RX0	N
ATOM	833	H	SER	244	20.298	3.026	33.545	1.00	0.00	RX0	H
ATOM	834	CA	SER	244	20.722	2.464	35.548	1.00	0.00	RX0	C
ATOM	835	CB	SER	244	22.206	2.694	35.312	1.00	0.00	RX0	C
ATOM	836	OG	SER	244	22.699	1.649	34.472	1.00	0.00	RX0	O
ATOM	837	HG	SER	244	22.208	1.715	33.659	1.00	0.00	RX0	H
ATOM	838	C	SER	244	20.112	3.547	36.442	1.00	0.00	RX0	C
ATOM	839	O	SER	244	20.448	3.642	37.630	1.00	0.00	RX0	O
ATOM	840	N	MET	245	19.184	4.322	35.895	1.00	0.00	RX0	N
ATOM	841	H	MET	245	18.819	4.098	34.988	1.00	0.00	RX0	H
ATOM	842	CA	MET	245	18.600	5.504	36.550	1.00	0.00	RX0	C
ATOM	843	CB	MET	245	17.573	6.190	35.650	1.00	0.00	RX0	C
ATOM	844	CG	MET	245	16.861	7.317	36.403	1.00	0.00	RX0	C
ATOM	845	SD	MET	245	15.430	7.990	35.554	1.00	0.00	RX0	S
ATOM	846	CE	MET	245	16.283	8.492	34.063	1.00	0.00	RX0	C
ATOM	847	C	MET	245	17.925	5.204	37.895	1.00	0.00	RX0	C
ATOM	848	O	MET	245	18.105	5.946	38.853	1.00	0.00	RX0	O
ATOM	849	N	GLU	246	17.212	4.082	37.945	1.00	0.00	RX0	N
ATOM	850	H	GLU	246	17.124	3.488	37.138	1.00	0.00	RX0	H
ATOM	851	CA	GLU	246	16.494	3.692	39.178	1.00	0.00	RX0	C
ATOM	852	CB	GLU	246	15.180	2.971	38.900	1.00	0.00	RX0	C

ATOM	853	CG	GLU	246	14.148	3.743	38.077	1.00	0.00	RX0	C
ATOM	854	CD	GLU	246	12.954	2.839	37.828	1.00	0.00	RX0	C
ATOM	855	OE1	GLU	246	12.794	2.335	36.720	1.00	0.00	RX0	O
ATOM	856	OE2	GLU	246	12.191	2.591	38.753	1.00	0.00	RX0	O
ATOM	857	C	GLU	246	17.379	2.867	40.123	1.00	0.00	RX0	C
ATOM	858	O	GLU	246	16.897	2.295	41.108	1.00	0.00	RX0	O
ATOM	859	N	HIS	247	18.674	2.844	39.836	1.00	0.00	RX0	N
ATOM	860	H	HIS	247	19.039	3.308	39.030	1.00	0.00	RX0	H
ATOM	861	CA	HIS	247	19.658	2.055	40.601	1.00	0.00	RX0	C
ATOM	862	CB	HIS	247	20.165	0.854	39.809	1.00	0.00	RX0	C
ATOM	863	CG	HIS	247	19.053	-0.142	39.600	1.00	0.00	RX0	C
ATOM	864	ND1	HIS	247	17.925	-0.210	40.335	1.00	0.00	RX0	N
ATOM	865	HD1	HIS	247	17.633	0.389	41.058	1.00	0.00	RX0	H
ATOM	866	CD2	HIS	247	19.013	-1.137	38.626	1.00	0.00	RX0	C
ATOM	867	NE2	HIS	247	17.848	-1.806	38.776	1.00	0.00	RX0	N
ATOM	868	CE1	HIS	247	17.178	-1.242	39.830	1.00	0.00	RX0	C
ATOM	869	C	HIS	247	20.841	2.947	41.015	1.00	0.00	RX0	C
ATOM	870	O	HIS	247	21.962	2.788	40.490	1.00	0.00	RX0	O
ATOM	871	N	PRO	248	20.632	3.827	41.991	1.00	0.00	RX0	N
ATOM	872	CD	PRO	248	19.383	3.972	42.733	1.00	0.00	RX0	C
ATOM	873	CA	PRO	248	21.659	4.767	42.484	1.00	0.00	RX0	C
ATOM	874	CB	PRO	248	20.980	5.449	43.675	1.00	0.00	RX0	C
ATOM	875	CG	PRO	248	19.487	5.343	43.386	1.00	0.00	RX0	C
ATOM	876	C	PRO	248	22.939	4.013	42.877	1.00	0.00	RX0	C
ATOM	877	O	PRO	248	22.892	2.963	43.503	1.00	0.00	RX0	O
ATOM	878	N	GLY	249	24.055	4.541	42.350	1.00	0.00	RX0	N
ATOM	879	H	GLY	249	23.973	5.297	41.706	1.00	0.00	RX0	H
ATOM	880	CA	GLY	249	25.407	3.996	42.610	1.00	0.00	RX0	C
ATOM	881	C	GLY	249	25.783	2.749	41.794	1.00	0.00	RX0	C
ATOM	882	O	GLY	249	26.914	2.250	41.927	1.00	0.00	RX0	O
ATOM	883	N	LYS	250	24.877	2.267	40.961	1.00	0.00	RX0	N
ATOM	884	H	LYS	250	23.982	2.694	40.813	1.00	0.00	RX0	H
ATOM	885	CA	LYS	250	25.097	1.050	40.158	1.00	0.00	RX0	C
ATOM	886	CB	LYS	250	24.369	-0.138	40.786	1.00	0.00	RX0	C
ATOM	887	CG	LYS	250	25.230	-0.846	41.841	1.00	0.00	RX0	C
ATOM	888	CD	LYS	250	24.588	-2.110	42.416	1.00	0.00	RX0	C
ATOM	889	CE	LYS	250	25.528	-2.977	43.262	1.00	0.00	RX0	C
ATOM	890	NZ	LYS	250	26.641	-3.486	42.446	1.00	0.00	RX0	N
ATOM	891	HZ1	LYS	250	27.367	-3.907	43.069	1.00	0.00	RX0	H
ATOM	892	HZ2	LYS	250	26.340	-4.177	41.723	1.00	0.00	RX0	H
ATOM	893	HZ3	LYS	250	27.142	-2.686	42.014	1.00	0.00	RX0	H
ATOM	894	C	LYS	250	24.802	1.266	38.671	1.00	0.00	RX0	C
ATOM	895	O	LYS	250	24.040	2.166	38.282	1.00	0.00	RX0	O
ATOM	896	N	LEU	251	25.472	0.476	37.863	1.00	0.00	RX0	N
ATOM	897	H	LEU	251	26.046	-0.241	38.244	1.00	0.00	RX0	H
ATOM	898	CA	LEU	251	25.292	0.430	36.401	1.00	0.00	RX0	C
ATOM	899	CB	LEU	251	26.626	0.591	35.684	1.00	0.00	RX0	C
ATOM	900	CG	LEU	251	27.161	2.016	35.762	1.00	0.00	RX0	C
ATOM	901	CD1	LEU	251	28.584	2.114	35.218	1.00	0.00	RX0	C
ATOM	902	CD2	LEU	251	26.208	3.008	35.091	1.00	0.00	RX0	C
ATOM	903	C	LEU	251	24.646	-0.892	36.017	1.00	0.00	RX0	C
ATOM	904	O	LEU	251	25.224	-1.976	36.286	1.00	0.00	RX0	O
ATOM	905	N	LEU	252	23.437	-0.808	35.533	1.00	0.00	RX0	N
ATOM	906	H	LEU	252	23.077	0.097	35.320	1.00	0.00	RX0	H
ATOM	907	CA	LEU	252	22.659	-1.981	35.114	1.00	0.00	RX0	C
ATOM	908	CB	LEU	252	21.165	-1.732	35.294	1.00	0.00	RX0	C
ATOM	909	CG	LEU	252	20.359	-3.028	35.220	1.00	0.00	RX0	C
ATOM	910	CD1	LEU	252	20.904	-4.096	36.154	1.00	0.00	RX0	C
ATOM	911	CD2	LEU	252	18.897	-2.796	35.546	1.00	0.00	RX0	C
ATOM	912	C	LEU	252	23.007	-2.330	33.663	1.00	0.00	RX0	C
ATOM	913	O	LEU	252	22.274	-2.025	32.731	1.00	0.00	RX0	O

ATOM	914	N	PHE	253	24.144	-3.005	33.506	1.00	0.00	RX0	N
ATOM	915	H	PHE	253	24.622	-3.316	34.332	1.00	0.00	RX0	H
ATOM	916	CA	PHE	253	24.599	-3.457	32.174	1.00	0.00	RX0	C
ATOM	917	CB	PHE	253	25.968	-4.125	32.266	1.00	0.00	RX0	C
ATOM	918	CG	PHE	253	27.031	-3.098	32.554	1.00	0.00	RX0	C
ATOM	919	CD1	PHE	253	27.565	-2.351	31.511	1.00	0.00	RX0	C
ATOM	920	CD2	PHE	253	27.484	-2.906	33.853	1.00	0.00	RX0	C
ATOM	921	CE1	PHE	253	28.562	-1.419	31.764	1.00	0.00	RX0	C
ATOM	922	CE2	PHE	253	28.481	-1.973	34.104	1.00	0.00	RX0	C
ATOM	923	CZ	PHE	253	29.021	-1.231	33.061	1.00	0.00	RX0	C
ATOM	924	C	PHE	253	23.603	-4.446	31.564	1.00	0.00	RX0	C
ATOM	925	O	PHE	253	23.259	-4.379	30.390	1.00	0.00	RX0	O
ATOM	926	N	ALA	254	23.094	-5.300	32.445	1.00	0.00	RX0	N
ATOM	927	H	ALA	254	23.402	-5.311	33.402	1.00	0.00	RX0	H
ATOM	928	CA	ALA	254	22.050	-6.280	32.141	1.00	0.00	RX0	C
ATOM	929	CB	ALA	254	22.694	-7.552	31.598	1.00	0.00	RX0	C
ATOM	930	C	ALA	254	21.288	-6.584	33.440	1.00	0.00	RX0	C
ATOM	931	O	ALA	254	21.887	-6.418	34.526	1.00	0.00	RX0	O
ATOM	932	N	PRO	255	20.056	-7.064	33.372	1.00	0.00	RX0	N
ATOM	933	CD	PRO	255	19.307	-7.244	32.131	1.00	0.00	RX0	C
ATOM	934	CA	PRO	255	19.236	-7.444	34.545	1.00	0.00	RX0	C
ATOM	935	CB	PRO	255	17.990	-8.078	33.926	1.00	0.00	RX0	C
ATOM	936	CG	PRO	255	17.857	-7.406	32.567	1.00	0.00	RX0	C
ATOM	937	C	PRO	255	19.972	-8.395	35.506	1.00	0.00	RX0	C
ATOM	938	O	PRO	255	19.756	-8.342	36.714	1.00	0.00	RX0	O
ATOM	939	N	ASN	256	20.900	-9.179	34.970	1.00	0.00	RX0	N
ATOM	940	H	ASN	256	21.117	-9.128	33.994	1.00	0.00	RX0	H
ATOM	941	CA	ASN	256	21.722	-10.125	35.761	1.00	0.00	RX0	C
ATOM	942	CB	ASN	256	21.714	-11.528	35.151	1.00	0.00	RX0	C
ATOM	943	CG	ASN	256	22.404	-11.529	33.796	1.00	0.00	RX0	C
ATOM	944	OD1	ASN	256	22.264	-10.603	32.997	1.00	0.00	RX0	O
ATOM	945	ND2	ASN	256	23.063	-12.672	33.528	1.00	0.00	RX0	N
ATOM	946	HD21	ASN	256	23.224	-13.334	34.263	1.00	0.00	RX0	H
ATOM	947	HD22	ASN	256	23.407	-12.921	32.617	1.00	0.00	RX0	H
ATOM	948	C	ASN	256	23.191	-9.669	35.876	1.00	0.00	RX0	C
ATOM	949	O	ASN	256	24.101	-10.505	36.011	1.00	0.00	RX0	O
ATOM	950	N	LEU	257	23.439	-8.384	35.742	1.00	0.00	RX0	N
ATOM	951	H	LEU	257	22.690	-7.724	35.650	1.00	0.00	RX0	H
ATOM	952	CA	LEU	257	24.796	-7.805	35.783	1.00	0.00	RX0	C
ATOM	953	CB	LEU	257	25.513	-7.967	34.443	1.00	0.00	RX0	C
ATOM	954	CG	LEU	257	27.023	-7.756	34.564	1.00	0.00	RX0	C
ATOM	955	CD1	LEU	257	27.658	-8.790	35.495	1.00	0.00	RX0	C
ATOM	956	CD2	LEU	257	27.712	-7.721	33.200	1.00	0.00	RX0	C
ATOM	957	C	LEU	257	24.720	-6.327	36.168	1.00	0.00	RX0	C
ATOM	958	O	LEU	257	24.738	-5.412	35.328	1.00	0.00	RX0	O
ATOM	959	N	LEU	258	24.604	-6.148	37.469	1.00	0.00	RX0	N
ATOM	960	H	LEU	258	24.657	-6.942	38.073	1.00	0.00	RX0	H
ATOM	961	CA	LEU	258	24.482	-4.834	38.118	1.00	0.00	RX0	C
ATOM	962	CB	LEU	258	23.233	-4.901	38.998	1.00	0.00	RX0	C
ATOM	963	CG	LEU	258	22.854	-3.618	39.729	1.00	0.00	RX0	C
ATOM	964	CD1	LEU	258	22.664	-2.445	38.776	1.00	0.00	RX0	C
ATOM	965	CD2	LEU	258	21.629	-3.820	40.622	1.00	0.00	RX0	C
ATOM	966	C	LEU	258	25.743	-4.564	38.938	1.00	0.00	RX0	C
ATOM	967	O	LEU	258	26.013	-5.237	39.948	1.00	0.00	RX0	O
ATOM	968	N	LEU	259	26.528	-3.622	38.460	1.00	0.00	RX0	N
ATOM	969	H	LEU	259	26.212	-3.063	37.686	1.00	0.00	RX0	H
ATOM	970	CA	LEU	259	27.862	-3.349	39.027	1.00	0.00	RX0	C
ATOM	971	CB	LEU	259	28.938	-3.493	37.947	1.00	0.00	RX0	C
ATOM	972	CG	LEU	259	28.879	-4.809	37.167	1.00	0.00	RX0	C
ATOM	973	CD1	LEU	259	29.895	-4.832	36.024	1.00	0.00	RX0	C
ATOM	974	CD2	LEU	259	29.028	-6.032	38.071	1.00	0.00	RX0	C

ATOM	975	C	LEU	259	27.958	-1.956	39.652	1.00	0.00	RX0	C
ATOM	976	O	LEU	259	27.419	-0.984	39.137	1.00	0.00	RX0	O
ATOM	977	N	ASP	260	28.645	-1.912	40.785	1.00	0.00	RX0	N
ATOM	978	H	ASP	260	29.220	-2.691	41.047	1.00	0.00	RX0	H
ATOM	979	CA	ASP	260	29.043	-0.657	41.454	1.00	0.00	RX0	C
ATOM	980	CB	ASP	260	29.078	-0.878	42.962	1.00	0.00	RX0	C
ATOM	981	CG	ASP	260	29.749	-2.211	43.211	1.00	0.00	RX0	C
ATOM	982	OD1	ASP	260	30.976	-2.265	43.250	1.00	0.00	RX0	O
ATOM	983	OD2	ASP	260	29.031	-3.205	43.319	1.00	0.00	RX0	O
ATOM	984	C	ASP	260	30.443	-0.222	40.970	1.00	0.00	RX0	C
ATOM	985	O	ASP	260	31.127	-1.008	40.295	1.00	0.00	RX0	O
ATOM	986	N	ARG	261	30.963	0.873	41.504	1.00	0.00	RX0	N
ATOM	987	H	ARG	261	30.386	1.411	42.116	1.00	0.00	RX0	H
ATOM	988	CA	ARG	261	32.263	1.408	41.044	1.00	0.00	RX0	C
ATOM	989	CB	ARG	261	32.425	2.883	41.443	1.00	0.00	RX0	C
ATOM	990	CG	ARG	261	32.682	3.154	42.929	1.00	0.00	RX0	C
ATOM	991	CD	ARG	261	32.654	4.646	43.280	1.00	0.00	RX0	C
ATOM	992	NE	ARG	261	33.508	5.424	42.385	1.00	0.00	RX0	N
ATOM	993	HE	ARG	261	33.155	5.684	41.468	1.00	0.00	RX0	H
ATOM	994	CZ	ARG	261	34.773	5.817	42.710	1.00	0.00	RX0	C
ATOM	995	NH1	ARG	261	35.250	5.572	43.950	1.00	0.00	RX0	N
ATOM	996	HH11	ARG	261	36.173	5.847	44.224	1.00	0.00	RX0	H
ATOM	997	HH12	ARG	261	34.674	5.120	44.634	1.00	0.00	RX0	H
ATOM	998	NH2	ARG	261	35.518	6.448	41.783	1.00	0.00	RX0	N
ATOM	999	HH21	ARG	261	36.466	6.751	41.865	1.00	0.00	RX0	H
ATOM	1000	HH22	ARG	261	35.053	6.656	40.899	1.00	0.00	RX0	H
ATOM	1001	C	ARG	261	33.476	0.540	41.436	1.00	0.00	RX0	C
ATOM	1002	O	ARG	261	34.378	0.347	40.637	1.00	0.00	RX0	O
ATOM	1003	N	ASN	262	33.410	-0.067	42.632	1.00	0.00	RX0	N
ATOM	1004	H	ASN	262	32.533	-0.040	43.114	1.00	0.00	RX0	H
ATOM	1005	CA	ASN	262	34.456	-1.000	43.094	1.00	0.00	RX0	C
ATOM	1006	CB	ASN	262	34.109	-1.491	44.492	1.00	0.00	RX0	C
ATOM	1007	CG	ASN	262	34.998	-2.668	44.825	1.00	0.00	RX0	C
ATOM	1008	OD1	ASN	262	36.165	-2.491	45.176	1.00	0.00	RX0	O
ATOM	1009	ND2	ASN	262	34.375	-3.858	44.750	1.00	0.00	RX0	N
ATOM	1010	HD21	ASN	262	33.407	-3.903	44.484	1.00	0.00	RX0	H
ATOM	1011	HD22	ASN	262	34.825	-4.732	44.941	1.00	0.00	RX0	H
ATOM	1012	C	ASN	262	34.635	-2.233	42.204	1.00	0.00	RX0	C
ATOM	1013	O	ASN	262	35.755	-2.665	41.964	1.00	0.00	RX0	O
ATOM	1014	N	GLN	263	33.530	-2.679	41.603	1.00	0.00	RX0	N
ATOM	1015	H	GLN	263	32.637	-2.269	41.817	1.00	0.00	RX0	H
ATOM	1016	CA	GLN	263	33.559	-3.756	40.599	1.00	0.00	RX0	C
ATOM	1017	CB	GLN	263	32.198	-4.423	40.446	1.00	0.00	RX0	C
ATOM	1018	CG	GLN	263	31.924	-5.187	41.740	1.00	0.00	RX0	C
ATOM	1019	CD	GLN	263	30.708	-6.068	41.596	1.00	0.00	RX0	C
ATOM	1020	OE1	GLN	263	30.633	-6.946	40.746	1.00	0.00	RX0	O
ATOM	1021	NE2	GLN	263	29.770	-5.820	42.521	1.00	0.00	RX0	N
ATOM	1022	HE21	GLN	263	29.885	-5.009	43.108	1.00	0.00	RX0	H
ATOM	1023	HE22	GLN	263	28.984	-6.422	42.642	1.00	0.00	RX0	H
ATOM	1024	C	GLN	263	34.189	-3.308	39.265	1.00	0.00	RX0	C
ATOM	1025	O	GLN	263	34.644	-4.116	38.479	1.00	0.00	RX0	O
ATOM	1026	N	GLY	264	34.180	-1.978	39.039	1.00	0.00	RX0	N
ATOM	1027	H	GLY	264	33.763	-1.376	39.719	1.00	0.00	RX0	H
ATOM	1028	CA	GLY	264	34.831	-1.337	37.881	1.00	0.00	RX0	C
ATOM	1029	C	GLY	264	36.364	-1.310	37.978	1.00	0.00	RX0	C
ATOM	1030	O	GLY	264	37.050	-1.510	36.977	1.00	0.00	RX0	O
ATOM	1031	N	LYS	265	36.881	-1.155	39.202	1.00	0.00	RX0	N
ATOM	1032	H	LYS	265	36.232	-1.005	39.949	1.00	0.00	RX0	H
ATOM	1033	CA	LYS	265	38.336	-1.184	39.469	1.00	0.00	RX0	C
ATOM	1034	CB	LYS	265	38.673	-1.149	40.952	1.00	0.00	RX0	C
ATOM	1035	CG	LYS	265	38.098	-0.104	41.895	1.00	0.00	RX0	C

ATOM	1036	CD	LYS	265	38.477	-0.635	43.278	1.00	0.00	RX0	C
ATOM	1037	CE	LYS	265	37.932	0.106	44.492	1.00	0.00	RX0	C
ATOM	1038	NZ	LYS	265	38.088	-0.801	45.638	1.00	0.00	RX0	N
ATOM	1039	HZ1	LYS	265	37.663	-0.399	46.495	1.00	0.00	RX0	H
ATOM	1040	HZ2	LYS	265	37.583	-1.689	45.417	1.00	0.00	RX0	H
ATOM	1041	HZ3	LYS	265	39.089	-1.024	45.797	1.00	0.00	RX0	H
ATOM	1042	C	LYS	265	38.994	-2.500	39.030	1.00	0.00	RX0	C
ATOM	1043	O	LYS	265	40.184	-2.535	38.765	1.00	0.00	RX0	O
ATOM	1044	N	CYS	266	38.153	-3.548	38.917	1.00	0.00	RX0	N
ATOM	1045	H	CYS	266	37.197	-3.455	39.192	1.00	0.00	RX0	H
ATOM	1046	CA	CYS	266	38.538	-4.854	38.355	1.00	0.00	RX0	C
ATOM	1047	CB	CYS	266	37.315	-5.759	38.263	1.00	0.00	RX0	C
ATOM	1048	SG	CYS	266	36.554	-5.947	39.897	1.00	0.00	RX0	S
ATOM	1049	C	CYS	266	39.318	-4.732	37.033	1.00	0.00	RX0	C
ATOM	1050	O	CYS	266	40.108	-5.603	36.695	1.00	0.00	RX0	O
ATOM	1051	N	VAL	267	39.075	-3.636	36.304	1.00	0.00	RX0	N
ATOM	1052	H	VAL	267	38.469	-2.902	36.620	1.00	0.00	RX0	H
ATOM	1053	CA	VAL	267	39.804	-3.345	35.058	1.00	0.00	RX0	C
ATOM	1054	CB	VAL	267	38.857	-3.352	33.851	1.00	0.00	RX0	C
ATOM	1055	CG1	VAL	267	39.586	-3.008	32.549	1.00	0.00	RX0	C
ATOM	1056	CG2	VAL	267	38.127	-4.693	33.739	1.00	0.00	RX0	C
ATOM	1057	C	VAL	267	40.557	-2.013	35.191	1.00	0.00	RX0	C
ATOM	1058	O	VAL	267	39.969	-0.956	35.468	1.00	0.00	RX0	O
ATOM	1059	N	GLU	268	41.829	-2.078	34.821	1.00	0.00	RX0	N
ATOM	1060	H	GLU	268	42.209	-2.964	34.565	1.00	0.00	RX0	H
ATOM	1061	CA	GLU	268	42.726	-0.908	34.727	1.00	0.00	RX0	C
ATOM	1062	CB	GLU	268	44.097	-1.333	34.209	1.00	0.00	RX0	C
ATOM	1063	CG	GLU	268	45.139	-0.222	34.331	1.00	0.00	RX0	C
ATOM	1064	CD	GLU	268	46.503	-0.803	34.036	1.00	0.00	RX0	C
ATOM	1065	OE1	GLU	268	46.592	-2.016	33.857	1.00	0.00	RX0	O
ATOM	1066	OE2	GLU	268	47.471	-0.045	33.998	1.00	0.00	RX0	O
ATOM	1067	C	GLU	268	42.079	0.194	33.866	1.00	0.00	RX0	C
ATOM	1068	O	GLU	268	41.697	-0.027	32.727	1.00	0.00	RX0	O
ATOM	1069	N	GLY	269	41.924	1.355	34.524	1.00	0.00	RX0	N
ATOM	1070	H	GLY	269	42.194	1.397	35.484	1.00	0.00	RX0	H
ATOM	1071	CA	GLY	269	41.377	2.576	33.902	1.00	0.00	RX0	C
ATOM	1072	C	GLY	269	39.898	2.490	33.494	1.00	0.00	RX0	C
ATOM	1073	O	GLY	269	39.424	3.343	32.745	1.00	0.00	RX0	O
ATOM	1074	N	MET	270	39.146	1.598	34.134	1.00	0.00	RX0	N
ATOM	1075	H	MET	270	39.580	0.955	34.768	1.00	0.00	RX0	H
ATOM	1076	CA	MET	270	37.704	1.465	33.841	1.00	0.00	RX0	C
ATOM	1077	CB	MET	270	37.280	0.008	33.653	1.00	0.00	RX0	C
ATOM	1078	CG	MET	270	35.924	-0.098	32.950	1.00	0.00	RX0	C
ATOM	1079	SD	MET	270	35.418	-1.787	32.584	1.00	0.00	RX0	S
ATOM	1080	CE	MET	270	35.155	-2.355	34.269	1.00	0.00	RX0	C
ATOM	1081	C	MET	270	36.825	2.181	34.877	1.00	0.00	RX0	C
ATOM	1082	O	MET	270	35.781	2.734	34.514	1.00	0.00	RX0	O
ATOM	1083	N	VAL	271	37.310	2.290	36.107	1.00	0.00	RX0	N
ATOM	1084	H	VAL	271	38.234	1.949	36.273	1.00	0.00	RX0	H
ATOM	1085	CA	VAL	271	36.591	3.043	37.171	1.00	0.00	RX0	C
ATOM	1086	CB	VAL	271	37.245	2.850	38.551	1.00	0.00	RX0	C
ATOM	1087	CG1	VAL	271	38.732	3.187	38.566	1.00	0.00	RX0	C
ATOM	1088	CG2	VAL	271	36.450	3.550	39.653	1.00	0.00	RX0	C
ATOM	1089	C	VAL	271	36.343	4.502	36.749	1.00	0.00	RX0	C
ATOM	1090	O	VAL	271	35.261	5.071	37.095	1.00	0.00	RX0	O
ATOM	1091	N	GLU	272	37.228	5.074	36.011	1.00	0.00	RX0	N
ATOM	1092	H	GLU	272	38.101	4.600	35.880	1.00	0.00	RX0	H
ATOM	1093	CA	GLU	272	37.141	6.453	35.460	1.00	0.00	RX0	C
ATOM	1094	CB	GLU	272	38.432	6.787	34.703	1.00	0.00	RX0	C
ATOM	1095	CG	GLU	272	39.675	7.024	35.575	1.00	0.00	RX0	C
ATOM	1096	CD	GLU	272	40.034	5.799	36.396	1.00	0.00	RX0	C

ATOM	1097	OE1	GLU	272	40.065	4.697	35.847	1.00	0.00	RX0	O
ATOM	1098	OE2	GLU	272	40.246	5.941	37.596	1.00	0.00	RX0	O
ATOM	1099	C	GLU	272	35.927	6.588	34.526	1.00	0.00	RX0	C
ATOM	1100	O	GLU	272	35.142	7.521	34.681	1.00	0.00	RX0	O
ATOM	1101	N	ILE	273	35.702	5.552	33.729	1.00	0.00	RX0	N
ATOM	1102	H	ILE	273	36.304	4.758	33.824	1.00	0.00	RX0	H
ATOM	1103	CA	ILE	273	34.533	5.484	32.820	1.00	0.00	RX0	C
ATOM	1104	CB	ILE	273	34.734	4.376	31.780	1.00	0.00	RX0	C
ATOM	1105	CG2	ILE	273	33.622	4.407	30.730	1.00	0.00	RX0	C
ATOM	1106	CG1	ILE	273	36.126	4.433	31.145	1.00	0.00	RX0	C
ATOM	1107	CD1	ILE	273	36.338	5.657	30.253	1.00	0.00	RX0	C
ATOM	1108	C	ILE	273	33.249	5.235	33.628	1.00	0.00	RX0	C
ATOM	1109	O	ILE	273	32.257	5.951	33.452	1.00	0.00	RX0	O
ATOM	1110	N	PHE	274	33.320	4.302	34.575	1.00	0.00	RX0	N
ATOM	1111	H	PHE	274	34.190	3.821	34.693	1.00	0.00	RX0	H
ATOM	1112	CA	PHE	274	32.191	3.976	35.472	1.00	0.00	RX0	C
ATOM	1113	CB	PHE	274	32.611	2.940	36.517	1.00	0.00	RX0	C
ATOM	1114	CG	PHE	274	32.270	1.539	36.076	1.00	0.00	RX0	C
ATOM	1115	CD1	PHE	274	32.534	1.118	34.779	1.00	0.00	RX0	C
ATOM	1116	CD2	PHE	274	31.687	0.665	36.988	1.00	0.00	RX0	C
ATOM	1117	CE1	PHE	274	32.222	-0.181	34.398	1.00	0.00	RX0	C
ATOM	1118	CE2	PHE	274	31.375	-0.634	36.607	1.00	0.00	RX0	C
ATOM	1119	CZ	PHE	274	31.651	-1.058	35.314	1.00	0.00	RX0	C
ATOM	1120	C	PHE	274	31.669	5.203	36.222	1.00	0.00	RX0	C
ATOM	1121	O	PHE	274	30.484	5.521	36.143	1.00	0.00	RX0	O
ATOM	1122	N	ASP	275	32.607	5.983	36.757	1.00	0.00	RX0	N
ATOM	1123	H	ASP	275	33.558	5.682	36.810	1.00	0.00	RX0	H
ATOM	1124	CA	ASP	275	32.273	7.222	37.483	1.00	0.00	RX0	C
ATOM	1125	CB	ASP	275	33.488	7.804	38.195	1.00	0.00	RX0	C
ATOM	1126	CG	ASP	275	33.607	7.136	39.549	1.00	0.00	RX0	C
ATOM	1127	OD1	ASP	275	33.830	5.932	39.620	1.00	0.00	RX0	O
ATOM	1128	OD2	ASP	275	33.508	7.813	40.566	1.00	0.00	RX0	O
ATOM	1129	C	ASP	275	31.555	8.270	36.629	1.00	0.00	RX0	C
ATOM	1130	O	ASP	275	30.604	8.890	37.102	1.00	0.00	RX0	O
ATOM	1131	N	MET	276	31.921	8.336	35.353	1.00	0.00	RX0	N
ATOM	1132	H	MET	276	32.663	7.738	35.041	1.00	0.00	RX0	H
ATOM	1133	CA	MET	276	31.257	9.254	34.408	1.00	0.00	RX0	C
ATOM	1134	CB	MET	276	32.092	9.447	33.144	1.00	0.00	RX0	C
ATOM	1135	CG	MET	276	33.423	10.151	33.407	1.00	0.00	RX0	C
ATOM	1136	SD	MET	276	34.296	10.545	31.883	1.00	0.00	RX0	S
ATOM	1137	CE	MET	276	34.342	8.886	31.193	1.00	0.00	RX0	C
ATOM	1138	C	MET	276	29.833	8.798	34.061	1.00	0.00	RX0	C
ATOM	1139	O	MET	276	28.893	9.589	34.158	1.00	0.00	RX0	O
ATOM	1140	N	LEU	277	29.673	7.488	33.891	1.00	0.00	RX0	N
ATOM	1141	H	LEU	277	30.490	6.907	33.949	1.00	0.00	RX0	H
ATOM	1142	CA	LEU	277	28.362	6.866	33.606	1.00	0.00	RX0	C
ATOM	1143	CB	LEU	277	28.562	5.402	33.225	1.00	0.00	RX0	C
ATOM	1144	CG	LEU	277	29.433	5.222	31.983	1.00	0.00	RX0	C
ATOM	1145	CD1	LEU	277	29.925	3.783	31.834	1.00	0.00	RX0	C
ATOM	1146	CD2	LEU	277	28.732	5.724	30.722	1.00	0.00	RX0	C
ATOM	1147	C	LEU	277	27.393	6.992	34.787	1.00	0.00	RX0	C
ATOM	1148	O	LEU	277	26.257	7.447	34.627	1.00	0.00	RX0	O
ATOM	1149	N	LEU	278	27.939	6.795	35.983	1.00	0.00	RX0	N
ATOM	1150	H	LEU	278	28.900	6.515	36.019	1.00	0.00	RX0	H
ATOM	1151	CA	LEU	278	27.196	6.926	37.250	1.00	0.00	RX0	C
ATOM	1152	CB	LEU	278	28.067	6.470	38.419	1.00	0.00	RX0	C
ATOM	1153	CG	LEU	278	28.221	4.953	38.466	1.00	0.00	RX0	C
ATOM	1154	CD1	LEU	278	29.274	4.502	39.478	1.00	0.00	RX0	C
ATOM	1155	CD2	LEU	278	26.869	4.290	38.709	1.00	0.00	RX0	C
ATOM	1156	C	LEU	278	26.716	8.359	37.508	1.00	0.00	RX0	C
ATOM	1157	O	LEU	278	25.554	8.575	37.840	1.00	0.00	RX0	O

ATOM	1158	N	ALA	279	27.582	9.317	37.175	1.00	0.00	RX0	N
ATOM	1159	H	ALA	279	28.513	9.059	36.898	1.00	0.00	RX0	H
ATOM	1160	CA	ALA	279	27.275	10.754	37.296	1.00	0.00	RX0	C
ATOM	1161	CB	ALA	279	28.528	11.595	37.048	1.00	0.00	RX0	C
ATOM	1162	C	ALA	279	26.182	11.196	36.310	1.00	0.00	RX0	C
ATOM	1163	O	ALA	279	25.263	11.922	36.684	1.00	0.00	RX0	O
ATOM	1164	N	THR	280	26.210	10.618	35.109	1.00	0.00	RX0	N
ATOM	1165	H	THR	280	26.970	10.008	34.881	1.00	0.00	RX0	H
ATOM	1166	CA	THR	280	25.203	10.899	34.059	1.00	0.00	RX0	C
ATOM	1167	CB	THR	280	25.747	10.369	32.739	1.00	0.00	RX0	C
ATOM	1168	OG1	THR	280	27.060	10.907	32.531	1.00	0.00	RX0	O
ATOM	1169	HG1	THR	280	27.666	10.443	33.100	1.00	0.00	RX0	H
ATOM	1170	CG2	THR	280	24.828	10.718	31.566	1.00	0.00	RX0	C
ATOM	1171	C	THR	280	23.835	10.327	34.462	1.00	0.00	RX0	C
ATOM	1172	O	THR	280	22.822	11.023	34.397	1.00	0.00	RX0	O
ATOM	1173	N	SER	281	23.868	9.112	35.003	1.00	0.00	RX0	N
ATOM	1174	H	SER	281	24.739	8.619	35.048	1.00	0.00	RX0	H
ATOM	1175	CA	SER	281	22.669	8.413	35.507	1.00	0.00	RX0	C
ATOM	1176	CB	SER	281	23.145	6.984	35.866	1.00	0.00	RX0	C
ATOM	1177	OG	SER	281	22.382	6.337	36.903	1.00	0.00	RX0	O
ATOM	1178	HG	SER	281	22.560	5.405	36.792	1.00	0.00	RX0	H
ATOM	1179	C	SER	281	22.019	9.180	36.675	1.00	0.00	RX0	C
ATOM	1180	O	SER	281	20.814	9.399	36.693	1.00	0.00	RX0	O
ATOM	1181	N	SER	282	22.889	9.788	37.491	1.00	0.00	RX0	N
ATOM	1182	H	SER	282	23.868	9.614	37.378	1.00	0.00	RX0	H
ATOM	1183	CA	SER	282	22.489	10.660	38.613	1.00	0.00	RX0	C
ATOM	1184	CB	SER	282	23.674	10.723	39.557	1.00	0.00	RX0	C
ATOM	1185	OG	SER	282	23.944	9.344	39.873	1.00	0.00	RX0	O
ATOM	1186	HG	SER	282	24.724	9.120	39.367	1.00	0.00	RX0	H
ATOM	1187	C	SER	282	21.828	11.963	38.130	1.00	0.00	RX0	C
ATOM	1188	O	SER	282	20.788	12.371	38.639	1.00	0.00	RX0	O
ATOM	1189	N	ARG	283	22.365	12.516	37.039	1.00	0.00	RX0	N
ATOM	1190	H	ARG	283	23.190	12.111	36.638	1.00	0.00	RX0	H
ATOM	1191	CA	ARG	283	21.822	13.735	36.412	1.00	0.00	RX0	C
ATOM	1192	CB	ARG	283	22.815	14.202	35.348	1.00	0.00	RX0	C
ATOM	1193	CG	ARG	283	22.357	15.385	34.499	1.00	0.00	RX0	C
ATOM	1194	CD	ARG	283	22.169	16.683	35.281	1.00	0.00	RX0	C
ATOM	1195	NE	ARG	283	21.815	17.755	34.355	1.00	0.00	RX0	N
ATOM	1196	HE	ARG	283	22.260	17.706	33.454	1.00	0.00	RX0	H
ATOM	1197	CZ	ARG	283	20.931	18.723	34.731	1.00	0.00	RX0	C
ATOM	1198	NH1	ARG	283	20.417	18.717	35.983	1.00	0.00	RX0	N
ATOM	1199	HH11	ARG	283	19.755	19.410	36.287	1.00	0.00	RX0	H
ATOM	1200	HH12	ARG	283	20.674	18.019	36.658	1.00	0.00	RX0	H
ATOM	1201	NH2	ARG	283	20.576	19.668	33.839	1.00	0.00	RX0	N
ATOM	1202	HH21	ARG	283	19.889	20.378	34.055	1.00	0.00	RX0	H
ATOM	1203	HH22	ARG	283	20.966	19.700	32.915	1.00	0.00	RX0	H
ATOM	1204	C	ARG	283	20.432	13.477	35.812	1.00	0.00	RX0	C
ATOM	1205	O	ARG	283	19.498	14.246	36.035	1.00	0.00	RX0	O
ATOM	1206	N	PHE	284	20.293	12.325	35.169	1.00	0.00	RX0	N
ATOM	1207	H	PHE	284	21.097	11.740	35.042	1.00	0.00	RX0	H
ATOM	1208	CA	PHE	284	19.003	11.899	34.606	1.00	0.00	RX0	C
ATOM	1209	CB	PHE	284	19.206	10.636	33.776	1.00	0.00	RX0	C
ATOM	1210	CG	PHE	284	19.681	10.996	32.391	1.00	0.00	RX0	C
ATOM	1211	CD1	PHE	284	19.372	12.239	31.853	1.00	0.00	RX0	C
ATOM	1212	CD2	PHE	284	20.401	10.073	31.644	1.00	0.00	RX0	C
ATOM	1213	CE1	PHE	284	19.750	12.542	30.550	1.00	0.00	RX0	C
ATOM	1214	CE2	PHE	284	20.782	10.380	30.343	1.00	0.00	RX0	C
ATOM	1215	CZ	PHE	284	20.442	11.608	29.790	1.00	0.00	RX0	C
ATOM	1216	C	PHE	284	17.921	11.680	35.654	1.00	0.00	RX0	C
ATOM	1217	O	PHE	284	16.817	12.216	35.524	1.00	0.00	RX0	O
ATOM	1218	N	ARG	285	18.348	11.087	36.760	1.00	0.00	RX0	N

ATOM	1219	H	ARG	285	19.279	10.717	36.763	1.00	0.00	RX0	H
ATOM	1220	CA	ARG	285	17.485	10.841	37.923	1.00	0.00	RX0	C
ATOM	1221	CB	ARG	285	18.334	10.055	38.923	1.00	0.00	RX0	C
ATOM	1222	CG	ARG	285	17.771	9.784	40.319	1.00	0.00	RX0	C
ATOM	1223	CD	ARG	285	18.800	9.023	41.168	1.00	0.00	RX0	C
ATOM	1224	NE	ARG	285	19.213	7.813	40.462	1.00	0.00	RX0	N
ATOM	1225	HE	ARG	285	18.456	7.214	40.168	1.00	0.00	RX0	H
ATOM	1226	CZ	ARG	285	20.503	7.633	40.045	1.00	0.00	RX0	C
ATOM	1227	NH1	ARG	285	21.471	8.415	40.568	1.00	0.00	RX0	N
ATOM	1228	HH11	ARG	285	22.435	8.407	40.255	1.00	0.00	RX0	H
ATOM	1229	HH12	ARG	285	21.266	9.069	41.299	1.00	0.00	RX0	H
ATOM	1230	NH2	ARG	285	20.764	6.694	39.110	1.00	0.00	RX0	N
ATOM	1231	HH21	ARG	285	21.649	6.563	38.642	1.00	0.00	RX0	H
ATOM	1232	HH22	ARG	285	20.022	6.081	38.811	1.00	0.00	RX0	H
ATOM	1233	C	ARG	285	17.003	12.164	38.534	1.00	0.00	RX0	C
ATOM	1234	O	ARG	285	15.822	12.321	38.816	1.00	0.00	RX0	O
ATOM	1235	N	MET	286	17.922	13.130	38.591	1.00	0.00	RX0	N
ATOM	1236	H	MET	286	18.856	12.922	38.296	1.00	0.00	RX0	H
ATOM	1237	CA	MET	286	17.643	14.467	39.144	1.00	0.00	RX0	C
ATOM	1238	CB	MET	286	18.961	15.233	39.260	1.00	0.00	RX0	C
ATOM	1239	CG	MET	286	18.794	16.702	39.650	1.00	0.00	RX0	C
ATOM	1240	SD	MET	286	20.311	17.644	39.423	1.00	0.00	RX0	S
ATOM	1241	CE	MET	286	19.711	19.226	40.037	1.00	0.00	RX0	C
ATOM	1242	C	MET	286	16.681	15.257	38.243	1.00	0.00	RX0	C
ATOM	1243	O	MET	286	15.799	15.962	38.735	1.00	0.00	RX0	O
ATOM	1244	N	MET	287	16.911	15.161	36.942	1.00	0.00	RX0	N
ATOM	1245	H	MET	287	17.631	14.537	36.631	1.00	0.00	RX0	H
ATOM	1246	CA	MET	287	16.049	15.814	35.941	1.00	0.00	RX0	C
ATOM	1247	CB	MET	287	16.684	15.860	34.556	1.00	0.00	RX0	C
ATOM	1248	CG	MET	287	17.872	16.813	34.515	1.00	0.00	RX0	C
ATOM	1249	SD	MET	287	18.234	17.349	32.841	1.00	0.00	RX0	S
ATOM	1250	CE	MET	287	16.609	18.040	32.489	1.00	0.00	RX0	C
ATOM	1251	C	MET	287	14.674	15.156	35.849	1.00	0.00	RX0	C
ATOM	1252	O	MET	287	13.755	15.729	35.264	1.00	0.00	RX0	O
ATOM	1253	N	ASN	288	14.583	13.920	36.346	1.00	0.00	RX0	N
ATOM	1254	H	ASN	288	15.393	13.490	36.746	1.00	0.00	RX0	H
ATOM	1255	CA	ASN	288	13.391	13.066	36.242	1.00	0.00	RX0	C
ATOM	1256	CB	ASN	288	12.174	13.626	36.977	1.00	0.00	RX0	C
ATOM	1257	CG	ASN	288	11.006	12.682	36.758	1.00	0.00	RX0	C
ATOM	1258	OD1	ASN	288	11.159	11.475	36.606	1.00	0.00	RX0	O
ATOM	1259	ND2	ASN	288	9.814	13.306	36.738	1.00	0.00	RX0	N
ATOM	1260	HD21	ASN	288	9.755	14.294	36.871	1.00	0.00	RX0	H
ATOM	1261	HD22	ASN	288	8.966	12.797	36.586	1.00	0.00	RX0	H
ATOM	1262	C	ASN	288	13.076	12.836	34.753	1.00	0.00	RX0	C
ATOM	1263	O	ASN	288	11.986	13.121	34.256	1.00	0.00	RX0	O
ATOM	1264	N	LEU	289	14.125	12.439	34.028	1.00	0.00	RX0	N
ATOM	1265	H	LEU	289	14.954	12.171	34.521	1.00	0.00	RX0	H
ATOM	1266	CA	LEU	289	14.030	12.183	32.583	1.00	0.00	RX0	C
ATOM	1267	CB	LEU	289	15.380	11.666	32.074	1.00	0.00	RX0	C
ATOM	1268	CG	LEU	289	15.462	11.443	30.561	1.00	0.00	RX0	C
ATOM	1269	CD1	LEU	289	15.501	12.762	29.803	1.00	0.00	RX0	C
ATOM	1270	CD2	LEU	289	16.645	10.566	30.158	1.00	0.00	RX0	C
ATOM	1271	C	LEU	289	12.926	11.152	32.321	1.00	0.00	RX0	C
ATOM	1272	O	LEU	289	12.814	10.137	33.014	1.00	0.00	RX0	O
ATOM	1273	N	GLN	290	12.165	11.420	31.281	1.00	0.00	RX0	N
ATOM	1274	H	GLN	290	12.396	12.179	30.666	1.00	0.00	RX0	H
ATOM	1275	CA	GLN	290	11.011	10.584	30.916	1.00	0.00	RX0	C
ATOM	1276	CB	GLN	290	9.774	11.433	30.644	1.00	0.00	RX0	C
ATOM	1277	CG	GLN	290	9.292	12.062	31.949	1.00	0.00	RX0	C
ATOM	1278	CD	GLN	290	9.105	10.952	32.966	1.00	0.00	RX0	C
ATOM	1279	OE1	GLN	290	8.314	10.031	32.783	1.00	0.00	RX0	O

ATOM	1280	NE2	GLN	290	9.892	11.086	34.046	1.00	0.00	RX0	N
ATOM	1281	HE21	GLN	290	10.536	11.850	34.123	1.00	0.00	RX0	H
ATOM	1282	HE22	GLN	290	9.943	10.448	34.817	1.00	0.00	RX0	H
ATOM	1283	C	GLN	290	11.379	9.701	29.727	1.00	0.00	RX0	C
ATOM	1284	O	GLN	290	12.115	10.141	28.832	1.00	0.00	RX0	O
ATOM	1285	N	GLY	291	10.739	8.533	29.672	1.00	0.00	RX0	N
ATOM	1286	H	GLY	291	10.097	8.326	30.409	1.00	0.00	RX0	H
ATOM	1287	CA	GLY	291	10.952	7.542	28.589	1.00	0.00	RX0	C
ATOM	1288	C	GLY	291	10.769	8.134	27.181	1.00	0.00	RX0	C
ATOM	1289	O	GLY	291	11.559	7.865	26.269	1.00	0.00	RX0	O
ATOM	1290	N	GLU	292	9.854	9.086	27.078	1.00	0.00	RX0	N
ATOM	1291	H	GLU	292	9.225	9.227	27.846	1.00	0.00	RX0	H
ATOM	1292	CA	GLU	292	9.541	9.789	25.813	1.00	0.00	RX0	C
ATOM	1293	CB	GLU	292	8.231	10.572	25.945	1.00	0.00	RX0	C
ATOM	1294	CG	GLU	292	6.968	9.717	26.090	1.00	0.00	RX0	C
ATOM	1295	CD	GLU	292	7.056	8.868	27.338	1.00	0.00	RX0	C
ATOM	1296	OE1	GLU	292	7.220	9.407	28.430	1.00	0.00	RX0	O
ATOM	1297	OE2	GLU	292	7.022	7.649	27.233	1.00	0.00	RX0	O
ATOM	1298	C	GLU	292	10.671	10.744	25.400	1.00	0.00	RX0	C
ATOM	1299	O	GLU	292	11.065	10.802	24.241	1.00	0.00	RX0	O
ATOM	1300	N	GLU	293	11.241	11.417	26.395	1.00	0.00	RX0	N
ATOM	1301	H	GLU	293	10.968	11.204	27.333	1.00	0.00	RX0	H
ATOM	1302	CA	GLU	293	12.389	12.320	26.184	1.00	0.00	RX0	C
ATOM	1303	CB	GLU	293	12.615	13.166	27.433	1.00	0.00	RX0	C
ATOM	1304	CG	GLU	293	11.376	13.936	27.888	1.00	0.00	RX0	C
ATOM	1305	CD	GLU	293	11.649	14.513	29.261	1.00	0.00	RX0	C
ATOM	1306	OE1	GLU	293	12.536	14.022	29.948	1.00	0.00	RX0	O
ATOM	1307	OE2	GLU	293	10.976	15.451	29.670	1.00	0.00	RX0	O
ATOM	1308	C	GLU	293	13.669	11.544	25.843	1.00	0.00	RX0	C
ATOM	1309	O	GLU	293	14.364	11.888	24.886	1.00	0.00	RX0	O
ATOM	1310	N	PHE	294	13.855	10.409	26.517	1.00	0.00	RX0	N
ATOM	1311	H	PHE	294	13.197	10.198	27.243	1.00	0.00	RX0	H
ATOM	1312	CA	PHE	294	14.999	9.503	26.300	1.00	0.00	RX0	C
ATOM	1313	CB	PHE	294	14.903	8.311	27.251	1.00	0.00	RX0	C
ATOM	1314	CG	PHE	294	15.863	7.229	26.822	1.00	0.00	RX0	C
ATOM	1315	CD1	PHE	294	17.233	7.451	26.865	1.00	0.00	RX0	C
ATOM	1316	CD2	PHE	294	15.368	6.014	26.367	1.00	0.00	RX0	C
ATOM	1317	CE1	PHE	294	18.106	6.464	26.424	1.00	0.00	RX0	C
ATOM	1318	CE2	PHE	294	16.243	5.031	25.927	1.00	0.00	RX0	C
ATOM	1319	CZ	PHE	294	17.613	5.255	25.950	1.00	0.00	RX0	C
ATOM	1320	C	PHE	294	15.115	9.012	24.847	1.00	0.00	RX0	C
ATOM	1321	O	PHE	294	16.186	9.132	24.238	1.00	0.00	RX0	O
ATOM	1322	N	VAL	295	14.000	8.581	24.280	1.00	0.00	RX0	N
ATOM	1323	H	VAL	295	13.159	8.564	24.829	1.00	0.00	RX0	H
ATOM	1324	CA	VAL	295	13.976	8.026	22.907	1.00	0.00	RX0	C
ATOM	1325	CB	VAL	295	12.685	7.249	22.619	1.00	0.00	RX0	C
ATOM	1326	CG1	VAL	295	12.587	6.065	23.579	1.00	0.00	RX0	C
ATOM	1327	CG2	VAL	295	11.428	8.115	22.665	1.00	0.00	RX0	C
ATOM	1328	C	VAL	295	14.286	9.097	21.847	1.00	0.00	RX0	C
ATOM	1329	O	VAL	295	14.999	8.845	20.884	1.00	0.00	RX0	O
ATOM	1330	N	CYS	296	13.866	10.330	22.156	1.00	0.00	RX0	N
ATOM	1331	H	CYS	296	13.306	10.479	22.974	1.00	0.00	RX0	H
ATOM	1332	CA	CYS	296	14.167	11.498	21.314	1.00	0.00	RX0	C
ATOM	1333	CB	CYS	296	13.264	12.653	21.730	1.00	0.00	RX0	C
ATOM	1334	SG	CYS	296	11.518	12.259	21.468	1.00	0.00	RX0	S
ATOM	1335	C	CYS	296	15.661	11.843	21.352	1.00	0.00	RX0	C
ATOM	1336	O	CYS	296	16.290	11.969	20.305	1.00	0.00	RX0	O
ATOM	1337	N	LEU	297	16.247	11.750	22.547	1.00	0.00	RX0	N
ATOM	1338	H	LEU	297	15.681	11.545	23.350	1.00	0.00	RX0	H
ATOM	1339	CA	LEU	297	17.681	12.031	22.752	1.00	0.00	RX0	C
ATOM	1340	CB	LEU	297	18.016	12.112	24.240	1.00	0.00	RX0	C

ATOM	1341	CG	LEU	297	17.338	13.284	24.949	1.00	0.00	RX0	C
ATOM	1342	CD1	LEU	297	17.612	13.259	26.452	1.00	0.00	RX0	C
ATOM	1343	CD2	LEU	297	17.711	14.626	24.317	1.00	0.00	RX0	C
ATOM	1344	C	LEU	297	18.589	11.009	22.068	1.00	0.00	RX0	C
ATOM	1345	O	LEU	297	19.526	11.385	21.359	1.00	0.00	RX0	O
ATOM	1346	N	LYS	298	18.173	9.750	22.123	1.00	0.00	RX0	N
ATOM	1347	H	LYS	298	17.360	9.543	22.671	1.00	0.00	RX0	H
ATOM	1348	CA	LYS	298	18.945	8.654	21.515	1.00	0.00	RX0	C
ATOM	1349	CB	LYS	298	18.412	7.326	22.050	1.00	0.00	RX0	C
ATOM	1350	CG	LYS	298	19.287	6.119	21.720	1.00	0.00	RX0	C
ATOM	1351	CD	LYS	298	18.698	4.846	22.325	1.00	0.00	RX0	C
ATOM	1352	CE	LYS	298	17.224	4.725	21.947	1.00	0.00	RX0	C
ATOM	1353	NZ	LYS	298	16.675	3.435	22.364	1.00	0.00	RX0	N
ATOM	1354	HZ1	LYS	298	15.925	3.150	21.696	1.00	0.00	RX0	H
ATOM	1355	HZ2	LYS	298	17.358	2.666	22.454	1.00	0.00	RX0	H
ATOM	1356	HZ3	LYS	298	16.103	3.511	23.235	1.00	0.00	RX0	H
ATOM	1357	C	LYS	298	18.925	8.733	19.978	1.00	0.00	RX0	C
ATOM	1358	O	LYS	298	19.964	8.557	19.332	1.00	0.00	RX0	O
ATOM	1359	N	SER	299	17.791	9.152	19.431	1.00	0.00	RX0	N
ATOM	1360	H	SER	299	16.964	9.262	19.983	1.00	0.00	RX0	H
ATOM	1361	CA	SER	299	17.645	9.383	17.977	1.00	0.00	RX0	C
ATOM	1362	CB	SER	299	16.155	9.360	17.704	1.00	0.00	RX0	C
ATOM	1363	OG	SER	299	15.662	8.196	18.375	1.00	0.00	RX0	O
ATOM	1364	HG	SER	299	15.141	8.493	19.113	1.00	0.00	RX0	H
ATOM	1365	C	SER	299	18.416	10.621	17.504	1.00	0.00	RX0	C
ATOM	1366	O	SER	299	19.051	10.583	16.444	1.00	0.00	RX0	O
ATOM	1367	N	ILE	300	18.478	11.644	18.354	1.00	0.00	RX0	N
ATOM	1368	H	ILE	300	17.951	11.595	19.205	1.00	0.00	RX0	H
ATOM	1369	CA	ILE	300	19.283	12.860	18.096	1.00	0.00	RX0	C
ATOM	1370	CB	ILE	300	19.081	13.935	19.167	1.00	0.00	RX0	C
ATOM	1371	CG2	ILE	300	20.122	15.049	19.041	1.00	0.00	RX0	C
ATOM	1372	CG1	ILE	300	17.675	14.517	19.095	1.00	0.00	RX0	C
ATOM	1373	CD1	ILE	300	17.416	15.534	20.205	1.00	0.00	RX0	C
ATOM	1374	C	ILE	300	20.773	12.490	18.013	1.00	0.00	RX0	C
ATOM	1375	O	ILE	300	21.456	12.924	17.087	1.00	0.00	RX0	O
ATOM	1376	N	ILE	301	21.227	11.630	18.921	1.00	0.00	RX0	N
ATOM	1377	H	ILE	301	20.603	11.317	19.641	1.00	0.00	RX0	H
ATOM	1378	CA	ILE	301	22.629	11.156	18.925	1.00	0.00	RX0	C
ATOM	1379	CB	ILE	301	22.797	10.179	20.079	1.00	0.00	RX0	C
ATOM	1380	CG2	ILE	301	23.991	9.253	19.877	1.00	0.00	RX0	C
ATOM	1381	CG1	ILE	301	22.839	10.943	21.399	1.00	0.00	RX0	C
ATOM	1382	CD1	ILE	301	22.865	10.001	22.598	1.00	0.00	RX0	C
ATOM	1383	C	ILE	301	22.948	10.474	17.584	1.00	0.00	RX0	C
ATOM	1384	O	ILE	301	23.927	10.823	16.926	1.00	0.00	RX0	O
ATOM	1385	N	LEU	302	22.047	9.582	17.175	1.00	0.00	RX0	N
ATOM	1386	H	LEU	302	21.274	9.365	17.776	1.00	0.00	RX0	H
ATOM	1387	CA	LEU	302	22.205	8.836	15.917	1.00	0.00	RX0	C
ATOM	1388	CB	LEU	302	20.992	7.930	15.709	1.00	0.00	RX0	C
ATOM	1389	CG	LEU	302	21.000	7.210	14.360	1.00	0.00	RX0	C
ATOM	1390	CD1	LEU	302	22.181	6.249	14.226	1.00	0.00	RX0	C
ATOM	1391	CD2	LEU	302	19.664	6.527	14.079	1.00	0.00	RX0	C
ATOM	1392	C	LEU	302	22.391	9.757	14.699	1.00	0.00	RX0	C
ATOM	1393	O	LEU	302	23.285	9.544	13.882	1.00	0.00	RX0	O
ATOM	1394	N	LEU	303	21.581	10.806	14.658	1.00	0.00	RX0	N
ATOM	1395	H	LEU	303	20.928	10.941	15.407	1.00	0.00	RX0	H
ATOM	1396	CA	LEU	303	21.546	11.719	13.502	1.00	0.00	RX0	C
ATOM	1397	CB	LEU	303	20.124	12.231	13.311	1.00	0.00	RX0	C
ATOM	1398	CG	LEU	303	19.163	11.065	13.083	1.00	0.00	RX0	C
ATOM	1399	CD1	LEU	303	17.707	11.486	13.250	1.00	0.00	RX0	C
ATOM	1400	CD2	LEU	303	19.420	10.365	11.747	1.00	0.00	RX0	C
ATOM	1401	C	LEU	303	22.576	12.849	13.543	1.00	0.00	RX0	C

ATOM	1402	O	LEU	303	23.073	13.268	12.494	1.00	0.00	RX0	O
ATOM	1403	N	ASN	304	22.971	13.240	14.745	1.00	0.00	RX0	N
ATOM	1404	H	ASN	304	22.640	12.728	15.538	1.00	0.00	RX0	H
ATOM	1405	CA	ASN	304	23.832	14.419	14.940	1.00	0.00	RX0	C
ATOM	1406	CB	ASN	304	23.513	15.283	16.178	1.00	0.00	RX0	C
ATOM	1407	CG	ASN	304	24.444	14.918	17.330	1.00	0.00	RX0	C
ATOM	1408	OD1	ASN	304	25.479	15.522	17.614	1.00	0.00	RX0	O
ATOM	1409	ND2	ASN	304	24.054	13.795	17.941	1.00	0.00	RX0	N
ATOM	1410	HD21	ASN	304	23.106	13.492	17.819	1.00	0.00	RX0	H
ATOM	1411	HD22	ASN	304	24.664	13.152	18.416	1.00	0.00	RX0	H
ATOM	1412	C	ASN	304	25.330	14.097	14.987	1.00	0.00	RX0	C
ATOM	1413	O	ASN	304	26.132	14.783	14.344	1.00	0.00	RX0	O
ATOM	1414	N	SER	305	25.693	13.015	15.656	1.00	0.00	RX0	N
ATOM	1415	H	SER	305	25.041	12.399	16.107	1.00	0.00	RX0	H
ATOM	1416	CA	SER	305	27.104	12.765	16.012	1.00	0.00	RX0	C
ATOM	1417	CB	SER	305	27.112	11.600	16.993	1.00	0.00	RX0	C
ATOM	1418	OG	SER	305	26.159	11.929	18.015	1.00	0.00	RX0	O
ATOM	1419	HG	SER	305	26.334	11.314	18.735	1.00	0.00	RX0	H
ATOM	1420	C	SER	305	28.103	12.695	14.846	1.00	0.00	RX0	C
ATOM	1421	O	SER	305	29.198	13.228	14.946	1.00	0.00	RX0	O
ATOM	1422	N	GLY	306	27.634	12.177	13.693	1.00	0.00	RX0	N
ATOM	1423	H	GLY	306	26.686	11.866	13.632	1.00	0.00	RX0	H
ATOM	1424	CA	GLY	306	28.500	12.079	12.501	1.00	0.00	RX0	C
ATOM	1425	C	GLY	306	28.091	12.961	11.315	1.00	0.00	RX0	C
ATOM	1426	O	GLY	306	28.756	12.897	10.274	1.00	0.00	RX0	O
ATOM	1427	N	VAL	307	27.242	13.954	11.536	1.00	0.00	RX0	N
ATOM	1428	H	VAL	307	26.877	14.102	12.458	1.00	0.00	RX0	H
ATOM	1429	CA	VAL	307	26.760	14.815	10.435	1.00	0.00	RX0	C
ATOM	1430	CB	VAL	307	25.404	15.467	10.760	1.00	0.00	RX0	C
ATOM	1431	CG1	VAL	307	25.506	16.556	11.827	1.00	0.00	RX0	C
ATOM	1432	CG2	VAL	307	24.729	15.983	9.488	1.00	0.00	RX0	C
ATOM	1433	C	VAL	307	27.809	15.841	9.948	1.00	0.00	RX0	C
ATOM	1434	O	VAL	307	27.790	16.280	8.811	1.00	0.00	RX0	O
ATOM	1435	N	TYR	308	28.719	16.199	10.859	1.00	0.00	RX0	N
ATOM	1436	H	TYR	308	28.694	15.741	11.748	1.00	0.00	RX0	H
ATOM	1437	CA	TYR	308	29.761	17.211	10.584	1.00	0.00	RX0	C
ATOM	1438	CB	TYR	308	30.105	18.012	11.852	1.00	0.00	RX0	C
ATOM	1439	CG	TYR	308	29.005	18.957	12.316	1.00	0.00	RX0	C
ATOM	1440	CD1	TYR	308	27.892	19.260	11.498	1.00	0.00	RX0	C
ATOM	1441	CE1	TYR	308	26.924	20.170	11.958	1.00	0.00	RX0	C
ATOM	1442	CD2	TYR	308	29.151	19.539	13.592	1.00	0.00	RX0	C
ATOM	1443	CE2	TYR	308	28.191	20.458	14.049	1.00	0.00	RX0	C
ATOM	1444	CZ	TYR	308	27.096	20.774	13.220	1.00	0.00	RX0	C
ATOM	1445	OH	TYR	308	26.169	21.707	13.646	1.00	0.00	RX0	O
ATOM	1446	HH	TYR	308	26.522	22.202	14.377	1.00	0.00	RX0	H
ATOM	1447	C	TYR	308	31.032	16.660	9.940	1.00	0.00	RX0	C
ATOM	1448	O	TYR	308	31.912	17.411	9.537	1.00	0.00	RX0	O
ATOM	1449	N	THR	309	31.091	15.334	9.861	1.00	0.00	RX0	N
ATOM	1450	H	THR	309	30.366	14.752	10.231	1.00	0.00	RX0	H
ATOM	1451	CA	THR	309	32.303	14.643	9.386	1.00	0.00	RX0	C
ATOM	1452	CB	THR	309	32.714	13.705	10.518	1.00	0.00	RX0	C
ATOM	1453	OG1	THR	309	31.535	13.158	11.139	1.00	0.00	RX0	O
ATOM	1454	HG1	THR	309	31.204	12.465	10.566	1.00	0.00	RX0	H
ATOM	1455	CG2	THR	309	33.565	14.434	11.561	1.00	0.00	RX0	C
ATOM	1456	C	THR	309	32.183	13.866	8.071	1.00	0.00	RX0	C
ATOM	1457	O	THR	309	33.137	13.195	7.681	1.00	0.00	RX0	O
ATOM	1458	N	PHE	310	31.041	13.953	7.382	1.00	0.00	RX0	N
ATOM	1459	H	PHE	310	30.302	14.499	7.772	1.00	0.00	RX0	H
ATOM	1460	CA	PHE	310	30.972	13.467	5.988	1.00	0.00	RX0	C
ATOM	1461	CB	PHE	310	29.593	13.711	5.365	1.00	0.00	RX0	C
ATOM	1462	CG	PHE	310	28.510	12.911	6.054	1.00	0.00	RX0	C

ATOM	1463	CD1	PHE	310	28.433	11.526	5.877	1.00	0.00	RX0	C
ATOM	1464	CD2	PHE	310	27.565	13.563	6.863	1.00	0.00	RX0	C
ATOM	1465	CE1	PHE	310	27.394	10.847	6.529	1.00	0.00	RX0	C
ATOM	1466	CE2	PHE	310	26.525	12.883	7.512	1.00	0.00	RX0	C
ATOM	1467	CZ	PHE	310	26.447	11.499	7.333	1.00	0.00	RX0	C
ATOM	1468	C	PHE	310	32.019	14.245	5.180	1.00	0.00	RX0	C
ATOM	1469	O	PHE	310	32.102	15.468	5.301	1.00	0.00	RX0	O
ATOM	1470	N	LEU	311	32.854	13.508	4.462	1.00	0.00	RX0	N
ATOM	1471	H	LEU	311	32.754	12.518	4.532	1.00	0.00	RX0	H
ATOM	1472	CA	LEU	311	33.988	14.087	3.701	1.00	0.00	RX0	C
ATOM	1473	CB	LEU	311	34.870	13.014	3.057	1.00	0.00	RX0	C
ATOM	1474	CG	LEU	311	35.536	12.107	4.103	1.00	0.00	RX0	C
ATOM	1475	CD1	LEU	311	36.610	11.220	3.489	1.00	0.00	RX0	C
ATOM	1476	CD2	LEU	311	36.138	12.880	5.274	1.00	0.00	RX0	C
ATOM	1477	C	LEU	311	33.468	15.166	2.739	1.00	0.00	RX0	C
ATOM	1478	O	LEU	311	33.554	16.351	2.969	1.00	0.00	RX0	O
ATOM	1479	N	SER	312	32.784	14.619	1.713	1.00	0.00	RX0	N
ATOM	1480	H	SER	312	32.615	13.636	1.746	1.00	0.00	RX0	H
ATOM	1481	CA	SER	312	32.345	15.333	0.526	1.00	0.00	RX0	C
ATOM	1482	CB	SER	312	31.731	14.275	-0.419	1.00	0.00	RX0	C
ATOM	1483	OG	SER	312	31.698	12.970	0.199	1.00	0.00	RX0	O
ATOM	1484	HG	SER	312	32.468	12.491	-0.119	1.00	0.00	RX0	H
ATOM	1485	C	SER	312	31.423	16.517	0.816	1.00	0.00	RX0	C
ATOM	1486	O	SER	312	31.042	16.847	1.948	1.00	0.00	RX0	O
ATOM	1487	N	SER	313	30.837	16.923	-0.276	1.00	0.00	RX0	N
ATOM	1488	H	SER	313	31.316	16.683	-1.120	1.00	0.00	RX0	H
ATOM	1489	CA	SER	313	29.838	17.983	-0.455	1.00	0.00	RX0	C
ATOM	1490	CB	SER	313	30.240	19.360	0.079	1.00	0.00	RX0	C
ATOM	1491	OG	SER	313	29.805	19.382	1.451	1.00	0.00	RX0	O
ATOM	1492	HG	SER	313	30.174	20.178	1.859	1.00	0.00	RX0	H
ATOM	1493	C	SER	313	29.353	17.880	-1.909	1.00	0.00	RX0	C
ATOM	1494	O	SER	313	29.168	18.836	-2.628	1.00	0.00	RX0	O
ATOM	1495	N	THR	314	29.278	16.602	-2.358	1.00	0.00	RX0	N
ATOM	1496	H	THR	314	29.408	15.853	-1.712	1.00	0.00	RX0	H
ATOM	1497	CA	THR	314	28.609	16.250	-3.614	1.00	0.00	RX0	C
ATOM	1498	CB	THR	314	28.700	14.743	-3.829	1.00	0.00	RX0	C
ATOM	1499	OG1	THR	314	28.418	14.047	-2.607	1.00	0.00	RX0	O
ATOM	1500	HG1	THR	314	28.802	13.182	-2.720	1.00	0.00	RX0	H
ATOM	1501	CG2	THR	314	30.091	14.359	-4.326	1.00	0.00	RX0	C
ATOM	1502	C	THR	314	27.167	16.747	-3.490	1.00	0.00	RX0	C
ATOM	1503	O	THR	314	26.675	16.978	-2.368	1.00	0.00	RX0	O
ATOM	1504	N	LEU	315	26.451	16.801	-4.589	1.00	0.00	RX0	N
ATOM	1505	H	LEU	315	26.872	16.517	-5.452	1.00	0.00	RX0	H
ATOM	1506	CA	LEU	315	25.050	17.256	-4.537	1.00	0.00	RX0	C
ATOM	1507	CB	LEU	315	24.429	17.394	-5.919	1.00	0.00	RX0	C
ATOM	1508	CG	LEU	315	23.041	18.039	-5.919	1.00	0.00	RX0	C
ATOM	1509	CD1	LEU	315	23.052	19.460	-5.347	1.00	0.00	RX0	C
ATOM	1510	CD2	LEU	315	22.411	17.986	-7.307	1.00	0.00	RX0	C
ATOM	1511	C	LEU	315	24.190	16.343	-3.639	1.00	0.00	RX0	C
ATOM	1512	O	LEU	315	23.484	16.800	-2.757	1.00	0.00	RX0	O
ATOM	1513	N	LYS	316	24.524	15.049	-3.730	1.00	0.00	RX0	N
ATOM	1514	H	LYS	316	25.159	14.808	-4.461	1.00	0.00	RX0	H
ATOM	1515	CA	LYS	316	23.912	13.990	-2.918	1.00	0.00	RX0	C
ATOM	1516	CB	LYS	316	24.370	12.624	-3.427	1.00	0.00	RX0	C
ATOM	1517	CG	LYS	316	23.207	11.697	-3.792	1.00	0.00	RX0	C
ATOM	1518	CD	LYS	316	22.509	11.046	-2.594	1.00	0.00	RX0	C
ATOM	1519	CE	LYS	316	21.003	11.325	-2.515	1.00	0.00	RX0	C
ATOM	1520	NZ	LYS	316	20.781	12.664	-1.963	1.00	0.00	RX0	N
ATOM	1521	HZ1	LYS	316	19.775	12.932	-2.004	1.00	0.00	RX0	H
ATOM	1522	HZ2	LYS	316	21.101	12.704	-0.969	1.00	0.00	RX0	H
ATOM	1523	HZ3	LYS	316	21.231	13.405	-2.542	1.00	0.00	RX0	H

ATOM	1524	C	LYS	316	24.213	14.187	-1.422	1.00	0.00	RX0	C
ATOM	1525	O	LYS	316	23.297	14.194	-0.611	1.00	0.00	RX0	O
ATOM	1526	N	SER	317	25.467	14.540	-1.122	1.00	0.00	RX0	N
ATOM	1527	H	SER	317	26.123	14.638	-1.870	1.00	0.00	RX0	H
ATOM	1528	CA	SER	317	25.934	14.787	0.260	1.00	0.00	RX0	C
ATOM	1529	CB	SER	317	27.456	14.870	0.363	1.00	0.00	RX0	C
ATOM	1530	OG	SER	317	27.916	14.102	1.485	1.00	0.00	RX0	O
ATOM	1531	HG	SER	317	27.140	13.796	1.948	1.00	0.00	RX0	H
ATOM	1532	C	SER	317	25.242	16.001	0.898	1.00	0.00	RX0	C
ATOM	1533	O	SER	317	24.716	15.915	2.007	1.00	0.00	RX0	O
ATOM	1534	N	LEU	318	25.067	17.043	0.088	1.00	0.00	RX0	N
ATOM	1535	H	LEU	318	25.389	16.988	-0.858	1.00	0.00	RX0	H
ATOM	1536	CA	LEU	318	24.405	18.288	0.525	1.00	0.00	RX0	C
ATOM	1537	CB	LEU	318	24.588	19.376	-0.529	1.00	0.00	RX0	C
ATOM	1538	CG	LEU	318	26.051	19.799	-0.641	1.00	0.00	RX0	C
ATOM	1539	CD1	LEU	318	26.290	20.734	-1.827	1.00	0.00	RX0	C
ATOM	1540	CD2	LEU	318	26.559	20.386	0.677	1.00	0.00	RX0	C
ATOM	1541	C	LEU	318	22.918	18.061	0.823	1.00	0.00	RX0	C
ATOM	1542	O	LEU	318	22.412	18.453	1.877	1.00	0.00	RX0	O
ATOM	1543	N	GLU	319	22.299	17.244	-0.024	1.00	0.00	RX0	N
ATOM	1544	H	GLU	319	22.781	16.938	-0.848	1.00	0.00	RX0	H
ATOM	1545	CA	GLU	319	20.900	16.808	0.157	1.00	0.00	RX0	C
ATOM	1546	CB	GLU	319	20.421	15.896	-0.967	1.00	0.00	RX0	C
ATOM	1547	CG	GLU	319	20.405	16.352	-2.422	1.00	0.00	RX0	C
ATOM	1548	CD	GLU	319	20.185	15.081	-3.218	1.00	0.00	RX0	C
ATOM	1549	OE1	GLU	319	21.081	14.648	-3.937	1.00	0.00	RX0	O
ATOM	1550	OE2	GLU	319	19.178	14.413	-3.002	1.00	0.00	RX0	O
ATOM	1551	C	GLU	319	20.725	15.924	1.402	1.00	0.00	RX0	C
ATOM	1552	O	GLU	319	19.808	16.153	2.196	1.00	0.00	RX0	O
ATOM	1553	N	GLU	320	21.706	15.058	1.645	1.00	0.00	RX0	N
ATOM	1554	H	GLU	320	22.418	14.937	0.954	1.00	0.00	RX0	H
ATOM	1555	CA	GLU	320	21.731	14.149	2.812	1.00	0.00	RX0	C
ATOM	1556	CB	GLU	320	22.903	13.146	2.852	1.00	0.00	RX0	C
ATOM	1557	CG	GLU	320	23.205	12.241	1.647	1.00	0.00	RX0	C
ATOM	1558	CD	GLU	320	22.022	11.402	1.197	1.00	0.00	RX0	C
ATOM	1559	OE1	GLU	320	22.055	10.184	1.374	1.00	0.00	RX0	O
ATOM	1560	OE2	GLU	320	21.098	11.954	0.603	1.00	0.00	RX0	O
ATOM	1561	C	GLU	320	21.765	14.931	4.131	1.00	0.00	RX0	C
ATOM	1562	O	GLU	320	20.881	14.754	4.973	1.00	0.00	RX0	O
ATOM	1563	N	LYS	321	22.647	15.926	4.188	1.00	0.00	RX0	N
ATOM	1564	H	LYS	321	23.238	16.061	3.390	1.00	0.00	RX0	H
ATOM	1565	CA	LYS	321	22.801	16.771	5.391	1.00	0.00	RX0	C
ATOM	1566	CB	LYS	321	24.059	17.672	5.203	1.00	0.00	RX0	C
ATOM	1567	CG	LYS	321	25.405	16.979	4.841	1.00	0.00	RX0	C
ATOM	1568	CD	LYS	321	26.582	17.898	4.391	1.00	0.00	RX0	C
ATOM	1569	CE	LYS	321	27.854	17.152	3.893	1.00	0.00	RX0	C
ATOM	1570	NZ	LYS	321	28.973	18.030	3.470	1.00	0.00	RX0	N
ATOM	1571	HZ1	LYS	321	29.825	17.519	3.143	1.00	0.00	RX0	H
ATOM	1572	HZ2	LYS	321	28.744	18.690	2.695	1.00	0.00	RX0	H
ATOM	1573	HZ3	LYS	321	29.259	18.693	4.223	1.00	0.00	RX0	H
ATOM	1574	C	LYS	321	21.549	17.612	5.656	1.00	0.00	RX0	C
ATOM	1575	O	LYS	321	21.102	17.724	6.798	1.00	0.00	RX0	O
ATOM	1576	N	ASP	322	20.935	18.099	4.575	1.00	0.00	RX0	N
ATOM	1577	H	ASP	322	21.322	17.950	3.662	1.00	0.00	RX0	H
ATOM	1578	CA	ASP	322	19.715	18.915	4.684	1.00	0.00	RX0	C
ATOM	1579	CB	ASP	322	19.380	19.446	3.289	1.00	0.00	RX0	C
ATOM	1580	CG	ASP	322	17.987	20.032	3.247	1.00	0.00	RX0	C
ATOM	1581	OD1	ASP	322	17.580	20.690	4.194	1.00	0.00	RX0	O
ATOM	1582	OD2	ASP	322	17.284	19.806	2.268	1.00	0.00	RX0	O
ATOM	1583	C	ASP	322	18.561	18.097	5.282	1.00	0.00	RX0	C
ATOM	1584	O	ASP	322	17.955	18.512	6.263	1.00	0.00	RX0	O

ATOM	1585	N	HIS	323	18.423	16.872	4.772	1.00	0.00	RX0	N
ATOM	1586	H	HIS	323	19.032	16.611	4.017	1.00	0.00	RX0	H
ATOM	1587	CA	HIS	323	17.423	15.916	5.274	1.00	0.00	RX0	C
ATOM	1588	CB	HIS	323	17.452	14.613	4.472	1.00	0.00	RX0	C
ATOM	1589	CG	HIS	323	16.262	13.775	4.875	1.00	0.00	RX0	C
ATOM	1590	ND1	HIS	323	16.260	12.432	4.968	1.00	0.00	RX0	N
ATOM	1591	HD1	HIS	323	17.005	11.815	4.795	1.00	0.00	RX0	H
ATOM	1592	CD2	HIS	323	14.988	14.246	5.193	1.00	0.00	RX0	C
ATOM	1593	NE2	HIS	323	14.211	13.179	5.481	1.00	0.00	RX0	N
ATOM	1594	CE1	HIS	323	14.993	12.056	5.343	1.00	0.00	RX0	C
ATOM	1595	C	HIS	323	17.630	15.607	6.766	1.00	0.00	RX0	C
ATOM	1596	O	HIS	323	16.677	15.663	7.540	1.00	0.00	RX0	O
ATOM	1597	N	ILE	324	18.888	15.420	7.164	1.00	0.00	RX0	N
ATOM	1598	H	ILE	324	19.613	15.436	6.471	1.00	0.00	RX0	H
ATOM	1599	CA	ILE	324	19.235	15.107	8.570	1.00	0.00	RX0	C
ATOM	1600	CB	ILE	324	20.719	14.765	8.705	1.00	0.00	RX0	C
ATOM	1601	CG2	ILE	324	21.120	14.618	10.172	1.00	0.00	RX0	C
ATOM	1602	CG1	ILE	324	21.045	13.500	7.912	1.00	0.00	RX0	C
ATOM	1603	CD1	ILE	324	22.535	13.158	7.917	1.00	0.00	RX0	C
ATOM	1604	C	ILE	324	18.844	16.271	9.496	1.00	0.00	RX0	C
ATOM	1605	O	ILE	324	18.200	16.053	10.527	1.00	0.00	RX0	O
ATOM	1606	N	HIS	325	19.159	17.485	9.071	1.00	0.00	RX0	N
ATOM	1607	H	HIS	325	19.599	17.591	8.177	1.00	0.00	RX0	H
ATOM	1608	CA	HIS	325	18.840	18.694	9.856	1.00	0.00	RX0	C
ATOM	1609	CB	HIS	325	19.552	19.922	9.296	1.00	0.00	RX0	C
ATOM	1610	CG	HIS	325	21.008	19.876	9.686	1.00	0.00	RX0	C
ATOM	1611	ND1	HIS	325	21.981	19.404	8.887	1.00	0.00	RX0	N
ATOM	1612	HD1	HIS	325	21.852	19.029	7.988	1.00	0.00	RX0	H
ATOM	1613	CD2	HIS	325	21.578	20.294	10.891	1.00	0.00	RX0	C
ATOM	1614	NE2	HIS	325	22.912	20.068	10.807	1.00	0.00	RX0	N
ATOM	1615	CE1	HIS	325	23.161	19.521	9.574	1.00	0.00	RX0	C
ATOM	1616	C	HIS	325	17.335	18.955	9.948	1.00	0.00	RX0	C
ATOM	1617	O	HIS	325	16.820	19.290	11.021	1.00	0.00	RX0	O
ATOM	1618	N	ARG	326	16.631	18.584	8.887	1.00	0.00	RX0	N
ATOM	1619	H	ARG	326	17.126	18.351	8.048	1.00	0.00	RX0	H
ATOM	1620	CA	ARG	326	15.160	18.594	8.873	1.00	0.00	RX0	C
ATOM	1621	CB	ARG	326	14.643	18.439	7.444	1.00	0.00	RX0	C
ATOM	1622	CG	ARG	326	14.661	19.775	6.698	1.00	0.00	RX0	C
ATOM	1623	CD	ARG	326	14.093	19.702	5.277	1.00	0.00	RX0	C
ATOM	1624	NE	ARG	326	15.070	19.203	4.312	1.00	0.00	RX0	N
ATOM	1625	HE	ARG	326	15.849	19.821	4.103	1.00	0.00	RX0	H
ATOM	1626	CZ	ARG	326	14.934	17.990	3.709	1.00	0.00	RX0	C
ATOM	1627	NH1	ARG	326	13.909	17.191	4.069	1.00	0.00	RX0	N
ATOM	1628	HH11	ARG	326	13.755	16.288	3.659	1.00	0.00	RX0	H
ATOM	1629	HH12	ARG	326	13.263	17.483	4.778	1.00	0.00	RX0	H
ATOM	1630	NH2	ARG	326	15.819	17.608	2.766	1.00	0.00	RX0	N
ATOM	1631	HH21	ARG	326	15.824	16.730	2.287	1.00	0.00	RX0	H
ATOM	1632	HH22	ARG	326	16.549	18.263	2.506	1.00	0.00	RX0	H
ATOM	1633	C	ARG	326	14.537	17.576	9.843	1.00	0.00	RX0	C
ATOM	1634	O	ARG	326	13.617	17.928	10.589	1.00	0.00	RX0	O
ATOM	1635	N	VAL	327	15.171	16.419	9.987	1.00	0.00	RX0	N
ATOM	1636	H	VAL	327	15.982	16.249	9.423	1.00	0.00	RX0	H
ATOM	1637	CA	VAL	327	14.709	15.376	10.935	1.00	0.00	RX0	C
ATOM	1638	CB	VAL	327	15.279	13.994	10.608	1.00	0.00	RX0	C
ATOM	1639	CG1	VAL	327	14.752	12.958	11.601	1.00	0.00	RX0	C
ATOM	1640	CG2	VAL	327	14.944	13.577	9.176	1.00	0.00	RX0	C
ATOM	1641	C	VAL	327	15.041	15.781	12.381	1.00	0.00	RX0	C
ATOM	1642	O	VAL	327	14.187	15.661	13.270	1.00	0.00	RX0	O
ATOM	1643	N	LEU	328	16.212	16.371	12.574	1.00	0.00	RX0	N
ATOM	1644	H	LEU	328	16.818	16.508	11.789	1.00	0.00	RX0	H
ATOM	1645	CA	LEU	328	16.631	16.899	13.887	1.00	0.00	RX0	C

ATOM	1646	CB	LEU	328	18.069	17.409	13.827	1.00	0.00	RX0	C
ATOM	1647	CG	LEU	328	19.081	16.266	13.760	1.00	0.00	RX0	C
ATOM	1648	CD1	LEU	328	20.500	16.769	13.491	1.00	0.00	RX0	C
ATOM	1649	CD2	LEU	328	19.006	15.392	15.012	1.00	0.00	RX0	C
ATOM	1650	C	LEU	328	15.692	18.005	14.386	1.00	0.00	RX0	C
ATOM	1651	O	LEU	328	15.231	17.955	15.519	1.00	0.00	RX0	O
ATOM	1652	N	ASP	329	15.222	18.821	13.436	1.00	0.00	RX0	N
ATOM	1653	H	ASP	329	15.683	18.906	12.550	1.00	0.00	RX0	H
ATOM	1654	CA	ASP	329	14.223	19.870	13.722	1.00	0.00	RX0	C
ATOM	1655	CB	ASP	329	14.063	20.825	12.536	1.00	0.00	RX0	C
ATOM	1656	CG	ASP	329	15.306	21.684	12.379	1.00	0.00	RX0	C
ATOM	1657	OD1	ASP	329	16.113	21.733	13.308	1.00	0.00	RX0	O
ATOM	1658	OD2	ASP	329	15.461	22.307	11.328	1.00	0.00	RX0	O
ATOM	1659	C	ASP	329	12.864	19.312	14.154	1.00	0.00	RX0	C
ATOM	1660	O	ASP	329	12.272	19.788	15.128	1.00	0.00	RX0	O
ATOM	1661	N	LYS	330	12.463	18.218	13.512	1.00	0.00	RX0	N
ATOM	1662	H	LYS	330	13.022	17.921	12.736	1.00	0.00	RX0	H
ATOM	1663	CA	LYS	330	11.217	17.525	13.871	1.00	0.00	RX0	C
ATOM	1664	CB	LYS	330	10.799	16.459	12.861	1.00	0.00	RX0	C
ATOM	1665	CG	LYS	330	9.624	15.604	13.368	1.00	0.00	RX0	C
ATOM	1666	CD	LYS	330	8.369	16.381	13.799	1.00	0.00	RX0	C
ATOM	1667	CE	LYS	330	7.826	17.307	12.720	1.00	0.00	RX0	C
ATOM	1668	NZ	LYS	330	7.437	16.476	11.581	1.00	0.00	RX0	N
ATOM	1669	HZ1	LYS	330	7.123	17.094	10.812	1.00	0.00	RX0	H
ATOM	1670	HZ2	LYS	330	8.248	15.891	11.281	1.00	0.00	RX0	H
ATOM	1671	HZ3	LYS	330	6.658	15.856	11.884	1.00	0.00	RX0	H
ATOM	1672	C	LYS	330	11.283	16.915	15.278	1.00	0.00	RX0	C
ATOM	1673	O	LYS	330	10.354	17.095	16.067	1.00	0.00	RX0	O
ATOM	1674	N	ILE	331	12.441	16.375	15.625	1.00	0.00	RX0	N
ATOM	1675	H	ILE	331	13.185	16.377	14.951	1.00	0.00	RX0	H
ATOM	1676	CA	ILE	331	12.656	15.802	16.972	1.00	0.00	RX0	C
ATOM	1677	CB	ILE	331	13.953	14.997	17.053	1.00	0.00	RX0	C
ATOM	1678	CG2	ILE	331	14.062	14.330	18.421	1.00	0.00	RX0	C
ATOM	1679	CG1	ILE	331	14.043	13.952	15.943	1.00	0.00	RX0	C
ATOM	1680	CD1	ILE	331	15.359	13.174	15.984	1.00	0.00	RX0	C
ATOM	1681	C	ILE	331	12.642	16.922	18.027	1.00	0.00	RX0	C
ATOM	1682	O	ILE	331	12.078	16.732	19.120	1.00	0.00	RX0	O
ATOM	1683	N	THR	332	13.158	18.084	17.675	1.00	0.00	RX0	N
ATOM	1684	H	THR	332	13.573	18.203	16.772	1.00	0.00	RX0	H
ATOM	1685	CA	THR	332	13.155	19.265	18.570	1.00	0.00	RX0	C
ATOM	1686	CB	THR	332	14.034	20.316	17.914	1.00	0.00	RX0	C
ATOM	1687	OG1	THR	332	15.302	19.711	17.628	1.00	0.00	RX0	O
ATOM	1688	HG1	THR	332	15.248	19.358	16.743	1.00	0.00	RX0	H
ATOM	1689	CG2	THR	332	14.205	21.555	18.795	1.00	0.00	RX0	C
ATOM	1690	C	THR	332	11.706	19.696	18.836	1.00	0.00	RX0	C
ATOM	1691	O	THR	332	11.302	19.832	19.995	1.00	0.00	RX0	O
ATOM	1692	N	ASP	333	10.912	19.714	17.772	1.00	0.00	RX0	N
ATOM	1693	H	ASP	333	11.280	19.673	16.837	1.00	0.00	RX0	H
ATOM	1694	CA	ASP	333	9.466	20.017	17.863	1.00	0.00	RX0	C
ATOM	1695	CB	ASP	333	8.671	19.793	16.567	1.00	0.00	RX0	C
ATOM	1696	CG	ASP	333	9.100	20.578	15.354	1.00	0.00	RX0	C
ATOM	1697	OD1	ASP	333	9.509	21.724	15.506	1.00	0.00	RX0	O
ATOM	1698	OD2	ASP	333	8.970	20.039	14.251	1.00	0.00	RX0	O
ATOM	1699	C	ASP	333	8.732	19.000	18.747	1.00	0.00	RX0	C
ATOM	1700	O	ASP	333	7.880	19.374	19.559	1.00	0.00	RX0	O
ATOM	1701	N	THR	334	9.187	17.759	18.682	1.00	0.00	RX0	N
ATOM	1702	H	THR	334	9.941	17.583	18.048	1.00	0.00	RX0	H
ATOM	1703	CA	THR	334	8.631	16.638	19.462	1.00	0.00	RX0	C
ATOM	1704	CB	THR	334	9.175	15.372	18.822	1.00	0.00	RX0	C
ATOM	1705	OG1	THR	334	8.792	15.338	17.438	1.00	0.00	RX0	O
ATOM	1706	HG1	THR	334	9.149	16.124	17.032	1.00	0.00	RX0	H

ATOM	1707	CG2	THR	334	8.722	14.119	19.558	1.00	0.00	RX0	C
ATOM	1708	C	THR	334	8.961	16.782	20.953	1.00	0.00	RX0	C
ATOM	1709	O	THR	334	8.059	16.689	21.789	1.00	0.00	RX0	O
ATOM	1710	N	LEU	335	10.217	17.081	21.263	1.00	0.00	RX0	N
ATOM	1711	H	LEU	335	10.880	17.204	20.523	1.00	0.00	RX0	H
ATOM	1712	CA	LEU	335	10.648	17.349	22.650	1.00	0.00	RX0	C
ATOM	1713	CB	LEU	335	12.150	17.611	22.696	1.00	0.00	RX0	C
ATOM	1714	CG	LEU	335	12.959	16.321	22.777	1.00	0.00	RX0	C
ATOM	1715	CD1	LEU	335	14.457	16.569	22.602	1.00	0.00	RX0	C
ATOM	1716	CD2	LEU	335	12.654	15.571	24.075	1.00	0.00	RX0	C
ATOM	1717	C	LEU	335	9.903	18.522	23.297	1.00	0.00	RX0	C
ATOM	1718	O	LEU	335	9.384	18.379	24.399	1.00	0.00	RX0	O
ATOM	1719	N	ILE	336	9.668	19.567	22.501	1.00	0.00	RX0	N
ATOM	1720	H	ILE	336	10.071	19.579	21.582	1.00	0.00	RX0	H
ATOM	1721	CA	ILE	336	8.897	20.743	22.962	1.00	0.00	RX0	C
ATOM	1722	CB	ILE	336	9.048	21.912	21.989	1.00	0.00	RX0	C
ATOM	1723	CG2	ILE	336	8.089	23.057	22.324	1.00	0.00	RX0	C
ATOM	1724	CG1	ILE	336	10.500	22.389	21.997	1.00	0.00	RX0	C
ATOM	1725	CD1	ILE	336	10.928	22.872	23.385	1.00	0.00	RX0	C
ATOM	1726	C	ILE	336	7.427	20.365	23.180	1.00	0.00	RX0	C
ATOM	1727	O	ILE	336	6.836	20.730	24.203	1.00	0.00	RX0	O
ATOM	1728	N	HIS	337	6.891	19.571	22.265	1.00	0.00	RX0	N
ATOM	1729	H	HIS	337	7.438	19.318	21.465	1.00	0.00	RX0	H
ATOM	1730	CA	HIS	337	5.501	19.096	22.359	1.00	0.00	RX0	C
ATOM	1731	CB	HIS	337	5.079	18.326	21.117	1.00	0.00	RX0	C
ATOM	1732	CG	HIS	337	3.599	18.064	21.217	1.00	0.00	RX0	C
ATOM	1733	ND1	HIS	337	2.655	18.986	20.955	1.00	0.00	RX0	N
ATOM	1734	HD1	HIS	337	2.812	19.908	20.658	1.00	0.00	RX0	H
ATOM	1735	CD2	HIS	337	2.974	16.880	21.607	1.00	0.00	RX0	C
ATOM	1736	NE2	HIS	337	1.634	17.097	21.584	1.00	0.00	RX0	N
ATOM	1737	CE1	HIS	337	1.439	18.395	21.181	1.00	0.00	RX0	C
ATOM	1738	C	HIS	337	5.301	18.263	23.631	1.00	0.00	RX0	C
ATOM	1739	O	HIS	337	4.339	18.490	24.365	1.00	0.00	RX0	O
ATOM	1740	N	LEU	338	6.274	17.405	23.914	1.00	0.00	RX0	N
ATOM	1741	H	LEU	338	7.051	17.336	23.285	1.00	0.00	RX0	H
ATOM	1742	CA	LEU	338	6.246	16.526	25.097	1.00	0.00	RX0	C
ATOM	1743	CB	LEU	338	7.441	15.576	25.089	1.00	0.00	RX0	C
ATOM	1744	CG	LEU	338	7.341	14.533	23.980	1.00	0.00	RX0	C
ATOM	1745	CD1	LEU	338	8.645	13.751	23.809	1.00	0.00	RX0	C
ATOM	1746	CD2	LEU	338	6.123	13.630	24.180	1.00	0.00	RX0	C
ATOM	1747	C	LEU	338	6.240	17.331	26.400	1.00	0.00	RX0	C
ATOM	1748	O	LEU	338	5.410	17.098	27.277	1.00	0.00	RX0	O
ATOM	1749	N	MET	339	7.027	18.402	26.394	1.00	0.00	RX0	N
ATOM	1750	H	MET	339	7.614	18.550	25.594	1.00	0.00	RX0	H
ATOM	1751	CA	MET	339	7.168	19.308	27.550	1.00	0.00	RX0	C
ATOM	1752	CB	MET	339	8.407	20.188	27.400	1.00	0.00	RX0	C
ATOM	1753	CG	MET	339	9.705	19.384	27.373	1.00	0.00	RX0	C
ATOM	1754	SD	MET	339	11.142	20.416	27.053	1.00	0.00	RX0	S
ATOM	1755	CE	MET	339	12.273	19.098	26.588	1.00	0.00	RX0	C
ATOM	1756	C	MET	339	5.924	20.183	27.753	1.00	0.00	RX0	C
ATOM	1757	O	MET	339	5.433	20.322	28.878	1.00	0.00	RX0	O
ATOM	1758	N	ALA	340	5.370	20.672	26.646	1.00	0.00	RX0	N
ATOM	1759	H	ALA	340	5.814	20.472	25.772	1.00	0.00	RX0	H
ATOM	1760	CA	ALA	340	4.120	21.456	26.636	1.00	0.00	RX0	C
ATOM	1761	CB	ALA	340	3.835	21.992	25.232	1.00	0.00	RX0	C
ATOM	1762	C	ALA	340	2.921	20.619	27.100	1.00	0.00	RX0	C
ATOM	1763	O	ALA	340	2.176	21.064	27.967	1.00	0.00	RX0	O
ATOM	1764	N	LYS	341	2.865	19.360	26.655	1.00	0.00	RX0	N
ATOM	1765	H	LYS	341	3.552	19.058	25.993	1.00	0.00	RX0	H
ATOM	1766	CA	LYS	341	1.846	18.397	27.117	1.00	0.00	RX0	C
ATOM	1767	CB	LYS	341	1.972	17.108	26.280	1.00	0.00	RX0	C

ATOM	1768	CG	LYS	341	0.692	16.267	26.163	1.00	0.00	RX0	C
ATOM	1769	CD	LYS	341	0.377	15.796	24.728	1.00	0.00	RX0	C
ATOM	1770	CE	LYS	341	1.354	14.786	24.103	1.00	0.00	RX0	C
ATOM	1771	NZ	LYS	341	1.028	14.577	22.681	1.00	0.00	RX0	N
ATOM	1772	HZ1	LYS	341	1.751	14.035	22.156	1.00	0.00	RX0	H
ATOM	1773	HZ2	LYS	341	0.171	14.020	22.476	1.00	0.00	RX0	H
ATOM	1774	HZ3	LYS	341	0.963	15.462	22.141	1.00	0.00	RX0	H
ATOM	1775	C	LYS	341	1.927	18.179	28.637	1.00	0.00	RX0	C
ATOM	1776	O	LYS	341	0.908	18.032	29.304	1.00	0.00	RX0	O
ATOM	1777	N	ALA	342	3.166	18.103	29.120	1.00	0.00	RX0	N
ATOM	1778	H	ALA	342	3.940	18.176	28.488	1.00	0.00	RX0	H
ATOM	1779	CA	ALA	342	3.459	17.942	30.556	1.00	0.00	RX0	C
ATOM	1780	CB	ALA	342	4.953	17.706	30.786	1.00	0.00	RX0	C
ATOM	1781	C	ALA	342	3.007	19.166	31.371	1.00	0.00	RX0	C
ATOM	1782	O	ALA	342	2.879	19.099	32.588	1.00	0.00	RX0	O
ATOM	1783	N	GLY	343	2.900	20.305	30.669	1.00	0.00	RX0	N
ATOM	1784	H	GLY	343	3.152	20.317	29.702	1.00	0.00	RX0	H
ATOM	1785	CA	GLY	343	2.393	21.560	31.244	1.00	0.00	RX0	C
ATOM	1786	C	GLY	343	3.505	22.433	31.829	1.00	0.00	RX0	C
ATOM	1787	O	GLY	343	3.244	23.282	32.678	1.00	0.00	RX0	O
ATOM	1788	N	LEU	344	4.736	22.226	31.356	1.00	0.00	RX0	N
ATOM	1789	H	LEU	344	4.854	21.555	30.622	1.00	0.00	RX0	H
ATOM	1790	CA	LEU	344	5.823	23.174	31.634	1.00	0.00	RX0	C
ATOM	1791	CB	LEU	344	7.150	22.610	31.139	1.00	0.00	RX0	C
ATOM	1792	CG	LEU	344	7.551	21.336	31.878	1.00	0.00	RX0	C
ATOM	1793	CD1	LEU	344	8.831	20.737	31.301	1.00	0.00	RX0	C
ATOM	1794	CD2	LEU	344	7.656	21.564	33.387	1.00	0.00	RX0	C
ATOM	1795	C	LEU	344	5.508	24.488	30.929	1.00	0.00	RX0	C
ATOM	1796	O	LEU	344	4.977	24.500	29.792	1.00	0.00	RX0	O
ATOM	1797	N	THR	345	5.822	25.577	31.575	1.00	0.00	RX0	N
ATOM	1798	H	THR	345	6.293	25.483	32.451	1.00	0.00	RX0	H
ATOM	1799	CA	THR	345	5.705	26.912	30.948	1.00	0.00	RX0	C
ATOM	1800	CB	THR	345	5.997	27.964	32.011	1.00	0.00	RX0	C
ATOM	1801	OG1	THR	345	7.172	27.606	32.737	1.00	0.00	RX0	O
ATOM	1802	HG1	THR	345	6.902	26.963	33.389	1.00	0.00	RX0	H
ATOM	1803	CG2	THR	345	4.820	28.123	32.973	1.00	0.00	RX0	C
ATOM	1804	C	THR	345	6.639	26.971	29.731	1.00	0.00	RX0	C
ATOM	1805	O	THR	345	7.615	26.233	29.623	1.00	0.00	RX0	O
ATOM	1806	N	LEU	346	6.390	27.971	28.898	1.00	0.00	RX0	N
ATOM	1807	H	LEU	346	5.564	28.512	29.044	1.00	0.00	RX0	H
ATOM	1808	CA	LEU	346	7.214	28.242	27.708	1.00	0.00	RX0	C
ATOM	1809	CB	LEU	346	6.672	29.451	26.948	1.00	0.00	RX0	C
ATOM	1810	CG	LEU	346	7.379	29.658	25.607	1.00	0.00	RX0	C
ATOM	1811	CD1	LEU	346	7.248	28.432	24.700	1.00	0.00	RX0	C
ATOM	1812	CD2	LEU	346	6.916	30.939	24.913	1.00	0.00	RX0	C
ATOM	1813	C	LEU	346	8.705	28.433	28.051	1.00	0.00	RX0	C
ATOM	1814	O	LEU	346	9.594	27.857	27.432	1.00	0.00	RX0	O
ATOM	1815	N	GLN	347	8.927	29.083	29.198	1.00	0.00	RX0	N
ATOM	1816	H	GLN	347	8.145	29.438	29.706	1.00	0.00	RX0	H
ATOM	1817	CA	GLN	347	10.275	29.275	29.754	1.00	0.00	RX0	C
ATOM	1818	CB	GLN	347	10.218	30.283	30.897	1.00	0.00	RX0	C
ATOM	1819	CG	GLN	347	11.598	30.620	31.458	1.00	0.00	RX0	C
ATOM	1820	CD	GLN	347	11.432	31.586	32.608	1.00	0.00	RX0	C
ATOM	1821	OE1	GLN	347	10.473	31.517	33.366	1.00	0.00	RX0	O
ATOM	1822	NE2	GLN	347	12.414	32.500	32.688	1.00	0.00	RX0	N
ATOM	1823	HE21	GLN	347	13.166	32.510	32.028	1.00	0.00	RX0	H
ATOM	1824	HE22	GLN	347	12.405	33.195	33.408	1.00	0.00	RX0	H
ATOM	1825	C	GLN	347	10.911	27.956	30.229	1.00	0.00	RX0	C
ATOM	1826	O	GLN	347	12.052	27.652	29.875	1.00	0.00	RX0	O
ATOM	1827	N	GLN	348	10.120	27.140	30.905	1.00	0.00	RX0	N
ATOM	1828	H	GLN	348	9.171	27.398	31.088	1.00	0.00	RX0	H

ATOM	1829	CA	GLN	348	10.582	25.823	31.394	1.00	0.00	RX0	C
ATOM	1830	CB	GLN	348	9.593	25.218	32.377	1.00	0.00	RX0	C
ATOM	1831	CG	GLN	348	9.648	25.885	33.748	1.00	0.00	RX0	C
ATOM	1832	CD	GLN	348	8.479	25.377	34.558	1.00	0.00	RX0	C
ATOM	1833	OE1	GLN	348	7.355	25.288	34.068	1.00	0.00	RX0	O
ATOM	1834	NE2	GLN	348	8.798	25.044	35.819	1.00	0.00	RX0	N
ATOM	1835	HE21	GLN	348	9.737	25.160	36.148	1.00	0.00	RX0	H
ATOM	1836	HE22	GLN	348	8.119	24.682	36.459	1.00	0.00	RX0	H
ATOM	1837	C	GLN	348	10.871	24.847	30.248	1.00	0.00	RX0	C
ATOM	1838	O	GLN	348	11.861	24.115	30.296	1.00	0.00	RX0	O
ATOM	1839	N	GLN	349	10.121	24.983	29.160	1.00	0.00	RX0	N
ATOM	1840	H	GLN	349	9.375	25.648	29.187	1.00	0.00	RX0	H
ATOM	1841	CA	GLN	349	10.299	24.172	27.940	1.00	0.00	RX0	C
ATOM	1842	CB	GLN	349	9.205	24.481	26.921	1.00	0.00	RX0	C
ATOM	1843	CG	GLN	349	7.821	24.032	27.384	1.00	0.00	RX0	C
ATOM	1844	CD	GLN	349	6.787	24.591	26.435	1.00	0.00	RX0	C
ATOM	1845	OE1	GLN	349	7.076	24.921	25.291	1.00	0.00	RX0	O
ATOM	1846	NE2	GLN	349	5.564	24.698	26.977	1.00	0.00	RX0	N
ATOM	1847	HE21	GLN	349	5.398	24.431	27.933	1.00	0.00	RX0	H
ATOM	1848	HE22	GLN	349	4.780	25.035	26.458	1.00	0.00	RX0	H
ATOM	1849	C	GLN	349	11.682	24.372	27.306	1.00	0.00	RX0	C
ATOM	1850	O	GLN	349	12.447	23.422	27.181	1.00	0.00	RX0	O
ATOM	1851	N	HIS	350	12.056	25.641	27.113	1.00	0.00	RX0	N
ATOM	1852	H	HIS	350	11.422	26.375	27.365	1.00	0.00	RX0	H
ATOM	1853	CA	HIS	350	13.344	25.953	26.463	1.00	0.00	RX0	C
ATOM	1854	CB	HIS	350	13.379	27.327	25.780	1.00	0.00	RX0	C
ATOM	1855	CG	HIS	350	13.575	28.469	26.745	1.00	0.00	RX0	C
ATOM	1856	ND1	HIS	350	12.559	29.184	27.254	1.00	0.00	RX0	N
ATOM	1857	HD1	HIS	350	11.601	29.024	27.096	1.00	0.00	RX0	H
ATOM	1858	CD2	HIS	350	14.783	28.993	27.219	1.00	0.00	RX0	C
ATOM	1859	NE2	HIS	350	14.483	30.040	28.024	1.00	0.00	RX0	N
ATOM	1860	CE1	HIS	350	13.118	30.158	28.042	1.00	0.00	RX0	C
ATOM	1861	C	HIS	350	14.540	25.670	27.386	1.00	0.00	RX0	C
ATOM	1862	O	HIS	350	15.573	25.182	26.934	1.00	0.00	RX0	O
ATOM	1863	N	GLN	351	14.320	25.854	28.691	1.00	0.00	RX0	N
ATOM	1864	H	GLN	351	13.441	26.232	28.990	1.00	0.00	RX0	H
ATOM	1865	CA	GLN	351	15.341	25.538	29.706	1.00	0.00	RX0	C
ATOM	1866	CB	GLN	351	14.916	26.060	31.073	1.00	0.00	RX0	C
ATOM	1867	CG	GLN	351	14.891	27.583	31.151	1.00	0.00	RX0	C
ATOM	1868	CD	GLN	351	14.203	27.976	32.439	1.00	0.00	RX0	C
ATOM	1869	OE1	GLN	351	13.357	27.260	32.965	1.00	0.00	RX0	O
ATOM	1870	NE2	GLN	351	14.628	29.153	32.931	1.00	0.00	RX0	N
ATOM	1871	HE21	GLN	351	15.325	29.678	32.441	1.00	0.00	RX0	H
ATOM	1872	HE22	GLN	351	14.266	29.516	33.790	1.00	0.00	RX0	H
ATOM	1873	C	GLN	351	15.597	24.030	29.805	1.00	0.00	RX0	C
ATOM	1874	O	GLN	351	16.752	23.608	29.740	1.00	0.00	RX0	O
ATOM	1875	N	ARG	352	14.527	23.242	29.744	1.00	0.00	RX0	N
ATOM	1876	H	ARG	352	13.617	23.654	29.656	1.00	0.00	RX0	H
ATOM	1877	CA	ARG	352	14.627	21.772	29.816	1.00	0.00	RX0	C
ATOM	1878	CB	ARG	352	13.310	21.110	30.247	1.00	0.00	RX0	C
ATOM	1879	CG	ARG	352	13.414	19.586	30.434	1.00	0.00	RX0	C
ATOM	1880	CD	ARG	352	12.214	18.995	31.185	1.00	0.00	RX0	C
ATOM	1881	NE	ARG	352	12.181	17.530	31.160	1.00	0.00	RX0	N
ATOM	1882	HE	ARG	352	11.954	17.067	30.288	1.00	0.00	RX0	H
ATOM	1883	CZ	ARG	352	12.325	16.774	32.292	1.00	0.00	RX0	C
ATOM	1884	NH1	ARG	352	12.649	17.373	33.456	1.00	0.00	RX0	N
ATOM	1885	HH11	ARG	352	12.834	16.826	34.286	1.00	0.00	RX0	H
ATOM	1886	HH12	ARG	352	12.729	18.367	33.534	1.00	0.00	RX0	H
ATOM	1887	NH2	ARG	352	12.137	15.440	32.233	1.00	0.00	RX0	N
ATOM	1888	HH21	ARG	352	12.188	14.803	33.010	1.00	0.00	RX0	H
ATOM	1889	HH22	ARG	352	11.921	15.024	31.327	1.00	0.00	RX0	H

ATOM	1890	C	ARG	352	15.192	21.188	28.514	1.00	0.00	RX0	C
ATOM	1891	O	ARG	352	16.048	20.305	28.551	1.00	0.00	RX0	O
ATOM	1892	N	LEU	353	14.843	21.816	27.391	1.00	0.00	RX0	N
ATOM	1893	H	LEU	353	14.132	22.520	27.429	1.00	0.00	RX0	H
ATOM	1894	CA	LEU	353	15.400	21.446	26.079	1.00	0.00	RX0	C
ATOM	1895	CB	LEU	353	14.765	22.302	24.985	1.00	0.00	RX0	C
ATOM	1896	CG	LEU	353	15.234	21.922	23.581	1.00	0.00	RX0	C
ATOM	1897	CD1	LEU	353	14.794	20.509	23.199	1.00	0.00	RX0	C
ATOM	1898	CD2	LEU	353	14.809	22.959	22.542	1.00	0.00	RX0	C
ATOM	1899	C	LEU	353	16.930	21.610	26.063	1.00	0.00	RX0	C
ATOM	1900	O	LEU	353	17.658	20.692	25.694	1.00	0.00	RX0	O
ATOM	1901	N	ALA	354	17.375	22.739	26.614	1.00	0.00	RX0	N
ATOM	1902	H	ALA	354	16.705	23.421	26.916	1.00	0.00	RX0	H
ATOM	1903	CA	ALA	354	18.808	23.073	26.716	1.00	0.00	RX0	C
ATOM	1904	CB	ALA	354	18.993	24.518	27.181	1.00	0.00	RX0	C
ATOM	1905	C	ALA	354	19.540	22.141	27.690	1.00	0.00	RX0	C
ATOM	1906	O	ALA	354	20.574	21.567	27.334	1.00	0.00	RX0	O
ATOM	1907	N	GLN	355	18.893	21.844	28.811	1.00	0.00	RX0	N
ATOM	1908	H	GLN	355	18.018	22.306	28.967	1.00	0.00	RX0	H
ATOM	1909	CA	GLN	355	19.427	20.920	29.833	1.00	0.00	RX0	C
ATOM	1910	CB	GLN	355	18.564	20.902	31.085	1.00	0.00	RX0	C
ATOM	1911	CG	GLN	355	18.673	22.212	31.858	1.00	0.00	RX0	C
ATOM	1912	CD	GLN	355	17.985	22.040	33.190	1.00	0.00	RX0	C
ATOM	1913	OE1	GLN	355	18.452	21.294	34.051	1.00	0.00	RX0	O
ATOM	1914	NE2	GLN	355	16.862	22.766	33.308	1.00	0.00	RX0	N
ATOM	1915	HE21	GLN	355	16.568	23.334	32.536	1.00	0.00	RX0	H
ATOM	1916	HE22	GLN	355	16.298	22.781	34.134	1.00	0.00	RX0	H
ATOM	1917	C	GLN	355	19.631	19.504	29.273	1.00	0.00	RX0	C
ATOM	1918	O	GLN	355	20.705	18.919	29.430	1.00	0.00	RX0	O
ATOM	1919	N	LEU	356	18.681	19.086	28.443	1.00	0.00	RX0	N
ATOM	1920	H	LEU	356	17.883	19.669	28.276	1.00	0.00	RX0	H
ATOM	1921	CA	LEU	356	18.716	17.770	27.778	1.00	0.00	RX0	C
ATOM	1922	CB	LEU	356	17.350	17.404	27.202	1.00	0.00	RX0	C
ATOM	1923	CG	LEU	356	16.352	17.081	28.310	1.00	0.00	RX0	C
ATOM	1924	CD1	LEU	356	15.007	16.608	27.758	1.00	0.00	RX0	C
ATOM	1925	CD2	LEU	356	16.947	16.088	29.305	1.00	0.00	RX0	C
ATOM	1926	C	LEU	356	19.795	17.661	26.699	1.00	0.00	RX0	C
ATOM	1927	O	LEU	356	20.593	16.720	26.700	1.00	0.00	RX0	O
ATOM	1928	N	LEU	357	19.916	18.722	25.913	1.00	0.00	RX0	N
ATOM	1929	H	LEU	357	19.280	19.489	26.028	1.00	0.00	RX0	H
ATOM	1930	CA	LEU	357	20.885	18.765	24.803	1.00	0.00	RX0	C
ATOM	1931	CB	LEU	357	20.530	19.866	23.804	1.00	0.00	RX0	C
ATOM	1932	CG	LEU	357	19.160	19.676	23.150	1.00	0.00	RX0	C
ATOM	1933	CD1	LEU	357	18.824	20.831	22.207	1.00	0.00	RX0	C
ATOM	1934	CD2	LEU	357	19.026	18.317	22.465	1.00	0.00	RX0	C
ATOM	1935	C	LEU	357	22.335	18.932	25.264	1.00	0.00	RX0	C
ATOM	1936	O	LEU	357	23.247	18.345	24.683	1.00	0.00	RX0	O
ATOM	1937	N	LEU	358	22.501	19.595	26.404	1.00	0.00	RX0	N
ATOM	1938	H	LEU	358	21.707	20.033	26.830	1.00	0.00	RX0	H
ATOM	1939	CA	LEU	358	23.826	19.754	27.029	1.00	0.00	RX0	C
ATOM	1940	CB	LEU	358	23.803	20.839	28.104	1.00	0.00	RX0	C
ATOM	1941	CG	LEU	358	23.627	22.236	27.508	1.00	0.00	RX0	C
ATOM	1942	CD1	LEU	358	23.474	23.302	28.594	1.00	0.00	RX0	C
ATOM	1943	CD2	LEU	358	24.737	22.570	26.511	1.00	0.00	RX0	C
ATOM	1944	C	LEU	358	24.390	18.455	27.609	1.00	0.00	RX0	C
ATOM	1945	O	LEU	358	25.603	18.227	27.557	1.00	0.00	RX0	O
ATOM	1946	N	ILE	359	23.510	17.559	28.043	1.00	0.00	RX0	N
ATOM	1947	H	ILE	359	22.534	17.781	27.999	1.00	0.00	RX0	H
ATOM	1948	CA	ILE	359	23.928	16.212	28.495	1.00	0.00	RX0	C
ATOM	1949	CB	ILE	359	22.752	15.476	29.133	1.00	0.00	RX0	C
ATOM	1950	CG2	ILE	359	23.132	14.061	29.570	1.00	0.00	RX0	C

ATOM	1951	CG1	ILE	359	22.236	16.302	30.312	1.00	0.00	RX0	C
ATOM	1952	CD1	ILE	359	20.903	15.805	30.866	1.00	0.00	RX0	C
ATOM	1953	C	ILE	359	24.559	15.428	27.334	1.00	0.00	RX0	C
ATOM	1954	O	ILE	359	25.552	14.723	27.543	1.00	0.00	RX0	O
ATOM	1955	N	LEU	360	24.038	15.620	26.133	1.00	0.00	RX0	N
ATOM	1956	H	LEU	360	23.266	16.250	26.029	1.00	0.00	RX0	H
ATOM	1957	CA	LEU	360	24.601	14.974	24.928	1.00	0.00	RX0	C
ATOM	1958	CB	LEU	360	23.757	15.275	23.689	1.00	0.00	RX0	C
ATOM	1959	CG	LEU	360	22.272	14.949	23.883	1.00	0.00	RX0	C
ATOM	1960	CD1	LEU	360	21.461	15.293	22.637	1.00	0.00	RX0	C
ATOM	1961	CD2	LEU	360	22.034	13.505	24.323	1.00	0.00	RX0	C
ATOM	1962	C	LEU	360	26.079	15.308	24.694	1.00	0.00	RX0	C
ATOM	1963	O	LEU	360	26.843	14.455	24.246	1.00	0.00	RX0	O
ATOM	1964	N	SER	361	26.491	16.472	25.202	1.00	0.00	RX0	N
ATOM	1965	H	SER	361	25.850	17.142	25.585	1.00	0.00	RX0	H
ATOM	1966	CA	SER	361	27.906	16.894	25.202	1.00	0.00	RX0	C
ATOM	1967	CB	SER	361	27.873	18.374	25.514	1.00	0.00	RX0	C
ATOM	1968	OG	SER	361	26.623	18.831	24.978	1.00	0.00	RX0	O
ATOM	1969	HG	SER	361	26.656	18.692	24.040	1.00	0.00	RX0	H
ATOM	1970	C	SER	361	28.769	15.985	26.099	1.00	0.00	RX0	C
ATOM	1971	O	SER	361	29.797	15.456	25.681	1.00	0.00	RX0	O
ATOM	1972	N	HIS	362	28.237	15.704	27.286	1.00	0.00	RX0	N
ATOM	1973	H	HIS	362	27.305	16.021	27.457	1.00	0.00	RX0	H
ATOM	1974	CA	HIS	362	28.854	14.793	28.272	1.00	0.00	RX0	C
ATOM	1975	CB	HIS	362	28.099	14.895	29.600	1.00	0.00	RX0	C
ATOM	1976	CG	HIS	362	28.137	16.318	30.111	1.00	0.00	RX0	C
ATOM	1977	ND1	HIS	362	27.354	17.322	29.658	1.00	0.00	RX0	N
ATOM	1978	HD1	HIS	362	26.674	17.295	28.947	1.00	0.00	RX0	H
ATOM	1979	CD2	HIS	362	28.970	16.823	31.113	1.00	0.00	RX0	C
ATOM	1980	NE2	HIS	362	28.682	18.140	31.259	1.00	0.00	RX0	N
ATOM	1981	CE1	HIS	362	27.690	18.446	30.365	1.00	0.00	RX0	C
ATOM	1982	C	HIS	362	28.890	13.339	27.777	1.00	0.00	RX0	C
ATOM	1983	O	HIS	362	29.902	12.656	27.936	1.00	0.00	RX0	O
ATOM	1984	N	ILE	363	27.856	12.940	27.039	1.00	0.00	RX0	N
ATOM	1985	H	ILE	363	27.102	13.584	26.897	1.00	0.00	RX0	H
ATOM	1986	CA	ILE	363	27.773	11.584	26.446	1.00	0.00	RX0	C
ATOM	1987	CB	ILE	363	26.355	11.165	26.052	1.00	0.00	RX0	C
ATOM	1988	CG2	ILE	363	26.273	9.639	25.980	1.00	0.00	RX0	C
ATOM	1989	CG1	ILE	363	25.348	11.639	27.096	1.00	0.00	RX0	C
ATOM	1990	CD1	ILE	363	23.922	11.194	26.780	1.00	0.00	RX0	C
ATOM	1991	C	ILE	363	28.830	11.416	25.343	1.00	0.00	RX0	C
ATOM	1992	O	ILE	363	29.487	10.370	25.263	1.00	0.00	RX0	O
ATOM	1993	N	ARG	364	29.035	12.469	24.564	1.00	0.00	RX0	N
ATOM	1994	H	ARG	364	28.381	13.228	24.611	1.00	0.00	RX0	H
ATOM	1995	CA	ARG	364	30.102	12.498	23.547	1.00	0.00	RX0	C
ATOM	1996	CB	ARG	364	29.999	13.843	22.816	1.00	0.00	RX0	C
ATOM	1997	CG	ARG	364	30.595	13.993	21.410	1.00	0.00	RX0	C
ATOM	1998	CD	ARG	364	29.865	13.191	20.325	1.00	0.00	RX0	C
ATOM	1999	NE	ARG	364	28.426	13.149	20.576	1.00	0.00	RX0	N
ATOM	2000	HE	ARG	364	28.102	12.472	21.256	1.00	0.00	RX0	H
ATOM	2001	CZ	ARG	364	27.473	13.876	19.923	1.00	0.00	RX0	C
ATOM	2002	NH1	ARG	364	27.799	14.714	18.913	1.00	0.00	RX0	N
ATOM	2003	HH11	ARG	364	27.085	15.209	18.393	1.00	0.00	RX0	H
ATOM	2004	HH12	ARG	364	28.751	14.853	18.633	1.00	0.00	RX0	H
ATOM	2005	NH2	ARG	364	26.192	13.725	20.310	1.00	0.00	RX0	N
ATOM	2006	HH21	ARG	364	25.428	14.250	19.933	1.00	0.00	RX0	H
ATOM	2007	HH22	ARG	364	25.997	13.020	21.017	1.00	0.00	RX0	H
ATOM	2008	C	ARG	364	31.469	12.319	24.223	1.00	0.00	RX0	C
ATOM	2009	O	ARG	364	32.264	11.465	23.831	1.00	0.00	RX0	O
ATOM	2010	N	HIS	365	31.645	13.058	25.316	1.00	0.00	RX0	N
ATOM	2011	H	HIS	365	30.915	13.695	25.573	1.00	0.00	RX0	H

ATOM	2012	CA	HIS	365	32.879	13.027	26.115	1.00	0.00	RX0	C
ATOM	2013	CB	HIS	365	32.829	14.044	27.255	1.00	0.00	RX0	C
ATOM	2014	CG	HIS	365	34.197	14.150	27.886	1.00	0.00	RX0	C
ATOM	2015	ND1	HIS	365	35.202	14.871	27.355	1.00	0.00	RX0	N
ATOM	2016	HD1	HIS	365	35.169	15.402	26.531	1.00	0.00	RX0	H
ATOM	2017	CD2	HIS	365	34.646	13.554	29.069	1.00	0.00	RX0	C
ATOM	2018	NE2	HIS	365	35.940	13.925	29.242	1.00	0.00	RX0	N
ATOM	2019	CE1	HIS	365	36.280	14.736	28.189	1.00	0.00	RX0	C
ATOM	2020	C	HIS	365	33.175	11.617	26.649	1.00	0.00	RX0	C
ATOM	2021	O	HIS	365	34.274	11.105	26.425	1.00	0.00	RX0	O
ATOM	2022	N	MET	366	32.138	10.946	27.137	1.00	0.00	RX0	N
ATOM	2023	H	MET	366	31.255	11.416	27.204	1.00	0.00	RX0	H
ATOM	2024	CA	MET	366	32.267	9.572	27.662	1.00	0.00	RX0	C
ATOM	2025	CB	MET	366	31.018	9.146	28.435	1.00	0.00	RX0	C
ATOM	2026	CG	MET	366	30.743	10.023	29.655	1.00	0.00	RX0	C
ATOM	2027	SD	MET	366	29.413	9.385	30.686	1.00	0.00	RX0	S
ATOM	2028	CE	MET	366	28.112	9.370	29.446	1.00	0.00	RX0	C
ATOM	2029	C	MET	366	32.567	8.557	26.555	1.00	0.00	RX0	C
ATOM	2030	O	MET	366	33.398	7.674	26.741	1.00	0.00	RX0	O
ATOM	2031	N	SER	367	31.985	8.782	25.378	1.00	0.00	RX0	N
ATOM	2032	H	SER	367	31.333	9.533	25.277	1.00	0.00	RX0	H
ATOM	2033	CA	SER	367	32.231	7.934	24.196	1.00	0.00	RX0	C
ATOM	2034	CB	SER	367	31.168	8.259	23.165	1.00	0.00	RX0	C
ATOM	2035	OG	SER	367	29.908	8.085	23.815	1.00	0.00	RX0	O
ATOM	2036	HG	SER	367	29.671	8.920	24.206	1.00	0.00	RX0	H
ATOM	2037	C	SER	367	33.681	8.046	23.711	1.00	0.00	RX0	C
ATOM	2038	O	SER	367	34.361	7.038	23.567	1.00	0.00	RX0	O
ATOM	2039	N	ASN	368	34.193	9.278	23.688	1.00	0.00	RX0	N
ATOM	2040	H	ASN	368	33.596	10.051	23.913	1.00	0.00	RX0	H
ATOM	2041	CA	ASN	368	35.583	9.549	23.266	1.00	0.00	RX0	C
ATOM	2042	CB	ASN	368	35.863	11.036	23.063	1.00	0.00	RX0	C
ATOM	2043	CG	ASN	368	36.469	11.218	21.684	1.00	0.00	RX0	C
ATOM	2044	OD1	ASN	368	35.809	10.987	20.677	1.00	0.00	RX0	O
ATOM	2045	ND2	ASN	368	37.733	11.687	21.672	1.00	0.00	RX0	N
ATOM	2046	HD21	ASN	368	38.277	11.805	22.506	1.00	0.00	RX0	H
ATOM	2047	HD22	ASN	368	38.170	11.925	20.803	1.00	0.00	RX0	H
ATOM	2048	C	ASN	368	36.615	8.948	24.226	1.00	0.00	RX0	C
ATOM	2049	O	ASN	368	37.514	8.215	23.806	1.00	0.00	RX0	O
ATOM	2050	N	LYS	369	36.353	9.119	25.515	1.00	0.00	RX0	N
ATOM	2051	H	LYS	369	35.553	9.668	25.769	1.00	0.00	RX0	H
ATOM	2052	CA	LYS	369	37.205	8.554	26.580	1.00	0.00	RX0	C
ATOM	2053	CB	LYS	369	36.876	9.122	27.964	1.00	0.00	RX0	C
ATOM	2054	CG	LYS	369	36.982	10.645	28.081	1.00	0.00	RX0	C
ATOM	2055	CD	LYS	369	38.295	11.211	27.541	1.00	0.00	RX0	C
ATOM	2056	CE	LYS	369	39.549	10.668	28.228	1.00	0.00	RX0	C
ATOM	2057	NZ	LYS	369	40.700	11.000	27.385	1.00	0.00	RX0	N
ATOM	2058	HZ1	LYS	369	41.170	11.891	27.609	1.00	0.00	RX0	H
ATOM	2059	HZ2	LYS	369	41.387	10.214	27.344	1.00	0.00	RX0	H
ATOM	2060	HZ3	LYS	369	40.400	11.014	26.384	1.00	0.00	RX0	H
ATOM	2061	C	LYS	369	37.117	7.021	26.620	1.00	0.00	RX0	C
ATOM	2062	O	LYS	369	38.123	6.337	26.771	1.00	0.00	RX0	O
ATOM	2063	N	GLY	370	35.908	6.527	26.299	1.00	0.00	RX0	N
ATOM	2064	H	GLY	370	35.156	7.164	26.130	1.00	0.00	RX0	H
ATOM	2065	CA	GLY	370	35.597	5.090	26.225	1.00	0.00	RX0	C
ATOM	2066	C	GLY	370	36.324	4.429	25.048	1.00	0.00	RX0	C
ATOM	2067	O	GLY	370	36.946	3.386	25.214	1.00	0.00	RX0	O
ATOM	2068	N	MET	371	36.380	5.143	23.928	1.00	0.00	RX0	N
ATOM	2069	H	MET	371	35.916	6.029	23.909	1.00	0.00	RX0	H
ATOM	2070	CA	MET	371	37.103	4.709	22.719	1.00	0.00	RX0	C
ATOM	2071	CB	MET	371	36.782	5.582	21.504	1.00	0.00	RX0	C
ATOM	2072	CG	MET	371	35.348	5.424	20.998	1.00	0.00	RX0	C

ATOM	2073	SD	MET	371	34.939	3.734	20.528	1.00	0.00	RX0	S
ATOM	2074	CE	MET	371	35.955	3.618	19.048	1.00	0.00	RX0	C
ATOM	2075	C	MET	371	38.619	4.671	22.937	1.00	0.00	RX0	C
ATOM	2076	O	MET	371	39.258	3.669	22.631	1.00	0.00	RX0	O
ATOM	2077	N	GLU	372	39.117	5.692	23.643	1.00	0.00	RX0	N
ATOM	2078	H	GLU	372	38.529	6.477	23.850	1.00	0.00	RX0	H
ATOM	2079	CA	GLU	372	40.525	5.740	24.082	1.00	0.00	RX0	C
ATOM	2080	CB	GLU	372	40.818	7.039	24.835	1.00	0.00	RX0	C
ATOM	2081	CG	GLU	372	40.693	8.307	23.987	1.00	0.00	RX0	C
ATOM	2082	CD	GLU	372	40.876	9.510	24.888	1.00	0.00	RX0	C
ATOM	2083	OE1	GLU	372	40.106	10.467	24.794	1.00	0.00	RX0	O
ATOM	2084	OE2	GLU	372	41.766	9.490	25.735	1.00	0.00	RX0	O
ATOM	2085	C	GLU	372	40.884	4.541	24.968	1.00	0.00	RX0	C
ATOM	2086	O	GLU	372	41.865	3.853	24.723	1.00	0.00	RX0	O
ATOM	2087	N	HIS	373	40.006	4.291	25.945	1.00	0.00	RX0	N
ATOM	2088	H	HIS	373	39.222	4.904	26.042	1.00	0.00	RX0	H
ATOM	2089	CA	HIS	373	40.194	3.212	26.919	1.00	0.00	RX0	C
ATOM	2090	CB	HIS	373	39.264	3.435	28.121	1.00	0.00	RX0	C
ATOM	2091	CG	HIS	373	38.386	2.246	28.433	1.00	0.00	RX0	C
ATOM	2092	ND1	HIS	373	37.277	1.933	27.736	1.00	0.00	RX0	N
ATOM	2093	HD1	HIS	373	36.944	2.396	26.934	1.00	0.00	RX0	H
ATOM	2094	CD2	HIS	373	38.533	1.330	29.480	1.00	0.00	RX0	C
ATOM	2095	NE2	HIS	373	37.493	0.461	29.406	1.00	0.00	RX0	N
ATOM	2096	CE1	HIS	373	36.723	0.833	28.335	1.00	0.00	RX0	C
ATOM	2097	C	HIS	373	40.078	1.832	26.262	1.00	0.00	RX0	C
ATOM	2098	O	HIS	373	40.934	1.001	26.470	1.00	0.00	RX0	O
ATOM	2099	N	LEU	374	39.111	1.679	25.351	1.00	0.00	RX0	N
ATOM	2100	H	LEU	374	38.558	2.474	25.114	1.00	0.00	RX0	H
ATOM	2101	CA	LEU	374	38.919	0.408	24.629	1.00	0.00	RX0	C
ATOM	2102	CB	LEU	374	37.660	0.451	23.762	1.00	0.00	RX0	C
ATOM	2103	CG	LEU	374	36.364	0.297	24.555	1.00	0.00	RX0	C
ATOM	2104	CD1	LEU	374	35.135	0.610	23.701	1.00	0.00	RX0	C
ATOM	2105	CD2	LEU	374	36.272	-1.079	25.217	1.00	0.00	RX0	C
ATOM	2106	C	LEU	374	40.113	0.051	23.744	1.00	0.00	RX0	C
ATOM	2107	O	LEU	374	40.555	-1.109	23.754	1.00	0.00	RX0	O
ATOM	2108	N	TYR	375	40.664	1.068	23.103	1.00	0.00	RX0	N
ATOM	2109	H	TYR	375	40.302	1.989	23.265	1.00	0.00	RX0	H
ATOM	2110	CA	TYR	375	41.854	0.983	22.222	1.00	0.00	RX0	C
ATOM	2111	CB	TYR	375	42.044	2.271	21.421	1.00	0.00	RX0	C
ATOM	2112	CG	TYR	375	41.095	2.371	20.253	1.00	0.00	RX0	C
ATOM	2113	CD1	TYR	375	40.656	1.200	19.604	1.00	0.00	RX0	C
ATOM	2114	CE1	TYR	375	39.837	1.321	18.468	1.00	0.00	RX0	C
ATOM	2115	CD2	TYR	375	40.704	3.655	19.825	1.00	0.00	RX0	C
ATOM	2116	CE2	TYR	375	39.884	3.776	18.692	1.00	0.00	RX0	C
ATOM	2117	CZ	TYR	375	39.477	2.608	18.018	1.00	0.00	RX0	C
ATOM	2118	OH	TYR	375	38.710	2.729	16.876	1.00	0.00	RX0	O
ATOM	2119	HH	TYR	375	38.410	3.623	16.781	1.00	0.00	RX0	H
ATOM	2120	C	TYR	375	43.165	0.750	22.977	1.00	0.00	RX0	C
ATOM	2121	O	TYR	375	44.176	1.452	22.753	1.00	0.00	RX0	O
ATOM	2122	N	SER	376	43.162	-0.226	23.845	1.00	0.00	RX0	N
ATOM	2123	H	SER	376	42.296	-0.612	24.146	1.00	0.00	RX0	H
ATOM	2124	CA	SER	376	44.345	-0.646	24.634	1.00	0.00	RX0	C
ATOM	2125	CB	SER	376	44.680	0.426	25.682	1.00	0.00	RX0	C
ATOM	2126	OG	SER	376	44.037	1.668	25.377	1.00	0.00	RX0	O
ATOM	2127	HG	SER	376	44.377	1.963	24.536	1.00	0.00	RX0	H
ATOM	2128	C	SER	376	44.183	-1.992	25.350	1.00	0.00	RX0	C
ATOM	2129	O	SER	376	44.985	-2.369	26.199	1.00	0.00	RX0	O
ATOM	2130	N	MET	377	43.156	-2.746	24.938	1.00	0.00	RX0	N
ATOM	2131	H	MET	377	42.560	-2.429	24.199	1.00	0.00	RX0	H
ATOM	2132	CA	MET	377	42.915	-4.112	25.422	1.00	0.00	RX0	C
ATOM	2133	CB	MET	377	41.735	-4.070	26.399	1.00	0.00	RX0	C

ATOM	2134	CG	MET	377	42.038	-3.407	27.743	1.00	0.00	RX0	C
ATOM	2135	SD	MET	377	40.549	-3.243	28.736	1.00	0.00	RX0	S
ATOM	2136	CE	MET	377	39.730	-2.014	27.707	1.00	0.00	RX0	C
ATOM	2137	C	MET	377	42.647	-5.080	24.257	1.00	0.00	RX0	C
ATOM	2138	O	MET	377	43.092	-4.854	23.130	1.00	0.00	RX0	O
ATOM	2139	N	LYS	378	41.856	-6.107	24.527	1.00	0.00	RX0	N
ATOM	2140	H	LYS	378	41.482	-6.207	25.443	1.00	0.00	RX0	H
ATOM	2141	CA	LYS	378	41.570	-7.191	23.576	1.00	0.00	RX0	C
ATOM	2142	CB	LYS	378	41.441	-8.519	24.363	1.00	0.00	RX0	C
ATOM	2143	CG	LYS	378	40.450	-8.515	25.553	1.00	0.00	RX0	C
ATOM	2144	CD	LYS	378	40.408	-9.804	26.402	1.00	0.00	RX0	C
ATOM	2145	CE	LYS	378	39.453	-9.725	27.613	1.00	0.00	RX0	C
ATOM	2146	NZ	LYS	378	39.491	-10.965	28.417	1.00	0.00	RX0	N
ATOM	2147	HZ1	LYS	378	38.898	-10.894	29.278	1.00	0.00	RX0	H
ATOM	2148	HZ2	LYS	378	39.147	-11.762	27.844	1.00	0.00	RX0	H
ATOM	2149	HZ3	LYS	378	40.468	-11.160	28.709	1.00	0.00	RX0	H
ATOM	2150	C	LYS	378	40.316	-6.920	22.720	1.00	0.00	RX0	C
ATOM	2151	O	LYS	378	40.245	-7.352	21.611	1.00	0.00	RX0	O
ATOM	2152	N	CYS	379	39.320	-6.266	23.410	1.00	0.00	RX0	N
ATOM	2153	H	CYS	379	39.565	-5.888	24.297	1.00	0.00	RX0	H
ATOM	2154	CA	CYS	379	37.939	-6.053	22.985	1.00	0.00	RX0	C
ATOM	2155	CB	CYS	379	37.612	-4.559	23.097	1.00	0.00	RX0	C
ATOM	2156	SG	CYS	379	35.839	-4.200	23.180	1.00	0.00	RX0	S
ATOM	2157	C	CYS	379	37.599	-6.654	21.614	1.00	0.00	RX0	C
ATOM	2158	O	CYS	379	37.115	-7.773	21.559	1.00	0.00	RX0	O
ATOM	2159	N	LYS	380	38.012	-5.930	20.563	1.00	0.00	RX0	N
ATOM	2160	H	LYS	380	38.382	-5.003	20.639	1.00	0.00	RX0	H
ATOM	2161	CA	LYS	380	37.949	-6.383	19.165	1.00	0.00	RX0	C
ATOM	2162	CB	LYS	380	36.479	-6.763	18.911	1.00	0.00	RX0	C
ATOM	2163	CG	LYS	380	35.905	-7.108	17.535	1.00	0.00	RX0	C
ATOM	2164	CD	LYS	380	34.395	-7.306	17.730	1.00	0.00	RX0	C
ATOM	2165	CE	LYS	380	33.570	-7.597	16.475	1.00	0.00	RX0	C
ATOM	2166	NZ	LYS	380	32.156	-7.708	16.858	1.00	0.00	RX0	N
ATOM	2167	HZ1	LYS	380	31.504	-7.910	16.081	1.00	0.00	RX0	H
ATOM	2168	HZ2	LYS	380	31.976	-8.445	17.577	1.00	0.00	RX0	H
ATOM	2169	HZ3	LYS	380	31.793	-6.865	17.355	1.00	0.00	RX0	H
ATOM	2170	C	LYS	380	38.311	-5.215	18.248	1.00	0.00	RX0	C
ATOM	2171	O	LYS	380	37.971	-4.057	18.518	1.00	0.00	RX0	O
ATOM	2172	N	ASN	381	38.881	-5.592	17.123	1.00	0.00	RX0	N
ATOM	2173	H	ASN	381	39.194	-6.538	17.053	1.00	0.00	RX0	H
ATOM	2174	CA	ASN	381	39.208	-4.700	15.996	1.00	0.00	RX0	C
ATOM	2175	CB	ASN	381	40.328	-3.703	16.345	1.00	0.00	RX0	C
ATOM	2176	CG	ASN	381	40.428	-2.627	15.271	1.00	0.00	RX0	C
ATOM	2177	OD1	ASN	381	39.460	-1.968	14.917	1.00	0.00	RX0	O
ATOM	2178	ND2	ASN	381	41.647	-2.483	14.742	1.00	0.00	RX0	N
ATOM	2179	HD21	ASN	381	42.462	-3.006	15.002	1.00	0.00	RX0	H
ATOM	2180	HD22	ASN	381	41.755	-1.821	14.005	1.00	0.00	RX0	H
ATOM	2181	C	ASN	381	39.620	-5.523	14.759	1.00	0.00	RX0	C
ATOM	2182	O	ASN	381	40.286	-5.062	13.834	1.00	0.00	RX0	O
ATOM	2183	N	VAL	382	39.176	-6.776	14.737	1.00	0.00	RX0	N
ATOM	2184	H	VAL	382	38.427	-7.000	15.352	1.00	0.00	RX0	H
ATOM	2185	CA	VAL	382	39.338	-7.659	13.572	1.00	0.00	RX0	C
ATOM	2186	CB	VAL	382	39.226	-9.119	14.010	1.00	0.00	RX0	C
ATOM	2187	CG1	VAL	382	39.385	-10.078	12.828	1.00	0.00	RX0	C
ATOM	2188	CG2	VAL	382	40.220	-9.411	15.135	1.00	0.00	RX0	C
ATOM	2189	C	VAL	382	38.195	-7.277	12.635	1.00	0.00	RX0	C
ATOM	2190	O	VAL	382	37.049	-7.276	13.059	1.00	0.00	RX0	O
ATOM	2191	N	VAL	383	38.551	-7.063	11.363	1.00	0.00	RX0	N
ATOM	2192	H	VAL	383	39.526	-7.051	11.156	1.00	0.00	RX0	H
ATOM	2193	CA	VAL	383	37.600	-6.539	10.366	1.00	0.00	RX0	C
ATOM	2194	CB	VAL	383	36.433	-7.502	10.106	1.00	0.00	RX0	C

ATOM	2195	CG1	VAL	383	35.393	-6.884	9.167	1.00	0.00	RX0	C
ATOM	2196	CG2	VAL	383	36.949	-8.847	9.590	1.00	0.00	RX0	C
ATOM	2197	C	VAL	383	37.102	-5.173	10.877	1.00	0.00	RX0	C
ATOM	2198	O	VAL	383	36.355	-5.097	11.860	1.00	0.00	RX0	O
ATOM	2199	N	PRO	384	37.530	-4.088	10.236	1.00	0.00	RX0	N
ATOM	2200	CD	PRO	384	38.342	-4.088	9.028	1.00	0.00	RX0	C
ATOM	2201	CA	PRO	384	37.142	-2.731	10.663	1.00	0.00	RX0	C
ATOM	2202	CB	PRO	384	37.724	-1.843	9.553	1.00	0.00	RX0	C
ATOM	2203	CG	PRO	384	37.979	-2.767	8.362	1.00	0.00	RX0	C
ATOM	2204	C	PRO	384	35.624	-2.628	10.828	1.00	0.00	RX0	C
ATOM	2205	O	PRO	384	34.847	-3.282	10.112	1.00	0.00	RX0	O
ATOM	2206	N	LEU	385	35.228	-1.769	11.750	1.00	0.00	RX0	N
ATOM	2207	H	LEU	385	35.906	-1.205	12.219	1.00	0.00	RX0	H
ATOM	2208	CA	LEU	385	33.812	-1.621	12.127	1.00	0.00	RX0	C
ATOM	2209	CB	LEU	385	33.671	-0.680	13.323	1.00	0.00	RX0	C
ATOM	2210	CG	LEU	385	32.275	-0.733	13.943	1.00	0.00	RX0	C
ATOM	2211	CD1	LEU	385	31.904	-2.145	14.400	1.00	0.00	RX0	C
ATOM	2212	CD2	LEU	385	32.117	0.289	15.065	1.00	0.00	RX0	C
ATOM	2213	C	LEU	385	32.896	-1.213	10.964	1.00	0.00	RX0	C
ATOM	2214	O	LEU	385	31.809	-1.761	10.815	1.00	0.00	RX0	O
ATOM	2215	N	TYR	386	33.449	-0.406	10.055	1.00	0.00	RX0	N
ATOM	2216	H	TYR	386	34.201	0.166	10.391	1.00	0.00	RX0	H
ATOM	2217	CA	TYR	386	32.718	0.069	8.867	1.00	0.00	RX0	C
ATOM	2218	CB	TYR	386	33.624	0.936	7.963	1.00	0.00	RX0	C
ATOM	2219	CG	TYR	386	32.855	1.688	6.882	1.00	0.00	RX0	C
ATOM	2220	CD1	TYR	386	32.587	3.063	7.064	1.00	0.00	RX0	C
ATOM	2221	CE1	TYR	386	31.869	3.768	6.080	1.00	0.00	RX0	C
ATOM	2222	CD2	TYR	386	32.430	1.014	5.715	1.00	0.00	RX0	C
ATOM	2223	CE2	TYR	386	31.706	1.715	4.732	1.00	0.00	RX0	C
ATOM	2224	CZ	TYR	386	31.427	3.085	4.927	1.00	0.00	RX0	C
ATOM	2225	OH	TYR	386	30.708	3.782	3.969	1.00	0.00	RX0	O
ATOM	2226	HH	TYR	386	30.406	3.202	3.275	1.00	0.00	RX0	H
ATOM	2227	C	TYR	386	32.144	-1.103	8.049	1.00	0.00	RX0	C
ATOM	2228	O	TYR	386	30.957	-1.141	7.757	1.00	0.00	RX0	O
ATOM	2229	N	ASP	387	32.969	-2.141	7.903	1.00	0.00	RX0	N
ATOM	2230	H	ASP	387	33.842	-2.142	8.392	1.00	0.00	RX0	H
ATOM	2231	CA	ASP	387	32.619	-3.323	7.093	1.00	0.00	RX0	C
ATOM	2232	CB	ASP	387	33.878	-4.092	6.719	1.00	0.00	RX0	C
ATOM	2233	CG	ASP	387	33.640	-4.872	5.445	1.00	0.00	RX0	C
ATOM	2234	OD1	ASP	387	33.791	-6.087	5.454	1.00	0.00	RX0	O
ATOM	2235	OD2	ASP	387	33.327	-4.276	4.418	1.00	0.00	RX0	O
ATOM	2236	C	ASP	387	31.563	-4.207	7.765	1.00	0.00	RX0	C
ATOM	2237	O	ASP	387	30.619	-4.649	7.114	1.00	0.00	RX0	O
ATOM	2238	N	LEU	388	31.677	-4.332	9.088	1.00	0.00	RX0	N
ATOM	2239	H	LEU	388	32.445	-3.871	9.536	1.00	0.00	RX0	H
ATOM	2240	CA	LEU	388	30.691	-5.072	9.891	1.00	0.00	RX0	C
ATOM	2241	CB	LEU	388	31.205	-5.219	11.324	1.00	0.00	RX0	C
ATOM	2242	CG	LEU	388	30.278	-6.048	12.214	1.00	0.00	RX0	C
ATOM	2243	CD1	LEU	388	30.088	-7.467	11.675	1.00	0.00	RX0	C
ATOM	2244	CD2	LEU	388	30.738	-6.043	13.673	1.00	0.00	RX0	C
ATOM	2245	C	LEU	388	29.315	-4.386	9.871	1.00	0.00	RX0	C
ATOM	2246	O	LEU	388	28.299	-5.023	9.588	1.00	0.00	RX0	O
ATOM	2247	N	LEU	389	29.339	-3.070	10.049	1.00	0.00	RX0	N
ATOM	2248	H	LEU	389	30.226	-2.625	10.188	1.00	0.00	RX0	H
ATOM	2249	CA	LEU	389	28.129	-2.229	9.970	1.00	0.00	RX0	C
ATOM	2250	CB	LEU	389	28.427	-0.786	10.359	1.00	0.00	RX0	C
ATOM	2251	CG	LEU	389	28.860	-0.632	11.813	1.00	0.00	RX0	C
ATOM	2252	CD1	LEU	389	29.250	0.810	12.111	1.00	0.00	RX0	C
ATOM	2253	CD2	LEU	389	27.803	-1.135	12.793	1.00	0.00	RX0	C
ATOM	2254	C	LEU	389	27.489	-2.268	8.583	1.00	0.00	RX0	C
ATOM	2255	O	LEU	389	26.284	-2.473	8.458	1.00	0.00	RX0	O

ATOM	2256	N	LEU	390	28.350	-2.246	7.565	1.00	0.00	RX0	N
ATOM	2257	H	LEU	390	29.326	-2.139	7.759	1.00	0.00	RX0	H
ATOM	2258	CA	LEU	390	27.929	-2.335	6.162	1.00	0.00	RX0	C
ATOM	2259	CB	LEU	390	29.166	-2.238	5.277	1.00	0.00	RX0	C
ATOM	2260	CG	LEU	390	28.844	-2.140	3.793	1.00	0.00	RX0	C
ATOM	2261	CD1	LEU	390	28.538	-0.697	3.398	1.00	0.00	RX0	C
ATOM	2262	CD2	LEU	390	29.931	-2.795	2.943	1.00	0.00	RX0	C
ATOM	2263	C	LEU	390	27.202	-3.653	5.862	1.00	0.00	RX0	C
ATOM	2264	O	LEU	390	26.123	-3.627	5.302	1.00	0.00	RX0	O
ATOM	2265	N	GLU	391	27.733	-4.754	6.404	1.00	0.00	RX0	N
ATOM	2266	H	GLU	391	28.650	-4.708	6.804	1.00	0.00	RX0	H
ATOM	2267	CA	GLU	391	27.124	-6.090	6.264	1.00	0.00	RX0	C
ATOM	2268	CB	GLU	391	28.039	-7.170	6.854	1.00	0.00	RX0	C
ATOM	2269	CG	GLU	391	28.355	-8.303	5.865	1.00	0.00	RX0	C
ATOM	2270	CD	GLU	391	29.445	-7.901	4.883	1.00	0.00	RX0	C
ATOM	2271	OE1	GLU	391	30.451	-8.592	4.780	1.00	0.00	RX0	O
ATOM	2272	OE2	GLU	391	29.350	-6.869	4.232	1.00	0.00	RX0	O
ATOM	2273	C	GLU	391	25.713	-6.159	6.867	1.00	0.00	RX0	C
ATOM	2274	O	GLU	391	24.778	-6.600	6.214	1.00	0.00	RX0	O
ATOM	2275	N	MET	392	25.589	-5.588	8.070	1.00	0.00	RX0	N
ATOM	2276	H	MET	392	26.415	-5.175	8.462	1.00	0.00	RX0	H
ATOM	2277	CA	MET	392	24.313	-5.555	8.807	1.00	0.00	RX0	C
ATOM	2278	CB	MET	392	24.545	-5.230	10.282	1.00	0.00	RX0	C
ATOM	2279	CG	MET	392	25.246	-6.385	11.000	1.00	0.00	RX0	C
ATOM	2280	SD	MET	392	25.411	-6.129	12.774	1.00	0.00	RX0	S
ATOM	2281	CE	MET	392	26.588	-4.772	12.719	1.00	0.00	RX0	C
ATOM	2282	C	MET	392	23.266	-4.630	8.173	1.00	0.00	RX0	C
ATOM	2283	O	MET	392	22.107	-5.006	8.028	1.00	0.00	RX0	O
ATOM	2284	N	LEU	393	23.721	-3.452	7.754	1.00	0.00	RX0	N
ATOM	2285	H	LEU	393	24.706	-3.280	7.791	1.00	0.00	RX0	H
ATOM	2286	CA	LEU	393	22.852	-2.457	7.099	1.00	0.00	RX0	C
ATOM	2287	CB	LEU	393	23.509	-1.081	7.154	1.00	0.00	RX0	C
ATOM	2288	CG	LEU	393	23.439	-0.452	8.543	1.00	0.00	RX0	C
ATOM	2289	CD1	LEU	393	24.493	0.636	8.723	1.00	0.00	RX0	C
ATOM	2290	CD2	LEU	393	22.032	0.058	8.856	1.00	0.00	RX0	C
ATOM	2291	C	LEU	393	22.462	-2.815	5.663	1.00	0.00	RX0	C
ATOM	2292	O	LEU	393	21.313	-2.601	5.257	1.00	0.00	RX0	O
ATOM	2293	N	ASP	394	23.400	-3.384	4.923	1.00	0.00	RX0	N
ATOM	2294	H	ASP	394	24.268	-3.665	5.328	1.00	0.00	RX0	H
ATOM	2295	CA	ASP	394	23.187	-3.768	3.516	1.00	0.00	RX0	C
ATOM	2296	CB	ASP	394	24.214	-3.604	2.407	1.00	0.00	RX0	C
ATOM	2297	CG	ASP	394	23.377	-3.478	1.116	1.00	0.00	RX0	C
ATOM	2298	OD1	ASP	394	22.141	-3.384	1.181	1.00	0.00	RX0	O
ATOM	2299	OD2	ASP	394	23.953	-3.452	0.031	1.00	0.00	RX0	O
ATOM	2300	C	ASP	394	22.492	-5.132	3.432	1.00	0.00	RX0	C
ATOM	2301	O	ASP	394	23.034	-6.108	2.888	1.00	0.00	RX0	O
ATOM	2302	N	ALA	395	21.256	-5.134	3.874	1.00	0.00	RX0	N
ATOM	2303	H	ALA	395	20.895	-4.232	4.113	1.00	0.00	RX0	H
ATOM	2304	CA	ALA	395	20.412	-6.338	3.880	1.00	0.00	RX0	C
ATOM	2305	CB	ALA	395	19.421	-6.294	5.044	1.00	0.00	RX0	C
ATOM	2306	C	ALA	395	19.639	-6.454	2.565	1.00	0.00	RX0	C
ATOM	2307	O	ALA	395	19.303	-5.463	1.911	1.00	0.00	RX0	O
ATOM	2308	N	HIS	396	19.441	-7.696	2.156	1.00	0.00	RX0	N
ATOM	2309	H	HIS	396	19.740	-8.409	2.788	1.00	0.00	RX0	H
ATOM	2310	CA	HIS	396	18.536	-7.999	1.035	1.00	0.00	RX0	C
ATOM	2311	CB	HIS	396	18.854	-9.400	0.508	1.00	0.00	RX0	C
ATOM	2312	CG	HIS	396	19.020	-10.348	1.676	1.00	0.00	RX0	C
ATOM	2313	ND1	HIS	396	20.216	-10.780	2.116	1.00	0.00	RX0	N
ATOM	2314	HD1	HIS	396	21.099	-10.558	1.744	1.00	0.00	RX0	H
ATOM	2315	CD2	HIS	396	18.024	-10.900	2.489	1.00	0.00	RX0	C
ATOM	2316	NE2	HIS	396	18.632	-11.666	3.423	1.00	0.00	RX0	N

ATOM	2317	CE1	HIS	396	19.984	-11.595	3.195	1.00	0.00	RX0	C
ATOM	2318	C	HIS	396	17.077	-7.909	1.523	1.00	0.00	RX0	C
ATOM	2319	O	HIS	396	16.821	-7.755	2.721	1.00	0.00	RX0	O
ATOM	2320	N	ARG	397	16.142	-8.174	0.629	1.00	0.00	RX0	N
ATOM	2321	H	ARG	397	16.384	-8.371	-0.320	1.00	0.00	RX0	H
ATOM	2322	CA	ARG	397	14.704	-8.151	0.982	1.00	0.00	RX0	C
ATOM	2323	CB	ARG	397	14.152	-6.914	0.225	1.00	0.00	RX0	C
ATOM	2324	CG	ARG	397	14.958	-5.598	0.515	1.00	0.00	RX0	C
ATOM	2325	CD	ARG	397	16.050	-5.127	-0.497	1.00	0.00	RX0	C
ATOM	2326	NE	ARG	397	17.296	-4.619	0.129	1.00	0.00	RX0	N
ATOM	2327	HE	ARG	397	17.477	-4.806	1.105	1.00	0.00	RX0	H
ATOM	2328	CZ	ARG	397	18.306	-4.030	-0.614	1.00	0.00	RX0	C
ATOM	2329	NH1	ARG	397	18.052	-3.676	-1.894	1.00	0.00	RX0	N
ATOM	2330	HH11	ARG	397	18.769	-3.285	-2.479	1.00	0.00	RX0	H
ATOM	2331	HH12	ARG	397	17.142	-3.788	-2.302	1.00	0.00	RX0	H
ATOM	2332	NH2	ARG	397	19.542	-3.822	-0.080	1.00	0.00	RX0	N
ATOM	2333	HH21	ARG	397	20.331	-3.365	-0.515	1.00	0.00	RX0	H
ATOM	2334	HH22	ARG	397	19.788	-4.157	0.846	1.00	0.00	RX0	H
ATOM	2335	C	ARG	397	14.082	-9.540	0.774	1.00	0.00	RX0	C
ATOM	2336	O	ARG	397	12.875	-9.706	0.613	1.00	0.00	RX0	O
ATOM	2337	N	LEU	398	14.938	-10.556	0.835	1.00	0.00	RX0	N
ATOM	2338	H	LEU	398	15.870	-10.365	1.130	1.00	0.00	RX0	H
ATOM	2339	CA	LEU	398	14.565	-11.951	0.542	1.00	0.00	RX0	C
ATOM	2340	CB	LEU	398	15.800	-12.794	0.229	1.00	0.00	RX0	C
ATOM	2341	CG	LEU	398	16.579	-12.267	-0.977	1.00	0.00	RX0	C
ATOM	2342	CD1	LEU	398	17.868	-13.057	-1.205	1.00	0.00	RX0	C
ATOM	2343	CD2	LEU	398	15.711	-12.204	-2.236	1.00	0.00	RX0	C
ATOM	2344	C	LEU	398	13.757	-12.584	1.680	1.00	0.00	RX0	C
ATOM	2345	O	LEU	398	12.772	-13.259	1.439	1.00	0.00	RX0	O
ATOM	2346	N	HIS	399	14.100	-12.169	2.904	1.00	0.00	RX0	N
ATOM	2347	H	HIS	399	14.845	-11.516	3.013	1.00	0.00	RX0	H
ATOM	2348	CA	HIS	399	13.392	-12.616	4.118	1.00	0.00	RX0	C
ATOM	2349	CB	HIS	399	14.353	-12.618	5.306	1.00	0.00	RX0	C
ATOM	2350	CG	HIS	399	15.413	-13.664	5.054	1.00	0.00	RX0	C
ATOM	2351	ND1	HIS	399	16.718	-13.393	4.850	1.00	0.00	RX0	N
ATOM	2352	HD1	HIS	399	17.157	-12.514	4.841	1.00	0.00	RX0	H
ATOM	2353	CD2	HIS	399	15.225	-15.046	4.972	1.00	0.00	RX0	C
ATOM	2354	NE2	HIS	399	16.432	-15.606	4.715	1.00	0.00	RX0	N
ATOM	2355	CE1	HIS	399	17.349	-14.591	4.641	1.00	0.00	RX0	C
ATOM	2356	C	HIS	399	12.131	-11.786	4.401	1.00	0.00	RX0	C
ATOM	2357	O	HIS	399	11.630	-11.743	5.524	1.00	0.00	RX0	O
ATOM	2358	N	ALA	400	11.619	-11.143	3.351	1.00	0.00	RX0	N
ATOM	2359	H	ALA	400	11.998	-11.316	2.444	1.00	0.00	RX0	H
ATOM	2360	CA	ALA	400	10.356	-10.385	3.402	1.00	0.00	RX0	C
ATOM	2361	CB	ALA	400	10.144	-9.552	2.138	1.00	0.00	RX0	C
ATOM	2362	C	ALA	400	9.147	-11.323	3.579	1.00	0.00	RX0	C
ATOM	2363	O	ALA	400	8.508	-11.220	4.647	1.00	0.00	RX0	O
ATOM	2364	N	SER	22	-16.808	-6.482	42.503	1.00	0.00	RX1	N
ATOM	2365	H	SER	22	-16.388	-6.484	41.593	1.00	0.00	RX1	H
ATOM	2366	CA	SER	22	-16.954	-7.844	43.019	1.00	0.00	RX1	C
ATOM	2367	CB	SER	22	-18.369	-8.334	42.663	1.00	0.00	RX1	C
ATOM	2368	OG	SER	22	-18.748	-9.459	43.463	1.00	0.00	RX1	O
ATOM	2369	HG	SER	22	-18.072	-10.112	43.298	1.00	0.00	RX1	H
ATOM	2370	C	SER	22	-15.852	-8.761	42.510	1.00	0.00	RX1	C
ATOM	2371	O	SER	22	-16.031	-9.964	42.353	1.00	0.00	RX1	O
ATOM	2372	N	THR	23	-14.716	-8.119	42.226	1.00	0.00	RX1	N
ATOM	2373	H	THR	23	-14.568	-7.141	42.364	1.00	0.00	RX1	H
ATOM	2374	CA	THR	23	-13.675	-8.798	41.466	1.00	0.00	RX1	C
ATOM	2375	CB	THR	23	-14.064	-8.416	40.046	1.00	0.00	RX1	C
ATOM	2376	OG1	THR	23	-15.114	-7.431	40.142	1.00	0.00	RX1	O
ATOM	2377	HG1	THR	23	-15.290	-7.157	39.251	1.00	0.00	RX1	H

ATOM	2378	CG2	THR	23	-14.546	-9.605	39.211	1.00	0.00	RX1	C
ATOM	2379	C	THR	23	-12.316	-8.272	41.883	1.00	0.00	RX1	C
ATOM	2380	O	THR	23	-12.198	-7.174	42.414	1.00	0.00	RX1	O
ATOM	2381	N	GLN	24	-11.287	-9.085	41.603	1.00	0.00	RX1	N
ATOM	2382	H	GLN	24	-11.453	-9.984	41.197	1.00	0.00	RX1	H
ATOM	2383	CA	GLN	24	-9.930	-8.550	41.711	1.00	0.00	RX1	C
ATOM	2384	CB	GLN	24	-8.913	-9.699	41.721	1.00	0.00	RX1	C
ATOM	2385	CG	GLN	24	-7.452	-9.337	41.413	1.00	0.00	RX1	C
ATOM	2386	CD	GLN	24	-6.830	-8.405	42.433	1.00	0.00	RX1	C
ATOM	2387	OE1	GLN	24	-7.487	-7.575	43.053	1.00	0.00	RX1	O
ATOM	2388	NE2	GLN	24	-5.501	-8.574	42.548	1.00	0.00	RX1	N
ATOM	2389	HE21	GLN	24	-5.008	-9.280	42.027	1.00	0.00	RX1	H
ATOM	2390	HE22	GLN	24	-4.944	-7.998	43.146	1.00	0.00	RX1	H
ATOM	2391	C	GLN	24	-9.660	-7.544	40.603	1.00	0.00	RX1	C
ATOM	2392	O	GLN	24	-9.600	-7.883	39.425	1.00	0.00	RX1	O
ATOM	2393	N	VAL	25	-9.546	-6.286	41.035	1.00	0.00	RX1	N
ATOM	2394	H	VAL	25	-9.471	-6.071	42.011	1.00	0.00	RX1	H
ATOM	2395	CA	VAL	25	-9.387	-5.206	40.066	1.00	0.00	RX1	C
ATOM	2396	CB	VAL	25	-10.612	-4.283	40.116	1.00	0.00	RX1	C
ATOM	2397	CG1	VAL	25	-10.431	-3.053	39.229	1.00	0.00	RX1	C
ATOM	2398	CG2	VAL	25	-11.898	-5.037	39.774	1.00	0.00	RX1	C
ATOM	2399	C	VAL	25	-8.127	-4.427	40.384	1.00	0.00	RX1	C
ATOM	2400	O	VAL	25	-7.909	-4.012	41.515	1.00	0.00	RX1	O
ATOM	2401	N	CYS	26	-7.309	-4.236	39.347	1.00	0.00	RX1	N
ATOM	2402	H	CYS	26	-7.515	-4.521	38.407	1.00	0.00	RX1	H
ATOM	2403	CA	CYS	26	-6.153	-3.376	39.574	1.00	0.00	RX1	C
ATOM	2404	CB	CYS	26	-4.884	-4.214	39.735	1.00	0.00	RX1	C
ATOM	2405	SG	CYS	26	-4.418	-5.109	38.234	1.00	0.00	RX1	S
ATOM	2406	C	CYS	26	-6.051	-2.369	38.456	1.00	0.00	RX1	C
ATOM	2407	O	CYS	26	-6.747	-2.476	37.455	1.00	0.00	RX1	O
ATOM	2408	N	THR	27	-5.174	-1.380	38.644	1.00	0.00	RX1	N
ATOM	2409	H	THR	27	-4.622	-1.223	39.460	1.00	0.00	RX1	H
ATOM	2410	CA	THR	27	-4.887	-0.550	37.480	1.00	0.00	RX1	C
ATOM	2411	CB	THR	27	-4.301	0.712	38.073	1.00	0.00	RX1	C
ATOM	2412	OG1	THR	27	-4.917	0.904	39.354	1.00	0.00	RX1	O
ATOM	2413	HG1	THR	27	-4.734	1.804	39.596	1.00	0.00	RX1	H
ATOM	2414	CG2	THR	27	-4.455	1.940	37.174	1.00	0.00	RX1	C
ATOM	2415	C	THR	27	-3.927	-1.271	36.558	1.00	0.00	RX1	C
ATOM	2416	O	THR	27	-3.163	-2.119	36.999	1.00	0.00	RX1	O
ATOM	2417	N	GLY	28	-3.996	-0.912	35.278	1.00	0.00	RX1	N
ATOM	2418	H	GLY	28	-4.555	-0.133	34.993	1.00	0.00	RX1	H
ATOM	2419	CA	GLY	28	-2.938	-1.409	34.410	1.00	0.00	RX1	C
ATOM	2420	C	GLY	28	-1.810	-0.416	34.395	1.00	0.00	RX1	C
ATOM	2421	O	GLY	28	-1.886	0.626	35.041	1.00	0.00	RX1	O
ATOM	2422	N	THR	29	-0.774	-0.750	33.630	1.00	0.00	RX1	N
ATOM	2423	H	THR	29	-0.715	-1.584	33.075	1.00	0.00	RX1	H
ATOM	2424	CA	THR	29	0.125	0.359	33.360	1.00	0.00	RX1	C
ATOM	2425	CB	THR	29	1.554	-0.176	33.336	1.00	0.00	RX1	C
ATOM	2426	OG1	THR	29	1.614	-1.403	32.607	1.00	0.00	RX1	O
ATOM	2427	HG1	THR	29	1.603	-1.130	31.684	1.00	0.00	RX1	H
ATOM	2428	CG2	THR	29	2.078	-0.400	34.754	1.00	0.00	RX1	C
ATOM	2429	C	THR	29	-0.301	1.092	32.103	1.00	0.00	RX1	C
ATOM	2430	O	THR	29	-1.182	0.640	31.375	1.00	0.00	RX1	O
ATOM	2431	N	ASP	30	0.353	2.242	31.908	1.00	0.00	RX1	N
ATOM	2432	H	ASP	30	1.026	2.627	32.535	1.00	0.00	RX1	H
ATOM	2433	CA	ASP	30	0.286	2.872	30.598	1.00	0.00	RX1	C
ATOM	2434	CB	ASP	30	-0.788	3.957	30.512	1.00	0.00	RX1	C
ATOM	2435	CG	ASP	30	-1.039	4.302	29.052	1.00	0.00	RX1	C
ATOM	2436	OD1	ASP	30	-0.424	3.699	28.175	1.00	0.00	RX1	O
ATOM	2437	OD2	ASP	30	-1.877	5.156	28.783	1.00	0.00	RX1	O
ATOM	2438	C	ASP	30	1.654	3.421	30.266	1.00	0.00	RX1	C

ATOM	2439	O	ASP	30	2.172	4.320	30.933	1.00	0.00	RX1	O
ATOM	2440	N	MET	31	2.260	2.796	29.255	1.00	0.00	RX1	N
ATOM	2441	H	MET	31	1.803	2.004	28.845	1.00	0.00	RX1	H
ATOM	2442	CA	MET	31	3.618	3.182	28.900	1.00	0.00	RX1	C
ATOM	2443	CB	MET	31	4.638	2.505	29.825	1.00	0.00	RX1	C
ATOM	2444	CG	MET	31	4.890	1.018	29.590	1.00	0.00	RX1	C
ATOM	2445	SD	MET	31	6.083	0.362	30.764	1.00	0.00	RX1	S
ATOM	2446	CE	MET	31	5.171	0.758	32.262	1.00	0.00	RX1	C
ATOM	2447	C	MET	31	4.021	3.034	27.448	1.00	0.00	RX1	C
ATOM	2448	O	MET	31	5.061	3.541	27.049	1.00	0.00	RX1	O
ATOM	2449	N	LYS	32	3.201	2.306	26.673	1.00	0.00	RX1	N
ATOM	2450	H	LYS	32	2.394	1.872	27.074	1.00	0.00	RX1	H
ATOM	2451	CA	LYS	32	3.691	1.847	25.368	1.00	0.00	RX1	C
ATOM	2452	CB	LYS	32	3.731	2.962	24.310	1.00	0.00	RX1	C
ATOM	2453	CG	LYS	32	2.329	3.376	23.856	1.00	0.00	RX1	C
ATOM	2454	CD	LYS	32	2.260	3.678	22.356	1.00	0.00	RX1	C
ATOM	2455	CE	LYS	32	0.824	3.898	21.869	1.00	0.00	RX1	C
ATOM	2456	NZ	LYS	32	0.805	4.083	20.415	1.00	0.00	RX1	N
ATOM	2457	HZ1	LYS	32	0.776	3.191	19.880	1.00	0.00	RX1	H
ATOM	2458	HZ2	LYS	32	-0.074	4.542	20.096	1.00	0.00	RX1	H
ATOM	2459	HZ3	LYS	32	1.602	4.639	20.050	1.00	0.00	RX1	H
ATOM	2460	C	LYS	32	5.011	1.095	25.508	1.00	0.00	RX1	C
ATOM	2461	O	LYS	32	5.265	0.486	26.540	1.00	0.00	RX1	O
ATOM	2462	N	LEU	33	5.879	1.161	24.491	1.00	0.00	RX1	N
ATOM	2463	H	LEU	33	5.759	1.674	23.642	1.00	0.00	RX1	H
ATOM	2464	CA	LEU	33	7.134	0.454	24.746	1.00	0.00	RX1	C
ATOM	2465	CB	LEU	33	7.694	-0.138	23.459	1.00	0.00	RX1	C
ATOM	2466	CG	LEU	33	6.662	-0.965	22.702	1.00	0.00	RX1	C
ATOM	2467	CD1	LEU	33	7.073	-1.309	21.275	1.00	0.00	RX1	C
ATOM	2468	CD2	LEU	33	6.322	-2.222	23.474	1.00	0.00	RX1	C
ATOM	2469	C	LEU	33	8.201	1.251	25.483	1.00	0.00	RX1	C
ATOM	2470	O	LEU	33	9.393	1.061	25.280	1.00	0.00	RX1	O
ATOM	2471	N	ARG	34	7.737	2.154	26.368	1.00	0.00	RX1	N
ATOM	2472	H	ARG	34	6.759	2.232	26.565	1.00	0.00	RX1	H
ATOM	2473	CA	ARG	34	8.680	2.932	27.172	1.00	0.00	RX1	C
ATOM	2474	CB	ARG	34	7.887	3.906	28.057	1.00	0.00	RX1	C
ATOM	2475	CG	ARG	34	8.605	4.615	29.205	1.00	0.00	RX1	C
ATOM	2476	CD	ARG	34	7.776	5.766	29.795	1.00	0.00	RX1	C
ATOM	2477	NE	ARG	34	6.424	5.364	30.195	1.00	0.00	RX1	N
ATOM	2478	HE	ARG	34	6.327	4.432	30.555	1.00	0.00	RX1	H
ATOM	2479	CZ	ARG	34	5.416	6.293	30.104	1.00	0.00	RX1	C
ATOM	2480	NH1	ARG	34	5.672	7.500	29.573	1.00	0.00	RX1	N
ATOM	2481	HH11	ARG	34	5.048	8.281	29.576	1.00	0.00	RX1	H
ATOM	2482	HH12	ARG	34	6.530	7.710	29.065	1.00	0.00	RX1	H
ATOM	2483	NH2	ARG	34	4.164	6.011	30.534	1.00	0.00	RX1	N
ATOM	2484	HH21	ARG	34	3.417	6.678	30.483	1.00	0.00	RX1	H
ATOM	2485	HH22	ARG	34	3.875	5.115	30.900	1.00	0.00	RX1	H
ATOM	2486	C	ARG	34	9.667	2.080	27.952	1.00	0.00	RX1	C
ATOM	2487	O	ARG	34	9.364	1.492	28.984	1.00	0.00	RX1	O
ATOM	2488	N	LEU	35	10.885	2.067	27.387	1.00	0.00	RX1	N
ATOM	2489	H	LEU	35	10.971	2.445	26.465	1.00	0.00	RX1	H
ATOM	2490	CA	LEU	35	12.027	1.416	28.024	1.00	0.00	RX1	C
ATOM	2491	CB	LEU	35	13.288	1.838	27.256	1.00	0.00	RX1	C
ATOM	2492	CG	LEU	35	14.534	0.984	27.499	1.00	0.00	RX1	C
ATOM	2493	CD1	LEU	35	14.333	-0.452	27.032	1.00	0.00	RX1	C
ATOM	2494	CD2	LEU	35	15.775	1.578	26.834	1.00	0.00	RX1	C
ATOM	2495	C	LEU	35	12.131	1.760	29.506	1.00	0.00	RX1	C
ATOM	2496	O	LEU	35	12.194	2.925	29.874	1.00	0.00	RX1	O
ATOM	2497	N	PRO	36	12.116	0.713	30.359	1.00	0.00	RX1	N
ATOM	2498	CD	PRO	36	11.971	-0.690	30.009	1.00	0.00	RX1	C
ATOM	2499	CA	PRO	36	12.204	0.950	31.805	1.00	0.00	RX1	C

ATOM	2500	CB	PRO	36	12.139	-0.470	32.371	1.00	0.00	RX1	C
ATOM	2501	CG	PRO	36	11.475	-1.326	31.295	1.00	0.00	RX1	C
ATOM	2502	C	PRO	36	13.476	1.673	32.222	1.00	0.00	RX1	C
ATOM	2503	O	PRO	36	14.541	1.430	31.667	1.00	0.00	RX1	O
ATOM	2504	N	ALA	37	13.318	2.546	33.233	1.00	0.00	RX1	N
ATOM	2505	H	ALA	37	12.455	2.652	33.725	1.00	0.00	RX1	H
ATOM	2506	CA	ALA	37	14.518	3.175	33.787	1.00	0.00	RX1	C
ATOM	2507	CB	ALA	37	14.175	4.520	34.427	1.00	0.00	RX1	C
ATOM	2508	C	ALA	37	15.273	2.290	34.771	1.00	0.00	RX1	C
ATOM	2509	O	ALA	37	16.403	2.564	35.173	1.00	0.00	RX1	O
ATOM	2510	N	SER	38	14.601	1.162	35.062	1.00	0.00	RX1	N
ATOM	2511	H	SER	38	13.604	1.164	35.159	1.00	0.00	RX1	H
ATOM	2512	CA	SER	38	15.295	-0.086	35.334	1.00	0.00	RX1	C
ATOM	2513	CB	SER	38	15.983	-0.048	36.696	1.00	0.00	RX1	C
ATOM	2514	OG	SER	38	15.058	0.229	37.741	1.00	0.00	RX1	O
ATOM	2515	HG	SER	38	14.313	0.700	37.365	1.00	0.00	RX1	H
ATOM	2516	C	SER	38	14.394	-1.287	35.077	1.00	0.00	RX1	C
ATOM	2517	O	SER	38	13.246	-1.355	35.500	1.00	0.00	RX1	O
ATOM	2518	N	PRO	39	14.954	-2.271	34.339	1.00	0.00	RX1	N
ATOM	2519	CD	PRO	39	16.176	-2.147	33.563	1.00	0.00	RX1	C
ATOM	2520	CA	PRO	39	14.342	-3.605	34.262	1.00	0.00	RX1	C
ATOM	2521	CB	PRO	39	15.487	-4.450	33.704	1.00	0.00	RX1	C
ATOM	2522	CG	PRO	39	16.315	-3.488	32.855	1.00	0.00	RX1	C
ATOM	2523	C	PRO	39	13.804	-4.158	35.577	1.00	0.00	RX1	C
ATOM	2524	O	PRO	39	12.689	-4.662	35.654	1.00	0.00	RX1	O
ATOM	2525	N	GLU	40	14.663	-4.050	36.608	1.00	0.00	RX1	N
ATOM	2526	H	GLU	40	15.541	-3.578	36.534	1.00	0.00	RX1	H
ATOM	2527	CA	GLU	40	14.301	-4.646	37.894	1.00	0.00	RX1	C
ATOM	2528	CB	GLU	40	15.480	-4.563	38.862	1.00	0.00	RX1	C
ATOM	2529	CG	GLU	40	16.729	-5.240	38.293	1.00	0.00	RX1	C
ATOM	2530	CD	GLU	40	17.863	-5.134	39.292	1.00	0.00	RX1	C
ATOM	2531	OE1	GLU	40	17.587	-4.983	40.481	1.00	0.00	RX1	O
ATOM	2532	OE2	GLU	40	19.019	-5.203	38.876	1.00	0.00	RX1	O
ATOM	2533	C	GLU	40	13.031	-4.090	38.514	1.00	0.00	RX1	C
ATOM	2534	O	GLU	40	12.109	-4.828	38.853	1.00	0.00	RX1	O
ATOM	2535	N	THR	41	12.988	-2.746	38.607	1.00	0.00	RX1	N
ATOM	2536	H	THR	41	13.666	-2.103	38.244	1.00	0.00	RX1	H
ATOM	2537	CA	THR	41	11.770	-2.156	39.166	1.00	0.00	RX1	C
ATOM	2538	CB	THR	41	11.999	-0.659	39.237	1.00	0.00	RX1	C
ATOM	2539	OG1	THR	41	12.534	-0.224	37.987	1.00	0.00	RX1	O
ATOM	2540	HG1	THR	41	12.441	0.737	37.995	1.00	0.00	RX1	H
ATOM	2541	CG2	THR	41	12.960	-0.302	40.374	1.00	0.00	RX1	C
ATOM	2542	C	THR	41	10.523	-2.534	38.386	1.00	0.00	RX1	C
ATOM	2543	O	THR	41	9.531	-3.009	38.928	1.00	0.00	RX1	O
ATOM	2544	N	HIS	42	10.658	-2.353	37.061	1.00	0.00	RX1	N
ATOM	2545	H	HIS	42	11.499	-1.902	36.744	1.00	0.00	RX1	H
ATOM	2546	CA	HIS	42	9.589	-2.729	36.136	1.00	0.00	RX1	C
ATOM	2547	CB	HIS	42	10.097	-2.639	34.701	1.00	0.00	RX1	C
ATOM	2548	CG	HIS	42	8.971	-2.835	33.714	1.00	0.00	RX1	C
ATOM	2549	ND1	HIS	42	7.881	-2.048	33.652	1.00	0.00	RX1	N
ATOM	2550	HD1	HIS	42	7.673	-1.285	34.230	1.00	0.00	RX1	H
ATOM	2551	CD2	HIS	42	8.886	-3.802	32.708	1.00	0.00	RX1	C
ATOM	2552	NE2	HIS	42	7.730	-3.585	32.036	1.00	0.00	RX1	N
ATOM	2553	CE1	HIS	42	7.111	-2.508	32.614	1.00	0.00	RX1	C
ATOM	2554	C	HIS	42	8.979	-4.101	36.392	1.00	0.00	RX1	C
ATOM	2555	O	HIS	42	7.784	-4.238	36.619	1.00	0.00	RX1	O
ATOM	2556	N	LEU	43	9.868	-5.108	36.364	1.00	0.00	RX1	N
ATOM	2557	H	LEU	43	10.845	-4.909	36.259	1.00	0.00	RX1	H
ATOM	2558	CA	LEU	43	9.415	-6.490	36.533	1.00	0.00	RX1	C
ATOM	2559	CB	LEU	43	10.624	-7.425	36.549	1.00	0.00	RX1	C
ATOM	2560	CG	LEU	43	10.269	-8.877	36.880	1.00	0.00	RX1	C

ATOM	2561	CD1	LEU	43	9.333	-9.509	35.849	1.00	0.00	RX1	C
ATOM	2562	CD2	LEU	43	11.523	-9.713	37.135	1.00	0.00	RX1	C
ATOM	2563	C	LEU	43	8.559	-6.720	37.769	1.00	0.00	RX1	C
ATOM	2564	O	LEU	43	7.419	-7.179	37.712	1.00	0.00	RX1	O
ATOM	2565	N	ASP	44	9.169	-6.387	38.909	1.00	0.00	RX1	N
ATOM	2566	H	ASP	44	10.066	-5.940	38.954	1.00	0.00	RX1	H
ATOM	2567	CA	ASP	44	8.444	-6.790	40.106	1.00	0.00	RX1	C
ATOM	2568	CB	ASP	44	9.402	-7.015	41.272	1.00	0.00	RX1	C
ATOM	2569	CG	ASP	44	9.694	-8.501	41.353	1.00	0.00	RX1	C
ATOM	2570	OD1	ASP	44	9.697	-9.050	42.450	1.00	0.00	RX1	O
ATOM	2571	OD2	ASP	44	9.837	-9.153	40.318	1.00	0.00	RX1	O
ATOM	2572	C	ASP	44	7.263	-5.910	40.456	1.00	0.00	RX1	C
ATOM	2573	O	ASP	44	6.269	-6.348	41.028	1.00	0.00	RX1	O
ATOM	2574	N	MET	45	7.362	-4.653	39.987	1.00	0.00	RX1	N
ATOM	2575	H	MET	45	8.198	-4.335	39.534	1.00	0.00	RX1	H
ATOM	2576	CA	MET	45	6.167	-3.809	40.000	1.00	0.00	RX1	C
ATOM	2577	CB	MET	45	6.512	-2.390	39.536	1.00	0.00	RX1	C
ATOM	2578	CG	MET	45	5.333	-1.417	39.435	1.00	0.00	RX1	C
ATOM	2579	SD	MET	45	4.389	-1.591	37.912	1.00	0.00	RX1	S
ATOM	2580	CE	MET	45	5.727	-1.225	36.763	1.00	0.00	RX1	C
ATOM	2581	C	MET	45	5.007	-4.406	39.215	1.00	0.00	RX1	C
ATOM	2582	O	MET	45	3.873	-4.430	39.678	1.00	0.00	RX1	O
ATOM	2583	N	LEU	46	5.342	-4.947	38.028	1.00	0.00	RX1	N
ATOM	2584	H	LEU	46	6.282	-4.913	37.680	1.00	0.00	RX1	H
ATOM	2585	CA	LEU	46	4.293	-5.629	37.267	1.00	0.00	RX1	C
ATOM	2586	CB	LEU	46	4.799	-6.189	35.940	1.00	0.00	RX1	C
ATOM	2587	CG	LEU	46	5.225	-5.141	34.918	1.00	0.00	RX1	C
ATOM	2588	CD1	LEU	46	5.633	-5.816	33.612	1.00	0.00	RX1	C
ATOM	2589	CD2	LEU	46	4.159	-4.069	34.694	1.00	0.00	RX1	C
ATOM	2590	C	LEU	46	3.617	-6.757	38.019	1.00	0.00	RX1	C
ATOM	2591	O	LEU	46	2.404	-6.909	37.985	1.00	0.00	RX1	O
ATOM	2592	N	ARG	47	4.445	-7.530	38.742	1.00	0.00	RX1	N
ATOM	2593	H	ARG	47	5.432	-7.352	38.744	1.00	0.00	RX1	H
ATOM	2594	CA	ARG	47	3.822	-8.567	39.568	1.00	0.00	RX1	C
ATOM	2595	CB	ARG	47	4.887	-9.464	40.194	1.00	0.00	RX1	C
ATOM	2596	CG	ARG	47	4.327	-10.795	40.710	1.00	0.00	RX1	C
ATOM	2597	CD	ARG	47	5.403	-11.806	41.124	1.00	0.00	RX1	C
ATOM	2598	NE	ARG	47	6.403	-11.967	40.068	1.00	0.00	RX1	N
ATOM	2599	HE	ARG	47	6.148	-12.394	39.192	1.00	0.00	RX1	H
ATOM	2600	CZ	ARG	47	7.609	-11.359	40.257	1.00	0.00	RX1	C
ATOM	2601	NH1	ARG	47	7.909	-10.858	41.469	1.00	0.00	RX1	N
ATOM	2602	HH11	ARG	47	8.760	-10.313	41.600	1.00	0.00	RX1	H
ATOM	2603	HH12	ARG	47	7.340	-10.953	42.286	1.00	0.00	RX1	H
ATOM	2604	NH2	ARG	47	8.467	-11.236	39.230	1.00	0.00	RX1	N
ATOM	2605	HH21	ARG	47	9.299	-10.667	39.357	1.00	0.00	RX1	H
ATOM	2606	HH22	ARG	47	8.297	-11.665	38.337	1.00	0.00	RX1	H
ATOM	2607	C	ARG	47	2.873	-8.024	40.621	1.00	0.00	RX1	C
ATOM	2608	O	ARG	47	1.710	-8.400	40.708	1.00	0.00	RX1	O
ATOM	2609	N	HIS	48	3.429	-7.082	41.400	1.00	0.00	RX1	N
ATOM	2610	H	HIS	48	4.371	-6.783	41.232	1.00	0.00	RX1	H
ATOM	2611	CA	HIS	48	2.652	-6.478	42.483	1.00	0.00	RX1	C
ATOM	2612	CB	HIS	48	3.497	-5.429	43.209	1.00	0.00	RX1	C
ATOM	2613	CG	HIS	48	2.972	-5.152	44.603	1.00	0.00	RX1	C
ATOM	2614	ND1	HIS	48	1.672	-5.050	44.945	1.00	0.00	RX1	N
ATOM	2615	HD1	HIS	48	0.889	-5.156	44.357	1.00	0.00	RX1	H
ATOM	2616	CD2	HIS	48	3.745	-4.951	45.750	1.00	0.00	RX1	C
ATOM	2617	NE2	HIS	48	2.898	-4.728	46.784	1.00	0.00	RX1	N
ATOM	2618	CE1	HIS	48	1.622	-4.788	46.289	1.00	0.00	RX1	C
ATOM	2619	C	HIS	48	1.328	-5.873	42.038	1.00	0.00	RX1	C
ATOM	2620	O	HIS	48	0.316	-5.948	42.723	1.00	0.00	RX1	O
ATOM	2621	N	LEU	49	1.393	-5.260	40.852	1.00	0.00	RX1	N

ATOM	2622	H	LEU	49	2.241	-5.255	40.321	1.00	0.00	RX1	H
ATOM	2623	CA	LEU	49	0.189	-4.637	40.320	1.00	0.00	RX1	C
ATOM	2624	CB	LEU	49	0.609	-3.610	39.269	1.00	0.00	RX1	C
ATOM	2625	CG	LEU	49	-0.524	-2.723	38.762	1.00	0.00	RX1	C
ATOM	2626	CD1	LEU	49	-1.235	-1.983	39.897	1.00	0.00	RX1	C
ATOM	2627	CD2	LEU	49	-0.022	-1.764	37.683	1.00	0.00	RX1	C
ATOM	2628	C	LEU	49	-0.809	-5.637	39.756	1.00	0.00	RX1	C
ATOM	2629	O	LEU	49	-1.977	-5.675	40.112	1.00	0.00	RX1	O
ATOM	2630	N	TYR	50	-0.282	-6.446	38.832	1.00	0.00	RX1	N
ATOM	2631	H	TYR	50	0.706	-6.491	38.684	1.00	0.00	RX1	H
ATOM	2632	CA	TYR	50	-1.184	-7.259	38.021	1.00	0.00	RX1	C
ATOM	2633	CB	TYR	50	-0.512	-7.648	36.712	1.00	0.00	RX1	C
ATOM	2634	CG	TYR	50	-0.285	-6.473	35.800	1.00	0.00	RX1	C
ATOM	2635	CD1	TYR	50	-1.195	-5.425	35.760	1.00	0.00	RX1	C
ATOM	2636	CE1	TYR	50	-1.031	-4.409	34.828	1.00	0.00	RX1	C
ATOM	2637	CD2	TYR	50	0.824	-6.467	34.962	1.00	0.00	RX1	C
ATOM	2638	CE2	TYR	50	0.985	-5.451	34.030	1.00	0.00	RX1	C
ATOM	2639	CZ	TYR	50	0.039	-4.440	33.943	1.00	0.00	RX1	C
ATOM	2640	OH	TYR	50	0.152	-3.475	32.967	1.00	0.00	RX1	O
ATOM	2641	HH	TYR	50	1.008	-3.050	33.039	1.00	0.00	RX1	H
ATOM	2642	C	TYR	50	-1.697	-8.543	38.647	1.00	0.00	RX1	C
ATOM	2643	O	TYR	50	-2.575	-9.199	38.098	1.00	0.00	RX1	O
ATOM	2644	N	GLN	51	-1.095	-8.913	39.788	1.00	0.00	RX1	N
ATOM	2645	H	GLN	51	-0.386	-8.330	40.190	1.00	0.00	RX1	H
ATOM	2646	CA	GLN	51	-1.322	-10.252	40.339	1.00	0.00	RX1	C
ATOM	2647	CB	GLN	51	-0.629	-10.383	41.692	1.00	0.00	RX1	C
ATOM	2648	CG	GLN	51	-0.367	-11.844	42.051	1.00	0.00	RX1	C
ATOM	2649	CD	GLN	51	0.785	-11.906	43.027	1.00	0.00	RX1	C
ATOM	2650	OE1	GLN	51	1.879	-12.360	42.703	1.00	0.00	RX1	O
ATOM	2651	NE2	GLN	51	0.483	-11.419	44.244	1.00	0.00	RX1	N
ATOM	2652	HE21	GLN	51	-0.431	-11.061	44.436	1.00	0.00	RX1	H
ATOM	2653	HE22	GLN	51	1.160	-11.399	44.979	1.00	0.00	RX1	H
ATOM	2654	C	GLN	51	-2.752	-10.787	40.369	1.00	0.00	RX1	C
ATOM	2655	O	GLN	51	-3.580	-10.402	41.188	1.00	0.00	RX1	O
ATOM	2656	N	GLY	52	-2.991	-11.719	39.429	1.00	0.00	RX1	N
ATOM	2657	H	GLY	52	-2.319	-11.891	38.705	1.00	0.00	RX1	H
ATOM	2658	CA	GLY	52	-4.292	-12.391	39.375	1.00	0.00	RX1	C
ATOM	2659	C	GLY	52	-5.473	-11.506	39.000	1.00	0.00	RX1	C
ATOM	2660	O	GLY	52	-6.616	-11.759	39.360	1.00	0.00	RX1	O
ATOM	2661	N	CYS	53	-5.152	-10.432	38.269	1.00	0.00	RX1	N
ATOM	2662	H	CYS	53	-4.222	-10.260	37.941	1.00	0.00	RX1	H
ATOM	2663	CA	CYS	53	-6.206	-9.450	38.036	1.00	0.00	RX1	C
ATOM	2664	CB	CYS	53	-5.610	-8.137	37.551	1.00	0.00	RX1	C
ATOM	2665	SG	CYS	53	-6.839	-6.817	37.641	1.00	0.00	RX1	S
ATOM	2666	C	CYS	53	-7.343	-9.893	37.139	1.00	0.00	RX1	C
ATOM	2667	O	CYS	53	-7.176	-10.250	35.978	1.00	0.00	RX1	O
ATOM	2668	N	GLN	54	-8.543	-9.825	37.717	1.00	0.00	RX1	N
ATOM	2669	H	GLN	54	-8.652	-9.424	38.627	1.00	0.00	RX1	H
ATOM	2670	CA	GLN	54	-9.693	-10.110	36.870	1.00	0.00	RX1	C
ATOM	2671	CB	GLN	54	-10.906	-10.469	37.710	1.00	0.00	RX1	C
ATOM	2672	CG	GLN	54	-10.595	-11.636	38.638	1.00	0.00	RX1	C
ATOM	2673	CD	GLN	54	-11.868	-12.024	39.345	1.00	0.00	RX1	C
ATOM	2674	OE1	GLN	54	-12.120	-11.620	40.475	1.00	0.00	RX1	O
ATOM	2675	NE2	GLN	54	-12.669	-12.814	38.611	1.00	0.00	RX1	N
ATOM	2676	HE21	GLN	54	-12.425	-13.073	37.671	1.00	0.00	RX1	H
ATOM	2677	HE22	GLN	54	-13.536	-13.162	38.968	1.00	0.00	RX1	H
ATOM	2678	C	GLN	54	-10.018	-8.984	35.912	1.00	0.00	RX1	C
ATOM	2679	O	GLN	54	-10.239	-9.199	34.727	1.00	0.00	RX1	O
ATOM	2680	N	VAL	55	-10.037	-7.773	36.485	1.00	0.00	RX1	N
ATOM	2681	H	VAL	55	-9.794	-7.629	37.448	1.00	0.00	RX1	H
ATOM	2682	CA	VAL	55	-10.273	-6.608	35.637	1.00	0.00	RX1	C

ATOM	2683	CB	VAL	55	-11.571	-5.894	36.027	1.00	0.00	RX1	C
ATOM	2684	CG1	VAL	55	-11.841	-4.715	35.089	1.00	0.00	RX1	C
ATOM	2685	CG2	VAL	55	-12.755	-6.861	36.087	1.00	0.00	RX1	C
ATOM	2686	C	VAL	55	-9.105	-5.648	35.729	1.00	0.00	RX1	C
ATOM	2687	O	VAL	55	-8.958	-4.902	36.692	1.00	0.00	RX1	O
ATOM	2688	N	VAL	56	-8.267	-5.706	34.690	1.00	0.00	RX1	N
ATOM	2689	H	VAL	56	-8.451	-6.248	33.870	1.00	0.00	RX1	H
ATOM	2690	CA	VAL	56	-7.177	-4.745	34.711	1.00	0.00	RX1	C
ATOM	2691	CB	VAL	56	-5.837	-5.393	34.326	1.00	0.00	RX1	C
ATOM	2692	CG1	VAL	56	-5.708	-5.775	32.856	1.00	0.00	RX1	C
ATOM	2693	CG2	VAL	56	-4.689	-4.511	34.787	1.00	0.00	RX1	C
ATOM	2694	C	VAL	56	-7.504	-3.459	33.963	1.00	0.00	RX1	C
ATOM	2695	O	VAL	56	-7.633	-3.371	32.744	1.00	0.00	RX1	O
ATOM	2696	N	GLN	57	-7.692	-2.446	34.809	1.00	0.00	RX1	N
ATOM	2697	H	GLN	57	-7.494	-2.585	35.779	1.00	0.00	RX1	H
ATOM	2698	CA	GLN	57	-8.078	-1.136	34.304	1.00	0.00	RX1	C
ATOM	2699	CB	GLN	57	-8.892	-0.389	35.351	1.00	0.00	RX1	C
ATOM	2700	CG	GLN	57	-9.970	-1.318	35.895	1.00	0.00	RX1	C
ATOM	2701	CD	GLN	57	-11.118	-0.502	36.439	1.00	0.00	RX1	C
ATOM	2702	OE1	GLN	57	-12.215	-0.495	35.888	1.00	0.00	RX1	O
ATOM	2703	NE2	GLN	57	-10.810	0.186	37.552	1.00	0.00	RX1	N
ATOM	2704	HE21	GLN	57	-9.892	0.117	37.945	1.00	0.00	RX1	H
ATOM	2705	HE22	GLN	57	-11.479	0.771	38.008	1.00	0.00	RX1	H
ATOM	2706	C	GLN	57	-6.903	-0.319	33.822	1.00	0.00	RX1	C
ATOM	2707	O	GLN	57	-6.334	0.505	34.530	1.00	0.00	RX1	O
ATOM	2708	N	GLY	58	-6.548	-0.629	32.577	1.00	0.00	RX1	N
ATOM	2709	H	GLY	58	-6.973	-1.387	32.075	1.00	0.00	RX1	H
ATOM	2710	CA	GLY	58	-5.407	0.011	31.938	1.00	0.00	RX1	C
ATOM	2711	C	GLY	58	-4.877	-0.965	30.918	1.00	0.00	RX1	C
ATOM	2712	O	GLY	58	-5.584	-1.895	30.537	1.00	0.00	RX1	O
ATOM	2713	N	ASN	59	-3.631	-0.739	30.493	1.00	0.00	RX1	N
ATOM	2714	H	ASN	59	-3.013	-0.062	30.902	1.00	0.00	RX1	H
ATOM	2715	CA	ASN	59	-3.087	-1.745	29.584	1.00	0.00	RX1	C
ATOM	2716	CB	ASN	59	-1.925	-1.226	28.748	1.00	0.00	RX1	C
ATOM	2717	CG	ASN	59	-2.289	-0.002	27.954	1.00	0.00	RX1	C
ATOM	2718	OD1	ASN	59	-3.263	0.019	27.201	1.00	0.00	RX1	O
ATOM	2719	ND2	ASN	59	-1.445	1.014	28.180	1.00	0.00	RX1	N
ATOM	2720	HD21	ASN	59	-0.618	0.853	28.732	1.00	0.00	RX1	H
ATOM	2721	HD22	ASN	59	-1.507	1.970	27.875	1.00	0.00	RX1	H
ATOM	2722	C	ASN	59	-2.569	-2.931	30.364	1.00	0.00	RX1	C
ATOM	2723	O	ASN	59	-2.456	-2.890	31.583	1.00	0.00	RX1	O
ATOM	2724	N	LEU	60	-2.244	-3.983	29.608	1.00	0.00	RX1	N
ATOM	2725	H	LEU	60	-2.355	-3.963	28.613	1.00	0.00	RX1	H
ATOM	2726	CA	LEU	60	-1.500	-5.071	30.227	1.00	0.00	RX1	C
ATOM	2727	CB	LEU	60	-2.219	-6.395	29.997	1.00	0.00	RX1	C
ATOM	2728	CG	LEU	60	-1.690	-7.527	30.871	1.00	0.00	RX1	C
ATOM	2729	CD1	LEU	60	-1.842	-7.198	32.353	1.00	0.00	RX1	C
ATOM	2730	CD2	LEU	60	-2.346	-8.861	30.525	1.00	0.00	RX1	C
ATOM	2731	C	LEU	60	-0.113	-5.117	29.631	1.00	0.00	RX1	C
ATOM	2732	O	LEU	60	0.160	-5.827	28.667	1.00	0.00	RX1	O
ATOM	2733	N	GLU	61	0.737	-4.271	30.210	1.00	0.00	RX1	N
ATOM	2734	H	GLU	61	0.514	-3.819	31.076	1.00	0.00	RX1	H
ATOM	2735	CA	GLU	61	2.055	-4.145	29.601	1.00	0.00	RX1	C
ATOM	2736	CB	GLU	61	2.510	-2.696	29.668	1.00	0.00	RX1	C
ATOM	2737	CG	GLU	61	1.503	-1.934	28.817	1.00	0.00	RX1	C
ATOM	2738	CD	GLU	61	1.608	-0.445	29.005	1.00	0.00	RX1	C
ATOM	2739	OE1	GLU	61	1.807	-0.003	30.130	1.00	0.00	RX1	O
ATOM	2740	OE2	GLU	61	1.451	0.277	28.027	1.00	0.00	RX1	O
ATOM	2741	C	GLU	61	3.054	-5.124	30.159	1.00	0.00	RX1	C
ATOM	2742	O	GLU	61	3.360	-5.168	31.342	1.00	0.00	RX1	O
ATOM	2743	N	LEU	62	3.484	-5.960	29.215	1.00	0.00	RX1	N

ATOM	2744	H	LEU	62	3.296	-5.770	28.252	1.00	0.00	RX1	H
ATOM	2745	CA	LEU	62	4.346	-7.095	29.512	1.00	0.00	RX1	C
ATOM	2746	CB	LEU	62	3.606	-8.398	29.234	1.00	0.00	RX1	C
ATOM	2747	CG	LEU	62	2.306	-8.491	30.024	1.00	0.00	RX1	C
ATOM	2748	CD1	LEU	62	1.464	-9.684	29.579	1.00	0.00	RX1	C
ATOM	2749	CD2	LEU	62	2.560	-8.461	31.532	1.00	0.00	RX1	C
ATOM	2750	C	LEU	62	5.574	-7.009	28.642	1.00	0.00	RX1	C
ATOM	2751	O	LEU	62	5.712	-7.659	27.607	1.00	0.00	RX1	O
ATOM	2752	N	THR	63	6.441	-6.112	29.097	1.00	0.00	RX1	N
ATOM	2753	H	THR	63	6.309	-5.571	29.926	1.00	0.00	RX1	H
ATOM	2754	CA	THR	63	7.494	-5.694	28.196	1.00	0.00	RX1	C
ATOM	2755	CB	THR	63	7.103	-4.254	27.948	1.00	0.00	RX1	C
ATOM	2756	OG1	THR	63	5.726	-4.111	28.339	1.00	0.00	RX1	O
ATOM	2757	HG1	THR	63	5.508	-3.194	28.217	1.00	0.00	RX1	H
ATOM	2758	CG2	THR	63	7.294	-3.855	26.489	1.00	0.00	RX1	C
ATOM	2759	C	THR	63	8.874	-5.876	28.782	1.00	0.00	RX1	C
ATOM	2760	O	THR	63	9.097	-5.716	29.974	1.00	0.00	RX1	O
ATOM	2761	N	TYR	64	9.804	-6.234	27.878	1.00	0.00	RX1	N
ATOM	2762	H	TYR	64	9.502	-6.473	26.954	1.00	0.00	RX1	H
ATOM	2763	CA	TYR	64	11.221	-6.335	28.250	1.00	0.00	RX1	C
ATOM	2764	CB	TYR	64	11.795	-4.977	28.658	1.00	0.00	RX1	C
ATOM	2765	CG	TYR	64	11.608	-3.938	27.583	1.00	0.00	RX1	C
ATOM	2766	CD1	TYR	64	12.454	-3.922	26.484	1.00	0.00	RX1	C
ATOM	2767	CE1	TYR	64	12.317	-2.926	25.527	1.00	0.00	RX1	C
ATOM	2768	CD2	TYR	64	10.606	-2.982	27.707	1.00	0.00	RX1	C
ATOM	2769	CE2	TYR	64	10.469	-1.984	26.752	1.00	0.00	RX1	C
ATOM	2770	CZ	TYR	64	11.337	-1.952	25.670	1.00	0.00	RX1	C
ATOM	2771	OH	TYR	64	11.242	-0.954	24.731	1.00	0.00	RX1	O
ATOM	2772	HH	TYR	64	10.525	-0.354	24.918	1.00	0.00	RX1	H
ATOM	2773	C	TYR	64	11.545	-7.319	29.366	1.00	0.00	RX1	C
ATOM	2774	O	TYR	64	12.585	-7.244	30.011	1.00	0.00	RX1	O
ATOM	2775	N	LEU	65	10.597	-8.236	29.592	1.00	0.00	RX1	N
ATOM	2776	H	LEU	65	9.819	-8.366	28.976	1.00	0.00	RX1	H
ATOM	2777	CA	LEU	65	10.710	-9.034	30.807	1.00	0.00	RX1	C
ATOM	2778	CB	LEU	65	9.372	-9.701	31.128	1.00	0.00	RX1	C
ATOM	2779	CG	LEU	65	8.298	-8.641	31.375	1.00	0.00	RX1	C
ATOM	2780	CD1	LEU	65	6.900	-9.237	31.513	1.00	0.00	RX1	C
ATOM	2781	CD2	LEU	65	8.666	-7.747	32.558	1.00	0.00	RX1	C
ATOM	2782	C	LEU	65	11.859	-10.020	30.768	1.00	0.00	RX1	C
ATOM	2783	O	LEU	65	12.048	-10.746	29.796	1.00	0.00	RX1	O
ATOM	2784	N	PRO	66	12.649	-9.980	31.871	1.00	0.00	RX1	N
ATOM	2785	CD	PRO	66	12.474	-9.066	32.994	1.00	0.00	RX1	C
ATOM	2786	CA	PRO	66	13.821	-10.850	32.020	1.00	0.00	RX1	C
ATOM	2787	CB	PRO	66	14.227	-10.584	33.472	1.00	0.00	RX1	C
ATOM	2788	CG	PRO	66	13.794	-9.147	33.746	1.00	0.00	RX1	C
ATOM	2789	C	PRO	66	13.596	-12.323	31.712	1.00	0.00	RX1	C
ATOM	2790	O	PRO	66	12.548	-12.897	31.975	1.00	0.00	RX1	O
ATOM	2791	N	THR	67	14.659	-12.908	31.142	1.00	0.00	RX1	N
ATOM	2792	H	THR	67	15.506	-12.394	31.015	1.00	0.00	RX1	H
ATOM	2793	CA	THR	67	14.615	-14.249	30.556	1.00	0.00	RX1	C
ATOM	2794	CB	THR	67	16.073	-14.589	30.331	1.00	0.00	RX1	C
ATOM	2795	OG1	THR	67	16.770	-13.365	30.063	1.00	0.00	RX1	O
ATOM	2796	HG1	THR	67	16.811	-13.275	29.109	1.00	0.00	RX1	H
ATOM	2797	CG2	THR	67	16.294	-15.623	29.232	1.00	0.00	RX1	C
ATOM	2798	C	THR	67	13.851	-15.325	31.317	1.00	0.00	RX1	C
ATOM	2799	O	THR	67	13.001	-16.040	30.798	1.00	0.00	RX1	O
ATOM	2800	N	ASN	68	14.213	-15.399	32.604	1.00	0.00	RX1	N
ATOM	2801	H	ASN	68	14.790	-14.699	33.023	1.00	0.00	RX1	H
ATOM	2802	CA	ASN	68	13.602	-16.458	33.406	1.00	0.00	RX1	C
ATOM	2803	CB	ASN	68	14.640	-17.379	34.053	1.00	0.00	RX1	C
ATOM	2804	CG	ASN	68	15.513	-18.058	33.022	1.00	0.00	RX1	C

ATOM	2805	OD1	ASN	68	15.060	-18.685	32.069	1.00	0.00	RX1	O
ATOM	2806	ND2	ASN	68	16.824	-17.890	33.266	1.00	0.00	RX1	N
ATOM	2807	HD21	ASN	68	17.150	-17.385	34.066	1.00	0.00	RX1	H
ATOM	2808	HD22	ASN	68	17.510	-18.284	32.648	1.00	0.00	RX1	H
ATOM	2809	C	ASN	68	12.759	-15.878	34.519	1.00	0.00	RX1	C
ATOM	2810	O	ASN	68	12.752	-16.346	35.651	1.00	0.00	RX1	O
ATOM	2811	N	ALA	69	12.090	-14.774	34.170	1.00	0.00	RX1	N
ATOM	2812	H	ALA	69	12.003	-14.473	33.218	1.00	0.00	RX1	H
ATOM	2813	CA	ALA	69	11.389	-14.070	35.235	1.00	0.00	RX1	C
ATOM	2814	CB	ALA	69	11.047	-12.651	34.798	1.00	0.00	RX1	C
ATOM	2815	C	ALA	69	10.116	-14.757	35.681	1.00	0.00	RX1	C
ATOM	2816	O	ALA	69	9.397	-15.376	34.907	1.00	0.00	RX1	O
ATOM	2817	N	SER	70	9.840	-14.594	36.984	1.00	0.00	RX1	N
ATOM	2818	H	SER	70	10.402	-14.027	37.586	1.00	0.00	RX1	H
ATOM	2819	CA	SER	70	8.545	-15.101	37.424	1.00	0.00	RX1	C
ATOM	2820	CB	SER	70	8.744	-15.189	38.913	1.00	0.00	RX1	C
ATOM	2821	OG	SER	70	10.162	-15.075	39.105	1.00	0.00	RX1	O
ATOM	2822	HG	SER	70	10.545	-15.884	38.782	1.00	0.00	RX1	H
ATOM	2823	C	SER	70	7.399	-14.225	36.949	1.00	0.00	RX1	C
ATOM	2824	O	SER	70	7.089	-13.178	37.515	1.00	0.00	RX1	O
ATOM	2825	N	LEU	71	6.799	-14.735	35.865	1.00	0.00	RX1	N
ATOM	2826	H	LEU	71	7.200	-15.541	35.424	1.00	0.00	RX1	H
ATOM	2827	CA	LEU	71	5.601	-14.116	35.301	1.00	0.00	RX1	C
ATOM	2828	CB	LEU	71	5.630	-14.197	33.778	1.00	0.00	RX1	C
ATOM	2829	CG	LEU	71	6.061	-12.894	33.108	1.00	0.00	RX1	C
ATOM	2830	CD1	LEU	71	7.446	-12.424	33.551	1.00	0.00	RX1	C
ATOM	2831	CD2	LEU	71	5.948	-13.004	31.591	1.00	0.00	RX1	C
ATOM	2832	C	LEU	71	4.293	-14.687	35.820	1.00	0.00	RX1	C
ATOM	2833	O	LEU	71	3.218	-14.421	35.303	1.00	0.00	RX1	O
ATOM	2834	N	SER	72	4.443	-15.484	36.888	1.00	0.00	RX1	N
ATOM	2835	H	SER	72	5.359	-15.684	37.220	1.00	0.00	RX1	H
ATOM	2836	CA	SER	72	3.346	-16.164	37.582	1.00	0.00	RX1	C
ATOM	2837	CB	SER	72	3.928	-16.539	38.936	1.00	0.00	RX1	C
ATOM	2838	OG	SER	72	5.356	-16.623	38.774	1.00	0.00	RX1	O
ATOM	2839	HG	SER	72	5.633	-17.308	39.375	1.00	0.00	RX1	H
ATOM	2840	C	SER	72	2.016	-15.418	37.628	1.00	0.00	RX1	C
ATOM	2841	O	SER	72	0.966	-15.909	37.239	1.00	0.00	RX1	O
ATOM	2842	N	PHE	73	2.139	-14.162	38.085	1.00	0.00	RX1	N
ATOM	2843	H	PHE	73	3.043	-13.847	38.363	1.00	0.00	RX1	H
ATOM	2844	CA	PHE	73	0.998	-13.247	38.198	1.00	0.00	RX1	C
ATOM	2845	CB	PHE	73	1.495	-11.839	38.535	1.00	0.00	RX1	C
ATOM	2846	CG	PHE	73	2.339	-11.221	37.441	1.00	0.00	RX1	C
ATOM	2847	CD1	PHE	73	1.789	-10.256	36.609	1.00	0.00	RX1	C
ATOM	2848	CD2	PHE	73	3.671	-11.583	37.281	1.00	0.00	RX1	C
ATOM	2849	CE1	PHE	73	2.572	-9.641	35.639	1.00	0.00	RX1	C
ATOM	2850	CE2	PHE	73	4.453	-10.969	36.312	1.00	0.00	RX1	C
ATOM	2851	CZ	PHE	73	3.908	-9.989	35.494	1.00	0.00	RX1	C
ATOM	2852	C	PHE	73	-0.025	-13.190	37.065	1.00	0.00	RX1	C
ATOM	2853	O	PHE	73	-1.191	-12.871	37.277	1.00	0.00	RX1	O
ATOM	2854	N	LEU	74	0.471	-13.493	35.858	1.00	0.00	RX1	N
ATOM	2855	H	LEU	74	1.394	-13.865	35.756	1.00	0.00	RX1	H
ATOM	2856	CA	LEU	74	-0.376	-13.411	34.672	1.00	0.00	RX1	C
ATOM	2857	CB	LEU	74	0.488	-13.483	33.423	1.00	0.00	RX1	C
ATOM	2858	CG	LEU	74	1.320	-12.225	33.250	1.00	0.00	RX1	C
ATOM	2859	CD1	LEU	74	2.343	-12.381	32.134	1.00	0.00	RX1	C
ATOM	2860	CD2	LEU	74	0.420	-11.013	33.040	1.00	0.00	RX1	C
ATOM	2861	C	LEU	74	-1.500	-14.421	34.571	1.00	0.00	RX1	C
ATOM	2862	O	LEU	74	-2.499	-14.175	33.905	1.00	0.00	RX1	O
ATOM	2863	N	GLN	75	-1.310	-15.553	35.274	1.00	0.00	RX1	N
ATOM	2864	H	GLN	75	-0.455	-15.677	35.780	1.00	0.00	RX1	H
ATOM	2865	CA	GLN	75	-2.229	-16.691	35.146	1.00	0.00	RX1	C

```
ATOM   2866  CB  GLN    75      -2.010 -17.664  36.306  1.00  0.00      RX1  C
ATOM   2867  CG  GLN    75      -2.078 -16.980  37.674  1.00  0.00      RX1  C
ATOM   2868  CD  GLN    75      -2.084 -18.018  38.773  1.00  0.00      RX1  C
ATOM   2869  OE1 GLN    75      -1.052 -18.519  39.211  1.00  0.00      RX1  O
ATOM   2870  NE2 GLN    75      -3.318 -18.296  39.221  1.00  0.00      RX1  N
ATOM   2871 HE21 GLN    75      -4.120 -17.852  38.799  1.00  0.00      RX1  H
ATOM   2872 HE22 GLN    75      -3.502 -18.922  39.974  1.00  0.00      RX1  H
ATOM   2873  C   GLN    75      -3.717 -16.390  34.974  1.00  0.00      RX1  C
ATOM   2874  O   GLN    75      -4.410 -16.992  34.161  1.00  0.00      RX1  O
ATOM   2875  N   ASP    76      -4.172 -15.417  35.774  1.00  0.00      RX1  N
ATOM   2876  H   ASP    76      -3.550 -14.862  36.322  1.00  0.00      RX1  H
ATOM   2877  CA  ASP    76      -5.616 -15.242  35.865  1.00  0.00      RX1  C
ATOM   2878  CB  ASP    76      -6.081 -15.320  37.322  1.00  0.00      RX1  C
ATOM   2879  CG  ASP    76      -5.872 -16.710  37.889  1.00  0.00      RX1  C
ATOM   2880  OD1 ASP    76      -5.553 -17.635  37.153  1.00  0.00      RX1  O
ATOM   2881  OD2 ASP    76      -6.071 -16.917  39.079  1.00  0.00      RX1  O
ATOM   2882  C   ASP    76      -6.156 -13.973  35.251  1.00  0.00      RX1  C
ATOM   2883  O   ASP    76      -7.290 -13.588  35.518  1.00  0.00      RX1  O
ATOM   2884  N   ILE    77      -5.310 -13.305  34.453  1.00  0.00      RX1  N
ATOM   2885  H   ILE    77      -4.449 -13.698  34.121  1.00  0.00      RX1  H
ATOM   2886  CA  ILE    77      -5.753 -11.970  34.056  1.00  0.00      RX1  C
ATOM   2887  CB  ILE    77      -4.574 -11.061  33.742  1.00  0.00      RX1  C
ATOM   2888  CG2 ILE    77      -5.037  -9.650  33.373  1.00  0.00      RX1  C
ATOM   2889  CG1 ILE    77      -3.657 -11.051  34.961  1.00  0.00      RX1  C
ATOM   2890  CD1 ILE    77      -2.564 -10.001  34.839  1.00  0.00      RX1  C
ATOM   2891  C   ILE    77      -6.824 -11.931  32.983  1.00  0.00      RX1  C
ATOM   2892  O   ILE    77      -6.597 -12.117  31.796  1.00  0.00      RX1  O
ATOM   2893  N   GLN    78      -8.034 -11.699  33.497  1.00  0.00      RX1  N
ATOM   2894  H   GLN    78      -8.078 -11.432  34.462  1.00  0.00      RX1  H
ATOM   2895  CA  GLN    78      -9.208 -11.953  32.671  1.00  0.00      RX1  C
ATOM   2896  CB  GLN    78     -10.382 -12.296  33.575  1.00  0.00      RX1  C
ATOM   2897  CG  GLN    78     -10.167 -13.674  34.189  1.00  0.00      RX1  C
ATOM   2898  CD  GLN    78     -10.773 -13.716  35.569  1.00  0.00      RX1  C
ATOM   2899  OE1 GLN    78     -11.932 -13.370  35.786  1.00  0.00      RX1  O
ATOM   2900  NE2 GLN    78      -9.912 -14.161  36.498  1.00  0.00      RX1  N
ATOM   2901 HE21 GLN    78      -8.954 -14.296  36.217  1.00  0.00      RX1  H
ATOM   2902 HE22 GLN    78     -10.114 -14.344  37.459  1.00  0.00      RX1  H
ATOM   2903  C   GLN    78      -9.556 -10.909  31.631  1.00  0.00      RX1  C
ATOM   2904  O   GLN    78      -9.885 -11.237  30.497  1.00  0.00      RX1  O
ATOM   2905  N   GLU    79      -9.494  -9.640  32.034  1.00  0.00      RX1  N
ATOM   2906  H   GLU    79      -9.263  -9.337  32.962  1.00  0.00      RX1  H
ATOM   2907  CA  GLU    79      -9.784  -8.656  30.997  1.00  0.00      RX1  C
ATOM   2908  CB  GLU    79     -11.257  -8.262  30.972  1.00  0.00      RX1  C
ATOM   2909  CG  GLU    79     -11.780  -7.587  32.230  1.00  0.00      RX1  C
ATOM   2910  CD  GLU    79     -13.239  -7.258  32.015  1.00  0.00      RX1  C
ATOM   2911  OE1 GLU    79     -13.615  -6.114  32.221  1.00  0.00      RX1  O
ATOM   2912  OE2 GLU    79     -14.009  -8.122  31.598  1.00  0.00      RX1  O
ATOM   2913  C   GLU    79      -8.918  -7.434  31.060  1.00  0.00      RX1  C
ATOM   2914  O   GLU    79      -8.597  -6.921  32.123  1.00  0.00      RX1  O
ATOM   2915  N   VAL    80      -8.544  -7.008  29.853  1.00  0.00      RX1  N
ATOM   2916  H   VAL    80      -8.922  -7.421  29.020  1.00  0.00      RX1  H
ATOM   2917  CA  VAL    80      -7.676  -5.842  29.758  1.00  0.00      RX1  C
ATOM   2918  CB  VAL    80      -6.478  -6.183  28.883  1.00  0.00      RX1  C
ATOM   2919  CG1 VAL    80      -5.534  -4.990  28.747  1.00  0.00      RX1  C
ATOM   2920  CG2 VAL    80      -5.793  -7.447  29.398  1.00  0.00      RX1  C
ATOM   2921  C   VAL    80      -8.443  -4.699  29.146  1.00  0.00      RX1  C
ATOM   2922  O   VAL    80      -8.992  -4.845  28.064  1.00  0.00      RX1  O
ATOM   2923  N   GLN    81      -8.479  -3.565  29.855  1.00  0.00      RX1  N
ATOM   2924  H   GLN    81      -7.967  -3.461  30.713  1.00  0.00      RX1  H
ATOM   2925  CA  GLN    81      -9.224  -2.469  29.234  1.00  0.00      RX1  C
ATOM   2926  CB  GLN    81      -9.656  -1.461  30.290  1.00  0.00      RX1  C
```

ATOM	2927	CG	GLN	81	-10.602	-2.116	31.298	1.00	0.00	RX1	C
ATOM	2928	CD	GLN	81	-11.016	-1.086	32.321	1.00	0.00	RX1	C
ATOM	2929	OE1	GLN	81	-10.467	0.008	32.374	1.00	0.00	RX1	O
ATOM	2930	NE2	GLN	81	-11.991	-1.506	33.146	1.00	0.00	RX1	N
ATOM	2931	HE21	GLN	81	-12.421	-2.404	33.044	1.00	0.00	RX1	H
ATOM	2932	HE22	GLN	81	-12.309	-0.946	33.919	1.00	0.00	RX1	H
ATOM	2933	C	GLN	81	-8.524	-1.810	28.054	1.00	0.00	RX1	C
ATOM	2934	O	GLN	81	-9.115	-1.514	27.018	1.00	0.00	RX1	O
ATOM	2935	N	GLY	82	-7.221	-1.599	28.256	1.00	0.00	RX1	N
ATOM	2936	H	GLY	82	-6.764	-1.958	29.069	1.00	0.00	RX1	H
ATOM	2937	CA	GLY	82	-6.430	-1.050	27.161	1.00	0.00	RX1	C
ATOM	2938	C	GLY	82	-5.999	-2.132	26.196	1.00	0.00	RX1	C
ATOM	2939	O	GLY	82	-6.748	-3.054	25.884	1.00	0.00	RX1	O
ATOM	2940	N	TYR	83	-4.754	-1.975	25.743	1.00	0.00	RX1	N
ATOM	2941	H	TYR	83	-4.152	-1.268	26.120	1.00	0.00	RX1	H
ATOM	2942	CA	TYR	83	-4.212	-3.022	24.884	1.00	0.00	RX1	C
ATOM	2943	CB	TYR	83	-3.418	-2.418	23.715	1.00	0.00	RX1	C
ATOM	2944	CG	TYR	83	-2.357	-1.449	24.178	1.00	0.00	RX1	C
ATOM	2945	CD1	TYR	83	-1.259	-1.889	24.906	1.00	0.00	RX1	C
ATOM	2946	CE1	TYR	83	-0.311	-0.979	25.350	1.00	0.00	RX1	C
ATOM	2947	CD2	TYR	83	-2.480	-0.102	23.861	1.00	0.00	RX1	C
ATOM	2948	CE2	TYR	83	-1.528	0.810	24.297	1.00	0.00	RX1	C
ATOM	2949	CZ	TYR	83	-0.453	0.370	25.056	1.00	0.00	RX1	C
ATOM	2950	OH	TYR	83	0.475	1.270	25.524	1.00	0.00	RX1	O
ATOM	2951	HH	TYR	83	0.741	0.982	26.398	1.00	0.00	RX1	H
ATOM	2952	C	TYR	83	-3.401	-4.034	25.675	1.00	0.00	RX1	C
ATOM	2953	O	TYR	83	-2.990	-3.779	26.803	1.00	0.00	RX1	O
ATOM	2954	N	VAL	84	-3.183	-5.195	25.048	1.00	0.00	RX1	N
ATOM	2955	H	VAL	84	-3.429	-5.317	24.085	1.00	0.00	RX1	H
ATOM	2956	CA	VAL	84	-2.225	-6.111	25.662	1.00	0.00	RX1	C
ATOM	2957	CB	VAL	84	-2.708	-7.557	25.562	1.00	0.00	RX1	C
ATOM	2958	CG1	VAL	84	-1.778	-8.515	26.306	1.00	0.00	RX1	C
ATOM	2959	CG2	VAL	84	-4.138	-7.675	26.070	1.00	0.00	RX1	C
ATOM	2960	C	VAL	84	-0.901	-5.950	24.952	1.00	0.00	RX1	C
ATOM	2961	O	VAL	84	-0.842	-5.949	23.729	1.00	0.00	RX1	O
ATOM	2962	N	LEU	85	0.141	-5.777	25.765	1.00	0.00	RX1	N
ATOM	2963	H	LEU	85	0.084	-5.874	26.761	1.00	0.00	RX1	H
ATOM	2964	CA	LEU	85	1.411	-5.400	25.166	1.00	0.00	RX1	C
ATOM	2965	CB	LEU	85	1.741	-3.985	25.610	1.00	0.00	RX1	C
ATOM	2966	CG	LEU	85	3.003	-3.433	24.969	1.00	0.00	RX1	C
ATOM	2967	CD1	LEU	85	2.933	-3.505	23.446	1.00	0.00	RX1	C
ATOM	2968	CD2	LEU	85	3.309	-2.036	25.497	1.00	0.00	RX1	C
ATOM	2969	C	LEU	85	2.528	-6.353	25.527	1.00	0.00	RX1	C
ATOM	2970	O	LEU	85	3.317	-6.120	26.432	1.00	0.00	RX1	O
ATOM	2971	N	ILE	86	2.557	-7.460	24.790	1.00	0.00	RX1	N
ATOM	2972	H	ILE	86	2.034	-7.516	23.938	1.00	0.00	RX1	H
ATOM	2973	CA	ILE	86	3.612	-8.411	25.113	1.00	0.00	RX1	C
ATOM	2974	CB	ILE	86	3.066	-9.834	25.062	1.00	0.00	RX1	C
ATOM	2975	CG2	ILE	86	4.054	-10.803	25.701	1.00	0.00	RX1	C
ATOM	2976	CG1	ILE	86	1.692	-9.915	25.722	1.00	0.00	RX1	C
ATOM	2977	CD1	ILE	86	1.101	-11.325	25.686	1.00	0.00	RX1	C
ATOM	2978	C	ILE	86	4.791	-8.250	24.171	1.00	0.00	RX1	C
ATOM	2979	O	ILE	86	4.795	-8.762	23.057	1.00	0.00	RX1	O
ATOM	2980	N	ALA	87	5.786	-7.489	24.638	1.00	0.00	RX1	N
ATOM	2981	H	ALA	87	5.820	-7.129	25.576	1.00	0.00	RX1	H
ATOM	2982	CA	ALA	87	6.825	-7.168	23.665	1.00	0.00	RX1	C
ATOM	2983	CB	ALA	87	6.514	-5.826	23.010	1.00	0.00	RX1	C
ATOM	2984	C	ALA	87	8.239	-7.145	24.208	1.00	0.00	RX1	C
ATOM	2985	O	ALA	87	8.474	-6.934	25.392	1.00	0.00	RX1	O
ATOM	2986	N	HIS	88	9.177	-7.374	23.269	1.00	0.00	RX1	N
ATOM	2987	H	HIS	88	8.864	-7.541	22.332	1.00	0.00	RX1	H

ATOM	2988	CA	HIS	88	10.621	-7.379	23.552	1.00	0.00	RX1	C
ATOM	2989	CB	HIS	88	11.189	-6.000	23.880	1.00	0.00	RX1	C
ATOM	2990	CG	HIS	88	10.602	-4.918	23.018	1.00	0.00	RX1	C
ATOM	2991	ND1	HIS	88	10.932	-4.686	21.736	1.00	0.00	RX1	N
ATOM	2992	HD1	HIS	88	11.573	-5.176	21.181	1.00	0.00	RX1	H
ATOM	2993	CD2	HIS	88	9.647	-3.985	23.417	1.00	0.00	RX1	C
ATOM	2994	NE2	HIS	88	9.402	-3.185	22.360	1.00	0.00	RX1	N
ATOM	2995	CE1	HIS	88	10.191	-3.608	21.322	1.00	0.00	RX1	C
ATOM	2996	C	HIS	88	11.055	-8.304	24.671	1.00	0.00	RX1	C
ATOM	2997	O	HIS	88	11.956	-8.019	25.453	1.00	0.00	RX1	O
ATOM	2998	N	ASN	89	10.334	-9.421	24.745	1.00	0.00	RX1	N
ATOM	2999	H	ASN	89	9.701	-9.706	24.027	1.00	0.00	RX1	H
ATOM	3000	CA	ASN	89	10.531	-10.233	25.935	1.00	0.00	RX1	C
ATOM	3001	CB	ASN	89	9.263	-10.979	26.319	1.00	0.00	RX1	C
ATOM	3002	CG	ASN	89	8.318	-10.000	26.962	1.00	0.00	RX1	C
ATOM	3003	OD1	ASN	89	8.557	-9.500	28.056	1.00	0.00	RX1	O
ATOM	3004	ND2	ASN	89	7.240	-9.737	26.214	1.00	0.00	RX1	N
ATOM	3005	HD21	ASN	89	7.149	-10.150	25.303	1.00	0.00	RX1	H
ATOM	3006	HD22	ASN	89	6.541	-9.101	26.554	1.00	0.00	RX1	H
ATOM	3007	C	ASN	89	11.661	-11.210	25.827	1.00	0.00	RX1	C
ATOM	3008	O	ASN	89	11.777	-11.974	24.879	1.00	0.00	RX1	O
ATOM	3009	N	GLN	90	12.475	-11.147	26.884	1.00	0.00	RX1	N
ATOM	3010	H	GLN	90	12.283	-10.516	27.637	1.00	0.00	RX1	H
ATOM	3011	CA	GLN	90	13.459	-12.201	27.090	1.00	0.00	RX1	C
ATOM	3012	CB	GLN	90	14.563	-11.648	27.981	1.00	0.00	RX1	C
ATOM	3013	CG	GLN	90	15.603	-10.821	27.242	1.00	0.00	RX1	C
ATOM	3014	CD	GLN	90	16.650	-11.760	26.696	1.00	0.00	RX1	C
ATOM	3015	OE1	GLN	90	17.109	-12.667	27.390	1.00	0.00	RX1	O
ATOM	3016	NE2	GLN	90	16.996	-11.493	25.428	1.00	0.00	RX1	N
ATOM	3017	HE21	GLN	90	16.600	-10.720	24.936	1.00	0.00	RX1	H
ATOM	3018	HE22	GLN	90	17.616	-12.067	24.883	1.00	0.00	RX1	H
ATOM	3019	C	GLN	90	12.835	-13.439	27.714	1.00	0.00	RX1	C
ATOM	3020	O	GLN	90	13.316	-14.558	27.570	1.00	0.00	RX1	O
ATOM	3021	N	VAL	91	11.736	-13.175	28.442	1.00	0.00	RX1	N
ATOM	3022	H	VAL	91	11.431	-12.234	28.580	1.00	0.00	RX1	H
ATOM	3023	CA	VAL	91	11.042	-14.268	29.111	1.00	0.00	RX1	C
ATOM	3024	CB	VAL	91	9.976	-13.732	30.075	1.00	0.00	RX1	C
ATOM	3025	CG1	VAL	91	8.827	-13.056	29.331	1.00	0.00	RX1	C
ATOM	3026	CG2	VAL	91	9.500	-14.817	31.045	1.00	0.00	RX1	C
ATOM	3027	C	VAL	91	10.488	-15.319	28.165	1.00	0.00	RX1	C
ATOM	3028	O	VAL	91	9.826	-15.044	27.171	1.00	0.00	RX1	O
ATOM	3029	N	ARG	92	10.823	-16.555	28.541	1.00	0.00	RX1	N
ATOM	3030	H	ARG	92	11.350	-16.650	29.388	1.00	0.00	RX1	H
ATOM	3031	CA	ARG	92	10.551	-17.663	27.630	1.00	0.00	RX1	C
ATOM	3032	CB	ARG	92	11.312	-18.901	28.109	1.00	0.00	RX1	C
ATOM	3033	CG	ARG	92	12.755	-18.547	28.491	1.00	0.00	RX1	C
ATOM	3034	CD	ARG	92	13.602	-19.767	28.864	1.00	0.00	RX1	C
ATOM	3035	NE	ARG	92	14.959	-19.413	29.291	1.00	0.00	RX1	N
ATOM	3036	HE	ARG	92	15.107	-19.408	30.290	1.00	0.00	RX1	H
ATOM	3037	CZ	ARG	92	15.939	-19.106	28.386	1.00	0.00	RX1	C
ATOM	3038	NH1	ARG	92	15.628	-18.968	27.079	1.00	0.00	RX1	N
ATOM	3039	HH11	ARG	92	16.308	-18.700	26.379	1.00	0.00	RX1	H
ATOM	3040	HH12	ARG	92	14.694	-19.095	26.741	1.00	0.00	RX1	H
ATOM	3041	NH2	ARG	92	17.210	-18.935	28.805	1.00	0.00	RX1	N
ATOM	3042	HH21	ARG	92	17.954	-18.695	28.167	1.00	0.00	RX1	H
ATOM	3043	HH22	ARG	92	17.477	-19.062	29.770	1.00	0.00	RX1	H
ATOM	3044	C	ARG	92	9.076	-17.938	27.343	1.00	0.00	RX1	C
ATOM	3045	O	ARG	92	8.675	-18.196	26.213	1.00	0.00	RX1	O
ATOM	3046	N	GLN	93	8.266	-17.850	28.409	1.00	0.00	RX1	N
ATOM	3047	H	GLN	93	8.578	-17.568	29.315	1.00	0.00	RX1	H
ATOM	3048	CA	GLN	93	6.834	-18.067	28.204	1.00	0.00	RX1	C

ATOM	3049	CB	GLN	93	6.380	-19.422	28.756	1.00	0.00	RX1	C
ATOM	3050	CG	GLN	93	6.308	-20.571	27.745	1.00	0.00	RX1	C
ATOM	3051	CD	GLN	93	7.678	-21.125	27.410	1.00	0.00	RX1	C
ATOM	3052	OE1	GLN	93	8.676	-20.851	28.070	1.00	0.00	RX1	O
ATOM	3053	NE2	GLN	93	7.662	-21.966	26.362	1.00	0.00	RX1	N
ATOM	3054	HE21	GLN	93	6.823	-22.139	25.827	1.00	0.00	RX1	H
ATOM	3055	HE22	GLN	93	8.464	-22.469	26.052	1.00	0.00	RX1	H
ATOM	3056	C	GLN	93	6.006	-16.989	28.871	1.00	0.00	RX1	C
ATOM	3057	O	GLN	93	6.401	-16.421	29.882	1.00	0.00	RX1	O
ATOM	3058	N	VAL	94	4.823	-16.757	28.278	1.00	0.00	RX1	N
ATOM	3059	H	VAL	94	4.551	-17.226	27.437	1.00	0.00	RX1	H
ATOM	3060	CA	VAL	94	3.831	-15.939	28.981	1.00	0.00	RX1	C
ATOM	3061	CB	VAL	94	3.435	-14.718	28.141	1.00	0.00	RX1	C
ATOM	3062	CG1	VAL	94	2.235	-13.975	28.729	1.00	0.00	RX1	C
ATOM	3063	CG2	VAL	94	4.627	-13.774	27.991	1.00	0.00	RX1	C
ATOM	3064	C	VAL	94	2.607	-16.766	29.354	1.00	0.00	RX1	C
ATOM	3065	O	VAL	94	1.767	-17.080	28.516	1.00	0.00	RX1	O
ATOM	3066	N	PRO	95	2.550	-17.117	30.660	1.00	0.00	RX1	N
ATOM	3067	CD	PRO	95	3.559	-16.828	31.672	1.00	0.00	RX1	C
ATOM	3068	CA	PRO	95	1.408	-17.876	31.179	1.00	0.00	RX1	C
ATOM	3069	CB	PRO	95	2.018	-18.492	32.441	1.00	0.00	RX1	C
ATOM	3070	CG	PRO	95	3.003	-17.438	32.954	1.00	0.00	RX1	C
ATOM	3071	C	PRO	95	0.212	-16.976	31.471	1.00	0.00	RX1	C
ATOM	3072	O	PRO	95	0.010	-16.485	32.576	1.00	0.00	RX1	O
ATOM	3073	N	LEU	96	-0.571	-16.749	30.418	1.00	0.00	RX1	N
ATOM	3074	H	LEU	96	-0.464	-17.249	29.555	1.00	0.00	RX1	H
ATOM	3075	CA	LEU	96	-1.721	-15.868	30.598	1.00	0.00	RX1	C
ATOM	3076	CB	LEU	96	-1.613	-14.696	29.632	1.00	0.00	RX1	C
ATOM	3077	CG	LEU	96	-1.735	-13.301	30.241	1.00	0.00	RX1	C
ATOM	3078	CD1	LEU	96	-1.477	-12.233	29.180	1.00	0.00	RX1	C
ATOM	3079	CD2	LEU	96	-3.066	-13.073	30.954	1.00	0.00	RX1	C
ATOM	3080	C	LEU	96	-3.024	-16.612	30.377	1.00	0.00	RX1	C
ATOM	3081	O	LEU	96	-3.922	-16.184	29.660	1.00	0.00	RX1	O
ATOM	3082	N	GLN	97	-3.073	-17.787	31.017	1.00	0.00	RX1	N
ATOM	3083	H	GLN	97	-2.319	-18.036	31.628	1.00	0.00	RX1	H
ATOM	3084	CA	GLN	97	-4.119	-18.759	30.697	1.00	0.00	RX1	C
ATOM	3085	CB	GLN	97	-4.023	-19.968	31.630	1.00	0.00	RX1	C
ATOM	3086	CG	GLN	97	-2.978	-21.014	31.223	1.00	0.00	RX1	C
ATOM	3087	CD	GLN	97	-1.570	-20.458	31.268	1.00	0.00	RX1	C
ATOM	3088	OE1	GLN	97	-1.233	-19.614	32.095	1.00	0.00	RX1	O
ATOM	3089	NE2	GLN	97	-0.761	-21.002	30.349	1.00	0.00	RX1	N
ATOM	3090	HE21	GLN	97	-1.143	-21.614	29.642	1.00	0.00	RX1	H
ATOM	3091	HE22	GLN	97	0.227	-20.851	30.260	1.00	0.00	RX1	H
ATOM	3092	C	GLN	97	-5.540	-18.217	30.692	1.00	0.00	RX1	C
ATOM	3093	O	GLN	97	-6.321	-18.422	29.766	1.00	0.00	RX1	O
ATOM	3094	N	ARG	98	-5.852	-17.499	31.773	1.00	0.00	RX1	N
ATOM	3095	H	ARG	98	-5.166	-17.267	32.464	1.00	0.00	RX1	H
ATOM	3096	CA	ARG	98	-7.214	-16.991	31.855	1.00	0.00	RX1	C
ATOM	3097	CB	ARG	98	-7.777	-17.165	33.266	1.00	0.00	RX1	C
ATOM	3098	CG	ARG	98	-7.951	-18.657	33.555	1.00	0.00	RX1	C
ATOM	3099	CD	ARG	98	-8.714	-18.977	34.841	1.00	0.00	RX1	C
ATOM	3100	NE	ARG	98	-7.921	-18.740	36.045	1.00	0.00	RX1	N
ATOM	3101	HE	ARG	98	-7.103	-18.144	35.993	1.00	0.00	RX1	H
ATOM	3102	CZ	ARG	98	-8.300	-19.327	37.218	1.00	0.00	RX1	C
ATOM	3103	NH1	ARG	98	-9.384	-20.136	37.230	1.00	0.00	RX1	N
ATOM	3104	HH11	ARG	98	-9.706	-20.584	38.067	1.00	0.00	RX1	H
ATOM	3105	HH12	ARG	98	-9.907	-20.315	36.393	1.00	0.00	RX1	H
ATOM	3106	NH2	ARG	98	-7.599	-19.095	38.344	1.00	0.00	RX1	N
ATOM	3107	HH21	ARG	98	-7.786	-19.481	39.247	1.00	0.00	RX1	H
ATOM	3108	HH22	ARG	98	-6.804	-18.456	38.310	1.00	0.00	RX1	H
ATOM	3109	C	ARG	98	-7.455	-15.603	31.293	1.00	0.00	RX1	C

ATOM	3110	O	ARG	98	-8.392	-14.918	31.687	1.00	0.00	RX1	O
ATOM	3111	N	LEU	99	-6.611	-15.230	30.314	1.00	0.00	RX1	N
ATOM	3112	H	LEU	99	-5.855	-15.809	30.008	1.00	0.00	RX1	H
ATOM	3113	CA	LEU	99	-6.992	-14.068	29.513	1.00	0.00	RX1	C
ATOM	3114	CB	LEU	99	-5.874	-13.665	28.550	1.00	0.00	RX1	C
ATOM	3115	CG	LEU	99	-6.191	-12.412	27.726	1.00	0.00	RX1	C
ATOM	3116	CD1	LEU	99	-6.563	-11.211	28.598	1.00	0.00	RX1	C
ATOM	3117	CD2	LEU	99	-5.056	-12.073	26.760	1.00	0.00	RX1	C
ATOM	3118	C	LEU	99	-8.267	-14.348	28.751	1.00	0.00	RX1	C
ATOM	3119	O	LEU	99	-8.335	-15.247	27.930	1.00	0.00	RX1	O
ATOM	3120	N	ARG	100	-9.284	-13.569	29.103	1.00	0.00	RX1	N
ATOM	3121	H	ARG	100	-9.123	-12.818	29.742	1.00	0.00	RX1	H
ATOM	3122	CA	ARG	100	-10.598	-13.797	28.517	1.00	0.00	RX1	C
ATOM	3123	CB	ARG	100	-11.622	-13.766	29.660	1.00	0.00	RX1	C
ATOM	3124	CG	ARG	100	-13.072	-13.453	29.295	1.00	0.00	RX1	C
ATOM	3125	CD	ARG	100	-13.988	-13.443	30.528	1.00	0.00	RX1	C
ATOM	3126	NE	ARG	100	-13.438	-12.643	31.629	1.00	0.00	RX1	N
ATOM	3127	HE	ARG	100	-12.825	-13.136	32.257	1.00	0.00	RX1	H
ATOM	3128	CZ	ARG	100	-13.727	-11.309	31.740	1.00	0.00	RX1	C
ATOM	3129	NH1	ARG	100	-14.533	-10.718	30.837	1.00	0.00	RX1	N
ATOM	3130	HH11	ARG	100	-14.685	-9.719	30.893	1.00	0.00	RX1	H
ATOM	3131	HH12	ARG	100	-14.979	-11.221	30.094	1.00	0.00	RX1	H
ATOM	3132	NH2	ARG	100	-13.213	-10.570	32.746	1.00	0.00	RX1	N
ATOM	3133	HH21	ARG	100	-13.381	-9.570	32.769	1.00	0.00	RX1	H
ATOM	3134	HH22	ARG	100	-12.651	-10.948	33.487	1.00	0.00	RX1	H
ATOM	3135	C	ARG	100	-10.893	-12.820	27.392	1.00	0.00	RX1	C
ATOM	3136	O	ARG	100	-11.257	-13.198	26.286	1.00	0.00	RX1	O
ATOM	3137	N	ILE	101	-10.718	-11.531	27.713	1.00	0.00	RX1	N
ATOM	3138	H	ILE	101	-10.358	-11.215	28.594	1.00	0.00	RX1	H
ATOM	3139	CA	ILE	101	-11.106	-10.553	26.703	1.00	0.00	RX1	C
ATOM	3140	CB	ILE	101	-12.601	-10.227	26.851	1.00	0.00	RX1	C
ATOM	3141	CG2	ILE	101	-12.917	-9.620	28.207	1.00	0.00	RX1	C
ATOM	3142	CG1	ILE	101	-13.156	-9.346	25.741	1.00	0.00	RX1	C
ATOM	3143	CD1	ILE	101	-14.657	-9.157	25.947	1.00	0.00	RX1	C
ATOM	3144	C	ILE	101	-10.216	-9.324	26.701	1.00	0.00	RX1	C
ATOM	3145	O	ILE	101	-9.916	-8.720	27.728	1.00	0.00	RX1	O
ATOM	3146	N	VAL	102	-9.788	-8.987	25.481	1.00	0.00	RX1	N
ATOM	3147	H	VAL	102	-10.160	-9.452	24.673	1.00	0.00	RX1	H
ATOM	3148	CA	VAL	102	-9.120	-7.697	25.345	1.00	0.00	RX1	C
ATOM	3149	CB	VAL	102	-8.068	-7.764	24.238	1.00	0.00	RX1	C
ATOM	3150	CG1	VAL	102	-7.258	-6.468	24.142	1.00	0.00	RX1	C
ATOM	3151	CG2	VAL	102	-7.181	-8.995	24.417	1.00	0.00	RX1	C
ATOM	3152	C	VAL	102	-10.185	-6.678	25.003	1.00	0.00	RX1	C
ATOM	3153	O	VAL	102	-11.075	-6.956	24.212	1.00	0.00	RX1	O
ATOM	3154	N	ARG	103	-10.093	-5.507	25.634	1.00	0.00	RX1	N
ATOM	3155	H	ARG	103	-9.340	-5.288	26.256	1.00	0.00	RX1	H
ATOM	3156	CA	ARG	103	-11.084	-4.505	25.264	1.00	0.00	RX1	C
ATOM	3157	CB	ARG	103	-11.607	-3.759	26.493	1.00	0.00	RX1	C
ATOM	3158	CG	ARG	103	-12.243	-4.720	27.504	1.00	0.00	RX1	C
ATOM	3159	CD	ARG	103	-12.870	-3.992	28.695	1.00	0.00	RX1	C
ATOM	3160	NE	ARG	103	-13.561	-4.914	29.597	1.00	0.00	RX1	N
ATOM	3161	HE	ARG	103	-13.040	-5.362	30.340	1.00	0.00	RX1	H
ATOM	3162	CZ	ARG	103	-14.898	-5.152	29.456	1.00	0.00	RX1	C
ATOM	3163	NH1	ARG	103	-15.587	-4.515	28.482	1.00	0.00	RX1	N
ATOM	3164	HH11	ARG	103	-16.570	-4.651	28.338	1.00	0.00	RX1	H
ATOM	3165	HH12	ARG	103	-15.126	-3.873	27.862	1.00	0.00	RX1	H
ATOM	3166	NH2	ARG	103	-15.508	-6.015	30.296	1.00	0.00	RX1	N
ATOM	3167	HH21	ARG	103	-16.478	-6.252	30.278	1.00	0.00	RX1	H
ATOM	3168	HH22	ARG	103	-14.948	-6.463	31.023	1.00	0.00	RX1	H
ATOM	3169	C	ARG	103	-10.606	-3.572	24.169	1.00	0.00	RX1	C
ATOM	3170	O	ARG	103	-11.326	-3.286	23.220	1.00	0.00	RX1	O

ATOM	3171	N	GLY	104	-9.338	-3.156	24.311	1.00	0.00	RX1	N
ATOM	3172	H	GLY	104	-8.805	-3.291	25.149	1.00	0.00	RX1	H
ATOM	3173	CA	GLY	104	-8.759	-2.404	23.199	1.00	0.00	RX1	C
ATOM	3174	C	GLY	104	-9.223	-0.965	23.106	1.00	0.00	RX1	C
ATOM	3175	O	GLY	104	-9.523	-0.427	22.049	1.00	0.00	RX1	O
ATOM	3176	N	THR	105	-9.249	-0.345	24.294	1.00	0.00	RX1	N
ATOM	3177	H	THR	105	-8.984	-0.809	25.138	1.00	0.00	RX1	H
ATOM	3178	CA	THR	105	-9.631	1.069	24.299	1.00	0.00	RX1	C
ATOM	3179	CB	THR	105	-10.040	1.413	25.726	1.00	0.00	RX1	C
ATOM	3180	OG1	THR	105	-10.911	0.378	26.210	1.00	0.00	RX1	O
ATOM	3181	HG1	THR	105	-10.360	-0.374	26.389	1.00	0.00	RX1	H
ATOM	3182	CG2	THR	105	-10.712	2.783	25.836	1.00	0.00	RX1	C
ATOM	3183	C	THR	105	-8.583	2.002	23.687	1.00	0.00	RX1	C
ATOM	3184	O	THR	105	-8.880	3.054	23.135	1.00	0.00	RX1	O
ATOM	3185	N	GLN	106	-7.325	1.552	23.800	1.00	0.00	RX1	N
ATOM	3186	H	GLN	106	-7.085	0.638	24.122	1.00	0.00	RX1	H
ATOM	3187	CA	GLN	106	-6.247	2.329	23.196	1.00	0.00	RX1	C
ATOM	3188	CB	GLN	106	-5.488	3.093	24.287	1.00	0.00	RX1	C
ATOM	3189	CG	GLN	106	-4.831	2.190	25.334	1.00	0.00	RX1	C
ATOM	3190	CD	GLN	106	-4.626	2.970	26.618	1.00	0.00	RX1	C
ATOM	3191	OE1	GLN	106	-5.584	3.210	27.348	1.00	0.00	RX1	O
ATOM	3192	NE2	GLN	106	-3.356	3.363	26.838	1.00	0.00	RX1	N
ATOM	3193	HE21	GLN	106	-2.585	3.112	26.252	1.00	0.00	RX1	H
ATOM	3194	HE22	GLN	106	-3.055	3.933	27.617	1.00	0.00	RX1	H
ATOM	3195	C	GLN	106	-5.357	1.414	22.385	1.00	0.00	RX1	C
ATOM	3196	O	GLN	106	-5.426	0.200	22.537	1.00	0.00	RX1	O
ATOM	3197	N	LEU	107	-4.583	2.037	21.482	1.00	0.00	RX1	N
ATOM	3198	H	LEU	107	-4.388	3.016	21.503	1.00	0.00	RX1	H
ATOM	3199	CA	LEU	107	-4.007	1.228	20.410	1.00	0.00	RX1	C
ATOM	3200	CB	LEU	107	-4.536	1.684	19.048	1.00	0.00	RX1	C
ATOM	3201	CG	LEU	107	-6.059	1.631	18.914	1.00	0.00	RX1	C
ATOM	3202	CD1	LEU	107	-6.519	2.239	17.589	1.00	0.00	RX1	C
ATOM	3203	CD2	LEU	107	-6.617	0.221	19.118	1.00	0.00	RX1	C
ATOM	3204	C	LEU	107	-2.490	1.191	20.374	1.00	0.00	RX1	C
ATOM	3205	O	LEU	107	-1.798	1.929	21.067	1.00	0.00	RX1	O
ATOM	3206	N	PHE	108	-2.024	0.284	19.506	1.00	0.00	RX1	N
ATOM	3207	H	PHE	108	-2.648	-0.325	19.017	1.00	0.00	RX1	H
ATOM	3208	CA	PHE	108	-0.598	0.080	19.293	1.00	0.00	RX1	C
ATOM	3209	CB	PHE	108	-0.176	-1.151	20.064	1.00	0.00	RX1	C
ATOM	3210	CG	PHE	108	1.105	-0.841	20.770	1.00	0.00	RX1	C
ATOM	3211	CD1	PHE	108	1.056	-0.323	22.052	1.00	0.00	RX1	C
ATOM	3212	CD2	PHE	108	2.317	-1.056	20.135	1.00	0.00	RX1	C
ATOM	3213	CE1	PHE	108	2.234	0.013	22.695	1.00	0.00	RX1	C
ATOM	3214	CE2	PHE	108	3.492	-0.717	20.784	1.00	0.00	RX1	C
ATOM	3215	CZ	PHE	108	3.451	-0.166	22.057	1.00	0.00	RX1	C
ATOM	3216	C	PHE	108	-0.304	-0.083	17.815	1.00	0.00	RX1	C
ATOM	3217	O	PHE	108	-1.198	-0.506	17.082	1.00	0.00	RX1	O
ATOM	3218	N	GLU	109	0.931	0.309	17.406	1.00	0.00	RX1	N
ATOM	3219	H	GLU	109	1.625	0.561	18.081	1.00	0.00	RX1	H
ATOM	3220	CA	GLU	109	1.266	0.582	15.993	1.00	0.00	RX1	C
ATOM	3221	CB	GLU	109	2.124	-0.542	15.357	1.00	0.00	RX1	C
ATOM	3222	CG	GLU	109	3.501	-0.681	16.052	1.00	0.00	RX1	C
ATOM	3223	CD	GLU	109	4.443	-1.715	15.418	1.00	0.00	RX1	C
ATOM	3224	OE1	GLU	109	4.076	-2.877	15.274	1.00	0.00	RX1	O
ATOM	3225	OE2	GLU	109	5.595	-1.395	15.120	1.00	0.00	RX1	O
ATOM	3226	C	GLU	109	0.067	1.061	15.168	1.00	0.00	RX1	C
ATOM	3227	O	GLU	109	-0.538	0.383	14.340	1.00	0.00	RX1	O
ATOM	3228	N	ASP	110	-0.309	2.284	15.580	1.00	0.00	RX1	N
ATOM	3229	H	ASP	110	0.385	2.843	16.032	1.00	0.00	RX1	H
ATOM	3230	CA	ASP	110	-1.647	2.490	16.150	1.00	0.00	RX1	C
ATOM	3231	CB	ASP	110	-1.767	3.891	16.750	1.00	0.00	RX1	C

ATOM	3232	CG	ASP	110	-0.959	3.964	18.033	1.00	0.00	RX1	C
ATOM	3233	OD1	ASP	110	-1.139	4.905	18.800	1.00	0.00	RX1	O
ATOM	3234	OD2	ASP	110	-0.144	3.079	18.289	1.00	0.00	RX1	O
ATOM	3235	C	ASP	110	-2.900	2.123	15.385	1.00	0.00	RX1	C
ATOM	3236	O	ASP	110	-3.661	2.951	14.905	1.00	0.00	RX1	O
ATOM	3237	N	ASN	111	-3.110	0.802	15.357	1.00	0.00	RX1	N
ATOM	3238	H	ASN	111	-2.365	0.192	15.640	1.00	0.00	RX1	H
ATOM	3239	CA	ASN	111	-4.384	0.263	14.873	1.00	0.00	RX1	C
ATOM	3240	CB	ASN	111	-4.299	-0.263	13.436	1.00	0.00	RX1	C
ATOM	3241	CG	ASN	111	-3.991	0.806	12.411	1.00	0.00	RX1	C
ATOM	3242	OD1	ASN	111	-4.867	1.283	11.697	1.00	0.00	RX1	O
ATOM	3243	ND2	ASN	111	-2.681	1.113	12.329	1.00	0.00	RX1	N
ATOM	3244	HD21	ASN	111	-2.002	0.706	12.951	1.00	0.00	RX1	H
ATOM	3245	HD22	ASN	111	-2.348	1.780	11.665	1.00	0.00	RX1	H
ATOM	3246	C	ASN	111	-4.862	-0.907	15.718	1.00	0.00	RX1	C
ATOM	3247	O	ASN	111	-5.905	-1.513	15.492	1.00	0.00	RX1	O
ATOM	3248	N	TYR	112	-4.007	-1.269	16.684	1.00	0.00	RX1	N
ATOM	3249	H	TYR	112	-3.207	-0.745	16.978	1.00	0.00	RX1	H
ATOM	3250	CA	TYR	112	-4.176	-2.619	17.202	1.00	0.00	RX1	C
ATOM	3251	CB	TYR	112	-3.008	-3.482	16.765	1.00	0.00	RX1	C
ATOM	3252	CG	TYR	112	-2.837	-3.464	15.265	1.00	0.00	RX1	C
ATOM	3253	CD1	TYR	112	-3.649	-4.259	14.470	1.00	0.00	RX1	C
ATOM	3254	CE1	TYR	112	-3.493	-4.254	13.092	1.00	0.00	RX1	C
ATOM	3255	CD2	TYR	112	-1.861	-2.667	14.677	1.00	0.00	RX1	C
ATOM	3256	CE2	TYR	112	-1.706	-2.657	13.296	1.00	0.00	RX1	C
ATOM	3257	CZ	TYR	112	-2.533	-3.443	12.503	1.00	0.00	RX1	C
ATOM	3258	OH	TYR	112	-2.414	-3.417	11.130	1.00	0.00	RX1	O
ATOM	3259	HH	TYR	112	-1.703	-2.835	10.893	1.00	0.00	RX1	H
ATOM	3260	C	TYR	112	-4.296	-2.696	18.704	1.00	0.00	RX1	C
ATOM	3261	O	TYR	112	-3.825	-1.829	19.425	1.00	0.00	RX1	O
ATOM	3262	N	ALA	113	-4.940	-3.784	19.135	1.00	0.00	RX1	N
ATOM	3263	H	ALA	113	-5.272	-4.474	18.494	1.00	0.00	RX1	H
ATOM	3264	CA	ALA	113	-5.056	-4.019	20.569	1.00	0.00	RX1	C
ATOM	3265	CB	ALA	113	-6.504	-4.321	20.937	1.00	0.00	RX1	C
ATOM	3266	C	ALA	113	-4.193	-5.162	21.070	1.00	0.00	RX1	C
ATOM	3267	O	ALA	113	-3.689	-5.161	22.187	1.00	0.00	RX1	O
ATOM	3268	N	LEU	114	-4.036	-6.168	20.201	1.00	0.00	RX1	N
ATOM	3269	H	LEU	114	-4.374	-6.134	19.260	1.00	0.00	RX1	H
ATOM	3270	CA	LEU	114	-3.199	-7.274	20.654	1.00	0.00	RX1	C
ATOM	3271	CB	LEU	114	-3.786	-8.612	20.215	1.00	0.00	RX1	C
ATOM	3272	CG	LEU	114	-3.084	-9.787	20.895	1.00	0.00	RX1	C
ATOM	3273	CD1	LEU	114	-3.283	-9.773	22.411	1.00	0.00	RX1	C
ATOM	3274	CD2	LEU	114	-3.489	-11.119	20.275	1.00	0.00	RX1	C
ATOM	3275	C	LEU	114	-1.767	-7.137	20.177	1.00	0.00	RX1	C
ATOM	3276	O	LEU	114	-1.362	-7.715	19.175	1.00	0.00	RX1	O
ATOM	3277	N	ALA	115	-1.030	-6.313	20.922	1.00	0.00	RX1	N
ATOM	3278	H	ALA	115	-1.335	-5.991	21.823	1.00	0.00	RX1	H
ATOM	3279	CA	ALA	115	0.322	-6.014	20.472	1.00	0.00	RX1	C
ATOM	3280	CB	ALA	115	0.674	-4.571	20.791	1.00	0.00	RX1	C
ATOM	3281	C	ALA	115	1.380	-6.910	21.076	1.00	0.00	RX1	C
ATOM	3282	O	ALA	115	1.876	-6.707	22.178	1.00	0.00	RX1	O
ATOM	3283	N	VAL	116	1.710	-7.930	20.288	1.00	0.00	RX1	N
ATOM	3284	H	VAL	116	1.343	-8.008	19.359	1.00	0.00	RX1	H
ATOM	3285	CA	VAL	116	2.817	-8.782	20.700	1.00	0.00	RX1	C
ATOM	3286	CB	VAL	116	2.349	-10.235	20.754	1.00	0.00	RX1	C
ATOM	3287	CG1	VAL	116	3.453	-11.158	21.262	1.00	0.00	RX1	C
ATOM	3288	CG2	VAL	116	1.064	-10.365	21.574	1.00	0.00	RX1	C
ATOM	3289	C	VAL	116	3.973	-8.596	19.732	1.00	0.00	RX1	C
ATOM	3290	O	VAL	116	3.904	-8.997	18.577	1.00	0.00	RX1	O
ATOM	3291	N	LEU	117	5.010	-7.910	20.230	1.00	0.00	RX1	N
ATOM	3292	H	LEU	117	5.118	-7.757	21.214	1.00	0.00	RX1	H

ATOM	3293	CA	LEU	117	5.980	-7.352	19.283	1.00	0.00	RX1	C
ATOM	3294	CB	LEU	117	5.874	-5.829	19.251	1.00	0.00	RX1	C
ATOM	3295	CG	LEU	117	4.506	-5.298	18.853	1.00	0.00	RX1	C
ATOM	3296	CD1	LEU	117	4.424	-3.782	19.014	1.00	0.00	RX1	C
ATOM	3297	CD2	LEU	117	4.146	-5.741	17.442	1.00	0.00	RX1	C
ATOM	3298	C	LEU	117	7.423	-7.676	19.595	1.00	0.00	RX1	C
ATOM	3299	O	LEU	117	7.800	-7.834	20.751	1.00	0.00	RX1	O
ATOM	3300	N	ASP	118	8.221	-7.715	18.512	1.00	0.00	RX1	N
ATOM	3301	H	ASP	118	7.797	-7.693	17.604	1.00	0.00	RX1	H
ATOM	3302	CA	ASP	118	9.689	-7.642	18.621	1.00	0.00	RX1	C
ATOM	3303	CB	ASP	118	10.154	-6.187	18.821	1.00	0.00	RX1	C
ATOM	3304	CG	ASP	118	9.823	-5.261	17.655	1.00	0.00	RX1	C
ATOM	3305	OD1	ASP	118	10.571	-5.219	16.683	1.00	0.00	RX1	O
ATOM	3306	OD2	ASP	118	8.839	-4.523	17.710	1.00	0.00	RX1	O
ATOM	3307	C	ASP	118	10.314	-8.530	19.698	1.00	0.00	RX1	C
ATOM	3308	O	ASP	118	11.147	-8.113	20.499	1.00	0.00	RX1	O
ATOM	3309	N	ASN	119	9.832	-9.782	19.731	1.00	0.00	RX1	N
ATOM	3310	H	ASN	119	9.277	-10.121	18.968	1.00	0.00	RX1	H
ATOM	3311	CA	ASN	119	10.020	-10.531	20.980	1.00	0.00	RX1	C
ATOM	3312	CB	ASN	119	8.838	-11.447	21.285	1.00	0.00	RX1	C
ATOM	3313	CG	ASN	119	7.847	-10.745	22.192	1.00	0.00	RX1	C
ATOM	3314	OD1	ASN	119	8.096	-10.469	23.364	1.00	0.00	RX1	O
ATOM	3315	ND2	ASN	119	6.687	-10.467	21.584	1.00	0.00	RX1	N
ATOM	3316	HD21	ASN	119	6.570	-10.729	20.621	1.00	0.00	RX1	H
ATOM	3317	HD22	ASN	119	5.932	-9.986	22.035	1.00	0.00	RX1	H
ATOM	3318	C	ASN	119	11.305	-11.312	21.206	1.00	0.00	RX1	C
ATOM	3319	O	ASN	119	11.319	-12.539	21.210	1.00	0.00	RX1	O
ATOM	3320	N	GLY	120	12.362	-10.532	21.479	1.00	0.00	RX1	N
ATOM	3321	H	GLY	120	12.326	-9.562	21.237	1.00	0.00	RX1	H
ATOM	3322	CA	GLY	120	13.552	-11.119	22.103	1.00	0.00	RX1	C
ATOM	3323	C	GLY	120	14.513	-11.850	21.179	1.00	0.00	RX1	C
ATOM	3324	O	GLY	120	14.439	-11.758	19.958	1.00	0.00	RX1	O
ATOM	3325	N	ASP	121	15.441	-12.567	21.837	1.00	0.00	RX1	N
ATOM	3326	H	ASP	121	15.390	-12.663	22.832	1.00	0.00	RX1	H
ATOM	3327	CA	ASP	121	16.464	-13.316	21.099	1.00	0.00	RX1	C
ATOM	3328	CB	ASP	121	17.490	-13.982	22.032	1.00	0.00	RX1	C
ATOM	3329	CG	ASP	121	18.364	-13.051	22.861	1.00	0.00	RX1	C
ATOM	3330	OD1	ASP	121	18.734	-11.977	22.403	1.00	0.00	RX1	O
ATOM	3331	OD2	ASP	121	18.728	-13.423	23.976	1.00	0.00	RX1	O
ATOM	3332	C	ASP	121	15.872	-14.425	20.234	1.00	0.00	RX1	C
ATOM	3333	O	ASP	121	15.037	-15.211	20.670	1.00	0.00	RX1	O
ATOM	3334	N	PRO	122	16.335	-14.474	18.965	1.00	0.00	RX1	N
ATOM	3335	CD	PRO	122	17.239	-13.515	18.350	1.00	0.00	RX1	C
ATOM	3336	CA	PRO	122	15.862	-15.506	18.031	1.00	0.00	RX1	C
ATOM	3337	CB	PRO	122	16.425	-15.018	16.688	1.00	0.00	RX1	C
ATOM	3338	CG	PRO	122	16.829	-13.556	16.886	1.00	0.00	RX1	C
ATOM	3339	C	PRO	122	16.308	-16.942	18.317	1.00	0.00	RX1	C
ATOM	3340	O	PRO	122	17.149	-17.494	17.616	1.00	0.00	RX1	O
ATOM	3341	N	LEU	123	15.708	-17.566	19.342	1.00	0.00	RX1	N
ATOM	3342	H	LEU	123	15.026	-17.103	19.914	1.00	0.00	RX1	H
ATOM	3343	CA	LEU	123	15.967	-19.003	19.449	1.00	0.00	RX1	C
ATOM	3344	CB	LEU	123	15.797	-19.465	20.902	1.00	0.00	RX1	C
ATOM	3345	CG	LEU	123	16.005	-20.949	21.222	1.00	0.00	RX1	C
ATOM	3346	CD1	LEU	123	17.449	-21.405	21.015	1.00	0.00	RX1	C
ATOM	3347	CD2	LEU	123	15.498	-21.289	22.624	1.00	0.00	RX1	C
ATOM	3348	C	LEU	123	15.108	-19.801	18.476	1.00	0.00	RX1	C
ATOM	3349	O	LEU	123	13.993	-19.428	18.124	1.00	0.00	RX1	O
ATOM	3350	N	ASN	124	15.710	-20.909	18.028	1.00	0.00	RX1	N
ATOM	3351	H	ASN	124	16.581	-21.209	18.417	1.00	0.00	RX1	H
ATOM	3352	CA	ASN	124	14.968	-21.846	17.189	1.00	0.00	RX1	C
ATOM	3353	CB	ASN	124	15.929	-22.569	16.243	1.00	0.00	RX1	C

ATOM	3354	CG	ASN	124	16.743	-23.553	17.060	1.00	0.00	RX1	C
ATOM	3355	OD1	ASN	124	17.233	-23.236	18.140	1.00	0.00	RX1	O
ATOM	3356	ND2	ASN	124	16.829	-24.782	16.518	1.00	0.00	RX1	N
ATOM	3357	HD21	ASN	124	16.484	-24.978	15.598	1.00	0.00	RX1	H
ATOM	3358	HD22	ASN	124	17.257	-25.534	17.028	1.00	0.00	RX1	H
ATOM	3359	C	ASN	124	14.199	-22.873	18.019	1.00	0.00	RX1	C
ATOM	3360	O	ASN	124	13.952	-22.673	19.202	1.00	0.00	RX1	O
ATOM	3361	N	ASN	125	13.831	-23.995	17.368	1.00	0.00	RX1	N
ATOM	3362	H	ASN	125	13.929	-24.080	16.377	1.00	0.00	RX1	H
ATOM	3363	CA	ASN	125	13.040	-25.005	18.081	1.00	0.00	RX1	C
ATOM	3364	CB	ASN	125	12.661	-26.203	17.214	1.00	0.00	RX1	C
ATOM	3365	CG	ASN	125	11.484	-25.813	16.361	1.00	0.00	RX1	C
ATOM	3366	OD1	ASN	125	11.648	-25.114	15.368	1.00	0.00	RX1	O
ATOM	3367	ND2	ASN	125	10.295	-26.241	16.818	1.00	0.00	RX1	N
ATOM	3368	HD21	ASN	125	10.213	-26.868	17.598	1.00	0.00	RX1	H
ATOM	3369	HD22	ASN	125	9.425	-25.955	16.408	1.00	0.00	RX1	H
ATOM	3370	C	ASN	125	13.569	-25.555	19.386	1.00	0.00	RX1	C
ATOM	3371	O	ASN	125	12.780	-25.992	20.219	1.00	0.00	RX1	O
ATOM	3372	N	THR	126	14.905	-25.527	19.537	1.00	0.00	RX1	N
ATOM	3373	H	THR	126	15.521	-25.128	18.861	1.00	0.00	RX1	H
ATOM	3374	CA	THR	126	15.537	-26.054	20.753	1.00	0.00	RX1	C
ATOM	3375	CB	THR	126	16.947	-25.510	20.671	1.00	0.00	RX1	C
ATOM	3376	OG1	THR	126	17.331	-25.557	19.288	1.00	0.00	RX1	O
ATOM	3377	HG1	THR	126	17.781	-24.727	19.133	1.00	0.00	RX1	H
ATOM	3378	CG2	THR	126	17.948	-26.239	21.568	1.00	0.00	RX1	C
ATOM	3379	C	THR	126	14.799	-25.717	22.046	1.00	0.00	RX1	C
ATOM	3380	O	THR	126	14.252	-24.631	22.215	1.00	0.00	RX1	O
ATOM	3381	N	THR	127	14.757	-26.724	22.934	1.00	0.00	RX1	N
ATOM	3382	H	THR	127	15.137	-27.636	22.776	1.00	0.00	RX1	H
ATOM	3383	CA	THR	127	14.120	-26.468	24.225	1.00	0.00	RX1	C
ATOM	3384	CB	THR	127	14.231	-27.803	24.928	1.00	0.00	RX1	C
ATOM	3385	OG1	THR	127	14.143	-28.812	23.912	1.00	0.00	RX1	O
ATOM	3386	HG1	THR	127	14.272	-29.643	24.353	1.00	0.00	RX1	H
ATOM	3387	CG2	THR	127	13.196	-28.008	26.037	1.00	0.00	RX1	C
ATOM	3388	C	THR	127	14.820	-25.328	24.959	1.00	0.00	RX1	C
ATOM	3389	O	THR	127	16.042	-25.249	24.982	1.00	0.00	RX1	O
ATOM	3390	N	PRO	128	13.999	-24.394	25.487	1.00	0.00	RX1	N
ATOM	3391	CD	PRO	128	12.541	-24.387	25.488	1.00	0.00	RX1	C
ATOM	3392	CA	PRO	128	14.587	-23.187	26.066	1.00	0.00	RX1	C
ATOM	3393	CB	PRO	128	13.385	-22.239	26.091	1.00	0.00	RX1	C
ATOM	3394	CG	PRO	128	12.173	-23.146	26.293	1.00	0.00	RX1	C
ATOM	3395	C	PRO	128	15.219	-23.438	27.427	1.00	0.00	RX1	C
ATOM	3396	O	PRO	128	14.631	-23.201	28.474	1.00	0.00	RX1	O
ATOM	3397	N	VAL	129	16.470	-23.914	27.367	1.00	0.00	RX1	N
ATOM	3398	H	VAL	129	16.929	-24.006	26.477	1.00	0.00	RX1	H
ATOM	3399	CA	VAL	129	17.211	-24.148	28.605	1.00	0.00	RX1	C
ATOM	3400	CB	VAL	129	18.591	-24.735	28.297	1.00	0.00	RX1	C
ATOM	3401	CG1	VAL	129	19.420	-24.938	29.568	1.00	0.00	RX1	C
ATOM	3402	CG2	VAL	129	18.455	-26.019	27.478	1.00	0.00	RX1	C
ATOM	3403	C	VAL	129	17.341	-22.876	29.426	1.00	0.00	RX1	C
ATOM	3404	O	VAL	129	17.864	-21.863	28.975	1.00	0.00	RX1	O
ATOM	3405	N	THR	130	16.835	-22.990	30.663	1.00	0.00	RX1	N
ATOM	3406	H	THR	130	16.367	-23.835	30.918	1.00	0.00	RX1	H
ATOM	3407	CA	THR	130	16.752	-21.863	31.591	1.00	0.00	RX1	C
ATOM	3408	CB	THR	130	16.499	-22.509	32.940	1.00	0.00	RX1	C
ATOM	3409	OG1	THR	130	15.943	-23.811	32.700	1.00	0.00	RX1	O
ATOM	3410	HG1	THR	130	15.589	-24.110	33.529	1.00	0.00	RX1	H
ATOM	3411	CG2	THR	130	15.594	-21.667	33.841	1.00	0.00	RX1	C
ATOM	3412	C	THR	130	17.935	-20.903	31.569	1.00	0.00	RX1	C
ATOM	3413	O	THR	130	17.804	-19.703	31.348	1.00	0.00	RX1	O
ATOM	3414	N	GLY	131	19.116	-21.506	31.762	1.00	0.00	RX1	N

```
ATOM   3415  H   GLY   131      19.168 -22.500  31.835  1.00  0.00      RX1  H
ATOM   3416  CA  GLY   131      20.312 -20.697  31.559  1.00  0.00      RX1  C
ATOM   3417  C   GLY   131      20.659 -20.555  30.089  1.00  0.00      RX1  C
ATOM   3418  O   GLY   131      20.280 -19.607  29.411  1.00  0.00      RX1  O
ATOM   3419  N   ALA   132      21.415 -21.565  29.634  1.00  0.00      RX1  N
ATOM   3420  H   ALA   132      21.595 -22.364  30.204  1.00  0.00      RX1  H
ATOM   3421  CA  ALA   132      21.991 -21.494  28.293  1.00  0.00      RX1  C
ATOM   3422  CB  ALA   132      23.178 -22.452  28.176  1.00  0.00      RX1  C
ATOM   3423  C   ALA   132      21.036 -21.766  27.141  1.00  0.00      RX1  C
ATOM   3424  O   ALA   132      21.041 -22.818  26.516  1.00  0.00      RX1  O
ATOM   3425  N   SER   133      20.240 -20.734  26.864  1.00  0.00      RX1  N
ATOM   3426  H   SER   133      20.169 -19.954  27.491  1.00  0.00      RX1  H
ATOM   3427  CA  SER   133      19.441 -20.645  25.646  1.00  0.00      RX1  C
ATOM   3428  CB  SER   133      18.193 -21.527  25.724  1.00  0.00      RX1  C
ATOM   3429  OG  SER   133      18.503 -22.920  25.625  1.00  0.00      RX1  O
ATOM   3430  HG  SER   133      19.442 -23.014  25.760  1.00  0.00      RX1  H
ATOM   3431  C   SER   133      19.001 -19.207  25.511  1.00  0.00      RX1  C
ATOM   3432  O   SER   133      18.540 -18.618  26.487  1.00  0.00      RX1  O
ATOM   3433  N   PRO   134      19.152 -18.661  24.280  1.00  0.00      RX1  N
ATOM   3434  CD  PRO   134      19.791 -19.304  23.139  1.00  0.00      RX1  C
ATOM   3435  CA  PRO   134      18.647 -17.314  23.975  1.00  0.00      RX1  C
ATOM   3436  CB  PRO   134      18.694 -17.313  22.445  1.00  0.00      RX1  C
ATOM   3437  CG  PRO   134      19.885 -18.200  22.094  1.00  0.00      RX1  C
ATOM   3438  C   PRO   134      17.272 -17.020  24.560  1.00  0.00      RX1  C
ATOM   3439  O   PRO   134      16.465 -17.923  24.776  1.00  0.00      RX1  O
ATOM   3440  N   GLY   135      17.072 -15.740  24.874  1.00  0.00      RX1  N
ATOM   3441  H   GLY   135      17.692 -15.000  24.599  1.00  0.00      RX1  H
ATOM   3442  CA  GLY   135      15.822 -15.384  25.526  1.00  0.00      RX1  C
ATOM   3443  C   GLY   135      14.902 -14.585  24.637  1.00  0.00      RX1  C
ATOM   3444  O   GLY   135      14.923 -13.360  24.597  1.00  0.00      RX1  O
ATOM   3445  N   GLY   136      14.079 -15.342  23.919  1.00  0.00      RX1  N
ATOM   3446  H   GLY   136      14.097 -16.343  23.893  1.00  0.00      RX1  H
ATOM   3447  CA  GLY   136      12.951 -14.671  23.297  1.00  0.00      RX1  C
ATOM   3448  C   GLY   136      11.668 -15.239  23.846  1.00  0.00      RX1  C
ATOM   3449  O   GLY   136      11.667 -16.158  24.661  1.00  0.00      RX1  O
ATOM   3450  N   LEU   137      10.572 -14.682  23.331  1.00  0.00      RX1  N
ATOM   3451  H   LEU   137      10.653 -13.989  22.612  1.00  0.00      RX1  H
ATOM   3452  CA  LEU   137       9.293 -15.265  23.717  1.00  0.00      RX1  C
ATOM   3453  CB  LEU   137       8.230 -14.175  23.723  1.00  0.00      RX1  C
ATOM   3454  CG  LEU   137       6.851 -14.664  24.148  1.00  0.00      RX1  C
ATOM   3455  CD1 LEU   137       6.869 -15.291  25.539  1.00  0.00      RX1  C
ATOM   3456  CD2 LEU   137       5.817 -13.551  24.028  1.00  0.00      RX1  C
ATOM   3457  C   LEU   137       8.906 -16.420  22.810  1.00  0.00      RX1  C
ATOM   3458  O   LEU   137       8.940 -16.318  21.592  1.00  0.00      RX1  O
ATOM   3459  N   ARG   138       8.561 -17.540  23.461  1.00  0.00      RX1  N
ATOM   3460  H   ARG   138       8.491 -17.533  24.460  1.00  0.00      RX1  H
ATOM   3461  CA  ARG   138       8.310 -18.761  22.701  1.00  0.00      RX1  C
ATOM   3462  CB  ARG   138       8.777 -19.974  23.510  1.00  0.00      RX1  C
ATOM   3463  CG  ARG   138       9.056 -21.177  22.618  1.00  0.00      RX1  C
ATOM   3464  CD  ARG   138       9.577 -22.400  23.363  1.00  0.00      RX1  C
ATOM   3465  NE  ARG   138       9.465 -23.596  22.531  1.00  0.00      RX1  N
ATOM   3466  HE  ARG   138       8.533 -23.974  22.406  1.00  0.00      RX1  H
ATOM   3467  CZ  ARG   138      10.563 -24.178  21.966  1.00  0.00      RX1  C
ATOM   3468  NH1 ARG   138      11.781 -23.614  22.124  1.00  0.00      RX1  N
ATOM   3469 HH11 ARG   138      12.632 -24.045  21.781  1.00  0.00      RX1  H
ATOM   3470 HH12 ARG   138      11.905 -22.739  22.594  1.00  0.00      RX1  H
ATOM   3471  NH2 ARG   138      10.407 -25.316  21.262  1.00  0.00      RX1  N
ATOM   3472 HH21 ARG   138      11.182 -25.818  20.857  1.00  0.00      RX1  H
ATOM   3473 HH22 ARG   138       9.478 -25.696  21.119  1.00  0.00      RX1  H
ATOM   3474  C   ARG   138       6.893 -18.961  22.190  1.00  0.00      RX1  C
ATOM   3475  O   ARG   138       6.663 -19.483  21.099  1.00  0.00      RX1  O
```

ATOM	3476	N	GLU	139	5.947	-18.546	23.046	1.00	0.00	RX1	N
ATOM	3477	H	GLU	139	6.135	-18.085	23.914	1.00	0.00	RX1	H
ATOM	3478	CA	GLU	139	4.542	-18.840	22.761	1.00	0.00	RX1	C
ATOM	3479	CB	GLU	139	4.263	-20.310	23.009	1.00	0.00	RX1	C
ATOM	3480	CG	GLU	139	4.518	-20.669	24.462	1.00	0.00	RX1	C
ATOM	3481	CD	GLU	139	5.038	-22.080	24.486	1.00	0.00	RX1	C
ATOM	3482	OE1	GLU	139	6.046	-22.327	23.833	1.00	0.00	RX1	O
ATOM	3483	OE2	GLU	139	4.457	-22.920	25.165	1.00	0.00	RX1	O
ATOM	3484	C	GLU	139	3.621	-17.952	23.569	1.00	0.00	RX1	C
ATOM	3485	O	GLU	139	4.046	-17.275	24.501	1.00	0.00	RX1	O
ATOM	3486	N	LEU	140	2.350	-17.968	23.155	1.00	0.00	RX1	N
ATOM	3487	H	LEU	140	1.996	-18.652	22.510	1.00	0.00	RX1	H
ATOM	3488	CA	LEU	140	1.402	-17.072	23.804	1.00	0.00	RX1	C
ATOM	3489	CB	LEU	140	0.717	-16.188	22.768	1.00	0.00	RX1	C
ATOM	3490	CG	LEU	140	1.628	-15.122	22.176	1.00	0.00	RX1	C
ATOM	3491	CD1	LEU	140	0.936	-14.360	21.046	1.00	0.00	RX1	C
ATOM	3492	CD2	LEU	140	2.158	-14.190	23.264	1.00	0.00	RX1	C
ATOM	3493	C	LEU	140	0.330	-17.852	24.518	1.00	0.00	RX1	C
ATOM	3494	O	LEU	140	-0.710	-18.147	23.953	1.00	0.00	RX1	O
ATOM	3495	N	GLN	141	0.610	-18.192	25.778	1.00	0.00	RX1	N
ATOM	3496	H	GLN	141	1.363	-17.786	26.298	1.00	0.00	RX1	H
ATOM	3497	CA	GLN	141	-0.350	-19.065	26.453	1.00	0.00	RX1	C
ATOM	3498	CB	GLN	141	0.371	-19.856	27.528	1.00	0.00	RX1	C
ATOM	3499	CG	GLN	141	1.544	-20.659	26.976	1.00	0.00	RX1	C
ATOM	3500	CD	GLN	141	2.273	-21.290	28.139	1.00	0.00	RX1	C
ATOM	3501	OE1	GLN	141	2.042	-20.960	29.300	1.00	0.00	RX1	O
ATOM	3502	NE2	GLN	141	3.175	-22.215	27.767	1.00	0.00	RX1	N
ATOM	3503	HE21	GLN	141	3.370	-22.411	26.798	1.00	0.00	RX1	H
ATOM	3504	HE22	GLN	141	3.706	-22.749	28.421	1.00	0.00	RX1	H
ATOM	3505	C	GLN	141	-1.553	-18.332	27.027	1.00	0.00	RX1	C
ATOM	3506	O	GLN	141	-1.681	-18.132	28.227	1.00	0.00	RX1	O
ATOM	3507	N	LEU	142	-2.424	-17.901	26.105	1.00	0.00	RX1	N
ATOM	3508	H	LEU	142	-2.306	-18.148	25.141	1.00	0.00	RX1	H
ATOM	3509	CA	LEU	142	-3.535	-17.052	26.537	1.00	0.00	RX1	C
ATOM	3510	CB	LEU	142	-3.620	-15.756	25.716	1.00	0.00	RX1	C
ATOM	3511	CG	LEU	142	-2.330	-15.204	25.088	1.00	0.00	RX1	C
ATOM	3512	CD1	LEU	142	-2.641	-14.058	24.124	1.00	0.00	RX1	C
ATOM	3513	CD2	LEU	142	-1.269	-14.774	26.099	1.00	0.00	RX1	C
ATOM	3514	C	LEU	142	-4.875	-17.759	26.421	1.00	0.00	RX1	C
ATOM	3515	O	LEU	142	-5.820	-17.242	25.838	1.00	0.00	RX1	O
ATOM	3516	N	ARG	143	-4.912	-19.001	26.942	1.00	0.00	RX1	N
ATOM	3517	H	ARG	143	-4.133	-19.342	27.471	1.00	0.00	RX1	H
ATOM	3518	CA	ARG	143	-5.944	-19.926	26.454	1.00	0.00	RX1	C
ATOM	3519	CB	ARG	143	-5.855	-21.301	27.118	1.00	0.00	RX1	C
ATOM	3520	CG	ARG	143	-6.456	-21.394	28.516	1.00	0.00	RX1	C
ATOM	3521	CD	ARG	143	-6.318	-22.798	29.085	1.00	0.00	RX1	C
ATOM	3522	NE	ARG	143	-4.911	-23.161	29.211	1.00	0.00	RX1	N
ATOM	3523	HE	ARG	143	-4.196	-22.547	28.843	1.00	0.00	RX1	H
ATOM	3524	CZ	ARG	143	-4.597	-24.344	29.802	1.00	0.00	RX1	C
ATOM	3525	NH1	ARG	143	-5.583	-25.167	30.208	1.00	0.00	RX1	N
ATOM	3526	HH11	ARG	143	-5.351	-26.072	30.616	1.00	0.00	RX1	H
ATOM	3527	HH12	ARG	143	-6.557	-24.974	30.106	1.00	0.00	RX1	H
ATOM	3528	NH2	ARG	143	-3.309	-24.683	29.973	1.00	0.00	RX1	N
ATOM	3529	HH21	ARG	143	-3.078	-25.536	30.465	1.00	0.00	RX1	H
ATOM	3530	HH22	ARG	143	-2.548	-24.121	29.614	1.00	0.00	RX1	H
ATOM	3531	C	ARG	143	-7.397	-19.481	26.327	1.00	0.00	RX1	C
ATOM	3532	O	ARG	143	-8.106	-19.864	25.397	1.00	0.00	RX1	O
ATOM	3533	N	SER	144	-7.816	-18.667	27.303	1.00	0.00	RX1	N
ATOM	3534	H	SER	144	-7.174	-18.227	27.932	1.00	0.00	RX1	H
ATOM	3535	CA	SER	144	-9.236	-18.342	27.356	1.00	0.00	RX1	C
ATOM	3536	CB	SER	144	-9.583	-18.076	28.815	1.00	0.00	RX1	C

ATOM	3537	OG	SER	144	-8.958	-19.091	29.612	1.00	0.00	RX1	O
ATOM	3538	HG	SER	144	-8.021	-18.972	29.503	1.00	0.00	RX1	H
ATOM	3539	C	SER	144	-9.722	-17.275	26.382	1.00	0.00	RX1	C
ATOM	3540	O	SER	144	-10.916	-16.993	26.294	1.00	0.00	RX1	O
ATOM	3541	N	LEU	145	-8.751	-16.702	25.647	1.00	0.00	RX1	N
ATOM	3542	H	LEU	145	-7.791	-16.964	25.754	1.00	0.00	RX1	H
ATOM	3543	CA	LEU	145	-9.076	-15.544	24.822	1.00	0.00	RX1	C
ATOM	3544	CB	LEU	145	-7.803	-15.009	24.166	1.00	0.00	RX1	C
ATOM	3545	CG	LEU	145	-7.950	-13.623	23.537	1.00	0.00	RX1	C
ATOM	3546	CD1	LEU	145	-8.508	-12.599	24.522	1.00	0.00	RX1	C
ATOM	3547	CD2	LEU	145	-6.639	-13.146	22.911	1.00	0.00	RX1	C
ATOM	3548	C	LEU	145	-10.187	-15.802	23.826	1.00	0.00	RX1	C
ATOM	3549	O	LEU	145	-10.126	-16.673	22.965	1.00	0.00	RX1	O
ATOM	3550	N	THR	146	-11.240	-15.017	24.043	1.00	0.00	RX1	N
ATOM	3551	H	THR	146	-11.284	-14.354	24.791	1.00	0.00	RX1	H
ATOM	3552	CA	THR	146	-12.446	-15.259	23.273	1.00	0.00	RX1	C
ATOM	3553	CB	THR	146	-13.507	-15.616	24.294	1.00	0.00	RX1	C
ATOM	3554	OG1	THR	146	-12.996	-15.319	25.600	1.00	0.00	RX1	O
ATOM	3555	HG1	THR	146	-12.295	-15.942	25.775	1.00	0.00	RX1	H
ATOM	3556	CG2	THR	146	-13.911	-17.088	24.201	1.00	0.00	RX1	C
ATOM	3557	C	THR	146	-12.831	-14.108	22.370	1.00	0.00	RX1	C
ATOM	3558	O	THR	146	-13.160	-14.311	21.210	1.00	0.00	RX1	O
ATOM	3559	N	GLU	147	-12.733	-12.894	22.943	1.00	0.00	RX1	N
ATOM	3560	H	GLU	147	-12.411	-12.759	23.881	1.00	0.00	RX1	H
ATOM	3561	CA	GLU	147	-12.969	-11.711	22.116	1.00	0.00	RX1	C
ATOM	3562	CB	GLU	147	-14.350	-11.088	22.381	1.00	0.00	RX1	C
ATOM	3563	CG	GLU	147	-15.588	-11.856	21.892	1.00	0.00	RX1	C
ATOM	3564	CD	GLU	147	-15.759	-11.740	20.385	1.00	0.00	RX1	C
ATOM	3565	OE1	GLU	147	-16.861	-11.476	19.908	1.00	0.00	RX1	O
ATOM	3566	OE2	GLU	147	-14.804	-11.926	19.648	1.00	0.00	RX1	O
ATOM	3567	C	GLU	147	-11.903	-10.650	22.320	1.00	0.00	RX1	C
ATOM	3568	O	GLU	147	-11.306	-10.515	23.385	1.00	0.00	RX1	O
ATOM	3569	N	ILE	148	-11.714	-9.880	21.246	1.00	0.00	RX1	N
ATOM	3570	H	ILE	148	-12.169	-10.140	20.390	1.00	0.00	RX1	H
ATOM	3571	CA	ILE	148	-10.937	-8.643	21.294	1.00	0.00	RX1	C
ATOM	3572	CB	ILE	148	-9.681	-8.740	20.423	1.00	0.00	RX1	C
ATOM	3573	CG2	ILE	148	-8.928	-7.407	20.402	1.00	0.00	RX1	C
ATOM	3574	CG1	ILE	148	-8.778	-9.892	20.860	1.00	0.00	RX1	C
ATOM	3575	CD1	ILE	148	-7.577	-10.073	19.933	1.00	0.00	RX1	C
ATOM	3576	C	ILE	148	-11.831	-7.542	20.767	1.00	0.00	RX1	C
ATOM	3577	O	ILE	148	-12.053	-7.434	19.564	1.00	0.00	RX1	O
ATOM	3578	N	LEU	149	-12.370	-6.771	21.728	1.00	0.00	RX1	N
ATOM	3579	H	LEU	149	-12.075	-6.909	22.671	1.00	0.00	RX1	H
ATOM	3580	CA	LEU	149	-13.449	-5.832	21.420	1.00	0.00	RX1	C
ATOM	3581	CB	LEU	149	-13.911	-5.044	22.646	1.00	0.00	RX1	C
ATOM	3582	CG	LEU	149	-14.500	-5.907	23.760	1.00	0.00	RX1	C
ATOM	3583	CD1	LEU	149	-15.027	-5.049	24.908	1.00	0.00	RX1	C
ATOM	3584	CD2	LEU	149	-15.585	-6.847	23.245	1.00	0.00	RX1	C
ATOM	3585	C	LEU	149	-13.198	-4.884	20.270	1.00	0.00	RX1	C
ATOM	3586	O	LEU	149	-13.901	-4.914	19.265	1.00	0.00	RX1	O
ATOM	3587	N	LYS	150	-12.184	-4.029	20.457	1.00	0.00	RX1	N
ATOM	3588	H	LYS	150	-11.611	-3.987	21.281	1.00	0.00	RX1	H
ATOM	3589	CA	LYS	150	-11.810	-3.161	19.347	1.00	0.00	RX1	C
ATOM	3590	CB	LYS	150	-12.420	-1.759	19.502	1.00	0.00	RX1	C
ATOM	3591	CG	LYS	150	-13.947	-1.828	19.365	1.00	0.00	RX1	C
ATOM	3592	CD	LYS	150	-14.730	-0.531	19.527	1.00	0.00	RX1	C
ATOM	3593	CE	LYS	150	-16.239	-0.806	19.465	1.00	0.00	RX1	C
ATOM	3594	NZ	LYS	150	-16.596	-1.470	18.203	1.00	0.00	RX1	N
ATOM	3595	HZ1	LYS	150	-17.628	-1.519	18.080	1.00	0.00	RX1	H
ATOM	3596	HZ2	LYS	150	-16.196	-0.967	17.387	1.00	0.00	RX1	H
ATOM	3597	HZ3	LYS	150	-16.261	-2.455	18.150	1.00	0.00	RX1	H

ATOM	3598	C	LYS	150	-10.307	-3.135	19.182	1.00	0.00	RX1	C
ATOM	3599	O	LYS	150	-9.560	-3.320	20.133	1.00	0.00	RX1	O
ATOM	3600	N	GLY	151	-9.900	-2.918	17.925	1.00	0.00	RX1	N
ATOM	3601	H	GLY	151	-10.548	-3.003	17.167	1.00	0.00	RX1	H
ATOM	3602	CA	GLY	151	-8.463	-2.940	17.668	1.00	0.00	RX1	C
ATOM	3603	C	GLY	151	-7.997	-4.309	17.219	1.00	0.00	RX1	C
ATOM	3604	O	GLY	151	-8.504	-5.337	17.651	1.00	0.00	RX1	O
ATOM	3605	N	GLY	152	-7.025	-4.280	16.301	1.00	0.00	RX1	N
ATOM	3606	H	GLY	152	-6.621	-3.416	15.992	1.00	0.00	RX1	H
ATOM	3607	CA	GLY	152	-6.680	-5.559	15.685	1.00	0.00	RX1	C
ATOM	3608	C	GLY	152	-5.574	-6.342	16.360	1.00	0.00	RX1	C
ATOM	3609	O	GLY	152	-5.078	-6.010	17.433	1.00	0.00	RX1	O
ATOM	3610	N	VAL	153	-5.197	-7.409	15.652	1.00	0.00	RX1	N
ATOM	3611	H	VAL	153	-5.554	-7.534	14.725	1.00	0.00	RX1	H
ATOM	3612	CA	VAL	153	-4.064	-8.219	16.079	1.00	0.00	RX1	C
ATOM	3613	CB	VAL	153	-4.323	-9.661	15.637	1.00	0.00	RX1	C
ATOM	3614	CG1	VAL	153	-3.062	-10.515	15.542	1.00	0.00	RX1	C
ATOM	3615	CG2	VAL	153	-5.369	-10.280	16.562	1.00	0.00	RX1	C
ATOM	3616	C	VAL	153	-2.774	-7.664	15.500	1.00	0.00	RX1	C
ATOM	3617	O	VAL	153	-2.697	-7.306	14.331	1.00	0.00	RX1	O
ATOM	3618	N	LEU	154	-1.781	-7.592	16.391	1.00	0.00	RX1	N
ATOM	3619	H	LEU	154	-1.876	-7.923	17.332	1.00	0.00	RX1	H
ATOM	3620	CA	LEU	154	-0.482	-7.030	16.038	1.00	0.00	RX1	C
ATOM	3621	CB	LEU	154	-0.360	-5.634	16.624	1.00	0.00	RX1	C
ATOM	3622	CG	LEU	154	0.907	-4.853	16.291	1.00	0.00	RX1	C
ATOM	3623	CD1	LEU	154	1.123	-4.669	14.793	1.00	0.00	RX1	C
ATOM	3624	CD2	LEU	154	0.937	-3.539	17.063	1.00	0.00	RX1	C
ATOM	3625	C	LEU	154	0.612	-7.900	16.602	1.00	0.00	RX1	C
ATOM	3626	O	LEU	154	1.350	-7.544	17.512	1.00	0.00	RX1	O
ATOM	3627	N	ILE	155	0.675	-9.099	16.040	1.00	0.00	RX1	N
ATOM	3628	H	ILE	155	0.180	-9.323	15.200	1.00	0.00	RX1	H
ATOM	3629	CA	ILE	155	1.744	-9.936	16.552	1.00	0.00	RX1	C
ATOM	3630	CB	ILE	155	1.242	-11.348	16.816	1.00	0.00	RX1	C
ATOM	3631	CG2	ILE	155	2.252	-12.094	17.672	1.00	0.00	RX1	C
ATOM	3632	CG1	ILE	155	-0.107	-11.332	17.526	1.00	0.00	RX1	C
ATOM	3633	CD1	ILE	155	-0.612	-12.749	17.789	1.00	0.00	RX1	C
ATOM	3634	C	ILE	155	2.937	-9.913	15.616	1.00	0.00	RX1	C
ATOM	3635	O	ILE	155	3.291	-10.895	14.971	1.00	0.00	RX1	O
ATOM	3636	N	GLN	156	3.521	-8.713	15.539	1.00	0.00	RX1	N
ATOM	3637	H	GLN	156	3.335	-8.045	16.263	1.00	0.00	RX1	H
ATOM	3638	CA	GLN	156	4.536	-8.456	14.521	1.00	0.00	RX1	C
ATOM	3639	CB	GLN	156	4.199	-7.120	13.850	1.00	0.00	RX1	C
ATOM	3640	CG	GLN	156	4.132	-7.149	12.319	1.00	0.00	RX1	C
ATOM	3641	CD	GLN	156	5.493	-6.839	11.732	1.00	0.00	RX1	C
ATOM	3642	OE1	GLN	156	5.980	-5.714	11.835	1.00	0.00	RX1	O
ATOM	3643	NE2	GLN	156	6.089	-7.877	11.116	1.00	0.00	RX1	N
ATOM	3644	HE21	GLN	156	5.702	-8.804	11.047	1.00	0.00	RX1	H
ATOM	3645	HE22	GLN	156	6.985	-7.809	10.678	1.00	0.00	RX1	H
ATOM	3646	C	GLN	156	5.967	-8.527	15.037	1.00	0.00	RX1	C
ATOM	3647	O	GLN	156	6.262	-8.146	16.165	1.00	0.00	RX1	O
ATOM	3648	N	ARG	157	6.839	-9.031	14.143	1.00	0.00	RX1	N
ATOM	3649	H	ARG	157	6.486	-9.513	13.338	1.00	0.00	RX1	H
ATOM	3650	CA	ARG	157	8.281	-9.155	14.390	1.00	0.00	RX1	C
ATOM	3651	CB	ARG	157	8.909	-7.774	14.596	1.00	0.00	RX1	C
ATOM	3652	CG	ARG	157	8.545	-6.880	13.410	1.00	0.00	RX1	C
ATOM	3653	CD	ARG	157	8.773	-5.386	13.631	1.00	0.00	RX1	C
ATOM	3654	NE	ARG	157	8.200	-4.954	14.902	1.00	0.00	RX1	N
ATOM	3655	HE	ARG	157	8.766	-5.155	15.716	1.00	0.00	RX1	H
ATOM	3656	CZ	ARG	157	7.018	-4.275	15.009	1.00	0.00	RX1	C
ATOM	3657	NH1	ARG	157	6.240	-4.034	13.937	1.00	0.00	RX1	N
ATOM	3658	HH11	ARG	157	5.438	-3.421	14.060	1.00	0.00	RX1	H

```
ATOM   3659 HH12 ARG   157       6.393  -4.437  13.025  1.00  0.00      RX1  H
ATOM   3660  NH2 ARG   157       6.629  -3.830  16.215  1.00  0.00      RX1  N
ATOM   3661 HH21 ARG   157       5.782  -3.272  16.286  1.00  0.00      RX1  H
ATOM   3662 HH22 ARG   157       7.162  -4.039  17.050  1.00  0.00      RX1  H
ATOM   3663  C   ARG   157       8.623 -10.139  15.495  1.00  0.00      RX1  C
ATOM   3664  O   ARG   157       9.182  -9.809  16.534  1.00  0.00      RX1  O
ATOM   3665  N   ASN   158       8.230 -11.394  15.238  1.00  0.00      RX1  N
ATOM   3666  H   ASN   158       7.825 -11.665  14.359  1.00  0.00      RX1  H
ATOM   3667  CA  ASN   158       8.333 -12.330  16.360  1.00  0.00      RX1  C
ATOM   3668  CB  ASN   158       6.950 -12.689  16.876  1.00  0.00      RX1  C
ATOM   3669  CG  ASN   158       6.417 -11.557  17.714  1.00  0.00      RX1  C
ATOM   3670  OD1 ASN   158       6.864 -11.290  18.825  1.00  0.00      RX1  O
ATOM   3671  ND2 ASN   158       5.434 -10.891  17.100  1.00  0.00      RX1  N
ATOM   3672 HD21 ASN   158       5.072 -11.189  16.212  1.00  0.00      RX1  H
ATOM   3673 HD22 ASN   158       5.032 -10.059  17.493  1.00  0.00      RX1  H
ATOM   3674  C   ASN   158       9.095 -13.616  16.114  1.00  0.00      RX1  C
ATOM   3675  O   ASN   158       8.526 -14.634  15.736  1.00  0.00      RX1  O
ATOM   3676  N   PRO   159      10.419 -13.547  16.400  1.00  0.00      RX1  N
ATOM   3677  CD  PRO   159      11.110 -12.410  16.997  1.00  0.00      RX1  C
ATOM   3678  CA  PRO   159      11.307 -14.667  16.067  1.00  0.00      RX1  C
ATOM   3679  CB  PRO   159      12.692 -14.071  16.348  1.00  0.00      RX1  C
ATOM   3680  CG  PRO   159      12.479 -12.957  17.374  1.00  0.00      RX1  C
ATOM   3681  C   PRO   159      11.010 -15.979  16.785  1.00  0.00      RX1  C
ATOM   3682  O   PRO   159      10.621 -16.977  16.189  1.00  0.00      RX1  O
ATOM   3683  N   GLN   160      11.193 -15.963  18.113  1.00  0.00      RX1  N
ATOM   3684  H   GLN   160      11.402 -15.126  18.617  1.00  0.00      RX1  H
ATOM   3685  CA  GLN   160      11.016 -17.228  18.827  1.00  0.00      RX1  C
ATOM   3686  CB  GLN   160      11.762 -17.127  20.158  1.00  0.00      RX1  C
ATOM   3687  CG  GLN   160      12.115 -18.462  20.813  1.00  0.00      RX1  C
ATOM   3688  CD  GLN   160      12.653 -18.185  22.199  1.00  0.00      RX1  C
ATOM   3689  OE1 GLN   160      13.789 -17.780  22.412  1.00  0.00      RX1  O
ATOM   3690  NE2 GLN   160      11.740 -18.384  23.149  1.00  0.00      RX1  N
ATOM   3691 HE21 GLN   160      10.856 -18.769  22.910  1.00  0.00      RX1  H
ATOM   3692 HE22 GLN   160      11.869 -18.076  24.097  1.00  0.00      RX1  H
ATOM   3693  C   GLN   160       9.557 -17.663  19.006  1.00  0.00      RX1  C
ATOM   3694  O   GLN   160       9.238 -18.687  19.606  1.00  0.00      RX1  O
ATOM   3695  N   LEU   161       8.670 -16.813  18.471  1.00  0.00      RX1  N
ATOM   3696  H   LEU   161       8.908 -16.182  17.735  1.00  0.00      RX1  H
ATOM   3697  CA  LEU   161       7.268 -16.960  18.814  1.00  0.00      RX1  C
ATOM   3698  CB  LEU   161       6.653 -15.576  18.956  1.00  0.00      RX1  C
ATOM   3699  CG  LEU   161       5.240 -15.592  19.519  1.00  0.00      RX1  C
ATOM   3700  CD1 LEU   161       5.249 -15.932  20.997  1.00  0.00      RX1  C
ATOM   3701  CD2 LEU   161       4.524 -14.272  19.298  1.00  0.00      RX1  C
ATOM   3702  C   LEU   161       6.505 -17.768  17.793  1.00  0.00      RX1  C
ATOM   3703  O   LEU   161       6.465 -17.457  16.608  1.00  0.00      RX1  O
ATOM   3704  N   CYS   162       5.880 -18.820  18.316  1.00  0.00      RX1  N
ATOM   3705  H   CYS   162       5.899 -18.998  19.301  1.00  0.00      RX1  H
ATOM   3706  CA  CYS   162       4.928 -19.495  17.448  1.00  0.00      RX1  C
ATOM   3707  CB  CYS   162       5.209 -20.987  17.431  1.00  0.00      RX1  C
ATOM   3708  SG  CYS   162       6.732 -21.353  16.535  1.00  0.00      RX1  S
ATOM   3709  C   CYS   162       3.499 -19.165  17.825  1.00  0.00      RX1  C
ATOM   3710  O   CYS   162       3.256 -18.362  18.717  1.00  0.00      RX1  O
ATOM   3711  N   TYR   163       2.572 -19.825  17.102  1.00  0.00      RX1  N
ATOM   3712  H   TYR   163       2.818 -20.373  16.303  1.00  0.00      RX1  H
ATOM   3713  CA  TYR   163       1.142 -19.809  17.435  1.00  0.00      RX1  C
ATOM   3714  CB  TYR   163       0.902 -20.249  18.883  1.00  0.00      RX1  C
ATOM   3715  CG  TYR   163       1.535 -21.610  19.052  1.00  0.00      RX1  C
ATOM   3716  CD1 TYR   163       1.016 -22.701  18.369  1.00  0.00      RX1  C
ATOM   3717  CE1 TYR   163       1.631 -23.942  18.451  1.00  0.00      RX1  C
ATOM   3718  CD2 TYR   163       2.652 -21.769  19.861  1.00  0.00      RX1  C
ATOM   3719  CE2 TYR   163       3.274 -23.008  19.938  1.00  0.00      RX1  C
```

ATOM	3720	CZ	TYR	163	2.774	-24.089	19.223	1.00	0.00	RX1	C
ATOM	3721	OH	TYR	163	3.418	-25.307	19.268	1.00	0.00	RX1	O
ATOM	3722	HH	TYR	163	4.057	-25.279	19.980	1.00	0.00	RX1	H
ATOM	3723	C	TYR	163	0.323	-18.591	17.037	1.00	0.00	RX1	C
ATOM	3724	O	TYR	163	-0.896	-18.556	17.142	1.00	0.00	RX1	O
ATOM	3725	N	GLN	164	1.051	-17.610	16.471	1.00	0.00	RX1	N
ATOM	3726	H	GLN	164	2.038	-17.621	16.613	1.00	0.00	RX1	H
ATOM	3727	CA	GLN	164	0.393	-16.495	15.776	1.00	0.00	RX1	C
ATOM	3728	CB	GLN	164	1.395	-15.615	15.017	1.00	0.00	RX1	C
ATOM	3729	CG	GLN	164	2.575	-15.037	15.798	1.00	0.00	RX1	C
ATOM	3730	CD	GLN	164	3.798	-15.915	15.663	1.00	0.00	RX1	C
ATOM	3731	OE1	GLN	164	3.739	-17.118	15.896	1.00	0.00	RX1	O
ATOM	3732	NE2	GLN	164	4.916	-15.260	15.319	1.00	0.00	RX1	N
ATOM	3733	HE21	GLN	164	4.821	-14.346	14.908	1.00	0.00	RX1	H
ATOM	3734	HE22	GLN	164	5.843	-15.631	15.427	1.00	0.00	RX1	H
ATOM	3735	C	GLN	164	-0.668	-16.906	14.759	1.00	0.00	RX1	C
ATOM	3736	O	GLN	164	-1.673	-16.242	14.558	1.00	0.00	RX1	O
ATOM	3737	N	ASP	165	-0.339	-18.023	14.106	1.00	0.00	RX1	N
ATOM	3738	H	ASP	165	0.421	-18.584	14.423	1.00	0.00	RX1	H
ATOM	3739	CA	ASP	165	-1.145	-18.599	13.032	1.00	0.00	RX1	C
ATOM	3740	CB	ASP	165	-0.240	-19.292	11.992	1.00	0.00	RX1	C
ATOM	3741	CG	ASP	165	0.995	-19.976	12.587	1.00	0.00	RX1	C
ATOM	3742	OD1	ASP	165	1.010	-20.366	13.753	1.00	0.00	RX1	O
ATOM	3743	OD2	ASP	165	2.014	-20.057	11.907	1.00	0.00	RX1	O
ATOM	3744	C	ASP	165	-2.230	-19.546	13.513	1.00	0.00	RX1	C
ATOM	3745	O	ASP	165	-3.397	-19.436	13.155	1.00	0.00	RX1	O
ATOM	3746	N	THR	166	-1.793	-20.488	14.359	1.00	0.00	RX1	N
ATOM	3747	H	THR	166	-0.834	-20.550	14.642	1.00	0.00	RX1	H
ATOM	3748	CA	THR	166	-2.692	-21.524	14.868	1.00	0.00	RX1	C
ATOM	3749	CB	THR	166	-1.828	-22.402	15.749	1.00	0.00	RX1	C
ATOM	3750	OG1	THR	166	-0.949	-21.551	16.480	1.00	0.00	RX1	O
ATOM	3751	HG1	THR	166	-1.284	-21.527	17.376	1.00	0.00	RX1	H
ATOM	3752	CG2	THR	166	-1.002	-23.388	14.922	1.00	0.00	RX1	C
ATOM	3753	C	THR	166	-3.933	-20.983	15.559	1.00	0.00	RX1	C
ATOM	3754	O	THR	166	-5.050	-21.477	15.426	1.00	0.00	RX1	O
ATOM	3755	N	ILE	167	-3.687	-19.905	16.313	1.00	0.00	RX1	N
ATOM	3756	H	ILE	167	-2.780	-19.477	16.344	1.00	0.00	RX1	H
ATOM	3757	CA	ILE	167	-4.855	-19.234	16.863	1.00	0.00	RX1	C
ATOM	3758	CB	ILE	167	-4.493	-18.429	18.099	1.00	0.00	RX1	C
ATOM	3759	CG2	ILE	167	-5.748	-17.803	18.702	1.00	0.00	RX1	C
ATOM	3760	CG1	ILE	167	-3.806	-19.337	19.110	1.00	0.00	RX1	C
ATOM	3761	CD1	ILE	167	-4.770	-20.422	19.589	1.00	0.00	RX1	C
ATOM	3762	C	ILE	167	-5.556	-18.377	15.829	1.00	0.00	RX1	C
ATOM	3763	O	ILE	167	-5.259	-17.212	15.609	1.00	0.00	RX1	O
ATOM	3764	N	LEU	168	-6.542	-19.024	15.198	1.00	0.00	RX1	N
ATOM	3765	H	LEU	168	-6.628	-20.009	15.357	1.00	0.00	RX1	H
ATOM	3766	CA	LEU	168	-7.302	-18.305	14.182	1.00	0.00	RX1	C
ATOM	3767	CB	LEU	168	-8.188	-19.285	13.415	1.00	0.00	RX1	C
ATOM	3768	CG	LEU	168	-8.784	-18.690	12.142	1.00	0.00	RX1	C
ATOM	3769	CD1	LEU	168	-7.700	-18.179	11.192	1.00	0.00	RX1	C
ATOM	3770	CD2	LEU	168	-9.740	-19.669	11.459	1.00	0.00	RX1	C
ATOM	3771	C	LEU	168	-8.085	-17.101	14.704	1.00	0.00	RX1	C
ATOM	3772	O	LEU	168	-9.225	-17.187	15.154	1.00	0.00	RX1	O
ATOM	3773	N	TRP	169	-7.407	-15.948	14.576	1.00	0.00	RX1	N
ATOM	3774	H	TRP	169	-6.429	-16.043	14.369	1.00	0.00	RX1	H
ATOM	3775	CA	TRP	169	-7.945	-14.671	15.057	1.00	0.00	RX1	C
ATOM	3776	CB	TRP	169	-6.938	-13.544	14.838	1.00	0.00	RX1	C
ATOM	3777	CG	TRP	169	-5.657	-13.916	15.536	1.00	0.00	RX1	C
ATOM	3778	CD2	TRP	169	-5.414	-14.021	16.953	1.00	0.00	RX1	C
ATOM	3779	CE2	TRP	169	-4.078	-14.451	17.124	1.00	0.00	RX1	C
ATOM	3780	CE3	TRP	169	-6.216	-13.798	18.066	1.00	0.00	RX1	C

ATOM	3781	CD1	TRP	169	-4.449	-14.280	14.930	1.00	0.00	RX1	C
ATOM	3782	NE1	TRP	169	-3.516	-14.598	15.861	1.00	0.00	RX1	N
ATOM	3783	HE1	TRP	169	-2.623	-14.951	15.651	1.00	0.00	RX1	H
ATOM	3784	CZ2	TRP	169	-3.580	-14.648	18.405	1.00	0.00	RX1	C
ATOM	3785	CZ3	TRP	169	-5.704	-13.997	19.341	1.00	0.00	RX1	C
ATOM	3786	CH2	TRP	169	-4.393	-14.424	19.509	1.00	0.00	RX1	C
ATOM	3787	C	TRP	169	-9.304	-14.278	14.515	1.00	0.00	RX1	C
ATOM	3788	O	TRP	169	-10.076	-13.578	15.154	1.00	0.00	RX1	O
ATOM	3789	N	LYS	170	-9.601	-14.839	13.330	1.00	0.00	RX1	N
ATOM	3790	H	LYS	170	-8.880	-15.357	12.874	1.00	0.00	RX1	H
ATOM	3791	CA	LYS	170	-10.940	-14.772	12.731	1.00	0.00	RX1	C
ATOM	3792	CB	LYS	170	-10.972	-15.726	11.532	1.00	0.00	RX1	C
ATOM	3793	CG	LYS	170	-12.206	-15.660	10.623	1.00	0.00	RX1	C
ATOM	3794	CD	LYS	170	-12.729	-17.053	10.251	1.00	0.00	RX1	C
ATOM	3795	CE	LYS	170	-13.977	-17.502	11.030	1.00	0.00	RX1	C
ATOM	3796	NZ	LYS	170	-13.788	-17.360	12.478	1.00	0.00	RX1	N
ATOM	3797	HZ1	LYS	170	-14.438	-17.955	13.038	1.00	0.00	RX1	H
ATOM	3798	HZ2	LYS	170	-12.855	-17.705	12.796	1.00	0.00	RX1	H
ATOM	3799	HZ3	LYS	170	-13.898	-16.373	12.790	1.00	0.00	RX1	H
ATOM	3800	C	LYS	170	-12.119	-15.088	13.659	1.00	0.00	RX1	C
ATOM	3801	O	LYS	170	-13.281	-14.863	13.336	1.00	0.00	RX1	O
ATOM	3802	N	ASP	171	-11.803	-15.693	14.801	1.00	0.00	RX1	N
ATOM	3803	H	ASP	171	-10.874	-15.996	15.019	1.00	0.00	RX1	H
ATOM	3804	CA	ASP	171	-12.892	-15.994	15.722	1.00	0.00	RX1	C
ATOM	3805	CB	ASP	171	-12.809	-17.472	16.120	1.00	0.00	RX1	C
ATOM	3806	CG	ASP	171	-12.812	-18.329	14.860	1.00	0.00	RX1	C
ATOM	3807	OD1	ASP	171	-13.821	-18.930	14.523	1.00	0.00	RX1	O
ATOM	3808	OD2	ASP	171	-11.821	-18.382	14.139	1.00	0.00	RX1	O
ATOM	3809	C	ASP	171	-12.917	-15.050	16.909	1.00	0.00	RX1	C
ATOM	3810	O	ASP	171	-13.969	-14.658	17.406	1.00	0.00	RX1	O
ATOM	3811	N	ILE	172	-11.686	-14.684	17.305	1.00	0.00	RX1	N
ATOM	3812	H	ILE	172	-10.893	-14.883	16.733	1.00	0.00	RX1	H
ATOM	3813	CA	ILE	172	-11.481	-13.832	18.477	1.00	0.00	RX1	C
ATOM	3814	CB	ILE	172	-10.115	-14.171	19.079	1.00	0.00	RX1	C
ATOM	3815	CG2	ILE	172	-9.859	-13.483	20.416	1.00	0.00	RX1	C
ATOM	3816	CG1	ILE	172	-9.986	-15.682	19.237	1.00	0.00	RX1	C
ATOM	3817	CD1	ILE	172	-8.715	-16.036	20.002	1.00	0.00	RX1	C
ATOM	3818	C	ILE	172	-11.616	-12.338	18.186	1.00	0.00	RX1	C
ATOM	3819	O	ILE	172	-11.702	-11.491	19.065	1.00	0.00	RX1	O
ATOM	3820	N	PHE	173	-11.660	-12.016	16.885	1.00	0.00	RX1	N
ATOM	3821	H	PHE	173	-11.536	-12.690	16.160	1.00	0.00	RX1	H
ATOM	3822	CA	PHE	173	-12.087	-10.650	16.593	1.00	0.00	RX1	C
ATOM	3823	CB	PHE	173	-11.944	-10.342	15.106	1.00	0.00	RX1	C
ATOM	3824	CG	PHE	173	-10.511	-10.032	14.763	1.00	0.00	RX1	C
ATOM	3825	CD1	PHE	173	-9.792	-9.141	15.548	1.00	0.00	RX1	C
ATOM	3826	CD2	PHE	173	-9.920	-10.627	13.657	1.00	0.00	RX1	C
ATOM	3827	CE1	PHE	173	-8.484	-8.824	15.208	1.00	0.00	RX1	C
ATOM	3828	CE2	PHE	173	-8.611	-10.309	13.319	1.00	0.00	RX1	C
ATOM	3829	CZ	PHE	173	-7.900	-9.392	14.082	1.00	0.00	RX1	C
ATOM	3830	C	PHE	173	-13.523	-10.436	17.006	1.00	0.00	RX1	C
ATOM	3831	O	PHE	173	-14.418	-11.174	16.596	1.00	0.00	RX1	O
ATOM	3832	N	HIS	174	-13.699	-9.401	17.841	1.00	0.00	RX1	N
ATOM	3833	H	HIS	174	-12.933	-8.853	18.177	1.00	0.00	RX1	H
ATOM	3834	CA	HIS	174	-15.055	-9.147	18.312	1.00	0.00	RX1	C
ATOM	3835	CB	HIS	174	-15.061	-7.995	19.304	1.00	0.00	RX1	C
ATOM	3836	CG	HIS	174	-16.427	-7.725	19.880	1.00	0.00	RX1	C
ATOM	3837	ND1	HIS	174	-17.232	-8.666	20.404	1.00	0.00	RX1	N
ATOM	3838	HD1	HIS	174	-17.024	-9.626	20.514	1.00	0.00	RX1	H
ATOM	3839	CD2	HIS	174	-17.067	-6.487	19.963	1.00	0.00	RX1	C
ATOM	3840	NE2	HIS	174	-18.275	-6.696	20.537	1.00	0.00	RX1	N
ATOM	3841	CE1	HIS	174	-18.378	-8.037	20.813	1.00	0.00	RX1	C

ATOM	3842	C	HIS	174	-16.029	-8.912	17.188	1.00	0.00	RX1	C
ATOM	3843	O	HIS	174	-15.714	-8.360	16.142	1.00	0.00	RX1	O
ATOM	3844	N	LYS	175	-17.252	-9.365	17.453	1.00	0.00	RX1	N
ATOM	3845	H	LYS	175	-17.438	-9.767	18.353	1.00	0.00	RX1	H
ATOM	3846	CA	LYS	175	-18.237	-9.275	16.380	1.00	0.00	RX1	C
ATOM	3847	CB	LYS	175	-19.374	-10.254	16.683	1.00	0.00	RX1	C
ATOM	3848	CG	LYS	175	-18.819	-11.590	17.213	1.00	0.00	RX1	C
ATOM	3849	CD	LYS	175	-17.891	-12.343	16.246	1.00	0.00	RX1	C
ATOM	3850	CE	LYS	175	-17.106	-13.503	16.886	1.00	0.00	RX1	C
ATOM	3851	NZ	LYS	175	-16.001	-13.014	17.721	1.00	0.00	RX1	N
ATOM	3852	HZ1	LYS	175	-16.298	-12.565	18.616	1.00	0.00	RX1	H
ATOM	3853	HZ2	LYS	175	-15.325	-13.759	17.990	1.00	0.00	RX1	H
ATOM	3854	HZ3	LYS	175	-15.432	-12.294	17.240	1.00	0.00	RX1	H
ATOM	3855	C	LYS	175	-18.681	-7.847	16.040	1.00	0.00	RX1	C
ATOM	3856	O	LYS	175	-19.304	-7.573	15.025	1.00	0.00	RX1	O
ATOM	3857	N	ASN	176	-18.271	-6.924	16.928	1.00	0.00	RX1	N
ATOM	3858	H	ASN	176	-17.832	-7.206	17.777	1.00	0.00	RX1	H
ATOM	3859	CA	ASN	176	-18.310	-5.502	16.578	1.00	0.00	RX1	C
ATOM	3860	CB	ASN	176	-19.270	-4.713	17.471	1.00	0.00	RX1	C
ATOM	3861	CG	ASN	176	-19.418	-3.303	16.923	1.00	0.00	RX1	C
ATOM	3862	OD1	ASN	176	-18.957	-2.319	17.503	1.00	0.00	RX1	O
ATOM	3863	ND2	ASN	176	-20.103	-3.256	15.765	1.00	0.00	RX1	N
ATOM	3864	HD21	ASN	176	-20.456	-4.096	15.344	1.00	0.00	RX1	H
ATOM	3865	HD22	ASN	176	-20.300	-2.401	15.284	1.00	0.00	RX1	H
ATOM	3866	C	ASN	176	-16.923	-4.855	16.584	1.00	0.00	RX1	C
ATOM	3867	O	ASN	176	-16.712	-3.741	17.059	1.00	0.00	RX1	O
ATOM	3868	N	ASN	177	-15.955	-5.602	16.045	1.00	0.00	RX1	N
ATOM	3869	H	ASN	177	-16.105	-6.494	15.612	1.00	0.00	RX1	H
ATOM	3870	CA	ASN	177	-14.636	-4.977	15.965	1.00	0.00	RX1	C
ATOM	3871	CB	ASN	177	-13.504	-5.948	16.271	1.00	0.00	RX1	C
ATOM	3872	CG	ASN	177	-12.235	-5.162	16.528	1.00	0.00	RX1	C
ATOM	3873	OD1	ASN	177	-12.083	-3.993	16.166	1.00	0.00	RX1	O
ATOM	3874	ND2	ASN	177	-11.315	-5.881	17.193	1.00	0.00	RX1	N
ATOM	3875	HD21	ASN	177	-11.572	-6.739	17.648	1.00	0.00	RX1	H
ATOM	3876	HD22	ASN	177	-10.354	-5.622	17.321	1.00	0.00	RX1	H
ATOM	3877	C	ASN	177	-14.389	-4.340	14.618	1.00	0.00	RX1	C
ATOM	3878	O	ASN	177	-13.951	-4.969	13.666	1.00	0.00	RX1	O
ATOM	3879	N	GLN	178	-14.681	-3.033	14.584	1.00	0.00	RX1	N
ATOM	3880	H	GLN	178	-15.005	-2.572	15.409	1.00	0.00	RX1	H
ATOM	3881	CA	GLN	178	-14.436	-2.315	13.332	1.00	0.00	RX1	C
ATOM	3882	CB	GLN	178	-15.041	-0.903	13.372	1.00	0.00	RX1	C
ATOM	3883	CG	GLN	178	-14.249	0.170	14.133	1.00	0.00	RX1	C
ATOM	3884	CD	GLN	178	-14.126	-0.178	15.602	1.00	0.00	RX1	C
ATOM	3885	OE1	GLN	178	-15.039	-0.723	16.223	1.00	0.00	RX1	O
ATOM	3886	NE2	GLN	178	-12.935	0.154	16.126	1.00	0.00	RX1	N
ATOM	3887	HE21	GLN	178	-12.251	0.608	15.550	1.00	0.00	RX1	H
ATOM	3888	HE22	GLN	178	-12.665	-0.012	17.075	1.00	0.00	RX1	H
ATOM	3889	C	GLN	178	-12.981	-2.293	12.875	1.00	0.00	RX1	C
ATOM	3890	O	GLN	178	-12.666	-2.199	11.699	1.00	0.00	RX1	O
ATOM	3891	N	LEU	179	-12.095	-2.394	13.876	1.00	0.00	RX1	N
ATOM	3892	H	LEU	179	-12.361	-2.637	14.809	1.00	0.00	RX1	H
ATOM	3893	CA	LEU	179	-10.691	-2.512	13.510	1.00	0.00	RX1	C
ATOM	3894	CB	LEU	179	-9.811	-1.591	14.351	1.00	0.00	RX1	C
ATOM	3895	CG	LEU	179	-10.062	-0.107	14.097	1.00	0.00	RX1	C
ATOM	3896	CD1	LEU	179	-9.215	0.766	15.022	1.00	0.00	RX1	C
ATOM	3897	CD2	LEU	179	-9.874	0.260	12.625	1.00	0.00	RX1	C
ATOM	3898	C	LEU	179	-10.214	-3.938	13.665	1.00	0.00	RX1	C
ATOM	3899	O	LEU	179	-9.296	-4.232	14.418	1.00	0.00	RX1	O
ATOM	3900	N	ALA	180	-10.874	-4.826	12.912	1.00	0.00	RX1	N
ATOM	3901	H	ALA	180	-11.591	-4.529	12.280	1.00	0.00	RX1	H
ATOM	3902	CA	ALA	180	-10.442	-6.224	12.944	1.00	0.00	RX1	C

ATOM	3903	CB	ALA	180	-11.591	-7.146	12.537	1.00	0.00	RX1	C
ATOM	3904	C	ALA	180	-9.244	-6.503	12.044	1.00	0.00	RX1	C
ATOM	3905	O	ALA	180	-9.267	-7.339	11.151	1.00	0.00	RX1	O
ATOM	3906	N	LEU	181	-8.191	-5.718	12.298	1.00	0.00	RX1	N
ATOM	3907	H	LEU	181	-8.144	-5.197	13.151	1.00	0.00	RX1	H
ATOM	3908	CA	LEU	181	-7.066	-5.753	11.372	1.00	0.00	RX1	C
ATOM	3909	CB	LEU	181	-6.428	-4.370	11.281	1.00	0.00	RX1	C
ATOM	3910	CG	LEU	181	-7.441	-3.281	10.926	1.00	0.00	RX1	C
ATOM	3911	CD1	LEU	181	-6.816	-1.888	10.988	1.00	0.00	RX1	C
ATOM	3912	CD2	LEU	181	-8.121	-3.543	9.580	1.00	0.00	RX1	C
ATOM	3913	C	LEU	181	-6.053	-6.816	11.740	1.00	0.00	RX1	C
ATOM	3914	O	LEU	181	-5.922	-7.213	12.893	1.00	0.00	RX1	O
ATOM	3915	N	THR	182	-5.364	-7.283	10.700	1.00	0.00	RX1	N
ATOM	3916	H	THR	182	-5.513	-6.961	9.765	1.00	0.00	RX1	H
ATOM	3917	CA	THR	182	-4.695	-8.568	10.848	1.00	0.00	RX1	C
ATOM	3918	CB	THR	182	-5.472	-9.383	9.842	1.00	0.00	RX1	C
ATOM	3919	OG1	THR	182	-6.573	-8.552	9.424	1.00	0.00	RX1	O
ATOM	3920	HG1	THR	182	-7.286	-8.681	10.044	1.00	0.00	RX1	H
ATOM	3921	CG2	THR	182	-5.982	-10.709	10.407	1.00	0.00	RX1	C
ATOM	3922	C	THR	182	-3.188	-8.563	10.672	1.00	0.00	RX1	C
ATOM	3923	O	THR	182	-2.649	-9.223	9.791	1.00	0.00	RX1	O
ATOM	3924	N	LEU	183	-2.498	-7.824	11.554	1.00	0.00	RX1	N
ATOM	3925	H	LEU	183	-2.917	-7.386	12.352	1.00	0.00	RX1	H
ATOM	3926	CA	LEU	183	-1.046	-7.988	11.484	1.00	0.00	RX1	C
ATOM	3927	CB	LEU	183	-0.288	-6.728	11.891	1.00	0.00	RX1	C
ATOM	3928	CG	LEU	183	-0.121	-5.791	10.695	1.00	0.00	RX1	C
ATOM	3929	CD1	LEU	183	0.788	-4.602	11.012	1.00	0.00	RX1	C
ATOM	3930	CD2	LEU	183	0.363	-6.551	9.459	1.00	0.00	RX1	C
ATOM	3931	C	LEU	183	-0.528	-9.206	12.225	1.00	0.00	RX1	C
ATOM	3932	O	LEU	183	0.039	-9.151	13.311	1.00	0.00	RX1	O
ATOM	3933	N	ILE	184	-0.769	-10.336	11.558	1.00	0.00	RX1	N
ATOM	3934	H	ILE	184	-1.145	-10.286	10.630	1.00	0.00	RX1	H
ATOM	3935	CA	ILE	184	-0.245	-11.604	12.048	1.00	0.00	RX1	C
ATOM	3936	CB	ILE	184	-1.182	-12.741	11.648	1.00	0.00	RX1	C
ATOM	3937	CG2	ILE	184	-0.666	-14.085	12.159	1.00	0.00	RX1	C
ATOM	3938	CG1	ILE	184	-2.621	-12.463	12.073	1.00	0.00	RX1	C
ATOM	3939	CD1	ILE	184	-3.573	-13.525	11.519	1.00	0.00	RX1	C
ATOM	3940	C	ILE	184	1.110	-11.842	11.414	1.00	0.00	RX1	C
ATOM	3941	O	ILE	184	1.212	-12.046	10.212	1.00	0.00	RX1	O
ATOM	3942	N	ASP	185	2.144	-11.779	12.254	1.00	0.00	RX1	N
ATOM	3943	H	ASP	185	2.091	-11.696	13.250	1.00	0.00	RX1	H
ATOM	3944	CA	ASP	185	3.458	-11.989	11.663	1.00	0.00	RX1	C
ATOM	3945	CB	ASP	185	4.356	-10.821	12.030	1.00	0.00	RX1	C
ATOM	3946	CG	ASP	185	5.828	-11.135	11.899	1.00	0.00	RX1	C
ATOM	3947	OD1	ASP	185	6.408	-10.850	10.858	1.00	0.00	RX1	O
ATOM	3948	OD2	ASP	185	6.417	-11.604	12.868	1.00	0.00	RX1	O
ATOM	3949	C	ASP	185	4.042	-13.321	12.067	1.00	0.00	RX1	C
ATOM	3950	O	ASP	185	3.821	-13.844	13.155	1.00	0.00	RX1	O
ATOM	3951	N	THR	186	4.788	-13.854	11.104	1.00	0.00	RX1	N
ATOM	3952	H	THR	186	4.871	-13.428	10.205	1.00	0.00	RX1	H
ATOM	3953	CA	THR	186	5.407	-15.151	11.311	1.00	0.00	RX1	C
ATOM	3954	CB	THR	186	4.582	-15.957	10.342	1.00	0.00	RX1	C
ATOM	3955	OG1	THR	186	3.806	-14.990	9.612	1.00	0.00	RX1	O
ATOM	3956	HG1	THR	186	3.150	-15.477	9.131	1.00	0.00	RX1	H
ATOM	3957	CG2	THR	186	3.637	-16.930	11.049	1.00	0.00	RX1	C
ATOM	3958	C	THR	186	6.900	-15.124	11.032	1.00	0.00	RX1	C
ATOM	3959	O	THR	186	7.482	-16.086	10.542	1.00	0.00	RX1	O
ATOM	3960	N	ASN	187	7.501	-13.969	11.374	1.00	0.00	RX1	N
ATOM	3961	H	ASN	187	6.975	-13.223	11.792	1.00	0.00	RX1	H
ATOM	3962	CA	ASN	187	8.951	-13.803	11.229	1.00	0.00	RX1	C
ATOM	3963	CB	ASN	187	9.349	-12.346	11.423	1.00	0.00	RX1	C

```
ATOM   3964  CG  ASN   187       9.995 -11.836  10.163  1.00  0.00      RX1  C
ATOM   3965  OD1 ASN   187      11.195 -11.964   9.941  1.00  0.00      RX1  O
ATOM   3966  ND2 ASN   187       9.108 -11.247   9.341  1.00  0.00      RX1  N
ATOM   3967 HD21 ASN   187       8.157 -11.142   9.661  1.00  0.00      RX1  H
ATOM   3968 HD22 ASN   187       9.346 -10.922   8.426  1.00  0.00      RX1  H
ATOM   3969  C   ASN   187       9.751 -14.597  12.239  1.00  0.00      RX1  C
ATOM   3970  O   ASN   187      10.260 -14.078  13.226  1.00  0.00      RX1  O
ATOM   3971  N   ARG   188       9.794 -15.901  11.964  1.00  0.00      RX1  N
ATOM   3972  H   ARG   188       9.463 -16.252  11.087  1.00  0.00      RX1  H
ATOM   3973  CA  ARG   188      10.239 -16.818  13.002  1.00  0.00      RX1  C
ATOM   3974  CB  ARG   188       9.270 -17.984  13.113  1.00  0.00      RX1  C
ATOM   3975  CG  ARG   188       7.872 -17.624  13.593  1.00  0.00      RX1  C
ATOM   3976  CD  ARG   188       6.990 -18.867  13.537  1.00  0.00      RX1  C
ATOM   3977  NE  ARG   188       5.677 -18.605  14.104  1.00  0.00      RX1  N
ATOM   3978  HE  ARG   188       5.655 -18.007  14.919  1.00  0.00      RX1  H
ATOM   3979  CZ  ARG   188       4.589 -19.216  13.562  1.00  0.00      RX1  C
ATOM   3980  NH1 ARG   188       4.707 -20.028  12.493  1.00  0.00      RX1  N
ATOM   3981 HH11 ARG   188       3.860 -20.402  12.077  1.00  0.00      RX1  H
ATOM   3982 HH12 ARG   188       5.587 -20.270  12.081  1.00  0.00      RX1  H
ATOM   3983  NH2 ARG   188       3.385 -18.993  14.094  1.00  0.00      RX1  N
ATOM   3984 HH21 ARG   188       2.569 -19.465  13.716  1.00  0.00      RX1  H
ATOM   3985 HH22 ARG   188       3.253 -18.351  14.854  1.00  0.00      RX1  H
ATOM   3986  C   ARG   188      11.620 -17.392  12.805  1.00  0.00      RX1  C
ATOM   3987  O   ARG   188      12.076 -17.665  11.704  1.00  0.00      RX1  O
ATOM   3988  N   SER   189      12.233 -17.633  13.965  1.00  0.00      RX1  N
ATOM   3989  H   SER   189      11.880 -17.319  14.844  1.00  0.00      RX1  H
ATOM   3990  CA  SER   189      13.373 -18.532  14.025  1.00  0.00      RX1  C
ATOM   3991  CB  SER   189      14.299 -17.945  15.081  1.00  0.00      RX1  C
ATOM   3992  OG  SER   189      13.494 -17.368  16.120  1.00  0.00      RX1  O
ATOM   3993  HG  SER   189      13.471 -18.025  16.817  1.00  0.00      RX1  H
ATOM   3994  C   SER   189      12.973 -19.978  14.298  1.00  0.00      RX1  C
ATOM   3995  O   SER   189      13.771 -20.900  14.174  1.00  0.00      RX1  O
ATOM   3996  N   ARG   190      11.698 -20.151  14.691  1.00  0.00      RX1  N
ATOM   3997  H   ARG   190      11.025 -19.415  14.762  1.00  0.00      RX1  H
ATOM   3998  CA  ARG   190      11.280 -21.521  14.965  1.00  0.00      RX1  C
ATOM   3999  CB  ARG   190      11.192 -21.795  16.466  1.00  0.00      RX1  C
ATOM   4000  CG  ARG   190       9.994 -21.168  17.174  1.00  0.00      RX1  C
ATOM   4001  CD  ARG   190       9.772 -21.880  18.508  1.00  0.00      RX1  C
ATOM   4002  NE  ARG   190       8.567 -21.437  19.209  1.00  0.00      RX1  N
ATOM   4003  HE  ARG   190       8.532 -20.472  19.510  1.00  0.00      RX1  H
ATOM   4004  CZ  ARG   190       7.679 -22.410  19.575  1.00  0.00      RX1  C
ATOM   4005  NH1 ARG   190       7.753 -23.626  18.995  1.00  0.00      RX1  N
ATOM   4006 HH11 ARG   190       7.262 -24.401  19.436  1.00  0.00      RX1  H
ATOM   4007 HH12 ARG   190       8.259 -23.817  18.144  1.00  0.00      RX1  H
ATOM   4008  NH2 ARG   190       6.745 -22.165  20.518  1.00  0.00      RX1  N
ATOM   4009 HH21 ARG   190       6.151 -22.915  20.856  1.00  0.00      RX1  H
ATOM   4010 HH22 ARG   190       6.615 -21.256  20.935  1.00  0.00      RX1  H
ATOM   4011  C   ARG   190       9.986 -21.936  14.294  1.00  0.00      RX1  C
ATOM   4012  O   ARG   190       9.186 -21.121  13.857  1.00  0.00      RX1  O
ATOM   4013  N   ALA   191       9.803 -23.257  14.265  1.00  0.00      RX1  N
ATOM   4014  H   ALA   191      10.486 -23.873  14.652  1.00  0.00      RX1  H
ATOM   4015  CA  ALA   191       8.490 -23.780  13.921  1.00  0.00      RX1  C
ATOM   4016  CB  ALA   191       8.623 -25.046  13.074  1.00  0.00      RX1  C
ATOM   4017  C   ALA   191       7.686 -24.106  15.167  1.00  0.00      RX1  C
ATOM   4018  O   ALA   191       8.204 -24.209  16.280  1.00  0.00      RX1  O
ATOM   4019  N   CYS   192       6.380 -24.270  14.917  1.00  0.00      RX1  N
ATOM   4020  H   CYS   192       6.027 -24.247  13.985  1.00  0.00      RX1  H
ATOM   4021  CA  CYS   192       5.502 -24.737  15.988  1.00  0.00      RX1  C
ATOM   4022  CB  CYS   192       4.061 -24.457  15.571  1.00  0.00      RX1  C
ATOM   4023  SG  CYS   192       3.918 -22.791  14.870  1.00  0.00      RX1  S
ATOM   4024  C   CYS   192       5.740 -26.203  16.285  1.00  0.00      RX1  C
```

ATOM	4025	O	CYS	192	6.375	-26.908	15.509	1.00	0.00	RX1	O
ATOM	4026	N	HIS	193	5.221	-26.629	17.436	1.00	0.00	RX1	N
ATOM	4027	H	HIS	193	4.596	-26.073	17.988	1.00	0.00	RX1	H
ATOM	4028	CA	HIS	193	5.238	-28.064	17.690	1.00	0.00	RX1	C
ATOM	4029	CB	HIS	193	5.475	-28.305	19.188	1.00	0.00	RX1	C
ATOM	4030	CG	HIS	193	6.955	-28.221	19.489	1.00	0.00	RX1	C
ATOM	4031	ND1	HIS	193	7.502	-28.595	20.659	1.00	0.00	RX1	N
ATOM	4032	HD1	HIS	193	7.015	-28.917	21.447	1.00	0.00	RX1	H
ATOM	4033	CD2	HIS	193	7.978	-27.768	18.649	1.00	0.00	RX1	C
ATOM	4034	NE2	HIS	193	9.152	-27.869	19.322	1.00	0.00	RX1	N
ATOM	4035	CE1	HIS	193	8.853	-28.382	20.562	1.00	0.00	RX1	C
ATOM	4036	C	HIS	193	3.921	-28.647	17.200	1.00	0.00	RX1	C
ATOM	4037	O	HIS	193	2.940	-27.925	17.077	1.00	0.00	RX1	O
ATOM	4038	N	PRO	194	3.920	-29.962	16.863	1.00	0.00	RX1	N
ATOM	4039	CD	PRO	194	5.051	-30.884	16.869	1.00	0.00	RX1	C
ATOM	4040	CA	PRO	194	2.680	-30.589	16.382	1.00	0.00	RX1	C
ATOM	4041	CB	PRO	194	3.061	-32.071	16.312	1.00	0.00	RX1	C
ATOM	4042	CG	PRO	194	4.565	-32.072	16.047	1.00	0.00	RX1	C
ATOM	4043	C	PRO	194	1.440	-30.327	17.228	1.00	0.00	RX1	C
ATOM	4044	O	PRO	194	1.343	-30.720	18.384	1.00	0.00	RX1	O
ATOM	4045	N	CYS	195	0.487	-29.646	16.574	1.00	0.00	RX1	N
ATOM	4046	H	CYS	195	0.640	-29.311	15.647	1.00	0.00	RX1	H
ATOM	4047	CA	CYS	195	-0.775	-29.328	17.242	1.00	0.00	RX1	C
ATOM	4048	CB	CYS	195	-1.634	-28.559	16.244	1.00	0.00	RX1	C
ATOM	4049	SG	CYS	195	-0.630	-27.396	15.284	1.00	0.00	RX1	S
ATOM	4050	C	CYS	195	-1.512	-30.547	17.767	1.00	0.00	RX1	C
ATOM	4051	O	CYS	195	-1.735	-31.521	17.057	1.00	0.00	RX1	O
ATOM	4052	N	SER	196	-1.882	-30.461	19.048	1.00	0.00	RX1	N
ATOM	4053	H	SER	196	-1.782	-29.634	19.597	1.00	0.00	RX1	H
ATOM	4054	CA	SER	196	-2.615	-31.597	19.595	1.00	0.00	RX1	C
ATOM	4055	CB	SER	196	-2.454	-31.517	21.110	1.00	0.00	RX1	C
ATOM	4056	OG	SER	196	-3.060	-30.311	21.572	1.00	0.00	RX1	O
ATOM	4057	HG	SER	196	-2.354	-29.697	21.774	1.00	0.00	RX1	H
ATOM	4058	C	SER	196	-4.071	-31.606	19.133	1.00	0.00	RX1	C
ATOM	4059	O	SER	196	-4.614	-30.569	18.769	1.00	0.00	RX1	O
ATOM	4060	N	PRO	197	-4.730	-32.793	19.183	1.00	0.00	RX1	N
ATOM	4061	CD	PRO	197	-4.193	-34.115	19.485	1.00	0.00	RX1	C
ATOM	4062	CA	PRO	197	-6.176	-32.800	18.915	1.00	0.00	RX1	C
ATOM	4063	CB	PRO	197	-6.516	-34.295	18.976	1.00	0.00	RX1	C
ATOM	4064	CG	PRO	197	-5.428	-34.934	19.839	1.00	0.00	RX1	C
ATOM	4065	C	PRO	197	-6.998	-31.934	19.872	1.00	0.00	RX1	C
ATOM	4066	O	PRO	197	-8.033	-31.383	19.518	1.00	0.00	RX1	O
ATOM	4067	N	MET	198	-6.475	-31.796	21.108	1.00	0.00	RX1	N
ATOM	4068	H	MET	198	-5.601	-32.214	21.345	1.00	0.00	RX1	H
ATOM	4069	CA	MET	198	-7.136	-30.887	22.053	1.00	0.00	RX1	C
ATOM	4070	CB	MET	198	-6.505	-31.002	23.437	1.00	0.00	RX1	C
ATOM	4071	CG	MET	198	-6.474	-32.440	23.953	1.00	0.00	RX1	C
ATOM	4072	SD	MET	198	-8.110	-33.189	23.995	1.00	0.00	RX1	S
ATOM	4073	CE	MET	198	-7.622	-34.814	24.595	1.00	0.00	RX1	C
ATOM	4074	C	MET	198	-7.138	-29.436	21.593	1.00	0.00	RX1	C
ATOM	4075	O	MET	198	-8.051	-28.648	21.831	1.00	0.00	RX1	O
ATOM	4076	N	CYS	199	-6.089	-29.141	20.817	1.00	0.00	RX1	N
ATOM	4077	H	CYS	199	-5.329	-29.774	20.660	1.00	0.00	RX1	H
ATOM	4078	CA	CYS	199	-6.109	-27.929	20.011	1.00	0.00	RX1	C
ATOM	4079	CB	CYS	199	-4.674	-27.556	19.690	1.00	0.00	RX1	C
ATOM	4080	SG	CYS	199	-3.651	-27.527	21.178	1.00	0.00	RX1	S
ATOM	4081	C	CYS	199	-6.945	-28.043	18.746	1.00	0.00	RX1	C
ATOM	4082	O	CYS	199	-6.498	-27.726	17.654	1.00	0.00	RX1	O
ATOM	4083	N	LYS	200	-8.204	-28.482	18.936	1.00	0.00	RX1	N
ATOM	4084	H	LYS	200	-8.466	-28.845	19.828	1.00	0.00	RX1	H
ATOM	4085	CA	LYS	200	-9.173	-28.532	17.838	1.00	0.00	RX1	C

```
ATOM   4086  CB  LYS   200      -10.608 -28.724  18.365  1.00  0.00       RX1  C
ATOM   4087  CG  LYS   200      -11.226 -27.536  19.124  1.00  0.00       RX1  C
ATOM   4088  CD  LYS   200      -10.670 -27.328  20.535  1.00  0.00       RX1  C
ATOM   4089  CE  LYS   200      -11.015 -25.977  21.155  1.00  0.00       RX1  C
ATOM   4090  NZ  LYS   200      -10.332 -25.862  22.451  1.00  0.00       RX1  N
ATOM   4091  HZ1 LYS   200      -10.272 -24.852  22.710  1.00  0.00       RX1  H
ATOM   4092  HZ2 LYS   200      -10.862 -26.371  23.185  1.00  0.00       RX1  H
ATOM   4093  HZ3 LYS   200       -9.378 -26.267  22.376  1.00  0.00       RX1  H
ATOM   4094  C   LYS   200       -9.114 -27.367  16.865  1.00  0.00       RX1  C
ATOM   4095  O   LYS   200       -9.074 -26.197  17.235  1.00  0.00       RX1  O
ATOM   4096  N   GLY   201       -9.061 -27.763  15.591  1.00  0.00       RX1  N
ATOM   4097  H   GLY   201       -9.010 -28.735  15.368  1.00  0.00       RX1  H
ATOM   4098  CA  GLY   201       -8.913 -26.752  14.549  1.00  0.00       RX1  C
ATOM   4099  C   GLY   201       -7.538 -26.106  14.494  1.00  0.00       RX1  C
ATOM   4100  O   GLY   201       -7.380 -25.004  13.987  1.00  0.00       RX1  O
ATOM   4101  N   SER   202       -6.561 -26.858  15.035  1.00  0.00       RX1  N
ATOM   4102  H   SER   202       -6.771 -27.703  15.521  1.00  0.00       RX1  H
ATOM   4103  CA  SER   202       -5.156 -26.438  15.107  1.00  0.00       RX1  C
ATOM   4104  CB  SER   202       -4.654 -26.167  13.690  1.00  0.00       RX1  C
ATOM   4105  OG  SER   202       -5.497 -26.899  12.788  1.00  0.00       RX1  O
ATOM   4106  HG  SER   202       -6.120 -26.250  12.479  1.00  0.00       RX1  H
ATOM   4107  C   SER   202       -4.847 -25.344  16.120  1.00  0.00       RX1  C
ATOM   4108  O   SER   202       -3.765 -24.777  16.163  1.00  0.00       RX1  O
ATOM   4109  N   ARG   203       -5.857 -25.048  16.947  1.00  0.00       RX1  N
ATOM   4110  H   ARG   203       -6.642 -25.662  17.036  1.00  0.00       RX1  H
ATOM   4111  CA  ARG   203       -5.732 -23.838  17.752  1.00  0.00       RX1  C
ATOM   4112  CB  ARG   203       -7.108 -23.235  18.002  1.00  0.00       RX1  C
ATOM   4113  CG  ARG   203       -7.922 -23.009  16.728  1.00  0.00       RX1  C
ATOM   4114  CD  ARG   203       -9.273 -22.379  17.059  1.00  0.00       RX1  C
ATOM   4115  NE  ARG   203      -10.162 -22.327  15.903  1.00  0.00       RX1  N
ATOM   4116  HE  ARG   203      -10.389 -23.221  15.506  1.00  0.00       RX1  H
ATOM   4117  CZ  ARG   203      -10.666 -21.126  15.485  1.00  0.00       RX1  C
ATOM   4118  NH1 ARG   203      -10.215 -19.968  16.019  1.00  0.00       RX1  N
ATOM   4119 HH11 ARG   203      -10.523 -19.076  15.652  1.00  0.00       RX1  H
ATOM   4120 HH12 ARG   203       -9.552 -19.926  16.782  1.00  0.00       RX1  H
ATOM   4121  NH2 ARG   203      -11.622 -21.109  14.534  1.00  0.00       RX1  N
ATOM   4122 HH21 ARG   203      -12.047 -20.228  14.262  1.00  0.00       RX1  H
ATOM   4123 HH22 ARG   203      -11.962 -21.934  14.079  1.00  0.00       RX1  H
ATOM   4124  C   ARG   203       -4.979 -24.018  19.063  1.00  0.00       RX1  C
ATOM   4125  O   ARG   203       -5.566 -24.076  20.141  1.00  0.00       RX1  O
ATOM   4126  N   CYS   204       -3.650 -24.104  18.922  1.00  0.00       RX1  N
ATOM   4127  H   CYS   204       -3.227 -24.063  18.014  1.00  0.00       RX1  H
ATOM   4128  CA  CYS   204       -2.796 -24.118  20.111  1.00  0.00       RX1  C
ATOM   4129  CB  CYS   204       -1.699 -25.159  19.928  1.00  0.00       RX1  C
ATOM   4130  SG  CYS   204       -1.969 -26.096  18.407  1.00  0.00       RX1  S
ATOM   4131  C   CYS   204       -2.160 -22.774  20.371  1.00  0.00       RX1  C
ATOM   4132  O   CYS   204       -1.808 -22.054  19.446  1.00  0.00       RX1  O
ATOM   4133  N   TRP   205       -2.051 -22.481  21.673  1.00  0.00       RX1  N
ATOM   4134  H   TRP   205       -2.356 -23.148  22.353  1.00  0.00       RX1  H
ATOM   4135  CA  TRP   205       -1.409 -21.250  22.137  1.00  0.00       RX1  C
ATOM   4136  CB  TRP   205       -2.053 -20.793  23.436  1.00  0.00       RX1  C
ATOM   4137  CG  TRP   205       -3.454 -20.279  23.251  1.00  0.00       RX1  C
ATOM   4138  CD2 TRP   205       -3.884 -18.974  22.810  1.00  0.00       RX1  C
ATOM   4139  CE2 TRP   205       -5.295 -18.945  22.888  1.00  0.00       RX1  C
ATOM   4140  CE3 TRP   205       -3.199 -17.847  22.379  1.00  0.00       RX1  C
ATOM   4141  CD1 TRP   205       -4.631 -20.969  23.552  1.00  0.00       RX1  C
ATOM   4142  NE1 TRP   205       -5.720 -20.185  23.344  1.00  0.00       RX1  N
ATOM   4143  HE1 TRP   205       -6.649 -20.417  23.557  1.00  0.00       RX1  H
ATOM   4144  CZ2 TRP   205       -5.984 -17.794  22.529  1.00  0.00       RX1  C
ATOM   4145  CZ3 TRP   205       -3.898 -16.703  22.021  1.00  0.00       RX1  C
ATOM   4146  CH2 TRP   205       -5.285 -16.674  22.102  1.00  0.00       RX1  C
```

ATOM	4147	C	TRP	205	0.074	-21.424	22.426	1.00	0.00	RX1	C
ATOM	4148	O	TRP	205	0.902	-20.535	22.254	1.00	0.00	RX1	O
ATOM	4149	N	GLY	206	0.355	-22.638	22.913	1.00	0.00	RX1	N
ATOM	4150	H	GLY	206	-0.318	-23.374	22.968	1.00	0.00	RX1	H
ATOM	4151	CA	GLY	206	1.727	-22.972	23.267	1.00	0.00	RX1	C
ATOM	4152	C	GLY	206	2.063	-24.333	22.713	1.00	0.00	RX1	C
ATOM	4153	O	GLY	206	1.248	-24.909	21.998	1.00	0.00	RX1	O
ATOM	4154	N	GLU	207	3.250	-24.840	23.084	1.00	0.00	RX1	N
ATOM	4155	H	GLU	207	3.843	-24.309	23.701	1.00	0.00	RX1	H
ATOM	4156	CA	GLU	207	3.585	-26.224	22.717	1.00	0.00	RX1	C
ATOM	4157	CB	GLU	207	5.059	-26.545	22.998	1.00	0.00	RX1	C
ATOM	4158	CG	GLU	207	6.083	-25.416	22.834	1.00	0.00	RX1	C
ATOM	4159	CD	GLU	207	6.524	-25.194	21.400	1.00	0.00	RX1	C
ATOM	4160	OE1	GLU	207	5.745	-24.743	20.573	1.00	0.00	RX1	O
ATOM	4161	OE2	GLU	207	7.694	-25.396	21.103	1.00	0.00	RX1	O
ATOM	4162	C	GLU	207	2.739	-27.216	23.512	1.00	0.00	RX1	C
ATOM	4163	O	GLU	207	3.176	-27.779	24.508	1.00	0.00	RX1	O
ATOM	4164	N	SER	208	1.478	-27.365	23.087	1.00	0.00	RX1	N
ATOM	4165	H	SER	208	1.125	-26.933	22.255	1.00	0.00	RX1	H
ATOM	4166	CA	SER	208	0.561	-27.818	24.126	1.00	0.00	RX1	C
ATOM	4167	CB	SER	208	0.103	-26.561	24.881	1.00	0.00	RX1	C
ATOM	4168	OG	SER	208	-0.266	-26.862	26.235	1.00	0.00	RX1	O
ATOM	4169	HG	SER	208	0.528	-26.727	26.748	1.00	0.00	RX1	H
ATOM	4170	C	SER	208	-0.591	-28.705	23.692	1.00	0.00	RX1	C
ATOM	4171	O	SER	208	-1.069	-28.703	22.561	1.00	0.00	RX1	O
ATOM	4172	N	SER	209	-1.038	-29.448	24.707	1.00	0.00	RX1	N
ATOM	4173	H	SER	209	-0.498	-29.579	25.536	1.00	0.00	RX1	H
ATOM	4174	CA	SER	209	-2.352	-30.081	24.679	1.00	0.00	RX1	C
ATOM	4175	CB	SER	209	-1.988	-31.488	25.079	1.00	0.00	RX1	C
ATOM	4176	OG	SER	209	-0.549	-31.483	25.089	1.00	0.00	RX1	O
ATOM	4177	HG	SER	209	-0.276	-32.381	25.223	1.00	0.00	RX1	H
ATOM	4178	C	SER	209	-3.362	-29.391	25.589	1.00	0.00	RX1	C
ATOM	4179	O	SER	209	-4.533	-29.739	25.636	1.00	0.00	RX1	O
ATOM	4180	N	GLU	210	-2.826	-28.418	26.339	1.00	0.00	RX1	N
ATOM	4181	H	GLU	210	-1.908	-28.060	26.173	1.00	0.00	RX1	H
ATOM	4182	CA	GLU	210	-3.585	-27.801	27.419	1.00	0.00	RX1	C
ATOM	4183	CB	GLU	210	-2.686	-27.854	28.655	1.00	0.00	RX1	C
ATOM	4184	CG	GLU	210	-3.242	-28.671	29.821	1.00	0.00	RX1	C
ATOM	4185	CD	GLU	210	-4.190	-27.799	30.606	1.00	0.00	RX1	C
ATOM	4186	OE1	GLU	210	-3.723	-26.966	31.378	1.00	0.00	RX1	O
ATOM	4187	OE2	GLU	210	-5.397	-27.892	30.419	1.00	0.00	RX1	O
ATOM	4188	C	GLU	210	-3.995	-26.382	27.061	1.00	0.00	RX1	C
ATOM	4189	O	GLU	210	-5.156	-25.985	27.085	1.00	0.00	RX1	O
ATOM	4190	N	ASP	211	-2.966	-25.613	26.672	1.00	0.00	RX1	N
ATOM	4191	H	ASP	211	-2.028	-25.959	26.638	1.00	0.00	RX1	H
ATOM	4192	CA	ASP	211	-3.270	-24.274	26.169	1.00	0.00	RX1	C
ATOM	4193	CB	ASP	211	-2.070	-23.333	26.288	1.00	0.00	RX1	C
ATOM	4194	CG	ASP	211	-2.037	-22.730	27.677	1.00	0.00	RX1	C
ATOM	4195	OD1	ASP	211	-2.665	-21.698	27.898	1.00	0.00	RX1	O
ATOM	4196	OD2	ASP	211	-1.398	-23.295	28.555	1.00	0.00	RX1	O
ATOM	4197	C	ASP	211	-3.807	-24.278	24.757	1.00	0.00	RX1	C
ATOM	4198	O	ASP	211	-3.093	-24.153	23.765	1.00	0.00	RX1	O
ATOM	4199	N	CYS	212	-5.131	-24.439	24.725	1.00	0.00	RX1	N
ATOM	4200	H	CYS	212	-5.650	-24.552	25.576	1.00	0.00	RX1	H
ATOM	4201	CA	CYS	212	-5.829	-24.511	23.448	1.00	0.00	RX1	C
ATOM	4202	CB	CYS	212	-6.337	-25.936	23.283	1.00	0.00	RX1	C
ATOM	4203	SG	CYS	212	-5.100	-27.151	23.792	1.00	0.00	RX1	S
ATOM	4204	C	CYS	212	-6.952	-23.495	23.434	1.00	0.00	RX1	C
ATOM	4205	O	CYS	212	-7.514	-23.187	24.476	1.00	0.00	RX1	O
ATOM	4206	N	GLN	213	-7.274	-22.972	22.238	1.00	0.00	RX1	N
ATOM	4207	H	GLN	213	-6.768	-23.247	21.418	1.00	0.00	RX1	H

```
ATOM   4208  CA  GLN   213      -8.230 -21.858  22.212  1.00  0.00      RX1  C
ATOM   4209  CB  GLN   213      -8.440 -21.338  20.790  1.00  0.00      RX1  C
ATOM   4210  CG  GLN   213      -8.871 -19.870  20.744  1.00  0.00      RX1  C
ATOM   4211  CD  GLN   213      -9.496 -19.550  19.401  1.00  0.00      RX1  C
ATOM   4212  OE1 GLN   213      -8.861 -19.548  18.348  1.00  0.00      RX1  O
ATOM   4213  NE2 GLN   213     -10.806 -19.277  19.492  1.00  0.00      RX1  N
ATOM   4214 HE21 GLN   213     -11.250 -19.378  20.389  1.00  0.00      RX1  H
ATOM   4215 HE22 GLN   213     -11.393 -18.969  18.746  1.00  0.00      RX1  H
ATOM   4216  C   GLN   213      -9.578 -22.140  22.858  1.00  0.00      RX1  C
ATOM   4217  O   GLN   213     -10.185 -23.191  22.642  1.00  0.00      RX1  O
ATOM   4218  N   SER   214     -10.019 -21.148  23.640  1.00  0.00      RX1  N
ATOM   4219  H   SER   214      -9.453 -20.347  23.844  1.00  0.00      RX1  H
ATOM   4220  CA  SER   214     -11.420 -21.140  24.048  1.00  0.00      RX1  C
ATOM   4221  CB  SER   214     -11.500 -20.355  25.343  1.00  0.00      RX1  C
ATOM   4222  OG  SER   214     -10.535 -20.954  26.213  1.00  0.00      RX1  O
ATOM   4223  HG  SER   214      -9.672 -20.746  25.868  1.00  0.00      RX1  H
ATOM   4224  C   SER   214     -12.329 -20.659  22.931  1.00  0.00      RX1  C
ATOM   4225  O   SER   214     -11.932 -20.593  21.769  1.00  0.00      RX1  O
TER
HEADER lig.000.00.pdb
ATOM      1  N   ASP   985      23.255  24.361  13.670  1.00  0.00      LX0  N
ATOM      2  H   ASP   985      23.910  23.740  14.112  0.00  0.00      LX0  H
ATOM      3  CA  ASP   985      23.424  25.771  14.025  1.00  0.00      LX0  C
ATOM      4  CB  ASP   985      22.095  26.383  14.489  1.00  0.00      LX0  C
ATOM      5  CG  ASP   985      21.878  27.707  13.773  1.00  0.00      LX0  C
ATOM      6  OD1 ASP   985      21.015  27.770  12.899  1.00  0.00      LX0  O
ATOM      7  OD2 ASP   985      22.581  28.668  14.073  1.00  0.00      LX0  O
ATOM      8  C   ASP   985      24.523  25.957  15.053  1.00  0.00      LX0  C
ATOM      9  O   ASP   985      25.166  24.991  15.446  1.00  0.00      LX0  O
ATOM     10  N   VAL   986      24.711  27.227  15.454  1.00  0.00      LX0  N
ATOM     11  H   VAL   986      24.102  27.909  15.041  0.00  0.00      LX0  H
ATOM     12  CA  VAL   986      25.633  27.617  16.523  1.00  0.00      LX0  C
ATOM     13  CB  VAL   986      24.925  27.635  17.893  1.00  0.00      LX0  C
ATOM     14  CG1 VAL   986      25.772  28.338  18.963  1.00  0.00      LX0  C
ATOM     15  CG2 VAL   986      23.543  28.288  17.797  1.00  0.00      LX0  C
ATOM     16  C   VAL   986      26.960  26.868  16.584  1.00  0.00      LX0  C
ATOM     17  O   VAL   986      27.196  25.984  17.400  1.00  0.00      LX0  O
ATOM     18  N   TYR   987      27.859  27.314  15.691  1.00  0.00      LX0  N
ATOM     19  H   TYR   987      27.616  28.039  15.051  0.00  0.00      LX0  H
ATOM     20  CA  TYR   987      29.230  26.835  15.860  1.00  0.00      LX0  C
ATOM     21  CB  TYR   987      30.099  27.152  14.630  1.00  0.00      LX0  C
ATOM     22  CG  TYR   987      31.484  26.547  14.770  1.00  0.00      LX0  C
ATOM     23  CD1 TYR   987      31.699  25.209  14.378  1.00  0.00      LX0  C
ATOM     24  CE1 TYR   987      32.979  24.648  14.532  1.00  0.00      LX0  C
ATOM     25  CD2 TYR   987      32.526  27.339  15.300  1.00  0.00      LX0  C
ATOM     26  CE2 TYR   987      33.802  26.777  15.462  1.00  0.00      LX0  C
ATOM     27  CZ  TYR   987      34.011  25.439  15.078  1.00  0.00      LX0  C
ATOM     28  OH  TYR   987      35.266  24.887  15.245  1.00  0.00      LX0  O
ATOM     29  HH  TYR   987      35.324  24.623  16.172  0.00  0.00      LX0  H
ATOM     30  C   TYR   987      29.855  27.376  17.135  1.00  0.00      LX0  C
ATOM     31  O   TYR   987      30.350  28.493  17.203  1.00  0.00      LX0  O
ATOM     32  N   VAL   988      29.808  26.506  18.145  1.00  0.00      LX0  N
ATOM     33  H   VAL   988      29.275  25.670  18.013  0.00  0.00      LX0  H
ATOM     34  CA  VAL   988      30.588  26.782  19.346  1.00  0.00      LX0  C
ATOM     35  CB  VAL   988      30.135  25.831  20.479  1.00  0.00      LX0  C
ATOM     36  CG1 VAL   988      30.253  24.351  20.095  1.00  0.00      LX0  C
ATOM     37  CG2 VAL   988      30.816  26.137  21.817  1.00  0.00      LX0  C
ATOM     38  C   VAL   988      32.081  26.691  19.045  1.00  0.00      LX0  C
ATOM     39  O   VAL   988      32.544  25.740  18.428  1.00  0.00      LX0  O
ATOM     40  N   PRO   989      32.826  27.736  19.474  1.00  0.00      LX0  N
ATOM     41  CD  PRO   989      32.347  29.014  19.986  1.00  0.00      LX0  C
```

ATOM	42	CA	PRO	989	34.289	27.647	19.431	1.00	0.00	LX0	C
ATOM	43	CB	PRO	989	34.725	28.994	20.017	1.00	0.00	LX0	C
ATOM	44	CG	PRO	989	33.543	29.937	19.810	1.00	0.00	LX0	C
ATOM	45	C	PRO	989	34.809	26.466	20.237	1.00	0.00	LX0	C
ATOM	46	O	PRO	989	34.824	26.483	21.463	1.00	0.00	LX0	O
ATOM	47	N	ASP	990	35.198	25.434	19.486	1.00	0.00	LX0	N
ATOM	48	H	ASP	990	35.150	25.475	18.490	0.00	0.00	LX0	H
ATOM	49	CA	ASP	990	35.824	24.282	20.126	1.00	0.00	LX0	C
ATOM	50	CB	ASP	990	35.587	23.001	19.304	1.00	0.00	LX0	C
ATOM	51	CG	ASP	990	35.827	23.190	17.813	1.00	0.00	LX0	C
ATOM	52	OD1	ASP	990	36.867	23.693	17.397	1.00	0.00	LX0	O
ATOM	53	OD2	ASP	990	34.961	22.832	17.026	1.00	0.00	LX0	O
ATOM	54	C	ASP	990	37.292	24.540	20.402	1.00	0.00	LX0	C
ATOM	55	O	ASP	990	37.786	25.653	20.244	1.00	0.00	LX0	O
ATOM	56	N	GLU	991	37.992	23.477	20.825	1.00	0.00	LX0	N
ATOM	57	H	GLU	991	37.596	22.554	20.868	0.00	0.00	LX0	H
ATOM	58	CA	GLU	991	39.378	23.669	21.246	1.00	0.00	LX0	C
ATOM	59	CB	GLU	991	39.891	22.449	22.032	1.00	0.00	LX0	C
ATOM	60	CG	GLU	991	40.085	21.117	21.278	1.00	0.00	LX0	C
ATOM	61	CD	GLU	991	38.778	20.426	20.902	1.00	0.00	LX0	C
ATOM	62	OE1	GLU	991	37.755	20.664	21.544	1.00	0.00	LX0	O
ATOM	63	OE2	GLU	991	38.791	19.632	19.963	1.00	0.00	LX0	O
ATOM	64	C	GLU	991	40.374	24.112	20.179	1.00	0.00	LX0	C
ATOM	65	O	GLU	991	41.542	24.358	20.456	1.00	0.00	LX0	O
ATOM	66	N	TRP	992	39.866	24.226	18.943	1.00	0.00	LX0	N
ATOM	67	H	TRP	992	38.900	24.033	18.762	0.00	0.00	LX0	H
ATOM	68	CA	TRP	992	40.750	24.687	17.879	1.00	0.00	LX0	C
ATOM	69	CB	TRP	992	40.538	23.871	16.594	1.00	0.00	LX0	C
ATOM	70	CG	TRP	992	40.719	22.388	16.845	1.00	0.00	LX0	C
ATOM	71	CD2	TRP	992	41.776	21.515	16.390	1.00	0.00	LX0	C
ATOM	72	CE2	TRP	992	41.495	20.200	16.902	1.00	0.00	LX0	C
ATOM	73	CE3	TRP	992	42.919	21.727	15.591	1.00	0.00	LX0	C
ATOM	74	CD1	TRP	992	39.874	21.561	17.599	1.00	0.00	LX0	C
ATOM	75	NE1	TRP	992	40.321	20.284	17.645	1.00	0.00	LX0	N
ATOM	76	HE1	TRP	992	39.879	19.590	18.193	0.00	0.00	LX0	H
ATOM	77	CZ2	TRP	992	42.372	19.132	16.616	1.00	0.00	LX0	C
ATOM	78	CZ3	TRP	992	43.785	20.648	15.311	1.00	0.00	LX0	C
ATOM	79	CH2	TRP	992	43.512	19.358	15.816	1.00	0.00	LX0	C
ATOM	80	C	TRP	992	40.621	26.171	17.581	1.00	0.00	LX0	C
ATOM	81	O	TRP	992	41.170	26.661	16.602	1.00	0.00	LX0	O
ATOM	82	N	GLU	993	39.859	26.870	18.440	1.00	0.00	LX0	N
ATOM	83	H	GLU	993	39.407	26.432	19.218	0.00	0.00	LX0	H
ATOM	84	CA	GLU	993	39.717	28.310	18.219	1.00	0.00	LX0	C
ATOM	85	CB	GLU	993	38.576	28.853	19.085	1.00	0.00	LX0	C
ATOM	86	CG	GLU	993	38.201	30.328	18.865	1.00	0.00	LX0	C
ATOM	87	CD	GLU	993	37.645	30.608	17.474	1.00	0.00	LX0	C
ATOM	88	OE1	GLU	993	37.108	29.716	16.820	1.00	0.00	LX0	O
ATOM	89	OE2	GLU	993	37.707	31.753	17.045	1.00	0.00	LX0	O
ATOM	90	C	GLU	993	41.012	29.083	18.428	1.00	0.00	LX0	C
ATOM	91	O	GLU	993	41.723	28.936	19.416	1.00	0.00	LX0	O
ATOM	92	N	VAL	994	41.305	29.898	17.415	1.00	0.00	LX0	N
ATOM	93	H	VAL	994	40.603	30.042	16.714	0.00	0.00	LX0	H
ATOM	94	CA	VAL	994	42.587	30.583	17.353	1.00	0.00	LX0	C
ATOM	95	CB	VAL	994	43.278	30.225	16.023	1.00	0.00	LX0	C
ATOM	96	CG1	VAL	994	44.476	31.110	15.675	1.00	0.00	LX0	C
ATOM	97	CG2	VAL	994	43.685	28.753	16.026	1.00	0.00	LX0	C
ATOM	98	C	VAL	994	42.390	32.075	17.516	1.00	0.00	LX0	C
ATOM	99	O	VAL	994	41.632	32.722	16.805	1.00	0.00	LX0	O
ATOM	100	N	ALA	995	43.123	32.615	18.499	1.00	0.00	LX0	N
ATOM	101	H	ALA	995	43.751	32.048	19.027	0.00	0.00	LX0	H
ATOM	102	CA	ALA	995	43.028	34.062	18.655	1.00	0.00	LX0	C

ATOM	103	CB	ALA	995	43.722	34.520	19.937	1.00	0.00	LX0	C
ATOM	104	C	ALA	995	43.589	34.820	17.462	1.00	0.00	LX0	C
ATOM	105	O	ALA	995	44.556	34.431	16.818	1.00	0.00	LX0	O
ATOM	106	N	ARG	996	42.909	35.938	17.175	1.00	0.00	LX0	N
ATOM	107	H	ARG	996	42.140	36.203	17.754	0.00	0.00	LX0	H
ATOM	108	CA	ARG	996	43.214	36.606	15.911	1.00	0.00	LX0	C
ATOM	109	CB	ARG	996	42.043	37.526	15.527	1.00	0.00	LX0	C
ATOM	110	CG	ARG	996	41.985	37.896	14.038	1.00	0.00	LX0	C
ATOM	111	CD	ARG	996	40.659	38.525	13.599	1.00	0.00	LX0	C
ATOM	112	NE	ARG	996	39.576	37.542	13.638	1.00	0.00	LX0	N
ATOM	113	HE	ARG	996	39.277	37.157	14.518	0.00	0.00	LX0	H
ATOM	114	CZ	ARG	996	38.958	37.122	12.514	1.00	0.00	LX0	C
ATOM	115	NH1	ARG	996	39.226	37.663	11.332	1.00	0.00	LX0	N
ATOM	116	HH11	ARG	996	38.758	37.301	10.519	0.00	0.00	LX0	H
ATOM	117	HH12	ARG	996	39.865	38.426	11.235	0.00	0.00	LX0	H
ATOM	118	NH2	ARG	996	38.056	36.159	12.592	1.00	0.00	LX0	N
ATOM	119	HH21	ARG	996	37.546	35.852	11.780	0.00	0.00	LX0	H
ATOM	120	HH22	ARG	996	37.837	35.744	13.480	0.00	0.00	LX0	H
ATOM	121	C	ARG	996	44.593	37.262	15.798	1.00	0.00	LX0	C
ATOM	122	O	ARG	996	45.031	37.651	14.723	1.00	0.00	LX0	O
ATOM	123	N	GLU	997	45.297	37.324	16.945	1.00	0.00	LX0	N
ATOM	124	H	GLU	997	44.948	36.911	17.783	0.00	0.00	LX0	H
ATOM	125	CA	GLU	997	46.687	37.790	16.864	1.00	0.00	LX0	C
ATOM	126	CB	GLU	997	47.293	38.055	18.254	1.00	0.00	LX0	C
ATOM	127	CG	GLU	997	47.026	37.050	19.389	1.00	0.00	LX0	C
ATOM	128	CD	GLU	997	47.622	35.672	19.148	1.00	0.00	LX0	C
ATOM	129	OE1	GLU	997	48.752	35.547	18.685	1.00	0.00	LX0	O
ATOM	130	OE2	GLU	997	46.963	34.686	19.449	1.00	0.00	LX0	O
ATOM	131	C	GLU	997	47.612	36.941	16.001	1.00	0.00	LX0	C
ATOM	132	O	GLU	997	48.646	37.383	15.514	1.00	0.00	LX0	O
ATOM	133	N	LYS	998	47.156	35.702	15.767	1.00	0.00	LX0	N
ATOM	134	H	LYS	998	46.329	35.370	16.224	0.00	0.00	LX0	H
ATOM	135	CA	LYS	998	47.967	34.856	14.899	1.00	0.00	LX0	C
ATOM	136	CB	LYS	998	47.664	33.374	15.130	1.00	0.00	LX0	C
ATOM	137	CG	LYS	998	47.602	33.138	16.628	1.00	0.00	LX0	C
ATOM	138	CD	LYS	998	47.883	31.736	17.144	1.00	0.00	LX0	C
ATOM	139	CE	LYS	998	48.067	31.774	18.663	1.00	0.00	LX0	C
ATOM	140	NZ	LYS	998	48.941	32.906	19.013	1.00	0.00	LX0	N
ATOM	141	HZ1	LYS	998	49.226	32.895	20.004	0.00	0.00	LX0	H
ATOM	142	HZ2	LYS	998	49.774	32.944	18.391	0.00	0.00	LX0	H
ATOM	143	HZ3	LYS	998	48.416	33.804	18.876	0.00	0.00	LX0	H
ATOM	144	C	LYS	998	47.926	35.169	13.419	1.00	0.00	LX0	C
ATOM	145	O	LYS	998	48.689	34.611	12.641	1.00	0.00	LX0	O
ATOM	146	N	ILE	999	46.970	36.025	13.044	1.00	0.00	LX0	N
ATOM	147	H	ILE	999	46.500	36.622	13.697	0.00	0.00	LX0	H
ATOM	148	CA	ILE	999	46.609	36.007	11.630	1.00	0.00	LX0	C
ATOM	149	CB	ILE	999	45.079	36.108	11.481	1.00	0.00	LX0	C
ATOM	150	CG2	ILE	999	44.622	35.818	10.050	1.00	0.00	LX0	C
ATOM	151	CG1	ILE	999	44.360	35.194	12.485	1.00	0.00	LX0	C
ATOM	152	CD1	ILE	999	44.671	33.704	12.327	1.00	0.00	LX0	C
ATOM	153	C	ILE	999	47.355	37.021	10.774	1.00	0.00	LX0	C
ATOM	154	O	ILE	999	46.812	37.996	10.265	1.00	0.00	LX0	O
ATOM	155	N	THR	1000	48.648	36.733	10.607	1.00	0.00	LX0	N
ATOM	156	H	THR	1000	49.076	35.929	11.027	0.00	0.00	LX0	H
ATOM	157	CA	THR	1000	49.414	37.618	9.737	1.00	0.00	LX0	C
ATOM	158	CB	THR	1000	50.894	37.629	10.151	1.00	0.00	LX0	C
ATOM	159	OG1	THR	1000	51.113	36.855	11.344	1.00	0.00	LX0	O
ATOM	160	HG1	THR	1000	51.172	35.955	11.026	0.00	0.00	LX0	H
ATOM	161	CG2	THR	1000	51.408	39.059	10.322	1.00	0.00	LX0	C
ATOM	162	C	THR	1000	49.243	37.297	8.254	1.00	0.00	LX0	C
ATOM	163	O	THR	1000	50.091	36.696	7.605	1.00	0.00	LX0	O

ATOM	164	N	MET	1001	48.075	37.700	7.723	1.00	0.00	LX0	N
ATOM	165	H	MET	1001	47.430	38.201	8.305	0.00	0.00	LX0	H
ATOM	166	CA	MET	1001	47.849	37.418	6.301	1.00	0.00	LX0	C
ATOM	167	CB	MET	1001	46.434	37.797	5.864	1.00	0.00	LX0	C
ATOM	168	CG	MET	1001	45.379	36.859	6.451	1.00	0.00	LX0	C
ATOM	169	SD	MET	1001	43.704	37.230	5.905	1.00	0.00	LX0	S
ATOM	170	CE	MET	1001	43.545	38.861	6.650	1.00	0.00	LX0	C
ATOM	171	C	MET	1001	48.883	38.052	5.386	1.00	0.00	LX0	C
ATOM	172	O	MET	1001	49.332	39.171	5.596	1.00	0.00	LX0	O
ATOM	173	N	SER	1002	49.274	37.256	4.387	1.00	0.00	LX0	N
ATOM	174	H	SER	1002	48.861	36.357	4.238	0.00	0.00	LX0	H
ATOM	175	CA	SER	1002	50.467	37.667	3.660	1.00	0.00	LX0	C
ATOM	176	CB	SER	1002	51.616	36.724	4.051	1.00	0.00	LX0	C
ATOM	177	OG	SER	1002	52.894	37.204	3.587	1.00	0.00	LX0	O
ATOM	178	HG	SER	1002	53.105	37.922	4.183	0.00	0.00	LX0	H
ATOM	179	C	SER	1002	50.277	37.809	2.155	1.00	0.00	LX0	C
ATOM	180	O	SER	1002	50.932	38.628	1.519	1.00	0.00	LX0	O
ATOM	181	N	ARG	1003	49.361	36.991	1.606	1.00	0.00	LX0	N
ATOM	182	H	ARG	1003	48.929	36.258	2.138	0.00	0.00	LX0	H
ATOM	183	CA	ARG	1003	48.942	37.165	0.210	1.00	0.00	LX0	C
ATOM	184	CB	ARG	1003	50.053	36.791	-0.787	1.00	0.00	LX0	C
ATOM	185	CG	ARG	1003	50.440	35.309	-0.832	1.00	0.00	LX0	C
ATOM	186	CD	ARG	1003	51.915	35.111	-1.195	1.00	0.00	LX0	C
ATOM	187	NE	ARG	1003	52.669	34.640	-0.032	1.00	0.00	LX0	N
ATOM	188	HE	ARG	1003	52.746	33.641	0.094	0.00	0.00	LX0	H
ATOM	189	CZ	ARG	1003	53.081	35.462	0.955	1.00	0.00	LX0	C
ATOM	190	NH1	ARG	1003	52.975	36.783	0.853	1.00	0.00	LX0	N
ATOM	191	HH11	ARG	1003	53.182	37.365	1.645	0.00	0.00	LX0	H
ATOM	192	HH12	ARG	1003	52.654	37.221	0.014	0.00	0.00	LX0	H
ATOM	193	NH2	ARG	1003	53.574	34.950	2.069	1.00	0.00	LX0	N
ATOM	194	HH21	ARG	1003	53.824	35.535	2.844	0.00	0.00	LX0	H
ATOM	195	HH22	ARG	1003	53.671	33.950	2.162	0.00	0.00	LX0	H
ATOM	196	C	ARG	1003	47.699	36.349	-0.052	1.00	0.00	LX0	C
ATOM	197	O	ARG	1003	47.330	35.520	0.770	1.00	0.00	LX0	O
ATOM	198	N	GLU	1004	47.083	36.598	-1.211	1.00	0.00	LX0	N
ATOM	199	H	GLU	1004	47.433	37.254	-1.877	0.00	0.00	LX0	H
ATOM	200	CA	GLU	1004	46.004	35.689	-1.578	1.00	0.00	LX0	C
ATOM	201	CB	GLU	1004	44.894	36.424	-2.327	1.00	0.00	LX0	C
ATOM	202	CG	GLU	1004	44.499	37.726	-1.630	1.00	0.00	LX0	C
ATOM	203	CD	GLU	1004	43.155	38.212	-2.126	1.00	0.00	LX0	C
ATOM	204	OE1	GLU	1004	43.008	38.427	-3.327	1.00	0.00	LX0	O
ATOM	205	OE2	GLU	1004	42.256	38.370	-1.302	1.00	0.00	LX0	O
ATOM	206	C	GLU	1004	46.538	34.533	-2.395	1.00	0.00	LX0	C
ATOM	207	O	GLU	1004	47.655	34.581	-2.896	1.00	0.00	LX0	O
ATOM	208	N	LEU	1005	45.703	33.495	-2.486	1.00	0.00	LX0	N
ATOM	209	H	LEU	1005	44.824	33.548	-2.013	0.00	0.00	LX0	H
ATOM	210	CA	LEU	1005	46.075	32.332	-3.288	1.00	0.00	LX0	C
ATOM	211	CB	LEU	1005	46.236	31.076	-2.424	1.00	0.00	LX0	C
ATOM	212	CG	LEU	1005	47.371	31.119	-1.399	1.00	0.00	LX0	C
ATOM	213	CD1	LEU	1005	47.351	29.877	-0.507	1.00	0.00	LX0	C
ATOM	214	CD2	LEU	1005	48.741	31.326	-2.048	1.00	0.00	LX0	C
ATOM	215	C	LEU	1005	45.066	32.053	-4.381	1.00	0.00	LX0	C
ATOM	216	O	LEU	1005	45.399	31.882	-5.546	1.00	0.00	LX0	O
ATOM	217	N	GLY	1006	43.799	32.004	-3.949	1.00	0.00	LX0	N
ATOM	218	H	GLY	1006	43.547	32.156	-2.991	0.00	0.00	LX0	H
ATOM	219	CA	GLY	1006	42.768	31.684	-4.930	1.00	0.00	LX0	C
ATOM	220	C	GLY	1006	41.427	31.517	-4.258	1.00	0.00	LX0	C
ATOM	221	O	GLY	1006	41.328	31.540	-3.038	1.00	0.00	LX0	O
ATOM	222	N	GLN	1007	40.398	31.371	-5.100	1.00	0.00	LX0	N
ATOM	223	H	GLN	1007	40.565	31.246	-6.075	0.00	0.00	LX0	H
ATOM	224	CA	GLN	1007	39.052	31.261	-4.540	1.00	0.00	LX0	C

ATOM	225	CB	GLN	1007	38.031	31.467	-5.670	1.00	0.00	LX0	C
ATOM	226	CG	GLN	1007	36.559	31.604	-5.256	1.00	0.00	LX0	C
ATOM	227	CD	GLN	1007	36.369	32.840	-4.400	1.00	0.00	LX0	C
ATOM	228	OE1	GLN	1007	36.827	33.929	-4.709	1.00	0.00	LX0	O
ATOM	229	NE2	GLN	1007	35.665	32.627	-3.288	1.00	0.00	LX0	N
ATOM	230	HE21	GLN	1007	35.339	31.712	-3.041	0.00	0.00	LX0	H
ATOM	231	HE22	GLN	1007	35.470	33.411	-2.705	0.00	0.00	LX0	H
ATOM	232	C	GLN	1007	38.829	29.942	-3.815	1.00	0.00	LX0	C
ATOM	233	O	GLN	1007	39.350	28.906	-4.202	1.00	0.00	LX0	O
ATOM	234	N	GLY	1008	38.019	30.036	-2.757	1.00	0.00	LX0	N
ATOM	235	H	GLY	1008	37.714	30.921	-2.402	0.00	0.00	LX0	H
ATOM	236	CA	GLY	1008	37.499	28.824	-2.141	1.00	0.00	LX0	C
ATOM	237	C	GLY	1008	36.015	28.994	-1.904	1.00	0.00	LX0	C
ATOM	238	O	GLY	1008	35.473	30.092	-1.993	1.00	0.00	LX0	O
ATOM	239	N	SER	1009	35.378	27.867	-1.574	1.00	0.00	LX0	N
ATOM	240	H	SER	1009	35.847	26.983	-1.586	0.00	0.00	LX0	H
ATOM	241	CA	SER	1009	33.916	27.814	-1.535	1.00	0.00	LX0	C
ATOM	242	CB	SER	1009	33.524	26.368	-1.224	1.00	0.00	LX0	C
ATOM	243	OG	SER	1009	34.476	25.494	-1.850	1.00	0.00	LX0	O
ATOM	244	HG	SER	1009	34.187	25.416	-2.767	0.00	0.00	LX0	H
ATOM	245	C	SER	1009	33.145	28.821	-0.676	1.00	0.00	LX0	C
ATOM	246	O	SER	1009	31.947	29.048	-0.838	1.00	0.00	LX0	O
ATOM	247	N	PHE	1010	33.876	29.427	0.273	1.00	0.00	LX0	N
ATOM	248	H	PHE	1010	34.841	29.206	0.394	0.00	0.00	LX0	H
ATOM	249	CA	PHE	1010	33.208	30.440	1.091	1.00	0.00	LX0	C
ATOM	250	CB	PHE	1010	32.857	29.872	2.469	1.00	0.00	LX0	C
ATOM	251	CG	PHE	1010	31.613	29.015	2.395	1.00	0.00	LX0	C
ATOM	252	CD1	PHE	1010	30.355	29.607	2.641	1.00	0.00	LX0	C
ATOM	253	CD2	PHE	1010	31.721	27.641	2.085	1.00	0.00	LX0	C
ATOM	254	CE1	PHE	1010	29.193	28.814	2.583	1.00	0.00	LX0	C
ATOM	255	CE2	PHE	1010	30.561	26.846	2.029	1.00	0.00	LX0	C
ATOM	256	CZ	PHE	1010	29.308	27.441	2.283	1.00	0.00	LX0	C
ATOM	257	C	PHE	1010	33.960	31.754	1.225	1.00	0.00	LX0	C
ATOM	258	O	PHE	1010	33.623	32.609	2.037	1.00	0.00	LX0	O
ATOM	259	N	GLY	1011	34.999	31.888	0.392	1.00	0.00	LX0	N
ATOM	260	H	GLY	1011	35.231	31.217	-0.314	0.00	0.00	LX0	H
ATOM	261	CA	GLY	1011	35.818	33.086	0.527	1.00	0.00	LX0	C
ATOM	262	C	GLY	1011	37.178	32.847	-0.080	1.00	0.00	LX0	C
ATOM	263	O	GLY	1011	37.435	31.797	-0.657	1.00	0.00	LX0	O
ATOM	264	N	MET	1012	38.032	33.865	0.044	1.00	0.00	LX0	N
ATOM	265	H	MET	1012	37.823	34.605	0.685	0.00	0.00	LX0	H
ATOM	266	CA	MET	1012	39.360	33.703	-0.540	1.00	0.00	LX0	C
ATOM	267	CB	MET	1012	39.969	35.086	-0.794	1.00	0.00	LX0	C
ATOM	268	CG	MET	1012	41.210	35.093	-1.691	1.00	0.00	LX0	C
ATOM	269	SD	MET	1012	40.854	34.609	-3.386	1.00	0.00	LX0	S
ATOM	270	CE	MET	1012	39.906	36.062	-3.863	1.00	0.00	LX0	C
ATOM	271	C	MET	1012	40.246	32.851	0.355	1.00	0.00	LX0	C
ATOM	272	O	MET	1012	40.127	32.871	1.576	1.00	0.00	LX0	O
ATOM	273	N	VAL	1013	41.123	32.087	-0.294	1.00	0.00	LX0	N
ATOM	274	H	VAL	1013	41.169	32.128	-1.291	0.00	0.00	LX0	H
ATOM	275	CA	VAL	1013	42.162	31.408	0.467	1.00	0.00	LX0	C
ATOM	276	CB	VAL	1013	42.420	30.008	-0.108	1.00	0.00	LX0	C
ATOM	277	CG1	VAL	1013	43.427	29.219	0.735	1.00	0.00	LX0	C
ATOM	278	CG2	VAL	1013	41.111	29.236	-0.289	1.00	0.00	LX0	C
ATOM	279	C	VAL	1013	43.416	32.256	0.413	1.00	0.00	LX0	C
ATOM	280	O	VAL	1013	43.821	32.711	-0.650	1.00	0.00	LX0	O
ATOM	281	N	TYR	1014	43.989	32.464	1.597	1.00	0.00	LX0	N
ATOM	282	H	TYR	1014	43.600	32.033	2.408	0.00	0.00	LX0	H
ATOM	283	CA	TYR	1014	45.191	33.279	1.715	1.00	0.00	LX0	C
ATOM	284	CB	TYR	1014	45.018	34.312	2.838	1.00	0.00	LX0	C
ATOM	285	CG	TYR	1014	43.983	35.354	2.487	1.00	0.00	LX0	C

ATOM	286	CD1	TYR	1014	44.424	36.600	2.004	1.00	0.00	LX0	C
ATOM	287	CE1	TYR	1014	43.471	37.564	1.640	1.00	0.00	LX0	C
ATOM	288	CD2	TYR	1014	42.613	35.060	2.653	1.00	0.00	LX0	C
ATOM	289	CE2	TYR	1014	41.661	36.022	2.281	1.00	0.00	LX0	C
ATOM	290	CZ	TYR	1014	42.103	37.251	1.756	1.00	0.00	LX0	C
ATOM	291	OH	TYR	1014	41.166	38.168	1.331	1.00	0.00	LX0	O
ATOM	292	HH	TYR	1014	41.419	38.448	0.445	0.00	0.00	LX0	H
ATOM	293	C	TYR	1014	46.373	32.408	2.068	1.00	0.00	LX0	C
ATOM	294	O	TYR	1014	46.222	31.401	2.749	1.00	0.00	LX0	O
ATOM	295	N	GLU	1015	47.557	32.861	1.635	1.00	0.00	LX0	N
ATOM	296	H	GLU	1015	47.592	33.668	1.047	0.00	0.00	LX0	H
ATOM	297	CA	GLU	1015	48.711	32.450	2.426	1.00	0.00	LX0	C
ATOM	298	CB	GLU	1015	50.015	32.394	1.622	1.00	0.00	LX0	C
ATOM	299	CG	GLU	1015	51.159	31.813	2.469	1.00	0.00	LX0	C
ATOM	300	CD	GLU	1015	52.519	31.944	1.802	1.00	0.00	LX0	C
ATOM	301	OE1	GLU	1015	52.655	31.732	0.606	1.00	0.00	LX0	O
ATOM	302	OE2	GLU	1015	53.485	32.253	2.485	1.00	0.00	LX0	O
ATOM	303	C	GLU	1015	48.865	33.436	3.561	1.00	0.00	LX0	C
ATOM	304	O	GLU	1015	49.151	34.616	3.371	1.00	0.00	LX0	O
ATOM	305	N	GLY	1016	48.629	32.906	4.755	1.00	0.00	LX0	N
ATOM	306	H	GLY	1016	48.444	31.924	4.837	0.00	0.00	LX0	H
ATOM	307	CA	GLY	1016	48.985	33.702	5.913	1.00	0.00	LX0	C
ATOM	308	C	GLY	1016	50.220	33.112	6.536	1.00	0.00	LX0	C
ATOM	309	O	GLY	1016	50.589	31.976	6.269	1.00	0.00	LX0	O
ATOM	310	N	VAL	1017	50.835	33.929	7.378	1.00	0.00	LX0	N
ATOM	311	H	VAL	1017	50.512	34.860	7.546	0.00	0.00	LX0	H
ATOM	312	CA	VAL	1017	51.840	33.374	8.266	1.00	0.00	LX0	C
ATOM	313	CB	VAL	1017	53.197	34.039	8.001	1.00	0.00	LX0	C
ATOM	314	CG1	VAL	1017	53.821	33.467	6.729	1.00	0.00	LX0	C
ATOM	315	CG2	VAL	1017	53.117	35.567	7.920	1.00	0.00	LX0	C
ATOM	316	C	VAL	1017	51.316	33.566	9.675	1.00	0.00	LX0	C
ATOM	317	O	VAL	1017	50.629	34.542	9.961	1.00	0.00	LX0	O
ATOM	318	N	ALA	1018	51.560	32.562	10.515	1.00	0.00	LX0	N
ATOM	319	H	ALA	1018	52.148	31.797	10.255	0.00	0.00	LX0	H
ATOM	320	CA	ALA	1018	50.758	32.548	11.730	1.00	0.00	LX0	C
ATOM	321	CB	ALA	1018	49.796	31.363	11.735	1.00	0.00	LX0	C
ATOM	322	C	ALA	1018	51.559	32.534	13.004	1.00	0.00	LX0	C
ATOM	323	O	ALA	1018	52.488	31.763	13.188	1.00	0.00	LX0	O
ATOM	324	N	LYS	1019	51.137	33.439	13.893	1.00	0.00	LX0	N
ATOM	325	H	LYS	1019	50.338	33.983	13.630	0.00	0.00	LX0	H
ATOM	326	CA	LYS	1019	51.830	33.614	15.168	1.00	0.00	LX0	C
ATOM	327	CB	LYS	1019	51.467	34.991	15.730	1.00	0.00	LX0	C
ATOM	328	CG	LYS	1019	52.519	35.676	16.608	1.00	0.00	LX0	C
ATOM	329	CD	LYS	1019	53.818	36.030	15.874	1.00	0.00	LX0	C
ATOM	330	CE	LYS	1019	53.596	36.744	14.535	1.00	0.00	LX0	C
ATOM	331	NZ	LYS	1019	53.956	35.821	13.457	1.00	0.00	LX0	N
ATOM	332	HZ1	LYS	1019	53.484	36.001	12.543	0.00	0.00	LX0	H
ATOM	333	HZ2	LYS	1019	54.956	35.913	13.181	0.00	0.00	LX0	H
ATOM	334	HZ3	LYS	1019	53.835	34.812	13.674	0.00	0.00	LX0	H
ATOM	335	C	LYS	1019	51.618	32.512	16.198	1.00	0.00	LX0	C
ATOM	336	O	LYS	1019	50.927	32.693	17.198	1.00	0.00	LX0	O
ATOM	337	N	GLY	1020	52.247	31.367	15.921	1.00	0.00	LX0	N
ATOM	338	H	GLY	1020	52.779	31.237	15.078	0.00	0.00	LX0	H
ATOM	339	CA	GLY	1020	52.135	30.274	16.879	1.00	0.00	LX0	C
ATOM	340	C	GLY	1020	50.913	29.405	16.688	1.00	0.00	LX0	C
ATOM	341	O	GLY	1020	50.022	29.376	17.530	1.00	0.00	LX0	O
ATOM	342	N	VAL	1021	50.886	28.706	15.539	1.00	0.00	LX0	N
ATOM	343	H	VAL	1021	51.644	28.775	14.886	0.00	0.00	LX0	H
ATOM	344	CA	VAL	1021	49.830	27.693	15.485	1.00	0.00	LX0	C
ATOM	345	CB	VAL	1021	48.856	27.847	14.298	1.00	0.00	LX0	C
ATOM	346	CG1	VAL	1021	48.052	29.141	14.414	1.00	0.00	LX0	C

ATOM	347	CG2	VAL	1021	49.515	27.708	12.929	1.00	0.00	LX0	C
ATOM	348	C	VAL	1021	50.321	26.263	15.624	1.00	0.00	LX0	C
ATOM	349	O	VAL	1021	49.741	25.455	16.341	1.00	0.00	LX0	O
ATOM	350	N	VAL	1022	51.439	25.960	14.941	1.00	0.00	LX0	N
ATOM	351	H	VAL	1022	51.949	26.645	14.416	0.00	0.00	LX0	H
ATOM	352	CA	VAL	1022	51.996	24.638	15.227	1.00	0.00	LX0	C
ATOM	353	CB	VAL	1022	52.720	24.039	14.011	1.00	0.00	LX0	C
ATOM	354	CG1	VAL	1022	53.270	22.634	14.287	1.00	0.00	LX0	C
ATOM	355	CG2	VAL	1022	51.769	23.988	12.818	1.00	0.00	LX0	C
ATOM	356	C	VAL	1022	52.877	24.705	16.458	1.00	0.00	LX0	C
ATOM	357	O	VAL	1022	54.093	24.817	16.399	1.00	0.00	LX0	O
ATOM	358	N	LYS	1023	52.160	24.672	17.597	1.00	0.00	LX0	N
ATOM	359	H	LYS	1023	51.166	24.666	17.478	0.00	0.00	LX0	H
ATOM	360	CA	LYS	1023	52.773	25.091	18.857	1.00	0.00	LX0	C
ATOM	361	CB	LYS	1023	53.858	24.099	19.320	1.00	0.00	LX0	C
ATOM	362	CG	LYS	1023	53.278	22.766	19.790	1.00	0.00	LX0	C
ATOM	363	CD	LYS	1023	52.376	22.954	21.008	1.00	0.00	LX0	C
ATOM	364	CE	LYS	1023	51.776	21.646	21.517	1.00	0.00	LX0	C
ATOM	365	NZ	LYS	1023	50.929	21.942	22.679	1.00	0.00	LX0	N
ATOM	366	HZ1	LYS	1023	50.517	21.061	23.046	0.00	0.00	LX0	H
ATOM	367	HZ2	LYS	1023	51.509	22.392	23.417	0.00	0.00	LX0	H
ATOM	368	HZ3	LYS	1023	50.169	22.592	22.393	0.00	0.00	LX0	H
ATOM	369	C	LYS	1023	53.223	26.546	18.783	1.00	0.00	LX0	C
ATOM	370	O	LYS	1023	52.634	27.332	18.052	1.00	0.00	LX0	O
ATOM	371	N	ASP	1024	54.253	26.867	19.568	1.00	0.00	LX0	N
ATOM	372	H	ASP	1024	54.780	26.194	20.091	0.00	0.00	LX0	H
ATOM	373	CA	ASP	1024	54.798	28.224	19.637	1.00	0.00	LX0	C
ATOM	374	CB	ASP	1024	55.909	28.266	20.698	1.00	0.00	LX0	C
ATOM	375	CG	ASP	1024	56.789	27.033	20.574	1.00	0.00	LX0	C
ATOM	376	OD1	ASP	1024	57.709	27.046	19.765	1.00	0.00	LX0	O
ATOM	377	OD2	ASP	1024	56.493	26.039	21.240	1.00	0.00	LX0	O
ATOM	378	C	ASP	1024	55.270	28.836	18.325	1.00	0.00	LX0	C
ATOM	379	O	ASP	1024	55.218	30.051	18.158	1.00	0.00	LX0	O
ATOM	380	N	GLU	1025	55.690	27.945	17.402	1.00	0.00	LX0	N
ATOM	381	H	GLU	1025	55.823	27.013	17.738	0.00	0.00	LX0	H
ATOM	382	CA	GLU	1025	56.155	28.317	16.055	1.00	0.00	LX0	C
ATOM	383	CB	GLU	1025	55.921	27.144	15.096	1.00	0.00	LX0	C
ATOM	384	CG	GLU	1025	56.929	27.035	13.944	1.00	0.00	LX0	C
ATOM	385	CD	GLU	1025	56.689	28.105	12.899	1.00	0.00	LX0	C
ATOM	386	OE1	GLU	1025	57.307	29.164	12.971	1.00	0.00	LX0	O
ATOM	387	OE2	GLU	1025	55.882	27.874	12.009	1.00	0.00	LX0	O
ATOM	388	C	GLU	1025	55.653	29.645	15.482	1.00	0.00	LX0	C
ATOM	389	O	GLU	1025	54.529	29.791	15.006	1.00	0.00	LX0	O
ATOM	390	N	PRO	1026	56.553	30.649	15.608	1.00	0.00	LX0	N
ATOM	391	CD	PRO	1026	57.938	30.509	16.052	1.00	0.00	LX0	C
ATOM	392	CA	PRO	1026	56.173	32.049	15.399	1.00	0.00	LX0	C
ATOM	393	CB	PRO	1026	57.496	32.797	15.593	1.00	0.00	LX0	C
ATOM	394	CG	PRO	1026	58.319	31.906	16.520	1.00	0.00	LX0	C
ATOM	395	C	PRO	1026	55.471	32.438	14.108	1.00	0.00	LX0	C
ATOM	396	O	PRO	1026	54.734	33.425	14.089	1.00	0.00	LX0	O
ATOM	397	N	GLU	1027	55.762	31.691	13.035	1.00	0.00	LX0	N
ATOM	398	H	GLU	1027	56.283	30.834	13.096	0.00	0.00	LX0	H
ATOM	399	CA	GLU	1027	55.346	32.212	11.739	1.00	0.00	LX0	C
ATOM	400	CB	GLU	1027	56.478	33.056	11.136	1.00	0.00	LX0	C
ATOM	401	CG	GLU	1027	56.003	34.198	10.229	1.00	0.00	LX0	C
ATOM	402	CD	GLU	1027	55.144	35.175	11.013	1.00	0.00	LX0	C
ATOM	403	OE1	GLU	1027	53.925	35.003	11.077	1.00	0.00	LX0	O
ATOM	404	OE2	GLU	1027	55.688	36.106	11.603	1.00	0.00	LX0	O
ATOM	405	C	GLU	1027	54.824	31.162	10.775	1.00	0.00	LX0	C
ATOM	406	O	GLU	1027	55.133	31.134	9.588	1.00	0.00	LX0	O
ATOM	407	N	THR	1028	53.984	30.285	11.339	1.00	0.00	LX0	N

ATOM	408	H	THR	1028	53.768	30.342	12.316	0.00	0.00	LX0	H
ATOM	409	CA	THR	1028	53.538	29.154	10.531	1.00	0.00	LX0	C
ATOM	410	CB	THR	1028	52.682	28.208	11.370	1.00	0.00	LX0	C
ATOM	411	OG1	THR	1028	53.119	28.169	12.739	1.00	0.00	LX0	O
ATOM	412	HG1	THR	1028	54.081	28.111	12.699	0.00	0.00	LX0	H
ATOM	413	CG2	THR	1028	52.677	26.810	10.751	1.00	0.00	LX0	C
ATOM	414	C	THR	1028	52.822	29.507	9.239	1.00	0.00	LX0	C
ATOM	415	O	THR	1028	51.764	30.125	9.232	1.00	0.00	LX0	O
ATOM	416	N	ARG	1029	53.447	29.082	8.135	1.00	0.00	LX0	N
ATOM	417	H	ARG	1029	54.319	28.607	8.235	0.00	0.00	LX0	H
ATOM	418	CA	ARG	1029	52.794	29.315	6.849	1.00	0.00	LX0	C
ATOM	419	CB	ARG	1029	53.797	29.116	5.716	1.00	0.00	LX0	C
ATOM	420	CG	ARG	1029	55.027	30.020	5.836	1.00	0.00	LX0	C
ATOM	421	CD	ARG	1029	55.982	29.903	4.647	1.00	0.00	LX0	C
ATOM	422	NE	ARG	1029	55.307	30.270	3.403	1.00	0.00	LX0	N
ATOM	423	HE	ARG	1029	54.729	31.092	3.385	0.00	0.00	LX0	H
ATOM	424	CZ	ARG	1029	55.378	29.504	2.298	1.00	0.00	LX0	C
ATOM	425	NH1	ARG	1029	56.127	28.408	2.292	1.00	0.00	LX0	N
ATOM	426	HH11	ARG	1029	56.102	27.820	1.472	0.00	0.00	LX0	H
ATOM	427	HH12	ARG	1029	56.693	28.148	3.070	0.00	0.00	LX0	H
ATOM	428	NH2	ARG	1029	54.683	29.822	1.213	1.00	0.00	LX0	N
ATOM	429	HH21	ARG	1029	54.706	29.220	0.407	0.00	0.00	LX0	H
ATOM	430	HH22	ARG	1029	54.095	30.648	1.183	0.00	0.00	LX0	H
ATOM	431	C	ARG	1029	51.562	28.442	6.664	1.00	0.00	LX0	C
ATOM	432	O	ARG	1029	51.629	27.219	6.706	1.00	0.00	LX0	O
ATOM	433	N	VAL	1030	50.429	29.133	6.501	1.00	0.00	LX0	N
ATOM	434	H	VAL	1030	50.473	30.129	6.430	0.00	0.00	LX0	H
ATOM	435	CA	VAL	1030	49.135	28.457	6.550	1.00	0.00	LX0	C
ATOM	436	CB	VAL	1030	48.476	28.652	7.925	1.00	0.00	LX0	C
ATOM	437	CG1	VAL	1030	49.149	27.799	8.997	1.00	0.00	LX0	C
ATOM	438	CG2	VAL	1030	48.418	30.126	8.335	1.00	0.00	LX0	C
ATOM	439	C	VAL	1030	48.190	28.915	5.453	1.00	0.00	LX0	C
ATOM	440	O	VAL	1030	48.188	30.066	5.034	1.00	0.00	LX0	O
ATOM	441	N	ALA	1031	47.373	27.954	5.007	1.00	0.00	LX0	N
ATOM	442	H	ALA	1031	47.383	27.062	5.457	0.00	0.00	LX0	H
ATOM	443	CA	ALA	1031	46.310	28.308	4.073	1.00	0.00	LX0	C
ATOM	444	CB	ALA	1031	45.962	27.128	3.164	1.00	0.00	LX0	C
ATOM	445	C	ALA	1031	45.061	28.752	4.804	1.00	0.00	LX0	C
ATOM	446	O	ALA	1031	44.252	27.961	5.277	1.00	0.00	LX0	O
ATOM	447	N	ILE	1032	44.947	30.078	4.894	1.00	0.00	LX0	N
ATOM	448	H	ILE	1032	45.616	30.645	4.410	0.00	0.00	LX0	H
ATOM	449	CA	ILE	1032	43.770	30.623	5.560	1.00	0.00	LX0	C
ATOM	450	CB	ILE	1032	44.101	31.975	6.205	1.00	0.00	LX0	C
ATOM	451	CG2	ILE	1032	42.908	32.550	6.978	1.00	0.00	LX0	C
ATOM	452	CG1	ILE	1032	45.341	31.836	7.090	1.00	0.00	LX0	C
ATOM	453	CD1	ILE	1032	45.783	33.147	7.735	1.00	0.00	LX0	C
ATOM	454	C	ILE	1032	42.588	30.740	4.618	1.00	0.00	LX0	C
ATOM	455	O	ILE	1032	42.469	31.677	3.841	1.00	0.00	LX0	O
ATOM	456	N	LYS	1033	41.710	29.742	4.711	1.00	0.00	LX0	N
ATOM	457	H	LYS	1033	41.847	29.051	5.423	0.00	0.00	LX0	H
ATOM	458	CA	LYS	1033	40.477	29.887	3.941	1.00	0.00	LX0	C
ATOM	459	CB	LYS	1033	39.827	28.524	3.691	1.00	0.00	LX0	C
ATOM	460	CG	LYS	1033	40.842	27.467	3.247	1.00	0.00	LX0	C
ATOM	461	CD	LYS	1033	40.223	26.146	2.788	1.00	0.00	LX0	C
ATOM	462	CE	LYS	1033	39.336	25.459	3.829	1.00	0.00	LX0	C
ATOM	463	NZ	LYS	1033	38.969	24.127	3.338	1.00	0.00	LX0	N
ATOM	464	HZ1	LYS	1033	38.163	23.665	3.794	0.00	0.00	LX0	H
ATOM	465	HZ2	LYS	1033	38.840	24.109	2.303	0.00	0.00	LX0	H
ATOM	466	HZ3	LYS	1033	39.768	23.451	3.394	0.00	0.00	LX0	H
ATOM	467	C	LYS	1033	39.516	30.813	4.658	1.00	0.00	LX0	C
ATOM	468	O	LYS	1033	39.347	30.724	5.868	1.00	0.00	LX0	O

ATOM	469	N	THR	1034	38.905	31.716	3.894	1.00	0.00	LX0	N
ATOM	470	H	THR	1034	39.059	31.800	2.910	0.00	0.00	LX0	H
ATOM	471	CA	THR	1034	37.952	32.557	4.607	1.00	0.00	LX0	C
ATOM	472	CB	THR	1034	38.134	34.028	4.220	1.00	0.00	LX0	C
ATOM	473	OG1	THR	1034	38.115	34.191	2.800	1.00	0.00	LX0	O
ATOM	474	HG1	THR	1034	38.986	33.928	2.510	0.00	0.00	LX0	H
ATOM	475	CG2	THR	1034	39.439	34.589	4.777	1.00	0.00	LX0	C
ATOM	476	C	THR	1034	36.516	32.116	4.428	1.00	0.00	LX0	C
ATOM	477	O	THR	1034	36.155	31.434	3.474	1.00	0.00	LX0	O
ATOM	478	N	VAL	1035	35.714	32.571	5.396	1.00	0.00	LX0	N
ATOM	479	H	VAL	1035	36.119	32.993	6.206	0.00	0.00	LX0	H
ATOM	480	CA	VAL	1035	34.300	32.759	5.108	1.00	0.00	LX0	C
ATOM	481	CB	VAL	1035	33.390	32.069	6.128	1.00	0.00	LX0	C
ATOM	482	CG1	VAL	1035	31.991	31.891	5.541	1.00	0.00	LX0	C
ATOM	483	CG2	VAL	1035	33.966	30.758	6.656	1.00	0.00	LX0	C
ATOM	484	C	VAL	1035	34.078	34.254	5.145	1.00	0.00	LX0	C
ATOM	485	O	VAL	1035	34.259	34.885	6.186	1.00	0.00	LX0	O
ATOM	486	N	ASN	1036	33.771	34.771	3.949	1.00	0.00	LX0	N
ATOM	487	H	ASN	1036	33.518	34.124	3.226	0.00	0.00	LX0	H
ATOM	488	CA	ASN	1036	33.911	36.196	3.638	1.00	0.00	LX0	C
ATOM	489	CB	ASN	1036	33.640	36.419	2.136	1.00	0.00	LX0	C
ATOM	490	CG	ASN	1036	32.157	36.590	1.836	1.00	0.00	LX0	C
ATOM	491	OD1	ASN	1036	31.303	35.897	2.367	1.00	0.00	LX0	O
ATOM	492	ND2	ASN	1036	31.880	37.561	0.970	1.00	0.00	LX0	N
ATOM	493	HD21	ASN	1036	32.575	38.250	0.726	0.00	0.00	LX0	H
ATOM	494	HD22	ASN	1036	30.971	37.694	0.586	0.00	0.00	LX0	H
ATOM	495	C	ASN	1036	33.147	37.180	4.521	1.00	0.00	LX0	C
ATOM	496	O	ASN	1036	32.471	36.818	5.479	1.00	0.00	LX0	O
ATOM	497	N	GLU	1037	33.263	38.462	4.147	1.00	0.00	LX0	N
ATOM	498	H	GLU	1037	33.801	38.696	3.332	0.00	0.00	LX0	H
ATOM	499	CA	GLU	1037	32.431	39.474	4.804	1.00	0.00	LX0	C
ATOM	500	CB	GLU	1037	32.701	40.917	4.329	1.00	0.00	LX0	C
ATOM	501	CG	GLU	1037	33.617	41.163	3.117	1.00	0.00	LX0	C
ATOM	502	CD	GLU	1037	33.166	40.355	1.914	1.00	0.00	LX0	C
ATOM	503	OE1	GLU	1037	33.946	39.531	1.450	1.00	0.00	LX0	O
ATOM	504	OE2	GLU	1037	32.021	40.479	1.490	1.00	0.00	LX0	O
ATOM	505	C	GLU	1037	30.936	39.198	4.737	1.00	0.00	LX0	C
ATOM	506	O	GLU	1037	30.278	39.081	5.764	1.00	0.00	LX0	O
ATOM	507	N	ALA	1038	30.437	39.060	3.502	1.00	0.00	LX0	N
ATOM	508	H	ALA	1038	31.052	39.230	2.725	0.00	0.00	LX0	H
ATOM	509	CA	ALA	1038	28.990	38.951	3.303	1.00	0.00	LX0	C
ATOM	510	CB	ALA	1038	28.661	38.915	1.809	1.00	0.00	LX0	C
ATOM	511	C	ALA	1038	28.266	37.795	3.983	1.00	0.00	LX0	C
ATOM	512	O	ALA	1038	27.193	37.969	4.545	1.00	0.00	LX0	O
ATOM	513	N	ALA	1039	28.897	36.610	3.894	1.00	0.00	LX0	N
ATOM	514	H	ALA	1039	29.759	36.562	3.388	0.00	0.00	LX0	H
ATOM	515	CA	ALA	1039	28.238	35.352	4.277	1.00	0.00	LX0	C
ATOM	516	CB	ALA	1039	29.286	34.250	4.416	1.00	0.00	LX0	C
ATOM	517	C	ALA	1039	27.396	35.355	5.548	1.00	0.00	LX0	C
ATOM	518	O	ALA	1039	27.751	35.957	6.564	1.00	0.00	LX0	O
ATOM	519	N	SER	1040	26.265	34.642	5.466	1.00	0.00	LX0	N
ATOM	520	H	SER	1040	26.063	34.123	4.627	0.00	0.00	LX0	H
ATOM	521	CA	SER	1040	25.423	34.582	6.659	1.00	0.00	LX0	C
ATOM	522	CB	SER	1040	24.061	33.949	6.351	1.00	0.00	LX0	C
ATOM	523	OG	SER	1040	24.204	32.543	6.110	1.00	0.00	LX0	O
ATOM	524	HG	SER	1040	24.370	32.480	5.163	0.00	0.00	LX0	H
ATOM	525	C	SER	1040	26.085	33.852	7.814	1.00	0.00	LX0	C
ATOM	526	O	SER	1040	27.114	33.199	7.669	1.00	0.00	LX0	O
ATOM	527	N	MET	1041	25.432	33.954	8.984	1.00	0.00	LX0	N
ATOM	528	H	MET	1041	24.605	34.508	9.067	0.00	0.00	LX0	H
ATOM	529	CA	MET	1041	25.950	33.150	10.089	1.00	0.00	LX0	C

ATOM	530	CB	MET	1041	25.259	33.497	11.420	1.00	0.00	LX0	C
ATOM	531	CG	MET	1041	23.811	33.024	11.611	1.00	0.00	LX0	C
ATOM	532	SD	MET	1041	22.666	33.671	10.381	1.00	0.00	LX0	S
ATOM	533	CE	MET	1041	21.201	32.779	10.925	1.00	0.00	LX0	C
ATOM	534	C	MET	1041	25.971	31.654	9.807	1.00	0.00	LX0	C
ATOM	535	O	MET	1041	26.930	30.964	10.124	1.00	0.00	LX0	O
ATOM	536	N	ARG	1042	24.893	31.187	9.147	1.00	0.00	LX0	N
ATOM	537	H	ARG	1042	24.180	31.799	8.810	0.00	0.00	LX0	H
ATOM	538	CA	ARG	1042	24.878	29.756	8.852	1.00	0.00	LX0	C
ATOM	539	CB	ARG	1042	23.498	29.258	8.438	1.00	0.00	LX0	C
ATOM	540	CG	ARG	1042	22.590	29.086	9.655	1.00	0.00	LX0	C
ATOM	541	CD	ARG	1042	21.765	27.800	9.573	1.00	0.00	LX0	C
ATOM	542	NE	ARG	1042	22.638	26.626	9.541	1.00	0.00	LX0	N
ATOM	543	HE	ARG	1042	23.370	26.548	8.855	0.00	0.00	LX0	H
ATOM	544	CZ	ARG	1042	22.507	25.608	10.414	1.00	0.00	LX0	C
ATOM	545	NH1	ARG	1042	21.577	25.640	11.357	1.00	0.00	LX0	N
ATOM	546	HH11	ARG	1042	21.393	24.862	11.955	0.00	0.00	LX0	H
ATOM	547	HH12	ARG	1042	21.066	26.489	11.552	0.00	0.00	LX0	H
ATOM	548	NH2	ARG	1042	23.333	24.573	10.338	1.00	0.00	LX0	N
ATOM	549	HH21	ARG	1042	23.292	23.789	10.953	0.00	0.00	LX0	H
ATOM	550	HH22	ARG	1042	24.072	24.571	9.639	0.00	0.00	LX0	H
ATOM	551	C	ARG	1042	25.932	29.312	7.867	1.00	0.00	LX0	C
ATOM	552	O	ARG	1042	26.593	28.307	8.064	1.00	0.00	LX0	O
ATOM	553	N	GLU	1043	26.128	30.144	6.833	1.00	0.00	LX0	N
ATOM	554	H	GLU	1043	25.530	30.934	6.686	0.00	0.00	LX0	H
ATOM	555	CA	GLU	1043	27.237	29.848	5.918	1.00	0.00	LX0	C
ATOM	556	CB	GLU	1043	27.327	30.953	4.878	1.00	0.00	LX0	C
ATOM	557	CG	GLU	1043	26.145	30.914	3.910	1.00	0.00	LX0	C
ATOM	558	CD	GLU	1043	25.996	32.267	3.251	1.00	0.00	LX0	C
ATOM	559	OE1	GLU	1043	24.947	32.882	3.415	1.00	0.00	LX0	O
ATOM	560	OE2	GLU	1043	26.933	32.729	2.613	1.00	0.00	LX0	O
ATOM	561	C	GLU	1043	28.581	29.645	6.607	1.00	0.00	LX0	C
ATOM	562	O	GLU	1043	29.365	28.757	6.287	1.00	0.00	LX0	O
ATOM	563	N	ARG	1044	28.784	30.496	7.626	1.00	0.00	LX0	N
ATOM	564	H	ARG	1044	28.098	31.203	7.810	0.00	0.00	LX0	H
ATOM	565	CA	ARG	1044	29.967	30.306	8.467	1.00	0.00	LX0	C
ATOM	566	CB	ARG	1044	30.154	31.489	9.414	1.00	0.00	LX0	C
ATOM	567	CG	ARG	1044	30.084	32.777	8.608	1.00	0.00	LX0	C
ATOM	568	CD	ARG	1044	30.105	34.067	9.415	1.00	0.00	LX0	C
ATOM	569	NE	ARG	1044	29.749	35.170	8.528	1.00	0.00	LX0	N
ATOM	570	HE	ARG	1044	28.780	35.374	8.362	0.00	0.00	LX0	H
ATOM	571	CZ	ARG	1044	30.657	35.769	7.736	1.00	0.00	LX0	C
ATOM	572	NH1	ARG	1044	31.945	35.457	7.772	1.00	0.00	LX0	N
ATOM	573	HH11	ARG	1044	32.581	35.902	7.128	0.00	0.00	LX0	H
ATOM	574	HH12	ARG	1044	32.312	34.777	8.413	0.00	0.00	LX0	H
ATOM	575	NH2	ARG	1044	30.238	36.680	6.882	1.00	0.00	LX0	N
ATOM	576	HH21	ARG	1044	30.890	37.164	6.290	0.00	0.00	LX0	H
ATOM	577	HH22	ARG	1044	29.258	36.889	6.785	0.00	0.00	LX0	H
ATOM	578	C	ARG	1044	29.966	29.003	9.239	1.00	0.00	LX0	C
ATOM	579	O	ARG	1044	30.944	28.269	9.242	1.00	0.00	LX0	O
ATOM	580	N	ILE	1045	28.814	28.740	9.878	1.00	0.00	LX0	N
ATOM	581	H	ILE	1045	28.048	29.375	9.773	0.00	0.00	LX0	H
ATOM	582	CA	ILE	1045	28.664	27.490	10.628	1.00	0.00	LX0	C
ATOM	583	CB	ILE	1045	27.270	27.422	11.279	1.00	0.00	LX0	C
ATOM	584	CG2	ILE	1045	27.008	26.109	12.026	1.00	0.00	LX0	C
ATOM	585	CG1	ILE	1045	27.082	28.621	12.209	1.00	0.00	LX0	C
ATOM	586	CD1	ILE	1045	25.620	28.862	12.577	1.00	0.00	LX0	C
ATOM	587	C	ILE	1045	28.973	26.259	9.788	1.00	0.00	LX0	C
ATOM	588	O	ILE	1045	29.883	25.503	10.095	1.00	0.00	LX0	O
ATOM	589	N	GLU	1046	28.233	26.126	8.681	1.00	0.00	LX0	N
ATOM	590	H	GLU	1046	27.422	26.696	8.539	0.00	0.00	LX0	H

ATOM	591	CA	GLU	1046	28.492	25.007	7.779	1.00	0.00	LX0	C
ATOM	592	CB	GLU	1046	27.487	25.005	6.628	1.00	0.00	LX0	C
ATOM	593	CG	GLU	1046	26.276	24.107	6.914	1.00	0.00	LX0	C
ATOM	594	CD	GLU	1046	25.587	24.515	8.203	1.00	0.00	LX0	C
ATOM	595	OE1	GLU	1046	25.726	23.818	9.203	1.00	0.00	LX0	O
ATOM	596	OE2	GLU	1046	24.897	25.527	8.220	1.00	0.00	LX0	O
ATOM	597	C	GLU	1046	29.915	24.879	7.284	1.00	0.00	LX0	C
ATOM	598	O	GLU	1046	30.513	23.814	7.353	1.00	0.00	LX0	O
ATOM	599	N	PHE	1047	30.476	26.012	6.832	1.00	0.00	LX0	N
ATOM	600	H	PHE	1047	29.962	26.872	6.787	0.00	0.00	LX0	H
ATOM	601	CA	PHE	1047	31.871	25.894	6.403	1.00	0.00	LX0	C
ATOM	602	CB	PHE	1047	32.345	27.171	5.704	1.00	0.00	LX0	C
ATOM	603	CG	PHE	1047	33.612	26.919	4.910	1.00	0.00	LX0	C
ATOM	604	CD1	PHE	1047	33.693	25.811	4.035	1.00	0.00	LX0	C
ATOM	605	CD2	PHE	1047	34.693	27.815	5.047	1.00	0.00	LX0	C
ATOM	606	CE1	PHE	1047	34.861	25.610	3.277	1.00	0.00	LX0	C
ATOM	607	CE2	PHE	1047	35.862	27.619	4.289	1.00	0.00	LX0	C
ATOM	608	CZ	PHE	1047	35.929	26.522	3.407	1.00	0.00	LX0	C
ATOM	609	C	PHE	1047	32.840	25.475	7.500	1.00	0.00	LX0	C
ATOM	610	O	PHE	1047	33.778	24.715	7.290	1.00	0.00	LX0	O
ATOM	611	N	LEU	1048	32.551	25.984	8.702	1.00	0.00	LX0	N
ATOM	612	H	LEU	1048	31.755	26.582	8.815	0.00	0.00	LX0	H
ATOM	613	CA	LEU	1048	33.353	25.593	9.860	1.00	0.00	LX0	C
ATOM	614	CB	LEU	1048	33.057	26.532	11.034	1.00	0.00	LX0	C
ATOM	615	CG	LEU	1048	33.896	27.821	11.128	1.00	0.00	LX0	C
ATOM	616	CD1	LEU	1048	34.249	28.482	9.794	1.00	0.00	LX0	C
ATOM	617	CD2	LEU	1048	33.227	28.826	12.065	1.00	0.00	LX0	C
ATOM	618	C	LEU	1048	33.145	24.135	10.247	1.00	0.00	LX0	C
ATOM	619	O	LEU	1048	34.047	23.453	10.721	1.00	0.00	LX0	O
ATOM	620	N	ASN	1049	31.917	23.671	9.981	1.00	0.00	LX0	N
ATOM	621	H	ASN	1049	31.237	24.299	9.603	0.00	0.00	LX0	H
ATOM	622	CA	ASN	1049	31.566	22.270	10.217	1.00	0.00	LX0	C
ATOM	623	CB	ASN	1049	30.064	22.028	10.016	1.00	0.00	LX0	C
ATOM	624	CG	ASN	1049	29.265	22.610	11.163	1.00	0.00	LX0	C
ATOM	625	OD1	ASN	1049	29.700	22.636	12.307	1.00	0.00	LX0	O
ATOM	626	ND2	ASN	1049	28.039	23.028	10.828	1.00	0.00	LX0	N
ATOM	627	HD21	ASN	1049	27.660	23.075	9.899	0.00	0.00	LX0	H
ATOM	628	HD22	ASN	1049	27.413	23.324	11.546	0.00	0.00	LX0	H
ATOM	629	C	ASN	1049	32.359	21.341	9.325	1.00	0.00	LX0	C
ATOM	630	O	ASN	1049	33.103	20.483	9.780	1.00	0.00	LX0	O
ATOM	631	N	GLU	1050	32.224	21.595	8.016	1.00	0.00	LX0	N
ATOM	632	H	GLU	1050	31.616	22.326	7.704	0.00	0.00	LX0	H
ATOM	633	CA	GLU	1050	32.966	20.777	7.057	1.00	0.00	LX0	C
ATOM	634	CB	GLU	1050	32.441	21.040	5.642	1.00	0.00	LX0	C
ATOM	635	CG	GLU	1050	30.934	20.736	5.567	1.00	0.00	LX0	C
ATOM	636	CD	GLU	1050	30.342	20.838	4.166	1.00	0.00	LX0	C
ATOM	637	OE1	GLU	1050	31.019	21.239	3.224	1.00	0.00	LX0	O
ATOM	638	OE2	GLU	1050	29.183	20.469	4.005	1.00	0.00	LX0	O
ATOM	639	C	GLU	1050	34.480	20.900	7.174	1.00	0.00	LX0	C
ATOM	640	O	GLU	1050	35.241	19.960	6.981	1.00	0.00	LX0	O
ATOM	641	N	ALA	1051	34.899	22.103	7.596	1.00	0.00	LX0	N
ATOM	642	H	ALA	1051	34.256	22.869	7.633	0.00	0.00	LX0	H
ATOM	643	CA	ALA	1051	36.303	22.231	7.986	1.00	0.00	LX0	C
ATOM	644	CB	ALA	1051	36.642	23.678	8.335	1.00	0.00	LX0	C
ATOM	645	C	ALA	1051	36.691	21.351	9.167	1.00	0.00	LX0	C
ATOM	646	O	ALA	1051	37.763	20.759	9.216	1.00	0.00	LX0	O
ATOM	647	N	SER	1052	35.754	21.268	10.123	1.00	0.00	LX0	N
ATOM	648	H	SER	1052	34.874	21.730	10.008	0.00	0.00	LX0	H
ATOM	649	CA	SER	1052	36.003	20.451	11.308	1.00	0.00	LX0	C
ATOM	650	CB	SER	1052	34.904	20.644	12.357	1.00	0.00	LX0	C
ATOM	651	OG	SER	1052	34.988	21.981	12.880	1.00	0.00	LX0	O

ATOM	652	HG	SER	1052	34.557	22.531	12.223	0.00	0.00	LX0	H
ATOM	653	C	SER	1052	36.299	18.979	11.085	1.00	0.00	LX0	C
ATOM	654	O	SER	1052	36.936	18.352	11.923	1.00	0.00	LX0	O
ATOM	655	N	VAL	1053	35.903	18.455	9.907	1.00	0.00	LX0	N
ATOM	656	H	VAL	1053	35.314	18.981	9.290	0.00	0.00	LX0	H
ATOM	657	CA	VAL	1053	36.364	17.093	9.594	1.00	0.00	LX0	C
ATOM	658	CB	VAL	1053	35.914	16.620	8.201	1.00	0.00	LX0	C
ATOM	659	CG1	VAL	1053	36.031	15.101	8.120	1.00	0.00	LX0	C
ATOM	660	CG2	VAL	1053	34.503	17.052	7.814	1.00	0.00	LX0	C
ATOM	661	C	VAL	1053	37.884	16.944	9.703	1.00	0.00	LX0	C
ATOM	662	O	VAL	1053	38.443	16.020	10.281	1.00	0.00	LX0	O
ATOM	663	N	MET	1054	38.543	17.975	9.153	1.00	0.00	LX0	N
ATOM	664	H	MET	1054	38.029	18.751	8.782	0.00	0.00	LX0	H
ATOM	665	CA	MET	1054	40.003	17.984	9.181	1.00	0.00	LX0	C
ATOM	666	CB	MET	1054	40.535	19.090	8.279	1.00	0.00	LX0	C
ATOM	667	CG	MET	1054	40.595	18.684	6.814	1.00	0.00	LX0	C
ATOM	668	SD	MET	1054	41.719	17.305	6.585	1.00	0.00	LX0	S
ATOM	669	CE	MET	1054	41.790	17.392	4.797	1.00	0.00	LX0	C
ATOM	670	C	MET	1054	40.672	18.070	10.544	1.00	0.00	LX0	C
ATOM	671	O	MET	1054	41.887	17.928	10.657	1.00	0.00	LX0	O
ATOM	672	N	LYS	1055	39.860	18.299	11.589	1.00	0.00	LX0	N
ATOM	673	H	LYS	1055	38.868	18.348	11.469	0.00	0.00	LX0	H
ATOM	674	CA	LYS	1055	40.452	18.261	12.925	1.00	0.00	LX0	C
ATOM	675	CB	LYS	1055	39.437	18.707	13.979	1.00	0.00	LX0	C
ATOM	676	CG	LYS	1055	39.152	20.200	13.850	1.00	0.00	LX0	C
ATOM	677	CD	LYS	1055	37.946	20.694	14.648	1.00	0.00	LX0	C
ATOM	678	CE	LYS	1055	37.785	22.204	14.470	1.00	0.00	LX0	C
ATOM	679	NZ	LYS	1055	36.484	22.651	14.965	1.00	0.00	LX0	N
ATOM	680	HZ1	LYS	1055	36.383	23.683	14.901	0.00	0.00	LX0	H
ATOM	681	HZ2	LYS	1055	35.681	22.233	14.452	0.00	0.00	LX0	H
ATOM	682	HZ3	LYS	1055	36.371	22.463	15.989	0.00	0.00	LX0	H
ATOM	683	C	LYS	1055	41.032	16.901	13.265	1.00	0.00	LX0	C
ATOM	684	O	LYS	1055	42.213	16.774	13.568	1.00	0.00	LX0	O
ATOM	685	N	GLU	1056	40.151	15.893	13.145	1.00	0.00	LX0	N
ATOM	686	H	GLU	1056	39.232	16.081	12.795	0.00	0.00	LX0	H
ATOM	687	CA	GLU	1056	40.495	14.540	13.594	1.00	0.00	LX0	C
ATOM	688	CB	GLU	1056	39.336	13.586	13.286	1.00	0.00	LX0	C
ATOM	689	CG	GLU	1056	39.446	12.253	14.034	1.00	0.00	LX0	C
ATOM	690	CD	GLU	1056	38.405	11.275	13.533	1.00	0.00	LX0	C
ATOM	691	OE1	GLU	1056	37.217	11.473	13.774	1.00	0.00	LX0	O
ATOM	692	OE2	GLU	1056	38.776	10.282	12.917	1.00	0.00	LX0	O
ATOM	693	C	GLU	1056	41.798	13.974	13.044	1.00	0.00	LX0	C
ATOM	694	O	GLU	1056	42.599	13.369	13.743	1.00	0.00	LX0	O
ATOM	695	N	PHE	1057	41.961	14.206	11.732	1.00	0.00	LX0	N
ATOM	696	H	PHE	1057	41.259	14.739	11.264	0.00	0.00	LX0	H
ATOM	697	CA	PHE	1057	43.021	13.513	10.999	1.00	0.00	LX0	C
ATOM	698	CB	PHE	1057	42.758	13.579	9.495	1.00	0.00	LX0	C
ATOM	699	CG	PHE	1057	41.374	13.073	9.192	1.00	0.00	LX0	C
ATOM	700	CD1	PHE	1057	41.055	11.719	9.436	1.00	0.00	LX0	C
ATOM	701	CD2	PHE	1057	40.421	13.979	8.683	1.00	0.00	LX0	C
ATOM	702	CE1	PHE	1057	39.748	11.269	9.182	1.00	0.00	LX0	C
ATOM	703	CE2	PHE	1057	39.116	13.526	8.427	1.00	0.00	LX0	C
ATOM	704	CZ	PHE	1057	38.792	12.180	8.683	1.00	0.00	LX0	C
ATOM	705	C	PHE	1057	44.450	13.944	11.268	1.00	0.00	LX0	C
ATOM	706	O	PHE	1057	45.089	14.590	10.448	1.00	0.00	LX0	O
ATOM	707	N	ASN	1058	44.954	13.568	12.441	1.00	0.00	LX0	N
ATOM	708	H	ASN	1058	44.434	12.928	13.017	0.00	0.00	LX0	H
ATOM	709	CA	ASN	1058	46.266	14.104	12.777	1.00	0.00	LX0	C
ATOM	710	CB	ASN	1058	46.307	14.574	14.230	1.00	0.00	LX0	C
ATOM	711	CG	ASN	1058	47.243	15.761	14.315	1.00	0.00	LX0	C
ATOM	712	OD1	ASN	1058	46.864	16.899	14.055	1.00	0.00	LX0	O

ATOM	713	ND2	ASN	1058	48.488	15.458	14.684	1.00	0.00	LX0 N
ATOM	714	HD21	ASN	1058	48.745	14.490	14.760	0.00	0.00	LX0 H
ATOM	715	HD22	ASN	1058	49.160	16.174	14.858	0.00	0.00	LX0 H
ATOM	716	C	ASN	1058	47.456	13.226	12.437	1.00	0.00	LX0 C
ATOM	717	O	ASN	1058	48.370	13.044	13.232	1.00	0.00	LX0 O
ATOM	718	N	CYS	1059	47.417	12.701	11.208	1.00	0.00	LX0 N
ATOM	719	H	CYS	1059	46.687	12.955	10.571	0.00	0.00	LX0 H
ATOM	720	CA	CYS	1059	48.597	11.980	10.743	1.00	0.00	LX0 C
ATOM	721	CB	CYS	1059	48.333	10.477	10.619	1.00	0.00	LX0 C
ATOM	722	SG	CYS	1059	49.849	9.510	10.415	1.00	0.00	LX0 S
ATOM	723	C	CYS	1059	49.062	12.590	9.440	1.00	0.00	LX0 C
ATOM	724	O	CYS	1059	48.331	13.346	8.811	1.00	0.00	LX0 O
ATOM	725	N	HIS	1060	50.322	12.286	9.089	1.00	0.00	LX0 N
ATOM	726	H	HIS	1060	50.812	11.544	9.551	0.00	0.00	LX0 H
ATOM	727	CA	HIS	1060	51.021	13.167	8.153	1.00	0.00	LX0 C
ATOM	728	CB	HIS	1060	52.435	12.653	7.886	1.00	0.00	LX0 C
ATOM	729	CG	HIS	1060	53.361	13.804	7.570	1.00	0.00	LX0 C
ATOM	730	ND1	HIS	1060	54.236	14.306	8.461	1.00	0.00	LX0 N
ATOM	731	HD1	HIS	1060	54.392	13.982	9.371	0.00	0.00	LX0 H
ATOM	732	CD2	HIS	1060	53.468	14.526	6.378	1.00	0.00	LX0 C
ATOM	733	NE2	HIS	1060	54.419	15.471	6.571	1.00	0.00	LX0 N
ATOM	734	CE1	HIS	1060	54.894	15.338	7.847	1.00	0.00	LX0 C
ATOM	735	C	HIS	1060	50.322	13.494	6.846	1.00	0.00	LX0 C
ATOM	736	O	HIS	1060	50.165	14.655	6.482	1.00	0.00	LX0 O
ATOM	737	N	HIS	1061	49.916	12.425	6.147	1.00	0.00	LX0 N
ATOM	738	H	HIS	1061	49.969	11.496	6.515	0.00	0.00	LX0 H
ATOM	739	CA	HIS	1061	49.501	12.659	4.764	1.00	0.00	LX0 C
ATOM	740	CB	HIS	1061	49.877	11.465	3.879	1.00	0.00	LX0 C
ATOM	741	CG	HIS	1061	51.339	11.138	4.085	1.00	0.00	LX0 C
ATOM	742	ND1	HIS	1061	51.771	10.030	4.710	1.00	0.00	LX0 N
ATOM	743	HD1	HIS	1061	51.206	9.299	5.048	0.00	0.00	LX0 H
ATOM	744	CD2	HIS	1061	52.450	11.904	3.724	1.00	0.00	LX0 C
ATOM	745	NE2	HIS	1061	53.553	11.240	4.147	1.00	0.00	LX0 N
ATOM	746	CE1	HIS	1061	53.136	10.085	4.755	1.00	0.00	LX0 C
ATOM	747	C	HIS	1061	48.060	13.096	4.550	1.00	0.00	LX0 C
ATOM	748	O	HIS	1061	47.471	12.909	3.493	1.00	0.00	LX0 O
ATOM	749	N	VAL	1062	47.512	13.721	5.597	1.00	0.00	LX0 N
ATOM	750	H	VAL	1062	48.007	13.868	6.454	0.00	0.00	LX0 H
ATOM	751	CA	VAL	1062	46.261	14.437	5.375	1.00	0.00	LX0 C
ATOM	752	CB	VAL	1062	45.166	13.924	6.330	1.00	0.00	LX0 C
ATOM	753	CG1	VAL	1062	43.765	14.313	5.849	1.00	0.00	LX0 C
ATOM	754	CG2	VAL	1062	45.242	12.408	6.526	1.00	0.00	LX0 C
ATOM	755	C	VAL	1062	46.550	15.914	5.589	1.00	0.00	LX0 C
ATOM	756	O	VAL	1062	47.525	16.264	6.252	1.00	0.00	LX0 O
ATOM	757	N	VAL	1063	45.696	16.772	5.009	1.00	0.00	LX0 N
ATOM	758	H	VAL	1063	44.983	16.434	4.391	0.00	0.00	LX0 H
ATOM	759	CA	VAL	1063	45.817	18.188	5.368	1.00	0.00	LX0 C
ATOM	760	CB	VAL	1063	44.907	19.054	4.473	1.00	0.00	LX0 C
ATOM	761	CG1	VAL	1063	44.948	20.556	4.780	1.00	0.00	LX0 C
ATOM	762	CG2	VAL	1063	45.238	18.809	3.005	1.00	0.00	LX0 C
ATOM	763	C	VAL	1063	45.543	18.419	6.850	1.00	0.00	LX0 C
ATOM	764	O	VAL	1063	44.697	17.788	7.483	1.00	0.00	LX0 O
ATOM	765	N	ARG	1064	46.314	19.349	7.409	1.00	0.00	LX0 N
ATOM	766	H	ARG	1064	47.062	19.775	6.892	0.00	0.00	LX0 H
ATOM	767	CA	ARG	1064	45.976	19.690	8.778	1.00	0.00	LX0 C
ATOM	768	CB	ARG	1064	47.231	19.809	9.644	1.00	0.00	LX0 C
ATOM	769	CG	ARG	1064	48.055	18.515	9.677	1.00	0.00	LX0 C
ATOM	770	CD	ARG	1064	47.233	17.267	10.023	1.00	0.00	LX0 C
ATOM	771	NE	ARG	1064	46.541	17.411	11.304	1.00	0.00	LX0 N
ATOM	772	HE	ARG	1064	47.081	17.498	12.147	0.00	0.00	LX0 H
ATOM	773	CZ	ARG	1064	45.194	17.383	11.390	1.00	0.00	LX0 C

ATOM	774	NH1	ARG	1064	44.436	17.279	10.299	1.00	0.00	LX0	N
ATOM	775	HH11	ARG	1064	43.435	17.293	10.369	0.00	0.00	LX0	H
ATOM	776	HH12	ARG	1064	44.830	17.173	9.379	0.00	0.00	LX0	H
ATOM	777	NH2	ARG	1064	44.643	17.456	12.596	1.00	0.00	LX0	N
ATOM	778	HH21	ARG	1064	43.650	17.417	12.755	0.00	0.00	LX0	H
ATOM	779	HH22	ARG	1064	45.242	17.531	13.402	0.00	0.00	LX0	H
ATOM	780	C	ARG	1064	45.078	20.896	8.910	1.00	0.00	LX0	C
ATOM	781	O	ARG	1064	45.374	21.988	8.445	1.00	0.00	LX0	O
ATOM	782	N	LEU	1065	43.955	20.660	9.603	1.00	0.00	LX0	N
ATOM	783	H	LEU	1065	43.727	19.748	9.937	0.00	0.00	LX0	H
ATOM	784	CA	LEU	1065	43.328	21.850	10.166	1.00	0.00	LX0	C
ATOM	785	CB	LEU	1065	41.840	21.632	10.447	1.00	0.00	LX0	C
ATOM	786	CG	LEU	1065	41.091	22.885	10.917	1.00	0.00	LX0	C
ATOM	787	CD1	LEU	1065	41.247	24.048	9.949	1.00	0.00	LX0	C
ATOM	788	CD2	LEU	1065	39.613	22.610	11.158	1.00	0.00	LX0	C
ATOM	789	C	LEU	1065	44.065	22.208	11.432	1.00	0.00	LX0	C
ATOM	790	O	LEU	1065	44.124	21.429	12.372	1.00	0.00	LX0	O
ATOM	791	N	LEU	1066	44.657	23.397	11.374	1.00	0.00	LX0	N
ATOM	792	H	LEU	1066	44.554	23.966	10.558	0.00	0.00	LX0	H
ATOM	793	CA	LEU	1066	45.396	23.872	12.535	1.00	0.00	LX0	C
ATOM	794	CB	LEU	1066	46.647	24.615	12.068	1.00	0.00	LX0	C
ATOM	795	CG	LEU	1066	47.568	23.680	11.279	1.00	0.00	LX0	C
ATOM	796	CD1	LEU	1066	48.640	24.437	10.499	1.00	0.00	LX0	C
ATOM	797	CD2	LEU	1066	48.163	22.589	12.171	1.00	0.00	LX0	C
ATOM	798	C	LEU	1066	44.549	24.704	13.475	1.00	0.00	LX0	C
ATOM	799	O	LEU	1066	44.800	24.772	14.669	1.00	0.00	LX0	O
ATOM	800	N	GLY	1067	43.508	25.314	12.889	1.00	0.00	LX0	N
ATOM	801	H	GLY	1067	43.377	25.333	11.896	0.00	0.00	LX0	H
ATOM	802	CA	GLY	1067	42.588	26.005	13.782	1.00	0.00	LX0	C
ATOM	803	C	GLY	1067	41.416	26.624	13.061	1.00	0.00	LX0	C
ATOM	804	O	GLY	1067	41.330	26.611	11.839	1.00	0.00	LX0	O
ATOM	805	N	VAL	1068	40.509	27.161	13.873	1.00	0.00	LX0	N
ATOM	806	H	VAL	1068	40.679	27.192	14.862	0.00	0.00	LX0	H
ATOM	807	CA	VAL	1068	39.362	27.899	13.357	1.00	0.00	LX0	C
ATOM	808	CB	VAL	1068	38.068	27.170	13.770	1.00	0.00	LX0	C
ATOM	809	CG1	VAL	1068	36.791	27.942	13.437	1.00	0.00	LX0	C
ATOM	810	CG2	VAL	1068	38.020	25.772	13.152	1.00	0.00	LX0	C
ATOM	811	C	VAL	1068	39.456	29.276	13.982	1.00	0.00	LX0	C
ATOM	812	O	VAL	1068	39.992	29.404	15.070	1.00	0.00	LX0	O
ATOM	813	N	VAL	1069	38.968	30.293	13.263	1.00	0.00	LX0	N
ATOM	814	H	VAL	1069	38.600	30.126	12.350	0.00	0.00	LX0	H
ATOM	815	CA	VAL	1069	38.801	31.574	13.944	1.00	0.00	LX0	C
ATOM	816	CB	VAL	1069	39.837	32.633	13.527	1.00	0.00	LX0	C
ATOM	817	CG1	VAL	1069	39.764	33.856	14.448	1.00	0.00	LX0	C
ATOM	818	CG2	VAL	1069	41.263	32.087	13.478	1.00	0.00	LX0	C
ATOM	819	C	VAL	1069	37.396	32.095	13.714	1.00	0.00	LX0	C
ATOM	820	O	VAL	1069	37.134	32.905	12.825	1.00	0.00	LX0	O
ATOM	821	N	SER	1070	36.493	31.576	14.548	1.00	0.00	LX0	N
ATOM	822	H	SER	1070	36.842	31.008	15.302	0.00	0.00	LX0	H
ATOM	823	CA	SER	1070	35.124	32.083	14.538	1.00	0.00	LX0	C
ATOM	824	CB	SER	1070	34.233	31.186	15.406	1.00	0.00	LX0	C
ATOM	825	OG	SER	1070	34.677	31.198	16.769	1.00	0.00	LX0	O
ATOM	826	HG	SER	1070	35.509	30.716	16.798	0.00	0.00	LX0	H
ATOM	827	C	SER	1070	35.029	33.538	14.976	1.00	0.00	LX0	C
ATOM	828	O	SER	1070	34.211	34.324	14.510	1.00	0.00	LX0	O
ATOM	829	N	GLN	1071	35.942	33.877	15.898	1.00	0.00	LX0	N
ATOM	830	H	GLN	1071	36.574	33.177	16.243	0.00	0.00	LX0	H
ATOM	831	CA	GLN	1071	35.908	35.227	16.445	1.00	0.00	LX0	C
ATOM	832	CB	GLN	1071	36.777	35.329	17.704	1.00	0.00	LX0	C
ATOM	833	CG	GLN	1071	36.478	34.266	18.769	1.00	0.00	LX0	C
ATOM	834	CD	GLN	1071	35.011	34.293	19.150	1.00	0.00	LX0	C

ATOM	835	OE1	GLN	1071	34.503	35.245	19.724	1.00	0.00	LX0	O
ATOM	836	NE2	GLN	1071	34.339	33.199	18.786	1.00	0.00	LX0	N
ATOM	837	HE21	GLN	1071	34.779	32.451	18.280	0.00	0.00	LX0	H
ATOM	838	HE22	GLN	1071	33.371	33.117	19.005	0.00	0.00	LX0	H
ATOM	839	C	GLN	1071	36.272	36.309	15.449	1.00	0.00	LX0	C
ATOM	840	O	GLN	1071	37.437	36.567	15.156	1.00	0.00	LX0	O
ATOM	841	N	GLY	1072	35.207	36.951	14.959	1.00	0.00	LX0	N
ATOM	842	H	GLY	1072	34.324	36.481	15.028	0.00	0.00	LX0	H
ATOM	843	CA	GLY	1072	35.402	38.179	14.196	1.00	0.00	LX0	C
ATOM	844	C	GLY	1072	35.374	38.003	12.691	1.00	0.00	LX0	C
ATOM	845	O	GLY	1072	35.513	36.918	12.140	1.00	0.00	LX0	O
ATOM	846	N	GLN	1073	35.184	39.155	12.041	1.00	0.00	LX0	N
ATOM	847	H	GLN	1073	35.183	40.016	12.547	0.00	0.00	LX0	H
ATOM	848	CA	GLN	1073	35.195	39.149	10.581	1.00	0.00	LX0	C
ATOM	849	CB	GLN	1073	34.276	40.260	10.065	1.00	0.00	LX0	C
ATOM	850	CG	GLN	1073	32.786	39.937	10.204	1.00	0.00	LX0	C
ATOM	851	CD	GLN	1073	32.331	39.052	9.057	1.00	0.00	LX0	C
ATOM	852	OE1	GLN	1073	32.886	38.001	8.760	1.00	0.00	LX0	O
ATOM	853	NE2	GLN	1073	31.286	39.559	8.401	1.00	0.00	LX0	N
ATOM	854	HE21	GLN	1073	30.852	40.411	8.690	0.00	0.00	LX0	H
ATOM	855	HE22	GLN	1073	30.912	39.112	7.588	0.00	0.00	LX0	H
ATOM	856	C	GLN	1073	36.601	39.314	10.025	1.00	0.00	LX0	C
ATOM	857	O	GLN	1073	37.421	40.017	10.602	1.00	0.00	LX0	O
ATOM	858	N	PRO	1074	36.872	38.636	8.884	1.00	0.00	LX0	N
ATOM	859	CD	PRO	1074	38.016	38.904	8.018	1.00	0.00	LX0	C
ATOM	860	CA	PRO	1074	36.050	37.514	8.419	1.00	0.00	LX0	C
ATOM	861	CB	PRO	1074	36.502	37.380	6.962	1.00	0.00	LX0	C
ATOM	862	CG	PRO	1074	37.973	37.797	6.969	1.00	0.00	LX0	C
ATOM	863	C	PRO	1074	36.353	36.287	9.265	1.00	0.00	LX0	C
ATOM	864	O	PRO	1074	37.370	36.220	9.955	1.00	0.00	LX0	O
ATOM	865	N	THR	1075	35.426	35.334	9.200	1.00	0.00	LX0	N
ATOM	866	H	THR	1075	34.646	35.426	8.581	0.00	0.00	LX0	H
ATOM	867	CA	THR	1075	35.666	34.084	9.916	1.00	0.00	LX0	C
ATOM	868	CB	THR	1075	34.334	33.354	10.023	1.00	0.00	LX0	C
ATOM	869	OG1	THR	1075	33.550	33.632	8.851	1.00	0.00	LX0	O
ATOM	870	HG1	THR	1075	34.116	33.414	8.112	0.00	0.00	LX0	H
ATOM	871	CG2	THR	1075	33.565	33.789	11.270	1.00	0.00	LX0	C
ATOM	872	C	THR	1075	36.705	33.258	9.184	1.00	0.00	LX0	C
ATOM	873	O	THR	1075	36.736	33.252	7.958	1.00	0.00	LX0	O
ATOM	874	N	LEU	1076	37.582	32.620	9.963	1.00	0.00	LX0	N
ATOM	875	H	LEU	1076	37.464	32.568	10.955	0.00	0.00	LX0	H
ATOM	876	CA	LEU	1076	38.741	32.032	9.298	1.00	0.00	LX0	C
ATOM	877	CB	LEU	1076	40.027	32.691	9.796	1.00	0.00	LX0	C
ATOM	878	CG	LEU	1076	40.010	34.220	9.762	1.00	0.00	LX0	C
ATOM	879	CD1	LEU	1076	41.120	34.811	10.617	1.00	0.00	LX0	C
ATOM	880	CD2	LEU	1076	40.046	34.780	8.345	1.00	0.00	LX0	C
ATOM	881	C	LEU	1076	38.836	30.538	9.497	1.00	0.00	LX0	C
ATOM	882	O	LEU	1076	38.383	29.990	10.494	1.00	0.00	LX0	O
ATOM	883	N	VAL	1077	39.473	29.912	8.506	1.00	0.00	LX0	N
ATOM	884	H	VAL	1077	39.742	30.422	7.688	0.00	0.00	LX0	H
ATOM	885	CA	VAL	1077	39.806	28.497	8.598	1.00	0.00	LX0	C
ATOM	886	CB	VAL	1077	39.028	27.697	7.541	1.00	0.00	LX0	C
ATOM	887	CG1	VAL	1077	39.373	26.210	7.590	1.00	0.00	LX0	C
ATOM	888	CG2	VAL	1077	37.518	27.913	7.663	1.00	0.00	LX0	C
ATOM	889	C	VAL	1077	41.302	28.362	8.386	1.00	0.00	LX0	C
ATOM	890	O	VAL	1077	41.827	28.646	7.318	1.00	0.00	LX0	O
ATOM	891	N	ILE	1078	41.973	27.959	9.463	1.00	0.00	LX0	N
ATOM	892	H	ILE	1078	41.485	27.629	10.272	0.00	0.00	LX0	H
ATOM	893	CA	ILE	1078	43.431	27.912	9.396	1.00	0.00	LX0	C
ATOM	894	CB	ILE	1078	44.045	28.345	10.740	1.00	0.00	LX0	C
ATOM	895	CG2	ILE	1078	45.567	28.491	10.653	1.00	0.00	LX0	C

ATOM	896	CG1	ILE	1078	43.381	29.612	11.290	1.00	0.00	LX0	C
ATOM	897	CD1	ILE	1078	43.547	30.827	10.377	1.00	0.00	LX0	C
ATOM	898	C	ILE	1078	43.908	26.525	9.014	1.00	0.00	LX0	C
ATOM	899	O	ILE	1078	44.066	25.651	9.859	1.00	0.00	LX0	O
ATOM	900	N	MET	1079	44.107	26.347	7.705	1.00	0.00	LX0	N
ATOM	901	H	MET	1079	44.002	27.096	7.047	0.00	0.00	LX0	H
ATOM	902	CA	MET	1079	44.639	25.051	7.295	1.00	0.00	LX0	C
ATOM	903	CB	MET	1079	43.871	24.496	6.094	1.00	0.00	LX0	C
ATOM	904	CG	MET	1079	42.520	23.935	6.527	1.00	0.00	LX0	C
ATOM	905	SD	MET	1079	41.588	23.150	5.208	1.00	0.00	LX0	S
ATOM	906	CE	MET	1079	40.260	22.489	6.229	1.00	0.00	LX0	C
ATOM	907	C	MET	1079	46.126	25.072	7.020	1.00	0.00	LX0	C
ATOM	908	O	MET	1079	46.768	26.113	6.980	1.00	0.00	LX0	O
ATOM	909	N	GLU	1080	46.638	23.856	6.820	1.00	0.00	LX0	N
ATOM	910	H	GLU	1080	46.042	23.066	6.961	0.00	0.00	LX0	H
ATOM	911	CA	GLU	1080	48.019	23.649	6.396	1.00	0.00	LX0	C
ATOM	912	CB	GLU	1080	48.251	22.144	6.467	1.00	0.00	LX0	C
ATOM	913	CG	GLU	1080	49.659	21.584	6.273	1.00	0.00	LX0	C
ATOM	914	CD	GLU	1080	49.575	20.072	6.382	1.00	0.00	LX0	C
ATOM	915	OE1	GLU	1080	48.721	19.470	5.737	1.00	0.00	LX0	O
ATOM	916	OE2	GLU	1080	50.356	19.474	7.116	1.00	0.00	LX0	O
ATOM	917	C	GLU	1080	48.264	24.220	5.007	1.00	0.00	LX0	C
ATOM	918	O	GLU	1080	47.359	24.323	4.186	1.00	0.00	LX0	O
ATOM	919	N	LEU	1081	49.516	24.634	4.780	1.00	0.00	LX0	N
ATOM	920	H	LEU	1081	50.249	24.465	5.439	0.00	0.00	LX0	H
ATOM	921	CA	LEU	1081	49.758	25.287	3.499	1.00	0.00	LX0	C
ATOM	922	CB	LEU	1081	50.879	26.310	3.630	1.00	0.00	LX0	C
ATOM	923	CG	LEU	1081	50.956	27.270	2.445	1.00	0.00	LX0	C
ATOM	924	CD1	LEU	1081	49.667	28.054	2.205	1.00	0.00	LX0	C
ATOM	925	CD2	LEU	1081	52.153	28.191	2.587	1.00	0.00	LX0	C
ATOM	926	C	LEU	1081	49.982	24.350	2.326	1.00	0.00	LX0	C
ATOM	927	O	LEU	1081	51.073	23.868	2.041	1.00	0.00	LX0	O
ATOM	928	N	MET	1082	48.865	24.137	1.626	1.00	0.00	LX0	N
ATOM	929	H	MET	1082	48.009	24.534	1.959	0.00	0.00	LX0	H
ATOM	930	CA	MET	1082	48.949	23.293	0.437	1.00	0.00	LX0	C
ATOM	931	CB	MET	1082	47.650	22.507	0.245	1.00	0.00	LX0	C
ATOM	932	CG	MET	1082	47.082	21.940	1.552	1.00	0.00	LX0	C
ATOM	933	SD	MET	1082	48.264	20.978	2.514	1.00	0.00	LX0	S
ATOM	934	CE	MET	1082	48.500	19.615	1.367	1.00	0.00	LX0	C
ATOM	935	C	MET	1082	49.338	24.051	-0.821	1.00	0.00	LX0	C
ATOM	936	O	MET	1082	48.576	24.209	-1.768	1.00	0.00	LX0	O
ATOM	937	N	THR	1083	50.577	24.549	-0.781	1.00	0.00	LX0	N
ATOM	938	H	THR	1083	51.170	24.402	0.015	0.00	0.00	LX0	H
ATOM	939	CA	THR	1083	50.985	25.539	-1.776	1.00	0.00	LX0	C
ATOM	940	CB	THR	1083	52.172	26.333	-1.246	1.00	0.00	LX0	C
ATOM	941	OG1	THR	1083	52.525	25.881	0.067	1.00	0.00	LX0	O
ATOM	942	HG1	THR	1083	53.338	26.339	0.276	0.00	0.00	LX0	H
ATOM	943	CG2	THR	1083	51.907	27.840	-1.275	1.00	0.00	LX0	C
ATOM	944	C	THR	1083	51.279	25.086	-3.199	1.00	0.00	LX0	C
ATOM	945	O	THR	1083	51.962	25.776	-3.951	1.00	0.00	LX0	O
ATOM	946	N	ARG	1084	50.765	23.906	-3.558	1.00	0.00	LX0	N
ATOM	947	H	ARG	1084	50.302	23.297	-2.909	0.00	0.00	LX0	H
ATOM	948	CA	ARG	1084	50.780	23.614	-4.989	1.00	0.00	LX0	C
ATOM	949	CB	ARG	1084	51.574	22.347	-5.310	1.00	0.00	LX0	C
ATOM	950	CG	ARG	1084	52.980	22.332	-4.713	1.00	0.00	LX0	C
ATOM	951	CD	ARG	1084	53.863	23.472	-5.214	1.00	0.00	LX0	C
ATOM	952	NE	ARG	1084	55.105	23.508	-4.452	1.00	0.00	LX0	N
ATOM	953	HE	ARG	1084	55.677	22.686	-4.442	0.00	0.00	LX0	H
ATOM	954	CZ	ARG	1084	55.454	24.593	-3.734	1.00	0.00	LX0	C
ATOM	955	NH1	ARG	1084	54.669	25.663	-3.643	1.00	0.00	LX0	N
ATOM	956	HH11	ARG	1084	54.966	26.425	-3.050	0.00	0.00	LX0	H

ATOM	957	HH12	ARG	1084	53.780	25.731	-4.111	0.00	0.00	LX0	H
ATOM	958	NH2	ARG	1084	56.599	24.588	-3.073	1.00	0.00	LX0	N
ATOM	959	HH21	ARG	1084	56.879	25.416	-2.571	0.00	0.00	LX0	H
ATOM	960	HH22	ARG	1084	57.172	23.766	-2.998	0.00	0.00	LX0	H
ATOM	961	C	ARG	1084	49.394	23.524	-5.594	1.00	0.00	LX0	C
ATOM	962	O	ARG	1084	49.233	23.317	-6.788	1.00	0.00	LX0	O
ATOM	963	N	GLY	1085	48.391	23.691	-4.716	1.00	0.00	LX0	N
ATOM	964	H	GLY	1085	48.545	23.784	-3.732	0.00	0.00	LX0	H
ATOM	965	CA	GLY	1085	47.044	23.453	-5.215	1.00	0.00	LX0	C
ATOM	966	C	GLY	1085	46.753	21.969	-5.265	1.00	0.00	LX0	C
ATOM	967	O	GLY	1085	47.338	21.173	-4.536	1.00	0.00	LX0	O
ATOM	968	N	ASP	1086	45.821	21.640	-6.154	1.00	0.00	LX0	N
ATOM	969	H	ASP	1086	45.429	22.285	-6.814	0.00	0.00	LX0	H
ATOM	970	CA	ASP	1086	45.478	20.235	-6.317	1.00	0.00	LX0	C
ATOM	971	CB	ASP	1086	44.028	20.106	-6.805	1.00	0.00	LX0	C
ATOM	972	CG	ASP	1086	43.828	20.716	-8.184	1.00	0.00	LX0	C
ATOM	973	OD1	ASP	1086	44.074	21.903	-8.366	1.00	0.00	LX0	O
ATOM	974	OD2	ASP	1086	43.442	19.996	-9.093	1.00	0.00	LX0	O
ATOM	975	C	ASP	1086	46.445	19.516	-7.233	1.00	0.00	LX0	C
ATOM	976	O	ASP	1086	47.066	20.090	-8.123	1.00	0.00	LX0	O
ATOM	977	N	LEU	1087	46.542	18.202	-6.990	1.00	0.00	LX0	N
ATOM	978	H	LEU	1087	46.015	17.795	-6.243	0.00	0.00	LX0	H
ATOM	979	CA	LEU	1087	47.412	17.398	-7.843	1.00	0.00	LX0	C
ATOM	980	CB	LEU	1087	47.481	15.949	-7.348	1.00	0.00	LX0	C
ATOM	981	CG	LEU	1087	48.440	15.051	-8.141	1.00	0.00	LX0	C
ATOM	982	CD1	LEU	1087	49.889	15.538	-8.076	1.00	0.00	LX0	C
ATOM	983	CD2	LEU	1087	48.299	13.580	-7.753	1.00	0.00	LX0	C
ATOM	984	C	LEU	1087	46.995	17.440	-9.297	1.00	0.00	LX0	C
ATOM	985	O	LEU	1087	47.811	17.430	-10.205	1.00	0.00	LX0	O
ATOM	986	N	LYS	1088	45.672	17.504	-9.483	1.00	0.00	LX0	N
ATOM	987	H	LYS	1088	45.064	17.626	-8.698	0.00	0.00	LX0	H
ATOM	988	CA	LYS	1088	45.168	17.505	-10.850	1.00	0.00	LX0	C
ATOM	989	CB	LYS	1088	43.649	17.425	-10.781	1.00	0.00	LX0	C
ATOM	990	CG	LYS	1088	42.959	17.158	-12.098	1.00	0.00	LX0	C
ATOM	991	CD	LYS	1088	41.627	16.466	-11.878	1.00	0.00	LX0	C
ATOM	992	CE	LYS	1088	41.127	15.991	-13.227	1.00	0.00	LX0	C
ATOM	993	NZ	LYS	1088	40.054	15.018	-13.077	1.00	0.00	LX0	N
ATOM	994	HZ1	LYS	1088	39.738	14.734	-14.022	0.00	0.00	LX0	H
ATOM	995	HZ2	LYS	1088	40.437	14.161	-12.614	0.00	0.00	LX0	H
ATOM	996	HZ3	LYS	1088	39.245	15.414	-12.548	0.00	0.00	LX0	H
ATOM	997	C	LYS	1088	45.675	18.659	-11.697	1.00	0.00	LX0	C
ATOM	998	O	LYS	1088	46.229	18.488	-12.779	1.00	0.00	LX0	O
ATOM	999	N	SER	1089	45.507	19.853	-11.131	1.00	0.00	LX0	N
ATOM	1000	H	SER	1089	45.031	19.967	-10.255	0.00	0.00	LX0	H
ATOM	1001	CA	SER	1089	46.025	21.005	-11.851	1.00	0.00	LX0	C
ATOM	1002	CB	SER	1089	45.379	22.268	-11.297	1.00	0.00	LX0	C
ATOM	1003	OG	SER	1089	43.967	22.025	-11.210	1.00	0.00	LX0	O
ATOM	1004	HG	SER	1089	43.807	21.808	-10.280	0.00	0.00	LX0	H
ATOM	1005	C	SER	1089	47.541	21.057	-11.921	1.00	0.00	LX0	C
ATOM	1006	O	SER	1089	48.113	21.398	-12.947	1.00	0.00	LX0	O
ATOM	1007	N	TYR	1090	48.171	20.607	-10.820	1.00	0.00	LX0	N
ATOM	1008	H	TYR	1090	47.648	20.422	-9.984	0.00	0.00	LX0	H
ATOM	1009	CA	TYR	1090	49.628	20.439	-10.843	1.00	0.00	LX0	C
ATOM	1010	CB	TYR	1090	50.098	19.889	-9.487	1.00	0.00	LX0	C
ATOM	1011	CG	TYR	1090	51.605	19.794	-9.410	1.00	0.00	LX0	C
ATOM	1012	CD1	TYR	1090	52.366	20.971	-9.258	1.00	0.00	LX0	C
ATOM	1013	CE1	TYR	1090	53.766	20.873	-9.201	1.00	0.00	LX0	C
ATOM	1014	CD2	TYR	1090	52.206	18.521	-9.495	1.00	0.00	LX0	C
ATOM	1015	CE2	TYR	1090	53.605	18.424	-9.437	1.00	0.00	LX0	C
ATOM	1016	CZ	TYR	1090	54.367	19.602	-9.294	1.00	0.00	LX0	C
ATOM	1017	OH	TYR	1090	55.743	19.512	-9.242	1.00	0.00	LX0	O

ATOM	1018	HH	TYR	1090	56.011	18.612	-9.375	0.00	0.00	LX0	H
ATOM	1019	C	TYR	1090	50.144	19.596	-12.011	1.00	0.00	LX0	C
ATOM	1020	O	TYR	1090	51.097	19.942	-12.703	1.00	0.00	LX0	O
ATOM	1021	N	LEU	1091	49.437	18.478	-12.225	1.00	0.00	LX0	N
ATOM	1022	H	LEU	1091	48.667	18.273	-11.622	0.00	0.00	LX0	H
ATOM	1023	CA	LEU	1091	49.752	17.616	-13.363	1.00	0.00	LX0	C
ATOM	1024	CB	LEU	1091	48.896	16.354	-13.326	1.00	0.00	LX0	C
ATOM	1025	CG	LEU	1091	49.164	15.424	-12.145	1.00	0.00	LX0	C
ATOM	1026	CD1	LEU	1091	48.007	14.446	-11.953	1.00	0.00	LX0	C
ATOM	1027	CD2	LEU	1091	50.519	14.722	-12.242	1.00	0.00	LX0	C
ATOM	1028	C	LEU	1091	49.561	18.314	-14.695	1.00	0.00	LX0	C
ATOM	1029	O	LEU	1091	50.348	18.187	-15.624	1.00	0.00	LX0	O
ATOM	1030	N	ARG	1092	48.479	19.102	-14.736	1.00	0.00	LX0	N
ATOM	1031	H	ARG	1092	47.867	19.150	-13.944	0.00	0.00	LX0	H
ATOM	1032	CA	ARG	1092	48.251	19.908	-15.933	1.00	0.00	LX0	C
ATOM	1033	CB	ARG	1092	46.857	20.537	-15.865	1.00	0.00	LX0	C
ATOM	1034	CG	ARG	1092	45.828	19.411	-15.969	1.00	0.00	LX0	C
ATOM	1035	CD	ARG	1092	44.358	19.760	-15.726	1.00	0.00	LX0	C
ATOM	1036	NE	ARG	1092	43.578	18.591	-16.120	1.00	0.00	LX0	N
ATOM	1037	HE	ARG	1092	44.029	17.944	-16.741	0.00	0.00	LX0	H
ATOM	1038	CZ	ARG	1092	42.327	18.294	-15.724	1.00	0.00	LX0	C
ATOM	1039	NH1	ARG	1092	41.661	19.058	-14.861	1.00	0.00	LX0	N
ATOM	1040	HH11	ARG	1092	40.709	18.871	-14.623	0.00	0.00	LX0	H
ATOM	1041	HH12	ARG	1092	42.098	19.835	-14.386	0.00	0.00	LX0	H
ATOM	1042	NH2	ARG	1092	41.781	17.183	-16.208	1.00	0.00	LX0	N
ATOM	1043	HH21	ARG	1092	40.846	16.870	-16.005	0.00	0.00	LX0	H
ATOM	1044	HH22	ARG	1092	42.338	16.580	-16.790	0.00	0.00	LX0	H
ATOM	1045	C	ARG	1092	49.360	20.905	-16.251	1.00	0.00	LX0	C
ATOM	1046	O	ARG	1092	49.741	21.082	-17.400	1.00	0.00	LX0	O
ATOM	1047	N	SER	1093	49.924	21.495	-15.187	1.00	0.00	LX0	N
ATOM	1048	H	SER	1093	49.557	21.401	-14.259	0.00	0.00	LX0	H
ATOM	1049	CA	SER	1093	51.097	22.338	-15.425	1.00	0.00	LX0	C
ATOM	1050	CB	SER	1093	51.358	23.234	-14.211	1.00	0.00	LX0	C
ATOM	1051	OG	SER	1093	50.873	22.609	-13.016	1.00	0.00	LX0	O
ATOM	1052	HG	SER	1093	51.313	21.767	-12.944	0.00	0.00	LX0	H
ATOM	1053	C	SER	1093	52.372	21.623	-15.861	1.00	0.00	LX0	C
ATOM	1054	O	SER	1093	53.343	22.241	-16.275	1.00	0.00	LX0	O
ATOM	1055	N	LEU	1094	52.336	20.286	-15.761	1.00	0.00	LX0	N
ATOM	1056	H	LEU	1094	51.524	19.802	-15.436	0.00	0.00	LX0	H
ATOM	1057	CA	LEU	1094	53.485	19.535	-16.263	1.00	0.00	LX0	C
ATOM	1058	CB	LEU	1094	53.710	18.275	-15.422	1.00	0.00	LX0	C
ATOM	1059	CG	LEU	1094	53.851	18.532	-13.919	1.00	0.00	LX0	C
ATOM	1060	CD1	LEU	1094	53.829	17.225	-13.125	1.00	0.00	LX0	C
ATOM	1061	CD2	LEU	1094	55.074	19.387	-13.581	1.00	0.00	LX0	C
ATOM	1062	C	LEU	1094	53.387	19.163	-17.736	1.00	0.00	LX0	C
ATOM	1063	O	LEU	1094	54.238	18.474	-18.285	1.00	0.00	LX0	O
ATOM	1064	N	ARG	1095	52.294	19.622	-18.366	1.00	0.00	LX0	N
ATOM	1065	H	ARG	1095	51.651	20.248	-17.926	0.00	0.00	LX0	H
ATOM	1066	CA	ARG	1095	52.173	19.308	-19.787	1.00	0.00	LX0	C
ATOM	1067	CB	ARG	1095	50.739	19.538	-20.262	1.00	0.00	LX0	C
ATOM	1068	CG	ARG	1095	49.787	18.549	-19.605	1.00	0.00	LX0	C
ATOM	1069	CD	ARG	1095	48.366	18.652	-20.144	1.00	0.00	LX0	C
ATOM	1070	NE	ARG	1095	47.512	17.735	-19.403	1.00	0.00	LX0	N
ATOM	1071	HE	ARG	1095	47.653	17.629	-18.413	0.00	0.00	LX0	H
ATOM	1072	CZ	ARG	1095	46.536	17.021	-19.989	1.00	0.00	LX0	C
ATOM	1073	NH1	ARG	1095	46.319	17.041	-21.300	1.00	0.00	LX0	N
ATOM	1074	HH11	ARG	1095	45.590	16.446	-21.682	0.00	0.00	LX0	H
ATOM	1075	HH12	ARG	1095	46.854	17.603	-21.924	0.00	0.00	LX0	H
ATOM	1076	NH2	ARG	1095	45.770	16.283	-19.219	1.00	0.00	LX0	N
ATOM	1077	HH21	ARG	1095	44.991	15.788	-19.623	0.00	0.00	LX0	H
ATOM	1078	HH22	ARG	1095	45.950	16.207	-18.235	0.00	0.00	LX0	H

ATOM	1079	C	ARG	1095	53.144	20.087	-20.651	1.00	0.00	LX0	C
ATOM	1080	O	ARG	1095	53.477	21.232	-20.372	1.00	0.00	LX0	O
ATOM	1081	N	PRO	1096	53.591	19.430	-21.747	1.00	0.00	LX0	N
ATOM	1082	CD	PRO	1096	53.367	18.035	-22.121	1.00	0.00	LX0	C
ATOM	1083	CA	PRO	1096	54.394	20.164	-22.724	1.00	0.00	LX0	C
ATOM	1084	CB	PRO	1096	54.753	19.079	-23.749	1.00	0.00	LX0	C
ATOM	1085	CG	PRO	1096	53.682	17.999	-23.609	1.00	0.00	LX0	C
ATOM	1086	C	PRO	1096	53.658	21.347	-23.329	1.00	0.00	LX0	C
ATOM	1087	O	PRO	1096	52.808	21.199	-24.197	1.00	0.00	LX0	O
ATOM	1088	N	GLU	1097	54.072	22.537	-22.865	1.00	0.00	LX0	N
ATOM	1089	H	GLU	1097	54.598	22.578	-22.015	0.00	0.00	LX0	H
ATOM	1090	CA	GLU	1097	53.695	23.749	-23.598	1.00	0.00	LX0	C
ATOM	1091	CB	GLU	1097	54.171	24.980	-22.831	1.00	0.00	LX0	C
ATOM	1092	CG	GLU	1097	53.567	26.285	-23.354	1.00	0.00	LX0	C
ATOM	1093	CD	GLU	1097	54.232	27.454	-22.662	1.00	0.00	LX0	C
ATOM	1094	OE1	GLU	1097	55.312	27.851	-23.094	1.00	0.00	LX0	O
ATOM	1095	OE2	GLU	1097	53.669	27.965	-21.697	1.00	0.00	LX0	O
ATOM	1096	C	GLU	1097	54.231	23.736	-25.027	1.00	0.00	LX0	C
ATOM	1097	O	GLU	1097	53.613	24.201	-25.978	1.00	0.00	LX0	O
ATOM	1098	N	MET	1098	55.406	23.087	-25.123	1.00	0.00	LX0	N
ATOM	1099	H	MET	1098	55.967	23.051	-24.293	0.00	0.00	LX0	H
ATOM	1100	CA	MET	1098	55.975	22.596	-26.379	1.00	0.00	LX0	C
ATOM	1101	CB	MET	1098	54.984	21.747	-27.200	1.00	0.00	LX0	C
ATOM	1102	CG	MET	1098	55.654	20.967	-28.335	1.00	0.00	LX0	C
ATOM	1103	SD	MET	1098	56.869	19.786	-27.722	1.00	0.00	LX0	S
ATOM	1104	CE	MET	1098	57.647	19.390	-29.295	1.00	0.00	LX0	C
ATOM	1105	C	MET	1098	56.677	23.631	-27.232	1.00	0.00	LX0	C
ATOM	1106	O	MET	1098	57.829	23.446	-27.607	1.00	0.00	LX0	O
ATOM	1107	N	GLU	1099	55.958	24.727	-27.526	1.00	0.00	LX0	N
ATOM	1108	H	GLU	1099	55.026	24.834	-27.176	0.00	0.00	LX0	H
ATOM	1109	CA	GLU	1099	56.559	25.707	-28.434	1.00	0.00	LX0	C
ATOM	1110	CB	GLU	1099	55.575	26.831	-28.775	1.00	0.00	LX0	C
ATOM	1111	CG	GLU	1099	56.096	27.801	-29.851	1.00	0.00	LX0	C
ATOM	1112	CD	GLU	1099	56.529	27.041	-31.095	1.00	0.00	LX0	C
ATOM	1113	OE1	GLU	1099	55.679	26.725	-31.921	1.00	0.00	LX0	O
ATOM	1114	OE2	GLU	1099	57.719	26.761	-31.230	1.00	0.00	LX0	O
ATOM	1115	C	GLU	1099	57.911	26.245	-27.987	1.00	0.00	LX0	C
ATOM	1116	O	GLU	1099	58.063	26.889	-26.955	1.00	0.00	LX0	O
ATOM	1117	N	ASN	1100	58.907	25.857	-28.799	1.00	0.00	LX0	N
ATOM	1118	H	ASN	1100	58.587	25.570	-29.707	0.00	0.00	LX0	H
ATOM	1119	CA	ASN	1100	60.329	26.054	-28.493	1.00	0.00	LX0	C
ATOM	1120	CB	ASN	1100	60.808	27.420	-28.993	1.00	0.00	LX0	C
ATOM	1121	CG	ASN	1100	61.462	27.256	-30.349	1.00	0.00	LX0	C
ATOM	1122	OD1	ASN	1100	62.676	27.179	-30.484	1.00	0.00	LX0	O
ATOM	1123	ND2	ASN	1100	60.605	27.211	-31.372	1.00	0.00	LX0	N
ATOM	1124	HD21	ASN	1100	59.608	27.264	-31.242	0.00	0.00	LX0	H
ATOM	1125	HD22	ASN	1100	60.947	27.110	-32.301	0.00	0.00	LX0	H
ATOM	1126	C	ASN	1100	60.789	25.818	-27.057	1.00	0.00	LX0	C
ATOM	1127	O	ASN	1100	61.751	26.412	-26.582	1.00	0.00	LX0	O
ATOM	1128	N	ASN	1101	60.069	24.919	-26.367	1.00	0.00	LX0	N
ATOM	1129	H	ASN	1101	59.303	24.428	-26.787	0.00	0.00	LX0	H
ATOM	1130	CA	ASN	1101	60.402	24.780	-24.951	1.00	0.00	LX0	C
ATOM	1131	CB	ASN	1101	59.465	25.590	-24.029	1.00	0.00	LX0	C
ATOM	1132	CG	ASN	1101	58.007	25.153	-24.035	1.00	0.00	LX0	C
ATOM	1133	OD1	ASN	1101	57.616	24.083	-23.580	1.00	0.00	LX0	O
ATOM	1134	ND2	ASN	1101	57.196	26.129	-24.438	1.00	0.00	LX0	N
ATOM	1135	HD21	ASN	1101	57.511	26.837	-25.073	0.00	0.00	LX0	H
ATOM	1136	HD22	ASN	1101	56.269	26.281	-24.081	0.00	0.00	LX0	H
ATOM	1137	C	ASN	1101	60.614	23.364	-24.459	1.00	0.00	LX0	C
ATOM	1138	O	ASN	1101	59.769	22.482	-24.556	1.00	0.00	LX0	O
ATOM	1139	N	PRO	1102	61.841	23.157	-23.922	1.00	0.00	LX0	N

ATOM	1140	CD	PRO	1102	62.979	24.070	-23.930	1.00	0.00	LX0	C
ATOM	1141	CA	PRO	1102	62.147	21.881	-23.271	1.00	0.00	LX0	C
ATOM	1142	CB	PRO	1102	63.658	21.973	-23.015	1.00	0.00	LX0	C
ATOM	1143	CG	PRO	1102	64.175	23.130	-23.872	1.00	0.00	LX0	C
ATOM	1144	C	PRO	1102	61.365	21.698	-21.979	1.00	0.00	LX0	C
ATOM	1145	O	PRO	1102	61.810	22.067	-20.899	1.00	0.00	LX0	O
ATOM	1146	N	VAL	1103	60.177	21.098	-22.135	1.00	0.00	LX0	N
ATOM	1147	H	VAL	1103	59.821	20.972	-23.062	0.00	0.00	LX0	H
ATOM	1148	CA	VAL	1103	59.424	20.736	-20.935	1.00	0.00	LX0	C
ATOM	1149	CB	VAL	1103	58.035	20.203	-21.341	1.00	0.00	LX0	C
ATOM	1150	CG1	VAL	1103	58.130	18.858	-22.071	1.00	0.00	LX0	C
ATOM	1151	CG2	VAL	1103	57.048	20.178	-20.169	1.00	0.00	LX0	C
ATOM	1152	C	VAL	1103	60.190	19.757	-20.045	1.00	0.00	LX0	C
ATOM	1153	O	VAL	1103	60.992	18.956	-20.514	1.00	0.00	LX0	O
ATOM	1154	N	LEU	1104	59.927	19.873	-18.736	1.00	0.00	LX0	N
ATOM	1155	H	LEU	1104	59.211	20.494	-18.427	0.00	0.00	LX0	H
ATOM	1156	CA	LEU	1104	60.540	18.890	-17.849	1.00	0.00	LX0	C
ATOM	1157	CB	LEU	1104	60.482	19.338	-16.382	1.00	0.00	LX0	C
ATOM	1158	CG	LEU	1104	61.494	20.421	-15.969	1.00	0.00	LX0	C
ATOM	1159	CD1	LEU	1104	62.925	20.032	-16.348	1.00	0.00	LX0	C
ATOM	1160	CD2	LEU	1104	61.122	21.829	-16.443	1.00	0.00	LX0	C
ATOM	1161	C	LEU	1104	59.892	17.530	-18.009	1.00	0.00	LX0	C
ATOM	1162	O	LEU	1104	58.758	17.403	-18.453	1.00	0.00	LX0	O
ATOM	1163	N	ALA	1105	60.676	16.512	-17.635	1.00	0.00	LX0	N
ATOM	1164	H	ALA	1105	61.557	16.690	-17.206	0.00	0.00	LX0	H
ATOM	1165	CA	ALA	1105	60.120	15.166	-17.723	1.00	0.00	LX0	C
ATOM	1166	CB	ALA	1105	61.239	14.129	-17.573	1.00	0.00	LX0	C
ATOM	1167	C	ALA	1105	59.067	14.922	-16.654	1.00	0.00	LX0	C
ATOM	1168	O	ALA	1105	59.222	15.335	-15.510	1.00	0.00	LX0	O
ATOM	1169	N	PRO	1106	57.980	14.223	-17.063	1.00	0.00	LX0	N
ATOM	1170	CD	PRO	1106	57.654	13.801	-18.422	1.00	0.00	LX0	C
ATOM	1171	CA	PRO	1106	57.009	13.767	-16.062	1.00	0.00	LX0	C
ATOM	1172	CB	PRO	1106	55.937	13.096	-16.938	1.00	0.00	LX0	C
ATOM	1173	CG	PRO	1106	56.648	12.676	-18.223	1.00	0.00	LX0	C
ATOM	1174	C	PRO	1106	57.681	12.838	-15.053	1.00	0.00	LX0	C
ATOM	1175	O	PRO	1106	58.660	12.168	-15.367	1.00	0.00	LX0	O
ATOM	1176	N	PRO	1107	57.148	12.851	-13.804	1.00	0.00	LX0	N
ATOM	1177	CD	PRO	1107	56.005	13.634	-13.342	1.00	0.00	LX0	C
ATOM	1178	CA	PRO	1107	57.750	12.045	-12.735	1.00	0.00	LX0	C
ATOM	1179	CB	PRO	1107	56.738	12.191	-11.594	1.00	0.00	LX0	C
ATOM	1180	CG	PRO	1107	56.090	13.555	-11.823	1.00	0.00	LX0	C
ATOM	1181	C	PRO	1107	58.027	10.596	-13.101	1.00	0.00	LX0	C
ATOM	1182	O	PRO	1107	57.236	9.929	-13.756	1.00	0.00	LX0	O
ATOM	1183	N	SER	1108	59.199	10.141	-12.636	1.00	0.00	LX0	N
ATOM	1184	H	SER	1108	59.802	10.685	-12.055	0.00	0.00	LX0	H
ATOM	1185	CA	SER	1108	59.529	8.734	-12.835	1.00	0.00	LX0	C
ATOM	1186	CB	SER	1108	60.970	8.509	-12.374	1.00	0.00	LX0	C
ATOM	1187	OG	SER	1108	61.191	9.205	-11.139	1.00	0.00	LX0	O
ATOM	1188	HG	SER	1108	61.683	8.600	-10.587	0.00	0.00	LX0	H
ATOM	1189	C	SER	1108	58.570	7.833	-12.082	1.00	0.00	LX0	C
ATOM	1190	O	SER	1108	57.846	8.286	-11.197	1.00	0.00	LX0	O
ATOM	1191	N	LEU	1109	58.617	6.534	-12.451	1.00	0.00	LX0	N
ATOM	1192	H	LEU	1109	59.216	6.242	-13.192	0.00	0.00	LX0	H
ATOM	1193	CA	LEU	1109	57.760	5.568	-11.759	1.00	0.00	LX0	C
ATOM	1194	CB	LEU	1109	58.045	4.140	-12.238	1.00	0.00	LX0	C
ATOM	1195	CG	LEU	1109	56.966	3.125	-11.833	1.00	0.00	LX0	C
ATOM	1196	CD1	LEU	1109	55.564	3.525	-12.300	1.00	0.00	LX0	C
ATOM	1197	CD2	LEU	1109	57.332	1.710	-12.279	1.00	0.00	LX0	C
ATOM	1198	C	LEU	1109	57.794	5.717	-10.247	1.00	0.00	LX0	C
ATOM	1199	O	LEU	1109	56.762	5.916	-9.629	1.00	0.00	LX0	O
ATOM	1200	N	SER	1110	59.027	5.791	-9.733	1.00	0.00	LX0	N

ATOM	1201	H	SER	1110	59.760	5.289	-10.194	0.00	0.00	LX0	H
ATOM	1202	CA	SER	1110	59.293	6.266	-8.372	1.00	0.00	LX0	C
ATOM	1203	CB	SER	1110	60.728	6.793	-8.298	1.00	0.00	LX0	C
ATOM	1204	OG	SER	1110	61.490	6.293	-9.410	1.00	0.00	LX0	O
ATOM	1205	HG	SER	1110	61.486	5.335	-9.297	0.00	0.00	LX0	H
ATOM	1206	C	SER	1110	58.317	7.263	-7.759	1.00	0.00	LX0	C
ATOM	1207	O	SER	1110	57.592	6.959	-6.823	1.00	0.00	LX0	O
ATOM	1208	N	LYS	1111	58.293	8.487	-8.322	1.00	0.00	LX0	N
ATOM	1209	H	LYS	1111	58.728	8.652	-9.208	0.00	0.00	LX0	H
ATOM	1210	CA	LYS	1111	57.379	9.432	-7.676	1.00	0.00	LX0	C
ATOM	1211	CB	LYS	1111	57.810	10.899	-7.855	1.00	0.00	LX0	C
ATOM	1212	CG	LYS	1111	57.777	11.824	-6.608	1.00	0.00	LX0	C
ATOM	1213	CD	LYS	1111	56.412	12.155	-5.968	1.00	0.00	LX0	C
ATOM	1214	CE	LYS	1111	56.367	13.326	-4.955	1.00	0.00	LX0	C
ATOM	1215	NZ	LYS	1111	56.618	12.972	-3.546	1.00	0.00	LX0	N
ATOM	1216	HZ1	LYS	1111	56.608	13.833	-2.966	0.00	0.00	LX0	H
ATOM	1217	HZ2	LYS	1111	55.856	12.384	-3.140	0.00	0.00	LX0	H
ATOM	1218	HZ3	LYS	1111	57.546	12.507	-3.388	0.00	0.00	LX0	H
ATOM	1219	C	LYS	1111	55.915	9.227	-8.030	1.00	0.00	LX0	C
ATOM	1220	O	LYS	1111	55.032	9.710	-7.342	1.00	0.00	LX0	O
ATOM	1221	N	MET	1112	55.658	8.471	-9.108	1.00	0.00	LX0	N
ATOM	1222	H	MET	1112	56.392	8.050	-9.647	0.00	0.00	LX0	H
ATOM	1223	CA	MET	1112	54.247	8.134	-9.316	1.00	0.00	LX0	C
ATOM	1224	CB	MET	1112	53.992	7.674	-10.752	1.00	0.00	LX0	C
ATOM	1225	CG	MET	1112	54.263	8.793	-11.761	1.00	0.00	LX0	C
ATOM	1226	SD	MET	1112	53.724	8.395	-13.432	1.00	0.00	LX0	S
ATOM	1227	CE	MET	1112	54.956	7.143	-13.809	1.00	0.00	LX0	C
ATOM	1228	C	MET	1112	53.698	7.139	-8.300	1.00	0.00	LX0	C
ATOM	1229	O	MET	1112	52.583	7.251	-7.805	1.00	0.00	LX0	O
ATOM	1230	N	ILE	1113	54.572	6.179	-7.976	1.00	0.00	LX0	N
ATOM	1231	H	ILE	1113	55.474	6.195	-8.400	0.00	0.00	LX0	H
ATOM	1232	CA	ILE	1113	54.320	5.205	-6.918	1.00	0.00	LX0	C
ATOM	1233	CB	ILE	1113	55.443	4.154	-6.958	1.00	0.00	LX0	C
ATOM	1234	CG2	ILE	1113	55.529	3.288	-5.703	1.00	0.00	LX0	C
ATOM	1235	CG1	ILE	1113	55.321	3.301	-8.223	1.00	0.00	LX0	C
ATOM	1236	CD1	ILE	1113	54.018	2.501	-8.295	1.00	0.00	LX0	C
ATOM	1237	C	ILE	1113	54.216	5.894	-5.570	1.00	0.00	LX0	C
ATOM	1238	O	ILE	1113	53.292	5.684	-4.795	1.00	0.00	LX0	O
ATOM	1239	N	GLN	1114	55.187	6.793	-5.363	1.00	0.00	LX0	N
ATOM	1240	H	GLN	1114	55.971	6.802	-5.980	0.00	0.00	LX0	H
ATOM	1241	CA	GLN	1114	55.144	7.650	-4.185	1.00	0.00	LX0	C
ATOM	1242	CB	GLN	1114	56.341	8.598	-4.179	1.00	0.00	LX0	C
ATOM	1243	CG	GLN	1114	56.324	9.642	-3.063	1.00	0.00	LX0	C
ATOM	1244	CD	GLN	1114	56.556	9.048	-1.690	1.00	0.00	LX0	C
ATOM	1245	OE1	GLN	1114	56.922	7.893	-1.523	1.00	0.00	LX0	O
ATOM	1246	NE2	GLN	1114	56.351	9.908	-0.696	1.00	0.00	LX0	N
ATOM	1247	HE21	GLN	1114	56.059	10.846	-0.908	0.00	0.00	LX0	H
ATOM	1248	HE22	GLN	1114	56.499	9.655	0.254	0.00	0.00	LX0	H
ATOM	1249	C	GLN	1114	53.827	8.383	-4.034	1.00	0.00	LX0	C
ATOM	1250	O	GLN	1114	53.157	8.227	-3.030	1.00	0.00	LX0	O
ATOM	1251	N	MET	1115	53.450	9.126	-5.090	1.00	0.00	LX0	N
ATOM	1252	H	MET	1115	54.075	9.222	-5.859	0.00	0.00	LX0	H
ATOM	1253	CA	MET	1115	52.164	9.829	-5.071	1.00	0.00	LX0	C
ATOM	1254	CB	MET	1115	51.903	10.572	-6.382	1.00	0.00	LX0	C
ATOM	1255	CG	MET	1115	52.700	11.870	-6.481	1.00	0.00	LX0	C
ATOM	1256	SD	MET	1115	52.346	12.840	-7.956	1.00	0.00	LX0	S
ATOM	1257	CE	MET	1115	53.399	11.952	-9.109	1.00	0.00	LX0	C
ATOM	1258	C	MET	1115	50.988	8.935	-4.749	1.00	0.00	LX0	C
ATOM	1259	O	MET	1115	50.137	9.245	-3.927	1.00	0.00	LX0	O
ATOM	1260	N	ALA	1116	51.011	7.761	-5.402	1.00	0.00	LX0	N
ATOM	1261	H	ALA	1116	51.714	7.589	-6.093	0.00	0.00	LX0	H

ATOM	1262	CA	ALA	1116	50.021	6.743	-5.058	1.00	0.00	LX0	C
ATOM	1263	CB	ALA	1116	50.313	5.435	-5.797	1.00	0.00	LX0	C
ATOM	1264	C	ALA	1116	49.942	6.461	-3.567	1.00	0.00	LX0	C
ATOM	1265	O	ALA	1116	48.877	6.446	-2.968	1.00	0.00	LX0	O
ATOM	1266	N	GLY	1117	51.128	6.282	-2.978	1.00	0.00	LX0	N
ATOM	1267	H	GLY	1117	51.974	6.362	-3.510	0.00	0.00	LX0	H
ATOM	1268	CA	GLY	1117	51.166	6.081	-1.535	1.00	0.00	LX0	C
ATOM	1269	C	GLY	1117	50.759	7.285	-0.705	1.00	0.00	LX0	C
ATOM	1270	O	GLY	1117	50.072	7.139	0.291	1.00	0.00	LX0	O
ATOM	1271	N	GLU	1118	51.187	8.477	-1.148	1.00	0.00	LX0	N
ATOM	1272	H	GLU	1118	51.683	8.524	-2.011	0.00	0.00	LX0	H
ATOM	1273	CA	GLU	1118	50.892	9.713	-0.417	1.00	0.00	LX0	C
ATOM	1274	CB	GLU	1118	51.580	10.898	-1.123	1.00	0.00	LX0	C
ATOM	1275	CG	GLU	1118	53.114	10.801	-1.132	1.00	0.00	LX0	C
ATOM	1276	CD	GLU	1118	53.801	11.851	-2.010	1.00	0.00	LX0	C
ATOM	1277	OE1	GLU	1118	53.324	12.171	-3.091	1.00	0.00	LX0	O
ATOM	1278	OE2	GLU	1118	54.862	12.336	-1.639	1.00	0.00	LX0	O
ATOM	1279	C	GLU	1118	49.392	9.916	-0.245	1.00	0.00	LX0	C
ATOM	1280	O	GLU	1118	48.857	10.131	0.839	1.00	0.00	LX0	O
ATOM	1281	N	ILE	1119	48.722	9.729	-1.389	1.00	0.00	LX0	N
ATOM	1282	H	ILE	1119	49.256	9.590	-2.225	0.00	0.00	LX0	H
ATOM	1283	CA	ILE	1119	47.260	9.709	-1.390	1.00	0.00	LX0	C
ATOM	1284	CB	ILE	1119	46.770	9.669	-2.846	1.00	0.00	LX0	C
ATOM	1285	CG2	ILE	1119	45.244	9.619	-2.974	1.00	0.00	LX0	C
ATOM	1286	CG1	ILE	1119	47.364	10.843	-3.628	1.00	0.00	LX0	C
ATOM	1287	CD1	ILE	1119	47.288	10.650	-5.142	1.00	0.00	LX0	C
ATOM	1288	C	ILE	1119	46.685	8.559	-0.566	1.00	0.00	LX0	C
ATOM	1289	O	ILE	1119	45.828	8.726	0.295	1.00	0.00	LX0	O
ATOM	1290	N	ALA	1120	47.210	7.360	-0.863	1.00	0.00	LX0	N
ATOM	1291	H	ALA	1120	47.936	7.294	-1.549	0.00	0.00	LX0	H
ATOM	1292	CA	ALA	1120	46.675	6.166	-0.209	1.00	0.00	LX0	C
ATOM	1293	CB	ALA	1120	47.307	4.885	-0.754	1.00	0.00	LX0	C
ATOM	1294	C	ALA	1120	46.799	6.139	1.301	1.00	0.00	LX0	C
ATOM	1295	O	ALA	1120	45.983	5.536	1.981	1.00	0.00	LX0	O
ATOM	1296	N	ASP	1121	47.829	6.832	1.799	1.00	0.00	LX0	N
ATOM	1297	H	ASP	1121	48.456	7.305	1.182	0.00	0.00	LX0	H
ATOM	1298	CA	ASP	1121	48.035	6.920	3.242	1.00	0.00	LX0	C
ATOM	1299	CB	ASP	1121	49.380	7.592	3.521	1.00	0.00	LX0	C
ATOM	1300	CG	ASP	1121	49.751	7.470	4.982	1.00	0.00	LX0	C
ATOM	1301	OD1	ASP	1121	50.206	6.407	5.385	1.00	0.00	LX0	O
ATOM	1302	OD2	ASP	1121	49.605	8.444	5.718	1.00	0.00	LX0	O
ATOM	1303	C	ASP	1121	46.894	7.658	3.909	1.00	0.00	LX0	C
ATOM	1304	O	ASP	1121	46.199	7.152	4.784	1.00	0.00	LX0	O
ATOM	1305	N	GLY	1122	46.665	8.868	3.368	1.00	0.00	LX0	N
ATOM	1306	H	GLY	1122	47.298	9.221	2.676	0.00	0.00	LX0	H
ATOM	1307	CA	GLY	1122	45.503	9.634	3.816	1.00	0.00	LX0	C
ATOM	1308	C	GLY	1122	44.200	8.853	3.739	1.00	0.00	LX0	C
ATOM	1309	O	GLY	1122	43.428	8.771	4.686	1.00	0.00	LX0	O
ATOM	1310	N	MET	1123	44.012	8.233	2.562	1.00	0.00	LX0	N
ATOM	1311	H	MET	1123	44.688	8.375	1.835	0.00	0.00	LX0	H
ATOM	1312	CA	MET	1123	42.830	7.388	2.374	1.00	0.00	LX0	C
ATOM	1313	CB	MET	1123	42.791	6.811	0.960	1.00	0.00	LX0	C
ATOM	1314	CG	MET	1123	42.664	7.844	-0.160	1.00	0.00	LX0	C
ATOM	1315	SD	MET	1123	41.171	8.842	-0.043	1.00	0.00	LX0	S
ATOM	1316	CE	MET	1123	39.964	7.522	-0.216	1.00	0.00	LX0	C
ATOM	1317	C	MET	1123	42.678	6.260	3.385	1.00	0.00	LX0	C
ATOM	1318	O	MET	1123	41.600	5.969	3.891	1.00	0.00	LX0	O
ATOM	1319	N	ALA	1124	43.827	5.636	3.673	1.00	0.00	LX0	N
ATOM	1320	H	ALA	1124	44.685	5.965	3.278	0.00	0.00	LX0	H
ATOM	1321	CA	ALA	1124	43.833	4.532	4.625	1.00	0.00	LX0	C
ATOM	1322	CB	ALA	1124	45.204	3.862	4.668	1.00	0.00	LX0	C

ATOM	1323	C	ALA	1124	43.461	4.977	6.020	1.00	0.00	LX0	C
ATOM	1324	O	ALA	1124	42.691	4.332	6.716	1.00	0.00	LX0	O
ATOM	1325	N	TYR	1125	44.007	6.150	6.374	1.00	0.00	LX0	N
ATOM	1326	H	TYR	1125	44.668	6.584	5.761	0.00	0.00	LX0	H
ATOM	1327	CA	TYR	1125	43.630	6.786	7.633	1.00	0.00	LX0	C
ATOM	1328	CB	TYR	1125	44.402	8.105	7.764	1.00	0.00	LX0	C
ATOM	1329	CG	TYR	1125	44.312	8.782	9.119	1.00	0.00	LX0	C
ATOM	1330	CD1	TYR	1125	43.661	8.190	10.228	1.00	0.00	LX0	C
ATOM	1331	CE1	TYR	1125	43.611	8.885	11.448	1.00	0.00	LX0	C
ATOM	1332	CD2	TYR	1125	44.924	10.043	9.216	1.00	0.00	LX0	C
ATOM	1333	CE2	TYR	1125	44.885	10.729	10.436	1.00	0.00	LX0	C
ATOM	1334	CZ	TYR	1125	44.228	10.148	11.535	1.00	0.00	LX0	C
ATOM	1335	OH	TYR	1125	44.200	10.859	12.719	1.00	0.00	LX0	O
ATOM	1336	HH	TYR	1125	43.623	10.417	13.336	0.00	0.00	LX0	H
ATOM	1337	C	TYR	1125	42.131	6.987	7.750	1.00	0.00	LX0	C
ATOM	1338	O	TYR	1125	41.493	6.552	8.700	1.00	0.00	LX0	O
ATOM	1339	N	LEU	1126	41.591	7.625	6.695	1.00	0.00	LX0	N
ATOM	1340	H	LEU	1126	42.199	7.959	5.972	0.00	0.00	LX0	H
ATOM	1341	CA	LEU	1126	40.140	7.822	6.634	1.00	0.00	LX0	C
ATOM	1342	CB	LEU	1126	39.714	8.361	5.262	1.00	0.00	LX0	C
ATOM	1343	CG	LEU	1126	40.448	9.615	4.779	1.00	0.00	LX0	C
ATOM	1344	CD1	LEU	1126	40.136	9.918	3.315	1.00	0.00	LX0	C
ATOM	1345	CD2	LEU	1126	40.205	10.827	5.673	1.00	0.00	LX0	C
ATOM	1346	C	LEU	1126	39.367	6.545	6.924	1.00	0.00	LX0	C
ATOM	1347	O	LEU	1126	38.536	6.454	7.820	1.00	0.00	LX0	O
ATOM	1348	N	ASN	1127	39.720	5.529	6.126	1.00	0.00	LX0	N
ATOM	1349	H	ASN	1127	40.445	5.645	5.444	0.00	0.00	LX0	H
ATOM	1350	CA	ASN	1127	38.953	4.298	6.262	1.00	0.00	LX0	C
ATOM	1351	CB	ASN	1127	39.156	3.384	5.056	1.00	0.00	LX0	C
ATOM	1352	CG	ASN	1127	38.110	2.287	5.094	1.00	0.00	LX0	C
ATOM	1353	OD1	ASN	1127	38.416	1.107	4.984	1.00	0.00	LX0	O
ATOM	1354	ND2	ASN	1127	36.857	2.720	5.273	1.00	0.00	LX0	N
ATOM	1355	HD21	ASN	1127	36.640	3.702	5.286	0.00	0.00	LX0	H
ATOM	1356	HD22	ASN	1127	36.129	2.061	5.436	0.00	0.00	LX0	H
ATOM	1357	C	ASN	1127	39.104	3.556	7.574	1.00	0.00	LX0	C
ATOM	1358	O	ASN	1127	38.152	3.032	8.133	1.00	0.00	LX0	O
ATOM	1359	N	ALA	1128	40.339	3.573	8.079	1.00	0.00	LX0	N
ATOM	1360	H	ALA	1128	41.074	4.082	7.632	0.00	0.00	LX0	H
ATOM	1361	CA	ALA	1128	40.531	2.953	9.385	1.00	0.00	LX0	C
ATOM	1362	CB	ALA	1128	41.962	2.439	9.536	1.00	0.00	LX0	C
ATOM	1363	C	ALA	1128	40.195	3.866	10.552	1.00	0.00	LX0	C
ATOM	1364	O	ALA	1128	40.648	3.665	11.679	1.00	0.00	LX0	O
ATOM	1365	N	ASN	1129	39.368	4.877	10.238	1.00	0.00	LX0	N
ATOM	1366	H	ASN	1129	39.160	5.119	9.290	0.00	0.00	LX0	H
ATOM	1367	CA	ASN	1129	38.881	5.749	11.294	1.00	0.00	LX0	C
ATOM	1368	CB	ASN	1129	39.937	6.801	11.661	1.00	0.00	LX0	C
ATOM	1369	CG	ASN	1129	39.795	7.176	13.122	1.00	0.00	LX0	C
ATOM	1370	OD1	ASN	1129	38.707	7.263	13.682	1.00	0.00	LX0	O
ATOM	1371	ND2	ASN	1129	40.964	7.344	13.744	1.00	0.00	LX0	N
ATOM	1372	HD21	ASN	1129	41.849	7.217	13.283	0.00	0.00	LX0	H
ATOM	1373	HD22	ASN	1129	41.010	7.597	14.709	0.00	0.00	LX0	H
ATOM	1374	C	ASN	1129	37.560	6.424	10.957	1.00	0.00	LX0	C
ATOM	1375	O	ASN	1129	37.423	7.622	11.149	1.00	0.00	LX0	O
ATOM	1376	N	LYS	1130	36.587	5.610	10.478	1.00	0.00	LX0	N
ATOM	1377	H	LYS	1130	36.819	4.657	10.276	0.00	0.00	LX0	H
ATOM	1378	CA	LYS	1130	35.176	6.013	10.259	1.00	0.00	LX0	C
ATOM	1379	CB	LYS	1130	34.601	6.976	11.321	1.00	0.00	LX0	C
ATOM	1380	CG	LYS	1130	34.690	6.546	12.787	1.00	0.00	LX0	C
ATOM	1381	CD	LYS	1130	34.506	7.738	13.736	1.00	0.00	LX0	C
ATOM	1382	CE	LYS	1130	35.810	8.335	14.294	1.00	0.00	LX0	C
ATOM	1383	NZ	LYS	1130	36.695	8.890	13.261	1.00	0.00	LX0	N

ATOM	1384	HZ1	LYS	1130	37.698	8.715	13.496	0.00	0.00	LX0	H
ATOM	1385	HZ2	LYS	1130	36.572	8.500	12.309	0.00	0.00	LX0	H
ATOM	1386	HZ3	LYS	1130	36.675	9.932	13.211	0.00	0.00	LX0	H
ATOM	1387	C	LYS	1130	34.775	6.580	8.899	1.00	0.00	LX0	C
ATOM	1388	O	LYS	1130	33.652	7.040	8.709	1.00	0.00	LX0	O
ATOM	1389	N	PHE	1131	35.722	6.600	7.951	1.00	0.00	LX0	N
ATOM	1390	H	PHE	1131	36.629	6.197	8.081	0.00	0.00	LX0	H
ATOM	1391	CA	PHE	1131	35.380	7.360	6.744	1.00	0.00	LX0	C
ATOM	1392	CB	PHE	1131	36.166	8.680	6.650	1.00	0.00	LX0	C
ATOM	1393	CG	PHE	1131	35.982	9.545	7.874	1.00	0.00	LX0	C
ATOM	1394	CD1	PHE	1131	36.867	9.402	8.963	1.00	0.00	LX0	C
ATOM	1395	CD2	PHE	1131	34.930	10.481	7.912	1.00	0.00	LX0	C
ATOM	1396	CE1	PHE	1131	36.686	10.193	10.115	1.00	0.00	LX0	C
ATOM	1397	CE2	PHE	1131	34.759	11.275	9.061	1.00	0.00	LX0	C
ATOM	1398	CZ	PHE	1131	35.631	11.123	10.156	1.00	0.00	LX0	C
ATOM	1399	C	PHE	1131	35.588	6.599	5.451	1.00	0.00	LX0	C
ATOM	1400	O	PHE	1131	36.436	5.720	5.342	1.00	0.00	LX0	O
ATOM	1401	N	VAL	1132	34.791	7.015	4.450	1.00	0.00	LX0	N
ATOM	1402	H	VAL	1132	34.066	7.682	4.629	0.00	0.00	LX0	H
ATOM	1403	CA	VAL	1132	35.149	6.769	3.048	1.00	0.00	LX0	C
ATOM	1404	CB	VAL	1132	34.393	5.582	2.405	1.00	0.00	LX0	C
ATOM	1405	CG1	VAL	1132	34.446	4.357	3.314	1.00	0.00	LX0	C
ATOM	1406	CG2	VAL	1132	32.957	5.889	1.977	1.00	0.00	LX0	C
ATOM	1407	C	VAL	1132	34.918	8.075	2.301	1.00	0.00	LX0	C
ATOM	1408	O	VAL	1132	34.342	8.992	2.879	1.00	0.00	LX0	O
ATOM	1409	N	HIS	1133	35.413	8.174	1.061	1.00	0.00	LX0	N
ATOM	1410	H	HIS	1133	35.612	7.373	0.489	0.00	0.00	LX0	H
ATOM	1411	CA	HIS	1133	35.487	9.535	0.535	1.00	0.00	LX0	C
ATOM	1412	CB	HIS	1133	36.778	9.738	-0.266	1.00	0.00	LX0	C
ATOM	1413	CG	HIS	1133	37.278	11.161	-0.148	1.00	0.00	LX0	C
ATOM	1414	ND1	HIS	1133	36.503	12.256	-0.260	1.00	0.00	LX0	N
ATOM	1415	HD1	HIS	1133	35.543	12.264	-0.459	0.00	0.00	LX0	H
ATOM	1416	CD2	HIS	1133	38.593	11.571	0.081	1.00	0.00	LX0	C
ATOM	1417	NE2	HIS	1133	38.594	12.926	0.103	1.00	0.00	LX0	N
ATOM	1418	CE1	HIS	1133	37.309	13.350	-0.106	1.00	0.00	LX0	C
ATOM	1419	C	HIS	1133	34.261	10.035	-0.209	1.00	0.00	LX0	C
ATOM	1420	O	HIS	1133	33.907	11.214	-0.143	1.00	0.00	LX0	O
ATOM	1421	N	ARG	1134	33.620	9.082	-0.916	1.00	0.00	LX0	N
ATOM	1422	H	ARG	1134	34.090	8.197	-1.025	0.00	0.00	LX0	H
ATOM	1423	CA	ARG	1134	32.408	9.360	-1.702	1.00	0.00	LX0	C
ATOM	1424	CB	ARG	1134	31.267	9.846	-0.787	1.00	0.00	LX0	C
ATOM	1425	CG	ARG	1134	29.840	9.754	-1.339	1.00	0.00	LX0	C
ATOM	1426	CD	ARG	1134	29.011	11.021	-1.100	1.00	0.00	LX0	C
ATOM	1427	NE	ARG	1134	29.002	11.403	0.309	1.00	0.00	LX0	N
ATOM	1428	HE	ARG	1134	29.806	11.903	0.647	0.00	0.00	LX0	H
ATOM	1429	CZ	ARG	1134	28.012	10.985	1.129	1.00	0.00	LX0	C
ATOM	1430	NH1	ARG	1134	26.941	10.360	0.650	1.00	0.00	LX0	N
ATOM	1431	HH11	ARG	1134	26.276	9.994	1.324	0.00	0.00	LX0	H
ATOM	1432	HH12	ARG	1134	26.775	10.212	-0.319	0.00	0.00	LX0	H
ATOM	1433	NH2	ARG	1134	28.104	11.178	2.436	1.00	0.00	LX0	N
ATOM	1434	HH21	ARG	1134	27.360	10.803	3.019	0.00	0.00	LX0	H
ATOM	1435	HH22	ARG	1134	28.867	11.649	2.867	0.00	0.00	LX0	H
ATOM	1436	C	ARG	1134	32.619	10.314	-2.884	1.00	0.00	LX0	C
ATOM	1437	O	ARG	1134	31.677	10.793	-3.512	1.00	0.00	LX0	O
ATOM	1438	N	ASP	1135	33.907	10.596	-3.134	1.00	0.00	LX0	N
ATOM	1439	H	ASP	1135	34.626	10.023	-2.732	0.00	0.00	LX0	H
ATOM	1440	CA	ASP	1135	34.277	11.733	-3.979	1.00	0.00	LX0	C
ATOM	1441	CB	ASP	1135	33.783	13.071	-3.396	1.00	0.00	LX0	C
ATOM	1442	CG	ASP	1135	34.030	14.214	-4.366	1.00	0.00	LX0	C
ATOM	1443	OD1	ASP	1135	33.337	14.329	-5.365	1.00	0.00	LX0	O
ATOM	1444	OD2	ASP	1135	34.947	14.991	-4.154	1.00	0.00	LX0	O

ATOM	1445	C	ASP	1135	35.778	11.773	-4.106	1.00	0.00	LX0	C
ATOM	1446	O	ASP	1135	36.493	11.904	-3.121	1.00	0.00	LX0	O
ATOM	1447	N	LEU	1136	36.228	11.618	-5.357	1.00	0.00	LX0	N
ATOM	1448	H	LEU	1136	35.601	11.484	-6.123	0.00	0.00	LX0	H
ATOM	1449	CA	LEU	1136	37.671	11.544	-5.538	1.00	0.00	LX0	C
ATOM	1450	CB	LEU	1136	38.152	10.117	-5.219	1.00	0.00	LX0	C
ATOM	1451	CG	LEU	1136	39.545	10.001	-4.589	1.00	0.00	LX0	C
ATOM	1452	CD1	LEU	1136	39.734	10.922	-3.389	1.00	0.00	LX0	C
ATOM	1453	CD2	LEU	1136	39.879	8.558	-4.222	1.00	0.00	LX0	C
ATOM	1454	C	LEU	1136	38.067	12.002	-6.927	1.00	0.00	LX0	C
ATOM	1455	O	LEU	1136	37.210	12.209	-7.787	1.00	0.00	LX0	O
ATOM	1456	N	ALA	1137	39.396	12.149	-7.064	1.00	0.00	LX0	N
ATOM	1457	H	ALA	1137	39.938	12.071	-6.226	0.00	0.00	LX0	H
ATOM	1458	CA	ALA	1137	40.186	12.385	-8.276	1.00	0.00	LX0	C
ATOM	1459	CB	ALA	1137	39.574	13.397	-9.234	1.00	0.00	LX0	C
ATOM	1460	C	ALA	1137	41.477	12.992	-7.800	1.00	0.00	LX0	C
ATOM	1461	O	ALA	1137	41.627	13.240	-6.608	1.00	0.00	LX0	O
ATOM	1462	N	ALA	1138	42.377	13.276	-8.750	1.00	0.00	LX0	N
ATOM	1463	H	ALA	1138	42.208	13.048	-9.714	0.00	0.00	LX0	H
ATOM	1464	CA	ALA	1138	43.584	13.985	-8.329	1.00	0.00	LX0	C
ATOM	1465	CB	ALA	1138	44.596	14.028	-9.473	1.00	0.00	LX0	C
ATOM	1466	C	ALA	1138	43.336	15.390	-7.777	1.00	0.00	LX0	C
ATOM	1467	O	ALA	1138	44.138	15.949	-7.040	1.00	0.00	LX0	O
ATOM	1468	N	ARG	1139	42.148	15.926	-8.125	1.00	0.00	LX0	N
ATOM	1469	H	ARG	1139	41.572	15.468	-8.800	0.00	0.00	LX0	H
ATOM	1470	CA	ARG	1139	41.732	17.191	-7.507	1.00	0.00	LX0	C
ATOM	1471	CB	ARG	1139	40.436	17.719	-8.151	1.00	0.00	LX0	C
ATOM	1472	CG	ARG	1139	39.229	16.850	-7.798	1.00	0.00	LX0	C
ATOM	1473	CD	ARG	1139	37.846	17.355	-8.207	1.00	0.00	LX0	C
ATOM	1474	NE	ARG	1139	36.816	16.648	-7.444	1.00	0.00	LX0	N
ATOM	1475	HE	ARG	1139	36.459	17.058	-6.598	0.00	0.00	LX0	H
ATOM	1476	CZ	ARG	1139	36.453	15.379	-7.714	1.00	0.00	LX0	C
ATOM	1477	NH1	ARG	1139	36.802	14.780	-8.845	1.00	0.00	LX0	N
ATOM	1478	HH11	ARG	1139	36.635	13.801	-8.980	0.00	0.00	LX0	H
ATOM	1479	HH12	ARG	1139	37.260	15.286	-9.588	0.00	0.00	LX0	H
ATOM	1480	NH2	ARG	1139	35.737	14.711	-6.825	1.00	0.00	LX0	N
ATOM	1481	HH21	ARG	1139	35.447	13.769	-6.963	0.00	0.00	LX0	H
ATOM	1482	HH22	ARG	1139	35.437	15.142	-5.960	0.00	0.00	LX0	H
ATOM	1483	C	ARG	1139	41.604	17.133	-5.982	1.00	0.00	LX0	C
ATOM	1484	O	ARG	1139	41.813	18.096	-5.260	1.00	0.00	LX0	O
ATOM	1485	N	ASN	1140	41.275	15.920	-5.506	1.00	0.00	LX0	N
ATOM	1486	H	ASN	1140	41.201	15.126	-6.106	0.00	0.00	LX0	H
ATOM	1487	CA	ASN	1140	41.080	15.781	-4.063	1.00	0.00	LX0	C
ATOM	1488	CB	ASN	1140	40.082	14.666	-3.724	1.00	0.00	LX0	C
ATOM	1489	CG	ASN	1140	38.661	15.043	-4.092	1.00	0.00	LX0	C
ATOM	1490	OD1	ASN	1140	38.395	15.825	-4.993	1.00	0.00	LX0	O
ATOM	1491	ND2	ASN	1140	37.735	14.432	-3.357	1.00	0.00	LX0	N
ATOM	1492	HD21	ASN	1140	37.921	13.713	-2.686	0.00	0.00	LX0	H
ATOM	1493	HD22	ASN	1140	36.775	14.688	-3.495	0.00	0.00	LX0	H
ATOM	1494	C	ASN	1140	42.371	15.527	-3.314	1.00	0.00	LX0	C
ATOM	1495	O	ASN	1140	42.376	15.213	-2.131	1.00	0.00	LX0	O
ATOM	1496	N	CYS	1141	43.479	15.654	-4.051	1.00	0.00	LX0	N
ATOM	1497	H	CYS	1141	43.476	15.953	-5.006	0.00	0.00	LX0	H
ATOM	1498	CA	CYS	1141	44.751	15.392	-3.401	1.00	0.00	LX0	C
ATOM	1499	CB	CYS	1141	45.426	14.210	-4.086	1.00	0.00	LX0	C
ATOM	1500	SG	CYS	1141	44.304	12.802	-4.297	1.00	0.00	LX0	S
ATOM	1501	C	CYS	1141	45.609	16.633	-3.455	1.00	0.00	LX0	C
ATOM	1502	O	CYS	1141	46.088	17.030	-4.507	1.00	0.00	LX0	O
ATOM	1503	N	MET	1142	45.729	17.279	-2.294	1.00	0.00	LX0	N
ATOM	1504	H	MET	1142	45.577	16.768	-1.447	0.00	0.00	LX0	H
ATOM	1505	CA	MET	1142	46.415	18.566	-2.344	1.00	0.00	LX0	C

ATOM	1506	CB	MET	1142	45.809	19.540	-1.336	1.00	0.00	LX0	C
ATOM	1507	CG	MET	1142	44.355	19.918	-1.622	1.00	0.00	LX0	C
ATOM	1508	SD	MET	1142	44.143	20.862	-3.135	1.00	0.00	LX0	S
ATOM	1509	CE	MET	1142	42.343	20.896	-3.154	1.00	0.00	LX0	C
ATOM	1510	C	MET	1142	47.901	18.414	-2.112	1.00	0.00	LX0	C
ATOM	1511	O	MET	1142	48.346	17.604	-1.308	1.00	0.00	LX0	O
ATOM	1512	N	VAL	1143	48.657	19.220	-2.858	1.00	0.00	LX0	N
ATOM	1513	H	VAL	1143	48.239	19.912	-3.449	0.00	0.00	LX0	H
ATOM	1514	CA	VAL	1143	50.103	19.131	-2.693	1.00	0.00	LX0	C
ATOM	1515	CB	VAL	1143	50.796	19.090	-4.062	1.00	0.00	LX0	C
ATOM	1516	CG1	VAL	1143	52.242	18.609	-3.939	1.00	0.00	LX0	C
ATOM	1517	CG2	VAL	1143	50.013	18.272	-5.090	1.00	0.00	LX0	C
ATOM	1518	C	VAL	1143	50.603	20.302	-1.868	1.00	0.00	LX0	C
ATOM	1519	O	VAL	1143	50.254	21.453	-2.121	1.00	0.00	LX0	O
ATOM	1520	N	ALA	1144	51.407	19.977	-0.851	1.00	0.00	LX0	N
ATOM	1521	H	ALA	1144	51.743	19.040	-0.727	0.00	0.00	LX0	H
ATOM	1522	CA	ALA	1144	51.919	21.101	-0.073	1.00	0.00	LX0	C
ATOM	1523	CB	ALA	1144	52.090	20.713	1.400	1.00	0.00	LX0	C
ATOM	1524	C	ALA	1144	53.210	21.656	-0.651	1.00	0.00	LX0	C
ATOM	1525	O	ALA	1144	53.705	21.199	-1.676	1.00	0.00	LX0	O
ATOM	1526	N	GLU	1145	53.740	22.680	0.046	1.00	0.00	LX0	N
ATOM	1527	H	GLU	1145	53.230	23.037	0.831	0.00	0.00	LX0	H
ATOM	1528	CA	GLU	1145	55.003	23.304	-0.375	1.00	0.00	LX0	C
ATOM	1529	CB	GLU	1145	55.439	24.313	0.698	1.00	0.00	LX0	C
ATOM	1530	CG	GLU	1145	56.475	25.371	0.282	1.00	0.00	LX0	C
ATOM	1531	CD	GLU	1145	55.829	26.537	-0.455	1.00	0.00	LX0	C
ATOM	1532	OE1	GLU	1145	55.015	27.236	0.133	1.00	0.00	LX0	O
ATOM	1533	OE2	GLU	1145	56.161	26.784	-1.606	1.00	0.00	LX0	O
ATOM	1534	C	GLU	1145	56.139	22.319	-0.680	1.00	0.00	LX0	C
ATOM	1535	O	GLU	1145	56.932	22.473	-1.605	1.00	0.00	LX0	O
ATOM	1536	N	ASP	1146	56.152	21.292	0.171	1.00	0.00	LX0	N
ATOM	1537	H	ASP	1146	55.377	21.147	0.789	0.00	0.00	LX0	H
ATOM	1538	CA	ASP	1146	57.130	20.206	0.153	1.00	0.00	LX0	C
ATOM	1539	CB	ASP	1146	56.923	19.347	1.411	1.00	0.00	LX0	C
ATOM	1540	CG	ASP	1146	55.490	18.825	1.552	1.00	0.00	LX0	C
ATOM	1541	OD1	ASP	1146	54.653	19.063	0.684	1.00	0.00	LX0	O
ATOM	1542	OD2	ASP	1146	55.207	18.167	2.543	1.00	0.00	LX0	O
ATOM	1543	C	ASP	1146	57.183	19.317	-1.087	1.00	0.00	LX0	C
ATOM	1544	O	ASP	1146	58.221	18.737	-1.382	1.00	0.00	LX0	O
ATOM	1545	N	PHE	1147	56.031	19.250	-1.787	1.00	0.00	LX0	N
ATOM	1546	H	PHE	1147	55.259	19.727	-1.360	0.00	0.00	LX0	H
ATOM	1547	CA	PHE	1147	55.779	18.289	-2.874	1.00	0.00	LX0	C
ATOM	1548	CB	PHE	1147	56.865	18.230	-3.967	1.00	0.00	LX0	C
ATOM	1549	CG	PHE	1147	57.039	19.552	-4.680	1.00	0.00	LX0	C
ATOM	1550	CD1	PHE	1147	56.231	19.845	-5.801	1.00	0.00	LX0	C
ATOM	1551	CD2	PHE	1147	58.024	20.460	-4.235	1.00	0.00	LX0	C
ATOM	1552	CE1	PHE	1147	56.437	21.046	-6.507	1.00	0.00	LX0	C
ATOM	1553	CE2	PHE	1147	58.230	21.663	-4.938	1.00	0.00	LX0	C
ATOM	1554	CZ	PHE	1147	57.446	21.935	-6.078	1.00	0.00	LX0	C
ATOM	1555	C	PHE	1147	55.418	16.880	-2.411	1.00	0.00	LX0	C
ATOM	1556	O	PHE	1147	55.691	15.877	-3.069	1.00	0.00	LX0	O
ATOM	1557	N	THR	1148	54.734	16.868	-1.265	1.00	0.00	LX0	N
ATOM	1558	H	THR	1148	54.629	17.688	-0.702	0.00	0.00	LX0	H
ATOM	1559	CA	THR	1148	54.036	15.658	-0.853	1.00	0.00	LX0	C
ATOM	1560	CB	THR	1148	54.203	15.481	0.658	1.00	0.00	LX0	C
ATOM	1561	OG1	THR	1148	55.561	15.729	1.037	1.00	0.00	LX0	O
ATOM	1562	HG1	THR	1148	55.539	16.570	1.498	0.00	0.00	LX0	H
ATOM	1563	CG2	THR	1148	53.744	14.108	1.150	1.00	0.00	LX0	C
ATOM	1564	C	THR	1148	52.568	15.773	-1.231	1.00	0.00	LX0	C
ATOM	1565	O	THR	1148	51.934	16.807	-1.028	1.00	0.00	LX0	O
ATOM	1566	N	VAL	1149	52.049	14.693	-1.828	1.00	0.00	LX0	N

ATOM	1567	H	VAL	1149	52.590	13.857	-1.970	0.00	0.00	LX0	H
ATOM	1568	CA	VAL	1149	50.660	14.795	-2.270	1.00	0.00	LX0	C
ATOM	1569	CB	VAL	1149	50.500	14.216	-3.676	1.00	0.00	LX0	C
ATOM	1570	CG1	VAL	1149	49.095	14.476	-4.213	1.00	0.00	LX0	C
ATOM	1571	CG2	VAL	1149	51.559	14.792	-4.618	1.00	0.00	LX0	C
ATOM	1572	C	VAL	1149	49.661	14.202	-1.291	1.00	0.00	LX0	C
ATOM	1573	O	VAL	1149	49.323	13.025	-1.300	1.00	0.00	LX0	O
ATOM	1574	N	LYS	1150	49.206	15.096	-0.416	1.00	0.00	LX0	N
ATOM	1575	H	LYS	1150	49.375	16.069	-0.581	0.00	0.00	LX0	H
ATOM	1576	CA	LYS	1150	48.311	14.627	0.634	1.00	0.00	LX0	C
ATOM	1577	CB	LYS	1150	48.337	15.602	1.800	1.00	0.00	LX0	C
ATOM	1578	CG	LYS	1150	49.715	15.883	2.389	1.00	0.00	LX0	C
ATOM	1579	CD	LYS	1150	49.476	16.660	3.673	1.00	0.00	LX0	C
ATOM	1580	CE	LYS	1150	50.697	17.000	4.516	1.00	0.00	LX0	C
ATOM	1581	NZ	LYS	1150	50.171	17.252	5.860	1.00	0.00	LX0	N
ATOM	1582	HZ1	LYS	1150	50.799	17.861	6.425	0.00	0.00	LX0	H
ATOM	1583	HZ2	LYS	1150	49.304	17.828	5.794	0.00	0.00	LX0	H
ATOM	1584	HZ3	LYS	1150	49.934	16.368	6.354	0.00	0.00	LX0	H
ATOM	1585	C	LYS	1150	46.870	14.451	0.193	1.00	0.00	LX0	C
ATOM	1586	O	LYS	1150	46.424	14.996	-0.811	1.00	0.00	LX0	O
ATOM	1587	N	ILE	1151	46.129	13.714	1.036	1.00	0.00	LX0	N
ATOM	1588	H	ILE	1151	46.568	13.278	1.824	0.00	0.00	LX0	H
ATOM	1589	CA	ILE	1151	44.676	13.823	0.920	1.00	0.00	LX0	C
ATOM	1590	CB	ILE	1151	43.984	12.667	1.660	1.00	0.00	LX0	C
ATOM	1591	CG2	ILE	1151	42.545	12.939	2.122	1.00	0.00	LX0	C
ATOM	1592	CG1	ILE	1151	44.022	11.459	0.726	1.00	0.00	LX0	C
ATOM	1593	CD1	ILE	1151	43.245	11.709	-0.572	1.00	0.00	LX0	C
ATOM	1594	C	ILE	1151	44.193	15.194	1.348	1.00	0.00	LX0	C
ATOM	1595	O	ILE	1151	44.573	15.733	2.384	1.00	0.00	LX0	O
ATOM	1596	N	GLY	1152	43.404	15.743	0.421	1.00	0.00	LX0	N
ATOM	1597	H	GLY	1152	43.010	15.178	-0.306	0.00	0.00	LX0	H
ATOM	1598	CA	GLY	1152	43.100	17.159	0.482	1.00	0.00	LX0	C
ATOM	1599	C	GLY	1152	41.769	17.512	1.094	1.00	0.00	LX0	C
ATOM	1600	O	GLY	1152	41.173	16.777	1.867	1.00	0.00	LX0	O
ATOM	1601	N	ASP	1153	41.381	18.735	0.716	1.00	0.00	LX0	N
ATOM	1602	H	ASP	1153	41.861	19.178	-0.036	0.00	0.00	LX0	H
ATOM	1603	CA	ASP	1153	40.351	19.477	1.434	1.00	0.00	LX0	C
ATOM	1604	CB	ASP	1153	40.221	20.849	0.779	1.00	0.00	LX0	C
ATOM	1605	CG	ASP	1153	39.994	21.953	1.792	1.00	0.00	LX0	C
ATOM	1606	OD1	ASP	1153	39.609	21.696	2.933	1.00	0.00	LX0	O
ATOM	1607	OD2	ASP	1153	40.191	23.109	1.439	1.00	0.00	LX0	O
ATOM	1608	C	ASP	1153	38.996	18.806	1.558	1.00	0.00	LX0	C
ATOM	1609	O	ASP	1153	38.508	18.155	0.642	1.00	0.00	LX0	O
ATOM	1610	N	PHE	1154	38.411	18.996	2.747	1.00	0.00	LX0	N
ATOM	1611	H	PHE	1154	38.795	19.699	3.348	0.00	0.00	LX0	H
ATOM	1612	CA	PHE	1154	37.091	18.408	2.942	1.00	0.00	LX0	C
ATOM	1613	CB	PHE	1154	37.010	17.599	4.245	1.00	0.00	LX0	C
ATOM	1614	CG	PHE	1154	37.886	16.362	4.195	1.00	0.00	LX0	C
ATOM	1615	CD1	PHE	1154	38.040	15.631	2.993	1.00	0.00	LX0	C
ATOM	1616	CD2	PHE	1154	38.539	15.953	5.376	1.00	0.00	LX0	C
ATOM	1617	CE1	PHE	1154	38.875	14.498	2.969	1.00	0.00	LX0	C
ATOM	1618	CE2	PHE	1154	39.372	14.818	5.354	1.00	0.00	LX0	C
ATOM	1619	CZ	PHE	1154	39.541	14.108	4.148	1.00	0.00	LX0	C
ATOM	1620	C	PHE	1154	35.989	19.436	2.858	1.00	0.00	LX0	C
ATOM	1621	O	PHE	1154	36.206	20.642	2.933	1.00	0.00	LX0	O
ATOM	1622	N	GLY	1155	34.781	18.896	2.660	1.00	0.00	LX0	N
ATOM	1623	H	GLY	1155	34.644	17.906	2.667	0.00	0.00	LX0	H
ATOM	1624	CA	GLY	1155	33.664	19.774	2.346	1.00	0.00	LX0	C
ATOM	1625	C	GLY	1155	33.803	20.377	0.967	1.00	0.00	LX0	C
ATOM	1626	O	GLY	1155	33.593	19.720	-0.048	1.00	0.00	LX0	O
ATOM	1627	N	MET	1156	34.216	21.658	0.994	1.00	0.00	LX0	N

ATOM	1628	H	MET	1156	34.349	22.029	1.911	0.00	0.00	LX0	H
ATOM	1629	CA	MET	1156	34.603	22.418	-0.203	1.00	0.00	LX0	C
ATOM	1630	CB	MET	1156	36.131	22.424	-0.337	1.00	0.00	LX0	C
ATOM	1631	CG	MET	1156	36.835	23.159	0.799	1.00	0.00	LX0	C
ATOM	1632	SD	MET	1156	36.832	24.949	0.622	1.00	0.00	LX0	S
ATOM	1633	CE	MET	1156	37.937	25.050	-0.795	1.00	0.00	LX0	C
ATOM	1634	C	MET	1156	33.897	22.038	-1.502	1.00	0.00	LX0	C
ATOM	1635	O	MET	1156	32.775	22.464	-1.730	1.00	0.00	LX0	O
ATOM	1636	N	THR	1157	34.590	21.202	-2.309	1.00	0.00	LX0	N
ATOM	1637	H	THR	1157	35.428	20.788	-1.965	0.00	0.00	LX0	H
ATOM	1638	CA	THR	1157	34.136	20.733	-3.623	1.00	0.00	LX0	C
ATOM	1639	CB	THR	1157	33.185	19.531	-3.489	1.00	0.00	LX0	C
ATOM	1640	OG1	THR	1157	32.194	19.753	-2.480	1.00	0.00	LX0	O
ATOM	1641	HG1	THR	1157	32.673	19.904	-1.668	0.00	0.00	LX0	H
ATOM	1642	CG2	THR	1157	33.970	18.265	-3.139	1.00	0.00	LX0	C
ATOM	1643	C	THR	1157	33.673	21.763	-4.650	1.00	0.00	LX0	C
ATOM	1644	O	THR	1157	34.360	21.994	-5.637	1.00	0.00	LX0	O
ATOM	1645	N	ARG	1158	32.507	22.368	-4.377	1.00	0.00	LX0	N
ATOM	1646	H	ARG	1158	32.072	22.145	-3.504	0.00	0.00	LX0	H
ATOM	1647	CA	ARG	1158	32.022	23.445	-5.242	1.00	0.00	LX0	C
ATOM	1648	CB	ARG	1158	30.634	23.935	-4.801	1.00	0.00	LX0	C
ATOM	1649	CG	ARG	1158	30.499	24.243	-3.303	1.00	0.00	LX0	C
ATOM	1650	CD	ARG	1158	29.330	25.177	-2.979	1.00	0.00	LX0	C
ATOM	1651	NE	ARG	1158	29.658	26.540	-3.390	1.00	0.00	LX0	N
ATOM	1652	HE	ARG	1158	30.026	26.725	-4.313	0.00	0.00	LX0	H
ATOM	1653	CZ	ARG	1158	29.800	27.548	-2.507	1.00	0.00	LX0	C
ATOM	1654	NH1	ARG	1158	29.500	27.389	-1.222	1.00	0.00	LX0	N
ATOM	1655	HH11	ARG	1158	29.698	28.124	-0.571	0.00	0.00	LX0	H
ATOM	1656	HH12	ARG	1158	29.100	26.534	-0.898	0.00	0.00	LX0	H
ATOM	1657	NH2	ARG	1158	30.282	28.704	-2.934	1.00	0.00	LX0	N
ATOM	1658	HH21	ARG	1158	30.420	29.500	-2.349	0.00	0.00	LX0	H
ATOM	1659	HH22	ARG	1158	30.602	28.735	-3.896	0.00	0.00	LX0	H
ATOM	1660	C	ARG	1158	32.965	24.632	-5.383	1.00	0.00	LX0	C
ATOM	1661	O	ARG	1158	33.787	24.911	-4.512	1.00	0.00	LX0	O
ATOM	1662	N	ASP	1159	32.766	25.294	-6.539	1.00	0.00	LX0	N
ATOM	1663	H	ASP	1159	32.240	24.801	-7.233	0.00	0.00	LX0	H
ATOM	1664	CA	ASP	1159	33.498	26.489	-6.984	1.00	0.00	LX0	C
ATOM	1665	CB	ASP	1159	33.287	27.775	-6.151	1.00	0.00	LX0	C
ATOM	1666	CG	ASP	1159	32.088	27.738	-5.224	1.00	0.00	LX0	C
ATOM	1667	OD1	ASP	1159	30.983	28.136	-5.591	1.00	0.00	LX0	O
ATOM	1668	OD2	ASP	1159	32.266	27.346	-4.083	1.00	0.00	LX0	O
ATOM	1669	C	ASP	1159	34.984	26.262	-7.187	1.00	0.00	LX0	C
ATOM	1670	O	ASP	1159	35.801	27.178	-7.153	1.00	0.00	LX0	O
ATOM	1671	N	ILE	1160	35.305	24.974	-7.369	1.00	0.00	LX0	N
ATOM	1672	H	ILE	1160	34.559	24.322	-7.530	0.00	0.00	LX0	H
ATOM	1673	CA	ILE	1160	36.709	24.601	-7.488	1.00	0.00	LX0	C
ATOM	1674	CB	ILE	1160	37.186	23.776	-6.268	1.00	0.00	LX0	C
ATOM	1675	CG2	ILE	1160	38.658	23.361	-6.391	1.00	0.00	LX0	C
ATOM	1676	CG1	ILE	1160	36.934	24.485	-4.931	1.00	0.00	LX0	C
ATOM	1677	CD1	ILE	1160	37.750	25.767	-4.739	1.00	0.00	LX0	C
ATOM	1678	C	ILE	1160	36.955	23.835	-8.775	1.00	0.00	LX0	C
ATOM	1679	O	ILE	1160	37.850	24.140	-9.556	1.00	0.00	LX0	O
ATOM	1680	N	TYR	1161	36.143	22.783	-8.945	1.00	0.00	LX0	N
ATOM	1681	H	TYR	1161	35.311	22.677	-8.390	0.00	0.00	LX0	H
ATOM	1682	CA	TYR	1161	36.531	21.822	-9.971	1.00	0.00	LX0	C
ATOM	1683	CB	TYR	1161	37.519	20.797	-9.406	1.00	0.00	LX0	C
ATOM	1684	CG	TYR	1161	38.743	20.722	-10.288	1.00	0.00	LX0	C
ATOM	1685	CD1	TYR	1161	38.717	19.919	-11.447	1.00	0.00	LX0	C
ATOM	1686	CE1	TYR	1161	39.855	19.877	-12.271	1.00	0.00	LX0	C
ATOM	1687	CD2	TYR	1161	39.882	21.468	-9.924	1.00	0.00	LX0	C
ATOM	1688	CE2	TYR	1161	41.017	21.425	-10.749	1.00	0.00	LX0	C

ATOM	1689	CZ	TYR	1161	40.990	20.635	-11.916	1.00	0.00	LX0	C
ATOM	1690	OH	TYR	1161	42.103	20.604	-12.743	1.00	0.00	LX0	O
ATOM	1691	HH	TYR	1161	42.796	21.081	-12.272	0.00	0.00	LX0	H
ATOM	1692	C	TYR	1161	35.361	21.136	-10.633	1.00	0.00	LX0	C
ATOM	1693	O	TYR	1161	35.302	19.923	-10.793	1.00	0.00	LX0	O
ATOM	1694	N	GLU	1162	34.418	21.984	-11.040	1.00	0.00	LX0	N
ATOM	1695	H	GLU	1162	34.492	22.947	-10.765	0.00	0.00	LX0	H
ATOM	1696	CA	GLU	1162	33.138	21.460	-11.516	1.00	0.00	LX0	C
ATOM	1697	CB	GLU	1162	32.118	22.602	-11.608	1.00	0.00	LX0	C
ATOM	1698	CG	GLU	1162	31.653	23.164	-10.248	1.00	0.00	LX0	C
ATOM	1699	CD	GLU	1162	32.818	23.684	-9.420	1.00	0.00	LX0	C
ATOM	1700	OE1	GLU	1162	33.098	23.115	-8.371	1.00	0.00	LX0	O
ATOM	1701	OE2	GLU	1162	33.495	24.612	-9.848	1.00	0.00	LX0	O
ATOM	1702	C	GLU	1162	33.201	20.637	-12.795	1.00	0.00	LX0	C
ATOM	1703	O	GLU	1162	32.318	19.850	-13.110	1.00	0.00	LX0	O
ATOM	1704	N	THR	1163	34.319	20.808	-13.515	1.00	0.00	LX0	N
ATOM	1705	H	THR	1163	35.039	21.435	-13.218	0.00	0.00	LX0	H
ATOM	1706	CA	THR	1163	34.579	19.954	-14.676	1.00	0.00	LX0	C
ATOM	1707	CB	THR	1163	35.771	20.542	-15.425	1.00	0.00	LX0	C
ATOM	1708	OG1	THR	1163	36.652	21.196	-14.496	1.00	0.00	LX0	O
ATOM	1709	HG1	THR	1163	37.305	21.653	-15.011	0.00	0.00	LX0	H
ATOM	1710	CG2	THR	1163	35.309	21.541	-16.487	1.00	0.00	LX0	C
ATOM	1711	C	THR	1163	34.782	18.470	-14.370	1.00	0.00	LX0	C
ATOM	1712	O	THR	1163	34.679	17.600	-15.231	1.00	0.00	LX0	O
ATOM	1713	N	ASP	1164	35.053	18.225	-13.084	1.00	0.00	LX0	N
ATOM	1714	H	ASP	1164	35.182	18.979	-12.440	0.00	0.00	LX0	H
ATOM	1715	CA	ASP	1164	35.121	16.862	-12.569	1.00	0.00	LX0	C
ATOM	1716	CB	ASP	1164	36.144	16.807	-11.439	1.00	0.00	LX0	C
ATOM	1717	CG	ASP	1164	37.513	16.343	-11.900	1.00	0.00	LX0	C
ATOM	1718	OD1	ASP	1164	37.970	16.713	-12.977	1.00	0.00	LX0	O
ATOM	1719	OD2	ASP	1164	38.146	15.583	-11.176	1.00	0.00	LX0	O
ATOM	1720	C	ASP	1164	33.792	16.319	-12.062	1.00	0.00	LX0	C
ATOM	1721	O	ASP	1164	33.710	15.259	-11.449	1.00	0.00	LX0	O
ATOM	1722	N	TYR	1165	32.733	17.095	-12.311	1.00	0.00	LX0	N
ATOM	1723	H	TYR	1165	32.771	17.931	-12.860	0.00	0.00	LX0	H
ATOM	1724	CA	TYR	1165	31.458	16.675	-11.749	1.00	0.00	LX0	C
ATOM	1725	CB	TYR	1165	31.005	17.666	-10.673	1.00	0.00	LX0	C
ATOM	1726	CG	TYR	1165	31.854	17.516	-9.433	1.00	0.00	LX0	C
ATOM	1727	CD1	TYR	1165	32.941	18.390	-9.224	1.00	0.00	LX0	C
ATOM	1728	CE1	TYR	1165	33.748	18.213	-8.087	1.00	0.00	LX0	C
ATOM	1729	CD2	TYR	1165	31.534	16.491	-8.520	1.00	0.00	LX0	C
ATOM	1730	CE2	TYR	1165	32.337	16.321	-7.383	1.00	0.00	LX0	C
ATOM	1731	CZ	TYR	1165	33.445	17.170	-7.188	1.00	0.00	LX0	C
ATOM	1732	OH	TYR	1165	34.270	16.970	-6.097	1.00	0.00	LX0	O
ATOM	1733	HH	TYR	1165	33.916	16.267	-5.539	0.00	0.00	LX0	H
ATOM	1734	C	TYR	1165	30.401	16.519	-12.816	1.00	0.00	LX0	C
ATOM	1735	O	TYR	1165	30.506	17.049	-13.915	1.00	0.00	LX0	O
ATOM	1736	N	TYR	1166	29.365	15.758	-12.437	1.00	0.00	LX0	N
ATOM	1737	H	TYR	1166	29.395	15.235	-11.582	0.00	0.00	LX0	H
ATOM	1738	CA	TYR	1166	28.196	15.657	-13.305	1.00	0.00	LX0	C
ATOM	1739	CB	TYR	1166	28.503	14.768	-14.519	1.00	0.00	LX0	C
ATOM	1740	CG	TYR	1166	27.323	14.670	-15.453	1.00	0.00	LX0	C
ATOM	1741	CD1	TYR	1166	26.997	15.757	-16.292	1.00	0.00	LX0	C
ATOM	1742	CE1	TYR	1166	25.853	15.667	-17.103	1.00	0.00	LX0	C
ATOM	1743	CD2	TYR	1166	26.563	13.487	-15.430	1.00	0.00	LX0	C
ATOM	1744	CE2	TYR	1166	25.417	13.409	-16.227	1.00	0.00	LX0	C
ATOM	1745	CZ	TYR	1166	25.072	14.495	-17.053	1.00	0.00	LX0	C
ATOM	1746	OH	TYR	1166	23.941	14.413	-17.836	1.00	0.00	LX0	O
ATOM	1747	HH	TYR	1166	23.593	13.518	-17.785	0.00	0.00	LX0	H
ATOM	1748	C	TYR	1166	27.008	15.143	-12.519	1.00	0.00	LX0	C
ATOM	1749	O	TYR	1166	27.145	14.405	-11.553	1.00	0.00	LX0	O

ATOM	1750	N	ARG	1167	25.826	15.578	-12.967	1.00	0.00	LX0	N
ATOM	1751	H	ARG	1167	25.783	16.127	-13.801	0.00	0.00	LX0	H
ATOM	1752	CA	ARG	1167	24.612	15.106	-12.313	1.00	0.00	LX0	C
ATOM	1753	CB	ARG	1167	23.529	16.185	-12.469	1.00	0.00	LX0	C
ATOM	1754	CG	ARG	1167	22.376	16.004	-11.486	1.00	0.00	LX0	C
ATOM	1755	CD	ARG	1167	21.216	16.996	-11.593	1.00	0.00	LX0	C
ATOM	1756	NE	ARG	1167	20.190	16.557	-10.652	1.00	0.00	LX0	N
ATOM	1757	HE	ARG	1167	19.923	15.592	-10.684	0.00	0.00	LX0	H
ATOM	1758	CZ	ARG	1167	19.721	17.302	-9.635	1.00	0.00	LX0	C
ATOM	1759	NH1	ARG	1167	19.908	18.621	-9.619	1.00	0.00	LX0	N
ATOM	1760	HH11	ARG	1167	19.588	19.191	-8.862	0.00	0.00	LX0	H
ATOM	1761	HH12	ARG	1167	20.378	19.064	-10.383	0.00	0.00	LX0	H
ATOM	1762	NH2	ARG	1167	19.084	16.677	-8.647	1.00	0.00	LX0	N
ATOM	1763	HH21	ARG	1167	18.661	17.150	-7.873	0.00	0.00	LX0	H
ATOM	1764	HH22	ARG	1167	19.042	15.669	-8.666	0.00	0.00	LX0	H
ATOM	1765	C	ARG	1167	24.151	13.733	-12.800	1.00	0.00	LX0	C
ATOM	1766	O	ARG	1167	23.156	13.630	-13.502	1.00	0.00	LX0	O
ATOM	1767	N	LYS	1168	24.922	12.697	-12.415	1.00	0.00	LX0	N
ATOM	1768	H	LYS	1168	25.633	12.889	-11.736	0.00	0.00	LX0	H
ATOM	1769	CA	LYS	1168	24.781	11.353	-13.004	1.00	0.00	LX0	C
ATOM	1770	CB	LYS	1168	25.490	10.281	-12.151	1.00	0.00	LX0	C
ATOM	1771	CG	LYS	1168	24.790	9.820	-10.864	1.00	0.00	LX0	C
ATOM	1772	CD	LYS	1168	25.172	8.389	-10.473	1.00	0.00	LX0	C
ATOM	1773	CE	LYS	1168	24.251	7.800	-9.397	1.00	0.00	LX0	C
ATOM	1774	NZ	LYS	1168	24.519	6.369	-9.216	1.00	0.00	LX0	N
ATOM	1775	HZ1	LYS	1168	23.835	5.932	-8.563	0.00	0.00	LX0	H
ATOM	1776	HZ2	LYS	1168	24.409	5.849	-10.116	0.00	0.00	LX0	H
ATOM	1777	HZ3	LYS	1168	25.477	6.186	-8.856	0.00	0.00	LX0	H
ATOM	1778	C	LYS	1168	23.370	10.884	-13.339	1.00	0.00	LX0	C
ATOM	1779	O	LYS	1168	22.475	10.940	-12.500	1.00	0.00	LX0	O
ATOM	1780	N	GLY	1169	23.203	10.422	-14.588	1.00	0.00	LX0	N
ATOM	1781	H	GLY	1169	23.918	10.517	-15.289	0.00	0.00	LX0	H
ATOM	1782	CA	GLY	1169	21.901	9.898	-15.011	1.00	0.00	LX0	C
ATOM	1783	C	GLY	1169	20.785	10.931	-14.973	1.00	0.00	LX0	C
ATOM	1784	O	GLY	1169	19.612	10.634	-14.737	1.00	0.00	LX0	O
ATOM	1785	N	GLY	1170	21.211	12.193	-15.126	1.00	0.00	LX0	N
ATOM	1786	H	GLY	1170	22.120	12.315	-15.537	0.00	0.00	LX0	H
ATOM	1787	CA	GLY	1170	20.352	13.323	-14.763	1.00	0.00	LX0	C
ATOM	1788	C	GLY	1170	19.663	13.170	-13.408	1.00	0.00	LX0	C
ATOM	1789	O	GLY	1170	18.454	13.348	-13.284	1.00	0.00	LX0	O
ATOM	1790	N	LYS	1171	20.455	12.761	-12.403	1.00	0.00	LX0	N
ATOM	1791	H	LYS	1171	21.428	12.610	-12.607	0.00	0.00	LX0	H
ATOM	1792	CA	LYS	1171	19.870	12.475	-11.087	1.00	0.00	LX0	C
ATOM	1793	CB	LYS	1171	19.896	10.979	-10.713	1.00	0.00	LX0	C
ATOM	1794	CG	LYS	1171	19.311	9.926	-11.670	1.00	0.00	LX0	C
ATOM	1795	CD	LYS	1171	17.789	9.698	-11.663	1.00	0.00	LX0	C
ATOM	1796	CE	LYS	1171	16.918	10.807	-12.265	1.00	0.00	LX0	C
ATOM	1797	NZ	LYS	1171	17.287	11.052	-13.666	1.00	0.00	LX0	N
ATOM	1798	HZ1	LYS	1171	17.452	12.074	-13.790	0.00	0.00	LX0	H
ATOM	1799	HZ2	LYS	1171	18.183	10.572	-13.903	0.00	0.00	LX0	H
ATOM	1800	HZ3	LYS	1171	16.553	10.733	-14.324	0.00	0.00	LX0	H
ATOM	1801	C	LYS	1171	20.538	13.257	-9.972	1.00	0.00	LX0	C
ATOM	1802	O	LYS	1171	19.940	14.155	-9.384	1.00	0.00	LX0	O
ATOM	1803	N	GLY	1172	21.806	12.897	-9.718	1.00	0.00	LX0	N
ATOM	1804	H	GLY	1172	22.268	12.202	-10.274	0.00	0.00	LX0	H
ATOM	1805	CA	GLY	1172	22.511	13.511	-8.592	1.00	0.00	LX0	C
ATOM	1806	C	GLY	1172	23.958	13.788	-8.947	1.00	0.00	LX0	C
ATOM	1807	O	GLY	1172	24.586	13.025	-9.666	1.00	0.00	LX0	O
ATOM	1808	N	LEU	1173	24.451	14.935	-8.462	1.00	0.00	LX0	N
ATOM	1809	H	LEU	1173	23.945	15.450	-7.774	0.00	0.00	LX0	H
ATOM	1810	CA	LEU	1173	25.803	15.308	-8.877	1.00	0.00	LX0	C

ATOM	1811	CB	LEU	1173	25.902	16.834	-9.033	1.00	0.00	LX0	C
ATOM	1812	CG	LEU	1173	27.266	17.426	-9.395	1.00	0.00	LX0	C
ATOM	1813	CD1	LEU	1173	27.133	18.532	-10.441	1.00	0.00	LX0	C
ATOM	1814	CD2	LEU	1173	28.026	17.913	-8.157	1.00	0.00	LX0	C
ATOM	1815	C	LEU	1173	26.896	14.701	-8.018	1.00	0.00	LX0	C
ATOM	1816	O	LEU	1173	26.912	14.825	-6.795	1.00	0.00	LX0	O
ATOM	1817	N	LEU	1174	27.779	14.020	-8.760	1.00	0.00	LX0	N
ATOM	1818	H	LEU	1174	27.670	14.043	-9.756	0.00	0.00	LX0	H
ATOM	1819	CA	LEU	1174	28.831	13.148	-8.247	1.00	0.00	LX0	C
ATOM	1820	CB	LEU	1174	28.230	11.739	-8.133	1.00	0.00	LX0	C
ATOM	1821	CG	LEU	1174	27.332	11.528	-6.908	1.00	0.00	LX0	C
ATOM	1822	CD1	LEU	1174	26.301	10.423	-7.126	1.00	0.00	LX0	C
ATOM	1823	CD2	LEU	1174	28.146	11.298	-5.635	1.00	0.00	LX0	C
ATOM	1824	C	LEU	1174	29.993	13.201	-9.244	1.00	0.00	LX0	C
ATOM	1825	O	LEU	1174	29.798	13.616	-10.385	1.00	0.00	LX0	O
ATOM	1826	N	PRO	1175	31.216	12.801	-8.809	1.00	0.00	LX0	N
ATOM	1827	CD	PRO	1175	31.601	12.370	-7.465	1.00	0.00	LX0	C
ATOM	1828	CA	PRO	1175	32.357	12.824	-9.738	1.00	0.00	LX0	C
ATOM	1829	CB	PRO	1175	33.543	12.797	-8.771	1.00	0.00	LX0	C
ATOM	1830	CG	PRO	1175	33.056	11.940	-7.604	1.00	0.00	LX0	C
ATOM	1831	C	PRO	1175	32.361	11.644	-10.704	1.00	0.00	LX0	C
ATOM	1832	O	PRO	1175	33.112	10.687	-10.563	1.00	0.00	LX0	O
ATOM	1833	N	VAL	1176	31.477	11.742	-11.706	1.00	0.00	LX0	N
ATOM	1834	H	VAL	1176	30.926	12.577	-11.739	0.00	0.00	LX0	H
ATOM	1835	CA	VAL	1176	31.125	10.535	-12.465	1.00	0.00	LX0	C
ATOM	1836	CB	VAL	1176	30.092	10.860	-13.552	1.00	0.00	LX0	C
ATOM	1837	CG1	VAL	1176	28.841	11.428	-12.884	1.00	0.00	LX0	C
ATOM	1838	CG2	VAL	1176	30.616	11.780	-14.659	1.00	0.00	LX0	C
ATOM	1839	C	VAL	1176	32.242	9.639	-13.000	1.00	0.00	LX0	C
ATOM	1840	O	VAL	1176	32.223	8.429	-12.833	1.00	0.00	LX0	O
ATOM	1841	N	ARG	1177	33.241	10.279	-13.629	1.00	0.00	LX0	N
ATOM	1842	H	ARG	1177	33.273	11.276	-13.605	0.00	0.00	LX0	H
ATOM	1843	CA	ARG	1177	34.290	9.475	-14.268	1.00	0.00	LX0	C
ATOM	1844	CB	ARG	1177	35.101	10.331	-15.246	1.00	0.00	LX0	C
ATOM	1845	CG	ARG	1177	34.247	11.077	-16.271	1.00	0.00	LX0	C
ATOM	1846	CD	ARG	1177	35.077	11.900	-17.255	1.00	0.00	LX0	C
ATOM	1847	NE	ARG	1177	34.217	12.506	-18.266	1.00	0.00	LX0	N
ATOM	1848	HE	ARG	1177	33.417	13.037	-17.974	0.00	0.00	LX0	H
ATOM	1849	CZ	ARG	1177	34.434	12.354	-19.590	1.00	0.00	LX0	C
ATOM	1850	NH1	ARG	1177	35.448	11.643	-20.065	1.00	0.00	LX0	N
ATOM	1851	HH11	ARG	1177	35.635	11.594	-21.054	0.00	0.00	LX0	H
ATOM	1852	HH12	ARG	1177	36.056	11.123	-19.456	0.00	0.00	LX0	H
ATOM	1853	NH2	ARG	1177	33.584	12.928	-20.424	1.00	0.00	LX0	N
ATOM	1854	HH21	ARG	1177	33.660	12.804	-21.422	0.00	0.00	LX0	H
ATOM	1855	HH22	ARG	1177	32.824	13.472	-20.056	0.00	0.00	LX0	H
ATOM	1856	C	ARG	1177	35.232	8.748	-13.317	1.00	0.00	LX0	C
ATOM	1857	O	ARG	1177	36.092	7.978	-13.716	1.00	0.00	LX0	O
ATOM	1858	N	TRP	1178	35.042	9.060	-12.030	1.00	0.00	LX0	N
ATOM	1859	H	TRP	1178	34.290	9.648	-11.737	0.00	0.00	LX0	H
ATOM	1860	CA	TRP	1178	35.928	8.516	-11.012	1.00	0.00	LX0	C
ATOM	1861	CB	TRP	1178	36.429	9.667	-10.126	1.00	0.00	LX0	C
ATOM	1862	CG	TRP	1178	37.176	10.646	-11.002	1.00	0.00	LX0	C
ATOM	1863	CD2	TRP	1178	36.650	11.704	-11.837	1.00	0.00	LX0	C
ATOM	1864	CE2	TRP	1178	37.753	12.262	-12.561	1.00	0.00	LX0	C
ATOM	1865	CE3	TRP	1178	35.348	12.190	-12.076	1.00	0.00	LX0	C
ATOM	1866	CD1	TRP	1178	38.556	10.650	-11.236	1.00	0.00	LX0	C
ATOM	1867	NE1	TRP	1178	38.897	11.597	-12.145	1.00	0.00	LX0	N
ATOM	1868	HE1	TRP	1178	39.807	11.762	-12.480	0.00	0.00	LX0	H
ATOM	1869	CZ2	TRP	1178	37.537	13.299	-13.492	1.00	0.00	LX0	C
ATOM	1870	CZ3	TRP	1178	35.141	13.222	-13.011	1.00	0.00	LX0	C
ATOM	1871	CH2	TRP	1178	36.228	13.765	-13.726	1.00	0.00	LX0	C

```
ATOM   1872  C   TRP  1178      35.256   7.441 -10.185  1.00  0.00      LX0  C
ATOM   1873  O   TRP  1178      35.839   6.867  -9.273  1.00  0.00      LX0  O
ATOM   1874  N   MET  1179      33.974   7.220 -10.504  1.00  0.00      LX0  N
ATOM   1875  H   MET  1179      33.561   7.546 -11.355  0.00  0.00      LX0  H
ATOM   1876  CA  MET  1179      33.221   6.368  -9.596  1.00  0.00      LX0  C
ATOM   1877  CB  MET  1179      31.791   6.884  -9.474  1.00  0.00      LX0  C
ATOM   1878  CG  MET  1179      31.745   8.289  -8.878  1.00  0.00      LX0  C
ATOM   1879  SD  MET  1179      30.166   9.110  -9.133  1.00  0.00      LX0  S
ATOM   1880  CE  MET  1179      29.127   7.945  -8.246  1.00  0.00      LX0  C
ATOM   1881  C   MET  1179      33.248   4.907  -9.972  1.00  0.00      LX0  C
ATOM   1882  O   MET  1179      33.316   4.514 -11.129  1.00  0.00      LX0  O
ATOM   1883  N   SER  1180      33.187   4.121  -8.901  1.00  0.00      LX0  N
ATOM   1884  H   SER  1180      33.172   4.512  -7.980  0.00  0.00      LX0  H
ATOM   1885  CA  SER  1180      33.086   2.672  -9.016  1.00  0.00      LX0  C
ATOM   1886  CB  SER  1180      33.280   2.132  -7.599  1.00  0.00      LX0  C
ATOM   1887  OG  SER  1180      32.939   3.134  -6.635  1.00  0.00      LX0  O
ATOM   1888  HG  SER  1180      32.018   2.992  -6.416  0.00  0.00      LX0  H
ATOM   1889  C   SER  1180      31.781   2.215  -9.662  1.00  0.00      LX0  C
ATOM   1890  O   SER  1180      30.790   2.936  -9.622  1.00  0.00      LX0  O
ATOM   1891  N   PRO  1181      31.805   0.993 -10.267  1.00  0.00      LX0  N
ATOM   1892  CD  PRO  1181      32.935   0.072 -10.336  1.00  0.00      LX0  C
ATOM   1893  CA  PRO  1181      30.631   0.454 -10.967  1.00  0.00      LX0  C
ATOM   1894  CB  PRO  1181      30.990  -1.028 -11.113  1.00  0.00      LX0  C
ATOM   1895  CG  PRO  1181      32.499  -1.031 -11.288  1.00  0.00      LX0  C
ATOM   1896  C   PRO  1181      29.289   0.686 -10.298  1.00  0.00      LX0  C
ATOM   1897  O   PRO  1181      28.428   1.405 -10.795  1.00  0.00      LX0  O
ATOM   1898  N   GLU  1182      29.165   0.050  -9.124  1.00  0.00      LX0  N
ATOM   1899  H   GLU  1182      29.908  -0.531  -8.787  0.00  0.00      LX0  H
ATOM   1900  CA  GLU  1182      27.971   0.200  -8.296  1.00  0.00      LX0  C
ATOM   1901  CB  GLU  1182      28.100  -0.653  -7.013  1.00  0.00      LX0  C
ATOM   1902  CG  GLU  1182      29.293  -0.440  -6.054  1.00  0.00      LX0  C
ATOM   1903  CD  GLU  1182      30.638  -0.832  -6.659  1.00  0.00      LX0  C
ATOM   1904  OE1 GLU  1182      30.713  -1.763  -7.454  1.00  0.00      LX0  O
ATOM   1905  OE2 GLU  1182      31.637  -0.197  -6.352  1.00  0.00      LX0  O
ATOM   1906  C   GLU  1182      27.593   1.653  -8.044  1.00  0.00      LX0  C
ATOM   1907  O   GLU  1182      26.524   2.139  -8.388  1.00  0.00      LX0  O
ATOM   1908  N   SER  1183      28.582   2.388  -7.523  1.00  0.00      LX0  N
ATOM   1909  H   SER  1183      29.440   1.962  -7.231  0.00  0.00      LX0  H
ATOM   1910  CA  SER  1183      28.369   3.817  -7.315  1.00  0.00      LX0  C
ATOM   1911  CB  SER  1183      29.672   4.411  -6.789  1.00  0.00      LX0  C
ATOM   1912  OG  SER  1183      30.330   3.439  -5.959  1.00  0.00      LX0  O
ATOM   1913  HG  SER  1183      29.793   3.323  -5.172  0.00  0.00      LX0  H
ATOM   1914  C   SER  1183      27.799   4.593  -8.508  1.00  0.00      LX0  C
ATOM   1915  O   SER  1183      26.922   5.452  -8.382  1.00  0.00      LX0  O
ATOM   1916  N   LEU  1184      28.303   4.228  -9.698  1.00  0.00      LX0  N
ATOM   1917  H   LEU  1184      28.967   3.480  -9.761  0.00  0.00      LX0  H
ATOM   1918  CA  LEU  1184      27.719   4.824 -10.893  1.00  0.00      LX0  C
ATOM   1919  CB  LEU  1184      28.609   4.651 -12.122  1.00  0.00      LX0  C
ATOM   1920  CG  LEU  1184      29.624   5.789 -12.237  1.00  0.00      LX0  C
ATOM   1921  CD1 LEU  1184      30.474   5.662 -13.495  1.00  0.00      LX0  C
ATOM   1922  CD2 LEU  1184      28.960   7.165 -12.164  1.00  0.00      LX0  C
ATOM   1923  C   LEU  1184      26.293   4.408 -11.175  1.00  0.00      LX0  C
ATOM   1924  O   LEU  1184      25.442   5.266 -11.377  1.00  0.00      LX0  O
ATOM   1925  N   LYS  1185      26.036   3.091 -11.116  1.00  0.00      LX0  N
ATOM   1926  H   LYS  1185      26.761   2.453 -10.846  0.00  0.00      LX0  H
ATOM   1927  CA  LYS  1185      24.619   2.721 -11.175  1.00  0.00      LX0  C
ATOM   1928  CB  LYS  1185      24.377   1.236 -11.518  1.00  0.00      LX0  C
ATOM   1929  CG  LYS  1185      25.426   0.205 -11.086  1.00  0.00      LX0  C
ATOM   1930  CD  LYS  1185      24.880  -1.224 -11.150  1.00  0.00      LX0  C
ATOM   1931  CE  LYS  1185      25.903  -2.309 -10.798  1.00  0.00      LX0  C
ATOM   1932  NZ  LYS  1185      26.772  -2.594 -11.944  1.00  0.00      LX0  N
```

```
ATOM   1933  HZ1 LYS  1185      27.284  -1.767 -12.303  0.00  0.00      LX0  H
ATOM   1934  HZ2 LYS  1185      27.462  -3.344 -11.748  0.00  0.00      LX0  H
ATOM   1935  HZ3 LYS  1185      26.225  -2.926 -12.773  0.00  0.00      LX0  H
ATOM   1936  C   LYS  1185      23.843   3.183  -9.947  1.00  0.00      LX0  C
ATOM   1937  O   LYS  1185      23.300   4.285  -9.928  1.00  0.00      LX0  O
ATOM   1938  N   ASP  1186      23.847   2.340  -8.914  1.00  0.00      LX0  N
ATOM   1939  H   ASP  1186      24.432   1.523  -8.892  0.00  0.00      LX0  H
ATOM   1940  CA  ASP  1186      23.216   2.719  -7.654  1.00  0.00      LX0  C
ATOM   1941  CB  ASP  1186      23.099   1.483  -6.733  1.00  0.00      LX0  C
ATOM   1942  CG  ASP  1186      24.386   0.666  -6.711  1.00  0.00      LX0  C
ATOM   1943  OD1 ASP  1186      25.211   0.881  -5.826  1.00  0.00      LX0  O
ATOM   1944  OD2 ASP  1186      24.575  -0.156  -7.605  1.00  0.00      LX0  O
ATOM   1945  C   ASP  1186      23.869   3.928  -6.987  1.00  0.00      LX0  C
ATOM   1946  O   ASP  1186      23.449   5.069  -7.176  1.00  0.00      LX0  O
ATOM   1947  N   GLY  1187      24.919   3.668  -6.211  1.00  0.00      LX0  N
ATOM   1948  H   GLY  1187      25.322   2.752  -6.276  0.00  0.00      LX0  H
ATOM   1949  CA  GLY  1187      25.446   4.728  -5.362  1.00  0.00      LX0  C
ATOM   1950  C   GLY  1187      25.879   4.256  -3.987  1.00  0.00      LX0  C
ATOM   1951  O   GLY  1187      25.946   5.024  -3.035  1.00  0.00      LX0  O
ATOM   1952  N   VAL  1188      26.200   2.956  -3.913  1.00  0.00      LX0  N
ATOM   1953  H   VAL  1188      26.068   2.320  -4.677  0.00  0.00      LX0  H
ATOM   1954  CA  VAL  1188      26.839   2.491  -2.685  1.00  0.00      LX0  C
ATOM   1955  CB  VAL  1188      26.793   0.953  -2.638  1.00  0.00      LX0  C
ATOM   1956  CG1 VAL  1188      27.619   0.339  -1.506  1.00  0.00      LX0  C
ATOM   1957  CG2 VAL  1188      25.338   0.483  -2.556  1.00  0.00      LX0  C
ATOM   1958  C   VAL  1188      28.258   3.032  -2.574  1.00  0.00      LX0  C
ATOM   1959  O   VAL  1188      29.039   2.991  -3.519  1.00  0.00      LX0  O
ATOM   1960  N   PHE  1189      28.547   3.556  -1.375  1.00  0.00      LX0  N
ATOM   1961  H   PHE  1189      27.855   3.603  -0.657  0.00  0.00      LX0  H
ATOM   1962  CA  PHE  1189      29.931   3.921  -1.096  1.00  0.00      LX0  C
ATOM   1963  CB  PHE  1189      30.118   5.435  -0.953  1.00  0.00      LX0  C
ATOM   1964  CG  PHE  1189      29.887   6.146  -2.267  1.00  0.00      LX0  C
ATOM   1965  CD1 PHE  1189      28.591   6.604  -2.593  1.00  0.00      LX0  C
ATOM   1966  CD2 PHE  1189      30.975   6.355  -3.142  1.00  0.00      LX0  C
ATOM   1967  CE1 PHE  1189      28.383   7.294  -3.803  1.00  0.00      LX0  C
ATOM   1968  CE2 PHE  1189      30.771   7.048  -4.351  1.00  0.00      LX0  C
ATOM   1969  CZ  PHE  1189      29.477   7.517  -4.663  1.00  0.00      LX0  C
ATOM   1970  C   PHE  1189      30.429   3.194   0.134  1.00  0.00      LX0  C
ATOM   1971  O   PHE  1189      29.722   2.971   1.112  1.00  0.00      LX0  O
ATOM   1972  N   THR  1190      31.678   2.769  -0.004  1.00  0.00      LX0  N
ATOM   1973  H   THR  1190      32.235   3.133  -0.751  0.00  0.00      LX0  H
ATOM   1974  CA  THR  1190      32.323   1.835   0.906  1.00  0.00      LX0  C
ATOM   1975  CB  THR  1190      32.126   0.413   0.374  1.00  0.00      LX0  C
ATOM   1976  OG1 THR  1190      32.473   0.367  -1.015  1.00  0.00      LX0  O
ATOM   1977  HG1 THR  1190      31.756   0.781  -1.489  0.00  0.00      LX0  H
ATOM   1978  CG2 THR  1190      30.719  -0.134   0.564  1.00  0.00      LX0  C
ATOM   1979  C   THR  1190      33.797   2.175   0.850  1.00  0.00      LX0  C
ATOM   1980  O   THR  1190      34.203   3.018   0.067  1.00  0.00      LX0  O
ATOM   1981  N   THR  1191      34.612   1.442   1.624  1.00  0.00      LX0  N
ATOM   1982  H   THR  1191      34.274   0.824   2.331  0.00  0.00      LX0  H
ATOM   1983  CA  THR  1191      36.039   1.566   1.298  1.00  0.00      LX0  C
ATOM   1984  CB  THR  1191      36.870   0.802   2.347  1.00  0.00      LX0  C
ATOM   1985  OG1 THR  1191      38.261   1.110   2.252  1.00  0.00      LX0  O
ATOM   1986  HG1 THR  1191      38.631   0.852   3.096  0.00  0.00      LX0  H
ATOM   1987  CG2 THR  1191      36.638  -0.710   2.389  1.00  0.00      LX0  C
ATOM   1988  C   THR  1191      36.332   1.156  -0.149  1.00  0.00      LX0  C
ATOM   1989  O   THR  1191      37.110   1.740  -0.891  1.00  0.00      LX0  O
ATOM   1990  N   TYR  1192      35.557   0.135  -0.539  1.00  0.00      LX0  N
ATOM   1991  H   TYR  1192      34.884  -0.238   0.093  0.00  0.00      LX0  H
ATOM   1992  CA  TYR  1192      35.678  -0.439  -1.869  1.00  0.00      LX0  C
ATOM   1993  CB  TYR  1192      34.789  -1.680  -1.965  1.00  0.00      LX0  C
```

ATOM	1994	CG	TYR	1192	34.966	-2.583	-0.758	1.00	0.00	LX0	C
ATOM	1995	CD1	TYR	1192	36.161	-3.319	-0.604	1.00	0.00	LX0	C
ATOM	1996	CE1	TYR	1192	36.303	-4.166	0.509	1.00	0.00	LX0	C
ATOM	1997	CD2	TYR	1192	33.921	-2.666	0.185	1.00	0.00	LX0	C
ATOM	1998	CE2	TYR	1192	34.069	-3.500	1.304	1.00	0.00	LX0	C
ATOM	1999	CZ	TYR	1192	35.253	-4.251	1.448	1.00	0.00	LX0	C
ATOM	2000	OH	TYR	1192	35.387	-5.100	2.529	1.00	0.00	LX0	O
ATOM	2001	HH	TYR	1192	34.823	-4.833	3.262	0.00	0.00	LX0	H
ATOM	2002	C	TYR	1192	35.395	0.520	-3.015	1.00	0.00	LX0	C
ATOM	2003	O	TYR	1192	35.970	0.396	-4.091	1.00	0.00	LX0	O
ATOM	2004	N	SER	1193	34.494	1.489	-2.771	1.00	0.00	LX0	N
ATOM	2005	H	SER	1193	34.055	1.619	-1.881	0.00	0.00	LX0	H
ATOM	2006	CA	SER	1193	34.318	2.475	-3.839	1.00	0.00	LX0	C
ATOM	2007	CB	SER	1193	33.120	3.393	-3.572	1.00	0.00	LX0	C
ATOM	2008	OG	SER	1193	33.086	3.806	-2.201	1.00	0.00	LX0	O
ATOM	2009	HG	SER	1193	33.646	4.585	-2.095	0.00	0.00	LX0	H
ATOM	2010	C	SER	1193	35.573	3.286	-4.056	1.00	0.00	LX0	C
ATOM	2011	O	SER	1193	36.177	3.321	-5.122	1.00	0.00	LX0	O
ATOM	2012	N	ASP	1194	35.978	3.886	-2.935	1.00	0.00	LX0	N
ATOM	2013	H	ASP	1194	35.486	3.797	-2.066	0.00	0.00	LX0	H
ATOM	2014	CA	ASP	1194	37.077	4.829	-3.034	1.00	0.00	LX0	C
ATOM	2015	CB	ASP	1194	37.059	5.745	-1.807	1.00	0.00	LX0	C
ATOM	2016	CG	ASP	1194	35.733	6.499	-1.742	1.00	0.00	LX0	C
ATOM	2017	OD1	ASP	1194	35.613	7.564	-2.341	1.00	0.00	LX0	O
ATOM	2018	OD2	ASP	1194	34.814	6.036	-1.073	1.00	0.00	LX0	O
ATOM	2019	C	ASP	1194	38.428	4.201	-3.352	1.00	0.00	LX0	C
ATOM	2020	O	ASP	1194	39.334	4.862	-3.838	1.00	0.00	LX0	O
ATOM	2021	N	VAL	1195	38.511	2.869	-3.154	1.00	0.00	LX0	N
ATOM	2022	H	VAL	1195	37.810	2.419	-2.598	0.00	0.00	LX0	H
ATOM	2023	CA	VAL	1195	39.632	2.130	-3.752	1.00	0.00	LX0	C
ATOM	2024	CB	VAL	1195	39.683	0.672	-3.250	1.00	0.00	LX0	C
ATOM	2025	CG1	VAL	1195	40.795	-0.140	-3.920	1.00	0.00	LX0	C
ATOM	2026	CG2	VAL	1195	39.857	0.607	-1.733	1.00	0.00	LX0	C
ATOM	2027	C	VAL	1195	39.648	2.184	-5.281	1.00	0.00	LX0	C
ATOM	2028	O	VAL	1195	40.652	2.507	-5.906	1.00	0.00	LX0	O
ATOM	2029	N	TRP	1196	38.476	1.883	-5.871	1.00	0.00	LX0	N
ATOM	2030	H	TRP	1196	37.667	1.724	-5.302	0.00	0.00	LX0	H
ATOM	2031	CA	TRP	1196	38.365	2.000	-7.332	1.00	0.00	LX0	C
ATOM	2032	CB	TRP	1196	36.947	1.580	-7.761	1.00	0.00	LX0	C
ATOM	2033	CG	TRP	1196	36.704	1.632	-9.259	1.00	0.00	LX0	C
ATOM	2034	CD2	TRP	1196	36.487	0.525	-10.162	1.00	0.00	LX0	C
ATOM	2035	CE2	TRP	1196	36.284	1.075	-11.474	1.00	0.00	LX0	C
ATOM	2036	CE3	TRP	1196	36.434	-0.872	-9.973	1.00	0.00	LX0	C
ATOM	2037	CD1	TRP	1196	36.612	2.777	-10.070	1.00	0.00	LX0	C
ATOM	2038	NE1	TRP	1196	36.368	2.457	-11.368	1.00	0.00	LX0	N
ATOM	2039	HE1	TRP	1196	36.255	3.097	-12.106	0.00	0.00	LX0	H
ATOM	2040	CZ2	TRP	1196	36.046	0.217	-12.570	1.00	0.00	LX0	C
ATOM	2041	CZ3	TRP	1196	36.189	-1.718	-11.077	1.00	0.00	LX0	C
ATOM	2042	CH2	TRP	1196	36.001	-1.178	-12.367	1.00	0.00	LX0	C
ATOM	2043	C	TRP	1196	38.731	3.404	-7.800	1.00	0.00	LX0	C
ATOM	2044	O	TRP	1196	39.569	3.618	-8.673	1.00	0.00	LX0	O
ATOM	2045	N	SER	1197	38.091	4.361	-7.116	1.00	0.00	LX0	N
ATOM	2046	H	SER	1197	37.350	4.141	-6.475	0.00	0.00	LX0	H
ATOM	2047	CA	SER	1197	38.397	5.763	-7.368	1.00	0.00	LX0	C
ATOM	2048	CB	SER	1197	37.486	6.606	-6.486	1.00	0.00	LX0	C
ATOM	2049	OG	SER	1197	36.149	6.118	-6.637	1.00	0.00	LX0	O
ATOM	2050	HG	SER	1197	35.928	6.266	-7.553	0.00	0.00	LX0	H
ATOM	2051	C	SER	1197	39.866	6.144	-7.240	1.00	0.00	LX0	C
ATOM	2052	O	SER	1197	40.401	6.903	-8.036	1.00	0.00	LX0	O
ATOM	2053	N	PHE	1198	40.524	5.534	-6.242	1.00	0.00	LX0	N
ATOM	2054	H	PHE	1198	40.023	4.952	-5.602	0.00	0.00	LX0	H

ATOM	2055	CA	PHE	1198	41.970	5.700	-6.093	1.00	0.00	LX0	C
ATOM	2056	CB	PHE	1198	42.430	4.985	-4.814	1.00	0.00	LX0	C
ATOM	2057	CG	PHE	1198	43.932	4.980	-4.670	1.00	0.00	LX0	C
ATOM	2058	CD1	PHE	1198	44.607	6.158	-4.286	1.00	0.00	LX0	C
ATOM	2059	CD2	PHE	1198	44.632	3.783	-4.934	1.00	0.00	LX0	C
ATOM	2060	CE1	PHE	1198	46.010	6.139	-4.174	1.00	0.00	LX0	C
ATOM	2061	CE2	PHE	1198	46.033	3.764	-4.820	1.00	0.00	LX0	C
ATOM	2062	CZ	PHE	1198	46.707	4.942	-4.442	1.00	0.00	LX0	C
ATOM	2063	C	PHE	1198	42.747	5.255	-7.323	1.00	0.00	LX0	C
ATOM	2064	O	PHE	1198	43.617	5.955	-7.828	1.00	0.00	LX0	O
ATOM	2065	N	GLY	1199	42.353	4.075	-7.827	1.00	0.00	LX0	N
ATOM	2066	H	GLY	1199	41.658	3.540	-7.339	0.00	0.00	LX0	H
ATOM	2067	CA	GLY	1199	42.936	3.626	-9.093	1.00	0.00	LX0	C
ATOM	2068	C	GLY	1199	42.772	4.637	-10.219	1.00	0.00	LX0	C
ATOM	2069	O	GLY	1199	43.674	4.917	-11.001	1.00	0.00	LX0	O
ATOM	2070	N	VAL	1200	41.563	5.219	-10.231	1.00	0.00	LX0	N
ATOM	2071	H	VAL	1200	40.887	4.967	-9.537	0.00	0.00	LX0	H
ATOM	2072	CA	VAL	1200	41.313	6.278	-11.209	1.00	0.00	LX0	C
ATOM	2073	CB	VAL	1200	39.820	6.640	-11.243	1.00	0.00	LX0	C
ATOM	2074	CG1	VAL	1200	39.509	7.559	-12.417	1.00	0.00	LX0	C
ATOM	2075	CG2	VAL	1200	38.933	5.398	-11.327	1.00	0.00	LX0	C
ATOM	2076	C	VAL	1200	42.199	7.511	-11.020	1.00	0.00	LX0	C
ATOM	2077	O	VAL	1200	42.691	8.107	-11.969	1.00	0.00	LX0	O
ATOM	2078	N	VAL	1201	42.452	7.840	-9.742	1.00	0.00	LX0	N
ATOM	2079	H	VAL	1201	42.008	7.324	-9.009	0.00	0.00	LX0	H
ATOM	2080	CA	VAL	1201	43.423	8.906	-9.463	1.00	0.00	LX0	C
ATOM	2081	CB	VAL	1201	43.547	9.170	-7.952	1.00	0.00	LX0	C
ATOM	2082	CG1	VAL	1201	44.506	10.320	-7.639	1.00	0.00	LX0	C
ATOM	2083	CG2	VAL	1201	42.183	9.432	-7.320	1.00	0.00	LX0	C
ATOM	2084	C	VAL	1201	44.793	8.621	-10.067	1.00	0.00	LX0	C
ATOM	2085	O	VAL	1201	45.448	9.484	-10.638	1.00	0.00	LX0	O
ATOM	2086	N	LEU	1202	45.183	7.343	-9.954	1.00	0.00	LX0	N
ATOM	2087	H	LEU	1202	44.598	6.696	-9.460	0.00	0.00	LX0	H
ATOM	2088	CA	LEU	1202	46.460	6.947	-10.554	1.00	0.00	LX0	C
ATOM	2089	CB	LEU	1202	46.805	5.496	-10.222	1.00	0.00	LX0	C
ATOM	2090	CG	LEU	1202	46.615	5.136	-8.751	1.00	0.00	LX0	C
ATOM	2091	CD1	LEU	1202	46.867	3.652	-8.501	1.00	0.00	LX0	C
ATOM	2092	CD2	LEU	1202	47.423	6.043	-7.828	1.00	0.00	LX0	C
ATOM	2093	C	LEU	1202	46.507	7.152	-12.057	1.00	0.00	LX0	C
ATOM	2094	O	LEU	1202	47.478	7.634	-12.630	1.00	0.00	LX0	O
ATOM	2095	N	TRP	1203	45.363	6.809	-12.668	1.00	0.00	LX0	N
ATOM	2096	H	TRP	1203	44.644	6.358	-12.134	0.00	0.00	LX0	H
ATOM	2097	CA	TRP	1203	45.166	7.124	-14.084	1.00	0.00	LX0	C
ATOM	2098	CB	TRP	1203	43.769	6.655	-14.495	1.00	0.00	LX0	C
ATOM	2099	CG	TRP	1203	43.540	6.774	-15.980	1.00	0.00	LX0	C
ATOM	2100	CD2	TRP	1203	43.121	7.928	-16.743	1.00	0.00	LX0	C
ATOM	2101	CE2	TRP	1203	43.017	7.511	-18.111	1.00	0.00	LX0	C
ATOM	2102	CE3	TRP	1203	42.825	9.263	-16.393	1.00	0.00	LX0	C
ATOM	2103	CD1	TRP	1203	43.672	5.740	-16.912	1.00	0.00	LX0	C
ATOM	2104	NE1	TRP	1203	43.364	6.167	-18.163	1.00	0.00	LX0	N
ATOM	2105	HE1	TRP	1203	43.356	5.595	-18.961	0.00	0.00	LX0	H
ATOM	2106	CZ2	TRP	1203	42.618	8.433	-19.102	1.00	0.00	LX0	C
ATOM	2107	CZ3	TRP	1203	42.432	10.177	-17.392	1.00	0.00	LX0	C
ATOM	2108	CH2	TRP	1203	42.324	9.764	-18.738	1.00	0.00	LX0	C
ATOM	2109	C	TRP	1203	45.384	8.598	-14.402	1.00	0.00	LX0	C
ATOM	2110	O	TRP	1203	46.055	8.972	-15.360	1.00	0.00	LX0	O
ATOM	2111	N	GLU	1204	44.806	9.432	-13.523	1.00	0.00	LX0	N
ATOM	2112	H	GLU	1204	44.240	9.068	-12.781	0.00	0.00	LX0	H
ATOM	2113	CA	GLU	1204	45.023	10.866	-13.706	1.00	0.00	LX0	C
ATOM	2114	CB	GLU	1204	44.178	11.719	-12.758	1.00	0.00	LX0	C
ATOM	2115	CG	GLU	1204	42.713	11.293	-12.766	1.00	0.00	LX0	C

ATOM	2116	CD	GLU	1204	41.808	12.499	-12.678	1.00	0.00	LX0	C
ATOM	2117	OE1	GLU	1204	41.100	12.765	-13.639	1.00	0.00	LX0	O
ATOM	2118	OE2	GLU	1204	41.739	13.157	-11.647	1.00	0.00	LX0	O
ATOM	2119	C	GLU	1204	46.482	11.265	-13.633	1.00	0.00	LX0	C
ATOM	2120	O	GLU	1204	46.989	11.978	-14.485	1.00	0.00	LX0	O
ATOM	2121	N	ILE	1205	47.172	10.719	-12.620	1.00	0.00	LX0	N
ATOM	2122	H	ILE	1205	46.683	10.152	-11.956	0.00	0.00	LX0	H
ATOM	2123	CA	ILE	1205	48.620	10.952	-12.549	1.00	0.00	LX0	C
ATOM	2124	CB	ILE	1205	49.241	10.155	-11.388	1.00	0.00	LX0	C
ATOM	2125	CG2	ILE	1205	50.749	10.391	-11.277	1.00	0.00	LX0	C
ATOM	2126	CG1	ILE	1205	48.535	10.465	-10.065	1.00	0.00	LX0	C
ATOM	2127	CD1	ILE	1205	48.997	9.570	-8.913	1.00	0.00	LX0	C
ATOM	2128	C	ILE	1205	49.333	10.660	-13.867	1.00	0.00	LX0	C
ATOM	2129	O	ILE	1205	50.034	11.484	-14.444	1.00	0.00	LX0	O
ATOM	2130	N	ALA	1206	49.054	9.443	-14.346	1.00	0.00	LX0	N
ATOM	2131	H	ALA	1206	48.433	8.843	-13.836	0.00	0.00	LX0	H
ATOM	2132	CA	ALA	1206	49.684	9.005	-15.585	1.00	0.00	LX0	C
ATOM	2133	CB	ALA	1206	49.461	7.506	-15.765	1.00	0.00	LX0	C
ATOM	2134	C	ALA	1206	49.271	9.741	-16.856	1.00	0.00	LX0	C
ATOM	2135	O	ALA	1206	49.930	9.650	-17.882	1.00	0.00	LX0	O
ATOM	2136	N	THR	1207	48.158	10.483	-16.766	1.00	0.00	LX0	N
ATOM	2137	H	THR	1207	47.631	10.553	-15.919	0.00	0.00	LX0	H
ATOM	2138	CA	THR	1207	47.786	11.249	-17.955	1.00	0.00	LX0	C
ATOM	2139	CB	THR	1207	46.290	11.120	-18.233	1.00	0.00	LX0	C
ATOM	2140	OG1	THR	1207	45.534	11.183	-17.013	1.00	0.00	LX0	O
ATOM	2141	HG1	THR	1207	45.658	10.336	-16.586	0.00	0.00	LX0	H
ATOM	2142	CG2	THR	1207	45.959	9.860	-19.020	1.00	0.00	LX0	C
ATOM	2143	C	THR	1207	48.138	12.722	-17.915	1.00	0.00	LX0	C
ATOM	2144	O	THR	1207	47.700	13.499	-18.757	1.00	0.00	LX0	O
ATOM	2145	N	LEU	1208	48.874	13.112	-16.852	1.00	0.00	LX0	N
ATOM	2146	H	LEU	1208	49.277	12.433	-16.234	0.00	0.00	LX0	H
ATOM	2147	CA	LEU	1208	48.934	14.544	-16.526	1.00	0.00	LX0	C
ATOM	2148	CB	LEU	1208	49.888	15.311	-17.453	1.00	0.00	LX0	C
ATOM	2149	CG	LEU	1208	51.323	14.772	-17.422	1.00	0.00	LX0	C
ATOM	2150	CD1	LEU	1208	52.213	15.479	-18.444	1.00	0.00	LX0	C
ATOM	2151	CD2	LEU	1208	51.931	14.806	-16.018	1.00	0.00	LX0	C
ATOM	2152	C	LEU	1208	47.548	15.182	-16.443	1.00	0.00	LX0	C
ATOM	2153	O	LEU	1208	47.241	16.263	-16.941	1.00	0.00	LX0	O
ATOM	2154	N	ALA	1209	46.709	14.369	-15.786	1.00	0.00	LX0	N
ATOM	2155	H	ALA	1209	47.115	13.524	-15.445	0.00	0.00	LX0	H
ATOM	2156	CA	ALA	1209	45.296	14.609	-15.527	1.00	0.00	LX0	C
ATOM	2157	CB	ALA	1209	45.126	15.710	-14.488	1.00	0.00	LX0	C
ATOM	2158	C	ALA	1209	44.450	14.909	-16.743	1.00	0.00	LX0	C
ATOM	2159	O	ALA	1209	44.110	16.055	-17.027	1.00	0.00	LX0	O
ATOM	2160	N	GLU	1210	44.101	13.840	-17.465	1.00	0.00	LX0	N
ATOM	2161	H	GLU	1210	44.402	12.919	-17.204	0.00	0.00	LX0	H
ATOM	2162	CA	GLU	1210	43.030	14.077	-18.433	1.00	0.00	LX0	C
ATOM	2163	CB	GLU	1210	43.138	13.130	-19.636	1.00	0.00	LX0	C
ATOM	2164	CG	GLU	1210	44.333	13.399	-20.569	1.00	0.00	LX0	C
ATOM	2165	CD	GLU	1210	44.248	14.739	-21.304	1.00	0.00	LX0	C
ATOM	2166	OE1	GLU	1210	44.877	14.885	-22.342	1.00	0.00	LX0	O
ATOM	2167	OE2	GLU	1210	43.613	15.680	-20.833	1.00	0.00	LX0	O
ATOM	2168	C	GLU	1210	41.671	14.062	-17.756	1.00	0.00	LX0	C
ATOM	2169	O	GLU	1210	41.598	14.303	-16.557	1.00	0.00	LX0	O
ATOM	2170	N	GLN	1211	40.611	13.782	-18.524	1.00	0.00	LX0	N
ATOM	2171	H	GLN	1211	40.633	13.706	-19.525	0.00	0.00	LX0	H
ATOM	2172	CA	GLN	1211	39.433	13.281	-17.829	1.00	0.00	LX0	C
ATOM	2173	CB	GLN	1211	38.177	14.102	-18.165	1.00	0.00	LX0	C
ATOM	2174	CG	GLN	1211	38.226	15.610	-17.862	1.00	0.00	LX0	C
ATOM	2175	CD	GLN	1211	38.335	15.904	-16.373	1.00	0.00	LX0	C
ATOM	2176	OE1	GLN	1211	39.412	15.931	-15.790	1.00	0.00	LX0	O

ATOM	2177	NE2	GLN	1211	37.179	16.188	-15.773	1.00	0.00	LX0	N
ATOM	2178	HE21	GLN	1211	36.278	16.237	-16.205	0.00	0.00	LX0	H
ATOM	2179	HE22	GLN	1211	37.203	16.399	-14.791	0.00	0.00	LX0	H
ATOM	2180	C	GLN	1211	39.261	11.833	-18.253	1.00	0.00	LX0	C
ATOM	2181	O	GLN	1211	39.291	11.521	-19.435	1.00	0.00	LX0	O
ATOM	2182	N	PRO	1212	39.117	10.937	-17.253	1.00	0.00	LX0	N
ATOM	2183	CD	PRO	1212	39.122	11.231	-15.828	1.00	0.00	LX0	C
ATOM	2184	CA	PRO	1212	38.972	9.502	-17.541	1.00	0.00	LX0	C
ATOM	2185	CB	PRO	1212	38.601	8.940	-16.168	1.00	0.00	LX0	C
ATOM	2186	CG	PRO	1212	39.290	9.870	-15.177	1.00	0.00	LX0	C
ATOM	2187	C	PRO	1212	37.951	9.188	-18.620	1.00	0.00	LX0	C
ATOM	2188	O	PRO	1212	36.920	9.851	-18.703	1.00	0.00	LX0	O
ATOM	2189	N	TYR	1213	38.279	8.178	-19.454	1.00	0.00	LX0	N
ATOM	2190	H	TYR	1213	39.165	7.707	-19.399	0.00	0.00	LX0	H
ATOM	2191	CA	TYR	1213	37.404	7.815	-20.578	1.00	0.00	LX0	C
ATOM	2192	CB	TYR	1213	36.051	7.231	-20.121	1.00	0.00	LX0	C
ATOM	2193	CG	TYR	1213	36.209	6.165	-19.059	1.00	0.00	LX0	C
ATOM	2194	CD1	TYR	1213	36.411	4.826	-19.448	1.00	0.00	LX0	C
ATOM	2195	CE1	TYR	1213	36.540	3.842	-18.454	1.00	0.00	LX0	C
ATOM	2196	CD2	TYR	1213	36.138	6.543	-17.702	1.00	0.00	LX0	C
ATOM	2197	CE2	TYR	1213	36.285	5.563	-16.709	1.00	0.00	LX0	C
ATOM	2198	CZ	TYR	1213	36.482	4.223	-17.098	1.00	0.00	LX0	C
ATOM	2199	OH	TYR	1213	36.622	3.261	-16.117	1.00	0.00	LX0	O
ATOM	2200	HH	TYR	1213	36.279	2.434	-16.447	0.00	0.00	LX0	H
ATOM	2201	C	TYR	1213	37.178	8.998	-21.504	1.00	0.00	LX0	C
ATOM	2202	O	TYR	1213	36.056	9.382	-21.826	1.00	0.00	LX0	O
ATOM	2203	N	GLN	1214	38.325	9.616	-21.849	1.00	0.00	LX0	N
ATOM	2204	H	GLN	1214	39.168	9.130	-21.609	0.00	0.00	LX0	H
ATOM	2205	CA	GLN	1214	38.347	11.011	-22.312	1.00	0.00	LX0	C
ATOM	2206	CB	GLN	1214	39.685	11.309	-23.013	1.00	0.00	LX0	C
ATOM	2207	CG	GLN	1214	39.930	12.765	-23.448	1.00	0.00	LX0	C
ATOM	2208	CD	GLN	1214	40.314	13.669	-22.289	1.00	0.00	LX0	C
ATOM	2209	OE1	GLN	1214	39.607	13.844	-21.306	1.00	0.00	LX0	O
ATOM	2210	NE2	GLN	1214	41.493	14.267	-22.468	1.00	0.00	LX0	N
ATOM	2211	HE21	GLN	1214	42.086	14.106	-23.255	0.00	0.00	LX0	H
ATOM	2212	HE22	GLN	1214	41.887	14.900	-21.796	0.00	0.00	LX0	H
ATOM	2213	C	GLN	1214	37.149	11.466	-23.138	1.00	0.00	LX0	C
ATOM	2214	O	GLN	1214	36.283	12.200	-22.670	1.00	0.00	LX0	O
ATOM	2215	N	GLY	1215	37.128	10.963	-24.382	1.00	0.00	LX0	N
ATOM	2216	H	GLY	1215	37.780	10.247	-24.627	0.00	0.00	LX0	H
ATOM	2217	CA	GLY	1215	36.179	11.503	-25.355	1.00	0.00	LX0	C
ATOM	2218	C	GLY	1215	34.716	11.116	-25.203	1.00	0.00	LX0	C
ATOM	2219	O	GLY	1215	33.866	11.570	-25.958	1.00	0.00	LX0	O
ATOM	2220	N	LEU	1216	34.435	10.251	-24.217	1.00	0.00	LX0	N
ATOM	2221	H	LEU	1216	35.118	9.984	-23.535	0.00	0.00	LX0	H
ATOM	2222	CA	LEU	1216	33.018	9.942	-24.043	1.00	0.00	LX0	C
ATOM	2223	CB	LEU	1216	32.834	8.697	-23.176	1.00	0.00	LX0	C
ATOM	2224	CG	LEU	1216	33.378	7.404	-23.784	1.00	0.00	LX0	C
ATOM	2225	CD1	LEU	1216	33.254	6.237	-22.803	1.00	0.00	LX0	C
ATOM	2226	CD2	LEU	1216	32.728	7.082	-25.132	1.00	0.00	LX0	C
ATOM	2227	C	LEU	1216	32.265	11.107	-23.430	1.00	0.00	LX0	C
ATOM	2228	O	LEU	1216	32.826	11.920	-22.701	1.00	0.00	LX0	O
ATOM	2229	N	SER	1217	30.962	11.152	-23.728	1.00	0.00	LX0	N
ATOM	2230	H	SER	1217	30.555	10.461	-24.325	0.00	0.00	LX0	H
ATOM	2231	CA	SER	1217	30.158	12.079	-22.939	1.00	0.00	LX0	C
ATOM	2232	CB	SER	1217	28.828	12.365	-23.638	1.00	0.00	LX0	C
ATOM	2233	OG	SER	1217	28.124	11.142	-23.866	1.00	0.00	LX0	O
ATOM	2234	HG	SER	1217	28.487	10.777	-24.671	0.00	0.00	LX0	H
ATOM	2235	C	SER	1217	29.953	11.518	-21.544	1.00	0.00	LX0	C
ATOM	2236	O	SER	1217	30.132	10.327	-21.313	1.00	0.00	LX0	O
ATOM	2237	N	ASN	1218	29.589	12.409	-20.610	1.00	0.00	LX0	N

ATOM	2238	H	ASN	1218	29.433	13.363	-20.858	0.00	0.00	LX0	H
ATOM	2239	CA	ASN	1218	29.571	11.937	-19.220	1.00	0.00	LX0	C
ATOM	2240	CB	ASN	1218	29.337	13.091	-18.240	1.00	0.00	LX0	C
ATOM	2241	CG	ASN	1218	30.592	13.946	-18.131	1.00	0.00	LX0	C
ATOM	2242	OD1	ASN	1218	31.640	13.660	-18.701	1.00	0.00	LX0	O
ATOM	2243	ND2	ASN	1218	30.448	15.034	-17.371	1.00	0.00	LX0	N
ATOM	2244	HD21	ASN	1218	29.582	15.268	-16.932	0.00	0.00	LX0	H
ATOM	2245	HD22	ASN	1218	31.218	15.654	-17.216	0.00	0.00	LX0	H
ATOM	2246	C	ASN	1218	28.645	10.758	-18.950	1.00	0.00	LX0	C
ATOM	2247	O	ASN	1218	29.013	9.801	-18.281	1.00	0.00	LX0	O
ATOM	2248	N	GLU	1219	27.455	10.833	-19.569	1.00	0.00	LX0	N
ATOM	2249	H	GLU	1219	27.173	11.651	-20.064	0.00	0.00	LX0	H
ATOM	2250	CA	GLU	1219	26.550	9.687	-19.438	1.00	0.00	LX0	C
ATOM	2251	CB	GLU	1219	25.138	10.031	-19.927	1.00	0.00	LX0	C
ATOM	2252	CG	GLU	1219	24.495	11.278	-19.295	1.00	0.00	LX0	C
ATOM	2253	CD	GLU	1219	24.093	11.101	-17.832	1.00	0.00	LX0	C
ATOM	2254	OE1	GLU	1219	24.799	10.448	-17.069	1.00	0.00	LX0	O
ATOM	2255	OE2	GLU	1219	23.083	11.676	-17.432	1.00	0.00	LX0	O
ATOM	2256	C	GLU	1219	27.040	8.405	-20.096	1.00	0.00	LX0	C
ATOM	2257	O	GLU	1219	26.785	7.296	-19.645	1.00	0.00	LX0	O
ATOM	2258	N	GLN	1220	27.803	8.587	-21.190	1.00	0.00	LX0	N
ATOM	2259	H	GLN	1220	28.032	9.504	-21.515	0.00	0.00	LX0	H
ATOM	2260	CA	GLN	1220	28.444	7.393	-21.748	1.00	0.00	LX0	C
ATOM	2261	CB	GLN	1220	29.105	7.685	-23.090	1.00	0.00	LX0	C
ATOM	2262	CG	GLN	1220	28.119	7.869	-24.239	1.00	0.00	LX0	C
ATOM	2263	CD	GLN	1220	28.897	8.322	-25.455	1.00	0.00	LX0	C
ATOM	2264	OE1	GLN	1220	29.442	9.417	-25.498	1.00	0.00	LX0	O
ATOM	2265	NE2	GLN	1220	28.939	7.433	-26.449	1.00	0.00	LX0	N
ATOM	2266	HE21	GLN	1220	28.490	6.545	-26.387	0.00	0.00	LX0	H
ATOM	2267	HE22	GLN	1220	29.443	7.683	-27.275	0.00	0.00	LX0	H
ATOM	2268	C	GLN	1220	29.456	6.768	-20.806	1.00	0.00	LX0	C
ATOM	2269	O	GLN	1220	29.538	5.553	-20.670	1.00	0.00	LX0	O
ATOM	2270	N	VAL	1221	30.188	7.664	-20.118	1.00	0.00	LX0	N
ATOM	2271	H	VAL	1221	30.073	8.641	-20.300	0.00	0.00	LX0	H
ATOM	2272	CA	VAL	1221	31.082	7.181	-19.065	1.00	0.00	LX0	C
ATOM	2273	CB	VAL	1221	31.846	8.333	-18.397	1.00	0.00	LX0	C
ATOM	2274	CG1	VAL	1221	32.785	7.821	-17.303	1.00	0.00	LX0	C
ATOM	2275	CG2	VAL	1221	32.607	9.167	-19.427	1.00	0.00	LX0	C
ATOM	2276	C	VAL	1221	30.349	6.343	-18.032	1.00	0.00	LX0	C
ATOM	2277	O	VAL	1221	30.755	5.232	-17.724	1.00	0.00	LX0	O
ATOM	2278	N	LEU	1222	29.206	6.889	-17.567	1.00	0.00	LX0	N
ATOM	2279	H	LEU	1222	28.973	7.834	-17.807	0.00	0.00	LX0	H
ATOM	2280	CA	LEU	1222	28.344	6.072	-16.705	1.00	0.00	LX0	C
ATOM	2281	CB	LEU	1222	26.973	6.704	-16.465	1.00	0.00	LX0	C
ATOM	2282	CG	LEU	1222	26.863	7.607	-15.245	1.00	0.00	LX0	C
ATOM	2283	CD1	LEU	1222	27.432	9.003	-15.490	1.00	0.00	LX0	C
ATOM	2284	CD2	LEU	1222	25.420	7.641	-14.747	1.00	0.00	LX0	C
ATOM	2285	C	LEU	1222	28.102	4.678	-17.247	1.00	0.00	LX0	C
ATOM	2286	O	LEU	1222	28.440	3.675	-16.635	1.00	0.00	LX0	O
ATOM	2287	N	ARG	1223	27.528	4.668	-18.458	1.00	0.00	LX0	N
ATOM	2288	H	ARG	1223	27.345	5.546	-18.904	0.00	0.00	LX0	H
ATOM	2289	CA	ARG	1223	27.165	3.389	-19.068	1.00	0.00	LX0	C
ATOM	2290	CB	ARG	1223	26.494	3.624	-20.422	1.00	0.00	LX0	C
ATOM	2291	CG	ARG	1223	25.273	4.537	-20.296	1.00	0.00	LX0	C
ATOM	2292	CD	ARG	1223	24.614	4.864	-21.635	1.00	0.00	LX0	C
ATOM	2293	NE	ARG	1223	23.982	3.680	-22.216	1.00	0.00	LX0	N
ATOM	2294	HE	ARG	1223	24.578	2.895	-22.396	0.00	0.00	LX0	H
ATOM	2295	CZ	ARG	1223	22.654	3.668	-22.467	1.00	0.00	LX0	C
ATOM	2296	NH1	ARG	1223	21.905	4.740	-22.196	1.00	0.00	LX0	N
ATOM	2297	HH11	ARG	1223	20.921	4.767	-22.377	0.00	0.00	LX0	H
ATOM	2298	HH12	ARG	1223	22.326	5.556	-21.794	0.00	0.00	LX0	H

ATOM	2299	NH2	ARG	1223	22.099	2.574	-22.988	1.00	0.00	LX0	N
ATOM	2300	HH21	ARG	1223	21.121	2.524	-23.194	0.00	0.00	LX0	H
ATOM	2301	HH22	ARG	1223	22.660	1.768	-23.185	0.00	0.00	LX0	H
ATOM	2302	C	ARG	1223	28.290	2.373	-19.184	1.00	0.00	LX0	C
ATOM	2303	O	ARG	1223	28.080	1.171	-19.099	1.00	0.00	LX0	O
ATOM	2304	N	PHE	1224	29.498	2.918	-19.369	1.00	0.00	LX0	N
ATOM	2305	H	PHE	1224	29.600	3.913	-19.423	0.00	0.00	LX0	H
ATOM	2306	CA	PHE	1224	30.657	2.037	-19.411	1.00	0.00	LX0	C
ATOM	2307	CB	PHE	1224	31.800	2.791	-20.104	1.00	0.00	LX0	C
ATOM	2308	CG	PHE	1224	32.872	1.872	-20.648	1.00	0.00	LX0	C
ATOM	2309	CD1	PHE	1224	32.522	0.727	-21.399	1.00	0.00	LX0	C
ATOM	2310	CD2	PHE	1224	34.223	2.202	-20.415	1.00	0.00	LX0	C
ATOM	2311	CE1	PHE	1224	33.537	-0.091	-21.931	1.00	0.00	LX0	C
ATOM	2312	CE2	PHE	1224	35.241	1.388	-20.949	1.00	0.00	LX0	C
ATOM	2313	CZ	PHE	1224	34.887	0.251	-21.705	1.00	0.00	LX0	C
ATOM	2314	C	PHE	1224	31.057	1.495	-18.043	1.00	0.00	LX0	C
ATOM	2315	O	PHE	1224	31.199	0.298	-17.813	1.00	0.00	LX0	O
ATOM	2316	N	VAL	1225	31.242	2.445	-17.118	1.00	0.00	LX0	N
ATOM	2317	H	VAL	1225	30.973	3.391	-17.305	0.00	0.00	LX0	H
ATOM	2318	CA	VAL	1225	31.824	2.027	-15.843	1.00	0.00	LX0	C
ATOM	2319	CB	VAL	1225	32.460	3.204	-15.091	1.00	0.00	LX0	C
ATOM	2320	CG1	VAL	1225	33.388	2.740	-13.963	1.00	0.00	LX0	C
ATOM	2321	CG2	VAL	1225	33.207	4.127	-16.046	1.00	0.00	LX0	C
ATOM	2322	C	VAL	1225	30.877	1.251	-14.945	1.00	0.00	LX0	C
ATOM	2323	O	VAL	1225	31.301	0.423	-14.149	1.00	0.00	LX0	O
ATOM	2324	N	MET	1226	29.566	1.505	-15.132	1.00	0.00	LX0	N
ATOM	2325	H	MET	1226	29.297	2.221	-15.777	0.00	0.00	LX0	H
ATOM	2326	CA	MET	1226	28.567	0.778	-14.337	1.00	0.00	LX0	C
ATOM	2327	CB	MET	1226	27.135	1.028	-14.814	1.00	0.00	LX0	C
ATOM	2328	CG	MET	1226	26.605	2.436	-14.550	1.00	0.00	LX0	C
ATOM	2329	SD	MET	1226	24.854	2.624	-14.931	1.00	0.00	LX0	S
ATOM	2330	CE	MET	1226	24.855	1.900	-16.578	1.00	0.00	LX0	C
ATOM	2331	C	MET	1226	28.780	-0.722	-14.256	1.00	0.00	LX0	C
ATOM	2332	O	MET	1226	28.656	-1.328	-13.194	1.00	0.00	LX0	O
ATOM	2333	N	GLU	1227	29.144	-1.281	-15.422	1.00	0.00	LX0	N
ATOM	2334	H	GLU	1227	29.224	-0.738	-16.258	0.00	0.00	LX0	H
ATOM	2335	CA	GLU	1227	29.413	-2.714	-15.409	1.00	0.00	LX0	C
ATOM	2336	CB	GLU	1227	28.473	-3.473	-16.359	1.00	0.00	LX0	C
ATOM	2337	CG	GLU	1227	26.981	-3.106	-16.252	1.00	0.00	LX0	C
ATOM	2338	CD	GLU	1227	26.483	-3.137	-14.813	1.00	0.00	LX0	C
ATOM	2339	OE1	GLU	1227	26.671	-4.128	-14.111	1.00	0.00	LX0	O
ATOM	2340	OE2	GLU	1227	25.931	-2.141	-14.357	1.00	0.00	LX0	O
ATOM	2341	C	GLU	1227	30.870	-3.083	-15.633	1.00	0.00	LX0	C
ATOM	2342	O	GLU	1227	31.215	-4.063	-16.280	1.00	0.00	LX0	O
ATOM	2343	N	GLY	1228	31.730	-2.250	-15.032	1.00	0.00	LX0	N
ATOM	2344	H	GLY	1228	31.390	-1.429	-14.574	0.00	0.00	LX0	H
ATOM	2345	CA	GLY	1228	33.148	-2.604	-15.003	1.00	0.00	LX0	C
ATOM	2346	C	GLY	1228	33.958	-2.294	-16.249	1.00	0.00	LX0	C
ATOM	2347	O	GLY	1228	34.997	-2.892	-16.503	1.00	0.00	LX0	O
ATOM	2348	N	GLY	1229	33.470	-1.307	-17.014	1.00	0.00	LX0	N
ATOM	2349	H	GLY	1229	32.598	-0.858	-16.818	0.00	0.00	LX0	H
ATOM	2350	CA	GLY	1229	34.301	-0.867	-18.133	1.00	0.00	LX0	C
ATOM	2351	C	GLY	1229	35.576	-0.180	-17.677	1.00	0.00	LX0	C
ATOM	2352	O	GLY	1229	35.556	0.892	-17.084	1.00	0.00	LX0	O
ATOM	2353	N	LEU	1230	36.687	-0.874	-17.955	1.00	0.00	LX0	N
ATOM	2354	H	LEU	1230	36.604	-1.742	-18.440	0.00	0.00	LX0	H
ATOM	2355	CA	LEU	1230	37.974	-0.358	-17.491	1.00	0.00	LX0	C
ATOM	2356	CB	LEU	1230	39.027	-1.471	-17.473	1.00	0.00	LX0	C
ATOM	2357	CG	LEU	1230	38.665	-2.649	-16.564	1.00	0.00	LX0	C
ATOM	2358	CD1	LEU	1230	39.721	-3.753	-16.629	1.00	0.00	LX0	C
ATOM	2359	CD2	LEU	1230	38.392	-2.212	-15.124	1.00	0.00	LX0	C

ATOM	2360	C	LEU	1230	38.479	0.847	-18.263	1.00	0.00	LX0	C
ATOM	2361	O	LEU	1230	37.981	1.189	-19.328	1.00	0.00	LX0	O
ATOM	2362	N	LEU	1231	39.487	1.476	-17.646	1.00	0.00	LX0	N
ATOM	2363	H	LEU	1231	39.889	1.080	-16.825	0.00	0.00	LX0	H
ATOM	2364	CA	LEU	1231	40.029	2.710	-18.204	1.00	0.00	LX0	C
ATOM	2365	CB	LEU	1231	40.704	3.491	-17.080	1.00	0.00	LX0	C
ATOM	2366	CG	LEU	1231	39.818	4.613	-16.555	1.00	0.00	LX0	C
ATOM	2367	CD1	LEU	1231	40.192	5.085	-15.157	1.00	0.00	LX0	C
ATOM	2368	CD2	LEU	1231	39.807	5.775	-17.533	1.00	0.00	LX0	C
ATOM	2369	C	LEU	1231	40.980	2.531	-19.371	1.00	0.00	LX0	C
ATOM	2370	O	LEU	1231	41.538	1.462	-19.601	1.00	0.00	LX0	O
ATOM	2371	N	ASP	1232	41.155	3.664	-20.068	1.00	0.00	LX0	N
ATOM	2372	H	ASP	1232	40.610	4.480	-19.865	0.00	0.00	LX0	H
ATOM	2373	CA	ASP	1232	42.165	3.797	-21.120	1.00	0.00	LX0	C
ATOM	2374	CB	ASP	1232	42.190	5.236	-21.688	1.00	0.00	LX0	C
ATOM	2375	CG	ASP	1232	40.848	5.970	-21.638	1.00	0.00	LX0	C
ATOM	2376	OD1	ASP	1232	40.171	6.051	-22.658	1.00	0.00	LX0	O
ATOM	2377	OD2	ASP	1232	40.492	6.490	-20.581	1.00	0.00	LX0	O
ATOM	2378	C	ASP	1232	43.551	3.504	-20.560	1.00	0.00	LX0	C
ATOM	2379	O	ASP	1232	43.805	3.757	-19.389	1.00	0.00	LX0	O
ATOM	2380	N	LYS	1233	44.464	3.000	-21.407	1.00	0.00	LX0	N
ATOM	2381	H	LYS	1233	44.255	2.825	-22.367	0.00	0.00	LX0	H
ATOM	2382	CA	LYS	1233	45.827	3.081	-20.878	1.00	0.00	LX0	C
ATOM	2383	CB	LYS	1233	46.698	1.834	-21.119	1.00	0.00	LX0	C
ATOM	2384	CG	LYS	1233	47.772	1.784	-20.019	1.00	0.00	LX0	C
ATOM	2385	CD	LYS	1233	49.000	0.883	-20.179	1.00	0.00	LX0	C
ATOM	2386	CE	LYS	1233	50.101	1.454	-21.080	1.00	0.00	LX0	C
ATOM	2387	NZ	LYS	1233	51.386	0.822	-20.749	1.00	0.00	LX0	N
ATOM	2388	HZ1	LYS	1233	52.118	0.961	-21.464	0.00	0.00	LX0	H
ATOM	2389	HZ2	LYS	1233	51.777	1.156	-19.842	0.00	0.00	LX0	H
ATOM	2390	HZ3	LYS	1233	51.303	-0.212	-20.610	0.00	0.00	LX0	H
ATOM	2391	C	LYS	1233	46.555	4.293	-21.418	1.00	0.00	LX0	C
ATOM	2392	O	LYS	1233	46.699	4.448	-22.624	1.00	0.00	LX0	O
ATOM	2393	N	PRO	1234	47.035	5.151	-20.489	1.00	0.00	LX0	N
ATOM	2394	CD	PRO	1234	46.791	5.131	-19.050	1.00	0.00	LX0	C
ATOM	2395	CA	PRO	1234	47.928	6.235	-20.910	1.00	0.00	LX0	C
ATOM	2396	CB	PRO	1234	48.319	6.885	-19.580	1.00	0.00	LX0	C
ATOM	2397	CG	PRO	1234	47.215	6.513	-18.591	1.00	0.00	LX0	C
ATOM	2398	C	PRO	1234	49.139	5.701	-21.660	1.00	0.00	LX0	C
ATOM	2399	O	PRO	1234	49.800	4.764	-21.212	1.00	0.00	LX0	O
ATOM	2400	N	ASP	1235	49.398	6.331	-22.817	1.00	0.00	LX0	N
ATOM	2401	H	ASP	1235	48.834	7.091	-23.130	0.00	0.00	LX0	H
ATOM	2402	CA	ASP	1235	50.591	5.924	-23.559	1.00	0.00	LX0	C
ATOM	2403	CB	ASP	1235	50.682	6.653	-24.909	1.00	0.00	LX0	C
ATOM	2404	CG	ASP	1235	51.919	6.203	-25.671	1.00	0.00	LX0	C
ATOM	2405	OD1	ASP	1235	52.702	7.056	-26.070	1.00	0.00	LX0	O
ATOM	2406	OD2	ASP	1235	52.147	5.000	-25.785	1.00	0.00	LX0	O
ATOM	2407	C	ASP	1235	51.850	6.103	-22.728	1.00	0.00	LX0	C
ATOM	2408	O	ASP	1235	51.920	6.950	-21.845	1.00	0.00	LX0	O
ATOM	2409	N	ASN	1236	52.791	5.182	-22.982	1.00	0.00	LX0	N
ATOM	2410	H	ASN	1236	52.656	4.693	-23.848	0.00	0.00	LX0	H
ATOM	2411	CA	ASN	1236	54.064	5.127	-22.260	1.00	0.00	LX0	C
ATOM	2412	CB	ASN	1236	54.995	6.266	-22.691	1.00	0.00	LX0	C
ATOM	2413	CG	ASN	1236	55.830	5.810	-23.872	1.00	0.00	LX0	C
ATOM	2414	OD1	ASN	1236	56.988	5.438	-23.739	1.00	0.00	LX0	O
ATOM	2415	ND2	ASN	1236	55.199	5.846	-25.048	1.00	0.00	LX0	N
ATOM	2416	HD21	ASN	1236	54.243	6.140	-25.177	0.00	0.00	LX0	H
ATOM	2417	HD22	ASN	1236	55.668	5.566	-25.880	0.00	0.00	LX0	H
ATOM	2418	C	ASN	1236	54.036	4.976	-20.744	1.00	0.00	LX0	C
ATOM	2419	O	ASN	1236	55.070	4.844	-20.104	1.00	0.00	LX0	O
ATOM	2420	N	CYS	1237	52.818	4.938	-20.173	1.00	0.00	LX0	N

ATOM	2421	H	CYS	1237	51.994	5.127	-20.708	0.00	0.00	LX0	H
ATOM	2422	CA	CYS	1237	52.727	4.676	-18.738	1.00	0.00	LX0	C
ATOM	2423	CB	CYS	1237	51.289	4.860	-18.252	1.00	0.00	LX0	C
ATOM	2424	SG	CYS	1237	51.055	4.491	-16.492	1.00	0.00	LX0	S
ATOM	2425	C	CYS	1237	53.219	3.284	-18.395	1.00	0.00	LX0	C
ATOM	2426	O	CYS	1237	52.716	2.290	-18.922	1.00	0.00	LX0	O
ATOM	2427	N	PRO	1238	54.237	3.254	-17.498	1.00	0.00	LX0	N
ATOM	2428	CD	PRO	1238	54.891	4.403	-16.878	1.00	0.00	LX0	C
ATOM	2429	CA	PRO	1238	54.816	1.981	-17.068	1.00	0.00	LX0	C
ATOM	2430	CB	PRO	1238	55.890	2.406	-16.062	1.00	0.00	LX0	C
ATOM	2431	CG	PRO	1238	56.232	3.851	-16.417	1.00	0.00	LX0	C
ATOM	2432	C	PRO	1238	53.781	1.060	-16.458	1.00	0.00	LX0	C
ATOM	2433	O	PRO	1238	53.163	1.353	-15.440	1.00	0.00	LX0	O
ATOM	2434	N	ASP	1239	53.624	-0.077	-17.139	1.00	0.00	LX0	N
ATOM	2435	H	ASP	1239	54.084	-0.213	-18.019	0.00	0.00	LX0	H
ATOM	2436	CA	ASP	1239	52.488	-0.949	-16.849	1.00	0.00	LX0	C
ATOM	2437	CB	ASP	1239	52.455	-2.105	-17.848	1.00	0.00	LX0	C
ATOM	2438	CG	ASP	1239	52.160	-1.527	-19.220	1.00	0.00	LX0	C
ATOM	2439	OD1	ASP	1239	53.027	-0.886	-19.812	1.00	0.00	LX0	O
ATOM	2440	OD2	ASP	1239	51.034	-1.644	-19.688	1.00	0.00	LX0	O
ATOM	2441	C	ASP	1239	52.305	-1.405	-15.415	1.00	0.00	LX0	C
ATOM	2442	O	ASP	1239	51.194	-1.656	-14.979	1.00	0.00	LX0	O
ATOM	2443	N	MET	1240	53.420	-1.417	-14.660	1.00	0.00	LX0	N
ATOM	2444	H	MET	1240	54.297	-1.266	-15.111	0.00	0.00	LX0	H
ATOM	2445	CA	MET	1240	53.313	-1.625	-13.209	1.00	0.00	LX0	C
ATOM	2446	CB	MET	1240	54.692	-1.424	-12.565	1.00	0.00	LX0	C
ATOM	2447	CG	MET	1240	54.820	-1.834	-11.091	1.00	0.00	LX0	C
ATOM	2448	SD	MET	1240	53.942	-0.757	-9.944	1.00	0.00	LX0	S
ATOM	2449	CE	MET	1240	54.359	-1.613	-8.415	1.00	0.00	LX0	C
ATOM	2450	C	MET	1240	52.236	-0.779	-12.526	1.00	0.00	LX0	C
ATOM	2451	O	MET	1240	51.387	-1.266	-11.788	1.00	0.00	LX0	O
ATOM	2452	N	LEU	1241	52.271	0.524	-12.854	1.00	0.00	LX0	N
ATOM	2453	H	LEU	1241	52.931	0.861	-13.526	0.00	0.00	LX0	H
ATOM	2454	CA	LEU	1241	51.234	1.396	-12.301	1.00	0.00	LX0	C
ATOM	2455	CB	LEU	1241	51.564	2.862	-12.609	1.00	0.00	LX0	C
ATOM	2456	CG	LEU	1241	50.720	3.891	-11.845	1.00	0.00	LX0	C
ATOM	2457	CD1	LEU	1241	50.847	3.748	-10.326	1.00	0.00	LX0	C
ATOM	2458	CD2	LEU	1241	51.020	5.318	-12.306	1.00	0.00	LX0	C
ATOM	2459	C	LEU	1241	49.828	1.019	-12.757	1.00	0.00	LX0	C
ATOM	2460	O	LEU	1241	48.871	1.018	-11.995	1.00	0.00	LX0	O
ATOM	2461	N	PHE	1242	49.754	0.650	-14.044	1.00	0.00	LX0	N
ATOM	2462	H	PHE	1242	50.591	0.587	-14.587	0.00	0.00	LX0	H
ATOM	2463	CA	PHE	1242	48.470	0.186	-14.574	1.00	0.00	LX0	C
ATOM	2464	CB	PHE	1242	48.514	0.210	-16.106	1.00	0.00	LX0	C
ATOM	2465	CG	PHE	1242	47.126	0.267	-16.708	1.00	0.00	LX0	C
ATOM	2466	CD1	PHE	1242	46.334	1.423	-16.527	1.00	0.00	LX0	C
ATOM	2467	CD2	PHE	1242	46.652	-0.829	-17.460	1.00	0.00	LX0	C
ATOM	2468	CE1	PHE	1242	45.054	1.488	-17.110	1.00	0.00	LX0	C
ATOM	2469	CE2	PHE	1242	45.373	-0.764	-18.048	1.00	0.00	LX0	C
ATOM	2470	CZ	PHE	1242	44.587	0.394	-17.868	1.00	0.00	LX0	C
ATOM	2471	C	PHE	1242	48.006	-1.168	-14.034	1.00	0.00	LX0	C
ATOM	2472	O	PHE	1242	46.838	-1.542	-14.103	1.00	0.00	LX0	O
ATOM	2473	N	GLU	1243	48.975	-1.892	-13.459	1.00	0.00	LX0	N
ATOM	2474	H	GLU	1243	49.931	-1.599	-13.494	0.00	0.00	LX0	H
ATOM	2475	CA	GLU	1243	48.608	-3.098	-12.734	1.00	0.00	LX0	C
ATOM	2476	CB	GLU	1243	49.807	-4.045	-12.605	1.00	0.00	LX0	C
ATOM	2477	CG	GLU	1243	49.424	-5.504	-12.320	1.00	0.00	LX0	C
ATOM	2478	CD	GLU	1243	48.500	-6.030	-13.407	1.00	0.00	LX0	C
ATOM	2479	OE1	GLU	1243	48.831	-5.955	-14.588	1.00	0.00	LX0	O
ATOM	2480	OE2	GLU	1243	47.401	-6.467	-13.093	1.00	0.00	LX0	O
ATOM	2481	C	GLU	1243	47.969	-2.758	-11.409	1.00	0.00	LX0	C

ATOM	2482	O	GLU	1243	46.857	-3.167	-11.108	1.00	0.00	LX0	O
ATOM	2483	N	LEU	1244	48.681	-1.890	-10.668	1.00	0.00	LX0	N
ATOM	2484	H	LEU	1244	49.604	-1.643	-10.969	0.00	0.00	LX0	H
ATOM	2485	CA	LEU	1244	48.109	-1.353	-9.429	1.00	0.00	LX0	C
ATOM	2486	CB	LEU	1244	49.042	-0.285	-8.852	1.00	0.00	LX0	C
ATOM	2487	CG	LEU	1244	48.666	0.163	-7.438	1.00	0.00	LX0	C
ATOM	2488	CD1	LEU	1244	48.786	-0.976	-6.423	1.00	0.00	LX0	C
ATOM	2489	CD2	LEU	1244	49.449	1.407	-7.020	1.00	0.00	LX0	C
ATOM	2490	C	LEU	1244	46.686	-0.819	-9.579	1.00	0.00	LX0	C
ATOM	2491	O	LEU	1244	45.776	-1.142	-8.824	1.00	0.00	LX0	O
ATOM	2492	N	MET	1245	46.523	-0.025	-10.651	1.00	0.00	LX0	N
ATOM	2493	H	MET	1245	47.336	0.248	-11.166	0.00	0.00	LX0	H
ATOM	2494	CA	MET	1245	45.182	0.452	-10.996	1.00	0.00	LX0	C
ATOM	2495	CB	MET	1245	45.194	1.228	-12.312	1.00	0.00	LX0	C
ATOM	2496	CG	MET	1245	46.072	2.475	-12.348	1.00	0.00	LX0	C
ATOM	2497	SD	MET	1245	45.927	3.334	-13.923	1.00	0.00	LX0	S
ATOM	2498	CE	MET	1245	47.549	4.108	-13.954	1.00	0.00	LX0	C
ATOM	2499	C	MET	1245	44.143	-0.655	-11.093	1.00	0.00	LX0	C
ATOM	2500	O	MET	1245	43.089	-0.621	-10.474	1.00	0.00	LX0	O
ATOM	2501	N	ARG	1246	44.500	-1.667	-11.895	1.00	0.00	LX0	N
ATOM	2502	H	ARG	1246	45.413	-1.698	-12.306	0.00	0.00	LX0	H
ATOM	2503	CA	ARG	1246	43.564	-2.779	-12.047	1.00	0.00	LX0	C
ATOM	2504	CB	ARG	1246	43.943	-3.608	-13.263	1.00	0.00	LX0	C
ATOM	2505	CG	ARG	1246	43.402	-3.004	-14.562	1.00	0.00	LX0	C
ATOM	2506	CD	ARG	1246	44.261	-3.294	-15.799	1.00	0.00	LX0	C
ATOM	2507	NE	ARG	1246	44.636	-4.704	-15.914	1.00	0.00	LX0	N
ATOM	2508	HE	ARG	1246	43.944	-5.365	-16.201	0.00	0.00	LX0	H
ATOM	2509	CZ	ARG	1246	45.873	-5.090	-15.523	1.00	0.00	LX0	C
ATOM	2510	NH1	ARG	1246	46.765	-4.202	-15.105	1.00	0.00	LX0	N
ATOM	2511	HH11	ARG	1246	47.664	-4.560	-14.814	0.00	0.00	LX0	H
ATOM	2512	HH12	ARG	1246	46.592	-3.216	-15.034	0.00	0.00	LX0	H
ATOM	2513	NH2	ARG	1246	46.209	-6.372	-15.516	1.00	0.00	LX0	N
ATOM	2514	HH21	ARG	1246	47.084	-6.602	-15.048	0.00	0.00	LX0	H
ATOM	2515	HH22	ARG	1246	45.656	-7.107	-15.893	0.00	0.00	LX0	H
ATOM	2516	C	ARG	1246	43.358	-3.645	-10.816	1.00	0.00	LX0	C
ATOM	2517	O	ARG	1246	42.308	-4.247	-10.638	1.00	0.00	LX0	O
ATOM	2518	N	MET	1247	44.366	-3.637	-9.930	1.00	0.00	LX0	N
ATOM	2519	H	MET	1247	45.225	-3.175	-10.153	0.00	0.00	LX0	H
ATOM	2520	CA	MET	1247	44.144	-4.239	-8.613	1.00	0.00	LX0	C
ATOM	2521	CB	MET	1247	45.403	-4.153	-7.749	1.00	0.00	LX0	C
ATOM	2522	CG	MET	1247	46.575	-4.983	-8.276	1.00	0.00	LX0	C
ATOM	2523	SD	MET	1247	48.099	-4.687	-7.360	1.00	0.00	LX0	S
ATOM	2524	CE	MET	1247	47.499	-5.058	-5.702	1.00	0.00	LX0	C
ATOM	2525	C	MET	1247	42.977	-3.580	-7.903	1.00	0.00	LX0	C
ATOM	2526	O	MET	1247	42.029	-4.222	-7.473	1.00	0.00	LX0	O
ATOM	2527	N	CYS	1248	43.059	-2.241	-7.880	1.00	0.00	LX0	N
ATOM	2528	H	CYS	1248	43.884	-1.783	-8.222	0.00	0.00	LX0	H
ATOM	2529	CA	CYS	1248	41.916	-1.469	-7.387	1.00	0.00	LX0	C
ATOM	2530	CB	CYS	1248	42.249	0.022	-7.411	1.00	0.00	LX0	C
ATOM	2531	SG	CYS	1248	43.803	0.416	-6.569	1.00	0.00	LX0	S
ATOM	2532	C	CYS	1248	40.615	-1.718	-8.141	1.00	0.00	LX0	C
ATOM	2533	O	CYS	1248	39.522	-1.736	-7.586	1.00	0.00	LX0	O
ATOM	2534	N	TRP	1249	40.785	-1.917	-9.453	1.00	0.00	LX0	N
ATOM	2535	H	TRP	1249	41.708	-1.910	-9.838	0.00	0.00	LX0	H
ATOM	2536	CA	TRP	1249	39.619	-2.070	-10.317	1.00	0.00	LX0	C
ATOM	2537	CB	TRP	1249	39.906	-1.529	-11.720	1.00	0.00	LX0	C
ATOM	2538	CG	TRP	1249	40.262	-0.060	-11.695	1.00	0.00	LX0	C
ATOM	2539	CD2	TRP	1249	41.054	0.660	-12.659	1.00	0.00	LX0	C
ATOM	2540	CE2	TRP	1249	41.128	2.024	-12.218	1.00	0.00	LX0	C
ATOM	2541	CE3	TRP	1249	41.699	0.265	-13.851	1.00	0.00	LX0	C
ATOM	2542	CD1	TRP	1249	39.899	0.898	-10.735	1.00	0.00	LX0	C

ATOM	2543	NE1	TRP	1249	40.401	2.123	-11.033	1.00	0.00	LX0	N
ATOM	2544	HE1	TRP	1249	40.244	2.930	-10.492	0.00	0.00	LX0	H
ATOM	2545	CZ2	TRP	1249	41.856	2.962	-12.978	1.00	0.00	LX0	C
ATOM	2546	CZ3	TRP	1249	42.420	1.215	-14.603	1.00	0.00	LX0	C
ATOM	2547	CH2	TRP	1249	42.501	2.556	-14.166	1.00	0.00	LX0	C
ATOM	2548	C	TRP	1249	39.025	-3.465	-10.408	1.00	0.00	LX0	C
ATOM	2549	O	TRP	1249	38.823	-4.022	-11.480	1.00	0.00	LX0	O
ATOM	2550	N	GLN	1250	38.708	-4.007	-9.228	1.00	0.00	LX0	N
ATOM	2551	H	GLN	1250	38.832	-3.473	-8.390	0.00	0.00	LX0	H
ATOM	2552	CA	GLN	1250	37.934	-5.242	-9.288	1.00	0.00	LX0	C
ATOM	2553	CB	GLN	1250	38.153	-6.073	-8.024	1.00	0.00	LX0	C
ATOM	2554	CG	GLN	1250	39.624	-6.404	-7.766	1.00	0.00	LX0	C
ATOM	2555	CD	GLN	1250	40.178	-7.197	-8.929	1.00	0.00	LX0	C
ATOM	2556	OE1	GLN	1250	39.697	-8.267	-9.276	1.00	0.00	LX0	O
ATOM	2557	NE2	GLN	1250	41.199	-6.601	-9.547	1.00	0.00	LX0	N
ATOM	2558	HE21	GLN	1250	41.531	-5.700	-9.256	0.00	0.00	LX0	H
ATOM	2559	HE22	GLN	1250	41.630	-7.034	-10.333	0.00	0.00	LX0	H
ATOM	2560	C	GLN	1250	36.464	-4.943	-9.469	1.00	0.00	LX0	C
ATOM	2561	O	GLN	1250	35.949	-3.987	-8.903	1.00	0.00	LX0	O
ATOM	2562	N	TYR	1251	35.783	-5.789	-10.262	1.00	0.00	LX0	N
ATOM	2563	H	TYR	1251	36.268	-6.515	-10.748	0.00	0.00	LX0	H
ATOM	2564	CA	TYR	1251	34.352	-5.507	-10.413	1.00	0.00	LX0	C
ATOM	2565	CB	TYR	1251	33.694	-6.408	-11.474	1.00	0.00	LX0	C
ATOM	2566	CG	TYR	1251	32.247	-5.999	-11.678	1.00	0.00	LX0	C
ATOM	2567	CD1	TYR	1251	31.964	-4.796	-12.354	1.00	0.00	LX0	C
ATOM	2568	CE1	TYR	1251	30.628	-4.385	-12.488	1.00	0.00	LX0	C
ATOM	2569	CD2	TYR	1251	31.220	-6.819	-11.164	1.00	0.00	LX0	C
ATOM	2570	CE2	TYR	1251	29.883	-6.405	-11.295	1.00	0.00	LX0	C
ATOM	2571	CZ	TYR	1251	29.605	-5.185	-11.945	1.00	0.00	LX0	C
ATOM	2572	OH	TYR	1251	28.297	-4.752	-12.048	1.00	0.00	LX0	O
ATOM	2573	HH	TYR	1251	27.990	-4.903	-12.948	0.00	0.00	LX0	H
ATOM	2574	C	TYR	1251	33.594	-5.574	-9.095	1.00	0.00	LX0	C
ATOM	2575	O	TYR	1251	32.959	-4.625	-8.657	1.00	0.00	LX0	O
ATOM	2576	N	ASN	1252	33.723	-6.754	-8.469	1.00	0.00	LX0	N
ATOM	2577	H	ASN	1252	34.358	-7.426	-8.844	0.00	0.00	LX0	H
ATOM	2578	CA	ASN	1252	33.073	-6.964	-7.173	1.00	0.00	LX0	C
ATOM	2579	CB	ASN	1252	33.342	-8.419	-6.735	1.00	0.00	LX0	C
ATOM	2580	CG	ASN	1252	32.956	-8.700	-5.288	1.00	0.00	LX0	C
ATOM	2581	OD1	ASN	1252	32.163	-8.006	-4.669	1.00	0.00	LX0	O
ATOM	2582	ND2	ASN	1252	33.621	-9.720	-4.747	1.00	0.00	LX0	N
ATOM	2583	HD21	ASN	1252	34.213	-10.324	-5.277	0.00	0.00	LX0	H
ATOM	2584	HD22	ASN	1252	33.570	-9.872	-3.757	0.00	0.00	LX0	H
ATOM	2585	C	ASN	1252	33.555	-5.950	-6.144	1.00	0.00	LX0	C
ATOM	2586	O	ASN	1252	34.751	-5.835	-5.902	1.00	0.00	LX0	O
ATOM	2587	N	PRO	1253	32.585	-5.222	-5.536	1.00	0.00	LX0	N
ATOM	2588	CD	PRO	1253	31.155	-5.211	-5.833	1.00	0.00	LX0	C
ATOM	2589	CA	PRO	1253	32.939	-4.339	-4.422	1.00	0.00	LX0	C
ATOM	2590	CB	PRO	1253	31.576	-3.811	-3.945	1.00	0.00	LX0	C
ATOM	2591	CG	PRO	1253	30.518	-4.749	-4.530	1.00	0.00	LX0	C
ATOM	2592	C	PRO	1253	33.778	-5.035	-3.360	1.00	0.00	LX0	C
ATOM	2593	O	PRO	1253	34.871	-4.600	-3.026	1.00	0.00	LX0	O
ATOM	2594	N	LYS	1254	33.262	-6.172	-2.873	1.00	0.00	LX0	N
ATOM	2595	H	LYS	1254	32.441	-6.568	-3.290	0.00	0.00	LX0	H
ATOM	2596	CA	LYS	1254	34.092	-6.925	-1.934	1.00	0.00	LX0	C
ATOM	2597	CB	LYS	1254	33.251	-7.708	-0.924	1.00	0.00	LX0	C
ATOM	2598	CG	LYS	1254	32.552	-6.758	0.043	1.00	0.00	LX0	C
ATOM	2599	CD	LYS	1254	32.402	-7.307	1.462	1.00	0.00	LX0	C
ATOM	2600	CE	LYS	1254	31.918	-6.179	2.370	1.00	0.00	LX0	C
ATOM	2601	NZ	LYS	1254	31.918	-6.571	3.783	1.00	0.00	LX0	N
ATOM	2602	HZ1	LYS	1254	31.829	-5.730	4.385	0.00	0.00	LX0	H
ATOM	2603	HZ2	LYS	1254	31.121	-7.199	4.029	0.00	0.00	LX0	H

ATOM	2604	HZ3	LYS	1254	32.809	-6.979	4.121	0.00	0.00	LX0	H
ATOM	2605	C	LYS	1254	35.128	-7.827	-2.577	1.00	0.00	LX0	C
ATOM	2606	O	LYS	1254	35.120	-9.042	-2.427	1.00	0.00	LX0	O
ATOM	2607	N	MET	1255	36.034	-7.154	-3.294	1.00	0.00	LX0	N
ATOM	2608	H	MET	1255	35.851	-6.199	-3.524	0.00	0.00	LX0	H
ATOM	2609	CA	MET	1255	37.261	-7.791	-3.765	1.00	0.00	LX0	C
ATOM	2610	CB	MET	1255	37.020	-8.530	-5.083	1.00	0.00	LX0	C
ATOM	2611	CG	MET	1255	37.180	-10.048	-4.972	1.00	0.00	LX0	C
ATOM	2612	SD	MET	1255	38.856	-10.582	-4.586	1.00	0.00	LX0	S
ATOM	2613	CE	MET	1255	39.653	-10.075	-6.120	1.00	0.00	LX0	C
ATOM	2614	C	MET	1255	38.387	-6.788	-3.947	1.00	0.00	LX0	C
ATOM	2615	O	MET	1255	39.438	-7.090	-4.493	1.00	0.00	LX0	O
ATOM	2616	N	ARG	1256	38.105	-5.551	-3.510	1.00	0.00	LX0	N
ATOM	2617	H	ARG	1256	37.325	-5.343	-2.922	0.00	0.00	LX0	H
ATOM	2618	CA	ARG	1256	39.105	-4.522	-3.768	1.00	0.00	LX0	C
ATOM	2619	CB	ARG	1256	38.411	-3.205	-4.134	1.00	0.00	LX0	C
ATOM	2620	CG	ARG	1256	37.394	-3.431	-5.254	1.00	0.00	LX0	C
ATOM	2621	CD	ARG	1256	36.682	-2.169	-5.728	1.00	0.00	LX0	C
ATOM	2622	NE	ARG	1256	35.567	-2.535	-6.596	1.00	0.00	LX0	N
ATOM	2623	HE	ARG	1256	35.683	-3.331	-7.192	0.00	0.00	LX0	H
ATOM	2624	CZ	ARG	1256	34.408	-1.854	-6.602	1.00	0.00	LX0	C
ATOM	2625	NH1	ARG	1256	34.266	-0.750	-5.884	1.00	0.00	LX0	N
ATOM	2626	HH11	ARG	1256	33.366	-0.295	-5.869	0.00	0.00	LX0	H
ATOM	2627	HH12	ARG	1256	35.029	-0.358	-5.361	0.00	0.00	LX0	H
ATOM	2628	NH2	ARG	1256	33.386	-2.296	-7.319	1.00	0.00	LX0	N
ATOM	2629	HH21	ARG	1256	32.493	-1.817	-7.283	0.00	0.00	LX0	H
ATOM	2630	HH22	ARG	1256	33.434	-3.115	-7.899	0.00	0.00	LX0	H
ATOM	2631	C	ARG	1256	40.043	-4.380	-2.586	1.00	0.00	LX0	C
ATOM	2632	O	ARG	1256	39.596	-4.235	-1.454	1.00	0.00	LX0	O
ATOM	2633	N	PRO	1257	41.359	-4.465	-2.889	1.00	0.00	LX0	N
ATOM	2634	CD	PRO	1257	41.938	-4.600	-4.221	1.00	0.00	LX0	C
ATOM	2635	CA	PRO	1257	42.371	-4.423	-1.826	1.00	0.00	LX0	C
ATOM	2636	CB	PRO	1257	43.668	-4.686	-2.604	1.00	0.00	LX0	C
ATOM	2637	CG	PRO	1257	43.397	-4.214	-4.030	1.00	0.00	LX0	C
ATOM	2638	C	PRO	1257	42.368	-3.095	-1.092	1.00	0.00	LX0	C
ATOM	2639	O	PRO	1257	42.016	-2.058	-1.647	1.00	0.00	LX0	O
ATOM	2640	N	SER	1258	42.759	-3.151	0.186	1.00	0.00	LX0	N
ATOM	2641	H	SER	1258	43.107	-4.009	0.582	0.00	0.00	LX0	H
ATOM	2642	CA	SER	1258	42.757	-1.872	0.884	1.00	0.00	LX0	C
ATOM	2643	CB	SER	1258	42.764	-2.059	2.406	1.00	0.00	LX0	C
ATOM	2644	OG	SER	1258	44.076	-2.415	2.863	1.00	0.00	LX0	O
ATOM	2645	HG	SER	1258	44.080	-3.378	2.837	0.00	0.00	LX0	H
ATOM	2646	C	SER	1258	43.894	-0.961	0.465	1.00	0.00	LX0	C
ATOM	2647	O	SER	1258	44.903	-1.373	-0.098	1.00	0.00	LX0	O
ATOM	2648	N	PHE	1259	43.722	0.315	0.842	1.00	0.00	LX0	N
ATOM	2649	H	PHE	1259	42.834	0.596	1.202	0.00	0.00	LX0	H
ATOM	2650	CA	PHE	1259	44.831	1.255	0.667	1.00	0.00	LX0	C
ATOM	2651	CB	PHE	1259	44.437	2.649	1.160	1.00	0.00	LX0	C
ATOM	2652	CG	PHE	1259	43.114	3.090	0.574	1.00	0.00	LX0	C
ATOM	2653	CD1	PHE	1259	43.043	3.490	-0.779	1.00	0.00	LX0	C
ATOM	2654	CD2	PHE	1259	41.969	3.098	1.401	1.00	0.00	LX0	C
ATOM	2655	CE1	PHE	1259	41.807	3.908	-1.310	1.00	0.00	LX0	C
ATOM	2656	CE2	PHE	1259	40.733	3.515	0.870	1.00	0.00	LX0	C
ATOM	2657	CZ	PHE	1259	40.667	3.919	-0.479	1.00	0.00	LX0	C
ATOM	2658	C	PHE	1259	46.133	0.810	1.327	1.00	0.00	LX0	C
ATOM	2659	O	PHE	1259	47.227	1.005	0.813	1.00	0.00	LX0	O
ATOM	2660	N	LEU	1260	45.961	0.159	2.491	1.00	0.00	LX0	N
ATOM	2661	H	LEU	1260	45.043	-0.075	2.810	0.00	0.00	LX0	H
ATOM	2662	CA	LEU	1260	47.138	-0.372	3.182	1.00	0.00	LX0	C
ATOM	2663	CB	LEU	1260	46.746	-0.890	4.565	1.00	0.00	LX0	C
ATOM	2664	CG	LEU	1260	46.294	0.224	5.511	1.00	0.00	LX0	C

ATOM	2665	CD1	LEU	1260	45.491	-0.321	6.691	1.00	0.00	LX0	C
ATOM	2666	CD2	LEU	1260	47.467	1.094	5.966	1.00	0.00	LX0	C
ATOM	2667	C	LEU	1260	47.860	-1.453	2.398	1.00	0.00	LX0	C
ATOM	2668	O	LEU	1260	49.084	-1.498	2.316	1.00	0.00	LX0	O
ATOM	2669	N	GLU	1261	47.041	-2.317	1.785	1.00	0.00	LX0	N
ATOM	2670	H	GLU	1261	46.045	-2.250	1.892	0.00	0.00	LX0	H
ATOM	2671	CA	GLU	1261	47.626	-3.336	0.913	1.00	0.00	LX0	C
ATOM	2672	CB	GLU	1261	46.547	-4.322	0.491	1.00	0.00	LX0	C
ATOM	2673	CG	GLU	1261	46.054	-5.129	1.694	1.00	0.00	LX0	C
ATOM	2674	CD	GLU	1261	44.579	-5.427	1.540	1.00	0.00	LX0	C
ATOM	2675	OE1	GLU	1261	43.835	-5.187	2.484	1.00	0.00	LX0	O
ATOM	2676	OE2	GLU	1261	44.156	-5.842	0.468	1.00	0.00	LX0	O
ATOM	2677	C	GLU	1261	48.352	-2.757	-0.288	1.00	0.00	LX0	C
ATOM	2678	O	GLU	1261	49.446	-3.180	-0.650	1.00	0.00	LX0	O
ATOM	2679	N	ILE	1262	47.724	-1.710	-0.846	1.00	0.00	LX0	N
ATOM	2680	H	ILE	1262	46.795	-1.495	-0.536	0.00	0.00	LX0	H
ATOM	2681	CA	ILE	1262	48.375	-0.933	-1.904	1.00	0.00	LX0	C
ATOM	2682	CB	ILE	1262	47.429	0.191	-2.362	1.00	0.00	LX0	C
ATOM	2683	CG2	ILE	1262	48.084	1.200	-3.311	1.00	0.00	LX0	C
ATOM	2684	CG1	ILE	1262	46.167	-0.432	-2.969	1.00	0.00	LX0	C
ATOM	2685	CD1	ILE	1262	45.014	0.558	-3.141	1.00	0.00	LX0	C
ATOM	2686	C	ILE	1262	49.758	-0.419	-1.510	1.00	0.00	LX0	C
ATOM	2687	O	ILE	1262	50.757	-0.672	-2.171	1.00	0.00	LX0	O
ATOM	2688	N	ILE	1263	49.805	0.266	-0.353	1.00	0.00	LX0	N
ATOM	2689	H	ILE	1263	48.956	0.468	0.137	0.00	0.00	LX0	H
ATOM	2690	CA	ILE	1263	51.126	0.715	0.108	1.00	0.00	LX0	C
ATOM	2691	CB	ILE	1263	51.015	1.564	1.385	1.00	0.00	LX0	C
ATOM	2692	CG2	ILE	1263	52.378	2.126	1.812	1.00	0.00	LX0	C
ATOM	2693	CG1	ILE	1263	49.998	2.695	1.199	1.00	0.00	LX0	C
ATOM	2694	CD1	ILE	1263	49.666	3.434	2.496	1.00	0.00	LX0	C
ATOM	2695	C	ILE	1263	52.118	-0.435	0.277	1.00	0.00	LX0	C
ATOM	2696	O	ILE	1263	53.269	-0.388	-0.142	1.00	0.00	LX0	O
ATOM	2697	N	SER	1264	51.585	-1.520	0.848	1.00	0.00	LX0	N
ATOM	2698	H	SER	1264	50.638	-1.490	1.165	0.00	0.00	LX0	H
ATOM	2699	CA	SER	1264	52.397	-2.731	0.962	1.00	0.00	LX0	C
ATOM	2700	CB	SER	1264	51.629	-3.792	1.749	1.00	0.00	LX0	C
ATOM	2701	OG	SER	1264	51.182	-3.224	2.988	1.00	0.00	LX0	O
ATOM	2702	HG	SER	1264	50.384	-2.735	2.793	0.00	0.00	LX0	H
ATOM	2703	C	SER	1264	52.941	-3.301	-0.347	1.00	0.00	LX0	C
ATOM	2704	O	SER	1264	54.009	-3.907	-0.388	1.00	0.00	LX0	O
ATOM	2705	N	SER	1265	52.182	-3.071	-1.423	1.00	0.00	LX0	N
ATOM	2706	H	SER	1265	51.336	-2.534	-1.396	0.00	0.00	LX0	H
ATOM	2707	CA	SER	1265	52.687	-3.538	-2.710	1.00	0.00	LX0	C
ATOM	2708	CB	SER	1265	51.526	-4.039	-3.577	1.00	0.00	LX0	C
ATOM	2709	OG	SER	1265	50.309	-3.361	-3.238	1.00	0.00	LX0	O
ATOM	2710	HG	SER	1265	50.050	-3.654	-2.369	0.00	0.00	LX0	H
ATOM	2711	C	SER	1265	53.572	-2.551	-3.458	1.00	0.00	LX0	C
ATOM	2712	O	SER	1265	54.242	-2.906	-4.417	1.00	0.00	LX0	O
ATOM	2713	N	ILE	1266	53.553	-1.295	-2.979	1.00	0.00	LX0	N
ATOM	2714	H	ILE	1266	52.930	-1.035	-2.240	0.00	0.00	LX0	H
ATOM	2715	CA	ILE	1266	54.338	-0.298	-3.707	1.00	0.00	LX0	C
ATOM	2716	CB	ILE	1266	53.428	0.818	-4.233	1.00	0.00	LX0	C
ATOM	2717	CG2	ILE	1266	52.387	0.242	-5.192	1.00	0.00	LX0	C
ATOM	2718	CG1	ILE	1266	52.816	1.648	-3.099	1.00	0.00	LX0	C
ATOM	2719	CD1	ILE	1266	51.909	2.774	-3.589	1.00	0.00	LX0	C
ATOM	2720	C	ILE	1266	55.536	0.293	-2.980	1.00	0.00	LX0	C
ATOM	2721	O	ILE	1266	56.330	1.032	-3.550	1.00	0.00	LX0	O
ATOM	2722	N	LYS	1267	55.642	-0.056	-1.683	1.00	0.00	LX0	N
ATOM	2723	H	LYS	1267	54.907	-0.603	-1.286	0.00	0.00	LX0	H
ATOM	2724	CA	LYS	1267	56.665	0.552	-0.821	1.00	0.00	LX0	C
ATOM	2725	CB	LYS	1267	56.599	-0.081	0.576	1.00	0.00	LX0	C

ATOM	2726	CG	LYS	1267	57.102	-1.521	0.576	1.00	0.00	LX0	C
ATOM	2727	CD	LYS	1267	56.361	-2.480	1.502	1.00	0.00	LX0	C
ATOM	2728	CE	LYS	1267	56.806	-3.924	1.246	1.00	0.00	LX0	C
ATOM	2729	NZ	LYS	1267	56.640	-4.251	-0.178	1.00	0.00	LX0	N
ATOM	2730	HZ1	LYS	1267	57.095	-5.144	-0.435	0.00	0.00	LX0	H
ATOM	2731	HZ2	LYS	1267	55.653	-4.222	-0.500	0.00	0.00	LX0	H
ATOM	2732	HZ3	LYS	1267	57.180	-3.574	-0.766	0.00	0.00	LX0	H
ATOM	2733	C	LYS	1267	58.095	0.622	-1.367	1.00	0.00	LX0	C
ATOM	2734	O	LYS	1267	58.880	1.499	-1.030	1.00	0.00	LX0	O
ATOM	2735	N	GLU	1268	58.369	-0.339	-2.255	1.00	0.00	LX0	N
ATOM	2736	H	GLU	1268	57.675	-1.039	-2.427	0.00	0.00	LX0	H
ATOM	2737	CA	GLU	1268	59.639	-0.472	-2.956	1.00	0.00	LX0	C
ATOM	2738	CB	GLU	1268	59.490	-1.617	-3.966	1.00	0.00	LX0	C
ATOM	2739	CG	GLU	1268	59.552	-3.054	-3.401	1.00	0.00	LX0	C
ATOM	2740	CD	GLU	1268	58.519	-3.375	-2.320	1.00	0.00	LX0	C
ATOM	2741	OE1	GLU	1268	57.344	-3.026	-2.434	1.00	0.00	LX0	O
ATOM	2742	OE2	GLU	1268	58.881	-3.998	-1.326	1.00	0.00	LX0	O
ATOM	2743	C	GLU	1268	60.191	0.798	-3.604	1.00	0.00	LX0	C
ATOM	2744	O	GLU	1268	61.379	1.078	-3.513	1.00	0.00	LX0	O
ATOM	2745	N	GLU	1269	59.295	1.565	-4.254	1.00	0.00	LX0	N
ATOM	2746	H	GLU	1269	58.318	1.341	-4.248	0.00	0.00	LX0	H
ATOM	2747	CA	GLU	1269	59.820	2.794	-4.864	1.00	0.00	LX0	C
ATOM	2748	CB	GLU	1269	59.392	2.963	-6.327	1.00	0.00	LX0	C
ATOM	2749	CG	GLU	1269	60.319	2.313	-7.362	1.00	0.00	LX0	C
ATOM	2750	CD	GLU	1269	60.037	2.888	-8.745	1.00	0.00	LX0	C
ATOM	2751	OE1	GLU	1269	58.921	2.744	-9.237	1.00	0.00	LX0	O
ATOM	2752	OE2	GLU	1269	60.928	3.507	-9.330	1.00	0.00	LX0	O
ATOM	2753	C	GLU	1269	59.504	4.081	-4.117	1.00	0.00	LX0	C
ATOM	2754	O	GLU	1269	59.555	5.175	-4.667	1.00	0.00	LX0	O
ATOM	2755	N	MET	1270	59.140	3.923	-2.837	1.00	0.00	LX0	N
ATOM	2756	H	MET	1270	59.267	3.051	-2.362	0.00	0.00	LX0	H
ATOM	2757	CA	MET	1270	58.735	5.143	-2.141	1.00	0.00	LX0	C
ATOM	2758	CB	MET	1270	57.869	4.814	-0.924	1.00	0.00	LX0	C
ATOM	2759	CG	MET	1270	56.643	3.970	-1.268	1.00	0.00	LX0	C
ATOM	2760	SD	MET	1270	55.418	4.784	-2.292	1.00	0.00	LX0	S
ATOM	2761	CE	MET	1270	54.672	5.758	-0.984	1.00	0.00	LX0	C
ATOM	2762	C	MET	1270	59.906	6.004	-1.711	1.00	0.00	LX0	C
ATOM	2763	O	MET	1270	60.962	5.512	-1.328	1.00	0.00	LX0	O
ATOM	2764	N	GLU	1271	59.661	7.324	-1.746	1.00	0.00	LX0	N
ATOM	2765	H	GLU	1271	58.752	7.619	-2.041	0.00	0.00	LX0	H
ATOM	2766	CA	GLU	1271	60.586	8.249	-1.085	1.00	0.00	LX0	C
ATOM	2767	CB	GLU	1271	60.103	9.695	-1.247	1.00	0.00	LX0	C
ATOM	2768	CG	GLU	1271	60.058	10.193	-2.700	1.00	0.00	LX0	C
ATOM	2769	CD	GLU	1271	59.233	11.472	-2.834	1.00	0.00	LX0	C
ATOM	2770	OE1	GLU	1271	58.445	11.804	-1.956	1.00	0.00	LX0	O
ATOM	2771	OE2	GLU	1271	59.310	12.131	-3.863	1.00	0.00	LX0	O
ATOM	2772	C	GLU	1271	60.680	7.898	0.393	1.00	0.00	LX0	C
ATOM	2773	O	GLU	1271	59.698	7.947	1.123	1.00	0.00	LX0	O
ATOM	2774	N	PRO	1272	61.902	7.492	0.818	1.00	0.00	LX0	N
ATOM	2775	CD	PRO	1272	63.159	7.604	0.084	1.00	0.00	LX0	C
ATOM	2776	CA	PRO	1272	62.061	6.790	2.100	1.00	0.00	LX0	C
ATOM	2777	CB	PRO	1272	63.576	6.848	2.315	1.00	0.00	LX0	C
ATOM	2778	CG	PRO	1272	64.147	6.775	0.899	1.00	0.00	LX0	C
ATOM	2779	C	PRO	1272	61.227	7.278	3.276	1.00	0.00	LX0	C
ATOM	2780	O	PRO	1272	60.487	6.514	3.893	1.00	0.00	LX0	O
ATOM	2781	N	GLY	1273	61.377	8.591	3.533	1.00	0.00	LX0	N
ATOM	2782	H	GLY	1273	61.925	9.139	2.905	0.00	0.00	LX0	H
ATOM	2783	CA	GLY	1273	60.780	9.207	4.722	1.00	0.00	LX0	C
ATOM	2784	C	GLY	1273	59.273	9.072	4.876	1.00	0.00	LX0	C
ATOM	2785	O	GLY	1273	58.728	9.164	5.970	1.00	0.00	LX0	O
ATOM	2786	N	PHE	1274	58.616	8.822	3.723	1.00	0.00	LX0	N

ATOM	2787	H	PHE	1274	59.118	8.769	2.859	0.00	0.00	LX0 H
ATOM	2788	CA	PHE	1274	57.174	8.559	3.719	1.00	0.00	LX0 C
ATOM	2789	CB	PHE	1274	56.769	7.979	2.351	1.00	0.00	LX0 C
ATOM	2790	CG	PHE	1274	55.315	7.557	2.292	1.00	0.00	LX0 C
ATOM	2791	CD1	PHE	1274	54.330	8.494	1.913	1.00	0.00	LX0 C
ATOM	2792	CD2	PHE	1274	54.970	6.226	2.619	1.00	0.00	LX0 C
ATOM	2793	CE1	PHE	1274	52.980	8.097	1.881	1.00	0.00	LX0 C
ATOM	2794	CE2	PHE	1274	53.620	5.829	2.590	1.00	0.00	LX0 C
ATOM	2795	CZ	PHE	1274	52.639	6.772	2.226	1.00	0.00	LX0 C
ATOM	2796	C	PHE	1274	56.739	7.639	4.846	1.00	0.00	LX0 C
ATOM	2797	O	PHE	1274	55.830	7.923	5.615	1.00	0.00	LX0 O
ATOM	2798	N	ARG	1275	57.445	6.498	4.908	1.00	0.00	LX0 N
ATOM	2799	H	ARG	1275	58.292	6.394	4.385	0.00	0.00	LX0 H
ATOM	2800	CA	ARG	1275	56.919	5.532	5.863	1.00	0.00	LX0 C
ATOM	2801	CB	ARG	1275	57.401	4.108	5.624	1.00	0.00	LX0 C
ATOM	2802	CG	ARG	1275	57.534	3.613	4.179	1.00	0.00	LX0 C
ATOM	2803	CD	ARG	1275	58.996	3.314	3.809	1.00	0.00	LX0 C
ATOM	2804	NE	ARG	1275	59.652	2.541	4.869	1.00	0.00	LX0 N
ATOM	2805	HE	ARG	1275	59.342	1.611	5.072	0.00	0.00	LX0 H
ATOM	2806	CZ	ARG	1275	60.471	3.174	5.737	1.00	0.00	LX0 C
ATOM	2807	NH1	ARG	1275	60.888	4.406	5.497	1.00	0.00	LX0 N
ATOM	2808	HH11	ARG	1275	61.366	4.924	6.229	0.00	0.00	LX0 H
ATOM	2809	HH12	ARG	1275	60.718	4.912	4.649	0.00	0.00	LX0 H
ATOM	2810	NH2	ARG	1275	60.807	2.587	6.875	1.00	0.00	LX0 N
ATOM	2811	HH21	ARG	1275	61.370	3.108	7.533	0.00	0.00	LX0 H
ATOM	2812	HH22	ARG	1275	60.472	1.670	7.111	0.00	0.00	LX0 H
ATOM	2813	C	ARG	1275	57.098	5.914	7.319	1.00	0.00	LX0 C
ATOM	2814	O	ARG	1275	56.210	5.685	8.128	1.00	0.00	LX0 O
ATOM	2815	N	GLU	1276	58.240	6.554	7.625	1.00	0.00	LX0 N
ATOM	2816	H	GLU	1276	58.985	6.705	6.971	0.00	0.00	LX0 H
ATOM	2817	CA	GLU	1276	58.413	7.027	9.003	1.00	0.00	LX0 C
ATOM	2818	CB	GLU	1276	59.765	7.725	9.190	1.00	0.00	LX0 C
ATOM	2819	CG	GLU	1276	60.987	6.802	9.329	1.00	0.00	LX0 C
ATOM	2820	CD	GLU	1276	61.283	6.036	8.051	1.00	0.00	LX0 C
ATOM	2821	OE1	GLU	1276	61.188	6.606	6.967	1.00	0.00	LX0 O
ATOM	2822	OE2	GLU	1276	61.601	4.851	8.125	1.00	0.00	LX0 O
ATOM	2823	C	GLU	1276	57.300	7.937	9.510	1.00	0.00	LX0 C
ATOM	2824	O	GLU	1276	56.950	7.943	10.683	1.00	0.00	LX0 O
ATOM	2825	N	VAL	1277	56.738	8.700	8.561	1.00	0.00	LX0 N
ATOM	2826	H	VAL	1277	57.068	8.667	7.615	0.00	0.00	LX0 H
ATOM	2827	CA	VAL	1277	55.623	9.551	8.976	1.00	0.00	LX0 C
ATOM	2828	CB	VAL	1277	55.845	10.987	8.480	1.00	0.00	LX0 C
ATOM	2829	CG1	VAL	1277	57.036	11.627	9.195	1.00	0.00	LX0 C
ATOM	2830	CG2	VAL	1277	55.992	11.062	6.957	1.00	0.00	LX0 C
ATOM	2831	C	VAL	1277	54.227	9.049	8.615	1.00	0.00	LX0 C
ATOM	2832	O	VAL	1277	53.231	9.739	8.797	1.00	0.00	LX0 O
ATOM	2833	N	SER	1278	54.192	7.828	8.070	1.00	0.00	LX0 N
ATOM	2834	H	SER	1278	55.022	7.278	8.030	0.00	0.00	LX0 H
ATOM	2835	CA	SER	1278	52.926	7.372	7.500	1.00	0.00	LX0 C
ATOM	2836	CB	SER	1278	53.206	6.311	6.431	1.00	0.00	LX0 C
ATOM	2837	OG	SER	1278	53.781	5.132	7.022	1.00	0.00	LX0 O
ATOM	2838	HG	SER	1278	54.501	5.429	7.572	0.00	0.00	LX0 H
ATOM	2839	C	SER	1278	51.912	6.852	8.504	1.00	0.00	LX0 C
ATOM	2840	O	SER	1278	52.258	6.279	9.534	1.00	0.00	LX0 O
ATOM	2841	N	PHE	1279	50.635	7.002	8.119	1.00	0.00	LX0 N
ATOM	2842	H	PHE	1279	50.433	7.440	7.237	0.00	0.00	LX0 H
ATOM	2843	CA	PHE	1279	49.584	6.233	8.784	1.00	0.00	LX0 C
ATOM	2844	CB	PHE	1279	48.211	6.578	8.184	1.00	0.00	LX0 C
ATOM	2845	CG	PHE	1279	47.121	5.668	8.708	1.00	0.00	LX0 C
ATOM	2846	CD1	PHE	1279	46.507	5.952	9.945	1.00	0.00	LX0 C
ATOM	2847	CD2	PHE	1279	46.743	4.541	7.944	1.00	0.00	LX0 C

ATOM	2848	CE1	PHE	1279	45.494	5.099	10.421	1.00	0.00	LX0 C
ATOM	2849	CE2	PHE	1279	45.736	3.684	8.424	1.00	0.00	LX0 C
ATOM	2850	CZ	PHE	1279	45.121	3.974	9.658	1.00	0.00	LX0 C
ATOM	2851	C	PHE	1279	49.867	4.740	8.723	1.00	0.00	LX0 C
ATOM	2852	O	PHE	1279	49.721	4.025	9.706	1.00	0.00	LX0 O
ATOM	2853	N	TYR	1280	50.339	4.321	7.538	1.00	0.00	LX0 N
ATOM	2854	H	TYR	1280	50.331	4.984	6.783	0.00	0.00	LX0 H
ATOM	2855	CA	TYR	1280	50.802	2.947	7.322	1.00	0.00	LX0 C
ATOM	2856	CB	TYR	1280	51.519	2.913	5.964	1.00	0.00	LX0 C
ATOM	2857	CG	TYR	1280	51.996	1.537	5.555	1.00	0.00	LX0 C
ATOM	2858	CD1	TYR	1280	53.384	1.298	5.511	1.00	0.00	LX0 C
ATOM	2859	CE1	TYR	1280	53.848	0.045	5.080	1.00	0.00	LX0 C
ATOM	2860	CD2	TYR	1280	51.057	0.545	5.205	1.00	0.00	LX0 C
ATOM	2861	CE2	TYR	1280	51.522	-0.708	4.775	1.00	0.00	LX0 C
ATOM	2862	CZ	TYR	1280	52.912	-0.939	4.708	1.00	0.00	LX0 C
ATOM	2863	OH	TYR	1280	53.377	-2.160	4.263	1.00	0.00	LX0 O
ATOM	2864	HH	TYR	1280	52.626	-2.702	4.021	0.00	0.00	LX0 H
ATOM	2865	C	TYR	1280	51.647	2.362	8.454	1.00	0.00	LX0 C
ATOM	2866	O	TYR	1280	51.416	1.252	8.918	1.00	0.00	LX0 O
ATOM	2867	N	TYR	1281	52.624	3.177	8.885	1.00	0.00	LX0 N
ATOM	2868	H	TYR	1281	52.755	4.063	8.437	0.00	0.00	LX0 H
ATOM	2869	CA	TYR	1281	53.442	2.771	10.032	1.00	0.00	LX0 C
ATOM	2870	CB	TYR	1281	54.825	3.430	9.967	1.00	0.00	LX0 C
ATOM	2871	CG	TYR	1281	55.880	2.641	9.211	1.00	0.00	LX0 C
ATOM	2872	CD1	TYR	1281	55.536	1.679	8.235	1.00	0.00	LX0 C
ATOM	2873	CE1	TYR	1281	56.559	0.996	7.553	1.00	0.00	LX0 C
ATOM	2874	CD2	TYR	1281	57.226	2.921	9.521	1.00	0.00	LX0 C
ATOM	2875	CE2	TYR	1281	58.249	2.245	8.838	1.00	0.00	LX0 C
ATOM	2876	CZ	TYR	1281	57.904	1.296	7.855	1.00	0.00	LX0 C
ATOM	2877	OH	TYR	1281	58.918	0.650	7.163	1.00	0.00	LX0 O
ATOM	2878	HH	TYR	1281	58.645	-0.254	7.031	0.00	0.00	LX0 H
ATOM	2879	C	TYR	1281	52.849	3.073	11.402	1.00	0.00	LX0 C
ATOM	2880	O	TYR	1281	53.211	2.467	12.401	1.00	0.00	LX0 O
ATOM	2881	N	SER	1282	51.967	4.080	11.423	1.00	0.00	LX0 N
ATOM	2882	H	SER	1282	51.688	4.523	10.571	0.00	0.00	LX0 H
ATOM	2883	CA	SER	1282	51.537	4.590	12.722	1.00	0.00	LX0 C
ATOM	2884	CB	SER	1282	50.781	5.907	12.528	1.00	0.00	LX0 C
ATOM	2885	OG	SER	1282	49.491	5.658	11.955	1.00	0.00	LX0 O
ATOM	2886	HG	SER	1282	49.607	4.966	11.305	0.00	0.00	LX0 H
ATOM	2887	C	SER	1282	50.709	3.634	13.567	1.00	0.00	LX0 C
ATOM	2888	O	SER	1282	50.028	2.746	13.073	1.00	0.00	LX0 O
ATOM	2889	N	GLU	1283	50.735	3.930	14.875	1.00	0.00	LX0 N
ATOM	2890	H	GLU	1283	51.400	4.591	15.216	0.00	0.00	LX0 H
ATOM	2891	CA	GLU	1283	49.838	3.263	15.824	1.00	0.00	LX0 C
ATOM	2892	CB	GLU	1283	50.123	3.870	17.196	1.00	0.00	LX0 C
ATOM	2893	CG	GLU	1283	49.513	3.160	18.402	1.00	0.00	LX0 C
ATOM	2894	CD	GLU	1283	50.022	3.855	19.647	1.00	0.00	LX0 C
ATOM	2895	OE1	GLU	1283	50.840	3.268	20.352	1.00	0.00	LX0 O
ATOM	2896	OE2	GLU	1283	49.619	4.991	19.893	1.00	0.00	LX0 O
ATOM	2897	C	GLU	1283	48.354	3.323	15.451	1.00	0.00	LX0 C
ATOM	2898	O	GLU	1283	47.579	2.394	15.648	1.00	0.00	LX0 O
ATOM	2899	N	GLU	1284	47.999	4.467	14.830	1.00	0.00	LX0 N
ATOM	2900	H	GLU	1284	48.679	5.186	14.708	0.00	0.00	LX0 H
ATOM	2901	CA	GLU	1284	46.632	4.640	14.328	1.00	0.00	LX0 C
ATOM	2902	CB	GLU	1284	46.477	6.035	13.714	1.00	0.00	LX0 C
ATOM	2903	CG	GLU	1284	45.364	6.908	14.314	1.00	0.00	LX0 C
ATOM	2904	CD	GLU	1284	43.986	6.297	14.105	1.00	0.00	LX0 C
ATOM	2905	OE1	GLU	1284	43.563	6.097	12.971	1.00	0.00	LX0 O
ATOM	2906	OE2	GLU	1284	43.303	6.004	15.079	1.00	0.00	LX0 O
ATOM	2907	C	GLU	1284	46.163	3.562	13.351	1.00	0.00	LX0 C
ATOM	2908	O	GLU	1284	44.977	3.265	13.217	1.00	0.00	LX0 O

```
ATOM   2909  N   ASN  1285      47.157   2.949  12.688  1.00  0.00      LX0  N
ATOM   2910  H   ASN  1285      48.107   3.214  12.851  0.00  0.00      LX0  H
ATOM   2911  CA  ASN  1285      46.894   1.733  11.916  1.00  0.00      LX0  C
ATOM   2912  CB  ASN  1285      48.019   1.528  10.897  1.00  0.00      LX0  C
ATOM   2913  CG  ASN  1285      47.708   0.379   9.964  1.00  0.00      LX0  C
ATOM   2914  OD1 ASN  1285      46.580  -0.084   9.847  1.00  0.00      LX0  O
ATOM   2915  ND2 ASN  1285      48.771  -0.061   9.287  1.00  0.00      LX0  N
ATOM   2916 HD21 ASN  1285      49.673   0.365   9.411  0.00  0.00      LX0  H
ATOM   2917 HD22 ASN  1285      48.662  -0.827   8.661  0.00  0.00      LX0  H
ATOM   2918  C   ASN  1285      46.710   0.515  12.815  1.00  0.00      LX0  C
ATOM   2919  O   ASN  1285      47.533  -0.386  12.922  1.00  0.00      LX0  O
ATOM   2920  N   LYS  1286      45.561   0.556  13.499  1.00  0.00      LX0  N
ATOM   2921  H   LYS  1286      44.953   1.314  13.274  0.00  0.00      LX0  H
ATOM   2922  CA  LYS  1286      45.356  -0.324  14.650  1.00  0.00      LX0  C
ATOM   2923  CB  LYS  1286      44.017  -0.005  15.334  1.00  0.00      LX0  C
ATOM   2924  CG  LYS  1286      43.602   1.473  15.374  1.00  0.00      LX0  C
ATOM   2925  CD  LYS  1286      42.199   1.661  14.782  1.00  0.00      LX0  C
ATOM   2926  CE  LYS  1286      41.700   3.114  14.721  1.00  0.00      LX0  C
ATOM   2927  NZ  LYS  1286      42.403   3.899  13.696  1.00  0.00      LX0  N
ATOM   2928  HZ1 LYS  1286      42.451   4.906  13.980  0.00  0.00      LX0  H
ATOM   2929  HZ2 LYS  1286      43.405   3.646  13.587  0.00  0.00      LX0  H
ATOM   2930  HZ3 LYS  1286      41.931   3.875  12.771  0.00  0.00      LX0  H
ATOM   2931  C   LYS  1286      45.453  -1.814  14.343  1.00  0.00      LX0  C
ATOM   2932  O   LYS  1286      44.488  -2.567  14.465  1.00  0.00      LX0  O
TER
```
