# Supplementary material for: Structural analysis of hubs in human NR-RTK network
Source: Biol Direct. 2011 Oct 5;6:49. doi: 10.1186/1745-6150-6-49 (PMC3220635; doi:10.1186/1745-6150-6-49)
Supplement: Additional file 13 — ESR1-IGF1R-EGFR. ESR1-IGF1R-EGFR complex structure. [file 1745-6150-6-49-S13.PDF]

```
HEADER ESR1-EGFR-IGF1R
REMARK original generated coordinate pdb file
ATOM      1  N   ALA   156      10.627  12.174   8.322  1.00  0.00       RX0  N
ATOM      2  H   ALA   156      11.205  11.359   8.277  1.00  0.00       RX0  H
ATOM      3  CA  ALA   156       9.864  12.527   9.538  1.00  0.00       RX0  C
ATOM      4  CB  ALA   156      10.757  12.403  10.765  1.00  0.00       RX0  C
ATOM      5  C   ALA   156       9.377  13.991   9.496  1.00  0.00       RX0  C
ATOM      6  O   ALA   156       9.121  14.644  10.500  1.00  0.00       RX0  O
ATOM      7  N   LEU   157       9.039  14.416   8.289  1.00  0.00       RX0  N
ATOM      8  H   LEU   157       9.136  13.769   7.530  1.00  0.00       RX0  H
ATOM      9  CA  LEU   157       8.850  15.849   7.979  1.00  0.00       RX0  C
ATOM     10  CB  LEU   157       9.724  16.244   6.794  1.00  0.00       RX0  C
ATOM     11  CG  LEU   157      11.132  15.663   6.878  1.00  0.00       RX0  C
ATOM     12  CD1 LEU   157      11.919  15.947   5.603  1.00  0.00       RX0  C
ATOM     13  CD2 LEU   157      11.867  16.113   8.138  1.00  0.00       RX0  C
ATOM     14  C   LEU   157       7.387  16.184   7.642  1.00  0.00       RX0  C
ATOM     15  O   LEU   157       7.075  17.216   7.039  1.00  0.00       RX0  O
ATOM     16  N   SER   158       6.522  15.244   7.955  1.00  0.00       RX0  N
ATOM     17  H   SER   158       6.875  14.326   8.099  1.00  0.00       RX0  H
ATOM     18  CA  SER   158       5.051  15.362   7.847  1.00  0.00       RX0  C
ATOM     19  CB  SER   158       4.729  14.894   6.436  1.00  0.00       RX0  C
ATOM     20  OG  SER   158       5.939  15.033   5.680  1.00  0.00       RX0  O
ATOM     21  HG  SER   158       6.098  15.973   5.622  1.00  0.00       RX0  H
ATOM     22  C   SER   158       4.335  14.560   8.949  1.00  0.00       RX0  C
ATOM     23  O   SER   158       3.148  14.670   9.188  1.00  0.00       RX0  O
ATOM     24  N   LEU   159       5.132  13.681   9.591  1.00  0.00       RX0  N
ATOM     25  H   LEU   159       6.083  13.586   9.324  1.00  0.00       RX0  H
ATOM     26  CA  LEU   159       4.759  12.951  10.797  1.00  0.00       RX0  C
ATOM     27  CB  LEU   159       5.860  11.985  11.247  1.00  0.00       RX0  C
ATOM     28  CG  LEU   159       5.905  10.626  10.538  1.00  0.00       RX0  C
ATOM     29  CD1 LEU   159       6.368  10.706   9.082  1.00  0.00       RX0  C
ATOM     30  CD2 LEU   159       6.746   9.631  11.339  1.00  0.00       RX0  C
ATOM     31  C   LEU   159       4.518  13.965  11.920  1.00  0.00       RX0  C
ATOM     32  O   LEU   159       5.291  14.932  12.058  1.00  0.00       RX0  O
ATOM     33  N   THR   160       3.434  13.807  12.646  1.00  0.00       RX0  N
ATOM     34  H   THR   160       2.847  13.019  12.460  1.00  0.00       RX0  H
ATOM     35  CA  THR   160       3.156  14.665  13.825  1.00  0.00       RX0  C
ATOM     36  CB  THR   160       1.666  14.648  14.226  1.00  0.00       RX0  C
ATOM     37  OG1 THR   160       1.372  15.747  15.093  1.00  0.00       RX0  O
ATOM     38  HG1 THR   160       0.424  15.812  15.136  1.00  0.00       RX0  H
ATOM     39  CG2 THR   160       1.177  13.340  14.832  1.00  0.00       RX0  C
ATOM     40  C   THR   160       4.203  14.411  14.921  1.00  0.00       RX0  C
ATOM     41  O   THR   160       4.902  13.383  14.913  1.00  0.00       RX0  O
ATOM     42  N   ALA   161       4.153  15.229  15.953  1.00  0.00       RX0  N
ATOM     43  H   ALA   161       3.461  15.953  15.917  1.00  0.00       RX0  H
ATOM     44  CA  ALA   161       4.942  15.044  17.184  1.00  0.00       RX0  C
ATOM     45  CB  ALA   161       4.755  16.234  18.117  1.00  0.00       RX0  C
ATOM     46  C   ALA   161       4.543  13.746  17.920  1.00  0.00       RX0  C
ATOM     47  O   ALA   161       5.387  12.950  18.288  1.00  0.00       RX0  O
ATOM     48  N   ASP   162       3.226  13.461  17.917  1.00  0.00       RX0  N
ATOM     49  H   ASP   162       2.570  14.188  17.721  1.00  0.00       RX0  H
ATOM     50  CA  ASP   162       2.690  12.196  18.469  1.00  0.00       RX0  C
ATOM     51  CB  ASP   162       1.201  12.325  18.828  1.00  0.00       RX0  C
ATOM     52  CG  ASP   162       1.056  13.198  20.078  1.00  0.00       RX0  C
ATOM     53  OD1 ASP   162       1.808  14.154  20.248  1.00  0.00       RX0  O
ATOM     54  OD2 ASP   162       0.203  12.928  20.922  1.00  0.00       RX0  O
ATOM     55  C   ASP   162       3.088  10.948  17.668  1.00  0.00       RX0  C
ATOM     56  O   ASP   162       3.397   9.903  18.257  1.00  0.00       RX0  O
ATOM     57  N   GLN   163       3.164  11.087  16.353  1.00  0.00       RX0  N
ATOM     58  H   GLN   163       3.079  12.014  15.999  1.00  0.00       RX0  H
ATOM     59  CA  GLN   163       3.593   9.998  15.449  1.00  0.00       RX0  C
```

ATOM	60	CB	GLN	163	3.250	10.287	13.995	1.00	0.00	RX0	C
ATOM	61	CG	GLN	163	1.824	9.881	13.632	1.00	0.00	RX0	C
ATOM	62	CD	GLN	163	1.555	10.381	12.235	1.00	0.00	RX0	C
ATOM	63	OE1	GLN	163	2.081	11.412	11.828	1.00	0.00	RX0	O
ATOM	64	NE2	GLN	163	0.722	9.603	11.524	1.00	0.00	RX0	N
ATOM	65	HE21	GLN	163	0.334	8.771	11.924	1.00	0.00	RX0	H
ATOM	66	HE22	GLN	163	0.465	9.829	10.584	1.00	0.00	RX0	H
ATOM	67	C	GLN	163	5.089	9.698	15.572	1.00	0.00	RX0	C
ATOM	68	O	GLN	163	5.477	8.537	15.545	1.00	0.00	RX0	O
ATOM	69	N	MET	164	5.882	10.740	15.840	1.00	0.00	RX0	N
ATOM	70	H	MET	164	5.492	11.661	15.891	1.00	0.00	RX0	H
ATOM	71	CA	MET	164	7.331	10.587	16.060	1.00	0.00	RX0	C
ATOM	72	CB	MET	164	8.015	11.955	16.082	1.00	0.00	RX0	C
ATOM	73	CG	MET	164	9.451	11.879	16.606	1.00	0.00	RX0	C
ATOM	74	SD	MET	164	10.536	10.864	15.595	1.00	0.00	RX0	S
ATOM	75	CE	MET	164	11.023	12.135	14.424	1.00	0.00	RX0	C
ATOM	76	C	MET	164	7.610	9.825	17.366	1.00	0.00	RX0	C
ATOM	77	O	MET	164	8.404	8.887	17.381	1.00	0.00	RX0	O
ATOM	78	N	VAL	165	6.828	10.145	18.396	1.00	0.00	RX0	N
ATOM	79	H	VAL	165	6.165	10.888	18.278	1.00	0.00	RX0	H
ATOM	80	CA	VAL	165	6.992	9.563	19.744	1.00	0.00	RX0	C
ATOM	81	CB	VAL	165	6.101	10.259	20.778	1.00	0.00	RX0	C
ATOM	82	CG1	VAL	165	6.181	9.567	22.138	1.00	0.00	RX0	C
ATOM	83	CG2	VAL	165	6.451	11.732	20.918	1.00	0.00	RX0	C
ATOM	84	C	VAL	165	6.649	8.067	19.731	1.00	0.00	RX0	C
ATOM	85	O	VAL	165	7.442	7.255	20.191	1.00	0.00	RX0	O
ATOM	86	N	SER	166	5.467	7.742	19.205	1.00	0.00	RX0	N
ATOM	87	H	SER	166	4.814	8.431	18.872	1.00	0.00	RX0	H
ATOM	88	CA	SER	166	5.029	6.335	19.106	1.00	0.00	RX0	C
ATOM	89	CB	SER	166	3.569	6.385	18.712	1.00	0.00	RX0	C
ATOM	90	OG	SER	166	3.007	7.453	19.477	1.00	0.00	RX0	O
ATOM	91	HG	SER	166	3.346	7.365	20.358	1.00	0.00	RX0	H
ATOM	92	C	SER	166	5.941	5.501	18.195	1.00	0.00	RX0	C
ATOM	93	O	SER	166	6.295	4.379	18.542	1.00	0.00	RX0	O
ATOM	94	N	ALA	167	6.456	6.129	17.133	1.00	0.00	RX0	N
ATOM	95	H	ALA	167	6.171	7.064	16.912	1.00	0.00	RX0	H
ATOM	96	CA	ALA	167	7.397	5.466	16.208	1.00	0.00	RX0	C
ATOM	97	CB	ALA	167	7.731	6.357	15.012	1.00	0.00	RX0	C
ATOM	98	C	ALA	167	8.706	5.103	16.927	1.00	0.00	RX0	C
ATOM	99	O	ALA	167	9.113	3.946	16.932	1.00	0.00	RX0	O
ATOM	100	N	LEU	168	9.179	6.054	17.734	1.00	0.00	RX0	N
ATOM	101	H	LEU	168	8.733	6.951	17.738	1.00	0.00	RX0	H
ATOM	102	CA	LEU	168	10.385	5.875	18.562	1.00	0.00	RX0	C
ATOM	103	CB	LEU	168	10.907	7.213	19.074	1.00	0.00	RX0	C
ATOM	104	CG	LEU	168	11.571	8.041	17.978	1.00	0.00	RX0	C
ATOM	105	CD1	LEU	168	12.102	9.369	18.519	1.00	0.00	RX0	C
ATOM	106	CD2	LEU	168	12.653	7.238	17.254	1.00	0.00	RX0	C
ATOM	107	C	LEU	168	10.197	4.896	19.724	1.00	0.00	RX0	C
ATOM	108	O	LEU	168	11.077	4.078	19.994	1.00	0.00	RX0	O
ATOM	109	N	LEU	169	9.007	4.918	20.317	1.00	0.00	RX0	N
ATOM	110	H	LEU	169	8.333	5.589	20.011	1.00	0.00	RX0	H
ATOM	111	CA	LEU	169	8.640	3.970	21.384	1.00	0.00	RX0	C
ATOM	112	CB	LEU	169	7.358	4.393	22.101	1.00	0.00	RX0	C
ATOM	113	CG	LEU	169	7.538	5.639	22.970	1.00	0.00	RX0	C
ATOM	114	CD1	LEU	169	6.226	6.046	23.641	1.00	0.00	RX0	C
ATOM	115	CD2	LEU	169	8.667	5.470	23.988	1.00	0.00	RX0	C
ATOM	116	C	LEU	169	8.505	2.536	20.864	1.00	0.00	RX0	C
ATOM	117	O	LEU	169	9.003	1.602	21.486	1.00	0.00	RX0	O
ATOM	118	N	ASP	170	7.977	2.423	19.645	1.00	0.00	RX0	N
ATOM	119	H	ASP	170	7.672	3.241	19.159	1.00	0.00	RX0	H
ATOM	120	CA	ASP	170	7.822	1.133	18.952	1.00	0.00	RX0	C

ATOM	121	CB	ASP	170	6.882	1.394	17.760	1.00	0.00	RX0	C
ATOM	122	CG	ASP	170	6.638	0.241	16.795	1.00	0.00	RX0	C
ATOM	123	OD1	ASP	170	7.092	-0.878	17.000	1.00	0.00	RX0	O
ATOM	124	OD2	ASP	170	6.018	0.474	15.761	1.00	0.00	RX0	O
ATOM	125	C	ASP	170	9.164	0.506	18.541	1.00	0.00	RX0	C
ATOM	126	O	ASP	170	9.313	-0.704	18.571	1.00	0.00	RX0	O
ATOM	127	N	ALA	171	10.119	1.387	18.228	1.00	0.00	RX0	N
ATOM	128	H	ALA	171	9.894	2.361	18.290	1.00	0.00	RX0	H
ATOM	129	CA	ALA	171	11.447	1.008	17.717	1.00	0.00	RX0	C
ATOM	130	CB	ALA	171	12.102	2.218	17.053	1.00	0.00	RX0	C
ATOM	131	C	ALA	171	12.418	0.479	18.779	1.00	0.00	RX0	C
ATOM	132	O	ALA	171	13.427	-0.136	18.431	1.00	0.00	RX0	O
ATOM	133	N	GLU	172	12.125	0.739	20.058	1.00	0.00	RX0	N
ATOM	134	H	GLU	172	11.281	1.230	20.277	1.00	0.00	RX0	H
ATOM	135	CA	GLU	172	13.017	0.374	21.170	1.00	0.00	RX0	C
ATOM	136	CB	GLU	172	12.365	0.709	22.510	1.00	0.00	RX0	C
ATOM	137	CG	GLU	172	12.323	2.227	22.680	1.00	0.00	RX0	C
ATOM	138	CD	GLU	172	13.728	2.778	22.513	1.00	0.00	RX0	C
ATOM	139	OE1	GLU	172	14.554	2.616	23.410	1.00	0.00	RX0	O
ATOM	140	OE2	GLU	172	14.031	3.386	21.485	1.00	0.00	RX0	O
ATOM	141	C	GLU	172	13.554	-1.065	21.099	1.00	0.00	RX0	C
ATOM	142	O	GLU	172	12.785	-2.004	20.837	1.00	0.00	RX0	O
ATOM	143	N	PRO	173	14.865	-1.209	21.269	1.00	0.00	RX0	N
ATOM	144	CD	PRO	173	15.789	-0.095	21.453	1.00	0.00	RX0	C
ATOM	145	CA	PRO	173	15.538	-2.517	21.328	1.00	0.00	RX0	C
ATOM	146	CB	PRO	173	17.014	-2.108	21.199	1.00	0.00	RX0	C
ATOM	147	CG	PRO	173	17.103	-0.741	21.869	1.00	0.00	RX0	C
ATOM	148	C	PRO	173	15.206	-3.249	22.640	1.00	0.00	RX0	C
ATOM	149	O	PRO	173	14.829	-2.595	23.631	1.00	0.00	RX0	O
ATOM	150	N	PRO	174	15.294	-4.574	22.646	1.00	0.00	RX0	N
ATOM	151	CD	PRO	174	15.605	-5.388	21.475	1.00	0.00	RX0	C
ATOM	152	CA	PRO	174	15.084	-5.400	23.852	1.00	0.00	RX0	C
ATOM	153	CB	PRO	174	14.968	-6.812	23.273	1.00	0.00	RX0	C
ATOM	154	CG	PRO	174	15.853	-6.784	22.031	1.00	0.00	RX0	C
ATOM	155	C	PRO	174	16.250	-5.248	24.838	1.00	0.00	RX0	C
ATOM	156	O	PRO	174	17.379	-4.922	24.444	1.00	0.00	RX0	O
ATOM	157	N	ILE	175	15.956	-5.464	26.106	1.00	0.00	RX0	N
ATOM	158	H	ILE	175	15.039	-5.788	26.328	1.00	0.00	RX0	H
ATOM	159	CA	ILE	175	16.988	-5.556	27.159	1.00	0.00	RX0	C
ATOM	160	CB	ILE	175	16.446	-5.212	28.551	1.00	0.00	RX0	C
ATOM	161	CG2	ILE	175	17.623	-5.099	29.520	1.00	0.00	RX0	C
ATOM	162	CG1	ILE	175	15.624	-3.918	28.562	1.00	0.00	RX0	C
ATOM	163	CD1	ILE	175	14.111	-4.134	28.454	1.00	0.00	RX0	C
ATOM	164	C	ILE	175	17.586	-6.969	27.112	1.00	0.00	RX0	C
ATOM	165	O	ILE	175	16.886	-7.963	27.343	1.00	0.00	RX0	O
ATOM	166	N	LEU	176	18.884	-7.017	26.884	1.00	0.00	RX0	N
ATOM	167	H	LEU	176	19.406	-6.165	26.837	1.00	0.00	RX0	H
ATOM	168	CA	LEU	176	19.617	-8.291	26.770	1.00	0.00	RX0	C
ATOM	169	CB	LEU	176	20.650	-8.225	25.648	1.00	0.00	RX0	C
ATOM	170	CG	LEU	176	20.014	-7.994	24.277	1.00	0.00	RX0	C
ATOM	171	CD1	LEU	176	21.072	-7.939	23.178	1.00	0.00	RX0	C
ATOM	172	CD2	LEU	176	18.931	-9.028	23.962	1.00	0.00	RX0	C
ATOM	173	C	LEU	176	20.277	-8.690	28.089	1.00	0.00	RX0	C
ATOM	174	O	LEU	176	20.563	-7.852	28.952	1.00	0.00	RX0	O
ATOM	175	N	TYR	177	20.459	-9.989	28.237	1.00	0.00	RX0	N
ATOM	176	H	TYR	177	20.224	-10.606	27.490	1.00	0.00	RX0	H
ATOM	177	CA	TYR	177	21.114	-10.573	29.420	1.00	0.00	RX0	C
ATOM	178	CB	TYR	177	20.420	-11.864	29.855	1.00	0.00	RX0	C
ATOM	179	CG	TYR	177	19.153	-11.561	30.618	1.00	0.00	RX0	C
ATOM	180	CD1	TYR	177	18.030	-11.069	29.963	1.00	0.00	RX0	C
ATOM	181	CE1	TYR	177	16.866	-10.811	30.678	1.00	0.00	RX0	C

ATOM	182	CD2	TYR	177	19.114	-11.791	31.988	1.00	0.00	RX0	C
ATOM	183	CE2	TYR	177	17.949	-11.541	32.702	1.00	0.00	RX0	C
ATOM	184	CZ	TYR	177	16.825	-11.049	32.047	1.00	0.00	RX0	C
ATOM	185	OH	TYR	177	15.672	-10.793	32.758	1.00	0.00	RX0	O
ATOM	186	HH	TYR	177	15.780	-11.075	33.656	1.00	0.00	RX0	H
ATOM	187	C	TYR	177	22.589	-10.858	29.163	1.00	0.00	RX0	C
ATOM	188	O	TYR	177	22.985	-11.163	28.046	1.00	0.00	RX0	O
ATOM	189	N	SER	178	23.381	-10.750	30.220	1.00	0.00	RX0	N
ATOM	190	H	SER	178	23.026	-10.499	31.124	1.00	0.00	RX0	H
ATOM	191	CA	SER	178	24.788	-11.188	30.183	1.00	0.00	RX0	C
ATOM	192	CB	SER	178	25.491	-10.620	31.400	1.00	0.00	RX0	C
ATOM	193	OG	SER	178	25.214	-9.220	31.413	1.00	0.00	RX0	O
ATOM	194	HG	SER	178	25.601	-8.861	30.625	1.00	0.00	RX0	H
ATOM	195	C	SER	178	24.834	-12.718	30.070	1.00	0.00	RX0	C
ATOM	196	O	SER	178	23.999	-13.413	30.674	1.00	0.00	RX0	O
ATOM	197	N	GLU	179	25.827	-13.218	29.362	1.00	0.00	RX0	N
ATOM	198	H	GLU	179	26.527	-12.634	28.946	1.00	0.00	RX0	H
ATOM	199	CA	GLU	179	26.033	-14.670	29.175	1.00	0.00	RX0	C
ATOM	200	CB	GLU	179	26.338	-15.014	27.717	1.00	0.00	RX0	C
ATOM	201	CG	GLU	179	25.803	-14.020	26.689	1.00	0.00	RX0	C
ATOM	202	CD	GLU	179	26.818	-12.927	26.376	1.00	0.00	RX0	C
ATOM	203	OE1	GLU	179	27.297	-12.894	25.254	1.00	0.00	RX0	O
ATOM	204	OE2	GLU	179	27.047	-12.031	27.189	1.00	0.00	RX0	O
ATOM	205	C	GLU	179	27.192	-15.208	30.012	1.00	0.00	RX0	C
ATOM	206	O	GLU	179	28.361	-15.227	29.589	1.00	0.00	RX0	O
ATOM	207	N	TYR	180	26.873	-15.475	31.254	1.00	0.00	RX0	N
ATOM	208	H	TYR	180	25.923	-15.392	31.565	1.00	0.00	RX0	H
ATOM	209	CA	TYR	180	27.735	-16.233	32.177	1.00	0.00	RX0	C
ATOM	210	CB	TYR	180	28.493	-15.322	33.155	1.00	0.00	RX0	C
ATOM	211	CG	TYR	180	27.551	-14.674	34.143	1.00	0.00	RX0	C
ATOM	212	CD1	TYR	180	26.926	-13.474	33.829	1.00	0.00	RX0	C
ATOM	213	CE1	TYR	180	26.019	-12.914	34.720	1.00	0.00	RX0	C
ATOM	214	CD2	TYR	180	27.305	-15.287	35.367	1.00	0.00	RX0	C
ATOM	215	CE2	TYR	180	26.387	-14.736	36.250	1.00	0.00	RX0	C
ATOM	216	CZ	TYR	180	25.728	-13.560	35.916	1.00	0.00	RX0	C
ATOM	217	OH	TYR	180	24.775	-13.043	36.766	1.00	0.00	RX0	O
ATOM	218	HH	TYR	180	24.680	-12.110	36.591	1.00	0.00	RX0	H
ATOM	219	C	TYR	180	26.838	-17.226	32.909	1.00	0.00	RX0	C
ATOM	220	O	TYR	180	25.642	-16.953	33.094	1.00	0.00	RX0	O
ATOM	221	N	ASP	181	27.404	-18.345	33.318	1.00	0.00	RX0	N
ATOM	222	H	ASP	181	28.392	-18.460	33.264	1.00	0.00	RX0	H
ATOM	223	CA	ASP	181	26.630	-19.347	34.059	1.00	0.00	RX0	C
ATOM	224	CB	ASP	181	27.318	-20.705	34.095	1.00	0.00	RX0	C
ATOM	225	CG	ASP	181	26.530	-21.558	35.064	1.00	0.00	RX0	C
ATOM	226	OD1	ASP	181	25.309	-21.579	34.972	1.00	0.00	RX0	O
ATOM	227	OD2	ASP	181	27.124	-22.166	35.944	1.00	0.00	RX0	O
ATOM	228	C	ASP	181	26.420	-18.851	35.504	1.00	0.00	RX0	C
ATOM	229	O	ASP	181	27.391	-18.832	36.273	1.00	0.00	RX0	O
ATOM	230	N	PRO	182	25.185	-18.489	35.856	1.00	0.00	RX0	N
ATOM	231	CD	PRO	182	24.009	-18.601	34.995	1.00	0.00	RX0	C
ATOM	232	CA	PRO	182	24.825	-17.989	37.201	1.00	0.00	RX0	C
ATOM	233	CB	PRO	182	23.394	-17.487	36.998	1.00	0.00	RX0	C
ATOM	234	CG	PRO	182	22.821	-18.409	35.928	1.00	0.00	RX0	C
ATOM	235	C	PRO	182	24.941	-19.052	38.308	1.00	0.00	RX0	C
ATOM	236	O	PRO	182	24.654	-18.763	39.474	1.00	0.00	RX0	O
ATOM	237	N	THR	183	25.345	-20.259	37.948	1.00	0.00	RX0	N
ATOM	238	H	THR	183	25.557	-20.521	37.004	1.00	0.00	RX0	H
ATOM	239	CA	THR	183	25.568	-21.363	38.913	1.00	0.00	RX0	C
ATOM	240	CB	THR	183	24.916	-22.567	38.265	1.00	0.00	RX0	C
ATOM	241	OG1	THR	183	23.895	-22.083	37.381	1.00	0.00	RX0	O
ATOM	242	HG1	THR	183	24.333	-22.012	36.531	1.00	0.00	RX0	H

ATOM	243	CG2	THR	183	24.350	-23.555	39.287	1.00	0.00	RX0	C
ATOM	244	C	THR	183	27.063	-21.532	39.218	1.00	0.00	RX0	C
ATOM	245	O	THR	183	27.455	-22.345	40.058	1.00	0.00	RX0	O
ATOM	246	N	ARG	184	27.887	-20.699	38.573	1.00	0.00	RX0	N
ATOM	247	H	ARG	184	27.521	-20.001	37.955	1.00	0.00	RX0	H
ATOM	248	CA	ARG	184	29.343	-20.701	38.681	1.00	0.00	RX0	C
ATOM	249	CB	ARG	184	29.931	-20.824	37.277	1.00	0.00	RX0	C
ATOM	250	CG	ARG	184	30.403	-22.249	36.987	1.00	0.00	RX0	C
ATOM	251	CD	ARG	184	30.847	-22.420	35.535	1.00	0.00	RX0	C
ATOM	252	NE	ARG	184	31.591	-21.248	35.074	1.00	0.00	RX0	N
ATOM	253	HE	ARG	184	31.027	-20.552	34.622	1.00	0.00	RX0	H
ATOM	254	CZ	ARG	184	32.926	-21.112	35.340	1.00	0.00	RX0	C
ATOM	255	NH1	ARG	184	33.591	-22.072	36.012	1.00	0.00	RX0	N
ATOM	256	HH11	ARG	184	34.565	-21.893	36.260	1.00	0.00	RX0	H
ATOM	257	HH12	ARG	184	33.190	-22.939	36.304	1.00	0.00	RX0	H
ATOM	258	NH2	ARG	184	33.584	-20.010	34.934	1.00	0.00	RX0	N
ATOM	259	HH21	ARG	184	34.579	-19.960	35.131	1.00	0.00	RX0	H
ATOM	260	HH22	ARG	184	33.168	-19.238	34.455	1.00	0.00	RX0	H
ATOM	261	C	ARG	184	29.836	-19.410	39.407	1.00	0.00	RX0	C
ATOM	262	O	ARG	184	29.116	-18.390	39.334	1.00	0.00	RX0	O
ATOM	263	N	PRO	185	30.940	-19.460	40.113	1.00	0.00	RX0	N
ATOM	264	CD	PRO	185	31.742	-20.664	40.297	1.00	0.00	RX0	C
ATOM	265	CA	PRO	185	31.574	-18.285	40.781	1.00	0.00	RX0	C
ATOM	266	CB	PRO	185	32.858	-18.870	41.383	1.00	0.00	RX0	C
ATOM	267	CG	PRO	185	33.142	-20.138	40.582	1.00	0.00	RX0	C
ATOM	268	C	PRO	185	31.820	-17.125	39.813	1.00	0.00	RX0	C
ATOM	269	O	PRO	185	31.836	-17.275	38.592	1.00	0.00	RX0	O
ATOM	270	N	PHE	186	32.164	-15.998	40.422	1.00	0.00	RX0	N
ATOM	271	H	PHE	186	32.239	-15.993	41.417	1.00	0.00	RX0	H
ATOM	272	CA	PHE	186	32.333	-14.726	39.697	1.00	0.00	RX0	C
ATOM	273	CB	PHE	186	31.517	-13.614	40.357	1.00	0.00	RX0	C
ATOM	274	CG	PHE	186	31.420	-12.438	39.413	1.00	0.00	RX0	C
ATOM	275	CD1	PHE	186	30.869	-12.612	38.148	1.00	0.00	RX0	C
ATOM	276	CD2	PHE	186	31.883	-11.187	39.803	1.00	0.00	RX0	C
ATOM	277	CE1	PHE	186	30.784	-11.536	37.272	1.00	0.00	RX0	C
ATOM	278	CE2	PHE	186	31.797	-10.111	38.927	1.00	0.00	RX0	C
ATOM	279	CZ	PHE	186	31.249	-10.285	37.661	1.00	0.00	RX0	C
ATOM	280	C	PHE	186	33.791	-14.305	39.507	1.00	0.00	RX0	C
ATOM	281	O	PHE	186	34.127	-13.678	38.496	1.00	0.00	RX0	O
ATOM	282	N	SER	187	34.655	-14.802	40.380	1.00	0.00	RX0	N
ATOM	283	H	SER	187	34.309	-15.353	41.134	1.00	0.00	RX0	H
ATOM	284	CA	SER	187	36.113	-14.542	40.380	1.00	0.00	RX0	C
ATOM	285	CB	SER	187	36.607	-15.270	41.616	1.00	0.00	RX0	C
ATOM	286	OG	SER	187	35.486	-15.358	42.513	1.00	0.00	RX0	O
ATOM	287	HG	SER	187	35.854	-15.393	43.389	1.00	0.00	RX0	H
ATOM	288	C	SER	187	36.764	-14.980	39.057	1.00	0.00	RX0	C
ATOM	289	O	SER	187	37.834	-14.531	38.683	1.00	0.00	RX0	O
ATOM	290	N	GLU	188	36.054	-15.878	38.369	1.00	0.00	RX0	N
ATOM	291	H	GLU	188	35.124	-16.105	38.646	1.00	0.00	RX0	H
ATOM	292	CA	GLU	188	36.561	-16.586	37.191	1.00	0.00	RX0	C
ATOM	293	CB	GLU	188	36.166	-18.054	37.349	1.00	0.00	RX0	C
ATOM	294	CG	GLU	188	36.934	-19.058	36.493	1.00	0.00	RX0	C
ATOM	295	CD	GLU	188	36.130	-20.337	36.479	1.00	0.00	RX0	C
ATOM	296	OE1	GLU	188	35.420	-20.610	37.438	1.00	0.00	RX0	O
ATOM	297	OE2	GLU	188	36.102	-21.018	35.460	1.00	0.00	RX0	O
ATOM	298	C	GLU	188	36.028	-16.018	35.856	1.00	0.00	RX0	C
ATOM	299	O	GLU	188	36.494	-16.416	34.788	1.00	0.00	RX0	O
ATOM	300	N	ALA	189	35.058	-15.107	35.914	1.00	0.00	RX0	N
ATOM	301	H	ALA	189	34.830	-14.681	36.792	1.00	0.00	RX0	H
ATOM	302	CA	ALA	189	34.543	-14.432	34.708	1.00	0.00	RX0	C
ATOM	303	CB	ALA	189	33.067	-14.081	34.891	1.00	0.00	RX0	C

ATOM	304	C	ALA	189	35.336	-13.151	34.407	1.00	0.00	RX0	C
ATOM	305	O	ALA	189	35.533	-12.292	35.270	1.00	0.00	RX0	O
ATOM	306	N	SER	190	35.819	-13.065	33.173	1.00	0.00	RX0	N
ATOM	307	H	SER	190	35.645	-13.797	32.514	1.00	0.00	RX0	H
ATOM	308	CA	SER	190	36.430	-11.825	32.646	1.00	0.00	RX0	C
ATOM	309	CB	SER	190	37.075	-12.288	31.361	1.00	0.00	RX0	C
ATOM	310	OG	SER	190	37.255	-13.700	31.523	1.00	0.00	RX0	O
ATOM	311	HG	SER	190	37.798	-13.823	32.296	1.00	0.00	RX0	H
ATOM	312	C	SER	190	35.341	-10.761	32.513	1.00	0.00	RX0	C
ATOM	313	O	SER	190	34.465	-10.869	31.639	1.00	0.00	RX0	O
ATOM	314	N	MET	191	35.401	-9.751	33.361	1.00	0.00	RX0	N
ATOM	315	H	MET	191	36.053	-9.819	34.117	1.00	0.00	RX0	H
ATOM	316	CA	MET	191	34.414	-8.652	33.337	1.00	0.00	RX0	C
ATOM	317	CB	MET	191	34.644	-7.665	34.478	1.00	0.00	RX0	C
ATOM	318	CG	MET	191	33.549	-6.599	34.500	1.00	0.00	RX0	C
ATOM	319	SD	MET	191	33.642	-5.546	35.950	1.00	0.00	RX0	S
ATOM	320	CE	MET	191	33.304	-6.813	37.184	1.00	0.00	RX0	C
ATOM	321	C	MET	191	34.384	-7.939	31.976	1.00	0.00	RX0	C
ATOM	322	O	MET	191	33.329	-7.841	31.363	1.00	0.00	RX0	O
ATOM	323	N	MET	192	35.577	-7.654	31.438	1.00	0.00	RX0	N
ATOM	324	H	MET	192	36.392	-7.771	32.002	1.00	0.00	RX0	H
ATOM	325	CA	MET	192	35.697	-7.082	30.089	1.00	0.00	RX0	C
ATOM	326	CB	MET	192	37.141	-6.660	29.807	1.00	0.00	RX0	C
ATOM	327	CG	MET	192	37.302	-5.956	28.455	1.00	0.00	RX0	C
ATOM	328	SD	MET	192	36.227	-4.522	28.268	1.00	0.00	RX0	S
ATOM	329	CE	MET	192	36.847	-3.530	29.637	1.00	0.00	RX0	C
ATOM	330	C	MET	192	35.151	-8.021	28.999	1.00	0.00	RX0	C
ATOM	331	O	MET	192	34.484	-7.587	28.093	1.00	0.00	RX0	O
ATOM	332	N	GLY	193	35.358	-9.342	29.220	1.00	0.00	RX0	N
ATOM	333	H	GLY	193	35.762	-9.623	30.085	1.00	0.00	RX0	H
ATOM	334	CA	GLY	193	34.804	-10.378	28.330	1.00	0.00	RX0	C
ATOM	335	C	GLY	193	33.267	-10.338	28.334	1.00	0.00	RX0	C
ATOM	336	O	GLY	193	32.637	-10.184	27.296	1.00	0.00	RX0	O
ATOM	337	N	LEU	194	32.696	-10.293	29.537	1.00	0.00	RX0	N
ATOM	338	H	LEU	194	33.270	-10.317	30.352	1.00	0.00	RX0	H
ATOM	339	CA	LEU	194	31.235	-10.169	29.722	1.00	0.00	RX0	C
ATOM	340	CB	LEU	194	30.847	-10.225	31.198	1.00	0.00	RX0	C
ATOM	341	CG	LEU	194	31.128	-11.571	31.858	1.00	0.00	RX0	C
ATOM	342	CD1	LEU	194	30.686	-11.564	33.322	1.00	0.00	RX0	C
ATOM	343	CD2	LEU	194	30.507	-12.727	31.073	1.00	0.00	RX0	C
ATOM	344	C	LEU	194	30.647	-8.891	29.116	1.00	0.00	RX0	C
ATOM	345	O	LEU	194	29.706	-8.959	28.317	1.00	0.00	RX0	O
ATOM	346	N	LEU	195	31.327	-7.782	29.364	1.00	0.00	RX0	N
ATOM	347	H	LEU	195	32.142	-7.847	29.936	1.00	0.00	RX0	H
ATOM	348	CA	LEU	195	30.920	-6.462	28.846	1.00	0.00	RX0	C
ATOM	349	CB	LEU	195	31.731	-5.338	29.493	1.00	0.00	RX0	C
ATOM	350	CG	LEU	195	31.533	-5.240	31.006	1.00	0.00	RX0	C
ATOM	351	CD1	LEU	195	32.368	-4.112	31.613	1.00	0.00	RX0	C
ATOM	352	CD2	LEU	195	30.057	-5.129	31.385	1.00	0.00	RX0	C
ATOM	353	C	LEU	195	31.020	-6.357	27.321	1.00	0.00	RX0	C
ATOM	354	O	LEU	195	30.051	-5.942	26.671	1.00	0.00	RX0	O
ATOM	355	N	THR	196	32.075	-6.931	26.767	1.00	0.00	RX0	N
ATOM	356	H	THR	196	32.801	-7.329	27.327	1.00	0.00	RX0	H
ATOM	357	CA	THR	196	32.335	-6.901	25.309	1.00	0.00	RX0	C
ATOM	358	CB	THR	196	33.785	-7.289	25.034	1.00	0.00	RX0	C
ATOM	359	OG1	THR	196	34.653	-6.360	25.697	1.00	0.00	RX0	O
ATOM	360	HG1	THR	196	34.321	-5.497	25.488	1.00	0.00	RX0	H
ATOM	361	CG2	THR	196	34.094	-7.338	23.536	1.00	0.00	RX0	C
ATOM	362	C	THR	196	31.317	-7.765	24.552	1.00	0.00	RX0	C
ATOM	363	O	THR	196	30.772	-7.327	23.532	1.00	0.00	RX0	O
ATOM	364	N	ASN	197	31.003	-8.928	25.107	1.00	0.00	RX0	N

ATOM	365	H	ASN	197	31.431	-9.188	25.975	1.00	0.00	RX0	H
ATOM	366	CA	ASN	197	30.010	-9.840	24.504	1.00	0.00	RX0	C
ATOM	367	CB	ASN	197	30.004	-11.200	25.193	1.00	0.00	RX0	C
ATOM	368	CG	ASN	197	31.069	-12.094	24.601	1.00	0.00	RX0	C
ATOM	369	OD1	ASN	197	31.024	-12.516	23.450	1.00	0.00	RX0	O
ATOM	370	ND2	ASN	197	32.059	-12.361	25.470	1.00	0.00	RX0	N
ATOM	371	HD21	ASN	197	32.042	-11.906	26.363	1.00	0.00	RX0	H
ATOM	372	HD22	ASN	197	32.800	-12.990	25.242	1.00	0.00	RX0	H
ATOM	373	C	ASN	197	28.594	-9.255	24.528	1.00	0.00	RX0	C
ATOM	374	O	ASN	197	27.900	-9.272	23.514	1.00	0.00	RX0	O
ATOM	375	N	LEU	198	28.277	-8.575	25.633	1.00	0.00	RX0	N
ATOM	376	H	LEU	198	28.904	-8.607	26.414	1.00	0.00	RX0	H
ATOM	377	CA	LEU	198	27.002	-7.850	25.760	1.00	0.00	RX0	C
ATOM	378	CB	LEU	198	26.869	-7.327	27.187	1.00	0.00	RX0	C
ATOM	379	CG	LEU	198	25.549	-6.619	27.480	1.00	0.00	RX0	C
ATOM	380	CD1	LEU	198	24.331	-7.525	27.286	1.00	0.00	RX0	C
ATOM	381	CD2	LEU	198	25.580	-6.005	28.875	1.00	0.00	RX0	C
ATOM	382	C	LEU	198	26.885	-6.719	24.724	1.00	0.00	RX0	C
ATOM	383	O	LEU	198	25.930	-6.676	23.947	1.00	0.00	RX0	O
ATOM	384	N	ALA	199	27.942	-5.914	24.641	1.00	0.00	RX0	N
ATOM	385	H	ALA	199	28.701	-6.064	25.276	1.00	0.00	RX0	H
ATOM	386	CA	ALA	199	28.029	-4.784	23.694	1.00	0.00	RX0	C
ATOM	387	CB	ALA	199	29.343	-4.024	23.882	1.00	0.00	RX0	C
ATOM	388	C	ALA	199	27.921	-5.230	22.227	1.00	0.00	RX0	C
ATOM	389	O	ALA	199	27.138	-4.660	21.467	1.00	0.00	RX0	O
ATOM	390	N	ASP	200	28.555	-6.360	21.908	1.00	0.00	RX0	N
ATOM	391	H	ASP	200	29.140	-6.810	22.584	1.00	0.00	RX0	H
ATOM	392	CA	ASP	200	28.494	-6.940	20.550	1.00	0.00	RX0	C
ATOM	393	CB	ASP	200	29.540	-8.047	20.368	1.00	0.00	RX0	C
ATOM	394	CG	ASP	200	30.215	-7.932	19.006	1.00	0.00	RX0	C
ATOM	395	OD1	ASP	200	30.643	-6.842	18.627	1.00	0.00	RX0	O
ATOM	396	OD2	ASP	200	30.378	-8.940	18.320	1.00	0.00	RX0	O
ATOM	397	C	ASP	200	27.084	-7.410	20.171	1.00	0.00	RX0	C
ATOM	398	O	ASP	200	26.604	-7.102	19.080	1.00	0.00	RX0	O
ATOM	399	N	ARG	201	26.390	-7.998	21.143	1.00	0.00	RX0	N
ATOM	400	H	ARG	201	26.849	-8.155	22.021	1.00	0.00	RX0	H
ATOM	401	CA	ARG	201	24.992	-8.434	20.957	1.00	0.00	RX0	C
ATOM	402	CB	ARG	201	24.569	-9.432	22.014	1.00	0.00	RX0	C
ATOM	403	CG	ARG	201	25.122	-10.813	21.677	1.00	0.00	RX0	C
ATOM	404	CD	ARG	201	24.655	-11.855	22.684	1.00	0.00	RX0	C
ATOM	405	NE	ARG	201	25.165	-11.529	24.009	1.00	0.00	RX0	N
ATOM	406	HE	ARG	201	26.149	-11.709	24.172	1.00	0.00	RX0	H
ATOM	407	CZ	ARG	201	24.339	-11.088	24.995	1.00	0.00	RX0	C
ATOM	408	NH1	ARG	201	23.022	-10.932	24.753	1.00	0.00	RX0	N
ATOM	409	HH11	ARG	201	22.396	-10.653	25.483	1.00	0.00	RX0	H
ATOM	410	HH12	ARG	201	22.640	-11.097	23.840	1.00	0.00	RX0	H
ATOM	411	NH2	ARG	201	24.845	-10.826	26.207	1.00	0.00	RX0	N
ATOM	412	HH21	ARG	201	24.291	-10.539	26.994	1.00	0.00	RX0	H
ATOM	413	HH22	ARG	201	25.832	-10.986	26.383	1.00	0.00	RX0	H
ATOM	414	C	ARG	201	23.991	-7.279	20.827	1.00	0.00	RX0	C
ATOM	415	O	ARG	201	23.123	-7.308	19.955	1.00	0.00	RX0	O
ATOM	416	N	GLU	202	24.240	-6.201	21.568	1.00	0.00	RX0	N
ATOM	417	H	GLU	202	25.009	-6.215	22.213	1.00	0.00	RX0	H
ATOM	418	CA	GLU	202	23.401	-4.988	21.493	1.00	0.00	RX0	C
ATOM	419	CB	GLU	202	23.558	-4.076	22.732	1.00	0.00	RX0	C
ATOM	420	CG	GLU	202	22.912	-4.683	23.996	1.00	0.00	RX0	C
ATOM	421	CD	GLU	202	22.861	-3.724	25.189	1.00	0.00	RX0	C
ATOM	422	OE1	GLU	202	21.832	-3.088	25.422	1.00	0.00	RX0	O
ATOM	423	OE2	GLU	202	23.821	-3.656	25.951	1.00	0.00	RX0	O
ATOM	424	C	GLU	202	23.526	-4.262	20.149	1.00	0.00	RX0	C
ATOM	425	O	GLU	202	22.539	-3.754	19.625	1.00	0.00	RX0	O

ATOM	426	N	LEU	203	24.712	-4.358	19.546	1.00	0.00	RX0	N
ATOM	427	H	LEU	203	25.464	-4.809	20.035	1.00	0.00	RX0	H
ATOM	428	CA	LEU	203	25.004	-3.680	18.270	1.00	0.00	RX0	C
ATOM	429	CB	LEU	203	26.480	-3.870	17.917	1.00	0.00	RX0	C
ATOM	430	CG	LEU	203	26.923	-3.121	16.658	1.00	0.00	RX0	C
ATOM	431	CD1	LEU	203	26.716	-1.611	16.782	1.00	0.00	RX0	C
ATOM	432	CD2	LEU	203	28.362	-3.470	16.276	1.00	0.00	RX0	C
ATOM	433	C	LEU	203	24.099	-4.160	17.127	1.00	0.00	RX0	C
ATOM	434	O	LEU	203	23.593	-3.349	16.346	1.00	0.00	RX0	O
ATOM	435	N	VAL	204	23.782	-5.447	17.151	1.00	0.00	RX0	N
ATOM	436	H	VAL	204	24.176	-6.003	17.886	1.00	0.00	RX0	H
ATOM	437	CA	VAL	204	22.925	-6.083	16.127	1.00	0.00	RX0	C
ATOM	438	CB	VAL	204	22.899	-7.597	16.333	1.00	0.00	RX0	C
ATOM	439	CG1	VAL	204	22.010	-8.279	15.292	1.00	0.00	RX0	C
ATOM	440	CG2	VAL	204	24.321	-8.163	16.349	1.00	0.00	RX0	C
ATOM	441	C	VAL	204	21.502	-5.497	16.213	1.00	0.00	RX0	C
ATOM	442	O	VAL	204	20.938	-5.041	15.221	1.00	0.00	RX0	O
ATOM	443	N	HIS	205	21.015	-5.393	17.448	1.00	0.00	RX0	N
ATOM	444	H	HIS	205	21.604	-5.675	18.208	1.00	0.00	RX0	H
ATOM	445	CA	HIS	205	19.703	-4.786	17.746	1.00	0.00	RX0	C
ATOM	446	CB	HIS	205	19.275	-5.097	19.181	1.00	0.00	RX0	C
ATOM	447	CG	HIS	205	19.003	-6.576	19.316	1.00	0.00	RX0	C
ATOM	448	ND1	HIS	205	17.808	-7.141	19.061	1.00	0.00	RX0	N
ATOM	449	HD1	HIS	205	16.991	-6.677	18.782	1.00	0.00	RX0	H
ATOM	450	CD2	HIS	205	19.899	-7.578	19.701	1.00	0.00	RX0	C
ATOM	451	NE2	HIS	205	19.227	-8.756	19.676	1.00	0.00	RX0	N
ATOM	452	CE1	HIS	205	17.942	-8.488	19.282	1.00	0.00	RX0	C
ATOM	453	C	HIS	205	19.668	-3.277	17.476	1.00	0.00	RX0	C
ATOM	454	O	HIS	205	18.642	-2.756	17.030	1.00	0.00	RX0	O
ATOM	455	N	MET	206	20.820	-2.627	17.609	1.00	0.00	RX0	N
ATOM	456	H	MET	206	21.610	-3.125	17.973	1.00	0.00	RX0	H
ATOM	457	CA	MET	206	20.969	-1.185	17.340	1.00	0.00	RX0	C
ATOM	458	CB	MET	206	22.357	-0.694	17.746	1.00	0.00	RX0	C
ATOM	459	CG	MET	206	22.541	0.805	17.511	1.00	0.00	RX0	C
ATOM	460	SD	MET	206	24.241	1.327	17.775	1.00	0.00	RX0	S
ATOM	461	CE	MET	206	24.495	0.519	19.360	1.00	0.00	RX0	C
ATOM	462	C	MET	206	20.721	-0.870	15.856	1.00	0.00	RX0	C
ATOM	463	O	MET	206	20.035	0.103	15.544	1.00	0.00	RX0	O
ATOM	464	N	ILE	207	21.183	-1.758	14.977	1.00	0.00	RX0	N
ATOM	465	H	ILE	207	21.731	-2.517	15.339	1.00	0.00	RX0	H
ATOM	466	CA	ILE	207	20.975	-1.628	13.516	1.00	0.00	RX0	C
ATOM	467	CB	ILE	207	21.671	-2.791	12.802	1.00	0.00	RX0	C
ATOM	468	CG2	ILE	207	21.419	-2.773	11.296	1.00	0.00	RX0	C
ATOM	469	CG1	ILE	207	23.164	-2.810	13.124	1.00	0.00	RX0	C
ATOM	470	CD1	ILE	207	23.907	-1.604	12.553	1.00	0.00	RX0	C
ATOM	471	C	ILE	207	19.470	-1.621	13.197	1.00	0.00	RX0	C
ATOM	472	O	ILE	207	18.988	-0.755	12.467	1.00	0.00	RX0	O
ATOM	473	N	ASN	208	18.761	-2.558	13.816	1.00	0.00	RX0	N
ATOM	474	H	ASN	208	19.238	-3.129	14.488	1.00	0.00	RX0	H
ATOM	475	CA	ASN	208	17.313	-2.734	13.586	1.00	0.00	RX0	C
ATOM	476	CB	ASN	208	16.798	-4.069	14.120	1.00	0.00	RX0	C
ATOM	477	CG	ASN	208	17.005	-5.134	13.055	1.00	0.00	RX0	C
ATOM	478	OD1	ASN	208	17.462	-4.872	11.939	1.00	0.00	RX0	O
ATOM	479	ND2	ASN	208	16.648	-6.364	13.464	1.00	0.00	RX0	N
ATOM	480	HD21	ASN	208	16.282	-6.505	14.386	1.00	0.00	RX0	H
ATOM	481	HD22	ASN	208	16.732	-7.180	12.891	1.00	0.00	RX0	H
ATOM	482	C	ASN	208	16.516	-1.532	14.103	1.00	0.00	RX0	C
ATOM	483	O	ASN	208	15.637	-1.017	13.417	1.00	0.00	RX0	O
ATOM	484	N	TRP	209	16.982	-1.008	15.238	1.00	0.00	RX0	N
ATOM	485	H	TRP	209	17.726	-1.474	15.722	1.00	0.00	RX0	H
ATOM	486	CA	TRP	209	16.425	0.201	15.864	1.00	0.00	RX0	C

ATOM	487	CB	TRP	209	17.092	0.419	17.231	1.00	0.00	RX0	C
ATOM	488	CG	TRP	209	16.695	1.748	17.837	1.00	0.00	RX0	C
ATOM	489	CD2	TRP	209	17.414	2.999	17.806	1.00	0.00	RX0	C
ATOM	490	CE2	TRP	209	16.635	3.960	18.495	1.00	0.00	RX0	C
ATOM	491	CE3	TRP	209	18.635	3.370	17.254	1.00	0.00	RX0	C
ATOM	492	CD1	TRP	209	15.527	2.029	18.553	1.00	0.00	RX0	C
ATOM	493	NE1	TRP	209	15.479	3.327	18.949	1.00	0.00	RX0	N
ATOM	494	HE1	TRP	209	14.722	3.719	19.452	1.00	0.00	RX0	H
ATOM	495	CZ2	TRP	209	17.098	5.266	18.598	1.00	0.00	RX0	C
ATOM	496	CZ3	TRP	209	19.087	4.678	17.371	1.00	0.00	RX0	C
ATOM	497	CH2	TRP	209	18.318	5.624	18.036	1.00	0.00	RX0	C
ATOM	498	C	TRP	209	16.619	1.438	14.972	1.00	0.00	RX0	C
ATOM	499	O	TRP	209	15.652	2.120	14.634	1.00	0.00	RX0	O
ATOM	500	N	ALA	210	17.853	1.624	14.503	1.00	0.00	RX0	N
ATOM	501	H	ALA	210	18.567	0.988	14.791	1.00	0.00	RX0	H
ATOM	502	CA	ALA	210	18.233	2.756	13.635	1.00	0.00	RX0	C
ATOM	503	CB	ALA	210	19.715	2.665	13.271	1.00	0.00	RX0	C
ATOM	504	C	ALA	210	17.400	2.800	12.347	1.00	0.00	RX0	C
ATOM	505	O	ALA	210	16.892	3.855	11.979	1.00	0.00	RX0	O
ATOM	506	N	LYS	211	17.095	1.613	11.820	1.00	0.00	RX0	N
ATOM	507	H	LYS	211	17.503	0.799	12.236	1.00	0.00	RX0	H
ATOM	508	CA	LYS	211	16.258	1.472	10.614	1.00	0.00	RX0	C
ATOM	509	CB	LYS	211	16.405	0.069	10.023	1.00	0.00	RX0	C
ATOM	510	CG	LYS	211	17.792	-0.091	9.390	1.00	0.00	RX0	C
ATOM	511	CD	LYS	211	18.066	-1.498	8.858	1.00	0.00	RX0	C
ATOM	512	CE	LYS	211	17.860	-2.513	9.975	1.00	0.00	RX0	C
ATOM	513	NZ	LYS	211	18.340	-3.853	9.613	1.00	0.00	RX0	N
ATOM	514	HZ1	LYS	211	18.154	-4.480	10.430	1.00	0.00	RX0	H
ATOM	515	HZ2	LYS	211	17.829	-4.209	8.783	1.00	0.00	RX0	H
ATOM	516	HZ3	LYS	211	19.361	-3.837	9.413	1.00	0.00	RX0	H
ATOM	517	C	LYS	211	14.795	1.898	10.823	1.00	0.00	RX0	C
ATOM	518	O	LYS	211	14.129	2.337	9.881	1.00	0.00	RX0	O
ATOM	519	N	ARG	212	14.357	1.870	12.071	1.00	0.00	RX0	N
ATOM	520	H	ARG	212	14.980	1.608	12.810	1.00	0.00	RX0	H
ATOM	521	CA	ARG	212	13.005	2.320	12.466	1.00	0.00	RX0	C
ATOM	522	CB	ARG	212	12.393	1.398	13.520	1.00	0.00	RX0	C
ATOM	523	CG	ARG	212	12.553	-0.087	13.194	1.00	0.00	RX0	C
ATOM	524	CD	ARG	212	11.674	-0.983	14.070	1.00	0.00	RX0	C
ATOM	525	NE	ARG	212	10.269	-0.778	13.726	1.00	0.00	RX0	N
ATOM	526	HE	ARG	212	10.081	-0.538	12.770	1.00	0.00	RX0	H
ATOM	527	CZ	ARG	212	9.293	-0.886	14.677	1.00	0.00	RX0	C
ATOM	528	NH1	ARG	212	9.608	-1.222	15.943	1.00	0.00	RX0	N
ATOM	529	HH11	ARG	212	8.886	-1.243	16.664	1.00	0.00	RX0	H
ATOM	530	HH12	ARG	212	10.531	-1.439	16.262	1.00	0.00	RX0	H
ATOM	531	NH2	ARG	212	8.015	-0.642	14.337	1.00	0.00	RX0	N
ATOM	532	HH21	ARG	212	7.295	-0.636	15.061	1.00	0.00	RX0	H
ATOM	533	HH22	ARG	212	7.693	-0.422	13.419	1.00	0.00	RX0	H
ATOM	534	C	ARG	212	12.933	3.790	12.894	1.00	0.00	RX0	C
ATOM	535	O	ARG	212	11.827	4.344	12.989	1.00	0.00	RX0	O
ATOM	536	N	VAL	213	14.074	4.417	13.148	1.00	0.00	RX0	N
ATOM	537	H	VAL	213	14.933	3.928	12.994	1.00	0.00	RX0	H
ATOM	538	CA	VAL	213	14.155	5.874	13.374	1.00	0.00	RX0	C
ATOM	539	CB	VAL	213	15.581	6.281	13.748	1.00	0.00	RX0	C
ATOM	540	CG1	VAL	213	15.751	7.800	13.838	1.00	0.00	RX0	C
ATOM	541	CG2	VAL	213	15.978	5.583	15.048	1.00	0.00	RX0	C
ATOM	542	C	VAL	213	13.672	6.590	12.095	1.00	0.00	RX0	C
ATOM	543	O	VAL	213	14.288	6.431	11.023	1.00	0.00	RX0	O
ATOM	544	N	PRO	214	12.622	7.395	12.213	1.00	0.00	RX0	N
ATOM	545	CD	PRO	214	11.896	7.609	13.458	1.00	0.00	RX0	C
ATOM	546	CA	PRO	214	12.035	8.145	11.084	1.00	0.00	RX0	C
ATOM	547	CB	PRO	214	10.927	8.954	11.761	1.00	0.00	RX0	C

ATOM	548	CG	PRO	214	10.552	8.163	13.010	1.00	0.00	RX0	C
ATOM	549	C	PRO	214	13.102	9.001	10.387	1.00	0.00	RX0	C
ATOM	550	O	PRO	214	13.853	9.727	11.025	1.00	0.00	RX0	O
ATOM	551	N	GLY	215	13.244	8.730	9.080	1.00	0.00	RX0	N
ATOM	552	H	GLY	215	12.749	7.982	8.636	1.00	0.00	RX0	H
ATOM	553	CA	GLY	215	14.194	9.473	8.227	1.00	0.00	RX0	C
ATOM	554	C	GLY	215	15.511	8.732	7.950	1.00	0.00	RX0	C
ATOM	555	O	GLY	215	16.085	8.889	6.862	1.00	0.00	RX0	O
ATOM	556	N	PHE	216	15.917	7.845	8.848	1.00	0.00	RX0	N
ATOM	557	H	PHE	216	15.330	7.654	9.637	1.00	0.00	RX0	H
ATOM	558	CA	PHE	216	17.224	7.160	8.764	1.00	0.00	RX0	C
ATOM	559	CB	PHE	216	17.447	6.293	9.998	1.00	0.00	RX0	C
ATOM	560	CG	PHE	216	18.882	5.831	10.061	1.00	0.00	RX0	C
ATOM	561	CD1	PHE	216	19.889	6.745	10.349	1.00	0.00	RX0	C
ATOM	562	CD2	PHE	216	19.197	4.495	9.846	1.00	0.00	RX0	C
ATOM	563	CE1	PHE	216	21.206	6.316	10.460	1.00	0.00	RX0	C
ATOM	564	CE2	PHE	216	20.513	4.065	9.958	1.00	0.00	RX0	C
ATOM	565	CZ	PHE	216	21.514	4.972	10.285	1.00	0.00	RX0	C
ATOM	566	C	PHE	216	17.435	6.347	7.474	1.00	0.00	RX0	C
ATOM	567	O	PHE	216	18.358	6.641	6.711	1.00	0.00	RX0	O
ATOM	568	N	VAL	217	16.482	5.483	7.151	1.00	0.00	RX0	N
ATOM	569	H	VAL	217	15.672	5.449	7.735	1.00	0.00	RX0	H
ATOM	570	CA	VAL	217	16.570	4.608	5.958	1.00	0.00	RX0	C
ATOM	571	CB	VAL	217	15.527	3.496	6.007	1.00	0.00	RX0	C
ATOM	572	CG1	VAL	217	15.894	2.482	7.081	1.00	0.00	RX0	C
ATOM	573	CG2	VAL	217	14.110	4.054	6.166	1.00	0.00	RX0	C
ATOM	574	C	VAL	217	16.469	5.342	4.608	1.00	0.00	RX0	C
ATOM	575	O	VAL	217	16.660	4.747	3.556	1.00	0.00	RX0	O
ATOM	576	N	ASP	218	16.058	6.613	4.671	1.00	0.00	RX0	N
ATOM	577	H	ASP	218	15.769	7.062	5.519	1.00	0.00	RX0	H
ATOM	578	CA	ASP	218	16.006	7.470	3.472	1.00	0.00	RX0	C
ATOM	579	CB	ASP	218	15.082	8.664	3.756	1.00	0.00	RX0	C
ATOM	580	CG	ASP	218	13.735	8.229	4.336	1.00	0.00	RX0	C
ATOM	581	OD1	ASP	218	12.733	8.343	3.634	1.00	0.00	RX0	O
ATOM	582	OD2	ASP	218	13.673	7.811	5.497	1.00	0.00	RX0	O
ATOM	583	C	ASP	218	17.401	7.924	3.023	1.00	0.00	RX0	C
ATOM	584	O	ASP	218	17.595	8.369	1.896	1.00	0.00	RX0	O
ATOM	585	N	LEU	219	18.344	7.857	3.967	1.00	0.00	RX0	N
ATOM	586	H	LEU	219	18.097	7.492	4.865	1.00	0.00	RX0	H
ATOM	587	CA	LEU	219	19.767	8.083	3.697	1.00	0.00	RX0	C
ATOM	588	CB	LEU	219	20.574	8.223	4.989	1.00	0.00	RX0	C
ATOM	589	CG	LEU	219	19.967	9.163	6.038	1.00	0.00	RX0	C
ATOM	590	CD1	LEU	219	20.830	9.193	7.294	1.00	0.00	RX0	C
ATOM	591	CD2	LEU	219	19.660	10.568	5.515	1.00	0.00	RX0	C
ATOM	592	C	LEU	219	20.350	6.970	2.832	1.00	0.00	RX0	C
ATOM	593	O	LEU	219	19.843	5.836	2.789	1.00	0.00	RX0	O
ATOM	594	N	THR	220	21.442	7.293	2.191	1.00	0.00	RX0	N
ATOM	595	H	THR	220	21.758	8.249	2.293	1.00	0.00	RX0	H
ATOM	596	CA	THR	220	22.263	6.312	1.453	1.00	0.00	RX0	C
ATOM	597	CB	THR	220	23.368	7.123	0.759	1.00	0.00	RX0	C
ATOM	598	OG1	THR	220	22.845	7.995	-0.258	1.00	0.00	RX0	O
ATOM	599	HG1	THR	220	22.331	8.649	0.236	1.00	0.00	RX0	H
ATOM	600	CG2	THR	220	24.444	6.226	0.165	1.00	0.00	RX0	C
ATOM	601	C	THR	220	22.829	5.303	2.454	1.00	0.00	RX0	C
ATOM	602	O	THR	220	23.112	5.646	3.611	1.00	0.00	RX0	O
ATOM	603	N	LEU	221	23.130	4.116	1.957	1.00	0.00	RX0	N
ATOM	604	H	LEU	221	22.821	3.899	1.033	1.00	0.00	RX0	H
ATOM	605	CA	LEU	221	23.750	3.065	2.781	1.00	0.00	RX0	C
ATOM	606	CB	LEU	221	23.956	1.866	1.863	1.00	0.00	RX0	C
ATOM	607	CG	LEU	221	24.844	0.769	2.433	1.00	0.00	RX0	C
ATOM	608	CD1	LEU	221	24.200	0.068	3.629	1.00	0.00	RX0	C

ATOM	609	CD2	LEU	221	25.274	-0.198	1.331	1.00	0.00	RX0	C
ATOM	610	C	LEU	221	25.092	3.509	3.394	1.00	0.00	RX0	C
ATOM	611	O	LEU	221	25.324	3.347	4.578	1.00	0.00	RX0	O
ATOM	612	N	HIS	222	25.854	4.270	2.593	1.00	0.00	RX0	N
ATOM	613	H	HIS	222	25.558	4.420	1.655	1.00	0.00	RX0	H
ATOM	614	CA	HIS	222	27.131	4.847	3.045	1.00	0.00	RX0	C
ATOM	615	CB	HIS	222	27.831	5.664	1.958	1.00	0.00	RX0	C
ATOM	616	CG	HIS	222	28.885	6.534	2.615	1.00	0.00	RX0	C
ATOM	617	ND1	HIS	222	29.835	6.071	3.451	1.00	0.00	RX0	N
ATOM	618	HD1	HIS	222	29.949	5.147	3.769	1.00	0.00	RX0	H
ATOM	619	CD2	HIS	222	29.036	7.920	2.499	1.00	0.00	RX0	C
ATOM	620	NE2	HIS	222	30.086	8.288	3.274	1.00	0.00	RX0	N
ATOM	621	CE1	HIS	222	30.575	7.150	3.860	1.00	0.00	RX0	C
ATOM	622	C	HIS	222	26.942	5.765	4.264	1.00	0.00	RX0	C
ATOM	623	O	HIS	222	27.673	5.642	5.246	1.00	0.00	RX0	O
ATOM	624	N	ASP	223	25.958	6.658	4.167	1.00	0.00	RX0	N
ATOM	625	H	ASP	223	25.441	6.798	3.322	1.00	0.00	RX0	H
ATOM	626	CA	ASP	223	25.694	7.636	5.242	1.00	0.00	RX0	C
ATOM	627	CB	ASP	223	24.850	8.750	4.600	1.00	0.00	RX0	C
ATOM	628	CG	ASP	223	25.559	9.249	3.329	1.00	0.00	RX0	C
ATOM	629	OD1	ASP	223	26.181	10.310	3.370	1.00	0.00	RX0	O
ATOM	630	OD2	ASP	223	25.515	8.574	2.294	1.00	0.00	RX0	O
ATOM	631	C	ASP	223	25.148	6.996	6.516	1.00	0.00	RX0	C
ATOM	632	O	ASP	223	25.558	7.375	7.616	1.00	0.00	RX0	O
ATOM	633	N	GLN	224	24.393	5.921	6.332	1.00	0.00	RX0	N
ATOM	634	H	GLN	224	24.139	5.700	5.388	1.00	0.00	RX0	H
ATOM	635	CA	GLN	224	23.868	5.118	7.452	1.00	0.00	RX0	C
ATOM	636	CB	GLN	224	22.850	4.090	6.957	1.00	0.00	RX0	C
ATOM	637	CG	GLN	224	21.598	4.755	6.381	1.00	0.00	RX0	C
ATOM	638	CD	GLN	224	20.562	3.698	6.063	1.00	0.00	RX0	C
ATOM	639	OE1	GLN	224	20.438	2.694	6.760	1.00	0.00	RX0	O
ATOM	640	NE2	GLN	224	19.829	3.971	4.970	1.00	0.00	RX0	N
ATOM	641	HE21	GLN	224	19.963	4.814	4.438	1.00	0.00	RX0	H
ATOM	642	HE22	GLN	224	19.102	3.383	4.617	1.00	0.00	RX0	H
ATOM	643	C	GLN	224	25.003	4.453	8.243	1.00	0.00	RX0	C
ATOM	644	O	GLN	224	25.073	4.591	9.468	1.00	0.00	RX0	O
ATOM	645	N	VAL	225	25.993	3.956	7.505	1.00	0.00	RX0	N
ATOM	646	H	VAL	225	25.898	3.990	6.507	1.00	0.00	RX0	H
ATOM	647	CA	VAL	225	27.191	3.319	8.093	1.00	0.00	RX0	C
ATOM	648	CB	VAL	225	28.036	2.536	7.097	1.00	0.00	RX0	C
ATOM	649	CG1	VAL	225	29.089	1.752	7.876	1.00	0.00	RX0	C
ATOM	650	CG2	VAL	225	27.195	1.574	6.264	1.00	0.00	RX0	C
ATOM	651	C	VAL	225	28.021	4.368	8.852	1.00	0.00	RX0	C
ATOM	652	O	VAL	225	28.415	4.141	9.995	1.00	0.00	RX0	O
ATOM	653	N	HIS	226	28.182	5.534	8.231	1.00	0.00	RX0	N
ATOM	654	H	HIS	226	27.811	5.642	7.306	1.00	0.00	RX0	H
ATOM	655	CA	HIS	226	28.959	6.641	8.815	1.00	0.00	RX0	C
ATOM	656	CB	HIS	226	29.130	7.826	7.865	1.00	0.00	RX0	C
ATOM	657	CG	HIS	226	30.044	8.850	8.511	1.00	0.00	RX0	C
ATOM	658	ND1	HIS	226	31.283	8.576	8.961	1.00	0.00	RX0	N
ATOM	659	HD1	HIS	226	31.728	7.700	8.965	1.00	0.00	RX0	H
ATOM	660	CD2	HIS	226	29.788	10.208	8.736	1.00	0.00	RX0	C
ATOM	661	NE2	HIS	226	30.883	10.752	9.323	1.00	0.00	RX0	N
ATOM	662	CE1	HIS	226	31.803	9.744	9.460	1.00	0.00	RX0	C
ATOM	663	C	HIS	226	28.363	7.118	10.150	1.00	0.00	RX0	C
ATOM	664	O	HIS	226	29.071	7.189	11.155	1.00	0.00	RX0	O
ATOM	665	N	LEU	227	27.047	7.317	10.166	1.00	0.00	RX0	N
ATOM	666	H	LEU	227	26.529	7.184	9.317	1.00	0.00	RX0	H
ATOM	667	CA	LEU	227	26.344	7.787	11.375	1.00	0.00	RX0	C
ATOM	668	CB	LEU	227	24.875	8.093	11.088	1.00	0.00	RX0	C
ATOM	669	CG	LEU	227	24.672	9.393	10.314	1.00	0.00	RX0	C

ATOM	670	CD1	LEU	227	23.189	9.689	10.107	1.00	0.00	RX0	C
ATOM	671	CD2	LEU	227	25.369	10.571	10.991	1.00	0.00	RX0	C
ATOM	672	C	LEU	227	26.435	6.799	12.540	1.00	0.00	RX0	C
ATOM	673	O	LEU	227	26.853	7.165	13.635	1.00	0.00	RX0	O
ATOM	674	N	LEU	228	26.270	5.522	12.200	1.00	0.00	RX0	N
ATOM	675	H	LEU	228	26.041	5.298	11.249	1.00	0.00	RX0	H
ATOM	676	CA	LEU	228	26.384	4.431	13.181	1.00	0.00	RX0	C
ATOM	677	CB	LEU	228	25.763	3.155	12.619	1.00	0.00	RX0	C
ATOM	678	CG	LEU	228	24.258	3.144	12.878	1.00	0.00	RX0	C
ATOM	679	CD1	LEU	228	23.516	2.133	12.010	1.00	0.00	RX0	C
ATOM	680	CD2	LEU	228	23.964	2.939	14.364	1.00	0.00	RX0	C
ATOM	681	C	LEU	228	27.805	4.188	13.685	1.00	0.00	RX0	C
ATOM	682	O	LEU	228	28.004	4.019	14.891	1.00	0.00	RX0	O
ATOM	683	N	GLU	229	28.784	4.376	12.809	1.00	0.00	RX0	N
ATOM	684	H	GLU	229	28.582	4.585	11.851	1.00	0.00	RX0	H
ATOM	685	CA	GLU	229	30.199	4.229	13.197	1.00	0.00	RX0	C
ATOM	686	CB	GLU	229	31.138	4.111	11.991	1.00	0.00	RX0	C
ATOM	687	CG	GLU	229	32.476	3.493	12.416	1.00	0.00	RX0	C
ATOM	688	CD	GLU	229	33.303	3.113	11.204	1.00	0.00	RX0	C
ATOM	689	OE1	GLU	229	33.162	3.763	10.171	1.00	0.00	RX0	O
ATOM	690	OE2	GLU	229	34.078	2.159	11.296	1.00	0.00	RX0	O
ATOM	691	C	GLU	229	30.618	5.338	14.175	1.00	0.00	RX0	C
ATOM	692	O	GLU	229	31.393	5.088	15.099	1.00	0.00	RX0	O
ATOM	693	N	CYS	230	30.060	6.523	13.970	1.00	0.00	RX0	N
ATOM	694	H	CYS	230	29.474	6.650	13.166	1.00	0.00	RX0	H
ATOM	695	CA	CYS	230	30.321	7.692	14.829	1.00	0.00	RX0	C
ATOM	696	CB	CYS	230	30.021	8.970	14.056	1.00	0.00	RX0	C
ATOM	697	SG	CYS	230	31.103	9.150	12.618	1.00	0.00	RX0	S
ATOM	698	C	CYS	230	29.592	7.653	16.182	1.00	0.00	RX0	C
ATOM	699	O	CYS	230	30.123	8.119	17.188	1.00	0.00	RX0	O
ATOM	700	N	ALA	231	28.434	7.000	16.215	1.00	0.00	RX0	N
ATOM	701	H	ALA	231	28.113	6.533	15.388	1.00	0.00	RX0	H
ATOM	702	CA	ALA	231	27.494	7.141	17.345	1.00	0.00	RX0	C
ATOM	703	CB	ALA	231	26.145	7.669	16.853	1.00	0.00	RX0	C
ATOM	704	C	ALA	231	27.249	5.885	18.186	1.00	0.00	RX0	C
ATOM	705	O	ALA	231	26.768	6.021	19.321	1.00	0.00	RX0	O
ATOM	706	N	TRP	232	27.687	4.719	17.731	1.00	0.00	RX0	N
ATOM	707	H	TRP	232	28.111	4.687	16.822	1.00	0.00	RX0	H
ATOM	708	CA	TRP	232	27.348	3.433	18.379	1.00	0.00	RX0	C
ATOM	709	CB	TRP	232	27.969	2.235	17.648	1.00	0.00	RX0	C
ATOM	710	CG	TRP	232	29.473	2.274	17.763	1.00	0.00	RX0	C
ATOM	711	CD2	TRP	232	30.326	1.536	18.664	1.00	0.00	RX0	C
ATOM	712	CE2	TRP	232	31.659	1.940	18.415	1.00	0.00	RX0	C
ATOM	713	CE3	TRP	232	30.065	0.588	19.644	1.00	0.00	RX0	C
ATOM	714	CD1	TRP	232	30.343	3.076	17.019	1.00	0.00	RX0	C
ATOM	715	NE1	TRP	232	31.631	2.887	17.396	1.00	0.00	RX0	N
ATOM	716	HE1	TRP	232	32.395	3.350	16.986	1.00	0.00	RX0	H
ATOM	717	CZ2	TRP	232	32.694	1.382	19.155	1.00	0.00	RX0	C
ATOM	718	CZ3	TRP	232	31.110	0.039	20.378	1.00	0.00	RX0	C
ATOM	719	CH2	TRP	232	32.420	0.435	20.134	1.00	0.00	RX0	C
ATOM	720	C	TRP	232	27.676	3.373	19.884	1.00	0.00	RX0	C
ATOM	721	O	TRP	232	26.862	2.914	20.672	1.00	0.00	RX0	O
ATOM	722	N	LEU	233	28.801	3.989	20.277	1.00	0.00	RX0	N
ATOM	723	H	LEU	233	29.366	4.448	19.592	1.00	0.00	RX0	H
ATOM	724	CA	LEU	233	29.211	3.960	21.691	1.00	0.00	RX0	C
ATOM	725	CB	LEU	233	30.721	4.150	21.830	1.00	0.00	RX0	C
ATOM	726	CG	LEU	233	31.205	3.778	23.232	1.00	0.00	RX0	C
ATOM	727	CD1	LEU	233	30.810	2.349	23.606	1.00	0.00	RX0	C
ATOM	728	CD2	LEU	233	32.704	4.013	23.404	1.00	0.00	RX0	C
ATOM	729	C	LEU	233	28.415	4.936	22.566	1.00	0.00	RX0	C
ATOM	730	O	LEU	233	27.943	4.566	23.634	1.00	0.00	RX0	O

ATOM	731	N	GLU	234	28.150	6.122	22.016	1.00	0.00	RX0	N
ATOM	732	H	GLU	234	28.468	6.304	21.087	1.00	0.00	RX0	H
ATOM	733	CA	GLU	234	27.227	7.090	22.644	1.00	0.00	RX0	C
ATOM	734	CB	GLU	234	27.079	8.297	21.705	1.00	0.00	RX0	C
ATOM	735	CG	GLU	234	27.727	9.635	22.078	1.00	0.00	RX0	C
ATOM	736	CD	GLU	234	27.558	10.620	20.927	1.00	0.00	RX0	C
ATOM	737	OE1	GLU	234	27.147	11.761	21.131	1.00	0.00	RX0	O
ATOM	738	OE2	GLU	234	27.893	10.276	19.803	1.00	0.00	RX0	O
ATOM	739	C	GLU	234	25.830	6.482	22.841	1.00	0.00	RX0	C
ATOM	740	O	GLU	234	25.253	6.598	23.926	1.00	0.00	RX0	O
ATOM	741	N	ILE	235	25.389	5.711	21.848	1.00	0.00	RX0	N
ATOM	742	H	ILE	235	25.961	5.635	21.030	1.00	0.00	RX0	H
ATOM	743	CA	ILE	235	24.069	5.045	21.863	1.00	0.00	RX0	C
ATOM	744	CB	ILE	235	23.697	4.487	20.486	1.00	0.00	RX0	C
ATOM	745	CG2	ILE	235	22.411	3.660	20.552	1.00	0.00	RX0	C
ATOM	746	CG1	ILE	235	23.559	5.618	19.467	1.00	0.00	RX0	C
ATOM	747	CD1	ILE	235	23.221	5.103	18.067	1.00	0.00	RX0	C
ATOM	748	C	ILE	235	24.018	3.945	22.939	1.00	0.00	RX0	C
ATOM	749	O	ILE	235	23.068	3.902	23.724	1.00	0.00	RX0	O
ATOM	750	N	LEU	236	25.072	3.140	23.020	1.00	0.00	RX0	N
ATOM	751	H	LEU	236	25.811	3.233	22.349	1.00	0.00	RX0	H
ATOM	752	CA	LEU	236	25.176	2.114	24.078	1.00	0.00	RX0	C
ATOM	753	CB	LEU	236	26.441	1.278	23.892	1.00	0.00	RX0	C
ATOM	754	CG	LEU	236	26.335	0.305	22.721	1.00	0.00	RX0	C
ATOM	755	CD1	LEU	236	27.663	-0.393	22.429	1.00	0.00	RX0	C
ATOM	756	CD2	LEU	236	25.199	-0.693	22.939	1.00	0.00	RX0	C
ATOM	757	C	LEU	236	25.182	2.733	25.479	1.00	0.00	RX0	C
ATOM	758	O	LEU	236	24.381	2.362	26.336	1.00	0.00	RX0	O
ATOM	759	N	MET	237	25.933	3.823	25.600	1.00	0.00	RX0	N
ATOM	760	H	MET	237	26.455	4.133	24.803	1.00	0.00	RX0	H
ATOM	761	CA	MET	237	26.132	4.523	26.881	1.00	0.00	RX0	C
ATOM	762	CB	MET	237	27.279	5.530	26.801	1.00	0.00	RX0	C
ATOM	763	CG	MET	237	28.652	4.858	26.814	1.00	0.00	RX0	C
ATOM	764	SD	MET	237	29.997	6.051	26.781	1.00	0.00	RX0	S
ATOM	765	CE	MET	237	31.348	4.910	27.115	1.00	0.00	RX0	C
ATOM	766	C	MET	237	24.875	5.215	27.409	1.00	0.00	RX0	C
ATOM	767	O	MET	237	24.517	5.003	28.572	1.00	0.00	RX0	O
ATOM	768	N	ILE	238	24.128	5.878	26.531	1.00	0.00	RX0	N
ATOM	769	H	ILE	238	24.455	5.959	25.586	1.00	0.00	RX0	H
ATOM	770	CA	ILE	238	22.871	6.546	26.925	1.00	0.00	RX0	C
ATOM	771	CB	ILE	238	22.351	7.525	25.856	1.00	0.00	RX0	C
ATOM	772	CG2	ILE	238	21.927	6.839	24.558	1.00	0.00	RX0	C
ATOM	773	CG1	ILE	238	21.225	8.384	26.435	1.00	0.00	RX0	C
ATOM	774	CD1	ILE	238	20.621	9.345	25.411	1.00	0.00	RX0	C
ATOM	775	C	ILE	238	21.800	5.514	27.357	1.00	0.00	RX0	C
ATOM	776	O	ILE	238	21.031	5.731	28.268	1.00	0.00	RX0	O
ATOM	777	N	GLY	239	21.845	4.355	26.660	1.00	0.00	RX0	N
ATOM	778	H	GLY	239	22.520	4.244	25.926	1.00	0.00	RX0	H
ATOM	779	CA	GLY	239	20.969	3.213	26.975	1.00	0.00	RX0	C
ATOM	780	C	GLY	239	21.301	2.634	28.356	1.00	0.00	RX0	C
ATOM	781	O	GLY	239	20.417	2.460	29.193	1.00	0.00	RX0	O
ATOM	782	N	LEU	240	22.605	2.583	28.639	1.00	0.00	RX0	N
ATOM	783	H	LEU	240	23.260	2.803	27.913	1.00	0.00	RX0	H
ATOM	784	CA	LEU	240	23.124	2.082	29.919	1.00	0.00	RX0	C
ATOM	785	CB	LEU	240	24.644	1.943	29.849	1.00	0.00	RX0	C
ATOM	786	CG	LEU	240	25.286	1.611	31.197	1.00	0.00	RX0	C
ATOM	787	CD1	LEU	240	24.821	0.265	31.750	1.00	0.00	RX0	C
ATOM	788	CD2	LEU	240	26.807	1.710	31.126	1.00	0.00	RX0	C
ATOM	789	C	LEU	240	22.728	2.995	31.086	1.00	0.00	RX0	C
ATOM	790	O	LEU	240	22.214	2.535	32.097	1.00	0.00	RX0	O
ATOM	791	N	VAL	241	22.901	4.295	30.880	1.00	0.00	RX0	N

ATOM	792	H	VAL	241	23.278	4.586	29.998	1.00	0.00	RX0	H
ATOM	793	CA	VAL	241	22.596	5.307	31.912	1.00	0.00	RX0	C
ATOM	794	CB	VAL	241	23.253	6.670	31.661	1.00	0.00	RX0	C
ATOM	795	CG1	VAL	241	24.772	6.505	31.604	1.00	0.00	RX0	C
ATOM	796	CG2	VAL	241	22.706	7.399	30.440	1.00	0.00	RX0	C
ATOM	797	C	VAL	241	21.084	5.392	32.193	1.00	0.00	RX0	C
ATOM	798	O	VAL	241	20.670	5.516	33.338	1.00	0.00	RX0	O
ATOM	799	N	TRP	242	20.290	5.181	31.134	1.00	0.00	RX0	N
ATOM	800	H	TRP	242	20.699	5.072	30.225	1.00	0.00	RX0	H
ATOM	801	CA	TRP	242	18.822	5.192	31.222	1.00	0.00	RX0	C
ATOM	802	CB	TRP	242	18.253	5.150	29.800	1.00	0.00	RX0	C
ATOM	803	CG	TRP	242	16.828	4.653	29.775	1.00	0.00	RX0	C
ATOM	804	CD2	TRP	242	15.635	5.323	30.232	1.00	0.00	RX0	C
ATOM	805	CE2	TRP	242	14.546	4.450	30.011	1.00	0.00	RX0	C
ATOM	806	CE3	TRP	242	15.413	6.569	30.801	1.00	0.00	RX0	C
ATOM	807	CD1	TRP	242	16.388	3.410	29.295	1.00	0.00	RX0	C
ATOM	808	NE1	TRP	242	15.042	3.286	29.432	1.00	0.00	RX0	N
ATOM	809	HE1	TRP	242	14.503	2.510	29.171	1.00	0.00	RX0	H
ATOM	810	CZ2	TRP	242	13.265	4.846	30.375	1.00	0.00	RX0	C
ATOM	811	CZ3	TRP	242	14.128	6.956	31.158	1.00	0.00	RX0	C
ATOM	812	CH2	TRP	242	13.059	6.094	30.950	1.00	0.00	RX0	C
ATOM	813	C	TRP	242	18.281	4.038	32.076	1.00	0.00	RX0	C
ATOM	814	O	TRP	242	17.477	4.269	32.979	1.00	0.00	RX0	O
ATOM	815	N	ARG	243	18.818	2.841	31.865	1.00	0.00	RX0	N
ATOM	816	H	ARG	243	19.515	2.737	31.151	1.00	0.00	RX0	H
ATOM	817	CA	ARG	243	18.359	1.661	32.627	1.00	0.00	RX0	C
ATOM	818	CB	ARG	243	18.486	0.403	31.737	1.00	0.00	RX0	C
ATOM	819	CG	ARG	243	19.891	-0.064	31.298	1.00	0.00	RX0	C
ATOM	820	CD	ARG	243	19.831	-1.101	30.153	1.00	0.00	RX0	C
ATOM	821	NE	ARG	243	21.115	-1.760	29.864	1.00	0.00	RX0	N
ATOM	822	HE	ARG	243	21.665	-2.016	30.672	1.00	0.00	RX0	H
ATOM	823	CZ	ARG	243	21.432	-2.131	28.570	1.00	0.00	RX0	C
ATOM	824	NH1	ARG	243	20.666	-1.725	27.538	1.00	0.00	RX0	N
ATOM	825	HH11	ARG	243	20.910	-2.039	26.602	1.00	0.00	RX0	H
ATOM	826	HH12	ARG	243	19.866	-1.135	27.640	1.00	0.00	RX0	H
ATOM	827	NH2	ARG	243	22.498	-2.912	28.304	1.00	0.00	RX0	N
ATOM	828	HH21	ARG	243	22.779	-3.128	27.347	1.00	0.00	RX0	H
ATOM	829	HH22	ARG	243	23.055	-3.343	29.023	1.00	0.00	RX0	H
ATOM	830	C	ARG	243	19.043	1.510	33.998	1.00	0.00	RX0	C
ATOM	831	O	ARG	243	18.610	0.722	34.836	1.00	0.00	RX0	O
ATOM	832	N	SER	244	20.027	2.366	34.245	1.00	0.00	RX0	N
ATOM	833	H	SER	244	20.316	3.011	33.538	1.00	0.00	RX0	H
ATOM	834	CA	SER	244	20.722	2.464	35.548	1.00	0.00	RX0	C
ATOM	835	CB	SER	244	22.206	2.696	35.311	1.00	0.00	RX0	C
ATOM	836	OG	SER	244	22.700	1.647	34.476	1.00	0.00	RX0	O
ATOM	837	HG	SER	244	22.226	1.717	33.654	1.00	0.00	RX0	H
ATOM	838	C	SER	244	20.112	3.547	36.442	1.00	0.00	RX0	C
ATOM	839	O	SER	244	20.448	3.642	37.630	1.00	0.00	RX0	O
ATOM	840	N	MET	245	19.184	4.322	35.895	1.00	0.00	RX0	N
ATOM	841	H	MET	245	18.885	4.152	34.954	1.00	0.00	RX0	H
ATOM	842	CA	MET	245	18.600	5.504	36.550	1.00	0.00	RX0	C
ATOM	843	CB	MET	245	17.597	6.196	35.632	1.00	0.00	RX0	C
ATOM	844	CG	MET	245	17.042	7.471	36.267	1.00	0.00	RX0	C
ATOM	845	SD	MET	245	15.742	8.234	35.294	1.00	0.00	RX0	S
ATOM	846	CE	MET	245	16.554	8.093	33.701	1.00	0.00	RX0	C
ATOM	847	C	MET	245	17.925	5.204	37.895	1.00	0.00	RX0	C
ATOM	848	O	MET	245	18.105	5.946	38.853	1.00	0.00	RX0	O
ATOM	849	N	GLU	246	17.212	4.082	37.945	1.00	0.00	RX0	N
ATOM	850	H	GLU	246	17.124	3.484	37.147	1.00	0.00	RX0	H
ATOM	851	CA	GLU	246	16.494	3.692	39.178	1.00	0.00	RX0	C
ATOM	852	CB	GLU	246	15.245	2.900	38.818	1.00	0.00	RX0	C

ATOM	853	CG	GLU	246	14.342	3.654	37.846	1.00	0.00	RX0	C
ATOM	854	CD	GLU	246	13.166	2.767	37.508	1.00	0.00	RX0	C
ATOM	855	OE1	GLU	246	13.010	1.737	38.160	1.00	0.00	RX0	O
ATOM	856	OE2	GLU	246	12.419	3.103	36.592	1.00	0.00	RX0	O
ATOM	857	C	GLU	246	17.379	2.867	40.123	1.00	0.00	RX0	C
ATOM	858	O	GLU	246	16.897	2.295	41.108	1.00	0.00	RX0	O
ATOM	859	N	HIS	247	18.674	2.844	39.836	1.00	0.00	RX0	N
ATOM	860	H	HIS	247	19.044	3.323	39.041	1.00	0.00	RX0	H
ATOM	861	CA	HIS	247	19.658	2.055	40.601	1.00	0.00	RX0	C
ATOM	862	CB	HIS	247	20.174	0.865	39.788	1.00	0.00	RX0	C
ATOM	863	CG	HIS	247	19.080	-0.136	39.486	1.00	0.00	RX0	C
ATOM	864	ND1	HIS	247	17.865	-0.169	40.072	1.00	0.00	RX0	N
ATOM	865	HD1	HIS	247	17.500	0.452	40.744	1.00	0.00	RX0	H
ATOM	866	CD2	HIS	247	19.154	-1.186	38.567	1.00	0.00	RX0	C
ATOM	867	NE2	HIS	247	17.977	-1.853	38.605	1.00	0.00	RX0	N
ATOM	868	CE1	HIS	247	17.181	-1.226	39.529	1.00	0.00	RX0	C
ATOM	869	C	HIS	247	20.841	2.947	41.015	1.00	0.00	RX0	C
ATOM	870	O	HIS	247	21.962	2.788	40.490	1.00	0.00	RX0	O
ATOM	871	N	PRO	248	20.632	3.827	41.991	1.00	0.00	RX0	N
ATOM	872	CD	PRO	248	19.384	3.973	42.733	1.00	0.00	RX0	C
ATOM	873	CA	PRO	248	21.659	4.767	42.484	1.00	0.00	RX0	C
ATOM	874	CB	PRO	248	20.980	5.449	43.675	1.00	0.00	RX0	C
ATOM	875	CG	PRO	248	19.487	5.344	43.385	1.00	0.00	RX0	C
ATOM	876	C	PRO	248	22.939	4.013	42.877	1.00	0.00	RX0	C
ATOM	877	O	PRO	248	22.892	2.963	43.503	1.00	0.00	RX0	O
ATOM	878	N	GLY	249	24.055	4.541	42.350	1.00	0.00	RX0	N
ATOM	879	H	GLY	249	23.973	5.299	41.708	1.00	0.00	RX0	H
ATOM	880	CA	GLY	249	25.407	3.996	42.610	1.00	0.00	RX0	C
ATOM	881	C	GLY	249	25.783	2.749	41.794	1.00	0.00	RX0	C
ATOM	882	O	GLY	249	26.914	2.250	41.927	1.00	0.00	RX0	O
ATOM	883	N	LYS	250	24.877	2.267	40.961	1.00	0.00	RX0	N
ATOM	884	H	LYS	250	23.983	2.697	40.813	1.00	0.00	RX0	H
ATOM	885	CA	LYS	250	25.097	1.050	40.158	1.00	0.00	RX0	C
ATOM	886	CB	LYS	250	24.364	-0.145	40.770	1.00	0.00	RX0	C
ATOM	887	CG	LYS	250	25.218	-0.871	41.818	1.00	0.00	RX0	C
ATOM	888	CD	LYS	250	24.578	-2.155	42.351	1.00	0.00	RX0	C
ATOM	889	CE	LYS	250	25.501	-3.025	43.213	1.00	0.00	RX0	C
ATOM	890	NZ	LYS	250	26.641	-3.521	42.428	1.00	0.00	RX0	N
ATOM	891	HZ1	LYS	250	27.339	-3.959	43.071	1.00	0.00	RX0	H
ATOM	892	HZ2	LYS	250	26.372	-4.196	41.677	1.00	0.00	RX0	H
ATOM	893	HZ3	LYS	250	27.172	-2.716	42.043	1.00	0.00	RX0	H
ATOM	894	C	LYS	250	24.802	1.266	38.671	1.00	0.00	RX0	C
ATOM	895	O	LYS	250	24.040	2.166	38.282	1.00	0.00	RX0	O
ATOM	896	N	LEU	251	25.472	0.476	37.863	1.00	0.00	RX0	N
ATOM	897	H	LEU	251	26.053	-0.236	38.243	1.00	0.00	RX0	H
ATOM	898	CA	LEU	251	25.292	0.430	36.401	1.00	0.00	RX0	C
ATOM	899	CB	LEU	251	26.626	0.591	35.684	1.00	0.00	RX0	C
ATOM	900	CG	LEU	251	27.161	2.016	35.763	1.00	0.00	RX0	C
ATOM	901	CD1	LEU	251	28.584	2.115	35.218	1.00	0.00	RX0	C
ATOM	902	CD2	LEU	251	26.208	3.009	35.093	1.00	0.00	RX0	C
ATOM	903	C	LEU	251	24.646	-0.892	36.017	1.00	0.00	RX0	C
ATOM	904	O	LEU	251	25.224	-1.976	36.286	1.00	0.00	RX0	O
ATOM	905	N	LEU	252	23.437	-0.808	35.533	1.00	0.00	RX0	N
ATOM	906	H	LEU	252	23.084	0.097	35.312	1.00	0.00	RX0	H
ATOM	907	CA	LEU	252	22.659	-1.981	35.114	1.00	0.00	RX0	C
ATOM	908	CB	LEU	252	21.157	-1.712	35.229	1.00	0.00	RX0	C
ATOM	909	CG	LEU	252	20.297	-2.956	34.971	1.00	0.00	RX0	C
ATOM	910	CD1	LEU	252	20.445	-3.994	36.082	1.00	0.00	RX0	C
ATOM	911	CD2	LEU	252	18.831	-2.606	34.722	1.00	0.00	RX0	C
ATOM	912	C	LEU	252	23.007	-2.330	33.663	1.00	0.00	RX0	C
ATOM	913	O	LEU	252	22.274	-2.025	32.731	1.00	0.00	RX0	O

ATOM	914	N	PHE	253	24.144	-3.005	33.506	1.00	0.00	RX0	N
ATOM	915	H	PHE	253	24.631	-3.308	34.330	1.00	0.00	RX0	H
ATOM	916	CA	PHE	253	24.599	-3.457	32.174	1.00	0.00	RX0	C
ATOM	917	CB	PHE	253	25.968	-4.124	32.271	1.00	0.00	RX0	C
ATOM	918	CG	PHE	253	27.031	-3.098	32.561	1.00	0.00	RX0	C
ATOM	919	CD1	PHE	253	27.563	-2.348	31.518	1.00	0.00	RX0	C
ATOM	920	CD2	PHE	253	27.486	-2.909	33.860	1.00	0.00	RX0	C
ATOM	921	CE1	PHE	253	28.561	-1.416	31.772	1.00	0.00	RX0	C
ATOM	922	CE2	PHE	253	28.483	-1.977	34.112	1.00	0.00	RX0	C
ATOM	923	CZ	PHE	253	29.021	-1.232	33.069	1.00	0.00	RX0	C
ATOM	924	C	PHE	253	23.603	-4.446	31.564	1.00	0.00	RX0	C
ATOM	925	O	PHE	253	23.259	-4.379	30.390	1.00	0.00	RX0	O
ATOM	926	N	ALA	254	23.094	-5.300	32.445	1.00	0.00	RX0	N
ATOM	927	H	ALA	254	23.408	-5.312	33.400	1.00	0.00	RX0	H
ATOM	928	CA	ALA	254	22.050	-6.280	32.141	1.00	0.00	RX0	C
ATOM	929	CB	ALA	254	22.697	-7.549	31.592	1.00	0.00	RX0	C
ATOM	930	C	ALA	254	21.288	-6.584	33.440	1.00	0.00	RX0	C
ATOM	931	O	ALA	254	21.887	-6.418	34.526	1.00	0.00	RX0	O
ATOM	932	N	PRO	255	20.056	-7.064	33.372	1.00	0.00	RX0	N
ATOM	933	CD	PRO	255	19.307	-7.244	32.131	1.00	0.00	RX0	C
ATOM	934	CA	PRO	255	19.236	-7.444	34.545	1.00	0.00	RX0	C
ATOM	935	CB	PRO	255	17.987	-8.068	33.924	1.00	0.00	RX0	C
ATOM	936	CG	PRO	255	17.856	-7.385	32.569	1.00	0.00	RX0	C
ATOM	937	C	PRO	255	19.972	-8.395	35.506	1.00	0.00	RX0	C
ATOM	938	O	PRO	255	19.756	-8.342	36.714	1.00	0.00	RX0	O
ATOM	939	N	ASN	256	20.900	-9.179	34.970	1.00	0.00	RX0	N
ATOM	940	H	ASN	256	21.113	-9.138	33.993	1.00	0.00	RX0	H
ATOM	941	CA	ASN	256	21.722	-10.125	35.761	1.00	0.00	RX0	C
ATOM	942	CB	ASN	256	21.709	-11.529	35.151	1.00	0.00	RX0	C
ATOM	943	CG	ASN	256	22.401	-11.535	33.797	1.00	0.00	RX0	C
ATOM	944	OD1	ASN	256	22.276	-10.604	33.001	1.00	0.00	RX0	O
ATOM	945	ND2	ASN	256	23.048	-12.684	33.529	1.00	0.00	RX0	N
ATOM	946	HD21	ASN	256	23.187	-13.356	34.262	1.00	0.00	RX0	H
ATOM	947	HD22	ASN	256	23.402	-12.936	32.623	1.00	0.00	RX0	H
ATOM	948	C	ASN	256	23.191	-9.669	35.876	1.00	0.00	RX0	C
ATOM	949	O	ASN	256	24.101	-10.505	36.011	1.00	0.00	RX0	O
ATOM	950	N	LEU	257	23.439	-8.384	35.742	1.00	0.00	RX0	N
ATOM	951	H	LEU	257	22.692	-7.727	35.628	1.00	0.00	RX0	H
ATOM	952	CA	LEU	257	24.796	-7.805	35.783	1.00	0.00	RX0	C
ATOM	953	CB	LEU	257	25.514	-7.967	34.444	1.00	0.00	RX0	C
ATOM	954	CG	LEU	257	27.024	-7.753	34.566	1.00	0.00	RX0	C
ATOM	955	CD1	LEU	257	27.659	-8.784	35.499	1.00	0.00	RX0	C
ATOM	956	CD2	LEU	257	27.714	-7.721	33.202	1.00	0.00	RX0	C
ATOM	957	C	LEU	257	24.720	-6.327	36.168	1.00	0.00	RX0	C
ATOM	958	O	LEU	257	24.738	-5.412	35.328	1.00	0.00	RX0	O
ATOM	959	N	LEU	258	24.604	-6.148	37.469	1.00	0.00	RX0	N
ATOM	960	H	LEU	258	24.689	-6.942	38.069	1.00	0.00	RX0	H
ATOM	961	CA	LEU	258	24.482	-4.834	38.118	1.00	0.00	RX0	C
ATOM	962	CB	LEU	258	23.219	-4.908	38.976	1.00	0.00	RX0	C
ATOM	963	CG	LEU	258	22.797	-3.619	39.669	1.00	0.00	RX0	C
ATOM	964	CD1	LEU	258	22.652	-2.466	38.686	1.00	0.00	RX0	C
ATOM	965	CD2	LEU	258	21.526	-3.817	40.496	1.00	0.00	RX0	C
ATOM	966	C	LEU	258	25.743	-4.564	38.938	1.00	0.00	RX0	C
ATOM	967	O	LEU	258	26.013	-5.237	39.948	1.00	0.00	RX0	O
ATOM	968	N	LEU	259	26.528	-3.622	38.460	1.00	0.00	RX0	N
ATOM	969	H	LEU	259	26.219	-3.062	37.684	1.00	0.00	RX0	H
ATOM	970	CA	LEU	259	27.862	-3.349	39.027	1.00	0.00	RX0	C
ATOM	971	CB	LEU	259	28.938	-3.493	37.948	1.00	0.00	RX0	C
ATOM	972	CG	LEU	259	28.885	-4.814	37.174	1.00	0.00	RX0	C
ATOM	973	CD1	LEU	259	29.898	-4.837	36.030	1.00	0.00	RX0	C
ATOM	974	CD2	LEU	259	29.042	-6.031	38.085	1.00	0.00	RX0	C

ATOM	975	C	LEU	259	27.958	-1.956	39.652	1.00	0.00	RX0	C
ATOM	976	O	LEU	259	27.419	-0.984	39.137	1.00	0.00	RX0	O
ATOM	977	N	ASP	260	28.645	-1.912	40.785	1.00	0.00	RX0	N
ATOM	978	H	ASP	260	29.224	-2.688	41.046	1.00	0.00	RX0	H
ATOM	979	CA	ASP	260	29.043	-0.657	41.454	1.00	0.00	RX0	C
ATOM	980	CB	ASP	260	29.066	-0.872	42.962	1.00	0.00	RX0	C
ATOM	981	CG	ASP	260	29.706	-2.217	43.215	1.00	0.00	RX0	C
ATOM	982	OD1	ASP	260	30.929	-2.309	43.201	1.00	0.00	RX0	O
ATOM	983	OD2	ASP	260	28.966	-3.188	43.371	1.00	0.00	RX0	O
ATOM	984	C	ASP	260	30.443	-0.222	40.970	1.00	0.00	RX0	C
ATOM	985	O	ASP	260	31.127	-1.008	40.295	1.00	0.00	RX0	O
ATOM	986	N	ARG	261	30.963	0.873	41.504	1.00	0.00	RX0	N
ATOM	987	H	ARG	261	30.394	1.401	42.133	1.00	0.00	RX0	H
ATOM	988	CA	ARG	261	32.263	1.408	41.044	1.00	0.00	RX0	C
ATOM	989	CB	ARG	261	32.418	2.888	41.427	1.00	0.00	RX0	C
ATOM	990	CG	ARG	261	32.634	3.173	42.915	1.00	0.00	RX0	C
ATOM	991	CD	ARG	261	32.505	4.659	43.268	1.00	0.00	RX0	C
ATOM	992	NE	ARG	261	33.380	5.504	42.456	1.00	0.00	RX0	N
ATOM	993	HE	ARG	261	33.195	5.603	41.465	1.00	0.00	RX0	H
ATOM	994	CZ	ARG	261	34.369	6.255	43.023	1.00	0.00	RX0	C
ATOM	995	NH1	ARG	261	34.577	6.210	44.353	1.00	0.00	RX0	N
ATOM	996	HH11	ARG	261	35.362	6.654	44.788	1.00	0.00	RX0	H
ATOM	997	HH12	ARG	261	33.921	5.749	44.971	1.00	0.00	RX0	H
ATOM	998	NH2	ARG	261	35.114	7.051	42.240	1.00	0.00	RX0	N
ATOM	999	HH21	ARG	261	35.842	7.672	42.554	1.00	0.00	RX0	H
ATOM	1000	HH22	ARG	261	34.921	7.067	41.241	1.00	0.00	RX0	H
ATOM	1001	C	ARG	261	33.476	0.540	41.436	1.00	0.00	RX0	C
ATOM	1002	O	ARG	261	34.378	0.347	40.637	1.00	0.00	RX0	O
ATOM	1003	N	ASN	262	33.410	-0.067	42.632	1.00	0.00	RX0	N
ATOM	1004	H	ASN	262	32.547	-0.022	43.136	1.00	0.00	RX0	H
ATOM	1005	CA	ASN	262	34.456	-1.000	43.094	1.00	0.00	RX0	C
ATOM	1006	CB	ASN	262	34.228	-1.478	44.526	1.00	0.00	RX0	C
ATOM	1007	CG	ASN	262	35.360	-2.419	44.910	1.00	0.00	RX0	C
ATOM	1008	OD1	ASN	262	36.457	-1.991	45.276	1.00	0.00	RX0	O
ATOM	1009	ND2	ASN	262	35.029	-3.722	44.850	1.00	0.00	RX0	N
ATOM	1010	HD21	ASN	262	34.107	-4.012	44.585	1.00	0.00	RX0	H
ATOM	1011	HD22	ASN	262	35.694	-4.444	45.050	1.00	0.00	RX0	H
ATOM	1012	C	ASN	262	34.635	-2.233	42.204	1.00	0.00	RX0	C
ATOM	1013	O	ASN	262	35.755	-2.665	41.964	1.00	0.00	RX0	O
ATOM	1014	N	GLN	263	33.530	-2.679	41.603	1.00	0.00	RX0	N
ATOM	1015	H	GLN	263	32.639	-2.273	41.829	1.00	0.00	RX0	H
ATOM	1016	CA	GLN	263	33.559	-3.756	40.599	1.00	0.00	RX0	C
ATOM	1017	CB	GLN	263	32.199	-4.425	40.454	1.00	0.00	RX0	C
ATOM	1018	CG	GLN	263	31.959	-5.203	41.746	1.00	0.00	RX0	C
ATOM	1019	CD	GLN	263	30.709	-6.041	41.654	1.00	0.00	RX0	C
ATOM	1020	OE1	GLN	263	30.571	-6.924	40.817	1.00	0.00	RX0	O
ATOM	1021	NE2	GLN	263	29.820	-5.756	42.615	1.00	0.00	RX0	N
ATOM	1022	HE21	GLN	263	29.969	-4.928	43.172	1.00	0.00	RX0	H
ATOM	1023	HE22	GLN	263	29.035	-6.344	42.799	1.00	0.00	RX0	H
ATOM	1024	C	GLN	263	34.189	-3.308	39.265	1.00	0.00	RX0	C
ATOM	1025	O	GLN	263	34.644	-4.116	38.479	1.00	0.00	RX0	O
ATOM	1026	N	GLY	264	34.180	-1.978	39.039	1.00	0.00	RX0	N
ATOM	1027	H	GLY	264	33.772	-1.378	39.727	1.00	0.00	RX0	H
ATOM	1028	CA	GLY	264	34.831	-1.337	37.881	1.00	0.00	RX0	C
ATOM	1029	C	GLY	264	36.364	-1.310	37.978	1.00	0.00	RX0	C
ATOM	1030	O	GLY	264	37.050	-1.510	36.977	1.00	0.00	RX0	O
ATOM	1031	N	LYS	265	36.881	-1.155	39.202	1.00	0.00	RX0	N
ATOM	1032	H	LYS	265	36.240	-1.023	39.960	1.00	0.00	RX0	H
ATOM	1033	CA	LYS	265	38.336	-1.184	39.469	1.00	0.00	RX0	C
ATOM	1034	CB	LYS	265	38.641	-1.158	40.946	1.00	0.00	RX0	C
ATOM	1035	CG	LYS	265	38.266	-0.001	41.852	1.00	0.00	RX0	C

ATOM	1036	CD	LYS	265	38.527	-0.700	43.173	1.00	0.00	RX0	C
ATOM	1037	CE	LYS	265	38.668	0.052	44.483	1.00	0.00	RX0	C
ATOM	1038	NZ	LYS	265	38.986	-1.015	45.439	1.00	0.00	RX0	N
ATOM	1039	HZ1	LYS	265	39.150	-0.690	46.403	1.00	0.00	RX0	H
ATOM	1040	HZ2	LYS	265	38.235	-1.740	45.418	1.00	0.00	RX0	H
ATOM	1041	HZ3	LYS	265	39.855	-1.509	45.114	1.00	0.00	RX0	H
ATOM	1042	C	LYS	265	38.994	-2.500	39.030	1.00	0.00	RX0	C
ATOM	1043	O	LYS	265	40.184	-2.535	38.765	1.00	0.00	RX0	O
ATOM	1044	N	CYS	266	38.153	-3.548	38.917	1.00	0.00	RX0	N
ATOM	1045	H	CYS	266	37.204	-3.462	39.219	1.00	0.00	RX0	H
ATOM	1046	CA	CYS	266	38.538	-4.854	38.355	1.00	0.00	RX0	C
ATOM	1047	CB	CYS	266	37.317	-5.761	38.255	1.00	0.00	RX0	C
ATOM	1048	SG	CYS	266	36.544	-5.961	39.881	1.00	0.00	RX0	S
ATOM	1049	C	CYS	266	39.318	-4.732	37.033	1.00	0.00	RX0	C
ATOM	1050	O	CYS	266	40.108	-5.603	36.695	1.00	0.00	RX0	O
ATOM	1051	N	VAL	267	39.075	-3.636	36.304	1.00	0.00	RX0	N
ATOM	1052	H	VAL	267	38.451	-2.914	36.612	1.00	0.00	RX0	H
ATOM	1053	CA	VAL	267	39.804	-3.345	35.058	1.00	0.00	RX0	C
ATOM	1054	CB	VAL	267	38.873	-3.370	33.840	1.00	0.00	RX0	C
ATOM	1055	CG1	VAL	267	39.618	-3.024	32.549	1.00	0.00	RX0	C
ATOM	1056	CG2	VAL	267	38.173	-4.727	33.724	1.00	0.00	RX0	C
ATOM	1057	C	VAL	267	40.557	-2.013	35.191	1.00	0.00	RX0	C
ATOM	1058	O	VAL	267	39.969	-0.956	35.468	1.00	0.00	RX0	O
ATOM	1059	N	GLU	268	41.829	-2.078	34.821	1.00	0.00	RX0	N
ATOM	1060	H	GLU	268	42.182	-2.951	34.508	1.00	0.00	RX0	H
ATOM	1061	CA	GLU	268	42.726	-0.908	34.727	1.00	0.00	RX0	C
ATOM	1062	CB	GLU	268	44.146	-1.279	34.345	1.00	0.00	RX0	C
ATOM	1063	CG	GLU	268	45.130	-0.114	34.302	1.00	0.00	RX0	C
ATOM	1064	CD	GLU	268	45.073	0.480	32.919	1.00	0.00	RX0	C
ATOM	1065	OE1	GLU	268	45.155	1.695	32.780	1.00	0.00	RX0	O
ATOM	1066	OE2	GLU	268	44.943	-0.281	31.966	1.00	0.00	RX0	O
ATOM	1067	C	GLU	268	42.079	0.194	33.866	1.00	0.00	RX0	C
ATOM	1068	O	GLU	268	41.697	-0.027	32.727	1.00	0.00	RX0	O
ATOM	1069	N	GLY	269	41.924	1.355	34.524	1.00	0.00	RX0	N
ATOM	1070	H	GLY	269	42.293	1.347	35.443	1.00	0.00	RX0	H
ATOM	1071	CA	GLY	269	41.377	2.576	33.902	1.00	0.00	RX0	C
ATOM	1072	C	GLY	269	39.898	2.490	33.494	1.00	0.00	RX0	C
ATOM	1073	O	GLY	269	39.424	3.343	32.745	1.00	0.00	RX0	O
ATOM	1074	N	MET	270	39.146	1.598	34.134	1.00	0.00	RX0	N
ATOM	1075	H	MET	270	39.574	0.912	34.724	1.00	0.00	RX0	H
ATOM	1076	CA	MET	270	37.704	1.465	33.841	1.00	0.00	RX0	C
ATOM	1077	CB	MET	270	37.285	0.007	33.653	1.00	0.00	RX0	C
ATOM	1078	CG	MET	270	35.929	-0.107	32.950	1.00	0.00	RX0	C
ATOM	1079	SD	MET	270	35.436	-1.799	32.579	1.00	0.00	RX0	S
ATOM	1080	CE	MET	270	35.185	-2.377	34.263	1.00	0.00	RX0	C
ATOM	1081	C	MET	270	36.825	2.181	34.877	1.00	0.00	RX0	C
ATOM	1082	O	MET	270	35.781	2.734	34.514	1.00	0.00	RX0	O
ATOM	1083	N	VAL	271	37.310	2.290	36.107	1.00	0.00	RX0	N
ATOM	1084	H	VAL	271	38.202	1.882	36.287	1.00	0.00	RX0	H
ATOM	1085	CA	VAL	271	36.591	3.043	37.171	1.00	0.00	RX0	C
ATOM	1086	CB	VAL	271	37.209	2.863	38.571	1.00	0.00	RX0	C
ATOM	1087	CG1	VAL	271	38.641	3.372	38.684	1.00	0.00	RX0	C
ATOM	1088	CG2	VAL	271	36.293	3.444	39.650	1.00	0.00	RX0	C
ATOM	1089	C	VAL	271	36.343	4.502	36.749	1.00	0.00	RX0	C
ATOM	1090	O	VAL	271	35.261	5.071	37.095	1.00	0.00	RX0	O
ATOM	1091	N	GLU	272	37.228	5.074	36.011	1.00	0.00	RX0	N
ATOM	1092	H	GLU	272	38.057	4.559	35.799	1.00	0.00	RX0	H
ATOM	1093	CA	GLU	272	37.141	6.453	35.460	1.00	0.00	RX0	C
ATOM	1094	CB	GLU	272	38.460	6.661	34.699	1.00	0.00	RX0	C
ATOM	1095	CG	GLU	272	39.788	6.764	35.491	1.00	0.00	RX0	C
ATOM	1096	CD	GLU	272	40.252	5.536	36.292	1.00	0.00	RX0	C

ATOM	1097	OE1	GLU	272	39.988	4.386	35.945	1.00	0.00	RX0	O
ATOM	1098	OE2	GLU	272	40.950	5.714	37.284	1.00	0.00	RX0	O
ATOM	1099	C	GLU	272	35.927	6.588	34.526	1.00	0.00	RX0	C
ATOM	1100	O	GLU	272	35.142	7.521	34.681	1.00	0.00	RX0	O
ATOM	1101	N	ILE	273	35.702	5.552	33.729	1.00	0.00	RX0	N
ATOM	1102	H	ILE	273	36.300	4.754	33.810	1.00	0.00	RX0	H
ATOM	1103	CA	ILE	273	34.533	5.484	32.820	1.00	0.00	RX0	C
ATOM	1104	CB	ILE	273	34.734	4.378	31.778	1.00	0.00	RX0	C
ATOM	1105	CG2	ILE	273	33.625	4.413	30.726	1.00	0.00	RX0	C
ATOM	1106	CG1	ILE	273	36.128	4.433	31.147	1.00	0.00	RX0	C
ATOM	1107	CD1	ILE	273	36.348	5.662	30.262	1.00	0.00	RX0	C
ATOM	1108	C	ILE	273	33.249	5.235	33.628	1.00	0.00	RX0	C
ATOM	1109	O	ILE	273	32.257	5.951	33.452	1.00	0.00	RX0	O
ATOM	1110	N	PHE	274	33.320	4.302	34.575	1.00	0.00	RX0	N
ATOM	1111	H	PHE	274	34.183	3.805	34.677	1.00	0.00	RX0	H
ATOM	1112	CA	PHE	274	32.191	3.976	35.472	1.00	0.00	RX0	C
ATOM	1113	CB	PHE	274	32.610	2.939	36.515	1.00	0.00	RX0	C
ATOM	1114	CG	PHE	274	32.277	1.537	36.069	1.00	0.00	RX0	C
ATOM	1115	CD1	PHE	274	32.554	1.119	34.773	1.00	0.00	RX0	C
ATOM	1116	CD2	PHE	274	31.688	0.660	36.973	1.00	0.00	RX0	C
ATOM	1117	CE1	PHE	274	32.249	-0.180	34.387	1.00	0.00	RX0	C
ATOM	1118	CE2	PHE	274	31.384	-0.639	36.587	1.00	0.00	RX0	C
ATOM	1119	CZ	PHE	274	31.673	-1.061	35.296	1.00	0.00	RX0	C
ATOM	1120	C	PHE	274	31.669	5.203	36.222	1.00	0.00	RX0	C
ATOM	1121	O	PHE	274	30.484	5.521	36.143	1.00	0.00	RX0	O
ATOM	1122	N	ASP	275	32.607	5.983	36.757	1.00	0.00	RX0	N
ATOM	1123	H	ASP	275	33.566	5.702	36.757	1.00	0.00	RX0	H
ATOM	1124	CA	ASP	275	32.273	7.222	37.483	1.00	0.00	RX0	C
ATOM	1125	CB	ASP	275	33.561	7.868	37.988	1.00	0.00	RX0	C
ATOM	1126	CG	ASP	275	33.994	7.316	39.326	1.00	0.00	RX0	C
ATOM	1127	OD1	ASP	275	33.169	7.198	40.225	1.00	0.00	RX0	O
ATOM	1128	OD2	ASP	275	35.180	7.055	39.508	1.00	0.00	RX0	O
ATOM	1129	C	ASP	275	31.555	8.270	36.629	1.00	0.00	RX0	C
ATOM	1130	O	ASP	275	30.604	8.890	37.102	1.00	0.00	RX0	O
ATOM	1131	N	MET	276	31.921	8.336	35.353	1.00	0.00	RX0	N
ATOM	1132	H	MET	276	32.656	7.734	35.033	1.00	0.00	RX0	H
ATOM	1133	CA	MET	276	31.257	9.254	34.408	1.00	0.00	RX0	C
ATOM	1134	CB	MET	276	32.115	9.443	33.158	1.00	0.00	RX0	C
ATOM	1135	CG	MET	276	33.493	10.022	33.483	1.00	0.00	RX0	C
ATOM	1136	SD	MET	276	34.428	10.477	32.013	1.00	0.00	RX0	S
ATOM	1137	CE	MET	276	34.358	8.889	31.176	1.00	0.00	RX0	C
ATOM	1138	C	MET	276	29.833	8.798	34.061	1.00	0.00	RX0	C
ATOM	1139	O	MET	276	28.893	9.589	34.158	1.00	0.00	RX0	O
ATOM	1140	N	LEU	277	29.673	7.488	33.891	1.00	0.00	RX0	N
ATOM	1141	H	LEU	277	30.490	6.905	33.934	1.00	0.00	RX0	H
ATOM	1142	CA	LEU	277	28.362	6.866	33.606	1.00	0.00	RX0	C
ATOM	1143	CB	LEU	277	28.562	5.402	33.224	1.00	0.00	RX0	C
ATOM	1144	CG	LEU	277	29.434	5.224	31.982	1.00	0.00	RX0	C
ATOM	1145	CD1	LEU	277	29.924	3.785	31.831	1.00	0.00	RX0	C
ATOM	1146	CD2	LEU	277	28.736	5.730	30.722	1.00	0.00	RX0	C
ATOM	1147	C	LEU	277	27.393	6.992	34.787	1.00	0.00	RX0	C
ATOM	1148	O	LEU	277	26.257	7.447	34.627	1.00	0.00	RX0	O
ATOM	1149	N	LEU	278	27.939	6.795	35.983	1.00	0.00	RX0	N
ATOM	1150	H	LEU	278	28.894	6.498	36.015	1.00	0.00	RX0	H
ATOM	1151	CA	LEU	278	27.196	6.926	37.250	1.00	0.00	RX0	C
ATOM	1152	CB	LEU	278	28.068	6.474	38.420	1.00	0.00	RX0	C
ATOM	1153	CG	LEU	278	28.224	4.958	38.472	1.00	0.00	RX0	C
ATOM	1154	CD1	LEU	278	29.274	4.512	39.491	1.00	0.00	RX0	C
ATOM	1155	CD2	LEU	278	26.873	4.292	38.711	1.00	0.00	RX0	C
ATOM	1156	C	LEU	278	26.716	8.359	37.508	1.00	0.00	RX0	C
ATOM	1157	O	LEU	278	25.554	8.575	37.840	1.00	0.00	RX0	O

ATOM	1158	N	ALA	279	27.582	9.317	37.175	1.00	0.00	RX0	N
ATOM	1159	H	ALA	279	28.504	9.048	36.884	1.00	0.00	RX0	H
ATOM	1160	CA	ALA	279	27.275	10.754	37.296	1.00	0.00	RX0	C
ATOM	1161	CB	ALA	279	28.517	11.573	37.005	1.00	0.00	RX0	C
ATOM	1162	C	ALA	279	26.182	11.196	36.310	1.00	0.00	RX0	C
ATOM	1163	O	ALA	279	25.263	11.922	36.684	1.00	0.00	RX0	O
ATOM	1164	N	THR	280	26.210	10.618	35.109	1.00	0.00	RX0	N
ATOM	1165	H	THR	280	26.967	10.007	34.873	1.00	0.00	RX0	H
ATOM	1166	CA	THR	280	25.203	10.899	34.059	1.00	0.00	RX0	C
ATOM	1167	CB	THR	280	25.745	10.370	32.738	1.00	0.00	RX0	C
ATOM	1168	OG1	THR	280	27.064	10.894	32.536	1.00	0.00	RX0	O
ATOM	1169	HG1	THR	280	27.666	10.407	33.088	1.00	0.00	RX0	H
ATOM	1170	CG2	THR	280	24.834	10.735	31.564	1.00	0.00	RX0	C
ATOM	1171	C	THR	280	23.835	10.327	34.462	1.00	0.00	RX0	C
ATOM	1172	O	THR	280	22.822	11.023	34.397	1.00	0.00	RX0	O
ATOM	1173	N	SER	281	23.868	9.112	35.003	1.00	0.00	RX0	N
ATOM	1174	H	SER	281	24.739	8.618	35.045	1.00	0.00	RX0	H
ATOM	1175	CA	SER	281	22.669	8.413	35.507	1.00	0.00	RX0	C
ATOM	1176	CB	SER	281	23.146	6.984	35.864	1.00	0.00	RX0	C
ATOM	1177	OG	SER	281	22.392	6.339	36.910	1.00	0.00	RX0	O
ATOM	1178	HG	SER	281	22.603	5.411	36.825	1.00	0.00	RX0	H
ATOM	1179	C	SER	281	22.019	9.180	36.675	1.00	0.00	RX0	C
ATOM	1180	O	SER	281	20.814	9.399	36.693	1.00	0.00	RX0	O
ATOM	1181	N	SER	282	22.889	9.788	37.491	1.00	0.00	RX0	N
ATOM	1182	H	SER	282	23.868	9.621	37.372	1.00	0.00	RX0	H
ATOM	1183	CA	SER	282	22.489	10.660	38.613	1.00	0.00	RX0	C
ATOM	1184	CB	SER	282	23.673	10.726	39.558	1.00	0.00	RX0	C
ATOM	1185	OG	SER	282	23.943	9.349	39.880	1.00	0.00	RX0	O
ATOM	1186	HG	SER	282	24.706	9.113	39.353	1.00	0.00	RX0	H
ATOM	1187	C	SER	282	21.828	11.963	38.130	1.00	0.00	RX0	C
ATOM	1188	O	SER	282	20.788	12.371	38.639	1.00	0.00	RX0	O
ATOM	1189	N	ARG	283	22.365	12.516	37.039	1.00	0.00	RX0	N
ATOM	1190	H	ARG	283	23.190	12.114	36.634	1.00	0.00	RX0	H
ATOM	1191	CA	ARG	283	21.822	13.735	36.412	1.00	0.00	RX0	C
ATOM	1192	CB	ARG	283	22.797	14.200	35.339	1.00	0.00	RX0	C
ATOM	1193	CG	ARG	283	22.190	15.088	34.254	1.00	0.00	RX0	C
ATOM	1194	CD	ARG	283	21.692	16.456	34.721	1.00	0.00	RX0	C
ATOM	1195	NE	ARG	283	21.968	17.404	33.650	1.00	0.00	RX0	N
ATOM	1196	HE	ARG	283	22.691	17.136	32.997	1.00	0.00	RX0	H
ATOM	1197	CZ	ARG	283	21.442	18.653	33.582	1.00	0.00	RX0	C
ATOM	1198	NH1	ARG	283	20.529	19.048	34.493	1.00	0.00	RX0	N
ATOM	1199	HH11	ARG	283	20.088	19.956	34.454	1.00	0.00	RX0	H
ATOM	1200	HH12	ARG	283	20.251	18.448	35.244	1.00	0.00	RX0	H
ATOM	1201	NH2	ARG	283	21.865	19.462	32.591	1.00	0.00	RX0	N
ATOM	1202	HH21	ARG	283	21.513	20.385	32.439	1.00	0.00	RX0	H
ATOM	1203	HH22	ARG	283	22.603	19.131	31.981	1.00	0.00	RX0	H
ATOM	1204	C	ARG	283	20.432	13.477	35.812	1.00	0.00	RX0	C
ATOM	1205	O	ARG	283	19.498	14.246	36.035	1.00	0.00	RX0	O
ATOM	1206	N	PHE	284	20.293	12.325	35.169	1.00	0.00	RX0	N
ATOM	1207	H	PHE	284	21.101	11.747	35.034	1.00	0.00	RX0	H
ATOM	1208	CA	PHE	284	19.003	11.899	34.606	1.00	0.00	RX0	C
ATOM	1209	CB	PHE	284	19.202	10.639	33.771	1.00	0.00	RX0	C
ATOM	1210	CG	PHE	284	19.670	11.010	32.387	1.00	0.00	RX0	C
ATOM	1211	CD1	PHE	284	19.161	12.145	31.767	1.00	0.00	RX0	C
ATOM	1212	CD2	PHE	284	20.586	10.206	31.722	1.00	0.00	RX0	C
ATOM	1213	CE1	PHE	284	19.533	12.448	30.463	1.00	0.00	RX0	C
ATOM	1214	CE2	PHE	284	20.961	10.515	30.420	1.00	0.00	RX0	C
ATOM	1215	CZ	PHE	284	20.421	11.624	29.783	1.00	0.00	RX0	C
ATOM	1216	C	PHE	284	17.921	11.680	35.654	1.00	0.00	RX0	C
ATOM	1217	O	PHE	284	16.817	12.216	35.524	1.00	0.00	RX0	O
ATOM	1218	N	ARG	285	18.348	11.087	36.760	1.00	0.00	RX0	N

ATOM	1219	H	ARG	285	19.281	10.722	36.764	1.00	0.00	RX0	H
ATOM	1220	CA	ARG	285	17.485	10.841	37.923	1.00	0.00	RX0	C
ATOM	1221	CB	ARG	285	18.333	10.059	38.927	1.00	0.00	RX0	C
ATOM	1222	CG	ARG	285	17.764	9.790	40.321	1.00	0.00	RX0	C
ATOM	1223	CD	ARG	285	18.789	9.028	41.174	1.00	0.00	RX0	C
ATOM	1224	NE	ARG	285	19.207	7.821	40.465	1.00	0.00	RX0	N
ATOM	1225	HE	ARG	285	18.452	7.223	40.166	1.00	0.00	RX0	H
ATOM	1226	CZ	ARG	285	20.500	7.648	40.054	1.00	0.00	RX0	C
ATOM	1227	NH1	ARG	285	21.464	8.428	40.587	1.00	0.00	RX0	N
ATOM	1228	HH11	ARG	285	22.429	8.423	40.279	1.00	0.00	RX0	H
ATOM	1229	HH12	ARG	285	21.253	9.077	41.321	1.00	0.00	RX0	H
ATOM	1230	NH2	ARG	285	20.766	6.715	39.115	1.00	0.00	RX0	N
ATOM	1231	HH21	ARG	285	21.654	6.585	38.652	1.00	0.00	RX0	H
ATOM	1232	HH22	ARG	285	20.025	6.105	38.807	1.00	0.00	RX0	H
ATOM	1233	C	ARG	285	17.003	12.164	38.534	1.00	0.00	RX0	C
ATOM	1234	O	ARG	285	15.822	12.321	38.816	1.00	0.00	RX0	O
ATOM	1235	N	MET	286	17.922	13.130	38.591	1.00	0.00	RX0	N
ATOM	1236	H	MET	286	18.854	12.929	38.285	1.00	0.00	RX0	H
ATOM	1237	CA	MET	286	17.643	14.467	39.144	1.00	0.00	RX0	C
ATOM	1238	CB	MET	286	18.955	15.221	39.361	1.00	0.00	RX0	C
ATOM	1239	CG	MET	286	18.774	16.547	40.099	1.00	0.00	RX0	C
ATOM	1240	SD	MET	286	20.350	17.356	40.414	1.00	0.00	RX0	S
ATOM	1241	CE	MET	286	19.733	18.775	41.334	1.00	0.00	RX0	C
ATOM	1242	C	MET	286	16.681	15.257	38.243	1.00	0.00	RX0	C
ATOM	1243	O	MET	286	15.799	15.962	38.735	1.00	0.00	RX0	O
ATOM	1244	N	MET	287	16.911	15.161	36.942	1.00	0.00	RX0	N
ATOM	1245	H	MET	287	17.642	14.551	36.631	1.00	0.00	RX0	H
ATOM	1246	CA	MET	287	16.049	15.814	35.941	1.00	0.00	RX0	C
ATOM	1247	CB	MET	287	16.683	15.860	34.556	1.00	0.00	RX0	C
ATOM	1248	CG	MET	287	17.834	16.856	34.498	1.00	0.00	RX0	C
ATOM	1249	SD	MET	287	18.235	17.300	32.805	1.00	0.00	RX0	S
ATOM	1250	CE	MET	287	16.618	17.959	32.370	1.00	0.00	RX0	C
ATOM	1251	C	MET	287	14.674	15.156	35.849	1.00	0.00	RX0	C
ATOM	1252	O	MET	287	13.755	15.729	35.264	1.00	0.00	RX0	O
ATOM	1253	N	ASN	288	14.583	13.920	36.346	1.00	0.00	RX0	N
ATOM	1254	H	ASN	288	15.394	13.492	36.747	1.00	0.00	RX0	H
ATOM	1255	CA	ASN	288	13.391	13.066	36.242	1.00	0.00	RX0	C
ATOM	1256	CB	ASN	288	12.161	13.612	36.968	1.00	0.00	RX0	C
ATOM	1257	CG	ASN	288	11.009	12.652	36.725	1.00	0.00	RX0	C
ATOM	1258	OD1	ASN	288	11.180	11.440	36.649	1.00	0.00	RX0	O
ATOM	1259	ND2	ASN	288	9.819	13.265	36.583	1.00	0.00	RX0	N
ATOM	1260	HD21	ASN	288	9.739	14.257	36.672	1.00	0.00	RX0	H
ATOM	1261	HD22	ASN	288	8.994	12.742	36.364	1.00	0.00	RX0	H
ATOM	1262	C	ASN	288	13.076	12.836	34.753	1.00	0.00	RX0	C
ATOM	1263	O	ASN	288	11.986	13.121	34.256	1.00	0.00	RX0	O
ATOM	1264	N	LEU	289	14.125	12.439	34.028	1.00	0.00	RX0	N
ATOM	1265	H	LEU	289	14.957	12.176	34.521	1.00	0.00	RX0	H
ATOM	1266	CA	LEU	289	14.030	12.183	32.583	1.00	0.00	RX0	C
ATOM	1267	CB	LEU	289	15.371	11.626	32.097	1.00	0.00	RX0	C
ATOM	1268	CG	LEU	289	15.404	11.210	30.623	1.00	0.00	RX0	C
ATOM	1269	CD1	LEU	289	15.553	12.412	29.698	1.00	0.00	RX0	C
ATOM	1270	CD2	LEU	289	16.473	10.155	30.341	1.00	0.00	RX0	C
ATOM	1271	C	LEU	289	12.926	11.152	32.321	1.00	0.00	RX0	C
ATOM	1272	O	LEU	289	12.814	10.137	33.014	1.00	0.00	RX0	O
ATOM	1273	N	GLN	290	12.165	11.420	31.281	1.00	0.00	RX0	N
ATOM	1274	H	GLN	290	12.394	12.178	30.664	1.00	0.00	RX0	H
ATOM	1275	CA	GLN	290	11.011	10.584	30.916	1.00	0.00	RX0	C
ATOM	1276	CB	GLN	290	9.778	11.452	30.673	1.00	0.00	RX0	C
ATOM	1277	CG	GLN	290	9.410	12.301	31.899	1.00	0.00	RX0	C
ATOM	1278	CD	GLN	290	8.986	11.428	33.072	1.00	0.00	RX0	C
ATOM	1279	OE1	GLN	290	7.827	11.055	33.214	1.00	0.00	RX0	O

ATOM	1280	NE2	GLN	290	9.974	11.155	33.940	1.00	0.00	RX0	N
ATOM	1281	HE21	GLN	290	10.899	11.514	33.798	1.00	0.00	RX0	H
ATOM	1282	HE22	GLN	290	9.881	10.606	34.773	1.00	0.00	RX0	H
ATOM	1283	C	GLN	290	11.379	9.701	29.727	1.00	0.00	RX0	C
ATOM	1284	O	GLN	290	12.115	10.141	28.832	1.00	0.00	RX0	O
ATOM	1285	N	GLY	291	10.739	8.533	29.672	1.00	0.00	RX0	N
ATOM	1286	H	GLY	291	10.027	8.353	30.351	1.00	0.00	RX0	H
ATOM	1287	CA	GLY	291	10.952	7.542	28.589	1.00	0.00	RX0	C
ATOM	1288	C	GLY	291	10.769	8.134	27.181	1.00	0.00	RX0	C
ATOM	1289	O	GLY	291	11.559	7.865	26.269	1.00	0.00	RX0	O
ATOM	1290	N	GLU	292	9.854	9.086	27.078	1.00	0.00	RX0	N
ATOM	1291	H	GLU	292	9.255	9.248	27.866	1.00	0.00	RX0	H
ATOM	1292	CA	GLU	292	9.541	9.789	25.813	1.00	0.00	RX0	C
ATOM	1293	CB	GLU	292	8.215	10.552	25.943	1.00	0.00	RX0	C
ATOM	1294	CG	GLU	292	6.976	9.706	26.279	1.00	0.00	RX0	C
ATOM	1295	CD	GLU	292	7.096	9.117	27.672	1.00	0.00	RX0	C
ATOM	1296	OE1	GLU	292	7.474	9.845	28.589	1.00	0.00	RX0	O
ATOM	1297	OE2	GLU	292	6.914	7.913	27.824	1.00	0.00	RX0	O
ATOM	1298	C	GLU	292	10.671	10.744	25.400	1.00	0.00	RX0	C
ATOM	1299	O	GLU	292	11.065	10.802	24.241	1.00	0.00	RX0	O
ATOM	1300	N	GLU	293	11.241	11.417	26.395	1.00	0.00	RX0	N
ATOM	1301	H	GLU	293	10.951	11.211	27.330	1.00	0.00	RX0	H
ATOM	1302	CA	GLU	293	12.389	12.320	26.184	1.00	0.00	RX0	C
ATOM	1303	CB	GLU	293	12.636	13.156	27.436	1.00	0.00	RX0	C
ATOM	1304	CG	GLU	293	11.406	13.908	27.934	1.00	0.00	RX0	C
ATOM	1305	CD	GLU	293	11.724	14.502	29.290	1.00	0.00	RX0	C
ATOM	1306	OE1	GLU	293	12.643	14.032	29.954	1.00	0.00	RX0	O
ATOM	1307	OE2	GLU	293	11.052	15.435	29.708	1.00	0.00	RX0	O
ATOM	1308	C	GLU	293	13.669	11.544	25.843	1.00	0.00	RX0	C
ATOM	1309	O	GLU	293	14.364	11.888	24.886	1.00	0.00	RX0	O
ATOM	1310	N	PHE	294	13.855	10.409	26.517	1.00	0.00	RX0	N
ATOM	1311	H	PHE	294	13.195	10.199	27.241	1.00	0.00	RX0	H
ATOM	1312	CA	PHE	294	14.999	9.503	26.300	1.00	0.00	RX0	C
ATOM	1313	CB	PHE	294	14.905	8.307	27.251	1.00	0.00	RX0	C
ATOM	1314	CG	PHE	294	15.869	7.228	26.816	1.00	0.00	RX0	C
ATOM	1315	CD1	PHE	294	17.240	7.455	26.841	1.00	0.00	RX0	C
ATOM	1316	CD2	PHE	294	15.376	6.005	26.373	1.00	0.00	RX0	C
ATOM	1317	CE1	PHE	294	18.113	6.469	26.397	1.00	0.00	RX0	C
ATOM	1318	CE2	PHE	294	16.251	5.020	25.931	1.00	0.00	RX0	C
ATOM	1319	CZ	PHE	294	17.620	5.256	25.934	1.00	0.00	RX0	C
ATOM	1320	C	PHE	294	15.115	9.012	24.847	1.00	0.00	RX0	C
ATOM	1321	O	PHE	294	16.186	9.132	24.238	1.00	0.00	RX0	O
ATOM	1322	N	VAL	295	14.000	8.581	24.280	1.00	0.00	RX0	N
ATOM	1323	H	VAL	295	13.158	8.569	24.828	1.00	0.00	RX0	H
ATOM	1324	CA	VAL	295	13.976	8.026	22.907	1.00	0.00	RX0	C
ATOM	1325	CB	VAL	295	12.686	7.251	22.616	1.00	0.00	RX0	C
ATOM	1326	CG1	VAL	295	12.586	6.064	23.571	1.00	0.00	RX0	C
ATOM	1327	CG2	VAL	295	11.430	8.119	22.660	1.00	0.00	RX0	C
ATOM	1328	C	VAL	295	14.286	9.097	21.847	1.00	0.00	RX0	C
ATOM	1329	O	VAL	295	14.999	8.845	20.884	1.00	0.00	RX0	O
ATOM	1330	N	CYS	296	13.866	10.330	22.156	1.00	0.00	RX0	N
ATOM	1331	H	CYS	296	13.306	10.480	22.974	1.00	0.00	RX0	H
ATOM	1332	CA	CYS	296	14.167	11.498	21.314	1.00	0.00	RX0	C
ATOM	1333	CB	CYS	296	13.264	12.653	21.730	1.00	0.00	RX0	C
ATOM	1334	SG	CYS	296	11.518	12.259	21.464	1.00	0.00	RX0	S
ATOM	1335	C	CYS	296	15.661	11.843	21.352	1.00	0.00	RX0	C
ATOM	1336	O	CYS	296	16.290	11.969	20.305	1.00	0.00	RX0	O
ATOM	1337	N	LEU	297	16.247	11.750	22.547	1.00	0.00	RX0	N
ATOM	1338	H	LEU	297	15.681	11.549	23.351	1.00	0.00	RX0	H
ATOM	1339	CA	LEU	297	17.681	12.031	22.752	1.00	0.00	RX0	C
ATOM	1340	CB	LEU	297	18.019	12.109	24.239	1.00	0.00	RX0	C

ATOM	1341	CG	LEU	297	17.349	13.281	24.952	1.00	0.00	RX0	C
ATOM	1342	CD1	LEU	297	17.645	13.256	26.450	1.00	0.00	RX0	C
ATOM	1343	CD2	LEU	297	17.712	14.623	24.315	1.00	0.00	RX0	C
ATOM	1344	C	LEU	297	18.589	11.009	22.068	1.00	0.00	RX0	C
ATOM	1345	O	LEU	297	19.526	11.385	21.359	1.00	0.00	RX0	O
ATOM	1346	N	LYS	298	18.173	9.750	22.123	1.00	0.00	RX0	N
ATOM	1347	H	LYS	298	17.359	9.545	22.671	1.00	0.00	RX0	H
ATOM	1348	CA	LYS	298	18.945	8.654	21.515	1.00	0.00	RX0	C
ATOM	1349	CB	LYS	298	18.431	7.321	22.046	1.00	0.00	RX0	C
ATOM	1350	CG	LYS	298	19.254	6.122	21.582	1.00	0.00	RX0	C
ATOM	1351	CD	LYS	298	18.613	4.835	22.087	1.00	0.00	RX0	C
ATOM	1352	CE	LYS	298	17.097	5.003	22.048	1.00	0.00	RX0	C
ATOM	1353	NZ	LYS	298	16.428	3.710	22.156	1.00	0.00	RX0	N
ATOM	1354	HZ1	LYS	298	15.435	3.866	22.452	1.00	0.00	RX0	H
ATOM	1355	HZ2	LYS	298	16.294	3.228	21.246	1.00	0.00	RX0	H
ATOM	1356	HZ3	LYS	298	16.786	3.059	22.876	1.00	0.00	RX0	H
ATOM	1357	C	LYS	298	18.925	8.733	19.978	1.00	0.00	RX0	C
ATOM	1358	O	LYS	298	19.964	8.557	19.332	1.00	0.00	RX0	O
ATOM	1359	N	SER	299	17.791	9.152	19.431	1.00	0.00	RX0	N
ATOM	1360	H	SER	299	16.963	9.263	19.981	1.00	0.00	RX0	H
ATOM	1361	CA	SER	299	17.645	9.383	17.977	1.00	0.00	RX0	C
ATOM	1362	CB	SER	299	16.155	9.359	17.704	1.00	0.00	RX0	C
ATOM	1363	OG	SER	299	15.664	8.202	18.388	1.00	0.00	RX0	O
ATOM	1364	HG	SER	299	15.121	8.510	19.107	1.00	0.00	RX0	H
ATOM	1365	C	SER	299	18.416	10.621	17.504	1.00	0.00	RX0	C
ATOM	1366	O	SER	299	19.051	10.583	16.444	1.00	0.00	RX0	O
ATOM	1367	N	ILE	300	18.478	11.644	18.354	1.00	0.00	RX0	N
ATOM	1368	H	ILE	300	17.952	11.595	19.206	1.00	0.00	RX0	H
ATOM	1369	CA	ILE	300	19.283	12.860	18.096	1.00	0.00	RX0	C
ATOM	1370	CB	ILE	300	19.082	13.934	19.169	1.00	0.00	RX0	C
ATOM	1371	CG2	ILE	300	20.125	15.047	19.044	1.00	0.00	RX0	C
ATOM	1372	CG1	ILE	300	17.677	14.518	19.099	1.00	0.00	RX0	C
ATOM	1373	CD1	ILE	300	17.421	15.535	20.210	1.00	0.00	RX0	C
ATOM	1374	C	ILE	300	20.773	12.490	18.013	1.00	0.00	RX0	C
ATOM	1375	O	ILE	300	21.456	12.924	17.087	1.00	0.00	RX0	O
ATOM	1376	N	ILE	301	21.227	11.630	18.921	1.00	0.00	RX0	N
ATOM	1377	H	ILE	301	20.605	11.322	19.645	1.00	0.00	RX0	H
ATOM	1378	CA	ILE	301	22.629	11.156	18.925	1.00	0.00	RX0	C
ATOM	1379	CB	ILE	301	22.879	10.197	20.085	1.00	0.00	RX0	C
ATOM	1380	CG2	ILE	301	24.188	9.443	19.883	1.00	0.00	RX0	C
ATOM	1381	CG1	ILE	301	22.865	10.943	21.416	1.00	0.00	RX0	C
ATOM	1382	CD1	ILE	301	23.238	10.027	22.580	1.00	0.00	RX0	C
ATOM	1383	C	ILE	301	22.948	10.474	17.584	1.00	0.00	RX0	C
ATOM	1384	O	ILE	301	23.927	10.823	16.926	1.00	0.00	RX0	O
ATOM	1385	N	LEU	302	22.047	9.582	17.175	1.00	0.00	RX0	N
ATOM	1386	H	LEU	302	21.274	9.368	17.776	1.00	0.00	RX0	H
ATOM	1387	CA	LEU	302	22.205	8.836	15.917	1.00	0.00	RX0	C
ATOM	1388	CB	LEU	302	20.991	7.931	15.713	1.00	0.00	RX0	C
ATOM	1389	CG	LEU	302	20.996	7.203	14.369	1.00	0.00	RX0	C
ATOM	1390	CD1	LEU	302	22.180	6.247	14.235	1.00	0.00	RX0	C
ATOM	1391	CD2	LEU	302	19.662	6.513	14.100	1.00	0.00	RX0	C
ATOM	1392	C	LEU	302	22.391	9.757	14.699	1.00	0.00	RX0	C
ATOM	1393	O	LEU	302	23.285	9.544	13.882	1.00	0.00	RX0	O
ATOM	1394	N	LEU	303	21.581	10.806	14.658	1.00	0.00	RX0	N
ATOM	1395	H	LEU	303	20.934	10.946	15.411	1.00	0.00	RX0	H
ATOM	1396	CA	LEU	303	21.546	11.719	13.502	1.00	0.00	RX0	C
ATOM	1397	CB	LEU	303	20.125	12.233	13.312	1.00	0.00	RX0	C
ATOM	1398	CG	LEU	303	19.160	11.068	13.088	1.00	0.00	RX0	C
ATOM	1399	CD1	LEU	303	17.706	11.493	13.263	1.00	0.00	RX0	C
ATOM	1400	CD2	LEU	303	19.406	10.369	11.750	1.00	0.00	RX0	C
ATOM	1401	C	LEU	303	22.576	12.849	13.543	1.00	0.00	RX0	C

ATOM	1402	O	LEU	303	23.073	13.268	12.494	1.00	0.00	RX0	O
ATOM	1403	N	ASN	304	22.971	13.240	14.745	1.00	0.00	RX0	N
ATOM	1404	H	ASN	304	22.645	12.730	15.542	1.00	0.00	RX0	H
ATOM	1405	CA	ASN	304	23.832	14.419	14.940	1.00	0.00	RX0	C
ATOM	1406	CB	ASN	304	23.517	15.269	16.186	1.00	0.00	RX0	C
ATOM	1407	CG	ASN	304	24.491	14.934	17.314	1.00	0.00	RX0	C
ATOM	1408	OD1	ASN	304	25.573	15.496	17.483	1.00	0.00	RX0	O
ATOM	1409	ND2	ASN	304	24.058	13.907	18.055	1.00	0.00	RX0	N
ATOM	1410	HD21	ASN	304	23.123	13.573	17.915	1.00	0.00	RX0	H
ATOM	1411	HD22	ASN	304	24.633	13.403	18.706	1.00	0.00	RX0	H
ATOM	1412	C	ASN	304	25.330	14.097	14.987	1.00	0.00	RX0	C
ATOM	1413	O	ASN	304	26.132	14.783	14.344	1.00	0.00	RX0	O
ATOM	1414	N	SER	305	25.693	13.015	15.656	1.00	0.00	RX0	N
ATOM	1415	H	SER	305	25.043	12.410	16.124	1.00	0.00	RX0	H
ATOM	1416	CA	SER	305	27.104	12.765	16.012	1.00	0.00	RX0	C
ATOM	1417	CB	SER	305	27.097	11.539	16.916	1.00	0.00	RX0	C
ATOM	1418	OG	SER	305	26.245	11.840	18.035	1.00	0.00	RX0	O
ATOM	1419	HG	SER	305	26.528	11.227	18.725	1.00	0.00	RX0	H
ATOM	1420	C	SER	305	28.103	12.695	14.846	1.00	0.00	RX0	C
ATOM	1421	O	SER	305	29.198	13.228	14.946	1.00	0.00	RX0	O
ATOM	1422	N	GLY	306	27.634	12.177	13.693	1.00	0.00	RX0	N
ATOM	1423	H	GLY	306	26.686	11.864	13.637	1.00	0.00	RX0	H
ATOM	1424	CA	GLY	306	28.500	12.079	12.501	1.00	0.00	RX0	C
ATOM	1425	C	GLY	306	28.091	12.961	11.315	1.00	0.00	RX0	C
ATOM	1426	O	GLY	306	28.756	12.897	10.274	1.00	0.00	RX0	O
ATOM	1427	N	VAL	307	27.242	13.954	11.536	1.00	0.00	RX0	N
ATOM	1428	H	VAL	307	26.878	14.102	12.459	1.00	0.00	RX0	H
ATOM	1429	CA	VAL	307	26.760	14.815	10.435	1.00	0.00	RX0	C
ATOM	1430	CB	VAL	307	25.385	15.435	10.732	1.00	0.00	RX0	C
ATOM	1431	CG1	VAL	307	25.433	16.506	11.816	1.00	0.00	RX0	C
ATOM	1432	CG2	VAL	307	24.732	15.947	9.447	1.00	0.00	RX0	C
ATOM	1433	C	VAL	307	27.809	15.841	9.948	1.00	0.00	RX0	C
ATOM	1434	O	VAL	307	27.790	16.280	8.811	1.00	0.00	RX0	O
ATOM	1435	N	TYR	308	28.719	16.199	10.859	1.00	0.00	RX0	N
ATOM	1436	H	TYR	308	28.740	15.716	11.734	1.00	0.00	RX0	H
ATOM	1437	CA	TYR	308	29.761	17.211	10.584	1.00	0.00	RX0	C
ATOM	1438	CB	TYR	308	30.019	18.047	11.838	1.00	0.00	RX0	C
ATOM	1439	CG	TYR	308	28.717	18.722	12.209	1.00	0.00	RX0	C
ATOM	1440	CD1	TYR	308	27.974	19.373	11.229	1.00	0.00	RX0	C
ATOM	1441	CE1	TYR	308	26.746	19.943	11.544	1.00	0.00	RX0	C
ATOM	1442	CD2	TYR	308	28.249	18.681	13.517	1.00	0.00	RX0	C
ATOM	1443	CE2	TYR	308	27.023	19.258	13.834	1.00	0.00	RX0	C
ATOM	1444	CZ	TYR	308	26.261	19.871	12.844	1.00	0.00	RX0	C
ATOM	1445	OH	TYR	308	25.023	20.397	13.155	1.00	0.00	RX0	O
ATOM	1446	HH	TYR	308	24.463	20.404	12.382	1.00	0.00	RX0	H
ATOM	1447	C	TYR	308	31.032	16.660	9.940	1.00	0.00	RX0	C
ATOM	1448	O	TYR	308	31.912	17.411	9.537	1.00	0.00	RX0	O
ATOM	1449	N	THR	309	31.091	15.334	9.861	1.00	0.00	RX0	N
ATOM	1450	H	THR	309	30.343	14.756	10.183	1.00	0.00	RX0	H
ATOM	1451	CA	THR	309	32.303	14.643	9.386	1.00	0.00	RX0	C
ATOM	1452	CB	THR	309	32.581	13.746	10.570	1.00	0.00	RX0	C
ATOM	1453	OG1	THR	309	31.326	13.575	11.255	1.00	0.00	RX0	O
ATOM	1454	HG1	THR	309	30.865	12.881	10.781	1.00	0.00	RX0	H
ATOM	1455	CG2	THR	309	33.612	14.353	11.525	1.00	0.00	RX0	C
ATOM	1456	C	THR	309	32.183	13.866	8.071	1.00	0.00	RX0	C
ATOM	1457	O	THR	309	33.137	13.195	7.681	1.00	0.00	RX0	O
ATOM	1458	N	PHE	310	31.041	13.953	7.382	1.00	0.00	RX0	N
ATOM	1459	H	PHE	310	30.302	14.512	7.750	1.00	0.00	RX0	H
ATOM	1460	CA	PHE	310	30.972	13.467	5.988	1.00	0.00	RX0	C
ATOM	1461	CB	PHE	310	29.585	13.698	5.381	1.00	0.00	RX0	C
ATOM	1462	CG	PHE	310	28.524	12.872	6.072	1.00	0.00	RX0	C

ATOM	1463	CD1	PHE	310	28.409	11.515	5.796	1.00	0.00	RX0	C
ATOM	1464	CD2	PHE	310	27.647	13.473	6.968	1.00	0.00	RX0	C
ATOM	1465	CE1	PHE	310	27.411	10.764	6.409	1.00	0.00	RX0	C
ATOM	1466	CE2	PHE	310	26.651	12.720	7.580	1.00	0.00	RX0	C
ATOM	1467	CZ	PHE	310	26.531	11.365	7.300	1.00	0.00	RX0	C
ATOM	1468	C	PHE	310	32.019	14.245	5.180	1.00	0.00	RX0	C
ATOM	1469	O	PHE	310	32.102	15.468	5.301	1.00	0.00	RX0	O
ATOM	1470	N	LEU	311	32.854	13.508	4.462	1.00	0.00	RX0	N
ATOM	1471	H	LEU	311	32.721	12.519	4.489	1.00	0.00	RX0	H
ATOM	1472	CA	LEU	311	33.988	14.087	3.701	1.00	0.00	RX0	C
ATOM	1473	CB	LEU	311	34.699	13.044	2.846	1.00	0.00	RX0	C
ATOM	1474	CG	LEU	311	35.055	11.738	3.536	1.00	0.00	RX0	C
ATOM	1475	CD1	LEU	311	35.445	10.704	2.481	1.00	0.00	RX0	C
ATOM	1476	CD2	LEU	311	36.109	11.920	4.629	1.00	0.00	RX0	C
ATOM	1477	C	LEU	311	33.468	15.166	2.739	1.00	0.00	RX0	C
ATOM	1478	O	LEU	311	33.554	16.351	2.969	1.00	0.00	RX0	O
ATOM	1479	N	SER	312	32.784	14.619	1.713	1.00	0.00	RX0	N
ATOM	1480	H	SER	312	32.594	13.639	1.722	1.00	0.00	RX0	H
ATOM	1481	CA	SER	312	32.345	15.333	0.526	1.00	0.00	RX0	C
ATOM	1482	CB	SER	312	31.800	14.212	-0.340	1.00	0.00	RX0	C
ATOM	1483	OG	SER	312	32.565	13.049	0.003	1.00	0.00	RX0	O
ATOM	1484	HG	SER	312	32.635	12.510	-0.776	1.00	0.00	RX0	H
ATOM	1485	C	SER	312	31.423	16.517	0.816	1.00	0.00	RX0	C
ATOM	1486	O	SER	312	31.042	16.847	1.948	1.00	0.00	RX0	O
ATOM	1487	N	SER	313	30.837	16.923	-0.276	1.00	0.00	RX0	N
ATOM	1488	H	SER	313	31.202	16.611	-1.154	1.00	0.00	RX0	H
ATOM	1489	CA	SER	313	29.838	17.983	-0.455	1.00	0.00	RX0	C
ATOM	1490	CB	SER	313	30.328	19.323	0.101	1.00	0.00	RX0	C
ATOM	1491	OG	SER	313	30.293	19.216	1.541	1.00	0.00	RX0	O
ATOM	1492	HG	SER	313	31.116	18.790	1.778	1.00	0.00	RX0	H
ATOM	1493	C	SER	313	29.353	17.880	-1.909	1.00	0.00	RX0	C
ATOM	1494	O	SER	313	29.168	18.836	-2.628	1.00	0.00	RX0	O
ATOM	1495	N	THR	314	29.278	16.602	-2.358	1.00	0.00	RX0	N
ATOM	1496	H	THR	314	29.451	15.827	-1.755	1.00	0.00	RX0	H
ATOM	1497	CA	THR	314	28.609	16.250	-3.614	1.00	0.00	RX0	C
ATOM	1498	CB	THR	314	28.739	14.741	-3.676	1.00	0.00	RX0	C
ATOM	1499	OG1	THR	314	29.866	14.370	-2.867	1.00	0.00	RX0	O
ATOM	1500	HG1	THR	314	30.111	13.495	-3.159	1.00	0.00	RX0	H
ATOM	1501	CG2	THR	314	28.868	14.206	-5.104	1.00	0.00	RX0	C
ATOM	1502	C	THR	314	27.167	16.747	-3.490	1.00	0.00	RX0	C
ATOM	1503	O	THR	314	26.675	16.978	-2.368	1.00	0.00	RX0	O
ATOM	1504	N	LEU	315	26.451	16.801	-4.589	1.00	0.00	RX0	N
ATOM	1505	H	LEU	315	26.870	16.632	-5.479	1.00	0.00	RX0	H
ATOM	1506	CA	LEU	315	25.050	17.256	-4.537	1.00	0.00	RX0	C
ATOM	1507	CB	LEU	315	24.457	17.341	-5.942	1.00	0.00	RX0	C
ATOM	1508	CG	LEU	315	23.041	17.921	-5.938	1.00	0.00	RX0	C
ATOM	1509	CD1	LEU	315	23.000	19.325	-5.329	1.00	0.00	RX0	C
ATOM	1510	CD2	LEU	315	22.401	17.873	-7.325	1.00	0.00	RX0	C
ATOM	1511	C	LEU	315	24.190	16.343	-3.639	1.00	0.00	RX0	C
ATOM	1512	O	LEU	315	23.484	16.800	-2.757	1.00	0.00	RX0	O
ATOM	1513	N	LYS	316	24.524	15.049	-3.730	1.00	0.00	RX0	N
ATOM	1514	H	LYS	316	25.054	14.788	-4.530	1.00	0.00	RX0	H
ATOM	1515	CA	LYS	316	23.912	13.990	-2.918	1.00	0.00	RX0	C
ATOM	1516	CB	LYS	316	24.477	12.675	-3.445	1.00	0.00	RX0	C
ATOM	1517	CG	LYS	316	23.946	11.374	-2.851	1.00	0.00	RX0	C
ATOM	1518	CD	LYS	316	24.485	10.224	-3.703	1.00	0.00	RX0	C
ATOM	1519	CE	LYS	316	23.953	8.841	-3.338	1.00	0.00	RX0	C
ATOM	1520	NZ	LYS	316	24.600	8.306	-2.136	1.00	0.00	RX0	N
ATOM	1521	HZ1	LYS	316	24.431	7.278	-2.141	1.00	0.00	RX0	H
ATOM	1522	HZ2	LYS	316	25.634	8.441	-2.145	1.00	0.00	RX0	H
ATOM	1523	HZ3	LYS	316	24.153	8.677	-1.264	1.00	0.00	RX0	H

ATOM	1524	C	LYS	316	24.213	14.187	-1.422	1.00	0.00	RX0	C
ATOM	1525	O	LYS	316	23.297	14.194	-0.611	1.00	0.00	RX0	O
ATOM	1526	N	SER	317	25.467	14.540	-1.122	1.00	0.00	RX0	N
ATOM	1527	H	SER	317	26.083	14.748	-1.875	1.00	0.00	RX0	H
ATOM	1528	CA	SER	317	25.934	14.787	0.260	1.00	0.00	RX0	C
ATOM	1529	CB	SER	317	27.465	14.869	0.203	1.00	0.00	RX0	C
ATOM	1530	OG	SER	317	28.086	14.565	1.458	1.00	0.00	RX0	O
ATOM	1531	HG	SER	317	27.713	13.731	1.743	1.00	0.00	RX0	H
ATOM	1532	C	SER	317	25.242	16.001	0.898	1.00	0.00	RX0	C
ATOM	1533	O	SER	317	24.716	15.915	2.007	1.00	0.00	RX0	O
ATOM	1534	N	LEU	318	25.067	17.043	0.088	1.00	0.00	RX0	N
ATOM	1535	H	LEU	318	25.333	16.974	-0.876	1.00	0.00	RX0	H
ATOM	1536	CA	LEU	318	24.405	18.288	0.525	1.00	0.00	RX0	C
ATOM	1537	CB	LEU	318	24.578	19.374	-0.533	1.00	0.00	RX0	C
ATOM	1538	CG	LEU	318	26.043	19.771	-0.695	1.00	0.00	RX0	C
ATOM	1539	CD1	LEU	318	26.252	20.715	-1.879	1.00	0.00	RX0	C
ATOM	1540	CD2	LEU	318	26.614	20.332	0.608	1.00	0.00	RX0	C
ATOM	1541	C	LEU	318	22.918	18.061	0.823	1.00	0.00	RX0	C
ATOM	1542	O	LEU	318	22.412	18.453	1.877	1.00	0.00	RX0	O
ATOM	1543	N	GLU	319	22.299	17.244	-0.024	1.00	0.00	RX0	N
ATOM	1544	H	GLU	319	22.760	16.925	-0.856	1.00	0.00	RX0	H
ATOM	1545	CA	GLU	319	20.900	16.808	0.157	1.00	0.00	RX0	C
ATOM	1546	CB	GLU	319	20.424	16.034	-1.076	1.00	0.00	RX0	C
ATOM	1547	CG	GLU	319	20.412	16.853	-2.369	1.00	0.00	RX0	C
ATOM	1548	CD	GLU	319	20.170	15.932	-3.551	1.00	0.00	RX0	C
ATOM	1549	OE1	GLU	319	21.133	15.562	-4.227	1.00	0.00	RX0	O
ATOM	1550	OE2	GLU	319	19.015	15.591	-3.797	1.00	0.00	RX0	O
ATOM	1551	C	GLU	319	20.725	15.924	1.402	1.00	0.00	RX0	C
ATOM	1552	O	GLU	319	19.808	16.153	2.196	1.00	0.00	RX0	O
ATOM	1553	N	GLU	320	21.706	15.058	1.645	1.00	0.00	RX0	N
ATOM	1554	H	GLU	320	22.390	14.915	0.929	1.00	0.00	RX0	H
ATOM	1555	CA	GLU	320	21.731	14.149	2.812	1.00	0.00	RX0	C
ATOM	1556	CB	GLU	320	22.929	13.188	2.783	1.00	0.00	RX0	C
ATOM	1557	CG	GLU	320	22.997	12.144	1.657	1.00	0.00	RX0	C
ATOM	1558	CD	GLU	320	22.096	10.944	1.914	1.00	0.00	RX0	C
ATOM	1559	OE1	GLU	320	21.766	10.677	3.065	1.00	0.00	RX0	O
ATOM	1560	OE2	GLU	320	21.749	10.250	0.957	1.00	0.00	RX0	O
ATOM	1561	C	GLU	320	21.765	14.931	4.131	1.00	0.00	RX0	C
ATOM	1562	O	GLU	320	20.881	14.754	4.973	1.00	0.00	RX0	O
ATOM	1563	N	LYS	321	22.647	15.926	4.188	1.00	0.00	RX0	N
ATOM	1564	H	LYS	321	23.235	16.046	3.385	1.00	0.00	RX0	H
ATOM	1565	CA	LYS	321	22.801	16.771	5.391	1.00	0.00	RX0	C
ATOM	1566	CB	LYS	321	24.079	17.636	5.229	1.00	0.00	RX0	C
ATOM	1567	CG	LYS	321	25.364	16.844	4.876	1.00	0.00	RX0	C
ATOM	1568	CD	LYS	321	26.622	17.657	4.464	1.00	0.00	RX0	C
ATOM	1569	CE	LYS	321	27.755	16.777	3.875	1.00	0.00	RX0	C
ATOM	1570	NZ	LYS	321	29.000	17.509	3.535	1.00	0.00	RX0	N
ATOM	1571	HZ1	LYS	321	29.688	16.866	3.079	1.00	0.00	RX0	H
ATOM	1572	HZ2	LYS	321	28.858	18.294	2.864	1.00	0.00	RX0	H
ATOM	1573	HZ3	LYS	321	29.457	17.872	4.394	1.00	0.00	RX0	H
ATOM	1574	C	LYS	321	21.549	17.612	5.656	1.00	0.00	RX0	C
ATOM	1575	O	LYS	321	21.102	17.724	6.798	1.00	0.00	RX0	O
ATOM	1576	N	ASP	322	20.935	18.099	4.575	1.00	0.00	RX0	N
ATOM	1577	H	ASP	322	21.315	17.937	3.661	1.00	0.00	RX0	H
ATOM	1578	CA	ASP	322	19.715	18.915	4.684	1.00	0.00	RX0	C
ATOM	1579	CB	ASP	322	19.398	19.467	3.291	1.00	0.00	RX0	C
ATOM	1580	CG	ASP	322	18.073	20.196	3.260	1.00	0.00	RX0	C
ATOM	1581	OD1	ASP	322	17.637	20.706	4.281	1.00	0.00	RX0	O
ATOM	1582	OD2	ASP	322	17.436	20.216	2.212	1.00	0.00	RX0	O
ATOM	1583	C	ASP	322	18.561	18.097	5.282	1.00	0.00	RX0	C
ATOM	1584	O	ASP	322	17.955	18.512	6.263	1.00	0.00	RX0	O

ATOM	1585	N	HIS	323	18.423	16.872	4.772	1.00	0.00	RX0	N
ATOM	1586	H	HIS	323	19.042	16.601	4.030	1.00	0.00	RX0	H
ATOM	1587	CA	HIS	323	17.423	15.916	5.274	1.00	0.00	RX0	C
ATOM	1588	CB	HIS	323	17.473	14.619	4.463	1.00	0.00	RX0	C
ATOM	1589	CG	HIS	323	16.343	13.714	4.894	1.00	0.00	RX0	C
ATOM	1590	ND1	HIS	323	16.419	12.372	4.950	1.00	0.00	RX0	N
ATOM	1591	HD1	HIS	323	17.191	11.805	4.726	1.00	0.00	RX0	H
ATOM	1592	CD2	HIS	323	15.058	14.104	5.279	1.00	0.00	RX0	C
ATOM	1593	NE2	HIS	323	14.356	12.987	5.568	1.00	0.00	RX0	N
ATOM	1594	CE1	HIS	323	15.193	11.915	5.367	1.00	0.00	RX0	C
ATOM	1595	C	HIS	323	17.630	15.607	6.766	1.00	0.00	RX0	C
ATOM	1596	O	HIS	323	16.677	15.663	7.540	1.00	0.00	RX0	O
ATOM	1597	N	ILE	324	18.888	15.420	7.164	1.00	0.00	RX0	N
ATOM	1598	H	ILE	324	19.612	15.422	6.469	1.00	0.00	RX0	H
ATOM	1599	CA	ILE	324	19.235	15.107	8.570	1.00	0.00	RX0	C
ATOM	1600	CB	ILE	324	20.717	14.755	8.704	1.00	0.00	RX0	C
ATOM	1601	CG2	ILE	324	21.120	14.611	10.171	1.00	0.00	RX0	C
ATOM	1602	CG1	ILE	324	21.026	13.481	7.916	1.00	0.00	RX0	C
ATOM	1603	CD1	ILE	324	22.512	13.117	7.919	1.00	0.00	RX0	C
ATOM	1604	C	ILE	324	18.844	16.271	9.496	1.00	0.00	RX0	C
ATOM	1605	O	ILE	324	18.200	16.053	10.527	1.00	0.00	RX0	O
ATOM	1606	N	HIS	325	19.159	17.485	9.071	1.00	0.00	RX0	N
ATOM	1607	H	HIS	325	19.588	17.590	8.171	1.00	0.00	RX0	H
ATOM	1608	CA	HIS	325	18.840	18.694	9.856	1.00	0.00	RX0	C
ATOM	1609	CB	HIS	325	19.599	19.920	9.346	1.00	0.00	RX0	C
ATOM	1610	CG	HIS	325	21.054	19.816	9.746	1.00	0.00	RX0	C
ATOM	1611	ND1	HIS	325	22.002	19.272	8.966	1.00	0.00	RX0	N
ATOM	1612	HD1	HIS	325	21.851	18.879	8.079	1.00	0.00	RX0	H
ATOM	1613	CD2	HIS	325	21.651	20.243	10.938	1.00	0.00	RX0	C
ATOM	1614	NE2	HIS	325	22.977	19.949	10.863	1.00	0.00	RX0	N
ATOM	1615	CE1	HIS	325	23.188	19.350	9.647	1.00	0.00	RX0	C
ATOM	1616	C	HIS	325	17.335	18.955	9.948	1.00	0.00	RX0	C
ATOM	1617	O	HIS	325	16.820	19.290	11.021	1.00	0.00	RX0	O
ATOM	1618	N	ARG	326	16.631	18.584	8.887	1.00	0.00	RX0	N
ATOM	1619	H	ARG	326	17.126	18.354	8.047	1.00	0.00	RX0	H
ATOM	1620	CA	ARG	326	15.160	18.594	8.873	1.00	0.00	RX0	C
ATOM	1621	CB	ARG	326	14.626	18.340	7.461	1.00	0.00	RX0	C
ATOM	1622	CG	ARG	326	14.922	19.417	6.417	1.00	0.00	RX0	C
ATOM	1623	CD	ARG	326	14.301	19.067	5.059	1.00	0.00	RX0	C
ATOM	1624	NE	ARG	326	15.310	18.948	4.008	1.00	0.00	RX0	N
ATOM	1625	HE	ARG	326	15.954	19.729	3.907	1.00	0.00	RX0	H
ATOM	1626	CZ	ARG	326	15.388	17.836	3.219	1.00	0.00	RX0	C
ATOM	1627	NH1	ARG	326	14.523	16.819	3.427	1.00	0.00	RX0	N
ATOM	1628	HH11	ARG	326	14.534	15.980	2.876	1.00	0.00	RX0	H
ATOM	1629	HH12	ARG	326	13.837	16.873	4.156	1.00	0.00	RX0	H
ATOM	1630	NH2	ARG	326	16.325	17.759	2.252	1.00	0.00	RX0	N
ATOM	1631	HH21	ARG	326	16.494	16.973	1.654	1.00	0.00	RX0	H
ATOM	1632	HH22	ARG	326	16.926	18.563	2.103	1.00	0.00	RX0	H
ATOM	1633	C	ARG	326	14.537	17.576	9.843	1.00	0.00	RX0	C
ATOM	1634	O	ARG	326	13.617	17.928	10.589	1.00	0.00	RX0	O
ATOM	1635	N	VAL	327	15.171	16.419	9.987	1.00	0.00	RX0	N
ATOM	1636	H	VAL	327	15.980	16.247	9.420	1.00	0.00	RX0	H
ATOM	1637	CA	VAL	327	14.709	15.376	10.935	1.00	0.00	RX0	C
ATOM	1638	CB	VAL	327	15.274	13.994	10.603	1.00	0.00	RX0	C
ATOM	1639	CG1	VAL	327	14.755	12.957	11.599	1.00	0.00	RX0	C
ATOM	1640	CG2	VAL	327	14.921	13.583	9.174	1.00	0.00	RX0	C
ATOM	1641	C	VAL	327	15.041	15.781	12.381	1.00	0.00	RX0	C
ATOM	1642	O	VAL	327	14.187	15.661	13.270	1.00	0.00	RX0	O
ATOM	1643	N	LEU	328	16.212	16.371	12.574	1.00	0.00	RX0	N
ATOM	1644	H	LEU	328	16.818	16.506	11.789	1.00	0.00	RX0	H
ATOM	1645	CA	LEU	328	16.631	16.899	13.887	1.00	0.00	RX0	C

ATOM	1646	CB	LEU	328	18.069	17.410	13.827	1.00	0.00	RX0	C
ATOM	1647	CG	LEU	328	19.082	16.269	13.760	1.00	0.00	RX0	C
ATOM	1648	CD1	LEU	328	20.501	16.773	13.490	1.00	0.00	RX0	C
ATOM	1649	CD2	LEU	328	19.010	15.396	15.013	1.00	0.00	RX0	C
ATOM	1650	C	LEU	328	15.692	18.005	14.386	1.00	0.00	RX0	C
ATOM	1651	O	LEU	328	15.231	17.955	15.519	1.00	0.00	RX0	O
ATOM	1652	N	ASP	329	15.222	18.821	13.436	1.00	0.00	RX0	N
ATOM	1653	H	ASP	329	15.679	18.898	12.547	1.00	0.00	RX0	H
ATOM	1654	CA	ASP	329	14.223	19.870	13.722	1.00	0.00	RX0	C
ATOM	1655	CB	ASP	329	14.102	20.859	12.561	1.00	0.00	RX0	C
ATOM	1656	CG	ASP	329	15.317	21.771	12.556	1.00	0.00	RX0	C
ATOM	1657	OD1	ASP	329	16.069	21.759	13.533	1.00	0.00	RX0	O
ATOM	1658	OD2	ASP	329	15.505	22.497	11.580	1.00	0.00	RX0	O
ATOM	1659	C	ASP	329	12.864	19.312	14.154	1.00	0.00	RX0	C
ATOM	1660	O	ASP	329	12.272	19.788	15.128	1.00	0.00	RX0	O
ATOM	1661	N	LYS	330	12.463	18.218	13.512	1.00	0.00	RX0	N
ATOM	1662	H	LYS	330	13.021	17.920	12.735	1.00	0.00	RX0	H
ATOM	1663	CA	LYS	330	11.217	17.525	13.871	1.00	0.00	RX0	C
ATOM	1664	CB	LYS	330	10.799	16.459	12.860	1.00	0.00	RX0	C
ATOM	1665	CG	LYS	330	9.622	15.607	13.367	1.00	0.00	RX0	C
ATOM	1666	CD	LYS	330	8.368	16.387	13.795	1.00	0.00	RX0	C
ATOM	1667	CE	LYS	330	7.827	17.311	12.712	1.00	0.00	RX0	C
ATOM	1668	NZ	LYS	330	7.437	16.477	11.575	1.00	0.00	RX0	N
ATOM	1669	HZ1	LYS	330	7.124	17.093	10.804	1.00	0.00	RX0	H
ATOM	1670	HZ2	LYS	330	8.247	15.890	11.278	1.00	0.00	RX0	H
ATOM	1671	HZ3	LYS	330	6.657	15.858	11.880	1.00	0.00	RX0	H
ATOM	1672	C	LYS	330	11.283	16.915	15.278	1.00	0.00	RX0	C
ATOM	1673	O	LYS	330	10.354	17.095	16.067	1.00	0.00	RX0	O
ATOM	1674	N	ILE	331	12.441	16.375	15.625	1.00	0.00	RX0	N
ATOM	1675	H	ILE	331	13.184	16.376	14.950	1.00	0.00	RX0	H
ATOM	1676	CA	ILE	331	12.656	15.802	16.972	1.00	0.00	RX0	C
ATOM	1677	CB	ILE	331	13.953	14.997	17.053	1.00	0.00	RX0	C
ATOM	1678	CG2	ILE	331	14.064	14.332	18.422	1.00	0.00	RX0	C
ATOM	1679	CG1	ILE	331	14.043	13.951	15.944	1.00	0.00	RX0	C
ATOM	1680	CD1	ILE	331	15.358	13.172	15.987	1.00	0.00	RX0	C
ATOM	1681	C	ILE	331	12.642	16.922	18.027	1.00	0.00	RX0	C
ATOM	1682	O	ILE	331	12.078	16.732	19.120	1.00	0.00	RX0	O
ATOM	1683	N	THR	332	13.158	18.084	17.675	1.00	0.00	RX0	N
ATOM	1684	H	THR	332	13.571	18.203	16.771	1.00	0.00	RX0	H
ATOM	1685	CA	THR	332	13.155	19.265	18.570	1.00	0.00	RX0	C
ATOM	1686	CB	THR	332	14.035	20.319	17.917	1.00	0.00	RX0	C
ATOM	1687	OG1	THR	332	15.304	19.716	17.627	1.00	0.00	RX0	O
ATOM	1688	HG1	THR	332	15.246	19.366	16.741	1.00	0.00	RX0	H
ATOM	1689	CG2	THR	332	14.206	21.554	18.803	1.00	0.00	RX0	C
ATOM	1690	C	THR	332	11.706	19.696	18.836	1.00	0.00	RX0	C
ATOM	1691	O	THR	332	11.302	19.832	19.995	1.00	0.00	RX0	O
ATOM	1692	N	ASP	333	10.912	19.714	17.772	1.00	0.00	RX0	N
ATOM	1693	H	ASP	333	11.280	19.673	16.837	1.00	0.00	RX0	H
ATOM	1694	CA	ASP	333	9.466	20.017	17.863	1.00	0.00	RX0	C
ATOM	1695	CB	ASP	333	8.671	19.791	16.567	1.00	0.00	RX0	C
ATOM	1696	CG	ASP	333	9.096	20.579	15.355	1.00	0.00	RX0	C
ATOM	1697	OD1	ASP	333	9.499	21.728	15.507	1.00	0.00	RX0	O
ATOM	1698	OD2	ASP	333	8.970	20.039	14.251	1.00	0.00	RX0	O
ATOM	1699	C	ASP	333	8.732	19.000	18.747	1.00	0.00	RX0	C
ATOM	1700	O	ASP	333	7.880	19.374	19.559	1.00	0.00	RX0	O
ATOM	1701	N	THR	334	9.187	17.759	18.682	1.00	0.00	RX0	N
ATOM	1702	H	THR	334	9.940	17.583	18.048	1.00	0.00	RX0	H
ATOM	1703	CA	THR	334	8.631	16.638	19.462	1.00	0.00	RX0	C
ATOM	1704	CB	THR	334	9.177	15.373	18.821	1.00	0.00	RX0	C
ATOM	1705	OG1	THR	334	8.793	15.339	17.438	1.00	0.00	RX0	O
ATOM	1706	HG1	THR	334	9.148	16.125	17.032	1.00	0.00	RX0	H

ATOM	1707	CG2	THR	334	8.726	14.119	19.558	1.00	0.00	RX0	C
ATOM	1708	C	THR	334	8.961	16.782	20.953	1.00	0.00	RX0	C
ATOM	1709	O	THR	334	8.059	16.689	21.789	1.00	0.00	RX0	O
ATOM	1710	N	LEU	335	10.217	17.081	21.263	1.00	0.00	RX0	N
ATOM	1711	H	LEU	335	10.880	17.204	20.523	1.00	0.00	RX0	H
ATOM	1712	CA	LEU	335	10.648	17.349	22.650	1.00	0.00	RX0	C
ATOM	1713	CB	LEU	335	12.150	17.613	22.696	1.00	0.00	RX0	C
ATOM	1714	CG	LEU	335	12.962	16.326	22.779	1.00	0.00	RX0	C
ATOM	1715	CD1	LEU	335	14.460	16.579	22.609	1.00	0.00	RX0	C
ATOM	1716	CD2	LEU	335	12.656	15.573	24.074	1.00	0.00	RX0	C
ATOM	1717	C	LEU	335	9.903	18.522	23.297	1.00	0.00	RX0	C
ATOM	1718	O	LEU	335	9.384	18.379	24.399	1.00	0.00	RX0	O
ATOM	1719	N	ILE	336	9.668	19.567	22.501	1.00	0.00	RX0	N
ATOM	1720	H	ILE	336	10.072	19.580	21.582	1.00	0.00	RX0	H
ATOM	1721	CA	ILE	336	8.897	20.743	22.962	1.00	0.00	RX0	C
ATOM	1722	CB	ILE	336	9.048	21.913	21.990	1.00	0.00	RX0	C
ATOM	1723	CG2	ILE	336	8.095	23.060	22.331	1.00	0.00	RX0	C
ATOM	1724	CG1	ILE	336	10.503	22.385	21.990	1.00	0.00	RX0	C
ATOM	1725	CD1	ILE	336	10.942	22.864	23.376	1.00	0.00	RX0	C
ATOM	1726	C	ILE	336	7.427	20.365	23.180	1.00	0.00	RX0	C
ATOM	1727	O	ILE	336	6.836	20.730	24.203	1.00	0.00	RX0	O
ATOM	1728	N	HIS	337	6.891	19.571	22.265	1.00	0.00	RX0	N
ATOM	1729	H	HIS	337	7.438	19.318	21.465	1.00	0.00	RX0	H
ATOM	1730	CA	HIS	337	5.501	19.096	22.359	1.00	0.00	RX0	C
ATOM	1731	CB	HIS	337	5.081	18.332	21.113	1.00	0.00	RX0	C
ATOM	1732	CG	HIS	337	3.596	18.091	21.195	1.00	0.00	RX0	C
ATOM	1733	ND1	HIS	337	2.668	19.012	20.880	1.00	0.00	RX0	N
ATOM	1734	HD1	HIS	337	2.840	19.919	20.548	1.00	0.00	RX0	H
ATOM	1735	CD2	HIS	337	2.950	16.931	21.621	1.00	0.00	RX0	C
ATOM	1736	NE2	HIS	337	1.613	17.160	21.566	1.00	0.00	RX0	N
ATOM	1737	CE1	HIS	337	1.441	18.444	21.107	1.00	0.00	RX0	C
ATOM	1738	C	HIS	337	5.301	18.263	23.631	1.00	0.00	RX0	C
ATOM	1739	O	HIS	337	4.339	18.490	24.365	1.00	0.00	RX0	O
ATOM	1740	N	LEU	338	6.274	17.405	23.914	1.00	0.00	RX0	N
ATOM	1741	H	LEU	338	7.051	17.336	23.286	1.00	0.00	RX0	H
ATOM	1742	CA	LEU	338	6.246	16.526	25.097	1.00	0.00	RX0	C
ATOM	1743	CB	LEU	338	7.441	15.575	25.089	1.00	0.00	RX0	C
ATOM	1744	CG	LEU	338	7.342	14.531	23.982	1.00	0.00	RX0	C
ATOM	1745	CD1	LEU	338	8.646	13.749	23.814	1.00	0.00	RX0	C
ATOM	1746	CD2	LEU	338	6.125	13.627	24.183	1.00	0.00	RX0	C
ATOM	1747	C	LEU	338	6.240	17.331	26.400	1.00	0.00	RX0	C
ATOM	1748	O	LEU	338	5.410	17.098	27.277	1.00	0.00	RX0	O
ATOM	1749	N	MET	339	7.027	18.402	26.394	1.00	0.00	RX0	N
ATOM	1750	H	MET	339	7.615	18.548	25.595	1.00	0.00	RX0	H
ATOM	1751	CA	MET	339	7.168	19.308	27.550	1.00	0.00	RX0	C
ATOM	1752	CB	MET	339	8.404	20.191	27.399	1.00	0.00	RX0	C
ATOM	1753	CG	MET	339	9.705	19.393	27.370	1.00	0.00	RX0	C
ATOM	1754	SD	MET	339	11.134	20.436	27.051	1.00	0.00	RX0	S
ATOM	1755	CE	MET	339	12.269	19.129	26.567	1.00	0.00	RX0	C
ATOM	1756	C	MET	339	5.924	20.183	27.753	1.00	0.00	RX0	C
ATOM	1757	O	MET	339	5.433	20.322	28.878	1.00	0.00	RX0	O
ATOM	1758	N	ALA	340	5.370	20.672	26.646	1.00	0.00	RX0	N
ATOM	1759	H	ALA	340	5.814	20.472	25.772	1.00	0.00	RX0	H
ATOM	1760	CA	ALA	340	4.120	21.456	26.636	1.00	0.00	RX0	C
ATOM	1761	CB	ALA	340	3.835	21.993	25.233	1.00	0.00	RX0	C
ATOM	1762	C	ALA	340	2.921	20.619	27.100	1.00	0.00	RX0	C
ATOM	1763	O	ALA	340	2.176	21.064	27.967	1.00	0.00	RX0	O
ATOM	1764	N	LYS	341	2.865	19.360	26.655	1.00	0.00	RX0	N
ATOM	1765	H	LYS	341	3.552	19.058	25.994	1.00	0.00	RX0	H
ATOM	1766	CA	LYS	341	1.846	18.397	27.117	1.00	0.00	RX0	C
ATOM	1767	CB	LYS	341	1.979	17.108	26.280	1.00	0.00	RX0	C

ATOM	1768	CG	LYS	341	0.705	16.260	26.147	1.00	0.00	RX0	C
ATOM	1769	CD	LYS	341	0.384	15.817	24.703	1.00	0.00	RX0	C
ATOM	1770	CE	LYS	341	1.352	14.815	24.048	1.00	0.00	RX0	C
ATOM	1771	NZ	LYS	341	1.002	14.625	22.627	1.00	0.00	RX0	N
ATOM	1772	HZ1	LYS	341	1.715	14.095	22.076	1.00	0.00	RX0	H
ATOM	1773	HZ2	LYS	341	0.150	14.057	22.436	1.00	0.00	RX0	H
ATOM	1774	HZ3	LYS	341	0.918	15.517	22.101	1.00	0.00	RX0	H
ATOM	1775	C	LYS	341	1.927	18.179	28.637	1.00	0.00	RX0	C
ATOM	1776	O	LYS	341	0.908	18.032	29.304	1.00	0.00	RX0	O
ATOM	1777	N	ALA	342	3.166	18.103	29.120	1.00	0.00	RX0	N
ATOM	1778	H	ALA	342	3.940	18.170	28.488	1.00	0.00	RX0	H
ATOM	1779	CA	ALA	342	3.459	17.942	30.556	1.00	0.00	RX0	C
ATOM	1780	CB	ALA	342	4.953	17.706	30.786	1.00	0.00	RX0	C
ATOM	1781	C	ALA	342	3.007	19.166	31.371	1.00	0.00	RX0	C
ATOM	1782	O	ALA	342	2.879	19.099	32.588	1.00	0.00	RX0	O
ATOM	1783	N	GLY	343	2.900	20.305	30.669	1.00	0.00	RX0	N
ATOM	1784	H	GLY	343	3.153	20.317	29.702	1.00	0.00	RX0	H
ATOM	1785	CA	GLY	343	2.393	21.560	31.244	1.00	0.00	RX0	C
ATOM	1786	C	GLY	343	3.505	22.433	31.829	1.00	0.00	RX0	C
ATOM	1787	O	GLY	343	3.244	23.282	32.678	1.00	0.00	RX0	O
ATOM	1788	N	LEU	344	4.736	22.226	31.356	1.00	0.00	RX0	N
ATOM	1789	H	LEU	344	4.854	21.555	30.623	1.00	0.00	RX0	H
ATOM	1790	CA	LEU	344	5.823	23.174	31.634	1.00	0.00	RX0	C
ATOM	1791	CB	LEU	344	7.151	22.611	31.138	1.00	0.00	RX0	C
ATOM	1792	CG	LEU	344	7.553	21.337	31.877	1.00	0.00	RX0	C
ATOM	1793	CD1	LEU	344	8.836	20.741	31.301	1.00	0.00	RX0	C
ATOM	1794	CD2	LEU	344	7.655	21.562	33.386	1.00	0.00	RX0	C
ATOM	1795	C	LEU	344	5.508	24.488	30.929	1.00	0.00	RX0	C
ATOM	1796	O	LEU	344	4.977	24.500	29.792	1.00	0.00	RX0	O
ATOM	1797	N	THR	345	5.822	25.577	31.575	1.00	0.00	RX0	N
ATOM	1798	H	THR	345	6.294	25.483	32.450	1.00	0.00	RX0	H
ATOM	1799	CA	THR	345	5.705	26.912	30.948	1.00	0.00	RX0	C
ATOM	1800	CB	THR	345	5.998	27.964	32.011	1.00	0.00	RX0	C
ATOM	1801	OG1	THR	345	7.168	27.600	32.742	1.00	0.00	RX0	O
ATOM	1802	HG1	THR	345	6.893	26.958	33.393	1.00	0.00	RX0	H
ATOM	1803	CG2	THR	345	4.818	28.128	32.969	1.00	0.00	RX0	C
ATOM	1804	C	THR	345	6.639	26.971	29.731	1.00	0.00	RX0	C
ATOM	1805	O	THR	345	7.615	26.233	29.623	1.00	0.00	RX0	O
ATOM	1806	N	LEU	346	6.390	27.971	28.898	1.00	0.00	RX0	N
ATOM	1807	H	LEU	346	5.564	28.513	29.044	1.00	0.00	RX0	H
ATOM	1808	CA	LEU	346	7.214	28.242	27.708	1.00	0.00	RX0	C
ATOM	1809	CB	LEU	346	6.673	29.452	26.948	1.00	0.00	RX0	C
ATOM	1810	CG	LEU	346	7.384	29.662	25.610	1.00	0.00	RX0	C
ATOM	1811	CD1	LEU	346	7.257	28.439	24.699	1.00	0.00	RX0	C
ATOM	1812	CD2	LEU	346	6.923	30.945	24.918	1.00	0.00	RX0	C
ATOM	1813	C	LEU	346	8.705	28.433	28.051	1.00	0.00	RX0	C
ATOM	1814	O	LEU	346	9.594	27.857	27.432	1.00	0.00	RX0	O
ATOM	1815	N	GLN	347	8.927	29.083	29.198	1.00	0.00	RX0	N
ATOM	1816	H	GLN	347	8.146	29.439	29.706	1.00	0.00	RX0	H
ATOM	1817	CA	GLN	347	10.275	29.275	29.754	1.00	0.00	RX0	C
ATOM	1818	CB	GLN	347	10.215	30.281	30.898	1.00	0.00	RX0	C
ATOM	1819	CG	GLN	347	11.589	30.595	31.488	1.00	0.00	RX0	C
ATOM	1820	CD	GLN	347	11.407	31.571	32.626	1.00	0.00	RX0	C
ATOM	1821	OE1	GLN	347	10.327	31.700	33.188	1.00	0.00	RX0	O
ATOM	1822	NE2	GLN	347	12.520	32.262	32.926	1.00	0.00	RX0	N
ATOM	1823	HE21	GLN	347	13.367	32.117	32.415	1.00	0.00	RX0	H
ATOM	1824	HE22	GLN	347	12.515	32.941	33.661	1.00	0.00	RX0	H
ATOM	1825	C	GLN	347	10.911	27.956	30.229	1.00	0.00	RX0	C
ATOM	1826	O	GLN	347	12.052	27.652	29.875	1.00	0.00	RX0	O
ATOM	1827	N	GLN	348	10.120	27.140	30.905	1.00	0.00	RX0	N
ATOM	1828	H	GLN	348	9.171	27.398	31.088	1.00	0.00	RX0	H

ATOM	1829	CA	GLN	348	10.582	25.823	31.394	1.00	0.00	RX0	C
ATOM	1830	CB	GLN	348	9.592	25.217	32.376	1.00	0.00	RX0	C
ATOM	1831	CG	GLN	348	9.644	25.881	33.748	1.00	0.00	RX0	C
ATOM	1832	CD	GLN	348	8.476	25.366	34.557	1.00	0.00	RX0	C
ATOM	1833	OE1	GLN	348	7.351	25.282	34.069	1.00	0.00	RX0	O
ATOM	1834	NE2	GLN	348	8.798	25.021	35.814	1.00	0.00	RX0	N
ATOM	1835	HE21	GLN	348	9.738	25.133	36.142	1.00	0.00	RX0	H
ATOM	1836	HE22	GLN	348	8.119	24.655	36.451	1.00	0.00	RX0	H
ATOM	1837	C	GLN	348	10.871	24.847	30.248	1.00	0.00	RX0	C
ATOM	1838	O	GLN	348	11.861	24.115	30.296	1.00	0.00	RX0	O
ATOM	1839	N	GLN	349	10.121	24.983	29.160	1.00	0.00	RX0	N
ATOM	1840	H	GLN	349	9.376	25.649	29.187	1.00	0.00	RX0	H
ATOM	1841	CA	GLN	349	10.299	24.172	27.940	1.00	0.00	RX0	C
ATOM	1842	CB	GLN	349	9.206	24.481	26.921	1.00	0.00	RX0	C
ATOM	1843	CG	GLN	349	7.821	24.034	27.384	1.00	0.00	RX0	C
ATOM	1844	CD	GLN	349	6.788	24.596	26.437	1.00	0.00	RX0	C
ATOM	1845	OE1	GLN	349	7.077	24.932	25.294	1.00	0.00	RX0	O
ATOM	1846	NE2	GLN	349	5.563	24.699	26.979	1.00	0.00	RX0	N
ATOM	1847	HE21	GLN	349	5.398	24.429	27.933	1.00	0.00	RX0	H
ATOM	1848	HE22	GLN	349	4.780	25.038	26.461	1.00	0.00	RX0	H
ATOM	1849	C	GLN	349	11.682	24.372	27.306	1.00	0.00	RX0	C
ATOM	1850	O	GLN	349	12.447	23.422	27.181	1.00	0.00	RX0	O
ATOM	1851	N	HIS	350	12.056	25.641	27.113	1.00	0.00	RX0	N
ATOM	1852	H	HIS	350	11.422	26.375	27.365	1.00	0.00	RX0	H
ATOM	1853	CA	HIS	350	13.344	25.953	26.463	1.00	0.00	RX0	C
ATOM	1854	CB	HIS	350	13.377	27.324	25.778	1.00	0.00	RX0	C
ATOM	1855	CG	HIS	350	13.537	28.475	26.738	1.00	0.00	RX0	C
ATOM	1856	ND1	HIS	350	12.508	29.224	27.167	1.00	0.00	RX0	N
ATOM	1857	HD1	HIS	350	11.557	29.071	26.964	1.00	0.00	RX0	H
ATOM	1858	CD2	HIS	350	14.725	28.987	27.270	1.00	0.00	RX0	C
ATOM	1859	NE2	HIS	350	14.401	30.065	28.022	1.00	0.00	RX0	N
ATOM	1860	CE1	HIS	350	13.040	30.210	27.958	1.00	0.00	RX0	C
ATOM	1861	C	HIS	350	14.540	25.670	27.386	1.00	0.00	RX0	C
ATOM	1862	O	HIS	350	15.573	25.182	26.934	1.00	0.00	RX0	O
ATOM	1863	N	GLN	351	14.320	25.854	28.691	1.00	0.00	RX0	N
ATOM	1864	H	GLN	351	13.439	26.227	28.992	1.00	0.00	RX0	H
ATOM	1865	CA	GLN	351	15.341	25.538	29.706	1.00	0.00	RX0	C
ATOM	1866	CB	GLN	351	14.923	26.078	31.068	1.00	0.00	RX0	C
ATOM	1867	CG	GLN	351	14.911	27.603	31.110	1.00	0.00	RX0	C
ATOM	1868	CD	GLN	351	14.257	28.048	32.399	1.00	0.00	RX0	C
ATOM	1869	OE1	GLN	351	13.311	27.442	32.892	1.00	0.00	RX0	O
ATOM	1870	NE2	GLN	351	14.830	29.140	32.933	1.00	0.00	RX0	N
ATOM	1871	HE21	GLN	351	15.600	29.578	32.466	1.00	0.00	RX0	H
ATOM	1872	HE22	GLN	351	14.509	29.522	33.800	1.00	0.00	RX0	H
ATOM	1873	C	GLN	351	15.597	24.030	29.805	1.00	0.00	RX0	C
ATOM	1874	O	GLN	351	16.752	23.608	29.740	1.00	0.00	RX0	O
ATOM	1875	N	ARG	352	14.527	23.242	29.744	1.00	0.00	RX0	N
ATOM	1876	H	ARG	352	13.618	23.654	29.656	1.00	0.00	RX0	H
ATOM	1877	CA	ARG	352	14.627	21.772	29.816	1.00	0.00	RX0	C
ATOM	1878	CB	ARG	352	13.311	21.110	30.247	1.00	0.00	RX0	C
ATOM	1879	CG	ARG	352	13.423	19.588	30.437	1.00	0.00	RX0	C
ATOM	1880	CD	ARG	352	12.234	18.992	31.201	1.00	0.00	RX0	C
ATOM	1881	NE	ARG	352	12.217	17.527	31.180	1.00	0.00	RX0	N
ATOM	1882	HE	ARG	352	12.008	17.059	30.306	1.00	0.00	RX0	H
ATOM	1883	CZ	ARG	352	12.350	16.775	32.316	1.00	0.00	RX0	C
ATOM	1884	NH1	ARG	352	12.647	17.383	33.482	1.00	0.00	RX0	N
ATOM	1885	HH11	ARG	352	12.826	16.839	34.316	1.00	0.00	RX0	H
ATOM	1886	HH12	ARG	352	12.713	18.378	33.558	1.00	0.00	RX0	H
ATOM	1887	NH2	ARG	352	12.180	15.439	32.257	1.00	0.00	RX0	N
ATOM	1888	HH21	ARG	352	12.218	14.805	33.038	1.00	0.00	RX0	H
ATOM	1889	HH22	ARG	352	11.987	15.017	31.348	1.00	0.00	RX0	H

ATOM	1890	C	ARG	352	15.192	21.188	28.514	1.00	0.00	RX0	C
ATOM	1891	O	ARG	352	16.048	20.305	28.551	1.00	0.00	RX0	O
ATOM	1892	N	LEU	353	14.843	21.816	27.391	1.00	0.00	RX0	N
ATOM	1893	H	LEU	353	14.136	22.524	27.429	1.00	0.00	RX0	H
ATOM	1894	CA	LEU	353	15.400	21.446	26.079	1.00	0.00	RX0	C
ATOM	1895	CB	LEU	353	14.770	22.309	24.988	1.00	0.00	RX0	C
ATOM	1896	CG	LEU	353	15.253	21.951	23.583	1.00	0.00	RX0	C
ATOM	1897	CD1	LEU	353	14.801	20.551	23.166	1.00	0.00	RX0	C
ATOM	1898	CD2	LEU	353	14.859	23.015	22.559	1.00	0.00	RX0	C
ATOM	1899	C	LEU	353	16.930	21.610	26.063	1.00	0.00	RX0	C
ATOM	1900	O	LEU	353	17.658	20.692	25.694	1.00	0.00	RX0	O
ATOM	1901	N	ALA	354	17.375	22.739	26.614	1.00	0.00	RX0	N
ATOM	1902	H	ALA	354	16.709	23.430	26.903	1.00	0.00	RX0	H
ATOM	1903	CA	ALA	354	18.808	23.073	26.716	1.00	0.00	RX0	C
ATOM	1904	CB	ALA	354	18.995	24.521	27.171	1.00	0.00	RX0	C
ATOM	1905	C	ALA	354	19.540	22.141	27.690	1.00	0.00	RX0	C
ATOM	1906	O	ALA	354	20.574	21.567	27.334	1.00	0.00	RX0	O
ATOM	1907	N	GLN	355	18.893	21.844	28.811	1.00	0.00	RX0	N
ATOM	1908	H	GLN	355	18.021	22.311	28.969	1.00	0.00	RX0	H
ATOM	1909	CA	GLN	355	19.427	20.920	29.833	1.00	0.00	RX0	C
ATOM	1910	CB	GLN	355	18.596	20.908	31.105	1.00	0.00	RX0	C
ATOM	1911	CG	GLN	355	18.777	22.208	31.881	1.00	0.00	RX0	C
ATOM	1912	CD	GLN	355	18.347	21.969	33.305	1.00	0.00	RX0	C
ATOM	1913	OE1	GLN	355	18.955	21.178	34.024	1.00	0.00	RX0	O
ATOM	1914	NE2	GLN	355	17.273	22.687	33.672	1.00	0.00	RX0	N
ATOM	1915	HE21	GLN	355	16.847	23.306	33.009	1.00	0.00	RX0	H
ATOM	1916	HE22	GLN	355	16.874	22.639	34.588	1.00	0.00	RX0	H
ATOM	1917	C	GLN	355	19.631	19.504	29.273	1.00	0.00	RX0	C
ATOM	1918	O	GLN	355	20.705	18.919	29.430	1.00	0.00	RX0	O
ATOM	1919	N	LEU	356	18.681	19.086	28.443	1.00	0.00	RX0	N
ATOM	1920	H	LEU	356	17.886	19.672	28.276	1.00	0.00	RX0	H
ATOM	1921	CA	LEU	356	18.716	17.770	27.778	1.00	0.00	RX0	C
ATOM	1922	CB	LEU	356	17.350	17.404	27.199	1.00	0.00	RX0	C
ATOM	1923	CG	LEU	356	16.344	17.038	28.289	1.00	0.00	RX0	C
ATOM	1924	CD1	LEU	356	14.985	16.649	27.706	1.00	0.00	RX0	C
ATOM	1925	CD2	LEU	356	16.896	15.951	29.210	1.00	0.00	RX0	C
ATOM	1926	C	LEU	356	19.795	17.661	26.699	1.00	0.00	RX0	C
ATOM	1927	O	LEU	356	20.593	16.720	26.700	1.00	0.00	RX0	O
ATOM	1928	N	LEU	357	19.916	18.722	25.913	1.00	0.00	RX0	N
ATOM	1929	H	LEU	357	19.287	19.495	26.033	1.00	0.00	RX0	H
ATOM	1930	CA	LEU	357	20.885	18.765	24.803	1.00	0.00	RX0	C
ATOM	1931	CB	LEU	357	20.542	19.882	23.817	1.00	0.00	RX0	C
ATOM	1932	CG	LEU	357	19.173	19.723	23.154	1.00	0.00	RX0	C
ATOM	1933	CD1	LEU	357	18.838	20.928	22.275	1.00	0.00	RX0	C
ATOM	1934	CD2	LEU	357	19.041	18.402	22.400	1.00	0.00	RX0	C
ATOM	1935	C	LEU	357	22.335	18.932	25.264	1.00	0.00	RX0	C
ATOM	1936	O	LEU	357	23.247	18.345	24.683	1.00	0.00	RX0	O
ATOM	1937	N	LEU	358	22.501	19.595	26.404	1.00	0.00	RX0	N
ATOM	1938	H	LEU	358	21.707	20.036	26.828	1.00	0.00	RX0	H
ATOM	1939	CA	LEU	358	23.826	19.754	27.029	1.00	0.00	RX0	C
ATOM	1940	CB	LEU	358	23.812	20.848	28.095	1.00	0.00	RX0	C
ATOM	1941	CG	LEU	358	23.690	22.246	27.485	1.00	0.00	RX0	C
ATOM	1942	CD1	LEU	358	23.565	23.326	28.559	1.00	0.00	RX0	C
ATOM	1943	CD2	LEU	358	24.824	22.536	26.499	1.00	0.00	RX0	C
ATOM	1944	C	LEU	358	24.390	18.455	27.609	1.00	0.00	RX0	C
ATOM	1945	O	LEU	358	25.603	18.227	27.557	1.00	0.00	RX0	O
ATOM	1946	N	ILE	359	23.510	17.559	28.043	1.00	0.00	RX0	N
ATOM	1947	H	ILE	359	22.535	17.787	28.021	1.00	0.00	RX0	H
ATOM	1948	CA	ILE	359	23.928	16.212	28.495	1.00	0.00	RX0	C
ATOM	1949	CB	ILE	359	22.743	15.448	29.085	1.00	0.00	RX0	C
ATOM	1950	CG2	ILE	359	23.151	14.056	29.569	1.00	0.00	RX0	C

ATOM	1951	CG1	ILE	359	22.106	16.258	30.208	1.00	0.00	RX0	C
ATOM	1952	CD1	ILE	359	20.755	15.702	30.650	1.00	0.00	RX0	C
ATOM	1953	C	ILE	359	24.559	15.428	27.334	1.00	0.00	RX0	C
ATOM	1954	O	ILE	359	25.552	14.723	27.543	1.00	0.00	RX0	O
ATOM	1955	N	LEU	360	24.038	15.620	26.133	1.00	0.00	RX0	N
ATOM	1956	H	LEU	360	23.286	16.272	26.024	1.00	0.00	RX0	H
ATOM	1957	CA	LEU	360	24.601	14.974	24.928	1.00	0.00	RX0	C
ATOM	1958	CB	LEU	360	23.755	15.280	23.692	1.00	0.00	RX0	C
ATOM	1959	CG	LEU	360	22.268	14.966	23.889	1.00	0.00	RX0	C
ATOM	1960	CD1	LEU	360	21.451	15.341	22.655	1.00	0.00	RX0	C
ATOM	1961	CD2	LEU	360	22.016	13.517	24.305	1.00	0.00	RX0	C
ATOM	1962	C	LEU	360	26.079	15.308	24.694	1.00	0.00	RX0	C
ATOM	1963	O	LEU	360	26.843	14.455	24.246	1.00	0.00	RX0	O
ATOM	1964	N	SER	361	26.491	16.472	25.202	1.00	0.00	RX0	N
ATOM	1965	H	SER	361	25.804	17.143	25.486	1.00	0.00	RX0	H
ATOM	1966	CA	SER	361	27.906	16.894	25.202	1.00	0.00	RX0	C
ATOM	1967	CB	SER	361	27.951	18.365	25.573	1.00	0.00	RX0	C
ATOM	1968	OG	SER	361	26.682	18.912	25.224	1.00	0.00	RX0	O
ATOM	1969	HG	SER	361	26.281	19.138	26.057	1.00	0.00	RX0	H
ATOM	1970	C	SER	361	28.769	15.985	26.099	1.00	0.00	RX0	C
ATOM	1971	O	SER	361	29.797	15.456	25.681	1.00	0.00	RX0	O
ATOM	1972	N	HIS	362	28.237	15.704	27.286	1.00	0.00	RX0	N
ATOM	1973	H	HIS	362	27.314	16.040	27.487	1.00	0.00	RX0	H
ATOM	1974	CA	HIS	362	28.854	14.793	28.272	1.00	0.00	RX0	C
ATOM	1975	CB	HIS	362	28.306	15.107	29.659	1.00	0.00	RX0	C
ATOM	1976	CG	HIS	362	28.654	16.572	29.788	1.00	0.00	RX0	C
ATOM	1977	ND1	HIS	362	27.766	17.564	29.964	1.00	0.00	RX0	N
ATOM	1978	HD1	HIS	362	26.810	17.484	30.194	1.00	0.00	RX0	H
ATOM	1979	CD2	HIS	362	29.925	17.137	29.629	1.00	0.00	RX0	C
ATOM	1980	NE2	HIS	362	29.788	18.483	29.695	1.00	0.00	RX0	N
ATOM	1981	CE1	HIS	362	28.457	18.746	29.902	1.00	0.00	RX0	C
ATOM	1982	C	HIS	362	28.890	13.339	27.777	1.00	0.00	RX0	C
ATOM	1983	O	HIS	362	29.902	12.656	27.936	1.00	0.00	RX0	O
ATOM	1984	N	ILE	363	27.856	12.940	27.039	1.00	0.00	RX0	N
ATOM	1985	H	ILE	363	27.087	13.573	26.930	1.00	0.00	RX0	H
ATOM	1986	CA	ILE	363	27.773	11.584	26.446	1.00	0.00	RX0	C
ATOM	1987	CB	ILE	363	26.352	11.273	25.973	1.00	0.00	RX0	C
ATOM	1988	CG2	ILE	363	26.229	9.835	25.467	1.00	0.00	RX0	C
ATOM	1989	CG1	ILE	363	25.376	11.509	27.126	1.00	0.00	RX0	C
ATOM	1990	CD1	ILE	363	23.926	11.227	26.742	1.00	0.00	RX0	C
ATOM	1991	C	ILE	363	28.830	11.416	25.343	1.00	0.00	RX0	C
ATOM	1992	O	ILE	363	29.487	10.370	25.263	1.00	0.00	RX0	O
ATOM	1993	N	ARG	364	29.035	12.469	24.564	1.00	0.00	RX0	N
ATOM	1994	H	ARG	364	28.383	13.229	24.616	1.00	0.00	RX0	H
ATOM	1995	CA	ARG	364	30.102	12.498	23.547	1.00	0.00	RX0	C
ATOM	1996	CB	ARG	364	30.030	13.803	22.758	1.00	0.00	RX0	C
ATOM	1997	CG	ARG	364	31.095	13.937	21.669	1.00	0.00	RX0	C
ATOM	1998	CD	ARG	364	31.042	12.852	20.590	1.00	0.00	RX0	C
ATOM	1999	NE	ARG	364	29.686	12.769	20.074	1.00	0.00	RX0	N
ATOM	2000	HE	ARG	364	29.056	12.110	20.512	1.00	0.00	RX0	H
ATOM	2001	CZ	ARG	364	29.207	13.658	19.165	1.00	0.00	RX0	C
ATOM	2002	NH1	ARG	364	30.062	14.374	18.393	1.00	0.00	RX0	N
ATOM	2003	HH11	ARG	364	29.743	15.017	17.693	1.00	0.00	RX0	H
ATOM	2004	HH12	ARG	364	31.071	14.282	18.458	1.00	0.00	RX0	H
ATOM	2005	NH2	ARG	364	27.865	13.784	19.084	1.00	0.00	RX0	N
ATOM	2006	HH21	ARG	364	27.367	14.428	18.490	1.00	0.00	RX0	H
ATOM	2007	HH22	ARG	364	27.295	13.160	19.647	1.00	0.00	RX0	H
ATOM	2008	C	ARG	364	31.469	12.319	24.223	1.00	0.00	RX0	C
ATOM	2009	O	ARG	364	32.264	11.465	23.831	1.00	0.00	RX0	O
ATOM	2010	N	HIS	365	31.645	13.058	25.316	1.00	0.00	RX0	N
ATOM	2011	H	HIS	365	30.876	13.621	25.625	1.00	0.00	RX0	H

ATOM	2012	CA	HIS	365	32.879	13.027	26.115	1.00	0.00	RX0	C
ATOM	2013	CB	HIS	365	32.922	14.090	27.216	1.00	0.00	RX0	C
ATOM	2014	CG	HIS	365	34.315	14.126	27.812	1.00	0.00	RX0	C
ATOM	2015	ND1	HIS	365	35.379	14.733	27.241	1.00	0.00	RX0	N
ATOM	2016	HD1	HIS	365	35.434	15.235	26.397	1.00	0.00	RX0	H
ATOM	2017	CD2	HIS	365	34.731	13.549	29.016	1.00	0.00	RX0	C
ATOM	2018	NE2	HIS	365	36.053	13.812	29.166	1.00	0.00	RX0	N
ATOM	2019	CE1	HIS	365	36.449	14.539	28.074	1.00	0.00	RX0	C
ATOM	2020	C	HIS	365	33.175	11.617	26.649	1.00	0.00	RX0	C
ATOM	2021	O	HIS	365	34.274	11.105	26.425	1.00	0.00	RX0	O
ATOM	2022	N	MET	366	32.138	10.946	27.137	1.00	0.00	RX0	N
ATOM	2023	H	MET	366	31.259	11.417	27.230	1.00	0.00	RX0	H
ATOM	2024	CA	MET	366	32.267	9.572	27.662	1.00	0.00	RX0	C
ATOM	2025	CB	MET	366	31.018	9.148	28.437	1.00	0.00	RX0	C
ATOM	2026	CG	MET	366	30.746	10.021	29.660	1.00	0.00	RX0	C
ATOM	2027	SD	MET	366	29.397	9.394	30.673	1.00	0.00	RX0	S
ATOM	2028	CE	MET	366	28.116	9.379	29.413	1.00	0.00	RX0	C
ATOM	2029	C	MET	366	32.567	8.557	26.555	1.00	0.00	RX0	C
ATOM	2030	O	MET	366	33.398	7.674	26.741	1.00	0.00	RX0	O
ATOM	2031	N	SER	367	31.985	8.782	25.378	1.00	0.00	RX0	N
ATOM	2032	H	SER	367	31.307	9.514	25.288	1.00	0.00	RX0	H
ATOM	2033	CA	SER	367	32.231	7.934	24.196	1.00	0.00	RX0	C
ATOM	2034	CB	SER	367	31.203	8.411	23.160	1.00	0.00	RX0	C
ATOM	2035	OG	SER	367	30.783	7.381	22.262	1.00	0.00	RX0	O
ATOM	2036	HG	SER	367	30.177	7.809	21.666	1.00	0.00	RX0	H
ATOM	2037	C	SER	367	33.681	8.046	23.711	1.00	0.00	RX0	C
ATOM	2038	O	SER	367	34.361	7.038	23.567	1.00	0.00	RX0	O
ATOM	2039	N	ASN	368	34.193	9.278	23.688	1.00	0.00	RX0	N
ATOM	2040	H	ASN	368	33.604	10.033	23.985	1.00	0.00	RX0	H
ATOM	2041	CA	ASN	368	35.583	9.549	23.266	1.00	0.00	RX0	C
ATOM	2042	CB	ASN	368	35.829	11.030	23.062	1.00	0.00	RX0	C
ATOM	2043	CG	ASN	368	35.884	11.343	21.580	1.00	0.00	RX0	C
ATOM	2044	OD1	ASN	368	36.465	12.345	21.174	1.00	0.00	RX0	O
ATOM	2045	ND2	ASN	368	35.181	10.520	20.779	1.00	0.00	RX0	N
ATOM	2046	HD21	ASN	368	34.661	9.716	21.071	1.00	0.00	RX0	H
ATOM	2047	HD22	ASN	368	35.157	10.670	19.783	1.00	0.00	RX0	H
ATOM	2048	C	ASN	368	36.615	8.948	24.226	1.00	0.00	RX0	C
ATOM	2049	O	ASN	368	37.514	8.215	23.806	1.00	0.00	RX0	O
ATOM	2050	N	LYS	369	36.353	9.119	25.515	1.00	0.00	RX0	N
ATOM	2051	H	LYS	369	35.562	9.675	25.782	1.00	0.00	RX0	H
ATOM	2052	CA	LYS	369	37.205	8.554	26.580	1.00	0.00	RX0	C
ATOM	2053	CB	LYS	369	36.868	9.110	27.966	1.00	0.00	RX0	C
ATOM	2054	CG	LYS	369	37.123	10.609	28.129	1.00	0.00	RX0	C
ATOM	2055	CD	LYS	369	38.519	11.024	27.666	1.00	0.00	RX0	C
ATOM	2056	CE	LYS	369	39.677	10.324	28.385	1.00	0.00	RX0	C
ATOM	2057	NZ	LYS	369	40.883	10.509	27.569	1.00	0.00	RX0	N
ATOM	2058	HZ1	LYS	369	41.627	11.059	28.026	1.00	0.00	RX0	H
ATOM	2059	HZ2	LYS	369	41.279	9.602	27.235	1.00	0.00	RX0	H
ATOM	2060	HZ3	LYS	369	40.635	10.942	26.651	1.00	0.00	RX0	H
ATOM	2061	C	LYS	369	37.117	7.021	26.620	1.00	0.00	RX0	C
ATOM	2062	O	LYS	369	38.123	6.337	26.771	1.00	0.00	RX0	O
ATOM	2063	N	GLY	370	35.908	6.527	26.299	1.00	0.00	RX0	N
ATOM	2064	H	GLY	370	35.153	7.164	26.142	1.00	0.00	RX0	H
ATOM	2065	CA	GLY	370	35.597	5.090	26.225	1.00	0.00	RX0	C
ATOM	2066	C	GLY	370	36.324	4.429	25.048	1.00	0.00	RX0	C
ATOM	2067	O	GLY	370	36.946	3.386	25.214	1.00	0.00	RX0	O
ATOM	2068	N	MET	371	36.380	5.143	23.928	1.00	0.00	RX0	N
ATOM	2069	H	MET	371	35.906	6.023	23.910	1.00	0.00	RX0	H
ATOM	2070	CA	MET	371	37.103	4.709	22.719	1.00	0.00	RX0	C
ATOM	2071	CB	MET	371	36.783	5.589	21.509	1.00	0.00	RX0	C
ATOM	2072	CG	MET	371	35.370	5.379	20.965	1.00	0.00	RX0	C

ATOM	2073	SD	MET	371	35.068	3.678	20.454	1.00	0.00	RX0	S
ATOM	2074	CE	MET	371	36.235	3.605	19.085	1.00	0.00	RX0	C
ATOM	2075	C	MET	371	38.619	4.671	22.937	1.00	0.00	RX0	C
ATOM	2076	O	MET	371	39.258	3.669	22.631	1.00	0.00	RX0	O
ATOM	2077	N	GLU	372	39.117	5.692	23.643	1.00	0.00	RX0	N
ATOM	2078	H	GLU	372	38.524	6.472	23.853	1.00	0.00	RX0	H
ATOM	2079	CA	GLU	372	40.525	5.740	24.082	1.00	0.00	RX0	C
ATOM	2080	CB	GLU	372	40.833	7.016	24.865	1.00	0.00	RX0	C
ATOM	2081	CG	GLU	372	40.644	8.305	24.073	1.00	0.00	RX0	C
ATOM	2082	CD	GLU	372	41.052	9.479	24.937	1.00	0.00	RX0	C
ATOM	2083	OE1	GLU	372	40.334	10.470	24.994	1.00	0.00	RX0	O
ATOM	2084	OE2	GLU	372	42.081	9.407	25.604	1.00	0.00	RX0	O
ATOM	2085	C	GLU	372	40.884	4.541	24.968	1.00	0.00	RX0	C
ATOM	2086	O	GLU	372	41.865	3.853	24.723	1.00	0.00	RX0	O
ATOM	2087	N	HIS	373	40.006	4.291	25.945	1.00	0.00	RX0	N
ATOM	2088	H	HIS	373	39.216	4.899	26.041	1.00	0.00	RX0	H
ATOM	2089	CA	HIS	373	40.194	3.212	26.919	1.00	0.00	RX0	C
ATOM	2090	CB	HIS	373	39.259	3.439	28.116	1.00	0.00	RX0	C
ATOM	2091	CG	HIS	373	38.395	2.242	28.438	1.00	0.00	RX0	C
ATOM	2092	ND1	HIS	373	37.287	1.915	27.748	1.00	0.00	RX0	N
ATOM	2093	HD1	HIS	373	36.940	2.373	26.949	1.00	0.00	RX0	H
ATOM	2094	CD2	HIS	373	38.560	1.332	29.488	1.00	0.00	RX0	C
ATOM	2095	NE2	HIS	373	37.530	0.451	29.422	1.00	0.00	RX0	N
ATOM	2096	CE1	HIS	373	36.749	0.810	28.355	1.00	0.00	RX0	C
ATOM	2097	C	HIS	373	40.078	1.832	26.262	1.00	0.00	RX0	C
ATOM	2098	O	HIS	373	40.934	1.001	26.470	1.00	0.00	RX0	O
ATOM	2099	N	LEU	374	39.111	1.679	25.351	1.00	0.00	RX0	N
ATOM	2100	H	LEU	374	38.544	2.467	25.124	1.00	0.00	RX0	H
ATOM	2101	CA	LEU	374	38.919	0.408	24.629	1.00	0.00	RX0	C
ATOM	2102	CB	LEU	374	37.660	0.451	23.762	1.00	0.00	RX0	C
ATOM	2103	CG	LEU	374	36.366	0.289	24.558	1.00	0.00	RX0	C
ATOM	2104	CD1	LEU	374	35.134	0.593	23.704	1.00	0.00	RX0	C
ATOM	2105	CD2	LEU	374	36.285	-1.086	25.221	1.00	0.00	RX0	C
ATOM	2106	C	LEU	374	40.113	0.051	23.744	1.00	0.00	RX0	C
ATOM	2107	O	LEU	374	40.555	-1.109	23.754	1.00	0.00	RX0	O
ATOM	2108	N	TYR	375	40.664	1.068	23.103	1.00	0.00	RX0	N
ATOM	2109	H	TYR	375	40.277	1.984	23.240	1.00	0.00	RX0	H
ATOM	2110	CA	TYR	375	41.854	0.983	22.222	1.00	0.00	RX0	C
ATOM	2111	CB	TYR	375	41.926	2.183	21.277	1.00	0.00	RX0	C
ATOM	2112	CG	TYR	375	41.008	1.903	20.111	1.00	0.00	RX0	C
ATOM	2113	CD1	TYR	375	40.520	0.614	19.930	1.00	0.00	RX0	C
ATOM	2114	CE1	TYR	375	39.719	0.318	18.837	1.00	0.00	RX0	C
ATOM	2115	CD2	TYR	375	40.665	2.906	19.211	1.00	0.00	RX0	C
ATOM	2116	CE2	TYR	375	39.866	2.607	18.111	1.00	0.00	RX0	C
ATOM	2117	CZ	TYR	375	39.406	1.308	17.916	1.00	0.00	RX0	C
ATOM	2118	OH	TYR	375	38.645	0.985	16.809	1.00	0.00	RX0	O
ATOM	2119	HH	TYR	375	38.562	1.751	16.251	1.00	0.00	RX0	H
ATOM	2120	C	TYR	375	43.165	0.750	22.977	1.00	0.00	RX0	C
ATOM	2121	O	TYR	375	44.176	1.452	22.753	1.00	0.00	RX0	O
ATOM	2122	N	SER	376	43.162	-0.226	23.845	1.00	0.00	RX0	N
ATOM	2123	H	SER	376	42.305	-0.665	24.096	1.00	0.00	RX0	H
ATOM	2124	CA	SER	376	44.345	-0.646	24.634	1.00	0.00	RX0	C
ATOM	2125	CB	SER	376	44.679	0.525	25.571	1.00	0.00	RX0	C
ATOM	2126	OG	SER	376	43.614	0.777	26.492	1.00	0.00	RX0	O
ATOM	2127	HG	SER	376	42.856	1.110	26.017	1.00	0.00	RX0	H
ATOM	2128	C	SER	376	44.183	-1.992	25.350	1.00	0.00	RX0	C
ATOM	2129	O	SER	376	44.985	-2.369	26.199	1.00	0.00	RX0	O
ATOM	2130	N	MET	377	43.156	-2.746	24.938	1.00	0.00	RX0	N
ATOM	2131	H	MET	377	42.543	-2.439	24.208	1.00	0.00	RX0	H
ATOM	2132	CA	MET	377	42.915	-4.112	25.422	1.00	0.00	RX0	C
ATOM	2133	CB	MET	377	41.770	-4.073	26.439	1.00	0.00	RX0	C

ATOM	2134	CG	MET	377	42.193	-3.552	27.813	1.00	0.00	RX0	C
ATOM	2135	SD	MET	377	40.786	-3.356	28.911	1.00	0.00	RX0	S
ATOM	2136	CE	MET	377	40.021	-1.973	28.049	1.00	0.00	RX0	C
ATOM	2137	C	MET	377	42.647	-5.080	24.257	1.00	0.00	RX0	C
ATOM	2138	O	MET	377	43.092	-4.854	23.130	1.00	0.00	RX0	O
ATOM	2139	N	LYS	378	41.856	-6.107	24.527	1.00	0.00	RX0	N
ATOM	2140	H	LYS	378	41.433	-6.173	25.424	1.00	0.00	RX0	H
ATOM	2141	CA	LYS	378	41.570	-7.191	23.576	1.00	0.00	RX0	C
ATOM	2142	CB	LYS	378	41.571	-8.552	24.304	1.00	0.00	RX0	C
ATOM	2143	CG	LYS	378	41.101	-8.570	25.769	1.00	0.00	RX0	C
ATOM	2144	CD	LYS	378	42.132	-9.190	26.733	1.00	0.00	RX0	C
ATOM	2145	CE	LYS	378	41.668	-9.198	28.199	1.00	0.00	RX0	C
ATOM	2146	NZ	LYS	378	42.699	-9.742	29.102	1.00	0.00	RX0	N
ATOM	2147	HZ1	LYS	378	42.377	-9.667	30.098	1.00	0.00	RX0	H
ATOM	2148	HZ2	LYS	378	42.909	-10.742	28.932	1.00	0.00	RX0	H
ATOM	2149	HZ3	LYS	378	43.579	-9.194	29.087	1.00	0.00	RX0	H
ATOM	2150	C	LYS	378	40.316	-6.920	22.720	1.00	0.00	RX0	C
ATOM	2151	O	LYS	378	40.245	-7.352	21.611	1.00	0.00	RX0	O
ATOM	2152	N	CYS	379	39.320	-6.266	23.410	1.00	0.00	RX0	N
ATOM	2153	H	CYS	379	39.553	-5.918	24.312	1.00	0.00	RX0	H
ATOM	2154	CA	CYS	379	37.939	-6.053	22.985	1.00	0.00	RX0	C
ATOM	2155	CB	CYS	379	37.616	-4.558	23.097	1.00	0.00	RX0	C
ATOM	2156	SG	CYS	379	35.849	-4.172	23.037	1.00	0.00	RX0	S
ATOM	2157	C	CYS	379	37.599	-6.654	21.614	1.00	0.00	RX0	C
ATOM	2158	O	CYS	379	37.115	-7.773	21.559	1.00	0.00	RX0	O
ATOM	2159	N	LYS	380	38.012	-5.930	20.563	1.00	0.00	RX0	N
ATOM	2160	H	LYS	380	38.378	-5.003	20.645	1.00	0.00	RX0	H
ATOM	2161	CA	LYS	380	37.949	-6.383	19.165	1.00	0.00	RX0	C
ATOM	2162	CB	LYS	380	36.477	-6.760	18.910	1.00	0.00	RX0	C
ATOM	2163	CG	LYS	380	35.914	-7.192	17.553	1.00	0.00	RX0	C
ATOM	2164	CD	LYS	380	34.399	-7.385	17.742	1.00	0.00	RX0	C
ATOM	2165	CE	LYS	380	33.590	-7.785	16.504	1.00	0.00	RX0	C
ATOM	2166	NZ	LYS	380	32.168	-7.874	16.866	1.00	0.00	RX0	N
ATOM	2167	HZ1	LYS	380	31.534	-8.121	16.087	1.00	0.00	RX0	H
ATOM	2168	HZ2	LYS	380	31.966	-8.567	17.623	1.00	0.00	RX0	H
ATOM	2169	HZ3	LYS	380	31.795	-7.004	17.306	1.00	0.00	RX0	H
ATOM	2170	C	LYS	380	38.311	-5.215	18.248	1.00	0.00	RX0	C
ATOM	2171	O	LYS	380	37.971	-4.057	18.518	1.00	0.00	RX0	O
ATOM	2172	N	ASN	381	38.881	-5.592	17.123	1.00	0.00	RX0	N
ATOM	2173	H	ASN	381	39.193	-6.540	17.051	1.00	0.00	RX0	H
ATOM	2174	CA	ASN	381	39.208	-4.700	15.996	1.00	0.00	RX0	C
ATOM	2175	CB	ASN	381	40.397	-3.831	16.414	1.00	0.00	RX0	C
ATOM	2176	CG	ASN	381	40.177	-2.361	16.127	1.00	0.00	RX0	C
ATOM	2177	OD1	ASN	381	41.068	-1.664	15.643	1.00	0.00	RX0	O
ATOM	2178	ND2	ASN	381	38.976	-1.902	16.523	1.00	0.00	RX0	N
ATOM	2179	HD21	ASN	381	38.313	-2.488	17.000	1.00	0.00	RX0	H
ATOM	2180	HD22	ASN	381	38.696	-0.942	16.408	1.00	0.00	RX0	H
ATOM	2181	C	ASN	381	39.620	-5.523	14.759	1.00	0.00	RX0	C
ATOM	2182	O	ASN	381	40.286	-5.062	13.834	1.00	0.00	RX0	O
ATOM	2183	N	VAL	382	39.176	-6.776	14.737	1.00	0.00	RX0	N
ATOM	2184	H	VAL	382	38.452	-7.045	15.367	1.00	0.00	RX0	H
ATOM	2185	CA	VAL	382	39.338	-7.659	13.572	1.00	0.00	RX0	C
ATOM	2186	CB	VAL	382	39.240	-9.119	14.008	1.00	0.00	RX0	C
ATOM	2187	CG1	VAL	382	39.315	-10.058	12.804	1.00	0.00	RX0	C
ATOM	2188	CG2	VAL	382	40.306	-9.433	15.060	1.00	0.00	RX0	C
ATOM	2189	C	VAL	382	38.195	-7.277	12.635	1.00	0.00	RX0	C
ATOM	2190	O	VAL	382	37.049	-7.276	13.059	1.00	0.00	RX0	O
ATOM	2191	N	VAL	383	38.551	-7.063	11.363	1.00	0.00	RX0	N
ATOM	2192	H	VAL	383	39.526	-7.053	11.154	1.00	0.00	RX0	H
ATOM	2193	CA	VAL	383	37.600	-6.539	10.366	1.00	0.00	RX0	C
ATOM	2194	CB	VAL	383	36.434	-7.503	10.109	1.00	0.00	RX0	C

ATOM	2195	CG1	VAL	383	35.389	-6.884	9.176	1.00	0.00	RX0	C
ATOM	2196	CG2	VAL	383	36.949	-8.845	9.586	1.00	0.00	RX0	C
ATOM	2197	C	VAL	383	37.102	-5.173	10.877	1.00	0.00	RX0	C
ATOM	2198	O	VAL	383	36.355	-5.097	11.860	1.00	0.00	RX0	O
ATOM	2199	N	PRO	384	37.530	-4.088	10.236	1.00	0.00	RX0	N
ATOM	2200	CD	PRO	384	38.375	-4.092	9.051	1.00	0.00	RX0	C
ATOM	2201	CA	PRO	384	37.142	-2.731	10.663	1.00	0.00	RX0	C
ATOM	2202	CB	PRO	384	37.746	-1.834	9.573	1.00	0.00	RX0	C
ATOM	2203	CG	PRO	384	38.095	-2.747	8.398	1.00	0.00	RX0	C
ATOM	2204	C	PRO	384	35.624	-2.628	10.828	1.00	0.00	RX0	C
ATOM	2205	O	PRO	384	34.847	-3.282	10.112	1.00	0.00	RX0	O
ATOM	2206	N	LEU	385	35.228	-1.769	11.750	1.00	0.00	RX0	N
ATOM	2207	H	LEU	385	35.902	-1.158	12.168	1.00	0.00	RX0	H
ATOM	2208	CA	LEU	385	33.812	-1.621	12.127	1.00	0.00	RX0	C
ATOM	2209	CB	LEU	385	33.681	-0.665	13.311	1.00	0.00	RX0	C
ATOM	2210	CG	LEU	385	32.288	-0.701	13.939	1.00	0.00	RX0	C
ATOM	2211	CD1	LEU	385	31.910	-2.105	14.416	1.00	0.00	RX0	C
ATOM	2212	CD2	LEU	385	32.144	0.336	15.050	1.00	0.00	RX0	C
ATOM	2213	C	LEU	385	32.896	-1.213	10.964	1.00	0.00	RX0	C
ATOM	2214	O	LEU	385	31.809	-1.761	10.815	1.00	0.00	RX0	O
ATOM	2215	N	TYR	386	33.449	-0.406	10.055	1.00	0.00	RX0	N
ATOM	2216	H	TYR	386	34.242	0.136	10.338	1.00	0.00	RX0	H
ATOM	2217	CA	TYR	386	32.718	0.069	8.867	1.00	0.00	RX0	C
ATOM	2218	CB	TYR	386	33.617	0.938	7.971	1.00	0.00	RX0	C
ATOM	2219	CG	TYR	386	32.773	1.626	6.918	1.00	0.00	RX0	C
ATOM	2220	CD1	TYR	386	32.412	2.959	7.078	1.00	0.00	RX0	C
ATOM	2221	CE1	TYR	386	31.522	3.552	6.188	1.00	0.00	RX0	C
ATOM	2222	CD2	TYR	386	32.325	0.921	5.806	1.00	0.00	RX0	C
ATOM	2223	CE2	TYR	386	31.416	1.499	4.934	1.00	0.00	RX0	C
ATOM	2224	CZ	TYR	386	30.983	2.800	5.150	1.00	0.00	RX0	C
ATOM	2225	OH	TYR	386	30.005	3.329	4.328	1.00	0.00	RX0	O
ATOM	2226	HH	TYR	386	29.562	2.605	3.904	1.00	0.00	RX0	H
ATOM	2227	C	TYR	386	32.144	-1.103	8.049	1.00	0.00	RX0	C
ATOM	2228	O	TYR	386	30.957	-1.141	7.757	1.00	0.00	RX0	O
ATOM	2229	N	ASP	387	32.969	-2.141	7.903	1.00	0.00	RX0	N
ATOM	2230	H	ASP	387	33.935	-2.074	8.166	1.00	0.00	RX0	H
ATOM	2231	CA	ASP	387	32.619	-3.323	7.093	1.00	0.00	RX0	C
ATOM	2232	CB	ASP	387	33.887	-4.119	6.767	1.00	0.00	RX0	C
ATOM	2233	CG	ASP	387	34.885	-3.254	6.012	1.00	0.00	RX0	C
ATOM	2234	OD1	ASP	387	35.420	-2.308	6.593	1.00	0.00	RX0	O
ATOM	2235	OD2	ASP	387	35.141	-3.535	4.843	1.00	0.00	RX0	O
ATOM	2236	C	ASP	387	31.563	-4.207	7.765	1.00	0.00	RX0	C
ATOM	2237	O	ASP	387	30.619	-4.649	7.114	1.00	0.00	RX0	O
ATOM	2238	N	LEU	388	31.677	-4.332	9.088	1.00	0.00	RX0	N
ATOM	2239	H	LEU	388	32.458	-3.877	9.521	1.00	0.00	RX0	H
ATOM	2240	CA	LEU	388	30.691	-5.072	9.891	1.00	0.00	RX0	C
ATOM	2241	CB	LEU	388	31.209	-5.214	11.323	1.00	0.00	RX0	C
ATOM	2242	CG	LEU	388	30.279	-6.016	12.235	1.00	0.00	RX0	C
ATOM	2243	CD1	LEU	388	30.063	-7.442	11.726	1.00	0.00	RX0	C
ATOM	2244	CD2	LEU	388	30.754	-5.993	13.689	1.00	0.00	RX0	C
ATOM	2245	C	LEU	388	29.315	-4.386	9.871	1.00	0.00	RX0	C
ATOM	2246	O	LEU	388	28.299	-5.023	9.588	1.00	0.00	RX0	O
ATOM	2247	N	LEU	389	29.339	-3.070	10.049	1.00	0.00	RX0	N
ATOM	2248	H	LEU	389	30.232	-2.632	10.172	1.00	0.00	RX0	H
ATOM	2249	CA	LEU	389	28.129	-2.229	9.970	1.00	0.00	RX0	C
ATOM	2250	CB	LEU	389	28.420	-0.791	10.382	1.00	0.00	RX0	C
ATOM	2251	CG	LEU	389	28.861	-0.648	11.835	1.00	0.00	RX0	C
ATOM	2252	CD1	LEU	389	29.249	0.793	12.142	1.00	0.00	RX0	C
ATOM	2253	CD2	LEU	389	27.812	-1.163	12.817	1.00	0.00	RX0	C
ATOM	2254	C	LEU	389	27.489	-2.268	8.583	1.00	0.00	RX0	C
ATOM	2255	O	LEU	389	26.284	-2.473	8.458	1.00	0.00	RX0	O

ATOM	2256	N	LEU	390	28.350	-2.246	7.565	1.00	0.00	RX0	N
ATOM	2257	H	LEU	390	29.328	-2.148	7.759	1.00	0.00	RX0	H
ATOM	2258	CA	LEU	390	27.929	-2.335	6.162	1.00	0.00	RX0	C
ATOM	2259	CB	LEU	390	29.173	-2.201	5.280	1.00	0.00	RX0	C
ATOM	2260	CG	LEU	390	28.926	-2.033	3.783	1.00	0.00	RX0	C
ATOM	2261	CD1	LEU	390	28.342	-0.661	3.458	1.00	0.00	RX0	C
ATOM	2262	CD2	LEU	390	30.200	-2.291	2.978	1.00	0.00	RX0	C
ATOM	2263	C	LEU	390	27.202	-3.653	5.862	1.00	0.00	RX0	C
ATOM	2264	O	LEU	390	26.123	-3.627	5.302	1.00	0.00	RX0	O
ATOM	2265	N	GLU	391	27.733	-4.754	6.404	1.00	0.00	RX0	N
ATOM	2266	H	GLU	391	28.627	-4.705	6.857	1.00	0.00	RX0	H
ATOM	2267	CA	GLU	391	27.124	-6.090	6.264	1.00	0.00	RX0	C
ATOM	2268	CB	GLU	391	27.946	-7.175	6.963	1.00	0.00	RX0	C
ATOM	2269	CG	GLU	391	29.309	-7.503	6.360	1.00	0.00	RX0	C
ATOM	2270	CD	GLU	391	29.921	-8.632	7.167	1.00	0.00	RX0	C
ATOM	2271	OE1	GLU	391	30.332	-8.392	8.302	1.00	0.00	RX0	O
ATOM	2272	OE2	GLU	391	29.971	-9.755	6.664	1.00	0.00	RX0	O
ATOM	2273	C	GLU	391	25.713	-6.159	6.867	1.00	0.00	RX0	C
ATOM	2274	O	GLU	391	24.778	-6.600	6.214	1.00	0.00	RX0	O
ATOM	2275	N	MET	392	25.589	-5.588	8.070	1.00	0.00	RX0	N
ATOM	2276	H	MET	392	26.425	-5.212	8.479	1.00	0.00	RX0	H
ATOM	2277	CA	MET	392	24.313	-5.555	8.807	1.00	0.00	RX0	C
ATOM	2278	CB	MET	392	24.543	-5.227	10.282	1.00	0.00	RX0	C
ATOM	2279	CG	MET	392	25.224	-6.387	11.011	1.00	0.00	RX0	C
ATOM	2280	SD	MET	392	25.375	-6.117	12.785	1.00	0.00	RX0	S
ATOM	2281	CE	MET	392	26.593	-4.797	12.729	1.00	0.00	RX0	C
ATOM	2282	C	MET	392	23.266	-4.630	8.173	1.00	0.00	RX0	C
ATOM	2283	O	MET	392	22.107	-5.006	8.028	1.00	0.00	RX0	O
ATOM	2284	N	LEU	393	23.721	-3.452	7.754	1.00	0.00	RX0	N
ATOM	2285	H	LEU	393	24.707	-3.282	7.795	1.00	0.00	RX0	H
ATOM	2286	CA	LEU	393	22.852	-2.457	7.099	1.00	0.00	RX0	C
ATOM	2287	CB	LEU	393	23.510	-1.081	7.149	1.00	0.00	RX0	C
ATOM	2288	CG	LEU	393	23.445	-0.453	8.539	1.00	0.00	RX0	C
ATOM	2289	CD1	LEU	393	24.505	0.629	8.723	1.00	0.00	RX0	C
ATOM	2290	CD2	LEU	393	22.041	0.067	8.852	1.00	0.00	RX0	C
ATOM	2291	C	LEU	393	22.462	-2.815	5.663	1.00	0.00	RX0	C
ATOM	2292	O	LEU	393	21.313	-2.601	5.257	1.00	0.00	RX0	O
ATOM	2293	N	ASP	394	23.400	-3.384	4.923	1.00	0.00	RX0	N
ATOM	2294	H	ASP	394	24.268	-3.663	5.329	1.00	0.00	RX0	H
ATOM	2295	CA	ASP	394	23.187	-3.768	3.516	1.00	0.00	RX0	C
ATOM	2296	CB	ASP	394	24.204	-3.577	2.400	1.00	0.00	RX0	C
ATOM	2297	CG	ASP	394	23.353	-3.454	1.117	1.00	0.00	RX0	C
ATOM	2298	OD1	ASP	394	22.120	-3.344	1.199	1.00	0.00	RX0	O
ATOM	2299	OD2	ASP	394	23.913	-3.448	0.023	1.00	0.00	RX0	O
ATOM	2300	C	ASP	394	22.492	-5.132	3.432	1.00	0.00	RX0	C
ATOM	2301	O	ASP	394	23.034	-6.108	2.888	1.00	0.00	RX0	O
ATOM	2302	N	ALA	395	21.256	-5.134	3.874	1.00	0.00	RX0	N
ATOM	2303	H	ALA	395	20.894	-4.232	4.111	1.00	0.00	RX0	H
ATOM	2304	CA	ALA	395	20.412	-6.338	3.880	1.00	0.00	RX0	C
ATOM	2305	CB	ALA	395	19.421	-6.294	5.044	1.00	0.00	RX0	C
ATOM	2306	C	ALA	395	19.639	-6.454	2.565	1.00	0.00	RX0	C
ATOM	2307	O	ALA	395	19.303	-5.463	1.911	1.00	0.00	RX0	O
ATOM	2308	N	HIS	396	19.441	-7.696	2.156	1.00	0.00	RX0	N
ATOM	2309	H	HIS	396	19.738	-8.410	2.787	1.00	0.00	RX0	H
ATOM	2310	CA	HIS	396	18.536	-7.999	1.035	1.00	0.00	RX0	C
ATOM	2311	CB	HIS	396	18.852	-9.400	0.507	1.00	0.00	RX0	C
ATOM	2312	CG	HIS	396	19.008	-10.355	1.671	1.00	0.00	RX0	C
ATOM	2313	ND1	HIS	396	20.198	-10.819	2.097	1.00	0.00	RX0	N
ATOM	2314	HD1	HIS	396	21.081	-10.617	1.715	1.00	0.00	RX0	H
ATOM	2315	CD2	HIS	396	18.008	-10.887	2.492	1.00	0.00	RX0	C
ATOM	2316	NE2	HIS	396	18.606	-11.673	3.416	1.00	0.00	RX0	N

ATOM	2317	CE1	HIS	396	19.957	-11.634	3.174	1.00	0.00	RX0	C
ATOM	2318	C	HIS	396	17.077	-7.909	1.523	1.00	0.00	RX0	C
ATOM	2319	O	HIS	396	16.821	-7.755	2.721	1.00	0.00	RX0	O
ATOM	2320	N	ARG	397	16.142	-8.174	0.629	1.00	0.00	RX0	N
ATOM	2321	H	ARG	397	16.383	-8.382	-0.318	1.00	0.00	RX0	H
ATOM	2322	CA	ARG	397	14.704	-8.151	0.982	1.00	0.00	RX0	C
ATOM	2323	CB	ARG	397	14.154	-6.952	0.167	1.00	0.00	RX0	C
ATOM	2324	CG	ARG	397	14.968	-5.623	0.344	1.00	0.00	RX0	C
ATOM	2325	CD	ARG	397	16.140	-5.292	-0.636	1.00	0.00	RX0	C
ATOM	2326	NE	ARG	397	17.365	-4.789	0.030	1.00	0.00	RX0	N
ATOM	2327	HE	ARG	397	17.538	-5.056	0.988	1.00	0.00	RX0	H
ATOM	2328	CZ	ARG	397	18.362	-4.096	-0.636	1.00	0.00	RX0	C
ATOM	2329	NH1	ARG	397	18.153	-3.727	-1.919	1.00	0.00	RX0	N
ATOM	2330	HH11	ARG	397	18.855	-3.240	-2.446	1.00	0.00	RX0	H
ATOM	2331	HH12	ARG	397	17.289	-3.924	-2.387	1.00	0.00	RX0	H
ATOM	2332	NH2	ARG	397	19.538	-3.796	-0.022	1.00	0.00	RX0	N
ATOM	2333	HH21	ARG	397	20.325	-3.294	-0.405	1.00	0.00	RX0	H
ATOM	2334	HH22	ARG	397	19.737	-4.105	0.922	1.00	0.00	RX0	H
ATOM	2335	C	ARG	397	14.082	-9.540	0.774	1.00	0.00	RX0	C
ATOM	2336	O	ARG	397	12.875	-9.706	0.613	1.00	0.00	RX0	O
ATOM	2337	N	LEU	398	14.938	-10.556	0.835	1.00	0.00	RX0	N
ATOM	2338	H	LEU	398	15.871	-10.365	1.127	1.00	0.00	RX0	H
ATOM	2339	CA	LEU	398	14.565	-11.951	0.542	1.00	0.00	RX0	C
ATOM	2340	CB	LEU	398	15.800	-12.794	0.230	1.00	0.00	RX0	C
ATOM	2341	CG	LEU	398	16.579	-12.269	-0.977	1.00	0.00	RX0	C
ATOM	2342	CD1	LEU	398	17.869	-13.058	-1.203	1.00	0.00	RX0	C
ATOM	2343	CD2	LEU	398	15.711	-12.210	-2.236	1.00	0.00	RX0	C
ATOM	2344	C	LEU	398	13.757	-12.584	1.680	1.00	0.00	RX0	C
ATOM	2345	O	LEU	398	12.772	-13.259	1.439	1.00	0.00	RX0	O
ATOM	2346	N	HIS	399	14.100	-12.169	2.904	1.00	0.00	RX0	N
ATOM	2347	H	HIS	399	14.844	-11.514	3.011	1.00	0.00	RX0	H
ATOM	2348	CA	HIS	399	13.392	-12.616	4.118	1.00	0.00	RX0	C
ATOM	2349	CB	HIS	399	14.353	-12.614	5.307	1.00	0.00	RX0	C
ATOM	2350	CG	HIS	399	15.403	-13.673	5.070	1.00	0.00	RX0	C
ATOM	2351	ND1	HIS	399	16.706	-13.416	4.840	1.00	0.00	RX0	N
ATOM	2352	HD1	HIS	399	17.151	-12.541	4.797	1.00	0.00	RX0	H
ATOM	2353	CD2	HIS	399	15.205	-15.056	5.033	1.00	0.00	RX0	C
ATOM	2354	NE2	HIS	399	16.405	-15.631	4.778	1.00	0.00	RX0	N
ATOM	2355	CE1	HIS	399	17.328	-14.625	4.659	1.00	0.00	RX0	C
ATOM	2356	C	HIS	399	12.131	-11.786	4.401	1.00	0.00	RX0	C
ATOM	2357	O	HIS	399	11.630	-11.743	5.524	1.00	0.00	RX0	O
ATOM	2358	N	ALA	400	11.619	-11.143	3.351	1.00	0.00	RX0	N
ATOM	2359	H	ALA	400	11.998	-11.314	2.443	1.00	0.00	RX0	H
ATOM	2360	CA	ALA	400	10.356	-10.385	3.402	1.00	0.00	RX0	C
ATOM	2361	CB	ALA	400	10.144	-9.553	2.138	1.00	0.00	RX0	C
ATOM	2362	C	ALA	400	9.147	-11.323	3.579	1.00	0.00	RX0	C
ATOM	2363	O	ALA	400	8.508	-11.220	4.647	1.00	0.00	RX0	O
ATOM	2364	N	GLU	26	48.123	22.246	20.338	1.00	0.00	RX1	N
ATOM	2365	H	GLU	26	47.765	22.590	21.207	1.00	0.00	RX1	H
ATOM	2366	CA	GLU	26	49.563	21.985	20.384	1.00	0.00	RX1	C
ATOM	2367	CB	GLU	26	50.352	23.288	20.441	1.00	0.00	RX1	C
ATOM	2368	CG	GLU	26	50.169	24.204	19.237	1.00	0.00	RX1	C
ATOM	2369	CD	GLU	26	51.049	25.412	19.464	1.00	0.00	RX1	C
ATOM	2370	OE1	GLU	26	51.449	26.046	18.490	1.00	0.00	RX1	O
ATOM	2371	OE2	GLU	26	51.347	25.700	20.623	1.00	0.00	RX1	O
ATOM	2372	C	GLU	26	49.990	21.137	21.567	1.00	0.00	RX1	C
ATOM	2373	O	GLU	26	49.196	20.776	22.437	1.00	0.00	RX1	O
ATOM	2374	N	GLU	27	51.289	20.806	21.561	1.00	0.00	RX1	N
ATOM	2375	H	GLU	27	51.933	21.188	20.899	1.00	0.00	RX1	H
ATOM	2376	CA	GLU	27	51.758	19.965	22.657	1.00	0.00	RX1	C
ATOM	2377	CB	GLU	27	52.957	19.104	22.251	1.00	0.00	RX1	C

ATOM	2378	CG	GLU	27	52.881	18.490	20.849	1.00	0.00	RX1	C
ATOM	2379	CD	GLU	27	51.610	17.685	20.649	1.00	0.00	RX1	C
ATOM	2380	OE1	GLU	27	51.084	17.108	21.597	1.00	0.00	RX1	O
ATOM	2381	OE2	GLU	27	51.111	17.660	19.532	1.00	0.00	RX1	O
ATOM	2382	C	GLU	27	52.082	20.753	23.908	1.00	0.00	RX1	C
ATOM	2383	O	GLU	27	53.220	21.103	24.199	1.00	0.00	RX1	O
ATOM	2384	N	LYS	28	51.000	21.021	24.653	1.00	0.00	RX1	N
ATOM	2385	H	LYS	28	50.121	20.661	24.341	1.00	0.00	RX1	H
ATOM	2386	CA	LYS	28	51.192	21.622	25.973	1.00	0.00	RX1	C
ATOM	2387	CB	LYS	28	49.840	21.929	26.620	1.00	0.00	RX1	C
ATOM	2388	CG	LYS	28	48.806	22.650	25.744	1.00	0.00	RX1	C
ATOM	2389	CD	LYS	28	47.498	22.890	26.513	1.00	0.00	RX1	C
ATOM	2390	CE	LYS	28	46.369	23.527	25.693	1.00	0.00	RX1	C
ATOM	2391	NZ	LYS	28	45.230	23.826	26.576	1.00	0.00	RX1	N
ATOM	2392	HZ1	LYS	28	44.487	24.344	26.067	1.00	0.00	RX1	H
ATOM	2393	HZ2	LYS	28	44.824	22.943	26.960	1.00	0.00	RX1	H
ATOM	2394	HZ3	LYS	28	45.558	24.421	27.366	1.00	0.00	RX1	H
ATOM	2395	C	LYS	28	51.989	20.677	26.860	1.00	0.00	RX1	C
ATOM	2396	O	LYS	28	51.841	19.464	26.759	1.00	0.00	RX1	O
ATOM	2397	N	LYS	29	52.865	21.249	27.696	1.00	0.00	RX1	N
ATOM	2398	H	LYS	29	52.808	22.212	27.968	1.00	0.00	RX1	H
ATOM	2399	CA	LYS	29	53.744	20.316	28.398	1.00	0.00	RX1	C
ATOM	2400	CB	LYS	29	55.014	21.011	28.892	1.00	0.00	RX1	C
ATOM	2401	CG	LYS	29	55.446	22.112	27.919	1.00	0.00	RX1	C
ATOM	2402	CD	LYS	29	56.683	22.891	28.364	1.00	0.00	RX1	C
ATOM	2403	CE	LYS	29	56.664	23.350	29.827	1.00	0.00	RX1	C
ATOM	2404	NZ	LYS	29	55.502	24.181	30.161	1.00	0.00	RX1	N
ATOM	2405	HZ1	LYS	29	55.658	24.670	31.069	1.00	0.00	RX1	H
ATOM	2406	HZ2	LYS	29	55.212	24.891	29.457	1.00	0.00	RX1	H
ATOM	2407	HZ3	LYS	29	54.636	23.619	30.294	1.00	0.00	RX1	H
ATOM	2408	C	LYS	29	53.017	19.595	29.512	1.00	0.00	RX1	C
ATOM	2409	O	LYS	29	52.391	20.188	30.385	1.00	0.00	RX1	O
ATOM	2410	N	VAL	30	53.096	18.269	29.393	1.00	0.00	RX1	N
ATOM	2411	H	VAL	30	53.706	17.850	28.725	1.00	0.00	RX1	H
ATOM	2412	CA	VAL	30	52.361	17.444	30.340	1.00	0.00	RX1	C
ATOM	2413	CB	VAL	30	51.748	16.240	29.623	1.00	0.00	RX1	C
ATOM	2414	CG1	VAL	30	50.782	16.702	28.532	1.00	0.00	RX1	C
ATOM	2415	CG2	VAL	30	52.829	15.308	29.071	1.00	0.00	RX1	C
ATOM	2416	C	VAL	30	53.222	16.998	31.503	1.00	0.00	RX1	C
ATOM	2417	O	VAL	30	54.444	16.972	31.418	1.00	0.00	RX1	O
ATOM	2418	N	CYS	31	52.530	16.620	32.579	1.00	0.00	RX1	N
ATOM	2419	H	CYS	31	51.533	16.712	32.642	1.00	0.00	RX1	H
ATOM	2420	CA	CYS	31	53.248	15.982	33.675	1.00	0.00	RX1	C
ATOM	2421	CB	CYS	31	53.494	16.983	34.799	1.00	0.00	RX1	C
ATOM	2422	SG	CYS	31	51.946	17.578	35.525	1.00	0.00	RX1	S
ATOM	2423	C	CYS	31	52.474	14.781	34.159	1.00	0.00	RX1	C
ATOM	2424	O	CYS	31	51.272	14.671	33.944	1.00	0.00	RX1	O
ATOM	2425	N	GLN	32	53.211	13.878	34.818	1.00	0.00	RX1	N
ATOM	2426	H	GLN	32	54.173	14.075	35.009	1.00	0.00	RX1	H
ATOM	2427	CA	GLN	32	52.601	12.612	35.225	1.00	0.00	RX1	C
ATOM	2428	CB	GLN	32	53.624	11.648	35.844	1.00	0.00	RX1	C
ATOM	2429	CG	GLN	32	54.779	11.207	34.933	1.00	0.00	RX1	C
ATOM	2430	CD	GLN	32	55.979	12.126	35.077	1.00	0.00	RX1	C
ATOM	2431	OE1	GLN	32	55.886	13.346	34.996	1.00	0.00	RX1	O
ATOM	2432	NE2	GLN	32	57.126	11.467	35.304	1.00	0.00	RX1	N
ATOM	2433	HE21	GLN	32	57.146	10.463	35.339	1.00	0.00	RX1	H
ATOM	2434	HE22	GLN	32	57.991	11.948	35.442	1.00	0.00	RX1	H
ATOM	2435	C	GLN	32	51.422	12.779	36.171	1.00	0.00	RX1	C
ATOM	2436	O	GLN	32	50.364	12.185	36.006	1.00	0.00	RX1	O
ATOM	2437	N	GLY	33	51.643	13.657	37.154	1.00	0.00	RX1	N
ATOM	2438	H	GLY	33	52.558	14.016	37.326	1.00	0.00	RX1	H

ATOM	2439	CA	GLY	33	50.589	13.819	38.149	1.00	0.00	RX1	C
ATOM	2440	C	GLY	33	50.900	13.023	39.395	1.00	0.00	RX1	C
ATOM	2441	O	GLY	33	51.757	12.148	39.393	1.00	0.00	RX1	O
ATOM	2442	N	THR	34	50.194	13.380	40.466	1.00	0.00	RX1	N
ATOM	2443	H	THR	34	49.498	14.100	40.497	1.00	0.00	RX1	H
ATOM	2444	CA	THR	34	50.445	12.650	41.700	1.00	0.00	RX1	C
ATOM	2445	CB	THR	34	50.593	13.717	42.777	1.00	0.00	RX1	C
ATOM	2446	OG1	THR	34	49.571	14.707	42.600	1.00	0.00	RX1	O
ATOM	2447	HG1	THR	34	48.825	14.325	43.066	1.00	0.00	RX1	H
ATOM	2448	CG2	THR	34	51.978	14.362	42.761	1.00	0.00	RX1	C
ATOM	2449	C	THR	34	49.373	11.610	41.990	1.00	0.00	RX1	C
ATOM	2450	O	THR	34	48.304	11.587	41.381	1.00	0.00	RX1	O
ATOM	2451	N	SER	35	49.720	10.726	42.935	1.00	0.00	RX1	N
ATOM	2452	H	SER	35	50.623	10.702	43.356	1.00	0.00	RX1	H
ATOM	2453	CA	SER	35	48.747	9.749	43.421	1.00	0.00	RX1	C
ATOM	2454	CB	SER	35	48.833	8.523	42.515	1.00	0.00	RX1	C
ATOM	2455	OG	SER	35	48.976	8.962	41.157	1.00	0.00	RX1	O
ATOM	2456	HG	SER	35	48.396	9.712	41.059	1.00	0.00	RX1	H
ATOM	2457	C	SER	35	48.985	9.439	44.888	1.00	0.00	RX1	C
ATOM	2458	O	SER	35	49.517	8.407	45.272	1.00	0.00	RX1	O
ATOM	2459	N	ASN	36	48.606	10.434	45.688	1.00	0.00	RX1	N
ATOM	2460	H	ASN	36	48.097	11.200	45.283	1.00	0.00	RX1	H
ATOM	2461	CA	ASN	36	48.909	10.410	47.115	1.00	0.00	RX1	C
ATOM	2462	CB	ASN	36	49.864	11.549	47.500	1.00	0.00	RX1	C
ATOM	2463	CG	ASN	36	49.130	12.722	48.124	1.00	0.00	RX1	C
ATOM	2464	OD1	ASN	36	48.795	12.724	49.301	1.00	0.00	RX1	O
ATOM	2465	ND2	ASN	36	48.935	13.755	47.294	1.00	0.00	RX1	N
ATOM	2466	HD21	ASN	36	49.077	13.660	46.302	1.00	0.00	RX1	H
ATOM	2467	HD22	ASN	36	48.621	14.662	47.595	1.00	0.00	RX1	H
ATOM	2468	C	ASN	36	47.669	10.387	47.991	1.00	0.00	RX1	C
ATOM	2469	O	ASN	36	47.633	9.767	49.046	1.00	0.00	RX1	O
ATOM	2470	N	LYS	37	46.654	11.125	47.508	1.00	0.00	RX1	N
ATOM	2471	H	LYS	37	46.745	11.553	46.604	1.00	0.00	RX1	H
ATOM	2472	CA	LYS	37	45.391	11.309	48.227	1.00	0.00	RX1	C
ATOM	2473	CB	LYS	37	44.310	10.334	47.745	1.00	0.00	RX1	C
ATOM	2474	CG	LYS	37	44.550	8.841	47.975	1.00	0.00	RX1	C
ATOM	2475	CD	LYS	37	43.266	8.002	47.978	1.00	0.00	RX1	C
ATOM	2476	CE	LYS	37	42.377	8.185	49.221	1.00	0.00	RX1	C
ATOM	2477	NZ	LYS	37	41.522	9.375	49.141	1.00	0.00	RX1	N
ATOM	2478	HZ1	LYS	37	40.848	9.404	49.936	1.00	0.00	RX1	H
ATOM	2479	HZ2	LYS	37	42.035	10.281	49.112	1.00	0.00	RX1	H
ATOM	2480	HZ3	LYS	37	40.870	9.328	48.329	1.00	0.00	RX1	H
ATOM	2481	C	LYS	37	45.418	11.399	49.754	1.00	0.00	RX1	C
ATOM	2482	O	LYS	37	44.701	10.687	50.452	1.00	0.00	RX1	O
ATOM	2483	N	LEU	38	46.268	12.347	50.201	1.00	0.00	RX1	N
ATOM	2484	H	LEU	38	46.866	12.777	49.523	1.00	0.00	RX1	H
ATOM	2485	CA	LEU	38	46.573	12.679	51.602	1.00	0.00	RX1	C
ATOM	2486	CB	LEU	38	45.354	12.647	52.533	1.00	0.00	RX1	C
ATOM	2487	CG	LEU	38	44.379	13.787	52.249	1.00	0.00	RX1	C
ATOM	2488	CD1	LEU	38	43.028	13.583	52.935	1.00	0.00	RX1	C
ATOM	2489	CD2	LEU	38	45.006	15.130	52.609	1.00	0.00	RX1	C
ATOM	2490	C	LEU	38	47.741	11.943	52.239	1.00	0.00	RX1	C
ATOM	2491	O	LEU	38	48.049	12.124	53.413	1.00	0.00	RX1	O
ATOM	2492	N	THR	39	48.417	11.135	51.419	1.00	0.00	RX1	N
ATOM	2493	H	THR	39	48.237	11.109	50.437	1.00	0.00	RX1	H
ATOM	2494	CA	THR	39	49.677	10.590	51.912	1.00	0.00	RX1	C
ATOM	2495	CB	THR	39	49.934	9.279	51.189	1.00	0.00	RX1	C
ATOM	2496	OG1	THR	39	48.731	8.500	51.216	1.00	0.00	RX1	O
ATOM	2497	HG1	THR	39	48.137	8.914	50.596	1.00	0.00	RX1	H
ATOM	2498	CG2	THR	39	51.107	8.507	51.797	1.00	0.00	RX1	C
ATOM	2499	C	THR	39	50.808	11.598	51.783	1.00	0.00	RX1	C

ATOM	2500	O	THR	39	51.340	11.850	50.710	1.00	0.00	RX1	O
ATOM	2501	N	GLN	40	51.148	12.172	52.949	1.00	0.00	RX1	N
ATOM	2502	H	GLN	40	50.656	11.903	53.776	1.00	0.00	RX1	H
ATOM	2503	CA	GLN	40	52.225	13.165	52.974	1.00	0.00	RX1	C
ATOM	2504	CB	GLN	40	52.405	13.691	54.400	1.00	0.00	RX1	C
ATOM	2505	CG	GLN	40	53.010	15.097	54.475	1.00	0.00	RX1	C
ATOM	2506	CD	GLN	40	53.022	15.574	55.917	1.00	0.00	RX1	C
ATOM	2507	OE1	GLN	40	54.002	16.109	56.426	1.00	0.00	RX1	O
ATOM	2508	NE2	GLN	40	51.857	15.368	56.563	1.00	0.00	RX1	N
ATOM	2509	HE21	GLN	40	51.056	14.953	56.131	1.00	0.00	RX1	H
ATOM	2510	HE22	GLN	40	51.761	15.637	57.521	1.00	0.00	RX1	H
ATOM	2511	C	GLN	40	53.534	12.666	52.373	1.00	0.00	RX1	C
ATOM	2512	O	GLN	40	54.169	11.742	52.865	1.00	0.00	RX1	O
ATOM	2513	N	LEU	41	53.884	13.312	51.253	1.00	0.00	RX1	N
ATOM	2514	H	LEU	41	53.316	14.058	50.915	1.00	0.00	RX1	H
ATOM	2515	CA	LEU	41	55.099	12.924	50.540	1.00	0.00	RX1	C
ATOM	2516	CB	LEU	41	55.010	13.367	49.080	1.00	0.00	RX1	C
ATOM	2517	CG	LEU	41	53.800	12.778	48.354	1.00	0.00	RX1	C
ATOM	2518	CD1	LEU	41	53.638	13.363	46.949	1.00	0.00	RX1	C
ATOM	2519	CD2	LEU	41	53.836	11.249	48.337	1.00	0.00	RX1	C
ATOM	2520	C	LEU	41	56.362	13.472	51.174	1.00	0.00	RX1	C
ATOM	2521	O	LEU	41	56.964	14.429	50.696	1.00	0.00	RX1	O
ATOM	2522	N	GLY	42	56.722	12.802	52.274	1.00	0.00	RX1	N
ATOM	2523	H	GLY	42	56.088	12.136	52.671	1.00	0.00	RX1	H
ATOM	2524	CA	GLY	42	57.779	13.340	53.120	1.00	0.00	RX1	C
ATOM	2525	C	GLY	42	57.152	14.191	54.201	1.00	0.00	RX1	C
ATOM	2526	O	GLY	42	56.016	13.965	54.599	1.00	0.00	RX1	O
ATOM	2527	N	THR	43	57.926	15.185	54.642	1.00	0.00	RX1	N
ATOM	2528	H	THR	43	58.824	15.362	54.232	1.00	0.00	RX1	H
ATOM	2529	CA	THR	43	57.350	16.178	55.539	1.00	0.00	RX1	C
ATOM	2530	CB	THR	43	58.506	16.959	56.164	1.00	0.00	RX1	C
ATOM	2531	OG1	THR	43	59.267	17.635	55.154	1.00	0.00	RX1	O
ATOM	2532	HG1	THR	43	59.928	17.019	54.823	1.00	0.00	RX1	H
ATOM	2533	CG2	THR	43	59.407	16.054	57.005	1.00	0.00	RX1	C
ATOM	2534	C	THR	43	56.381	17.086	54.796	1.00	0.00	RX1	C
ATOM	2535	O	THR	43	56.148	16.938	53.602	1.00	0.00	RX1	O
ATOM	2536	N	PHE	44	55.880	18.091	55.529	1.00	0.00	RX1	N
ATOM	2537	H	PHE	44	55.961	18.070	56.523	1.00	0.00	RX1	H
ATOM	2538	CA	PHE	44	55.132	19.148	54.848	1.00	0.00	RX1	C
ATOM	2539	CB	PHE	44	54.634	20.172	55.863	1.00	0.00	RX1	C
ATOM	2540	CG	PHE	44	53.691	19.518	56.842	1.00	0.00	RX1	C
ATOM	2541	CD1	PHE	44	52.372	19.283	56.477	1.00	0.00	RX1	C
ATOM	2542	CD2	PHE	44	54.137	19.156	58.107	1.00	0.00	RX1	C
ATOM	2543	CE1	PHE	44	51.497	18.691	57.379	1.00	0.00	RX1	C
ATOM	2544	CE2	PHE	44	53.263	18.559	59.008	1.00	0.00	RX1	C
ATOM	2545	CZ	PHE	44	51.942	18.328	58.644	1.00	0.00	RX1	C
ATOM	2546	C	PHE	44	55.927	19.850	53.757	1.00	0.00	RX1	C
ATOM	2547	O	PHE	44	55.455	20.084	52.649	1.00	0.00	RX1	O
ATOM	2548	N	GLU	45	57.181	20.173	54.119	1.00	0.00	RX1	N
ATOM	2549	H	GLU	45	57.606	19.860	54.966	1.00	0.00	RX1	H
ATOM	2550	CA	GLU	45	57.962	20.883	53.110	1.00	0.00	RX1	C
ATOM	2551	CB	GLU	45	59.164	21.537	53.795	1.00	0.00	RX1	C
ATOM	2552	CG	GLU	45	59.947	22.522	52.924	1.00	0.00	RX1	C
ATOM	2553	CD	GLU	45	59.206	23.837	52.858	1.00	0.00	RX1	C
ATOM	2554	OE1	GLU	45	58.345	23.993	51.998	1.00	0.00	RX1	O
ATOM	2555	OE2	GLU	45	59.489	24.706	53.679	1.00	0.00	RX1	O
ATOM	2556	C	GLU	45	58.451	19.975	51.998	1.00	0.00	RX1	C
ATOM	2557	O	GLU	45	58.420	20.328	50.825	1.00	0.00	RX1	O
ATOM	2558	N	ASP	46	58.802	18.737	52.386	1.00	0.00	RX1	N
ATOM	2559	H	ASP	46	58.905	18.484	53.350	1.00	0.00	RX1	H
ATOM	2560	CA	ASP	46	59.116	17.762	51.334	1.00	0.00	RX1	C

ATOM	2561	CB	ASP	46	59.415	16.366	51.855	1.00	0.00	RX1	C
ATOM	2562	CG	ASP	46	60.767	16.258	52.493	1.00	0.00	RX1	C
ATOM	2563	OD1	ASP	46	61.752	16.546	51.817	1.00	0.00	RX1	O
ATOM	2564	OD2	ASP	46	60.820	15.857	53.655	1.00	0.00	RX1	O
ATOM	2565	C	ASP	46	57.966	17.536	50.371	1.00	0.00	RX1	C
ATOM	2566	O	ASP	46	58.152	17.362	49.170	1.00	0.00	RX1	O
ATOM	2567	N	HIS	47	56.752	17.595	50.944	1.00	0.00	RX1	N
ATOM	2568	H	HIS	47	56.670	17.703	51.935	1.00	0.00	RX1	H
ATOM	2569	CA	HIS	47	55.557	17.512	50.111	1.00	0.00	RX1	C
ATOM	2570	CB	HIS	47	54.264	17.359	50.920	1.00	0.00	RX1	C
ATOM	2571	CG	HIS	47	53.116	16.873	50.050	1.00	0.00	RX1	C
ATOM	2572	ND1	HIS	47	53.008	17.032	48.713	1.00	0.00	RX1	N
ATOM	2573	HD1	HIS	47	53.627	17.496	48.110	1.00	0.00	RX1	H
ATOM	2574	CD2	HIS	47	51.983	16.182	50.490	1.00	0.00	RX1	C
ATOM	2575	NE2	HIS	47	51.199	15.929	49.414	1.00	0.00	RX1	N
ATOM	2576	CE1	HIS	47	51.832	16.454	48.318	1.00	0.00	RX1	C
ATOM	2577	C	HIS	47	55.433	18.667	49.145	1.00	0.00	RX1	C
ATOM	2578	O	HIS	47	55.242	18.461	47.952	1.00	0.00	RX1	O
ATOM	2579	N	PHE	48	55.597	19.884	49.688	1.00	0.00	RX1	N
ATOM	2580	H	PHE	48	55.738	19.982	50.677	1.00	0.00	RX1	H
ATOM	2581	CA	PHE	48	55.651	21.071	48.828	1.00	0.00	RX1	C
ATOM	2582	CB	PHE	48	55.984	22.311	49.667	1.00	0.00	RX1	C
ATOM	2583	CG	PHE	48	56.607	23.389	48.806	1.00	0.00	RX1	C
ATOM	2584	CD1	PHE	48	55.851	24.059	47.852	1.00	0.00	RX1	C
ATOM	2585	CD2	PHE	48	57.952	23.708	48.961	1.00	0.00	RX1	C
ATOM	2586	CE1	PHE	48	56.438	25.031	47.051	1.00	0.00	RX1	C
ATOM	2587	CE2	PHE	48	58.540	24.682	48.164	1.00	0.00	RX1	C
ATOM	2588	CZ	PHE	48	57.783	25.342	47.204	1.00	0.00	RX1	C
ATOM	2589	C	PHE	48	56.645	20.918	47.678	1.00	0.00	RX1	C
ATOM	2590	O	PHE	48	56.348	21.118	46.506	1.00	0.00	RX1	O
ATOM	2591	N	LEU	49	57.848	20.497	48.073	1.00	0.00	RX1	N
ATOM	2592	H	LEU	49	58.035	20.370	49.046	1.00	0.00	RX1	H
ATOM	2593	CA	LEU	49	58.889	20.292	47.078	1.00	0.00	RX1	C
ATOM	2594	CB	LEU	49	60.221	20.074	47.793	1.00	0.00	RX1	C
ATOM	2595	CG	LEU	49	61.434	20.324	46.898	1.00	0.00	RX1	C
ATOM	2596	CD1	LEU	49	61.376	21.705	46.248	1.00	0.00	RX1	C
ATOM	2597	CD2	LEU	49	62.744	20.109	47.656	1.00	0.00	RX1	C
ATOM	2598	C	LEU	49	58.578	19.184	46.086	1.00	0.00	RX1	C
ATOM	2599	O	LEU	49	58.958	19.231	44.927	1.00	0.00	RX1	O
ATOM	2600	N	SER	50	57.838	18.179	46.566	1.00	0.00	RX1	N
ATOM	2601	H	SER	50	57.519	18.156	47.514	1.00	0.00	RX1	H
ATOM	2602	CA	SER	50	57.388	17.147	45.634	1.00	0.00	RX1	C
ATOM	2603	CB	SER	50	56.982	15.926	46.444	1.00	0.00	RX1	C
ATOM	2604	OG	SER	50	58.080	15.638	47.317	1.00	0.00	RX1	O
ATOM	2605	HG	SER	50	57.744	15.767	48.208	1.00	0.00	RX1	H
ATOM	2606	C	SER	50	56.354	17.623	44.633	1.00	0.00	RX1	C
ATOM	2607	O	SER	50	56.392	17.282	43.459	1.00	0.00	RX1	O
ATOM	2608	N	LEU	51	55.465	18.486	45.144	1.00	0.00	RX1	N
ATOM	2609	H	LEU	51	55.523	18.775	46.100	1.00	0.00	RX1	H
ATOM	2610	CA	LEU	51	54.486	19.128	44.272	1.00	0.00	RX1	C
ATOM	2611	CB	LEU	51	53.578	20.022	45.116	1.00	0.00	RX1	C
ATOM	2612	CG	LEU	51	52.165	20.137	44.555	1.00	0.00	RX1	C
ATOM	2613	CD1	LEU	51	51.468	18.781	44.587	1.00	0.00	RX1	C
ATOM	2614	CD2	LEU	51	51.346	21.210	45.270	1.00	0.00	RX1	C
ATOM	2615	C	LEU	51	55.164	19.921	43.166	1.00	0.00	RX1	C
ATOM	2616	O	LEU	51	54.865	19.816	41.985	1.00	0.00	RX1	O
ATOM	2617	N	GLN	52	56.173	20.680	43.623	1.00	0.00	RX1	N
ATOM	2618	H	GLN	52	56.334	20.746	44.609	1.00	0.00	RX1	H
ATOM	2619	CA	GLN	52	57.030	21.392	42.679	1.00	0.00	RX1	C
ATOM	2620	CB	GLN	52	58.090	22.167	43.455	1.00	0.00	RX1	C
ATOM	2621	CG	GLN	52	58.911	23.110	42.580	1.00	0.00	RX1	C

ATOM	2622	CD	GLN	52	60.013	23.711	43.423	1.00	0.00	RX1	C
ATOM	2623	OE1	GLN	52	61.191	23.448	43.219	1.00	0.00	RX1	O
ATOM	2624	NE2	GLN	52	59.565	24.524	44.398	1.00	0.00	RX1	N
ATOM	2625	HE21	GLN	52	58.591	24.710	44.529	1.00	0.00	RX1	H
ATOM	2626	HE22	GLN	52	60.204	24.964	45.029	1.00	0.00	RX1	H
ATOM	2627	C	GLN	52	57.671	20.493	41.631	1.00	0.00	RX1	C
ATOM	2628	O	GLN	52	57.540	20.699	40.433	1.00	0.00	RX1	O
ATOM	2629	N	ARG	53	58.348	19.458	42.151	1.00	0.00	RX1	N
ATOM	2630	H	ARG	53	58.386	19.348	43.142	1.00	0.00	RX1	H
ATOM	2631	CA	ARG	53	59.037	18.505	41.279	1.00	0.00	RX1	C
ATOM	2632	CB	ARG	53	59.729	17.428	42.137	1.00	0.00	RX1	C
ATOM	2633	CG	ARG	53	60.943	17.957	42.921	1.00	0.00	RX1	C
ATOM	2634	CD	ARG	53	61.595	16.971	43.911	1.00	0.00	RX1	C
ATOM	2635	NE	ARG	53	60.788	16.739	45.115	1.00	0.00	RX1	N
ATOM	2636	HE	ARG	53	59.800	16.634	44.968	1.00	0.00	RX1	H
ATOM	2637	CZ	ARG	53	61.383	16.659	46.351	1.00	0.00	RX1	C
ATOM	2638	NH1	ARG	53	62.723	16.810	46.449	1.00	0.00	RX1	N
ATOM	2639	HH11	ARG	53	63.199	16.762	47.333	1.00	0.00	RX1	H
ATOM	2640	HH12	ARG	53	63.292	16.980	45.641	1.00	0.00	RX1	H
ATOM	2641	NH2	ARG	53	60.639	16.436	47.460	1.00	0.00	RX1	N
ATOM	2642	HH21	ARG	53	61.011	16.408	48.396	1.00	0.00	RX1	H
ATOM	2643	HH22	ARG	53	59.641	16.278	47.406	1.00	0.00	RX1	H
ATOM	2644	C	ARG	53	58.164	17.879	40.201	1.00	0.00	RX1	C
ATOM	2645	O	ARG	53	58.568	17.725	39.058	1.00	0.00	RX1	O
ATOM	2646	N	MET	54	56.938	17.536	40.618	1.00	0.00	RX1	N
ATOM	2647	H	MET	54	56.625	17.735	41.547	1.00	0.00	RX1	H
ATOM	2648	CA	MET	54	56.041	16.913	39.649	1.00	0.00	RX1	C
ATOM	2649	CB	MET	54	54.983	16.091	40.387	1.00	0.00	RX1	C
ATOM	2650	CG	MET	54	54.026	15.353	39.449	1.00	0.00	RX1	C
ATOM	2651	SD	MET	54	54.834	14.152	38.381	1.00	0.00	RX1	S
ATOM	2652	CE	MET	54	55.273	12.947	39.643	1.00	0.00	RX1	C
ATOM	2653	C	MET	54	55.400	17.883	38.664	1.00	0.00	RX1	C
ATOM	2654	O	MET	54	55.177	17.574	37.501	1.00	0.00	RX1	O
ATOM	2655	N	PHE	55	55.078	19.074	39.184	1.00	0.00	RX1	N
ATOM	2656	H	PHE	55	55.343	19.365	40.106	1.00	0.00	RX1	H
ATOM	2657	CA	PHE	55	54.252	19.935	38.340	1.00	0.00	RX1	C
ATOM	2658	CB	PHE	55	52.989	20.366	39.082	1.00	0.00	RX1	C
ATOM	2659	CG	PHE	55	52.215	19.156	39.535	1.00	0.00	RX1	C
ATOM	2660	CD1	PHE	55	51.555	18.371	38.601	1.00	0.00	RX1	C
ATOM	2661	CD2	PHE	55	52.157	18.827	40.883	1.00	0.00	RX1	C
ATOM	2662	CE1	PHE	55	50.830	17.263	39.016	1.00	0.00	RX1	C
ATOM	2663	CE2	PHE	55	51.433	17.716	41.295	1.00	0.00	RX1	C
ATOM	2664	CZ	PHE	55	50.763	16.937	40.363	1.00	0.00	RX1	C
ATOM	2665	C	PHE	55	54.942	21.166	37.786	1.00	0.00	RX1	C
ATOM	2666	O	PHE	55	54.300	22.098	37.308	1.00	0.00	RX1	O
ATOM	2667	N	ASN	56	56.280	21.162	37.887	1.00	0.00	RX1	N
ATOM	2668	H	ASN	56	56.773	20.394	38.298	1.00	0.00	RX1	H
ATOM	2669	CA	ASN	56	56.976	22.399	37.531	1.00	0.00	RX1	C
ATOM	2670	CB	ASN	56	58.480	22.290	37.709	1.00	0.00	RX1	C
ATOM	2671	CG	ASN	56	59.071	23.495	37.010	1.00	0.00	RX1	C
ATOM	2672	OD1	ASN	56	58.750	24.638	37.325	1.00	0.00	RX1	O
ATOM	2673	ND2	ASN	56	59.956	23.180	36.048	1.00	0.00	RX1	N
ATOM	2674	HD21	ASN	56	60.123	22.218	35.827	1.00	0.00	RX1	H
ATOM	2675	HD22	ASN	56	60.452	23.876	35.529	1.00	0.00	RX1	H
ATOM	2676	C	ASN	56	56.761	22.887	36.110	1.00	0.00	RX1	C
ATOM	2677	O	ASN	56	57.025	22.198	35.134	1.00	0.00	RX1	O
ATOM	2678	N	ASN	57	56.215	24.114	36.060	1.00	0.00	RX1	N
ATOM	2679	H	ASN	57	56.134	24.609	36.922	1.00	0.00	RX1	H
ATOM	2680	CA	ASN	57	55.801	24.738	34.798	1.00	0.00	RX1	C
ATOM	2681	CB	ASN	57	57.011	25.338	34.077	1.00	0.00	RX1	C
ATOM	2682	CG	ASN	57	56.561	26.210	32.920	1.00	0.00	RX1	C

ATOM	2683	OD1	ASN	57	56.738	25.878	31.749	1.00	0.00	RX1	O
ATOM	2684	ND2	ASN	57	56.008	27.372	33.305	1.00	0.00	RX1	N
ATOM	2685	HD21	ASN	57	55.789	27.571	34.263	1.00	0.00	RX1	H
ATOM	2686	HD22	ASN	57	55.745	28.075	32.644	1.00	0.00	RX1	H
ATOM	2687	C	ASN	57	54.976	23.858	33.860	1.00	0.00	RX1	C
ATOM	2688	O	ASN	57	55.079	23.923	32.639	1.00	0.00	RX1	O
ATOM	2689	N	CYS	58	54.144	23.018	34.481	1.00	0.00	RX1	N
ATOM	2690	H	CYS	58	54.001	23.012	35.472	1.00	0.00	RX1	H
ATOM	2691	CA	CYS	58	53.319	22.171	33.627	1.00	0.00	RX1	C
ATOM	2692	CB	CYS	58	52.972	20.892	34.381	1.00	0.00	RX1	C
ATOM	2693	SG	CYS	58	51.987	19.748	33.389	1.00	0.00	RX1	S
ATOM	2694	C	CYS	58	52.076	22.891	33.155	1.00	0.00	RX1	C
ATOM	2695	O	CYS	58	51.507	23.704	33.872	1.00	0.00	RX1	O
ATOM	2696	N	GLU	59	51.669	22.551	31.931	1.00	0.00	RX1	N
ATOM	2697	H	GLU	59	52.188	21.937	31.332	1.00	0.00	RX1	H
ATOM	2698	CA	GLU	59	50.374	23.050	31.493	1.00	0.00	RX1	C
ATOM	2699	CB	GLU	59	50.386	23.554	30.044	1.00	0.00	RX1	C
ATOM	2700	CG	GLU	59	51.185	24.835	29.766	1.00	0.00	RX1	C
ATOM	2701	CD	GLU	59	52.655	24.529	29.573	1.00	0.00	RX1	C
ATOM	2702	OE1	GLU	59	53.443	25.438	29.334	1.00	0.00	RX1	O
ATOM	2703	OE2	GLU	59	53.034	23.371	29.648	1.00	0.00	RX1	O
ATOM	2704	C	GLU	59	49.281	22.017	31.662	1.00	0.00	RX1	C
ATOM	2705	O	GLU	59	48.160	22.347	32.032	1.00	0.00	RX1	O
ATOM	2706	N	VAL	60	49.643	20.756	31.368	1.00	0.00	RX1	N
ATOM	2707	H	VAL	60	50.585	20.504	31.132	1.00	0.00	RX1	H
ATOM	2708	CA	VAL	60	48.641	19.699	31.507	1.00	0.00	RX1	C
ATOM	2709	CB	VAL	60	48.284	19.061	30.163	1.00	0.00	RX1	C
ATOM	2710	CG1	VAL	60	47.173	18.021	30.335	1.00	0.00	RX1	C
ATOM	2711	CG2	VAL	60	47.909	20.109	29.124	1.00	0.00	RX1	C
ATOM	2712	C	VAL	60	49.060	18.607	32.471	1.00	0.00	RX1	C
ATOM	2713	O	VAL	60	49.901	17.762	32.187	1.00	0.00	RX1	O
ATOM	2714	N	VAL	61	48.396	18.632	33.626	1.00	0.00	RX1	N
ATOM	2715	H	VAL	61	47.656	19.291	33.753	1.00	0.00	RX1	H
ATOM	2716	CA	VAL	61	48.519	17.467	34.493	1.00	0.00	RX1	C
ATOM	2717	CB	VAL	61	48.010	17.811	35.887	1.00	0.00	RX1	C
ATOM	2718	CG1	VAL	61	48.160	16.624	36.830	1.00	0.00	RX1	C
ATOM	2719	CG2	VAL	61	48.696	19.066	36.417	1.00	0.00	RX1	C
ATOM	2720	C	VAL	61	47.726	16.305	33.920	1.00	0.00	RX1	C
ATOM	2721	O	VAL	61	46.531	16.418	33.675	1.00	0.00	RX1	O
ATOM	2722	N	LEU	62	48.436	15.191	33.711	1.00	0.00	RX1	N
ATOM	2723	H	LEU	62	49.410	15.146	33.937	1.00	0.00	RX1	H
ATOM	2724	CA	LEU	62	47.713	14.008	33.250	1.00	0.00	RX1	C
ATOM	2725	CB	LEU	62	48.669	13.008	32.606	1.00	0.00	RX1	C
ATOM	2726	CG	LEU	62	49.376	13.585	31.381	1.00	0.00	RX1	C
ATOM	2727	CD1	LEU	62	50.440	12.628	30.846	1.00	0.00	RX1	C
ATOM	2728	CD2	LEU	62	48.379	14.012	30.302	1.00	0.00	RX1	C
ATOM	2729	C	LEU	62	46.929	13.339	34.361	1.00	0.00	RX1	C
ATOM	2730	O	LEU	62	45.711	13.222	34.322	1.00	0.00	RX1	O
ATOM	2731	N	GLY	63	47.695	12.915	35.373	1.00	0.00	RX1	N
ATOM	2732	H	GLY	63	48.688	13.038	35.384	1.00	0.00	RX1	H
ATOM	2733	CA	GLY	63	47.024	12.349	36.536	1.00	0.00	RX1	C
ATOM	2734	C	GLY	63	46.425	13.421	37.420	1.00	0.00	RX1	C
ATOM	2735	O	GLY	63	45.772	14.358	36.973	1.00	0.00	RX1	O
ATOM	2736	N	ASN	64	46.686	13.237	38.716	1.00	0.00	RX1	N
ATOM	2737	H	ASN	64	47.350	12.573	39.058	1.00	0.00	RX1	H
ATOM	2738	CA	ASN	64	46.015	14.150	39.633	1.00	0.00	RX1	C
ATOM	2739	CB	ASN	64	45.703	13.512	40.976	1.00	0.00	RX1	C
ATOM	2740	CG	ASN	64	44.862	12.279	40.820	1.00	0.00	RX1	C
ATOM	2741	OD1	ASN	64	43.887	12.244	40.073	1.00	0.00	RX1	O
ATOM	2742	ND2	ASN	64	45.297	11.270	41.593	1.00	0.00	RX1	N
ATOM	2743	HD21	ASN	64	46.115	11.418	42.161	1.00	0.00	RX1	H

ATOM	2744	HD22	ASN	64	44.837	10.385	41.664	1.00	0.00	RX1	H
ATOM	2745	C	ASN	64	46.856	15.350	39.957	1.00	0.00	RX1	C
ATOM	2746	O	ASN	64	48.080	15.297	39.959	1.00	0.00	RX1	O
ATOM	2747	N	LEU	65	46.139	16.428	40.264	1.00	0.00	RX1	N
ATOM	2748	H	LEU	65	45.139	16.396	40.248	1.00	0.00	RX1	H
ATOM	2749	CA	LEU	65	46.812	17.552	40.892	1.00	0.00	RX1	C
ATOM	2750	CB	LEU	65	46.392	18.834	40.179	1.00	0.00	RX1	C
ATOM	2751	CG	LEU	65	47.065	20.101	40.698	1.00	0.00	RX1	C
ATOM	2752	CD1	LEU	65	48.586	19.983	40.719	1.00	0.00	RX1	C
ATOM	2753	CD2	LEU	65	46.603	21.322	39.907	1.00	0.00	RX1	C
ATOM	2754	C	LEU	65	46.469	17.568	42.368	1.00	0.00	RX1	C
ATOM	2755	O	LEU	65	45.474	18.137	42.806	1.00	0.00	RX1	O
ATOM	2756	N	GLU	66	47.321	16.874	43.128	1.00	0.00	RX1	N
ATOM	2757	H	GLU	66	48.172	16.474	42.779	1.00	0.00	RX1	H
ATOM	2758	CA	GLU	66	47.011	16.847	44.554	1.00	0.00	RX1	C
ATOM	2759	CB	GLU	66	47.229	15.448	45.098	1.00	0.00	RX1	C
ATOM	2760	CG	GLU	66	46.316	14.418	44.444	1.00	0.00	RX1	C
ATOM	2761	CD	GLU	66	46.986	13.073	44.557	1.00	0.00	RX1	C
ATOM	2762	OE1	GLU	66	48.209	13.039	44.549	1.00	0.00	RX1	O
ATOM	2763	OE2	GLU	66	46.308	12.058	44.661	1.00	0.00	RX1	O
ATOM	2764	C	GLU	66	47.803	17.854	45.347	1.00	0.00	RX1	C
ATOM	2765	O	GLU	66	48.867	17.590	45.894	1.00	0.00	RX1	O
ATOM	2766	N	ILE	67	47.215	19.049	45.377	1.00	0.00	RX1	N
ATOM	2767	H	ILE	67	46.266	19.099	45.062	1.00	0.00	RX1	H
ATOM	2768	CA	ILE	67	47.745	20.075	46.266	1.00	0.00	RX1	C
ATOM	2769	CB	ILE	67	47.139	21.423	45.895	1.00	0.00	RX1	C
ATOM	2770	CG2	ILE	67	47.773	22.571	46.683	1.00	0.00	RX1	C
ATOM	2771	CG1	ILE	67	47.222	21.625	44.385	1.00	0.00	RX1	C
ATOM	2772	CD1	ILE	67	46.387	22.817	43.930	1.00	0.00	RX1	C
ATOM	2773	C	ILE	67	47.422	19.725	47.708	1.00	0.00	RX1	C
ATOM	2774	O	ILE	67	46.374	20.068	48.244	1.00	0.00	RX1	O
ATOM	2775	N	THR	68	48.367	19.002	48.307	1.00	0.00	RX1	N
ATOM	2776	H	THR	68	49.146	18.616	47.814	1.00	0.00	RX1	H
ATOM	2777	CA	THR	68	48.135	18.633	49.693	1.00	0.00	RX1	C
ATOM	2778	CB	THR	68	47.851	17.137	49.661	1.00	0.00	RX1	C
ATOM	2779	OG1	THR	68	48.439	16.569	48.483	1.00	0.00	RX1	O
ATOM	2780	HG1	THR	68	49.379	16.602	48.633	1.00	0.00	RX1	H
ATOM	2781	CG2	THR	68	46.354	16.860	49.641	1.00	0.00	RX1	C
ATOM	2782	C	THR	68	49.274	19.029	50.609	1.00	0.00	RX1	C
ATOM	2783	O	THR	68	50.440	18.997	50.233	1.00	0.00	RX1	O
ATOM	2784	N	TYR	69	48.872	19.400	51.839	1.00	0.00	RX1	N
ATOM	2785	H	TYR	69	47.888	19.534	51.981	1.00	0.00	RX1	H
ATOM	2786	CA	TYR	69	49.812	19.678	52.935	1.00	0.00	RX1	C
ATOM	2787	CB	TYR	69	50.663	18.452	53.275	1.00	0.00	RX1	C
ATOM	2788	CG	TYR	69	49.807	17.324	53.789	1.00	0.00	RX1	C
ATOM	2789	CD1	TYR	69	49.310	17.374	55.084	1.00	0.00	RX1	C
ATOM	2790	CE1	TYR	69	48.586	16.304	55.591	1.00	0.00	RX1	C
ATOM	2791	CD2	TYR	69	49.536	16.226	52.982	1.00	0.00	RX1	C
ATOM	2792	CE2	TYR	69	48.808	15.157	53.487	1.00	0.00	RX1	C
ATOM	2793	CZ	TYR	69	48.348	15.188	54.798	1.00	0.00	RX1	C
ATOM	2794	OH	TYR	69	47.665	14.112	55.325	1.00	0.00	RX1	O
ATOM	2795	HH	TYR	69	47.706	13.376	54.718	1.00	0.00	RX1	H
ATOM	2796	C	TYR	69	50.730	20.887	52.808	1.00	0.00	RX1	C
ATOM	2797	O	TYR	69	51.596	21.118	53.647	1.00	0.00	RX1	O
ATOM	2798	N	VAL	70	50.522	21.664	51.742	1.00	0.00	RX1	N
ATOM	2799	H	VAL	70	49.745	21.509	51.133	1.00	0.00	RX1	H
ATOM	2800	CA	VAL	70	51.399	22.814	51.539	1.00	0.00	RX1	C
ATOM	2801	CB	VAL	70	51.256	23.322	50.107	1.00	0.00	RX1	C
ATOM	2802	CG1	VAL	70	52.177	24.510	49.856	1.00	0.00	RX1	C
ATOM	2803	CG2	VAL	70	51.497	22.186	49.111	1.00	0.00	RX1	C
ATOM	2804	C	VAL	70	51.154	23.922	52.555	1.00	0.00	RX1	C

ATOM	2805	O	VAL	70	50.046	24.414	52.742	1.00	0.00	RX1	O
ATOM	2806	N	GLN	71	52.248	24.263	53.243	1.00	0.00	RX1	N
ATOM	2807	H	GLN	71	53.153	23.940	52.969	1.00	0.00	RX1	H
ATOM	2808	CA	GLN	71	52.084	25.202	54.345	1.00	0.00	RX1	C
ATOM	2809	CB	GLN	71	53.092	24.902	55.455	1.00	0.00	RX1	C
ATOM	2810	CG	GLN	71	52.988	23.476	55.995	1.00	0.00	RX1	C
ATOM	2811	CD	GLN	71	51.656	23.285	56.687	1.00	0.00	RX1	C
ATOM	2812	OE1	GLN	71	51.320	23.980	57.643	1.00	0.00	RX1	O
ATOM	2813	NE2	GLN	71	50.900	22.318	56.144	1.00	0.00	RX1	N
ATOM	2814	HE21	GLN	71	51.263	21.800	55.365	1.00	0.00	RX1	H
ATOM	2815	HE22	GLN	71	49.983	22.068	56.456	1.00	0.00	RX1	H
ATOM	2816	C	GLN	71	52.143	26.675	53.977	1.00	0.00	RX1	C
ATOM	2817	O	GLN	71	52.620	27.078	52.922	1.00	0.00	RX1	O
ATOM	2818	N	ARG	72	51.624	27.448	54.944	1.00	0.00	RX1	N
ATOM	2819	H	ARG	72	51.225	26.932	55.704	1.00	0.00	RX1	H
ATOM	2820	CA	ARG	72	51.413	28.898	54.916	1.00	0.00	RX1	C
ATOM	2821	CB	ARG	72	51.825	29.502	56.261	1.00	0.00	RX1	C
ATOM	2822	CG	ARG	72	50.842	30.566	56.757	1.00	0.00	RX1	C
ATOM	2823	CD	ARG	72	49.529	29.967	57.270	1.00	0.00	RX1	C
ATOM	2824	NE	ARG	72	48.530	31.010	57.484	1.00	0.00	RX1	N
ATOM	2825	HE	ARG	72	48.533	31.792	56.843	1.00	0.00	RX1	H
ATOM	2826	CZ	ARG	72	47.524	30.873	58.397	1.00	0.00	RX1	C
ATOM	2827	NH1	ARG	72	47.541	29.836	59.257	1.00	0.00	RX1	N
ATOM	2828	HH11	ARG	72	46.812	29.676	59.942	1.00	0.00	RX1	H
ATOM	2829	HH12	ARG	72	48.275	29.159	59.272	1.00	0.00	RX1	H
ATOM	2830	NH2	ARG	72	46.528	31.776	58.430	1.00	0.00	RX1	N
ATOM	2831	HH21	ARG	72	45.790	31.725	59.115	1.00	0.00	RX1	H
ATOM	2832	HH22	ARG	72	46.479	32.525	57.742	1.00	0.00	RX1	H
ATOM	2833	C	ARG	72	51.954	29.745	53.769	1.00	0.00	RX1	C
ATOM	2834	O	ARG	72	51.216	30.427	53.070	1.00	0.00	RX1	O
ATOM	2835	N	ASN	73	53.286	29.707	53.633	1.00	0.00	RX1	N
ATOM	2836	H	ASN	73	53.829	28.980	54.051	1.00	0.00	RX1	H
ATOM	2837	CA	ASN	73	53.894	30.737	52.790	1.00	0.00	RX1	C
ATOM	2838	CB	ASN	73	55.139	31.324	53.445	1.00	0.00	RX1	C
ATOM	2839	CG	ASN	73	54.827	32.716	53.943	1.00	0.00	RX1	C
ATOM	2840	OD1	ASN	73	54.765	32.961	55.142	1.00	0.00	RX1	O
ATOM	2841	ND2	ASN	73	54.624	33.617	52.966	1.00	0.00	RX1	N
ATOM	2842	HD21	ASN	73	54.708	33.357	52.001	1.00	0.00	RX1	H
ATOM	2843	HD22	ASN	73	54.375	34.559	53.188	1.00	0.00	RX1	H
ATOM	2844	C	ASN	73	54.242	30.339	51.372	1.00	0.00	RX1	C
ATOM	2845	O	ASN	73	54.742	31.147	50.596	1.00	0.00	RX1	O
ATOM	2846	N	TYR	74	54.015	29.058	51.053	1.00	0.00	RX1	N
ATOM	2847	H	TYR	74	53.459	28.431	51.604	1.00	0.00	RX1	H
ATOM	2848	CA	TYR	74	54.563	28.661	49.757	1.00	0.00	RX1	C
ATOM	2849	CB	TYR	74	54.910	27.172	49.724	1.00	0.00	RX1	C
ATOM	2850	CG	TYR	74	55.509	26.725	51.036	1.00	0.00	RX1	C
ATOM	2851	CD1	TYR	74	55.005	25.585	51.645	1.00	0.00	RX1	C
ATOM	2852	CE1	TYR	74	55.496	25.174	52.874	1.00	0.00	RX1	C
ATOM	2853	CD2	TYR	74	56.542	27.435	51.639	1.00	0.00	RX1	C
ATOM	2854	CE2	TYR	74	57.029	27.029	52.875	1.00	0.00	RX1	C
ATOM	2855	CZ	TYR	74	56.499	25.904	53.495	1.00	0.00	RX1	C
ATOM	2856	OH	TYR	74	56.970	25.503	54.724	1.00	0.00	RX1	O
ATOM	2857	HH	TYR	74	57.843	25.141	54.541	1.00	0.00	RX1	H
ATOM	2858	C	TYR	74	53.691	29.014	48.564	1.00	0.00	RX1	C
ATOM	2859	O	TYR	74	52.785	28.269	48.207	1.00	0.00	RX1	O
ATOM	2860	N	ASP	75	54.028	30.146	47.915	1.00	0.00	RX1	N
ATOM	2861	H	ASP	75	54.748	30.737	48.279	1.00	0.00	RX1	H
ATOM	2862	CA	ASP	75	53.456	30.313	46.573	1.00	0.00	RX1	C
ATOM	2863	CB	ASP	75	53.739	31.690	45.920	1.00	0.00	RX1	C
ATOM	2864	CG	ASP	75	53.104	31.844	44.522	1.00	0.00	RX1	C
ATOM	2865	OD1	ASP	75	52.553	32.895	44.197	1.00	0.00	RX1	O

ATOM	2866	OD2	ASP	75	53.180	30.942	43.696	1.00	0.00	RX1	O
ATOM	2867	C	ASP	75	53.904	29.198	45.635	1.00	0.00	RX1	C
ATOM	2868	O	ASP	75	55.071	28.987	45.315	1.00	0.00	RX1	O
ATOM	2869	N	LEU	76	52.876	28.467	45.217	1.00	0.00	RX1	N
ATOM	2870	H	LEU	76	51.958	28.751	45.485	1.00	0.00	RX1	H
ATOM	2871	CA	LEU	76	53.114	27.370	44.293	1.00	0.00	RX1	C
ATOM	2872	CB	LEU	76	51.985	26.364	44.471	1.00	0.00	RX1	C
ATOM	2873	CG	LEU	76	51.832	25.929	45.926	1.00	0.00	RX1	C
ATOM	2874	CD1	LEU	76	50.435	25.402	46.251	1.00	0.00	RX1	C
ATOM	2875	CD2	LEU	76	52.916	24.931	46.304	1.00	0.00	RX1	C
ATOM	2876	C	LEU	76	53.181	27.832	42.849	1.00	0.00	RX1	C
ATOM	2877	O	LEU	76	52.312	27.538	42.038	1.00	0.00	RX1	O
ATOM	2878	N	SER	77	54.256	28.568	42.530	1.00	0.00	RX1	N
ATOM	2879	H	SER	77	54.901	28.804	43.260	1.00	0.00	RX1	H
ATOM	2880	CA	SER	77	54.307	29.188	41.203	1.00	0.00	RX1	C
ATOM	2881	CB	SER	77	55.545	30.074	41.163	1.00	0.00	RX1	C
ATOM	2882	OG	SER	77	55.303	31.166	42.067	1.00	0.00	RX1	O
ATOM	2883	HG	SER	77	55.048	30.791	42.915	1.00	0.00	RX1	H
ATOM	2884	C	SER	77	54.067	28.306	39.993	1.00	0.00	RX1	C
ATOM	2885	O	SER	77	53.460	28.725	39.019	1.00	0.00	RX1	O
ATOM	2886	N	PHE	78	54.507	27.047	40.118	1.00	0.00	RX1	N
ATOM	2887	H	PHE	78	54.986	26.774	40.949	1.00	0.00	RX1	H
ATOM	2888	CA	PHE	78	54.202	26.085	39.056	1.00	0.00	RX1	C
ATOM	2889	CB	PHE	78	54.814	24.718	39.381	1.00	0.00	RX1	C
ATOM	2890	CG	PHE	78	54.414	24.218	40.749	1.00	0.00	RX1	C
ATOM	2891	CD1	PHE	78	53.304	23.397	40.890	1.00	0.00	RX1	C
ATOM	2892	CD2	PHE	78	55.166	24.566	41.866	1.00	0.00	RX1	C
ATOM	2893	CE1	PHE	78	52.947	22.920	42.143	1.00	0.00	RX1	C
ATOM	2894	CE2	PHE	78	54.807	24.095	43.122	1.00	0.00	RX1	C
ATOM	2895	CZ	PHE	78	53.700	23.269	43.257	1.00	0.00	RX1	C
ATOM	2896	C	PHE	78	52.735	25.998	38.639	1.00	0.00	RX1	C
ATOM	2897	O	PHE	78	52.419	25.930	37.457	1.00	0.00	RX1	O
ATOM	2898	N	LEU	79	51.853	26.079	39.657	1.00	0.00	RX1	N
ATOM	2899	H	LEU	79	52.166	26.232	40.595	1.00	0.00	RX1	H
ATOM	2900	CA	LEU	79	50.410	26.082	39.390	1.00	0.00	RX1	C
ATOM	2901	CB	LEU	79	49.589	26.201	40.668	1.00	0.00	RX1	C
ATOM	2902	CG	LEU	79	49.746	25.099	41.704	1.00	0.00	RX1	C
ATOM	2903	CD1	LEU	79	48.821	25.379	42.883	1.00	0.00	RX1	C
ATOM	2904	CD2	LEU	79	49.499	23.706	41.136	1.00	0.00	RX1	C
ATOM	2905	C	LEU	79	49.928	27.181	38.463	1.00	0.00	RX1	C
ATOM	2906	O	LEU	79	48.951	27.035	37.740	1.00	0.00	RX1	O
ATOM	2907	N	LYS	80	50.675	28.295	38.486	1.00	0.00	RX1	N
ATOM	2908	H	LYS	80	51.514	28.347	39.026	1.00	0.00	RX1	H
ATOM	2909	CA	LYS	80	50.301	29.386	37.589	1.00	0.00	RX1	C
ATOM	2910	CB	LYS	80	51.032	30.679	37.997	1.00	0.00	RX1	C
ATOM	2911	CG	LYS	80	50.783	31.043	39.472	1.00	0.00	RX1	C
ATOM	2912	CD	LYS	80	51.224	32.454	39.905	1.00	0.00	RX1	C
ATOM	2913	CE	LYS	80	52.707	32.667	40.241	1.00	0.00	RX1	C
ATOM	2914	NZ	LYS	80	53.008	32.398	41.658	1.00	0.00	RX1	N
ATOM	2915	HZ1	LYS	80	54.032	32.304	41.827	1.00	0.00	RX1	H
ATOM	2916	HZ2	LYS	80	52.674	33.108	42.347	1.00	0.00	RX1	H
ATOM	2917	HZ3	LYS	80	52.615	31.511	42.035	1.00	0.00	RX1	H
ATOM	2918	C	LYS	80	50.426	29.075	36.097	1.00	0.00	RX1	C
ATOM	2919	O	LYS	80	49.932	29.813	35.254	1.00	0.00	RX1	O
ATOM	2920	N	THR	81	51.084	27.940	35.810	1.00	0.00	RX1	N
ATOM	2921	H	THR	81	51.443	27.335	36.516	1.00	0.00	RX1	H
ATOM	2922	CA	THR	81	51.117	27.466	34.426	1.00	0.00	RX1	C
ATOM	2923	CB	THR	81	52.437	26.746	34.230	1.00	0.00	RX1	C
ATOM	2924	OG1	THR	81	53.424	27.277	35.133	1.00	0.00	RX1	O
ATOM	2925	HG1	THR	81	53.182	26.920	35.983	1.00	0.00	RX1	H
ATOM	2926	CG2	THR	81	52.873	26.808	32.766	1.00	0.00	RX1	C

ATOM	2927	C	THR	81	49.937	26.583	34.025	1.00	0.00	RX1	C
ATOM	2928	O	THR	81	49.553	26.482	32.863	1.00	0.00	RX1	O
ATOM	2929	N	ILE	82	49.399	25.907	35.051	1.00	0.00	RX1	N
ATOM	2930	H	ILE	82	49.510	26.198	36.000	1.00	0.00	RX1	H
ATOM	2931	CA	ILE	82	48.530	24.775	34.743	1.00	0.00	RX1	C
ATOM	2932	CB	ILE	82	48.375	23.871	35.965	1.00	0.00	RX1	C
ATOM	2933	CG2	ILE	82	47.527	22.641	35.633	1.00	0.00	RX1	C
ATOM	2934	CG1	ILE	82	49.757	23.490	36.498	1.00	0.00	RX1	C
ATOM	2935	CD1	ILE	82	49.712	22.495	37.653	1.00	0.00	RX1	C
ATOM	2936	C	ILE	82	47.184	25.182	34.181	1.00	0.00	RX1	C
ATOM	2937	O	ILE	82	46.357	25.801	34.834	1.00	0.00	RX1	O
ATOM	2938	N	GLN	83	47.016	24.791	32.916	1.00	0.00	RX1	N
ATOM	2939	H	GLN	83	47.720	24.217	32.497	1.00	0.00	RX1	H
ATOM	2940	CA	GLN	83	45.750	25.060	32.245	1.00	0.00	RX1	C
ATOM	2941	CB	GLN	83	45.969	25.253	30.753	1.00	0.00	RX1	C
ATOM	2942	CG	GLN	83	47.072	26.236	30.389	1.00	0.00	RX1	C
ATOM	2943	CD	GLN	83	47.268	26.137	28.895	1.00	0.00	RX1	C
ATOM	2944	OE1	GLN	83	46.416	25.618	28.170	1.00	0.00	RX1	O
ATOM	2945	NE2	GLN	83	48.440	26.642	28.477	1.00	0.00	RX1	N
ATOM	2946	HE21	GLN	83	49.088	26.998	29.156	1.00	0.00	RX1	H
ATOM	2947	HE22	GLN	83	48.738	26.680	27.523	1.00	0.00	RX1	H
ATOM	2948	C	GLN	83	44.753	23.932	32.416	1.00	0.00	RX1	C
ATOM	2949	O	GLN	83	43.549	24.134	32.535	1.00	0.00	RX1	O
ATOM	2950	N	GLU	84	45.316	22.720	32.356	1.00	0.00	RX1	N
ATOM	2951	H	GLU	84	46.304	22.554	32.383	1.00	0.00	RX1	H
ATOM	2952	CA	GLU	84	44.456	21.553	32.240	1.00	0.00	RX1	C
ATOM	2953	CB	GLU	84	44.512	20.984	30.818	1.00	0.00	RX1	C
ATOM	2954	CG	GLU	84	44.376	22.009	29.686	1.00	0.00	RX1	C
ATOM	2955	CD	GLU	84	44.148	21.290	28.375	1.00	0.00	RX1	C
ATOM	2956	OE1	GLU	84	44.512	20.128	28.272	1.00	0.00	RX1	O
ATOM	2957	OE2	GLU	84	43.550	21.854	27.461	1.00	0.00	RX1	O
ATOM	2958	C	GLU	84	44.875	20.473	33.209	1.00	0.00	RX1	C
ATOM	2959	O	GLU	84	46.050	20.160	33.331	1.00	0.00	RX1	O
ATOM	2960	N	VAL	85	43.873	19.896	33.874	1.00	0.00	RX1	N
ATOM	2961	H	VAL	85	42.921	20.197	33.778	1.00	0.00	RX1	H
ATOM	2962	CA	VAL	85	44.150	18.658	34.601	1.00	0.00	RX1	C
ATOM	2963	CB	VAL	85	43.848	18.842	36.094	1.00	0.00	RX1	C
ATOM	2964	CG1	VAL	85	44.174	17.590	36.913	1.00	0.00	RX1	C
ATOM	2965	CG2	VAL	85	44.572	20.072	36.641	1.00	0.00	RX1	C
ATOM	2966	C	VAL	85	43.266	17.584	33.999	1.00	0.00	RX1	C
ATOM	2967	O	VAL	85	42.156	17.881	33.572	1.00	0.00	RX1	O
ATOM	2968	N	ALA	86	43.772	16.347	33.948	1.00	0.00	RX1	N
ATOM	2969	H	ALA	86	44.694	16.128	34.278	1.00	0.00	RX1	H
ATOM	2970	CA	ALA	86	42.861	15.303	33.496	1.00	0.00	RX1	C
ATOM	2971	CB	ALA	86	43.525	14.381	32.473	1.00	0.00	RX1	C
ATOM	2972	C	ALA	86	42.243	14.500	34.624	1.00	0.00	RX1	C
ATOM	2973	O	ALA	86	41.042	14.248	34.623	1.00	0.00	RX1	O
ATOM	2974	N	GLY	87	43.092	14.142	35.600	1.00	0.00	RX1	N
ATOM	2975	H	GLY	87	44.074	14.345	35.571	1.00	0.00	RX1	H
ATOM	2976	CA	GLY	87	42.538	13.507	36.796	1.00	0.00	RX1	C
ATOM	2977	C	GLY	87	41.838	14.489	37.721	1.00	0.00	RX1	C
ATOM	2978	O	GLY	87	41.345	15.535	37.302	1.00	0.00	RX1	O
ATOM	2979	N	TYR	88	41.811	14.105	39.004	1.00	0.00	RX1	N
ATOM	2980	H	TYR	88	42.343	13.313	39.314	1.00	0.00	RX1	H
ATOM	2981	CA	TYR	88	41.168	15.001	39.962	1.00	0.00	RX1	C
ATOM	2982	CB	TYR	88	40.401	14.220	41.042	1.00	0.00	RX1	C
ATOM	2983	CG	TYR	88	41.295	13.363	41.911	1.00	0.00	RX1	C
ATOM	2984	CD1	TYR	88	42.124	13.937	42.869	1.00	0.00	RX1	C
ATOM	2985	CE1	TYR	88	42.924	13.137	43.675	1.00	0.00	RX1	C
ATOM	2986	CD2	TYR	88	41.265	11.981	41.767	1.00	0.00	RX1	C
ATOM	2987	CE2	TYR	88	42.058	11.180	42.580	1.00	0.00	RX1	C

ATOM	2988	CZ	TYR	88	42.891	11.756	43.531	1.00	0.00	RX1	C
ATOM	2989	OH	TYR	88	43.684	10.955	44.328	1.00	0.00	RX1	O
ATOM	2990	HH	TYR	88	44.505	11.414	44.510	1.00	0.00	RX1	H
ATOM	2991	C	TYR	88	42.117	16.031	40.551	1.00	0.00	RX1	C
ATOM	2992	O	TYR	88	43.333	15.910	40.464	1.00	0.00	RX1	O
ATOM	2993	N	VAL	89	41.512	17.056	41.167	1.00	0.00	RX1	N
ATOM	2994	H	VAL	89	40.524	17.088	41.321	1.00	0.00	RX1	H
ATOM	2995	CA	VAL	89	42.347	18.026	41.870	1.00	0.00	RX1	C
ATOM	2996	CB	VAL	89	42.197	19.432	41.292	1.00	0.00	RX1	C
ATOM	2997	CG1	VAL	89	43.120	20.417	42.010	1.00	0.00	RX1	C
ATOM	2998	CG2	VAL	89	42.457	19.433	39.790	1.00	0.00	RX1	C
ATOM	2999	C	VAL	89	42.029	18.028	43.351	1.00	0.00	RX1	C
ATOM	3000	O	VAL	89	41.018	18.548	43.813	1.00	0.00	RX1	O
ATOM	3001	N	LEU	90	42.944	17.393	44.083	1.00	0.00	RX1	N
ATOM	3002	H	LEU	90	43.841	17.188	43.688	1.00	0.00	RX1	H
ATOM	3003	CA	LEU	90	42.740	17.380	45.524	1.00	0.00	RX1	C
ATOM	3004	CB	LEU	90	43.207	16.045	46.099	1.00	0.00	RX1	C
ATOM	3005	CG	LEU	90	43.165	15.980	47.624	1.00	0.00	RX1	C
ATOM	3006	CD1	LEU	90	41.770	16.255	48.181	1.00	0.00	RX1	C
ATOM	3007	CD2	LEU	90	43.739	14.663	48.139	1.00	0.00	RX1	C
ATOM	3008	C	LEU	90	43.471	18.536	46.169	1.00	0.00	RX1	C
ATOM	3009	O	LEU	90	44.691	18.589	46.177	1.00	0.00	RX1	O
ATOM	3010	N	ILE	91	42.670	19.456	46.706	1.00	0.00	RX1	N
ATOM	3011	H	ILE	91	41.678	19.329	46.752	1.00	0.00	RX1	H
ATOM	3012	CA	ILE	91	43.281	20.535	47.472	1.00	0.00	RX1	C
ATOM	3013	CB	ILE	91	42.745	21.883	46.990	1.00	0.00	RX1	C
ATOM	3014	CG2	ILE	91	43.369	23.039	47.767	1.00	0.00	RX1	C
ATOM	3015	CG1	ILE	91	42.948	22.041	45.485	1.00	0.00	RX1	C
ATOM	3016	CD1	ILE	91	42.504	23.414	44.979	1.00	0.00	RX1	C
ATOM	3017	C	ILE	91	42.963	20.340	48.941	1.00	0.00	RX1	C
ATOM	3018	O	ILE	91	41.900	20.730	49.411	1.00	0.00	RX1	O
ATOM	3019	N	ALA	92	43.902	19.702	49.646	1.00	0.00	RX1	N
ATOM	3020	H	ALA	92	44.804	19.462	49.277	1.00	0.00	RX1	H
ATOM	3021	CA	ALA	92	43.571	19.423	51.039	1.00	0.00	RX1	C
ATOM	3022	CB	ALA	92	43.041	18.001	51.209	1.00	0.00	RX1	C
ATOM	3023	C	ALA	92	44.699	19.647	52.021	1.00	0.00	RX1	C
ATOM	3024	O	ALA	92	45.875	19.465	51.723	1.00	0.00	RX1	O
ATOM	3025	N	LEU	93	44.267	20.045	53.233	1.00	0.00	RX1	N
ATOM	3026	H	LEU	93	43.288	20.233	53.336	1.00	0.00	RX1	H
ATOM	3027	CA	LEU	93	45.195	20.254	54.354	1.00	0.00	RX1	C
ATOM	3028	CB	LEU	93	45.693	18.915	54.904	1.00	0.00	RX1	C
ATOM	3029	CG	LEU	93	44.618	18.200	55.723	1.00	0.00	RX1	C
ATOM	3030	CD1	LEU	93	44.994	16.754	56.044	1.00	0.00	RX1	C
ATOM	3031	CD2	LEU	93	44.284	18.977	56.997	1.00	0.00	RX1	C
ATOM	3032	C	LEU	93	46.350	21.204	54.083	1.00	0.00	RX1	C
ATOM	3033	O	LEU	93	47.459	21.079	54.591	1.00	0.00	RX1	O
ATOM	3034	N	ASN	94	46.025	22.184	53.238	1.00	0.00	RX1	N
ATOM	3035	H	ASN	94	45.085	22.308	52.923	1.00	0.00	RX1	H
ATOM	3036	CA	ASN	94	47.020	23.212	52.969	1.00	0.00	RX1	C
ATOM	3037	CB	ASN	94	46.956	23.728	51.531	1.00	0.00	RX1	C
ATOM	3038	CG	ASN	94	47.110	22.611	50.527	1.00	0.00	RX1	C
ATOM	3039	OD1	ASN	94	48.195	22.107	50.254	1.00	0.00	RX1	O
ATOM	3040	ND2	ASN	94	45.949	22.262	49.964	1.00	0.00	RX1	N
ATOM	3041	HD21	ASN	94	45.089	22.672	50.278	1.00	0.00	RX1	H
ATOM	3042	HD22	ASN	94	45.905	21.567	49.245	1.00	0.00	RX1	H
ATOM	3043	C	ASN	94	46.763	24.384	53.873	1.00	0.00	RX1	C
ATOM	3044	O	ASN	94	45.625	24.782	54.094	1.00	0.00	RX1	O
ATOM	3045	N	THR	95	47.862	24.926	54.390	1.00	0.00	RX1	N
ATOM	3046	H	THR	95	48.795	24.596	54.238	1.00	0.00	RX1	H
ATOM	3047	CA	THR	95	47.675	26.158	55.140	1.00	0.00	RX1	C
ATOM	3048	CB	THR	95	48.441	25.965	56.431	1.00	0.00	RX1	C

ATOM	3049	OG1	THR	95	49.739	25.468	56.116	1.00	0.00	RX1	O
ATOM	3050	HG1	THR	95	50.051	25.055	56.921	1.00	0.00	RX1	H
ATOM	3051	CG2	THR	95	47.746	24.940	57.320	1.00	0.00	RX1	C
ATOM	3052	C	THR	95	48.068	27.401	54.361	1.00	0.00	RX1	C
ATOM	3053	O	THR	95	47.858	28.523	54.798	1.00	0.00	RX1	O
ATOM	3054	N	VAL	96	48.646	27.158	53.167	1.00	0.00	RX1	N
ATOM	3055	H	VAL	96	48.817	26.224	52.854	1.00	0.00	RX1	H
ATOM	3056	CA	VAL	96	48.960	28.304	52.313	1.00	0.00	RX1	C
ATOM	3057	CB	VAL	96	49.801	27.874	51.101	1.00	0.00	RX1	C
ATOM	3058	CG1	VAL	96	49.164	26.719	50.335	1.00	0.00	RX1	C
ATOM	3059	CG2	VAL	96	50.130	29.065	50.199	1.00	0.00	RX1	C
ATOM	3060	C	VAL	96	47.742	29.131	51.917	1.00	0.00	RX1	C
ATOM	3061	O	VAL	96	46.811	28.670	51.265	1.00	0.00	RX1	O
ATOM	3062	N	GLU	97	47.799	30.388	52.379	1.00	0.00	RX1	N
ATOM	3063	H	GLU	97	48.601	30.692	52.895	1.00	0.00	RX1	H
ATOM	3064	CA	GLU	97	46.607	31.230	52.270	1.00	0.00	RX1	C
ATOM	3065	CB	GLU	97	46.732	32.448	53.188	1.00	0.00	RX1	C
ATOM	3066	CG	GLU	97	46.864	31.928	54.620	1.00	0.00	RX1	C
ATOM	3067	CD	GLU	97	46.940	33.034	55.652	1.00	0.00	RX1	C
ATOM	3068	OE1	GLU	97	45.917	33.379	56.238	1.00	0.00	RX1	O
ATOM	3069	OE2	GLU	97	48.042	33.471	55.968	1.00	0.00	RX1	O
ATOM	3070	C	GLU	97	46.173	31.579	50.861	1.00	0.00	RX1	C
ATOM	3071	O	GLU	97	44.996	31.752	50.568	1.00	0.00	RX1	O
ATOM	3072	N	ARG	98	47.174	31.661	49.981	1.00	0.00	RX1	N
ATOM	3073	H	ARG	98	48.120	31.444	50.217	1.00	0.00	RX1	H
ATOM	3074	CA	ARG	98	46.801	31.876	48.590	1.00	0.00	RX1	C
ATOM	3075	CB	ARG	98	47.396	33.177	48.054	1.00	0.00	RX1	C
ATOM	3076	CG	ARG	98	46.862	33.529	46.663	1.00	0.00	RX1	C
ATOM	3077	CD	ARG	98	47.613	34.701	46.027	1.00	0.00	RX1	C
ATOM	3078	NE	ARG	98	46.927	35.179	44.827	1.00	0.00	RX1	N
ATOM	3079	HE	ARG	98	46.111	35.740	44.996	1.00	0.00	RX1	H
ATOM	3080	CZ	ARG	98	47.375	34.827	43.583	1.00	0.00	RX1	C
ATOM	3081	NH1	ARG	98	48.470	34.049	43.448	1.00	0.00	RX1	N
ATOM	3082	HH11	ARG	98	48.750	33.766	42.517	1.00	0.00	RX1	H
ATOM	3083	HH12	ARG	98	49.029	33.730	44.219	1.00	0.00	RX1	H
ATOM	3084	NH2	ARG	98	46.716	35.260	42.488	1.00	0.00	RX1	N
ATOM	3085	HH21	ARG	98	47.025	34.979	41.557	1.00	0.00	RX1	H
ATOM	3086	HH22	ARG	98	45.911	35.854	42.518	1.00	0.00	RX1	H
ATOM	3087	C	ARG	98	47.275	30.723	47.735	1.00	0.00	RX1	C
ATOM	3088	O	ARG	98	48.468	30.552	47.525	1.00	0.00	RX1	O
ATOM	3089	N	ILE	99	46.309	29.939	47.243	1.00	0.00	RX1	N
ATOM	3090	H	ILE	99	45.343	30.193	47.336	1.00	0.00	RX1	H
ATOM	3091	CA	ILE	99	46.754	28.879	46.338	1.00	0.00	RX1	C
ATOM	3092	CB	ILE	99	46.016	27.567	46.610	1.00	0.00	RX1	C
ATOM	3093	CG2	ILE	99	46.315	26.500	45.555	1.00	0.00	RX1	C
ATOM	3094	CG1	ILE	99	46.399	27.078	48.002	1.00	0.00	RX1	C
ATOM	3095	CD1	ILE	99	45.795	25.721	48.346	1.00	0.00	RX1	C
ATOM	3096	C	ILE	99	46.642	29.312	44.887	1.00	0.00	RX1	C
ATOM	3097	O	ILE	99	45.567	29.570	44.358	1.00	0.00	RX1	O
ATOM	3098	N	PRO	100	47.829	29.428	44.252	1.00	0.00	RX1	N
ATOM	3099	CD	PRO	100	49.136	29.130	44.807	1.00	0.00	RX1	C
ATOM	3100	CA	PRO	100	47.909	30.045	42.926	1.00	0.00	RX1	C
ATOM	3101	CB	PRO	100	49.386	30.445	42.837	1.00	0.00	RX1	C
ATOM	3102	CG	PRO	100	49.976	30.253	44.232	1.00	0.00	RX1	C
ATOM	3103	C	PRO	100	47.506	29.158	41.756	1.00	0.00	RX1	C
ATOM	3104	O	PRO	100	48.214	29.087	40.759	1.00	0.00	RX1	O
ATOM	3105	N	LEU	101	46.339	28.505	41.869	1.00	0.00	RX1	N
ATOM	3106	H	LEU	101	45.721	28.685	42.636	1.00	0.00	RX1	H
ATOM	3107	CA	LEU	101	45.871	27.734	40.713	1.00	0.00	RX1	C
ATOM	3108	CB	LEU	101	44.938	26.616	41.169	1.00	0.00	RX1	C
ATOM	3109	CG	LEU	101	45.404	25.219	40.762	1.00	0.00	RX1	C

ATOM	3110	CD1	LEU	101	44.314	24.187	41.052	1.00	0.00	RX1	C
ATOM	3111	CD2	LEU	101	45.896	25.155	39.314	1.00	0.00	RX1	C
ATOM	3112	C	LEU	101	45.154	28.600	39.686	1.00	0.00	RX1	C
ATOM	3113	O	LEU	101	44.080	28.306	39.180	1.00	0.00	RX1	O
ATOM	3114	N	GLU	102	45.779	29.751	39.451	1.00	0.00	RX1	N
ATOM	3115	H	GLU	102	46.756	29.850	39.642	1.00	0.00	RX1	H
ATOM	3116	CA	GLU	102	44.958	30.858	38.990	1.00	0.00	RX1	C
ATOM	3117	CB	GLU	102	45.502	32.155	39.578	1.00	0.00	RX1	C
ATOM	3118	CG	GLU	102	46.950	32.461	39.209	1.00	0.00	RX1	C
ATOM	3119	CD	GLU	102	47.334	33.737	39.918	1.00	0.00	RX1	C
ATOM	3120	OE1	GLU	102	46.696	34.762	39.691	1.00	0.00	RX1	O
ATOM	3121	OE2	GLU	102	48.232	33.709	40.751	1.00	0.00	RX1	O
ATOM	3122	C	GLU	102	44.730	30.931	37.493	1.00	0.00	RX1	C
ATOM	3123	O	GLU	102	43.797	31.553	36.999	1.00	0.00	RX1	O
ATOM	3124	N	ASN	103	45.623	30.243	36.771	1.00	0.00	RX1	N
ATOM	3125	H	ASN	103	46.311	29.646	37.182	1.00	0.00	RX1	H
ATOM	3126	CA	ASN	103	45.402	30.262	35.325	1.00	0.00	RX1	C
ATOM	3127	CB	ASN	103	46.691	30.526	34.550	1.00	0.00	RX1	C
ATOM	3128	CG	ASN	103	47.055	31.993	34.612	1.00	0.00	RX1	C
ATOM	3129	OD1	ASN	103	46.207	32.885	34.672	1.00	0.00	RX1	O
ATOM	3130	ND2	ASN	103	48.383	32.196	34.606	1.00	0.00	RX1	N
ATOM	3131	HD21	ASN	103	48.995	31.395	34.603	1.00	0.00	RX1	H
ATOM	3132	HD22	ASN	103	48.822	33.094	34.602	1.00	0.00	RX1	H
ATOM	3133	C	ASN	103	44.769	28.997	34.780	1.00	0.00	RX1	C
ATOM	3134	O	ASN	103	44.711	28.777	33.575	1.00	0.00	RX1	O
ATOM	3135	N	LEU	104	44.282	28.183	35.734	1.00	0.00	RX1	N
ATOM	3136	H	LEU	104	44.257	28.452	36.695	1.00	0.00	RX1	H
ATOM	3137	CA	LEU	104	43.594	26.948	35.365	1.00	0.00	RX1	C
ATOM	3138	CB	LEU	104	43.215	26.216	36.654	1.00	0.00	RX1	C
ATOM	3139	CG	LEU	104	42.644	24.807	36.496	1.00	0.00	RX1	C
ATOM	3140	CD1	LEU	104	43.639	23.842	35.855	1.00	0.00	RX1	C
ATOM	3141	CD2	LEU	104	42.137	24.273	37.834	1.00	0.00	RX1	C
ATOM	3142	C	LEU	104	42.374	27.242	34.520	1.00	0.00	RX1	C
ATOM	3143	O	LEU	104	41.721	28.256	34.722	1.00	0.00	RX1	O
ATOM	3144	N	GLN	105	42.102	26.337	33.572	1.00	0.00	RX1	N
ATOM	3145	H	GLN	105	42.690	25.540	33.442	1.00	0.00	RX1	H
ATOM	3146	CA	GLN	105	40.871	26.495	32.803	1.00	0.00	RX1	C
ATOM	3147	CB	GLN	105	41.157	26.902	31.363	1.00	0.00	RX1	C
ATOM	3148	CG	GLN	105	41.972	28.184	31.219	1.00	0.00	RX1	C
ATOM	3149	CD	GLN	105	42.072	28.524	29.751	1.00	0.00	RX1	C
ATOM	3150	OE1	GLN	105	41.104	28.924	29.116	1.00	0.00	RX1	O
ATOM	3151	NE2	GLN	105	43.301	28.327	29.241	1.00	0.00	RX1	N
ATOM	3152	HE21	GLN	105	44.046	28.023	29.837	1.00	0.00	RX1	H
ATOM	3153	HE22	GLN	105	43.495	28.478	28.271	1.00	0.00	RX1	H
ATOM	3154	C	GLN	105	39.960	25.286	32.811	1.00	0.00	RX1	C
ATOM	3155	O	GLN	105	38.741	25.429	32.809	1.00	0.00	RX1	O
ATOM	3156	N	ILE	106	40.592	24.097	32.830	1.00	0.00	RX1	N
ATOM	3157	H	ILE	106	41.589	23.988	32.847	1.00	0.00	RX1	H
ATOM	3158	CA	ILE	106	39.775	22.885	32.846	1.00	0.00	RX1	C
ATOM	3159	CB	ILE	106	39.603	22.317	31.428	1.00	0.00	RX1	C
ATOM	3160	CG2	ILE	106	40.930	21.860	30.845	1.00	0.00	RX1	C
ATOM	3161	CG1	ILE	106	38.589	21.178	31.361	1.00	0.00	RX1	C
ATOM	3162	CD1	ILE	106	38.538	20.578	29.958	1.00	0.00	RX1	C
ATOM	3163	C	ILE	106	40.278	21.825	33.814	1.00	0.00	RX1	C
ATOM	3164	O	ILE	106	41.462	21.512	33.886	1.00	0.00	RX1	O
ATOM	3165	N	ILE	107	39.306	21.259	34.536	1.00	0.00	RX1	N
ATOM	3166	H	ILE	107	38.357	21.571	34.447	1.00	0.00	RX1	H
ATOM	3167	CA	ILE	107	39.558	19.970	35.168	1.00	0.00	RX1	C
ATOM	3168	CB	ILE	107	39.169	20.026	36.645	1.00	0.00	RX1	C
ATOM	3169	CG2	ILE	107	39.437	18.700	37.361	1.00	0.00	RX1	C
ATOM	3170	CG1	ILE	107	39.868	21.200	37.328	1.00	0.00	RX1	C

ATOM	3171	CD1	ILE	107	39.398	21.408	38.766	1.00	0.00	RX1	C
ATOM	3172	C	ILE	107	38.732	18.931	34.432	1.00	0.00	RX1	C
ATOM	3173	O	ILE	107	37.547	19.119	34.182	1.00	0.00	RX1	O
ATOM	3174	N	ARG	108	39.405	17.840	34.058	1.00	0.00	RX1	N
ATOM	3175	H	ARG	108	40.373	17.716	34.280	1.00	0.00	RX1	H
ATOM	3176	CA	ARG	108	38.641	16.795	33.385	1.00	0.00	RX1	C
ATOM	3177	CB	ARG	108	39.459	16.167	32.258	1.00	0.00	RX1	C
ATOM	3178	CG	ARG	108	39.758	17.209	31.179	1.00	0.00	RX1	C
ATOM	3179	CD	ARG	108	40.650	16.708	30.044	1.00	0.00	RX1	C
ATOM	3180	NE	ARG	108	40.731	17.710	28.980	1.00	0.00	RX1	N
ATOM	3181	HE	ARG	108	39.903	17.862	28.428	1.00	0.00	RX1	H
ATOM	3182	CZ	ARG	108	41.872	18.437	28.790	1.00	0.00	RX1	C
ATOM	3183	NH1	ARG	108	42.965	18.216	29.548	1.00	0.00	RX1	N
ATOM	3184	HH11	ARG	108	43.803	18.756	29.362	1.00	0.00	RX1	H
ATOM	3185	HH12	ARG	108	42.996	17.546	30.293	1.00	0.00	RX1	H
ATOM	3186	NH2	ARG	108	41.896	19.381	27.834	1.00	0.00	RX1	N
ATOM	3187	HH21	ARG	108	42.724	19.953	27.684	1.00	0.00	RX1	H
ATOM	3188	HH22	ARG	108	41.099	19.553	27.248	1.00	0.00	RX1	H
ATOM	3189	C	ARG	108	38.057	15.757	34.324	1.00	0.00	RX1	C
ATOM	3190	O	ARG	108	37.073	15.102	34.005	1.00	0.00	RX1	O
ATOM	3191	N	GLY	109	38.680	15.668	35.513	1.00	0.00	RX1	N
ATOM	3192	H	GLY	109	39.603	16.025	35.653	1.00	0.00	RX1	H
ATOM	3193	CA	GLY	109	38.041	14.942	36.611	1.00	0.00	RX1	C
ATOM	3194	C	GLY	109	37.736	13.486	36.333	1.00	0.00	RX1	C
ATOM	3195	O	GLY	109	36.690	12.945	36.669	1.00	0.00	RX1	O
ATOM	3196	N	ASN	110	38.718	12.868	35.672	1.00	0.00	RX1	N
ATOM	3197	H	ASN	110	39.601	13.320	35.540	1.00	0.00	RX1	H
ATOM	3198	CA	ASN	110	38.570	11.433	35.457	1.00	0.00	RX1	C
ATOM	3199	CB	ASN	110	39.391	10.971	34.246	1.00	0.00	RX1	C
ATOM	3200	CG	ASN	110	38.688	11.199	32.908	1.00	0.00	RX1	C
ATOM	3201	OD1	ASN	110	38.999	10.536	31.920	1.00	0.00	RX1	O
ATOM	3202	ND2	ASN	110	37.733	12.155	32.894	1.00	0.00	RX1	N
ATOM	3203	HD21	ASN	110	37.510	12.758	33.665	1.00	0.00	RX1	H
ATOM	3204	HD22	ASN	110	37.177	12.321	32.078	1.00	0.00	RX1	H
ATOM	3205	C	ASN	110	38.999	10.713	36.720	1.00	0.00	RX1	C
ATOM	3206	O	ASN	110	40.007	11.067	37.317	1.00	0.00	RX1	O
ATOM	3207	N	MET	111	38.152	9.734	37.096	1.00	0.00	RX1	N
ATOM	3208	H	MET	111	37.344	9.593	36.528	1.00	0.00	RX1	H
ATOM	3209	CA	MET	111	38.158	9.099	38.421	1.00	0.00	RX1	C
ATOM	3210	CB	MET	111	39.482	8.437	38.816	1.00	0.00	RX1	C
ATOM	3211	CG	MET	111	39.286	7.209	39.718	1.00	0.00	RX1	C
ATOM	3212	SD	MET	111	38.504	7.495	41.317	1.00	0.00	RX1	S
ATOM	3213	CE	MET	111	39.847	8.394	42.105	1.00	0.00	RX1	C
ATOM	3214	C	MET	111	37.701	10.017	39.531	1.00	0.00	RX1	C
ATOM	3215	O	MET	111	38.342	10.988	39.916	1.00	0.00	RX1	O
ATOM	3216	N	TYR	112	36.522	9.659	40.040	1.00	0.00	RX1	N
ATOM	3217	H	TYR	112	36.067	8.806	39.776	1.00	0.00	RX1	H
ATOM	3218	CA	TYR	112	35.967	10.575	41.023	1.00	0.00	RX1	C
ATOM	3219	CB	TYR	112	34.448	10.506	41.062	1.00	0.00	RX1	C
ATOM	3220	CG	TYR	112	33.792	10.950	39.775	1.00	0.00	RX1	C
ATOM	3221	CD1	TYR	112	34.525	11.362	38.665	1.00	0.00	RX1	C
ATOM	3222	CE1	TYR	112	33.876	11.669	37.475	1.00	0.00	RX1	C
ATOM	3223	CD2	TYR	112	32.407	10.920	39.710	1.00	0.00	RX1	C
ATOM	3224	CE2	TYR	112	31.757	11.251	38.533	1.00	0.00	RX1	C
ATOM	3225	CZ	TYR	112	32.491	11.593	37.407	1.00	0.00	RX1	C
ATOM	3226	OH	TYR	112	31.819	11.841	36.228	1.00	0.00	RX1	O
ATOM	3227	HH	TYR	112	31.045	11.287	36.243	1.00	0.00	RX1	H
ATOM	3228	C	TYR	112	36.501	10.365	42.423	1.00	0.00	RX1	C
ATOM	3229	O	TYR	112	36.274	9.346	43.079	1.00	0.00	RX1	O
ATOM	3230	N	TYR	113	37.219	11.409	42.851	1.00	0.00	RX1	N
ATOM	3231	H	TYR	113	37.270	12.200	42.237	1.00	0.00	RX1	H

ATOM	3232	CA	TYR	113	37.724	11.471	44.217	1.00	0.00	RX1	C
ATOM	3233	CB	TYR	113	38.455	12.797	44.430	1.00	0.00	RX1	C
ATOM	3234	CG	TYR	113	39.382	12.644	45.606	1.00	0.00	RX1	C
ATOM	3235	CD1	TYR	113	40.326	11.629	45.587	1.00	0.00	RX1	C
ATOM	3236	CE1	TYR	113	41.156	11.433	46.680	1.00	0.00	RX1	C
ATOM	3237	CD2	TYR	113	39.290	13.491	46.702	1.00	0.00	RX1	C
ATOM	3238	CE2	TYR	113	40.110	13.281	47.804	1.00	0.00	RX1	C
ATOM	3239	CZ	TYR	113	41.023	12.234	47.805	1.00	0.00	RX1	C
ATOM	3240	OH	TYR	113	41.788	11.969	48.928	1.00	0.00	RX1	O
ATOM	3241	HH	TYR	113	41.639	12.670	49.557	1.00	0.00	RX1	H
ATOM	3242	C	TYR	113	36.624	11.288	45.247	1.00	0.00	RX1	C
ATOM	3243	O	TYR	113	35.522	11.802	45.098	1.00	0.00	RX1	O
ATOM	3244	N	GLU	114	36.973	10.456	46.244	1.00	0.00	RX1	N
ATOM	3245	H	GLU	114	37.929	10.166	46.328	1.00	0.00	RX1	H
ATOM	3246	CA	GLU	114	36.077	9.951	47.293	1.00	0.00	RX1	C
ATOM	3247	CB	GLU	114	36.291	10.679	48.621	1.00	0.00	RX1	C
ATOM	3248	CG	GLU	114	37.743	11.062	48.926	1.00	0.00	RX1	C
ATOM	3249	CD	GLU	114	38.702	9.881	48.918	1.00	0.00	RX1	C
ATOM	3250	OE1	GLU	114	39.177	9.482	47.855	1.00	0.00	RX1	O
ATOM	3251	OE2	GLU	114	39.080	9.413	49.986	1.00	0.00	RX1	O
ATOM	3252	C	GLU	114	34.593	9.826	46.994	1.00	0.00	RX1	C
ATOM	3253	O	GLU	114	33.738	10.082	47.834	1.00	0.00	RX1	O
ATOM	3254	N	ASN	115	34.350	9.353	45.753	1.00	0.00	RX1	N
ATOM	3255	H	ASN	115	35.126	9.377	45.124	1.00	0.00	RX1	H
ATOM	3256	CA	ASN	115	33.006	9.234	45.174	1.00	0.00	RX1	C
ATOM	3257	CB	ASN	115	31.934	8.565	46.057	1.00	0.00	RX1	C
ATOM	3258	CG	ASN	115	32.439	7.312	46.740	1.00	0.00	RX1	C
ATOM	3259	OD1	ASN	115	32.649	6.264	46.131	1.00	0.00	RX1	O
ATOM	3260	ND2	ASN	115	32.624	7.479	48.060	1.00	0.00	RX1	N
ATOM	3261	HD21	ASN	115	32.551	8.417	48.419	1.00	0.00	RX1	H
ATOM	3262	HD22	ASN	115	32.848	6.750	48.706	1.00	0.00	RX1	H
ATOM	3263	C	ASN	115	32.468	10.578	44.741	1.00	0.00	RX1	C
ATOM	3264	O	ASN	115	32.398	11.537	45.491	1.00	0.00	RX1	O
ATOM	3265	N	SER	116	32.099	10.602	43.456	1.00	0.00	RX1	N
ATOM	3266	H	SER	116	32.199	9.782	42.899	1.00	0.00	RX1	H
ATOM	3267	CA	SER	116	31.484	11.787	42.856	1.00	0.00	RX1	C
ATOM	3268	CB	SER	116	30.156	11.958	43.581	1.00	0.00	RX1	C
ATOM	3269	OG	SER	116	29.670	10.625	43.810	1.00	0.00	RX1	O
ATOM	3270	HG	SER	116	29.426	10.293	42.958	1.00	0.00	RX1	H
ATOM	3271	C	SER	116	32.314	13.049	42.639	1.00	0.00	RX1	C
ATOM	3272	O	SER	116	31.908	13.884	41.839	1.00	0.00	RX1	O
ATOM	3273	N	TYR	117	33.453	13.199	43.334	1.00	0.00	RX1	N
ATOM	3274	H	TYR	117	33.845	12.519	43.960	1.00	0.00	RX1	H
ATOM	3275	CA	TYR	117	34.091	14.509	43.193	1.00	0.00	RX1	C
ATOM	3276	CB	TYR	117	34.494	15.067	44.553	1.00	0.00	RX1	C
ATOM	3277	CG	TYR	117	33.312	14.967	45.475	1.00	0.00	RX1	C
ATOM	3278	CD1	TYR	117	32.115	15.578	45.125	1.00	0.00	RX1	C
ATOM	3279	CE1	TYR	117	31.018	15.464	45.965	1.00	0.00	RX1	C
ATOM	3280	CD2	TYR	117	33.425	14.253	46.660	1.00	0.00	RX1	C
ATOM	3281	CE2	TYR	117	32.328	14.142	47.502	1.00	0.00	RX1	C
ATOM	3282	CZ	TYR	117	31.130	14.754	47.154	1.00	0.00	RX1	C
ATOM	3283	OH	TYR	117	30.052	14.680	48.010	1.00	0.00	RX1	O
ATOM	3284	HH	TYR	117	29.265	14.664	47.459	1.00	0.00	RX1	H
ATOM	3285	C	TYR	117	35.297	14.553	42.280	1.00	0.00	RX1	C
ATOM	3286	O	TYR	117	35.845	13.536	41.881	1.00	0.00	RX1	O
ATOM	3287	N	ALA	118	35.701	15.791	41.981	1.00	0.00	RX1	N
ATOM	3288	H	ALA	118	35.173	16.586	42.282	1.00	0.00	RX1	H
ATOM	3289	CA	ALA	118	36.977	15.984	41.298	1.00	0.00	RX1	C
ATOM	3290	CB	ALA	118	36.788	16.412	39.846	1.00	0.00	RX1	C
ATOM	3291	C	ALA	118	37.783	17.067	41.970	1.00	0.00	RX1	C
ATOM	3292	O	ALA	118	38.919	16.876	42.383	1.00	0.00	RX1	O

ATOM	3293	N	LEU	119	37.131	18.233	42.065	1.00	0.00	RX1	N
ATOM	3294	H	LEU	119	36.166	18.323	41.816	1.00	0.00	RX1	H
ATOM	3295	CA	LEU	119	37.802	19.313	42.776	1.00	0.00	RX1	C
ATOM	3296	CB	LEU	119	37.409	20.655	42.161	1.00	0.00	RX1	C
ATOM	3297	CG	LEU	119	38.078	21.858	42.827	1.00	0.00	RX1	C
ATOM	3298	CD1	LEU	119	39.601	21.757	42.809	1.00	0.00	RX1	C
ATOM	3299	CD2	LEU	119	37.597	23.174	42.220	1.00	0.00	RX1	C
ATOM	3300	C	LEU	119	37.445	19.254	44.245	1.00	0.00	RX1	C
ATOM	3301	O	LEU	119	36.378	19.687	44.660	1.00	0.00	RX1	O
ATOM	3302	N	ALA	120	38.365	18.659	45.006	1.00	0.00	RX1	N
ATOM	3303	H	ALA	120	39.260	18.403	44.632	1.00	0.00	RX1	H
ATOM	3304	CA	ALA	120	38.048	18.484	46.417	1.00	0.00	RX1	C
ATOM	3305	CB	ALA	120	38.227	17.022	46.821	1.00	0.00	RX1	C
ATOM	3306	C	ALA	120	38.882	19.374	47.315	1.00	0.00	RX1	C
ATOM	3307	O	ALA	120	40.008	19.063	47.682	1.00	0.00	RX1	O
ATOM	3308	N	VAL	121	38.271	20.515	47.641	1.00	0.00	RX1	N
ATOM	3309	H	VAL	121	37.317	20.665	47.375	1.00	0.00	RX1	H
ATOM	3310	CA	VAL	121	38.947	21.484	48.500	1.00	0.00	RX1	C
ATOM	3311	CB	VAL	121	38.584	22.896	48.041	1.00	0.00	RX1	C
ATOM	3312	CG1	VAL	121	39.351	23.979	48.798	1.00	0.00	RX1	C
ATOM	3313	CG2	VAL	121	38.792	23.009	46.531	1.00	0.00	RX1	C
ATOM	3314	C	VAL	121	38.624	21.261	49.974	1.00	0.00	RX1	C
ATOM	3315	O	VAL	121	37.726	21.863	50.559	1.00	0.00	RX1	O
ATOM	3316	N	LEU	122	39.398	20.329	50.539	1.00	0.00	RX1	N
ATOM	3317	H	LEU	122	40.212	19.995	50.058	1.00	0.00	RX1	H
ATOM	3318	CA	LEU	122	39.092	19.852	51.886	1.00	0.00	RX1	C
ATOM	3319	CB	LEU	122	39.106	18.323	51.906	1.00	0.00	RX1	C
ATOM	3320	CG	LEU	122	38.351	17.666	50.751	1.00	0.00	RX1	C
ATOM	3321	CD1	LEU	122	38.567	16.152	50.730	1.00	0.00	RX1	C
ATOM	3322	CD2	LEU	122	36.868	18.029	50.746	1.00	0.00	RX1	C
ATOM	3323	C	LEU	122	40.048	20.358	52.951	1.00	0.00	RX1	C
ATOM	3324	O	LEU	122	41.259	20.393	52.770	1.00	0.00	RX1	O
ATOM	3325	N	SER	123	39.453	20.718	54.095	1.00	0.00	RX1	N
ATOM	3326	H	SER	123	38.458	20.664	54.174	1.00	0.00	RX1	H
ATOM	3327	CA	SER	123	40.235	20.831	55.331	1.00	0.00	RX1	C
ATOM	3328	CB	SER	123	40.451	19.387	55.735	1.00	0.00	RX1	C
ATOM	3329	OG	SER	123	39.294	18.680	55.258	1.00	0.00	RX1	O
ATOM	3330	HG	SER	123	39.431	17.768	55.482	1.00	0.00	RX1	H
ATOM	3331	C	SER	123	41.477	21.713	55.289	1.00	0.00	RX1	C
ATOM	3332	O	SER	123	42.517	21.427	55.866	1.00	0.00	RX1	O
ATOM	3333	N	ASN	124	41.330	22.810	54.542	1.00	0.00	RX1	N
ATOM	3334	H	ASN	124	40.421	23.086	54.239	1.00	0.00	RX1	H
ATOM	3335	CA	ASN	124	42.530	23.610	54.296	1.00	0.00	RX1	C
ATOM	3336	CB	ASN	124	42.474	24.281	52.927	1.00	0.00	RX1	C
ATOM	3337	CG	ASN	124	42.662	23.261	51.834	1.00	0.00	RX1	C
ATOM	3338	OD1	ASN	124	43.739	22.710	51.635	1.00	0.00	RX1	O
ATOM	3339	ND2	ASN	124	41.542	23.015	51.141	1.00	0.00	RX1	N
ATOM	3340	HD21	ASN	124	40.707	23.542	51.291	1.00	0.00	RX1	H
ATOM	3341	HD22	ASN	124	41.524	22.276	50.462	1.00	0.00	RX1	H
ATOM	3342	C	ASN	124	42.742	24.681	55.335	1.00	0.00	RX1	C
ATOM	3343	O	ASN	124	42.397	25.834	55.107	1.00	0.00	RX1	O
ATOM	3344	N	TYR	125	43.289	24.254	56.485	1.00	0.00	RX1	N
ATOM	3345	H	TYR	125	43.581	23.307	56.640	1.00	0.00	RX1	H
ATOM	3346	CA	TYR	125	43.436	25.208	57.583	1.00	0.00	RX1	C
ATOM	3347	CB	TYR	125	42.088	25.460	58.255	1.00	0.00	RX1	C
ATOM	3348	CG	TYR	125	41.550	24.190	58.867	1.00	0.00	RX1	C
ATOM	3349	CD1	TYR	125	41.898	23.849	60.165	1.00	0.00	RX1	C
ATOM	3350	CE1	TYR	125	41.322	22.748	60.777	1.00	0.00	RX1	C
ATOM	3351	CD2	TYR	125	40.690	23.373	58.145	1.00	0.00	RX1	C
ATOM	3352	CE2	TYR	125	40.103	22.274	58.756	1.00	0.00	RX1	C
ATOM	3353	CZ	TYR	125	40.374	22.005	60.090	1.00	0.00	RX1	C

ATOM	3354	OH	TYR	125	39.658	21.037	60.767	1.00	0.00	RX1	O
ATOM	3355	HH	TYR	125	39.563	20.285	60.185	1.00	0.00	RX1	H
ATOM	3356	C	TYR	125	44.482	24.817	58.608	1.00	0.00	RX1	C
ATOM	3357	O	TYR	125	44.837	23.652	58.736	1.00	0.00	RX1	O
ATOM	3358	N	ASP	126	44.939	25.842	59.342	1.00	0.00	RX1	N
ATOM	3359	H	ASP	126	44.651	26.792	59.212	1.00	0.00	RX1	H
ATOM	3360	CA	ASP	126	45.790	25.579	60.499	1.00	0.00	RX1	C
ATOM	3361	CB	ASP	126	46.870	26.656	60.650	1.00	0.00	RX1	C
ATOM	3362	CG	ASP	126	46.328	27.938	61.256	1.00	0.00	RX1	C
ATOM	3363	OD1	ASP	126	47.113	28.707	61.790	1.00	0.00	RX1	O
ATOM	3364	OD2	ASP	126	45.137	28.199	61.194	1.00	0.00	RX1	O
ATOM	3365	C	ASP	126	44.988	25.405	61.781	1.00	0.00	RX1	C
ATOM	3366	O	ASP	126	43.763	25.362	61.775	1.00	0.00	RX1	O
ATOM	3367	N	ALA	127	45.729	25.348	62.903	1.00	0.00	RX1	N
ATOM	3368	H	ALA	127	46.721	25.451	62.858	1.00	0.00	RX1	H
ATOM	3369	CA	ALA	127	45.062	25.277	64.204	1.00	0.00	RX1	C
ATOM	3370	CB	ALA	127	46.097	25.259	65.329	1.00	0.00	RX1	C
ATOM	3371	C	ALA	127	44.067	26.397	64.479	1.00	0.00	RX1	C
ATOM	3372	O	ALA	127	43.054	26.217	65.142	1.00	0.00	RX1	O
ATOM	3373	N	ASN	128	44.387	27.567	63.908	1.00	0.00	RX1	N
ATOM	3374	H	ASN	128	45.125	27.645	63.234	1.00	0.00	RX1	H
ATOM	3375	CA	ASN	128	43.501	28.717	64.099	1.00	0.00	RX1	C
ATOM	3376	CB	ASN	128	44.231	30.054	63.933	1.00	0.00	RX1	C
ATOM	3377	CG	ASN	128	45.350	30.210	64.935	1.00	0.00	RX1	C
ATOM	3378	OD1	ASN	128	45.148	30.533	66.101	1.00	0.00	RX1	O
ATOM	3379	ND2	ASN	128	46.563	29.976	64.405	1.00	0.00	RX1	N
ATOM	3380	HD21	ASN	128	46.645	29.694	63.442	1.00	0.00	RX1	H
ATOM	3381	HD22	ASN	128	47.410	30.066	64.925	1.00	0.00	RX1	H
ATOM	3382	C	ASN	128	42.329	28.753	63.132	1.00	0.00	RX1	C
ATOM	3383	O	ASN	128	41.688	29.780	62.959	1.00	0.00	RX1	O
ATOM	3384	N	LYS	129	42.092	27.602	62.467	1.00	0.00	RX1	N
ATOM	3385	H	LYS	129	42.684	26.810	62.610	1.00	0.00	RX1	H
ATOM	3386	CA	LYS	129	41.087	27.536	61.403	1.00	0.00	RX1	C
ATOM	3387	CB	LYS	129	39.665	27.600	61.974	1.00	0.00	RX1	C
ATOM	3388	CG	LYS	129	39.223	26.385	62.803	1.00	0.00	RX1	C
ATOM	3389	CD	LYS	129	38.985	25.114	61.976	1.00	0.00	RX1	C
ATOM	3390	CE	LYS	129	38.366	23.966	62.787	1.00	0.00	RX1	C
ATOM	3391	NZ	LYS	129	38.115	22.787	61.938	1.00	0.00	RX1	N
ATOM	3392	HZ1	LYS	129	37.402	22.168	62.363	1.00	0.00	RX1	H
ATOM	3393	HZ2	LYS	129	37.768	23.057	60.993	1.00	0.00	RX1	H
ATOM	3394	HZ3	LYS	129	38.976	22.216	61.791	1.00	0.00	RX1	H
ATOM	3395	C	LYS	129	41.307	28.548	60.284	1.00	0.00	RX1	C
ATOM	3396	O	LYS	129	40.381	29.102	59.704	1.00	0.00	RX1	O
ATOM	3397	N	THR	130	42.598	28.752	59.994	1.00	0.00	RX1	N
ATOM	3398	H	THR	130	43.341	28.290	60.476	1.00	0.00	RX1	H
ATOM	3399	CA	THR	130	42.926	29.603	58.858	1.00	0.00	RX1	C
ATOM	3400	CB	THR	130	43.452	30.962	59.356	1.00	0.00	RX1	C
ATOM	3401	OG1	THR	130	44.616	30.816	60.190	1.00	0.00	RX1	O
ATOM	3402	HG1	THR	130	44.526	29.983	60.657	1.00	0.00	RX1	H
ATOM	3403	CG2	THR	130	42.376	31.776	60.077	1.00	0.00	RX1	C
ATOM	3404	C	THR	130	43.877	28.896	57.914	1.00	0.00	RX1	C
ATOM	3405	O	THR	130	44.992	28.540	58.274	1.00	0.00	RX1	O
ATOM	3406	N	GLY	131	43.399	28.679	56.694	1.00	0.00	RX1	N
ATOM	3407	H	GLY	131	42.449	28.845	56.418	1.00	0.00	RX1	H
ATOM	3408	CA	GLY	131	44.362	28.174	55.726	1.00	0.00	RX1	C
ATOM	3409	C	GLY	131	44.146	28.821	54.391	1.00	0.00	RX1	C
ATOM	3410	O	GLY	131	44.401	29.999	54.195	1.00	0.00	RX1	O
ATOM	3411	N	LEU	132	43.623	28.005	53.473	1.00	0.00	RX1	N
ATOM	3412	H	LEU	132	43.279	27.111	53.762	1.00	0.00	RX1	H
ATOM	3413	CA	LEU	132	43.321	28.589	52.170	1.00	0.00	RX1	C
ATOM	3414	CB	LEU	132	42.908	27.485	51.199	1.00	0.00	RX1	C

ATOM	3415	CG	LEU	132	42.424	27.979	49.837	1.00	0.00	RX1	C
ATOM	3416	CD1	LEU	132	43.429	28.902	49.154	1.00	0.00	RX1	C
ATOM	3417	CD2	LEU	132	42.017	26.808	48.946	1.00	0.00	RX1	C
ATOM	3418	C	LEU	132	42.267	29.680	52.255	1.00	0.00	RX1	C
ATOM	3419	O	LEU	132	41.098	29.428	52.507	1.00	0.00	RX1	O
ATOM	3420	N	LYS	133	42.757	30.901	52.027	1.00	0.00	RX1	N
ATOM	3421	H	LYS	133	43.732	31.013	51.839	1.00	0.00	RX1	H
ATOM	3422	CA	LYS	133	41.857	32.040	51.940	1.00	0.00	RX1	C
ATOM	3423	CB	LYS	133	42.507	33.277	52.552	1.00	0.00	RX1	C
ATOM	3424	CG	LYS	133	41.544	34.461	52.553	1.00	0.00	RX1	C
ATOM	3425	CD	LYS	133	42.192	35.756	53.028	1.00	0.00	RX1	C
ATOM	3426	CE	LYS	133	41.176	36.894	53.085	1.00	0.00	RX1	C
ATOM	3427	NZ	LYS	133	40.592	37.139	51.763	1.00	0.00	RX1	N
ATOM	3428	HZ1	LYS	133	39.853	37.876	51.792	1.00	0.00	RX1	H
ATOM	3429	HZ2	LYS	133	41.315	37.457	51.085	1.00	0.00	RX1	H
ATOM	3430	HZ3	LYS	133	40.209	36.290	51.295	1.00	0.00	RX1	H
ATOM	3431	C	LYS	133	41.443	32.326	50.510	1.00	0.00	RX1	C
ATOM	3432	O	LYS	133	40.284	32.217	50.137	1.00	0.00	RX1	O
ATOM	3433	N	GLU	134	42.448	32.704	49.713	1.00	0.00	RX1	N
ATOM	3434	H	GLU	134	43.407	32.653	49.994	1.00	0.00	RX1	H
ATOM	3435	CA	GLU	134	42.094	33.011	48.336	1.00	0.00	RX1	C
ATOM	3436	CB	GLU	134	42.616	34.376	47.892	1.00	0.00	RX1	C
ATOM	3437	CG	GLU	134	41.499	35.411	47.719	1.00	0.00	RX1	C
ATOM	3438	CD	GLU	134	41.144	36.049	49.048	1.00	0.00	RX1	C
ATOM	3439	OE1	GLU	134	40.012	35.934	49.507	1.00	0.00	RX1	O
ATOM	3440	OE2	GLU	134	41.980	36.745	49.610	1.00	0.00	RX1	O
ATOM	3441	C	GLU	134	42.534	31.946	47.362	1.00	0.00	RX1	C
ATOM	3442	O	GLU	134	43.708	31.617	47.214	1.00	0.00	RX1	O
ATOM	3443	N	LEU	135	41.508	31.435	46.674	1.00	0.00	RX1	N
ATOM	3444	H	LEU	135	40.573	31.738	46.864	1.00	0.00	RX1	H
ATOM	3445	CA	LEU	135	41.768	30.511	45.573	1.00	0.00	RX1	C
ATOM	3446	CB	LEU	135	41.084	29.184	45.904	1.00	0.00	RX1	C
ATOM	3447	CG	LEU	135	41.277	28.051	44.900	1.00	0.00	RX1	C
ATOM	3448	CD1	LEU	135	42.750	27.741	44.644	1.00	0.00	RX1	C
ATOM	3449	CD2	LEU	135	40.498	26.808	45.330	1.00	0.00	RX1	C
ATOM	3450	C	LEU	135	41.278	31.103	44.256	1.00	0.00	RX1	C
ATOM	3451	O	LEU	135	40.154	30.881	43.823	1.00	0.00	RX1	O
ATOM	3452	N	PRO	136	42.153	31.926	43.630	1.00	0.00	RX1	N
ATOM	3453	CD	PRO	136	43.530	32.205	43.998	1.00	0.00	RX1	C
ATOM	3454	CA	PRO	136	41.697	32.751	42.508	1.00	0.00	RX1	C
ATOM	3455	CB	PRO	136	42.749	33.869	42.461	1.00	0.00	RX1	C
ATOM	3456	CG	PRO	136	43.660	33.686	43.679	1.00	0.00	RX1	C
ATOM	3457	C	PRO	136	41.627	32.027	41.170	1.00	0.00	RX1	C
ATOM	3458	O	PRO	136	42.344	32.369	40.238	1.00	0.00	RX1	O
ATOM	3459	N	MET	137	40.742	31.028	41.073	1.00	0.00	RX1	N
ATOM	3460	H	MET	137	40.101	30.830	41.818	1.00	0.00	RX1	H
ATOM	3461	CA	MET	137	40.697	30.297	39.802	1.00	0.00	RX1	C
ATOM	3462	CB	MET	137	40.296	28.841	40.027	1.00	0.00	RX1	C
ATOM	3463	CG	MET	137	41.264	28.107	40.951	1.00	0.00	RX1	C
ATOM	3464	SD	MET	137	40.810	26.385	41.199	1.00	0.00	RX1	S
ATOM	3465	CE	MET	137	39.152	26.671	41.832	1.00	0.00	RX1	C
ATOM	3466	C	MET	137	39.823	30.924	38.722	1.00	0.00	RX1	C
ATOM	3467	O	MET	137	38.961	30.296	38.125	1.00	0.00	RX1	O
ATOM	3468	N	ARG	138	40.094	32.213	38.478	1.00	0.00	RX1	N
ATOM	3469	H	ARG	138	40.894	32.614	38.925	1.00	0.00	RX1	H
ATOM	3470	CA	ARG	138	39.264	33.000	37.557	1.00	0.00	RX1	C
ATOM	3471	CB	ARG	138	39.622	34.486	37.517	1.00	0.00	RX1	C
ATOM	3472	CG	ARG	138	40.837	34.863	36.668	1.00	0.00	RX1	C
ATOM	3473	CD	ARG	138	42.100	34.310	37.302	1.00	0.00	RX1	C
ATOM	3474	NE	ARG	138	43.298	34.490	36.504	1.00	0.00	RX1	N
ATOM	3475	HE	ARG	138	43.251	34.193	35.541	1.00	0.00	RX1	H

ATOM	3476	CZ	ARG	138	44.394	34.740	37.269	1.00	0.00	RX1	C
ATOM	3477	NH1	ARG	138	44.220	35.176	38.537	1.00	0.00	RX1	N
ATOM	3478	HH11	ARG	138	45.016	35.258	39.152	1.00	0.00	RX1	H
ATOM	3479	HH12	ARG	138	43.319	35.422	38.928	1.00	0.00	RX1	H
ATOM	3480	NH2	ARG	138	45.634	34.532	36.780	1.00	0.00	RX1	N
ATOM	3481	HH21	ARG	138	46.445	34.716	37.348	1.00	0.00	RX1	H
ATOM	3482	HH22	ARG	138	45.785	34.160	35.853	1.00	0.00	RX1	H
ATOM	3483	C	ARG	138	39.089	32.487	36.139	1.00	0.00	RX1	C
ATOM	3484	O	ARG	138	38.121	32.803	35.457	1.00	0.00	RX1	O
ATOM	3485	N	ASN	139	40.095	31.718	35.708	1.00	0.00	RX1	N
ATOM	3486	H	ASN	139	40.837	31.414	36.304	1.00	0.00	RX1	H
ATOM	3487	CA	ASN	139	40.012	31.268	34.322	1.00	0.00	RX1	C
ATOM	3488	CB	ASN	139	41.369	31.130	33.614	1.00	0.00	RX1	C
ATOM	3489	CG	ASN	139	42.293	32.323	33.689	1.00	0.00	RX1	C
ATOM	3490	OD1	ASN	139	41.934	33.441	34.045	1.00	0.00	RX1	O
ATOM	3491	ND2	ASN	139	43.553	31.998	33.348	1.00	0.00	RX1	N
ATOM	3492	HD21	ASN	139	43.746	31.063	33.036	1.00	0.00	RX1	H
ATOM	3493	HD22	ASN	139	44.334	32.624	33.412	1.00	0.00	RX1	H
ATOM	3494	C	ASN	139	39.386	29.899	34.172	1.00	0.00	RX1	C
ATOM	3495	O	ASN	139	39.085	29.447	33.071	1.00	0.00	RX1	O
ATOM	3496	N	LEU	140	39.195	29.258	35.342	1.00	0.00	RX1	N
ATOM	3497	H	LEU	140	39.280	29.719	36.224	1.00	0.00	RX1	H
ATOM	3498	CA	LEU	140	38.647	27.909	35.348	1.00	0.00	RX1	C
ATOM	3499	CB	LEU	140	38.838	27.282	36.732	1.00	0.00	RX1	C
ATOM	3500	CG	LEU	140	38.174	25.916	36.933	1.00	0.00	RX1	C
ATOM	3501	CD1	LEU	140	38.748	24.833	36.028	1.00	0.00	RX1	C
ATOM	3502	CD2	LEU	140	38.199	25.484	38.397	1.00	0.00	RX1	C
ATOM	3503	C	LEU	140	37.195	27.972	34.965	1.00	0.00	RX1	C
ATOM	3504	O	LEU	140	36.338	28.318	35.760	1.00	0.00	RX1	O
ATOM	3505	N	GLN	141	36.965	27.655	33.693	1.00	0.00	RX1	N
ATOM	3506	H	GLN	141	37.705	27.336	33.098	1.00	0.00	RX1	H
ATOM	3507	CA	GLN	141	35.581	27.722	33.256	1.00	0.00	RX1	C
ATOM	3508	CB	GLN	141	35.448	28.600	32.025	1.00	0.00	RX1	C
ATOM	3509	CG	GLN	141	35.845	30.041	32.311	1.00	0.00	RX1	C
ATOM	3510	CD	GLN	141	36.155	30.698	30.992	1.00	0.00	RX1	C
ATOM	3511	OE1	GLN	141	35.277	31.206	30.297	1.00	0.00	RX1	O
ATOM	3512	NE2	GLN	141	37.463	30.637	30.686	1.00	0.00	RX1	N
ATOM	3513	HE21	GLN	141	38.103	30.203	31.332	1.00	0.00	RX1	H
ATOM	3514	HE22	GLN	141	37.852	30.988	29.834	1.00	0.00	RX1	H
ATOM	3515	C	GLN	141	34.969	26.364	33.028	1.00	0.00	RX1	C
ATOM	3516	O	GLN	141	33.765	26.253	32.834	1.00	0.00	RX1	O
ATOM	3517	N	GLU	142	35.832	25.340	33.046	1.00	0.00	RX1	N
ATOM	3518	H	GLU	142	36.814	25.405	33.234	1.00	0.00	RX1	H
ATOM	3519	CA	GLU	142	35.296	24.020	32.754	1.00	0.00	RX1	C
ATOM	3520	CB	GLU	142	35.695	23.590	31.348	1.00	0.00	RX1	C
ATOM	3521	CG	GLU	142	34.790	22.487	30.807	1.00	0.00	RX1	C
ATOM	3522	CD	GLU	142	33.494	23.122	30.369	1.00	0.00	RX1	C
ATOM	3523	OE1	GLU	142	33.469	23.701	29.289	1.00	0.00	RX1	O
ATOM	3524	OE2	GLU	142	32.517	23.101	31.116	1.00	0.00	RX1	O
ATOM	3525	C	GLU	142	35.754	22.977	33.745	1.00	0.00	RX1	C
ATOM	3526	O	GLU	142	36.941	22.781	33.970	1.00	0.00	RX1	O
ATOM	3527	N	ILE	143	34.765	22.287	34.310	1.00	0.00	RX1	N
ATOM	3528	H	ILE	143	33.807	22.489	34.095	1.00	0.00	RX1	H
ATOM	3529	CA	ILE	143	35.091	20.990	34.891	1.00	0.00	RX1	C
ATOM	3530	CB	ILE	143	34.921	20.962	36.413	1.00	0.00	RX1	C
ATOM	3531	CG2	ILE	143	35.253	19.574	36.961	1.00	0.00	RX1	C
ATOM	3532	CG1	ILE	143	35.751	22.044	37.104	1.00	0.00	RX1	C
ATOM	3533	CD1	ILE	143	35.575	22.033	38.623	1.00	0.00	RX1	C
ATOM	3534	C	ILE	143	34.171	19.990	34.231	1.00	0.00	RX1	C
ATOM	3535	O	ILE	143	32.966	19.999	34.451	1.00	0.00	RX1	O
ATOM	3536	N	LEU	144	34.787	19.174	33.367	1.00	0.00	RX1	N

ATOM	3537	H	LEU	144	35.787	19.133	33.359	1.00	0.00	RX1	H
ATOM	3538	CA	LEU	144	33.955	18.248	32.603	1.00	0.00	RX1	C
ATOM	3539	CB	LEU	144	34.744	17.634	31.446	1.00	0.00	RX1	C
ATOM	3540	CG	LEU	144	34.903	18.595	30.270	1.00	0.00	RX1	C
ATOM	3541	CD1	LEU	144	35.802	18.017	29.178	1.00	0.00	RX1	C
ATOM	3542	CD2	LEU	144	33.545	19.022	29.712	1.00	0.00	RX1	C
ATOM	3543	C	LEU	144	33.346	17.157	33.453	1.00	0.00	RX1	C
ATOM	3544	O	LEU	144	32.137	16.984	33.516	1.00	0.00	RX1	O
ATOM	3545	N	HIS	145	34.250	16.424	34.110	1.00	0.00	RX1	N
ATOM	3546	H	HIS	145	35.235	16.592	34.135	1.00	0.00	RX1	H
ATOM	3547	CA	HIS	145	33.732	15.385	34.981	1.00	0.00	RX1	C
ATOM	3548	CB	HIS	145	34.018	13.992	34.418	1.00	0.00	RX1	C
ATOM	3549	CG	HIS	145	32.981	13.689	33.363	1.00	0.00	RX1	C
ATOM	3550	ND1	HIS	145	31.811	13.087	33.633	1.00	0.00	RX1	N
ATOM	3551	HD1	HIS	145	31.534	12.763	34.519	1.00	0.00	RX1	H
ATOM	3552	CD2	HIS	145	33.027	13.984	31.998	1.00	0.00	RX1	C
ATOM	3553	NE2	HIS	145	31.865	13.554	31.444	1.00	0.00	RX1	N
ATOM	3554	CE1	HIS	145	31.115	13.004	32.454	1.00	0.00	RX1	C
ATOM	3555	C	HIS	145	34.222	15.558	36.394	1.00	0.00	RX1	C
ATOM	3556	O	HIS	145	35.178	16.275	36.665	1.00	0.00	RX1	O
ATOM	3557	N	GLY	146	33.482	14.891	37.285	1.00	0.00	RX1	N
ATOM	3558	H	GLY	146	32.711	14.347	36.964	1.00	0.00	RX1	H
ATOM	3559	CA	GLY	146	33.684	15.146	38.703	1.00	0.00	RX1	C
ATOM	3560	C	GLY	146	32.978	16.400	39.179	1.00	0.00	RX1	C
ATOM	3561	O	GLY	146	32.979	17.446	38.546	1.00	0.00	RX1	O
ATOM	3562	N	ALA	147	32.340	16.233	40.337	1.00	0.00	RX1	N
ATOM	3563	H	ALA	147	32.414	15.380	40.849	1.00	0.00	RX1	H
ATOM	3564	CA	ALA	147	31.667	17.379	40.932	1.00	0.00	RX1	C
ATOM	3565	CB	ALA	147	30.465	16.924	41.758	1.00	0.00	RX1	C
ATOM	3566	C	ALA	147	32.606	18.182	41.810	1.00	0.00	RX1	C
ATOM	3567	O	ALA	147	33.765	17.828	42.018	1.00	0.00	RX1	O
ATOM	3568	N	VAL	148	32.060	19.292	42.316	1.00	0.00	RX1	N
ATOM	3569	H	VAL	148	31.081	19.481	42.267	1.00	0.00	RX1	H
ATOM	3570	CA	VAL	148	32.908	20.089	43.190	1.00	0.00	RX1	C
ATOM	3571	CB	VAL	148	32.792	21.570	42.803	1.00	0.00	RX1	C
ATOM	3572	CG1	VAL	148	31.451	22.176	43.222	1.00	0.00	RX1	C
ATOM	3573	CG2	VAL	148	33.991	22.372	43.296	1.00	0.00	RX1	C
ATOM	3574	C	VAL	148	32.593	19.827	44.657	1.00	0.00	RX1	C
ATOM	3575	O	VAL	148	31.449	19.586	45.030	1.00	0.00	RX1	O
ATOM	3576	N	ARG	149	33.666	19.858	45.452	1.00	0.00	RX1	N
ATOM	3577	H	ARG	149	34.586	20.081	45.124	1.00	0.00	RX1	H
ATOM	3578	CA	ARG	149	33.524	19.642	46.884	1.00	0.00	RX1	C
ATOM	3579	CB	ARG	149	34.056	18.260	47.267	1.00	0.00	RX1	C
ATOM	3580	CG	ARG	149	33.723	17.895	48.714	1.00	0.00	RX1	C
ATOM	3581	CD	ARG	149	32.244	17.566	48.889	1.00	0.00	RX1	C
ATOM	3582	NE	ARG	149	31.793	17.806	50.255	1.00	0.00	RX1	N
ATOM	3583	HE	ARG	149	32.067	18.681	50.680	1.00	0.00	RX1	H
ATOM	3584	CZ	ARG	149	30.923	16.979	50.902	1.00	0.00	RX1	C
ATOM	3585	NH1	ARG	149	30.505	15.834	50.323	1.00	0.00	RX1	N
ATOM	3586	HH11	ARG	149	29.885	15.193	50.776	1.00	0.00	RX1	H
ATOM	3587	HH12	ARG	149	30.760	15.568	49.383	1.00	0.00	RX1	H
ATOM	3588	NH2	ARG	149	30.473	17.337	52.119	1.00	0.00	RX1	N
ATOM	3589	HH21	ARG	149	29.901	16.778	52.715	1.00	0.00	RX1	H
ATOM	3590	HH22	ARG	149	30.691	18.272	52.456	1.00	0.00	RX1	H
ATOM	3591	C	ARG	149	34.286	20.708	47.641	1.00	0.00	RX1	C
ATOM	3592	O	ARG	149	35.471	20.923	47.416	1.00	0.00	RX1	O
ATOM	3593	N	PHE	150	33.573	21.360	48.555	1.00	0.00	RX1	N
ATOM	3594	H	PHE	150	32.618	21.127	48.759	1.00	0.00	RX1	H
ATOM	3595	CA	PHE	150	34.286	22.213	49.499	1.00	0.00	RX1	C
ATOM	3596	CB	PHE	150	33.847	23.669	49.377	1.00	0.00	RX1	C
ATOM	3597	CG	PHE	150	34.325	24.249	48.073	1.00	0.00	RX1	C

ATOM	3598	CD1	PHE	150	35.591	24.810	47.996	1.00	0.00	RX1	C
ATOM	3599	CD2	PHE	150	33.500	24.227	46.957	1.00	0.00	RX1	C
ATOM	3600	CE1	PHE	150	36.043	25.338	46.795	1.00	0.00	RX1	C
ATOM	3601	CE2	PHE	150	33.950	24.758	45.758	1.00	0.00	RX1	C
ATOM	3602	CZ	PHE	150	35.226	25.298	45.673	1.00	0.00	RX1	C
ATOM	3603	C	PHE	150	33.909	21.741	50.868	1.00	0.00	RX1	C
ATOM	3604	O	PHE	150	32.728	21.605	51.134	1.00	0.00	RX1	O
ATOM	3605	N	SER	151	34.917	21.492	51.706	1.00	0.00	RX1	N
ATOM	3606	H	SER	151	35.891	21.558	51.480	1.00	0.00	RX1	H
ATOM	3607	CA	SER	151	34.538	21.078	53.053	1.00	0.00	RX1	C
ATOM	3608	CB	SER	151	34.342	19.564	53.043	1.00	0.00	RX1	C
ATOM	3609	OG	SER	151	33.472	19.233	51.957	1.00	0.00	RX1	O
ATOM	3610	HG	SER	151	32.721	19.830	52.080	1.00	0.00	RX1	H
ATOM	3611	C	SER	151	35.538	21.546	54.080	1.00	0.00	RX1	C
ATOM	3612	O	SER	151	36.735	21.612	53.815	1.00	0.00	RX1	O
ATOM	3613	N	ASN	152	34.973	21.903	55.250	1.00	0.00	RX1	N
ATOM	3614	H	ASN	152	33.980	21.789	55.304	1.00	0.00	RX1	H
ATOM	3615	CA	ASN	152	35.685	22.610	56.327	1.00	0.00	RX1	C
ATOM	3616	CB	ASN	152	35.746	21.852	57.650	1.00	0.00	RX1	C
ATOM	3617	CG	ASN	152	36.054	22.864	58.747	1.00	0.00	RX1	C
ATOM	3618	OD1	ASN	152	36.891	22.666	59.632	1.00	0.00	RX1	O
ATOM	3619	ND2	ASN	152	35.266	23.956	58.702	1.00	0.00	RX1	N
ATOM	3620	HD21	ASN	152	34.648	24.154	57.932	1.00	0.00	RX1	H
ATOM	3621	HD22	ASN	152	35.248	24.663	59.407	1.00	0.00	RX1	H
ATOM	3622	C	ASN	152	37.056	23.195	56.041	1.00	0.00	RX1	C
ATOM	3623	O	ASN	152	38.097	22.712	56.465	1.00	0.00	RX1	O
ATOM	3624	N	ASN	153	36.979	24.296	55.302	1.00	0.00	RX1	N
ATOM	3625	H	ASN	153	36.083	24.697	55.089	1.00	0.00	RX1	H
ATOM	3626	CA	ASN	153	38.173	25.029	54.890	1.00	0.00	RX1	C
ATOM	3627	CB	ASN	153	38.312	24.948	53.369	1.00	0.00	RX1	C
ATOM	3628	CG	ASN	153	36.979	25.265	52.710	1.00	0.00	RX1	C
ATOM	3629	OD1	ASN	153	36.187	26.089	53.163	1.00	0.00	RX1	O
ATOM	3630	ND2	ASN	153	36.744	24.515	51.617	1.00	0.00	RX1	N
ATOM	3631	HD21	ASN	153	37.355	23.772	51.326	1.00	0.00	RX1	H
ATOM	3632	HD22	ASN	153	35.927	24.663	51.060	1.00	0.00	RX1	H
ATOM	3633	C	ASN	153	38.171	26.491	55.337	1.00	0.00	RX1	C
ATOM	3634	O	ASN	153	38.179	27.408	54.525	1.00	0.00	RX1	O
ATOM	3635	N	PRO	154	38.138	26.728	56.675	1.00	0.00	RX1	N
ATOM	3636	CD	PRO	154	38.179	25.815	57.804	1.00	0.00	RX1	C
ATOM	3637	CA	PRO	154	38.039	28.119	57.106	1.00	0.00	RX1	C
ATOM	3638	CB	PRO	154	37.770	28.019	58.610	1.00	0.00	RX1	C
ATOM	3639	CG	PRO	154	37.412	26.563	58.881	1.00	0.00	RX1	C
ATOM	3640	C	PRO	154	39.260	28.946	56.742	1.00	0.00	RX1	C
ATOM	3641	O	PRO	154	40.385	28.462	56.671	1.00	0.00	RX1	O
ATOM	3642	N	ALA	155	38.898	30.212	56.467	1.00	0.00	RX1	N
ATOM	3643	H	ALA	155	37.921	30.388	56.559	1.00	0.00	RX1	H
ATOM	3644	CA	ALA	155	39.661	31.258	55.776	1.00	0.00	RX1	C
ATOM	3645	CB	ALA	155	41.184	31.105	55.781	1.00	0.00	RX1	C
ATOM	3646	C	ALA	155	39.194	31.447	54.349	1.00	0.00	RX1	C
ATOM	3647	O	ALA	155	39.238	32.555	53.833	1.00	0.00	RX1	O
ATOM	3648	N	LEU	156	38.676	30.351	53.762	1.00	0.00	RX1	N
ATOM	3649	H	LEU	156	38.741	29.437	54.165	1.00	0.00	RX1	H
ATOM	3650	CA	LEU	156	38.206	30.465	52.381	1.00	0.00	RX1	C
ATOM	3651	CB	LEU	156	37.722	29.107	51.864	1.00	0.00	RX1	C
ATOM	3652	CG	LEU	156	37.627	29.009	50.339	1.00	0.00	RX1	C
ATOM	3653	CD1	LEU	156	38.917	29.439	49.646	1.00	0.00	RX1	C
ATOM	3654	CD2	LEU	156	37.242	27.601	49.894	1.00	0.00	RX1	C
ATOM	3655	C	LEU	156	37.183	31.557	52.130	1.00	0.00	RX1	C
ATOM	3656	O	LEU	156	36.055	31.560	52.616	1.00	0.00	RX1	O
ATOM	3657	N	CYS	157	37.667	32.505	51.336	1.00	0.00	RX1	N
ATOM	3658	H	CYS	157	38.571	32.422	50.912	1.00	0.00	RX1	H

ATOM	3659	CA	CYS	157	36.819	33.590	50.888	1.00	0.00	RX1	C
ATOM	3660	CB	CYS	157	37.450	34.922	51.284	1.00	0.00	RX1	C
ATOM	3661	SG	CYS	157	37.868	35.089	53.036	1.00	0.00	RX1	S
ATOM	3662	C	CYS	157	36.737	33.497	49.387	1.00	0.00	RX1	C
ATOM	3663	O	CYS	157	37.494	32.770	48.755	1.00	0.00	RX1	O
ATOM	3664	N	ASN	158	35.805	34.283	48.834	1.00	0.00	RX1	N
ATOM	3665	H	ASN	158	35.047	34.647	49.383	1.00	0.00	RX1	H
ATOM	3666	CA	ASN	158	35.785	34.598	47.402	1.00	0.00	RX1	C
ATOM	3667	CB	ASN	158	37.115	35.195	46.958	1.00	0.00	RX1	C
ATOM	3668	CG	ASN	158	36.973	36.683	47.138	1.00	0.00	RX1	C
ATOM	3669	OD1	ASN	158	35.965	37.260	46.741	1.00	0.00	RX1	O
ATOM	3670	ND2	ASN	158	38.000	37.271	47.770	1.00	0.00	RX1	N
ATOM	3671	HD21	ASN	158	38.775	36.733	48.124	1.00	0.00	RX1	H
ATOM	3672	HD22	ASN	158	38.027	38.265	47.911	1.00	0.00	RX1	H
ATOM	3673	C	ASN	158	35.258	33.540	46.453	1.00	0.00	RX1	C
ATOM	3674	O	ASN	158	34.420	33.798	45.598	1.00	0.00	RX1	O
ATOM	3675	N	VAL	159	35.720	32.306	46.687	1.00	0.00	RX1	N
ATOM	3676	H	VAL	159	36.418	32.158	47.386	1.00	0.00	RX1	H
ATOM	3677	CA	VAL	159	35.114	31.164	46.002	1.00	0.00	RX1	C
ATOM	3678	CB	VAL	159	35.974	29.921	46.233	1.00	0.00	RX1	C
ATOM	3679	CG1	VAL	159	35.550	28.771	45.325	1.00	0.00	RX1	C
ATOM	3680	CG2	VAL	159	37.451	30.258	46.037	1.00	0.00	RX1	C
ATOM	3681	C	VAL	159	33.653	30.928	46.393	1.00	0.00	RX1	C
ATOM	3682	O	VAL	159	32.868	30.319	45.675	1.00	0.00	RX1	O
ATOM	3683	N	GLU	160	33.314	31.489	47.571	1.00	0.00	RX1	N
ATOM	3684	H	GLU	160	33.956	32.078	48.056	1.00	0.00	RX1	H
ATOM	3685	CA	GLU	160	31.962	31.383	48.125	1.00	0.00	RX1	C
ATOM	3686	CB	GLU	160	31.903	32.167	49.457	1.00	0.00	RX1	C
ATOM	3687	CG	GLU	160	31.562	33.678	49.482	1.00	0.00	RX1	C
ATOM	3688	CD	GLU	160	32.560	34.564	48.747	1.00	0.00	RX1	C
ATOM	3689	OE1	GLU	160	33.186	35.441	49.331	1.00	0.00	RX1	O
ATOM	3690	OE2	GLU	160	32.697	34.434	47.549	1.00	0.00	RX1	O
ATOM	3691	C	GLU	160	30.808	31.735	47.188	1.00	0.00	RX1	C
ATOM	3692	O	GLU	160	29.683	31.274	47.325	1.00	0.00	RX1	O
ATOM	3693	N	SER	161	31.150	32.586	46.217	1.00	0.00	RX1	N
ATOM	3694	H	SER	161	32.102	32.872	46.141	1.00	0.00	RX1	H
ATOM	3695	CA	SER	161	30.109	33.146	45.370	1.00	0.00	RX1	C
ATOM	3696	CB	SER	161	30.377	34.657	45.282	1.00	0.00	RX1	C
ATOM	3697	OG	SER	161	31.782	34.935	45.086	1.00	0.00	RX1	O
ATOM	3698	HG	SER	161	32.253	34.383	45.710	1.00	0.00	RX1	H
ATOM	3699	C	SER	161	29.931	32.478	44.020	1.00	0.00	RX1	C
ATOM	3700	O	SER	161	29.047	32.835	43.250	1.00	0.00	RX1	O
ATOM	3701	N	ILE	162	30.834	31.533	43.722	1.00	0.00	RX1	N
ATOM	3702	H	ILE	162	31.406	31.096	44.420	1.00	0.00	RX1	H
ATOM	3703	CA	ILE	162	30.836	31.061	42.339	1.00	0.00	RX1	C
ATOM	3704	CB	ILE	162	32.191	30.431	41.964	1.00	0.00	RX1	C
ATOM	3705	CG2	ILE	162	32.073	29.515	40.745	1.00	0.00	RX1	C
ATOM	3706	CG1	ILE	162	33.239	31.505	41.652	1.00	0.00	RX1	C
ATOM	3707	CD1	ILE	162	33.761	32.332	42.825	1.00	0.00	RX1	C
ATOM	3708	C	ILE	162	29.670	30.139	42.031	1.00	0.00	RX1	C
ATOM	3709	O	ILE	162	29.474	29.092	42.636	1.00	0.00	RX1	O
ATOM	3710	N	GLN	163	28.907	30.557	41.010	1.00	0.00	RX1	N
ATOM	3711	H	GLN	163	29.076	31.456	40.596	1.00	0.00	RX1	H
ATOM	3712	CA	GLN	163	27.954	29.588	40.479	1.00	0.00	RX1	C
ATOM	3713	CB	GLN	163	26.828	30.263	39.694	1.00	0.00	RX1	C
ATOM	3714	CG	GLN	163	27.307	31.014	38.458	1.00	0.00	RX1	C
ATOM	3715	CD	GLN	163	26.140	31.684	37.795	1.00	0.00	RX1	C
ATOM	3716	OE1	GLN	163	24.981	31.334	38.030	1.00	0.00	RX1	O
ATOM	3717	NE2	GLN	163	26.515	32.709	37.020	1.00	0.00	RX1	N
ATOM	3718	HE21	GLN	163	27.486	32.817	36.780	1.00	0.00	RX1	H
ATOM	3719	HE22	GLN	163	25.899	33.428	36.687	1.00	0.00	RX1	H

ATOM	3720	C	GLN	163	28.641	28.472	39.703	1.00	0.00	RX1	C
ATOM	3721	O	GLN	163	28.912	28.537	38.508	1.00	0.00	RX1	O
ATOM	3722	N	TRP	164	28.925	27.410	40.467	1.00	0.00	RX1	N
ATOM	3723	H	TRP	164	28.794	27.492	41.459	1.00	0.00	RX1	H
ATOM	3724	CA	TRP	164	29.737	26.333	39.901	1.00	0.00	RX1	C
ATOM	3725	CB	TRP	164	30.026	25.272	40.963	1.00	0.00	RX1	C
ATOM	3726	CG	TRP	164	31.140	25.790	41.840	1.00	0.00	RX1	C
ATOM	3727	CD2	TRP	164	32.552	25.797	41.545	1.00	0.00	RX1	C
ATOM	3728	CE2	TRP	164	33.213	26.418	42.628	1.00	0.00	RX1	C
ATOM	3729	CE3	TRP	164	33.289	25.333	40.463	1.00	0.00	RX1	C
ATOM	3730	CD1	TRP	164	31.027	26.405	43.095	1.00	0.00	RX1	C
ATOM	3731	NE1	TRP	164	32.248	26.780	43.564	1.00	0.00	RX1	N
ATOM	3732	HE1	TRP	164	32.411	27.246	44.412	1.00	0.00	RX1	H
ATOM	3733	CZ2	TRP	164	34.594	26.562	42.593	1.00	0.00	RX1	C
ATOM	3734	CZ3	TRP	164	34.670	25.482	40.442	1.00	0.00	RX1	C
ATOM	3735	CH2	TRP	164	35.320	26.096	41.504	1.00	0.00	RX1	C
ATOM	3736	C	TRP	164	29.268	25.706	38.598	1.00	0.00	RX1	C
ATOM	3737	O	TRP	164	30.069	25.214	37.820	1.00	0.00	RX1	O
ATOM	3738	N	ARG	165	27.950	25.787	38.355	1.00	0.00	RX1	N
ATOM	3739	H	ARG	165	27.387	26.244	39.038	1.00	0.00	RX1	H
ATOM	3740	CA	ARG	165	27.416	25.239	37.101	1.00	0.00	RX1	C
ATOM	3741	CB	ARG	165	25.906	25.456	37.066	1.00	0.00	RX1	C
ATOM	3742	CG	ARG	165	25.188	25.234	38.396	1.00	0.00	RX1	C
ATOM	3743	CD	ARG	165	23.817	25.916	38.416	1.00	0.00	RX1	C
ATOM	3744	NE	ARG	165	23.942	27.372	38.287	1.00	0.00	RX1	N
ATOM	3745	HE	ARG	165	24.587	27.719	37.598	1.00	0.00	RX1	H
ATOM	3746	CZ	ARG	165	23.133	28.184	39.034	1.00	0.00	RX1	C
ATOM	3747	NH1	ARG	165	22.246	27.623	39.888	1.00	0.00	RX1	N
ATOM	3748	HH11	ARG	165	21.630	28.168	40.465	1.00	0.00	RX1	H
ATOM	3749	HH12	ARG	165	22.165	26.626	39.983	1.00	0.00	RX1	H
ATOM	3750	NH2	ARG	165	23.228	29.529	38.915	1.00	0.00	RX1	N
ATOM	3751	HH21	ARG	165	22.632	30.166	39.409	1.00	0.00	RX1	H
ATOM	3752	HH22	ARG	165	23.913	29.983	38.323	1.00	0.00	RX1	H
ATOM	3753	C	ARG	165	27.978	25.818	35.802	1.00	0.00	RX1	C
ATOM	3754	O	ARG	165	27.978	25.183	34.755	1.00	0.00	RX1	O
ATOM	3755	N	ASP	166	28.472	27.067	35.900	1.00	0.00	RX1	N
ATOM	3756	H	ASP	166	28.529	27.563	36.767	1.00	0.00	RX1	H
ATOM	3757	CA	ASP	166	29.155	27.586	34.709	1.00	0.00	RX1	C
ATOM	3758	CB	ASP	166	29.434	29.082	34.861	1.00	0.00	RX1	C
ATOM	3759	CG	ASP	166	29.745	29.718	33.519	1.00	0.00	RX1	C
ATOM	3760	OD1	ASP	166	30.869	29.606	33.042	1.00	0.00	RX1	O
ATOM	3761	OD2	ASP	166	28.858	30.347	32.948	1.00	0.00	RX1	O
ATOM	3762	C	ASP	166	30.443	26.824	34.423	1.00	0.00	RX1	C
ATOM	3763	O	ASP	166	30.807	26.473	33.300	1.00	0.00	RX1	O
ATOM	3764	N	ILE	167	31.096	26.540	35.555	1.00	0.00	RX1	N
ATOM	3765	H	ILE	167	30.639	26.649	36.435	1.00	0.00	RX1	H
ATOM	3766	CA	ILE	167	32.386	25.870	35.501	1.00	0.00	RX1	C
ATOM	3767	CB	ILE	167	33.146	26.098	36.805	1.00	0.00	RX1	C
ATOM	3768	CG2	ILE	167	34.595	25.653	36.653	1.00	0.00	RX1	C
ATOM	3769	CG1	ILE	167	33.001	27.538	37.304	1.00	0.00	RX1	C
ATOM	3770	CD1	ILE	167	33.437	28.595	36.290	1.00	0.00	RX1	C
ATOM	3771	C	ILE	167	32.222	24.387	35.223	1.00	0.00	RX1	C
ATOM	3772	O	ILE	167	32.422	23.903	34.115	1.00	0.00	RX1	O
ATOM	3773	N	VAL	168	31.801	23.689	36.290	1.00	0.00	RX1	N
ATOM	3774	H	VAL	168	31.458	24.214	37.065	1.00	0.00	RX1	H
ATOM	3775	CA	VAL	168	31.528	22.254	36.198	1.00	0.00	RX1	C
ATOM	3776	CB	VAL	168	31.106	21.705	37.573	1.00	0.00	RX1	C
ATOM	3777	CG1	VAL	168	31.407	20.213	37.698	1.00	0.00	RX1	C
ATOM	3778	CG2	VAL	168	31.792	22.448	38.719	1.00	0.00	RX1	C
ATOM	3779	C	VAL	168	30.467	22.004	35.135	1.00	0.00	RX1	C
ATOM	3780	O	VAL	168	29.859	22.952	34.647	1.00	0.00	RX1	O

ATOM	3781	N	SER	169	30.265	20.738	34.762	1.00	0.00	RX1	N
ATOM	3782	H	SER	169	30.847	19.980	35.061	1.00	0.00	RX1	H
ATOM	3783	CA	SER	169	29.227	20.508	33.765	1.00	0.00	RX1	C
ATOM	3784	CB	SER	169	29.260	19.024	33.395	1.00	0.00	RX1	C
ATOM	3785	OG	SER	169	28.348	18.280	34.211	1.00	0.00	RX1	O
ATOM	3786	HG	SER	169	27.826	17.799	33.564	1.00	0.00	RX1	H
ATOM	3787	C	SER	169	27.850	21.026	34.176	1.00	0.00	RX1	C
ATOM	3788	O	SER	169	27.594	21.377	35.325	1.00	0.00	RX1	O
ATOM	3789	N	SER	170	26.941	21.002	33.198	1.00	0.00	RX1	N
ATOM	3790	H	SER	170	27.156	20.585	32.316	1.00	0.00	RX1	H
ATOM	3791	CA	SER	170	25.551	21.342	33.500	1.00	0.00	RX1	C
ATOM	3792	CB	SER	170	24.923	21.486	32.123	1.00	0.00	RX1	C
ATOM	3793	OG	SER	170	25.721	20.724	31.204	1.00	0.00	RX1	O
ATOM	3794	HG	SER	170	25.575	19.797	31.433	1.00	0.00	RX1	H
ATOM	3795	C	SER	170	24.814	20.356	34.410	1.00	0.00	RX1	C
ATOM	3796	O	SER	170	23.624	20.482	34.678	1.00	0.00	RX1	O
ATOM	3797	N	ASP	171	25.572	19.335	34.828	1.00	0.00	RX1	N
ATOM	3798	H	ASP	171	26.558	19.317	34.679	1.00	0.00	RX1	H
ATOM	3799	CA	ASP	171	24.963	18.055	35.147	1.00	0.00	RX1	C
ATOM	3800	CB	ASP	171	25.315	17.069	34.026	1.00	0.00	RX1	C
ATOM	3801	CG	ASP	171	25.301	17.799	32.691	1.00	0.00	RX1	C
ATOM	3802	OD1	ASP	171	24.240	18.083	32.141	1.00	0.00	RX1	O
ATOM	3803	OD2	ASP	171	26.368	18.150	32.213	1.00	0.00	RX1	O
ATOM	3804	C	ASP	171	25.416	17.568	36.508	1.00	0.00	RX1	C
ATOM	3805	O	ASP	171	24.673	16.977	37.288	1.00	0.00	RX1	O
ATOM	3806	N	PHE	172	26.676	17.939	36.806	1.00	0.00	RX1	N
ATOM	3807	H	PHE	172	27.269	18.345	36.106	1.00	0.00	RX1	H
ATOM	3808	CA	PHE	172	27.210	17.702	38.151	1.00	0.00	RX1	C
ATOM	3809	CB	PHE	172	28.714	17.916	38.192	1.00	0.00	RX1	C
ATOM	3810	CG	PHE	172	29.369	16.726	37.554	1.00	0.00	RX1	C
ATOM	3811	CD1	PHE	172	29.556	15.572	38.299	1.00	0.00	RX1	C
ATOM	3812	CD2	PHE	172	29.759	16.775	36.225	1.00	0.00	RX1	C
ATOM	3813	CE1	PHE	172	30.091	14.448	37.691	1.00	0.00	RX1	C
ATOM	3814	CE2	PHE	172	30.280	15.648	35.612	1.00	0.00	RX1	C
ATOM	3815	CZ	PHE	172	30.417	14.477	36.341	1.00	0.00	RX1	C
ATOM	3816	C	PHE	172	26.576	18.458	39.302	1.00	0.00	RX1	C
ATOM	3817	O	PHE	172	26.931	18.276	40.460	1.00	0.00	RX1	O
ATOM	3818	N	LEU	173	25.564	19.274	38.953	1.00	0.00	RX1	N
ATOM	3819	H	LEU	173	25.379	19.446	37.989	1.00	0.00	RX1	H
ATOM	3820	CA	LEU	173	24.668	19.767	40.003	1.00	0.00	RX1	C
ATOM	3821	CB	LEU	173	23.574	20.686	39.434	1.00	0.00	RX1	C
ATOM	3822	CG	LEU	173	23.006	20.365	38.044	1.00	0.00	RX1	C
ATOM	3823	CD1	LEU	173	22.162	19.091	37.988	1.00	0.00	RX1	C
ATOM	3824	CD2	LEU	173	22.224	21.560	37.497	1.00	0.00	RX1	C
ATOM	3825	C	LEU	173	24.119	18.701	40.950	1.00	0.00	RX1	C
ATOM	3826	O	LEU	173	23.851	18.950	42.116	1.00	0.00	RX1	O
ATOM	3827	N	SER	174	24.032	17.482	40.393	1.00	0.00	RX1	N
ATOM	3828	H	SER	174	24.313	17.316	39.448	1.00	0.00	RX1	H
ATOM	3829	CA	SER	174	23.660	16.301	41.170	1.00	0.00	RX1	C
ATOM	3830	CB	SER	174	23.751	15.154	40.179	1.00	0.00	RX1	C
ATOM	3831	OG	SER	174	24.860	15.439	39.321	1.00	0.00	RX1	O
ATOM	3832	HG	SER	174	24.513	15.575	38.443	1.00	0.00	RX1	H
ATOM	3833	C	SER	174	24.453	16.052	42.454	1.00	0.00	RX1	C
ATOM	3834	O	SER	174	23.950	15.494	43.425	1.00	0.00	RX1	O
ATOM	3835	N	ASN	175	25.720	16.500	42.434	1.00	0.00	RX1	N
ATOM	3836	H	ASN	175	26.143	16.931	41.634	1.00	0.00	RX1	H
ATOM	3837	CA	ASN	175	26.458	16.404	43.691	1.00	0.00	RX1	C
ATOM	3838	CB	ASN	175	27.115	15.050	43.917	1.00	0.00	RX1	C
ATOM	3839	CG	ASN	175	27.054	14.823	45.410	1.00	0.00	RX1	C
ATOM	3840	OD1	ASN	175	28.052	14.679	46.110	1.00	0.00	RX1	O
ATOM	3841	ND2	ASN	175	25.787	14.789	45.866	1.00	0.00	RX1	N

ATOM	3842	HD21	ASN	175	25.012	14.899	45.233	1.00	0.00	RX1	H
ATOM	3843	HD22	ASN	175	25.569	14.651	46.832	1.00	0.00	RX1	H
ATOM	3844	C	ASN	175	27.463	17.505	43.936	1.00	0.00	RX1	C
ATOM	3845	O	ASN	175	28.583	17.281	44.375	1.00	0.00	RX1	O
ATOM	3846	N	MET	176	27.014	18.729	43.631	1.00	0.00	RX1	N
ATOM	3847	H	MET	176	26.066	18.870	43.343	1.00	0.00	RX1	H
ATOM	3848	CA	MET	176	27.912	19.848	43.920	1.00	0.00	RX1	C
ATOM	3849	CB	MET	176	27.654	21.027	42.970	1.00	0.00	RX1	C
ATOM	3850	CG	MET	176	26.236	21.605	42.992	1.00	0.00	RX1	C
ATOM	3851	SD	MET	176	26.035	22.980	41.847	1.00	0.00	RX1	S
ATOM	3852	CE	MET	176	24.351	23.424	42.300	1.00	0.00	RX1	C
ATOM	3853	C	MET	176	27.929	20.249	45.393	1.00	0.00	RX1	C
ATOM	3854	O	MET	176	27.337	21.229	45.830	1.00	0.00	RX1	O
ATOM	3855	N	SER	177	28.630	19.411	46.162	1.00	0.00	RX1	N
ATOM	3856	H	SER	177	29.216	18.708	45.753	1.00	0.00	RX1	H
ATOM	3857	CA	SER	177	28.652	19.654	47.599	1.00	0.00	RX1	C
ATOM	3858	CB	SER	177	28.814	18.289	48.278	1.00	0.00	RX1	C
ATOM	3859	OG	SER	177	28.318	18.277	49.625	1.00	0.00	RX1	O
ATOM	3860	HG	SER	177	28.846	18.912	50.119	1.00	0.00	RX1	H
ATOM	3861	C	SER	177	29.670	20.715	47.974	1.00	0.00	RX1	C
ATOM	3862	O	SER	177	30.817	20.472	48.328	1.00	0.00	RX1	O
ATOM	3863	N	MET	178	29.171	21.943	47.850	1.00	0.00	RX1	N
ATOM	3864	H	MET	178	28.214	22.044	47.571	1.00	0.00	RX1	H
ATOM	3865	CA	MET	178	29.995	23.083	48.226	1.00	0.00	RX1	C
ATOM	3866	CB	MET	178	29.819	24.203	47.191	1.00	0.00	RX1	C
ATOM	3867	CG	MET	178	28.360	24.542	46.863	1.00	0.00	RX1	C
ATOM	3868	SD	MET	178	28.182	25.705	45.499	1.00	0.00	RX1	S
ATOM	3869	CE	MET	178	28.885	27.159	46.293	1.00	0.00	RX1	C
ATOM	3870	C	MET	178	29.741	23.535	49.655	1.00	0.00	RX1	C
ATOM	3871	O	MET	178	29.245	24.625	49.917	1.00	0.00	RX1	O
ATOM	3872	N	ASP	179	30.098	22.634	50.580	1.00	0.00	RX1	N
ATOM	3873	H	ASP	179	30.586	21.780	50.376	1.00	0.00	RX1	H
ATOM	3874	CA	ASP	179	29.912	22.944	51.997	1.00	0.00	RX1	C
ATOM	3875	CB	ASP	179	29.606	21.696	52.853	1.00	0.00	RX1	C
ATOM	3876	CG	ASP	179	30.305	20.422	52.400	1.00	0.00	RX1	C
ATOM	3877	OD1	ASP	179	31.071	19.852	53.172	1.00	0.00	RX1	O
ATOM	3878	OD2	ASP	179	30.035	19.930	51.305	1.00	0.00	RX1	O
ATOM	3879	C	ASP	179	30.981	23.853	52.589	1.00	0.00	RX1	C
ATOM	3880	O	ASP	179	31.887	23.499	53.338	1.00	0.00	RX1	O
ATOM	3881	N	PHE	180	30.789	25.119	52.187	1.00	0.00	RX1	N
ATOM	3882	H	PHE	180	29.990	25.300	51.612	1.00	0.00	RX1	H
ATOM	3883	CA	PHE	180	31.547	26.215	52.777	1.00	0.00	RX1	C
ATOM	3884	CB	PHE	180	31.334	27.521	52.006	1.00	0.00	RX1	C
ATOM	3885	CG	PHE	180	31.991	27.520	50.647	1.00	0.00	RX1	C
ATOM	3886	CD1	PHE	180	31.298	27.067	49.532	1.00	0.00	RX1	C
ATOM	3887	CD2	PHE	180	33.284	28.010	50.508	1.00	0.00	RX1	C
ATOM	3888	CE1	PHE	180	31.884	27.136	48.274	1.00	0.00	RX1	C
ATOM	3889	CE2	PHE	180	33.866	28.081	49.248	1.00	0.00	RX1	C
ATOM	3890	CZ	PHE	180	33.162	27.658	48.129	1.00	0.00	RX1	C
ATOM	3891	C	PHE	180	31.121	26.474	54.208	1.00	0.00	RX1	C
ATOM	3892	O	PHE	180	30.089	26.023	54.688	1.00	0.00	RX1	O
ATOM	3893	N	GLN	181	31.960	27.284	54.851	1.00	0.00	RX1	N
ATOM	3894	H	GLN	181	32.771	27.667	54.408	1.00	0.00	RX1	H
ATOM	3895	CA	GLN	181	31.594	27.852	56.140	1.00	0.00	RX1	C
ATOM	3896	CB	GLN	181	32.261	27.112	57.303	1.00	0.00	RX1	C
ATOM	3897	CG	GLN	181	33.761	27.389	57.448	1.00	0.00	RX1	C
ATOM	3898	CD	GLN	181	34.548	26.732	56.337	1.00	0.00	RX1	C
ATOM	3899	OE1	GLN	181	34.815	25.537	56.399	1.00	0.00	RX1	O
ATOM	3900	NE2	GLN	181	34.933	27.566	55.351	1.00	0.00	RX1	N
ATOM	3901	HE21	GLN	181	34.653	28.531	55.345	1.00	0.00	RX1	H
ATOM	3902	HE22	GLN	181	35.494	27.271	54.567	1.00	0.00	RX1	H

ATOM	3903	C	GLN	181	32.041	29.294	56.125	1.00	0.00	RX1	C
ATOM	3904	O	GLN	181	32.829	29.675	55.266	1.00	0.00	RX1	O
ATOM	3905	N	ASN	182	31.563	30.063	57.117	1.00	0.00	RX1	N
ATOM	3906	H	ASN	182	30.977	29.689	57.833	1.00	0.00	RX1	H
ATOM	3907	CA	ASN	182	32.157	31.398	57.219	1.00	0.00	RX1	C
ATOM	3908	CB	ASN	182	31.473	32.209	58.316	1.00	0.00	RX1	C
ATOM	3909	CG	ASN	182	32.213	33.520	58.415	1.00	0.00	RX1	C
ATOM	3910	OD1	ASN	182	32.391	34.209	57.417	1.00	0.00	RX1	O
ATOM	3911	ND2	ASN	182	32.703	33.783	59.637	1.00	0.00	RX1	N
ATOM	3912	HD21	ASN	182	32.455	33.265	60.455	1.00	0.00	RX1	H
ATOM	3913	HD22	ASN	182	33.395	34.505	59.728	1.00	0.00	RX1	H
ATOM	3914	C	ASN	182	33.654	31.351	57.497	1.00	0.00	RX1	C
ATOM	3915	O	ASN	182	34.156	30.495	58.217	1.00	0.00	RX1	O
ATOM	3916	N	HIS	183	34.337	32.298	56.860	1.00	0.00	RX1	N
ATOM	3917	H	HIS	183	33.831	33.064	56.455	1.00	0.00	RX1	H
ATOM	3918	CA	HIS	183	35.771	32.405	57.090	1.00	0.00	RX1	C
ATOM	3919	CB	HIS	183	36.475	32.948	55.850	1.00	0.00	RX1	C
ATOM	3920	CG	HIS	183	35.645	34.005	55.168	1.00	0.00	RX1	C
ATOM	3921	ND1	HIS	183	34.926	33.756	54.061	1.00	0.00	RX1	N
ATOM	3922	HD1	HIS	183	34.897	32.899	53.572	1.00	0.00	RX1	H
ATOM	3923	CD2	HIS	183	35.486	35.348	55.523	1.00	0.00	RX1	C
ATOM	3924	NE2	HIS	183	34.657	35.908	54.609	1.00	0.00	RX1	N
ATOM	3925	CE1	HIS	183	34.309	34.927	53.712	1.00	0.00	RX1	C
ATOM	3926	C	HIS	183	36.091	33.246	58.307	1.00	0.00	RX1	C
ATOM	3927	O	HIS	183	35.306	34.080	58.734	1.00	0.00	RX1	O
ATOM	3928	N	LEU	184	37.302	33.012	58.831	1.00	0.00	RX1	N
ATOM	3929	H	LEU	184	37.939	32.349	58.443	1.00	0.00	RX1	H
ATOM	3930	CA	LEU	184	37.778	33.894	59.900	1.00	0.00	RX1	C
ATOM	3931	CB	LEU	184	38.500	33.082	60.973	1.00	0.00	RX1	C
ATOM	3932	CG	LEU	184	37.623	31.983	61.575	1.00	0.00	RX1	C
ATOM	3933	CD1	LEU	184	38.397	31.147	62.593	1.00	0.00	RX1	C
ATOM	3934	CD2	LEU	184	36.324	32.538	62.164	1.00	0.00	RX1	C
ATOM	3935	C	LEU	184	38.679	34.994	59.374	1.00	0.00	RX1	C
ATOM	3936	O	LEU	184	39.742	35.289	59.912	1.00	0.00	RX1	O
ATOM	3937	N	GLY	185	38.216	35.548	58.252	1.00	0.00	RX1	N
ATOM	3938	H	GLY	185	37.266	35.423	57.965	1.00	0.00	RX1	H
ATOM	3939	CA	GLY	185	39.010	36.535	57.540	1.00	0.00	RX1	C
ATOM	3940	C	GLY	185	38.089	37.558	56.936	1.00	0.00	RX1	C
ATOM	3941	O	GLY	185	37.180	38.070	57.587	1.00	0.00	RX1	O
ATOM	3942	N	SER	186	38.341	37.808	55.644	1.00	0.00	RX1	N
ATOM	3943	H	SER	186	39.097	37.413	55.128	1.00	0.00	RX1	H
ATOM	3944	CA	SER	186	37.446	38.724	54.951	1.00	0.00	RX1	C
ATOM	3945	CB	SER	186	37.943	40.147	55.214	1.00	0.00	RX1	C
ATOM	3946	OG	SER	186	38.468	40.220	56.545	1.00	0.00	RX1	O
ATOM	3947	HG	SER	186	37.789	39.870	57.120	1.00	0.00	RX1	H
ATOM	3948	C	SER	186	37.326	38.421	53.482	1.00	0.00	RX1	C
ATOM	3949	O	SER	186	38.306	38.180	52.783	1.00	0.00	RX1	O
ATOM	3950	N	CYS	187	36.075	38.470	53.041	1.00	0.00	RX1	N
ATOM	3951	H	CYS	187	35.304	38.639	53.656	1.00	0.00	RX1	H
ATOM	3952	CA	CYS	187	35.793	38.349	51.615	1.00	0.00	RX1	C
ATOM	3953	CB	CYS	187	34.386	37.780	51.461	1.00	0.00	RX1	C
ATOM	3954	SG	CYS	187	33.262	38.490	52.691	1.00	0.00	RX1	S
ATOM	3955	C	CYS	187	35.943	39.663	50.873	1.00	0.00	RX1	C
ATOM	3956	O	CYS	187	34.978	40.224	50.372	1.00	0.00	RX1	O
ATOM	3957	N	GLN	188	37.200	40.149	50.815	1.00	0.00	RX1	N
ATOM	3958	H	GLN	188	37.976	39.619	51.163	1.00	0.00	RX1	H
ATOM	3959	CA	GLN	188	37.399	41.356	50.012	1.00	0.00	RX1	C
ATOM	3960	CB	GLN	188	38.797	41.959	50.223	1.00	0.00	RX1	C
ATOM	3961	CG	GLN	188	39.957	41.352	49.425	1.00	0.00	RX1	C
ATOM	3962	CD	GLN	188	40.133	39.881	49.739	1.00	0.00	RX1	C
ATOM	3963	OE1	GLN	188	39.970	39.425	50.870	1.00	0.00	RX1	O

ATOM	3964	NE2	GLN	188	40.505	39.164	48.671	1.00	0.00	RX1	N
ATOM	3965	HE21	GLN	188	40.536	39.576	47.761	1.00	0.00	RX1	H
ATOM	3966	HE22	GLN	188	40.780	38.198	48.736	1.00	0.00	RX1	H
ATOM	3967	C	GLN	188	37.054	41.116	48.552	1.00	0.00	RX1	C
ATOM	3968	O	GLN	188	37.513	40.159	47.937	1.00	0.00	RX1	O
ATOM	3969	N	LYS	189	36.149	41.978	48.079	1.00	0.00	RX1	N
ATOM	3970	H	LYS	189	35.907	42.813	48.567	1.00	0.00	RX1	H
ATOM	3971	CA	LYS	189	35.421	41.561	46.891	1.00	0.00	RX1	C
ATOM	3972	CB	LYS	189	33.911	41.758	47.081	1.00	0.00	RX1	C
ATOM	3973	CG	LYS	189	33.082	40.570	46.563	1.00	0.00	RX1	C
ATOM	3974	CD	LYS	189	33.548	39.201	47.086	1.00	0.00	RX1	C
ATOM	3975	CE	LYS	189	32.676	38.042	46.580	1.00	0.00	RX1	C
ATOM	3976	NZ	LYS	189	33.368	36.748	46.660	1.00	0.00	RX1	N
ATOM	3977	HZ1	LYS	189	32.913	36.060	46.025	1.00	0.00	RX1	H
ATOM	3978	HZ2	LYS	189	34.361	36.838	46.355	1.00	0.00	RX1	H
ATOM	3979	HZ3	LYS	189	33.319	36.312	47.613	1.00	0.00	RX1	H
ATOM	3980	C	LYS	189	36.020	42.033	45.573	1.00	0.00	RX1	C
ATOM	3981	O	LYS	189	37.231	41.965	45.384	1.00	0.00	RX1	O
ATOM	3982	N	CYS	190	35.152	42.474	44.649	1.00	0.00	RX1	N
ATOM	3983	H	CYS	190	34.190	42.717	44.768	1.00	0.00	RX1	H
ATOM	3984	CA	CYS	190	35.659	42.594	43.289	1.00	0.00	RX1	C
ATOM	3985	CB	CYS	190	35.343	41.309	42.530	1.00	0.00	RX1	C
ATOM	3986	SG	CYS	190	35.596	39.831	43.544	1.00	0.00	RX1	S
ATOM	3987	C	CYS	190	35.127	43.813	42.580	1.00	0.00	RX1	C
ATOM	3988	O	CYS	190	34.271	44.519	43.099	1.00	0.00	RX1	O
ATOM	3989	N	ASP	191	35.683	44.014	41.378	1.00	0.00	RX1	N
ATOM	3990	H	ASP	191	36.299	43.352	40.959	1.00	0.00	RX1	H
ATOM	3991	CA	ASP	191	35.187	45.062	40.489	1.00	0.00	RX1	C
ATOM	3992	CB	ASP	191	36.155	45.142	39.299	1.00	0.00	RX1	C
ATOM	3993	CG	ASP	191	35.789	46.287	38.378	1.00	0.00	RX1	C
ATOM	3994	OD1	ASP	191	36.250	47.400	38.619	1.00	0.00	RX1	O
ATOM	3995	OD2	ASP	191	35.027	46.073	37.439	1.00	0.00	RX1	O
ATOM	3996	C	ASP	191	33.763	44.745	40.042	1.00	0.00	RX1	C
ATOM	3997	O	ASP	191	33.383	43.579	39.964	1.00	0.00	RX1	O
ATOM	3998	N	PRO	192	32.966	45.807	39.761	1.00	0.00	RX1	N
ATOM	3999	CD	PRO	192	33.209	47.195	40.145	1.00	0.00	RX1	C
ATOM	4000	CA	PRO	192	31.696	45.641	39.039	1.00	0.00	RX1	C
ATOM	4001	CB	PRO	192	31.284	47.088	38.751	1.00	0.00	RX1	C
ATOM	4002	CG	PRO	192	31.877	47.893	39.904	1.00	0.00	RX1	C
ATOM	4003	C	PRO	192	31.639	44.745	37.796	1.00	0.00	RX1	C
ATOM	4004	O	PRO	192	30.553	44.498	37.287	1.00	0.00	RX1	O
ATOM	4005	N	SER	193	32.794	44.227	37.337	1.00	0.00	RX1	N
ATOM	4006	H	SER	193	33.706	44.503	37.653	1.00	0.00	RX1	H
ATOM	4007	CA	SER	193	32.757	43.123	36.374	1.00	0.00	RX1	C
ATOM	4008	CB	SER	193	34.205	42.732	36.079	1.00	0.00	RX1	C
ATOM	4009	OG	SER	193	34.925	42.556	37.305	1.00	0.00	RX1	O
ATOM	4010	HG	SER	193	35.459	41.772	37.162	1.00	0.00	RX1	H
ATOM	4011	C	SER	193	31.889	41.941	36.797	1.00	0.00	RX1	C
ATOM	4012	O	SER	193	31.277	41.257	35.984	1.00	0.00	RX1	O
ATOM	4013	N	CYS	194	31.855	41.753	38.130	1.00	0.00	RX1	N
ATOM	4014	H	CYS	194	32.448	42.319	38.708	1.00	0.00	RX1	H
ATOM	4015	CA	CYS	194	30.999	40.735	38.745	1.00	0.00	RX1	C
ATOM	4016	CB	CYS	194	30.930	40.963	40.249	1.00	0.00	RX1	C
ATOM	4017	SG	CYS	194	32.556	40.954	41.028	1.00	0.00	RX1	S
ATOM	4018	C	CYS	194	29.582	40.624	38.211	1.00	0.00	RX1	C
ATOM	4019	O	CYS	194	28.756	41.516	38.363	1.00	0.00	RX1	O
ATOM	4020	N	PRO	195	29.317	39.445	37.603	1.00	0.00	RX1	N
ATOM	4021	CD	PRO	195	30.283	38.422	37.241	1.00	0.00	RX1	C
ATOM	4022	CA	PRO	195	27.937	39.093	37.268	1.00	0.00	RX1	C
ATOM	4023	CB	PRO	195	28.139	37.881	36.344	1.00	0.00	RX1	C
ATOM	4024	CG	PRO	195	29.634	37.798	36.019	1.00	0.00	RX1	C

ATOM	4025	C	PRO	195	27.158	38.756	38.541	1.00	0.00	RX1	C
ATOM	4026	O	PRO	195	27.320	39.382	39.581	1.00	0.00	RX1	O
ATOM	4027	N	ASN	196	26.321	37.709	38.461	1.00	0.00	RX1	N
ATOM	4028	H	ASN	196	26.217	37.124	37.655	1.00	0.00	RX1	H
ATOM	4029	CA	ASN	196	25.666	37.280	39.699	1.00	0.00	RX1	C
ATOM	4030	CB	ASN	196	24.419	36.439	39.403	1.00	0.00	RX1	C
ATOM	4031	CG	ASN	196	24.780	35.166	38.658	1.00	0.00	RX1	C
ATOM	4032	OD1	ASN	196	25.277	35.198	37.533	1.00	0.00	RX1	O
ATOM	4033	ND2	ASN	196	24.439	34.041	39.310	1.00	0.00	RX1	N
ATOM	4034	HD21	ASN	196	24.149	34.103	40.266	1.00	0.00	RX1	H
ATOM	4035	HD22	ASN	196	24.506	33.138	38.883	1.00	0.00	RX1	H
ATOM	4036	C	ASN	196	26.579	36.535	40.665	1.00	0.00	RX1	C
ATOM	4037	O	ASN	196	26.589	35.314	40.747	1.00	0.00	RX1	O
ATOM	4038	N	GLY	197	27.344	37.340	41.412	1.00	0.00	RX1	N
ATOM	4039	H	GLY	197	27.410	38.313	41.181	1.00	0.00	RX1	H
ATOM	4040	CA	GLY	197	28.333	36.728	42.290	1.00	0.00	RX1	C
ATOM	4041	C	GLY	197	29.571	36.332	41.515	1.00	0.00	RX1	C
ATOM	4042	O	GLY	197	30.151	37.123	40.780	1.00	0.00	RX1	O
ATOM	4043	N	SER	198	29.939	35.058	41.711	1.00	0.00	RX1	N
ATOM	4044	H	SER	198	29.313	34.438	42.184	1.00	0.00	RX1	H
ATOM	4045	CA	SER	198	31.048	34.476	40.958	1.00	0.00	RX1	C
ATOM	4046	CB	SER	198	30.486	34.163	39.588	1.00	0.00	RX1	C
ATOM	4047	OG	SER	198	29.408	33.228	39.718	1.00	0.00	RX1	O
ATOM	4048	HG	SER	198	29.144	33.072	38.812	1.00	0.00	RX1	H
ATOM	4049	C	SER	198	32.356	35.253	40.915	1.00	0.00	RX1	C
ATOM	4050	O	SER	198	32.966	35.461	39.869	1.00	0.00	RX1	O
ATOM	4051	N	CYS	199	32.762	35.673	42.117	1.00	0.00	RX1	N
ATOM	4052	H	CYS	199	32.308	35.441	42.981	1.00	0.00	RX1	H
ATOM	4053	CA	CYS	199	33.836	36.654	42.140	1.00	0.00	RX1	C
ATOM	4054	CB	CYS	199	33.169	37.994	42.412	1.00	0.00	RX1	C
ATOM	4055	SG	CYS	199	31.717	37.808	43.478	1.00	0.00	RX1	S
ATOM	4056	C	CYS	199	34.975	36.315	43.081	1.00	0.00	RX1	C
ATOM	4057	O	CYS	199	34.865	36.402	44.301	1.00	0.00	RX1	O
ATOM	4058	N	TRP	200	36.094	35.910	42.449	1.00	0.00	RX1	N
ATOM	4059	H	TRP	200	36.251	36.098	41.477	1.00	0.00	RX1	H
ATOM	4060	CA	TRP	200	37.188	35.318	43.225	1.00	0.00	RX1	C
ATOM	4061	CB	TRP	200	38.032	34.302	42.431	1.00	0.00	RX1	C
ATOM	4062	CG	TRP	200	37.306	33.460	41.401	1.00	0.00	RX1	C
ATOM	4063	CD2	TRP	200	37.114	32.029	41.408	1.00	0.00	RX1	C
ATOM	4064	CE2	TRP	200	36.480	31.671	40.194	1.00	0.00	RX1	C
ATOM	4065	CE3	TRP	200	37.431	31.042	42.331	1.00	0.00	RX1	C
ATOM	4066	CD1	TRP	200	36.764	33.878	40.177	1.00	0.00	RX1	C
ATOM	4067	NE1	TRP	200	36.278	32.832	39.460	1.00	0.00	RX1	N
ATOM	4068	HE1	TRP	200	35.874	32.878	38.568	1.00	0.00	RX1	H
ATOM	4069	CZ2	TRP	200	36.187	30.337	39.935	1.00	0.00	RX1	C
ATOM	4070	CZ3	TRP	200	37.130	29.714	42.061	1.00	0.00	RX1	C
ATOM	4071	CH2	TRP	200	36.512	29.361	40.867	1.00	0.00	RX1	C
ATOM	4072	C	TRP	200	38.173	36.329	43.815	1.00	0.00	RX1	C
ATOM	4073	O	TRP	200	39.368	36.068	43.913	1.00	0.00	RX1	O
ATOM	4074	N	GLY	201	37.641	37.501	44.191	1.00	0.00	RX1	N
ATOM	4075	H	GLY	201	36.658	37.668	44.134	1.00	0.00	RX1	H
ATOM	4076	CA	GLY	201	38.546	38.606	44.511	1.00	0.00	RX1	C
ATOM	4077	C	GLY	201	38.849	39.412	43.263	1.00	0.00	RX1	C
ATOM	4078	O	GLY	201	38.935	38.860	42.177	1.00	0.00	RX1	O
ATOM	4079	N	ALA	202	38.987	40.736	43.455	1.00	0.00	RX1	N
ATOM	4080	H	ALA	202	38.787	41.111	44.363	1.00	0.00	RX1	H
ATOM	4081	CA	ALA	202	39.102	41.642	42.303	1.00	0.00	RX1	C
ATOM	4082	CB	ALA	202	39.366	43.074	42.776	1.00	0.00	RX1	C
ATOM	4083	C	ALA	202	40.126	41.300	41.227	1.00	0.00	RX1	C
ATOM	4084	O	ALA	202	39.784	40.998	40.090	1.00	0.00	RX1	O
ATOM	4085	N	GLY	203	41.407	41.380	41.643	1.00	0.00	RX1	N

ATOM	4086	H	GLY	203	41.605	41.687	42.570	1.00	0.00	RX1	H
ATOM	4087	CA	GLY	203	42.495	41.199	40.677	1.00	0.00	RX1	C
ATOM	4088	C	GLY	203	42.370	42.105	39.461	1.00	0.00	RX1	C
ATOM	4089	O	GLY	203	41.797	43.183	39.532	1.00	0.00	RX1	O
ATOM	4090	N	GLU	204	42.896	41.587	38.346	1.00	0.00	RX1	N
ATOM	4091	H	GLU	204	43.306	40.678	38.301	1.00	0.00	RX1	H
ATOM	4092	CA	GLU	204	42.389	42.086	37.074	1.00	0.00	RX1	C
ATOM	4093	CB	GLU	204	43.548	42.364	36.121	1.00	0.00	RX1	C
ATOM	4094	CG	GLU	204	43.086	42.848	34.747	1.00	0.00	RX1	C
ATOM	4095	CD	GLU	204	44.291	42.976	33.844	1.00	0.00	RX1	C
ATOM	4096	OE1	GLU	204	44.207	42.552	32.694	1.00	0.00	RX1	O
ATOM	4097	OE2	GLU	204	45.312	43.488	34.300	1.00	0.00	RX1	O
ATOM	4098	C	GLU	204	41.529	40.957	36.555	1.00	0.00	RX1	C
ATOM	4099	O	GLU	204	41.901	39.798	36.707	1.00	0.00	RX1	O
ATOM	4100	N	GLU	205	40.343	41.346	36.046	1.00	0.00	RX1	N
ATOM	4101	H	GLU	205	40.209	42.321	35.879	1.00	0.00	RX1	H
ATOM	4102	CA	GLU	205	39.210	40.423	35.880	1.00	0.00	RX1	C
ATOM	4103	CB	GLU	205	38.724	40.301	34.435	1.00	0.00	RX1	C
ATOM	4104	CG	GLU	205	37.189	40.356	34.351	1.00	0.00	RX1	C
ATOM	4105	CD	GLU	205	36.555	39.449	35.393	1.00	0.00	RX1	C
ATOM	4106	OE1	GLU	205	36.425	38.253	35.152	1.00	0.00	RX1	O
ATOM	4107	OE2	GLU	205	36.227	39.926	36.474	1.00	0.00	RX1	O
ATOM	4108	C	GLU	205	39.309	39.046	36.516	1.00	0.00	RX1	C
ATOM	4109	O	GLU	205	39.486	38.009	35.874	1.00	0.00	RX1	O
ATOM	4110	N	ASN	206	39.185	39.083	37.839	1.00	0.00	RX1	N
ATOM	4111	H	ASN	206	38.972	39.925	38.341	1.00	0.00	RX1	H
ATOM	4112	CA	ASN	206	39.348	37.823	38.540	1.00	0.00	RX1	C
ATOM	4113	CB	ASN	206	40.437	38.011	39.600	1.00	0.00	RX1	C
ATOM	4114	CG	ASN	206	40.918	36.729	40.242	1.00	0.00	RX1	C
ATOM	4115	OD1	ASN	206	41.860	36.071	39.796	1.00	0.00	RX1	O
ATOM	4116	ND2	ASN	206	40.306	36.488	41.399	1.00	0.00	RX1	N
ATOM	4117	HD21	ASN	206	39.515	37.077	41.603	1.00	0.00	RX1	H
ATOM	4118	HD22	ASN	206	40.517	35.819	42.112	1.00	0.00	RX1	H
ATOM	4119	C	ASN	206	38.008	37.265	39.013	1.00	0.00	RX1	C
ATOM	4120	O	ASN	206	37.841	36.744	40.110	1.00	0.00	RX1	O
ATOM	4121	N	CYS	207	37.045	37.369	38.081	1.00	0.00	RX1	N
ATOM	4122	H	CYS	207	37.123	37.927	37.248	1.00	0.00	RX1	H
ATOM	4123	CA	CYS	207	35.750	36.726	38.278	1.00	0.00	RX1	C
ATOM	4124	CB	CYS	207	34.696	37.827	38.382	1.00	0.00	RX1	C
ATOM	4125	SG	CYS	207	35.116	38.978	39.717	1.00	0.00	RX1	S
ATOM	4126	C	CYS	207	35.466	35.681	37.202	1.00	0.00	RX1	C
ATOM	4127	O	CYS	207	36.349	35.319	36.426	1.00	0.00	RX1	O
ATOM	4128	N	GLN	208	34.225	35.164	37.211	1.00	0.00	RX1	N
ATOM	4129	H	GLN	208	33.532	35.532	37.836	1.00	0.00	RX1	H
ATOM	4130	CA	GLN	208	33.830	34.116	36.263	1.00	0.00	RX1	C
ATOM	4131	CB	GLN	208	32.712	33.313	36.938	1.00	0.00	RX1	C
ATOM	4132	CG	GLN	208	31.949	32.260	36.128	1.00	0.00	RX1	C
ATOM	4133	CD	GLN	208	30.769	31.764	36.955	1.00	0.00	RX1	C
ATOM	4134	OE1	GLN	208	29.733	32.413	37.083	1.00	0.00	RX1	O
ATOM	4135	NE2	GLN	208	30.971	30.565	37.521	1.00	0.00	RX1	N
ATOM	4136	HE21	GLN	208	31.867	30.131	37.449	1.00	0.00	RX1	H
ATOM	4137	HE22	GLN	208	30.251	30.051	37.993	1.00	0.00	RX1	H
ATOM	4138	C	GLN	208	33.381	34.673	34.917	1.00	0.00	RX1	C
ATOM	4139	O	GLN	208	32.543	35.560	34.838	1.00	0.00	RX1	O
ATOM	4140	N	LYS	209	33.950	34.092	33.847	1.00	0.00	RX1	N
ATOM	4141	H	LYS	209	34.514	33.274	33.953	1.00	0.00	RX1	H
ATOM	4142	CA	LYS	209	33.724	34.686	32.522	1.00	0.00	RX1	C
ATOM	4143	CB	LYS	209	34.875	34.366	31.559	1.00	0.00	RX1	C
ATOM	4144	CG	LYS	209	36.279	34.306	32.168	1.00	0.00	RX1	C
ATOM	4145	CD	LYS	209	36.692	35.584	32.891	1.00	0.00	RX1	C
ATOM	4146	CE	LYS	209	38.079	35.474	33.520	1.00	0.00	RX1	C

ATOM	4147	NZ	LYS	209	38.099	36.364	34.677	1.00	0.00	RX1	N
ATOM	4148	HZ1	LYS	209	39.049	36.571	35.046	1.00	0.00	RX1	H
ATOM	4149	HZ2	LYS	209	37.666	37.285	34.455	1.00	0.00	RX1	H
ATOM	4150	HZ3	LYS	209	37.514	35.969	35.439	1.00	0.00	RX1	H
ATOM	4151	C	LYS	209	32.417	34.329	31.803	1.00	0.00	RX1	C
ATOM	4152	O	LYS	209	32.347	34.373	30.574	1.00	0.00	RX1	O
ATOM	4153	N	LEU	210	31.401	33.947	32.609	1.00	0.00	RX1	N
ATOM	4154	H	LEU	210	31.502	34.133	33.585	1.00	0.00	RX1	H
ATOM	4155	CA	LEU	210	30.161	33.291	32.151	1.00	0.00	RX1	C
ATOM	4156	CB	LEU	210	28.939	34.191	32.351	1.00	0.00	RX1	C
ATOM	4157	CG	LEU	210	28.167	33.924	33.644	1.00	0.00	RX1	C
ATOM	4158	CD1	LEU	210	28.944	34.384	34.868	1.00	0.00	RX1	C
ATOM	4159	CD2	LEU	210	26.768	34.540	33.620	1.00	0.00	RX1	C
ATOM	4160	C	LEU	210	30.108	32.756	30.730	1.00	0.00	RX1	C
ATOM	4161	O	LEU	210	29.826	33.478	29.776	1.00	0.00	RX1	O
ATOM	4162	N	THR	211	30.398	31.463	30.618	1.00	0.00	RX1	N
ATOM	4163	H	THR	211	30.651	30.895	31.408	1.00	0.00	RX1	H
ATOM	4164	CA	THR	211	30.364	30.894	29.274	1.00	0.00	RX1	C
ATOM	4165	CB	THR	211	31.842	30.703	28.974	1.00	0.00	RX1	C
ATOM	4166	OG1	THR	211	32.505	30.624	30.246	1.00	0.00	RX1	O
ATOM	4167	HG1	THR	211	33.434	30.791	30.065	1.00	0.00	RX1	H
ATOM	4168	CG2	THR	211	32.460	31.840	28.158	1.00	0.00	RX1	C
ATOM	4169	C	THR	211	29.513	29.637	29.155	1.00	0.00	RX1	C
ATOM	4170	O	THR	211	29.570	28.917	28.165	1.00	0.00	RX1	O
ATOM	4171	N	LYS	212	28.739	29.381	30.221	1.00	0.00	RX1	N
ATOM	4172	H	LYS	212	28.767	29.970	31.031	1.00	0.00	RX1	H
ATOM	4173	CA	LYS	212	27.888	28.191	30.245	1.00	0.00	RX1	C
ATOM	4174	CB	LYS	212	28.456	27.204	31.263	1.00	0.00	RX1	C
ATOM	4175	CG	LYS	212	27.889	25.784	31.299	1.00	0.00	RX1	C
ATOM	4176	CD	LYS	212	28.739	24.789	30.507	1.00	0.00	RX1	C
ATOM	4177	CE	LYS	212	29.318	23.644	31.353	1.00	0.00	RX1	C
ATOM	4178	NZ	LYS	212	30.304	24.114	32.339	1.00	0.00	RX1	N
ATOM	4179	HZ1	LYS	212	29.977	23.900	33.302	1.00	0.00	RX1	H
ATOM	4180	HZ2	LYS	212	31.224	23.631	32.207	1.00	0.00	RX1	H
ATOM	4181	HZ3	LYS	212	30.468	25.138	32.296	1.00	0.00	RX1	H
ATOM	4182	C	LYS	212	26.450	28.540	30.599	1.00	0.00	RX1	C
ATOM	4183	O	LYS	212	25.498	28.180	29.921	1.00	0.00	RX1	O
ATOM	4184	N	ILE	213	26.327	29.293	31.710	1.00	0.00	RX1	N
ATOM	4185	H	ILE	213	27.147	29.586	32.208	1.00	0.00	RX1	H
ATOM	4186	CA	ILE	213	24.985	29.642	32.193	1.00	0.00	RX1	C
ATOM	4187	CB	ILE	213	25.095	30.474	33.477	1.00	0.00	RX1	C
ATOM	4188	CG2	ILE	213	23.741	31.022	33.932	1.00	0.00	RX1	C
ATOM	4189	CG1	ILE	213	25.748	29.651	34.583	1.00	0.00	RX1	C
ATOM	4190	CD1	ILE	213	24.908	28.428	34.942	1.00	0.00	RX1	C
ATOM	4191	C	ILE	213	24.131	30.374	31.165	1.00	0.00	RX1	C
ATOM	4192	O	ILE	213	22.964	30.079	30.951	1.00	0.00	RX1	O
ATOM	4193	N	ILE	214	24.785	31.347	30.523	1.00	0.00	RX1	N
ATOM	4194	H	ILE	214	25.771	31.453	30.632	1.00	0.00	RX1	H
ATOM	4195	CA	ILE	214	24.032	32.085	29.511	1.00	0.00	RX1	C
ATOM	4196	CB	ILE	214	24.289	33.595	29.625	1.00	0.00	RX1	C
ATOM	4197	CG2	ILE	214	23.352	34.175	30.685	1.00	0.00	RX1	C
ATOM	4198	CG1	ILE	214	25.745	33.959	29.944	1.00	0.00	RX1	C
ATOM	4199	CD1	ILE	214	26.780	33.619	28.873	1.00	0.00	RX1	C
ATOM	4200	C	ILE	214	24.199	31.587	28.086	1.00	0.00	RX1	C
ATOM	4201	O	ILE	214	24.311	32.355	27.137	1.00	0.00	RX1	O
ATOM	4202	N	CYS	215	24.213	30.251	27.971	1.00	0.00	RX1	N
ATOM	4203	H	CYS	215	24.042	29.640	28.745	1.00	0.00	RX1	H
ATOM	4204	CA	CYS	215	24.211	29.717	26.613	1.00	0.00	RX1	C
ATOM	4205	CB	CYS	215	24.591	28.235	26.634	1.00	0.00	RX1	C
ATOM	4206	SG	CYS	215	26.319	27.971	27.105	1.00	0.00	RX1	S
ATOM	4207	C	CYS	215	22.893	29.948	25.905	1.00	0.00	RX1	C

ATOM	4208	O	CYS	215	21.827	29.957	26.508	1.00	0.00	RX1	O
ATOM	4209	N	ALA	216	23.015	30.149	24.584	1.00	0.00	RX1	N
ATOM	4210	H	ALA	216	23.899	30.086	24.128	1.00	0.00	RX1	H
ATOM	4211	CA	ALA	216	21.783	30.292	23.817	1.00	0.00	RX1	C
ATOM	4212	CB	ALA	216	22.088	30.712	22.379	1.00	0.00	RX1	C
ATOM	4213	C	ALA	216	20.986	29.002	23.801	1.00	0.00	RX1	C
ATOM	4214	O	ALA	216	21.529	27.917	23.968	1.00	0.00	RX1	O
ATOM	4215	N	GLN	217	19.670	29.178	23.591	1.00	0.00	RX1	N
ATOM	4216	H	GLN	217	19.347	30.112	23.457	1.00	0.00	RX1	H
ATOM	4217	CA	GLN	217	18.735	28.048	23.682	1.00	0.00	RX1	C
ATOM	4218	CB	GLN	217	17.333	28.513	23.302	1.00	0.00	RX1	C
ATOM	4219	CG	GLN	217	16.857	29.662	24.191	1.00	0.00	RX1	C
ATOM	4220	CD	GLN	217	15.551	30.195	23.648	1.00	0.00	RX1	C
ATOM	4221	OE1	GLN	217	15.215	30.000	22.487	1.00	0.00	RX1	O
ATOM	4222	NE2	GLN	217	14.835	30.892	24.549	1.00	0.00	RX1	N
ATOM	4223	HE21	GLN	217	15.153	30.995	25.494	1.00	0.00	RX1	H
ATOM	4224	HE22	GLN	217	13.967	31.320	24.297	1.00	0.00	RX1	H
ATOM	4225	C	GLN	217	19.126	26.810	22.883	1.00	0.00	RX1	C
ATOM	4226	O	GLN	217	18.953	25.674	23.305	1.00	0.00	RX1	O
ATOM	4227	N	GLN	218	19.691	27.103	21.705	1.00	0.00	RX1	N
ATOM	4228	H	GLN	218	19.871	28.041	21.415	1.00	0.00	RX1	H
ATOM	4229	CA	GLN	218	20.459	26.060	21.042	1.00	0.00	RX1	C
ATOM	4230	CB	GLN	218	19.887	25.814	19.643	1.00	0.00	RX1	C
ATOM	4231	CG	GLN	218	20.656	24.776	18.821	1.00	0.00	RX1	C
ATOM	4232	CD	GLN	218	20.702	23.462	19.573	1.00	0.00	RX1	C
ATOM	4233	OE1	GLN	218	21.548	23.246	20.434	1.00	0.00	RX1	O
ATOM	4234	NE2	GLN	218	19.748	22.596	19.190	1.00	0.00	RX1	N
ATOM	4235	HE21	GLN	218	19.090	22.827	18.471	1.00	0.00	RX1	H
ATOM	4236	HE22	GLN	218	19.664	21.691	19.609	1.00	0.00	RX1	H
ATOM	4237	C	GLN	218	21.896	26.539	20.989	1.00	0.00	RX1	C
ATOM	4238	O	GLN	218	22.144	27.692	20.648	1.00	0.00	RX1	O
ATOM	4239	N	CYS	219	22.818	25.644	21.371	1.00	0.00	RX1	N
ATOM	4240	H	CYS	219	22.607	24.684	21.579	1.00	0.00	RX1	H
ATOM	4241	CA	CYS	219	24.204	26.094	21.501	1.00	0.00	RX1	C
ATOM	4242	CB	CYS	219	24.350	26.912	22.788	1.00	0.00	RX1	C
ATOM	4243	SG	CYS	219	25.738	28.078	22.772	1.00	0.00	RX1	S
ATOM	4244	C	CYS	219	25.189	24.950	21.454	1.00	0.00	RX1	C
ATOM	4245	O	CYS	219	24.996	23.909	22.068	1.00	0.00	RX1	O
ATOM	4246	N	SER	220	26.268	25.180	20.699	1.00	0.00	RX1	N
ATOM	4247	H	SER	220	26.430	26.034	20.207	1.00	0.00	RX1	H
ATOM	4248	CA	SER	220	27.291	24.146	20.589	1.00	0.00	RX1	C
ATOM	4249	CB	SER	220	27.936	24.389	19.237	1.00	0.00	RX1	C
ATOM	4250	OG	SER	220	27.786	25.782	18.939	1.00	0.00	RX1	O
ATOM	4251	HG	SER	220	28.681	26.123	18.921	1.00	0.00	RX1	H
ATOM	4252	C	SER	220	28.275	24.104	21.748	1.00	0.00	RX1	C
ATOM	4253	O	SER	220	29.450	24.431	21.629	1.00	0.00	RX1	O
ATOM	4254	N	GLY	221	27.737	23.665	22.891	1.00	0.00	RX1	N
ATOM	4255	H	GLY	221	26.747	23.532	22.975	1.00	0.00	RX1	H
ATOM	4256	CA	GLY	221	28.595	23.647	24.067	1.00	0.00	RX1	C
ATOM	4257	C	GLY	221	28.581	24.979	24.781	1.00	0.00	RX1	C
ATOM	4258	O	GLY	221	27.563	25.408	25.305	1.00	0.00	RX1	O
ATOM	4259	N	ARG	222	29.762	25.605	24.790	1.00	0.00	RX1	N
ATOM	4260	H	ARG	222	30.494	25.299	24.183	1.00	0.00	RX1	H
ATOM	4261	CA	ARG	222	29.832	26.889	25.485	1.00	0.00	RX1	C
ATOM	4262	CB	ARG	222	31.277	27.217	25.873	1.00	0.00	RX1	C
ATOM	4263	CG	ARG	222	31.998	26.144	26.690	1.00	0.00	RX1	C
ATOM	4264	CD	ARG	222	31.459	25.961	28.107	1.00	0.00	RX1	C
ATOM	4265	NE	ARG	222	31.635	27.163	28.919	1.00	0.00	RX1	N
ATOM	4266	HE	ARG	222	31.357	28.027	28.487	1.00	0.00	RX1	H
ATOM	4267	CZ	ARG	222	32.013	27.026	30.224	1.00	0.00	RX1	C
ATOM	4268	NH1	ARG	222	32.384	25.819	30.670	1.00	0.00	RX1	N

ATOM	4269	HH11	ARG	222	32.696	25.684	31.620	1.00	0.00	RX1	H
ATOM	4270	HH12	ARG	222	32.440	24.979	30.101	1.00	0.00	RX1	H
ATOM	4271	NH2	ARG	222	32.013	28.074	31.072	1.00	0.00	RX1	N
ATOM	4272	HH21	ARG	222	32.082	27.959	32.072	1.00	0.00	RX1	H
ATOM	4273	HH22	ARG	222	31.929	29.037	30.783	1.00	0.00	RX1	H
ATOM	4274	C	ARG	222	29.270	28.019	24.641	1.00	0.00	RX1	C
ATOM	4275	O	ARG	222	29.220	27.943	23.418	1.00	0.00	RX1	O
ATOM	4276	N	CYS	223	28.895	29.091	25.338	1.00	0.00	RX1	N
ATOM	4277	H	CYS	223	28.925	29.118	26.339	1.00	0.00	RX1	H
ATOM	4278	CA	CYS	223	28.680	30.340	24.618	1.00	0.00	RX1	C
ATOM	4279	CB	CYS	223	27.364	30.947	25.082	1.00	0.00	RX1	C
ATOM	4280	SG	CYS	223	27.278	30.962	26.890	1.00	0.00	RX1	S
ATOM	4281	C	CYS	223	29.835	31.265	24.910	1.00	0.00	RX1	C
ATOM	4282	O	CYS	223	30.153	31.534	26.063	1.00	0.00	RX1	O
ATOM	4283	N	ARG	224	30.492	31.728	23.840	1.00	0.00	RX1	N
ATOM	4284	H	ARG	224	30.129	31.664	22.908	1.00	0.00	RX1	H
ATOM	4285	CA	ARG	224	31.565	32.647	24.199	1.00	0.00	RX1	C
ATOM	4286	CB	ARG	224	32.809	32.418	23.322	1.00	0.00	RX1	C
ATOM	4287	CG	ARG	224	32.792	33.023	21.923	1.00	0.00	RX1	C
ATOM	4288	CD	ARG	224	33.606	32.246	20.882	1.00	0.00	RX1	C
ATOM	4289	NE	ARG	224	33.854	33.097	19.723	1.00	0.00	RX1	N
ATOM	4290	HE	ARG	224	34.753	33.550	19.684	1.00	0.00	RX1	H
ATOM	4291	CZ	ARG	224	32.766	33.477	18.999	1.00	0.00	RX1	C
ATOM	4292	NH1	ARG	224	31.666	32.705	18.984	1.00	0.00	RX1	N
ATOM	4293	HH11	ARG	224	30.821	33.088	18.585	1.00	0.00	RX1	H
ATOM	4294	HH12	ARG	224	31.608	31.779	19.372	1.00	0.00	RX1	H
ATOM	4295	NH2	ARG	224	32.772	34.640	18.323	1.00	0.00	RX1	N
ATOM	4296	HH21	ARG	224	31.876	34.994	17.999	1.00	0.00	RX1	H
ATOM	4297	HH22	ARG	224	33.588	35.198	18.173	1.00	0.00	RX1	H
ATOM	4298	C	ARG	224	31.038	34.072	24.318	1.00	0.00	RX1	C
ATOM	4299	O	ARG	224	31.215	34.754	25.333	1.00	0.00	RX1	O
ATOM	4300	N	GLY	225	30.284	34.426	23.255	1.00	0.00	RX1	N
ATOM	4301	H	GLY	225	30.169	33.815	22.469	1.00	0.00	RX1	H
ATOM	4302	CA	GLY	225	29.409	35.589	23.293	1.00	0.00	RX1	C
ATOM	4303	C	GLY	225	28.295	35.349	24.287	1.00	0.00	RX1	C
ATOM	4304	O	GLY	225	27.863	34.230	24.536	1.00	0.00	RX1	O
ATOM	4305	N	LYS	226	27.922	36.451	24.935	1.00	0.00	RX1	N
ATOM	4306	H	LYS	226	28.069	37.372	24.578	1.00	0.00	RX1	H
ATOM	4307	CA	LYS	226	27.487	36.211	26.305	1.00	0.00	RX1	C
ATOM	4308	CB	LYS	226	28.280	37.100	27.263	1.00	0.00	RX1	C
ATOM	4309	CG	LYS	226	29.719	37.223	26.758	1.00	0.00	RX1	C
ATOM	4310	CD	LYS	226	30.753	37.499	27.839	1.00	0.00	RX1	C
ATOM	4311	CE	LYS	226	30.778	36.379	28.874	1.00	0.00	RX1	C
ATOM	4312	NZ	LYS	226	31.105	35.070	28.279	1.00	0.00	RX1	N
ATOM	4313	HZ1	LYS	226	31.885	34.656	28.838	1.00	0.00	RX1	H
ATOM	4314	HZ2	LYS	226	30.302	34.424	28.438	1.00	0.00	RX1	H
ATOM	4315	HZ3	LYS	226	31.329	35.099	27.259	1.00	0.00	RX1	H
ATOM	4316	C	LYS	226	25.995	36.260	26.557	1.00	0.00	RX1	C
ATOM	4317	O	LYS	226	25.536	37.017	27.403	1.00	0.00	RX1	O
ATOM	4318	N	SER	227	25.308	35.418	25.759	1.00	0.00	RX1	N
ATOM	4319	H	SER	227	25.846	34.826	25.155	1.00	0.00	RX1	H
ATOM	4320	CA	SER	227	23.852	35.179	25.685	1.00	0.00	RX1	C
ATOM	4321	CB	SER	227	22.970	36.205	26.426	1.00	0.00	RX1	C
ATOM	4322	OG	SER	227	23.368	37.549	26.122	1.00	0.00	RX1	O
ATOM	4323	HG	SER	227	24.011	37.772	26.789	1.00	0.00	RX1	H
ATOM	4324	C	SER	227	23.284	34.868	24.299	1.00	0.00	RX1	C
ATOM	4325	O	SER	227	22.542	33.904	24.145	1.00	0.00	RX1	O
ATOM	4326	N	PRO	228	23.627	35.680	23.254	1.00	0.00	RX1	N
ATOM	4327	CD	PRO	228	24.447	36.891	23.194	1.00	0.00	RX1	C
ATOM	4328	CA	PRO	228	23.116	35.301	21.934	1.00	0.00	RX1	C
ATOM	4329	CB	PRO	228	23.385	36.557	21.102	1.00	0.00	RX1	C

ATOM	4330	CG	PRO	228	24.660	37.137	21.707	1.00	0.00	RX1	C
ATOM	4331	C	PRO	228	23.852	34.083	21.405	1.00	0.00	RX1	C
ATOM	4332	O	PRO	228	24.735	33.525	22.048	1.00	0.00	RX1	O
ATOM	4333	N	SER	229	23.490	33.724	20.171	1.00	0.00	RX1	N
ATOM	4334	H	SER	229	22.768	34.194	19.666	1.00	0.00	RX1	H
ATOM	4335	CA	SER	229	24.078	32.538	19.552	1.00	0.00	RX1	C
ATOM	4336	CB	SER	229	23.085	32.195	18.461	1.00	0.00	RX1	C
ATOM	4337	OG	SER	229	21.846	32.808	18.857	1.00	0.00	RX1	O
ATOM	4338	HG	SER	229	21.168	32.362	18.364	1.00	0.00	RX1	H
ATOM	4339	C	SER	229	25.538	32.604	19.108	1.00	0.00	RX1	C
ATOM	4340	O	SER	229	25.947	31.913	18.184	1.00	0.00	RX1	O
ATOM	4341	N	ASP	230	26.323	33.453	19.793	1.00	0.00	RX1	N
ATOM	4342	H	ASP	230	26.017	33.862	20.649	1.00	0.00	RX1	H
ATOM	4343	CA	ASP	230	27.753	33.470	19.502	1.00	0.00	RX1	C
ATOM	4344	CB	ASP	230	28.315	34.859	19.789	1.00	0.00	RX1	C
ATOM	4345	CG	ASP	230	29.727	34.950	19.262	1.00	0.00	RX1	C
ATOM	4346	OD1	ASP	230	30.617	35.323	20.016	1.00	0.00	RX1	O
ATOM	4347	OD2	ASP	230	29.961	34.619	18.100	1.00	0.00	RX1	O
ATOM	4348	C	ASP	230	28.461	32.360	20.269	1.00	0.00	RX1	C
ATOM	4349	O	ASP	230	29.135	32.511	21.287	1.00	0.00	RX1	O
ATOM	4350	N	CYS	231	28.186	31.174	19.726	1.00	0.00	RX1	N
ATOM	4351	H	CYS	231	27.720	31.130	18.839	1.00	0.00	RX1	H
ATOM	4352	CA	CYS	231	28.554	29.946	20.413	1.00	0.00	RX1	C
ATOM	4353	CB	CYS	231	27.648	28.844	19.879	1.00	0.00	RX1	C
ATOM	4354	SG	CYS	231	27.523	28.911	18.075	1.00	0.00	RX1	S
ATOM	4355	C	CYS	231	30.026	29.619	20.294	1.00	0.00	RX1	C
ATOM	4356	O	CYS	231	30.798	30.285	19.612	1.00	0.00	RX1	O
ATOM	4357	N	CYS	232	30.388	28.553	20.999	1.00	0.00	RX1	N
ATOM	4358	H	CYS	232	29.751	28.024	21.563	1.00	0.00	RX1	H
ATOM	4359	CA	CYS	232	31.713	28.005	20.773	1.00	0.00	RX1	C
ATOM	4360	CB	CYS	232	32.269	27.556	22.118	1.00	0.00	RX1	C
ATOM	4361	SG	CYS	232	32.471	28.964	23.238	1.00	0.00	RX1	S
ATOM	4362	C	CYS	232	31.636	26.879	19.769	1.00	0.00	RX1	C
ATOM	4363	O	CYS	232	30.560	26.472	19.347	1.00	0.00	RX1	O
ATOM	4364	N	HIS	233	32.820	26.371	19.401	1.00	0.00	RX1	N
ATOM	4365	H	HIS	233	33.674	26.690	19.811	1.00	0.00	RX1	H
ATOM	4366	CA	HIS	233	32.772	25.062	18.758	1.00	0.00	RX1	C
ATOM	4367	CB	HIS	233	34.109	24.807	18.050	1.00	0.00	RX1	C
ATOM	4368	CG	HIS	233	34.223	23.411	17.480	1.00	0.00	RX1	C
ATOM	4369	ND1	HIS	233	35.259	22.586	17.734	1.00	0.00	RX1	N
ATOM	4370	HD1	HIS	233	36.081	22.800	18.236	1.00	0.00	RX1	H
ATOM	4371	CD2	HIS	233	33.328	22.758	16.628	1.00	0.00	RX1	C
ATOM	4372	NE2	HIS	233	33.834	21.526	16.381	1.00	0.00	RX1	N
ATOM	4373	CE1	HIS	233	35.022	21.416	17.056	1.00	0.00	RX1	C
ATOM	4374	C	HIS	233	32.507	24.028	19.835	1.00	0.00	RX1	C
ATOM	4375	O	HIS	233	32.921	24.203	20.972	1.00	0.00	RX1	O
ATOM	4376	N	ASN	234	31.855	22.935	19.411	1.00	0.00	RX1	N
ATOM	4377	H	ASN	234	31.429	22.977	18.509	1.00	0.00	RX1	H
ATOM	4378	CA	ASN	234	31.745	21.704	20.207	1.00	0.00	RX1	C
ATOM	4379	CB	ASN	234	31.528	20.519	19.268	1.00	0.00	RX1	C
ATOM	4380	CG	ASN	234	31.560	19.187	19.997	1.00	0.00	RX1	C
ATOM	4381	OD1	ASN	234	31.280	19.045	21.185	1.00	0.00	RX1	O
ATOM	4382	ND2	ASN	234	31.890	18.185	19.166	1.00	0.00	RX1	N
ATOM	4383	HD21	ASN	234	32.076	18.358	18.197	1.00	0.00	RX1	H
ATOM	4384	HD22	ASN	234	31.965	17.217	19.423	1.00	0.00	RX1	H
ATOM	4385	C	ASN	234	32.930	21.441	21.142	1.00	0.00	RX1	C
ATOM	4386	O	ASN	234	32.800	21.250	22.344	1.00	0.00	RX1	O
ATOM	4387	N	GLN	235	34.124	21.470	20.536	1.00	0.00	RX1	N
ATOM	4388	H	GLN	235	34.222	21.774	19.590	1.00	0.00	RX1	H
ATOM	4389	CA	GLN	235	35.287	21.084	21.336	1.00	0.00	RX1	C
ATOM	4390	CB	GLN	235	36.363	20.509	20.430	1.00	0.00	RX1	C

ATOM	4391	CG	GLN	235	36.514	19.016	20.673	1.00	0.00	RX1	C
ATOM	4392	CD	GLN	235	35.167	18.346	20.509	1.00	0.00	RX1	C
ATOM	4393	OE1	GLN	235	34.376	18.222	21.444	1.00	0.00	RX1	O
ATOM	4394	NE2	GLN	235	34.963	17.887	19.267	1.00	0.00	RX1	N
ATOM	4395	HE21	GLN	235	35.714	17.982	18.604	1.00	0.00	RX1	H
ATOM	4396	HE22	GLN	235	34.141	17.406	18.952	1.00	0.00	RX1	H
ATOM	4397	C	GLN	235	35.894	22.098	22.281	1.00	0.00	RX1	C
ATOM	4398	O	GLN	235	36.884	21.831	22.956	1.00	0.00	RX1	O
ATOM	4399	N	CYS	236	35.270	23.271	22.291	1.00	0.00	RX1	N
ATOM	4400	H	CYS	236	34.368	23.413	21.888	1.00	0.00	RX1	H
ATOM	4401	CA	CYS	236	35.844	24.348	23.077	1.00	0.00	RX1	C
ATOM	4402	CB	CYS	236	35.475	25.662	22.428	1.00	0.00	RX1	C
ATOM	4403	SG	CYS	236	35.834	25.637	20.658	1.00	0.00	RX1	S
ATOM	4404	C	CYS	236	35.405	24.321	24.512	1.00	0.00	RX1	C
ATOM	4405	O	CYS	236	34.414	24.931	24.892	1.00	0.00	RX1	O
ATOM	4406	N	ALA	237	36.200	23.595	25.303	1.00	0.00	RX1	N
ATOM	4407	H	ALA	237	37.075	23.251	24.956	1.00	0.00	RX1	H
ATOM	4408	CA	ALA	237	35.963	23.730	26.733	1.00	0.00	RX1	C
ATOM	4409	CB	ALA	237	36.782	22.720	27.528	1.00	0.00	RX1	C
ATOM	4410	C	ALA	237	36.388	25.111	27.176	1.00	0.00	RX1	C
ATOM	4411	O	ALA	237	37.291	25.703	26.586	1.00	0.00	RX1	O
ATOM	4412	N	ALA	238	35.668	25.596	28.202	1.00	0.00	RX1	N
ATOM	4413	H	ALA	238	34.924	25.017	28.550	1.00	0.00	RX1	H
ATOM	4414	CA	ALA	238	35.775	26.982	28.671	1.00	0.00	RX1	C
ATOM	4415	CB	ALA	238	37.210	27.371	29.053	1.00	0.00	RX1	C
ATOM	4416	C	ALA	238	35.196	28.024	27.723	1.00	0.00	RX1	C
ATOM	4417	O	ALA	238	34.243	28.722	28.052	1.00	0.00	RX1	O
ATOM	4418	N	GLY	239	35.803	28.088	26.532	1.00	0.00	RX1	N
ATOM	4419	H	GLY	239	36.569	27.493	26.272	1.00	0.00	RX1	H
ATOM	4420	CA	GLY	239	35.335	29.003	25.500	1.00	0.00	RX1	C
ATOM	4421	C	GLY	239	36.248	28.885	24.298	1.00	0.00	RX1	C
ATOM	4422	O	GLY	239	37.057	27.965	24.208	1.00	0.00	RX1	O
ATOM	4423	N	CYS	240	36.108	29.851	23.382	1.00	0.00	RX1	N
ATOM	4424	H	CYS	240	35.482	30.629	23.439	1.00	0.00	RX1	H
ATOM	4425	CA	CYS	240	36.998	29.823	22.224	1.00	0.00	RX1	C
ATOM	4426	CB	CYS	240	36.485	28.850	21.168	1.00	0.00	RX1	C
ATOM	4427	SG	CYS	240	34.774	29.146	20.673	1.00	0.00	RX1	S
ATOM	4428	C	CYS	240	37.202	31.193	21.633	1.00	0.00	RX1	C
ATOM	4429	O	CYS	240	36.560	32.158	22.030	1.00	0.00	RX1	O
ATOM	4430	N	THR	241	38.107	31.221	20.652	1.00	0.00	RX1	N
ATOM	4431	H	THR	241	38.632	30.413	20.399	1.00	0.00	RX1	H
ATOM	4432	CA	THR	241	38.284	32.445	19.884	1.00	0.00	RX1	C
ATOM	4433	CB	THR	241	39.718	32.319	19.418	1.00	0.00	RX1	C
ATOM	4434	OG1	THR	241	40.401	31.498	20.380	1.00	0.00	RX1	O
ATOM	4435	HG1	THR	241	40.364	30.594	20.080	1.00	0.00	RX1	H
ATOM	4436	CG2	THR	241	40.405	33.674	19.240	1.00	0.00	RX1	C
ATOM	4437	C	THR	241	37.251	32.541	18.770	1.00	0.00	RX1	C
ATOM	4438	O	THR	241	36.415	33.437	18.707	1.00	0.00	RX1	O
ATOM	4439	N	GLY	242	37.325	31.524	17.903	1.00	0.00	RX1	N
ATOM	4440	H	GLY	242	37.933	30.736	18.011	1.00	0.00	RX1	H
ATOM	4441	CA	GLY	242	36.265	31.383	16.916	1.00	0.00	RX1	C
ATOM	4442	C	GLY	242	35.516	30.104	17.212	1.00	0.00	RX1	C
ATOM	4443	O	GLY	242	36.034	29.212	17.872	1.00	0.00	RX1	O
ATOM	4444	N	PRO	243	34.264	30.036	16.714	1.00	0.00	RX1	N
ATOM	4445	CD	PRO	243	33.559	31.085	15.990	1.00	0.00	RX1	C
ATOM	4446	CA	PRO	243	33.471	28.817	16.905	1.00	0.00	RX1	C
ATOM	4447	CB	PRO	243	32.056	29.346	16.666	1.00	0.00	RX1	C
ATOM	4448	CG	PRO	243	32.227	30.444	15.614	1.00	0.00	RX1	C
ATOM	4449	C	PRO	243	33.859	27.703	15.936	1.00	0.00	RX1	C
ATOM	4450	O	PRO	243	33.043	27.185	15.184	1.00	0.00	RX1	O
ATOM	4451	N	ARG	244	35.149	27.354	15.966	1.00	0.00	RX1	N

ATOM	4452	H	ARG	244	35.817	27.678	16.643	1.00	0.00	RX1	H
ATOM	4453	CA	ARG	244	35.651	26.458	14.935	1.00	0.00	RX1	C
ATOM	4454	CB	ARG	244	36.279	27.321	13.835	1.00	0.00	RX1	C
ATOM	4455	CG	ARG	244	36.193	26.901	12.359	1.00	0.00	RX1	C
ATOM	4456	CD	ARG	244	36.562	25.455	12.001	1.00	0.00	RX1	C
ATOM	4457	NE	ARG	244	37.971	25.047	11.819	1.00	0.00	RX1	N
ATOM	4458	HE	ARG	244	38.023	24.255	11.201	1.00	0.00	RX1	H
ATOM	4459	CZ	ARG	244	39.128	25.379	12.495	1.00	0.00	RX1	C
ATOM	4460	NH1	ARG	244	39.264	26.499	13.244	1.00	0.00	RX1	N
ATOM	4461	HH11	ARG	244	40.080	26.727	13.801	1.00	0.00	RX1	H
ATOM	4462	HH12	ARG	244	38.572	27.221	13.348	1.00	0.00	RX1	H
ATOM	4463	NH2	ARG	244	40.159	24.507	12.418	1.00	0.00	RX1	N
ATOM	4464	HH21	ARG	244	41.030	24.708	12.888	1.00	0.00	RX1	H
ATOM	4465	HH22	ARG	244	40.115	23.604	11.960	1.00	0.00	RX1	H
ATOM	4466	C	ARG	244	36.632	25.490	15.572	1.00	0.00	RX1	C
ATOM	4467	O	ARG	244	37.384	25.847	16.465	1.00	0.00	RX1	O
ATOM	4468	N	GLU	245	36.583	24.249	15.063	1.00	0.00	RX1	N
ATOM	4469	H	GLU	245	35.843	24.050	14.423	1.00	0.00	RX1	H
ATOM	4470	CA	GLU	245	37.523	23.165	15.379	1.00	0.00	RX1	C
ATOM	4471	CB	GLU	245	37.975	22.525	14.070	1.00	0.00	RX1	C
ATOM	4472	CG	GLU	245	38.955	21.365	14.182	1.00	0.00	RX1	C
ATOM	4473	CD	GLU	245	39.409	21.011	12.785	1.00	0.00	RX1	C
ATOM	4474	OE1	GLU	245	38.909	20.042	12.232	1.00	0.00	RX1	O
ATOM	4475	OE2	GLU	245	40.284	21.683	12.246	1.00	0.00	RX1	O
ATOM	4476	C	GLU	245	38.698	23.426	16.325	1.00	0.00	RX1	C
ATOM	4477	O	GLU	245	38.728	22.943	17.448	1.00	0.00	RX1	O
ATOM	4478	N	SER	246	39.689	24.170	15.815	1.00	0.00	RX1	N
ATOM	4479	H	SER	246	39.627	24.714	14.983	1.00	0.00	RX1	H
ATOM	4480	CA	SER	246	40.932	24.265	16.575	1.00	0.00	RX1	C
ATOM	4481	CB	SER	246	42.045	24.454	15.559	1.00	0.00	RX1	C
ATOM	4482	OG	SER	246	41.525	25.239	14.480	1.00	0.00	RX1	O
ATOM	4483	HG	SER	246	41.647	26.139	14.800	1.00	0.00	RX1	H
ATOM	4484	C	SER	246	40.975	25.304	17.675	1.00	0.00	RX1	C
ATOM	4485	O	SER	246	41.835	25.273	18.545	1.00	0.00	RX1	O
ATOM	4486	N	ASP	247	40.046	26.257	17.577	1.00	0.00	RX1	N
ATOM	4487	H	ASP	247	39.233	26.180	16.999	1.00	0.00	RX1	H
ATOM	4488	CA	ASP	247	40.353	27.567	18.149	1.00	0.00	RX1	C
ATOM	4489	CB	ASP	247	39.748	28.657	17.249	1.00	0.00	RX1	C
ATOM	4490	CG	ASP	247	39.798	28.282	15.765	1.00	0.00	RX1	C
ATOM	4491	OD1	ASP	247	40.785	27.726	15.285	1.00	0.00	RX1	O
ATOM	4492	OD2	ASP	247	38.824	28.531	15.063	1.00	0.00	RX1	O
ATOM	4493	C	ASP	247	39.936	27.722	19.605	1.00	0.00	RX1	C
ATOM	4494	O	ASP	247	39.497	28.777	20.061	1.00	0.00	RX1	O
ATOM	4495	N	CYS	248	40.054	26.589	20.309	1.00	0.00	RX1	N
ATOM	4496	H	CYS	248	40.626	25.853	19.949	1.00	0.00	RX1	H
ATOM	4497	CA	CYS	248	39.542	26.460	21.668	1.00	0.00	RX1	C
ATOM	4498	CB	CYS	248	39.365	24.970	21.949	1.00	0.00	RX1	C
ATOM	4499	SG	CYS	248	38.545	24.111	20.582	1.00	0.00	RX1	S
ATOM	4500	C	CYS	248	40.468	27.085	22.687	1.00	0.00	RX1	C
ATOM	4501	O	CYS	248	41.654	27.250	22.443	1.00	0.00	RX1	O
ATOM	4502	N	LEU	249	39.895	27.392	23.861	1.00	0.00	RX1	N
ATOM	4503	H	LEU	249	38.914	27.277	24.032	1.00	0.00	RX1	H
ATOM	4504	CA	LEU	249	40.813	27.724	24.952	1.00	0.00	RX1	C
ATOM	4505	CB	LEU	249	40.092	28.519	26.039	1.00	0.00	RX1	C
ATOM	4506	CG	LEU	249	39.537	29.857	25.551	1.00	0.00	RX1	C
ATOM	4507	CD1	LEU	249	38.805	30.599	26.671	1.00	0.00	RX1	C
ATOM	4508	CD2	LEU	249	40.614	30.723	24.895	1.00	0.00	RX1	C
ATOM	4509	C	LEU	249	41.436	26.471	25.548	1.00	0.00	RX1	C
ATOM	4510	O	LEU	249	42.648	26.284	25.631	1.00	0.00	RX1	O
ATOM	4511	N	VAL	250	40.522	25.579	25.946	1.00	0.00	RX1	N
ATOM	4512	H	VAL	250	39.529	25.714	25.884	1.00	0.00	RX1	H

ATOM	4513	CA	VAL	250	41.001	24.270	26.369	1.00	0.00	RX1	C
ATOM	4514	CB	VAL	250	41.009	24.137	27.893	1.00	0.00	RX1	C
ATOM	4515	CG1	VAL	250	42.232	24.826	28.499	1.00	0.00	RX1	C
ATOM	4516	CG2	VAL	250	39.719	24.665	28.505	1.00	0.00	RX1	C
ATOM	4517	C	VAL	250	40.205	23.176	25.691	1.00	0.00	RX1	C
ATOM	4518	O	VAL	250	39.110	23.394	25.187	1.00	0.00	RX1	O
ATOM	4519	N	CYS	251	40.828	21.995	25.642	1.00	0.00	RX1	N
ATOM	4520	H	CYS	251	41.688	21.821	26.127	1.00	0.00	RX1	H
ATOM	4521	CA	CYS	251	40.239	21.010	24.736	1.00	0.00	RX1	C
ATOM	4522	CB	CYS	251	41.384	20.313	24.008	1.00	0.00	RX1	C
ATOM	4523	SG	CYS	251	42.752	21.475	23.765	1.00	0.00	RX1	S
ATOM	4524	C	CYS	251	39.284	20.042	25.408	1.00	0.00	RX1	C
ATOM	4525	O	CYS	251	39.625	19.405	26.401	1.00	0.00	RX1	O
ATOM	4526	N	ARG	252	38.083	19.935	24.821	1.00	0.00	RX1	N
ATOM	4527	H	ARG	252	37.837	20.518	24.042	1.00	0.00	RX1	H
ATOM	4528	CA	ARG	252	37.157	18.917	25.327	1.00	0.00	RX1	C
ATOM	4529	CB	ARG	252	35.707	19.187	24.926	1.00	0.00	RX1	C
ATOM	4530	CG	ARG	252	35.051	20.293	25.743	1.00	0.00	RX1	C
ATOM	4531	CD	ARG	252	33.553	20.456	25.498	1.00	0.00	RX1	C
ATOM	4532	NE	ARG	252	32.842	19.238	25.877	1.00	0.00	RX1	N
ATOM	4533	HE	ARG	252	32.887	18.994	26.852	1.00	0.00	RX1	H
ATOM	4534	CZ	ARG	252	32.201	18.538	24.896	1.00	0.00	RX1	C
ATOM	4535	NH1	ARG	252	32.172	19.039	23.647	1.00	0.00	RX1	N
ATOM	4536	HH11	ARG	252	31.747	18.602	22.842	1.00	0.00	RX1	H
ATOM	4537	HH12	ARG	252	32.583	19.930	23.412	1.00	0.00	RX1	H
ATOM	4538	NH2	ARG	252	31.613	17.362	25.197	1.00	0.00	RX1	N
ATOM	4539	HH21	ARG	252	31.116	16.806	24.522	1.00	0.00	RX1	H
ATOM	4540	HH22	ARG	252	31.631	16.979	26.126	1.00	0.00	RX1	H
ATOM	4541	C	ARG	252	37.470	17.489	24.919	1.00	0.00	RX1	C
ATOM	4542	O	ARG	252	37.023	16.540	25.554	1.00	0.00	RX1	O
ATOM	4543	N	LYS	253	38.188	17.383	23.787	1.00	0.00	RX1	N
ATOM	4544	H	LYS	253	38.665	18.135	23.333	1.00	0.00	RX1	H
ATOM	4545	CA	LYS	253	38.225	16.104	23.082	1.00	0.00	RX1	C
ATOM	4546	CB	LYS	253	36.891	15.857	22.382	1.00	0.00	RX1	C
ATOM	4547	CG	LYS	253	36.051	14.776	23.055	1.00	0.00	RX1	C
ATOM	4548	CD	LYS	253	34.595	14.856	22.616	1.00	0.00	RX1	C
ATOM	4549	CE	LYS	253	34.422	15.055	21.108	1.00	0.00	RX1	C
ATOM	4550	NZ	LYS	253	34.650	13.845	20.311	1.00	0.00	RX1	N
ATOM	4551	HZ1	LYS	253	34.235	13.985	19.366	1.00	0.00	RX1	H
ATOM	4552	HZ2	LYS	253	35.670	13.646	20.166	1.00	0.00	RX1	H
ATOM	4553	HZ3	LYS	253	34.219	12.997	20.712	1.00	0.00	RX1	H
ATOM	4554	C	LYS	253	39.392	16.032	22.114	1.00	0.00	RX1	C
ATOM	4555	O	LYS	253	40.248	16.905	22.125	1.00	0.00	RX1	O
ATOM	4556	N	PHE	254	39.362	14.959	21.295	1.00	0.00	RX1	N
ATOM	4557	H	PHE	254	38.606	14.309	21.359	1.00	0.00	RX1	H
ATOM	4558	CA	PHE	254	40.411	14.619	20.326	1.00	0.00	RX1	C
ATOM	4559	CB	PHE	254	39.872	13.628	19.288	1.00	0.00	RX1	C
ATOM	4560	CG	PHE	254	39.713	12.232	19.863	1.00	0.00	RX1	C
ATOM	4561	CD1	PHE	254	39.830	11.137	19.018	1.00	0.00	RX1	C
ATOM	4562	CD2	PHE	254	39.450	12.015	21.211	1.00	0.00	RX1	C
ATOM	4563	CE1	PHE	254	39.676	9.845	19.507	1.00	0.00	RX1	C
ATOM	4564	CE2	PHE	254	39.294	10.726	21.704	1.00	0.00	RX1	C
ATOM	4565	CZ	PHE	254	39.402	9.637	20.851	1.00	0.00	RX1	C
ATOM	4566	C	PHE	254	41.147	15.763	19.648	1.00	0.00	RX1	C
ATOM	4567	O	PHE	254	40.665	16.446	18.746	1.00	0.00	RX1	O
ATOM	4568	N	ARG	255	42.373	15.924	20.146	1.00	0.00	RX1	N
ATOM	4569	H	ARG	255	42.777	15.204	20.712	1.00	0.00	RX1	H
ATOM	4570	CA	ARG	255	43.197	17.012	19.648	1.00	0.00	RX1	C
ATOM	4571	CB	ARG	255	44.014	17.602	20.801	1.00	0.00	RX1	C
ATOM	4572	CG	ARG	255	44.448	19.056	20.593	1.00	0.00	RX1	C
ATOM	4573	CD	ARG	255	45.935	19.235	20.900	1.00	0.00	RX1	C

ATOM	4574	NE	ARG	255	46.680	18.377	19.989	1.00	0.00	RX1	N
ATOM	4575	HE	ARG	255	46.231	18.123	19.122	1.00	0.00	RX1	H
ATOM	4576	CZ	ARG	255	47.954	17.960	20.203	1.00	0.00	RX1	C
ATOM	4577	NH1	ARG	255	48.650	18.393	21.266	1.00	0.00	RX1	N
ATOM	4578	HH11	ARG	255	49.584	18.030	21.426	1.00	0.00	RX1	H
ATOM	4579	HH12	ARG	255	48.325	19.091	21.912	1.00	0.00	RX1	H
ATOM	4580	NH2	ARG	255	48.511	17.111	19.324	1.00	0.00	RX1	N
ATOM	4581	HH21	ARG	255	49.494	16.874	19.412	1.00	0.00	RX1	H
ATOM	4582	HH22	ARG	255	47.991	16.727	18.557	1.00	0.00	RX1	H
ATOM	4583	C	ARG	255	44.081	16.553	18.498	1.00	0.00	RX1	C
ATOM	4584	O	ARG	255	45.300	16.441	18.602	1.00	0.00	RX1	O
ATOM	4585	N	ASP	256	43.387	16.261	17.389	1.00	0.00	RX1	N
ATOM	4586	H	ASP	256	42.425	16.529	17.366	1.00	0.00	RX1	H
ATOM	4587	CA	ASP	256	44.083	15.801	16.188	1.00	0.00	RX1	C
ATOM	4588	CB	ASP	256	43.095	15.517	15.064	1.00	0.00	RX1	C
ATOM	4589	CG	ASP	256	43.878	14.979	13.890	1.00	0.00	RX1	C
ATOM	4590	OD1	ASP	256	44.902	14.352	14.114	1.00	0.00	RX1	O
ATOM	4591	OD2	ASP	256	43.488	15.170	12.742	1.00	0.00	RX1	O
ATOM	4592	C	ASP	256	45.125	16.776	15.685	1.00	0.00	RX1	C
ATOM	4593	O	ASP	256	44.803	17.865	15.216	1.00	0.00	RX1	O
ATOM	4594	N	GLU	257	46.393	16.350	15.831	1.00	0.00	RX1	N
ATOM	4595	H	GLU	257	46.531	15.383	16.061	1.00	0.00	RX1	H
ATOM	4596	CA	GLU	257	47.510	17.254	15.543	1.00	0.00	RX1	C
ATOM	4597	CB	GLU	257	47.863	17.149	14.057	1.00	0.00	RX1	C
ATOM	4598	CG	GLU	257	48.404	15.730	13.836	1.00	0.00	RX1	C
ATOM	4599	CD	GLU	257	48.523	15.380	12.368	1.00	0.00	RX1	C
ATOM	4600	OE1	GLU	257	47.848	15.978	11.538	1.00	0.00	RX1	O
ATOM	4601	OE2	GLU	257	49.237	14.444	12.029	1.00	0.00	RX1	O
ATOM	4602	C	GLU	257	47.284	18.650	16.127	1.00	0.00	RX1	C
ATOM	4603	O	GLU	257	47.105	18.760	17.332	1.00	0.00	RX1	O
ATOM	4604	N	ALA	258	47.241	19.698	15.291	1.00	0.00	RX1	N
ATOM	4605	H	ALA	258	47.249	19.605	14.297	1.00	0.00	RX1	H
ATOM	4606	CA	ALA	258	46.999	20.994	15.934	1.00	0.00	RX1	C
ATOM	4607	CB	ALA	258	47.859	22.079	15.285	1.00	0.00	RX1	C
ATOM	4608	C	ALA	258	45.545	21.461	15.940	1.00	0.00	RX1	C
ATOM	4609	O	ALA	258	45.247	22.646	15.871	1.00	0.00	RX1	O
ATOM	4610	N	THR	259	44.625	20.486	15.977	1.00	0.00	RX1	N
ATOM	4611	H	THR	259	44.842	19.525	16.151	1.00	0.00	RX1	H
ATOM	4612	CA	THR	259	43.215	20.856	15.865	1.00	0.00	RX1	C
ATOM	4613	CB	THR	259	42.753	20.713	14.398	1.00	0.00	RX1	C
ATOM	4614	OG1	THR	259	42.978	19.390	13.890	1.00	0.00	RX1	O
ATOM	4615	HG1	THR	259	43.740	19.040	14.357	1.00	0.00	RX1	H
ATOM	4616	CG2	THR	259	43.391	21.727	13.445	1.00	0.00	RX1	C
ATOM	4617	C	THR	259	42.357	20.081	16.849	1.00	0.00	RX1	C
ATOM	4618	O	THR	259	42.701	18.972	17.231	1.00	0.00	RX1	O
ATOM	4619	N	CYS	260	41.222	20.674	17.238	1.00	0.00	RX1	N
ATOM	4620	H	CYS	260	40.888	21.568	16.945	1.00	0.00	RX1	H
ATOM	4621	CA	CYS	260	40.336	19.858	18.063	1.00	0.00	RX1	C
ATOM	4622	CB	CYS	260	39.937	20.649	19.304	1.00	0.00	RX1	C
ATOM	4623	SG	CYS	260	41.383	21.309	20.170	1.00	0.00	RX1	S
ATOM	4624	C	CYS	260	39.161	19.325	17.265	1.00	0.00	RX1	C
ATOM	4625	O	CYS	260	38.110	19.940	17.122	1.00	0.00	RX1	O
ATOM	4626	N	LYS	261	39.424	18.140	16.699	1.00	0.00	RX1	N
ATOM	4627	H	LYS	261	40.188	17.612	17.075	1.00	0.00	RX1	H
ATOM	4628	CA	LYS	261	38.394	17.487	15.892	1.00	0.00	RX1	C
ATOM	4629	CB	LYS	261	39.002	16.499	14.898	1.00	0.00	RX1	C
ATOM	4630	CG	LYS	261	40.039	17.230	14.041	1.00	0.00	RX1	C
ATOM	4631	CD	LYS	261	40.122	16.721	12.601	1.00	0.00	RX1	C
ATOM	4632	CE	LYS	261	41.269	17.357	11.806	1.00	0.00	RX1	C
ATOM	4633	NZ	LYS	261	41.299	18.811	12.010	1.00	0.00	RX1	N
ATOM	4634	HZ1	LYS	261	41.966	19.275	11.372	1.00	0.00	RX1	H

ATOM	4635	HZ2	LYS	261	40.364	19.253	11.884	1.00	0.00	RX1	H
ATOM	4636	HZ3	LYS	261	41.618	19.016	12.978	1.00	0.00	RX1	H
ATOM	4637	C	LYS	261	37.316	16.906	16.787	1.00	0.00	RX1	C
ATOM	4638	O	LYS	261	37.332	17.070	18.001	1.00	0.00	RX1	O
ATOM	4639	N	ASP	262	36.375	16.190	16.153	1.00	0.00	RX1	N
ATOM	4640	H	ASP	262	36.378	16.045	15.165	1.00	0.00	RX1	H
ATOM	4641	CA	ASP	262	35.537	15.404	17.049	1.00	0.00	RX1	C
ATOM	4642	CB	ASP	262	34.114	15.164	16.540	1.00	0.00	RX1	C
ATOM	4643	CG	ASP	262	33.278	14.725	17.732	1.00	0.00	RX1	C
ATOM	4644	OD1	ASP	262	32.750	15.563	18.452	1.00	0.00	RX1	O
ATOM	4645	OD2	ASP	262	33.195	13.538	18.012	1.00	0.00	RX1	O
ATOM	4646	C	ASP	262	36.204	14.118	17.486	1.00	0.00	RX1	C
ATOM	4647	O	ASP	262	36.540	13.959	18.653	1.00	0.00	RX1	O
ATOM	4648	N	THR	263	36.393	13.232	16.508	1.00	0.00	RX1	N
ATOM	4649	H	THR	263	36.162	13.372	15.545	1.00	0.00	RX1	H
ATOM	4650	CA	THR	263	37.048	11.965	16.797	1.00	0.00	RX1	C
ATOM	4651	CB	THR	263	35.926	10.962	17.090	1.00	0.00	RX1	C
ATOM	4652	OG1	THR	263	35.039	11.513	18.079	1.00	0.00	RX1	O
ATOM	4653	HG1	THR	263	34.410	12.060	17.606	1.00	0.00	RX1	H
ATOM	4654	CG2	THR	263	36.446	9.594	17.541	1.00	0.00	RX1	C
ATOM	4655	C	THR	263	37.900	11.636	15.581	1.00	0.00	RX1	C
ATOM	4656	O	THR	263	37.621	12.152	14.504	1.00	0.00	RX1	O
ATOM	4657	N	CYS	264	38.950	10.821	15.789	1.00	0.00	RX1	N
ATOM	4658	H	CYS	264	39.144	10.436	16.690	1.00	0.00	RX1	H
ATOM	4659	CA	CYS	264	39.869	10.479	14.692	1.00	0.00	RX1	C
ATOM	4660	CB	CYS	264	40.799	9.355	15.141	1.00	0.00	RX1	C
ATOM	4661	SG	CYS	264	41.478	9.618	16.791	1.00	0.00	RX1	S
ATOM	4662	C	CYS	264	39.229	10.064	13.375	1.00	0.00	RX1	C
ATOM	4663	O	CYS	264	38.508	9.076	13.313	1.00	0.00	RX1	O
ATOM	4664	N	PRO	265	39.549	10.832	12.304	1.00	0.00	RX1	N
ATOM	4665	CD	PRO	265	40.237	12.116	12.337	1.00	0.00	RX1	C
ATOM	4666	CA	PRO	265	39.190	10.396	10.948	1.00	0.00	RX1	C
ATOM	4667	CB	PRO	265	39.809	11.496	10.075	1.00	0.00	RX1	C
ATOM	4668	CG	PRO	265	39.924	12.724	10.977	1.00	0.00	RX1	C
ATOM	4669	C	PRO	265	39.722	9.008	10.598	1.00	0.00	RX1	C
ATOM	4670	O	PRO	265	40.927	8.775	10.571	1.00	0.00	RX1	O
ATOM	4671	N	PRO	266	38.769	8.082	10.336	1.00	0.00	RX1	N
ATOM	4672	CD	PRO	266	37.329	8.305	10.324	1.00	0.00	RX1	C
ATOM	4673	CA	PRO	266	39.138	6.686	10.067	1.00	0.00	RX1	C
ATOM	4674	CB	PRO	266	37.782	6.025	9.792	1.00	0.00	RX1	C
ATOM	4675	CG	PRO	266	36.748	6.907	10.492	1.00	0.00	RX1	C
ATOM	4676	C	PRO	266	40.115	6.515	8.916	1.00	0.00	RX1	C
ATOM	4677	O	PRO	266	40.218	7.352	8.029	1.00	0.00	RX1	O
ATOM	4678	N	LEU	267	40.843	5.387	8.974	1.00	0.00	RX1	N
ATOM	4679	H	LEU	267	40.715	4.730	9.716	1.00	0.00	RX1	H
ATOM	4680	CA	LEU	267	41.831	5.131	7.920	1.00	0.00	RX1	C
ATOM	4681	CB	LEU	267	42.709	3.934	8.285	1.00	0.00	RX1	C
ATOM	4682	CG	LEU	267	44.034	4.290	8.963	1.00	0.00	RX1	C
ATOM	4683	CD1	LEU	267	43.863	5.031	10.291	1.00	0.00	RX1	C
ATOM	4684	CD2	LEU	267	44.907	3.046	9.114	1.00	0.00	RX1	C
ATOM	4685	C	LEU	267	41.234	4.910	6.539	1.00	0.00	RX1	C
ATOM	4686	O	LEU	267	41.750	5.351	5.518	1.00	0.00	RX1	O
ATOM	4687	N	MET	268	40.105	4.193	6.560	1.00	0.00	RX1	N
ATOM	4688	H	MET	268	39.611	3.963	7.396	1.00	0.00	RX1	H
ATOM	4689	CA	MET	268	39.387	4.042	5.303	1.00	0.00	RX1	C
ATOM	4690	CB	MET	268	39.121	2.567	5.003	1.00	0.00	RX1	C
ATOM	4691	CG	MET	268	40.381	1.699	5.058	1.00	0.00	RX1	C
ATOM	4692	SD	MET	268	41.661	2.200	3.895	1.00	0.00	RX1	S
ATOM	4693	CE	MET	268	40.846	1.690	2.373	1.00	0.00	RX1	C
ATOM	4694	C	MET	268	38.088	4.800	5.418	1.00	0.00	RX1	C
ATOM	4695	O	MET	268	37.581	4.992	6.517	1.00	0.00	RX1	O

ATOM	4696	N	LEU	269	37.594	5.234	4.260	1.00	0.00	RX1	N
ATOM	4697	H	LEU	269	37.979	4.929	3.388	1.00	0.00	RX1	H
ATOM	4698	CA	LEU	269	36.271	5.846	4.227	1.00	0.00	RX1	C
ATOM	4699	CB	LEU	269	36.259	7.323	3.823	1.00	0.00	RX1	C
ATOM	4700	CG	LEU	269	37.260	8.308	4.432	1.00	0.00	RX1	C
ATOM	4701	CD1	LEU	269	37.421	8.206	5.951	1.00	0.00	RX1	C
ATOM	4702	CD2	LEU	269	38.559	8.317	3.637	1.00	0.00	RX1	C
ATOM	4703	C	LEU	269	35.475	5.094	3.187	1.00	0.00	RX1	C
ATOM	4704	O	LEU	269	35.918	4.066	2.692	1.00	0.00	RX1	O
ATOM	4705	N	TYR	270	34.312	5.644	2.839	1.00	0.00	RX1	N
ATOM	4706	H	TYR	270	33.937	6.523	3.138	1.00	0.00	RX1	H
ATOM	4707	CA	TYR	270	33.545	4.963	1.812	1.00	0.00	RX1	C
ATOM	4708	CB	TYR	270	32.540	4.094	2.543	1.00	0.00	RX1	C
ATOM	4709	CG	TYR	270	31.802	3.119	1.664	1.00	0.00	RX1	C
ATOM	4710	CD1	TYR	270	32.366	1.886	1.365	1.00	0.00	RX1	C
ATOM	4711	CE1	TYR	270	31.651	0.960	0.617	1.00	0.00	RX1	C
ATOM	4712	CD2	TYR	270	30.535	3.440	1.198	1.00	0.00	RX1	C
ATOM	4713	CE2	TYR	270	29.811	2.509	0.469	1.00	0.00	RX1	C
ATOM	4714	CZ	TYR	270	30.372	1.274	0.174	1.00	0.00	RX1	C
ATOM	4715	OH	TYR	270	29.655	0.372	-0.581	1.00	0.00	RX1	O
ATOM	4716	HH	TYR	270	30.212	0.052	-1.289	1.00	0.00	RX1	H
ATOM	4717	C	TYR	270	32.905	6.008	0.935	1.00	0.00	RX1	C
ATOM	4718	O	TYR	270	32.478	7.048	1.415	1.00	0.00	RX1	O
ATOM	4719	N	ASN	271	32.868	5.702	-0.359	1.00	0.00	RX1	N
ATOM	4720	H	ASN	271	33.283	4.853	-0.672	1.00	0.00	RX1	H
ATOM	4721	CA	ASN	271	32.446	6.742	-1.285	1.00	0.00	RX1	C
ATOM	4722	CB	ASN	271	32.988	6.464	-2.676	1.00	0.00	RX1	C
ATOM	4723	CG	ASN	271	33.123	7.795	-3.365	1.00	0.00	RX1	C
ATOM	4724	OD1	ASN	271	32.174	8.329	-3.939	1.00	0.00	RX1	O
ATOM	4725	ND2	ASN	271	34.363	8.293	-3.283	1.00	0.00	RX1	N
ATOM	4726	HD21	ASN	271	35.016	7.786	-2.691	1.00	0.00	RX1	H
ATOM	4727	HD22	ASN	271	34.730	9.117	-3.707	1.00	0.00	RX1	H
ATOM	4728	C	ASN	271	30.952	6.952	-1.359	1.00	0.00	RX1	C
ATOM	4729	O	ASN	271	30.204	6.061	-1.753	1.00	0.00	RX1	O
ATOM	4730	N	PRO	272	30.522	8.192	-1.022	1.00	0.00	RX1	N
ATOM	4731	CD	PRO	272	31.339	9.314	-0.575	1.00	0.00	RX1	C
ATOM	4732	CA	PRO	272	29.093	8.512	-1.109	1.00	0.00	RX1	C
ATOM	4733	CB	PRO	272	29.041	9.964	-0.615	1.00	0.00	RX1	C
ATOM	4734	CG	PRO	272	30.448	10.523	-0.825	1.00	0.00	RX1	C
ATOM	4735	C	PRO	272	28.509	8.314	-2.498	1.00	0.00	RX1	C
ATOM	4736	O	PRO	272	27.334	8.008	-2.676	1.00	0.00	RX1	O
ATOM	4737	N	THR	273	29.383	8.508	-3.487	1.00	0.00	RX1	N
ATOM	4738	H	THR	273	30.343	8.734	-3.335	1.00	0.00	RX1	H
ATOM	4739	CA	THR	273	28.938	8.311	-4.856	1.00	0.00	RX1	C
ATOM	4740	CB	THR	273	29.724	9.337	-5.643	1.00	0.00	RX1	C
ATOM	4741	OG1	THR	273	30.344	10.214	-4.686	1.00	0.00	RX1	O
ATOM	4742	HG1	THR	273	31.207	9.828	-4.539	1.00	0.00	RX1	H
ATOM	4743	CG2	THR	273	28.848	10.113	-6.627	1.00	0.00	RX1	C
ATOM	4744	C	THR	273	29.087	6.872	-5.320	1.00	0.00	RX1	C
ATOM	4745	O	THR	273	28.119	6.174	-5.591	1.00	0.00	RX1	O
ATOM	4746	N	THR	274	30.357	6.457	-5.392	1.00	0.00	RX1	N
ATOM	4747	H	THR	274	31.132	7.040	-5.144	1.00	0.00	RX1	H
ATOM	4748	CA	THR	274	30.651	5.194	-6.061	1.00	0.00	RX1	C
ATOM	4749	CB	THR	274	31.936	5.530	-6.779	1.00	0.00	RX1	C
ATOM	4750	OG1	THR	274	31.988	6.965	-6.850	1.00	0.00	RX1	O
ATOM	4751	HG1	THR	274	32.785	7.177	-7.321	1.00	0.00	RX1	H
ATOM	4752	CG2	THR	274	32.044	4.894	-8.167	1.00	0.00	RX1	C
ATOM	4753	C	THR	274	30.712	3.956	-5.183	1.00	0.00	RX1	C
ATOM	4754	O	THR	274	31.144	2.895	-5.619	1.00	0.00	RX1	O
ATOM	4755	N	TYR	275	30.271	4.133	-3.923	1.00	0.00	RX1	N
ATOM	4756	H	TYR	275	29.969	5.032	-3.603	1.00	0.00	RX1	H

ATOM	4757	CA	TYR	275	30.132	3.002	-2.997	1.00	0.00	RX1	C
ATOM	4758	CB	TYR	275	28.867	2.215	-3.325	1.00	0.00	RX1	C
ATOM	4759	CG	TYR	275	27.707	3.170	-3.247	1.00	0.00	RX1	C
ATOM	4760	CD1	TYR	275	27.649	4.086	-2.205	1.00	0.00	RX1	C
ATOM	4761	CE1	TYR	275	26.595	4.983	-2.132	1.00	0.00	RX1	C
ATOM	4762	CD2	TYR	275	26.708	3.141	-4.211	1.00	0.00	RX1	C
ATOM	4763	CE2	TYR	275	25.645	4.030	-4.128	1.00	0.00	RX1	C
ATOM	4764	CZ	TYR	275	25.585	4.946	-3.085	1.00	0.00	RX1	C
ATOM	4765	OH	TYR	275	24.505	5.807	-2.994	1.00	0.00	RX1	O
ATOM	4766	HH	TYR	275	23.842	5.483	-3.598	1.00	0.00	RX1	H
ATOM	4767	C	TYR	275	31.333	2.076	-2.869	1.00	0.00	RX1	C
ATOM	4768	O	TYR	275	31.232	0.858	-2.771	1.00	0.00	RX1	O
ATOM	4769	N	GLN	276	32.495	2.732	-2.878	1.00	0.00	RX1	N
ATOM	4770	H	GLN	276	32.541	3.724	-2.784	1.00	0.00	RX1	H
ATOM	4771	CA	GLN	276	33.732	1.967	-2.809	1.00	0.00	RX1	C
ATOM	4772	CB	GLN	276	34.551	2.309	-4.052	1.00	0.00	RX1	C
ATOM	4773	CG	GLN	276	35.069	1.076	-4.790	1.00	0.00	RX1	C
ATOM	4774	CD	GLN	276	33.960	0.056	-4.976	1.00	0.00	RX1	C
ATOM	4775	OE1	GLN	276	34.131	-1.109	-4.639	1.00	0.00	RX1	O
ATOM	4776	NE2	GLN	276	32.823	0.532	-5.523	1.00	0.00	RX1	N
ATOM	4777	HE21	GLN	276	32.660	1.488	-5.781	1.00	0.00	RX1	H
ATOM	4778	HE22	GLN	276	32.036	-0.071	-5.650	1.00	0.00	RX1	H
ATOM	4779	C	GLN	276	34.447	2.291	-1.519	1.00	0.00	RX1	C
ATOM	4780	O	GLN	276	34.010	3.175	-0.798	1.00	0.00	RX1	O
ATOM	4781	N	MET	277	35.521	1.551	-1.229	1.00	0.00	RX1	N
ATOM	4782	H	MET	277	35.945	0.914	-1.870	1.00	0.00	RX1	H
ATOM	4783	CA	MET	277	36.208	1.904	0.011	1.00	0.00	RX1	C
ATOM	4784	CB	MET	277	36.706	0.638	0.709	1.00	0.00	RX1	C
ATOM	4785	CG	MET	277	37.027	0.851	2.189	1.00	0.00	RX1	C
ATOM	4786	SD	MET	277	35.563	1.154	3.194	1.00	0.00	RX1	S
ATOM	4787	CE	MET	277	34.850	-0.497	3.121	1.00	0.00	RX1	C
ATOM	4788	C	MET	277	37.341	2.882	-0.244	1.00	0.00	RX1	C
ATOM	4789	O	MET	277	38.370	2.532	-0.812	1.00	0.00	RX1	O
ATOM	4790	N	ASP	278	37.081	4.127	0.164	1.00	0.00	RX1	N
ATOM	4791	H	ASP	278	36.285	4.303	0.740	1.00	0.00	RX1	H
ATOM	4792	CA	ASP	278	38.067	5.180	-0.077	1.00	0.00	RX1	C
ATOM	4793	CB	ASP	278	37.446	6.578	0.019	1.00	0.00	RX1	C
ATOM	4794	CG	ASP	278	36.301	6.802	-0.951	1.00	0.00	RX1	C
ATOM	4795	OD1	ASP	278	36.260	6.190	-2.016	1.00	0.00	RX1	O
ATOM	4796	OD2	ASP	278	35.440	7.621	-0.645	1.00	0.00	RX1	O
ATOM	4797	C	ASP	278	39.205	5.108	0.923	1.00	0.00	RX1	C
ATOM	4798	O	ASP	278	39.110	4.463	1.961	1.00	0.00	RX1	O
ATOM	4799	N	VAL	279	40.294	5.807	0.588	1.00	0.00	RX1	N
ATOM	4800	H	VAL	279	40.311	6.426	-0.197	1.00	0.00	RX1	H
ATOM	4801	CA	VAL	279	41.414	5.793	1.527	1.00	0.00	RX1	C
ATOM	4802	CB	VAL	279	42.638	5.137	0.868	1.00	0.00	RX1	C
ATOM	4803	CG1	VAL	279	43.078	5.898	-0.384	1.00	0.00	RX1	C
ATOM	4804	CG2	VAL	279	43.781	4.915	1.862	1.00	0.00	RX1	C
ATOM	4805	C	VAL	279	41.710	7.192	2.047	1.00	0.00	RX1	C
ATOM	4806	O	VAL	279	41.611	8.171	1.317	1.00	0.00	RX1	O
ATOM	4807	N	ASN	280	42.038	7.252	3.344	1.00	0.00	RX1	N
ATOM	4808	H	ASN	280	42.177	6.427	3.898	1.00	0.00	RX1	H
ATOM	4809	CA	ASN	280	42.264	8.559	3.962	1.00	0.00	RX1	C
ATOM	4810	CB	ASN	280	41.647	8.555	5.353	1.00	0.00	RX1	C
ATOM	4811	CG	ASN	280	41.786	9.904	6.008	1.00	0.00	RX1	C
ATOM	4812	OD1	ASN	280	42.166	10.904	5.406	1.00	0.00	RX1	O
ATOM	4813	ND2	ASN	280	41.476	9.860	7.311	1.00	0.00	RX1	N
ATOM	4814	HD21	ASN	280	41.065	9.028	7.695	1.00	0.00	RX1	H
ATOM	4815	HD22	ASN	280	41.688	10.602	7.952	1.00	0.00	RX1	H
ATOM	4816	C	ASN	280	43.729	8.944	4.055	1.00	0.00	RX1	C
ATOM	4817	O	ASN	280	44.498	8.316	4.770	1.00	0.00	RX1	O

ATOM	4818	N	PRO	281	44.094	10.034	3.334	1.00	0.00	RX1	N
ATOM	4819	CD	PRO	281	43.275	10.754	2.367	1.00	0.00	RX1	C
ATOM	4820	CA	PRO	281	45.434	10.611	3.505	1.00	0.00	RX1	C
ATOM	4821	CB	PRO	281	45.367	11.867	2.629	1.00	0.00	RX1	C
ATOM	4822	CG	PRO	281	44.290	11.577	1.584	1.00	0.00	RX1	C
ATOM	4823	C	PRO	281	45.783	10.925	4.956	1.00	0.00	RX1	C
ATOM	4824	O	PRO	281	46.828	10.557	5.476	1.00	0.00	RX1	O
ATOM	4825	N	GLU	282	44.828	11.607	5.600	1.00	0.00	RX1	N
ATOM	4826	H	GLU	282	43.927	11.750	5.189	1.00	0.00	RX1	H
ATOM	4827	CA	GLU	282	44.983	11.881	7.024	1.00	0.00	RX1	C
ATOM	4828	CB	GLU	282	44.254	13.170	7.403	1.00	0.00	RX1	C
ATOM	4829	CG	GLU	282	45.000	14.441	6.998	1.00	0.00	RX1	C
ATOM	4830	CD	GLU	282	46.224	14.603	7.875	1.00	0.00	RX1	C
ATOM	4831	OE1	GLU	282	47.205	15.195	7.438	1.00	0.00	RX1	O
ATOM	4832	OE2	GLU	282	46.214	14.139	9.011	1.00	0.00	RX1	O
ATOM	4833	C	GLU	282	44.450	10.740	7.869	1.00	0.00	RX1	C
ATOM	4834	O	GLU	282	43.571	10.908	8.709	1.00	0.00	RX1	O
ATOM	4835	N	GLY	283	45.001	9.548	7.589	1.00	0.00	RX1	N
ATOM	4836	H	GLY	283	45.775	9.493	6.956	1.00	0.00	RX1	H
ATOM	4837	CA	GLY	283	44.567	8.370	8.336	1.00	0.00	RX1	C
ATOM	4838	C	GLY	283	44.944	8.471	9.798	1.00	0.00	RX1	C
ATOM	4839	O	GLY	283	46.106	8.401	10.178	1.00	0.00	RX1	O
ATOM	4840	N	LYS	284	43.910	8.697	10.612	1.00	0.00	RX1	N
ATOM	4841	H	LYS	284	42.948	8.672	10.334	1.00	0.00	RX1	H
ATOM	4842	CA	LYS	284	44.297	9.162	11.931	1.00	0.00	RX1	C
ATOM	4843	CB	LYS	284	43.428	10.337	12.346	1.00	0.00	RX1	C
ATOM	4844	CG	LYS	284	44.332	11.523	12.641	1.00	0.00	RX1	C
ATOM	4845	CD	LYS	284	45.342	11.723	11.516	1.00	0.00	RX1	C
ATOM	4846	CE	LYS	284	46.370	12.805	11.814	1.00	0.00	RX1	C
ATOM	4847	NZ	LYS	284	45.794	14.145	11.653	1.00	0.00	RX1	N
ATOM	4848	HZ1	LYS	284	46.506	14.862	11.900	1.00	0.00	RX1	H
ATOM	4849	HZ2	LYS	284	45.504	14.307	10.667	1.00	0.00	RX1	H
ATOM	4850	HZ3	LYS	284	44.989	14.289	12.304	1.00	0.00	RX1	H
ATOM	4851	C	LYS	284	44.438	8.131	13.020	1.00	0.00	RX1	C
ATOM	4852	O	LYS	284	43.517	7.792	13.759	1.00	0.00	RX1	O
ATOM	4853	N	TYR	285	45.686	7.654	13.081	1.00	0.00	RX1	N
ATOM	4854	H	TYR	285	46.385	8.094	12.512	1.00	0.00	RX1	H
ATOM	4855	CA	TYR	285	46.041	6.680	14.107	1.00	0.00	RX1	C
ATOM	4856	CB	TYR	285	47.498	6.257	13.953	1.00	0.00	RX1	C
ATOM	4857	CG	TYR	285	47.698	5.534	12.640	1.00	0.00	RX1	C
ATOM	4858	CD1	TYR	285	47.974	6.243	11.477	1.00	0.00	RX1	C
ATOM	4859	CE1	TYR	285	48.204	5.572	10.283	1.00	0.00	RX1	C
ATOM	4860	CD2	TYR	285	47.620	4.149	12.595	1.00	0.00	RX1	C
ATOM	4861	CE2	TYR	285	47.850	3.473	11.403	1.00	0.00	RX1	C
ATOM	4862	CZ	TYR	285	48.152	4.184	10.249	1.00	0.00	RX1	C
ATOM	4863	OH	TYR	285	48.402	3.511	9.071	1.00	0.00	RX1	O
ATOM	4864	HH	TYR	285	48.157	2.599	9.169	1.00	0.00	RX1	H
ATOM	4865	C	TYR	285	45.760	7.205	15.501	1.00	0.00	RX1	C
ATOM	4866	O	TYR	285	46.195	8.273	15.922	1.00	0.00	RX1	O
ATOM	4867	N	SER	286	44.928	6.422	16.177	1.00	0.00	RX1	N
ATOM	4868	H	SER	286	44.642	5.515	15.860	1.00	0.00	RX1	H
ATOM	4869	CA	SER	286	44.336	6.967	17.385	1.00	0.00	RX1	C
ATOM	4870	CB	SER	286	42.934	6.409	17.315	1.00	0.00	RX1	C
ATOM	4871	OG	SER	286	42.736	6.059	15.932	1.00	0.00	RX1	O
ATOM	4872	HG	SER	286	42.943	6.829	15.403	1.00	0.00	RX1	H
ATOM	4873	C	SER	286	45.141	6.683	18.637	1.00	0.00	RX1	C
ATOM	4874	O	SER	286	44.925	5.703	19.343	1.00	0.00	RX1	O
ATOM	4875	N	PHE	287	46.092	7.594	18.891	1.00	0.00	RX1	N
ATOM	4876	H	PHE	287	46.198	8.414	18.324	1.00	0.00	RX1	H
ATOM	4877	CA	PHE	287	46.815	7.475	20.154	1.00	0.00	RX1	C
ATOM	4878	CB	PHE	287	48.244	7.997	20.006	1.00	0.00	RX1	C

ATOM	4879	CG	PHE	287	49.088	7.499	21.154	1.00	0.00	RX1	C
ATOM	4880	CD1	PHE	287	49.072	6.150	21.490	1.00	0.00	RX1	C
ATOM	4881	CD2	PHE	287	49.885	8.384	21.870	1.00	0.00	RX1	C
ATOM	4882	CE1	PHE	287	49.857	5.682	22.537	1.00	0.00	RX1	C
ATOM	4883	CE2	PHE	287	50.670	7.916	22.917	1.00	0.00	RX1	C
ATOM	4884	CZ	PHE	287	50.659	6.566	23.248	1.00	0.00	RX1	C
ATOM	4885	C	PHE	287	46.070	8.168	21.285	1.00	0.00	RX1	C
ATOM	4886	O	PHE	287	46.431	9.225	21.792	1.00	0.00	RX1	O
ATOM	4887	N	GLY	288	44.953	7.513	21.635	1.00	0.00	RX1	N
ATOM	4888	H	GLY	288	44.694	6.685	21.135	1.00	0.00	RX1	H
ATOM	4889	CA	GLY	288	44.012	8.192	22.522	1.00	0.00	RX1	C
ATOM	4890	C	GLY	288	43.456	9.444	21.865	1.00	0.00	RX1	C
ATOM	4891	O	GLY	288	43.365	9.534	20.646	1.00	0.00	RX1	O
ATOM	4892	N	ALA	289	43.161	10.431	22.724	1.00	0.00	RX1	N
ATOM	4893	H	ALA	289	43.132	10.242	23.709	1.00	0.00	RX1	H
ATOM	4894	CA	ALA	289	42.758	11.736	22.198	1.00	0.00	RX1	C
ATOM	4895	CB	ALA	289	42.407	12.689	23.342	1.00	0.00	RX1	C
ATOM	4896	C	ALA	289	43.787	12.429	21.317	1.00	0.00	RX1	C
ATOM	4897	O	ALA	289	43.469	13.324	20.539	1.00	0.00	RX1	O
ATOM	4898	N	THR	290	45.040	11.988	21.474	1.00	0.00	RX1	N
ATOM	4899	H	THR	290	45.344	11.259	22.086	1.00	0.00	RX1	H
ATOM	4900	CA	THR	290	46.054	12.540	20.592	1.00	0.00	RX1	C
ATOM	4901	CB	THR	290	47.305	12.411	21.420	1.00	0.00	RX1	C
ATOM	4902	OG1	THR	290	46.893	12.455	22.797	1.00	0.00	RX1	O
ATOM	4903	HG1	THR	290	47.631	12.125	23.292	1.00	0.00	RX1	H
ATOM	4904	CG2	THR	290	48.350	13.482	21.099	1.00	0.00	RX1	C
ATOM	4905	C	THR	290	46.095	11.806	19.264	1.00	0.00	RX1	C
ATOM	4906	O	THR	290	46.851	10.869	19.030	1.00	0.00	RX1	O
ATOM	4907	N	CYS	291	45.217	12.289	18.382	1.00	0.00	RX1	N
ATOM	4908	H	CYS	291	44.607	13.048	18.611	1.00	0.00	RX1	H
ATOM	4909	CA	CYS	291	45.254	11.716	17.042	1.00	0.00	RX1	C
ATOM	4910	CB	CYS	291	43.983	12.118	16.315	1.00	0.00	RX1	C
ATOM	4911	SG	CYS	291	42.624	12.406	17.476	1.00	0.00	RX1	S
ATOM	4912	C	CYS	291	46.523	12.100	16.305	1.00	0.00	RX1	C
ATOM	4913	O	CYS	291	46.935	13.257	16.294	1.00	0.00	RX1	O
ATOM	4914	N	VAL	292	47.147	11.059	15.742	1.00	0.00	RX1	N
ATOM	4915	H	VAL	292	46.754	10.137	15.718	1.00	0.00	RX1	H
ATOM	4916	CA	VAL	292	48.444	11.255	15.101	1.00	0.00	RX1	C
ATOM	4917	CB	VAL	292	49.576	10.703	15.975	1.00	0.00	RX1	C
ATOM	4918	CG1	VAL	292	49.817	11.568	17.214	1.00	0.00	RX1	C
ATOM	4919	CG2	VAL	292	49.317	9.241	16.338	1.00	0.00	RX1	C
ATOM	4920	C	VAL	292	48.471	10.600	13.733	1.00	0.00	RX1	C
ATOM	4921	O	VAL	292	47.624	9.781	13.396	1.00	0.00	RX1	O
ATOM	4922	N	LYS	293	49.487	10.991	12.948	1.00	0.00	RX1	N
ATOM	4923	H	LYS	293	50.131	11.700	13.235	1.00	0.00	RX1	H
ATOM	4924	CA	LYS	293	49.538	10.390	11.616	1.00	0.00	RX1	C
ATOM	4925	CB	LYS	293	50.135	11.369	10.600	1.00	0.00	RX1	C
ATOM	4926	CG	LYS	293	49.185	11.616	9.422	1.00	0.00	RX1	C
ATOM	4927	CD	LYS	293	49.735	12.596	8.381	1.00	0.00	RX1	C
ATOM	4928	CE	LYS	293	50.013	13.999	8.933	1.00	0.00	RX1	C
ATOM	4929	NZ	LYS	293	48.769	14.634	9.377	1.00	0.00	RX1	N
ATOM	4930	HZ1	LYS	293	48.825	15.008	10.352	1.00	0.00	RX1	H
ATOM	4931	HZ2	LYS	293	48.450	15.385	8.730	1.00	0.00	RX1	H
ATOM	4932	HZ3	LYS	293	47.963	13.980	9.326	1.00	0.00	RX1	H
ATOM	4933	C	LYS	293	50.170	9.006	11.508	1.00	0.00	RX1	C
ATOM	4934	O	LYS	293	50.137	8.382	10.457	1.00	0.00	RX1	O
ATOM	4935	N	LYS	294	50.729	8.539	12.647	1.00	0.00	RX1	N
ATOM	4936	H	LYS	294	50.807	9.080	13.485	1.00	0.00	RX1	H
ATOM	4937	CA	LYS	294	51.031	7.110	12.806	1.00	0.00	RX1	C
ATOM	4938	CB	LYS	294	52.113	6.590	11.852	1.00	0.00	RX1	C
ATOM	4939	CG	LYS	294	53.511	7.173	12.036	1.00	0.00	RX1	C

ATOM	4940	CD	LYS	294	54.460	6.550	11.012	1.00	0.00	RX1	C
ATOM	4941	CE	LYS	294	55.896	7.060	11.100	1.00	0.00	RX1	C
ATOM	4942	NZ	LYS	294	56.693	6.397	10.057	1.00	0.00	RX1	N
ATOM	4943	HZ1	LYS	294	57.675	6.737	10.093	1.00	0.00	RX1	H
ATOM	4944	HZ2	LYS	294	56.673	5.368	10.210	1.00	0.00	RX1	H
ATOM	4945	HZ3	LYS	294	56.285	6.612	9.125	1.00	0.00	RX1	H
ATOM	4946	C	LYS	294	51.376	6.742	14.232	1.00	0.00	RX1	C
ATOM	4947	O	LYS	294	51.809	7.574	15.018	1.00	0.00	RX1	O
ATOM	4948	N	CYS	295	51.145	5.456	14.527	1.00	0.00	RX1	N
ATOM	4949	H	CYS	295	50.820	4.808	13.841	1.00	0.00	RX1	H
ATOM	4950	CA	CYS	295	51.380	4.970	15.888	1.00	0.00	RX1	C
ATOM	4951	CB	CYS	295	50.796	3.566	16.010	1.00	0.00	RX1	C
ATOM	4952	SG	CYS	295	49.181	3.454	15.214	1.00	0.00	RX1	S
ATOM	4953	C	CYS	295	52.838	4.970	16.322	1.00	0.00	RX1	C
ATOM	4954	O	CYS	295	53.737	4.674	15.541	1.00	0.00	RX1	O
ATOM	4955	N	PRO	296	53.048	5.295	17.622	1.00	0.00	RX1	N
ATOM	4956	CD	PRO	296	52.073	5.856	18.549	1.00	0.00	RX1	C
ATOM	4957	CA	PRO	296	54.360	5.054	18.237	1.00	0.00	RX1	C
ATOM	4958	CB	PRO	296	54.129	5.473	19.694	1.00	0.00	RX1	C
ATOM	4959	CG	PRO	296	52.933	6.425	19.669	1.00	0.00	RX1	C
ATOM	4960	C	PRO	296	54.785	3.597	18.127	1.00	0.00	RX1	C
ATOM	4961	O	PRO	296	54.009	2.692	18.404	1.00	0.00	RX1	O
ATOM	4962	N	ARG	297	56.056	3.420	17.713	1.00	0.00	RX1	N
ATOM	4963	H	ARG	297	56.585	4.240	17.505	1.00	0.00	RX1	H
ATOM	4964	CA	ARG	297	56.608	2.092	17.402	1.00	0.00	RX1	C
ATOM	4965	CB	ARG	297	58.138	2.138	17.474	1.00	0.00	RX1	C
ATOM	4966	CG	ARG	297	58.751	0.740	17.335	1.00	0.00	RX1	C
ATOM	4967	CD	ARG	297	60.019	0.537	18.164	1.00	0.00	RX1	C
ATOM	4968	NE	ARG	297	61.134	1.294	17.610	1.00	0.00	RX1	N
ATOM	4969	HE	ARG	297	61.064	1.522	16.635	1.00	0.00	RX1	H
ATOM	4970	CZ	ARG	297	62.231	1.510	18.393	1.00	0.00	RX1	C
ATOM	4971	NH1	ARG	297	62.217	1.164	19.699	1.00	0.00	RX1	N
ATOM	4972	HH11	ARG	297	63.017	1.322	20.299	1.00	0.00	RX1	H
ATOM	4973	HH12	ARG	297	61.431	0.749	20.177	1.00	0.00	RX1	H
ATOM	4974	NH2	ARG	297	63.327	2.070	17.841	1.00	0.00	RX1	N
ATOM	4975	HH21	ARG	297	64.167	2.164	18.394	1.00	0.00	RX1	H
ATOM	4976	HH22	ARG	297	63.367	2.390	16.892	1.00	0.00	RX1	H
ATOM	4977	C	ARG	297	56.156	0.918	18.266	1.00	0.00	RX1	C
ATOM	4978	O	ARG	297	55.831	-0.159	17.786	1.00	0.00	RX1	O
ATOM	4979	N	ASN	298	56.240	1.159	19.580	1.00	0.00	RX1	N
ATOM	4980	H	ASN	298	56.245	2.083	19.963	1.00	0.00	RX1	H
ATOM	4981	CA	ASN	298	56.072	0.010	20.463	1.00	0.00	RX1	C
ATOM	4982	CB	ASN	298	56.715	0.226	21.832	1.00	0.00	RX1	C
ATOM	4983	CG	ASN	298	55.866	1.179	22.642	1.00	0.00	RX1	C
ATOM	4984	OD1	ASN	298	55.196	2.054	22.103	1.00	0.00	RX1	O
ATOM	4985	ND2	ASN	298	55.964	0.985	23.965	1.00	0.00	RX1	N
ATOM	4986	HD21	ASN	298	56.542	0.253	24.340	1.00	0.00	RX1	H
ATOM	4987	HD22	ASN	298	55.549	1.603	24.630	1.00	0.00	RX1	H
ATOM	4988	C	ASN	298	54.645	-0.466	20.634	1.00	0.00	RX1	C
ATOM	4989	O	ASN	298	54.409	-1.601	21.033	1.00	0.00	RX1	O
ATOM	4990	N	TYR	299	53.699	0.440	20.360	1.00	0.00	RX1	N
ATOM	4991	H	TYR	299	53.912	1.293	19.882	1.00	0.00	RX1	H
ATOM	4992	CA	TYR	299	52.310	0.035	20.536	1.00	0.00	RX1	C
ATOM	4993	CB	TYR	299	51.402	1.263	20.658	1.00	0.00	RX1	C
ATOM	4994	CG	TYR	299	51.310	1.676	22.109	1.00	0.00	RX1	C
ATOM	4995	CD1	TYR	299	52.313	2.431	22.707	1.00	0.00	RX1	C
ATOM	4996	CE1	TYR	299	52.243	2.746	24.060	1.00	0.00	RX1	C
ATOM	4997	CD2	TYR	299	50.212	1.281	22.859	1.00	0.00	RX1	C
ATOM	4998	CE2	TYR	299	50.135	1.604	24.207	1.00	0.00	RX1	C
ATOM	4999	CZ	TYR	299	51.160	2.314	24.818	1.00	0.00	RX1	C
ATOM	5000	OH	TYR	299	51.095	2.567	26.175	1.00	0.00	RX1	O

ATOM	5001	HH	TYR	299	51.916	2.948	26.483	1.00	0.00	RX1	H
ATOM	5002	C	TYR	299	51.846	-0.891	19.434	1.00	0.00	RX1	C
ATOM	5003	O	TYR	299	52.315	-0.836	18.306	1.00	0.00	RX1	O
ATOM	5004	N	VAL	300	50.918	-1.773	19.828	1.00	0.00	RX1	N
ATOM	5005	H	VAL	300	50.432	-1.639	20.690	1.00	0.00	RX1	H
ATOM	5006	CA	VAL	300	50.330	-2.625	18.799	1.00	0.00	RX1	C
ATOM	5007	CB	VAL	300	49.468	-3.735	19.395	1.00	0.00	RX1	C
ATOM	5008	CG1	VAL	300	49.096	-4.751	18.325	1.00	0.00	RX1	C
ATOM	5009	CG2	VAL	300	50.105	-4.400	20.600	1.00	0.00	RX1	C
ATOM	5010	C	VAL	300	49.462	-1.778	17.899	1.00	0.00	RX1	C
ATOM	5011	O	VAL	300	48.564	-1.075	18.346	1.00	0.00	RX1	O
ATOM	5012	N	VAL	301	49.776	-1.857	16.614	1.00	0.00	RX1	N
ATOM	5013	H	VAL	301	50.454	-2.521	16.292	1.00	0.00	RX1	H
ATOM	5014	CA	VAL	301	48.888	-1.138	15.716	1.00	0.00	RX1	C
ATOM	5015	CB	VAL	301	49.703	-0.633	14.524	1.00	0.00	RX1	C
ATOM	5016	CG1	VAL	301	48.936	0.351	13.639	1.00	0.00	RX1	C
ATOM	5017	CG2	VAL	301	51.015	-0.039	15.033	1.00	0.00	RX1	C
ATOM	5018	C	VAL	301	47.760	-2.073	15.312	1.00	0.00	RX1	C
ATOM	5019	O	VAL	301	47.937	-3.284	15.240	1.00	0.00	RX1	O
ATOM	5020	N	THR	302	46.592	-1.497	15.040	1.00	0.00	RX1	N
ATOM	5021	H	THR	302	46.390	-0.527	15.183	1.00	0.00	RX1	H
ATOM	5022	CA	THR	302	45.722	-2.319	14.215	1.00	0.00	RX1	C
ATOM	5023	CB	THR	302	44.328	-2.399	14.831	1.00	0.00	RX1	C
ATOM	5024	OG1	THR	302	43.713	-1.111	14.831	1.00	0.00	RX1	O
ATOM	5025	HG1	THR	302	42.825	-1.242	15.165	1.00	0.00	RX1	H
ATOM	5026	CG2	THR	302	44.371	-2.971	16.248	1.00	0.00	RX1	C
ATOM	5027	C	THR	302	45.721	-1.753	12.815	1.00	0.00	RX1	C
ATOM	5028	O	THR	302	45.963	-0.568	12.622	1.00	0.00	RX1	O
ATOM	5029	N	ASP	303	45.380	-2.612	11.849	1.00	0.00	RX1	N
ATOM	5030	H	ASP	303	45.325	-3.605	11.981	1.00	0.00	RX1	H
ATOM	5031	CA	ASP	303	45.184	-2.090	10.492	1.00	0.00	RX1	C
ATOM	5032	CB	ASP	303	44.934	-3.251	9.522	1.00	0.00	RX1	C
ATOM	5033	CG	ASP	303	46.001	-4.325	9.678	1.00	0.00	RX1	C
ATOM	5034	OD1	ASP	303	46.904	-4.416	8.852	1.00	0.00	RX1	O
ATOM	5035	OD2	ASP	303	45.926	-5.127	10.605	1.00	0.00	RX1	O
ATOM	5036	C	ASP	303	44.078	-1.037	10.387	1.00	0.00	RX1	C
ATOM	5037	O	ASP	303	44.025	-0.215	9.483	1.00	0.00	RX1	O
ATOM	5038	N	HIS	304	43.194	-1.086	11.401	1.00	0.00	RX1	N
ATOM	5039	H	HIS	304	43.303	-1.769	12.119	1.00	0.00	RX1	H
ATOM	5040	CA	HIS	304	42.159	-0.062	11.545	1.00	0.00	RX1	C
ATOM	5041	CB	HIS	304	41.193	-0.562	12.618	1.00	0.00	RX1	C
ATOM	5042	CG	HIS	304	39.819	0.062	12.554	1.00	0.00	RX1	C
ATOM	5043	ND1	HIS	304	39.518	1.262	12.023	1.00	0.00	RX1	N
ATOM	5044	HD1	HIS	304	40.153	1.906	11.635	1.00	0.00	RX1	H
ATOM	5045	CD2	HIS	304	38.645	-0.524	13.033	1.00	0.00	RX1	C
ATOM	5046	NE2	HIS	304	37.626	0.330	12.781	1.00	0.00	RX1	N
ATOM	5047	CE1	HIS	304	38.161	1.433	12.159	1.00	0.00	RX1	C
ATOM	5048	C	HIS	304	42.684	1.322	11.912	1.00	0.00	RX1	C
ATOM	5049	O	HIS	304	42.097	2.340	11.558	1.00	0.00	RX1	O
ATOM	5050	N	GLY	305	43.804	1.306	12.653	1.00	0.00	RX1	N
ATOM	5051	H	GLY	305	44.260	0.459	12.930	1.00	0.00	RX1	H
ATOM	5052	CA	GLY	305	44.390	2.576	13.073	1.00	0.00	RX1	C
ATOM	5053	C	GLY	305	44.567	2.746	14.573	1.00	0.00	RX1	C
ATOM	5054	O	GLY	305	45.022	3.775	15.061	1.00	0.00	RX1	O
ATOM	5055	N	SER	306	44.179	1.695	15.297	1.00	0.00	RX1	N
ATOM	5056	H	SER	306	43.814	0.843	14.926	1.00	0.00	RX1	H
ATOM	5057	CA	SER	306	44.220	1.804	16.750	1.00	0.00	RX1	C
ATOM	5058	CB	SER	306	43.133	0.843	17.179	1.00	0.00	RX1	C
ATOM	5059	OG	SER	306	42.189	0.829	16.092	1.00	0.00	RX1	O
ATOM	5060	HG	SER	306	41.604	0.087	16.226	1.00	0.00	RX1	H
ATOM	5061	C	SER	306	45.608	1.572	17.325	1.00	0.00	RX1	C

ATOM	5062	O	SER	306	46.316	0.664	16.908	1.00	0.00	RX1	O
ATOM	5063	N	CYS	307	45.965	2.423	18.299	1.00	0.00	RX1	N
ATOM	5064	H	CYS	307	45.371	3.157	18.631	1.00	0.00	RX1	H
ATOM	5065	CA	CYS	307	47.267	2.233	18.942	1.00	0.00	RX1	C
ATOM	5066	CB	CYS	307	47.893	3.600	19.189	1.00	0.00	RX1	C
ATOM	5067	SG	CYS	307	47.770	4.686	17.745	1.00	0.00	RX1	S
ATOM	5068	C	CYS	307	47.181	1.423	20.224	1.00	0.00	RX1	C
ATOM	5069	O	CYS	307	47.406	1.921	21.320	1.00	0.00	RX1	O
ATOM	5070	N	VAL	308	46.791	0.156	20.050	1.00	0.00	RX1	N
ATOM	5071	H	VAL	308	46.933	-0.274	19.155	1.00	0.00	RX1	H
ATOM	5072	CA	VAL	308	46.493	-0.617	21.253	1.00	0.00	RX1	C
ATOM	5073	CB	VAL	308	45.532	-1.769	20.931	1.00	0.00	RX1	C
ATOM	5074	CG1	VAL	308	44.239	-1.235	20.321	1.00	0.00	RX1	C
ATOM	5075	CG2	VAL	308	46.154	-2.798	19.997	1.00	0.00	RX1	C
ATOM	5076	C	VAL	308	47.716	-1.085	22.038	1.00	0.00	RX1	C
ATOM	5077	O	VAL	308	48.827	-1.201	21.541	1.00	0.00	RX1	O
ATOM	5078	N	ARG	309	47.453	-1.372	23.320	1.00	0.00	RX1	N
ATOM	5079	H	ARG	309	46.529	-1.267	23.669	1.00	0.00	RX1	H
ATOM	5080	CA	ARG	309	48.500	-1.989	24.139	1.00	0.00	RX1	C
ATOM	5081	CB	ARG	309	48.227	-1.852	25.632	1.00	0.00	RX1	C
ATOM	5082	CG	ARG	309	48.129	-0.455	26.231	1.00	0.00	RX1	C
ATOM	5083	CD	ARG	309	47.664	-0.635	27.673	1.00	0.00	RX1	C
ATOM	5084	NE	ARG	309	47.459	0.614	28.399	1.00	0.00	RX1	N
ATOM	5085	HE	ARG	309	48.135	1.347	28.277	1.00	0.00	RX1	H
ATOM	5086	CZ	ARG	309	46.493	0.565	29.367	1.00	0.00	RX1	C
ATOM	5087	NH1	ARG	309	45.605	-0.450	29.365	1.00	0.00	RX1	N
ATOM	5088	HH11	ARG	309	44.979	-0.541	30.157	1.00	0.00	RX1	H
ATOM	5089	HH12	ARG	309	45.520	-1.124	28.626	1.00	0.00	RX1	H
ATOM	5090	NH2	ARG	309	46.436	1.507	30.329	1.00	0.00	RX1	N
ATOM	5091	HH21	ARG	309	45.779	1.402	31.103	1.00	0.00	RX1	H
ATOM	5092	HH22	ARG	309	47.020	2.317	30.367	1.00	0.00	RX1	H
ATOM	5093	C	ARG	309	48.659	-3.479	23.897	1.00	0.00	RX1	C
ATOM	5094	O	ARG	309	49.714	-4.062	24.123	1.00	0.00	RX1	O
ATOM	5095	N	ALA	310	47.529	-4.077	23.501	1.00	0.00	RX1	N
ATOM	5096	H	ALA	310	46.762	-3.611	23.066	1.00	0.00	RX1	H
ATOM	5097	CA	ALA	310	47.413	-5.515	23.695	1.00	0.00	RX1	C
ATOM	5098	CB	ALA	310	46.292	-5.809	24.688	1.00	0.00	RX1	C
ATOM	5099	C	ALA	310	47.085	-6.235	22.412	1.00	0.00	RX1	C
ATOM	5100	O	ALA	310	46.782	-5.633	21.391	1.00	0.00	RX1	O
ATOM	5101	N	CYS	311	47.127	-7.568	22.518	1.00	0.00	RX1	N
ATOM	5102	H	CYS	311	47.329	-8.022	23.383	1.00	0.00	RX1	H
ATOM	5103	CA	CYS	311	46.545	-8.331	21.419	1.00	0.00	RX1	C
ATOM	5104	CB	CYS	311	47.023	-9.776	21.511	1.00	0.00	RX1	C
ATOM	5105	SG	CYS	311	48.818	-9.881	21.723	1.00	0.00	RX1	S
ATOM	5106	C	CYS	311	45.031	-8.251	21.464	1.00	0.00	RX1	C
ATOM	5107	O	CYS	311	44.430	-8.296	22.532	1.00	0.00	RX1	O
ATOM	5108	N	GLY	312	44.443	-8.158	20.259	1.00	0.00	RX1	N
ATOM	5109	H	GLY	312	44.979	-8.123	19.421	1.00	0.00	RX1	H
ATOM	5110	CA	GLY	312	42.994	-8.351	20.186	1.00	0.00	RX1	C
ATOM	5111	C	GLY	312	42.613	-9.754	20.631	1.00	0.00	RX1	C
ATOM	5112	O	GLY	312	43.466	-10.629	20.712	1.00	0.00	RX1	O
ATOM	5113	N	ALA	313	41.316	-9.929	20.924	1.00	0.00	RX1	N
ATOM	5114	H	ALA	313	40.699	-9.147	20.819	1.00	0.00	RX1	H
ATOM	5115	CA	ALA	313	40.854	-11.164	21.569	1.00	0.00	RX1	C
ATOM	5116	CB	ALA	313	39.327	-11.239	21.518	1.00	0.00	RX1	C
ATOM	5117	C	ALA	313	41.423	-12.481	21.049	1.00	0.00	RX1	C
ATOM	5118	O	ALA	313	42.020	-13.261	21.781	1.00	0.00	RX1	O
ATOM	5119	N	ASP	314	41.210	-12.681	19.747	1.00	0.00	RX1	N
ATOM	5120	H	ASP	314	40.822	-11.979	19.145	1.00	0.00	RX1	H
ATOM	5121	CA	ASP	314	41.675	-13.884	19.049	1.00	0.00	RX1	C
ATOM	5122	CB	ASP	314	40.613	-14.347	18.046	1.00	0.00	RX1	C

ATOM	5123	CG	ASP	314	40.283	-13.257	17.035	1.00	0.00	RX1	C
ATOM	5124	OD1	ASP	314	40.938	-12.219	17.012	1.00	0.00	RX1	O
ATOM	5125	OD2	ASP	314	39.358	-13.449	16.253	1.00	0.00	RX1	O
ATOM	5126	C	ASP	314	43.029	-13.728	18.363	1.00	0.00	RX1	C
ATOM	5127	O	ASP	314	43.508	-14.570	17.610	1.00	0.00	RX1	O
ATOM	5128	N	SER	315	43.620	-12.565	18.627	1.00	0.00	RX1	N
ATOM	5129	H	SER	315	43.301	-11.907	19.306	1.00	0.00	RX1	H
ATOM	5130	CA	SER	315	44.762	-12.165	17.828	1.00	0.00	RX1	C
ATOM	5131	CB	SER	315	44.415	-10.745	17.433	1.00	0.00	RX1	C
ATOM	5132	OG	SER	315	43.040	-10.544	17.791	1.00	0.00	RX1	O
ATOM	5133	HG	SER	315	42.516	-11.178	17.296	1.00	0.00	RX1	H
ATOM	5134	C	SER	315	46.077	-12.350	18.558	1.00	0.00	RX1	C
ATOM	5135	O	SER	315	46.123	-12.502	19.772	1.00	0.00	RX1	O
ATOM	5136	N	TYR	316	47.159	-12.311	17.771	1.00	0.00	RX1	N
ATOM	5137	H	TYR	316	47.117	-12.171	16.778	1.00	0.00	RX1	H
ATOM	5138	CA	TYR	316	48.450	-12.219	18.441	1.00	0.00	RX1	C
ATOM	5139	CB	TYR	316	49.328	-13.476	18.309	1.00	0.00	RX1	C
ATOM	5140	CG	TYR	316	50.371	-13.434	19.412	1.00	0.00	RX1	C
ATOM	5141	CD1	TYR	316	49.972	-13.588	20.734	1.00	0.00	RX1	C
ATOM	5142	CE1	TYR	316	50.891	-13.441	21.767	1.00	0.00	RX1	C
ATOM	5143	CD2	TYR	316	51.714	-13.195	19.130	1.00	0.00	RX1	C
ATOM	5144	CE2	TYR	316	52.633	-13.034	20.166	1.00	0.00	RX1	C
ATOM	5145	CZ	TYR	316	52.220	-13.142	21.490	1.00	0.00	RX1	C
ATOM	5146	OH	TYR	316	53.106	-12.967	22.537	1.00	0.00	RX1	O
ATOM	5147	HH	TYR	316	53.688	-12.217	22.382	1.00	0.00	RX1	H
ATOM	5148	C	TYR	316	49.176	-10.973	17.978	1.00	0.00	RX1	C
ATOM	5149	O	TYR	316	48.931	-10.471	16.887	1.00	0.00	RX1	O
ATOM	5150	N	GLU	317	50.066	-10.490	18.856	1.00	0.00	RX1	N
ATOM	5151	H	GLU	317	50.232	-10.938	19.733	1.00	0.00	RX1	H
ATOM	5152	CA	GLU	317	50.935	-9.390	18.451	1.00	0.00	RX1	C
ATOM	5153	CB	GLU	317	51.510	-8.690	19.683	1.00	0.00	RX1	C
ATOM	5154	CG	GLU	317	52.071	-7.315	19.332	1.00	0.00	RX1	C
ATOM	5155	CD	GLU	317	53.056	-6.846	20.387	1.00	0.00	RX1	C
ATOM	5156	OE1	GLU	317	54.119	-6.361	20.027	1.00	0.00	RX1	O
ATOM	5157	OE2	GLU	317	52.790	-6.925	21.579	1.00	0.00	RX1	O
ATOM	5158	C	GLU	317	52.066	-9.879	17.560	1.00	0.00	RX1	C
ATOM	5159	O	GLU	317	53.079	-10.398	18.025	1.00	0.00	RX1	O
ATOM	5160	N	MET	318	51.835	-9.712	16.258	1.00	0.00	RX1	N
ATOM	5161	H	MET	318	51.050	-9.185	15.923	1.00	0.00	RX1	H
ATOM	5162	CA	MET	318	52.824	-10.178	15.296	1.00	0.00	RX1	C
ATOM	5163	CB	MET	318	52.166	-11.175	14.340	1.00	0.00	RX1	C
ATOM	5164	CG	MET	318	53.163	-12.089	13.627	1.00	0.00	RX1	C
ATOM	5165	SD	MET	318	54.019	-13.168	14.786	1.00	0.00	RX1	S
ATOM	5166	CE	MET	318	52.582	-14.036	15.433	1.00	0.00	RX1	C
ATOM	5167	C	MET	318	53.421	-9.012	14.538	1.00	0.00	RX1	C
ATOM	5168	O	MET	318	52.756	-8.020	14.273	1.00	0.00	RX1	O
ATOM	5169	N	GLU	319	54.704	-9.148	14.208	1.00	0.00	RX1	N
ATOM	5170	H	GLU	319	55.185	-10.019	14.325	1.00	0.00	RX1	H
ATOM	5171	CA	GLU	319	55.277	-8.134	13.329	1.00	0.00	RX1	C
ATOM	5172	CB	GLU	319	56.812	-8.083	13.409	1.00	0.00	RX1	C
ATOM	5173	CG	GLU	319	57.560	-9.421	13.329	1.00	0.00	RX1	C
ATOM	5174	CD	GLU	319	57.516	-10.117	14.677	1.00	0.00	RX1	C
ATOM	5175	OE1	GLU	319	58.171	-9.660	15.606	1.00	0.00	RX1	O
ATOM	5176	OE2	GLU	319	56.780	-11.085	14.836	1.00	0.00	RX1	O
ATOM	5177	C	GLU	319	54.805	-8.326	11.901	1.00	0.00	RX1	C
ATOM	5178	O	GLU	319	54.775	-9.433	11.377	1.00	0.00	RX1	O
ATOM	5179	N	GLU	320	54.394	-7.198	11.314	1.00	0.00	RX1	N
ATOM	5180	H	GLU	320	54.446	-6.307	11.769	1.00	0.00	RX1	H
ATOM	5181	CA	GLU	320	53.867	-7.304	9.958	1.00	0.00	RX1	C
ATOM	5182	CB	GLU	320	52.342	-7.184	9.989	1.00	0.00	RX1	C
ATOM	5183	CG	GLU	320	51.635	-7.643	8.710	1.00	0.00	RX1	C

ATOM	5184	CD	GLU	320	50.151	-7.393	8.877	1.00	0.00	RX1	C
ATOM	5185	OE1	GLU	320	49.545	-6.745	8.028	1.00	0.00	RX1	O
ATOM	5186	OE2	GLU	320	49.589	-7.786	9.894	1.00	0.00	RX1	O
ATOM	5187	C	GLU	320	54.545	-6.304	9.034	1.00	0.00	RX1	C
ATOM	5188	O	GLU	320	55.643	-6.543	8.544	1.00	0.00	RX1	O
ATOM	5189	N	ASP	321	53.897	-5.148	8.839	1.00	0.00	RX1	N
ATOM	5190	H	ASP	321	53.058	-4.872	9.310	1.00	0.00	RX1	H
ATOM	5191	CA	ASP	321	54.538	-4.101	8.042	1.00	0.00	RX1	C
ATOM	5192	CB	ASP	321	53.515	-3.247	7.274	1.00	0.00	RX1	C
ATOM	5193	CG	ASP	321	52.172	-3.130	7.980	1.00	0.00	RX1	C
ATOM	5194	OD1	ASP	321	51.148	-3.177	7.306	1.00	0.00	RX1	O
ATOM	5195	OD2	ASP	321	52.110	-3.024	9.201	1.00	0.00	RX1	O
ATOM	5196	C	ASP	321	55.489	-3.235	8.850	1.00	0.00	RX1	C
ATOM	5197	O	ASP	321	55.346	-2.031	9.027	1.00	0.00	RX1	O
ATOM	5198	N	GLY	322	56.514	-3.930	9.366	1.00	0.00	RX1	N
ATOM	5199	H	GLY	322	56.551	-4.922	9.236	1.00	0.00	RX1	H
ATOM	5200	CA	GLY	322	57.465	-3.241	10.234	1.00	0.00	RX1	C
ATOM	5201	C	GLY	322	56.992	-3.135	11.672	1.00	0.00	RX1	C
ATOM	5202	O	GLY	322	57.605	-3.645	12.600	1.00	0.00	RX1	O
ATOM	5203	N	VAL	323	55.858	-2.440	11.817	1.00	0.00	RX1	N
ATOM	5204	H	VAL	323	55.366	-2.127	11.002	1.00	0.00	RX1	H
ATOM	5205	CA	VAL	323	55.246	-2.399	13.142	1.00	0.00	RX1	C
ATOM	5206	CB	VAL	323	54.204	-1.284	13.209	1.00	0.00	RX1	C
ATOM	5207	CG1	VAL	323	54.873	0.086	13.090	1.00	0.00	RX1	C
ATOM	5208	CG2	VAL	323	53.116	-1.488	12.154	1.00	0.00	RX1	C
ATOM	5209	C	VAL	323	54.642	-3.734	13.542	1.00	0.00	RX1	C
ATOM	5210	O	VAL	323	54.389	-4.606	12.716	1.00	0.00	RX1	O
ATOM	5211	N	ARG	324	54.427	-3.857	14.858	1.00	0.00	RX1	N
ATOM	5212	H	ARG	324	54.528	-3.096	15.498	1.00	0.00	RX1	H
ATOM	5213	CA	ARG	324	53.743	-5.073	15.277	1.00	0.00	RX1	C
ATOM	5214	CB	ARG	324	54.340	-5.656	16.561	1.00	0.00	RX1	C
ATOM	5215	CG	ARG	324	55.871	-5.646	16.568	1.00	0.00	RX1	C
ATOM	5216	CD	ARG	324	56.501	-6.533	17.651	1.00	0.00	RX1	C
ATOM	5217	NE	ARG	324	56.607	-7.924	17.208	1.00	0.00	RX1	N
ATOM	5218	HE	ARG	324	57.252	-8.139	16.460	1.00	0.00	RX1	H
ATOM	5219	CZ	ARG	324	55.863	-8.924	17.762	1.00	0.00	RX1	C
ATOM	5220	NH1	ARG	324	55.016	-8.652	18.768	1.00	0.00	RX1	N
ATOM	5221	HH11	ARG	324	54.421	-9.366	19.154	1.00	0.00	RX1	H
ATOM	5222	HH12	ARG	324	54.908	-7.728	19.164	1.00	0.00	RX1	H
ATOM	5223	NH2	ARG	324	55.969	-10.179	17.291	1.00	0.00	RX1	N
ATOM	5224	HH21	ARG	324	55.411	-10.943	17.622	1.00	0.00	RX1	H
ATOM	5225	HH22	ARG	324	56.629	-10.393	16.542	1.00	0.00	RX1	H
ATOM	5226	C	ARG	324	52.257	-4.824	15.410	1.00	0.00	RX1	C
ATOM	5227	O	ARG	324	51.805	-3.972	16.167	1.00	0.00	RX1	O
ATOM	5228	N	LYS	325	51.517	-5.568	14.592	1.00	0.00	RX1	N
ATOM	5229	H	LYS	325	51.908	-6.344	14.094	1.00	0.00	RX1	H
ATOM	5230	CA	LYS	325	50.073	-5.409	14.640	1.00	0.00	RX1	C
ATOM	5231	CB	LYS	325	49.527	-5.112	13.247	1.00	0.00	RX1	C
ATOM	5232	CG	LYS	325	50.023	-3.748	12.775	1.00	0.00	RX1	C
ATOM	5233	CD	LYS	325	49.471	-3.378	11.409	1.00	0.00	RX1	C
ATOM	5234	CE	LYS	325	49.733	-4.563	10.501	1.00	0.00	RX1	C
ATOM	5235	NZ	LYS	325	49.589	-4.209	9.089	1.00	0.00	RX1	N
ATOM	5236	HZ1	LYS	325	49.816	-5.036	8.497	1.00	0.00	RX1	H
ATOM	5237	HZ2	LYS	325	48.623	-3.897	8.862	1.00	0.00	RX1	H
ATOM	5238	HZ3	LYS	325	50.294	-3.479	8.826	1.00	0.00	RX1	H
ATOM	5239	C	LYS	325	49.413	-6.622	15.244	1.00	0.00	RX1	C
ATOM	5240	O	LYS	325	50.047	-7.638	15.499	1.00	0.00	RX1	O
ATOM	5241	N	CYS	326	48.104	-6.494	15.465	1.00	0.00	RX1	N
ATOM	5242	H	CYS	326	47.585	-5.655	15.290	1.00	0.00	RX1	H
ATOM	5243	CA	CYS	326	47.452	-7.724	15.890	1.00	0.00	RX1	C
ATOM	5244	CB	CYS	326	46.817	-7.528	17.265	1.00	0.00	RX1	C

ATOM	5245	SG	CYS	326	45.802	-6.035	17.385	1.00	0.00	RX1	S
ATOM	5246	C	CYS	326	46.495	-8.269	14.859	1.00	0.00	RX1	C
ATOM	5247	O	CYS	326	45.454	-7.695	14.569	1.00	0.00	RX1	O
ATOM	5248	N	LYS	327	46.906	-9.416	14.307	1.00	0.00	RX1	N
ATOM	5249	H	LYS	327	47.736	-9.885	14.617	1.00	0.00	RX1	H
ATOM	5250	CA	LYS	327	45.934	-10.129	13.488	1.00	0.00	RX1	C
ATOM	5251	CB	LYS	327	46.414	-10.321	12.048	1.00	0.00	RX1	C
ATOM	5252	CG	LYS	327	46.270	-9.052	11.204	1.00	0.00	RX1	C
ATOM	5253	CD	LYS	327	46.485	-9.330	9.715	1.00	0.00	RX1	C
ATOM	5254	CE	LYS	327	46.239	-8.106	8.830	1.00	0.00	RX1	C
ATOM	5255	NZ	LYS	327	47.208	-7.060	9.150	1.00	0.00	RX1	N
ATOM	5256	HZ1	LYS	327	47.810	-6.838	8.331	1.00	0.00	RX1	H
ATOM	5257	HZ2	LYS	327	46.761	-6.171	9.462	1.00	0.00	RX1	H
ATOM	5258	HZ3	LYS	327	47.892	-7.372	9.869	1.00	0.00	RX1	H
ATOM	5259	C	LYS	327	45.590	-11.453	14.123	1.00	0.00	RX1	C
ATOM	5260	O	LYS	327	46.321	-11.970	14.964	1.00	0.00	RX1	O
ATOM	5261	N	LYS	328	44.424	-11.958	13.712	1.00	0.00	RX1	N
ATOM	5262	H	LYS	328	43.940	-11.599	12.917	1.00	0.00	RX1	H
ATOM	5263	CA	LYS	328	43.929	-13.131	14.416	1.00	0.00	RX1	C
ATOM	5264	CB	LYS	328	42.408	-13.234	14.325	1.00	0.00	RX1	C
ATOM	5265	CG	LYS	328	41.852	-13.493	12.928	1.00	0.00	RX1	C
ATOM	5266	CD	LYS	328	40.326	-13.638	12.899	1.00	0.00	RX1	C
ATOM	5267	CE	LYS	328	39.755	-15.033	13.190	1.00	0.00	RX1	C
ATOM	5268	NZ	LYS	328	39.917	-15.488	14.578	1.00	0.00	RX1	N
ATOM	5269	HZ1	LYS	328	39.446	-16.418	14.686	1.00	0.00	RX1	H
ATOM	5270	HZ2	LYS	328	40.909	-15.632	14.838	1.00	0.00	RX1	H
ATOM	5271	HZ3	LYS	328	39.489	-14.827	15.257	1.00	0.00	RX1	H
ATOM	5272	C	LYS	328	44.567	-14.439	14.020	1.00	0.00	RX1	C
ATOM	5273	O	LYS	328	44.895	-14.696	12.868	1.00	0.00	RX1	O
ATOM	5274	N	CYS	329	44.684	-15.279	15.045	1.00	0.00	RX1	N
ATOM	5275	H	CYS	329	44.383	-15.023	15.966	1.00	0.00	RX1	H
ATOM	5276	CA	CYS	329	44.708	-16.696	14.723	1.00	0.00	RX1	C
ATOM	5277	CB	CYS	329	45.827	-17.356	15.525	1.00	0.00	RX1	C
ATOM	5278	SG	CYS	329	45.981	-16.721	17.213	1.00	0.00	RX1	S
ATOM	5279	C	CYS	329	43.312	-17.220	15.009	1.00	0.00	RX1	C
ATOM	5280	O	CYS	329	42.367	-16.436	15.061	1.00	0.00	RX1	O
ATOM	5281	N	GLU	330	43.187	-18.539	15.212	1.00	0.00	RX1	N
ATOM	5282	H	GLU	330	43.910	-19.224	15.186	1.00	0.00	RX1	H
ATOM	5283	CA	GLU	330	41.882	-18.889	15.753	1.00	0.00	RX1	C
ATOM	5284	CB	GLU	330	41.192	-19.954	14.899	1.00	0.00	RX1	C
ATOM	5285	CG	GLU	330	39.677	-19.886	15.094	1.00	0.00	RX1	C
ATOM	5286	CD	GLU	330	39.264	-18.442	14.896	1.00	0.00	RX1	C
ATOM	5287	OE1	GLU	330	39.048	-17.725	15.871	1.00	0.00	RX1	O
ATOM	5288	OE2	GLU	330	39.210	-17.996	13.760	1.00	0.00	RX1	O
ATOM	5289	C	GLU	330	41.936	-19.277	17.212	1.00	0.00	RX1	C
ATOM	5290	O	GLU	330	42.902	-19.886	17.676	1.00	0.00	RX1	O
ATOM	5291	N	GLY	331	40.884	-18.850	17.922	1.00	0.00	RX1	N
ATOM	5292	H	GLY	331	40.178	-18.326	17.437	1.00	0.00	RX1	H
ATOM	5293	CA	GLY	331	40.904	-18.909	19.378	1.00	0.00	RX1	C
ATOM	5294	C	GLY	331	41.735	-17.776	19.951	1.00	0.00	RX1	C
ATOM	5295	O	GLY	331	42.633	-17.260	19.297	1.00	0.00	RX1	O
ATOM	5296	N	PRO	332	41.417	-17.408	21.212	1.00	0.00	RX1	N
ATOM	5297	CD	PRO	332	40.377	-18.006	22.037	1.00	0.00	RX1	C
ATOM	5298	CA	PRO	332	42.108	-16.292	21.870	1.00	0.00	RX1	C
ATOM	5299	CB	PRO	332	41.573	-16.376	23.304	1.00	0.00	RX1	C
ATOM	5300	CG	PRO	332	40.184	-16.998	23.164	1.00	0.00	RX1	C
ATOM	5301	C	PRO	332	43.629	-16.280	21.797	1.00	0.00	RX1	C
ATOM	5302	O	PRO	332	44.291	-17.271	21.483	1.00	0.00	RX1	O
ATOM	5303	N	CYS	333	44.169	-15.099	22.134	1.00	0.00	RX1	N
ATOM	5304	H	CYS	333	43.569	-14.313	22.298	1.00	0.00	RX1	H
ATOM	5305	CA	CYS	333	45.617	-14.970	22.309	1.00	0.00	RX1	C

ATOM	5306	CB	CYS	333	45.906	-13.560	22.825	1.00	0.00	RX1	C
ATOM	5307	SG	CYS	333	47.672	-13.209	23.000	1.00	0.00	RX1	S
ATOM	5308	C	CYS	333	46.155	-16.054	23.230	1.00	0.00	RX1	C
ATOM	5309	O	CYS	333	45.471	-16.511	24.138	1.00	0.00	RX1	O
ATOM	5310	N	ARG	334	47.372	-16.512	22.904	1.00	0.00	RX1	N
ATOM	5311	H	ARG	334	47.984	-15.968	22.330	1.00	0.00	RX1	H
ATOM	5312	CA	ARG	334	47.821	-17.751	23.542	1.00	0.00	RX1	C
ATOM	5313	CB	ARG	334	49.046	-18.327	22.829	1.00	0.00	RX1	C
ATOM	5314	CG	ARG	334	48.793	-19.186	21.579	1.00	0.00	RX1	C
ATOM	5315	CD	ARG	334	48.262	-18.474	20.327	1.00	0.00	RX1	C
ATOM	5316	NE	ARG	334	46.805	-18.329	20.319	1.00	0.00	RX1	N
ATOM	5317	HE	ARG	334	46.357	-17.663	20.929	1.00	0.00	RX1	H
ATOM	5318	CZ	ARG	334	46.063	-19.073	19.447	1.00	0.00	RX1	C
ATOM	5319	NH1	ARG	334	46.656	-20.027	18.696	1.00	0.00	RX1	N
ATOM	5320	HH11	ARG	334	46.127	-20.593	18.057	1.00	0.00	RX1	H
ATOM	5321	HH12	ARG	334	47.642	-20.205	18.752	1.00	0.00	RX1	H
ATOM	5322	NH2	ARG	334	44.746	-18.840	19.342	1.00	0.00	RX1	N
ATOM	5323	HH21	ARG	334	44.122	-19.347	18.735	1.00	0.00	RX1	H
ATOM	5324	HH22	ARG	334	44.300	-18.097	19.869	1.00	0.00	RX1	H
ATOM	5325	C	ARG	334	48.104	-17.628	25.028	1.00	0.00	RX1	C
ATOM	5326	O	ARG	334	49.198	-17.274	25.444	1.00	0.00	RX1	O
ATOM	5327	N	LYS	335	47.054	-17.956	25.802	1.00	0.00	RX1	N
ATOM	5328	H	LYS	335	46.191	-18.126	25.325	1.00	0.00	RX1	H
ATOM	5329	CA	LYS	335	47.050	-17.851	27.263	1.00	0.00	RX1	C
ATOM	5330	CB	LYS	335	47.083	-19.237	27.942	1.00	0.00	RX1	C
ATOM	5331	CG	LYS	335	48.411	-19.970	28.169	1.00	0.00	RX1	C
ATOM	5332	CD	LYS	335	49.214	-20.354	26.927	1.00	0.00	RX1	C
ATOM	5333	CE	LYS	335	50.548	-20.998	27.310	1.00	0.00	RX1	C
ATOM	5334	NZ	LYS	335	51.281	-20.112	28.221	1.00	0.00	RX1	N
ATOM	5335	HZ1	LYS	335	52.069	-20.607	28.686	1.00	0.00	RX1	H
ATOM	5336	HZ2	LYS	335	51.661	-19.260	27.761	1.00	0.00	RX1	H
ATOM	5337	HZ3	LYS	335	50.675	-19.785	28.997	1.00	0.00	RX1	H
ATOM	5338	C	LYS	335	47.949	-16.785	27.879	1.00	0.00	RX1	C
ATOM	5339	O	LYS	335	49.098	-16.976	28.272	1.00	0.00	RX1	O
ATOM	5340	N	VAL	336	47.333	-15.600	27.869	1.00	0.00	RX1	N
ATOM	5341	H	VAL	336	46.360	-15.504	27.658	1.00	0.00	RX1	H
ATOM	5342	CA	VAL	336	48.014	-14.416	28.369	1.00	0.00	RX1	C
ATOM	5343	CB	VAL	336	48.258	-13.400	27.243	1.00	0.00	RX1	C
ATOM	5344	CG1	VAL	336	49.106	-14.009	26.128	1.00	0.00	RX1	C
ATOM	5345	CG2	VAL	336	46.953	-12.821	26.694	1.00	0.00	RX1	C
ATOM	5346	C	VAL	336	47.178	-13.796	29.466	1.00	0.00	RX1	C
ATOM	5347	O	VAL	336	45.957	-13.905	29.465	1.00	0.00	RX1	O
ATOM	5348	N	CYS	337	47.876	-13.133	30.386	1.00	0.00	RX1	N
ATOM	5349	H	CYS	337	48.864	-12.986	30.325	1.00	0.00	RX1	H
ATOM	5350	CA	CYS	337	47.102	-12.279	31.278	1.00	0.00	RX1	C
ATOM	5351	CB	CYS	337	47.409	-12.648	32.726	1.00	0.00	RX1	C
ATOM	5352	SG	CYS	337	47.155	-14.410	33.047	1.00	0.00	RX1	S
ATOM	5353	C	CYS	337	47.469	-10.855	30.957	1.00	0.00	RX1	C
ATOM	5354	O	CYS	337	48.463	-10.625	30.278	1.00	0.00	RX1	O
ATOM	5355	N	ASN	338	46.667	-9.907	31.465	1.00	0.00	RX1	N
ATOM	5356	H	ASN	338	45.824	-10.112	31.969	1.00	0.00	RX1	H
ATOM	5357	CA	ASN	338	47.160	-8.535	31.331	1.00	0.00	RX1	C
ATOM	5358	CB	ASN	338	46.075	-7.495	31.582	1.00	0.00	RX1	C
ATOM	5359	CG	ASN	338	45.441	-7.036	30.278	1.00	0.00	RX1	C
ATOM	5360	OD1	ASN	338	46.075	-6.533	29.361	1.00	0.00	RX1	O
ATOM	5361	ND2	ASN	338	44.105	-7.181	30.250	1.00	0.00	RX1	N
ATOM	5362	HD21	ASN	338	43.640	-7.710	30.971	1.00	0.00	RX1	H
ATOM	5363	HD22	ASN	338	43.537	-6.724	29.568	1.00	0.00	RX1	H
ATOM	5364	C	ASN	338	48.390	-8.281	32.191	1.00	0.00	RX1	C
ATOM	5365	O	ASN	338	49.501	-8.172	31.693	1.00	0.00	RX1	O
ATOM	5366	N	GLY	339	48.168	-8.266	33.513	1.00	0.00	RX1	N

ATOM	5367	H	GLY	339	47.280	-8.281	33.979	1.00	0.00	RX1	H
ATOM	5368	CA	GLY	339	49.336	-8.177	34.384	1.00	0.00	RX1	C
ATOM	5369	C	GLY	339	48.896	-7.957	35.810	1.00	0.00	RX1	C
ATOM	5370	O	GLY	339	47.811	-8.365	36.196	1.00	0.00	RX1	O
ATOM	5371	N	ILE	340	49.762	-7.281	36.570	1.00	0.00	RX1	N
ATOM	5372	H	ILE	340	50.594	-6.895	36.166	1.00	0.00	RX1	H
ATOM	5373	CA	ILE	340	49.316	-6.799	37.874	1.00	0.00	RX1	C
ATOM	5374	CB	ILE	340	50.353	-7.143	38.944	1.00	0.00	RX1	C
ATOM	5375	CG2	ILE	340	50.075	-6.420	40.261	1.00	0.00	RX1	C
ATOM	5376	CG1	ILE	340	50.397	-8.652	39.161	1.00	0.00	RX1	C
ATOM	5377	CD1	ILE	340	49.109	-9.151	39.820	1.00	0.00	RX1	C
ATOM	5378	C	ILE	340	49.117	-5.304	37.765	1.00	0.00	RX1	C
ATOM	5379	O	ILE	340	49.992	-4.586	37.297	1.00	0.00	RX1	O
ATOM	5380	N	GLY	341	47.922	-4.872	38.174	1.00	0.00	RX1	N
ATOM	5381	H	GLY	341	47.211	-5.477	38.532	1.00	0.00	RX1	H
ATOM	5382	CA	GLY	341	47.557	-3.506	37.827	1.00	0.00	RX1	C
ATOM	5383	C	GLY	341	46.781	-3.477	36.525	1.00	0.00	RX1	C
ATOM	5384	O	GLY	341	45.565	-3.338	36.493	1.00	0.00	RX1	O
ATOM	5385	N	ILE	342	47.547	-3.622	35.438	1.00	0.00	RX1	N
ATOM	5386	H	ILE	342	48.532	-3.779	35.520	1.00	0.00	RX1	H
ATOM	5387	CA	ILE	342	46.902	-3.475	34.134	1.00	0.00	RX1	C
ATOM	5388	CB	ILE	342	47.970	-3.394	33.048	1.00	0.00	RX1	C
ATOM	5389	CG2	ILE	342	48.620	-4.759	32.896	1.00	0.00	RX1	C
ATOM	5390	CG1	ILE	342	47.437	-2.811	31.737	1.00	0.00	RX1	C
ATOM	5391	CD1	ILE	342	48.495	-2.674	30.653	1.00	0.00	RX1	C
ATOM	5392	C	ILE	342	45.818	-4.511	33.817	1.00	0.00	RX1	C
ATOM	5393	O	ILE	342	45.839	-5.648	34.281	1.00	0.00	RX1	O
ATOM	5394	N	GLY	343	44.857	-4.054	32.995	1.00	0.00	RX1	N
ATOM	5395	H	GLY	343	44.883	-3.096	32.708	1.00	0.00	RX1	H
ATOM	5396	CA	GLY	343	43.763	-4.913	32.561	1.00	0.00	RX1	C
ATOM	5397	C	GLY	343	42.936	-5.463	33.691	1.00	0.00	RX1	C
ATOM	5398	O	GLY	343	42.386	-4.723	34.488	1.00	0.00	RX1	O
ATOM	5399	N	GLU	344	42.863	-6.787	33.744	1.00	0.00	RX1	N
ATOM	5400	H	GLU	344	43.436	-7.375	33.178	1.00	0.00	RX1	H
ATOM	5401	CA	GLU	344	41.945	-7.365	34.719	1.00	0.00	RX1	C
ATOM	5402	CB	GLU	344	41.402	-8.718	34.215	1.00	0.00	RX1	C
ATOM	5403	CG	GLU	344	42.380	-9.706	33.549	1.00	0.00	RX1	C
ATOM	5404	CD	GLU	344	42.651	-9.366	32.087	1.00	0.00	RX1	C
ATOM	5405	OE1	GLU	344	43.650	-9.805	31.526	1.00	0.00	RX1	O
ATOM	5406	OE2	GLU	344	41.869	-8.663	31.458	1.00	0.00	RX1	O
ATOM	5407	C	GLU	344	42.447	-7.412	36.167	1.00	0.00	RX1	C
ATOM	5408	O	GLU	344	42.300	-8.424	36.841	1.00	0.00	RX1	O
ATOM	5409	N	PHE	345	43.070	-6.295	36.614	1.00	0.00	RX1	N
ATOM	5410	H	PHE	345	43.103	-5.459	36.070	1.00	0.00	RX1	H
ATOM	5411	CA	PHE	345	43.817	-6.338	37.869	1.00	0.00	RX1	C
ATOM	5412	CB	PHE	345	45.195	-6.991	37.666	1.00	0.00	RX1	C
ATOM	5413	CG	PHE	345	45.144	-8.468	37.329	1.00	0.00	RX1	C
ATOM	5414	CD1	PHE	345	45.127	-9.413	38.347	1.00	0.00	RX1	C
ATOM	5415	CD2	PHE	345	45.138	-8.892	36.001	1.00	0.00	RX1	C
ATOM	5416	CE1	PHE	345	45.129	-10.768	38.035	1.00	0.00	RX1	C
ATOM	5417	CE2	PHE	345	45.144	-10.246	35.691	1.00	0.00	RX1	C
ATOM	5418	CZ	PHE	345	45.146	-11.187	36.711	1.00	0.00	RX1	C
ATOM	5419	C	PHE	345	44.054	-5.007	38.590	1.00	0.00	RX1	C
ATOM	5420	O	PHE	345	44.967	-4.929	39.407	1.00	0.00	RX1	O
ATOM	5421	N	LYS	346	43.248	-3.959	38.298	1.00	0.00	RX1	N
ATOM	5422	H	LYS	346	42.452	-4.060	37.701	1.00	0.00	RX1	H
ATOM	5423	CA	LYS	346	43.650	-2.643	38.847	1.00	0.00	RX1	C
ATOM	5424	CB	LYS	346	42.845	-1.483	38.226	1.00	0.00	RX1	C
ATOM	5425	CG	LYS	346	43.139	-0.038	38.678	1.00	0.00	RX1	C
ATOM	5426	CD	LYS	346	42.303	1.020	37.927	1.00	0.00	RX1	C
ATOM	5427	CE	LYS	346	42.594	2.483	38.315	1.00	0.00	RX1	C

ATOM	5428	NZ	LYS	346	42.017	3.450	37.358	1.00	0.00	RX1	N
ATOM	5429	HZ1	LYS	346	42.089	4.431	37.711	1.00	0.00	RX1	H
ATOM	5430	HZ2	LYS	346	42.472	3.463	36.427	1.00	0.00	RX1	H
ATOM	5431	HZ3	LYS	346	40.988	3.392	37.184	1.00	0.00	RX1	H
ATOM	5432	C	LYS	346	43.739	-2.522	40.368	1.00	0.00	RX1	C
ATOM	5433	O	LYS	346	44.583	-1.809	40.898	1.00	0.00	RX1	O
ATOM	5434	N	ASP	347	42.874	-3.283	41.058	1.00	0.00	RX1	N
ATOM	5435	H	ASP	347	42.226	-3.877	40.585	1.00	0.00	RX1	H
ATOM	5436	CA	ASP	347	42.937	-3.283	42.529	1.00	0.00	RX1	C
ATOM	5437	CB	ASP	347	41.574	-3.721	43.086	1.00	0.00	RX1	C
ATOM	5438	CG	ASP	347	41.078	-2.829	44.217	1.00	0.00	RX1	C
ATOM	5439	OD1	ASP	347	40.204	-3.248	44.974	1.00	0.00	RX1	O
ATOM	5440	OD2	ASP	347	41.472	-1.669	44.316	1.00	0.00	RX1	O
ATOM	5441	C	ASP	347	44.058	-4.141	43.133	1.00	0.00	RX1	C
ATOM	5442	O	ASP	347	43.896	-4.846	44.121	1.00	0.00	RX1	O
ATOM	5443	N	SER	348	45.228	-4.087	42.473	1.00	0.00	RX1	N
ATOM	5444	H	SER	348	45.404	-3.447	41.725	1.00	0.00	RX1	H
ATOM	5445	CA	SER	348	46.289	-5.003	42.886	1.00	0.00	RX1	C
ATOM	5446	CB	SER	348	46.291	-6.225	41.973	1.00	0.00	RX1	C
ATOM	5447	OG	SER	348	44.945	-6.521	41.592	1.00	0.00	RX1	O
ATOM	5448	HG	SER	348	44.708	-5.815	41.001	1.00	0.00	RX1	H
ATOM	5449	C	SER	348	47.646	-4.338	42.982	1.00	0.00	RX1	C
ATOM	5450	O	SER	348	48.355	-4.181	41.998	1.00	0.00	RX1	O
ATOM	5451	N	LEU	349	47.996	-3.963	44.223	1.00	0.00	RX1	N
ATOM	5452	H	LEU	349	47.377	-4.118	44.993	1.00	0.00	RX1	H
ATOM	5453	CA	LEU	349	49.277	-3.264	44.368	1.00	0.00	RX1	C
ATOM	5454	CB	LEU	349	49.330	-2.574	45.737	1.00	0.00	RX1	C
ATOM	5455	CG	LEU	349	49.189	-3.528	46.930	1.00	0.00	RX1	C
ATOM	5456	CD1	LEU	349	50.515	-3.753	47.659	1.00	0.00	RX1	C
ATOM	5457	CD2	LEU	349	48.084	-3.080	47.888	1.00	0.00	RX1	C
ATOM	5458	C	LEU	349	50.544	-4.076	44.106	1.00	0.00	RX1	C
ATOM	5459	O	LEU	349	51.583	-3.543	43.744	1.00	0.00	RX1	O
ATOM	5460	N	SER	350	50.416	-5.396	44.298	1.00	0.00	RX1	N
ATOM	5461	H	SER	350	49.578	-5.860	44.579	1.00	0.00	RX1	H
ATOM	5462	CA	SER	350	51.573	-6.245	44.026	1.00	0.00	RX1	C
ATOM	5463	CB	SER	350	52.472	-6.251	45.274	1.00	0.00	RX1	C
ATOM	5464	OG	SER	350	53.720	-6.922	45.040	1.00	0.00	RX1	O
ATOM	5465	HG	SER	350	53.550	-7.857	45.167	1.00	0.00	RX1	H
ATOM	5466	C	SER	350	51.109	-7.627	43.647	1.00	0.00	RX1	C
ATOM	5467	O	SER	350	50.028	-8.068	44.032	1.00	0.00	RX1	O
ATOM	5468	N	ILE	351	51.998	-8.309	42.913	1.00	0.00	RX1	N
ATOM	5469	H	ILE	351	52.859	-7.862	42.663	1.00	0.00	RX1	H
ATOM	5470	CA	ILE	351	51.852	-9.756	42.807	1.00	0.00	RX1	C
ATOM	5471	CB	ILE	351	52.813	-10.280	41.729	1.00	0.00	RX1	C
ATOM	5472	CG2	ILE	351	54.269	-10.142	42.172	1.00	0.00	RX1	C
ATOM	5473	CG1	ILE	351	52.465	-11.690	41.248	1.00	0.00	RX1	C
ATOM	5474	CD1	ILE	351	53.315	-12.105	40.046	1.00	0.00	RX1	C
ATOM	5475	C	ILE	351	52.086	-10.384	44.177	1.00	0.00	RX1	C
ATOM	5476	O	ILE	351	52.758	-9.803	45.024	1.00	0.00	RX1	O
ATOM	5477	N	ASN	352	51.438	-11.539	44.371	1.00	0.00	RX1	N
ATOM	5478	H	ASN	352	50.966	-12.041	43.643	1.00	0.00	RX1	H
ATOM	5479	CA	ASN	352	51.326	-12.151	45.695	1.00	0.00	RX1	C
ATOM	5480	CB	ASN	352	50.355	-11.372	46.580	1.00	0.00	RX1	C
ATOM	5481	CG	ASN	352	48.985	-11.384	45.937	1.00	0.00	RX1	C
ATOM	5482	OD1	ASN	352	48.278	-12.383	45.931	1.00	0.00	RX1	O
ATOM	5483	ND2	ASN	352	48.633	-10.203	45.396	1.00	0.00	RX1	N
ATOM	5484	HD21	ASN	352	49.269	-9.426	45.342	1.00	0.00	RX1	H
ATOM	5485	HD22	ASN	352	47.724	-10.027	45.017	1.00	0.00	RX1	H
ATOM	5486	C	ASN	352	50.828	-13.567	45.499	1.00	0.00	RX1	C
ATOM	5487	O	ASN	352	50.340	-13.888	44.419	1.00	0.00	RX1	O
ATOM	5488	N	ALA	353	50.958	-14.394	46.552	1.00	0.00	RX1	N

ATOM	5489	H	ALA	353	51.277	-14.088	47.452	1.00	0.00	RX1	H
ATOM	5490	CA	ALA	353	50.550	-15.790	46.364	1.00	0.00	RX1	C
ATOM	5491	CB	ALA	353	50.821	-16.614	47.621	1.00	0.00	RX1	C
ATOM	5492	C	ALA	353	49.090	-15.954	45.977	1.00	0.00	RX1	C
ATOM	5493	O	ALA	353	48.727	-16.666	45.045	1.00	0.00	RX1	O
ATOM	5494	N	THR	354	48.264	-15.192	46.709	1.00	0.00	RX1	N
ATOM	5495	H	THR	354	48.588	-14.592	47.440	1.00	0.00	RX1	H
ATOM	5496	CA	THR	354	46.823	-15.196	46.466	1.00	0.00	RX1	C
ATOM	5497	CB	THR	354	46.257	-14.152	47.403	1.00	0.00	RX1	C
ATOM	5498	OG1	THR	354	47.145	-14.054	48.521	1.00	0.00	RX1	O
ATOM	5499	HG1	THR	354	46.660	-13.566	49.177	1.00	0.00	RX1	H
ATOM	5500	CG2	THR	354	44.828	-14.465	47.849	1.00	0.00	RX1	C
ATOM	5501	C	THR	354	46.381	-15.023	45.019	1.00	0.00	RX1	C
ATOM	5502	O	THR	354	45.337	-15.523	44.603	1.00	0.00	RX1	O
ATOM	5503	N	ASN	355	47.217	-14.311	44.246	1.00	0.00	RX1	N
ATOM	5504	H	ASN	355	48.048	-13.887	44.618	1.00	0.00	RX1	H
ATOM	5505	CA	ASN	355	46.834	-14.216	42.843	1.00	0.00	RX1	C
ATOM	5506	CB	ASN	355	46.423	-12.806	42.446	1.00	0.00	RX1	C
ATOM	5507	CG	ASN	355	45.177	-12.968	41.608	1.00	0.00	RX1	C
ATOM	5508	OD1	ASN	355	44.539	-14.022	41.619	1.00	0.00	RX1	O
ATOM	5509	ND2	ASN	355	44.822	-11.859	40.938	1.00	0.00	RX1	N
ATOM	5510	HD21	ASN	355	45.385	-11.030	40.966	1.00	0.00	RX1	H
ATOM	5511	HD22	ASN	355	43.986	-11.788	40.392	1.00	0.00	RX1	H
ATOM	5512	C	ASN	355	47.737	-14.824	41.792	1.00	0.00	RX1	C
ATOM	5513	O	ASN	355	47.308	-15.035	40.662	1.00	0.00	RX1	O
ATOM	5514	N	ILE	356	48.985	-15.148	42.191	1.00	0.00	RX1	N
ATOM	5515	H	ILE	356	49.284	-15.018	43.139	1.00	0.00	RX1	H
ATOM	5516	CA	ILE	356	49.937	-15.644	41.184	1.00	0.00	RX1	C
ATOM	5517	CB	ILE	356	51.337	-15.872	41.775	1.00	0.00	RX1	C
ATOM	5518	CG2	ILE	356	51.384	-17.110	42.673	1.00	0.00	RX1	C
ATOM	5519	CG1	ILE	356	52.383	-15.908	40.654	1.00	0.00	RX1	C
ATOM	5520	CD1	ILE	356	53.825	-16.083	41.133	1.00	0.00	RX1	C
ATOM	5521	C	ILE	356	49.479	-16.851	40.361	1.00	0.00	RX1	C
ATOM	5522	O	ILE	356	49.859	-17.033	39.213	1.00	0.00	RX1	O
ATOM	5523	N	LYS	357	48.573	-17.632	40.982	1.00	0.00	RX1	N
ATOM	5524	H	LYS	357	48.468	-17.468	41.961	1.00	0.00	RX1	H
ATOM	5525	CA	LYS	357	47.905	-18.740	40.280	1.00	0.00	RX1	C
ATOM	5526	CB	LYS	357	46.600	-19.126	40.980	1.00	0.00	RX1	C
ATOM	5527	CG	LYS	357	46.599	-19.014	42.503	1.00	0.00	RX1	C
ATOM	5528	CD	LYS	357	45.267	-18.414	42.949	1.00	0.00	RX1	C
ATOM	5529	CE	LYS	357	44.945	-17.258	42.002	1.00	0.00	RX1	C
ATOM	5530	NZ	LYS	357	43.909	-16.359	42.520	1.00	0.00	RX1	N
ATOM	5531	HZ1	LYS	357	43.845	-15.540	41.874	1.00	0.00	RX1	H
ATOM	5532	HZ2	LYS	357	42.988	-16.827	42.579	1.00	0.00	RX1	H
ATOM	5533	HZ3	LYS	357	44.207	-15.995	43.451	1.00	0.00	RX1	H
ATOM	5534	C	LYS	357	47.549	-18.483	38.817	1.00	0.00	RX1	C
ATOM	5535	O	LYS	357	47.748	-19.319	37.949	1.00	0.00	RX1	O
ATOM	5536	N	HIS	358	47.005	-17.273	38.581	1.00	0.00	RX1	N
ATOM	5537	H	HIS	358	47.009	-16.560	39.285	1.00	0.00	RX1	H
ATOM	5538	CA	HIS	358	46.552	-16.972	37.216	1.00	0.00	RX1	C
ATOM	5539	CB	HIS	358	45.842	-15.612	37.135	1.00	0.00	RX1	C
ATOM	5540	CG	HIS	358	44.607	-15.538	38.007	1.00	0.00	RX1	C
ATOM	5541	ND1	HIS	358	43.901	-14.406	38.187	1.00	0.00	RX1	N
ATOM	5542	HD1	HIS	358	44.077	-13.538	37.763	1.00	0.00	RX1	H
ATOM	5543	CD2	HIS	358	44.011	-16.552	38.762	1.00	0.00	RX1	C
ATOM	5544	NE2	HIS	358	42.946	-16.020	39.411	1.00	0.00	RX1	N
ATOM	5545	CE1	HIS	358	42.876	-14.696	39.050	1.00	0.00	RX1	C
ATOM	5546	C	HIS	358	47.684	-16.968	36.198	1.00	0.00	RX1	C
ATOM	5547	O	HIS	358	47.566	-17.397	35.059	1.00	0.00	RX1	O
ATOM	5548	N	PHE	359	48.822	-16.469	36.696	1.00	0.00	RX1	N
ATOM	5549	H	PHE	359	48.921	-16.292	37.674	1.00	0.00	RX1	H

ATOM	5550	CA	PHE	359	50.002	-16.350	35.846	1.00	0.00	RX1	C
ATOM	5551	CB	PHE	359	50.960	-15.312	36.436	1.00	0.00	RX1	C
ATOM	5552	CG	PHE	359	50.212	-14.030	36.713	1.00	0.00	RX1	C
ATOM	5553	CD1	PHE	359	49.925	-13.156	35.672	1.00	0.00	RX1	C
ATOM	5554	CD2	PHE	359	49.803	-13.726	38.007	1.00	0.00	RX1	C
ATOM	5555	CE1	PHE	359	49.216	-11.986	35.921	1.00	0.00	RX1	C
ATOM	5556	CE2	PHE	359	49.092	-12.560	38.256	1.00	0.00	RX1	C
ATOM	5557	CZ	PHE	359	48.792	-11.694	37.211	1.00	0.00	RX1	C
ATOM	5558	C	PHE	359	50.740	-17.655	35.590	1.00	0.00	RX1	C
ATOM	5559	O	PHE	359	51.715	-17.696	34.850	1.00	0.00	RX1	O
ATOM	5560	N	LYS	360	50.246	-18.732	36.241	1.00	0.00	RX1	N
ATOM	5561	H	LYS	360	49.397	-18.663	36.765	1.00	0.00	RX1	H
ATOM	5562	CA	LYS	360	51.002	-19.988	36.295	1.00	0.00	RX1	C
ATOM	5563	CB	LYS	360	50.232	-21.042	37.096	1.00	0.00	RX1	C
ATOM	5564	CG	LYS	360	51.155	-22.048	37.786	1.00	0.00	RX1	C
ATOM	5565	CD	LYS	360	50.448	-22.892	38.846	1.00	0.00	RX1	C
ATOM	5566	CE	LYS	360	51.434	-23.712	39.684	1.00	0.00	RX1	C
ATOM	5567	NZ	LYS	360	52.357	-22.813	40.382	1.00	0.00	RX1	N
ATOM	5568	HZ1	LYS	360	53.015	-22.292	39.777	1.00	0.00	RX1	H
ATOM	5569	HZ2	LYS	360	51.884	-22.107	40.986	1.00	0.00	RX1	H
ATOM	5570	HZ3	LYS	360	52.962	-23.296	41.084	1.00	0.00	RX1	H
ATOM	5571	C	LYS	360	51.523	-20.551	34.981	1.00	0.00	RX1	C
ATOM	5572	O	LYS	360	52.523	-21.259	34.933	1.00	0.00	RX1	O
ATOM	5573	N	ASN	361	50.810	-20.189	33.907	1.00	0.00	RX1	N
ATOM	5574	H	ASN	361	49.998	-19.608	33.969	1.00	0.00	RX1	H
ATOM	5575	CA	ASN	361	51.431	-20.432	32.612	1.00	0.00	RX1	C
ATOM	5576	CB	ASN	361	51.054	-21.788	32.033	1.00	0.00	RX1	C
ATOM	5577	CG	ASN	361	52.144	-22.186	31.063	1.00	0.00	RX1	C
ATOM	5578	OD1	ASN	361	52.214	-21.749	29.917	1.00	0.00	RX1	O
ATOM	5579	ND2	ASN	361	52.999	-23.078	31.592	1.00	0.00	RX1	N
ATOM	5580	HD21	ASN	361	52.903	-23.338	32.556	1.00	0.00	RX1	H
ATOM	5581	HD22	ASN	361	53.748	-23.492	31.076	1.00	0.00	RX1	H
ATOM	5582	C	ASN	361	51.131	-19.342	31.607	1.00	0.00	RX1	C
ATOM	5583	O	ASN	361	50.428	-19.553	30.624	1.00	0.00	RX1	O
ATOM	5584	N	CYS	362	51.687	-18.160	31.889	1.00	0.00	RX1	N
ATOM	5585	H	CYS	362	52.309	-18.048	32.669	1.00	0.00	RX1	H
ATOM	5586	CA	CYS	362	51.514	-17.068	30.929	1.00	0.00	RX1	C
ATOM	5587	CB	CYS	362	51.939	-15.753	31.577	1.00	0.00	RX1	C
ATOM	5588	SG	CYS	362	50.772	-15.209	32.845	1.00	0.00	RX1	S
ATOM	5589	C	CYS	362	52.238	-17.302	29.609	1.00	0.00	RX1	C
ATOM	5590	O	CYS	362	52.792	-18.373	29.366	1.00	0.00	RX1	O
ATOM	5591	N	THR	363	52.204	-16.264	28.762	1.00	0.00	RX1	N
ATOM	5592	H	THR	363	51.670	-15.432	28.911	1.00	0.00	RX1	H
ATOM	5593	CA	THR	363	53.054	-16.321	27.575	1.00	0.00	RX1	C
ATOM	5594	CB	THR	363	52.249	-16.930	26.437	1.00	0.00	RX1	C
ATOM	5595	OG1	THR	363	51.305	-17.863	26.973	1.00	0.00	RX1	O
ATOM	5596	HG1	THR	363	50.456	-17.412	26.924	1.00	0.00	RX1	H
ATOM	5597	CG2	THR	363	53.145	-17.582	25.383	1.00	0.00	RX1	C
ATOM	5598	C	THR	363	53.566	-14.931	27.248	1.00	0.00	RX1	C
ATOM	5599	O	THR	363	54.721	-14.580	27.448	1.00	0.00	RX1	O
ATOM	5600	N	SER	364	52.610	-14.116	26.799	1.00	0.00	RX1	N
ATOM	5601	H	SER	364	51.656	-14.376	26.677	1.00	0.00	RX1	H
ATOM	5602	CA	SER	364	52.875	-12.697	26.944	1.00	0.00	RX1	C
ATOM	5603	CB	SER	364	52.438	-11.992	25.650	1.00	0.00	RX1	C
ATOM	5604	OG	SER	364	52.956	-10.660	25.580	1.00	0.00	RX1	O
ATOM	5605	HG	SER	364	53.444	-10.612	24.755	1.00	0.00	RX1	H
ATOM	5606	C	SER	364	52.144	-12.240	28.192	1.00	0.00	RX1	C
ATOM	5607	O	SER	364	51.263	-12.931	28.705	1.00	0.00	RX1	O
ATOM	5608	N	ILE	365	52.572	-11.070	28.649	1.00	0.00	RX1	N
ATOM	5609	H	ILE	365	53.302	-10.588	28.157	1.00	0.00	RX1	H
ATOM	5610	CA	ILE	365	51.907	-10.323	29.700	1.00	0.00	RX1	C

ATOM	5611	CB	ILE	365	52.837	-10.191	30.912	1.00	0.00	RX1	C
ATOM	5612	CG2	ILE	365	52.547	-8.965	31.776	1.00	0.00	RX1	C
ATOM	5613	CG1	ILE	365	52.744	-11.465	31.749	1.00	0.00	RX1	C
ATOM	5614	CD1	ILE	365	51.345	-11.644	32.339	1.00	0.00	RX1	C
ATOM	5615	C	ILE	365	51.587	-8.994	29.065	1.00	0.00	RX1	C
ATOM	5616	O	ILE	365	52.454	-8.261	28.597	1.00	0.00	RX1	O
ATOM	5617	N	SER	366	50.282	-8.757	29.005	1.00	0.00	RX1	N
ATOM	5618	H	SER	366	49.654	-9.295	29.568	1.00	0.00	RX1	H
ATOM	5619	CA	SER	366	49.788	-7.550	28.366	1.00	0.00	RX1	C
ATOM	5620	CB	SER	366	48.414	-7.971	27.831	1.00	0.00	RX1	C
ATOM	5621	OG	SER	366	47.706	-6.909	27.197	1.00	0.00	RX1	O
ATOM	5622	HG	SER	366	47.337	-6.409	27.929	1.00	0.00	RX1	H
ATOM	5623	C	SER	366	49.849	-6.378	29.330	1.00	0.00	RX1	C
ATOM	5624	O	SER	366	48.858	-5.918	29.882	1.00	0.00	RX1	O
ATOM	5625	N	GLY	367	51.092	-5.933	29.515	1.00	0.00	RX1	N
ATOM	5626	H	GLY	367	51.866	-6.353	29.039	1.00	0.00	RX1	H
ATOM	5627	CA	GLY	367	51.321	-4.888	30.493	1.00	0.00	RX1	C
ATOM	5628	C	GLY	367	52.369	-5.281	31.500	1.00	0.00	RX1	C
ATOM	5629	O	GLY	367	53.406	-5.840	31.154	1.00	0.00	RX1	O
ATOM	5630	N	ASP	368	52.055	-4.926	32.744	1.00	0.00	RX1	N
ATOM	5631	H	ASP	368	51.173	-4.512	32.962	1.00	0.00	RX1	H
ATOM	5632	CA	ASP	368	53.114	-4.742	33.726	1.00	0.00	RX1	C
ATOM	5633	CB	ASP	368	52.952	-3.359	34.340	1.00	0.00	RX1	C
ATOM	5634	CG	ASP	368	52.460	-2.390	33.284	1.00	0.00	RX1	C
ATOM	5635	OD1	ASP	368	53.251	-1.948	32.461	1.00	0.00	RX1	O
ATOM	5636	OD2	ASP	368	51.277	-2.073	33.273	1.00	0.00	RX1	O
ATOM	5637	C	ASP	368	53.110	-5.775	34.828	1.00	0.00	RX1	C
ATOM	5638	O	ASP	368	52.099	-6.401	35.132	1.00	0.00	RX1	O
ATOM	5639	N	LEU	369	54.286	-5.909	35.455	1.00	0.00	RX1	N
ATOM	5640	H	LEU	369	55.092	-5.396	35.150	1.00	0.00	RX1	H
ATOM	5641	CA	LEU	369	54.334	-6.709	36.676	1.00	0.00	RX1	C
ATOM	5642	CB	LEU	369	55.131	-7.999	36.465	1.00	0.00	RX1	C
ATOM	5643	CG	LEU	369	54.423	-9.015	35.562	1.00	0.00	RX1	C
ATOM	5644	CD1	LEU	369	55.314	-10.217	35.245	1.00	0.00	RX1	C
ATOM	5645	CD2	LEU	369	53.078	-9.456	36.145	1.00	0.00	RX1	C
ATOM	5646	C	LEU	369	54.885	-5.926	37.851	1.00	0.00	RX1	C
ATOM	5647	O	LEU	369	56.040	-5.515	37.884	1.00	0.00	RX1	O
ATOM	5648	N	HIS	370	53.980	-5.732	38.820	1.00	0.00	RX1	N
ATOM	5649	H	HIS	370	53.089	-6.179	38.771	1.00	0.00	RX1	H
ATOM	5650	CA	HIS	370	54.364	-5.029	40.045	1.00	0.00	RX1	C
ATOM	5651	CB	HIS	370	53.170	-4.247	40.596	1.00	0.00	RX1	C
ATOM	5652	CG	HIS	370	52.850	-3.042	39.744	1.00	0.00	RX1	C
ATOM	5653	ND1	HIS	370	51.952	-3.022	38.738	1.00	0.00	RX1	N
ATOM	5654	HD1	HIS	370	51.389	-3.756	38.402	1.00	0.00	RX1	H
ATOM	5655	CD2	HIS	370	53.406	-1.768	39.869	1.00	0.00	RX1	C
ATOM	5656	NE2	HIS	370	52.837	-0.973	38.932	1.00	0.00	RX1	N
ATOM	5657	CE1	HIS	370	51.939	-1.747	38.236	1.00	0.00	RX1	C
ATOM	5658	C	HIS	370	54.847	-5.990	41.115	1.00	0.00	RX1	C
ATOM	5659	O	HIS	370	54.076	-6.800	41.618	1.00	0.00	RX1	O
ATOM	5660	N	ILE	371	56.139	-5.873	41.449	1.00	0.00	RX1	N
ATOM	5661	H	ILE	371	56.767	-5.218	41.023	1.00	0.00	RX1	H
ATOM	5662	CA	ILE	371	56.651	-6.689	42.552	1.00	0.00	RX1	C
ATOM	5663	CB	ILE	371	57.767	-7.627	42.081	1.00	0.00	RX1	C
ATOM	5664	CG2	ILE	371	58.102	-8.651	43.167	1.00	0.00	RX1	C
ATOM	5665	CG1	ILE	371	57.408	-8.302	40.756	1.00	0.00	RX1	C
ATOM	5666	CD1	ILE	371	58.487	-9.279	40.295	1.00	0.00	RX1	C
ATOM	5667	C	ILE	371	57.140	-5.818	43.700	1.00	0.00	RX1	C
ATOM	5668	O	ILE	371	58.318	-5.495	43.830	1.00	0.00	RX1	O
ATOM	5669	N	LEU	372	56.153	-5.425	44.513	1.00	0.00	RX1	N
ATOM	5670	H	LEU	372	55.259	-5.875	44.464	1.00	0.00	RX1	H
ATOM	5671	CA	LEU	372	56.435	-4.515	45.626	1.00	0.00	RX1	C

ATOM	5672	CB	LEU	372	55.145	-3.785	46.030	1.00	0.00	RX1	C
ATOM	5673	CG	LEU	372	54.894	-2.450	45.318	1.00	0.00	RX1	C
ATOM	5674	CD1	LEU	372	54.779	-2.576	43.799	1.00	0.00	RX1	C
ATOM	5675	CD2	LEU	372	53.686	-1.730	45.915	1.00	0.00	RX1	C
ATOM	5676	C	LEU	372	57.017	-5.273	46.811	1.00	0.00	RX1	C
ATOM	5677	O	LEU	372	56.721	-6.449	46.992	1.00	0.00	RX1	O
ATOM	5678	N	PRO	373	57.853	-4.582	47.639	1.00	0.00	RX1	N
ATOM	5679	CD	PRO	373	58.225	-3.174	47.539	1.00	0.00	RX1	C
ATOM	5680	CA	PRO	373	58.527	-5.247	48.767	1.00	0.00	RX1	C
ATOM	5681	CB	PRO	373	59.075	-4.068	49.574	1.00	0.00	RX1	C
ATOM	5682	CG	PRO	373	59.382	-3.011	48.517	1.00	0.00	RX1	C
ATOM	5683	C	PRO	373	57.696	-6.224	49.586	1.00	0.00	RX1	C
ATOM	5684	O	PRO	373	58.096	-7.344	49.888	1.00	0.00	RX1	O
ATOM	5685	N	VAL	374	56.488	-5.743	49.917	1.00	0.00	RX1	N
ATOM	5686	H	VAL	374	56.194	-4.846	49.594	1.00	0.00	RX1	H
ATOM	5687	CA	VAL	374	55.619	-6.542	50.778	1.00	0.00	RX1	C
ATOM	5688	CB	VAL	374	54.366	-5.735	51.135	1.00	0.00	RX1	C
ATOM	5689	CG1	VAL	374	53.382	-5.654	49.965	1.00	0.00	RX1	C
ATOM	5690	CG2	VAL	374	53.739	-6.239	52.434	1.00	0.00	RX1	C
ATOM	5691	C	VAL	374	55.285	-7.953	50.286	1.00	0.00	RX1	C
ATOM	5692	O	VAL	374	54.981	-8.847	51.066	1.00	0.00	RX1	O
ATOM	5693	N	ALA	375	55.407	-8.145	48.957	1.00	0.00	RX1	N
ATOM	5694	H	ALA	375	55.709	-7.407	48.353	1.00	0.00	RX1	H
ATOM	5695	CA	ALA	375	55.167	-9.473	48.388	1.00	0.00	RX1	C
ATOM	5696	CB	ALA	375	55.520	-9.496	46.899	1.00	0.00	RX1	C
ATOM	5697	C	ALA	375	55.919	-10.606	49.070	1.00	0.00	RX1	C
ATOM	5698	O	ALA	375	55.385	-11.679	49.318	1.00	0.00	RX1	O
ATOM	5699	N	PHE	376	57.189	-10.319	49.397	1.00	0.00	RX1	N
ATOM	5700	H	PHE	376	57.567	-9.393	49.320	1.00	0.00	RX1	H
ATOM	5701	CA	PHE	376	57.946	-11.402	50.025	1.00	0.00	RX1	C
ATOM	5702	CB	PHE	376	59.361	-11.460	49.456	1.00	0.00	RX1	C
ATOM	5703	CG	PHE	376	59.248	-11.780	47.986	1.00	0.00	RX1	C
ATOM	5704	CD1	PHE	376	59.613	-10.839	47.035	1.00	0.00	RX1	C
ATOM	5705	CD2	PHE	376	58.761	-13.015	47.584	1.00	0.00	RX1	C
ATOM	5706	CE1	PHE	376	59.473	-11.121	45.682	1.00	0.00	RX1	C
ATOM	5707	CE2	PHE	376	58.625	-13.300	46.231	1.00	0.00	RX1	C
ATOM	5708	CZ	PHE	376	58.970	-12.349	45.279	1.00	0.00	RX1	C
ATOM	5709	C	PHE	376	57.948	-11.404	51.543	1.00	0.00	RX1	C
ATOM	5710	O	PHE	376	58.837	-11.936	52.200	1.00	0.00	RX1	O
ATOM	5711	N	ARG	377	56.888	-10.781	52.074	1.00	0.00	RX1	N
ATOM	5712	H	ARG	377	56.219	-10.312	51.499	1.00	0.00	RX1	H
ATOM	5713	CA	ARG	377	56.607	-10.904	53.502	1.00	0.00	RX1	C
ATOM	5714	CB	ARG	377	56.935	-9.607	54.245	1.00	0.00	RX1	C
ATOM	5715	CG	ARG	377	58.439	-9.407	54.441	1.00	0.00	RX1	C
ATOM	5716	CD	ARG	377	59.068	-10.640	55.098	1.00	0.00	RX1	C
ATOM	5717	NE	ARG	377	60.470	-10.422	55.445	1.00	0.00	RX1	N
ATOM	5718	HE	ARG	377	60.628	-9.869	56.267	1.00	0.00	RX1	H
ATOM	5719	CZ	ARG	377	61.473	-10.980	54.696	1.00	0.00	RX1	C
ATOM	5720	NH1	ARG	377	61.209	-11.663	53.564	1.00	0.00	RX1	N
ATOM	5721	HH11	ARG	377	61.990	-12.029	53.024	1.00	0.00	RX1	H
ATOM	5722	HH12	ARG	377	60.284	-11.829	53.196	1.00	0.00	RX1	H
ATOM	5723	NH2	ARG	377	62.748	-10.846	55.104	1.00	0.00	RX1	N
ATOM	5724	HH21	ARG	377	63.468	-11.321	54.569	1.00	0.00	RX1	H
ATOM	5725	HH22	ARG	377	63.041	-10.323	55.904	1.00	0.00	RX1	H
ATOM	5726	C	ARG	377	55.181	-11.331	53.793	1.00	0.00	RX1	C
ATOM	5727	O	ARG	377	54.899	-11.978	54.791	1.00	0.00	RX1	O
ATOM	5728	N	GLY	378	54.302	-10.963	52.852	1.00	0.00	RX1	N
ATOM	5729	H	GLY	378	54.540	-10.323	52.126	1.00	0.00	RX1	H
ATOM	5730	CA	GLY	378	52.886	-11.193	53.094	1.00	0.00	RX1	C
ATOM	5731	C	GLY	378	52.224	-9.918	53.558	1.00	0.00	RX1	C
ATOM	5732	O	GLY	378	52.617	-9.319	54.553	1.00	0.00	RX1	O

ATOM	5733	N	ASP	379	51.229	-9.490	52.771	1.00	0.00	RX1	N
ATOM	5734	H	ASP	379	50.752	-10.092	52.129	1.00	0.00	RX1	H
ATOM	5735	CA	ASP	379	50.632	-8.234	53.207	1.00	0.00	RX1	C
ATOM	5736	CB	ASP	379	50.141	-7.369	52.043	1.00	0.00	RX1	C
ATOM	5737	CG	ASP	379	49.586	-6.057	52.572	1.00	0.00	RX1	C
ATOM	5738	OD1	ASP	379	50.077	-5.554	53.577	1.00	0.00	RX1	O
ATOM	5739	OD2	ASP	379	48.639	-5.537	51.994	1.00	0.00	RX1	O
ATOM	5740	C	ASP	379	49.544	-8.444	54.231	1.00	0.00	RX1	C
ATOM	5741	O	ASP	379	48.627	-9.247	54.088	1.00	0.00	RX1	O
ATOM	5742	N	SER	380	49.703	-7.661	55.300	1.00	0.00	RX1	N
ATOM	5743	H	SER	380	50.372	-6.914	55.269	1.00	0.00	RX1	H
ATOM	5744	CA	SER	380	48.674	-7.693	56.326	1.00	0.00	RX1	C
ATOM	5745	CB	SER	380	49.354	-7.028	57.499	1.00	0.00	RX1	C
ATOM	5746	OG	SER	380	50.761	-7.166	57.256	1.00	0.00	RX1	O
ATOM	5747	HG	SER	380	50.911	-8.064	56.981	1.00	0.00	RX1	H
ATOM	5748	C	SER	380	47.364	-7.072	55.873	1.00	0.00	RX1	C
ATOM	5749	O	SER	380	46.286	-7.575	56.166	1.00	0.00	RX1	O
ATOM	5750	N	PHE	381	47.494	-5.964	55.124	1.00	0.00	RX1	N
ATOM	5751	H	PHE	381	48.376	-5.696	54.720	1.00	0.00	RX1	H
ATOM	5752	CA	PHE	381	46.279	-5.252	54.734	1.00	0.00	RX1	C
ATOM	5753	CB	PHE	381	46.640	-3.872	54.181	1.00	0.00	RX1	C
ATOM	5754	CG	PHE	381	45.388	-3.154	53.740	1.00	0.00	RX1	C
ATOM	5755	CD1	PHE	381	44.418	-2.807	54.673	1.00	0.00	RX1	C
ATOM	5756	CD2	PHE	381	45.205	-2.844	52.398	1.00	0.00	RX1	C
ATOM	5757	CE1	PHE	381	43.260	-2.159	54.262	1.00	0.00	RX1	C
ATOM	5758	CE2	PHE	381	44.046	-2.196	51.986	1.00	0.00	RX1	C
ATOM	5759	CZ	PHE	381	43.073	-1.857	52.918	1.00	0.00	RX1	C
ATOM	5760	C	PHE	381	45.408	-6.026	53.757	1.00	0.00	RX1	C
ATOM	5761	O	PHE	381	44.196	-6.114	53.893	1.00	0.00	RX1	O
ATOM	5762	N	THR	382	46.092	-6.616	52.773	1.00	0.00	RX1	N
ATOM	5763	H	THR	382	47.088	-6.544	52.697	1.00	0.00	RX1	H
ATOM	5764	CA	THR	382	45.328	-7.416	51.820	1.00	0.00	RX1	C
ATOM	5765	CB	THR	382	45.867	-7.194	50.402	1.00	0.00	RX1	C
ATOM	5766	OG1	THR	382	47.292	-7.327	50.346	1.00	0.00	RX1	O
ATOM	5767	HG1	THR	382	47.660	-6.562	50.791	1.00	0.00	RX1	H
ATOM	5768	CG2	THR	382	45.456	-5.817	49.878	1.00	0.00	RX1	C
ATOM	5769	C	THR	382	45.145	-8.876	52.209	1.00	0.00	RX1	C
ATOM	5770	O	THR	382	44.614	-9.677	51.450	1.00	0.00	RX1	O
ATOM	5771	N	HIS	383	45.642	-9.195	53.422	1.00	0.00	RX1	N
ATOM	5772	H	HIS	383	46.135	-8.508	53.951	1.00	0.00	RX1	H
ATOM	5773	CA	HIS	383	45.620	-10.574	53.925	1.00	0.00	RX1	C
ATOM	5774	CB	HIS	383	44.200	-11.061	54.249	1.00	0.00	RX1	C
ATOM	5775	CG	HIS	383	43.421	-10.112	55.135	1.00	0.00	RX1	C
ATOM	5776	ND1	HIS	383	43.941	-9.094	55.847	1.00	0.00	RX1	N
ATOM	5777	HD1	HIS	383	44.879	-8.804	55.888	1.00	0.00	RX1	H
ATOM	5778	CD2	HIS	383	42.040	-10.140	55.356	1.00	0.00	RX1	C
ATOM	5779	NE2	HIS	383	41.735	-9.130	56.207	1.00	0.00	RX1	N
ATOM	5780	CE1	HIS	383	42.907	-8.485	56.507	1.00	0.00	RX1	C
ATOM	5781	C	HIS	383	46.276	-11.581	52.988	1.00	0.00	RX1	C
ATOM	5782	O	HIS	383	45.825	-12.708	52.817	1.00	0.00	RX1	O
ATOM	5783	N	THR	384	47.354	-11.115	52.349	1.00	0.00	RX1	N
ATOM	5784	H	THR	384	47.813	-10.261	52.600	1.00	0.00	RX1	H
ATOM	5785	CA	THR	384	47.930	-11.967	51.318	1.00	0.00	RX1	C
ATOM	5786	CB	THR	384	48.253	-10.949	50.248	1.00	0.00	RX1	C
ATOM	5787	OG1	THR	384	48.393	-9.699	50.932	1.00	0.00	RX1	O
ATOM	5788	HG1	THR	384	48.043	-9.004	50.376	1.00	0.00	RX1	H
ATOM	5789	CG2	THR	384	47.141	-10.822	49.207	1.00	0.00	RX1	C
ATOM	5790	C	THR	384	49.115	-12.790	51.789	1.00	0.00	RX1	C
ATOM	5791	O	THR	384	50.139	-12.266	52.213	1.00	0.00	RX1	O
ATOM	5792	N	PRO	385	48.955	-14.133	51.690	1.00	0.00	RX1	N
ATOM	5793	CD	PRO	385	47.716	-14.843	51.407	1.00	0.00	RX1	C

ATOM	5794	CA	PRO	385	50.099	-15.032	51.877	1.00	0.00	RX1	C
ATOM	5795	CB	PRO	385	49.519	-16.388	51.452	1.00	0.00	RX1	C
ATOM	5796	CG	PRO	385	48.027	-16.291	51.756	1.00	0.00	RX1	C
ATOM	5797	C	PRO	385	51.329	-14.632	51.068	1.00	0.00	RX1	C
ATOM	5798	O	PRO	385	51.232	-14.198	49.920	1.00	0.00	RX1	O
ATOM	5799	N	PRO	386	52.502	-14.795	51.730	1.00	0.00	RX1	N
ATOM	5800	CD	PRO	386	52.660	-15.280	53.095	1.00	0.00	RX1	C
ATOM	5801	CA	PRO	386	53.777	-14.479	51.079	1.00	0.00	RX1	C
ATOM	5802	CB	PRO	386	54.811	-14.972	52.096	1.00	0.00	RX1	C
ATOM	5803	CG	PRO	386	54.102	-14.939	53.448	1.00	0.00	RX1	C
ATOM	5804	C	PRO	386	53.939	-15.149	49.731	1.00	0.00	RX1	C
ATOM	5805	O	PRO	386	53.560	-16.295	49.521	1.00	0.00	RX1	O
ATOM	5806	N	LEU	387	54.522	-14.373	48.817	1.00	0.00	RX1	N
ATOM	5807	H	LEU	387	54.903	-13.483	49.068	1.00	0.00	RX1	H
ATOM	5808	CA	LEU	387	54.832	-14.978	47.532	1.00	0.00	RX1	C
ATOM	5809	CB	LEU	387	55.068	-13.882	46.495	1.00	0.00	RX1	C
ATOM	5810	CG	LEU	387	55.216	-14.437	45.082	1.00	0.00	RX1	C
ATOM	5811	CD1	LEU	387	54.089	-15.410	44.747	1.00	0.00	RX1	C
ATOM	5812	CD2	LEU	387	55.331	-13.320	44.047	1.00	0.00	RX1	C
ATOM	5813	C	LEU	387	55.998	-15.943	47.645	1.00	0.00	RX1	C
ATOM	5814	O	LEU	387	57.127	-15.560	47.919	1.00	0.00	RX1	O
ATOM	5815	N	ASP	388	55.654	-17.219	47.442	1.00	0.00	RX1	N
ATOM	5816	H	ASP	388	54.707	-17.467	47.248	1.00	0.00	RX1	H
ATOM	5817	CA	ASP	388	56.668	-18.265	47.571	1.00	0.00	RX1	C
ATOM	5818	CB	ASP	388	55.983	-19.626	47.423	1.00	0.00	RX1	C
ATOM	5819	CG	ASP	388	56.988	-20.758	47.375	1.00	0.00	RX1	C
ATOM	5820	OD1	ASP	388	57.988	-20.733	48.082	1.00	0.00	RX1	O
ATOM	5821	OD2	ASP	388	56.790	-21.680	46.600	1.00	0.00	RX1	O
ATOM	5822	C	ASP	388	57.865	-18.105	46.635	1.00	0.00	RX1	C
ATOM	5823	O	ASP	388	57.754	-18.038	45.412	1.00	0.00	RX1	O
ATOM	5824	N	PRO	389	59.057	-18.061	47.283	1.00	0.00	RX1	N
ATOM	5825	CD	PRO	389	59.232	-17.940	48.727	1.00	0.00	RX1	C
ATOM	5826	CA	PRO	389	60.327	-18.110	46.554	1.00	0.00	RX1	C
ATOM	5827	CB	PRO	389	61.358	-18.186	47.686	1.00	0.00	RX1	C
ATOM	5828	CG	PRO	389	60.687	-17.530	48.891	1.00	0.00	RX1	C
ATOM	5829	C	PRO	389	60.511	-19.238	45.541	1.00	0.00	RX1	C
ATOM	5830	O	PRO	389	61.426	-19.185	44.722	1.00	0.00	RX1	O
ATOM	5831	N	GLN	390	59.669	-20.276	45.643	1.00	0.00	RX1	N
ATOM	5832	H	GLN	390	58.928	-20.315	46.320	1.00	0.00	RX1	H
ATOM	5833	CA	GLN	390	59.714	-21.312	44.614	1.00	0.00	RX1	C
ATOM	5834	CB	GLN	390	59.483	-22.678	45.245	1.00	0.00	RX1	C
ATOM	5835	CG	GLN	390	60.325	-22.821	46.511	1.00	0.00	RX1	C
ATOM	5836	CD	GLN	390	59.787	-23.964	47.339	1.00	0.00	RX1	C
ATOM	5837	OE1	GLN	390	60.438	-24.991	47.513	1.00	0.00	RX1	O
ATOM	5838	NE2	GLN	390	58.568	-23.722	47.845	1.00	0.00	RX1	N
ATOM	5839	HE21	GLN	390	58.111	-22.836	47.666	1.00	0.00	RX1	H
ATOM	5840	HE22	GLN	390	58.045	-24.361	48.402	1.00	0.00	RX1	H
ATOM	5841	C	GLN	390	58.734	-21.031	43.490	1.00	0.00	RX1	C
ATOM	5842	O	GLN	390	59.100	-21.007	42.320	1.00	0.00	RX1	O
ATOM	5843	N	GLU	391	57.481	-20.748	43.901	1.00	0.00	RX1	N
ATOM	5844	H	GLU	391	57.231	-20.862	44.865	1.00	0.00	RX1	H
ATOM	5845	CA	GLU	391	56.444	-20.355	42.938	1.00	0.00	RX1	C
ATOM	5846	CB	GLU	391	55.151	-19.938	43.643	1.00	0.00	RX1	C
ATOM	5847	CG	GLU	391	54.230	-21.110	43.997	1.00	0.00	RX1	C
ATOM	5848	CD	GLU	391	53.585	-21.677	42.744	1.00	0.00	RX1	C
ATOM	5849	OE1	GLU	391	52.464	-21.288	42.415	1.00	0.00	RX1	O
ATOM	5850	OE2	GLU	391	54.177	-22.532	42.087	1.00	0.00	RX1	O
ATOM	5851	C	GLU	391	56.853	-19.279	41.949	1.00	0.00	RX1	C
ATOM	5852	O	GLU	391	56.430	-19.264	40.802	1.00	0.00	RX1	O
ATOM	5853	N	LEU	392	57.756	-18.399	42.414	1.00	0.00	RX1	N
ATOM	5854	H	LEU	392	57.945	-18.396	43.398	1.00	0.00	RX1	H

ATOM	5855	CA	LEU	392	58.410	-17.457	41.497	1.00	0.00	RX1	C
ATOM	5856	CB	LEU	392	59.699	-16.930	42.113	1.00	0.00	RX1	C
ATOM	5857	CG	LEU	392	59.435	-15.820	43.117	1.00	0.00	RX1	C
ATOM	5858	CD1	LEU	392	60.737	-15.316	43.728	1.00	0.00	RX1	C
ATOM	5859	CD2	LEU	392	58.627	-14.687	42.485	1.00	0.00	RX1	C
ATOM	5860	C	LEU	392	58.715	-17.933	40.082	1.00	0.00	RX1	C
ATOM	5861	O	LEU	392	58.459	-17.235	39.107	1.00	0.00	RX1	O
ATOM	5862	N	ASP	393	59.264	-19.156	40.002	1.00	0.00	RX1	N
ATOM	5863	H	ASP	393	59.353	-19.745	40.808	1.00	0.00	RX1	H
ATOM	5864	CA	ASP	393	59.715	-19.635	38.694	1.00	0.00	RX1	C
ATOM	5865	CB	ASP	393	60.588	-20.882	38.852	1.00	0.00	RX1	C
ATOM	5866	CG	ASP	393	61.907	-20.682	38.125	1.00	0.00	RX1	C
ATOM	5867	OD1	ASP	393	62.513	-21.660	37.693	1.00	0.00	RX1	O
ATOM	5868	OD2	ASP	393	62.358	-19.548	37.981	1.00	0.00	RX1	O
ATOM	5869	C	ASP	393	58.645	-19.813	37.615	1.00	0.00	RX1	C
ATOM	5870	O	ASP	393	58.928	-19.940	36.430	1.00	0.00	RX1	O
ATOM	5871	N	ILE	394	57.371	-19.720	38.060	1.00	0.00	RX1	N
ATOM	5872	H	ILE	394	57.207	-19.687	39.048	1.00	0.00	RX1	H
ATOM	5873	CA	ILE	394	56.240	-19.481	37.145	1.00	0.00	RX1	C
ATOM	5874	CB	ILE	394	55.039	-18.987	37.957	1.00	0.00	RX1	C
ATOM	5875	CG2	ILE	394	54.044	-18.186	37.121	1.00	0.00	RX1	C
ATOM	5876	CG1	ILE	394	54.354	-20.136	38.688	1.00	0.00	RX1	C
ATOM	5877	CD1	ILE	394	53.261	-19.593	39.607	1.00	0.00	RX1	C
ATOM	5878	C	ILE	394	56.543	-18.478	36.035	1.00	0.00	RX1	C
ATOM	5879	O	ILE	394	56.191	-18.640	34.869	1.00	0.00	RX1	O
ATOM	5880	N	LEU	395	57.227	-17.411	36.468	1.00	0.00	RX1	N
ATOM	5881	H	LEU	395	57.580	-17.385	37.405	1.00	0.00	RX1	H
ATOM	5882	CA	LEU	395	57.480	-16.300	35.560	1.00	0.00	RX1	C
ATOM	5883	CB	LEU	395	57.904	-15.088	36.384	1.00	0.00	RX1	C
ATOM	5884	CG	LEU	395	56.822	-14.777	37.426	1.00	0.00	RX1	C
ATOM	5885	CD1	LEU	395	57.325	-13.899	38.571	1.00	0.00	RX1	C
ATOM	5886	CD2	LEU	395	55.554	-14.221	36.775	1.00	0.00	RX1	C
ATOM	5887	C	LEU	395	58.398	-16.603	34.384	1.00	0.00	RX1	C
ATOM	5888	O	LEU	395	58.479	-15.847	33.426	1.00	0.00	RX1	O
ATOM	5889	N	LYS	396	59.007	-17.802	34.434	1.00	0.00	RX1	N
ATOM	5890	H	LYS	396	58.965	-18.382	35.248	1.00	0.00	RX1	H
ATOM	5891	CA	LYS	396	59.678	-18.303	33.235	1.00	0.00	RX1	C
ATOM	5892	CB	LYS	396	60.455	-19.583	33.532	1.00	0.00	RX1	C
ATOM	5893	CG	LYS	396	61.795	-19.380	34.238	1.00	0.00	RX1	C
ATOM	5894	CD	LYS	396	62.512	-20.723	34.382	1.00	0.00	RX1	C
ATOM	5895	CE	LYS	396	63.970	-20.615	34.830	1.00	0.00	RX1	C
ATOM	5896	NZ	LYS	396	64.080	-20.014	36.160	1.00	0.00	RX1	N
ATOM	5897	HZ1	LYS	396	65.078	-19.862	36.410	1.00	0.00	RX1	H
ATOM	5898	HZ2	LYS	396	63.596	-19.096	36.224	1.00	0.00	RX1	H
ATOM	5899	HZ3	LYS	396	63.607	-20.591	36.891	1.00	0.00	RX1	H
ATOM	5900	C	LYS	396	58.778	-18.556	32.031	1.00	0.00	RX1	C
ATOM	5901	O	LYS	396	59.250	-18.788	30.925	1.00	0.00	RX1	O
ATOM	5902	N	THR	397	57.461	-18.510	32.273	1.00	0.00	RX1	N
ATOM	5903	H	THR	397	57.078	-18.317	33.175	1.00	0.00	RX1	H
ATOM	5904	CA	THR	397	56.563	-18.580	31.122	1.00	0.00	RX1	C
ATOM	5905	CB	THR	397	55.226	-19.169	31.579	1.00	0.00	RX1	C
ATOM	5906	OG1	THR	397	54.648	-18.417	32.656	1.00	0.00	RX1	O
ATOM	5907	HG1	THR	397	55.074	-18.704	33.460	1.00	0.00	RX1	H
ATOM	5908	CG2	THR	397	55.417	-20.621	32.011	1.00	0.00	RX1	C
ATOM	5909	C	THR	397	56.427	-17.271	30.353	1.00	0.00	RX1	C
ATOM	5910	O	THR	397	55.959	-17.217	29.221	1.00	0.00	RX1	O
ATOM	5911	N	VAL	398	56.857	-16.199	31.032	1.00	0.00	RX1	N
ATOM	5912	H	VAL	398	57.397	-16.261	31.871	1.00	0.00	RX1	H
ATOM	5913	CA	VAL	398	56.676	-14.884	30.437	1.00	0.00	RX1	C
ATOM	5914	CB	VAL	398	56.593	-13.823	31.528	1.00	0.00	RX1	C
ATOM	5915	CG1	VAL	398	56.277	-12.467	30.911	1.00	0.00	RX1	C

ATOM	5916	CG2	VAL	398	55.573	-14.218	32.598	1.00	0.00	RX1	C
ATOM	5917	C	VAL	398	57.750	-14.542	29.420	1.00	0.00	RX1	C
ATOM	5918	O	VAL	398	58.837	-14.074	29.727	1.00	0.00	RX1	O
ATOM	5919	N	LYS	399	57.361	-14.793	28.170	1.00	0.00	RX1	N
ATOM	5920	H	LYS	399	56.446	-15.173	28.025	1.00	0.00	RX1	H
ATOM	5921	CA	LYS	399	58.193	-14.385	27.042	1.00	0.00	RX1	C
ATOM	5922	CB	LYS	399	57.677	-15.053	25.763	1.00	0.00	RX1	C
ATOM	5923	CG	LYS	399	58.393	-16.365	25.437	1.00	0.00	RX1	C
ATOM	5924	CD	LYS	399	58.342	-17.398	26.560	1.00	0.00	RX1	C
ATOM	5925	CE	LYS	399	59.604	-18.254	26.581	1.00	0.00	RX1	C
ATOM	5926	NZ	LYS	399	60.762	-17.381	26.789	1.00	0.00	RX1	N
ATOM	5927	HZ1	LYS	399	60.925	-16.761	25.964	1.00	0.00	RX1	H
ATOM	5928	HZ2	LYS	399	60.663	-16.726	27.591	1.00	0.00	RX1	H
ATOM	5929	HZ3	LYS	399	61.668	-17.880	26.902	1.00	0.00	RX1	H
ATOM	5930	C	LYS	399	58.230	-12.879	26.865	1.00	0.00	RX1	C
ATOM	5931	O	LYS	399	59.274	-12.258	26.697	1.00	0.00	RX1	O
ATOM	5932	N	GLU	400	57.016	-12.313	26.920	1.00	0.00	RX1	N
ATOM	5933	H	GLU	400	56.182	-12.831	27.121	1.00	0.00	RX1	H
ATOM	5934	CA	GLU	400	56.956	-10.863	26.782	1.00	0.00	RX1	C
ATOM	5935	CB	GLU	400	56.190	-10.459	25.510	1.00	0.00	RX1	C
ATOM	5936	CG	GLU	400	56.423	-11.341	24.274	1.00	0.00	RX1	C
ATOM	5937	CD	GLU	400	55.698	-10.774	23.060	1.00	0.00	RX1	C
ATOM	5938	OE1	GLU	400	56.299	-10.649	21.996	1.00	0.00	RX1	O
ATOM	5939	OE2	GLU	400	54.531	-10.415	23.162	1.00	0.00	RX1	O
ATOM	5940	C	GLU	400	56.288	-10.222	27.977	1.00	0.00	RX1	C
ATOM	5941	O	GLU	400	55.202	-10.616	28.374	1.00	0.00	RX1	O
ATOM	5942	N	ILE	401	56.954	-9.203	28.515	1.00	0.00	RX1	N
ATOM	5943	H	ILE	401	57.855	-8.952	28.173	1.00	0.00	RX1	H
ATOM	5944	CA	ILE	401	56.246	-8.207	29.310	1.00	0.00	RX1	C
ATOM	5945	CB	ILE	401	57.071	-7.830	30.538	1.00	0.00	RX1	C
ATOM	5946	CG2	ILE	401	56.507	-6.609	31.261	1.00	0.00	RX1	C
ATOM	5947	CG1	ILE	401	57.180	-9.025	31.475	1.00	0.00	RX1	C
ATOM	5948	CD1	ILE	401	57.949	-8.690	32.746	1.00	0.00	RX1	C
ATOM	5949	C	ILE	401	56.065	-7.004	28.414	1.00	0.00	RX1	C
ATOM	5950	O	ILE	401	57.023	-6.411	27.936	1.00	0.00	RX1	O
ATOM	5951	N	THR	402	54.805	-6.669	28.155	1.00	0.00	RX1	N
ATOM	5952	H	THR	402	54.005	-7.085	28.590	1.00	0.00	RX1	H
ATOM	5953	CA	THR	402	54.694	-5.562	27.213	1.00	0.00	RX1	C
ATOM	5954	CB	THR	402	53.533	-5.831	26.265	1.00	0.00	RX1	C
ATOM	5955	OG1	THR	402	52.402	-6.295	26.994	1.00	0.00	RX1	O
ATOM	5956	HG1	THR	402	52.537	-7.234	27.107	1.00	0.00	RX1	H
ATOM	5957	CG2	THR	402	53.925	-6.886	25.228	1.00	0.00	RX1	C
ATOM	5958	C	THR	402	54.722	-4.174	27.833	1.00	0.00	RX1	C
ATOM	5959	O	THR	402	54.973	-3.175	27.169	1.00	0.00	RX1	O
ATOM	5960	N	GLY	403	54.492	-4.153	29.149	1.00	0.00	RX1	N
ATOM	5961	H	GLY	403	54.348	-4.986	29.685	1.00	0.00	RX1	H
ATOM	5962	CA	GLY	403	54.703	-2.904	29.867	1.00	0.00	RX1	C
ATOM	5963	C	GLY	403	56.044	-2.908	30.565	1.00	0.00	RX1	C
ATOM	5964	O	GLY	403	57.082	-3.059	29.927	1.00	0.00	RX1	O
ATOM	5965	N	PHE	404	55.974	-2.759	31.891	1.00	0.00	RX1	N
ATOM	5966	H	PHE	404	55.086	-2.688	32.352	1.00	0.00	RX1	H
ATOM	5967	CA	PHE	404	57.207	-2.716	32.667	1.00	0.00	RX1	C
ATOM	5968	CB	PHE	404	57.370	-1.358	33.359	1.00	0.00	RX1	C
ATOM	5969	CG	PHE	404	56.238	-1.067	34.315	1.00	0.00	RX1	C
ATOM	5970	CD1	PHE	404	56.159	-1.708	35.546	1.00	0.00	RX1	C
ATOM	5971	CD2	PHE	404	55.275	-0.133	33.961	1.00	0.00	RX1	C
ATOM	5972	CE1	PHE	404	55.121	-1.410	36.418	1.00	0.00	RX1	C
ATOM	5973	CE2	PHE	404	54.242	0.177	34.833	1.00	0.00	RX1	C
ATOM	5974	CZ	PHE	404	54.169	-0.462	36.062	1.00	0.00	RX1	C
ATOM	5975	C	PHE	404	57.370	-3.858	33.655	1.00	0.00	RX1	C
ATOM	5976	O	PHE	404	56.433	-4.555	34.027	1.00	0.00	RX1	O

ATOM	5977	N	LEU	405	58.623	-3.987	34.096	1.00	0.00	RX1	N
ATOM	5978	H	LEU	405	59.349	-3.377	33.779	1.00	0.00	RX1	H
ATOM	5979	CA	LEU	405	58.937	-4.930	35.156	1.00	0.00	RX1	C
ATOM	5980	CB	LEU	405	59.937	-5.951	34.622	1.00	0.00	RX1	C
ATOM	5981	CG	LEU	405	60.279	-7.052	35.621	1.00	0.00	RX1	C
ATOM	5982	CD1	LEU	405	59.027	-7.699	36.215	1.00	0.00	RX1	C
ATOM	5983	CD2	LEU	405	61.235	-8.078	35.010	1.00	0.00	RX1	C
ATOM	5984	C	LEU	405	59.469	-4.209	36.381	1.00	0.00	RX1	C
ATOM	5985	O	LEU	405	60.589	-3.708	36.411	1.00	0.00	RX1	O
ATOM	5986	N	LEU	406	58.593	-4.167	37.392	1.00	0.00	RX1	N
ATOM	5987	H	LEU	406	57.731	-4.679	37.362	1.00	0.00	RX1	H
ATOM	5988	CA	LEU	406	58.963	-3.482	38.626	1.00	0.00	RX1	C
ATOM	5989	CB	LEU	406	57.773	-2.643	39.101	1.00	0.00	RX1	C
ATOM	5990	CG	LEU	406	58.052	-1.773	40.329	1.00	0.00	RX1	C
ATOM	5991	CD1	LEU	406	59.125	-0.724	40.063	1.00	0.00	RX1	C
ATOM	5992	CD2	LEU	406	56.781	-1.130	40.874	1.00	0.00	RX1	C
ATOM	5993	C	LEU	406	59.407	-4.453	39.706	1.00	0.00	RX1	C
ATOM	5994	O	LEU	406	58.644	-4.823	40.591	1.00	0.00	RX1	O
ATOM	5995	N	ILE	407	60.676	-4.860	39.604	1.00	0.00	RX1	N
ATOM	5996	H	ILE	407	61.308	-4.401	38.977	1.00	0.00	RX1	H
ATOM	5997	CA	ILE	407	61.173	-5.692	40.696	1.00	0.00	RX1	C
ATOM	5998	CB	ILE	407	62.184	-6.732	40.226	1.00	0.00	RX1	C
ATOM	5999	CG2	ILE	407	62.447	-7.737	41.344	1.00	0.00	RX1	C
ATOM	6000	CG1	ILE	407	61.705	-7.454	38.975	1.00	0.00	RX1	C
ATOM	6001	CD1	ILE	407	62.659	-8.579	38.581	1.00	0.00	RX1	C
ATOM	6002	C	ILE	407	61.762	-4.868	41.827	1.00	0.00	RX1	C
ATOM	6003	O	ILE	407	62.943	-4.537	41.871	1.00	0.00	RX1	O
ATOM	6004	N	GLN	408	60.857	-4.558	42.760	1.00	0.00	RX1	N
ATOM	6005	H	GLN	408	59.904	-4.864	42.688	1.00	0.00	RX1	H
ATOM	6006	CA	GLN	408	61.351	-3.954	43.994	1.00	0.00	RX1	C
ATOM	6007	CB	GLN	408	60.351	-2.940	44.527	1.00	0.00	RX1	C
ATOM	6008	CG	GLN	408	60.052	-1.831	43.529	1.00	0.00	RX1	C
ATOM	6009	CD	GLN	408	58.964	-0.947	44.095	1.00	0.00	RX1	C
ATOM	6010	OE1	GLN	408	58.095	-1.387	44.839	1.00	0.00	RX1	O
ATOM	6011	NE2	GLN	408	59.064	0.335	43.706	1.00	0.00	RX1	N
ATOM	6012	HE21	GLN	408	59.803	0.623	43.096	1.00	0.00	RX1	H
ATOM	6013	HE22	GLN	408	58.407	1.023	44.015	1.00	0.00	RX1	H
ATOM	6014	C	GLN	408	61.665	-4.976	45.070	1.00	0.00	RX1	C
ATOM	6015	O	GLN	408	62.455	-4.747	45.976	1.00	0.00	RX1	O
ATOM	6016	N	ALA	409	60.994	-6.125	44.939	1.00	0.00	RX1	N
ATOM	6017	H	ALA	409	60.353	-6.311	44.193	1.00	0.00	RX1	H
ATOM	6018	CA	ALA	409	61.252	-7.157	45.932	1.00	0.00	RX1	C
ATOM	6019	CB	ALA	409	59.975	-7.503	46.678	1.00	0.00	RX1	C
ATOM	6020	C	ALA	409	61.773	-8.428	45.317	1.00	0.00	RX1	C
ATOM	6021	O	ALA	409	61.387	-8.810	44.221	1.00	0.00	RX1	O
ATOM	6022	N	TRP	410	62.643	-9.075	46.098	1.00	0.00	RX1	N
ATOM	6023	H	TRP	410	62.963	-8.725	46.981	1.00	0.00	RX1	H
ATOM	6024	CA	TRP	410	63.157	-10.397	45.750	1.00	0.00	RX1	C
ATOM	6025	CB	TRP	410	64.373	-10.246	44.839	1.00	0.00	RX1	C
ATOM	6026	CG	TRP	410	64.400	-11.233	43.696	1.00	0.00	RX1	C
ATOM	6027	CD2	TRP	410	63.356	-11.597	42.765	1.00	0.00	RX1	C
ATOM	6028	CE2	TRP	410	63.911	-12.516	41.844	1.00	0.00	RX1	C
ATOM	6029	CE3	TRP	410	62.024	-11.221	42.631	1.00	0.00	RX1	C
ATOM	6030	CD1	TRP	410	65.521	-11.962	43.283	1.00	0.00	RX1	C
ATOM	6031	NE1	TRP	410	65.244	-12.721	42.194	1.00	0.00	RX1	N
ATOM	6032	HE1	TRP	410	65.899	-13.287	41.727	1.00	0.00	RX1	H
ATOM	6033	CZ2	TRP	410	63.119	-13.021	40.819	1.00	0.00	RX1	C
ATOM	6034	CZ3	TRP	410	61.243	-11.735	41.604	1.00	0.00	RX1	C
ATOM	6035	CH2	TRP	410	61.792	-12.631	40.697	1.00	0.00	RX1	C
ATOM	6036	C	TRP	410	63.561	-11.030	47.061	1.00	0.00	RX1	C
ATOM	6037	O	TRP	410	64.080	-10.339	47.929	1.00	0.00	RX1	O

ATOM	6038	N	PRO	411	63.278	-12.344	47.209	1.00	0.00	RX1	N
ATOM	6039	CD	PRO	411	62.621	-13.218	46.252	1.00	0.00	RX1	C
ATOM	6040	CA	PRO	411	63.618	-13.014	48.467	1.00	0.00	RX1	C
ATOM	6041	CB	PRO	411	63.170	-14.456	48.221	1.00	0.00	RX1	C
ATOM	6042	CG	PRO	411	62.125	-14.370	47.113	1.00	0.00	RX1	C
ATOM	6043	C	PRO	411	65.093	-12.931	48.799	1.00	0.00	RX1	C
ATOM	6044	O	PRO	411	65.956	-13.026	47.934	1.00	0.00	RX1	O
ATOM	6045	N	GLU	412	65.334	-12.792	50.105	1.00	0.00	RX1	N
ATOM	6046	H	GLU	412	64.556	-12.641	50.719	1.00	0.00	RX1	H
ATOM	6047	CA	GLU	412	66.698	-12.666	50.620	1.00	0.00	RX1	C
ATOM	6048	CB	GLU	412	66.706	-12.721	52.152	1.00	0.00	RX1	C
ATOM	6049	CG	GLU	412	65.836	-11.661	52.844	1.00	0.00	RX1	C
ATOM	6050	CD	GLU	412	64.369	-12.061	52.867	1.00	0.00	RX1	C
ATOM	6051	OE1	GLU	412	63.980	-12.858	53.708	1.00	0.00	RX1	O
ATOM	6052	OE2	GLU	412	63.576	-11.534	52.097	1.00	0.00	RX1	O
ATOM	6053	C	GLU	412	67.687	-13.670	50.047	1.00	0.00	RX1	C
ATOM	6054	O	GLU	412	68.778	-13.337	49.597	1.00	0.00	RX1	O
ATOM	6055	N	ASN	413	67.235	-14.930	50.039	1.00	0.00	RX1	N
ATOM	6056	H	ASN	413	66.325	-15.132	50.397	1.00	0.00	RX1	H
ATOM	6057	CA	ASN	413	68.095	-15.958	49.452	1.00	0.00	RX1	C
ATOM	6058	CB	ASN	413	67.925	-17.320	50.127	1.00	0.00	RX1	C
ATOM	6059	CG	ASN	413	68.666	-17.347	51.444	1.00	0.00	RX1	C
ATOM	6060	OD1	ASN	413	68.288	-16.686	52.403	1.00	0.00	RX1	O
ATOM	6061	ND2	ASN	413	69.741	-18.158	51.449	1.00	0.00	RX1	N
ATOM	6062	HD21	ASN	413	70.015	-18.677	50.640	1.00	0.00	RX1	H
ATOM	6063	HD22	ASN	413	70.291	-18.255	52.279	1.00	0.00	RX1	H
ATOM	6064	C	ASN	413	67.902	-16.146	47.958	1.00	0.00	RX1	C
ATOM	6065	O	ASN	413	67.621	-17.244	47.477	1.00	0.00	RX1	O
ATOM	6066	N	ARG	414	68.066	-15.028	47.237	1.00	0.00	RX1	N
ATOM	6067	H	ARG	414	68.241	-14.135	47.662	1.00	0.00	RX1	H
ATOM	6068	CA	ARG	414	68.056	-15.093	45.779	1.00	0.00	RX1	C
ATOM	6069	CB	ARG	414	66.712	-14.662	45.195	1.00	0.00	RX1	C
ATOM	6070	CG	ARG	414	65.593	-15.688	45.362	1.00	0.00	RX1	C
ATOM	6071	CD	ARG	414	65.974	-17.031	44.740	1.00	0.00	RX1	C
ATOM	6072	NE	ARG	414	64.845	-17.958	44.736	1.00	0.00	RX1	N
ATOM	6073	HE	ARG	414	64.130	-17.801	44.039	1.00	0.00	RX1	H
ATOM	6074	CZ	ARG	414	64.836	-18.997	45.618	1.00	0.00	RX1	C
ATOM	6075	NH1	ARG	414	65.821	-19.095	46.543	1.00	0.00	RX1	N
ATOM	6076	HH11	ARG	414	65.888	-19.861	47.185	1.00	0.00	RX1	H
ATOM	6077	HH12	ARG	414	66.538	-18.387	46.641	1.00	0.00	RX1	H
ATOM	6078	NH2	ARG	414	63.841	-19.908	45.546	1.00	0.00	RX1	N
ATOM	6079	HH21	ARG	414	63.792	-20.733	46.113	1.00	0.00	RX1	H
ATOM	6080	HH22	ARG	414	63.071	-19.774	44.899	1.00	0.00	RX1	H
ATOM	6081	C	ARG	414	69.157	-14.261	45.162	1.00	0.00	RX1	C
ATOM	6082	O	ARG	414	69.205	-13.041	45.262	1.00	0.00	RX1	O
ATOM	6083	N	THR	415	70.050	-15.002	44.506	1.00	0.00	RX1	N
ATOM	6084	H	THR	415	70.029	-16.000	44.492	1.00	0.00	RX1	H
ATOM	6085	CA	THR	415	71.195	-14.343	43.893	1.00	0.00	RX1	C
ATOM	6086	CB	THR	415	72.319	-15.281	44.254	1.00	0.00	RX1	C
ATOM	6087	OG1	THR	415	71.763	-16.246	45.165	1.00	0.00	RX1	O
ATOM	6088	HG1	THR	415	72.498	-16.763	45.474	1.00	0.00	RX1	H
ATOM	6089	CG2	THR	415	73.499	-14.548	44.895	1.00	0.00	RX1	C
ATOM	6090	C	THR	415	71.058	-14.030	42.411	1.00	0.00	RX1	C
ATOM	6091	O	THR	415	71.885	-13.329	41.840	1.00	0.00	RX1	O
ATOM	6092	N	ASP	416	69.982	-14.568	41.823	1.00	0.00	RX1	N
ATOM	6093	H	ASP	416	69.247	-15.037	42.310	1.00	0.00	RX1	H
ATOM	6094	CA	ASP	416	69.769	-14.367	40.392	1.00	0.00	RX1	C
ATOM	6095	CB	ASP	416	70.225	-15.619	39.633	1.00	0.00	RX1	C
ATOM	6096	CG	ASP	416	70.207	-15.387	38.135	1.00	0.00	RX1	C
ATOM	6097	OD1	ASP	416	71.225	-14.993	37.578	1.00	0.00	RX1	O
ATOM	6098	OD2	ASP	416	69.176	-15.614	37.514	1.00	0.00	RX1	O

ATOM	6099	C	ASP	416	68.301	-14.054	40.163	1.00	0.00	RX1	C
ATOM	6100	O	ASP	416	67.453	-14.318	41.018	1.00	0.00	RX1	O
ATOM	6101	N	LEU	417	68.012	-13.463	38.997	1.00	0.00	RX1	N
ATOM	6102	H	LEU	417	68.701	-13.469	38.267	1.00	0.00	RX1	H
ATOM	6103	CA	LEU	417	66.607	-13.149	38.749	1.00	0.00	RX1	C
ATOM	6104	CB	LEU	417	66.427	-11.991	37.764	1.00	0.00	RX1	C
ATOM	6105	CG	LEU	417	66.658	-10.623	38.414	1.00	0.00	RX1	C
ATOM	6106	CD1	LEU	417	66.323	-9.466	37.473	1.00	0.00	RX1	C
ATOM	6107	CD2	LEU	417	65.878	-10.478	39.716	1.00	0.00	RX1	C
ATOM	6108	C	LEU	417	65.711	-14.309	38.353	1.00	0.00	RX1	C
ATOM	6109	O	LEU	417	64.519	-14.110	38.176	1.00	0.00	RX1	O
ATOM	6110	N	HIS	418	66.314	-15.514	38.269	1.00	0.00	RX1	N
ATOM	6111	H	HIS	418	67.317	-15.531	38.263	1.00	0.00	RX1	H
ATOM	6112	CA	HIS	418	65.645	-16.817	38.093	1.00	0.00	RX1	C
ATOM	6113	CB	HIS	418	65.810	-17.707	39.338	1.00	0.00	RX1	C
ATOM	6114	CG	HIS	418	64.949	-17.241	40.486	1.00	0.00	RX1	C
ATOM	6115	ND1	HIS	418	65.049	-16.022	41.045	1.00	0.00	RX1	N
ATOM	6116	HD1	HIS	418	65.714	-15.332	40.822	1.00	0.00	RX1	H
ATOM	6117	CD2	HIS	418	63.933	-17.954	41.132	1.00	0.00	RX1	C
ATOM	6118	NE2	HIS	418	63.419	-17.143	42.090	1.00	0.00	RX1	N
ATOM	6119	CE1	HIS	418	64.102	-15.956	42.029	1.00	0.00	RX1	C
ATOM	6120	C	HIS	418	64.244	-16.913	37.481	1.00	0.00	RX1	C
ATOM	6121	O	HIS	418	64.059	-17.444	36.389	1.00	0.00	RX1	O
ATOM	6122	N	ALA	419	63.252	-16.359	38.190	1.00	0.00	RX1	N
ATOM	6123	H	ALA	419	63.471	-15.844	39.017	1.00	0.00	RX1	H
ATOM	6124	CA	ALA	419	61.904	-16.297	37.628	1.00	0.00	RX1	C
ATOM	6125	CB	ALA	419	61.020	-15.455	38.541	1.00	0.00	RX1	C
ATOM	6126	C	ALA	419	61.860	-15.697	36.229	1.00	0.00	RX1	C
ATOM	6127	O	ALA	419	61.233	-16.199	35.309	1.00	0.00	RX1	O
ATOM	6128	N	PHE	420	62.603	-14.594	36.098	1.00	0.00	RX1	N
ATOM	6129	H	PHE	420	63.177	-14.263	36.847	1.00	0.00	RX1	H
ATOM	6130	CA	PHE	420	62.603	-13.928	34.801	1.00	0.00	RX1	C
ATOM	6131	CB	PHE	420	62.542	-12.419	35.003	1.00	0.00	RX1	C
ATOM	6132	CG	PHE	420	61.248	-12.035	35.673	1.00	0.00	RX1	C
ATOM	6133	CD1	PHE	420	60.068	-12.023	34.940	1.00	0.00	RX1	C
ATOM	6134	CD2	PHE	420	61.238	-11.681	37.016	1.00	0.00	RX1	C
ATOM	6135	CE1	PHE	420	58.881	-11.627	35.540	1.00	0.00	RX1	C
ATOM	6136	CE2	PHE	420	60.050	-11.289	37.618	1.00	0.00	RX1	C
ATOM	6137	CZ	PHE	420	58.876	-11.247	36.876	1.00	0.00	RX1	C
ATOM	6138	C	PHE	420	63.754	-14.284	33.873	1.00	0.00	RX1	C
ATOM	6139	O	PHE	420	64.124	-13.519	32.992	1.00	0.00	RX1	O
ATOM	6140	N	GLU	421	64.311	-15.491	34.076	1.00	0.00	RX1	N
ATOM	6141	H	GLU	421	63.996	-16.102	34.804	1.00	0.00	RX1	H
ATOM	6142	CA	GLU	421	65.384	-15.898	33.158	1.00	0.00	RX1	C
ATOM	6143	CB	GLU	421	65.950	-17.259	33.534	1.00	0.00	RX1	C
ATOM	6144	CG	GLU	421	66.732	-17.244	34.835	1.00	0.00	RX1	C
ATOM	6145	CD	GLU	421	67.221	-18.645	35.123	1.00	0.00	RX1	C
ATOM	6146	OE1	GLU	421	68.153	-19.081	34.464	1.00	0.00	RX1	O
ATOM	6147	OE2	GLU	421	66.680	-19.307	35.999	1.00	0.00	RX1	O
ATOM	6148	C	GLU	421	64.948	-15.995	31.710	1.00	0.00	RX1	C
ATOM	6149	O	GLU	421	65.613	-15.570	30.771	1.00	0.00	RX1	O
ATOM	6150	N	ASN	422	63.770	-16.621	31.587	1.00	0.00	RX1	N
ATOM	6151	H	ASN	422	63.202	-16.751	32.399	1.00	0.00	RX1	H
ATOM	6152	CA	ASN	422	63.259	-17.012	30.274	1.00	0.00	RX1	C
ATOM	6153	CB	ASN	422	62.533	-18.346	30.427	1.00	0.00	RX1	C
ATOM	6154	CG	ASN	422	62.127	-18.934	29.093	1.00	0.00	RX1	C
ATOM	6155	OD1	ASN	422	62.681	-18.653	28.035	1.00	0.00	RX1	O
ATOM	6156	ND2	ASN	422	61.143	-19.841	29.204	1.00	0.00	RX1	N
ATOM	6157	HD21	ASN	422	60.617	-19.862	30.060	1.00	0.00	RX1	H
ATOM	6158	HD22	ASN	422	60.876	-20.487	28.491	1.00	0.00	RX1	H
ATOM	6159	C	ASN	422	62.363	-15.950	29.654	1.00	0.00	RX1	C

ATOM	6160	O	ASN	422	61.324	-16.240	29.070	1.00	0.00	RX1	O
ATOM	6161	N	LEU	423	62.816	-14.707	29.868	1.00	0.00	RX1	N
ATOM	6162	H	LEU	423	63.748	-14.544	30.194	1.00	0.00	RX1	H
ATOM	6163	CA	LEU	423	62.075	-13.524	29.448	1.00	0.00	RX1	C
ATOM	6164	CB	LEU	423	62.060	-12.531	30.612	1.00	0.00	RX1	C
ATOM	6165	CG	LEU	423	60.841	-11.621	30.808	1.00	0.00	RX1	C
ATOM	6166	CD1	LEU	423	61.160	-10.548	31.846	1.00	0.00	RX1	C
ATOM	6167	CD2	LEU	423	60.308	-10.979	29.531	1.00	0.00	RX1	C
ATOM	6168	C	LEU	423	62.826	-12.906	28.290	1.00	0.00	RX1	C
ATOM	6169	O	LEU	423	64.009	-12.613	28.413	1.00	0.00	RX1	O
ATOM	6170	N	GLU	424	62.114	-12.718	27.181	1.00	0.00	RX1	N
ATOM	6171	H	GLU	424	61.159	-13.010	27.089	1.00	0.00	RX1	H
ATOM	6172	CA	GLU	424	62.810	-12.174	26.023	1.00	0.00	RX1	C
ATOM	6173	CB	GLU	424	62.471	-12.947	24.744	1.00	0.00	RX1	C
ATOM	6174	CG	GLU	424	63.092	-14.337	24.588	1.00	0.00	RX1	C
ATOM	6175	CD	GLU	424	62.208	-15.385	25.220	1.00	0.00	RX1	C
ATOM	6176	OE1	GLU	424	61.267	-15.033	25.922	1.00	0.00	RX1	O
ATOM	6177	OE2	GLU	424	62.424	-16.574	24.998	1.00	0.00	RX1	O
ATOM	6178	C	GLU	424	62.559	-10.704	25.778	1.00	0.00	RX1	C
ATOM	6179	O	GLU	424	63.386	-9.990	25.220	1.00	0.00	RX1	O
ATOM	6180	N	ILE	425	61.351	-10.277	26.157	1.00	0.00	RX1	N
ATOM	6181	H	ILE	425	60.703	-10.849	26.665	1.00	0.00	RX1	H
ATOM	6182	CA	ILE	425	60.891	-8.993	25.635	1.00	0.00	RX1	C
ATOM	6183	CB	ILE	425	59.865	-9.307	24.547	1.00	0.00	RX1	C
ATOM	6184	CG2	ILE	425	59.067	-8.068	24.174	1.00	0.00	RX1	C
ATOM	6185	CG1	ILE	425	60.532	-9.992	23.350	1.00	0.00	RX1	C
ATOM	6186	CD1	ILE	425	59.631	-10.865	22.483	1.00	0.00	RX1	C
ATOM	6187	C	ILE	425	60.290	-8.118	26.721	1.00	0.00	RX1	C
ATOM	6188	O	ILE	425	59.483	-8.588	27.506	1.00	0.00	RX1	O
ATOM	6189	N	ILE	426	60.682	-6.834	26.720	1.00	0.00	RX1	N
ATOM	6190	H	ILE	426	61.402	-6.497	26.111	1.00	0.00	RX1	H
ATOM	6191	CA	ILE	426	59.973	-5.832	27.518	1.00	0.00	RX1	C
ATOM	6192	CB	ILE	426	60.780	-5.439	28.763	1.00	0.00	RX1	C
ATOM	6193	CG2	ILE	426	60.082	-4.322	29.537	1.00	0.00	RX1	C
ATOM	6194	CG1	ILE	426	61.030	-6.648	29.666	1.00	0.00	RX1	C
ATOM	6195	CD1	ILE	426	61.828	-6.301	30.919	1.00	0.00	RX1	C
ATOM	6196	C	ILE	426	59.667	-4.609	26.659	1.00	0.00	RX1	C
ATOM	6197	O	ILE	426	60.560	-3.920	26.178	1.00	0.00	RX1	O
ATOM	6198	N	ARG	427	58.360	-4.381	26.449	1.00	0.00	RX1	N
ATOM	6199	H	ARG	427	57.678	-4.912	26.953	1.00	0.00	RX1	H
ATOM	6200	CA	ARG	427	58.007	-3.335	25.486	1.00	0.00	RX1	C
ATOM	6201	CB	ARG	427	56.855	-3.758	24.565	1.00	0.00	RX1	C
ATOM	6202	CG	ARG	427	57.289	-4.930	23.691	1.00	0.00	RX1	C
ATOM	6203	CD	ARG	427	56.394	-5.285	22.502	1.00	0.00	RX1	C
ATOM	6204	NE	ARG	427	57.126	-6.227	21.662	1.00	0.00	RX1	N
ATOM	6205	HE	ARG	427	58.033	-5.933	21.333	1.00	0.00	RX1	H
ATOM	6206	CZ	ARG	427	56.774	-7.541	21.606	1.00	0.00	RX1	C
ATOM	6207	NH1	ARG	427	55.530	-7.947	21.944	1.00	0.00	RX1	N
ATOM	6208	HH11	ARG	427	55.330	-8.925	22.145	1.00	0.00	RX1	H
ATOM	6209	HH12	ARG	427	54.735	-7.324	21.986	1.00	0.00	RX1	H
ATOM	6210	NH2	ARG	427	57.714	-8.427	21.223	1.00	0.00	RX1	N
ATOM	6211	HH21	ARG	427	57.497	-9.416	21.220	1.00	0.00	RX1	H
ATOM	6212	HH22	ARG	427	58.652	-8.136	20.974	1.00	0.00	RX1	H
ATOM	6213	C	ARG	427	57.778	-1.932	26.010	1.00	0.00	RX1	C
ATOM	6214	O	ARG	427	57.709	-0.985	25.234	1.00	0.00	RX1	O
ATOM	6215	N	GLY	428	57.653	-1.815	27.342	1.00	0.00	RX1	N
ATOM	6216	H	GLY	428	57.624	-2.611	27.950	1.00	0.00	RX1	H
ATOM	6217	CA	GLY	428	57.533	-0.473	27.918	1.00	0.00	RX1	C
ATOM	6218	C	GLY	428	56.382	0.375	27.396	1.00	0.00	RX1	C
ATOM	6219	O	GLY	428	56.510	1.577	27.174	1.00	0.00	RX1	O
ATOM	6220	N	ARG	429	55.243	-0.308	27.198	1.00	0.00	RX1	N

ATOM	6221	H	ARG	429	55.213	-1.284	27.417	1.00	0.00	RX1	H
ATOM	6222	CA	ARG	429	54.045	0.417	26.775	1.00	0.00	RX1	C
ATOM	6223	CB	ARG	429	52.965	-0.560	26.319	1.00	0.00	RX1	C
ATOM	6224	CG	ARG	429	53.393	-1.204	25.009	1.00	0.00	RX1	C
ATOM	6225	CD	ARG	429	52.411	-2.216	24.431	1.00	0.00	RX1	C
ATOM	6226	NE	ARG	429	52.904	-2.569	23.109	1.00	0.00	RX1	N
ATOM	6227	HE	ARG	429	53.383	-1.840	22.611	1.00	0.00	RX1	H
ATOM	6228	CZ	ARG	429	52.814	-3.815	22.573	1.00	0.00	RX1	C
ATOM	6229	NH1	ARG	429	52.095	-4.783	23.173	1.00	0.00	RX1	N
ATOM	6230	HH11	ARG	429	52.123	-5.730	22.816	1.00	0.00	RX1	H
ATOM	6231	HH12	ARG	429	51.478	-4.605	23.952	1.00	0.00	RX1	H
ATOM	6232	NH2	ARG	429	53.460	-4.048	21.419	1.00	0.00	RX1	N
ATOM	6233	HH21	ARG	429	53.468	-4.963	20.983	1.00	0.00	RX1	H
ATOM	6234	HH22	ARG	429	53.965	-3.318	20.942	1.00	0.00	RX1	H
ATOM	6235	C	ARG	429	53.510	1.327	27.855	1.00	0.00	RX1	C
ATOM	6236	O	ARG	429	53.404	2.538	27.697	1.00	0.00	RX1	O
ATOM	6237	N	THR	430	53.217	0.670	28.980	1.00	0.00	RX1	N
ATOM	6238	H	THR	430	53.434	-0.297	29.099	1.00	0.00	RX1	H
ATOM	6239	CA	THR	430	53.141	1.466	30.193	1.00	0.00	RX1	C
ATOM	6240	CB	THR	430	51.953	0.964	31.042	1.00	0.00	RX1	C
ATOM	6241	OG1	THR	430	52.280	0.731	32.412	1.00	0.00	RX1	O
ATOM	6242	HG1	THR	430	52.252	-0.226	32.552	1.00	0.00	RX1	H
ATOM	6243	CG2	THR	430	51.272	-0.240	30.394	1.00	0.00	RX1	C
ATOM	6244	C	THR	430	54.529	1.435	30.808	1.00	0.00	RX1	C
ATOM	6245	O	THR	430	55.351	0.591	30.457	1.00	0.00	RX1	O
ATOM	6246	N	LYS	431	54.790	2.456	31.633	1.00	0.00	RX1	N
ATOM	6247	H	LYS	431	54.070	3.029	32.026	1.00	0.00	RX1	H
ATOM	6248	CA	LYS	431	56.159	2.675	32.088	1.00	0.00	RX1	C
ATOM	6249	CB	LYS	431	56.871	3.740	31.243	1.00	0.00	RX1	C
ATOM	6250	CG	LYS	431	56.715	3.699	29.719	1.00	0.00	RX1	C
ATOM	6251	CD	LYS	431	57.410	4.889	29.051	1.00	0.00	RX1	C
ATOM	6252	CE	LYS	431	57.011	5.124	27.590	1.00	0.00	RX1	C
ATOM	6253	NZ	LYS	431	57.458	4.018	26.742	1.00	0.00	RX1	N
ATOM	6254	HZ1	LYS	431	57.099	4.094	25.766	1.00	0.00	RX1	H
ATOM	6255	HZ2	LYS	431	58.488	4.016	26.580	1.00	0.00	RX1	H
ATOM	6256	HZ3	LYS	431	57.164	3.088	27.095	1.00	0.00	RX1	H
ATOM	6257	C	LYS	431	56.117	3.176	33.516	1.00	0.00	RX1	C
ATOM	6258	O	LYS	431	55.366	4.095	33.825	1.00	0.00	RX1	O
ATOM	6259	N	GLN	432	56.939	2.570	34.385	1.00	0.00	RX1	N
ATOM	6260	H	GLN	432	57.596	1.889	34.058	1.00	0.00	RX1	H
ATOM	6261	CA	GLN	432	56.938	3.073	35.759	1.00	0.00	RX1	C
ATOM	6262	CB	GLN	432	57.783	2.199	36.684	1.00	0.00	RX1	C
ATOM	6263	CG	GLN	432	56.931	1.305	37.583	1.00	0.00	RX1	C
ATOM	6264	CD	GLN	432	55.967	2.150	38.392	1.00	0.00	RX1	C
ATOM	6265	OE1	GLN	432	56.222	3.319	38.677	1.00	0.00	RX1	O
ATOM	6266	NE2	GLN	432	54.839	1.505	38.738	1.00	0.00	RX1	N
ATOM	6267	HE21	GLN	432	54.654	0.553	38.474	1.00	0.00	RX1	H
ATOM	6268	HE22	GLN	432	54.107	1.927	39.273	1.00	0.00	RX1	H
ATOM	6269	C	GLN	432	57.385	4.516	35.861	1.00	0.00	RX1	C
ATOM	6270	O	GLN	432	58.322	4.942	35.197	1.00	0.00	RX1	O
ATOM	6271	N	HIS	433	56.617	5.267	36.667	1.00	0.00	RX1	N
ATOM	6272	H	HIS	433	55.926	4.804	37.225	1.00	0.00	RX1	H
ATOM	6273	CA	HIS	433	56.779	6.725	36.742	1.00	0.00	RX1	C
ATOM	6274	CB	HIS	433	58.053	7.110	37.500	1.00	0.00	RX1	C
ATOM	6275	CG	HIS	433	57.900	6.876	38.985	1.00	0.00	RX1	C
ATOM	6276	ND1	HIS	433	57.494	5.720	39.548	1.00	0.00	RX1	N
ATOM	6277	HD1	HIS	433	57.230	4.889	39.094	1.00	0.00	RX1	H
ATOM	6278	CD2	HIS	433	58.155	7.799	40.003	1.00	0.00	RX1	C
ATOM	6279	NE2	HIS	433	57.900	7.187	41.184	1.00	0.00	RX1	N
ATOM	6280	CE1	HIS	433	57.493	5.908	40.905	1.00	0.00	RX1	C
ATOM	6281	C	HIS	433	56.724	7.464	35.407	1.00	0.00	RX1	C

ATOM	6282	O	HIS	433	57.241	8.565	35.258	1.00	0.00	RX1	O
ATOM	6283	N	GLY	434	56.087	6.811	34.422	1.00	0.00	RX1	N
ATOM	6284	H	GLY	434	55.711	5.891	34.542	1.00	0.00	RX1	H
ATOM	6285	CA	GLY	434	56.061	7.414	33.090	1.00	0.00	RX1	C
ATOM	6286	C	GLY	434	57.313	7.194	32.249	1.00	0.00	RX1	C
ATOM	6287	O	GLY	434	57.375	7.575	31.087	1.00	0.00	RX1	O
ATOM	6288	N	GLN	435	58.317	6.561	32.875	1.00	0.00	RX1	N
ATOM	6289	H	GLN	435	58.226	6.200	33.801	1.00	0.00	RX1	H
ATOM	6290	CA	GLN	435	59.588	6.453	32.168	1.00	0.00	RX1	C
ATOM	6291	CB	GLN	435	60.621	7.318	32.889	1.00	0.00	RX1	C
ATOM	6292	CG	GLN	435	61.868	7.583	32.050	1.00	0.00	RX1	C
ATOM	6293	CD	GLN	435	62.951	8.185	32.922	1.00	0.00	RX1	C
ATOM	6294	OE1	GLN	435	63.661	7.493	33.651	1.00	0.00	RX1	O
ATOM	6295	NE2	GLN	435	63.037	9.522	32.813	1.00	0.00	RX1	N
ATOM	6296	HE21	GLN	435	62.426	10.012	32.190	1.00	0.00	RX1	H
ATOM	6297	HE22	GLN	435	63.701	10.063	33.328	1.00	0.00	RX1	H
ATOM	6298	C	GLN	435	60.112	5.032	31.996	1.00	0.00	RX1	C
ATOM	6299	O	GLN	435	60.468	4.598	30.907	1.00	0.00	RX1	O
ATOM	6300	N	PHE	436	60.181	4.326	33.130	1.00	0.00	RX1	N
ATOM	6301	H	PHE	436	59.707	4.614	33.960	1.00	0.00	RX1	H
ATOM	6302	CA	PHE	436	60.967	3.091	33.122	1.00	0.00	RX1	C
ATOM	6303	CB	PHE	436	61.462	2.702	34.517	1.00	0.00	RX1	C
ATOM	6304	CG	PHE	436	61.663	3.889	35.425	1.00	0.00	RX1	C
ATOM	6305	CD1	PHE	436	60.908	3.982	36.588	1.00	0.00	RX1	C
ATOM	6306	CD2	PHE	436	62.603	4.869	35.130	1.00	0.00	RX1	C
ATOM	6307	CE1	PHE	436	61.085	5.047	37.460	1.00	0.00	RX1	C
ATOM	6308	CE2	PHE	436	62.778	5.934	36.006	1.00	0.00	RX1	C
ATOM	6309	CZ	PHE	436	62.023	6.027	37.168	1.00	0.00	RX1	C
ATOM	6310	C	PHE	436	60.241	1.876	32.585	1.00	0.00	RX1	C
ATOM	6311	O	PHE	436	59.091	1.632	32.924	1.00	0.00	RX1	O
ATOM	6312	N	SER	437	60.964	1.098	31.777	1.00	0.00	RX1	N
ATOM	6313	H	SER	437	61.880	1.309	31.435	1.00	0.00	RX1	H
ATOM	6314	CA	SER	437	60.484	-0.258	31.549	1.00	0.00	RX1	C
ATOM	6315	CB	SER	437	60.721	-0.583	30.092	1.00	0.00	RX1	C
ATOM	6316	OG	SER	437	61.907	0.103	29.689	1.00	0.00	RX1	O
ATOM	6317	HG	SER	437	61.940	-0.016	28.740	1.00	0.00	RX1	H
ATOM	6318	C	SER	437	61.137	-1.264	32.477	1.00	0.00	RX1	C
ATOM	6319	O	SER	437	60.503	-2.181	32.980	1.00	0.00	RX1	O
ATOM	6320	N	LEU	438	62.435	-1.049	32.722	1.00	0.00	RX1	N
ATOM	6321	H	LEU	438	62.926	-0.247	32.373	1.00	0.00	RX1	H
ATOM	6322	CA	LEU	438	63.027	-1.907	33.743	1.00	0.00	RX1	C
ATOM	6323	CB	LEU	438	64.357	-2.480	33.265	1.00	0.00	RX1	C
ATOM	6324	CG	LEU	438	64.232	-3.923	32.786	1.00	0.00	RX1	C
ATOM	6325	CD1	LEU	438	65.526	-4.423	32.146	1.00	0.00	RX1	C
ATOM	6326	CD2	LEU	438	63.763	-4.841	33.916	1.00	0.00	RX1	C
ATOM	6327	C	LEU	438	63.235	-1.162	35.035	1.00	0.00	RX1	C
ATOM	6328	O	LEU	438	63.777	-0.064	35.043	1.00	0.00	RX1	O
ATOM	6329	N	ALA	439	62.799	-1.800	36.123	1.00	0.00	RX1	N
ATOM	6330	H	ALA	439	62.279	-2.659	36.102	1.00	0.00	RX1	H
ATOM	6331	CA	ALA	439	63.035	-1.168	37.414	1.00	0.00	RX1	C
ATOM	6332	CB	ALA	439	61.836	-0.311	37.806	1.00	0.00	RX1	C
ATOM	6333	C	ALA	439	63.312	-2.195	38.489	1.00	0.00	RX1	C
ATOM	6334	O	ALA	439	62.410	-2.795	39.059	1.00	0.00	RX1	O
ATOM	6335	N	VAL	440	64.617	-2.400	38.701	1.00	0.00	RX1	N
ATOM	6336	H	VAL	440	65.302	-1.762	38.342	1.00	0.00	RX1	H
ATOM	6337	CA	VAL	440	65.061	-3.453	39.613	1.00	0.00	RX1	C
ATOM	6338	CB	VAL	440	65.811	-4.529	38.821	1.00	0.00	RX1	C
ATOM	6339	CG1	VAL	440	66.471	-5.552	39.739	1.00	0.00	RX1	C
ATOM	6340	CG2	VAL	440	64.909	-5.188	37.776	1.00	0.00	RX1	C
ATOM	6341	C	VAL	440	65.960	-2.838	40.671	1.00	0.00	RX1	C
ATOM	6342	O	VAL	440	67.013	-2.287	40.359	1.00	0.00	RX1	O

ATOM	6343	N	VAL	441	65.489	-2.901	41.923	1.00	0.00	RX1	N
ATOM	6344	H	VAL	441	64.687	-3.451	42.177	1.00	0.00	RX1	H
ATOM	6345	CA	VAL	441	66.225	-2.138	42.932	1.00	0.00	RX1	C
ATOM	6346	CB	VAL	441	65.473	-0.850	43.301	1.00	0.00	RX1	C
ATOM	6347	CG1	VAL	441	65.468	0.168	42.155	1.00	0.00	RX1	C
ATOM	6348	CG2	VAL	441	64.063	-1.163	43.801	1.00	0.00	RX1	C
ATOM	6349	C	VAL	441	66.596	-2.912	44.185	1.00	0.00	RX1	C
ATOM	6350	O	VAL	441	65.851	-3.754	44.663	1.00	0.00	RX1	O
ATOM	6351	N	SER	442	67.777	-2.559	44.718	1.00	0.00	RX1	N
ATOM	6352	H	SER	442	68.300	-1.869	44.219	1.00	0.00	RX1	H
ATOM	6353	CA	SER	442	68.218	-2.985	46.060	1.00	0.00	RX1	C
ATOM	6354	CB	SER	442	67.201	-2.420	47.046	1.00	0.00	RX1	C
ATOM	6355	OG	SER	442	66.648	-1.236	46.454	1.00	0.00	RX1	O
ATOM	6356	HG	SER	442	65.764	-1.478	46.203	1.00	0.00	RX1	H
ATOM	6357	C	SER	442	68.607	-4.444	46.310	1.00	0.00	RX1	C
ATOM	6358	O	SER	442	69.171	-4.807	47.346	1.00	0.00	RX1	O
ATOM	6359	N	LEU	443	68.268	-5.283	45.324	1.00	0.00	RX1	N
ATOM	6360	H	LEU	443	67.962	-4.911	44.445	1.00	0.00	RX1	H
ATOM	6361	CA	LEU	443	68.195	-6.724	45.554	1.00	0.00	RX1	C
ATOM	6362	CB	LEU	443	67.639	-7.441	44.328	1.00	0.00	RX1	C
ATOM	6363	CG	LEU	443	66.321	-6.878	43.808	1.00	0.00	RX1	C
ATOM	6364	CD1	LEU	443	65.859	-7.669	42.591	1.00	0.00	RX1	C
ATOM	6365	CD2	LEU	443	65.241	-6.793	44.888	1.00	0.00	RX1	C
ATOM	6366	C	LEU	443	69.476	-7.416	45.988	1.00	0.00	RX1	C
ATOM	6367	O	LEU	443	70.561	-6.844	46.062	1.00	0.00	RX1	O
ATOM	6368	N	ASN	444	69.288	-8.712	46.286	1.00	0.00	RX1	N
ATOM	6369	H	ASN	444	68.398	-9.157	46.176	1.00	0.00	RX1	H
ATOM	6370	CA	ASN	444	70.450	-9.527	46.645	1.00	0.00	RX1	C
ATOM	6371	CB	ASN	444	70.100	-10.579	47.695	1.00	0.00	RX1	C
ATOM	6372	CG	ASN	444	70.326	-9.989	49.066	1.00	0.00	RX1	C
ATOM	6373	OD1	ASN	444	70.595	-8.795	49.212	1.00	0.00	RX1	O
ATOM	6374	ND2	ASN	444	70.197	-10.877	50.064	1.00	0.00	RX1	N
ATOM	6375	HD21	ASN	444	69.943	-11.827	49.844	1.00	0.00	RX1	H
ATOM	6376	HD22	ASN	444	70.317	-10.666	51.033	1.00	0.00	RX1	H
ATOM	6377	C	ASN	444	71.124	-10.211	45.475	1.00	0.00	RX1	C
ATOM	6378	O	ASN	444	72.080	-10.964	45.623	1.00	0.00	RX1	O
ATOM	6379	N	ILE	445	70.586	-9.899	44.288	1.00	0.00	RX1	N
ATOM	6380	H	ILE	445	69.871	-9.213	44.202	1.00	0.00	RX1	H
ATOM	6381	CA	ILE	445	71.143	-10.501	43.085	1.00	0.00	RX1	C
ATOM	6382	CB	ILE	445	70.241	-10.206	41.884	1.00	0.00	RX1	C
ATOM	6383	CG2	ILE	445	68.885	-10.874	42.095	1.00	0.00	RX1	C
ATOM	6384	CG1	ILE	445	70.095	-8.706	41.609	1.00	0.00	RX1	C
ATOM	6385	CD1	ILE	445	69.269	-8.409	40.359	1.00	0.00	RX1	C
ATOM	6386	C	ILE	445	72.589	-10.126	42.811	1.00	0.00	RX1	C
ATOM	6387	O	ILE	445	72.963	-8.962	42.733	1.00	0.00	RX1	O
ATOM	6388	N	THR	446	73.397	-11.180	42.681	1.00	0.00	RX1	N
ATOM	6389	H	THR	446	73.045	-12.113	42.720	1.00	0.00	RX1	H
ATOM	6390	CA	THR	446	74.719	-10.926	42.133	1.00	0.00	RX1	C
ATOM	6391	CB	THR	446	75.751	-11.819	42.832	1.00	0.00	RX1	C
ATOM	6392	OG1	THR	446	76.994	-11.113	42.949	1.00	0.00	RX1	O
ATOM	6393	HG1	THR	446	76.723	-10.197	42.897	1.00	0.00	RX1	H
ATOM	6394	CG2	THR	446	75.912	-13.197	42.187	1.00	0.00	RX1	C
ATOM	6395	C	THR	446	74.736	-10.974	40.617	1.00	0.00	RX1	C
ATOM	6396	O	THR	446	75.682	-10.500	39.997	1.00	0.00	RX1	O
ATOM	6397	N	SER	447	73.665	-11.552	40.066	1.00	0.00	RX1	N
ATOM	6398	H	SER	447	72.940	-12.037	40.556	1.00	0.00	RX1	H
ATOM	6399	CA	SER	447	73.546	-11.564	38.620	1.00	0.00	RX1	C
ATOM	6400	CB	SER	447	73.762	-13.025	38.316	1.00	0.00	RX1	C
ATOM	6401	OG	SER	447	73.886	-13.657	39.599	1.00	0.00	RX1	O
ATOM	6402	HG	SER	447	73.064	-14.127	39.708	1.00	0.00	RX1	H
ATOM	6403	C	SER	447	72.205	-11.009	38.201	1.00	0.00	RX1	C

ATOM	6404	O	SER	447	71.204	-11.131	38.897	1.00	0.00	RX1	O
ATOM	6405	N	LEU	448	72.244	-10.348	37.041	1.00	0.00	RX1	N
ATOM	6406	H	LEU	448	73.062	-10.388	36.467	1.00	0.00	RX1	H
ATOM	6407	CA	LEU	448	71.013	-9.761	36.530	1.00	0.00	RX1	C
ATOM	6408	CB	LEU	448	71.336	-8.693	35.488	1.00	0.00	RX1	C
ATOM	6409	CG	LEU	448	70.972	-7.284	35.954	1.00	0.00	RX1	C
ATOM	6410	CD1	LEU	448	71.405	-6.219	34.946	1.00	0.00	RX1	C
ATOM	6411	CD2	LEU	448	69.493	-7.167	36.322	1.00	0.00	RX1	C
ATOM	6412	C	LEU	448	70.027	-10.768	35.973	1.00	0.00	RX1	C
ATOM	6413	O	LEU	448	68.830	-10.534	35.943	1.00	0.00	RX1	O
ATOM	6414	N	GLY	449	70.568	-11.909	35.525	1.00	0.00	RX1	N
ATOM	6415	H	GLY	449	71.562	-12.014	35.458	1.00	0.00	RX1	H
ATOM	6416	CA	GLY	449	69.691	-13.061	35.295	1.00	0.00	RX1	C
ATOM	6417	C	GLY	449	68.619	-12.999	34.211	1.00	0.00	RX1	C
ATOM	6418	O	GLY	449	67.924	-13.976	33.957	1.00	0.00	RX1	O
ATOM	6419	N	LEU	450	68.512	-11.837	33.547	1.00	0.00	RX1	N
ATOM	6420	H	LEU	450	69.098	-11.068	33.790	1.00	0.00	RX1	H
ATOM	6421	CA	LEU	450	67.549	-11.706	32.451	1.00	0.00	RX1	C
ATOM	6422	CB	LEU	450	67.172	-10.239	32.256	1.00	0.00	RX1	C
ATOM	6423	CG	LEU	450	66.528	-9.609	33.489	1.00	0.00	RX1	C
ATOM	6424	CD1	LEU	450	66.479	-8.085	33.401	1.00	0.00	RX1	C
ATOM	6425	CD2	LEU	450	65.148	-10.200	33.753	1.00	0.00	RX1	C
ATOM	6426	C	LEU	450	68.086	-12.263	31.144	1.00	0.00	RX1	C
ATOM	6427	O	LEU	450	68.160	-11.597	30.123	1.00	0.00	RX1	O
ATOM	6428	N	ARG	451	68.508	-13.526	31.235	1.00	0.00	RX1	N
ATOM	6429	H	ARG	451	68.252	-14.035	32.056	1.00	0.00	RX1	H
ATOM	6430	CA	ARG	451	69.370	-14.067	30.185	1.00	0.00	RX1	C
ATOM	6431	CB	ARG	451	70.063	-15.347	30.669	1.00	0.00	RX1	C
ATOM	6432	CG	ARG	451	69.192	-16.220	31.572	1.00	0.00	RX1	C
ATOM	6433	CD	ARG	451	69.982	-17.302	32.309	1.00	0.00	RX1	C
ATOM	6434	NE	ARG	451	71.083	-16.743	33.089	1.00	0.00	RX1	N
ATOM	6435	HE	ARG	451	71.904	-16.405	32.605	1.00	0.00	RX1	H
ATOM	6436	CZ	ARG	451	71.020	-16.512	34.436	1.00	0.00	RX1	C
ATOM	6437	NH1	ARG	451	69.962	-16.916	35.165	1.00	0.00	RX1	N
ATOM	6438	HH11	ARG	451	69.859	-16.621	36.129	1.00	0.00	RX1	H
ATOM	6439	HH12	ARG	451	69.235	-17.512	34.801	1.00	0.00	RX1	H
ATOM	6440	NH2	ARG	451	72.032	-15.852	35.022	1.00	0.00	RX1	N
ATOM	6441	HH21	ARG	451	72.021	-15.624	36.006	1.00	0.00	RX1	H
ATOM	6442	HH22	ARG	451	72.827	-15.580	34.453	1.00	0.00	RX1	H
ATOM	6443	C	ARG	451	68.768	-14.196	28.795	1.00	0.00	RX1	C
ATOM	6444	O	ARG	451	69.468	-14.186	27.793	1.00	0.00	RX1	O
ATOM	6445	N	SER	452	67.431	-14.277	28.760	1.00	0.00	RX1	N
ATOM	6446	H	SER	452	66.842	-14.220	29.565	1.00	0.00	RX1	H
ATOM	6447	CA	SER	452	66.853	-14.327	27.418	1.00	0.00	RX1	C
ATOM	6448	CB	SER	452	65.731	-15.356	27.456	1.00	0.00	RX1	C
ATOM	6449	OG	SER	452	66.182	-16.452	28.268	1.00	0.00	RX1	O
ATOM	6450	HG	SER	452	66.242	-16.109	29.152	1.00	0.00	RX1	H
ATOM	6451	C	SER	452	66.496	-12.979	26.795	1.00	0.00	RX1	C
ATOM	6452	O	SER	452	65.930	-12.902	25.709	1.00	0.00	RX1	O
ATOM	6453	N	LEU	453	66.829	-11.901	27.529	1.00	0.00	RX1	N
ATOM	6454	H	LEU	453	67.409	-11.965	28.341	1.00	0.00	RX1	H
ATOM	6455	CA	LEU	453	66.297	-10.601	27.127	1.00	0.00	RX1	C
ATOM	6456	CB	LEU	453	66.455	-9.592	28.266	1.00	0.00	RX1	C
ATOM	6457	CG	LEU	453	65.390	-8.494	28.285	1.00	0.00	RX1	C
ATOM	6458	CD1	LEU	453	63.990	-9.070	28.487	1.00	0.00	RX1	C
ATOM	6459	CD2	LEU	453	65.689	-7.433	29.343	1.00	0.00	RX1	C
ATOM	6460	C	LEU	453	66.839	-10.050	25.817	1.00	0.00	RX1	C
ATOM	6461	O	LEU	453	67.898	-9.443	25.738	1.00	0.00	RX1	O
ATOM	6462	N	LYS	454	66.031	-10.279	24.780	1.00	0.00	RX1	N
ATOM	6463	H	LYS	454	65.183	-10.781	24.956	1.00	0.00	RX1	H
ATOM	6464	CA	LYS	454	66.361	-9.763	23.455	1.00	0.00	RX1	C

ATOM	6465	CB	LYS	454	66.120	-10.823	22.376	1.00	0.00	RX1	C
ATOM	6466	CG	LYS	454	64.651	-11.258	22.381	1.00	0.00	RX1	C
ATOM	6467	CD	LYS	454	64.053	-11.701	21.042	1.00	0.00	RX1	C
ATOM	6468	CE	LYS	454	63.908	-10.567	20.019	1.00	0.00	RX1	C
ATOM	6469	NZ	LYS	454	63.279	-9.395	20.641	1.00	0.00	RX1	N
ATOM	6470	HZ1	LYS	454	62.703	-8.827	19.980	1.00	0.00	RX1	H
ATOM	6471	HZ2	LYS	454	62.597	-9.644	21.385	1.00	0.00	RX1	H
ATOM	6472	HZ3	LYS	454	64.000	-8.763	21.048	1.00	0.00	RX1	H
ATOM	6473	C	LYS	454	65.566	-8.535	23.027	1.00	0.00	RX1	C
ATOM	6474	O	LYS	454	65.420	-8.290	21.829	1.00	0.00	RX1	O
ATOM	6475	N	GLU	455	65.002	-7.802	24.000	1.00	0.00	RX1	N
ATOM	6476	H	GLU	455	65.023	-8.061	24.968	1.00	0.00	RX1	H
ATOM	6477	CA	GLU	455	64.172	-6.660	23.606	1.00	0.00	RX1	C
ATOM	6478	CB	GLU	455	62.854	-7.142	22.990	1.00	0.00	RX1	C
ATOM	6479	CG	GLU	455	62.304	-6.279	21.852	1.00	0.00	RX1	C
ATOM	6480	CD	GLU	455	61.068	-6.942	21.260	1.00	0.00	RX1	C
ATOM	6481	OE1	GLU	455	61.178	-8.051	20.743	1.00	0.00	RX1	O
ATOM	6482	OE2	GLU	455	59.990	-6.358	21.311	1.00	0.00	RX1	O
ATOM	6483	C	GLU	455	63.871	-5.751	24.771	1.00	0.00	RX1	C
ATOM	6484	O	GLU	455	63.208	-6.154	25.716	1.00	0.00	RX1	O
ATOM	6485	N	ILE	456	64.339	-4.504	24.655	1.00	0.00	RX1	N
ATOM	6486	H	ILE	456	65.002	-4.198	23.967	1.00	0.00	RX1	H
ATOM	6487	CA	ILE	456	63.612	-3.486	25.404	1.00	0.00	RX1	C
ATOM	6488	CB	ILE	456	64.426	-2.864	26.539	1.00	0.00	RX1	C
ATOM	6489	CG2	ILE	456	63.524	-1.961	27.383	1.00	0.00	RX1	C
ATOM	6490	CG1	ILE	456	65.130	-3.909	27.404	1.00	0.00	RX1	C
ATOM	6491	CD1	ILE	456	66.040	-3.266	28.450	1.00	0.00	RX1	C
ATOM	6492	C	ILE	456	63.131	-2.432	24.425	1.00	0.00	RX1	C
ATOM	6493	O	ILE	456	63.833	-1.505	24.030	1.00	0.00	RX1	O
ATOM	6494	N	SER	457	61.892	-2.664	23.991	1.00	0.00	RX1	N
ATOM	6495	H	SER	457	61.313	-3.355	24.431	1.00	0.00	RX1	H
ATOM	6496	CA	SER	457	61.392	-1.936	22.828	1.00	0.00	RX1	C
ATOM	6497	CB	SER	457	60.188	-2.745	22.388	1.00	0.00	RX1	C
ATOM	6498	OG	SER	457	60.349	-4.029	23.007	1.00	0.00	RX1	O
ATOM	6499	HG	SER	457	60.233	-4.697	22.329	1.00	0.00	RX1	H
ATOM	6500	C	SER	457	61.166	-0.441	23.028	1.00	0.00	RX1	C
ATOM	6501	O	SER	457	61.145	0.339	22.077	1.00	0.00	RX1	O
ATOM	6502	N	ASP	458	61.018	-0.093	24.319	1.00	0.00	RX1	N
ATOM	6503	H	ASP	458	61.031	-0.764	25.065	1.00	0.00	RX1	H
ATOM	6504	CA	ASP	458	60.781	1.277	24.776	1.00	0.00	RX1	C
ATOM	6505	CB	ASP	458	59.356	1.656	24.345	1.00	0.00	RX1	C
ATOM	6506	CG	ASP	458	58.929	3.093	24.599	1.00	0.00	RX1	C
ATOM	6507	OD1	ASP	458	59.658	3.883	25.186	1.00	0.00	RX1	O
ATOM	6508	OD2	ASP	458	57.795	3.426	24.275	1.00	0.00	RX1	O
ATOM	6509	C	ASP	458	60.983	1.275	26.288	1.00	0.00	RX1	C
ATOM	6510	O	ASP	458	60.986	0.220	26.915	1.00	0.00	RX1	O
ATOM	6511	N	GLY	459	61.179	2.474	26.845	1.00	0.00	RX1	N
ATOM	6512	H	GLY	459	61.215	3.282	26.255	1.00	0.00	RX1	H
ATOM	6513	CA	GLY	459	61.293	2.617	28.292	1.00	0.00	RX1	C
ATOM	6514	C	GLY	459	62.735	2.740	28.743	1.00	0.00	RX1	C
ATOM	6515	O	GLY	459	63.654	2.288	28.070	1.00	0.00	RX1	O
ATOM	6516	N	ASP	460	62.887	3.425	29.881	1.00	0.00	RX1	N
ATOM	6517	H	ASP	460	62.081	3.675	30.416	1.00	0.00	RX1	H
ATOM	6518	CA	ASP	460	64.220	3.683	30.433	1.00	0.00	RX1	C
ATOM	6519	CB	ASP	460	64.191	5.127	30.966	1.00	0.00	RX1	C
ATOM	6520	CG	ASP	460	65.517	5.762	31.375	1.00	0.00	RX1	C
ATOM	6521	OD1	ASP	460	65.629	6.981	31.365	1.00	0.00	RX1	O
ATOM	6522	OD2	ASP	460	66.432	5.092	31.820	1.00	0.00	RX1	O
ATOM	6523	C	ASP	460	64.496	2.633	31.503	1.00	0.00	RX1	C
ATOM	6524	O	ASP	460	63.594	1.942	31.967	1.00	0.00	RX1	O
ATOM	6525	N	VAL	461	65.771	2.504	31.855	1.00	0.00	RX1	N

ATOM	6526	H	VAL	461	66.468	3.137	31.506	1.00	0.00	RX1	H
ATOM	6527	CA	VAL	461	66.145	1.555	32.888	1.00	0.00	RX1	C
ATOM	6528	CB	VAL	461	67.290	0.698	32.342	1.00	0.00	RX1	C
ATOM	6529	CG1	VAL	461	68.082	-0.051	33.413	1.00	0.00	RX1	C
ATOM	6530	CG2	VAL	461	66.725	-0.249	31.285	1.00	0.00	RX1	C
ATOM	6531	C	VAL	461	66.514	2.253	34.185	1.00	0.00	RX1	C
ATOM	6532	O	VAL	461	67.233	3.244	34.224	1.00	0.00	RX1	O
ATOM	6533	N	ILE	462	65.999	1.659	35.263	1.00	0.00	RX1	N
ATOM	6534	H	ILE	462	65.330	0.920	35.179	1.00	0.00	RX1	H
ATOM	6535	CA	ILE	462	66.539	1.944	36.583	1.00	0.00	RX1	C
ATOM	6536	CB	ILE	462	65.701	2.980	37.345	1.00	0.00	RX1	C
ATOM	6537	CG2	ILE	462	64.240	2.570	37.500	1.00	0.00	RX1	C
ATOM	6538	CG1	ILE	462	66.363	3.329	38.678	1.00	0.00	RX1	C
ATOM	6539	CD1	ILE	462	65.619	4.440	39.418	1.00	0.00	RX1	C
ATOM	6540	C	ILE	462	66.775	0.658	37.360	1.00	0.00	RX1	C
ATOM	6541	O	ILE	462	65.949	0.129	38.096	1.00	0.00	RX1	O
ATOM	6542	N	ILE	463	67.982	0.144	37.126	1.00	0.00	RX1	N
ATOM	6543	H	ILE	463	68.678	0.657	36.620	1.00	0.00	RX1	H
ATOM	6544	CA	ILE	463	68.376	-1.003	37.931	1.00	0.00	RX1	C
ATOM	6545	CB	ILE	463	68.816	-2.162	37.036	1.00	0.00	RX1	C
ATOM	6546	CG2	ILE	463	69.293	-3.361	37.849	1.00	0.00	RX1	C
ATOM	6547	CG1	ILE	463	67.660	-2.558	36.117	1.00	0.00	RX1	C
ATOM	6548	CD1	ILE	463	67.986	-3.769	35.248	1.00	0.00	RX1	C
ATOM	6549	C	ILE	463	69.443	-0.558	38.908	1.00	0.00	RX1	C
ATOM	6550	O	ILE	463	70.635	-0.570	38.634	1.00	0.00	RX1	O
ATOM	6551	N	SER	464	68.938	-0.083	40.047	1.00	0.00	RX1	N
ATOM	6552	H	SER	464	67.993	-0.265	40.328	1.00	0.00	RX1	H
ATOM	6553	CA	SER	464	69.837	0.620	40.955	1.00	0.00	RX1	C
ATOM	6554	CB	SER	464	69.536	2.115	40.883	1.00	0.00	RX1	C
ATOM	6555	OG	SER	464	69.935	2.637	39.612	1.00	0.00	RX1	O
ATOM	6556	HG	SER	464	69.584	2.057	38.943	1.00	0.00	RX1	H
ATOM	6557	C	SER	464	69.769	0.104	42.377	1.00	0.00	RX1	C
ATOM	6558	O	SER	464	68.845	-0.595	42.772	1.00	0.00	RX1	O
ATOM	6559	N	GLY	465	70.801	0.475	43.145	1.00	0.00	RX1	N
ATOM	6560	H	GLY	465	71.593	0.927	42.732	1.00	0.00	RX1	H
ATOM	6561	CA	GLY	465	70.805	0.099	44.558	1.00	0.00	RX1	C
ATOM	6562	C	GLY	465	71.111	-1.366	44.828	1.00	0.00	RX1	C
ATOM	6563	O	GLY	465	71.079	-1.848	45.955	1.00	0.00	RX1	O
ATOM	6564	N	ASN	466	71.384	-2.090	43.737	1.00	0.00	RX1	N
ATOM	6565	H	ASN	466	71.619	-1.662	42.863	1.00	0.00	RX1	H
ATOM	6566	CA	ASN	466	71.558	-3.527	43.909	1.00	0.00	RX1	C
ATOM	6567	CB	ASN	466	71.187	-4.353	42.674	1.00	0.00	RX1	C
ATOM	6568	CG	ASN	466	69.778	-4.067	42.209	1.00	0.00	RX1	C
ATOM	6569	OD1	ASN	466	68.806	-4.637	42.696	1.00	0.00	RX1	O
ATOM	6570	ND2	ASN	466	69.717	-3.139	41.243	1.00	0.00	RX1	N
ATOM	6571	HD21	ASN	466	70.566	-2.734	40.889	1.00	0.00	RX1	H
ATOM	6572	HD22	ASN	466	68.842	-2.818	40.868	1.00	0.00	RX1	H
ATOM	6573	C	ASN	466	72.969	-3.843	44.335	1.00	0.00	RX1	C
ATOM	6574	O	ASN	466	73.875	-4.112	43.554	1.00	0.00	RX1	O
ATOM	6575	N	LYS	467	73.088	-3.807	45.665	1.00	0.00	RX1	N
ATOM	6576	H	LYS	467	72.284	-3.380	46.080	1.00	0.00	RX1	H
ATOM	6577	CA	LYS	467	74.330	-4.073	46.404	1.00	0.00	RX1	C
ATOM	6578	CB	LYS	467	73.989	-4.460	47.846	1.00	0.00	RX1	C
ATOM	6579	CG	LYS	467	72.712	-3.791	48.359	1.00	0.00	RX1	C
ATOM	6580	CD	LYS	467	72.176	-4.381	49.661	1.00	0.00	RX1	C
ATOM	6581	CE	LYS	467	71.991	-5.901	49.612	1.00	0.00	RX1	C
ATOM	6582	NZ	LYS	467	71.157	-6.329	48.479	1.00	0.00	RX1	N
ATOM	6583	HZ1	LYS	467	70.647	-7.189	48.762	1.00	0.00	RX1	H
ATOM	6584	HZ2	LYS	467	70.465	-5.614	48.164	1.00	0.00	RX1	H
ATOM	6585	HZ3	LYS	467	71.738	-6.616	47.658	1.00	0.00	RX1	H
ATOM	6586	C	LYS	467	75.222	-5.162	45.822	1.00	0.00	RX1	C

ATOM	6587	O	LYS	467	76.441	-5.086	45.782	1.00	0.00	RX1	O
ATOM	6588	N	ASN	468	74.529	-6.220	45.392	1.00	0.00	RX1	N
ATOM	6589	H	ASN	468	73.530	-6.214	45.357	1.00	0.00	RX1	H
ATOM	6590	CA	ASN	468	75.264	-7.429	45.040	1.00	0.00	RX1	C
ATOM	6591	CB	ASN	468	74.499	-8.683	45.486	1.00	0.00	RX1	C
ATOM	6592	CG	ASN	468	73.921	-8.575	46.889	1.00	0.00	RX1	C
ATOM	6593	OD1	ASN	468	73.116	-7.696	47.212	1.00	0.00	RX1	O
ATOM	6594	ND2	ASN	468	74.357	-9.543	47.713	1.00	0.00	RX1	N
ATOM	6595	HD21	ASN	468	74.978	-10.249	47.370	1.00	0.00	RX1	H
ATOM	6596	HD22	ASN	468	74.074	-9.618	48.670	1.00	0.00	RX1	H
ATOM	6597	C	ASN	468	75.616	-7.545	43.561	1.00	0.00	RX1	C
ATOM	6598	O	ASN	468	76.415	-8.384	43.155	1.00	0.00	RX1	O
ATOM	6599	N	LEU	469	74.975	-6.670	42.772	1.00	0.00	RX1	N
ATOM	6600	H	LEU	469	74.471	-5.909	43.181	1.00	0.00	RX1	H
ATOM	6601	CA	LEU	469	74.882	-6.855	41.324	1.00	0.00	RX1	C
ATOM	6602	CB	LEU	469	73.767	-5.960	40.797	1.00	0.00	RX1	C
ATOM	6603	CG	LEU	469	73.329	-6.238	39.366	1.00	0.00	RX1	C
ATOM	6604	CD1	LEU	469	72.918	-7.693	39.178	1.00	0.00	RX1	C
ATOM	6605	CD2	LEU	469	72.233	-5.269	38.936	1.00	0.00	RX1	C
ATOM	6606	C	LEU	469	76.140	-6.667	40.495	1.00	0.00	RX1	C
ATOM	6607	O	LEU	469	76.518	-5.564	40.111	1.00	0.00	RX1	O
ATOM	6608	N	CYS	470	76.752	-7.814	40.194	1.00	0.00	RX1	N
ATOM	6609	H	CYS	470	76.347	-8.681	40.483	1.00	0.00	RX1	H
ATOM	6610	CA	CYS	470	77.765	-7.787	39.144	1.00	0.00	RX1	C
ATOM	6611	CB	CYS	470	78.635	-9.031	39.257	1.00	0.00	RX1	C
ATOM	6612	SG	CYS	470	79.625	-8.995	40.768	1.00	0.00	RX1	S
ATOM	6613	C	CYS	470	77.121	-7.669	37.775	1.00	0.00	RX1	C
ATOM	6614	O	CYS	470	75.903	-7.682	37.649	1.00	0.00	RX1	O
ATOM	6615	N	TYR	471	77.987	-7.553	36.751	1.00	0.00	RX1	N
ATOM	6616	H	TYR	471	78.968	-7.500	36.940	1.00	0.00	RX1	H
ATOM	6617	CA	TYR	471	77.554	-7.646	35.349	1.00	0.00	RX1	C
ATOM	6618	CB	TYR	471	76.906	-9.003	35.032	1.00	0.00	RX1	C
ATOM	6619	CG	TYR	471	77.876	-10.132	35.294	1.00	0.00	RX1	C
ATOM	6620	CD1	TYR	471	77.662	-10.970	36.406	1.00	0.00	RX1	C
ATOM	6621	CE1	TYR	471	78.556	-12.026	36.643	1.00	0.00	RX1	C
ATOM	6622	CD2	TYR	471	78.963	-10.320	34.417	1.00	0.00	RX1	C
ATOM	6623	CE2	TYR	471	79.847	-11.386	34.648	1.00	0.00	RX1	C
ATOM	6624	CZ	TYR	471	79.632	-12.228	35.757	1.00	0.00	RX1	C
ATOM	6625	OH	TYR	471	80.498	-13.279	35.994	1.00	0.00	RX1	O
ATOM	6626	HH	TYR	471	81.123	-13.359	35.274	1.00	0.00	RX1	H
ATOM	6627	C	TYR	471	76.735	-6.513	34.742	1.00	0.00	RX1	C
ATOM	6628	O	TYR	471	76.800	-6.286	33.541	1.00	0.00	RX1	O
ATOM	6629	N	ALA	472	75.996	-5.775	35.594	1.00	0.00	RX1	N
ATOM	6630	H	ALA	472	75.901	-6.105	36.532	1.00	0.00	RX1	H
ATOM	6631	CA	ALA	472	75.125	-4.693	35.114	1.00	0.00	RX1	C
ATOM	6632	CB	ALA	472	74.602	-3.869	36.291	1.00	0.00	RX1	C
ATOM	6633	C	ALA	472	75.713	-3.726	34.093	1.00	0.00	RX1	C
ATOM	6634	O	ALA	472	75.047	-3.235	33.191	1.00	0.00	RX1	O
ATOM	6635	N	ASN	473	77.013	-3.469	34.274	1.00	0.00	RX1	N
ATOM	6636	H	ASN	473	77.533	-4.007	34.937	1.00	0.00	RX1	H
ATOM	6637	CA	ASN	473	77.718	-2.623	33.308	1.00	0.00	RX1	C
ATOM	6638	CB	ASN	473	78.886	-1.763	33.759	1.00	0.00	RX1	C
ATOM	6639	CG	ASN	473	79.091	-0.511	32.912	1.00	0.00	RX1	C
ATOM	6640	OD1	ASN	473	80.218	-0.144	32.615	1.00	0.00	RX1	O
ATOM	6641	ND2	ASN	473	77.982	0.170	32.602	1.00	0.00	RX1	N
ATOM	6642	HD21	ASN	473	77.043	-0.090	32.803	1.00	0.00	RX1	H
ATOM	6643	HD22	ASN	473	78.069	1.053	32.121	1.00	0.00	RX1	H
ATOM	6644	C	ASN	473	77.843	-3.222	31.911	1.00	0.00	RX1	C
ATOM	6645	O	ASN	473	77.713	-2.551	30.897	1.00	0.00	RX1	O
ATOM	6646	N	THR	474	78.099	-4.535	31.899	1.00	0.00	RX1	N
ATOM	6647	H	THR	474	78.098	-5.037	32.764	1.00	0.00	RX1	H

ATOM	6648	CA	THR	474	78.426	-5.183	30.630	1.00	0.00	RX1	C
ATOM	6649	CB	THR	474	78.990	-6.577	30.942	1.00	0.00	RX1	C
ATOM	6650	OG1	THR	474	77.948	-7.495	31.301	1.00	0.00	RX1	O
ATOM	6651	HG1	THR	474	77.521	-7.164	32.089	1.00	0.00	RX1	H
ATOM	6652	CG2	THR	474	80.028	-6.559	32.056	1.00	0.00	RX1	C
ATOM	6653	C	THR	474	77.294	-5.325	29.617	1.00	0.00	RX1	C
ATOM	6654	O	THR	474	77.494	-5.712	28.474	1.00	0.00	RX1	O
ATOM	6655	N	ILE	475	76.077	-5.000	30.083	1.00	0.00	RX1	N
ATOM	6656	H	ILE	475	75.981	-4.568	30.979	1.00	0.00	RX1	H
ATOM	6657	CA	ILE	475	74.892	-5.293	29.271	1.00	0.00	RX1	C
ATOM	6658	CB	ILE	475	73.636	-5.037	30.116	1.00	0.00	RX1	C
ATOM	6659	CG2	ILE	475	72.341	-5.436	29.406	1.00	0.00	RX1	C
ATOM	6660	CG1	ILE	475	73.749	-5.747	31.464	1.00	0.00	RX1	C
ATOM	6661	CD1	ILE	475	73.762	-7.265	31.323	1.00	0.00	RX1	C
ATOM	6662	C	ILE	475	74.819	-4.573	27.920	1.00	0.00	RX1	C
ATOM	6663	O	ILE	475	74.088	-4.966	27.022	1.00	0.00	RX1	O
ATOM	6664	N	ASN	476	75.586	-3.467	27.820	1.00	0.00	RX1	N
ATOM	6665	H	ASN	476	76.215	-3.230	28.560	1.00	0.00	RX1	H
ATOM	6666	CA	ASN	476	75.464	-2.597	26.639	1.00	0.00	RX1	C
ATOM	6667	CB	ASN	476	76.066	-3.161	25.362	1.00	0.00	RX1	C
ATOM	6668	CG	ASN	476	75.916	-2.104	24.283	1.00	0.00	RX1	C
ATOM	6669	OD1	ASN	476	75.657	-0.928	24.519	1.00	0.00	RX1	O
ATOM	6670	ND2	ASN	476	76.137	-2.574	23.056	1.00	0.00	RX1	N
ATOM	6671	HD21	ASN	476	76.221	-3.567	22.933	1.00	0.00	RX1	H
ATOM	6672	HD22	ASN	476	76.190	-1.948	22.283	1.00	0.00	RX1	H
ATOM	6673	C	ASN	476	74.035	-2.146	26.386	1.00	0.00	RX1	C
ATOM	6674	O	ASN	476	73.373	-2.403	25.387	1.00	0.00	RX1	O
ATOM	6675	N	TRP	477	73.584	-1.426	27.413	1.00	0.00	RX1	N
ATOM	6676	H	TRP	477	74.208	-1.223	28.166	1.00	0.00	RX1	H
ATOM	6677	CA	TRP	477	72.176	-1.062	27.481	1.00	0.00	RX1	C
ATOM	6678	CB	TRP	477	71.975	-0.191	28.706	1.00	0.00	RX1	C
ATOM	6679	CG	TRP	477	72.115	-1.034	29.945	1.00	0.00	RX1	C
ATOM	6680	CD2	TRP	477	71.119	-1.896	30.531	1.00	0.00	RX1	C
ATOM	6681	CE2	TRP	477	71.684	-2.464	31.695	1.00	0.00	RX1	C
ATOM	6682	CE3	TRP	477	69.823	-2.228	30.159	1.00	0.00	RX1	C
ATOM	6683	CD1	TRP	477	73.235	-1.133	30.784	1.00	0.00	RX1	C
ATOM	6684	NE1	TRP	477	72.980	-1.975	31.818	1.00	0.00	RX1	N
ATOM	6685	HE1	TRP	477	73.620	-2.222	32.522	1.00	0.00	RX1	H
ATOM	6686	CZ2	TRP	477	70.935	-3.349	32.459	1.00	0.00	RX1	C
ATOM	6687	CZ3	TRP	477	69.085	-3.117	30.931	1.00	0.00	RX1	C
ATOM	6688	CH2	TRP	477	69.639	-3.674	32.076	1.00	0.00	RX1	C
ATOM	6689	C	TRP	477	71.551	-0.432	26.245	1.00	0.00	RX1	C
ATOM	6690	O	TRP	477	70.404	-0.699	25.917	1.00	0.00	RX1	O
ATOM	6691	N	LYS	478	72.335	0.409	25.550	1.00	0.00	RX1	N
ATOM	6692	H	LYS	478	73.317	0.473	25.730	1.00	0.00	RX1	H
ATOM	6693	CA	LYS	478	71.718	1.028	24.375	1.00	0.00	RX1	C
ATOM	6694	CB	LYS	478	72.460	2.302	23.959	1.00	0.00	RX1	C
ATOM	6695	CG	LYS	478	71.680	3.608	24.198	1.00	0.00	RX1	C
ATOM	6696	CD	LYS	478	70.866	4.154	23.008	1.00	0.00	RX1	C
ATOM	6697	CE	LYS	478	69.606	3.379	22.599	1.00	0.00	RX1	C
ATOM	6698	NZ	LYS	478	68.557	3.507	23.619	1.00	0.00	RX1	N
ATOM	6699	HZ1	LYS	478	67.968	2.647	23.640	1.00	0.00	RX1	H
ATOM	6700	HZ2	LYS	478	68.938	3.629	24.571	1.00	0.00	RX1	H
ATOM	6701	HZ3	LYS	478	67.889	4.275	23.401	1.00	0.00	RX1	H
ATOM	6702	C	LYS	478	71.460	0.113	23.187	1.00	0.00	RX1	C
ATOM	6703	O	LYS	478	70.625	0.407	22.343	1.00	0.00	RX1	O
ATOM	6704	N	LYS	479	72.183	-1.017	23.179	1.00	0.00	RX1	N
ATOM	6705	H	LYS	479	72.843	-1.225	23.904	1.00	0.00	RX1	H
ATOM	6706	CA	LYS	479	71.844	-2.065	22.215	1.00	0.00	RX1	C
ATOM	6707	CB	LYS	479	72.988	-3.092	22.153	1.00	0.00	RX1	C
ATOM	6708	CG	LYS	479	72.824	-4.443	21.426	1.00	0.00	RX1	C

ATOM	6709	CD	LYS	479	72.060	-5.510	22.230	1.00	0.00	RX1	C
ATOM	6710	CE	LYS	479	72.412	-6.959	21.892	1.00	0.00	RX1	C
ATOM	6711	NZ	LYS	479	73.737	-7.265	22.440	1.00	0.00	RX1	N
ATOM	6712	HZ1	LYS	479	73.965	-8.281	22.447	1.00	0.00	RX1	H
ATOM	6713	HZ2	LYS	479	74.501	-6.761	21.938	1.00	0.00	RX1	H
ATOM	6714	HZ3	LYS	479	73.841	-6.887	23.401	1.00	0.00	RX1	H
ATOM	6715	C	LYS	479	70.508	-2.717	22.529	1.00	0.00	RX1	C
ATOM	6716	O	LYS	479	69.721	-3.047	21.652	1.00	0.00	RX1	O
ATOM	6717	N	LEU	480	70.292	-2.907	23.838	1.00	0.00	RX1	N
ATOM	6718	H	LEU	480	70.892	-2.523	24.540	1.00	0.00	RX1	H
ATOM	6719	CA	LEU	480	69.089	-3.658	24.175	1.00	0.00	RX1	C
ATOM	6720	CB	LEU	480	69.293	-4.411	25.480	1.00	0.00	RX1	C
ATOM	6721	CG	LEU	480	68.282	-5.543	25.575	1.00	0.00	RX1	C
ATOM	6722	CD1	LEU	480	68.387	-6.477	24.369	1.00	0.00	RX1	C
ATOM	6723	CD2	LEU	480	68.401	-6.287	26.896	1.00	0.00	RX1	C
ATOM	6724	C	LEU	480	67.811	-2.844	24.214	1.00	0.00	RX1	C
ATOM	6725	O	LEU	480	66.773	-3.219	23.677	1.00	0.00	RX1	O
ATOM	6726	N	PHE	481	67.932	-1.688	24.879	1.00	0.00	RX1	N
ATOM	6727	H	PHE	481	68.805	-1.380	25.251	1.00	0.00	RX1	H
ATOM	6728	CA	PHE	481	66.809	-0.777	24.748	1.00	0.00	RX1	C
ATOM	6729	CB	PHE	481	66.497	-0.022	26.052	1.00	0.00	RX1	C
ATOM	6730	CG	PHE	481	67.635	0.709	26.726	1.00	0.00	RX1	C
ATOM	6731	CD1	PHE	481	68.375	1.684	26.073	1.00	0.00	RX1	C
ATOM	6732	CD2	PHE	481	67.900	0.440	28.060	1.00	0.00	RX1	C
ATOM	6733	CE1	PHE	481	69.330	2.426	26.752	1.00	0.00	RX1	C
ATOM	6734	CE2	PHE	481	68.852	1.182	28.746	1.00	0.00	RX1	C
ATOM	6735	CZ	PHE	481	69.551	2.192	28.099	1.00	0.00	RX1	C
ATOM	6736	C	PHE	481	66.903	0.086	23.509	1.00	0.00	RX1	C
ATOM	6737	O	PHE	481	67.655	1.049	23.413	1.00	0.00	RX1	O
ATOM	6738	N	GLY	482	66.121	-0.331	22.514	1.00	0.00	RX1	N
ATOM	6739	H	GLY	482	65.415	-1.021	22.693	1.00	0.00	RX1	H
ATOM	6740	CA	GLY	482	66.387	0.225	21.189	1.00	0.00	RX1	C
ATOM	6741	C	GLY	482	65.627	1.491	20.835	1.00	0.00	RX1	C
ATOM	6742	O	GLY	482	65.007	1.587	19.781	1.00	0.00	RX1	O
ATOM	6743	N	THR	483	65.684	2.473	21.739	1.00	0.00	RX1	N
ATOM	6744	H	THR	483	66.121	2.372	22.632	1.00	0.00	RX1	H
ATOM	6745	CA	THR	483	64.861	3.657	21.497	1.00	0.00	RX1	C
ATOM	6746	CB	THR	483	63.543	3.380	22.229	1.00	0.00	RX1	C
ATOM	6747	OG1	THR	483	63.236	1.986	22.064	1.00	0.00	RX1	O
ATOM	6748	HG1	THR	483	62.386	1.838	22.478	1.00	0.00	RX1	H
ATOM	6749	CG2	THR	483	62.366	4.244	21.767	1.00	0.00	RX1	C
ATOM	6750	C	THR	483	65.579	4.947	21.890	1.00	0.00	RX1	C
ATOM	6751	O	THR	483	66.585	4.944	22.599	1.00	0.00	RX1	O
ATOM	6752	N	SER	484	65.049	6.059	21.370	1.00	0.00	RX1	N
ATOM	6753	H	SER	484	64.249	6.045	20.774	1.00	0.00	RX1	H
ATOM	6754	CA	SER	484	65.528	7.375	21.780	1.00	0.00	RX1	C
ATOM	6755	CB	SER	484	64.833	8.297	20.797	1.00	0.00	RX1	C
ATOM	6756	OG	SER	484	64.539	7.484	19.651	1.00	0.00	RX1	O
ATOM	6757	HG	SER	484	64.391	8.086	18.929	1.00	0.00	RX1	H
ATOM	6758	C	SER	484	65.271	7.661	23.255	1.00	0.00	RX1	C
ATOM	6759	O	SER	484	64.563	6.923	23.930	1.00	0.00	RX1	O
ATOM	6760	N	GLY	485	65.873	8.761	23.737	1.00	0.00	RX1	N
ATOM	6761	H	GLY	485	66.530	9.278	23.191	1.00	0.00	RX1	H
ATOM	6762	CA	GLY	485	65.589	9.151	25.120	1.00	0.00	RX1	C
ATOM	6763	C	GLY	485	66.385	8.388	26.167	1.00	0.00	RX1	C
ATOM	6764	O	GLY	485	67.351	8.897	26.731	1.00	0.00	RX1	O
ATOM	6765	N	GLN	486	65.926	7.143	26.367	1.00	0.00	RX1	N
ATOM	6766	H	GLN	486	65.123	6.912	25.814	1.00	0.00	RX1	H
ATOM	6767	CA	GLN	486	66.433	6.152	27.329	1.00	0.00	RX1	C
ATOM	6768	CB	GLN	486	66.385	4.766	26.701	1.00	0.00	RX1	C
ATOM	6769	CG	GLN	486	65.029	4.377	26.127	1.00	0.00	RX1	C

ATOM	6770	CD	GLN	486	65.237	3.168	25.249	1.00	0.00	RX1	C
ATOM	6771	OE1	GLN	486	66.208	3.095	24.496	1.00	0.00	RX1	O
ATOM	6772	NE2	GLN	486	64.270	2.245	25.349	1.00	0.00	RX1	N
ATOM	6773	HE21	GLN	486	63.642	2.320	26.130	1.00	0.00	RX1	H
ATOM	6774	HE22	GLN	486	64.156	1.450	24.750	1.00	0.00	RX1	H
ATOM	6775	C	GLN	486	67.831	6.336	27.902	1.00	0.00	RX1	C
ATOM	6776	O	GLN	486	68.795	6.672	27.210	1.00	0.00	RX1	O
ATOM	6777	N	LYS	487	67.903	6.076	29.208	1.00	0.00	RX1	N
ATOM	6778	H	LYS	487	67.120	5.792	29.773	1.00	0.00	RX1	H
ATOM	6779	CA	LYS	487	69.195	6.005	29.876	1.00	0.00	RX1	C
ATOM	6780	CB	LYS	487	69.324	7.138	30.896	1.00	0.00	RX1	C
ATOM	6781	CG	LYS	487	69.476	8.482	30.190	1.00	0.00	RX1	C
ATOM	6782	CD	LYS	487	70.793	8.532	29.418	1.00	0.00	RX1	C
ATOM	6783	CE	LYS	487	70.756	9.529	28.263	1.00	0.00	RX1	C
ATOM	6784	NZ	LYS	487	69.758	9.070	27.290	1.00	0.00	RX1	N
ATOM	6785	HZ1	LYS	487	68.783	9.237	27.620	1.00	0.00	RX1	H
ATOM	6786	HZ2	LYS	487	69.859	9.550	26.378	1.00	0.00	RX1	H
ATOM	6787	HZ3	LYS	487	69.815	8.039	27.141	1.00	0.00	RX1	H
ATOM	6788	C	LYS	487	69.351	4.664	30.556	1.00	0.00	RX1	C
ATOM	6789	O	LYS	487	68.497	3.794	30.452	1.00	0.00	RX1	O
ATOM	6790	N	THR	488	70.482	4.536	31.257	1.00	0.00	RX1	N
ATOM	6791	H	THR	488	71.228	5.197	31.321	1.00	0.00	RX1	H
ATOM	6792	CA	THR	488	70.537	3.406	32.166	1.00	0.00	RX1	C
ATOM	6793	CB	THR	488	71.352	2.268	31.524	1.00	0.00	RX1	C
ATOM	6794	OG1	THR	488	71.151	1.038	32.231	1.00	0.00	RX1	O
ATOM	6795	HG1	THR	488	71.893	0.481	32.029	1.00	0.00	RX1	H
ATOM	6796	CG2	THR	488	72.838	2.586	31.315	1.00	0.00	RX1	C
ATOM	6797	C	THR	488	70.985	3.858	33.536	1.00	0.00	RX1	C
ATOM	6798	O	THR	488	72.142	4.118	33.847	1.00	0.00	RX1	O
ATOM	6799	N	LYS	489	69.951	3.991	34.358	1.00	0.00	RX1	N
ATOM	6800	H	LYS	489	69.049	3.671	34.067	1.00	0.00	RX1	H
ATOM	6801	CA	LYS	489	70.210	4.306	35.749	1.00	0.00	RX1	C
ATOM	6802	CB	LYS	489	69.024	5.109	36.274	1.00	0.00	RX1	C
ATOM	6803	CG	LYS	489	68.798	6.269	35.294	1.00	0.00	RX1	C
ATOM	6804	CD	LYS	489	67.616	7.182	35.614	1.00	0.00	RX1	C
ATOM	6805	CE	LYS	489	66.242	6.535	35.426	1.00	0.00	RX1	C
ATOM	6806	NZ	LYS	489	65.935	6.295	34.011	1.00	0.00	RX1	N
ATOM	6807	HZ1	LYS	489	66.285	5.381	33.642	1.00	0.00	RX1	H
ATOM	6808	HZ2	LYS	489	64.907	6.329	33.846	1.00	0.00	RX1	H
ATOM	6809	HZ3	LYS	489	66.294	6.997	33.330	1.00	0.00	RX1	H
ATOM	6810	C	LYS	489	70.535	3.027	36.499	1.00	0.00	RX1	C
ATOM	6811	O	LYS	489	69.691	2.381	37.110	1.00	0.00	RX1	O
ATOM	6812	N	ILE	490	71.821	2.678	36.375	1.00	0.00	RX1	N
ATOM	6813	H	ILE	490	72.421	3.256	35.816	1.00	0.00	RX1	H
ATOM	6814	CA	ILE	490	72.329	1.493	37.066	1.00	0.00	RX1	C
ATOM	6815	CB	ILE	490	72.932	0.466	36.081	1.00	0.00	RX1	C
ATOM	6816	CG2	ILE	490	71.832	-0.473	35.585	1.00	0.00	RX1	C
ATOM	6817	CG1	ILE	490	73.648	1.073	34.869	1.00	0.00	RX1	C
ATOM	6818	CD1	ILE	490	74.811	2.001	35.194	1.00	0.00	RX1	C
ATOM	6819	C	ILE	490	73.262	1.833	38.219	1.00	0.00	RX1	C
ATOM	6820	O	ILE	490	74.331	1.262	38.413	1.00	0.00	RX1	O
ATOM	6821	N	ILE	491	72.819	2.855	38.959	1.00	0.00	RX1	N
ATOM	6822	H	ILE	491	71.848	3.095	38.939	1.00	0.00	RX1	H
ATOM	6823	CA	ILE	491	73.720	3.390	39.972	1.00	0.00	RX1	C
ATOM	6824	CB	ILE	491	73.430	4.886	40.172	1.00	0.00	RX1	C
ATOM	6825	CG2	ILE	491	73.573	5.626	38.838	1.00	0.00	RX1	C
ATOM	6826	CG1	ILE	491	72.062	5.145	40.816	1.00	0.00	RX1	C
ATOM	6827	CD1	ILE	491	71.820	6.619	41.144	1.00	0.00	RX1	C
ATOM	6828	C	ILE	491	73.644	2.622	41.277	1.00	0.00	RX1	C
ATOM	6829	O	ILE	491	72.715	1.866	41.525	1.00	0.00	RX1	O
ATOM	6830	N	SER	492	74.681	2.832	42.108	1.00	0.00	RX1	N

ATOM	6831	H	SER	492	75.385	3.497	41.839	1.00	0.00	RX1	H
ATOM	6832	CA	SER	492	74.708	2.193	43.430	1.00	0.00	RX1	C
ATOM	6833	CB	SER	492	73.729	2.924	44.370	1.00	0.00	RX1	C
ATOM	6834	OG	SER	492	73.872	2.486	45.727	1.00	0.00	RX1	O
ATOM	6835	HG	SER	492	73.880	1.532	45.697	1.00	0.00	RX1	H
ATOM	6836	C	SER	492	74.520	0.677	43.437	1.00	0.00	RX1	C
ATOM	6837	O	SER	492	73.983	0.089	44.370	1.00	0.00	RX1	O
ATOM	6838	N	ASN	493	74.983	0.072	42.337	1.00	0.00	RX1	N
ATOM	6839	H	ASN	493	75.500	0.590	41.655	1.00	0.00	RX1	H
ATOM	6840	CA	ASN	493	75.051	-1.386	42.354	1.00	0.00	RX1	C
ATOM	6841	CB	ASN	493	74.829	-1.973	40.958	1.00	0.00	RX1	C
ATOM	6842	CG	ASN	493	73.419	-1.705	40.478	1.00	0.00	RX1	C
ATOM	6843	OD1	ASN	493	72.422	-2.016	41.117	1.00	0.00	RX1	O
ATOM	6844	ND2	ASN	493	73.364	-1.133	39.284	1.00	0.00	RX1	N
ATOM	6845	HD21	ASN	493	74.176	-0.746	38.839	1.00	0.00	RX1	H
ATOM	6846	HD22	ASN	493	72.472	-1.024	38.843	1.00	0.00	RX1	H
ATOM	6847	C	ASN	493	76.417	-1.763	42.885	1.00	0.00	RX1	C
ATOM	6848	O	ASN	493	77.223	-0.876	43.133	1.00	0.00	RX1	O
ATOM	6849	N	ARG	494	76.660	-3.082	42.999	1.00	0.00	RX1	N
ATOM	6850	H	ARG	494	75.863	-3.679	42.898	1.00	0.00	RX1	H
ATOM	6851	CA	ARG	494	77.973	-3.650	43.364	1.00	0.00	RX1	C
ATOM	6852	CB	ARG	494	78.085	-5.014	42.692	1.00	0.00	RX1	C
ATOM	6853	CG	ARG	494	79.301	-5.897	42.954	1.00	0.00	RX1	C
ATOM	6854	CD	ARG	494	79.278	-6.615	44.302	1.00	0.00	RX1	C
ATOM	6855	NE	ARG	494	80.375	-7.578	44.356	1.00	0.00	RX1	N
ATOM	6856	HE	ARG	494	81.301	-7.240	44.554	1.00	0.00	RX1	H
ATOM	6857	CZ	ARG	494	80.181	-8.866	43.950	1.00	0.00	RX1	C
ATOM	6858	NH1	ARG	494	78.950	-9.314	43.626	1.00	0.00	RX1	N
ATOM	6859	HH11	ARG	494	78.803	-10.267	43.318	1.00	0.00	RX1	H
ATOM	6860	HH12	ARG	494	78.132	-8.728	43.653	1.00	0.00	RX1	H
ATOM	6861	NH2	ARG	494	81.234	-9.689	43.863	1.00	0.00	RX1	N
ATOM	6862	HH21	ARG	494	81.118	-10.633	43.527	1.00	0.00	RX1	H
ATOM	6863	HH22	ARG	494	82.181	-9.425	44.105	1.00	0.00	RX1	H
ATOM	6864	C	ARG	494	79.211	-2.819	43.031	1.00	0.00	RX1	C
ATOM	6865	O	ARG	494	80.107	-2.619	43.844	1.00	0.00	RX1	O
ATOM	6866	N	GLY	495	79.218	-2.360	41.772	1.00	0.00	RX1	N
ATOM	6867	H	GLY	495	78.430	-2.521	41.183	1.00	0.00	RX1	H
ATOM	6868	CA	GLY	495	80.376	-1.593	41.337	1.00	0.00	RX1	C
ATOM	6869	C	GLY	495	81.327	-2.472	40.562	1.00	0.00	RX1	C
ATOM	6870	O	GLY	495	81.571	-3.625	40.901	1.00	0.00	RX1	O
ATOM	6871	N	GLU	496	81.833	-1.883	39.469	1.00	0.00	RX1	N
ATOM	6872	H	GLU	496	81.639	-0.917	39.304	1.00	0.00	RX1	H
ATOM	6873	CA	GLU	496	82.713	-2.635	38.570	1.00	0.00	RX1	C
ATOM	6874	CB	GLU	496	83.203	-1.619	37.527	1.00	0.00	RX1	C
ATOM	6875	CG	GLU	496	84.101	-2.069	36.369	1.00	0.00	RX1	C
ATOM	6876	CD	GLU	496	85.464	-2.522	36.825	1.00	0.00	RX1	C
ATOM	6877	OE1	GLU	496	85.847	-3.644	36.513	1.00	0.00	RX1	O
ATOM	6878	OE2	GLU	496	86.158	-1.773	37.511	1.00	0.00	RX1	O
ATOM	6879	C	GLU	496	83.933	-3.202	39.293	1.00	0.00	RX1	C
ATOM	6880	O	GLU	496	84.275	-4.379	39.220	1.00	0.00	RX1	O
ATOM	6881	N	ASN	497	84.517	-2.303	40.097	1.00	0.00	RX1	N
ATOM	6882	H	ASN	497	84.178	-1.362	40.084	1.00	0.00	RX1	H
ATOM	6883	CA	ASN	497	85.539	-2.705	41.067	1.00	0.00	RX1	C
ATOM	6884	CB	ASN	497	85.884	-1.525	42.000	1.00	0.00	RX1	C
ATOM	6885	CG	ASN	497	84.650	-0.978	42.710	1.00	0.00	RX1	C
ATOM	6886	OD1	ASN	497	83.576	-0.858	42.134	1.00	0.00	RX1	O
ATOM	6887	ND2	ASN	497	84.835	-0.681	43.998	1.00	0.00	RX1	N
ATOM	6888	HD21	ASN	497	85.715	-0.749	44.461	1.00	0.00	RX1	H
ATOM	6889	HD22	ASN	497	84.017	-0.403	44.501	1.00	0.00	RX1	H
ATOM	6890	C	ASN	497	85.190	-3.957	41.866	1.00	0.00	RX1	C
ATOM	6891	O	ASN	497	85.930	-4.931	41.888	1.00	0.00	RX1	O

ATOM	6892	N	SER	498	84.008	-3.909	42.490	1.00	0.00	RX1	N
ATOM	6893	H	SER	498	83.382	-3.131	42.411	1.00	0.00	RX1	H
ATOM	6894	CA	SER	498	83.604	-5.019	43.347	1.00	0.00	RX1	C
ATOM	6895	CB	SER	498	82.608	-4.389	44.291	1.00	0.00	RX1	C
ATOM	6896	OG	SER	498	82.825	-2.975	44.203	1.00	0.00	RX1	O
ATOM	6897	HG	SER	498	81.949	-2.581	44.229	1.00	0.00	RX1	H
ATOM	6898	C	SER	498	83.119	-6.262	42.609	1.00	0.00	RX1	C
ATOM	6899	O	SER	498	82.976	-7.345	43.168	1.00	0.00	RX1	O
ATOM	6900	N	CYS	499	82.887	-6.053	41.306	1.00	0.00	RX1	N
ATOM	6901	H	CYS	499	82.949	-5.119	40.948	1.00	0.00	RX1	H
ATOM	6902	CA	CYS	499	82.579	-7.153	40.397	1.00	0.00	RX1	C
ATOM	6903	CB	CYS	499	82.037	-6.570	39.102	1.00	0.00	RX1	C
ATOM	6904	SG	CYS	499	81.334	-7.739	37.922	1.00	0.00	RX1	S
ATOM	6905	C	CYS	499	83.820	-7.990	40.125	1.00	0.00	RX1	C
ATOM	6906	O	CYS	499	83.886	-9.190	40.388	1.00	0.00	RX1	O
ATOM	6907	N	LYS	500	84.858	-7.281	39.643	1.00	0.00	RX1	N
ATOM	6908	H	LYS	500	84.781	-6.296	39.464	1.00	0.00	RX1	H
ATOM	6909	CA	LYS	500	86.116	-8.010	39.477	1.00	0.00	RX1	C
ATOM	6910	CB	LYS	500	87.103	-7.320	38.530	1.00	0.00	RX1	C
ATOM	6911	CG	LYS	500	87.552	-5.895	38.851	1.00	0.00	RX1	C
ATOM	6912	CD	LYS	500	88.739	-5.535	37.950	1.00	0.00	RX1	C
ATOM	6913	CE	LYS	500	89.164	-4.066	37.972	1.00	0.00	RX1	C
ATOM	6914	NZ	LYS	500	88.155	-3.257	37.286	1.00	0.00	RX1	N
ATOM	6915	HZ1	LYS	500	88.372	-2.250	37.181	1.00	0.00	RX1	H
ATOM	6916	HZ2	LYS	500	87.847	-3.630	36.364	1.00	0.00	RX1	H
ATOM	6917	HZ3	LYS	500	87.231	-3.252	37.780	1.00	0.00	RX1	H
ATOM	6918	C	LYS	500	86.771	-8.464	40.772	1.00	0.00	RX1	C
ATOM	6919	O	LYS	500	87.477	-9.462	40.814	1.00	0.00	RX1	O
ATOM	6920	N	ALA	501	86.437	-7.739	41.852	1.00	0.00	RX1	N
ATOM	6921	H	ALA	501	85.925	-6.885	41.758	1.00	0.00	RX1	H
ATOM	6922	CA	ALA	501	86.867	-8.190	43.177	1.00	0.00	RX1	C
ATOM	6923	CB	ALA	501	86.647	-7.100	44.226	1.00	0.00	RX1	C
ATOM	6924	C	ALA	501	86.232	-9.478	43.690	1.00	0.00	RX1	C
ATOM	6925	O	ALA	501	86.464	-9.910	44.811	1.00	0.00	RX1	O
ATOM	6926	N	THR	502	85.424	-10.105	42.828	1.00	0.00	RX1	N
ATOM	6927	H	THR	502	85.045	-9.704	41.994	1.00	0.00	RX1	H
ATOM	6928	CA	THR	502	85.110	-11.496	43.138	1.00	0.00	RX1	C
ATOM	6929	CB	THR	502	83.796	-11.494	43.897	1.00	0.00	RX1	C
ATOM	6930	OG1	THR	502	83.700	-10.257	44.625	1.00	0.00	RX1	O
ATOM	6931	HG1	THR	502	84.522	-10.203	45.116	1.00	0.00	RX1	H
ATOM	6932	CG2	THR	502	83.649	-12.693	44.836	1.00	0.00	RX1	C
ATOM	6933	C	THR	502	85.123	-12.375	41.898	1.00	0.00	RX1	C
ATOM	6934	O	THR	502	84.412	-13.363	41.774	1.00	0.00	RX1	O
ATOM	6935	N	GLY	503	85.961	-11.930	40.946	1.00	0.00	RX1	N
ATOM	6936	H	GLY	503	86.538	-11.128	41.101	1.00	0.00	RX1	H
ATOM	6937	CA	GLY	503	86.101	-12.667	39.691	1.00	0.00	RX1	C
ATOM	6938	C	GLY	503	84.857	-12.779	38.823	1.00	0.00	RX1	C
ATOM	6939	O	GLY	503	84.805	-13.568	37.889	1.00	0.00	RX1	O
ATOM	6940	N	GLN	504	83.850	-11.953	39.146	1.00	0.00	RX1	N
ATOM	6941	H	GLN	504	83.960	-11.186	39.779	1.00	0.00	RX1	H
ATOM	6942	CA	GLN	504	82.604	-12.112	38.403	1.00	0.00	RX1	C
ATOM	6943	CB	GLN	504	81.414	-11.767	39.296	1.00	0.00	RX1	C
ATOM	6944	CG	GLN	504	81.300	-12.655	40.534	1.00	0.00	RX1	C
ATOM	6945	CD	GLN	504	80.117	-12.194	41.359	1.00	0.00	RX1	C
ATOM	6946	OE1	GLN	504	80.254	-11.707	42.481	1.00	0.00	RX1	O
ATOM	6947	NE2	GLN	504	78.938	-12.349	40.736	1.00	0.00	RX1	N
ATOM	6948	HE21	GLN	504	78.883	-12.772	39.829	1.00	0.00	RX1	H
ATOM	6949	HE22	GLN	504	78.081	-12.048	41.159	1.00	0.00	RX1	H
ATOM	6950	C	GLN	504	82.565	-11.291	37.130	1.00	0.00	RX1	C
ATOM	6951	O	GLN	504	81.782	-10.363	36.962	1.00	0.00	RX1	O
ATOM	6952	N	VAL	505	83.486	-11.673	36.241	1.00	0.00	RX1	N

ATOM	6953	H	VAL	505	83.993	-12.514	36.439	1.00	0.00	RX1	H
ATOM	6954	CA	VAL	505	83.558	-11.009	34.947	1.00	0.00	RX1	C
ATOM	6955	CB	VAL	505	84.966	-10.446	34.691	1.00	0.00	RX1	C
ATOM	6956	CG1	VAL	505	85.216	-9.216	35.565	1.00	0.00	RX1	C
ATOM	6957	CG2	VAL	505	86.060	-11.509	34.848	1.00	0.00	RX1	C
ATOM	6958	C	VAL	505	83.118	-11.968	33.858	1.00	0.00	RX1	C
ATOM	6959	O	VAL	505	82.656	-13.065	34.145	1.00	0.00	RX1	O
ATOM	6960	N	CYS	506	83.277	-11.519	32.603	1.00	0.00	RX1	N
ATOM	6961	H	CYS	506	83.506	-10.561	32.461	1.00	0.00	RX1	H
ATOM	6962	CA	CYS	506	82.812	-12.395	31.524	1.00	0.00	RX1	C
ATOM	6963	CB	CYS	506	81.314	-12.223	31.314	1.00	0.00	RX1	C
ATOM	6964	SG	CYS	506	80.873	-10.501	30.967	1.00	0.00	RX1	S
ATOM	6965	C	CYS	506	83.530	-12.258	30.191	1.00	0.00	RX1	C
ATOM	6966	O	CYS	506	83.309	-13.026	29.258	1.00	0.00	RX1	O
ATOM	6967	N	HIS	507	84.382	-11.220	30.122	1.00	0.00	RX1	N
ATOM	6968	H	HIS	507	84.635	-10.710	30.938	1.00	0.00	RX1	H
ATOM	6969	CA	HIS	507	84.982	-10.877	28.832	1.00	0.00	RX1	C
ATOM	6970	CB	HIS	507	85.642	-9.492	28.860	1.00	0.00	RX1	C
ATOM	6971	CG	HIS	507	86.114	-9.108	27.473	1.00	0.00	RX1	C
ATOM	6972	ND1	HIS	507	87.195	-9.642	26.868	1.00	0.00	RX1	N
ATOM	6973	HD1	HIS	507	87.795	-10.325	27.245	1.00	0.00	RX1	H
ATOM	6974	CD2	HIS	507	85.520	-8.197	26.600	1.00	0.00	RX1	C
ATOM	6975	NE2	HIS	507	86.255	-8.194	25.461	1.00	0.00	RX1	N
ATOM	6976	CE1	HIS	507	87.283	-9.082	25.624	1.00	0.00	RX1	C
ATOM	6977	C	HIS	507	86.022	-11.881	28.357	1.00	0.00	RX1	C
ATOM	6978	O	HIS	507	87.210	-11.772	28.636	1.00	0.00	RX1	O
ATOM	6979	N	ALA	508	85.485	-12.855	27.618	1.00	0.00	RX1	N
ATOM	6980	H	ALA	508	84.510	-12.748	27.405	1.00	0.00	RX1	H
ATOM	6981	CA	ALA	508	86.247	-13.965	27.054	1.00	0.00	RX1	C
ATOM	6982	CB	ALA	508	86.892	-14.845	28.135	1.00	0.00	RX1	C
ATOM	6983	C	ALA	508	85.289	-14.809	26.244	1.00	0.00	RX1	C
ATOM	6984	O	ALA	508	85.382	-14.915	25.030	1.00	0.00	RX1	O
ATOM	6985	N	LEU	509	84.319	-15.366	26.990	1.00	0.00	RX1	N
ATOM	6986	H	LEU	509	84.279	-15.174	27.970	1.00	0.00	RX1	H
ATOM	6987	CA	LEU	509	83.209	-16.007	26.288	1.00	0.00	RX1	C
ATOM	6988	CB	LEU	509	82.599	-17.170	27.092	1.00	0.00	RX1	C
ATOM	6989	CG	LEU	509	81.830	-16.839	28.382	1.00	0.00	RX1	C
ATOM	6990	CD1	LEU	509	80.752	-17.883	28.662	1.00	0.00	RX1	C
ATOM	6991	CD2	LEU	509	82.733	-16.624	29.600	1.00	0.00	RX1	C
ATOM	6992	C	LEU	509	82.159	-14.991	25.879	1.00	0.00	RX1	C
ATOM	6993	O	LEU	509	81.599	-15.000	24.790	1.00	0.00	RX1	O
ATOM	6994	N	CYS	510	81.957	-14.052	26.810	1.00	0.00	RX1	N
ATOM	6995	H	CYS	510	82.449	-14.008	27.677	1.00	0.00	RX1	H
ATOM	6996	CA	CYS	510	81.157	-12.913	26.398	1.00	0.00	RX1	C
ATOM	6997	CB	CYS	510	80.443	-12.315	27.597	1.00	0.00	RX1	C
ATOM	6998	SG	CYS	510	79.609	-13.583	28.583	1.00	0.00	RX1	S
ATOM	6999	C	CYS	510	82.042	-11.900	25.716	1.00	0.00	RX1	C
ATOM	7000	O	CYS	510	83.259	-11.890	25.885	1.00	0.00	RX1	O
ATOM	7001	N	SER	511	81.380	-11.083	24.902	1.00	0.00	RX1	N
ATOM	7002	H	SER	511	80.378	-11.109	24.913	1.00	0.00	RX1	H
ATOM	7003	CA	SER	511	82.122	-10.074	24.159	1.00	0.00	RX1	C
ATOM	7004	CB	SER	511	81.312	-9.882	22.878	1.00	0.00	RX1	C
ATOM	7005	OG	SER	511	80.059	-9.255	23.172	1.00	0.00	RX1	O
ATOM	7006	HG	SER	511	79.697	-9.676	23.953	1.00	0.00	RX1	H
ATOM	7007	C	SER	511	82.302	-8.826	25.031	1.00	0.00	RX1	C
ATOM	7008	O	SER	511	81.939	-8.866	26.201	1.00	0.00	RX1	O
ATOM	7009	N	PRO	512	82.811	-7.691	24.460	1.00	0.00	RX1	N
ATOM	7010	CD	PRO	512	83.660	-7.539	23.279	1.00	0.00	RX1	C
ATOM	7011	CA	PRO	512	82.558	-6.397	25.118	1.00	0.00	RX1	C
ATOM	7012	CB	PRO	512	83.023	-5.395	24.063	1.00	0.00	RX1	C
ATOM	7013	CG	PRO	512	84.195	-6.122	23.414	1.00	0.00	RX1	C

ATOM	7014	C	PRO	512	81.123	-6.192	25.598	1.00	0.00	RX1	C
ATOM	7015	O	PRO	512	80.870	-5.668	26.676	1.00	0.00	RX1	O
ATOM	7016	N	GLU	513	80.186	-6.680	24.762	1.00	0.00	RX1	N
ATOM	7017	H	GLU	513	80.428	-7.136	23.906	1.00	0.00	RX1	H
ATOM	7018	CA	GLU	513	78.872	-6.901	25.354	1.00	0.00	RX1	C
ATOM	7019	CB	GLU	513	77.754	-7.036	24.319	1.00	0.00	RX1	C
ATOM	7020	CG	GLU	513	77.724	-6.004	23.193	1.00	0.00	RX1	C
ATOM	7021	CD	GLU	513	76.385	-6.099	22.488	1.00	0.00	RX1	C
ATOM	7022	OE1	GLU	513	75.450	-5.452	22.924	1.00	0.00	RX1	O
ATOM	7023	OE2	GLU	513	76.224	-6.828	21.513	1.00	0.00	RX1	O
ATOM	7024	C	GLU	513	78.903	-8.174	26.170	1.00	0.00	RX1	C
ATOM	7025	O	GLU	513	78.991	-9.280	25.639	1.00	0.00	RX1	O
ATOM	7026	N	GLY	514	78.887	-7.937	27.480	1.00	0.00	RX1	N
ATOM	7027	H	GLY	514	78.750	-6.992	27.780	1.00	0.00	RX1	H
ATOM	7028	CA	GLY	514	79.091	-9.026	28.421	1.00	0.00	RX1	C
ATOM	7029	C	GLY	514	77.885	-9.914	28.611	1.00	0.00	RX1	C
ATOM	7030	O	GLY	514	77.452	-10.621	27.707	1.00	0.00	RX1	O
ATOM	7031	N	CYS	515	77.372	-9.878	29.843	1.00	0.00	RX1	N
ATOM	7032	H	CYS	515	77.680	-9.232	30.546	1.00	0.00	RX1	H
ATOM	7033	CA	CYS	515	76.399	-10.911	30.179	1.00	0.00	RX1	C
ATOM	7034	CB	CYS	515	77.125	-12.221	30.491	1.00	0.00	RX1	C
ATOM	7035	SG	CYS	515	78.219	-12.038	31.911	1.00	0.00	RX1	S
ATOM	7036	C	CYS	515	75.516	-10.515	31.337	1.00	0.00	RX1	C
ATOM	7037	O	CYS	515	75.826	-9.604	32.092	1.00	0.00	RX1	O
ATOM	7038	N	TRP	516	74.408	-11.255	31.460	1.00	0.00	RX1	N
ATOM	7039	H	TRP	516	74.203	-12.006	30.828	1.00	0.00	RX1	H
ATOM	7040	CA	TRP	516	73.555	-11.040	32.626	1.00	0.00	RX1	C
ATOM	7041	CB	TRP	516	72.113	-11.461	32.341	1.00	0.00	RX1	C
ATOM	7042	CG	TRP	516	71.550	-10.763	31.130	1.00	0.00	RX1	C
ATOM	7043	CD2	TRP	516	70.826	-9.519	31.095	1.00	0.00	RX1	C
ATOM	7044	CE2	TRP	516	70.461	-9.276	29.753	1.00	0.00	RX1	C
ATOM	7045	CE3	TRP	516	70.460	-8.615	32.083	1.00	0.00	RX1	C
ATOM	7046	CD1	TRP	516	71.586	-11.204	29.799	1.00	0.00	RX1	C
ATOM	7047	NE1	TRP	516	70.943	-10.328	28.982	1.00	0.00	RX1	N
ATOM	7048	HE1	TRP	516	70.813	-10.442	28.016	1.00	0.00	RX1	H
ATOM	7049	CZ2	TRP	516	69.738	-8.134	29.441	1.00	0.00	RX1	C
ATOM	7050	CZ3	TRP	516	69.738	-7.475	31.757	1.00	0.00	RX1	C
ATOM	7051	CH2	TRP	516	69.375	-7.236	30.438	1.00	0.00	RX1	C
ATOM	7052	C	TRP	516	74.039	-11.733	33.892	1.00	0.00	RX1	C
ATOM	7053	O	TRP	516	73.531	-11.494	34.982	1.00	0.00	RX1	O
ATOM	7054	N	GLY	517	75.040	-12.601	33.719	1.00	0.00	RX1	N
ATOM	7055	H	GLY	517	75.443	-12.868	32.843	1.00	0.00	RX1	H
ATOM	7056	CA	GLY	517	75.476	-13.401	34.852	1.00	0.00	RX1	C
ATOM	7057	C	GLY	517	76.643	-14.278	34.458	1.00	0.00	RX1	C
ATOM	7058	O	GLY	517	77.130	-14.224	33.337	1.00	0.00	RX1	O
ATOM	7059	N	PRO	518	77.104	-15.079	35.443	1.00	0.00	RX1	N
ATOM	7060	CD	PRO	518	76.569	-15.184	36.791	1.00	0.00	RX1	C
ATOM	7061	CA	PRO	518	78.304	-15.894	35.225	1.00	0.00	RX1	C
ATOM	7062	CB	PRO	518	78.605	-16.426	36.634	1.00	0.00	RX1	C
ATOM	7063	CG	PRO	518	77.791	-15.575	37.611	1.00	0.00	RX1	C
ATOM	7064	C	PRO	518	78.156	-17.033	34.222	1.00	0.00	RX1	C
ATOM	7065	O	PRO	518	79.130	-17.645	33.798	1.00	0.00	RX1	O
ATOM	7066	N	GLU	519	76.895	-17.356	33.914	1.00	0.00	RX1	N
ATOM	7067	H	GLU	519	76.114	-16.731	33.983	1.00	0.00	RX1	H
ATOM	7068	CA	GLU	519	76.699	-18.620	33.219	1.00	0.00	RX1	C
ATOM	7069	CB	GLU	519	75.296	-19.139	33.537	1.00	0.00	RX1	C
ATOM	7070	CG	GLU	519	75.054	-19.231	35.043	1.00	0.00	RX1	C
ATOM	7071	CD	GLU	519	73.653	-19.745	35.284	1.00	0.00	RX1	C
ATOM	7072	OE1	GLU	519	72.800	-18.978	35.716	1.00	0.00	RX1	O
ATOM	7073	OE2	GLU	519	73.391	-20.913	35.016	1.00	0.00	RX1	O
ATOM	7074	C	GLU	519	76.936	-18.505	31.724	1.00	0.00	RX1	C

ATOM	7075	O	GLU	519	76.662	-17.489	31.099	1.00	0.00	RX1	O
ATOM	7076	N	PRO	520	77.433	-19.615	31.122	1.00	0.00	RX1	N
ATOM	7077	CD	PRO	520	77.896	-20.828	31.785	1.00	0.00	RX1	C
ATOM	7078	CA	PRO	520	77.610	-19.661	29.660	1.00	0.00	RX1	C
ATOM	7079	CB	PRO	520	78.129	-21.084	29.429	1.00	0.00	RX1	C
ATOM	7080	CG	PRO	520	78.791	-21.489	30.744	1.00	0.00	RX1	C
ATOM	7081	C	PRO	520	76.408	-19.336	28.769	1.00	0.00	RX1	C
ATOM	7082	O	PRO	520	76.535	-19.282	27.553	1.00	0.00	RX1	O
ATOM	7083	N	ARG	521	75.242	-19.157	29.408	1.00	0.00	RX1	N
ATOM	7084	H	ARG	521	75.212	-19.072	30.400	1.00	0.00	RX1	H
ATOM	7085	CA	ARG	521	74.018	-18.849	28.672	1.00	0.00	RX1	C
ATOM	7086	CB	ARG	521	72.908	-19.788	29.150	1.00	0.00	RX1	C
ATOM	7087	CG	ARG	521	72.741	-19.747	30.668	1.00	0.00	RX1	C
ATOM	7088	CD	ARG	521	71.839	-20.850	31.224	1.00	0.00	RX1	C
ATOM	7089	NE	ARG	521	71.803	-20.765	32.681	1.00	0.00	RX1	N
ATOM	7090	HE	ARG	521	72.669	-20.773	33.204	1.00	0.00	RX1	H
ATOM	7091	CZ	ARG	521	70.637	-20.540	33.344	1.00	0.00	RX1	C
ATOM	7092	NH1	ARG	521	69.470	-20.582	32.671	1.00	0.00	RX1	N
ATOM	7093	HH11	ARG	521	68.627	-20.301	33.157	1.00	0.00	RX1	H
ATOM	7094	HH12	ARG	521	69.402	-20.854	31.712	1.00	0.00	RX1	H
ATOM	7095	NH2	ARG	521	70.654	-20.251	34.657	1.00	0.00	RX1	N
ATOM	7096	HH21	ARG	521	69.816	-20.037	35.174	1.00	0.00	RX1	H
ATOM	7097	HH22	ARG	521	71.542	-20.197	35.158	1.00	0.00	RX1	H
ATOM	7098	C	ARG	521	73.589	-17.387	28.761	1.00	0.00	RX1	C
ATOM	7099	O	ARG	521	72.578	-16.979	28.210	1.00	0.00	RX1	O
ATOM	7100	N	ASP	522	74.395	-16.610	29.498	1.00	0.00	RX1	N
ATOM	7101	H	ASP	522	75.278	-16.918	29.854	1.00	0.00	RX1	H
ATOM	7102	CA	ASP	522	73.984	-15.248	29.846	1.00	0.00	RX1	C
ATOM	7103	CB	ASP	522	74.636	-14.857	31.169	1.00	0.00	RX1	C
ATOM	7104	CG	ASP	522	73.789	-15.181	32.380	1.00	0.00	RX1	C
ATOM	7105	OD1	ASP	522	74.163	-16.037	33.172	1.00	0.00	RX1	O
ATOM	7106	OD2	ASP	522	72.775	-14.532	32.589	1.00	0.00	RX1	O
ATOM	7107	C	ASP	522	74.345	-14.160	28.843	1.00	0.00	RX1	C
ATOM	7108	O	ASP	522	74.287	-12.973	29.146	1.00	0.00	RX1	O
ATOM	7109	N	CYS	523	74.816	-14.581	27.664	1.00	0.00	RX1	N
ATOM	7110	H	CYS	523	74.661	-15.502	27.311	1.00	0.00	RX1	H
ATOM	7111	CA	CYS	523	75.593	-13.593	26.913	1.00	0.00	RX1	C
ATOM	7112	CB	CYS	523	76.688	-14.329	26.154	1.00	0.00	RX1	C
ATOM	7113	SG	CYS	523	77.457	-15.563	27.235	1.00	0.00	RX1	S
ATOM	7114	C	CYS	523	74.822	-12.609	26.052	1.00	0.00	RX1	C
ATOM	7115	O	CYS	523	74.041	-12.965	25.182	1.00	0.00	RX1	O
ATOM	7116	N	VAL	524	75.116	-11.328	26.322	1.00	0.00	RX1	N
ATOM	7117	H	VAL	524	75.790	-11.150	27.041	1.00	0.00	RX1	H
ATOM	7118	CA	VAL	524	74.542	-10.246	25.518	1.00	0.00	RX1	C
ATOM	7119	CB	VAL	524	74.636	-8.923	26.291	1.00	0.00	RX1	C
ATOM	7120	CG1	VAL	524	73.765	-7.831	25.672	1.00	0.00	RX1	C
ATOM	7121	CG2	VAL	524	74.238	-9.131	27.752	1.00	0.00	RX1	C
ATOM	7122	C	VAL	524	75.147	-10.148	24.116	1.00	0.00	RX1	C
ATOM	7123	O	VAL	524	74.515	-9.691	23.162	1.00	0.00	RX1	O
ATOM	7124	N	SER	525	76.400	-10.630	24.039	1.00	0.00	RX1	N
ATOM	7125	H	SER	525	76.932	-10.816	24.869	1.00	0.00	RX1	H
ATOM	7126	CA	SER	525	76.989	-11.012	22.756	1.00	0.00	RX1	C
ATOM	7127	CB	SER	525	77.325	-9.834	21.832	1.00	0.00	RX1	C
ATOM	7128	OG	SER	525	77.505	-10.307	20.489	1.00	0.00	RX1	O
ATOM	7129	HG	SER	525	77.731	-9.550	19.959	1.00	0.00	RX1	H
ATOM	7130	C	SER	525	78.200	-11.886	23.012	1.00	0.00	RX1	C
ATOM	7131	O	SER	525	78.712	-11.952	24.127	1.00	0.00	RX1	O
ATOM	7132	N	CYS	526	78.606	-12.603	21.959	1.00	0.00	RX1	N
ATOM	7133	H	CYS	526	78.306	-12.350	21.037	1.00	0.00	RX1	H
ATOM	7134	CA	CYS	526	79.325	-13.839	22.258	1.00	0.00	RX1	C
ATOM	7135	CB	CYS	526	78.423	-15.027	21.931	1.00	0.00	RX1	C

ATOM	7136	SG	CYS	526	76.745	-14.823	22.582	1.00	0.00	RX1	S
ATOM	7137	C	CYS	526	80.674	-14.005	21.587	1.00	0.00	RX1	C
ATOM	7138	O	CYS	526	81.061	-13.280	20.670	1.00	0.00	RX1	O
ATOM	7139	N	ARG	527	81.391	-15.028	22.067	1.00	0.00	RX1	N
ATOM	7140	H	ARG	527	81.105	-15.530	22.887	1.00	0.00	RX1	H
ATOM	7141	CA	ARG	527	82.610	-15.376	21.357	1.00	0.00	RX1	C
ATOM	7142	CB	ARG	527	83.909	-15.401	22.131	1.00	0.00	RX1	C
ATOM	7143	CG	ARG	527	84.387	-13.937	22.070	1.00	0.00	RX1	C
ATOM	7144	CD	ARG	527	85.240	-13.438	20.873	1.00	0.00	RX1	C
ATOM	7145	NE	ARG	527	84.819	-13.783	19.504	1.00	0.00	RX1	N
ATOM	7146	HE	ARG	527	85.327	-14.522	19.042	1.00	0.00	RX1	H
ATOM	7147	CZ	ARG	527	83.942	-13.055	18.760	1.00	0.00	RX1	C
ATOM	7148	NH1	ARG	527	83.184	-12.120	19.318	1.00	0.00	RX1	N
ATOM	7149	HH11	ARG	527	82.492	-11.615	18.787	1.00	0.00	RX1	H
ATOM	7150	HH12	ARG	527	83.252	-11.917	20.290	1.00	0.00	RX1	H
ATOM	7151	NH2	ARG	527	83.824	-13.301	17.460	1.00	0.00	RX1	N
ATOM	7152	HH21	ARG	527	83.153	-12.842	16.862	1.00	0.00	RX1	H
ATOM	7153	HH22	ARG	527	84.411	-13.988	17.015	1.00	0.00	RX1	H
ATOM	7154	C	ARG	527	82.403	-16.323	20.195	1.00	0.00	RX1	C
ATOM	7155	O	ARG	527	82.634	-17.522	20.259	1.00	0.00	RX1	O
ATOM	7156	N	ASN	528	81.997	-15.671	19.091	1.00	0.00	RX1	N
ATOM	7157	H	ASN	528	81.687	-14.726	19.208	1.00	0.00	RX1	H
ATOM	7158	CA	ASN	528	81.832	-16.345	17.795	1.00	0.00	RX1	C
ATOM	7159	CB	ASN	528	83.043	-17.201	17.327	1.00	0.00	RX1	C
ATOM	7160	CG	ASN	528	84.391	-16.649	17.742	1.00	0.00	RX1	C
ATOM	7161	OD1	ASN	528	84.904	-15.678	17.205	1.00	0.00	RX1	O
ATOM	7162	ND2	ASN	528	84.942	-17.319	18.752	1.00	0.00	RX1	N
ATOM	7163	HD21	ASN	528	84.444	-18.037	19.243	1.00	0.00	RX1	H
ATOM	7164	HD22	ASN	528	85.863	-17.095	19.081	1.00	0.00	RX1	H
ATOM	7165	C	ASN	528	80.577	-17.204	17.785	1.00	0.00	RX1	C
ATOM	7166	O	ASN	528	79.723	-17.061	18.653	1.00	0.00	RX1	O
ATOM	7167	N	VAL	529	80.470	-18.080	16.766	1.00	0.00	RX1	N
ATOM	7168	H	VAL	529	81.178	-18.178	16.070	1.00	0.00	RX1	H
ATOM	7169	CA	VAL	529	79.255	-18.896	16.648	1.00	0.00	RX1	C
ATOM	7170	CB	VAL	529	79.309	-19.781	15.398	1.00	0.00	RX1	C
ATOM	7171	CG1	VAL	529	78.055	-20.653	15.288	1.00	0.00	RX1	C
ATOM	7172	CG2	VAL	529	79.527	-18.945	14.134	1.00	0.00	RX1	C
ATOM	7173	C	VAL	529	78.966	-19.741	17.883	1.00	0.00	RX1	C
ATOM	7174	O	VAL	529	79.672	-20.689	18.202	1.00	0.00	RX1	O
ATOM	7175	N	SER	530	77.900	-19.308	18.557	1.00	0.00	RX1	N
ATOM	7176	H	SER	530	77.296	-18.602	18.192	1.00	0.00	RX1	H
ATOM	7177	CA	SER	530	77.496	-19.938	19.806	1.00	0.00	RX1	C
ATOM	7178	CB	SER	530	77.291	-18.805	20.797	1.00	0.00	RX1	C
ATOM	7179	OG	SER	530	78.514	-18.062	20.897	1.00	0.00	RX1	O
ATOM	7180	HG	SER	530	78.786	-17.841	20.011	1.00	0.00	RX1	H
ATOM	7181	C	SER	530	76.275	-20.808	19.564	1.00	0.00	RX1	C
ATOM	7182	O	SER	530	75.555	-20.609	18.590	1.00	0.00	RX1	O
ATOM	7183	N	ARG	531	76.104	-21.827	20.415	1.00	0.00	RX1	N
ATOM	7184	H	ARG	531	76.552	-21.896	21.315	1.00	0.00	RX1	H
ATOM	7185	CA	ARG	531	75.233	-22.908	19.961	1.00	0.00	RX1	C
ATOM	7186	CB	ARG	531	76.062	-24.160	19.664	1.00	0.00	RX1	C
ATOM	7187	CG	ARG	531	77.153	-23.949	18.610	1.00	0.00	RX1	C
ATOM	7188	CD	ARG	531	78.453	-24.649	19.007	1.00	0.00	RX1	C
ATOM	7189	NE	ARG	531	78.900	-24.136	20.299	1.00	0.00	RX1	N
ATOM	7190	HE	ARG	531	78.342	-24.329	21.121	1.00	0.00	RX1	H
ATOM	7191	CZ	ARG	531	79.927	-23.245	20.397	1.00	0.00	RX1	C
ATOM	7192	NH1	ARG	531	80.651	-22.935	19.304	1.00	0.00	RX1	N
ATOM	7193	HH11	ARG	531	81.358	-22.224	19.328	1.00	0.00	RX1	H
ATOM	7194	HH12	ARG	531	80.478	-23.369	18.419	1.00	0.00	RX1	H
ATOM	7195	NH2	ARG	531	80.189	-22.683	21.588	1.00	0.00	RX1	N
ATOM	7196	HH21	ARG	531	80.965	-22.074	21.792	1.00	0.00	RX1	H

```
ATOM   7197 HH22 ARG   531      79.563 -22.866  22.370  1.00  0.00      RX1  H
ATOM   7198  C   ARG   531      74.127 -23.252  20.931  1.00  0.00      RX1  C
ATOM   7199  O   ARG   531      74.334 -23.656  22.065  1.00  0.00      RX1  O
ATOM   7200  N   GLY   532      72.898 -23.078  20.426  1.00  0.00      RX1  N
ATOM   7201  H   GLY   532      72.801 -22.659  19.524  1.00  0.00      RX1  H
ATOM   7202  CA  GLY   532      71.748 -23.632  21.149  1.00  0.00      RX1  C
ATOM   7203  C   GLY   532      71.453 -23.110  22.555  1.00  0.00      RX1  C
ATOM   7204  O   GLY   532      70.622 -23.682  23.258  1.00  0.00      RX1  O
ATOM   7205  N   ARG   533      72.124 -21.979  22.877  1.00  0.00      RX1  N
ATOM   7206  H   ARG   533      72.830 -21.684  22.237  1.00  0.00      RX1  H
ATOM   7207  CA  ARG   533      72.135 -21.248  24.158  1.00  0.00      RX1  C
ATOM   7208  CB  ARG   533      70.862 -21.327  25.000  1.00  0.00      RX1  C
ATOM   7209  CG  ARG   533      69.616 -20.771  24.325  1.00  0.00      RX1  C
ATOM   7210  CD  ARG   533      68.393 -21.269  25.090  1.00  0.00      RX1  C
ATOM   7211  NE  ARG   533      67.197 -21.249  24.255  1.00  0.00      RX1  N
ATOM   7212  HE  ARG   533      66.614 -20.437  24.373  1.00  0.00      RX1  H
ATOM   7213  CZ  ARG   533      66.961 -22.305  23.415  1.00  0.00      RX1  C
ATOM   7214  NH1 ARG   533      67.869 -23.309  23.303  1.00  0.00      RX1  N
ATOM   7215 HH11 ARG   533      67.714 -24.136  22.758  1.00  0.00      RX1  H
ATOM   7216 HH12 ARG   533      68.776 -23.280  23.755  1.00  0.00      RX1  H
ATOM   7217  NH2 ARG   533      65.810 -22.327  22.705  1.00  0.00      RX1  N
ATOM   7218 HH21 ARG   533      65.574 -23.069  22.072  1.00  0.00      RX1  H
ATOM   7219 HH22 ARG   533      65.137 -21.584  22.788  1.00  0.00      RX1  H
ATOM   7220  C   ARG   533      73.306 -21.540  25.078  1.00  0.00      RX1  C
ATOM   7221  O   ARG   533      73.239 -21.284  26.273  1.00  0.00      RX1  O
ATOM   7222  N   GLU   534      74.397 -22.038  24.488  1.00  0.00      RX1  N
ATOM   7223  H   GLU   534      74.488 -22.350  23.542  1.00  0.00      RX1  H
ATOM   7224  CA  GLU   534      75.636 -21.705  25.177  1.00  0.00      RX1  C
ATOM   7225  CB  GLU   534      76.422 -22.948  25.630  1.00  0.00      RX1  C
ATOM   7226  CG  GLU   534      76.873 -23.944  24.557  1.00  0.00      RX1  C
ATOM   7227  CD  GLU   534      77.955 -23.349  23.681  1.00  0.00      RX1  C
ATOM   7228  OE1 GLU   534      77.676 -23.032  22.537  1.00  0.00      RX1  O
ATOM   7229  OE2 GLU   534      79.090 -23.196  24.118  1.00  0.00      RX1  O
ATOM   7230  C   GLU   534      76.438 -20.752  24.322  1.00  0.00      RX1  C
ATOM   7231  O   GLU   534      76.171 -20.592  23.134  1.00  0.00      RX1  O
ATOM   7232  N   CYS   535      77.399 -20.107  24.989  1.00  0.00      RX1  N
ATOM   7233  H   CYS   535      77.479 -20.181  25.983  1.00  0.00      RX1  H
ATOM   7234  CA  CYS   535      78.203 -19.122  24.276  1.00  0.00      RX1  C
ATOM   7235  CB  CYS   535      77.679 -17.735  24.625  1.00  0.00      RX1  C
ATOM   7236  SG  CYS   535      75.877 -17.610  24.468  1.00  0.00      RX1  S
ATOM   7237  C   CYS   535      79.685 -19.258  24.552  1.00  0.00      RX1  C
ATOM   7238  O   CYS   535      80.440 -18.295  24.603  1.00  0.00      RX1  O
ATOM   7239  N   VAL   536      80.069 -20.520  24.774  1.00  0.00      RX1  N
ATOM   7240  H   VAL   536      79.450 -21.297  24.628  1.00  0.00      RX1  H
ATOM   7241  CA  VAL   536      81.465 -20.756  25.125  1.00  0.00      RX1  C
ATOM   7242  CB  VAL   536      81.596 -21.182  26.588  1.00  0.00      RX1  C
ATOM   7243  CG1 VAL   536      80.845 -22.485  26.862  1.00  0.00      RX1  C
ATOM   7244  CG2 VAL   536      83.069 -21.263  26.992  1.00  0.00      RX1  C
ATOM   7245  C   VAL   536      82.136 -21.761  24.208  1.00  0.00      RX1  C
ATOM   7246  O   VAL   536      82.164 -21.631  22.985  1.00  0.00      RX1  O
TER
HEADER lig.000.00.pdb
ATOM      1  N   ASP   985      85.671   8.876 -14.776  1.00  0.00      LX0  N
ATOM      2  H   ASP   985      85.532   8.042 -15.315  0.00  0.00      LX0  H
ATOM      3  CA  ASP   985      84.439   9.621 -14.523  1.00  0.00      LX0  C
ATOM      4  CB  ASP   985      84.624  11.094 -14.935  1.00  0.00      LX0  C
ATOM      5  CG  ASP   985      83.574  11.999 -14.305  1.00  0.00      LX0  C
ATOM      6  OD1 ASP   985      82.961  11.623 -13.311  1.00  0.00      LX0  O
ATOM      7  OD2 ASP   985      83.348  13.098 -14.797  1.00  0.00      LX0  O
ATOM      8  C   ASP   985      83.277   8.939 -15.234  1.00  0.00      LX0  C
ATOM      9  O   ASP   985      83.481   7.929 -15.897  1.00  0.00      LX0  O
```

ATOM	10	N	VAL	986	82.067	9.506	-15.063	1.00	0.00	LX0	N
ATOM	11	H	VAL	986	82.046	10.351	-14.527	0.00	0.00	LX0	H
ATOM	12	CA	VAL	986	80.853	8.978	-15.687	1.00	0.00	LX0	C
ATOM	13	CB	VAL	986	80.804	9.275	-17.201	1.00	0.00	LX0	C
ATOM	14	CG1	VAL	986	79.429	8.959	-17.802	1.00	0.00	LX0	C
ATOM	15	CG2	VAL	986	81.189	10.730	-17.489	1.00	0.00	LX0	C
ATOM	16	C	VAL	986	80.593	7.511	-15.370	1.00	0.00	LX0	C
ATOM	17	O	VAL	986	80.763	6.599	-16.169	1.00	0.00	LX0	O
ATOM	18	N	TYR	987	80.144	7.335	-14.116	1.00	0.00	LX0	N
ATOM	19	H	TYR	987	80.035	8.128	-13.521	0.00	0.00	LX0	H
ATOM	20	CA	TYR	987	79.825	5.980	-13.668	1.00	0.00	LX0	C
ATOM	21	CB	TYR	987	79.502	5.971	-12.165	1.00	0.00	LX0	C
ATOM	22	CG	TYR	987	79.405	4.553	-11.636	1.00	0.00	LX0	C
ATOM	23	CD1	TYR	987	80.582	3.893	-11.230	1.00	0.00	LX0	C
ATOM	24	CE1	TYR	987	80.494	2.575	-10.751	1.00	0.00	LX0	C
ATOM	25	CD2	TYR	987	78.141	3.928	-11.567	1.00	0.00	LX0	C
ATOM	26	CE2	TYR	987	78.053	2.609	-11.094	1.00	0.00	LX0	C
ATOM	27	CZ	TYR	987	79.232	1.951	-10.693	1.00	0.00	LX0	C
ATOM	28	OH	TYR	987	79.150	0.654	-10.226	1.00	0.00	LX0	O
ATOM	29	HH	TYR	987	79.112	0.080	-11.001	0.00	0.00	LX0	H
ATOM	30	C	TYR	987	78.725	5.301	-14.473	1.00	0.00	LX0	C
ATOM	31	O	TYR	987	77.536	5.472	-14.240	1.00	0.00	LX0	O
ATOM	32	N	VAL	988	79.200	4.484	-15.415	1.00	0.00	LX0	N
ATOM	33	H	VAL	988	80.178	4.532	-15.617	0.00	0.00	LX0	H
ATOM	34	CA	VAL	988	78.300	3.522	-16.042	1.00	0.00	LX0	C
ATOM	35	CB	VAL	988	78.966	2.970	-17.326	1.00	0.00	LX0	C
ATOM	36	CG1	VAL	988	80.331	2.325	-17.057	1.00	0.00	LX0	C
ATOM	37	CG2	VAL	988	78.041	2.047	-18.126	1.00	0.00	LX0	C
ATOM	38	C	VAL	988	77.920	2.430	-15.045	1.00	0.00	LX0	C
ATOM	39	O	VAL	988	78.769	1.897	-14.341	1.00	0.00	LX0	O
ATOM	40	N	PRO	989	76.603	2.129	-14.976	1.00	0.00	LX0	N
ATOM	41	CD	PRO	989	75.494	2.867	-15.570	1.00	0.00	LX0	C
ATOM	42	CA	PRO	989	76.166	0.954	-14.213	1.00	0.00	LX0	C
ATOM	43	CB	PRO	989	74.647	0.966	-14.415	1.00	0.00	LX0	C
ATOM	44	CG	PRO	989	74.289	2.408	-14.763	1.00	0.00	LX0	C
ATOM	45	C	PRO	989	76.807	-0.335	-14.710	1.00	0.00	LX0	C
ATOM	46	O	PRO	989	76.428	-0.890	-15.735	1.00	0.00	LX0	O
ATOM	47	N	ASP	990	77.812	-0.761	-13.941	1.00	0.00	LX0	N
ATOM	48	H	ASP	990	78.106	-0.227	-13.150	0.00	0.00	LX0	H
ATOM	49	CA	ASP	990	78.423	-2.062	-14.197	1.00	0.00	LX0	C
ATOM	50	CB	ASP	990	79.871	-2.090	-13.677	1.00	0.00	LX0	C
ATOM	51	CG	ASP	990	79.999	-1.531	-12.268	1.00	0.00	LX0	C
ATOM	52	OD1	ASP	990	79.342	-1.995	-11.341	1.00	0.00	LX0	O
ATOM	53	OD2	ASP	990	80.768	-0.601	-12.070	1.00	0.00	LX0	O
ATOM	54	C	ASP	990	77.582	-3.193	-13.633	1.00	0.00	LX0	C
ATOM	55	O	ASP	990	76.454	-2.993	-13.190	1.00	0.00	LX0	O
ATOM	56	N	GLU	991	78.161	-4.403	-13.657	1.00	0.00	LX0	N
ATOM	57	H	GLU	991	79.117	-4.538	-13.934	0.00	0.00	LX0	H
ATOM	58	CA	GLU	991	77.380	-5.565	-13.240	1.00	0.00	LX0	C
ATOM	59	CB	GLU	991	78.077	-6.878	-13.643	1.00	0.00	LX0	C
ATOM	60	CG	GLU	991	79.372	-7.278	-12.908	1.00	0.00	LX0	C
ATOM	61	CD	GLU	991	80.585	-6.433	-13.284	1.00	0.00	LX0	C
ATOM	62	OE1	GLU	991	80.595	-5.821	-14.352	1.00	0.00	LX0	O
ATOM	63	OE2	GLU	991	81.535	-6.404	-12.503	1.00	0.00	LX0	O
ATOM	64	C	GLU	991	76.919	-5.597	-11.787	1.00	0.00	LX0	C
ATOM	65	O	GLU	991	76.144	-6.453	-11.377	1.00	0.00	LX0	O
ATOM	66	N	TRP	992	77.406	-4.612	-11.019	1.00	0.00	LX0	N
ATOM	67	H	TRP	992	78.021	-3.916	-11.396	0.00	0.00	LX0	H
ATOM	68	CA	TRP	992	76.976	-4.559	-9.627	1.00	0.00	LX0	C
ATOM	69	CB	TRP	992	78.104	-4.067	-8.708	1.00	0.00	LX0	C
ATOM	70	CG	TRP	992	79.327	-4.955	-8.801	1.00	0.00	LX0	C

ATOM	71	CD2	TRP	992	79.937	-5.762	-7.769	1.00	0.00	LX0	C
ATOM	72	CE2	TRP	992	81.074	-6.420	-8.356	1.00	0.00	LX0	C
ATOM	73	CE3	TRP	992	79.624	-5.979	-6.410	1.00	0.00	LX0	C
ATOM	74	CD1	TRP	992	80.124	-5.167	-9.933	1.00	0.00	LX0	C
ATOM	75	NE1	TRP	992	81.143	-6.023	-9.688	1.00	0.00	LX0	N
ATOM	76	HE1	TRP	992	81.759	-6.318	-10.403	0.00	0.00	LX0	H
ATOM	77	CZ2	TRP	992	81.870	-7.281	-7.571	1.00	0.00	LX0	C
ATOM	78	CZ3	TRP	992	80.431	-6.841	-5.639	1.00	0.00	LX0	C
ATOM	79	CH2	TRP	992	81.546	-7.488	-6.213	1.00	0.00	LX0	C
ATOM	80	C	TRP	992	75.729	-3.723	-9.397	1.00	0.00	LX0	C
ATOM	81	O	TRP	992	75.350	-3.485	-8.257	1.00	0.00	LX0	O
ATOM	82	N	GLU	993	75.113	-3.274	-10.505	1.00	0.00	LX0	N
ATOM	83	H	GLU	993	75.483	-3.456	-11.418	0.00	0.00	LX0	H
ATOM	84	CA	GLU	993	73.897	-2.472	-10.359	1.00	0.00	LX0	C
ATOM	85	CB	GLU	993	73.582	-1.747	-11.671	1.00	0.00	LX0	C
ATOM	86	CG	GLU	993	72.446	-0.710	-11.604	1.00	0.00	LX0	C
ATOM	87	CD	GLU	993	72.761	0.480	-10.706	1.00	0.00	LX0	C
ATOM	88	OE1	GLU	993	73.925	0.807	-10.477	1.00	0.00	LX0	O
ATOM	89	OE2	GLU	993	71.824	1.129	-10.256	1.00	0.00	LX0	O
ATOM	90	C	GLU	993	72.697	-3.239	-9.818	1.00	0.00	LX0	C
ATOM	91	O	GLU	993	71.908	-3.874	-10.513	1.00	0.00	LX0	O
ATOM	92	N	VAL	994	72.609	-3.149	-8.493	1.00	0.00	LX0	N
ATOM	93	H	VAL	994	73.302	-2.578	-8.053	0.00	0.00	LX0	H
ATOM	94	CA	VAL	994	71.494	-3.713	-7.750	1.00	0.00	LX0	C
ATOM	95	CB	VAL	994	71.888	-3.763	-6.264	1.00	0.00	LX0	C
ATOM	96	CG1	VAL	994	70.736	-4.055	-5.304	1.00	0.00	LX0	C
ATOM	97	CG2	VAL	994	73.010	-4.784	-6.080	1.00	0.00	LX0	C
ATOM	98	C	VAL	994	70.213	-2.937	-8.002	1.00	0.00	LX0	C
ATOM	99	O	VAL	994	69.973	-1.855	-7.481	1.00	0.00	LX0	O
ATOM	100	N	ALA	995	69.368	-3.569	-8.828	1.00	0.00	LX0	N
ATOM	101	H	ALA	995	69.712	-4.359	-9.334	0.00	0.00	LX0	H
ATOM	102	CA	ALA	995	68.051	-2.976	-9.061	1.00	0.00	LX0	C
ATOM	103	CB	ALA	995	67.191	-3.898	-9.925	1.00	0.00	LX0	C
ATOM	104	C	ALA	995	67.298	-2.645	-7.780	1.00	0.00	LX0	C
ATOM	105	O	ALA	995	67.328	-3.363	-6.788	1.00	0.00	LX0	O
ATOM	106	N	ARG	996	66.623	-1.488	-7.836	1.00	0.00	LX0	N
ATOM	107	H	ARG	996	66.641	-0.961	-8.682	0.00	0.00	LX0	H
ATOM	108	CA	ARG	996	66.112	-0.944	-6.576	1.00	0.00	LX0	C
ATOM	109	CB	ARG	996	65.776	0.545	-6.761	1.00	0.00	LX0	C
ATOM	110	CG	ARG	996	65.788	1.359	-5.459	1.00	0.00	LX0	C
ATOM	111	CD	ARG	996	65.757	2.878	-5.661	1.00	0.00	LX0	C
ATOM	112	NE	ARG	996	67.009	3.358	-6.247	1.00	0.00	LX0	N
ATOM	113	HE	ARG	996	67.291	3.040	-7.159	0.00	0.00	LX0	H
ATOM	114	CZ	ARG	996	67.803	4.244	-5.609	1.00	0.00	LX0	C
ATOM	115	NH1	ARG	996	67.435	4.802	-4.462	1.00	0.00	LX0	N
ATOM	116	HH11	ARG	996	68.067	5.442	-4.013	0.00	0.00	LX0	H
ATOM	117	HH12	ARG	996	66.546	4.615	-4.046	0.00	0.00	LX0	H
ATOM	118	NH2	ARG	996	68.967	4.578	-6.144	1.00	0.00	LX0	N
ATOM	119	HH21	ARG	996	69.552	5.277	-5.718	0.00	0.00	LX0	H
ATOM	120	HH22	ARG	996	69.266	4.169	-7.011	0.00	0.00	LX0	H
ATOM	121	C	ARG	996	65.023	-1.747	-5.858	1.00	0.00	LX0	C
ATOM	122	O	ARG	996	64.704	-1.498	-4.701	1.00	0.00	LX0	O
ATOM	123	N	GLU	997	64.490	-2.764	-6.565	1.00	0.00	LX0	N
ATOM	124	H	GLU	997	64.845	-2.989	-7.469	0.00	0.00	LX0	H
ATOM	125	CA	GLU	997	63.580	-3.681	-5.866	1.00	0.00	LX0	C
ATOM	126	CB	GLU	997	62.876	-4.647	-6.835	1.00	0.00	LX0	C
ATOM	127	CG	GLU	997	63.698	-5.316	-7.953	1.00	0.00	LX0	C
ATOM	128	CD	GLU	997	64.750	-6.298	-7.459	1.00	0.00	LX0	C
ATOM	129	OE1	GLU	997	64.541	-7.011	-6.481	1.00	0.00	LX0	O
ATOM	130	OE2	GLU	997	65.806	-6.382	-8.072	1.00	0.00	LX0	O
ATOM	131	C	GLU	997	64.183	-4.417	-4.677	1.00	0.00	LX0	C

ATOM	132	O	GLU	997	63.505	-4.843	-3.749	1.00	0.00	LX0	O
ATOM	133	N	LYS	998	65.522	-4.482	-4.704	1.00	0.00	LX0	N
ATOM	134	H	LYS	998	66.024	-4.163	-5.509	0.00	0.00	LX0	H
ATOM	135	CA	LYS	998	66.180	-5.113	-3.567	1.00	0.00	LX0	C
ATOM	136	CB	LYS	998	67.605	-5.551	-3.914	1.00	0.00	LX0	C
ATOM	137	CG	LYS	998	67.590	-6.206	-5.285	1.00	0.00	LX0	C
ATOM	138	CD	LYS	998	68.646	-7.261	-5.577	1.00	0.00	LX0	C
ATOM	139	CE	LYS	998	68.217	-8.080	-6.798	1.00	0.00	LX0	C
ATOM	140	NZ	LYS	998	66.796	-8.439	-6.654	1.00	0.00	LX0	N
ATOM	141	HZ1	LYS	998	66.485	-9.115	-7.368	0.00	0.00	LX0	H
ATOM	142	HZ2	LYS	998	66.589	-8.789	-5.696	0.00	0.00	LX0	H
ATOM	143	HZ3	LYS	998	66.208	-7.583	-6.803	0.00	0.00	LX0	H
ATOM	144	C	LYS	998	66.182	-4.333	-2.270	1.00	0.00	LX0	C
ATOM	145	O	LYS	998	66.566	-4.848	-1.229	1.00	0.00	LX0	O
ATOM	146	N	ILE	999	65.795	-3.058	-2.365	1.00	0.00	LX0	N
ATOM	147	H	ILE	999	65.312	-2.699	-3.165	0.00	0.00	LX0	H
ATOM	148	CA	ILE	999	66.146	-2.212	-1.232	1.00	0.00	LX0	C
ATOM	149	CB	ILE	999	66.633	-0.840	-1.731	1.00	0.00	LX0	C
ATOM	150	CG2	ILE	999	67.281	-0.026	-0.611	1.00	0.00	LX0	C
ATOM	151	CG1	ILE	999	67.598	-0.987	-2.916	1.00	0.00	LX0	C
ATOM	152	CD1	ILE	999	68.885	-1.748	-2.588	1.00	0.00	LX0	C
ATOM	153	C	ILE	999	65.068	-2.112	-0.162	1.00	0.00	LX0	C
ATOM	154	O	ILE	999	64.385	-1.107	0.010	1.00	0.00	LX0	O
ATOM	155	N	THR	1000	64.961	-3.211	0.589	1.00	0.00	LX0	N
ATOM	156	H	THR	1000	65.541	-4.017	0.445	0.00	0.00	LX0	H
ATOM	157	CA	THR	1000	64.061	-3.168	1.736	1.00	0.00	LX0	C
ATOM	158	CB	THR	1000	63.598	-4.591	2.090	1.00	0.00	LX0	C
ATOM	159	OG1	THR	1000	64.300	-5.579	1.312	1.00	0.00	LX0	O
ATOM	160	HG1	THR	1000	65.074	-5.770	1.855	0.00	0.00	LX0	H
ATOM	161	CG2	THR	1000	62.090	-4.741	1.897	1.00	0.00	LX0	C
ATOM	162	C	THR	1000	64.674	-2.441	2.930	1.00	0.00	LX0	C
ATOM	163	O	THR	1000	65.190	-3.024	3.874	1.00	0.00	LX0	O
ATOM	164	N	MET	1001	64.630	-1.101	2.847	1.00	0.00	LX0	N
ATOM	165	H	MET	1001	64.210	-0.684	2.038	0.00	0.00	LX0	H
ATOM	166	CA	MET	1001	65.204	-0.337	3.960	1.00	0.00	LX0	C
ATOM	167	CB	MET	1001	65.185	1.164	3.675	1.00	0.00	LX0	C
ATOM	168	CG	MET	1001	66.178	1.546	2.579	1.00	0.00	LX0	C
ATOM	169	SD	MET	1001	66.194	3.306	2.207	1.00	0.00	LX0	S
ATOM	170	CE	MET	1001	64.519	3.441	1.562	1.00	0.00	LX0	C
ATOM	171	C	MET	1001	64.552	-0.628	5.299	1.00	0.00	LX0	C
ATOM	172	O	MET	1001	63.345	-0.800	5.404	1.00	0.00	LX0	O
ATOM	173	N	SER	1002	65.417	-0.709	6.316	1.00	0.00	LX0	N
ATOM	174	H	SER	1002	66.401	-0.568	6.203	0.00	0.00	LX0	H
ATOM	175	CA	SER	1002	64.863	-1.200	7.569	1.00	0.00	LX0	C
ATOM	176	CB	SER	1002	65.402	-2.616	7.815	1.00	0.00	LX0	C
ATOM	177	OG	SER	1002	64.644	-3.309	8.824	1.00	0.00	LX0	O
ATOM	178	HG	SER	1002	63.838	-3.566	8.377	0.00	0.00	LX0	H
ATOM	179	C	SER	1002	65.039	-0.257	8.752	1.00	0.00	LX0	C
ATOM	180	O	SER	1002	64.182	-0.185	9.626	1.00	0.00	LX0	O
ATOM	181	N	ARG	1003	66.172	0.468	8.751	1.00	0.00	LX0	N
ATOM	182	H	ARG	1003	66.901	0.301	8.081	0.00	0.00	LX0	H
ATOM	183	CA	ARG	1003	66.380	1.535	9.736	1.00	0.00	LX0	C
ATOM	184	CB	ARG	1003	66.561	0.984	11.162	1.00	0.00	LX0	C
ATOM	185	CG	ARG	1003	67.833	0.163	11.398	1.00	0.00	LX0	C
ATOM	186	CD	ARG	1003	67.636	-0.917	12.466	1.00	0.00	LX0	C
ATOM	187	NE	ARG	1003	67.688	-2.250	11.862	1.00	0.00	LX0	N
ATOM	188	HE	ARG	1003	68.589	-2.705	11.820	0.00	0.00	LX0	H
ATOM	189	CZ	ARG	1003	66.642	-2.791	11.203	1.00	0.00	LX0	C
ATOM	190	NH1	ARG	1003	65.446	-2.210	11.198	1.00	0.00	LX0	N
ATOM	191	HH11	ARG	1003	64.717	-2.579	10.611	0.00	0.00	LX0	H
ATOM	192	HH12	ARG	1003	65.257	-1.388	11.732	0.00	0.00	LX0	H

ATOM	193	NH2	ARG	1003	66.805	-3.909	10.516	1.00	0.00	LX0	N
ATOM	194	HH21	ARG	1003	66.062	-4.294	9.964	0.00	0.00	LX0	H
ATOM	195	HH22	ARG	1003	67.701	-4.372	10.516	0.00	0.00	LX0	H
ATOM	196	C	ARG	1003	67.569	2.380	9.343	1.00	0.00	LX0	C
ATOM	197	O	ARG	1003	68.334	2.000	8.465	1.00	0.00	LX0	O
ATOM	198	N	GLU	1004	67.703	3.519	10.029	1.00	0.00	LX0	N
ATOM	199	H	GLU	1004	67.077	3.784	10.758	0.00	0.00	LX0	H
ATOM	200	CA	GLU	1004	68.959	4.240	9.856	1.00	0.00	LX0	C
ATOM	201	CB	GLU	1004	68.741	5.750	9.932	1.00	0.00	LX0	C
ATOM	202	CG	GLU	1004	67.546	6.197	9.089	1.00	0.00	LX0	C
ATOM	203	CD	GLU	1004	67.571	7.695	8.884	1.00	0.00	LX0	C
ATOM	204	OE1	GLU	1004	67.467	8.427	9.864	1.00	0.00	LX0	O
ATOM	205	OE2	GLU	1004	67.690	8.120	7.736	1.00	0.00	LX0	O
ATOM	206	C	GLU	1004	69.959	3.787	10.896	1.00	0.00	LX0	C
ATOM	207	O	GLU	1004	69.589	3.194	11.903	1.00	0.00	LX0	O
ATOM	208	N	LEU	1005	71.228	4.071	10.598	1.00	0.00	LX0	N
ATOM	209	H	LEU	1005	71.430	4.540	9.738	0.00	0.00	LX0	H
ATOM	210	CA	LEU	1005	72.275	3.761	11.567	1.00	0.00	LX0	C
ATOM	211	CB	LEU	1005	73.326	2.817	10.975	1.00	0.00	LX0	C
ATOM	212	CG	LEU	1005	72.811	1.427	10.602	1.00	0.00	LX0	C
ATOM	213	CD1	LEU	1005	73.885	0.622	9.871	1.00	0.00	LX0	C
ATOM	214	CD2	LEU	1005	72.253	0.666	11.807	1.00	0.00	LX0	C
ATOM	215	C	LEU	1005	72.957	5.016	12.069	1.00	0.00	LX0	C
ATOM	216	O	LEU	1005	73.110	5.244	13.261	1.00	0.00	LX0	O
ATOM	217	N	GLY	1006	73.367	5.831	11.090	1.00	0.00	LX0	N
ATOM	218	H	GLY	1006	73.231	5.622	10.119	0.00	0.00	LX0	H
ATOM	219	CA	GLY	1006	74.063	7.050	11.482	1.00	0.00	LX0	C
ATOM	220	C	GLY	1006	74.582	7.779	10.268	1.00	0.00	LX0	C
ATOM	221	O	GLY	1006	74.509	7.279	9.152	1.00	0.00	LX0	O
ATOM	222	N	GLN	1007	75.092	8.989	10.528	1.00	0.00	LX0	N
ATOM	223	H	GLN	1007	75.209	9.287	11.472	0.00	0.00	LX0	H
ATOM	224	CA	GLN	1007	75.593	9.788	9.412	1.00	0.00	LX0	C
ATOM	225	CB	GLN	1007	75.855	11.221	9.901	1.00	0.00	LX0	C
ATOM	226	CG	GLN	1007	76.254	12.251	8.834	1.00	0.00	LX0	C
ATOM	227	CD	GLN	1007	75.138	12.419	7.821	1.00	0.00	LX0	C
ATOM	228	OE1	GLN	1007	73.963	12.489	8.145	1.00	0.00	LX0	O
ATOM	229	NE2	GLN	1007	75.557	12.479	6.558	1.00	0.00	LX0	N
ATOM	230	HE21	GLN	1007	76.524	12.352	6.320	0.00	0.00	LX0	H
ATOM	231	HE22	GLN	1007	74.883	12.655	5.846	0.00	0.00	LX0	H
ATOM	232	C	GLN	1007	76.831	9.177	8.778	1.00	0.00	LX0	C
ATOM	233	O	GLN	1007	77.686	8.622	9.455	1.00	0.00	LX0	O
ATOM	234	N	GLY	1008	76.882	9.311	7.451	1.00	0.00	LX0	N
ATOM	235	H	GLY	1008	76.124	9.692	6.920	0.00	0.00	LX0	H
ATOM	236	CA	GLY	1008	78.108	8.957	6.758	1.00	0.00	LX0	C
ATOM	237	C	GLY	1008	78.501	10.075	5.821	1.00	0.00	LX0	C
ATOM	238	O	GLY	1008	77.745	11.005	5.560	1.00	0.00	LX0	O
ATOM	239	N	SER	1009	79.722	9.941	5.304	1.00	0.00	LX0	N
ATOM	240	H	SER	1009	80.318	9.180	5.568	0.00	0.00	LX0	H
ATOM	241	CA	SER	1009	80.346	11.032	4.558	1.00	0.00	LX0	C
ATOM	242	CB	SER	1009	81.754	10.579	4.186	1.00	0.00	LX0	C
ATOM	243	OG	SER	1009	82.223	9.678	5.198	1.00	0.00	LX0	O
ATOM	244	HG	SER	1009	82.678	10.226	5.840	0.00	0.00	LX0	H
ATOM	245	C	SER	1009	79.612	11.602	3.345	1.00	0.00	LX0	C
ATOM	246	O	SER	1009	79.885	12.709	2.885	1.00	0.00	LX0	O
ATOM	247	N	PHE	1010	78.679	10.797	2.816	1.00	0.00	LX0	N
ATOM	248	H	PHE	1010	78.500	9.893	3.201	0.00	0.00	LX0	H
ATOM	249	CA	PHE	1010	77.929	11.301	1.665	1.00	0.00	LX0	C
ATOM	250	CB	PHE	1010	78.329	10.549	0.390	1.00	0.00	LX0	C
ATOM	251	CG	PHE	1010	79.694	10.976	-0.104	1.00	0.00	LX0	C
ATOM	252	CD1	PHE	1010	79.776	11.887	-1.179	1.00	0.00	LX0	C
ATOM	253	CD2	PHE	1010	80.862	10.461	0.499	1.00	0.00	LX0	C

ATOM	254	CE1	PHE	1010	81.039	12.286	-1.658	1.00	0.00	LX0	C
ATOM	255	CE2	PHE	1010	82.127	10.858	0.025	1.00	0.00	LX0	C
ATOM	256	CZ	PHE	1010	82.202	11.766	-1.052	1.00	0.00	LX0	C
ATOM	257	C	PHE	1010	76.419	11.286	1.845	1.00	0.00	LX0	C
ATOM	258	O	PHE	1010	75.659	11.603	0.937	1.00	0.00	LX0	O
ATOM	259	N	GLY	1011	76.012	10.898	3.060	1.00	0.00	LX0	N
ATOM	260	H	GLY	1011	76.637	10.733	3.825	0.00	0.00	LX0	H
ATOM	261	CA	GLY	1011	74.587	10.688	3.282	1.00	0.00	LX0	C
ATOM	262	C	GLY	1011	74.415	9.742	4.444	1.00	0.00	LX0	C
ATOM	263	O	GLY	1011	75.384	9.368	5.093	1.00	0.00	LX0	O
ATOM	264	N	MET	1012	73.157	9.381	4.705	1.00	0.00	LX0	N
ATOM	265	H	MET	1012	72.438	9.573	4.036	0.00	0.00	LX0	H
ATOM	266	CA	MET	1012	72.939	8.494	5.846	1.00	0.00	LX0	C
ATOM	267	CB	MET	1012	71.467	8.574	6.263	1.00	0.00	LX0	C
ATOM	268	CG	MET	1012	71.140	7.981	7.637	1.00	0.00	LX0	C
ATOM	269	SD	MET	1012	71.842	8.930	8.994	1.00	0.00	LX0	S
ATOM	270	CE	MET	1012	70.806	10.393	8.835	1.00	0.00	LX0	C
ATOM	271	C	MET	1012	73.359	7.066	5.524	1.00	0.00	LX0	C
ATOM	272	O	MET	1012	73.269	6.620	4.385	1.00	0.00	LX0	O
ATOM	273	N	VAL	1013	73.834	6.374	6.560	1.00	0.00	LX0	N
ATOM	274	H	VAL	1013	73.892	6.794	7.465	0.00	0.00	LX0	H
ATOM	275	CA	VAL	1013	74.017	4.937	6.399	1.00	0.00	LX0	C
ATOM	276	CB	VAL	1013	75.347	4.481	7.020	1.00	0.00	LX0	C
ATOM	277	CG1	VAL	1013	75.604	2.985	6.800	1.00	0.00	LX0	C
ATOM	278	CG2	VAL	1013	76.510	5.322	6.487	1.00	0.00	LX0	C
ATOM	279	C	VAL	1013	72.836	4.227	7.029	1.00	0.00	LX0	C
ATOM	280	O	VAL	1013	72.452	4.516	8.155	1.00	0.00	LX0	O
ATOM	281	N	TYR	1014	72.260	3.317	6.241	1.00	0.00	LX0	N
ATOM	282	H	TYR	1014	72.674	3.126	5.351	0.00	0.00	LX0	H
ATOM	283	CA	TYR	1014	71.086	2.580	6.696	1.00	0.00	LX0	C
ATOM	284	CB	TYR	1014	70.011	2.566	5.601	1.00	0.00	LX0	C
ATOM	285	CG	TYR	1014	69.427	3.938	5.360	1.00	0.00	LX0	C
ATOM	286	CD1	TYR	1014	68.177	4.242	5.932	1.00	0.00	LX0	C
ATOM	287	CE1	TYR	1014	67.640	5.525	5.749	1.00	0.00	LX0	C
ATOM	288	CD2	TYR	1014	70.134	4.871	4.573	1.00	0.00	LX0	C
ATOM	289	CE2	TYR	1014	69.599	6.157	4.397	1.00	0.00	LX0	C
ATOM	290	CZ	TYR	1014	68.373	6.476	5.012	1.00	0.00	LX0	C
ATOM	291	OH	TYR	1014	67.886	7.759	4.901	1.00	0.00	LX0	O
ATOM	292	HH	TYR	1014	67.658	8.058	5.787	0.00	0.00	LX0	H
ATOM	293	C	TYR	1014	71.442	1.143	7.004	1.00	0.00	LX0	C
ATOM	294	O	TYR	1014	72.321	0.573	6.369	1.00	0.00	LX0	O
ATOM	295	N	GLU	1015	70.686	0.564	7.947	1.00	0.00	LX0	N
ATOM	296	H	GLU	1015	70.022	1.113	8.453	0.00	0.00	LX0	H
ATOM	297	CA	GLU	1015	70.527	-0.882	7.831	1.00	0.00	LX0	C
ATOM	298	CB	GLU	1015	70.307	-1.593	9.175	1.00	0.00	LX0	C
ATOM	299	CG	GLU	1015	70.274	-3.123	9.006	1.00	0.00	LX0	C
ATOM	300	CD	GLU	1015	69.980	-3.878	10.298	1.00	0.00	LX0	C
ATOM	301	OE1	GLU	1015	70.386	-3.466	11.376	1.00	0.00	LX0	O
ATOM	302	OE2	GLU	1015	69.330	-4.912	10.243	1.00	0.00	LX0	O
ATOM	303	C	GLU	1015	69.361	-1.148	6.906	1.00	0.00	LX0	C
ATOM	304	O	GLU	1015	68.188	-0.990	7.242	1.00	0.00	LX0	O
ATOM	305	N	GLY	1016	69.740	-1.532	5.693	1.00	0.00	LX0	N
ATOM	306	H	GLY	1016	70.712	-1.700	5.511	0.00	0.00	LX0	H
ATOM	307	CA	GLY	1016	68.701	-2.100	4.857	1.00	0.00	LX0	C
ATOM	308	C	GLY	1016	68.763	-3.598	4.995	1.00	0.00	LX0	C
ATOM	309	O	GLY	1016	69.701	-4.148	5.556	1.00	0.00	LX0	O
ATOM	310	N	VAL	1017	67.737	-4.224	4.444	1.00	0.00	LX0	N
ATOM	311	H	VAL	1017	66.956	-3.741	4.045	0.00	0.00	LX0	H
ATOM	312	CA	VAL	1017	67.828	-5.653	4.201	1.00	0.00	LX0	C
ATOM	313	CB	VAL	1017	66.919	-6.417	5.176	1.00	0.00	LX0	C
ATOM	314	CG1	VAL	1017	67.622	-6.627	6.516	1.00	0.00	LX0	C

ATOM	315	CG2	VAL	1017	65.563	-5.737	5.391	1.00	0.00	LX0	C
ATOM	316	C	VAL	1017	67.471	-5.827	2.738	1.00	0.00	LX0	C
ATOM	317	O	VAL	1017	66.765	-4.992	2.180	1.00	0.00	LX0	O
ATOM	318	N	ALA	1018	68.045	-6.850	2.103	1.00	0.00	LX0	N
ATOM	319	H	ALA	1018	68.597	-7.520	2.598	0.00	0.00	LX0	H
ATOM	320	CA	ALA	1018	68.029	-6.765	0.649	1.00	0.00	LX0	C
ATOM	321	CB	ALA	1018	69.404	-6.367	0.119	1.00	0.00	LX0	C
ATOM	322	C	ALA	1018	67.560	-7.999	-0.081	1.00	0.00	LX0	C
ATOM	323	O	ALA	1018	67.987	-9.116	0.170	1.00	0.00	LX0	O
ATOM	324	N	LYS	1019	66.651	-7.721	-1.026	1.00	0.00	LX0	N
ATOM	325	H	LYS	1019	66.449	-6.751	-1.156	0.00	0.00	LX0	H
ATOM	326	CA	LYS	1019	65.990	-8.755	-1.821	1.00	0.00	LX0	C
ATOM	327	CB	LYS	1019	64.731	-8.151	-2.448	1.00	0.00	LX0	C
ATOM	328	CG	LYS	1019	63.505	-9.060	-2.552	1.00	0.00	LX0	C
ATOM	329	CD	LYS	1019	62.865	-9.441	-1.211	1.00	0.00	LX0	C
ATOM	330	CE	LYS	1019	62.588	-8.256	-0.276	1.00	0.00	LX0	C
ATOM	331	NZ	LYS	1019	63.661	-8.145	0.716	1.00	0.00	LX0	N
ATOM	332	HZ1	LYS	1019	63.989	-7.173	0.888	0.00	0.00	LX0	H
ATOM	333	HZ2	LYS	1019	63.327	-8.439	1.656	0.00	0.00	LX0	H
ATOM	334	HZ3	LYS	1019	64.481	-8.763	0.549	0.00	0.00	LX0	H
ATOM	335	C	LYS	1019	66.841	-9.455	-2.870	1.00	0.00	LX0	C
ATOM	336	O	LYS	1019	66.676	-9.258	-4.072	1.00	0.00	LX0	O
ATOM	337	N	GLY	1020	67.749	-10.295	-2.370	1.00	0.00	LX0	N
ATOM	338	H	GLY	1020	67.905	-10.396	-1.381	0.00	0.00	LX0	H
ATOM	339	CA	GLY	1020	68.544	-11.069	-3.313	1.00	0.00	LX0	C
ATOM	340	C	GLY	1020	69.837	-10.397	-3.711	1.00	0.00	LX0	C
ATOM	341	O	GLY	1020	70.058	-10.085	-4.876	1.00	0.00	LX0	O
ATOM	342	N	VAL	1021	70.682	-10.160	-2.693	1.00	0.00	LX0	N
ATOM	343	H	VAL	1021	70.438	-10.444	-1.763	0.00	0.00	LX0	H
ATOM	344	CA	VAL	1021	72.007	-9.695	-3.106	1.00	0.00	LX0	C
ATOM	345	CB	VAL	1021	72.412	-8.341	-2.493	1.00	0.00	LX0	C
ATOM	346	CG1	VAL	1021	71.514	-7.222	-3.017	1.00	0.00	LX0	C
ATOM	347	CG2	VAL	1021	72.475	-8.347	-0.968	1.00	0.00	LX0	C
ATOM	348	C	VAL	1021	73.106	-10.733	-2.968	1.00	0.00	LX0	C
ATOM	349	O	VAL	1021	73.915	-10.932	-3.868	1.00	0.00	LX0	O
ATOM	350	N	VAL	1022	73.104	-11.432	-1.820	1.00	0.00	LX0	N
ATOM	351	H	VAL	1022	72.404	-11.306	-1.114	0.00	0.00	LX0	H
ATOM	352	CA	VAL	1022	74.050	-12.545	-1.767	1.00	0.00	LX0	C
ATOM	353	CB	VAL	1022	74.562	-12.791	-0.339	1.00	0.00	LX0	C
ATOM	354	CG1	VAL	1022	75.603	-13.916	-0.273	1.00	0.00	LX0	C
ATOM	355	CG2	VAL	1022	75.163	-11.502	0.221	1.00	0.00	LX0	C
ATOM	356	C	VAL	1022	73.431	-13.779	-2.395	1.00	0.00	LX0	C
ATOM	357	O	VAL	1022	72.922	-14.680	-1.744	1.00	0.00	LX0	O
ATOM	358	N	LYS	1023	73.472	-13.732	-3.740	1.00	0.00	LX0	N
ATOM	359	H	LYS	1023	73.850	-12.891	-4.129	0.00	0.00	LX0	H
ATOM	360	CA	LYS	1023	72.624	-14.622	-4.532	1.00	0.00	LX0	C
ATOM	361	CB	LYS	1023	73.103	-16.085	-4.456	1.00	0.00	LX0	C
ATOM	362	CG	LYS	1023	74.498	-16.278	-5.053	1.00	0.00	LX0	C
ATOM	363	CD	LYS	1023	74.547	-15.880	-6.529	1.00	0.00	LX0	C
ATOM	364	CE	LYS	1023	75.940	-16.022	-7.138	1.00	0.00	LX0	C
ATOM	365	NZ	LYS	1023	75.893	-15.575	-8.537	1.00	0.00	LX0	N
ATOM	366	HZ1	LYS	1023	76.833	-15.679	-8.970	0.00	0.00	LX0	H
ATOM	367	HZ2	LYS	1023	75.202	-16.151	-9.058	0.00	0.00	LX0	H
ATOM	368	HZ3	LYS	1023	75.605	-14.575	-8.569	0.00	0.00	LX0	H
ATOM	369	C	LYS	1023	71.149	-14.393	-4.218	1.00	0.00	LX0	C
ATOM	370	O	LYS	1023	70.753	-13.256	-3.997	1.00	0.00	LX0	O
ATOM	371	N	ASP	1024	70.367	-15.476	-4.229	1.00	0.00	LX0	N
ATOM	372	H	ASP	1024	70.710	-16.416	-4.281	0.00	0.00	LX0	H
ATOM	373	CA	ASP	1024	68.921	-15.381	-4.018	1.00	0.00	LX0	C
ATOM	374	CB	ASP	1024	68.288	-16.765	-4.221	1.00	0.00	LX0	C
ATOM	375	CG	ASP	1024	69.117	-17.817	-3.503	1.00	0.00	LX0	C

ATOM	376	OD1	ASP	1024	68.905	-18.020	-2.313	1.00	0.00	LX0	O
ATOM	377	OD2	ASP	1024	70.019	-18.372	-4.131	1.00	0.00	LX0	O
ATOM	378	C	ASP	1024	68.469	-14.758	-2.703	1.00	0.00	LX0	C
ATOM	379	O	ASP	1024	67.448	-14.079	-2.658	1.00	0.00	LX0	O
ATOM	380	N	GLU	1025	69.293	-14.992	-1.662	1.00	0.00	LX0	N
ATOM	381	H	GLU	1025	69.979	-15.702	-1.822	0.00	0.00	LX0	H
ATOM	382	CA	GLU	1025	69.082	-14.465	-0.304	1.00	0.00	LX0	C
ATOM	383	CB	GLU	1025	70.444	-14.305	0.379	1.00	0.00	LX0	C
ATOM	384	CG	GLU	1025	70.467	-14.664	1.870	1.00	0.00	LX0	C
ATOM	385	CD	GLU	1025	69.890	-13.549	2.716	1.00	0.00	LX0	C
ATOM	386	OE1	GLU	1025	68.672	-13.439	2.819	1.00	0.00	LX0	O
ATOM	387	OE2	GLU	1025	70.669	-12.789	3.280	1.00	0.00	LX0	O
ATOM	388	C	GLU	1025	68.204	-13.219	-0.153	1.00	0.00	LX0	C
ATOM	389	O	GLU	1025	68.617	-12.080	-0.358	1.00	0.00	LX0	O
ATOM	390	N	PRO	1026	66.917	-13.506	0.168	1.00	0.00	LX0	N
ATOM	391	CD	PRO	1026	66.391	-14.821	0.518	1.00	0.00	LX0	C
ATOM	392	CA	PRO	1026	65.862	-12.491	0.070	1.00	0.00	LX0	C
ATOM	393	CB	PRO	1026	64.586	-13.280	0.381	1.00	0.00	LX0	C
ATOM	394	CG	PRO	1026	64.928	-14.742	0.106	1.00	0.00	LX0	C
ATOM	395	C	PRO	1026	65.978	-11.251	0.940	1.00	0.00	LX0	C
ATOM	396	O	PRO	1026	65.311	-10.250	0.676	1.00	0.00	LX0	O
ATOM	397	N	GLU	1027	66.784	-11.363	1.999	1.00	0.00	LX0	N
ATOM	398	H	GLU	1027	67.366	-12.164	2.155	0.00	0.00	LX0	H
ATOM	399	CA	GLU	1027	66.740	-10.287	2.977	1.00	0.00	LX0	C
ATOM	400	CB	GLU	1027	65.636	-10.578	4.005	1.00	0.00	LX0	C
ATOM	401	CG	GLU	1027	64.961	-9.332	4.592	1.00	0.00	LX0	C
ATOM	402	CD	GLU	1027	64.313	-8.489	3.504	1.00	0.00	LX0	C
ATOM	403	OE1	GLU	1027	65.008	-7.745	2.810	1.00	0.00	LX0	O
ATOM	404	OE2	GLU	1027	63.101	-8.572	3.328	1.00	0.00	LX0	O
ATOM	405	C	GLU	1027	68.080	-9.952	3.606	1.00	0.00	LX0	C
ATOM	406	O	GLU	1027	68.212	-9.690	4.798	1.00	0.00	LX0	O
ATOM	407	N	THR	1028	69.096	-9.942	2.732	1.00	0.00	LX0	N
ATOM	408	H	THR	1028	68.952	-10.202	1.773	0.00	0.00	LX0	H
ATOM	409	CA	THR	1028	70.443	-9.717	3.251	1.00	0.00	LX0	C
ATOM	410	CB	THR	1028	71.461	-9.831	2.119	1.00	0.00	LX0	C
ATOM	411	OG1	THR	1028	71.090	-10.859	1.190	1.00	0.00	LX0	O
ATOM	412	HG1	THR	1028	70.829	-11.611	1.735	0.00	0.00	LX0	H
ATOM	413	CG2	THR	1028	72.871	-10.062	2.665	1.00	0.00	LX0	C
ATOM	414	C	THR	1028	70.639	-8.405	3.994	1.00	0.00	LX0	C
ATOM	415	O	THR	1028	70.384	-7.329	3.467	1.00	0.00	LX0	O
ATOM	416	N	ARG	1029	71.118	-8.532	5.239	1.00	0.00	LX0	N
ATOM	417	H	ARG	1029	71.270	-9.452	5.597	0.00	0.00	LX0	H
ATOM	418	CA	ARG	1029	71.430	-7.308	5.977	1.00	0.00	LX0	C
ATOM	419	CB	ARG	1029	71.708	-7.623	7.450	1.00	0.00	LX0	C
ATOM	420	CG	ARG	1029	70.566	-8.377	8.140	1.00	0.00	LX0	C
ATOM	421	CD	ARG	1029	70.799	-8.602	9.637	1.00	0.00	LX0	C
ATOM	422	NE	ARG	1029	70.827	-7.335	10.367	1.00	0.00	LX0	N
ATOM	423	HE	ARG	1029	70.276	-6.565	10.028	0.00	0.00	LX0	H
ATOM	424	CZ	ARG	1029	71.546	-7.167	11.495	1.00	0.00	LX0	C
ATOM	425	NH1	ARG	1029	72.284	-8.166	11.967	1.00	0.00	LX0	N
ATOM	426	HH11	ARG	1029	72.845	-8.013	12.791	0.00	0.00	LX0	H
ATOM	427	HH12	ARG	1029	72.308	-9.056	11.521	0.00	0.00	LX0	H
ATOM	428	NH2	ARG	1029	71.536	-5.998	12.131	1.00	0.00	LX0	N
ATOM	429	HH21	ARG	1029	72.099	-5.864	12.953	0.00	0.00	LX0	H
ATOM	430	HH22	ARG	1029	70.982	-5.220	11.787	0.00	0.00	LX0	H
ATOM	431	C	ARG	1029	72.580	-6.527	5.356	1.00	0.00	LX0	C
ATOM	432	O	ARG	1029	73.704	-7.006	5.241	1.00	0.00	LX0	O
ATOM	433	N	VAL	1030	72.231	-5.308	4.932	1.00	0.00	LX0	N
ATOM	434	H	VAL	1030	71.304	-4.976	5.102	0.00	0.00	LX0	H
ATOM	435	CA	VAL	1030	73.186	-4.502	4.178	1.00	0.00	LX0	C
ATOM	436	CB	VAL	1030	72.797	-4.433	2.690	1.00	0.00	LX0	C

ATOM	437	CG1	VAL	1030	72.985	-5.788	2.012	1.00	0.00	LX0	C
ATOM	438	CG2	VAL	1030	71.385	-3.888	2.462	1.00	0.00	LX0	C
ATOM	439	C	VAL	1030	73.379	-3.108	4.745	1.00	0.00	LX0	C
ATOM	440	O	VAL	1030	72.442	-2.424	5.143	1.00	0.00	LX0	O
ATOM	441	N	ALA	1031	74.656	-2.710	4.757	1.00	0.00	LX0	N
ATOM	442	H	ALA	1031	75.341	-3.307	4.342	0.00	0.00	LX0	H
ATOM	443	CA	ALA	1031	74.967	-1.329	5.111	1.00	0.00	LX0	C
ATOM	444	CB	ALA	1031	76.383	-1.214	5.678	1.00	0.00	LX0	C
ATOM	445	C	ALA	1031	74.854	-0.426	3.904	1.00	0.00	LX0	C
ATOM	446	O	ALA	1031	75.710	-0.390	3.029	1.00	0.00	LX0	O
ATOM	447	N	ILE	1032	73.734	0.294	3.879	1.00	0.00	LX0	N
ATOM	448	H	ILE	1032	73.110	0.250	4.661	0.00	0.00	LX0	H
ATOM	449	CA	ILE	1032	73.552	1.198	2.749	1.00	0.00	LX0	C
ATOM	450	CB	ILE	1032	72.066	1.353	2.415	1.00	0.00	LX0	C
ATOM	451	CG2	ILE	1032	71.863	2.185	1.145	1.00	0.00	LX0	C
ATOM	452	CG1	ILE	1032	71.381	-0.011	2.321	1.00	0.00	LX0	C
ATOM	453	CD1	ILE	1032	69.877	0.110	2.088	1.00	0.00	LX0	C
ATOM	454	C	ILE	1032	74.178	2.552	3.008	1.00	0.00	LX0	C
ATOM	455	O	ILE	1032	73.636	3.369	3.741	1.00	0.00	LX0	O
ATOM	456	N	LYS	1033	75.333	2.769	2.377	1.00	0.00	LX0	N
ATOM	457	H	LYS	1033	75.676	2.076	1.740	0.00	0.00	LX0	H
ATOM	458	CA	LYS	1033	75.854	4.132	2.438	1.00	0.00	LX0	C
ATOM	459	CB	LYS	1033	77.381	4.144	2.298	1.00	0.00	LX0	C
ATOM	460	CG	LYS	1033	78.104	3.080	3.132	1.00	0.00	LX0	C
ATOM	461	CD	LYS	1033	79.631	3.231	3.179	1.00	0.00	LX0	C
ATOM	462	CE	LYS	1033	80.333	3.283	1.816	1.00	0.00	LX0	C
ATOM	463	NZ	LYS	1033	81.795	3.259	1.967	1.00	0.00	LX0	N
ATOM	464	HZ1	LYS	1033	82.285	3.711	1.163	0.00	0.00	LX0	H
ATOM	465	HZ2	LYS	1033	82.135	3.689	2.851	0.00	0.00	LX0	H
ATOM	466	HZ3	LYS	1033	82.169	2.292	2.051	0.00	0.00	LX0	H
ATOM	467	C	LYS	1033	75.207	4.977	1.356	1.00	0.00	LX0	C
ATOM	468	O	LYS	1033	75.265	4.638	0.180	1.00	0.00	LX0	O
ATOM	469	N	THR	1034	74.549	6.063	1.771	1.00	0.00	LX0	N
ATOM	470	H	THR	1034	74.499	6.347	2.729	0.00	0.00	LX0	H
ATOM	471	CA	THR	1034	73.936	6.824	0.686	1.00	0.00	LX0	C
ATOM	472	CB	THR	1034	72.540	7.321	1.076	1.00	0.00	LX0	C
ATOM	473	OG1	THR	1034	72.587	8.143	2.244	1.00	0.00	LX0	O
ATOM	474	HG1	THR	1034	72.644	7.529	2.972	0.00	0.00	LX0	H
ATOM	475	CG2	THR	1034	71.585	6.155	1.312	1.00	0.00	LX0	C
ATOM	476	C	THR	1034	74.790	7.964	0.173	1.00	0.00	LX0	C
ATOM	477	O	THR	1034	75.657	8.488	0.863	1.00	0.00	LX0	O
ATOM	478	N	VAL	1035	74.467	8.341	-1.072	1.00	0.00	LX0	N
ATOM	479	H	VAL	1035	73.848	7.774	-1.614	0.00	0.00	LX0	H
ATOM	480	CA	VAL	1035	74.753	9.713	-1.472	1.00	0.00	LX0	C
ATOM	481	CB	VAL	1035	75.478	9.818	-2.815	1.00	0.00	LX0	C
ATOM	482	CG1	VAL	1035	76.236	11.143	-2.886	1.00	0.00	LX0	C
ATOM	483	CG2	VAL	1035	76.355	8.608	-3.126	1.00	0.00	LX0	C
ATOM	484	C	VAL	1035	73.422	10.419	-1.576	1.00	0.00	LX0	C
ATOM	485	O	VAL	1035	72.577	10.042	-2.387	1.00	0.00	LX0	O
ATOM	486	N	ASN	1036	73.273	11.400	-0.680	1.00	0.00	LX0	N
ATOM	487	H	ASN	1036	74.086	11.675	-0.163	0.00	0.00	LX0	H
ATOM	488	CA	ASN	1036	71.965	11.965	-0.345	1.00	0.00	LX0	C
ATOM	489	CB	ASN	1036	72.125	12.965	0.818	1.00	0.00	LX0	C
ATOM	490	CG	ASN	1036	72.411	14.383	0.342	1.00	0.00	LX0	C
ATOM	491	OD1	ASN	1036	73.219	14.625	-0.543	1.00	0.00	LX0	O
ATOM	492	ND2	ASN	1036	71.696	15.319	0.963	1.00	0.00	LX0	N
ATOM	493	HD21	ASN	1036	70.897	15.068	1.527	0.00	0.00	LX0	H
ATOM	494	HD22	ASN	1036	71.871	16.292	0.850	0.00	0.00	LX0	H
ATOM	495	C	ASN	1036	71.123	12.520	-1.491	1.00	0.00	LX0	C
ATOM	496	O	ASN	1036	71.511	12.527	-2.656	1.00	0.00	LX0	O
ATOM	497	N	GLU	1037	69.939	13.008	-1.102	1.00	0.00	LX0	N

ATOM	498	H	GLU	1037	69.706	12.999	-0.126	0.00	0.00	LX0 H
ATOM	499	CA	GLU	1037	69.101	13.732	-2.059	1.00	0.00	LX0 C
ATOM	500	CB	GLU	1037	67.728	14.150	-1.489	1.00	0.00	LX0 C
ATOM	501	CG	GLU	1037	67.450	14.005	0.019	1.00	0.00	LX0 C
ATOM	502	CD	GLU	1037	68.526	14.681	0.852	1.00	0.00	LX0 C
ATOM	503	OE1	GLU	1037	69.204	13.980	1.596	1.00	0.00	LX0 O
ATOM	504	OE2	GLU	1037	68.747	15.878	0.709	1.00	0.00	LX0 O
ATOM	505	C	GLU	1037	69.770	14.945	-2.688	1.00	0.00	LX0 C
ATOM	506	O	GLU	1037	69.910	15.022	-3.903	1.00	0.00	LX0 O
ATOM	507	N	ALA	1038	70.196	15.868	-1.814	1.00	0.00	LX0 N
ATOM	508	H	ALA	1038	69.993	15.722	-0.841	0.00	0.00	LX0 H
ATOM	509	CA	ALA	1038	70.679	17.169	-2.282	1.00	0.00	LX0 C
ATOM	510	CB	ALA	1038	70.925	18.105	-1.098	1.00	0.00	LX0 C
ATOM	511	C	ALA	1038	71.914	17.190	-3.170	1.00	0.00	LX0 C
ATOM	512	O	ALA	1038	72.108	18.112	-3.953	1.00	0.00	LX0 O
ATOM	513	N	ALA	1039	72.747	16.144	-3.019	1.00	0.00	LX0 N
ATOM	514	H	ALA	1039	72.592	15.486	-2.282	0.00	0.00	LX0 H
ATOM	515	CA	ALA	1039	73.946	16.059	-3.860	1.00	0.00	LX0 C
ATOM	516	CB	ALA	1039	74.666	14.738	-3.589	1.00	0.00	LX0 C
ATOM	517	C	ALA	1039	73.651	16.165	-5.352	1.00	0.00	LX0 C
ATOM	518	O	ALA	1039	72.590	15.765	-5.822	1.00	0.00	LX0 O
ATOM	519	N	SER	1040	74.610	16.731	-6.086	1.00	0.00	LX0 N
ATOM	520	H	SER	1040	75.535	16.837	-5.715	0.00	0.00	LX0 H
ATOM	521	CA	SER	1040	74.300	16.879	-7.501	1.00	0.00	LX0 C
ATOM	522	CB	SER	1040	75.013	18.116	-8.076	1.00	0.00	LX0 C
ATOM	523	OG	SER	1040	76.442	17.984	-8.018	1.00	0.00	LX0 O
ATOM	524	HG	SER	1040	76.692	17.941	-7.093	0.00	0.00	LX0 H
ATOM	525	C	SER	1040	74.536	15.598	-8.288	1.00	0.00	LX0 C
ATOM	526	O	SER	1040	73.617	14.847	-8.604	1.00	0.00	LX0 O
ATOM	527	N	MET	1041	75.824	15.374	-8.569	1.00	0.00	LX0 N
ATOM	528	H	MET	1041	76.480	15.963	-8.091	0.00	0.00	LX0 H
ATOM	529	CA	MET	1041	76.241	14.209	-9.338	1.00	0.00	LX0 C
ATOM	530	CB	MET	1041	75.931	14.339	-10.843	1.00	0.00	LX0 C
ATOM	531	CG	MET	1041	76.766	15.342	-11.652	1.00	0.00	LX0 C
ATOM	532	SD	MET	1041	76.531	17.054	-11.156	1.00	0.00	LX0 S
ATOM	533	CE	MET	1041	77.831	17.788	-12.159	1.00	0.00	LX0 C
ATOM	534	C	MET	1041	77.689	13.832	-9.103	1.00	0.00	LX0 C
ATOM	535	O	MET	1041	78.041	12.661	-9.106	1.00	0.00	LX0 O
ATOM	536	N	ARG	1042	78.526	14.864	-8.891	1.00	0.00	LX0 N
ATOM	537	H	ARG	1042	78.157	15.788	-8.781	0.00	0.00	LX0 H
ATOM	538	CA	ARG	1042	79.954	14.580	-8.715	1.00	0.00	LX0 C
ATOM	539	CB	ARG	1042	80.732	15.892	-8.610	1.00	0.00	LX0 C
ATOM	540	CG	ARG	1042	81.153	16.420	-9.981	1.00	0.00	LX0 C
ATOM	541	CD	ARG	1042	82.637	16.176	-10.291	1.00	0.00	LX0 C
ATOM	542	NE	ARG	1042	83.033	14.781	-10.099	1.00	0.00	LX0 N
ATOM	543	HE	ARG	1042	83.271	14.428	-9.185	0.00	0.00	LX0 H
ATOM	544	CZ	ARG	1042	83.107	13.896	-11.112	1.00	0.00	LX0 C
ATOM	545	NH1	ARG	1042	82.806	14.256	-12.353	1.00	0.00	LX0 N
ATOM	546	HH11	ARG	1042	82.882	13.569	-13.098	0.00	0.00	LX0 H
ATOM	547	HH12	ARG	1042	82.518	15.175	-12.594	0.00	0.00	LX0 H
ATOM	548	NH2	ARG	1042	83.489	12.655	-10.867	1.00	0.00	LX0 N
ATOM	549	HH21	ARG	1042	83.520	11.982	-11.613	0.00	0.00	LX0 H
ATOM	550	HH22	ARG	1042	83.758	12.352	-9.935	0.00	0.00	LX0 H
ATOM	551	C	ARG	1042	80.240	13.677	-7.532	1.00	0.00	LX0 C
ATOM	552	O	ARG	1042	80.807	12.601	-7.642	1.00	0.00	LX0 O
ATOM	553	N	GLU	1043	79.732	14.160	-6.394	1.00	0.00	LX0 N
ATOM	554	H	GLU	1043	79.228	15.020	-6.472	0.00	0.00	LX0 H
ATOM	555	CA	GLU	1043	79.739	13.411	-5.134	1.00	0.00	LX0 C
ATOM	556	CB	GLU	1043	78.820	14.047	-4.076	1.00	0.00	LX0 C
ATOM	557	CG	GLU	1043	78.524	15.555	-4.150	1.00	0.00	LX0 C
ATOM	558	CD	GLU	1043	77.629	15.922	-5.333	1.00	0.00	LX0 C

ATOM	559	OE1	GLU	1043	77.060	15.046	-5.985	1.00	0.00	LX0	O
ATOM	560	OE2	GLU	1043	77.525	17.101	-5.635	1.00	0.00	LX0	O
ATOM	561	C	GLU	1043	79.286	11.973	-5.305	1.00	0.00	LX0	C
ATOM	562	O	GLU	1043	79.884	11.018	-4.821	1.00	0.00	LX0	O
ATOM	563	N	ARG	1044	78.180	11.875	-6.062	1.00	0.00	LX0	N
ATOM	564	H	ARG	1044	77.768	12.730	-6.383	0.00	0.00	LX0	H
ATOM	565	CA	ARG	1044	77.647	10.550	-6.374	1.00	0.00	LX0	C
ATOM	566	CB	ARG	1044	76.347	10.638	-7.176	1.00	0.00	LX0	C
ATOM	567	CG	ARG	1044	75.356	11.648	-6.604	1.00	0.00	LX0	C
ATOM	568	CD	ARG	1044	74.003	11.607	-7.314	1.00	0.00	LX0	C
ATOM	569	NE	ARG	1044	73.098	12.625	-6.785	1.00	0.00	LX0	N
ATOM	570	HE	ARG	1044	73.037	13.497	-7.286	0.00	0.00	LX0	H
ATOM	571	CZ	ARG	1044	72.443	12.453	-5.616	1.00	0.00	LX0	C
ATOM	572	NH1	ARG	1044	72.531	11.329	-4.916	1.00	0.00	LX0	N
ATOM	573	HH11	ARG	1044	72.097	11.277	-4.007	0.00	0.00	LX0	H
ATOM	574	HH12	ARG	1044	73.030	10.528	-5.259	0.00	0.00	LX0	H
ATOM	575	NH2	ARG	1044	71.709	13.436	-5.137	1.00	0.00	LX0	N
ATOM	576	HH21	ARG	1044	71.204	13.316	-4.277	0.00	0.00	LX0	H
ATOM	577	HH22	ARG	1044	71.659	14.335	-5.587	0.00	0.00	LX0	H
ATOM	578	C	ARG	1044	78.632	9.645	-7.086	1.00	0.00	LX0	C
ATOM	579	O	ARG	1044	78.863	8.510	-6.689	1.00	0.00	LX0	O
ATOM	580	N	ILE	1045	79.225	10.209	-8.150	1.00	0.00	LX0	N
ATOM	581	H	ILE	1045	79.059	11.175	-8.356	0.00	0.00	LX0	H
ATOM	582	CA	ILE	1045	80.209	9.431	-8.902	1.00	0.00	LX0	C
ATOM	583	CB	ILE	1045	80.662	10.183	-10.166	1.00	0.00	LX0	C
ATOM	584	CG2	ILE	1045	81.685	9.386	-10.984	1.00	0.00	LX0	C
ATOM	585	CG1	ILE	1045	79.448	10.545	-11.028	1.00	0.00	LX0	C
ATOM	586	CD1	ILE	1045	79.773	11.524	-12.157	1.00	0.00	LX0	C
ATOM	587	C	ILE	1045	81.382	8.992	-8.043	1.00	0.00	LX0	C
ATOM	588	O	ILE	1045	81.732	7.822	-8.012	1.00	0.00	LX0	O
ATOM	589	N	GLU	1046	81.929	9.952	-7.287	1.00	0.00	LX0	N
ATOM	590	H	GLU	1046	81.689	10.914	-7.430	0.00	0.00	LX0	H
ATOM	591	CA	GLU	1046	83.038	9.584	-6.407	1.00	0.00	LX0	C
ATOM	592	CB	GLU	1046	83.651	10.825	-5.760	1.00	0.00	LX0	C
ATOM	593	CG	GLU	1046	84.823	11.388	-6.575	1.00	0.00	LX0	C
ATOM	594	CD	GLU	1046	84.375	11.790	-7.968	1.00	0.00	LX0	C
ATOM	595	OE1	GLU	1046	84.471	10.982	-8.889	1.00	0.00	LX0	O
ATOM	596	OE2	GLU	1046	83.928	12.918	-8.141	1.00	0.00	LX0	O
ATOM	597	C	GLU	1046	82.742	8.508	-5.383	1.00	0.00	LX0	C
ATOM	598	O	GLU	1046	83.536	7.605	-5.149	1.00	0.00	LX0	O
ATOM	599	N	PHE	1047	81.538	8.598	-4.808	1.00	0.00	LX0	N
ATOM	600	H	PHE	1047	80.928	9.363	-5.026	0.00	0.00	LX0	H
ATOM	601	CA	PHE	1047	81.167	7.533	-3.877	1.00	0.00	LX0	C
ATOM	602	CB	PHE	1047	79.941	7.973	-3.081	1.00	0.00	LX0	C
ATOM	603	CG	PHE	1047	79.958	7.480	-1.650	1.00	0.00	LX0	C
ATOM	604	CD1	PHE	1047	81.168	7.412	-0.922	1.00	0.00	LX0	C
ATOM	605	CD2	PHE	1047	78.730	7.131	-1.048	1.00	0.00	LX0	C
ATOM	606	CE1	PHE	1047	81.140	7.037	0.435	1.00	0.00	LX0	C
ATOM	607	CE2	PHE	1047	78.699	6.757	0.307	1.00	0.00	LX0	C
ATOM	608	CZ	PHE	1047	79.903	6.734	1.039	1.00	0.00	LX0	C
ATOM	609	C	PHE	1047	80.966	6.176	-4.544	1.00	0.00	LX0	C
ATOM	610	O	PHE	1047	81.332	5.120	-4.046	1.00	0.00	LX0	O
ATOM	611	N	LEU	1048	80.405	6.260	-5.756	1.00	0.00	LX0	N
ATOM	612	H	LEU	1048	80.116	7.151	-6.111	0.00	0.00	LX0	H
ATOM	613	CA	LEU	1048	80.271	5.053	-6.575	1.00	0.00	LX0	C
ATOM	614	CB	LEU	1048	79.370	5.359	-7.776	1.00	0.00	LX0	C
ATOM	615	CG	LEU	1048	77.846	5.234	-7.577	1.00	0.00	LX0	C
ATOM	616	CD1	LEU	1048	77.307	5.639	-6.201	1.00	0.00	LX0	C
ATOM	617	CD2	LEU	1048	77.109	5.977	-8.690	1.00	0.00	LX0	C
ATOM	618	C	LEU	1048	81.618	4.485	-7.019	1.00	0.00	LX0	C
ATOM	619	O	LEU	1048	81.801	3.288	-7.224	1.00	0.00	LX0	O

ATOM	620	N	ASN	1049	82.581	5.412	-7.116	1.00	0.00	LX0	N
ATOM	621	H	ASN	1049	82.339	6.372	-6.981	0.00	0.00	LX0	H
ATOM	622	CA	ASN	1049	83.963	5.035	-7.399	1.00	0.00	LX0	C
ATOM	623	CB	ASN	1049	84.813	6.254	-7.785	1.00	0.00	LX0	C
ATOM	624	CG	ASN	1049	84.496	6.687	-9.204	1.00	0.00	LX0	C
ATOM	625	OD1	ASN	1049	84.242	5.870	-10.079	1.00	0.00	LX0	O
ATOM	626	ND2	ASN	1049	84.544	8.008	-9.414	1.00	0.00	LX0	N
ATOM	627	HD21	ASN	1049	84.701	8.720	-8.725	0.00	0.00	LX0	H
ATOM	628	HD22	ASN	1049	84.394	8.348	-10.339	0.00	0.00	LX0	H
ATOM	629	C	ASN	1049	84.584	4.291	-6.237	1.00	0.00	LX0	C
ATOM	630	O	ASN	1049	85.179	3.234	-6.401	1.00	0.00	LX0	O
ATOM	631	N	GLU	1050	84.356	4.848	-5.033	1.00	0.00	LX0	N
ATOM	632	H	GLU	1050	83.895	5.735	-4.982	0.00	0.00	LX0	H
ATOM	633	CA	GLU	1050	84.754	4.154	-3.804	1.00	0.00	LX0	C
ATOM	634	CB	GLU	1050	84.300	4.958	-2.584	1.00	0.00	LX0	C
ATOM	635	CG	GLU	1050	84.685	4.297	-1.261	1.00	0.00	LX0	C
ATOM	636	CD	GLU	1050	83.481	4.267	-0.348	1.00	0.00	LX0	C
ATOM	637	OE1	GLU	1050	82.565	3.489	-0.598	1.00	0.00	LX0	O
ATOM	638	OE2	GLU	1050	83.452	4.994	0.642	1.00	0.00	LX0	O
ATOM	639	C	GLU	1050	84.228	2.725	-3.742	1.00	0.00	LX0	C
ATOM	640	O	GLU	1050	84.973	1.755	-3.642	1.00	0.00	LX0	O
ATOM	641	N	ALA	1051	82.899	2.641	-3.914	1.00	0.00	LX0	N
ATOM	642	H	ALA	1051	82.362	3.482	-3.813	0.00	0.00	LX0	H
ATOM	643	CA	ALA	1051	82.251	1.338	-4.061	1.00	0.00	LX0	C
ATOM	644	CB	ALA	1051	80.792	1.533	-4.472	1.00	0.00	LX0	C
ATOM	645	C	ALA	1051	82.926	0.422	-5.074	1.00	0.00	LX0	C
ATOM	646	O	ALA	1051	83.151	-0.764	-4.856	1.00	0.00	LX0	O
ATOM	647	N	SER	1052	83.279	1.044	-6.204	1.00	0.00	LX0	N
ATOM	648	H	SER	1052	83.087	2.018	-6.325	0.00	0.00	LX0	H
ATOM	649	CA	SER	1052	83.991	0.274	-7.215	1.00	0.00	LX0	C
ATOM	650	CB	SER	1052	83.936	1.004	-8.554	1.00	0.00	LX0	C
ATOM	651	OG	SER	1052	82.549	1.141	-8.903	1.00	0.00	LX0	O
ATOM	652	HG	SER	1052	82.267	1.968	-8.503	0.00	0.00	LX0	H
ATOM	653	C	SER	1052	85.373	-0.238	-6.847	1.00	0.00	LX0	C
ATOM	654	O	SER	1052	85.770	-1.322	-7.251	1.00	0.00	LX0	O
ATOM	655	N	VAL	1053	86.067	0.533	-6.001	1.00	0.00	LX0	N
ATOM	656	H	VAL	1053	85.698	1.414	-5.696	0.00	0.00	LX0	H
ATOM	657	CA	VAL	1053	87.299	-0.032	-5.445	1.00	0.00	LX0	C
ATOM	658	CB	VAL	1053	88.103	1.058	-4.715	1.00	0.00	LX0	C
ATOM	659	CG1	VAL	1053	89.486	0.563	-4.296	1.00	0.00	LX0	C
ATOM	660	CG2	VAL	1053	88.234	2.321	-5.570	1.00	0.00	LX0	C
ATOM	661	C	VAL	1053	87.002	-1.228	-4.539	1.00	0.00	LX0	C
ATOM	662	O	VAL	1053	87.596	-2.298	-4.611	1.00	0.00	LX0	O
ATOM	663	N	MET	1054	85.974	-1.010	-3.705	1.00	0.00	LX0	N
ATOM	664	H	MET	1054	85.538	-0.108	-3.699	0.00	0.00	LX0	H
ATOM	665	CA	MET	1054	85.530	-2.081	-2.810	1.00	0.00	LX0	C
ATOM	666	CB	MET	1054	84.486	-1.546	-1.834	1.00	0.00	LX0	C
ATOM	667	CG	MET	1054	85.109	-0.523	-0.886	1.00	0.00	LX0	C
ATOM	668	SD	MET	1054	86.340	-1.281	0.181	1.00	0.00	LX0	S
ATOM	669	CE	MET	1054	85.221	-2.101	1.325	1.00	0.00	LX0	C
ATOM	670	C	MET	1054	85.058	-3.366	-3.476	1.00	0.00	LX0	C
ATOM	671	O	MET	1054	85.081	-4.445	-2.884	1.00	0.00	LX0	O
ATOM	672	N	LYS	1055	84.672	-3.228	-4.758	1.00	0.00	LX0	N
ATOM	673	H	LYS	1055	84.549	-2.304	-5.125	0.00	0.00	LX0	H
ATOM	674	CA	LYS	1055	84.441	-4.425	-5.574	1.00	0.00	LX0	C
ATOM	675	CB	LYS	1055	84.123	-4.060	-7.028	1.00	0.00	LX0	C
ATOM	676	CG	LYS	1055	82.799	-3.310	-7.147	1.00	0.00	LX0	C
ATOM	677	CD	LYS	1055	82.515	-2.675	-8.509	1.00	0.00	LX0	C
ATOM	678	CE	LYS	1055	81.218	-1.866	-8.428	1.00	0.00	LX0	C
ATOM	679	NZ	LYS	1055	81.061	-0.965	-9.570	1.00	0.00	LX0	N
ATOM	680	HZ1	LYS	1055	80.155	-0.462	-9.520	0.00	0.00	LX0	H

ATOM	681	HZ2	LYS	1055	81.799	-0.235	-9.647	0.00	0.00	LX0	H
ATOM	682	HZ3	LYS	1055	80.992	-1.471	-10.484	0.00	0.00	LX0	H
ATOM	683	C	LYS	1055	85.593	-5.413	-5.527	1.00	0.00	LX0	C
ATOM	684	O	LYS	1055	85.416	-6.570	-5.165	1.00	0.00	LX0	O
ATOM	685	N	GLU	1056	86.784	-4.886	-5.853	1.00	0.00	LX0	N
ATOM	686	H	GLU	1056	86.877	-3.899	-6.006	0.00	0.00	LX0	H
ATOM	687	CA	GLU	1056	87.950	-5.760	-6.005	1.00	0.00	LX0	C
ATOM	688	CB	GLU	1056	89.145	-4.960	-6.521	1.00	0.00	LX0	C
ATOM	689	CG	GLU	1056	90.143	-5.854	-7.258	1.00	0.00	LX0	C
ATOM	690	CD	GLU	1056	91.541	-5.306	-7.091	1.00	0.00	LX0	C
ATOM	691	OE1	GLU	1056	92.070	-4.691	-8.013	1.00	0.00	LX0	O
ATOM	692	OE2	GLU	1056	92.145	-5.536	-6.049	1.00	0.00	LX0	O
ATOM	693	C	GLU	1056	88.363	-6.555	-4.770	1.00	0.00	LX0	C
ATOM	694	O	GLU	1056	88.959	-7.623	-4.840	1.00	0.00	LX0	O
ATOM	695	N	PHE	1057	88.016	-5.989	-3.605	1.00	0.00	LX0	N
ATOM	696	H	PHE	1057	87.518	-5.123	-3.610	0.00	0.00	LX0	H
ATOM	697	CA	PHE	1057	88.498	-6.620	-2.377	1.00	0.00	LX0	C
ATOM	698	CB	PHE	1057	88.587	-5.594	-1.247	1.00	0.00	LX0	C
ATOM	699	CG	PHE	1057	89.413	-4.402	-1.666	1.00	0.00	LX0	C
ATOM	700	CD1	PHE	1057	90.714	-4.586	-2.185	1.00	0.00	LX0	C
ATOM	701	CD2	PHE	1057	88.854	-3.116	-1.524	1.00	0.00	LX0	C
ATOM	702	CE1	PHE	1057	91.467	-3.462	-2.569	1.00	0.00	LX0	C
ATOM	703	CE2	PHE	1057	89.608	-1.993	-1.907	1.00	0.00	LX0	C
ATOM	704	CZ	PHE	1057	90.907	-2.177	-2.425	1.00	0.00	LX0	C
ATOM	705	C	PHE	1057	87.735	-7.847	-1.906	1.00	0.00	LX0	C
ATOM	706	O	PHE	1057	87.132	-7.860	-0.840	1.00	0.00	LX0	O
ATOM	707	N	ASN	1058	87.748	-8.888	-2.741	1.00	0.00	LX0	N
ATOM	708	H	ASN	1058	88.391	-8.931	-3.512	0.00	0.00	LX0	H
ATOM	709	CA	ASN	1058	86.790	-9.950	-2.464	1.00	0.00	LX0	C
ATOM	710	CB	ASN	1058	86.166	-10.469	-3.758	1.00	0.00	LX0	C
ATOM	711	CG	ASN	1058	84.766	-10.949	-3.441	1.00	0.00	LX0	C
ATOM	712	OD1	ASN	1058	83.838	-10.161	-3.296	1.00	0.00	LX0	O
ATOM	713	ND2	ASN	1058	84.646	-12.272	-3.329	1.00	0.00	LX0	N
ATOM	714	HD21	ASN	1058	85.479	-12.832	-3.338	0.00	0.00	LX0	H
ATOM	715	HD22	ASN	1058	83.752	-12.698	-3.213	0.00	0.00	LX0	H
ATOM	716	C	ASN	1058	87.264	-11.088	-1.583	1.00	0.00	LX0	C
ATOM	717	O	ASN	1058	87.113	-12.259	-1.911	1.00	0.00	LX0	O
ATOM	718	N	CYS	1059	87.827	-10.702	-0.435	1.00	0.00	LX0	N
ATOM	719	H	CYS	1059	87.834	-9.738	-0.163	0.00	0.00	LX0	H
ATOM	720	CA	CYS	1059	88.154	-11.773	0.500	1.00	0.00	LX0	C
ATOM	721	CB	CYS	1059	89.651	-11.840	0.782	1.00	0.00	LX0	C
ATOM	722	SG	CYS	1059	90.126	-13.401	1.565	1.00	0.00	LX0	S
ATOM	723	C	CYS	1059	87.370	-11.616	1.779	1.00	0.00	LX0	C
ATOM	724	O	CYS	1059	86.846	-10.546	2.066	1.00	0.00	LX0	O
ATOM	725	N	HIS	1060	87.294	-12.730	2.529	1.00	0.00	LX0	N
ATOM	726	H	HIS	1060	87.815	-13.542	2.263	0.00	0.00	LX0	H
ATOM	727	CA	HIS	1060	86.394	-12.765	3.683	1.00	0.00	LX0	C
ATOM	728	CB	HIS	1060	86.529	-14.096	4.429	1.00	0.00	LX0	C
ATOM	729	CG	HIS	1060	85.268	-14.416	5.202	1.00	0.00	LX0	C
ATOM	730	ND1	HIS	1060	84.431	-15.412	4.858	1.00	0.00	LX0	N
ATOM	731	HD1	HIS	1060	84.550	-16.041	4.118	0.00	0.00	LX0	H
ATOM	732	CD2	HIS	1060	84.758	-13.778	6.338	1.00	0.00	LX0	C
ATOM	733	NE2	HIS	1060	83.602	-14.403	6.666	1.00	0.00	LX0	N
ATOM	734	CE1	HIS	1060	83.400	-15.409	5.759	1.00	0.00	LX0	C
ATOM	735	C	HIS	1060	86.535	-11.595	4.638	1.00	0.00	LX0	C
ATOM	736	O	HIS	1060	85.582	-10.870	4.903	1.00	0.00	LX0	O
ATOM	737	N	HIS	1061	87.772	-11.423	5.127	1.00	0.00	LX0	N
ATOM	738	H	HIS	1061	88.539	-12.007	4.856	0.00	0.00	LX0	H
ATOM	739	CA	HIS	1061	87.942	-10.396	6.152	1.00	0.00	LX0	C
ATOM	740	CB	HIS	1061	89.034	-10.796	7.150	1.00	0.00	LX0	C
ATOM	741	CG	HIS	1061	88.693	-12.146	7.738	1.00	0.00	LX0	C

ATOM	742	ND1	HIS	1061	89.393	-13.263	7.481	1.00	0.00	LX0 N
ATOM	743	HD1	HIS	1061	90.214	-13.325	6.941	0.00	0.00	LX0 H
ATOM	744	CD2	HIS	1061	87.625	-12.471	8.579	1.00	0.00	LX0 C
ATOM	745	NE2	HIS	1061	87.693	-13.804	8.815	1.00	0.00	LX0 N
ATOM	746	CE1	HIS	1061	88.781	-14.293	8.140	1.00	0.00	LX0 C
ATOM	747	C	HIS	1061	88.125	-8.970	5.659	1.00	0.00	LX0 C
ATOM	748	O	HIS	1061	88.718	-8.131	6.324	1.00	0.00	LX0 O
ATOM	749	N	VAL	1062	87.565	-8.708	4.471	1.00	0.00	LX0 N
ATOM	750	H	VAL	1062	87.083	-9.400	3.932	0.00	0.00	LX0 H
ATOM	751	CA	VAL	1062	87.385	-7.305	4.112	1.00	0.00	LX0 C
ATOM	752	CB	VAL	1062	88.004	-7.010	2.731	1.00	0.00	LX0 C
ATOM	753	CG1	VAL	1062	88.143	-5.504	2.482	1.00	0.00	LX0 C
ATOM	754	CG2	VAL	1062	89.355	-7.707	2.544	1.00	0.00	LX0 C
ATOM	755	C	VAL	1062	85.886	-7.042	4.116	1.00	0.00	LX0 C
ATOM	756	O	VAL	1062	85.094	-7.971	3.962	1.00	0.00	LX0 O
ATOM	757	N	VAL	1063	85.511	-5.767	4.298	1.00	0.00	LX0 N
ATOM	758	H	VAL	1063	86.188	-5.072	4.555	0.00	0.00	LX0 H
ATOM	759	CA	VAL	1063	84.113	-5.423	4.034	1.00	0.00	LX0 C
ATOM	760	CB	VAL	1063	83.833	-3.969	4.453	1.00	0.00	LX0 C
ATOM	761	CG1	VAL	1063	82.418	-3.470	4.127	1.00	0.00	LX0 C
ATOM	762	CG2	VAL	1063	84.103	-3.829	5.947	1.00	0.00	LX0 C
ATOM	763	C	VAL	1063	83.743	-5.686	2.580	1.00	0.00	LX0 C
ATOM	764	O	VAL	1063	84.510	-5.465	1.642	1.00	0.00	LX0 O
ATOM	765	N	ARG	1064	82.537	-6.225	2.418	1.00	0.00	LX0 N
ATOM	766	H	ARG	1064	81.943	-6.440	3.199	0.00	0.00	LX0 H
ATOM	767	CA	ARG	1064	82.168	-6.468	1.038	1.00	0.00	LX0 C
ATOM	768	CB	ARG	1064	81.634	-7.888	0.844	1.00	0.00	LX0 C
ATOM	769	CG	ARG	1064	82.694	-8.946	1.173	1.00	0.00	LX0 C
ATOM	770	CD	ARG	1064	84.041	-8.684	0.486	1.00	0.00	LX0 C
ATOM	771	NE	ARG	1064	83.894	-8.569	-0.964	1.00	0.00	LX0 N
ATOM	772	HE	ARG	1064	83.533	-9.354	-1.478	0.00	0.00	LX0 H
ATOM	773	CZ	ARG	1064	84.286	-7.469	-1.644	1.00	0.00	LX0 C
ATOM	774	NH1	ARG	1064	84.746	-6.383	-1.021	1.00	0.00	LX0 N
ATOM	775	HH11	ARG	1064	85.032	-5.576	-1.548	0.00	0.00	LX0 H
ATOM	776	HH12	ARG	1064	84.836	-6.339	-0.017	0.00	0.00	LX0 H
ATOM	777	NH2	ARG	1064	84.205	-7.503	-2.968	1.00	0.00	LX0 N
ATOM	778	HH21	ARG	1064	84.525	-6.760	-3.564	0.00	0.00	LX0 H
ATOM	779	HH22	ARG	1064	83.831	-8.326	-3.413	0.00	0.00	LX0 H
ATOM	780	C	ARG	1064	81.257	-5.428	0.445	1.00	0.00	LX0 C
ATOM	781	O	ARG	1064	80.270	-5.006	1.029	1.00	0.00	LX0 O
ATOM	782	N	LEU	1065	81.624	-5.039	-0.782	1.00	0.00	LX0 N
ATOM	783	H	LEU	1065	82.425	-5.426	-1.226	0.00	0.00	LX0 H
ATOM	784	CA	LEU	1065	80.561	-4.405	-1.548	1.00	0.00	LX0 C
ATOM	785	CB	LEU	1065	81.104	-3.567	-2.705	1.00	0.00	LX0 C
ATOM	786	CG	LEU	1065	79.979	-2.758	-3.358	1.00	0.00	LX0 C
ATOM	787	CD1	LEU	1065	79.602	-1.542	-2.524	1.00	0.00	LX0 C
ATOM	788	CD2	LEU	1065	80.264	-2.388	-4.803	1.00	0.00	LX0 C
ATOM	789	C	LEU	1065	79.667	-5.497	-2.087	1.00	0.00	LX0 C
ATOM	790	O	LEU	1065	80.148	-6.481	-2.632	1.00	0.00	LX0 O
ATOM	791	N	LEU	1066	78.371	-5.281	-1.883	1.00	0.00	LX0 N
ATOM	792	H	LEU	1066	78.081	-4.469	-1.379	0.00	0.00	LX0 H
ATOM	793	CA	LEU	1066	77.407	-6.189	-2.487	1.00	0.00	LX0 C
ATOM	794	CB	LEU	1066	76.369	-6.616	-1.447	1.00	0.00	LX0 C
ATOM	795	CG	LEU	1066	77.017	-7.307	-0.240	1.00	0.00	LX0 C
ATOM	796	CD1	LEU	1066	76.008	-7.590	0.870	1.00	0.00	LX0 C
ATOM	797	CD2	LEU	1066	77.786	-8.571	-0.635	1.00	0.00	LX0 C
ATOM	798	C	LEU	1066	76.768	-5.617	-3.740	1.00	0.00	LX0 C
ATOM	799	O	LEU	1066	76.318	-6.343	-4.614	1.00	0.00	LX0 O
ATOM	800	N	GLY	1067	76.780	-4.276	-3.810	1.00	0.00	LX0 N
ATOM	801	H	GLY	1067	77.058	-3.689	-3.047	0.00	0.00	LX0 H
ATOM	802	CA	GLY	1067	76.337	-3.680	-5.065	1.00	0.00	LX0 C

ATOM	803	C	GLY	1067	76.309	-2.169	-5.028	1.00	0.00	LX0	C
ATOM	804	O	GLY	1067	76.647	-1.537	-4.034	1.00	0.00	LX0	O
ATOM	805	N	VAL	1068	75.896	-1.619	-6.168	1.00	0.00	LX0	N
ATOM	806	H	VAL	1068	75.580	-2.205	-6.917	0.00	0.00	LX0	H
ATOM	807	CA	VAL	1068	75.715	-0.179	-6.321	1.00	0.00	LX0	C
ATOM	808	CB	VAL	1068	76.696	0.344	-7.389	1.00	0.00	LX0	C
ATOM	809	CG1	VAL	1068	76.529	1.833	-7.694	1.00	0.00	LX0	C
ATOM	810	CG2	VAL	1068	78.143	0.042	-6.999	1.00	0.00	LX0	C
ATOM	811	C	VAL	1068	74.276	0.006	-6.764	1.00	0.00	LX0	C
ATOM	812	O	VAL	1068	73.731	-0.876	-7.407	1.00	0.00	LX0	O
ATOM	813	N	VAL	1069	73.678	1.141	-6.384	1.00	0.00	LX0	N
ATOM	814	H	VAL	1069	74.161	1.784	-5.794	0.00	0.00	LX0	H
ATOM	815	CA	VAL	1069	72.410	1.486	-7.022	1.00	0.00	LX0	C
ATOM	816	CB	VAL	1069	71.185	1.291	-6.113	1.00	0.00	LX0	C
ATOM	817	CG1	VAL	1069	69.899	1.344	-6.945	1.00	0.00	LX0	C
ATOM	818	CG2	VAL	1069	71.243	0.010	-5.284	1.00	0.00	LX0	C
ATOM	819	C	VAL	1069	72.460	2.919	-7.512	1.00	0.00	LX0	C
ATOM	820	O	VAL	1069	71.989	3.852	-6.860	1.00	0.00	LX0	O
ATOM	821	N	SER	1070	73.078	3.060	-8.687	1.00	0.00	LX0	N
ATOM	822	H	SER	1070	73.292	2.215	-9.189	0.00	0.00	LX0	H
ATOM	823	CA	SER	1070	73.130	4.371	-9.326	1.00	0.00	LX0	C
ATOM	824	CB	SER	1070	74.048	4.314	-10.554	1.00	0.00	LX0	C
ATOM	825	OG	SER	1070	73.526	3.413	-11.541	1.00	0.00	LX0	O
ATOM	826	HG	SER	1070	73.612	2.523	-11.186	0.00	0.00	LX0	H
ATOM	827	C	SER	1070	71.754	4.904	-9.697	1.00	0.00	LX0	C
ATOM	828	O	SER	1070	71.439	6.085	-9.595	1.00	0.00	LX0	O
ATOM	829	N	GLN	1071	70.920	3.949	-10.126	1.00	0.00	LX0	N
ATOM	830	H	GLN	1071	71.242	3.000	-10.171	0.00	0.00	LX0	H
ATOM	831	CA	GLN	1071	69.600	4.343	-10.594	1.00	0.00	LX0	C
ATOM	832	CB	GLN	1071	68.951	3.203	-11.387	1.00	0.00	LX0	C
ATOM	833	CG	GLN	1071	69.828	2.664	-12.525	1.00	0.00	LX0	C
ATOM	834	CD	GLN	1071	70.208	3.786	-13.471	1.00	0.00	LX0	C
ATOM	835	OE1	GLN	1071	69.378	4.386	-14.140	1.00	0.00	LX0	O
ATOM	836	NE2	GLN	1071	71.512	4.062	-13.487	1.00	0.00	LX0	N
ATOM	837	HE21	GLN	1071	72.157	3.568	-12.895	0.00	0.00	LX0	H
ATOM	838	HE22	GLN	1071	71.859	4.776	-14.088	0.00	0.00	LX0	H
ATOM	839	C	GLN	1071	68.687	4.842	-9.497	1.00	0.00	LX0	C
ATOM	840	O	GLN	1071	68.184	4.089	-8.667	1.00	0.00	LX0	O
ATOM	841	N	GLY	1072	68.484	6.162	-9.549	1.00	0.00	LX0	N
ATOM	842	H	GLY	1072	69.138	6.705	-10.078	0.00	0.00	LX0	H
ATOM	843	CA	GLY	1072	67.447	6.756	-8.713	1.00	0.00	LX0	C
ATOM	844	C	GLY	1072	67.958	7.427	-7.455	1.00	0.00	LX0	C
ATOM	845	O	GLY	1072	69.037	7.156	-6.942	1.00	0.00	LX0	O
ATOM	846	N	GLN	1073	67.105	8.335	-6.971	1.00	0.00	LX0	N
ATOM	847	H	GLN	1073	66.203	8.435	-7.386	0.00	0.00	LX0	H
ATOM	848	CA	GLN	1073	67.422	8.998	-5.710	1.00	0.00	LX0	C
ATOM	849	CB	GLN	1073	66.744	10.370	-5.678	1.00	0.00	LX0	C
ATOM	850	CG	GLN	1073	67.427	11.418	-6.560	1.00	0.00	LX0	C
ATOM	851	CD	GLN	1073	68.582	12.058	-5.813	1.00	0.00	LX0	C
ATOM	852	OE1	GLN	1073	69.452	11.410	-5.242	1.00	0.00	LX0	O
ATOM	853	NE2	GLN	1073	68.524	13.391	-5.822	1.00	0.00	LX0	N
ATOM	854	HE21	GLN	1073	67.815	13.880	-6.327	0.00	0.00	LX0	H
ATOM	855	HE22	GLN	1073	69.183	13.944	-5.311	0.00	0.00	LX0	H
ATOM	856	C	GLN	1073	66.986	8.163	-4.515	1.00	0.00	LX0	C
ATOM	857	O	GLN	1073	65.967	7.486	-4.567	1.00	0.00	LX0	O
ATOM	858	N	PRO	1074	67.790	8.216	-3.427	1.00	0.00	LX0	N
ATOM	859	CD	PRO	1074	67.391	7.794	-2.088	1.00	0.00	LX0	C
ATOM	860	CA	PRO	1074	69.183	8.670	-3.497	1.00	0.00	LX0	C
ATOM	861	CB	PRO	1074	69.487	8.946	-2.023	1.00	0.00	LX0	C
ATOM	862	CG	PRO	1074	68.659	7.922	-1.249	1.00	0.00	LX0	C
ATOM	863	C	PRO	1074	70.037	7.563	-4.102	1.00	0.00	LX0	C

ATOM	864	O	PRO	1074	69.609	6.414	-4.213	1.00	0.00	LX0	O
ATOM	865	N	THR	1075	71.244	7.952	-4.508	1.00	0.00	LX0	N
ATOM	866	H	THR	1075	71.583	8.872	-4.310	0.00	0.00	LX0	H
ATOM	867	CA	THR	1075	72.135	6.939	-5.067	1.00	0.00	LX0	C
ATOM	868	CB	THR	1075	73.219	7.663	-5.853	1.00	0.00	LX0	C
ATOM	869	OG1	THR	1075	73.572	8.872	-5.161	1.00	0.00	LX0	O
ATOM	870	HG1	THR	1075	73.847	8.582	-4.293	0.00	0.00	LX0	H
ATOM	871	CG2	THR	1075	72.745	7.995	-7.267	1.00	0.00	LX0	C
ATOM	872	C	THR	1075	72.718	6.094	-3.953	1.00	0.00	LX0	C
ATOM	873	O	THR	1075	73.102	6.620	-2.914	1.00	0.00	LX0	O
ATOM	874	N	LEU	1076	72.714	4.778	-4.177	1.00	0.00	LX0	N
ATOM	875	H	LEU	1076	72.512	4.393	-5.079	0.00	0.00	LX0	H
ATOM	876	CA	LEU	1076	73.044	3.928	-3.039	1.00	0.00	LX0	C
ATOM	877	CB	LEU	1076	71.930	2.909	-2.787	1.00	0.00	LX0	C
ATOM	878	CG	LEU	1076	70.508	3.475	-2.750	1.00	0.00	LX0	C
ATOM	879	CD1	LEU	1076	69.463	2.365	-2.725	1.00	0.00	LX0	C
ATOM	880	CD2	LEU	1076	70.288	4.450	-1.599	1.00	0.00	LX0	C
ATOM	881	C	LEU	1076	74.352	3.193	-3.220	1.00	0.00	LX0	C
ATOM	882	O	LEU	1076	74.709	2.784	-4.317	1.00	0.00	LX0	O
ATOM	883	N	VAL	1077	75.029	3.016	-2.087	1.00	0.00	LX0	N
ATOM	884	H	VAL	1077	74.743	3.463	-1.237	0.00	0.00	LX0	H
ATOM	885	CA	VAL	1077	76.139	2.075	-2.041	1.00	0.00	LX0	C
ATOM	886	CB	VAL	1077	77.406	2.791	-1.547	1.00	0.00	LX0	C
ATOM	887	CG1	VAL	1077	78.579	1.838	-1.330	1.00	0.00	LX0	C
ATOM	888	CG2	VAL	1077	77.791	3.927	-2.498	1.00	0.00	LX0	C
ATOM	889	C	VAL	1077	75.728	0.942	-1.119	1.00	0.00	LX0	C
ATOM	890	O	VAL	1077	75.391	1.150	0.040	1.00	0.00	LX0	O
ATOM	891	N	ILE	1078	75.708	-0.259	-1.701	1.00	0.00	LX0	N
ATOM	892	H	ILE	1078	76.091	-0.396	-2.616	0.00	0.00	LX0	H
ATOM	893	CA	ILE	1078	75.207	-1.395	-0.932	1.00	0.00	LX0	C
ATOM	894	CB	ILE	1078	74.256	-2.245	-1.794	1.00	0.00	LX0	C
ATOM	895	CG2	ILE	1078	73.665	-3.431	-1.025	1.00	0.00	LX0	C
ATOM	896	CG1	ILE	1078	73.162	-1.381	-2.421	1.00	0.00	LX0	C
ATOM	897	CD1	ILE	1078	72.245	-0.731	-1.384	1.00	0.00	LX0	C
ATOM	898	C	ILE	1078	76.353	-2.234	-0.417	1.00	0.00	LX0	C
ATOM	899	O	ILE	1078	76.966	-3.000	-1.152	1.00	0.00	LX0	O
ATOM	900	N	MET	1079	76.630	-2.054	0.873	1.00	0.00	LX0	N
ATOM	901	H	MET	1079	76.105	-1.420	1.443	0.00	0.00	LX0	H
ATOM	902	CA	MET	1079	77.682	-2.884	1.447	1.00	0.00	LX0	C
ATOM	903	CB	MET	1079	78.718	-2.021	2.167	1.00	0.00	LX0	C
ATOM	904	CG	MET	1079	79.549	-1.241	1.158	1.00	0.00	LX0	C
ATOM	905	SD	MET	1079	80.897	-0.318	1.891	1.00	0.00	LX0	S
ATOM	906	CE	MET	1079	81.702	0.128	0.347	1.00	0.00	LX0	C
ATOM	907	C	MET	1079	77.158	-3.962	2.365	1.00	0.00	LX0	C
ATOM	908	O	MET	1079	75.982	-4.022	2.698	1.00	0.00	LX0	O
ATOM	909	N	GLU	1080	78.106	-4.806	2.771	1.00	0.00	LX0	N
ATOM	910	H	GLU	1080	79.029	-4.683	2.409	0.00	0.00	LX0	H
ATOM	911	CA	GLU	1080	77.889	-5.799	3.817	1.00	0.00	LX0	C
ATOM	912	CB	GLU	1080	79.181	-6.611	3.857	1.00	0.00	LX0	C
ATOM	913	CG	GLU	1080	79.349	-7.725	4.886	1.00	0.00	LX0	C
ATOM	914	CD	GLU	1080	80.765	-8.253	4.751	1.00	0.00	LX0	C
ATOM	915	OE1	GLU	1080	81.711	-7.489	4.930	1.00	0.00	LX0	O
ATOM	916	OE2	GLU	1080	80.945	-9.432	4.459	1.00	0.00	LX0	O
ATOM	917	C	GLU	1080	77.592	-5.103	5.135	1.00	0.00	LX0	C
ATOM	918	O	GLU	1080	78.175	-4.071	5.448	1.00	0.00	LX0	O
ATOM	919	N	LEU	1081	76.639	-5.671	5.889	1.00	0.00	LX0	N
ATOM	920	H	LEU	1081	76.170	-6.514	5.623	0.00	0.00	LX0	H
ATOM	921	CA	LEU	1081	76.363	-4.992	7.151	1.00	0.00	LX0	C
ATOM	922	CB	LEU	1081	74.966	-5.300	7.676	1.00	0.00	LX0	C
ATOM	923	CG	LEU	1081	74.571	-4.397	8.844	1.00	0.00	LX0	C
ATOM	924	CD1	LEU	1081	74.345	-2.945	8.434	1.00	0.00	LX0	C

ATOM	925	CD2	LEU	1081	73.378	-4.965	9.584	1.00	0.00	LX0	C
ATOM	926	C	LEU	1081	77.388	-5.225	8.241	1.00	0.00	LX0	C
ATOM	927	O	LEU	1081	77.330	-6.155	9.035	1.00	0.00	LX0	O
ATOM	928	N	MET	1082	78.317	-4.274	8.274	1.00	0.00	LX0	N
ATOM	929	H	MET	1082	78.350	-3.602	7.532	0.00	0.00	LX0	H
ATOM	930	CA	MET	1082	79.183	-4.236	9.445	1.00	0.00	LX0	C
ATOM	931	CB	MET	1082	80.573	-3.760	9.039	1.00	0.00	LX0	C
ATOM	932	CG	MET	1082	81.114	-4.582	7.867	1.00	0.00	LX0	C
ATOM	933	SD	MET	1082	81.294	-6.331	8.244	1.00	0.00	LX0	S
ATOM	934	CE	MET	1082	82.590	-6.165	9.474	1.00	0.00	LX0	C
ATOM	935	C	MET	1082	78.574	-3.384	10.538	1.00	0.00	LX0	C
ATOM	936	O	MET	1082	78.969	-2.249	10.784	1.00	0.00	LX0	O
ATOM	937	N	THR	1083	77.545	-3.985	11.156	1.00	0.00	LX0	N
ATOM	938	H	THR	1083	77.285	-4.923	10.913	0.00	0.00	LX0	H
ATOM	939	CA	THR	1083	76.627	-3.241	12.022	1.00	0.00	LX0	C
ATOM	940	CB	THR	1083	75.777	-4.226	12.825	1.00	0.00	LX0	C
ATOM	941	OG1	THR	1083	75.657	-5.478	12.137	1.00	0.00	LX0	O
ATOM	942	HG1	THR	1083	75.117	-6.021	12.713	0.00	0.00	LX0	H
ATOM	943	CG2	THR	1083	74.405	-3.638	13.165	1.00	0.00	LX0	C
ATOM	944	C	THR	1083	77.221	-2.215	12.974	1.00	0.00	LX0	C
ATOM	945	O	THR	1083	76.667	-1.155	13.232	1.00	0.00	LX0	O
ATOM	946	N	ARG	1084	78.364	-2.615	13.540	1.00	0.00	LX0	N
ATOM	947	H	ARG	1084	78.905	-3.367	13.156	0.00	0.00	LX0	H
ATOM	948	CA	ARG	1084	78.794	-1.872	14.715	1.00	0.00	LX0	C
ATOM	949	CB	ARG	1084	79.174	-2.873	15.795	1.00	0.00	LX0	C
ATOM	950	CG	ARG	1084	77.908	-3.652	16.123	1.00	0.00	LX0	C
ATOM	951	CD	ARG	1084	78.171	-5.000	16.757	1.00	0.00	LX0	C
ATOM	952	NE	ARG	1084	76.903	-5.672	16.997	1.00	0.00	LX0	N
ATOM	953	HE	ARG	1084	76.288	-5.888	16.230	0.00	0.00	LX0	H
ATOM	954	CZ	ARG	1084	76.556	-6.062	18.232	1.00	0.00	LX0	C
ATOM	955	NH1	ARG	1084	77.339	-5.783	19.264	1.00	0.00	LX0	N
ATOM	956	HH11	ARG	1084	77.088	-6.070	20.194	0.00	0.00	LX0	H
ATOM	957	HH12	ARG	1084	78.194	-5.288	19.134	0.00	0.00	LX0	H
ATOM	958	NH2	ARG	1084	75.425	-6.728	18.405	1.00	0.00	LX0	N
ATOM	959	HH21	ARG	1084	75.167	-7.085	19.299	0.00	0.00	LX0	H
ATOM	960	HH22	ARG	1084	74.834	-6.876	17.599	0.00	0.00	LX0	H
ATOM	961	C	ARG	1084	79.856	-0.828	14.475	1.00	0.00	LX0	C
ATOM	962	O	ARG	1084	80.537	-0.389	15.392	1.00	0.00	LX0	O
ATOM	963	N	GLY	1085	79.958	-0.433	13.198	1.00	0.00	LX0	N
ATOM	964	H	GLY	1085	79.458	-0.898	12.465	0.00	0.00	LX0	H
ATOM	965	CA	GLY	1085	80.894	0.650	12.922	1.00	0.00	LX0	C
ATOM	966	C	GLY	1085	82.334	0.235	13.128	1.00	0.00	LX0	C
ATOM	967	O	GLY	1085	82.750	-0.851	12.736	1.00	0.00	LX0	O
ATOM	968	N	ASP	1086	83.073	1.158	13.739	1.00	0.00	LX0	N
ATOM	969	H	ASP	1086	82.701	1.974	14.188	0.00	0.00	LX0	H
ATOM	970	CA	ASP	1086	84.512	0.967	13.779	1.00	0.00	LX0	C
ATOM	971	CB	ASP	1086	85.205	2.317	13.559	1.00	0.00	LX0	C
ATOM	972	CG	ASP	1086	84.996	3.249	14.739	1.00	0.00	LX0	C
ATOM	973	OD1	ASP	1086	83.864	3.628	15.016	1.00	0.00	LX0	O
ATOM	974	OD2	ASP	1086	85.968	3.571	15.406	1.00	0.00	LX0	O
ATOM	975	C	ASP	1086	85.026	0.270	15.023	1.00	0.00	LX0	C
ATOM	976	O	ASP	1086	84.476	0.339	16.118	1.00	0.00	LX0	O
ATOM	977	N	LEU	1087	86.166	-0.398	14.799	1.00	0.00	LX0	N
ATOM	978	H	LEU	1087	86.507	-0.413	13.859	0.00	0.00	LX0	H
ATOM	979	CA	LEU	1087	86.880	-1.076	15.881	1.00	0.00	LX0	C
ATOM	980	CB	LEU	1087	88.144	-1.731	15.309	1.00	0.00	LX0	C
ATOM	981	CG	LEU	1087	89.175	-2.310	16.290	1.00	0.00	LX0	C
ATOM	982	CD1	LEU	1087	88.677	-3.526	17.066	1.00	0.00	LX0	C
ATOM	983	CD2	LEU	1087	90.482	-2.632	15.578	1.00	0.00	LX0	C
ATOM	984	C	LEU	1087	87.210	-0.150	17.034	1.00	0.00	LX0	C
ATOM	985	O	LEU	1087	87.196	-0.525	18.198	1.00	0.00	LX0	O

ATOM	986	N	LYS	1088	87.516	1.094	16.657	1.00	0.00	LX0	N
ATOM	987	H	LYS	1088	87.306	1.423	15.735	0.00	0.00	LX0	H
ATOM	988	CA	LYS	1088	87.983	2.007	17.686	1.00	0.00	LX0	C
ATOM	989	CB	LYS	1088	88.611	3.201	16.985	1.00	0.00	LX0	C
ATOM	990	CG	LYS	1088	89.403	4.095	17.904	1.00	0.00	LX0	C
ATOM	991	CD	LYS	1088	90.531	4.795	17.174	1.00	0.00	LX0	C
ATOM	992	CE	LYS	1088	91.412	5.433	18.229	1.00	0.00	LX0	C
ATOM	993	NZ	LYS	1088	92.582	6.058	17.627	1.00	0.00	LX0	N
ATOM	994	HZ1	LYS	1088	93.024	6.664	18.347	0.00	0.00	LX0	H
ATOM	995	HZ2	LYS	1088	93.256	5.297	17.379	0.00	0.00	LX0	H
ATOM	996	HZ3	LYS	1088	92.311	6.639	16.800	0.00	0.00	LX0	H
ATOM	997	C	LYS	1088	86.923	2.375	18.710	1.00	0.00	LX0	C
ATOM	998	O	LYS	1088	87.118	2.268	19.917	1.00	0.00	LX0	O
ATOM	999	N	SER	1089	85.763	2.763	18.180	1.00	0.00	LX0	N
ATOM	1000	H	SER	1089	85.644	2.859	17.188	0.00	0.00	LX0	H
ATOM	1001	CA	SER	1089	84.654	3.041	19.084	1.00	0.00	LX0	C
ATOM	1002	CB	SER	1089	83.566	3.771	18.307	1.00	0.00	LX0	C
ATOM	1003	OG	SER	1089	84.198	4.825	17.567	1.00	0.00	LX0	O
ATOM	1004	HG	SER	1089	84.271	4.494	16.661	0.00	0.00	LX0	H
ATOM	1005	C	SER	1089	84.161	1.807	19.821	1.00	0.00	LX0	C
ATOM	1006	O	SER	1089	83.881	1.826	21.014	1.00	0.00	LX0	O
ATOM	1007	N	TYR	1090	84.173	0.694	19.069	1.00	0.00	LX0	N
ATOM	1008	H	TYR	1090	84.324	0.765	18.078	0.00	0.00	LX0	H
ATOM	1009	CA	TYR	1090	83.967	-0.616	19.686	1.00	0.00	LX0	C
ATOM	1010	CB	TYR	1090	84.127	-1.662	18.569	1.00	0.00	LX0	C
ATOM	1011	CG	TYR	1090	84.442	-3.068	19.021	1.00	0.00	LX0	C
ATOM	1012	CD1	TYR	1090	83.624	-3.717	19.965	1.00	0.00	LX0	C
ATOM	1013	CE1	TYR	1090	83.942	-5.030	20.338	1.00	0.00	LX0	C
ATOM	1014	CD2	TYR	1090	85.564	-3.699	18.451	1.00	0.00	LX0	C
ATOM	1015	CE2	TYR	1090	85.878	-5.015	18.823	1.00	0.00	LX0	C
ATOM	1016	CZ	TYR	1090	85.059	-5.667	19.763	1.00	0.00	LX0	C
ATOM	1017	OH	TYR	1090	85.349	-6.965	20.133	1.00	0.00	LX0	O
ATOM	1018	HH	TYR	1090	86.006	-7.324	19.543	0.00	0.00	LX0	H
ATOM	1019	C	TYR	1090	84.811	-0.858	20.941	1.00	0.00	LX0	C
ATOM	1020	O	TYR	1090	84.299	-1.251	21.982	1.00	0.00	LX0	O
ATOM	1021	N	LEU	1091	86.111	-0.554	20.820	1.00	0.00	LX0	N
ATOM	1022	H	LEU	1091	86.464	-0.244	19.937	0.00	0.00	LX0	H
ATOM	1023	CA	LEU	1091	86.979	-0.656	21.997	1.00	0.00	LX0	C
ATOM	1024	CB	LEU	1091	88.437	-0.439	21.602	1.00	0.00	LX0	C
ATOM	1025	CG	LEU	1091	88.942	-1.442	20.570	1.00	0.00	LX0	C
ATOM	1026	CD1	LEU	1091	90.190	-0.921	19.859	1.00	0.00	LX0	C
ATOM	1027	CD2	LEU	1091	89.108	-2.844	21.157	1.00	0.00	LX0	C
ATOM	1028	C	LEU	1091	86.615	0.311	23.110	1.00	0.00	LX0	C
ATOM	1029	O	LEU	1091	86.603	-0.016	24.291	1.00	0.00	LX0	O
ATOM	1030	N	ARG	1092	86.294	1.536	22.666	1.00	0.00	LX0	N
ATOM	1031	H	ARG	1092	86.331	1.721	21.682	0.00	0.00	LX0	H
ATOM	1032	CA	ARG	1092	85.894	2.572	23.619	1.00	0.00	LX0	C
ATOM	1033	CB	ARG	1092	85.689	3.903	22.883	1.00	0.00	LX0	C
ATOM	1034	CG	ARG	1092	87.054	4.442	22.450	1.00	0.00	LX0	C
ATOM	1035	CD	ARG	1092	87.102	5.680	21.545	1.00	0.00	LX0	C
ATOM	1036	NE	ARG	1092	88.489	6.140	21.532	1.00	0.00	LX0	N
ATOM	1037	HE	ARG	1092	89.048	5.878	22.323	0.00	0.00	LX0	H
ATOM	1038	CZ	ARG	1092	89.107	6.841	20.559	1.00	0.00	LX0	C
ATOM	1039	NH1	ARG	1092	88.485	7.203	19.440	1.00	0.00	LX0	N
ATOM	1040	HH11	ARG	1092	88.950	7.741	18.735	0.00	0.00	LX0	H
ATOM	1041	HH12	ARG	1092	87.533	6.928	19.240	0.00	0.00	LX0	H
ATOM	1042	NH2	ARG	1092	90.390	7.138	20.745	1.00	0.00	LX0	N
ATOM	1043	HH21	ARG	1092	90.948	7.675	20.100	0.00	0.00	LX0	H
ATOM	1044	HH22	ARG	1092	90.858	6.777	21.559	0.00	0.00	LX0	H
ATOM	1045	C	ARG	1092	84.728	2.203	24.529	1.00	0.00	LX0	C
ATOM	1046	O	ARG	1092	84.680	2.603	25.685	1.00	0.00	LX0	O

ATOM	1047	N	SER	1093	83.833	1.362	23.985	1.00	0.00	LX0	N
ATOM	1048	H	SER	1093	83.828	1.208	22.995	0.00	0.00	LX0	H
ATOM	1049	CA	SER	1093	82.748	0.818	24.810	1.00	0.00	LX0	C
ATOM	1050	CB	SER	1093	81.926	-0.197	24.017	1.00	0.00	LX0	C
ATOM	1051	OG	SER	1093	81.946	0.136	22.625	1.00	0.00	LX0	O
ATOM	1052	HG	SER	1093	82.691	-0.337	22.266	0.00	0.00	LX0	H
ATOM	1053	C	SER	1093	83.126	0.194	26.150	1.00	0.00	LX0	C
ATOM	1054	O	SER	1093	82.353	0.199	27.096	1.00	0.00	LX0	O
ATOM	1055	N	LEU	1094	84.347	-0.363	26.201	1.00	0.00	LX0	N
ATOM	1056	H	LEU	1094	85.000	-0.272	25.447	0.00	0.00	LX0	H
ATOM	1057	CA	LEU	1094	84.710	-1.028	27.452	1.00	0.00	LX0	C
ATOM	1058	CB	LEU	1094	85.695	-2.165	27.211	1.00	0.00	LX0	C
ATOM	1059	CG	LEU	1094	85.056	-3.440	26.681	1.00	0.00	LX0	C
ATOM	1060	CD1	LEU	1094	86.110	-4.527	26.468	1.00	0.00	LX0	C
ATOM	1061	CD2	LEU	1094	83.922	-3.912	27.592	1.00	0.00	LX0	C
ATOM	1062	C	LEU	1094	85.254	-0.149	28.559	1.00	0.00	LX0	C
ATOM	1063	O	LEU	1094	85.696	-0.638	29.593	1.00	0.00	LX0	O
ATOM	1064	N	ARG	1095	85.241	1.168	28.315	1.00	0.00	LX0	N
ATOM	1065	H	ARG	1095	84.780	1.548	27.513	0.00	0.00	LX0	H
ATOM	1066	CA	ARG	1095	85.690	2.014	29.417	1.00	0.00	LX0	C
ATOM	1067	CB	ARG	1095	85.938	3.436	28.927	1.00	0.00	LX0	C
ATOM	1068	CG	ARG	1095	87.059	3.428	27.898	1.00	0.00	LX0	C
ATOM	1069	CD	ARG	1095	87.348	4.799	27.307	1.00	0.00	LX0	C
ATOM	1070	NE	ARG	1095	88.369	4.656	26.280	1.00	0.00	LX0	N
ATOM	1071	HE	ARG	1095	88.242	3.977	25.550	0.00	0.00	LX0	H
ATOM	1072	CZ	ARG	1095	89.514	5.357	26.313	1.00	0.00	LX0	C
ATOM	1073	NH1	ARG	1095	89.793	6.231	27.275	1.00	0.00	LX0	N
ATOM	1074	HH11	ARG	1095	90.692	6.704	27.258	0.00	0.00	LX0	H
ATOM	1075	HH12	ARG	1095	89.163	6.429	28.019	0.00	0.00	LX0	H
ATOM	1076	NH2	ARG	1095	90.380	5.159	25.345	1.00	0.00	LX0	N
ATOM	1077	HH21	ARG	1095	91.213	5.723	25.308	0.00	0.00	LX0	H
ATOM	1078	HH22	ARG	1095	90.224	4.457	24.646	0.00	0.00	LX0	H
ATOM	1079	C	ARG	1095	84.710	1.996	30.570	1.00	0.00	LX0	C
ATOM	1080	O	ARG	1095	83.517	2.192	30.386	1.00	0.00	LX0	O
ATOM	1081	N	PRO	1096	85.254	1.732	31.780	1.00	0.00	LX0	N
ATOM	1082	CD	PRO	1096	86.659	1.511	32.104	1.00	0.00	LX0	C
ATOM	1083	CA	PRO	1096	84.375	1.644	32.945	1.00	0.00	LX0	C
ATOM	1084	CB	PRO	1096	85.347	1.323	34.090	1.00	0.00	LX0	C
ATOM	1085	CG	PRO	1096	86.729	1.762	33.604	1.00	0.00	LX0	C
ATOM	1086	C	PRO	1096	83.552	2.898	33.154	1.00	0.00	LX0	C
ATOM	1087	O	PRO	1096	84.062	3.980	33.422	1.00	0.00	LX0	O
ATOM	1088	N	GLU	1097	82.237	2.673	33.032	1.00	0.00	LX0	N
ATOM	1089	H	GLU	1097	81.911	1.778	32.717	0.00	0.00	LX0	H
ATOM	1090	CA	GLU	1097	81.276	3.756	33.236	1.00	0.00	LX0	C
ATOM	1091	CB	GLU	1097	79.919	3.219	32.789	1.00	0.00	LX0	C
ATOM	1092	CG	GLU	1097	78.828	4.230	32.438	1.00	0.00	LX0	C
ATOM	1093	CD	GLU	1097	77.521	3.469	32.298	1.00	0.00	LX0	C
ATOM	1094	OE1	GLU	1097	77.342	2.756	31.315	1.00	0.00	LX0	O
ATOM	1095	OE2	GLU	1097	76.689	3.564	33.194	1.00	0.00	LX0	O
ATOM	1096	C	GLU	1097	81.283	4.316	34.665	1.00	0.00	LX0	C
ATOM	1097	O	GLU	1097	80.810	5.408	34.962	1.00	0.00	LX0	O
ATOM	1098	N	MET	1098	81.914	3.507	35.536	1.00	0.00	LX0	N
ATOM	1099	H	MET	1098	81.896	2.534	35.312	0.00	0.00	LX0	H
ATOM	1100	CA	MET	1098	82.475	3.924	36.824	1.00	0.00	LX0	C
ATOM	1101	CB	MET	1098	83.413	5.134	36.693	1.00	0.00	LX0	C
ATOM	1102	CG	MET	1098	84.431	5.233	37.832	1.00	0.00	LX0	C
ATOM	1103	SD	MET	1098	85.512	3.794	37.917	1.00	0.00	LX0	S
ATOM	1104	CE	MET	1098	86.424	4.249	39.401	1.00	0.00	LX0	C
ATOM	1105	C	MET	1098	81.520	4.065	37.995	1.00	0.00	LX0	C
ATOM	1106	O	MET	1098	81.792	3.555	39.076	1.00	0.00	LX0	O
ATOM	1107	N	GLU	1099	80.395	4.758	37.756	1.00	0.00	LX0	N

ATOM	1108	H	GLU	1099	80.220	5.123	36.841	0.00	0.00	LX0	H
ATOM	1109	CA	GLU	1099	79.418	4.859	38.844	1.00	0.00	LX0	C
ATOM	1110	CB	GLU	1099	78.460	6.043	38.616	1.00	0.00	LX0	C
ATOM	1111	CG	GLU	1099	77.195	6.153	39.498	1.00	0.00	LX0	C
ATOM	1112	CD	GLU	1099	77.419	5.768	40.955	1.00	0.00	LX0	C
ATOM	1113	OE1	GLU	1099	78.389	6.211	41.563	1.00	0.00	LX0	O
ATOM	1114	OE2	GLU	1099	76.628	4.986	41.480	1.00	0.00	LX0	O
ATOM	1115	C	GLU	1099	78.722	3.547	39.170	1.00	0.00	LX0	C
ATOM	1116	O	GLU	1099	77.705	3.151	38.615	1.00	0.00	LX0	O
ATOM	1117	N	ASN	1100	79.392	2.866	40.112	1.00	0.00	LX0	N
ATOM	1118	H	ASN	1100	80.231	3.314	40.422	0.00	0.00	LX0	H
ATOM	1119	CA	ASN	1100	78.917	1.626	40.724	1.00	0.00	LX0	C
ATOM	1120	CB	ASN	1100	78.023	1.931	41.921	1.00	0.00	LX0	C
ATOM	1121	CG	ASN	1100	78.867	2.485	43.051	1.00	0.00	LX0	C
ATOM	1122	OD1	ASN	1100	79.608	1.779	43.723	1.00	0.00	LX0	O
ATOM	1123	ND2	ASN	1100	78.747	3.800	43.230	1.00	0.00	LX0	N
ATOM	1124	HD21	ASN	1100	78.174	4.379	42.634	0.00	0.00	LX0	H
ATOM	1125	HD22	ASN	1100	79.246	4.270	43.950	0.00	0.00	LX0	H
ATOM	1126	C	ASN	1100	78.313	0.597	39.784	1.00	0.00	LX0	C
ATOM	1127	O	ASN	1100	77.341	-0.098	40.059	1.00	0.00	LX0	O
ATOM	1128	N	ASN	1101	79.004	0.507	38.641	1.00	0.00	LX0	N
ATOM	1129	H	ASN	1101	79.763	1.141	38.475	0.00	0.00	LX0	H
ATOM	1130	CA	ASN	1101	78.485	-0.261	37.512	1.00	0.00	LX0	C
ATOM	1131	CB	ASN	1101	77.906	0.663	36.428	1.00	0.00	LX0	C
ATOM	1132	CG	ASN	1101	78.821	1.839	36.137	1.00	0.00	LX0	C
ATOM	1133	OD1	ASN	1101	79.980	1.879	36.532	1.00	0.00	LX0	O
ATOM	1134	ND2	ASN	1101	78.229	2.844	35.498	1.00	0.00	LX0	N
ATOM	1135	HD21	ASN	1101	77.345	2.788	35.023	0.00	0.00	LX0	H
ATOM	1136	HD22	ASN	1101	78.658	3.738	35.408	0.00	0.00	LX0	H
ATOM	1137	C	ASN	1101	79.489	-1.246	36.935	1.00	0.00	LX0	C
ATOM	1138	O	ASN	1101	80.487	-0.916	36.313	1.00	0.00	LX0	O
ATOM	1139	N	PRO	1102	79.187	-2.532	37.209	1.00	0.00	LX0	N
ATOM	1140	CD	PRO	1102	78.044	-2.967	37.994	1.00	0.00	LX0	C
ATOM	1141	CA	PRO	1102	80.098	-3.645	36.883	1.00	0.00	LX0	C
ATOM	1142	CB	PRO	1102	79.469	-4.795	37.649	1.00	0.00	LX0	C
ATOM	1143	CG	PRO	1102	78.630	-4.145	38.731	1.00	0.00	LX0	C
ATOM	1144	C	PRO	1102	80.347	-4.013	35.429	1.00	0.00	LX0	C
ATOM	1145	O	PRO	1102	79.555	-4.739	34.833	1.00	0.00	LX0	O
ATOM	1146	N	VAL	1103	81.472	-3.505	34.898	1.00	0.00	LX0	N
ATOM	1147	H	VAL	1103	81.986	-2.826	35.416	0.00	0.00	LX0	H
ATOM	1148	CA	VAL	1103	81.919	-3.953	33.579	1.00	0.00	LX0	C
ATOM	1149	CB	VAL	1103	82.037	-2.756	32.593	1.00	0.00	LX0	C
ATOM	1150	CG1	VAL	1103	83.121	-1.734	32.930	1.00	0.00	LX0	C
ATOM	1151	CG2	VAL	1103	82.003	-3.177	31.120	1.00	0.00	LX0	C
ATOM	1152	C	VAL	1103	83.070	-4.973	33.599	1.00	0.00	LX0	C
ATOM	1153	O	VAL	1103	82.911	-6.023	34.208	1.00	0.00	LX0	O
ATOM	1154	N	LEU	1104	84.185	-4.709	32.892	1.00	0.00	LX0	N
ATOM	1155	H	LEU	1104	84.441	-3.800	32.572	0.00	0.00	LX0	H
ATOM	1156	CA	LEU	1104	85.027	-5.833	32.497	1.00	0.00	LX0	C
ATOM	1157	CB	LEU	1104	84.753	-6.203	31.031	1.00	0.00	LX0	C
ATOM	1158	CG	LEU	1104	83.390	-6.870	30.841	1.00	0.00	LX0	C
ATOM	1159	CD1	LEU	1104	82.977	-7.026	29.377	1.00	0.00	LX0	C
ATOM	1160	CD2	LEU	1104	83.322	-8.186	31.608	1.00	0.00	LX0	C
ATOM	1161	C	LEU	1104	86.509	-5.614	32.699	1.00	0.00	LX0	C
ATOM	1162	O	LEU	1104	86.995	-4.522	32.960	1.00	0.00	LX0	O
ATOM	1163	N	ALA	1105	87.210	-6.746	32.543	1.00	0.00	LX0	N
ATOM	1164	H	ALA	1105	86.725	-7.573	32.275	0.00	0.00	LX0	H
ATOM	1165	CA	ALA	1105	88.660	-6.680	32.391	1.00	0.00	LX0	C
ATOM	1166	CB	ALA	1105	89.248	-8.066	32.695	1.00	0.00	LX0	C
ATOM	1167	C	ALA	1105	88.982	-6.286	30.954	1.00	0.00	LX0	C
ATOM	1168	O	ALA	1105	88.178	-6.532	30.060	1.00	0.00	LX0	O

ATOM	1169	N	PRO	1106	90.176	-5.669	30.750	1.00	0.00	LX0	N
ATOM	1170	CD	PRO	1106	91.147	-5.247	31.757	1.00	0.00	LX0	C
ATOM	1171	CA	PRO	1106	90.622	-5.391	29.376	1.00	0.00	LX0	C
ATOM	1172	CB	PRO	1106	91.980	-4.703	29.608	1.00	0.00	LX0	C
ATOM	1173	CG	PRO	1106	92.454	-5.151	30.987	1.00	0.00	LX0	C
ATOM	1174	C	PRO	1106	90.684	-6.660	28.529	1.00	0.00	LX0	C
ATOM	1175	O	PRO	1106	90.823	-7.760	29.054	1.00	0.00	LX0	O
ATOM	1176	N	PRO	1107	90.541	-6.476	27.191	1.00	0.00	LX0	N
ATOM	1177	CD	PRO	1107	90.366	-5.211	26.484	1.00	0.00	LX0	C
ATOM	1178	CA	PRO	1107	90.508	-7.623	26.279	1.00	0.00	LX0	C
ATOM	1179	CB	PRO	1107	90.573	-6.965	24.898	1.00	0.00	LX0	C
ATOM	1180	CG	PRO	1107	89.903	-5.609	25.089	1.00	0.00	LX0	C
ATOM	1181	C	PRO	1107	91.550	-8.703	26.511	1.00	0.00	LX0	C
ATOM	1182	O	PRO	1107	92.755	-8.489	26.530	1.00	0.00	LX0	O
ATOM	1183	N	SER	1108	90.991	-9.900	26.695	1.00	0.00	LX0	N
ATOM	1184	H	SER	1108	90.001	-10.009	26.647	0.00	0.00	LX0	H
ATOM	1185	CA	SER	1108	91.803	-11.078	26.972	1.00	0.00	LX0	C
ATOM	1186	CB	SER	1108	90.841	-12.219	27.345	1.00	0.00	LX0	C
ATOM	1187	OG	SER	1108	89.471	-11.804	27.152	1.00	0.00	LX0	O
ATOM	1188	HG	SER	1108	88.986	-12.054	27.939	0.00	0.00	LX0	H
ATOM	1189	C	SER	1108	92.729	-11.438	25.814	1.00	0.00	LX0	C
ATOM	1190	O	SER	1108	92.368	-11.267	24.655	1.00	0.00	LX0	O
ATOM	1191	N	LEU	1109	93.933	-11.941	26.168	1.00	0.00	LX0	N
ATOM	1192	H	LEU	1109	94.154	-12.012	27.139	0.00	0.00	LX0	H
ATOM	1193	CA	LEU	1109	94.976	-12.182	25.156	1.00	0.00	LX0	C
ATOM	1194	CB	LEU	1109	96.151	-12.959	25.761	1.00	0.00	LX0	C
ATOM	1195	CG	LEU	1109	97.371	-13.106	24.838	1.00	0.00	LX0	C
ATOM	1196	CD1	LEU	1109	97.914	-11.768	24.327	1.00	0.00	LX0	C
ATOM	1197	CD2	LEU	1109	98.459	-13.960	25.489	1.00	0.00	LX0	C
ATOM	1198	C	LEU	1109	94.511	-12.799	23.843	1.00	0.00	LX0	C
ATOM	1199	O	LEU	1109	94.527	-12.150	22.805	1.00	0.00	LX0	O
ATOM	1200	N	SER	1110	94.014	-14.037	23.951	1.00	0.00	LX0	N
ATOM	1201	H	SER	1110	94.152	-14.592	24.771	0.00	0.00	LX0	H
ATOM	1202	CA	SER	1110	93.516	-14.735	22.764	1.00	0.00	LX0	C
ATOM	1203	CB	SER	1110	92.947	-16.087	23.192	1.00	0.00	LX0	C
ATOM	1204	OG	SER	1110	93.492	-16.439	24.472	1.00	0.00	LX0	O
ATOM	1205	HG	SER	1110	94.399	-16.718	24.292	0.00	0.00	LX0	H
ATOM	1206	C	SER	1110	92.503	-13.968	21.927	1.00	0.00	LX0	C
ATOM	1207	O	SER	1110	92.425	-14.065	20.713	1.00	0.00	LX0	O
ATOM	1208	N	LYS	1111	91.715	-13.156	22.647	1.00	0.00	LX0	N
ATOM	1209	H	LYS	1111	91.940	-12.989	23.605	0.00	0.00	LX0	H
ATOM	1210	CA	LYS	1111	90.713	-12.353	21.948	1.00	0.00	LX0	C
ATOM	1211	CB	LYS	1111	89.663	-11.865	22.951	1.00	0.00	LX0	C
ATOM	1212	CG	LYS	1111	89.118	-13.040	23.768	1.00	0.00	LX0	C
ATOM	1213	CD	LYS	1111	88.350	-14.028	22.895	1.00	0.00	LX0	C
ATOM	1214	CE	LYS	1111	88.173	-15.408	23.525	1.00	0.00	LX0	C
ATOM	1215	NZ	LYS	1111	87.103	-16.096	22.799	1.00	0.00	LX0	N
ATOM	1216	HZ1	LYS	1111	86.172	-15.851	23.190	0.00	0.00	LX0	H
ATOM	1217	HZ2	LYS	1111	87.138	-15.845	21.784	0.00	0.00	LX0	H
ATOM	1218	HZ3	LYS	1111	87.235	-17.130	22.749	0.00	0.00	LX0	H
ATOM	1219	C	LYS	1111	91.324	-11.211	21.155	1.00	0.00	LX0	C
ATOM	1220	O	LYS	1111	90.923	-10.894	20.041	1.00	0.00	LX0	O
ATOM	1221	N	MET	1112	92.363	-10.628	21.772	1.00	0.00	LX0	N
ATOM	1222	H	MET	1112	92.672	-10.966	22.665	0.00	0.00	LX0	H
ATOM	1223	CA	MET	1112	93.139	-9.643	21.022	1.00	0.00	LX0	C
ATOM	1224	CB	MET	1112	94.153	-8.926	21.912	1.00	0.00	LX0	C
ATOM	1225	CG	MET	1112	93.488	-8.172	23.059	1.00	0.00	LX0	C
ATOM	1226	SD	MET	1112	94.631	-7.152	24.001	1.00	0.00	LX0	S
ATOM	1227	CE	MET	1112	95.644	-8.477	24.668	1.00	0.00	LX0	C
ATOM	1228	C	MET	1112	93.842	-10.237	19.819	1.00	0.00	LX0	C
ATOM	1229	O	MET	1112	93.891	-9.643	18.752	1.00	0.00	LX0	O

ATOM	1230	N	ILE	1113	94.348	-11.461	20.032	1.00	0.00	LX0	N
ATOM	1231	H	ILE	1113	94.292	-11.867	20.944	0.00	0.00	LX0	H
ATOM	1232	CA	ILE	1113	94.958	-12.196	18.924	1.00	0.00	LX0	C
ATOM	1233	CB	ILE	1113	95.587	-13.500	19.439	1.00	0.00	LX0	C
ATOM	1234	CG2	ILE	1113	96.121	-14.391	18.315	1.00	0.00	LX0	C
ATOM	1235	CG1	ILE	1113	96.680	-13.191	20.467	1.00	0.00	LX0	C
ATOM	1236	CD1	ILE	1113	97.858	-12.404	19.888	1.00	0.00	LX0	C
ATOM	1237	C	ILE	1113	93.990	-12.436	17.776	1.00	0.00	LX0	C
ATOM	1238	O	ILE	1113	94.302	-12.171	16.624	1.00	0.00	LX0	O
ATOM	1239	N	GLN	1114	92.779	-12.884	18.154	1.00	0.00	LX0	N
ATOM	1240	H	GLN	1114	92.649	-13.171	19.101	0.00	0.00	LX0	H
ATOM	1241	CA	GLN	1114	91.702	-13.037	17.176	1.00	0.00	LX0	C
ATOM	1242	CB	GLN	1114	90.404	-13.469	17.879	1.00	0.00	LX0	C
ATOM	1243	CG	GLN	1114	89.159	-13.629	16.989	1.00	0.00	LX0	C
ATOM	1244	CD	GLN	1114	89.373	-14.683	15.919	1.00	0.00	LX0	C
ATOM	1245	OE1	GLN	1114	90.092	-15.660	16.087	1.00	0.00	LX0	O
ATOM	1246	NE2	GLN	1114	88.705	-14.440	14.792	1.00	0.00	LX0	N
ATOM	1247	HE21	GLN	1114	88.171	-13.591	14.691	0.00	0.00	LX0	H
ATOM	1248	HE22	GLN	1114	88.727	-15.065	14.019	0.00	0.00	LX0	H
ATOM	1249	C	GLN	1114	91.513	-11.805	16.308	1.00	0.00	LX0	C
ATOM	1250	O	GLN	1114	91.718	-11.839	15.102	1.00	0.00	LX0	O
ATOM	1251	N	MET	1115	91.193	-10.697	16.997	1.00	0.00	LX0	N
ATOM	1252	H	MET	1115	91.051	-10.752	17.987	0.00	0.00	LX0	H
ATOM	1253	CA	MET	1115	90.987	-9.458	16.244	1.00	0.00	LX0	C
ATOM	1254	CB	MET	1115	90.544	-8.333	17.182	1.00	0.00	LX0	C
ATOM	1255	CG	MET	1115	89.178	-8.632	17.808	1.00	0.00	LX0	C
ATOM	1256	SD	MET	1115	88.600	-7.344	18.929	1.00	0.00	LX0	S
ATOM	1257	CE	MET	1115	89.804	-7.594	20.242	1.00	0.00	LX0	C
ATOM	1258	C	MET	1115	92.173	-9.042	15.387	1.00	0.00	LX0	C
ATOM	1259	O	MET	1115	92.060	-8.651	14.233	1.00	0.00	LX0	O
ATOM	1260	N	ALA	1116	93.356	-9.197	16.000	1.00	0.00	LX0	N
ATOM	1261	H	ALA	1116	93.377	-9.536	16.940	0.00	0.00	LX0	H
ATOM	1262	CA	ALA	1116	94.590	-8.934	15.262	1.00	0.00	LX0	C
ATOM	1263	CB	ALA	1116	95.813	-9.236	16.129	1.00	0.00	LX0	C
ATOM	1264	C	ALA	1116	94.715	-9.730	13.976	1.00	0.00	LX0	C
ATOM	1265	O	ALA	1116	95.128	-9.227	12.940	1.00	0.00	LX0	O
ATOM	1266	N	GLY	1117	94.318	-11.003	14.086	1.00	0.00	LX0	N
ATOM	1267	H	GLY	1117	93.926	-11.338	14.945	0.00	0.00	LX0	H
ATOM	1268	CA	GLY	1117	94.356	-11.865	12.913	1.00	0.00	LX0	C
ATOM	1269	C	GLY	1117	93.377	-11.445	11.842	1.00	0.00	LX0	C
ATOM	1270	O	GLY	1117	93.727	-11.361	10.675	1.00	0.00	LX0	O
ATOM	1271	N	GLU	1118	92.146	-11.154	12.286	1.00	0.00	LX0	N
ATOM	1272	H	GLU	1118	91.939	-11.238	13.264	0.00	0.00	LX0	H
ATOM	1273	CA	GLU	1118	91.110	-10.712	11.346	1.00	0.00	LX0	C
ATOM	1274	CB	GLU	1118	89.816	-10.438	12.111	1.00	0.00	LX0	C
ATOM	1275	CG	GLU	1118	89.326	-11.647	12.911	1.00	0.00	LX0	C
ATOM	1276	CD	GLU	1118	88.250	-11.250	13.909	1.00	0.00	LX0	C
ATOM	1277	OE1	GLU	1118	88.403	-10.250	14.602	1.00	0.00	LX0	O
ATOM	1278	OE2	GLU	1118	87.257	-11.959	14.013	1.00	0.00	LX0	O
ATOM	1279	C	GLU	1118	91.527	-9.500	10.521	1.00	0.00	LX0	C
ATOM	1280	O	GLU	1118	91.478	-9.471	9.294	1.00	0.00	LX0	O
ATOM	1281	N	ILE	1119	92.025	-8.507	11.274	1.00	0.00	LX0	N
ATOM	1282	H	ILE	1119	91.962	-8.599	12.271	0.00	0.00	LX0	H
ATOM	1283	CA	ILE	1119	92.606	-7.327	10.630	1.00	0.00	LX0	C
ATOM	1284	CB	ILE	1119	93.061	-6.326	11.703	1.00	0.00	LX0	C
ATOM	1285	CG2	ILE	1119	93.735	-5.078	11.122	1.00	0.00	LX0	C
ATOM	1286	CG1	ILE	1119	91.890	-5.948	12.610	1.00	0.00	LX0	C
ATOM	1287	CD1	ILE	1119	92.370	-5.325	13.919	1.00	0.00	LX0	C
ATOM	1288	C	ILE	1119	93.747	-7.674	9.679	1.00	0.00	LX0	C
ATOM	1289	O	ILE	1119	93.780	-7.278	8.520	1.00	0.00	LX0	O
ATOM	1290	N	ALA	1120	94.691	-8.457	10.222	1.00	0.00	LX0	N

ATOM	1291	H	ALA	1120	94.586	-8.791	11.159	0.00	0.00	LX0	H
ATOM	1292	CA	ALA	1120	95.861	-8.803	9.417	1.00	0.00	LX0	C
ATOM	1293	CB	ALA	1120	96.880	-9.597	10.235	1.00	0.00	LX0	C
ATOM	1294	C	ALA	1120	95.550	-9.565	8.141	1.00	0.00	LX0	C
ATOM	1295	O	ALA	1120	96.225	-9.406	7.134	1.00	0.00	LX0	O
ATOM	1296	N	ASP	1121	94.480	-10.367	8.217	1.00	0.00	LX0	N
ATOM	1297	H	ASP	1121	93.975	-10.438	9.076	0.00	0.00	LX0	H
ATOM	1298	CA	ASP	1121	94.019	-11.120	7.053	1.00	0.00	LX0	C
ATOM	1299	CB	ASP	1121	92.902	-12.075	7.482	1.00	0.00	LX0	C
ATOM	1300	CG	ASP	1121	92.586	-13.068	6.386	1.00	0.00	LX0	C
ATOM	1301	OD1	ASP	1121	93.319	-14.041	6.247	1.00	0.00	LX0	O
ATOM	1302	OD2	ASP	1121	91.600	-12.880	5.677	1.00	0.00	LX0	O
ATOM	1303	C	ASP	1121	93.564	-10.202	5.937	1.00	0.00	LX0	C
ATOM	1304	O	ASP	1121	94.062	-10.224	4.816	1.00	0.00	LX0	O
ATOM	1305	N	GLY	1122	92.633	-9.312	6.326	1.00	0.00	LX0	N
ATOM	1306	H	GLY	1122	92.257	-9.374	7.254	0.00	0.00	LX0	H
ATOM	1307	CA	GLY	1122	92.178	-8.310	5.362	1.00	0.00	LX0	C
ATOM	1308	C	GLY	1122	93.305	-7.497	4.740	1.00	0.00	LX0	C
ATOM	1309	O	GLY	1122	93.396	-7.316	3.531	1.00	0.00	LX0	O
ATOM	1310	N	MET	1123	94.197	-7.044	5.637	1.00	0.00	LX0	N
ATOM	1311	H	MET	1123	94.034	-7.227	6.608	0.00	0.00	LX0	H
ATOM	1312	CA	MET	1123	95.384	-6.318	5.176	1.00	0.00	LX0	C
ATOM	1313	CB	MET	1123	96.243	-5.867	6.356	1.00	0.00	LX0	C
ATOM	1314	CG	MET	1123	95.573	-4.849	7.276	1.00	0.00	LX0	C
ATOM	1315	SD	MET	1123	95.137	-3.322	6.432	1.00	0.00	LX0	S
ATOM	1316	CE	MET	1123	96.806	-2.780	6.046	1.00	0.00	LX0	C
ATOM	1317	C	MET	1123	96.253	-7.098	4.207	1.00	0.00	LX0	C
ATOM	1318	O	MET	1123	96.733	-6.595	3.197	1.00	0.00	LX0	O
ATOM	1319	N	ALA	1124	96.420	-8.380	4.558	1.00	0.00	LX0	N
ATOM	1320	H	ALA	1124	95.963	-8.744	5.370	0.00	0.00	LX0	H
ATOM	1321	CA	ALA	1124	97.217	-9.257	3.711	1.00	0.00	LX0	C
ATOM	1322	CB	ALA	1124	97.379	-10.631	4.358	1.00	0.00	LX0	C
ATOM	1323	C	ALA	1124	96.633	-9.410	2.324	1.00	0.00	LX0	C
ATOM	1324	O	ALA	1124	97.341	-9.340	1.329	1.00	0.00	LX0	O
ATOM	1325	N	TYR	1125	95.294	-9.548	2.293	1.00	0.00	LX0	N
ATOM	1326	H	TYR	1125	94.783	-9.652	3.148	0.00	0.00	LX0	H
ATOM	1327	CA	TYR	1125	94.627	-9.508	0.993	1.00	0.00	LX0	C
ATOM	1328	CB	TYR	1125	93.111	-9.707	1.125	1.00	0.00	LX0	C
ATOM	1329	CG	TYR	1125	92.441	-9.685	-0.238	1.00	0.00	LX0	C
ATOM	1330	CD1	TYR	1125	92.454	-10.845	-1.040	1.00	0.00	LX0	C
ATOM	1331	CE1	TYR	1125	91.785	-10.831	-2.276	1.00	0.00	LX0	C
ATOM	1332	CD2	TYR	1125	91.821	-8.495	-0.669	1.00	0.00	LX0	C
ATOM	1333	CE2	TYR	1125	91.175	-8.476	-1.915	1.00	0.00	LX0	C
ATOM	1334	CZ	TYR	1125	91.130	-9.653	-2.686	1.00	0.00	LX0	C
ATOM	1335	OH	TYR	1125	90.399	-9.666	-3.858	1.00	0.00	LX0	O
ATOM	1336	HH	TYR	1125	90.385	-8.802	-4.271	0.00	0.00	LX0	H
ATOM	1337	C	TYR	1125	94.932	-8.243	0.217	1.00	0.00	LX0	C
ATOM	1338	O	TYR	1125	95.336	-8.293	-0.934	1.00	0.00	LX0	O
ATOM	1339	N	LEU	1126	94.743	-7.102	0.903	1.00	0.00	LX0	N
ATOM	1340	H	LEU	1126	94.412	-7.144	1.848	0.00	0.00	LX0	H
ATOM	1341	CA	LEU	1126	94.993	-5.832	0.215	1.00	0.00	LX0	C
ATOM	1342	CB	LEU	1126	94.853	-4.636	1.163	1.00	0.00	LX0	C
ATOM	1343	CG	LEU	1126	93.535	-4.558	1.939	1.00	0.00	LX0	C
ATOM	1344	CD1	LEU	1126	93.558	-3.411	2.947	1.00	0.00	LX0	C
ATOM	1345	CD2	LEU	1126	92.307	-4.494	1.032	1.00	0.00	LX0	C
ATOM	1346	C	LEU	1126	96.352	-5.785	-0.463	1.00	0.00	LX0	C
ATOM	1347	O	LEU	1126	96.478	-5.620	-1.670	1.00	0.00	LX0	O
ATOM	1348	N	ASN	1127	97.372	-5.993	0.379	1.00	0.00	LX0	N
ATOM	1349	H	ASN	1127	97.196	-6.197	1.345	0.00	0.00	LX0	H
ATOM	1350	CA	ASN	1127	98.716	-5.847	-0.170	1.00	0.00	LX0	C
ATOM	1351	CB	ASN	1127	99.738	-5.660	0.951	1.00	0.00	LX0	C

ATOM	1352	CG	ASN	1127	100.987	-4.999	0.398	1.00	0.00	LX0	C
ATOM	1353	OD1	ASN	1127	102.106	-5.442	0.627	1.00	0.00	LX0	O
ATOM	1354	ND2	ASN	1127	100.759	-3.909	-0.343	1.00	0.00	LX0	N
ATOM	1355	HD21	ASN	1127	99.831	-3.548	-0.471	0.00	0.00	LX0	H
ATOM	1356	HD22	ASN	1127	101.521	-3.451	-0.793	0.00	0.00	LX0	H
ATOM	1357	C	ASN	1127	99.126	-6.907	-1.175	1.00	0.00	LX0	C
ATOM	1358	O	ASN	1127	99.638	-6.628	-2.253	1.00	0.00	LX0	O
ATOM	1359	N	ALA	1128	98.822	-8.158	-0.800	1.00	0.00	LX0	N
ATOM	1360	H	ALA	1128	98.333	-8.333	0.056	0.00	0.00	LX0	H
ATOM	1361	CA	ALA	1128	99.093	-9.238	-1.746	1.00	0.00	LX0	C
ATOM	1362	CB	ALA	1128	99.122	-10.596	-1.044	1.00	0.00	LX0	C
ATOM	1363	C	ALA	1128	98.138	-9.306	-2.929	1.00	0.00	LX0	C
ATOM	1364	O	ALA	1128	98.257	-10.160	-3.806	1.00	0.00	LX0	O
ATOM	1365	N	ASN	1129	97.204	-8.350	-2.942	1.00	0.00	LX0	N
ATOM	1366	H	ASN	1129	96.970	-7.827	-2.119	0.00	0.00	LX0	H
ATOM	1367	CA	ASN	1129	96.461	-8.088	-4.164	1.00	0.00	LX0	C
ATOM	1368	CB	ASN	1129	95.020	-8.598	-4.048	1.00	0.00	LX0	C
ATOM	1369	CG	ASN	1129	94.471	-8.908	-5.425	1.00	0.00	LX0	C
ATOM	1370	OD1	ASN	1129	94.562	-8.126	-6.363	1.00	0.00	LX0	O
ATOM	1371	ND2	ASN	1129	93.911	-10.111	-5.518	1.00	0.00	LX0	N
ATOM	1372	HD21	ASN	1129	93.923	-10.761	-4.751	0.00	0.00	LX0	H
ATOM	1373	HD22	ASN	1129	93.484	-10.437	-6.358	0.00	0.00	LX0	H
ATOM	1374	C	ASN	1129	96.526	-6.616	-4.539	1.00	0.00	LX0	C
ATOM	1375	O	ASN	1129	95.528	-5.955	-4.802	1.00	0.00	LX0	O
ATOM	1376	N	LYS	1130	97.789	-6.135	-4.586	1.00	0.00	LX0	N
ATOM	1377	H	LYS	1130	98.489	-6.671	-4.110	0.00	0.00	LX0	H
ATOM	1378	CA	LYS	1130	98.177	-4.812	-5.111	1.00	0.00	LX0	C
ATOM	1379	CB	LYS	1130	97.418	-4.378	-6.379	1.00	0.00	LX0	C
ATOM	1380	CG	LYS	1130	97.481	-5.321	-7.580	1.00	0.00	LX0	C
ATOM	1381	CD	LYS	1130	96.364	-5.019	-8.587	1.00	0.00	LX0	C
ATOM	1382	CE	LYS	1130	95.105	-5.888	-8.434	1.00	0.00	LX0	C
ATOM	1383	NZ	LYS	1130	94.404	-5.700	-7.155	1.00	0.00	LX0	N
ATOM	1384	HZ1	LYS	1130	93.923	-6.584	-6.883	0.00	0.00	LX0	H
ATOM	1385	HZ2	LYS	1130	94.985	-5.454	-6.335	0.00	0.00	LX0	H
ATOM	1386	HZ3	LYS	1130	93.599	-5.034	-7.221	0.00	0.00	LX0	H
ATOM	1387	C	LYS	1130	98.114	-3.646	-4.139	1.00	0.00	LX0	C
ATOM	1388	O	LYS	1130	98.999	-2.802	-4.086	1.00	0.00	LX0	O
ATOM	1389	N	PHE	1131	96.988	-3.584	-3.425	1.00	0.00	LX0	N
ATOM	1390	H	PHE	1131	96.421	-4.401	-3.300	0.00	0.00	LX0	H
ATOM	1391	CA	PHE	1131	96.702	-2.335	-2.728	1.00	0.00	LX0	C
ATOM	1392	CB	PHE	1131	95.210	-2.241	-2.401	1.00	0.00	LX0	C
ATOM	1393	CG	PHE	1131	94.424	-1.905	-3.650	1.00	0.00	LX0	C
ATOM	1394	CD1	PHE	1131	94.146	-2.908	-4.605	1.00	0.00	LX0	C
ATOM	1395	CD2	PHE	1131	93.979	-0.578	-3.835	1.00	0.00	LX0	C
ATOM	1396	CE1	PHE	1131	93.414	-2.579	-5.762	1.00	0.00	LX0	C
ATOM	1397	CE2	PHE	1131	93.244	-0.249	-4.990	1.00	0.00	LX0	C
ATOM	1398	CZ	PHE	1131	92.967	-1.253	-5.942	1.00	0.00	LX0	C
ATOM	1399	C	PHE	1131	97.544	-2.082	-1.496	1.00	0.00	LX0	C
ATOM	1400	O	PHE	1131	97.977	-2.979	-0.785	1.00	0.00	LX0	O
ATOM	1401	N	VAL	1132	97.749	-0.783	-1.269	1.00	0.00	LX0	N
ATOM	1402	H	VAL	1132	97.396	-0.089	-1.894	0.00	0.00	LX0	H
ATOM	1403	CA	VAL	1132	98.403	-0.407	-0.025	1.00	0.00	LX0	C
ATOM	1404	CB	VAL	1132	99.780	0.220	-0.321	1.00	0.00	LX0	C
ATOM	1405	CG1	VAL	1132	99.711	1.418	-1.275	1.00	0.00	LX0	C
ATOM	1406	CG2	VAL	1132	100.546	0.525	0.964	1.00	0.00	LX0	C
ATOM	1407	C	VAL	1132	97.474	0.484	0.780	1.00	0.00	LX0	C
ATOM	1408	O	VAL	1132	96.993	1.520	0.322	1.00	0.00	LX0	O
ATOM	1409	N	HIS	1133	97.172	0.003	1.988	1.00	0.00	LX0	N
ATOM	1410	H	HIS	1133	97.725	-0.713	2.423	0.00	0.00	LX0	H
ATOM	1411	CA	HIS	1133	96.171	0.750	2.735	1.00	0.00	LX0	C
ATOM	1412	CB	HIS	1133	95.482	-0.093	3.807	1.00	0.00	LX0	C

ATOM	1413	CG	HIS	1133	94.121	0.502	4.079	1.00	0.00	LX0	C
ATOM	1414	ND1	HIS	1133	93.712	0.946	5.279	1.00	0.00	LX0	N
ATOM	1415	HD1	HIS	1133	94.239	0.977	6.108	0.00	0.00	LX0	H
ATOM	1416	CD2	HIS	1133	93.095	0.708	3.155	1.00	0.00	LX0	C
ATOM	1417	NE2	HIS	1133	92.063	1.286	3.813	1.00	0.00	LX0	N
ATOM	1418	CE1	HIS	1133	92.440	1.431	5.123	1.00	0.00	LX0	C
ATOM	1419	C	HIS	1133	96.666	2.066	3.293	1.00	0.00	LX0	C
ATOM	1420	O	HIS	1133	95.933	3.050	3.247	1.00	0.00	LX0	O
ATOM	1421	N	ARG	1134	97.938	2.057	3.743	1.00	0.00	LX0	N
ATOM	1422	H	ARG	1134	98.409	1.168	3.815	0.00	0.00	LX0	H
ATOM	1423	CA	ARG	1134	98.681	3.225	4.244	1.00	0.00	LX0	C
ATOM	1424	CB	ARG	1134	98.698	4.400	3.254	1.00	0.00	LX0	C
ATOM	1425	CG	ARG	1134	99.164	4.050	1.838	1.00	0.00	LX0	C
ATOM	1426	CD	ARG	1134	98.336	4.783	0.778	1.00	0.00	LX0	C
ATOM	1427	NE	ARG	1134	96.933	4.401	0.930	1.00	0.00	LX0	N
ATOM	1428	HE	ARG	1134	96.747	3.411	0.940	0.00	0.00	LX0	H
ATOM	1429	CZ	ARG	1134	95.974	5.304	1.225	1.00	0.00	LX0	C
ATOM	1430	NH1	ARG	1134	96.204	6.606	1.066	1.00	0.00	LX0	N
ATOM	1431	HH11	ARG	1134	95.523	7.298	1.305	0.00	0.00	LX0	H
ATOM	1432	HH12	ARG	1134	97.085	6.909	0.701	0.00	0.00	LX0	H
ATOM	1433	NH2	ARG	1134	94.808	4.875	1.698	1.00	0.00	LX0	N
ATOM	1434	HH21	ARG	1134	94.009	5.459	1.829	0.00	0.00	LX0	H
ATOM	1435	HH22	ARG	1134	94.745	3.910	1.979	0.00	0.00	LX0	H
ATOM	1436	C	ARG	1134	98.254	3.721	5.614	1.00	0.00	LX0	C
ATOM	1437	O	ARG	1134	99.040	3.860	6.539	1.00	0.00	LX0	O
ATOM	1438	N	ASP	1135	96.953	4.009	5.688	1.00	0.00	LX0	N
ATOM	1439	H	ASP	1135	96.344	3.800	4.927	0.00	0.00	LX0	H
ATOM	1440	CA	ASP	1135	96.372	4.427	6.952	1.00	0.00	LX0	C
ATOM	1441	CB	ASP	1135	95.332	5.519	6.647	1.00	0.00	LX0	C
ATOM	1442	CG	ASP	1135	94.464	5.909	7.835	1.00	0.00	LX0	C
ATOM	1443	OD1	ASP	1135	94.806	5.621	8.981	1.00	0.00	LX0	O
ATOM	1444	OD2	ASP	1135	93.393	6.463	7.612	1.00	0.00	LX0	O
ATOM	1445	C	ASP	1135	95.779	3.217	7.642	1.00	0.00	LX0	C
ATOM	1446	O	ASP	1135	94.935	2.509	7.097	1.00	0.00	LX0	O
ATOM	1447	N	LEU	1136	96.258	3.006	8.868	1.00	0.00	LX0	N
ATOM	1448	H	LEU	1136	96.974	3.604	9.226	0.00	0.00	LX0	H
ATOM	1449	CA	LEU	1136	95.630	1.962	9.664	1.00	0.00	LX0	C
ATOM	1450	CB	LEU	1136	96.271	0.592	9.401	1.00	0.00	LX0	C
ATOM	1451	CG	LEU	1136	95.445	-0.583	9.935	1.00	0.00	LX0	C
ATOM	1452	CD1	LEU	1136	94.082	-0.684	9.250	1.00	0.00	LX0	C
ATOM	1453	CD2	LEU	1136	96.220	-1.897	9.871	1.00	0.00	LX0	C
ATOM	1454	C	LEU	1136	95.615	2.313	11.135	1.00	0.00	LX0	C
ATOM	1455	O	LEU	1136	96.377	1.799	11.942	1.00	0.00	LX0	O
ATOM	1456	N	ALA	1137	94.684	3.219	11.452	1.00	0.00	LX0	N
ATOM	1457	H	ALA	1137	94.153	3.654	10.725	0.00	0.00	LX0	H
ATOM	1458	CA	ALA	1137	94.306	3.276	12.862	1.00	0.00	LX0	C
ATOM	1459	CB	ALA	1137	93.759	4.651	13.218	1.00	0.00	LX0	C
ATOM	1460	C	ALA	1137	93.225	2.251	13.128	1.00	0.00	LX0	C
ATOM	1461	O	ALA	1137	92.654	1.706	12.191	1.00	0.00	LX0	O
ATOM	1462	N	ALA	1138	92.937	2.021	14.421	1.00	0.00	LX0	N
ATOM	1463	H	ALA	1138	93.434	2.501	15.151	0.00	0.00	LX0	H
ATOM	1464	CA	ALA	1138	91.943	0.989	14.741	1.00	0.00	LX0	C
ATOM	1465	CB	ALA	1138	91.740	0.882	16.252	1.00	0.00	LX0	C
ATOM	1466	C	ALA	1138	90.586	1.172	14.076	1.00	0.00	LX0	C
ATOM	1467	O	ALA	1138	89.937	0.237	13.629	1.00	0.00	LX0	O
ATOM	1468	N	ARG	1139	90.200	2.455	13.987	1.00	0.00	LX0	N
ATOM	1469	H	ARG	1139	90.778	3.146	14.417	0.00	0.00	LX0	H
ATOM	1470	CA	ARG	1139	88.944	2.808	13.312	1.00	0.00	LX0	C
ATOM	1471	CB	ARG	1139	88.755	4.328	13.327	1.00	0.00	LX0	C
ATOM	1472	CG	ARG	1139	89.959	4.981	12.663	1.00	0.00	LX0	C
ATOM	1473	CD	ARG	1139	89.773	6.401	12.157	1.00	0.00	LX0	C

ATOM	1474	NE	ARG	1139	90.696	6.569	11.042	1.00	0.00	LX0	N
ATOM	1475	HE	ARG	1139	90.438	6.167	10.157	0.00	0.00	LX0	H
ATOM	1476	CZ	ARG	1139	91.962	6.986	11.216	1.00	0.00	LX0	C
ATOM	1477	NH1	ARG	1139	92.381	7.473	12.374	1.00	0.00	LX0	N
ATOM	1478	HH11	ARG	1139	93.234	7.991	12.472	0.00	0.00	LX0	H
ATOM	1479	HH12	ARG	1139	91.846	7.359	13.220	0.00	0.00	LX0	H
ATOM	1480	NH2	ARG	1139	92.799	6.880	10.205	1.00	0.00	LX0	N
ATOM	1481	HH21	ARG	1139	93.788	7.024	10.254	0.00	0.00	LX0	H
ATOM	1482	HH22	ARG	1139	92.487	6.597	9.290	0.00	0.00	LX0	H
ATOM	1483	C	ARG	1139	88.768	2.276	11.886	1.00	0.00	LX0	C
ATOM	1484	O	ARG	1139	87.667	2.124	11.379	1.00	0.00	LX0	O
ATOM	1485	N	ASN	1140	89.913	1.983	11.250	1.00	0.00	LX0	N
ATOM	1486	H	ASN	1140	90.795	2.040	11.715	0.00	0.00	LX0	H
ATOM	1487	CA	ASN	1140	89.836	1.535	9.859	1.00	0.00	LX0	C
ATOM	1488	CB	ASN	1140	91.152	1.791	9.116	1.00	0.00	LX0	C
ATOM	1489	CG	ASN	1140	91.485	3.269	9.011	1.00	0.00	LX0	C
ATOM	1490	OD1	ASN	1140	90.870	4.151	9.598	1.00	0.00	LX0	O
ATOM	1491	ND2	ASN	1140	92.531	3.515	8.228	1.00	0.00	LX0	N
ATOM	1492	HD21	ASN	1140	93.065	2.815	7.754	0.00	0.00	LX0	H
ATOM	1493	HD22	ASN	1140	92.828	4.464	8.099	0.00	0.00	LX0	H
ATOM	1494	C	ASN	1140	89.434	0.077	9.699	1.00	0.00	LX0	C
ATOM	1495	O	ASN	1140	89.406	-0.471	8.603	1.00	0.00	LX0	O
ATOM	1496	N	CYS	1141	89.124	-0.546	10.841	1.00	0.00	LX0	N
ATOM	1497	H	CYS	1141	89.170	-0.092	11.731	0.00	0.00	LX0	H
ATOM	1498	CA	CYS	1141	88.579	-1.892	10.774	1.00	0.00	LX0	C
ATOM	1499	CB	CYS	1141	89.429	-2.805	11.651	1.00	0.00	LX0	C
ATOM	1500	SG	CYS	1141	91.199	-2.623	11.309	1.00	0.00	LX0	S
ATOM	1501	C	CYS	1141	87.133	-1.865	11.228	1.00	0.00	LX0	C
ATOM	1502	O	CYS	1141	86.806	-1.192	12.195	1.00	0.00	LX0	O
ATOM	1503	N	MET	1142	86.268	-2.565	10.484	1.00	0.00	LX0	N
ATOM	1504	H	MET	1142	86.607	-3.249	9.837	0.00	0.00	LX0	H
ATOM	1505	CA	MET	1142	84.858	-2.482	10.865	1.00	0.00	LX0	C
ATOM	1506	CB	MET	1142	83.965	-2.125	9.673	1.00	0.00	LX0	C
ATOM	1507	CG	MET	1142	84.236	-0.739	9.082	1.00	0.00	LX0	C
ATOM	1508	SD	MET	1142	83.989	0.616	10.236	1.00	0.00	LX0	S
ATOM	1509	CE	MET	1142	84.760	1.919	9.264	1.00	0.00	LX0	C
ATOM	1510	C	MET	1142	84.365	-3.746	11.537	1.00	0.00	LX0	C
ATOM	1511	O	MET	1142	84.957	-4.813	11.418	1.00	0.00	LX0	O
ATOM	1512	N	VAL	1143	83.261	-3.575	12.273	1.00	0.00	LX0	N
ATOM	1513	H	VAL	1143	82.798	-2.687	12.285	0.00	0.00	LX0	H
ATOM	1514	CA	VAL	1143	82.855	-4.651	13.170	1.00	0.00	LX0	C
ATOM	1515	CB	VAL	1143	82.893	-4.165	14.624	1.00	0.00	LX0	C
ATOM	1516	CG1	VAL	1143	82.882	-5.349	15.578	1.00	0.00	LX0	C
ATOM	1517	CG2	VAL	1143	84.106	-3.287	14.920	1.00	0.00	LX0	C
ATOM	1518	C	VAL	1143	81.496	-5.252	12.842	1.00	0.00	LX0	C
ATOM	1519	O	VAL	1143	80.492	-4.565	12.673	1.00	0.00	LX0	O
ATOM	1520	N	ALA	1144	81.521	-6.587	12.753	1.00	0.00	LX0	N
ATOM	1521	H	ALA	1144	82.382	-7.064	12.938	0.00	0.00	LX0	H
ATOM	1522	CA	ALA	1144	80.284	-7.328	12.530	1.00	0.00	LX0	C
ATOM	1523	CB	ALA	1144	80.599	-8.695	11.917	1.00	0.00	LX0	C
ATOM	1524	C	ALA	1144	79.494	-7.527	13.812	1.00	0.00	LX0	C
ATOM	1525	O	ALA	1144	79.952	-7.227	14.907	1.00	0.00	LX0	O
ATOM	1526	N	GLU	1145	78.277	-8.065	13.625	1.00	0.00	LX0	N
ATOM	1527	H	GLU	1145	77.975	-8.242	12.690	0.00	0.00	LX0	H
ATOM	1528	CA	GLU	1145	77.336	-8.243	14.736	1.00	0.00	LX0	C
ATOM	1529	CB	GLU	1145	76.094	-8.965	14.201	1.00	0.00	LX0	C
ATOM	1530	CG	GLU	1145	74.804	-8.749	15.001	1.00	0.00	LX0	C
ATOM	1531	CD	GLU	1145	74.331	-7.312	14.875	1.00	0.00	LX0	C
ATOM	1532	OE1	GLU	1145	73.819	-6.942	13.824	1.00	0.00	LX0	O
ATOM	1533	OE2	GLU	1145	74.462	-6.562	15.833	1.00	0.00	LX0	O
ATOM	1534	C	GLU	1145	77.872	-8.922	16.002	1.00	0.00	LX0	C

ATOM	1535	O	GLU	1145	77.591	-8.542	17.133	1.00	0.00	LX0	O
ATOM	1536	N	ASP	1146	78.672	-9.963	15.752	1.00	0.00	LX0	N
ATOM	1537	H	ASP	1146	78.969	-10.215	14.828	0.00	0.00	LX0	H
ATOM	1538	CA	ASP	1146	79.231	-10.744	16.857	1.00	0.00	LX0	C
ATOM	1539	CB	ASP	1146	79.468	-12.175	16.362	1.00	0.00	LX0	C
ATOM	1540	CG	ASP	1146	80.688	-12.195	15.455	1.00	0.00	LX0	C
ATOM	1541	OD1	ASP	1146	80.691	-11.510	14.434	1.00	0.00	LX0	O
ATOM	1542	OD2	ASP	1146	81.675	-12.811	15.834	1.00	0.00	LX0	O
ATOM	1543	C	ASP	1146	80.523	-10.188	17.454	1.00	0.00	LX0	C
ATOM	1544	O	ASP	1146	81.052	-10.697	18.440	1.00	0.00	LX0	O
ATOM	1545	N	PHE	1147	81.025	-9.151	16.758	1.00	0.00	LX0	N
ATOM	1546	H	PHE	1147	80.489	-8.799	15.993	0.00	0.00	LX0	H
ATOM	1547	CA	PHE	1147	82.371	-8.606	16.928	1.00	0.00	LX0	C
ATOM	1548	CB	PHE	1147	82.749	-8.279	18.383	1.00	0.00	LX0	C
ATOM	1549	CG	PHE	1147	81.775	-7.343	19.065	1.00	0.00	LX0	C
ATOM	1550	CD1	PHE	1147	81.443	-6.099	18.489	1.00	0.00	LX0	C
ATOM	1551	CD2	PHE	1147	81.246	-7.721	20.314	1.00	0.00	LX0	C
ATOM	1552	CE1	PHE	1147	80.642	-5.190	19.206	1.00	0.00	LX0	C
ATOM	1553	CE2	PHE	1147	80.442	-6.817	21.033	1.00	0.00	LX0	C
ATOM	1554	CZ	PHE	1147	80.181	-5.544	20.489	1.00	0.00	LX0	C
ATOM	1555	C	PHE	1147	83.510	-9.361	16.253	1.00	0.00	LX0	C
ATOM	1556	O	PHE	1147	84.635	-9.331	16.731	1.00	0.00	LX0	O
ATOM	1557	N	THR	1148	83.197	-9.979	15.101	1.00	0.00	LX0	N
ATOM	1558	H	THR	1148	82.250	-10.174	14.841	0.00	0.00	LX0	H
ATOM	1559	CA	THR	1148	84.285	-10.253	14.153	1.00	0.00	LX0	C
ATOM	1560	CB	THR	1148	83.776	-11.226	13.071	1.00	0.00	LX0	C
ATOM	1561	OG1	THR	1148	83.366	-12.466	13.661	1.00	0.00	LX0	O
ATOM	1562	HG1	THR	1148	82.548	-12.306	14.138	0.00	0.00	LX0	H
ATOM	1563	CG2	THR	1148	84.777	-11.485	11.939	1.00	0.00	LX0	C
ATOM	1564	C	THR	1148	84.764	-8.938	13.535	1.00	0.00	LX0	C
ATOM	1565	O	THR	1148	83.951	-8.127	13.094	1.00	0.00	LX0	O
ATOM	1566	N	VAL	1149	86.085	-8.720	13.563	1.00	0.00	LX0	N
ATOM	1567	H	VAL	1149	86.717	-9.435	13.887	0.00	0.00	LX0	H
ATOM	1568	CA	VAL	1149	86.558	-7.400	13.142	1.00	0.00	LX0	C
ATOM	1569	CB	VAL	1149	87.352	-6.709	14.259	1.00	0.00	LX0	C
ATOM	1570	CG1	VAL	1149	87.667	-5.262	13.880	1.00	0.00	LX0	C
ATOM	1571	CG2	VAL	1149	86.611	-6.762	15.595	1.00	0.00	LX0	C
ATOM	1572	C	VAL	1149	87.332	-7.393	11.833	1.00	0.00	LX0	C
ATOM	1573	O	VAL	1149	88.530	-7.629	11.748	1.00	0.00	LX0	O
ATOM	1574	N	LYS	1150	86.571	-7.093	10.778	1.00	0.00	LX0	N
ATOM	1575	H	LYS	1150	85.681	-6.667	10.948	0.00	0.00	LX0	H
ATOM	1576	CA	LYS	1150	87.226	-7.105	9.474	1.00	0.00	LX0	C
ATOM	1577	CB	LYS	1150	86.200	-7.259	8.365	1.00	0.00	LX0	C
ATOM	1578	CG	LYS	1150	85.332	-8.509	8.392	1.00	0.00	LX0	C
ATOM	1579	CD	LYS	1150	84.536	-8.452	7.098	1.00	0.00	LX0	C
ATOM	1580	CE	LYS	1150	83.585	-9.601	6.805	1.00	0.00	LX0	C
ATOM	1581	NZ	LYS	1150	83.340	-9.527	5.363	1.00	0.00	LX0	N
ATOM	1582	HZ1	LYS	1150	82.493	-10.062	5.078	0.00	0.00	LX0	H
ATOM	1583	HZ2	LYS	1150	83.087	-8.551	5.100	0.00	0.00	LX0	H
ATOM	1584	HZ3	LYS	1150	84.180	-9.804	4.813	0.00	0.00	LX0	H
ATOM	1585	C	LYS	1150	88.009	-5.841	9.180	1.00	0.00	LX0	C
ATOM	1586	O	LYS	1150	87.809	-4.799	9.793	1.00	0.00	LX0	O
ATOM	1587	N	ILE	1151	88.857	-5.950	8.146	1.00	0.00	LX0	N
ATOM	1588	H	ILE	1151	88.985	-6.837	7.702	0.00	0.00	LX0	H
ATOM	1589	CA	ILE	1151	89.309	-4.715	7.507	1.00	0.00	LX0	C
ATOM	1590	CB	ILE	1151	90.444	-5.016	6.517	1.00	0.00	LX0	C
ATOM	1591	CG2	ILE	1151	90.595	-4.039	5.341	1.00	0.00	LX0	C
ATOM	1592	CG1	ILE	1151	91.723	-5.066	7.343	1.00	0.00	LX0	C
ATOM	1593	CD1	ILE	1151	92.009	-3.730	8.034	1.00	0.00	LX0	C
ATOM	1594	C	ILE	1151	88.150	-3.962	6.888	1.00	0.00	LX0	C
ATOM	1595	O	ILE	1151	87.252	-4.536	6.281	1.00	0.00	LX0	O

ATOM	1596	N	GLY	1152	88.189	-2.660	7.182	1.00	0.00	LX0	N
ATOM	1597	H	GLY	1152	89.011	-2.207	7.532	0.00	0.00	LX0	H
ATOM	1598	CA	GLY	1152	86.962	-1.903	7.053	1.00	0.00	LX0	C
ATOM	1599	C	GLY	1152	86.670	-1.281	5.715	1.00	0.00	LX0	C
ATOM	1600	O	GLY	1152	86.997	-1.779	4.644	1.00	0.00	LX0	O
ATOM	1601	N	ASP	1153	85.966	-0.162	5.881	1.00	0.00	LX0	N
ATOM	1602	H	ASP	1153	85.924	0.263	6.782	0.00	0.00	LX0	H
ATOM	1603	CA	ASP	1153	85.421	0.554	4.742	1.00	0.00	LX0	C
ATOM	1604	CB	ASP	1153	84.131	1.235	5.215	1.00	0.00	LX0	C
ATOM	1605	CG	ASP	1153	83.329	1.858	4.091	1.00	0.00	LX0	C
ATOM	1606	OD1	ASP	1153	83.392	1.394	2.954	1.00	0.00	LX0	O
ATOM	1607	OD2	ASP	1153	82.620	2.820	4.356	1.00	0.00	LX0	O
ATOM	1608	C	ASP	1153	86.452	1.523	4.201	1.00	0.00	LX0	C
ATOM	1609	O	ASP	1153	87.372	1.936	4.900	1.00	0.00	LX0	O
ATOM	1610	N	PHE	1154	86.289	1.817	2.912	1.00	0.00	LX0	N
ATOM	1611	H	PHE	1154	85.402	1.591	2.505	0.00	0.00	LX0	H
ATOM	1612	CA	PHE	1154	87.245	2.718	2.286	1.00	0.00	LX0	C
ATOM	1613	CB	PHE	1154	87.668	2.154	0.921	1.00	0.00	LX0	C
ATOM	1614	CG	PHE	1154	88.767	1.113	1.045	1.00	0.00	LX0	C
ATOM	1615	CD1	PHE	1154	88.641	0.017	1.931	1.00	0.00	LX0	C
ATOM	1616	CD2	PHE	1154	89.915	1.256	0.236	1.00	0.00	LX0	C
ATOM	1617	CE1	PHE	1154	89.659	-0.954	1.994	1.00	0.00	LX0	C
ATOM	1618	CE2	PHE	1154	90.937	0.288	0.297	1.00	0.00	LX0	C
ATOM	1619	CZ	PHE	1154	90.792	-0.813	1.167	1.00	0.00	LX0	C
ATOM	1620	C	PHE	1154	86.641	4.106	2.192	1.00	0.00	LX0	C
ATOM	1621	O	PHE	1154	85.633	4.395	2.820	1.00	0.00	LX0	O
ATOM	1622	N	GLY	1155	87.294	4.956	1.376	1.00	0.00	LX0	N
ATOM	1623	H	GLY	1155	88.154	4.672	0.963	0.00	0.00	LX0	H
ATOM	1624	CA	GLY	1155	86.689	6.245	1.033	1.00	0.00	LX0	C
ATOM	1625	C	GLY	1155	86.288	7.116	2.207	1.00	0.00	LX0	C
ATOM	1626	O	GLY	1155	87.120	7.798	2.793	1.00	0.00	LX0	O
ATOM	1627	N	MET	1156	84.976	7.054	2.503	1.00	0.00	LX0	N
ATOM	1628	H	MET	1156	84.462	6.375	1.966	0.00	0.00	LX0	H
ATOM	1629	CA	MET	1156	84.335	7.739	3.635	1.00	0.00	LX0	C
ATOM	1630	CB	MET	1156	83.687	6.698	4.553	1.00	0.00	LX0	C
ATOM	1631	CG	MET	1156	82.466	6.035	3.922	1.00	0.00	LX0	C
ATOM	1632	SD	MET	1156	80.917	6.874	4.286	1.00	0.00	LX0	S
ATOM	1633	CE	MET	1156	80.790	6.349	6.003	1.00	0.00	LX0	C
ATOM	1634	C	MET	1156	85.162	8.749	4.430	1.00	0.00	LX0	C
ATOM	1635	O	MET	1156	85.117	9.942	4.159	1.00	0.00	LX0	O
ATOM	1636	N	THR	1157	85.922	8.200	5.402	1.00	0.00	LX0	N
ATOM	1637	H	THR	1157	85.964	7.204	5.438	0.00	0.00	LX0	H
ATOM	1638	CA	THR	1157	86.776	8.959	6.324	1.00	0.00	LX0	C
ATOM	1639	CB	THR	1157	88.142	9.274	5.692	1.00	0.00	LX0	C
ATOM	1640	OG1	THR	1157	87.991	9.914	4.418	1.00	0.00	LX0	O
ATOM	1641	HG1	THR	1157	87.630	9.253	3.829	0.00	0.00	LX0	H
ATOM	1642	CG2	THR	1157	88.988	8.006	5.552	1.00	0.00	LX0	C
ATOM	1643	C	THR	1157	86.182	10.172	7.033	1.00	0.00	LX0	C
ATOM	1644	O	THR	1157	85.835	10.100	8.204	1.00	0.00	LX0	O
ATOM	1645	N	ARG	1158	86.098	11.277	6.277	1.00	0.00	LX0	N
ATOM	1646	H	ARG	1158	86.305	11.152	5.305	0.00	0.00	LX0	H
ATOM	1647	CA	ARG	1158	85.553	12.514	6.835	1.00	0.00	LX0	C
ATOM	1648	CB	ARG	1158	85.693	13.675	5.846	1.00	0.00	LX0	C
ATOM	1649	CG	ARG	1158	85.281	13.328	4.411	1.00	0.00	LX0	C
ATOM	1650	CD	ARG	1158	84.739	14.531	3.640	1.00	0.00	LX0	C
ATOM	1651	NE	ARG	1158	83.431	14.904	4.173	1.00	0.00	LX0	N
ATOM	1652	HE	ARG	1158	83.354	15.325	5.087	0.00	0.00	LX0	H
ATOM	1653	CZ	ARG	1158	82.289	14.485	3.594	1.00	0.00	LX0	C
ATOM	1654	NH1	ARG	1158	82.301	13.843	2.430	1.00	0.00	LX0	N
ATOM	1655	HH11	ARG	1158	81.432	13.489	2.070	0.00	0.00	LX0	H
ATOM	1656	HH12	ARG	1158	83.151	13.696	1.928	0.00	0.00	LX0	H

ATOM	1657	NH2	ARG	1158	81.134	14.694	4.199	1.00	0.00	LX0	N
ATOM	1658	HH21	ARG	1158	80.260	14.396	3.814	0.00	0.00	LX0	H
ATOM	1659	HH22	ARG	1158	81.110	15.123	5.121	0.00	0.00	LX0	H
ATOM	1660	C	ARG	1158	84.112	12.407	7.307	1.00	0.00	LX0	C
ATOM	1661	O	ARG	1158	83.349	11.577	6.819	1.00	0.00	LX0	O
ATOM	1662	N	ASP	1159	83.803	13.301	8.263	1.00	0.00	LX0	N
ATOM	1663	H	ASP	1159	84.572	13.786	8.685	0.00	0.00	LX0	H
ATOM	1664	CA	ASP	1159	82.502	13.395	8.941	1.00	0.00	LX0	C
ATOM	1665	CB	ASP	1159	81.281	13.452	7.996	1.00	0.00	LX0	C
ATOM	1666	CG	ASP	1159	81.217	14.686	7.104	1.00	0.00	LX0	C
ATOM	1667	OD1	ASP	1159	82.242	15.243	6.716	1.00	0.00	LX0	O
ATOM	1668	OD2	ASP	1159	80.119	15.051	6.691	1.00	0.00	LX0	O
ATOM	1669	C	ASP	1159	82.291	12.274	9.945	1.00	0.00	LX0	C
ATOM	1670	O	ASP	1159	81.181	11.993	10.385	1.00	0.00	LX0	O
ATOM	1671	N	ILE	1160	83.410	11.600	10.253	1.00	0.00	LX0	N
ATOM	1672	H	ILE	1160	84.297	11.997	9.999	0.00	0.00	LX0	H
ATOM	1673	CA	ILE	1160	83.312	10.395	11.071	1.00	0.00	LX0	C
ATOM	1674	CB	ILE	1160	83.551	9.114	10.234	1.00	0.00	LX0	C
ATOM	1675	CG2	ILE	1160	83.405	7.843	11.082	1.00	0.00	LX0	C
ATOM	1676	CG1	ILE	1160	82.685	9.033	8.969	1.00	0.00	LX0	C
ATOM	1677	CD1	ILE	1160	81.192	8.829	9.239	1.00	0.00	LX0	C
ATOM	1678	C	ILE	1160	84.280	10.444	12.242	1.00	0.00	LX0	C
ATOM	1679	O	ILE	1160	83.920	10.234	13.395	1.00	0.00	LX0	O
ATOM	1680	N	TYR	1161	85.551	10.686	11.891	1.00	0.00	LX0	N
ATOM	1681	H	TYR	1161	85.776	11.018	10.968	0.00	0.00	LX0	H
ATOM	1682	CA	TYR	1161	86.574	10.430	12.900	1.00	0.00	LX0	C
ATOM	1683	CB	TYR	1161	87.007	8.959	12.862	1.00	0.00	LX0	C
ATOM	1684	CG	TYR	1161	86.827	8.319	14.221	1.00	0.00	LX0	C
ATOM	1685	CD1	TYR	1161	87.675	8.696	15.280	1.00	0.00	LX0	C
ATOM	1686	CE1	TYR	1161	87.519	8.087	16.535	1.00	0.00	LX0	C
ATOM	1687	CD2	TYR	1161	85.814	7.353	14.391	1.00	0.00	LX0	C
ATOM	1688	CE2	TYR	1161	85.655	6.747	15.649	1.00	0.00	LX0	C
ATOM	1689	CZ	TYR	1161	86.512	7.116	16.706	1.00	0.00	LX0	C
ATOM	1690	OH	TYR	1161	86.372	6.522	17.951	1.00	0.00	LX0	O
ATOM	1691	HH	TYR	1161	85.636	5.906	17.874	0.00	0.00	LX0	H
ATOM	1692	C	TYR	1161	87.773	11.352	12.813	1.00	0.00	LX0	C
ATOM	1693	O	TYR	1161	88.929	10.951	12.888	1.00	0.00	LX0	O
ATOM	1694	N	GLU	1162	87.448	12.635	12.666	1.00	0.00	LX0	N
ATOM	1695	H	GLU	1162	86.484	12.867	12.503	0.00	0.00	LX0	H
ATOM	1696	CA	GLU	1162	88.496	13.606	12.348	1.00	0.00	LX0	C
ATOM	1697	CB	GLU	1162	87.861	14.927	11.894	1.00	0.00	LX0	C
ATOM	1698	CG	GLU	1162	87.153	14.866	10.525	1.00	0.00	LX0	C
ATOM	1699	CD	GLU	1162	86.050	13.821	10.513	1.00	0.00	LX0	C
ATOM	1700	OE1	GLU	1162	86.206	12.800	9.857	1.00	0.00	LX0	O
ATOM	1701	OE2	GLU	1162	85.059	13.981	11.214	1.00	0.00	LX0	O
ATOM	1702	C	GLU	1162	89.527	13.818	13.446	1.00	0.00	LX0	C
ATOM	1703	O	GLU	1162	90.688	14.128	13.210	1.00	0.00	LX0	O
ATOM	1704	N	THR	1163	89.067	13.577	14.682	1.00	0.00	LX0	N
ATOM	1705	H	THR	1163	88.122	13.285	14.833	0.00	0.00	LX0	H
ATOM	1706	CA	THR	1163	89.976	13.609	15.830	1.00	0.00	LX0	C
ATOM	1707	CB	THR	1163	89.120	13.572	17.091	1.00	0.00	LX0	C
ATOM	1708	OG1	THR	1163	87.959	12.759	16.853	1.00	0.00	LX0	O
ATOM	1709	HG1	THR	1163	87.436	12.789	17.645	0.00	0.00	LX0	H
ATOM	1710	CG2	THR	1163	88.688	14.979	17.507	1.00	0.00	LX0	C
ATOM	1711	C	THR	1163	91.043	12.515	15.856	1.00	0.00	LX0	C
ATOM	1712	O	THR	1163	91.986	12.524	16.642	1.00	0.00	LX0	O
ATOM	1713	N	ASP	1164	90.843	11.564	14.938	1.00	0.00	LX0	N
ATOM	1714	H	ASP	1164	90.042	11.586	14.340	0.00	0.00	LX0	H
ATOM	1715	CA	ASP	1164	91.806	10.493	14.737	1.00	0.00	LX0	C
ATOM	1716	CB	ASP	1164	90.997	9.208	14.595	1.00	0.00	LX0	C
ATOM	1717	CG	ASP	1164	91.451	8.121	15.545	1.00	0.00	LX0	C

ATOM	1718	OD1	ASP	1164	91.171	8.206	16.735	1.00	0.00	LX0	O
ATOM	1719	OD2	ASP	1164	92.052	7.150	15.100	1.00	0.00	LX0	O
ATOM	1720	C	ASP	1164	92.725	10.745	13.539	1.00	0.00	LX0	C
ATOM	1721	O	ASP	1164	93.396	9.859	13.011	1.00	0.00	LX0	O
ATOM	1722	N	TYR	1165	92.727	12.020	13.121	1.00	0.00	LX0	N
ATOM	1723	H	TYR	1165	92.153	12.721	13.548	0.00	0.00	LX0	H
ATOM	1724	CA	TYR	1165	93.631	12.474	12.070	1.00	0.00	LX0	C
ATOM	1725	CB	TYR	1165	92.857	12.802	10.788	1.00	0.00	LX0	C
ATOM	1726	CG	TYR	1165	92.405	11.558	10.061	1.00	0.00	LX0	C
ATOM	1727	CD1	TYR	1165	91.101	11.068	10.277	1.00	0.00	LX0	C
ATOM	1728	CE1	TYR	1165	90.688	9.912	9.595	1.00	0.00	LX0	C
ATOM	1729	CD2	TYR	1165	93.302	10.931	9.173	1.00	0.00	LX0	C
ATOM	1730	CE2	TYR	1165	92.888	9.776	8.490	1.00	0.00	LX0	C
ATOM	1731	CZ	TYR	1165	91.592	9.273	8.723	1.00	0.00	LX0	C
ATOM	1732	OH	TYR	1165	91.202	8.101	8.103	1.00	0.00	LX0	O
ATOM	1733	HH	TYR	1165	91.928	7.746	7.588	0.00	0.00	LX0	H
ATOM	1734	C	TYR	1165	94.347	13.723	12.548	1.00	0.00	LX0	C
ATOM	1735	O	TYR	1165	94.052	14.255	13.611	1.00	0.00	LX0	O
ATOM	1736	N	TYR	1166	95.290	14.178	11.709	1.00	0.00	LX0	N
ATOM	1737	H	TYR	1166	95.594	13.633	10.925	0.00	0.00	LX0	H
ATOM	1738	CA	TYR	1166	95.927	15.471	11.940	1.00	0.00	LX0	C
ATOM	1739	CB	TYR	1166	96.911	15.396	13.116	1.00	0.00	LX0	C
ATOM	1740	CG	TYR	1166	97.663	16.694	13.312	1.00	0.00	LX0	C
ATOM	1741	CD1	TYR	1166	96.977	17.852	13.736	1.00	0.00	LX0	C
ATOM	1742	CE1	TYR	1166	97.697	19.047	13.897	1.00	0.00	LX0	C
ATOM	1743	CD2	TYR	1166	99.046	16.700	13.048	1.00	0.00	LX0	C
ATOM	1744	CE2	TYR	1166	99.760	17.893	13.209	1.00	0.00	LX0	C
ATOM	1745	CZ	TYR	1166	99.081	19.052	13.633	1.00	0.00	LX0	C
ATOM	1746	OH	TYR	1166	99.784	20.223	13.803	1.00	0.00	LX0	O
ATOM	1747	HH	TYR	1166	100.699	20.074	13.551	0.00	0.00	LX0	H
ATOM	1748	C	TYR	1166	96.617	15.950	10.679	1.00	0.00	LX0	C
ATOM	1749	O	TYR	1166	97.173	15.186	9.905	1.00	0.00	LX0	O
ATOM	1750	N	ARG	1167	96.555	17.268	10.494	1.00	0.00	LX0	N
ATOM	1751	H	ARG	1167	96.147	17.853	11.193	0.00	0.00	LX0	H
ATOM	1752	CA	ARG	1167	97.220	17.827	9.325	1.00	0.00	LX0	C
ATOM	1753	CB	ARG	1167	96.461	19.111	8.970	1.00	0.00	LX0	C
ATOM	1754	CG	ARG	1167	96.890	19.825	7.693	1.00	0.00	LX0	C
ATOM	1755	CD	ARG	1167	96.043	21.068	7.407	1.00	0.00	LX0	C
ATOM	1756	NE	ARG	1167	96.736	21.937	6.459	1.00	0.00	LX0	N
ATOM	1757	HE	ARG	1167	97.457	22.537	6.813	0.00	0.00	LX0	H
ATOM	1758	CZ	ARG	1167	96.579	21.836	5.125	1.00	0.00	LX0	C
ATOM	1759	NH1	ARG	1167	95.605	21.092	4.605	1.00	0.00	LX0	N
ATOM	1760	HH11	ARG	1167	95.523	20.954	3.618	0.00	0.00	LX0	H
ATOM	1761	HH12	ARG	1167	94.941	20.645	5.205	0.00	0.00	LX0	H
ATOM	1762	NH2	ARG	1167	97.437	22.484	4.345	1.00	0.00	LX0	N
ATOM	1763	HH21	ARG	1167	97.364	22.481	3.348	0.00	0.00	LX0	H
ATOM	1764	HH22	ARG	1167	98.204	22.972	4.775	0.00	0.00	LX0	H
ATOM	1765	C	ARG	1167	98.723	18.024	9.535	1.00	0.00	LX0	C
ATOM	1766	O	ARG	1167	99.158	19.105	9.912	1.00	0.00	LX0	O
ATOM	1767	N	LYS	1168	99.500	16.942	9.298	1.00	0.00	LX0	N
ATOM	1768	H	LYS	1168	99.056	16.128	8.914	0.00	0.00	LX0	H
ATOM	1769	CA	LYS	1168	100.926	16.988	9.669	1.00	0.00	LX0	C
ATOM	1770	CB	LYS	1168	101.699	15.682	9.359	1.00	0.00	LX0	C
ATOM	1771	CG	LYS	1168	102.407	15.512	8.004	1.00	0.00	LX0	C
ATOM	1772	CD	LYS	1168	103.607	14.547	8.050	1.00	0.00	LX0	C
ATOM	1773	CE	LYS	1168	104.472	14.535	6.776	1.00	0.00	LX0	C
ATOM	1774	NZ	LYS	1168	105.637	13.645	6.910	1.00	0.00	LX0	N
ATOM	1775	HZ1	LYS	1168	106.284	13.734	6.095	0.00	0.00	LX0	H
ATOM	1776	HZ2	LYS	1168	106.223	13.887	7.739	0.00	0.00	LX0	H
ATOM	1777	HZ3	LYS	1168	105.363	12.644	6.986	0.00	0.00	LX0	H
ATOM	1778	C	LYS	1168	101.689	18.194	9.145	1.00	0.00	LX0	C

ATOM	1779	O	LYS	1168	101.650	18.500	7.956	1.00	0.00	LX0	O
ATOM	1780	N	GLY	1169	102.344	18.892	10.083	1.00	0.00	LX0	N
ATOM	1781	H	GLY	1169	102.345	18.614	11.050	0.00	0.00	LX0	H
ATOM	1782	CA	GLY	1169	103.086	20.102	9.729	1.00	0.00	LX0	C
ATOM	1783	C	GLY	1169	102.297	21.149	8.956	1.00	0.00	LX0	C
ATOM	1784	O	GLY	1169	102.829	21.875	8.115	1.00	0.00	LX0	O
ATOM	1785	N	GLY	1170	100.983	21.183	9.232	1.00	0.00	LX0	N
ATOM	1786	H	GLY	1170	100.611	20.548	9.916	0.00	0.00	LX0	H
ATOM	1787	CA	GLY	1170	100.110	22.096	8.494	1.00	0.00	LX0	C
ATOM	1788	C	GLY	1170	99.930	21.784	7.009	1.00	0.00	LX0	C
ATOM	1789	O	GLY	1170	99.477	22.613	6.221	1.00	0.00	LX0	O
ATOM	1790	N	LYS	1171	100.311	20.554	6.645	1.00	0.00	LX0	N
ATOM	1791	H	LYS	1171	100.581	19.900	7.352	0.00	0.00	LX0	H
ATOM	1792	CA	LYS	1171	100.287	20.178	5.234	1.00	0.00	LX0	C
ATOM	1793	CB	LYS	1171	101.630	19.578	4.795	1.00	0.00	LX0	C
ATOM	1794	CG	LYS	1171	102.899	20.408	5.040	1.00	0.00	LX0	C
ATOM	1795	CD	LYS	1171	103.027	21.692	4.208	1.00	0.00	LX0	C
ATOM	1796	CE	LYS	1171	102.776	22.988	4.988	1.00	0.00	LX0	C
ATOM	1797	NZ	LYS	1171	103.740	23.109	6.091	1.00	0.00	LX0	N
ATOM	1798	HZ1	LYS	1171	103.539	23.957	6.654	0.00	0.00	LX0	H
ATOM	1799	HZ2	LYS	1171	103.629	22.294	6.733	0.00	0.00	LX0	H
ATOM	1800	HZ3	LYS	1171	104.716	23.133	5.739	0.00	0.00	LX0	H
ATOM	1801	C	LYS	1171	99.149	19.235	4.875	1.00	0.00	LX0	C
ATOM	1802	O	LYS	1171	98.184	19.625	4.228	1.00	0.00	LX0	O
ATOM	1803	N	GLY	1172	99.302	17.976	5.309	1.00	0.00	LX0	N
ATOM	1804	H	GLY	1172	100.025	17.720	5.953	0.00	0.00	LX0	H
ATOM	1805	CA	GLY	1172	98.363	16.975	4.805	1.00	0.00	LX0	C
ATOM	1806	C	GLY	1172	97.642	16.250	5.917	1.00	0.00	LX0	C
ATOM	1807	O	GLY	1172	98.177	16.072	7.003	1.00	0.00	LX0	O
ATOM	1808	N	LEU	1173	96.394	15.869	5.610	1.00	0.00	LX0	N
ATOM	1809	H	LEU	1173	96.063	15.926	4.670	0.00	0.00	LX0	H
ATOM	1810	CA	LEU	1173	95.606	15.189	6.637	1.00	0.00	LX0	C
ATOM	1811	CB	LEU	1173	94.110	15.373	6.367	1.00	0.00	LX0	C
ATOM	1812	CG	LEU	1173	93.227	15.051	7.576	1.00	0.00	LX0	C
ATOM	1813	CD1	LEU	1173	93.440	16.049	8.716	1.00	0.00	LX0	C
ATOM	1814	CD2	LEU	1173	91.752	14.932	7.193	1.00	0.00	LX0	C
ATOM	1815	C	LEU	1173	95.967	13.721	6.770	1.00	0.00	LX0	C
ATOM	1816	O	LEU	1173	95.488	12.858	6.049	1.00	0.00	LX0	O
ATOM	1817	N	LEU	1174	96.874	13.496	7.717	1.00	0.00	LX0	N
ATOM	1818	H	LEU	1174	97.201	14.258	8.277	0.00	0.00	LX0	H
ATOM	1819	CA	LEU	1174	97.419	12.161	7.919	1.00	0.00	LX0	C
ATOM	1820	CB	LEU	1174	98.921	12.222	7.617	1.00	0.00	LX0	C
ATOM	1821	CG	LEU	1174	99.224	12.510	6.142	1.00	0.00	LX0	C
ATOM	1822	CD1	LEU	1174	100.702	12.808	5.903	1.00	0.00	LX0	C
ATOM	1823	CD2	LEU	1174	98.722	11.393	5.225	1.00	0.00	LX0	C
ATOM	1824	C	LEU	1174	97.130	11.671	9.330	1.00	0.00	LX0	C
ATOM	1825	O	LEU	1174	97.006	12.453	10.266	1.00	0.00	LX0	O
ATOM	1826	N	PRO	1175	96.998	10.337	9.489	1.00	0.00	LX0	N
ATOM	1827	CD	PRO	1175	97.005	9.307	8.453	1.00	0.00	LX0	C
ATOM	1828	CA	PRO	1175	96.784	9.795	10.838	1.00	0.00	LX0	C
ATOM	1829	CB	PRO	1175	96.258	8.395	10.518	1.00	0.00	LX0	C
ATOM	1830	CG	PRO	1175	96.995	8.004	9.237	1.00	0.00	LX0	C
ATOM	1831	C	PRO	1175	98.061	9.773	11.677	1.00	0.00	LX0	C
ATOM	1832	O	PRO	1175	98.641	8.729	11.942	1.00	0.00	LX0	O
ATOM	1833	N	VAL	1176	98.491	10.972	12.099	1.00	0.00	LX0	N
ATOM	1834	H	VAL	1176	97.947	11.776	11.856	0.00	0.00	LX0	H
ATOM	1835	CA	VAL	1176	99.871	11.081	12.590	1.00	0.00	LX0	C
ATOM	1836	CB	VAL	1176	100.235	12.536	12.917	1.00	0.00	LX0	C
ATOM	1837	CG1	VAL	1176	100.003	13.401	11.679	1.00	0.00	LX0	C
ATOM	1838	CG2	VAL	1176	99.534	13.094	14.160	1.00	0.00	LX0	C
ATOM	1839	C	VAL	1176	100.320	10.132	13.695	1.00	0.00	LX0	C

ATOM	1840	O	VAL	1176	101.408	9.578	13.662	1.00	0.00	LX0	O
ATOM	1841	N	ARG	1177	99.419	9.932	14.670	1.00	0.00	LX0	N
ATOM	1842	H	ARG	1177	98.505	10.322	14.580	0.00	0.00	LX0	H
ATOM	1843	CA	ARG	1177	99.803	9.090	15.807	1.00	0.00	LX0	C
ATOM	1844	CB	ARG	1177	98.820	9.293	16.962	1.00	0.00	LX0	C
ATOM	1845	CG	ARG	1177	98.685	10.753	17.397	1.00	0.00	LX0	C
ATOM	1846	CD	ARG	1177	97.734	10.937	18.578	1.00	0.00	LX0	C
ATOM	1847	NE	ARG	1177	97.743	12.322	19.044	1.00	0.00	LX0	N
ATOM	1848	HE	ARG	1177	97.484	13.060	18.415	0.00	0.00	LX0	H
ATOM	1849	CZ	ARG	1177	98.129	12.656	20.294	1.00	0.00	LX0	C
ATOM	1850	NH1	ARG	1177	98.531	11.747	21.170	1.00	0.00	LX0	N
ATOM	1851	HH11	ARG	1177	98.758	12.015	22.113	0.00	0.00	LX0	H
ATOM	1852	HH12	ARG	1177	98.627	10.778	20.917	0.00	0.00	LX0	H
ATOM	1853	NH2	ARG	1177	98.117	13.932	20.640	1.00	0.00	LX0	N
ATOM	1854	HH21	ARG	1177	98.436	14.240	21.546	0.00	0.00	LX0	H
ATOM	1855	HH22	ARG	1177	97.812	14.618	19.973	0.00	0.00	LX0	H
ATOM	1856	C	ARG	1177	99.965	7.607	15.494	1.00	0.00	LX0	C
ATOM	1857	O	ARG	1177	100.413	6.814	16.309	1.00	0.00	LX0	O
ATOM	1858	N	TRP	1178	99.548	7.275	14.268	1.00	0.00	LX0	N
ATOM	1859	H	TRP	1178	99.240	7.966	13.618	0.00	0.00	LX0	H
ATOM	1860	CA	TRP	1178	99.554	5.890	13.822	1.00	0.00	LX0	C
ATOM	1861	CB	TRP	1178	98.165	5.556	13.264	1.00	0.00	LX0	C
ATOM	1862	CG	TRP	1178	97.128	5.818	14.332	1.00	0.00	LX0	C
ATOM	1863	CD2	TRP	1178	96.470	7.058	14.688	1.00	0.00	LX0	C
ATOM	1864	CE2	TRP	1178	95.625	6.785	15.812	1.00	0.00	LX0	C
ATOM	1865	CE3	TRP	1178	96.533	8.370	14.175	1.00	0.00	LX0	C
ATOM	1866	CD1	TRP	1178	96.642	4.875	15.242	1.00	0.00	LX0	C
ATOM	1867	NE1	TRP	1178	95.763	5.436	16.108	1.00	0.00	LX0	N
ATOM	1868	HE1	TRP	1178	95.321	4.945	16.836	0.00	0.00	LX0	H
ATOM	1869	CZ2	TRP	1178	94.872	7.822	16.401	1.00	0.00	LX0	C
ATOM	1870	CZ3	TRP	1178	95.779	9.402	14.770	1.00	0.00	LX0	C
ATOM	1871	CH2	TRP	1178	94.954	9.129	15.881	1.00	0.00	LX0	C
ATOM	1872	C	TRP	1178	100.625	5.632	12.779	1.00	0.00	LX0	C
ATOM	1873	O	TRP	1178	100.772	4.532	12.258	1.00	0.00	LX0	O
ATOM	1874	N	MET	1179	101.340	6.717	12.448	1.00	0.00	LX0	N
ATOM	1875	H	MET	1179	101.357	7.543	13.012	0.00	0.00	LX0	H
ATOM	1876	CA	MET	1179	102.211	6.601	11.288	1.00	0.00	LX0	C
ATOM	1877	CB	MET	1179	102.239	7.918	10.520	1.00	0.00	LX0	C
ATOM	1878	CG	MET	1179	100.932	8.173	9.776	1.00	0.00	LX0	C
ATOM	1879	SD	MET	1179	100.737	9.888	9.271	1.00	0.00	LX0	S
ATOM	1880	CE	MET	1179	102.199	10.031	8.237	1.00	0.00	LX0	C
ATOM	1881	C	MET	1179	103.608	6.142	11.615	1.00	0.00	LX0	C
ATOM	1882	O	MET	1179	104.143	6.323	12.701	1.00	0.00	LX0	O
ATOM	1883	N	SER	1180	104.167	5.527	10.577	1.00	0.00	LX0	N
ATOM	1884	H	SER	1180	103.654	5.385	9.731	0.00	0.00	LX0	H
ATOM	1885	CA	SER	1180	105.545	5.062	10.611	1.00	0.00	LX0	C
ATOM	1886	CB	SER	1180	105.700	4.129	9.410	1.00	0.00	LX0	C
ATOM	1887	OG	SER	1180	104.690	4.398	8.430	1.00	0.00	LX0	O
ATOM	1888	HG	SER	1180	105.107	4.947	7.765	0.00	0.00	LX0	H
ATOM	1889	C	SER	1180	106.558	6.202	10.636	1.00	0.00	LX0	C
ATOM	1890	O	SER	1180	106.278	7.295	10.157	1.00	0.00	LX0	O
ATOM	1891	N	PRO	1181	107.752	5.920	11.228	1.00	0.00	LX0	N
ATOM	1892	CD	PRO	1181	108.162	4.641	11.799	1.00	0.00	LX0	C
ATOM	1893	CA	PRO	1181	108.798	6.941	11.379	1.00	0.00	LX0	C
ATOM	1894	CB	PRO	1181	110.020	6.102	11.758	1.00	0.00	LX0	C
ATOM	1895	CG	PRO	1181	109.451	4.936	12.549	1.00	0.00	LX0	C
ATOM	1896	C	PRO	1181	109.036	7.831	10.173	1.00	0.00	LX0	C
ATOM	1897	O	PRO	1181	108.822	9.038	10.201	1.00	0.00	LX0	O
ATOM	1898	N	GLU	1182	109.473	7.162	9.098	1.00	0.00	LX0	N
ATOM	1899	H	GLU	1182	109.647	6.178	9.155	0.00	0.00	LX0	H
ATOM	1900	CA	GLU	1182	109.686	7.834	7.818	1.00	0.00	LX0	C

ATOM	1901	CB	GLU	1182	110.216	6.829	6.772	1.00	0.00	LX0	C
ATOM	1902	CG	GLU	1182	109.414	5.547	6.455	1.00	0.00	LX0	C
ATOM	1903	CD	GLU	1182	109.341	4.568	7.624	1.00	0.00	LX0	C
ATOM	1904	OE1	GLU	1182	110.288	4.439	8.393	1.00	0.00	LX0	O
ATOM	1905	OE2	GLU	1182	108.316	3.927	7.796	1.00	0.00	LX0	O
ATOM	1906	C	GLU	1182	108.485	8.648	7.354	1.00	0.00	LX0	C
ATOM	1907	O	GLU	1182	108.535	9.853	7.140	1.00	0.00	LX0	O
ATOM	1908	N	SER	1183	107.338	7.959	7.324	1.00	0.00	LX0	N
ATOM	1909	H	SER	1183	107.324	6.970	7.475	0.00	0.00	LX0	H
ATOM	1910	CA	SER	1183	106.102	8.667	7.000	1.00	0.00	LX0	C
ATOM	1911	CB	SER	1183	104.948	7.673	7.092	1.00	0.00	LX0	C
ATOM	1912	OG	SER	1183	105.414	6.369	6.709	1.00	0.00	LX0	O
ATOM	1913	HG	SER	1183	105.613	6.403	5.771	0.00	0.00	LX0	H
ATOM	1914	C	SER	1183	105.837	9.955	7.792	1.00	0.00	LX0	C
ATOM	1915	O	SER	1183	105.363	10.970	7.276	1.00	0.00	LX0	O
ATOM	1916	N	LEU	1184	106.205	9.891	9.081	1.00	0.00	LX0	N
ATOM	1917	H	LEU	1184	106.633	9.063	9.451	0.00	0.00	LX0	H
ATOM	1918	CA	LEU	1184	106.114	11.113	9.871	1.00	0.00	LX0	C
ATOM	1919	CB	LEU	1184	106.105	10.826	11.371	1.00	0.00	LX0	C
ATOM	1920	CG	LEU	1184	104.694	10.436	11.808	1.00	0.00	LX0	C
ATOM	1921	CD1	LEU	1184	104.620	10.108	13.291	1.00	0.00	LX0	C
ATOM	1922	CD2	LEU	1184	103.668	11.508	11.438	1.00	0.00	LX0	C
ATOM	1923	C	LEU	1184	107.104	12.191	9.497	1.00	0.00	LX0	C
ATOM	1924	O	LEU	1184	106.697	13.328	9.279	1.00	0.00	LX0	O
ATOM	1925	N	LYS	1185	108.383	11.800	9.356	1.00	0.00	LX0	N
ATOM	1926	H	LYS	1185	108.624	10.834	9.466	0.00	0.00	LX0	H
ATOM	1927	CA	LYS	1185	109.291	12.783	8.760	1.00	0.00	LX0	C
ATOM	1928	CB	LYS	1185	110.786	12.464	8.975	1.00	0.00	LX0	C
ATOM	1929	CG	LYS	1185	111.219	10.996	9.054	1.00	0.00	LX0	C
ATOM	1930	CD	LYS	1185	112.715	10.835	8.779	1.00	0.00	LX0	C
ATOM	1931	CE	LYS	1185	113.236	9.404	8.941	1.00	0.00	LX0	C
ATOM	1932	NZ	LYS	1185	113.425	9.090	10.361	1.00	0.00	LX0	N
ATOM	1933	HZ1	LYS	1185	112.555	9.154	10.922	0.00	0.00	LX0	H
ATOM	1934	HZ2	LYS	1185	113.851	8.156	10.514	0.00	0.00	LX0	H
ATOM	1935	HZ3	LYS	1185	114.074	9.772	10.819	0.00	0.00	LX0	H
ATOM	1936	C	LYS	1185	108.955	13.081	7.304	1.00	0.00	LX0	C
ATOM	1937	O	LYS	1185	108.145	13.961	7.020	1.00	0.00	LX0	O
ATOM	1938	N	ASP	1186	109.558	12.304	6.399	1.00	0.00	LX0	N
ATOM	1939	H	ASP	1186	110.071	11.478	6.655	0.00	0.00	LX0	H
ATOM	1940	CA	ASP	1186	109.240	12.451	4.983	1.00	0.00	LX0	C
ATOM	1941	CB	ASP	1186	110.216	11.605	4.130	1.00	0.00	LX0	C
ATOM	1942	CG	ASP	1186	110.412	10.202	4.695	1.00	0.00	LX0	C
ATOM	1943	OD1	ASP	1186	109.731	9.282	4.250	1.00	0.00	LX0	O
ATOM	1944	OD2	ASP	1186	111.225	10.043	5.602	1.00	0.00	LX0	O
ATOM	1945	C	ASP	1186	107.767	12.201	4.674	1.00	0.00	LX0	C
ATOM	1946	O	ASP	1186	106.951	13.122	4.651	1.00	0.00	LX0	O
ATOM	1947	N	GLY	1187	107.426	10.933	4.464	1.00	0.00	LX0	N
ATOM	1948	H	GLY	1187	108.092	10.222	4.701	0.00	0.00	LX0	H
ATOM	1949	CA	GLY	1187	106.076	10.661	3.990	1.00	0.00	LX0	C
ATOM	1950	C	GLY	1187	105.969	9.481	3.047	1.00	0.00	LX0	C
ATOM	1951	O	GLY	1187	104.951	9.284	2.396	1.00	0.00	LX0	O
ATOM	1952	N	VAL	1188	107.045	8.680	3.001	1.00	0.00	LX0	N
ATOM	1953	H	VAL	1188	107.885	8.862	3.519	0.00	0.00	LX0	H
ATOM	1954	CA	VAL	1188	106.922	7.459	2.208	1.00	0.00	LX0	C
ATOM	1955	CB	VAL	1188	108.299	6.790	2.041	1.00	0.00	LX0	C
ATOM	1956	CG1	VAL	1188	108.240	5.451	1.296	1.00	0.00	LX0	C
ATOM	1957	CG2	VAL	1188	109.264	7.750	1.341	1.00	0.00	LX0	C
ATOM	1958	C	VAL	1188	105.897	6.505	2.804	1.00	0.00	LX0	C
ATOM	1959	O	VAL	1188	105.932	6.166	3.982	1.00	0.00	LX0	O
ATOM	1960	N	PHE	1189	104.973	6.102	1.927	1.00	0.00	LX0	N
ATOM	1961	H	PHE	1189	104.975	6.452	0.992	0.00	0.00	LX0	H

ATOM	1962	CA	PHE	1189	104.048	5.051	2.326	1.00	0.00	LX0	C
ATOM	1963	CB	PHE	1189	102.597	5.505	2.166	1.00	0.00	LX0	C
ATOM	1964	CG	PHE	1189	102.225	6.551	3.192	1.00	0.00	LX0	C
ATOM	1965	CD1	PHE	1189	102.157	7.907	2.806	1.00	0.00	LX0	C
ATOM	1966	CD2	PHE	1189	101.938	6.153	4.516	1.00	0.00	LX0	C
ATOM	1967	CE1	PHE	1189	101.787	8.879	3.755	1.00	0.00	LX0	C
ATOM	1968	CE2	PHE	1189	101.566	7.122	5.466	1.00	0.00	LX0	C
ATOM	1969	CZ	PHE	1189	101.490	8.475	5.073	1.00	0.00	LX0	C
ATOM	1970	C	PHE	1189	104.289	3.819	1.487	1.00	0.00	LX0	C
ATOM	1971	O	PHE	1189	104.362	3.882	0.267	1.00	0.00	LX0	O
ATOM	1972	N	THR	1190	104.432	2.700	2.194	1.00	0.00	LX0	N
ATOM	1973	H	THR	1190	104.311	2.740	3.189	0.00	0.00	LX0	H
ATOM	1974	CA	THR	1190	104.550	1.403	1.532	1.00	0.00	LX0	C
ATOM	1975	CB	THR	1190	106.024	0.985	1.427	1.00	0.00	LX0	C
ATOM	1976	OG1	THR	1190	106.651	1.097	2.711	1.00	0.00	LX0	O
ATOM	1977	HG1	THR	1190	106.755	2.030	2.877	0.00	0.00	LX0	H
ATOM	1978	CG2	THR	1190	106.819	1.748	0.366	1.00	0.00	LX0	C
ATOM	1979	C	THR	1190	103.799	0.405	2.391	1.00	0.00	LX0	C
ATOM	1980	O	THR	1190	103.212	0.782	3.392	1.00	0.00	LX0	O
ATOM	1981	N	THR	1191	103.919	-0.886	2.045	1.00	0.00	LX0	N
ATOM	1982	H	THR	1191	104.302	-1.153	1.165	0.00	0.00	LX0	H
ATOM	1983	CA	THR	1191	103.459	-1.896	3.008	1.00	0.00	LX0	C
ATOM	1984	CB	THR	1191	103.688	-3.288	2.382	1.00	0.00	LX0	C
ATOM	1985	OG1	THR	1191	102.917	-4.307	3.018	1.00	0.00	LX0	O
ATOM	1986	HG1	THR	1191	102.748	-4.959	2.340	0.00	0.00	LX0	H
ATOM	1987	CG2	THR	1191	105.164	-3.687	2.281	1.00	0.00	LX0	C
ATOM	1988	C	THR	1191	104.060	-1.743	4.419	1.00	0.00	LX0	C
ATOM	1989	O	THR	1191	103.489	-2.089	5.446	1.00	0.00	LX0	O
ATOM	1990	N	TYR	1192	105.263	-1.136	4.435	1.00	0.00	LX0	N
ATOM	1991	H	TYR	1192	105.692	-0.780	3.606	0.00	0.00	LX0	H
ATOM	1992	CA	TYR	1192	105.896	-0.929	5.731	1.00	0.00	LX0	C
ATOM	1993	CB	TYR	1192	107.378	-0.591	5.564	1.00	0.00	LX0	C
ATOM	1994	CG	TYR	1192	108.097	-1.607	4.702	1.00	0.00	LX0	C
ATOM	1995	CD1	TYR	1192	108.086	-2.975	5.053	1.00	0.00	LX0	C
ATOM	1996	CE1	TYR	1192	108.823	-3.885	4.276	1.00	0.00	LX0	C
ATOM	1997	CD2	TYR	1192	108.787	-1.138	3.568	1.00	0.00	LX0	C
ATOM	1998	CE2	TYR	1192	109.517	-2.047	2.787	1.00	0.00	LX0	C
ATOM	1999	CZ	TYR	1192	109.544	-3.404	3.163	1.00	0.00	LX0	C
ATOM	2000	OH	TYR	1192	110.314	-4.276	2.415	1.00	0.00	LX0	O
ATOM	2001	HH	TYR	1192	110.242	-5.144	2.801	0.00	0.00	LX0	H
ATOM	2002	C	TYR	1192	105.228	0.096	6.633	1.00	0.00	LX0	C
ATOM	2003	O	TYR	1192	105.407	0.064	7.847	1.00	0.00	LX0	O
ATOM	2004	N	SER	1193	104.453	1.007	6.018	1.00	0.00	LX0	N
ATOM	2005	H	SER	1193	104.288	1.015	5.033	0.00	0.00	LX0	H
ATOM	2006	CA	SER	1193	103.631	1.850	6.890	1.00	0.00	LX0	C
ATOM	2007	CB	SER	1193	103.023	3.042	6.138	1.00	0.00	LX0	C
ATOM	2008	OG	SER	1193	103.197	2.896	4.722	1.00	0.00	LX0	O
ATOM	2009	HG	SER	1193	102.557	2.234	4.442	0.00	0.00	LX0	H
ATOM	2010	C	SER	1193	102.549	1.034	7.559	1.00	0.00	LX0	C
ATOM	2011	O	SER	1193	102.451	0.938	8.774	1.00	0.00	LX0	O
ATOM	2012	N	ASP	1194	101.805	0.364	6.672	1.00	0.00	LX0	N
ATOM	2013	H	ASP	1194	101.910	0.514	5.687	0.00	0.00	LX0	H
ATOM	2014	CA	ASP	1194	100.705	-0.521	7.041	1.00	0.00	LX0	C
ATOM	2015	CB	ASP	1194	100.309	-1.352	5.813	1.00	0.00	LX0	C
ATOM	2016	CG	ASP	1194	99.796	-0.503	4.657	1.00	0.00	LX0	C
ATOM	2017	OD1	ASP	1194	100.418	0.491	4.285	1.00	0.00	LX0	O
ATOM	2018	OD2	ASP	1194	98.758	-0.844	4.107	1.00	0.00	LX0	O
ATOM	2019	C	ASP	1194	101.008	-1.424	8.227	1.00	0.00	LX0	C
ATOM	2020	O	ASP	1194	100.258	-1.513	9.192	1.00	0.00	LX0	O
ATOM	2021	N	VAL	1195	102.185	-2.069	8.137	1.00	0.00	LX0	N
ATOM	2022	H	VAL	1195	102.700	-1.990	7.281	0.00	0.00	LX0	H

ATOM	2023	CA	VAL	1195	102.613	-2.915	9.256	1.00	0.00	LX0	C
ATOM	2024	CB	VAL	1195	103.871	-3.717	8.882	1.00	0.00	LX0	C
ATOM	2025	CG1	VAL	1195	104.298	-4.668	10.004	1.00	0.00	LX0	C
ATOM	2026	CG2	VAL	1195	103.661	-4.491	7.578	1.00	0.00	LX0	C
ATOM	2027	C	VAL	1195	102.802	-2.171	10.578	1.00	0.00	LX0	C
ATOM	2028	O	VAL	1195	102.324	-2.582	11.629	1.00	0.00	LX0	O
ATOM	2029	N	TRP	1196	103.512	-1.033	10.484	1.00	0.00	LX0	N
ATOM	2030	H	TRP	1196	103.766	-0.682	9.581	0.00	0.00	LX0	H
ATOM	2031	CA	TRP	1196	103.713	-0.220	11.689	1.00	0.00	LX0	C
ATOM	2032	CB	TRP	1196	104.564	1.005	11.320	1.00	0.00	LX0	C
ATOM	2033	CG	TRP	1196	104.860	1.906	12.502	1.00	0.00	LX0	C
ATOM	2034	CD2	TRP	1196	106.085	2.006	13.258	1.00	0.00	LX0	C
ATOM	2035	CE2	TRP	1196	105.885	3.014	14.261	1.00	0.00	LX0	C
ATOM	2036	CE3	TRP	1196	107.328	1.344	13.165	1.00	0.00	LX0	C
ATOM	2037	CD1	TRP	1196	103.998	2.853	13.080	1.00	0.00	LX0	C
ATOM	2038	NE1	TRP	1196	104.595	3.507	14.111	1.00	0.00	LX0	N
ATOM	2039	HE1	TRP	1196	104.191	4.231	14.638	0.00	0.00	LX0	H
ATOM	2040	CZ2	TRP	1196	106.928	3.334	15.156	1.00	0.00	LX0	C
ATOM	2041	CZ3	TRP	1196	108.364	1.680	14.062	1.00	0.00	LX0	C
ATOM	2042	CH2	TRP	1196	108.164	2.664	15.054	1.00	0.00	LX0	C
ATOM	2043	C	TRP	1196	102.392	0.166	12.347	1.00	0.00	LX0	C
ATOM	2044	O	TRP	1196	102.156	-0.041	13.535	1.00	0.00	LX0	O
ATOM	2045	N	SER	1197	101.521	0.701	11.483	1.00	0.00	LX0	N
ATOM	2046	H	SER	1197	101.775	0.903	10.535	0.00	0.00	LX0	H
ATOM	2047	CA	SER	1197	100.182	1.059	11.925	1.00	0.00	LX0	C
ATOM	2048	CB	SER	1197	99.457	1.731	10.767	1.00	0.00	LX0	C
ATOM	2049	OG	SER	1197	100.320	2.720	10.194	1.00	0.00	LX0	O
ATOM	2050	HG	SER	1197	100.533	3.326	10.900	0.00	0.00	LX0	H
ATOM	2051	C	SER	1197	99.393	-0.086	12.539	1.00	0.00	LX0	C
ATOM	2052	O	SER	1197	98.748	0.077	13.562	1.00	0.00	LX0	O
ATOM	2053	N	PHE	1198	99.539	-1.281	11.940	1.00	0.00	LX0	N
ATOM	2054	H	PHE	1198	100.040	-1.340	11.076	0.00	0.00	LX0	H
ATOM	2055	CA	PHE	1198	98.966	-2.480	12.561	1.00	0.00	LX0	C
ATOM	2056	CB	PHE	1198	99.286	-3.713	11.702	1.00	0.00	LX0	C
ATOM	2057	CG	PHE	1198	98.707	-4.980	12.293	1.00	0.00	LX0	C
ATOM	2058	CD1	PHE	1198	97.338	-5.274	12.117	1.00	0.00	LX0	C
ATOM	2059	CD2	PHE	1198	99.554	-5.844	13.021	1.00	0.00	LX0	C
ATOM	2060	CE1	PHE	1198	96.807	-6.448	12.685	1.00	0.00	LX0	C
ATOM	2061	CE2	PHE	1198	99.024	-7.017	13.589	1.00	0.00	LX0	C
ATOM	2062	CZ	PHE	1198	97.655	-7.306	13.416	1.00	0.00	LX0	C
ATOM	2063	C	PHE	1198	99.388	-2.685	14.011	1.00	0.00	LX0	C
ATOM	2064	O	PHE	1198	98.580	-2.931	14.900	1.00	0.00	LX0	O
ATOM	2065	N	GLY	1199	100.704	-2.525	14.224	1.00	0.00	LX0	N
ATOM	2066	H	GLY	1199	101.311	-2.343	13.445	0.00	0.00	LX0	H
ATOM	2067	CA	GLY	1199	101.194	-2.580	15.603	1.00	0.00	LX0	C
ATOM	2068	C	GLY	1199	100.523	-1.565	16.515	1.00	0.00	LX0	C
ATOM	2069	O	GLY	1199	100.130	-1.840	17.644	1.00	0.00	LX0	O
ATOM	2070	N	VAL	1200	100.372	-0.364	15.937	1.00	0.00	LX0	N
ATOM	2071	H	VAL	1200	100.662	-0.231	14.987	0.00	0.00	LX0	H
ATOM	2072	CA	VAL	1200	99.661	0.675	16.678	1.00	0.00	LX0	C
ATOM	2073	CB	VAL	1200	99.761	2.027	15.958	1.00	0.00	LX0	C
ATOM	2074	CG1	VAL	1200	99.211	3.144	16.833	1.00	0.00	LX0	C
ATOM	2075	CG2	VAL	1200	101.197	2.363	15.557	1.00	0.00	LX0	C
ATOM	2076	C	VAL	1200	98.214	0.310	17.008	1.00	0.00	LX0	C
ATOM	2077	O	VAL	1200	97.736	0.536	18.110	1.00	0.00	LX0	O
ATOM	2078	N	VAL	1201	97.550	-0.338	16.035	1.00	0.00	LX0	N
ATOM	2079	H	VAL	1201	97.998	-0.477	15.154	0.00	0.00	LX0	H
ATOM	2080	CA	VAL	1201	96.201	-0.859	16.278	1.00	0.00	LX0	C
ATOM	2081	CB	VAL	1201	95.661	-1.598	15.040	1.00	0.00	LX0	C
ATOM	2082	CG1	VAL	1201	94.294	-2.247	15.273	1.00	0.00	LX0	C
ATOM	2083	CG2	VAL	1201	95.600	-0.663	13.837	1.00	0.00	LX0	C

ATOM	2084	C	VAL	1201	96.139	-1.753	17.505	1.00	0.00	LX0	C
ATOM	2085	O	VAL	1201	95.237	-1.662	18.324	1.00	0.00	LX0	O
ATOM	2086	N	LEU	1202	97.172	-2.599	17.623	1.00	0.00	LX0	N
ATOM	2087	H	LEU	1202	97.872	-2.616	16.905	0.00	0.00	LX0	H
ATOM	2088	CA	LEU	1202	97.234	-3.461	18.807	1.00	0.00	LX0	C
ATOM	2089	CB	LEU	1202	98.426	-4.410	18.710	1.00	0.00	LX0	C
ATOM	2090	CG	LEU	1202	98.514	-5.149	17.376	1.00	0.00	LX0	C
ATOM	2091	CD1	LEU	1202	99.839	-5.892	17.235	1.00	0.00	LX0	C
ATOM	2092	CD2	LEU	1202	97.304	-6.046	17.132	1.00	0.00	LX0	C
ATOM	2093	C	LEU	1202	97.271	-2.690	20.120	1.00	0.00	LX0	C
ATOM	2094	O	LEU	1202	96.558	-2.980	21.076	1.00	0.00	LX0	O
ATOM	2095	N	TRP	1203	98.113	-1.643	20.094	1.00	0.00	LX0	N
ATOM	2096	H	TRP	1203	98.675	-1.490	19.277	0.00	0.00	LX0	H
ATOM	2097	CA	TRP	1203	98.133	-0.696	21.213	1.00	0.00	LX0	C
ATOM	2098	CB	TRP	1203	99.194	0.378	20.927	1.00	0.00	LX0	C
ATOM	2099	CG	TRP	1203	99.356	1.373	22.056	1.00	0.00	LX0	C
ATOM	2100	CD2	TRP	1203	98.564	2.551	22.329	1.00	0.00	LX0	C
ATOM	2101	CE2	TRP	1203	99.126	3.181	23.487	1.00	0.00	LX0	C
ATOM	2102	CE3	TRP	1203	97.436	3.120	21.700	1.00	0.00	LX0	C
ATOM	2103	CD1	TRP	1203	100.341	1.355	23.056	1.00	0.00	LX0	C
ATOM	2104	NE1	TRP	1203	100.208	2.415	23.899	1.00	0.00	LX0	N
ATOM	2105	HE1	TRP	1203	100.782	2.622	24.682	0.00	0.00	LX0	H
ATOM	2106	CZ2	TRP	1203	98.554	4.368	23.992	1.00	0.00	LX0	C
ATOM	2107	CZ3	TRP	1203	96.868	4.302	22.216	1.00	0.00	LX0	C
ATOM	2108	CH2	TRP	1203	97.427	4.924	23.354	1.00	0.00	LX0	C
ATOM	2109	C	TRP	1203	96.760	-0.100	21.511	1.00	0.00	LX0	C
ATOM	2110	O	TRP	1203	96.324	-0.003	22.655	1.00	0.00	LX0	O
ATOM	2111	N	GLU	1204	96.069	0.271	20.419	1.00	0.00	LX0	N
ATOM	2112	H	GLU	1204	96.482	0.183	19.512	0.00	0.00	LX0	H
ATOM	2113	CA	GLU	1204	94.720	0.804	20.608	1.00	0.00	LX0	C
ATOM	2114	CB	GLU	1204	94.107	1.364	19.316	1.00	0.00	LX0	C
ATOM	2115	CG	GLU	1204	95.052	2.285	18.540	1.00	0.00	LX0	C
ATOM	2116	CD	GLU	1204	94.324	3.505	18.014	1.00	0.00	LX0	C
ATOM	2117	OE1	GLU	1204	94.438	4.560	18.620	1.00	0.00	LX0	O
ATOM	2118	OE2	GLU	1204	93.699	3.467	16.958	1.00	0.00	LX0	O
ATOM	2119	C	GLU	1204	93.776	-0.186	21.263	1.00	0.00	LX0	C
ATOM	2120	O	GLU	1204	93.065	0.135	22.203	1.00	0.00	LX0	O
ATOM	2121	N	ILE	1205	93.835	-1.436	20.779	1.00	0.00	LX0	N
ATOM	2122	H	ILE	1205	94.455	-1.629	20.020	0.00	0.00	LX0	H
ATOM	2123	CA	ILE	1205	93.015	-2.480	21.405	1.00	0.00	LX0	C
ATOM	2124	CB	ILE	1205	93.257	-3.843	20.729	1.00	0.00	LX0	C
ATOM	2125	CG2	ILE	1205	92.424	-4.956	21.373	1.00	0.00	LX0	C
ATOM	2126	CG1	ILE	1205	92.985	-3.769	19.222	1.00	0.00	LX0	C
ATOM	2127	CD1	ILE	1205	93.373	-5.044	18.469	1.00	0.00	LX0	C
ATOM	2128	C	ILE	1205	93.210	-2.554	22.915	1.00	0.00	LX0	C
ATOM	2129	O	ILE	1205	92.277	-2.496	23.708	1.00	0.00	LX0	O
ATOM	2130	N	ALA	1206	94.498	-2.624	23.271	1.00	0.00	LX0	N
ATOM	2131	H	ALA	1206	95.213	-2.642	22.568	0.00	0.00	LX0	H
ATOM	2132	CA	ALA	1206	94.812	-2.689	24.694	1.00	0.00	LX0	C
ATOM	2133	CB	ALA	1206	96.293	-3.011	24.877	1.00	0.00	LX0	C
ATOM	2134	C	ALA	1206	94.435	-1.457	25.512	1.00	0.00	LX0	C
ATOM	2135	O	ALA	1206	94.134	-1.546	26.694	1.00	0.00	LX0	O
ATOM	2136	N	THR	1207	94.440	-0.293	24.844	1.00	0.00	LX0	N
ATOM	2137	H	THR	1207	94.631	-0.252	23.862	0.00	0.00	LX0	H
ATOM	2138	CA	THR	1207	94.083	0.908	25.603	1.00	0.00	LX0	C
ATOM	2139	CB	THR	1207	94.849	2.127	25.093	1.00	0.00	LX0	C
ATOM	2140	OG1	THR	1207	94.876	2.156	23.658	1.00	0.00	LX0	O
ATOM	2141	HG1	THR	1207	95.548	1.528	23.397	0.00	0.00	LX0	H
ATOM	2142	CG2	THR	1207	96.252	2.211	25.677	1.00	0.00	LX0	C
ATOM	2143	C	THR	1207	92.610	1.264	25.641	1.00	0.00	LX0	C
ATOM	2144	O	THR	1207	92.242	2.369	26.025	1.00	0.00	LX0	O

ATOM	2145	N	LEU	1208	91.766	0.326	25.158	1.00	0.00	LX0	N
ATOM	2146	H	LEU	1208	92.108	-0.582	24.910	0.00	0.00	LX0	H
ATOM	2147	CA	LEU	1208	90.366	0.697	24.898	1.00	0.00	LX0	C
ATOM	2148	CB	LEU	1208	89.557	0.815	26.196	1.00	0.00	LX0	C
ATOM	2149	CG	LEU	1208	89.679	-0.445	27.054	1.00	0.00	LX0	C
ATOM	2150	CD1	LEU	1208	89.016	-0.278	28.421	1.00	0.00	LX0	C
ATOM	2151	CD2	LEU	1208	89.188	-1.684	26.306	1.00	0.00	LX0	C
ATOM	2152	C	LEU	1208	90.254	1.944	24.030	1.00	0.00	LX0	C
ATOM	2153	O	LEU	1208	89.473	2.873	24.232	1.00	0.00	LX0	O
ATOM	2154	N	ALA	1209	91.170	1.901	23.054	1.00	0.00	LX0	N
ATOM	2155	H	ALA	1209	91.719	1.068	23.017	0.00	0.00	LX0	H
ATOM	2156	CA	ALA	1209	91.427	2.940	22.070	1.00	0.00	LX0	C
ATOM	2157	CB	ALA	1209	90.292	2.980	21.058	1.00	0.00	LX0	C
ATOM	2158	C	ALA	1209	91.685	4.320	22.633	1.00	0.00	LX0	C
ATOM	2159	O	ALA	1209	90.828	5.201	22.607	1.00	0.00	LX0	O
ATOM	2160	N	GLU	1210	92.905	4.498	23.153	1.00	0.00	LX0	N
ATOM	2161	H	GLU	1210	93.588	3.762	23.149	0.00	0.00	LX0	H
ATOM	2162	CA	GLU	1210	93.249	5.894	23.421	1.00	0.00	LX0	C
ATOM	2163	CB	GLU	1210	94.275	6.024	24.556	1.00	0.00	LX0	C
ATOM	2164	CG	GLU	1210	93.726	5.738	25.965	1.00	0.00	LX0	C
ATOM	2165	CD	GLU	1210	92.682	6.756	26.430	1.00	0.00	LX0	C
ATOM	2166	OE1	GLU	1210	92.497	6.913	27.627	1.00	0.00	LX0	O
ATOM	2167	OE2	GLU	1210	91.987	7.367	25.620	1.00	0.00	LX0	O
ATOM	2168	C	GLU	1210	93.653	6.622	22.154	1.00	0.00	LX0	C
ATOM	2169	O	GLU	1210	93.331	6.165	21.064	1.00	0.00	LX0	O
ATOM	2170	N	GLN	1211	94.353	7.752	22.311	1.00	0.00	LX0	N
ATOM	2171	H	GLN	1211	94.537	8.192	23.193	0.00	0.00	LX0	H
ATOM	2172	CA	GLN	1211	95.129	8.169	21.152	1.00	0.00	LX0	C
ATOM	2173	CB	GLN	1211	94.896	9.650	20.816	1.00	0.00	LX0	C
ATOM	2174	CG	GLN	1211	93.438	10.068	20.563	1.00	0.00	LX0	C
ATOM	2175	CD	GLN	1211	92.843	9.368	19.353	1.00	0.00	LX0	C
ATOM	2176	OE1	GLN	1211	92.484	8.196	19.383	1.00	0.00	LX0	O
ATOM	2177	NE2	GLN	1211	92.690	10.146	18.281	1.00	0.00	LX0	N
ATOM	2178	HE21	GLN	1211	92.928	11.115	18.200	0.00	0.00	LX0	H
ATOM	2179	HE22	GLN	1211	92.264	9.718	17.479	0.00	0.00	LX0	H
ATOM	2180	C	GLN	1211	96.586	7.891	21.465	1.00	0.00	LX0	C
ATOM	2181	O	GLN	1211	97.081	8.219	22.537	1.00	0.00	LX0	O
ATOM	2182	N	PRO	1212	97.275	7.234	20.509	1.00	0.00	LX0	N
ATOM	2183	CD	PRO	1212	96.765	6.753	19.237	1.00	0.00	LX0	C
ATOM	2184	CA	PRO	1212	98.686	6.895	20.722	1.00	0.00	LX0	C
ATOM	2185	CB	PRO	1212	99.100	6.303	19.377	1.00	0.00	LX0	C
ATOM	2186	CG	PRO	1212	97.807	5.738	18.803	1.00	0.00	LX0	C
ATOM	2187	C	PRO	1212	99.520	8.084	21.143	1.00	0.00	LX0	C
ATOM	2188	O	PRO	1212	99.293	9.207	20.701	1.00	0.00	LX0	O
ATOM	2189	N	TYR	1213	100.465	7.800	22.057	1.00	0.00	LX0	N
ATOM	2190	H	TYR	1213	100.570	6.852	22.360	0.00	0.00	LX0	H
ATOM	2191	CA	TYR	1213	101.300	8.868	22.621	1.00	0.00	LX0	C
ATOM	2192	CB	TYR	1213	102.276	9.459	21.580	1.00	0.00	LX0	C
ATOM	2193	CG	TYR	1213	103.017	8.391	20.800	1.00	0.00	LX0	C
ATOM	2194	CD1	TYR	1213	104.241	7.897	21.295	1.00	0.00	LX0	C
ATOM	2195	CE1	TYR	1213	104.930	6.919	20.557	1.00	0.00	LX0	C
ATOM	2196	CD2	TYR	1213	102.468	7.927	19.586	1.00	0.00	LX0	C
ATOM	2197	CE2	TYR	1213	103.148	6.939	18.856	1.00	0.00	LX0	C
ATOM	2198	CZ	TYR	1213	104.373	6.450	19.350	1.00	0.00	LX0	C
ATOM	2199	OH	TYR	1213	105.041	5.481	18.629	1.00	0.00	LX0	O
ATOM	2200	HH	TYR	1213	105.979	5.633	18.721	0.00	0.00	LX0	H
ATOM	2201	C	TYR	1213	100.467	9.974	23.263	1.00	0.00	LX0	C
ATOM	2202	O	TYR	1213	100.707	11.165	23.092	1.00	0.00	LX0	O
ATOM	2203	N	GLN	1214	99.426	9.503	23.981	1.00	0.00	LX0	N
ATOM	2204	H	GLN	1214	99.321	8.510	24.036	0.00	0.00	LX0	H
ATOM	2205	CA	GLN	1214	98.313	10.339	24.452	1.00	0.00	LX0	C

ATOM	2206	CB	GLN	1214	97.692	9.705	25.709	1.00	0.00	LX0	C
ATOM	2207	CG	GLN	1214	96.357	10.298	26.189	1.00	0.00	LX0	C
ATOM	2208	CD	GLN	1214	95.192	9.848	25.326	1.00	0.00	LX0	C
ATOM	2209	OE1	GLN	1214	95.156	10.003	24.113	1.00	0.00	LX0	O
ATOM	2210	NE2	GLN	1214	94.217	9.275	26.029	1.00	0.00	LX0	N
ATOM	2211	HE21	GLN	1214	94.249	9.109	27.014	0.00	0.00	LX0	H
ATOM	2212	HE22	GLN	1214	93.382	8.915	25.604	0.00	0.00	LX0	H
ATOM	2213	C	GLN	1214	98.585	11.830	24.625	1.00	0.00	LX0	C
ATOM	2214	O	GLN	1214	98.240	12.640	23.771	1.00	0.00	LX0	O
ATOM	2215	N	GLY	1215	99.248	12.143	25.751	1.00	0.00	LX0	N
ATOM	2216	H	GLY	1215	99.621	11.416	26.324	0.00	0.00	LX0	H
ATOM	2217	CA	GLY	1215	99.347	13.543	26.161	1.00	0.00	LX0	C
ATOM	2218	C	GLY	1215	100.262	14.456	25.359	1.00	0.00	LX0	C
ATOM	2219	O	GLY	1215	100.335	15.651	25.614	1.00	0.00	LX0	O
ATOM	2220	N	LEU	1216	100.974	13.868	24.385	1.00	0.00	LX0	N
ATOM	2221	H	LEU	1216	100.815	12.919	24.113	0.00	0.00	LX0	H
ATOM	2222	CA	LEU	1216	101.760	14.777	23.554	1.00	0.00	LX0	C
ATOM	2223	CB	LEU	1216	102.782	14.006	22.718	1.00	0.00	LX0	C
ATOM	2224	CG	LEU	1216	103.865	13.287	23.524	1.00	0.00	LX0	C
ATOM	2225	CD1	LEU	1216	104.763	12.447	22.614	1.00	0.00	LX0	C
ATOM	2226	CD2	LEU	1216	104.679	14.254	24.387	1.00	0.00	LX0	C
ATOM	2227	C	LEU	1216	100.865	15.586	22.638	1.00	0.00	LX0	C
ATOM	2228	O	LEU	1216	99.792	15.142	22.239	1.00	0.00	LX0	O
ATOM	2229	N	SER	1217	101.346	16.787	22.295	1.00	0.00	LX0	N
ATOM	2230	H	SER	1217	102.230	17.097	22.643	0.00	0.00	LX0	H
ATOM	2231	CA	SER	1217	100.638	17.475	21.220	1.00	0.00	LX0	C
ATOM	2232	CB	SER	1217	101.030	18.954	21.180	1.00	0.00	LX0	C
ATOM	2233	OG	SER	1217	102.444	19.082	21.015	1.00	0.00	LX0	O
ATOM	2234	HG	SER	1217	102.818	19.000	21.891	0.00	0.00	LX0	H
ATOM	2235	C	SER	1217	100.907	16.782	19.898	1.00	0.00	LX0	C
ATOM	2236	O	SER	1217	101.857	16.017	19.768	1.00	0.00	LX0	O
ATOM	2237	N	ASN	1218	100.033	17.051	18.918	1.00	0.00	LX0	N
ATOM	2238	H	ASN	1218	99.297	17.710	19.065	0.00	0.00	LX0	H
ATOM	2239	CA	ASN	1218	100.152	16.246	17.699	1.00	0.00	LX0	C
ATOM	2240	CB	ASN	1218	98.977	16.491	16.749	1.00	0.00	LX0	C
ATOM	2241	CG	ASN	1218	97.728	15.799	17.274	1.00	0.00	LX0	C
ATOM	2242	OD1	ASN	1218	97.746	15.049	18.243	1.00	0.00	LX0	O
ATOM	2243	ND2	ASN	1218	96.614	16.086	16.599	1.00	0.00	LX0	N
ATOM	2244	HD21	ASN	1218	96.610	16.714	15.824	0.00	0.00	LX0	H
ATOM	2245	HD22	ASN	1218	95.746	15.663	16.859	0.00	0.00	LX0	H
ATOM	2246	C	ASN	1218	101.497	16.345	16.994	1.00	0.00	LX0	C
ATOM	2247	O	ASN	1218	102.097	15.341	16.634	1.00	0.00	LX0	O
ATOM	2248	N	GLU	1219	101.985	17.595	16.892	1.00	0.00	LX0	N
ATOM	2249	H	GLU	1219	101.429	18.389	17.128	0.00	0.00	LX0	H
ATOM	2250	CA	GLU	1219	103.345	17.775	16.370	1.00	0.00	LX0	C
ATOM	2251	CB	GLU	1219	103.695	19.257	16.231	1.00	0.00	LX0	C
ATOM	2252	CG	GLU	1219	102.926	19.994	15.128	1.00	0.00	LX0	C
ATOM	2253	CD	GLU	1219	103.262	19.448	13.747	1.00	0.00	LX0	C
ATOM	2254	OE1	GLU	1219	104.435	19.230	13.453	1.00	0.00	LX0	O
ATOM	2255	OE2	GLU	1219	102.343	19.255	12.957	1.00	0.00	LX0	O
ATOM	2256	C	GLU	1219	104.435	17.070	17.164	1.00	0.00	LX0	C
ATOM	2257	O	GLU	1219	105.379	16.506	16.626	1.00	0.00	LX0	O
ATOM	2258	N	GLN	1220	104.260	17.084	18.499	1.00	0.00	LX0	N
ATOM	2259	H	GLN	1220	103.455	17.509	18.912	0.00	0.00	LX0	H
ATOM	2260	CA	GLN	1220	105.213	16.304	19.293	1.00	0.00	LX0	C
ATOM	2261	CB	GLN	1220	104.988	16.490	20.789	1.00	0.00	LX0	C
ATOM	2262	CG	GLN	1220	105.466	17.835	21.328	1.00	0.00	LX0	C
ATOM	2263	CD	GLN	1220	105.032	17.941	22.774	1.00	0.00	LX0	C
ATOM	2264	OE1	GLN	1220	103.852	18.034	23.087	1.00	0.00	LX0	O
ATOM	2265	NE2	GLN	1220	106.033	17.919	23.656	1.00	0.00	LX0	N
ATOM	2266	HE21	GLN	1220	106.986	17.830	23.375	0.00	0.00	LX0	H

ATOM	2267	HE22	GLN	1220	105.802	17.994	24.625	0.00	0.00	LX0	H
ATOM	2268	C	GLN	1220	105.214	14.823	18.961	1.00	0.00	LX0	C
ATOM	2269	O	GLN	1220	106.258	14.187	18.907	1.00	0.00	LX0	O
ATOM	2270	N	VAL	1221	103.997	14.315	18.697	1.00	0.00	LX0	N
ATOM	2271	H	VAL	1221	103.189	14.902	18.760	0.00	0.00	LX0	H
ATOM	2272	CA	VAL	1221	103.911	12.929	18.237	1.00	0.00	LX0	C
ATOM	2273	CB	VAL	1221	102.457	12.469	18.072	1.00	0.00	LX0	C
ATOM	2274	CG1	VAL	1221	102.393	10.991	17.681	1.00	0.00	LX0	C
ATOM	2275	CG2	VAL	1221	101.637	12.738	19.332	1.00	0.00	LX0	C
ATOM	2276	C	VAL	1221	104.702	12.684	16.963	1.00	0.00	LX0	C
ATOM	2277	O	VAL	1221	105.507	11.764	16.896	1.00	0.00	LX0	O
ATOM	2278	N	LEU	1222	104.486	13.587	15.979	1.00	0.00	LX0	N
ATOM	2279	H	LEU	1222	103.779	14.287	16.101	0.00	0.00	LX0	H
ATOM	2280	CA	LEU	1222	105.311	13.527	14.763	1.00	0.00	LX0	C
ATOM	2281	CB	LEU	1222	105.212	14.790	13.897	1.00	0.00	LX0	C
ATOM	2282	CG	LEU	1222	104.068	14.919	12.896	1.00	0.00	LX0	C
ATOM	2283	CD1	LEU	1222	102.746	15.336	13.533	1.00	0.00	LX0	C
ATOM	2284	CD2	LEU	1222	104.467	15.886	11.784	1.00	0.00	LX0	C
ATOM	2285	C	LEU	1222	106.785	13.345	15.076	1.00	0.00	LX0	C
ATOM	2286	O	LEU	1222	107.422	12.361	14.726	1.00	0.00	LX0	O
ATOM	2287	N	ARG	1223	107.279	14.358	15.800	1.00	0.00	LX0	N
ATOM	2288	H	ARG	1223	106.638	15.065	16.106	0.00	0.00	LX0	H
ATOM	2289	CA	ARG	1223	108.708	14.433	16.092	1.00	0.00	LX0	C
ATOM	2290	CB	ARG	1223	108.998	15.738	16.833	1.00	0.00	LX0	C
ATOM	2291	CG	ARG	1223	108.502	16.937	16.019	1.00	0.00	LX0	C
ATOM	2292	CD	ARG	1223	108.459	18.247	16.804	1.00	0.00	LX0	C
ATOM	2293	NE	ARG	1223	109.802	18.747	17.092	1.00	0.00	LX0	N
ATOM	2294	HE	ARG	1223	110.391	18.161	17.651	0.00	0.00	LX0	H
ATOM	2295	CZ	ARG	1223	110.191	19.944	16.601	1.00	0.00	LX0	C
ATOM	2296	NH1	ARG	1223	109.365	20.665	15.838	1.00	0.00	LX0	N
ATOM	2297	HH11	ARG	1223	109.616	21.560	15.469	0.00	0.00	LX0	H
ATOM	2298	HH12	ARG	1223	108.453	20.313	15.610	0.00	0.00	LX0	H
ATOM	2299	NH2	ARG	1223	111.411	20.398	16.889	1.00	0.00	LX0	N
ATOM	2300	HH21	ARG	1223	111.740	21.280	16.550	0.00	0.00	LX0	H
ATOM	2301	HH22	ARG	1223	112.032	19.855	17.457	0.00	0.00	LX0	H
ATOM	2302	C	ARG	1223	109.278	13.228	16.820	1.00	0.00	LX0	C
ATOM	2303	O	ARG	1223	110.383	12.776	16.555	1.00	0.00	LX0	O
ATOM	2304	N	PHE	1224	108.456	12.717	17.746	1.00	0.00	LX0	N
ATOM	2305	H	PHE	1224	107.548	13.116	17.889	0.00	0.00	LX0	H
ATOM	2306	CA	PHE	1224	108.879	11.535	18.490	1.00	0.00	LX0	C
ATOM	2307	CB	PHE	1224	107.910	11.326	19.662	1.00	0.00	LX0	C
ATOM	2308	CG	PHE	1224	108.400	10.292	20.653	1.00	0.00	LX0	C
ATOM	2309	CD1	PHE	1224	109.663	10.438	21.269	1.00	0.00	LX0	C
ATOM	2310	CD2	PHE	1224	107.561	9.201	20.962	1.00	0.00	LX0	C
ATOM	2311	CE1	PHE	1224	110.087	9.485	22.215	1.00	0.00	LX0	C
ATOM	2312	CE2	PHE	1224	107.981	8.248	21.910	1.00	0.00	LX0	C
ATOM	2313	CZ	PHE	1224	109.239	8.402	22.530	1.00	0.00	LX0	C
ATOM	2314	C	PHE	1224	109.004	10.296	17.615	1.00	0.00	LX0	C
ATOM	2315	O	PHE	1224	109.994	9.571	17.621	1.00	0.00	LX0	O
ATOM	2316	N	VAL	1225	107.938	10.080	16.835	1.00	0.00	LX0	N
ATOM	2317	H	VAL	1225	107.189	10.744	16.774	0.00	0.00	LX0	H
ATOM	2318	CA	VAL	1225	107.956	8.843	16.060	1.00	0.00	LX0	C
ATOM	2319	CB	VAL	1225	106.540	8.420	15.646	1.00	0.00	LX0	C
ATOM	2320	CG1	VAL	1225	106.489	7.005	15.062	1.00	0.00	LX0	C
ATOM	2321	CG2	VAL	1225	105.579	8.542	16.826	1.00	0.00	LX0	C
ATOM	2322	C	VAL	1225	108.919	8.866	14.882	1.00	0.00	LX0	C
ATOM	2323	O	VAL	1225	109.432	7.831	14.479	1.00	0.00	LX0	O
ATOM	2324	N	MET	1226	109.198	10.089	14.381	1.00	0.00	LX0	N
ATOM	2325	H	MET	1226	108.675	10.873	14.720	0.00	0.00	LX0	H
ATOM	2326	CA	MET	1226	110.148	10.244	13.266	1.00	0.00	LX0	C
ATOM	2327	CB	MET	1226	110.634	11.684	13.090	1.00	0.00	LX0	C

ATOM	2328	CG	MET	1226	109.591	12.703	12.640	1.00	0.00	LX0	C
ATOM	2329	SD	MET	1226	110.300	14.298	12.190	1.00	0.00	LX0	S
ATOM	2330	CE	MET	1226	111.472	14.479	13.545	1.00	0.00	LX0	C
ATOM	2331	C	MET	1226	111.387	9.365	13.316	1.00	0.00	LX0	C
ATOM	2332	O	MET	1226	111.750	8.712	12.339	1.00	0.00	LX0	O
ATOM	2333	N	GLU	1227	112.005	9.368	14.509	1.00	0.00	LX0	N
ATOM	2334	H	GLU	1227	111.655	9.918	15.267	0.00	0.00	LX0	H
ATOM	2335	CA	GLU	1227	113.214	8.558	14.627	1.00	0.00	LX0	C
ATOM	2336	CB	GLU	1227	114.430	9.408	15.023	1.00	0.00	LX0	C
ATOM	2337	CG	GLU	1227	114.638	10.692	14.201	1.00	0.00	LX0	C
ATOM	2338	CD	GLU	1227	114.581	10.423	12.704	1.00	0.00	LX0	C
ATOM	2339	OE1	GLU	1227	115.292	9.558	12.199	1.00	0.00	LX0	O
ATOM	2340	OE2	GLU	1227	113.781	11.055	12.022	1.00	0.00	LX0	O
ATOM	2341	C	GLU	1227	113.071	7.333	15.512	1.00	0.00	LX0	C
ATOM	2342	O	GLU	1227	113.976	6.921	16.226	1.00	0.00	LX0	O
ATOM	2343	N	GLY	1228	111.874	6.741	15.416	1.00	0.00	LX0	N
ATOM	2344	H	GLY	1228	111.156	7.161	14.863	0.00	0.00	LX0	H
ATOM	2345	CA	GLY	1228	111.669	5.473	16.109	1.00	0.00	LX0	C
ATOM	2346	C	GLY	1228	111.266	5.569	17.568	1.00	0.00	LX0	C
ATOM	2347	O	GLY	1228	111.511	4.669	18.363	1.00	0.00	LX0	O
ATOM	2348	N	GLY	1229	110.597	6.683	17.898	1.00	0.00	LX0	N
ATOM	2349	H	GLY	1229	110.432	7.428	17.251	0.00	0.00	LX0	H
ATOM	2350	CA	GLY	1229	110.014	6.723	19.236	1.00	0.00	LX0	C
ATOM	2351	C	GLY	1229	108.830	5.783	19.363	1.00	0.00	LX0	C
ATOM	2352	O	GLY	1229	107.728	6.063	18.906	1.00	0.00	LX0	O
ATOM	2353	N	LEU	1230	109.130	4.631	19.976	1.00	0.00	LX0	N
ATOM	2354	H	LEU	1230	110.075	4.486	20.266	0.00	0.00	LX0	H
ATOM	2355	CA	LEU	1230	108.104	3.596	20.101	1.00	0.00	LX0	C
ATOM	2356	CB	LEU	1230	108.730	2.257	20.506	1.00	0.00	LX0	C
ATOM	2357	CG	LEU	1230	109.906	1.792	19.641	1.00	0.00	LX0	C
ATOM	2358	CD1	LEU	1230	110.481	0.469	20.151	1.00	0.00	LX0	C
ATOM	2359	CD2	LEU	1230	109.558	1.715	18.155	1.00	0.00	LX0	C
ATOM	2360	C	LEU	1230	106.993	3.952	21.072	1.00	0.00	LX0	C
ATOM	2361	O	LEU	1230	107.095	4.890	21.853	1.00	0.00	LX0	O
ATOM	2362	N	LEU	1231	105.931	3.140	20.983	1.00	0.00	LX0	N
ATOM	2363	H	LEU	1231	105.930	2.377	20.343	0.00	0.00	LX0	H
ATOM	2364	CA	LEU	1231	104.889	3.256	21.996	1.00	0.00	LX0	C
ATOM	2365	CB	LEU	1231	103.559	2.758	21.437	1.00	0.00	LX0	C
ATOM	2366	CG	LEU	1231	102.715	3.862	20.815	1.00	0.00	LX0	C
ATOM	2367	CD1	LEU	1231	101.661	3.324	19.852	1.00	0.00	LX0	C
ATOM	2368	CD2	LEU	1231	102.108	4.754	21.893	1.00	0.00	LX0	C
ATOM	2369	C	LEU	1231	105.239	2.510	23.267	1.00	0.00	LX0	C
ATOM	2370	O	LEU	1231	106.196	1.745	23.339	1.00	0.00	LX0	O
ATOM	2371	N	ASP	1232	104.400	2.799	24.263	1.00	0.00	LX0	N
ATOM	2372	H	ASP	1232	103.606	3.392	24.119	0.00	0.00	LX0	H
ATOM	2373	CA	ASP	1232	104.471	2.141	25.560	1.00	0.00	LX0	C
ATOM	2374	CB	ASP	1232	103.786	3.048	26.597	1.00	0.00	LX0	C
ATOM	2375	CG	ASP	1232	102.340	3.297	26.194	1.00	0.00	LX0	C
ATOM	2376	OD1	ASP	1232	102.089	4.214	25.415	1.00	0.00	LX0	O
ATOM	2377	OD2	ASP	1232	101.473	2.542	26.621	1.00	0.00	LX0	O
ATOM	2378	C	ASP	1232	103.834	0.758	25.531	1.00	0.00	LX0	C
ATOM	2379	O	ASP	1232	103.357	0.279	24.507	1.00	0.00	LX0	O
ATOM	2380	N	LYS	1233	103.835	0.136	26.721	1.00	0.00	LX0	N
ATOM	2381	H	LYS	1233	104.214	0.580	27.529	0.00	0.00	LX0	H
ATOM	2382	CA	LYS	1233	102.891	-0.962	26.864	1.00	0.00	LX0	C
ATOM	2383	CB	LYS	1233	103.547	-2.256	27.354	1.00	0.00	LX0	C
ATOM	2384	CG	LYS	1233	102.574	-3.426	27.163	1.00	0.00	LX0	C
ATOM	2385	CD	LYS	1233	102.907	-4.711	27.910	1.00	0.00	LX0	C
ATOM	2386	CE	LYS	1233	102.747	-4.599	29.427	1.00	0.00	LX0	C
ATOM	2387	NZ	LYS	1233	102.793	-5.952	29.990	1.00	0.00	LX0	N
ATOM	2388	HZ1	LYS	1233	102.663	-5.980	31.014	0.00	0.00	LX0	H

ATOM	2389	HZ2	LYS	1233	102.079	-6.564	29.541	0.00	0.00	LX0	H
ATOM	2390	HZ3	LYS	1233	103.698	-6.430	29.773	0.00	0.00	LX0	H
ATOM	2391	C	LYS	1233	101.809	-0.581	27.850	1.00	0.00	LX0	C
ATOM	2392	O	LYS	1233	102.093	-0.315	29.013	1.00	0.00	LX0	O
ATOM	2393	N	PRO	1234	100.551	-0.615	27.359	1.00	0.00	LX0	N
ATOM	2394	CD	PRO	1234	100.161	-0.831	25.971	1.00	0.00	LX0	C
ATOM	2395	CA	PRO	1234	99.411	-0.432	28.261	1.00	0.00	LX0	C
ATOM	2396	CB	PRO	1234	98.217	-0.708	27.347	1.00	0.00	LX0	C
ATOM	2397	CG	PRO	1234	98.710	-0.391	25.936	1.00	0.00	LX0	C
ATOM	2398	C	PRO	1234	99.446	-1.371	29.456	1.00	0.00	LX0	C
ATOM	2399	O	PRO	1234	99.866	-2.525	29.364	1.00	0.00	LX0	O
ATOM	2400	N	ASP	1235	98.967	-0.818	30.583	1.00	0.00	LX0	N
ATOM	2401	H	ASP	1235	98.648	0.127	30.577	0.00	0.00	LX0	H
ATOM	2402	CA	ASP	1235	98.838	-1.646	31.783	1.00	0.00	LX0	C
ATOM	2403	CB	ASP	1235	98.275	-0.815	32.948	1.00	0.00	LX0	C
ATOM	2404	CG	ASP	1235	98.091	-1.672	34.192	1.00	0.00	LX0	C
ATOM	2405	OD1	ASP	1235	96.988	-1.700	34.724	1.00	0.00	LX0	O
ATOM	2406	OD2	ASP	1235	99.021	-2.379	34.576	1.00	0.00	LX0	O
ATOM	2407	C	ASP	1235	98.013	-2.897	31.520	1.00	0.00	LX0	C
ATOM	2408	O	ASP	1235	97.157	-2.931	30.642	1.00	0.00	LX0	O
ATOM	2409	N	ASN	1236	98.385	-3.948	32.266	1.00	0.00	LX0	N
ATOM	2410	H	ASN	1236	98.976	-3.707	33.040	0.00	0.00	LX0	H
ATOM	2411	CA	ASN	1236	97.735	-5.258	32.184	1.00	0.00	LX0	C
ATOM	2412	CB	ASN	1236	96.293	-5.202	32.704	1.00	0.00	LX0	C
ATOM	2413	CG	ASN	1236	96.265	-5.561	34.176	1.00	0.00	LX0	C
ATOM	2414	OD1	ASN	1236	95.834	-6.637	34.568	1.00	0.00	LX0	O
ATOM	2415	ND2	ASN	1236	96.742	-4.619	34.995	1.00	0.00	LX0	N
ATOM	2416	HD21	ASN	1236	97.073	-3.715	34.694	0.00	0.00	LX0	H
ATOM	2417	HD22	ASN	1236	96.781	-4.777	35.976	0.00	0.00	LX0	H
ATOM	2418	C	ASN	1236	97.821	-6.030	30.875	1.00	0.00	LX0	C
ATOM	2419	O	ASN	1236	97.584	-7.230	30.838	1.00	0.00	LX0	O
ATOM	2420	N	CYS	1237	98.214	-5.333	29.794	1.00	0.00	LX0	N
ATOM	2421	H	CYS	1237	98.348	-4.344	29.850	0.00	0.00	LX0	H
ATOM	2422	CA	CYS	1237	98.353	-6.020	28.512	1.00	0.00	LX0	C
ATOM	2423	CB	CYS	1237	98.689	-5.007	27.414	1.00	0.00	LX0	C
ATOM	2424	SG	CYS	1237	98.783	-5.730	25.754	1.00	0.00	LX0	S
ATOM	2425	C	CYS	1237	99.383	-7.139	28.545	1.00	0.00	LX0	C
ATOM	2426	O	CYS	1237	100.554	-6.928	28.860	1.00	0.00	LX0	O
ATOM	2427	N	PRO	1238	98.898	-8.360	28.220	1.00	0.00	LX0	N
ATOM	2428	CD	PRO	1238	97.508	-8.711	27.951	1.00	0.00	LX0	C
ATOM	2429	CA	PRO	1238	99.796	-9.514	28.152	1.00	0.00	LX0	C
ATOM	2430	CB	PRO	1238	98.846	-10.674	27.841	1.00	0.00	LX0	C
ATOM	2431	CG	PRO	1238	97.457	-10.198	28.265	1.00	0.00	LX0	C
ATOM	2432	C	PRO	1238	100.885	-9.343	27.109	1.00	0.00	LX0	C
ATOM	2433	O	PRO	1238	100.642	-9.138	25.924	1.00	0.00	LX0	O
ATOM	2434	N	ASP	1239	102.114	-9.437	27.622	1.00	0.00	LX0	N
ATOM	2435	H	ASP	1239	102.241	-9.541	28.611	0.00	0.00	LX0	H
ATOM	2436	CA	ASP	1239	103.287	-9.020	26.854	1.00	0.00	LX0	C
ATOM	2437	CB	ASP	1239	104.536	-9.114	27.734	1.00	0.00	LX0	C
ATOM	2438	CG	ASP	1239	104.384	-8.139	28.888	1.00	0.00	LX0	C
ATOM	2439	OD1	ASP	1239	103.572	-8.372	29.782	1.00	0.00	LX0	O
ATOM	2440	OD2	ASP	1239	105.018	-7.090	28.876	1.00	0.00	LX0	O
ATOM	2441	C	ASP	1239	103.492	-9.652	25.490	1.00	0.00	LX0	C
ATOM	2442	O	ASP	1239	104.084	-9.059	24.600	1.00	0.00	LX0	O
ATOM	2443	N	MET	1240	102.907	-10.853	25.320	1.00	0.00	LX0	N
ATOM	2444	H	MET	1240	102.500	-11.296	26.115	0.00	0.00	LX0	H
ATOM	2445	CA	MET	1240	102.861	-11.460	23.983	1.00	0.00	LX0	C
ATOM	2446	CB	MET	1240	101.984	-12.718	24.036	1.00	0.00	LX0	C
ATOM	2447	CG	MET	1240	102.007	-13.626	22.798	1.00	0.00	LX0	C
ATOM	2448	SD	MET	1240	101.219	-12.919	21.341	1.00	0.00	LX0	S
ATOM	2449	CE	MET	1240	101.424	-14.326	20.236	1.00	0.00	LX0	C

ATOM	2450	C	MET	1240	102.422	-10.501	22.876	1.00	0.00	LX0	C
ATOM	2451	O	MET	1240	103.069	-10.342	21.845	1.00	0.00	LX0	O
ATOM	2452	N	LEU	1241	101.305	-9.809	23.165	1.00	0.00	LX0	N
ATOM	2453	H	LEU	1241	100.855	-9.906	24.054	0.00	0.00	LX0	H
ATOM	2454	CA	LEU	1241	100.859	-8.830	22.175	1.00	0.00	LX0	C
ATOM	2455	CB	LEU	1241	99.467	-8.292	22.523	1.00	0.00	LX0	C
ATOM	2456	CG	LEU	1241	98.792	-7.579	21.343	1.00	0.00	LX0	C
ATOM	2457	CD1	LEU	1241	98.521	-8.531	20.176	1.00	0.00	LX0	C
ATOM	2458	CD2	LEU	1241	97.534	-6.822	21.764	1.00	0.00	LX0	C
ATOM	2459	C	LEU	1241	101.850	-7.695	21.952	1.00	0.00	LX0	C
ATOM	2460	O	LEU	1241	102.061	-7.229	20.842	1.00	0.00	LX0	O
ATOM	2461	N	PHE	1242	102.488	-7.295	23.061	1.00	0.00	LX0	N
ATOM	2462	H	PHE	1242	102.333	-7.782	23.920	0.00	0.00	LX0	H
ATOM	2463	CA	PHE	1242	103.530	-6.271	22.960	1.00	0.00	LX0	C
ATOM	2464	CB	PHE	1242	103.865	-5.748	24.360	1.00	0.00	LX0	C
ATOM	2465	CG	PHE	1242	104.690	-4.477	24.337	1.00	0.00	LX0	C
ATOM	2466	CD1	PHE	1242	104.285	-3.377	23.547	1.00	0.00	LX0	C
ATOM	2467	CD2	PHE	1242	105.848	-4.406	25.140	1.00	0.00	LX0	C
ATOM	2468	CE1	PHE	1242	105.034	-2.185	23.577	1.00	0.00	LX0	C
ATOM	2469	CE2	PHE	1242	106.596	-3.213	25.177	1.00	0.00	LX0	C
ATOM	2470	CZ	PHE	1242	106.174	-2.110	24.403	1.00	0.00	LX0	C
ATOM	2471	C	PHE	1242	104.784	-6.707	22.209	1.00	0.00	LX0	C
ATOM	2472	O	PHE	1242	105.550	-5.899	21.688	1.00	0.00	LX0	O
ATOM	2473	N	GLU	1243	104.962	-8.033	22.145	1.00	0.00	LX0	N
ATOM	2474	H	GLU	1243	104.376	-8.667	22.652	0.00	0.00	LX0	H
ATOM	2475	CA	GLU	1243	106.016	-8.514	21.263	1.00	0.00	LX0	C
ATOM	2476	CB	GLU	1243	106.413	-9.952	21.613	1.00	0.00	LX0	C
ATOM	2477	CG	GLU	1243	107.830	-10.328	21.151	1.00	0.00	LX0	C
ATOM	2478	CD	GLU	1243	108.855	-9.360	21.722	1.00	0.00	LX0	C
ATOM	2479	OE1	GLU	1243	108.888	-9.135	22.930	1.00	0.00	LX0	O
ATOM	2480	OE2	GLU	1243	109.599	-8.761	20.957	1.00	0.00	LX0	O
ATOM	2481	C	GLU	1243	105.660	-8.317	19.805	1.00	0.00	LX0	C
ATOM	2482	O	GLU	1243	106.378	-7.685	19.042	1.00	0.00	LX0	O
ATOM	2483	N	LEU	1244	104.448	-8.798	19.476	1.00	0.00	LX0	N
ATOM	2484	H	LEU	1244	103.935	-9.328	20.155	0.00	0.00	LX0	H
ATOM	2485	CA	LEU	1244	103.926	-8.547	18.128	1.00	0.00	LX0	C
ATOM	2486	CB	LEU	1244	102.508	-9.111	18.004	1.00	0.00	LX0	C
ATOM	2487	CG	LEU	1244	102.000	-9.179	16.561	1.00	0.00	LX0	C
ATOM	2488	CD1	LEU	1244	102.836	-10.131	15.702	1.00	0.00	LX0	C
ATOM	2489	CD2	LEU	1244	100.507	-9.505	16.505	1.00	0.00	LX0	C
ATOM	2490	C	LEU	1244	103.981	-7.082	17.693	1.00	0.00	LX0	C
ATOM	2491	O	LEU	1244	104.434	-6.734	16.609	1.00	0.00	LX0	O
ATOM	2492	N	MET	1245	103.554	-6.223	18.637	1.00	0.00	LX0	N
ATOM	2493	H	MET	1245	103.130	-6.603	19.457	0.00	0.00	LX0	H
ATOM	2494	CA	MET	1245	103.669	-4.778	18.425	1.00	0.00	LX0	C
ATOM	2495	CB	MET	1245	103.300	-3.989	19.682	1.00	0.00	LX0	C
ATOM	2496	CG	MET	1245	101.865	-4.124	20.186	1.00	0.00	LX0	C
ATOM	2497	SD	MET	1245	101.593	-3.110	21.648	1.00	0.00	LX0	S
ATOM	2498	CE	MET	1245	100.131	-3.936	22.289	1.00	0.00	LX0	C
ATOM	2499	C	MET	1245	105.055	-4.341	17.989	1.00	0.00	LX0	C
ATOM	2500	O	MET	1245	105.240	-3.683	16.976	1.00	0.00	LX0	O
ATOM	2501	N	ARG	1246	106.040	-4.757	18.799	1.00	0.00	LX0	N
ATOM	2502	H	ARG	1246	105.843	-5.375	19.563	0.00	0.00	LX0	H
ATOM	2503	CA	ARG	1246	107.409	-4.366	18.469	1.00	0.00	LX0	C
ATOM	2504	CB	ARG	1246	108.309	-4.566	19.677	1.00	0.00	LX0	C
ATOM	2505	CG	ARG	1246	108.231	-3.389	20.654	1.00	0.00	LX0	C
ATOM	2506	CD	ARG	1246	108.420	-3.785	22.123	1.00	0.00	LX0	C
ATOM	2507	NE	ARG	1246	109.584	-4.645	22.342	1.00	0.00	LX0	N
ATOM	2508	HE	ARG	1246	110.497	-4.239	22.309	0.00	0.00	LX0	H
ATOM	2509	CZ	ARG	1246	109.400	-5.977	22.494	1.00	0.00	LX0	C
ATOM	2510	NH1	ARG	1246	108.182	-6.502	22.494	1.00	0.00	LX0	N

ATOM	2511	HH11	ARG	1246	108.118	-7.504	22.602	0.00	0.00	LX0	H
ATOM	2512	HH12	ARG	1246	107.338	-5.975	22.366	0.00	0.00	LX0	H
ATOM	2513	NH2	ARG	1246	110.435	-6.796	22.615	1.00	0.00	LX0	N
ATOM	2514	HH21	ARG	1246	110.227	-7.791	22.558	0.00	0.00	LX0	H
ATOM	2515	HH22	ARG	1246	111.380	-6.505	22.719	0.00	0.00	LX0	H
ATOM	2516	C	ARG	1246	107.999	-5.006	17.226	1.00	0.00	LX0	C
ATOM	2517	O	ARG	1246	108.869	-4.439	16.577	1.00	0.00	LX0	O
ATOM	2518	N	MET	1247	107.454	-6.181	16.873	1.00	0.00	LX0	N
ATOM	2519	H	MET	1247	106.802	-6.636	17.480	0.00	0.00	LX0	H
ATOM	2520	CA	MET	1247	107.785	-6.729	15.555	1.00	0.00	LX0	C
ATOM	2521	CB	MET	1247	107.108	-8.085	15.343	1.00	0.00	LX0	C
ATOM	2522	CG	MET	1247	107.609	-9.178	16.288	1.00	0.00	LX0	C
ATOM	2523	SD	MET	1247	106.653	-10.700	16.160	1.00	0.00	LX0	S
ATOM	2524	CE	MET	1247	106.872	-11.013	14.400	1.00	0.00	LX0	C
ATOM	2525	C	MET	1247	107.403	-5.770	14.441	1.00	0.00	LX0	C
ATOM	2526	O	MET	1247	108.193	-5.416	13.577	1.00	0.00	LX0	O
ATOM	2527	N	CYS	1248	106.152	-5.301	14.549	1.00	0.00	LX0	N
ATOM	2528	H	CYS	1248	105.537	-5.652	15.260	0.00	0.00	LX0	H
ATOM	2529	CA	CYS	1248	105.732	-4.243	13.631	1.00	0.00	LX0	C
ATOM	2530	CB	CYS	1248	104.245	-3.960	13.819	1.00	0.00	LX0	C
ATOM	2531	SG	CYS	1248	103.221	-5.451	13.712	1.00	0.00	LX0	S
ATOM	2532	C	CYS	1248	106.529	-2.950	13.757	1.00	0.00	LX0	C
ATOM	2533	O	CYS	1248	106.805	-2.244	12.794	1.00	0.00	LX0	O
ATOM	2534	N	TRP	1249	106.904	-2.671	15.011	1.00	0.00	LX0	N
ATOM	2535	H	TRP	1249	106.677	-3.309	15.744	0.00	0.00	LX0	H
ATOM	2536	CA	TRP	1249	107.596	-1.420	15.301	1.00	0.00	LX0	C
ATOM	2537	CB	TRP	1249	107.244	-0.902	16.698	1.00	0.00	LX0	C
ATOM	2538	CG	TRP	1249	105.773	-0.578	16.817	1.00	0.00	LX0	C
ATOM	2539	CD2	TRP	1249	104.990	-0.536	18.026	1.00	0.00	LX0	C
ATOM	2540	CE2	TRP	1249	103.649	-0.190	17.652	1.00	0.00	LX0	C
ATOM	2541	CE3	TRP	1249	105.311	-0.762	19.382	1.00	0.00	LX0	C
ATOM	2542	CD1	TRP	1249	104.876	-0.253	15.787	1.00	0.00	LX0	C
ATOM	2543	NE1	TRP	1249	103.627	-0.025	16.268	1.00	0.00	LX0	N
ATOM	2544	HE1	TRP	1249	102.858	0.221	15.708	0.00	0.00	LX0	H
ATOM	2545	CZ2	TRP	1249	102.654	-0.082	18.647	1.00	0.00	LX0	C
ATOM	2546	CZ3	TRP	1249	104.307	-0.648	20.365	1.00	0.00	LX0	C
ATOM	2547	CH2	TRP	1249	102.985	-0.314	19.998	1.00	0.00	LX0	C
ATOM	2548	C	TRP	1249	109.101	-1.446	15.121	1.00	0.00	LX0	C
ATOM	2549	O	TRP	1249	109.873	-1.025	15.974	1.00	0.00	LX0	O
ATOM	2550	N	GLN	1250	109.501	-1.931	13.944	1.00	0.00	LX0	N
ATOM	2551	H	GLN	1250	108.825	-2.204	13.258	0.00	0.00	LX0	H
ATOM	2552	CA	GLN	1250	110.917	-1.760	13.650	1.00	0.00	LX0	C
ATOM	2553	CB	GLN	1250	111.386	-2.841	12.678	1.00	0.00	LX0	C
ATOM	2554	CG	GLN	1250	111.297	-4.246	13.277	1.00	0.00	LX0	C
ATOM	2555	CD	GLN	1250	112.201	-4.328	14.489	1.00	0.00	LX0	C
ATOM	2556	OE1	GLN	1250	113.402	-4.099	14.422	1.00	0.00	LX0	O
ATOM	2557	NE2	GLN	1250	111.565	-4.634	15.620	1.00	0.00	LX0	N
ATOM	2558	HE21	GLN	1250	110.568	-4.747	15.641	0.00	0.00	LX0	H
ATOM	2559	HE22	GLN	1250	112.086	-4.731	16.463	0.00	0.00	LX0	H
ATOM	2560	C	GLN	1250	111.188	-0.382	13.096	1.00	0.00	LX0	C
ATOM	2561	O	GLN	1250	110.446	0.114	12.258	1.00	0.00	LX0	O
ATOM	2562	N	TYR	1251	112.286	0.230	13.574	1.00	0.00	LX0	N
ATOM	2563	H	TYR	1251	112.793	-0.192	14.326	0.00	0.00	LX0	H
ATOM	2564	CA	TYR	1251	112.613	1.538	12.994	1.00	0.00	LX0	C
ATOM	2565	CB	TYR	1251	113.833	2.180	13.684	1.00	0.00	LX0	C
ATOM	2566	CG	TYR	1251	114.169	3.521	13.055	1.00	0.00	LX0	C
ATOM	2567	CD1	TYR	1251	113.193	4.538	13.030	1.00	0.00	LX0	C
ATOM	2568	CE1	TYR	1251	113.481	5.751	12.388	1.00	0.00	LX0	C
ATOM	2569	CD2	TYR	1251	115.446	3.711	12.486	1.00	0.00	LX0	C
ATOM	2570	CE2	TYR	1251	115.738	4.930	11.847	1.00	0.00	LX0	C
ATOM	2571	CZ	TYR	1251	114.743	5.928	11.793	1.00	0.00	LX0	C

ATOM	2572	OH	TYR	1251	114.995	7.112	11.129	1.00	0.00	LX0	O
ATOM	2573	HH	TYR	1251	115.484	7.700	11.716	0.00	0.00	LX0	H
ATOM	2574	C	TYR	1251	112.796	1.469	11.485	1.00	0.00	LX0	C
ATOM	2575	O	TYR	1251	112.072	2.085	10.715	1.00	0.00	LX0	O
ATOM	2576	N	ASN	1252	113.782	0.640	11.108	1.00	0.00	LX0	N
ATOM	2577	H	ASN	1252	114.268	0.129	11.814	0.00	0.00	LX0	H
ATOM	2578	CA	ASN	1252	114.017	0.384	9.684	1.00	0.00	LX0	C
ATOM	2579	CB	ASN	1252	115.225	-0.559	9.553	1.00	0.00	LX0	C
ATOM	2580	CG	ASN	1252	115.586	-0.884	8.109	1.00	0.00	LX0	C
ATOM	2581	OD1	ASN	1252	114.816	-0.731	7.171	1.00	0.00	LX0	O
ATOM	2582	ND2	ASN	1252	116.824	-1.363	7.968	1.00	0.00	LX0	N
ATOM	2583	HD21	ASN	1252	117.435	-1.488	8.748	0.00	0.00	LX0	H
ATOM	2584	HD22	ASN	1252	117.139	-1.605	7.052	0.00	0.00	LX0	H
ATOM	2585	C	ASN	1252	112.780	-0.192	9.009	1.00	0.00	LX0	C
ATOM	2586	O	ASN	1252	112.309	-1.263	9.377	1.00	0.00	LX0	O
ATOM	2587	N	PRO	1253	112.267	0.551	7.998	1.00	0.00	LX0	N
ATOM	2588	CD	PRO	1253	112.767	1.829	7.497	1.00	0.00	LX0	C
ATOM	2589	CA	PRO	1253	111.079	0.084	7.280	1.00	0.00	LX0	C
ATOM	2590	CB	PRO	1253	110.876	1.160	6.203	1.00	0.00	LX0	C
ATOM	2591	CG	PRO	1253	112.204	1.907	6.085	1.00	0.00	LX0	C
ATOM	2592	C	PRO	1253	111.178	-1.339	6.755	1.00	0.00	LX0	C
ATOM	2593	O	PRO	1253	110.264	-2.139	6.889	1.00	0.00	LX0	O
ATOM	2594	N	LYS	1254	112.342	-1.650	6.169	1.00	0.00	LX0	N
ATOM	2595	H	LYS	1254	113.127	-1.030	6.222	0.00	0.00	LX0	H
ATOM	2596	CA	LYS	1254	112.453	-2.996	5.614	1.00	0.00	LX0	C
ATOM	2597	CB	LYS	1254	113.518	-3.057	4.522	1.00	0.00	LX0	C
ATOM	2598	CG	LYS	1254	113.104	-2.242	3.301	1.00	0.00	LX0	C
ATOM	2599	CD	LYS	1254	113.834	-2.697	2.042	1.00	0.00	LX0	C
ATOM	2600	CE	LYS	1254	113.249	-2.057	0.785	1.00	0.00	LX0	C
ATOM	2601	NZ	LYS	1254	113.868	-2.677	-0.394	1.00	0.00	LX0	N
ATOM	2602	HZ1	LYS	1254	113.483	-2.245	-1.258	0.00	0.00	LX0	H
ATOM	2603	HZ2	LYS	1254	114.898	-2.538	-0.360	0.00	0.00	LX0	H
ATOM	2604	HZ3	LYS	1254	113.653	-3.695	-0.388	0.00	0.00	LX0	H
ATOM	2605	C	LYS	1254	112.639	-4.142	6.593	1.00	0.00	LX0	C
ATOM	2606	O	LYS	1254	112.819	-5.281	6.188	1.00	0.00	LX0	O
ATOM	2607	N	MET	1255	112.586	-3.807	7.889	1.00	0.00	LX0	N
ATOM	2608	H	MET	1255	112.416	-2.873	8.201	0.00	0.00	LX0	H
ATOM	2609	CA	MET	1255	112.586	-4.912	8.841	1.00	0.00	LX0	C
ATOM	2610	CB	MET	1255	113.545	-4.628	9.999	1.00	0.00	LX0	C
ATOM	2611	CG	MET	1255	115.005	-4.458	9.574	1.00	0.00	LX0	C
ATOM	2612	SD	MET	1255	115.711	-5.924	8.803	1.00	0.00	LX0	S
ATOM	2613	CE	MET	1255	115.565	-7.049	10.203	1.00	0.00	LX0	C
ATOM	2614	C	MET	1255	111.206	-5.263	9.372	1.00	0.00	LX0	C
ATOM	2615	O	MET	1255	111.060	-6.089	10.262	1.00	0.00	LX0	O
ATOM	2616	N	ARG	1256	110.194	-4.579	8.814	1.00	0.00	LX0	N
ATOM	2617	H	ARG	1256	110.321	-3.975	8.028	0.00	0.00	LX0	H
ATOM	2618	CA	ARG	1256	108.846	-4.883	9.292	1.00	0.00	LX0	C
ATOM	2619	CB	ARG	1256	107.960	-3.638	9.168	1.00	0.00	LX0	C
ATOM	2620	CG	ARG	1256	108.622	-2.426	9.828	1.00	0.00	LX0	C
ATOM	2621	CD	ARG	1256	107.825	-1.127	9.728	1.00	0.00	LX0	C
ATOM	2622	NE	ARG	1256	108.690	-0.005	10.087	1.00	0.00	LX0	N
ATOM	2623	HE	ARG	1256	109.387	-0.161	10.789	0.00	0.00	LX0	H
ATOM	2624	CZ	ARG	1256	108.617	1.182	9.458	1.00	0.00	LX0	C
ATOM	2625	NH1	ARG	1256	107.625	1.437	8.618	1.00	0.00	LX0	N
ATOM	2626	HH11	ARG	1256	107.630	2.309	8.113	0.00	0.00	LX0	H
ATOM	2627	HH12	ARG	1256	106.866	0.794	8.479	0.00	0.00	LX0	H
ATOM	2628	NH2	ARG	1256	109.553	2.101	9.658	1.00	0.00	LX0	N
ATOM	2629	HH21	ARG	1256	109.523	2.978	9.148	0.00	0.00	LX0	H
ATOM	2630	HH22	ARG	1256	110.331	1.970	10.281	0.00	0.00	LX0	H
ATOM	2631	C	ARG	1256	108.255	-6.087	8.574	1.00	0.00	LX0	C
ATOM	2632	O	ARG	1256	108.196	-6.123	7.350	1.00	0.00	LX0	O

ATOM	2633	N	PRO	1257	107.849	-7.090	9.390	1.00	0.00	LX0	N
ATOM	2634	CD	PRO	1257	107.855	-7.076	10.849	1.00	0.00	LX0	C
ATOM	2635	CA	PRO	1257	107.348	-8.354	8.835	1.00	0.00	LX0	C
ATOM	2636	CB	PRO	1257	107.243	-9.232	10.089	1.00	0.00	LX0	C
ATOM	2637	CG	PRO	1257	106.988	-8.263	11.240	1.00	0.00	LX0	C
ATOM	2638	C	PRO	1257	106.019	-8.180	8.122	1.00	0.00	LX0	C
ATOM	2639	O	PRO	1257	105.270	-7.246	8.390	1.00	0.00	LX0	O
ATOM	2640	N	SER	1258	105.733	-9.105	7.199	1.00	0.00	LX0	N
ATOM	2641	H	SER	1258	106.339	-9.896	7.053	0.00	0.00	LX0	H
ATOM	2642	CA	SER	1258	104.433	-8.945	6.556	1.00	0.00	LX0	C
ATOM	2643	CB	SER	1258	104.363	-9.703	5.225	1.00	0.00	LX0	C
ATOM	2644	OG	SER	1258	104.153	-11.104	5.453	1.00	0.00	LX0	O
ATOM	2645	HG	SER	1258	105.042	-11.474	5.485	0.00	0.00	LX0	H
ATOM	2646	C	SER	1258	103.274	-9.348	7.448	1.00	0.00	LX0	C
ATOM	2647	O	SER	1258	103.415	-10.061	8.436	1.00	0.00	LX0	O
ATOM	2648	N	PHE	1259	102.080	-8.923	7.004	1.00	0.00	LX0	N
ATOM	2649	H	PHE	1259	102.043	-8.261	6.257	0.00	0.00	LX0	H
ATOM	2650	CA	PHE	1259	100.872	-9.433	7.656	1.00	0.00	LX0	C
ATOM	2651	CB	PHE	1259	99.619	-8.825	7.027	1.00	0.00	LX0	C
ATOM	2652	CG	PHE	1259	99.659	-7.321	7.150	1.00	0.00	LX0	C
ATOM	2653	CD1	PHE	1259	99.390	-6.722	8.400	1.00	0.00	LX0	C
ATOM	2654	CD2	PHE	1259	99.973	-6.544	6.014	1.00	0.00	LX0	C
ATOM	2655	CE1	PHE	1259	99.446	-5.321	8.517	1.00	0.00	LX0	C
ATOM	2656	CE2	PHE	1259	100.030	-5.143	6.131	1.00	0.00	LX0	C
ATOM	2657	CZ	PHE	1259	99.768	-4.549	7.383	1.00	0.00	LX0	C
ATOM	2658	C	PHE	1259	100.764	-10.950	7.678	1.00	0.00	LX0	C
ATOM	2659	O	PHE	1259	100.280	-11.552	8.626	1.00	0.00	LX0	O
ATOM	2660	N	LEU	1260	101.276	-11.555	6.594	1.00	0.00	LX0	N
ATOM	2661	H	LEU	1260	101.774	-11.035	5.900	0.00	0.00	LX0	H
ATOM	2662	CA	LEU	1260	101.267	-13.016	6.554	1.00	0.00	LX0	C
ATOM	2663	CB	LEU	1260	101.666	-13.505	5.164	1.00	0.00	LX0	C
ATOM	2664	CG	LEU	1260	100.631	-13.115	4.111	1.00	0.00	LX0	C
ATOM	2665	CD1	LEU	1260	101.178	-13.268	2.692	1.00	0.00	LX0	C
ATOM	2666	CD2	LEU	1260	99.312	-13.862	4.324	1.00	0.00	LX0	C
ATOM	2667	C	LEU	1260	102.141	-13.650	7.618	1.00	0.00	LX0	C
ATOM	2668	O	LEU	1260	101.778	-14.629	8.263	1.00	0.00	LX0	O
ATOM	2669	N	GLU	1261	103.306	-13.019	7.809	1.00	0.00	LX0	N
ATOM	2670	H	GLU	1261	103.567	-12.233	7.243	0.00	0.00	LX0	H
ATOM	2671	CA	GLU	1261	104.172	-13.473	8.898	1.00	0.00	LX0	C
ATOM	2672	CB	GLU	1261	105.522	-12.780	8.790	1.00	0.00	LX0	C
ATOM	2673	CG	GLU	1261	106.257	-13.238	7.527	1.00	0.00	LX0	C
ATOM	2674	CD	GLU	1261	107.002	-12.073	6.913	1.00	0.00	LX0	C
ATOM	2675	OE1	GLU	1261	106.843	-11.843	5.719	1.00	0.00	LX0	O
ATOM	2676	OE2	GLU	1261	107.700	-11.363	7.624	1.00	0.00	LX0	O
ATOM	2677	C	GLU	1261	103.552	-13.300	10.274	1.00	0.00	LX0	C
ATOM	2678	O	GLU	1261	103.616	-14.181	11.125	1.00	0.00	LX0	O
ATOM	2679	N	ILE	1262	102.877	-12.149	10.427	1.00	0.00	LX0	N
ATOM	2680	H	ILE	1262	102.958	-11.455	9.708	0.00	0.00	LX0	H
ATOM	2681	CA	ILE	1262	102.081	-11.911	11.635	1.00	0.00	LX0	C
ATOM	2682	CB	ILE	1262	101.417	-10.524	11.549	1.00	0.00	LX0	C
ATOM	2683	CG2	ILE	1262	100.392	-10.267	12.659	1.00	0.00	LX0	C
ATOM	2684	CG1	ILE	1262	102.500	-9.440	11.515	1.00	0.00	LX0	C
ATOM	2685	CD1	ILE	1262	101.966	-8.049	11.172	1.00	0.00	LX0	C
ATOM	2686	C	ILE	1262	101.076	-13.024	11.927	1.00	0.00	LX0	C
ATOM	2687	O	ILE	1262	101.068	-13.627	12.993	1.00	0.00	LX0	O
ATOM	2688	N	ILE	1263	100.252	-13.333	10.910	1.00	0.00	LX0	N
ATOM	2689	H	ILE	1263	100.286	-12.799	10.064	0.00	0.00	LX0	H
ATOM	2690	CA	ILE	1263	99.314	-14.443	11.119	1.00	0.00	LX0	C
ATOM	2691	CB	ILE	1263	98.369	-14.625	9.920	1.00	0.00	LX0	C
ATOM	2692	CG2	ILE	1263	97.303	-15.689	10.208	1.00	0.00	LX0	C
ATOM	2693	CG1	ILE	1263	97.714	-13.300	9.524	1.00	0.00	LX0	C

ATOM	2694	CD1	ILE	1263	96.979	-13.367	8.184	1.00	0.00	LX0	C
ATOM	2695	C	ILE	1263	100.024	-15.748	11.467	1.00	0.00	LX0	C
ATOM	2696	O	ILE	1263	99.664	-16.475	12.383	1.00	0.00	LX0	O
ATOM	2697	N	SER	1264	101.116	-15.975	10.729	1.00	0.00	LX0	N
ATOM	2698	H	SER	1264	101.357	-15.339	9.997	0.00	0.00	LX0	H
ATOM	2699	CA	SER	1264	101.941	-17.142	11.034	1.00	0.00	LX0	C
ATOM	2700	CB	SER	1264	103.044	-17.283	9.987	1.00	0.00	LX0	C
ATOM	2701	OG	SER	1264	102.448	-17.293	8.681	1.00	0.00	LX0	O
ATOM	2702	HG	SER	1264	102.324	-16.378	8.436	0.00	0.00	LX0	H
ATOM	2703	C	SER	1264	102.503	-17.222	12.450	1.00	0.00	LX0	C
ATOM	2704	O	SER	1264	102.750	-18.307	12.971	1.00	0.00	LX0	O
ATOM	2705	N	SER	1265	102.677	-16.047	13.065	1.00	0.00	LX0	N
ATOM	2706	H	SER	1265	102.448	-15.167	12.643	0.00	0.00	LX0	H
ATOM	2707	CA	SER	1265	103.136	-16.073	14.450	1.00	0.00	LX0	C
ATOM	2708	CB	SER	1265	104.152	-14.949	14.682	1.00	0.00	LX0	C
ATOM	2709	OG	SER	1265	103.868	-13.829	13.833	1.00	0.00	LX0	O
ATOM	2710	HG	SER	1265	104.080	-14.087	12.941	0.00	0.00	LX0	H
ATOM	2711	C	SER	1265	102.035	-16.071	15.501	1.00	0.00	LX0	C
ATOM	2712	O	SER	1265	102.260	-16.416	16.653	1.00	0.00	LX0	O
ATOM	2713	N	ILE	1266	100.828	-15.676	15.063	1.00	0.00	LX0	N
ATOM	2714	H	ILE	1266	100.703	-15.336	14.130	0.00	0.00	LX0	H
ATOM	2715	CA	ILE	1266	99.757	-15.624	16.060	1.00	0.00	LX0	C
ATOM	2716	CB	ILE	1266	99.126	-14.227	16.115	1.00	0.00	LX0	C
ATOM	2717	CG2	ILE	1266	100.184	-13.190	16.490	1.00	0.00	LX0	C
ATOM	2718	CG1	ILE	1266	98.366	-13.871	14.831	1.00	0.00	LX0	C
ATOM	2719	CD1	ILE	1266	97.668	-12.514	14.904	1.00	0.00	LX0	C
ATOM	2720	C	ILE	1266	98.681	-16.694	15.953	1.00	0.00	LX0	C
ATOM	2721	O	ILE	1266	97.817	-16.821	16.812	1.00	0.00	LX0	O
ATOM	2722	N	LYS	1267	98.760	-17.470	14.856	1.00	0.00	LX0	N
ATOM	2723	H	LYS	1267	99.467	-17.268	14.179	0.00	0.00	LX0	H
ATOM	2724	CA	LYS	1267	97.699	-18.440	14.560	1.00	0.00	LX0	C
ATOM	2725	CB	LYS	1267	98.023	-19.186	13.257	1.00	0.00	LX0	C
ATOM	2726	CG	LYS	1267	99.171	-20.178	13.424	1.00	0.00	LX0	C
ATOM	2727	CD	LYS	1267	100.097	-20.329	12.221	1.00	0.00	LX0	C
ATOM	2728	CE	LYS	1267	101.294	-21.224	12.567	1.00	0.00	LX0	C
ATOM	2729	NZ	LYS	1267	101.970	-20.717	13.770	1.00	0.00	LX0	N
ATOM	2730	HZ1	LYS	1267	102.624	-21.407	14.181	0.00	0.00	LX0	H
ATOM	2731	HZ2	LYS	1267	102.425	-19.793	13.648	0.00	0.00	LX0	H
ATOM	2732	HZ3	LYS	1267	101.284	-20.618	14.554	0.00	0.00	LX0	H
ATOM	2733	C	LYS	1267	97.305	-19.387	15.692	1.00	0.00	LX0	C
ATOM	2734	O	LYS	1267	96.176	-19.850	15.782	1.00	0.00	LX0	O
ATOM	2735	N	GLU	1268	98.292	-19.613	16.568	1.00	0.00	LX0	N
ATOM	2736	H	GLU	1268	99.205	-19.251	16.376	0.00	0.00	LX0	H
ATOM	2737	CA	GLU	1268	98.134	-20.437	17.760	1.00	0.00	LX0	C
ATOM	2738	CB	GLU	1268	99.457	-20.411	18.536	1.00	0.00	LX0	C
ATOM	2739	CG	GLU	1268	100.589	-21.313	17.999	1.00	0.00	LX0	C
ATOM	2740	CD	GLU	1268	101.003	-21.033	16.556	1.00	0.00	LX0	C
ATOM	2741	OE1	GLU	1268	101.122	-19.881	16.140	1.00	0.00	LX0	O
ATOM	2742	OE2	GLU	1268	101.221	-21.984	15.810	1.00	0.00	LX0	O
ATOM	2743	C	GLU	1268	96.950	-20.075	18.652	1.00	0.00	LX0	C
ATOM	2744	O	GLU	1268	96.247	-20.947	19.147	1.00	0.00	LX0	O
ATOM	2745	N	GLU	1269	96.741	-18.758	18.827	1.00	0.00	LX0	N
ATOM	2746	H	GLU	1269	97.300	-18.070	18.361	0.00	0.00	LX0	H
ATOM	2747	CA	GLU	1269	95.565	-18.395	19.624	1.00	0.00	LX0	C
ATOM	2748	CB	GLU	1269	95.864	-17.341	20.695	1.00	0.00	LX0	C
ATOM	2749	CG	GLU	1269	96.720	-17.787	21.885	1.00	0.00	LX0	C
ATOM	2750	CD	GLU	1269	96.541	-16.805	23.036	1.00	0.00	LX0	C
ATOM	2751	OE1	GLU	1269	96.695	-15.603	22.837	1.00	0.00	LX0	O
ATOM	2752	OE2	GLU	1269	96.208	-17.238	24.137	1.00	0.00	LX0	O
ATOM	2753	C	GLU	1269	94.359	-17.930	18.822	1.00	0.00	LX0	C
ATOM	2754	O	GLU	1269	93.425	-17.340	19.353	1.00	0.00	LX0	O

ATOM	2755	N	MET	1270	94.403	-18.194	17.507	1.00	0.00	LX0	N
ATOM	2756	H	MET	1270	95.089	-18.814	17.128	0.00	0.00	LX0	H
ATOM	2757	CA	MET	1270	93.231	-17.773	16.740	1.00	0.00	LX0	C
ATOM	2758	CB	MET	1270	93.539	-17.698	15.244	1.00	0.00	LX0	C
ATOM	2759	CG	MET	1270	94.639	-16.700	14.879	1.00	0.00	LX0	C
ATOM	2760	SD	MET	1270	94.242	-14.998	15.290	1.00	0.00	LX0	S
ATOM	2761	CE	MET	1270	92.766	-14.852	14.279	1.00	0.00	LX0	C
ATOM	2762	C	MET	1270	92.062	-18.707	16.960	1.00	0.00	LX0	C
ATOM	2763	O	MET	1270	92.199	-19.923	16.884	1.00	0.00	LX0	O
ATOM	2764	N	GLU	1271	90.895	-18.101	17.225	1.00	0.00	LX0	N
ATOM	2765	H	GLU	1271	90.821	-17.104	17.169	0.00	0.00	LX0	H
ATOM	2766	CA	GLU	1271	89.716	-18.954	17.363	1.00	0.00	LX0	C
ATOM	2767	CB	GLU	1271	88.542	-18.175	17.991	1.00	0.00	LX0	C
ATOM	2768	CG	GLU	1271	88.935	-17.599	19.365	1.00	0.00	LX0	C
ATOM	2769	CD	GLU	1271	87.735	-17.135	20.183	1.00	0.00	LX0	C
ATOM	2770	OE1	GLU	1271	87.302	-15.992	20.059	1.00	0.00	LX0	O
ATOM	2771	OE2	GLU	1271	87.237	-17.888	21.015	1.00	0.00	LX0	O
ATOM	2772	C	GLU	1271	89.398	-19.659	16.043	1.00	0.00	LX0	C
ATOM	2773	O	GLU	1271	89.160	-19.050	15.004	1.00	0.00	LX0	O
ATOM	2774	N	PRO	1272	89.490	-21.014	16.114	1.00	0.00	LX0	N
ATOM	2775	CD	PRO	1272	89.412	-21.814	17.334	1.00	0.00	LX0	C
ATOM	2776	CA	PRO	1272	89.783	-21.847	14.939	1.00	0.00	LX0	C
ATOM	2777	CB	PRO	1272	89.321	-23.230	15.401	1.00	0.00	LX0	C
ATOM	2778	CG	PRO	1272	89.703	-23.243	16.881	1.00	0.00	LX0	C
ATOM	2779	C	PRO	1272	89.285	-21.412	13.570	1.00	0.00	LX0	C
ATOM	2780	O	PRO	1272	90.077	-21.229	12.648	1.00	0.00	LX0	O
ATOM	2781	N	GLY	1273	87.949	-21.258	13.496	1.00	0.00	LX0	N
ATOM	2782	H	GLY	1273	87.425	-21.397	14.332	0.00	0.00	LX0	H
ATOM	2783	CA	GLY	1273	87.254	-20.991	12.230	1.00	0.00	LX0	C
ATOM	2784	C	GLY	1273	87.838	-19.915	11.326	1.00	0.00	LX0	C
ATOM	2785	O	GLY	1273	87.707	-19.957	10.108	1.00	0.00	LX0	O
ATOM	2786	N	PHE	1274	88.516	-18.948	11.980	1.00	0.00	LX0	N
ATOM	2787	H	PHE	1274	88.554	-18.960	12.981	0.00	0.00	LX0	H
ATOM	2788	CA	PHE	1274	89.249	-17.919	11.237	1.00	0.00	LX0	C
ATOM	2789	CB	PHE	1274	90.198	-17.184	12.201	1.00	0.00	LX0	C
ATOM	2790	CG	PHE	1274	91.145	-16.248	11.474	1.00	0.00	LX0	C
ATOM	2791	CD1	PHE	1274	90.695	-14.981	11.045	1.00	0.00	LX0	C
ATOM	2792	CD2	PHE	1274	92.472	-16.672	11.232	1.00	0.00	LX0	C
ATOM	2793	CE1	PHE	1274	91.581	-14.134	10.352	1.00	0.00	LX0	C
ATOM	2794	CE2	PHE	1274	93.358	-15.826	10.540	1.00	0.00	LX0	C
ATOM	2795	CZ	PHE	1274	92.901	-14.566	10.104	1.00	0.00	LX0	C
ATOM	2796	C	PHE	1274	90.000	-18.444	10.019	1.00	0.00	LX0	C
ATOM	2797	O	PHE	1274	89.865	-17.952	8.905	1.00	0.00	LX0	O
ATOM	2798	N	ARG	1275	90.824	-19.472	10.284	1.00	0.00	LX0	N
ATOM	2799	H	ARG	1275	90.751	-19.972	11.149	0.00	0.00	LX0	H
ATOM	2800	CA	ARG	1275	91.684	-19.825	9.161	1.00	0.00	LX0	C
ATOM	2801	CB	ARG	1275	92.949	-20.586	9.546	1.00	0.00	LX0	C
ATOM	2802	CG	ARG	1275	93.678	-20.223	10.846	1.00	0.00	LX0	C
ATOM	2803	CD	ARG	1275	93.524	-21.311	11.921	1.00	0.00	LX0	C
ATOM	2804	NE	ARG	1275	93.784	-22.638	11.353	1.00	0.00	LX0	N
ATOM	2805	HE	ARG	1275	94.697	-22.872	11.013	0.00	0.00	LX0	H
ATOM	2806	CZ	ARG	1275	92.741	-23.439	11.044	1.00	0.00	LX0	C
ATOM	2807	NH1	ARG	1275	91.523	-23.153	11.472	1.00	0.00	LX0	N
ATOM	2808	HH11	ARG	1275	90.730	-23.677	11.115	0.00	0.00	LX0	H
ATOM	2809	HH12	ARG	1275	91.305	-22.402	12.099	0.00	0.00	LX0	H
ATOM	2810	NH2	ARG	1275	92.923	-24.483	10.252	1.00	0.00	LX0	N
ATOM	2811	HH21	ARG	1275	92.115	-25.029	9.987	0.00	0.00	LX0	H
ATOM	2812	HH22	ARG	1275	93.828	-24.704	9.877	0.00	0.00	LX0	H
ATOM	2813	C	ARG	1275	90.986	-20.518	8.007	1.00	0.00	LX0	C
ATOM	2814	O	ARG	1275	91.349	-20.321	6.855	1.00	0.00	LX0	O
ATOM	2815	N	GLU	1276	89.939	-21.290	8.337	1.00	0.00	LX0	N

ATOM	2816	H	GLU	1276	89.674	-21.493	9.283	0.00	0.00	LX0	H
ATOM	2817	CA	GLU	1276	89.157	-21.887	7.250	1.00	0.00	LX0	C
ATOM	2818	CB	GLU	1276	88.025	-22.762	7.801	1.00	0.00	LX0	C
ATOM	2819	CG	GLU	1276	88.438	-24.147	8.328	1.00	0.00	LX0	C
ATOM	2820	CD	GLU	1276	89.327	-24.065	9.558	1.00	0.00	LX0	C
ATOM	2821	OE1	GLU	1276	89.065	-23.256	10.445	1.00	0.00	LX0	O
ATOM	2822	OE2	GLU	1276	90.305	-24.806	9.637	1.00	0.00	LX0	O
ATOM	2823	C	GLU	1276	88.592	-20.881	6.255	1.00	0.00	LX0	C
ATOM	2824	O	GLU	1276	88.458	-21.146	5.067	1.00	0.00	LX0	O
ATOM	2825	N	VAL	1277	88.287	-19.689	6.789	1.00	0.00	LX0	N
ATOM	2826	H	VAL	1277	88.416	-19.514	7.768	0.00	0.00	LX0	H
ATOM	2827	CA	VAL	1277	87.803	-18.658	5.872	1.00	0.00	LX0	C
ATOM	2828	CB	VAL	1277	86.499	-18.040	6.400	1.00	0.00	LX0	C
ATOM	2829	CG1	VAL	1277	85.372	-19.075	6.393	1.00	0.00	LX0	C
ATOM	2830	CG2	VAL	1277	86.673	-17.397	7.780	1.00	0.00	LX0	C
ATOM	2831	C	VAL	1277	88.815	-17.576	5.509	1.00	0.00	LX0	C
ATOM	2832	O	VAL	1277	88.462	-16.507	5.026	1.00	0.00	LX0	O
ATOM	2833	N	SER	1278	90.089	-17.874	5.784	1.00	0.00	LX0	N
ATOM	2834	H	SER	1278	90.354	-18.807	6.020	0.00	0.00	LX0	H
ATOM	2835	CA	SER	1278	91.041	-16.772	5.684	1.00	0.00	LX0	C
ATOM	2836	CB	SER	1278	92.120	-16.930	6.759	1.00	0.00	LX0	C
ATOM	2837	OG	SER	1278	92.911	-18.106	6.513	1.00	0.00	LX0	O
ATOM	2838	HG	SER	1278	92.300	-18.835	6.470	0.00	0.00	LX0	H
ATOM	2839	C	SER	1278	91.670	-16.593	4.313	1.00	0.00	LX0	C
ATOM	2840	O	SER	1278	91.845	-17.544	3.553	1.00	0.00	LX0	O
ATOM	2841	N	PHE	1279	92.077	-15.341	4.054	1.00	0.00	LX0	N
ATOM	2842	H	PHE	1279	91.930	-14.620	4.740	0.00	0.00	LX0	H
ATOM	2843	CA	PHE	1279	93.030	-15.098	2.972	1.00	0.00	LX0	C
ATOM	2844	CB	PHE	1279	93.363	-13.600	2.884	1.00	0.00	LX0	C
ATOM	2845	CG	PHE	1279	94.465	-13.318	1.886	1.00	0.00	LX0	C
ATOM	2846	CD1	PHE	1279	94.162	-13.251	0.511	1.00	0.00	LX0	C
ATOM	2847	CD2	PHE	1279	95.782	-13.124	2.356	1.00	0.00	LX0	C
ATOM	2848	CE1	PHE	1279	95.190	-12.964	-0.406	1.00	0.00	LX0	C
ATOM	2849	CE2	PHE	1279	96.811	-12.846	1.437	1.00	0.00	LX0	C
ATOM	2850	CZ	PHE	1279	96.502	-12.763	0.065	1.00	0.00	LX0	C
ATOM	2851	C	PHE	1279	94.285	-15.935	3.153	1.00	0.00	LX0	C
ATOM	2852	O	PHE	1279	94.767	-16.567	2.223	1.00	0.00	LX0	O
ATOM	2853	N	TYR	1280	94.744	-15.963	4.413	1.00	0.00	LX0	N
ATOM	2854	H	TYR	1280	94.303	-15.346	5.073	0.00	0.00	LX0	H
ATOM	2855	CA	TYR	1280	95.876	-16.803	4.812	1.00	0.00	LX0	C
ATOM	2856	CB	TYR	1280	95.971	-16.751	6.343	1.00	0.00	LX0	C
ATOM	2857	CG	TYR	1280	97.200	-17.433	6.902	1.00	0.00	LX0	C
ATOM	2858	CD1	TYR	1280	97.019	-18.593	7.682	1.00	0.00	LX0	C
ATOM	2859	CE1	TYR	1280	98.140	-19.205	8.265	1.00	0.00	LX0	C
ATOM	2860	CD2	TYR	1280	98.478	-16.887	6.661	1.00	0.00	LX0	C
ATOM	2861	CE2	TYR	1280	99.599	-17.500	7.246	1.00	0.00	LX0	C
ATOM	2862	CZ	TYR	1280	99.414	-18.645	8.050	1.00	0.00	LX0	C
ATOM	2863	OH	TYR	1280	100.506	-19.238	8.653	1.00	0.00	LX0	O
ATOM	2864	HH	TYR	1280	101.282	-18.709	8.471	0.00	0.00	LX0	H
ATOM	2865	C	TYR	1280	95.863	-18.235	4.272	1.00	0.00	LX0	C
ATOM	2866	O	TYR	1280	96.879	-18.760	3.830	1.00	0.00	LX0	O
ATOM	2867	N	TYR	1281	94.665	-18.837	4.312	1.00	0.00	LX0	N
ATOM	2868	H	TYR	1281	93.883	-18.355	4.714	0.00	0.00	LX0	H
ATOM	2869	CA	TYR	1281	94.535	-20.179	3.738	1.00	0.00	LX0	C
ATOM	2870	CB	TYR	1281	93.498	-21.000	4.512	1.00	0.00	LX0	C
ATOM	2871	CG	TYR	1281	94.048	-21.747	5.715	1.00	0.00	LX0	C
ATOM	2872	CD1	TYR	1281	95.237	-21.344	6.364	1.00	0.00	LX0	C
ATOM	2873	CE1	TYR	1281	95.680	-22.059	7.490	1.00	0.00	LX0	C
ATOM	2874	CD2	TYR	1281	93.307	-22.859	6.165	1.00	0.00	LX0	C
ATOM	2875	CE2	TYR	1281	93.745	-23.570	7.293	1.00	0.00	LX0	C
ATOM	2876	CZ	TYR	1281	94.920	-23.154	7.951	1.00	0.00	LX0	C

```
ATOM   2877  OH  TYR  1281      95.326 -23.835   9.089  1.00  0.00      LX0  O
ATOM   2878  HH  TYR  1281      96.280 -23.833   9.102  0.00  0.00      LX0  H
ATOM   2879  C   TYR  1281      94.174 -20.236   2.260  1.00  0.00      LX0  C
ATOM   2880  O   TYR  1281      94.297 -21.269   1.614  1.00  0.00      LX0  O
ATOM   2881  N   SER  1282      93.668 -19.106   1.757  1.00  0.00      LX0  N
ATOM   2882  H   SER  1282      93.636 -18.275   2.311  0.00  0.00      LX0  H
ATOM   2883  CA  SER  1282      93.099 -19.157   0.414  1.00  0.00      LX0  C
ATOM   2884  CB  SER  1282      92.238 -17.912   0.182  1.00  0.00      LX0  C
ATOM   2885  OG  SER  1282      93.068 -16.755   0.014  1.00  0.00      LX0  O
ATOM   2886  HG  SER  1282      93.709 -16.765   0.723  0.00  0.00      LX0  H
ATOM   2887  C   SER  1282      94.104 -19.332  -0.716  1.00  0.00      LX0  C
ATOM   2888  O   SER  1282      95.293 -19.071  -0.582  1.00  0.00      LX0  O
ATOM   2889  N   GLU  1283      93.542 -19.706  -1.877  1.00  0.00      LX0  N
ATOM   2890  H   GLU  1283      92.591 -20.009  -1.900  0.00  0.00      LX0  H
ATOM   2891  CA  GLU  1283      94.325 -19.676  -3.117  1.00  0.00      LX0  C
ATOM   2892  CB  GLU  1283      93.429 -20.163  -4.253  1.00  0.00      LX0  C
ATOM   2893  CG  GLU  1283      94.142 -20.424  -5.579  1.00  0.00      LX0  C
ATOM   2894  CD  GLU  1283      93.117 -20.922  -6.575  1.00  0.00      LX0  C
ATOM   2895  OE1 GLU  1283      93.022 -22.133  -6.762  1.00  0.00      LX0  O
ATOM   2896  OE2 GLU  1283      92.410 -20.096  -7.149  1.00  0.00      LX0  O
ATOM   2897  C   GLU  1283      94.939 -18.309  -3.421  1.00  0.00      LX0  C
ATOM   2898  O   GLU  1283      96.074 -18.168  -3.860  1.00  0.00      LX0  O
ATOM   2899  N   GLU  1284      94.142 -17.279  -3.079  1.00  0.00      LX0  N
ATOM   2900  H   GLU  1284      93.229 -17.452  -2.716  0.00  0.00      LX0  H
ATOM   2901  CA  GLU  1284      94.634 -15.905  -3.219  1.00  0.00      LX0  C
ATOM   2902  CB  GLU  1284      93.513 -14.906  -2.916  1.00  0.00      LX0  C
ATOM   2903  CG  GLU  1284      93.179 -13.933  -4.058  1.00  0.00      LX0  C
ATOM   2904  CD  GLU  1284      94.353 -13.029  -4.399  1.00  0.00      LX0  C
ATOM   2905  OE1 GLU  1284      94.769 -12.218  -3.576  1.00  0.00      LX0  O
ATOM   2906  OE2 GLU  1284      94.875 -13.110  -5.504  1.00  0.00      LX0  O
ATOM   2907  C   GLU  1284      95.892 -15.568  -2.422  1.00  0.00      LX0  C
ATOM   2908  O   GLU  1284      96.567 -14.576  -2.665  1.00  0.00      LX0  O
ATOM   2909  N   ASN  1285      96.228 -16.459  -1.477  1.00  0.00      LX0  N
ATOM   2910  H   ASN  1285      95.668 -17.266  -1.294  0.00  0.00      LX0  H
ATOM   2911  CA  ASN  1285      97.509 -16.289  -0.791  1.00  0.00      LX0  C
ATOM   2912  CB  ASN  1285      97.429 -16.897   0.610  1.00  0.00      LX0  C
ATOM   2913  CG  ASN  1285      98.477 -16.280   1.510  1.00  0.00      LX0  C
ATOM   2914  OD1 ASN  1285      98.982 -15.192   1.274  1.00  0.00      LX0  O
ATOM   2915  ND2 ASN  1285      98.789 -17.029   2.570  1.00  0.00      LX0  N
ATOM   2916 HD21 ASN  1285      98.295 -17.881   2.761  0.00  0.00      LX0  H
ATOM   2917 HD22 ASN  1285      99.516 -16.716   3.174  0.00  0.00      LX0  H
ATOM   2918  C   ASN  1285      98.733 -16.813  -1.543  1.00  0.00      LX0  C
ATOM   2919  O   ASN  1285      99.810 -16.996  -0.990  1.00  0.00      LX0  O
ATOM   2920  N   LYS  1286      98.539 -17.065  -2.853  1.00  0.00      LX0  N
ATOM   2921  H   LYS  1286      97.613 -17.046  -3.230  0.00  0.00      LX0  H
ATOM   2922  CA  LYS  1286      99.659 -17.464  -3.717  1.00  0.00      LX0  C
ATOM   2923  CB  LYS  1286     100.538 -16.276  -4.161  1.00  0.00      LX0  C
ATOM   2924  CG  LYS  1286      99.863 -15.225  -5.056  1.00  0.00      LX0  C
ATOM   2925  CD  LYS  1286      99.080 -14.160  -4.286  1.00  0.00      LX0  C
ATOM   2926  CE  LYS  1286      98.125 -13.347  -5.164  1.00  0.00      LX0  C
ATOM   2927  NZ  LYS  1286      97.207 -12.586  -4.310  1.00  0.00      LX0  N
ATOM   2928  HZ1 LYS  1286      96.313 -12.377  -4.810  0.00  0.00      LX0  H
ATOM   2929  HZ2 LYS  1286      96.862 -13.154  -3.507  0.00  0.00      LX0  H
ATOM   2930  HZ3 LYS  1286      97.604 -11.694  -3.964  0.00  0.00      LX0  H
ATOM   2931  C   LYS  1286     100.544 -18.574  -3.168  1.00  0.00      LX0  C
ATOM   2932  O   LYS  1286     101.177 -19.327  -3.901  1.00  0.00      LX0  O
TER
```
