# Supplementary material for: Structural analysis of hubs in human NR-RTK network
Source: Biol Direct. 2011 Oct 5;6:49. doi: 10.1186/1745-6150-6-49 (PMC3220635; doi:10.1186/1745-6150-6-49)
Supplement: Additional file 14 — ESR1-EGFR-PGR. ESR1-EGFR-PGR complex structure. [file 1745-6150-6-49-S14.PDF]

```
HEADER ESR1-EGFR-PGR
REMARK original generated coordinate pdb file
ATOM      1  N   ALA   156      10.627  12.174   8.322  1.00  0.00      RX0  N
ATOM      2  H   ALA   156      11.189  11.348   8.269  1.00  0.00      RX0  H
ATOM      3  CA  ALA   156       9.864  12.527   9.538  1.00  0.00      RX0  C
ATOM      4  CB  ALA   156      10.757  12.402  10.765  1.00  0.00      RX0  C
ATOM      5  C   ALA   156       9.377  13.991   9.496  1.00  0.00      RX0  C
ATOM      6  O   ALA   156       9.121  14.644  10.500  1.00  0.00      RX0  O
ATOM      7  N   LEU   157       9.039  14.416   8.289  1.00  0.00      RX0  N
ATOM      8  H   LEU   157       9.137  13.769   7.529  1.00  0.00      RX0  H
ATOM      9  CA  LEU   157       8.850  15.849   7.979  1.00  0.00      RX0  C
ATOM     10  CB  LEU   157       9.718  16.248   6.792  1.00  0.00      RX0  C
ATOM     11  CG  LEU   157      11.129  15.675   6.861  1.00  0.00      RX0  C
ATOM     12  CD1 LEU   157      11.904  15.986   5.585  1.00  0.00      RX0  C
ATOM     13  CD2 LEU   157      11.869  16.112   8.122  1.00  0.00      RX0  C
ATOM     14  C   LEU   157       7.387  16.184   7.642  1.00  0.00      RX0  C
ATOM     15  O   LEU   157       7.075  17.216   7.039  1.00  0.00      RX0  O
ATOM     16  N   SER   158       6.522  15.244   7.955  1.00  0.00      RX0  N
ATOM     17  H   SER   158       6.875  14.326   8.099  1.00  0.00      RX0  H
ATOM     18  CA  SER   158       5.051  15.362   7.847  1.00  0.00      RX0  C
ATOM     19  CB  SER   158       4.728  14.895   6.436  1.00  0.00      RX0  C
ATOM     20  OG  SER   158       5.938  15.034   5.680  1.00  0.00      RX0  O
ATOM     21  HG  SER   158       6.098  15.974   5.623  1.00  0.00      RX0  H
ATOM     22  C   SER   158       4.335  14.560   8.949  1.00  0.00      RX0  C
ATOM     23  O   SER   158       3.148  14.670   9.188  1.00  0.00      RX0  O
ATOM     24  N   LEU   159       5.132  13.681   9.591  1.00  0.00      RX0  N
ATOM     25  H   LEU   159       6.083  13.586   9.324  1.00  0.00      RX0  H
ATOM     26  CA  LEU   159       4.759  12.951  10.797  1.00  0.00      RX0  C
ATOM     27  CB  LEU   159       5.860  11.985  11.247  1.00  0.00      RX0  C
ATOM     28  CG  LEU   159       5.904  10.626  10.539  1.00  0.00      RX0  C
ATOM     29  CD1 LEU   159       6.366  10.705   9.082  1.00  0.00      RX0  C
ATOM     30  CD2 LEU   159       6.746   9.630  11.339  1.00  0.00      RX0  C
ATOM     31  C   LEU   159       4.518  13.965  11.920  1.00  0.00      RX0  C
ATOM     32  O   LEU   159       5.291  14.932  12.058  1.00  0.00      RX0  O
ATOM     33  N   THR   160       3.434  13.807  12.646  1.00  0.00      RX0  N
ATOM     34  H   THR   160       2.847  13.019  12.459  1.00  0.00      RX0  H
ATOM     35  CA  THR   160       3.156  14.665  13.825  1.00  0.00      RX0  C
ATOM     36  CB  THR   160       1.666  14.648  14.226  1.00  0.00      RX0  C
ATOM     37  OG1 THR   160       1.372  15.748  15.093  1.00  0.00      RX0  O
ATOM     38  HG1 THR   160       0.424  15.813  15.135  1.00  0.00      RX0  H
ATOM     39  CG2 THR   160       1.177  13.341  14.833  1.00  0.00      RX0  C
ATOM     40  C   THR   160       4.203  14.411  14.921  1.00  0.00      RX0  C
ATOM     41  O   THR   160       4.902  13.383  14.913  1.00  0.00      RX0  O
ATOM     42  N   ALA   161       4.153  15.229  15.953  1.00  0.00      RX0  N
ATOM     43  H   ALA   161       3.461  15.953  15.916  1.00  0.00      RX0  H
ATOM     44  CA  ALA   161       4.942  15.044  17.184  1.00  0.00      RX0  C
ATOM     45  CB  ALA   161       4.755  16.234  18.117  1.00  0.00      RX0  C
ATOM     46  C   ALA   161       4.543  13.746  17.920  1.00  0.00      RX0  C
ATOM     47  O   ALA   161       5.387  12.950  18.288  1.00  0.00      RX0  O
ATOM     48  N   ASP   162       3.226  13.461  17.917  1.00  0.00      RX0  N
ATOM     49  H   ASP   162       2.570  14.188  17.721  1.00  0.00      RX0  H
ATOM     50  CA  ASP   162       2.690  12.196  18.469  1.00  0.00      RX0  C
ATOM     51  CB  ASP   162       1.202  12.325  18.828  1.00  0.00      RX0  C
ATOM     52  CG  ASP   162       1.056  13.197  20.078  1.00  0.00      RX0  C
ATOM     53  OD1 ASP   162       1.810  14.153  20.249  1.00  0.00      RX0  O
ATOM     54  OD2 ASP   162       0.202  12.929  20.921  1.00  0.00      RX0  O
ATOM     55  C   ASP   162       3.088  10.948  17.668  1.00  0.00      RX0  C
ATOM     56  O   ASP   162       3.397   9.903  18.257  1.00  0.00      RX0  O
ATOM     57  N   GLN   163       3.164  11.087  16.353  1.00  0.00      RX0  N
ATOM     58  H   GLN   163       3.079  12.014  15.999  1.00  0.00      RX0  H
ATOM     59  CA  GLN   163       3.593   9.998  15.449  1.00  0.00      RX0  C
```

ATOM	60	CB	GLN	163	3.250	10.287	13.995	1.00	0.00	RX0	C
ATOM	61	CG	GLN	163	1.824	9.882	13.632	1.00	0.00	RX0	C
ATOM	62	CD	GLN	163	1.556	10.381	12.234	1.00	0.00	RX0	C
ATOM	63	OE1	GLN	163	2.082	11.412	11.827	1.00	0.00	RX0	O
ATOM	64	NE2	GLN	163	0.724	9.602	11.523	1.00	0.00	RX0	N
ATOM	65	HE21	GLN	163	0.335	8.771	11.923	1.00	0.00	RX0	H
ATOM	66	HE22	GLN	163	0.468	9.827	10.582	1.00	0.00	RX0	H
ATOM	67	C	GLN	163	5.089	9.698	15.572	1.00	0.00	RX0	C
ATOM	68	O	GLN	163	5.477	8.537	15.545	1.00	0.00	RX0	O
ATOM	69	N	MET	164	5.882	10.740	15.840	1.00	0.00	RX0	N
ATOM	70	H	MET	164	5.492	11.661	15.891	1.00	0.00	RX0	H
ATOM	71	CA	MET	164	7.331	10.587	16.060	1.00	0.00	RX0	C
ATOM	72	CB	MET	164	8.014	11.955	16.082	1.00	0.00	RX0	C
ATOM	73	CG	MET	164	9.451	11.879	16.607	1.00	0.00	RX0	C
ATOM	74	SD	MET	164	10.538	10.866	15.596	1.00	0.00	RX0	S
ATOM	75	CE	MET	164	11.021	12.137	14.424	1.00	0.00	RX0	C
ATOM	76	C	MET	164	7.610	9.825	17.366	1.00	0.00	RX0	C
ATOM	77	O	MET	164	8.404	8.887	17.381	1.00	0.00	RX0	O
ATOM	78	N	VAL	165	6.828	10.145	18.396	1.00	0.00	RX0	N
ATOM	79	H	VAL	165	6.165	10.888	18.278	1.00	0.00	RX0	H
ATOM	80	CA	VAL	165	6.992	9.563	19.744	1.00	0.00	RX0	C
ATOM	81	CB	VAL	165	6.101	10.259	20.778	1.00	0.00	RX0	C
ATOM	82	CG1	VAL	165	6.181	9.567	22.138	1.00	0.00	RX0	C
ATOM	83	CG2	VAL	165	6.451	11.732	20.918	1.00	0.00	RX0	C
ATOM	84	C	VAL	165	6.649	8.067	19.731	1.00	0.00	RX0	C
ATOM	85	O	VAL	165	7.442	7.255	20.191	1.00	0.00	RX0	O
ATOM	86	N	SER	166	5.467	7.742	19.205	1.00	0.00	RX0	N
ATOM	87	H	SER	166	4.817	8.431	18.868	1.00	0.00	RX0	H
ATOM	88	CA	SER	166	5.029	6.335	19.106	1.00	0.00	RX0	C
ATOM	89	CB	SER	166	3.570	6.384	18.711	1.00	0.00	RX0	C
ATOM	90	OG	SER	166	3.005	7.450	19.477	1.00	0.00	RX0	O
ATOM	91	HG	SER	166	3.365	7.379	20.351	1.00	0.00	RX0	H
ATOM	92	C	SER	166	5.941	5.501	18.195	1.00	0.00	RX0	C
ATOM	93	O	SER	166	6.295	4.379	18.542	1.00	0.00	RX0	O
ATOM	94	N	ALA	167	6.456	6.129	17.133	1.00	0.00	RX0	N
ATOM	95	H	ALA	167	6.173	7.064	16.910	1.00	0.00	RX0	H
ATOM	96	CA	ALA	167	7.397	5.466	16.208	1.00	0.00	RX0	C
ATOM	97	CB	ALA	167	7.731	6.357	15.012	1.00	0.00	RX0	C
ATOM	98	C	ALA	167	8.706	5.103	16.927	1.00	0.00	RX0	C
ATOM	99	O	ALA	167	9.113	3.946	16.932	1.00	0.00	RX0	O
ATOM	100	N	LEU	168	9.179	6.054	17.734	1.00	0.00	RX0	N
ATOM	101	H	LEU	168	8.734	6.951	17.734	1.00	0.00	RX0	H
ATOM	102	CA	LEU	168	10.385	5.875	18.562	1.00	0.00	RX0	C
ATOM	103	CB	LEU	168	10.907	7.213	19.074	1.00	0.00	RX0	C
ATOM	104	CG	LEU	168	11.571	8.041	17.978	1.00	0.00	RX0	C
ATOM	105	CD1	LEU	168	12.103	9.369	18.519	1.00	0.00	RX0	C
ATOM	106	CD2	LEU	168	12.653	7.238	17.254	1.00	0.00	RX0	C
ATOM	107	C	LEU	168	10.197	4.896	19.724	1.00	0.00	RX0	C
ATOM	108	O	LEU	168	11.077	4.078	19.994	1.00	0.00	RX0	O
ATOM	109	N	LEU	169	9.007	4.918	20.317	1.00	0.00	RX0	N
ATOM	110	H	LEU	169	8.332	5.585	20.005	1.00	0.00	RX0	H
ATOM	111	CA	LEU	169	8.640	3.970	21.384	1.00	0.00	RX0	C
ATOM	112	CB	LEU	169	7.359	4.393	22.101	1.00	0.00	RX0	C
ATOM	113	CG	LEU	169	7.539	5.639	22.970	1.00	0.00	RX0	C
ATOM	114	CD1	LEU	169	6.227	6.046	23.641	1.00	0.00	RX0	C
ATOM	115	CD2	LEU	169	8.667	5.470	23.988	1.00	0.00	RX0	C
ATOM	116	C	LEU	169	8.505	2.536	20.864	1.00	0.00	RX0	C
ATOM	117	O	LEU	169	9.003	1.602	21.486	1.00	0.00	RX0	O
ATOM	118	N	ASP	170	7.977	2.423	19.645	1.00	0.00	RX0	N
ATOM	119	H	ASP	170	7.693	3.240	19.144	1.00	0.00	RX0	H
ATOM	120	CA	ASP	170	7.822	1.133	18.952	1.00	0.00	RX0	C

ATOM	121	CB	ASP	170	6.847	1.297	17.776	1.00	0.00	RX0	C
ATOM	122	CG	ASP	170	7.245	0.478	16.561	1.00	0.00	RX0	C
ATOM	123	OD1	ASP	170	7.077	-0.739	16.542	1.00	0.00	RX0	O
ATOM	124	OD2	ASP	170	7.722	1.054	15.594	1.00	0.00	RX0	O
ATOM	125	C	ASP	170	9.164	0.506	18.541	1.00	0.00	RX0	C
ATOM	126	O	ASP	170	9.313	-0.704	18.571	1.00	0.00	RX0	O
ATOM	127	N	ALA	171	10.119	1.387	18.228	1.00	0.00	RX0	N
ATOM	128	H	ALA	171	9.856	2.351	18.225	1.00	0.00	RX0	H
ATOM	129	CA	ALA	171	11.447	1.008	17.717	1.00	0.00	RX0	C
ATOM	130	CB	ALA	171	12.101	2.214	17.043	1.00	0.00	RX0	C
ATOM	131	C	ALA	171	12.418	0.479	18.779	1.00	0.00	RX0	C
ATOM	132	O	ALA	171	13.427	-0.136	18.431	1.00	0.00	RX0	O
ATOM	133	N	GLU	172	12.125	0.739	20.058	1.00	0.00	RX0	N
ATOM	134	H	GLU	172	11.284	1.236	20.271	1.00	0.00	RX0	H
ATOM	135	CA	GLU	172	13.017	0.374	21.170	1.00	0.00	RX0	C
ATOM	136	CB	GLU	172	12.364	0.707	22.510	1.00	0.00	RX0	C
ATOM	137	CG	GLU	172	12.326	2.226	22.684	1.00	0.00	RX0	C
ATOM	138	CD	GLU	172	13.734	2.773	22.523	1.00	0.00	RX0	C
ATOM	139	OE1	GLU	172	14.558	2.605	23.422	1.00	0.00	RX0	O
ATOM	140	OE2	GLU	172	14.042	3.383	21.498	1.00	0.00	RX0	O
ATOM	141	C	GLU	172	13.554	-1.065	21.099	1.00	0.00	RX0	C
ATOM	142	O	GLU	172	12.785	-2.004	20.837	1.00	0.00	RX0	O
ATOM	143	N	PRO	173	14.865	-1.209	21.269	1.00	0.00	RX0	N
ATOM	144	CD	PRO	173	15.788	-0.095	21.452	1.00	0.00	RX0	C
ATOM	145	CA	PRO	173	15.538	-2.517	21.328	1.00	0.00	RX0	C
ATOM	146	CB	PRO	173	17.014	-2.108	21.199	1.00	0.00	RX0	C
ATOM	147	CG	PRO	173	17.102	-0.740	21.868	1.00	0.00	RX0	C
ATOM	148	C	PRO	173	15.206	-3.249	22.640	1.00	0.00	RX0	C
ATOM	149	O	PRO	173	14.829	-2.595	23.631	1.00	0.00	RX0	O
ATOM	150	N	PRO	174	15.294	-4.574	22.646	1.00	0.00	RX0	N
ATOM	151	CD	PRO	174	15.595	-5.390	21.471	1.00	0.00	RX0	C
ATOM	152	CA	PRO	174	15.084	-5.400	23.852	1.00	0.00	RX0	C
ATOM	153	CB	PRO	174	14.972	-6.811	23.270	1.00	0.00	RX0	C
ATOM	154	CG	PRO	174	15.859	-6.779	22.028	1.00	0.00	RX0	C
ATOM	155	C	PRO	174	16.250	-5.248	24.838	1.00	0.00	RX0	C
ATOM	156	O	PRO	174	17.379	-4.922	24.444	1.00	0.00	RX0	O
ATOM	157	N	ILE	175	15.956	-5.464	26.106	1.00	0.00	RX0	N
ATOM	158	H	ILE	175	15.040	-5.794	26.327	1.00	0.00	RX0	H
ATOM	159	CA	ILE	175	16.988	-5.556	27.159	1.00	0.00	RX0	C
ATOM	160	CB	ILE	175	16.446	-5.212	28.551	1.00	0.00	RX0	C
ATOM	161	CG2	ILE	175	17.622	-5.098	29.520	1.00	0.00	RX0	C
ATOM	162	CG1	ILE	175	15.621	-3.919	28.562	1.00	0.00	RX0	C
ATOM	163	CD1	ILE	175	14.109	-4.138	28.452	1.00	0.00	RX0	C
ATOM	164	C	ILE	175	17.586	-6.969	27.112	1.00	0.00	RX0	C
ATOM	165	O	ILE	175	16.886	-7.963	27.343	1.00	0.00	RX0	O
ATOM	166	N	LEU	176	18.884	-7.017	26.884	1.00	0.00	RX0	N
ATOM	167	H	LEU	176	19.373	-6.149	26.801	1.00	0.00	RX0	H
ATOM	168	CA	LEU	176	19.617	-8.291	26.770	1.00	0.00	RX0	C
ATOM	169	CB	LEU	176	20.637	-8.226	25.635	1.00	0.00	RX0	C
ATOM	170	CG	LEU	176	19.985	-7.994	24.269	1.00	0.00	RX0	C
ATOM	171	CD1	LEU	176	21.040	-7.832	23.178	1.00	0.00	RX0	C
ATOM	172	CD2	LEU	176	18.969	-9.081	23.909	1.00	0.00	RX0	C
ATOM	173	C	LEU	176	20.277	-8.690	28.089	1.00	0.00	RX0	C
ATOM	174	O	LEU	176	20.563	-7.852	28.952	1.00	0.00	RX0	O
ATOM	175	N	TYR	177	20.459	-9.989	28.237	1.00	0.00	RX0	N
ATOM	176	H	TYR	177	20.174	-10.588	27.494	1.00	0.00	RX0	H
ATOM	177	CA	TYR	177	21.114	-10.573	29.420	1.00	0.00	RX0	C
ATOM	178	CB	TYR	177	20.418	-11.865	29.850	1.00	0.00	RX0	C
ATOM	179	CG	TYR	177	19.139	-11.582	30.604	1.00	0.00	RX0	C
ATOM	180	CD1	TYR	177	17.984	-11.180	29.901	1.00	0.00	RX0	C
ATOM	181	CE1	TYR	177	16.800	-10.938	30.617	1.00	0.00	RX0	C

ATOM	182	CD2	TYR	177	19.140	-11.745	32.004	1.00	0.00	RX0	C
ATOM	183	CE2	TYR	177	17.955	-11.512	32.719	1.00	0.00	RX0	C
ATOM	184	CZ	TYR	177	16.802	-11.108	32.016	1.00	0.00	RX0	C
ATOM	185	OH	TYR	177	15.642	-10.866	32.723	1.00	0.00	RX0	O
ATOM	186	HH	TYR	177	15.759	-11.124	33.628	1.00	0.00	RX0	H
ATOM	187	C	TYR	177	22.589	-10.858	29.163	1.00	0.00	RX0	C
ATOM	188	O	TYR	177	22.985	-11.163	28.046	1.00	0.00	RX0	O
ATOM	189	N	SER	178	23.381	-10.750	30.220	1.00	0.00	RX0	N
ATOM	190	H	SER	178	23.007	-10.480	31.110	1.00	0.00	RX0	H
ATOM	191	CA	SER	178	24.788	-11.188	30.183	1.00	0.00	RX0	C
ATOM	192	CB	SER	178	25.524	-10.668	31.407	1.00	0.00	RX0	C
ATOM	193	OG	SER	178	25.393	-9.246	31.428	1.00	0.00	RX0	O
ATOM	194	HG	SER	178	25.551	-8.943	30.542	1.00	0.00	RX0	H
ATOM	195	C	SER	178	24.834	-12.718	30.070	1.00	0.00	RX0	C
ATOM	196	O	SER	178	23.999	-13.413	30.674	1.00	0.00	RX0	O
ATOM	197	N	GLU	179	25.827	-13.218	29.362	1.00	0.00	RX0	N
ATOM	198	H	GLU	179	26.459	-12.605	28.888	1.00	0.00	RX0	H
ATOM	199	CA	GLU	179	26.033	-14.670	29.175	1.00	0.00	RX0	C
ATOM	200	CB	GLU	179	26.250	-14.982	27.706	1.00	0.00	RX0	C
ATOM	201	CG	GLU	179	24.995	-14.623	26.929	1.00	0.00	RX0	C
ATOM	202	CD	GLU	179	25.395	-14.068	25.590	1.00	0.00	RX0	C
ATOM	203	OE1	GLU	179	25.016	-14.635	24.575	1.00	0.00	RX0	O
ATOM	204	OE2	GLU	179	26.012	-13.008	25.556	1.00	0.00	RX0	O
ATOM	205	C	GLU	179	27.192	-15.208	30.012	1.00	0.00	RX0	C
ATOM	206	O	GLU	179	28.361	-15.227	29.589	1.00	0.00	RX0	O
ATOM	207	N	TYR	180	26.873	-15.475	31.254	1.00	0.00	RX0	N
ATOM	208	H	TYR	180	25.929	-15.316	31.547	1.00	0.00	RX0	H
ATOM	209	CA	TYR	180	27.735	-16.233	32.177	1.00	0.00	RX0	C
ATOM	210	CB	TYR	180	28.500	-15.311	33.151	1.00	0.00	RX0	C
ATOM	211	CG	TYR	180	27.583	-14.643	34.154	1.00	0.00	RX0	C
ATOM	212	CD1	TYR	180	26.977	-13.427	33.857	1.00	0.00	RX0	C
ATOM	213	CE1	TYR	180	26.079	-12.866	34.757	1.00	0.00	RX0	C
ATOM	214	CD2	TYR	180	27.336	-15.262	35.375	1.00	0.00	RX0	C
ATOM	215	CE2	TYR	180	26.427	-14.709	36.266	1.00	0.00	RX0	C
ATOM	216	CZ	TYR	180	25.779	-13.524	35.944	1.00	0.00	RX0	C
ATOM	217	OH	TYR	180	24.828	-13.013	36.800	1.00	0.00	RX0	O
ATOM	218	HH	TYR	180	24.722	-12.082	36.622	1.00	0.00	RX0	H
ATOM	219	C	TYR	180	26.838	-17.226	32.909	1.00	0.00	RX0	C
ATOM	220	O	TYR	180	25.642	-16.953	33.094	1.00	0.00	RX0	O
ATOM	221	N	ASP	181	27.404	-18.345	33.318	1.00	0.00	RX0	N
ATOM	222	H	ASP	181	28.390	-18.452	33.222	1.00	0.00	RX0	H
ATOM	223	CA	ASP	181	26.630	-19.347	34.059	1.00	0.00	RX0	C
ATOM	224	CB	ASP	181	27.168	-20.785	34.051	1.00	0.00	RX0	C
ATOM	225	CG	ASP	181	26.225	-21.667	34.874	1.00	0.00	RX0	C
ATOM	226	OD1	ASP	181	25.010	-21.609	34.698	1.00	0.00	RX0	O
ATOM	227	OD2	ASP	181	26.685	-22.357	35.767	1.00	0.00	RX0	O
ATOM	228	C	ASP	181	26.420	-18.851	35.504	1.00	0.00	RX0	C
ATOM	229	O	ASP	181	27.391	-18.832	36.273	1.00	0.00	RX0	O
ATOM	230	N	PRO	182	25.185	-18.489	35.856	1.00	0.00	RX0	N
ATOM	231	CD	PRO	182	24.006	-18.567	34.991	1.00	0.00	RX0	C
ATOM	232	CA	PRO	182	24.825	-17.989	37.201	1.00	0.00	RX0	C
ATOM	233	CB	PRO	182	23.399	-17.467	37.004	1.00	0.00	RX0	C
ATOM	234	CG	PRO	182	22.813	-18.351	35.911	1.00	0.00	RX0	C
ATOM	235	C	PRO	182	24.941	-19.052	38.308	1.00	0.00	RX0	C
ATOM	236	O	PRO	182	24.654	-18.763	39.474	1.00	0.00	RX0	O
ATOM	237	N	THR	183	25.345	-20.259	37.948	1.00	0.00	RX0	N
ATOM	238	H	THR	183	25.571	-20.461	36.994	1.00	0.00	RX0	H
ATOM	239	CA	THR	183	25.568	-21.363	38.913	1.00	0.00	RX0	C
ATOM	240	CB	THR	183	25.055	-22.691	38.366	1.00	0.00	RX0	C
ATOM	241	OG1	THR	183	24.172	-22.485	37.257	1.00	0.00	RX0	O
ATOM	242	HG1	THR	183	24.786	-22.309	36.531	1.00	0.00	RX0	H

ATOM	243	CG2	THR	183	24.419	-23.542	39.462	1.00	0.00	RX0	C
ATOM	244	C	THR	183	27.063	-21.532	39.218	1.00	0.00	RX0	C
ATOM	245	O	THR	183	27.455	-22.345	40.058	1.00	0.00	RX0	O
ATOM	246	N	ARG	184	27.887	-20.699	38.573	1.00	0.00	RX0	N
ATOM	247	H	ARG	184	27.513	-19.988	37.976	1.00	0.00	RX0	H
ATOM	248	CA	ARG	184	29.343	-20.701	38.681	1.00	0.00	RX0	C
ATOM	249	CB	ARG	184	29.912	-20.806	37.267	1.00	0.00	RX0	C
ATOM	250	CG	ARG	184	30.289	-22.242	36.900	1.00	0.00	RX0	C
ATOM	251	CD	ARG	184	30.752	-22.389	35.446	1.00	0.00	RX0	C
ATOM	252	NE	ARG	184	31.593	-21.269	35.015	1.00	0.00	RX0	N
ATOM	253	HE	ARG	184	31.127	-20.537	34.519	1.00	0.00	RX0	H
ATOM	254	CZ	ARG	184	32.905	-21.155	35.326	1.00	0.00	RX0	C
ATOM	255	NH1	ARG	184	33.525	-22.098	36.043	1.00	0.00	RX0	N
ATOM	256	HH11	ARG	184	34.491	-21.929	36.323	1.00	0.00	RX0	H
ATOM	257	HH12	ARG	184	33.080	-22.940	36.329	1.00	0.00	RX0	H
ATOM	258	NH2	ARG	184	33.570	-20.076	34.906	1.00	0.00	RX0	N
ATOM	259	HH21	ARG	184	34.558	-20.021	35.127	1.00	0.00	RX0	H
ATOM	260	HH22	ARG	184	33.155	-19.326	34.396	1.00	0.00	RX0	H
ATOM	261	C	ARG	184	29.836	-19.410	39.407	1.00	0.00	RX0	C
ATOM	262	O	ARG	184	29.116	-18.390	39.334	1.00	0.00	RX0	O
ATOM	263	N	PRO	185	30.940	-19.460	40.113	1.00	0.00	RX0	N
ATOM	264	CD	PRO	185	31.754	-20.660	40.299	1.00	0.00	RX0	C
ATOM	265	CA	PRO	185	31.574	-18.285	40.781	1.00	0.00	RX0	C
ATOM	266	CB	PRO	185	32.859	-18.864	41.386	1.00	0.00	RX0	C
ATOM	267	CG	PRO	185	33.151	-20.127	40.582	1.00	0.00	RX0	C
ATOM	268	C	PRO	185	31.820	-17.125	39.813	1.00	0.00	RX0	C
ATOM	269	O	PRO	185	31.836	-17.275	38.592	1.00	0.00	RX0	O
ATOM	270	N	PHE	186	32.164	-15.998	40.422	1.00	0.00	RX0	N
ATOM	271	H	PHE	186	32.240	-15.992	41.417	1.00	0.00	RX0	H
ATOM	272	CA	PHE	186	32.333	-14.726	39.697	1.00	0.00	RX0	C
ATOM	273	CB	PHE	186	31.517	-13.614	40.357	1.00	0.00	RX0	C
ATOM	274	CG	PHE	186	31.416	-12.440	39.411	1.00	0.00	RX0	C
ATOM	275	CD1	PHE	186	30.859	-12.616	38.149	1.00	0.00	RX0	C
ATOM	276	CD2	PHE	186	31.881	-11.188	39.796	1.00	0.00	RX0	C
ATOM	277	CE1	PHE	186	30.770	-11.542	37.271	1.00	0.00	RX0	C
ATOM	278	CE2	PHE	186	31.791	-10.114	38.918	1.00	0.00	RX0	C
ATOM	279	CZ	PHE	186	31.237	-10.291	37.655	1.00	0.00	RX0	C
ATOM	280	C	PHE	186	33.791	-14.305	39.507	1.00	0.00	RX0	C
ATOM	281	O	PHE	186	34.127	-13.678	38.496	1.00	0.00	RX0	O
ATOM	282	N	SER	187	34.655	-14.802	40.380	1.00	0.00	RX0	N
ATOM	283	H	SER	187	34.309	-15.353	41.134	1.00	0.00	RX0	H
ATOM	284	CA	SER	187	36.113	-14.542	40.380	1.00	0.00	RX0	C
ATOM	285	CB	SER	187	36.607	-15.270	41.616	1.00	0.00	RX0	C
ATOM	286	OG	SER	187	35.486	-15.356	42.514	1.00	0.00	RX0	O
ATOM	287	HG	SER	187	35.854	-15.390	43.389	1.00	0.00	RX0	H
ATOM	288	C	SER	187	36.764	-14.980	39.057	1.00	0.00	RX0	C
ATOM	289	O	SER	187	37.834	-14.531	38.683	1.00	0.00	RX0	O
ATOM	290	N	GLU	188	36.054	-15.878	38.369	1.00	0.00	RX0	N
ATOM	291	H	GLU	188	35.124	-16.106	38.645	1.00	0.00	RX0	H
ATOM	292	CA	GLU	188	36.561	-16.586	37.191	1.00	0.00	RX0	C
ATOM	293	CB	GLU	188	36.151	-18.049	37.358	1.00	0.00	RX0	C
ATOM	294	CG	GLU	188	36.890	-19.077	36.504	1.00	0.00	RX0	C
ATOM	295	CD	GLU	188	36.068	-20.346	36.519	1.00	0.00	RX0	C
ATOM	296	OE1	GLU	188	35.316	-20.564	37.461	1.00	0.00	RX0	O
ATOM	297	OE2	GLU	188	36.067	-21.074	35.532	1.00	0.00	RX0	O
ATOM	298	C	GLU	188	36.028	-16.018	35.856	1.00	0.00	RX0	C
ATOM	299	O	GLU	188	36.494	-16.416	34.788	1.00	0.00	RX0	O
ATOM	300	N	ALA	189	35.058	-15.107	35.914	1.00	0.00	RX0	N
ATOM	301	H	ALA	189	34.831	-14.681	36.792	1.00	0.00	RX0	H
ATOM	302	CA	ALA	189	34.543	-14.432	34.708	1.00	0.00	RX0	C
ATOM	303	CB	ALA	189	33.067	-14.081	34.891	1.00	0.00	RX0	C

ATOM	304	C	ALA	189	35.336	-13.151	34.407	1.00	0.00	RX0	C
ATOM	305	O	ALA	189	35.533	-12.292	35.270	1.00	0.00	RX0	O
ATOM	306	N	SER	190	35.819	-13.065	33.173	1.00	0.00	RX0	N
ATOM	307	H	SER	190	35.632	-13.799	32.519	1.00	0.00	RX0	H
ATOM	308	CA	SER	190	36.430	-11.825	32.646	1.00	0.00	RX0	C
ATOM	309	CB	SER	190	37.068	-12.294	31.358	1.00	0.00	RX0	C
ATOM	310	OG	SER	190	37.208	-13.714	31.504	1.00	0.00	RX0	O
ATOM	311	HG	SER	190	37.767	-13.863	32.260	1.00	0.00	RX0	H
ATOM	312	C	SER	190	35.341	-10.761	32.513	1.00	0.00	RX0	C
ATOM	313	O	SER	190	34.465	-10.869	31.639	1.00	0.00	RX0	O
ATOM	314	N	MET	191	35.401	-9.751	33.361	1.00	0.00	RX0	N
ATOM	315	H	MET	191	36.054	-9.818	34.117	1.00	0.00	RX0	H
ATOM	316	CA	MET	191	34.414	-8.652	33.337	1.00	0.00	RX0	C
ATOM	317	CB	MET	191	34.645	-7.665	34.478	1.00	0.00	RX0	C
ATOM	318	CG	MET	191	33.550	-6.599	34.501	1.00	0.00	RX0	C
ATOM	319	SD	MET	191	33.644	-5.547	35.952	1.00	0.00	RX0	S
ATOM	320	CE	MET	191	33.307	-6.816	37.185	1.00	0.00	RX0	C
ATOM	321	C	MET	191	34.384	-7.939	31.976	1.00	0.00	RX0	C
ATOM	322	O	MET	191	33.329	-7.841	31.363	1.00	0.00	RX0	O
ATOM	323	N	MET	192	35.577	-7.654	31.438	1.00	0.00	RX0	N
ATOM	324	H	MET	192	36.392	-7.770	32.003	1.00	0.00	RX0	H
ATOM	325	CA	MET	192	35.697	-7.082	30.089	1.00	0.00	RX0	C
ATOM	326	CB	MET	192	37.141	-6.660	29.807	1.00	0.00	RX0	C
ATOM	327	CG	MET	192	37.303	-5.959	28.455	1.00	0.00	RX0	C
ATOM	328	SD	MET	192	36.227	-4.526	28.263	1.00	0.00	RX0	S
ATOM	329	CE	MET	192	36.844	-3.531	29.631	1.00	0.00	RX0	C
ATOM	330	C	MET	192	35.151	-8.021	28.999	1.00	0.00	RX0	C
ATOM	331	O	MET	192	34.484	-7.587	28.093	1.00	0.00	RX0	O
ATOM	332	N	GLY	193	35.358	-9.342	29.220	1.00	0.00	RX0	N
ATOM	333	H	GLY	193	35.765	-9.622	30.084	1.00	0.00	RX0	H
ATOM	334	CA	GLY	193	34.804	-10.378	28.330	1.00	0.00	RX0	C
ATOM	335	C	GLY	193	33.267	-10.338	28.334	1.00	0.00	RX0	C
ATOM	336	O	GLY	193	32.637	-10.184	27.296	1.00	0.00	RX0	O
ATOM	337	N	LEU	194	32.696	-10.293	29.537	1.00	0.00	RX0	N
ATOM	338	H	LEU	194	33.271	-10.315	30.351	1.00	0.00	RX0	H
ATOM	339	CA	LEU	194	31.235	-10.169	29.722	1.00	0.00	RX0	C
ATOM	340	CB	LEU	194	30.848	-10.221	31.199	1.00	0.00	RX0	C
ATOM	341	CG	LEU	194	31.152	-11.554	31.873	1.00	0.00	RX0	C
ATOM	342	CD1	LEU	194	30.712	-11.537	33.337	1.00	0.00	RX0	C
ATOM	343	CD2	LEU	194	30.550	-12.731	31.103	1.00	0.00	RX0	C
ATOM	344	C	LEU	194	30.647	-8.891	29.116	1.00	0.00	RX0	C
ATOM	345	O	LEU	194	29.706	-8.959	28.317	1.00	0.00	RX0	O
ATOM	346	N	LEU	195	31.327	-7.782	29.364	1.00	0.00	RX0	N
ATOM	347	H	LEU	195	32.140	-7.844	29.940	1.00	0.00	RX0	H
ATOM	348	CA	LEU	195	30.920	-6.462	28.846	1.00	0.00	RX0	C
ATOM	349	CB	LEU	195	31.732	-5.338	29.492	1.00	0.00	RX0	C
ATOM	350	CG	LEU	195	31.536	-5.239	31.005	1.00	0.00	RX0	C
ATOM	351	CD1	LEU	195	32.375	-4.113	31.610	1.00	0.00	RX0	C
ATOM	352	CD2	LEU	195	30.061	-5.123	31.386	1.00	0.00	RX0	C
ATOM	353	C	LEU	195	31.020	-6.357	27.321	1.00	0.00	RX0	C
ATOM	354	O	LEU	195	30.051	-5.942	26.671	1.00	0.00	RX0	O
ATOM	355	N	THR	196	32.075	-6.931	26.767	1.00	0.00	RX0	N
ATOM	356	H	THR	196	32.806	-7.316	27.329	1.00	0.00	RX0	H
ATOM	357	CA	THR	196	32.335	-6.901	25.309	1.00	0.00	RX0	C
ATOM	358	CB	THR	196	33.785	-7.289	25.033	1.00	0.00	RX0	C
ATOM	359	OG1	THR	196	34.653	-6.365	25.700	1.00	0.00	RX0	O
ATOM	360	HG1	THR	196	34.338	-5.498	25.478	1.00	0.00	RX0	H
ATOM	361	CG2	THR	196	34.094	-7.333	23.535	1.00	0.00	RX0	C
ATOM	362	C	THR	196	31.317	-7.765	24.552	1.00	0.00	RX0	C
ATOM	363	O	THR	196	30.772	-7.327	23.532	1.00	0.00	RX0	O
ATOM	364	N	ASN	197	31.003	-8.928	25.107	1.00	0.00	RX0	N

ATOM	365	H	ASN	197	31.424	-9.172	25.983	1.00	0.00	RX0	H
ATOM	366	CA	ASN	197	30.010	-9.840	24.504	1.00	0.00	RX0	C
ATOM	367	CB	ASN	197	29.972	-11.199	25.205	1.00	0.00	RX0	C
ATOM	368	CG	ASN	197	30.958	-12.149	24.562	1.00	0.00	RX0	C
ATOM	369	OD1	ASN	197	30.661	-12.862	23.611	1.00	0.00	RX0	O
ATOM	370	ND2	ASN	197	32.168	-12.130	25.148	1.00	0.00	RX0	N
ATOM	371	HD21	ASN	197	32.340	-11.513	25.920	1.00	0.00	RX0	H
ATOM	372	HD22	ASN	197	32.904	-12.723	24.824	1.00	0.00	RX0	H
ATOM	373	C	ASN	197	28.594	-9.255	24.528	1.00	0.00	RX0	C
ATOM	374	O	ASN	197	27.900	-9.272	23.514	1.00	0.00	RX0	O
ATOM	375	N	LEU	198	28.277	-8.575	25.633	1.00	0.00	RX0	N
ATOM	376	H	LEU	198	28.914	-8.570	26.406	1.00	0.00	RX0	H
ATOM	377	CA	LEU	198	27.002	-7.850	25.760	1.00	0.00	RX0	C
ATOM	378	CB	LEU	198	26.850	-7.337	27.188	1.00	0.00	RX0	C
ATOM	379	CG	LEU	198	25.448	-6.824	27.498	1.00	0.00	RX0	C
ATOM	380	CD1	LEU	198	24.371	-7.879	27.236	1.00	0.00	RX0	C
ATOM	381	CD2	LEU	198	25.382	-6.287	28.924	1.00	0.00	RX0	C
ATOM	382	C	LEU	198	26.885	-6.719	24.724	1.00	0.00	RX0	C
ATOM	383	O	LEU	198	25.930	-6.676	23.947	1.00	0.00	RX0	O
ATOM	384	N	ALA	199	27.942	-5.914	24.641	1.00	0.00	RX0	N
ATOM	385	H	ALA	199	28.697	-6.058	25.282	1.00	0.00	RX0	H
ATOM	386	CA	ALA	199	28.029	-4.784	23.694	1.00	0.00	RX0	C
ATOM	387	CB	ALA	199	29.343	-4.024	23.882	1.00	0.00	RX0	C
ATOM	388	C	ALA	199	27.921	-5.230	22.227	1.00	0.00	RX0	C
ATOM	389	O	ALA	199	27.138	-4.660	21.467	1.00	0.00	RX0	O
ATOM	390	N	ASP	200	28.555	-6.360	21.908	1.00	0.00	RX0	N
ATOM	391	H	ASP	200	29.135	-6.812	22.587	1.00	0.00	RX0	H
ATOM	392	CA	ASP	200	28.494	-6.940	20.550	1.00	0.00	RX0	C
ATOM	393	CB	ASP	200	29.537	-8.052	20.395	1.00	0.00	RX0	C
ATOM	394	CG	ASP	200	30.133	-8.075	18.994	1.00	0.00	RX0	C
ATOM	395	OD1	ASP	200	30.151	-7.055	18.308	1.00	0.00	RX0	O
ATOM	396	OD2	ASP	200	30.635	-9.115	18.579	1.00	0.00	RX0	O
ATOM	397	C	ASP	200	27.084	-7.410	20.171	1.00	0.00	RX0	C
ATOM	398	O	ASP	200	26.604	-7.102	19.080	1.00	0.00	RX0	O
ATOM	399	N	ARG	201	26.390	-7.998	21.143	1.00	0.00	RX0	N
ATOM	400	H	ARG	201	26.865	-8.179	22.010	1.00	0.00	RX0	H
ATOM	401	CA	ARG	201	24.992	-8.434	20.957	1.00	0.00	RX0	C
ATOM	402	CB	ARG	201	24.558	-9.494	21.951	1.00	0.00	RX0	C
ATOM	403	CG	ARG	201	24.994	-10.866	21.432	1.00	0.00	RX0	C
ATOM	404	CD	ARG	201	24.121	-12.016	21.940	1.00	0.00	RX0	C
ATOM	405	NE	ARG	201	24.226	-12.163	23.386	1.00	0.00	RX0	N
ATOM	406	HE	ARG	201	25.026	-12.660	23.760	1.00	0.00	RX0	H
ATOM	407	CZ	ARG	201	23.330	-11.649	24.247	1.00	0.00	RX0	C
ATOM	408	NH1	ARG	201	22.198	-11.106	23.803	1.00	0.00	RX0	N
ATOM	409	HH11	ARG	201	21.527	-10.705	24.422	1.00	0.00	RX0	H
ATOM	410	HH12	ARG	201	22.015	-11.095	22.819	1.00	0.00	RX0	H
ATOM	411	NH2	ARG	201	23.613	-11.693	25.538	1.00	0.00	RX0	N
ATOM	412	HH21	ARG	201	23.064	-11.335	26.297	1.00	0.00	RX0	H
ATOM	413	HH22	ARG	201	24.483	-12.142	25.780	1.00	0.00	RX0	H
ATOM	414	C	ARG	201	23.991	-7.279	20.827	1.00	0.00	RX0	C
ATOM	415	O	ARG	201	23.123	-7.308	19.955	1.00	0.00	RX0	O
ATOM	416	N	GLU	202	24.240	-6.201	21.568	1.00	0.00	RX0	N
ATOM	417	H	GLU	202	25.005	-6.220	22.214	1.00	0.00	RX0	H
ATOM	418	CA	GLU	202	23.401	-4.988	21.493	1.00	0.00	RX0	C
ATOM	419	CB	GLU	202	23.561	-4.071	22.728	1.00	0.00	RX0	C
ATOM	420	CG	GLU	202	22.933	-4.674	24.003	1.00	0.00	RX0	C
ATOM	421	CD	GLU	202	22.871	-3.701	25.184	1.00	0.00	RX0	C
ATOM	422	OE1	GLU	202	21.830	-3.085	25.416	1.00	0.00	RX0	O
ATOM	423	OE2	GLU	202	23.835	-3.601	25.937	1.00	0.00	RX0	O
ATOM	424	C	GLU	202	23.526	-4.262	20.149	1.00	0.00	RX0	C
ATOM	425	O	GLU	202	22.539	-3.754	19.625	1.00	0.00	RX0	O

ATOM	426	N	LEU	203	24.712	-4.358	19.546	1.00	0.00	RX0	N
ATOM	427	H	LEU	203	25.464	-4.805	20.039	1.00	0.00	RX0	H
ATOM	428	CA	LEU	203	25.004	-3.680	18.270	1.00	0.00	RX0	C
ATOM	429	CB	LEU	203	26.480	-3.879	17.921	1.00	0.00	RX0	C
ATOM	430	CG	LEU	203	26.934	-3.146	16.658	1.00	0.00	RX0	C
ATOM	431	CD1	LEU	203	26.736	-1.633	16.765	1.00	0.00	RX0	C
ATOM	432	CD2	LEU	203	28.375	-3.507	16.292	1.00	0.00	RX0	C
ATOM	433	C	LEU	203	24.099	-4.160	17.127	1.00	0.00	RX0	C
ATOM	434	O	LEU	203	23.593	-3.349	16.346	1.00	0.00	RX0	O
ATOM	435	N	VAL	204	23.782	-5.447	17.151	1.00	0.00	RX0	N
ATOM	436	H	VAL	204	24.203	-6.000	17.876	1.00	0.00	RX0	H
ATOM	437	CA	VAL	204	22.925	-6.083	16.127	1.00	0.00	RX0	C
ATOM	438	CB	VAL	204	22.916	-7.610	16.293	1.00	0.00	RX0	C
ATOM	439	CG1	VAL	204	22.087	-8.301	15.205	1.00	0.00	RX0	C
ATOM	440	CG2	VAL	204	24.342	-8.164	16.335	1.00	0.00	RX0	C
ATOM	441	C	VAL	204	21.502	-5.497	16.213	1.00	0.00	RX0	C
ATOM	442	O	VAL	204	20.938	-5.041	15.221	1.00	0.00	RX0	O
ATOM	443	N	HIS	205	21.015	-5.393	17.448	1.00	0.00	RX0	N
ATOM	444	H	HIS	205	21.605	-5.676	18.204	1.00	0.00	RX0	H
ATOM	445	CA	HIS	205	19.703	-4.786	17.746	1.00	0.00	RX0	C
ATOM	446	CB	HIS	205	19.261	-5.115	19.171	1.00	0.00	RX0	C
ATOM	447	CG	HIS	205	19.006	-6.600	19.257	1.00	0.00	RX0	C
ATOM	448	ND1	HIS	205	17.896	-7.194	18.781	1.00	0.00	RX0	N
ATOM	449	HD1	HIS	205	17.130	-6.754	18.364	1.00	0.00	RX0	H
ATOM	450	CD2	HIS	205	19.847	-7.578	19.791	1.00	0.00	RX0	C
ATOM	451	NE2	HIS	205	19.228	-8.770	19.628	1.00	0.00	RX0	N
ATOM	452	CE1	HIS	205	18.030	-8.538	19.006	1.00	0.00	RX0	C
ATOM	453	C	HIS	205	19.668	-3.277	17.476	1.00	0.00	RX0	C
ATOM	454	O	HIS	205	18.642	-2.756	17.030	1.00	0.00	RX0	O
ATOM	455	N	MET	206	20.820	-2.627	17.609	1.00	0.00	RX0	N
ATOM	456	H	MET	206	21.597	-3.127	18.003	1.00	0.00	RX0	H
ATOM	457	CA	MET	206	20.969	-1.185	17.340	1.00	0.00	RX0	C
ATOM	458	CB	MET	206	22.356	-0.691	17.759	1.00	0.00	RX0	C
ATOM	459	CG	MET	206	22.536	0.825	17.632	1.00	0.00	RX0	C
ATOM	460	SD	MET	206	24.244	1.329	17.878	1.00	0.00	RX0	S
ATOM	461	CE	MET	206	24.535	0.437	19.410	1.00	0.00	RX0	C
ATOM	462	C	MET	206	20.721	-0.870	15.856	1.00	0.00	RX0	C
ATOM	463	O	MET	206	20.035	0.103	15.544	1.00	0.00	RX0	O
ATOM	464	N	ILE	207	21.183	-1.758	14.977	1.00	0.00	RX0	N
ATOM	465	H	ILE	207	21.763	-2.488	15.349	1.00	0.00	RX0	H
ATOM	466	CA	ILE	207	20.975	-1.628	13.516	1.00	0.00	RX0	C
ATOM	467	CB	ILE	207	21.679	-2.772	12.767	1.00	0.00	RX0	C
ATOM	468	CG2	ILE	207	21.468	-2.687	11.252	1.00	0.00	RX0	C
ATOM	469	CG1	ILE	207	23.165	-2.852	13.127	1.00	0.00	RX0	C
ATOM	470	CD1	ILE	207	23.981	-1.641	12.672	1.00	0.00	RX0	C
ATOM	471	C	ILE	207	19.470	-1.621	13.197	1.00	0.00	RX0	C
ATOM	472	O	ILE	207	18.988	-0.755	12.467	1.00	0.00	RX0	O
ATOM	473	N	ASN	208	18.761	-2.558	13.816	1.00	0.00	RX0	N
ATOM	474	H	ASN	208	19.258	-3.126	14.474	1.00	0.00	RX0	H
ATOM	475	CA	ASN	208	17.313	-2.734	13.586	1.00	0.00	RX0	C
ATOM	476	CB	ASN	208	16.795	-4.032	14.208	1.00	0.00	RX0	C
ATOM	477	CG	ASN	208	16.532	-5.064	13.127	1.00	0.00	RX0	C
ATOM	478	OD1	ASN	208	16.236	-4.768	11.973	1.00	0.00	RX0	O
ATOM	479	ND2	ASN	208	16.664	-6.324	13.550	1.00	0.00	RX0	N
ATOM	480	HD21	ASN	208	16.886	-6.535	14.501	1.00	0.00	RX0	H
ATOM	481	HD22	ASN	208	16.541	-7.074	12.901	1.00	0.00	RX0	H
ATOM	482	C	ASN	208	16.516	-1.532	14.103	1.00	0.00	RX0	C
ATOM	483	O	ASN	208	15.637	-1.017	13.417	1.00	0.00	RX0	O
ATOM	484	N	TRP	209	16.982	-1.008	15.238	1.00	0.00	RX0	N
ATOM	485	H	TRP	209	17.728	-1.465	15.726	1.00	0.00	RX0	H
ATOM	486	CA	TRP	209	16.425	0.201	15.864	1.00	0.00	RX0	C

ATOM	487	CB	TRP	209	17.092	0.419	17.231	1.00	0.00	RX0	C
ATOM	488	CG	TRP	209	16.694	1.748	17.837	1.00	0.00	RX0	C
ATOM	489	CD2	TRP	209	17.412	3.000	17.806	1.00	0.00	RX0	C
ATOM	490	CE2	TRP	209	16.632	3.960	18.496	1.00	0.00	RX0	C
ATOM	491	CE3	TRP	209	18.632	3.372	17.255	1.00	0.00	RX0	C
ATOM	492	CD1	TRP	209	15.525	2.028	18.553	1.00	0.00	RX0	C
ATOM	493	NE1	TRP	209	15.477	3.326	18.949	1.00	0.00	RX0	N
ATOM	494	HE1	TRP	209	14.719	3.717	19.451	1.00	0.00	RX0	H
ATOM	495	CZ2	TRP	209	17.094	5.267	18.598	1.00	0.00	RX0	C
ATOM	496	CZ3	TRP	209	19.084	4.681	17.371	1.00	0.00	RX0	C
ATOM	497	CH2	TRP	209	18.314	5.626	18.037	1.00	0.00	RX0	C
ATOM	498	C	TRP	209	16.619	1.438	14.972	1.00	0.00	RX0	C
ATOM	499	O	TRP	209	15.652	2.120	14.634	1.00	0.00	RX0	O
ATOM	500	N	ALA	210	17.853	1.624	14.503	1.00	0.00	RX0	N
ATOM	501	H	ALA	210	18.567	0.986	14.788	1.00	0.00	RX0	H
ATOM	502	CA	ALA	210	18.233	2.756	13.635	1.00	0.00	RX0	C
ATOM	503	CB	ALA	210	19.715	2.666	13.274	1.00	0.00	RX0	C
ATOM	504	C	ALA	210	17.400	2.800	12.347	1.00	0.00	RX0	C
ATOM	505	O	ALA	210	16.892	3.855	11.979	1.00	0.00	RX0	O
ATOM	506	N	LYS	211	17.095	1.613	11.820	1.00	0.00	RX0	N
ATOM	507	H	LYS	211	17.538	0.807	12.221	1.00	0.00	RX0	H
ATOM	508	CA	LYS	211	16.258	1.472	10.614	1.00	0.00	RX0	C
ATOM	509	CB	LYS	211	16.340	0.055	10.046	1.00	0.00	RX0	C
ATOM	510	CG	LYS	211	17.712	-0.268	9.440	1.00	0.00	RX0	C
ATOM	511	CD	LYS	211	17.810	-1.713	8.944	1.00	0.00	RX0	C
ATOM	512	CE	LYS	211	17.447	-2.667	10.076	1.00	0.00	RX0	C
ATOM	513	NZ	LYS	211	17.575	-4.081	9.710	1.00	0.00	RX0	N
ATOM	514	HZ1	LYS	211	17.332	-4.667	10.542	1.00	0.00	RX0	H
ATOM	515	HZ2	LYS	211	16.947	-4.367	8.931	1.00	0.00	RX0	H
ATOM	516	HZ3	LYS	211	18.552	-4.328	9.465	1.00	0.00	RX0	H
ATOM	517	C	LYS	211	14.795	1.898	10.823	1.00	0.00	RX0	C
ATOM	518	O	LYS	211	14.129	2.337	9.881	1.00	0.00	RX0	O
ATOM	519	N	ARG	212	14.357	1.870	12.071	1.00	0.00	RX0	N
ATOM	520	H	ARG	212	14.985	1.607	12.802	1.00	0.00	RX0	H
ATOM	521	CA	ARG	212	13.005	2.320	12.466	1.00	0.00	RX0	C
ATOM	522	CB	ARG	212	12.390	1.353	13.487	1.00	0.00	RX0	C
ATOM	523	CG	ARG	212	12.668	-0.114	13.129	1.00	0.00	RX0	C
ATOM	524	CD	ARG	212	11.951	-1.158	13.989	1.00	0.00	RX0	C
ATOM	525	NE	ARG	212	10.558	-1.275	13.571	1.00	0.00	RX0	N
ATOM	526	HE	ARG	212	10.342	-1.393	12.598	1.00	0.00	RX0	H
ATOM	527	CZ	ARG	212	9.538	-1.121	14.435	1.00	0.00	RX0	C
ATOM	528	NH1	ARG	212	9.741	-1.044	15.744	1.00	0.00	RX0	N
ATOM	529	HH11	ARG	212	8.940	-0.856	16.335	1.00	0.00	RX0	H
ATOM	530	HH12	ARG	212	10.630	-1.146	16.181	1.00	0.00	RX0	H
ATOM	531	NH2	ARG	212	8.305	-1.027	13.968	1.00	0.00	RX0	N
ATOM	532	HH21	ARG	212	7.594	-0.746	14.637	1.00	0.00	RX0	H
ATOM	533	HH22	ARG	212	8.069	-1.178	13.010	1.00	0.00	RX0	H
ATOM	534	C	ARG	212	12.933	3.790	12.894	1.00	0.00	RX0	C
ATOM	535	O	ARG	212	11.827	4.344	12.989	1.00	0.00	RX0	O
ATOM	536	N	VAL	213	14.074	4.417	13.148	1.00	0.00	RX0	N
ATOM	537	H	VAL	213	14.931	3.924	12.996	1.00	0.00	RX0	H
ATOM	538	CA	VAL	213	14.155	5.874	13.374	1.00	0.00	RX0	C
ATOM	539	CB	VAL	213	15.582	6.281	13.746	1.00	0.00	RX0	C
ATOM	540	CG1	VAL	213	15.751	7.800	13.837	1.00	0.00	RX0	C
ATOM	541	CG2	VAL	213	15.983	5.583	15.043	1.00	0.00	RX0	C
ATOM	542	C	VAL	213	13.672	6.590	12.095	1.00	0.00	RX0	C
ATOM	543	O	VAL	213	14.288	6.431	11.023	1.00	0.00	RX0	O
ATOM	544	N	PRO	214	12.622	7.395	12.213	1.00	0.00	RX0	N
ATOM	545	CD	PRO	214	11.895	7.607	13.458	1.00	0.00	RX0	C
ATOM	546	CA	PRO	214	12.035	8.145	11.084	1.00	0.00	RX0	C
ATOM	547	CB	PRO	214	10.927	8.954	11.761	1.00	0.00	RX0	C

ATOM	548	CG	PRO	214	10.552	8.162	13.010	1.00	0.00	RX0	C
ATOM	549	C	PRO	214	13.102	9.001	10.387	1.00	0.00	RX0	C
ATOM	550	O	PRO	214	13.853	9.727	11.025	1.00	0.00	RX0	O
ATOM	551	N	GLY	215	13.244	8.730	9.080	1.00	0.00	RX0	N
ATOM	552	H	GLY	215	12.749	7.982	8.637	1.00	0.00	RX0	H
ATOM	553	CA	GLY	215	14.194	9.473	8.227	1.00	0.00	RX0	C
ATOM	554	C	GLY	215	15.511	8.732	7.950	1.00	0.00	RX0	C
ATOM	555	O	GLY	215	16.085	8.889	6.862	1.00	0.00	RX0	O
ATOM	556	N	PHE	216	15.917	7.845	8.848	1.00	0.00	RX0	N
ATOM	557	H	PHE	216	15.329	7.652	9.636	1.00	0.00	RX0	H
ATOM	558	CA	PHE	216	17.224	7.160	8.764	1.00	0.00	RX0	C
ATOM	559	CB	PHE	216	17.455	6.293	9.996	1.00	0.00	RX0	C
ATOM	560	CG	PHE	216	18.893	5.838	10.048	1.00	0.00	RX0	C
ATOM	561	CD1	PHE	216	19.904	6.766	10.272	1.00	0.00	RX0	C
ATOM	562	CD2	PHE	216	19.207	4.494	9.888	1.00	0.00	RX0	C
ATOM	563	CE1	PHE	216	21.223	6.344	10.375	1.00	0.00	RX0	C
ATOM	564	CE2	PHE	216	20.526	4.072	9.993	1.00	0.00	RX0	C
ATOM	565	CZ	PHE	216	21.531	4.994	10.259	1.00	0.00	RX0	C
ATOM	566	C	PHE	216	17.435	6.347	7.474	1.00	0.00	RX0	C
ATOM	567	O	PHE	216	18.358	6.641	6.711	1.00	0.00	RX0	O
ATOM	568	N	VAL	217	16.482	5.483	7.151	1.00	0.00	RX0	N
ATOM	569	H	VAL	217	15.674	5.446	7.737	1.00	0.00	RX0	H
ATOM	570	CA	VAL	217	16.570	4.608	5.958	1.00	0.00	RX0	C
ATOM	571	CB	VAL	217	15.533	3.489	6.010	1.00	0.00	RX0	C
ATOM	572	CG1	VAL	217	15.915	2.471	7.075	1.00	0.00	RX0	C
ATOM	573	CG2	VAL	217	14.114	4.038	6.181	1.00	0.00	RX0	C
ATOM	574	C	VAL	217	16.469	5.342	4.608	1.00	0.00	RX0	C
ATOM	575	O	VAL	217	16.660	4.747	3.556	1.00	0.00	RX0	O
ATOM	576	N	ASP	218	16.058	6.613	4.671	1.00	0.00	RX0	N
ATOM	577	H	ASP	218	15.765	7.061	5.519	1.00	0.00	RX0	H
ATOM	578	CA	ASP	218	16.006	7.470	3.472	1.00	0.00	RX0	C
ATOM	579	CB	ASP	218	15.097	8.676	3.760	1.00	0.00	RX0	C
ATOM	580	CG	ASP	218	13.745	8.267	4.346	1.00	0.00	RX0	C
ATOM	581	OD1	ASP	218	12.737	8.444	3.665	1.00	0.00	RX0	O
ATOM	582	OD2	ASP	218	13.684	7.811	5.493	1.00	0.00	RX0	O
ATOM	583	C	ASP	218	17.401	7.924	3.023	1.00	0.00	RX0	C
ATOM	584	O	ASP	218	17.595	8.369	1.896	1.00	0.00	RX0	O
ATOM	585	N	LEU	219	18.344	7.857	3.967	1.00	0.00	RX0	N
ATOM	586	H	LEU	219	18.097	7.481	4.860	1.00	0.00	RX0	H
ATOM	587	CA	LEU	219	19.767	8.083	3.697	1.00	0.00	RX0	C
ATOM	588	CB	LEU	219	20.530	8.282	5.005	1.00	0.00	RX0	C
ATOM	589	CG	LEU	219	19.877	9.383	5.848	1.00	0.00	RX0	C
ATOM	590	CD1	LEU	219	20.434	9.424	7.269	1.00	0.00	RX0	C
ATOM	591	CD2	LEU	219	19.925	10.749	5.159	1.00	0.00	RX0	C
ATOM	592	C	LEU	219	20.350	6.970	2.832	1.00	0.00	RX0	C
ATOM	593	O	LEU	219	19.843	5.836	2.789	1.00	0.00	RX0	O
ATOM	594	N	THR	220	21.442	7.293	2.191	1.00	0.00	RX0	N
ATOM	595	H	THR	220	21.770	8.241	2.280	1.00	0.00	RX0	H
ATOM	596	CA	THR	220	22.263	6.312	1.453	1.00	0.00	RX0	C
ATOM	597	CB	THR	220	23.370	7.128	0.784	1.00	0.00	RX0	C
ATOM	598	OG1	THR	220	22.857	8.029	-0.208	1.00	0.00	RX0	O
ATOM	599	HG1	THR	220	22.440	8.758	0.282	1.00	0.00	RX0	H
ATOM	600	CG2	THR	220	24.446	6.246	0.166	1.00	0.00	RX0	C
ATOM	601	C	THR	220	22.829	5.303	2.454	1.00	0.00	RX0	C
ATOM	602	O	THR	220	23.112	5.646	3.611	1.00	0.00	RX0	O
ATOM	603	N	LEU	221	23.130	4.116	1.957	1.00	0.00	RX0	N
ATOM	604	H	LEU	221	22.824	3.900	1.032	1.00	0.00	RX0	H
ATOM	605	CA	LEU	221	23.750	3.065	2.781	1.00	0.00	RX0	C
ATOM	606	CB	LEU	221	23.961	1.864	1.867	1.00	0.00	RX0	C
ATOM	607	CG	LEU	221	24.875	0.786	2.436	1.00	0.00	RX0	C
ATOM	608	CD1	LEU	221	24.248	0.068	3.630	1.00	0.00	RX0	C

ATOM	609	CD2	LEU	221	25.331	-0.167	1.332	1.00	0.00	RX0	C
ATOM	610	C	LEU	221	25.092	3.509	3.394	1.00	0.00	RX0	C
ATOM	611	O	LEU	221	25.324	3.347	4.578	1.00	0.00	RX0	O
ATOM	612	N	HIS	222	25.854	4.270	2.593	1.00	0.00	RX0	N
ATOM	613	H	HIS	222	25.551	4.429	1.658	1.00	0.00	RX0	H
ATOM	614	CA	HIS	222	27.131	4.847	3.045	1.00	0.00	RX0	C
ATOM	615	CB	HIS	222	27.831	5.668	1.961	1.00	0.00	RX0	C
ATOM	616	CG	HIS	222	28.895	6.524	2.621	1.00	0.00	RX0	C
ATOM	617	ND1	HIS	222	29.826	6.051	3.473	1.00	0.00	RX0	N
ATOM	618	HD1	HIS	222	29.916	5.128	3.804	1.00	0.00	RX0	H
ATOM	619	CD2	HIS	222	29.075	7.905	2.493	1.00	0.00	RX0	C
ATOM	620	NE2	HIS	222	30.124	8.259	3.275	1.00	0.00	RX0	N
ATOM	621	CE1	HIS	222	30.584	7.118	3.879	1.00	0.00	RX0	C
ATOM	622	C	HIS	222	26.942	5.765	4.264	1.00	0.00	RX0	C
ATOM	623	O	HIS	222	27.673	5.642	5.246	1.00	0.00	RX0	O
ATOM	624	N	ASP	223	25.958	6.658	4.167	1.00	0.00	RX0	N
ATOM	625	H	ASP	223	25.455	6.813	3.316	1.00	0.00	RX0	H
ATOM	626	CA	ASP	223	25.694	7.636	5.242	1.00	0.00	RX0	C
ATOM	627	CB	ASP	223	24.872	8.762	4.597	1.00	0.00	RX0	C
ATOM	628	CG	ASP	223	25.615	9.246	3.341	1.00	0.00	RX0	C
ATOM	629	OD1	ASP	223	26.300	10.265	3.408	1.00	0.00	RX0	O
ATOM	630	OD2	ASP	223	25.537	8.593	2.294	1.00	0.00	RX0	O
ATOM	631	C	ASP	223	25.148	6.996	6.516	1.00	0.00	RX0	C
ATOM	632	O	ASP	223	25.558	7.375	7.616	1.00	0.00	RX0	O
ATOM	633	N	GLN	224	24.393	5.921	6.332	1.00	0.00	RX0	N
ATOM	634	H	GLN	224	24.146	5.694	5.387	1.00	0.00	RX0	H
ATOM	635	CA	GLN	224	23.868	5.118	7.452	1.00	0.00	RX0	C
ATOM	636	CB	GLN	224	22.846	4.094	6.957	1.00	0.00	RX0	C
ATOM	637	CG	GLN	224	21.597	4.765	6.377	1.00	0.00	RX0	C
ATOM	638	CD	GLN	224	20.553	3.713	6.073	1.00	0.00	RX0	C
ATOM	639	OE1	GLN	224	20.407	2.729	6.793	1.00	0.00	RX0	O
ATOM	640	NE2	GLN	224	19.836	3.965	4.964	1.00	0.00	RX0	N
ATOM	641	HE21	GLN	224	19.982	4.793	4.413	1.00	0.00	RX0	H
ATOM	642	HE22	GLN	224	19.110	3.371	4.619	1.00	0.00	RX0	H
ATOM	643	C	GLN	224	25.003	4.453	8.243	1.00	0.00	RX0	C
ATOM	644	O	GLN	224	25.073	4.591	9.468	1.00	0.00	RX0	O
ATOM	645	N	VAL	225	25.993	3.956	7.505	1.00	0.00	RX0	N
ATOM	646	H	VAL	225	25.898	3.987	6.507	1.00	0.00	RX0	H
ATOM	647	CA	VAL	225	27.191	3.319	8.093	1.00	0.00	RX0	C
ATOM	648	CB	VAL	225	28.034	2.537	7.095	1.00	0.00	RX0	C
ATOM	649	CG1	VAL	225	29.091	1.755	7.870	1.00	0.00	RX0	C
ATOM	650	CG2	VAL	225	27.191	1.573	6.267	1.00	0.00	RX0	C
ATOM	651	C	VAL	225	28.021	4.368	8.852	1.00	0.00	RX0	C
ATOM	652	O	VAL	225	28.415	4.141	9.995	1.00	0.00	RX0	O
ATOM	653	N	HIS	226	28.182	5.534	8.231	1.00	0.00	RX0	N
ATOM	654	H	HIS	226	27.810	5.643	7.306	1.00	0.00	RX0	H
ATOM	655	CA	HIS	226	28.959	6.641	8.815	1.00	0.00	RX0	C
ATOM	656	CB	HIS	226	29.128	7.822	7.862	1.00	0.00	RX0	C
ATOM	657	CG	HIS	226	30.084	8.805	8.502	1.00	0.00	RX0	C
ATOM	658	ND1	HIS	226	31.323	8.477	8.914	1.00	0.00	RX0	N
ATOM	659	HD1	HIS	226	31.738	7.587	8.879	1.00	0.00	RX0	H
ATOM	660	CD2	HIS	226	29.871	10.162	8.771	1.00	0.00	RX0	C
ATOM	661	NE2	HIS	226	30.996	10.652	9.350	1.00	0.00	RX0	N
ATOM	662	CE1	HIS	226	31.889	9.612	9.436	1.00	0.00	RX0	C
ATOM	663	C	HIS	226	28.363	7.118	10.150	1.00	0.00	RX0	C
ATOM	664	O	HIS	226	29.071	7.189	11.155	1.00	0.00	RX0	O
ATOM	665	N	LEU	227	27.047	7.317	10.166	1.00	0.00	RX0	N
ATOM	666	H	LEU	227	26.530	7.184	9.317	1.00	0.00	RX0	H
ATOM	667	CA	LEU	227	26.344	7.787	11.375	1.00	0.00	RX0	C
ATOM	668	CB	LEU	227	24.875	8.082	11.076	1.00	0.00	RX0	C
ATOM	669	CG	LEU	227	24.666	9.356	10.259	1.00	0.00	RX0	C

ATOM	670	CD1	LEU	227	23.198	9.546	9.885	1.00	0.00	RX0	C
ATOM	671	CD2	LEU	227	25.220	10.587	10.976	1.00	0.00	RX0	C
ATOM	672	C	LEU	227	26.435	6.799	12.540	1.00	0.00	RX0	C
ATOM	673	O	LEU	227	26.853	7.165	13.635	1.00	0.00	RX0	O
ATOM	674	N	LEU	228	26.270	5.522	12.200	1.00	0.00	RX0	N
ATOM	675	H	LEU	228	26.041	5.297	11.249	1.00	0.00	RX0	H
ATOM	676	CA	LEU	228	26.384	4.431	13.181	1.00	0.00	RX0	C
ATOM	677	CB	LEU	228	25.764	3.153	12.622	1.00	0.00	RX0	C
ATOM	678	CG	LEU	228	24.263	3.123	12.903	1.00	0.00	RX0	C
ATOM	679	CD1	LEU	228	23.524	2.094	12.053	1.00	0.00	RX0	C
ATOM	680	CD2	LEU	228	23.992	2.925	14.395	1.00	0.00	RX0	C
ATOM	681	C	LEU	228	27.805	4.188	13.685	1.00	0.00	RX0	C
ATOM	682	O	LEU	228	28.004	4.019	14.891	1.00	0.00	RX0	O
ATOM	683	N	GLU	229	28.784	4.376	12.809	1.00	0.00	RX0	N
ATOM	684	H	GLU	229	28.582	4.586	11.851	1.00	0.00	RX0	H
ATOM	685	CA	GLU	229	30.199	4.229	13.197	1.00	0.00	RX0	C
ATOM	686	CB	GLU	229	31.137	4.120	11.989	1.00	0.00	RX0	C
ATOM	687	CG	GLU	229	32.483	3.515	12.407	1.00	0.00	RX0	C
ATOM	688	CD	GLU	229	33.298	3.125	11.192	1.00	0.00	RX0	C
ATOM	689	OE1	GLU	229	33.155	3.773	10.157	1.00	0.00	RX0	O
ATOM	690	OE2	GLU	229	34.067	2.166	11.282	1.00	0.00	RX0	O
ATOM	691	C	GLU	229	30.618	5.338	14.175	1.00	0.00	RX0	C
ATOM	692	O	GLU	229	31.393	5.088	15.099	1.00	0.00	RX0	O
ATOM	693	N	CYS	230	30.060	6.523	13.970	1.00	0.00	RX0	N
ATOM	694	H	CYS	230	29.476	6.652	13.165	1.00	0.00	RX0	H
ATOM	695	CA	CYS	230	30.321	7.692	14.829	1.00	0.00	RX0	C
ATOM	696	CB	CYS	230	30.006	8.968	14.059	1.00	0.00	RX0	C
ATOM	697	SG	CYS	230	31.042	9.141	12.588	1.00	0.00	RX0	S
ATOM	698	C	CYS	230	29.592	7.653	16.182	1.00	0.00	RX0	C
ATOM	699	O	CYS	230	30.123	8.119	17.188	1.00	0.00	RX0	O
ATOM	700	N	ALA	231	28.434	7.000	16.215	1.00	0.00	RX0	N
ATOM	701	H	ALA	231	28.113	6.533	15.388	1.00	0.00	RX0	H
ATOM	702	CA	ALA	231	27.494	7.141	17.345	1.00	0.00	RX0	C
ATOM	703	CB	ALA	231	26.145	7.669	16.853	1.00	0.00	RX0	C
ATOM	704	C	ALA	231	27.249	5.885	18.186	1.00	0.00	RX0	C
ATOM	705	O	ALA	231	26.768	6.021	19.321	1.00	0.00	RX0	O
ATOM	706	N	TRP	232	27.687	4.719	17.731	1.00	0.00	RX0	N
ATOM	707	H	TRP	232	28.111	4.687	16.822	1.00	0.00	RX0	H
ATOM	708	CA	TRP	232	27.348	3.433	18.379	1.00	0.00	RX0	C
ATOM	709	CB	TRP	232	27.969	2.235	17.648	1.00	0.00	RX0	C
ATOM	710	CG	TRP	232	29.473	2.274	17.765	1.00	0.00	RX0	C
ATOM	711	CD2	TRP	232	30.325	1.534	18.665	1.00	0.00	RX0	C
ATOM	712	CE2	TRP	232	31.658	1.938	18.419	1.00	0.00	RX0	C
ATOM	713	CE3	TRP	232	30.062	0.583	19.643	1.00	0.00	RX0	C
ATOM	714	CD1	TRP	232	30.344	3.078	17.024	1.00	0.00	RX0	C
ATOM	715	NE1	TRP	232	31.632	2.888	17.403	1.00	0.00	RX0	N
ATOM	716	HE1	TRP	232	32.396	3.352	16.995	1.00	0.00	RX0	H
ATOM	717	CZ2	TRP	232	32.692	1.379	19.160	1.00	0.00	RX0	C
ATOM	718	CZ3	TRP	232	31.106	0.032	20.376	1.00	0.00	RX0	C
ATOM	719	CH2	TRP	232	32.416	0.429	20.136	1.00	0.00	RX0	C
ATOM	720	C	TRP	232	27.676	3.373	19.884	1.00	0.00	RX0	C
ATOM	721	O	TRP	232	26.862	2.914	20.672	1.00	0.00	RX0	O
ATOM	722	N	LEU	233	28.801	3.989	20.277	1.00	0.00	RX0	N
ATOM	723	H	LEU	233	29.366	4.448	19.592	1.00	0.00	RX0	H
ATOM	724	CA	LEU	233	29.211	3.960	21.691	1.00	0.00	RX0	C
ATOM	725	CB	LEU	233	30.721	4.151	21.830	1.00	0.00	RX0	C
ATOM	726	CG	LEU	233	31.205	3.779	23.232	1.00	0.00	RX0	C
ATOM	727	CD1	LEU	233	30.810	2.349	23.606	1.00	0.00	RX0	C
ATOM	728	CD2	LEU	233	32.704	4.014	23.404	1.00	0.00	RX0	C
ATOM	729	C	LEU	233	28.415	4.936	22.566	1.00	0.00	RX0	C
ATOM	730	O	LEU	233	27.943	4.566	23.634	1.00	0.00	RX0	O

ATOM	731	N	GLU	234	28.150	6.122	22.016	1.00	0.00	RX0	N
ATOM	732	H	GLU	234	28.469	6.305	21.088	1.00	0.00	RX0	H
ATOM	733	CA	GLU	234	27.227	7.090	22.644	1.00	0.00	RX0	C
ATOM	734	CB	GLU	234	27.083	8.295	21.702	1.00	0.00	RX0	C
ATOM	735	CG	GLU	234	27.708	9.642	22.087	1.00	0.00	RX0	C
ATOM	736	CD	GLU	234	27.550	10.624	20.931	1.00	0.00	RX0	C
ATOM	737	OE1	GLU	234	27.133	11.764	21.129	1.00	0.00	RX0	O
ATOM	738	OE2	GLU	234	27.900	10.279	19.812	1.00	0.00	RX0	O
ATOM	739	C	GLU	234	25.830	6.482	22.841	1.00	0.00	RX0	C
ATOM	740	O	GLU	234	25.253	6.598	23.926	1.00	0.00	RX0	O
ATOM	741	N	ILE	235	25.389	5.711	21.848	1.00	0.00	RX0	N
ATOM	742	H	ILE	235	25.961	5.635	21.030	1.00	0.00	RX0	H
ATOM	743	CA	ILE	235	24.069	5.045	21.863	1.00	0.00	RX0	C
ATOM	744	CB	ILE	235	23.698	4.486	20.486	1.00	0.00	RX0	C
ATOM	745	CG2	ILE	235	22.415	3.655	20.553	1.00	0.00	RX0	C
ATOM	746	CG1	ILE	235	23.557	5.617	19.467	1.00	0.00	RX0	C
ATOM	747	CD1	ILE	235	23.219	5.102	18.068	1.00	0.00	RX0	C
ATOM	748	C	ILE	235	24.018	3.945	22.939	1.00	0.00	RX0	C
ATOM	749	O	ILE	235	23.068	3.902	23.724	1.00	0.00	RX0	O
ATOM	750	N	LEU	236	25.072	3.140	23.020	1.00	0.00	RX0	N
ATOM	751	H	LEU	236	25.810	3.232	22.348	1.00	0.00	RX0	H
ATOM	752	CA	LEU	236	25.176	2.114	24.078	1.00	0.00	RX0	C
ATOM	753	CB	LEU	236	26.446	1.285	23.897	1.00	0.00	RX0	C
ATOM	754	CG	LEU	236	26.342	0.290	22.745	1.00	0.00	RX0	C
ATOM	755	CD1	LEU	236	27.678	-0.392	22.449	1.00	0.00	RX0	C
ATOM	756	CD2	LEU	236	25.226	-0.722	22.999	1.00	0.00	RX0	C
ATOM	757	C	LEU	236	25.182	2.733	25.479	1.00	0.00	RX0	C
ATOM	758	O	LEU	236	24.381	2.362	26.336	1.00	0.00	RX0	O
ATOM	759	N	MET	237	25.933	3.823	25.600	1.00	0.00	RX0	N
ATOM	760	H	MET	237	26.455	4.134	24.803	1.00	0.00	RX0	H
ATOM	761	CA	MET	237	26.132	4.523	26.881	1.00	0.00	RX0	C
ATOM	762	CB	MET	237	27.279	5.530	26.801	1.00	0.00	RX0	C
ATOM	763	CG	MET	237	28.652	4.858	26.814	1.00	0.00	RX0	C
ATOM	764	SD	MET	237	29.998	6.051	26.781	1.00	0.00	RX0	S
ATOM	765	CE	MET	237	31.348	4.909	27.115	1.00	0.00	RX0	C
ATOM	766	C	MET	237	24.875	5.215	27.409	1.00	0.00	RX0	C
ATOM	767	O	MET	237	24.517	5.003	28.572	1.00	0.00	RX0	O
ATOM	768	N	ILE	238	24.128	5.878	26.531	1.00	0.00	RX0	N
ATOM	769	H	ILE	238	24.455	5.959	25.586	1.00	0.00	RX0	H
ATOM	770	CA	ILE	238	22.871	6.546	26.925	1.00	0.00	RX0	C
ATOM	771	CB	ILE	238	22.351	7.525	25.857	1.00	0.00	RX0	C
ATOM	772	CG2	ILE	238	21.927	6.840	24.558	1.00	0.00	RX0	C
ATOM	773	CG1	ILE	238	21.224	8.384	26.436	1.00	0.00	RX0	C
ATOM	774	CD1	ILE	238	20.621	9.346	25.412	1.00	0.00	RX0	C
ATOM	775	C	ILE	238	21.800	5.514	27.357	1.00	0.00	RX0	C
ATOM	776	O	ILE	238	21.031	5.731	28.268	1.00	0.00	RX0	O
ATOM	777	N	GLY	239	21.845	4.355	26.660	1.00	0.00	RX0	N
ATOM	778	H	GLY	239	22.520	4.244	25.926	1.00	0.00	RX0	H
ATOM	779	CA	GLY	239	20.969	3.213	26.975	1.00	0.00	RX0	C
ATOM	780	C	GLY	239	21.301	2.634	28.356	1.00	0.00	RX0	C
ATOM	781	O	GLY	239	20.417	2.460	29.193	1.00	0.00	RX0	O
ATOM	782	N	LEU	240	22.605	2.583	28.639	1.00	0.00	RX0	N
ATOM	783	H	LEU	240	23.260	2.803	27.913	1.00	0.00	RX0	H
ATOM	784	CA	LEU	240	23.124	2.082	29.919	1.00	0.00	RX0	C
ATOM	785	CB	LEU	240	24.644	1.943	29.849	1.00	0.00	RX0	C
ATOM	786	CG	LEU	240	25.286	1.611	31.196	1.00	0.00	RX0	C
ATOM	787	CD1	LEU	240	24.820	0.265	31.750	1.00	0.00	RX0	C
ATOM	788	CD2	LEU	240	26.807	1.709	31.126	1.00	0.00	RX0	C
ATOM	789	C	LEU	240	22.728	2.995	31.086	1.00	0.00	RX0	C
ATOM	790	O	LEU	240	22.214	2.535	32.097	1.00	0.00	RX0	O
ATOM	791	N	VAL	241	22.901	4.295	30.880	1.00	0.00	RX0	N

ATOM	792	H	VAL	241	23.278	4.586	29.998	1.00	0.00	RX0	H
ATOM	793	CA	VAL	241	22.596	5.307	31.912	1.00	0.00	RX0	C
ATOM	794	CB	VAL	241	23.253	6.671	31.660	1.00	0.00	RX0	C
ATOM	795	CG1	VAL	241	24.771	6.505	31.603	1.00	0.00	RX0	C
ATOM	796	CG2	VAL	241	22.706	7.399	30.440	1.00	0.00	RX0	C
ATOM	797	C	VAL	241	21.084	5.392	32.193	1.00	0.00	RX0	C
ATOM	798	O	VAL	241	20.670	5.516	33.338	1.00	0.00	RX0	O
ATOM	799	N	TRP	242	20.290	5.181	31.134	1.00	0.00	RX0	N
ATOM	800	H	TRP	242	20.699	5.072	30.225	1.00	0.00	RX0	H
ATOM	801	CA	TRP	242	18.822	5.192	31.222	1.00	0.00	RX0	C
ATOM	802	CB	TRP	242	18.253	5.150	29.800	1.00	0.00	RX0	C
ATOM	803	CG	TRP	242	16.828	4.653	29.776	1.00	0.00	RX0	C
ATOM	804	CD2	TRP	242	15.635	5.323	30.232	1.00	0.00	RX0	C
ATOM	805	CE2	TRP	242	14.546	4.450	30.012	1.00	0.00	RX0	C
ATOM	806	CE3	TRP	242	15.413	6.569	30.802	1.00	0.00	RX0	C
ATOM	807	CD1	TRP	242	16.388	3.410	29.295	1.00	0.00	RX0	C
ATOM	808	NE1	TRP	242	15.042	3.287	29.432	1.00	0.00	RX0	N
ATOM	809	HE1	TRP	242	14.503	2.510	29.171	1.00	0.00	RX0	H
ATOM	810	CZ2	TRP	242	13.265	4.846	30.375	1.00	0.00	RX0	C
ATOM	811	CZ3	TRP	242	14.128	6.956	31.158	1.00	0.00	RX0	C
ATOM	812	CH2	TRP	242	13.059	6.094	30.951	1.00	0.00	RX0	C
ATOM	813	C	TRP	242	18.281	4.038	32.076	1.00	0.00	RX0	C
ATOM	814	O	TRP	242	17.477	4.269	32.979	1.00	0.00	RX0	O
ATOM	815	N	ARG	243	18.818	2.841	31.865	1.00	0.00	RX0	N
ATOM	816	H	ARG	243	19.515	2.737	31.151	1.00	0.00	RX0	H
ATOM	817	CA	ARG	243	18.359	1.661	32.627	1.00	0.00	RX0	C
ATOM	818	CB	ARG	243	18.488	0.403	31.737	1.00	0.00	RX0	C
ATOM	819	CG	ARG	243	19.894	-0.062	31.299	1.00	0.00	RX0	C
ATOM	820	CD	ARG	243	19.837	-1.097	30.152	1.00	0.00	RX0	C
ATOM	821	NE	ARG	243	21.121	-1.755	29.863	1.00	0.00	RX0	N
ATOM	822	HE	ARG	243	21.670	-2.012	30.672	1.00	0.00	RX0	H
ATOM	823	CZ	ARG	243	21.441	-2.124	28.569	1.00	0.00	RX0	C
ATOM	824	NH1	ARG	243	20.677	-1.717	27.536	1.00	0.00	RX0	N
ATOM	825	HH11	ARG	243	20.923	-2.030	26.600	1.00	0.00	RX0	H
ATOM	826	HH12	ARG	243	19.877	-1.126	27.637	1.00	0.00	RX0	H
ATOM	827	NH2	ARG	243	22.509	-2.903	28.305	1.00	0.00	RX0	N
ATOM	828	HH21	ARG	243	22.794	-3.115	27.347	1.00	0.00	RX0	H
ATOM	829	HH22	ARG	243	23.064	-3.336	29.025	1.00	0.00	RX0	H
ATOM	830	C	ARG	243	19.043	1.510	33.998	1.00	0.00	RX0	C
ATOM	831	O	ARG	243	18.610	0.722	34.836	1.00	0.00	RX0	O
ATOM	832	N	SER	244	20.027	2.366	34.245	1.00	0.00	RX0	N
ATOM	833	H	SER	244	20.316	3.010	33.538	1.00	0.00	RX0	H
ATOM	834	CA	SER	244	20.722	2.464	35.548	1.00	0.00	RX0	C
ATOM	835	CB	SER	244	22.206	2.697	35.311	1.00	0.00	RX0	C
ATOM	836	OG	SER	244	22.700	1.647	34.476	1.00	0.00	RX0	O
ATOM	837	HG	SER	244	22.226	1.717	33.654	1.00	0.00	RX0	H
ATOM	838	C	SER	244	20.112	3.547	36.442	1.00	0.00	RX0	C
ATOM	839	O	SER	244	20.448	3.642	37.630	1.00	0.00	RX0	O
ATOM	840	N	MET	245	19.184	4.322	35.895	1.00	0.00	RX0	N
ATOM	841	H	MET	245	18.884	4.151	34.954	1.00	0.00	RX0	H
ATOM	842	CA	MET	245	18.600	5.504	36.550	1.00	0.00	RX0	C
ATOM	843	CB	MET	245	17.597	6.196	35.632	1.00	0.00	RX0	C
ATOM	844	CG	MET	245	17.043	7.471	36.267	1.00	0.00	RX0	C
ATOM	845	SD	MET	245	15.741	8.234	35.295	1.00	0.00	RX0	S
ATOM	846	CE	MET	245	16.554	8.094	33.702	1.00	0.00	RX0	C
ATOM	847	C	MET	245	17.925	5.204	37.895	1.00	0.00	RX0	C
ATOM	848	O	MET	245	18.105	5.946	38.853	1.00	0.00	RX0	O
ATOM	849	N	GLU	246	17.212	4.082	37.945	1.00	0.00	RX0	N
ATOM	850	H	GLU	246	17.124	3.485	37.147	1.00	0.00	RX0	H
ATOM	851	CA	GLU	246	16.494	3.692	39.178	1.00	0.00	RX0	C
ATOM	852	CB	GLU	246	15.244	2.901	38.818	1.00	0.00	RX0	C

ATOM	853	CG	GLU	246	14.342	3.656	37.846	1.00	0.00	RX0	C
ATOM	854	CD	GLU	246	13.164	2.773	37.508	1.00	0.00	RX0	C
ATOM	855	OE1	GLU	246	12.967	1.778	38.202	1.00	0.00	RX0	O
ATOM	856	OE2	GLU	246	12.457	3.077	36.551	1.00	0.00	RX0	O
ATOM	857	C	GLU	246	17.379	2.867	40.123	1.00	0.00	RX0	C
ATOM	858	O	GLU	246	16.897	2.295	41.108	1.00	0.00	RX0	O
ATOM	859	N	HIS	247	18.674	2.844	39.836	1.00	0.00	RX0	N
ATOM	860	H	HIS	247	19.044	3.323	39.041	1.00	0.00	RX0	H
ATOM	861	CA	HIS	247	19.658	2.055	40.601	1.00	0.00	RX0	C
ATOM	862	CB	HIS	247	20.174	0.865	39.788	1.00	0.00	RX0	C
ATOM	863	CG	HIS	247	19.080	-0.136	39.487	1.00	0.00	RX0	C
ATOM	864	ND1	HIS	247	17.866	-0.169	40.074	1.00	0.00	RX0	N
ATOM	865	HD1	HIS	247	17.501	0.451	40.747	1.00	0.00	RX0	H
ATOM	866	CD2	HIS	247	19.153	-1.186	38.568	1.00	0.00	RX0	C
ATOM	867	NE2	HIS	247	17.976	-1.853	38.607	1.00	0.00	RX0	N
ATOM	868	CE1	HIS	247	17.181	-1.226	39.532	1.00	0.00	RX0	C
ATOM	869	C	HIS	247	20.841	2.947	41.015	1.00	0.00	RX0	C
ATOM	870	O	HIS	247	21.962	2.788	40.490	1.00	0.00	RX0	O
ATOM	871	N	PRO	248	20.632	3.827	41.991	1.00	0.00	RX0	N
ATOM	872	CD	PRO	248	19.384	3.973	42.733	1.00	0.00	RX0	C
ATOM	873	CA	PRO	248	21.659	4.767	42.484	1.00	0.00	RX0	C
ATOM	874	CB	PRO	248	20.980	5.449	43.675	1.00	0.00	RX0	C
ATOM	875	CG	PRO	248	19.487	5.344	43.385	1.00	0.00	RX0	C
ATOM	876	C	PRO	248	22.939	4.013	42.877	1.00	0.00	RX0	C
ATOM	877	O	PRO	248	22.892	2.963	43.503	1.00	0.00	RX0	O
ATOM	878	N	GLY	249	24.055	4.541	42.350	1.00	0.00	RX0	N
ATOM	879	H	GLY	249	23.973	5.299	41.708	1.00	0.00	RX0	H
ATOM	880	CA	GLY	249	25.407	3.996	42.610	1.00	0.00	RX0	C
ATOM	881	C	GLY	249	25.783	2.749	41.794	1.00	0.00	RX0	C
ATOM	882	O	GLY	249	26.914	2.250	41.927	1.00	0.00	RX0	O
ATOM	883	N	LYS	250	24.877	2.267	40.961	1.00	0.00	RX0	N
ATOM	884	H	LYS	250	23.983	2.697	40.813	1.00	0.00	RX0	H
ATOM	885	CA	LYS	250	25.097	1.050	40.158	1.00	0.00	RX0	C
ATOM	886	CB	LYS	250	24.363	-0.145	40.770	1.00	0.00	RX0	C
ATOM	887	CG	LYS	250	25.218	-0.872	41.817	1.00	0.00	RX0	C
ATOM	888	CD	LYS	250	24.576	-2.156	42.350	1.00	0.00	RX0	C
ATOM	889	CE	LYS	250	25.499	-3.027	43.212	1.00	0.00	RX0	C
ATOM	890	NZ	LYS	250	26.639	-3.523	42.426	1.00	0.00	RX0	N
ATOM	891	HZ1	LYS	250	27.337	-3.962	43.069	1.00	0.00	RX0	H
ATOM	892	HZ2	LYS	250	26.370	-4.198	41.675	1.00	0.00	RX0	H
ATOM	893	HZ3	LYS	250	27.170	-2.718	42.042	1.00	0.00	RX0	H
ATOM	894	C	LYS	250	24.802	1.266	38.671	1.00	0.00	RX0	C
ATOM	895	O	LYS	250	24.040	2.166	38.282	1.00	0.00	RX0	O
ATOM	896	N	LEU	251	25.472	0.476	37.863	1.00	0.00	RX0	N
ATOM	897	H	LEU	251	26.053	-0.236	38.243	1.00	0.00	RX0	H
ATOM	898	CA	LEU	251	25.292	0.430	36.401	1.00	0.00	RX0	C
ATOM	899	CB	LEU	251	26.626	0.592	35.684	1.00	0.00	RX0	C
ATOM	900	CG	LEU	251	27.161	2.017	35.763	1.00	0.00	RX0	C
ATOM	901	CD1	LEU	251	28.584	2.115	35.219	1.00	0.00	RX0	C
ATOM	902	CD2	LEU	251	26.208	3.009	35.093	1.00	0.00	RX0	C
ATOM	903	C	LEU	251	24.646	-0.892	36.017	1.00	0.00	RX0	C
ATOM	904	O	LEU	251	25.224	-1.976	36.286	1.00	0.00	RX0	O
ATOM	905	N	LEU	252	23.437	-0.808	35.533	1.00	0.00	RX0	N
ATOM	906	H	LEU	252	23.084	0.097	35.312	1.00	0.00	RX0	H
ATOM	907	CA	LEU	252	22.659	-1.981	35.114	1.00	0.00	RX0	C
ATOM	908	CB	LEU	252	21.157	-1.712	35.229	1.00	0.00	RX0	C
ATOM	909	CG	LEU	252	20.297	-2.955	34.971	1.00	0.00	RX0	C
ATOM	910	CD1	LEU	252	20.445	-3.994	36.082	1.00	0.00	RX0	C
ATOM	911	CD2	LEU	252	18.831	-2.605	34.722	1.00	0.00	RX0	C
ATOM	912	C	LEU	252	23.007	-2.330	33.663	1.00	0.00	RX0	C
ATOM	913	O	LEU	252	22.274	-2.025	32.731	1.00	0.00	RX0	O

ATOM	914	N	PHE	253	24.144	-3.005	33.506	1.00	0.00	RX0	N
ATOM	915	H	PHE	253	24.631	-3.308	34.330	1.00	0.00	RX0	H
ATOM	916	CA	PHE	253	24.599	-3.457	32.174	1.00	0.00	RX0	C
ATOM	917	CB	PHE	253	25.968	-4.125	32.266	1.00	0.00	RX0	C
ATOM	918	CG	PHE	253	27.031	-3.100	32.565	1.00	0.00	RX0	C
ATOM	919	CD1	PHE	253	27.564	-2.344	31.528	1.00	0.00	RX0	C
ATOM	920	CD2	PHE	253	27.482	-2.921	33.866	1.00	0.00	RX0	C
ATOM	921	CE1	PHE	253	28.561	-1.414	31.790	1.00	0.00	RX0	C
ATOM	922	CE2	PHE	253	28.479	-1.989	34.126	1.00	0.00	RX0	C
ATOM	923	CZ	PHE	253	29.019	-1.238	33.089	1.00	0.00	RX0	C
ATOM	924	C	PHE	253	23.603	-4.446	31.564	1.00	0.00	RX0	C
ATOM	925	O	PHE	253	23.259	-4.379	30.390	1.00	0.00	RX0	O
ATOM	926	N	ALA	254	23.094	-5.300	32.445	1.00	0.00	RX0	N
ATOM	927	H	ALA	254	23.409	-5.311	33.399	1.00	0.00	RX0	H
ATOM	928	CA	ALA	254	22.050	-6.280	32.141	1.00	0.00	RX0	C
ATOM	929	CB	ALA	254	22.712	-7.553	31.609	1.00	0.00	RX0	C
ATOM	930	C	ALA	254	21.288	-6.584	33.440	1.00	0.00	RX0	C
ATOM	931	O	ALA	254	21.887	-6.418	34.526	1.00	0.00	RX0	O
ATOM	932	N	PRO	255	20.056	-7.064	33.372	1.00	0.00	RX0	N
ATOM	933	CD	PRO	255	19.310	-7.247	32.129	1.00	0.00	RX0	C
ATOM	934	CA	PRO	255	19.236	-7.444	34.545	1.00	0.00	RX0	C
ATOM	935	CB	PRO	255	17.988	-8.071	33.923	1.00	0.00	RX0	C
ATOM	936	CG	PRO	255	17.858	-7.394	32.565	1.00	0.00	RX0	C
ATOM	937	C	PRO	255	19.972	-8.395	35.506	1.00	0.00	RX0	C
ATOM	938	O	PRO	255	19.756	-8.342	36.714	1.00	0.00	RX0	O
ATOM	939	N	ASN	256	20.900	-9.179	34.970	1.00	0.00	RX0	N
ATOM	940	H	ASN	256	21.113	-9.138	33.993	1.00	0.00	RX0	H
ATOM	941	CA	ASN	256	21.722	-10.125	35.761	1.00	0.00	RX0	C
ATOM	942	CB	ASN	256	21.713	-11.527	35.148	1.00	0.00	RX0	C
ATOM	943	CG	ASN	256	22.420	-11.529	33.801	1.00	0.00	RX0	C
ATOM	944	OD1	ASN	256	22.291	-10.603	32.999	1.00	0.00	RX0	O
ATOM	945	ND2	ASN	256	23.084	-12.671	33.547	1.00	0.00	RX0	N
ATOM	946	HD21	ASN	256	23.231	-13.329	34.289	1.00	0.00	RX0	H
ATOM	947	HD22	ASN	256	23.441	-12.924	32.642	1.00	0.00	RX0	H
ATOM	948	C	ASN	256	23.191	-9.669	35.876	1.00	0.00	RX0	C
ATOM	949	O	ASN	256	24.101	-10.505	36.011	1.00	0.00	RX0	O
ATOM	950	N	LEU	257	23.439	-8.384	35.742	1.00	0.00	RX0	N
ATOM	951	H	LEU	257	22.692	-7.726	35.629	1.00	0.00	RX0	H
ATOM	952	CA	LEU	257	24.796	-7.805	35.783	1.00	0.00	RX0	C
ATOM	953	CB	LEU	257	25.523	-7.966	34.449	1.00	0.00	RX0	C
ATOM	954	CG	LEU	257	27.032	-7.755	34.595	1.00	0.00	RX0	C
ATOM	955	CD1	LEU	257	27.652	-8.799	35.525	1.00	0.00	RX0	C
ATOM	956	CD2	LEU	257	27.745	-7.704	33.243	1.00	0.00	RX0	C
ATOM	957	C	LEU	257	24.720	-6.327	36.168	1.00	0.00	RX0	C
ATOM	958	O	LEU	257	24.738	-5.412	35.328	1.00	0.00	RX0	O
ATOM	959	N	LEU	258	24.604	-6.148	37.469	1.00	0.00	RX0	N
ATOM	960	H	LEU	258	24.690	-6.942	38.069	1.00	0.00	RX0	H
ATOM	961	CA	LEU	258	24.482	-4.834	38.118	1.00	0.00	RX0	C
ATOM	962	CB	LEU	258	23.219	-4.908	38.976	1.00	0.00	RX0	C
ATOM	963	CG	LEU	258	22.796	-3.619	39.668	1.00	0.00	RX0	C
ATOM	964	CD1	LEU	258	22.652	-2.466	38.686	1.00	0.00	RX0	C
ATOM	965	CD2	LEU	258	21.525	-3.817	40.495	1.00	0.00	RX0	C
ATOM	966	C	LEU	258	25.743	-4.564	38.938	1.00	0.00	RX0	C
ATOM	967	O	LEU	258	26.013	-5.237	39.948	1.00	0.00	RX0	O
ATOM	968	N	LEU	259	26.528	-3.622	38.460	1.00	0.00	RX0	N
ATOM	969	H	LEU	259	26.219	-3.062	37.684	1.00	0.00	RX0	H
ATOM	970	CA	LEU	259	27.862	-3.349	39.027	1.00	0.00	RX0	C
ATOM	971	CB	LEU	259	28.938	-3.494	37.948	1.00	0.00	RX0	C
ATOM	972	CG	LEU	259	28.888	-4.817	37.179	1.00	0.00	RX0	C
ATOM	973	CD1	LEU	259	29.903	-4.843	36.036	1.00	0.00	RX0	C
ATOM	974	CD2	LEU	259	29.046	-6.031	38.094	1.00	0.00	RX0	C

ATOM	975	C	LEU	259	27.958	-1.956	39.652	1.00	0.00	RX0	C
ATOM	976	O	LEU	259	27.419	-0.984	39.137	1.00	0.00	RX0	O
ATOM	977	N	ASP	260	28.645	-1.912	40.785	1.00	0.00	RX0	N
ATOM	978	H	ASP	260	29.224	-2.688	41.046	1.00	0.00	RX0	H
ATOM	979	CA	ASP	260	29.043	-0.657	41.454	1.00	0.00	RX0	C
ATOM	980	CB	ASP	260	29.065	-0.872	42.962	1.00	0.00	RX0	C
ATOM	981	CG	ASP	260	29.704	-2.218	43.214	1.00	0.00	RX0	C
ATOM	982	OD1	ASP	260	30.927	-2.312	43.199	1.00	0.00	RX0	O
ATOM	983	OD2	ASP	260	28.962	-3.188	43.371	1.00	0.00	RX0	O
ATOM	984	C	ASP	260	30.443	-0.222	40.970	1.00	0.00	RX0	C
ATOM	985	O	ASP	260	31.127	-1.008	40.295	1.00	0.00	RX0	O
ATOM	986	N	ARG	261	30.963	0.873	41.504	1.00	0.00	RX0	N
ATOM	987	H	ARG	261	30.394	1.402	42.133	1.00	0.00	RX0	H
ATOM	988	CA	ARG	261	32.263	1.408	41.044	1.00	0.00	RX0	C
ATOM	989	CB	ARG	261	32.420	2.887	41.431	1.00	0.00	RX0	C
ATOM	990	CG	ARG	261	32.645	3.168	42.919	1.00	0.00	RX0	C
ATOM	991	CD	ARG	261	32.523	4.653	43.277	1.00	0.00	RX0	C
ATOM	992	NE	ARG	261	33.395	5.496	42.461	1.00	0.00	RX0	N
ATOM	993	HE	ARG	261	33.211	5.588	41.469	1.00	0.00	RX0	H
ATOM	994	CZ	ARG	261	34.380	6.255	43.024	1.00	0.00	RX0	C
ATOM	995	NH1	ARG	261	34.585	6.221	44.356	1.00	0.00	RX0	N
ATOM	996	HH11	ARG	261	35.367	6.672	44.788	1.00	0.00	RX0	H
ATOM	997	HH12	ARG	261	33.930	5.762	44.976	1.00	0.00	RX0	H
ATOM	998	NH2	ARG	261	35.125	7.047	42.237	1.00	0.00	RX0	N
ATOM	999	HH21	ARG	261	35.852	7.672	42.549	1.00	0.00	RX0	H
ATOM	1000	HH22	ARG	261	34.934	7.057	41.239	1.00	0.00	RX0	H
ATOM	1001	C	ARG	261	33.476	0.540	41.436	1.00	0.00	RX0	C
ATOM	1002	O	ARG	261	34.378	0.347	40.637	1.00	0.00	RX0	O
ATOM	1003	N	ASN	262	33.410	-0.067	42.632	1.00	0.00	RX0	N
ATOM	1004	H	ASN	262	32.547	-0.022	43.136	1.00	0.00	RX0	H
ATOM	1005	CA	ASN	262	34.456	-1.000	43.094	1.00	0.00	RX0	C
ATOM	1006	CB	ASN	262	34.228	-1.477	44.526	1.00	0.00	RX0	C
ATOM	1007	CG	ASN	262	35.360	-2.419	44.910	1.00	0.00	RX0	C
ATOM	1008	OD1	ASN	262	36.457	-1.990	45.276	1.00	0.00	RX0	O
ATOM	1009	ND2	ASN	262	35.029	-3.722	44.851	1.00	0.00	RX0	N
ATOM	1010	HD21	ASN	262	34.107	-4.012	44.585	1.00	0.00	RX0	H
ATOM	1011	HD22	ASN	262	35.694	-4.443	45.052	1.00	0.00	RX0	H
ATOM	1012	C	ASN	262	34.635	-2.233	42.204	1.00	0.00	RX0	C
ATOM	1013	O	ASN	262	35.755	-2.665	41.964	1.00	0.00	RX0	O
ATOM	1014	N	GLN	263	33.530	-2.679	41.603	1.00	0.00	RX0	N
ATOM	1015	H	GLN	263	32.639	-2.273	41.829	1.00	0.00	RX0	H
ATOM	1016	CA	GLN	263	33.559	-3.756	40.599	1.00	0.00	RX0	C
ATOM	1017	CB	GLN	263	32.199	-4.426	40.455	1.00	0.00	RX0	C
ATOM	1018	CG	GLN	263	31.963	-5.205	41.747	1.00	0.00	RX0	C
ATOM	1019	CD	GLN	263	30.713	-6.043	41.658	1.00	0.00	RX0	C
ATOM	1020	OE1	GLN	263	30.575	-6.930	40.825	1.00	0.00	RX0	O
ATOM	1021	NE2	GLN	263	29.824	-5.754	42.618	1.00	0.00	RX0	N
ATOM	1022	HE21	GLN	263	29.972	-4.923	43.172	1.00	0.00	RX0	H
ATOM	1023	HE22	GLN	263	29.039	-6.340	42.804	1.00	0.00	RX0	H
ATOM	1024	C	GLN	263	34.189	-3.308	39.265	1.00	0.00	RX0	C
ATOM	1025	O	GLN	263	34.644	-4.116	38.479	1.00	0.00	RX0	O
ATOM	1026	N	GLY	264	34.180	-1.978	39.039	1.00	0.00	RX0	N
ATOM	1027	H	GLY	264	33.772	-1.378	39.727	1.00	0.00	RX0	H
ATOM	1028	CA	GLY	264	34.831	-1.337	37.881	1.00	0.00	RX0	C
ATOM	1029	C	GLY	264	36.364	-1.310	37.978	1.00	0.00	RX0	C
ATOM	1030	O	GLY	264	37.050	-1.510	36.977	1.00	0.00	RX0	O
ATOM	1031	N	LYS	265	36.881	-1.155	39.202	1.00	0.00	RX0	N
ATOM	1032	H	LYS	265	36.240	-1.023	39.960	1.00	0.00	RX0	H
ATOM	1033	CA	LYS	265	38.336	-1.184	39.469	1.00	0.00	RX0	C
ATOM	1034	CB	LYS	265	38.640	-1.158	40.946	1.00	0.00	RX0	C
ATOM	1035	CG	LYS	265	38.265	-0.000	41.851	1.00	0.00	RX0	C

ATOM	1036	CD	LYS	265	38.525	-0.698	43.172	1.00	0.00	RX0	C
ATOM	1037	CE	LYS	265	38.667	0.054	44.482	1.00	0.00	RX0	C
ATOM	1038	NZ	LYS	265	38.986	-1.012	45.438	1.00	0.00	RX0	N
ATOM	1039	HZ1	LYS	265	39.150	-0.687	46.402	1.00	0.00	RX0	H
ATOM	1040	HZ2	LYS	265	38.235	-1.738	45.417	1.00	0.00	RX0	H
ATOM	1041	HZ3	LYS	265	39.854	-1.506	45.113	1.00	0.00	RX0	H
ATOM	1042	C	LYS	265	38.994	-2.500	39.030	1.00	0.00	RX0	C
ATOM	1043	O	LYS	265	40.184	-2.535	38.765	1.00	0.00	RX0	O
ATOM	1044	N	CYS	266	38.153	-3.548	38.917	1.00	0.00	RX0	N
ATOM	1045	H	CYS	266	37.204	-3.462	39.220	1.00	0.00	RX0	H
ATOM	1046	CA	CYS	266	38.538	-4.854	38.355	1.00	0.00	RX0	C
ATOM	1047	CB	CYS	266	37.317	-5.761	38.254	1.00	0.00	RX0	C
ATOM	1048	SG	CYS	266	36.544	-5.962	39.881	1.00	0.00	RX0	S
ATOM	1049	C	CYS	266	39.318	-4.732	37.033	1.00	0.00	RX0	C
ATOM	1050	O	CYS	266	40.108	-5.603	36.695	1.00	0.00	RX0	O
ATOM	1051	N	VAL	267	39.075	-3.636	36.304	1.00	0.00	RX0	N
ATOM	1052	H	VAL	267	38.451	-2.914	36.612	1.00	0.00	RX0	H
ATOM	1053	CA	VAL	267	39.804	-3.345	35.058	1.00	0.00	RX0	C
ATOM	1054	CB	VAL	267	38.872	-3.369	33.841	1.00	0.00	RX0	C
ATOM	1055	CG1	VAL	267	39.617	-3.023	32.549	1.00	0.00	RX0	C
ATOM	1056	CG2	VAL	267	38.173	-4.726	33.724	1.00	0.00	RX0	C
ATOM	1057	C	VAL	267	40.557	-2.013	35.191	1.00	0.00	RX0	C
ATOM	1058	O	VAL	267	39.969	-0.956	35.468	1.00	0.00	RX0	O
ATOM	1059	N	GLU	268	41.829	-2.078	34.821	1.00	0.00	RX0	N
ATOM	1060	H	GLU	268	42.181	-2.951	34.508	1.00	0.00	RX0	H
ATOM	1061	CA	GLU	268	42.726	-0.908	34.727	1.00	0.00	RX0	C
ATOM	1062	CB	GLU	268	44.146	-1.278	34.344	1.00	0.00	RX0	C
ATOM	1063	CG	GLU	268	45.131	-0.114	34.301	1.00	0.00	RX0	C
ATOM	1064	CD	GLU	268	45.074	0.480	32.918	1.00	0.00	RX0	C
ATOM	1065	OE1	GLU	268	45.156	1.695	32.779	1.00	0.00	RX0	O
ATOM	1066	OE2	GLU	268	44.945	-0.281	31.966	1.00	0.00	RX0	O
ATOM	1067	C	GLU	268	42.079	0.194	33.866	1.00	0.00	RX0	C
ATOM	1068	O	GLU	268	41.697	-0.027	32.727	1.00	0.00	RX0	O
ATOM	1069	N	GLY	269	41.924	1.355	34.524	1.00	0.00	RX0	N
ATOM	1070	H	GLY	269	42.291	1.347	35.443	1.00	0.00	RX0	H
ATOM	1071	CA	GLY	269	41.377	2.576	33.902	1.00	0.00	RX0	C
ATOM	1072	C	GLY	269	39.898	2.490	33.494	1.00	0.00	RX0	C
ATOM	1073	O	GLY	269	39.424	3.343	32.745	1.00	0.00	RX0	O
ATOM	1074	N	MET	270	39.146	1.598	34.134	1.00	0.00	RX0	N
ATOM	1075	H	MET	270	39.574	0.912	34.724	1.00	0.00	RX0	H
ATOM	1076	CA	MET	270	37.704	1.465	33.841	1.00	0.00	RX0	C
ATOM	1077	CB	MET	270	37.285	0.007	33.653	1.00	0.00	RX0	C
ATOM	1078	CG	MET	270	35.930	-0.107	32.949	1.00	0.00	RX0	C
ATOM	1079	SD	MET	270	35.437	-1.800	32.579	1.00	0.00	RX0	S
ATOM	1080	CE	MET	270	35.185	-2.377	34.263	1.00	0.00	RX0	C
ATOM	1081	C	MET	270	36.825	2.181	34.877	1.00	0.00	RX0	C
ATOM	1082	O	MET	270	35.781	2.734	34.514	1.00	0.00	RX0	O
ATOM	1083	N	VAL	271	37.310	2.290	36.107	1.00	0.00	RX0	N
ATOM	1084	H	VAL	271	38.202	1.882	36.287	1.00	0.00	RX0	H
ATOM	1085	CA	VAL	271	36.591	3.043	37.171	1.00	0.00	RX0	C
ATOM	1086	CB	VAL	271	37.209	2.863	38.571	1.00	0.00	RX0	C
ATOM	1087	CG1	VAL	271	38.641	3.372	38.684	1.00	0.00	RX0	C
ATOM	1088	CG2	VAL	271	36.293	3.444	39.650	1.00	0.00	RX0	C
ATOM	1089	C	VAL	271	36.343	4.502	36.749	1.00	0.00	RX0	C
ATOM	1090	O	VAL	271	35.261	5.071	37.095	1.00	0.00	RX0	O
ATOM	1091	N	GLU	272	37.228	5.074	36.011	1.00	0.00	RX0	N
ATOM	1092	H	GLU	272	38.057	4.558	35.798	1.00	0.00	RX0	H
ATOM	1093	CA	GLU	272	37.141	6.453	35.460	1.00	0.00	RX0	C
ATOM	1094	CB	GLU	272	38.460	6.661	34.699	1.00	0.00	RX0	C
ATOM	1095	CG	GLU	272	39.788	6.765	35.491	1.00	0.00	RX0	C
ATOM	1096	CD	GLU	272	40.252	5.536	36.292	1.00	0.00	RX0	C

ATOM	1097	OE1	GLU	272	39.987	4.386	35.945	1.00	0.00	RX0	O
ATOM	1098	OE2	GLU	272	40.949	5.714	37.285	1.00	0.00	RX0	O
ATOM	1099	C	GLU	272	35.927	6.588	34.526	1.00	0.00	RX0	C
ATOM	1100	O	GLU	272	35.142	7.521	34.681	1.00	0.00	RX0	O
ATOM	1101	N	ILE	273	35.702	5.552	33.729	1.00	0.00	RX0	N
ATOM	1102	H	ILE	273	36.300	4.754	33.810	1.00	0.00	RX0	H
ATOM	1103	CA	ILE	273	34.533	5.484	32.820	1.00	0.00	RX0	C
ATOM	1104	CB	ILE	273	34.734	4.378	31.778	1.00	0.00	RX0	C
ATOM	1105	CG2	ILE	273	33.625	4.413	30.726	1.00	0.00	RX0	C
ATOM	1106	CG1	ILE	273	36.129	4.433	31.147	1.00	0.00	RX0	C
ATOM	1107	CD1	ILE	273	36.348	5.662	30.263	1.00	0.00	RX0	C
ATOM	1108	C	ILE	273	33.249	5.235	33.628	1.00	0.00	RX0	C
ATOM	1109	O	ILE	273	32.257	5.951	33.452	1.00	0.00	RX0	O
ATOM	1110	N	PHE	274	33.320	4.302	34.575	1.00	0.00	RX0	N
ATOM	1111	H	PHE	274	34.183	3.805	34.677	1.00	0.00	RX0	H
ATOM	1112	CA	PHE	274	32.191	3.976	35.472	1.00	0.00	RX0	C
ATOM	1113	CB	PHE	274	32.611	2.939	36.516	1.00	0.00	RX0	C
ATOM	1114	CG	PHE	274	32.278	1.537	36.071	1.00	0.00	RX0	C
ATOM	1115	CD1	PHE	274	32.556	1.118	34.776	1.00	0.00	RX0	C
ATOM	1116	CD2	PHE	274	31.688	0.661	36.975	1.00	0.00	RX0	C
ATOM	1117	CE1	PHE	274	32.252	-0.181	34.391	1.00	0.00	RX0	C
ATOM	1118	CE2	PHE	274	31.384	-0.639	36.590	1.00	0.00	RX0	C
ATOM	1119	CZ	PHE	274	31.675	-1.061	35.299	1.00	0.00	RX0	C
ATOM	1120	C	PHE	274	31.669	5.203	36.222	1.00	0.00	RX0	C
ATOM	1121	O	PHE	274	30.484	5.521	36.143	1.00	0.00	RX0	O
ATOM	1122	N	ASP	275	32.607	5.983	36.757	1.00	0.00	RX0	N
ATOM	1123	H	ASP	275	33.566	5.702	36.757	1.00	0.00	RX0	H
ATOM	1124	CA	ASP	275	32.273	7.222	37.483	1.00	0.00	RX0	C
ATOM	1125	CB	ASP	275	33.560	7.869	37.990	1.00	0.00	RX0	C
ATOM	1126	CG	ASP	275	33.998	7.311	39.325	1.00	0.00	RX0	C
ATOM	1127	OD1	ASP	275	33.175	7.185	40.225	1.00	0.00	RX0	O
ATOM	1128	OD2	ASP	275	35.185	7.054	39.504	1.00	0.00	RX0	O
ATOM	1129	C	ASP	275	31.555	8.270	36.629	1.00	0.00	RX0	C
ATOM	1130	O	ASP	275	30.604	8.890	37.102	1.00	0.00	RX0	O
ATOM	1131	N	MET	276	31.921	8.336	35.353	1.00	0.00	RX0	N
ATOM	1132	H	MET	276	32.656	7.734	35.033	1.00	0.00	RX0	H
ATOM	1133	CA	MET	276	31.257	9.254	34.408	1.00	0.00	RX0	C
ATOM	1134	CB	MET	276	32.115	9.443	33.158	1.00	0.00	RX0	C
ATOM	1135	CG	MET	276	33.493	10.023	33.483	1.00	0.00	RX0	C
ATOM	1136	SD	MET	276	34.428	10.478	32.014	1.00	0.00	RX0	S
ATOM	1137	CE	MET	276	34.358	8.890	31.176	1.00	0.00	RX0	C
ATOM	1138	C	MET	276	29.833	8.798	34.061	1.00	0.00	RX0	C
ATOM	1139	O	MET	276	28.893	9.589	34.158	1.00	0.00	RX0	O
ATOM	1140	N	LEU	277	29.673	7.488	33.891	1.00	0.00	RX0	N
ATOM	1141	H	LEU	277	30.490	6.905	33.934	1.00	0.00	RX0	H
ATOM	1142	CA	LEU	277	28.362	6.866	33.606	1.00	0.00	RX0	C
ATOM	1143	CB	LEU	277	28.562	5.402	33.224	1.00	0.00	RX0	C
ATOM	1144	CG	LEU	277	29.434	5.224	31.982	1.00	0.00	RX0	C
ATOM	1145	CD1	LEU	277	29.924	3.785	31.831	1.00	0.00	RX0	C
ATOM	1146	CD2	LEU	277	28.736	5.730	30.722	1.00	0.00	RX0	C
ATOM	1147	C	LEU	277	27.393	6.992	34.787	1.00	0.00	RX0	C
ATOM	1148	O	LEU	277	26.257	7.447	34.627	1.00	0.00	RX0	O
ATOM	1149	N	LEU	278	27.939	6.795	35.983	1.00	0.00	RX0	N
ATOM	1150	H	LEU	278	28.895	6.498	36.015	1.00	0.00	RX0	H
ATOM	1151	CA	LEU	278	27.196	6.926	37.250	1.00	0.00	RX0	C
ATOM	1152	CB	LEU	278	28.068	6.474	38.420	1.00	0.00	RX0	C
ATOM	1153	CG	LEU	278	28.225	4.958	38.472	1.00	0.00	RX0	C
ATOM	1154	CD1	LEU	278	29.276	4.514	39.490	1.00	0.00	RX0	C
ATOM	1155	CD2	LEU	278	26.875	4.291	38.711	1.00	0.00	RX0	C
ATOM	1156	C	LEU	278	26.716	8.359	37.508	1.00	0.00	RX0	C
ATOM	1157	O	LEU	278	25.554	8.575	37.840	1.00	0.00	RX0	O

ATOM	1158	N	ALA	279	27.582	9.317	37.175	1.00	0.00	RX0	N
ATOM	1159	H	ALA	279	28.504	9.048	36.884	1.00	0.00	RX0	H
ATOM	1160	CA	ALA	279	27.275	10.754	37.296	1.00	0.00	RX0	C
ATOM	1161	CB	ALA	279	28.517	11.573	37.005	1.00	0.00	RX0	C
ATOM	1162	C	ALA	279	26.182	11.196	36.310	1.00	0.00	RX0	C
ATOM	1163	O	ALA	279	25.263	11.922	36.684	1.00	0.00	RX0	O
ATOM	1164	N	THR	280	26.210	10.618	35.109	1.00	0.00	RX0	N
ATOM	1165	H	THR	280	26.966	10.007	34.873	1.00	0.00	RX0	H
ATOM	1166	CA	THR	280	25.203	10.899	34.059	1.00	0.00	RX0	C
ATOM	1167	CB	THR	280	25.745	10.369	32.738	1.00	0.00	RX0	C
ATOM	1168	OG1	THR	280	27.064	10.894	32.536	1.00	0.00	RX0	O
ATOM	1169	HG1	THR	280	27.666	10.406	33.087	1.00	0.00	RX0	H
ATOM	1170	CG2	THR	280	24.834	10.734	31.564	1.00	0.00	RX0	C
ATOM	1171	C	THR	280	23.835	10.327	34.462	1.00	0.00	RX0	C
ATOM	1172	O	THR	280	22.822	11.023	34.397	1.00	0.00	RX0	O
ATOM	1173	N	SER	281	23.868	9.112	35.003	1.00	0.00	RX0	N
ATOM	1174	H	SER	281	24.739	8.619	35.046	1.00	0.00	RX0	H
ATOM	1175	CA	SER	281	22.669	8.413	35.507	1.00	0.00	RX0	C
ATOM	1176	CB	SER	281	23.146	6.984	35.864	1.00	0.00	RX0	C
ATOM	1177	OG	SER	281	22.394	6.341	36.913	1.00	0.00	RX0	O
ATOM	1178	HG	SER	281	22.606	5.413	36.831	1.00	0.00	RX0	H
ATOM	1179	C	SER	281	22.019	9.180	36.675	1.00	0.00	RX0	C
ATOM	1180	O	SER	281	20.814	9.399	36.693	1.00	0.00	RX0	O
ATOM	1181	N	SER	282	22.889	9.788	37.491	1.00	0.00	RX0	N
ATOM	1182	H	SER	282	23.868	9.621	37.371	1.00	0.00	RX0	H
ATOM	1183	CA	SER	282	22.489	10.660	38.613	1.00	0.00	RX0	C
ATOM	1184	CB	SER	282	23.673	10.725	39.558	1.00	0.00	RX0	C
ATOM	1185	OG	SER	282	23.941	9.348	39.880	1.00	0.00	RX0	O
ATOM	1186	HG	SER	282	24.704	9.111	39.353	1.00	0.00	RX0	H
ATOM	1187	C	SER	282	21.828	11.963	38.130	1.00	0.00	RX0	C
ATOM	1188	O	SER	282	20.788	12.371	38.639	1.00	0.00	RX0	O
ATOM	1189	N	ARG	283	22.365	12.516	37.039	1.00	0.00	RX0	N
ATOM	1190	H	ARG	283	23.191	12.115	36.635	1.00	0.00	RX0	H
ATOM	1191	CA	ARG	283	21.822	13.735	36.412	1.00	0.00	RX0	C
ATOM	1192	CB	ARG	283	22.796	14.199	35.338	1.00	0.00	RX0	C
ATOM	1193	CG	ARG	283	22.189	15.088	34.255	1.00	0.00	RX0	C
ATOM	1194	CD	ARG	283	21.690	16.456	34.724	1.00	0.00	RX0	C
ATOM	1195	NE	ARG	283	21.964	17.404	33.653	1.00	0.00	RX0	N
ATOM	1196	HE	ARG	283	22.686	17.135	32.999	1.00	0.00	RX0	H
ATOM	1197	CZ	ARG	283	21.439	18.654	33.583	1.00	0.00	RX0	C
ATOM	1198	NH1	ARG	283	20.527	19.051	34.493	1.00	0.00	RX0	N
ATOM	1199	HH11	ARG	283	20.087	19.959	34.453	1.00	0.00	RX0	H
ATOM	1200	HH12	ARG	283	20.249	18.453	35.246	1.00	0.00	RX0	H
ATOM	1201	NH2	ARG	283	21.862	19.460	32.590	1.00	0.00	RX0	N
ATOM	1202	HH21	ARG	283	21.510	20.383	32.436	1.00	0.00	RX0	H
ATOM	1203	HH22	ARG	283	22.600	19.127	31.980	1.00	0.00	RX0	H
ATOM	1204	C	ARG	283	20.432	13.477	35.812	1.00	0.00	RX0	C
ATOM	1205	O	ARG	283	19.498	14.246	36.035	1.00	0.00	RX0	O
ATOM	1206	N	PHE	284	20.293	12.325	35.169	1.00	0.00	RX0	N
ATOM	1207	H	PHE	284	21.101	11.747	35.034	1.00	0.00	RX0	H
ATOM	1208	CA	PHE	284	19.003	11.899	34.606	1.00	0.00	RX0	C
ATOM	1209	CB	PHE	284	19.203	10.640	33.772	1.00	0.00	RX0	C
ATOM	1210	CG	PHE	284	19.670	11.011	32.387	1.00	0.00	RX0	C
ATOM	1211	CD1	PHE	284	19.162	12.146	31.768	1.00	0.00	RX0	C
ATOM	1212	CD2	PHE	284	20.585	10.205	31.721	1.00	0.00	RX0	C
ATOM	1213	CE1	PHE	284	19.534	12.450	30.464	1.00	0.00	RX0	C
ATOM	1214	CE2	PHE	284	20.961	10.515	30.419	1.00	0.00	RX0	C
ATOM	1215	CZ	PHE	284	20.420	11.624	29.783	1.00	0.00	RX0	C
ATOM	1216	C	PHE	284	17.921	11.680	35.654	1.00	0.00	RX0	C
ATOM	1217	O	PHE	284	16.817	12.216	35.524	1.00	0.00	RX0	O
ATOM	1218	N	ARG	285	18.348	11.087	36.760	1.00	0.00	RX0	N

ATOM	1219	H	ARG	285	19.281	10.722	36.764	1.00	0.00	RX0	H
ATOM	1220	CA	ARG	285	17.485	10.841	37.923	1.00	0.00	RX0	C
ATOM	1221	CB	ARG	285	18.332	10.060	38.927	1.00	0.00	RX0	C
ATOM	1222	CG	ARG	285	17.761	9.789	40.320	1.00	0.00	RX0	C
ATOM	1223	CD	ARG	285	18.785	9.029	41.174	1.00	0.00	RX0	C
ATOM	1224	NE	ARG	285	19.205	7.822	40.466	1.00	0.00	RX0	N
ATOM	1225	HE	ARG	285	18.451	7.223	40.165	1.00	0.00	RX0	H
ATOM	1226	CZ	ARG	285	20.497	7.650	40.055	1.00	0.00	RX0	C
ATOM	1227	NH1	ARG	285	21.461	8.430	40.588	1.00	0.00	RX0	N
ATOM	1228	HH11	ARG	285	22.426	8.427	40.281	1.00	0.00	RX0	H
ATOM	1229	HH12	ARG	285	21.249	9.080	41.323	1.00	0.00	RX0	H
ATOM	1230	NH2	ARG	285	20.764	6.718	39.115	1.00	0.00	RX0	N
ATOM	1231	HH21	ARG	285	21.653	6.589	38.652	1.00	0.00	RX0	H
ATOM	1232	HH22	ARG	285	20.024	6.107	38.808	1.00	0.00	RX0	H
ATOM	1233	C	ARG	285	17.003	12.164	38.534	1.00	0.00	RX0	C
ATOM	1234	O	ARG	285	15.822	12.321	38.816	1.00	0.00	RX0	O
ATOM	1235	N	MET	286	17.922	13.130	38.591	1.00	0.00	RX0	N
ATOM	1236	H	MET	286	18.854	12.929	38.285	1.00	0.00	RX0	H
ATOM	1237	CA	MET	286	17.643	14.467	39.144	1.00	0.00	RX0	C
ATOM	1238	CB	MET	286	18.955	15.221	39.360	1.00	0.00	RX0	C
ATOM	1239	CG	MET	286	18.776	16.547	40.100	1.00	0.00	RX0	C
ATOM	1240	SD	MET	286	20.352	17.354	40.413	1.00	0.00	RX0	S
ATOM	1241	CE	MET	286	19.739	18.772	41.336	1.00	0.00	RX0	C
ATOM	1242	C	MET	286	16.681	15.257	38.243	1.00	0.00	RX0	C
ATOM	1243	O	MET	286	15.799	15.962	38.735	1.00	0.00	RX0	O
ATOM	1244	N	MET	287	16.911	15.161	36.942	1.00	0.00	RX0	N
ATOM	1245	H	MET	287	17.642	14.551	36.631	1.00	0.00	RX0	H
ATOM	1246	CA	MET	287	16.049	15.814	35.941	1.00	0.00	RX0	C
ATOM	1247	CB	MET	287	16.683	15.860	34.555	1.00	0.00	RX0	C
ATOM	1248	CG	MET	287	17.835	16.855	34.497	1.00	0.00	RX0	C
ATOM	1249	SD	MET	287	18.235	17.299	32.803	1.00	0.00	RX0	S
ATOM	1250	CE	MET	287	16.617	17.959	32.370	1.00	0.00	RX0	C
ATOM	1251	C	MET	287	14.674	15.156	35.849	1.00	0.00	RX0	C
ATOM	1252	O	MET	287	13.755	15.729	35.264	1.00	0.00	RX0	O
ATOM	1253	N	ASN	288	14.583	13.920	36.346	1.00	0.00	RX0	N
ATOM	1254	H	ASN	288	15.394	13.492	36.747	1.00	0.00	RX0	H
ATOM	1255	CA	ASN	288	13.391	13.066	36.242	1.00	0.00	RX0	C
ATOM	1256	CB	ASN	288	12.161	13.612	36.968	1.00	0.00	RX0	C
ATOM	1257	CG	ASN	288	11.009	12.652	36.725	1.00	0.00	RX0	C
ATOM	1258	OD1	ASN	288	11.180	11.440	36.649	1.00	0.00	RX0	O
ATOM	1259	ND2	ASN	288	9.819	13.264	36.583	1.00	0.00	RX0	N
ATOM	1260	HD21	ASN	288	9.739	14.256	36.672	1.00	0.00	RX0	H
ATOM	1261	HD22	ASN	288	8.994	12.742	36.364	1.00	0.00	RX0	H
ATOM	1262	C	ASN	288	13.076	12.836	34.753	1.00	0.00	RX0	C
ATOM	1263	O	ASN	288	11.986	13.121	34.256	1.00	0.00	RX0	O
ATOM	1264	N	LEU	289	14.125	12.439	34.028	1.00	0.00	RX0	N
ATOM	1265	H	LEU	289	14.957	12.176	34.521	1.00	0.00	RX0	H
ATOM	1266	CA	LEU	289	14.030	12.183	32.583	1.00	0.00	RX0	C
ATOM	1267	CB	LEU	289	15.371	11.626	32.097	1.00	0.00	RX0	C
ATOM	1268	CG	LEU	289	15.404	11.209	30.624	1.00	0.00	RX0	C
ATOM	1269	CD1	LEU	289	15.553	12.412	29.698	1.00	0.00	RX0	C
ATOM	1270	CD2	LEU	289	16.474	10.155	30.342	1.00	0.00	RX0	C
ATOM	1271	C	LEU	289	12.926	11.152	32.321	1.00	0.00	RX0	C
ATOM	1272	O	LEU	289	12.814	10.137	33.014	1.00	0.00	RX0	O
ATOM	1273	N	GLN	290	12.165	11.420	31.281	1.00	0.00	RX0	N
ATOM	1274	H	GLN	290	12.394	12.178	30.664	1.00	0.00	RX0	H
ATOM	1275	CA	GLN	290	11.011	10.584	30.916	1.00	0.00	RX0	C
ATOM	1276	CB	GLN	290	9.778	11.452	30.673	1.00	0.00	RX0	C
ATOM	1277	CG	GLN	290	9.411	12.301	31.899	1.00	0.00	RX0	C
ATOM	1278	CD	GLN	290	8.986	11.428	33.072	1.00	0.00	RX0	C
ATOM	1279	OE1	GLN	290	7.827	11.055	33.214	1.00	0.00	RX0	O

ATOM	1280	NE2	GLN	290	9.975	11.155	33.940	1.00	0.00	RX0	N
ATOM	1281	HE21	GLN	290	10.899	11.513	33.798	1.00	0.00	RX0	H
ATOM	1282	HE22	GLN	290	9.881	10.606	34.773	1.00	0.00	RX0	H
ATOM	1283	C	GLN	290	11.379	9.701	29.727	1.00	0.00	RX0	C
ATOM	1284	O	GLN	290	12.115	10.141	28.832	1.00	0.00	RX0	O
ATOM	1285	N	GLY	291	10.739	8.533	29.672	1.00	0.00	RX0	N
ATOM	1286	H	GLY	291	10.027	8.353	30.351	1.00	0.00	RX0	H
ATOM	1287	CA	GLY	291	10.952	7.542	28.589	1.00	0.00	RX0	C
ATOM	1288	C	GLY	291	10.769	8.134	27.181	1.00	0.00	RX0	C
ATOM	1289	O	GLY	291	11.559	7.865	26.269	1.00	0.00	RX0	O
ATOM	1290	N	GLU	292	9.854	9.086	27.078	1.00	0.00	RX0	N
ATOM	1291	H	GLU	292	9.255	9.248	27.866	1.00	0.00	RX0	H
ATOM	1292	CA	GLU	292	9.541	9.789	25.813	1.00	0.00	RX0	C
ATOM	1293	CB	GLU	292	8.215	10.552	25.943	1.00	0.00	RX0	C
ATOM	1294	CG	GLU	292	6.976	9.706	26.280	1.00	0.00	RX0	C
ATOM	1295	CD	GLU	292	7.097	9.118	27.674	1.00	0.00	RX0	C
ATOM	1296	OE1	GLU	292	7.474	9.847	28.589	1.00	0.00	RX0	O
ATOM	1297	OE2	GLU	292	6.914	7.914	27.826	1.00	0.00	RX0	O
ATOM	1298	C	GLU	292	10.671	10.744	25.400	1.00	0.00	RX0	C
ATOM	1299	O	GLU	292	11.065	10.802	24.241	1.00	0.00	RX0	O
ATOM	1300	N	GLU	293	11.241	11.417	26.395	1.00	0.00	RX0	N
ATOM	1301	H	GLU	293	10.951	11.211	27.330	1.00	0.00	RX0	H
ATOM	1302	CA	GLU	293	12.389	12.320	26.184	1.00	0.00	RX0	C
ATOM	1303	CB	GLU	293	12.636	13.156	27.436	1.00	0.00	RX0	C
ATOM	1304	CG	GLU	293	11.406	13.908	27.934	1.00	0.00	RX0	C
ATOM	1305	CD	GLU	293	11.724	14.502	29.290	1.00	0.00	RX0	C
ATOM	1306	OE1	GLU	293	12.643	14.032	29.954	1.00	0.00	RX0	O
ATOM	1307	OE2	GLU	293	11.052	15.435	29.708	1.00	0.00	RX0	O
ATOM	1308	C	GLU	293	13.669	11.544	25.843	1.00	0.00	RX0	C
ATOM	1309	O	GLU	293	14.364	11.888	24.886	1.00	0.00	RX0	O
ATOM	1310	N	PHE	294	13.855	10.409	26.517	1.00	0.00	RX0	N
ATOM	1311	H	PHE	294	13.195	10.199	27.241	1.00	0.00	RX0	H
ATOM	1312	CA	PHE	294	14.999	9.503	26.300	1.00	0.00	RX0	C
ATOM	1313	CB	PHE	294	14.905	8.307	27.251	1.00	0.00	RX0	C
ATOM	1314	CG	PHE	294	15.869	7.228	26.816	1.00	0.00	RX0	C
ATOM	1315	CD1	PHE	294	17.240	7.455	26.841	1.00	0.00	RX0	C
ATOM	1316	CD2	PHE	294	15.377	6.005	26.373	1.00	0.00	RX0	C
ATOM	1317	CE1	PHE	294	18.114	6.470	26.397	1.00	0.00	RX0	C
ATOM	1318	CE2	PHE	294	16.251	5.020	25.931	1.00	0.00	RX0	C
ATOM	1319	CZ	PHE	294	17.620	5.256	25.934	1.00	0.00	RX0	C
ATOM	1320	C	PHE	294	15.115	9.012	24.847	1.00	0.00	RX0	C
ATOM	1321	O	PHE	294	16.186	9.132	24.238	1.00	0.00	RX0	O
ATOM	1322	N	VAL	295	14.000	8.581	24.280	1.00	0.00	RX0	N
ATOM	1323	H	VAL	295	13.158	8.569	24.828	1.00	0.00	RX0	H
ATOM	1324	CA	VAL	295	13.976	8.026	22.907	1.00	0.00	RX0	C
ATOM	1325	CB	VAL	295	12.686	7.251	22.616	1.00	0.00	RX0	C
ATOM	1326	CG1	VAL	295	12.587	6.063	23.571	1.00	0.00	RX0	C
ATOM	1327	CG2	VAL	295	11.430	8.119	22.661	1.00	0.00	RX0	C
ATOM	1328	C	VAL	295	14.286	9.097	21.847	1.00	0.00	RX0	C
ATOM	1329	O	VAL	295	14.999	8.845	20.884	1.00	0.00	RX0	O
ATOM	1330	N	CYS	296	13.866	10.330	22.156	1.00	0.00	RX0	N
ATOM	1331	H	CYS	296	13.306	10.480	22.974	1.00	0.00	RX0	H
ATOM	1332	CA	CYS	296	14.167	11.498	21.314	1.00	0.00	RX0	C
ATOM	1333	CB	CYS	296	13.264	12.653	21.730	1.00	0.00	RX0	C
ATOM	1334	SG	CYS	296	11.518	12.259	21.463	1.00	0.00	RX0	S
ATOM	1335	C	CYS	296	15.661	11.843	21.352	1.00	0.00	RX0	C
ATOM	1336	O	CYS	296	16.290	11.969	20.305	1.00	0.00	RX0	O
ATOM	1337	N	LEU	297	16.247	11.750	22.547	1.00	0.00	RX0	N
ATOM	1338	H	LEU	297	15.681	11.549	23.351	1.00	0.00	RX0	H
ATOM	1339	CA	LEU	297	17.681	12.031	22.752	1.00	0.00	RX0	C
ATOM	1340	CB	LEU	297	18.019	12.109	24.239	1.00	0.00	RX0	C

ATOM	1341	CG	LEU	297	17.349	13.281	24.951	1.00	0.00	RX0	C
ATOM	1342	CD1	LEU	297	17.646	13.258	26.450	1.00	0.00	RX0	C
ATOM	1343	CD2	LEU	297	17.713	14.623	24.314	1.00	0.00	RX0	C
ATOM	1344	C	LEU	297	18.589	11.009	22.068	1.00	0.00	RX0	C
ATOM	1345	O	LEU	297	19.526	11.385	21.359	1.00	0.00	RX0	O
ATOM	1346	N	LYS	298	18.173	9.750	22.123	1.00	0.00	RX0	N
ATOM	1347	H	LYS	298	17.359	9.545	22.671	1.00	0.00	RX0	H
ATOM	1348	CA	LYS	298	18.945	8.654	21.515	1.00	0.00	RX0	C
ATOM	1349	CB	LYS	298	18.432	7.320	22.046	1.00	0.00	RX0	C
ATOM	1350	CG	LYS	298	19.253	6.121	21.581	1.00	0.00	RX0	C
ATOM	1351	CD	LYS	298	18.616	4.834	22.088	1.00	0.00	RX0	C
ATOM	1352	CE	LYS	298	17.099	4.998	22.051	1.00	0.00	RX0	C
ATOM	1353	NZ	LYS	298	16.433	3.704	22.168	1.00	0.00	RX0	N
ATOM	1354	HZ1	LYS	298	15.445	3.857	22.481	1.00	0.00	RX0	H
ATOM	1355	HZ2	LYS	298	16.279	3.227	21.258	1.00	0.00	RX0	H
ATOM	1356	HZ3	LYS	298	16.807	3.049	22.876	1.00	0.00	RX0	H
ATOM	1357	C	LYS	298	18.925	8.733	19.978	1.00	0.00	RX0	C
ATOM	1358	O	LYS	298	19.964	8.557	19.332	1.00	0.00	RX0	O
ATOM	1359	N	SER	299	17.791	9.152	19.431	1.00	0.00	RX0	N
ATOM	1360	H	SER	299	16.962	9.262	19.980	1.00	0.00	RX0	H
ATOM	1361	CA	SER	299	17.645	9.383	17.977	1.00	0.00	RX0	C
ATOM	1362	CB	SER	299	16.155	9.359	17.705	1.00	0.00	RX0	C
ATOM	1363	OG	SER	299	15.665	8.202	18.390	1.00	0.00	RX0	O
ATOM	1364	HG	SER	299	15.119	8.509	19.107	1.00	0.00	RX0	H
ATOM	1365	C	SER	299	18.416	10.621	17.504	1.00	0.00	RX0	C
ATOM	1366	O	SER	299	19.051	10.583	16.444	1.00	0.00	RX0	O
ATOM	1367	N	ILE	300	18.478	11.644	18.354	1.00	0.00	RX0	N
ATOM	1368	H	ILE	300	17.952	11.595	19.206	1.00	0.00	RX0	H
ATOM	1369	CA	ILE	300	19.283	12.860	18.096	1.00	0.00	RX0	C
ATOM	1370	CB	ILE	300	19.082	13.934	19.169	1.00	0.00	RX0	C
ATOM	1371	CG2	ILE	300	20.126	15.045	19.048	1.00	0.00	RX0	C
ATOM	1372	CG1	ILE	300	17.678	14.519	19.098	1.00	0.00	RX0	C
ATOM	1373	CD1	ILE	300	17.421	15.535	20.210	1.00	0.00	RX0	C
ATOM	1374	C	ILE	300	20.773	12.490	18.013	1.00	0.00	RX0	C
ATOM	1375	O	ILE	300	21.456	12.924	17.087	1.00	0.00	RX0	O
ATOM	1376	N	ILE	301	21.227	11.630	18.921	1.00	0.00	RX0	N
ATOM	1377	H	ILE	301	20.604	11.321	19.644	1.00	0.00	RX0	H
ATOM	1378	CA	ILE	301	22.629	11.156	18.925	1.00	0.00	RX0	C
ATOM	1379	CB	ILE	301	22.879	10.198	20.085	1.00	0.00	RX0	C
ATOM	1380	CG2	ILE	301	24.187	9.442	19.884	1.00	0.00	RX0	C
ATOM	1381	CG1	ILE	301	22.866	10.946	21.416	1.00	0.00	RX0	C
ATOM	1382	CD1	ILE	301	23.237	10.031	22.581	1.00	0.00	RX0	C
ATOM	1383	C	ILE	301	22.948	10.474	17.584	1.00	0.00	RX0	C
ATOM	1384	O	ILE	301	23.927	10.823	16.926	1.00	0.00	RX0	O
ATOM	1385	N	LEU	302	22.047	9.582	17.175	1.00	0.00	RX0	N
ATOM	1386	H	LEU	302	21.274	9.368	17.776	1.00	0.00	RX0	H
ATOM	1387	CA	LEU	302	22.205	8.836	15.917	1.00	0.00	RX0	C
ATOM	1388	CB	LEU	302	20.995	7.927	15.705	1.00	0.00	RX0	C
ATOM	1389	CG	LEU	302	21.010	7.211	14.353	1.00	0.00	RX0	C
ATOM	1390	CD1	LEU	302	22.190	6.249	14.221	1.00	0.00	RX0	C
ATOM	1391	CD2	LEU	302	19.675	6.531	14.061	1.00	0.00	RX0	C
ATOM	1392	C	LEU	302	22.391	9.757	14.699	1.00	0.00	RX0	C
ATOM	1393	O	LEU	302	23.285	9.544	13.882	1.00	0.00	RX0	O
ATOM	1394	N	LEU	303	21.581	10.806	14.658	1.00	0.00	RX0	N
ATOM	1395	H	LEU	303	20.934	10.946	15.411	1.00	0.00	RX0	H
ATOM	1396	CA	LEU	303	21.546	11.719	13.502	1.00	0.00	RX0	C
ATOM	1397	CB	LEU	303	20.125	12.234	13.313	1.00	0.00	RX0	C
ATOM	1398	CG	LEU	303	19.157	11.071	13.098	1.00	0.00	RX0	C
ATOM	1399	CD1	LEU	303	17.704	11.500	13.274	1.00	0.00	RX0	C
ATOM	1400	CD2	LEU	303	19.401	10.362	11.764	1.00	0.00	RX0	C
ATOM	1401	C	LEU	303	22.576	12.849	13.543	1.00	0.00	RX0	C

ATOM	1402	O	LEU	303	23.073	13.268	12.494	1.00	0.00	RX0	O
ATOM	1403	N	ASN	304	22.971	13.240	14.745	1.00	0.00	RX0	N
ATOM	1404	H	ASN	304	22.645	12.730	15.542	1.00	0.00	RX0	H
ATOM	1405	CA	ASN	304	23.832	14.419	14.940	1.00	0.00	RX0	C
ATOM	1406	CB	ASN	304	23.519	15.268	16.187	1.00	0.00	RX0	C
ATOM	1407	CG	ASN	304	24.491	14.932	17.316	1.00	0.00	RX0	C
ATOM	1408	OD1	ASN	304	25.574	15.492	17.485	1.00	0.00	RX0	O
ATOM	1409	ND2	ASN	304	24.056	13.906	18.057	1.00	0.00	RX0	N
ATOM	1410	HD21	ASN	304	23.121	13.574	17.918	1.00	0.00	RX0	H
ATOM	1411	HD22	ASN	304	24.631	13.402	18.708	1.00	0.00	RX0	H
ATOM	1412	C	ASN	304	25.330	14.097	14.987	1.00	0.00	RX0	C
ATOM	1413	O	ASN	304	26.132	14.783	14.344	1.00	0.00	RX0	O
ATOM	1414	N	SER	305	25.693	13.015	15.656	1.00	0.00	RX0	N
ATOM	1415	H	SER	305	25.042	12.410	16.124	1.00	0.00	RX0	H
ATOM	1416	CA	SER	305	27.104	12.765	16.012	1.00	0.00	RX0	C
ATOM	1417	CB	SER	305	27.098	11.540	16.918	1.00	0.00	RX0	C
ATOM	1418	OG	SER	305	26.248	11.839	18.039	1.00	0.00	RX0	O
ATOM	1419	HG	SER	305	26.534	11.228	18.730	1.00	0.00	RX0	H
ATOM	1420	C	SER	305	28.103	12.695	14.846	1.00	0.00	RX0	C
ATOM	1421	O	SER	305	29.198	13.228	14.946	1.00	0.00	RX0	O
ATOM	1422	N	GLY	306	27.634	12.177	13.693	1.00	0.00	RX0	N
ATOM	1423	H	GLY	306	26.686	11.864	13.638	1.00	0.00	RX0	H
ATOM	1424	CA	GLY	306	28.500	12.079	12.501	1.00	0.00	RX0	C
ATOM	1425	C	GLY	306	28.091	12.961	11.315	1.00	0.00	RX0	C
ATOM	1426	O	GLY	306	28.756	12.897	10.274	1.00	0.00	RX0	O
ATOM	1427	N	VAL	307	27.242	13.954	11.536	1.00	0.00	RX0	N
ATOM	1428	H	VAL	307	26.879	14.103	12.459	1.00	0.00	RX0	H
ATOM	1429	CA	VAL	307	26.760	14.815	10.435	1.00	0.00	RX0	C
ATOM	1430	CB	VAL	307	25.386	15.437	10.733	1.00	0.00	RX0	C
ATOM	1431	CG1	VAL	307	25.436	16.501	11.823	1.00	0.00	RX0	C
ATOM	1432	CG2	VAL	307	24.735	15.959	9.452	1.00	0.00	RX0	C
ATOM	1433	C	VAL	307	27.809	15.841	9.948	1.00	0.00	RX0	C
ATOM	1434	O	VAL	307	27.790	16.280	8.811	1.00	0.00	RX0	O
ATOM	1435	N	TYR	308	28.719	16.199	10.859	1.00	0.00	RX0	N
ATOM	1436	H	TYR	308	28.741	15.716	11.733	1.00	0.00	RX0	H
ATOM	1437	CA	TYR	308	29.761	17.211	10.584	1.00	0.00	RX0	C
ATOM	1438	CB	TYR	308	30.019	18.046	11.839	1.00	0.00	RX0	C
ATOM	1439	CG	TYR	308	28.717	18.720	12.211	1.00	0.00	RX0	C
ATOM	1440	CD1	TYR	308	27.974	19.369	11.231	1.00	0.00	RX0	C
ATOM	1441	CE1	TYR	308	26.745	19.938	11.545	1.00	0.00	RX0	C
ATOM	1442	CD2	TYR	308	28.250	18.680	13.519	1.00	0.00	RX0	C
ATOM	1443	CE2	TYR	308	27.024	19.256	13.836	1.00	0.00	RX0	C
ATOM	1444	CZ	TYR	308	26.261	19.867	12.846	1.00	0.00	RX0	C
ATOM	1445	OH	TYR	308	25.023	20.392	13.156	1.00	0.00	RX0	O
ATOM	1446	HH	TYR	308	24.466	20.405	12.382	1.00	0.00	RX0	H
ATOM	1447	C	TYR	308	31.032	16.660	9.940	1.00	0.00	RX0	C
ATOM	1448	O	TYR	308	31.912	17.411	9.537	1.00	0.00	RX0	O
ATOM	1449	N	THR	309	31.091	15.334	9.861	1.00	0.00	RX0	N
ATOM	1450	H	THR	309	30.345	14.755	10.187	1.00	0.00	RX0	H
ATOM	1451	CA	THR	309	32.303	14.643	9.386	1.00	0.00	RX0	C
ATOM	1452	CB	THR	309	32.588	13.748	10.570	1.00	0.00	RX0	C
ATOM	1453	OG1	THR	309	31.336	13.579	11.262	1.00	0.00	RX0	O
ATOM	1454	HG1	THR	309	30.886	12.864	10.809	1.00	0.00	RX0	H
ATOM	1455	CG2	THR	309	33.625	14.355	11.518	1.00	0.00	RX0	C
ATOM	1456	C	THR	309	32.183	13.866	8.071	1.00	0.00	RX0	C
ATOM	1457	O	THR	309	33.137	13.195	7.681	1.00	0.00	RX0	O
ATOM	1458	N	PHE	310	31.041	13.953	7.382	1.00	0.00	RX0	N
ATOM	1459	H	PHE	310	30.301	14.511	7.751	1.00	0.00	RX0	H
ATOM	1460	CA	PHE	310	30.972	13.467	5.988	1.00	0.00	RX0	C
ATOM	1461	CB	PHE	310	29.599	13.731	5.369	1.00	0.00	RX0	C
ATOM	1462	CG	PHE	310	28.524	12.923	6.052	1.00	0.00	RX0	C

ATOM	1463	CD1	PHE	310	28.406	11.565	5.785	1.00	0.00	RX0	C
ATOM	1464	CD2	PHE	310	27.644	13.539	6.933	1.00	0.00	RX0	C
ATOM	1465	CE1	PHE	310	27.402	10.825	6.397	1.00	0.00	RX0	C
ATOM	1466	CE2	PHE	310	26.640	12.797	7.543	1.00	0.00	RX0	C
ATOM	1467	CZ	PHE	310	26.518	11.439	7.275	1.00	0.00	RX0	C
ATOM	1468	C	PHE	310	32.019	14.245	5.180	1.00	0.00	RX0	C
ATOM	1469	O	PHE	310	32.102	15.468	5.301	1.00	0.00	RX0	O
ATOM	1470	N	LEU	311	32.854	13.508	4.462	1.00	0.00	RX0	N
ATOM	1471	H	LEU	311	32.721	12.519	4.489	1.00	0.00	RX0	H
ATOM	1472	CA	LEU	311	33.988	14.087	3.701	1.00	0.00	RX0	C
ATOM	1473	CB	LEU	311	34.699	13.043	2.847	1.00	0.00	RX0	C
ATOM	1474	CG	LEU	311	35.054	11.737	3.537	1.00	0.00	RX0	C
ATOM	1475	CD1	LEU	311	35.444	10.703	2.483	1.00	0.00	RX0	C
ATOM	1476	CD2	LEU	311	36.107	11.920	4.631	1.00	0.00	RX0	C
ATOM	1477	C	LEU	311	33.468	15.166	2.739	1.00	0.00	RX0	C
ATOM	1478	O	LEU	311	33.554	16.351	2.969	1.00	0.00	RX0	O
ATOM	1479	N	SER	312	32.784	14.619	1.713	1.00	0.00	RX0	N
ATOM	1480	H	SER	312	32.593	13.640	1.725	1.00	0.00	RX0	H
ATOM	1481	CA	SER	312	32.345	15.333	0.526	1.00	0.00	RX0	C
ATOM	1482	CB	SER	312	31.802	14.211	-0.339	1.00	0.00	RX0	C
ATOM	1483	OG	SER	312	32.570	13.050	0.008	1.00	0.00	RX0	O
ATOM	1484	HG	SER	312	32.579	12.473	-0.746	1.00	0.00	RX0	H
ATOM	1485	C	SER	312	31.423	16.517	0.816	1.00	0.00	RX0	C
ATOM	1486	O	SER	312	31.042	16.847	1.948	1.00	0.00	RX0	O
ATOM	1487	N	SER	313	30.837	16.923	-0.276	1.00	0.00	RX0	N
ATOM	1488	H	SER	313	31.202	16.610	-1.154	1.00	0.00	RX0	H
ATOM	1489	CA	SER	313	29.838	17.983	-0.455	1.00	0.00	RX0	C
ATOM	1490	CB	SER	313	30.333	19.323	0.095	1.00	0.00	RX0	C
ATOM	1491	OG	SER	313	30.307	19.221	1.535	1.00	0.00	RX0	O
ATOM	1492	HG	SER	313	31.127	18.788	1.771	1.00	0.00	RX0	H
ATOM	1493	C	SER	313	29.353	17.880	-1.909	1.00	0.00	RX0	C
ATOM	1494	O	SER	313	29.168	18.836	-2.628	1.00	0.00	RX0	O
ATOM	1495	N	THR	314	29.278	16.602	-2.358	1.00	0.00	RX0	N
ATOM	1496	H	THR	314	29.449	15.828	-1.754	1.00	0.00	RX0	H
ATOM	1497	CA	THR	314	28.609	16.250	-3.614	1.00	0.00	RX0	C
ATOM	1498	CB	THR	314	28.738	14.740	-3.676	1.00	0.00	RX0	C
ATOM	1499	OG1	THR	314	29.860	14.368	-2.862	1.00	0.00	RX0	O
ATOM	1500	HG1	THR	314	30.100	13.490	-3.148	1.00	0.00	RX0	H
ATOM	1501	CG2	THR	314	28.873	14.207	-5.103	1.00	0.00	RX0	C
ATOM	1502	C	THR	314	27.167	16.747	-3.490	1.00	0.00	RX0	C
ATOM	1503	O	THR	314	26.675	16.978	-2.368	1.00	0.00	RX0	O
ATOM	1504	N	LEU	315	26.451	16.801	-4.589	1.00	0.00	RX0	N
ATOM	1505	H	LEU	315	26.870	16.630	-5.479	1.00	0.00	RX0	H
ATOM	1506	CA	LEU	315	25.050	17.256	-4.537	1.00	0.00	RX0	C
ATOM	1507	CB	LEU	315	24.462	17.340	-5.945	1.00	0.00	RX0	C
ATOM	1508	CG	LEU	315	23.053	17.934	-5.951	1.00	0.00	RX0	C
ATOM	1509	CD1	LEU	315	23.023	19.341	-5.349	1.00	0.00	RX0	C
ATOM	1510	CD2	LEU	315	22.422	17.888	-7.343	1.00	0.00	RX0	C
ATOM	1511	C	LEU	315	24.190	16.343	-3.639	1.00	0.00	RX0	C
ATOM	1512	O	LEU	315	23.484	16.800	-2.757	1.00	0.00	RX0	O
ATOM	1513	N	LYS	316	24.524	15.049	-3.730	1.00	0.00	RX0	N
ATOM	1514	H	LYS	316	25.052	14.787	-4.530	1.00	0.00	RX0	H
ATOM	1515	CA	LYS	316	23.912	13.990	-2.918	1.00	0.00	RX0	C
ATOM	1516	CB	LYS	316	24.490	12.681	-3.440	1.00	0.00	RX0	C
ATOM	1517	CG	LYS	316	23.973	11.393	-2.814	1.00	0.00	RX0	C
ATOM	1518	CD	LYS	316	24.481	10.233	-3.666	1.00	0.00	RX0	C
ATOM	1519	CE	LYS	316	23.906	8.877	-3.280	1.00	0.00	RX0	C
ATOM	1520	NZ	LYS	316	24.596	8.318	-2.114	1.00	0.00	RX0	N
ATOM	1521	HZ1	LYS	316	24.488	7.283	-2.168	1.00	0.00	RX0	H
ATOM	1522	HZ2	LYS	316	25.617	8.523	-2.116	1.00	0.00	RX0	H
ATOM	1523	HZ3	LYS	316	24.133	8.635	-1.228	1.00	0.00	RX0	H

ATOM	1524	C	LYS	316	24.213	14.187	-1.422	1.00	0.00	RX0	C
ATOM	1525	O	LYS	316	23.297	14.194	-0.611	1.00	0.00	RX0	O
ATOM	1526	N	SER	317	25.467	14.540	-1.122	1.00	0.00	RX0	N
ATOM	1527	H	SER	317	26.084	14.743	-1.876	1.00	0.00	RX0	H
ATOM	1528	CA	SER	317	25.934	14.787	0.260	1.00	0.00	RX0	C
ATOM	1529	CB	SER	317	27.466	14.861	0.212	1.00	0.00	RX0	C
ATOM	1530	OG	SER	317	28.075	14.543	1.469	1.00	0.00	RX0	O
ATOM	1531	HG	SER	317	27.692	13.710	1.746	1.00	0.00	RX0	H
ATOM	1532	C	SER	317	25.242	16.001	0.898	1.00	0.00	RX0	C
ATOM	1533	O	SER	317	24.716	15.915	2.007	1.00	0.00	RX0	O
ATOM	1534	N	LEU	318	25.067	17.043	0.088	1.00	0.00	RX0	N
ATOM	1535	H	LEU	318	25.331	16.972	-0.876	1.00	0.00	RX0	H
ATOM	1536	CA	LEU	318	24.405	18.288	0.525	1.00	0.00	RX0	C
ATOM	1537	CB	LEU	318	24.579	19.374	-0.532	1.00	0.00	RX0	C
ATOM	1538	CG	LEU	318	26.044	19.771	-0.694	1.00	0.00	RX0	C
ATOM	1539	CD1	LEU	318	26.253	20.715	-1.878	1.00	0.00	RX0	C
ATOM	1540	CD2	LEU	318	26.617	20.329	0.609	1.00	0.00	RX0	C
ATOM	1541	C	LEU	318	22.918	18.061	0.823	1.00	0.00	RX0	C
ATOM	1542	O	LEU	318	22.412	18.453	1.877	1.00	0.00	RX0	O
ATOM	1543	N	GLU	319	22.299	17.244	-0.024	1.00	0.00	RX0	N
ATOM	1544	H	GLU	319	22.760	16.923	-0.856	1.00	0.00	RX0	H
ATOM	1545	CA	GLU	319	20.900	16.808	0.157	1.00	0.00	RX0	C
ATOM	1546	CB	GLU	319	20.423	16.038	-1.079	1.00	0.00	RX0	C
ATOM	1547	CG	GLU	319	20.412	16.866	-2.367	1.00	0.00	RX0	C
ATOM	1548	CD	GLU	319	20.163	15.955	-3.556	1.00	0.00	RX0	C
ATOM	1549	OE1	GLU	319	21.117	15.624	-4.264	1.00	0.00	RX0	O
ATOM	1550	OE2	GLU	319	19.011	15.586	-3.778	1.00	0.00	RX0	O
ATOM	1551	C	GLU	319	20.725	15.924	1.402	1.00	0.00	RX0	C
ATOM	1552	O	GLU	319	19.808	16.153	2.196	1.00	0.00	RX0	O
ATOM	1553	N	GLU	320	21.706	15.058	1.645	1.00	0.00	RX0	N
ATOM	1554	H	GLU	320	22.387	14.912	0.926	1.00	0.00	RX0	H
ATOM	1555	CA	GLU	320	21.731	14.149	2.812	1.00	0.00	RX0	C
ATOM	1556	CB	GLU	320	22.878	13.108	2.809	1.00	0.00	RX0	C
ATOM	1557	CG	GLU	320	23.173	12.192	1.597	1.00	0.00	RX0	C
ATOM	1558	CD	GLU	320	22.269	10.980	1.382	1.00	0.00	RX0	C
ATOM	1559	OE1	GLU	320	21.852	10.338	2.343	1.00	0.00	RX0	O
ATOM	1560	OE2	GLU	320	22.045	10.623	0.224	1.00	0.00	RX0	O
ATOM	1561	C	GLU	320	21.765	14.931	4.131	1.00	0.00	RX0	C
ATOM	1562	O	GLU	320	20.881	14.754	4.973	1.00	0.00	RX0	O
ATOM	1563	N	LYS	321	22.647	15.926	4.188	1.00	0.00	RX0	N
ATOM	1564	H	LYS	321	23.231	16.050	3.382	1.00	0.00	RX0	H
ATOM	1565	CA	LYS	321	22.801	16.771	5.391	1.00	0.00	RX0	C
ATOM	1566	CB	LYS	321	24.081	17.632	5.232	1.00	0.00	RX0	C
ATOM	1567	CG	LYS	321	25.363	16.833	4.883	1.00	0.00	RX0	C
ATOM	1568	CD	LYS	321	26.627	17.640	4.479	1.00	0.00	RX0	C
ATOM	1569	CE	LYS	321	27.754	16.756	3.886	1.00	0.00	RX0	C
ATOM	1570	NZ	LYS	321	29.004	17.482	3.552	1.00	0.00	RX0	N
ATOM	1571	HZ1	LYS	321	29.685	16.842	3.081	1.00	0.00	RX0	H
ATOM	1572	HZ2	LYS	321	28.863	18.279	2.896	1.00	0.00	RX0	H
ATOM	1573	HZ3	LYS	321	29.469	17.827	4.414	1.00	0.00	RX0	H
ATOM	1574	C	LYS	321	21.549	17.612	5.656	1.00	0.00	RX0	C
ATOM	1575	O	LYS	321	21.102	17.724	6.798	1.00	0.00	RX0	O
ATOM	1576	N	ASP	322	20.935	18.099	4.575	1.00	0.00	RX0	N
ATOM	1577	H	ASP	322	21.316	17.940	3.660	1.00	0.00	RX0	H
ATOM	1578	CA	ASP	322	19.715	18.915	4.684	1.00	0.00	RX0	C
ATOM	1579	CB	ASP	322	19.398	19.467	3.291	1.00	0.00	RX0	C
ATOM	1580	CG	ASP	322	18.080	20.207	3.268	1.00	0.00	RX0	C
ATOM	1581	OD1	ASP	322	17.653	20.718	4.293	1.00	0.00	RX0	O
ATOM	1582	OD2	ASP	322	17.438	20.232	2.223	1.00	0.00	RX0	O
ATOM	1583	C	ASP	322	18.561	18.097	5.282	1.00	0.00	RX0	C
ATOM	1584	O	ASP	322	17.955	18.512	6.263	1.00	0.00	RX0	O

ATOM	1585	N	HIS	323	18.423	16.872	4.772	1.00	0.00	RX0	N
ATOM	1586	H	HIS	323	19.039	16.603	4.026	1.00	0.00	RX0	H
ATOM	1587	CA	HIS	323	17.423	15.916	5.274	1.00	0.00	RX0	C
ATOM	1588	CB	HIS	323	17.460	14.613	4.473	1.00	0.00	RX0	C
ATOM	1589	CG	HIS	323	16.308	13.739	4.911	1.00	0.00	RX0	C
ATOM	1590	ND1	HIS	323	16.363	12.401	5.039	1.00	0.00	RX0	N
ATOM	1591	HD1	HIS	323	17.131	11.811	4.871	1.00	0.00	RX0	H
ATOM	1592	CD2	HIS	323	15.017	14.164	5.232	1.00	0.00	RX0	C
ATOM	1593	NE2	HIS	323	14.290	13.073	5.556	1.00	0.00	RX0	N
ATOM	1594	CE1	HIS	323	15.117	11.981	5.438	1.00	0.00	RX0	C
ATOM	1595	C	HIS	323	17.630	15.607	6.766	1.00	0.00	RX0	C
ATOM	1596	O	HIS	323	16.677	15.663	7.540	1.00	0.00	RX0	O
ATOM	1597	N	ILE	324	18.888	15.420	7.164	1.00	0.00	RX0	N
ATOM	1598	H	ILE	324	19.612	15.430	6.470	1.00	0.00	RX0	H
ATOM	1599	CA	ILE	324	19.235	15.107	8.570	1.00	0.00	RX0	C
ATOM	1600	CB	ILE	324	20.719	14.763	8.704	1.00	0.00	RX0	C
ATOM	1601	CG2	ILE	324	21.124	14.625	10.170	1.00	0.00	RX0	C
ATOM	1602	CG1	ILE	324	21.037	13.490	7.918	1.00	0.00	RX0	C
ATOM	1603	CD1	ILE	324	22.525	13.138	7.922	1.00	0.00	RX0	C
ATOM	1604	C	ILE	324	18.844	16.271	9.496	1.00	0.00	RX0	C
ATOM	1605	O	ILE	324	18.200	16.053	10.527	1.00	0.00	RX0	O
ATOM	1606	N	HIS	325	19.159	17.485	9.071	1.00	0.00	RX0	N
ATOM	1607	H	HIS	325	19.587	17.591	8.171	1.00	0.00	RX0	H
ATOM	1608	CA	HIS	325	18.840	18.694	9.856	1.00	0.00	RX0	C
ATOM	1609	CB	HIS	325	19.597	19.921	9.344	1.00	0.00	RX0	C
ATOM	1610	CG	HIS	325	21.052	19.821	9.747	1.00	0.00	RX0	C
ATOM	1611	ND1	HIS	325	22.002	19.274	8.970	1.00	0.00	RX0	N
ATOM	1612	HD1	HIS	325	21.851	18.879	8.083	1.00	0.00	RX0	H
ATOM	1613	CD2	HIS	325	21.647	20.253	10.938	1.00	0.00	RX0	C
ATOM	1614	NE2	HIS	325	22.973	19.960	10.866	1.00	0.00	RX0	N
ATOM	1615	CE1	HIS	325	23.186	19.358	9.651	1.00	0.00	RX0	C
ATOM	1616	C	HIS	325	17.335	18.955	9.948	1.00	0.00	RX0	C
ATOM	1617	O	HIS	325	16.820	19.290	11.021	1.00	0.00	RX0	O
ATOM	1618	N	ARG	326	16.631	18.584	8.887	1.00	0.00	RX0	N
ATOM	1619	H	ARG	326	17.125	18.355	8.047	1.00	0.00	RX0	H
ATOM	1620	CA	ARG	326	15.160	18.594	8.873	1.00	0.00	RX0	C
ATOM	1621	CB	ARG	326	14.631	18.343	7.458	1.00	0.00	RX0	C
ATOM	1622	CG	ARG	326	14.903	19.442	6.431	1.00	0.00	RX0	C
ATOM	1623	CD	ARG	326	14.292	19.102	5.066	1.00	0.00	RX0	C
ATOM	1624	NE	ARG	326	15.313	18.966	4.027	1.00	0.00	RX0	N
ATOM	1625	HE	ARG	326	15.978	19.731	3.946	1.00	0.00	RX0	H
ATOM	1626	CZ	ARG	326	15.361	17.872	3.211	1.00	0.00	RX0	C
ATOM	1627	NH1	ARG	326	14.458	16.881	3.382	1.00	0.00	RX0	N
ATOM	1628	HH11	ARG	326	14.446	16.057	2.811	1.00	0.00	RX0	H
ATOM	1629	HH12	ARG	326	13.765	16.942	4.104	1.00	0.00	RX0	H
ATOM	1630	NH2	ARG	326	16.305	17.787	2.251	1.00	0.00	RX0	N
ATOM	1631	HH21	ARG	326	16.455	17.010	1.636	1.00	0.00	RX0	H
ATOM	1632	HH22	ARG	326	16.930	18.576	2.124	1.00	0.00	RX0	H
ATOM	1633	C	ARG	326	14.537	17.576	9.843	1.00	0.00	RX0	C
ATOM	1634	O	ARG	326	13.617	17.928	10.589	1.00	0.00	RX0	O
ATOM	1635	N	VAL	327	15.171	16.419	9.987	1.00	0.00	RX0	N
ATOM	1636	H	VAL	327	15.979	16.247	9.419	1.00	0.00	RX0	H
ATOM	1637	CA	VAL	327	14.709	15.376	10.935	1.00	0.00	RX0	C
ATOM	1638	CB	VAL	327	15.274	13.994	10.602	1.00	0.00	RX0	C
ATOM	1639	CG1	VAL	327	14.756	12.956	11.598	1.00	0.00	RX0	C
ATOM	1640	CG2	VAL	327	14.921	13.584	9.173	1.00	0.00	RX0	C
ATOM	1641	C	VAL	327	15.041	15.781	12.381	1.00	0.00	RX0	C
ATOM	1642	O	VAL	327	14.187	15.661	13.270	1.00	0.00	RX0	O
ATOM	1643	N	LEU	328	16.212	16.371	12.574	1.00	0.00	RX0	N
ATOM	1644	H	LEU	328	16.818	16.506	11.789	1.00	0.00	RX0	H
ATOM	1645	CA	LEU	328	16.631	16.899	13.887	1.00	0.00	RX0	C

ATOM	1646	CB	LEU	328	18.069	17.410	13.827	1.00	0.00	RX0	C
ATOM	1647	CG	LEU	328	19.083	16.269	13.759	1.00	0.00	RX0	C
ATOM	1648	CD1	LEU	328	20.501	16.774	13.488	1.00	0.00	RX0	C
ATOM	1649	CD2	LEU	328	19.012	15.397	15.013	1.00	0.00	RX0	C
ATOM	1650	C	LEU	328	15.692	18.005	14.386	1.00	0.00	RX0	C
ATOM	1651	O	LEU	328	15.231	17.955	15.519	1.00	0.00	RX0	O
ATOM	1652	N	ASP	329	15.222	18.821	13.436	1.00	0.00	RX0	N
ATOM	1653	H	ASP	329	15.678	18.897	12.547	1.00	0.00	RX0	H
ATOM	1654	CA	ASP	329	14.223	19.870	13.722	1.00	0.00	RX0	C
ATOM	1655	CB	ASP	329	14.101	20.858	12.561	1.00	0.00	RX0	C
ATOM	1656	CG	ASP	329	15.316	21.770	12.556	1.00	0.00	RX0	C
ATOM	1657	OD1	ASP	329	16.067	21.760	13.532	1.00	0.00	RX0	O
ATOM	1658	OD2	ASP	329	15.504	22.496	11.579	1.00	0.00	RX0	O
ATOM	1659	C	ASP	329	12.864	19.312	14.154	1.00	0.00	RX0	C
ATOM	1660	O	ASP	329	12.272	19.788	15.128	1.00	0.00	RX0	O
ATOM	1661	N	LYS	330	12.463	18.218	13.512	1.00	0.00	RX0	N
ATOM	1662	H	LYS	330	13.021	17.920	12.735	1.00	0.00	RX0	H
ATOM	1663	CA	LYS	330	11.217	17.525	13.871	1.00	0.00	RX0	C
ATOM	1664	CB	LYS	330	10.799	16.459	12.860	1.00	0.00	RX0	C
ATOM	1665	CG	LYS	330	9.621	15.608	13.367	1.00	0.00	RX0	C
ATOM	1666	CD	LYS	330	8.368	16.389	13.794	1.00	0.00	RX0	C
ATOM	1667	CE	LYS	330	7.827	17.312	12.710	1.00	0.00	RX0	C
ATOM	1668	NZ	LYS	330	7.437	16.477	11.575	1.00	0.00	RX0	N
ATOM	1669	HZ1	LYS	330	7.124	17.093	10.803	1.00	0.00	RX0	H
ATOM	1670	HZ2	LYS	330	8.247	15.890	11.278	1.00	0.00	RX0	H
ATOM	1671	HZ3	LYS	330	6.657	15.859	11.880	1.00	0.00	RX0	H
ATOM	1672	C	LYS	330	11.283	16.915	15.278	1.00	0.00	RX0	C
ATOM	1673	O	LYS	330	10.354	17.095	16.067	1.00	0.00	RX0	O
ATOM	1674	N	ILE	331	12.441	16.375	15.625	1.00	0.00	RX0	N
ATOM	1675	H	ILE	331	13.184	16.376	14.950	1.00	0.00	RX0	H
ATOM	1676	CA	ILE	331	12.656	15.802	16.972	1.00	0.00	RX0	C
ATOM	1677	CB	ILE	331	13.953	14.997	17.053	1.00	0.00	RX0	C
ATOM	1678	CG2	ILE	331	14.064	14.332	18.422	1.00	0.00	RX0	C
ATOM	1679	CG1	ILE	331	14.042	13.951	15.945	1.00	0.00	RX0	C
ATOM	1680	CD1	ILE	331	15.356	13.170	15.989	1.00	0.00	RX0	C
ATOM	1681	C	ILE	331	12.642	16.922	18.027	1.00	0.00	RX0	C
ATOM	1682	O	ILE	331	12.078	16.732	19.120	1.00	0.00	RX0	O
ATOM	1683	N	THR	332	13.158	18.084	17.675	1.00	0.00	RX0	N
ATOM	1684	H	THR	332	13.570	18.203	16.771	1.00	0.00	RX0	H
ATOM	1685	CA	THR	332	13.155	19.265	18.570	1.00	0.00	RX0	C
ATOM	1686	CB	THR	332	14.034	20.319	17.917	1.00	0.00	RX0	C
ATOM	1687	OG1	THR	332	15.304	19.716	17.627	1.00	0.00	RX0	O
ATOM	1688	HG1	THR	332	15.246	19.366	16.742	1.00	0.00	RX0	H
ATOM	1689	CG2	THR	332	14.206	21.554	18.803	1.00	0.00	RX0	C
ATOM	1690	C	THR	332	11.706	19.696	18.836	1.00	0.00	RX0	C
ATOM	1691	O	THR	332	11.302	19.832	19.995	1.00	0.00	RX0	O
ATOM	1692	N	ASP	333	10.912	19.714	17.772	1.00	0.00	RX0	N
ATOM	1693	H	ASP	333	11.280	19.673	16.837	1.00	0.00	RX0	H
ATOM	1694	CA	ASP	333	9.466	20.017	17.863	1.00	0.00	RX0	C
ATOM	1695	CB	ASP	333	8.671	19.792	16.567	1.00	0.00	RX0	C
ATOM	1696	CG	ASP	333	9.097	20.579	15.355	1.00	0.00	RX0	C
ATOM	1697	OD1	ASP	333	9.500	21.727	15.507	1.00	0.00	RX0	O
ATOM	1698	OD2	ASP	333	8.970	20.039	14.251	1.00	0.00	RX0	O
ATOM	1699	C	ASP	333	8.732	19.000	18.747	1.00	0.00	RX0	C
ATOM	1700	O	ASP	333	7.880	19.374	19.559	1.00	0.00	RX0	O
ATOM	1701	N	THR	334	9.187	17.759	18.682	1.00	0.00	RX0	N
ATOM	1702	H	THR	334	9.940	17.583	18.048	1.00	0.00	RX0	H
ATOM	1703	CA	THR	334	8.631	16.638	19.462	1.00	0.00	RX0	C
ATOM	1704	CB	THR	334	9.177	15.373	18.821	1.00	0.00	RX0	C
ATOM	1705	OG1	THR	334	8.793	15.339	17.438	1.00	0.00	RX0	O
ATOM	1706	HG1	THR	334	9.149	16.125	17.032	1.00	0.00	RX0	H

ATOM	1707	CG2	THR	334	8.726	14.119	19.558	1.00	0.00	RX0	C
ATOM	1708	C	THR	334	8.961	16.782	20.953	1.00	0.00	RX0	C
ATOM	1709	O	THR	334	8.059	16.689	21.789	1.00	0.00	RX0	O
ATOM	1710	N	LEU	335	10.217	17.081	21.263	1.00	0.00	RX0	N
ATOM	1711	H	LEU	335	10.880	17.204	20.523	1.00	0.00	RX0	H
ATOM	1712	CA	LEU	335	10.648	17.349	22.650	1.00	0.00	RX0	C
ATOM	1713	CB	LEU	335	12.150	17.613	22.696	1.00	0.00	RX0	C
ATOM	1714	CG	LEU	335	12.962	16.326	22.779	1.00	0.00	RX0	C
ATOM	1715	CD1	LEU	335	14.460	16.579	22.610	1.00	0.00	RX0	C
ATOM	1716	CD2	LEU	335	12.655	15.573	24.074	1.00	0.00	RX0	C
ATOM	1717	C	LEU	335	9.903	18.522	23.297	1.00	0.00	RX0	C
ATOM	1718	O	LEU	335	9.384	18.379	24.399	1.00	0.00	RX0	O
ATOM	1719	N	ILE	336	9.668	19.567	22.501	1.00	0.00	RX0	N
ATOM	1720	H	ILE	336	10.072	19.580	21.582	1.00	0.00	RX0	H
ATOM	1721	CA	ILE	336	8.897	20.743	22.962	1.00	0.00	RX0	C
ATOM	1722	CB	ILE	336	9.048	21.913	21.990	1.00	0.00	RX0	C
ATOM	1723	CG2	ILE	336	8.095	23.060	22.331	1.00	0.00	RX0	C
ATOM	1724	CG1	ILE	336	10.503	22.385	21.990	1.00	0.00	RX0	C
ATOM	1725	CD1	ILE	336	10.942	22.864	23.376	1.00	0.00	RX0	C
ATOM	1726	C	ILE	336	7.427	20.365	23.180	1.00	0.00	RX0	C
ATOM	1727	O	ILE	336	6.836	20.730	24.203	1.00	0.00	RX0	O
ATOM	1728	N	HIS	337	6.891	19.571	22.265	1.00	0.00	RX0	N
ATOM	1729	H	HIS	337	7.438	19.318	21.465	1.00	0.00	RX0	H
ATOM	1730	CA	HIS	337	5.501	19.096	22.359	1.00	0.00	RX0	C
ATOM	1731	CB	HIS	337	5.081	18.332	21.113	1.00	0.00	RX0	C
ATOM	1732	CG	HIS	337	3.596	18.091	21.195	1.00	0.00	RX0	C
ATOM	1733	ND1	HIS	337	2.668	19.011	20.879	1.00	0.00	RX0	N
ATOM	1734	HD1	HIS	337	2.840	19.919	20.547	1.00	0.00	RX0	H
ATOM	1735	CD2	HIS	337	2.950	16.931	21.621	1.00	0.00	RX0	C
ATOM	1736	NE2	HIS	337	1.613	17.160	21.566	1.00	0.00	RX0	N
ATOM	1737	CE1	HIS	337	1.441	18.443	21.106	1.00	0.00	RX0	C
ATOM	1738	C	HIS	337	5.301	18.263	23.631	1.00	0.00	RX0	C
ATOM	1739	O	HIS	337	4.339	18.490	24.365	1.00	0.00	RX0	O
ATOM	1740	N	LEU	338	6.274	17.405	23.914	1.00	0.00	RX0	N
ATOM	1741	H	LEU	338	7.051	17.336	23.286	1.00	0.00	RX0	H
ATOM	1742	CA	LEU	338	6.246	16.526	25.097	1.00	0.00	RX0	C
ATOM	1743	CB	LEU	338	7.441	15.575	25.089	1.00	0.00	RX0	C
ATOM	1744	CG	LEU	338	7.342	14.531	23.982	1.00	0.00	RX0	C
ATOM	1745	CD1	LEU	338	8.646	13.749	23.814	1.00	0.00	RX0	C
ATOM	1746	CD2	LEU	338	6.125	13.627	24.183	1.00	0.00	RX0	C
ATOM	1747	C	LEU	338	6.240	17.331	26.400	1.00	0.00	RX0	C
ATOM	1748	O	LEU	338	5.410	17.098	27.277	1.00	0.00	RX0	O
ATOM	1749	N	MET	339	7.027	18.402	26.394	1.00	0.00	RX0	N
ATOM	1750	H	MET	339	7.615	18.548	25.595	1.00	0.00	RX0	H
ATOM	1751	CA	MET	339	7.168	19.308	27.550	1.00	0.00	RX0	C
ATOM	1752	CB	MET	339	8.404	20.191	27.399	1.00	0.00	RX0	C
ATOM	1753	CG	MET	339	9.705	19.394	27.370	1.00	0.00	RX0	C
ATOM	1754	SD	MET	339	11.134	20.436	27.051	1.00	0.00	RX0	S
ATOM	1755	CE	MET	339	12.269	19.129	26.566	1.00	0.00	RX0	C
ATOM	1756	C	MET	339	5.924	20.183	27.753	1.00	0.00	RX0	C
ATOM	1757	O	MET	339	5.433	20.322	28.878	1.00	0.00	RX0	O
ATOM	1758	N	ALA	340	5.370	20.672	26.646	1.00	0.00	RX0	N
ATOM	1759	H	ALA	340	5.814	20.472	25.772	1.00	0.00	RX0	H
ATOM	1760	CA	ALA	340	4.120	21.456	26.636	1.00	0.00	RX0	C
ATOM	1761	CB	ALA	340	3.835	21.993	25.233	1.00	0.00	RX0	C
ATOM	1762	C	ALA	340	2.921	20.619	27.100	1.00	0.00	RX0	C
ATOM	1763	O	ALA	340	2.176	21.064	27.967	1.00	0.00	RX0	O
ATOM	1764	N	LYS	341	2.865	19.360	26.655	1.00	0.00	RX0	N
ATOM	1765	H	LYS	341	3.552	19.058	25.994	1.00	0.00	RX0	H
ATOM	1766	CA	LYS	341	1.846	18.397	27.117	1.00	0.00	RX0	C
ATOM	1767	CB	LYS	341	1.979	17.108	26.280	1.00	0.00	RX0	C

ATOM	1768	CG	LYS	341	0.705	16.260	26.147	1.00	0.00	RX0	C
ATOM	1769	CD	LYS	341	0.383	15.817	24.704	1.00	0.00	RX0	C
ATOM	1770	CE	LYS	341	1.352	14.815	24.049	1.00	0.00	RX0	C
ATOM	1771	NZ	LYS	341	1.002	14.625	22.628	1.00	0.00	RX0	N
ATOM	1772	HZ1	LYS	341	1.715	14.095	22.076	1.00	0.00	RX0	H
ATOM	1773	HZ2	LYS	341	0.149	14.058	22.436	1.00	0.00	RX0	H
ATOM	1774	HZ3	LYS	341	0.917	15.516	22.102	1.00	0.00	RX0	H
ATOM	1775	C	LYS	341	1.927	18.179	28.637	1.00	0.00	RX0	C
ATOM	1776	O	LYS	341	0.908	18.032	29.304	1.00	0.00	RX0	O
ATOM	1777	N	ALA	342	3.166	18.103	29.120	1.00	0.00	RX0	N
ATOM	1778	H	ALA	342	3.940	18.170	28.488	1.00	0.00	RX0	H
ATOM	1779	CA	ALA	342	3.459	17.942	30.556	1.00	0.00	RX0	C
ATOM	1780	CB	ALA	342	4.953	17.706	30.786	1.00	0.00	RX0	C
ATOM	1781	C	ALA	342	3.007	19.166	31.371	1.00	0.00	RX0	C
ATOM	1782	O	ALA	342	2.879	19.099	32.588	1.00	0.00	RX0	O
ATOM	1783	N	GLY	343	2.900	20.305	30.669	1.00	0.00	RX0	N
ATOM	1784	H	GLY	343	3.153	20.317	29.702	1.00	0.00	RX0	H
ATOM	1785	CA	GLY	343	2.393	21.560	31.244	1.00	0.00	RX0	C
ATOM	1786	C	GLY	343	3.505	22.433	31.829	1.00	0.00	RX0	C
ATOM	1787	O	GLY	343	3.244	23.282	32.678	1.00	0.00	RX0	O
ATOM	1788	N	LEU	344	4.736	22.226	31.356	1.00	0.00	RX0	N
ATOM	1789	H	LEU	344	4.854	21.555	30.623	1.00	0.00	RX0	H
ATOM	1790	CA	LEU	344	5.823	23.174	31.634	1.00	0.00	RX0	C
ATOM	1791	CB	LEU	344	7.151	22.611	31.138	1.00	0.00	RX0	C
ATOM	1792	CG	LEU	344	7.553	21.337	31.877	1.00	0.00	RX0	C
ATOM	1793	CD1	LEU	344	8.836	20.741	31.301	1.00	0.00	RX0	C
ATOM	1794	CD2	LEU	344	7.655	21.562	33.386	1.00	0.00	RX0	C
ATOM	1795	C	LEU	344	5.508	24.488	30.929	1.00	0.00	RX0	C
ATOM	1796	O	LEU	344	4.977	24.500	29.792	1.00	0.00	RX0	O
ATOM	1797	N	THR	345	5.822	25.577	31.575	1.00	0.00	RX0	N
ATOM	1798	H	THR	345	6.294	25.483	32.450	1.00	0.00	RX0	H
ATOM	1799	CA	THR	345	5.705	26.912	30.948	1.00	0.00	RX0	C
ATOM	1800	CB	THR	345	5.998	27.964	32.011	1.00	0.00	RX0	C
ATOM	1801	OG1	THR	345	7.168	27.599	32.742	1.00	0.00	RX0	O
ATOM	1802	HG1	THR	345	6.893	26.957	33.393	1.00	0.00	RX0	H
ATOM	1803	CG2	THR	345	4.818	28.128	32.969	1.00	0.00	RX0	C
ATOM	1804	C	THR	345	6.639	26.971	29.731	1.00	0.00	RX0	C
ATOM	1805	O	THR	345	7.615	26.233	29.623	1.00	0.00	RX0	O
ATOM	1806	N	LEU	346	6.390	27.971	28.898	1.00	0.00	RX0	N
ATOM	1807	H	LEU	346	5.564	28.513	29.045	1.00	0.00	RX0	H
ATOM	1808	CA	LEU	346	7.214	28.242	27.708	1.00	0.00	RX0	C
ATOM	1809	CB	LEU	346	6.673	29.452	26.948	1.00	0.00	RX0	C
ATOM	1810	CG	LEU	346	7.384	29.662	25.610	1.00	0.00	RX0	C
ATOM	1811	CD1	LEU	346	7.257	28.439	24.699	1.00	0.00	RX0	C
ATOM	1812	CD2	LEU	346	6.923	30.945	24.918	1.00	0.00	RX0	C
ATOM	1813	C	LEU	346	8.705	28.433	28.051	1.00	0.00	RX0	C
ATOM	1814	O	LEU	346	9.594	27.857	27.432	1.00	0.00	RX0	O
ATOM	1815	N	GLN	347	8.927	29.083	29.198	1.00	0.00	RX0	N
ATOM	1816	H	GLN	347	8.146	29.440	29.706	1.00	0.00	RX0	H
ATOM	1817	CA	GLN	347	10.275	29.275	29.754	1.00	0.00	RX0	C
ATOM	1818	CB	GLN	347	10.215	30.280	30.899	1.00	0.00	RX0	C
ATOM	1819	CG	GLN	347	11.588	30.594	31.490	1.00	0.00	RX0	C
ATOM	1820	CD	GLN	347	11.405	31.568	32.629	1.00	0.00	RX0	C
ATOM	1821	OE1	GLN	347	10.323	31.701	33.186	1.00	0.00	RX0	O
ATOM	1822	NE2	GLN	347	12.520	32.254	32.935	1.00	0.00	RX0	N
ATOM	1823	HE21	GLN	347	13.369	32.106	32.428	1.00	0.00	RX0	H
ATOM	1824	HE22	GLN	347	12.514	32.931	33.672	1.00	0.00	RX0	H
ATOM	1825	C	GLN	347	10.911	27.956	30.229	1.00	0.00	RX0	C
ATOM	1826	O	GLN	347	12.052	27.652	29.875	1.00	0.00	RX0	O
ATOM	1827	N	GLN	348	10.120	27.140	30.905	1.00	0.00	RX0	N
ATOM	1828	H	GLN	348	9.171	27.398	31.088	1.00	0.00	RX0	H

ATOM	1829	CA	GLN	348	10.582	25.823	31.394	1.00	0.00	RX0	C
ATOM	1830	CB	GLN	348	9.592	25.216	32.376	1.00	0.00	RX0	C
ATOM	1831	CG	GLN	348	9.644	25.880	33.748	1.00	0.00	RX0	C
ATOM	1832	CD	GLN	348	8.476	25.365	34.557	1.00	0.00	RX0	C
ATOM	1833	OE1	GLN	348	7.351	25.283	34.068	1.00	0.00	RX0	O
ATOM	1834	NE2	GLN	348	8.799	25.020	35.814	1.00	0.00	RX0	N
ATOM	1835	HE21	GLN	348	9.738	25.132	36.142	1.00	0.00	RX0	H
ATOM	1836	HE22	GLN	348	8.119	24.654	36.451	1.00	0.00	RX0	H
ATOM	1837	C	GLN	348	10.871	24.847	30.248	1.00	0.00	RX0	C
ATOM	1838	O	GLN	348	11.861	24.115	30.296	1.00	0.00	RX0	O
ATOM	1839	N	GLN	349	10.121	24.983	29.160	1.00	0.00	RX0	N
ATOM	1840	H	GLN	349	9.376	25.649	29.187	1.00	0.00	RX0	H
ATOM	1841	CA	GLN	349	10.299	24.172	27.940	1.00	0.00	RX0	C
ATOM	1842	CB	GLN	349	9.206	24.481	26.921	1.00	0.00	RX0	C
ATOM	1843	CG	GLN	349	7.821	24.034	27.384	1.00	0.00	RX0	C
ATOM	1844	CD	GLN	349	6.788	24.596	26.437	1.00	0.00	RX0	C
ATOM	1845	OE1	GLN	349	7.077	24.932	25.294	1.00	0.00	RX0	O
ATOM	1846	NE2	GLN	349	5.563	24.699	26.979	1.00	0.00	RX0	N
ATOM	1847	HE21	GLN	349	5.398	24.429	27.933	1.00	0.00	RX0	H
ATOM	1848	HE22	GLN	349	4.780	25.038	26.461	1.00	0.00	RX0	H
ATOM	1849	C	GLN	349	11.682	24.372	27.306	1.00	0.00	RX0	C
ATOM	1850	O	GLN	349	12.447	23.422	27.181	1.00	0.00	RX0	O
ATOM	1851	N	HIS	350	12.056	25.641	27.113	1.00	0.00	RX0	N
ATOM	1852	H	HIS	350	11.422	26.375	27.365	1.00	0.00	RX0	H
ATOM	1853	CA	HIS	350	13.344	25.953	26.463	1.00	0.00	RX0	C
ATOM	1854	CB	HIS	350	13.376	27.324	25.779	1.00	0.00	RX0	C
ATOM	1855	CG	HIS	350	13.537	28.475	26.739	1.00	0.00	RX0	C
ATOM	1856	ND1	HIS	350	12.509	29.224	27.168	1.00	0.00	RX0	N
ATOM	1857	HD1	HIS	350	11.557	29.071	26.965	1.00	0.00	RX0	H
ATOM	1858	CD2	HIS	350	14.726	28.986	27.271	1.00	0.00	RX0	C
ATOM	1859	NE2	HIS	350	14.402	30.065	28.022	1.00	0.00	RX0	N
ATOM	1860	CE1	HIS	350	13.041	30.210	27.959	1.00	0.00	RX0	C
ATOM	1861	C	HIS	350	14.540	25.670	27.386	1.00	0.00	RX0	C
ATOM	1862	O	HIS	350	15.573	25.182	26.934	1.00	0.00	RX0	O
ATOM	1863	N	GLN	351	14.320	25.854	28.691	1.00	0.00	RX0	N
ATOM	1864	H	GLN	351	13.439	26.227	28.992	1.00	0.00	RX0	H
ATOM	1865	CA	GLN	351	15.341	25.538	29.706	1.00	0.00	RX0	C
ATOM	1866	CB	GLN	351	14.923	26.079	31.068	1.00	0.00	RX0	C
ATOM	1867	CG	GLN	351	14.911	27.604	31.109	1.00	0.00	RX0	C
ATOM	1868	CD	GLN	351	14.260	28.049	32.398	1.00	0.00	RX0	C
ATOM	1869	OE1	GLN	351	13.313	27.446	32.892	1.00	0.00	RX0	O
ATOM	1870	NE2	GLN	351	14.836	29.141	32.932	1.00	0.00	RX0	N
ATOM	1871	HE21	GLN	351	15.606	29.577	32.464	1.00	0.00	RX0	H
ATOM	1872	HE22	GLN	351	14.516	29.523	33.799	1.00	0.00	RX0	H
ATOM	1873	C	GLN	351	15.597	24.030	29.805	1.00	0.00	RX0	C
ATOM	1874	O	GLN	351	16.752	23.608	29.740	1.00	0.00	RX0	O
ATOM	1875	N	ARG	352	14.527	23.242	29.744	1.00	0.00	RX0	N
ATOM	1876	H	ARG	352	13.618	23.654	29.656	1.00	0.00	RX0	H
ATOM	1877	CA	ARG	352	14.627	21.772	29.816	1.00	0.00	RX0	C
ATOM	1878	CB	ARG	352	13.311	21.110	30.247	1.00	0.00	RX0	C
ATOM	1879	CG	ARG	352	13.423	19.588	30.437	1.00	0.00	RX0	C
ATOM	1880	CD	ARG	352	12.234	18.992	31.201	1.00	0.00	RX0	C
ATOM	1881	NE	ARG	352	12.217	17.527	31.180	1.00	0.00	RX0	N
ATOM	1882	HE	ARG	352	12.008	17.059	30.306	1.00	0.00	RX0	H
ATOM	1883	CZ	ARG	352	12.350	16.775	32.316	1.00	0.00	RX0	C
ATOM	1884	NH1	ARG	352	12.647	17.383	33.482	1.00	0.00	RX0	N
ATOM	1885	HH11	ARG	352	12.825	16.839	34.316	1.00	0.00	RX0	H
ATOM	1886	HH12	ARG	352	12.713	18.378	33.558	1.00	0.00	RX0	H
ATOM	1887	NH2	ARG	352	12.180	15.439	32.257	1.00	0.00	RX0	N
ATOM	1888	HH21	ARG	352	12.218	14.805	33.038	1.00	0.00	RX0	H
ATOM	1889	HH22	ARG	352	11.987	15.017	31.348	1.00	0.00	RX0	H

ATOM	1890	C	ARG	352	15.192	21.188	28.514	1.00	0.00	RX0	C
ATOM	1891	O	ARG	352	16.048	20.305	28.551	1.00	0.00	RX0	O
ATOM	1892	N	LEU	353	14.843	21.816	27.391	1.00	0.00	RX0	N
ATOM	1893	H	LEU	353	14.136	22.524	27.429	1.00	0.00	RX0	H
ATOM	1894	CA	LEU	353	15.400	21.446	26.079	1.00	0.00	RX0	C
ATOM	1895	CB	LEU	353	14.770	22.309	24.988	1.00	0.00	RX0	C
ATOM	1896	CG	LEU	353	15.252	21.951	23.583	1.00	0.00	RX0	C
ATOM	1897	CD1	LEU	353	14.800	20.551	23.166	1.00	0.00	RX0	C
ATOM	1898	CD2	LEU	353	14.859	23.015	22.559	1.00	0.00	RX0	C
ATOM	1899	C	LEU	353	16.930	21.610	26.063	1.00	0.00	RX0	C
ATOM	1900	O	LEU	353	17.658	20.692	25.694	1.00	0.00	RX0	O
ATOM	1901	N	ALA	354	17.375	22.739	26.614	1.00	0.00	RX0	N
ATOM	1902	H	ALA	354	16.709	23.430	26.903	1.00	0.00	RX0	H
ATOM	1903	CA	ALA	354	18.808	23.073	26.716	1.00	0.00	RX0	C
ATOM	1904	CB	ALA	354	18.995	24.521	27.171	1.00	0.00	RX0	C
ATOM	1905	C	ALA	354	19.540	22.141	27.690	1.00	0.00	RX0	C
ATOM	1906	O	ALA	354	20.574	21.567	27.334	1.00	0.00	RX0	O
ATOM	1907	N	GLN	355	18.893	21.844	28.811	1.00	0.00	RX0	N
ATOM	1908	H	GLN	355	18.021	22.311	28.969	1.00	0.00	RX0	H
ATOM	1909	CA	GLN	355	19.427	20.920	29.833	1.00	0.00	RX0	C
ATOM	1910	CB	GLN	355	18.596	20.909	31.105	1.00	0.00	RX0	C
ATOM	1911	CG	GLN	355	18.778	22.209	31.880	1.00	0.00	RX0	C
ATOM	1912	CD	GLN	355	18.344	21.971	33.304	1.00	0.00	RX0	C
ATOM	1913	OE1	GLN	355	18.952	21.181	34.025	1.00	0.00	RX0	O
ATOM	1914	NE2	GLN	355	17.268	22.687	33.666	1.00	0.00	RX0	N
ATOM	1915	HE21	GLN	355	16.842	23.305	33.002	1.00	0.00	RX0	H
ATOM	1916	HE22	GLN	355	16.867	22.640	34.582	1.00	0.00	RX0	H
ATOM	1917	C	GLN	355	19.631	19.504	29.273	1.00	0.00	RX0	C
ATOM	1918	O	GLN	355	20.705	18.919	29.430	1.00	0.00	RX0	O
ATOM	1919	N	LEU	356	18.681	19.086	28.443	1.00	0.00	RX0	N
ATOM	1920	H	LEU	356	17.885	19.672	28.276	1.00	0.00	RX0	H
ATOM	1921	CA	LEU	356	18.716	17.770	27.778	1.00	0.00	RX0	C
ATOM	1922	CB	LEU	356	17.350	17.404	27.199	1.00	0.00	RX0	C
ATOM	1923	CG	LEU	356	16.344	17.038	28.289	1.00	0.00	RX0	C
ATOM	1924	CD1	LEU	356	14.985	16.649	27.706	1.00	0.00	RX0	C
ATOM	1925	CD2	LEU	356	16.895	15.952	29.210	1.00	0.00	RX0	C
ATOM	1926	C	LEU	356	19.795	17.661	26.699	1.00	0.00	RX0	C
ATOM	1927	O	LEU	356	20.593	16.720	26.700	1.00	0.00	RX0	O
ATOM	1928	N	LEU	357	19.916	18.722	25.913	1.00	0.00	RX0	N
ATOM	1929	H	LEU	357	19.287	19.494	26.032	1.00	0.00	RX0	H
ATOM	1930	CA	LEU	357	20.885	18.765	24.803	1.00	0.00	RX0	C
ATOM	1931	CB	LEU	357	20.542	19.882	23.817	1.00	0.00	RX0	C
ATOM	1932	CG	LEU	357	19.173	19.723	23.155	1.00	0.00	RX0	C
ATOM	1933	CD1	LEU	357	18.837	20.927	22.275	1.00	0.00	RX0	C
ATOM	1934	CD2	LEU	357	19.040	18.401	22.400	1.00	0.00	RX0	C
ATOM	1935	C	LEU	357	22.335	18.932	25.264	1.00	0.00	RX0	C
ATOM	1936	O	LEU	357	23.247	18.345	24.683	1.00	0.00	RX0	O
ATOM	1937	N	LEU	358	22.501	19.595	26.404	1.00	0.00	RX0	N
ATOM	1938	H	LEU	358	21.707	20.037	26.828	1.00	0.00	RX0	H
ATOM	1939	CA	LEU	358	23.826	19.754	27.029	1.00	0.00	RX0	C
ATOM	1940	CB	LEU	358	23.812	20.848	28.095	1.00	0.00	RX0	C
ATOM	1941	CG	LEU	358	23.687	22.246	27.487	1.00	0.00	RX0	C
ATOM	1942	CD1	LEU	358	23.560	23.325	28.563	1.00	0.00	RX0	C
ATOM	1943	CD2	LEU	358	24.822	22.540	26.503	1.00	0.00	RX0	C
ATOM	1944	C	LEU	358	24.390	18.455	27.609	1.00	0.00	RX0	C
ATOM	1945	O	LEU	358	25.603	18.227	27.557	1.00	0.00	RX0	O
ATOM	1946	N	ILE	359	23.510	17.559	28.043	1.00	0.00	RX0	N
ATOM	1947	H	ILE	359	22.535	17.791	28.030	1.00	0.00	RX0	H
ATOM	1948	CA	ILE	359	23.928	16.212	28.495	1.00	0.00	RX0	C
ATOM	1949	CB	ILE	359	22.743	15.448	29.084	1.00	0.00	RX0	C
ATOM	1950	CG2	ILE	359	23.151	14.057	29.570	1.00	0.00	RX0	C

ATOM	1951	CG1	ILE	359	22.105	16.258	30.207	1.00	0.00	RX0	C
ATOM	1952	CD1	ILE	359	20.753	15.703	30.647	1.00	0.00	RX0	C
ATOM	1953	C	ILE	359	24.559	15.428	27.334	1.00	0.00	RX0	C
ATOM	1954	O	ILE	359	25.552	14.723	27.543	1.00	0.00	RX0	O
ATOM	1955	N	LEU	360	24.038	15.620	26.133	1.00	0.00	RX0	N
ATOM	1956	H	LEU	360	23.273	16.258	26.029	1.00	0.00	RX0	H
ATOM	1957	CA	LEU	360	24.601	14.974	24.928	1.00	0.00	RX0	C
ATOM	1958	CB	LEU	360	23.755	15.280	23.692	1.00	0.00	RX0	C
ATOM	1959	CG	LEU	360	22.268	14.966	23.890	1.00	0.00	RX0	C
ATOM	1960	CD1	LEU	360	21.451	15.342	22.656	1.00	0.00	RX0	C
ATOM	1961	CD2	LEU	360	22.017	13.517	24.306	1.00	0.00	RX0	C
ATOM	1962	C	LEU	360	26.079	15.308	24.694	1.00	0.00	RX0	C
ATOM	1963	O	LEU	360	26.843	14.455	24.246	1.00	0.00	RX0	O
ATOM	1964	N	SER	361	26.491	16.472	25.202	1.00	0.00	RX0	N
ATOM	1965	H	SER	361	25.821	17.134	25.535	1.00	0.00	RX0	H
ATOM	1966	CA	SER	361	27.906	16.894	25.202	1.00	0.00	RX0	C
ATOM	1967	CB	SER	361	27.909	18.363	25.575	1.00	0.00	RX0	C
ATOM	1968	OG	SER	361	26.676	18.886	25.078	1.00	0.00	RX0	O
ATOM	1969	HG	SER	361	26.186	19.158	25.847	1.00	0.00	RX0	H
ATOM	1970	C	SER	361	28.769	15.985	26.099	1.00	0.00	RX0	C
ATOM	1971	O	SER	361	29.797	15.456	25.681	1.00	0.00	RX0	O
ATOM	1972	N	HIS	362	28.237	15.704	27.286	1.00	0.00	RX0	N
ATOM	1973	H	HIS	362	27.316	16.045	27.490	1.00	0.00	RX0	H
ATOM	1974	CA	HIS	362	28.854	14.793	28.272	1.00	0.00	RX0	C
ATOM	1975	CB	HIS	362	28.304	15.107	29.659	1.00	0.00	RX0	C
ATOM	1976	CG	HIS	362	28.650	16.572	29.789	1.00	0.00	RX0	C
ATOM	1977	ND1	HIS	362	27.759	17.563	29.964	1.00	0.00	RX0	N
ATOM	1978	HD1	HIS	362	26.804	17.481	30.193	1.00	0.00	RX0	H
ATOM	1979	CD2	HIS	362	29.920	17.140	29.632	1.00	0.00	RX0	C
ATOM	1980	NE2	HIS	362	29.781	18.486	29.699	1.00	0.00	RX0	N
ATOM	1981	CE1	HIS	362	28.448	18.745	29.904	1.00	0.00	RX0	C
ATOM	1982	C	HIS	362	28.890	13.339	27.777	1.00	0.00	RX0	C
ATOM	1983	O	HIS	362	29.902	12.656	27.936	1.00	0.00	RX0	O
ATOM	1984	N	ILE	363	27.856	12.940	27.039	1.00	0.00	RX0	N
ATOM	1985	H	ILE	363	27.086	13.573	26.932	1.00	0.00	RX0	H
ATOM	1986	CA	ILE	363	27.773	11.584	26.446	1.00	0.00	RX0	C
ATOM	1987	CB	ILE	363	26.352	11.271	25.975	1.00	0.00	RX0	C
ATOM	1988	CG2	ILE	363	26.229	9.832	25.474	1.00	0.00	RX0	C
ATOM	1989	CG1	ILE	363	25.376	11.510	27.127	1.00	0.00	RX0	C
ATOM	1990	CD1	ILE	363	23.927	11.225	26.744	1.00	0.00	RX0	C
ATOM	1991	C	ILE	363	28.830	11.416	25.343	1.00	0.00	RX0	C
ATOM	1992	O	ILE	363	29.487	10.370	25.263	1.00	0.00	RX0	O
ATOM	1993	N	ARG	364	29.035	12.469	24.564	1.00	0.00	RX0	N
ATOM	1994	H	ARG	364	28.383	13.229	24.616	1.00	0.00	RX0	H
ATOM	1995	CA	ARG	364	30.102	12.498	23.547	1.00	0.00	RX0	C
ATOM	1996	CB	ARG	364	30.031	13.803	22.758	1.00	0.00	RX0	C
ATOM	1997	CG	ARG	364	31.093	13.935	21.667	1.00	0.00	RX0	C
ATOM	1998	CD	ARG	364	31.032	12.852	20.587	1.00	0.00	RX0	C
ATOM	1999	NE	ARG	364	29.675	12.775	20.072	1.00	0.00	RX0	N
ATOM	2000	HE	ARG	364	29.044	12.115	20.506	1.00	0.00	RX0	H
ATOM	2001	CZ	ARG	364	29.197	13.670	19.169	1.00	0.00	RX0	C
ATOM	2002	NH1	ARG	364	30.054	14.393	18.404	1.00	0.00	RX0	N
ATOM	2003	HH11	ARG	364	29.735	15.042	17.710	1.00	0.00	RX0	H
ATOM	2004	HH12	ARG	364	31.063	14.300	18.469	1.00	0.00	RX0	H
ATOM	2005	NH2	ARG	364	27.855	13.796	19.084	1.00	0.00	RX0	N
ATOM	2006	HH21	ARG	364	27.358	14.443	18.493	1.00	0.00	RX0	H
ATOM	2007	HH22	ARG	364	27.283	13.169	19.642	1.00	0.00	RX0	H
ATOM	2008	C	ARG	364	31.469	12.319	24.223	1.00	0.00	RX0	C
ATOM	2009	O	ARG	364	32.264	11.465	23.831	1.00	0.00	RX0	O
ATOM	2010	N	HIS	365	31.645	13.058	25.316	1.00	0.00	RX0	N
ATOM	2011	H	HIS	365	30.875	13.620	25.625	1.00	0.00	RX0	H

ATOM	2012	CA	HIS	365	32.879	13.027	26.115	1.00	0.00	RX0	C
ATOM	2013	CB	HIS	365	32.922	14.091	27.215	1.00	0.00	RX0	C
ATOM	2014	CG	HIS	365	34.315	14.126	27.811	1.00	0.00	RX0	C
ATOM	2015	ND1	HIS	365	35.381	14.729	27.237	1.00	0.00	RX0	N
ATOM	2016	HD1	HIS	365	35.436	15.228	26.391	1.00	0.00	RX0	H
ATOM	2017	CD2	HIS	365	34.732	13.553	29.017	1.00	0.00	RX0	C
ATOM	2018	NE2	HIS	365	36.054	13.815	29.165	1.00	0.00	RX0	N
ATOM	2019	CE1	HIS	365	36.451	14.537	28.071	1.00	0.00	RX0	C
ATOM	2020	C	HIS	365	33.175	11.617	26.649	1.00	0.00	RX0	C
ATOM	2021	O	HIS	365	34.274	11.105	26.425	1.00	0.00	RX0	O
ATOM	2022	N	MET	366	32.138	10.946	27.137	1.00	0.00	RX0	N
ATOM	2023	H	MET	366	31.258	11.417	27.230	1.00	0.00	RX0	H
ATOM	2024	CA	MET	366	32.267	9.572	27.662	1.00	0.00	RX0	C
ATOM	2025	CB	MET	366	31.019	9.148	28.437	1.00	0.00	RX0	C
ATOM	2026	CG	MET	366	30.746	10.021	29.660	1.00	0.00	RX0	C
ATOM	2027	SD	MET	366	29.398	9.394	30.674	1.00	0.00	RX0	S
ATOM	2028	CE	MET	366	28.117	9.377	29.413	1.00	0.00	RX0	C
ATOM	2029	C	MET	366	32.567	8.557	26.555	1.00	0.00	RX0	C
ATOM	2030	O	MET	366	33.398	7.674	26.741	1.00	0.00	RX0	O
ATOM	2031	N	SER	367	31.985	8.782	25.378	1.00	0.00	RX0	N
ATOM	2032	H	SER	367	31.307	9.514	25.288	1.00	0.00	RX0	H
ATOM	2033	CA	SER	367	32.231	7.934	24.196	1.00	0.00	RX0	C
ATOM	2034	CB	SER	367	31.202	8.410	23.160	1.00	0.00	RX0	C
ATOM	2035	OG	SER	367	30.787	7.382	22.258	1.00	0.00	RX0	O
ATOM	2036	HG	SER	367	30.182	7.812	21.662	1.00	0.00	RX0	H
ATOM	2037	C	SER	367	33.681	8.046	23.711	1.00	0.00	RX0	C
ATOM	2038	O	SER	367	34.361	7.038	23.567	1.00	0.00	RX0	O
ATOM	2039	N	ASN	368	34.193	9.278	23.688	1.00	0.00	RX0	N
ATOM	2040	H	ASN	368	33.604	10.033	23.985	1.00	0.00	RX0	H
ATOM	2041	CA	ASN	368	35.583	9.549	23.266	1.00	0.00	RX0	C
ATOM	2042	CB	ASN	368	35.829	11.030	23.064	1.00	0.00	RX0	C
ATOM	2043	CG	ASN	368	35.888	11.344	21.583	1.00	0.00	RX0	C
ATOM	2044	OD1	ASN	368	36.474	12.343	21.178	1.00	0.00	RX0	O
ATOM	2045	ND2	ASN	368	35.183	10.525	20.780	1.00	0.00	RX0	N
ATOM	2046	HD21	ASN	368	34.658	9.723	21.071	1.00	0.00	RX0	H
ATOM	2047	HD22	ASN	368	35.161	10.674	19.784	1.00	0.00	RX0	H
ATOM	2048	C	ASN	368	36.615	8.948	24.226	1.00	0.00	RX0	C
ATOM	2049	O	ASN	368	37.514	8.215	23.806	1.00	0.00	RX0	O
ATOM	2050	N	LYS	369	36.353	9.119	25.515	1.00	0.00	RX0	N
ATOM	2051	H	LYS	369	35.562	9.674	25.782	1.00	0.00	RX0	H
ATOM	2052	CA	LYS	369	37.205	8.554	26.580	1.00	0.00	RX0	C
ATOM	2053	CB	LYS	369	36.868	9.110	27.966	1.00	0.00	RX0	C
ATOM	2054	CG	LYS	369	37.123	10.609	28.129	1.00	0.00	RX0	C
ATOM	2055	CD	LYS	369	38.519	11.024	27.665	1.00	0.00	RX0	C
ATOM	2056	CE	LYS	369	39.678	10.324	28.385	1.00	0.00	RX0	C
ATOM	2057	NZ	LYS	369	40.883	10.507	27.568	1.00	0.00	RX0	N
ATOM	2058	HZ1	LYS	369	41.629	11.055	28.024	1.00	0.00	RX0	H
ATOM	2059	HZ2	LYS	369	41.277	9.600	27.232	1.00	0.00	RX0	H
ATOM	2060	HZ3	LYS	369	40.635	10.942	26.650	1.00	0.00	RX0	H
ATOM	2061	C	LYS	369	37.117	7.021	26.620	1.00	0.00	RX0	C
ATOM	2062	O	LYS	369	38.123	6.337	26.771	1.00	0.00	RX0	O
ATOM	2063	N	GLY	370	35.908	6.527	26.299	1.00	0.00	RX0	N
ATOM	2064	H	GLY	370	35.153	7.164	26.141	1.00	0.00	RX0	H
ATOM	2065	CA	GLY	370	35.597	5.090	26.225	1.00	0.00	RX0	C
ATOM	2066	C	GLY	370	36.324	4.429	25.048	1.00	0.00	RX0	C
ATOM	2067	O	GLY	370	36.946	3.386	25.214	1.00	0.00	RX0	O
ATOM	2068	N	MET	371	36.380	5.143	23.928	1.00	0.00	RX0	N
ATOM	2069	H	MET	371	35.906	6.023	23.910	1.00	0.00	RX0	H
ATOM	2070	CA	MET	371	37.103	4.709	22.719	1.00	0.00	RX0	C
ATOM	2071	CB	MET	371	36.783	5.589	21.509	1.00	0.00	RX0	C
ATOM	2072	CG	MET	371	35.370	5.380	20.965	1.00	0.00	RX0	C

ATOM	2073	SD	MET	371	35.065	3.681	20.454	1.00	0.00	RX0	S
ATOM	2074	CE	MET	371	36.234	3.605	19.087	1.00	0.00	RX0	C
ATOM	2075	C	MET	371	38.619	4.671	22.937	1.00	0.00	RX0	C
ATOM	2076	O	MET	371	39.258	3.669	22.631	1.00	0.00	RX0	O
ATOM	2077	N	GLU	372	39.117	5.692	23.643	1.00	0.00	RX0	N
ATOM	2078	H	GLU	372	38.524	6.472	23.853	1.00	0.00	RX0	H
ATOM	2079	CA	GLU	372	40.525	5.740	24.082	1.00	0.00	RX0	C
ATOM	2080	CB	GLU	372	40.833	7.016	24.865	1.00	0.00	RX0	C
ATOM	2081	CG	GLU	372	40.644	8.305	24.072	1.00	0.00	RX0	C
ATOM	2082	CD	GLU	372	41.052	9.479	24.935	1.00	0.00	RX0	C
ATOM	2083	OE1	GLU	372	40.333	10.470	24.994	1.00	0.00	RX0	O
ATOM	2084	OE2	GLU	372	42.081	9.408	25.602	1.00	0.00	RX0	O
ATOM	2085	C	GLU	372	40.884	4.541	24.968	1.00	0.00	RX0	C
ATOM	2086	O	GLU	372	41.865	3.853	24.723	1.00	0.00	RX0	O
ATOM	2087	N	HIS	373	40.006	4.291	25.945	1.00	0.00	RX0	N
ATOM	2088	H	HIS	373	39.216	4.898	26.041	1.00	0.00	RX0	H
ATOM	2089	CA	HIS	373	40.194	3.212	26.919	1.00	0.00	RX0	C
ATOM	2090	CB	HIS	373	39.259	3.439	28.116	1.00	0.00	RX0	C
ATOM	2091	CG	HIS	373	38.396	2.242	28.438	1.00	0.00	RX0	C
ATOM	2092	ND1	HIS	373	37.287	1.914	27.748	1.00	0.00	RX0	N
ATOM	2093	HD1	HIS	373	36.940	2.372	26.949	1.00	0.00	RX0	H
ATOM	2094	CD2	HIS	373	38.560	1.332	29.488	1.00	0.00	RX0	C
ATOM	2095	NE2	HIS	373	37.531	0.451	29.423	1.00	0.00	RX0	N
ATOM	2096	CE1	HIS	373	36.750	0.809	28.355	1.00	0.00	RX0	C
ATOM	2097	C	HIS	373	40.078	1.832	26.262	1.00	0.00	RX0	C
ATOM	2098	O	HIS	373	40.934	1.001	26.470	1.00	0.00	RX0	O
ATOM	2099	N	LEU	374	39.111	1.679	25.351	1.00	0.00	RX0	N
ATOM	2100	H	LEU	374	38.544	2.468	25.124	1.00	0.00	RX0	H
ATOM	2101	CA	LEU	374	38.919	0.408	24.629	1.00	0.00	RX0	C
ATOM	2102	CB	LEU	374	37.660	0.451	23.762	1.00	0.00	RX0	C
ATOM	2103	CG	LEU	374	36.366	0.289	24.558	1.00	0.00	RX0	C
ATOM	2104	CD1	LEU	374	35.134	0.593	23.705	1.00	0.00	RX0	C
ATOM	2105	CD2	LEU	374	36.285	-1.086	25.222	1.00	0.00	RX0	C
ATOM	2106	C	LEU	374	40.113	0.051	23.744	1.00	0.00	RX0	C
ATOM	2107	O	LEU	374	40.555	-1.109	23.754	1.00	0.00	RX0	O
ATOM	2108	N	TYR	375	40.664	1.068	23.103	1.00	0.00	RX0	N
ATOM	2109	H	TYR	375	40.277	1.984	23.240	1.00	0.00	RX0	H
ATOM	2110	CA	TYR	375	41.854	0.983	22.222	1.00	0.00	RX0	C
ATOM	2111	CB	TYR	375	41.926	2.183	21.277	1.00	0.00	RX0	C
ATOM	2112	CG	TYR	375	41.009	1.896	20.112	1.00	0.00	RX0	C
ATOM	2113	CD1	TYR	375	40.527	0.605	19.934	1.00	0.00	RX0	C
ATOM	2114	CE1	TYR	375	39.725	0.303	18.843	1.00	0.00	RX0	C
ATOM	2115	CD2	TYR	375	40.662	2.897	19.210	1.00	0.00	RX0	C
ATOM	2116	CE2	TYR	375	39.863	2.592	18.112	1.00	0.00	RX0	C
ATOM	2117	CZ	TYR	375	39.407	1.291	17.921	1.00	0.00	RX0	C
ATOM	2118	OH	TYR	375	38.647	0.963	16.815	1.00	0.00	RX0	O
ATOM	2119	HH	TYR	375	38.571	1.726	16.250	1.00	0.00	RX0	H
ATOM	2120	C	TYR	375	43.165	0.750	22.977	1.00	0.00	RX0	C
ATOM	2121	O	TYR	375	44.176	1.452	22.753	1.00	0.00	RX0	O
ATOM	2122	N	SER	376	43.162	-0.226	23.845	1.00	0.00	RX0	N
ATOM	2123	H	SER	376	42.305	-0.666	24.097	1.00	0.00	RX0	H
ATOM	2124	CA	SER	376	44.345	-0.646	24.634	1.00	0.00	RX0	C
ATOM	2125	CB	SER	376	44.678	0.524	25.572	1.00	0.00	RX0	C
ATOM	2126	OG	SER	376	43.615	0.773	26.495	1.00	0.00	RX0	O
ATOM	2127	HG	SER	376	42.855	1.105	26.022	1.00	0.00	RX0	H
ATOM	2128	C	SER	376	44.183	-1.992	25.350	1.00	0.00	RX0	C
ATOM	2129	O	SER	376	44.985	-2.369	26.199	1.00	0.00	RX0	O
ATOM	2130	N	MET	377	43.156	-2.746	24.938	1.00	0.00	RX0	N
ATOM	2131	H	MET	377	42.543	-2.439	24.208	1.00	0.00	RX0	H
ATOM	2132	CA	MET	377	42.915	-4.112	25.422	1.00	0.00	RX0	C
ATOM	2133	CB	MET	377	41.770	-4.074	26.439	1.00	0.00	RX0	C

ATOM	2134	CG	MET	377	42.193	-3.552	27.813	1.00	0.00	RX0	C
ATOM	2135	SD	MET	377	40.787	-3.356	28.912	1.00	0.00	RX0	S
ATOM	2136	CE	MET	377	40.021	-1.973	28.050	1.00	0.00	RX0	C
ATOM	2137	C	MET	377	42.647	-5.080	24.257	1.00	0.00	RX0	C
ATOM	2138	O	MET	377	43.092	-4.854	23.130	1.00	0.00	RX0	O
ATOM	2139	N	LYS	378	41.856	-6.107	24.527	1.00	0.00	RX0	N
ATOM	2140	H	LYS	378	41.434	-6.174	25.424	1.00	0.00	RX0	H
ATOM	2141	CA	LYS	378	41.570	-7.191	23.576	1.00	0.00	RX0	C
ATOM	2142	CB	LYS	378	41.572	-8.553	24.302	1.00	0.00	RX0	C
ATOM	2143	CG	LYS	378	41.101	-8.576	25.767	1.00	0.00	RX0	C
ATOM	2144	CD	LYS	378	42.142	-9.174	26.733	1.00	0.00	RX0	C
ATOM	2145	CE	LYS	378	41.673	-9.198	28.197	1.00	0.00	RX0	C
ATOM	2146	NZ	LYS	378	42.709	-9.726	29.101	1.00	0.00	RX0	N
ATOM	2147	HZ1	LYS	378	42.385	-9.659	30.096	1.00	0.00	RX0	H
ATOM	2148	HZ2	LYS	378	42.936	-10.723	28.930	1.00	0.00	RX0	H
ATOM	2149	HZ3	LYS	378	43.581	-9.164	29.088	1.00	0.00	RX0	H
ATOM	2150	C	LYS	378	40.316	-6.920	22.720	1.00	0.00	RX0	C
ATOM	2151	O	LYS	378	40.245	-7.352	21.611	1.00	0.00	RX0	O
ATOM	2152	N	CYS	379	39.320	-6.266	23.410	1.00	0.00	RX0	N
ATOM	2153	H	CYS	379	39.553	-5.917	24.312	1.00	0.00	RX0	H
ATOM	2154	CA	CYS	379	37.939	-6.053	22.985	1.00	0.00	RX0	C
ATOM	2155	CB	CYS	379	37.616	-4.558	23.097	1.00	0.00	RX0	C
ATOM	2156	SG	CYS	379	35.848	-4.174	23.049	1.00	0.00	RX0	S
ATOM	2157	C	CYS	379	37.599	-6.654	21.614	1.00	0.00	RX0	C
ATOM	2158	O	CYS	379	37.115	-7.773	21.559	1.00	0.00	RX0	O
ATOM	2159	N	LYS	380	38.012	-5.930	20.563	1.00	0.00	RX0	N
ATOM	2160	H	LYS	380	38.378	-5.003	20.645	1.00	0.00	RX0	H
ATOM	2161	CA	LYS	380	37.949	-6.383	19.165	1.00	0.00	RX0	C
ATOM	2162	CB	LYS	380	36.461	-6.779	18.906	1.00	0.00	RX0	C
ATOM	2163	CG	LYS	380	35.816	-7.145	17.547	1.00	0.00	RX0	C
ATOM	2164	CD	LYS	380	34.297	-7.442	17.718	1.00	0.00	RX0	C
ATOM	2165	CE	LYS	380	33.471	-7.766	16.448	1.00	0.00	RX0	C
ATOM	2166	NZ	LYS	380	32.047	-8.039	16.749	1.00	0.00	RX0	N
ATOM	2167	HZ1	LYS	380	31.449	-8.240	15.917	1.00	0.00	RX0	H
ATOM	2168	HZ2	LYS	380	31.908	-8.845	17.396	1.00	0.00	RX0	H
ATOM	2169	HZ3	LYS	380	31.569	-7.275	17.274	1.00	0.00	RX0	H
ATOM	2170	C	LYS	380	38.311	-5.215	18.248	1.00	0.00	RX0	C
ATOM	2171	O	LYS	380	37.971	-4.057	18.518	1.00	0.00	RX0	O
ATOM	2172	N	ASN	381	38.881	-5.592	17.123	1.00	0.00	RX0	N
ATOM	2173	H	ASN	381	39.190	-6.541	17.049	1.00	0.00	RX0	H
ATOM	2174	CA	ASN	381	39.208	-4.700	15.996	1.00	0.00	RX0	C
ATOM	2175	CB	ASN	381	40.400	-3.834	16.410	1.00	0.00	RX0	C
ATOM	2176	CG	ASN	381	40.177	-2.364	16.128	1.00	0.00	RX0	C
ATOM	2177	OD1	ASN	381	41.067	-1.663	15.648	1.00	0.00	RX0	O
ATOM	2178	ND2	ASN	381	38.974	-1.910	16.524	1.00	0.00	RX0	N
ATOM	2179	HD21	ASN	381	38.311	-2.498	16.998	1.00	0.00	RX0	H
ATOM	2180	HD22	ASN	381	38.694	-0.950	16.411	1.00	0.00	RX0	H
ATOM	2181	C	ASN	381	39.620	-5.523	14.759	1.00	0.00	RX0	C
ATOM	2182	O	ASN	381	40.286	-5.062	13.834	1.00	0.00	RX0	O
ATOM	2183	N	VAL	382	39.176	-6.776	14.737	1.00	0.00	RX0	N
ATOM	2184	H	VAL	382	38.450	-7.045	15.365	1.00	0.00	RX0	H
ATOM	2185	CA	VAL	382	39.338	-7.659	13.572	1.00	0.00	RX0	C
ATOM	2186	CB	VAL	382	39.241	-9.119	14.009	1.00	0.00	RX0	C
ATOM	2187	CG1	VAL	382	39.314	-10.060	12.807	1.00	0.00	RX0	C
ATOM	2188	CG2	VAL	382	40.309	-9.430	15.059	1.00	0.00	RX0	C
ATOM	2189	C	VAL	382	38.195	-7.277	12.635	1.00	0.00	RX0	C
ATOM	2190	O	VAL	382	37.049	-7.276	13.059	1.00	0.00	RX0	O
ATOM	2191	N	VAL	383	38.551	-7.063	11.363	1.00	0.00	RX0	N
ATOM	2192	H	VAL	383	39.526	-7.051	11.156	1.00	0.00	RX0	H
ATOM	2193	CA	VAL	383	37.600	-6.539	10.366	1.00	0.00	RX0	C
ATOM	2194	CB	VAL	383	36.434	-7.503	10.110	1.00	0.00	RX0	C

ATOM	2195	CG1	VAL	383	35.389	-6.885	9.178	1.00	0.00	RX0	C
ATOM	2196	CG2	VAL	383	36.949	-8.846	9.587	1.00	0.00	RX0	C
ATOM	2197	C	VAL	383	37.102	-5.173	10.877	1.00	0.00	RX0	C
ATOM	2198	O	VAL	383	36.355	-5.097	11.860	1.00	0.00	RX0	O
ATOM	2199	N	PRO	384	37.530	-4.088	10.236	1.00	0.00	RX0	N
ATOM	2200	CD	PRO	384	38.371	-4.092	9.049	1.00	0.00	RX0	C
ATOM	2201	CA	PRO	384	37.142	-2.731	10.663	1.00	0.00	RX0	C
ATOM	2202	CB	PRO	384	37.743	-1.835	9.571	1.00	0.00	RX0	C
ATOM	2203	CG	PRO	384	38.082	-2.749	8.394	1.00	0.00	RX0	C
ATOM	2204	C	PRO	384	35.624	-2.628	10.828	1.00	0.00	RX0	C
ATOM	2205	O	PRO	384	34.847	-3.282	10.112	1.00	0.00	RX0	O
ATOM	2206	N	LEU	385	35.228	-1.769	11.750	1.00	0.00	RX0	N
ATOM	2207	H	LEU	385	35.903	-1.161	12.171	1.00	0.00	RX0	H
ATOM	2208	CA	LEU	385	33.812	-1.621	12.127	1.00	0.00	RX0	C
ATOM	2209	CB	LEU	385	33.685	-0.660	13.308	1.00	0.00	RX0	C
ATOM	2210	CG	LEU	385	32.291	-0.680	13.934	1.00	0.00	RX0	C
ATOM	2211	CD1	LEU	385	31.902	-2.075	14.427	1.00	0.00	RX0	C
ATOM	2212	CD2	LEU	385	32.152	0.372	15.031	1.00	0.00	RX0	C
ATOM	2213	C	LEU	385	32.896	-1.213	10.964	1.00	0.00	RX0	C
ATOM	2214	O	LEU	385	31.809	-1.761	10.815	1.00	0.00	RX0	O
ATOM	2215	N	TYR	386	33.449	-0.406	10.055	1.00	0.00	RX0	N
ATOM	2216	H	TYR	386	34.240	0.139	10.340	1.00	0.00	RX0	H
ATOM	2217	CA	TYR	386	32.718	0.069	8.867	1.00	0.00	RX0	C
ATOM	2218	CB	TYR	386	33.622	0.932	7.969	1.00	0.00	RX0	C
ATOM	2219	CG	TYR	386	32.782	1.614	6.910	1.00	0.00	RX0	C
ATOM	2220	CD1	TYR	386	32.412	2.945	7.069	1.00	0.00	RX0	C
ATOM	2221	CE1	TYR	386	31.520	3.532	6.177	1.00	0.00	RX0	C
ATOM	2222	CD2	TYR	386	32.345	0.907	5.795	1.00	0.00	RX0	C
ATOM	2223	CE2	TYR	386	31.433	1.478	4.921	1.00	0.00	RX0	C
ATOM	2224	CZ	TYR	386	30.988	2.775	5.138	1.00	0.00	RX0	C
ATOM	2225	OH	TYR	386	30.003	3.296	4.319	1.00	0.00	RX0	O
ATOM	2226	HH	TYR	386	29.568	2.568	3.892	1.00	0.00	RX0	H
ATOM	2227	C	TYR	386	32.144	-1.103	8.049	1.00	0.00	RX0	C
ATOM	2228	O	TYR	386	30.957	-1.141	7.757	1.00	0.00	RX0	O
ATOM	2229	N	ASP	387	32.969	-2.141	7.903	1.00	0.00	RX0	N
ATOM	2230	H	ASP	387	33.935	-2.070	8.163	1.00	0.00	RX0	H
ATOM	2231	CA	ASP	387	32.619	-3.323	7.093	1.00	0.00	RX0	C
ATOM	2232	CB	ASP	387	33.875	-4.141	6.786	1.00	0.00	RX0	C
ATOM	2233	CG	ASP	387	34.875	-3.315	6.000	1.00	0.00	RX0	C
ATOM	2234	OD1	ASP	387	35.409	-2.347	6.544	1.00	0.00	RX0	O
ATOM	2235	OD2	ASP	387	35.131	-3.649	4.846	1.00	0.00	RX0	O
ATOM	2236	C	ASP	387	31.563	-4.207	7.765	1.00	0.00	RX0	C
ATOM	2237	O	ASP	387	30.619	-4.649	7.114	1.00	0.00	RX0	O
ATOM	2238	N	LEU	388	31.677	-4.332	9.088	1.00	0.00	RX0	N
ATOM	2239	H	LEU	388	32.442	-3.853	9.525	1.00	0.00	RX0	H
ATOM	2240	CA	LEU	388	30.691	-5.072	9.891	1.00	0.00	RX0	C
ATOM	2241	CB	LEU	388	31.226	-5.198	11.319	1.00	0.00	RX0	C
ATOM	2242	CG	LEU	388	30.363	-6.039	12.260	1.00	0.00	RX0	C
ATOM	2243	CD1	LEU	388	30.258	-7.496	11.805	1.00	0.00	RX0	C
ATOM	2244	CD2	LEU	388	30.840	-5.907	13.706	1.00	0.00	RX0	C
ATOM	2245	C	LEU	388	29.315	-4.386	9.871	1.00	0.00	RX0	C
ATOM	2246	O	LEU	388	28.299	-5.023	9.588	1.00	0.00	RX0	O
ATOM	2247	N	LEU	389	29.339	-3.070	10.049	1.00	0.00	RX0	N
ATOM	2248	H	LEU	389	30.231	-2.637	10.179	1.00	0.00	RX0	H
ATOM	2249	CA	LEU	389	28.129	-2.229	9.970	1.00	0.00	RX0	C
ATOM	2250	CB	LEU	389	28.422	-0.788	10.367	1.00	0.00	RX0	C
ATOM	2251	CG	LEU	389	28.890	-0.618	11.808	1.00	0.00	RX0	C
ATOM	2252	CD1	LEU	389	29.332	0.817	12.060	1.00	0.00	RX0	C
ATOM	2253	CD2	LEU	389	27.839	-1.059	12.824	1.00	0.00	RX0	C
ATOM	2254	C	LEU	389	27.489	-2.268	8.583	1.00	0.00	RX0	C
ATOM	2255	O	LEU	389	26.284	-2.473	8.458	1.00	0.00	RX0	O

ATOM	2256	N	LEU	390	28.350	-2.246	7.565	1.00	0.00	RX0	N
ATOM	2257	H	LEU	390	29.321	-2.131	7.771	1.00	0.00	RX0	H
ATOM	2258	CA	LEU	390	27.929	-2.335	6.162	1.00	0.00	RX0	C
ATOM	2259	CB	LEU	390	29.174	-2.204	5.281	1.00	0.00	RX0	C
ATOM	2260	CG	LEU	390	28.929	-2.027	3.785	1.00	0.00	RX0	C
ATOM	2261	CD1	LEU	390	28.369	-0.643	3.467	1.00	0.00	RX0	C
ATOM	2262	CD2	LEU	390	30.199	-2.301	2.977	1.00	0.00	RX0	C
ATOM	2263	C	LEU	390	27.202	-3.653	5.862	1.00	0.00	RX0	C
ATOM	2264	O	LEU	390	26.123	-3.627	5.302	1.00	0.00	RX0	O
ATOM	2265	N	GLU	391	27.733	-4.754	6.404	1.00	0.00	RX0	N
ATOM	2266	H	GLU	391	28.619	-4.677	6.871	1.00	0.00	RX0	H
ATOM	2267	CA	GLU	391	27.124	-6.090	6.264	1.00	0.00	RX0	C
ATOM	2268	CB	GLU	391	28.103	-7.112	6.884	1.00	0.00	RX0	C
ATOM	2269	CG	GLU	391	27.752	-8.614	6.881	1.00	0.00	RX0	C
ATOM	2270	CD	GLU	391	27.098	-9.039	8.187	1.00	0.00	RX0	C
ATOM	2271	OE1	GLU	391	27.549	-9.997	8.815	1.00	0.00	RX0	O
ATOM	2272	OE2	GLU	391	26.122	-8.414	8.578	1.00	0.00	RX0	O
ATOM	2273	C	GLU	391	25.713	-6.159	6.867	1.00	0.00	RX0	C
ATOM	2274	O	GLU	391	24.778	-6.600	6.214	1.00	0.00	RX0	O
ATOM	2275	N	MET	392	25.589	-5.588	8.070	1.00	0.00	RX0	N
ATOM	2276	H	MET	392	26.454	-5.364	8.525	1.00	0.00	RX0	H
ATOM	2277	CA	MET	392	24.313	-5.555	8.807	1.00	0.00	RX0	C
ATOM	2278	CB	MET	392	24.554	-5.160	10.264	1.00	0.00	RX0	C
ATOM	2279	CG	MET	392	25.334	-6.218	11.040	1.00	0.00	RX0	C
ATOM	2280	SD	MET	392	25.421	-5.883	12.807	1.00	0.00	RX0	S
ATOM	2281	CE	MET	392	26.748	-4.676	12.764	1.00	0.00	RX0	C
ATOM	2282	C	MET	392	23.266	-4.630	8.173	1.00	0.00	RX0	C
ATOM	2283	O	MET	392	22.107	-5.006	8.028	1.00	0.00	RX0	O
ATOM	2284	N	LEU	393	23.721	-3.452	7.754	1.00	0.00	RX0	N
ATOM	2285	H	LEU	393	24.706	-3.291	7.829	1.00	0.00	RX0	H
ATOM	2286	CA	LEU	393	22.852	-2.457	7.099	1.00	0.00	RX0	C
ATOM	2287	CB	LEU	393	23.506	-1.078	7.150	1.00	0.00	RX0	C
ATOM	2288	CG	LEU	393	23.415	-0.427	8.529	1.00	0.00	RX0	C
ATOM	2289	CD1	LEU	393	24.502	0.625	8.730	1.00	0.00	RX0	C
ATOM	2290	CD2	LEU	393	22.019	0.140	8.793	1.00	0.00	RX0	C
ATOM	2291	C	LEU	393	22.462	-2.815	5.663	1.00	0.00	RX0	C
ATOM	2292	O	LEU	393	21.313	-2.601	5.257	1.00	0.00	RX0	O
ATOM	2293	N	ASP	394	23.400	-3.384	4.923	1.00	0.00	RX0	N
ATOM	2294	H	ASP	394	24.261	-3.668	5.337	1.00	0.00	RX0	H
ATOM	2295	CA	ASP	394	23.187	-3.768	3.516	1.00	0.00	RX0	C
ATOM	2296	CB	ASP	394	24.205	-3.553	2.407	1.00	0.00	RX0	C
ATOM	2297	CG	ASP	394	23.356	-3.359	1.134	1.00	0.00	RX0	C
ATOM	2298	OD1	ASP	394	22.124	-3.239	1.225	1.00	0.00	RX0	O
ATOM	2299	OD2	ASP	394	23.917	-3.308	0.041	1.00	0.00	RX0	O
ATOM	2300	C	ASP	394	22.492	-5.132	3.432	1.00	0.00	RX0	C
ATOM	2301	O	ASP	394	23.034	-6.108	2.888	1.00	0.00	RX0	O
ATOM	2302	N	ALA	395	21.256	-5.134	3.874	1.00	0.00	RX0	N
ATOM	2303	H	ALA	395	20.921	-4.236	4.161	1.00	0.00	RX0	H
ATOM	2304	CA	ALA	395	20.412	-6.338	3.880	1.00	0.00	RX0	C
ATOM	2305	CB	ALA	395	19.459	-6.295	5.060	1.00	0.00	RX0	C
ATOM	2306	C	ALA	395	19.639	-6.454	2.565	1.00	0.00	RX0	C
ATOM	2307	O	ALA	395	19.303	-5.463	1.911	1.00	0.00	RX0	O
ATOM	2308	N	HIS	396	19.441	-7.696	2.156	1.00	0.00	RX0	N
ATOM	2309	H	HIS	396	19.789	-8.400	2.774	1.00	0.00	RX0	H
ATOM	2310	CA	HIS	396	18.536	-7.999	1.035	1.00	0.00	RX0	C
ATOM	2311	CB	HIS	396	18.861	-9.433	0.577	1.00	0.00	RX0	C
ATOM	2312	CG	HIS	396	18.943	-10.372	1.775	1.00	0.00	RX0	C
ATOM	2313	ND1	HIS	396	20.092	-10.707	2.394	1.00	0.00	RX0	N
ATOM	2314	HD1	HIS	396	21.004	-10.424	2.167	1.00	0.00	RX0	H
ATOM	2315	CD2	HIS	396	17.891	-11.002	2.449	1.00	0.00	RX0	C
ATOM	2316	NE2	HIS	396	18.412	-11.714	3.474	1.00	0.00	RX0	N

ATOM	2317	CE1	HIS	396	19.773	-11.533	3.443	1.00	0.00	RX0	C
ATOM	2318	C	HIS	396	17.077	-7.909	1.523	1.00	0.00	RX0	C
ATOM	2319	O	HIS	396	16.821	-7.755	2.721	1.00	0.00	RX0	O
ATOM	2320	N	ARG	397	16.142	-8.174	0.629	1.00	0.00	RX0	N
ATOM	2321	H	ARG	397	16.413	-8.333	-0.319	1.00	0.00	RX0	H
ATOM	2322	CA	ARG	397	14.704	-8.151	0.982	1.00	0.00	RX0	C
ATOM	2323	CB	ARG	397	14.117	-6.929	0.227	1.00	0.00	RX0	C
ATOM	2324	CG	ARG	397	14.906	-5.589	0.444	1.00	0.00	RX0	C
ATOM	2325	CD	ARG	397	16.073	-5.228	-0.530	1.00	0.00	RX0	C
ATOM	2326	NE	ARG	397	17.279	-4.675	0.128	1.00	0.00	RX0	N
ATOM	2327	HE	ARG	397	17.517	-4.941	1.070	1.00	0.00	RX0	H
ATOM	2328	CZ	ARG	397	18.234	-3.941	-0.522	1.00	0.00	RX0	C
ATOM	2329	NH1	ARG	397	18.015	-3.508	-1.761	1.00	0.00	RX0	N
ATOM	2330	HH11	ARG	397	18.725	-2.997	-2.247	1.00	0.00	RX0	H
ATOM	2331	HH12	ARG	397	17.144	-3.685	-2.219	1.00	0.00	RX0	H
ATOM	2332	NH2	ARG	397	19.403	-3.658	0.067	1.00	0.00	RX0	N
ATOM	2333	HH21	ARG	397	20.149	-3.109	-0.323	1.00	0.00	RX0	H
ATOM	2334	HH22	ARG	397	19.623	-4.031	0.979	1.00	0.00	RX0	H
ATOM	2335	C	ARG	397	14.082	-9.540	0.774	1.00	0.00	RX0	C
ATOM	2336	O	ARG	397	12.875	-9.706	0.613	1.00	0.00	RX0	O
ATOM	2337	N	LEU	398	14.938	-10.556	0.835	1.00	0.00	RX0	N
ATOM	2338	H	LEU	398	15.878	-10.339	1.081	1.00	0.00	RX0	H
ATOM	2339	CA	LEU	398	14.565	-11.951	0.542	1.00	0.00	RX0	C
ATOM	2340	CB	LEU	398	15.788	-12.807	0.210	1.00	0.00	RX0	C
ATOM	2341	CG	LEU	398	16.569	-12.312	-1.010	1.00	0.00	RX0	C
ATOM	2342	CD1	LEU	398	17.867	-13.097	-1.200	1.00	0.00	RX0	C
ATOM	2343	CD2	LEU	398	15.717	-12.298	-2.281	1.00	0.00	RX0	C
ATOM	2344	C	LEU	398	13.757	-12.584	1.680	1.00	0.00	RX0	C
ATOM	2345	O	LEU	398	12.772	-13.259	1.439	1.00	0.00	RX0	O
ATOM	2346	N	HIS	399	14.100	-12.169	2.904	1.00	0.00	RX0	N
ATOM	2347	H	HIS	399	14.856	-11.523	2.990	1.00	0.00	RX0	H
ATOM	2348	CA	HIS	399	13.392	-12.616	4.118	1.00	0.00	RX0	C
ATOM	2349	CB	HIS	399	14.412	-12.677	5.245	1.00	0.00	RX0	C
ATOM	2350	CG	HIS	399	15.296	-13.867	4.942	1.00	0.00	RX0	C
ATOM	2351	ND1	HIS	399	16.630	-13.818	4.769	1.00	0.00	RX0	N
ATOM	2352	HD1	HIS	399	17.211	-13.024	4.795	1.00	0.00	RX0	H
ATOM	2353	CD2	HIS	399	14.876	-15.189	4.772	1.00	0.00	RX0	C
ATOM	2354	NE2	HIS	399	15.965	-15.946	4.497	1.00	0.00	RX0	N
ATOM	2355	CE1	HIS	399	17.049	-15.096	4.496	1.00	0.00	RX0	C
ATOM	2356	C	HIS	399	12.131	-11.786	4.401	1.00	0.00	RX0	C
ATOM	2357	O	HIS	399	11.630	-11.743	5.524	1.00	0.00	RX0	O
ATOM	2358	N	ALA	400	11.619	-11.143	3.351	1.00	0.00	RX0	N
ATOM	2359	H	ALA	400	12.047	-11.257	2.457	1.00	0.00	RX0	H
ATOM	2360	CA	ALA	400	10.356	-10.385	3.402	1.00	0.00	RX0	C
ATOM	2361	CB	ALA	400	10.146	-9.561	2.134	1.00	0.00	RX0	C
ATOM	2362	C	ALA	400	9.147	-11.323	3.579	1.00	0.00	RX0	C
ATOM	2363	O	ALA	400	8.508	-11.220	4.647	1.00	0.00	RX0	O
ATOM	2364	N	GLU	26	48.123	22.246	20.338	1.00	0.00	RX1	N
ATOM	2365	H	GLU	26	47.724	22.311	21.249	1.00	0.00	RX1	H
ATOM	2366	CA	GLU	26	49.563	21.985	20.384	1.00	0.00	RX1	C
ATOM	2367	CB	GLU	26	50.346	23.292	20.419	1.00	0.00	RX1	C
ATOM	2368	CG	GLU	26	50.107	24.195	19.213	1.00	0.00	RX1	C
ATOM	2369	CD	GLU	26	50.964	25.427	19.390	1.00	0.00	RX1	C
ATOM	2370	OE1	GLU	26	51.306	26.059	18.392	1.00	0.00	RX1	O
ATOM	2371	OE2	GLU	26	51.301	25.738	20.532	1.00	0.00	RX1	O
ATOM	2372	C	GLU	26	49.990	21.137	21.567	1.00	0.00	RX1	C
ATOM	2373	O	GLU	26	49.196	20.776	22.437	1.00	0.00	RX1	O
ATOM	2374	N	GLU	27	51.289	20.806	21.561	1.00	0.00	RX1	N
ATOM	2375	H	GLU	27	51.932	21.186	20.897	1.00	0.00	RX1	H
ATOM	2376	CA	GLU	27	51.758	19.965	22.657	1.00	0.00	RX1	C
ATOM	2377	CB	GLU	27	52.960	19.105	22.256	1.00	0.00	RX1	C

ATOM	2378	CG	GLU	27	52.880	18.472	20.863	1.00	0.00	RX1	C
ATOM	2379	CD	GLU	27	51.603	17.675	20.677	1.00	0.00	RX1	C
ATOM	2380	OE1	GLU	27	51.088	17.095	21.629	1.00	0.00	RX1	O
ATOM	2381	OE2	GLU	27	51.087	17.660	19.568	1.00	0.00	RX1	O
ATOM	2382	C	GLU	27	52.082	20.753	23.908	1.00	0.00	RX1	C
ATOM	2383	O	GLU	27	53.220	21.103	24.199	1.00	0.00	RX1	O
ATOM	2384	N	LYS	28	51.000	21.021	24.653	1.00	0.00	RX1	N
ATOM	2385	H	LYS	28	50.122	20.659	24.342	1.00	0.00	RX1	H
ATOM	2386	CA	LYS	28	51.192	21.622	25.973	1.00	0.00	RX1	C
ATOM	2387	CB	LYS	28	49.839	21.928	26.620	1.00	0.00	RX1	C
ATOM	2388	CG	LYS	28	48.804	22.646	25.744	1.00	0.00	RX1	C
ATOM	2389	CD	LYS	28	47.496	22.884	26.513	1.00	0.00	RX1	C
ATOM	2390	CE	LYS	28	46.364	23.520	25.695	1.00	0.00	RX1	C
ATOM	2391	NZ	LYS	28	45.227	23.818	26.580	1.00	0.00	RX1	N
ATOM	2392	HZ1	LYS	28	44.485	24.339	26.073	1.00	0.00	RX1	H
ATOM	2393	HZ2	LYS	28	44.820	22.935	26.962	1.00	0.00	RX1	H
ATOM	2394	HZ3	LYS	28	45.557	24.411	27.371	1.00	0.00	RX1	H
ATOM	2395	C	LYS	28	51.989	20.677	26.860	1.00	0.00	RX1	C
ATOM	2396	O	LYS	28	51.841	19.464	26.759	1.00	0.00	RX1	O
ATOM	2397	N	LYS	29	52.865	21.249	27.696	1.00	0.00	RX1	N
ATOM	2398	H	LYS	29	52.808	22.212	27.968	1.00	0.00	RX1	H
ATOM	2399	CA	LYS	29	53.744	20.316	28.398	1.00	0.00	RX1	C
ATOM	2400	CB	LYS	29	55.015	21.010	28.892	1.00	0.00	RX1	C
ATOM	2401	CG	LYS	29	55.447	22.111	27.920	1.00	0.00	RX1	C
ATOM	2402	CD	LYS	29	56.684	22.890	28.366	1.00	0.00	RX1	C
ATOM	2403	CE	LYS	29	56.664	23.349	29.828	1.00	0.00	RX1	C
ATOM	2404	NZ	LYS	29	55.501	24.181	30.161	1.00	0.00	RX1	N
ATOM	2405	HZ1	LYS	29	55.657	24.670	31.068	1.00	0.00	RX1	H
ATOM	2406	HZ2	LYS	29	55.211	24.889	29.456	1.00	0.00	RX1	H
ATOM	2407	HZ3	LYS	29	54.636	23.618	30.294	1.00	0.00	RX1	H
ATOM	2408	C	LYS	29	53.017	19.595	29.512	1.00	0.00	RX1	C
ATOM	2409	O	LYS	29	52.391	20.188	30.385	1.00	0.00	RX1	O
ATOM	2410	N	VAL	30	53.096	18.269	29.393	1.00	0.00	RX1	N
ATOM	2411	H	VAL	30	53.706	17.850	28.725	1.00	0.00	RX1	H
ATOM	2412	CA	VAL	30	52.361	17.444	30.340	1.00	0.00	RX1	C
ATOM	2413	CB	VAL	30	51.748	16.241	29.623	1.00	0.00	RX1	C
ATOM	2414	CG1	VAL	30	50.782	16.701	28.531	1.00	0.00	RX1	C
ATOM	2415	CG2	VAL	30	52.829	15.308	29.072	1.00	0.00	RX1	C
ATOM	2416	C	VAL	30	53.222	16.998	31.503	1.00	0.00	RX1	C
ATOM	2417	O	VAL	30	54.444	16.972	31.418	1.00	0.00	RX1	O
ATOM	2418	N	CYS	31	52.530	16.620	32.579	1.00	0.00	RX1	N
ATOM	2419	H	CYS	31	51.533	16.712	32.641	1.00	0.00	RX1	H
ATOM	2420	CA	CYS	31	53.248	15.982	33.675	1.00	0.00	RX1	C
ATOM	2421	CB	CYS	31	53.494	16.983	34.799	1.00	0.00	RX1	C
ATOM	2422	SG	CYS	31	51.947	17.579	35.525	1.00	0.00	RX1	S
ATOM	2423	C	CYS	31	52.474	14.781	34.159	1.00	0.00	RX1	C
ATOM	2424	O	CYS	31	51.272	14.671	33.944	1.00	0.00	RX1	O
ATOM	2425	N	GLN	32	53.211	13.878	34.818	1.00	0.00	RX1	N
ATOM	2426	H	GLN	32	54.174	14.075	35.009	1.00	0.00	RX1	H
ATOM	2427	CA	GLN	32	52.601	12.612	35.225	1.00	0.00	RX1	C
ATOM	2428	CB	GLN	32	53.624	11.648	35.844	1.00	0.00	RX1	C
ATOM	2429	CG	GLN	32	54.779	11.207	34.934	1.00	0.00	RX1	C
ATOM	2430	CD	GLN	32	55.979	12.126	35.077	1.00	0.00	RX1	C
ATOM	2431	OE1	GLN	32	55.886	13.346	34.997	1.00	0.00	RX1	O
ATOM	2432	NE2	GLN	32	57.126	11.467	35.304	1.00	0.00	RX1	N
ATOM	2433	HE21	GLN	32	57.146	10.463	35.339	1.00	0.00	RX1	H
ATOM	2434	HE22	GLN	32	57.990	11.948	35.442	1.00	0.00	RX1	H
ATOM	2435	C	GLN	32	51.422	12.779	36.171	1.00	0.00	RX1	C
ATOM	2436	O	GLN	32	50.364	12.185	36.006	1.00	0.00	RX1	O
ATOM	2437	N	GLY	33	51.643	13.657	37.154	1.00	0.00	RX1	N
ATOM	2438	H	GLY	33	52.558	14.017	37.326	1.00	0.00	RX1	H

ATOM	2439	CA	GLY	33	50.589	13.819	38.149	1.00	0.00	RX1	C
ATOM	2440	C	GLY	33	50.900	13.023	39.395	1.00	0.00	RX1	C
ATOM	2441	O	GLY	33	51.757	12.148	39.393	1.00	0.00	RX1	O
ATOM	2442	N	THR	34	50.194	13.380	40.466	1.00	0.00	RX1	N
ATOM	2443	H	THR	34	49.498	14.100	40.497	1.00	0.00	RX1	H
ATOM	2444	CA	THR	34	50.445	12.650	41.700	1.00	0.00	RX1	C
ATOM	2445	CB	THR	34	50.592	13.717	42.777	1.00	0.00	RX1	C
ATOM	2446	OG1	THR	34	49.570	14.707	42.600	1.00	0.00	RX1	O
ATOM	2447	HG1	THR	34	48.824	14.323	43.066	1.00	0.00	RX1	H
ATOM	2448	CG2	THR	34	51.978	14.363	42.761	1.00	0.00	RX1	C
ATOM	2449	C	THR	34	49.373	11.610	41.990	1.00	0.00	RX1	C
ATOM	2450	O	THR	34	48.304	11.587	41.381	1.00	0.00	RX1	O
ATOM	2451	N	SER	35	49.720	10.726	42.935	1.00	0.00	RX1	N
ATOM	2452	H	SER	35	50.623	10.702	43.357	1.00	0.00	RX1	H
ATOM	2453	CA	SER	35	48.747	9.749	43.421	1.00	0.00	RX1	C
ATOM	2454	CB	SER	35	48.832	8.523	42.515	1.00	0.00	RX1	C
ATOM	2455	OG	SER	35	48.969	8.960	41.157	1.00	0.00	RX1	O
ATOM	2456	HG	SER	35	48.389	9.711	41.061	1.00	0.00	RX1	H
ATOM	2457	C	SER	35	48.985	9.439	44.888	1.00	0.00	RX1	C
ATOM	2458	O	SER	35	49.517	8.407	45.272	1.00	0.00	RX1	O
ATOM	2459	N	ASN	36	48.606	10.434	45.688	1.00	0.00	RX1	N
ATOM	2460	H	ASN	36	48.097	11.200	45.283	1.00	0.00	RX1	H
ATOM	2461	CA	ASN	36	48.909	10.410	47.115	1.00	0.00	RX1	C
ATOM	2462	CB	ASN	36	49.863	11.550	47.500	1.00	0.00	RX1	C
ATOM	2463	CG	ASN	36	49.129	12.723	48.125	1.00	0.00	RX1	C
ATOM	2464	OD1	ASN	36	48.796	12.726	49.302	1.00	0.00	RX1	O
ATOM	2465	ND2	ASN	36	48.935	13.755	47.294	1.00	0.00	RX1	N
ATOM	2466	HD21	ASN	36	49.080	13.660	46.303	1.00	0.00	RX1	H
ATOM	2467	HD22	ASN	36	48.622	14.662	47.595	1.00	0.00	RX1	H
ATOM	2468	C	ASN	36	47.669	10.387	47.991	1.00	0.00	RX1	C
ATOM	2469	O	ASN	36	47.633	9.767	49.046	1.00	0.00	RX1	O
ATOM	2470	N	LYS	37	46.654	11.125	47.508	1.00	0.00	RX1	N
ATOM	2471	H	LYS	37	46.745	11.553	46.605	1.00	0.00	RX1	H
ATOM	2472	CA	LYS	37	45.391	11.309	48.227	1.00	0.00	RX1	C
ATOM	2473	CB	LYS	37	44.309	10.334	47.745	1.00	0.00	RX1	C
ATOM	2474	CG	LYS	37	44.550	8.841	47.976	1.00	0.00	RX1	C
ATOM	2475	CD	LYS	37	43.265	8.002	47.978	1.00	0.00	RX1	C
ATOM	2476	CE	LYS	37	42.377	8.186	49.221	1.00	0.00	RX1	C
ATOM	2477	NZ	LYS	37	41.522	9.376	49.141	1.00	0.00	RX1	N
ATOM	2478	HZ1	LYS	37	40.848	9.406	49.935	1.00	0.00	RX1	H
ATOM	2479	HZ2	LYS	37	42.035	10.282	49.111	1.00	0.00	RX1	H
ATOM	2480	HZ3	LYS	37	40.871	9.329	48.328	1.00	0.00	RX1	H
ATOM	2481	C	LYS	37	45.418	11.399	49.754	1.00	0.00	RX1	C
ATOM	2482	O	LYS	37	44.701	10.687	50.452	1.00	0.00	RX1	O
ATOM	2483	N	LEU	38	46.268	12.347	50.201	1.00	0.00	RX1	N
ATOM	2484	H	LEU	38	46.866	12.776	49.523	1.00	0.00	RX1	H
ATOM	2485	CA	LEU	38	46.573	12.679	51.602	1.00	0.00	RX1	C
ATOM	2486	CB	LEU	38	45.354	12.647	52.533	1.00	0.00	RX1	C
ATOM	2487	CG	LEU	38	44.379	13.787	52.249	1.00	0.00	RX1	C
ATOM	2488	CD1	LEU	38	43.029	13.584	52.937	1.00	0.00	RX1	C
ATOM	2489	CD2	LEU	38	45.006	15.131	52.605	1.00	0.00	RX1	C
ATOM	2490	C	LEU	38	47.741	11.943	52.239	1.00	0.00	RX1	C
ATOM	2491	O	LEU	38	48.049	12.124	53.413	1.00	0.00	RX1	O
ATOM	2492	N	THR	39	48.417	11.135	51.419	1.00	0.00	RX1	N
ATOM	2493	H	THR	39	48.237	11.109	50.437	1.00	0.00	RX1	H
ATOM	2494	CA	THR	39	49.677	10.590	51.912	1.00	0.00	RX1	C
ATOM	2495	CB	THR	39	49.933	9.279	51.188	1.00	0.00	RX1	C
ATOM	2496	OG1	THR	39	48.729	8.501	51.217	1.00	0.00	RX1	O
ATOM	2497	HG1	THR	39	48.134	8.916	50.599	1.00	0.00	RX1	H
ATOM	2498	CG2	THR	39	51.106	8.506	51.795	1.00	0.00	RX1	C
ATOM	2499	C	THR	39	50.808	11.598	51.783	1.00	0.00	RX1	C

ATOM	2500	O	THR	39	51.340	11.850	50.710	1.00	0.00	RX1	O
ATOM	2501	N	GLN	40	51.148	12.172	52.949	1.00	0.00	RX1	N
ATOM	2502	H	GLN	40	50.656	11.904	53.776	1.00	0.00	RX1	H
ATOM	2503	CA	GLN	40	52.225	13.165	52.974	1.00	0.00	RX1	C
ATOM	2504	CB	GLN	40	52.406	13.690	54.400	1.00	0.00	RX1	C
ATOM	2505	CG	GLN	40	53.012	15.095	54.476	1.00	0.00	RX1	C
ATOM	2506	CD	GLN	40	53.024	15.572	55.918	1.00	0.00	RX1	C
ATOM	2507	OE1	GLN	40	54.004	16.108	56.426	1.00	0.00	RX1	O
ATOM	2508	NE2	GLN	40	51.859	15.365	56.564	1.00	0.00	RX1	N
ATOM	2509	HE21	GLN	40	51.058	14.950	56.133	1.00	0.00	RX1	H
ATOM	2510	HE22	GLN	40	51.765	15.633	57.523	1.00	0.00	RX1	H
ATOM	2511	C	GLN	40	53.534	12.666	52.373	1.00	0.00	RX1	C
ATOM	2512	O	GLN	40	54.169	11.742	52.865	1.00	0.00	RX1	O
ATOM	2513	N	LEU	41	53.884	13.312	51.253	1.00	0.00	RX1	N
ATOM	2514	H	LEU	41	53.316	14.058	50.914	1.00	0.00	RX1	H
ATOM	2515	CA	LEU	41	55.099	12.924	50.540	1.00	0.00	RX1	C
ATOM	2516	CB	LEU	41	55.010	13.367	49.080	1.00	0.00	RX1	C
ATOM	2517	CG	LEU	41	53.799	12.778	48.354	1.00	0.00	RX1	C
ATOM	2518	CD1	LEU	41	53.638	13.362	46.949	1.00	0.00	RX1	C
ATOM	2519	CD2	LEU	41	53.835	11.248	48.338	1.00	0.00	RX1	C
ATOM	2520	C	LEU	41	56.362	13.472	51.174	1.00	0.00	RX1	C
ATOM	2521	O	LEU	41	56.964	14.429	50.696	1.00	0.00	RX1	O
ATOM	2522	N	GLY	42	56.722	12.802	52.274	1.00	0.00	RX1	N
ATOM	2523	H	GLY	42	56.088	12.136	52.671	1.00	0.00	RX1	H
ATOM	2524	CA	GLY	42	57.779	13.340	53.120	1.00	0.00	RX1	C
ATOM	2525	C	GLY	42	57.152	14.191	54.201	1.00	0.00	RX1	C
ATOM	2526	O	GLY	42	56.016	13.965	54.599	1.00	0.00	RX1	O
ATOM	2527	N	THR	43	57.926	15.185	54.642	1.00	0.00	RX1	N
ATOM	2528	H	THR	43	58.822	15.364	54.229	1.00	0.00	RX1	H
ATOM	2529	CA	THR	43	57.350	16.178	55.539	1.00	0.00	RX1	C
ATOM	2530	CB	THR	43	58.513	16.956	56.154	1.00	0.00	RX1	C
ATOM	2531	OG1	THR	43	59.271	17.622	55.135	1.00	0.00	RX1	O
ATOM	2532	HG1	THR	43	59.933	17.005	54.808	1.00	0.00	RX1	H
ATOM	2533	CG2	THR	43	59.416	16.048	56.989	1.00	0.00	RX1	C
ATOM	2534	C	THR	43	56.381	17.086	54.796	1.00	0.00	RX1	C
ATOM	2535	O	THR	43	56.148	16.938	53.602	1.00	0.00	RX1	O
ATOM	2536	N	PHE	44	55.880	18.091	55.529	1.00	0.00	RX1	N
ATOM	2537	H	PHE	44	55.960	18.070	56.523	1.00	0.00	RX1	H
ATOM	2538	CA	PHE	44	55.132	19.148	54.848	1.00	0.00	RX1	C
ATOM	2539	CB	PHE	44	54.636	20.173	55.863	1.00	0.00	RX1	C
ATOM	2540	CG	PHE	44	53.694	19.520	56.844	1.00	0.00	RX1	C
ATOM	2541	CD1	PHE	44	52.375	19.279	56.480	1.00	0.00	RX1	C
ATOM	2542	CD2	PHE	44	54.140	19.163	58.111	1.00	0.00	RX1	C
ATOM	2543	CE1	PHE	44	51.502	18.687	57.383	1.00	0.00	RX1	C
ATOM	2544	CE2	PHE	44	53.268	18.567	59.013	1.00	0.00	RX1	C
ATOM	2545	CZ	PHE	44	51.947	18.331	58.650	1.00	0.00	RX1	C
ATOM	2546	C	PHE	44	55.927	19.850	53.757	1.00	0.00	RX1	C
ATOM	2547	O	PHE	44	55.455	20.084	52.649	1.00	0.00	RX1	O
ATOM	2548	N	GLU	45	57.181	20.173	54.119	1.00	0.00	RX1	N
ATOM	2549	H	GLU	45	57.606	19.860	54.966	1.00	0.00	RX1	H
ATOM	2550	CA	GLU	45	57.962	20.883	53.110	1.00	0.00	RX1	C
ATOM	2551	CB	GLU	45	59.166	21.536	53.785	1.00	0.00	RX1	C
ATOM	2552	CG	GLU	45	59.920	22.527	52.899	1.00	0.00	RX1	C
ATOM	2553	CD	GLU	45	59.135	23.816	52.824	1.00	0.00	RX1	C
ATOM	2554	OE1	GLU	45	58.271	23.937	51.964	1.00	0.00	RX1	O
ATOM	2555	OE2	GLU	45	59.390	24.701	53.637	1.00	0.00	RX1	O
ATOM	2556	C	GLU	45	58.451	19.975	51.998	1.00	0.00	RX1	C
ATOM	2557	O	GLU	45	58.420	20.328	50.825	1.00	0.00	RX1	O
ATOM	2558	N	ASP	46	58.802	18.737	52.386	1.00	0.00	RX1	N
ATOM	2559	H	ASP	46	58.904	18.484	53.350	1.00	0.00	RX1	H
ATOM	2560	CA	ASP	46	59.116	17.762	51.334	1.00	0.00	RX1	C

ATOM	2561	CB	ASP	46	59.408	16.365	51.855	1.00	0.00	RX1	C
ATOM	2562	CG	ASP	46	60.778	16.252	52.456	1.00	0.00	RX1	C
ATOM	2563	OD1	ASP	46	61.748	16.502	51.742	1.00	0.00	RX1	O
ATOM	2564	OD2	ASP	46	60.859	15.875	53.624	1.00	0.00	RX1	O
ATOM	2565	C	ASP	46	57.966	17.536	50.371	1.00	0.00	RX1	C
ATOM	2566	O	ASP	46	58.152	17.362	49.170	1.00	0.00	RX1	O
ATOM	2567	N	HIS	47	56.752	17.595	50.944	1.00	0.00	RX1	N
ATOM	2568	H	HIS	47	56.669	17.706	51.935	1.00	0.00	RX1	H
ATOM	2569	CA	HIS	47	55.557	17.512	50.111	1.00	0.00	RX1	C
ATOM	2570	CB	HIS	47	54.264	17.360	50.920	1.00	0.00	RX1	C
ATOM	2571	CG	HIS	47	53.116	16.873	50.050	1.00	0.00	RX1	C
ATOM	2572	ND1	HIS	47	53.007	17.033	48.714	1.00	0.00	RX1	N
ATOM	2573	HD1	HIS	47	53.625	17.498	48.110	1.00	0.00	RX1	H
ATOM	2574	CD2	HIS	47	51.984	16.180	50.490	1.00	0.00	RX1	C
ATOM	2575	NE2	HIS	47	51.200	15.926	49.414	1.00	0.00	RX1	N
ATOM	2576	CE1	HIS	47	51.833	16.453	48.319	1.00	0.00	RX1	C
ATOM	2577	C	HIS	47	55.433	18.667	49.145	1.00	0.00	RX1	C
ATOM	2578	O	HIS	47	55.242	18.461	47.952	1.00	0.00	RX1	O
ATOM	2579	N	PHE	48	55.597	19.884	49.688	1.00	0.00	RX1	N
ATOM	2580	H	PHE	48	55.739	19.983	50.677	1.00	0.00	RX1	H
ATOM	2581	CA	PHE	48	55.651	21.071	48.828	1.00	0.00	RX1	C
ATOM	2582	CB	PHE	48	55.982	22.311	49.667	1.00	0.00	RX1	C
ATOM	2583	CG	PHE	48	56.614	23.383	48.805	1.00	0.00	RX1	C
ATOM	2584	CD1	PHE	48	55.860	24.066	47.860	1.00	0.00	RX1	C
ATOM	2585	CD2	PHE	48	57.964	23.681	48.951	1.00	0.00	RX1	C
ATOM	2586	CE1	PHE	48	56.456	25.031	47.056	1.00	0.00	RX1	C
ATOM	2587	CE2	PHE	48	58.561	24.647	48.151	1.00	0.00	RX1	C
ATOM	2588	CZ	PHE	48	57.806	25.321	47.200	1.00	0.00	RX1	C
ATOM	2589	C	PHE	48	56.645	20.918	47.678	1.00	0.00	RX1	C
ATOM	2590	O	PHE	48	56.348	21.118	46.506	1.00	0.00	RX1	O
ATOM	2591	N	LEU	49	57.848	20.497	48.073	1.00	0.00	RX1	N
ATOM	2592	H	LEU	49	58.037	20.377	49.047	1.00	0.00	RX1	H
ATOM	2593	CA	LEU	49	58.889	20.292	47.078	1.00	0.00	RX1	C
ATOM	2594	CB	LEU	49	60.225	20.079	47.788	1.00	0.00	RX1	C
ATOM	2595	CG	LEU	49	61.429	20.340	46.885	1.00	0.00	RX1	C
ATOM	2596	CD1	LEU	49	61.354	21.721	46.234	1.00	0.00	RX1	C
ATOM	2597	CD2	LEU	49	62.747	20.138	47.633	1.00	0.00	RX1	C
ATOM	2598	C	LEU	49	58.578	19.184	46.086	1.00	0.00	RX1	C
ATOM	2599	O	LEU	49	58.958	19.231	44.927	1.00	0.00	RX1	O
ATOM	2600	N	SER	50	57.838	18.179	46.566	1.00	0.00	RX1	N
ATOM	2601	H	SER	50	57.519	18.156	47.514	1.00	0.00	RX1	H
ATOM	2602	CA	SER	50	57.388	17.147	45.634	1.00	0.00	RX1	C
ATOM	2603	CB	SER	50	56.983	15.926	46.444	1.00	0.00	RX1	C
ATOM	2604	OG	SER	50	58.081	15.639	47.317	1.00	0.00	RX1	O
ATOM	2605	HG	SER	50	57.744	15.764	48.208	1.00	0.00	RX1	H
ATOM	2606	C	SER	50	56.354	17.623	44.633	1.00	0.00	RX1	C
ATOM	2607	O	SER	50	56.392	17.282	43.459	1.00	0.00	RX1	O
ATOM	2608	N	LEU	51	55.465	18.486	45.144	1.00	0.00	RX1	N
ATOM	2609	H	LEU	51	55.524	18.776	46.100	1.00	0.00	RX1	H
ATOM	2610	CA	LEU	51	54.486	19.128	44.272	1.00	0.00	RX1	C
ATOM	2611	CB	LEU	51	53.579	20.023	45.116	1.00	0.00	RX1	C
ATOM	2612	CG	LEU	51	52.164	20.131	44.559	1.00	0.00	RX1	C
ATOM	2613	CD1	LEU	51	51.473	18.772	44.596	1.00	0.00	RX1	C
ATOM	2614	CD2	LEU	51	51.343	21.203	45.273	1.00	0.00	RX1	C
ATOM	2615	C	LEU	51	55.164	19.921	43.166	1.00	0.00	RX1	C
ATOM	2616	O	LEU	51	54.865	19.816	41.985	1.00	0.00	RX1	O
ATOM	2617	N	GLN	52	56.173	20.680	43.623	1.00	0.00	RX1	N
ATOM	2618	H	GLN	52	56.334	20.746	44.609	1.00	0.00	RX1	H
ATOM	2619	CA	GLN	52	57.030	21.392	42.679	1.00	0.00	RX1	C
ATOM	2620	CB	GLN	52	58.092	22.171	43.450	1.00	0.00	RX1	C
ATOM	2621	CG	GLN	52	58.903	23.113	42.565	1.00	0.00	RX1	C

ATOM	2622	CD	GLN	52	60.009	23.723	43.396	1.00	0.00	RX1	C
ATOM	2623	OE1	GLN	52	61.188	23.480	43.169	1.00	0.00	RX1	O
ATOM	2624	NE2	GLN	52	59.565	24.519	44.385	1.00	0.00	RX1	N
ATOM	2625	HE21	GLN	52	58.590	24.688	44.534	1.00	0.00	RX1	H
ATOM	2626	HE22	GLN	52	60.208	24.964	45.010	1.00	0.00	RX1	H
ATOM	2627	C	GLN	52	57.671	20.493	41.631	1.00	0.00	RX1	C
ATOM	2628	O	GLN	52	57.540	20.699	40.433	1.00	0.00	RX1	O
ATOM	2629	N	ARG	53	58.348	19.458	42.151	1.00	0.00	RX1	N
ATOM	2630	H	ARG	53	58.386	19.348	43.142	1.00	0.00	RX1	H
ATOM	2631	CA	ARG	53	59.037	18.505	41.279	1.00	0.00	RX1	C
ATOM	2632	CB	ARG	53	59.725	17.427	42.139	1.00	0.00	RX1	C
ATOM	2633	CG	ARG	53	60.936	17.949	42.933	1.00	0.00	RX1	C
ATOM	2634	CD	ARG	53	61.584	16.962	43.926	1.00	0.00	RX1	C
ATOM	2635	NE	ARG	53	60.781	16.736	45.134	1.00	0.00	RX1	N
ATOM	2636	HE	ARG	53	59.793	16.628	44.990	1.00	0.00	RX1	H
ATOM	2637	CZ	ARG	53	61.378	16.662	46.370	1.00	0.00	RX1	C
ATOM	2638	NH1	ARG	53	62.717	16.816	46.466	1.00	0.00	RX1	N
ATOM	2639	HH11	ARG	53	63.196	16.772	47.349	1.00	0.00	RX1	H
ATOM	2640	HH12	ARG	53	63.284	16.983	45.655	1.00	0.00	RX1	H
ATOM	2641	NH2	ARG	53	60.636	16.444	47.482	1.00	0.00	RX1	N
ATOM	2642	HH21	ARG	53	61.011	16.419	48.417	1.00	0.00	RX1	H
ATOM	2643	HH22	ARG	53	59.638	16.287	47.431	1.00	0.00	RX1	H
ATOM	2644	C	ARG	53	58.164	17.879	40.201	1.00	0.00	RX1	C
ATOM	2645	O	ARG	53	58.568	17.725	39.058	1.00	0.00	RX1	O
ATOM	2646	N	MET	54	56.938	17.536	40.618	1.00	0.00	RX1	N
ATOM	2647	H	MET	54	56.625	17.735	41.547	1.00	0.00	RX1	H
ATOM	2648	CA	MET	54	56.041	16.913	39.649	1.00	0.00	RX1	C
ATOM	2649	CB	MET	54	54.983	16.091	40.387	1.00	0.00	RX1	C
ATOM	2650	CG	MET	54	54.026	15.353	39.449	1.00	0.00	RX1	C
ATOM	2651	SD	MET	54	54.834	14.152	38.381	1.00	0.00	RX1	S
ATOM	2652	CE	MET	54	55.272	12.947	39.642	1.00	0.00	RX1	C
ATOM	2653	C	MET	54	55.400	17.883	38.664	1.00	0.00	RX1	C
ATOM	2654	O	MET	54	55.177	17.574	37.501	1.00	0.00	RX1	O
ATOM	2655	N	PHE	55	55.078	19.074	39.184	1.00	0.00	RX1	N
ATOM	2656	H	PHE	55	55.343	19.365	40.105	1.00	0.00	RX1	H
ATOM	2657	CA	PHE	55	54.252	19.935	38.340	1.00	0.00	RX1	C
ATOM	2658	CB	PHE	55	52.989	20.365	39.082	1.00	0.00	RX1	C
ATOM	2659	CG	PHE	55	52.214	19.157	39.537	1.00	0.00	RX1	C
ATOM	2660	CD1	PHE	55	51.557	18.370	38.604	1.00	0.00	RX1	C
ATOM	2661	CD2	PHE	55	52.155	18.831	40.885	1.00	0.00	RX1	C
ATOM	2662	CE1	PHE	55	50.833	17.262	39.020	1.00	0.00	RX1	C
ATOM	2663	CE2	PHE	55	51.432	17.720	41.298	1.00	0.00	RX1	C
ATOM	2664	CZ	PHE	55	50.764	16.938	40.367	1.00	0.00	RX1	C
ATOM	2665	C	PHE	55	54.942	21.166	37.786	1.00	0.00	RX1	C
ATOM	2666	O	PHE	55	54.300	22.098	37.308	1.00	0.00	RX1	O
ATOM	2667	N	ASN	56	56.280	21.162	37.887	1.00	0.00	RX1	N
ATOM	2668	H	ASN	56	56.773	20.394	38.298	1.00	0.00	RX1	H
ATOM	2669	CA	ASN	56	56.976	22.399	37.531	1.00	0.00	RX1	C
ATOM	2670	CB	ASN	56	58.480	22.288	37.710	1.00	0.00	RX1	C
ATOM	2671	CG	ASN	56	59.074	23.493	37.012	1.00	0.00	RX1	C
ATOM	2672	OD1	ASN	56	58.749	24.637	37.319	1.00	0.00	RX1	O
ATOM	2673	ND2	ASN	56	59.968	23.176	36.059	1.00	0.00	RX1	N
ATOM	2674	HD21	ASN	56	60.142	22.213	35.844	1.00	0.00	RX1	H
ATOM	2675	HD22	ASN	56	60.467	23.870	35.541	1.00	0.00	RX1	H
ATOM	2676	C	ASN	56	56.761	22.887	36.110	1.00	0.00	RX1	C
ATOM	2677	O	ASN	56	57.025	22.198	35.134	1.00	0.00	RX1	O
ATOM	2678	N	ASN	57	56.215	24.114	36.060	1.00	0.00	RX1	N
ATOM	2679	H	ASN	57	56.134	24.608	36.922	1.00	0.00	RX1	H
ATOM	2680	CA	ASN	57	55.801	24.738	34.798	1.00	0.00	RX1	C
ATOM	2681	CB	ASN	57	57.011	25.337	34.076	1.00	0.00	RX1	C
ATOM	2682	CG	ASN	57	56.560	26.210	32.921	1.00	0.00	RX1	C

ATOM	2683	OD1	ASN	57	56.736	25.880	31.750	1.00	0.00	RX1	O
ATOM	2684	ND2	ASN	57	56.008	27.372	33.310	1.00	0.00	RX1	N
ATOM	2685	HD21	ASN	57	55.791	27.569	34.269	1.00	0.00	RX1	H
ATOM	2686	HD22	ASN	57	55.745	28.077	32.651	1.00	0.00	RX1	H
ATOM	2687	C	ASN	57	54.976	23.858	33.860	1.00	0.00	RX1	C
ATOM	2688	O	ASN	57	55.079	23.923	32.639	1.00	0.00	RX1	O
ATOM	2689	N	CYS	58	54.144	23.018	34.481	1.00	0.00	RX1	N
ATOM	2690	H	CYS	58	54.002	23.012	35.472	1.00	0.00	RX1	H
ATOM	2691	CA	CYS	58	53.319	22.171	33.627	1.00	0.00	RX1	C
ATOM	2692	CB	CYS	58	52.973	20.892	34.381	1.00	0.00	RX1	C
ATOM	2693	SG	CYS	58	51.987	19.749	33.390	1.00	0.00	RX1	S
ATOM	2694	C	CYS	58	52.076	22.891	33.155	1.00	0.00	RX1	C
ATOM	2695	O	CYS	58	51.507	23.704	33.872	1.00	0.00	RX1	O
ATOM	2696	N	GLU	59	51.669	22.551	31.931	1.00	0.00	RX1	N
ATOM	2697	H	GLU	59	52.187	21.936	31.332	1.00	0.00	RX1	H
ATOM	2698	CA	GLU	59	50.374	23.050	31.493	1.00	0.00	RX1	C
ATOM	2699	CB	GLU	59	50.386	23.554	30.044	1.00	0.00	RX1	C
ATOM	2700	CG	GLU	59	51.187	24.834	29.766	1.00	0.00	RX1	C
ATOM	2701	CD	GLU	59	52.655	24.526	29.568	1.00	0.00	RX1	C
ATOM	2702	OE1	GLU	59	53.443	25.434	29.322	1.00	0.00	RX1	O
ATOM	2703	OE2	GLU	59	53.034	23.368	29.649	1.00	0.00	RX1	O
ATOM	2704	C	GLU	59	49.281	22.017	31.662	1.00	0.00	RX1	C
ATOM	2705	O	GLU	59	48.160	22.347	32.032	1.00	0.00	RX1	O
ATOM	2706	N	VAL	60	49.643	20.756	31.368	1.00	0.00	RX1	N
ATOM	2707	H	VAL	60	50.585	20.505	31.132	1.00	0.00	RX1	H
ATOM	2708	CA	VAL	60	48.641	19.699	31.507	1.00	0.00	RX1	C
ATOM	2709	CB	VAL	60	48.285	19.060	30.163	1.00	0.00	RX1	C
ATOM	2710	CG1	VAL	60	47.175	18.020	30.335	1.00	0.00	RX1	C
ATOM	2711	CG2	VAL	60	47.909	20.108	29.124	1.00	0.00	RX1	C
ATOM	2712	C	VAL	60	49.060	18.607	32.471	1.00	0.00	RX1	C
ATOM	2713	O	VAL	60	49.901	17.762	32.187	1.00	0.00	RX1	O
ATOM	2714	N	VAL	61	48.396	18.632	33.626	1.00	0.00	RX1	N
ATOM	2715	H	VAL	61	47.655	19.291	33.753	1.00	0.00	RX1	H
ATOM	2716	CA	VAL	61	48.519	17.467	34.493	1.00	0.00	RX1	C
ATOM	2717	CB	VAL	61	48.011	17.811	35.887	1.00	0.00	RX1	C
ATOM	2718	CG1	VAL	61	48.161	16.623	36.830	1.00	0.00	RX1	C
ATOM	2719	CG2	VAL	61	48.697	19.065	36.417	1.00	0.00	RX1	C
ATOM	2720	C	VAL	61	47.726	16.305	33.920	1.00	0.00	RX1	C
ATOM	2721	O	VAL	61	46.531	16.418	33.675	1.00	0.00	RX1	O
ATOM	2722	N	LEU	62	48.436	15.191	33.711	1.00	0.00	RX1	N
ATOM	2723	H	LEU	62	49.410	15.146	33.937	1.00	0.00	RX1	H
ATOM	2724	CA	LEU	62	47.713	14.008	33.250	1.00	0.00	RX1	C
ATOM	2725	CB	LEU	62	48.669	13.008	32.605	1.00	0.00	RX1	C
ATOM	2726	CG	LEU	62	49.376	13.584	31.381	1.00	0.00	RX1	C
ATOM	2727	CD1	LEU	62	50.440	12.626	30.846	1.00	0.00	RX1	C
ATOM	2728	CD2	LEU	62	48.379	14.011	30.301	1.00	0.00	RX1	C
ATOM	2729	C	LEU	62	46.929	13.339	34.361	1.00	0.00	RX1	C
ATOM	2730	O	LEU	62	45.711	13.222	34.322	1.00	0.00	RX1	O
ATOM	2731	N	GLY	63	47.695	12.915	35.373	1.00	0.00	RX1	N
ATOM	2732	H	GLY	63	48.688	13.038	35.384	1.00	0.00	RX1	H
ATOM	2733	CA	GLY	63	47.024	12.349	36.536	1.00	0.00	RX1	C
ATOM	2734	C	GLY	63	46.425	13.421	37.420	1.00	0.00	RX1	C
ATOM	2735	O	GLY	63	45.772	14.358	36.973	1.00	0.00	RX1	O
ATOM	2736	N	ASN	64	46.686	13.237	38.716	1.00	0.00	RX1	N
ATOM	2737	H	ASN	64	47.350	12.573	39.059	1.00	0.00	RX1	H
ATOM	2738	CA	ASN	64	46.015	14.150	39.633	1.00	0.00	RX1	C
ATOM	2739	CB	ASN	64	45.702	13.512	40.976	1.00	0.00	RX1	C
ATOM	2740	CG	ASN	64	44.861	12.279	40.819	1.00	0.00	RX1	C
ATOM	2741	OD1	ASN	64	43.886	12.244	40.072	1.00	0.00	RX1	O
ATOM	2742	ND2	ASN	64	45.295	11.269	41.591	1.00	0.00	RX1	N
ATOM	2743	HD21	ASN	64	46.113	11.417	42.160	1.00	0.00	RX1	H

ATOM	2744	HD22	ASN	64	44.835	10.384	41.660	1.00	0.00	RX1	H
ATOM	2745	C	ASN	64	46.856	15.350	39.957	1.00	0.00	RX1	C
ATOM	2746	O	ASN	64	48.080	15.297	39.959	1.00	0.00	RX1	O
ATOM	2747	N	LEU	65	46.139	16.428	40.264	1.00	0.00	RX1	N
ATOM	2748	H	LEU	65	45.139	16.396	40.248	1.00	0.00	RX1	H
ATOM	2749	CA	LEU	65	46.812	17.552	40.892	1.00	0.00	RX1	C
ATOM	2750	CB	LEU	65	46.392	18.834	40.179	1.00	0.00	RX1	C
ATOM	2751	CG	LEU	65	47.064	20.101	40.698	1.00	0.00	RX1	C
ATOM	2752	CD1	LEU	65	48.585	19.984	40.720	1.00	0.00	RX1	C
ATOM	2753	CD2	LEU	65	46.602	21.322	39.907	1.00	0.00	RX1	C
ATOM	2754	C	LEU	65	46.469	17.568	42.368	1.00	0.00	RX1	C
ATOM	2755	O	LEU	65	45.474	18.137	42.806	1.00	0.00	RX1	O
ATOM	2756	N	GLU	66	47.321	16.874	43.128	1.00	0.00	RX1	N
ATOM	2757	H	GLU	66	48.172	16.474	42.779	1.00	0.00	RX1	H
ATOM	2758	CA	GLU	66	47.011	16.847	44.554	1.00	0.00	RX1	C
ATOM	2759	CB	GLU	66	47.228	15.447	45.096	1.00	0.00	RX1	C
ATOM	2760	CG	GLU	66	46.315	14.417	44.443	1.00	0.00	RX1	C
ATOM	2761	CD	GLU	66	46.986	13.072	44.557	1.00	0.00	RX1	C
ATOM	2762	OE1	GLU	66	48.209	13.039	44.548	1.00	0.00	RX1	O
ATOM	2763	OE2	GLU	66	46.308	12.056	44.662	1.00	0.00	RX1	O
ATOM	2764	C	GLU	66	47.803	17.854	45.347	1.00	0.00	RX1	C
ATOM	2765	O	GLU	66	48.867	17.590	45.894	1.00	0.00	RX1	O
ATOM	2766	N	ILE	67	47.215	19.049	45.377	1.00	0.00	RX1	N
ATOM	2767	H	ILE	67	46.266	19.098	45.062	1.00	0.00	RX1	H
ATOM	2768	CA	ILE	67	47.745	20.075	46.266	1.00	0.00	RX1	C
ATOM	2769	CB	ILE	67	47.138	21.422	45.895	1.00	0.00	RX1	C
ATOM	2770	CG2	ILE	67	47.771	22.571	46.681	1.00	0.00	RX1	C
ATOM	2771	CG1	ILE	67	47.220	21.622	44.384	1.00	0.00	RX1	C
ATOM	2772	CD1	ILE	67	46.386	22.813	43.927	1.00	0.00	RX1	C
ATOM	2773	C	ILE	67	47.422	19.725	47.708	1.00	0.00	RX1	C
ATOM	2774	O	ILE	67	46.374	20.068	48.244	1.00	0.00	RX1	O
ATOM	2775	N	THR	68	48.367	19.002	48.307	1.00	0.00	RX1	N
ATOM	2776	H	THR	68	49.145	18.615	47.814	1.00	0.00	RX1	H
ATOM	2777	CA	THR	68	48.135	18.633	49.693	1.00	0.00	RX1	C
ATOM	2778	CB	THR	68	47.850	17.137	49.661	1.00	0.00	RX1	C
ATOM	2779	OG1	THR	68	48.441	16.568	48.486	1.00	0.00	RX1	O
ATOM	2780	HG1	THR	68	49.380	16.598	48.641	1.00	0.00	RX1	H
ATOM	2781	CG2	THR	68	46.353	16.862	49.636	1.00	0.00	RX1	C
ATOM	2782	C	THR	68	49.274	19.029	50.609	1.00	0.00	RX1	C
ATOM	2783	O	THR	68	50.440	18.997	50.233	1.00	0.00	RX1	O
ATOM	2784	N	TYR	69	48.872	19.400	51.839	1.00	0.00	RX1	N
ATOM	2785	H	TYR	69	47.888	19.534	51.981	1.00	0.00	RX1	H
ATOM	2786	CA	TYR	69	49.812	19.678	52.935	1.00	0.00	RX1	C
ATOM	2787	CB	TYR	69	50.664	18.453	53.275	1.00	0.00	RX1	C
ATOM	2788	CG	TYR	69	49.807	17.324	53.789	1.00	0.00	RX1	C
ATOM	2789	CD1	TYR	69	49.310	17.374	55.084	1.00	0.00	RX1	C
ATOM	2790	CE1	TYR	69	48.584	16.304	55.589	1.00	0.00	RX1	C
ATOM	2791	CD2	TYR	69	49.536	16.226	52.982	1.00	0.00	RX1	C
ATOM	2792	CE2	TYR	69	48.806	15.158	53.486	1.00	0.00	RX1	C
ATOM	2793	CZ	TYR	69	48.346	15.189	54.796	1.00	0.00	RX1	C
ATOM	2794	OH	TYR	69	47.660	14.115	55.321	1.00	0.00	RX1	O
ATOM	2795	HH	TYR	69	47.702	13.378	54.715	1.00	0.00	RX1	H
ATOM	2796	C	TYR	69	50.730	20.887	52.808	1.00	0.00	RX1	C
ATOM	2797	O	TYR	69	51.596	21.118	53.647	1.00	0.00	RX1	O
ATOM	2798	N	VAL	70	50.522	21.664	51.742	1.00	0.00	RX1	N
ATOM	2799	H	VAL	70	49.745	21.510	51.132	1.00	0.00	RX1	H
ATOM	2800	CA	VAL	70	51.399	22.814	51.539	1.00	0.00	RX1	C
ATOM	2801	CB	VAL	70	51.257	23.324	50.108	1.00	0.00	RX1	C
ATOM	2802	CG1	VAL	70	52.173	24.517	49.861	1.00	0.00	RX1	C
ATOM	2803	CG2	VAL	70	51.506	22.191	49.111	1.00	0.00	RX1	C
ATOM	2804	C	VAL	70	51.154	23.922	52.555	1.00	0.00	RX1	C

ATOM	2805	O	VAL	70	50.046	24.414	52.742	1.00	0.00	RX1	O
ATOM	2806	N	GLN	71	52.248	24.263	53.243	1.00	0.00	RX1	N
ATOM	2807	H	GLN	71	53.154	23.941	52.968	1.00	0.00	RX1	H
ATOM	2808	CA	GLN	71	52.084	25.202	54.345	1.00	0.00	RX1	C
ATOM	2809	CB	GLN	71	53.095	24.898	55.451	1.00	0.00	RX1	C
ATOM	2810	CG	GLN	71	52.986	23.469	55.983	1.00	0.00	RX1	C
ATOM	2811	CD	GLN	71	51.654	23.283	56.675	1.00	0.00	RX1	C
ATOM	2812	OE1	GLN	71	51.320	23.984	57.627	1.00	0.00	RX1	O
ATOM	2813	NE2	GLN	71	50.894	22.318	56.136	1.00	0.00	RX1	N
ATOM	2814	HE21	GLN	71	51.261	21.787	55.367	1.00	0.00	RX1	H
ATOM	2815	HE22	GLN	71	49.971	22.081	56.440	1.00	0.00	RX1	H
ATOM	2816	C	GLN	71	52.143	26.675	53.977	1.00	0.00	RX1	C
ATOM	2817	O	GLN	71	52.620	27.078	52.922	1.00	0.00	RX1	O
ATOM	2818	N	ARG	72	51.624	27.448	54.944	1.00	0.00	RX1	N
ATOM	2819	H	ARG	72	51.223	26.931	55.702	1.00	0.00	RX1	H
ATOM	2820	CA	ARG	72	51.413	28.898	54.916	1.00	0.00	RX1	C
ATOM	2821	CB	ARG	72	51.823	29.501	56.262	1.00	0.00	RX1	C
ATOM	2822	CG	ARG	72	50.837	30.562	56.760	1.00	0.00	RX1	C
ATOM	2823	CD	ARG	72	49.525	29.961	57.275	1.00	0.00	RX1	C
ATOM	2824	NE	ARG	72	48.525	31.004	57.486	1.00	0.00	RX1	N
ATOM	2825	HE	ARG	72	48.527	31.784	56.842	1.00	0.00	RX1	H
ATOM	2826	CZ	ARG	72	47.519	30.870	58.399	1.00	0.00	RX1	C
ATOM	2827	NH1	ARG	72	47.534	29.836	59.261	1.00	0.00	RX1	N
ATOM	2828	HH11	ARG	72	46.804	29.679	59.945	1.00	0.00	RX1	H
ATOM	2829	HH12	ARG	72	48.267	29.158	59.277	1.00	0.00	RX1	H
ATOM	2830	NH2	ARG	72	46.525	31.776	58.431	1.00	0.00	RX1	N
ATOM	2831	HH21	ARG	72	45.787	31.728	59.116	1.00	0.00	RX1	H
ATOM	2832	HH22	ARG	72	46.479	32.522	57.740	1.00	0.00	RX1	H
ATOM	2833	C	ARG	72	51.954	29.745	53.769	1.00	0.00	RX1	C
ATOM	2834	O	ARG	72	51.216	30.427	53.070	1.00	0.00	RX1	O
ATOM	2835	N	ASN	73	53.286	29.707	53.633	1.00	0.00	RX1	N
ATOM	2836	H	ASN	73	53.828	28.976	54.045	1.00	0.00	RX1	H
ATOM	2837	CA	ASN	73	53.894	30.737	52.790	1.00	0.00	RX1	C
ATOM	2838	CB	ASN	73	55.122	31.349	53.460	1.00	0.00	RX1	C
ATOM	2839	CG	ASN	73	55.018	32.863	53.431	1.00	0.00	RX1	C
ATOM	2840	OD1	ASN	73	55.089	33.511	54.470	1.00	0.00	RX1	O
ATOM	2841	ND2	ASN	73	54.824	33.407	52.219	1.00	0.00	RX1	N
ATOM	2842	HD21	ASN	73	54.825	32.847	51.386	1.00	0.00	RX1	H
ATOM	2843	HD22	ASN	73	54.671	34.389	52.112	1.00	0.00	RX1	H
ATOM	2844	C	ASN	73	54.242	30.339	51.372	1.00	0.00	RX1	C
ATOM	2845	O	ASN	73	54.742	31.147	50.596	1.00	0.00	RX1	O
ATOM	2846	N	TYR	74	54.015	29.058	51.053	1.00	0.00	RX1	N
ATOM	2847	H	TYR	74	53.462	28.429	51.604	1.00	0.00	RX1	H
ATOM	2848	CA	TYR	74	54.563	28.661	49.757	1.00	0.00	RX1	C
ATOM	2849	CB	TYR	74	54.912	27.174	49.708	1.00	0.00	RX1	C
ATOM	2850	CG	TYR	74	55.519	26.726	51.013	1.00	0.00	RX1	C
ATOM	2851	CD1	TYR	74	55.018	25.587	51.626	1.00	0.00	RX1	C
ATOM	2852	CE1	TYR	74	55.500	25.194	52.864	1.00	0.00	RX1	C
ATOM	2853	CD2	TYR	74	56.552	27.441	51.609	1.00	0.00	RX1	C
ATOM	2854	CE2	TYR	74	57.031	27.052	52.853	1.00	0.00	RX1	C
ATOM	2855	CZ	TYR	74	56.491	25.939	53.487	1.00	0.00	RX1	C
ATOM	2856	OH	TYR	74	56.938	25.565	54.734	1.00	0.00	RX1	O
ATOM	2857	HH	TYR	74	57.808	25.189	54.572	1.00	0.00	RX1	H
ATOM	2858	C	TYR	74	53.691	29.014	48.564	1.00	0.00	RX1	C
ATOM	2859	O	TYR	74	52.785	28.269	48.207	1.00	0.00	RX1	O
ATOM	2860	N	ASP	75	54.028	30.146	47.915	1.00	0.00	RX1	N
ATOM	2861	H	ASP	75	54.754	30.732	48.273	1.00	0.00	RX1	H
ATOM	2862	CA	ASP	75	53.456	30.313	46.573	1.00	0.00	RX1	C
ATOM	2863	CB	ASP	75	53.739	31.690	45.922	1.00	0.00	RX1	C
ATOM	2864	CG	ASP	75	53.106	31.846	44.522	1.00	0.00	RX1	C
ATOM	2865	OD1	ASP	75	52.559	32.900	44.197	1.00	0.00	RX1	O

ATOM	2866	OD2	ASP	75	53.177	30.943	43.697	1.00	0.00	RX1	O
ATOM	2867	C	ASP	75	53.904	29.198	45.635	1.00	0.00	RX1	C
ATOM	2868	O	ASP	75	55.071	28.987	45.315	1.00	0.00	RX1	O
ATOM	2869	N	LEU	76	52.876	28.467	45.217	1.00	0.00	RX1	N
ATOM	2870	H	LEU	76	51.958	28.751	45.485	1.00	0.00	RX1	H
ATOM	2871	CA	LEU	76	53.114	27.370	44.293	1.00	0.00	RX1	C
ATOM	2872	CB	LEU	76	51.987	26.363	44.471	1.00	0.00	RX1	C
ATOM	2873	CG	LEU	76	51.835	25.927	45.926	1.00	0.00	RX1	C
ATOM	2874	CD1	LEU	76	50.442	25.390	46.250	1.00	0.00	RX1	C
ATOM	2875	CD2	LEU	76	52.927	24.938	46.306	1.00	0.00	RX1	C
ATOM	2876	C	LEU	76	53.181	27.832	42.849	1.00	0.00	RX1	C
ATOM	2877	O	LEU	76	52.312	27.538	42.038	1.00	0.00	RX1	O
ATOM	2878	N	SER	77	54.256	28.568	42.530	1.00	0.00	RX1	N
ATOM	2879	H	SER	77	54.902	28.804	43.260	1.00	0.00	RX1	H
ATOM	2880	CA	SER	77	54.307	29.188	41.203	1.00	0.00	RX1	C
ATOM	2881	CB	SER	77	55.544	30.075	41.164	1.00	0.00	RX1	C
ATOM	2882	OG	SER	77	55.301	31.167	42.067	1.00	0.00	RX1	O
ATOM	2883	HG	SER	77	55.045	30.791	42.914	1.00	0.00	RX1	H
ATOM	2884	C	SER	77	54.067	28.306	39.993	1.00	0.00	RX1	C
ATOM	2885	O	SER	77	53.460	28.725	39.019	1.00	0.00	RX1	O
ATOM	2886	N	PHE	78	54.507	27.047	40.118	1.00	0.00	RX1	N
ATOM	2887	H	PHE	78	54.986	26.773	40.949	1.00	0.00	RX1	H
ATOM	2888	CA	PHE	78	54.202	26.085	39.056	1.00	0.00	RX1	C
ATOM	2889	CB	PHE	78	54.813	24.718	39.381	1.00	0.00	RX1	C
ATOM	2890	CG	PHE	78	54.413	24.219	40.750	1.00	0.00	RX1	C
ATOM	2891	CD1	PHE	78	53.299	23.402	40.892	1.00	0.00	RX1	C
ATOM	2892	CD2	PHE	78	55.166	24.563	41.867	1.00	0.00	RX1	C
ATOM	2893	CE1	PHE	78	52.941	22.926	42.145	1.00	0.00	RX1	C
ATOM	2894	CE2	PHE	78	54.807	24.093	43.122	1.00	0.00	RX1	C
ATOM	2895	CZ	PHE	78	53.697	23.270	43.258	1.00	0.00	RX1	C
ATOM	2896	C	PHE	78	52.735	25.998	38.639	1.00	0.00	RX1	C
ATOM	2897	O	PHE	78	52.419	25.930	37.457	1.00	0.00	RX1	O
ATOM	2898	N	LEU	79	51.853	26.079	39.657	1.00	0.00	RX1	N
ATOM	2899	H	LEU	79	52.166	26.233	40.594	1.00	0.00	RX1	H
ATOM	2900	CA	LEU	79	50.410	26.082	39.390	1.00	0.00	RX1	C
ATOM	2901	CB	LEU	79	49.589	26.203	40.668	1.00	0.00	RX1	C
ATOM	2902	CG	LEU	79	49.741	25.101	41.704	1.00	0.00	RX1	C
ATOM	2903	CD1	LEU	79	48.819	25.386	42.884	1.00	0.00	RX1	C
ATOM	2904	CD2	LEU	79	49.487	23.710	41.136	1.00	0.00	RX1	C
ATOM	2905	C	LEU	79	49.928	27.181	38.463	1.00	0.00	RX1	C
ATOM	2906	O	LEU	79	48.951	27.035	37.740	1.00	0.00	RX1	O
ATOM	2907	N	LYS	80	50.675	28.295	38.486	1.00	0.00	RX1	N
ATOM	2908	H	LYS	80	51.513	28.348	39.027	1.00	0.00	RX1	H
ATOM	2909	CA	LYS	80	50.301	29.386	37.589	1.00	0.00	RX1	C
ATOM	2910	CB	LYS	80	51.031	30.679	37.998	1.00	0.00	RX1	C
ATOM	2911	CG	LYS	80	50.783	31.042	39.473	1.00	0.00	RX1	C
ATOM	2912	CD	LYS	80	51.221	32.454	39.906	1.00	0.00	RX1	C
ATOM	2913	CE	LYS	80	52.703	32.670	40.242	1.00	0.00	RX1	C
ATOM	2914	NZ	LYS	80	53.006	32.400	41.658	1.00	0.00	RX1	N
ATOM	2915	HZ1	LYS	80	54.030	32.305	41.827	1.00	0.00	RX1	H
ATOM	2916	HZ2	LYS	80	52.672	33.110	42.347	1.00	0.00	RX1	H
ATOM	2917	HZ3	LYS	80	52.612	31.513	42.035	1.00	0.00	RX1	H
ATOM	2918	C	LYS	80	50.426	29.075	36.097	1.00	0.00	RX1	C
ATOM	2919	O	LYS	80	49.932	29.813	35.254	1.00	0.00	RX1	O
ATOM	2920	N	THR	81	51.084	27.940	35.810	1.00	0.00	RX1	N
ATOM	2921	H	THR	81	51.442	27.335	36.516	1.00	0.00	RX1	H
ATOM	2922	CA	THR	81	51.117	27.466	34.426	1.00	0.00	RX1	C
ATOM	2923	CB	THR	81	52.437	26.747	34.229	1.00	0.00	RX1	C
ATOM	2924	OG1	THR	81	53.424	27.277	35.133	1.00	0.00	RX1	O
ATOM	2925	HG1	THR	81	53.181	26.922	35.983	1.00	0.00	RX1	H
ATOM	2926	CG2	THR	81	52.874	26.809	32.765	1.00	0.00	RX1	C

ATOM	2927	C	THR	81	49.937	26.583	34.025	1.00	0.00	RX1	C
ATOM	2928	O	THR	81	49.553	26.482	32.863	1.00	0.00	RX1	O
ATOM	2929	N	ILE	82	49.399	25.907	35.051	1.00	0.00	RX1	N
ATOM	2930	H	ILE	82	49.510	26.198	36.000	1.00	0.00	RX1	H
ATOM	2931	CA	ILE	82	48.530	24.775	34.743	1.00	0.00	RX1	C
ATOM	2932	CB	ILE	82	48.375	23.870	35.964	1.00	0.00	RX1	C
ATOM	2933	CG2	ILE	82	47.527	22.641	35.633	1.00	0.00	RX1	C
ATOM	2934	CG1	ILE	82	49.757	23.490	36.498	1.00	0.00	RX1	C
ATOM	2935	CD1	ILE	82	49.712	22.496	37.654	1.00	0.00	RX1	C
ATOM	2936	C	ILE	82	47.184	25.182	34.181	1.00	0.00	RX1	C
ATOM	2937	O	ILE	82	46.357	25.801	34.834	1.00	0.00	RX1	O
ATOM	2938	N	GLN	83	47.016	24.791	32.916	1.00	0.00	RX1	N
ATOM	2939	H	GLN	83	47.719	24.216	32.497	1.00	0.00	RX1	H
ATOM	2940	CA	GLN	83	45.750	25.060	32.245	1.00	0.00	RX1	C
ATOM	2941	CB	GLN	83	45.969	25.253	30.753	1.00	0.00	RX1	C
ATOM	2942	CG	GLN	83	47.078	26.229	30.389	1.00	0.00	RX1	C
ATOM	2943	CD	GLN	83	47.272	26.129	28.895	1.00	0.00	RX1	C
ATOM	2944	OE1	GLN	83	46.421	25.607	28.172	1.00	0.00	RX1	O
ATOM	2945	NE2	GLN	83	48.441	26.637	28.474	1.00	0.00	RX1	N
ATOM	2946	HE21	GLN	83	49.090	26.997	29.151	1.00	0.00	RX1	H
ATOM	2947	HE22	GLN	83	48.738	26.675	27.519	1.00	0.00	RX1	H
ATOM	2948	C	GLN	83	44.753	23.932	32.416	1.00	0.00	RX1	C
ATOM	2949	O	GLN	83	43.549	24.134	32.535	1.00	0.00	RX1	O
ATOM	2950	N	GLU	84	45.316	22.720	32.356	1.00	0.00	RX1	N
ATOM	2951	H	GLU	84	46.304	22.554	32.383	1.00	0.00	RX1	H
ATOM	2952	CA	GLU	84	44.456	21.553	32.240	1.00	0.00	RX1	C
ATOM	2953	CB	GLU	84	44.512	20.983	30.819	1.00	0.00	RX1	C
ATOM	2954	CG	GLU	84	44.374	22.006	29.685	1.00	0.00	RX1	C
ATOM	2955	CD	GLU	84	44.146	21.284	28.375	1.00	0.00	RX1	C
ATOM	2956	OE1	GLU	84	44.512	20.123	28.273	1.00	0.00	RX1	O
ATOM	2957	OE2	GLU	84	43.545	21.846	27.461	1.00	0.00	RX1	O
ATOM	2958	C	GLU	84	44.875	20.473	33.209	1.00	0.00	RX1	C
ATOM	2959	O	GLU	84	46.050	20.160	33.331	1.00	0.00	RX1	O
ATOM	2960	N	VAL	85	43.873	19.896	33.874	1.00	0.00	RX1	N
ATOM	2961	H	VAL	85	42.921	20.197	33.778	1.00	0.00	RX1	H
ATOM	2962	CA	VAL	85	44.150	18.658	34.601	1.00	0.00	RX1	C
ATOM	2963	CB	VAL	85	43.848	18.842	36.093	1.00	0.00	RX1	C
ATOM	2964	CG1	VAL	85	44.174	17.590	36.912	1.00	0.00	RX1	C
ATOM	2965	CG2	VAL	85	44.572	20.072	36.640	1.00	0.00	RX1	C
ATOM	2966	C	VAL	85	43.266	17.584	33.999	1.00	0.00	RX1	C
ATOM	2967	O	VAL	85	42.156	17.881	33.572	1.00	0.00	RX1	O
ATOM	2968	N	ALA	86	43.772	16.347	33.948	1.00	0.00	RX1	N
ATOM	2969	H	ALA	86	44.694	16.128	34.277	1.00	0.00	RX1	H
ATOM	2970	CA	ALA	86	42.861	15.303	33.496	1.00	0.00	RX1	C
ATOM	2971	CB	ALA	86	43.525	14.381	32.473	1.00	0.00	RX1	C
ATOM	2972	C	ALA	86	42.243	14.500	34.624	1.00	0.00	RX1	C
ATOM	2973	O	ALA	86	41.042	14.248	34.623	1.00	0.00	RX1	O
ATOM	2974	N	GLY	87	43.092	14.142	35.600	1.00	0.00	RX1	N
ATOM	2975	H	GLY	87	44.074	14.344	35.570	1.00	0.00	RX1	H
ATOM	2976	CA	GLY	87	42.538	13.507	36.796	1.00	0.00	RX1	C
ATOM	2977	C	GLY	87	41.838	14.489	37.721	1.00	0.00	RX1	C
ATOM	2978	O	GLY	87	41.345	15.535	37.302	1.00	0.00	RX1	O
ATOM	2979	N	TYR	88	41.811	14.105	39.004	1.00	0.00	RX1	N
ATOM	2980	H	TYR	88	42.344	13.313	39.314	1.00	0.00	RX1	H
ATOM	2981	CA	TYR	88	41.168	15.001	39.962	1.00	0.00	RX1	C
ATOM	2982	CB	TYR	88	40.401	14.220	41.041	1.00	0.00	RX1	C
ATOM	2983	CG	TYR	88	41.294	13.363	41.911	1.00	0.00	RX1	C
ATOM	2984	CD1	TYR	88	42.124	13.937	42.869	1.00	0.00	RX1	C
ATOM	2985	CE1	TYR	88	42.925	13.137	43.675	1.00	0.00	RX1	C
ATOM	2986	CD2	TYR	88	41.264	11.981	41.768	1.00	0.00	RX1	C
ATOM	2987	CE2	TYR	88	42.057	11.179	42.580	1.00	0.00	RX1	C

ATOM	2988	CZ	TYR	88	42.891	11.756	43.531	1.00	0.00	RX1	C
ATOM	2989	OH	TYR	88	43.682	10.954	44.329	1.00	0.00	RX1	O
ATOM	2990	HH	TYR	88	44.503	11.414	44.511	1.00	0.00	RX1	H
ATOM	2991	C	TYR	88	42.117	16.031	40.551	1.00	0.00	RX1	C
ATOM	2992	O	TYR	88	43.333	15.910	40.464	1.00	0.00	RX1	O
ATOM	2993	N	VAL	89	41.512	17.056	41.167	1.00	0.00	RX1	N
ATOM	2994	H	VAL	89	40.524	17.088	41.321	1.00	0.00	RX1	H
ATOM	2995	CA	VAL	89	42.347	18.026	41.870	1.00	0.00	RX1	C
ATOM	2996	CB	VAL	89	42.197	19.432	41.292	1.00	0.00	RX1	C
ATOM	2997	CG1	VAL	89	43.119	20.417	42.010	1.00	0.00	RX1	C
ATOM	2998	CG2	VAL	89	42.457	19.433	39.790	1.00	0.00	RX1	C
ATOM	2999	C	VAL	89	42.029	18.028	43.351	1.00	0.00	RX1	C
ATOM	3000	O	VAL	89	41.018	18.548	43.813	1.00	0.00	RX1	O
ATOM	3001	N	LEU	90	42.944	17.393	44.083	1.00	0.00	RX1	N
ATOM	3002	H	LEU	90	43.842	17.189	43.688	1.00	0.00	RX1	H
ATOM	3003	CA	LEU	90	42.740	17.380	45.524	1.00	0.00	RX1	C
ATOM	3004	CB	LEU	90	43.207	16.045	46.098	1.00	0.00	RX1	C
ATOM	3005	CG	LEU	90	43.164	15.979	47.624	1.00	0.00	RX1	C
ATOM	3006	CD1	LEU	90	41.770	16.255	48.181	1.00	0.00	RX1	C
ATOM	3007	CD2	LEU	90	43.738	14.662	48.138	1.00	0.00	RX1	C
ATOM	3008	C	LEU	90	43.471	18.536	46.169	1.00	0.00	RX1	C
ATOM	3009	O	LEU	90	44.691	18.589	46.177	1.00	0.00	RX1	O
ATOM	3010	N	ILE	91	42.670	19.456	46.706	1.00	0.00	RX1	N
ATOM	3011	H	ILE	91	41.678	19.329	46.752	1.00	0.00	RX1	H
ATOM	3012	CA	ILE	91	43.281	20.535	47.472	1.00	0.00	RX1	C
ATOM	3013	CB	ILE	91	42.745	21.883	46.990	1.00	0.00	RX1	C
ATOM	3014	CG2	ILE	91	43.369	23.038	47.768	1.00	0.00	RX1	C
ATOM	3015	CG1	ILE	91	42.946	22.041	45.486	1.00	0.00	RX1	C
ATOM	3016	CD1	ILE	91	42.503	23.414	44.981	1.00	0.00	RX1	C
ATOM	3017	C	ILE	91	42.963	20.340	48.941	1.00	0.00	RX1	C
ATOM	3018	O	ILE	91	41.900	20.730	49.411	1.00	0.00	RX1	O
ATOM	3019	N	ALA	92	43.902	19.702	49.646	1.00	0.00	RX1	N
ATOM	3020	H	ALA	92	44.804	19.462	49.276	1.00	0.00	RX1	H
ATOM	3021	CA	ALA	92	43.571	19.423	51.039	1.00	0.00	RX1	C
ATOM	3022	CB	ALA	92	43.041	18.001	51.209	1.00	0.00	RX1	C
ATOM	3023	C	ALA	92	44.699	19.647	52.021	1.00	0.00	RX1	C
ATOM	3024	O	ALA	92	45.875	19.465	51.723	1.00	0.00	RX1	O
ATOM	3025	N	LEU	93	44.267	20.045	53.233	1.00	0.00	RX1	N
ATOM	3026	H	LEU	93	43.288	20.232	53.336	1.00	0.00	RX1	H
ATOM	3027	CA	LEU	93	45.195	20.254	54.354	1.00	0.00	RX1	C
ATOM	3028	CB	LEU	93	45.693	18.915	54.904	1.00	0.00	RX1	C
ATOM	3029	CG	LEU	93	44.617	18.202	55.725	1.00	0.00	RX1	C
ATOM	3030	CD1	LEU	93	44.990	16.755	56.044	1.00	0.00	RX1	C
ATOM	3031	CD2	LEU	93	44.288	18.979	57.000	1.00	0.00	RX1	C
ATOM	3032	C	LEU	93	46.350	21.204	54.083	1.00	0.00	RX1	C
ATOM	3033	O	LEU	93	47.459	21.079	54.591	1.00	0.00	RX1	O
ATOM	3034	N	ASN	94	46.025	22.184	53.238	1.00	0.00	RX1	N
ATOM	3035	H	ASN	94	45.085	22.308	52.923	1.00	0.00	RX1	H
ATOM	3036	CA	ASN	94	47.020	23.212	52.969	1.00	0.00	RX1	C
ATOM	3037	CB	ASN	94	46.955	23.728	51.531	1.00	0.00	RX1	C
ATOM	3038	CG	ASN	94	47.109	22.610	50.527	1.00	0.00	RX1	C
ATOM	3039	OD1	ASN	94	48.193	22.105	50.255	1.00	0.00	RX1	O
ATOM	3040	ND2	ASN	94	45.948	22.261	49.964	1.00	0.00	RX1	N
ATOM	3041	HD21	ASN	94	45.088	22.672	50.278	1.00	0.00	RX1	H
ATOM	3042	HD22	ASN	94	45.903	21.565	49.247	1.00	0.00	RX1	H
ATOM	3043	C	ASN	94	46.763	24.384	53.873	1.00	0.00	RX1	C
ATOM	3044	O	ASN	94	45.625	24.782	54.094	1.00	0.00	RX1	O
ATOM	3045	N	THR	95	47.862	24.926	54.390	1.00	0.00	RX1	N
ATOM	3046	H	THR	95	48.795	24.598	54.235	1.00	0.00	RX1	H
ATOM	3047	CA	THR	95	47.675	26.158	55.140	1.00	0.00	RX1	C
ATOM	3048	CB	THR	95	48.440	25.968	56.433	1.00	0.00	RX1	C

ATOM	3049	OG1	THR	95	49.746	25.492	56.120	1.00	0.00	RX1	O
ATOM	3050	HG1	THR	95	50.061	25.077	56.923	1.00	0.00	RX1	H
ATOM	3051	CG2	THR	95	47.757	24.929	57.315	1.00	0.00	RX1	C
ATOM	3052	C	THR	95	48.068	27.401	54.361	1.00	0.00	RX1	C
ATOM	3053	O	THR	95	47.858	28.523	54.798	1.00	0.00	RX1	O
ATOM	3054	N	VAL	96	48.646	27.158	53.167	1.00	0.00	RX1	N
ATOM	3055	H	VAL	96	48.818	26.224	52.854	1.00	0.00	RX1	H
ATOM	3056	CA	VAL	96	48.960	28.304	52.313	1.00	0.00	RX1	C
ATOM	3057	CB	VAL	96	49.799	27.873	51.101	1.00	0.00	RX1	C
ATOM	3058	CG1	VAL	96	49.159	26.719	50.335	1.00	0.00	RX1	C
ATOM	3059	CG2	VAL	96	50.131	29.063	50.199	1.00	0.00	RX1	C
ATOM	3060	C	VAL	96	47.742	29.131	51.917	1.00	0.00	RX1	C
ATOM	3061	O	VAL	96	46.811	28.670	51.265	1.00	0.00	RX1	O
ATOM	3062	N	GLU	97	47.799	30.388	52.379	1.00	0.00	RX1	N
ATOM	3063	H	GLU	97	48.601	30.692	52.895	1.00	0.00	RX1	H
ATOM	3064	CA	GLU	97	46.607	31.230	52.270	1.00	0.00	RX1	C
ATOM	3065	CB	GLU	97	46.731	32.448	53.189	1.00	0.00	RX1	C
ATOM	3066	CG	GLU	97	46.869	31.925	54.620	1.00	0.00	RX1	C
ATOM	3067	CD	GLU	97	46.935	33.029	55.654	1.00	0.00	RX1	C
ATOM	3068	OE1	GLU	97	45.907	33.371	56.233	1.00	0.00	RX1	O
ATOM	3069	OE2	GLU	97	48.034	33.466	55.981	1.00	0.00	RX1	O
ATOM	3070	C	GLU	97	46.173	31.579	50.861	1.00	0.00	RX1	C
ATOM	3071	O	GLU	97	44.996	31.752	50.568	1.00	0.00	RX1	O
ATOM	3072	N	ARG	98	47.174	31.661	49.981	1.00	0.00	RX1	N
ATOM	3073	H	ARG	98	48.120	31.445	50.217	1.00	0.00	RX1	H
ATOM	3074	CA	ARG	98	46.801	31.876	48.590	1.00	0.00	RX1	C
ATOM	3075	CB	ARG	98	47.396	33.177	48.055	1.00	0.00	RX1	C
ATOM	3076	CG	ARG	98	46.861	33.529	46.665	1.00	0.00	RX1	C
ATOM	3077	CD	ARG	98	47.610	34.704	46.032	1.00	0.00	RX1	C
ATOM	3078	NE	ARG	98	46.926	35.181	44.831	1.00	0.00	RX1	N
ATOM	3079	HE	ARG	98	46.107	35.738	44.998	1.00	0.00	RX1	H
ATOM	3080	CZ	ARG	98	47.377	34.828	43.588	1.00	0.00	RX1	C
ATOM	3081	NH1	ARG	98	48.474	34.053	43.456	1.00	0.00	RX1	N
ATOM	3082	HH11	ARG	98	48.756	33.770	42.525	1.00	0.00	RX1	H
ATOM	3083	HH12	ARG	98	49.033	33.736	44.227	1.00	0.00	RX1	H
ATOM	3084	NH2	ARG	98	46.716	35.258	42.493	1.00	0.00	RX1	N
ATOM	3085	HH21	ARG	98	47.027	34.979	41.563	1.00	0.00	RX1	H
ATOM	3086	HH22	ARG	98	45.910	35.849	42.523	1.00	0.00	RX1	H
ATOM	3087	C	ARG	98	47.275	30.723	47.735	1.00	0.00	RX1	C
ATOM	3088	O	ARG	98	48.468	30.552	47.525	1.00	0.00	RX1	O
ATOM	3089	N	ILE	99	46.309	29.939	47.243	1.00	0.00	RX1	N
ATOM	3090	H	ILE	99	45.343	30.194	47.335	1.00	0.00	RX1	H
ATOM	3091	CA	ILE	99	46.754	28.879	46.338	1.00	0.00	RX1	C
ATOM	3092	CB	ILE	99	46.015	27.568	46.609	1.00	0.00	RX1	C
ATOM	3093	CG2	ILE	99	46.316	26.500	45.557	1.00	0.00	RX1	C
ATOM	3094	CG1	ILE	99	46.394	27.080	48.004	1.00	0.00	RX1	C
ATOM	3095	CD1	ILE	99	45.789	25.723	48.346	1.00	0.00	RX1	C
ATOM	3096	C	ILE	99	46.642	29.312	44.887	1.00	0.00	RX1	C
ATOM	3097	O	ILE	99	45.567	29.570	44.358	1.00	0.00	RX1	O
ATOM	3098	N	PRO	100	47.829	29.428	44.252	1.00	0.00	RX1	N
ATOM	3099	CD	PRO	100	49.135	29.130	44.808	1.00	0.00	RX1	C
ATOM	3100	CA	PRO	100	47.909	30.045	42.926	1.00	0.00	RX1	C
ATOM	3101	CB	PRO	100	49.386	30.444	42.838	1.00	0.00	RX1	C
ATOM	3102	CG	PRO	100	49.975	30.254	44.233	1.00	0.00	RX1	C
ATOM	3103	C	PRO	100	47.506	29.158	41.756	1.00	0.00	RX1	C
ATOM	3104	O	PRO	100	48.214	29.087	40.759	1.00	0.00	RX1	O
ATOM	3105	N	LEU	101	46.339	28.505	41.869	1.00	0.00	RX1	N
ATOM	3106	H	LEU	101	45.721	28.685	42.635	1.00	0.00	RX1	H
ATOM	3107	CA	LEU	101	45.871	27.734	40.713	1.00	0.00	RX1	C
ATOM	3108	CB	LEU	101	44.938	26.617	41.169	1.00	0.00	RX1	C
ATOM	3109	CG	LEU	101	45.401	25.220	40.762	1.00	0.00	RX1	C

ATOM	3110	CD1	LEU	101	44.311	24.189	41.052	1.00	0.00	RX1	C
ATOM	3111	CD2	LEU	101	45.893	25.156	39.314	1.00	0.00	RX1	C
ATOM	3112	C	LEU	101	45.154	28.600	39.686	1.00	0.00	RX1	C
ATOM	3113	O	LEU	101	44.080	28.306	39.180	1.00	0.00	RX1	O
ATOM	3114	N	GLU	102	45.779	29.751	39.451	1.00	0.00	RX1	N
ATOM	3115	H	GLU	102	46.757	29.849	39.641	1.00	0.00	RX1	H
ATOM	3116	CA	GLU	102	44.958	30.858	38.990	1.00	0.00	RX1	C
ATOM	3117	CB	GLU	102	45.503	32.153	39.579	1.00	0.00	RX1	C
ATOM	3118	CG	GLU	102	46.955	32.453	39.221	1.00	0.00	RX1	C
ATOM	3119	CD	GLU	102	47.335	33.733	39.925	1.00	0.00	RX1	C
ATOM	3120	OE1	GLU	102	46.693	34.755	39.696	1.00	0.00	RX1	O
ATOM	3121	OE2	GLU	102	48.234	33.711	40.758	1.00	0.00	RX1	O
ATOM	3122	C	GLU	102	44.730	30.931	37.493	1.00	0.00	RX1	C
ATOM	3123	O	GLU	102	43.797	31.553	36.999	1.00	0.00	RX1	O
ATOM	3124	N	ASN	103	45.623	30.243	36.771	1.00	0.00	RX1	N
ATOM	3125	H	ASN	103	46.312	29.647	37.182	1.00	0.00	RX1	H
ATOM	3126	CA	ASN	103	45.402	30.262	35.325	1.00	0.00	RX1	C
ATOM	3127	CB	ASN	103	46.687	30.536	34.547	1.00	0.00	RX1	C
ATOM	3128	CG	ASN	103	47.070	31.998	34.635	1.00	0.00	RX1	C
ATOM	3129	OD1	ASN	103	46.237	32.901	34.740	1.00	0.00	RX1	O
ATOM	3130	ND2	ASN	103	48.400	32.184	34.591	1.00	0.00	RX1	N
ATOM	3131	HD21	ASN	103	49.001	31.376	34.560	1.00	0.00	RX1	H
ATOM	3132	HD22	ASN	103	48.850	33.076	34.588	1.00	0.00	RX1	H
ATOM	3133	C	ASN	103	44.769	28.997	34.780	1.00	0.00	RX1	C
ATOM	3134	O	ASN	103	44.711	28.777	33.575	1.00	0.00	RX1	O
ATOM	3135	N	LEU	104	44.282	28.183	35.734	1.00	0.00	RX1	N
ATOM	3136	H	LEU	104	44.257	28.452	36.695	1.00	0.00	RX1	H
ATOM	3137	CA	LEU	104	43.594	26.948	35.365	1.00	0.00	RX1	C
ATOM	3138	CB	LEU	104	43.215	26.216	36.654	1.00	0.00	RX1	C
ATOM	3139	CG	LEU	104	42.644	24.807	36.496	1.00	0.00	RX1	C
ATOM	3140	CD1	LEU	104	43.638	23.841	35.855	1.00	0.00	RX1	C
ATOM	3141	CD2	LEU	104	42.138	24.273	37.835	1.00	0.00	RX1	C
ATOM	3142	C	LEU	104	42.374	27.242	34.520	1.00	0.00	RX1	C
ATOM	3143	O	LEU	104	41.721	28.256	34.722	1.00	0.00	RX1	O
ATOM	3144	N	GLN	105	42.102	26.337	33.572	1.00	0.00	RX1	N
ATOM	3145	H	GLN	105	42.690	25.540	33.441	1.00	0.00	RX1	H
ATOM	3146	CA	GLN	105	40.871	26.495	32.803	1.00	0.00	RX1	C
ATOM	3147	CB	GLN	105	41.161	26.901	31.363	1.00	0.00	RX1	C
ATOM	3148	CG	GLN	105	41.971	28.186	31.223	1.00	0.00	RX1	C
ATOM	3149	CD	GLN	105	42.110	28.504	29.755	1.00	0.00	RX1	C
ATOM	3150	OE1	GLN	105	41.155	28.879	29.084	1.00	0.00	RX1	O
ATOM	3151	NE2	GLN	105	43.356	28.315	29.286	1.00	0.00	RX1	N
ATOM	3152	HE21	GLN	105	44.085	28.035	29.912	1.00	0.00	RX1	H
ATOM	3153	HE22	GLN	105	43.577	28.448	28.319	1.00	0.00	RX1	H
ATOM	3154	C	GLN	105	39.960	25.286	32.811	1.00	0.00	RX1	C
ATOM	3155	O	GLN	105	38.741	25.429	32.809	1.00	0.00	RX1	O
ATOM	3156	N	ILE	106	40.592	24.097	32.830	1.00	0.00	RX1	N
ATOM	3157	H	ILE	106	41.589	23.988	32.847	1.00	0.00	RX1	H
ATOM	3158	CA	ILE	106	39.775	22.885	32.846	1.00	0.00	RX1	C
ATOM	3159	CB	ILE	106	39.602	22.316	31.428	1.00	0.00	RX1	C
ATOM	3160	CG2	ILE	106	40.929	21.860	30.845	1.00	0.00	RX1	C
ATOM	3161	CG1	ILE	106	38.589	21.177	31.361	1.00	0.00	RX1	C
ATOM	3162	CD1	ILE	106	38.536	20.574	29.960	1.00	0.00	RX1	C
ATOM	3163	C	ILE	106	40.278	21.825	33.814	1.00	0.00	RX1	C
ATOM	3164	O	ILE	106	41.462	21.512	33.886	1.00	0.00	RX1	O
ATOM	3165	N	ILE	107	39.306	21.259	34.536	1.00	0.00	RX1	N
ATOM	3166	H	ILE	107	38.357	21.571	34.447	1.00	0.00	RX1	H
ATOM	3167	CA	ILE	107	39.558	19.970	35.168	1.00	0.00	RX1	C
ATOM	3168	CB	ILE	107	39.169	20.026	36.645	1.00	0.00	RX1	C
ATOM	3169	CG2	ILE	107	39.438	18.700	37.360	1.00	0.00	RX1	C
ATOM	3170	CG1	ILE	107	39.868	21.200	37.327	1.00	0.00	RX1	C

ATOM	3171	CD1	ILE	107	39.397	21.409	38.765	1.00	0.00	RX1	C
ATOM	3172	C	ILE	107	38.732	18.931	34.432	1.00	0.00	RX1	C
ATOM	3173	O	ILE	107	37.547	19.119	34.182	1.00	0.00	RX1	O
ATOM	3174	N	ARG	108	39.405	17.840	34.058	1.00	0.00	RX1	N
ATOM	3175	H	ARG	108	40.373	17.716	34.279	1.00	0.00	RX1	H
ATOM	3176	CA	ARG	108	38.641	16.795	33.385	1.00	0.00	RX1	C
ATOM	3177	CB	ARG	108	39.459	16.166	32.259	1.00	0.00	RX1	C
ATOM	3178	CG	ARG	108	39.760	17.206	31.179	1.00	0.00	RX1	C
ATOM	3179	CD	ARG	108	40.652	16.701	30.045	1.00	0.00	RX1	C
ATOM	3180	NE	ARG	108	40.735	17.701	28.980	1.00	0.00	RX1	N
ATOM	3181	HE	ARG	108	39.906	17.852	28.428	1.00	0.00	RX1	H
ATOM	3182	CZ	ARG	108	41.875	18.428	28.789	1.00	0.00	RX1	C
ATOM	3183	NH1	ARG	108	42.968	18.208	29.547	1.00	0.00	RX1	N
ATOM	3184	HH11	ARG	108	43.805	18.749	29.360	1.00	0.00	RX1	H
ATOM	3185	HH12	ARG	108	42.999	17.538	30.292	1.00	0.00	RX1	H
ATOM	3186	NH2	ARG	108	41.899	19.372	27.833	1.00	0.00	RX1	N
ATOM	3187	HH21	ARG	108	42.725	19.945	27.681	1.00	0.00	RX1	H
ATOM	3188	HH22	ARG	108	41.101	19.541	27.246	1.00	0.00	RX1	H
ATOM	3189	C	ARG	108	38.057	15.757	34.324	1.00	0.00	RX1	C
ATOM	3190	O	ARG	108	37.073	15.102	34.005	1.00	0.00	RX1	O
ATOM	3191	N	GLY	109	38.680	15.668	35.513	1.00	0.00	RX1	N
ATOM	3192	H	GLY	109	39.603	16.025	35.653	1.00	0.00	RX1	H
ATOM	3193	CA	GLY	109	38.041	14.942	36.611	1.00	0.00	RX1	C
ATOM	3194	C	GLY	109	37.736	13.486	36.333	1.00	0.00	RX1	C
ATOM	3195	O	GLY	109	36.690	12.945	36.669	1.00	0.00	RX1	O
ATOM	3196	N	ASN	110	38.718	12.868	35.672	1.00	0.00	RX1	N
ATOM	3197	H	ASN	110	39.602	13.320	35.541	1.00	0.00	RX1	H
ATOM	3198	CA	ASN	110	38.570	11.433	35.457	1.00	0.00	RX1	C
ATOM	3199	CB	ASN	110	39.391	10.972	34.246	1.00	0.00	RX1	C
ATOM	3200	CG	ASN	110	38.687	11.200	32.909	1.00	0.00	RX1	C
ATOM	3201	OD1	ASN	110	38.997	10.539	31.919	1.00	0.00	RX1	O
ATOM	3202	ND2	ASN	110	37.732	12.155	32.894	1.00	0.00	RX1	N
ATOM	3203	HD21	ASN	110	37.509	12.758	33.666	1.00	0.00	RX1	H
ATOM	3204	HD22	ASN	110	37.176	12.323	32.079	1.00	0.00	RX1	H
ATOM	3205	C	ASN	110	38.999	10.713	36.720	1.00	0.00	RX1	C
ATOM	3206	O	ASN	110	40.007	11.067	37.317	1.00	0.00	RX1	O
ATOM	3207	N	MET	111	38.152	9.734	37.096	1.00	0.00	RX1	N
ATOM	3208	H	MET	111	37.344	9.592	36.528	1.00	0.00	RX1	H
ATOM	3209	CA	MET	111	38.158	9.099	38.421	1.00	0.00	RX1	C
ATOM	3210	CB	MET	111	39.482	8.438	38.816	1.00	0.00	RX1	C
ATOM	3211	CG	MET	111	39.287	7.209	39.718	1.00	0.00	RX1	C
ATOM	3212	SD	MET	111	38.504	7.495	41.317	1.00	0.00	RX1	S
ATOM	3213	CE	MET	111	39.847	8.394	42.106	1.00	0.00	RX1	C
ATOM	3214	C	MET	111	37.701	10.017	39.531	1.00	0.00	RX1	C
ATOM	3215	O	MET	111	38.342	10.988	39.916	1.00	0.00	RX1	O
ATOM	3216	N	TYR	112	36.522	9.659	40.040	1.00	0.00	RX1	N
ATOM	3217	H	TYR	112	36.067	8.806	39.775	1.00	0.00	RX1	H
ATOM	3218	CA	TYR	112	35.967	10.575	41.023	1.00	0.00	RX1	C
ATOM	3219	CB	TYR	112	34.448	10.505	41.061	1.00	0.00	RX1	C
ATOM	3220	CG	TYR	112	33.792	10.950	39.774	1.00	0.00	RX1	C
ATOM	3221	CD1	TYR	112	34.524	11.362	38.665	1.00	0.00	RX1	C
ATOM	3222	CE1	TYR	112	33.874	11.669	37.475	1.00	0.00	RX1	C
ATOM	3223	CD2	TYR	112	32.407	10.920	39.711	1.00	0.00	RX1	C
ATOM	3224	CE2	TYR	112	31.756	11.250	38.535	1.00	0.00	RX1	C
ATOM	3225	CZ	TYR	112	32.489	11.592	37.409	1.00	0.00	RX1	C
ATOM	3226	OH	TYR	112	31.818	11.840	36.230	1.00	0.00	RX1	O
ATOM	3227	HH	TYR	112	31.045	11.285	36.244	1.00	0.00	RX1	H
ATOM	3228	C	TYR	112	36.501	10.365	42.423	1.00	0.00	RX1	C
ATOM	3229	O	TYR	112	36.274	9.346	43.079	1.00	0.00	RX1	O
ATOM	3230	N	TYR	113	37.219	11.409	42.851	1.00	0.00	RX1	N
ATOM	3231	H	TYR	113	37.270	12.200	42.238	1.00	0.00	RX1	H

ATOM	3232	CA	TYR	113	37.724	11.471	44.217	1.00	0.00	RX1	C
ATOM	3233	CB	TYR	113	38.455	12.797	44.430	1.00	0.00	RX1	C
ATOM	3234	CG	TYR	113	39.381	12.644	45.606	1.00	0.00	RX1	C
ATOM	3235	CD1	TYR	113	40.326	11.630	45.587	1.00	0.00	RX1	C
ATOM	3236	CE1	TYR	113	41.156	11.434	46.680	1.00	0.00	RX1	C
ATOM	3237	CD2	TYR	113	39.288	13.491	46.702	1.00	0.00	RX1	C
ATOM	3238	CE2	TYR	113	40.109	13.282	47.805	1.00	0.00	RX1	C
ATOM	3239	CZ	TYR	113	41.022	12.235	47.805	1.00	0.00	RX1	C
ATOM	3240	OH	TYR	113	41.787	11.970	48.928	1.00	0.00	RX1	O
ATOM	3241	HH	TYR	113	41.639	12.671	49.557	1.00	0.00	RX1	H
ATOM	3242	C	TYR	113	36.624	11.288	45.247	1.00	0.00	RX1	C
ATOM	3243	O	TYR	113	35.522	11.802	45.098	1.00	0.00	RX1	O
ATOM	3244	N	GLU	114	36.973	10.456	46.244	1.00	0.00	RX1	N
ATOM	3245	H	GLU	114	37.929	10.166	46.328	1.00	0.00	RX1	H
ATOM	3246	CA	GLU	114	36.077	9.951	47.293	1.00	0.00	RX1	C
ATOM	3247	CB	GLU	114	36.291	10.679	48.621	1.00	0.00	RX1	C
ATOM	3248	CG	GLU	114	37.742	11.063	48.926	1.00	0.00	RX1	C
ATOM	3249	CD	GLU	114	38.702	9.882	48.918	1.00	0.00	RX1	C
ATOM	3250	OE1	GLU	114	39.177	9.483	47.855	1.00	0.00	RX1	O
ATOM	3251	OE2	GLU	114	39.079	9.414	49.986	1.00	0.00	RX1	O
ATOM	3252	C	GLU	114	34.593	9.826	46.994	1.00	0.00	RX1	C
ATOM	3253	O	GLU	114	33.738	10.082	47.834	1.00	0.00	RX1	O
ATOM	3254	N	ASN	115	34.350	9.353	45.753	1.00	0.00	RX1	N
ATOM	3255	H	ASN	115	35.126	9.379	45.125	1.00	0.00	RX1	H
ATOM	3256	CA	ASN	115	33.006	9.234	45.174	1.00	0.00	RX1	C
ATOM	3257	CB	ASN	115	31.933	8.567	46.058	1.00	0.00	RX1	C
ATOM	3258	CG	ASN	115	32.438	7.317	46.745	1.00	0.00	RX1	C
ATOM	3259	OD1	ASN	115	32.659	6.270	46.138	1.00	0.00	RX1	O
ATOM	3260	ND2	ASN	115	32.608	7.484	48.067	1.00	0.00	RX1	N
ATOM	3261	HD21	ASN	115	32.530	8.423	48.423	1.00	0.00	RX1	H
ATOM	3262	HD22	ASN	115	32.827	6.756	48.715	1.00	0.00	RX1	H
ATOM	3263	C	ASN	115	32.468	10.578	44.741	1.00	0.00	RX1	C
ATOM	3264	O	ASN	115	32.398	11.537	45.491	1.00	0.00	RX1	O
ATOM	3265	N	SER	116	32.099	10.602	43.456	1.00	0.00	RX1	N
ATOM	3266	H	SER	116	32.210	9.784	42.897	1.00	0.00	RX1	H
ATOM	3267	CA	SER	116	31.484	11.787	42.856	1.00	0.00	RX1	C
ATOM	3268	CB	SER	116	30.154	11.974	43.571	1.00	0.00	RX1	C
ATOM	3269	OG	SER	116	29.663	10.646	43.809	1.00	0.00	RX1	O
ATOM	3270	HG	SER	116	29.431	10.297	42.960	1.00	0.00	RX1	H
ATOM	3271	C	SER	116	32.314	13.049	42.639	1.00	0.00	RX1	C
ATOM	3272	O	SER	116	31.908	13.884	41.839	1.00	0.00	RX1	O
ATOM	3273	N	TYR	117	33.453	13.199	43.334	1.00	0.00	RX1	N
ATOM	3274	H	TYR	117	33.845	12.520	43.960	1.00	0.00	RX1	H
ATOM	3275	CA	TYR	117	34.091	14.509	43.193	1.00	0.00	RX1	C
ATOM	3276	CB	TYR	117	34.494	15.067	44.553	1.00	0.00	RX1	C
ATOM	3277	CG	TYR	117	33.312	14.967	45.474	1.00	0.00	RX1	C
ATOM	3278	CD1	TYR	117	32.115	15.577	45.123	1.00	0.00	RX1	C
ATOM	3279	CE1	TYR	117	31.017	15.463	45.963	1.00	0.00	RX1	C
ATOM	3280	CD2	TYR	117	33.425	14.254	46.661	1.00	0.00	RX1	C
ATOM	3281	CE2	TYR	117	32.327	14.143	47.501	1.00	0.00	RX1	C
ATOM	3282	CZ	TYR	117	31.130	14.755	47.152	1.00	0.00	RX1	C
ATOM	3283	OH	TYR	117	30.052	14.681	48.008	1.00	0.00	RX1	O
ATOM	3284	HH	TYR	117	29.266	14.667	47.457	1.00	0.00	RX1	H
ATOM	3285	C	TYR	117	35.297	14.553	42.280	1.00	0.00	RX1	C
ATOM	3286	O	TYR	117	35.845	13.536	41.881	1.00	0.00	RX1	O
ATOM	3287	N	ALA	118	35.701	15.791	41.981	1.00	0.00	RX1	N
ATOM	3288	H	ALA	118	35.173	16.586	42.281	1.00	0.00	RX1	H
ATOM	3289	CA	ALA	118	36.977	15.984	41.298	1.00	0.00	RX1	C
ATOM	3290	CB	ALA	118	36.789	16.411	39.846	1.00	0.00	RX1	C
ATOM	3291	C	ALA	118	37.783	17.067	41.970	1.00	0.00	RX1	C
ATOM	3292	O	ALA	118	38.919	16.876	42.383	1.00	0.00	RX1	O

ATOM	3293	N	LEU	119	37.131	18.233	42.065	1.00	0.00	RX1	N
ATOM	3294	H	LEU	119	36.165	18.323	41.816	1.00	0.00	RX1	H
ATOM	3295	CA	LEU	119	37.802	19.313	42.776	1.00	0.00	RX1	C
ATOM	3296	CB	LEU	119	37.410	20.655	42.160	1.00	0.00	RX1	C
ATOM	3297	CG	LEU	119	38.077	21.859	42.827	1.00	0.00	RX1	C
ATOM	3298	CD1	LEU	119	39.601	21.759	42.811	1.00	0.00	RX1	C
ATOM	3299	CD2	LEU	119	37.596	23.174	42.219	1.00	0.00	RX1	C
ATOM	3300	C	LEU	119	37.445	19.254	44.245	1.00	0.00	RX1	C
ATOM	3301	O	LEU	119	36.378	19.687	44.660	1.00	0.00	RX1	O
ATOM	3302	N	ALA	120	38.365	18.659	45.006	1.00	0.00	RX1	N
ATOM	3303	H	ALA	120	39.260	18.403	44.632	1.00	0.00	RX1	H
ATOM	3304	CA	ALA	120	38.048	18.484	46.417	1.00	0.00	RX1	C
ATOM	3305	CB	ALA	120	38.227	17.022	46.821	1.00	0.00	RX1	C
ATOM	3306	C	ALA	120	38.882	19.374	47.315	1.00	0.00	RX1	C
ATOM	3307	O	ALA	120	40.008	19.063	47.682	1.00	0.00	RX1	O
ATOM	3308	N	VAL	121	38.271	20.515	47.641	1.00	0.00	RX1	N
ATOM	3309	H	VAL	121	37.317	20.665	47.375	1.00	0.00	RX1	H
ATOM	3310	CA	VAL	121	38.947	21.484	48.500	1.00	0.00	RX1	C
ATOM	3311	CB	VAL	121	38.584	22.896	48.041	1.00	0.00	RX1	C
ATOM	3312	CG1	VAL	121	39.351	23.979	48.799	1.00	0.00	RX1	C
ATOM	3313	CG2	VAL	121	38.793	23.010	46.531	1.00	0.00	RX1	C
ATOM	3314	C	VAL	121	38.624	21.261	49.974	1.00	0.00	RX1	C
ATOM	3315	O	VAL	121	37.726	21.863	50.559	1.00	0.00	RX1	O
ATOM	3316	N	LEU	122	39.398	20.329	50.539	1.00	0.00	RX1	N
ATOM	3317	H	LEU	122	40.212	19.995	50.057	1.00	0.00	RX1	H
ATOM	3318	CA	LEU	122	39.092	19.852	51.886	1.00	0.00	RX1	C
ATOM	3319	CB	LEU	122	39.106	18.323	51.906	1.00	0.00	RX1	C
ATOM	3320	CG	LEU	122	38.351	17.665	50.751	1.00	0.00	RX1	C
ATOM	3321	CD1	LEU	122	38.567	16.151	50.732	1.00	0.00	RX1	C
ATOM	3322	CD2	LEU	122	36.868	18.028	50.747	1.00	0.00	RX1	C
ATOM	3323	C	LEU	122	40.048	20.358	52.951	1.00	0.00	RX1	C
ATOM	3324	O	LEU	122	41.259	20.393	52.770	1.00	0.00	RX1	O
ATOM	3325	N	SER	123	39.453	20.718	54.095	1.00	0.00	RX1	N
ATOM	3326	H	SER	123	38.458	20.664	54.174	1.00	0.00	RX1	H
ATOM	3327	CA	SER	123	40.235	20.831	55.331	1.00	0.00	RX1	C
ATOM	3328	CB	SER	123	40.449	19.387	55.735	1.00	0.00	RX1	C
ATOM	3329	OG	SER	123	39.291	18.682	55.258	1.00	0.00	RX1	O
ATOM	3330	HG	SER	123	39.426	17.770	55.483	1.00	0.00	RX1	H
ATOM	3331	C	SER	123	41.477	21.713	55.289	1.00	0.00	RX1	C
ATOM	3332	O	SER	123	42.517	21.427	55.866	1.00	0.00	RX1	O
ATOM	3333	N	ASN	124	41.330	22.810	54.542	1.00	0.00	RX1	N
ATOM	3334	H	ASN	124	40.421	23.087	54.239	1.00	0.00	RX1	H
ATOM	3335	CA	ASN	124	42.530	23.610	54.296	1.00	0.00	RX1	C
ATOM	3336	CB	ASN	124	42.473	24.281	52.927	1.00	0.00	RX1	C
ATOM	3337	CG	ASN	124	42.661	23.261	51.834	1.00	0.00	RX1	C
ATOM	3338	OD1	ASN	124	43.738	22.710	51.635	1.00	0.00	RX1	O
ATOM	3339	ND2	ASN	124	41.541	23.015	51.141	1.00	0.00	RX1	N
ATOM	3340	HD21	ASN	124	40.706	23.542	51.292	1.00	0.00	RX1	H
ATOM	3341	HD22	ASN	124	41.523	22.275	50.463	1.00	0.00	RX1	H
ATOM	3342	C	ASN	124	42.742	24.681	55.335	1.00	0.00	RX1	C
ATOM	3343	O	ASN	124	42.397	25.834	55.107	1.00	0.00	RX1	O
ATOM	3344	N	TYR	125	43.289	24.254	56.485	1.00	0.00	RX1	N
ATOM	3345	H	TYR	125	43.582	23.307	56.641	1.00	0.00	RX1	H
ATOM	3346	CA	TYR	125	43.436	25.208	57.583	1.00	0.00	RX1	C
ATOM	3347	CB	TYR	125	42.088	25.460	58.255	1.00	0.00	RX1	C
ATOM	3348	CG	TYR	125	41.550	24.190	58.867	1.00	0.00	RX1	C
ATOM	3349	CD1	TYR	125	41.898	23.848	60.165	1.00	0.00	RX1	C
ATOM	3350	CE1	TYR	125	41.322	22.747	60.777	1.00	0.00	RX1	C
ATOM	3351	CD2	TYR	125	40.690	23.374	58.145	1.00	0.00	RX1	C
ATOM	3352	CE2	TYR	125	40.102	22.275	58.756	1.00	0.00	RX1	C
ATOM	3353	CZ	TYR	125	40.373	22.005	60.089	1.00	0.00	RX1	C

ATOM	3354	OH	TYR	125	39.656	21.036	60.767	1.00	0.00	RX1	O
ATOM	3355	HH	TYR	125	39.561	20.285	60.183	1.00	0.00	RX1	H
ATOM	3356	C	TYR	125	44.482	24.817	58.608	1.00	0.00	RX1	C
ATOM	3357	O	TYR	125	44.837	23.652	58.736	1.00	0.00	RX1	O
ATOM	3358	N	ASP	126	44.939	25.842	59.342	1.00	0.00	RX1	N
ATOM	3359	H	ASP	126	44.652	26.792	59.212	1.00	0.00	RX1	H
ATOM	3360	CA	ASP	126	45.790	25.579	60.499	1.00	0.00	RX1	C
ATOM	3361	CB	ASP	126	46.871	26.655	60.649	1.00	0.00	RX1	C
ATOM	3362	CG	ASP	126	46.329	27.937	61.258	1.00	0.00	RX1	C
ATOM	3363	OD1	ASP	126	47.113	28.703	61.796	1.00	0.00	RX1	O
ATOM	3364	OD2	ASP	126	45.138	28.199	61.194	1.00	0.00	RX1	O
ATOM	3365	C	ASP	126	44.988	25.405	61.781	1.00	0.00	RX1	C
ATOM	3366	O	ASP	126	43.763	25.362	61.775	1.00	0.00	RX1	O
ATOM	3367	N	ALA	127	45.729	25.348	62.903	1.00	0.00	RX1	N
ATOM	3368	H	ALA	127	46.721	25.451	62.858	1.00	0.00	RX1	H
ATOM	3369	CA	ALA	127	45.062	25.277	64.204	1.00	0.00	RX1	C
ATOM	3370	CB	ALA	127	46.097	25.259	65.329	1.00	0.00	RX1	C
ATOM	3371	C	ALA	127	44.067	26.397	64.479	1.00	0.00	RX1	C
ATOM	3372	O	ALA	127	43.054	26.217	65.142	1.00	0.00	RX1	O
ATOM	3373	N	ASN	128	44.387	27.567	63.908	1.00	0.00	RX1	N
ATOM	3374	H	ASN	128	45.125	27.645	63.234	1.00	0.00	RX1	H
ATOM	3375	CA	ASN	128	43.501	28.717	64.099	1.00	0.00	RX1	C
ATOM	3376	CB	ASN	128	44.231	30.054	63.934	1.00	0.00	RX1	C
ATOM	3377	CG	ASN	128	45.349	30.211	64.937	1.00	0.00	RX1	C
ATOM	3378	OD1	ASN	128	45.146	30.534	66.103	1.00	0.00	RX1	O
ATOM	3379	ND2	ASN	128	46.563	29.975	64.409	1.00	0.00	RX1	N
ATOM	3380	HD21	ASN	128	46.646	29.693	63.445	1.00	0.00	RX1	H
ATOM	3381	HD22	ASN	128	47.409	30.063	64.930	1.00	0.00	RX1	H
ATOM	3382	C	ASN	128	42.329	28.753	63.132	1.00	0.00	RX1	C
ATOM	3383	O	ASN	128	41.688	29.780	62.959	1.00	0.00	RX1	O
ATOM	3384	N	LYS	129	42.092	27.602	62.467	1.00	0.00	RX1	N
ATOM	3385	H	LYS	129	42.684	26.810	62.610	1.00	0.00	RX1	H
ATOM	3386	CA	LYS	129	41.087	27.536	61.403	1.00	0.00	RX1	C
ATOM	3387	CB	LYS	129	39.664	27.600	61.974	1.00	0.00	RX1	C
ATOM	3388	CG	LYS	129	39.223	26.385	62.803	1.00	0.00	RX1	C
ATOM	3389	CD	LYS	129	38.985	25.115	61.976	1.00	0.00	RX1	C
ATOM	3390	CE	LYS	129	38.366	23.966	62.786	1.00	0.00	RX1	C
ATOM	3391	NZ	LYS	129	38.114	22.788	61.938	1.00	0.00	RX1	N
ATOM	3392	HZ1	LYS	129	37.401	22.168	62.362	1.00	0.00	RX1	H
ATOM	3393	HZ2	LYS	129	37.767	23.057	60.992	1.00	0.00	RX1	H
ATOM	3394	HZ3	LYS	129	38.975	22.217	61.791	1.00	0.00	RX1	H
ATOM	3395	C	LYS	129	41.307	28.548	60.284	1.00	0.00	RX1	C
ATOM	3396	O	LYS	129	40.381	29.102	59.704	1.00	0.00	RX1	O
ATOM	3397	N	THR	130	42.598	28.752	59.994	1.00	0.00	RX1	N
ATOM	3398	H	THR	130	43.341	28.290	60.476	1.00	0.00	RX1	H
ATOM	3399	CA	THR	130	42.926	29.603	58.858	1.00	0.00	RX1	C
ATOM	3400	CB	THR	130	43.451	30.961	59.356	1.00	0.00	RX1	C
ATOM	3401	OG1	THR	130	44.615	30.815	60.191	1.00	0.00	RX1	O
ATOM	3402	HG1	THR	130	44.523	29.983	60.658	1.00	0.00	RX1	H
ATOM	3403	CG2	THR	130	42.375	31.775	60.078	1.00	0.00	RX1	C
ATOM	3404	C	THR	130	43.877	28.896	57.914	1.00	0.00	RX1	C
ATOM	3405	O	THR	130	44.992	28.540	58.274	1.00	0.00	RX1	O
ATOM	3406	N	GLY	131	43.399	28.679	56.694	1.00	0.00	RX1	N
ATOM	3407	H	GLY	131	42.449	28.846	56.418	1.00	0.00	RX1	H
ATOM	3408	CA	GLY	131	44.362	28.174	55.726	1.00	0.00	RX1	C
ATOM	3409	C	GLY	131	44.146	28.821	54.391	1.00	0.00	RX1	C
ATOM	3410	O	GLY	131	44.401	29.999	54.195	1.00	0.00	RX1	O
ATOM	3411	N	LEU	132	43.623	28.005	53.473	1.00	0.00	RX1	N
ATOM	3412	H	LEU	132	43.279	27.111	53.762	1.00	0.00	RX1	H
ATOM	3413	CA	LEU	132	43.321	28.589	52.170	1.00	0.00	RX1	C
ATOM	3414	CB	LEU	132	42.909	27.485	51.199	1.00	0.00	RX1	C

ATOM	3415	CG	LEU	132	42.423	27.979	49.837	1.00	0.00	RX1	C
ATOM	3416	CD1	LEU	132	43.425	28.905	49.154	1.00	0.00	RX1	C
ATOM	3417	CD2	LEU	132	42.018	26.807	48.946	1.00	0.00	RX1	C
ATOM	3418	C	LEU	132	42.267	29.680	52.255	1.00	0.00	RX1	C
ATOM	3419	O	LEU	132	41.098	29.428	52.507	1.00	0.00	RX1	O
ATOM	3420	N	LYS	133	42.757	30.901	52.027	1.00	0.00	RX1	N
ATOM	3421	H	LYS	133	43.732	31.013	51.838	1.00	0.00	RX1	H
ATOM	3422	CA	LYS	133	41.857	32.040	51.940	1.00	0.00	RX1	C
ATOM	3423	CB	LYS	133	42.507	33.277	52.552	1.00	0.00	RX1	C
ATOM	3424	CG	LYS	133	41.544	34.460	52.554	1.00	0.00	RX1	C
ATOM	3425	CD	LYS	133	42.191	35.755	53.029	1.00	0.00	RX1	C
ATOM	3426	CE	LYS	133	41.176	36.894	53.085	1.00	0.00	RX1	C
ATOM	3427	NZ	LYS	133	40.592	37.139	51.762	1.00	0.00	RX1	N
ATOM	3428	HZ1	LYS	133	39.854	37.877	51.792	1.00	0.00	RX1	H
ATOM	3429	HZ2	LYS	133	41.316	37.458	51.084	1.00	0.00	RX1	H
ATOM	3430	HZ3	LYS	133	40.210	36.290	51.294	1.00	0.00	RX1	H
ATOM	3431	C	LYS	133	41.443	32.326	50.510	1.00	0.00	RX1	C
ATOM	3432	O	LYS	133	40.284	32.217	50.137	1.00	0.00	RX1	O
ATOM	3433	N	GLU	134	42.448	32.704	49.713	1.00	0.00	RX1	N
ATOM	3434	H	GLU	134	43.407	32.654	49.994	1.00	0.00	RX1	H
ATOM	3435	CA	GLU	134	42.094	33.011	48.336	1.00	0.00	RX1	C
ATOM	3436	CB	GLU	134	42.617	34.376	47.891	1.00	0.00	RX1	C
ATOM	3437	CG	GLU	134	41.500	35.411	47.719	1.00	0.00	RX1	C
ATOM	3438	CD	GLU	134	41.145	36.049	49.047	1.00	0.00	RX1	C
ATOM	3439	OE1	GLU	134	40.012	35.934	49.506	1.00	0.00	RX1	O
ATOM	3440	OE2	GLU	134	41.981	36.744	49.610	1.00	0.00	RX1	O
ATOM	3441	C	GLU	134	42.534	31.946	47.362	1.00	0.00	RX1	C
ATOM	3442	O	GLU	134	43.708	31.617	47.214	1.00	0.00	RX1	O
ATOM	3443	N	LEU	135	41.508	31.435	46.674	1.00	0.00	RX1	N
ATOM	3444	H	LEU	135	40.573	31.739	46.863	1.00	0.00	RX1	H
ATOM	3445	CA	LEU	135	41.768	30.511	45.573	1.00	0.00	RX1	C
ATOM	3446	CB	LEU	135	41.084	29.184	45.904	1.00	0.00	RX1	C
ATOM	3447	CG	LEU	135	41.278	28.050	44.900	1.00	0.00	RX1	C
ATOM	3448	CD1	LEU	135	42.751	27.742	44.643	1.00	0.00	RX1	C
ATOM	3449	CD2	LEU	135	40.500	26.807	45.331	1.00	0.00	RX1	C
ATOM	3450	C	LEU	135	41.278	31.103	44.256	1.00	0.00	RX1	C
ATOM	3451	O	LEU	135	40.154	30.881	43.823	1.00	0.00	RX1	O
ATOM	3452	N	PRO	136	42.153	31.926	43.630	1.00	0.00	RX1	N
ATOM	3453	CD	PRO	136	43.530	32.205	43.998	1.00	0.00	RX1	C
ATOM	3454	CA	PRO	136	41.697	32.751	42.508	1.00	0.00	RX1	C
ATOM	3455	CB	PRO	136	42.750	33.869	42.461	1.00	0.00	RX1	C
ATOM	3456	CG	PRO	136	43.660	33.686	43.679	1.00	0.00	RX1	C
ATOM	3457	C	PRO	136	41.627	32.027	41.170	1.00	0.00	RX1	C
ATOM	3458	O	PRO	136	42.344	32.369	40.238	1.00	0.00	RX1	O
ATOM	3459	N	MET	137	40.742	31.028	41.073	1.00	0.00	RX1	N
ATOM	3460	H	MET	137	40.100	30.831	41.818	1.00	0.00	RX1	H
ATOM	3461	CA	MET	137	40.697	30.297	39.802	1.00	0.00	RX1	C
ATOM	3462	CB	MET	137	40.297	28.841	40.027	1.00	0.00	RX1	C
ATOM	3463	CG	MET	137	41.266	28.108	40.951	1.00	0.00	RX1	C
ATOM	3464	SD	MET	137	40.813	26.386	41.200	1.00	0.00	RX1	S
ATOM	3465	CE	MET	137	39.155	26.671	41.833	1.00	0.00	RX1	C
ATOM	3466	C	MET	137	39.823	30.924	38.722	1.00	0.00	RX1	C
ATOM	3467	O	MET	137	38.961	30.296	38.125	1.00	0.00	RX1	O
ATOM	3468	N	ARG	138	40.094	32.213	38.478	1.00	0.00	RX1	N
ATOM	3469	H	ARG	138	40.892	32.616	38.927	1.00	0.00	RX1	H
ATOM	3470	CA	ARG	138	39.264	33.000	37.557	1.00	0.00	RX1	C
ATOM	3471	CB	ARG	138	39.635	34.484	37.514	1.00	0.00	RX1	C
ATOM	3472	CG	ARG	138	40.857	34.858	36.671	1.00	0.00	RX1	C
ATOM	3473	CD	ARG	138	42.118	34.284	37.292	1.00	0.00	RX1	C
ATOM	3474	NE	ARG	138	43.326	34.497	36.515	1.00	0.00	RX1	N
ATOM	3475	HE	ARG	138	43.304	34.218	35.547	1.00	0.00	RX1	H

ATOM	3476	CZ	ARG	138	44.406	34.748	37.304	1.00	0.00	RX1	C
ATOM	3477	NH1	ARG	138	44.204	35.169	38.573	1.00	0.00	RX1	N
ATOM	3478	HH11	ARG	138	44.989	35.249	39.201	1.00	0.00	RX1	H
ATOM	3479	HH12	ARG	138	43.294	35.404	38.950	1.00	0.00	RX1	H
ATOM	3480	NH2	ARG	138	45.657	34.555	36.841	1.00	0.00	RX1	N
ATOM	3481	HH21	ARG	138	46.453	34.741	37.432	1.00	0.00	RX1	H
ATOM	3482	HH22	ARG	138	45.836	34.192	35.914	1.00	0.00	RX1	H
ATOM	3483	C	ARG	138	39.089	32.487	36.139	1.00	0.00	RX1	C
ATOM	3484	O	ARG	138	38.121	32.803	35.457	1.00	0.00	RX1	O
ATOM	3485	N	ASN	139	40.095	31.718	35.708	1.00	0.00	RX1	N
ATOM	3486	H	ASN	139	40.834	31.410	36.306	1.00	0.00	RX1	H
ATOM	3487	CA	ASN	139	40.012	31.268	34.322	1.00	0.00	RX1	C
ATOM	3488	CB	ASN	139	41.378	31.160	33.627	1.00	0.00	RX1	C
ATOM	3489	CG	ASN	139	42.221	32.416	33.642	1.00	0.00	RX1	C
ATOM	3490	OD1	ASN	139	41.784	33.520	33.954	1.00	0.00	RX1	O
ATOM	3491	ND2	ASN	139	43.499	32.171	33.303	1.00	0.00	RX1	N
ATOM	3492	HD21	ASN	139	43.759	31.241	33.031	1.00	0.00	RX1	H
ATOM	3493	HD22	ASN	139	44.229	32.857	33.318	1.00	0.00	RX1	H
ATOM	3494	C	ASN	139	39.386	29.899	34.172	1.00	0.00	RX1	C
ATOM	3495	O	ASN	139	39.085	29.447	33.071	1.00	0.00	RX1	O
ATOM	3496	N	LEU	140	39.195	29.258	35.342	1.00	0.00	RX1	N
ATOM	3497	H	LEU	140	39.279	29.719	36.224	1.00	0.00	RX1	H
ATOM	3498	CA	LEU	140	38.647	27.909	35.348	1.00	0.00	RX1	C
ATOM	3499	CB	LEU	140	38.838	27.282	36.733	1.00	0.00	RX1	C
ATOM	3500	CG	LEU	140	38.175	25.916	36.933	1.00	0.00	RX1	C
ATOM	3501	CD1	LEU	140	38.749	24.834	36.028	1.00	0.00	RX1	C
ATOM	3502	CD2	LEU	140	38.200	25.485	38.397	1.00	0.00	RX1	C
ATOM	3503	C	LEU	140	37.195	27.972	34.965	1.00	0.00	RX1	C
ATOM	3504	O	LEU	140	36.338	28.318	35.760	1.00	0.00	RX1	O
ATOM	3505	N	GLN	141	36.965	27.655	33.693	1.00	0.00	RX1	N
ATOM	3506	H	GLN	141	37.705	27.336	33.098	1.00	0.00	RX1	H
ATOM	3507	CA	GLN	141	35.581	27.722	33.256	1.00	0.00	RX1	C
ATOM	3508	CB	GLN	141	35.449	28.600	32.025	1.00	0.00	RX1	C
ATOM	3509	CG	GLN	141	35.849	30.041	32.312	1.00	0.00	RX1	C
ATOM	3510	CD	GLN	141	36.157	30.700	30.994	1.00	0.00	RX1	C
ATOM	3511	OE1	GLN	141	35.278	31.204	30.298	1.00	0.00	RX1	O
ATOM	3512	NE2	GLN	141	37.466	30.646	30.690	1.00	0.00	RX1	N
ATOM	3513	HE21	GLN	141	38.104	30.214	31.338	1.00	0.00	RX1	H
ATOM	3514	HE22	GLN	141	37.856	31.002	29.841	1.00	0.00	RX1	H
ATOM	3515	C	GLN	141	34.969	26.364	33.028	1.00	0.00	RX1	C
ATOM	3516	O	GLN	141	33.765	26.253	32.834	1.00	0.00	RX1	O
ATOM	3517	N	GLU	142	35.832	25.340	33.046	1.00	0.00	RX1	N
ATOM	3518	H	GLU	142	36.814	25.405	33.234	1.00	0.00	RX1	H
ATOM	3519	CA	GLU	142	35.296	24.020	32.754	1.00	0.00	RX1	C
ATOM	3520	CB	GLU	142	35.695	23.590	31.349	1.00	0.00	RX1	C
ATOM	3521	CG	GLU	142	34.792	22.485	30.810	1.00	0.00	RX1	C
ATOM	3522	CD	GLU	142	33.495	23.117	30.371	1.00	0.00	RX1	C
ATOM	3523	OE1	GLU	142	33.470	23.696	29.290	1.00	0.00	RX1	O
ATOM	3524	OE2	GLU	142	32.519	23.097	31.118	1.00	0.00	RX1	O
ATOM	3525	C	GLU	142	35.754	22.977	33.745	1.00	0.00	RX1	C
ATOM	3526	O	GLU	142	36.941	22.781	33.970	1.00	0.00	RX1	O
ATOM	3527	N	ILE	143	34.765	22.287	34.310	1.00	0.00	RX1	N
ATOM	3528	H	ILE	143	33.807	22.489	34.095	1.00	0.00	RX1	H
ATOM	3529	CA	ILE	143	35.091	20.990	34.891	1.00	0.00	RX1	C
ATOM	3530	CB	ILE	143	34.921	20.963	36.413	1.00	0.00	RX1	C
ATOM	3531	CG2	ILE	143	35.251	19.573	36.960	1.00	0.00	RX1	C
ATOM	3532	CG1	ILE	143	35.752	22.044	37.103	1.00	0.00	RX1	C
ATOM	3533	CD1	ILE	143	35.574	22.036	38.622	1.00	0.00	RX1	C
ATOM	3534	C	ILE	143	34.171	19.990	34.231	1.00	0.00	RX1	C
ATOM	3535	O	ILE	143	32.966	19.999	34.451	1.00	0.00	RX1	O
ATOM	3536	N	LEU	144	34.787	19.174	33.367	1.00	0.00	RX1	N

ATOM	3537	H	LEU	144	35.787	19.134	33.359	1.00	0.00	RX1	H
ATOM	3538	CA	LEU	144	33.955	18.248	32.603	1.00	0.00	RX1	C
ATOM	3539	CB	LEU	144	34.743	17.633	31.447	1.00	0.00	RX1	C
ATOM	3540	CG	LEU	144	34.898	18.594	30.269	1.00	0.00	RX1	C
ATOM	3541	CD1	LEU	144	35.792	18.014	29.174	1.00	0.00	RX1	C
ATOM	3542	CD2	LEU	144	33.537	19.021	29.717	1.00	0.00	RX1	C
ATOM	3543	C	LEU	144	33.346	17.157	33.453	1.00	0.00	RX1	C
ATOM	3544	O	LEU	144	32.137	16.984	33.516	1.00	0.00	RX1	O
ATOM	3545	N	HIS	145	34.250	16.424	34.110	1.00	0.00	RX1	N
ATOM	3546	H	HIS	145	35.236	16.592	34.135	1.00	0.00	RX1	H
ATOM	3547	CA	HIS	145	33.732	15.385	34.981	1.00	0.00	RX1	C
ATOM	3548	CB	HIS	145	34.017	13.992	34.417	1.00	0.00	RX1	C
ATOM	3549	CG	HIS	145	32.979	13.689	33.364	1.00	0.00	RX1	C
ATOM	3550	ND1	HIS	145	31.809	13.087	33.636	1.00	0.00	RX1	N
ATOM	3551	HD1	HIS	145	31.534	12.762	34.521	1.00	0.00	RX1	H
ATOM	3552	CD2	HIS	145	33.023	13.984	31.999	1.00	0.00	RX1	C
ATOM	3553	NE2	HIS	145	31.860	13.554	31.447	1.00	0.00	RX1	N
ATOM	3554	CE1	HIS	145	31.112	13.003	32.457	1.00	0.00	RX1	C
ATOM	3555	C	HIS	145	34.222	15.558	36.394	1.00	0.00	RX1	C
ATOM	3556	O	HIS	145	35.178	16.275	36.665	1.00	0.00	RX1	O
ATOM	3557	N	GLY	146	33.482	14.891	37.285	1.00	0.00	RX1	N
ATOM	3558	H	GLY	146	32.711	14.346	36.965	1.00	0.00	RX1	H
ATOM	3559	CA	GLY	146	33.684	15.146	38.703	1.00	0.00	RX1	C
ATOM	3560	C	GLY	146	32.978	16.400	39.179	1.00	0.00	RX1	C
ATOM	3561	O	GLY	146	32.979	17.446	38.546	1.00	0.00	RX1	O
ATOM	3562	N	ALA	147	32.340	16.233	40.337	1.00	0.00	RX1	N
ATOM	3563	H	ALA	147	32.415	15.380	40.850	1.00	0.00	RX1	H
ATOM	3564	CA	ALA	147	31.667	17.379	40.932	1.00	0.00	RX1	C
ATOM	3565	CB	ALA	147	30.465	16.924	41.758	1.00	0.00	RX1	C
ATOM	3566	C	ALA	147	32.606	18.182	41.810	1.00	0.00	RX1	C
ATOM	3567	O	ALA	147	33.765	17.828	42.018	1.00	0.00	RX1	O
ATOM	3568	N	VAL	148	32.060	19.292	42.316	1.00	0.00	RX1	N
ATOM	3569	H	VAL	148	31.081	19.481	42.266	1.00	0.00	RX1	H
ATOM	3570	CA	VAL	148	32.908	20.089	43.190	1.00	0.00	RX1	C
ATOM	3571	CB	VAL	148	32.792	21.570	42.804	1.00	0.00	RX1	C
ATOM	3572	CG1	VAL	148	31.451	22.176	43.223	1.00	0.00	RX1	C
ATOM	3573	CG2	VAL	148	33.992	22.372	43.296	1.00	0.00	RX1	C
ATOM	3574	C	VAL	148	32.593	19.827	44.657	1.00	0.00	RX1	C
ATOM	3575	O	VAL	148	31.449	19.586	45.030	1.00	0.00	RX1	O
ATOM	3576	N	ARG	149	33.666	19.858	45.452	1.00	0.00	RX1	N
ATOM	3577	H	ARG	149	34.587	20.081	45.124	1.00	0.00	RX1	H
ATOM	3578	CA	ARG	149	33.524	19.642	46.884	1.00	0.00	RX1	C
ATOM	3579	CB	ARG	149	34.056	18.259	47.267	1.00	0.00	RX1	C
ATOM	3580	CG	ARG	149	33.724	17.893	48.713	1.00	0.00	RX1	C
ATOM	3581	CD	ARG	149	32.244	17.565	48.887	1.00	0.00	RX1	C
ATOM	3582	NE	ARG	149	31.793	17.804	50.253	1.00	0.00	RX1	N
ATOM	3583	HE	ARG	149	32.068	18.678	50.679	1.00	0.00	RX1	H
ATOM	3584	CZ	ARG	149	30.921	16.978	50.900	1.00	0.00	RX1	C
ATOM	3585	NH1	ARG	149	30.502	15.834	50.320	1.00	0.00	RX1	N
ATOM	3586	HH11	ARG	149	29.881	15.194	50.773	1.00	0.00	RX1	H
ATOM	3587	HH12	ARG	149	30.757	15.569	49.379	1.00	0.00	RX1	H
ATOM	3588	NH2	ARG	149	30.472	17.334	52.118	1.00	0.00	RX1	N
ATOM	3589	HH21	ARG	149	29.900	16.776	52.714	1.00	0.00	RX1	H
ATOM	3590	HH22	ARG	149	30.693	18.269	52.454	1.00	0.00	RX1	H
ATOM	3591	C	ARG	149	34.286	20.708	47.641	1.00	0.00	RX1	C
ATOM	3592	O	ARG	149	35.471	20.923	47.416	1.00	0.00	RX1	O
ATOM	3593	N	PHE	150	33.573	21.360	48.555	1.00	0.00	RX1	N
ATOM	3594	H	PHE	150	32.618	21.126	48.759	1.00	0.00	RX1	H
ATOM	3595	CA	PHE	150	34.286	22.213	49.499	1.00	0.00	RX1	C
ATOM	3596	CB	PHE	150	33.847	23.669	49.377	1.00	0.00	RX1	C
ATOM	3597	CG	PHE	150	34.324	24.249	48.074	1.00	0.00	RX1	C

ATOM	3598	CD1	PHE	150	35.591	24.810	47.996	1.00	0.00	RX1	C
ATOM	3599	CD2	PHE	150	33.499	24.228	46.957	1.00	0.00	RX1	C
ATOM	3600	CE1	PHE	150	36.043	25.338	46.794	1.00	0.00	RX1	C
ATOM	3601	CE2	PHE	150	33.949	24.759	45.758	1.00	0.00	RX1	C
ATOM	3602	CZ	PHE	150	35.226	25.298	45.672	1.00	0.00	RX1	C
ATOM	3603	C	PHE	150	33.909	21.741	50.868	1.00	0.00	RX1	C
ATOM	3604	O	PHE	150	32.728	21.605	51.134	1.00	0.00	RX1	O
ATOM	3605	N	SER	151	34.917	21.492	51.706	1.00	0.00	RX1	N
ATOM	3606	H	SER	151	35.891	21.559	51.481	1.00	0.00	RX1	H
ATOM	3607	CA	SER	151	34.538	21.078	53.053	1.00	0.00	RX1	C
ATOM	3608	CB	SER	151	34.341	19.564	53.044	1.00	0.00	RX1	C
ATOM	3609	OG	SER	151	33.471	19.232	51.958	1.00	0.00	RX1	O
ATOM	3610	HG	SER	151	32.720	19.829	52.081	1.00	0.00	RX1	H
ATOM	3611	C	SER	151	35.538	21.546	54.080	1.00	0.00	RX1	C
ATOM	3612	O	SER	151	36.735	21.612	53.815	1.00	0.00	RX1	O
ATOM	3613	N	ASN	152	34.973	21.903	55.250	1.00	0.00	RX1	N
ATOM	3614	H	ASN	152	33.980	21.790	55.304	1.00	0.00	RX1	H
ATOM	3615	CA	ASN	152	35.685	22.610	56.327	1.00	0.00	RX1	C
ATOM	3616	CB	ASN	152	35.747	21.852	57.650	1.00	0.00	RX1	C
ATOM	3617	CG	ASN	152	36.054	22.863	58.747	1.00	0.00	RX1	C
ATOM	3618	OD1	ASN	152	36.891	22.666	59.632	1.00	0.00	RX1	O
ATOM	3619	ND2	ASN	152	35.264	23.954	58.703	1.00	0.00	RX1	N
ATOM	3620	HD21	ASN	152	34.645	24.151	57.933	1.00	0.00	RX1	H
ATOM	3621	HD22	ASN	152	35.244	24.660	59.409	1.00	0.00	RX1	H
ATOM	3622	C	ASN	152	37.056	23.195	56.041	1.00	0.00	RX1	C
ATOM	3623	O	ASN	152	38.097	22.712	56.465	1.00	0.00	RX1	O
ATOM	3624	N	ASN	153	36.979	24.296	55.302	1.00	0.00	RX1	N
ATOM	3625	H	ASN	153	36.083	24.697	55.089	1.00	0.00	RX1	H
ATOM	3626	CA	ASN	153	38.173	25.029	54.890	1.00	0.00	RX1	C
ATOM	3627	CB	ASN	153	38.312	24.947	53.369	1.00	0.00	RX1	C
ATOM	3628	CG	ASN	153	36.979	25.265	52.710	1.00	0.00	RX1	C
ATOM	3629	OD1	ASN	153	36.188	26.088	53.163	1.00	0.00	RX1	O
ATOM	3630	ND2	ASN	153	36.744	24.515	51.616	1.00	0.00	RX1	N
ATOM	3631	HD21	ASN	153	37.356	23.773	51.324	1.00	0.00	RX1	H
ATOM	3632	HD22	ASN	153	35.927	24.663	51.060	1.00	0.00	RX1	H
ATOM	3633	C	ASN	153	38.171	26.491	55.337	1.00	0.00	RX1	C
ATOM	3634	O	ASN	153	38.179	27.408	54.525	1.00	0.00	RX1	O
ATOM	3635	N	PRO	154	38.138	26.728	56.675	1.00	0.00	RX1	N
ATOM	3636	CD	PRO	154	38.179	25.815	57.804	1.00	0.00	RX1	C
ATOM	3637	CA	PRO	154	38.039	28.119	57.106	1.00	0.00	RX1	C
ATOM	3638	CB	PRO	154	37.770	28.019	58.610	1.00	0.00	RX1	C
ATOM	3639	CG	PRO	154	37.411	26.563	58.880	1.00	0.00	RX1	C
ATOM	3640	C	PRO	154	39.260	28.946	56.742	1.00	0.00	RX1	C
ATOM	3641	O	PRO	154	40.385	28.462	56.671	1.00	0.00	RX1	O
ATOM	3642	N	ALA	155	38.898	30.212	56.467	1.00	0.00	RX1	N
ATOM	3643	H	ALA	155	37.921	30.387	56.557	1.00	0.00	RX1	H
ATOM	3644	CA	ALA	155	39.661	31.258	55.776	1.00	0.00	RX1	C
ATOM	3645	CB	ALA	155	41.184	31.105	55.781	1.00	0.00	RX1	C
ATOM	3646	C	ALA	155	39.194	31.447	54.349	1.00	0.00	RX1	C
ATOM	3647	O	ALA	155	39.238	32.555	53.833	1.00	0.00	RX1	O
ATOM	3648	N	LEU	156	38.676	30.351	53.762	1.00	0.00	RX1	N
ATOM	3649	H	LEU	156	38.740	29.437	54.165	1.00	0.00	RX1	H
ATOM	3650	CA	LEU	156	38.206	30.465	52.381	1.00	0.00	RX1	C
ATOM	3651	CB	LEU	156	37.723	29.107	51.864	1.00	0.00	RX1	C
ATOM	3652	CG	LEU	156	37.627	29.008	50.339	1.00	0.00	RX1	C
ATOM	3653	CD1	LEU	156	38.917	29.438	49.646	1.00	0.00	RX1	C
ATOM	3654	CD2	LEU	156	37.241	27.600	49.895	1.00	0.00	RX1	C
ATOM	3655	C	LEU	156	37.183	31.557	52.130	1.00	0.00	RX1	C
ATOM	3656	O	LEU	156	36.055	31.560	52.616	1.00	0.00	RX1	O
ATOM	3657	N	CYS	157	37.667	32.505	51.336	1.00	0.00	RX1	N
ATOM	3658	H	CYS	157	38.570	32.421	50.911	1.00	0.00	RX1	H

ATOM	3659	CA	CYS	157	36.819	33.590	50.888	1.00	0.00	RX1	C
ATOM	3660	CB	CYS	157	37.450	34.922	51.284	1.00	0.00	RX1	C
ATOM	3661	SG	CYS	157	37.868	35.089	53.036	1.00	0.00	RX1	S
ATOM	3662	C	CYS	157	36.737	33.497	49.387	1.00	0.00	RX1	C
ATOM	3663	O	CYS	157	37.494	32.770	48.755	1.00	0.00	RX1	O
ATOM	3664	N	ASN	158	35.805	34.283	48.834	1.00	0.00	RX1	N
ATOM	3665	H	ASN	158	35.047	34.648	49.383	1.00	0.00	RX1	H
ATOM	3666	CA	ASN	158	35.785	34.598	47.402	1.00	0.00	RX1	C
ATOM	3667	CB	ASN	158	37.115	35.195	46.958	1.00	0.00	RX1	C
ATOM	3668	CG	ASN	158	36.972	36.683	47.138	1.00	0.00	RX1	C
ATOM	3669	OD1	ASN	158	35.964	37.259	46.740	1.00	0.00	RX1	O
ATOM	3670	ND2	ASN	158	37.999	37.272	47.770	1.00	0.00	RX1	N
ATOM	3671	HD21	ASN	158	38.774	36.734	48.124	1.00	0.00	RX1	H
ATOM	3672	HD22	ASN	158	38.027	38.265	47.911	1.00	0.00	RX1	H
ATOM	3673	C	ASN	158	35.258	33.540	46.453	1.00	0.00	RX1	C
ATOM	3674	O	ASN	158	34.420	33.798	45.598	1.00	0.00	RX1	O
ATOM	3675	N	VAL	159	35.720	32.306	46.687	1.00	0.00	RX1	N
ATOM	3676	H	VAL	159	36.418	32.159	47.385	1.00	0.00	RX1	H
ATOM	3677	CA	VAL	159	35.114	31.164	46.002	1.00	0.00	RX1	C
ATOM	3678	CB	VAL	159	35.973	29.921	46.234	1.00	0.00	RX1	C
ATOM	3679	CG1	VAL	159	35.550	28.771	45.325	1.00	0.00	RX1	C
ATOM	3680	CG2	VAL	159	37.451	30.258	46.038	1.00	0.00	RX1	C
ATOM	3681	C	VAL	159	33.653	30.928	46.393	1.00	0.00	RX1	C
ATOM	3682	O	VAL	159	32.868	30.319	45.675	1.00	0.00	RX1	O
ATOM	3683	N	GLU	160	33.314	31.489	47.571	1.00	0.00	RX1	N
ATOM	3684	H	GLU	160	33.956	32.078	48.056	1.00	0.00	RX1	H
ATOM	3685	CA	GLU	160	31.962	31.383	48.125	1.00	0.00	RX1	C
ATOM	3686	CB	GLU	160	31.903	32.166	49.457	1.00	0.00	RX1	C
ATOM	3687	CG	GLU	160	31.562	33.678	49.483	1.00	0.00	RX1	C
ATOM	3688	CD	GLU	160	32.560	34.565	48.747	1.00	0.00	RX1	C
ATOM	3689	OE1	GLU	160	33.186	35.442	49.331	1.00	0.00	RX1	O
ATOM	3690	OE2	GLU	160	32.697	34.435	47.550	1.00	0.00	RX1	O
ATOM	3691	C	GLU	160	30.808	31.735	47.188	1.00	0.00	RX1	C
ATOM	3692	O	GLU	160	29.683	31.274	47.325	1.00	0.00	RX1	O
ATOM	3693	N	SER	161	31.150	32.586	46.217	1.00	0.00	RX1	N
ATOM	3694	H	SER	161	32.102	32.873	46.140	1.00	0.00	RX1	H
ATOM	3695	CA	SER	161	30.109	33.146	45.370	1.00	0.00	RX1	C
ATOM	3696	CB	SER	161	30.377	34.657	45.283	1.00	0.00	RX1	C
ATOM	3697	OG	SER	161	31.781	34.935	45.087	1.00	0.00	RX1	O
ATOM	3698	HG	SER	161	32.253	34.383	45.710	1.00	0.00	RX1	H
ATOM	3699	C	SER	161	29.931	32.478	44.020	1.00	0.00	RX1	C
ATOM	3700	O	SER	161	29.047	32.835	43.250	1.00	0.00	RX1	O
ATOM	3701	N	ILE	162	30.834	31.533	43.722	1.00	0.00	RX1	N
ATOM	3702	H	ILE	162	31.406	31.096	44.420	1.00	0.00	RX1	H
ATOM	3703	CA	ILE	162	30.836	31.061	42.339	1.00	0.00	RX1	C
ATOM	3704	CB	ILE	162	32.191	30.431	41.965	1.00	0.00	RX1	C
ATOM	3705	CG2	ILE	162	32.073	29.511	40.750	1.00	0.00	RX1	C
ATOM	3706	CG1	ILE	162	33.238	31.506	41.652	1.00	0.00	RX1	C
ATOM	3707	CD1	ILE	162	33.763	32.332	42.824	1.00	0.00	RX1	C
ATOM	3708	C	ILE	162	29.670	30.139	42.031	1.00	0.00	RX1	C
ATOM	3709	O	ILE	162	29.474	29.092	42.636	1.00	0.00	RX1	O
ATOM	3710	N	GLN	163	28.907	30.557	41.010	1.00	0.00	RX1	N
ATOM	3711	H	GLN	163	29.077	31.455	40.595	1.00	0.00	RX1	H
ATOM	3712	CA	GLN	163	27.954	29.588	40.479	1.00	0.00	RX1	C
ATOM	3713	CB	GLN	163	26.831	30.265	39.685	1.00	0.00	RX1	C
ATOM	3714	CG	GLN	163	27.307	31.024	38.450	1.00	0.00	RX1	C
ATOM	3715	CD	GLN	163	26.140	31.689	37.777	1.00	0.00	RX1	C
ATOM	3716	OE1	GLN	163	24.981	31.324	37.992	1.00	0.00	RX1	O
ATOM	3717	NE2	GLN	163	26.516	32.724	37.014	1.00	0.00	RX1	N
ATOM	3718	HE21	GLN	163	27.492	32.849	36.803	1.00	0.00	RX1	H
ATOM	3719	HE22	GLN	163	25.899	33.434	36.659	1.00	0.00	RX1	H

ATOM	3720	C	GLN	163	28.641	28.472	39.703	1.00	0.00	RX1	C
ATOM	3721	O	GLN	163	28.912	28.537	38.508	1.00	0.00	RX1	O
ATOM	3722	N	TRP	164	28.925	27.410	40.467	1.00	0.00	RX1	N
ATOM	3723	H	TRP	164	28.793	27.491	41.459	1.00	0.00	RX1	H
ATOM	3724	CA	TRP	164	29.737	26.333	39.901	1.00	0.00	RX1	C
ATOM	3725	CB	TRP	164	30.027	25.272	40.963	1.00	0.00	RX1	C
ATOM	3726	CG	TRP	164	31.141	25.790	41.840	1.00	0.00	RX1	C
ATOM	3727	CD2	TRP	164	32.552	25.797	41.544	1.00	0.00	RX1	C
ATOM	3728	CE2	TRP	164	33.213	26.418	42.628	1.00	0.00	RX1	C
ATOM	3729	CE3	TRP	164	33.289	25.335	40.461	1.00	0.00	RX1	C
ATOM	3730	CD1	TRP	164	31.028	26.404	43.095	1.00	0.00	RX1	C
ATOM	3731	NE1	TRP	164	32.248	26.780	43.564	1.00	0.00	RX1	N
ATOM	3732	HE1	TRP	164	32.412	27.245	44.412	1.00	0.00	RX1	H
ATOM	3733	CZ2	TRP	164	34.594	26.563	42.592	1.00	0.00	RX1	C
ATOM	3734	CZ3	TRP	164	34.670	25.484	40.440	1.00	0.00	RX1	C
ATOM	3735	CH2	TRP	164	35.320	26.098	41.503	1.00	0.00	RX1	C
ATOM	3736	C	TRP	164	29.268	25.706	38.598	1.00	0.00	RX1	C
ATOM	3737	O	TRP	164	30.069	25.214	37.820	1.00	0.00	RX1	O
ATOM	3738	N	ARG	165	27.950	25.787	38.355	1.00	0.00	RX1	N
ATOM	3739	H	ARG	165	27.383	26.241	39.036	1.00	0.00	RX1	H
ATOM	3740	CA	ARG	165	27.416	25.239	37.101	1.00	0.00	RX1	C
ATOM	3741	CB	ARG	165	25.905	25.453	37.062	1.00	0.00	RX1	C
ATOM	3742	CG	ARG	165	25.181	25.234	38.389	1.00	0.00	RX1	C
ATOM	3743	CD	ARG	165	23.811	25.919	38.399	1.00	0.00	RX1	C
ATOM	3744	NE	ARG	165	23.939	27.376	38.273	1.00	0.00	RX1	N
ATOM	3745	HE	ARG	165	24.607	27.723	37.606	1.00	0.00	RX1	H
ATOM	3746	CZ	ARG	165	23.106	28.194	38.986	1.00	0.00	RX1	C
ATOM	3747	NH1	ARG	165	22.195	27.637	39.817	1.00	0.00	RX1	N
ATOM	3748	HH11	ARG	165	21.563	28.185	40.373	1.00	0.00	RX1	H
ATOM	3749	HH12	ARG	165	22.115	26.641	39.911	1.00	0.00	RX1	H
ATOM	3750	NH2	ARG	165	23.200	29.538	38.860	1.00	0.00	RX1	N
ATOM	3751	HH21	ARG	165	22.597	30.177	39.342	1.00	0.00	RX1	H
ATOM	3752	HH22	ARG	165	23.893	29.990	38.274	1.00	0.00	RX1	H
ATOM	3753	C	ARG	165	27.978	25.818	35.802	1.00	0.00	RX1	C
ATOM	3754	O	ARG	165	27.978	25.183	34.755	1.00	0.00	RX1	O
ATOM	3755	N	ASP	166	28.472	27.067	35.900	1.00	0.00	RX1	N
ATOM	3756	H	ASP	166	28.528	27.563	36.767	1.00	0.00	RX1	H
ATOM	3757	CA	ASP	166	29.155	27.586	34.709	1.00	0.00	RX1	C
ATOM	3758	CB	ASP	166	29.436	29.082	34.862	1.00	0.00	RX1	C
ATOM	3759	CG	ASP	166	29.747	29.717	33.519	1.00	0.00	RX1	C
ATOM	3760	OD1	ASP	166	30.871	29.603	33.042	1.00	0.00	RX1	O
ATOM	3761	OD2	ASP	166	28.861	30.346	32.947	1.00	0.00	RX1	O
ATOM	3762	C	ASP	166	30.443	26.824	34.423	1.00	0.00	RX1	C
ATOM	3763	O	ASP	166	30.807	26.473	33.300	1.00	0.00	RX1	O
ATOM	3764	N	ILE	167	31.096	26.540	35.555	1.00	0.00	RX1	N
ATOM	3765	H	ILE	167	30.640	26.650	36.435	1.00	0.00	RX1	H
ATOM	3766	CA	ILE	167	32.386	25.870	35.501	1.00	0.00	RX1	C
ATOM	3767	CB	ILE	167	33.145	26.096	36.806	1.00	0.00	RX1	C
ATOM	3768	CG2	ILE	167	34.594	25.652	36.651	1.00	0.00	RX1	C
ATOM	3769	CG1	ILE	167	32.999	27.535	37.308	1.00	0.00	RX1	C
ATOM	3770	CD1	ILE	167	33.442	28.592	36.298	1.00	0.00	RX1	C
ATOM	3771	C	ILE	167	32.222	24.387	35.223	1.00	0.00	RX1	C
ATOM	3772	O	ILE	167	32.422	23.903	34.115	1.00	0.00	RX1	O
ATOM	3773	N	VAL	168	31.801	23.689	36.290	1.00	0.00	RX1	N
ATOM	3774	H	VAL	168	31.457	24.214	37.065	1.00	0.00	RX1	H
ATOM	3775	CA	VAL	168	31.528	22.254	36.198	1.00	0.00	RX1	C
ATOM	3776	CB	VAL	168	31.106	21.705	37.573	1.00	0.00	RX1	C
ATOM	3777	CG1	VAL	168	31.406	20.214	37.698	1.00	0.00	RX1	C
ATOM	3778	CG2	VAL	168	31.791	22.448	38.719	1.00	0.00	RX1	C
ATOM	3779	C	VAL	168	30.467	22.004	35.135	1.00	0.00	RX1	C
ATOM	3780	O	VAL	168	29.859	22.952	34.647	1.00	0.00	RX1	O

ATOM	3781	N	SER	169	30.265	20.738	34.762	1.00	0.00	RX1	N
ATOM	3782	H	SER	169	30.848	19.981	35.061	1.00	0.00	RX1	H
ATOM	3783	CA	SER	169	29.227	20.508	33.765	1.00	0.00	RX1	C
ATOM	3784	CB	SER	169	29.259	19.024	33.395	1.00	0.00	RX1	C
ATOM	3785	OG	SER	169	28.348	18.280	34.211	1.00	0.00	RX1	O
ATOM	3786	HG	SER	169	27.826	17.800	33.564	1.00	0.00	RX1	H
ATOM	3787	C	SER	169	27.850	21.026	34.176	1.00	0.00	RX1	C
ATOM	3788	O	SER	169	27.594	21.377	35.325	1.00	0.00	RX1	O
ATOM	3789	N	SER	170	26.941	21.002	33.198	1.00	0.00	RX1	N
ATOM	3790	H	SER	170	27.155	20.584	32.317	1.00	0.00	RX1	H
ATOM	3791	CA	SER	170	25.551	21.342	33.500	1.00	0.00	RX1	C
ATOM	3792	CB	SER	170	24.921	21.487	32.124	1.00	0.00	RX1	C
ATOM	3793	OG	SER	170	25.718	20.725	31.204	1.00	0.00	RX1	O
ATOM	3794	HG	SER	170	25.573	19.798	31.433	1.00	0.00	RX1	H
ATOM	3795	C	SER	170	24.814	20.356	34.410	1.00	0.00	RX1	C
ATOM	3796	O	SER	170	23.624	20.482	34.678	1.00	0.00	RX1	O
ATOM	3797	N	ASP	171	25.572	19.335	34.828	1.00	0.00	RX1	N
ATOM	3798	H	ASP	171	26.558	19.317	34.679	1.00	0.00	RX1	H
ATOM	3799	CA	ASP	171	24.963	18.055	35.147	1.00	0.00	RX1	C
ATOM	3800	CB	ASP	171	25.312	17.068	34.026	1.00	0.00	RX1	C
ATOM	3801	CG	ASP	171	25.297	17.797	32.690	1.00	0.00	RX1	C
ATOM	3802	OD1	ASP	171	24.236	18.076	32.139	1.00	0.00	RX1	O
ATOM	3803	OD2	ASP	171	26.363	18.152	32.214	1.00	0.00	RX1	O
ATOM	3804	C	ASP	171	25.416	17.568	36.508	1.00	0.00	RX1	C
ATOM	3805	O	ASP	171	24.673	16.977	37.288	1.00	0.00	RX1	O
ATOM	3806	N	PHE	172	26.676	17.939	36.806	1.00	0.00	RX1	N
ATOM	3807	H	PHE	172	27.269	18.345	36.106	1.00	0.00	RX1	H
ATOM	3808	CA	PHE	172	27.210	17.702	38.151	1.00	0.00	RX1	C
ATOM	3809	CB	PHE	172	28.713	17.916	38.192	1.00	0.00	RX1	C
ATOM	3810	CG	PHE	172	29.369	16.726	37.554	1.00	0.00	RX1	C
ATOM	3811	CD1	PHE	172	29.556	15.571	38.300	1.00	0.00	RX1	C
ATOM	3812	CD2	PHE	172	29.759	16.775	36.226	1.00	0.00	RX1	C
ATOM	3813	CE1	PHE	172	30.091	14.447	37.692	1.00	0.00	RX1	C
ATOM	3814	CE2	PHE	172	30.280	15.647	35.612	1.00	0.00	RX1	C
ATOM	3815	CZ	PHE	172	30.418	14.477	36.342	1.00	0.00	RX1	C
ATOM	3816	C	PHE	172	26.576	18.458	39.302	1.00	0.00	RX1	C
ATOM	3817	O	PHE	172	26.931	18.276	40.460	1.00	0.00	RX1	O
ATOM	3818	N	LEU	173	25.564	19.274	38.953	1.00	0.00	RX1	N
ATOM	3819	H	LEU	173	25.379	19.446	37.989	1.00	0.00	RX1	H
ATOM	3820	CA	LEU	173	24.668	19.767	40.003	1.00	0.00	RX1	C
ATOM	3821	CB	LEU	173	23.574	20.686	39.434	1.00	0.00	RX1	C
ATOM	3822	CG	LEU	173	23.005	20.365	38.044	1.00	0.00	RX1	C
ATOM	3823	CD1	LEU	173	22.162	19.090	37.988	1.00	0.00	RX1	C
ATOM	3824	CD2	LEU	173	22.224	21.560	37.497	1.00	0.00	RX1	C
ATOM	3825	C	LEU	173	24.119	18.701	40.950	1.00	0.00	RX1	C
ATOM	3826	O	LEU	173	23.851	18.950	42.116	1.00	0.00	RX1	O
ATOM	3827	N	SER	174	24.032	17.482	40.393	1.00	0.00	RX1	N
ATOM	3828	H	SER	174	24.313	17.318	39.448	1.00	0.00	RX1	H
ATOM	3829	CA	SER	174	23.660	16.301	41.170	1.00	0.00	RX1	C
ATOM	3830	CB	SER	174	23.752	15.155	40.180	1.00	0.00	RX1	C
ATOM	3831	OG	SER	174	24.860	15.442	39.323	1.00	0.00	RX1	O
ATOM	3832	HG	SER	174	24.513	15.574	38.444	1.00	0.00	RX1	H
ATOM	3833	C	SER	174	24.453	16.052	42.454	1.00	0.00	RX1	C
ATOM	3834	O	SER	174	23.950	15.494	43.425	1.00	0.00	RX1	O
ATOM	3835	N	ASN	175	25.720	16.500	42.434	1.00	0.00	RX1	N
ATOM	3836	H	ASN	175	26.142	16.932	41.634	1.00	0.00	RX1	H
ATOM	3837	CA	ASN	175	26.458	16.404	43.691	1.00	0.00	RX1	C
ATOM	3838	CB	ASN	175	27.113	15.049	43.918	1.00	0.00	RX1	C
ATOM	3839	CG	ASN	175	27.053	14.828	45.412	1.00	0.00	RX1	C
ATOM	3840	OD1	ASN	175	28.051	14.683	46.111	1.00	0.00	RX1	O
ATOM	3841	ND2	ASN	175	25.787	14.801	45.870	1.00	0.00	RX1	N

ATOM	3842	HD21	ASN	175	25.011	14.910	45.238	1.00	0.00	RX1	H
ATOM	3843	HD22	ASN	175	25.570	14.672	46.837	1.00	0.00	RX1	H
ATOM	3844	C	ASN	175	27.463	17.505	43.936	1.00	0.00	RX1	C
ATOM	3845	O	ASN	175	28.583	17.281	44.375	1.00	0.00	RX1	O
ATOM	3846	N	MET	176	27.014	18.729	43.631	1.00	0.00	RX1	N
ATOM	3847	H	MET	176	26.066	18.870	43.342	1.00	0.00	RX1	H
ATOM	3848	CA	MET	176	27.912	19.848	43.920	1.00	0.00	RX1	C
ATOM	3849	CB	MET	176	27.654	21.027	42.970	1.00	0.00	RX1	C
ATOM	3850	CG	MET	176	26.236	21.605	42.992	1.00	0.00	RX1	C
ATOM	3851	SD	MET	176	26.035	22.979	41.845	1.00	0.00	RX1	S
ATOM	3852	CE	MET	176	24.352	23.423	42.298	1.00	0.00	RX1	C
ATOM	3853	C	MET	176	27.929	20.249	45.393	1.00	0.00	RX1	C
ATOM	3854	O	MET	176	27.337	21.229	45.830	1.00	0.00	RX1	O
ATOM	3855	N	SER	177	28.630	19.411	46.162	1.00	0.00	RX1	N
ATOM	3856	H	SER	177	29.216	18.708	45.753	1.00	0.00	RX1	H
ATOM	3857	CA	SER	177	28.652	19.654	47.599	1.00	0.00	RX1	C
ATOM	3858	CB	SER	177	28.813	18.289	48.277	1.00	0.00	RX1	C
ATOM	3859	OG	SER	177	28.320	18.277	49.625	1.00	0.00	RX1	O
ATOM	3860	HG	SER	177	28.849	18.912	50.117	1.00	0.00	RX1	H
ATOM	3861	C	SER	177	29.670	20.715	47.974	1.00	0.00	RX1	C
ATOM	3862	O	SER	177	30.817	20.472	48.328	1.00	0.00	RX1	O
ATOM	3863	N	MET	178	29.171	21.943	47.850	1.00	0.00	RX1	N
ATOM	3864	H	MET	178	28.214	22.044	47.572	1.00	0.00	RX1	H
ATOM	3865	CA	MET	178	29.995	23.083	48.226	1.00	0.00	RX1	C
ATOM	3866	CB	MET	178	29.818	24.204	47.191	1.00	0.00	RX1	C
ATOM	3867	CG	MET	178	28.360	24.542	46.863	1.00	0.00	RX1	C
ATOM	3868	SD	MET	178	28.181	25.706	45.499	1.00	0.00	RX1	S
ATOM	3869	CE	MET	178	28.885	27.160	46.293	1.00	0.00	RX1	C
ATOM	3870	C	MET	178	29.741	23.535	49.655	1.00	0.00	RX1	C
ATOM	3871	O	MET	178	29.245	24.625	49.917	1.00	0.00	RX1	O
ATOM	3872	N	ASP	179	30.098	22.634	50.580	1.00	0.00	RX1	N
ATOM	3873	H	ASP	179	30.586	21.780	50.376	1.00	0.00	RX1	H
ATOM	3874	CA	ASP	179	29.912	22.944	51.997	1.00	0.00	RX1	C
ATOM	3875	CB	ASP	179	29.606	21.696	52.853	1.00	0.00	RX1	C
ATOM	3876	CG	ASP	179	30.306	20.421	52.400	1.00	0.00	RX1	C
ATOM	3877	OD1	ASP	179	31.070	19.850	53.173	1.00	0.00	RX1	O
ATOM	3878	OD2	ASP	179	30.037	19.930	51.305	1.00	0.00	RX1	O
ATOM	3879	C	ASP	179	30.981	23.853	52.589	1.00	0.00	RX1	C
ATOM	3880	O	ASP	179	31.887	23.499	53.338	1.00	0.00	RX1	O
ATOM	3881	N	PHE	180	30.789	25.119	52.187	1.00	0.00	RX1	N
ATOM	3882	H	PHE	180	29.989	25.300	51.613	1.00	0.00	RX1	H
ATOM	3883	CA	PHE	180	31.547	26.215	52.777	1.00	0.00	RX1	C
ATOM	3884	CB	PHE	180	31.333	27.521	52.006	1.00	0.00	RX1	C
ATOM	3885	CG	PHE	180	31.991	27.520	50.647	1.00	0.00	RX1	C
ATOM	3886	CD1	PHE	180	31.298	27.067	49.532	1.00	0.00	RX1	C
ATOM	3887	CD2	PHE	180	33.284	28.010	50.507	1.00	0.00	RX1	C
ATOM	3888	CE1	PHE	180	31.884	27.136	48.274	1.00	0.00	RX1	C
ATOM	3889	CE2	PHE	180	33.866	28.080	49.248	1.00	0.00	RX1	C
ATOM	3890	CZ	PHE	180	33.162	27.658	48.128	1.00	0.00	RX1	C
ATOM	3891	C	PHE	180	31.121	26.474	54.208	1.00	0.00	RX1	C
ATOM	3892	O	PHE	180	30.089	26.023	54.688	1.00	0.00	RX1	O
ATOM	3893	N	GLN	181	31.960	27.284	54.851	1.00	0.00	RX1	N
ATOM	3894	H	GLN	181	32.770	27.669	54.407	1.00	0.00	RX1	H
ATOM	3895	CA	GLN	181	31.594	27.852	56.140	1.00	0.00	RX1	C
ATOM	3896	CB	GLN	181	32.260	27.112	57.303	1.00	0.00	RX1	C
ATOM	3897	CG	GLN	181	33.761	27.389	57.448	1.00	0.00	RX1	C
ATOM	3898	CD	GLN	181	34.548	26.731	56.338	1.00	0.00	RX1	C
ATOM	3899	OE1	GLN	181	34.814	25.536	56.400	1.00	0.00	RX1	O
ATOM	3900	NE2	GLN	181	34.933	27.564	55.352	1.00	0.00	RX1	N
ATOM	3901	HE21	GLN	181	34.653	28.530	55.347	1.00	0.00	RX1	H
ATOM	3902	HE22	GLN	181	35.493	27.271	54.567	1.00	0.00	RX1	H

ATOM	3903	C	GLN	181	32.041	29.294	56.125	1.00	0.00	RX1	C
ATOM	3904	O	GLN	181	32.829	29.675	55.266	1.00	0.00	RX1	O
ATOM	3905	N	ASN	182	31.563	30.063	57.117	1.00	0.00	RX1	N
ATOM	3906	H	ASN	182	30.977	29.689	57.833	1.00	0.00	RX1	H
ATOM	3907	CA	ASN	182	32.157	31.398	57.219	1.00	0.00	RX1	C
ATOM	3908	CB	ASN	182	31.473	32.209	58.316	1.00	0.00	RX1	C
ATOM	3909	CG	ASN	182	32.211	33.521	58.413	1.00	0.00	RX1	C
ATOM	3910	OD1	ASN	182	32.389	34.209	57.414	1.00	0.00	RX1	O
ATOM	3911	ND2	ASN	182	32.700	33.788	59.635	1.00	0.00	RX1	N
ATOM	3912	HD21	ASN	182	32.454	33.271	60.454	1.00	0.00	RX1	H
ATOM	3913	HD22	ASN	182	33.389	34.513	59.726	1.00	0.00	RX1	H
ATOM	3914	C	ASN	182	33.654	31.351	57.497	1.00	0.00	RX1	C
ATOM	3915	O	ASN	182	34.156	30.495	58.217	1.00	0.00	RX1	O
ATOM	3916	N	HIS	183	34.337	32.298	56.860	1.00	0.00	RX1	N
ATOM	3917	H	HIS	183	33.831	33.064	56.455	1.00	0.00	RX1	H
ATOM	3918	CA	HIS	183	35.771	32.405	57.090	1.00	0.00	RX1	C
ATOM	3919	CB	HIS	183	36.475	32.948	55.850	1.00	0.00	RX1	C
ATOM	3920	CG	HIS	183	35.646	34.005	55.168	1.00	0.00	RX1	C
ATOM	3921	ND1	HIS	183	34.926	33.755	54.061	1.00	0.00	RX1	N
ATOM	3922	HD1	HIS	183	34.896	32.898	53.572	1.00	0.00	RX1	H
ATOM	3923	CD2	HIS	183	35.487	35.347	55.522	1.00	0.00	RX1	C
ATOM	3924	NE2	HIS	183	34.658	35.908	54.608	1.00	0.00	RX1	N
ATOM	3925	CE1	HIS	183	34.309	34.927	53.712	1.00	0.00	RX1	C
ATOM	3926	C	HIS	183	36.091	33.246	58.307	1.00	0.00	RX1	C
ATOM	3927	O	HIS	183	35.306	34.080	58.734	1.00	0.00	RX1	O
ATOM	3928	N	LEU	184	37.302	33.012	58.831	1.00	0.00	RX1	N
ATOM	3929	H	LEU	184	37.939	32.349	58.443	1.00	0.00	RX1	H
ATOM	3930	CA	LEU	184	37.778	33.894	59.900	1.00	0.00	RX1	C
ATOM	3931	CB	LEU	184	38.500	33.082	60.974	1.00	0.00	RX1	C
ATOM	3932	CG	LEU	184	37.624	31.983	61.575	1.00	0.00	RX1	C
ATOM	3933	CD1	LEU	184	38.398	31.148	62.594	1.00	0.00	RX1	C
ATOM	3934	CD2	LEU	184	36.324	32.538	62.164	1.00	0.00	RX1	C
ATOM	3935	C	LEU	184	38.679	34.994	59.374	1.00	0.00	RX1	C
ATOM	3936	O	LEU	184	39.742	35.289	59.912	1.00	0.00	RX1	O
ATOM	3937	N	GLY	185	38.216	35.548	58.252	1.00	0.00	RX1	N
ATOM	3938	H	GLY	185	37.266	35.423	57.965	1.00	0.00	RX1	H
ATOM	3939	CA	GLY	185	39.010	36.535	57.540	1.00	0.00	RX1	C
ATOM	3940	C	GLY	185	38.089	37.558	56.936	1.00	0.00	RX1	C
ATOM	3941	O	GLY	185	37.180	38.070	57.587	1.00	0.00	RX1	O
ATOM	3942	N	SER	186	38.341	37.808	55.644	1.00	0.00	RX1	N
ATOM	3943	H	SER	186	39.096	37.412	55.128	1.00	0.00	RX1	H
ATOM	3944	CA	SER	186	37.446	38.724	54.951	1.00	0.00	RX1	C
ATOM	3945	CB	SER	186	37.937	40.148	55.214	1.00	0.00	RX1	C
ATOM	3946	OG	SER	186	38.458	40.226	56.546	1.00	0.00	RX1	O
ATOM	3947	HG	SER	186	37.780	39.873	57.120	1.00	0.00	RX1	H
ATOM	3948	C	SER	186	37.326	38.421	53.482	1.00	0.00	RX1	C
ATOM	3949	O	SER	186	38.306	38.180	52.783	1.00	0.00	RX1	O
ATOM	3950	N	CYS	187	36.075	38.470	53.041	1.00	0.00	RX1	N
ATOM	3951	H	CYS	187	35.304	38.639	53.656	1.00	0.00	RX1	H
ATOM	3952	CA	CYS	187	35.793	38.349	51.615	1.00	0.00	RX1	C
ATOM	3953	CB	CYS	187	34.386	37.780	51.461	1.00	0.00	RX1	C
ATOM	3954	SG	CYS	187	33.264	38.489	52.695	1.00	0.00	RX1	S
ATOM	3955	C	CYS	187	35.943	39.663	50.873	1.00	0.00	RX1	C
ATOM	3956	O	CYS	187	34.978	40.224	50.372	1.00	0.00	RX1	O
ATOM	3957	N	GLN	188	37.200	40.149	50.815	1.00	0.00	RX1	N
ATOM	3958	H	GLN	188	37.976	39.619	51.163	1.00	0.00	RX1	H
ATOM	3959	CA	GLN	188	37.399	41.356	50.012	1.00	0.00	RX1	C
ATOM	3960	CB	GLN	188	38.797	41.959	50.221	1.00	0.00	RX1	C
ATOM	3961	CG	GLN	188	39.956	41.351	49.422	1.00	0.00	RX1	C
ATOM	3962	CD	GLN	188	40.133	39.881	49.738	1.00	0.00	RX1	C
ATOM	3963	OE1	GLN	188	39.970	39.426	50.870	1.00	0.00	RX1	O

ATOM	3964	NE2	GLN	188	40.505	39.163	48.671	1.00	0.00	RX1	N
ATOM	3965	HE21	GLN	188	40.538	39.576	47.761	1.00	0.00	RX1	H
ATOM	3966	HE22	GLN	188	40.779	38.198	48.735	1.00	0.00	RX1	H
ATOM	3967	C	GLN	188	37.054	41.116	48.552	1.00	0.00	RX1	C
ATOM	3968	O	GLN	188	37.513	40.159	47.937	1.00	0.00	RX1	O
ATOM	3969	N	LYS	189	36.149	41.978	48.079	1.00	0.00	RX1	N
ATOM	3970	H	LYS	189	35.906	42.813	48.566	1.00	0.00	RX1	H
ATOM	3971	CA	LYS	189	35.421	41.561	46.891	1.00	0.00	RX1	C
ATOM	3972	CB	LYS	189	33.911	41.758	47.081	1.00	0.00	RX1	C
ATOM	3973	CG	LYS	189	33.082	40.570	46.563	1.00	0.00	RX1	C
ATOM	3974	CD	LYS	189	33.548	39.201	47.085	1.00	0.00	RX1	C
ATOM	3975	CE	LYS	189	32.675	38.043	46.578	1.00	0.00	RX1	C
ATOM	3976	NZ	LYS	189	33.367	36.749	46.660	1.00	0.00	RX1	N
ATOM	3977	HZ1	LYS	189	32.913	36.060	46.025	1.00	0.00	RX1	H
ATOM	3978	HZ2	LYS	189	34.361	36.839	46.354	1.00	0.00	RX1	H
ATOM	3979	HZ3	LYS	189	33.318	36.313	47.613	1.00	0.00	RX1	H
ATOM	3980	C	LYS	189	36.020	42.033	45.573	1.00	0.00	RX1	C
ATOM	3981	O	LYS	189	37.231	41.965	45.384	1.00	0.00	RX1	O
ATOM	3982	N	CYS	190	35.152	42.474	44.649	1.00	0.00	RX1	N
ATOM	3983	H	CYS	190	34.190	42.717	44.769	1.00	0.00	RX1	H
ATOM	3984	CA	CYS	190	35.659	42.594	43.289	1.00	0.00	RX1	C
ATOM	3985	CB	CYS	190	35.343	41.309	42.530	1.00	0.00	RX1	C
ATOM	3986	SG	CYS	190	35.596	39.831	43.544	1.00	0.00	RX1	S
ATOM	3987	C	CYS	190	35.127	43.813	42.580	1.00	0.00	RX1	C
ATOM	3988	O	CYS	190	34.271	44.519	43.099	1.00	0.00	RX1	O
ATOM	3989	N	ASP	191	35.683	44.014	41.378	1.00	0.00	RX1	N
ATOM	3990	H	ASP	191	36.300	43.352	40.959	1.00	0.00	RX1	H
ATOM	3991	CA	ASP	191	35.187	45.062	40.489	1.00	0.00	RX1	C
ATOM	3992	CB	ASP	191	36.155	45.141	39.298	1.00	0.00	RX1	C
ATOM	3993	CG	ASP	191	35.790	46.286	38.378	1.00	0.00	RX1	C
ATOM	3994	OD1	ASP	191	36.252	47.399	38.617	1.00	0.00	RX1	O
ATOM	3995	OD2	ASP	191	35.026	46.073	37.439	1.00	0.00	RX1	O
ATOM	3996	C	ASP	191	33.763	44.745	40.042	1.00	0.00	RX1	C
ATOM	3997	O	ASP	191	33.383	43.579	39.964	1.00	0.00	RX1	O
ATOM	3998	N	PRO	192	32.966	45.807	39.761	1.00	0.00	RX1	N
ATOM	3999	CD	PRO	192	33.209	47.195	40.145	1.00	0.00	RX1	C
ATOM	4000	CA	PRO	192	31.696	45.641	39.039	1.00	0.00	RX1	C
ATOM	4001	CB	PRO	192	31.284	47.088	38.750	1.00	0.00	RX1	C
ATOM	4002	CG	PRO	192	31.877	47.893	39.903	1.00	0.00	RX1	C
ATOM	4003	C	PRO	192	31.639	44.745	37.796	1.00	0.00	RX1	C
ATOM	4004	O	PRO	192	30.553	44.498	37.287	1.00	0.00	RX1	O
ATOM	4005	N	SER	193	32.794	44.227	37.337	1.00	0.00	RX1	N
ATOM	4006	H	SER	193	33.706	44.502	37.653	1.00	0.00	RX1	H
ATOM	4007	CA	SER	193	32.757	43.123	36.374	1.00	0.00	RX1	C
ATOM	4008	CB	SER	193	34.206	42.733	36.078	1.00	0.00	RX1	C
ATOM	4009	OG	SER	193	34.926	42.550	37.303	1.00	0.00	RX1	O
ATOM	4010	HG	SER	193	35.457	41.765	37.158	1.00	0.00	RX1	H
ATOM	4011	C	SER	193	31.889	41.941	36.797	1.00	0.00	RX1	C
ATOM	4012	O	SER	193	31.277	41.257	35.984	1.00	0.00	RX1	O
ATOM	4013	N	CYS	194	31.855	41.753	38.130	1.00	0.00	RX1	N
ATOM	4014	H	CYS	194	32.448	42.319	38.708	1.00	0.00	RX1	H
ATOM	4015	CA	CYS	194	30.999	40.735	38.745	1.00	0.00	RX1	C
ATOM	4016	CB	CYS	194	30.931	40.963	40.249	1.00	0.00	RX1	C
ATOM	4017	SG	CYS	194	32.556	40.955	41.028	1.00	0.00	RX1	S
ATOM	4018	C	CYS	194	29.582	40.624	38.211	1.00	0.00	RX1	C
ATOM	4019	O	CYS	194	28.756	41.516	38.363	1.00	0.00	RX1	O
ATOM	4020	N	PRO	195	29.317	39.445	37.603	1.00	0.00	RX1	N
ATOM	4021	CD	PRO	195	30.283	38.422	37.240	1.00	0.00	RX1	C
ATOM	4022	CA	PRO	195	27.937	39.093	37.268	1.00	0.00	RX1	C
ATOM	4023	CB	PRO	195	28.139	37.880	36.345	1.00	0.00	RX1	C
ATOM	4024	CG	PRO	195	29.634	37.797	36.019	1.00	0.00	RX1	C

ATOM	4025	C	PRO	195	27.158	38.756	38.541	1.00	0.00	RX1	C
ATOM	4026	O	PRO	195	27.320	39.382	39.581	1.00	0.00	RX1	O
ATOM	4027	N	ASN	196	26.321	37.709	38.461	1.00	0.00	RX1	N
ATOM	4028	H	ASN	196	26.215	37.124	37.655	1.00	0.00	RX1	H
ATOM	4029	CA	ASN	196	25.666	37.280	39.699	1.00	0.00	RX1	C
ATOM	4030	CB	ASN	196	24.416	36.441	39.411	1.00	0.00	RX1	C
ATOM	4031	CG	ASN	196	24.770	35.168	38.661	1.00	0.00	RX1	C
ATOM	4032	OD1	ASN	196	25.255	35.204	37.532	1.00	0.00	RX1	O
ATOM	4033	ND2	ASN	196	24.438	34.040	39.314	1.00	0.00	RX1	N
ATOM	4034	HD21	ASN	196	24.159	34.099	40.273	1.00	0.00	RX1	H
ATOM	4035	HD22	ASN	196	24.503	33.139	38.883	1.00	0.00	RX1	H
ATOM	4036	C	ASN	196	26.579	36.535	40.665	1.00	0.00	RX1	C
ATOM	4037	O	ASN	196	26.589	35.314	40.747	1.00	0.00	RX1	O
ATOM	4038	N	GLY	197	27.344	37.340	41.412	1.00	0.00	RX1	N
ATOM	4039	H	GLY	197	27.410	38.313	41.181	1.00	0.00	RX1	H
ATOM	4040	CA	GLY	197	28.333	36.728	42.290	1.00	0.00	RX1	C
ATOM	4041	C	GLY	197	29.571	36.332	41.515	1.00	0.00	RX1	C
ATOM	4042	O	GLY	197	30.151	37.123	40.780	1.00	0.00	RX1	O
ATOM	4043	N	SER	198	29.939	35.058	41.711	1.00	0.00	RX1	N
ATOM	4044	H	SER	198	29.312	34.438	42.183	1.00	0.00	RX1	H
ATOM	4045	CA	SER	198	31.048	34.476	40.958	1.00	0.00	RX1	C
ATOM	4046	CB	SER	198	30.485	34.166	39.588	1.00	0.00	RX1	C
ATOM	4047	OG	SER	198	29.405	33.234	39.721	1.00	0.00	RX1	O
ATOM	4048	HG	SER	198	29.145	33.072	38.816	1.00	0.00	RX1	H
ATOM	4049	C	SER	198	32.356	35.253	40.915	1.00	0.00	RX1	C
ATOM	4050	O	SER	198	32.966	35.461	39.869	1.00	0.00	RX1	O
ATOM	4051	N	CYS	199	32.762	35.673	42.117	1.00	0.00	RX1	N
ATOM	4052	H	CYS	199	32.309	35.441	42.981	1.00	0.00	RX1	H
ATOM	4053	CA	CYS	199	33.836	36.654	42.140	1.00	0.00	RX1	C
ATOM	4054	CB	CYS	199	33.169	37.994	42.412	1.00	0.00	RX1	C
ATOM	4055	SG	CYS	199	31.716	37.809	43.477	1.00	0.00	RX1	S
ATOM	4056	C	CYS	199	34.975	36.315	43.081	1.00	0.00	RX1	C
ATOM	4057	O	CYS	199	34.865	36.402	44.301	1.00	0.00	RX1	O
ATOM	4058	N	TRP	200	36.094	35.910	42.449	1.00	0.00	RX1	N
ATOM	4059	H	TRP	200	36.252	36.099	41.477	1.00	0.00	RX1	H
ATOM	4060	CA	TRP	200	37.188	35.318	43.225	1.00	0.00	RX1	C
ATOM	4061	CB	TRP	200	38.033	34.304	42.431	1.00	0.00	RX1	C
ATOM	4062	CG	TRP	200	37.308	33.461	41.400	1.00	0.00	RX1	C
ATOM	4063	CD2	TRP	200	37.115	32.031	41.407	1.00	0.00	RX1	C
ATOM	4064	CE2	TRP	200	36.483	31.672	40.194	1.00	0.00	RX1	C
ATOM	4065	CE3	TRP	200	37.432	31.045	42.331	1.00	0.00	RX1	C
ATOM	4066	CD1	TRP	200	36.768	33.879	40.175	1.00	0.00	RX1	C
ATOM	4067	NE1	TRP	200	36.282	32.833	39.459	1.00	0.00	RX1	N
ATOM	4068	HE1	TRP	200	35.878	32.879	38.566	1.00	0.00	RX1	H
ATOM	4069	CZ2	TRP	200	36.189	30.339	39.935	1.00	0.00	RX1	C
ATOM	4070	CZ3	TRP	200	37.131	29.716	42.062	1.00	0.00	RX1	C
ATOM	4071	CH2	TRP	200	36.513	29.363	40.869	1.00	0.00	RX1	C
ATOM	4072	C	TRP	200	38.173	36.329	43.815	1.00	0.00	RX1	C
ATOM	4073	O	TRP	200	39.368	36.068	43.913	1.00	0.00	RX1	O
ATOM	4074	N	GLY	201	37.641	37.501	44.191	1.00	0.00	RX1	N
ATOM	4075	H	GLY	201	36.658	37.668	44.134	1.00	0.00	RX1	H
ATOM	4076	CA	GLY	201	38.546	38.606	44.511	1.00	0.00	RX1	C
ATOM	4077	C	GLY	201	38.849	39.412	43.263	1.00	0.00	RX1	C
ATOM	4078	O	GLY	201	38.935	38.860	42.177	1.00	0.00	RX1	O
ATOM	4079	N	ALA	202	38.987	40.736	43.455	1.00	0.00	RX1	N
ATOM	4080	H	ALA	202	38.787	41.111	44.363	1.00	0.00	RX1	H
ATOM	4081	CA	ALA	202	39.102	41.642	42.303	1.00	0.00	RX1	C
ATOM	4082	CB	ALA	202	39.365	43.074	42.775	1.00	0.00	RX1	C
ATOM	4083	C	ALA	202	40.126	41.300	41.227	1.00	0.00	RX1	C
ATOM	4084	O	ALA	202	39.784	40.998	40.090	1.00	0.00	RX1	O
ATOM	4085	N	GLY	203	41.407	41.380	41.643	1.00	0.00	RX1	N

ATOM	4086	H	GLY	203	41.605	41.687	42.570	1.00	0.00	RX1	H
ATOM	4087	CA	GLY	203	42.495	41.199	40.677	1.00	0.00	RX1	C
ATOM	4088	C	GLY	203	42.370	42.105	39.461	1.00	0.00	RX1	C
ATOM	4089	O	GLY	203	41.797	43.183	39.532	1.00	0.00	RX1	O
ATOM	4090	N	GLU	204	42.896	41.587	38.346	1.00	0.00	RX1	N
ATOM	4091	H	GLU	204	43.307	40.678	38.301	1.00	0.00	RX1	H
ATOM	4092	CA	GLU	204	42.389	42.086	37.074	1.00	0.00	RX1	C
ATOM	4093	CB	GLU	204	43.549	42.362	36.122	1.00	0.00	RX1	C
ATOM	4094	CG	GLU	204	43.089	42.840	34.745	1.00	0.00	RX1	C
ATOM	4095	CD	GLU	204	44.296	42.965	33.844	1.00	0.00	RX1	C
ATOM	4096	OE1	GLU	204	44.154	42.747	32.643	1.00	0.00	RX1	O
ATOM	4097	OE2	GLU	204	45.374	43.264	34.352	1.00	0.00	RX1	O
ATOM	4098	C	GLU	204	41.529	40.957	36.555	1.00	0.00	RX1	C
ATOM	4099	O	GLU	204	41.901	39.798	36.707	1.00	0.00	RX1	O
ATOM	4100	N	GLU	205	40.343	41.346	36.046	1.00	0.00	RX1	N
ATOM	4101	H	GLU	205	40.209	42.321	35.878	1.00	0.00	RX1	H
ATOM	4102	CA	GLU	205	39.210	40.423	35.880	1.00	0.00	RX1	C
ATOM	4103	CB	GLU	205	38.727	40.300	34.434	1.00	0.00	RX1	C
ATOM	4104	CG	GLU	205	37.192	40.351	34.345	1.00	0.00	RX1	C
ATOM	4105	CD	GLU	205	36.558	39.443	35.387	1.00	0.00	RX1	C
ATOM	4106	OE1	GLU	205	36.435	38.245	35.148	1.00	0.00	RX1	O
ATOM	4107	OE2	GLU	205	36.223	39.921	36.465	1.00	0.00	RX1	O
ATOM	4108	C	GLU	205	39.309	39.046	36.516	1.00	0.00	RX1	C
ATOM	4109	O	GLU	205	39.486	38.009	35.874	1.00	0.00	RX1	O
ATOM	4110	N	ASN	206	39.185	39.083	37.839	1.00	0.00	RX1	N
ATOM	4111	H	ASN	206	38.972	39.925	38.341	1.00	0.00	RX1	H
ATOM	4112	CA	ASN	206	39.348	37.823	38.540	1.00	0.00	RX1	C
ATOM	4113	CB	ASN	206	40.435	38.006	39.602	1.00	0.00	RX1	C
ATOM	4114	CG	ASN	206	40.909	36.720	40.241	1.00	0.00	RX1	C
ATOM	4115	OD1	ASN	206	41.839	36.051	39.786	1.00	0.00	RX1	O
ATOM	4116	ND2	ASN	206	40.307	36.488	41.404	1.00	0.00	RX1	N
ATOM	4117	HD21	ASN	206	39.525	37.086	41.615	1.00	0.00	RX1	H
ATOM	4118	HD22	ASN	206	40.516	35.815	42.114	1.00	0.00	RX1	H
ATOM	4119	C	ASN	206	38.008	37.265	39.013	1.00	0.00	RX1	C
ATOM	4120	O	ASN	206	37.841	36.744	40.110	1.00	0.00	RX1	O
ATOM	4121	N	CYS	207	37.045	37.369	38.081	1.00	0.00	RX1	N
ATOM	4122	H	CYS	207	37.124	37.925	37.247	1.00	0.00	RX1	H
ATOM	4123	CA	CYS	207	35.750	36.726	38.278	1.00	0.00	RX1	C
ATOM	4124	CB	CYS	207	34.696	37.827	38.381	1.00	0.00	RX1	C
ATOM	4125	SG	CYS	207	35.115	38.982	39.713	1.00	0.00	RX1	S
ATOM	4126	C	CYS	207	35.466	35.681	37.202	1.00	0.00	RX1	C
ATOM	4127	O	CYS	207	36.349	35.319	36.426	1.00	0.00	RX1	O
ATOM	4128	N	GLN	208	34.225	35.164	37.211	1.00	0.00	RX1	N
ATOM	4129	H	GLN	208	33.532	35.532	37.836	1.00	0.00	RX1	H
ATOM	4130	CA	GLN	208	33.830	34.116	36.263	1.00	0.00	RX1	C
ATOM	4131	CB	GLN	208	32.714	33.314	36.939	1.00	0.00	RX1	C
ATOM	4132	CG	GLN	208	31.961	32.254	36.133	1.00	0.00	RX1	C
ATOM	4133	CD	GLN	208	30.782	31.762	36.963	1.00	0.00	RX1	C
ATOM	4134	OE1	GLN	208	29.748	32.414	37.091	1.00	0.00	RX1	O
ATOM	4135	NE2	GLN	208	30.982	30.565	37.531	1.00	0.00	RX1	N
ATOM	4136	HE21	GLN	208	31.881	30.134	37.464	1.00	0.00	RX1	H
ATOM	4137	HE22	GLN	208	30.262	30.049	38.001	1.00	0.00	RX1	H
ATOM	4138	C	GLN	208	33.381	34.673	34.917	1.00	0.00	RX1	C
ATOM	4139	O	GLN	208	32.543	35.560	34.838	1.00	0.00	RX1	O
ATOM	4140	N	LYS	209	33.950	34.092	33.847	1.00	0.00	RX1	N
ATOM	4141	H	LYS	209	34.515	33.275	33.954	1.00	0.00	RX1	H
ATOM	4142	CA	LYS	209	33.724	34.686	32.522	1.00	0.00	RX1	C
ATOM	4143	CB	LYS	209	34.877	34.368	31.560	1.00	0.00	RX1	C
ATOM	4144	CG	LYS	209	36.280	34.302	32.170	1.00	0.00	RX1	C
ATOM	4145	CD	LYS	209	36.699	35.577	32.897	1.00	0.00	RX1	C
ATOM	4146	CE	LYS	209	38.088	35.464	33.521	1.00	0.00	RX1	C

ATOM	4147	NZ	LYS	209	38.111	36.349	34.682	1.00	0.00	RX1	N
ATOM	4148	HZ1	LYS	209	39.063	36.557	35.047	1.00	0.00	RX1	H
ATOM	4149	HZ2	LYS	209	37.677	37.271	34.465	1.00	0.00	RX1	H
ATOM	4150	HZ3	LYS	209	37.530	35.949	35.444	1.00	0.00	RX1	H
ATOM	4151	C	LYS	209	32.417	34.329	31.803	1.00	0.00	RX1	C
ATOM	4152	O	LYS	209	32.347	34.373	30.574	1.00	0.00	RX1	O
ATOM	4153	N	LEU	210	31.401	33.947	32.609	1.00	0.00	RX1	N
ATOM	4154	H	LEU	210	31.502	34.132	33.585	1.00	0.00	RX1	H
ATOM	4155	CA	LEU	210	30.161	33.291	32.151	1.00	0.00	RX1	C
ATOM	4156	CB	LEU	210	28.938	34.189	32.352	1.00	0.00	RX1	C
ATOM	4157	CG	LEU	210	28.170	33.916	33.645	1.00	0.00	RX1	C
ATOM	4158	CD1	LEU	210	28.950	34.375	34.868	1.00	0.00	RX1	C
ATOM	4159	CD2	LEU	210	26.769	34.526	33.627	1.00	0.00	RX1	C
ATOM	4160	C	LEU	210	30.108	32.756	30.730	1.00	0.00	RX1	C
ATOM	4161	O	LEU	210	29.826	33.478	29.776	1.00	0.00	RX1	O
ATOM	4162	N	THR	211	30.398	31.463	30.618	1.00	0.00	RX1	N
ATOM	4163	H	THR	211	30.650	30.894	31.408	1.00	0.00	RX1	H
ATOM	4164	CA	THR	211	30.364	30.894	29.274	1.00	0.00	RX1	C
ATOM	4165	CB	THR	211	31.842	30.702	28.974	1.00	0.00	RX1	C
ATOM	4166	OG1	THR	211	32.505	30.622	30.247	1.00	0.00	RX1	O
ATOM	4167	HG1	THR	211	33.434	30.787	30.066	1.00	0.00	RX1	H
ATOM	4168	CG2	THR	211	32.460	31.840	28.160	1.00	0.00	RX1	C
ATOM	4169	C	THR	211	29.513	29.637	29.155	1.00	0.00	RX1	C
ATOM	4170	O	THR	211	29.570	28.917	28.165	1.00	0.00	RX1	O
ATOM	4171	N	LYS	212	28.739	29.381	30.221	1.00	0.00	RX1	N
ATOM	4172	H	LYS	212	28.766	29.970	31.030	1.00	0.00	RX1	H
ATOM	4173	CA	LYS	212	27.888	28.191	30.245	1.00	0.00	RX1	C
ATOM	4174	CB	LYS	212	28.455	27.205	31.264	1.00	0.00	RX1	C
ATOM	4175	CG	LYS	212	27.889	25.785	31.300	1.00	0.00	RX1	C
ATOM	4176	CD	LYS	212	28.739	24.791	30.505	1.00	0.00	RX1	C
ATOM	4177	CE	LYS	212	29.319	23.645	31.348	1.00	0.00	RX1	C
ATOM	4178	NZ	LYS	212	30.302	24.113	32.337	1.00	0.00	RX1	N
ATOM	4179	HZ1	LYS	212	29.972	23.894	33.299	1.00	0.00	RX1	H
ATOM	4180	HZ2	LYS	212	31.222	23.632	32.205	1.00	0.00	RX1	H
ATOM	4181	HZ3	LYS	212	30.463	25.138	32.299	1.00	0.00	RX1	H
ATOM	4182	C	LYS	212	26.450	28.540	30.599	1.00	0.00	RX1	C
ATOM	4183	O	LYS	212	25.498	28.180	29.921	1.00	0.00	RX1	O
ATOM	4184	N	ILE	213	26.327	29.293	31.710	1.00	0.00	RX1	N
ATOM	4185	H	ILE	213	27.147	29.586	32.209	1.00	0.00	RX1	H
ATOM	4186	CA	ILE	213	24.985	29.642	32.193	1.00	0.00	RX1	C
ATOM	4187	CB	ILE	213	25.097	30.473	33.476	1.00	0.00	RX1	C
ATOM	4188	CG2	ILE	213	23.744	31.026	33.930	1.00	0.00	RX1	C
ATOM	4189	CG1	ILE	213	25.744	29.645	34.582	1.00	0.00	RX1	C
ATOM	4190	CD1	ILE	213	24.899	28.424	34.934	1.00	0.00	RX1	C
ATOM	4191	C	ILE	213	24.131	30.374	31.165	1.00	0.00	RX1	C
ATOM	4192	O	ILE	213	22.964	30.079	30.951	1.00	0.00	RX1	O
ATOM	4193	N	ILE	214	24.785	31.347	30.523	1.00	0.00	RX1	N
ATOM	4194	H	ILE	214	25.771	31.454	30.633	1.00	0.00	RX1	H
ATOM	4195	CA	ILE	214	24.032	32.085	29.511	1.00	0.00	RX1	C
ATOM	4196	CB	ILE	214	24.287	33.596	29.625	1.00	0.00	RX1	C
ATOM	4197	CG2	ILE	214	23.347	34.174	30.683	1.00	0.00	RX1	C
ATOM	4198	CG1	ILE	214	25.743	33.961	29.946	1.00	0.00	RX1	C
ATOM	4199	CD1	ILE	214	26.779	33.622	28.876	1.00	0.00	RX1	C
ATOM	4200	C	ILE	214	24.199	31.587	28.086	1.00	0.00	RX1	C
ATOM	4201	O	ILE	214	24.311	32.355	27.137	1.00	0.00	RX1	O
ATOM	4202	N	CYS	215	24.213	30.251	27.971	1.00	0.00	RX1	N
ATOM	4203	H	CYS	215	24.042	29.640	28.745	1.00	0.00	RX1	H
ATOM	4204	CA	CYS	215	24.211	29.717	26.613	1.00	0.00	RX1	C
ATOM	4205	CB	CYS	215	24.592	28.236	26.634	1.00	0.00	RX1	C
ATOM	4206	SG	CYS	215	26.321	27.974	27.104	1.00	0.00	RX1	S
ATOM	4207	C	CYS	215	22.893	29.948	25.905	1.00	0.00	RX1	C

ATOM	4208	O	CYS	215	21.827	29.957	26.508	1.00	0.00	RX1	O
ATOM	4209	N	ALA	216	23.015	30.149	24.584	1.00	0.00	RX1	N
ATOM	4210	H	ALA	216	23.899	30.086	24.128	1.00	0.00	RX1	H
ATOM	4211	CA	ALA	216	21.783	30.292	23.817	1.00	0.00	RX1	C
ATOM	4212	CB	ALA	216	22.088	30.712	22.379	1.00	0.00	RX1	C
ATOM	4213	C	ALA	216	20.986	29.002	23.801	1.00	0.00	RX1	C
ATOM	4214	O	ALA	216	21.529	27.917	23.968	1.00	0.00	RX1	O
ATOM	4215	N	GLN	217	19.670	29.178	23.591	1.00	0.00	RX1	N
ATOM	4216	H	GLN	217	19.347	30.112	23.457	1.00	0.00	RX1	H
ATOM	4217	CA	GLN	217	18.735	28.048	23.682	1.00	0.00	RX1	C
ATOM	4218	CB	GLN	217	17.332	28.513	23.302	1.00	0.00	RX1	C
ATOM	4219	CG	GLN	217	16.856	29.663	24.192	1.00	0.00	RX1	C
ATOM	4220	CD	GLN	217	15.550	30.195	23.649	1.00	0.00	RX1	C
ATOM	4221	OE1	GLN	217	15.216	30.000	22.487	1.00	0.00	RX1	O
ATOM	4222	NE2	GLN	217	14.835	30.893	24.549	1.00	0.00	RX1	N
ATOM	4223	HE21	GLN	217	15.154	30.995	25.494	1.00	0.00	RX1	H
ATOM	4224	HE22	GLN	217	13.967	31.320	24.298	1.00	0.00	RX1	H
ATOM	4225	C	GLN	217	19.126	26.810	22.883	1.00	0.00	RX1	C
ATOM	4226	O	GLN	217	18.953	25.674	23.305	1.00	0.00	RX1	O
ATOM	4227	N	GLN	218	19.691	27.103	21.705	1.00	0.00	RX1	N
ATOM	4228	H	GLN	218	19.872	28.041	21.416	1.00	0.00	RX1	H
ATOM	4229	CA	GLN	218	20.459	26.060	21.042	1.00	0.00	RX1	C
ATOM	4230	CB	GLN	218	19.887	25.813	19.643	1.00	0.00	RX1	C
ATOM	4231	CG	GLN	218	20.656	24.774	18.822	1.00	0.00	RX1	C
ATOM	4232	CD	GLN	218	20.698	23.461	19.575	1.00	0.00	RX1	C
ATOM	4233	OE1	GLN	218	21.542	23.243	20.438	1.00	0.00	RX1	O
ATOM	4234	NE2	GLN	218	19.745	22.595	19.191	1.00	0.00	RX1	N
ATOM	4235	HE21	GLN	218	19.089	22.825	18.470	1.00	0.00	RX1	H
ATOM	4236	HE22	GLN	218	19.659	21.690	19.609	1.00	0.00	RX1	H
ATOM	4237	C	GLN	218	21.896	26.539	20.989	1.00	0.00	RX1	C
ATOM	4238	O	GLN	218	22.144	27.692	20.648	1.00	0.00	RX1	O
ATOM	4239	N	CYS	219	22.818	25.644	21.371	1.00	0.00	RX1	N
ATOM	4240	H	CYS	219	22.607	24.684	21.579	1.00	0.00	RX1	H
ATOM	4241	CA	CYS	219	24.204	26.094	21.501	1.00	0.00	RX1	C
ATOM	4242	CB	CYS	219	24.349	26.913	22.788	1.00	0.00	RX1	C
ATOM	4243	SG	CYS	219	25.733	28.083	22.770	1.00	0.00	RX1	S
ATOM	4244	C	CYS	219	25.189	24.950	21.454	1.00	0.00	RX1	C
ATOM	4245	O	CYS	219	24.996	23.909	22.068	1.00	0.00	RX1	O
ATOM	4246	N	SER	220	26.268	25.180	20.699	1.00	0.00	RX1	N
ATOM	4247	H	SER	220	26.430	26.035	20.208	1.00	0.00	RX1	H
ATOM	4248	CA	SER	220	27.291	24.146	20.589	1.00	0.00	RX1	C
ATOM	4249	CB	SER	220	27.936	24.389	19.237	1.00	0.00	RX1	C
ATOM	4250	OG	SER	220	27.785	25.782	18.938	1.00	0.00	RX1	O
ATOM	4251	HG	SER	220	28.680	26.124	18.922	1.00	0.00	RX1	H
ATOM	4252	C	SER	220	28.275	24.104	21.748	1.00	0.00	RX1	C
ATOM	4253	O	SER	220	29.450	24.431	21.629	1.00	0.00	RX1	O
ATOM	4254	N	GLY	221	27.737	23.665	22.891	1.00	0.00	RX1	N
ATOM	4255	H	GLY	221	26.747	23.531	22.975	1.00	0.00	RX1	H
ATOM	4256	CA	GLY	221	28.595	23.647	24.067	1.00	0.00	RX1	C
ATOM	4257	C	GLY	221	28.581	24.979	24.781	1.00	0.00	RX1	C
ATOM	4258	O	GLY	221	27.563	25.408	25.305	1.00	0.00	RX1	O
ATOM	4259	N	ARG	222	29.762	25.605	24.790	1.00	0.00	RX1	N
ATOM	4260	H	ARG	222	30.494	25.299	24.184	1.00	0.00	RX1	H
ATOM	4261	CA	ARG	222	29.832	26.889	25.485	1.00	0.00	RX1	C
ATOM	4262	CB	ARG	222	31.278	27.216	25.872	1.00	0.00	RX1	C
ATOM	4263	CG	ARG	222	31.998	26.143	26.689	1.00	0.00	RX1	C
ATOM	4264	CD	ARG	222	31.459	25.956	28.105	1.00	0.00	RX1	C
ATOM	4265	NE	ARG	222	31.632	27.158	28.919	1.00	0.00	RX1	N
ATOM	4266	HE	ARG	222	31.351	28.020	28.487	1.00	0.00	RX1	H
ATOM	4267	CZ	ARG	222	32.013	27.021	30.224	1.00	0.00	RX1	C
ATOM	4268	NH1	ARG	222	32.385	25.815	30.670	1.00	0.00	RX1	N

ATOM	4269	HH11	ARG	222	32.698	25.681	31.620	1.00	0.00	RX1	H
ATOM	4270	HH12	ARG	222	32.439	24.976	30.099	1.00	0.00	RX1	H
ATOM	4271	NH2	ARG	222	32.012	28.069	31.070	1.00	0.00	RX1	N
ATOM	4272	HH21	ARG	222	32.084	27.953	32.071	1.00	0.00	RX1	H
ATOM	4273	HH22	ARG	222	31.928	29.031	30.780	1.00	0.00	RX1	H
ATOM	4274	C	ARG	222	29.270	28.019	24.641	1.00	0.00	RX1	C
ATOM	4275	O	ARG	222	29.220	27.943	23.418	1.00	0.00	RX1	O
ATOM	4276	N	CYS	223	28.895	29.091	25.338	1.00	0.00	RX1	N
ATOM	4277	H	CYS	223	28.925	29.118	26.339	1.00	0.00	RX1	H
ATOM	4278	CA	CYS	223	28.680	30.340	24.618	1.00	0.00	RX1	C
ATOM	4279	CB	CYS	223	27.363	30.947	25.080	1.00	0.00	RX1	C
ATOM	4280	SG	CYS	223	27.277	30.970	26.888	1.00	0.00	RX1	S
ATOM	4281	C	CYS	223	29.835	31.265	24.910	1.00	0.00	RX1	C
ATOM	4282	O	CYS	223	30.153	31.534	26.063	1.00	0.00	RX1	O
ATOM	4283	N	ARG	224	30.492	31.728	23.840	1.00	0.00	RX1	N
ATOM	4284	H	ARG	224	30.129	31.664	22.909	1.00	0.00	RX1	H
ATOM	4285	CA	ARG	224	31.565	32.647	24.199	1.00	0.00	RX1	C
ATOM	4286	CB	ARG	224	32.808	32.419	23.320	1.00	0.00	RX1	C
ATOM	4287	CG	ARG	224	32.790	33.022	21.922	1.00	0.00	RX1	C
ATOM	4288	CD	ARG	224	33.606	32.247	20.881	1.00	0.00	RX1	C
ATOM	4289	NE	ARG	224	33.852	33.097	19.721	1.00	0.00	RX1	N
ATOM	4290	HE	ARG	224	34.750	33.552	19.683	1.00	0.00	RX1	H
ATOM	4291	CZ	ARG	224	32.764	33.477	18.998	1.00	0.00	RX1	C
ATOM	4292	NH1	ARG	224	31.665	32.704	18.983	1.00	0.00	RX1	N
ATOM	4293	HH11	ARG	224	30.819	33.086	18.584	1.00	0.00	RX1	H
ATOM	4294	HH12	ARG	224	31.609	31.778	19.372	1.00	0.00	RX1	H
ATOM	4295	NH2	ARG	224	32.769	34.640	18.321	1.00	0.00	RX1	N
ATOM	4296	HH21	ARG	224	31.873	34.993	17.997	1.00	0.00	RX1	H
ATOM	4297	HH22	ARG	224	33.585	35.197	18.171	1.00	0.00	RX1	H
ATOM	4298	C	ARG	224	31.038	34.072	24.318	1.00	0.00	RX1	C
ATOM	4299	O	ARG	224	31.215	34.754	25.333	1.00	0.00	RX1	O
ATOM	4300	N	GLY	225	30.284	34.426	23.255	1.00	0.00	RX1	N
ATOM	4301	H	GLY	225	30.168	33.815	22.469	1.00	0.00	RX1	H
ATOM	4302	CA	GLY	225	29.409	35.589	23.293	1.00	0.00	RX1	C
ATOM	4303	C	GLY	225	28.295	35.349	24.287	1.00	0.00	RX1	C
ATOM	4304	O	GLY	225	27.863	34.230	24.536	1.00	0.00	RX1	O
ATOM	4305	N	LYS	226	27.922	36.451	24.935	1.00	0.00	RX1	N
ATOM	4306	H	LYS	226	28.069	37.372	24.579	1.00	0.00	RX1	H
ATOM	4307	CA	LYS	226	27.487	36.211	26.305	1.00	0.00	RX1	C
ATOM	4308	CB	LYS	226	28.279	37.101	27.263	1.00	0.00	RX1	C
ATOM	4309	CG	LYS	226	29.718	37.222	26.758	1.00	0.00	RX1	C
ATOM	4310	CD	LYS	226	30.752	37.500	27.838	1.00	0.00	RX1	C
ATOM	4311	CE	LYS	226	30.778	36.380	28.874	1.00	0.00	RX1	C
ATOM	4312	NZ	LYS	226	31.105	35.072	28.280	1.00	0.00	RX1	N
ATOM	4313	HZ1	LYS	226	31.885	34.658	28.837	1.00	0.00	RX1	H
ATOM	4314	HZ2	LYS	226	30.302	34.425	28.440	1.00	0.00	RX1	H
ATOM	4315	HZ3	LYS	226	31.327	35.100	27.259	1.00	0.00	RX1	H
ATOM	4316	C	LYS	226	25.995	36.260	26.557	1.00	0.00	RX1	C
ATOM	4317	O	LYS	226	25.536	37.017	27.403	1.00	0.00	RX1	O
ATOM	4318	N	SER	227	25.308	35.418	25.759	1.00	0.00	RX1	N
ATOM	4319	H	SER	227	25.846	34.827	25.155	1.00	0.00	RX1	H
ATOM	4320	CA	SER	227	23.852	35.179	25.685	1.00	0.00	RX1	C
ATOM	4321	CB	SER	227	22.970	36.205	26.426	1.00	0.00	RX1	C
ATOM	4322	OG	SER	227	23.368	37.549	26.121	1.00	0.00	RX1	O
ATOM	4323	HG	SER	227	24.011	37.772	26.788	1.00	0.00	RX1	H
ATOM	4324	C	SER	227	23.284	34.868	24.299	1.00	0.00	RX1	C
ATOM	4325	O	SER	227	22.542	33.904	24.145	1.00	0.00	RX1	O
ATOM	4326	N	PRO	228	23.627	35.680	23.254	1.00	0.00	RX1	N
ATOM	4327	CD	PRO	228	24.447	36.891	23.194	1.00	0.00	RX1	C
ATOM	4328	CA	PRO	228	23.116	35.301	21.934	1.00	0.00	RX1	C
ATOM	4329	CB	PRO	228	23.385	36.557	21.103	1.00	0.00	RX1	C

ATOM	4330	CG	PRO	228	24.660	37.137	21.707	1.00	0.00	RX1	C
ATOM	4331	C	PRO	228	23.852	34.083	21.405	1.00	0.00	RX1	C
ATOM	4332	O	PRO	228	24.735	33.525	22.048	1.00	0.00	RX1	O
ATOM	4333	N	SER	229	23.490	33.724	20.171	1.00	0.00	RX1	N
ATOM	4334	H	SER	229	22.774	34.199	19.663	1.00	0.00	RX1	H
ATOM	4335	CA	SER	229	24.078	32.538	19.552	1.00	0.00	RX1	C
ATOM	4336	CB	SER	229	23.082	32.197	18.464	1.00	0.00	RX1	C
ATOM	4337	OG	SER	229	21.847	32.818	18.861	1.00	0.00	RX1	O
ATOM	4338	HG	SER	229	21.167	32.371	18.372	1.00	0.00	RX1	H
ATOM	4339	C	SER	229	25.538	32.604	19.108	1.00	0.00	RX1	C
ATOM	4340	O	SER	229	25.947	31.913	18.184	1.00	0.00	RX1	O
ATOM	4341	N	ASP	230	26.323	33.453	19.793	1.00	0.00	RX1	N
ATOM	4342	H	ASP	230	26.017	33.862	20.650	1.00	0.00	RX1	H
ATOM	4343	CA	ASP	230	27.753	33.470	19.502	1.00	0.00	RX1	C
ATOM	4344	CB	ASP	230	28.315	34.859	19.789	1.00	0.00	RX1	C
ATOM	4345	CG	ASP	230	29.727	34.950	19.261	1.00	0.00	RX1	C
ATOM	4346	OD1	ASP	230	30.617	35.325	20.015	1.00	0.00	RX1	O
ATOM	4347	OD2	ASP	230	29.960	34.617	18.100	1.00	0.00	RX1	O
ATOM	4348	C	ASP	230	28.461	32.360	20.269	1.00	0.00	RX1	C
ATOM	4349	O	ASP	230	29.135	32.511	21.287	1.00	0.00	RX1	O
ATOM	4350	N	CYS	231	28.186	31.174	19.726	1.00	0.00	RX1	N
ATOM	4351	H	CYS	231	27.720	31.130	18.839	1.00	0.00	RX1	H
ATOM	4352	CA	CYS	231	28.554	29.946	20.413	1.00	0.00	RX1	C
ATOM	4353	CB	CYS	231	27.649	28.844	19.877	1.00	0.00	RX1	C
ATOM	4354	SG	CYS	231	27.530	28.909	18.073	1.00	0.00	RX1	S
ATOM	4355	C	CYS	231	30.026	29.619	20.294	1.00	0.00	RX1	C
ATOM	4356	O	CYS	231	30.798	30.285	19.612	1.00	0.00	RX1	O
ATOM	4357	N	CYS	232	30.388	28.553	20.999	1.00	0.00	RX1	N
ATOM	4358	H	CYS	232	29.751	28.024	21.563	1.00	0.00	RX1	H
ATOM	4359	CA	CYS	232	31.713	28.005	20.773	1.00	0.00	RX1	C
ATOM	4360	CB	CYS	232	32.269	27.556	22.118	1.00	0.00	RX1	C
ATOM	4361	SG	CYS	232	32.471	28.963	23.238	1.00	0.00	RX1	S
ATOM	4362	C	CYS	232	31.636	26.879	19.769	1.00	0.00	RX1	C
ATOM	4363	O	CYS	232	30.560	26.472	19.347	1.00	0.00	RX1	O
ATOM	4364	N	HIS	233	32.820	26.371	19.401	1.00	0.00	RX1	N
ATOM	4365	H	HIS	233	33.674	26.690	19.811	1.00	0.00	RX1	H
ATOM	4366	CA	HIS	233	32.772	25.062	18.758	1.00	0.00	RX1	C
ATOM	4367	CB	HIS	233	34.109	24.806	18.050	1.00	0.00	RX1	C
ATOM	4368	CG	HIS	233	34.222	23.411	17.480	1.00	0.00	RX1	C
ATOM	4369	ND1	HIS	233	35.258	22.586	17.732	1.00	0.00	RX1	N
ATOM	4370	HD1	HIS	233	36.081	22.799	18.234	1.00	0.00	RX1	H
ATOM	4371	CD2	HIS	233	33.326	22.758	16.628	1.00	0.00	RX1	C
ATOM	4372	NE2	HIS	233	33.831	21.526	16.380	1.00	0.00	RX1	N
ATOM	4373	CE1	HIS	233	35.021	21.416	17.054	1.00	0.00	RX1	C
ATOM	4374	C	HIS	233	32.507	24.028	19.835	1.00	0.00	RX1	C
ATOM	4375	O	HIS	233	32.921	24.203	20.972	1.00	0.00	RX1	O
ATOM	4376	N	ASN	234	31.855	22.935	19.411	1.00	0.00	RX1	N
ATOM	4377	H	ASN	234	31.429	22.977	18.509	1.00	0.00	RX1	H
ATOM	4378	CA	ASN	234	31.745	21.704	20.207	1.00	0.00	RX1	C
ATOM	4379	CB	ASN	234	31.525	20.510	19.280	1.00	0.00	RX1	C
ATOM	4380	CG	ASN	234	31.544	19.190	20.032	1.00	0.00	RX1	C
ATOM	4381	OD1	ASN	234	31.243	19.069	21.217	1.00	0.00	RX1	O
ATOM	4382	ND2	ASN	234	31.887	18.172	19.226	1.00	0.00	RX1	N
ATOM	4383	HD21	ASN	234	32.087	18.322	18.256	1.00	0.00	RX1	H
ATOM	4384	HD22	ASN	234	31.966	17.211	19.505	1.00	0.00	RX1	H
ATOM	4385	C	ASN	234	32.930	21.441	21.142	1.00	0.00	RX1	C
ATOM	4386	O	ASN	234	32.800	21.250	22.344	1.00	0.00	RX1	O
ATOM	4387	N	GLN	235	34.124	21.470	20.536	1.00	0.00	RX1	N
ATOM	4388	H	GLN	235	34.222	21.774	19.590	1.00	0.00	RX1	H
ATOM	4389	CA	GLN	235	35.287	21.084	21.336	1.00	0.00	RX1	C
ATOM	4390	CB	GLN	235	36.360	20.504	20.429	1.00	0.00	RX1	C

ATOM	4391	CG	GLN	235	36.500	19.007	20.661	1.00	0.00	RX1	C
ATOM	4392	CD	GLN	235	35.150	18.343	20.492	1.00	0.00	RX1	C
ATOM	4393	OE1	GLN	235	34.354	18.224	21.423	1.00	0.00	RX1	O
ATOM	4394	NE2	GLN	235	34.952	17.875	19.253	1.00	0.00	RX1	N
ATOM	4395	HE21	GLN	235	35.704	17.970	18.592	1.00	0.00	RX1	H
ATOM	4396	HE22	GLN	235	34.135	17.386	18.936	1.00	0.00	RX1	H
ATOM	4397	C	GLN	235	35.894	22.098	22.281	1.00	0.00	RX1	C
ATOM	4398	O	GLN	235	36.884	21.831	22.956	1.00	0.00	RX1	O
ATOM	4399	N	CYS	236	35.270	23.271	22.291	1.00	0.00	RX1	N
ATOM	4400	H	CYS	236	34.367	23.413	21.889	1.00	0.00	RX1	H
ATOM	4401	CA	CYS	236	35.844	24.348	23.077	1.00	0.00	RX1	C
ATOM	4402	CB	CYS	236	35.474	25.662	22.428	1.00	0.00	RX1	C
ATOM	4403	SG	CYS	236	35.833	25.637	20.658	1.00	0.00	RX1	S
ATOM	4404	C	CYS	236	35.405	24.321	24.512	1.00	0.00	RX1	C
ATOM	4405	O	CYS	236	34.414	24.931	24.892	1.00	0.00	RX1	O
ATOM	4406	N	ALA	237	36.200	23.595	25.303	1.00	0.00	RX1	N
ATOM	4407	H	ALA	237	37.075	23.251	24.956	1.00	0.00	RX1	H
ATOM	4408	CA	ALA	237	35.963	23.730	26.733	1.00	0.00	RX1	C
ATOM	4409	CB	ALA	237	36.783	22.720	27.528	1.00	0.00	RX1	C
ATOM	4410	C	ALA	237	36.388	25.111	27.176	1.00	0.00	RX1	C
ATOM	4411	O	ALA	237	37.291	25.703	26.586	1.00	0.00	RX1	O
ATOM	4412	N	ALA	238	35.668	25.596	28.202	1.00	0.00	RX1	N
ATOM	4413	H	ALA	238	34.923	25.017	28.549	1.00	0.00	RX1	H
ATOM	4414	CA	ALA	238	35.775	26.982	28.671	1.00	0.00	RX1	C
ATOM	4415	CB	ALA	238	37.210	27.372	29.053	1.00	0.00	RX1	C
ATOM	4416	C	ALA	238	35.196	28.024	27.723	1.00	0.00	RX1	C
ATOM	4417	O	ALA	238	34.243	28.722	28.052	1.00	0.00	RX1	O
ATOM	4418	N	GLY	239	35.803	28.088	26.532	1.00	0.00	RX1	N
ATOM	4419	H	GLY	239	36.569	27.494	26.272	1.00	0.00	RX1	H
ATOM	4420	CA	GLY	239	35.335	29.003	25.500	1.00	0.00	RX1	C
ATOM	4421	C	GLY	239	36.248	28.885	24.298	1.00	0.00	RX1	C
ATOM	4422	O	GLY	239	37.057	27.965	24.208	1.00	0.00	RX1	O
ATOM	4423	N	CYS	240	36.108	29.851	23.382	1.00	0.00	RX1	N
ATOM	4424	H	CYS	240	35.481	30.629	23.439	1.00	0.00	RX1	H
ATOM	4425	CA	CYS	240	36.998	29.823	22.224	1.00	0.00	RX1	C
ATOM	4426	CB	CYS	240	36.485	28.850	21.168	1.00	0.00	RX1	C
ATOM	4427	SG	CYS	240	34.774	29.145	20.674	1.00	0.00	RX1	S
ATOM	4428	C	CYS	240	37.202	31.193	21.633	1.00	0.00	RX1	C
ATOM	4429	O	CYS	240	36.560	32.158	22.030	1.00	0.00	RX1	O
ATOM	4430	N	THR	241	38.107	31.221	20.652	1.00	0.00	RX1	N
ATOM	4431	H	THR	241	38.633	30.413	20.398	1.00	0.00	RX1	H
ATOM	4432	CA	THR	241	38.284	32.445	19.884	1.00	0.00	RX1	C
ATOM	4433	CB	THR	241	39.717	32.320	19.416	1.00	0.00	RX1	C
ATOM	4434	OG1	THR	241	40.405	31.505	20.379	1.00	0.00	RX1	O
ATOM	4435	HG1	THR	241	40.371	30.599	20.082	1.00	0.00	RX1	H
ATOM	4436	CG2	THR	241	40.400	33.676	19.229	1.00	0.00	RX1	C
ATOM	4437	C	THR	241	37.251	32.541	18.770	1.00	0.00	RX1	C
ATOM	4438	O	THR	241	36.415	33.437	18.707	1.00	0.00	RX1	O
ATOM	4439	N	GLY	242	37.325	31.524	17.903	1.00	0.00	RX1	N
ATOM	4440	H	GLY	242	37.932	30.735	18.012	1.00	0.00	RX1	H
ATOM	4441	CA	GLY	242	36.265	31.383	16.916	1.00	0.00	RX1	C
ATOM	4442	C	GLY	242	35.516	30.104	17.212	1.00	0.00	RX1	C
ATOM	4443	O	GLY	242	36.034	29.212	17.872	1.00	0.00	RX1	O
ATOM	4444	N	PRO	243	34.264	30.036	16.714	1.00	0.00	RX1	N
ATOM	4445	CD	PRO	243	33.560	31.085	15.991	1.00	0.00	RX1	C
ATOM	4446	CA	PRO	243	33.471	28.817	16.905	1.00	0.00	RX1	C
ATOM	4447	CB	PRO	243	32.056	29.346	16.666	1.00	0.00	RX1	C
ATOM	4448	CG	PRO	243	32.227	30.445	15.615	1.00	0.00	RX1	C
ATOM	4449	C	PRO	243	33.859	27.703	15.936	1.00	0.00	RX1	C
ATOM	4450	O	PRO	243	33.043	27.185	15.184	1.00	0.00	RX1	O
ATOM	4451	N	ARG	244	35.149	27.354	15.966	1.00	0.00	RX1	N

ATOM	4452	H	ARG	244	35.817	27.678	16.643	1.00	0.00	RX1	H
ATOM	4453	CA	ARG	244	35.651	26.458	14.935	1.00	0.00	RX1	C
ATOM	4454	CB	ARG	244	36.278	27.323	13.835	1.00	0.00	RX1	C
ATOM	4455	CG	ARG	244	36.191	26.904	12.359	1.00	0.00	RX1	C
ATOM	4456	CD	ARG	244	36.560	25.457	12.001	1.00	0.00	RX1	C
ATOM	4457	NE	ARG	244	37.970	25.049	11.819	1.00	0.00	RX1	N
ATOM	4458	HE	ARG	244	38.022	24.258	11.201	1.00	0.00	RX1	H
ATOM	4459	CZ	ARG	244	39.127	25.381	12.494	1.00	0.00	RX1	C
ATOM	4460	NH1	ARG	244	39.263	26.502	13.242	1.00	0.00	RX1	N
ATOM	4461	HH11	ARG	244	40.078	26.730	13.800	1.00	0.00	RX1	H
ATOM	4462	HH12	ARG	244	38.571	27.226	13.343	1.00	0.00	RX1	H
ATOM	4463	NH2	ARG	244	40.157	24.509	12.419	1.00	0.00	RX1	N
ATOM	4464	HH21	ARG	244	41.028	24.710	12.889	1.00	0.00	RX1	H
ATOM	4465	HH22	ARG	244	40.114	23.604	11.963	1.00	0.00	RX1	H
ATOM	4466	C	ARG	244	36.632	25.490	15.572	1.00	0.00	RX1	C
ATOM	4467	O	ARG	244	37.384	25.847	16.465	1.00	0.00	RX1	O
ATOM	4468	N	GLU	245	36.583	24.249	15.063	1.00	0.00	RX1	N
ATOM	4469	H	GLU	245	35.843	24.050	14.423	1.00	0.00	RX1	H
ATOM	4470	CA	GLU	245	37.523	23.165	15.379	1.00	0.00	RX1	C
ATOM	4471	CB	GLU	245	37.975	22.525	14.070	1.00	0.00	RX1	C
ATOM	4472	CG	GLU	245	38.956	21.366	14.182	1.00	0.00	RX1	C
ATOM	4473	CD	GLU	245	39.413	21.013	12.786	1.00	0.00	RX1	C
ATOM	4474	OE1	GLU	245	38.916	20.043	12.233	1.00	0.00	RX1	O
ATOM	4475	OE2	GLU	245	40.285	21.689	12.247	1.00	0.00	RX1	O
ATOM	4476	C	GLU	245	38.698	23.426	16.325	1.00	0.00	RX1	C
ATOM	4477	O	GLU	245	38.728	22.943	17.448	1.00	0.00	RX1	O
ATOM	4478	N	SER	246	39.689	24.170	15.815	1.00	0.00	RX1	N
ATOM	4479	H	SER	246	39.627	24.713	14.983	1.00	0.00	RX1	H
ATOM	4480	CA	SER	246	40.932	24.265	16.575	1.00	0.00	RX1	C
ATOM	4481	CB	SER	246	42.046	24.454	15.559	1.00	0.00	RX1	C
ATOM	4482	OG	SER	246	41.525	25.239	14.480	1.00	0.00	RX1	O
ATOM	4483	HG	SER	246	41.650	26.139	14.799	1.00	0.00	RX1	H
ATOM	4484	C	SER	246	40.975	25.304	17.675	1.00	0.00	RX1	C
ATOM	4485	O	SER	246	41.835	25.273	18.545	1.00	0.00	RX1	O
ATOM	4486	N	ASP	247	40.046	26.257	17.577	1.00	0.00	RX1	N
ATOM	4487	H	ASP	247	39.234	26.180	16.998	1.00	0.00	RX1	H
ATOM	4488	CA	ASP	247	40.353	27.567	18.149	1.00	0.00	RX1	C
ATOM	4489	CB	ASP	247	39.747	28.657	17.250	1.00	0.00	RX1	C
ATOM	4490	CG	ASP	247	39.797	28.284	15.765	1.00	0.00	RX1	C
ATOM	4491	OD1	ASP	247	40.783	27.725	15.285	1.00	0.00	RX1	O
ATOM	4492	OD2	ASP	247	38.824	28.537	15.063	1.00	0.00	RX1	O
ATOM	4493	C	ASP	247	39.936	27.722	19.605	1.00	0.00	RX1	C
ATOM	4494	O	ASP	247	39.497	28.777	20.061	1.00	0.00	RX1	O
ATOM	4495	N	CYS	248	40.054	26.589	20.309	1.00	0.00	RX1	N
ATOM	4496	H	CYS	248	40.627	25.852	19.950	1.00	0.00	RX1	H
ATOM	4497	CA	CYS	248	39.542	26.460	21.668	1.00	0.00	RX1	C
ATOM	4498	CB	CYS	248	39.365	24.970	21.949	1.00	0.00	RX1	C
ATOM	4499	SG	CYS	248	38.545	24.113	20.580	1.00	0.00	RX1	S
ATOM	4500	C	CYS	248	40.468	27.085	22.687	1.00	0.00	RX1	C
ATOM	4501	O	CYS	248	41.654	27.250	22.443	1.00	0.00	RX1	O
ATOM	4502	N	LEU	249	39.895	27.392	23.861	1.00	0.00	RX1	N
ATOM	4503	H	LEU	249	38.915	27.276	24.032	1.00	0.00	RX1	H
ATOM	4504	CA	LEU	249	40.813	27.724	24.952	1.00	0.00	RX1	C
ATOM	4505	CB	LEU	249	40.091	28.519	26.038	1.00	0.00	RX1	C
ATOM	4506	CG	LEU	249	39.537	29.858	25.550	1.00	0.00	RX1	C
ATOM	4507	CD1	LEU	249	38.805	30.599	26.669	1.00	0.00	RX1	C
ATOM	4508	CD2	LEU	249	40.614	30.723	24.894	1.00	0.00	RX1	C
ATOM	4509	C	LEU	249	41.436	26.471	25.548	1.00	0.00	RX1	C
ATOM	4510	O	LEU	249	42.648	26.284	25.631	1.00	0.00	RX1	O
ATOM	4511	N	VAL	250	40.522	25.579	25.946	1.00	0.00	RX1	N
ATOM	4512	H	VAL	250	39.529	25.714	25.885	1.00	0.00	RX1	H

ATOM	4513	CA	VAL	250	41.001	24.270	26.369	1.00	0.00	RX1	C
ATOM	4514	CB	VAL	250	41.010	24.137	27.893	1.00	0.00	RX1	C
ATOM	4515	CG1	VAL	250	42.234	24.825	28.499	1.00	0.00	RX1	C
ATOM	4516	CG2	VAL	250	39.720	24.667	28.504	1.00	0.00	RX1	C
ATOM	4517	C	VAL	250	40.205	23.176	25.691	1.00	0.00	RX1	C
ATOM	4518	O	VAL	250	39.110	23.394	25.187	1.00	0.00	RX1	O
ATOM	4519	N	CYS	251	40.828	21.995	25.642	1.00	0.00	RX1	N
ATOM	4520	H	CYS	251	41.687	21.820	26.128	1.00	0.00	RX1	H
ATOM	4521	CA	CYS	251	40.239	21.010	24.736	1.00	0.00	RX1	C
ATOM	4522	CB	CYS	251	41.384	20.313	24.008	1.00	0.00	RX1	C
ATOM	4523	SG	CYS	251	42.755	21.473	23.772	1.00	0.00	RX1	S
ATOM	4524	C	CYS	251	39.284	20.042	25.408	1.00	0.00	RX1	C
ATOM	4525	O	CYS	251	39.625	19.405	26.401	1.00	0.00	RX1	O
ATOM	4526	N	ARG	252	38.083	19.935	24.821	1.00	0.00	RX1	N
ATOM	4527	H	ARG	252	37.837	20.518	24.042	1.00	0.00	RX1	H
ATOM	4528	CA	ARG	252	37.157	18.917	25.327	1.00	0.00	RX1	C
ATOM	4529	CB	ARG	252	35.707	19.189	24.929	1.00	0.00	RX1	C
ATOM	4530	CG	ARG	252	35.050	20.279	25.766	1.00	0.00	RX1	C
ATOM	4531	CD	ARG	252	33.550	20.432	25.532	1.00	0.00	RX1	C
ATOM	4532	NE	ARG	252	32.855	19.199	25.893	1.00	0.00	RX1	N
ATOM	4533	HE	ARG	252	32.912	18.936	26.862	1.00	0.00	RX1	H
ATOM	4534	CZ	ARG	252	32.210	18.517	24.904	1.00	0.00	RX1	C
ATOM	4535	NH1	ARG	252	32.167	19.043	23.665	1.00	0.00	RX1	N
ATOM	4536	HH11	ARG	252	31.730	18.621	22.858	1.00	0.00	RX1	H
ATOM	4537	HH12	ARG	252	32.575	19.938	23.439	1.00	0.00	RX1	H
ATOM	4538	NH2	ARG	252	31.629	17.332	25.186	1.00	0.00	RX1	N
ATOM	4539	HH21	ARG	252	31.127	16.790	24.504	1.00	0.00	RX1	H
ATOM	4540	HH22	ARG	252	31.656	16.930	26.106	1.00	0.00	RX1	H
ATOM	4541	C	ARG	252	37.470	17.489	24.919	1.00	0.00	RX1	C
ATOM	4542	O	ARG	252	37.023	16.540	25.554	1.00	0.00	RX1	O
ATOM	4543	N	LYS	253	38.188	17.383	23.787	1.00	0.00	RX1	N
ATOM	4544	H	LYS	253	38.665	18.135	23.332	1.00	0.00	RX1	H
ATOM	4545	CA	LYS	253	38.225	16.104	23.082	1.00	0.00	RX1	C
ATOM	4546	CB	LYS	253	36.891	15.857	22.383	1.00	0.00	RX1	C
ATOM	4547	CG	LYS	253	36.052	14.776	23.058	1.00	0.00	RX1	C
ATOM	4548	CD	LYS	253	34.595	14.853	22.620	1.00	0.00	RX1	C
ATOM	4549	CE	LYS	253	34.423	15.050	21.112	1.00	0.00	RX1	C
ATOM	4550	NZ	LYS	253	34.657	13.841	20.315	1.00	0.00	RX1	N
ATOM	4551	HZ1	LYS	253	34.245	13.981	19.370	1.00	0.00	RX1	H
ATOM	4552	HZ2	LYS	253	35.677	13.644	20.173	1.00	0.00	RX1	H
ATOM	4553	HZ3	LYS	253	34.227	12.992	20.714	1.00	0.00	RX1	H
ATOM	4554	C	LYS	253	39.392	16.032	22.114	1.00	0.00	RX1	C
ATOM	4555	O	LYS	253	40.248	16.905	22.125	1.00	0.00	RX1	O
ATOM	4556	N	PHE	254	39.362	14.959	21.295	1.00	0.00	RX1	N
ATOM	4557	H	PHE	254	38.607	14.309	21.360	1.00	0.00	RX1	H
ATOM	4558	CA	PHE	254	40.411	14.619	20.326	1.00	0.00	RX1	C
ATOM	4559	CB	PHE	254	39.873	13.629	19.288	1.00	0.00	RX1	C
ATOM	4560	CG	PHE	254	39.716	12.232	19.862	1.00	0.00	RX1	C
ATOM	4561	CD1	PHE	254	39.831	11.138	19.017	1.00	0.00	RX1	C
ATOM	4562	CD2	PHE	254	39.455	12.015	21.210	1.00	0.00	RX1	C
ATOM	4563	CE1	PHE	254	39.678	9.845	19.505	1.00	0.00	RX1	C
ATOM	4564	CE2	PHE	254	39.300	10.725	21.704	1.00	0.00	RX1	C
ATOM	4565	CZ	PHE	254	39.406	9.636	20.850	1.00	0.00	RX1	C
ATOM	4566	C	PHE	254	41.147	15.763	19.648	1.00	0.00	RX1	C
ATOM	4567	O	PHE	254	40.665	16.446	18.746	1.00	0.00	RX1	O
ATOM	4568	N	ARG	255	42.373	15.924	20.146	1.00	0.00	RX1	N
ATOM	4569	H	ARG	255	42.777	15.203	20.711	1.00	0.00	RX1	H
ATOM	4570	CA	ARG	255	43.197	17.012	19.648	1.00	0.00	RX1	C
ATOM	4571	CB	ARG	255	44.013	17.599	20.803	1.00	0.00	RX1	C
ATOM	4572	CG	ARG	255	44.447	19.055	20.604	1.00	0.00	RX1	C
ATOM	4573	CD	ARG	255	45.933	19.230	20.914	1.00	0.00	RX1	C

ATOM	4574	NE	ARG	255	46.676	18.375	19.999	1.00	0.00	RX1	N
ATOM	4575	HE	ARG	255	46.226	18.126	19.130	1.00	0.00	RX1	H
ATOM	4576	CZ	ARG	255	47.947	17.948	20.211	1.00	0.00	RX1	C
ATOM	4577	NH1	ARG	255	48.648	18.374	21.275	1.00	0.00	RX1	N
ATOM	4578	HH11	ARG	255	49.582	18.007	21.428	1.00	0.00	RX1	H
ATOM	4579	HH12	ARG	255	48.328	19.069	21.926	1.00	0.00	RX1	H
ATOM	4580	NH2	ARG	255	48.496	17.096	19.329	1.00	0.00	RX1	N
ATOM	4581	HH21	ARG	255	49.478	16.855	19.413	1.00	0.00	RX1	H
ATOM	4582	HH22	ARG	255	47.970	16.715	18.565	1.00	0.00	RX1	H
ATOM	4583	C	ARG	255	44.081	16.553	18.498	1.00	0.00	RX1	C
ATOM	4584	O	ARG	255	45.300	16.441	18.602	1.00	0.00	RX1	O
ATOM	4585	N	ASP	256	43.387	16.261	17.389	1.00	0.00	RX1	N
ATOM	4586	H	ASP	256	42.425	16.529	17.366	1.00	0.00	RX1	H
ATOM	4587	CA	ASP	256	44.083	15.801	16.188	1.00	0.00	RX1	C
ATOM	4588	CB	ASP	256	43.092	15.523	15.066	1.00	0.00	RX1	C
ATOM	4589	CG	ASP	256	43.874	14.992	13.890	1.00	0.00	RX1	C
ATOM	4590	OD1	ASP	256	44.889	14.350	14.113	1.00	0.00	RX1	O
ATOM	4591	OD2	ASP	256	43.494	15.206	12.743	1.00	0.00	RX1	O
ATOM	4592	C	ASP	256	45.125	16.776	15.685	1.00	0.00	RX1	C
ATOM	4593	O	ASP	256	44.803	17.865	15.216	1.00	0.00	RX1	O
ATOM	4594	N	GLU	257	46.393	16.350	15.831	1.00	0.00	RX1	N
ATOM	4595	H	GLU	257	46.531	15.383	16.062	1.00	0.00	RX1	H
ATOM	4596	CA	GLU	257	47.510	17.254	15.543	1.00	0.00	RX1	C
ATOM	4597	CB	GLU	257	47.862	17.149	14.057	1.00	0.00	RX1	C
ATOM	4598	CG	GLU	257	48.394	15.728	13.837	1.00	0.00	RX1	C
ATOM	4599	CD	GLU	257	48.521	15.381	12.368	1.00	0.00	RX1	C
ATOM	4600	OE1	GLU	257	47.842	15.975	11.539	1.00	0.00	RX1	O
ATOM	4601	OE2	GLU	257	49.245	14.453	12.031	1.00	0.00	RX1	O
ATOM	4602	C	GLU	257	47.284	18.650	16.127	1.00	0.00	RX1	C
ATOM	4603	O	GLU	257	47.105	18.760	17.332	1.00	0.00	RX1	O
ATOM	4604	N	ALA	258	47.241	19.698	15.291	1.00	0.00	RX1	N
ATOM	4605	H	ALA	258	47.249	19.605	14.297	1.00	0.00	RX1	H
ATOM	4606	CA	ALA	258	46.999	20.994	15.934	1.00	0.00	RX1	C
ATOM	4607	CB	ALA	258	47.859	22.079	15.286	1.00	0.00	RX1	C
ATOM	4608	C	ALA	258	45.545	21.461	15.940	1.00	0.00	RX1	C
ATOM	4609	O	ALA	258	45.247	22.646	15.871	1.00	0.00	RX1	O
ATOM	4610	N	THR	259	44.625	20.486	15.977	1.00	0.00	RX1	N
ATOM	4611	H	THR	259	44.842	19.525	16.151	1.00	0.00	RX1	H
ATOM	4612	CA	THR	259	43.215	20.856	15.865	1.00	0.00	RX1	C
ATOM	4613	CB	THR	259	42.754	20.712	14.397	1.00	0.00	RX1	C
ATOM	4614	OG1	THR	259	42.983	19.391	13.889	1.00	0.00	RX1	O
ATOM	4615	HG1	THR	259	43.749	19.045	14.354	1.00	0.00	RX1	H
ATOM	4616	CG2	THR	259	43.391	21.729	13.446	1.00	0.00	RX1	C
ATOM	4617	C	THR	259	42.357	20.081	16.849	1.00	0.00	RX1	C
ATOM	4618	O	THR	259	42.701	18.972	17.231	1.00	0.00	RX1	O
ATOM	4619	N	CYS	260	41.222	20.674	17.238	1.00	0.00	RX1	N
ATOM	4620	H	CYS	260	40.889	21.568	16.945	1.00	0.00	RX1	H
ATOM	4621	CA	CYS	260	40.336	19.858	18.063	1.00	0.00	RX1	C
ATOM	4622	CB	CYS	260	39.936	20.648	19.304	1.00	0.00	RX1	C
ATOM	4623	SG	CYS	260	41.382	21.299	20.178	1.00	0.00	RX1	S
ATOM	4624	C	CYS	260	39.161	19.325	17.265	1.00	0.00	RX1	C
ATOM	4625	O	CYS	260	38.110	19.940	17.122	1.00	0.00	RX1	O
ATOM	4626	N	LYS	261	39.424	18.140	16.699	1.00	0.00	RX1	N
ATOM	4627	H	LYS	261	40.188	17.612	17.075	1.00	0.00	RX1	H
ATOM	4628	CA	LYS	261	38.394	17.487	15.892	1.00	0.00	RX1	C
ATOM	4629	CB	LYS	261	39.000	16.500	14.896	1.00	0.00	RX1	C
ATOM	4630	CG	LYS	261	40.032	17.232	14.035	1.00	0.00	RX1	C
ATOM	4631	CD	LYS	261	40.120	16.714	12.598	1.00	0.00	RX1	C
ATOM	4632	CE	LYS	261	41.267	17.350	11.802	1.00	0.00	RX1	C
ATOM	4633	NZ	LYS	261	41.304	18.802	12.015	1.00	0.00	RX1	N
ATOM	4634	HZ1	LYS	261	41.970	19.266	11.376	1.00	0.00	RX1	H

ATOM	4635	HZ2	LYS	261	40.370	19.249	11.895	1.00	0.00	RX1	H
ATOM	4636	HZ3	LYS	261	41.628	18.999	12.982	1.00	0.00	RX1	H
ATOM	4637	C	LYS	261	37.316	16.906	16.787	1.00	0.00	RX1	C
ATOM	4638	O	LYS	261	37.332	17.070	18.001	1.00	0.00	RX1	O
ATOM	4639	N	ASP	262	36.375	16.190	16.153	1.00	0.00	RX1	N
ATOM	4640	H	ASP	262	36.377	16.045	15.165	1.00	0.00	RX1	H
ATOM	4641	CA	ASP	262	35.537	15.404	17.049	1.00	0.00	RX1	C
ATOM	4642	CB	ASP	262	34.112	15.168	16.544	1.00	0.00	RX1	C
ATOM	4643	CG	ASP	262	33.279	14.731	17.739	1.00	0.00	RX1	C
ATOM	4644	OD1	ASP	262	32.751	15.571	18.457	1.00	0.00	RX1	O
ATOM	4645	OD2	ASP	262	33.198	13.544	18.021	1.00	0.00	RX1	O
ATOM	4646	C	ASP	262	36.204	14.118	17.486	1.00	0.00	RX1	C
ATOM	4647	O	ASP	262	36.540	13.959	18.653	1.00	0.00	RX1	O
ATOM	4648	N	THR	263	36.393	13.232	16.508	1.00	0.00	RX1	N
ATOM	4649	H	THR	263	36.164	13.372	15.544	1.00	0.00	RX1	H
ATOM	4650	CA	THR	263	37.048	11.965	16.797	1.00	0.00	RX1	C
ATOM	4651	CB	THR	263	35.926	10.962	17.091	1.00	0.00	RX1	C
ATOM	4652	OG1	THR	263	35.039	11.514	18.081	1.00	0.00	RX1	O
ATOM	4653	HG1	THR	263	34.410	12.061	17.608	1.00	0.00	RX1	H
ATOM	4654	CG2	THR	263	36.446	9.595	17.543	1.00	0.00	RX1	C
ATOM	4655	C	THR	263	37.900	11.636	15.581	1.00	0.00	RX1	C
ATOM	4656	O	THR	263	37.621	12.152	14.504	1.00	0.00	RX1	O
ATOM	4657	N	CYS	264	38.950	10.821	15.789	1.00	0.00	RX1	N
ATOM	4658	H	CYS	264	39.145	10.436	16.690	1.00	0.00	RX1	H
ATOM	4659	CA	CYS	264	39.869	10.479	14.692	1.00	0.00	RX1	C
ATOM	4660	CB	CYS	264	40.800	9.355	15.142	1.00	0.00	RX1	C
ATOM	4661	SG	CYS	264	41.486	9.624	16.789	1.00	0.00	RX1	S
ATOM	4662	C	CYS	264	39.229	10.064	13.375	1.00	0.00	RX1	C
ATOM	4663	O	CYS	264	38.508	9.076	13.313	1.00	0.00	RX1	O
ATOM	4664	N	PRO	265	39.549	10.832	12.304	1.00	0.00	RX1	N
ATOM	4665	CD	PRO	265	40.237	12.116	12.337	1.00	0.00	RX1	C
ATOM	4666	CA	PRO	265	39.190	10.396	10.948	1.00	0.00	RX1	C
ATOM	4667	CB	PRO	265	39.809	11.495	10.075	1.00	0.00	RX1	C
ATOM	4668	CG	PRO	265	39.925	12.724	10.977	1.00	0.00	RX1	C
ATOM	4669	C	PRO	265	39.722	9.008	10.598	1.00	0.00	RX1	C
ATOM	4670	O	PRO	265	40.927	8.775	10.571	1.00	0.00	RX1	O
ATOM	4671	N	PRO	266	38.769	8.082	10.336	1.00	0.00	RX1	N
ATOM	4672	CD	PRO	266	37.329	8.306	10.324	1.00	0.00	RX1	C
ATOM	4673	CA	PRO	266	39.138	6.686	10.067	1.00	0.00	RX1	C
ATOM	4674	CB	PRO	266	37.782	6.025	9.792	1.00	0.00	RX1	C
ATOM	4675	CG	PRO	266	36.749	6.907	10.491	1.00	0.00	RX1	C
ATOM	4676	C	PRO	266	40.115	6.515	8.916	1.00	0.00	RX1	C
ATOM	4677	O	PRO	266	40.218	7.352	8.029	1.00	0.00	RX1	O
ATOM	4678	N	LEU	267	40.843	5.387	8.974	1.00	0.00	RX1	N
ATOM	4679	H	LEU	267	40.715	4.730	9.716	1.00	0.00	RX1	H
ATOM	4680	CA	LEU	267	41.831	5.131	7.920	1.00	0.00	RX1	C
ATOM	4681	CB	LEU	267	42.709	3.934	8.284	1.00	0.00	RX1	C
ATOM	4682	CG	LEU	267	44.034	4.290	8.963	1.00	0.00	RX1	C
ATOM	4683	CD1	LEU	267	43.863	5.031	10.291	1.00	0.00	RX1	C
ATOM	4684	CD2	LEU	267	44.907	3.046	9.114	1.00	0.00	RX1	C
ATOM	4685	C	LEU	267	41.234	4.910	6.539	1.00	0.00	RX1	C
ATOM	4686	O	LEU	267	41.750	5.351	5.518	1.00	0.00	RX1	O
ATOM	4687	N	MET	268	40.105	4.193	6.560	1.00	0.00	RX1	N
ATOM	4688	H	MET	268	39.611	3.962	7.396	1.00	0.00	RX1	H
ATOM	4689	CA	MET	268	39.387	4.042	5.303	1.00	0.00	RX1	C
ATOM	4690	CB	MET	268	39.121	2.567	5.003	1.00	0.00	RX1	C
ATOM	4691	CG	MET	268	40.381	1.699	5.059	1.00	0.00	RX1	C
ATOM	4692	SD	MET	268	41.663	2.201	3.897	1.00	0.00	RX1	S
ATOM	4693	CE	MET	268	40.849	1.692	2.374	1.00	0.00	RX1	C
ATOM	4694	C	MET	268	38.088	4.800	5.418	1.00	0.00	RX1	C
ATOM	4695	O	MET	268	37.581	4.992	6.517	1.00	0.00	RX1	O

ATOM	4696	N	LEU	269	37.594	5.234	4.260	1.00	0.00	RX1	N
ATOM	4697	H	LEU	269	37.979	4.928	3.388	1.00	0.00	RX1	H
ATOM	4698	CA	LEU	269	36.271	5.846	4.227	1.00	0.00	RX1	C
ATOM	4699	CB	LEU	269	36.259	7.323	3.823	1.00	0.00	RX1	C
ATOM	4700	CG	LEU	269	37.261	8.308	4.432	1.00	0.00	RX1	C
ATOM	4701	CD1	LEU	269	37.422	8.206	5.950	1.00	0.00	RX1	C
ATOM	4702	CD2	LEU	269	38.561	8.317	3.637	1.00	0.00	RX1	C
ATOM	4703	C	LEU	269	35.475	5.094	3.187	1.00	0.00	RX1	C
ATOM	4704	O	LEU	269	35.918	4.066	2.692	1.00	0.00	RX1	O
ATOM	4705	N	TYR	270	34.312	5.644	2.839	1.00	0.00	RX1	N
ATOM	4706	H	TYR	270	33.937	6.523	3.138	1.00	0.00	RX1	H
ATOM	4707	CA	TYR	270	33.545	4.963	1.812	1.00	0.00	RX1	C
ATOM	4708	CB	TYR	270	32.540	4.095	2.545	1.00	0.00	RX1	C
ATOM	4709	CG	TYR	270	31.800	3.121	1.668	1.00	0.00	RX1	C
ATOM	4710	CD1	TYR	270	32.362	1.886	1.371	1.00	0.00	RX1	C
ATOM	4711	CE1	TYR	270	31.647	0.962	0.621	1.00	0.00	RX1	C
ATOM	4712	CD2	TYR	270	30.533	3.443	1.203	1.00	0.00	RX1	C
ATOM	4713	CE2	TYR	270	29.809	2.514	0.472	1.00	0.00	RX1	C
ATOM	4714	CZ	TYR	270	30.370	1.279	0.175	1.00	0.00	RX1	C
ATOM	4715	OH	TYR	270	29.654	0.380	-0.585	1.00	0.00	RX1	O
ATOM	4716	HH	TYR	270	30.217	0.053	-1.286	1.00	0.00	RX1	H
ATOM	4717	C	TYR	270	32.905	6.008	0.935	1.00	0.00	RX1	C
ATOM	4718	O	TYR	270	32.478	7.048	1.415	1.00	0.00	RX1	O
ATOM	4719	N	ASN	271	32.868	5.702	-0.359	1.00	0.00	RX1	N
ATOM	4720	H	ASN	271	33.282	4.852	-0.672	1.00	0.00	RX1	H
ATOM	4721	CA	ASN	271	32.446	6.742	-1.285	1.00	0.00	RX1	C
ATOM	4722	CB	ASN	271	32.988	6.463	-2.676	1.00	0.00	RX1	C
ATOM	4723	CG	ASN	271	33.124	7.795	-3.363	1.00	0.00	RX1	C
ATOM	4724	OD1	ASN	271	32.176	8.331	-3.936	1.00	0.00	RX1	O
ATOM	4725	ND2	ASN	271	34.364	8.292	-3.280	1.00	0.00	RX1	N
ATOM	4726	HD21	ASN	271	35.016	7.785	-2.687	1.00	0.00	RX1	H
ATOM	4727	HD22	ASN	271	34.732	9.118	-3.701	1.00	0.00	RX1	H
ATOM	4728	C	ASN	271	30.952	6.952	-1.359	1.00	0.00	RX1	C
ATOM	4729	O	ASN	271	30.204	6.061	-1.753	1.00	0.00	RX1	O
ATOM	4730	N	PRO	272	30.522	8.192	-1.022	1.00	0.00	RX1	N
ATOM	4731	CD	PRO	272	31.339	9.314	-0.574	1.00	0.00	RX1	C
ATOM	4732	CA	PRO	272	29.093	8.512	-1.109	1.00	0.00	RX1	C
ATOM	4733	CB	PRO	272	29.040	9.964	-0.614	1.00	0.00	RX1	C
ATOM	4734	CG	PRO	272	30.447	10.523	-0.824	1.00	0.00	RX1	C
ATOM	4735	C	PRO	272	28.509	8.314	-2.498	1.00	0.00	RX1	C
ATOM	4736	O	PRO	272	27.334	8.008	-2.676	1.00	0.00	RX1	O
ATOM	4737	N	THR	273	29.383	8.508	-3.487	1.00	0.00	RX1	N
ATOM	4738	H	THR	273	30.342	8.733	-3.333	1.00	0.00	RX1	H
ATOM	4739	CA	THR	273	28.938	8.311	-4.856	1.00	0.00	RX1	C
ATOM	4740	CB	THR	273	29.730	9.335	-5.640	1.00	0.00	RX1	C
ATOM	4741	OG1	THR	273	30.345	10.213	-4.679	1.00	0.00	RX1	O
ATOM	4742	HG1	THR	273	31.207	9.828	-4.528	1.00	0.00	RX1	H
ATOM	4743	CG2	THR	273	28.862	10.112	-6.631	1.00	0.00	RX1	C
ATOM	4744	C	THR	273	29.087	6.872	-5.320	1.00	0.00	RX1	C
ATOM	4745	O	THR	273	28.119	6.174	-5.591	1.00	0.00	RX1	O
ATOM	4746	N	THR	274	30.357	6.457	-5.392	1.00	0.00	RX1	N
ATOM	4747	H	THR	274	31.130	7.043	-5.143	1.00	0.00	RX1	H
ATOM	4748	CA	THR	274	30.651	5.194	-6.061	1.00	0.00	RX1	C
ATOM	4749	CB	THR	274	31.936	5.530	-6.778	1.00	0.00	RX1	C
ATOM	4750	OG1	THR	274	31.990	6.965	-6.842	1.00	0.00	RX1	O
ATOM	4751	HG1	THR	274	32.790	7.179	-7.307	1.00	0.00	RX1	H
ATOM	4752	CG2	THR	274	32.048	4.901	-8.169	1.00	0.00	RX1	C
ATOM	4753	C	THR	274	30.712	3.956	-5.183	1.00	0.00	RX1	C
ATOM	4754	O	THR	274	31.144	2.895	-5.619	1.00	0.00	RX1	O
ATOM	4755	N	TYR	275	30.271	4.133	-3.923	1.00	0.00	RX1	N
ATOM	4756	H	TYR	275	29.969	5.032	-3.603	1.00	0.00	RX1	H

ATOM	4757	CA	TYR	275	30.132	3.002	-2.997	1.00	0.00	RX1	C
ATOM	4758	CB	TYR	275	28.859	2.221	-3.312	1.00	0.00	RX1	C
ATOM	4759	CG	TYR	275	27.699	3.181	-3.267	1.00	0.00	RX1	C
ATOM	4760	CD1	TYR	275	27.612	4.113	-2.240	1.00	0.00	RX1	C
ATOM	4761	CE1	TYR	275	26.552	5.007	-2.209	1.00	0.00	RX1	C
ATOM	4762	CD2	TYR	275	26.725	3.138	-4.257	1.00	0.00	RX1	C
ATOM	4763	CE2	TYR	275	25.659	4.027	-4.217	1.00	0.00	RX1	C
ATOM	4764	CZ	TYR	275	25.568	4.958	-3.189	1.00	0.00	RX1	C
ATOM	4765	OH	TYR	275	24.489	5.824	-3.140	1.00	0.00	RX1	O
ATOM	4766	HH	TYR	275	23.894	5.550	-3.835	1.00	0.00	RX1	H
ATOM	4767	C	TYR	275	31.333	2.076	-2.869	1.00	0.00	RX1	C
ATOM	4768	O	TYR	275	31.232	0.858	-2.771	1.00	0.00	RX1	O
ATOM	4769	N	GLN	276	32.495	2.732	-2.878	1.00	0.00	RX1	N
ATOM	4770	H	GLN	276	32.540	3.724	-2.785	1.00	0.00	RX1	H
ATOM	4771	CA	GLN	276	33.732	1.967	-2.809	1.00	0.00	RX1	C
ATOM	4772	CB	GLN	276	34.550	2.308	-4.052	1.00	0.00	RX1	C
ATOM	4773	CG	GLN	276	35.068	1.075	-4.791	1.00	0.00	RX1	C
ATOM	4774	CD	GLN	276	33.957	0.056	-4.978	1.00	0.00	RX1	C
ATOM	4775	OE1	GLN	276	34.129	-1.110	-4.642	1.00	0.00	RX1	O
ATOM	4776	NE2	GLN	276	32.822	0.533	-5.524	1.00	0.00	RX1	N
ATOM	4777	HE21	GLN	276	32.660	1.489	-5.780	1.00	0.00	RX1	H
ATOM	4778	HE22	GLN	276	32.034	-0.070	-5.653	1.00	0.00	RX1	H
ATOM	4779	C	GLN	276	34.447	2.291	-1.519	1.00	0.00	RX1	C
ATOM	4780	O	GLN	276	34.010	3.175	-0.798	1.00	0.00	RX1	O
ATOM	4781	N	MET	277	35.521	1.551	-1.229	1.00	0.00	RX1	N
ATOM	4782	H	MET	277	35.944	0.913	-1.869	1.00	0.00	RX1	H
ATOM	4783	CA	MET	277	36.208	1.904	0.011	1.00	0.00	RX1	C
ATOM	4784	CB	MET	277	36.707	0.639	0.709	1.00	0.00	RX1	C
ATOM	4785	CG	MET	277	37.032	0.853	2.188	1.00	0.00	RX1	C
ATOM	4786	SD	MET	277	35.570	1.146	3.199	1.00	0.00	RX1	S
ATOM	4787	CE	MET	277	34.881	-0.516	3.149	1.00	0.00	RX1	C
ATOM	4788	C	MET	277	37.341	2.882	-0.244	1.00	0.00	RX1	C
ATOM	4789	O	MET	277	38.370	2.532	-0.812	1.00	0.00	RX1	O
ATOM	4790	N	ASP	278	37.081	4.127	0.164	1.00	0.00	RX1	N
ATOM	4791	H	ASP	278	36.284	4.303	0.740	1.00	0.00	RX1	H
ATOM	4792	CA	ASP	278	38.067	5.180	-0.077	1.00	0.00	RX1	C
ATOM	4793	CB	ASP	278	37.446	6.578	0.021	1.00	0.00	RX1	C
ATOM	4794	CG	ASP	278	36.301	6.804	-0.949	1.00	0.00	RX1	C
ATOM	4795	OD1	ASP	278	36.258	6.190	-2.013	1.00	0.00	RX1	O
ATOM	4796	OD2	ASP	278	35.444	7.627	-0.644	1.00	0.00	RX1	O
ATOM	4797	C	ASP	278	39.205	5.108	0.923	1.00	0.00	RX1	C
ATOM	4798	O	ASP	278	39.110	4.463	1.961	1.00	0.00	RX1	O
ATOM	4799	N	VAL	279	40.294	5.807	0.588	1.00	0.00	RX1	N
ATOM	4800	H	VAL	279	40.311	6.426	-0.197	1.00	0.00	RX1	H
ATOM	4801	CA	VAL	279	41.414	5.793	1.527	1.00	0.00	RX1	C
ATOM	4802	CB	VAL	279	42.638	5.138	0.868	1.00	0.00	RX1	C
ATOM	4803	CG1	VAL	279	43.078	5.898	-0.385	1.00	0.00	RX1	C
ATOM	4804	CG2	VAL	279	43.781	4.916	1.861	1.00	0.00	RX1	C
ATOM	4805	C	VAL	279	41.710	7.192	2.047	1.00	0.00	RX1	C
ATOM	4806	O	VAL	279	41.611	8.171	1.317	1.00	0.00	RX1	O
ATOM	4807	N	ASN	280	42.038	7.252	3.344	1.00	0.00	RX1	N
ATOM	4808	H	ASN	280	42.177	6.427	3.898	1.00	0.00	RX1	H
ATOM	4809	CA	ASN	280	42.264	8.559	3.962	1.00	0.00	RX1	C
ATOM	4810	CB	ASN	280	41.647	8.555	5.353	1.00	0.00	RX1	C
ATOM	4811	CG	ASN	280	41.785	9.903	6.008	1.00	0.00	RX1	C
ATOM	4812	OD1	ASN	280	42.165	10.904	5.406	1.00	0.00	RX1	O
ATOM	4813	ND2	ASN	280	41.476	9.859	7.310	1.00	0.00	RX1	N
ATOM	4814	HD21	ASN	280	41.064	9.027	7.695	1.00	0.00	RX1	H
ATOM	4815	HD22	ASN	280	41.688	10.602	7.950	1.00	0.00	RX1	H
ATOM	4816	C	ASN	280	43.729	8.944	4.055	1.00	0.00	RX1	C
ATOM	4817	O	ASN	280	44.498	8.316	4.770	1.00	0.00	RX1	O

ATOM	4818	N	PRO	281	44.094	10.034	3.334	1.00	0.00	RX1	N
ATOM	4819	CD	PRO	281	43.275	10.754	2.367	1.00	0.00	RX1	C
ATOM	4820	CA	PRO	281	45.434	10.611	3.505	1.00	0.00	RX1	C
ATOM	4821	CB	PRO	281	45.367	11.867	2.629	1.00	0.00	RX1	C
ATOM	4822	CG	PRO	281	44.290	11.576	1.584	1.00	0.00	RX1	C
ATOM	4823	C	PRO	281	45.783	10.925	4.956	1.00	0.00	RX1	C
ATOM	4824	O	PRO	281	46.828	10.557	5.476	1.00	0.00	RX1	O
ATOM	4825	N	GLU	282	44.828	11.607	5.600	1.00	0.00	RX1	N
ATOM	4826	H	GLU	282	43.927	11.752	5.188	1.00	0.00	RX1	H
ATOM	4827	CA	GLU	282	44.983	11.881	7.024	1.00	0.00	RX1	C
ATOM	4828	CB	GLU	282	44.253	13.170	7.402	1.00	0.00	RX1	C
ATOM	4829	CG	GLU	282	44.998	14.441	6.997	1.00	0.00	RX1	C
ATOM	4830	CD	GLU	282	46.221	14.604	7.876	1.00	0.00	RX1	C
ATOM	4831	OE1	GLU	282	47.202	15.198	7.441	1.00	0.00	RX1	O
ATOM	4832	OE2	GLU	282	46.209	14.140	9.012	1.00	0.00	RX1	O
ATOM	4833	C	GLU	282	44.450	10.740	7.869	1.00	0.00	RX1	C
ATOM	4834	O	GLU	282	43.571	10.908	8.709	1.00	0.00	RX1	O
ATOM	4835	N	GLY	283	45.001	9.548	7.589	1.00	0.00	RX1	N
ATOM	4836	H	GLY	283	45.775	9.492	6.955	1.00	0.00	RX1	H
ATOM	4837	CA	GLY	283	44.567	8.370	8.336	1.00	0.00	RX1	C
ATOM	4838	C	GLY	283	44.944	8.471	9.798	1.00	0.00	RX1	C
ATOM	4839	O	GLY	283	46.106	8.401	10.178	1.00	0.00	RX1	O
ATOM	4840	N	LYS	284	43.910	8.697	10.612	1.00	0.00	RX1	N
ATOM	4841	H	LYS	284	42.948	8.672	10.333	1.00	0.00	RX1	H
ATOM	4842	CA	LYS	284	44.297	9.162	11.931	1.00	0.00	RX1	C
ATOM	4843	CB	LYS	284	43.429	10.337	12.346	1.00	0.00	RX1	C
ATOM	4844	CG	LYS	284	44.332	11.524	12.642	1.00	0.00	RX1	C
ATOM	4845	CD	LYS	284	45.343	11.728	11.518	1.00	0.00	RX1	C
ATOM	4846	CE	LYS	284	46.365	12.817	11.816	1.00	0.00	RX1	C
ATOM	4847	NZ	LYS	284	45.781	14.153	11.651	1.00	0.00	RX1	N
ATOM	4848	HZ1	LYS	284	46.488	14.874	11.901	1.00	0.00	RX1	H
ATOM	4849	HZ2	LYS	284	45.494	14.314	10.664	1.00	0.00	RX1	H
ATOM	4850	HZ3	LYS	284	44.974	14.295	12.300	1.00	0.00	RX1	H
ATOM	4851	C	LYS	284	44.438	8.131	13.020	1.00	0.00	RX1	C
ATOM	4852	O	LYS	284	43.517	7.792	13.759	1.00	0.00	RX1	O
ATOM	4853	N	TYR	285	45.686	7.654	13.081	1.00	0.00	RX1	N
ATOM	4854	H	TYR	285	46.385	8.094	12.512	1.00	0.00	RX1	H
ATOM	4855	CA	TYR	285	46.041	6.680	14.107	1.00	0.00	RX1	C
ATOM	4856	CB	TYR	285	47.499	6.257	13.953	1.00	0.00	RX1	C
ATOM	4857	CG	TYR	285	47.698	5.536	12.640	1.00	0.00	RX1	C
ATOM	4858	CD1	TYR	285	47.973	6.245	11.477	1.00	0.00	RX1	C
ATOM	4859	CE1	TYR	285	48.202	5.574	10.283	1.00	0.00	RX1	C
ATOM	4860	CD2	TYR	285	47.621	4.150	12.595	1.00	0.00	RX1	C
ATOM	4861	CE2	TYR	285	47.850	3.474	11.404	1.00	0.00	RX1	C
ATOM	4862	CZ	TYR	285	48.151	4.186	10.249	1.00	0.00	RX1	C
ATOM	4863	OH	TYR	285	48.400	3.513	9.071	1.00	0.00	RX1	O
ATOM	4864	HH	TYR	285	48.161	2.600	9.171	1.00	0.00	RX1	H
ATOM	4865	C	TYR	285	45.760	7.205	15.501	1.00	0.00	RX1	C
ATOM	4866	O	TYR	285	46.195	8.273	15.922	1.00	0.00	RX1	O
ATOM	4867	N	SER	286	44.928	6.422	16.177	1.00	0.00	RX1	N
ATOM	4868	H	SER	286	44.637	5.515	15.864	1.00	0.00	RX1	H
ATOM	4869	CA	SER	286	44.336	6.967	17.385	1.00	0.00	RX1	C
ATOM	4870	CB	SER	286	42.934	6.411	17.315	1.00	0.00	RX1	C
ATOM	4871	OG	SER	286	42.745	6.056	15.934	1.00	0.00	RX1	O
ATOM	4872	HG	SER	286	42.944	6.827	15.404	1.00	0.00	RX1	H
ATOM	4873	C	SER	286	45.141	6.683	18.637	1.00	0.00	RX1	C
ATOM	4874	O	SER	286	44.925	5.703	19.343	1.00	0.00	RX1	O
ATOM	4875	N	PHE	287	46.092	7.594	18.891	1.00	0.00	RX1	N
ATOM	4876	H	PHE	287	46.198	8.414	18.325	1.00	0.00	RX1	H
ATOM	4877	CA	PHE	287	46.815	7.475	20.154	1.00	0.00	RX1	C
ATOM	4878	CB	PHE	287	48.243	7.996	20.006	1.00	0.00	RX1	C

ATOM	4879	CG	PHE	287	49.087	7.500	21.154	1.00	0.00	RX1	C
ATOM	4880	CD1	PHE	287	49.072	6.151	21.490	1.00	0.00	RX1	C
ATOM	4881	CD2	PHE	287	49.885	8.384	21.870	1.00	0.00	RX1	C
ATOM	4882	CE1	PHE	287	49.857	5.682	22.536	1.00	0.00	RX1	C
ATOM	4883	CE2	PHE	287	50.670	7.916	22.918	1.00	0.00	RX1	C
ATOM	4884	CZ	PHE	287	50.657	6.566	23.250	1.00	0.00	RX1	C
ATOM	4885	C	PHE	287	46.070	8.168	21.285	1.00	0.00	RX1	C
ATOM	4886	O	PHE	287	46.431	9.225	21.792	1.00	0.00	RX1	O
ATOM	4887	N	GLY	288	44.953	7.513	21.635	1.00	0.00	RX1	N
ATOM	4888	H	GLY	288	44.694	6.685	21.135	1.00	0.00	RX1	H
ATOM	4889	CA	GLY	288	44.012	8.192	22.522	1.00	0.00	RX1	C
ATOM	4890	C	GLY	288	43.456	9.444	21.865	1.00	0.00	RX1	C
ATOM	4891	O	GLY	288	43.365	9.534	20.646	1.00	0.00	RX1	O
ATOM	4892	N	ALA	289	43.161	10.431	22.724	1.00	0.00	RX1	N
ATOM	4893	H	ALA	289	43.131	10.242	23.709	1.00	0.00	RX1	H
ATOM	4894	CA	ALA	289	42.758	11.736	22.198	1.00	0.00	RX1	C
ATOM	4895	CB	ALA	289	42.407	12.689	23.342	1.00	0.00	RX1	C
ATOM	4896	C	ALA	289	43.787	12.429	21.317	1.00	0.00	RX1	C
ATOM	4897	O	ALA	289	43.469	13.324	20.539	1.00	0.00	RX1	O
ATOM	4898	N	THR	290	45.040	11.988	21.474	1.00	0.00	RX1	N
ATOM	4899	H	THR	290	45.344	11.259	22.086	1.00	0.00	RX1	H
ATOM	4900	CA	THR	290	46.054	12.540	20.592	1.00	0.00	RX1	C
ATOM	4901	CB	THR	290	47.306	12.412	21.420	1.00	0.00	RX1	C
ATOM	4902	OG1	THR	290	46.896	12.463	22.797	1.00	0.00	RX1	O
ATOM	4903	HG1	THR	290	47.633	12.132	23.292	1.00	0.00	RX1	H
ATOM	4904	CG2	THR	290	48.354	13.477	21.092	1.00	0.00	RX1	C
ATOM	4905	C	THR	290	46.095	11.806	19.264	1.00	0.00	RX1	C
ATOM	4906	O	THR	290	46.851	10.869	19.030	1.00	0.00	RX1	O
ATOM	4907	N	CYS	291	45.217	12.289	18.382	1.00	0.00	RX1	N
ATOM	4908	H	CYS	291	44.606	13.047	18.611	1.00	0.00	RX1	H
ATOM	4909	CA	CYS	291	45.254	11.716	17.042	1.00	0.00	RX1	C
ATOM	4910	CB	CYS	291	43.982	12.118	16.315	1.00	0.00	RX1	C
ATOM	4911	SG	CYS	291	42.626	12.412	17.477	1.00	0.00	RX1	S
ATOM	4912	C	CYS	291	46.523	12.100	16.305	1.00	0.00	RX1	C
ATOM	4913	O	CYS	291	46.935	13.257	16.294	1.00	0.00	RX1	O
ATOM	4914	N	VAL	292	47.147	11.059	15.742	1.00	0.00	RX1	N
ATOM	4915	H	VAL	292	46.754	10.136	15.718	1.00	0.00	RX1	H
ATOM	4916	CA	VAL	292	48.444	11.255	15.101	1.00	0.00	RX1	C
ATOM	4917	CB	VAL	292	49.577	10.704	15.975	1.00	0.00	RX1	C
ATOM	4918	CG1	VAL	292	49.818	11.568	17.215	1.00	0.00	RX1	C
ATOM	4919	CG2	VAL	292	49.317	9.241	16.338	1.00	0.00	RX1	C
ATOM	4920	C	VAL	292	48.471	10.600	13.733	1.00	0.00	RX1	C
ATOM	4921	O	VAL	292	47.624	9.781	13.396	1.00	0.00	RX1	O
ATOM	4922	N	LYS	293	49.487	10.991	12.948	1.00	0.00	RX1	N
ATOM	4923	H	LYS	293	50.131	11.699	13.235	1.00	0.00	RX1	H
ATOM	4924	CA	LYS	293	49.538	10.390	11.616	1.00	0.00	RX1	C
ATOM	4925	CB	LYS	293	50.137	11.370	10.603	1.00	0.00	RX1	C
ATOM	4926	CG	LYS	293	49.192	11.617	9.421	1.00	0.00	RX1	C
ATOM	4927	CD	LYS	293	49.745	12.601	8.384	1.00	0.00	RX1	C
ATOM	4928	CE	LYS	293	50.014	14.005	8.937	1.00	0.00	RX1	C
ATOM	4929	NZ	LYS	293	48.765	14.633	9.378	1.00	0.00	RX1	N
ATOM	4930	HZ1	LYS	293	48.815	15.002	10.355	1.00	0.00	RX1	H
ATOM	4931	HZ2	LYS	293	48.448	15.388	8.735	1.00	0.00	RX1	H
ATOM	4932	HZ3	LYS	293	47.960	13.979	9.318	1.00	0.00	RX1	H
ATOM	4933	C	LYS	293	50.170	9.006	11.508	1.00	0.00	RX1	C
ATOM	4934	O	LYS	293	50.137	8.382	10.457	1.00	0.00	RX1	O
ATOM	4935	N	LYS	294	50.729	8.539	12.647	1.00	0.00	RX1	N
ATOM	4936	H	LYS	294	50.807	9.079	13.485	1.00	0.00	RX1	H
ATOM	4937	CA	LYS	294	51.031	7.110	12.806	1.00	0.00	RX1	C
ATOM	4938	CB	LYS	294	52.117	6.597	11.854	1.00	0.00	RX1	C
ATOM	4939	CG	LYS	294	53.498	7.226	12.019	1.00	0.00	RX1	C

ATOM	4940	CD	LYS	294	54.451	6.638	10.981	1.00	0.00	RX1	C
ATOM	4941	CE	LYS	294	55.865	7.212	11.041	1.00	0.00	RX1	C
ATOM	4942	NZ	LYS	294	56.665	6.588	9.978	1.00	0.00	RX1	N
ATOM	4943	HZ1	LYS	294	57.632	6.969	9.997	1.00	0.00	RX1	H
ATOM	4944	HZ2	LYS	294	56.690	5.559	10.124	1.00	0.00	RX1	H
ATOM	4945	HZ3	LYS	294	56.228	6.793	9.056	1.00	0.00	RX1	H
ATOM	4946	C	LYS	294	51.376	6.742	14.232	1.00	0.00	RX1	C
ATOM	4947	O	LYS	294	51.809	7.574	15.018	1.00	0.00	RX1	O
ATOM	4948	N	CYS	295	51.145	5.456	14.527	1.00	0.00	RX1	N
ATOM	4949	H	CYS	295	50.821	4.808	13.840	1.00	0.00	RX1	H
ATOM	4950	CA	CYS	295	51.380	4.970	15.888	1.00	0.00	RX1	C
ATOM	4951	CB	CYS	295	50.796	3.566	16.009	1.00	0.00	RX1	C
ATOM	4952	SG	CYS	295	49.180	3.455	15.215	1.00	0.00	RX1	S
ATOM	4953	C	CYS	295	52.838	4.970	16.322	1.00	0.00	RX1	C
ATOM	4954	O	CYS	295	53.737	4.674	15.541	1.00	0.00	RX1	O
ATOM	4955	N	PRO	296	53.048	5.295	17.622	1.00	0.00	RX1	N
ATOM	4956	CD	PRO	296	52.073	5.856	18.549	1.00	0.00	RX1	C
ATOM	4957	CA	PRO	296	54.360	5.054	18.237	1.00	0.00	RX1	C
ATOM	4958	CB	PRO	296	54.129	5.473	19.694	1.00	0.00	RX1	C
ATOM	4959	CG	PRO	296	52.933	6.425	19.669	1.00	0.00	RX1	C
ATOM	4960	C	PRO	296	54.785	3.597	18.127	1.00	0.00	RX1	C
ATOM	4961	O	PRO	296	54.009	2.692	18.404	1.00	0.00	RX1	O
ATOM	4962	N	ARG	297	56.056	3.420	17.713	1.00	0.00	RX1	N
ATOM	4963	H	ARG	297	56.585	4.239	17.504	1.00	0.00	RX1	H
ATOM	4964	CA	ARG	297	56.608	2.092	17.402	1.00	0.00	RX1	C
ATOM	4965	CB	ARG	297	58.139	2.133	17.471	1.00	0.00	RX1	C
ATOM	4966	CG	ARG	297	58.741	0.728	17.344	1.00	0.00	RX1	C
ATOM	4967	CD	ARG	297	60.004	0.510	18.178	1.00	0.00	RX1	C
ATOM	4968	NE	ARG	297	61.146	1.199	17.591	1.00	0.00	RX1	N
ATOM	4969	HE	ARG	297	61.088	1.373	16.604	1.00	0.00	RX1	H
ATOM	4970	CZ	ARG	297	62.249	1.421	18.362	1.00	0.00	RX1	C
ATOM	4971	NH1	ARG	297	62.226	1.144	19.686	1.00	0.00	RX1	N
ATOM	4972	HH11	ARG	297	63.029	1.317	20.279	1.00	0.00	RX1	H
ATOM	4973	HH12	ARG	297	61.432	0.765	20.180	1.00	0.00	RX1	H
ATOM	4974	NH2	ARG	297	63.360	1.918	17.782	1.00	0.00	RX1	N
ATOM	4975	HH21	ARG	297	64.200	2.032	18.333	1.00	0.00	RX1	H
ATOM	4976	HH22	ARG	297	63.412	2.173	16.814	1.00	0.00	RX1	H
ATOM	4977	C	ARG	297	56.156	0.918	18.266	1.00	0.00	RX1	C
ATOM	4978	O	ARG	297	55.831	-0.159	17.786	1.00	0.00	RX1	O
ATOM	4979	N	ASN	298	56.240	1.159	19.580	1.00	0.00	RX1	N
ATOM	4980	H	ASN	298	56.245	2.082	19.963	1.00	0.00	RX1	H
ATOM	4981	CA	ASN	298	56.072	0.010	20.463	1.00	0.00	RX1	C
ATOM	4982	CB	ASN	298	56.715	0.226	21.831	1.00	0.00	RX1	C
ATOM	4983	CG	ASN	298	55.866	1.179	22.641	1.00	0.00	RX1	C
ATOM	4984	OD1	ASN	298	55.195	2.054	22.103	1.00	0.00	RX1	O
ATOM	4985	ND2	ASN	298	55.966	0.986	23.964	1.00	0.00	RX1	N
ATOM	4986	HD21	ASN	298	56.544	0.255	24.339	1.00	0.00	RX1	H
ATOM	4987	HD22	ASN	298	55.551	1.604	24.630	1.00	0.00	RX1	H
ATOM	4988	C	ASN	298	54.645	-0.466	20.634	1.00	0.00	RX1	C
ATOM	4989	O	ASN	298	54.409	-1.601	21.033	1.00	0.00	RX1	O
ATOM	4990	N	TYR	299	53.699	0.440	20.360	1.00	0.00	RX1	N
ATOM	4991	H	TYR	299	53.912	1.293	19.881	1.00	0.00	RX1	H
ATOM	4992	CA	TYR	299	52.310	0.035	20.536	1.00	0.00	RX1	C
ATOM	4993	CB	TYR	299	51.402	1.263	20.658	1.00	0.00	RX1	C
ATOM	4994	CG	TYR	299	51.310	1.677	22.109	1.00	0.00	RX1	C
ATOM	4995	CD1	TYR	299	52.313	2.432	22.706	1.00	0.00	RX1	C
ATOM	4996	CE1	TYR	299	52.243	2.747	24.060	1.00	0.00	RX1	C
ATOM	4997	CD2	TYR	299	50.212	1.281	22.860	1.00	0.00	RX1	C
ATOM	4998	CE2	TYR	299	50.136	1.604	24.207	1.00	0.00	RX1	C
ATOM	4999	CZ	TYR	299	51.161	2.315	24.818	1.00	0.00	RX1	C
ATOM	5000	OH	TYR	299	51.096	2.568	26.175	1.00	0.00	RX1	O

ATOM	5001	HH	TYR	299	51.917	2.949	26.483	1.00	0.00	RX1	H
ATOM	5002	C	TYR	299	51.846	-0.891	19.434	1.00	0.00	RX1	C
ATOM	5003	O	TYR	299	52.315	-0.836	18.306	1.00	0.00	RX1	O
ATOM	5004	N	VAL	300	50.918	-1.773	19.828	1.00	0.00	RX1	N
ATOM	5005	H	VAL	300	50.432	-1.638	20.689	1.00	0.00	RX1	H
ATOM	5006	CA	VAL	300	50.330	-2.625	18.799	1.00	0.00	RX1	C
ATOM	5007	CB	VAL	300	49.469	-3.735	19.395	1.00	0.00	RX1	C
ATOM	5008	CG1	VAL	300	49.100	-4.754	18.326	1.00	0.00	RX1	C
ATOM	5009	CG2	VAL	300	50.104	-4.396	20.603	1.00	0.00	RX1	C
ATOM	5010	C	VAL	300	49.462	-1.778	17.899	1.00	0.00	RX1	C
ATOM	5011	O	VAL	300	48.564	-1.075	18.346	1.00	0.00	RX1	O
ATOM	5012	N	VAL	301	49.776	-1.857	16.614	1.00	0.00	RX1	N
ATOM	5013	H	VAL	301	50.454	-2.521	16.292	1.00	0.00	RX1	H
ATOM	5014	CA	VAL	301	48.888	-1.138	15.716	1.00	0.00	RX1	C
ATOM	5015	CB	VAL	301	49.702	-0.632	14.524	1.00	0.00	RX1	C
ATOM	5016	CG1	VAL	301	48.935	0.353	13.641	1.00	0.00	RX1	C
ATOM	5017	CG2	VAL	301	51.016	-0.041	15.033	1.00	0.00	RX1	C
ATOM	5018	C	VAL	301	47.760	-2.073	15.312	1.00	0.00	RX1	C
ATOM	5019	O	VAL	301	47.937	-3.284	15.240	1.00	0.00	RX1	O
ATOM	5020	N	THR	302	46.592	-1.497	15.040	1.00	0.00	RX1	N
ATOM	5021	H	THR	302	46.389	-0.528	15.184	1.00	0.00	RX1	H
ATOM	5022	CA	THR	302	45.722	-2.319	14.215	1.00	0.00	RX1	C
ATOM	5023	CB	THR	302	44.328	-2.401	14.831	1.00	0.00	RX1	C
ATOM	5024	OG1	THR	302	43.710	-1.114	14.833	1.00	0.00	RX1	O
ATOM	5025	HG1	THR	302	42.825	-1.247	15.172	1.00	0.00	RX1	H
ATOM	5026	CG2	THR	302	44.368	-2.975	16.248	1.00	0.00	RX1	C
ATOM	5027	C	THR	302	45.721	-1.753	12.815	1.00	0.00	RX1	C
ATOM	5028	O	THR	302	45.963	-0.568	12.622	1.00	0.00	RX1	O
ATOM	5029	N	ASP	303	45.380	-2.612	11.849	1.00	0.00	RX1	N
ATOM	5030	H	ASP	303	45.327	-3.605	11.980	1.00	0.00	RX1	H
ATOM	5031	CA	ASP	303	45.184	-2.090	10.492	1.00	0.00	RX1	C
ATOM	5032	CB	ASP	303	44.934	-3.251	9.523	1.00	0.00	RX1	C
ATOM	5033	CG	ASP	303	46.001	-4.324	9.677	1.00	0.00	RX1	C
ATOM	5034	OD1	ASP	303	46.902	-4.416	8.849	1.00	0.00	RX1	O
ATOM	5035	OD2	ASP	303	45.928	-5.126	10.604	1.00	0.00	RX1	O
ATOM	5036	C	ASP	303	44.078	-1.037	10.387	1.00	0.00	RX1	C
ATOM	5037	O	ASP	303	44.025	-0.215	9.483	1.00	0.00	RX1	O
ATOM	5038	N	HIS	304	43.194	-1.086	11.401	1.00	0.00	RX1	N
ATOM	5039	H	HIS	304	43.303	-1.769	12.119	1.00	0.00	RX1	H
ATOM	5040	CA	HIS	304	42.159	-0.062	11.545	1.00	0.00	RX1	C
ATOM	5041	CB	HIS	304	41.193	-0.562	12.619	1.00	0.00	RX1	C
ATOM	5042	CG	HIS	304	39.820	0.064	12.555	1.00	0.00	RX1	C
ATOM	5043	ND1	HIS	304	39.519	1.264	12.023	1.00	0.00	RX1	N
ATOM	5044	HD1	HIS	304	40.155	1.908	11.634	1.00	0.00	RX1	H
ATOM	5045	CD2	HIS	304	38.645	-0.519	13.036	1.00	0.00	RX1	C
ATOM	5046	NE2	HIS	304	37.627	0.336	12.784	1.00	0.00	RX1	N
ATOM	5047	CE1	HIS	304	38.163	1.437	12.160	1.00	0.00	RX1	C
ATOM	5048	C	HIS	304	42.684	1.322	11.912	1.00	0.00	RX1	C
ATOM	5049	O	HIS	304	42.097	2.340	11.558	1.00	0.00	RX1	O
ATOM	5050	N	GLY	305	43.804	1.306	12.653	1.00	0.00	RX1	N
ATOM	5051	H	GLY	305	44.259	0.458	12.931	1.00	0.00	RX1	H
ATOM	5052	CA	GLY	305	44.390	2.576	13.073	1.00	0.00	RX1	C
ATOM	5053	C	GLY	305	44.567	2.746	14.573	1.00	0.00	RX1	C
ATOM	5054	O	GLY	305	45.022	3.775	15.061	1.00	0.00	RX1	O
ATOM	5055	N	SER	306	44.179	1.695	15.297	1.00	0.00	RX1	N
ATOM	5056	H	SER	306	43.811	0.844	14.926	1.00	0.00	RX1	H
ATOM	5057	CA	SER	306	44.220	1.804	16.750	1.00	0.00	RX1	C
ATOM	5058	CB	SER	306	43.133	0.843	17.178	1.00	0.00	RX1	C
ATOM	5059	OG	SER	306	42.187	0.833	16.094	1.00	0.00	RX1	O
ATOM	5060	HG	SER	306	41.603	0.089	16.226	1.00	0.00	RX1	H
ATOM	5061	C	SER	306	45.608	1.572	17.325	1.00	0.00	RX1	C

ATOM	5062	O	SER	306	46.316	0.664	16.908	1.00	0.00	RX1	O
ATOM	5063	N	CYS	307	45.965	2.423	18.299	1.00	0.00	RX1	N
ATOM	5064	H	CYS	307	45.371	3.157	18.631	1.00	0.00	RX1	H
ATOM	5065	CA	CYS	307	47.267	2.233	18.942	1.00	0.00	RX1	C
ATOM	5066	CB	CYS	307	47.892	3.600	19.190	1.00	0.00	RX1	C
ATOM	5067	SG	CYS	307	47.769	4.685	17.745	1.00	0.00	RX1	S
ATOM	5068	C	CYS	307	47.181	1.423	20.224	1.00	0.00	RX1	C
ATOM	5069	O	CYS	307	47.406	1.921	21.320	1.00	0.00	RX1	O
ATOM	5070	N	VAL	308	46.791	0.156	20.050	1.00	0.00	RX1	N
ATOM	5071	H	VAL	308	46.933	-0.274	19.155	1.00	0.00	RX1	H
ATOM	5072	CA	VAL	308	46.493	-0.617	21.253	1.00	0.00	RX1	C
ATOM	5073	CB	VAL	308	45.531	-1.768	20.931	1.00	0.00	RX1	C
ATOM	5074	CG1	VAL	308	44.241	-1.232	20.315	1.00	0.00	RX1	C
ATOM	5075	CG2	VAL	308	46.154	-2.800	20.001	1.00	0.00	RX1	C
ATOM	5076	C	VAL	308	47.716	-1.085	22.038	1.00	0.00	RX1	C
ATOM	5077	O	VAL	308	48.827	-1.201	21.541	1.00	0.00	RX1	O
ATOM	5078	N	ARG	309	47.453	-1.372	23.320	1.00	0.00	RX1	N
ATOM	5079	H	ARG	309	46.528	-1.267	23.668	1.00	0.00	RX1	H
ATOM	5080	CA	ARG	309	48.500	-1.989	24.139	1.00	0.00	RX1	C
ATOM	5081	CB	ARG	309	48.228	-1.851	25.632	1.00	0.00	RX1	C
ATOM	5082	CG	ARG	309	48.129	-0.453	26.230	1.00	0.00	RX1	C
ATOM	5083	CD	ARG	309	47.667	-0.633	27.673	1.00	0.00	RX1	C
ATOM	5084	NE	ARG	309	47.461	0.615	28.400	1.00	0.00	RX1	N
ATOM	5085	HE	ARG	309	48.136	1.350	28.277	1.00	0.00	RX1	H
ATOM	5086	CZ	ARG	309	46.496	0.565	29.368	1.00	0.00	RX1	C
ATOM	5087	NH1	ARG	309	45.609	-0.451	29.365	1.00	0.00	RX1	N
ATOM	5088	HH11	ARG	309	44.983	-0.543	30.158	1.00	0.00	RX1	H
ATOM	5089	HH12	ARG	309	45.524	-1.125	28.627	1.00	0.00	RX1	H
ATOM	5090	NH2	ARG	309	46.439	1.507	30.330	1.00	0.00	RX1	N
ATOM	5091	HH21	ARG	309	45.782	1.403	31.104	1.00	0.00	RX1	H
ATOM	5092	HH22	ARG	309	47.023	2.317	30.367	1.00	0.00	RX1	H
ATOM	5093	C	ARG	309	48.659	-3.479	23.897	1.00	0.00	RX1	C
ATOM	5094	O	ARG	309	49.714	-4.062	24.123	1.00	0.00	RX1	O
ATOM	5095	N	ALA	310	47.529	-4.077	23.501	1.00	0.00	RX1	N
ATOM	5096	H	ALA	310	46.762	-3.611	23.066	1.00	0.00	RX1	H
ATOM	5097	CA	ALA	310	47.413	-5.515	23.695	1.00	0.00	RX1	C
ATOM	5098	CB	ALA	310	46.291	-5.809	24.688	1.00	0.00	RX1	C
ATOM	5099	C	ALA	310	47.085	-6.235	22.412	1.00	0.00	RX1	C
ATOM	5100	O	ALA	310	46.782	-5.633	21.391	1.00	0.00	RX1	O
ATOM	5101	N	CYS	311	47.127	-7.568	22.518	1.00	0.00	RX1	N
ATOM	5102	H	CYS	311	47.330	-8.022	23.383	1.00	0.00	RX1	H
ATOM	5103	CA	CYS	311	46.545	-8.331	21.419	1.00	0.00	RX1	C
ATOM	5104	CB	CYS	311	47.023	-9.776	21.511	1.00	0.00	RX1	C
ATOM	5105	SG	CYS	311	48.818	-9.881	21.725	1.00	0.00	RX1	S
ATOM	5106	C	CYS	311	45.031	-8.251	21.464	1.00	0.00	RX1	C
ATOM	5107	O	CYS	311	44.430	-8.296	22.532	1.00	0.00	RX1	O
ATOM	5108	N	GLY	312	44.443	-8.158	20.259	1.00	0.00	RX1	N
ATOM	5109	H	GLY	312	44.980	-8.124	19.421	1.00	0.00	RX1	H
ATOM	5110	CA	GLY	312	42.994	-8.351	20.186	1.00	0.00	RX1	C
ATOM	5111	C	GLY	312	42.613	-9.754	20.631	1.00	0.00	RX1	C
ATOM	5112	O	GLY	312	43.466	-10.629	20.712	1.00	0.00	RX1	O
ATOM	5113	N	ALA	313	41.316	-9.929	20.924	1.00	0.00	RX1	N
ATOM	5114	H	ALA	313	40.701	-9.146	20.822	1.00	0.00	RX1	H
ATOM	5115	CA	ALA	313	40.854	-11.164	21.569	1.00	0.00	RX1	C
ATOM	5116	CB	ALA	313	39.327	-11.239	21.519	1.00	0.00	RX1	C
ATOM	5117	C	ALA	313	41.423	-12.481	21.049	1.00	0.00	RX1	C
ATOM	5118	O	ALA	313	42.020	-13.261	21.781	1.00	0.00	RX1	O
ATOM	5119	N	ASP	314	41.210	-12.681	19.747	1.00	0.00	RX1	N
ATOM	5120	H	ASP	314	40.809	-11.985	19.147	1.00	0.00	RX1	H
ATOM	5121	CA	ASP	314	41.675	-13.884	19.049	1.00	0.00	RX1	C
ATOM	5122	CB	ASP	314	40.614	-14.349	18.046	1.00	0.00	RX1	C

ATOM	5123	CG	ASP	314	40.284	-13.260	17.034	1.00	0.00	RX1	C
ATOM	5124	OD1	ASP	314	40.934	-12.219	17.015	1.00	0.00	RX1	O
ATOM	5125	OD2	ASP	314	39.363	-13.455	16.248	1.00	0.00	RX1	O
ATOM	5126	C	ASP	314	43.029	-13.728	18.363	1.00	0.00	RX1	C
ATOM	5127	O	ASP	314	43.508	-14.570	17.610	1.00	0.00	RX1	O
ATOM	5128	N	SER	315	43.620	-12.565	18.627	1.00	0.00	RX1	N
ATOM	5129	H	SER	315	43.314	-11.907	19.310	1.00	0.00	RX1	H
ATOM	5130	CA	SER	315	44.762	-12.165	17.828	1.00	0.00	RX1	C
ATOM	5131	CB	SER	315	44.412	-10.744	17.434	1.00	0.00	RX1	C
ATOM	5132	OG	SER	315	43.039	-10.537	17.797	1.00	0.00	RX1	O
ATOM	5133	HG	SER	315	42.506	-11.165	17.305	1.00	0.00	RX1	H
ATOM	5134	C	SER	315	46.077	-12.350	18.558	1.00	0.00	RX1	C
ATOM	5135	O	SER	315	46.123	-12.502	19.772	1.00	0.00	RX1	O
ATOM	5136	N	TYR	316	47.159	-12.311	17.771	1.00	0.00	RX1	N
ATOM	5137	H	TYR	316	47.116	-12.171	16.778	1.00	0.00	RX1	H
ATOM	5138	CA	TYR	316	48.450	-12.219	18.441	1.00	0.00	RX1	C
ATOM	5139	CB	TYR	316	49.328	-13.476	18.308	1.00	0.00	RX1	C
ATOM	5140	CG	TYR	316	50.371	-13.440	19.410	1.00	0.00	RX1	C
ATOM	5141	CD1	TYR	316	49.979	-13.626	20.730	1.00	0.00	RX1	C
ATOM	5142	CE1	TYR	316	50.899	-13.483	21.763	1.00	0.00	RX1	C
ATOM	5143	CD2	TYR	316	51.709	-13.175	19.129	1.00	0.00	RX1	C
ATOM	5144	CE2	TYR	316	52.629	-13.016	20.165	1.00	0.00	RX1	C
ATOM	5145	CZ	TYR	316	52.221	-13.154	21.488	1.00	0.00	RX1	C
ATOM	5146	OH	TYR	316	53.106	-12.978	22.536	1.00	0.00	RX1	O
ATOM	5147	HH	TYR	316	53.691	-12.231	22.380	1.00	0.00	RX1	H
ATOM	5148	C	TYR	316	49.176	-10.973	17.978	1.00	0.00	RX1	C
ATOM	5149	O	TYR	316	48.931	-10.471	16.887	1.00	0.00	RX1	O
ATOM	5150	N	GLU	317	50.066	-10.490	18.856	1.00	0.00	RX1	N
ATOM	5151	H	GLU	317	50.232	-10.938	19.733	1.00	0.00	RX1	H
ATOM	5152	CA	GLU	317	50.935	-9.390	18.451	1.00	0.00	RX1	C
ATOM	5153	CB	GLU	317	51.511	-8.691	19.684	1.00	0.00	RX1	C
ATOM	5154	CG	GLU	317	52.069	-7.313	19.337	1.00	0.00	RX1	C
ATOM	5155	CD	GLU	317	53.055	-6.845	20.392	1.00	0.00	RX1	C
ATOM	5156	OE1	GLU	317	54.117	-6.359	20.031	1.00	0.00	RX1	O
ATOM	5157	OE2	GLU	317	52.791	-6.924	21.584	1.00	0.00	RX1	O
ATOM	5158	C	GLU	317	52.066	-9.879	17.560	1.00	0.00	RX1	C
ATOM	5159	O	GLU	317	53.079	-10.398	18.025	1.00	0.00	RX1	O
ATOM	5160	N	MET	318	51.835	-9.712	16.258	1.00	0.00	RX1	N
ATOM	5161	H	MET	318	51.050	-9.185	15.923	1.00	0.00	RX1	H
ATOM	5162	CA	MET	318	52.824	-10.178	15.296	1.00	0.00	RX1	C
ATOM	5163	CB	MET	318	52.165	-11.173	14.340	1.00	0.00	RX1	C
ATOM	5164	CG	MET	318	53.160	-12.087	13.624	1.00	0.00	RX1	C
ATOM	5165	SD	MET	318	54.016	-13.170	14.779	1.00	0.00	RX1	S
ATOM	5166	CE	MET	318	52.577	-14.033	15.430	1.00	0.00	RX1	C
ATOM	5167	C	MET	318	53.421	-9.012	14.538	1.00	0.00	RX1	C
ATOM	5168	O	MET	318	52.756	-8.020	14.273	1.00	0.00	RX1	O
ATOM	5169	N	GLU	319	54.704	-9.148	14.208	1.00	0.00	RX1	N
ATOM	5170	H	GLU	319	55.185	-10.019	14.326	1.00	0.00	RX1	H
ATOM	5171	CA	GLU	319	55.277	-8.134	13.329	1.00	0.00	RX1	C
ATOM	5172	CB	GLU	319	56.812	-8.083	13.410	1.00	0.00	RX1	C
ATOM	5173	CG	GLU	319	57.560	-9.421	13.333	1.00	0.00	RX1	C
ATOM	5174	CD	GLU	319	57.514	-10.116	14.681	1.00	0.00	RX1	C
ATOM	5175	OE1	GLU	319	58.167	-9.658	15.612	1.00	0.00	RX1	O
ATOM	5176	OE2	GLU	319	56.778	-11.085	14.840	1.00	0.00	RX1	O
ATOM	5177	C	GLU	319	54.805	-8.326	11.901	1.00	0.00	RX1	C
ATOM	5178	O	GLU	319	54.775	-9.433	11.377	1.00	0.00	RX1	O
ATOM	5179	N	GLU	320	54.394	-7.198	11.314	1.00	0.00	RX1	N
ATOM	5180	H	GLU	320	54.447	-6.307	11.769	1.00	0.00	RX1	H
ATOM	5181	CA	GLU	320	53.867	-7.304	9.958	1.00	0.00	RX1	C
ATOM	5182	CB	GLU	320	52.342	-7.184	9.988	1.00	0.00	RX1	C
ATOM	5183	CG	GLU	320	51.635	-7.644	8.711	1.00	0.00	RX1	C

ATOM	5184	CD	GLU	320	50.151	-7.395	8.877	1.00	0.00	RX1	C
ATOM	5185	OE1	GLU	320	49.545	-6.747	8.027	1.00	0.00	RX1	O
ATOM	5186	OE2	GLU	320	49.588	-7.787	9.893	1.00	0.00	RX1	O
ATOM	5187	C	GLU	320	54.545	-6.304	9.034	1.00	0.00	RX1	C
ATOM	5188	O	GLU	320	55.643	-6.543	8.544	1.00	0.00	RX1	O
ATOM	5189	N	ASP	321	53.897	-5.148	8.839	1.00	0.00	RX1	N
ATOM	5190	H	ASP	321	53.058	-4.872	9.310	1.00	0.00	RX1	H
ATOM	5191	CA	ASP	321	54.538	-4.101	8.042	1.00	0.00	RX1	C
ATOM	5192	CB	ASP	321	53.515	-3.247	7.274	1.00	0.00	RX1	C
ATOM	5193	CG	ASP	321	52.170	-3.135	7.977	1.00	0.00	RX1	C
ATOM	5194	OD1	ASP	321	51.147	-3.189	7.302	1.00	0.00	RX1	O
ATOM	5195	OD2	ASP	321	52.106	-3.025	9.198	1.00	0.00	RX1	O
ATOM	5196	C	ASP	321	55.489	-3.235	8.850	1.00	0.00	RX1	C
ATOM	5197	O	ASP	321	55.346	-2.031	9.027	1.00	0.00	RX1	O
ATOM	5198	N	GLY	322	56.514	-3.930	9.366	1.00	0.00	RX1	N
ATOM	5199	H	GLY	322	56.550	-4.923	9.239	1.00	0.00	RX1	H
ATOM	5200	CA	GLY	322	57.465	-3.241	10.234	1.00	0.00	RX1	C
ATOM	5201	C	GLY	322	56.992	-3.135	11.672	1.00	0.00	RX1	C
ATOM	5202	O	GLY	322	57.605	-3.645	12.600	1.00	0.00	RX1	O
ATOM	5203	N	VAL	323	55.858	-2.440	11.817	1.00	0.00	RX1	N
ATOM	5204	H	VAL	323	55.367	-2.127	11.002	1.00	0.00	RX1	H
ATOM	5205	CA	VAL	323	55.246	-2.399	13.142	1.00	0.00	RX1	C
ATOM	5206	CB	VAL	323	54.204	-1.285	13.209	1.00	0.00	RX1	C
ATOM	5207	CG1	VAL	323	54.872	0.086	13.090	1.00	0.00	RX1	C
ATOM	5208	CG2	VAL	323	53.117	-1.489	12.153	1.00	0.00	RX1	C
ATOM	5209	C	VAL	323	54.642	-3.734	13.542	1.00	0.00	RX1	C
ATOM	5210	O	VAL	323	54.389	-4.606	12.716	1.00	0.00	RX1	O
ATOM	5211	N	ARG	324	54.427	-3.857	14.858	1.00	0.00	RX1	N
ATOM	5212	H	ARG	324	54.529	-3.096	15.498	1.00	0.00	RX1	H
ATOM	5213	CA	ARG	324	53.743	-5.073	15.277	1.00	0.00	RX1	C
ATOM	5214	CB	ARG	324	54.340	-5.656	16.561	1.00	0.00	RX1	C
ATOM	5215	CG	ARG	324	55.872	-5.642	16.573	1.00	0.00	RX1	C
ATOM	5216	CD	ARG	324	56.498	-6.529	17.657	1.00	0.00	RX1	C
ATOM	5217	NE	ARG	324	56.603	-7.920	17.213	1.00	0.00	RX1	N
ATOM	5218	HE	ARG	324	57.248	-8.136	16.464	1.00	0.00	RX1	H
ATOM	5219	CZ	ARG	324	55.858	-8.920	17.765	1.00	0.00	RX1	C
ATOM	5220	NH1	ARG	324	55.013	-8.649	18.771	1.00	0.00	RX1	N
ATOM	5221	HH11	ARG	324	54.417	-9.364	19.156	1.00	0.00	RX1	H
ATOM	5222	HH12	ARG	324	54.907	-7.725	19.168	1.00	0.00	RX1	H
ATOM	5223	NH2	ARG	324	55.962	-10.174	17.292	1.00	0.00	RX1	N
ATOM	5224	HH21	ARG	324	55.402	-10.937	17.622	1.00	0.00	RX1	H
ATOM	5225	HH22	ARG	324	56.623	-10.387	16.545	1.00	0.00	RX1	H
ATOM	5226	C	ARG	324	52.257	-4.824	15.410	1.00	0.00	RX1	C
ATOM	5227	O	ARG	324	51.805	-3.972	16.167	1.00	0.00	RX1	O
ATOM	5228	N	LYS	325	51.517	-5.568	14.592	1.00	0.00	RX1	N
ATOM	5229	H	LYS	325	51.908	-6.343	14.093	1.00	0.00	RX1	H
ATOM	5230	CA	LYS	325	50.073	-5.409	14.640	1.00	0.00	RX1	C
ATOM	5231	CB	LYS	325	49.527	-5.112	13.247	1.00	0.00	RX1	C
ATOM	5232	CG	LYS	325	50.023	-3.748	12.774	1.00	0.00	RX1	C
ATOM	5233	CD	LYS	325	49.469	-3.379	11.408	1.00	0.00	RX1	C
ATOM	5234	CE	LYS	325	49.731	-4.564	10.500	1.00	0.00	RX1	C
ATOM	5235	NZ	LYS	325	49.587	-4.211	9.088	1.00	0.00	RX1	N
ATOM	5236	HZ1	LYS	325	49.814	-5.038	8.497	1.00	0.00	RX1	H
ATOM	5237	HZ2	LYS	325	48.621	-3.899	8.860	1.00	0.00	RX1	H
ATOM	5238	HZ3	LYS	325	50.292	-3.482	8.824	1.00	0.00	RX1	H
ATOM	5239	C	LYS	325	49.413	-6.622	15.244	1.00	0.00	RX1	C
ATOM	5240	O	LYS	325	50.047	-7.638	15.499	1.00	0.00	RX1	O
ATOM	5241	N	CYS	326	48.104	-6.494	15.465	1.00	0.00	RX1	N
ATOM	5242	H	CYS	326	47.585	-5.655	15.290	1.00	0.00	RX1	H
ATOM	5243	CA	CYS	326	47.452	-7.724	15.890	1.00	0.00	RX1	C
ATOM	5244	CB	CYS	326	46.817	-7.529	17.265	1.00	0.00	RX1	C

ATOM	5245	SG	CYS	326	45.799	-6.039	17.387	1.00	0.00	RX1	S
ATOM	5246	C	CYS	326	46.495	-8.269	14.859	1.00	0.00	RX1	C
ATOM	5247	O	CYS	326	45.454	-7.695	14.569	1.00	0.00	RX1	O
ATOM	5248	N	LYS	327	46.906	-9.416	14.307	1.00	0.00	RX1	N
ATOM	5249	H	LYS	327	47.735	-9.886	14.617	1.00	0.00	RX1	H
ATOM	5250	CA	LYS	327	45.934	-10.129	13.488	1.00	0.00	RX1	C
ATOM	5251	CB	LYS	327	46.414	-10.321	12.048	1.00	0.00	RX1	C
ATOM	5252	CG	LYS	327	46.270	-9.052	11.204	1.00	0.00	RX1	C
ATOM	5253	CD	LYS	327	46.484	-9.330	9.714	1.00	0.00	RX1	C
ATOM	5254	CE	LYS	327	46.237	-8.105	8.830	1.00	0.00	RX1	C
ATOM	5255	NZ	LYS	327	47.208	-7.060	9.149	1.00	0.00	RX1	N
ATOM	5256	HZ1	LYS	327	47.810	-6.840	8.330	1.00	0.00	RX1	H
ATOM	5257	HZ2	LYS	327	46.761	-6.171	9.460	1.00	0.00	RX1	H
ATOM	5258	HZ3	LYS	327	47.891	-7.372	9.868	1.00	0.00	RX1	H
ATOM	5259	C	LYS	327	45.590	-11.453	14.123	1.00	0.00	RX1	C
ATOM	5260	O	LYS	327	46.321	-11.970	14.964	1.00	0.00	RX1	O
ATOM	5261	N	LYS	328	44.424	-11.958	13.712	1.00	0.00	RX1	N
ATOM	5262	H	LYS	328	43.940	-11.599	12.917	1.00	0.00	RX1	H
ATOM	5263	CA	LYS	328	43.929	-13.131	14.416	1.00	0.00	RX1	C
ATOM	5264	CB	LYS	328	42.408	-13.234	14.327	1.00	0.00	RX1	C
ATOM	5265	CG	LYS	328	41.853	-13.488	12.928	1.00	0.00	RX1	C
ATOM	5266	CD	LYS	328	40.329	-13.639	12.892	1.00	0.00	RX1	C
ATOM	5267	CE	LYS	328	39.759	-15.035	13.180	1.00	0.00	RX1	C
ATOM	5268	NZ	LYS	328	39.917	-15.490	14.568	1.00	0.00	RX1	N
ATOM	5269	HZ1	LYS	328	39.449	-16.422	14.673	1.00	0.00	RX1	H
ATOM	5270	HZ2	LYS	328	40.909	-15.632	14.832	1.00	0.00	RX1	H
ATOM	5271	HZ3	LYS	328	39.484	-14.833	15.248	1.00	0.00	RX1	H
ATOM	5272	C	LYS	328	44.567	-14.439	14.020	1.00	0.00	RX1	C
ATOM	5273	O	LYS	328	44.895	-14.696	12.868	1.00	0.00	RX1	O
ATOM	5274	N	CYS	329	44.684	-15.279	15.045	1.00	0.00	RX1	N
ATOM	5275	H	CYS	329	44.382	-15.023	15.966	1.00	0.00	RX1	H
ATOM	5276	CA	CYS	329	44.708	-16.696	14.723	1.00	0.00	RX1	C
ATOM	5277	CB	CYS	329	45.827	-17.356	15.526	1.00	0.00	RX1	C
ATOM	5278	SG	CYS	329	45.981	-16.719	17.213	1.00	0.00	RX1	S
ATOM	5279	C	CYS	329	43.312	-17.220	15.009	1.00	0.00	RX1	C
ATOM	5280	O	CYS	329	42.367	-16.436	15.061	1.00	0.00	RX1	O
ATOM	5281	N	GLU	330	43.187	-18.539	15.212	1.00	0.00	RX1	N
ATOM	5282	H	GLU	330	43.909	-19.224	15.185	1.00	0.00	RX1	H
ATOM	5283	CA	GLU	330	41.882	-18.889	15.753	1.00	0.00	RX1	C
ATOM	5284	CB	GLU	330	41.195	-19.955	14.898	1.00	0.00	RX1	C
ATOM	5285	CG	GLU	330	39.680	-19.887	15.093	1.00	0.00	RX1	C
ATOM	5286	CD	GLU	330	39.271	-18.442	14.891	1.00	0.00	RX1	C
ATOM	5287	OE1	GLU	330	39.057	-17.721	15.864	1.00	0.00	RX1	O
ATOM	5288	OE2	GLU	330	39.219	-17.999	13.753	1.00	0.00	RX1	O
ATOM	5289	C	GLU	330	41.936	-19.277	17.212	1.00	0.00	RX1	C
ATOM	5290	O	GLU	330	42.902	-19.886	17.676	1.00	0.00	RX1	O
ATOM	5291	N	GLY	331	40.884	-18.850	17.922	1.00	0.00	RX1	N
ATOM	5292	H	GLY	331	40.179	-18.323	17.437	1.00	0.00	RX1	H
ATOM	5293	CA	GLY	331	40.904	-18.909	19.378	1.00	0.00	RX1	C
ATOM	5294	C	GLY	331	41.735	-17.776	19.951	1.00	0.00	RX1	C
ATOM	5295	O	GLY	331	42.633	-17.260	19.297	1.00	0.00	RX1	O
ATOM	5296	N	PRO	332	41.417	-17.408	21.212	1.00	0.00	RX1	N
ATOM	5297	CD	PRO	332	40.377	-18.006	22.036	1.00	0.00	RX1	C
ATOM	5298	CA	PRO	332	42.108	-16.292	21.870	1.00	0.00	RX1	C
ATOM	5299	CB	PRO	332	41.573	-16.376	23.303	1.00	0.00	RX1	C
ATOM	5300	CG	PRO	332	40.185	-16.998	23.164	1.00	0.00	RX1	C
ATOM	5301	C	PRO	332	43.629	-16.280	21.797	1.00	0.00	RX1	C
ATOM	5302	O	PRO	332	44.291	-17.271	21.483	1.00	0.00	RX1	O
ATOM	5303	N	CYS	333	44.169	-15.099	22.134	1.00	0.00	RX1	N
ATOM	5304	H	CYS	333	43.570	-14.311	22.290	1.00	0.00	RX1	H
ATOM	5305	CA	CYS	333	45.617	-14.970	22.309	1.00	0.00	RX1	C

ATOM	5306	CB	CYS	333	45.909	-13.560	22.825	1.00	0.00	RX1	C
ATOM	5307	SG	CYS	333	47.676	-13.213	22.994	1.00	0.00	RX1	S
ATOM	5308	C	CYS	333	46.155	-16.054	23.230	1.00	0.00	RX1	C
ATOM	5309	O	CYS	333	45.471	-16.511	24.138	1.00	0.00	RX1	O
ATOM	5310	N	ARG	334	47.372	-16.512	22.904	1.00	0.00	RX1	N
ATOM	5311	H	ARG	334	47.984	-15.968	22.330	1.00	0.00	RX1	H
ATOM	5312	CA	ARG	334	47.821	-17.751	23.542	1.00	0.00	RX1	C
ATOM	5313	CB	ARG	334	49.046	-18.327	22.829	1.00	0.00	RX1	C
ATOM	5314	CG	ARG	334	48.793	-19.186	21.579	1.00	0.00	RX1	C
ATOM	5315	CD	ARG	334	48.261	-18.475	20.327	1.00	0.00	RX1	C
ATOM	5316	NE	ARG	334	46.805	-18.330	20.319	1.00	0.00	RX1	N
ATOM	5317	HE	ARG	334	46.357	-17.664	20.929	1.00	0.00	RX1	H
ATOM	5318	CZ	ARG	334	46.063	-19.073	19.447	1.00	0.00	RX1	C
ATOM	5319	NH1	ARG	334	46.656	-20.027	18.696	1.00	0.00	RX1	N
ATOM	5320	HH11	ARG	334	46.127	-20.592	18.057	1.00	0.00	RX1	H
ATOM	5321	HH12	ARG	334	47.642	-20.204	18.753	1.00	0.00	RX1	H
ATOM	5322	NH2	ARG	334	44.746	-18.839	19.342	1.00	0.00	RX1	N
ATOM	5323	HH21	ARG	334	44.122	-19.347	18.734	1.00	0.00	RX1	H
ATOM	5324	HH22	ARG	334	44.300	-18.096	19.869	1.00	0.00	RX1	H
ATOM	5325	C	ARG	334	48.104	-17.628	25.028	1.00	0.00	RX1	C
ATOM	5326	O	ARG	334	49.198	-17.274	25.444	1.00	0.00	RX1	O
ATOM	5327	N	LYS	335	47.054	-17.956	25.802	1.00	0.00	RX1	N
ATOM	5328	H	LYS	335	46.191	-18.125	25.325	1.00	0.00	RX1	H
ATOM	5329	CA	LYS	335	47.050	-17.851	27.263	1.00	0.00	RX1	C
ATOM	5330	CB	LYS	335	47.084	-19.237	27.942	1.00	0.00	RX1	C
ATOM	5331	CG	LYS	335	48.411	-19.970	28.169	1.00	0.00	RX1	C
ATOM	5332	CD	LYS	335	49.213	-20.355	26.927	1.00	0.00	RX1	C
ATOM	5333	CE	LYS	335	50.547	-20.998	27.310	1.00	0.00	RX1	C
ATOM	5334	NZ	LYS	335	51.281	-20.113	28.222	1.00	0.00	RX1	N
ATOM	5335	HZ1	LYS	335	52.070	-20.606	28.685	1.00	0.00	RX1	H
ATOM	5336	HZ2	LYS	335	51.660	-19.259	27.761	1.00	0.00	RX1	H
ATOM	5337	HZ3	LYS	335	50.675	-19.786	28.998	1.00	0.00	RX1	H
ATOM	5338	C	LYS	335	47.949	-16.785	27.879	1.00	0.00	RX1	C
ATOM	5339	O	LYS	335	49.098	-16.976	28.272	1.00	0.00	RX1	O
ATOM	5340	N	VAL	336	47.333	-15.600	27.869	1.00	0.00	RX1	N
ATOM	5341	H	VAL	336	46.361	-15.504	27.657	1.00	0.00	RX1	H
ATOM	5342	CA	VAL	336	48.014	-14.416	28.369	1.00	0.00	RX1	C
ATOM	5343	CB	VAL	336	48.258	-13.401	27.243	1.00	0.00	RX1	C
ATOM	5344	CG1	VAL	336	49.106	-14.010	26.128	1.00	0.00	RX1	C
ATOM	5345	CG2	VAL	336	46.952	-12.823	26.694	1.00	0.00	RX1	C
ATOM	5346	C	VAL	336	47.178	-13.796	29.466	1.00	0.00	RX1	C
ATOM	5347	O	VAL	336	45.957	-13.905	29.465	1.00	0.00	RX1	O
ATOM	5348	N	CYS	337	47.876	-13.133	30.386	1.00	0.00	RX1	N
ATOM	5349	H	CYS	337	48.864	-12.986	30.326	1.00	0.00	RX1	H
ATOM	5350	CA	CYS	337	47.102	-12.279	31.278	1.00	0.00	RX1	C
ATOM	5351	CB	CYS	337	47.409	-12.650	32.726	1.00	0.00	RX1	C
ATOM	5352	SG	CYS	337	47.153	-14.413	33.043	1.00	0.00	RX1	S
ATOM	5353	C	CYS	337	47.469	-10.855	30.957	1.00	0.00	RX1	C
ATOM	5354	O	CYS	337	48.463	-10.625	30.278	1.00	0.00	RX1	O
ATOM	5355	N	ASN	338	46.667	-9.907	31.465	1.00	0.00	RX1	N
ATOM	5356	H	ASN	338	45.824	-10.112	31.969	1.00	0.00	RX1	H
ATOM	5357	CA	ASN	338	47.160	-8.535	31.331	1.00	0.00	RX1	C
ATOM	5358	CB	ASN	338	46.075	-7.495	31.583	1.00	0.00	RX1	C
ATOM	5359	CG	ASN	338	45.442	-7.035	30.279	1.00	0.00	RX1	C
ATOM	5360	OD1	ASN	338	46.075	-6.532	29.360	1.00	0.00	RX1	O
ATOM	5361	ND2	ASN	338	44.105	-7.176	30.252	1.00	0.00	RX1	N
ATOM	5362	HD21	ASN	338	43.641	-7.705	30.974	1.00	0.00	RX1	H
ATOM	5363	HD22	ASN	338	43.538	-6.717	29.570	1.00	0.00	RX1	H
ATOM	5364	C	ASN	338	48.390	-8.281	32.191	1.00	0.00	RX1	C
ATOM	5365	O	ASN	338	49.501	-8.172	31.693	1.00	0.00	RX1	O
ATOM	5366	N	GLY	339	48.168	-8.266	33.513	1.00	0.00	RX1	N

ATOM	5367	H	GLY	339	47.281	-8.280	33.979	1.00	0.00	RX1	H
ATOM	5368	CA	GLY	339	49.336	-8.177	34.384	1.00	0.00	RX1	C
ATOM	5369	C	GLY	339	48.896	-7.957	35.810	1.00	0.00	RX1	C
ATOM	5370	O	GLY	339	47.811	-8.365	36.196	1.00	0.00	RX1	O
ATOM	5371	N	ILE	340	49.762	-7.281	36.570	1.00	0.00	RX1	N
ATOM	5372	H	ILE	340	50.594	-6.895	36.166	1.00	0.00	RX1	H
ATOM	5373	CA	ILE	340	49.316	-6.799	37.874	1.00	0.00	RX1	C
ATOM	5374	CB	ILE	340	50.353	-7.142	38.944	1.00	0.00	RX1	C
ATOM	5375	CG2	ILE	340	50.074	-6.419	40.260	1.00	0.00	RX1	C
ATOM	5376	CG1	ILE	340	50.400	-8.652	39.159	1.00	0.00	RX1	C
ATOM	5377	CD1	ILE	340	49.114	-9.153	39.820	1.00	0.00	RX1	C
ATOM	5378	C	ILE	340	49.117	-5.304	37.765	1.00	0.00	RX1	C
ATOM	5379	O	ILE	340	49.992	-4.586	37.297	1.00	0.00	RX1	O
ATOM	5380	N	GLY	341	47.922	-4.872	38.174	1.00	0.00	RX1	N
ATOM	5381	H	GLY	341	47.211	-5.477	38.532	1.00	0.00	RX1	H
ATOM	5382	CA	GLY	341	47.557	-3.506	37.827	1.00	0.00	RX1	C
ATOM	5383	C	GLY	341	46.781	-3.477	36.525	1.00	0.00	RX1	C
ATOM	5384	O	GLY	341	45.565	-3.338	36.493	1.00	0.00	RX1	O
ATOM	5385	N	ILE	342	47.547	-3.622	35.438	1.00	0.00	RX1	N
ATOM	5386	H	ILE	342	48.532	-3.779	35.520	1.00	0.00	RX1	H
ATOM	5387	CA	ILE	342	46.902	-3.475	34.134	1.00	0.00	RX1	C
ATOM	5388	CB	ILE	342	47.970	-3.395	33.048	1.00	0.00	RX1	C
ATOM	5389	CG2	ILE	342	48.621	-4.759	32.898	1.00	0.00	RX1	C
ATOM	5390	CG1	ILE	342	47.437	-2.813	31.737	1.00	0.00	RX1	C
ATOM	5391	CD1	ILE	342	48.495	-2.674	30.653	1.00	0.00	RX1	C
ATOM	5392	C	ILE	342	45.818	-4.511	33.817	1.00	0.00	RX1	C
ATOM	5393	O	ILE	342	45.839	-5.648	34.281	1.00	0.00	RX1	O
ATOM	5394	N	GLY	343	44.857	-4.054	32.995	1.00	0.00	RX1	N
ATOM	5395	H	GLY	343	44.883	-3.096	32.708	1.00	0.00	RX1	H
ATOM	5396	CA	GLY	343	43.763	-4.913	32.561	1.00	0.00	RX1	C
ATOM	5397	C	GLY	343	42.936	-5.463	33.691	1.00	0.00	RX1	C
ATOM	5398	O	GLY	343	42.386	-4.723	34.488	1.00	0.00	RX1	O
ATOM	5399	N	GLU	344	42.863	-6.787	33.744	1.00	0.00	RX1	N
ATOM	5400	H	GLU	344	43.436	-7.376	33.179	1.00	0.00	RX1	H
ATOM	5401	CA	GLU	344	41.945	-7.365	34.719	1.00	0.00	RX1	C
ATOM	5402	CB	GLU	344	41.398	-8.716	34.215	1.00	0.00	RX1	C
ATOM	5403	CG	GLU	344	42.372	-9.707	33.546	1.00	0.00	RX1	C
ATOM	5404	CD	GLU	344	42.648	-9.363	32.087	1.00	0.00	RX1	C
ATOM	5405	OE1	GLU	344	43.651	-9.799	31.529	1.00	0.00	RX1	O
ATOM	5406	OE2	GLU	344	41.867	-8.658	31.456	1.00	0.00	RX1	O
ATOM	5407	C	GLU	344	42.447	-7.412	36.167	1.00	0.00	RX1	C
ATOM	5408	O	GLU	344	42.300	-8.424	36.841	1.00	0.00	RX1	O
ATOM	5409	N	PHE	345	43.070	-6.295	36.614	1.00	0.00	RX1	N
ATOM	5410	H	PHE	345	43.104	-5.459	36.070	1.00	0.00	RX1	H
ATOM	5411	CA	PHE	345	43.817	-6.338	37.869	1.00	0.00	RX1	C
ATOM	5412	CB	PHE	345	45.197	-6.986	37.665	1.00	0.00	RX1	C
ATOM	5413	CG	PHE	345	45.153	-8.460	37.313	1.00	0.00	RX1	C
ATOM	5414	CD1	PHE	345	45.158	-9.414	38.322	1.00	0.00	RX1	C
ATOM	5415	CD2	PHE	345	45.136	-8.872	35.982	1.00	0.00	RX1	C
ATOM	5416	CE1	PHE	345	45.169	-10.766	37.997	1.00	0.00	RX1	C
ATOM	5417	CE2	PHE	345	45.152	-10.223	35.659	1.00	0.00	RX1	C
ATOM	5418	CZ	PHE	345	45.177	-11.174	36.669	1.00	0.00	RX1	C
ATOM	5419	C	PHE	345	44.054	-5.007	38.590	1.00	0.00	RX1	C
ATOM	5420	O	PHE	345	44.967	-4.929	39.407	1.00	0.00	RX1	O
ATOM	5421	N	LYS	346	43.248	-3.959	38.298	1.00	0.00	RX1	N
ATOM	5422	H	LYS	346	42.452	-4.060	37.702	1.00	0.00	RX1	H
ATOM	5423	CA	LYS	346	43.650	-2.643	38.847	1.00	0.00	RX1	C
ATOM	5424	CB	LYS	346	42.845	-1.483	38.226	1.00	0.00	RX1	C
ATOM	5425	CG	LYS	346	43.140	-0.038	38.678	1.00	0.00	RX1	C
ATOM	5426	CD	LYS	346	42.303	1.020	37.928	1.00	0.00	RX1	C
ATOM	5427	CE	LYS	346	42.594	2.483	38.315	1.00	0.00	RX1	C

ATOM	5428	NZ	LYS	346	42.016	3.450	37.359	1.00	0.00	RX1	N
ATOM	5429	HZ1	LYS	346	42.088	4.431	37.712	1.00	0.00	RX1	H
ATOM	5430	HZ2	LYS	346	42.470	3.464	36.428	1.00	0.00	RX1	H
ATOM	5431	HZ3	LYS	346	40.987	3.391	37.185	1.00	0.00	RX1	H
ATOM	5432	C	LYS	346	43.739	-2.522	40.368	1.00	0.00	RX1	C
ATOM	5433	O	LYS	346	44.583	-1.809	40.898	1.00	0.00	RX1	O
ATOM	5434	N	ASP	347	42.874	-3.283	41.058	1.00	0.00	RX1	N
ATOM	5435	H	ASP	347	42.226	-3.876	40.585	1.00	0.00	RX1	H
ATOM	5436	CA	ASP	347	42.937	-3.283	42.529	1.00	0.00	RX1	C
ATOM	5437	CB	ASP	347	41.574	-3.720	43.086	1.00	0.00	RX1	C
ATOM	5438	CG	ASP	347	41.077	-2.827	44.216	1.00	0.00	RX1	C
ATOM	5439	OD1	ASP	347	40.203	-3.245	44.973	1.00	0.00	RX1	O
ATOM	5440	OD2	ASP	347	41.471	-1.667	44.313	1.00	0.00	RX1	O
ATOM	5441	C	ASP	347	44.058	-4.141	43.133	1.00	0.00	RX1	C
ATOM	5442	O	ASP	347	43.896	-4.846	44.121	1.00	0.00	RX1	O
ATOM	5443	N	SER	348	45.228	-4.087	42.473	1.00	0.00	RX1	N
ATOM	5444	H	SER	348	45.405	-3.445	41.727	1.00	0.00	RX1	H
ATOM	5445	CA	SER	348	46.289	-5.003	42.886	1.00	0.00	RX1	C
ATOM	5446	CB	SER	348	46.289	-6.225	41.973	1.00	0.00	RX1	C
ATOM	5447	OG	SER	348	44.942	-6.516	41.592	1.00	0.00	RX1	O
ATOM	5448	HG	SER	348	44.706	-5.808	41.004	1.00	0.00	RX1	H
ATOM	5449	C	SER	348	47.646	-4.338	42.982	1.00	0.00	RX1	C
ATOM	5450	O	SER	348	48.355	-4.181	41.998	1.00	0.00	RX1	O
ATOM	5451	N	LEU	349	47.996	-3.963	44.223	1.00	0.00	RX1	N
ATOM	5452	H	LEU	349	47.378	-4.118	44.993	1.00	0.00	RX1	H
ATOM	5453	CA	LEU	349	49.277	-3.264	44.368	1.00	0.00	RX1	C
ATOM	5454	CB	LEU	349	49.332	-2.575	45.737	1.00	0.00	RX1	C
ATOM	5455	CG	LEU	349	49.203	-3.532	46.929	1.00	0.00	RX1	C
ATOM	5456	CD1	LEU	349	50.529	-3.728	47.667	1.00	0.00	RX1	C
ATOM	5457	CD2	LEU	349	48.081	-3.109	47.879	1.00	0.00	RX1	C
ATOM	5458	C	LEU	349	50.544	-4.076	44.106	1.00	0.00	RX1	C
ATOM	5459	O	LEU	349	51.583	-3.543	43.744	1.00	0.00	RX1	O
ATOM	5460	N	SER	350	50.416	-5.396	44.298	1.00	0.00	RX1	N
ATOM	5461	H	SER	350	49.578	-5.859	44.580	1.00	0.00	RX1	H
ATOM	5462	CA	SER	350	51.573	-6.245	44.026	1.00	0.00	RX1	C
ATOM	5463	CB	SER	350	52.470	-6.251	45.275	1.00	0.00	RX1	C
ATOM	5464	OG	SER	350	53.719	-6.922	45.043	1.00	0.00	RX1	O
ATOM	5465	HG	SER	350	53.550	-7.856	45.173	1.00	0.00	RX1	H
ATOM	5466	C	SER	350	51.109	-7.627	43.647	1.00	0.00	RX1	C
ATOM	5467	O	SER	350	50.028	-8.068	44.032	1.00	0.00	RX1	O
ATOM	5468	N	ILE	351	51.998	-8.309	42.913	1.00	0.00	RX1	N
ATOM	5469	H	ILE	351	52.860	-7.862	42.663	1.00	0.00	RX1	H
ATOM	5470	CA	ILE	351	51.852	-9.756	42.807	1.00	0.00	RX1	C
ATOM	5471	CB	ILE	351	52.813	-10.281	41.729	1.00	0.00	RX1	C
ATOM	5472	CG2	ILE	351	54.269	-10.142	42.172	1.00	0.00	RX1	C
ATOM	5473	CG1	ILE	351	52.464	-11.690	41.247	1.00	0.00	RX1	C
ATOM	5474	CD1	ILE	351	53.313	-12.104	40.044	1.00	0.00	RX1	C
ATOM	5475	C	ILE	351	52.086	-10.384	44.177	1.00	0.00	RX1	C
ATOM	5476	O	ILE	351	52.758	-9.803	45.024	1.00	0.00	RX1	O
ATOM	5477	N	ASN	352	51.438	-11.539	44.371	1.00	0.00	RX1	N
ATOM	5478	H	ASN	352	50.967	-12.041	43.643	1.00	0.00	RX1	H
ATOM	5479	CA	ASN	352	51.326	-12.151	45.695	1.00	0.00	RX1	C
ATOM	5480	CB	ASN	352	50.355	-11.370	46.578	1.00	0.00	RX1	C
ATOM	5481	CG	ASN	352	48.984	-11.383	45.936	1.00	0.00	RX1	C
ATOM	5482	OD1	ASN	352	48.278	-12.382	45.929	1.00	0.00	RX1	O
ATOM	5483	ND2	ASN	352	48.629	-10.201	45.399	1.00	0.00	RX1	N
ATOM	5484	HD21	ASN	352	49.265	-9.423	45.348	1.00	0.00	RX1	H
ATOM	5485	HD22	ASN	352	47.721	-10.027	45.021	1.00	0.00	RX1	H
ATOM	5486	C	ASN	352	50.828	-13.567	45.499	1.00	0.00	RX1	C
ATOM	5487	O	ASN	352	50.340	-13.888	44.419	1.00	0.00	RX1	O
ATOM	5488	N	ALA	353	50.958	-14.394	46.552	1.00	0.00	RX1	N

ATOM	5489	H	ALA	353	51.277	-14.088	47.452	1.00	0.00	RX1	H
ATOM	5490	CA	ALA	353	50.550	-15.790	46.364	1.00	0.00	RX1	C
ATOM	5491	CB	ALA	353	50.821	-16.614	47.621	1.00	0.00	RX1	C
ATOM	5492	C	ALA	353	49.090	-15.954	45.977	1.00	0.00	RX1	C
ATOM	5493	O	ALA	353	48.727	-16.666	45.045	1.00	0.00	RX1	O
ATOM	5494	N	THR	354	48.264	-15.192	46.709	1.00	0.00	RX1	N
ATOM	5495	H	THR	354	48.589	-14.590	47.438	1.00	0.00	RX1	H
ATOM	5496	CA	THR	354	46.823	-15.196	46.466	1.00	0.00	RX1	C
ATOM	5497	CB	THR	354	46.260	-14.151	47.404	1.00	0.00	RX1	C
ATOM	5498	OG1	THR	354	47.153	-14.055	48.520	1.00	0.00	RX1	O
ATOM	5499	HG1	THR	354	46.671	-13.566	49.178	1.00	0.00	RX1	H
ATOM	5500	CG2	THR	354	44.832	-14.460	47.854	1.00	0.00	RX1	C
ATOM	5501	C	THR	354	46.381	-15.023	45.019	1.00	0.00	RX1	C
ATOM	5502	O	THR	354	45.337	-15.523	44.603	1.00	0.00	RX1	O
ATOM	5503	N	ASN	355	47.217	-14.311	44.246	1.00	0.00	RX1	N
ATOM	5504	H	ASN	355	48.048	-13.887	44.618	1.00	0.00	RX1	H
ATOM	5505	CA	ASN	355	46.834	-14.216	42.843	1.00	0.00	RX1	C
ATOM	5506	CB	ASN	355	46.425	-12.807	42.446	1.00	0.00	RX1	C
ATOM	5507	CG	ASN	355	45.202	-12.976	41.575	1.00	0.00	RX1	C
ATOM	5508	OD1	ASN	355	44.550	-14.020	41.595	1.00	0.00	RX1	O
ATOM	5509	ND2	ASN	355	44.886	-11.882	40.864	1.00	0.00	RX1	N
ATOM	5510	HD21	ASN	355	45.461	-11.061	40.886	1.00	0.00	RX1	H
ATOM	5511	HD22	ASN	355	44.068	-11.814	40.291	1.00	0.00	RX1	H
ATOM	5512	C	ASN	355	47.737	-14.824	41.792	1.00	0.00	RX1	C
ATOM	5513	O	ASN	355	47.308	-15.035	40.662	1.00	0.00	RX1	O
ATOM	5514	N	ILE	356	48.985	-15.148	42.191	1.00	0.00	RX1	N
ATOM	5515	H	ILE	356	49.284	-15.017	43.139	1.00	0.00	RX1	H
ATOM	5516	CA	ILE	356	49.937	-15.644	41.184	1.00	0.00	RX1	C
ATOM	5517	CB	ILE	356	51.337	-15.872	41.775	1.00	0.00	RX1	C
ATOM	5518	CG2	ILE	356	51.383	-17.111	42.673	1.00	0.00	RX1	C
ATOM	5519	CG1	ILE	356	52.384	-15.907	40.654	1.00	0.00	RX1	C
ATOM	5520	CD1	ILE	356	53.825	-16.082	41.133	1.00	0.00	RX1	C
ATOM	5521	C	ILE	356	49.479	-16.851	40.361	1.00	0.00	RX1	C
ATOM	5522	O	ILE	356	49.859	-17.033	39.213	1.00	0.00	RX1	O
ATOM	5523	N	LYS	357	48.573	-17.632	40.982	1.00	0.00	RX1	N
ATOM	5524	H	LYS	357	48.469	-17.468	41.961	1.00	0.00	RX1	H
ATOM	5525	CA	LYS	357	47.905	-18.740	40.280	1.00	0.00	RX1	C
ATOM	5526	CB	LYS	357	46.599	-19.125	40.979	1.00	0.00	RX1	C
ATOM	5527	CG	LYS	357	46.597	-19.010	42.503	1.00	0.00	RX1	C
ATOM	5528	CD	LYS	357	45.264	-18.408	42.947	1.00	0.00	RX1	C
ATOM	5529	CE	LYS	357	44.946	-17.252	42.000	1.00	0.00	RX1	C
ATOM	5530	NZ	LYS	357	43.911	-16.350	42.515	1.00	0.00	RX1	N
ATOM	5531	HZ1	LYS	357	43.852	-15.530	41.870	1.00	0.00	RX1	H
ATOM	5532	HZ2	LYS	357	42.988	-16.816	42.570	1.00	0.00	RX1	H
ATOM	5533	HZ3	LYS	357	44.207	-15.989	43.448	1.00	0.00	RX1	H
ATOM	5534	C	LYS	357	47.549	-18.483	38.817	1.00	0.00	RX1	C
ATOM	5535	O	LYS	357	47.748	-19.319	37.949	1.00	0.00	RX1	O
ATOM	5536	N	HIS	358	47.005	-17.273	38.581	1.00	0.00	RX1	N
ATOM	5537	H	HIS	358	47.009	-16.560	39.285	1.00	0.00	RX1	H
ATOM	5538	CA	HIS	358	46.552	-16.972	37.216	1.00	0.00	RX1	C
ATOM	5539	CB	HIS	358	45.842	-15.612	37.133	1.00	0.00	RX1	C
ATOM	5540	CG	HIS	358	44.603	-15.537	37.999	1.00	0.00	RX1	C
ATOM	5541	ND1	HIS	358	43.885	-14.410	38.160	1.00	0.00	RX1	N
ATOM	5542	HD1	HIS	358	44.052	-13.547	37.722	1.00	0.00	RX1	H
ATOM	5543	CD2	HIS	358	44.015	-16.546	38.767	1.00	0.00	RX1	C
ATOM	5544	NE2	HIS	358	42.942	-16.016	39.405	1.00	0.00	RX1	N
ATOM	5545	CE1	HIS	358	42.860	-14.698	39.024	1.00	0.00	RX1	C
ATOM	5546	C	HIS	358	47.684	-16.968	36.198	1.00	0.00	RX1	C
ATOM	5547	O	HIS	358	47.566	-17.397	35.059	1.00	0.00	RX1	O
ATOM	5548	N	PHE	359	48.822	-16.469	36.696	1.00	0.00	RX1	N
ATOM	5549	H	PHE	359	48.921	-16.293	37.674	1.00	0.00	RX1	H

ATOM	5550	CA	PHE	359	50.002	-16.350	35.846	1.00	0.00	RX1	C
ATOM	5551	CB	PHE	359	50.961	-15.313	36.436	1.00	0.00	RX1	C
ATOM	5552	CG	PHE	359	50.213	-14.031	36.716	1.00	0.00	RX1	C
ATOM	5553	CD1	PHE	359	49.921	-13.157	35.676	1.00	0.00	RX1	C
ATOM	5554	CD2	PHE	359	49.809	-13.728	38.011	1.00	0.00	RX1	C
ATOM	5555	CE1	PHE	359	49.213	-11.988	35.928	1.00	0.00	RX1	C
ATOM	5556	CE2	PHE	359	49.098	-12.562	38.263	1.00	0.00	RX1	C
ATOM	5557	CZ	PHE	359	48.794	-11.695	37.219	1.00	0.00	RX1	C
ATOM	5558	C	PHE	359	50.740	-17.655	35.590	1.00	0.00	RX1	C
ATOM	5559	O	PHE	359	51.715	-17.696	34.850	1.00	0.00	RX1	O
ATOM	5560	N	LYS	360	50.246	-18.732	36.241	1.00	0.00	RX1	N
ATOM	5561	H	LYS	360	49.397	-18.663	36.766	1.00	0.00	RX1	H
ATOM	5562	CA	LYS	360	51.002	-19.988	36.295	1.00	0.00	RX1	C
ATOM	5563	CB	LYS	360	50.233	-21.042	37.096	1.00	0.00	RX1	C
ATOM	5564	CG	LYS	360	51.155	-22.047	37.787	1.00	0.00	RX1	C
ATOM	5565	CD	LYS	360	50.449	-22.890	38.848	1.00	0.00	RX1	C
ATOM	5566	CE	LYS	360	51.434	-23.712	39.685	1.00	0.00	RX1	C
ATOM	5567	NZ	LYS	360	52.359	-22.813	40.383	1.00	0.00	RX1	N
ATOM	5568	HZ1	LYS	360	53.016	-22.292	39.778	1.00	0.00	RX1	H
ATOM	5569	HZ2	LYS	360	51.886	-22.107	40.988	1.00	0.00	RX1	H
ATOM	5570	HZ3	LYS	360	52.963	-23.296	41.085	1.00	0.00	RX1	H
ATOM	5571	C	LYS	360	51.523	-20.551	34.981	1.00	0.00	RX1	C
ATOM	5572	O	LYS	360	52.523	-21.259	34.933	1.00	0.00	RX1	O
ATOM	5573	N	ASN	361	50.810	-20.189	33.907	1.00	0.00	RX1	N
ATOM	5574	H	ASN	361	49.997	-19.609	33.968	1.00	0.00	RX1	H
ATOM	5575	CA	ASN	361	51.431	-20.432	32.612	1.00	0.00	RX1	C
ATOM	5576	CB	ASN	361	51.054	-21.788	32.032	1.00	0.00	RX1	C
ATOM	5577	CG	ASN	361	52.144	-22.186	31.064	1.00	0.00	RX1	C
ATOM	5578	OD1	ASN	361	52.217	-21.748	29.918	1.00	0.00	RX1	O
ATOM	5579	ND2	ASN	361	52.998	-23.078	31.593	1.00	0.00	RX1	N
ATOM	5580	HD21	ASN	361	52.900	-23.340	32.556	1.00	0.00	RX1	H
ATOM	5581	HD22	ASN	361	53.748	-23.491	31.076	1.00	0.00	RX1	H
ATOM	5582	C	ASN	361	51.131	-19.342	31.607	1.00	0.00	RX1	C
ATOM	5583	O	ASN	361	50.428	-19.553	30.624	1.00	0.00	RX1	O
ATOM	5584	N	CYS	362	51.687	-18.160	31.889	1.00	0.00	RX1	N
ATOM	5585	H	CYS	362	52.310	-18.048	32.669	1.00	0.00	RX1	H
ATOM	5586	CA	CYS	362	51.514	-17.068	30.929	1.00	0.00	RX1	C
ATOM	5587	CB	CYS	362	51.939	-15.753	31.578	1.00	0.00	RX1	C
ATOM	5588	SG	CYS	362	50.772	-15.209	32.845	1.00	0.00	RX1	S
ATOM	5589	C	CYS	362	52.238	-17.302	29.609	1.00	0.00	RX1	C
ATOM	5590	O	CYS	362	52.792	-18.373	29.366	1.00	0.00	RX1	O
ATOM	5591	N	THR	363	52.204	-16.264	28.762	1.00	0.00	RX1	N
ATOM	5592	H	THR	363	51.669	-15.432	28.911	1.00	0.00	RX1	H
ATOM	5593	CA	THR	363	53.054	-16.321	27.575	1.00	0.00	RX1	C
ATOM	5594	CB	THR	363	52.249	-16.930	26.437	1.00	0.00	RX1	C
ATOM	5595	OG1	THR	363	51.304	-17.864	26.973	1.00	0.00	RX1	O
ATOM	5596	HG1	THR	363	50.455	-17.412	26.925	1.00	0.00	RX1	H
ATOM	5597	CG2	THR	363	53.144	-17.581	25.383	1.00	0.00	RX1	C
ATOM	5598	C	THR	363	53.566	-14.931	27.248	1.00	0.00	RX1	C
ATOM	5599	O	THR	363	54.721	-14.580	27.448	1.00	0.00	RX1	O
ATOM	5600	N	SER	364	52.610	-14.116	26.799	1.00	0.00	RX1	N
ATOM	5601	H	SER	364	51.656	-14.376	26.677	1.00	0.00	RX1	H
ATOM	5602	CA	SER	364	52.875	-12.697	26.944	1.00	0.00	RX1	C
ATOM	5603	CB	SER	364	52.438	-11.995	25.649	1.00	0.00	RX1	C
ATOM	5604	OG	SER	364	52.962	-10.666	25.571	1.00	0.00	RX1	O
ATOM	5605	HG	SER	364	53.446	-10.623	24.742	1.00	0.00	RX1	H
ATOM	5606	C	SER	364	52.144	-12.240	28.192	1.00	0.00	RX1	C
ATOM	5607	O	SER	364	51.263	-12.931	28.705	1.00	0.00	RX1	O
ATOM	5608	N	ILE	365	52.572	-11.070	28.649	1.00	0.00	RX1	N
ATOM	5609	H	ILE	365	53.303	-10.589	28.158	1.00	0.00	RX1	H
ATOM	5610	CA	ILE	365	51.907	-10.323	29.700	1.00	0.00	RX1	C

ATOM	5611	CB	ILE	365	52.836	-10.191	30.912	1.00	0.00	RX1	C
ATOM	5612	CG2	ILE	365	52.547	-8.965	31.775	1.00	0.00	RX1	C
ATOM	5613	CG1	ILE	365	52.744	-11.465	31.748	1.00	0.00	RX1	C
ATOM	5614	CD1	ILE	365	51.346	-11.645	32.340	1.00	0.00	RX1	C
ATOM	5615	C	ILE	365	51.587	-8.994	29.065	1.00	0.00	RX1	C
ATOM	5616	O	ILE	365	52.454	-8.261	28.597	1.00	0.00	RX1	O
ATOM	5617	N	SER	366	50.282	-8.757	29.005	1.00	0.00	RX1	N
ATOM	5618	H	SER	366	49.654	-9.295	29.568	1.00	0.00	RX1	H
ATOM	5619	CA	SER	366	49.788	-7.550	28.366	1.00	0.00	RX1	C
ATOM	5620	CB	SER	366	48.415	-7.973	27.831	1.00	0.00	RX1	C
ATOM	5621	OG	SER	366	47.705	-6.912	27.196	1.00	0.00	RX1	O
ATOM	5622	HG	SER	366	47.333	-6.414	27.928	1.00	0.00	RX1	H
ATOM	5623	C	SER	366	49.849	-6.378	29.330	1.00	0.00	RX1	C
ATOM	5624	O	SER	366	48.858	-5.918	29.882	1.00	0.00	RX1	O
ATOM	5625	N	GLY	367	51.092	-5.933	29.515	1.00	0.00	RX1	N
ATOM	5626	H	GLY	367	51.866	-6.353	29.040	1.00	0.00	RX1	H
ATOM	5627	CA	GLY	367	51.321	-4.888	30.493	1.00	0.00	RX1	C
ATOM	5628	C	GLY	367	52.369	-5.281	31.500	1.00	0.00	RX1	C
ATOM	5629	O	GLY	367	53.406	-5.840	31.154	1.00	0.00	RX1	O
ATOM	5630	N	ASP	368	52.055	-4.926	32.744	1.00	0.00	RX1	N
ATOM	5631	H	ASP	368	51.174	-4.510	32.962	1.00	0.00	RX1	H
ATOM	5632	CA	ASP	368	53.114	-4.742	33.726	1.00	0.00	RX1	C
ATOM	5633	CB	ASP	368	52.952	-3.358	34.339	1.00	0.00	RX1	C
ATOM	5634	CG	ASP	368	52.459	-2.391	33.282	1.00	0.00	RX1	C
ATOM	5635	OD1	ASP	368	53.250	-1.949	32.459	1.00	0.00	RX1	O
ATOM	5636	OD2	ASP	368	51.274	-2.077	33.270	1.00	0.00	RX1	O
ATOM	5637	C	ASP	368	53.110	-5.775	34.828	1.00	0.00	RX1	C
ATOM	5638	O	ASP	368	52.099	-6.401	35.132	1.00	0.00	RX1	O
ATOM	5639	N	LEU	369	54.286	-5.909	35.455	1.00	0.00	RX1	N
ATOM	5640	H	LEU	369	55.092	-5.397	35.149	1.00	0.00	RX1	H
ATOM	5641	CA	LEU	369	54.334	-6.709	36.676	1.00	0.00	RX1	C
ATOM	5642	CB	LEU	369	55.131	-7.999	36.465	1.00	0.00	RX1	C
ATOM	5643	CG	LEU	369	54.423	-9.015	35.562	1.00	0.00	RX1	C
ATOM	5644	CD1	LEU	369	55.314	-10.216	35.244	1.00	0.00	RX1	C
ATOM	5645	CD2	LEU	369	53.078	-9.457	36.144	1.00	0.00	RX1	C
ATOM	5646	C	LEU	369	54.885	-5.926	37.851	1.00	0.00	RX1	C
ATOM	5647	O	LEU	369	56.040	-5.515	37.884	1.00	0.00	RX1	O
ATOM	5648	N	HIS	370	53.980	-5.732	38.820	1.00	0.00	RX1	N
ATOM	5649	H	HIS	370	53.089	-6.178	38.771	1.00	0.00	RX1	H
ATOM	5650	CA	HIS	370	54.364	-5.029	40.045	1.00	0.00	RX1	C
ATOM	5651	CB	HIS	370	53.170	-4.247	40.597	1.00	0.00	RX1	C
ATOM	5652	CG	HIS	370	52.850	-3.042	39.745	1.00	0.00	RX1	C
ATOM	5653	ND1	HIS	370	51.952	-3.021	38.739	1.00	0.00	RX1	N
ATOM	5654	HD1	HIS	370	51.390	-3.755	38.401	1.00	0.00	RX1	H
ATOM	5655	CD2	HIS	370	53.405	-1.767	39.872	1.00	0.00	RX1	C
ATOM	5656	NE2	HIS	370	52.837	-0.972	38.935	1.00	0.00	RX1	N
ATOM	5657	CE1	HIS	370	51.939	-1.746	38.237	1.00	0.00	RX1	C
ATOM	5658	C	HIS	370	54.847	-5.990	41.115	1.00	0.00	RX1	C
ATOM	5659	O	HIS	370	54.076	-6.800	41.618	1.00	0.00	RX1	O
ATOM	5660	N	ILE	371	56.139	-5.873	41.449	1.00	0.00	RX1	N
ATOM	5661	H	ILE	371	56.767	-5.218	41.023	1.00	0.00	RX1	H
ATOM	5662	CA	ILE	371	56.651	-6.689	42.552	1.00	0.00	RX1	C
ATOM	5663	CB	ILE	371	57.767	-7.627	42.081	1.00	0.00	RX1	C
ATOM	5664	CG2	ILE	371	58.102	-8.651	43.167	1.00	0.00	RX1	C
ATOM	5665	CG1	ILE	371	57.409	-8.301	40.756	1.00	0.00	RX1	C
ATOM	5666	CD1	ILE	371	58.488	-9.277	40.294	1.00	0.00	RX1	C
ATOM	5667	C	ILE	371	57.140	-5.818	43.700	1.00	0.00	RX1	C
ATOM	5668	O	ILE	371	58.318	-5.495	43.830	1.00	0.00	RX1	O
ATOM	5669	N	LEU	372	56.153	-5.425	44.513	1.00	0.00	RX1	N
ATOM	5670	H	LEU	372	55.259	-5.875	44.465	1.00	0.00	RX1	H
ATOM	5671	CA	LEU	372	56.435	-4.515	45.626	1.00	0.00	RX1	C

ATOM	5672	CB	LEU	372	55.145	-3.784	46.030	1.00	0.00	RX1	C
ATOM	5673	CG	LEU	372	54.897	-2.450	45.316	1.00	0.00	RX1	C
ATOM	5674	CD1	LEU	372	54.780	-2.577	43.797	1.00	0.00	RX1	C
ATOM	5675	CD2	LEU	372	53.690	-1.726	45.913	1.00	0.00	RX1	C
ATOM	5676	C	LEU	372	57.017	-5.273	46.811	1.00	0.00	RX1	C
ATOM	5677	O	LEU	372	56.721	-6.449	46.992	1.00	0.00	RX1	O
ATOM	5678	N	PRO	373	57.853	-4.582	47.639	1.00	0.00	RX1	N
ATOM	5679	CD	PRO	373	58.225	-3.174	47.539	1.00	0.00	RX1	C
ATOM	5680	CA	PRO	373	58.527	-5.247	48.767	1.00	0.00	RX1	C
ATOM	5681	CB	PRO	373	59.076	-4.068	49.574	1.00	0.00	RX1	C
ATOM	5682	CG	PRO	373	59.382	-3.011	48.517	1.00	0.00	RX1	C
ATOM	5683	C	PRO	373	57.696	-6.224	49.586	1.00	0.00	RX1	C
ATOM	5684	O	PRO	373	58.096	-7.344	49.888	1.00	0.00	RX1	O
ATOM	5685	N	VAL	374	56.488	-5.743	49.917	1.00	0.00	RX1	N
ATOM	5686	H	VAL	374	56.194	-4.846	49.593	1.00	0.00	RX1	H
ATOM	5687	CA	VAL	374	55.619	-6.542	50.778	1.00	0.00	RX1	C
ATOM	5688	CB	VAL	374	54.365	-5.735	51.135	1.00	0.00	RX1	C
ATOM	5689	CG1	VAL	374	53.380	-5.658	49.967	1.00	0.00	RX1	C
ATOM	5690	CG2	VAL	374	53.741	-6.238	52.437	1.00	0.00	RX1	C
ATOM	5691	C	VAL	374	55.285	-7.953	50.286	1.00	0.00	RX1	C
ATOM	5692	O	VAL	374	54.981	-8.847	51.066	1.00	0.00	RX1	O
ATOM	5693	N	ALA	375	55.407	-8.145	48.957	1.00	0.00	RX1	N
ATOM	5694	H	ALA	375	55.708	-7.406	48.353	1.00	0.00	RX1	H
ATOM	5695	CA	ALA	375	55.167	-9.473	48.388	1.00	0.00	RX1	C
ATOM	5696	CB	ALA	375	55.519	-9.496	46.899	1.00	0.00	RX1	C
ATOM	5697	C	ALA	375	55.919	-10.606	49.070	1.00	0.00	RX1	C
ATOM	5698	O	ALA	375	55.385	-11.679	49.318	1.00	0.00	RX1	O
ATOM	5699	N	PHE	376	57.189	-10.319	49.397	1.00	0.00	RX1	N
ATOM	5700	H	PHE	376	57.567	-9.393	49.321	1.00	0.00	RX1	H
ATOM	5701	CA	PHE	376	57.946	-11.402	50.025	1.00	0.00	RX1	C
ATOM	5702	CB	PHE	376	59.361	-11.460	49.456	1.00	0.00	RX1	C
ATOM	5703	CG	PHE	376	59.249	-11.780	47.986	1.00	0.00	RX1	C
ATOM	5704	CD1	PHE	376	59.613	-10.840	47.035	1.00	0.00	RX1	C
ATOM	5705	CD2	PHE	376	58.763	-13.016	47.584	1.00	0.00	RX1	C
ATOM	5706	CE1	PHE	376	59.472	-11.121	45.682	1.00	0.00	RX1	C
ATOM	5707	CE2	PHE	376	58.625	-13.301	46.232	1.00	0.00	RX1	C
ATOM	5708	CZ	PHE	376	58.968	-12.349	45.280	1.00	0.00	RX1	C
ATOM	5709	C	PHE	376	57.948	-11.404	51.543	1.00	0.00	RX1	C
ATOM	5710	O	PHE	376	58.837	-11.936	52.200	1.00	0.00	RX1	O
ATOM	5711	N	ARG	377	56.888	-10.781	52.074	1.00	0.00	RX1	N
ATOM	5712	H	ARG	377	56.219	-10.312	51.499	1.00	0.00	RX1	H
ATOM	5713	CA	ARG	377	56.607	-10.904	53.502	1.00	0.00	RX1	C
ATOM	5714	CB	ARG	377	56.935	-9.607	54.245	1.00	0.00	RX1	C
ATOM	5715	CG	ARG	377	58.440	-9.408	54.443	1.00	0.00	RX1	C
ATOM	5716	CD	ARG	377	59.067	-10.643	55.099	1.00	0.00	RX1	C
ATOM	5717	NE	ARG	377	60.469	-10.427	55.448	1.00	0.00	RX1	N
ATOM	5718	HE	ARG	377	60.628	-9.876	56.271	1.00	0.00	RX1	H
ATOM	5719	CZ	ARG	377	61.472	-10.985	54.699	1.00	0.00	RX1	C
ATOM	5720	NH1	ARG	377	61.208	-11.667	53.566	1.00	0.00	RX1	N
ATOM	5721	HH11	ARG	377	61.988	-12.032	53.025	1.00	0.00	RX1	H
ATOM	5722	HH12	ARG	377	60.283	-11.831	53.197	1.00	0.00	RX1	H
ATOM	5723	NH2	ARG	377	62.747	-10.853	55.108	1.00	0.00	RX1	N
ATOM	5724	HH21	ARG	377	63.467	-11.328	54.572	1.00	0.00	RX1	H
ATOM	5725	HH22	ARG	377	63.040	-10.331	55.908	1.00	0.00	RX1	H
ATOM	5726	C	ARG	377	55.181	-11.331	53.793	1.00	0.00	RX1	C
ATOM	5727	O	ARG	377	54.899	-11.978	54.791	1.00	0.00	RX1	O
ATOM	5728	N	GLY	378	54.302	-10.963	52.852	1.00	0.00	RX1	N
ATOM	5729	H	GLY	378	54.540	-10.324	52.126	1.00	0.00	RX1	H
ATOM	5730	CA	GLY	378	52.886	-11.193	53.094	1.00	0.00	RX1	C
ATOM	5731	C	GLY	378	52.224	-9.918	53.558	1.00	0.00	RX1	C
ATOM	5732	O	GLY	378	52.617	-9.319	54.553	1.00	0.00	RX1	O

ATOM	5733	N	ASP	379	51.229	-9.490	52.771	1.00	0.00	RX1	N
ATOM	5734	H	ASP	379	50.752	-10.091	52.129	1.00	0.00	RX1	H
ATOM	5735	CA	ASP	379	50.632	-8.234	53.207	1.00	0.00	RX1	C
ATOM	5736	CB	ASP	379	50.140	-7.370	52.044	1.00	0.00	RX1	C
ATOM	5737	CG	ASP	379	49.586	-6.056	52.574	1.00	0.00	RX1	C
ATOM	5738	OD1	ASP	379	50.078	-5.553	53.578	1.00	0.00	RX1	O
ATOM	5739	OD2	ASP	379	48.639	-5.537	51.996	1.00	0.00	RX1	O
ATOM	5740	C	ASP	379	49.544	-8.444	54.231	1.00	0.00	RX1	C
ATOM	5741	O	ASP	379	48.627	-9.247	54.088	1.00	0.00	RX1	O
ATOM	5742	N	SER	380	49.703	-7.661	55.300	1.00	0.00	RX1	N
ATOM	5743	H	SER	380	50.368	-6.911	55.270	1.00	0.00	RX1	H
ATOM	5744	CA	SER	380	48.674	-7.693	56.326	1.00	0.00	RX1	C
ATOM	5745	CB	SER	380	49.356	-7.027	57.499	1.00	0.00	RX1	C
ATOM	5746	OG	SER	380	50.763	-7.156	57.247	1.00	0.00	RX1	O
ATOM	5747	HG	SER	380	50.920	-8.055	56.977	1.00	0.00	RX1	H
ATOM	5748	C	SER	380	47.364	-7.072	55.873	1.00	0.00	RX1	C
ATOM	5749	O	SER	380	46.286	-7.575	56.166	1.00	0.00	RX1	O
ATOM	5750	N	PHE	381	47.494	-5.964	55.124	1.00	0.00	RX1	N
ATOM	5751	H	PHE	381	48.375	-5.695	54.719	1.00	0.00	RX1	H
ATOM	5752	CA	PHE	381	46.279	-5.252	54.734	1.00	0.00	RX1	C
ATOM	5753	CB	PHE	381	46.640	-3.873	54.180	1.00	0.00	RX1	C
ATOM	5754	CG	PHE	381	45.390	-3.154	53.737	1.00	0.00	RX1	C
ATOM	5755	CD1	PHE	381	44.420	-2.803	54.669	1.00	0.00	RX1	C
ATOM	5756	CD2	PHE	381	45.207	-2.849	52.393	1.00	0.00	RX1	C
ATOM	5757	CE1	PHE	381	43.263	-2.155	54.255	1.00	0.00	RX1	C
ATOM	5758	CE2	PHE	381	44.049	-2.202	51.979	1.00	0.00	RX1	C
ATOM	5759	CZ	PHE	381	43.076	-1.858	52.910	1.00	0.00	RX1	C
ATOM	5760	C	PHE	381	45.408	-6.026	53.757	1.00	0.00	RX1	C
ATOM	5761	O	PHE	381	44.196	-6.114	53.893	1.00	0.00	RX1	O
ATOM	5762	N	THR	382	46.092	-6.616	52.773	1.00	0.00	RX1	N
ATOM	5763	H	THR	382	47.088	-6.544	52.697	1.00	0.00	RX1	H
ATOM	5764	CA	THR	382	45.328	-7.416	51.820	1.00	0.00	RX1	C
ATOM	5765	CB	THR	382	45.867	-7.192	50.403	1.00	0.00	RX1	C
ATOM	5766	OG1	THR	382	47.292	-7.328	50.347	1.00	0.00	RX1	O
ATOM	5767	HG1	THR	382	47.662	-6.564	50.792	1.00	0.00	RX1	H
ATOM	5768	CG2	THR	382	45.462	-5.811	49.885	1.00	0.00	RX1	C
ATOM	5769	C	THR	382	45.145	-8.876	52.209	1.00	0.00	RX1	C
ATOM	5770	O	THR	382	44.614	-9.677	51.450	1.00	0.00	RX1	O
ATOM	5771	N	HIS	383	45.642	-9.195	53.422	1.00	0.00	RX1	N
ATOM	5772	H	HIS	383	46.135	-8.507	53.951	1.00	0.00	RX1	H
ATOM	5773	CA	HIS	383	45.620	-10.574	53.925	1.00	0.00	RX1	C
ATOM	5774	CB	HIS	383	44.200	-11.061	54.249	1.00	0.00	RX1	C
ATOM	5775	CG	HIS	383	43.421	-10.114	55.138	1.00	0.00	RX1	C
ATOM	5776	ND1	HIS	383	43.942	-9.094	55.848	1.00	0.00	RX1	N
ATOM	5777	HD1	HIS	383	44.879	-8.803	55.886	1.00	0.00	RX1	H
ATOM	5778	CD2	HIS	383	42.042	-10.145	55.363	1.00	0.00	RX1	C
ATOM	5779	NE2	HIS	383	41.737	-9.136	56.214	1.00	0.00	RX1	N
ATOM	5780	CE1	HIS	383	42.908	-8.488	56.512	1.00	0.00	RX1	C
ATOM	5781	C	HIS	383	46.276	-11.581	52.988	1.00	0.00	RX1	C
ATOM	5782	O	HIS	383	45.825	-12.708	52.817	1.00	0.00	RX1	O
ATOM	5783	N	THR	384	47.354	-11.115	52.349	1.00	0.00	RX1	N
ATOM	5784	H	THR	384	47.817	-10.264	52.602	1.00	0.00	RX1	H
ATOM	5785	CA	THR	384	47.930	-11.967	51.318	1.00	0.00	RX1	C
ATOM	5786	CB	THR	384	48.253	-10.949	50.248	1.00	0.00	RX1	C
ATOM	5787	OG1	THR	384	48.393	-9.699	50.931	1.00	0.00	RX1	O
ATOM	5788	HG1	THR	384	48.039	-9.006	50.376	1.00	0.00	RX1	H
ATOM	5789	CG2	THR	384	47.142	-10.825	49.205	1.00	0.00	RX1	C
ATOM	5790	C	THR	384	49.115	-12.790	51.789	1.00	0.00	RX1	C
ATOM	5791	O	THR	384	50.139	-12.266	52.213	1.00	0.00	RX1	O
ATOM	5792	N	PRO	385	48.955	-14.133	51.690	1.00	0.00	RX1	N
ATOM	5793	CD	PRO	385	47.716	-14.843	51.408	1.00	0.00	RX1	C

ATOM	5794	CA	PRO	385	50.099	-15.032	51.877	1.00	0.00	RX1	C
ATOM	5795	CB	PRO	385	49.519	-16.388	51.453	1.00	0.00	RX1	C
ATOM	5796	CG	PRO	385	48.027	-16.291	51.758	1.00	0.00	RX1	C
ATOM	5797	C	PRO	385	51.329	-14.632	51.068	1.00	0.00	RX1	C
ATOM	5798	O	PRO	385	51.232	-14.198	49.920	1.00	0.00	RX1	O
ATOM	5799	N	PRO	386	52.502	-14.795	51.730	1.00	0.00	RX1	N
ATOM	5800	CD	PRO	386	52.659	-15.280	53.095	1.00	0.00	RX1	C
ATOM	5801	CA	PRO	386	53.777	-14.479	51.079	1.00	0.00	RX1	C
ATOM	5802	CB	PRO	386	54.811	-14.972	52.096	1.00	0.00	RX1	C
ATOM	5803	CG	PRO	386	54.101	-14.939	53.448	1.00	0.00	RX1	C
ATOM	5804	C	PRO	386	53.939	-15.149	49.731	1.00	0.00	RX1	C
ATOM	5805	O	PRO	386	53.560	-16.295	49.521	1.00	0.00	RX1	O
ATOM	5806	N	LEU	387	54.522	-14.373	48.817	1.00	0.00	RX1	N
ATOM	5807	H	LEU	387	54.902	-13.483	49.068	1.00	0.00	RX1	H
ATOM	5808	CA	LEU	387	54.832	-14.978	47.532	1.00	0.00	RX1	C
ATOM	5809	CB	LEU	387	55.068	-13.882	46.495	1.00	0.00	RX1	C
ATOM	5810	CG	LEU	387	55.217	-14.437	45.082	1.00	0.00	RX1	C
ATOM	5811	CD1	LEU	387	54.090	-15.408	44.746	1.00	0.00	RX1	C
ATOM	5812	CD2	LEU	387	55.334	-13.319	44.047	1.00	0.00	RX1	C
ATOM	5813	C	LEU	387	55.998	-15.943	47.645	1.00	0.00	RX1	C
ATOM	5814	O	LEU	387	57.127	-15.560	47.919	1.00	0.00	RX1	O
ATOM	5815	N	ASP	388	55.654	-17.219	47.442	1.00	0.00	RX1	N
ATOM	5816	H	ASP	388	54.707	-17.467	47.248	1.00	0.00	RX1	H
ATOM	5817	CA	ASP	388	56.668	-18.265	47.571	1.00	0.00	RX1	C
ATOM	5818	CB	ASP	388	55.983	-19.627	47.424	1.00	0.00	RX1	C
ATOM	5819	CG	ASP	388	56.989	-20.759	47.376	1.00	0.00	RX1	C
ATOM	5820	OD1	ASP	388	57.989	-20.734	48.083	1.00	0.00	RX1	O
ATOM	5821	OD2	ASP	388	56.790	-21.681	46.600	1.00	0.00	RX1	O
ATOM	5822	C	ASP	388	57.865	-18.105	46.635	1.00	0.00	RX1	C
ATOM	5823	O	ASP	388	57.754	-18.038	45.412	1.00	0.00	RX1	O
ATOM	5824	N	PRO	389	59.057	-18.061	47.283	1.00	0.00	RX1	N
ATOM	5825	CD	PRO	389	59.232	-17.941	48.727	1.00	0.00	RX1	C
ATOM	5826	CA	PRO	389	60.327	-18.110	46.554	1.00	0.00	RX1	C
ATOM	5827	CB	PRO	389	61.357	-18.186	47.686	1.00	0.00	RX1	C
ATOM	5828	CG	PRO	389	60.687	-17.530	48.890	1.00	0.00	RX1	C
ATOM	5829	C	PRO	389	60.511	-19.238	45.541	1.00	0.00	RX1	C
ATOM	5830	O	PRO	389	61.426	-19.185	44.722	1.00	0.00	RX1	O
ATOM	5831	N	GLN	390	59.669	-20.276	45.643	1.00	0.00	RX1	N
ATOM	5832	H	GLN	390	58.928	-20.316	46.320	1.00	0.00	RX1	H
ATOM	5833	CA	GLN	390	59.714	-21.312	44.614	1.00	0.00	RX1	C
ATOM	5834	CB	GLN	390	59.484	-22.679	45.245	1.00	0.00	RX1	C
ATOM	5835	CG	GLN	390	60.326	-22.821	46.510	1.00	0.00	RX1	C
ATOM	5836	CD	GLN	390	59.789	-23.965	47.338	1.00	0.00	RX1	C
ATOM	5837	OE1	GLN	390	60.439	-24.992	47.512	1.00	0.00	RX1	O
ATOM	5838	NE2	GLN	390	58.570	-23.722	47.845	1.00	0.00	RX1	N
ATOM	5839	HE21	GLN	390	58.113	-22.836	47.666	1.00	0.00	RX1	H
ATOM	5840	HE22	GLN	390	58.047	-24.362	48.402	1.00	0.00	RX1	H
ATOM	5841	C	GLN	390	58.734	-21.031	43.490	1.00	0.00	RX1	C
ATOM	5842	O	GLN	390	59.100	-21.007	42.320	1.00	0.00	RX1	O
ATOM	5843	N	GLU	391	57.481	-20.748	43.901	1.00	0.00	RX1	N
ATOM	5844	H	GLU	391	57.231	-20.862	44.865	1.00	0.00	RX1	H
ATOM	5845	CA	GLU	391	56.444	-20.355	42.938	1.00	0.00	RX1	C
ATOM	5846	CB	GLU	391	55.152	-19.937	43.643	1.00	0.00	RX1	C
ATOM	5847	CG	GLU	391	54.231	-21.110	43.998	1.00	0.00	RX1	C
ATOM	5848	CD	GLU	391	53.587	-21.677	42.744	1.00	0.00	RX1	C
ATOM	5849	OE1	GLU	391	52.466	-21.287	42.415	1.00	0.00	RX1	O
ATOM	5850	OE2	GLU	391	54.178	-22.532	42.088	1.00	0.00	RX1	O
ATOM	5851	C	GLU	391	56.853	-19.279	41.949	1.00	0.00	RX1	C
ATOM	5852	O	GLU	391	56.430	-19.264	40.802	1.00	0.00	RX1	O
ATOM	5853	N	LEU	392	57.756	-18.399	42.414	1.00	0.00	RX1	N
ATOM	5854	H	LEU	392	57.945	-18.396	43.398	1.00	0.00	RX1	H

ATOM	5855	CA	LEU	392	58.410	-17.457	41.497	1.00	0.00	RX1	C
ATOM	5856	CB	LEU	392	59.699	-16.930	42.112	1.00	0.00	RX1	C
ATOM	5857	CG	LEU	392	59.435	-15.818	43.116	1.00	0.00	RX1	C
ATOM	5858	CD1	LEU	392	60.738	-15.315	43.727	1.00	0.00	RX1	C
ATOM	5859	CD2	LEU	392	58.628	-14.685	42.483	1.00	0.00	RX1	C
ATOM	5860	C	LEU	392	58.715	-17.933	40.082	1.00	0.00	RX1	C
ATOM	5861	O	LEU	392	58.459	-17.235	39.107	1.00	0.00	RX1	O
ATOM	5862	N	ASP	393	59.264	-19.156	40.002	1.00	0.00	RX1	N
ATOM	5863	H	ASP	393	59.354	-19.745	40.808	1.00	0.00	RX1	H
ATOM	5864	CA	ASP	393	59.715	-19.635	38.694	1.00	0.00	RX1	C
ATOM	5865	CB	ASP	393	60.588	-20.882	38.852	1.00	0.00	RX1	C
ATOM	5866	CG	ASP	393	61.907	-20.683	38.125	1.00	0.00	RX1	C
ATOM	5867	OD1	ASP	393	62.511	-21.661	37.692	1.00	0.00	RX1	O
ATOM	5868	OD2	ASP	393	62.359	-19.550	37.983	1.00	0.00	RX1	O
ATOM	5869	C	ASP	393	58.645	-19.813	37.615	1.00	0.00	RX1	C
ATOM	5870	O	ASP	393	58.928	-19.940	36.430	1.00	0.00	RX1	O
ATOM	5871	N	ILE	394	57.371	-19.720	38.060	1.00	0.00	RX1	N
ATOM	5872	H	ILE	394	57.207	-19.687	39.048	1.00	0.00	RX1	H
ATOM	5873	CA	ILE	394	56.240	-19.481	37.145	1.00	0.00	RX1	C
ATOM	5874	CB	ILE	394	55.039	-18.986	37.957	1.00	0.00	RX1	C
ATOM	5875	CG2	ILE	394	54.044	-18.184	37.122	1.00	0.00	RX1	C
ATOM	5876	CG1	ILE	394	54.354	-20.134	38.688	1.00	0.00	RX1	C
ATOM	5877	CD1	ILE	394	53.261	-19.591	39.607	1.00	0.00	RX1	C
ATOM	5878	C	ILE	394	56.543	-18.478	36.035	1.00	0.00	RX1	C
ATOM	5879	O	ILE	394	56.191	-18.640	34.869	1.00	0.00	RX1	O
ATOM	5880	N	LEU	395	57.227	-17.411	36.468	1.00	0.00	RX1	N
ATOM	5881	H	LEU	395	57.580	-17.385	37.405	1.00	0.00	RX1	H
ATOM	5882	CA	LEU	395	57.480	-16.300	35.560	1.00	0.00	RX1	C
ATOM	5883	CB	LEU	395	57.904	-15.088	36.384	1.00	0.00	RX1	C
ATOM	5884	CG	LEU	395	56.822	-14.776	37.425	1.00	0.00	RX1	C
ATOM	5885	CD1	LEU	395	57.325	-13.900	38.571	1.00	0.00	RX1	C
ATOM	5886	CD2	LEU	395	55.555	-14.218	36.775	1.00	0.00	RX1	C
ATOM	5887	C	LEU	395	58.398	-16.603	34.384	1.00	0.00	RX1	C
ATOM	5888	O	LEU	395	58.479	-15.847	33.426	1.00	0.00	RX1	O
ATOM	5889	N	LYS	396	59.007	-17.802	34.434	1.00	0.00	RX1	N
ATOM	5890	H	LYS	396	58.966	-18.382	35.248	1.00	0.00	RX1	H
ATOM	5891	CA	LYS	396	59.678	-18.303	33.235	1.00	0.00	RX1	C
ATOM	5892	CB	LYS	396	60.456	-19.583	33.532	1.00	0.00	RX1	C
ATOM	5893	CG	LYS	396	61.795	-19.379	34.239	1.00	0.00	RX1	C
ATOM	5894	CD	LYS	396	62.512	-20.722	34.382	1.00	0.00	RX1	C
ATOM	5895	CE	LYS	396	63.970	-20.614	34.831	1.00	0.00	RX1	C
ATOM	5896	NZ	LYS	396	64.080	-20.014	36.161	1.00	0.00	RX1	N
ATOM	5897	HZ1	LYS	396	65.078	-19.861	36.411	1.00	0.00	RX1	H
ATOM	5898	HZ2	LYS	396	63.596	-19.096	36.226	1.00	0.00	RX1	H
ATOM	5899	HZ3	LYS	396	63.608	-20.592	36.892	1.00	0.00	RX1	H
ATOM	5900	C	LYS	396	58.778	-18.556	32.031	1.00	0.00	RX1	C
ATOM	5901	O	LYS	396	59.250	-18.788	30.925	1.00	0.00	RX1	O
ATOM	5902	N	THR	397	57.461	-18.510	32.273	1.00	0.00	RX1	N
ATOM	5903	H	THR	397	57.078	-18.316	33.175	1.00	0.00	RX1	H
ATOM	5904	CA	THR	397	56.563	-18.580	31.122	1.00	0.00	RX1	C
ATOM	5905	CB	THR	397	55.225	-19.169	31.579	1.00	0.00	RX1	C
ATOM	5906	OG1	THR	397	54.648	-18.417	32.656	1.00	0.00	RX1	O
ATOM	5907	HG1	THR	397	55.075	-18.703	33.460	1.00	0.00	RX1	H
ATOM	5908	CG2	THR	397	55.416	-20.621	32.011	1.00	0.00	RX1	C
ATOM	5909	C	THR	397	56.427	-17.271	30.353	1.00	0.00	RX1	C
ATOM	5910	O	THR	397	55.959	-17.217	29.221	1.00	0.00	RX1	O
ATOM	5911	N	VAL	398	56.857	-16.199	31.032	1.00	0.00	RX1	N
ATOM	5912	H	VAL	398	57.396	-16.261	31.872	1.00	0.00	RX1	H
ATOM	5913	CA	VAL	398	56.676	-14.884	30.437	1.00	0.00	RX1	C
ATOM	5914	CB	VAL	398	56.593	-13.823	31.528	1.00	0.00	RX1	C
ATOM	5915	CG1	VAL	398	56.277	-12.467	30.912	1.00	0.00	RX1	C

ATOM	5916	CG2	VAL	398	55.572	-14.218	32.597	1.00	0.00	RX1	C
ATOM	5917	C	VAL	398	57.750	-14.542	29.420	1.00	0.00	RX1	C
ATOM	5918	O	VAL	398	58.837	-14.074	29.727	1.00	0.00	RX1	O
ATOM	5919	N	LYS	399	57.361	-14.793	28.170	1.00	0.00	RX1	N
ATOM	5920	H	LYS	399	56.446	-15.173	28.025	1.00	0.00	RX1	H
ATOM	5921	CA	LYS	399	58.193	-14.385	27.042	1.00	0.00	RX1	C
ATOM	5922	CB	LYS	399	57.678	-15.053	25.763	1.00	0.00	RX1	C
ATOM	5923	CG	LYS	399	58.394	-16.365	25.438	1.00	0.00	RX1	C
ATOM	5924	CD	LYS	399	58.344	-17.398	26.562	1.00	0.00	RX1	C
ATOM	5925	CE	LYS	399	59.606	-18.254	26.580	1.00	0.00	RX1	C
ATOM	5926	NZ	LYS	399	60.764	-17.381	26.787	1.00	0.00	RX1	N
ATOM	5927	HZ1	LYS	399	60.926	-16.762	25.962	1.00	0.00	RX1	H
ATOM	5928	HZ2	LYS	399	60.666	-16.726	27.589	1.00	0.00	RX1	H
ATOM	5929	HZ3	LYS	399	61.669	-17.881	26.900	1.00	0.00	RX1	H
ATOM	5930	C	LYS	399	58.230	-12.879	26.865	1.00	0.00	RX1	C
ATOM	5931	O	LYS	399	59.274	-12.258	26.697	1.00	0.00	RX1	O
ATOM	5932	N	GLU	400	57.016	-12.313	26.920	1.00	0.00	RX1	N
ATOM	5933	H	GLU	400	56.182	-12.831	27.121	1.00	0.00	RX1	H
ATOM	5934	CA	GLU	400	56.956	-10.863	26.782	1.00	0.00	RX1	C
ATOM	5935	CB	GLU	400	56.192	-10.459	25.509	1.00	0.00	RX1	C
ATOM	5936	CG	GLU	400	56.424	-11.343	24.274	1.00	0.00	RX1	C
ATOM	5937	CD	GLU	400	55.705	-10.775	23.057	1.00	0.00	RX1	C
ATOM	5938	OE1	GLU	400	56.316	-10.636	22.000	1.00	0.00	RX1	O
ATOM	5939	OE2	GLU	400	54.534	-10.428	23.150	1.00	0.00	RX1	O
ATOM	5940	C	GLU	400	56.288	-10.222	27.977	1.00	0.00	RX1	C
ATOM	5941	O	GLU	400	55.202	-10.616	28.374	1.00	0.00	RX1	O
ATOM	5942	N	ILE	401	56.954	-9.203	28.515	1.00	0.00	RX1	N
ATOM	5943	H	ILE	401	57.856	-8.953	28.173	1.00	0.00	RX1	H
ATOM	5944	CA	ILE	401	56.246	-8.207	29.310	1.00	0.00	RX1	C
ATOM	5945	CB	ILE	401	57.071	-7.830	30.538	1.00	0.00	RX1	C
ATOM	5946	CG2	ILE	401	56.506	-6.609	31.261	1.00	0.00	RX1	C
ATOM	5947	CG1	ILE	401	57.181	-9.025	31.474	1.00	0.00	RX1	C
ATOM	5948	CD1	ILE	401	57.949	-8.690	32.745	1.00	0.00	RX1	C
ATOM	5949	C	ILE	401	56.065	-7.004	28.414	1.00	0.00	RX1	C
ATOM	5950	O	ILE	401	57.023	-6.411	27.936	1.00	0.00	RX1	O
ATOM	5951	N	THR	402	54.805	-6.669	28.155	1.00	0.00	RX1	N
ATOM	5952	H	THR	402	54.005	-7.084	28.590	1.00	0.00	RX1	H
ATOM	5953	CA	THR	402	54.694	-5.562	27.213	1.00	0.00	RX1	C
ATOM	5954	CB	THR	402	53.533	-5.831	26.265	1.00	0.00	RX1	C
ATOM	5955	OG1	THR	402	52.402	-6.297	26.994	1.00	0.00	RX1	O
ATOM	5956	HG1	THR	402	52.537	-7.235	27.107	1.00	0.00	RX1	H
ATOM	5957	CG2	THR	402	53.926	-6.886	25.228	1.00	0.00	RX1	C
ATOM	5958	C	THR	402	54.722	-4.174	27.833	1.00	0.00	RX1	C
ATOM	5959	O	THR	402	54.973	-3.175	27.169	1.00	0.00	RX1	O
ATOM	5960	N	GLY	403	54.492	-4.153	29.149	1.00	0.00	RX1	N
ATOM	5961	H	GLY	403	54.347	-4.986	29.685	1.00	0.00	RX1	H
ATOM	5962	CA	GLY	403	54.703	-2.904	29.867	1.00	0.00	RX1	C
ATOM	5963	C	GLY	403	56.044	-2.908	30.565	1.00	0.00	RX1	C
ATOM	5964	O	GLY	403	57.082	-3.059	29.927	1.00	0.00	RX1	O
ATOM	5965	N	PHE	404	55.974	-2.759	31.891	1.00	0.00	RX1	N
ATOM	5966	H	PHE	404	55.085	-2.688	32.352	1.00	0.00	RX1	H
ATOM	5967	CA	PHE	404	57.207	-2.716	32.667	1.00	0.00	RX1	C
ATOM	5968	CB	PHE	404	57.370	-1.358	33.359	1.00	0.00	RX1	C
ATOM	5969	CG	PHE	404	56.237	-1.068	34.315	1.00	0.00	RX1	C
ATOM	5970	CD1	PHE	404	56.160	-1.708	35.547	1.00	0.00	RX1	C
ATOM	5971	CD2	PHE	404	55.274	-0.135	33.960	1.00	0.00	RX1	C
ATOM	5972	CE1	PHE	404	55.122	-1.411	36.419	1.00	0.00	RX1	C
ATOM	5973	CE2	PHE	404	54.240	0.174	34.833	1.00	0.00	RX1	C
ATOM	5974	CZ	PHE	404	54.168	-0.464	36.062	1.00	0.00	RX1	C
ATOM	5975	C	PHE	404	57.370	-3.858	33.655	1.00	0.00	RX1	C
ATOM	5976	O	PHE	404	56.433	-4.555	34.027	1.00	0.00	RX1	O

ATOM	5977	N	LEU	405	58.623	-3.987	34.096	1.00	0.00	RX1	N
ATOM	5978	H	LEU	405	59.349	-3.377	33.780	1.00	0.00	RX1	H
ATOM	5979	CA	LEU	405	58.937	-4.930	35.156	1.00	0.00	RX1	C
ATOM	5980	CB	LEU	405	59.937	-5.951	34.622	1.00	0.00	RX1	C
ATOM	5981	CG	LEU	405	60.279	-7.052	35.620	1.00	0.00	RX1	C
ATOM	5982	CD1	LEU	405	59.026	-7.699	36.214	1.00	0.00	RX1	C
ATOM	5983	CD2	LEU	405	61.235	-8.078	35.009	1.00	0.00	RX1	C
ATOM	5984	C	LEU	405	59.469	-4.209	36.381	1.00	0.00	RX1	C
ATOM	5985	O	LEU	405	60.589	-3.708	36.411	1.00	0.00	RX1	O
ATOM	5986	N	LEU	406	58.593	-4.167	37.392	1.00	0.00	RX1	N
ATOM	5987	H	LEU	406	57.731	-4.679	37.362	1.00	0.00	RX1	H
ATOM	5988	CA	LEU	406	58.963	-3.482	38.626	1.00	0.00	RX1	C
ATOM	5989	CB	LEU	406	57.773	-2.643	39.101	1.00	0.00	RX1	C
ATOM	5990	CG	LEU	406	58.053	-1.773	40.329	1.00	0.00	RX1	C
ATOM	5991	CD1	LEU	406	59.125	-0.724	40.064	1.00	0.00	RX1	C
ATOM	5992	CD2	LEU	406	56.782	-1.130	40.875	1.00	0.00	RX1	C
ATOM	5993	C	LEU	406	59.407	-4.453	39.706	1.00	0.00	RX1	C
ATOM	5994	O	LEU	406	58.644	-4.823	40.591	1.00	0.00	RX1	O
ATOM	5995	N	ILE	407	60.676	-4.860	39.604	1.00	0.00	RX1	N
ATOM	5996	H	ILE	407	61.308	-4.402	38.976	1.00	0.00	RX1	H
ATOM	5997	CA	ILE	407	61.173	-5.692	40.696	1.00	0.00	RX1	C
ATOM	5998	CB	ILE	407	62.184	-6.732	40.226	1.00	0.00	RX1	C
ATOM	5999	CG2	ILE	407	62.447	-7.736	41.344	1.00	0.00	RX1	C
ATOM	6000	CG1	ILE	407	61.705	-7.453	38.975	1.00	0.00	RX1	C
ATOM	6001	CD1	ILE	407	62.659	-8.579	38.582	1.00	0.00	RX1	C
ATOM	6002	C	ILE	407	61.762	-4.868	41.827	1.00	0.00	RX1	C
ATOM	6003	O	ILE	407	62.943	-4.537	41.871	1.00	0.00	RX1	O
ATOM	6004	N	GLN	408	60.857	-4.558	42.760	1.00	0.00	RX1	N
ATOM	6005	H	GLN	408	59.904	-4.864	42.688	1.00	0.00	RX1	H
ATOM	6006	CA	GLN	408	61.351	-3.954	43.994	1.00	0.00	RX1	C
ATOM	6007	CB	GLN	408	60.351	-2.940	44.527	1.00	0.00	RX1	C
ATOM	6008	CG	GLN	408	60.052	-1.830	43.530	1.00	0.00	RX1	C
ATOM	6009	CD	GLN	408	58.965	-0.947	44.096	1.00	0.00	RX1	C
ATOM	6010	OE1	GLN	408	58.096	-1.387	44.841	1.00	0.00	RX1	O
ATOM	6011	NE2	GLN	408	59.063	0.336	43.706	1.00	0.00	RX1	N
ATOM	6012	HE21	GLN	408	59.802	0.624	43.096	1.00	0.00	RX1	H
ATOM	6013	HE22	GLN	408	58.407	1.023	44.015	1.00	0.00	RX1	H
ATOM	6014	C	GLN	408	61.665	-4.976	45.070	1.00	0.00	RX1	C
ATOM	6015	O	GLN	408	62.455	-4.747	45.976	1.00	0.00	RX1	O
ATOM	6016	N	ALA	409	60.994	-6.125	44.939	1.00	0.00	RX1	N
ATOM	6017	H	ALA	409	60.353	-6.311	44.194	1.00	0.00	RX1	H
ATOM	6018	CA	ALA	409	61.252	-7.157	45.932	1.00	0.00	RX1	C
ATOM	6019	CB	ALA	409	59.975	-7.503	46.678	1.00	0.00	RX1	C
ATOM	6020	C	ALA	409	61.773	-8.428	45.317	1.00	0.00	RX1	C
ATOM	6021	O	ALA	409	61.387	-8.810	44.221	1.00	0.00	RX1	O
ATOM	6022	N	TRP	410	62.643	-9.075	46.098	1.00	0.00	RX1	N
ATOM	6023	H	TRP	410	62.963	-8.726	46.982	1.00	0.00	RX1	H
ATOM	6024	CA	TRP	410	63.157	-10.397	45.750	1.00	0.00	RX1	C
ATOM	6025	CB	TRP	410	64.374	-10.246	44.840	1.00	0.00	RX1	C
ATOM	6026	CG	TRP	410	64.400	-11.234	43.697	1.00	0.00	RX1	C
ATOM	6027	CD2	TRP	410	63.355	-11.597	42.766	1.00	0.00	RX1	C
ATOM	6028	CE2	TRP	410	63.911	-12.517	41.845	1.00	0.00	RX1	C
ATOM	6029	CE3	TRP	410	62.024	-11.221	42.631	1.00	0.00	RX1	C
ATOM	6030	CD1	TRP	410	65.522	-11.962	43.284	1.00	0.00	RX1	C
ATOM	6031	NE1	TRP	410	65.244	-12.721	42.194	1.00	0.00	RX1	N
ATOM	6032	HE1	TRP	410	65.899	-13.288	41.728	1.00	0.00	RX1	H
ATOM	6033	CZ2	TRP	410	63.119	-13.020	40.820	1.00	0.00	RX1	C
ATOM	6034	CZ3	TRP	410	61.243	-11.734	41.605	1.00	0.00	RX1	C
ATOM	6035	CH2	TRP	410	61.791	-12.631	40.698	1.00	0.00	RX1	C
ATOM	6036	C	TRP	410	63.561	-11.030	47.061	1.00	0.00	RX1	C
ATOM	6037	O	TRP	410	64.080	-10.339	47.929	1.00	0.00	RX1	O

ATOM	6038	N	PRO	411	63.278	-12.344	47.209	1.00	0.00	RX1 N
ATOM	6039	CD	PRO	411	62.621	-13.218	46.252	1.00	0.00	RX1 C
ATOM	6040	CA	PRO	411	63.618	-13.014	48.467	1.00	0.00	RX1 C
ATOM	6041	CB	PRO	411	63.170	-14.456	48.221	1.00	0.00	RX1 C
ATOM	6042	CG	PRO	411	62.125	-14.370	47.113	1.00	0.00	RX1 C
ATOM	6043	C	PRO	411	65.093	-12.931	48.799	1.00	0.00	RX1 C
ATOM	6044	O	PRO	411	65.956	-13.026	47.934	1.00	0.00	RX1 O
ATOM	6045	N	GLU	412	65.334	-12.792	50.105	1.00	0.00	RX1 N
ATOM	6046	H	GLU	412	64.556	-12.642	50.719	1.00	0.00	RX1 H
ATOM	6047	CA	GLU	412	66.698	-12.666	50.620	1.00	0.00	RX1 C
ATOM	6048	CB	GLU	412	66.706	-12.721	52.152	1.00	0.00	RX1 C
ATOM	6049	CG	GLU	412	65.835	-11.663	52.844	1.00	0.00	RX1 C
ATOM	6050	CD	GLU	412	64.368	-12.066	52.868	1.00	0.00	RX1 C
ATOM	6051	OE1	GLU	412	63.981	-12.863	53.709	1.00	0.00	RX1 O
ATOM	6052	OE2	GLU	412	63.574	-11.540	52.098	1.00	0.00	RX1 O
ATOM	6053	C	GLU	412	67.687	-13.670	50.047	1.00	0.00	RX1 C
ATOM	6054	O	GLU	412	68.778	-13.337	49.597	1.00	0.00	RX1 O
ATOM	6055	N	ASN	413	67.235	-14.930	50.039	1.00	0.00	RX1 N
ATOM	6056	H	ASN	413	66.323	-15.131	50.394	1.00	0.00	RX1 H
ATOM	6057	CA	ASN	413	68.095	-15.958	49.452	1.00	0.00	RX1 C
ATOM	6058	CB	ASN	413	67.928	-17.319	50.131	1.00	0.00	RX1 C
ATOM	6059	CG	ASN	413	68.718	-17.356	51.420	1.00	0.00	RX1 C
ATOM	6060	OD1	ASN	413	68.405	-16.665	52.381	1.00	0.00	RX1 O
ATOM	6061	ND2	ASN	413	69.757	-18.211	51.399	1.00	0.00	RX1 N
ATOM	6062	HD21	ASN	413	69.979	-18.754	50.590	1.00	0.00	RX1 H
ATOM	6063	HD22	ASN	413	70.334	-18.317	52.209	1.00	0.00	RX1 H
ATOM	6064	C	ASN	413	67.902	-16.146	47.958	1.00	0.00	RX1 C
ATOM	6065	O	ASN	413	67.621	-17.244	47.477	1.00	0.00	RX1 O
ATOM	6066	N	ARG	414	68.066	-15.028	47.237	1.00	0.00	RX1 N
ATOM	6067	H	ARG	414	68.242	-14.135	47.662	1.00	0.00	RX1 H
ATOM	6068	CA	ARG	414	68.056	-15.093	45.779	1.00	0.00	RX1 C
ATOM	6069	CB	ARG	414	66.712	-14.662	45.195	1.00	0.00	RX1 C
ATOM	6070	CG	ARG	414	65.593	-15.687	45.362	1.00	0.00	RX1 C
ATOM	6071	CD	ARG	414	65.973	-17.030	44.739	1.00	0.00	RX1 C
ATOM	6072	NE	ARG	414	64.844	-17.958	44.735	1.00	0.00	RX1 N
ATOM	6073	HE	ARG	414	64.129	-17.800	44.038	1.00	0.00	RX1 H
ATOM	6074	CZ	ARG	414	64.836	-18.997	45.616	1.00	0.00	RX1 C
ATOM	6075	NH1	ARG	414	65.821	-19.096	46.542	1.00	0.00	RX1 N
ATOM	6076	HH11	ARG	414	65.889	-19.862	47.184	1.00	0.00	RX1 H
ATOM	6077	HH12	ARG	414	66.538	-18.387	46.640	1.00	0.00	RX1 H
ATOM	6078	NH2	ARG	414	63.841	-19.908	45.544	1.00	0.00	RX1 N
ATOM	6079	HH21	ARG	414	63.793	-20.733	46.111	1.00	0.00	RX1 H
ATOM	6080	HH22	ARG	414	63.072	-19.774	44.897	1.00	0.00	RX1 H
ATOM	6081	C	ARG	414	69.157	-14.261	45.162	1.00	0.00	RX1 C
ATOM	6082	O	ARG	414	69.205	-13.041	45.262	1.00	0.00	RX1 O
ATOM	6083	N	THR	415	70.050	-15.002	44.506	1.00	0.00	RX1 N
ATOM	6084	H	THR	415	70.029	-15.999	44.493	1.00	0.00	RX1 H
ATOM	6085	CA	THR	415	71.195	-14.343	43.893	1.00	0.00	RX1 C
ATOM	6086	CB	THR	415	72.317	-15.282	44.253	1.00	0.00	RX1 C
ATOM	6087	OG1	THR	415	71.763	-16.235	45.178	1.00	0.00	RX1 O
ATOM	6088	HG1	THR	415	72.501	-16.741	45.498	1.00	0.00	RX1 H
ATOM	6089	CG2	THR	415	73.508	-14.551	44.876	1.00	0.00	RX1 C
ATOM	6090	C	THR	415	71.058	-14.030	42.411	1.00	0.00	RX1 C
ATOM	6091	O	THR	415	71.885	-13.329	41.840	1.00	0.00	RX1 O
ATOM	6092	N	ASP	416	69.982	-14.568	41.823	1.00	0.00	RX1 N
ATOM	6093	H	ASP	416	69.247	-15.037	42.309	1.00	0.00	RX1 H
ATOM	6094	CA	ASP	416	69.769	-14.367	40.392	1.00	0.00	RX1 C
ATOM	6095	CB	ASP	416	70.228	-15.617	39.631	1.00	0.00	RX1 C
ATOM	6096	CG	ASP	416	70.208	-15.385	38.133	1.00	0.00	RX1 C
ATOM	6097	OD1	ASP	416	71.226	-14.992	37.575	1.00	0.00	RX1 O
ATOM	6098	OD2	ASP	416	69.176	-15.611	37.513	1.00	0.00	RX1 O

ATOM	6099	C	ASP	416	68.301	-14.054	40.163	1.00	0.00	RX1	C
ATOM	6100	O	ASP	416	67.453	-14.318	41.018	1.00	0.00	RX1	O
ATOM	6101	N	LEU	417	68.012	-13.463	38.997	1.00	0.00	RX1	N
ATOM	6102	H	LEU	417	68.701	-13.467	38.267	1.00	0.00	RX1	H
ATOM	6103	CA	LEU	417	66.607	-13.149	38.749	1.00	0.00	RX1	C
ATOM	6104	CB	LEU	417	66.426	-11.990	37.765	1.00	0.00	RX1	C
ATOM	6105	CG	LEU	417	66.659	-10.623	38.413	1.00	0.00	RX1	C
ATOM	6106	CD1	LEU	417	66.323	-9.465	37.473	1.00	0.00	RX1	C
ATOM	6107	CD2	LEU	417	65.881	-10.477	39.717	1.00	0.00	RX1	C
ATOM	6108	C	LEU	417	65.711	-14.309	38.353	1.00	0.00	RX1	C
ATOM	6109	O	LEU	417	64.519	-14.110	38.176	1.00	0.00	RX1	O
ATOM	6110	N	HIS	418	66.314	-15.514	38.269	1.00	0.00	RX1	N
ATOM	6111	H	HIS	418	67.317	-15.531	38.262	1.00	0.00	RX1	H
ATOM	6112	CA	HIS	418	65.645	-16.817	38.093	1.00	0.00	RX1	C
ATOM	6113	CB	HIS	418	65.810	-17.707	39.338	1.00	0.00	RX1	C
ATOM	6114	CG	HIS	418	64.949	-17.241	40.486	1.00	0.00	RX1	C
ATOM	6115	ND1	HIS	418	65.049	-16.023	41.045	1.00	0.00	RX1	N
ATOM	6116	HD1	HIS	418	65.714	-15.332	40.822	1.00	0.00	RX1	H
ATOM	6117	CD2	HIS	418	63.933	-17.954	41.132	1.00	0.00	RX1	C
ATOM	6118	NE2	HIS	418	63.419	-17.143	42.090	1.00	0.00	RX1	N
ATOM	6119	CE1	HIS	418	64.102	-15.956	42.029	1.00	0.00	RX1	C
ATOM	6120	C	HIS	418	64.244	-16.913	37.481	1.00	0.00	RX1	C
ATOM	6121	O	HIS	418	64.059	-17.444	36.389	1.00	0.00	RX1	O
ATOM	6122	N	ALA	419	63.252	-16.359	38.190	1.00	0.00	RX1	N
ATOM	6123	H	ALA	419	63.472	-15.844	39.017	1.00	0.00	RX1	H
ATOM	6124	CA	ALA	419	61.904	-16.297	37.628	1.00	0.00	RX1	C
ATOM	6125	CB	ALA	419	61.020	-15.455	38.541	1.00	0.00	RX1	C
ATOM	6126	C	ALA	419	61.860	-15.697	36.229	1.00	0.00	RX1	C
ATOM	6127	O	ALA	419	61.233	-16.199	35.309	1.00	0.00	RX1	O
ATOM	6128	N	PHE	420	62.603	-14.594	36.098	1.00	0.00	RX1	N
ATOM	6129	H	PHE	420	63.177	-14.263	36.847	1.00	0.00	RX1	H
ATOM	6130	CA	PHE	420	62.603	-13.928	34.801	1.00	0.00	RX1	C
ATOM	6131	CB	PHE	420	62.541	-12.420	35.003	1.00	0.00	RX1	C
ATOM	6132	CG	PHE	420	61.248	-12.035	35.673	1.00	0.00	RX1	C
ATOM	6133	CD1	PHE	420	60.068	-12.023	34.939	1.00	0.00	RX1	C
ATOM	6134	CD2	PHE	420	61.238	-11.681	37.016	1.00	0.00	RX1	C
ATOM	6135	CE1	PHE	420	58.881	-11.628	35.540	1.00	0.00	RX1	C
ATOM	6136	CE2	PHE	420	60.050	-11.290	37.617	1.00	0.00	RX1	C
ATOM	6137	CZ	PHE	420	58.875	-11.248	36.876	1.00	0.00	RX1	C
ATOM	6138	C	PHE	420	63.754	-14.284	33.873	1.00	0.00	RX1	C
ATOM	6139	O	PHE	420	64.124	-13.519	32.992	1.00	0.00	RX1	O
ATOM	6140	N	GLU	421	64.311	-15.491	34.076	1.00	0.00	RX1	N
ATOM	6141	H	GLU	421	63.996	-16.102	34.804	1.00	0.00	RX1	H
ATOM	6142	CA	GLU	421	65.384	-15.898	33.158	1.00	0.00	RX1	C
ATOM	6143	CB	GLU	421	65.951	-17.259	33.534	1.00	0.00	RX1	C
ATOM	6144	CG	GLU	421	66.733	-17.242	34.834	1.00	0.00	RX1	C
ATOM	6145	CD	GLU	421	67.222	-18.643	35.123	1.00	0.00	RX1	C
ATOM	6146	OE1	GLU	421	68.154	-19.080	34.465	1.00	0.00	RX1	O
ATOM	6147	OE2	GLU	421	66.679	-19.305	35.999	1.00	0.00	RX1	O
ATOM	6148	C	GLU	421	64.948	-15.995	31.710	1.00	0.00	RX1	C
ATOM	6149	O	GLU	421	65.613	-15.570	30.771	1.00	0.00	RX1	O
ATOM	6150	N	ASN	422	63.770	-16.621	31.587	1.00	0.00	RX1	N
ATOM	6151	H	ASN	422	63.203	-16.751	32.399	1.00	0.00	RX1	H
ATOM	6152	CA	ASN	422	63.259	-17.012	30.274	1.00	0.00	RX1	C
ATOM	6153	CB	ASN	422	62.533	-18.346	30.427	1.00	0.00	RX1	C
ATOM	6154	CG	ASN	422	62.127	-18.933	29.093	1.00	0.00	RX1	C
ATOM	6155	OD1	ASN	422	62.682	-18.652	28.036	1.00	0.00	RX1	O
ATOM	6156	ND2	ASN	422	61.143	-19.841	29.204	1.00	0.00	RX1	N
ATOM	6157	HD21	ASN	422	60.617	-19.862	30.060	1.00	0.00	RX1	H
ATOM	6158	HD22	ASN	422	60.877	-20.486	28.490	1.00	0.00	RX1	H
ATOM	6159	C	ASN	422	62.363	-15.950	29.654	1.00	0.00	RX1	C

```
ATOM   6160  O   ASN   422      61.324 -16.240  29.070  1.00  0.00      RX1  O
ATOM   6161  N   LEU   423      62.816 -14.707  29.868  1.00  0.00      RX1  N
ATOM   6162  H   LEU   423      63.748 -14.544  30.195  1.00  0.00      RX1  H
ATOM   6163  CA  LEU   423      62.075 -13.524  29.448  1.00  0.00      RX1  C
ATOM   6164  CB  LEU   423      62.060 -12.531  30.612  1.00  0.00      RX1  C
ATOM   6165  CG  LEU   423      60.841 -11.621  30.807  1.00  0.00      RX1  C
ATOM   6166  CD1 LEU   423      61.160 -10.548  31.846  1.00  0.00      RX1  C
ATOM   6167  CD2 LEU   423      60.308 -10.979  29.531  1.00  0.00      RX1  C
ATOM   6168  C   LEU   423      62.826 -12.906  28.290  1.00  0.00      RX1  C
ATOM   6169  O   LEU   423      64.009 -12.613  28.413  1.00  0.00      RX1  O
ATOM   6170  N   GLU   424      62.114 -12.718  27.181  1.00  0.00      RX1  N
ATOM   6171  H   GLU   424      61.158 -13.009  27.090  1.00  0.00      RX1  H
ATOM   6172  CA  GLU   424      62.810 -12.174  26.023  1.00  0.00      RX1  C
ATOM   6173  CB  GLU   424      62.472 -12.947  24.743  1.00  0.00      RX1  C
ATOM   6174  CG  GLU   424      63.094 -14.337  24.589  1.00  0.00      RX1  C
ATOM   6175  CD  GLU   424      62.207 -15.386  25.217  1.00  0.00      RX1  C
ATOM   6176  OE1 GLU   424      61.269 -15.035  25.922  1.00  0.00      RX1  O
ATOM   6177  OE2 GLU   424      62.420 -16.574  24.990  1.00  0.00      RX1  O
ATOM   6178  C   GLU   424      62.559 -10.704  25.778  1.00  0.00      RX1  C
ATOM   6179  O   GLU   424      63.386  -9.990  25.220  1.00  0.00      RX1  O
ATOM   6180  N   ILE   425      61.351 -10.277  26.157  1.00  0.00      RX1  N
ATOM   6181  H   ILE   425      60.703 -10.850  26.665  1.00  0.00      RX1  H
ATOM   6182  CA  ILE   425      60.891  -8.993  25.635  1.00  0.00      RX1  C
ATOM   6183  CB  ILE   425      59.865  -9.306  24.546  1.00  0.00      RX1  C
ATOM   6184  CG2 ILE   425      59.070  -8.066  24.170  1.00  0.00      RX1  C
ATOM   6185  CG1 ILE   425      60.534  -9.993  23.350  1.00  0.00      RX1  C
ATOM   6186  CD1 ILE   425      59.629 -10.851  22.472  1.00  0.00      RX1  C
ATOM   6187  C   ILE   425      60.290  -8.118  26.721  1.00  0.00      RX1  C
ATOM   6188  O   ILE   425      59.483  -8.588  27.506  1.00  0.00      RX1  O
ATOM   6189  N   ILE   426      60.682  -6.834  26.720  1.00  0.00      RX1  N
ATOM   6190  H   ILE   426      61.402  -6.497  26.112  1.00  0.00      RX1  H
ATOM   6191  CA  ILE   426      59.973  -5.832  27.518  1.00  0.00      RX1  C
ATOM   6192  CB  ILE   426      60.780  -5.439  28.762  1.00  0.00      RX1  C
ATOM   6193  CG2 ILE   426      60.082  -4.322  29.536  1.00  0.00      RX1  C
ATOM   6194  CG1 ILE   426      61.031  -6.648  29.665  1.00  0.00      RX1  C
ATOM   6195  CD1 ILE   426      61.829  -6.300  30.918  1.00  0.00      RX1  C
ATOM   6196  C   ILE   426      59.667  -4.609  26.659  1.00  0.00      RX1  C
ATOM   6197  O   ILE   426      60.560  -3.920  26.178  1.00  0.00      RX1  O
ATOM   6198  N   ARG   427      58.360  -4.381  26.449  1.00  0.00      RX1  N
ATOM   6199  H   ARG   427      57.678  -4.913  26.952  1.00  0.00      RX1  H
ATOM   6200  CA  ARG   427      58.007  -3.335  25.486  1.00  0.00      RX1  C
ATOM   6201  CB  ARG   427      56.854  -3.757  24.566  1.00  0.00      RX1  C
ATOM   6202  CG  ARG   427      57.286  -4.930  23.693  1.00  0.00      RX1  C
ATOM   6203  CD  ARG   427      56.392  -5.277  22.501  1.00  0.00      RX1  C
ATOM   6204  NE  ARG   427      57.125  -6.214  21.655  1.00  0.00      RX1  N
ATOM   6205  HE  ARG   427      58.029  -5.916  21.322  1.00  0.00      RX1  H
ATOM   6206  CZ  ARG   427      56.780  -7.530  21.600  1.00  0.00      RX1  C
ATOM   6207  NH1 ARG   427      55.539  -7.942  21.943  1.00  0.00      RX1  N
ATOM   6208 HH11 ARG   427      55.345  -8.922  22.144  1.00  0.00      RX1  H
ATOM   6209 HH12 ARG   427      54.741  -7.323  21.989  1.00  0.00      RX1  H
ATOM   6210  NH2 ARG   427      57.723  -8.411  21.213  1.00  0.00      RX1  N
ATOM   6211 HH21 ARG   427      57.511  -9.401  21.210  1.00  0.00      RX1  H
ATOM   6212 HH22 ARG   427      58.659  -8.115  20.962  1.00  0.00      RX1  H
ATOM   6213  C   ARG   427      57.778  -1.932  26.010  1.00  0.00      RX1  C
ATOM   6214  O   ARG   427      57.709  -0.985  25.234  1.00  0.00      RX1  O
ATOM   6215  N   GLY   428      57.653  -1.815  27.342  1.00  0.00      RX1  N
ATOM   6216  H   GLY   428      57.624  -2.611  27.950  1.00  0.00      RX1  H
ATOM   6217  CA  GLY   428      57.533  -0.473  27.918  1.00  0.00      RX1  C
ATOM   6218  C   GLY   428      56.382   0.375  27.396  1.00  0.00      RX1  C
ATOM   6219  O   GLY   428      56.510   1.577  27.174  1.00  0.00      RX1  O
ATOM   6220  N   ARG   429      55.243  -0.308  27.198  1.00  0.00      RX1  N
```

ATOM	6221	H	ARG	429	55.213	-1.284	27.417	1.00	0.00	RX1	H
ATOM	6222	CA	ARG	429	54.045	0.417	26.775	1.00	0.00	RX1	C
ATOM	6223	CB	ARG	429	52.964	-0.560	26.319	1.00	0.00	RX1	C
ATOM	6224	CG	ARG	429	53.390	-1.203	25.009	1.00	0.00	RX1	C
ATOM	6225	CD	ARG	429	52.406	-2.212	24.430	1.00	0.00	RX1	C
ATOM	6226	NE	ARG	429	52.897	-2.564	23.107	1.00	0.00	RX1	N
ATOM	6227	HE	ARG	429	53.375	-1.835	22.607	1.00	0.00	RX1	H
ATOM	6228	CZ	ARG	429	52.812	-3.812	22.575	1.00	0.00	RX1	C
ATOM	6229	NH1	ARG	429	52.096	-4.780	23.177	1.00	0.00	RX1	N
ATOM	6230	HH11	ARG	429	52.127	-5.728	22.823	1.00	0.00	RX1	H
ATOM	6231	HH12	ARG	429	51.478	-4.601	23.956	1.00	0.00	RX1	H
ATOM	6232	NH2	ARG	429	53.459	-4.047	21.423	1.00	0.00	RX1	N
ATOM	6233	HH21	ARG	429	53.469	-4.963	20.990	1.00	0.00	RX1	H
ATOM	6234	HH22	ARG	429	53.964	-3.316	20.946	1.00	0.00	RX1	H
ATOM	6235	C	ARG	429	53.510	1.327	27.855	1.00	0.00	RX1	C
ATOM	6236	O	ARG	429	53.404	2.538	27.697	1.00	0.00	RX1	O
ATOM	6237	N	THR	430	53.217	0.670	28.980	1.00	0.00	RX1	N
ATOM	6238	H	THR	430	53.436	-0.296	29.099	1.00	0.00	RX1	H
ATOM	6239	CA	THR	430	53.141	1.466	30.193	1.00	0.00	RX1	C
ATOM	6240	CB	THR	430	51.953	0.961	31.040	1.00	0.00	RX1	C
ATOM	6241	OG1	THR	430	52.277	0.728	32.409	1.00	0.00	RX1	O
ATOM	6242	HG1	THR	430	52.248	-0.230	32.548	1.00	0.00	RX1	H
ATOM	6243	CG2	THR	430	51.277	-0.245	30.389	1.00	0.00	RX1	C
ATOM	6244	C	THR	430	54.529	1.435	30.808	1.00	0.00	RX1	C
ATOM	6245	O	THR	430	55.351	0.591	30.457	1.00	0.00	RX1	O
ATOM	6246	N	LYS	431	54.790	2.456	31.633	1.00	0.00	RX1	N
ATOM	6247	H	LYS	431	54.070	3.029	32.026	1.00	0.00	RX1	H
ATOM	6248	CA	LYS	431	56.159	2.675	32.088	1.00	0.00	RX1	C
ATOM	6249	CB	LYS	431	56.871	3.740	31.244	1.00	0.00	RX1	C
ATOM	6250	CG	LYS	431	56.715	3.699	29.719	1.00	0.00	RX1	C
ATOM	6251	CD	LYS	431	57.410	4.889	29.051	1.00	0.00	RX1	C
ATOM	6252	CE	LYS	431	57.012	5.124	27.590	1.00	0.00	RX1	C
ATOM	6253	NZ	LYS	431	57.458	4.018	26.743	1.00	0.00	RX1	N
ATOM	6254	HZ1	LYS	431	57.099	4.094	25.766	1.00	0.00	RX1	H
ATOM	6255	HZ2	LYS	431	58.488	4.015	26.580	1.00	0.00	RX1	H
ATOM	6256	HZ3	LYS	431	57.164	3.088	27.096	1.00	0.00	RX1	H
ATOM	6257	C	LYS	431	56.117	3.176	33.516	1.00	0.00	RX1	C
ATOM	6258	O	LYS	431	55.366	4.095	33.825	1.00	0.00	RX1	O
ATOM	6259	N	GLN	432	56.939	2.570	34.385	1.00	0.00	RX1	N
ATOM	6260	H	GLN	432	57.596	1.889	34.058	1.00	0.00	RX1	H
ATOM	6261	CA	GLN	432	56.938	3.073	35.759	1.00	0.00	RX1	C
ATOM	6262	CB	GLN	432	57.781	2.198	36.685	1.00	0.00	RX1	C
ATOM	6263	CG	GLN	432	56.929	1.305	37.585	1.00	0.00	RX1	C
ATOM	6264	CD	GLN	432	55.965	2.151	38.393	1.00	0.00	RX1	C
ATOM	6265	OE1	GLN	432	56.221	3.320	38.677	1.00	0.00	RX1	O
ATOM	6266	NE2	GLN	432	54.838	1.505	38.741	1.00	0.00	RX1	N
ATOM	6267	HE21	GLN	432	54.654	0.553	38.479	1.00	0.00	RX1	H
ATOM	6268	HE22	GLN	432	54.107	1.928	39.278	1.00	0.00	RX1	H
ATOM	6269	C	GLN	432	57.385	4.516	35.861	1.00	0.00	RX1	C
ATOM	6270	O	GLN	432	58.322	4.942	35.197	1.00	0.00	RX1	O
ATOM	6271	N	HIS	433	56.617	5.267	36.667	1.00	0.00	RX1	N
ATOM	6272	H	HIS	433	55.926	4.804	37.226	1.00	0.00	RX1	H
ATOM	6273	CA	HIS	433	56.779	6.725	36.742	1.00	0.00	RX1	C
ATOM	6274	CB	HIS	433	58.053	7.111	37.500	1.00	0.00	RX1	C
ATOM	6275	CG	HIS	433	57.900	6.875	38.985	1.00	0.00	RX1	C
ATOM	6276	ND1	HIS	433	57.494	5.719	39.547	1.00	0.00	RX1	N
ATOM	6277	HD1	HIS	433	57.230	4.888	39.092	1.00	0.00	RX1	H
ATOM	6278	CD2	HIS	433	58.155	7.798	40.004	1.00	0.00	RX1	C
ATOM	6279	NE2	HIS	433	57.899	7.184	41.185	1.00	0.00	RX1	N
ATOM	6280	CE1	HIS	433	57.493	5.905	40.904	1.00	0.00	RX1	C
ATOM	6281	C	HIS	433	56.724	7.464	35.407	1.00	0.00	RX1	C

ATOM	6282	O	HIS	433	57.241	8.565	35.258	1.00	0.00	RX1	O
ATOM	6283	N	GLY	434	56.087	6.811	34.422	1.00	0.00	RX1	N
ATOM	6284	H	GLY	434	55.711	5.891	34.542	1.00	0.00	RX1	H
ATOM	6285	CA	GLY	434	56.061	7.414	33.090	1.00	0.00	RX1	C
ATOM	6286	C	GLY	434	57.313	7.194	32.249	1.00	0.00	RX1	C
ATOM	6287	O	GLY	434	57.375	7.575	31.087	1.00	0.00	RX1	O
ATOM	6288	N	GLN	435	58.317	6.561	32.875	1.00	0.00	RX1	N
ATOM	6289	H	GLN	435	58.226	6.200	33.800	1.00	0.00	RX1	H
ATOM	6290	CA	GLN	435	59.588	6.453	32.168	1.00	0.00	RX1	C
ATOM	6291	CB	GLN	435	60.621	7.319	32.888	1.00	0.00	RX1	C
ATOM	6292	CG	GLN	435	61.874	7.564	32.052	1.00	0.00	RX1	C
ATOM	6293	CD	GLN	435	62.952	8.184	32.916	1.00	0.00	RX1	C
ATOM	6294	OE1	GLN	435	63.671	7.506	33.650	1.00	0.00	RX1	O
ATOM	6295	NE2	GLN	435	63.020	9.520	32.794	1.00	0.00	RX1	N
ATOM	6296	HE21	GLN	435	62.399	9.993	32.167	1.00	0.00	RX1	H
ATOM	6297	HE22	GLN	435	63.676	10.079	33.301	1.00	0.00	RX1	H
ATOM	6298	C	GLN	435	60.112	5.032	31.996	1.00	0.00	RX1	C
ATOM	6299	O	GLN	435	60.468	4.598	30.907	1.00	0.00	RX1	O
ATOM	6300	N	PHE	436	60.181	4.326	33.130	1.00	0.00	RX1	N
ATOM	6301	H	PHE	436	59.706	4.614	33.960	1.00	0.00	RX1	H
ATOM	6302	CA	PHE	436	60.967	3.091	33.122	1.00	0.00	RX1	C
ATOM	6303	CB	PHE	436	61.464	2.703	34.517	1.00	0.00	RX1	C
ATOM	6304	CG	PHE	436	61.661	3.891	35.426	1.00	0.00	RX1	C
ATOM	6305	CD1	PHE	436	60.911	3.975	36.593	1.00	0.00	RX1	C
ATOM	6306	CD2	PHE	436	62.591	4.879	35.129	1.00	0.00	RX1	C
ATOM	6307	CE1	PHE	436	61.082	5.041	37.466	1.00	0.00	RX1	C
ATOM	6308	CE2	PHE	436	62.760	5.946	36.004	1.00	0.00	RX1	C
ATOM	6309	CZ	PHE	436	62.010	6.030	37.169	1.00	0.00	RX1	C
ATOM	6310	C	PHE	436	60.241	1.876	32.585	1.00	0.00	RX1	C
ATOM	6311	O	PHE	436	59.091	1.632	32.924	1.00	0.00	RX1	O
ATOM	6312	N	SER	437	60.964	1.098	31.777	1.00	0.00	RX1	N
ATOM	6313	H	SER	437	61.881	1.309	31.436	1.00	0.00	RX1	H
ATOM	6314	CA	SER	437	60.484	-0.258	31.549	1.00	0.00	RX1	C
ATOM	6315	CB	SER	437	60.721	-0.582	30.092	1.00	0.00	RX1	C
ATOM	6316	OG	SER	437	61.907	0.103	29.690	1.00	0.00	RX1	O
ATOM	6317	HG	SER	437	61.943	-0.020	28.741	1.00	0.00	RX1	H
ATOM	6318	C	SER	437	61.137	-1.264	32.477	1.00	0.00	RX1	C
ATOM	6319	O	SER	437	60.503	-2.181	32.980	1.00	0.00	RX1	O
ATOM	6320	N	LEU	438	62.435	-1.049	32.722	1.00	0.00	RX1	N
ATOM	6321	H	LEU	438	62.925	-0.246	32.374	1.00	0.00	RX1	H
ATOM	6322	CA	LEU	438	63.027	-1.907	33.743	1.00	0.00	RX1	C
ATOM	6323	CB	LEU	438	64.357	-2.481	33.265	1.00	0.00	RX1	C
ATOM	6324	CG	LEU	438	64.231	-3.924	32.787	1.00	0.00	RX1	C
ATOM	6325	CD1	LEU	438	65.526	-4.425	32.149	1.00	0.00	RX1	C
ATOM	6326	CD2	LEU	438	63.759	-4.841	33.916	1.00	0.00	RX1	C
ATOM	6327	C	LEU	438	63.235	-1.162	35.035	1.00	0.00	RX1	C
ATOM	6328	O	LEU	438	63.777	-0.064	35.043	1.00	0.00	RX1	O
ATOM	6329	N	ALA	439	62.799	-1.800	36.123	1.00	0.00	RX1	N
ATOM	6330	H	ALA	439	62.279	-2.659	36.102	1.00	0.00	RX1	H
ATOM	6331	CA	ALA	439	63.035	-1.168	37.414	1.00	0.00	RX1	C
ATOM	6332	CB	ALA	439	61.836	-0.311	37.806	1.00	0.00	RX1	C
ATOM	6333	C	ALA	439	63.312	-2.195	38.489	1.00	0.00	RX1	C
ATOM	6334	O	ALA	439	62.410	-2.795	39.059	1.00	0.00	RX1	O
ATOM	6335	N	VAL	440	64.617	-2.400	38.701	1.00	0.00	RX1	N
ATOM	6336	H	VAL	440	65.302	-1.762	38.343	1.00	0.00	RX1	H
ATOM	6337	CA	VAL	440	65.061	-3.453	39.613	1.00	0.00	RX1	C
ATOM	6338	CB	VAL	440	65.812	-4.528	38.821	1.00	0.00	RX1	C
ATOM	6339	CG1	VAL	440	66.475	-5.550	39.739	1.00	0.00	RX1	C
ATOM	6340	CG2	VAL	440	64.910	-5.188	37.776	1.00	0.00	RX1	C
ATOM	6341	C	VAL	440	65.960	-2.838	40.671	1.00	0.00	RX1	C
ATOM	6342	O	VAL	440	67.013	-2.287	40.359	1.00	0.00	RX1	O

ATOM	6343	N	VAL	441	65.489	-2.901	41.923	1.00	0.00	RX1	N
ATOM	6344	H	VAL	441	64.687	-3.451	42.177	1.00	0.00	RX1	H
ATOM	6345	CA	VAL	441	66.225	-2.138	42.932	1.00	0.00	RX1	C
ATOM	6346	CB	VAL	441	65.473	-0.850	43.301	1.00	0.00	RX1	C
ATOM	6347	CG1	VAL	441	65.468	0.168	42.155	1.00	0.00	RX1	C
ATOM	6348	CG2	VAL	441	64.063	-1.164	43.801	1.00	0.00	RX1	C
ATOM	6349	C	VAL	441	66.596	-2.912	44.185	1.00	0.00	RX1	C
ATOM	6350	O	VAL	441	65.851	-3.754	44.663	1.00	0.00	RX1	O
ATOM	6351	N	SER	442	67.777	-2.559	44.718	1.00	0.00	RX1	N
ATOM	6352	H	SER	442	68.300	-1.869	44.219	1.00	0.00	RX1	H
ATOM	6353	CA	SER	442	68.218	-2.985	46.060	1.00	0.00	RX1	C
ATOM	6354	CB	SER	442	67.203	-2.419	47.046	1.00	0.00	RX1	C
ATOM	6355	OG	SER	442	66.653	-1.232	46.457	1.00	0.00	RX1	O
ATOM	6356	HG	SER	442	65.771	-1.473	46.201	1.00	0.00	RX1	H
ATOM	6357	C	SER	442	68.607	-4.444	46.310	1.00	0.00	RX1	C
ATOM	6358	O	SER	442	69.171	-4.807	47.346	1.00	0.00	RX1	O
ATOM	6359	N	LEU	443	68.268	-5.283	45.324	1.00	0.00	RX1	N
ATOM	6360	H	LEU	443	67.963	-4.912	44.445	1.00	0.00	RX1	H
ATOM	6361	CA	LEU	443	68.195	-6.724	45.554	1.00	0.00	RX1	C
ATOM	6362	CB	LEU	443	67.640	-7.441	44.327	1.00	0.00	RX1	C
ATOM	6363	CG	LEU	443	66.322	-6.878	43.807	1.00	0.00	RX1	C
ATOM	6364	CD1	LEU	443	65.860	-7.668	42.590	1.00	0.00	RX1	C
ATOM	6365	CD2	LEU	443	65.242	-6.793	44.886	1.00	0.00	RX1	C
ATOM	6366	C	LEU	443	69.476	-7.416	45.988	1.00	0.00	RX1	C
ATOM	6367	O	LEU	443	70.561	-6.844	46.062	1.00	0.00	RX1	O
ATOM	6368	N	ASN	444	69.288	-8.712	46.286	1.00	0.00	RX1	N
ATOM	6369	H	ASN	444	68.398	-9.157	46.175	1.00	0.00	RX1	H
ATOM	6370	CA	ASN	444	70.450	-9.527	46.645	1.00	0.00	RX1	C
ATOM	6371	CB	ASN	444	70.099	-10.579	47.695	1.00	0.00	RX1	C
ATOM	6372	CG	ASN	444	70.327	-9.991	49.066	1.00	0.00	RX1	C
ATOM	6373	OD1	ASN	444	70.595	-8.797	49.212	1.00	0.00	RX1	O
ATOM	6374	ND2	ASN	444	70.201	-10.881	50.063	1.00	0.00	RX1	N
ATOM	6375	HD21	ASN	444	69.948	-11.830	49.843	1.00	0.00	RX1	H
ATOM	6376	HD22	ASN	444	70.323	-10.670	51.032	1.00	0.00	RX1	H
ATOM	6377	C	ASN	444	71.124	-10.211	45.475	1.00	0.00	RX1	C
ATOM	6378	O	ASN	444	72.080	-10.964	45.623	1.00	0.00	RX1	O
ATOM	6379	N	ILE	445	70.586	-9.899	44.288	1.00	0.00	RX1	N
ATOM	6380	H	ILE	445	69.872	-9.212	44.201	1.00	0.00	RX1	H
ATOM	6381	CA	ILE	445	71.143	-10.501	43.085	1.00	0.00	RX1	C
ATOM	6382	CB	ILE	445	70.242	-10.206	41.883	1.00	0.00	RX1	C
ATOM	6383	CG2	ILE	445	68.884	-10.872	42.095	1.00	0.00	RX1	C
ATOM	6384	CG1	ILE	445	70.098	-8.706	41.607	1.00	0.00	RX1	C
ATOM	6385	CD1	ILE	445	69.279	-8.409	40.352	1.00	0.00	RX1	C
ATOM	6386	C	ILE	445	72.589	-10.126	42.811	1.00	0.00	RX1	C
ATOM	6387	O	ILE	445	72.963	-8.962	42.733	1.00	0.00	RX1	O
ATOM	6388	N	THR	446	73.397	-11.180	42.681	1.00	0.00	RX1	N
ATOM	6389	H	THR	446	73.045	-12.113	42.718	1.00	0.00	RX1	H
ATOM	6390	CA	THR	446	74.719	-10.926	42.133	1.00	0.00	RX1	C
ATOM	6391	CB	THR	446	75.759	-11.812	42.830	1.00	0.00	RX1	C
ATOM	6392	OG1	THR	446	77.001	-11.102	42.944	1.00	0.00	RX1	O
ATOM	6393	HG1	THR	446	76.726	-10.188	42.897	1.00	0.00	RX1	H
ATOM	6394	CG2	THR	446	75.930	-13.188	42.185	1.00	0.00	RX1	C
ATOM	6395	C	THR	446	74.736	-10.974	40.617	1.00	0.00	RX1	C
ATOM	6396	O	THR	446	75.682	-10.500	39.997	1.00	0.00	RX1	O
ATOM	6397	N	SER	447	73.665	-11.552	40.066	1.00	0.00	RX1	N
ATOM	6398	H	SER	447	72.943	-12.041	40.556	1.00	0.00	RX1	H
ATOM	6399	CA	SER	447	73.546	-11.564	38.620	1.00	0.00	RX1	C
ATOM	6400	CB	SER	447	73.764	-13.025	38.320	1.00	0.00	RX1	C
ATOM	6401	OG	SER	447	73.900	-13.651	39.605	1.00	0.00	RX1	O
ATOM	6402	HG	SER	447	73.096	-14.154	39.706	1.00	0.00	RX1	H
ATOM	6403	C	SER	447	72.205	-11.009	38.201	1.00	0.00	RX1	C

```
ATOM   6404  O   SER   447      71.204 -11.131  38.897  1.00  0.00      RX1  O
ATOM   6405  N   LEU   448      72.244 -10.348  37.041  1.00  0.00      RX1  N
ATOM   6406  H   LEU   448      73.063 -10.386  36.468  1.00  0.00      RX1  H
ATOM   6407  CA  LEU   448      71.013  -9.761  36.530  1.00  0.00      RX1  C
ATOM   6408  CB  LEU   448      71.336  -8.693  35.488  1.00  0.00      RX1  C
ATOM   6409  CG  LEU   448      70.977  -7.284  35.958  1.00  0.00      RX1  C
ATOM   6410  CD1 LEU   448      71.407  -6.219  34.949  1.00  0.00      RX1  C
ATOM   6411  CD2 LEU   448      69.499  -7.166  36.330  1.00  0.00      RX1  C
ATOM   6412  C   LEU   448      70.027 -10.768  35.973  1.00  0.00      RX1  C
ATOM   6413  O   LEU   448      68.830 -10.534  35.943  1.00  0.00      RX1  O
ATOM   6414  N   GLY   449      70.568 -11.909  35.525  1.00  0.00      RX1  N
ATOM   6415  H   GLY   449      71.562 -12.014  35.460  1.00  0.00      RX1  H
ATOM   6416  CA  GLY   449      69.691 -13.061  35.295  1.00  0.00      RX1  C
ATOM   6417  C   GLY   449      68.619 -12.999  34.211  1.00  0.00      RX1  C
ATOM   6418  O   GLY   449      67.924 -13.976  33.957  1.00  0.00      RX1  O
ATOM   6419  N   LEU   450      68.512 -11.837  33.547  1.00  0.00      RX1  N
ATOM   6420  H   LEU   450      69.098 -11.068  33.790  1.00  0.00      RX1  H
ATOM   6421  CA  LEU   450      67.549 -11.706  32.451  1.00  0.00      RX1  C
ATOM   6422  CB  LEU   450      67.171 -10.239  32.256  1.00  0.00      RX1  C
ATOM   6423  CG  LEU   450      66.527  -9.610  33.489  1.00  0.00      RX1  C
ATOM   6424  CD1 LEU   450      66.476  -8.085  33.402  1.00  0.00      RX1  C
ATOM   6425  CD2 LEU   450      65.147 -10.201  33.754  1.00  0.00      RX1  C
ATOM   6426  C   LEU   450      68.086 -12.263  31.144  1.00  0.00      RX1  C
ATOM   6427  O   LEU   450      68.160 -11.597  30.123  1.00  0.00      RX1  O
ATOM   6428  N   ARG   451      68.508 -13.526  31.235  1.00  0.00      RX1  N
ATOM   6429  H   ARG   451      68.252 -14.035  32.056  1.00  0.00      RX1  H
ATOM   6430  CA  ARG   451      69.370 -14.067  30.185  1.00  0.00      RX1  C
ATOM   6431  CB  ARG   451      70.062 -15.347  30.669  1.00  0.00      RX1  C
ATOM   6432  CG  ARG   451      69.192 -16.218  31.575  1.00  0.00      RX1  C
ATOM   6433  CD  ARG   451      69.981 -17.301  32.310  1.00  0.00      RX1  C
ATOM   6434  NE  ARG   451      71.084 -16.743  33.089  1.00  0.00      RX1  N
ATOM   6435  HE  ARG   451      71.903 -16.405  32.604  1.00  0.00      RX1  H
ATOM   6436  CZ  ARG   451      71.022 -16.513  34.436  1.00  0.00      RX1  C
ATOM   6437  NH1 ARG   451      69.964 -16.916  35.165  1.00  0.00      RX1  N
ATOM   6438 HH11 ARG   451      69.862 -16.621  36.130  1.00  0.00      RX1  H
ATOM   6439 HH12 ARG   451      69.237 -17.511  34.801  1.00  0.00      RX1  H
ATOM   6440  NH2 ARG   451      72.035 -15.854  35.021  1.00  0.00      RX1  N
ATOM   6441 HH21 ARG   451      72.025 -15.625  36.005  1.00  0.00      RX1  H
ATOM   6442 HH22 ARG   451      72.830 -15.584  34.452  1.00  0.00      RX1  H
ATOM   6443  C   ARG   451      68.768 -14.196  28.795  1.00  0.00      RX1  C
ATOM   6444  O   ARG   451      69.468 -14.186  27.793  1.00  0.00      RX1  O
ATOM   6445  N   SER   452      67.431 -14.277  28.760  1.00  0.00      RX1  N
ATOM   6446  H   SER   452      66.842 -14.220  29.565  1.00  0.00      RX1  H
ATOM   6447  CA  SER   452      66.853 -14.327  27.418  1.00  0.00      RX1  C
ATOM   6448  CB  SER   452      65.732 -15.357  27.456  1.00  0.00      RX1  C
ATOM   6449  OG  SER   452      66.184 -16.452  28.269  1.00  0.00      RX1  O
ATOM   6450  HG  SER   452      66.243 -16.107  29.152  1.00  0.00      RX1  H
ATOM   6451  C   SER   452      66.496 -12.979  26.795  1.00  0.00      RX1  C
ATOM   6452  O   SER   452      65.930 -12.902  25.709  1.00  0.00      RX1  O
ATOM   6453  N   LEU   453      66.829 -11.901  27.529  1.00  0.00      RX1  N
ATOM   6454  H   LEU   453      67.412 -11.964  28.339  1.00  0.00      RX1  H
ATOM   6455  CA  LEU   453      66.297 -10.601  27.127  1.00  0.00      RX1  C
ATOM   6456  CB  LEU   453      66.454  -9.592  28.266  1.00  0.00      RX1  C
ATOM   6457  CG  LEU   453      65.388  -8.495  28.286  1.00  0.00      RX1  C
ATOM   6458  CD1 LEU   453      63.990  -9.072  28.490  1.00  0.00      RX1  C
ATOM   6459  CD2 LEU   453      65.687  -7.433  29.343  1.00  0.00      RX1  C
ATOM   6460  C   LEU   453      66.839 -10.050  25.817  1.00  0.00      RX1  C
ATOM   6461  O   LEU   453      67.898  -9.443  25.738  1.00  0.00      RX1  O
ATOM   6462  N   LYS   454      66.031 -10.279  24.780  1.00  0.00      RX1  N
ATOM   6463  H   LYS   454      65.183 -10.781  24.955  1.00  0.00      RX1  H
ATOM   6464  CA  LYS   454      66.361  -9.763  23.455  1.00  0.00      RX1  C
```

ATOM	6465	CB	LYS	454	66.117	-10.822	22.376	1.00	0.00	RX1	C
ATOM	6466	CG	LYS	454	64.645	-11.251	22.380	1.00	0.00	RX1	C
ATOM	6467	CD	LYS	454	64.046	-11.692	21.040	1.00	0.00	RX1	C
ATOM	6468	CE	LYS	454	63.907	-10.559	20.015	1.00	0.00	RX1	C
ATOM	6469	NZ	LYS	454	63.280	-9.383	20.634	1.00	0.00	RX1	N
ATOM	6470	HZ1	LYS	454	62.709	-8.814	19.971	1.00	0.00	RX1	H
ATOM	6471	HZ2	LYS	454	62.593	-9.628	21.374	1.00	0.00	RX1	H
ATOM	6472	HZ3	LYS	454	64.003	-8.755	21.043	1.00	0.00	RX1	H
ATOM	6473	C	LYS	454	65.566	-8.535	23.027	1.00	0.00	RX1	C
ATOM	6474	O	LYS	454	65.420	-8.290	21.829	1.00	0.00	RX1	O
ATOM	6475	N	GLU	455	65.002	-7.802	24.000	1.00	0.00	RX1	N
ATOM	6476	H	GLU	455	65.026	-8.059	24.968	1.00	0.00	RX1	H
ATOM	6477	CA	GLU	455	64.172	-6.660	23.606	1.00	0.00	RX1	C
ATOM	6478	CB	GLU	455	62.855	-7.141	22.989	1.00	0.00	RX1	C
ATOM	6479	CG	GLU	455	62.298	-6.269	21.860	1.00	0.00	RX1	C
ATOM	6480	CD	GLU	455	61.066	-6.933	21.262	1.00	0.00	RX1	C
ATOM	6481	OE1	GLU	455	61.179	-8.042	20.747	1.00	0.00	RX1	O
ATOM	6482	OE2	GLU	455	59.988	-6.348	21.305	1.00	0.00	RX1	O
ATOM	6483	C	GLU	455	63.871	-5.751	24.771	1.00	0.00	RX1	C
ATOM	6484	O	GLU	455	63.208	-6.154	25.716	1.00	0.00	RX1	O
ATOM	6485	N	ILE	456	64.339	-4.504	24.655	1.00	0.00	RX1	N
ATOM	6486	H	ILE	456	65.003	-4.199	23.968	1.00	0.00	RX1	H
ATOM	6487	CA	ILE	456	63.612	-3.486	25.404	1.00	0.00	RX1	C
ATOM	6488	CB	ILE	456	64.426	-2.864	26.538	1.00	0.00	RX1	C
ATOM	6489	CG2	ILE	456	63.525	-1.960	27.381	1.00	0.00	RX1	C
ATOM	6490	CG1	ILE	456	65.130	-3.909	27.403	1.00	0.00	RX1	C
ATOM	6491	CD1	ILE	456	66.039	-3.266	28.450	1.00	0.00	RX1	C
ATOM	6492	C	ILE	456	63.131	-2.432	24.425	1.00	0.00	RX1	C
ATOM	6493	O	ILE	456	63.833	-1.505	24.030	1.00	0.00	RX1	O
ATOM	6494	N	SER	457	61.892	-2.664	23.991	1.00	0.00	RX1	N
ATOM	6495	H	SER	457	61.312	-3.353	24.433	1.00	0.00	RX1	H
ATOM	6496	CA	SER	457	61.392	-1.936	22.828	1.00	0.00	RX1	C
ATOM	6497	CB	SER	457	60.188	-2.744	22.387	1.00	0.00	RX1	C
ATOM	6498	OG	SER	457	60.343	-4.026	23.012	1.00	0.00	RX1	O
ATOM	6499	HG	SER	457	60.225	-4.696	22.337	1.00	0.00	RX1	H
ATOM	6500	C	SER	457	61.166	-0.441	23.028	1.00	0.00	RX1	C
ATOM	6501	O	SER	457	61.145	0.339	22.077	1.00	0.00	RX1	O
ATOM	6502	N	ASP	458	61.018	-0.093	24.319	1.00	0.00	RX1	N
ATOM	6503	H	ASP	458	61.032	-0.764	25.065	1.00	0.00	RX1	H
ATOM	6504	CA	ASP	458	60.781	1.277	24.776	1.00	0.00	RX1	C
ATOM	6505	CB	ASP	458	59.356	1.656	24.345	1.00	0.00	RX1	C
ATOM	6506	CG	ASP	458	58.929	3.093	24.599	1.00	0.00	RX1	C
ATOM	6507	OD1	ASP	458	59.658	3.883	25.187	1.00	0.00	RX1	O
ATOM	6508	OD2	ASP	458	57.795	3.426	24.276	1.00	0.00	RX1	O
ATOM	6509	C	ASP	458	60.983	1.275	26.288	1.00	0.00	RX1	C
ATOM	6510	O	ASP	458	60.986	0.220	26.915	1.00	0.00	RX1	O
ATOM	6511	N	GLY	459	61.179	2.474	26.845	1.00	0.00	RX1	N
ATOM	6512	H	GLY	459	61.215	3.282	26.255	1.00	0.00	RX1	H
ATOM	6513	CA	GLY	459	61.293	2.617	28.292	1.00	0.00	RX1	C
ATOM	6514	C	GLY	459	62.735	2.740	28.743	1.00	0.00	RX1	C
ATOM	6515	O	GLY	459	63.654	2.288	28.070	1.00	0.00	RX1	O
ATOM	6516	N	ASP	460	62.887	3.425	29.881	1.00	0.00	RX1	N
ATOM	6517	H	ASP	460	62.080	3.675	30.415	1.00	0.00	RX1	H
ATOM	6518	CA	ASP	460	64.220	3.683	30.433	1.00	0.00	RX1	C
ATOM	6519	CB	ASP	460	64.193	5.127	30.964	1.00	0.00	RX1	C
ATOM	6520	CG	ASP	460	65.518	5.761	31.376	1.00	0.00	RX1	C
ATOM	6521	OD1	ASP	460	65.629	6.981	31.371	1.00	0.00	RX1	O
ATOM	6522	OD2	ASP	460	66.435	5.091	31.817	1.00	0.00	RX1	O
ATOM	6523	C	ASP	460	64.496	2.633	31.503	1.00	0.00	RX1	C
ATOM	6524	O	ASP	460	63.594	1.942	31.967	1.00	0.00	RX1	O
ATOM	6525	N	VAL	461	65.771	2.504	31.855	1.00	0.00	RX1	N

ATOM	6526	H	VAL	461	66.467	3.138	31.505	1.00	0.00	RX1	H
ATOM	6527	CA	VAL	461	66.145	1.555	32.888	1.00	0.00	RX1	C
ATOM	6528	CB	VAL	461	67.289	0.696	32.342	1.00	0.00	RX1	C
ATOM	6529	CG1	VAL	461	68.076	-0.057	33.412	1.00	0.00	RX1	C
ATOM	6530	CG2	VAL	461	66.723	-0.246	31.283	1.00	0.00	RX1	C
ATOM	6531	C	VAL	461	66.514	2.253	34.185	1.00	0.00	RX1	C
ATOM	6532	O	VAL	461	67.233	3.244	34.224	1.00	0.00	RX1	O
ATOM	6533	N	ILE	462	65.999	1.659	35.263	1.00	0.00	RX1	N
ATOM	6534	H	ILE	462	65.331	0.920	35.180	1.00	0.00	RX1	H
ATOM	6535	CA	ILE	462	66.539	1.944	36.583	1.00	0.00	RX1	C
ATOM	6536	CB	ILE	462	65.702	2.980	37.345	1.00	0.00	RX1	C
ATOM	6537	CG2	ILE	462	64.239	2.572	37.497	1.00	0.00	RX1	C
ATOM	6538	CG1	ILE	462	66.362	3.325	38.680	1.00	0.00	RX1	C
ATOM	6539	CD1	ILE	462	65.619	4.436	39.422	1.00	0.00	RX1	C
ATOM	6540	C	ILE	462	66.775	0.658	37.360	1.00	0.00	RX1	C
ATOM	6541	O	ILE	462	65.949	0.129	38.096	1.00	0.00	RX1	O
ATOM	6542	N	ILE	463	67.982	0.144	37.126	1.00	0.00	RX1	N
ATOM	6543	H	ILE	463	68.678	0.656	36.619	1.00	0.00	RX1	H
ATOM	6544	CA	ILE	463	68.376	-1.003	37.931	1.00	0.00	RX1	C
ATOM	6545	CB	ILE	463	68.824	-2.158	37.035	1.00	0.00	RX1	C
ATOM	6546	CG2	ILE	463	69.308	-3.356	37.846	1.00	0.00	RX1	C
ATOM	6547	CG1	ILE	463	67.669	-2.559	36.116	1.00	0.00	RX1	C
ATOM	6548	CD1	ILE	463	68.000	-3.768	35.246	1.00	0.00	RX1	C
ATOM	6549	C	ILE	463	69.443	-0.558	38.908	1.00	0.00	RX1	C
ATOM	6550	O	ILE	463	70.635	-0.570	38.634	1.00	0.00	RX1	O
ATOM	6551	N	SER	464	68.938	-0.083	40.047	1.00	0.00	RX1	N
ATOM	6552	H	SER	464	67.994	-0.266	40.328	1.00	0.00	RX1	H
ATOM	6553	CA	SER	464	69.837	0.620	40.955	1.00	0.00	RX1	C
ATOM	6554	CB	SER	464	69.540	2.115	40.878	1.00	0.00	RX1	C
ATOM	6555	OG	SER	464	69.961	2.632	39.612	1.00	0.00	RX1	O
ATOM	6556	HG	SER	464	69.559	2.090	38.940	1.00	0.00	RX1	H
ATOM	6557	C	SER	464	69.769	0.104	42.377	1.00	0.00	RX1	C
ATOM	6558	O	SER	464	68.845	-0.595	42.772	1.00	0.00	RX1	O
ATOM	6559	N	GLY	465	70.801	0.475	43.145	1.00	0.00	RX1	N
ATOM	6560	H	GLY	465	71.597	0.915	42.727	1.00	0.00	RX1	H
ATOM	6561	CA	GLY	465	70.805	0.099	44.558	1.00	0.00	RX1	C
ATOM	6562	C	GLY	465	71.111	-1.366	44.828	1.00	0.00	RX1	C
ATOM	6563	O	GLY	465	71.079	-1.848	45.955	1.00	0.00	RX1	O
ATOM	6564	N	ASN	466	71.384	-2.090	43.737	1.00	0.00	RX1	N
ATOM	6565	H	ASN	466	71.617	-1.662	42.863	1.00	0.00	RX1	H
ATOM	6566	CA	ASN	466	71.558	-3.527	43.909	1.00	0.00	RX1	C
ATOM	6567	CB	ASN	466	71.187	-4.353	42.673	1.00	0.00	RX1	C
ATOM	6568	CG	ASN	466	69.779	-4.065	42.209	1.00	0.00	RX1	C
ATOM	6569	OD1	ASN	466	68.806	-4.635	42.693	1.00	0.00	RX1	O
ATOM	6570	ND2	ASN	466	69.720	-3.135	41.244	1.00	0.00	RX1	N
ATOM	6571	HD21	ASN	466	70.570	-2.729	40.893	1.00	0.00	RX1	H
ATOM	6572	HD22	ASN	466	68.845	-2.814	40.868	1.00	0.00	RX1	H
ATOM	6573	C	ASN	466	72.969	-3.843	44.335	1.00	0.00	RX1	C
ATOM	6574	O	ASN	466	73.875	-4.112	43.554	1.00	0.00	RX1	O
ATOM	6575	N	LYS	467	73.088	-3.807	45.665	1.00	0.00	RX1	N
ATOM	6576	H	LYS	467	72.283	-3.382	46.080	1.00	0.00	RX1	H
ATOM	6577	CA	LYS	467	74.330	-4.073	46.404	1.00	0.00	RX1	C
ATOM	6578	CB	LYS	467	73.989	-4.461	47.846	1.00	0.00	RX1	C
ATOM	6579	CG	LYS	467	72.712	-3.792	48.359	1.00	0.00	RX1	C
ATOM	6580	CD	LYS	467	72.177	-4.382	49.660	1.00	0.00	RX1	C
ATOM	6581	CE	LYS	467	71.991	-5.901	49.611	1.00	0.00	RX1	C
ATOM	6582	NZ	LYS	467	71.157	-6.330	48.479	1.00	0.00	RX1	N
ATOM	6583	HZ1	LYS	467	70.647	-7.190	48.763	1.00	0.00	RX1	H
ATOM	6584	HZ2	LYS	467	70.465	-5.615	48.163	1.00	0.00	RX1	H
ATOM	6585	HZ3	LYS	467	71.737	-6.617	47.658	1.00	0.00	RX1	H
ATOM	6586	C	LYS	467	75.222	-5.162	45.822	1.00	0.00	RX1	C

ATOM	6587	O	LYS	467	76.441	-5.086	45.782	1.00	0.00	RX1	O
ATOM	6588	N	ASN	468	74.529	-6.220	45.392	1.00	0.00	RX1	N
ATOM	6589	H	ASN	468	73.530	-6.214	45.358	1.00	0.00	RX1	H
ATOM	6590	CA	ASN	468	75.264	-7.429	45.040	1.00	0.00	RX1	C
ATOM	6591	CB	ASN	468	74.498	-8.683	45.485	1.00	0.00	RX1	C
ATOM	6592	CG	ASN	468	73.918	-8.576	46.887	1.00	0.00	RX1	C
ATOM	6593	OD1	ASN	468	73.117	-7.695	47.212	1.00	0.00	RX1	O
ATOM	6594	ND2	ASN	468	74.347	-9.549	47.709	1.00	0.00	RX1	N
ATOM	6595	HD21	ASN	468	74.966	-10.258	47.366	1.00	0.00	RX1	H
ATOM	6596	HD22	ASN	468	74.062	-9.625	48.665	1.00	0.00	RX1	H
ATOM	6597	C	ASN	468	75.616	-7.545	43.561	1.00	0.00	RX1	C
ATOM	6598	O	ASN	468	76.415	-8.384	43.155	1.00	0.00	RX1	O
ATOM	6599	N	LEU	469	74.975	-6.670	42.772	1.00	0.00	RX1	N
ATOM	6600	H	LEU	469	74.484	-5.896	43.168	1.00	0.00	RX1	H
ATOM	6601	CA	LEU	469	74.882	-6.855	41.324	1.00	0.00	RX1	C
ATOM	6602	CB	LEU	469	73.783	-5.947	40.798	1.00	0.00	RX1	C
ATOM	6603	CG	LEU	469	73.362	-6.250	39.370	1.00	0.00	RX1	C
ATOM	6604	CD1	LEU	469	72.970	-7.713	39.213	1.00	0.00	RX1	C
ATOM	6605	CD2	LEU	469	72.255	-5.304	38.918	1.00	0.00	RX1	C
ATOM	6606	C	LEU	469	76.140	-6.667	40.495	1.00	0.00	RX1	C
ATOM	6607	O	LEU	469	76.518	-5.564	40.111	1.00	0.00	RX1	O
ATOM	6608	N	CYS	470	76.752	-7.814	40.194	1.00	0.00	RX1	N
ATOM	6609	H	CYS	470	76.338	-8.692	40.430	1.00	0.00	RX1	H
ATOM	6610	CA	CYS	470	77.765	-7.787	39.144	1.00	0.00	RX1	C
ATOM	6611	CB	CYS	470	78.627	-9.032	39.279	1.00	0.00	RX1	C
ATOM	6612	SG	CYS	470	79.571	-8.988	40.817	1.00	0.00	RX1	S
ATOM	6613	C	CYS	470	77.121	-7.669	37.775	1.00	0.00	RX1	C
ATOM	6614	O	CYS	470	75.903	-7.682	37.649	1.00	0.00	RX1	O
ATOM	6615	N	TYR	471	77.987	-7.553	36.751	1.00	0.00	RX1	N
ATOM	6616	H	TYR	471	78.966	-7.454	36.930	1.00	0.00	RX1	H
ATOM	6617	CA	TYR	471	77.554	-7.646	35.349	1.00	0.00	RX1	C
ATOM	6618	CB	TYR	471	76.884	-8.994	35.062	1.00	0.00	RX1	C
ATOM	6619	CG	TYR	471	77.854	-10.119	35.324	1.00	0.00	RX1	C
ATOM	6620	CD1	TYR	471	77.663	-10.970	36.405	1.00	0.00	RX1	C
ATOM	6621	CE1	TYR	471	78.551	-12.014	36.630	1.00	0.00	RX1	C
ATOM	6622	CD2	TYR	471	78.937	-10.307	34.476	1.00	0.00	RX1	C
ATOM	6623	CE2	TYR	471	79.815	-11.361	34.693	1.00	0.00	RX1	C
ATOM	6624	CZ	TYR	471	79.622	-12.218	35.768	1.00	0.00	RX1	C
ATOM	6625	OH	TYR	471	80.491	-13.268	35.993	1.00	0.00	RX1	O
ATOM	6626	HH	TYR	471	81.110	-13.337	35.266	1.00	0.00	RX1	H
ATOM	6627	C	TYR	471	76.735	-6.513	34.742	1.00	0.00	RX1	C
ATOM	6628	O	TYR	471	76.800	-6.286	33.541	1.00	0.00	RX1	O
ATOM	6629	N	ALA	472	75.996	-5.775	35.594	1.00	0.00	RX1	N
ATOM	6630	H	ALA	472	75.910	-6.082	36.542	1.00	0.00	RX1	H
ATOM	6631	CA	ALA	472	75.125	-4.693	35.114	1.00	0.00	RX1	C
ATOM	6632	CB	ALA	472	74.612	-3.865	36.292	1.00	0.00	RX1	C
ATOM	6633	C	ALA	472	75.713	-3.726	34.093	1.00	0.00	RX1	C
ATOM	6634	O	ALA	472	75.047	-3.235	33.191	1.00	0.00	RX1	O
ATOM	6635	N	ASN	473	77.013	-3.469	34.274	1.00	0.00	RX1	N
ATOM	6636	H	ASN	473	77.543	-4.004	34.932	1.00	0.00	RX1	H
ATOM	6637	CA	ASN	473	77.718	-2.623	33.308	1.00	0.00	RX1	C
ATOM	6638	CB	ASN	473	79.096	-2.194	33.822	1.00	0.00	RX1	C
ATOM	6639	CG	ASN	473	80.010	-3.398	33.909	1.00	0.00	RX1	C
ATOM	6640	OD1	ASN	473	79.597	-4.474	34.339	1.00	0.00	RX1	O
ATOM	6641	ND2	ASN	473	81.269	-3.161	33.503	1.00	0.00	RX1	N
ATOM	6642	HD21	ASN	473	81.544	-2.266	33.148	1.00	0.00	RX1	H
ATOM	6643	HD22	ASN	473	81.984	-3.861	33.553	1.00	0.00	RX1	H
ATOM	6644	C	ASN	473	77.843	-3.222	31.911	1.00	0.00	RX1	C
ATOM	6645	O	ASN	473	77.713	-2.551	30.897	1.00	0.00	RX1	O
ATOM	6646	N	THR	474	78.099	-4.535	31.899	1.00	0.00	RX1	N
ATOM	6647	H	THR	474	78.125	-5.064	32.749	1.00	0.00	RX1	H

ATOM	6648	CA	THR	474	78.426	-5.183	30.630	1.00	0.00	RX1	C
ATOM	6649	CB	THR	474	79.157	-6.507	30.905	1.00	0.00	RX1	C
ATOM	6650	OG1	THR	474	78.321	-7.469	31.564	1.00	0.00	RX1	O
ATOM	6651	HG1	THR	474	77.823	-7.019	32.239	1.00	0.00	RX1	H
ATOM	6652	CG2	THR	474	80.447	-6.287	31.693	1.00	0.00	RX1	C
ATOM	6653	C	THR	474	77.294	-5.325	29.617	1.00	0.00	RX1	C
ATOM	6654	O	THR	474	77.494	-5.712	28.474	1.00	0.00	RX1	O
ATOM	6655	N	ILE	475	76.077	-5.000	30.083	1.00	0.00	RX1	N
ATOM	6656	H	ILE	475	75.982	-4.587	30.988	1.00	0.00	RX1	H
ATOM	6657	CA	ILE	475	74.892	-5.293	29.271	1.00	0.00	RX1	C
ATOM	6658	CB	ILE	475	73.633	-5.037	30.113	1.00	0.00	RX1	C
ATOM	6659	CG2	ILE	475	72.340	-5.437	29.400	1.00	0.00	RX1	C
ATOM	6660	CG1	ILE	475	73.742	-5.747	31.462	1.00	0.00	RX1	C
ATOM	6661	CD1	ILE	475	73.761	-7.265	31.319	1.00	0.00	RX1	C
ATOM	6662	C	ILE	475	74.819	-4.573	27.920	1.00	0.00	RX1	C
ATOM	6663	O	ILE	475	74.088	-4.966	27.022	1.00	0.00	RX1	O
ATOM	6664	N	ASN	476	75.586	-3.467	27.820	1.00	0.00	RX1	N
ATOM	6665	H	ASN	476	76.220	-3.241	28.558	1.00	0.00	RX1	H
ATOM	6666	CA	ASN	476	75.464	-2.597	26.639	1.00	0.00	RX1	C
ATOM	6667	CB	ASN	476	76.075	-3.144	25.360	1.00	0.00	RX1	C
ATOM	6668	CG	ASN	476	75.941	-2.066	24.298	1.00	0.00	RX1	C
ATOM	6669	OD1	ASN	476	75.680	-0.894	24.574	1.00	0.00	RX1	O
ATOM	6670	ND2	ASN	476	76.184	-2.523	23.059	1.00	0.00	RX1	N
ATOM	6671	HD21	ASN	476	76.284	-3.518	22.934	1.00	0.00	RX1	H
ATOM	6672	HD22	ASN	476	76.253	-1.953	22.243	1.00	0.00	RX1	H
ATOM	6673	C	ASN	476	74.035	-2.146	26.386	1.00	0.00	RX1	C
ATOM	6674	O	ASN	476	73.373	-2.403	25.387	1.00	0.00	RX1	O
ATOM	6675	N	TRP	477	73.584	-1.426	27.413	1.00	0.00	RX1	N
ATOM	6676	H	TRP	477	74.204	-1.250	28.175	1.00	0.00	RX1	H
ATOM	6677	CA	TRP	477	72.176	-1.062	27.481	1.00	0.00	RX1	C
ATOM	6678	CB	TRP	477	71.975	-0.195	28.708	1.00	0.00	RX1	C
ATOM	6679	CG	TRP	477	72.104	-1.047	29.942	1.00	0.00	RX1	C
ATOM	6680	CD2	TRP	477	71.102	-1.909	30.517	1.00	0.00	RX1	C
ATOM	6681	CE2	TRP	477	71.657	-2.484	31.681	1.00	0.00	RX1	C
ATOM	6682	CE3	TRP	477	69.806	-2.232	30.136	1.00	0.00	RX1	C
ATOM	6683	CD1	TRP	477	73.218	-1.155	30.787	1.00	0.00	RX1	C
ATOM	6684	NE1	TRP	477	72.954	-2.001	31.814	1.00	0.00	RX1	N
ATOM	6685	HE1	TRP	477	73.588	-2.260	32.522	1.00	0.00	RX1	H
ATOM	6686	CZ2	TRP	477	70.900	-3.371	32.436	1.00	0.00	RX1	C
ATOM	6687	CZ3	TRP	477	69.060	-3.123	30.898	1.00	0.00	RX1	C
ATOM	6688	CH2	TRP	477	69.605	-3.689	32.044	1.00	0.00	RX1	C
ATOM	6689	C	TRP	477	71.551	-0.432	26.245	1.00	0.00	RX1	C
ATOM	6690	O	TRP	477	70.404	-0.699	25.917	1.00	0.00	RX1	O
ATOM	6691	N	LYS	478	72.335	0.409	25.550	1.00	0.00	RX1	N
ATOM	6692	H	LYS	478	73.321	0.450	25.712	1.00	0.00	RX1	H
ATOM	6693	CA	LYS	478	71.718	1.028	24.375	1.00	0.00	RX1	C
ATOM	6694	CB	LYS	478	72.459	2.302	23.959	1.00	0.00	RX1	C
ATOM	6695	CG	LYS	478	71.679	3.608	24.200	1.00	0.00	RX1	C
ATOM	6696	CD	LYS	478	70.865	4.155	23.012	1.00	0.00	RX1	C
ATOM	6697	CE	LYS	478	69.606	3.380	22.601	1.00	0.00	RX1	C
ATOM	6698	NZ	LYS	478	68.555	3.508	23.620	1.00	0.00	RX1	N
ATOM	6699	HZ1	LYS	478	67.966	2.647	23.639	1.00	0.00	RX1	H
ATOM	6700	HZ2	LYS	478	68.934	3.628	24.573	1.00	0.00	RX1	H
ATOM	6701	HZ3	LYS	478	67.888	4.276	23.401	1.00	0.00	RX1	H
ATOM	6702	C	LYS	478	71.460	0.113	23.187	1.00	0.00	RX1	C
ATOM	6703	O	LYS	478	70.625	0.407	22.343	1.00	0.00	RX1	O
ATOM	6704	N	LYS	479	72.183	-1.017	23.179	1.00	0.00	RX1	N
ATOM	6705	H	LYS	479	72.840	-1.232	23.903	1.00	0.00	RX1	H
ATOM	6706	CA	LYS	479	71.844	-2.065	22.215	1.00	0.00	RX1	C
ATOM	6707	CB	LYS	479	73.008	-3.076	22.191	1.00	0.00	RX1	C
ATOM	6708	CG	LYS	479	72.934	-4.427	21.451	1.00	0.00	RX1	C

ATOM	6709	CD	LYS	479	72.397	-5.599	22.292	1.00	0.00	RX1	C
ATOM	6710	CE	LYS	479	72.811	-7.004	21.805	1.00	0.00	RX1	C
ATOM	6711	NZ	LYS	479	74.234	-7.260	22.078	1.00	0.00	RX1	N
ATOM	6712	HZ1	LYS	479	74.514	-8.270	22.102	1.00	0.00	RX1	H
ATOM	6713	HZ2	LYS	479	74.872	-6.759	21.423	1.00	0.00	RX1	H
ATOM	6714	HZ3	LYS	479	74.513	-6.838	22.988	1.00	0.00	RX1	H
ATOM	6715	C	LYS	479	70.508	-2.717	22.529	1.00	0.00	RX1	C
ATOM	6716	O	LYS	479	69.721	-3.047	21.652	1.00	0.00	RX1	O
ATOM	6717	N	LEU	480	70.292	-2.907	23.838	1.00	0.00	RX1	N
ATOM	6718	H	LEU	480	70.905	-2.542	24.539	1.00	0.00	RX1	H
ATOM	6719	CA	LEU	480	69.089	-3.658	24.175	1.00	0.00	RX1	C
ATOM	6720	CB	LEU	480	69.293	-4.412	25.480	1.00	0.00	RX1	C
ATOM	6721	CG	LEU	480	68.284	-5.547	25.571	1.00	0.00	RX1	C
ATOM	6722	CD1	LEU	480	68.395	-6.480	24.366	1.00	0.00	RX1	C
ATOM	6723	CD2	LEU	480	68.400	-6.289	26.893	1.00	0.00	RX1	C
ATOM	6724	C	LEU	480	67.811	-2.844	24.214	1.00	0.00	RX1	C
ATOM	6725	O	LEU	480	66.773	-3.219	23.677	1.00	0.00	RX1	O
ATOM	6726	N	PHE	481	67.932	-1.688	24.879	1.00	0.00	RX1	N
ATOM	6727	H	PHE	481	68.807	-1.381	25.248	1.00	0.00	RX1	H
ATOM	6728	CA	PHE	481	66.809	-0.777	24.748	1.00	0.00	RX1	C
ATOM	6729	CB	PHE	481	66.497	-0.022	26.051	1.00	0.00	RX1	C
ATOM	6730	CG	PHE	481	67.635	0.709	26.726	1.00	0.00	RX1	C
ATOM	6731	CD1	PHE	481	68.375	1.684	26.073	1.00	0.00	RX1	C
ATOM	6732	CD2	PHE	481	67.901	0.439	28.060	1.00	0.00	RX1	C
ATOM	6733	CE1	PHE	481	69.330	2.427	26.753	1.00	0.00	RX1	C
ATOM	6734	CE2	PHE	481	68.853	1.180	28.746	1.00	0.00	RX1	C
ATOM	6735	CZ	PHE	481	69.552	2.191	28.100	1.00	0.00	RX1	C
ATOM	6736	C	PHE	481	66.903	0.086	23.509	1.00	0.00	RX1	C
ATOM	6737	O	PHE	481	67.655	1.049	23.413	1.00	0.00	RX1	O
ATOM	6738	N	GLY	482	66.121	-0.331	22.514	1.00	0.00	RX1	N
ATOM	6739	H	GLY	482	65.416	-1.022	22.692	1.00	0.00	RX1	H
ATOM	6740	CA	GLY	482	66.387	0.225	21.189	1.00	0.00	RX1	C
ATOM	6741	C	GLY	482	65.627	1.491	20.835	1.00	0.00	RX1	C
ATOM	6742	O	GLY	482	65.007	1.587	19.781	1.00	0.00	RX1	O
ATOM	6743	N	THR	483	65.684	2.473	21.739	1.00	0.00	RX1	N
ATOM	6744	H	THR	483	66.120	2.372	22.632	1.00	0.00	RX1	H
ATOM	6745	CA	THR	483	64.861	3.657	21.497	1.00	0.00	RX1	C
ATOM	6746	CB	THR	483	63.542	3.379	22.227	1.00	0.00	RX1	C
ATOM	6747	OG1	THR	483	63.234	1.985	22.058	1.00	0.00	RX1	O
ATOM	6748	HG1	THR	483	62.384	1.836	22.471	1.00	0.00	RX1	H
ATOM	6749	CG2	THR	483	62.366	4.243	21.764	1.00	0.00	RX1	C
ATOM	6750	C	THR	483	65.579	4.947	21.890	1.00	0.00	RX1	C
ATOM	6751	O	THR	483	66.585	4.944	22.599	1.00	0.00	RX1	O
ATOM	6752	N	SER	484	65.049	6.059	21.370	1.00	0.00	RX1	N
ATOM	6753	H	SER	484	64.244	6.042	20.780	1.00	0.00	RX1	H
ATOM	6754	CA	SER	484	65.528	7.375	21.780	1.00	0.00	RX1	C
ATOM	6755	CB	SER	484	64.830	8.294	20.796	1.00	0.00	RX1	C
ATOM	6756	OG	SER	484	64.531	7.475	19.654	1.00	0.00	RX1	O
ATOM	6757	HG	SER	484	64.326	8.075	18.946	1.00	0.00	RX1	H
ATOM	6758	C	SER	484	65.271	7.661	23.255	1.00	0.00	RX1	C
ATOM	6759	O	SER	484	64.563	6.923	23.930	1.00	0.00	RX1	O
ATOM	6760	N	GLY	485	65.873	8.761	23.737	1.00	0.00	RX1	N
ATOM	6761	H	GLY	485	66.529	9.278	23.191	1.00	0.00	RX1	H
ATOM	6762	CA	GLY	485	65.589	9.151	25.120	1.00	0.00	RX1	C
ATOM	6763	C	GLY	485	66.385	8.388	26.167	1.00	0.00	RX1	C
ATOM	6764	O	GLY	485	67.351	8.897	26.731	1.00	0.00	RX1	O
ATOM	6765	N	GLN	486	65.926	7.143	26.367	1.00	0.00	RX1	N
ATOM	6766	H	GLN	486	65.123	6.912	25.814	1.00	0.00	RX1	H
ATOM	6767	CA	GLN	486	66.433	6.152	27.329	1.00	0.00	RX1	C
ATOM	6768	CB	GLN	486	66.385	4.766	26.701	1.00	0.00	RX1	C
ATOM	6769	CG	GLN	486	65.029	4.378	26.128	1.00	0.00	RX1	C

ATOM	6770	CD	GLN	486	65.236	3.169	25.250	1.00	0.00	RX1	C
ATOM	6771	OE1	GLN	486	66.207	3.094	24.497	1.00	0.00	RX1	O
ATOM	6772	NE2	GLN	486	64.268	2.246	25.349	1.00	0.00	RX1	N
ATOM	6773	HE21	GLN	486	63.639	2.322	26.129	1.00	0.00	RX1	H
ATOM	6774	HE22	GLN	486	64.153	1.451	24.750	1.00	0.00	RX1	H
ATOM	6775	C	GLN	486	67.831	6.336	27.902	1.00	0.00	RX1	C
ATOM	6776	O	GLN	486	68.795	6.672	27.210	1.00	0.00	RX1	O
ATOM	6777	N	LYS	487	67.903	6.076	29.208	1.00	0.00	RX1	N
ATOM	6778	H	LYS	487	67.119	5.794	29.772	1.00	0.00	RX1	H
ATOM	6779	CA	LYS	487	69.195	6.005	29.876	1.00	0.00	RX1	C
ATOM	6780	CB	LYS	487	69.325	7.140	30.894	1.00	0.00	RX1	C
ATOM	6781	CG	LYS	487	69.475	8.483	30.184	1.00	0.00	RX1	C
ATOM	6782	CD	LYS	487	70.790	8.531	29.407	1.00	0.00	RX1	C
ATOM	6783	CE	LYS	487	70.753	9.533	28.256	1.00	0.00	RX1	C
ATOM	6784	NZ	LYS	487	69.752	9.081	27.283	1.00	0.00	RX1	N
ATOM	6785	HZ1	LYS	487	68.779	9.255	27.616	1.00	0.00	RX1	H
ATOM	6786	HZ2	LYS	487	69.855	9.564	26.373	1.00	0.00	RX1	H
ATOM	6787	HZ3	LYS	487	69.800	8.051	27.132	1.00	0.00	RX1	H
ATOM	6788	C	LYS	487	69.351	4.664	30.556	1.00	0.00	RX1	C
ATOM	6789	O	LYS	487	68.497	3.794	30.452	1.00	0.00	RX1	O
ATOM	6790	N	THR	488	70.482	4.536	31.257	1.00	0.00	RX1	N
ATOM	6791	H	THR	488	71.214	5.210	31.328	1.00	0.00	RX1	H
ATOM	6792	CA	THR	488	70.537	3.406	32.166	1.00	0.00	RX1	C
ATOM	6793	CB	THR	488	71.333	2.253	31.537	1.00	0.00	RX1	C
ATOM	6794	OG1	THR	488	71.152	1.047	32.287	1.00	0.00	RX1	O
ATOM	6795	HG1	THR	488	71.694	0.378	31.889	1.00	0.00	RX1	H
ATOM	6796	CG2	THR	488	72.812	2.578	31.308	1.00	0.00	RX1	C
ATOM	6797	C	THR	488	70.985	3.858	33.536	1.00	0.00	RX1	C
ATOM	6798	O	THR	488	72.142	4.118	33.847	1.00	0.00	RX1	O
ATOM	6799	N	LYS	489	69.951	3.991	34.358	1.00	0.00	RX1	N
ATOM	6800	H	LYS	489	69.048	3.672	34.070	1.00	0.00	RX1	H
ATOM	6801	CA	LYS	489	70.210	4.306	35.749	1.00	0.00	RX1	C
ATOM	6802	CB	LYS	489	69.023	5.107	36.276	1.00	0.00	RX1	C
ATOM	6803	CG	LYS	489	68.797	6.273	35.303	1.00	0.00	RX1	C
ATOM	6804	CD	LYS	489	67.609	7.178	35.627	1.00	0.00	RX1	C
ATOM	6805	CE	LYS	489	66.240	6.525	35.428	1.00	0.00	RX1	C
ATOM	6806	NZ	LYS	489	65.937	6.296	34.010	1.00	0.00	RX1	N
ATOM	6807	HZ1	LYS	489	66.294	5.390	33.633	1.00	0.00	RX1	H
ATOM	6808	HZ2	LYS	489	64.909	6.325	33.846	1.00	0.00	RX1	H
ATOM	6809	HZ3	LYS	489	66.289	7.011	33.339	1.00	0.00	RX1	H
ATOM	6810	C	LYS	489	70.535	3.027	36.499	1.00	0.00	RX1	C
ATOM	6811	O	LYS	489	69.691	2.381	37.110	1.00	0.00	RX1	O
ATOM	6812	N	ILE	490	71.821	2.678	36.375	1.00	0.00	RX1	N
ATOM	6813	H	ILE	490	72.436	3.264	35.843	1.00	0.00	RX1	H
ATOM	6814	CA	ILE	490	72.329	1.493	37.066	1.00	0.00	RX1	C
ATOM	6815	CB	ILE	490	72.957	0.504	36.065	1.00	0.00	RX1	C
ATOM	6816	CG2	ILE	490	71.848	-0.354	35.454	1.00	0.00	RX1	C
ATOM	6817	CG1	ILE	490	73.773	1.170	34.947	1.00	0.00	RX1	C
ATOM	6818	CD1	ILE	490	75.121	1.778	35.345	1.00	0.00	RX1	C
ATOM	6819	C	ILE	490	73.262	1.833	38.219	1.00	0.00	RX1	C
ATOM	6820	O	ILE	490	74.331	1.262	38.413	1.00	0.00	RX1	O
ATOM	6821	N	ILE	491	72.819	2.855	38.959	1.00	0.00	RX1	N
ATOM	6822	H	ILE	491	71.839	3.065	38.979	1.00	0.00	RX1	H
ATOM	6823	CA	ILE	491	73.720	3.390	39.972	1.00	0.00	RX1	C
ATOM	6824	CB	ILE	491	73.444	4.879	40.168	1.00	0.00	RX1	C
ATOM	6825	CG2	ILE	491	73.672	5.623	38.850	1.00	0.00	RX1	C
ATOM	6826	CG1	ILE	491	72.039	5.121	40.726	1.00	0.00	RX1	C
ATOM	6827	CD1	ILE	491	71.768	6.594	41.029	1.00	0.00	RX1	C
ATOM	6828	C	ILE	491	73.644	2.622	41.277	1.00	0.00	RX1	C
ATOM	6829	O	ILE	491	72.715	1.866	41.525	1.00	0.00	RX1	O
ATOM	6830	N	SER	492	74.681	2.832	42.108	1.00	0.00	RX1	N

ATOM	6831	H	SER	492	75.295	3.599	41.943	1.00	0.00	RX1	H
ATOM	6832	CA	SER	492	74.708	2.193	43.430	1.00	0.00	RX1	C
ATOM	6833	CB	SER	492	73.726	3.021	44.234	1.00	0.00	RX1	C
ATOM	6834	OG	SER	492	73.768	4.328	43.638	1.00	0.00	RX1	O
ATOM	6835	HG	SER	492	73.119	4.844	44.101	1.00	0.00	RX1	H
ATOM	6836	C	SER	492	74.520	0.677	43.437	1.00	0.00	RX1	C
ATOM	6837	O	SER	492	73.983	0.089	44.370	1.00	0.00	RX1	O
ATOM	6838	N	ASN	493	74.983	0.072	42.337	1.00	0.00	RX1	N
ATOM	6839	H	ASN	493	75.521	0.591	41.677	1.00	0.00	RX1	H
ATOM	6840	CA	ASN	493	75.051	-1.386	42.354	1.00	0.00	RX1	C
ATOM	6841	CB	ASN	493	74.849	-1.964	40.948	1.00	0.00	RX1	C
ATOM	6842	CG	ASN	493	73.429	-1.738	40.463	1.00	0.00	RX1	C
ATOM	6843	OD1	ASN	493	72.447	-2.009	41.147	1.00	0.00	RX1	O
ATOM	6844	ND2	ASN	493	73.360	-1.233	39.225	1.00	0.00	RX1	N
ATOM	6845	HD21	ASN	493	74.166	-0.906	38.726	1.00	0.00	RX1	H
ATOM	6846	HD22	ASN	493	72.474	-1.107	38.769	1.00	0.00	RX1	H
ATOM	6847	C	ASN	493	76.417	-1.763	42.885	1.00	0.00	RX1	C
ATOM	6848	O	ASN	493	77.223	-0.876	43.133	1.00	0.00	RX1	O
ATOM	6849	N	ARG	494	76.660	-3.082	42.999	1.00	0.00	RX1	N
ATOM	6850	H	ARG	494	75.874	-3.693	42.893	1.00	0.00	RX1	H
ATOM	6851	CA	ARG	494	77.973	-3.650	43.364	1.00	0.00	RX1	C
ATOM	6852	CB	ARG	494	78.094	-5.007	42.686	1.00	0.00	RX1	C
ATOM	6853	CG	ARG	494	79.317	-5.871	42.970	1.00	0.00	RX1	C
ATOM	6854	CD	ARG	494	79.279	-6.587	44.318	1.00	0.00	RX1	C
ATOM	6855	NE	ARG	494	80.380	-7.545	44.384	1.00	0.00	RX1	N
ATOM	6856	HE	ARG	494	81.303	-7.204	44.589	1.00	0.00	RX1	H
ATOM	6857	CZ	ARG	494	80.191	-8.834	43.977	1.00	0.00	RX1	C
ATOM	6858	NH1	ARG	494	78.960	-9.286	43.656	1.00	0.00	RX1	N
ATOM	6859	HH11	ARG	494	78.813	-10.243	43.361	1.00	0.00	RX1	H
ATOM	6860	HH12	ARG	494	78.142	-8.700	43.670	1.00	0.00	RX1	H
ATOM	6861	NH2	ARG	494	81.247	-9.653	43.886	1.00	0.00	RX1	N
ATOM	6862	HH21	ARG	494	81.134	-10.596	43.546	1.00	0.00	RX1	H
ATOM	6863	HH22	ARG	494	82.194	-9.388	44.126	1.00	0.00	RX1	H
ATOM	6864	C	ARG	494	79.211	-2.819	43.031	1.00	0.00	RX1	C
ATOM	6865	O	ARG	494	80.107	-2.619	43.844	1.00	0.00	RX1	O
ATOM	6866	N	GLY	495	79.218	-2.360	41.772	1.00	0.00	RX1	N
ATOM	6867	H	GLY	495	78.510	-2.640	41.129	1.00	0.00	RX1	H
ATOM	6868	CA	GLY	495	80.376	-1.593	41.337	1.00	0.00	RX1	C
ATOM	6869	C	GLY	495	81.327	-2.472	40.562	1.00	0.00	RX1	C
ATOM	6870	O	GLY	495	81.571	-3.625	40.901	1.00	0.00	RX1	O
ATOM	6871	N	GLU	496	81.833	-1.883	39.469	1.00	0.00	RX1	N
ATOM	6872	H	GLU	496	81.654	-0.915	39.306	1.00	0.00	RX1	H
ATOM	6873	CA	GLU	496	82.713	-2.635	38.570	1.00	0.00	RX1	C
ATOM	6874	CB	GLU	496	83.048	-1.742	37.368	1.00	0.00	RX1	C
ATOM	6875	CG	GLU	496	83.787	-2.416	36.210	1.00	0.00	RX1	C
ATOM	6876	CD	GLU	496	85.233	-2.628	36.591	1.00	0.00	RX1	C
ATOM	6877	OE1	GLU	496	85.828	-3.621	36.183	1.00	0.00	RX1	O
ATOM	6878	OE2	GLU	496	85.775	-1.817	37.341	1.00	0.00	RX1	O
ATOM	6879	C	GLU	496	83.933	-3.202	39.293	1.00	0.00	RX1	C
ATOM	6880	O	GLU	496	84.275	-4.379	39.220	1.00	0.00	RX1	O
ATOM	6881	N	ASN	497	84.517	-2.303	40.097	1.00	0.00	RX1	N
ATOM	6882	H	ASN	497	84.162	-1.369	40.110	1.00	0.00	RX1	H
ATOM	6883	CA	ASN	497	85.539	-2.705	41.067	1.00	0.00	RX1	C
ATOM	6884	CB	ASN	497	85.930	-1.545	41.994	1.00	0.00	RX1	C
ATOM	6885	CG	ASN	497	84.742	-1.140	42.838	1.00	0.00	RX1	C
ATOM	6886	OD1	ASN	497	83.651	-0.917	42.317	1.00	0.00	RX1	O
ATOM	6887	ND2	ASN	497	84.991	-1.118	44.157	1.00	0.00	RX1	N
ATOM	6888	HD21	ASN	497	85.908	-1.209	44.543	1.00	0.00	RX1	H
ATOM	6889	HD22	ASN	497	84.221	-1.051	44.796	1.00	0.00	RX1	H
ATOM	6890	C	ASN	497	85.190	-3.957	41.866	1.00	0.00	RX1	C
ATOM	6891	O	ASN	497	85.930	-4.931	41.888	1.00	0.00	RX1	O

ATOM	6892	N	SER	498	84.008	-3.909	42.490	1.00	0.00	RX1	N
ATOM	6893	H	SER	498	83.380	-3.131	42.426	1.00	0.00	RX1	H
ATOM	6894	CA	SER	498	83.604	-5.019	43.347	1.00	0.00	RX1	C
ATOM	6895	CB	SER	498	82.617	-4.407	44.316	1.00	0.00	RX1	C
ATOM	6896	OG	SER	498	82.816	-2.991	44.246	1.00	0.00	RX1	O
ATOM	6897	HG	SER	498	81.934	-2.615	44.230	1.00	0.00	RX1	H
ATOM	6898	C	SER	498	83.119	-6.262	42.609	1.00	0.00	RX1	C
ATOM	6899	O	SER	498	82.976	-7.345	43.168	1.00	0.00	RX1	O
ATOM	6900	N	CYS	499	82.887	-6.053	41.306	1.00	0.00	RX1	N
ATOM	6901	H	CYS	499	82.966	-5.134	40.915	1.00	0.00	RX1	H
ATOM	6902	CA	CYS	499	82.579	-7.153	40.397	1.00	0.00	RX1	C
ATOM	6903	CB	CYS	499	81.963	-6.522	39.142	1.00	0.00	RX1	C
ATOM	6904	SG	CYS	499	81.273	-7.645	37.908	1.00	0.00	RX1	S
ATOM	6905	C	CYS	499	83.820	-7.990	40.125	1.00	0.00	RX1	C
ATOM	6906	O	CYS	499	83.886	-9.190	40.388	1.00	0.00	RX1	O
ATOM	6907	N	LYS	500	84.858	-7.281	39.643	1.00	0.00	RX1	N
ATOM	6908	H	LYS	500	84.790	-6.292	39.488	1.00	0.00	RX1	H
ATOM	6909	CA	LYS	500	86.116	-8.010	39.477	1.00	0.00	RX1	C
ATOM	6910	CB	LYS	500	87.110	-7.278	38.572	1.00	0.00	RX1	C
ATOM	6911	CG	LYS	500	87.446	-5.839	38.956	1.00	0.00	RX1	C
ATOM	6912	CD	LYS	500	88.666	-5.352	38.173	1.00	0.00	RX1	C
ATOM	6913	CE	LYS	500	88.900	-3.846	38.280	1.00	0.00	RX1	C
ATOM	6914	NZ	LYS	500	87.840	-3.156	37.546	1.00	0.00	RX1	N
ATOM	6915	HZ1	LYS	500	87.890	-2.120	37.522	1.00	0.00	RX1	H
ATOM	6916	HZ2	LYS	500	87.705	-3.478	36.564	1.00	0.00	RX1	H
ATOM	6917	HZ3	LYS	500	86.875	-3.328	37.910	1.00	0.00	RX1	H
ATOM	6918	C	LYS	500	86.771	-8.464	40.772	1.00	0.00	RX1	C
ATOM	6919	O	LYS	500	87.477	-9.462	40.814	1.00	0.00	RX1	O
ATOM	6920	N	ALA	501	86.437	-7.739	41.852	1.00	0.00	RX1	N
ATOM	6921	H	ALA	501	85.920	-6.887	41.760	1.00	0.00	RX1	H
ATOM	6922	CA	ALA	501	86.867	-8.190	43.177	1.00	0.00	RX1	C
ATOM	6923	CB	ALA	501	86.648	-7.100	44.226	1.00	0.00	RX1	C
ATOM	6924	C	ALA	501	86.232	-9.478	43.690	1.00	0.00	RX1	C
ATOM	6925	O	ALA	501	86.464	-9.910	44.811	1.00	0.00	RX1	O
ATOM	6926	N	THR	502	85.424	-10.105	42.828	1.00	0.00	RX1	N
ATOM	6927	H	THR	502	85.046	-9.706	41.993	1.00	0.00	RX1	H
ATOM	6928	CA	THR	502	85.110	-11.496	43.138	1.00	0.00	RX1	C
ATOM	6929	CB	THR	502	83.797	-11.490	43.898	1.00	0.00	RX1	C
ATOM	6930	OG1	THR	502	83.709	-10.258	44.635	1.00	0.00	RX1	O
ATOM	6931	HG1	THR	502	84.531	-10.214	45.126	1.00	0.00	RX1	H
ATOM	6932	CG2	THR	502	83.643	-12.695	44.829	1.00	0.00	RX1	C
ATOM	6933	C	THR	502	85.123	-12.375	41.898	1.00	0.00	RX1	C
ATOM	6934	O	THR	502	84.412	-13.363	41.774	1.00	0.00	RX1	O
ATOM	6935	N	GLY	503	85.961	-11.930	40.946	1.00	0.00	RX1	N
ATOM	6936	H	GLY	503	86.540	-11.129	41.102	1.00	0.00	RX1	H
ATOM	6937	CA	GLY	503	86.101	-12.667	39.691	1.00	0.00	RX1	C
ATOM	6938	C	GLY	503	84.857	-12.779	38.823	1.00	0.00	RX1	C
ATOM	6939	O	GLY	503	84.805	-13.568	37.889	1.00	0.00	RX1	O
ATOM	6940	N	GLN	504	83.850	-11.953	39.146	1.00	0.00	RX1	N
ATOM	6941	H	GLN	504	83.960	-11.187	39.780	1.00	0.00	RX1	H
ATOM	6942	CA	GLN	504	82.604	-12.112	38.403	1.00	0.00	RX1	C
ATOM	6943	CB	GLN	504	81.416	-11.753	39.291	1.00	0.00	RX1	C
ATOM	6944	CG	GLN	504	81.309	-12.627	40.541	1.00	0.00	RX1	C
ATOM	6945	CD	GLN	504	80.137	-12.158	41.377	1.00	0.00	RX1	C
ATOM	6946	OE1	GLN	504	80.286	-11.683	42.502	1.00	0.00	RX1	O
ATOM	6947	NE2	GLN	504	78.952	-12.295	40.761	1.00	0.00	RX1	N
ATOM	6948	HE21	GLN	504	78.882	-12.709	39.852	1.00	0.00	RX1	H
ATOM	6949	HE22	GLN	504	78.101	-11.988	41.195	1.00	0.00	RX1	H
ATOM	6950	C	GLN	504	82.565	-11.291	37.130	1.00	0.00	RX1	C
ATOM	6951	O	GLN	504	81.782	-10.363	36.962	1.00	0.00	RX1	O
ATOM	6952	N	VAL	505	83.486	-11.673	36.241	1.00	0.00	RX1	N

ATOM	6953	H	VAL	505	83.979	-12.532	36.396	1.00	0.00	RX1	H
ATOM	6954	CA	VAL	505	83.558	-11.009	34.947	1.00	0.00	RX1	C
ATOM	6955	CB	VAL	505	84.978	-10.500	34.696	1.00	0.00	RX1	C
ATOM	6956	CG1	VAL	505	85.317	-9.363	35.662	1.00	0.00	RX1	C
ATOM	6957	CG2	VAL	505	85.995	-11.641	34.771	1.00	0.00	RX1	C
ATOM	6958	C	VAL	505	83.118	-11.968	33.858	1.00	0.00	RX1	C
ATOM	6959	O	VAL	505	82.656	-13.065	34.145	1.00	0.00	RX1	O
ATOM	6960	N	CYS	506	83.277	-11.519	32.603	1.00	0.00	RX1	N
ATOM	6961	H	CYS	506	83.502	-10.566	32.412	1.00	0.00	RX1	H
ATOM	6962	CA	CYS	506	82.812	-12.395	31.524	1.00	0.00	RX1	C
ATOM	6963	CB	CYS	506	81.328	-12.135	31.366	1.00	0.00	RX1	C
ATOM	6964	SG	CYS	506	81.027	-10.349	31.307	1.00	0.00	RX1	S
ATOM	6965	C	CYS	506	83.530	-12.258	30.191	1.00	0.00	RX1	C
ATOM	6966	O	CYS	506	83.309	-13.026	29.258	1.00	0.00	RX1	O
ATOM	6967	N	HIS	507	84.382	-11.220	30.122	1.00	0.00	RX1	N
ATOM	6968	H	HIS	507	84.694	-10.742	30.938	1.00	0.00	RX1	H
ATOM	6969	CA	HIS	507	84.982	-10.877	28.832	1.00	0.00	RX1	C
ATOM	6970	CB	HIS	507	85.582	-9.472	28.863	1.00	0.00	RX1	C
ATOM	6971	CG	HIS	507	86.121	-9.140	27.491	1.00	0.00	RX1	C
ATOM	6972	ND1	HIS	507	87.318	-9.550	27.036	1.00	0.00	RX1	N
ATOM	6973	HD1	HIS	507	87.950	-10.123	27.529	1.00	0.00	RX1	H
ATOM	6974	CD2	HIS	507	85.493	-8.397	26.491	1.00	0.00	RX1	C
ATOM	6975	NE2	HIS	507	86.326	-8.366	25.421	1.00	0.00	RX1	N
ATOM	6976	CE1	HIS	507	87.452	-9.075	25.758	1.00	0.00	RX1	C
ATOM	6977	C	HIS	507	86.022	-11.881	28.357	1.00	0.00	RX1	C
ATOM	6978	O	HIS	507	87.210	-11.772	28.636	1.00	0.00	RX1	O
ATOM	6979	N	ALA	508	85.485	-12.855	27.618	1.00	0.00	RX1	N
ATOM	6980	H	ALA	508	84.509	-12.799	27.388	1.00	0.00	RX1	H
ATOM	6981	CA	ALA	508	86.247	-13.965	27.054	1.00	0.00	RX1	C
ATOM	6982	CB	ALA	508	86.853	-14.848	28.150	1.00	0.00	RX1	C
ATOM	6983	C	ALA	508	85.289	-14.809	26.244	1.00	0.00	RX1	C
ATOM	6984	O	ALA	508	85.382	-14.915	25.030	1.00	0.00	RX1	O
ATOM	6985	N	LEU	509	84.319	-15.366	26.990	1.00	0.00	RX1	N
ATOM	6986	H	LEU	509	84.254	-15.158	27.965	1.00	0.00	RX1	H
ATOM	6987	CA	LEU	509	83.209	-16.007	26.288	1.00	0.00	RX1	C
ATOM	6988	CB	LEU	509	82.598	-17.171	27.086	1.00	0.00	RX1	C
ATOM	6989	CG	LEU	509	81.791	-16.824	28.346	1.00	0.00	RX1	C
ATOM	6990	CD1	LEU	509	80.716	-17.874	28.616	1.00	0.00	RX1	C
ATOM	6991	CD2	LEU	509	82.659	-16.579	29.583	1.00	0.00	RX1	C
ATOM	6992	C	LEU	509	82.159	-14.991	25.879	1.00	0.00	RX1	C
ATOM	6993	O	LEU	509	81.599	-15.000	24.790	1.00	0.00	RX1	O
ATOM	6994	N	CYS	510	81.957	-14.052	26.810	1.00	0.00	RX1	N
ATOM	6995	H	CYS	510	82.435	-14.007	27.686	1.00	0.00	RX1	H
ATOM	6996	CA	CYS	510	81.157	-12.913	26.398	1.00	0.00	RX1	C
ATOM	6997	CB	CYS	510	80.443	-12.360	27.617	1.00	0.00	RX1	C
ATOM	6998	SG	CYS	510	79.782	-13.703	28.633	1.00	0.00	RX1	S
ATOM	6999	C	CYS	510	82.042	-11.900	25.716	1.00	0.00	RX1	C
ATOM	7000	O	CYS	510	83.259	-11.890	25.885	1.00	0.00	RX1	O
ATOM	7001	N	SER	511	81.380	-11.083	24.902	1.00	0.00	RX1	N
ATOM	7002	H	SER	511	80.378	-11.087	24.907	1.00	0.00	RX1	H
ATOM	7003	CA	SER	511	82.122	-10.074	24.159	1.00	0.00	RX1	C
ATOM	7004	CB	SER	511	81.339	-9.927	22.858	1.00	0.00	RX1	C
ATOM	7005	OG	SER	511	79.994	-9.549	23.135	1.00	0.00	RX1	O
ATOM	7006	HG	SER	511	79.771	-9.802	24.031	1.00	0.00	RX1	H
ATOM	7007	C	SER	511	82.302	-8.826	25.031	1.00	0.00	RX1	C
ATOM	7008	O	SER	511	81.939	-8.866	26.201	1.00	0.00	RX1	O
ATOM	7009	N	PRO	512	82.811	-7.691	24.460	1.00	0.00	RX1	N
ATOM	7010	CD	PRO	512	83.606	-7.536	23.241	1.00	0.00	RX1	C
ATOM	7011	CA	PRO	512	82.558	-6.397	25.118	1.00	0.00	RX1	C
ATOM	7012	CB	PRO	512	82.996	-5.385	24.056	1.00	0.00	RX1	C
ATOM	7013	CG	PRO	512	84.107	-6.098	23.290	1.00	0.00	RX1	C

ATOM	7014	C	PRO	512	81.123	-6.192	25.598	1.00	0.00	RX1	C
ATOM	7015	O	PRO	512	80.870	-5.668	26.676	1.00	0.00	RX1	O
ATOM	7016	N	GLU	513	80.186	-6.680	24.762	1.00	0.00	RX1	N
ATOM	7017	H	GLU	513	80.409	-7.141	23.906	1.00	0.00	RX1	H
ATOM	7018	CA	GLU	513	78.872	-6.901	25.354	1.00	0.00	RX1	C
ATOM	7019	CB	GLU	513	77.794	-6.966	24.288	1.00	0.00	RX1	C
ATOM	7020	CG	GLU	513	77.963	-5.894	23.215	1.00	0.00	RX1	C
ATOM	7021	CD	GLU	513	76.650	-5.802	22.484	1.00	0.00	RX1	C
ATOM	7022	OE1	GLU	513	75.668	-5.451	23.117	1.00	0.00	RX1	O
ATOM	7023	OE2	GLU	513	76.562	-6.136	21.308	1.00	0.00	RX1	O
ATOM	7024	C	GLU	513	78.903	-8.174	26.170	1.00	0.00	RX1	C
ATOM	7025	O	GLU	513	78.991	-9.280	25.639	1.00	0.00	RX1	O
ATOM	7026	N	GLY	514	78.887	-7.937	27.480	1.00	0.00	RX1	N
ATOM	7027	H	GLY	514	78.716	-7.001	27.788	1.00	0.00	RX1	H
ATOM	7028	CA	GLY	514	79.091	-9.026	28.421	1.00	0.00	RX1	C
ATOM	7029	C	GLY	514	77.885	-9.914	28.611	1.00	0.00	RX1	C
ATOM	7030	O	GLY	514	77.452	-10.621	27.707	1.00	0.00	RX1	O
ATOM	7031	N	CYS	515	77.372	-9.878	29.843	1.00	0.00	RX1	N
ATOM	7032	H	CYS	515	77.661	-9.258	30.580	1.00	0.00	RX1	H
ATOM	7033	CA	CYS	515	76.399	-10.911	30.179	1.00	0.00	RX1	C
ATOM	7034	CB	CYS	515	77.150	-12.202	30.473	1.00	0.00	RX1	C
ATOM	7035	SG	CYS	515	78.243	-11.976	31.884	1.00	0.00	RX1	S
ATOM	7036	C	CYS	515	75.516	-10.515	31.337	1.00	0.00	RX1	C
ATOM	7037	O	CYS	515	75.826	-9.604	32.092	1.00	0.00	RX1	O
ATOM	7038	N	TRP	516	74.408	-11.255	31.460	1.00	0.00	RX1	N
ATOM	7039	H	TRP	516	74.203	-12.006	30.828	1.00	0.00	RX1	H
ATOM	7040	CA	TRP	516	73.555	-11.040	32.626	1.00	0.00	RX1	C
ATOM	7041	CB	TRP	516	72.113	-11.461	32.340	1.00	0.00	RX1	C
ATOM	7042	CG	TRP	516	71.550	-10.761	31.130	1.00	0.00	RX1	C
ATOM	7043	CD2	TRP	516	70.825	-9.517	31.097	1.00	0.00	RX1	C
ATOM	7044	CE2	TRP	516	70.461	-9.272	29.756	1.00	0.00	RX1	C
ATOM	7045	CE3	TRP	516	70.455	-8.617	32.088	1.00	0.00	RX1	C
ATOM	7046	CD1	TRP	516	71.590	-11.198	29.798	1.00	0.00	RX1	C
ATOM	7047	NE1	TRP	516	70.947	-10.321	28.982	1.00	0.00	RX1	N
ATOM	7048	HE1	TRP	516	70.820	-10.430	28.016	1.00	0.00	RX1	H
ATOM	7049	CZ2	TRP	516	69.738	-8.129	29.446	1.00	0.00	RX1	C
ATOM	7050	CZ3	TRP	516	69.732	-7.476	31.763	1.00	0.00	RX1	C
ATOM	7051	CH2	TRP	516	69.371	-7.235	30.445	1.00	0.00	RX1	C
ATOM	7052	C	TRP	516	74.039	-11.733	33.892	1.00	0.00	RX1	C
ATOM	7053	O	TRP	516	73.531	-11.494	34.982	1.00	0.00	RX1	O
ATOM	7054	N	GLY	517	75.040	-12.601	33.719	1.00	0.00	RX1	N
ATOM	7055	H	GLY	517	75.439	-12.871	32.842	1.00	0.00	RX1	H
ATOM	7056	CA	GLY	517	75.476	-13.401	34.852	1.00	0.00	RX1	C
ATOM	7057	C	GLY	517	76.643	-14.278	34.458	1.00	0.00	RX1	C
ATOM	7058	O	GLY	517	77.130	-14.224	33.337	1.00	0.00	RX1	O
ATOM	7059	N	PRO	518	77.104	-15.079	35.443	1.00	0.00	RX1	N
ATOM	7060	CD	PRO	518	76.570	-15.183	36.791	1.00	0.00	RX1	C
ATOM	7061	CA	PRO	518	78.304	-15.894	35.225	1.00	0.00	RX1	C
ATOM	7062	CB	PRO	518	78.606	-16.425	36.634	1.00	0.00	RX1	C
ATOM	7063	CG	PRO	518	77.793	-15.572	37.611	1.00	0.00	RX1	C
ATOM	7064	C	PRO	518	78.156	-17.033	34.222	1.00	0.00	RX1	C
ATOM	7065	O	PRO	518	79.130	-17.645	33.798	1.00	0.00	RX1	O
ATOM	7066	N	GLU	519	76.895	-17.356	33.914	1.00	0.00	RX1	N
ATOM	7067	H	GLU	519	76.114	-16.731	33.983	1.00	0.00	RX1	H
ATOM	7068	CA	GLU	519	76.699	-18.620	33.219	1.00	0.00	RX1	C
ATOM	7069	CB	GLU	519	75.296	-19.140	33.536	1.00	0.00	RX1	C
ATOM	7070	CG	GLU	519	75.054	-19.233	35.043	1.00	0.00	RX1	C
ATOM	7071	CD	GLU	519	73.653	-19.745	35.283	1.00	0.00	RX1	C
ATOM	7072	OE1	GLU	519	72.800	-18.979	35.716	1.00	0.00	RX1	O
ATOM	7073	OE2	GLU	519	73.391	-20.914	35.015	1.00	0.00	RX1	O
ATOM	7074	C	GLU	519	76.936	-18.505	31.724	1.00	0.00	RX1	C

ATOM	7075	O	GLU	519	76.662	-17.489	31.099	1.00	0.00	RX1	O
ATOM	7076	N	PRO	520	77.433	-19.615	31.122	1.00	0.00	RX1	N
ATOM	7077	CD	PRO	520	77.896	-20.828	31.785	1.00	0.00	RX1	C
ATOM	7078	CA	PRO	520	77.610	-19.661	29.660	1.00	0.00	RX1	C
ATOM	7079	CB	PRO	520	78.129	-21.084	29.429	1.00	0.00	RX1	C
ATOM	7080	CG	PRO	520	78.791	-21.489	30.745	1.00	0.00	RX1	C
ATOM	7081	C	PRO	520	76.408	-19.336	28.769	1.00	0.00	RX1	C
ATOM	7082	O	PRO	520	76.535	-19.282	27.553	1.00	0.00	RX1	O
ATOM	7083	N	ARG	521	75.242	-19.157	29.408	1.00	0.00	RX1	N
ATOM	7084	H	ARG	521	75.211	-19.074	30.400	1.00	0.00	RX1	H
ATOM	7085	CA	ARG	521	74.018	-18.849	28.672	1.00	0.00	RX1	C
ATOM	7086	CB	ARG	521	72.908	-19.788	29.150	1.00	0.00	RX1	C
ATOM	7087	CG	ARG	521	72.740	-19.747	30.668	1.00	0.00	RX1	C
ATOM	7088	CD	ARG	521	71.838	-20.850	31.223	1.00	0.00	RX1	C
ATOM	7089	NE	ARG	521	71.802	-20.766	32.680	1.00	0.00	RX1	N
ATOM	7090	HE	ARG	521	72.667	-20.775	33.202	1.00	0.00	RX1	H
ATOM	7091	CZ	ARG	521	70.635	-20.539	33.343	1.00	0.00	RX1	C
ATOM	7092	NH1	ARG	521	69.469	-20.579	32.669	1.00	0.00	RX1	N
ATOM	7093	HH11	ARG	521	68.627	-20.298	33.156	1.00	0.00	RX1	H
ATOM	7094	HH12	ARG	521	69.401	-20.850	31.709	1.00	0.00	RX1	H
ATOM	7095	NH2	ARG	521	70.653	-20.250	34.655	1.00	0.00	RX1	N
ATOM	7096	HH21	ARG	521	69.815	-20.035	35.174	1.00	0.00	RX1	H
ATOM	7097	HH22	ARG	521	71.541	-20.198	35.156	1.00	0.00	RX1	H
ATOM	7098	C	ARG	521	73.589	-17.387	28.761	1.00	0.00	RX1	C
ATOM	7099	O	ARG	521	72.578	-16.979	28.210	1.00	0.00	RX1	O
ATOM	7100	N	ASP	522	74.395	-16.610	29.498	1.00	0.00	RX1	N
ATOM	7101	H	ASP	522	75.277	-16.919	29.855	1.00	0.00	RX1	H
ATOM	7102	CA	ASP	522	73.984	-15.248	29.846	1.00	0.00	RX1	C
ATOM	7103	CB	ASP	522	74.638	-14.856	31.168	1.00	0.00	RX1	C
ATOM	7104	CG	ASP	522	73.793	-15.181	32.379	1.00	0.00	RX1	C
ATOM	7105	OD1	ASP	522	74.167	-16.036	33.171	1.00	0.00	RX1	O
ATOM	7106	OD2	ASP	522	72.777	-14.534	32.588	1.00	0.00	RX1	O
ATOM	7107	C	ASP	522	74.345	-14.160	28.843	1.00	0.00	RX1	C
ATOM	7108	O	ASP	522	74.287	-12.973	29.146	1.00	0.00	RX1	O
ATOM	7109	N	CYS	523	74.816	-14.581	27.664	1.00	0.00	RX1	N
ATOM	7110	H	CYS	523	74.655	-15.500	27.309	1.00	0.00	RX1	H
ATOM	7111	CA	CYS	523	75.593	-13.593	26.913	1.00	0.00	RX1	C
ATOM	7112	CB	CYS	523	76.687	-14.326	26.151	1.00	0.00	RX1	C
ATOM	7113	SG	CYS	523	77.481	-15.542	27.234	1.00	0.00	RX1	S
ATOM	7114	C	CYS	523	74.822	-12.609	26.052	1.00	0.00	RX1	C
ATOM	7115	O	CYS	523	74.041	-12.965	25.182	1.00	0.00	RX1	O
ATOM	7116	N	VAL	524	75.116	-11.328	26.322	1.00	0.00	RX1	N
ATOM	7117	H	VAL	524	75.805	-11.131	27.018	1.00	0.00	RX1	H
ATOM	7118	CA	VAL	524	74.542	-10.246	25.518	1.00	0.00	RX1	C
ATOM	7119	CB	VAL	524	74.671	-8.920	26.275	1.00	0.00	RX1	C
ATOM	7120	CG1	VAL	524	73.943	-7.784	25.559	1.00	0.00	RX1	C
ATOM	7121	CG2	VAL	524	74.171	-9.073	27.711	1.00	0.00	RX1	C
ATOM	7122	C	VAL	524	75.147	-10.148	24.116	1.00	0.00	RX1	C
ATOM	7123	O	VAL	524	74.515	-9.691	23.162	1.00	0.00	RX1	O
ATOM	7124	N	SER	525	76.400	-10.630	24.039	1.00	0.00	RX1	N
ATOM	7125	H	SER	525	76.968	-10.835	24.839	1.00	0.00	RX1	H
ATOM	7126	CA	SER	525	76.989	-11.012	22.756	1.00	0.00	RX1	C
ATOM	7127	CB	SER	525	77.371	-9.803	21.874	1.00	0.00	RX1	C
ATOM	7128	OG	SER	525	76.317	-8.846	21.699	1.00	0.00	RX1	O
ATOM	7129	HG	SER	525	76.771	-8.020	21.527	1.00	0.00	RX1	H
ATOM	7130	C	SER	525	78.200	-11.886	23.012	1.00	0.00	RX1	C
ATOM	7131	O	SER	525	78.712	-11.952	24.127	1.00	0.00	RX1	O
ATOM	7132	N	CYS	526	78.606	-12.603	21.959	1.00	0.00	RX1	N
ATOM	7133	H	CYS	526	78.291	-12.447	21.025	1.00	0.00	RX1	H
ATOM	7134	CA	CYS	526	79.325	-13.839	22.258	1.00	0.00	RX1	C
ATOM	7135	CB	CYS	526	78.387	-15.003	21.958	1.00	0.00	RX1	C

```
ATOM   7136  SG  CYS   526      76.690 -14.616  22.453  1.00  0.00      RX1  S
ATOM   7137  C   CYS   526      80.674 -14.005  21.587  1.00  0.00      RX1  C
ATOM   7138  O   CYS   526      81.061 -13.280  20.670  1.00  0.00      RX1  O
ATOM   7139  N   ARG   527      81.391 -15.028  22.067  1.00  0.00      RX1  N
ATOM   7140  H   ARG   527      81.100 -15.577  22.857  1.00  0.00      RX1  H
ATOM   7141  CA  ARG   527      82.610 -15.376  21.357  1.00  0.00      RX1  C
ATOM   7142  CB  ARG   527      83.651 -15.929  22.336  1.00  0.00      RX1  C
ATOM   7143  CG  ARG   527      85.072 -16.158  21.795  1.00  0.00      RX1  C
ATOM   7144  CD  ARG   527      85.830 -14.903  21.343  1.00  0.00      RX1  C
ATOM   7145  NE  ARG   527      85.320 -14.382  20.075  1.00  0.00      RX1  N
ATOM   7146  HE  ARG   527      85.417 -14.969  19.261  1.00  0.00      RX1  H
ATOM   7147  CZ  ARG   527      84.666 -13.187  20.042  1.00  0.00      RX1  C
ATOM   7148  NH1 ARG   527      84.573 -12.450  21.173  1.00  0.00      RX1  N
ATOM   7149 HH11 ARG   527      84.092 -11.572  21.208  1.00  0.00      RX1  H
ATOM   7150 HH12 ARG   527      84.983 -12.769  22.035  1.00  0.00      RX1  H
ATOM   7151  NH2 ARG   527      84.111 -12.788  18.878  1.00  0.00      RX1  N
ATOM   7152 HH21 ARG   527      83.613 -11.926  18.757  1.00  0.00      RX1  H
ATOM   7153 HH22 ARG   527      84.182 -13.396  18.071  1.00  0.00      RX1  H
ATOM   7154  C   ARG   527      82.403 -16.323  20.195  1.00  0.00      RX1  C
ATOM   7155  O   ARG   527      82.634 -17.522  20.259  1.00  0.00      RX1  O
ATOM   7156  N   ASN   528      81.997 -15.671  19.091  1.00  0.00      RX1  N
ATOM   7157  H   ASN   528      81.637 -14.747  19.225  1.00  0.00      RX1  H
ATOM   7158  CA  ASN   528      81.832 -16.345  17.795  1.00  0.00      RX1  C
ATOM   7159  CB  ASN   528      83.081 -17.125  17.340  1.00  0.00      RX1  C
ATOM   7160  CG  ASN   528      84.207 -16.184  16.950  1.00  0.00      RX1  C
ATOM   7161  OD1 ASN   528      84.358 -15.080  17.477  1.00  0.00      RX1  O
ATOM   7162  ND2 ASN   528      85.004 -16.683  15.988  1.00  0.00      RX1  N
ATOM   7163 HD21 ASN   528      84.849 -17.599  15.612  1.00  0.00      RX1  H
ATOM   7164 HD22 ASN   528      85.781 -16.177  15.611  1.00  0.00      RX1  H
ATOM   7165  C   ASN   528      80.577 -17.204  17.785  1.00  0.00      RX1  C
ATOM   7166  O   ASN   528      79.723 -17.061  18.653  1.00  0.00      RX1  O
ATOM   7167  N   VAL   529      80.470 -18.080  16.766  1.00  0.00      RX1  N
ATOM   7168  H   VAL   529      81.208 -18.234  16.115  1.00  0.00      RX1  H
ATOM   7169  CA  VAL   529      79.255 -18.896  16.648  1.00  0.00      RX1  C
ATOM   7170  CB  VAL   529      79.318 -19.779  15.399  1.00  0.00      RX1  C
ATOM   7171  CG1 VAL   529      78.049 -20.624  15.256  1.00  0.00      RX1  C
ATOM   7172  CG2 VAL   529      79.586 -18.933  14.153  1.00  0.00      RX1  C
ATOM   7173  C   VAL   529      78.966 -19.741  17.883  1.00  0.00      RX1  C
ATOM   7174  O   VAL   529      79.672 -20.689  18.202  1.00  0.00      RX1  O
ATOM   7175  N   SER   530      77.900 -19.308  18.557  1.00  0.00      RX1  N
ATOM   7176  H   SER   530      77.286 -18.612  18.191  1.00  0.00      RX1  H
ATOM   7177  CA  SER   530      77.496 -19.938  19.806  1.00  0.00      RX1  C
ATOM   7178  CB  SER   530      77.276 -18.803  20.794  1.00  0.00      RX1  C
ATOM   7179  OG  SER   530      78.480 -18.029  20.883  1.00  0.00      RX1  O
ATOM   7180  HG  SER   530      78.788 -17.879  19.995  1.00  0.00      RX1  H
ATOM   7181  C   SER   530      76.275 -20.808  19.564  1.00  0.00      RX1  C
ATOM   7182  O   SER   530      75.555 -20.609  18.590  1.00  0.00      RX1  O
ATOM   7183  N   ARG   531      76.104 -21.827  20.415  1.00  0.00      RX1  N
ATOM   7184  H   ARG   531      76.553 -21.897  21.314  1.00  0.00      RX1  H
ATOM   7185  CA  ARG   531      75.233 -22.908  19.961  1.00  0.00      RX1  C
ATOM   7186  CB  ARG   531      76.062 -24.160  19.662  1.00  0.00      RX1  C
ATOM   7187  CG  ARG   531      77.151 -23.947  18.606  1.00  0.00      RX1  C
ATOM   7188  CD  ARG   531      78.453 -24.643  19.003  1.00  0.00      RX1  C
ATOM   7189  NE  ARG   531      78.899 -24.131  20.296  1.00  0.00      RX1  N
ATOM   7190  HE  ARG   531      78.337 -24.322  21.116  1.00  0.00      RX1  H
ATOM   7191  CZ  ARG   531      79.931 -23.247  20.398  1.00  0.00      RX1  C
ATOM   7192  NH1 ARG   531      80.662 -22.941  19.309  1.00  0.00      RX1  N
ATOM   7193 HH11 ARG   531      81.379 -22.240  19.340  1.00  0.00      RX1  H
ATOM   7194 HH12 ARG   531      80.488 -23.370  18.422  1.00  0.00      RX1  H
ATOM   7195  NH2 ARG   531      80.192 -22.687  21.590  1.00  0.00      RX1  N
ATOM   7196 HH21 ARG   531      80.969 -22.080  21.797  1.00  0.00      RX1  H
```

```
ATOM   7197 HH22 ARG   531      79.563 -22.867  22.371  1.00  0.00      RX1  H
ATOM   7198  C   ARG   531      74.127 -23.252  20.931  1.00  0.00      RX1  C
ATOM   7199  O   ARG   531      74.334 -23.656  22.065  1.00  0.00      RX1  O
ATOM   7200  N   GLY   532      72.898 -23.078  20.426  1.00  0.00      RX1  N
ATOM   7201  H   GLY   532      72.801 -22.659  19.524  1.00  0.00      RX1  H
ATOM   7202  CA  GLY   532      71.748 -23.632  21.149  1.00  0.00      RX1  C
ATOM   7203  C   GLY   532      71.453 -23.110  22.555  1.00  0.00      RX1  C
ATOM   7204  O   GLY   532      70.622 -23.682  23.258  1.00  0.00      RX1  O
ATOM   7205  N   ARG   533      72.124 -21.979  22.877  1.00  0.00      RX1  N
ATOM   7206  H   ARG   533      72.831 -21.684  22.237  1.00  0.00      RX1  H
ATOM   7207  CA  ARG   533      72.135 -21.248  24.158  1.00  0.00      RX1  C
ATOM   7208  CB  ARG   533      70.862 -21.328  25.000  1.00  0.00      RX1  C
ATOM   7209  CG  ARG   533      69.616 -20.769  24.327  1.00  0.00      RX1  C
ATOM   7210  CD  ARG   533      68.393 -21.268  25.091  1.00  0.00      RX1  C
ATOM   7211  NE  ARG   533      67.197 -21.249  24.255  1.00  0.00      RX1  N
ATOM   7212  HE  ARG   533      66.613 -20.437  24.371  1.00  0.00      RX1  H
ATOM   7213  CZ  ARG   533      66.961 -22.304  23.415  1.00  0.00      RX1  C
ATOM   7214  NH1 ARG   533      67.869 -23.308  23.302  1.00  0.00      RX1  N
ATOM   7215 HH11 ARG   533      67.713 -24.134  22.757  1.00  0.00      RX1  H
ATOM   7216 HH12 ARG   533      68.775 -23.278  23.755  1.00  0.00      RX1  H
ATOM   7217  NH2 ARG   533      65.810 -22.324  22.704  1.00  0.00      RX1  N
ATOM   7218 HH21 ARG   533      65.573 -23.066  22.071  1.00  0.00      RX1  H
ATOM   7219 HH22 ARG   533      65.138 -21.581  22.788  1.00  0.00      RX1  H
ATOM   7220  C   ARG   533      73.306 -21.540  25.078  1.00  0.00      RX1  C
ATOM   7221  O   ARG   533      73.239 -21.284  26.273  1.00  0.00      RX1  O
ATOM   7222  N   GLU   534      74.397 -22.038  24.488  1.00  0.00      RX1  N
ATOM   7223  H   GLU   534      74.488 -22.350  23.542  1.00  0.00      RX1  H
ATOM   7224  CA  GLU   534      75.636 -21.705  25.177  1.00  0.00      RX1  C
ATOM   7225  CB  GLU   534      76.422 -22.948  25.629  1.00  0.00      RX1  C
ATOM   7226  CG  GLU   534      76.872 -23.944  24.555  1.00  0.00      RX1  C
ATOM   7227  CD  GLU   534      77.954 -23.349  23.679  1.00  0.00      RX1  C
ATOM   7228  OE1 GLU   534      77.674 -23.032  22.536  1.00  0.00      RX1  O
ATOM   7229  OE2 GLU   534      79.089 -23.198  24.117  1.00  0.00      RX1  O
ATOM   7230  C   GLU   534      76.438 -20.752  24.322  1.00  0.00      RX1  C
ATOM   7231  O   GLU   534      76.171 -20.592  23.134  1.00  0.00      RX1  O
ATOM   7232  N   CYS   535      77.399 -20.107  24.989  1.00  0.00      RX1  N
ATOM   7233  H   CYS   535      77.483 -20.184  25.982  1.00  0.00      RX1  H
ATOM   7234  CA  CYS   535      78.203 -19.122  24.276  1.00  0.00      RX1  C
ATOM   7235  CB  CYS   535      77.682 -17.733  24.622  1.00  0.00      RX1  C
ATOM   7236  SG  CYS   535      75.886 -17.587  24.428  1.00  0.00      RX1  S
ATOM   7237  C   CYS   535      79.685 -19.258  24.552  1.00  0.00      RX1  C
ATOM   7238  O   CYS   535      80.440 -18.295  24.603  1.00  0.00      RX1  O
ATOM   7239  N   VAL   536      80.069 -20.520  24.774  1.00  0.00      RX1  N
ATOM   7240  H   VAL   536      79.451 -21.298  24.629  1.00  0.00      RX1  H
ATOM   7241  CA  VAL   536      81.465 -20.756  25.125  1.00  0.00      RX1  C
ATOM   7242  CB  VAL   536      81.596 -21.183  26.588  1.00  0.00      RX1  C
ATOM   7243  CG1 VAL   536      80.845 -22.486  26.860  1.00  0.00      RX1  C
ATOM   7244  CG2 VAL   536      83.069 -21.264  26.991  1.00  0.00      RX1  C
ATOM   7245  C   VAL   536      82.136 -21.761  24.208  1.00  0.00      RX1  C
ATOM   7246  O   VAL   536      82.164 -21.631  22.985  1.00  0.00      RX1  O
TER
HEADER lig.000.01.pdb
ATOM      1  N   PRO   563      -0.556 -31.523  26.101  1.00  0.00      LX0  N
ATOM      2  CD  PRO   563      -1.189 -32.704  25.525  1.00  0.00      LX0  C
ATOM      3  CA  PRO   563       0.910 -31.594  26.123  1.00  0.00      LX0  C
ATOM      4  CB  PRO   563       1.151 -33.073  25.801  1.00  0.00      LX0  C
ATOM      5  CG  PRO   563      -0.027 -33.482  24.916  1.00  0.00      LX0  C
ATOM      6  C   PRO   563       1.573 -30.650  25.128  1.00  0.00      LX0  C
ATOM      7  O   PRO   563       2.786 -30.657  24.936  1.00  0.00      LX0  O
ATOM      8  N   GLN   564       0.721 -29.835  24.487  1.00  0.00      LX0  N
ATOM      9  H   GLN   564      -0.249 -29.819  24.725  0.00  0.00      LX0  H
```

ATOM	10	CA	GLN	564	1.284	-28.985	23.449	1.00	0.00	LX0	C
ATOM	11	CB	GLN	564	0.279	-28.733	22.327	1.00	0.00	LX0	C
ATOM	12	CG	GLN	564	0.655	-29.530	21.070	1.00	0.00	LX0	C
ATOM	13	CD	GLN	564	0.763	-31.013	21.388	1.00	0.00	LX0	C
ATOM	14	OE1	GLN	564	-0.220	-31.714	21.577	1.00	0.00	LX0	O
ATOM	15	NE2	GLN	564	2.018	-31.468	21.448	1.00	0.00	LX0	N
ATOM	16	HE21	GLN	564	2.809	-30.857	21.356	0.00	0.00	LX0	H
ATOM	17	HE22	GLN	564	2.169	-32.441	21.600	0.00	0.00	LX0	H
ATOM	18	C	GLN	564	1.989	-27.742	23.927	1.00	0.00	LX0	C
ATOM	19	O	GLN	564	1.437	-26.676	24.166	1.00	0.00	LX0	O
ATOM	20	N	LYS	565	3.293	-27.971	24.050	1.00	0.00	LX0	N
ATOM	21	H	LYS	565	3.596	-28.872	23.733	0.00	0.00	LX0	H
ATOM	22	CA	LYS	565	4.217	-26.894	24.366	1.00	0.00	LX0	C
ATOM	23	CB	LYS	565	5.557	-27.508	24.766	1.00	0.00	LX0	C
ATOM	24	CG	LYS	565	5.425	-28.524	25.903	1.00	0.00	LX0	C
ATOM	25	CD	LYS	565	6.491	-29.629	25.897	1.00	0.00	LX0	C
ATOM	26	CE	LYS	565	6.275	-30.742	24.857	1.00	0.00	LX0	C
ATOM	27	NZ	LYS	565	6.460	-30.246	23.491	1.00	0.00	LX0	N
ATOM	28	HZ1	LYS	565	6.412	-30.979	22.752	0.00	0.00	LX0	H
ATOM	29	HZ2	LYS	565	5.698	-29.610	23.177	0.00	0.00	LX0	H
ATOM	30	HZ3	LYS	565	7.361	-29.754	23.351	0.00	0.00	LX0	H
ATOM	31	C	LYS	565	4.386	-26.007	23.154	1.00	0.00	LX0	C
ATOM	32	O	LYS	565	5.212	-26.260	22.290	1.00	0.00	LX0	O
ATOM	33	N	ILE	566	3.558	-24.961	23.109	1.00	0.00	LX0	N
ATOM	34	H	ILE	566	2.857	-24.847	23.814	0.00	0.00	LX0	H
ATOM	35	CA	ILE	566	3.691	-24.091	21.945	1.00	0.00	LX0	C
ATOM	36	CB	ILE	566	2.458	-23.189	21.782	1.00	0.00	LX0	C
ATOM	37	CG2	ILE	566	2.540	-22.334	20.509	1.00	0.00	LX0	C
ATOM	38	CG1	ILE	566	1.175	-24.028	21.810	1.00	0.00	LX0	C
ATOM	39	CD1	ILE	566	-0.100	-23.183	21.817	1.00	0.00	LX0	C
ATOM	40	C	ILE	566	4.977	-23.280	21.953	1.00	0.00	LX0	C
ATOM	41	O	ILE	566	5.194	-22.392	22.769	1.00	0.00	LX0	O
ATOM	42	N	CYS	567	5.816	-23.617	20.969	1.00	0.00	LX0	N
ATOM	43	H	CYS	567	5.575	-24.415	20.416	0.00	0.00	LX0	H
ATOM	44	CA	CYS	567	6.943	-22.757	20.627	1.00	0.00	LX0	C
ATOM	45	CB	CYS	567	7.842	-23.406	19.574	1.00	0.00	LX0	C
ATOM	46	SG	CYS	567	9.245	-22.367	19.067	1.00	0.00	LX0	S
ATOM	47	C	CYS	567	6.496	-21.402	20.132	1.00	0.00	LX0	C
ATOM	48	O	CYS	567	6.318	-21.167	18.939	1.00	0.00	LX0	O
ATOM	49	N	LEU	568	6.345	-20.502	21.112	1.00	0.00	LX0	N
ATOM	50	H	LEU	568	6.444	-20.856	22.046	0.00	0.00	LX0	H
ATOM	51	CA	LEU	568	5.847	-19.152	20.836	1.00	0.00	LX0	C
ATOM	52	CB	LEU	568	5.940	-18.291	22.098	1.00	0.00	LX0	C
ATOM	53	CG	LEU	568	5.183	-18.873	23.296	1.00	0.00	LX0	C
ATOM	54	CD1	LEU	568	5.458	-18.082	24.575	1.00	0.00	LX0	C
ATOM	55	CD2	LEU	568	3.683	-19.020	23.024	1.00	0.00	LX0	C
ATOM	56	C	LEU	568	6.534	-18.458	19.672	1.00	0.00	LX0	C
ATOM	57	O	LEU	568	5.931	-17.771	18.849	1.00	0.00	LX0	O
ATOM	58	N	ILE	569	7.852	-18.705	19.612	1.00	0.00	LX0	N
ATOM	59	H	ILE	569	8.270	-19.279	20.317	0.00	0.00	LX0	H
ATOM	60	CA	ILE	569	8.602	-18.145	18.491	1.00	0.00	LX0	C
ATOM	61	CB	ILE	569	10.117	-18.316	18.694	1.00	0.00	LX0	C
ATOM	62	CG2	ILE	569	10.932	-17.691	17.554	1.00	0.00	LX0	C
ATOM	63	CG1	ILE	569	10.536	-17.740	20.049	1.00	0.00	LX0	C
ATOM	64	CD1	ILE	569	10.254	-16.243	20.177	1.00	0.00	LX0	C
ATOM	65	C	ILE	569	8.153	-18.659	17.131	1.00	0.00	LX0	C
ATOM	66	O	ILE	569	7.790	-17.879	16.256	1.00	0.00	LX0	O
ATOM	67	N	CYS	570	8.186	-19.990	16.989	1.00	0.00	LX0	N
ATOM	68	H	CYS	570	8.304	-20.594	17.779	0.00	0.00	LX0	H
ATOM	69	CA	CYS	570	7.988	-20.509	15.638	1.00	0.00	LX0	C
ATOM	70	CB	CYS	570	8.894	-21.714	15.389	1.00	0.00	LX0	C

ATOM	71	SG	CYS	570	8.315	-23.231	16.185	1.00	0.00	LX0	S
ATOM	72	C	CYS	570	6.561	-20.837	15.235	1.00	0.00	LX0	C
ATOM	73	O	CYS	570	6.288	-21.170	14.087	1.00	0.00	LX0	O
ATOM	74	N	GLY	571	5.662	-20.756	16.227	1.00	0.00	LX0	N
ATOM	75	H	GLY	571	5.956	-20.567	17.164	0.00	0.00	LX0	H
ATOM	76	CA	GLY	571	4.268	-21.100	15.950	1.00	0.00	LX0	C
ATOM	77	C	GLY	571	4.019	-22.583	15.714	1.00	0.00	LX0	C
ATOM	78	O	GLY	571	3.172	-22.974	14.915	1.00	0.00	LX0	O
ATOM	79	N	ASP	572	4.804	-23.392	16.438	1.00	0.00	LX0	N
ATOM	80	H	ASP	572	5.431	-23.032	17.130	0.00	0.00	LX0	H
ATOM	81	CA	ASP	572	4.651	-24.844	16.330	1.00	0.00	LX0	C
ATOM	82	CB	ASP	572	5.469	-25.419	15.167	1.00	0.00	LX0	C
ATOM	83	CG	ASP	572	4.500	-25.825	14.075	1.00	0.00	LX0	C
ATOM	84	OD1	ASP	572	3.606	-26.620	14.330	1.00	0.00	LX0	O
ATOM	85	OD2	ASP	572	4.549	-25.282	12.976	1.00	0.00	LX0	O
ATOM	86	C	ASP	572	4.993	-25.505	17.644	1.00	0.00	LX0	C
ATOM	87	O	ASP	572	5.097	-24.813	18.647	1.00	0.00	LX0	O
ATOM	88	N	GLU	573	5.162	-26.834	17.616	1.00	0.00	LX0	N
ATOM	89	H	GLU	573	5.048	-27.337	16.760	0.00	0.00	LX0	H
ATOM	90	CA	GLU	573	5.558	-27.532	18.842	1.00	0.00	LX0	C
ATOM	91	CB	GLU	573	5.434	-29.035	18.595	1.00	0.00	LX0	C
ATOM	92	CG	GLU	573	4.470	-29.753	19.547	1.00	0.00	LX0	C
ATOM	93	CD	GLU	573	5.081	-29.909	20.926	1.00	0.00	LX0	C
ATOM	94	OE1	GLU	573	4.443	-29.570	21.919	1.00	0.00	LX0	O
ATOM	95	OE2	GLU	573	6.204	-30.388	21.028	1.00	0.00	LX0	O
ATOM	96	C	GLU	573	6.948	-27.153	19.348	1.00	0.00	LX0	C
ATOM	97	O	GLU	573	7.791	-26.658	18.605	1.00	0.00	LX0	O
ATOM	98	N	ALA	574	7.147	-27.376	20.652	1.00	0.00	LX0	N
ATOM	99	H	ALA	574	6.407	-27.789	21.188	0.00	0.00	LX0	H
ATOM	100	CA	ALA	574	8.428	-26.995	21.237	1.00	0.00	LX0	C
ATOM	101	CB	ALA	574	8.266	-25.919	22.308	1.00	0.00	LX0	C
ATOM	102	C	ALA	574	9.147	-28.140	21.896	1.00	0.00	LX0	C
ATOM	103	O	ALA	574	8.590	-28.897	22.687	1.00	0.00	LX0	O
ATOM	104	N	SER	575	10.436	-28.207	21.562	1.00	0.00	LX0	N
ATOM	105	H	SER	575	10.867	-27.514	20.985	0.00	0.00	LX0	H
ATOM	106	CA	SER	575	11.272	-29.213	22.201	1.00	0.00	LX0	C
ATOM	107	CB	SER	575	12.490	-29.466	21.311	1.00	0.00	LX0	C
ATOM	108	OG	SER	575	12.849	-28.257	20.618	1.00	0.00	LX0	O
ATOM	109	HG	SER	575	13.382	-27.767	21.241	0.00	0.00	LX0	H
ATOM	110	C	SER	575	11.638	-28.893	23.645	1.00	0.00	LX0	C
ATOM	111	O	SER	575	11.931	-29.774	24.441	1.00	0.00	LX0	O
ATOM	112	N	GLY	576	11.580	-27.591	23.969	1.00	0.00	LX0	N
ATOM	113	H	GLY	576	11.417	-26.866	23.295	0.00	0.00	LX0	H
ATOM	114	CA	GLY	576	11.727	-27.270	25.384	1.00	0.00	LX0	C
ATOM	115	C	GLY	576	12.143	-25.836	25.596	1.00	0.00	LX0	C
ATOM	116	O	GLY	576	11.835	-24.958	24.802	1.00	0.00	LX0	O
ATOM	117	N	CYS	577	12.872	-25.634	26.699	1.00	0.00	LX0	N
ATOM	118	H	CYS	577	13.129	-26.403	27.281	0.00	0.00	LX0	H
ATOM	119	CA	CYS	577	13.450	-24.305	26.893	1.00	0.00	LX0	C
ATOM	120	CB	CYS	577	13.694	-24.051	28.383	1.00	0.00	LX0	C
ATOM	121	SG	CYS	577	14.541	-22.495	28.761	1.00	0.00	LX0	S
ATOM	122	C	CYS	577	14.735	-24.174	26.104	1.00	0.00	LX0	C
ATOM	123	O	CYS	577	15.769	-24.742	26.445	1.00	0.00	LX0	O
ATOM	124	N	HIS	578	14.637	-23.431	25.003	1.00	0.00	LX0	N
ATOM	125	H	HIS	578	13.767	-23.049	24.683	0.00	0.00	LX0	H
ATOM	126	CA	HIS	578	15.875	-23.172	24.281	1.00	0.00	LX0	C
ATOM	127	CB	HIS	578	15.863	-23.770	22.872	1.00	0.00	LX0	C
ATOM	128	CG	HIS	578	15.614	-25.263	22.895	1.00	0.00	LX0	C
ATOM	129	ND1	HIS	578	15.854	-26.076	23.944	1.00	0.00	LX0	N
ATOM	130	HD1	HIS	578	16.171	-25.804	24.835	0.00	0.00	LX0	H
ATOM	131	CD2	HIS	578	15.110	-26.033	21.846	1.00	0.00	LX0	C

ATOM	132	NE2	HIS	578	15.049	-27.317	22.277	1.00	0.00	LX0	N
ATOM	133	CE1	HIS	578	15.505	-27.345	23.569	1.00	0.00	LX0	C
ATOM	134	C	HIS	578	16.160	-21.694	24.241	1.00	0.00	LX0	C
ATOM	135	O	HIS	578	15.562	-20.924	23.502	1.00	0.00	LX0	O
ATOM	136	N	TYR	579	17.114	-21.342	25.117	1.00	0.00	LX0	N
ATOM	137	H	TYR	579	17.432	-22.065	25.728	0.00	0.00	LX0	H
ATOM	138	CA	TYR	579	17.528	-19.955	25.360	1.00	0.00	LX0	C
ATOM	139	CB	TYR	579	17.958	-19.175	24.101	1.00	0.00	LX0	C
ATOM	140	CG	TYR	579	18.922	-19.925	23.205	1.00	0.00	LX0	C
ATOM	141	CD1	TYR	579	20.032	-20.619	23.735	1.00	0.00	LX0	C
ATOM	142	CE1	TYR	579	20.878	-21.321	22.857	1.00	0.00	LX0	C
ATOM	143	CD2	TYR	579	18.662	-19.888	21.823	1.00	0.00	LX0	C
ATOM	144	CE2	TYR	579	19.511	-20.577	20.948	1.00	0.00	LX0	C
ATOM	145	CZ	TYR	579	20.596	-21.303	21.474	1.00	0.00	LX0	C
ATOM	146	OH	TYR	579	21.393	-22.025	20.607	1.00	0.00	LX0	O
ATOM	147	HH	TYR	579	21.069	-21.893	19.717	0.00	0.00	LX0	H
ATOM	148	C	TYR	579	16.544	-19.121	26.165	1.00	0.00	LX0	C
ATOM	149	O	TYR	579	16.928	-18.470	27.127	1.00	0.00	LX0	O
ATOM	150	N	GLY	580	15.275	-19.169	25.741	1.00	0.00	LX0	N
ATOM	151	H	GLY	580	15.022	-19.682	24.920	0.00	0.00	LX0	H
ATOM	152	CA	GLY	580	14.257	-18.451	26.501	1.00	0.00	LX0	C
ATOM	153	C	GLY	580	12.976	-19.247	26.603	1.00	0.00	LX0	C
ATOM	154	O	GLY	580	12.957	-20.375	27.081	1.00	0.00	LX0	O
ATOM	155	N	VAL	581	11.899	-18.613	26.107	1.00	0.00	LX0	N
ATOM	156	H	VAL	581	11.997	-17.686	25.738	0.00	0.00	LX0	H
ATOM	157	CA	VAL	581	10.607	-19.305	26.137	1.00	0.00	LX0	C
ATOM	158	CB	VAL	581	9.482	-18.393	25.627	1.00	0.00	LX0	C
ATOM	159	CG1	VAL	581	9.341	-17.146	26.502	1.00	0.00	LX0	C
ATOM	160	CG2	VAL	581	9.642	-18.075	24.136	1.00	0.00	LX0	C
ATOM	161	C	VAL	581	10.583	-20.624	25.374	1.00	0.00	LX0	C
ATOM	162	O	VAL	581	11.530	-20.990	24.689	1.00	0.00	LX0	O
ATOM	163	N	LEU	582	9.425	-21.302	25.491	1.00	0.00	LX0	N
ATOM	164	H	LEU	582	8.714	-20.946	26.093	0.00	0.00	LX0	H
ATOM	165	CA	LEU	582	9.213	-22.528	24.717	1.00	0.00	LX0	C
ATOM	166	CB	LEU	582	7.766	-22.998	24.845	1.00	0.00	LX0	C
ATOM	167	CG	LEU	582	7.402	-23.507	26.239	1.00	0.00	LX0	C
ATOM	168	CD1	LEU	582	5.899	-23.759	26.363	1.00	0.00	LX0	C
ATOM	169	CD2	LEU	582	8.225	-24.737	26.631	1.00	0.00	LX0	C
ATOM	170	C	LEU	582	9.581	-22.410	23.249	1.00	0.00	LX0	C
ATOM	171	O	LEU	582	9.047	-21.599	22.497	1.00	0.00	LX0	O
ATOM	172	N	THR	583	10.543	-23.260	22.897	1.00	0.00	LX0	N
ATOM	173	H	THR	583	10.980	-23.852	23.574	0.00	0.00	LX0	H
ATOM	174	CA	THR	583	11.133	-23.189	21.572	1.00	0.00	LX0	C
ATOM	175	CB	THR	583	12.404	-22.345	21.631	1.00	0.00	LX0	C
ATOM	176	OG1	THR	583	13.004	-22.464	22.921	1.00	0.00	LX0	O
ATOM	177	HG1	THR	583	12.539	-21.842	23.479	0.00	0.00	LX0	H
ATOM	178	CG2	THR	583	12.132	-20.874	21.325	1.00	0.00	LX0	C
ATOM	179	C	THR	583	11.413	-24.561	20.993	1.00	0.00	LX0	C
ATOM	180	O	THR	583	11.562	-25.567	21.682	1.00	0.00	LX0	O
ATOM	181	N	CYS	584	11.453	-24.559	19.659	1.00	0.00	LX0	N
ATOM	182	H	CYS	584	11.301	-23.719	19.142	0.00	0.00	LX0	H
ATOM	183	CA	CYS	584	11.863	-25.785	18.987	1.00	0.00	LX0	C
ATOM	184	CB	CYS	584	11.061	-25.945	17.695	1.00	0.00	LX0	C
ATOM	185	SG	CYS	584	11.295	-24.533	16.579	1.00	0.00	LX0	S
ATOM	186	C	CYS	584	13.351	-25.746	18.701	1.00	0.00	LX0	C
ATOM	187	O	CYS	584	13.974	-24.687	18.738	1.00	0.00	LX0	O
ATOM	188	N	GLY	585	13.895	-26.924	18.352	1.00	0.00	LX0	N
ATOM	189	H	GLY	585	13.386	-27.755	18.587	0.00	0.00	LX0	H
ATOM	190	CA	GLY	585	15.271	-26.973	17.841	1.00	0.00	LX0	C
ATOM	191	C	GLY	585	15.557	-25.975	16.722	1.00	0.00	LX0	C
ATOM	192	O	GLY	585	16.559	-25.269	16.696	1.00	0.00	LX0	O

ATOM	193	N	SER	586	14.569	-25.901	15.822	1.00	0.00	LX0	N
ATOM	194	H	SER	586	13.827	-26.573	15.809	0.00	0.00	LX0	H
ATOM	195	CA	SER	586	14.617	-24.942	14.718	1.00	0.00	LX0	C
ATOM	196	CB	SER	586	13.434	-25.231	13.792	1.00	0.00	LX0	C
ATOM	197	OG	SER	586	13.037	-26.600	13.971	1.00	0.00	LX0	O
ATOM	198	HG	SER	586	12.560	-26.858	13.189	0.00	0.00	LX0	H
ATOM	199	C	SER	586	14.685	-23.462	15.098	1.00	0.00	LX0	C
ATOM	200	O	SER	586	14.992	-22.585	14.295	1.00	0.00	LX0	O
ATOM	201	N	CYS	587	14.381	-23.199	16.376	1.00	0.00	LX0	N
ATOM	202	H	CYS	587	14.086	-23.936	16.982	0.00	0.00	LX0	H
ATOM	203	CA	CYS	587	14.704	-21.878	16.904	1.00	0.00	LX0	C
ATOM	204	CB	CYS	587	13.619	-21.382	17.854	1.00	0.00	LX0	C
ATOM	205	SG	CYS	587	12.076	-21.086	16.952	1.00	0.00	LX0	S
ATOM	206	C	CYS	587	16.077	-21.809	17.531	1.00	0.00	LX0	C
ATOM	207	O	CYS	587	16.841	-20.895	17.256	1.00	0.00	LX0	O
ATOM	208	N	LYS	588	16.381	-22.844	18.330	1.00	0.00	LX0	N
ATOM	209	H	LYS	588	15.685	-23.541	18.512	0.00	0.00	LX0	H
ATOM	210	CA	LYS	588	17.708	-22.958	18.946	1.00	0.00	LX0	C
ATOM	211	CB	LYS	588	17.818	-24.346	19.583	1.00	0.00	LX0	C
ATOM	212	CG	LYS	588	19.088	-24.632	20.384	1.00	0.00	LX0	C
ATOM	213	CD	LYS	588	19.109	-26.085	20.857	1.00	0.00	LX0	C
ATOM	214	CE	LYS	588	20.374	-26.449	21.634	1.00	0.00	LX0	C
ATOM	215	NZ	LYS	588	20.324	-27.876	21.980	1.00	0.00	LX0	N
ATOM	216	HZ1	LYS	588	21.169	-28.134	22.529	0.00	0.00	LX0	H
ATOM	217	HZ2	LYS	588	19.470	-28.068	22.542	0.00	0.00	LX0	H
ATOM	218	HZ3	LYS	588	20.295	-28.443	21.109	0.00	0.00	LX0	H
ATOM	219	C	LYS	588	18.883	-22.691	18.004	1.00	0.00	LX0	C
ATOM	220	O	LYS	588	19.792	-21.915	18.281	1.00	0.00	LX0	O
ATOM	221	N	VAL	589	18.806	-23.369	16.850	1.00	0.00	LX0	N
ATOM	222	H	VAL	589	18.043	-23.999	16.692	0.00	0.00	LX0	H
ATOM	223	CA	VAL	589	19.881	-23.161	15.880	1.00	0.00	LX0	C
ATOM	224	CB	VAL	589	19.922	-24.303	14.852	1.00	0.00	LX0	C
ATOM	225	CG1	VAL	589	20.262	-25.621	15.552	1.00	0.00	LX0	C
ATOM	226	CG2	VAL	589	18.638	-24.417	14.021	1.00	0.00	LX0	C
ATOM	227	C	VAL	589	19.884	-21.796	15.205	1.00	0.00	LX0	C
ATOM	228	O	VAL	589	20.925	-21.252	14.855	1.00	0.00	LX0	O
ATOM	229	N	PHE	590	18.662	-21.260	15.042	1.00	0.00	LX0	N
ATOM	230	H	PHE	590	17.878	-21.691	15.483	0.00	0.00	LX0	H
ATOM	231	CA	PHE	590	18.539	-19.962	14.384	1.00	0.00	LX0	C
ATOM	232	CB	PHE	590	17.063	-19.696	14.037	1.00	0.00	LX0	C
ATOM	233	CG	PHE	590	16.835	-18.285	13.531	1.00	0.00	LX0	C
ATOM	234	CD1	PHE	590	17.172	-17.949	12.202	1.00	0.00	LX0	C
ATOM	235	CD2	PHE	590	16.293	-17.321	14.412	1.00	0.00	LX0	C
ATOM	236	CE1	PHE	590	16.980	-16.628	11.753	1.00	0.00	LX0	C
ATOM	237	CE2	PHE	590	16.104	-16.000	13.966	1.00	0.00	LX0	C
ATOM	238	CZ	PHE	590	16.454	-15.665	12.642	1.00	0.00	LX0	C
ATOM	239	C	PHE	590	19.135	-18.842	15.214	1.00	0.00	LX0	C
ATOM	240	O	PHE	590	20.009	-18.110	14.776	1.00	0.00	LX0	O
ATOM	241	N	PHE	591	18.609	-18.747	16.444	1.00	0.00	LX0	N
ATOM	242	H	PHE	591	17.952	-19.429	16.762	0.00	0.00	LX0	H
ATOM	243	CA	PHE	591	18.966	-17.599	17.275	1.00	0.00	LX0	C
ATOM	244	CB	PHE	591	18.139	-17.624	18.564	1.00	0.00	LX0	C
ATOM	245	CG	PHE	591	18.337	-16.352	19.357	1.00	0.00	LX0	C
ATOM	246	CD1	PHE	591	17.990	-15.108	18.787	1.00	0.00	LX0	C
ATOM	247	CD2	PHE	591	18.881	-16.435	20.655	1.00	0.00	LX0	C
ATOM	248	CE1	PHE	591	18.208	-13.926	19.519	1.00	0.00	LX0	C
ATOM	249	CE2	PHE	591	19.100	-15.253	21.386	1.00	0.00	LX0	C
ATOM	250	CZ	PHE	591	18.769	-14.010	20.809	1.00	0.00	LX0	C
ATOM	251	C	PHE	591	20.454	-17.474	17.547	1.00	0.00	LX0	C
ATOM	252	O	PHE	591	21.018	-16.392	17.606	1.00	0.00	LX0	O
ATOM	253	N	LYS	592	21.072	-18.660	17.667	1.00	0.00	LX0	N

ATOM	254	H	LYS	592	20.544	-19.506	17.626	0.00	0.00	LX0	H
ATOM	255	CA	LYS	592	22.528	-18.659	17.752	1.00	0.00	LX0	C
ATOM	256	CB	LYS	592	22.999	-20.088	18.026	1.00	0.00	LX0	C
ATOM	257	CG	LYS	592	24.443	-20.192	18.513	1.00	0.00	LX0	C
ATOM	258	CD	LYS	592	24.891	-21.650	18.632	1.00	0.00	LX0	C
ATOM	259	CE	LYS	592	26.371	-21.814	18.994	1.00	0.00	LX0	C
ATOM	260	NZ	LYS	592	27.214	-21.201	17.964	1.00	0.00	LX0	N
ATOM	261	HZ1	LYS	592	28.142	-21.633	17.821	0.00	0.00	LX0	H
ATOM	262	HZ2	LYS	592	26.751	-21.103	17.033	0.00	0.00	LX0	H
ATOM	263	HZ3	LYS	592	27.415	-20.194	18.156	0.00	0.00	LX0	H
ATOM	264	C	LYS	592	23.191	-18.052	16.517	1.00	0.00	LX0	C
ATOM	265	O	LYS	592	23.871	-17.034	16.568	1.00	0.00	LX0	O
ATOM	266	N	ARG	593	22.922	-18.716	15.379	1.00	0.00	LX0	N
ATOM	267	H	ARG	593	22.262	-19.467	15.372	0.00	0.00	LX0	H
ATOM	268	CA	ARG	593	23.608	-18.302	14.153	1.00	0.00	LX0	C
ATOM	269	CB	ARG	593	23.257	-19.240	13.002	1.00	0.00	LX0	C
ATOM	270	CG	ARG	593	23.764	-20.664	13.223	1.00	0.00	LX0	C
ATOM	271	CD	ARG	593	23.365	-21.581	12.069	1.00	0.00	LX0	C
ATOM	272	NE	ARG	593	23.894	-22.930	12.265	1.00	0.00	LX0	N
ATOM	273	HE	ARG	593	24.618	-23.019	12.952	0.00	0.00	LX0	H
ATOM	274	CZ	ARG	593	23.451	-23.959	11.512	1.00	0.00	LX0	C
ATOM	275	NH1	ARG	593	22.499	-23.766	10.597	1.00	0.00	LX0	N
ATOM	276	HH11	ARG	593	22.153	-24.505	10.020	0.00	0.00	LX0	H
ATOM	277	HH12	ARG	593	22.111	-22.851	10.470	0.00	0.00	LX0	H
ATOM	278	NH2	ARG	593	23.976	-25.171	11.689	1.00	0.00	LX0	N
ATOM	279	HH21	ARG	593	23.677	-25.961	11.153	0.00	0.00	LX0	H
ATOM	280	HH22	ARG	593	24.694	-25.316	12.372	0.00	0.00	LX0	H
ATOM	281	C	ARG	593	23.390	-16.861	13.726	1.00	0.00	LX0	C
ATOM	282	O	ARG	593	24.292	-16.196	13.237	1.00	0.00	LX0	O
ATOM	283	N	ALA	594	22.149	-16.407	13.948	1.00	0.00	LX0	N
ATOM	284	H	ALA	594	21.483	-16.987	14.414	0.00	0.00	LX0	H
ATOM	285	CA	ALA	594	21.801	-15.045	13.546	1.00	0.00	LX0	C
ATOM	286	CB	ALA	594	20.284	-14.911	13.405	1.00	0.00	LX0	C
ATOM	287	C	ALA	594	22.314	-13.943	14.461	1.00	0.00	LX0	C
ATOM	288	O	ALA	594	22.036	-12.767	14.266	1.00	0.00	LX0	O
ATOM	289	N	MET	595	23.069	-14.376	15.482	1.00	0.00	LX0	N
ATOM	290	H	MET	595	23.147	-15.347	15.700	0.00	0.00	LX0	H
ATOM	291	CA	MET	595	23.864	-13.384	16.194	1.00	0.00	LX0	C
ATOM	292	CB	MET	595	23.549	-13.403	17.692	1.00	0.00	LX0	C
ATOM	293	CG	MET	595	22.060	-13.222	18.013	1.00	0.00	LX0	C
ATOM	294	SD	MET	595	21.373	-11.642	17.489	1.00	0.00	LX0	S
ATOM	295	CE	MET	595	22.186	-10.592	18.700	1.00	0.00	LX0	C
ATOM	296	C	MET	595	25.353	-13.559	15.942	1.00	0.00	LX0	C
ATOM	297	O	MET	595	26.119	-12.604	15.927	1.00	0.00	LX0	O
ATOM	298	N	GLU	596	25.733	-14.837	15.730	1.00	0.00	LX0	N
ATOM	299	H	GLU	596	25.062	-15.573	15.811	0.00	0.00	LX0	H
ATOM	300	CA	GLU	596	27.132	-15.138	15.396	1.00	0.00	LX0	C
ATOM	301	CB	GLU	596	27.362	-16.649	15.307	1.00	0.00	LX0	C
ATOM	302	CG	GLU	596	27.046	-17.370	16.615	1.00	0.00	LX0	C
ATOM	303	CD	GLU	596	27.363	-18.847	16.503	1.00	0.00	LX0	C
ATOM	304	OE1	GLU	596	26.529	-19.628	16.043	1.00	0.00	LX0	O
ATOM	305	OE2	GLU	596	28.417	-19.262	16.978	1.00	0.00	LX0	O
ATOM	306	C	GLU	596	27.604	-14.476	14.114	1.00	0.00	LX0	C
ATOM	307	O	GLU	596	28.631	-13.812	14.045	1.00	0.00	LX0	O
ATOM	308	N	GLY	597	26.766	-14.666	13.088	1.00	0.00	LX0	N
ATOM	309	H	GLY	597	25.919	-15.179	13.223	0.00	0.00	LX0	H
ATOM	310	CA	GLY	597	26.872	-13.721	11.988	1.00	0.00	LX0	C
ATOM	311	C	GLY	597	26.035	-12.533	12.389	1.00	0.00	LX0	C
ATOM	312	O	GLY	597	24.881	-12.687	12.764	1.00	0.00	LX0	O
ATOM	313	N	GLN	598	26.677	-11.359	12.357	1.00	0.00	LX0	N
ATOM	314	H	GLN	598	27.565	-11.266	11.911	0.00	0.00	LX0	H

ATOM	315	CA	GLN	598	25.949	-10.224	12.920	1.00	0.00	LX0	C
ATOM	316	CB	GLN	598	26.947	-9.194	13.502	1.00	0.00	LX0	C
ATOM	317	CG	GLN	598	27.908	-9.858	14.519	1.00	0.00	LX0	C
ATOM	318	CD	GLN	598	28.769	-8.880	15.322	1.00	0.00	LX0	C
ATOM	319	OE1	GLN	598	29.963	-8.678	15.094	1.00	0.00	LX0	O
ATOM	320	NE2	GLN	598	28.133	-8.337	16.365	1.00	0.00	LX0	N
ATOM	321	HE21	GLN	598	27.160	-8.471	16.536	0.00	0.00	LX0	H
ATOM	322	HE22	GLN	598	28.652	-7.816	17.050	0.00	0.00	LX0	H
ATOM	323	C	GLN	598	24.866	-9.661	11.990	1.00	0.00	LX0	C
ATOM	324	O	GLN	598	24.045	-8.831	12.371	1.00	0.00	LX0	O
ATOM	325	N	HIS	599	24.887	-10.213	10.757	1.00	0.00	LX0	N
ATOM	326	H	HIS	599	25.749	-10.607	10.441	0.00	0.00	LX0	H
ATOM	327	CA	HIS	599	23.882	-9.960	9.724	1.00	0.00	LX0	C
ATOM	328	CB	HIS	599	23.892	-11.067	8.665	1.00	0.00	LX0	C
ATOM	329	CG	HIS	599	23.400	-10.494	7.353	1.00	0.00	LX0	C
ATOM	330	ND1	HIS	599	23.886	-9.365	6.808	1.00	0.00	LX0	N
ATOM	331	HD1	HIS	599	24.620	-8.806	7.167	0.00	0.00	LX0	H
ATOM	332	CD2	HIS	599	22.398	-10.997	6.519	1.00	0.00	LX0	C
ATOM	333	NE2	HIS	599	22.283	-10.156	5.461	1.00	0.00	LX0	N
ATOM	334	CE1	HIS	599	23.201	-9.148	5.641	1.00	0.00	LX0	C
ATOM	335	C	HIS	599	22.468	-9.594	10.112	1.00	0.00	LX0	C
ATOM	336	O	HIS	599	21.710	-10.328	10.739	1.00	0.00	LX0	O
ATOM	337	N	ASN	600	22.149	-8.377	9.667	1.00	0.00	LX0	N
ATOM	338	H	ASN	600	22.782	-7.921	9.041	0.00	0.00	LX0	H
ATOM	339	CA	ASN	600	20.827	-7.852	9.967	1.00	0.00	LX0	C
ATOM	340	CB	ASN	600	20.987	-6.449	10.531	1.00	0.00	LX0	C
ATOM	341	CG	ASN	600	19.879	-6.133	11.495	1.00	0.00	LX0	C
ATOM	342	OD1	ASN	600	18.773	-5.757	11.119	1.00	0.00	LX0	O
ATOM	343	ND2	ASN	600	20.254	-6.252	12.767	1.00	0.00	LX0	N
ATOM	344	HD21	ASN	600	21.169	-6.605	12.975	0.00	0.00	LX0	H
ATOM	345	HD22	ASN	600	19.705	-5.991	13.558	0.00	0.00	LX0	H
ATOM	346	C	ASN	600	19.941	-7.900	8.737	1.00	0.00	LX0	C
ATOM	347	O	ASN	600	20.399	-8.201	7.645	1.00	0.00	LX0	O
ATOM	348	N	TYR	601	18.638	-7.644	8.936	1.00	0.00	LX0	N
ATOM	349	H	TYR	601	18.288	-7.335	9.819	0.00	0.00	LX0	H
ATOM	350	CA	TYR	601	17.770	-7.959	7.800	1.00	0.00	LX0	C
ATOM	351	CB	TYR	601	16.959	-9.241	8.057	1.00	0.00	LX0	C
ATOM	352	CG	TYR	601	17.817	-10.318	8.684	1.00	0.00	LX0	C
ATOM	353	CD1	TYR	601	18.661	-11.094	7.864	1.00	0.00	LX0	C
ATOM	354	CE1	TYR	601	19.535	-12.013	8.467	1.00	0.00	LX0	C
ATOM	355	CD2	TYR	601	17.770	-10.492	10.084	1.00	0.00	LX0	C
ATOM	356	CE2	TYR	601	18.647	-11.407	10.686	1.00	0.00	LX0	C
ATOM	357	CZ	TYR	601	19.539	-12.131	9.871	1.00	0.00	LX0	C
ATOM	358	OH	TYR	601	20.455	-12.966	10.468	1.00	0.00	LX0	O
ATOM	359	HH	TYR	601	21.084	-12.410	10.931	0.00	0.00	LX0	H
ATOM	360	C	TYR	601	16.863	-6.812	7.393	1.00	0.00	LX0	C
ATOM	361	O	TYR	601	16.659	-5.859	8.144	1.00	0.00	LX0	O
ATOM	362	N	LEU	602	16.325	-6.946	6.172	1.00	0.00	LX0	N
ATOM	363	H	LEU	602	16.548	-7.726	5.585	0.00	0.00	LX0	H
ATOM	364	CA	LEU	602	15.264	-6.041	5.736	1.00	0.00	LX0	C
ATOM	365	CB	LEU	602	15.697	-5.188	4.537	1.00	0.00	LX0	C
ATOM	366	CG	LEU	602	16.610	-4.014	4.898	1.00	0.00	LX0	C
ATOM	367	CD1	LEU	602	17.156	-3.317	3.650	1.00	0.00	LX0	C
ATOM	368	CD2	LEU	602	15.924	-3.027	5.845	1.00	0.00	LX0	C
ATOM	369	C	LEU	602	14.042	-6.856	5.379	1.00	0.00	LX0	C
ATOM	370	O	LEU	602	14.104	-8.077	5.283	1.00	0.00	LX0	O
ATOM	371	N	CYS	603	12.932	-6.130	5.206	1.00	0.00	LX0	N
ATOM	372	H	CYS	603	12.920	-5.130	5.262	0.00	0.00	LX0	H
ATOM	373	CA	CYS	603	11.667	-6.800	4.928	1.00	0.00	LX0	C
ATOM	374	CB	CYS	603	11.069	-7.345	6.231	1.00	0.00	LX0	C
ATOM	375	SG	CYS	603	9.556	-8.316	6.014	1.00	0.00	LX0	S

ATOM	376	C	CYS	603	10.733	-5.805	4.274	1.00	0.00	LX0	C
ATOM	377	O	CYS	603	11.014	-4.615	4.249	1.00	0.00	LX0	O
ATOM	378	N	ALA	604	9.615	-6.337	3.752	1.00	0.00	LX0	N
ATOM	379	H	ALA	604	9.470	-7.324	3.802	0.00	0.00	LX0	H
ATOM	380	CA	ALA	604	8.556	-5.426	3.312	1.00	0.00	LX0	C
ATOM	381	CB	ALA	604	7.627	-6.126	2.321	1.00	0.00	LX0	C
ATOM	382	C	ALA	604	7.729	-4.902	4.477	1.00	0.00	LX0	C
ATOM	383	O	ALA	604	7.103	-3.843	4.440	1.00	0.00	LX0	O
ATOM	384	N	GLY	605	7.760	-5.711	5.546	1.00	0.00	LX0	N
ATOM	385	H	GLY	605	8.252	-6.578	5.523	0.00	0.00	LX0	H
ATOM	386	CA	GLY	605	7.315	-5.185	6.827	1.00	0.00	LX0	C
ATOM	387	C	GLY	605	8.375	-4.283	7.417	1.00	0.00	LX0	C
ATOM	388	O	GLY	605	9.561	-4.456	7.157	1.00	0.00	LX0	O
ATOM	389	N	ARG	606	7.914	-3.303	8.202	1.00	0.00	LX0	N
ATOM	390	H	ARG	606	6.972	-3.352	8.552	0.00	0.00	LX0	H
ATOM	391	CA	ARG	606	8.848	-2.302	8.720	1.00	0.00	LX0	C
ATOM	392	CB	ARG	606	8.180	-0.934	8.903	1.00	0.00	LX0	C
ATOM	393	CG	ARG	606	7.543	-0.266	7.675	1.00	0.00	LX0	C
ATOM	394	CD	ARG	606	6.087	-0.656	7.388	1.00	0.00	LX0	C
ATOM	395	NE	ARG	606	5.965	-1.766	6.440	1.00	0.00	LX0	N
ATOM	396	HE	ARG	606	6.578	-1.792	5.646	0.00	0.00	LX0	H
ATOM	397	CZ	ARG	606	4.949	-2.649	6.550	1.00	0.00	LX0	C
ATOM	398	NH1	ARG	606	4.216	-2.721	7.655	1.00	0.00	LX0	N
ATOM	399	HH11	ARG	606	3.390	-3.273	7.739	0.00	0.00	LX0	H
ATOM	400	HH12	ARG	606	4.534	-2.268	8.499	0.00	0.00	LX0	H
ATOM	401	NH2	ARG	606	4.708	-3.475	5.540	1.00	0.00	LX0	N
ATOM	402	HH21	ARG	606	3.939	-4.112	5.541	0.00	0.00	LX0	H
ATOM	403	HH22	ARG	606	5.346	-3.480	4.760	0.00	0.00	LX0	H
ATOM	404	C	ARG	606	9.458	-2.756	10.033	1.00	0.00	LX0	C
ATOM	405	O	ARG	606	9.394	-2.107	11.073	1.00	0.00	LX0	O
ATOM	406	N	ASN	607	10.001	-3.981	9.933	1.00	0.00	LX0	N
ATOM	407	H	ASN	607	10.156	-4.334	9.009	0.00	0.00	LX0	H
ATOM	408	CA	ASN	607	10.219	-4.827	11.106	1.00	0.00	LX0	C
ATOM	409	CB	ASN	607	11.518	-4.483	11.850	1.00	0.00	LX0	C
ATOM	410	CG	ASN	607	12.025	-5.718	12.572	1.00	0.00	LX0	C
ATOM	411	OD1	ASN	607	11.279	-6.513	13.135	1.00	0.00	LX0	O
ATOM	412	ND2	ASN	607	13.344	-5.900	12.462	1.00	0.00	LX0	N
ATOM	413	HD21	ASN	607	13.976	-5.203	12.119	0.00	0.00	LX0	H
ATOM	414	HD22	ASN	607	13.689	-6.805	12.716	0.00	0.00	LX0	H
ATOM	415	C	ASN	607	9.015	-4.905	12.035	1.00	0.00	LX0	C
ATOM	416	O	ASN	607	9.109	-4.781	13.248	1.00	0.00	LX0	O
ATOM	417	N	ASP	608	7.864	-5.133	11.382	1.00	0.00	LX0	N
ATOM	418	H	ASP	608	7.813	-5.149	10.385	0.00	0.00	LX0	H
ATOM	419	CA	ASP	608	6.644	-5.397	12.139	1.00	0.00	LX0	C
ATOM	420	CB	ASP	608	5.469	-4.487	11.713	1.00	0.00	LX0	C
ATOM	421	CG	ASP	608	5.508	-4.083	10.244	1.00	0.00	LX0	C
ATOM	422	OD1	ASP	608	5.551	-2.892	9.960	1.00	0.00	LX0	O
ATOM	423	OD2	ASP	608	5.511	-4.945	9.372	1.00	0.00	LX0	O
ATOM	424	C	ASP	608	6.298	-6.879	12.154	1.00	0.00	LX0	C
ATOM	425	O	ASP	608	6.552	-7.548	13.149	1.00	0.00	LX0	O
ATOM	426	N	CYS	609	5.776	-7.365	11.015	1.00	0.00	LX0	N
ATOM	427	H	CYS	609	5.619	-6.692	10.289	0.00	0.00	LX0	H
ATOM	428	CA	CYS	609	5.513	-8.782	10.718	1.00	0.00	LX0	C
ATOM	429	CB	CYS	609	6.502	-9.300	9.673	1.00	0.00	LX0	C
ATOM	430	SG	CYS	609	6.538	-8.266	8.188	1.00	0.00	LX0	S
ATOM	431	C	CYS	609	5.343	-9.828	11.818	1.00	0.00	LX0	C
ATOM	432	O	CYS	609	6.171	-10.059	12.697	1.00	0.00	LX0	O
ATOM	433	N	ILE	610	4.193	-10.507	11.673	1.00	0.00	LX0	N
ATOM	434	H	ILE	610	3.595	-10.293	10.904	0.00	0.00	LX0	H
ATOM	435	CA	ILE	610	3.952	-11.697	12.488	1.00	0.00	LX0	C
ATOM	436	CB	ILE	610	2.480	-12.132	12.361	1.00	0.00	LX0	C

ATOM	437	CG2	ILE	610	2.149	-13.354	13.228	1.00	0.00	LX0	C
ATOM	438	CG1	ILE	610	1.541	-10.958	12.657	1.00	0.00	LX0	C
ATOM	439	CD1	ILE	610	0.072	-11.282	12.375	1.00	0.00	LX0	C
ATOM	440	C	ILE	610	4.887	-12.823	12.075	1.00	0.00	LX0	C
ATOM	441	O	ILE	610	5.078	-13.097	10.898	1.00	0.00	LX0	O
ATOM	442	N	VAL	611	5.487	-13.439	13.100	1.00	0.00	LX0	N
ATOM	443	H	VAL	611	5.284	-13.157	14.033	0.00	0.00	LX0	H
ATOM	444	CA	VAL	611	6.303	-14.606	12.787	1.00	0.00	LX0	C
ATOM	445	CB	VAL	611	7.723	-14.439	13.336	1.00	0.00	LX0	C
ATOM	446	CG1	VAL	611	8.594	-15.675	13.094	1.00	0.00	LX0	C
ATOM	447	CG2	VAL	611	8.359	-13.182	12.745	1.00	0.00	LX0	C
ATOM	448	C	VAL	611	5.643	-15.865	13.303	1.00	0.00	LX0	C
ATOM	449	O	VAL	611	5.319	-15.985	14.486	1.00	0.00	LX0	O
ATOM	450	N	ASP	612	5.464	-16.768	12.341	1.00	0.00	LX0	N
ATOM	451	H	ASP	612	5.748	-16.545	11.410	0.00	0.00	LX0	H
ATOM	452	CA	ASP	612	4.598	-17.932	12.493	1.00	0.00	LX0	C
ATOM	453	CB	ASP	612	3.194	-17.562	11.989	1.00	0.00	LX0	C
ATOM	454	CG	ASP	612	3.281	-16.893	10.627	1.00	0.00	LX0	C
ATOM	455	OD1	ASP	612	3.709	-17.517	9.657	1.00	0.00	LX0	O
ATOM	456	OD2	ASP	612	2.951	-15.721	10.538	1.00	0.00	LX0	O
ATOM	457	C	ASP	612	5.174	-19.106	11.717	1.00	0.00	LX0	C
ATOM	458	O	ASP	612	6.347	-19.102	11.357	1.00	0.00	LX0	O
ATOM	459	N	LYS	613	4.301	-20.096	11.440	1.00	0.00	LX0	N
ATOM	460	H	LYS	613	3.356	-20.008	11.745	0.00	0.00	LX0	H
ATOM	461	CA	LYS	613	4.730	-21.282	10.694	1.00	0.00	LX0	C
ATOM	462	CB	LYS	613	3.551	-22.209	10.393	1.00	0.00	LX0	C
ATOM	463	CG	LYS	613	2.797	-22.701	11.629	1.00	0.00	LX0	C
ATOM	464	CD	LYS	613	1.792	-23.803	11.279	1.00	0.00	LX0	C
ATOM	465	CE	LYS	613	1.020	-24.364	12.479	1.00	0.00	LX0	C
ATOM	466	NZ	LYS	613	1.950	-24.882	13.486	1.00	0.00	LX0	N
ATOM	467	HZ1	LYS	613	1.510	-25.468	14.218	0.00	0.00	LX0	H
ATOM	468	HZ2	LYS	613	2.708	-25.484	13.085	0.00	0.00	LX0	H
ATOM	469	HZ3	LYS	613	2.467	-24.115	13.958	0.00	0.00	LX0	H
ATOM	470	C	LYS	613	5.499	-21.035	9.405	1.00	0.00	LX0	C
ATOM	471	O	LYS	613	6.423	-21.764	9.063	1.00	0.00	LX0	O
ATOM	472	N	ILE	614	5.070	-19.978	8.705	1.00	0.00	LX0	N
ATOM	473	H	ILE	614	4.366	-19.376	9.088	0.00	0.00	LX0	H
ATOM	474	CA	ILE	614	5.782	-19.634	7.477	1.00	0.00	LX0	C
ATOM	475	CB	ILE	614	4.817	-18.965	6.485	1.00	0.00	LX0	C
ATOM	476	CG2	ILE	614	5.489	-18.682	5.136	1.00	0.00	LX0	C
ATOM	477	CG1	ILE	614	3.531	-19.783	6.331	1.00	0.00	LX0	C
ATOM	478	CD1	ILE	614	2.442	-19.038	5.555	1.00	0.00	LX0	C
ATOM	479	C	ILE	614	6.961	-18.723	7.774	1.00	0.00	LX0	C
ATOM	480	O	ILE	614	8.108	-18.928	7.380	1.00	0.00	LX0	O
ATOM	481	N	ARG	615	6.621	-17.663	8.517	1.00	0.00	LX0	N
ATOM	482	H	ARG	615	5.695	-17.580	8.896	0.00	0.00	LX0	H
ATOM	483	CA	ARG	615	7.641	-16.636	8.690	1.00	0.00	LX0	C
ATOM	484	CB	ARG	615	6.970	-15.301	8.987	1.00	0.00	LX0	C
ATOM	485	CG	ARG	615	6.498	-14.708	7.656	1.00	0.00	LX0	C
ATOM	486	CD	ARG	615	5.326	-13.739	7.786	1.00	0.00	LX0	C
ATOM	487	NE	ARG	615	4.152	-14.467	8.262	1.00	0.00	LX0	N
ATOM	488	HE	ARG	615	4.087	-14.689	9.245	0.00	0.00	LX0	H
ATOM	489	CZ	ARG	615	3.225	-14.959	7.418	1.00	0.00	LX0	C
ATOM	490	NH1	ARG	615	3.232	-14.617	6.130	1.00	0.00	LX0	N
ATOM	491	HH11	ARG	615	2.562	-14.981	5.486	0.00	0.00	LX0	H
ATOM	492	HH12	ARG	615	3.925	-13.977	5.797	0.00	0.00	LX0	H
ATOM	493	NH2	ARG	615	2.310	-15.795	7.887	1.00	0.00	LX0	N
ATOM	494	HH21	ARG	615	1.548	-16.166	7.364	0.00	0.00	LX0	H
ATOM	495	HH22	ARG	615	2.416	-16.085	8.853	0.00	0.00	LX0	H
ATOM	496	C	ARG	615	8.818	-16.967	9.593	1.00	0.00	LX0	C
ATOM	497	O	ARG	615	9.797	-16.236	9.660	1.00	0.00	LX0	O

ATOM	498	N	ARG	616	8.740	-18.152	10.216	1.00	0.00	LX0	N
ATOM	499	H	ARG	616	7.874	-18.655	10.241	0.00	0.00	LX0	H
ATOM	500	CA	ARG	616	9.965	-18.680	10.814	1.00	0.00	LX0	C
ATOM	501	CB	ARG	616	9.678	-19.937	11.641	1.00	0.00	LX0	C
ATOM	502	CG	ARG	616	9.046	-21.083	10.849	1.00	0.00	LX0	C
ATOM	503	CD	ARG	616	8.813	-22.312	11.716	1.00	0.00	LX0	C
ATOM	504	NE	ARG	616	8.108	-23.364	10.989	1.00	0.00	LX0	N
ATOM	505	HE	ARG	616	8.463	-23.664	10.102	0.00	0.00	LX0	H
ATOM	506	CZ	ARG	616	7.020	-23.929	11.548	1.00	0.00	LX0	C
ATOM	507	NH1	ARG	616	6.471	-23.408	12.638	1.00	0.00	LX0	N
ATOM	508	HH11	ARG	616	5.715	-23.901	13.082	0.00	0.00	LX0	H
ATOM	509	HH12	ARG	616	6.756	-22.531	13.036	0.00	0.00	LX0	H
ATOM	510	NH2	ARG	616	6.476	-25.020	11.019	1.00	0.00	LX0	N
ATOM	511	HH21	ARG	616	5.691	-25.444	11.493	0.00	0.00	LX0	H
ATOM	512	HH22	ARG	616	6.817	-25.436	10.178	0.00	0.00	LX0	H
ATOM	513	C	ARG	616	11.104	-18.907	9.825	1.00	0.00	LX0	C
ATOM	514	O	ARG	616	12.268	-18.652	10.116	1.00	0.00	LX0	O
ATOM	515	N	LYS	617	10.722	-19.375	8.625	1.00	0.00	LX0	N
ATOM	516	H	LYS	617	9.758	-19.558	8.418	0.00	0.00	LX0	H
ATOM	517	CA	LYS	617	11.739	-19.366	7.579	1.00	0.00	LX0	C
ATOM	518	CB	LYS	617	11.507	-20.492	6.552	1.00	0.00	LX0	C
ATOM	519	CG	LYS	617	12.332	-20.389	5.251	1.00	0.00	LX0	C
ATOM	520	CD	LYS	617	13.855	-20.405	5.451	1.00	0.00	LX0	C
ATOM	521	CE	LYS	617	14.638	-19.670	4.350	1.00	0.00	LX0	C
ATOM	522	NZ	LYS	617	14.309	-18.236	4.358	1.00	0.00	LX0	N
ATOM	523	HZ1	LYS	617	15.116	-17.641	4.078	0.00	0.00	LX0	H
ATOM	524	HZ2	LYS	617	13.526	-18.043	3.701	0.00	0.00	LX0	H
ATOM	525	HZ3	LYS	617	14.001	-17.924	5.303	0.00	0.00	LX0	H
ATOM	526	C	LYS	617	11.818	-18.015	6.900	1.00	0.00	LX0	C
ATOM	527	O	LYS	617	12.891	-17.551	6.519	1.00	0.00	LX0	O
ATOM	528	N	ASN	618	10.636	-17.417	6.712	1.00	0.00	LX0	N
ATOM	529	H	ASN	618	9.770	-17.809	7.028	0.00	0.00	LX0	H
ATOM	530	CA	ASN	618	10.745	-16.328	5.754	1.00	0.00	LX0	C
ATOM	531	CB	ASN	618	10.174	-16.711	4.395	1.00	0.00	LX0	C
ATOM	532	CG	ASN	618	11.274	-16.393	3.409	1.00	0.00	LX0	C
ATOM	533	OD1	ASN	618	12.224	-17.155	3.245	1.00	0.00	LX0	O
ATOM	534	ND2	ASN	618	11.140	-15.212	2.811	1.00	0.00	LX0	N
ATOM	535	HD21	ASN	618	10.369	-14.628	3.069	0.00	0.00	LX0	H
ATOM	536	HD22	ASN	618	11.790	-14.862	2.132	0.00	0.00	LX0	H
ATOM	537	C	ASN	618	10.398	-14.913	6.138	1.00	0.00	LX0	C
ATOM	538	O	ASN	618	10.115	-14.092	5.277	1.00	0.00	LX0	O
ATOM	539	N	CYS	619	10.514	-14.673	7.453	1.00	0.00	LX0	N
ATOM	540	H	CYS	619	10.539	-15.426	8.109	0.00	0.00	LX0	H
ATOM	541	CA	CYS	619	10.839	-13.319	7.903	1.00	0.00	LX0	C
ATOM	542	CB	CYS	619	9.573	-12.473	8.108	1.00	0.00	LX0	C
ATOM	543	SG	CYS	619	9.897	-10.728	8.495	1.00	0.00	LX0	S
ATOM	544	C	CYS	619	11.767	-13.218	9.119	1.00	0.00	LX0	C
ATOM	545	O	CYS	619	11.425	-12.611	10.128	1.00	0.00	LX0	O
ATOM	546	N	PRO	620	13.007	-13.789	9.015	1.00	0.00	LX0	N
ATOM	547	CD	PRO	620	13.506	-14.715	8.007	1.00	0.00	LX0	C
ATOM	548	CA	PRO	620	14.041	-13.526	10.028	1.00	0.00	LX0	C
ATOM	549	CB	PRO	620	15.312	-14.101	9.400	1.00	0.00	LX0	C
ATOM	550	CG	PRO	620	14.797	-15.279	8.585	1.00	0.00	LX0	C
ATOM	551	C	PRO	620	14.193	-12.091	10.508	1.00	0.00	LX0	C
ATOM	552	O	PRO	620	14.456	-11.850	11.677	1.00	0.00	LX0	O
ATOM	553	N	ALA	621	13.955	-11.145	9.580	1.00	0.00	LX0	N
ATOM	554	H	ALA	621	13.809	-11.416	8.630	0.00	0.00	LX0	H
ATOM	555	CA	ALA	621	13.926	-9.729	9.968	1.00	0.00	LX0	C
ATOM	556	CB	ALA	621	13.341	-8.885	8.833	1.00	0.00	LX0	C
ATOM	557	C	ALA	621	13.145	-9.426	11.240	1.00	0.00	LX0	C
ATOM	558	O	ALA	621	13.616	-8.767	12.162	1.00	0.00	LX0	O

ATOM	559	N	CYS	622	11.926	-9.978	11.242	1.00	0.00	LX0	N
ATOM	560	H	CYS	622	11.608	-10.538	10.474	0.00	0.00	LX0	H
ATOM	561	CA	CYS	622	11.117	-9.832	12.444	1.00	0.00	LX0	C
ATOM	562	CB	CYS	622	9.656	-9.641	12.057	1.00	0.00	LX0	C
ATOM	563	SG	CYS	622	9.410	-8.182	11.012	1.00	0.00	LX0	S
ATOM	564	C	CYS	622	11.272	-10.969	13.438	1.00	0.00	LX0	C
ATOM	565	O	CYS	622	10.974	-10.839	14.619	1.00	0.00	LX0	O
ATOM	566	N	ARG	623	11.766	-12.104	12.919	1.00	0.00	LX0	N
ATOM	567	H	ARG	623	11.970	-12.148	11.942	0.00	0.00	LX0	H
ATOM	568	CA	ARG	623	11.950	-13.268	13.783	1.00	0.00	LX0	C
ATOM	569	CB	ARG	623	12.246	-14.515	12.946	1.00	0.00	LX0	C
ATOM	570	CG	ARG	623	12.315	-15.797	13.775	1.00	0.00	LX0	C
ATOM	571	CD	ARG	623	12.605	-17.036	12.935	1.00	0.00	LX0	C
ATOM	572	NE	ARG	623	12.743	-18.200	13.805	1.00	0.00	LX0	N
ATOM	573	HE	ARG	623	12.413	-18.095	14.745	0.00	0.00	LX0	H
ATOM	574	CZ	ARG	623	13.360	-19.321	13.379	1.00	0.00	LX0	C
ATOM	575	NH1	ARG	623	13.763	-19.457	12.119	1.00	0.00	LX0	N
ATOM	576	HH11	ARG	623	14.315	-20.233	11.818	0.00	0.00	LX0	H
ATOM	577	HH12	ARG	623	13.497	-18.780	11.421	0.00	0.00	LX0	H
ATOM	578	NH2	ARG	623	13.564	-20.301	14.246	1.00	0.00	LX0	N
ATOM	579	HH21	ARG	623	14.080	-21.130	14.000	0.00	0.00	LX0	H
ATOM	580	HH22	ARG	623	13.212	-20.234	15.180	0.00	0.00	LX0	H
ATOM	581	C	ARG	623	12.990	-13.075	14.869	1.00	0.00	LX0	C
ATOM	582	O	ARG	623	12.752	-13.365	16.032	1.00	0.00	LX0	O
ATOM	583	N	LEU	624	14.144	-12.539	14.444	1.00	0.00	LX0	N
ATOM	584	H	LEU	624	14.262	-12.338	13.472	0.00	0.00	LX0	H
ATOM	585	CA	LEU	624	15.217	-12.251	15.400	1.00	0.00	LX0	C
ATOM	586	CB	LEU	624	16.393	-11.622	14.644	1.00	0.00	LX0	C
ATOM	587	CG	LEU	624	17.683	-11.478	15.456	1.00	0.00	LX0	C
ATOM	588	CD1	LEU	624	18.213	-12.832	15.929	1.00	0.00	LX0	C
ATOM	589	CD2	LEU	624	18.745	-10.691	14.688	1.00	0.00	LX0	C
ATOM	590	C	LEU	624	14.742	-11.362	16.541	1.00	0.00	LX0	C
ATOM	591	O	LEU	624	14.942	-11.613	17.725	1.00	0.00	LX0	O
ATOM	592	N	ARG	625	14.014	-10.323	16.095	1.00	0.00	LX0	N
ATOM	593	H	ARG	625	13.915	-10.198	15.109	0.00	0.00	LX0	H
ATOM	594	CA	ARG	625	13.323	-9.453	17.042	1.00	0.00	LX0	C
ATOM	595	CB	ARG	625	12.487	-8.437	16.265	1.00	0.00	LX0	C
ATOM	596	CG	ARG	625	11.839	-7.364	17.138	1.00	0.00	LX0	C
ATOM	597	CD	ARG	625	10.558	-6.835	16.498	1.00	0.00	LX0	C
ATOM	598	NE	ARG	625	9.545	-7.891	16.423	1.00	0.00	LX0	N
ATOM	599	HE	ARG	625	9.415	-8.479	17.227	0.00	0.00	LX0	H
ATOM	600	CZ	ARG	625	8.726	-7.961	15.356	1.00	0.00	LX0	C
ATOM	601	NH1	ARG	625	8.919	-7.175	14.316	1.00	0.00	LX0	N
ATOM	602	HH11	ARG	625	8.238	-7.157	13.583	0.00	0.00	LX0	H
ATOM	603	HH12	ARG	625	9.718	-6.568	14.211	0.00	0.00	LX0	H
ATOM	604	NH2	ARG	625	7.705	-8.812	15.328	1.00	0.00	LX0	N
ATOM	605	HH21	ARG	625	7.096	-8.827	14.521	0.00	0.00	LX0	H
ATOM	606	HH22	ARG	625	7.526	-9.430	16.091	0.00	0.00	LX0	H
ATOM	607	C	ARG	625	12.456	-10.225	18.030	1.00	0.00	LX0	C
ATOM	608	O	ARG	625	12.626	-10.136	19.238	1.00	0.00	LX0	O
ATOM	609	N	LYS	626	11.542	-11.021	17.446	1.00	0.00	LX0	N
ATOM	610	H	LYS	626	11.502	-11.049	16.447	0.00	0.00	LX0	H
ATOM	611	CA	LYS	626	10.624	-11.836	18.244	1.00	0.00	LX0	C
ATOM	612	CB	LYS	626	9.762	-12.700	17.312	1.00	0.00	LX0	C
ATOM	613	CG	LYS	626	8.516	-13.306	17.967	1.00	0.00	LX0	C
ATOM	614	CD	LYS	626	7.799	-14.313	17.062	1.00	0.00	LX0	C
ATOM	615	CE	LYS	626	6.532	-14.882	17.706	1.00	0.00	LX0	C
ATOM	616	NZ	LYS	626	6.024	-16.023	16.930	1.00	0.00	LX0	N
ATOM	617	HZ1	LYS	626	5.550	-16.693	17.573	0.00	0.00	LX0	H
ATOM	618	HZ2	LYS	626	5.371	-15.744	16.168	0.00	0.00	LX0	H
ATOM	619	HZ3	LYS	626	6.803	-16.539	16.475	0.00	0.00	LX0	H

ATOM	620	C	LYS	626	11.314	-12.689	19.301	1.00	0.00	LX0	C
ATOM	621	O	LYS	626	10.899	-12.768	20.450	1.00	0.00	LX0	O
ATOM	622	N	CYS	627	12.422	-13.303	18.860	1.00	0.00	LX0	N
ATOM	623	H	CYS	627	12.703	-13.190	17.907	0.00	0.00	LX0	H
ATOM	624	CA	CYS	627	13.221	-14.112	19.778	1.00	0.00	LX0	C
ATOM	625	CB	CYS	627	14.411	-14.722	19.038	1.00	0.00	LX0	C
ATOM	626	SG	CYS	627	13.925	-15.797	17.659	1.00	0.00	LX0	S
ATOM	627	C	CYS	627	13.682	-13.361	21.015	1.00	0.00	LX0	C
ATOM	628	O	CYS	627	13.499	-13.783	22.152	1.00	0.00	LX0	O
ATOM	629	N	CYS	628	14.263	-12.186	20.750	1.00	0.00	LX0	N
ATOM	630	H	CYS	628	14.364	-11.843	19.812	0.00	0.00	LX0	H
ATOM	631	CA	CYS	628	14.686	-11.419	21.918	1.00	0.00	LX0	C
ATOM	632	CB	CYS	628	15.705	-10.365	21.518	1.00	0.00	LX0	C
ATOM	633	SG	CYS	628	17.191	-11.108	20.811	1.00	0.00	LX0	S
ATOM	634	C	CYS	628	13.556	-10.820	22.739	1.00	0.00	LX0	C
ATOM	635	O	CYS	628	13.651	-10.680	23.950	1.00	0.00	LX0	O
ATOM	636	N	GLN	629	12.450	-10.517	22.037	1.00	0.00	LX0	N
ATOM	637	H	GLN	629	12.432	-10.660	21.048	0.00	0.00	LX0	H
ATOM	638	CA	GLN	629	11.253	-10.078	22.759	1.00	0.00	LX0	C
ATOM	639	CB	GLN	629	10.147	-9.668	21.784	1.00	0.00	LX0	C
ATOM	640	CG	GLN	629	10.516	-8.441	20.949	1.00	0.00	LX0	C
ATOM	641	CD	GLN	629	9.417	-8.149	19.948	1.00	0.00	LX0	C
ATOM	642	OE1	GLN	629	9.124	-8.914	19.035	1.00	0.00	LX0	O
ATOM	643	NE2	GLN	629	8.822	-6.969	20.141	1.00	0.00	LX0	N
ATOM	644	HE21	GLN	629	9.102	-6.369	20.890	0.00	0.00	LX0	H
ATOM	645	HE22	GLN	629	8.088	-6.665	19.535	0.00	0.00	LX0	H
ATOM	646	C	GLN	629	10.731	-11.120	23.733	1.00	0.00	LX0	C
ATOM	647	O	GLN	629	10.252	-10.821	24.817	1.00	0.00	LX0	O
ATOM	648	N	ALA	630	10.888	-12.379	23.305	1.00	0.00	LX0	N
ATOM	649	H	ALA	630	11.270	-12.576	22.401	0.00	0.00	LX0	H
ATOM	650	CA	ALA	630	10.533	-13.456	24.217	1.00	0.00	LX0	C
ATOM	651	CB	ALA	630	9.806	-14.552	23.448	1.00	0.00	LX0	C
ATOM	652	C	ALA	630	11.716	-14.028	24.987	1.00	0.00	LX0	C
ATOM	653	O	ALA	630	11.821	-15.223	25.249	1.00	0.00	LX0	O
ATOM	654	N	GLY	631	12.608	-13.091	25.353	1.00	0.00	LX0	N
ATOM	655	H	GLY	631	12.488	-12.147	25.047	0.00	0.00	LX0	H
ATOM	656	CA	GLY	631	13.657	-13.392	26.324	1.00	0.00	LX0	C
ATOM	657	C	GLY	631	14.578	-14.556	26.010	1.00	0.00	LX0	C
ATOM	658	O	GLY	631	15.030	-15.271	26.895	1.00	0.00	LX0	O
ATOM	659	N	MET	632	14.870	-14.719	24.711	1.00	0.00	LX0	N
ATOM	660	H	MET	632	14.451	-14.145	24.004	0.00	0.00	LX0	H
ATOM	661	CA	MET	632	15.835	-15.775	24.412	1.00	0.00	LX0	C
ATOM	662	CB	MET	632	15.694	-16.246	22.962	1.00	0.00	LX0	C
ATOM	663	CG	MET	632	14.345	-16.942	22.742	1.00	0.00	LX0	C
ATOM	664	SD	MET	632	14.075	-17.505	21.054	1.00	0.00	LX0	S
ATOM	665	CE	MET	632	15.363	-18.757	20.981	1.00	0.00	LX0	C
ATOM	666	C	MET	632	17.268	-15.428	24.789	1.00	0.00	LX0	C
ATOM	667	O	MET	632	18.002	-14.747	24.083	1.00	0.00	LX0	O
ATOM	668	N	VAL	633	17.644	-15.936	25.968	1.00	0.00	LX0	N
ATOM	669	H	VAL	633	16.991	-16.470	26.513	0.00	0.00	LX0	H
ATOM	670	CA	VAL	633	19.009	-15.699	26.426	1.00	0.00	LX0	C
ATOM	671	CB	VAL	633	19.097	-15.820	27.956	1.00	0.00	LX0	C
ATOM	672	CG1	VAL	633	20.500	-15.500	28.483	1.00	0.00	LX0	C
ATOM	673	CG2	VAL	633	18.036	-14.950	28.635	1.00	0.00	LX0	C
ATOM	674	C	VAL	633	20.012	-16.613	25.747	1.00	0.00	LX0	C
ATOM	675	O	VAL	633	20.092	-17.812	26.013	1.00	0.00	LX0	O
ATOM	676	N	LEU	634	20.798	-15.976	24.868	1.00	0.00	LX0	N
ATOM	677	H	LEU	634	20.591	-15.026	24.645	0.00	0.00	LX0	H
ATOM	678	CA	LEU	634	21.931	-16.687	24.276	1.00	0.00	LX0	C
ATOM	679	CB	LEU	634	22.474	-15.919	23.066	1.00	0.00	LX0	C
ATOM	680	CG	LEU	634	22.298	-16.664	21.734	1.00	0.00	LX0	C

ATOM	681	CD1	LEU	634	22.975	-15.903	20.594	1.00	0.00	LX0	C
ATOM	682	CD2	LEU	634	22.765	-18.125	21.776	1.00	0.00	LX0	C
ATOM	683	C	LEU	634	23.010	-17.113	25.277	1.00	0.00	LX0	C
ATOM	684	O	LEU	634	22.731	-17.305	26.459	1.00	0.00	LX0	O
ATOM	685	N	GLY	635	24.226	-17.364	24.769	1.00	0.00	LX0	N
ATOM	686	H	GLY	635	24.579	-16.796	24.022	0.00	0.00	LX0	H
ATOM	687	CA	GLY	635	25.098	-18.268	25.512	1.00	0.00	LX0	C
ATOM	688	C	GLY	635	24.565	-19.689	25.474	1.00	0.00	LX0	C
ATOM	689	O	GLY	635	23.360	-19.929	25.503	1.00	0.00	LX0	O
ATOM	690	N	GLY	636	25.518	-20.626	25.384	1.00	0.00	LX0	N
ATOM	691	H	GLY	636	26.478	-20.350	25.428	0.00	0.00	LX0	H
ATOM	692	CA	GLY	636	25.109	-22.019	25.212	1.00	0.00	LX0	C
ATOM	693	C	GLY	636	24.469	-22.647	26.439	1.00	0.00	LX0	C
ATOM	694	O	GLY	636	23.509	-22.148	27.020	1.00	0.00	LX0	O
ATOM	695	N	ARG	637	25.057	-23.791	26.813	1.00	0.00	LX0	N
ATOM	696	H	ARG	637	25.908	-24.096	26.389	0.00	0.00	LX0	H
ATOM	697	CA	ARG	637	24.493	-24.497	27.958	1.00	0.00	LX0	C
ATOM	698	CB	ARG	637	24.703	-26.012	27.806	1.00	0.00	LX0	C
ATOM	699	CG	ARG	637	23.617	-26.929	28.399	1.00	0.00	LX0	C
ATOM	700	CD	ARG	637	23.496	-26.910	29.927	1.00	0.00	LX0	C
ATOM	701	NE	ARG	637	22.534	-27.901	30.415	1.00	0.00	LX0	N
ATOM	702	HE	ARG	637	22.641	-28.839	30.082	0.00	0.00	LX0	H
ATOM	703	CZ	ARG	637	21.651	-27.573	31.386	1.00	0.00	LX0	C
ATOM	704	NH1	ARG	637	21.536	-26.316	31.794	1.00	0.00	LX0	N
ATOM	705	HH11	ARG	637	20.976	-26.031	32.581	0.00	0.00	LX0	H
ATOM	706	HH12	ARG	637	22.043	-25.572	31.344	0.00	0.00	LX0	H
ATOM	707	NH2	ARG	637	20.898	-28.517	31.944	1.00	0.00	LX0	N
ATOM	708	HH21	ARG	637	20.244	-28.287	32.666	0.00	0.00	LX0	H
ATOM	709	HH22	ARG	637	20.972	-29.472	31.658	0.00	0.00	LX0	H
ATOM	710	C	ARG	637	25.005	-23.969	29.286	1.00	0.00	LX0	C
ATOM	711	O	ARG	637	25.823	-24.576	29.961	1.00	0.00	LX0	O
ATOM	712	N	LYS	638	24.437	-22.811	29.648	1.00	0.00	LX0	N
ATOM	713	H	LYS	638	23.789	-22.405	29.006	0.00	0.00	LX0	H
ATOM	714	CA	LYS	638	24.516	-22.433	31.060	1.00	0.00	LX0	C
ATOM	715	CB	LYS	638	23.998	-21.004	31.318	1.00	0.00	LX0	C
ATOM	716	CG	LYS	638	22.621	-20.634	30.743	1.00	0.00	LX0	C
ATOM	717	CD	LYS	638	22.691	-20.077	29.317	1.00	0.00	LX0	C
ATOM	718	CE	LYS	638	21.331	-19.929	28.631	1.00	0.00	LX0	C
ATOM	719	NZ	LYS	638	21.556	-19.465	27.259	1.00	0.00	LX0	N
ATOM	720	HZ1	LYS	638	20.670	-19.235	26.763	0.00	0.00	LX0	H
ATOM	721	HZ2	LYS	638	22.090	-18.574	27.277	0.00	0.00	LX0	H
ATOM	722	HZ3	LYS	638	22.119	-20.136	26.690	0.00	0.00	LX0	H
ATOM	723	C	LYS	638	23.749	-23.454	31.879	1.00	0.00	LX0	C
ATOM	724	O	LYS	638	22.752	-24.000	31.411	1.00	0.00	LX0	O
ATOM	725	N	PHE	639	24.275	-23.751	33.066	1.00	0.00	LX0	N
ATOM	726	H	PHE	639	25.009	-23.188	33.456	0.00	0.00	LX0	H
ATOM	727	CA	PHE	639	23.684	-24.854	33.813	1.00	0.00	LX0	C
ATOM	728	CB	PHE	639	24.689	-25.424	34.819	1.00	0.00	LX0	C
ATOM	729	CG	PHE	639	25.920	-25.881	34.068	1.00	0.00	LX0	C
ATOM	730	CD1	PHE	639	25.850	-27.033	33.252	1.00	0.00	LX0	C
ATOM	731	CD2	PHE	639	27.116	-25.139	34.179	1.00	0.00	LX0	C
ATOM	732	CE1	PHE	639	26.985	-27.434	32.520	1.00	0.00	LX0	C
ATOM	733	CE2	PHE	639	28.252	-25.538	33.449	1.00	0.00	LX0	C
ATOM	734	CZ	PHE	639	28.171	-26.677	32.620	1.00	0.00	LX0	C
ATOM	735	C	PHE	639	22.349	-24.507	34.440	1.00	0.00	LX0	C
ATOM	736	O	PHE	639	21.326	-25.119	34.135	1.00	0.00	LX0	O
ATOM	737	N	LYS	640	22.413	-23.473	35.291	1.00	0.00	LX0	N
ATOM	738	H	LYS	640	23.319	-23.076	35.471	0.00	0.00	LX0	H
ATOM	739	CA	LYS	640	21.224	-22.862	35.890	1.00	0.00	LX0	C
ATOM	740	CB	LYS	640	20.421	-23.828	36.785	1.00	0.00	LX0	C
ATOM	741	CG	LYS	640	21.230	-24.654	37.793	1.00	0.00	LX0	C

```
ATOM    742  CD  LYS   640      20.422 -24.971  39.054  1.00  0.00      LX0  C
ATOM    743  CE  LYS   640      20.838 -24.143  40.281  1.00  0.00      LX0  C
ATOM    744  NZ  LYS   640      20.805 -22.696  40.008  1.00  0.00      LX0  N
ATOM    745  HZ1 LYS   640      21.052 -22.150  40.855  0.00  0.00      LX0  H
ATOM    746  HZ2 LYS   640      21.481 -22.443  39.252  0.00  0.00      LX0  H
ATOM    747  HZ3 LYS   640      19.871 -22.402  39.663  0.00  0.00      LX0  H
ATOM    748  C   LYS   640      21.557 -21.609  36.683  1.00  0.00      LX0  C
ATOM    749  O   LYS   640      21.204 -21.480  37.859  1.00  0.00      LX0  O
TER
```
