# Supplementary material for: Structural analysis of hubs in human NR-RTK network
Source: Biol Direct. 2011 Oct 5;6:49. doi: 10.1186/1745-6150-6-49 (PMC3220635; doi:10.1186/1745-6150-6-49)
Supplement: Additional file 15 — ESR1-EGFR-Erbb2. ESR1-EGFR-Erbb2 complex structure. [file 1745-6150-6-49-S15.PDF]

```
HEADER ESR1-EGFR-ERBB2
REMARK original generated coordinate pdb file
ATOM      1  N   ALA   156      10.627  12.174   8.322  1.00  0.00       RX0  N
ATOM      2  H   ALA   156      11.203  11.358   8.277  0.00  0.00       RX0  H
ATOM      3  CA  ALA   156       9.864  12.527   9.538  1.00  0.00       RX0  C
ATOM      4  CB  ALA   156      10.756  12.401  10.765  1.00  0.00       RX0  C
ATOM      5  C   ALA   156       9.377  13.991   9.496  1.00  0.00       RX0  C
ATOM      6  O   ALA   156       9.121  14.644  10.500  1.00  0.00       RX0  O
ATOM      7  N   LEU   157       9.039  14.416   8.289  1.00  0.00       RX0  N
ATOM      8  H   LEU   157       9.137  13.769   7.529  0.00  0.00       RX0  H
ATOM      9  CA  LEU   157       8.850  15.849   7.979  1.00  0.00       RX0  C
ATOM     10  CB  LEU   157       9.711  16.243   6.787  1.00  0.00       RX0  C
ATOM     11  CG  LEU   157      11.118  15.665   6.857  1.00  0.00       RX0  C
ATOM     12  CD1 LEU   157      11.864  15.911   5.553  1.00  0.00       RX0  C
ATOM     13  CD2 LEU   157      11.886  16.154   8.081  1.00  0.00       RX0  C
ATOM     14  C   LEU   157       7.387  16.184   7.642  1.00  0.00       RX0  C
ATOM     15  O   LEU   157       7.075  17.216   7.039  1.00  0.00       RX0  O
ATOM     16  N   SER   158       6.522  15.244   7.955  1.00  0.00       RX0  N
ATOM     17  H   SER   158       6.875  14.326   8.100  0.00  0.00       RX0  H
ATOM     18  CA  SER   158       5.051  15.362   7.847  1.00  0.00       RX0  C
ATOM     19  CB  SER   158       4.729  14.892   6.438  1.00  0.00       RX0  C
ATOM     20  OG  SER   158       5.938  15.040   5.682  1.00  0.00       RX0  O
ATOM     21  HG  SER   158       6.099  15.980   5.624  0.00  0.00       RX0  H
ATOM     22  C   SER   158       4.335  14.560   8.949  1.00  0.00       RX0  C
ATOM     23  O   SER   158       3.148  14.670   9.188  1.00  0.00       RX0  O
ATOM     24  N   LEU   159       5.132  13.681   9.591  1.00  0.00       RX0  N
ATOM     25  H   LEU   159       6.082  13.581   9.322  0.00  0.00       RX0  H
ATOM     26  CA  LEU   159       4.759  12.951  10.797  1.00  0.00       RX0  C
ATOM     27  CB  LEU   159       5.862  11.986  11.242  1.00  0.00       RX0  C
ATOM     28  CG  LEU   159       5.807  10.583  10.627  1.00  0.00       RX0  C
ATOM     29  CD1 LEU   159       6.083  10.564   9.121  1.00  0.00       RX0  C
ATOM     30  CD2 LEU   159       6.732   9.626  11.381  1.00  0.00       RX0  C
ATOM     31  C   LEU   159       4.518  13.965  11.920  1.00  0.00       RX0  C
ATOM     32  O   LEU   159       5.291  14.932  12.058  1.00  0.00       RX0  O
ATOM     33  N   THR   160       3.434  13.807  12.646  1.00  0.00       RX0  N
ATOM     34  H   THR   160       2.847  13.020  12.457  0.00  0.00       RX0  H
ATOM     35  CA  THR   160       3.156  14.665  13.825  1.00  0.00       RX0  C
ATOM     36  CB  THR   160       1.666  14.644  14.227  1.00  0.00       RX0  C
ATOM     37  OG1 THR   160       1.376  15.724  15.120  1.00  0.00       RX0  O
ATOM     38  HG1 THR   160       0.428  15.806  15.146  0.00  0.00       RX0  H
ATOM     39  CG2 THR   160       1.175  13.324  14.806  1.00  0.00       RX0  C
ATOM     40  C   THR   160       4.203  14.411  14.921  1.00  0.00       RX0  C
ATOM     41  O   THR   160       4.902  13.383  14.913  1.00  0.00       RX0  O
ATOM     42  N   ALA   161       4.153  15.229  15.953  1.00  0.00       RX0  N
ATOM     43  H   ALA   161       3.464  15.956  15.914  0.00  0.00       RX0  H
ATOM     44  CA  ALA   161       4.942  15.044  17.184  1.00  0.00       RX0  C
ATOM     45  CB  ALA   161       4.759  16.237  18.114  1.00  0.00       RX0  C
ATOM     46  C   ALA   161       4.543  13.746  17.920  1.00  0.00       RX0  C
ATOM     47  O   ALA   161       5.387  12.950  18.288  1.00  0.00       RX0  O
ATOM     48  N   ASP   162       3.226  13.461  17.917  1.00  0.00       RX0  N
ATOM     49  H   ASP   162       2.572  14.181  17.694  0.00  0.00       RX0  H
ATOM     50  CA  ASP   162       2.690  12.196  18.469  1.00  0.00       RX0  C
ATOM     51  CB  ASP   162       1.212  12.325  18.865  1.00  0.00       RX0  C
ATOM     52  CG  ASP   162       1.138  13.155  20.143  1.00  0.00       RX0  C
ATOM     53  OD1 ASP   162       2.122  13.794  20.503  1.00  0.00       RX0  O
ATOM     54  OD2 ASP   162       0.114  13.159  20.820  1.00  0.00       RX0  O
ATOM     55  C   ASP   162       3.088  10.948  17.668  1.00  0.00       RX0  C
ATOM     56  O   ASP   162       3.397   9.903  18.257  1.00  0.00       RX0  O
ATOM     57  N   GLN   163       3.164  11.087  16.353  1.00  0.00       RX0  N
ATOM     58  H   GLN   163       3.082  12.015  15.999  0.00  0.00       RX0  H
ATOM     59  CA  GLN   163       3.593   9.998  15.449  1.00  0.00       RX0  C
```

ATOM	60	CB	GLN	163	3.253	10.287	13.995	1.00	0.00	RX0	C
ATOM	61	CG	GLN	163	1.840	9.855	13.613	1.00	0.00	RX0	C
ATOM	62	CD	GLN	163	1.578	10.364	12.217	1.00	0.00	RX0	C
ATOM	63	OE1	GLN	163	2.095	11.405	11.827	1.00	0.00	RX0	O
ATOM	64	NE2	GLN	163	0.762	9.583	11.489	1.00	0.00	RX0	N
ATOM	65	HE21	GLN	163	0.376	8.743	11.873	0.00	0.00	RX0	H
ATOM	66	HE22	GLN	163	0.517	9.815	10.547	0.00	0.00	RX0	H
ATOM	67	C	GLN	163	5.089	9.698	15.572	1.00	0.00	RX0	C
ATOM	68	O	GLN	163	5.477	8.537	15.545	1.00	0.00	RX0	O
ATOM	69	N	MET	164	5.882	10.740	15.840	1.00	0.00	RX0	N
ATOM	70	H	MET	164	5.496	11.663	15.887	0.00	0.00	RX0	H
ATOM	71	CA	MET	164	7.331	10.587	16.060	1.00	0.00	RX0	C
ATOM	72	CB	MET	164	8.014	11.955	16.081	1.00	0.00	RX0	C
ATOM	73	CG	MET	164	9.451	11.879	16.606	1.00	0.00	RX0	C
ATOM	74	SD	MET	164	10.535	10.863	15.594	1.00	0.00	RX0	S
ATOM	75	CE	MET	164	11.027	12.134	14.426	1.00	0.00	RX0	C
ATOM	76	C	MET	164	7.610	9.825	17.366	1.00	0.00	RX0	C
ATOM	77	O	MET	164	8.404	8.887	17.381	1.00	0.00	RX0	O
ATOM	78	N	VAL	165	6.828	10.145	18.396	1.00	0.00	RX0	N
ATOM	79	H	VAL	165	6.185	10.906	18.283	0.00	0.00	RX0	H
ATOM	80	CA	VAL	165	6.992	9.563	19.744	1.00	0.00	RX0	C
ATOM	81	CB	VAL	165	6.101	10.259	20.779	1.00	0.00	RX0	C
ATOM	82	CG1	VAL	165	6.187	9.569	22.140	1.00	0.00	RX0	C
ATOM	83	CG2	VAL	165	6.447	11.733	20.917	1.00	0.00	RX0	C
ATOM	84	C	VAL	165	6.649	8.067	19.731	1.00	0.00	RX0	C
ATOM	85	O	VAL	165	7.442	7.255	20.191	1.00	0.00	RX0	O
ATOM	86	N	SER	166	5.467	7.742	19.205	1.00	0.00	RX0	N
ATOM	87	H	SER	166	4.846	8.462	18.896	0.00	0.00	RX0	H
ATOM	88	CA	SER	166	5.029	6.335	19.106	1.00	0.00	RX0	C
ATOM	89	CB	SER	166	3.584	6.425	18.661	1.00	0.00	RX0	C
ATOM	90	OG	SER	166	2.958	7.463	19.430	1.00	0.00	RX0	O
ATOM	91	HG	SER	166	2.945	8.260	18.909	0.00	0.00	RX0	H
ATOM	92	C	SER	166	5.941	5.501	18.195	1.00	0.00	RX0	C
ATOM	93	O	SER	166	6.295	4.379	18.542	1.00	0.00	RX0	O
ATOM	94	N	ALA	167	6.456	6.129	17.133	1.00	0.00	RX0	N
ATOM	95	H	ALA	167	6.180	7.067	16.916	0.00	0.00	RX0	H
ATOM	96	CA	ALA	167	7.397	5.466	16.208	1.00	0.00	RX0	C
ATOM	97	CB	ALA	167	7.731	6.357	15.012	1.00	0.00	RX0	C
ATOM	98	C	ALA	167	8.706	5.103	16.927	1.00	0.00	RX0	C
ATOM	99	O	ALA	167	9.113	3.946	16.932	1.00	0.00	RX0	O
ATOM	100	N	LEU	168	9.179	6.054	17.734	1.00	0.00	RX0	N
ATOM	101	H	LEU	168	8.732	6.951	17.741	0.00	0.00	RX0	H
ATOM	102	CA	LEU	168	10.385	5.875	18.562	1.00	0.00	RX0	C
ATOM	103	CB	LEU	168	10.907	7.214	19.074	1.00	0.00	RX0	C
ATOM	104	CG	LEU	168	11.570	8.041	17.977	1.00	0.00	RX0	C
ATOM	105	CD1	LEU	168	12.101	9.369	18.517	1.00	0.00	RX0	C
ATOM	106	CD2	LEU	168	12.651	7.238	17.253	1.00	0.00	RX0	C
ATOM	107	C	LEU	168	10.197	4.896	19.724	1.00	0.00	RX0	C
ATOM	108	O	LEU	168	11.077	4.078	19.994	1.00	0.00	RX0	O
ATOM	109	N	LEU	169	9.007	4.918	20.317	1.00	0.00	RX0	N
ATOM	110	H	LEU	169	8.330	5.588	20.013	0.00	0.00	RX0	H
ATOM	111	CA	LEU	169	8.640	3.970	21.384	1.00	0.00	RX0	C
ATOM	112	CB	LEU	169	7.358	4.394	22.102	1.00	0.00	RX0	C
ATOM	113	CG	LEU	169	7.541	5.638	22.971	1.00	0.00	RX0	C
ATOM	114	CD1	LEU	169	6.230	6.047	23.642	1.00	0.00	RX0	C
ATOM	115	CD2	LEU	169	8.669	5.467	23.989	1.00	0.00	RX0	C
ATOM	116	C	LEU	169	8.505	2.536	20.864	1.00	0.00	RX0	C
ATOM	117	O	LEU	169	9.003	1.602	21.486	1.00	0.00	RX0	O
ATOM	118	N	ASP	170	7.977	2.423	19.645	1.00	0.00	RX0	N
ATOM	119	H	ASP	170	7.667	3.242	19.164	0.00	0.00	RX0	H
ATOM	120	CA	ASP	170	7.822	1.133	18.952	1.00	0.00	RX0	C

ATOM	121	CB	ASP	170	6.871	1.411	17.766	1.00	0.00	RX0	C
ATOM	122	CG	ASP	170	6.675	0.319	16.726	1.00	0.00	RX0	C
ATOM	123	OD1	ASP	170	7.112	-0.812	16.886	1.00	0.00	RX0	O
ATOM	124	OD2	ASP	170	6.093	0.614	15.686	1.00	0.00	RX0	O
ATOM	125	C	ASP	170	9.164	0.506	18.541	1.00	0.00	RX0	C
ATOM	126	O	ASP	170	9.313	-0.704	18.571	1.00	0.00	RX0	O
ATOM	127	N	ALA	171	10.119	1.387	18.228	1.00	0.00	RX0	N
ATOM	128	H	ALA	171	9.894	2.361	18.290	0.00	0.00	RX0	H
ATOM	129	CA	ALA	171	11.447	1.008	17.717	1.00	0.00	RX0	C
ATOM	130	CB	ALA	171	12.102	2.218	17.051	1.00	0.00	RX0	C
ATOM	131	C	ALA	171	12.418	0.479	18.779	1.00	0.00	RX0	C
ATOM	132	O	ALA	171	13.427	-0.136	18.431	1.00	0.00	RX0	O
ATOM	133	N	GLU	172	12.125	0.739	20.058	1.00	0.00	RX0	N
ATOM	134	H	GLU	172	11.281	1.229	20.277	0.00	0.00	RX0	H
ATOM	135	CA	GLU	172	13.017	0.374	21.170	1.00	0.00	RX0	C
ATOM	136	CB	GLU	172	12.363	0.706	22.511	1.00	0.00	RX0	C
ATOM	137	CG	GLU	172	12.322	2.225	22.682	1.00	0.00	RX0	C
ATOM	138	CD	GLU	172	13.727	2.776	22.517	1.00	0.00	RX0	C
ATOM	139	OE1	GLU	172	14.551	2.618	23.419	1.00	0.00	RX0	O
ATOM	140	OE2	GLU	172	14.033	3.379	21.487	1.00	0.00	RX0	O
ATOM	141	C	GLU	172	13.554	-1.065	21.099	1.00	0.00	RX0	C
ATOM	142	O	GLU	172	12.785	-2.004	20.837	1.00	0.00	RX0	O
ATOM	143	N	PRO	173	14.865	-1.209	21.269	1.00	0.00	RX0	N
ATOM	144	CD	PRO	173	15.788	-0.095	21.452	1.00	0.00	RX0	C
ATOM	145	CA	PRO	173	15.538	-2.517	21.328	1.00	0.00	RX0	C
ATOM	146	CB	PRO	173	17.014	-2.107	21.200	1.00	0.00	RX0	C
ATOM	147	CG	PRO	173	17.102	-0.740	21.869	1.00	0.00	RX0	C
ATOM	148	C	PRO	173	15.206	-3.249	22.640	1.00	0.00	RX0	C
ATOM	149	O	PRO	173	14.829	-2.595	23.631	1.00	0.00	RX0	O
ATOM	150	N	PRO	174	15.294	-4.574	22.646	1.00	0.00	RX0	N
ATOM	151	CD	PRO	174	15.603	-5.387	21.474	1.00	0.00	RX0	C
ATOM	152	CA	PRO	174	15.084	-5.400	23.852	1.00	0.00	RX0	C
ATOM	153	CB	PRO	174	14.969	-6.812	23.272	1.00	0.00	RX0	C
ATOM	154	CG	PRO	174	15.854	-6.783	22.030	1.00	0.00	RX0	C
ATOM	155	C	PRO	174	16.250	-5.248	24.838	1.00	0.00	RX0	C
ATOM	156	O	PRO	174	17.379	-4.922	24.444	1.00	0.00	RX0	O
ATOM	157	N	ILE	175	15.956	-5.464	26.106	1.00	0.00	RX0	N
ATOM	158	H	ILE	175	15.038	-5.787	26.328	0.00	0.00	RX0	H
ATOM	159	CA	ILE	175	16.988	-5.556	27.159	1.00	0.00	RX0	C
ATOM	160	CB	ILE	175	16.446	-5.211	28.551	1.00	0.00	RX0	C
ATOM	161	CG2	ILE	175	17.623	-5.097	29.519	1.00	0.00	RX0	C
ATOM	162	CG1	ILE	175	15.623	-3.917	28.561	1.00	0.00	RX0	C
ATOM	163	CD1	ILE	175	14.110	-4.134	28.454	1.00	0.00	RX0	C
ATOM	164	C	ILE	175	17.586	-6.969	27.112	1.00	0.00	RX0	C
ATOM	165	O	ILE	175	16.886	-7.963	27.343	1.00	0.00	RX0	O
ATOM	166	N	LEU	176	18.884	-7.017	26.884	1.00	0.00	RX0	N
ATOM	167	H	LEU	176	19.405	-6.164	26.840	0.00	0.00	RX0	H
ATOM	168	CA	LEU	176	19.617	-8.291	26.770	1.00	0.00	RX0	C
ATOM	169	CB	LEU	176	20.627	-8.169	25.637	1.00	0.00	RX0	C
ATOM	170	CG	LEU	176	19.948	-7.963	24.281	1.00	0.00	RX0	C
ATOM	171	CD1	LEU	176	20.971	-7.824	23.156	1.00	0.00	RX0	C
ATOM	172	CD2	LEU	176	18.929	-9.063	23.979	1.00	0.00	RX0	C
ATOM	173	C	LEU	176	20.277	-8.690	28.089	1.00	0.00	RX0	C
ATOM	174	O	LEU	176	20.563	-7.852	28.952	1.00	0.00	RX0	O
ATOM	175	N	TYR	177	20.459	-9.989	28.237	1.00	0.00	RX0	N
ATOM	176	H	TYR	177	20.212	-10.612	27.498	0.00	0.00	RX0	H
ATOM	177	CA	TYR	177	21.114	-10.573	29.420	1.00	0.00	RX0	C
ATOM	178	CB	TYR	177	20.420	-11.864	29.855	1.00	0.00	RX0	C
ATOM	179	CG	TYR	177	19.152	-11.561	30.616	1.00	0.00	RX0	C
ATOM	180	CD1	TYR	177	18.029	-11.068	29.960	1.00	0.00	RX0	C
ATOM	181	CE1	TYR	177	16.865	-10.810	30.673	1.00	0.00	RX0	C

ATOM	182	CD2	TYR	177	19.111	-11.789	31.986	1.00	0.00	RX0	C
ATOM	183	CE2	TYR	177	17.945	-11.538	32.699	1.00	0.00	RX0	C
ATOM	184	CZ	TYR	177	16.823	-11.047	32.042	1.00	0.00	RX0	C
ATOM	185	OH	TYR	177	15.668	-10.789	32.752	1.00	0.00	RX0	O
ATOM	186	HH	TYR	177	15.775	-11.071	33.651	0.00	0.00	RX0	H
ATOM	187	C	TYR	177	22.589	-10.858	29.163	1.00	0.00	RX0	C
ATOM	188	O	TYR	177	22.985	-11.163	28.046	1.00	0.00	RX0	O
ATOM	189	N	SER	178	23.381	-10.750	30.220	1.00	0.00	RX0	N
ATOM	190	H	SER	178	23.021	-10.513	31.126	0.00	0.00	RX0	H
ATOM	191	CA	SER	178	24.788	-11.188	30.183	1.00	0.00	RX0	C
ATOM	192	CB	SER	178	25.477	-10.634	31.410	1.00	0.00	RX0	C
ATOM	193	OG	SER	178	25.119	-9.257	31.474	1.00	0.00	RX0	O
ATOM	194	HG	SER	178	25.265	-8.896	30.609	0.00	0.00	RX0	H
ATOM	195	C	SER	178	24.834	-12.718	30.070	1.00	0.00	RX0	C
ATOM	196	O	SER	178	23.999	-13.413	30.674	1.00	0.00	RX0	O
ATOM	197	N	GLU	179	25.827	-13.218	29.362	1.00	0.00	RX0	N
ATOM	198	H	GLU	179	26.496	-12.647	28.883	0.00	0.00	RX0	H
ATOM	199	CA	GLU	179	26.033	-14.670	29.175	1.00	0.00	RX0	C
ATOM	200	CB	GLU	179	26.388	-15.110	27.760	1.00	0.00	RX0	C
ATOM	201	CG	GLU	179	25.355	-14.852	26.684	1.00	0.00	RX0	C
ATOM	202	CD	GLU	179	25.987	-13.864	25.745	1.00	0.00	RX0	C
ATOM	203	OE1	GLU	179	25.681	-13.887	24.561	1.00	0.00	RX0	O
ATOM	204	OE2	GLU	179	26.724	-12.999	26.217	1.00	0.00	RX0	O
ATOM	205	C	GLU	179	27.192	-15.208	30.012	1.00	0.00	RX0	C
ATOM	206	O	GLU	179	28.361	-15.227	29.589	1.00	0.00	RX0	O
ATOM	207	N	TYR	180	26.873	-15.475	31.254	1.00	0.00	RX0	N
ATOM	208	H	TYR	180	25.920	-15.398	31.558	0.00	0.00	RX0	H
ATOM	209	CA	TYR	180	27.735	-16.233	32.177	1.00	0.00	RX0	C
ATOM	210	CB	TYR	180	28.502	-15.327	33.152	1.00	0.00	RX0	C
ATOM	211	CG	TYR	180	27.571	-14.678	34.149	1.00	0.00	RX0	C
ATOM	212	CD1	TYR	180	26.933	-13.484	33.836	1.00	0.00	RX0	C
ATOM	213	CE1	TYR	180	26.046	-12.916	34.741	1.00	0.00	RX0	C
ATOM	214	CD2	TYR	180	27.350	-15.282	35.382	1.00	0.00	RX0	C
ATOM	215	CE2	TYR	180	26.453	-14.721	36.280	1.00	0.00	RX0	C
ATOM	216	CZ	TYR	180	25.788	-13.547	35.952	1.00	0.00	RX0	C
ATOM	217	OH	TYR	180	24.866	-13.017	36.827	1.00	0.00	RX0	O
ATOM	218	HH	TYR	180	24.716	-12.101	36.606	0.00	0.00	RX0	H
ATOM	219	C	TYR	180	26.838	-17.226	32.909	1.00	0.00	RX0	C
ATOM	220	O	TYR	180	25.642	-16.953	33.094	1.00	0.00	RX0	O
ATOM	221	N	ASP	181	27.404	-18.345	33.318	1.00	0.00	RX0	N
ATOM	222	H	ASP	181	28.375	-18.503	33.158	0.00	0.00	RX0	H
ATOM	223	CA	ASP	181	26.630	-19.347	34.059	1.00	0.00	RX0	C
ATOM	224	CB	ASP	181	27.295	-20.716	34.075	1.00	0.00	RX0	C
ATOM	225	CG	ASP	181	26.456	-21.546	35.013	1.00	0.00	RX0	C
ATOM	226	OD1	ASP	181	25.252	-21.605	34.815	1.00	0.00	RX0	O
ATOM	227	OD2	ASP	181	26.982	-22.075	35.979	1.00	0.00	RX0	O
ATOM	228	C	ASP	181	26.420	-18.851	35.504	1.00	0.00	RX0	C
ATOM	229	O	ASP	181	27.391	-18.832	36.273	1.00	0.00	RX0	O
ATOM	230	N	PRO	182	25.185	-18.489	35.856	1.00	0.00	RX0	N
ATOM	231	CD	PRO	182	24.009	-18.604	34.996	1.00	0.00	RX0	C
ATOM	232	CA	PRO	182	24.825	-17.989	37.201	1.00	0.00	RX0	C
ATOM	233	CB	PRO	182	23.395	-17.484	36.997	1.00	0.00	RX0	C
ATOM	234	CG	PRO	182	22.821	-18.407	35.927	1.00	0.00	RX0	C
ATOM	235	C	PRO	182	24.941	-19.052	38.308	1.00	0.00	RX0	C
ATOM	236	O	PRO	182	24.654	-18.763	39.474	1.00	0.00	RX0	O
ATOM	237	N	THR	183	25.345	-20.259	37.948	1.00	0.00	RX0	N
ATOM	238	H	THR	183	25.518	-20.544	37.002	0.00	0.00	RX0	H
ATOM	239	CA	THR	183	25.568	-21.363	38.913	1.00	0.00	RX0	C
ATOM	240	CB	THR	183	24.919	-22.569	38.272	1.00	0.00	RX0	C
ATOM	241	OG1	THR	183	23.991	-22.089	37.292	1.00	0.00	RX0	O
ATOM	242	HG1	THR	183	24.480	-22.124	36.466	0.00	0.00	RX0	H

ATOM	243	CG2	THR	183	24.240	-23.487	39.286	1.00	0.00	RX0	C
ATOM	244	C	THR	183	27.063	-21.532	39.218	1.00	0.00	RX0	C
ATOM	245	O	THR	183	27.455	-22.345	40.058	1.00	0.00	RX0	O
ATOM	246	N	ARG	184	27.887	-20.699	38.573	1.00	0.00	RX0	N
ATOM	247	H	ARG	184	27.504	-20.029	37.938	0.00	0.00	RX0	H
ATOM	248	CA	ARG	184	29.343	-20.701	38.681	1.00	0.00	RX0	C
ATOM	249	CB	ARG	184	29.760	-21.043	37.252	1.00	0.00	RX0	C
ATOM	250	CG	ARG	184	31.205	-21.228	36.821	1.00	0.00	RX0	C
ATOM	251	CD	ARG	184	31.238	-20.748	35.368	1.00	0.00	RX0	C
ATOM	252	NE	ARG	184	30.416	-19.538	35.303	1.00	0.00	RX0	N
ATOM	253	HE	ARG	184	29.440	-19.698	35.124	0.00	0.00	RX0	H
ATOM	254	CZ	ARG	184	30.908	-18.451	35.960	1.00	0.00	RX0	C
ATOM	255	NH1	ARG	184	32.236	-18.309	36.116	1.00	0.00	RX0	N
ATOM	256	HH11	ARG	184	32.646	-17.604	36.696	0.00	0.00	RX0	H
ATOM	257	HH12	ARG	184	32.914	-18.966	35.729	0.00	0.00	RX0	H
ATOM	258	NH2	ARG	184	30.032	-17.591	36.520	1.00	0.00	RX0	N
ATOM	259	HH21	ARG	184	30.355	-16.821	37.078	0.00	0.00	RX0	H
ATOM	260	HH22	ARG	184	29.035	-17.740	36.460	0.00	0.00	RX0	H
ATOM	261	C	ARG	184	29.836	-19.410	39.407	1.00	0.00	RX0	C
ATOM	262	O	ARG	184	29.116	-18.390	39.334	1.00	0.00	RX0	O
ATOM	263	N	PRO	185	30.940	-19.460	40.113	1.00	0.00	RX0	N
ATOM	264	CD	PRO	185	31.731	-20.667	40.322	1.00	0.00	RX0	C
ATOM	265	CA	PRO	185	31.574	-18.285	40.781	1.00	0.00	RX0	C
ATOM	266	CB	PRO	185	32.854	-18.864	41.395	1.00	0.00	RX0	C
ATOM	267	CG	PRO	185	33.131	-20.150	40.624	1.00	0.00	RX0	C
ATOM	268	C	PRO	185	31.820	-17.125	39.813	1.00	0.00	RX0	C
ATOM	269	O	PRO	185	31.836	-17.275	38.592	1.00	0.00	RX0	O
ATOM	270	N	PHE	186	32.164	-15.998	40.422	1.00	0.00	RX0	N
ATOM	271	H	PHE	186	32.254	-15.995	41.417	0.00	0.00	RX0	H
ATOM	272	CA	PHE	186	32.333	-14.726	39.697	1.00	0.00	RX0	C
ATOM	273	CB	PHE	186	31.515	-13.614	40.355	1.00	0.00	RX0	C
ATOM	274	CG	PHE	186	31.412	-12.444	39.405	1.00	0.00	RX0	C
ATOM	275	CD1	PHE	186	30.861	-12.628	38.141	1.00	0.00	RX0	C
ATOM	276	CD2	PHE	186	31.869	-11.188	39.788	1.00	0.00	RX0	C
ATOM	277	CE1	PHE	186	30.769	-11.557	37.260	1.00	0.00	RX0	C
ATOM	278	CE2	PHE	186	31.777	-10.117	38.906	1.00	0.00	RX0	C
ATOM	279	CZ	PHE	186	31.228	-10.302	37.642	1.00	0.00	RX0	C
ATOM	280	C	PHE	186	33.791	-14.305	39.507	1.00	0.00	RX0	C
ATOM	281	O	PHE	186	34.127	-13.678	38.496	1.00	0.00	RX0	O
ATOM	282	N	SER	187	34.655	-14.802	40.380	1.00	0.00	RX0	N
ATOM	283	H	SER	187	34.313	-15.351	41.138	0.00	0.00	RX0	H
ATOM	284	CA	SER	187	36.113	-14.542	40.380	1.00	0.00	RX0	C
ATOM	285	CB	SER	187	36.609	-15.270	41.615	1.00	0.00	RX0	C
ATOM	286	OG	SER	187	35.488	-15.363	42.513	1.00	0.00	RX0	O
ATOM	287	HG	SER	187	35.862	-15.421	43.385	0.00	0.00	RX0	H
ATOM	288	C	SER	187	36.764	-14.980	39.057	1.00	0.00	RX0	C
ATOM	289	O	SER	187	37.834	-14.531	38.683	1.00	0.00	RX0	O
ATOM	290	N	GLU	188	36.054	-15.878	38.369	1.00	0.00	RX0	N
ATOM	291	H	GLU	188	35.136	-16.129	38.661	0.00	0.00	RX0	H
ATOM	292	CA	GLU	188	36.561	-16.586	37.191	1.00	0.00	RX0	C
ATOM	293	CB	GLU	188	36.206	-18.053	37.451	1.00	0.00	RX0	C
ATOM	294	CG	GLU	188	36.248	-19.054	36.302	1.00	0.00	RX0	C
ATOM	295	CD	GLU	188	34.866	-19.653	36.242	1.00	0.00	RX0	C
ATOM	296	OE1	GLU	188	34.242	-19.776	37.292	1.00	0.00	RX0	O
ATOM	297	OE2	GLU	188	34.359	-19.896	35.152	1.00	0.00	RX0	O
ATOM	298	C	GLU	188	36.028	-16.018	35.856	1.00	0.00	RX0	C
ATOM	299	O	GLU	188	36.494	-16.416	34.788	1.00	0.00	RX0	O
ATOM	300	N	ALA	189	35.058	-15.107	35.914	1.00	0.00	RX0	N
ATOM	301	H	ALA	189	34.851	-14.674	36.794	0.00	0.00	RX0	H
ATOM	302	CA	ALA	189	34.543	-14.432	34.708	1.00	0.00	RX0	C
ATOM	303	CB	ALA	189	33.066	-14.086	34.892	1.00	0.00	RX0	C

ATOM	304	C	ALA	189	35.336	-13.151	34.407	1.00	0.00	RX0	C
ATOM	305	O	ALA	189	35.533	-12.292	35.270	1.00	0.00	RX0	O
ATOM	306	N	SER	190	35.819	-13.065	33.173	1.00	0.00	RX0	N
ATOM	307	H	SER	190	35.619	-13.820	32.552	0.00	0.00	RX0	H
ATOM	308	CA	SER	190	36.430	-11.825	32.646	1.00	0.00	RX0	C
ATOM	309	CB	SER	190	37.098	-12.196	31.296	1.00	0.00	RX0	C
ATOM	310	OG	SER	190	38.258	-11.393	30.967	1.00	0.00	RX0	O
ATOM	311	HG	SER	190	38.786	-11.437	31.766	0.00	0.00	RX0	H
ATOM	312	C	SER	190	35.341	-10.761	32.513	1.00	0.00	RX0	C
ATOM	313	O	SER	190	34.465	-10.869	31.639	1.00	0.00	RX0	O
ATOM	314	N	MET	191	35.401	-9.751	33.361	1.00	0.00	RX0	N
ATOM	315	H	MET	191	36.038	-9.820	34.130	0.00	0.00	RX0	H
ATOM	316	CA	MET	191	34.414	-8.652	33.337	1.00	0.00	RX0	C
ATOM	317	CB	MET	191	34.643	-7.665	34.478	1.00	0.00	RX0	C
ATOM	318	CG	MET	191	33.550	-6.597	34.496	1.00	0.00	RX0	C
ATOM	319	SD	MET	191	33.642	-5.541	35.944	1.00	0.00	RX0	S
ATOM	320	CE	MET	191	33.287	-6.803	37.180	1.00	0.00	RX0	C
ATOM	321	C	MET	191	34.384	-7.939	31.976	1.00	0.00	RX0	C
ATOM	322	O	MET	191	33.329	-7.841	31.363	1.00	0.00	RX0	O
ATOM	323	N	MET	192	35.577	-7.654	31.438	1.00	0.00	RX0	N
ATOM	324	H	MET	192	36.383	-7.740	32.020	0.00	0.00	RX0	H
ATOM	325	CA	MET	192	35.697	-7.082	30.089	1.00	0.00	RX0	C
ATOM	326	CB	MET	192	37.136	-6.648	29.804	1.00	0.00	RX0	C
ATOM	327	CG	MET	192	37.280	-5.928	28.460	1.00	0.00	RX0	C
ATOM	328	SD	MET	192	36.169	-4.520	28.287	1.00	0.00	RX0	S
ATOM	329	CE	MET	192	36.763	-3.522	29.662	1.00	0.00	RX0	C
ATOM	330	C	MET	192	35.151	-8.021	28.999	1.00	0.00	RX0	C
ATOM	331	O	MET	192	34.484	-7.587	28.093	1.00	0.00	RX0	O
ATOM	332	N	GLY	193	35.358	-9.342	29.220	1.00	0.00	RX0	N
ATOM	333	H	GLY	193	35.698	-9.610	30.117	0.00	0.00	RX0	H
ATOM	334	CA	GLY	193	34.804	-10.378	28.330	1.00	0.00	RX0	C
ATOM	335	C	GLY	193	33.267	-10.338	28.334	1.00	0.00	RX0	C
ATOM	336	O	GLY	193	32.637	-10.184	27.296	1.00	0.00	RX0	O
ATOM	337	N	LEU	194	32.696	-10.293	29.537	1.00	0.00	RX0	N
ATOM	338	H	LEU	194	33.263	-10.302	30.357	0.00	0.00	RX0	H
ATOM	339	CA	LEU	194	31.235	-10.169	29.722	1.00	0.00	RX0	C
ATOM	340	CB	LEU	194	30.848	-10.223	31.199	1.00	0.00	RX0	C
ATOM	341	CG	LEU	194	31.145	-11.560	31.869	1.00	0.00	RX0	C
ATOM	342	CD1	LEU	194	30.705	-11.547	33.332	1.00	0.00	RX0	C
ATOM	343	CD2	LEU	194	30.538	-12.731	31.095	1.00	0.00	RX0	C
ATOM	344	C	LEU	194	30.647	-8.891	29.116	1.00	0.00	RX0	C
ATOM	345	O	LEU	194	29.706	-8.959	28.317	1.00	0.00	RX0	O
ATOM	346	N	LEU	195	31.327	-7.782	29.364	1.00	0.00	RX0	N
ATOM	347	H	LEU	195	32.135	-7.840	29.946	0.00	0.00	RX0	H
ATOM	348	CA	LEU	195	30.920	-6.462	28.846	1.00	0.00	RX0	C
ATOM	349	CB	LEU	195	31.731	-5.338	29.493	1.00	0.00	RX0	C
ATOM	350	CG	LEU	195	31.532	-5.239	31.006	1.00	0.00	RX0	C
ATOM	351	CD1	LEU	195	32.368	-4.111	31.612	1.00	0.00	RX0	C
ATOM	352	CD2	LEU	195	30.057	-5.129	31.385	1.00	0.00	RX0	C
ATOM	353	C	LEU	195	31.020	-6.357	27.321	1.00	0.00	RX0	C
ATOM	354	O	LEU	195	30.051	-5.942	26.671	1.00	0.00	RX0	O
ATOM	355	N	THR	196	32.075	-6.931	26.767	1.00	0.00	RX0	N
ATOM	356	H	THR	196	32.806	-7.314	27.329	0.00	0.00	RX0	H
ATOM	357	CA	THR	196	32.335	-6.901	25.309	1.00	0.00	RX0	C
ATOM	358	CB	THR	196	33.782	-7.303	25.031	1.00	0.00	RX0	C
ATOM	359	OG1	THR	196	34.666	-6.395	25.702	1.00	0.00	RX0	O
ATOM	360	HG1	THR	196	34.391	-5.524	25.443	0.00	0.00	RX0	H
ATOM	361	CG2	THR	196	34.089	-7.348	23.533	1.00	0.00	RX0	C
ATOM	362	C	THR	196	31.317	-7.765	24.552	1.00	0.00	RX0	C
ATOM	363	O	THR	196	30.772	-7.327	23.532	1.00	0.00	RX0	O
ATOM	364	N	ASN	197	31.003	-8.928	25.107	1.00	0.00	RX0	N

ATOM	365	H	ASN	197	31.428	-9.178	25.979	0.00	0.00	RX0	H
ATOM	366	CA	ASN	197	30.010	-9.840	24.504	1.00	0.00	RX0	C
ATOM	367	CB	ASN	197	29.976	-11.209	25.181	1.00	0.00	RX0	C
ATOM	368	CG	ASN	197	31.015	-12.117	24.560	1.00	0.00	RX0	C
ATOM	369	OD1	ASN	197	30.869	-12.644	23.464	1.00	0.00	RX0	O
ATOM	370	ND2	ASN	197	32.099	-12.277	25.339	1.00	0.00	RX0	N
ATOM	371	HD21	ASN	197	32.162	-11.760	26.196	0.00	0.00	RX0	H
ATOM	372	HD22	ASN	197	32.833	-12.897	25.068	0.00	0.00	RX0	H
ATOM	373	C	ASN	197	28.594	-9.255	24.528	1.00	0.00	RX0	C
ATOM	374	O	ASN	197	27.900	-9.272	23.514	1.00	0.00	RX0	O
ATOM	375	N	LEU	198	28.277	-8.575	25.633	1.00	0.00	RX0	N
ATOM	376	H	LEU	198	28.917	-8.571	26.404	0.00	0.00	RX0	H
ATOM	377	CA	LEU	198	27.002	-7.850	25.760	1.00	0.00	RX0	C
ATOM	378	CB	LEU	198	26.852	-7.338	27.189	1.00	0.00	RX0	C
ATOM	379	CG	LEU	198	25.457	-6.808	27.502	1.00	0.00	RX0	C
ATOM	380	CD1	LEU	198	24.366	-7.849	27.243	1.00	0.00	RX0	C
ATOM	381	CD2	LEU	198	25.402	-6.272	28.928	1.00	0.00	RX0	C
ATOM	382	C	LEU	198	26.885	-6.719	24.724	1.00	0.00	RX0	C
ATOM	383	O	LEU	198	25.930	-6.676	23.947	1.00	0.00	RX0	O
ATOM	384	N	ALA	199	27.942	-5.914	24.641	1.00	0.00	RX0	N
ATOM	385	H	ALA	199	28.700	-6.060	25.278	0.00	0.00	RX0	H
ATOM	386	CA	ALA	199	28.029	-4.784	23.694	1.00	0.00	RX0	C
ATOM	387	CB	ALA	199	29.343	-4.024	23.882	1.00	0.00	RX0	C
ATOM	388	C	ALA	199	27.921	-5.230	22.227	1.00	0.00	RX0	C
ATOM	389	O	ALA	199	27.138	-4.660	21.467	1.00	0.00	RX0	O
ATOM	390	N	ASP	200	28.555	-6.360	21.908	1.00	0.00	RX0	N
ATOM	391	H	ASP	200	29.141	-6.809	22.584	0.00	0.00	RX0	H
ATOM	392	CA	ASP	200	28.494	-6.940	20.550	1.00	0.00	RX0	C
ATOM	393	CB	ASP	200	29.524	-8.055	20.376	1.00	0.00	RX0	C
ATOM	394	CG	ASP	200	30.199	-7.944	19.017	1.00	0.00	RX0	C
ATOM	395	OD1	ASP	200	30.592	-6.851	18.615	1.00	0.00	RX0	O
ATOM	396	OD2	ASP	200	30.387	-8.955	18.345	1.00	0.00	RX0	O
ATOM	397	C	ASP	200	27.084	-7.410	20.171	1.00	0.00	RX0	C
ATOM	398	O	ASP	200	26.604	-7.102	19.080	1.00	0.00	RX0	O
ATOM	399	N	ARG	201	26.390	-7.998	21.143	1.00	0.00	RX0	N
ATOM	400	H	ARG	201	26.848	-8.160	22.020	0.00	0.00	RX0	H
ATOM	401	CA	ARG	201	24.992	-8.434	20.957	1.00	0.00	RX0	C
ATOM	402	CB	ARG	201	24.554	-9.410	22.036	1.00	0.00	RX0	C
ATOM	403	CG	ARG	201	24.000	-10.685	21.400	1.00	0.00	RX0	C
ATOM	404	CD	ARG	201	23.278	-11.575	22.408	1.00	0.00	RX0	C
ATOM	405	NE	ARG	201	24.105	-11.805	23.587	1.00	0.00	RX0	N
ATOM	406	HE	ARG	201	24.928	-12.393	23.511	0.00	0.00	RX0	H
ATOM	407	CZ	ARG	201	23.730	-11.284	24.786	1.00	0.00	RX0	C
ATOM	408	NH1	ARG	201	22.585	-10.589	24.881	1.00	0.00	RX0	N
ATOM	409	HH11	ARG	201	22.297	-10.216	25.765	0.00	0.00	RX0	H
ATOM	410	HH12	ARG	201	22.003	-10.424	24.081	0.00	0.00	RX0	H
ATOM	411	NH2	ARG	201	24.493	-11.462	25.868	1.00	0.00	RX0	N
ATOM	412	HH21	ARG	201	24.235	-11.173	26.795	0.00	0.00	RX0	H
ATOM	413	HH22	ARG	201	25.372	-11.960	25.777	0.00	0.00	RX0	H
ATOM	414	C	ARG	201	23.991	-7.279	20.827	1.00	0.00	RX0	C
ATOM	415	O	ARG	201	23.123	-7.308	19.955	1.00	0.00	RX0	O
ATOM	416	N	GLU	202	24.240	-6.201	21.568	1.00	0.00	RX0	N
ATOM	417	H	GLU	202	25.010	-6.216	22.212	0.00	0.00	RX0	H
ATOM	418	CA	GLU	202	23.401	-4.988	21.493	1.00	0.00	RX0	C
ATOM	419	CB	GLU	202	23.558	-4.078	22.732	1.00	0.00	RX0	C
ATOM	420	CG	GLU	202	22.918	-4.692	23.997	1.00	0.00	RX0	C
ATOM	421	CD	GLU	202	22.868	-3.742	25.196	1.00	0.00	RX0	C
ATOM	422	OE1	GLU	202	21.846	-3.093	25.423	1.00	0.00	RX0	O
ATOM	423	OE2	GLU	202	23.821	-3.695	25.968	1.00	0.00	RX0	O
ATOM	424	C	GLU	202	23.526	-4.262	20.149	1.00	0.00	RX0	C
ATOM	425	O	GLU	202	22.539	-3.754	19.625	1.00	0.00	RX0	O

ATOM	426	N	LEU	203	24.712	-4.358	19.546	1.00	0.00	RX0	N
ATOM	427	H	LEU	203	25.464	-4.810	20.035	0.00	0.00	RX0	H
ATOM	428	CA	LEU	203	25.004	-3.680	18.270	1.00	0.00	RX0	C
ATOM	429	CB	LEU	203	26.481	-3.870	17.916	1.00	0.00	RX0	C
ATOM	430	CG	LEU	203	26.921	-3.125	16.654	1.00	0.00	RX0	C
ATOM	431	CD1	LEU	203	26.716	-1.614	16.774	1.00	0.00	RX0	C
ATOM	432	CD2	LEU	203	28.358	-3.476	16.269	1.00	0.00	RX0	C
ATOM	433	C	LEU	203	24.099	-4.160	17.127	1.00	0.00	RX0	C
ATOM	434	O	LEU	203	23.593	-3.349	16.346	1.00	0.00	RX0	O
ATOM	435	N	VAL	204	23.782	-5.447	17.151	1.00	0.00	RX0	N
ATOM	436	H	VAL	204	24.176	-6.003	17.886	0.00	0.00	RX0	H
ATOM	437	CA	VAL	204	22.925	-6.083	16.127	1.00	0.00	RX0	C
ATOM	438	CB	VAL	204	22.898	-7.596	16.334	1.00	0.00	RX0	C
ATOM	439	CG1	VAL	204	22.008	-8.279	15.294	1.00	0.00	RX0	C
ATOM	440	CG2	VAL	204	24.319	-8.164	16.351	1.00	0.00	RX0	C
ATOM	441	C	VAL	204	21.502	-5.497	16.213	1.00	0.00	RX0	C
ATOM	442	O	VAL	204	20.938	-5.041	15.221	1.00	0.00	RX0	O
ATOM	443	N	HIS	205	21.015	-5.393	17.448	1.00	0.00	RX0	N
ATOM	444	H	HIS	205	21.605	-5.674	18.208	0.00	0.00	RX0	H
ATOM	445	CA	HIS	205	19.703	-4.786	17.746	1.00	0.00	RX0	C
ATOM	446	CB	HIS	205	19.274	-5.103	19.183	1.00	0.00	RX0	C
ATOM	447	CG	HIS	205	18.997	-6.582	19.306	1.00	0.00	RX0	C
ATOM	448	ND1	HIS	205	17.792	-7.136	19.082	1.00	0.00	RX0	N
ATOM	449	HD1	HIS	205	16.970	-6.665	18.835	0.00	0.00	RX0	H
ATOM	450	CD2	HIS	205	19.897	-7.593	19.652	1.00	0.00	RX0	C
ATOM	451	NE2	HIS	205	19.219	-8.766	19.635	1.00	0.00	RX0	N
ATOM	452	CE1	HIS	205	17.925	-8.485	19.284	1.00	0.00	RX0	C
ATOM	453	C	HIS	205	19.668	-3.277	17.476	1.00	0.00	RX0	C
ATOM	454	O	HIS	205	18.642	-2.756	17.030	1.00	0.00	RX0	O
ATOM	455	N	MET	206	20.820	-2.627	17.609	1.00	0.00	RX0	N
ATOM	456	H	MET	206	21.610	-3.124	17.973	0.00	0.00	RX0	H
ATOM	457	CA	MET	206	20.969	-1.185	17.340	1.00	0.00	RX0	C
ATOM	458	CB	MET	206	22.357	-0.694	17.746	1.00	0.00	RX0	C
ATOM	459	CG	MET	206	22.542	0.805	17.510	1.00	0.00	RX0	C
ATOM	460	SD	MET	206	24.242	1.327	17.772	1.00	0.00	RX0	S
ATOM	461	CE	MET	206	24.498	0.517	19.356	1.00	0.00	RX0	C
ATOM	462	C	MET	206	20.721	-0.870	15.856	1.00	0.00	RX0	C
ATOM	463	O	MET	206	20.035	0.103	15.544	1.00	0.00	RX0	O
ATOM	464	N	ILE	207	21.183	-1.758	14.977	1.00	0.00	RX0	N
ATOM	465	H	ILE	207	21.733	-2.516	15.339	0.00	0.00	RX0	H
ATOM	466	CA	ILE	207	20.975	-1.628	13.516	1.00	0.00	RX0	C
ATOM	467	CB	ILE	207	21.671	-2.790	12.801	1.00	0.00	RX0	C
ATOM	468	CG2	ILE	207	21.416	-2.771	11.295	1.00	0.00	RX0	C
ATOM	469	CG1	ILE	207	23.165	-2.809	13.121	1.00	0.00	RX0	C
ATOM	470	CD1	ILE	207	23.908	-1.598	12.558	1.00	0.00	RX0	C
ATOM	471	C	ILE	207	19.470	-1.621	13.197	1.00	0.00	RX0	C
ATOM	472	O	ILE	207	18.988	-0.755	12.467	1.00	0.00	RX0	O
ATOM	473	N	ASN	208	18.761	-2.558	13.816	1.00	0.00	RX0	N
ATOM	474	H	ASN	208	19.237	-3.125	14.491	0.00	0.00	RX0	H
ATOM	475	CA	ASN	208	17.313	-2.734	13.586	1.00	0.00	RX0	C
ATOM	476	CB	ASN	208	16.800	-4.069	14.125	1.00	0.00	RX0	C
ATOM	477	CG	ASN	208	17.108	-5.160	13.115	1.00	0.00	RX0	C
ATOM	478	OD1	ASN	208	17.511	-4.908	11.976	1.00	0.00	RX0	O
ATOM	479	ND2	ASN	208	16.907	-6.397	13.597	1.00	0.00	RX0	N
ATOM	480	HD21	ASN	208	16.578	-6.528	14.533	0.00	0.00	RX0	H
ATOM	481	HD22	ASN	208	17.079	-7.228	13.067	0.00	0.00	RX0	H
ATOM	482	C	ASN	208	16.516	-1.532	14.103	1.00	0.00	RX0	C
ATOM	483	O	ASN	208	15.637	-1.017	13.417	1.00	0.00	RX0	O
ATOM	484	N	TRP	209	16.982	-1.008	15.238	1.00	0.00	RX0	N
ATOM	485	H	TRP	209	17.725	-1.474	15.722	0.00	0.00	RX0	H
ATOM	486	CA	TRP	209	16.425	0.201	15.864	1.00	0.00	RX0	C

ATOM	487	CB	TRP	209	17.092	0.419	17.231	1.00	0.00	RX0	C
ATOM	488	CG	TRP	209	16.695	1.748	17.836	1.00	0.00	RX0	C
ATOM	489	CD2	TRP	209	17.416	2.999	17.808	1.00	0.00	RX0	C
ATOM	490	CE2	TRP	209	16.636	3.960	18.496	1.00	0.00	RX0	C
ATOM	491	CE3	TRP	209	18.636	3.369	17.256	1.00	0.00	RX0	C
ATOM	492	CD1	TRP	209	15.527	2.030	18.552	1.00	0.00	RX0	C
ATOM	493	NE1	TRP	209	15.480	3.327	18.950	1.00	0.00	RX0	N
ATOM	494	HE1	TRP	209	14.724	3.720	19.453	0.00	0.00	RX0	H
ATOM	495	CZ2	TRP	209	17.102	5.265	18.601	1.00	0.00	RX0	C
ATOM	496	CZ3	TRP	209	19.091	4.676	17.375	1.00	0.00	RX0	C
ATOM	497	CH2	TRP	209	18.322	5.623	18.040	1.00	0.00	RX0	C
ATOM	498	C	TRP	209	16.619	1.438	14.972	1.00	0.00	RX0	C
ATOM	499	O	TRP	209	15.652	2.120	14.634	1.00	0.00	RX0	O
ATOM	500	N	ALA	210	17.853	1.624	14.503	1.00	0.00	RX0	N
ATOM	501	H	ALA	210	18.568	0.988	14.791	0.00	0.00	RX0	H
ATOM	502	CA	ALA	210	18.233	2.756	13.635	1.00	0.00	RX0	C
ATOM	503	CB	ALA	210	19.715	2.665	13.273	1.00	0.00	RX0	C
ATOM	504	C	ALA	210	17.400	2.800	12.347	1.00	0.00	RX0	C
ATOM	505	O	ALA	210	16.892	3.855	11.979	1.00	0.00	RX0	O
ATOM	506	N	LYS	211	17.095	1.613	11.820	1.00	0.00	RX0	N
ATOM	507	H	LYS	211	17.504	0.799	12.235	0.00	0.00	RX0	H
ATOM	508	CA	LYS	211	16.258	1.472	10.614	1.00	0.00	RX0	C
ATOM	509	CB	LYS	211	16.399	0.065	10.029	1.00	0.00	RX0	C
ATOM	510	CG	LYS	211	17.791	-0.116	9.411	1.00	0.00	RX0	C
ATOM	511	CD	LYS	211	18.061	-1.526	8.882	1.00	0.00	RX0	C
ATOM	512	CE	LYS	211	17.834	-2.547	9.991	1.00	0.00	RX0	C
ATOM	513	NZ	LYS	211	18.356	-3.878	9.647	1.00	0.00	RX0	N
ATOM	514	HZ1	LYS	211	18.177	-4.501	10.467	0.00	0.00	RX0	H
ATOM	515	HZ2	LYS	211	17.869	-4.259	8.814	0.00	0.00	RX0	H
ATOM	516	HZ3	LYS	211	19.380	-3.835	9.465	0.00	0.00	RX0	H
ATOM	517	C	LYS	211	14.795	1.898	10.823	1.00	0.00	RX0	C
ATOM	518	O	LYS	211	14.129	2.337	9.881	1.00	0.00	RX0	O
ATOM	519	N	ARG	212	14.357	1.870	12.071	1.00	0.00	RX0	N
ATOM	520	H	ARG	212	14.980	1.608	12.810	0.00	0.00	RX0	H
ATOM	521	CA	ARG	212	13.005	2.320	12.466	1.00	0.00	RX0	C
ATOM	522	CB	ARG	212	12.385	1.387	13.505	1.00	0.00	RX0	C
ATOM	523	CG	ARG	212	12.545	-0.085	13.127	1.00	0.00	RX0	C
ATOM	524	CD	ARG	212	11.652	-1.012	13.953	1.00	0.00	RX0	C
ATOM	525	NE	ARG	212	10.252	-0.794	13.596	1.00	0.00	RX0	N
ATOM	526	HE	ARG	212	10.055	-0.618	12.627	0.00	0.00	RX0	H
ATOM	527	CZ	ARG	212	9.287	-0.817	14.561	1.00	0.00	RX0	C
ATOM	528	NH1	ARG	212	9.613	-1.084	15.841	1.00	0.00	RX0	N
ATOM	529	HH11	ARG	212	8.893	-1.084	16.562	0.00	0.00	RX0	H
ATOM	530	HH12	ARG	212	10.539	-1.276	16.166	0.00	0.00	RX0	H
ATOM	531	NH2	ARG	212	8.013	-0.563	14.218	1.00	0.00	RX0	N
ATOM	532	HH21	ARG	212	7.301	-0.515	14.948	0.00	0.00	RX0	H
ATOM	533	HH22	ARG	212	7.692	-0.376	13.292	0.00	0.00	RX0	H
ATOM	534	C	ARG	212	12.933	3.790	12.894	1.00	0.00	RX0	C
ATOM	535	O	ARG	212	11.827	4.344	12.989	1.00	0.00	RX0	O
ATOM	536	N	VAL	213	14.074	4.417	13.148	1.00	0.00	RX0	N
ATOM	537	H	VAL	213	14.933	3.928	12.994	0.00	0.00	RX0	H
ATOM	538	CA	VAL	213	14.155	5.874	13.374	1.00	0.00	RX0	C
ATOM	539	CB	VAL	213	15.581	6.281	13.749	1.00	0.00	RX0	C
ATOM	540	CG1	VAL	213	15.750	7.800	13.839	1.00	0.00	RX0	C
ATOM	541	CG2	VAL	213	15.978	5.584	15.048	1.00	0.00	RX0	C
ATOM	542	C	VAL	213	13.672	6.590	12.095	1.00	0.00	RX0	C
ATOM	543	O	VAL	213	14.288	6.431	11.023	1.00	0.00	RX0	O
ATOM	544	N	PRO	214	12.622	7.395	12.213	1.00	0.00	RX0	N
ATOM	545	CD	PRO	214	11.896	7.609	13.459	1.00	0.00	RX0	C
ATOM	546	CA	PRO	214	12.035	8.145	11.084	1.00	0.00	RX0	C
ATOM	547	CB	PRO	214	10.926	8.953	11.762	1.00	0.00	RX0	C

ATOM	548	CG	PRO	214	10.553	8.162	13.011	1.00	0.00	RX0	C
ATOM	549	C	PRO	214	13.102	9.001	10.387	1.00	0.00	RX0	C
ATOM	550	O	PRO	214	13.853	9.727	11.025	1.00	0.00	RX0	O
ATOM	551	N	GLY	215	13.244	8.730	9.080	1.00	0.00	RX0	N
ATOM	552	H	GLY	215	12.750	7.982	8.635	0.00	0.00	RX0	H
ATOM	553	CA	GLY	215	14.194	9.473	8.227	1.00	0.00	RX0	C
ATOM	554	C	GLY	215	15.511	8.732	7.950	1.00	0.00	RX0	C
ATOM	555	O	GLY	215	16.085	8.889	6.862	1.00	0.00	RX0	O
ATOM	556	N	PHE	216	15.917	7.845	8.848	1.00	0.00	RX0	N
ATOM	557	H	PHE	216	15.329	7.652	9.636	0.00	0.00	RX0	H
ATOM	558	CA	PHE	216	17.224	7.160	8.764	1.00	0.00	RX0	C
ATOM	559	CB	PHE	216	17.452	6.294	9.997	1.00	0.00	RX0	C
ATOM	560	CG	PHE	216	18.892	5.845	10.057	1.00	0.00	RX0	C
ATOM	561	CD1	PHE	216	19.896	6.774	10.304	1.00	0.00	RX0	C
ATOM	562	CD2	PHE	216	19.213	4.504	9.881	1.00	0.00	RX0	C
ATOM	563	CE1	PHE	216	21.216	6.357	10.415	1.00	0.00	RX0	C
ATOM	564	CE2	PHE	216	20.534	4.087	9.993	1.00	0.00	RX0	C
ATOM	565	CZ	PHE	216	21.532	5.010	10.281	1.00	0.00	RX0	C
ATOM	566	C	PHE	216	17.435	6.347	7.474	1.00	0.00	RX0	C
ATOM	567	O	PHE	216	18.358	6.641	6.711	1.00	0.00	RX0	O
ATOM	568	N	VAL	217	16.482	5.483	7.151	1.00	0.00	RX0	N
ATOM	569	H	VAL	217	15.672	5.451	7.735	0.00	0.00	RX0	H
ATOM	570	CA	VAL	217	16.570	4.608	5.958	1.00	0.00	RX0	C
ATOM	571	CB	VAL	217	15.531	3.492	6.008	1.00	0.00	RX0	C
ATOM	572	CG1	VAL	217	15.905	2.479	7.081	1.00	0.00	RX0	C
ATOM	573	CG2	VAL	217	14.112	4.044	6.170	1.00	0.00	RX0	C
ATOM	574	C	VAL	217	16.469	5.342	4.608	1.00	0.00	RX0	C
ATOM	575	O	VAL	217	16.660	4.747	3.556	1.00	0.00	RX0	O
ATOM	576	N	ASP	218	16.058	6.613	4.671	1.00	0.00	RX0	N
ATOM	577	H	ASP	218	15.769	7.063	5.519	0.00	0.00	RX0	H
ATOM	578	CA	ASP	218	16.006	7.470	3.472	1.00	0.00	RX0	C
ATOM	579	CB	ASP	218	15.073	8.654	3.759	1.00	0.00	RX0	C
ATOM	580	CG	ASP	218	13.734	8.188	4.333	1.00	0.00	RX0	C
ATOM	581	OD1	ASP	218	12.741	8.227	3.611	1.00	0.00	RX0	O
ATOM	582	OD2	ASP	218	13.668	7.812	5.508	1.00	0.00	RX0	O
ATOM	583	C	ASP	218	17.401	7.924	3.023	1.00	0.00	RX0	C
ATOM	584	O	ASP	218	17.595	8.369	1.896	1.00	0.00	RX0	O
ATOM	585	N	LEU	219	18.344	7.857	3.967	1.00	0.00	RX0	N
ATOM	586	H	LEU	219	18.095	7.489	4.863	0.00	0.00	RX0	H
ATOM	587	CA	LEU	219	19.767	8.083	3.697	1.00	0.00	RX0	C
ATOM	588	CB	LEU	219	20.539	8.275	5.000	1.00	0.00	RX0	C
ATOM	589	CG	LEU	219	19.911	9.375	5.859	1.00	0.00	RX0	C
ATOM	590	CD1	LEU	219	20.503	9.403	7.266	1.00	0.00	RX0	C
ATOM	591	CD2	LEU	219	19.954	10.742	5.176	1.00	0.00	RX0	C
ATOM	592	C	LEU	219	20.350	6.970	2.832	1.00	0.00	RX0	C
ATOM	593	O	LEU	219	19.843	5.836	2.789	1.00	0.00	RX0	O
ATOM	594	N	THR	220	21.442	7.293	2.191	1.00	0.00	RX0	N
ATOM	595	H	THR	220	21.728	8.256	2.238	0.00	0.00	RX0	H
ATOM	596	CA	THR	220	22.263	6.312	1.453	1.00	0.00	RX0	C
ATOM	597	CB	THR	220	23.340	6.881	0.506	1.00	0.00	RX0	C
ATOM	598	OG1	THR	220	24.636	6.946	1.104	1.00	0.00	RX0	O
ATOM	599	HG1	THR	220	24.726	7.824	1.486	0.00	0.00	RX0	H
ATOM	600	CG2	THR	220	22.940	8.191	-0.153	1.00	0.00	RX0	C
ATOM	601	C	THR	220	22.829	5.303	2.454	1.00	0.00	RX0	C
ATOM	602	O	THR	220	23.112	5.646	3.611	1.00	0.00	RX0	O
ATOM	603	N	LEU	221	23.130	4.116	1.957	1.00	0.00	RX0	N
ATOM	604	H	LEU	221	22.896	3.922	1.007	0.00	0.00	RX0	H
ATOM	605	CA	LEU	221	23.750	3.065	2.781	1.00	0.00	RX0	C
ATOM	606	CB	LEU	221	23.997	1.839	1.905	1.00	0.00	RX0	C
ATOM	607	CG	LEU	221	24.917	0.819	2.573	1.00	0.00	RX0	C
ATOM	608	CD1	LEU	221	24.204	0.053	3.684	1.00	0.00	RX0	C

ATOM	609	CD2	LEU	221	25.589	-0.096	1.551	1.00	0.00	RX0	C
ATOM	610	C	LEU	221	25.092	3.509	3.394	1.00	0.00	RX0	C
ATOM	611	O	LEU	221	25.324	3.347	4.578	1.00	0.00	RX0	O
ATOM	612	N	HIS	222	25.854	4.270	2.593	1.00	0.00	RX0	N
ATOM	613	H	HIS	222	25.506	4.508	1.688	0.00	0.00	RX0	H
ATOM	614	CA	HIS	222	27.131	4.847	3.045	1.00	0.00	RX0	C
ATOM	615	CB	HIS	222	27.804	5.609	1.883	1.00	0.00	RX0	C
ATOM	616	CG	HIS	222	28.896	6.545	2.370	1.00	0.00	RX0	C
ATOM	617	ND1	HIS	222	29.825	6.222	3.289	1.00	0.00	RX0	N
ATOM	618	HD1	HIS	222	29.942	5.360	3.747	0.00	0.00	RX0	H
ATOM	619	CD2	HIS	222	29.110	7.867	1.971	1.00	0.00	RX0	C
ATOM	620	NE2	HIS	222	30.180	8.330	2.662	1.00	0.00	RX0	N
ATOM	621	CE1	HIS	222	30.616	7.320	3.479	1.00	0.00	RX0	C
ATOM	622	C	HIS	222	26.942	5.765	4.264	1.00	0.00	RX0	C
ATOM	623	O	HIS	222	27.673	5.642	5.246	1.00	0.00	RX0	O
ATOM	624	N	ASP	223	25.958	6.658	4.167	1.00	0.00	RX0	N
ATOM	625	H	ASP	223	25.486	6.797	3.294	0.00	0.00	RX0	H
ATOM	626	CA	ASP	223	25.694	7.636	5.242	1.00	0.00	RX0	C
ATOM	627	CB	ASP	223	24.742	8.736	4.751	1.00	0.00	RX0	C
ATOM	628	CG	ASP	223	25.364	9.562	3.633	1.00	0.00	RX0	C
ATOM	629	OD1	ASP	223	25.576	10.758	3.824	1.00	0.00	RX0	O
ATOM	630	OD2	ASP	223	25.626	9.015	2.564	1.00	0.00	RX0	O
ATOM	631	C	ASP	223	25.148	6.996	6.516	1.00	0.00	RX0	C
ATOM	632	O	ASP	223	25.558	7.375	7.616	1.00	0.00	RX0	O
ATOM	633	N	GLN	224	24.393	5.921	6.332	1.00	0.00	RX0	N
ATOM	634	H	GLN	224	24.152	5.690	5.386	0.00	0.00	RX0	H
ATOM	635	CA	GLN	224	23.868	5.118	7.452	1.00	0.00	RX0	C
ATOM	636	CB	GLN	224	22.844	4.095	6.960	1.00	0.00	RX0	C
ATOM	637	CG	GLN	224	21.592	4.770	6.394	1.00	0.00	RX0	C
ATOM	638	CD	GLN	224	20.542	3.724	6.089	1.00	0.00	RX0	C
ATOM	639	OE1	GLN	224	20.373	2.750	6.818	1.00	0.00	RX0	O
ATOM	640	NE2	GLN	224	19.843	3.974	4.969	1.00	0.00	RX0	N
ATOM	641	HE21	GLN	224	20.016	4.793	4.413	0.00	0.00	RX0	H
ATOM	642	HE22	GLN	224	19.109	3.393	4.620	0.00	0.00	RX0	H
ATOM	643	C	GLN	224	25.003	4.453	8.243	1.00	0.00	RX0	C
ATOM	644	O	GLN	224	25.073	4.591	9.468	1.00	0.00	RX0	O
ATOM	645	N	VAL	225	25.993	3.956	7.505	1.00	0.00	RX0	N
ATOM	646	H	VAL	225	25.894	3.979	6.507	0.00	0.00	RX0	H
ATOM	647	CA	VAL	225	27.191	3.319	8.093	1.00	0.00	RX0	C
ATOM	648	CB	VAL	225	28.044	2.592	7.042	1.00	0.00	RX0	C
ATOM	649	CG1	VAL	225	29.242	1.895	7.688	1.00	0.00	RX0	C
ATOM	650	CG2	VAL	225	27.220	1.573	6.259	1.00	0.00	RX0	C
ATOM	651	C	VAL	225	28.021	4.368	8.852	1.00	0.00	RX0	C
ATOM	652	O	VAL	225	28.415	4.141	9.995	1.00	0.00	RX0	O
ATOM	653	N	HIS	226	28.182	5.534	8.231	1.00	0.00	RX0	N
ATOM	654	H	HIS	226	27.820	5.622	7.300	0.00	0.00	RX0	H
ATOM	655	CA	HIS	226	28.959	6.641	8.815	1.00	0.00	RX0	C
ATOM	656	CB	HIS	226	29.173	7.818	7.854	1.00	0.00	RX0	C
ATOM	657	CG	HIS	226	30.091	8.848	8.495	1.00	0.00	RX0	C
ATOM	658	ND1	HIS	226	31.284	8.556	9.051	1.00	0.00	RX0	N
ATOM	659	HD1	HIS	226	31.701	7.672	9.134	0.00	0.00	RX0	H
ATOM	660	CD2	HIS	226	29.870	10.222	8.636	1.00	0.00	RX0	C
ATOM	661	NE2	HIS	226	30.938	10.754	9.280	1.00	0.00	RX0	N
ATOM	662	CE1	HIS	226	31.809	9.727	9.535	1.00	0.00	RX0	C
ATOM	663	C	HIS	226	28.363	7.118	10.150	1.00	0.00	RX0	C
ATOM	664	O	HIS	226	29.071	7.189	11.155	1.00	0.00	RX0	O
ATOM	665	N	LEU	227	27.047	7.317	10.166	1.00	0.00	RX0	N
ATOM	666	H	LEU	227	26.524	7.188	9.319	0.00	0.00	RX0	H
ATOM	667	CA	LEU	227	26.344	7.787	11.375	1.00	0.00	RX0	C
ATOM	668	CB	LEU	227	24.875	8.089	11.079	1.00	0.00	RX0	C
ATOM	669	CG	LEU	227	24.676	9.364	10.262	1.00	0.00	RX0	C

ATOM	670	CD1	LEU	227	23.202	9.597	9.941	1.00	0.00	RX0	C
ATOM	671	CD2	LEU	227	25.291	10.581	10.951	1.00	0.00	RX0	C
ATOM	672	C	LEU	227	26.435	6.799	12.540	1.00	0.00	RX0	C
ATOM	673	O	LEU	227	26.853	7.165	13.635	1.00	0.00	RX0	O
ATOM	674	N	LEU	228	26.270	5.522	12.200	1.00	0.00	RX0	N
ATOM	675	H	LEU	228	26.040	5.299	11.249	0.00	0.00	RX0	H
ATOM	676	CA	LEU	228	26.384	4.431	13.181	1.00	0.00	RX0	C
ATOM	677	CB	LEU	228	25.764	3.155	12.619	1.00	0.00	RX0	C
ATOM	678	CG	LEU	228	24.258	3.145	12.877	1.00	0.00	RX0	C
ATOM	679	CD1	LEU	228	23.516	2.132	12.011	1.00	0.00	RX0	C
ATOM	680	CD2	LEU	228	23.963	2.944	14.363	1.00	0.00	RX0	C
ATOM	681	C	LEU	228	27.805	4.188	13.685	1.00	0.00	RX0	C
ATOM	682	O	LEU	228	28.004	4.019	14.891	1.00	0.00	RX0	O
ATOM	683	N	GLU	229	28.784	4.376	12.809	1.00	0.00	RX0	N
ATOM	684	H	GLU	229	28.577	4.590	11.853	0.00	0.00	RX0	H
ATOM	685	CA	GLU	229	30.199	4.229	13.197	1.00	0.00	RX0	C
ATOM	686	CB	GLU	229	31.151	4.095	11.988	1.00	0.00	RX0	C
ATOM	687	CG	GLU	229	32.523	3.513	12.391	1.00	0.00	RX0	C
ATOM	688	CD	GLU	229	33.413	3.134	11.207	1.00	0.00	RX0	C
ATOM	689	OE1	GLU	229	33.450	3.851	10.211	1.00	0.00	RX0	O
ATOM	690	OE2	GLU	229	34.109	2.121	11.292	1.00	0.00	RX0	O
ATOM	691	C	GLU	229	30.618	5.338	14.175	1.00	0.00	RX0	C
ATOM	692	O	GLU	229	31.393	5.088	15.099	1.00	0.00	RX0	O
ATOM	693	N	CYS	230	30.060	6.523	13.970	1.00	0.00	RX0	N
ATOM	694	H	CYS	230	29.477	6.654	13.165	0.00	0.00	RX0	H
ATOM	695	CA	CYS	230	30.321	7.692	14.829	1.00	0.00	RX0	C
ATOM	696	CB	CYS	230	30.006	8.968	14.060	1.00	0.00	RX0	C
ATOM	697	SG	CYS	230	31.044	9.131	12.592	1.00	0.00	RX0	S
ATOM	698	C	CYS	230	29.592	7.653	16.182	1.00	0.00	RX0	C
ATOM	699	O	CYS	230	30.123	8.119	17.188	1.00	0.00	RX0	O
ATOM	700	N	ALA	231	28.434	7.000	16.215	1.00	0.00	RX0	N
ATOM	701	H	ALA	231	28.113	6.534	15.388	0.00	0.00	RX0	H
ATOM	702	CA	ALA	231	27.494	7.141	17.345	1.00	0.00	RX0	C
ATOM	703	CB	ALA	231	26.145	7.668	16.853	1.00	0.00	RX0	C
ATOM	704	C	ALA	231	27.249	5.885	18.186	1.00	0.00	RX0	C
ATOM	705	O	ALA	231	26.768	6.021	19.321	1.00	0.00	RX0	O
ATOM	706	N	TRP	232	27.687	4.719	17.731	1.00	0.00	RX0	N
ATOM	707	H	TRP	232	28.111	4.687	16.823	0.00	0.00	RX0	H
ATOM	708	CA	TRP	232	27.348	3.433	18.379	1.00	0.00	RX0	C
ATOM	709	CB	TRP	232	27.970	2.236	17.648	1.00	0.00	RX0	C
ATOM	710	CG	TRP	232	29.474	2.277	17.763	1.00	0.00	RX0	C
ATOM	711	CD2	TRP	232	30.329	1.534	18.656	1.00	0.00	RX0	C
ATOM	712	CE2	TRP	232	31.661	1.944	18.412	1.00	0.00	RX0	C
ATOM	713	CE3	TRP	232	30.070	0.577	19.629	1.00	0.00	RX0	C
ATOM	714	CD1	TRP	232	30.342	3.088	17.026	1.00	0.00	RX0	C
ATOM	715	NE1	TRP	232	31.630	2.900	17.401	1.00	0.00	RX0	N
ATOM	716	HE1	TRP	232	32.392	3.374	16.998	0.00	0.00	RX0	H
ATOM	717	CZ2	TRP	232	32.697	1.382	19.147	1.00	0.00	RX0	C
ATOM	718	CZ3	TRP	232	31.116	0.025	20.358	1.00	0.00	RX0	C
ATOM	719	CH2	TRP	232	32.425	0.426	20.119	1.00	0.00	RX0	C
ATOM	720	C	TRP	232	27.676	3.373	19.884	1.00	0.00	RX0	C
ATOM	721	O	TRP	232	26.862	2.914	20.672	1.00	0.00	RX0	O
ATOM	722	N	LEU	233	28.801	3.989	20.277	1.00	0.00	RX0	N
ATOM	723	H	LEU	233	29.369	4.447	19.594	0.00	0.00	RX0	H
ATOM	724	CA	LEU	233	29.211	3.960	21.691	1.00	0.00	RX0	C
ATOM	725	CB	LEU	233	30.721	4.149	21.833	1.00	0.00	RX0	C
ATOM	726	CG	LEU	233	31.195	3.767	23.236	1.00	0.00	RX0	C
ATOM	727	CD1	LEU	233	30.800	2.333	23.594	1.00	0.00	RX0	C
ATOM	728	CD2	LEU	233	32.692	4.004	23.424	1.00	0.00	RX0	C
ATOM	729	C	LEU	233	28.415	4.936	22.566	1.00	0.00	RX0	C
ATOM	730	O	LEU	233	27.943	4.566	23.634	1.00	0.00	RX0	O

ATOM	731	N	GLU	234	28.150	6.122	22.016	1.00	0.00	RX0	N
ATOM	732	H	GLU	234	28.486	6.311	21.096	0.00	0.00	RX0	H
ATOM	733	CA	GLU	234	27.227	7.090	22.644	1.00	0.00	RX0	C
ATOM	734	CB	GLU	234	27.046	8.313	21.762	1.00	0.00	RX0	C
ATOM	735	CG	GLU	234	27.906	9.512	22.127	1.00	0.00	RX0	C
ATOM	736	CD	GLU	234	27.540	10.672	21.229	1.00	0.00	RX0	C
ATOM	737	OE1	GLU	234	27.081	11.697	21.716	1.00	0.00	RX0	O
ATOM	738	OE2	GLU	234	27.769	10.592	20.030	1.00	0.00	RX0	O
ATOM	739	C	GLU	234	25.830	6.482	22.841	1.00	0.00	RX0	C
ATOM	740	O	GLU	234	25.253	6.598	23.926	1.00	0.00	RX0	O
ATOM	741	N	ILE	235	25.389	5.711	21.848	1.00	0.00	RX0	N
ATOM	742	H	ILE	235	25.962	5.635	21.031	0.00	0.00	RX0	H
ATOM	743	CA	ILE	235	24.069	5.045	21.863	1.00	0.00	RX0	C
ATOM	744	CB	ILE	235	23.697	4.488	20.485	1.00	0.00	RX0	C
ATOM	745	CG2	ILE	235	22.411	3.661	20.550	1.00	0.00	RX0	C
ATOM	746	CG1	ILE	235	23.559	5.620	19.467	1.00	0.00	RX0	C
ATOM	747	CD1	ILE	235	23.223	5.107	18.067	1.00	0.00	RX0	C
ATOM	748	C	ILE	235	24.018	3.945	22.939	1.00	0.00	RX0	C
ATOM	749	O	ILE	235	23.068	3.902	23.724	1.00	0.00	RX0	O
ATOM	750	N	LEU	236	25.072	3.140	23.020	1.00	0.00	RX0	N
ATOM	751	H	LEU	236	25.812	3.233	22.350	0.00	0.00	RX0	H
ATOM	752	CA	LEU	236	25.176	2.114	24.078	1.00	0.00	RX0	C
ATOM	753	CB	LEU	236	26.442	1.277	23.891	1.00	0.00	RX0	C
ATOM	754	CG	LEU	236	26.336	0.307	22.719	1.00	0.00	RX0	C
ATOM	755	CD1	LEU	236	27.665	-0.389	22.425	1.00	0.00	RX0	C
ATOM	756	CD2	LEU	236	25.202	-0.693	22.935	1.00	0.00	RX0	C
ATOM	757	C	LEU	236	25.182	2.733	25.479	1.00	0.00	RX0	C
ATOM	758	O	LEU	236	24.381	2.362	26.336	1.00	0.00	RX0	O
ATOM	759	N	MET	237	25.933	3.823	25.600	1.00	0.00	RX0	N
ATOM	760	H	MET	237	26.455	4.135	24.804	0.00	0.00	RX0	H
ATOM	761	CA	MET	237	26.132	4.523	26.881	1.00	0.00	RX0	C
ATOM	762	CB	MET	237	27.280	5.529	26.802	1.00	0.00	RX0	C
ATOM	763	CG	MET	237	28.652	4.855	26.815	1.00	0.00	RX0	C
ATOM	764	SD	MET	237	29.999	6.047	26.797	1.00	0.00	RX0	S
ATOM	765	CE	MET	237	31.349	4.901	27.116	1.00	0.00	RX0	C
ATOM	766	C	MET	237	24.875	5.215	27.409	1.00	0.00	RX0	C
ATOM	767	O	MET	237	24.517	5.003	28.572	1.00	0.00	RX0	O
ATOM	768	N	ILE	238	24.128	5.878	26.531	1.00	0.00	RX0	N
ATOM	769	H	ILE	238	24.456	5.961	25.587	0.00	0.00	RX0	H
ATOM	770	CA	ILE	238	22.871	6.546	26.925	1.00	0.00	RX0	C
ATOM	771	CB	ILE	238	22.351	7.525	25.857	1.00	0.00	RX0	C
ATOM	772	CG2	ILE	238	21.924	6.840	24.559	1.00	0.00	RX0	C
ATOM	773	CG1	ILE	238	21.227	8.387	26.438	1.00	0.00	RX0	C
ATOM	774	CD1	ILE	238	20.623	9.348	25.413	1.00	0.00	RX0	C
ATOM	775	C	ILE	238	21.800	5.514	27.357	1.00	0.00	RX0	C
ATOM	776	O	ILE	238	21.031	5.731	28.268	1.00	0.00	RX0	O
ATOM	777	N	GLY	239	21.845	4.355	26.660	1.00	0.00	RX0	N
ATOM	778	H	GLY	239	22.520	4.244	25.926	0.00	0.00	RX0	H
ATOM	779	CA	GLY	239	20.969	3.213	26.975	1.00	0.00	RX0	C
ATOM	780	C	GLY	239	21.301	2.634	28.356	1.00	0.00	RX0	C
ATOM	781	O	GLY	239	20.417	2.460	29.193	1.00	0.00	RX0	O
ATOM	782	N	LEU	240	22.605	2.583	28.639	1.00	0.00	RX0	N
ATOM	783	H	LEU	240	23.260	2.804	27.913	0.00	0.00	RX0	H
ATOM	784	CA	LEU	240	23.124	2.082	29.919	1.00	0.00	RX0	C
ATOM	785	CB	LEU	240	24.645	1.943	29.850	1.00	0.00	RX0	C
ATOM	786	CG	LEU	240	25.285	1.615	31.199	1.00	0.00	RX0	C
ATOM	787	CD1	LEU	240	24.824	0.268	31.753	1.00	0.00	RX0	C
ATOM	788	CD2	LEU	240	26.806	1.719	31.131	1.00	0.00	RX0	C
ATOM	789	C	LEU	240	22.728	2.995	31.086	1.00	0.00	RX0	C
ATOM	790	O	LEU	240	22.214	2.535	32.097	1.00	0.00	RX0	O
ATOM	791	N	VAL	241	22.901	4.295	30.880	1.00	0.00	RX0	N

ATOM	792	H	VAL	241	23.279	4.586	29.998	0.00	0.00	RX0	H
ATOM	793	CA	VAL	241	22.596	5.307	31.912	1.00	0.00	RX0	C
ATOM	794	CB	VAL	241	23.252	6.671	31.661	1.00	0.00	RX0	C
ATOM	795	CG1	VAL	241	24.770	6.505	31.597	1.00	0.00	RX0	C
ATOM	796	CG2	VAL	241	22.699	7.406	30.447	1.00	0.00	RX0	C
ATOM	797	C	VAL	241	21.084	5.392	32.193	1.00	0.00	RX0	C
ATOM	798	O	VAL	241	20.670	5.516	33.338	1.00	0.00	RX0	O
ATOM	799	N	TRP	242	20.290	5.181	31.134	1.00	0.00	RX0	N
ATOM	800	H	TRP	242	20.699	5.072	30.225	0.00	0.00	RX0	H
ATOM	801	CA	TRP	242	18.822	5.192	31.222	1.00	0.00	RX0	C
ATOM	802	CB	TRP	242	18.253	5.150	29.801	1.00	0.00	RX0	C
ATOM	803	CG	TRP	242	16.826	4.658	29.777	1.00	0.00	RX0	C
ATOM	804	CD2	TRP	242	15.637	5.332	30.237	1.00	0.00	RX0	C
ATOM	805	CE2	TRP	242	14.543	4.466	30.014	1.00	0.00	RX0	C
ATOM	806	CE3	TRP	242	15.420	6.582	30.799	1.00	0.00	RX0	C
ATOM	807	CD1	TRP	242	16.380	3.416	29.301	1.00	0.00	RX0	C
ATOM	808	NE1	TRP	242	15.033	3.297	29.440	1.00	0.00	RX0	N
ATOM	809	HE1	TRP	242	14.491	2.523	29.181	0.00	0.00	RX0	H
ATOM	810	CZ2	TRP	242	13.265	4.867	30.382	1.00	0.00	RX0	C
ATOM	811	CZ3	TRP	242	14.137	6.975	31.159	1.00	0.00	RX0	C
ATOM	812	CH2	TRP	242	13.065	6.118	30.953	1.00	0.00	RX0	C
ATOM	813	C	TRP	242	18.281	4.038	32.076	1.00	0.00	RX0	C
ATOM	814	O	TRP	242	17.477	4.269	32.979	1.00	0.00	RX0	O
ATOM	815	N	ARG	243	18.818	2.841	31.865	1.00	0.00	RX0	N
ATOM	816	H	ARG	243	19.515	2.737	31.151	0.00	0.00	RX0	H
ATOM	817	CA	ARG	243	18.359	1.661	32.627	1.00	0.00	RX0	C
ATOM	818	CB	ARG	243	18.485	0.404	31.737	1.00	0.00	RX0	C
ATOM	819	CG	ARG	243	19.891	-0.063	31.299	1.00	0.00	RX0	C
ATOM	820	CD	ARG	243	19.833	-1.094	30.149	1.00	0.00	RX0	C
ATOM	821	NE	ARG	243	21.117	-1.754	29.862	1.00	0.00	RX0	N
ATOM	822	HE	ARG	243	21.665	-2.009	30.673	0.00	0.00	RX0	H
ATOM	823	CZ	ARG	243	21.436	-2.127	28.569	1.00	0.00	RX0	C
ATOM	824	NH1	ARG	243	20.672	-1.721	27.535	1.00	0.00	RX0	N
ATOM	825	HH11	ARG	243	20.917	-2.036	26.600	0.00	0.00	RX0	H
ATOM	826	HH12	ARG	243	19.872	-1.130	27.636	0.00	0.00	RX0	H
ATOM	827	NH2	ARG	243	22.500	-2.910	28.305	1.00	0.00	RX0	N
ATOM	828	HH21	ARG	243	22.778	-3.130	27.348	0.00	0.00	RX0	H
ATOM	829	HH22	ARG	243	23.059	-3.339	29.024	0.00	0.00	RX0	H
ATOM	830	C	ARG	243	19.043	1.510	33.998	1.00	0.00	RX0	C
ATOM	831	O	ARG	243	18.610	0.722	34.836	1.00	0.00	RX0	O
ATOM	832	N	SER	244	20.027	2.366	34.245	1.00	0.00	RX0	N
ATOM	833	H	SER	244	20.317	3.010	33.538	0.00	0.00	RX0	H
ATOM	834	CA	SER	244	20.722	2.464	35.548	1.00	0.00	RX0	C
ATOM	835	CB	SER	244	22.207	2.696	35.311	1.00	0.00	RX0	C
ATOM	836	OG	SER	244	22.699	1.646	34.476	1.00	0.00	RX0	O
ATOM	837	HG	SER	244	22.227	1.718	33.653	0.00	0.00	RX0	H
ATOM	838	C	SER	244	20.112	3.547	36.442	1.00	0.00	RX0	C
ATOM	839	O	SER	244	20.448	3.642	37.630	1.00	0.00	RX0	O
ATOM	840	N	MET	245	19.184	4.322	35.895	1.00	0.00	RX0	N
ATOM	841	H	MET	245	18.883	4.150	34.955	0.00	0.00	RX0	H
ATOM	842	CA	MET	245	18.600	5.504	36.550	1.00	0.00	RX0	C
ATOM	843	CB	MET	245	17.596	6.196	35.632	1.00	0.00	RX0	C
ATOM	844	CG	MET	245	17.042	7.471	36.266	1.00	0.00	RX0	C
ATOM	845	SD	MET	245	15.737	8.232	35.298	1.00	0.00	RX0	S
ATOM	846	CE	MET	245	16.551	8.108	33.704	1.00	0.00	RX0	C
ATOM	847	C	MET	245	17.925	5.204	37.895	1.00	0.00	RX0	C
ATOM	848	O	MET	245	18.105	5.946	38.853	1.00	0.00	RX0	O
ATOM	849	N	GLU	246	17.212	4.082	37.945	1.00	0.00	RX0	N
ATOM	850	H	GLU	246	17.124	3.482	37.148	0.00	0.00	RX0	H
ATOM	851	CA	GLU	246	16.494	3.692	39.178	1.00	0.00	RX0	C
ATOM	852	CB	GLU	246	15.244	2.900	38.818	1.00	0.00	RX0	C

ATOM	853	CG	GLU	246	14.334	3.660	37.857	1.00	0.00	RX0	C
ATOM	854	CD	GLU	246	13.172	2.763	37.503	1.00	0.00	RX0	C
ATOM	855	OE1	GLU	246	13.271	1.564	37.750	1.00	0.00	RX0	O
ATOM	856	OE2	GLU	246	12.176	3.264	36.989	1.00	0.00	RX0	O
ATOM	857	C	GLU	246	17.379	2.867	40.123	1.00	0.00	RX0	C
ATOM	858	O	GLU	246	16.897	2.295	41.108	1.00	0.00	RX0	O
ATOM	859	N	HIS	247	18.674	2.844	39.836	1.00	0.00	RX0	N
ATOM	860	H	HIS	247	19.044	3.322	39.040	0.00	0.00	RX0	H
ATOM	861	CA	HIS	247	19.658	2.055	40.601	1.00	0.00	RX0	C
ATOM	862	CB	HIS	247	20.176	0.861	39.785	1.00	0.00	RX0	C
ATOM	863	CG	HIS	247	19.083	-0.140	39.490	1.00	0.00	RX0	C
ATOM	864	ND1	HIS	247	17.867	-0.170	40.070	1.00	0.00	RX0	N
ATOM	865	HD1	HIS	247	17.497	0.454	40.734	0.00	0.00	RX0	H
ATOM	866	CD2	HIS	247	19.163	-1.196	38.583	1.00	0.00	RX0	C
ATOM	867	NE2	HIS	247	17.989	-1.866	38.618	1.00	0.00	RX0	N
ATOM	868	CE1	HIS	247	17.188	-1.233	39.532	1.00	0.00	RX0	C
ATOM	869	C	HIS	247	20.841	2.947	41.015	1.00	0.00	RX0	C
ATOM	870	O	HIS	247	21.962	2.788	40.490	1.00	0.00	RX0	O
ATOM	871	N	PRO	248	20.632	3.827	41.991	1.00	0.00	RX0	N
ATOM	872	CD	PRO	248	19.385	3.969	42.735	1.00	0.00	RX0	C
ATOM	873	CA	PRO	248	21.659	4.767	42.484	1.00	0.00	RX0	C
ATOM	874	CB	PRO	248	20.977	5.451	43.673	1.00	0.00	RX0	C
ATOM	875	CG	PRO	248	19.484	5.341	43.384	1.00	0.00	RX0	C
ATOM	876	C	PRO	248	22.939	4.013	42.877	1.00	0.00	RX0	C
ATOM	877	O	PRO	248	22.892	2.963	43.503	1.00	0.00	RX0	O
ATOM	878	N	GLY	249	24.055	4.541	42.350	1.00	0.00	RX0	N
ATOM	879	H	GLY	249	23.974	5.299	41.708	0.00	0.00	RX0	H
ATOM	880	CA	GLY	249	25.407	3.996	42.610	1.00	0.00	RX0	C
ATOM	881	C	GLY	249	25.783	2.749	41.794	1.00	0.00	RX0	C
ATOM	882	O	GLY	249	26.914	2.250	41.927	1.00	0.00	RX0	O
ATOM	883	N	LYS	250	24.877	2.267	40.961	1.00	0.00	RX0	N
ATOM	884	H	LYS	250	23.983	2.698	40.813	0.00	0.00	RX0	H
ATOM	885	CA	LYS	250	25.097	1.050	40.158	1.00	0.00	RX0	C
ATOM	886	CB	LYS	250	24.366	-0.144	40.774	1.00	0.00	RX0	C
ATOM	887	CG	LYS	250	25.218	-0.843	41.841	1.00	0.00	RX0	C
ATOM	888	CD	LYS	250	24.603	-2.133	42.386	1.00	0.00	RX0	C
ATOM	889	CE	LYS	250	25.542	-2.957	43.276	1.00	0.00	RX0	C
ATOM	890	NZ	LYS	250	26.700	-3.443	42.512	1.00	0.00	RX0	N
ATOM	891	HZ1	LYS	250	27.410	-3.852	43.162	0.00	0.00	RX0	H
ATOM	892	HZ2	LYS	250	26.445	-4.141	41.780	0.00	0.00	RX0	H
ATOM	893	HZ3	LYS	250	27.206	-2.637	42.097	0.00	0.00	RX0	H
ATOM	894	C	LYS	250	24.802	1.266	38.671	1.00	0.00	RX0	C
ATOM	895	O	LYS	250	24.040	2.166	38.282	1.00	0.00	RX0	O
ATOM	896	N	LEU	251	25.472	0.476	37.863	1.00	0.00	RX0	N
ATOM	897	H	LEU	251	26.054	-0.236	38.243	0.00	0.00	RX0	H
ATOM	898	CA	LEU	251	25.292	0.430	36.401	1.00	0.00	RX0	C
ATOM	899	CB	LEU	251	26.626	0.591	35.684	1.00	0.00	RX0	C
ATOM	900	CG	LEU	251	27.159	2.017	35.761	1.00	0.00	RX0	C
ATOM	901	CD1	LEU	251	28.581	2.117	35.215	1.00	0.00	RX0	C
ATOM	902	CD2	LEU	251	26.204	3.007	35.090	1.00	0.00	RX0	C
ATOM	903	C	LEU	251	24.646	-0.892	36.017	1.00	0.00	RX0	C
ATOM	904	O	LEU	251	25.224	-1.976	36.286	1.00	0.00	RX0	O
ATOM	905	N	LEU	252	23.437	-0.808	35.533	1.00	0.00	RX0	N
ATOM	906	H	LEU	252	23.083	0.097	35.313	0.00	0.00	RX0	H
ATOM	907	CA	LEU	252	22.659	-1.981	35.114	1.00	0.00	RX0	C
ATOM	908	CB	LEU	252	21.157	-1.711	35.228	1.00	0.00	RX0	C
ATOM	909	CG	LEU	252	20.301	-2.957	34.976	1.00	0.00	RX0	C
ATOM	910	CD1	LEU	252	20.456	-3.992	36.087	1.00	0.00	RX0	C
ATOM	911	CD2	LEU	252	18.833	-2.612	34.729	1.00	0.00	RX0	C
ATOM	912	C	LEU	252	23.007	-2.330	33.663	1.00	0.00	RX0	C
ATOM	913	O	LEU	252	22.274	-2.025	32.731	1.00	0.00	RX0	O

ATOM	914	N	PHE	253	24.144	-3.005	33.506	1.00	0.00	RX0	N
ATOM	915	H	PHE	253	24.631	-3.308	34.330	0.00	0.00	RX0	H
ATOM	916	CA	PHE	253	24.599	-3.457	32.174	1.00	0.00	RX0	C
ATOM	917	CB	PHE	253	25.968	-4.125	32.267	1.00	0.00	RX0	C
ATOM	918	CG	PHE	253	27.032	-3.097	32.546	1.00	0.00	RX0	C
ATOM	919	CD1	PHE	253	27.566	-2.360	31.496	1.00	0.00	RX0	C
ATOM	920	CD2	PHE	253	27.486	-2.895	33.843	1.00	0.00	RX0	C
ATOM	921	CE1	PHE	253	28.565	-1.426	31.740	1.00	0.00	RX0	C
ATOM	922	CE2	PHE	253	28.484	-1.960	34.086	1.00	0.00	RX0	C
ATOM	923	CZ	PHE	253	29.024	-1.229	33.035	1.00	0.00	RX0	C
ATOM	924	C	PHE	253	23.603	-4.446	31.564	1.00	0.00	RX0	C
ATOM	925	O	PHE	253	23.259	-4.379	30.390	1.00	0.00	RX0	O
ATOM	926	N	ALA	254	23.094	-5.300	32.445	1.00	0.00	RX0	N
ATOM	927	H	ALA	254	23.407	-5.310	33.400	0.00	0.00	RX0	H
ATOM	928	CA	ALA	254	22.050	-6.280	32.141	1.00	0.00	RX0	C
ATOM	929	CB	ALA	254	22.685	-7.547	31.581	1.00	0.00	RX0	C
ATOM	930	C	ALA	254	21.288	-6.584	33.440	1.00	0.00	RX0	C
ATOM	931	O	ALA	254	21.887	-6.418	34.526	1.00	0.00	RX0	O
ATOM	932	N	PRO	255	20.056	-7.064	33.372	1.00	0.00	RX0	N
ATOM	933	CD	PRO	255	19.305	-7.243	32.132	1.00	0.00	RX0	C
ATOM	934	CA	PRO	255	19.236	-7.444	34.545	1.00	0.00	RX0	C
ATOM	935	CB	PRO	255	17.988	-8.071	33.924	1.00	0.00	RX0	C
ATOM	936	CG	PRO	255	17.855	-7.387	32.571	1.00	0.00	RX0	C
ATOM	937	C	PRO	255	19.972	-8.395	35.506	1.00	0.00	RX0	C
ATOM	938	O	PRO	255	19.756	-8.342	36.714	1.00	0.00	RX0	O
ATOM	939	N	ASN	256	20.900	-9.179	34.970	1.00	0.00	RX0	N
ATOM	940	H	ASN	256	21.111	-9.140	33.992	0.00	0.00	RX0	H
ATOM	941	CA	ASN	256	21.722	-10.125	35.761	1.00	0.00	RX0	C
ATOM	942	CB	ASN	256	21.704	-11.531	35.156	1.00	0.00	RX0	C
ATOM	943	CG	ASN	256	22.391	-11.546	33.798	1.00	0.00	RX0	C
ATOM	944	OD1	ASN	256	22.268	-10.619	32.997	1.00	0.00	RX0	O
ATOM	945	ND2	ASN	256	23.031	-12.701	33.535	1.00	0.00	RX0	N
ATOM	946	HD21	ASN	256	23.160	-13.371	34.269	0.00	0.00	RX0	H
ATOM	947	HD22	ASN	256	23.383	-12.958	32.629	0.00	0.00	RX0	H
ATOM	948	C	ASN	256	23.191	-9.669	35.876	1.00	0.00	RX0	C
ATOM	949	O	ASN	256	24.101	-10.505	36.011	1.00	0.00	RX0	O
ATOM	950	N	LEU	257	23.439	-8.384	35.742	1.00	0.00	RX0	N
ATOM	951	H	LEU	257	22.692	-7.726	35.629	0.00	0.00	RX0	H
ATOM	952	CA	LEU	257	24.796	-7.805	35.783	1.00	0.00	RX0	C
ATOM	953	CB	LEU	257	25.512	-7.968	34.444	1.00	0.00	RX0	C
ATOM	954	CG	LEU	257	27.023	-7.765	34.561	1.00	0.00	RX0	C
ATOM	955	CD1	LEU	257	27.653	-8.797	35.498	1.00	0.00	RX0	C
ATOM	956	CD2	LEU	257	27.710	-7.747	33.196	1.00	0.00	RX0	C
ATOM	957	C	LEU	257	24.720	-6.327	36.168	1.00	0.00	RX0	C
ATOM	958	O	LEU	257	24.738	-5.412	35.328	1.00	0.00	RX0	O
ATOM	959	N	LEU	258	24.604	-6.148	37.469	1.00	0.00	RX0	N
ATOM	960	H	LEU	258	24.689	-6.942	38.070	0.00	0.00	RX0	H
ATOM	961	CA	LEU	258	24.482	-4.834	38.118	1.00	0.00	RX0	C
ATOM	962	CB	LEU	258	23.223	-4.901	38.983	1.00	0.00	RX0	C
ATOM	963	CG	LEU	258	22.825	-3.608	39.681	1.00	0.00	RX0	C
ATOM	964	CD1	LEU	258	22.649	-2.463	38.694	1.00	0.00	RX0	C
ATOM	965	CD2	LEU	258	21.582	-3.799	40.551	1.00	0.00	RX0	C
ATOM	966	C	LEU	258	25.743	-4.564	38.938	1.00	0.00	RX0	C
ATOM	967	O	LEU	258	26.013	-5.237	39.948	1.00	0.00	RX0	O
ATOM	968	N	LEU	259	26.528	-3.622	38.460	1.00	0.00	RX0	N
ATOM	969	H	LEU	259	26.218	-3.063	37.684	0.00	0.00	RX0	H
ATOM	970	CA	LEU	259	27.862	-3.349	39.027	1.00	0.00	RX0	C
ATOM	971	CB	LEU	259	28.937	-3.492	37.946	1.00	0.00	RX0	C
ATOM	972	CG	LEU	259	28.873	-4.805	37.160	1.00	0.00	RX0	C
ATOM	973	CD1	LEU	259	29.883	-4.824	36.013	1.00	0.00	RX0	C
ATOM	974	CD2	LEU	259	29.024	-6.032	38.058	1.00	0.00	RX0	C

ATOM	975	C	LEU	259	27.958	-1.956	39.652	1.00	0.00	RX0	C
ATOM	976	O	LEU	259	27.419	-0.984	39.137	1.00	0.00	RX0	O
ATOM	977	N	ASP	260	28.645	-1.912	40.785	1.00	0.00	RX0	N
ATOM	978	H	ASP	260	29.227	-2.687	41.044	0.00	0.00	RX0	H
ATOM	979	CA	ASP	260	29.043	-0.657	41.454	1.00	0.00	RX0	C
ATOM	980	CB	ASP	260	29.088	-0.861	42.964	1.00	0.00	RX0	C
ATOM	981	CG	ASP	260	29.787	-2.175	43.225	1.00	0.00	RX0	C
ATOM	982	OD1	ASP	260	31.014	-2.207	43.255	1.00	0.00	RX0	O
ATOM	983	OD2	ASP	260	29.088	-3.179	43.353	1.00	0.00	RX0	O
ATOM	984	C	ASP	260	30.443	-0.222	40.970	1.00	0.00	RX0	C
ATOM	985	O	ASP	260	31.127	-1.008	40.295	1.00	0.00	RX0	O
ATOM	986	N	ARG	261	30.963	0.873	41.504	1.00	0.00	RX0	N
ATOM	987	H	ARG	261	30.390	1.411	42.121	0.00	0.00	RX0	H
ATOM	988	CA	ARG	261	32.263	1.408	41.044	1.00	0.00	RX0	C
ATOM	989	CB	ARG	261	32.415	2.886	41.436	1.00	0.00	RX0	C
ATOM	990	CG	ARG	261	32.592	3.167	42.930	1.00	0.00	RX0	C
ATOM	991	CD	ARG	261	32.495	4.660	43.266	1.00	0.00	RX0	C
ATOM	992	NE	ARG	261	33.370	5.462	42.414	1.00	0.00	RX0	N
ATOM	993	HE	ARG	261	33.085	5.668	41.461	0.00	0.00	RX0	H
ATOM	994	CZ	ARG	261	34.563	5.966	42.842	1.00	0.00	RX0	C
ATOM	995	NH1	ARG	261	34.936	5.793	44.129	1.00	0.00	RX0	N
ATOM	996	HH11	ARG	261	35.802	6.154	44.481	0.00	0.00	RX0	H
ATOM	997	HH12	ARG	261	34.337	5.310	44.770	0.00	0.00	RX0	H
ATOM	998	NH2	ARG	261	35.340	6.632	41.968	1.00	0.00	RX0	N
ATOM	999	HH21	ARG	261	36.249	7.013	42.128	0.00	0.00	RX0	H
ATOM	1000	HH22	ARG	261	34.944	6.783	41.039	0.00	0.00	RX0	H
ATOM	1001	C	ARG	261	33.476	0.540	41.436	1.00	0.00	RX0	C
ATOM	1002	O	ARG	261	34.378	0.347	40.637	1.00	0.00	RX0	O
ATOM	1003	N	ASN	262	33.410	-0.067	42.632	1.00	0.00	RX0	N
ATOM	1004	H	ASN	262	32.530	-0.051	43.110	0.00	0.00	RX0	H
ATOM	1005	CA	ASN	262	34.456	-1.000	43.094	1.00	0.00	RX0	C
ATOM	1006	CB	ASN	262	34.128	-1.466	44.502	1.00	0.00	RX0	C
ATOM	1007	CG	ASN	262	35.097	-2.566	44.880	1.00	0.00	RX0	C
ATOM	1008	OD1	ASN	262	36.254	-2.301	45.220	1.00	0.00	RX0	O
ATOM	1009	ND2	ASN	262	34.550	-3.797	44.851	1.00	0.00	RX0	N
ATOM	1010	HD21	ASN	262	33.585	-3.908	44.589	0.00	0.00	RX0	H
ATOM	1011	HD22	ASN	262	35.054	-4.634	45.072	0.00	0.00	RX0	H
ATOM	1012	C	ASN	262	34.635	-2.233	42.204	1.00	0.00	RX0	C
ATOM	1013	O	ASN	262	35.755	-2.665	41.964	1.00	0.00	RX0	O
ATOM	1014	N	GLN	263	33.530	-2.679	41.603	1.00	0.00	RX0	N
ATOM	1015	H	GLN	263	32.638	-2.270	41.822	0.00	0.00	RX0	H
ATOM	1016	CA	GLN	263	33.559	-3.756	40.599	1.00	0.00	RX0	C
ATOM	1017	CB	GLN	263	32.198	-4.421	40.445	1.00	0.00	RX0	C
ATOM	1018	CG	GLN	263	31.923	-5.191	41.735	1.00	0.00	RX0	C
ATOM	1019	CD	GLN	263	30.696	-6.057	41.589	1.00	0.00	RX0	C
ATOM	1020	OE1	GLN	263	30.611	-6.935	40.741	1.00	0.00	RX0	O
ATOM	1021	NE2	GLN	263	29.759	-5.794	42.510	1.00	0.00	RX0	N
ATOM	1022	HE21	GLN	263	29.889	-4.988	43.100	0.00	0.00	RX0	H
ATOM	1023	HE22	GLN	263	28.965	-6.388	42.623	0.00	0.00	RX0	H
ATOM	1024	C	GLN	263	34.189	-3.308	39.265	1.00	0.00	RX0	C
ATOM	1025	O	GLN	263	34.644	-4.116	38.479	1.00	0.00	RX0	O
ATOM	1026	N	GLY	264	34.180	-1.978	39.039	1.00	0.00	RX0	N
ATOM	1027	H	GLY	264	33.764	-1.376	39.720	0.00	0.00	RX0	H
ATOM	1028	CA	GLY	264	34.831	-1.337	37.881	1.00	0.00	RX0	C
ATOM	1029	C	GLY	264	36.364	-1.310	37.978	1.00	0.00	RX0	C
ATOM	1030	O	GLY	264	37.050	-1.510	36.977	1.00	0.00	RX0	O
ATOM	1031	N	LYS	265	36.881	-1.155	39.202	1.00	0.00	RX0	N
ATOM	1032	H	LYS	265	36.232	-1.006	39.949	0.00	0.00	RX0	H
ATOM	1033	CA	LYS	265	38.336	-1.184	39.469	1.00	0.00	RX0	C
ATOM	1034	CB	LYS	265	38.667	-1.125	40.952	1.00	0.00	RX0	C
ATOM	1035	CG	LYS	265	38.110	-0.028	41.846	1.00	0.00	RX0	C

ATOM	1036	CD	LYS	265	38.495	-0.483	43.253	1.00	0.00	RX0	C
ATOM	1037	CE	LYS	265	37.943	0.321	44.422	1.00	0.00	RX0	C
ATOM	1038	NZ	LYS	265	38.108	-0.519	45.617	1.00	0.00	RX0	N
ATOM	1039	HZ1	LYS	265	37.645	-0.092	46.441	0.00	0.00	RX0	H
ATOM	1040	HZ2	LYS	265	37.645	-1.437	45.428	0.00	0.00	RX0	H
ATOM	1041	HZ3	LYS	265	39.114	-0.688	45.809	0.00	0.00	RX0	H
ATOM	1042	C	LYS	265	38.994	-2.500	39.030	1.00	0.00	RX0	C
ATOM	1043	O	LYS	265	40.184	-2.535	38.765	1.00	0.00	RX0	O
ATOM	1044	N	CYS	266	38.153	-3.548	38.917	1.00	0.00	RX0	N
ATOM	1045	H	CYS	266	37.198	-3.455	39.193	0.00	0.00	RX0	H
ATOM	1046	CA	CYS	266	38.538	-4.854	38.355	1.00	0.00	RX0	C
ATOM	1047	CB	CYS	266	37.314	-5.758	38.261	1.00	0.00	RX0	C
ATOM	1048	SG	CYS	266	36.552	-5.950	39.893	1.00	0.00	RX0	S
ATOM	1049	C	CYS	266	39.318	-4.732	37.033	1.00	0.00	RX0	C
ATOM	1050	O	CYS	266	40.108	-5.603	36.695	1.00	0.00	RX0	O
ATOM	1051	N	VAL	267	39.075	-3.636	36.304	1.00	0.00	RX0	N
ATOM	1052	H	VAL	267	38.469	-2.902	36.620	0.00	0.00	RX0	H
ATOM	1053	CA	VAL	267	39.804	-3.345	35.058	1.00	0.00	RX0	C
ATOM	1054	CB	VAL	267	38.857	-3.351	33.851	1.00	0.00	RX0	C
ATOM	1055	CG1	VAL	267	39.586	-3.008	32.550	1.00	0.00	RX0	C
ATOM	1056	CG2	VAL	267	38.126	-4.692	33.739	1.00	0.00	RX0	C
ATOM	1057	C	VAL	267	40.557	-2.013	35.191	1.00	0.00	RX0	C
ATOM	1058	O	VAL	267	39.969	-0.956	35.468	1.00	0.00	RX0	O
ATOM	1059	N	GLU	268	41.829	-2.078	34.821	1.00	0.00	RX0	N
ATOM	1060	H	GLU	268	42.209	-2.964	34.565	0.00	0.00	RX0	H
ATOM	1061	CA	GLU	268	42.726	-0.908	34.727	1.00	0.00	RX0	C
ATOM	1062	CB	GLU	268	44.098	-1.334	34.208	1.00	0.00	RX0	C
ATOM	1063	CG	GLU	268	45.140	-0.223	34.331	1.00	0.00	RX0	C
ATOM	1064	CD	GLU	268	46.503	-0.806	34.035	1.00	0.00	RX0	C
ATOM	1065	OE1	GLU	268	46.591	-2.019	33.856	1.00	0.00	RX0	O
ATOM	1066	OE2	GLU	268	47.472	-0.051	33.999	1.00	0.00	RX0	O
ATOM	1067	C	GLU	268	42.079	0.194	33.866	1.00	0.00	RX0	C
ATOM	1068	O	GLU	268	41.697	-0.027	32.727	1.00	0.00	RX0	O
ATOM	1069	N	GLY	269	41.924	1.355	34.524	1.00	0.00	RX0	N
ATOM	1070	H	GLY	269	42.193	1.398	35.484	0.00	0.00	RX0	H
ATOM	1071	CA	GLY	269	41.377	2.576	33.902	1.00	0.00	RX0	C
ATOM	1072	C	GLY	269	39.898	2.490	33.494	1.00	0.00	RX0	C
ATOM	1073	O	GLY	269	39.424	3.343	32.745	1.00	0.00	RX0	O
ATOM	1074	N	MET	270	39.146	1.598	34.134	1.00	0.00	RX0	N
ATOM	1075	H	MET	270	39.580	0.956	34.768	0.00	0.00	RX0	H
ATOM	1076	CA	MET	270	37.704	1.465	33.841	1.00	0.00	RX0	C
ATOM	1077	CB	MET	270	37.280	0.008	33.652	1.00	0.00	RX0	C
ATOM	1078	CG	MET	270	35.924	-0.098	32.948	1.00	0.00	RX0	C
ATOM	1079	SD	MET	270	35.417	-1.787	32.583	1.00	0.00	RX0	S
ATOM	1080	CE	MET	270	35.153	-2.354	34.269	1.00	0.00	RX0	C
ATOM	1081	C	MET	270	36.825	2.181	34.877	1.00	0.00	RX0	C
ATOM	1082	O	MET	270	35.781	2.734	34.514	1.00	0.00	RX0	O
ATOM	1083	N	VAL	271	37.310	2.290	36.107	1.00	0.00	RX0	N
ATOM	1084	H	VAL	271	38.234	1.949	36.273	0.00	0.00	RX0	H
ATOM	1085	CA	VAL	271	36.591	3.043	37.171	1.00	0.00	RX0	C
ATOM	1086	CB	VAL	271	37.248	2.855	38.550	1.00	0.00	RX0	C
ATOM	1087	CG1	VAL	271	38.734	3.200	38.561	1.00	0.00	RX0	C
ATOM	1088	CG2	VAL	271	36.453	3.556	39.653	1.00	0.00	RX0	C
ATOM	1089	C	VAL	271	36.343	4.502	36.749	1.00	0.00	RX0	C
ATOM	1090	O	VAL	271	35.261	5.071	37.095	1.00	0.00	RX0	O
ATOM	1091	N	GLU	272	37.228	5.074	36.011	1.00	0.00	RX0	N
ATOM	1092	H	GLU	272	38.100	4.599	35.878	0.00	0.00	RX0	H
ATOM	1093	CA	GLU	272	37.141	6.453	35.460	1.00	0.00	RX0	C
ATOM	1094	CB	GLU	272	38.430	6.788	34.701	1.00	0.00	RX0	C
ATOM	1095	CG	GLU	272	39.676	7.025	35.568	1.00	0.00	RX0	C
ATOM	1096	CD	GLU	272	40.050	5.795	36.375	1.00	0.00	RX0	C

ATOM	1097	OE1	GLU	272	40.058	4.695	35.823	1.00	0.00	RX0	O
ATOM	1098	OE2	GLU	272	40.301	5.932	37.568	1.00	0.00	RX0	O
ATOM	1099	C	GLU	272	35.927	6.588	34.526	1.00	0.00	RX0	C
ATOM	1100	O	GLU	272	35.142	7.521	34.681	1.00	0.00	RX0	O
ATOM	1101	N	ILE	273	35.702	5.552	33.729	1.00	0.00	RX0	N
ATOM	1102	H	ILE	273	36.304	4.758	33.824	0.00	0.00	RX0	H
ATOM	1103	CA	ILE	273	34.533	5.484	32.820	1.00	0.00	RX0	C
ATOM	1104	CB	ILE	273	34.734	4.376	31.780	1.00	0.00	RX0	C
ATOM	1105	CG2	ILE	273	33.622	4.404	30.731	1.00	0.00	RX0	C
ATOM	1106	CG1	ILE	273	36.127	4.434	31.145	1.00	0.00	RX0	C
ATOM	1107	CD1	ILE	273	36.338	5.657	30.252	1.00	0.00	RX0	C
ATOM	1108	C	ILE	273	33.249	5.235	33.628	1.00	0.00	RX0	C
ATOM	1109	O	ILE	273	32.257	5.951	33.452	1.00	0.00	RX0	O
ATOM	1110	N	PHE	274	33.320	4.302	34.575	1.00	0.00	RX0	N
ATOM	1111	H	PHE	274	34.190	3.821	34.693	0.00	0.00	RX0	H
ATOM	1112	CA	PHE	274	32.191	3.976	35.472	1.00	0.00	RX0	C
ATOM	1113	CB	PHE	274	32.611	2.939	36.515	1.00	0.00	RX0	C
ATOM	1114	CG	PHE	274	32.268	1.538	36.072	1.00	0.00	RX0	C
ATOM	1115	CD1	PHE	274	32.532	1.120	34.774	1.00	0.00	RX0	C
ATOM	1116	CD2	PHE	274	31.686	0.663	36.982	1.00	0.00	RX0	C
ATOM	1117	CE1	PHE	274	32.219	-0.178	34.390	1.00	0.00	RX0	C
ATOM	1118	CE2	PHE	274	31.374	-0.635	36.599	1.00	0.00	RX0	C
ATOM	1119	CZ	PHE	274	31.649	-1.057	35.304	1.00	0.00	RX0	C
ATOM	1120	C	PHE	274	31.669	5.203	36.222	1.00	0.00	RX0	C
ATOM	1121	O	PHE	274	30.484	5.521	36.143	1.00	0.00	RX0	O
ATOM	1122	N	ASP	275	32.607	5.983	36.757	1.00	0.00	RX0	N
ATOM	1123	H	ASP	275	33.560	5.685	36.802	0.00	0.00	RX0	H
ATOM	1124	CA	ASP	275	32.273	7.222	37.483	1.00	0.00	RX0	C
ATOM	1125	CB	ASP	275	33.488	7.801	38.198	1.00	0.00	RX0	C
ATOM	1126	CG	ASP	275	33.580	7.160	39.569	1.00	0.00	RX0	C
ATOM	1127	OD1	ASP	275	33.911	5.984	39.675	1.00	0.00	RX0	O
ATOM	1128	OD2	ASP	275	33.349	7.836	40.565	1.00	0.00	RX0	O
ATOM	1129	C	ASP	275	31.555	8.270	36.629	1.00	0.00	RX0	C
ATOM	1130	O	ASP	275	30.604	8.890	37.102	1.00	0.00	RX0	O
ATOM	1131	N	MET	276	31.921	8.336	35.353	1.00	0.00	RX0	N
ATOM	1132	H	MET	276	32.664	7.739	35.041	0.00	0.00	RX0	H
ATOM	1133	CA	MET	276	31.257	9.254	34.408	1.00	0.00	RX0	C
ATOM	1134	CB	MET	276	32.089	9.446	33.142	1.00	0.00	RX0	C
ATOM	1135	CG	MET	276	33.421	10.149	33.400	1.00	0.00	RX0	C
ATOM	1136	SD	MET	276	34.291	10.537	31.873	1.00	0.00	RX0	S
ATOM	1137	CE	MET	276	34.324	8.878	31.182	1.00	0.00	RX0	C
ATOM	1138	C	MET	276	29.833	8.798	34.061	1.00	0.00	RX0	C
ATOM	1139	O	MET	276	28.893	9.589	34.158	1.00	0.00	RX0	O
ATOM	1140	N	LEU	277	29.673	7.488	33.891	1.00	0.00	RX0	N
ATOM	1141	H	LEU	277	30.490	6.907	33.949	0.00	0.00	RX0	H
ATOM	1142	CA	LEU	277	28.362	6.866	33.606	1.00	0.00	RX0	C
ATOM	1143	CB	LEU	277	28.562	5.402	33.226	1.00	0.00	RX0	C
ATOM	1144	CG	LEU	277	29.433	5.219	31.985	1.00	0.00	RX0	C
ATOM	1145	CD1	LEU	277	29.931	3.781	31.844	1.00	0.00	RX0	C
ATOM	1146	CD2	LEU	277	28.730	5.711	30.722	1.00	0.00	RX0	C
ATOM	1147	C	LEU	277	27.393	6.992	34.787	1.00	0.00	RX0	C
ATOM	1148	O	LEU	277	26.257	7.447	34.627	1.00	0.00	RX0	O
ATOM	1149	N	LEU	278	27.939	6.795	35.983	1.00	0.00	RX0	N
ATOM	1150	H	LEU	278	28.900	6.516	36.019	0.00	0.00	RX0	H
ATOM	1151	CA	LEU	278	27.196	6.926	37.250	1.00	0.00	RX0	C
ATOM	1152	CB	LEU	278	28.067	6.469	38.419	1.00	0.00	RX0	C
ATOM	1153	CG	LEU	278	28.220	4.953	38.466	1.00	0.00	RX0	C
ATOM	1154	CD1	LEU	278	29.273	4.502	39.479	1.00	0.00	RX0	C
ATOM	1155	CD2	LEU	278	26.869	4.290	38.708	1.00	0.00	RX0	C
ATOM	1156	C	LEU	278	26.716	8.359	37.508	1.00	0.00	RX0	C
ATOM	1157	O	LEU	278	25.554	8.575	37.840	1.00	0.00	RX0	O

ATOM	1158	N	ALA	279	27.582	9.317	37.175	1.00	0.00	RX0	N
ATOM	1159	H	ALA	279	28.513	9.059	36.899	0.00	0.00	RX0	H
ATOM	1160	CA	ALA	279	27.275	10.754	37.296	1.00	0.00	RX0	C
ATOM	1161	CB	ALA	279	28.528	11.595	37.049	1.00	0.00	RX0	C
ATOM	1162	C	ALA	279	26.182	11.196	36.310	1.00	0.00	RX0	C
ATOM	1163	O	ALA	279	25.263	11.922	36.684	1.00	0.00	RX0	O
ATOM	1164	N	THR	280	26.210	10.618	35.109	1.00	0.00	RX0	N
ATOM	1165	H	THR	280	26.970	10.007	34.881	0.00	0.00	RX0	H
ATOM	1166	CA	THR	280	25.203	10.899	34.059	1.00	0.00	RX0	C
ATOM	1167	CB	THR	280	25.749	10.367	32.740	1.00	0.00	RX0	C
ATOM	1168	OG1	THR	280	27.060	10.906	32.532	1.00	0.00	RX0	O
ATOM	1169	HG1	THR	280	27.667	10.444	33.102	0.00	0.00	RX0	H
ATOM	1170	CG2	THR	280	24.830	10.713	31.565	1.00	0.00	RX0	C
ATOM	1171	C	THR	280	23.835	10.327	34.462	1.00	0.00	RX0	C
ATOM	1172	O	THR	280	22.822	11.023	34.397	1.00	0.00	RX0	O
ATOM	1173	N	SER	281	23.868	9.112	35.003	1.00	0.00	RX0	N
ATOM	1174	H	SER	281	24.739	8.619	35.047	0.00	0.00	RX0	H
ATOM	1175	CA	SER	281	22.669	8.413	35.507	1.00	0.00	RX0	C
ATOM	1176	CB	SER	281	23.141	6.983	35.867	1.00	0.00	RX0	C
ATOM	1177	OG	SER	281	22.390	6.348	36.921	1.00	0.00	RX0	O
ATOM	1178	HG	SER	281	22.596	5.419	36.842	0.00	0.00	RX0	H
ATOM	1179	C	SER	281	22.019	9.180	36.675	1.00	0.00	RX0	C
ATOM	1180	O	SER	281	20.814	9.399	36.693	1.00	0.00	RX0	O
ATOM	1181	N	SER	282	22.889	9.788	37.491	1.00	0.00	RX0	N
ATOM	1182	H	SER	282	23.869	9.619	37.375	0.00	0.00	RX0	H
ATOM	1183	CA	SER	282	22.489	10.660	38.613	1.00	0.00	RX0	C
ATOM	1184	CB	SER	282	23.673	10.724	39.559	1.00	0.00	RX0	C
ATOM	1185	OG	SER	282	23.940	9.345	39.877	1.00	0.00	RX0	O
ATOM	1186	HG	SER	282	24.718	9.118	39.368	0.00	0.00	RX0	H
ATOM	1187	C	SER	282	21.828	11.963	38.130	1.00	0.00	RX0	C
ATOM	1188	O	SER	282	20.788	12.371	38.639	1.00	0.00	RX0	O
ATOM	1189	N	ARG	283	22.365	12.516	37.039	1.00	0.00	RX0	N
ATOM	1190	H	ARG	283	23.191	12.112	36.639	0.00	0.00	RX0	H
ATOM	1191	CA	ARG	283	21.822	13.735	36.412	1.00	0.00	RX0	C
ATOM	1192	CB	ARG	283	22.811	14.212	35.349	1.00	0.00	RX0	C
ATOM	1193	CG	ARG	283	22.349	15.413	34.527	1.00	0.00	RX0	C
ATOM	1194	CD	ARG	283	22.165	16.694	35.339	1.00	0.00	RX0	C
ATOM	1195	NE	ARG	283	21.780	17.783	34.445	1.00	0.00	RX0	N
ATOM	1196	HE	ARG	283	22.214	17.766	33.537	0.00	0.00	RX0	H
ATOM	1197	CZ	ARG	283	20.880	18.721	34.858	1.00	0.00	RX0	C
ATOM	1198	NH1	ARG	283	20.384	18.670	36.116	1.00	0.00	RX0	N
ATOM	1199	HH11	ARG	283	19.714	19.340	36.451	0.00	0.00	RX0	H
ATOM	1200	HH12	ARG	283	20.666	17.958	36.767	0.00	0.00	RX0	H
ATOM	1201	NH2	ARG	283	20.493	19.681	33.994	1.00	0.00	RX0	N
ATOM	1202	HH21	ARG	283	19.792	20.372	34.229	0.00	0.00	RX0	H
ATOM	1203	HH22	ARG	283	20.873	19.744	33.067	0.00	0.00	RX0	H
ATOM	1204	C	ARG	283	20.432	13.477	35.812	1.00	0.00	RX0	C
ATOM	1205	O	ARG	283	19.498	14.246	36.035	1.00	0.00	RX0	O
ATOM	1206	N	PHE	284	20.293	12.325	35.169	1.00	0.00	RX0	N
ATOM	1207	H	PHE	284	21.098	11.742	35.040	0.00	0.00	RX0	H
ATOM	1208	CA	PHE	284	19.003	11.899	34.606	1.00	0.00	RX0	C
ATOM	1209	CB	PHE	284	19.202	10.643	33.765	1.00	0.00	RX0	C
ATOM	1210	CG	PHE	284	19.646	11.023	32.375	1.00	0.00	RX0	C
ATOM	1211	CD1	PHE	284	19.182	12.198	31.797	1.00	0.00	RX0	C
ATOM	1212	CD2	PHE	284	20.494	10.185	31.662	1.00	0.00	RX0	C
ATOM	1213	CE1	PHE	284	19.534	12.515	30.490	1.00	0.00	RX0	C
ATOM	1214	CE2	PHE	284	20.849	10.506	30.357	1.00	0.00	RX0	C
ATOM	1215	CZ	PHE	284	20.354	11.661	29.764	1.00	0.00	RX0	C
ATOM	1216	C	PHE	284	17.921	11.680	35.654	1.00	0.00	RX0	C
ATOM	1217	O	PHE	284	16.817	12.216	35.524	1.00	0.00	RX0	O
ATOM	1218	N	ARG	285	18.348	11.087	36.760	1.00	0.00	RX0	N

ATOM	1219	H	ARG	285	19.279	10.718	36.763	0.00	0.00	RX0	H
ATOM	1220	CA	ARG	285	17.485	10.841	37.923	1.00	0.00	RX0	C
ATOM	1221	CB	ARG	285	18.334	10.058	38.927	1.00	0.00	RX0	C
ATOM	1222	CG	ARG	285	17.765	9.787	40.321	1.00	0.00	RX0	C
ATOM	1223	CD	ARG	285	18.791	9.029	41.176	1.00	0.00	RX0	C
ATOM	1224	NE	ARG	285	19.213	7.820	40.470	1.00	0.00	RX0	N
ATOM	1225	HE	ARG	285	18.460	7.218	40.172	0.00	0.00	RX0	H
ATOM	1226	CZ	ARG	285	20.504	7.652	40.056	1.00	0.00	RX0	C
ATOM	1227	NH1	ARG	285	21.467	8.432	40.588	1.00	0.00	RX0	N
ATOM	1228	HH11	ARG	285	22.432	8.429	40.279	0.00	0.00	RX0	H
ATOM	1229	HH12	ARG	285	21.257	9.081	41.323	0.00	0.00	RX0	H
ATOM	1230	NH2	ARG	285	20.768	6.724	39.111	1.00	0.00	RX0	N
ATOM	1231	HH21	ARG	285	21.655	6.597	38.645	0.00	0.00	RX0	H
ATOM	1232	HH22	ARG	285	20.028	6.115	38.804	0.00	0.00	RX0	H
ATOM	1233	C	ARG	285	17.003	12.164	38.534	1.00	0.00	RX0	C
ATOM	1234	O	ARG	285	15.822	12.321	38.816	1.00	0.00	RX0	O
ATOM	1235	N	MET	286	17.922	13.130	38.591	1.00	0.00	RX0	N
ATOM	1236	H	MET	286	18.856	12.922	38.296	0.00	0.00	RX0	H
ATOM	1237	CA	MET	286	17.643	14.467	39.144	1.00	0.00	RX0	C
ATOM	1238	CB	MET	286	18.961	15.233	39.263	1.00	0.00	RX0	C
ATOM	1239	CG	MET	286	18.792	16.698	39.666	1.00	0.00	RX0	C
ATOM	1240	SD	MET	286	20.308	17.643	39.448	1.00	0.00	RX0	S
ATOM	1241	CE	MET	286	19.713	19.214	40.093	1.00	0.00	RX0	C
ATOM	1242	C	MET	286	16.681	15.257	38.243	1.00	0.00	RX0	C
ATOM	1243	O	MET	286	15.799	15.962	38.735	1.00	0.00	RX0	O
ATOM	1244	N	MET	287	16.911	15.161	36.942	1.00	0.00	RX0	N
ATOM	1245	H	MET	287	17.629	14.536	36.630	0.00	0.00	RX0	H
ATOM	1246	CA	MET	287	16.049	15.814	35.941	1.00	0.00	RX0	C
ATOM	1247	CB	MET	287	16.685	15.857	34.556	1.00	0.00	RX0	C
ATOM	1248	CG	MET	287	17.864	16.820	34.512	1.00	0.00	RX0	C
ATOM	1249	SD	MET	287	18.271	17.301	32.831	1.00	0.00	RX0	S
ATOM	1250	CE	MET	287	16.650	17.957	32.402	1.00	0.00	RX0	C
ATOM	1251	C	MET	287	14.674	15.156	35.849	1.00	0.00	RX0	C
ATOM	1252	O	MET	287	13.755	15.729	35.264	1.00	0.00	RX0	O
ATOM	1253	N	ASN	288	14.583	13.920	36.346	1.00	0.00	RX0	N
ATOM	1254	H	ASN	288	15.392	13.490	36.747	0.00	0.00	RX0	H
ATOM	1255	CA	ASN	288	13.391	13.066	36.242	1.00	0.00	RX0	C
ATOM	1256	CB	ASN	288	12.161	13.613	36.968	1.00	0.00	RX0	C
ATOM	1257	CG	ASN	288	11.008	12.656	36.723	1.00	0.00	RX0	C
ATOM	1258	OD1	ASN	288	11.175	11.443	36.649	1.00	0.00	RX0	O
ATOM	1259	ND2	ASN	288	9.819	13.272	36.578	1.00	0.00	RX0	N
ATOM	1260	HD21	ASN	288	9.742	14.264	36.666	0.00	0.00	RX0	H
ATOM	1261	HD22	ASN	288	8.993	12.752	36.357	0.00	0.00	RX0	H
ATOM	1262	C	ASN	288	13.076	12.836	34.753	1.00	0.00	RX0	C
ATOM	1263	O	ASN	288	11.986	13.121	34.256	1.00	0.00	RX0	O
ATOM	1264	N	LEU	289	14.125	12.439	34.028	1.00	0.00	RX0	N
ATOM	1265	H	LEU	289	14.956	12.174	34.521	0.00	0.00	RX0	H
ATOM	1266	CA	LEU	289	14.030	12.183	32.583	1.00	0.00	RX0	C
ATOM	1267	CB	LEU	289	15.372	11.628	32.098	1.00	0.00	RX0	C
ATOM	1268	CG	LEU	289	15.411	11.218	30.623	1.00	0.00	RX0	C
ATOM	1269	CD1	LEU	289	15.552	12.426	29.704	1.00	0.00	RX0	C
ATOM	1270	CD2	LEU	289	16.489	10.174	30.339	1.00	0.00	RX0	C
ATOM	1271	C	LEU	289	12.926	11.152	32.321	1.00	0.00	RX0	C
ATOM	1272	O	LEU	289	12.814	10.137	33.014	1.00	0.00	RX0	O
ATOM	1273	N	GLN	290	12.165	11.420	31.281	1.00	0.00	RX0	N
ATOM	1274	H	GLN	290	12.393	12.179	30.665	0.00	0.00	RX0	H
ATOM	1275	CA	GLN	290	11.011	10.584	30.916	1.00	0.00	RX0	C
ATOM	1276	CB	GLN	290	9.780	11.455	30.676	1.00	0.00	RX0	C
ATOM	1277	CG	GLN	290	9.419	12.312	31.898	1.00	0.00	RX0	C
ATOM	1278	CD	GLN	290	8.987	11.449	33.076	1.00	0.00	RX0	C
ATOM	1279	OE1	GLN	290	7.822	11.102	33.229	1.00	0.00	RX0	O

ATOM	1280	NE2	GLN	290	9.976	11.158	33.938	1.00	0.00	RX0	N
ATOM	1281	HE21	GLN	290	10.907	11.497	33.789	0.00	0.00	RX0	H
ATOM	1282	HE22	GLN	290	9.876	10.613	34.772	0.00	0.00	RX0	H
ATOM	1283	C	GLN	290	11.379	9.701	29.727	1.00	0.00	RX0	C
ATOM	1284	O	GLN	290	12.115	10.141	28.832	1.00	0.00	RX0	O
ATOM	1285	N	GLY	291	10.739	8.533	29.672	1.00	0.00	RX0	N
ATOM	1286	H	GLY	291	10.024	8.356	30.348	0.00	0.00	RX0	H
ATOM	1287	CA	GLY	291	10.952	7.542	28.589	1.00	0.00	RX0	C
ATOM	1288	C	GLY	291	10.769	8.134	27.181	1.00	0.00	RX0	C
ATOM	1289	O	GLY	291	11.559	7.865	26.269	1.00	0.00	RX0	O
ATOM	1290	N	GLU	292	9.854	9.086	27.078	1.00	0.00	RX0	N
ATOM	1291	H	GLU	292	9.263	9.256	27.871	0.00	0.00	RX0	H
ATOM	1292	CA	GLU	292	9.541	9.789	25.813	1.00	0.00	RX0	C
ATOM	1293	CB	GLU	292	8.213	10.550	25.940	1.00	0.00	RX0	C
ATOM	1294	CG	GLU	292	6.981	9.710	26.317	1.00	0.00	RX0	C
ATOM	1295	CD	GLU	292	7.114	9.178	27.730	1.00	0.00	RX0	C
ATOM	1296	OE1	GLU	292	7.509	9.940	28.609	1.00	0.00	RX0	O
ATOM	1297	OE2	GLU	292	6.931	7.984	27.935	1.00	0.00	RX0	O
ATOM	1298	C	GLU	292	10.671	10.744	25.400	1.00	0.00	RX0	C
ATOM	1299	O	GLU	292	11.065	10.802	24.241	1.00	0.00	RX0	O
ATOM	1300	N	GLU	293	11.241	11.417	26.395	1.00	0.00	RX0	N
ATOM	1301	H	GLU	293	10.948	11.213	27.330	0.00	0.00	RX0	H
ATOM	1302	CA	GLU	293	12.389	12.320	26.184	1.00	0.00	RX0	C
ATOM	1303	CB	GLU	293	12.637	13.156	27.435	1.00	0.00	RX0	C
ATOM	1304	CG	GLU	293	11.407	13.911	27.930	1.00	0.00	RX0	C
ATOM	1305	CD	GLU	293	11.725	14.507	29.284	1.00	0.00	RX0	C
ATOM	1306	OE1	GLU	293	12.638	14.033	29.952	1.00	0.00	RX0	O
ATOM	1307	OE2	GLU	293	11.059	15.448	29.696	1.00	0.00	RX0	O
ATOM	1308	C	GLU	293	13.669	11.544	25.843	1.00	0.00	RX0	C
ATOM	1309	O	GLU	293	14.364	11.888	24.886	1.00	0.00	RX0	O
ATOM	1310	N	PHE	294	13.855	10.409	26.517	1.00	0.00	RX0	N
ATOM	1311	H	PHE	294	13.194	10.200	27.241	0.00	0.00	RX0	H
ATOM	1312	CA	PHE	294	14.999	9.503	26.300	1.00	0.00	RX0	C
ATOM	1313	CB	PHE	294	14.905	8.309	27.252	1.00	0.00	RX0	C
ATOM	1314	CG	PHE	294	15.869	7.229	26.817	1.00	0.00	RX0	C
ATOM	1315	CD1	PHE	294	17.241	7.457	26.842	1.00	0.00	RX0	C
ATOM	1316	CD2	PHE	294	15.377	6.006	26.375	1.00	0.00	RX0	C
ATOM	1317	CE1	PHE	294	18.114	6.471	26.400	1.00	0.00	RX0	C
ATOM	1318	CE2	PHE	294	16.252	5.020	25.934	1.00	0.00	RX0	C
ATOM	1319	CZ	PHE	294	17.621	5.256	25.939	1.00	0.00	RX0	C
ATOM	1320	C	PHE	294	15.115	9.012	24.847	1.00	0.00	RX0	C
ATOM	1321	O	PHE	294	16.186	9.132	24.238	1.00	0.00	RX0	O
ATOM	1322	N	VAL	295	14.000	8.581	24.280	1.00	0.00	RX0	N
ATOM	1323	H	VAL	295	13.158	8.569	24.828	0.00	0.00	RX0	H
ATOM	1324	CA	VAL	295	13.976	8.026	22.907	1.00	0.00	RX0	C
ATOM	1325	CB	VAL	295	12.686	7.251	22.616	1.00	0.00	RX0	C
ATOM	1326	CG1	VAL	295	12.588	6.063	23.571	1.00	0.00	RX0	C
ATOM	1327	CG2	VAL	295	11.429	8.117	22.662	1.00	0.00	RX0	C
ATOM	1328	C	VAL	295	14.286	9.097	21.847	1.00	0.00	RX0	C
ATOM	1329	O	VAL	295	14.999	8.845	20.884	1.00	0.00	RX0	O
ATOM	1330	N	CYS	296	13.866	10.330	22.156	1.00	0.00	RX0	N
ATOM	1331	H	CYS	296	13.305	10.481	22.973	0.00	0.00	RX0	H
ATOM	1332	CA	CYS	296	14.167	11.498	21.314	1.00	0.00	RX0	C
ATOM	1333	CB	CYS	296	13.264	12.653	21.731	1.00	0.00	RX0	C
ATOM	1334	SG	CYS	296	11.519	12.259	21.463	1.00	0.00	RX0	S
ATOM	1335	C	CYS	296	15.661	11.843	21.352	1.00	0.00	RX0	C
ATOM	1336	O	CYS	296	16.290	11.969	20.305	1.00	0.00	RX0	O
ATOM	1337	N	LEU	297	16.247	11.750	22.547	1.00	0.00	RX0	N
ATOM	1338	H	LEU	297	15.681	11.550	23.351	0.00	0.00	RX0	H
ATOM	1339	CA	LEU	297	17.681	12.031	22.752	1.00	0.00	RX0	C
ATOM	1340	CB	LEU	297	18.017	12.109	24.240	1.00	0.00	RX0	C

ATOM	1341	CG	LEU	297	17.354	13.288	24.947	1.00	0.00	RX0	C
ATOM	1342	CD1	LEU	297	17.638	13.260	26.448	1.00	0.00	RX0	C
ATOM	1343	CD2	LEU	297	17.738	14.626	24.313	1.00	0.00	RX0	C
ATOM	1344	C	LEU	297	18.589	11.009	22.068	1.00	0.00	RX0	C
ATOM	1345	O	LEU	297	19.526	11.385	21.359	1.00	0.00	RX0	O
ATOM	1346	N	LYS	298	18.173	9.750	22.123	1.00	0.00	RX0	N
ATOM	1347	H	LYS	298	17.360	9.546	22.672	0.00	0.00	RX0	H
ATOM	1348	CA	LYS	298	18.945	8.654	21.515	1.00	0.00	RX0	C
ATOM	1349	CB	LYS	298	18.436	7.320	22.047	1.00	0.00	RX0	C
ATOM	1350	CG	LYS	298	19.259	6.120	21.585	1.00	0.00	RX0	C
ATOM	1351	CD	LYS	298	18.617	4.833	22.089	1.00	0.00	RX0	C
ATOM	1352	CE	LYS	298	17.100	5.005	22.052	1.00	0.00	RX0	C
ATOM	1353	NZ	LYS	298	16.429	3.713	22.164	1.00	0.00	RX0	N
ATOM	1354	HZ1	LYS	298	15.435	3.867	22.459	0.00	0.00	RX0	H
ATOM	1355	HZ2	LYS	298	16.299	3.227	21.256	0.00	0.00	RX0	H
ATOM	1356	HZ3	LYS	298	16.788	3.065	22.887	0.00	0.00	RX0	H
ATOM	1357	C	LYS	298	18.925	8.733	19.978	1.00	0.00	RX0	C
ATOM	1358	O	LYS	298	19.964	8.557	19.332	1.00	0.00	RX0	O
ATOM	1359	N	SER	299	17.791	9.152	19.431	1.00	0.00	RX0	N
ATOM	1360	H	SER	299	16.965	9.270	19.982	0.00	0.00	RX0	H
ATOM	1361	CA	SER	299	17.645	9.383	17.977	1.00	0.00	RX0	C
ATOM	1362	CB	SER	299	16.155	9.362	17.703	1.00	0.00	RX0	C
ATOM	1363	OG	SER	299	15.658	8.204	18.381	1.00	0.00	RX0	O
ATOM	1364	HG	SER	299	15.130	8.510	19.111	0.00	0.00	RX0	H
ATOM	1365	C	SER	299	18.416	10.621	17.504	1.00	0.00	RX0	C
ATOM	1366	O	SER	299	19.051	10.583	16.444	1.00	0.00	RX0	O
ATOM	1367	N	ILE	300	18.478	11.644	18.354	1.00	0.00	RX0	N
ATOM	1368	H	ILE	300	17.959	11.595	19.210	0.00	0.00	RX0	H
ATOM	1369	CA	ILE	300	19.283	12.860	18.096	1.00	0.00	RX0	C
ATOM	1370	CB	ILE	300	19.065	13.962	19.152	1.00	0.00	RX0	C
ATOM	1371	CG2	ILE	300	20.048	15.132	19.000	1.00	0.00	RX0	C
ATOM	1372	CG1	ILE	300	17.636	14.489	19.092	1.00	0.00	RX0	C
ATOM	1373	CD1	ILE	300	17.317	15.465	20.226	1.00	0.00	RX0	C
ATOM	1374	C	ILE	300	20.773	12.490	18.013	1.00	0.00	RX0	C
ATOM	1375	O	ILE	300	21.456	12.924	17.087	1.00	0.00	RX0	O
ATOM	1376	N	ILE	301	21.227	11.630	18.921	1.00	0.00	RX0	N
ATOM	1377	H	ILE	301	20.613	11.328	19.653	0.00	0.00	RX0	H
ATOM	1378	CA	ILE	301	22.629	11.156	18.925	1.00	0.00	RX0	C
ATOM	1379	CB	ILE	301	22.881	10.196	20.083	1.00	0.00	RX0	C
ATOM	1380	CG2	ILE	301	24.196	9.450	19.885	1.00	0.00	RX0	C
ATOM	1381	CG1	ILE	301	22.858	10.935	21.418	1.00	0.00	RX0	C
ATOM	1382	CD1	ILE	301	23.232	10.016	22.580	1.00	0.00	RX0	C
ATOM	1383	C	ILE	301	22.948	10.474	17.584	1.00	0.00	RX0	C
ATOM	1384	O	ILE	301	23.927	10.823	16.926	1.00	0.00	RX0	O
ATOM	1385	N	LEU	302	22.047	9.582	17.175	1.00	0.00	RX0	N
ATOM	1386	H	LEU	302	21.271	9.377	17.775	0.00	0.00	RX0	H
ATOM	1387	CA	LEU	302	22.205	8.836	15.917	1.00	0.00	RX0	C
ATOM	1388	CB	LEU	302	20.991	7.930	15.710	1.00	0.00	RX0	C
ATOM	1389	CG	LEU	302	20.997	7.210	14.361	1.00	0.00	RX0	C
ATOM	1390	CD1	LEU	302	22.179	6.252	14.223	1.00	0.00	RX0	C
ATOM	1391	CD2	LEU	302	19.662	6.524	14.084	1.00	0.00	RX0	C
ATOM	1392	C	LEU	302	22.391	9.757	14.699	1.00	0.00	RX0	C
ATOM	1393	O	LEU	302	23.285	9.544	13.882	1.00	0.00	RX0	O
ATOM	1394	N	LEU	303	21.581	10.806	14.658	1.00	0.00	RX0	N
ATOM	1395	H	LEU	303	20.946	10.955	15.419	0.00	0.00	RX0	H
ATOM	1396	CA	LEU	303	21.546	11.719	13.502	1.00	0.00	RX0	C
ATOM	1397	CB	LEU	303	20.125	12.232	13.311	1.00	0.00	RX0	C
ATOM	1398	CG	LEU	303	19.163	11.065	13.081	1.00	0.00	RX0	C
ATOM	1399	CD1	LEU	303	17.707	11.488	13.247	1.00	0.00	RX0	C
ATOM	1400	CD2	LEU	303	19.421	10.367	11.744	1.00	0.00	RX0	C
ATOM	1401	C	LEU	303	22.576	12.849	13.543	1.00	0.00	RX0	C

ATOM	1402	O	LEU	303	23.073	13.268	12.494	1.00	0.00	RX0	O
ATOM	1403	N	ASN	304	22.971	13.240	14.745	1.00	0.00	RX0	N
ATOM	1404	H	ASN	304	22.611	12.752	15.544	0.00	0.00	RX0	H
ATOM	1405	CA	ASN	304	23.832	14.419	14.940	1.00	0.00	RX0	C
ATOM	1406	CB	ASN	304	23.461	15.200	16.217	1.00	0.00	RX0	C
ATOM	1407	CG	ASN	304	24.564	16.177	16.628	1.00	0.00	RX0	C
ATOM	1408	OD1	ASN	304	25.271	15.982	17.617	1.00	0.00	RX0	O
ATOM	1409	ND2	ASN	304	24.683	17.226	15.820	1.00	0.00	RX0	N
ATOM	1410	HD21	ASN	304	24.226	17.305	14.935	0.00	0.00	RX0	H
ATOM	1411	HD22	ASN	304	25.232	18.015	16.109	0.00	0.00	RX0	H
ATOM	1412	C	ASN	304	25.330	14.097	14.987	1.00	0.00	RX0	C
ATOM	1413	O	ASN	304	26.132	14.783	14.344	1.00	0.00	RX0	O
ATOM	1414	N	SER	305	25.693	13.015	15.656	1.00	0.00	RX0	N
ATOM	1415	H	SER	305	25.011	12.440	16.119	0.00	0.00	RX0	H
ATOM	1416	CA	SER	305	27.104	12.765	16.012	1.00	0.00	RX0	C
ATOM	1417	CB	SER	305	27.177	11.534	16.905	1.00	0.00	RX0	C
ATOM	1418	OG	SER	305	26.270	11.745	17.993	1.00	0.00	RX0	O
ATOM	1419	HG	SER	305	26.529	11.117	18.672	0.00	0.00	RX0	H
ATOM	1420	C	SER	305	28.103	12.695	14.846	1.00	0.00	RX0	C
ATOM	1421	O	SER	305	29.198	13.228	14.946	1.00	0.00	RX0	O
ATOM	1422	N	GLY	306	27.634	12.177	13.693	1.00	0.00	RX0	N
ATOM	1423	H	GLY	306	26.687	11.865	13.640	0.00	0.00	RX0	H
ATOM	1424	CA	GLY	306	28.500	12.079	12.501	1.00	0.00	RX0	C
ATOM	1425	C	GLY	306	28.091	12.961	11.315	1.00	0.00	RX0	C
ATOM	1426	O	GLY	306	28.756	12.897	10.274	1.00	0.00	RX0	O
ATOM	1427	N	VAL	307	27.242	13.954	11.536	1.00	0.00	RX0	N
ATOM	1428	H	VAL	307	26.874	14.098	12.457	0.00	0.00	RX0	H
ATOM	1429	CA	VAL	307	26.760	14.815	10.435	1.00	0.00	RX0	C
ATOM	1430	CB	VAL	307	25.405	15.467	10.768	1.00	0.00	RX0	C
ATOM	1431	CG1	VAL	307	25.492	16.589	11.809	1.00	0.00	RX0	C
ATOM	1432	CG2	VAL	307	24.718	15.929	9.483	1.00	0.00	RX0	C
ATOM	1433	C	VAL	307	27.809	15.841	9.948	1.00	0.00	RX0	C
ATOM	1434	O	VAL	307	27.790	16.280	8.811	1.00	0.00	RX0	O
ATOM	1435	N	TYR	308	28.719	16.199	10.859	1.00	0.00	RX0	N
ATOM	1436	H	TYR	308	28.697	15.735	11.745	0.00	0.00	RX0	H
ATOM	1437	CA	TYR	308	29.761	17.211	10.584	1.00	0.00	RX0	C
ATOM	1438	CB	TYR	308	30.213	17.892	11.873	1.00	0.00	RX0	C
ATOM	1439	CG	TYR	308	29.212	18.872	12.432	1.00	0.00	RX0	C
ATOM	1440	CD1	TYR	308	28.298	19.535	11.586	1.00	0.00	RX0	C
ATOM	1441	CE1	TYR	308	27.443	20.497	12.137	1.00	0.00	RX0	C
ATOM	1442	CD2	TYR	308	29.263	19.117	13.816	1.00	0.00	RX0	C
ATOM	1443	CE2	TYR	308	28.412	20.084	14.366	1.00	0.00	RX0	C
ATOM	1444	CZ	TYR	308	27.528	20.773	13.515	1.00	0.00	RX0	C
ATOM	1445	OH	TYR	308	26.726	21.755	14.045	1.00	0.00	RX0	O
ATOM	1446	HH	TYR	308	26.537	21.516	14.951	0.00	0.00	RX0	H
ATOM	1447	C	TYR	308	31.032	16.660	9.940	1.00	0.00	RX0	C
ATOM	1448	O	TYR	308	31.912	17.411	9.537	1.00	0.00	RX0	O
ATOM	1449	N	THR	309	31.091	15.334	9.861	1.00	0.00	RX0	N
ATOM	1450	H	THR	309	30.362	14.750	10.219	0.00	0.00	RX0	H
ATOM	1451	CA	THR	309	32.303	14.643	9.386	1.00	0.00	RX0	C
ATOM	1452	CB	THR	309	32.714	13.687	10.503	1.00	0.00	RX0	C
ATOM	1453	OG1	THR	309	31.535	13.147	11.128	1.00	0.00	RX0	O
ATOM	1454	HG1	THR	309	31.179	12.483	10.535	0.00	0.00	RX0	H
ATOM	1455	CG2	THR	309	33.580	14.391	11.549	1.00	0.00	RX0	C
ATOM	1456	C	THR	309	32.183	13.866	8.071	1.00	0.00	RX0	C
ATOM	1457	O	THR	309	33.137	13.195	7.681	1.00	0.00	RX0	O
ATOM	1458	N	PHE	310	31.041	13.953	7.382	1.00	0.00	RX0	N
ATOM	1459	H	PHE	310	30.294	14.489	7.769	0.00	0.00	RX0	H
ATOM	1460	CA	PHE	310	30.972	13.467	5.988	1.00	0.00	RX0	C
ATOM	1461	CB	PHE	310	29.584	13.704	5.380	1.00	0.00	RX0	C
ATOM	1462	CG	PHE	310	28.520	12.871	6.060	1.00	0.00	RX0	C

ATOM	1463	CD1	PHE	310	28.387	11.522	5.752	1.00	0.00	RX0	C
ATOM	1464	CD2	PHE	310	27.662	13.457	6.984	1.00	0.00	RX0	C
ATOM	1465	CE1	PHE	310	27.397	10.765	6.366	1.00	0.00	RX0	C
ATOM	1466	CE2	PHE	310	26.671	12.698	7.594	1.00	0.00	RX0	C
ATOM	1467	CZ	PHE	310	26.535	11.351	7.285	1.00	0.00	RX0	C
ATOM	1468	C	PHE	310	32.019	14.245	5.180	1.00	0.00	RX0	C
ATOM	1469	O	PHE	310	32.102	15.468	5.301	1.00	0.00	RX0	O
ATOM	1470	N	LEU	311	32.854	13.508	4.462	1.00	0.00	RX0	N
ATOM	1471	H	LEU	311	32.743	12.519	4.542	0.00	0.00	RX0	H
ATOM	1472	CA	LEU	311	33.988	14.087	3.701	1.00	0.00	RX0	C
ATOM	1473	CB	LEU	311	35.000	13.088	3.115	1.00	0.00	RX0	C
ATOM	1474	CG	LEU	311	34.639	12.020	2.086	1.00	0.00	RX0	C
ATOM	1475	CD1	LEU	311	35.936	11.410	1.555	1.00	0.00	RX0	C
ATOM	1476	CD2	LEU	311	33.679	10.949	2.612	1.00	0.00	RX0	C
ATOM	1477	C	LEU	311	33.468	15.166	2.739	1.00	0.00	RX0	C
ATOM	1478	O	LEU	311	33.554	16.351	2.969	1.00	0.00	RX0	O
ATOM	1479	N	SER	312	32.784	14.619	1.713	1.00	0.00	RX0	N
ATOM	1480	H	SER	312	32.644	13.630	1.716	0.00	0.00	RX0	H
ATOM	1481	CA	SER	312	32.345	15.333	0.526	1.00	0.00	RX0	C
ATOM	1482	CB	SER	312	31.753	14.268	-0.400	1.00	0.00	RX0	C
ATOM	1483	OG	SER	312	32.598	13.111	-0.384	1.00	0.00	RX0	O
ATOM	1484	HG	SER	312	33.450	13.383	-0.704	0.00	0.00	RX0	H
ATOM	1485	C	SER	312	31.423	16.517	0.816	1.00	0.00	RX0	C
ATOM	1486	O	SER	312	31.042	16.847	1.948	1.00	0.00	RX0	O
ATOM	1487	N	SER	313	30.837	16.923	-0.276	1.00	0.00	RX0	N
ATOM	1488	H	SER	313	31.278	16.628	-1.123	0.00	0.00	RX0	H
ATOM	1489	CA	SER	313	29.838	17.983	-0.455	1.00	0.00	RX0	C
ATOM	1490	CB	SER	313	30.329	19.344	0.044	1.00	0.00	RX0	C
ATOM	1491	OG	SER	313	30.286	19.301	1.488	1.00	0.00	RX0	O
ATOM	1492	HG	SER	313	31.026	18.742	1.742	0.00	0.00	RX0	H
ATOM	1493	C	SER	313	29.353	17.880	-1.909	1.00	0.00	RX0	C
ATOM	1494	O	SER	313	29.168	18.836	-2.628	1.00	0.00	RX0	O
ATOM	1495	N	THR	314	29.278	16.602	-2.358	1.00	0.00	RX0	N
ATOM	1496	H	THR	314	29.449	15.843	-1.738	0.00	0.00	RX0	H
ATOM	1497	CA	THR	314	28.609	16.250	-3.614	1.00	0.00	RX0	C
ATOM	1498	CB	THR	314	28.736	14.740	-3.689	1.00	0.00	RX0	C
ATOM	1499	OG1	THR	314	29.803	14.363	-2.809	1.00	0.00	RX0	O
ATOM	1500	HG1	THR	314	29.974	13.438	-2.966	0.00	0.00	RX0	H
ATOM	1501	CG2	THR	314	28.972	14.231	-5.112	1.00	0.00	RX0	C
ATOM	1502	C	THR	314	27.167	16.747	-3.490	1.00	0.00	RX0	C
ATOM	1503	O	THR	314	26.675	16.978	-2.368	1.00	0.00	RX0	O
ATOM	1504	N	LEU	315	26.451	16.801	-4.589	1.00	0.00	RX0	N
ATOM	1505	H	LEU	315	26.877	16.634	-5.476	0.00	0.00	RX0	H
ATOM	1506	CA	LEU	315	25.050	17.256	-4.537	1.00	0.00	RX0	C
ATOM	1507	CB	LEU	315	24.471	17.353	-5.950	1.00	0.00	RX0	C
ATOM	1508	CG	LEU	315	23.065	17.958	-5.989	1.00	0.00	RX0	C
ATOM	1509	CD1	LEU	315	23.010	19.331	-5.320	1.00	0.00	RX0	C
ATOM	1510	CD2	LEU	315	22.505	17.983	-7.409	1.00	0.00	RX0	C
ATOM	1511	C	LEU	315	24.190	16.343	-3.639	1.00	0.00	RX0	C
ATOM	1512	O	LEU	315	23.484	16.800	-2.757	1.00	0.00	RX0	O
ATOM	1513	N	LYS	316	24.524	15.049	-3.730	1.00	0.00	RX0	N
ATOM	1514	H	LYS	316	25.057	14.796	-4.529	0.00	0.00	RX0	H
ATOM	1515	CA	LYS	316	23.912	13.990	-2.918	1.00	0.00	RX0	C
ATOM	1516	CB	LYS	316	24.520	12.671	-3.372	1.00	0.00	RX0	C
ATOM	1517	CG	LYS	316	23.439	11.612	-3.433	1.00	0.00	RX0	C
ATOM	1518	CD	LYS	316	22.747	11.377	-2.098	1.00	0.00	RX0	C
ATOM	1519	CE	LYS	316	21.214	11.328	-2.197	1.00	0.00	RX0	C
ATOM	1520	NZ	LYS	316	20.643	12.529	-1.571	1.00	0.00	RX0	N
ATOM	1521	HZ1	LYS	316	19.634	12.418	-1.349	0.00	0.00	RX0	H
ATOM	1522	HZ2	LYS	316	21.103	12.725	-0.654	0.00	0.00	RX0	H
ATOM	1523	HZ3	LYS	316	20.726	13.361	-2.192	0.00	0.00	RX0	H

ATOM	1524	C	LYS	316	24.213	14.187	-1.422	1.00	0.00	RX0	C
ATOM	1525	O	LYS	316	23.297	14.194	-0.611	1.00	0.00	RX0	O
ATOM	1526	N	SER	317	25.467	14.540	-1.122	1.00	0.00	RX0	N
ATOM	1527	H	SER	317	26.106	14.680	-1.874	0.00	0.00	RX0	H
ATOM	1528	CA	SER	317	25.934	14.787	0.260	1.00	0.00	RX0	C
ATOM	1529	CB	SER	317	27.454	14.908	0.247	1.00	0.00	RX0	C
ATOM	1530	OG	SER	317	27.982	15.008	1.571	1.00	0.00	RX0	O
ATOM	1531	HG	SER	317	27.704	14.217	2.029	0.00	0.00	RX0	H
ATOM	1532	C	SER	317	25.242	16.001	0.898	1.00	0.00	RX0	C
ATOM	1533	O	SER	317	24.716	15.915	2.007	1.00	0.00	RX0	O
ATOM	1534	N	LEU	318	25.067	17.043	0.088	1.00	0.00	RX0	N
ATOM	1535	H	LEU	318	25.397	16.974	-0.855	0.00	0.00	RX0	H
ATOM	1536	CA	LEU	318	24.405	18.288	0.525	1.00	0.00	RX0	C
ATOM	1537	CB	LEU	318	24.444	19.345	-0.584	1.00	0.00	RX0	C
ATOM	1538	CG	LEU	318	25.771	19.582	-1.297	1.00	0.00	RX0	C
ATOM	1539	CD1	LEU	318	25.574	20.376	-2.582	1.00	0.00	RX0	C
ATOM	1540	CD2	LEU	318	26.832	20.202	-0.407	1.00	0.00	RX0	C
ATOM	1541	C	LEU	318	22.918	18.061	0.823	1.00	0.00	RX0	C
ATOM	1542	O	LEU	318	22.412	18.453	1.877	1.00	0.00	RX0	O
ATOM	1543	N	GLU	319	22.299	17.244	-0.024	1.00	0.00	RX0	N
ATOM	1544	H	GLU	319	22.775	16.968	-0.863	0.00	0.00	RX0	H
ATOM	1545	CA	GLU	319	20.900	16.808	0.157	1.00	0.00	RX0	C
ATOM	1546	CB	GLU	319	20.422	15.902	-0.974	1.00	0.00	RX0	C
ATOM	1547	CG	GLU	319	20.481	16.397	-2.413	1.00	0.00	RX0	C
ATOM	1548	CD	GLU	319	20.401	15.142	-3.257	1.00	0.00	RX0	C
ATOM	1549	OE1	GLU	319	21.298	14.866	-4.043	1.00	0.00	RX0	O
ATOM	1550	OE2	GLU	319	19.488	14.355	-3.038	1.00	0.00	RX0	O
ATOM	1551	C	GLU	319	20.725	15.924	1.402	1.00	0.00	RX0	C
ATOM	1552	O	GLU	319	19.808	16.153	2.196	1.00	0.00	RX0	O
ATOM	1553	N	GLU	320	21.706	15.058	1.645	1.00	0.00	RX0	N
ATOM	1554	H	GLU	320	22.434	14.955	0.967	0.00	0.00	RX0	H
ATOM	1555	CA	GLU	320	21.731	14.149	2.812	1.00	0.00	RX0	C
ATOM	1556	CB	GLU	320	22.791	13.048	2.745	1.00	0.00	RX0	C
ATOM	1557	CG	GLU	320	22.665	12.092	1.551	1.00	0.00	RX0	C
ATOM	1558	CD	GLU	320	21.266	11.516	1.372	1.00	0.00	RX0	C
ATOM	1559	OE1	GLU	320	20.370	12.240	0.931	1.00	0.00	RX0	O
ATOM	1560	OE2	GLU	320	21.097	10.317	1.574	1.00	0.00	RX0	O
ATOM	1561	C	GLU	320	21.765	14.931	4.131	1.00	0.00	RX0	C
ATOM	1562	O	GLU	320	20.881	14.754	4.973	1.00	0.00	RX0	O
ATOM	1563	N	LYS	321	22.647	15.926	4.188	1.00	0.00	RX0	N
ATOM	1564	H	LYS	321	23.273	15.996	3.405	0.00	0.00	RX0	H
ATOM	1565	CA	LYS	321	22.801	16.771	5.391	1.00	0.00	RX0	C
ATOM	1566	CB	LYS	321	24.010	17.707	5.253	1.00	0.00	RX0	C
ATOM	1567	CG	LYS	321	25.340	17.054	4.853	1.00	0.00	RX0	C
ATOM	1568	CD	LYS	321	26.392	18.119	4.527	1.00	0.00	RX0	C
ATOM	1569	CE	LYS	321	27.578	17.649	3.670	1.00	0.00	RX0	C
ATOM	1570	NZ	LYS	321	28.380	18.822	3.281	1.00	0.00	RX0	N
ATOM	1571	HZ1	LYS	321	29.040	18.601	2.502	0.00	0.00	RX0	H
ATOM	1572	HZ2	LYS	321	27.748	19.596	2.979	0.00	0.00	RX0	H
ATOM	1573	HZ3	LYS	321	28.944	19.149	4.087	0.00	0.00	RX0	H
ATOM	1574	C	LYS	321	21.549	17.612	5.656	1.00	0.00	RX0	C
ATOM	1575	O	LYS	321	21.102	17.724	6.798	1.00	0.00	RX0	O
ATOM	1576	N	ASP	322	20.935	18.099	4.575	1.00	0.00	RX0	N
ATOM	1577	H	ASP	322	21.334	17.933	3.670	0.00	0.00	RX0	H
ATOM	1578	CA	ASP	322	19.715	18.915	4.684	1.00	0.00	RX0	C
ATOM	1579	CB	ASP	322	19.445	19.473	3.270	1.00	0.00	RX0	C
ATOM	1580	CG	ASP	322	18.205	20.344	3.150	1.00	0.00	RX0	C
ATOM	1581	OD1	ASP	322	17.803	20.962	4.125	1.00	0.00	RX0	O
ATOM	1582	OD2	ASP	322	17.597	20.358	2.080	1.00	0.00	RX0	O
ATOM	1583	C	ASP	322	18.561	18.097	5.282	1.00	0.00	RX0	C
ATOM	1584	O	ASP	322	17.955	18.512	6.263	1.00	0.00	RX0	O

ATOM	1585	N	HIS	323	18.423	16.872	4.772	1.00	0.00	RX0	N
ATOM	1586	H	HIS	323	19.053	16.589	4.045	0.00	0.00	RX0	H
ATOM	1587	CA	HIS	323	17.423	15.916	5.274	1.00	0.00	RX0	C
ATOM	1588	CB	HIS	323	17.459	14.623	4.469	1.00	0.00	RX0	C
ATOM	1589	CG	HIS	323	16.295	13.754	4.873	1.00	0.00	RX0	C
ATOM	1590	ND1	HIS	323	16.326	12.412	4.970	1.00	0.00	RX0	N
ATOM	1591	HD1	HIS	323	17.087	11.815	4.790	0.00	0.00	RX0	H
ATOM	1592	CD2	HIS	323	15.011	14.192	5.196	1.00	0.00	RX0	C
ATOM	1593	NE2	HIS	323	14.263	13.108	5.492	1.00	0.00	RX0	N
ATOM	1594	CE1	HIS	323	15.072	12.005	5.354	1.00	0.00	RX0	C
ATOM	1595	C	HIS	323	17.630	15.607	6.766	1.00	0.00	RX0	C
ATOM	1596	O	HIS	323	16.677	15.663	7.540	1.00	0.00	RX0	O
ATOM	1597	N	ILE	324	18.888	15.420	7.164	1.00	0.00	RX0	N
ATOM	1598	H	ILE	324	19.611	15.413	6.468	0.00	0.00	RX0	H
ATOM	1599	CA	ILE	324	19.235	15.107	8.570	1.00	0.00	RX0	C
ATOM	1600	CB	ILE	324	20.717	14.759	8.705	1.00	0.00	RX0	C
ATOM	1601	CG2	ILE	324	21.119	14.617	10.173	1.00	0.00	RX0	C
ATOM	1602	CG1	ILE	324	21.031	13.485	7.919	1.00	0.00	RX0	C
ATOM	1603	CD1	ILE	324	22.517	13.124	7.926	1.00	0.00	RX0	C
ATOM	1604	C	ILE	324	18.844	16.271	9.496	1.00	0.00	RX0	C
ATOM	1605	O	ILE	324	18.200	16.053	10.527	1.00	0.00	RX0	O
ATOM	1606	N	HIS	325	19.159	17.485	9.071	1.00	0.00	RX0	N
ATOM	1607	H	HIS	325	19.630	17.565	8.190	0.00	0.00	RX0	H
ATOM	1608	CA	HIS	325	18.840	18.694	9.856	1.00	0.00	RX0	C
ATOM	1609	CB	HIS	325	19.620	19.895	9.328	1.00	0.00	RX0	C
ATOM	1610	CG	HIS	325	21.080	19.673	9.638	1.00	0.00	RX0	C
ATOM	1611	ND1	HIS	325	21.996	19.382	8.705	1.00	0.00	RX0	N
ATOM	1612	HD1	HIS	325	21.807	19.230	7.755	0.00	0.00	RX0	H
ATOM	1613	CD2	HIS	325	21.707	19.705	10.886	1.00	0.00	RX0	C
ATOM	1614	NE2	HIS	325	23.019	19.429	10.678	1.00	0.00	RX0	N
ATOM	1615	CE1	HIS	325	23.197	19.230	9.337	1.00	0.00	RX0	C
ATOM	1616	C	HIS	325	17.335	18.955	9.948	1.00	0.00	RX0	C
ATOM	1617	O	HIS	325	16.820	19.290	11.021	1.00	0.00	RX0	O
ATOM	1618	N	ARG	326	16.631	18.584	8.887	1.00	0.00	RX0	N
ATOM	1619	H	ARG	326	17.135	18.363	8.051	0.00	0.00	RX0	H
ATOM	1620	CA	ARG	326	15.160	18.594	8.873	1.00	0.00	RX0	C
ATOM	1621	CB	ARG	326	14.658	18.473	7.433	1.00	0.00	RX0	C
ATOM	1622	CG	ARG	326	14.736	19.825	6.715	1.00	0.00	RX0	C
ATOM	1623	CD	ARG	326	14.246	19.800	5.264	1.00	0.00	RX0	C
ATOM	1624	NE	ARG	326	15.304	19.442	4.319	1.00	0.00	RX0	N
ATOM	1625	HE	ARG	326	16.049	20.110	4.174	0.00	0.00	RX0	H
ATOM	1626	CZ	ARG	326	15.300	18.272	3.632	1.00	0.00	RX0	C
ATOM	1627	NH1	ARG	326	14.356	17.351	3.906	1.00	0.00	RX0	N
ATOM	1628	HH11	ARG	326	14.296	16.471	3.427	0.00	0.00	RX0	H
ATOM	1629	HH12	ARG	326	13.675	17.523	4.623	0.00	0.00	RX0	H
ATOM	1630	NH2	ARG	326	16.239	18.057	2.692	1.00	0.00	RX0	N
ATOM	1631	HH21	ARG	326	16.361	17.208	2.175	0.00	0.00	RX0	H
ATOM	1632	HH22	ARG	326	16.889	18.805	2.475	0.00	0.00	RX0	H
ATOM	1633	C	ARG	326	14.537	17.576	9.843	1.00	0.00	RX0	C
ATOM	1634	O	ARG	326	13.617	17.928	10.589	1.00	0.00	RX0	O
ATOM	1635	N	VAL	327	15.171	16.419	9.987	1.00	0.00	RX0	N
ATOM	1636	H	VAL	327	15.977	16.251	9.418	0.00	0.00	RX0	H
ATOM	1637	CA	VAL	327	14.709	15.376	10.935	1.00	0.00	RX0	C
ATOM	1638	CB	VAL	327	15.271	13.993	10.599	1.00	0.00	RX0	C
ATOM	1639	CG1	VAL	327	14.752	12.956	11.595	1.00	0.00	RX0	C
ATOM	1640	CG2	VAL	327	14.913	13.587	9.170	1.00	0.00	RX0	C
ATOM	1641	C	VAL	327	15.041	15.781	12.381	1.00	0.00	RX0	C
ATOM	1642	O	VAL	327	14.187	15.661	13.270	1.00	0.00	RX0	O
ATOM	1643	N	LEU	328	16.212	16.371	12.574	1.00	0.00	RX0	N
ATOM	1644	H	LEU	328	16.824	16.468	11.786	0.00	0.00	RX0	H
ATOM	1645	CA	LEU	328	16.631	16.899	13.887	1.00	0.00	RX0	C

ATOM	1646	CB	LEU	328	18.070	17.415	13.831	1.00	0.00	RX0	C
ATOM	1647	CG	LEU	328	19.106	16.294	13.753	1.00	0.00	RX0	C
ATOM	1648	CD1	LEU	328	20.505	16.832	13.451	1.00	0.00	RX0	C
ATOM	1649	CD2	LEU	328	19.092	15.426	15.012	1.00	0.00	RX0	C
ATOM	1650	C	LEU	328	15.692	18.005	14.386	1.00	0.00	RX0	C
ATOM	1651	O	LEU	328	15.231	17.955	15.519	1.00	0.00	RX0	O
ATOM	1652	N	ASP	329	15.222	18.821	13.436	1.00	0.00	RX0	N
ATOM	1653	H	ASP	329	15.660	18.861	12.535	0.00	0.00	RX0	H
ATOM	1654	CA	ASP	329	14.223	19.870	13.722	1.00	0.00	RX0	C
ATOM	1655	CB	ASP	329	13.766	20.687	12.503	1.00	0.00	RX0	C
ATOM	1656	CG	ASP	329	14.815	21.488	11.776	1.00	0.00	RX0	C
ATOM	1657	OD1	ASP	329	15.449	22.349	12.371	1.00	0.00	RX0	O
ATOM	1658	OD2	ASP	329	14.936	21.314	10.569	1.00	0.00	RX0	O
ATOM	1659	C	ASP	329	12.864	19.312	14.154	1.00	0.00	RX0	C
ATOM	1660	O	ASP	329	12.272	19.788	15.128	1.00	0.00	RX0	O
ATOM	1661	N	LYS	330	12.463	18.218	13.512	1.00	0.00	RX0	N
ATOM	1662	H	LYS	330	13.004	17.954	12.711	0.00	0.00	RX0	H
ATOM	1663	CA	LYS	330	11.217	17.525	13.871	1.00	0.00	RX0	C
ATOM	1664	CB	LYS	330	10.801	16.460	12.859	1.00	0.00	RX0	C
ATOM	1665	CG	LYS	330	9.626	15.604	13.365	1.00	0.00	RX0	C
ATOM	1666	CD	LYS	330	8.370	16.379	13.795	1.00	0.00	RX0	C
ATOM	1667	CE	LYS	330	7.826	17.306	12.717	1.00	0.00	RX0	C
ATOM	1668	NZ	LYS	330	7.438	16.477	11.576	1.00	0.00	RX0	N
ATOM	1669	HZ1	LYS	330	7.128	17.097	10.807	0.00	0.00	RX0	H
ATOM	1670	HZ2	LYS	330	8.248	15.890	11.279	0.00	0.00	RX0	H
ATOM	1671	HZ3	LYS	330	6.656	15.859	11.877	0.00	0.00	RX0	H
ATOM	1672	C	LYS	330	11.283	16.915	15.278	1.00	0.00	RX0	C
ATOM	1673	O	LYS	330	10.354	17.095	16.067	1.00	0.00	RX0	O
ATOM	1674	N	ILE	331	12.441	16.375	15.625	1.00	0.00	RX0	N
ATOM	1675	H	ILE	331	13.181	16.371	14.947	0.00	0.00	RX0	H
ATOM	1676	CA	ILE	331	12.656	15.802	16.972	1.00	0.00	RX0	C
ATOM	1677	CB	ILE	331	13.948	14.989	17.055	1.00	0.00	RX0	C
ATOM	1678	CG2	ILE	331	14.031	14.300	18.415	1.00	0.00	RX0	C
ATOM	1679	CG1	ILE	331	14.047	13.959	15.933	1.00	0.00	RX0	C
ATOM	1680	CD1	ILE	331	15.359	13.176	15.977	1.00	0.00	RX0	C
ATOM	1681	C	ILE	331	12.642	16.922	18.027	1.00	0.00	RX0	C
ATOM	1682	O	ILE	331	12.078	16.732	19.120	1.00	0.00	RX0	O
ATOM	1683	N	THR	332	13.158	18.084	17.675	1.00	0.00	RX0	N
ATOM	1684	H	THR	332	13.547	18.205	16.761	0.00	0.00	RX0	H
ATOM	1685	CA	THR	332	13.155	19.265	18.570	1.00	0.00	RX0	C
ATOM	1686	CB	THR	332	14.024	20.345	17.925	1.00	0.00	RX0	C
ATOM	1687	OG1	THR	332	15.322	19.799	17.651	1.00	0.00	RX0	O
ATOM	1688	HG1	THR	332	15.256	19.302	16.840	0.00	0.00	RX0	H
ATOM	1689	CG2	THR	332	14.149	21.593	18.802	1.00	0.00	RX0	C
ATOM	1690	C	THR	332	11.706	19.696	18.836	1.00	0.00	RX0	C
ATOM	1691	O	THR	332	11.302	19.832	19.995	1.00	0.00	RX0	O
ATOM	1692	N	ASP	333	10.912	19.714	17.772	1.00	0.00	RX0	N
ATOM	1693	H	ASP	333	11.278	19.677	16.837	0.00	0.00	RX0	H
ATOM	1694	CA	ASP	333	9.466	20.017	17.863	1.00	0.00	RX0	C
ATOM	1695	CB	ASP	333	8.673	19.802	16.565	1.00	0.00	RX0	C
ATOM	1696	CG	ASP	333	9.132	20.568	15.350	1.00	0.00	RX0	C
ATOM	1697	OD1	ASP	333	9.569	21.705	15.498	1.00	0.00	RX0	O
ATOM	1698	OD2	ASP	333	9.001	20.025	14.249	1.00	0.00	RX0	O
ATOM	1699	C	ASP	333	8.732	19.000	18.747	1.00	0.00	RX0	C
ATOM	1700	O	ASP	333	7.880	19.374	19.559	1.00	0.00	RX0	O
ATOM	1701	N	THR	334	9.187	17.759	18.682	1.00	0.00	RX0	N
ATOM	1702	H	THR	334	9.940	17.582	18.047	0.00	0.00	RX0	H
ATOM	1703	CA	THR	334	8.631	16.638	19.462	1.00	0.00	RX0	C
ATOM	1704	CB	THR	334	9.175	15.373	18.822	1.00	0.00	RX0	C
ATOM	1705	OG1	THR	334	8.792	15.339	17.439	1.00	0.00	RX0	O
ATOM	1706	HG1	THR	334	9.147	16.125	17.032	0.00	0.00	RX0	H

ATOM	1707	CG2	THR	334	8.724	14.119	19.557	1.00	0.00	RX0	C
ATOM	1708	C	THR	334	8.961	16.782	20.953	1.00	0.00	RX0	C
ATOM	1709	O	THR	334	8.059	16.689	21.789	1.00	0.00	RX0	O
ATOM	1710	N	LEU	335	10.217	17.081	21.263	1.00	0.00	RX0	N
ATOM	1711	H	LEU	335	10.881	17.204	20.523	0.00	0.00	RX0	H
ATOM	1712	CA	LEU	335	10.648	17.349	22.650	1.00	0.00	RX0	C
ATOM	1713	CB	LEU	335	12.151	17.609	22.699	1.00	0.00	RX0	C
ATOM	1714	CG	LEU	335	12.956	16.319	22.801	1.00	0.00	RX0	C
ATOM	1715	CD1	LEU	335	14.458	16.566	22.665	1.00	0.00	RX0	C
ATOM	1716	CD2	LEU	335	12.618	15.568	24.088	1.00	0.00	RX0	C
ATOM	1717	C	LEU	335	9.903	18.522	23.297	1.00	0.00	RX0	C
ATOM	1718	O	LEU	335	9.384	18.379	24.399	1.00	0.00	RX0	O
ATOM	1719	N	ILE	336	9.668	19.567	22.501	1.00	0.00	RX0	N
ATOM	1720	H	ILE	336	10.073	19.580	21.583	0.00	0.00	RX0	H
ATOM	1721	CA	ILE	336	8.897	20.743	22.962	1.00	0.00	RX0	C
ATOM	1722	CB	ILE	336	9.048	21.912	21.989	1.00	0.00	RX0	C
ATOM	1723	CG2	ILE	336	8.091	23.058	22.326	1.00	0.00	RX0	C
ATOM	1724	CG1	ILE	336	10.501	22.387	21.993	1.00	0.00	RX0	C
ATOM	1725	CD1	ILE	336	10.934	22.866	23.381	1.00	0.00	RX0	C
ATOM	1726	C	ILE	336	7.427	20.365	23.180	1.00	0.00	RX0	C
ATOM	1727	O	ILE	336	6.836	20.730	24.203	1.00	0.00	RX0	O
ATOM	1728	N	HIS	337	6.891	19.571	22.265	1.00	0.00	RX0	N
ATOM	1729	H	HIS	337	7.439	19.317	21.465	0.00	0.00	RX0	H
ATOM	1730	CA	HIS	337	5.501	19.096	22.359	1.00	0.00	RX0	C
ATOM	1731	CB	HIS	337	5.093	18.324	21.116	1.00	0.00	RX0	C
ATOM	1732	CG	HIS	337	3.623	18.022	21.226	1.00	0.00	RX0	C
ATOM	1733	ND1	HIS	337	2.655	18.939	21.044	1.00	0.00	RX0	N
ATOM	1734	HD1	HIS	337	2.786	19.882	20.807	0.00	0.00	RX0	H
ATOM	1735	CD2	HIS	337	3.033	16.801	21.548	1.00	0.00	RX0	C
ATOM	1736	NE2	HIS	337	1.690	16.990	21.566	1.00	0.00	RX0	N
ATOM	1737	CE1	HIS	337	1.457	18.308	21.253	1.00	0.00	RX0	C
ATOM	1738	C	HIS	337	5.301	18.263	23.631	1.00	0.00	RX0	C
ATOM	1739	O	HIS	337	4.339	18.490	24.365	1.00	0.00	RX0	O
ATOM	1740	N	LEU	338	6.274	17.405	23.914	1.00	0.00	RX0	N
ATOM	1741	H	LEU	338	7.051	17.335	23.285	0.00	0.00	RX0	H
ATOM	1742	CA	LEU	338	6.246	16.526	25.097	1.00	0.00	RX0	C
ATOM	1743	CB	LEU	338	7.439	15.573	25.088	1.00	0.00	RX0	C
ATOM	1744	CG	LEU	338	7.334	14.530	23.978	1.00	0.00	RX0	C
ATOM	1745	CD1	LEU	338	8.634	13.740	23.811	1.00	0.00	RX0	C
ATOM	1746	CD2	LEU	338	6.111	13.634	24.177	1.00	0.00	RX0	C
ATOM	1747	C	LEU	338	6.240	17.331	26.400	1.00	0.00	RX0	C
ATOM	1748	O	LEU	338	5.410	17.098	27.277	1.00	0.00	RX0	O
ATOM	1749	N	MET	339	7.027	18.402	26.394	1.00	0.00	RX0	N
ATOM	1750	H	MET	339	7.615	18.548	25.595	0.00	0.00	RX0	H
ATOM	1751	CA	MET	339	7.168	19.308	27.550	1.00	0.00	RX0	C
ATOM	1752	CB	MET	339	8.406	20.190	27.400	1.00	0.00	RX0	C
ATOM	1753	CG	MET	339	9.705	19.389	27.372	1.00	0.00	RX0	C
ATOM	1754	SD	MET	339	11.138	20.428	27.053	1.00	0.00	RX0	S
ATOM	1755	CE	MET	339	12.272	19.115	26.581	1.00	0.00	RX0	C
ATOM	1756	C	MET	339	5.924	20.183	27.753	1.00	0.00	RX0	C
ATOM	1757	O	MET	339	5.433	20.322	28.878	1.00	0.00	RX0	O
ATOM	1758	N	ALA	340	5.370	20.672	26.646	1.00	0.00	RX0	N
ATOM	1759	H	ALA	340	5.814	20.472	25.772	0.00	0.00	RX0	H
ATOM	1760	CA	ALA	340	4.120	21.456	26.636	1.00	0.00	RX0	C
ATOM	1761	CB	ALA	340	3.834	21.992	25.232	1.00	0.00	RX0	C
ATOM	1762	C	ALA	340	2.921	20.619	27.100	1.00	0.00	RX0	C
ATOM	1763	O	ALA	340	2.176	21.064	27.967	1.00	0.00	RX0	O
ATOM	1764	N	LYS	341	2.865	19.360	26.655	1.00	0.00	RX0	N
ATOM	1765	H	LYS	341	3.556	19.056	25.998	0.00	0.00	RX0	H
ATOM	1766	CA	LYS	341	1.846	18.397	27.117	1.00	0.00	RX0	C
ATOM	1767	CB	LYS	341	1.960	17.112	26.273	1.00	0.00	RX0	C

ATOM	1768	CG	LYS	341	0.678	16.277	26.150	1.00	0.00	RX0	C
ATOM	1769	CD	LYS	341	0.415	15.760	24.722	1.00	0.00	RX0	C
ATOM	1770	CE	LYS	341	1.425	14.745	24.167	1.00	0.00	RX0	C
ATOM	1771	NZ	LYS	341	1.152	14.492	22.742	1.00	0.00	RX0	N
ATOM	1772	HZ1	LYS	341	1.872	13.902	22.269	0.00	0.00	RX0	H
ATOM	1773	HZ2	LYS	341	0.271	13.985	22.513	0.00	0.00	RX0	H
ATOM	1774	HZ3	LYS	341	1.151	15.359	22.171	0.00	0.00	RX0	H
ATOM	1775	C	LYS	341	1.927	18.179	28.637	1.00	0.00	RX0	C
ATOM	1776	O	LYS	341	0.908	18.032	29.304	1.00	0.00	RX0	O
ATOM	1777	N	ALA	342	3.166	18.103	29.120	1.00	0.00	RX0	N
ATOM	1778	H	ALA	342	3.940	18.169	28.489	0.00	0.00	RX0	H
ATOM	1779	CA	ALA	342	3.459	17.942	30.556	1.00	0.00	RX0	C
ATOM	1780	CB	ALA	342	4.954	17.707	30.786	1.00	0.00	RX0	C
ATOM	1781	C	ALA	342	3.007	19.166	31.371	1.00	0.00	RX0	C
ATOM	1782	O	ALA	342	2.879	19.099	32.588	1.00	0.00	RX0	O
ATOM	1783	N	GLY	343	2.900	20.305	30.669	1.00	0.00	RX0	N
ATOM	1784	H	GLY	343	3.152	20.317	29.702	0.00	0.00	RX0	H
ATOM	1785	CA	GLY	343	2.393	21.560	31.244	1.00	0.00	RX0	C
ATOM	1786	C	GLY	343	3.505	22.433	31.829	1.00	0.00	RX0	C
ATOM	1787	O	GLY	343	3.244	23.282	32.678	1.00	0.00	RX0	O
ATOM	1788	N	LEU	344	4.736	22.226	31.356	1.00	0.00	RX0	N
ATOM	1789	H	LEU	344	4.854	21.555	30.623	0.00	0.00	RX0	H
ATOM	1790	CA	LEU	344	5.823	23.174	31.634	1.00	0.00	RX0	C
ATOM	1791	CB	LEU	344	7.151	22.612	31.138	1.00	0.00	RX0	C
ATOM	1792	CG	LEU	344	7.559	21.341	31.879	1.00	0.00	RX0	C
ATOM	1793	CD1	LEU	344	8.843	20.750	31.303	1.00	0.00	RX0	C
ATOM	1794	CD2	LEU	344	7.661	21.571	33.388	1.00	0.00	RX0	C
ATOM	1795	C	LEU	344	5.508	24.488	30.929	1.00	0.00	RX0	C
ATOM	1796	O	LEU	344	4.977	24.500	29.792	1.00	0.00	RX0	O
ATOM	1797	N	THR	345	5.822	25.577	31.575	1.00	0.00	RX0	N
ATOM	1798	H	THR	345	6.307	25.495	32.445	0.00	0.00	RX0	H
ATOM	1799	CA	THR	345	5.705	26.912	30.948	1.00	0.00	RX0	C
ATOM	1800	CB	THR	345	6.002	27.967	32.008	1.00	0.00	RX0	C
ATOM	1801	OG1	THR	345	7.190	27.614	32.716	1.00	0.00	RX0	O
ATOM	1802	HG1	THR	345	6.928	26.978	33.377	0.00	0.00	RX0	H
ATOM	1803	CG2	THR	345	4.837	28.100	32.992	1.00	0.00	RX0	C
ATOM	1804	C	THR	345	6.639	26.971	29.731	1.00	0.00	RX0	C
ATOM	1805	O	THR	345	7.615	26.233	29.623	1.00	0.00	RX0	O
ATOM	1806	N	LEU	346	6.390	27.971	28.898	1.00	0.00	RX0	N
ATOM	1807	H	LEU	346	5.564	28.512	29.044	0.00	0.00	RX0	H
ATOM	1808	CA	LEU	346	7.214	28.242	27.708	1.00	0.00	RX0	C
ATOM	1809	CB	LEU	346	6.672	29.452	26.948	1.00	0.00	RX0	C
ATOM	1810	CG	LEU	346	7.380	29.659	25.608	1.00	0.00	RX0	C
ATOM	1811	CD1	LEU	346	7.249	28.434	24.699	1.00	0.00	RX0	C
ATOM	1812	CD2	LEU	346	6.917	30.941	24.915	1.00	0.00	RX0	C
ATOM	1813	C	LEU	346	8.705	28.433	28.051	1.00	0.00	RX0	C
ATOM	1814	O	LEU	346	9.594	27.857	27.432	1.00	0.00	RX0	O
ATOM	1815	N	GLN	347	8.927	29.083	29.198	1.00	0.00	RX0	N
ATOM	1816	H	GLN	347	8.145	29.436	29.708	0.00	0.00	RX0	H
ATOM	1817	CA	GLN	347	10.275	29.275	29.754	1.00	0.00	RX0	C
ATOM	1818	CB	GLN	347	10.216	30.281	30.899	1.00	0.00	RX0	C
ATOM	1819	CG	GLN	347	11.593	30.610	31.473	1.00	0.00	RX0	C
ATOM	1820	CD	GLN	347	11.418	31.573	32.624	1.00	0.00	RX0	C
ATOM	1821	OE1	GLN	347	10.428	31.537	33.343	1.00	0.00	RX0	O
ATOM	1822	NE2	GLN	347	12.431	32.448	32.750	1.00	0.00	RX0	N
ATOM	1823	HE21	GLN	347	13.208	32.434	32.119	0.00	0.00	RX0	H
ATOM	1824	HE22	GLN	347	12.422	33.138	33.475	0.00	0.00	RX0	H
ATOM	1825	C	GLN	347	10.911	27.956	30.229	1.00	0.00	RX0	C
ATOM	1826	O	GLN	347	12.052	27.652	29.875	1.00	0.00	RX0	O
ATOM	1827	N	GLN	348	10.120	27.140	30.905	1.00	0.00	RX0	N
ATOM	1828	H	GLN	348	9.172	27.401	31.088	0.00	0.00	RX0	H

ATOM	1829	CA	GLN	348	10.582	25.823	31.394	1.00	0.00	RX0	C
ATOM	1830	CB	GLN	348	9.587	25.222	32.375	1.00	0.00	RX0	C
ATOM	1831	CG	GLN	348	9.676	25.857	33.760	1.00	0.00	RX0	C
ATOM	1832	CD	GLN	348	8.487	25.391	34.568	1.00	0.00	RX0	C
ATOM	1833	OE1	GLN	348	7.365	25.330	34.072	1.00	0.00	RX0	O
ATOM	1834	NE2	GLN	348	8.789	25.054	35.831	1.00	0.00	RX0	N
ATOM	1835	HE21	GLN	348	9.724	25.166	36.172	0.00	0.00	RX0	H
ATOM	1836	HE22	GLN	348	8.099	24.687	36.456	0.00	0.00	RX0	H
ATOM	1837	C	GLN	348	10.871	24.847	30.248	1.00	0.00	RX0	C
ATOM	1838	O	GLN	348	11.861	24.115	30.296	1.00	0.00	RX0	O
ATOM	1839	N	GLN	349	10.121	24.983	29.160	1.00	0.00	RX0	N
ATOM	1840	H	GLN	349	9.376	25.650	29.185	0.00	0.00	RX0	H
ATOM	1841	CA	GLN	349	10.299	24.172	27.940	1.00	0.00	RX0	C
ATOM	1842	CB	GLN	349	9.205	24.481	26.921	1.00	0.00	RX0	C
ATOM	1843	CG	GLN	349	7.821	24.033	27.385	1.00	0.00	RX0	C
ATOM	1844	CD	GLN	349	6.787	24.589	26.434	1.00	0.00	RX0	C
ATOM	1845	OE1	GLN	349	7.075	24.916	25.289	1.00	0.00	RX0	O
ATOM	1846	NE2	GLN	349	5.564	24.698	26.978	1.00	0.00	RX0	N
ATOM	1847	HE21	GLN	349	5.399	24.434	27.934	0.00	0.00	RX0	H
ATOM	1848	HE22	GLN	349	4.780	25.034	26.459	0.00	0.00	RX0	H
ATOM	1849	C	GLN	349	11.682	24.372	27.306	1.00	0.00	RX0	C
ATOM	1850	O	GLN	349	12.447	23.422	27.181	1.00	0.00	RX0	O
ATOM	1851	N	HIS	350	12.056	25.641	27.113	1.00	0.00	RX0	N
ATOM	1852	H	HIS	350	11.422	26.375	27.365	0.00	0.00	RX0	H
ATOM	1853	CA	HIS	350	13.344	25.953	26.463	1.00	0.00	RX0	C
ATOM	1854	CB	HIS	350	13.379	27.327	25.782	1.00	0.00	RX0	C
ATOM	1855	CG	HIS	350	13.580	28.468	26.748	1.00	0.00	RX0	C
ATOM	1856	ND1	HIS	350	12.566	29.183	27.263	1.00	0.00	RX0	N
ATOM	1857	HD1	HIS	350	11.607	29.024	27.108	0.00	0.00	RX0	H
ATOM	1858	CD2	HIS	350	14.790	28.990	27.219	1.00	0.00	RX0	C
ATOM	1859	NE2	HIS	350	14.493	30.035	28.027	1.00	0.00	RX0	N
ATOM	1860	CE1	HIS	350	13.129	30.154	28.051	1.00	0.00	RX0	C
ATOM	1861	C	HIS	350	14.540	25.670	27.386	1.00	0.00	RX0	C
ATOM	1862	O	HIS	350	15.573	25.182	26.934	1.00	0.00	RX0	O
ATOM	1863	N	GLN	351	14.320	25.854	28.691	1.00	0.00	RX0	N
ATOM	1864	H	GLN	351	13.441	26.233	28.990	0.00	0.00	RX0	H
ATOM	1865	CA	GLN	351	15.341	25.538	29.706	1.00	0.00	RX0	C
ATOM	1866	CB	GLN	351	14.917	26.061	31.072	1.00	0.00	RX0	C
ATOM	1867	CG	GLN	351	14.895	27.584	31.148	1.00	0.00	RX0	C
ATOM	1868	CD	GLN	351	14.217	27.983	32.438	1.00	0.00	RX0	C
ATOM	1869	OE1	GLN	351	13.359	27.279	32.962	1.00	0.00	RX0	O
ATOM	1870	NE2	GLN	351	14.664	29.149	32.937	1.00	0.00	RX0	N
ATOM	1871	HE21	GLN	351	15.368	29.664	32.447	0.00	0.00	RX0	H
ATOM	1872	HE22	GLN	351	14.313	29.512	33.800	0.00	0.00	RX0	H
ATOM	1873	C	GLN	351	15.597	24.030	29.805	1.00	0.00	RX0	C
ATOM	1874	O	GLN	351	16.752	23.608	29.740	1.00	0.00	RX0	O
ATOM	1875	N	ARG	352	14.527	23.242	29.744	1.00	0.00	RX0	N
ATOM	1876	H	ARG	352	13.617	23.653	29.656	0.00	0.00	RX0	H
ATOM	1877	CA	ARG	352	14.627	21.772	29.816	1.00	0.00	RX0	C
ATOM	1878	CB	ARG	352	13.311	21.111	30.248	1.00	0.00	RX0	C
ATOM	1879	CG	ARG	352	13.425	19.590	30.445	1.00	0.00	RX0	C
ATOM	1880	CD	ARG	352	12.236	18.993	31.209	1.00	0.00	RX0	C
ATOM	1881	NE	ARG	352	12.219	17.527	31.185	1.00	0.00	RX0	N
ATOM	1882	HE	ARG	352	12.020	17.062	30.307	0.00	0.00	RX0	H
ATOM	1883	CZ	ARG	352	12.338	16.771	32.320	1.00	0.00	RX0	C
ATOM	1884	NH1	ARG	352	12.623	17.374	33.492	1.00	0.00	RX0	N
ATOM	1885	HH11	ARG	352	12.797	16.826	34.324	0.00	0.00	RX0	H
ATOM	1886	HH12	ARG	352	12.685	18.368	33.575	0.00	0.00	RX0	H
ATOM	1887	NH2	ARG	352	12.166	15.436	32.254	1.00	0.00	RX0	N
ATOM	1888	HH21	ARG	352	12.197	14.799	33.033	0.00	0.00	RX0	H
ATOM	1889	HH22	ARG	352	11.980	15.018	31.342	0.00	0.00	RX0	H

ATOM	1890	C	ARG	352	15.192	21.188	28.514	1.00	0.00	RX0	C
ATOM	1891	O	ARG	352	16.048	20.305	28.551	1.00	0.00	RX0	O
ATOM	1892	N	LEU	353	14.843	21.816	27.391	1.00	0.00	RX0	N
ATOM	1893	H	LEU	353	14.131	22.519	27.429	0.00	0.00	RX0	H
ATOM	1894	CA	LEU	353	15.400	21.446	26.079	1.00	0.00	RX0	C
ATOM	1895	CB	LEU	353	14.767	22.302	24.983	1.00	0.00	RX0	C
ATOM	1896	CG	LEU	353	15.246	21.929	23.581	1.00	0.00	RX0	C
ATOM	1897	CD1	LEU	353	14.814	20.516	23.189	1.00	0.00	RX0	C
ATOM	1898	CD2	LEU	353	14.825	22.969	22.544	1.00	0.00	RX0	C
ATOM	1899	C	LEU	353	16.930	21.610	26.063	1.00	0.00	RX0	C
ATOM	1900	O	LEU	353	17.658	20.692	25.694	1.00	0.00	RX0	O
ATOM	1901	N	ALA	354	17.375	22.739	26.614	1.00	0.00	RX0	N
ATOM	1902	H	ALA	354	16.704	23.421	26.916	0.00	0.00	RX0	H
ATOM	1903	CA	ALA	354	18.808	23.073	26.716	1.00	0.00	RX0	C
ATOM	1904	CB	ALA	354	18.994	24.518	27.181	1.00	0.00	RX0	C
ATOM	1905	C	ALA	354	19.540	22.141	27.690	1.00	0.00	RX0	C
ATOM	1906	O	ALA	354	20.574	21.567	27.334	1.00	0.00	RX0	O
ATOM	1907	N	GLN	355	18.893	21.844	28.811	1.00	0.00	RX0	N
ATOM	1908	H	GLN	355	18.017	22.305	28.966	0.00	0.00	RX0	H
ATOM	1909	CA	GLN	355	19.427	20.920	29.833	1.00	0.00	RX0	C
ATOM	1910	CB	GLN	355	18.564	20.902	31.082	1.00	0.00	RX0	C
ATOM	1911	CG	GLN	355	18.678	22.207	31.860	1.00	0.00	RX0	C
ATOM	1912	CD	GLN	355	17.945	22.052	33.168	1.00	0.00	RX0	C
ATOM	1913	OE1	GLN	355	18.384	21.322	34.057	1.00	0.00	RX0	O
ATOM	1914	NE2	GLN	355	16.818	22.776	33.235	1.00	0.00	RX0	N
ATOM	1915	HE21	GLN	355	16.551	23.327	32.442	0.00	0.00	RX0	H
ATOM	1916	HE22	GLN	355	16.223	22.802	34.038	0.00	0.00	RX0	H
ATOM	1917	C	GLN	355	19.631	19.504	29.273	1.00	0.00	RX0	C
ATOM	1918	O	GLN	355	20.705	18.919	29.430	1.00	0.00	RX0	O
ATOM	1919	N	LEU	356	18.681	19.086	28.443	1.00	0.00	RX0	N
ATOM	1920	H	LEU	356	17.883	19.669	28.276	0.00	0.00	RX0	H
ATOM	1921	CA	LEU	356	18.716	17.770	27.778	1.00	0.00	RX0	C
ATOM	1922	CB	LEU	356	17.348	17.401	27.205	1.00	0.00	RX0	C
ATOM	1923	CG	LEU	356	16.353	17.033	28.304	1.00	0.00	RX0	C
ATOM	1924	CD1	LEU	356	14.993	16.631	27.735	1.00	0.00	RX0	C
ATOM	1925	CD2	LEU	356	16.923	15.957	29.228	1.00	0.00	RX0	C
ATOM	1926	C	LEU	356	19.795	17.661	26.699	1.00	0.00	RX0	C
ATOM	1927	O	LEU	356	20.593	16.720	26.700	1.00	0.00	RX0	O
ATOM	1928	N	LEU	357	19.916	18.722	25.913	1.00	0.00	RX0	N
ATOM	1929	H	LEU	357	19.251	19.469	26.016	0.00	0.00	RX0	H
ATOM	1930	CA	LEU	357	20.885	18.765	24.803	1.00	0.00	RX0	C
ATOM	1931	CB	LEU	357	20.541	19.868	23.799	1.00	0.00	RX0	C
ATOM	1932	CG	LEU	357	19.184	19.706	23.107	1.00	0.00	RX0	C
ATOM	1933	CD1	LEU	357	18.889	20.890	22.187	1.00	0.00	RX0	C
ATOM	1934	CD2	LEU	357	19.044	18.370	22.377	1.00	0.00	RX0	C
ATOM	1935	C	LEU	357	22.335	18.932	25.264	1.00	0.00	RX0	C
ATOM	1936	O	LEU	357	23.247	18.345	24.683	1.00	0.00	RX0	O
ATOM	1937	N	LEU	358	22.501	19.595	26.404	1.00	0.00	RX0	N
ATOM	1938	H	LEU	358	21.697	20.050	26.793	0.00	0.00	RX0	H
ATOM	1939	CA	LEU	358	23.826	19.754	27.029	1.00	0.00	RX0	C
ATOM	1940	CB	LEU	358	23.796	20.795	28.157	1.00	0.00	RX0	C
ATOM	1941	CG	LEU	358	23.504	22.234	27.725	1.00	0.00	RX0	C
ATOM	1942	CD1	LEU	358	23.246	23.131	28.934	1.00	0.00	RX0	C
ATOM	1943	CD2	LEU	358	24.582	22.808	26.811	1.00	0.00	RX0	C
ATOM	1944	C	LEU	358	24.390	18.455	27.609	1.00	0.00	RX0	C
ATOM	1945	O	LEU	358	25.603	18.227	27.557	1.00	0.00	RX0	O
ATOM	1946	N	ILE	359	23.510	17.559	28.043	1.00	0.00	RX0	N
ATOM	1947	H	ILE	359	22.539	17.808	28.033	0.00	0.00	RX0	H
ATOM	1948	CA	ILE	359	23.928	16.212	28.495	1.00	0.00	RX0	C
ATOM	1949	CB	ILE	359	22.752	15.466	29.143	1.00	0.00	RX0	C
ATOM	1950	CG2	ILE	359	23.131	14.058	29.613	1.00	0.00	RX0	C

ATOM	1951	CG1	ILE	359	22.210	16.288	30.313	1.00	0.00	RX0	C
ATOM	1952	CD1	ILE	359	20.899	15.740	30.871	1.00	0.00	RX0	C
ATOM	1953	C	ILE	359	24.559	15.428	27.334	1.00	0.00	RX0	C
ATOM	1954	O	ILE	359	25.552	14.723	27.543	1.00	0.00	RX0	O
ATOM	1955	N	LEU	360	24.038	15.620	26.133	1.00	0.00	RX0	N
ATOM	1956	H	LEU	360	23.230	16.206	26.051	0.00	0.00	RX0	H
ATOM	1957	CA	LEU	360	24.601	14.974	24.928	1.00	0.00	RX0	C
ATOM	1958	CB	LEU	360	23.762	15.280	23.683	1.00	0.00	RX0	C
ATOM	1959	CG	LEU	360	22.266	14.974	23.839	1.00	0.00	RX0	C
ATOM	1960	CD1	LEU	360	21.492	15.361	22.582	1.00	0.00	RX0	C
ATOM	1961	CD2	LEU	360	21.983	13.526	24.243	1.00	0.00	RX0	C
ATOM	1962	C	LEU	360	26.079	15.308	24.694	1.00	0.00	RX0	C
ATOM	1963	O	LEU	360	26.843	14.455	24.246	1.00	0.00	RX0	O
ATOM	1964	N	SER	361	26.491	16.472	25.202	1.00	0.00	RX0	N
ATOM	1965	H	SER	361	25.797	17.100	25.554	0.00	0.00	RX0	H
ATOM	1966	CA	SER	361	27.906	16.894	25.202	1.00	0.00	RX0	C
ATOM	1967	CB	SER	361	28.039	18.361	25.614	1.00	0.00	RX0	C
ATOM	1968	OG	SER	361	29.181	18.959	24.994	1.00	0.00	RX0	O
ATOM	1969	HG	SER	361	28.997	19.894	24.926	0.00	0.00	RX0	H
ATOM	1970	C	SER	361	28.769	15.985	26.099	1.00	0.00	RX0	C
ATOM	1971	O	SER	361	29.797	15.456	25.681	1.00	0.00	RX0	O
ATOM	1972	N	HIS	362	28.237	15.704	27.286	1.00	0.00	RX0	N
ATOM	1973	H	HIS	362	27.319	16.056	27.459	0.00	0.00	RX0	H
ATOM	1974	CA	HIS	362	28.854	14.793	28.272	1.00	0.00	RX0	C
ATOM	1975	CB	HIS	362	28.099	14.887	29.600	1.00	0.00	RX0	C
ATOM	1976	CG	HIS	362	28.133	16.302	30.134	1.00	0.00	RX0	C
ATOM	1977	ND1	HIS	362	27.363	17.315	29.680	1.00	0.00	RX0	N
ATOM	1978	HD1	HIS	362	26.699	17.302	28.958	0.00	0.00	RX0	H
ATOM	1979	CD2	HIS	362	28.947	16.789	31.160	1.00	0.00	RX0	C
ATOM	1980	NE2	HIS	362	28.658	18.104	31.317	1.00	0.00	RX0	N
ATOM	1981	CE1	HIS	362	27.686	18.429	30.408	1.00	0.00	RX0	C
ATOM	1982	C	HIS	362	28.890	13.339	27.777	1.00	0.00	RX0	C
ATOM	1983	O	HIS	362	29.902	12.656	27.936	1.00	0.00	RX0	O
ATOM	1984	N	ILE	363	27.856	12.940	27.039	1.00	0.00	RX0	N
ATOM	1985	H	ILE	363	27.100	13.582	26.902	0.00	0.00	RX0	H
ATOM	1986	CA	ILE	363	27.773	11.584	26.446	1.00	0.00	RX0	C
ATOM	1987	CB	ILE	363	26.352	11.281	25.967	1.00	0.00	RX0	C
ATOM	1988	CG2	ILE	363	26.224	9.849	25.446	1.00	0.00	RX0	C
ATOM	1989	CG1	ILE	363	25.371	11.513	27.117	1.00	0.00	RX0	C
ATOM	1990	CD1	ILE	363	23.920	11.261	26.715	1.00	0.00	RX0	C
ATOM	1991	C	ILE	363	28.830	11.416	25.343	1.00	0.00	RX0	C
ATOM	1992	O	ILE	363	29.487	10.370	25.263	1.00	0.00	RX0	O
ATOM	1993	N	ARG	364	29.035	12.469	24.564	1.00	0.00	RX0	N
ATOM	1994	H	ARG	364	28.393	13.237	24.612	0.00	0.00	RX0	H
ATOM	1995	CA	ARG	364	30.102	12.498	23.547	1.00	0.00	RX0	C
ATOM	1996	CB	ARG	364	30.047	13.843	22.818	1.00	0.00	RX0	C
ATOM	1997	CG	ARG	364	31.032	14.075	21.661	1.00	0.00	RX0	C
ATOM	1998	CD	ARG	364	30.806	13.177	20.439	1.00	0.00	RX0	C
ATOM	1999	NE	ARG	364	29.378	12.974	20.216	1.00	0.00	RX0	N
ATOM	2000	HE	ARG	364	28.934	12.193	20.672	0.00	0.00	RX0	H
ATOM	2001	CZ	ARG	364	28.549	13.795	19.549	1.00	0.00	RX0	C
ATOM	2002	NH1	ARG	364	29.021	14.779	18.788	1.00	0.00	RX0	N
ATOM	2003	HH11	ARG	364	28.403	15.398	18.302	0.00	0.00	RX0	H
ATOM	2004	HH12	ARG	364	30.008	14.893	18.692	0.00	0.00	RX0	H
ATOM	2005	NH2	ARG	364	27.247	13.590	19.673	1.00	0.00	RX0	N
ATOM	2006	HH21	ARG	364	26.537	14.125	19.215	0.00	0.00	RX0	H
ATOM	2007	HH22	ARG	364	26.963	12.809	20.254	0.00	0.00	RX0	H
ATOM	2008	C	ARG	364	31.469	12.319	24.223	1.00	0.00	RX0	C
ATOM	2009	O	ARG	364	32.264	11.465	23.831	1.00	0.00	RX0	O
ATOM	2010	N	HIS	365	31.645	13.058	25.316	1.00	0.00	RX0	N
ATOM	2011	H	HIS	365	30.917	13.697	25.579	0.00	0.00	RX0	H

ATOM	2012	CA	HIS	365	32.879	13.027	26.115	1.00	0.00	RX0	C
ATOM	2013	CB	HIS	365	32.810	14.043	27.257	1.00	0.00	RX0	C
ATOM	2014	CG	HIS	365	34.161	14.168	27.922	1.00	0.00	RX0	C
ATOM	2015	ND1	HIS	365	35.113	15.021	27.503	1.00	0.00	RX0	N
ATOM	2016	HD1	HIS	365	35.041	15.654	26.759	0.00	0.00	RX0	H
ATOM	2017	CD2	HIS	365	34.643	13.464	29.030	1.00	0.00	RX0	C
ATOM	2018	NE2	HIS	365	35.903	13.905	29.272	1.00	0.00	RX0	N
ATOM	2019	CE1	HIS	365	36.191	14.862	28.335	1.00	0.00	RX0	C
ATOM	2020	C	HIS	365	33.175	11.617	26.649	1.00	0.00	RX0	C
ATOM	2021	O	HIS	365	34.274	11.105	26.425	1.00	0.00	RX0	O
ATOM	2022	N	MET	366	32.138	10.946	27.137	1.00	0.00	RX0	N
ATOM	2023	H	MET	366	31.253	11.413	27.198	0.00	0.00	RX0	H
ATOM	2024	CA	MET	366	32.267	9.572	27.662	1.00	0.00	RX0	C
ATOM	2025	CB	MET	366	31.014	9.141	28.426	1.00	0.00	RX0	C
ATOM	2026	CG	MET	366	30.714	10.014	29.643	1.00	0.00	RX0	C
ATOM	2027	SD	MET	366	29.363	9.368	30.641	1.00	0.00	RX0	S
ATOM	2028	CE	MET	366	28.099	9.320	29.363	1.00	0.00	RX0	C
ATOM	2029	C	MET	366	32.567	8.557	26.555	1.00	0.00	RX0	C
ATOM	2030	O	MET	366	33.398	7.674	26.741	1.00	0.00	RX0	O
ATOM	2031	N	SER	367	31.985	8.782	25.378	1.00	0.00	RX0	N
ATOM	2032	H	SER	367	31.318	9.523	25.275	0.00	0.00	RX0	H
ATOM	2033	CA	SER	367	32.231	7.934	24.196	1.00	0.00	RX0	C
ATOM	2034	CB	SER	367	31.256	8.430	23.128	1.00	0.00	RX0	C
ATOM	2035	OG	SER	367	30.643	7.364	22.401	1.00	0.00	RX0	O
ATOM	2036	HG	SER	367	30.109	7.806	21.750	0.00	0.00	RX0	H
ATOM	2037	C	SER	367	33.681	8.046	23.711	1.00	0.00	RX0	C
ATOM	2038	O	SER	367	34.361	7.038	23.567	1.00	0.00	RX0	O
ATOM	2039	N	ASN	368	34.193	9.278	23.688	1.00	0.00	RX0	N
ATOM	2040	H	ASN	368	33.584	10.046	23.895	0.00	0.00	RX0	H
ATOM	2041	CA	ASN	368	35.583	9.549	23.266	1.00	0.00	RX0	C
ATOM	2042	CB	ASN	368	35.870	11.050	23.183	1.00	0.00	RX0	C
ATOM	2043	CG	ASN	368	35.107	11.701	22.051	1.00	0.00	RX0	C
ATOM	2044	OD1	ASN	368	34.740	11.089	21.058	1.00	0.00	RX0	O
ATOM	2045	ND2	ASN	368	34.871	13.005	22.249	1.00	0.00	RX0	N
ATOM	2046	HD21	ASN	368	35.178	13.486	23.069	0.00	0.00	RX0	H
ATOM	2047	HD22	ASN	368	34.366	13.476	21.529	0.00	0.00	RX0	H
ATOM	2048	C	ASN	368	36.615	8.948	24.226	1.00	0.00	RX0	C
ATOM	2049	O	ASN	368	37.514	8.215	23.806	1.00	0.00	RX0	O
ATOM	2050	N	LYS	369	36.353	9.119	25.515	1.00	0.00	RX0	N
ATOM	2051	H	LYS	369	35.552	9.667	25.769	0.00	0.00	RX0	H
ATOM	2052	CA	LYS	369	37.205	8.554	26.580	1.00	0.00	RX0	C
ATOM	2053	CB	LYS	369	36.859	9.102	27.968	1.00	0.00	RX0	C
ATOM	2054	CG	LYS	369	37.002	10.617	28.123	1.00	0.00	RX0	C
ATOM	2055	CD	LYS	369	38.343	11.155	27.630	1.00	0.00	RX0	C
ATOM	2056	CE	LYS	369	39.564	10.553	28.332	1.00	0.00	RX0	C
ATOM	2057	NZ	LYS	369	40.741	10.893	27.524	1.00	0.00	RX0	N
ATOM	2058	HZ1	LYS	369	41.442	10.118	27.497	0.00	0.00	RX0	H
ATOM	2059	HZ2	LYS	369	40.467	10.902	26.514	0.00	0.00	RX0	H
ATOM	2060	HZ3	LYS	369	41.192	11.797	27.744	0.00	0.00	RX0	H
ATOM	2061	C	LYS	369	37.117	7.021	26.620	1.00	0.00	RX0	C
ATOM	2062	O	LYS	369	38.123	6.337	26.771	1.00	0.00	RX0	O
ATOM	2063	N	GLY	370	35.908	6.527	26.299	1.00	0.00	RX0	N
ATOM	2064	H	GLY	370	35.157	7.164	26.123	0.00	0.00	RX0	H
ATOM	2065	CA	GLY	370	35.597	5.090	26.225	1.00	0.00	RX0	C
ATOM	2066	C	GLY	370	36.324	4.429	25.048	1.00	0.00	RX0	C
ATOM	2067	O	GLY	370	36.946	3.386	25.214	1.00	0.00	RX0	O
ATOM	2068	N	MET	371	36.380	5.143	23.928	1.00	0.00	RX0	N
ATOM	2069	H	MET	371	35.913	6.027	23.905	0.00	0.00	RX0	H
ATOM	2070	CA	MET	371	37.103	4.709	22.719	1.00	0.00	RX0	C
ATOM	2071	CB	MET	371	36.776	5.585	21.507	1.00	0.00	RX0	C
ATOM	2072	CG	MET	371	35.342	5.410	21.004	1.00	0.00	RX0	C

ATOM	2073	SD	MET	371	34.976	3.723	20.493	1.00	0.00	RX0	S
ATOM	2074	CE	MET	371	35.997	3.679	19.011	1.00	0.00	RX0	C
ATOM	2075	C	MET	371	38.619	4.671	22.937	1.00	0.00	RX0	C
ATOM	2076	O	MET	371	39.258	3.669	22.631	1.00	0.00	RX0	O
ATOM	2077	N	GLU	372	39.117	5.692	23.643	1.00	0.00	RX0	N
ATOM	2078	H	GLU	372	38.530	6.479	23.845	0.00	0.00	RX0	H
ATOM	2079	CA	GLU	372	40.525	5.740	24.082	1.00	0.00	RX0	C
ATOM	2080	CB	GLU	372	40.827	7.026	24.847	1.00	0.00	RX0	C
ATOM	2081	CG	GLU	372	40.690	8.316	24.039	1.00	0.00	RX0	C
ATOM	2082	CD	GLU	372	40.918	9.477	24.984	1.00	0.00	RX0	C
ATOM	2083	OE1	GLU	372	40.218	10.484	24.895	1.00	0.00	RX0	O
ATOM	2084	OE2	GLU	372	41.767	9.370	25.866	1.00	0.00	RX0	O
ATOM	2085	C	GLU	372	40.884	4.541	24.968	1.00	0.00	RX0	C
ATOM	2086	O	GLU	372	41.865	3.853	24.723	1.00	0.00	RX0	O
ATOM	2087	N	HIS	373	40.006	4.291	25.945	1.00	0.00	RX0	N
ATOM	2088	H	HIS	373	39.223	4.906	26.041	0.00	0.00	RX0	H
ATOM	2089	CA	HIS	373	40.194	3.212	26.919	1.00	0.00	RX0	C
ATOM	2090	CB	HIS	373	39.267	3.433	28.126	1.00	0.00	RX0	C
ATOM	2091	CG	HIS	373	38.363	2.257	28.419	1.00	0.00	RX0	C
ATOM	2092	ND1	HIS	373	37.244	1.981	27.723	1.00	0.00	RX0	N
ATOM	2093	HD1	HIS	373	36.917	2.464	26.932	0.00	0.00	RX0	H
ATOM	2094	CD2	HIS	373	38.493	1.320	29.450	1.00	0.00	RX0	C
ATOM	2095	NE2	HIS	373	37.433	0.477	29.367	1.00	0.00	RX0	N
ATOM	2096	CE1	HIS	373	36.666	0.885	28.307	1.00	0.00	RX0	C
ATOM	2097	C	HIS	373	40.078	1.832	26.262	1.00	0.00	RX0	C
ATOM	2098	O	HIS	373	40.934	1.001	26.470	1.00	0.00	RX0	O
ATOM	2099	N	LEU	374	39.111	1.679	25.351	1.00	0.00	RX0	N
ATOM	2100	H	LEU	374	38.556	2.473	25.117	0.00	0.00	RX0	H
ATOM	2101	CA	LEU	374	38.919	0.408	24.629	1.00	0.00	RX0	C
ATOM	2102	CB	LEU	374	37.666	0.455	23.753	1.00	0.00	RX0	C
ATOM	2103	CG	LEU	374	36.361	0.297	24.531	1.00	0.00	RX0	C
ATOM	2104	CD1	LEU	374	35.143	0.607	23.659	1.00	0.00	RX0	C
ATOM	2105	CD2	LEU	374	36.264	-1.079	25.190	1.00	0.00	RX0	C
ATOM	2106	C	LEU	374	40.113	0.051	23.744	1.00	0.00	RX0	C
ATOM	2107	O	LEU	374	40.555	-1.109	23.754	1.00	0.00	RX0	O
ATOM	2108	N	TYR	375	40.664	1.068	23.103	1.00	0.00	RX0	N
ATOM	2109	H	TYR	375	40.303	1.989	23.271	0.00	0.00	RX0	H
ATOM	2110	CA	TYR	375	41.854	0.983	22.222	1.00	0.00	RX0	C
ATOM	2111	CB	TYR	375	41.908	2.152	21.240	1.00	0.00	RX0	C
ATOM	2112	CG	TYR	375	41.057	1.769	20.051	1.00	0.00	RX0	C
ATOM	2113	CD1	TYR	375	41.144	0.476	19.546	1.00	0.00	RX0	C
ATOM	2114	CE1	TYR	375	40.379	0.097	18.450	1.00	0.00	RX0	C
ATOM	2115	CD2	TYR	375	40.195	2.686	19.458	1.00	0.00	RX0	C
ATOM	2116	CE2	TYR	375	39.430	2.305	18.359	1.00	0.00	RX0	C
ATOM	2117	CZ	TYR	375	39.522	1.012	17.851	1.00	0.00	RX0	C
ATOM	2118	OH	TYR	375	38.770	0.627	16.755	1.00	0.00	RX0	O
ATOM	2119	HH	TYR	375	38.195	1.342	16.505	0.00	0.00	RX0	H
ATOM	2120	C	TYR	375	43.165	0.750	22.977	1.00	0.00	RX0	C
ATOM	2121	O	TYR	375	44.176	1.452	22.753	1.00	0.00	RX0	O
ATOM	2122	N	SER	376	43.162	-0.226	23.845	1.00	0.00	RX0	N
ATOM	2123	H	SER	376	42.296	-0.612	24.147	0.00	0.00	RX0	H
ATOM	2124	CA	SER	376	44.345	-0.646	24.634	1.00	0.00	RX0	C
ATOM	2125	CB	SER	376	44.674	0.412	25.699	1.00	0.00	RX0	C
ATOM	2126	OG	SER	376	44.136	1.694	25.358	1.00	0.00	RX0	O
ATOM	2127	HG	SER	376	44.472	1.926	24.498	0.00	0.00	RX0	H
ATOM	2128	C	SER	376	44.183	-1.992	25.350	1.00	0.00	RX0	C
ATOM	2129	O	SER	376	44.985	-2.369	26.199	1.00	0.00	RX0	O
ATOM	2130	N	MET	377	43.156	-2.746	24.938	1.00	0.00	RX0	N
ATOM	2131	H	MET	377	42.554	-2.426	24.205	0.00	0.00	RX0	H
ATOM	2132	CA	MET	377	42.915	-4.112	25.422	1.00	0.00	RX0	C
ATOM	2133	CB	MET	377	41.735	-4.071	26.400	1.00	0.00	RX0	C

ATOM	2134	CG	MET	377	42.039	-3.410	27.744	1.00	0.00	RX0	C
ATOM	2135	SD	MET	377	40.551	-3.246	28.739	1.00	0.00	RX0	S
ATOM	2136	CE	MET	377	39.729	-2.018	27.709	1.00	0.00	RX0	C
ATOM	2137	C	MET	377	42.647	-5.080	24.257	1.00	0.00	RX0	C
ATOM	2138	O	MET	377	43.092	-4.854	23.130	1.00	0.00	RX0	O
ATOM	2139	N	LYS	378	41.856	-6.107	24.527	1.00	0.00	RX0	N
ATOM	2140	H	LYS	378	41.482	-6.207	25.443	0.00	0.00	RX0	H
ATOM	2141	CA	LYS	378	41.570	-7.191	23.576	1.00	0.00	RX0	C
ATOM	2142	CB	LYS	378	41.440	-8.519	24.363	1.00	0.00	RX0	C
ATOM	2143	CG	LYS	378	40.449	-8.517	25.553	1.00	0.00	RX0	C
ATOM	2144	CD	LYS	378	40.402	-9.806	26.399	1.00	0.00	RX0	C
ATOM	2145	CE	LYS	378	39.446	-9.727	27.610	1.00	0.00	RX0	C
ATOM	2146	NZ	LYS	378	39.478	-10.969	28.411	1.00	0.00	RX0	N
ATOM	2147	HZ1	LYS	378	38.889	-10.897	29.275	0.00	0.00	RX0	H
ATOM	2148	HZ2	LYS	378	39.128	-11.762	27.838	0.00	0.00	RX0	H
ATOM	2149	HZ3	LYS	378	40.455	-11.171	28.700	0.00	0.00	RX0	H
ATOM	2150	C	LYS	378	40.316	-6.920	22.720	1.00	0.00	RX0	C
ATOM	2151	O	LYS	378	40.245	-7.352	21.611	1.00	0.00	RX0	O
ATOM	2152	N	CYS	379	39.320	-6.266	23.410	1.00	0.00	RX0	N
ATOM	2153	H	CYS	379	39.562	-5.893	24.300	0.00	0.00	RX0	H
ATOM	2154	CA	CYS	379	37.939	-6.053	22.985	1.00	0.00	RX0	C
ATOM	2155	CB	CYS	379	37.607	-4.561	23.107	1.00	0.00	RX0	C
ATOM	2156	SG	CYS	379	35.833	-4.201	23.140	1.00	0.00	RX0	S
ATOM	2157	C	CYS	379	37.599	-6.654	21.614	1.00	0.00	RX0	C
ATOM	2158	O	CYS	379	37.115	-7.773	21.559	1.00	0.00	RX0	O
ATOM	2159	N	LYS	380	38.012	-5.930	20.563	1.00	0.00	RX0	N
ATOM	2160	H	LYS	380	38.389	-5.013	20.698	0.00	0.00	RX0	H
ATOM	2161	CA	LYS	380	37.949	-6.383	19.165	1.00	0.00	RX0	C
ATOM	2162	CB	LYS	380	36.474	-6.757	18.914	1.00	0.00	RX0	C
ATOM	2163	CG	LYS	380	35.902	-7.187	17.560	1.00	0.00	RX0	C
ATOM	2164	CD	LYS	380	34.388	-7.372	17.756	1.00	0.00	RX0	C
ATOM	2165	CE	LYS	380	33.572	-7.762	16.520	1.00	0.00	RX0	C
ATOM	2166	NZ	LYS	380	32.154	-7.853	16.894	1.00	0.00	RX0	N
ATOM	2167	HZ1	LYS	380	31.511	-8.103	16.124	0.00	0.00	RX0	H
ATOM	2168	HZ2	LYS	380	31.964	-8.548	17.652	0.00	0.00	RX0	H
ATOM	2169	HZ3	LYS	380	31.782	-6.984	17.337	0.00	0.00	RX0	H
ATOM	2170	C	LYS	380	38.311	-5.215	18.248	1.00	0.00	RX0	C
ATOM	2171	O	LYS	380	37.971	-4.057	18.518	1.00	0.00	RX0	O
ATOM	2172	N	ASN	381	38.881	-5.592	17.123	1.00	0.00	RX0	N
ATOM	2173	H	ASN	381	39.164	-6.549	17.098	0.00	0.00	RX0	H
ATOM	2174	CA	ASN	381	39.208	-4.700	15.996	1.00	0.00	RX0	C
ATOM	2175	CB	ASN	381	40.305	-3.724	16.478	1.00	0.00	RX0	C
ATOM	2176	CG	ASN	381	40.750	-2.703	15.448	1.00	0.00	RX0	C
ATOM	2177	OD1	ASN	381	41.858	-2.780	14.929	1.00	0.00	RX0	O
ATOM	2178	ND2	ASN	381	39.881	-1.719	15.205	1.00	0.00	RX0	N
ATOM	2179	HD21	ASN	381	39.043	-1.585	15.738	0.00	0.00	RX0	H
ATOM	2180	HD22	ASN	381	40.072	-1.049	14.491	0.00	0.00	RX0	H
ATOM	2181	C	ASN	381	39.620	-5.523	14.759	1.00	0.00	RX0	C
ATOM	2182	O	ASN	381	40.286	-5.062	13.834	1.00	0.00	RX0	O
ATOM	2183	N	VAL	382	39.176	-6.776	14.737	1.00	0.00	RX0	N
ATOM	2184	H	VAL	382	38.462	-7.027	15.385	0.00	0.00	RX0	H
ATOM	2185	CA	VAL	382	39.338	-7.659	13.572	1.00	0.00	RX0	C
ATOM	2186	CB	VAL	382	39.232	-9.097	14.099	1.00	0.00	RX0	C
ATOM	2187	CG1	VAL	382	38.929	-10.147	13.033	1.00	0.00	RX0	C
ATOM	2188	CG2	VAL	382	40.493	-9.436	14.898	1.00	0.00	RX0	C
ATOM	2189	C	VAL	382	38.195	-7.277	12.635	1.00	0.00	RX0	C
ATOM	2190	O	VAL	382	37.049	-7.276	13.059	1.00	0.00	RX0	O
ATOM	2191	N	VAL	383	38.551	-7.063	11.363	1.00	0.00	RX0	N
ATOM	2192	H	VAL	383	39.520	-7.146	11.128	0.00	0.00	RX0	H
ATOM	2193	CA	VAL	383	37.600	-6.539	10.366	1.00	0.00	RX0	C
ATOM	2194	CB	VAL	383	36.422	-7.489	10.081	1.00	0.00	RX0	C

ATOM	2195	CG1	VAL	383	35.399	-6.889	9.114	1.00	0.00	RX0	C
ATOM	2196	CG2	VAL	383	36.914	-8.840	9.576	1.00	0.00	RX0	C
ATOM	2197	C	VAL	383	37.102	-5.173	10.877	1.00	0.00	RX0	C
ATOM	2198	O	VAL	383	36.355	-5.097	11.860	1.00	0.00	RX0	O
ATOM	2199	N	PRO	384	37.530	-4.088	10.236	1.00	0.00	RX0	N
ATOM	2200	CD	PRO	384	38.441	-4.081	9.095	1.00	0.00	RX0	C
ATOM	2201	CA	PRO	384	37.142	-2.731	10.663	1.00	0.00	RX0	C
ATOM	2202	CB	PRO	384	37.665	-1.867	9.512	1.00	0.00	RX0	C
ATOM	2203	CG	PRO	384	38.872	-2.627	8.965	1.00	0.00	RX0	C
ATOM	2204	C	PRO	384	35.624	-2.628	10.828	1.00	0.00	RX0	C
ATOM	2205	O	PRO	384	34.847	-3.282	10.112	1.00	0.00	RX0	O
ATOM	2206	N	LEU	385	35.228	-1.769	11.750	1.00	0.00	RX0	N
ATOM	2207	H	LEU	385	35.920	-1.205	12.194	0.00	0.00	RX0	H
ATOM	2208	CA	LEU	385	33.812	-1.621	12.127	1.00	0.00	RX0	C
ATOM	2209	CB	LEU	385	33.676	-0.666	13.313	1.00	0.00	RX0	C
ATOM	2210	CG	LEU	385	32.278	-0.695	13.929	1.00	0.00	RX0	C
ATOM	2211	CD1	LEU	385	31.892	-2.095	14.410	1.00	0.00	RX0	C
ATOM	2212	CD2	LEU	385	32.125	0.350	15.030	1.00	0.00	RX0	C
ATOM	2213	C	LEU	385	32.896	-1.213	10.964	1.00	0.00	RX0	C
ATOM	2214	O	LEU	385	31.809	-1.761	10.815	1.00	0.00	RX0	O
ATOM	2215	N	TYR	386	33.449	-0.406	10.055	1.00	0.00	RX0	N
ATOM	2216	H	TYR	386	34.232	0.136	10.363	0.00	0.00	RX0	H
ATOM	2217	CA	TYR	386	32.718	0.069	8.867	1.00	0.00	RX0	C
ATOM	2218	CB	TYR	386	33.636	0.933	7.988	1.00	0.00	RX0	C
ATOM	2219	CG	TYR	386	32.894	1.687	6.900	1.00	0.00	RX0	C
ATOM	2220	CD1	TYR	386	32.700	3.076	7.048	1.00	0.00	RX0	C
ATOM	2221	CE1	TYR	386	32.063	3.793	6.019	1.00	0.00	RX0	C
ATOM	2222	CD2	TYR	386	32.439	0.996	5.756	1.00	0.00	RX0	C
ATOM	2223	CE2	TYR	386	31.803	1.711	4.728	1.00	0.00	RX0	C
ATOM	2224	CZ	TYR	386	31.637	3.103	4.865	1.00	0.00	RX0	C
ATOM	2225	OH	TYR	386	31.047	3.815	3.839	1.00	0.00	RX0	O
ATOM	2226	HH	TYR	386	31.313	3.432	2.998	0.00	0.00	RX0	H
ATOM	2227	C	TYR	386	32.144	-1.103	8.049	1.00	0.00	RX0	C
ATOM	2228	O	TYR	386	30.957	-1.141	7.757	1.00	0.00	RX0	O
ATOM	2229	N	ASP	387	32.969	-2.141	7.903	1.00	0.00	RX0	N
ATOM	2230	H	ASP	387	33.929	-2.064	8.180	0.00	0.00	RX0	H
ATOM	2231	CA	ASP	387	32.619	-3.323	7.093	1.00	0.00	RX0	C
ATOM	2232	CB	ASP	387	33.843	-4.203	6.792	1.00	0.00	RX0	C
ATOM	2233	CG	ASP	387	35.155	-3.463	6.546	1.00	0.00	RX0	C
ATOM	2234	OD1	ASP	387	35.159	-2.311	6.115	1.00	0.00	RX0	O
ATOM	2235	OD2	ASP	387	36.198	-4.063	6.789	1.00	0.00	RX0	O
ATOM	2236	C	ASP	387	31.563	-4.207	7.765	1.00	0.00	RX0	C
ATOM	2237	O	ASP	387	30.619	-4.649	7.114	1.00	0.00	RX0	O
ATOM	2238	N	LEU	388	31.677	-4.332	9.088	1.00	0.00	RX0	N
ATOM	2239	H	LEU	388	32.453	-3.868	9.516	0.00	0.00	RX0	H
ATOM	2240	CA	LEU	388	30.691	-5.072	9.891	1.00	0.00	RX0	C
ATOM	2241	CB	LEU	388	31.207	-5.217	11.324	1.00	0.00	RX0	C
ATOM	2242	CG	LEU	388	30.284	-6.048	12.217	1.00	0.00	RX0	C
ATOM	2243	CD1	LEU	388	30.091	-7.466	11.678	1.00	0.00	RX0	C
ATOM	2244	CD2	LEU	388	30.751	-6.043	13.673	1.00	0.00	RX0	C
ATOM	2245	C	LEU	388	29.315	-4.386	9.871	1.00	0.00	RX0	C
ATOM	2246	O	LEU	388	28.299	-5.023	9.588	1.00	0.00	RX0	O
ATOM	2247	N	LEU	389	29.339	-3.070	10.049	1.00	0.00	RX0	N
ATOM	2248	H	LEU	389	30.229	-2.627	10.177	0.00	0.00	RX0	H
ATOM	2249	CA	LEU	389	28.129	-2.229	9.970	1.00	0.00	RX0	C
ATOM	2250	CB	LEU	389	28.428	-0.784	10.353	1.00	0.00	RX0	C
ATOM	2251	CG	LEU	389	28.867	-0.623	11.805	1.00	0.00	RX0	C
ATOM	2252	CD1	LEU	389	29.263	0.819	12.091	1.00	0.00	RX0	C
ATOM	2253	CD2	LEU	389	27.813	-1.119	12.792	1.00	0.00	RX0	C
ATOM	2254	C	LEU	389	27.489	-2.268	8.583	1.00	0.00	RX0	C
ATOM	2255	O	LEU	389	26.284	-2.473	8.458	1.00	0.00	RX0	O

ATOM	2256	N	LEU	390	28.350	-2.246	7.565	1.00	0.00	RX0	N
ATOM	2257	H	LEU	390	29.327	-2.140	7.756	0.00	0.00	RX0	H
ATOM	2258	CA	LEU	390	27.929	-2.335	6.162	1.00	0.00	RX0	C
ATOM	2259	CB	LEU	390	29.105	-2.072	5.214	1.00	0.00	RX0	C
ATOM	2260	CG	LEU	390	28.675	-1.758	3.777	1.00	0.00	RX0	C
ATOM	2261	CD1	LEU	390	29.417	-0.547	3.213	1.00	0.00	RX0	C
ATOM	2262	CD2	LEU	390	28.779	-2.975	2.855	1.00	0.00	RX0	C
ATOM	2263	C	LEU	390	27.202	-3.653	5.862	1.00	0.00	RX0	C
ATOM	2264	O	LEU	390	26.123	-3.627	5.302	1.00	0.00	RX0	O
ATOM	2265	N	GLU	391	27.733	-4.754	6.404	1.00	0.00	RX0	N
ATOM	2266	H	GLU	391	28.636	-4.701	6.835	0.00	0.00	RX0	H
ATOM	2267	CA	GLU	391	27.124	-6.090	6.264	1.00	0.00	RX0	C
ATOM	2268	CB	GLU	391	28.002	-7.120	6.978	1.00	0.00	RX0	C
ATOM	2269	CG	GLU	391	29.233	-7.584	6.201	1.00	0.00	RX0	C
ATOM	2270	CD	GLU	391	28.899	-8.865	5.466	1.00	0.00	RX0	C
ATOM	2271	OE1	GLU	391	28.126	-9.658	6.000	1.00	0.00	RX0	O
ATOM	2272	OE2	GLU	391	29.422	-9.077	4.374	1.00	0.00	RX0	O
ATOM	2273	C	GLU	391	25.713	-6.159	6.867	1.00	0.00	RX0	C
ATOM	2274	O	GLU	391	24.778	-6.600	6.214	1.00	0.00	RX0	O
ATOM	2275	N	MET	392	25.589	-5.588	8.070	1.00	0.00	RX0	N
ATOM	2276	H	MET	392	26.420	-5.194	8.468	0.00	0.00	RX0	H
ATOM	2277	CA	MET	392	24.313	-5.555	8.807	1.00	0.00	RX0	C
ATOM	2278	CB	MET	392	24.544	-5.227	10.282	1.00	0.00	RX0	C
ATOM	2279	CG	MET	392	25.241	-6.382	11.005	1.00	0.00	RX0	C
ATOM	2280	SD	MET	392	25.407	-6.114	12.777	1.00	0.00	RX0	S
ATOM	2281	CE	MET	392	26.596	-4.768	12.713	1.00	0.00	RX0	C
ATOM	2282	C	MET	392	23.266	-4.630	8.173	1.00	0.00	RX0	C
ATOM	2283	O	MET	392	22.107	-5.006	8.028	1.00	0.00	RX0	O
ATOM	2284	N	LEU	393	23.721	-3.452	7.754	1.00	0.00	RX0	N
ATOM	2285	H	LEU	393	24.708	-3.286	7.782	0.00	0.00	RX0	H
ATOM	2286	CA	LEU	393	22.852	-2.457	7.099	1.00	0.00	RX0	C
ATOM	2287	CB	LEU	393	23.510	-1.081	7.151	1.00	0.00	RX0	C
ATOM	2288	CG	LEU	393	23.446	-0.450	8.539	1.00	0.00	RX0	C
ATOM	2289	CD1	LEU	393	24.508	0.629	8.719	1.00	0.00	RX0	C
ATOM	2290	CD2	LEU	393	22.044	0.072	8.852	1.00	0.00	RX0	C
ATOM	2291	C	LEU	393	22.462	-2.815	5.663	1.00	0.00	RX0	C
ATOM	2292	O	LEU	393	21.313	-2.601	5.257	1.00	0.00	RX0	O
ATOM	2293	N	ASP	394	23.400	-3.384	4.923	1.00	0.00	RX0	N
ATOM	2294	H	ASP	394	24.270	-3.668	5.321	0.00	0.00	RX0	H
ATOM	2295	CA	ASP	394	23.187	-3.768	3.516	1.00	0.00	RX0	C
ATOM	2296	CB	ASP	394	24.399	-3.672	2.589	1.00	0.00	RX0	C
ATOM	2297	CG	ASP	394	23.851	-3.238	1.235	1.00	0.00	RX0	C
ATOM	2298	OD1	ASP	394	22.676	-2.860	1.168	1.00	0.00	RX0	O
ATOM	2299	OD2	ASP	394	24.600	-3.246	0.257	1.00	0.00	RX0	O
ATOM	2300	C	ASP	394	22.492	-5.132	3.432	1.00	0.00	RX0	C
ATOM	2301	O	ASP	394	23.034	-6.108	2.888	1.00	0.00	RX0	O
ATOM	2302	N	ALA	395	21.256	-5.134	3.874	1.00	0.00	RX0	N
ATOM	2303	H	ALA	395	20.888	-4.230	4.093	0.00	0.00	RX0	H
ATOM	2304	CA	ALA	395	20.412	-6.338	3.880	1.00	0.00	RX0	C
ATOM	2305	CB	ALA	395	19.422	-6.296	5.045	1.00	0.00	RX0	C
ATOM	2306	C	ALA	395	19.639	-6.454	2.565	1.00	0.00	RX0	C
ATOM	2307	O	ALA	395	19.303	-5.463	1.911	1.00	0.00	RX0	O
ATOM	2308	N	HIS	396	19.441	-7.696	2.156	1.00	0.00	RX0	N
ATOM	2309	H	HIS	396	19.743	-8.406	2.788	0.00	0.00	RX0	H
ATOM	2310	CA	HIS	396	18.536	-7.999	1.035	1.00	0.00	RX0	C
ATOM	2311	CB	HIS	396	18.859	-9.403	0.506	1.00	0.00	RX0	C
ATOM	2312	CG	HIS	396	19.039	-10.343	1.677	1.00	0.00	RX0	C
ATOM	2313	ND1	HIS	396	20.240	-10.718	2.150	1.00	0.00	RX0	N
ATOM	2314	HD1	HIS	396	21.121	-10.448	1.807	0.00	0.00	RX0	H
ATOM	2315	CD2	HIS	396	18.048	-10.945	2.457	1.00	0.00	RX0	C
ATOM	2316	NE2	HIS	396	18.662	-11.687	3.405	1.00	0.00	RX0	N

```
ATOM   2317  CE1 HIS   396      20.015 -11.549   3.217  1.00  0.00      RX0  C
ATOM   2318  C   HIS   396      17.077  -7.909   1.523  1.00  0.00      RX0  C
ATOM   2319  O   HIS   396      16.821  -7.755   2.721  1.00  0.00      RX0  O
ATOM   2320  N   ARG   397      16.142  -8.174   0.629  1.00  0.00      RX0  N
ATOM   2321  H   ARG   397      16.391  -8.365  -0.320  0.00  0.00      RX0  H
ATOM   2322  CA  ARG   397      14.704  -8.151   0.982  1.00  0.00      RX0  C
ATOM   2323  CB  ARG   397      14.045  -6.996   0.217  1.00  0.00      RX0  C
ATOM   2324  CG  ARG   397      14.838  -5.736   0.594  1.00  0.00      RX0  C
ATOM   2325  CD  ARG   397      14.657  -4.458  -0.221  1.00  0.00      RX0  C
ATOM   2326  NE  ARG   397      15.750  -3.541   0.121  1.00  0.00      RX0  N
ATOM   2327  HE  ARG   397      16.646  -3.978   0.271  0.00  0.00      RX0  H
ATOM   2328  CZ  ARG   397      15.550  -2.193   0.213  1.00  0.00      RX0  C
ATOM   2329  NH1 ARG   397      14.314  -1.690  -0.014  1.00  0.00      RX0  N
ATOM   2330 HH11 ARG   397      14.108  -0.708   0.041  0.00  0.00      RX0  H
ATOM   2331 HH12 ARG   397      13.545  -2.291  -0.250  0.00  0.00      RX0  H
ATOM   2332  NH2 ARG   397      16.594  -1.390   0.532  1.00  0.00      RX0  N
ATOM   2333 HH21 ARG   397      16.514  -0.392   0.618  0.00  0.00      RX0  H
ATOM   2334 HH22 ARG   397      17.513  -1.768   0.701  0.00  0.00      RX0  H
ATOM   2335  C   ARG   397      14.082  -9.540   0.774  1.00  0.00      RX0  C
ATOM   2336  O   ARG   397      12.875  -9.706   0.613  1.00  0.00      RX0  O
ATOM   2337  N   LEU   398      14.938 -10.556   0.835  1.00  0.00      RX0  N
ATOM   2338  H   LEU   398      15.873 -10.363   1.121  0.00  0.00      RX0  H
ATOM   2339  CA  LEU   398      14.565 -11.951   0.542  1.00  0.00      RX0  C
ATOM   2340  CB  LEU   398      15.799 -12.797   0.229  1.00  0.00      RX0  C
ATOM   2341  CG  LEU   398      16.581 -12.273  -0.976  1.00  0.00      RX0  C
ATOM   2342  CD1 LEU   398      17.867 -13.069  -1.202  1.00  0.00      RX0  C
ATOM   2343  CD2 LEU   398      15.716 -12.205  -2.236  1.00  0.00      RX0  C
ATOM   2344  C   LEU   398      13.757 -12.584   1.680  1.00  0.00      RX0  C
ATOM   2345  O   LEU   398      12.772 -13.259   1.439  1.00  0.00      RX0  O
ATOM   2346  N   HIS   399      14.100 -12.169   2.904  1.00  0.00      RX0  N
ATOM   2347  H   HIS   399      14.842 -11.513   3.010  0.00  0.00      RX0  H
ATOM   2348  CA  HIS   399      13.392 -12.616   4.118  1.00  0.00      RX0  C
ATOM   2349  CB  HIS   399      14.354 -12.614   5.303  1.00  0.00      RX0  C
ATOM   2350  CG  HIS   399      15.405 -13.673   5.065  1.00  0.00      RX0  C
ATOM   2351  ND1 HIS   399      16.706 -13.418   4.815  1.00  0.00      RX0  N
ATOM   2352  HD1 HIS   399      17.151 -12.542   4.758  0.00  0.00      RX0  H
ATOM   2353  CD2 HIS   399      15.208 -15.058   5.046  1.00  0.00      RX0  C
ATOM   2354  NE2 HIS   399      16.406 -15.634   4.779  1.00  0.00      RX0  N
ATOM   2355  CE1 HIS   399      17.327 -14.628   4.638  1.00  0.00      RX0  C
ATOM   2356  C   HIS   399      12.131 -11.786   4.401  1.00  0.00      RX0  C
ATOM   2357  O   HIS   399      11.630 -11.743   5.524  1.00  0.00      RX0  O
ATOM   2358  N   ALA   400      11.619 -11.143   3.351  1.00  0.00      RX0  N
ATOM   2359  H   ALA   400      11.998 -11.314   2.443  0.00  0.00      RX0  H
ATOM   2360  CA  ALA   400      10.356 -10.385   3.402  1.00  0.00      RX0  C
ATOM   2361  CB  ALA   400      10.144  -9.552   2.138  1.00  0.00      RX0  C
ATOM   2362  C   ALA   400       9.147 -11.323   3.579  1.00  0.00      RX0  C
ATOM   2363  O   ALA   400       8.508 -11.220   4.647  1.00  0.00      RX0  O
TER
HEADER lig.000.01.pdb
ATOM      1  N   GLU    26      60.738  22.011  -9.048  1.00  0.00      LX0  N
ATOM      2  H   GLU    26      61.679  21.675  -9.095  1.00  0.00      LX0  H
ATOM      3  CA  GLU    26      59.774  21.589 -10.066  1.00  0.00      LX0  C
ATOM      4  CB  GLU    26      59.832  20.080 -10.291  1.00  0.00      LX0  C
ATOM      5  CG  GLU    26      61.197  19.528 -10.698  1.00  0.00      LX0  C
ATOM      6  CD  GLU    26      61.082  18.024 -10.846  1.00  0.00      LX0  C
ATOM      7  OE1 GLU    26      61.255  17.534 -11.960  1.00  0.00      LX0  O
ATOM      8  OE2 GLU    26      60.810  17.350  -9.849  1.00  0.00      LX0  O
ATOM      9  C   GLU    26      58.345  21.943  -9.709  1.00  0.00      LX0  C
ATOM     10  O   GLU    26      57.989  22.117  -8.543  1.00  0.00      LX0  O
ATOM     11  N   GLU    27      57.517  22.009 -10.763  1.00  0.00      LX0  N
ATOM     12  H   GLU    27      57.867  21.879 -11.689  1.00  0.00      LX0  H
```

ATOM	13	CA	GLU	27	56.095	22.223	-10.502	1.00	0.00	LX0	C
ATOM	14	CB	GLU	27	55.378	22.937	-11.658	1.00	0.00	LX0	C
ATOM	15	CG	GLU	27	56.127	24.102	-12.322	1.00	0.00	LX0	C
ATOM	16	CD	GLU	27	56.752	25.034	-11.299	1.00	0.00	LX0	C
ATOM	17	OE1	GLU	27	56.062	25.566	-10.433	1.00	0.00	LX0	O
ATOM	18	OE2	GLU	27	57.963	25.201	-11.335	1.00	0.00	LX0	O
ATOM	19	C	GLU	27	55.373	20.944	-10.136	1.00	0.00	LX0	C
ATOM	20	O	GLU	27	54.536	20.421	-10.861	1.00	0.00	LX0	O
ATOM	21	N	LYS	28	55.770	20.455	-8.954	1.00	0.00	LX0	N
ATOM	22	H	LYS	28	56.410	21.001	-8.416	1.00	0.00	LX0	H
ATOM	23	CA	LYS	28	55.200	19.217	-8.438	1.00	0.00	LX0	C
ATOM	24	CB	LYS	28	55.900	18.843	-7.130	1.00	0.00	LX0	C
ATOM	25	CG	LYS	28	57.432	18.888	-7.144	1.00	0.00	LX0	C
ATOM	26	CD	LYS	28	58.019	18.475	-5.791	1.00	0.00	LX0	C
ATOM	27	CE	LYS	28	59.539	18.645	-5.693	1.00	0.00	LX0	C
ATOM	28	NZ	LYS	28	60.015	17.957	-4.485	1.00	0.00	LX0	N
ATOM	29	HZ1	LYS	28	61.049	18.049	-4.393	1.00	0.00	LX0	H
ATOM	30	HZ2	LYS	28	59.549	18.326	-3.627	1.00	0.00	LX0	H
ATOM	31	HZ3	LYS	28	59.813	16.941	-4.583	1.00	0.00	LX0	H
ATOM	32	C	LYS	28	53.709	19.383	-8.215	1.00	0.00	LX0	C
ATOM	33	O	LYS	28	53.274	20.265	-7.481	1.00	0.00	LX0	O
ATOM	34	N	LYS	29	52.941	18.547	-8.919	1.00	0.00	LX0	N
ATOM	35	H	LYS	29	53.341	17.750	-9.379	1.00	0.00	LX0	H
ATOM	36	CA	LYS	29	51.509	18.829	-8.938	1.00	0.00	LX0	C
ATOM	37	CB	LYS	29	50.821	18.097	-10.094	1.00	0.00	LX0	C
ATOM	38	CG	LYS	29	51.635	18.197	-11.391	1.00	0.00	LX0	C
ATOM	39	CD	LYS	29	51.068	17.400	-12.569	1.00	0.00	LX0	C
ATOM	40	CE	LYS	29	50.723	15.940	-12.253	1.00	0.00	LX0	C
ATOM	41	NZ	LYS	29	51.867	15.186	-11.725	1.00	0.00	LX0	N
ATOM	42	HZ1	LYS	29	51.602	14.805	-10.786	1.00	0.00	LX0	H
ATOM	43	HZ2	LYS	29	52.107	14.361	-12.307	1.00	0.00	LX0	H
ATOM	44	HZ3	LYS	29	52.712	15.755	-11.497	1.00	0.00	LX0	H
ATOM	45	C	LYS	29	50.845	18.540	-7.608	1.00	0.00	LX0	C
ATOM	46	O	LYS	29	51.117	17.546	-6.943	1.00	0.00	LX0	O
ATOM	47	N	VAL	30	49.997	19.497	-7.230	1.00	0.00	LX0	N
ATOM	48	H	VAL	30	49.734	20.219	-7.865	1.00	0.00	LX0	H
ATOM	49	CA	VAL	30	49.432	19.415	-5.890	1.00	0.00	LX0	C
ATOM	50	CB	VAL	30	49.513	20.783	-5.196	1.00	0.00	LX0	C
ATOM	51	CG1	VAL	30	50.970	21.209	-5.009	1.00	0.00	LX0	C
ATOM	52	CG2	VAL	30	48.703	21.861	-5.926	1.00	0.00	LX0	C
ATOM	53	C	VAL	30	48.012	18.887	-5.892	1.00	0.00	LX0	C
ATOM	54	O	VAL	30	47.307	18.969	-6.891	1.00	0.00	LX0	O
ATOM	55	N	CYS	31	47.615	18.373	-4.725	1.00	0.00	LX0	N
ATOM	56	H	CYS	31	48.272	18.245	-3.979	1.00	0.00	LX0	H
ATOM	57	CA	CYS	31	46.214	17.993	-4.583	1.00	0.00	LX0	C
ATOM	58	CB	CYS	31	46.026	16.489	-4.780	1.00	0.00	LX0	C
ATOM	59	SG	CYS	31	46.817	15.508	-3.476	1.00	0.00	LX0	S
ATOM	60	C	CYS	31	45.679	18.415	-3.235	1.00	0.00	LX0	C
ATOM	61	O	CYS	31	46.433	18.694	-2.308	1.00	0.00	LX0	O
ATOM	62	N	GLN	32	44.341	18.443	-3.154	1.00	0.00	LX0	N
ATOM	63	H	GLN	32	43.808	18.189	-3.963	1.00	0.00	LX0	H
ATOM	64	CA	GLN	32	43.704	18.848	-1.901	1.00	0.00	LX0	C
ATOM	65	CB	GLN	32	42.180	19.000	-2.046	1.00	0.00	LX0	C
ATOM	66	CG	GLN	32	41.683	20.024	-3.079	1.00	0.00	LX0	C
ATOM	67	CD	GLN	32	41.484	19.393	-4.448	1.00	0.00	LX0	C
ATOM	68	OE1	GLN	32	42.268	18.587	-4.926	1.00	0.00	LX0	O
ATOM	69	NE2	GLN	32	40.376	19.791	-5.072	1.00	0.00	LX0	N
ATOM	70	HE21	GLN	32	39.743	20.448	-4.659	1.00	0.00	LX0	H
ATOM	71	HE22	GLN	32	40.187	19.418	-5.977	1.00	0.00	LX0	H
ATOM	72	C	GLN	32	44.023	17.913	-0.746	1.00	0.00	LX0	C
ATOM	73	O	GLN	32	44.412	18.329	0.336	1.00	0.00	LX0	O

ATOM	74	N	GLY	33	43.875	16.619	-1.050	1.00	0.00	LX0	N
ATOM	75	H	GLY	33	43.486	16.364	-1.933	1.00	0.00	LX0	H
ATOM	76	CA	GLY	33	44.194	15.634	-0.023	1.00	0.00	LX0	C
ATOM	77	C	GLY	33	42.978	15.117	0.714	1.00	0.00	LX0	C
ATOM	78	O	GLY	33	42.059	15.858	1.053	1.00	0.00	LX0	O
ATOM	79	N	THR	34	42.989	13.795	0.930	1.00	0.00	LX0	N
ATOM	80	H	THR	34	43.834	13.265	0.853	1.00	0.00	LX0	H
ATOM	81	CA	THR	34	41.899	13.220	1.709	1.00	0.00	LX0	C
ATOM	82	CB	THR	34	41.916	11.694	1.580	1.00	0.00	LX0	C
ATOM	83	OG1	THR	34	43.263	11.207	1.501	1.00	0.00	LX0	O
ATOM	84	HG1	THR	34	43.199	10.265	1.664	1.00	0.00	LX0	H
ATOM	85	CG2	THR	34	41.124	11.231	0.357	1.00	0.00	LX0	C
ATOM	86	C	THR	34	42.004	13.654	3.155	1.00	0.00	LX0	C
ATOM	87	O	THR	34	42.912	13.278	3.879	1.00	0.00	LX0	O
ATOM	88	N	SER	35	41.048	14.511	3.517	1.00	0.00	LX0	N
ATOM	89	H	SER	35	40.353	14.812	2.867	1.00	0.00	LX0	H
ATOM	90	CA	SER	35	41.174	15.121	4.833	1.00	0.00	LX0	C
ATOM	91	CB	SER	35	41.604	16.582	4.661	1.00	0.00	LX0	C
ATOM	92	OG	SER	35	42.693	16.664	3.724	1.00	0.00	LX0	O
ATOM	93	HG	SER	35	42.312	16.532	2.856	1.00	0.00	LX0	H
ATOM	94	C	SER	35	39.903	14.988	5.649	1.00	0.00	LX0	C
ATOM	95	O	SER	35	39.109	15.913	5.755	1.00	0.00	LX0	O
ATOM	96	N	ASN	36	39.723	13.775	6.186	1.00	0.00	LX0	N
ATOM	97	H	ASN	36	40.477	13.110	6.157	1.00	0.00	LX0	H
ATOM	98	CA	ASN	36	38.469	13.489	6.883	1.00	0.00	LX0	C
ATOM	99	CB	ASN	36	37.403	12.954	5.915	1.00	0.00	LX0	C
ATOM	100	CG	ASN	36	37.579	11.535	5.368	1.00	0.00	LX0	C
ATOM	101	OD1	ASN	36	36.647	11.004	4.784	1.00	0.00	LX0	O
ATOM	102	ND2	ASN	36	38.744	10.907	5.551	1.00	0.00	LX0	N
ATOM	103	HD21	ASN	36	39.584	11.257	5.967	1.00	0.00	LX0	H
ATOM	104	HD22	ASN	36	38.750	9.957	5.227	1.00	0.00	LX0	H
ATOM	105	C	ASN	36	38.586	12.660	8.152	1.00	0.00	LX0	C
ATOM	106	O	ASN	36	37.704	11.892	8.526	1.00	0.00	LX0	O
ATOM	107	N	LYS	37	39.748	12.878	8.787	1.00	0.00	LX0	N
ATOM	108	H	LYS	37	40.419	13.430	8.290	1.00	0.00	LX0	H
ATOM	109	CA	LYS	37	40.245	12.229	10.002	1.00	0.00	LX0	C
ATOM	110	CB	LYS	37	40.198	13.183	11.202	1.00	0.00	LX0	C
ATOM	111	CG	LYS	37	38.813	13.505	11.764	1.00	0.00	LX0	C
ATOM	112	CD	LYS	37	38.850	13.814	13.266	1.00	0.00	LX0	C
ATOM	113	CE	LYS	37	39.048	12.595	14.187	1.00	0.00	LX0	C
ATOM	114	NZ	LYS	37	40.400	12.024	14.121	1.00	0.00	LX0	N
ATOM	115	HZ1	LYS	37	40.648	11.618	15.049	1.00	0.00	LX0	H
ATOM	116	HZ2	LYS	37	40.528	11.279	13.398	1.00	0.00	LX0	H
ATOM	117	HZ3	LYS	37	41.139	12.756	14.038	1.00	0.00	LX0	H
ATOM	118	C	LYS	37	39.811	10.820	10.392	1.00	0.00	LX0	C
ATOM	119	O	LYS	37	39.699	10.541	11.585	1.00	0.00	LX0	O
ATOM	120	N	LEU	38	39.618	9.985	9.343	1.00	0.00	LX0	N
ATOM	121	H	LEU	38	39.849	10.399	8.458	1.00	0.00	LX0	H
ATOM	122	CA	LEU	38	39.290	8.547	9.361	1.00	0.00	LX0	C
ATOM	123	CB	LEU	38	38.640	8.024	10.653	1.00	0.00	LX0	C
ATOM	124	CG	LEU	38	38.780	6.513	10.855	1.00	0.00	LX0	C
ATOM	125	CD1	LEU	38	40.246	6.082	10.933	1.00	0.00	LX0	C
ATOM	126	CD2	LEU	38	37.979	6.027	12.062	1.00	0.00	LX0	C
ATOM	127	C	LEU	38	38.398	8.118	8.206	1.00	0.00	LX0	C
ATOM	128	O	LEU	38	38.615	7.102	7.558	1.00	0.00	LX0	O
ATOM	129	N	THR	39	37.363	8.938	8.003	1.00	0.00	LX0	N
ATOM	130	H	THR	39	37.296	9.782	8.536	1.00	0.00	LX0	H
ATOM	131	CA	THR	39	36.221	8.546	7.172	1.00	0.00	LX0	C
ATOM	132	CB	THR	39	35.287	9.767	7.122	1.00	0.00	LX0	C
ATOM	133	OG1	THR	39	35.267	10.388	8.417	1.00	0.00	LX0	O
ATOM	134	HG1	THR	39	35.833	11.155	8.356	1.00	0.00	LX0	H

ATOM	135	CG2	THR	39	33.867	9.481	6.624	1.00	0.00	LX0	C
ATOM	136	C	THR	39	36.530	7.947	5.790	1.00	0.00	LX0	C
ATOM	137	O	THR	39	37.549	8.223	5.165	1.00	0.00	LX0	O
ATOM	138	N	GLN	40	35.602	7.066	5.373	1.00	0.00	LX0	N
ATOM	139	H	GLN	40	34.776	6.946	5.921	1.00	0.00	LX0	H
ATOM	140	CA	GLN	40	35.669	6.439	4.053	1.00	0.00	LX0	C
ATOM	141	CB	GLN	40	35.245	4.970	4.195	1.00	0.00	LX0	C
ATOM	142	CG	GLN	40	35.345	4.107	2.929	1.00	0.00	LX0	C
ATOM	143	CD	GLN	40	34.716	2.738	3.129	1.00	0.00	LX0	C
ATOM	144	OE1	GLN	40	33.963	2.246	2.304	1.00	0.00	LX0	O
ATOM	145	NE2	GLN	40	35.056	2.116	4.260	1.00	0.00	LX0	N
ATOM	146	HE21	GLN	40	34.720	1.183	4.391	1.00	0.00	LX0	H
ATOM	147	HE22	GLN	40	35.633	2.517	4.966	1.00	0.00	LX0	H
ATOM	148	C	GLN	40	34.730	7.158	3.100	1.00	0.00	LX0	C
ATOM	149	O	GLN	40	33.649	7.567	3.495	1.00	0.00	LX0	O
ATOM	150	N	LEU	41	35.155	7.270	1.833	1.00	0.00	LX0	N
ATOM	151	H	LEU	41	36.059	6.935	1.581	1.00	0.00	LX0	H
ATOM	152	CA	LEU	41	34.161	7.676	0.840	1.00	0.00	LX0	C
ATOM	153	CB	LEU	41	34.792	8.503	-0.294	1.00	0.00	LX0	C
ATOM	154	CG	LEU	41	33.818	9.299	-1.190	1.00	0.00	LX0	C
ATOM	155	CD1	LEU	41	34.483	10.544	-1.776	1.00	0.00	LX0	C
ATOM	156	CD2	LEU	41	33.182	8.475	-2.314	1.00	0.00	LX0	C
ATOM	157	C	LEU	41	33.427	6.487	0.268	1.00	0.00	LX0	C
ATOM	158	O	LEU	41	33.995	5.693	-0.469	1.00	0.00	LX0	O
ATOM	159	N	GLY	42	32.131	6.451	0.594	1.00	0.00	LX0	N
ATOM	160	H	GLY	42	31.788	7.075	1.297	1.00	0.00	LX0	H
ATOM	161	CA	GLY	42	31.235	5.545	-0.119	1.00	0.00	LX0	C
ATOM	162	C	GLY	42	31.517	4.067	0.050	1.00	0.00	LX0	C
ATOM	163	O	GLY	42	31.522	3.515	1.145	1.00	0.00	LX0	O
ATOM	164	N	THR	43	31.711	3.468	-1.123	1.00	0.00	LX0	N
ATOM	165	H	THR	43	31.668	3.992	-1.972	1.00	0.00	LX0	H
ATOM	166	CA	THR	43	32.022	2.050	-1.228	1.00	0.00	LX0	C
ATOM	167	CB	THR	43	31.691	1.629	-2.668	1.00	0.00	LX0	C
ATOM	168	OG1	THR	43	30.822	2.599	-3.284	1.00	0.00	LX0	O
ATOM	169	HG1	THR	43	31.268	2.939	-4.067	1.00	0.00	LX0	H
ATOM	170	CG2	THR	43	31.083	0.228	-2.736	1.00	0.00	LX0	C
ATOM	171	C	THR	43	33.491	1.800	-0.912	1.00	0.00	LX0	C
ATOM	172	O	THR	43	34.312	2.705	-1.002	1.00	0.00	LX0	O
ATOM	173	N	PHE	44	33.823	0.539	-0.598	1.00	0.00	LX0	N
ATOM	174	H	PHE	44	33.136	-0.185	-0.557	1.00	0.00	LX0	H
ATOM	175	CA	PHE	44	35.247	0.252	-0.376	1.00	0.00	LX0	C
ATOM	176	CB	PHE	44	35.424	-1.203	0.062	1.00	0.00	LX0	C
ATOM	177	CG	PHE	44	34.543	-1.527	1.248	1.00	0.00	LX0	C
ATOM	178	CD1	PHE	44	34.765	-0.883	2.485	1.00	0.00	LX0	C
ATOM	179	CD2	PHE	44	33.508	-2.475	1.093	1.00	0.00	LX0	C
ATOM	180	CE1	PHE	44	33.933	-1.184	3.579	1.00	0.00	LX0	C
ATOM	181	CE2	PHE	44	32.677	-2.781	2.187	1.00	0.00	LX0	C
ATOM	182	CZ	PHE	44	32.897	-2.128	3.418	1.00	0.00	LX0	C
ATOM	183	C	PHE	44	36.101	0.558	-1.602	1.00	0.00	LX0	C
ATOM	184	O	PHE	44	37.105	1.269	-1.577	1.00	0.00	LX0	O
ATOM	185	N	GLU	45	35.550	0.061	-2.718	1.00	0.00	LX0	N
ATOM	186	H	GLU	45	34.848	-0.649	-2.623	1.00	0.00	LX0	H
ATOM	187	CA	GLU	45	36.005	0.429	-4.057	1.00	0.00	LX0	C
ATOM	188	CB	GLU	45	35.059	-0.127	-5.129	1.00	0.00	LX0	C
ATOM	189	CG	GLU	45	35.053	-1.650	-5.334	1.00	0.00	LX0	C
ATOM	190	CD	GLU	45	34.583	-2.405	-4.102	1.00	0.00	LX0	C
ATOM	191	OE1	GLU	45	33.640	-1.966	-3.443	1.00	0.00	LX0	O
ATOM	192	OE2	GLU	45	35.190	-3.423	-3.785	1.00	0.00	LX0	O
ATOM	193	C	GLU	45	36.120	1.927	-4.242	1.00	0.00	LX0	C
ATOM	194	O	GLU	45	37.110	2.441	-4.740	1.00	0.00	LX0	O
ATOM	195	N	ASP	46	35.069	2.619	-3.782	1.00	0.00	LX0	N

ATOM	196	H	ASP	46	34.310	2.145	-3.344	1.00	0.00	LX0	H
ATOM	197	CA	ASP	46	35.097	4.075	-3.880	1.00	0.00	LX0	C
ATOM	198	CB	ASP	46	33.731	4.719	-3.685	1.00	0.00	LX0	C
ATOM	199	CG	ASP	46	33.074	4.930	-5.027	1.00	0.00	LX0	C
ATOM	200	OD1	ASP	46	32.186	4.153	-5.359	1.00	0.00	LX0	O
ATOM	201	OD2	ASP	46	33.445	5.872	-5.730	1.00	0.00	LX0	O
ATOM	202	C	ASP	46	36.098	4.791	-3.009	1.00	0.00	LX0	C
ATOM	203	O	ASP	46	36.506	5.900	-3.333	1.00	0.00	LX0	O
ATOM	204	N	HIS	47	36.548	4.145	-1.922	1.00	0.00	LX0	N
ATOM	205	H	HIS	47	36.143	3.284	-1.604	1.00	0.00	LX0	H
ATOM	206	CA	HIS	47	37.658	4.837	-1.276	1.00	0.00	LX0	C
ATOM	207	CB	HIS	47	37.841	4.523	0.210	1.00	0.00	LX0	C
ATOM	208	CG	HIS	47	38.326	5.786	0.899	1.00	0.00	LX0	C
ATOM	209	ND1	HIS	47	38.888	5.820	2.119	1.00	0.00	LX0	N
ATOM	210	HD1	HIS	47	39.097	5.057	2.700	1.00	0.00	LX0	H
ATOM	211	CD2	HIS	47	38.261	7.100	0.419	1.00	0.00	LX0	C
ATOM	212	NE2	HIS	47	38.783	7.915	1.360	1.00	0.00	LX0	N
ATOM	213	CE1	HIS	47	39.173	7.128	2.413	1.00	0.00	LX0	C
ATOM	214	C	HIS	47	38.961	4.749	-2.040	1.00	0.00	LX0	C
ATOM	215	O	HIS	47	39.641	5.747	-2.242	1.00	0.00	LX0	O
ATOM	216	N	PHE	48	39.224	3.537	-2.562	1.00	0.00	LX0	N
ATOM	217	H	PHE	48	38.625	2.765	-2.329	1.00	0.00	LX0	H
ATOM	218	CA	PHE	48	40.294	3.424	-3.565	1.00	0.00	LX0	C
ATOM	219	CB	PHE	48	40.336	1.967	-4.072	1.00	0.00	LX0	C
ATOM	220	CG	PHE	48	40.742	1.824	-5.528	1.00	0.00	LX0	C
ATOM	221	CD1	PHE	48	42.060	2.128	-5.938	1.00	0.00	LX0	C
ATOM	222	CD2	PHE	48	39.771	1.399	-6.460	1.00	0.00	LX0	C
ATOM	223	CE1	PHE	48	42.401	2.034	-7.301	1.00	0.00	LX0	C
ATOM	224	CE2	PHE	48	40.111	1.301	-7.823	1.00	0.00	LX0	C
ATOM	225	CZ	PHE	48	41.421	1.629	-8.231	1.00	0.00	LX0	C
ATOM	226	C	PHE	48	40.148	4.451	-4.688	1.00	0.00	LX0	C
ATOM	227	O	PHE	48	41.060	5.175	-5.070	1.00	0.00	LX0	O
ATOM	228	N	LEU	49	38.907	4.506	-5.174	1.00	0.00	LX0	N
ATOM	229	H	LEU	49	38.220	3.876	-4.820	1.00	0.00	LX0	H
ATOM	230	CA	LEU	49	38.608	5.419	-6.262	1.00	0.00	LX0	C
ATOM	231	CB	LEU	49	37.253	5.067	-6.876	1.00	0.00	LX0	C
ATOM	232	CG	LEU	49	37.097	5.497	-8.336	1.00	0.00	LX0	C
ATOM	233	CD1	LEU	49	38.227	4.962	-9.219	1.00	0.00	LX0	C
ATOM	234	CD2	LEU	49	35.726	5.106	-8.888	1.00	0.00	LX0	C
ATOM	235	C	LEU	49	38.720	6.881	-5.880	1.00	0.00	LX0	C
ATOM	236	O	LEU	49	39.001	7.726	-6.711	1.00	0.00	LX0	O
ATOM	237	N	SER	50	38.538	7.167	-4.587	1.00	0.00	LX0	N
ATOM	238	H	SER	50	38.297	6.449	-3.933	1.00	0.00	LX0	H
ATOM	239	CA	SER	50	38.816	8.532	-4.148	1.00	0.00	LX0	C
ATOM	240	CB	SER	50	38.119	8.829	-2.829	1.00	0.00	LX0	C
ATOM	241	OG	SER	50	36.742	8.469	-2.976	1.00	0.00	LX0	O
ATOM	242	HG	SER	50	36.662	7.600	-2.578	1.00	0.00	LX0	H
ATOM	243	C	SER	50	40.288	8.853	-4.074	1.00	0.00	LX0	C
ATOM	244	O	SER	50	40.728	9.930	-4.451	1.00	0.00	LX0	O
ATOM	245	N	LEU	51	41.048	7.840	-3.640	1.00	0.00	LX0	N
ATOM	246	H	LEU	51	40.642	6.986	-3.312	1.00	0.00	LX0	H
ATOM	247	CA	LEU	51	42.498	7.988	-3.723	1.00	0.00	LX0	C
ATOM	248	CB	LEU	51	43.199	6.800	-3.063	1.00	0.00	LX0	C
ATOM	249	CG	LEU	51	43.544	6.983	-1.580	1.00	0.00	LX0	C
ATOM	250	CD1	LEU	51	42.325	7.100	-0.660	1.00	0.00	LX0	C
ATOM	251	CD2	LEU	51	44.493	5.881	-1.116	1.00	0.00	LX0	C
ATOM	252	C	LEU	51	42.985	8.210	-5.148	1.00	0.00	LX0	C
ATOM	253	O	LEU	51	43.910	8.964	-5.406	1.00	0.00	LX0	O
ATOM	254	N	GLN	52	42.270	7.577	-6.089	1.00	0.00	LX0	N
ATOM	255	H	GLN	52	41.601	6.885	-5.814	1.00	0.00	LX0	H
ATOM	256	CA	GLN	52	42.525	7.929	-7.484	1.00	0.00	LX0	C

ATOM	257	CB	GLN	52	41.847	6.912	-8.402	1.00	0.00	LX0	C
ATOM	258	CG	GLN	52	42.368	6.950	-9.839	1.00	0.00	LX0	C
ATOM	259	CD	GLN	52	41.615	5.929	-10.662	1.00	0.00	LX0	C
ATOM	260	OE1	GLN	52	40.742	6.251	-11.452	1.00	0.00	LX0	O
ATOM	261	NE2	GLN	52	41.987	4.665	-10.442	1.00	0.00	LX0	N
ATOM	262	HE21	GLN	52	42.701	4.419	-9.789	1.00	0.00	LX0	H
ATOM	263	HE22	GLN	52	41.516	3.955	-10.963	1.00	0.00	LX0	H
ATOM	264	C	GLN	52	42.135	9.362	-7.835	1.00	0.00	LX0	C
ATOM	265	O	GLN	52	42.951	10.173	-8.246	1.00	0.00	LX0	O
ATOM	266	N	ARG	53	40.842	9.647	-7.613	1.00	0.00	LX0	N
ATOM	267	H	ARG	53	40.260	8.912	-7.278	1.00	0.00	LX0	H
ATOM	268	CA	ARG	53	40.266	10.962	-7.915	1.00	0.00	LX0	C
ATOM	269	CB	ARG	53	38.785	11.001	-7.487	1.00	0.00	LX0	C
ATOM	270	CG	ARG	53	37.869	10.089	-8.323	1.00	0.00	LX0	C
ATOM	271	CD	ARG	53	36.404	9.985	-7.854	1.00	0.00	LX0	C
ATOM	272	NE	ARG	53	36.265	9.327	-6.549	1.00	0.00	LX0	N
ATOM	273	HE	ARG	53	36.860	9.628	-5.803	1.00	0.00	LX0	H
ATOM	274	CZ	ARG	53	35.355	8.348	-6.321	1.00	0.00	LX0	C
ATOM	275	NH1	ARG	53	34.542	7.900	-7.272	1.00	0.00	LX0	N
ATOM	276	HH11	ARG	53	33.908	7.145	-7.039	1.00	0.00	LX0	H
ATOM	277	HH12	ARG	53	34.544	8.271	-8.197	1.00	0.00	LX0	H
ATOM	278	NH2	ARG	53	35.259	7.800	-5.121	1.00	0.00	LX0	N
ATOM	279	HH21	ARG	53	34.602	7.046	-4.974	1.00	0.00	LX0	H
ATOM	280	HH22	ARG	53	35.817	8.093	-4.340	1.00	0.00	LX0	H
ATOM	281	C	ARG	53	41.027	12.156	-7.345	1.00	0.00	LX0	C
ATOM	282	O	ARG	53	41.035	13.241	-7.908	1.00	0.00	LX0	O
ATOM	283	N	MET	54	41.668	11.898	-6.199	1.00	0.00	LX0	N
ATOM	284	H	MET	54	41.598	10.990	-5.788	1.00	0.00	LX0	H
ATOM	285	CA	MET	54	42.454	12.952	-5.571	1.00	0.00	LX0	C
ATOM	286	CB	MET	54	42.222	12.897	-4.059	1.00	0.00	LX0	C
ATOM	287	CG	MET	54	42.793	14.084	-3.285	1.00	0.00	LX0	C
ATOM	288	SD	MET	54	41.984	15.645	-3.664	1.00	0.00	LX0	S
ATOM	289	CE	MET	54	40.388	15.294	-2.908	1.00	0.00	LX0	C
ATOM	290	C	MET	54	43.940	12.906	-5.904	1.00	0.00	LX0	C
ATOM	291	O	MET	54	44.586	13.916	-6.142	1.00	0.00	LX0	O
ATOM	292	N	PHE	55	44.477	11.679	-5.873	1.00	0.00	LX0	N
ATOM	293	H	PHE	55	43.919	10.854	-5.794	1.00	0.00	LX0	H
ATOM	294	CA	PHE	55	45.936	11.592	-5.935	1.00	0.00	LX0	C
ATOM	295	CB	PHE	55	46.506	10.732	-4.799	1.00	0.00	LX0	C
ATOM	296	CG	PHE	55	45.960	11.159	-3.457	1.00	0.00	LX0	C
ATOM	297	CD1	PHE	55	46.398	12.368	-2.877	1.00	0.00	LX0	C
ATOM	298	CD2	PHE	55	45.015	10.337	-2.808	1.00	0.00	LX0	C
ATOM	299	CE1	PHE	55	45.867	12.770	-1.637	1.00	0.00	LX0	C
ATOM	300	CE2	PHE	55	44.482	10.737	-1.569	1.00	0.00	LX0	C
ATOM	301	CZ	PHE	55	44.910	11.954	-1.002	1.00	0.00	LX0	C
ATOM	302	C	PHE	55	46.503	11.099	-7.249	1.00	0.00	LX0	C
ATOM	303	O	PHE	55	47.674	10.741	-7.338	1.00	0.00	LX0	O
ATOM	304	N	ASN	56	45.641	11.077	-8.278	1.00	0.00	LX0	N
ATOM	305	H	ASN	56	44.681	11.333	-8.152	1.00	0.00	LX0	H
ATOM	306	CA	ASN	56	46.129	10.570	-9.563	1.00	0.00	LX0	C
ATOM	307	CB	ASN	56	45.010	10.473	-10.600	1.00	0.00	LX0	C
ATOM	308	CG	ASN	56	45.448	9.544	-11.714	1.00	0.00	LX0	C
ATOM	309	OD1	ASN	56	46.458	8.858	-11.632	1.00	0.00	LX0	O
ATOM	310	ND2	ASN	56	44.629	9.537	-12.769	1.00	0.00	LX0	N
ATOM	311	HD21	ASN	56	43.816	10.117	-12.794	1.00	0.00	LX0	H
ATOM	312	HD22	ASN	56	44.849	8.937	-13.535	1.00	0.00	LX0	H
ATOM	313	C	ASN	56	47.307	11.341	-10.130	1.00	0.00	LX0	C
ATOM	314	O	ASN	56	47.186	12.472	-10.581	1.00	0.00	LX0	O
ATOM	315	N	ASN	57	48.464	10.665	-10.047	1.00	0.00	LX0	N
ATOM	316	H	ASN	57	48.413	9.747	-9.661	1.00	0.00	LX0	H
ATOM	317	CA	ASN	57	49.742	11.285	-10.404	1.00	0.00	LX0	C

ATOM	318	CB	ASN	57	49.886	11.420	-11.933	1.00	0.00	LX0	C
ATOM	319	CG	ASN	57	51.320	11.728	-12.336	1.00	0.00	LX0	C
ATOM	320	OD1	ASN	57	51.657	12.802	-12.826	1.00	0.00	LX0	O
ATOM	321	ND2	ASN	57	52.166	10.714	-12.144	1.00	0.00	LX0	N
ATOM	322	HD21	ASN	57	51.910	9.891	-11.634	1.00	0.00	LX0	H
ATOM	323	HD22	ASN	57	53.102	10.797	-12.480	1.00	0.00	LX0	H
ATOM	324	C	ASN	57	50.027	12.593	-9.677	1.00	0.00	LX0	C
ATOM	325	O	ASN	57	50.534	13.550	-10.247	1.00	0.00	LX0	O
ATOM	326	N	CYS	58	49.667	12.595	-8.387	1.00	0.00	LX0	N
ATOM	327	H	CYS	58	49.291	11.775	-7.954	1.00	0.00	LX0	H
ATOM	328	CA	CYS	58	49.987	13.788	-7.606	1.00	0.00	LX0	C
ATOM	329	CB	CYS	58	48.880	14.073	-6.593	1.00	0.00	LX0	C
ATOM	330	SG	CYS	58	49.189	15.540	-5.577	1.00	0.00	LX0	S
ATOM	331	C	CYS	58	51.320	13.650	-6.906	1.00	0.00	LX0	C
ATOM	332	O	CYS	58	51.731	12.557	-6.537	1.00	0.00	LX0	O
ATOM	333	N	GLU	59	51.969	14.803	-6.739	1.00	0.00	LX0	N
ATOM	334	H	GLU	59	51.610	15.636	-7.167	1.00	0.00	LX0	H
ATOM	335	CA	GLU	59	53.285	14.801	-6.121	1.00	0.00	LX0	C
ATOM	336	CB	GLU	59	54.330	15.537	-6.972	1.00	0.00	LX0	C
ATOM	337	CG	GLU	59	54.645	14.996	-8.377	1.00	0.00	LX0	C
ATOM	338	CD	GLU	59	53.686	15.510	-9.443	1.00	0.00	LX0	C
ATOM	339	OE1	GLU	59	54.129	16.021	-10.464	1.00	0.00	LX0	O
ATOM	340	OE2	GLU	59	52.479	15.412	-9.289	1.00	0.00	LX0	O
ATOM	341	C	GLU	59	53.262	15.402	-4.735	1.00	0.00	LX0	C
ATOM	342	O	GLU	59	53.931	14.922	-3.827	1.00	0.00	LX0	O
ATOM	343	N	VAL	60	52.475	16.480	-4.608	1.00	0.00	LX0	N
ATOM	344	H	VAL	60	51.933	16.814	-5.382	1.00	0.00	LX0	H
ATOM	345	CA	VAL	60	52.366	17.111	-3.297	1.00	0.00	LX0	C
ATOM	346	CB	VAL	60	52.930	18.541	-3.285	1.00	0.00	LX0	C
ATOM	347	CG1	VAL	60	52.930	19.141	-1.872	1.00	0.00	LX0	C
ATOM	348	CG2	VAL	60	54.329	18.604	-3.890	1.00	0.00	LX0	C
ATOM	349	C	VAL	60	50.942	17.114	-2.787	1.00	0.00	LX0	C
ATOM	350	O	VAL	60	50.065	17.818	-3.275	1.00	0.00	LX0	O
ATOM	351	N	VAL	61	50.752	16.302	-1.748	1.00	0.00	LX0	N
ATOM	352	H	VAL	61	51.543	15.830	-1.356	1.00	0.00	LX0	H
ATOM	353	CA	VAL	61	49.477	16.413	-1.050	1.00	0.00	LX0	C
ATOM	354	CB	VAL	61	49.200	15.126	-0.270	1.00	0.00	LX0	C
ATOM	355	CG1	VAL	61	47.834	15.160	0.408	1.00	0.00	LX0	C
ATOM	356	CG2	VAL	61	49.338	13.901	-1.169	1.00	0.00	LX0	C
ATOM	357	C	VAL	61	49.518	17.612	-0.121	1.00	0.00	LX0	C
ATOM	358	O	VAL	61	50.380	17.702	0.743	1.00	0.00	LX0	O
ATOM	359	N	LEU	62	48.577	18.537	-0.345	1.00	0.00	LX0	N
ATOM	360	H	LEU	62	47.898	18.411	-1.071	1.00	0.00	LX0	H
ATOM	361	CA	LEU	62	48.563	19.702	0.538	1.00	0.00	LX0	C
ATOM	362	CB	LEU	62	47.826	20.871	-0.115	1.00	0.00	LX0	C
ATOM	363	CG	LEU	62	48.472	21.353	-1.414	1.00	0.00	LX0	C
ATOM	364	CD1	LEU	62	47.590	22.382	-2.121	1.00	0.00	LX0	C
ATOM	365	CD2	LEU	62	49.896	21.868	-1.193	1.00	0.00	LX0	C
ATOM	366	C	LEU	62	47.970	19.400	1.899	1.00	0.00	LX0	C
ATOM	367	O	LEU	62	48.598	19.554	2.940	1.00	0.00	LX0	O
ATOM	368	N	GLY	63	46.711	18.949	1.836	1.00	0.00	LX0	N
ATOM	369	H	GLY	63	46.245	18.803	0.963	1.00	0.00	LX0	H
ATOM	370	CA	GLY	63	46.085	18.504	3.072	1.00	0.00	LX0	C
ATOM	371	C	GLY	63	46.553	17.118	3.441	1.00	0.00	LX0	C
ATOM	372	O	GLY	63	47.715	16.758	3.279	1.00	0.00	LX0	O
ATOM	373	N	ASN	64	45.598	16.348	3.958	1.00	0.00	LX0	N
ATOM	374	H	ASN	64	44.628	16.596	3.915	1.00	0.00	LX0	H
ATOM	375	CA	ASN	64	46.053	15.060	4.462	1.00	0.00	LX0	C
ATOM	376	CB	ASN	64	45.193	14.536	5.606	1.00	0.00	LX0	C
ATOM	377	CG	ASN	64	45.116	15.523	6.740	1.00	0.00	LX0	C
ATOM	378	OD1	ASN	64	46.072	16.198	7.107	1.00	0.00	LX0	O

ATOM	379	ND2	ASN	64	43.902	15.583	7.285	1.00	0.00	LX0	N
ATOM	380	HD21	ASN	64	43.215	14.908	6.997	1.00	0.00	LX0	H
ATOM	381	HD22	ASN	64	43.677	16.235	8.003	1.00	0.00	LX0	H
ATOM	382	C	ASN	64	46.118	14.005	3.392	1.00	0.00	LX0	C
ATOM	383	O	ASN	64	45.396	14.010	2.402	1.00	0.00	LX0	O
ATOM	384	N	LEU	65	47.032	13.077	3.652	1.00	0.00	LX0	N
ATOM	385	H	LEU	65	47.606	13.168	4.465	1.00	0.00	LX0	H
ATOM	386	CA	LEU	65	46.992	11.839	2.899	1.00	0.00	LX0	C
ATOM	387	CB	LEU	65	48.410	11.506	2.434	1.00	0.00	LX0	C
ATOM	388	CG	LEU	65	48.584	10.254	1.572	1.00	0.00	LX0	C
ATOM	389	CD1	LEU	65	47.600	10.162	0.409	1.00	0.00	LX0	C
ATOM	390	CD2	LEU	65	50.023	10.164	1.078	1.00	0.00	LX0	C
ATOM	391	C	LEU	65	46.356	10.774	3.768	1.00	0.00	LX0	C
ATOM	392	O	LEU	65	47.007	9.944	4.395	1.00	0.00	LX0	O
ATOM	393	N	GLU	66	45.023	10.862	3.808	1.00	0.00	LX0	N
ATOM	394	H	GLU	66	44.517	11.548	3.276	1.00	0.00	LX0	H
ATOM	395	CA	GLU	66	44.332	9.799	4.521	1.00	0.00	LX0	C
ATOM	396	CB	GLU	66	43.072	10.316	5.202	1.00	0.00	LX0	C
ATOM	397	CG	GLU	66	43.439	11.282	6.330	1.00	0.00	LX0	C
ATOM	398	CD	GLU	66	42.206	11.907	6.943	1.00	0.00	LX0	C
ATOM	399	OE1	GLU	66	41.189	11.233	7.072	1.00	0.00	LX0	O
ATOM	400	OE2	GLU	66	42.246	13.086	7.287	1.00	0.00	LX0	O
ATOM	401	C	GLU	66	44.040	8.630	3.617	1.00	0.00	LX0	C
ATOM	402	O	GLU	66	43.251	8.687	2.679	1.00	0.00	LX0	O
ATOM	403	N	ILE	67	44.764	7.564	3.944	1.00	0.00	LX0	N
ATOM	404	H	ILE	67	45.367	7.625	4.741	1.00	0.00	LX0	H
ATOM	405	CA	ILE	67	44.494	6.272	3.338	1.00	0.00	LX0	C
ATOM	406	CB	ILE	67	45.815	5.599	2.943	1.00	0.00	LX0	C
ATOM	407	CG2	ILE	67	45.589	4.262	2.229	1.00	0.00	LX0	C
ATOM	408	CG1	ILE	67	46.682	6.555	2.121	1.00	0.00	LX0	C
ATOM	409	CD1	ILE	67	48.097	6.027	1.899	1.00	0.00	LX0	C
ATOM	410	C	ILE	67	43.759	5.453	4.373	1.00	0.00	LX0	C
ATOM	411	O	ILE	67	44.358	4.905	5.293	1.00	0.00	LX0	O
ATOM	412	N	THR	68	42.437	5.428	4.216	1.00	0.00	LX0	N
ATOM	413	H	THR	68	41.947	5.938	3.511	1.00	0.00	LX0	H
ATOM	414	CA	THR	68	41.689	4.676	5.211	1.00	0.00	LX0	C
ATOM	415	CB	THR	68	40.902	5.640	6.097	1.00	0.00	LX0	C
ATOM	416	OG1	THR	68	40.210	6.601	5.290	1.00	0.00	LX0	O
ATOM	417	HG1	THR	68	39.530	6.979	5.851	1.00	0.00	LX0	H
ATOM	418	CG2	THR	68	41.807	6.354	7.105	1.00	0.00	LX0	C
ATOM	419	C	THR	68	40.782	3.643	4.582	1.00	0.00	LX0	C
ATOM	420	O	THR	68	40.254	3.832	3.491	1.00	0.00	LX0	O
ATOM	421	N	TYR	69	40.617	2.532	5.320	1.00	0.00	LX0	N
ATOM	422	H	TYR	69	41.171	2.443	6.150	1.00	0.00	LX0	H
ATOM	423	CA	TYR	69	39.654	1.492	4.929	1.00	0.00	LX0	C
ATOM	424	CB	TYR	69	38.203	1.974	5.063	1.00	0.00	LX0	C
ATOM	425	CG	TYR	69	37.801	2.388	6.463	1.00	0.00	LX0	C
ATOM	426	CD1	TYR	69	37.404	1.401	7.388	1.00	0.00	LX0	C
ATOM	427	CE1	TYR	69	36.944	1.798	8.656	1.00	0.00	LX0	C
ATOM	428	CD2	TYR	69	37.787	3.759	6.792	1.00	0.00	LX0	C
ATOM	429	CE2	TYR	69	37.329	4.155	8.060	1.00	0.00	LX0	C
ATOM	430	CZ	TYR	69	36.903	3.171	8.975	1.00	0.00	LX0	C
ATOM	431	OH	TYR	69	36.453	3.568	10.217	1.00	0.00	LX0	O
ATOM	432	HH	TYR	69	35.529	3.313	10.344	1.00	0.00	LX0	H
ATOM	433	C	TYR	69	39.826	0.861	3.547	1.00	0.00	LX0	C
ATOM	434	O	TYR	69	38.927	0.224	3.011	1.00	0.00	LX0	O
ATOM	435	N	VAL	70	41.016	1.064	2.966	1.00	0.00	LX0	N
ATOM	436	H	VAL	70	41.732	1.570	3.443	1.00	0.00	LX0	H
ATOM	437	CA	VAL	70	41.237	0.492	1.641	1.00	0.00	LX0	C
ATOM	438	CB	VAL	70	42.420	1.192	0.951	1.00	0.00	LX0	C
ATOM	439	CG1	VAL	70	42.726	0.620	-0.434	1.00	0.00	LX0	C

```
ATOM    440  CG2 VAL    70      42.165   2.700   0.860  1.00  0.00      LX0  C
ATOM    441  C   VAL    70      41.407  -1.016   1.723  1.00  0.00      LX0  C
ATOM    442  O   VAL    70      42.326  -1.543   2.344  1.00  0.00      LX0  O
ATOM    443  N   GLN    71      40.428  -1.678   1.097  1.00  0.00      LX0  N
ATOM    444  H   GLN    71      39.716  -1.157   0.628  1.00  0.00      LX0  H
ATOM    445  CA  GLN    71      40.361  -3.130   1.210  1.00  0.00      LX0  C
ATOM    446  CB  GLN    71      38.950  -3.621   0.870  1.00  0.00      LX0  C
ATOM    447  CG  GLN    71      37.884  -3.194   1.887  1.00  0.00      LX0  C
ATOM    448  CD  GLN    71      37.989  -4.011   3.161  1.00  0.00      LX0  C
ATOM    449  OE1 GLN    71      38.673  -5.028   3.223  1.00  0.00      LX0  O
ATOM    450  NE2 GLN    71      37.271  -3.527   4.174  1.00  0.00      LX0  N
ATOM    451 HE21 GLN    71      36.704  -2.702   4.168  1.00  0.00      LX0  H
ATOM    452 HE22 GLN    71      37.193  -3.980   5.068  1.00  0.00      LX0  H
ATOM    453  C   GLN    71      41.425  -3.910   0.452  1.00  0.00      LX0  C
ATOM    454  O   GLN    71      42.089  -3.424  -0.458  1.00  0.00      LX0  O
ATOM    455  N   ARG    72      41.532  -5.170   0.906  1.00  0.00      LX0  N
ATOM    456  H   ARG    72      40.918  -5.366   1.673  1.00  0.00      LX0  H
ATOM    457  CA  ARG    72      42.622  -6.114   0.644  1.00  0.00      LX0  C
ATOM    458  CB  ARG    72      42.091  -7.554   0.691  1.00  0.00      LX0  C
ATOM    459  CG  ARG    72      43.195  -8.579   0.978  1.00  0.00      LX0  C
ATOM    460  CD  ARG    72      43.758  -8.418   2.390  1.00  0.00      LX0  C
ATOM    461  NE  ARG    72      45.047  -9.087   2.542  1.00  0.00      LX0  N
ATOM    462  HE  ARG    72      45.782  -8.969   1.864  1.00  0.00      LX0  H
ATOM    463  CZ  ARG    72      45.372  -9.724   3.681  1.00  0.00      LX0  C
ATOM    464  NH1 ARG    72      44.469  -9.918   4.630  1.00  0.00      LX0  N
ATOM    465 HH11 ARG    72      44.731 -10.303   5.523  1.00  0.00      LX0  H
ATOM    466 HH12 ARG    72      43.509  -9.672   4.522  1.00  0.00      LX0  H
ATOM    467  NH2 ARG    72      46.605 -10.162   3.865  1.00  0.00      LX0  N
ATOM    468 HH21 ARG    72      46.849 -10.666   4.693  1.00  0.00      LX0  H
ATOM    469 HH22 ARG    72      47.324  -9.976   3.169  1.00  0.00      LX0  H
ATOM    470  C   ARG    72      43.551  -5.932  -0.547  1.00  0.00      LX0  C
ATOM    471  O   ARG    72      44.768  -6.028  -0.425  1.00  0.00      LX0  O
ATOM    472  N   ASN    73      42.947  -5.723  -1.721  1.00  0.00      LX0  N
ATOM    473  H   ASN    73      41.967  -5.537  -1.788  1.00  0.00      LX0  H
ATOM    474  CA  ASN    73      43.850  -5.779  -2.865  1.00  0.00      LX0  C
ATOM    475  CB  ASN    73      43.924  -7.202  -3.426  1.00  0.00      LX0  C
ATOM    476  CG  ASN    73      45.375  -7.569  -3.677  1.00  0.00      LX0  C
ATOM    477  OD1 ASN    73      45.784  -7.916  -4.774  1.00  0.00      LX0  O
ATOM    478  ND2 ASN    73      46.145  -7.526  -2.583  1.00  0.00      LX0  N
ATOM    479 HD21 ASN    73      45.808  -7.212  -1.693  1.00  0.00      LX0  H
ATOM    480 HD22 ASN    73      47.091  -7.832  -2.664  1.00  0.00      LX0  H
ATOM    481  C   ASN    73      43.616  -4.761  -3.953  1.00  0.00      LX0  C
ATOM    482  O   ASN    73      43.744  -5.038  -5.137  1.00  0.00      LX0  O
ATOM    483  N   TYR    74      43.259  -3.547  -3.512  1.00  0.00      LX0  N
ATOM    484  H   TYR    74      43.206  -3.324  -2.537  1.00  0.00      LX0  H
ATOM    485  CA  TYR    74      43.283  -2.524  -4.556  1.00  0.00      LX0  C
ATOM    486  CB  TYR    74      42.360  -1.337  -4.267  1.00  0.00      LX0  C
ATOM    487  CG  TYR    74      40.996  -1.718  -3.735  1.00  0.00      LX0  C
ATOM    488  CD1 TYR    74      40.112  -2.528  -4.481  1.00  0.00      LX0  C
ATOM    489  CE1 TYR    74      38.843  -2.822  -3.945  1.00  0.00      LX0  C
ATOM    490  CD2 TYR    74      40.643  -1.200  -2.478  1.00  0.00      LX0  C
ATOM    491  CE2 TYR    74      39.378  -1.480  -1.951  1.00  0.00      LX0  C
ATOM    492  CZ  TYR    74      38.500  -2.298  -2.680  1.00  0.00      LX0  C
ATOM    493  OH  TYR    74      37.285  -2.584  -2.098  1.00  0.00      LX0  O
ATOM    494  HH  TYR    74      36.656  -2.911  -2.746  1.00  0.00      LX0  H
ATOM    495  C   TYR    74      44.685  -1.979  -4.724  1.00  0.00      LX0  C
ATOM    496  O   TYR    74      45.292  -1.524  -3.762  1.00  0.00      LX0  O
ATOM    497  N   ASP    75      45.174  -2.027  -5.971  1.00  0.00      LX0  N
ATOM    498  H   ASP    75      44.640  -2.413  -6.718  1.00  0.00      LX0  H
ATOM    499  CA  ASP    75      46.403  -1.264  -6.196  1.00  0.00      LX0  C
ATOM    500  CB  ASP    75      47.085  -1.661  -7.520  1.00  0.00      LX0  C
```

ATOM	501	CG	ASP	75	48.260	-0.742	-7.849	1.00	0.00	LX0	C
ATOM	502	OD1	ASP	75	49.221	-0.662	-7.086	1.00	0.00	LX0	O
ATOM	503	OD2	ASP	75	48.179	-0.024	-8.836	1.00	0.00	LX0	O
ATOM	504	C	ASP	75	46.126	0.228	-6.110	1.00	0.00	LX0	C
ATOM	505	O	ASP	75	45.037	0.714	-6.393	1.00	0.00	LX0	O
ATOM	506	N	LEU	76	47.172	0.919	-5.660	1.00	0.00	LX0	N
ATOM	507	H	LEU	76	48.033	0.411	-5.586	1.00	0.00	LX0	H
ATOM	508	CA	LEU	76	47.057	2.356	-5.478	1.00	0.00	LX0	C
ATOM	509	CB	LEU	76	47.035	2.705	-3.985	1.00	0.00	LX0	C
ATOM	510	CG	LEU	76	45.964	2.012	-3.136	1.00	0.00	LX0	C
ATOM	511	CD1	LEU	76	46.296	2.084	-1.645	1.00	0.00	LX0	C
ATOM	512	CD2	LEU	76	44.560	2.539	-3.428	1.00	0.00	LX0	C
ATOM	513	C	LEU	76	48.203	3.076	-6.162	1.00	0.00	LX0	C
ATOM	514	O	LEU	76	48.724	4.067	-5.667	1.00	0.00	LX0	O
ATOM	515	N	SER	77	48.618	2.536	-7.321	1.00	0.00	LX0	N
ATOM	516	H	SER	77	48.196	1.691	-7.668	1.00	0.00	LX0	H
ATOM	517	CA	SER	77	49.868	3.028	-7.906	1.00	0.00	LX0	C
ATOM	518	CB	SER	77	50.273	2.213	-9.129	1.00	0.00	LX0	C
ATOM	519	OG	SER	77	50.870	0.990	-8.663	1.00	0.00	LX0	O
ATOM	520	HG	SER	77	50.107	0.399	-8.527	1.00	0.00	LX0	H
ATOM	521	C	SER	77	50.062	4.512	-8.164	1.00	0.00	LX0	C
ATOM	522	O	SER	77	51.191	4.980	-8.226	1.00	0.00	LX0	O
ATOM	523	N	PHE	78	48.942	5.244	-8.254	1.00	0.00	LX0	N
ATOM	524	H	PHE	78	48.066	4.768	-8.231	1.00	0.00	LX0	H
ATOM	525	CA	PHE	78	49.040	6.710	-8.278	1.00	0.00	LX0	C
ATOM	526	CB	PHE	78	47.642	7.353	-8.351	1.00	0.00	LX0	C
ATOM	527	CG	PHE	78	46.615	6.582	-7.550	1.00	0.00	LX0	C
ATOM	528	CD1	PHE	78	46.543	6.747	-6.151	1.00	0.00	LX0	C
ATOM	529	CD2	PHE	78	45.752	5.689	-8.225	1.00	0.00	LX0	C
ATOM	530	CE1	PHE	78	45.613	5.990	-5.414	1.00	0.00	LX0	C
ATOM	531	CE2	PHE	78	44.821	4.933	-7.489	1.00	0.00	LX0	C
ATOM	532	CZ	PHE	78	44.764	5.089	-6.089	1.00	0.00	LX0	C
ATOM	533	C	PHE	78	49.898	7.333	-7.174	1.00	0.00	LX0	C
ATOM	534	O	PHE	78	50.643	8.284	-7.393	1.00	0.00	LX0	O
ATOM	535	N	LEU	79	49.812	6.696	-5.989	1.00	0.00	LX0	N
ATOM	536	H	LEU	79	49.187	5.920	-5.894	1.00	0.00	LX0	H
ATOM	537	CA	LEU	79	50.618	7.123	-4.840	1.00	0.00	LX0	C
ATOM	538	CB	LEU	79	50.301	6.295	-3.591	1.00	0.00	LX0	C
ATOM	539	CG	LEU	79	48.836	6.229	-3.148	1.00	0.00	LX0	C
ATOM	540	CD1	LEU	79	48.692	5.336	-1.919	1.00	0.00	LX0	C
ATOM	541	CD2	LEU	79	48.204	7.597	-2.901	1.00	0.00	LX0	C
ATOM	542	C	LEU	79	52.128	7.129	-5.053	1.00	0.00	LX0	C
ATOM	543	O	LEU	79	52.873	7.807	-4.358	1.00	0.00	LX0	O
ATOM	544	N	LYS	80	52.565	6.378	-6.082	1.00	0.00	LX0	N
ATOM	545	H	LYS	80	51.913	5.851	-6.625	1.00	0.00	LX0	H
ATOM	546	CA	LYS	80	53.986	6.424	-6.435	1.00	0.00	LX0	C
ATOM	547	CB	LYS	80	54.326	5.460	-7.567	1.00	0.00	LX0	C
ATOM	548	CG	LYS	80	54.348	4.012	-7.111	1.00	0.00	LX0	C
ATOM	549	CD	LYS	80	54.932	3.068	-8.162	1.00	0.00	LX0	C
ATOM	550	CE	LYS	80	53.892	2.138	-8.779	1.00	0.00	LX0	C
ATOM	551	NZ	LYS	80	53.226	1.363	-7.723	1.00	0.00	LX0	N
ATOM	552	HZ1	LYS	80	52.530	0.710	-8.156	1.00	0.00	LX0	H
ATOM	553	HZ2	LYS	80	53.934	0.870	-7.134	1.00	0.00	LX0	H
ATOM	554	HZ3	LYS	80	52.677	2.003	-7.121	1.00	0.00	LX0	H
ATOM	555	C	LYS	80	54.527	7.784	-6.825	1.00	0.00	LX0	C
ATOM	556	O	LYS	80	55.731	8.013	-6.814	1.00	0.00	LX0	O
ATOM	557	N	THR	81	53.593	8.664	-7.190	1.00	0.00	LX0	N
ATOM	558	H	THR	81	52.619	8.444	-7.154	1.00	0.00	LX0	H
ATOM	559	CA	THR	81	54.051	9.994	-7.563	1.00	0.00	LX0	C
ATOM	560	CB	THR	81	53.042	10.632	-8.519	1.00	0.00	LX0	C
ATOM	561	OG1	THR	81	52.325	9.633	-9.272	1.00	0.00	LX0	O

ATOM	562	HG1	THR	81	51.715	9.236	-8.653	1.00	0.00	LX0	H
ATOM	563	CG2	THR	81	53.739	11.626	-9.447	1.00	0.00	LX0	C
ATOM	564	C	THR	81	54.348	10.907	-6.378	1.00	0.00	LX0	C
ATOM	565	O	THR	81	55.082	11.886	-6.469	1.00	0.00	LX0	O
ATOM	566	N	ILE	82	53.737	10.536	-5.239	1.00	0.00	LX0	N
ATOM	567	H	ILE	82	53.298	9.645	-5.130	1.00	0.00	LX0	H
ATOM	568	CA	ILE	82	53.790	11.470	-4.121	1.00	0.00	LX0	C
ATOM	569	CB	ILE	82	52.711	11.151	-3.078	1.00	0.00	LX0	C
ATOM	570	CG2	ILE	82	52.666	12.217	-1.978	1.00	0.00	LX0	C
ATOM	571	CG1	ILE	82	51.347	10.995	-3.757	1.00	0.00	LX0	C
ATOM	572	CD1	ILE	82	50.223	10.634	-2.791	1.00	0.00	LX0	C
ATOM	573	C	ILE	82	55.164	11.582	-3.495	1.00	0.00	LX0	C
ATOM	574	O	ILE	82	55.693	10.675	-2.866	1.00	0.00	LX0	O
ATOM	575	N	GLN	83	55.719	12.773	-3.715	1.00	0.00	LX0	N
ATOM	576	H	GLN	83	55.185	13.443	-4.231	1.00	0.00	LX0	H
ATOM	577	CA	GLN	83	56.992	13.087	-3.085	1.00	0.00	LX0	C
ATOM	578	CB	GLN	83	57.783	14.069	-3.944	1.00	0.00	LX0	C
ATOM	579	CG	GLN	83	57.960	13.705	-5.419	1.00	0.00	LX0	C
ATOM	580	CD	GLN	83	58.795	14.796	-6.060	1.00	0.00	LX0	C
ATOM	581	OE1	GLN	83	59.563	15.484	-5.392	1.00	0.00	LX0	O
ATOM	582	NE2	GLN	83	58.609	14.940	-7.376	1.00	0.00	LX0	N
ATOM	583	HE21	GLN	83	57.938	14.391	-7.873	1.00	0.00	LX0	H
ATOM	584	HE22	GLN	83	59.144	15.599	-7.913	1.00	0.00	LX0	H
ATOM	585	C	GLN	83	56.812	13.691	-1.706	1.00	0.00	LX0	C
ATOM	586	O	GLN	83	57.517	13.373	-0.753	1.00	0.00	LX0	O
ATOM	587	N	GLU	84	55.848	14.622	-1.670	1.00	0.00	LX0	N
ATOM	588	H	GLU	84	55.220	14.756	-2.438	1.00	0.00	LX0	H
ATOM	589	CA	GLU	84	55.726	15.491	-0.508	1.00	0.00	LX0	C
ATOM	590	CB	GLU	84	56.142	16.928	-0.866	1.00	0.00	LX0	C
ATOM	591	CG	GLU	84	57.425	17.080	-1.705	1.00	0.00	LX0	C
ATOM	592	CD	GLU	84	57.869	18.532	-1.833	1.00	0.00	LX0	C
ATOM	593	OE1	GLU	84	57.043	19.432	-1.736	1.00	0.00	LX0	O
ATOM	594	OE2	GLU	84	59.062	18.787	-1.992	1.00	0.00	LX0	O
ATOM	595	C	GLU	84	54.309	15.510	0.023	1.00	0.00	LX0	C
ATOM	596	O	GLU	84	53.363	15.748	-0.715	1.00	0.00	LX0	O
ATOM	597	N	VAL	85	54.185	15.278	1.331	1.00	0.00	LX0	N
ATOM	598	H	VAL	85	54.972	15.025	1.899	1.00	0.00	LX0	H
ATOM	599	CA	VAL	85	52.886	15.583	1.930	1.00	0.00	LX0	C
ATOM	600	CB	VAL	85	52.319	14.361	2.675	1.00	0.00	LX0	C
ATOM	601	CG1	VAL	85	50.881	14.580	3.159	1.00	0.00	LX0	C
ATOM	602	CG2	VAL	85	52.386	13.110	1.803	1.00	0.00	LX0	C
ATOM	603	C	VAL	85	53.073	16.760	2.867	1.00	0.00	LX0	C
ATOM	604	O	VAL	85	54.131	16.903	3.465	1.00	0.00	LX0	O
ATOM	605	N	ALA	86	52.043	17.605	2.964	1.00	0.00	LX0	N
ATOM	606	H	ALA	86	51.219	17.465	2.413	1.00	0.00	LX0	H
ATOM	607	CA	ALA	86	52.114	18.640	3.986	1.00	0.00	LX0	C
ATOM	608	CB	ALA	86	51.695	19.998	3.421	1.00	0.00	LX0	C
ATOM	609	C	ALA	86	51.286	18.287	5.207	1.00	0.00	LX0	C
ATOM	610	O	ALA	86	51.746	18.362	6.341	1.00	0.00	LX0	O
ATOM	611	N	GLY	87	50.046	17.855	4.932	1.00	0.00	LX0	N
ATOM	612	H	GLY	87	49.668	17.875	4.003	1.00	0.00	LX0	H
ATOM	613	CA	GLY	87	49.246	17.332	6.037	1.00	0.00	LX0	C
ATOM	614	C	GLY	87	49.743	16.003	6.580	1.00	0.00	LX0	C
ATOM	615	O	GLY	87	50.834	15.536	6.270	1.00	0.00	LX0	O
ATOM	616	N	TYR	88	48.890	15.417	7.429	1.00	0.00	LX0	N
ATOM	617	H	TYR	88	47.952	15.757	7.511	1.00	0.00	LX0	H
ATOM	618	CA	TYR	88	49.323	14.149	8.006	1.00	0.00	LX0	C
ATOM	619	CB	TYR	88	48.778	13.977	9.436	1.00	0.00	LX0	C
ATOM	620	CG	TYR	88	47.266	13.956	9.524	1.00	0.00	LX0	C
ATOM	621	CD1	TYR	88	46.551	12.837	9.050	1.00	0.00	LX0	C
ATOM	622	CE1	TYR	88	45.152	12.827	9.153	1.00	0.00	LX0	C

ATOM	623	CD2	TYR	88	46.612	15.057	10.109	1.00	0.00	LX0	C
ATOM	624	CE2	TYR	88	45.214	15.040	10.234	1.00	0.00	LX0	C
ATOM	625	CZ	TYR	88	44.502	13.929	9.741	1.00	0.00	LX0	C
ATOM	626	OH	TYR	88	43.127	13.928	9.824	1.00	0.00	LX0	O
ATOM	627	HH	TYR	88	42.787	13.408	9.094	1.00	0.00	LX0	H
ATOM	628	C	TYR	88	49.004	12.966	7.113	1.00	0.00	LX0	C
ATOM	629	O	TYR	88	48.122	13.029	6.264	1.00	0.00	LX0	O
ATOM	630	N	VAL	89	49.751	11.880	7.333	1.00	0.00	LX0	N
ATOM	631	H	VAL	89	50.402	11.857	8.093	1.00	0.00	LX0	H
ATOM	632	CA	VAL	89	49.435	10.682	6.563	1.00	0.00	LX0	C
ATOM	633	CB	VAL	89	50.681	10.127	5.859	1.00	0.00	LX0	C
ATOM	634	CG1	VAL	89	50.333	8.961	4.930	1.00	0.00	LX0	C
ATOM	635	CG2	VAL	89	51.415	11.226	5.093	1.00	0.00	LX0	C
ATOM	636	C	VAL	89	48.780	9.636	7.445	1.00	0.00	LX0	C
ATOM	637	O	VAL	89	49.422	8.906	8.195	1.00	0.00	LX0	O
ATOM	638	N	LEU	90	47.450	9.621	7.341	1.00	0.00	LX0	N
ATOM	639	H	LEU	90	47.015	10.122	6.590	1.00	0.00	LX0	H
ATOM	640	CA	LEU	90	46.726	8.658	8.162	1.00	0.00	LX0	C
ATOM	641	CB	LEU	90	45.410	9.263	8.652	1.00	0.00	LX0	C
ATOM	642	CG	LEU	90	44.537	8.350	9.517	1.00	0.00	LX0	C
ATOM	643	CD1	LEU	90	45.224	7.942	10.818	1.00	0.00	LX0	C
ATOM	644	CD2	LEU	90	43.162	8.966	9.762	1.00	0.00	LX0	C
ATOM	645	C	LEU	90	46.493	7.370	7.406	1.00	0.00	LX0	C
ATOM	646	O	LEU	90	45.585	7.249	6.597	1.00	0.00	LX0	O
ATOM	647	N	ILE	91	47.366	6.410	7.707	1.00	0.00	LX0	N
ATOM	648	H	ILE	91	48.022	6.557	8.450	1.00	0.00	LX0	H
ATOM	649	CA	ILE	91	47.146	5.089	7.136	1.00	0.00	LX0	C
ATOM	650	CB	ILE	91	48.488	4.472	6.713	1.00	0.00	LX0	C
ATOM	651	CG2	ILE	91	48.326	3.060	6.145	1.00	0.00	LX0	C
ATOM	652	CG1	ILE	91	49.207	5.392	5.723	1.00	0.00	LX0	C
ATOM	653	CD1	ILE	91	50.588	4.881	5.304	1.00	0.00	LX0	C
ATOM	654	C	ILE	91	46.419	4.222	8.147	1.00	0.00	LX0	C
ATOM	655	O	ILE	91	47.028	3.595	9.007	1.00	0.00	LX0	O
ATOM	656	N	ALA	92	45.089	4.232	8.032	1.00	0.00	LX0	N
ATOM	657	H	ALA	92	44.630	4.717	7.282	1.00	0.00	LX0	H
ATOM	658	CA	ALA	92	44.362	3.482	9.048	1.00	0.00	LX0	C
ATOM	659	CB	ALA	92	43.720	4.422	10.068	1.00	0.00	LX0	C
ATOM	660	C	ALA	92	43.300	2.562	8.496	1.00	0.00	LX0	C
ATOM	661	O	ALA	92	42.631	2.853	7.511	1.00	0.00	LX0	O
ATOM	662	N	LEU	93	43.163	1.427	9.204	1.00	0.00	LX0	N
ATOM	663	H	LEU	93	43.820	1.253	9.939	1.00	0.00	LX0	H
ATOM	664	CA	LEU	93	42.093	0.467	8.912	1.00	0.00	LX0	C
ATOM	665	CB	LEU	93	40.734	1.027	9.350	1.00	0.00	LX0	C
ATOM	666	CG	LEU	93	40.635	1.191	10.870	1.00	0.00	LX0	C
ATOM	667	CD1	LEU	93	39.537	2.169	11.278	1.00	0.00	LX0	C
ATOM	668	CD2	LEU	93	40.472	-0.155	11.576	1.00	0.00	LX0	C
ATOM	669	C	LEU	93	42.062	-0.059	7.486	1.00	0.00	LX0	C
ATOM	670	O	LEU	93	41.032	-0.414	6.931	1.00	0.00	LX0	O
ATOM	671	N	ASN	94	43.262	-0.079	6.900	1.00	0.00	LX0	N
ATOM	672	H	ASN	94	44.078	0.148	7.430	1.00	0.00	LX0	H
ATOM	673	CA	ASN	94	43.355	-0.603	5.542	1.00	0.00	LX0	C
ATOM	674	CB	ASN	94	44.467	0.060	4.731	1.00	0.00	LX0	C
ATOM	675	CG	ASN	94	44.283	1.555	4.674	1.00	0.00	LX0	C
ATOM	676	OD1	ASN	94	43.470	2.099	3.941	1.00	0.00	LX0	O
ATOM	677	ND2	ASN	94	45.098	2.209	5.490	1.00	0.00	LX0	N
ATOM	678	HD21	ASN	94	45.710	1.721	6.113	1.00	0.00	LX0	H
ATOM	679	HD22	ASN	94	45.057	3.206	5.488	1.00	0.00	LX0	H
ATOM	680	C	ASN	94	43.665	-2.069	5.606	1.00	0.00	LX0	C
ATOM	681	O	ASN	94	44.506	-2.507	6.379	1.00	0.00	LX0	O
ATOM	682	N	THR	95	42.964	-2.817	4.762	1.00	0.00	LX0	N
ATOM	683	H	THR	95	42.284	-2.444	4.129	1.00	0.00	LX0	H

ATOM	684	CA	THR	95	43.336	-4.222	4.718	1.00	0.00	LX0	C
ATOM	685	CB	THR	95	42.058	-5.059	4.704	1.00	0.00	LX0	C
ATOM	686	OG1	THR	95	41.272	-4.729	3.564	1.00	0.00	LX0	O
ATOM	687	HG1	THR	95	40.374	-4.973	3.792	1.00	0.00	LX0	H
ATOM	688	CG2	THR	95	41.211	-4.793	5.949	1.00	0.00	LX0	C
ATOM	689	C	THR	95	44.280	-4.547	3.565	1.00	0.00	LX0	C
ATOM	690	O	THR	95	44.816	-5.641	3.457	1.00	0.00	LX0	O
ATOM	691	N	VAL	96	44.470	-3.534	2.699	1.00	0.00	LX0	N
ATOM	692	H	VAL	96	43.940	-2.691	2.792	1.00	0.00	LX0	H
ATOM	693	CA	VAL	96	45.344	-3.729	1.543	1.00	0.00	LX0	C
ATOM	694	CB	VAL	96	45.233	-2.519	0.593	1.00	0.00	LX0	C
ATOM	695	CG1	VAL	96	45.650	-1.213	1.267	1.00	0.00	LX0	C
ATOM	696	CG2	VAL	96	45.946	-2.750	-0.742	1.00	0.00	LX0	C
ATOM	697	C	VAL	96	46.790	-4.123	1.839	1.00	0.00	LX0	C
ATOM	698	O	VAL	96	47.526	-3.455	2.556	1.00	0.00	LX0	O
ATOM	699	N	GLU	97	47.145	-5.251	1.199	1.00	0.00	LX0	N
ATOM	700	H	GLU	97	46.411	-5.735	0.721	1.00	0.00	LX0	H
ATOM	701	CA	GLU	97	48.462	-5.877	1.346	1.00	0.00	LX0	C
ATOM	702	CB	GLU	97	48.587	-7.017	0.334	1.00	0.00	LX0	C
ATOM	703	CG	GLU	97	49.435	-8.210	0.792	1.00	0.00	LX0	C
ATOM	704	CD	GLU	97	48.617	-9.113	1.698	1.00	0.00	LX0	C
ATOM	705	OE1	GLU	97	49.032	-9.377	2.822	1.00	0.00	LX0	O
ATOM	706	OE2	GLU	97	47.556	-9.570	1.282	1.00	0.00	LX0	O
ATOM	707	C	GLU	97	49.661	-4.945	1.202	1.00	0.00	LX0	C
ATOM	708	O	GLU	97	50.631	-4.955	1.953	1.00	0.00	LX0	O
ATOM	709	N	ARG	98	49.568	-4.136	0.143	1.00	0.00	LX0	N
ATOM	710	H	ARG	98	48.734	-4.100	-0.407	1.00	0.00	LX0	H
ATOM	711	CA	ARG	98	50.716	-3.298	-0.175	1.00	0.00	LX0	C
ATOM	712	CB	ARG	98	51.465	-3.895	-1.372	1.00	0.00	LX0	C
ATOM	713	CG	ARG	98	52.786	-3.207	-1.731	1.00	0.00	LX0	C
ATOM	714	CD	ARG	98	53.428	-3.827	-2.977	1.00	0.00	LX0	C
ATOM	715	NE	ARG	98	54.789	-3.336	-3.211	1.00	0.00	LX0	N
ATOM	716	HE	ARG	98	55.531	-3.879	-2.811	1.00	0.00	LX0	H
ATOM	717	CZ	ARG	98	55.032	-2.210	-3.922	1.00	0.00	LX0	C
ATOM	718	NH1	ARG	98	54.033	-1.470	-4.390	1.00	0.00	LX0	N
ATOM	719	HH11	ARG	98	54.250	-0.618	-4.879	1.00	0.00	LX0	H
ATOM	720	HH12	ARG	98	53.079	-1.742	-4.264	1.00	0.00	LX0	H
ATOM	721	NH2	ARG	98	56.282	-1.829	-4.161	1.00	0.00	LX0	N
ATOM	722	HH21	ARG	98	56.464	-0.980	-4.680	1.00	0.00	LX0	H
ATOM	723	HH22	ARG	98	57.069	-2.358	-3.845	1.00	0.00	LX0	H
ATOM	724	C	ARG	98	50.248	-1.902	-0.493	1.00	0.00	LX0	C
ATOM	725	O	ARG	98	49.416	-1.713	-1.368	1.00	0.00	LX0	O
ATOM	726	N	ILE	99	50.814	-0.930	0.229	1.00	0.00	LX0	N
ATOM	727	H	ILE	99	51.530	-1.140	0.900	1.00	0.00	LX0	H
ATOM	728	CA	ILE	99	50.470	0.432	-0.168	1.00	0.00	LX0	C
ATOM	729	CB	ILE	99	50.062	1.280	1.047	1.00	0.00	LX0	C
ATOM	730	CG2	ILE	99	49.893	2.769	0.721	1.00	0.00	LX0	C
ATOM	731	CG1	ILE	99	48.770	0.704	1.628	1.00	0.00	LX0	C
ATOM	732	CD1	ILE	99	48.268	1.432	2.872	1.00	0.00	LX0	C
ATOM	733	C	ILE	99	51.597	1.061	-0.961	1.00	0.00	LX0	C
ATOM	734	O	ILE	99	52.687	1.303	-0.459	1.00	0.00	LX0	O
ATOM	735	N	PRO	100	51.308	1.310	-2.259	1.00	0.00	LX0	N
ATOM	736	CD	PRO	100	50.089	0.941	-2.961	1.00	0.00	LX0	C
ATOM	737	CA	PRO	100	52.302	1.902	-3.160	1.00	0.00	LX0	C
ATOM	738	CB	PRO	100	51.690	1.675	-4.549	1.00	0.00	LX0	C
ATOM	739	CG	PRO	100	50.594	0.632	-4.360	1.00	0.00	LX0	C
ATOM	740	C	PRO	100	52.664	3.369	-2.944	1.00	0.00	LX0	C
ATOM	741	O	PRO	100	52.866	4.100	-3.906	1.00	0.00	LX0	O
ATOM	742	N	LEU	101	52.818	3.773	-1.673	1.00	0.00	LX0	N
ATOM	743	H	LEU	101	52.715	3.126	-0.921	1.00	0.00	LX0	H
ATOM	744	CA	LEU	101	53.398	5.087	-1.380	1.00	0.00	LX0	C

ATOM	745	CB	LEU	101	53.017	5.492	0.038	1.00	0.00	LX0	C
ATOM	746	CG	LEU	101	52.046	6.661	0.138	1.00	0.00	LX0	C
ATOM	747	CD1	LEU	101	51.774	6.982	1.606	1.00	0.00	LX0	C
ATOM	748	CD2	LEU	101	52.522	7.887	-0.645	1.00	0.00	LX0	C
ATOM	749	C	LEU	101	54.916	5.093	-1.494	1.00	0.00	LX0	C
ATOM	750	O	LEU	101	55.647	5.782	-0.799	1.00	0.00	LX0	O
ATOM	751	N	GLU	102	55.379	4.221	-2.390	1.00	0.00	LX0	N
ATOM	752	H	GLU	102	54.767	3.978	-3.138	1.00	0.00	LX0	H
ATOM	753	CA	GLU	102	56.745	3.722	-2.282	1.00	0.00	LX0	C
ATOM	754	CB	GLU	102	56.858	2.437	-3.125	1.00	0.00	LX0	C
ATOM	755	CG	GLU	102	56.201	2.523	-4.511	1.00	0.00	LX0	C
ATOM	756	CD	GLU	102	56.136	1.180	-5.228	1.00	0.00	LX0	C
ATOM	757	OE1	GLU	102	57.169	0.529	-5.366	1.00	0.00	LX0	O
ATOM	758	OE2	GLU	102	55.052	0.782	-5.669	1.00	0.00	LX0	O
ATOM	759	C	GLU	102	57.832	4.761	-2.531	1.00	0.00	LX0	C
ATOM	760	O	GLU	102	58.976	4.631	-2.110	1.00	0.00	LX0	O
ATOM	761	N	ASN	103	57.411	5.830	-3.221	1.00	0.00	LX0	N
ATOM	762	H	ASN	103	56.446	5.960	-3.439	1.00	0.00	LX0	H
ATOM	763	CA	ASN	103	58.410	6.855	-3.511	1.00	0.00	LX0	C
ATOM	764	CB	ASN	103	58.321	7.341	-4.957	1.00	0.00	LX0	C
ATOM	765	CG	ASN	103	58.663	6.239	-5.939	1.00	0.00	LX0	C
ATOM	766	OD1	ASN	103	59.394	5.290	-5.666	1.00	0.00	LX0	O
ATOM	767	ND2	ASN	103	58.091	6.410	-7.131	1.00	0.00	LX0	N
ATOM	768	HD21	ASN	103	57.447	7.173	-7.246	1.00	0.00	LX0	H
ATOM	769	HD22	ASN	103	58.285	5.816	-7.908	1.00	0.00	LX0	H
ATOM	770	C	ASN	103	58.405	8.059	-2.590	1.00	0.00	LX0	C
ATOM	771	O	ASN	103	59.214	8.963	-2.775	1.00	0.00	LX0	O
ATOM	772	N	LEU	104	57.477	8.036	-1.609	1.00	0.00	LX0	N
ATOM	773	H	LEU	104	56.892	7.238	-1.467	1.00	0.00	LX0	H
ATOM	774	CA	LEU	104	57.319	9.180	-0.703	1.00	0.00	LX0	C
ATOM	775	CB	LEU	104	56.260	8.846	0.357	1.00	0.00	LX0	C
ATOM	776	CG	LEU	104	55.918	9.965	1.350	1.00	0.00	LX0	C
ATOM	777	CD1	LEU	104	55.378	11.213	0.656	1.00	0.00	LX0	C
ATOM	778	CD2	LEU	104	54.973	9.479	2.448	1.00	0.00	LX0	C
ATOM	779	C	LEU	104	58.621	9.597	-0.050	1.00	0.00	LX0	C
ATOM	780	O	LEU	104	59.367	8.754	0.429	1.00	0.00	LX0	O
ATOM	781	N	GLN	105	58.870	10.914	-0.083	1.00	0.00	LX0	N
ATOM	782	H	GLN	105	58.190	11.540	-0.466	1.00	0.00	LX0	H
ATOM	783	CA	GLN	105	60.135	11.394	0.470	1.00	0.00	LX0	C
ATOM	784	CB	GLN	105	60.850	12.326	-0.508	1.00	0.00	LX0	C
ATOM	785	CG	GLN	105	61.022	11.760	-1.917	1.00	0.00	LX0	C
ATOM	786	CD	GLN	105	61.840	12.728	-2.743	1.00	0.00	LX0	C
ATOM	787	OE1	GLN	105	62.886	13.211	-2.336	1.00	0.00	LX0	O
ATOM	788	NE2	GLN	105	61.303	13.009	-3.932	1.00	0.00	LX0	N
ATOM	789	HE21	GLN	105	60.461	12.569	-4.236	1.00	0.00	LX0	H
ATOM	790	HE22	GLN	105	61.754	13.678	-4.521	1.00	0.00	LX0	H
ATOM	791	C	GLN	105	60.014	12.087	1.813	1.00	0.00	LX0	C
ATOM	792	O	GLN	105	60.810	11.886	2.729	1.00	0.00	LX0	O
ATOM	793	N	ILE	106	58.977	12.938	1.869	1.00	0.00	LX0	N
ATOM	794	H	ILE	106	58.346	13.042	1.098	1.00	0.00	LX0	H
ATOM	795	CA	ILE	106	58.830	13.790	3.045	1.00	0.00	LX0	C
ATOM	796	CB	ILE	106	59.406	15.193	2.757	1.00	0.00	LX0	C
ATOM	797	CG2	ILE	106	58.699	15.842	1.572	1.00	0.00	LX0	C
ATOM	798	CG1	ILE	106	59.386	16.129	3.970	1.00	0.00	LX0	C
ATOM	799	CD1	ILE	106	59.894	17.532	3.636	1.00	0.00	LX0	C
ATOM	800	C	ILE	106	57.392	13.873	3.519	1.00	0.00	LX0	C
ATOM	801	O	ILE	106	56.455	14.046	2.745	1.00	0.00	LX0	O
ATOM	802	N	ILE	107	57.266	13.778	4.844	1.00	0.00	LX0	N
ATOM	803	H	ILE	107	58.070	13.576	5.409	1.00	0.00	LX0	H
ATOM	804	CA	ILE	107	56.049	14.316	5.435	1.00	0.00	LX0	C
ATOM	805	CB	ILE	107	55.425	13.306	6.403	1.00	0.00	LX0	C

ATOM	806	CG2	ILE	107	54.098	13.810	6.985	1.00	0.00	LX0	C
ATOM	807	CG1	ILE	107	55.265	11.949	5.713	1.00	0.00	LX0	C
ATOM	808	CD1	ILE	107	54.903	10.823	6.677	1.00	0.00	LX0	C
ATOM	809	C	ILE	107	56.423	15.606	6.136	1.00	0.00	LX0	C
ATOM	810	O	ILE	107	57.348	15.648	6.939	1.00	0.00	LX0	O
ATOM	811	N	ARG	108	55.701	16.669	5.767	1.00	0.00	LX0	N
ATOM	812	H	ARG	108	54.950	16.571	5.115	1.00	0.00	LX0	H
ATOM	813	CA	ARG	108	55.996	17.930	6.435	1.00	0.00	LX0	C
ATOM	814	CB	ARG	108	55.602	19.135	5.579	1.00	0.00	LX0	C
ATOM	815	CG	ARG	108	56.576	19.302	4.413	1.00	0.00	LX0	C
ATOM	816	CD	ARG	108	56.315	20.535	3.545	1.00	0.00	LX0	C
ATOM	817	NE	ARG	108	57.410	20.722	2.591	1.00	0.00	LX0	N
ATOM	818	HE	ARG	108	58.282	21.091	2.930	1.00	0.00	LX0	H
ATOM	819	CZ	ARG	108	57.288	20.345	1.301	1.00	0.00	LX0	C
ATOM	820	NH1	ARG	108	56.134	19.901	0.816	1.00	0.00	LX0	N
ATOM	821	HH11	ARG	108	56.097	19.640	-0.159	1.00	0.00	LX0	H
ATOM	822	HH12	ARG	108	55.316	19.823	1.383	1.00	0.00	LX0	H
ATOM	823	NH2	ARG	108	58.339	20.412	0.500	1.00	0.00	LX0	N
ATOM	824	HH21	ARG	108	58.262	20.103	-0.462	1.00	0.00	LX0	H
ATOM	825	HH22	ARG	108	59.215	20.755	0.844	1.00	0.00	LX0	H
ATOM	826	C	ARG	108	55.413	18.006	7.824	1.00	0.00	LX0	C
ATOM	827	O	ARG	108	56.127	18.244	8.782	1.00	0.00	LX0	O
ATOM	828	N	GLY	109	54.102	17.749	7.905	1.00	0.00	LX0	N
ATOM	829	H	GLY	109	53.524	17.657	7.093	1.00	0.00	LX0	H
ATOM	830	CA	GLY	109	53.531	17.745	9.248	1.00	0.00	LX0	C
ATOM	831	C	GLY	109	52.968	19.087	9.676	1.00	0.00	LX0	C
ATOM	832	O	GLY	109	53.021	19.498	10.827	1.00	0.00	LX0	O
ATOM	833	N	ASN	110	52.369	19.763	8.686	1.00	0.00	LX0	N
ATOM	834	H	ASN	110	52.460	19.451	7.739	1.00	0.00	LX0	H
ATOM	835	CA	ASN	110	51.547	20.928	9.024	1.00	0.00	LX0	C
ATOM	836	CB	ASN	110	51.006	21.636	7.769	1.00	0.00	LX0	C
ATOM	837	CG	ASN	110	52.096	22.009	6.778	1.00	0.00	LX0	C
ATOM	838	OD1	ASN	110	52.909	21.197	6.355	1.00	0.00	LX0	O
ATOM	839	ND2	ASN	110	52.037	23.272	6.352	1.00	0.00	LX0	N
ATOM	840	HD21	ASN	110	51.444	23.962	6.775	1.00	0.00	LX0	H
ATOM	841	HD22	ASN	110	52.626	23.558	5.602	1.00	0.00	LX0	H
ATOM	842	C	ASN	110	50.364	20.477	9.868	1.00	0.00	LX0	C
ATOM	843	O	ASN	110	50.084	20.934	10.975	1.00	0.00	LX0	O
ATOM	844	N	MET	111	49.682	19.485	9.284	1.00	0.00	LX0	N
ATOM	845	H	MET	111	50.034	19.075	8.443	1.00	0.00	LX0	H
ATOM	846	CA	MET	111	48.661	18.833	10.092	1.00	0.00	LX0	C
ATOM	847	CB	MET	111	47.501	18.360	9.220	1.00	0.00	LX0	C
ATOM	848	CG	MET	111	46.621	19.499	8.705	1.00	0.00	LX0	C
ATOM	849	SD	MET	111	45.749	20.340	10.038	1.00	0.00	LX0	S
ATOM	850	CE	MET	111	44.738	18.955	10.593	1.00	0.00	LX0	C
ATOM	851	C	MET	111	49.254	17.682	10.870	1.00	0.00	LX0	C
ATOM	852	O	MET	111	50.192	17.028	10.435	1.00	0.00	LX0	O
ATOM	853	N	TYR	112	48.669	17.492	12.056	1.00	0.00	LX0	N
ATOM	854	H	TYR	112	47.843	17.997	12.302	1.00	0.00	LX0	H
ATOM	855	CA	TYR	112	49.080	16.376	12.901	1.00	0.00	LX0	C
ATOM	856	CB	TYR	112	49.585	16.880	14.254	1.00	0.00	LX0	C
ATOM	857	CG	TYR	112	51.025	17.342	14.352	1.00	0.00	LX0	C
ATOM	858	CD1	TYR	112	51.773	17.761	13.232	1.00	0.00	LX0	C
ATOM	859	CE1	TYR	112	53.078	18.245	13.431	1.00	0.00	LX0	C
ATOM	860	CD2	TYR	112	51.576	17.364	15.648	1.00	0.00	LX0	C
ATOM	861	CE2	TYR	112	52.878	17.843	15.849	1.00	0.00	LX0	C
ATOM	862	CZ	TYR	112	53.612	18.285	14.735	1.00	0.00	LX0	C
ATOM	863	OH	TYR	112	54.893	18.763	14.930	1.00	0.00	LX0	O
ATOM	864	HH	TYR	112	55.017	18.961	15.852	1.00	0.00	LX0	H
ATOM	865	C	TYR	112	47.825	15.582	13.188	1.00	0.00	LX0	C
ATOM	866	O	TYR	112	46.805	16.177	13.517	1.00	0.00	LX0	O

ATOM	867	N	TYR	113	47.913	14.252	13.061	1.00	0.00	LX0	N
ATOM	868	H	TYR	113	48.811	13.835	12.923	1.00	0.00	LX0	H
ATOM	869	CA	TYR	113	46.747	13.464	13.456	1.00	0.00	LX0	C
ATOM	870	CB	TYR	113	46.824	12.036	12.892	1.00	0.00	LX0	C
ATOM	871	CG	TYR	113	45.469	11.372	13.004	1.00	0.00	LX0	C
ATOM	872	CD1	TYR	113	44.385	11.933	12.301	1.00	0.00	LX0	C
ATOM	873	CE1	TYR	113	43.105	11.390	12.464	1.00	0.00	LX0	C
ATOM	874	CD2	TYR	113	45.313	10.230	13.816	1.00	0.00	LX0	C
ATOM	875	CE2	TYR	113	44.027	9.682	13.982	1.00	0.00	LX0	C
ATOM	876	CZ	TYR	113	42.935	10.297	13.333	1.00	0.00	LX0	C
ATOM	877	OH	TYR	113	41.649	9.841	13.559	1.00	0.00	LX0	O
ATOM	878	HH	TYR	113	41.695	8.956	13.905	1.00	0.00	LX0	H
ATOM	879	C	TYR	113	46.585	13.459	14.962	1.00	0.00	LX0	C
ATOM	880	O	TYR	113	47.461	13.004	15.680	1.00	0.00	LX0	O
ATOM	881	N	GLU	114	45.455	14.052	15.387	1.00	0.00	LX0	N
ATOM	882	H	GLU	114	44.717	14.201	14.727	1.00	0.00	LX0	H
ATOM	883	CA	GLU	114	45.156	14.311	16.803	1.00	0.00	LX0	C
ATOM	884	CB	GLU	114	44.585	13.064	17.500	1.00	0.00	LX0	C
ATOM	885	CG	GLU	114	43.827	12.052	16.622	1.00	0.00	LX0	C
ATOM	886	CD	GLU	114	42.573	12.606	15.958	1.00	0.00	LX0	C
ATOM	887	OE1	GLU	114	42.655	13.216	14.891	1.00	0.00	LX0	O
ATOM	888	OE2	GLU	114	41.480	12.340	16.445	1.00	0.00	LX0	O
ATOM	889	C	GLU	114	46.268	14.960	17.632	1.00	0.00	LX0	C
ATOM	890	O	GLU	114	46.390	14.783	18.838	1.00	0.00	LX0	O
ATOM	891	N	ASN	115	47.078	15.751	16.900	1.00	0.00	LX0	N
ATOM	892	H	ASN	115	46.844	15.836	15.933	1.00	0.00	LX0	H
ATOM	893	CA	ASN	115	48.358	16.259	17.411	1.00	0.00	LX0	C
ATOM	894	CB	ASN	115	48.197	17.364	18.469	1.00	0.00	LX0	C
ATOM	895	CG	ASN	115	49.313	18.375	18.269	1.00	0.00	LX0	C
ATOM	896	OD1	ASN	115	49.480	18.953	17.199	1.00	0.00	LX0	O
ATOM	897	ND2	ASN	115	50.126	18.504	19.320	1.00	0.00	LX0	N
ATOM	898	HD21	ASN	115	49.956	18.071	20.204	1.00	0.00	LX0	H
ATOM	899	HD22	ASN	115	50.970	19.055	19.291	1.00	0.00	LX0	H
ATOM	900	C	ASN	115	49.366	15.174	17.789	1.00	0.00	LX0	C
ATOM	901	O	ASN	115	49.062	13.993	17.739	1.00	0.00	LX0	O
ATOM	902	N	SER	116	50.605	15.607	18.101	1.00	0.00	LX0	N
ATOM	903	H	SER	116	50.809	16.580	18.168	1.00	0.00	LX0	H
ATOM	904	CA	SER	116	51.743	14.683	18.165	1.00	0.00	LX0	C
ATOM	905	CB	SER	116	51.575	13.607	19.260	1.00	0.00	LX0	C
ATOM	906	OG	SER	116	52.746	12.791	19.376	1.00	0.00	LX0	O
ATOM	907	HG	SER	116	52.812	12.575	20.306	1.00	0.00	LX0	H
ATOM	908	C	SER	116	52.133	14.106	16.808	1.00	0.00	LX0	C
ATOM	909	O	SER	116	53.132	14.501	16.217	1.00	0.00	LX0	O
ATOM	910	N	TYR	117	51.324	13.157	16.327	1.00	0.00	LX0	N
ATOM	911	H	TYR	117	50.413	13.010	16.722	1.00	0.00	LX0	H
ATOM	912	CA	TYR	117	51.794	12.384	15.183	1.00	0.00	LX0	C
ATOM	913	CB	TYR	117	51.233	10.962	15.264	1.00	0.00	LX0	C
ATOM	914	CG	TYR	117	51.664	10.327	16.567	1.00	0.00	LX0	C
ATOM	915	CD1	TYR	117	52.997	9.895	16.699	1.00	0.00	LX0	C
ATOM	916	CE1	TYR	117	53.412	9.314	17.906	1.00	0.00	LX0	C
ATOM	917	CD2	TYR	117	50.730	10.191	17.616	1.00	0.00	LX0	C
ATOM	918	CE2	TYR	117	51.146	9.610	18.827	1.00	0.00	LX0	C
ATOM	919	CZ	TYR	117	52.480	9.171	18.950	1.00	0.00	LX0	C
ATOM	920	OH	TYR	117	52.888	8.560	20.121	1.00	0.00	LX0	O
ATOM	921	HH	TYR	117	53.356	9.225	20.637	1.00	0.00	LX0	H
ATOM	922	C	TYR	117	51.506	13.011	13.836	1.00	0.00	LX0	C
ATOM	923	O	TYR	117	50.552	13.755	13.662	1.00	0.00	LX0	O
ATOM	924	N	ALA	118	52.369	12.663	12.877	1.00	0.00	LX0	N
ATOM	925	H	ALA	118	53.155	12.098	13.123	1.00	0.00	LX0	H
ATOM	926	CA	ALA	118	52.067	13.009	11.492	1.00	0.00	LX0	C
ATOM	927	CB	ALA	118	53.202	13.808	10.853	1.00	0.00	LX0	C

ATOM	928	C	ALA	118	51.819	11.766	10.665	1.00	0.00	LX0	C
ATOM	929	O	ALA	118	50.823	11.631	9.966	1.00	0.00	LX0	O
ATOM	930	N	LEU	119	52.767	10.829	10.788	1.00	0.00	LX0	N
ATOM	931	H	LEU	119	53.530	10.950	11.423	1.00	0.00	LX0	H
ATOM	932	CA	LEU	119	52.471	9.535	10.181	1.00	0.00	LX0	C
ATOM	933	CB	LEU	119	53.774	8.872	9.721	1.00	0.00	LX0	C
ATOM	934	CG	LEU	119	53.624	7.541	8.971	1.00	0.00	LX0	C
ATOM	935	CD1	LEU	119	52.697	7.645	7.760	1.00	0.00	LX0	C
ATOM	936	CD2	LEU	119	54.982	6.968	8.567	1.00	0.00	LX0	C
ATOM	937	C	LEU	119	51.727	8.683	11.187	1.00	0.00	LX0	C
ATOM	938	O	LEU	119	52.151	8.570	12.329	1.00	0.00	LX0	O
ATOM	939	N	ALA	120	50.608	8.110	10.735	1.00	0.00	LX0	N
ATOM	940	H	ALA	120	50.274	8.304	9.810	1.00	0.00	LX0	H
ATOM	941	CA	ALA	120	49.845	7.289	11.668	1.00	0.00	LX0	C
ATOM	942	CB	ALA	120	48.698	8.095	12.281	1.00	0.00	LX0	C
ATOM	943	C	ALA	120	49.306	6.037	11.011	1.00	0.00	LX0	C
ATOM	944	O	ALA	120	48.259	6.026	10.376	1.00	0.00	LX0	O
ATOM	945	N	VAL	121	50.098	4.974	11.178	1.00	0.00	LX0	N
ATOM	946	H	VAL	121	50.914	5.063	11.750	1.00	0.00	LX0	H
ATOM	947	CA	VAL	121	49.751	3.695	10.564	1.00	0.00	LX0	C
ATOM	948	CB	VAL	121	51.013	3.066	9.952	1.00	0.00	LX0	C
ATOM	949	CG1	VAL	121	50.733	1.772	9.186	1.00	0.00	LX0	C
ATOM	950	CG2	VAL	121	51.723	4.077	9.052	1.00	0.00	LX0	C
ATOM	951	C	VAL	121	49.059	2.764	11.551	1.00	0.00	LX0	C
ATOM	952	O	VAL	121	49.679	1.940	12.219	1.00	0.00	LX0	O
ATOM	953	N	LEU	122	47.738	2.966	11.627	1.00	0.00	LX0	N
ATOM	954	H	LEU	122	47.308	3.565	10.947	1.00	0.00	LX0	H
ATOM	955	CA	LEU	122	46.944	2.316	12.671	1.00	0.00	LX0	C
ATOM	956	CB	LEU	122	46.067	3.354	13.377	1.00	0.00	LX0	C
ATOM	957	CG	LEU	122	46.783	4.648	13.772	1.00	0.00	LX0	C
ATOM	958	CD1	LEU	122	45.797	5.723	14.228	1.00	0.00	LX0	C
ATOM	959	CD2	LEU	122	47.882	4.409	14.803	1.00	0.00	LX0	C
ATOM	960	C	LEU	122	46.038	1.212	12.151	1.00	0.00	LX0	C
ATOM	961	O	LEU	122	45.374	1.369	11.134	1.00	0.00	LX0	O
ATOM	962	N	SER	123	46.009	0.100	12.905	1.00	0.00	LX0	N
ATOM	963	H	SER	123	46.637	0.030	13.681	1.00	0.00	LX0	H
ATOM	964	CA	SER	123	44.998	-0.941	12.671	1.00	0.00	LX0	C
ATOM	965	CB	SER	123	43.662	-0.543	13.294	1.00	0.00	LX0	C
ATOM	966	OG	SER	123	43.725	-0.653	14.721	1.00	0.00	LX0	O
ATOM	967	HG	SER	123	42.954	-1.176	14.941	1.00	0.00	LX0	H
ATOM	968	C	SER	123	44.799	-1.410	11.237	1.00	0.00	LX0	C
ATOM	969	O	SER	123	43.701	-1.442	10.699	1.00	0.00	LX0	O
ATOM	970	N	ASN	124	45.929	-1.761	10.621	1.00	0.00	LX0	N
ATOM	971	H	ASN	124	46.758	-1.899	11.161	1.00	0.00	LX0	H
ATOM	972	CA	ASN	124	45.809	-2.037	9.189	1.00	0.00	LX0	C
ATOM	973	CB	ASN	124	46.929	-1.365	8.396	1.00	0.00	LX0	C
ATOM	974	CG	ASN	124	46.738	0.131	8.396	1.00	0.00	LX0	C
ATOM	975	OD1	ASN	124	45.888	0.691	7.720	1.00	0.00	LX0	O
ATOM	976	ND2	ASN	124	47.571	0.773	9.208	1.00	0.00	LX0	N
ATOM	977	HD21	ASN	124	48.290	0.309	9.717	1.00	0.00	LX0	H
ATOM	978	HD22	ASN	124	47.422	1.760	9.288	1.00	0.00	LX0	H
ATOM	979	C	ASN	124	45.790	-3.511	8.871	1.00	0.00	LX0	C
ATOM	980	O	ASN	124	46.829	-4.080	8.553	1.00	0.00	LX0	O
ATOM	981	N	TYR	125	44.588	-4.104	9.001	1.00	0.00	LX0	N
ATOM	982	H	TYR	125	43.772	-3.567	9.224	1.00	0.00	LX0	H
ATOM	983	CA	TYR	125	44.476	-5.552	8.804	1.00	0.00	LX0	C
ATOM	984	CB	TYR	125	45.174	-6.319	9.935	1.00	0.00	LX0	C
ATOM	985	CG	TYR	125	44.520	-6.025	11.256	1.00	0.00	LX0	C
ATOM	986	CD1	TYR	125	43.467	-6.850	11.693	1.00	0.00	LX0	C
ATOM	987	CE1	TYR	125	42.867	-6.584	12.929	1.00	0.00	LX0	C
ATOM	988	CD2	TYR	125	44.982	-4.928	12.004	1.00	0.00	LX0	C

ATOM	989	CE2	TYR	125	44.388	-4.666	13.241	1.00	0.00	LX0	C
ATOM	990	CZ	TYR	125	43.366	-5.515	13.695	1.00	0.00	LX0	C
ATOM	991	OH	TYR	125	42.859	-5.308	14.959	1.00	0.00	LX0	O
ATOM	992	HH	TYR	125	42.148	-4.675	14.808	1.00	0.00	LX0	H
ATOM	993	C	TYR	125	43.054	-6.061	8.638	1.00	0.00	LX0	C
ATOM	994	O	TYR	125	42.092	-5.384	8.977	1.00	0.00	LX0	O
ATOM	995	N	ASP	126	42.989	-7.294	8.114	1.00	0.00	LX0	N
ATOM	996	H	ASP	126	43.820	-7.787	7.854	1.00	0.00	LX0	H
ATOM	997	CA	ASP	126	41.717	-7.967	7.862	1.00	0.00	LX0	C
ATOM	998	CB	ASP	126	41.883	-8.800	6.573	1.00	0.00	LX0	C
ATOM	999	CG	ASP	126	42.549	-10.155	6.813	1.00	0.00	LX0	C
ATOM	1000	OD1	ASP	126	41.983	-11.157	6.401	1.00	0.00	LX0	O
ATOM	1001	OD2	ASP	126	43.594	-10.237	7.450	1.00	0.00	LX0	O
ATOM	1002	C	ASP	126	41.170	-8.752	9.059	1.00	0.00	LX0	C
ATOM	1003	O	ASP	126	41.419	-8.422	10.214	1.00	0.00	LX0	O
ATOM	1004	N	ALA	127	40.428	-9.834	8.748	1.00	0.00	LX0	N
ATOM	1005	H	ALA	127	40.285	-10.098	7.795	1.00	0.00	LX0	H
ATOM	1006	CA	ALA	127	40.104	-10.797	9.796	1.00	0.00	LX0	C
ATOM	1007	CB	ALA	127	39.153	-11.870	9.260	1.00	0.00	LX0	C
ATOM	1008	C	ALA	127	41.328	-11.490	10.367	1.00	0.00	LX0	C
ATOM	1009	O	ALA	127	41.509	-11.635	11.569	1.00	0.00	LX0	O
ATOM	1010	N	ASN	128	42.186	-11.910	9.434	1.00	0.00	LX0	N
ATOM	1011	H	ASN	128	42.099	-11.590	8.486	1.00	0.00	LX0	H
ATOM	1012	CA	ASN	128	43.316	-12.757	9.817	1.00	0.00	LX0	C
ATOM	1013	CB	ASN	128	43.756	-13.640	8.643	1.00	0.00	LX0	C
ATOM	1014	CG	ASN	128	42.622	-14.519	8.158	1.00	0.00	LX0	C
ATOM	1015	OD1	ASN	128	42.341	-15.585	8.685	1.00	0.00	LX0	O
ATOM	1016	ND2	ASN	128	41.976	-14.026	7.097	1.00	0.00	LX0	N
ATOM	1017	HD21	ASN	128	42.208	-13.124	6.717	1.00	0.00	LX0	H
ATOM	1018	HD22	ASN	128	41.245	-14.563	6.684	1.00	0.00	LX0	H
ATOM	1019	C	ASN	128	44.524	-11.984	10.307	1.00	0.00	LX0	C
ATOM	1020	O	ASN	128	45.659	-12.383	10.085	1.00	0.00	LX0	O
ATOM	1021	N	LYS	129	44.252	-10.824	10.943	1.00	0.00	LX0	N
ATOM	1022	H	LYS	129	43.301	-10.639	11.185	1.00	0.00	LX0	H
ATOM	1023	CA	LYS	129	45.333	-9.917	11.348	1.00	0.00	LX0	C
ATOM	1024	CB	LYS	129	45.975	-10.364	12.673	1.00	0.00	LX0	C
ATOM	1025	CG	LYS	129	45.229	-10.040	13.976	1.00	0.00	LX0	C
ATOM	1026	CD	LYS	129	45.293	-8.561	14.382	1.00	0.00	LX0	C
ATOM	1027	CE	LYS	129	44.781	-8.285	15.803	1.00	0.00	LX0	C
ATOM	1028	NZ	LYS	129	44.767	-6.841	16.098	1.00	0.00	LX0	N
ATOM	1029	HZ1	LYS	129	43.956	-6.370	15.644	1.00	0.00	LX0	H
ATOM	1030	HZ2	LYS	129	44.696	-6.672	17.118	1.00	0.00	LX0	H
ATOM	1031	HZ3	LYS	129	45.629	-6.348	15.775	1.00	0.00	LX0	H
ATOM	1032	C	LYS	129	46.411	-9.658	10.295	1.00	0.00	LX0	C
ATOM	1033	O	LYS	129	47.585	-9.514	10.603	1.00	0.00	LX0	O
ATOM	1034	N	THR	130	45.966	-9.591	9.034	1.00	0.00	LX0	N
ATOM	1035	H	THR	130	45.007	-9.739	8.787	1.00	0.00	LX0	H
ATOM	1036	CA	THR	130	46.942	-9.233	8.012	1.00	0.00	LX0	C
ATOM	1037	CB	THR	130	47.311	-10.449	7.146	1.00	0.00	LX0	C
ATOM	1038	OG1	THR	130	46.148	-11.016	6.518	1.00	0.00	LX0	O
ATOM	1039	HG1	THR	130	45.486	-11.102	7.207	1.00	0.00	LX0	H
ATOM	1040	CG2	THR	130	48.089	-11.516	7.919	1.00	0.00	LX0	C
ATOM	1041	C	THR	130	46.413	-8.103	7.157	1.00	0.00	LX0	C
ATOM	1042	O	THR	130	45.295	-8.153	6.663	1.00	0.00	LX0	O
ATOM	1043	N	GLY	131	47.227	-7.068	7.008	1.00	0.00	LX0	N
ATOM	1044	H	GLY	131	48.051	-6.951	7.567	1.00	0.00	LX0	H
ATOM	1045	CA	GLY	131	46.764	-6.058	6.068	1.00	0.00	LX0	C
ATOM	1046	C	GLY	131	47.922	-5.412	5.375	1.00	0.00	LX0	C
ATOM	1047	O	GLY	131	48.439	-5.896	4.380	1.00	0.00	LX0	O
ATOM	1048	N	LEU	132	48.345	-4.302	5.982	1.00	0.00	LX0	N
ATOM	1049	H	LEU	132	47.937	-4.038	6.857	1.00	0.00	LX0	H

```
ATOM   1050  CA  LEU   132      49.520  -3.666   5.404  1.00  0.00      LX0  C
ATOM   1051  CB  LEU   132      49.626  -2.216   5.884  1.00  0.00      LX0  C
ATOM   1052  CG  LEU   132      50.795  -1.429   5.283  1.00  0.00      LX0  C
ATOM   1053  CD1 LEU   132      50.841  -1.523   3.759  1.00  0.00      LX0  C
ATOM   1054  CD2 LEU   132      50.798   0.016   5.772  1.00  0.00      LX0  C
ATOM   1055  C   LEU   132      50.792  -4.450   5.663  1.00  0.00      LX0  C
ATOM   1056  O   LEU   132      51.445  -4.304   6.687  1.00  0.00      LX0  O
ATOM   1057  N   LYS   133      51.115  -5.280   4.669  1.00  0.00      LX0  N
ATOM   1058  H   LYS   133      50.469  -5.385   3.907  1.00  0.00      LX0  H
ATOM   1059  CA  LYS   133      52.412  -5.939   4.673  1.00  0.00      LX0  C
ATOM   1060  CB  LYS   133      52.360  -7.204   3.806  1.00  0.00      LX0  C
ATOM   1061  CG  LYS   133      53.676  -7.980   3.825  1.00  0.00      LX0  C
ATOM   1062  CD  LYS   133      53.716  -9.166   2.866  1.00  0.00      LX0  C
ATOM   1063  CE  LYS   133      55.081  -9.858   2.884  1.00  0.00      LX0  C
ATOM   1064  NZ  LYS   133      56.123  -8.925   2.440  1.00  0.00      LX0  N
ATOM   1065  HZ1 LYS   133      57.068  -9.352   2.415  1.00  0.00      LX0  H
ATOM   1066  HZ2 LYS   133      55.922  -8.551   1.489  1.00  0.00      LX0  H
ATOM   1067  HZ3 LYS   133      56.185  -8.057   3.016  1.00  0.00      LX0  H
ATOM   1068  C   LYS   133      53.510  -5.001   4.213  1.00  0.00      LX0  C
ATOM   1069  O   LYS   133      54.417  -4.622   4.947  1.00  0.00      LX0  O
ATOM   1070  N   GLU   134      53.390  -4.642   2.933  1.00  0.00      LX0  N
ATOM   1071  H   GLU   134      52.556  -4.853   2.417  1.00  0.00      LX0  H
ATOM   1072  CA  GLU   134      54.481  -3.841   2.409  1.00  0.00      LX0  C
ATOM   1073  CB  GLU   134      54.900  -4.298   1.015  1.00  0.00      LX0  C
ATOM   1074  CG  GLU   134      56.355  -4.761   0.924  1.00  0.00      LX0  C
ATOM   1075  CD  GLU   134      56.473  -6.203   1.374  1.00  0.00      LX0  C
ATOM   1076  OE1 GLU   134      56.179  -7.096   0.588  1.00  0.00      LX0  O
ATOM   1077  OE2 GLU   134      56.899  -6.464   2.494  1.00  0.00      LX0  O
ATOM   1078  C   GLU   134      54.163  -2.369   2.398  1.00  0.00      LX0  C
ATOM   1079  O   GLU   134      53.351  -1.879   1.618  1.00  0.00      LX0  O
ATOM   1080  N   LEU   135      54.885  -1.678   3.286  1.00  0.00      LX0  N
ATOM   1081  H   LEU   135      55.446  -2.177   3.948  1.00  0.00      LX0  H
ATOM   1082  CA  LEU   135      54.944  -0.225   3.157  1.00  0.00      LX0  C
ATOM   1083  CB  LEU   135      54.580   0.421   4.495  1.00  0.00      LX0  C
ATOM   1084  CG  LEU   135      54.304   1.925   4.425  1.00  0.00      LX0  C
ATOM   1085  CD1 LEU   135      53.188   2.266   3.434  1.00  0.00      LX0  C
ATOM   1086  CD2 LEU   135      54.033   2.500   5.816  1.00  0.00      LX0  C
ATOM   1087  C   LEU   135      56.324   0.196   2.670  1.00  0.00      LX0  C
ATOM   1088  O   LEU   135      57.226   0.510   3.437  1.00  0.00      LX0  O
ATOM   1089  N   PRO   136      56.491   0.150   1.331  1.00  0.00      LX0  N
ATOM   1090  CD  PRO   136      55.487   0.001   0.291  1.00  0.00      LX0  C
ATOM   1091  CA  PRO   136      57.845   0.166   0.791  1.00  0.00      LX0  C
ATOM   1092  CB  PRO   136      57.694  -0.672  -0.486  1.00  0.00      LX0  C
ATOM   1093  CG  PRO   136      56.197  -0.796  -0.794  1.00  0.00      LX0  C
ATOM   1094  C   PRO   136      58.374   1.558   0.513  1.00  0.00      LX0  C
ATOM   1095  O   PRO   136      58.881   1.826  -0.571  1.00  0.00      LX0  O
ATOM   1096  N   MET   137      58.221   2.448   1.503  1.00  0.00      LX0  N
ATOM   1097  H   MET   137      57.973   2.130   2.421  1.00  0.00      LX0  H
ATOM   1098  CA  MET   137      58.536   3.848   1.213  1.00  0.00      LX0  C
ATOM   1099  CB  MET   137      57.740   4.796   2.116  1.00  0.00      LX0  C
ATOM   1100  CG  MET   137      56.240   4.502   2.099  1.00  0.00      LX0  C
ATOM   1101  SD  MET   137      55.234   5.788   2.860  1.00  0.00      LX0  S
ATOM   1102  CE  MET   137      55.863   5.677   4.538  1.00  0.00      LX0  C
ATOM   1103  C   MET   137      60.015   4.189   1.248  1.00  0.00      LX0  C
ATOM   1104  O   MET   137      60.508   4.864   2.140  1.00  0.00      LX0  O
ATOM   1105  N   ARG   138      60.722   3.701   0.218  1.00  0.00      LX0  N
ATOM   1106  H   ARG   138      60.200   3.280  -0.527  1.00  0.00      LX0  H
ATOM   1107  CA  ARG   138      62.185   3.816   0.222  1.00  0.00      LX0  C
ATOM   1108  CB  ARG   138      62.867   3.032  -0.913  1.00  0.00      LX0  C
ATOM   1109  CG  ARG   138      62.489   3.430  -2.347  1.00  0.00      LX0  C
ATOM   1110  CD  ARG   138      61.297   2.614  -2.831  1.00  0.00      LX0  C
```

ATOM	1111	NE	ARG	138	60.755	3.046	-4.114	1.00	0.00	LX0	N
ATOM	1112	HE	ARG	138	60.755	4.019	-4.362	1.00	0.00	LX0	H
ATOM	1113	CZ	ARG	138	59.994	2.169	-4.800	1.00	0.00	LX0	C
ATOM	1114	NH1	ARG	138	59.923	0.886	-4.457	1.00	0.00	LX0	N
ATOM	1115	HH11	ARG	138	59.250	0.291	-4.901	1.00	0.00	LX0	H
ATOM	1116	HH12	ARG	138	60.527	0.487	-3.753	1.00	0.00	LX0	H
ATOM	1117	NH2	ARG	138	59.276	2.606	-5.823	1.00	0.00	LX0	N
ATOM	1118	HH21	ARG	138	58.654	1.985	-6.306	1.00	0.00	LX0	H
ATOM	1119	HH22	ARG	138	59.340	3.573	-6.092	1.00	0.00	LX0	H
ATOM	1120	C	ARG	138	62.755	5.219	0.219	1.00	0.00	LX0	C
ATOM	1121	O	ARG	138	63.956	5.422	0.372	1.00	0.00	LX0	O
ATOM	1122	N	ASN	139	61.871	6.191	-0.011	1.00	0.00	LX0	N
ATOM	1123	H	ASN	139	60.887	6.028	-0.088	1.00	0.00	LX0	H
ATOM	1124	CA	ASN	139	62.434	7.531	-0.005	1.00	0.00	LX0	C
ATOM	1125	CB	ASN	139	62.087	8.302	-1.274	1.00	0.00	LX0	C
ATOM	1126	CG	ASN	139	62.672	7.632	-2.494	1.00	0.00	LX0	C
ATOM	1127	OD1	ASN	139	63.705	6.964	-2.456	1.00	0.00	LX0	O
ATOM	1128	ND2	ASN	139	61.945	7.834	-3.595	1.00	0.00	LX0	N
ATOM	1129	HD21	ASN	139	61.110	8.389	-3.524	1.00	0.00	LX0	H
ATOM	1130	HD22	ASN	139	62.220	7.450	-4.474	1.00	0.00	LX0	H
ATOM	1131	C	ASN	139	62.066	8.345	1.208	1.00	0.00	LX0	C
ATOM	1132	O	ASN	139	62.447	9.506	1.305	1.00	0.00	LX0	O
ATOM	1133	N	LEU	140	61.325	7.706	2.135	1.00	0.00	LX0	N
ATOM	1134	H	LEU	140	61.079	6.739	2.070	1.00	0.00	LX0	H
ATOM	1135	CA	LEU	140	60.948	8.475	3.315	1.00	0.00	LX0	C
ATOM	1136	CB	LEU	140	59.777	7.851	4.080	1.00	0.00	LX0	C
ATOM	1137	CG	LEU	140	59.229	8.798	5.159	1.00	0.00	LX0	C
ATOM	1138	CD1	LEU	140	58.618	10.064	4.559	1.00	0.00	LX0	C
ATOM	1139	CD2	LEU	140	58.261	8.106	6.114	1.00	0.00	LX0	C
ATOM	1140	C	LEU	140	62.141	8.692	4.211	1.00	0.00	LX0	C
ATOM	1141	O	LEU	140	62.556	7.847	4.992	1.00	0.00	LX0	O
ATOM	1142	N	GLN	141	62.696	9.881	3.995	1.00	0.00	LX0	N
ATOM	1143	H	GLN	141	62.271	10.463	3.297	1.00	0.00	LX0	H
ATOM	1144	CA	GLN	141	63.893	10.254	4.728	1.00	0.00	LX0	C
ATOM	1145	CB	GLN	141	65.025	10.549	3.744	1.00	0.00	LX0	C
ATOM	1146	CG	GLN	141	65.380	9.339	2.873	1.00	0.00	LX0	C
ATOM	1147	CD	GLN	141	65.906	9.800	1.528	1.00	0.00	LX0	C
ATOM	1148	OE1	GLN	141	67.028	9.524	1.128	1.00	0.00	LX0	O
ATOM	1149	NE2	GLN	141	65.028	10.509	0.812	1.00	0.00	LX0	N
ATOM	1150	HE21	GLN	141	64.079	10.608	1.122	1.00	0.00	LX0	H
ATOM	1151	HE22	GLN	141	65.313	10.936	-0.043	1.00	0.00	LX0	H
ATOM	1152	C	GLN	141	63.620	11.428	5.640	1.00	0.00	LX0	C
ATOM	1153	O	GLN	141	64.281	11.628	6.651	1.00	0.00	LX0	O
ATOM	1154	N	GLU	142	62.599	12.201	5.248	1.00	0.00	LX0	N
ATOM	1155	H	GLU	142	62.022	12.016	4.449	1.00	0.00	LX0	H
ATOM	1156	CA	GLU	142	62.315	13.351	6.089	1.00	0.00	LX0	C
ATOM	1157	CB	GLU	142	62.548	14.643	5.310	1.00	0.00	LX0	C
ATOM	1158	CG	GLU	142	62.573	15.896	6.189	1.00	0.00	LX0	C
ATOM	1159	CD	GLU	142	63.832	15.897	7.022	1.00	0.00	LX0	C
ATOM	1160	OE1	GLU	142	64.880	16.272	6.509	1.00	0.00	LX0	O
ATOM	1161	OE2	GLU	142	63.790	15.474	8.171	1.00	0.00	LX0	O
ATOM	1162	C	GLU	142	60.915	13.335	6.649	1.00	0.00	LX0	C
ATOM	1163	O	GLU	142	59.930	13.255	5.923	1.00	0.00	LX0	O
ATOM	1164	N	ILE	143	60.867	13.452	7.976	1.00	0.00	LX0	N
ATOM	1165	H	ILE	143	61.717	13.483	8.508	1.00	0.00	LX0	H
ATOM	1166	CA	ILE	143	59.628	13.985	8.522	1.00	0.00	LX0	C
ATOM	1167	CB	ILE	143	58.851	12.973	9.384	1.00	0.00	LX0	C
ATOM	1168	CG2	ILE	143	57.573	13.604	9.944	1.00	0.00	LX0	C
ATOM	1169	CG1	ILE	143	58.495	11.709	8.597	1.00	0.00	LX0	C
ATOM	1170	CD1	ILE	143	57.753	10.662	9.432	1.00	0.00	LX0	C
ATOM	1171	C	ILE	143	59.987	15.234	9.292	1.00	0.00	LX0	C

ATOM	1172	O	ILE	143	60.666	15.202	10.316	1.00	0.00	LX0	O
ATOM	1173	N	LEU	144	59.538	16.352	8.712	1.00	0.00	LX0	N
ATOM	1174	H	LEU	144	58.900	16.280	7.943	1.00	0.00	LX0	H
ATOM	1175	CA	LEU	144	59.893	17.625	9.330	1.00	0.00	LX0	C
ATOM	1176	CB	LEU	144	59.517	18.796	8.422	1.00	0.00	LX0	C
ATOM	1177	CG	LEU	144	60.485	19.069	7.272	1.00	0.00	LX0	C
ATOM	1178	CD1	LEU	144	59.975	20.205	6.384	1.00	0.00	LX0	C
ATOM	1179	CD2	LEU	144	61.905	19.348	7.770	1.00	0.00	LX0	C
ATOM	1180	C	LEU	144	59.273	17.804	10.700	1.00	0.00	LX0	C
ATOM	1181	O	LEU	144	59.938	18.140	11.674	1.00	0.00	LX0	O
ATOM	1182	N	HIS	145	57.958	17.566	10.720	1.00	0.00	LX0	N
ATOM	1183	H	HIS	145	57.454	17.300	9.895	1.00	0.00	LX0	H
ATOM	1184	CA	HIS	145	57.195	17.789	11.939	1.00	0.00	LX0	C
ATOM	1185	CB	HIS	145	56.400	19.104	11.878	1.00	0.00	LX0	C
ATOM	1186	CG	HIS	145	57.141	20.198	11.139	1.00	0.00	LX0	C
ATOM	1187	ND1	HIS	145	58.377	20.633	11.450	1.00	0.00	LX0	N
ATOM	1188	HD1	HIS	145	58.965	20.261	12.142	1.00	0.00	LX0	H
ATOM	1189	CD2	HIS	145	56.683	20.918	10.031	1.00	0.00	LX0	C
ATOM	1190	NE2	HIS	145	57.655	21.795	9.682	1.00	0.00	LX0	N
ATOM	1191	CE1	HIS	145	58.698	21.619	10.555	1.00	0.00	LX0	C
ATOM	1192	C	HIS	145	56.242	16.632	12.140	1.00	0.00	LX0	C
ATOM	1193	O	HIS	145	55.860	15.957	11.194	1.00	0.00	LX0	O
ATOM	1194	N	GLY	146	55.881	16.424	13.411	1.00	0.00	LX0	N
ATOM	1195	H	GLY	146	56.150	17.081	14.114	1.00	0.00	LX0	H
ATOM	1196	CA	GLY	146	55.036	15.269	13.702	1.00	0.00	LX0	C
ATOM	1197	C	GLY	146	55.797	13.986	13.957	1.00	0.00	LX0	C
ATOM	1198	O	GLY	146	56.588	13.516	13.154	1.00	0.00	LX0	O
ATOM	1199	N	ALA	147	55.522	13.434	15.144	1.00	0.00	LX0	N
ATOM	1200	H	ALA	147	54.832	13.858	15.729	1.00	0.00	LX0	H
ATOM	1201	CA	ALA	147	56.107	12.137	15.469	1.00	0.00	LX0	C
ATOM	1202	CB	ALA	147	55.888	11.824	16.950	1.00	0.00	LX0	C
ATOM	1203	C	ALA	147	55.527	11.014	14.625	1.00	0.00	LX0	C
ATOM	1204	O	ALA	147	54.577	11.193	13.870	1.00	0.00	LX0	O
ATOM	1205	N	VAL	148	56.139	9.838	14.775	1.00	0.00	LX0	N
ATOM	1206	H	VAL	148	56.849	9.687	15.461	1.00	0.00	LX0	H
ATOM	1207	CA	VAL	148	55.640	8.747	13.950	1.00	0.00	LX0	C
ATOM	1208	CB	VAL	148	56.787	8.197	13.086	1.00	0.00	LX0	C
ATOM	1209	CG1	VAL	148	57.994	7.776	13.922	1.00	0.00	LX0	C
ATOM	1210	CG2	VAL	148	56.316	7.100	12.136	1.00	0.00	LX0	C
ATOM	1211	C	VAL	148	54.930	7.683	14.771	1.00	0.00	LX0	C
ATOM	1212	O	VAL	148	55.403	7.237	15.808	1.00	0.00	LX0	O
ATOM	1213	N	ARG	149	53.748	7.323	14.267	1.00	0.00	LX0	N
ATOM	1214	H	ARG	149	53.417	7.722	13.412	1.00	0.00	LX0	H
ATOM	1215	CA	ARG	149	52.950	6.320	14.954	1.00	0.00	LX0	C
ATOM	1216	CB	ARG	149	51.574	6.895	15.301	1.00	0.00	LX0	C
ATOM	1217	CG	ARG	149	50.806	6.055	16.320	1.00	0.00	LX0	C
ATOM	1218	CD	ARG	149	51.503	6.062	17.674	1.00	0.00	LX0	C
ATOM	1219	NE	ARG	149	50.972	5.035	18.561	1.00	0.00	LX0	N
ATOM	1220	HE	ARG	149	50.499	4.243	18.161	1.00	0.00	LX0	H
ATOM	1221	CZ	ARG	149	51.351	5.021	19.853	1.00	0.00	LX0	C
ATOM	1222	NH1	ARG	149	51.991	6.046	20.408	1.00	0.00	LX0	N
ATOM	1223	HH11	ARG	149	52.413	5.910	21.308	1.00	0.00	LX0	H
ATOM	1224	HH12	ARG	149	52.111	6.938	19.961	1.00	0.00	LX0	H
ATOM	1225	NH2	ARG	149	51.115	3.951	20.592	1.00	0.00	LX0	N
ATOM	1226	HH21	ARG	149	51.334	3.915	21.564	1.00	0.00	LX0	H
ATOM	1227	HH22	ARG	149	50.778	3.094	20.178	1.00	0.00	LX0	H
ATOM	1228	C	ARG	149	52.782	5.080	14.107	1.00	0.00	LX0	C
ATOM	1229	O	ARG	149	52.326	5.135	12.971	1.00	0.00	LX0	O
ATOM	1230	N	PHE	150	53.145	3.953	14.717	1.00	0.00	LX0	N
ATOM	1231	H	PHE	150	53.536	3.971	15.639	1.00	0.00	LX0	H
ATOM	1232	CA	PHE	150	52.736	2.681	14.132	1.00	0.00	LX0	C

ATOM	1233	CB	PHE	150	53.949	1.882	13.646	1.00	0.00	LX0	C
ATOM	1234	CG	PHE	150	54.394	2.318	12.267	1.00	0.00	LX0	C
ATOM	1235	CD1	PHE	150	54.117	1.478	11.168	1.00	0.00	LX0	C
ATOM	1236	CD2	PHE	150	55.087	3.535	12.092	1.00	0.00	LX0	C
ATOM	1237	CE1	PHE	150	54.542	1.854	9.879	1.00	0.00	LX0	C
ATOM	1238	CE2	PHE	150	55.510	3.915	10.804	1.00	0.00	LX0	C
ATOM	1239	CZ	PHE	150	55.233	3.071	9.710	1.00	0.00	LX0	C
ATOM	1240	C	PHE	150	52.036	1.918	15.226	1.00	0.00	LX0	C
ATOM	1241	O	PHE	150	52.535	1.889	16.343	1.00	0.00	LX0	O
ATOM	1242	N	SER	151	50.884	1.333	14.879	1.00	0.00	LX0	N
ATOM	1243	H	SER	151	50.470	1.470	13.978	1.00	0.00	LX0	H
ATOM	1244	CA	SER	151	50.154	0.590	15.904	1.00	0.00	LX0	C
ATOM	1245	CB	SER	151	49.311	1.542	16.762	1.00	0.00	LX0	C
ATOM	1246	OG	SER	151	50.119	2.630	17.229	1.00	0.00	LX0	O
ATOM	1247	HG	SER	151	50.845	2.193	17.689	1.00	0.00	LX0	H
ATOM	1248	C	SER	151	49.241	-0.449	15.290	1.00	0.00	LX0	C
ATOM	1249	O	SER	151	48.473	-0.149	14.381	1.00	0.00	LX0	O
ATOM	1250	N	ASN	152	49.333	-1.680	15.831	1.00	0.00	LX0	N
ATOM	1251	H	ASN	152	50.088	-1.867	16.465	1.00	0.00	LX0	H
ATOM	1252	CA	ASN	152	48.349	-2.724	15.492	1.00	0.00	LX0	C
ATOM	1253	CB	ASN	152	47.032	-2.469	16.251	1.00	0.00	LX0	C
ATOM	1254	CG	ASN	152	45.972	-3.538	16.019	1.00	0.00	LX0	C
ATOM	1255	OD1	ASN	152	46.189	-4.750	16.033	1.00	0.00	LX0	O
ATOM	1256	ND2	ASN	152	44.762	-3.012	15.821	1.00	0.00	LX0	N
ATOM	1257	HD21	ASN	152	44.669	-2.013	15.782	1.00	0.00	LX0	H
ATOM	1258	HD22	ASN	152	43.930	-3.540	15.651	1.00	0.00	LX0	H
ATOM	1259	C	ASN	152	48.152	-2.936	13.994	1.00	0.00	LX0	C
ATOM	1260	O	ASN	152	47.059	-2.935	13.442	1.00	0.00	LX0	O
ATOM	1261	N	ASN	153	49.301	-3.107	13.343	1.00	0.00	LX0	N
ATOM	1262	H	ASN	153	50.153	-3.149	13.872	1.00	0.00	LX0	H
ATOM	1263	CA	ASN	153	49.304	-3.297	11.893	1.00	0.00	LX0	C
ATOM	1264	CB	ASN	153	49.948	-2.068	11.226	1.00	0.00	LX0	C
ATOM	1265	CG	ASN	153	51.280	-1.723	11.880	1.00	0.00	LX0	C
ATOM	1266	OD1	ASN	153	52.076	-2.568	12.268	1.00	0.00	LX0	O
ATOM	1267	ND2	ASN	153	51.475	-0.413	12.041	1.00	0.00	LX0	N
ATOM	1268	HD21	ASN	153	50.791	0.281	11.809	1.00	0.00	LX0	H
ATOM	1269	HD22	ASN	153	52.354	-0.142	12.426	1.00	0.00	LX0	H
ATOM	1270	C	ASN	153	49.964	-4.591	11.417	1.00	0.00	LX0	C
ATOM	1271	O	ASN	153	50.934	-4.579	10.669	1.00	0.00	LX0	O
ATOM	1272	N	PRO	154	49.431	-5.756	11.869	1.00	0.00	LX0	N
ATOM	1273	CD	PRO	154	48.271	-6.014	12.708	1.00	0.00	LX0	C
ATOM	1274	CA	PRO	154	50.112	-6.991	11.492	1.00	0.00	LX0	C
ATOM	1275	CB	PRO	154	49.481	-8.047	12.405	1.00	0.00	LX0	C
ATOM	1276	CG	PRO	154	48.650	-7.290	13.439	1.00	0.00	LX0	C
ATOM	1277	C	PRO	154	49.988	-7.290	10.007	1.00	0.00	LX0	C
ATOM	1278	O	PRO	154	49.039	-6.897	9.338	1.00	0.00	LX0	O
ATOM	1279	N	ALA	155	51.061	-7.972	9.568	1.00	0.00	LX0	N
ATOM	1280	H	ALA	155	51.703	-8.213	10.292	1.00	0.00	LX0	H
ATOM	1281	CA	ALA	155	51.509	-8.223	8.190	1.00	0.00	LX0	C
ATOM	1282	CB	ALA	155	50.466	-8.043	7.082	1.00	0.00	LX0	C
ATOM	1283	C	ALA	155	52.732	-7.399	7.850	1.00	0.00	LX0	C
ATOM	1284	O	ALA	155	53.592	-7.829	7.094	1.00	0.00	LX0	O
ATOM	1285	N	LEU	156	52.797	-6.219	8.493	1.00	0.00	LX0	N
ATOM	1286	H	LEU	156	52.006	-5.868	8.994	1.00	0.00	LX0	H
ATOM	1287	CA	LEU	156	53.915	-5.311	8.242	1.00	0.00	LX0	C
ATOM	1288	CB	LEU	156	53.840	-4.132	9.212	1.00	0.00	LX0	C
ATOM	1289	CG	LEU	156	54.642	-2.903	8.778	1.00	0.00	LX0	C
ATOM	1290	CD1	LEU	156	54.150	-2.320	7.453	1.00	0.00	LX0	C
ATOM	1291	CD2	LEU	156	54.663	-1.851	9.880	1.00	0.00	LX0	C
ATOM	1292	C	LEU	156	55.303	-5.928	8.242	1.00	0.00	LX0	C
ATOM	1293	O	LEU	156	55.717	-6.629	9.160	1.00	0.00	LX0	O

ATOM	1294	N	CYS	157	56.002	-5.616	7.151	1.00	0.00	LX0	N
ATOM	1295	H	CYS	157	55.579	-5.067	6.427	1.00	0.00	LX0	H
ATOM	1296	CA	CYS	157	57.382	-6.061	7.057	1.00	0.00	LX0	C
ATOM	1297	CB	CYS	157	57.619	-6.787	5.737	1.00	0.00	LX0	C
ATOM	1298	SG	CYS	157	56.598	-8.265	5.521	1.00	0.00	LX0	S
ATOM	1299	C	CYS	157	58.346	-4.905	7.144	1.00	0.00	LX0	C
ATOM	1300	O	CYS	157	58.213	-3.904	6.450	1.00	0.00	LX0	O
ATOM	1301	N	ASN	158	59.362	-5.109	7.996	1.00	0.00	LX0	N
ATOM	1302	H	ASN	158	59.255	-5.754	8.756	1.00	0.00	LX0	H
ATOM	1303	CA	ASN	158	60.661	-4.438	7.836	1.00	0.00	LX0	C
ATOM	1304	CB	ASN	158	61.258	-4.628	6.440	1.00	0.00	LX0	C
ATOM	1305	CG	ASN	158	61.612	-6.074	6.189	1.00	0.00	LX0	C
ATOM	1306	OD1	ASN	158	61.399	-6.955	7.017	1.00	0.00	LX0	O
ATOM	1307	ND2	ASN	158	62.137	-6.283	4.986	1.00	0.00	LX0	N
ATOM	1308	HD21	ASN	158	62.349	-5.511	4.383	1.00	0.00	LX0	H
ATOM	1309	HD22	ASN	158	62.357	-7.204	4.663	1.00	0.00	LX0	H
ATOM	1310	C	ASN	158	60.786	-2.996	8.286	1.00	0.00	LX0	C
ATOM	1311	O	ASN	158	61.858	-2.529	8.657	1.00	0.00	LX0	O
ATOM	1312	N	VAL	159	59.629	-2.314	8.311	1.00	0.00	LX0	N
ATOM	1313	H	VAL	159	58.822	-2.732	7.896	1.00	0.00	LX0	H
ATOM	1314	CA	VAL	159	59.615	-0.941	8.821	1.00	0.00	LX0	C
ATOM	1315	CB	VAL	159	58.224	-0.315	8.610	1.00	0.00	LX0	C
ATOM	1316	CG1	VAL	159	58.181	1.166	8.994	1.00	0.00	LX0	C
ATOM	1317	CG2	VAL	159	57.767	-0.491	7.158	1.00	0.00	LX0	C
ATOM	1318	C	VAL	159	60.109	-0.802	10.265	1.00	0.00	LX0	C
ATOM	1319	O	VAL	159	60.579	0.243	10.705	1.00	0.00	LX0	O
ATOM	1320	N	GLU	160	60.027	-1.939	10.980	1.00	0.00	LX0	N
ATOM	1321	H	GLU	160	59.717	-2.792	10.562	1.00	0.00	LX0	H
ATOM	1322	CA	GLU	160	60.561	-1.971	12.340	1.00	0.00	LX0	C
ATOM	1323	CB	GLU	160	60.215	-3.318	13.000	1.00	0.00	LX0	C
ATOM	1324	CG	GLU	160	61.132	-4.532	12.755	1.00	0.00	LX0	C
ATOM	1325	CD	GLU	160	61.245	-4.919	11.290	1.00	0.00	LX0	C
ATOM	1326	OE1	GLU	160	60.423	-5.681	10.787	1.00	0.00	LX0	O
ATOM	1327	OE2	GLU	160	62.210	-4.527	10.644	1.00	0.00	LX0	O
ATOM	1328	C	GLU	160	62.036	-1.603	12.485	1.00	0.00	LX0	C
ATOM	1329	O	GLU	160	62.497	-1.184	13.538	1.00	0.00	LX0	O
ATOM	1330	N	SER	161	62.757	-1.756	11.368	1.00	0.00	LX0	N
ATOM	1331	H	SER	161	62.335	-2.152	10.552	1.00	0.00	LX0	H
ATOM	1332	CA	SER	161	64.184	-1.474	11.432	1.00	0.00	LX0	C
ATOM	1333	CB	SER	161	64.936	-2.580	10.690	1.00	0.00	LX0	C
ATOM	1334	OG	SER	161	64.501	-2.664	9.323	1.00	0.00	LX0	O
ATOM	1335	HG	SER	161	63.587	-2.954	9.345	1.00	0.00	LX0	H
ATOM	1336	C	SER	161	64.620	-0.087	10.977	1.00	0.00	LX0	C
ATOM	1337	O	SER	161	65.806	0.205	10.879	1.00	0.00	LX0	O
ATOM	1338	N	ILE	162	63.628	0.768	10.674	1.00	0.00	LX0	N
ATOM	1339	H	ILE	162	62.669	0.541	10.848	1.00	0.00	LX0	H
ATOM	1340	CA	ILE	162	64.044	2.113	10.268	1.00	0.00	LX0	C
ATOM	1341	CB	ILE	162	62.941	2.814	9.436	1.00	0.00	LX0	C
ATOM	1342	CG2	ILE	162	63.036	4.344	9.423	1.00	0.00	LX0	C
ATOM	1343	CG1	ILE	162	62.987	2.340	7.982	1.00	0.00	LX0	C
ATOM	1344	CD1	ILE	162	62.366	0.977	7.709	1.00	0.00	LX0	C
ATOM	1345	C	ILE	162	64.489	2.930	11.472	1.00	0.00	LX0	C
ATOM	1346	O	ILE	162	63.831	2.987	12.504	1.00	0.00	LX0	O
ATOM	1347	N	GLN	163	65.655	3.566	11.312	1.00	0.00	LX0	N
ATOM	1348	H	GLN	163	66.101	3.555	10.414	1.00	0.00	LX0	H
ATOM	1349	CA	GLN	163	66.047	4.406	12.438	1.00	0.00	LX0	C
ATOM	1350	CB	GLN	163	67.565	4.419	12.630	1.00	0.00	LX0	C
ATOM	1351	CG	GLN	163	68.396	4.825	11.415	1.00	0.00	LX0	C
ATOM	1352	CD	GLN	163	69.851	4.634	11.766	1.00	0.00	LX0	C
ATOM	1353	OE1	GLN	163	70.407	5.280	12.641	1.00	0.00	LX0	O
ATOM	1354	NE2	GLN	163	70.439	3.683	11.045	1.00	0.00	LX0	N

ATOM	1355	HE21	GLN	163	69.949	3.167	10.347	1.00	0.00	LX0	H
ATOM	1356	HE22	GLN	163	71.411	3.473	11.178	1.00	0.00	LX0	H
ATOM	1357	C	GLN	163	65.414	5.784	12.396	1.00	0.00	LX0	C
ATOM	1358	O	GLN	163	65.879	6.726	11.768	1.00	0.00	LX0	O
ATOM	1359	N	TRP	164	64.285	5.855	13.109	1.00	0.00	LX0	N
ATOM	1360	H	TRP	164	63.949	4.996	13.502	1.00	0.00	LX0	H
ATOM	1361	CA	TRP	164	63.422	7.026	12.951	1.00	0.00	LX0	C
ATOM	1362	CB	TRP	164	62.076	6.790	13.637	1.00	0.00	LX0	C
ATOM	1363	CG	TRP	164	61.284	5.807	12.806	1.00	0.00	LX0	C
ATOM	1364	CD2	TRP	164	60.443	6.091	11.668	1.00	0.00	LX0	C
ATOM	1365	CE2	TRP	164	59.917	4.839	11.203	1.00	0.00	LX0	C
ATOM	1366	CE3	TRP	164	60.099	7.290	11.007	1.00	0.00	LX0	C
ATOM	1367	CD1	TRP	164	61.223	4.414	12.966	1.00	0.00	LX0	C
ATOM	1368	NE1	TRP	164	60.425	3.839	12.026	1.00	0.00	LX0	N
ATOM	1369	HE1	TRP	164	60.261	2.874	11.935	1.00	0.00	LX0	H
ATOM	1370	CZ2	TRP	164	59.051	4.814	10.091	1.00	0.00	LX0	C
ATOM	1371	CZ3	TRP	164	59.235	7.250	9.894	1.00	0.00	LX0	C
ATOM	1372	CH2	TRP	164	58.712	6.020	9.442	1.00	0.00	LX0	C
ATOM	1373	C	TRP	164	63.967	8.416	13.256	1.00	0.00	LX0	C
ATOM	1374	O	TRP	164	63.458	9.400	12.735	1.00	0.00	LX0	O
ATOM	1375	N	ARG	165	65.029	8.495	14.079	1.00	0.00	LX0	N
ATOM	1376	H	ARG	165	65.457	7.666	14.433	1.00	0.00	LX0	H
ATOM	1377	CA	ARG	165	65.584	9.843	14.278	1.00	0.00	LX0	C
ATOM	1378	CB	ARG	165	66.344	9.930	15.622	1.00	0.00	LX0	C
ATOM	1379	CG	ARG	165	66.739	11.355	16.056	1.00	0.00	LX0	C
ATOM	1380	CD	ARG	165	67.164	11.534	17.524	1.00	0.00	LX0	C
ATOM	1381	NE	ARG	165	66.028	11.761	18.429	1.00	0.00	LX0	N
ATOM	1382	HE	ARG	165	65.243	11.145	18.389	1.00	0.00	LX0	H
ATOM	1383	CZ	ARG	165	66.053	12.782	19.326	1.00	0.00	LX0	C
ATOM	1384	NH1	ARG	165	67.099	13.597	19.395	1.00	0.00	LX0	N
ATOM	1385	HH11	ARG	165	67.084	14.332	20.090	1.00	0.00	LX0	H
ATOM	1386	HH12	ARG	165	67.882	13.533	18.781	1.00	0.00	LX0	H
ATOM	1387	NH2	ARG	165	65.031	13.008	20.152	1.00	0.00	LX0	N
ATOM	1388	HH21	ARG	165	65.102	13.784	20.808	1.00	0.00	LX0	H
ATOM	1389	HH22	ARG	165	64.170	12.494	20.188	1.00	0.00	LX0	H
ATOM	1390	C	ARG	165	66.342	10.413	13.071	1.00	0.00	LX0	C
ATOM	1391	O	ARG	165	66.558	11.608	12.930	1.00	0.00	LX0	O
ATOM	1392	N	ASP	166	66.682	9.489	12.159	1.00	0.00	LX0	N
ATOM	1393	H	ASP	166	66.529	8.514	12.319	1.00	0.00	LX0	H
ATOM	1394	CA	ASP	166	67.158	9.907	10.837	1.00	0.00	LX0	C
ATOM	1395	CB	ASP	166	67.767	8.674	10.170	1.00	0.00	LX0	C
ATOM	1396	CG	ASP	166	68.525	8.948	8.888	1.00	0.00	LX0	C
ATOM	1397	OD1	ASP	166	67.907	9.197	7.857	1.00	0.00	LX0	O
ATOM	1398	OD2	ASP	166	69.746	8.856	8.904	1.00	0.00	LX0	O
ATOM	1399	C	ASP	166	66.058	10.533	9.984	1.00	0.00	LX0	C
ATOM	1400	O	ASP	166	66.257	11.370	9.109	1.00	0.00	LX0	O
ATOM	1401	N	ILE	167	64.839	10.090	10.301	1.00	0.00	LX0	N
ATOM	1402	H	ILE	167	64.694	9.482	11.078	1.00	0.00	LX0	H
ATOM	1403	CA	ILE	167	63.730	10.614	9.518	1.00	0.00	LX0	C
ATOM	1404	CB	ILE	167	62.647	9.533	9.344	1.00	0.00	LX0	C
ATOM	1405	CG2	ILE	167	61.650	9.912	8.250	1.00	0.00	LX0	C
ATOM	1406	CG1	ILE	167	63.247	8.147	9.085	1.00	0.00	LX0	C
ATOM	1407	CD1	ILE	167	64.025	8.042	7.774	1.00	0.00	LX0	C
ATOM	1408	C	ILE	167	63.179	11.895	10.125	1.00	0.00	LX0	C
ATOM	1409	O	ILE	167	63.347	13.000	9.617	1.00	0.00	LX0	O
ATOM	1410	N	VAL	168	62.509	11.688	11.266	1.00	0.00	LX0	N
ATOM	1411	H	VAL	168	62.623	10.800	11.712	1.00	0.00	LX0	H
ATOM	1412	CA	VAL	168	61.809	12.787	11.932	1.00	0.00	LX0	C
ATOM	1413	CB	VAL	168	60.787	12.211	12.937	1.00	0.00	LX0	C
ATOM	1414	CG1	VAL	168	59.661	13.195	13.256	1.00	0.00	LX0	C
ATOM	1415	CG2	VAL	168	60.183	10.884	12.469	1.00	0.00	LX0	C

ATOM	1416	C	VAL	168	62.812	13.741	12.585	1.00	0.00	LX0	C
ATOM	1417	O	VAL	168	64.014	13.505	12.517	1.00	0.00	LX0	O
ATOM	1418	N	SER	169	62.325	14.820	13.203	1.00	0.00	LX0	N
ATOM	1419	H	SER	169	61.356	15.060	13.171	1.00	0.00	LX0	H
ATOM	1420	CA	SER	169	63.265	15.636	13.967	1.00	0.00	LX0	C
ATOM	1421	CB	SER	169	63.334	17.014	13.307	1.00	0.00	LX0	C
ATOM	1422	OG	SER	169	63.369	16.822	11.876	1.00	0.00	LX0	O
ATOM	1423	HG	SER	169	62.481	17.036	11.581	1.00	0.00	LX0	H
ATOM	1424	C	SER	169	62.908	15.626	15.450	1.00	0.00	LX0	C
ATOM	1425	O	SER	169	61.784	15.282	15.808	1.00	0.00	LX0	O
ATOM	1426	N	SER	170	63.909	15.917	16.297	1.00	0.00	LX0	N
ATOM	1427	H	SER	170	64.740	16.401	16.016	1.00	0.00	LX0	H
ATOM	1428	CA	SER	170	63.841	15.515	17.708	1.00	0.00	LX0	C
ATOM	1429	CB	SER	170	65.081	16.020	18.435	1.00	0.00	LX0	C
ATOM	1430	OG	SER	170	66.199	15.984	17.537	1.00	0.00	LX0	O
ATOM	1431	HG	SER	170	66.410	16.918	17.421	1.00	0.00	LX0	H
ATOM	1432	C	SER	170	62.589	15.909	18.470	1.00	0.00	LX0	C
ATOM	1433	O	SER	170	61.843	15.076	18.976	1.00	0.00	LX0	O
ATOM	1434	N	ASP	171	62.373	17.232	18.453	1.00	0.00	LX0	N
ATOM	1435	H	ASP	171	63.069	17.806	18.012	1.00	0.00	LX0	H
ATOM	1436	CA	ASP	171	61.212	17.902	19.046	1.00	0.00	LX0	C
ATOM	1437	CB	ASP	171	61.179	19.318	18.461	1.00	0.00	LX0	C
ATOM	1438	CG	ASP	171	60.044	20.124	19.042	1.00	0.00	LX0	C
ATOM	1439	OD1	ASP	171	59.979	20.289	20.255	1.00	0.00	LX0	O
ATOM	1440	OD2	ASP	171	59.176	20.537	18.285	1.00	0.00	LX0	O
ATOM	1441	C	ASP	171	59.872	17.204	18.843	1.00	0.00	LX0	C
ATOM	1442	O	ASP	171	58.958	17.227	19.665	1.00	0.00	LX0	O
ATOM	1443	N	PHE	172	59.809	16.595	17.658	1.00	0.00	LX0	N
ATOM	1444	H	PHE	172	60.631	16.561	17.092	1.00	0.00	LX0	H
ATOM	1445	CA	PHE	172	58.586	15.934	17.246	1.00	0.00	LX0	C
ATOM	1446	CB	PHE	172	58.422	16.101	15.729	1.00	0.00	LX0	C
ATOM	1447	CG	PHE	172	58.794	17.510	15.312	1.00	0.00	LX0	C
ATOM	1448	CD1	PHE	172	57.856	18.554	15.451	1.00	0.00	LX0	C
ATOM	1449	CD2	PHE	172	60.090	17.764	14.812	1.00	0.00	LX0	C
ATOM	1450	CE1	PHE	172	58.226	19.869	15.110	1.00	0.00	LX0	C
ATOM	1451	CE2	PHE	172	60.466	19.080	14.481	1.00	0.00	LX0	C
ATOM	1452	CZ	PHE	172	59.531	20.121	14.643	1.00	0.00	LX0	C
ATOM	1453	C	PHE	172	58.596	14.476	17.659	1.00	0.00	LX0	C
ATOM	1454	O	PHE	172	57.722	13.977	18.358	1.00	0.00	LX0	O
ATOM	1455	N	LEU	173	59.655	13.792	17.192	1.00	0.00	LX0	N
ATOM	1456	H	LEU	173	60.407	14.284	16.751	1.00	0.00	LX0	H
ATOM	1457	CA	LEU	173	59.660	12.342	17.374	1.00	0.00	LX0	C
ATOM	1458	CB	LEU	173	60.763	11.668	16.553	1.00	0.00	LX0	C
ATOM	1459	CG	LEU	173	62.200	11.873	17.035	1.00	0.00	LX0	C
ATOM	1460	CD1	LEU	173	62.694	10.717	17.907	1.00	0.00	LX0	C
ATOM	1461	CD2	LEU	173	63.133	12.112	15.856	1.00	0.00	LX0	C
ATOM	1462	C	LEU	173	59.655	11.846	18.804	1.00	0.00	LX0	C
ATOM	1463	O	LEU	173	59.170	10.764	19.096	1.00	0.00	LX0	O
ATOM	1464	N	SER	174	60.187	12.679	19.702	1.00	0.00	LX0	N
ATOM	1465	H	SER	174	60.631	13.543	19.449	1.00	0.00	LX0	H
ATOM	1466	CA	SER	174	60.233	12.200	21.079	1.00	0.00	LX0	C
ATOM	1467	CB	SER	174	61.265	13.027	21.841	1.00	0.00	LX0	C
ATOM	1468	OG	SER	174	62.471	13.089	21.059	1.00	0.00	LX0	O
ATOM	1469	HG	SER	174	62.837	13.953	21.269	1.00	0.00	LX0	H
ATOM	1470	C	SER	174	58.897	12.057	21.814	1.00	0.00	LX0	C
ATOM	1471	O	SER	174	58.844	11.699	22.982	1.00	0.00	LX0	O
ATOM	1472	N	ASN	175	57.798	12.311	21.078	1.00	0.00	LX0	N
ATOM	1473	H	ASN	175	57.857	12.681	20.148	1.00	0.00	LX0	H
ATOM	1474	CA	ASN	175	56.510	11.922	21.652	1.00	0.00	LX0	C
ATOM	1475	CB	ASN	175	55.519	13.086	21.533	1.00	0.00	LX0	C
ATOM	1476	CG	ASN	175	54.349	12.895	22.483	1.00	0.00	LX0	C

ATOM	1477	OD1	ASN	175	53.205	12.720	22.087	1.00	0.00	LX0	O
ATOM	1478	ND2	ASN	175	54.681	12.956	23.776	1.00	0.00	LX0	N
ATOM	1479	HD21	ASN	175	55.622	13.103	24.076	1.00	0.00	LX0	H
ATOM	1480	HD22	ASN	175	53.948	12.856	24.445	1.00	0.00	LX0	H
ATOM	1481	C	ASN	175	55.932	10.618	21.098	1.00	0.00	LX0	C
ATOM	1482	O	ASN	175	54.756	10.302	21.246	1.00	0.00	LX0	O
ATOM	1483	N	MET	176	56.802	9.858	20.420	1.00	0.00	LX0	N
ATOM	1484	H	MET	176	57.774	10.085	20.350	1.00	0.00	LX0	H
ATOM	1485	CA	MET	176	56.270	8.626	19.844	1.00	0.00	LX0	C
ATOM	1486	CB	MET	176	56.962	8.303	18.510	1.00	0.00	LX0	C
ATOM	1487	CG	MET	176	58.402	7.789	18.634	1.00	0.00	LX0	C
ATOM	1488	SD	MET	176	59.230	7.592	17.052	1.00	0.00	LX0	S
ATOM	1489	CE	MET	176	60.816	7.016	17.674	1.00	0.00	LX0	C
ATOM	1490	C	MET	176	56.301	7.424	20.769	1.00	0.00	LX0	C
ATOM	1491	O	MET	176	57.222	7.223	21.549	1.00	0.00	LX0	O
ATOM	1492	N	SER	177	55.277	6.587	20.586	1.00	0.00	LX0	N
ATOM	1493	H	SER	177	54.473	6.856	20.056	1.00	0.00	LX0	H
ATOM	1494	CA	SER	177	55.603	5.177	20.738	1.00	0.00	LX0	C
ATOM	1495	CB	SER	177	54.948	4.539	21.980	1.00	0.00	LX0	C
ATOM	1496	OG	SER	177	53.524	4.418	21.852	1.00	0.00	LX0	O
ATOM	1497	HG	SER	177	53.392	3.527	21.503	1.00	0.00	LX0	H
ATOM	1498	C	SER	177	55.224	4.515	19.436	1.00	0.00	LX0	C
ATOM	1499	O	SER	177	54.285	4.947	18.776	1.00	0.00	LX0	O
ATOM	1500	N	MET	178	56.002	3.501	19.062	1.00	0.00	LX0	N
ATOM	1501	H	MET	178	56.761	3.157	19.610	1.00	0.00	LX0	H
ATOM	1502	CA	MET	178	55.628	2.872	17.805	1.00	0.00	LX0	C
ATOM	1503	CB	MET	178	56.518	3.372	16.664	1.00	0.00	LX0	C
ATOM	1504	CG	MET	178	58.027	3.212	16.857	1.00	0.00	LX0	C
ATOM	1505	SD	MET	178	58.955	4.200	15.674	1.00	0.00	LX0	S
ATOM	1506	CE	MET	178	57.887	3.966	14.244	1.00	0.00	LX0	C
ATOM	1507	C	MET	178	55.530	1.376	17.950	1.00	0.00	LX0	C
ATOM	1508	O	MET	178	56.488	0.614	17.901	1.00	0.00	LX0	O
ATOM	1509	N	ASP	179	54.281	1.018	18.211	1.00	0.00	LX0	N
ATOM	1510	H	ASP	179	53.534	1.678	18.112	1.00	0.00	LX0	H
ATOM	1511	CA	ASP	179	53.939	-0.319	18.669	1.00	0.00	LX0	C
ATOM	1512	CB	ASP	179	52.673	-0.311	19.555	1.00	0.00	LX0	C
ATOM	1513	CG	ASP	179	52.210	1.092	19.952	1.00	0.00	LX0	C
ATOM	1514	OD1	ASP	179	51.153	1.502	19.484	1.00	0.00	LX0	O
ATOM	1515	OD2	ASP	179	52.900	1.793	20.698	1.00	0.00	LX0	O
ATOM	1516	C	ASP	179	53.823	-1.295	17.515	1.00	0.00	LX0	C
ATOM	1517	O	ASP	179	52.753	-1.668	17.041	1.00	0.00	LX0	O
ATOM	1518	N	PHE	180	55.028	-1.682	17.068	1.00	0.00	LX0	N
ATOM	1519	H	PHE	180	55.833	-1.287	17.512	1.00	0.00	LX0	H
ATOM	1520	CA	PHE	180	55.123	-2.764	16.094	1.00	0.00	LX0	C
ATOM	1521	CB	PHE	180	56.536	-2.866	15.509	1.00	0.00	LX0	C
ATOM	1522	CG	PHE	180	56.894	-1.686	14.636	1.00	0.00	LX0	C
ATOM	1523	CD1	PHE	180	57.763	-0.695	15.141	1.00	0.00	LX0	C
ATOM	1524	CD2	PHE	180	56.383	-1.610	13.322	1.00	0.00	LX0	C
ATOM	1525	CE1	PHE	180	58.146	0.376	14.311	1.00	0.00	LX0	C
ATOM	1526	CE2	PHE	180	56.766	-0.538	12.493	1.00	0.00	LX0	C
ATOM	1527	CZ	PHE	180	57.650	0.440	12.992	1.00	0.00	LX0	C
ATOM	1528	C	PHE	180	54.774	-4.101	16.718	1.00	0.00	LX0	C
ATOM	1529	O	PHE	180	54.767	-4.274	17.930	1.00	0.00	LX0	O
ATOM	1530	N	GLN	181	54.515	-5.050	15.816	1.00	0.00	LX0	N
ATOM	1531	H	GLN	181	54.528	-4.841	14.837	1.00	0.00	LX0	H
ATOM	1532	CA	GLN	181	54.228	-6.411	16.248	1.00	0.00	LX0	C
ATOM	1533	CB	GLN	181	52.736	-6.591	16.576	1.00	0.00	LX0	C
ATOM	1534	CG	GLN	181	51.779	-6.630	15.374	1.00	0.00	LX0	C
ATOM	1535	CD	GLN	181	51.703	-5.293	14.663	1.00	0.00	LX0	C
ATOM	1536	OE1	GLN	181	50.955	-4.411	15.054	1.00	0.00	LX0	O
ATOM	1537	NE2	GLN	181	52.468	-5.190	13.569	1.00	0.00	LX0	N

ATOM	1538	HE21	GLN	181	53.107	-5.915	13.302	1.00	0.00	LX0	H
ATOM	1539	HE22	GLN	181	52.420	-4.362	13.002	1.00	0.00	LX0	H
ATOM	1540	C	GLN	181	54.644	-7.360	15.149	1.00	0.00	LX0	C
ATOM	1541	O	GLN	181	54.778	-6.951	14.001	1.00	0.00	LX0	O
ATOM	1542	N	ASN	182	54.819	-8.633	15.532	1.00	0.00	LX0	N
ATOM	1543	H	ASN	182	54.622	-8.910	16.469	1.00	0.00	LX0	H
ATOM	1544	CA	ASN	182	55.123	-9.595	14.472	1.00	0.00	LX0	C
ATOM	1545	CB	ASN	182	55.601	-10.931	15.060	1.00	0.00	LX0	C
ATOM	1546	CG	ASN	182	56.190	-11.785	13.950	1.00	0.00	LX0	C
ATOM	1547	OD1	ASN	182	56.797	-11.280	13.018	1.00	0.00	LX0	O
ATOM	1548	ND2	ASN	182	55.933	-13.090	14.051	1.00	0.00	LX0	N
ATOM	1549	HD21	ASN	182	55.461	-13.496	14.829	1.00	0.00	LX0	H
ATOM	1550	HD22	ASN	182	56.209	-13.661	13.279	1.00	0.00	LX0	H
ATOM	1551	C	ASN	182	53.954	-9.801	13.521	1.00	0.00	LX0	C
ATOM	1552	O	ASN	182	52.791	-9.684	13.891	1.00	0.00	LX0	O
ATOM	1553	N	HIS	183	54.320	-10.101	12.275	1.00	0.00	LX0	N
ATOM	1554	H	HIS	183	55.296	-10.252	12.097	1.00	0.00	LX0	H
ATOM	1555	CA	HIS	183	53.294	-10.512	11.324	1.00	0.00	LX0	C
ATOM	1556	CB	HIS	183	53.740	-10.229	9.884	1.00	0.00	LX0	C
ATOM	1557	CG	HIS	183	55.222	-10.442	9.697	1.00	0.00	LX0	C
ATOM	1558	ND1	HIS	183	56.114	-9.438	9.732	1.00	0.00	LX0	N
ATOM	1559	HD1	HIS	183	55.927	-8.474	9.821	1.00	0.00	LX0	H
ATOM	1560	CD2	HIS	183	55.899	-11.646	9.487	1.00	0.00	LX0	C
ATOM	1561	NE2	HIS	183	57.219	-11.352	9.399	1.00	0.00	LX0	N
ATOM	1562	CE1	HIS	183	57.350	-9.995	9.555	1.00	0.00	LX0	C
ATOM	1563	C	HIS	183	52.921	-11.969	11.488	1.00	0.00	LX0	C
ATOM	1564	O	HIS	183	53.690	-12.782	11.986	1.00	0.00	LX0	O
ATOM	1565	N	LEU	184	51.701	-12.276	11.028	1.00	0.00	LX0	N
ATOM	1566	H	LEU	184	51.103	-11.594	10.611	1.00	0.00	LX0	H
ATOM	1567	CA	LEU	184	51.359	-13.695	10.999	1.00	0.00	LX0	C
ATOM	1568	CB	LEU	184	49.846	-13.908	11.090	1.00	0.00	LX0	C
ATOM	1569	CG	LEU	184	49.222	-13.415	12.399	1.00	0.00	LX0	C
ATOM	1570	CD1	LEU	184	47.723	-13.710	12.436	1.00	0.00	LX0	C
ATOM	1571	CD2	LEU	184	49.929	-13.974	13.637	1.00	0.00	LX0	C
ATOM	1572	C	LEU	184	51.933	-14.385	9.779	1.00	0.00	LX0	C
ATOM	1573	O	LEU	184	51.297	-14.519	8.744	1.00	0.00	LX0	O
ATOM	1574	N	GLY	185	53.191	-14.795	9.950	1.00	0.00	LX0	N
ATOM	1575	H	GLY	185	53.682	-14.611	10.805	1.00	0.00	LX0	H
ATOM	1576	CA	GLY	185	53.846	-15.479	8.844	1.00	0.00	LX0	C
ATOM	1577	C	GLY	185	55.334	-15.239	8.896	1.00	0.00	LX0	C
ATOM	1578	O	GLY	185	55.968	-15.366	9.937	1.00	0.00	LX0	O
ATOM	1579	N	SER	186	55.868	-14.869	7.731	1.00	0.00	LX0	N
ATOM	1580	H	SER	186	55.327	-14.777	6.895	1.00	0.00	LX0	H
ATOM	1581	CA	SER	186	57.289	-14.548	7.687	1.00	0.00	LX0	C
ATOM	1582	CB	SER	186	58.114	-15.815	7.441	1.00	0.00	LX0	C
ATOM	1583	OG	SER	186	57.698	-16.846	8.347	1.00	0.00	LX0	O
ATOM	1584	HG	SER	186	57.421	-16.414	9.154	1.00	0.00	LX0	H
ATOM	1585	C	SER	186	57.537	-13.508	6.618	1.00	0.00	LX0	C
ATOM	1586	O	SER	186	56.720	-13.327	5.722	1.00	0.00	LX0	O
ATOM	1587	N	CYS	187	58.673	-12.820	6.743	1.00	0.00	LX0	N
ATOM	1588	H	CYS	187	59.331	-12.980	7.479	1.00	0.00	LX0	H
ATOM	1589	CA	CYS	187	58.902	-11.779	5.751	1.00	0.00	LX0	C
ATOM	1590	CB	CYS	187	58.929	-10.409	6.419	1.00	0.00	LX0	C
ATOM	1591	SG	CYS	187	60.168	-10.281	7.733	1.00	0.00	LX0	S
ATOM	1592	C	CYS	187	60.176	-11.985	4.976	1.00	0.00	LX0	C
ATOM	1593	O	CYS	187	61.100	-12.666	5.407	1.00	0.00	LX0	O
ATOM	1594	N	GLN	188	60.207	-11.315	3.819	1.00	0.00	LX0	N
ATOM	1595	H	GLN	188	59.405	-10.812	3.502	1.00	0.00	LX0	H
ATOM	1596	CA	GLN	188	61.501	-11.132	3.177	1.00	0.00	LX0	C
ATOM	1597	CB	GLN	188	61.328	-10.867	1.664	1.00	0.00	LX0	C
ATOM	1598	CG	GLN	188	60.878	-9.499	1.109	1.00	0.00	LX0	C

ATOM	1599	CD	GLN	188	59.445	-9.089	1.425	1.00	0.00	LX0	C
ATOM	1600	OE1	GLN	188	58.676	-9.726	2.143	1.00	0.00	LX0	O
ATOM	1601	NE2	GLN	188	59.106	-7.947	0.831	1.00	0.00	LX0	N
ATOM	1602	HE21	GLN	188	59.733	-7.473	0.210	1.00	0.00	LX0	H
ATOM	1603	HE22	GLN	188	58.215	-7.507	0.968	1.00	0.00	LX0	H
ATOM	1604	C	GLN	188	62.330	-10.106	3.944	1.00	0.00	LX0	C
ATOM	1605	O	GLN	188	62.241	-8.901	3.762	1.00	0.00	LX0	O
ATOM	1606	N	LYS	189	63.086	-10.662	4.903	1.00	0.00	LX0	N
ATOM	1607	H	LYS	189	63.057	-11.658	5.002	1.00	0.00	LX0	H
ATOM	1608	CA	LYS	189	63.565	-9.799	5.984	1.00	0.00	LX0	C
ATOM	1609	CB	LYS	189	64.129	-10.654	7.135	1.00	0.00	LX0	C
ATOM	1610	CG	LYS	189	64.378	-9.865	8.431	1.00	0.00	LX0	C
ATOM	1611	CD	LYS	189	63.132	-9.092	8.873	1.00	0.00	LX0	C
ATOM	1612	CE	LYS	189	63.454	-7.845	9.696	1.00	0.00	LX0	C
ATOM	1613	NZ	LYS	189	62.353	-6.893	9.531	1.00	0.00	LX0	N
ATOM	1614	HZ1	LYS	189	62.672	-5.916	9.699	1.00	0.00	LX0	H
ATOM	1615	HZ2	LYS	189	61.962	-6.946	8.571	1.00	0.00	LX0	H
ATOM	1616	HZ3	LYS	189	61.574	-7.000	10.217	1.00	0.00	LX0	H
ATOM	1617	C	LYS	189	64.484	-8.637	5.615	1.00	0.00	LX0	C
ATOM	1618	O	LYS	189	64.471	-7.598	6.264	1.00	0.00	LX0	O
ATOM	1619	N	CYS	190	65.279	-8.842	4.560	1.00	0.00	LX0	N
ATOM	1620	H	CYS	190	65.286	-9.675	4.010	1.00	0.00	LX0	H
ATOM	1621	CA	CYS	190	66.233	-7.777	4.268	1.00	0.00	LX0	C
ATOM	1622	CB	CYS	190	67.504	-7.961	5.104	1.00	0.00	LX0	C
ATOM	1623	SG	CYS	190	68.036	-9.692	5.212	1.00	0.00	LX0	S
ATOM	1624	C	CYS	190	66.571	-7.685	2.802	1.00	0.00	LX0	C
ATOM	1625	O	CYS	190	66.624	-8.684	2.095	1.00	0.00	LX0	O
ATOM	1626	N	ASP	191	66.799	-6.430	2.406	1.00	0.00	LX0	N
ATOM	1627	H	ASP	191	66.689	-5.669	3.040	1.00	0.00	LX0	H
ATOM	1628	CA	ASP	191	67.459	-6.152	1.135	1.00	0.00	LX0	C
ATOM	1629	CB	ASP	191	67.112	-4.718	0.730	1.00	0.00	LX0	C
ATOM	1630	CG	ASP	191	65.842	-4.733	-0.091	1.00	0.00	LX0	C
ATOM	1631	OD1	ASP	191	64.874	-4.083	0.283	1.00	0.00	LX0	O
ATOM	1632	OD2	ASP	191	65.815	-5.379	-1.131	1.00	0.00	LX0	O
ATOM	1633	C	ASP	191	68.958	-6.364	1.292	1.00	0.00	LX0	C
ATOM	1634	O	ASP	191	69.461	-6.327	2.411	1.00	0.00	LX0	O
ATOM	1635	N	PRO	192	69.660	-6.642	0.161	1.00	0.00	LX0	N
ATOM	1636	CD	PRO	192	69.148	-6.708	-1.208	1.00	0.00	LX0	C
ATOM	1637	CA	PRO	192	71.089	-6.981	0.229	1.00	0.00	LX0	C
ATOM	1638	CB	PRO	192	71.515	-6.966	-1.242	1.00	0.00	LX0	C
ATOM	1639	CG	PRO	192	70.259	-7.382	-2.002	1.00	0.00	LX0	C
ATOM	1640	C	PRO	192	71.988	-6.143	1.131	1.00	0.00	LX0	C
ATOM	1641	O	PRO	192	72.781	-6.676	1.899	1.00	0.00	LX0	O
ATOM	1642	N	SER	193	71.860	-4.821	0.994	1.00	0.00	LX0	N
ATOM	1643	H	SER	193	71.162	-4.405	0.402	1.00	0.00	LX0	H
ATOM	1644	CA	SER	193	72.792	-3.994	1.752	1.00	0.00	LX0	C
ATOM	1645	CB	SER	193	73.274	-2.814	0.907	1.00	0.00	LX0	C
ATOM	1646	OG	SER	193	73.520	-3.250	-0.436	1.00	0.00	LX0	O
ATOM	1647	HG	SER	193	72.647	-3.349	-0.825	1.00	0.00	LX0	H
ATOM	1648	C	SER	193	72.272	-3.529	3.098	1.00	0.00	LX0	C
ATOM	1649	O	SER	193	73.025	-3.408	4.056	1.00	0.00	LX0	O
ATOM	1650	N	CYS	194	70.948	-3.275	3.135	1.00	0.00	LX0	N
ATOM	1651	H	CYS	194	70.444	-3.350	2.269	1.00	0.00	LX0	H
ATOM	1652	CA	CYS	194	70.242	-2.982	4.395	1.00	0.00	LX0	C
ATOM	1653	CB	CYS	194	69.593	-4.255	4.934	1.00	0.00	LX0	C
ATOM	1654	SG	CYS	194	67.856	-4.420	4.457	1.00	0.00	LX0	S
ATOM	1655	C	CYS	194	71.018	-2.306	5.527	1.00	0.00	LX0	C
ATOM	1656	O	CYS	194	71.322	-2.922	6.544	1.00	0.00	LX0	O
ATOM	1657	N	PRO	195	71.318	-0.993	5.327	1.00	0.00	LX0	N
ATOM	1658	CD	PRO	195	70.754	-0.132	4.289	1.00	0.00	LX0	C
ATOM	1659	CA	PRO	195	72.306	-0.284	6.158	1.00	0.00	LX0	C

ATOM	1660	CB	PRO	195	71.992	1.184	5.856	1.00	0.00	LX0	C
ATOM	1661	CG	PRO	195	71.540	1.170	4.397	1.00	0.00	LX0	C
ATOM	1662	C	PRO	195	72.366	-0.630	7.642	1.00	0.00	LX0	C
ATOM	1663	O	PRO	195	73.375	-1.082	8.169	1.00	0.00	LX0	O
ATOM	1664	N	ASN	196	71.228	-0.407	8.305	1.00	0.00	LX0	N
ATOM	1665	H	ASN	196	70.444	0.024	7.856	1.00	0.00	LX0	H
ATOM	1666	CA	ASN	196	71.141	-0.915	9.673	1.00	0.00	LX0	C
ATOM	1667	CB	ASN	196	71.211	0.205	10.717	1.00	0.00	LX0	C
ATOM	1668	CG	ASN	196	72.556	0.906	10.689	1.00	0.00	LX0	C
ATOM	1669	OD1	ASN	196	72.650	2.105	10.465	1.00	0.00	LX0	O
ATOM	1670	ND2	ASN	196	73.607	0.123	10.944	1.00	0.00	LX0	N
ATOM	1671	HD21	ASN	196	73.524	-0.864	11.068	1.00	0.00	LX0	H
ATOM	1672	HD22	ASN	196	74.508	0.550	10.966	1.00	0.00	LX0	H
ATOM	1673	C	ASN	196	69.854	-1.685	9.824	1.00	0.00	LX0	C
ATOM	1674	O	ASN	196	68.998	-1.394	10.648	1.00	0.00	LX0	O
ATOM	1675	N	GLY	197	69.713	-2.653	8.910	1.00	0.00	LX0	N
ATOM	1676	H	GLY	197	70.462	-2.857	8.277	1.00	0.00	LX0	H
ATOM	1677	CA	GLY	197	68.347	-3.077	8.626	1.00	0.00	LX0	C
ATOM	1678	C	GLY	197	67.706	-2.022	7.748	1.00	0.00	LX0	C
ATOM	1679	O	GLY	197	67.900	-1.982	6.538	1.00	0.00	LX0	O
ATOM	1680	N	SER	198	66.996	-1.109	8.426	1.00	0.00	LX0	N
ATOM	1681	H	SER	198	66.972	-1.156	9.427	1.00	0.00	LX0	H
ATOM	1682	CA	SER	198	66.438	0.064	7.750	1.00	0.00	LX0	C
ATOM	1683	CB	SER	198	67.550	1.110	7.546	1.00	0.00	LX0	C
ATOM	1684	OG	SER	198	68.765	0.487	7.093	1.00	0.00	LX0	O
ATOM	1685	HG	SER	198	68.488	-0.135	6.422	1.00	0.00	LX0	H
ATOM	1686	C	SER	198	65.645	-0.212	6.476	1.00	0.00	LX0	C
ATOM	1687	O	SER	198	65.707	0.524	5.498	1.00	0.00	LX0	O
ATOM	1688	N	CYS	199	64.918	-1.334	6.510	1.00	0.00	LX0	N
ATOM	1689	H	CYS	199	64.781	-1.859	7.354	1.00	0.00	LX0	H
ATOM	1690	CA	CYS	199	64.329	-1.745	5.239	1.00	0.00	LX0	C
ATOM	1691	CB	CYS	199	64.606	-3.231	5.001	1.00	0.00	LX0	C
ATOM	1692	SG	CYS	199	64.578	-4.227	6.519	1.00	0.00	LX0	S
ATOM	1693	C	CYS	199	62.867	-1.373	5.090	1.00	0.00	LX0	C
ATOM	1694	O	CYS	199	62.005	-1.717	5.887	1.00	0.00	LX0	O
ATOM	1695	N	TRP	200	62.610	-0.599	4.031	1.00	0.00	LX0	N
ATOM	1696	H	TRP	200	63.296	-0.466	3.314	1.00	0.00	LX0	H
ATOM	1697	CA	TRP	200	61.222	-0.201	3.814	1.00	0.00	LX0	C
ATOM	1698	CB	TRP	200	61.174	1.104	3.025	1.00	0.00	LX0	C
ATOM	1699	CG	TRP	200	61.358	2.298	3.926	1.00	0.00	LX0	C
ATOM	1700	CD2	TRP	200	60.365	2.887	4.787	1.00	0.00	LX0	C
ATOM	1701	CE2	TRP	200	60.968	4.026	5.414	1.00	0.00	LX0	C
ATOM	1702	CE3	TRP	200	59.024	2.550	5.067	1.00	0.00	LX0	C
ATOM	1703	CD1	TRP	200	62.503	3.096	4.077	1.00	0.00	LX0	C
ATOM	1704	NE1	TRP	200	62.278	4.117	4.951	1.00	0.00	LX0	N
ATOM	1705	HE1	TRP	200	62.910	4.823	5.204	1.00	0.00	LX0	H
ATOM	1706	CZ2	TRP	200	60.215	4.807	6.314	1.00	0.00	LX0	C
ATOM	1707	CZ3	TRP	200	58.281	3.338	5.968	1.00	0.00	LX0	C
ATOM	1708	CH2	TRP	200	58.875	4.459	6.584	1.00	0.00	LX0	C
ATOM	1709	C	TRP	200	60.461	-1.281	3.073	1.00	0.00	LX0	C
ATOM	1710	O	TRP	200	60.305	-1.238	1.860	1.00	0.00	LX0	O
ATOM	1711	N	GLY	201	60.022	-2.284	3.844	1.00	0.00	LX0	N
ATOM	1712	H	GLY	201	60.262	-2.270	4.815	1.00	0.00	LX0	H
ATOM	1713	CA	GLY	201	59.679	-3.518	3.136	1.00	0.00	LX0	C
ATOM	1714	C	GLY	201	60.980	-4.161	2.693	1.00	0.00	LX0	C
ATOM	1715	O	GLY	201	62.009	-3.928	3.318	1.00	0.00	LX0	O
ATOM	1716	N	ALA	202	60.911	-4.929	1.601	1.00	0.00	LX0	N
ATOM	1717	H	ALA	202	60.043	-5.151	1.156	1.00	0.00	LX0	H
ATOM	1718	CA	ALA	202	62.162	-5.326	0.956	1.00	0.00	LX0	C
ATOM	1719	CB	ALA	202	62.880	-6.456	1.702	1.00	0.00	LX0	C
ATOM	1720	C	ALA	202	61.886	-5.800	-0.446	1.00	0.00	LX0	C

ATOM	1721	O	ALA	202	60.752	-6.153	-0.760	1.00	0.00	LX0	O
ATOM	1722	N	GLY	203	62.943	-5.803	-1.258	1.00	0.00	LX0	N
ATOM	1723	H	GLY	203	63.840	-5.506	-0.909	1.00	0.00	LX0	H
ATOM	1724	CA	GLY	203	62.804	-6.360	-2.599	1.00	0.00	LX0	C
ATOM	1725	C	GLY	203	63.927	-5.984	-3.548	1.00	0.00	LX0	C
ATOM	1726	O	GLY	203	64.461	-6.818	-4.268	1.00	0.00	LX0	O
ATOM	1727	N	GLU	204	64.222	-4.676	-3.539	1.00	0.00	LX0	N
ATOM	1728	H	GLU	204	63.838	-4.095	-2.825	1.00	0.00	LX0	H
ATOM	1729	CA	GLU	204	65.129	-4.128	-4.546	1.00	0.00	LX0	C
ATOM	1730	CB	GLU	204	64.312	-3.729	-5.784	1.00	0.00	LX0	C
ATOM	1731	CG	GLU	204	65.120	-3.456	-7.058	1.00	0.00	LX0	C
ATOM	1732	CD	GLU	204	64.175	-3.210	-8.223	1.00	0.00	LX0	C
ATOM	1733	OE1	GLU	204	63.423	-2.234	-8.189	1.00	0.00	LX0	O
ATOM	1734	OE2	GLU	204	64.193	-4.000	-9.166	1.00	0.00	LX0	O
ATOM	1735	C	GLU	204	65.934	-2.974	-3.968	1.00	0.00	LX0	C
ATOM	1736	O	GLU	204	65.810	-1.817	-4.350	1.00	0.00	LX0	O
ATOM	1737	N	GLU	205	66.734	-3.345	-2.951	1.00	0.00	LX0	N
ATOM	1738	H	GLU	205	66.715	-4.303	-2.654	1.00	0.00	LX0	H
ATOM	1739	CA	GLU	205	67.441	-2.360	-2.121	1.00	0.00	LX0	C
ATOM	1740	CB	GLU	205	68.754	-1.894	-2.759	1.00	0.00	LX0	C
ATOM	1741	CG	GLU	205	69.797	-1.459	-1.717	1.00	0.00	LX0	C
ATOM	1742	CD	GLU	205	70.222	-2.638	-0.857	1.00	0.00	LX0	C
ATOM	1743	OE1	GLU	205	69.992	-2.618	0.351	1.00	0.00	LX0	O
ATOM	1744	OE2	GLU	205	70.817	-3.572	-1.386	1.00	0.00	LX0	O
ATOM	1745	C	GLU	205	66.559	-1.209	-1.655	1.00	0.00	LX0	C
ATOM	1746	O	GLU	205	66.847	-0.016	-1.731	1.00	0.00	LX0	O
ATOM	1747	N	ASN	206	65.390	-1.660	-1.189	1.00	0.00	LX0	N
ATOM	1748	H	ASN	206	65.340	-2.639	-0.978	1.00	0.00	LX0	H
ATOM	1749	CA	ASN	206	64.277	-0.762	-0.906	1.00	0.00	LX0	C
ATOM	1750	CB	ASN	206	62.968	-1.544	-1.101	1.00	0.00	LX0	C
ATOM	1751	CG	ASN	206	61.807	-0.627	-1.435	1.00	0.00	LX0	C
ATOM	1752	OD1	ASN	206	61.397	-0.460	-2.579	1.00	0.00	LX0	O
ATOM	1753	ND2	ASN	206	61.265	-0.020	-0.386	1.00	0.00	LX0	N
ATOM	1754	HD21	ASN	206	61.578	-0.266	0.538	1.00	0.00	LX0	H
ATOM	1755	HD22	ASN	206	60.529	0.643	-0.504	1.00	0.00	LX0	H
ATOM	1756	C	ASN	206	64.384	-0.134	0.476	1.00	0.00	LX0	C
ATOM	1757	O	ASN	206	63.506	-0.251	1.324	1.00	0.00	LX0	O
ATOM	1758	N	CYS	207	65.522	0.537	0.685	1.00	0.00	LX0	N
ATOM	1759	H	CYS	207	66.177	0.656	-0.062	1.00	0.00	LX0	H
ATOM	1760	CA	CYS	207	65.849	0.905	2.057	1.00	0.00	LX0	C
ATOM	1761	CB	CYS	207	67.080	0.120	2.515	1.00	0.00	LX0	C
ATOM	1762	SG	CYS	207	66.776	-1.660	2.569	1.00	0.00	LX0	S
ATOM	1763	C	CYS	207	66.036	2.388	2.315	1.00	0.00	LX0	C
ATOM	1764	O	CYS	207	66.173	3.220	1.420	1.00	0.00	LX0	O
ATOM	1765	N	GLN	208	66.047	2.667	3.626	1.00	0.00	LX0	N
ATOM	1766	H	GLN	208	65.907	1.917	4.274	1.00	0.00	LX0	H
ATOM	1767	CA	GLN	208	66.420	3.977	4.144	1.00	0.00	LX0	C
ATOM	1768	CB	GLN	208	66.108	4.016	5.645	1.00	0.00	LX0	C
ATOM	1769	CG	GLN	208	66.544	5.288	6.376	1.00	0.00	LX0	C
ATOM	1770	CD	GLN	208	66.469	5.086	7.879	1.00	0.00	LX0	C
ATOM	1771	OE1	GLN	208	66.185	4.020	8.411	1.00	0.00	LX0	O
ATOM	1772	NE2	GLN	208	66.738	6.193	8.561	1.00	0.00	LX0	N
ATOM	1773	HE21	GLN	208	67.036	7.043	8.113	1.00	0.00	LX0	H
ATOM	1774	HE22	GLN	208	66.657	6.218	9.556	1.00	0.00	LX0	H
ATOM	1775	C	GLN	208	67.891	4.264	3.900	1.00	0.00	LX0	C
ATOM	1776	O	GLN	208	68.785	3.639	4.455	1.00	0.00	LX0	O
ATOM	1777	N	LYS	209	68.106	5.267	3.047	1.00	0.00	LX0	N
ATOM	1778	H	LYS	209	67.334	5.765	2.659	1.00	0.00	LX0	H
ATOM	1779	CA	LYS	209	69.479	5.747	2.979	1.00	0.00	LX0	C
ATOM	1780	CB	LYS	209	69.932	6.151	1.562	1.00	0.00	LX0	C
ATOM	1781	CG	LYS	209	68.961	6.939	0.674	1.00	0.00	LX0	C

ATOM	1782	CD	LYS	209	67.908	6.054	0.001	1.00	0.00	LX0	C
ATOM	1783	CE	LYS	209	66.870	6.832	-0.801	1.00	0.00	LX0	C
ATOM	1784	NZ	LYS	209	65.844	5.887	-1.248	1.00	0.00	LX0	N
ATOM	1785	HZ1	LYS	209	65.068	6.397	-1.718	1.00	0.00	LX0	H
ATOM	1786	HZ2	LYS	209	66.234	5.173	-1.890	1.00	0.00	LX0	H
ATOM	1787	HZ3	LYS	209	65.435	5.413	-0.413	1.00	0.00	LX0	H
ATOM	1788	C	LYS	209	69.706	6.843	3.994	1.00	0.00	LX0	C
ATOM	1789	O	LYS	209	69.201	7.955	3.891	1.00	0.00	LX0	O
ATOM	1790	N	LEU	210	70.485	6.438	5.007	1.00	0.00	LX0	N
ATOM	1791	H	LEU	210	70.752	5.475	5.026	1.00	0.00	LX0	H
ATOM	1792	CA	LEU	210	70.776	7.320	6.140	1.00	0.00	LX0	C
ATOM	1793	CB	LEU	210	71.863	6.710	7.024	1.00	0.00	LX0	C
ATOM	1794	CG	LEU	210	71.377	5.792	8.152	1.00	0.00	LX0	C
ATOM	1795	CD1	LEU	210	70.612	4.555	7.671	1.00	0.00	LX0	C
ATOM	1796	CD2	LEU	210	72.550	5.420	9.059	1.00	0.00	LX0	C
ATOM	1797	C	LEU	210	71.184	8.724	5.737	1.00	0.00	LX0	C
ATOM	1798	O	LEU	210	72.149	8.943	5.006	1.00	0.00	LX0	O
ATOM	1799	N	THR	211	70.380	9.664	6.217	1.00	0.00	LX0	N
ATOM	1800	H	THR	211	69.613	9.400	6.812	1.00	0.00	LX0	H
ATOM	1801	CA	THR	211	70.602	11.036	5.791	1.00	0.00	LX0	C
ATOM	1802	CB	THR	211	69.695	11.361	4.596	1.00	0.00	LX0	C
ATOM	1803	OG1	THR	211	68.474	10.616	4.705	1.00	0.00	LX0	O
ATOM	1804	HG1	THR	211	68.670	9.732	4.384	1.00	0.00	LX0	H
ATOM	1805	CG2	THR	211	70.357	11.111	3.239	1.00	0.00	LX0	C
ATOM	1806	C	THR	211	70.479	12.073	6.900	1.00	0.00	LX0	C
ATOM	1807	O	THR	211	70.543	13.267	6.644	1.00	0.00	LX0	O
ATOM	1808	N	LYS	212	70.326	11.589	8.139	1.00	0.00	LX0	N
ATOM	1809	H	LYS	212	70.112	10.620	8.256	1.00	0.00	LX0	H
ATOM	1810	CA	LYS	212	70.497	12.466	9.299	1.00	0.00	LX0	C
ATOM	1811	CB	LYS	212	69.177	12.691	10.041	1.00	0.00	LX0	C
ATOM	1812	CG	LYS	212	68.491	14.046	9.840	1.00	0.00	LX0	C
ATOM	1813	CD	LYS	212	67.091	14.051	10.470	1.00	0.00	LX0	C
ATOM	1814	CE	LYS	212	66.277	15.328	10.250	1.00	0.00	LX0	C
ATOM	1815	NZ	LYS	212	64.861	15.052	10.529	1.00	0.00	LX0	N
ATOM	1816	HZ1	LYS	212	64.363	15.912	10.841	1.00	0.00	LX0	H
ATOM	1817	HZ2	LYS	212	64.365	14.716	9.671	1.00	0.00	LX0	H
ATOM	1818	HZ3	LYS	212	64.767	14.320	11.260	1.00	0.00	LX0	H
ATOM	1819	C	LYS	212	71.532	11.941	10.282	1.00	0.00	LX0	C
ATOM	1820	O	LYS	212	72.413	12.660	10.729	1.00	0.00	LX0	O
ATOM	1821	N	ILE	213	71.393	10.644	10.616	1.00	0.00	LX0	N
ATOM	1822	H	ILE	213	70.694	10.079	10.171	1.00	0.00	LX0	H
ATOM	1823	CA	ILE	213	72.231	10.109	11.697	1.00	0.00	LX0	C
ATOM	1824	CB	ILE	213	71.818	8.664	12.046	1.00	0.00	LX0	C
ATOM	1825	CG2	ILE	213	72.774	7.960	13.020	1.00	0.00	LX0	C
ATOM	1826	CG1	ILE	213	70.392	8.651	12.601	1.00	0.00	LX0	C
ATOM	1827	CD1	ILE	213	70.215	9.443	13.901	1.00	0.00	LX0	C
ATOM	1828	C	ILE	213	73.729	10.226	11.456	1.00	0.00	LX0	C
ATOM	1829	O	ILE	213	74.489	10.684	12.299	1.00	0.00	LX0	O
ATOM	1830	N	ILE	214	74.131	9.809	10.250	1.00	0.00	LX0	N
ATOM	1831	H	ILE	214	73.456	9.545	9.564	1.00	0.00	LX0	H
ATOM	1832	CA	ILE	214	75.568	9.905	9.995	1.00	0.00	LX0	C
ATOM	1833	CB	ILE	214	76.082	8.690	9.198	1.00	0.00	LX0	C
ATOM	1834	CG2	ILE	214	76.200	7.505	10.158	1.00	0.00	LX0	C
ATOM	1835	CG1	ILE	214	75.227	8.303	7.984	1.00	0.00	LX0	C
ATOM	1836	CD1	ILE	214	75.420	9.162	6.737	1.00	0.00	LX0	C
ATOM	1837	C	ILE	214	76.041	11.242	9.441	1.00	0.00	LX0	C
ATOM	1838	O	ILE	214	76.780	11.340	8.467	1.00	0.00	LX0	O
ATOM	1839	N	CYS	215	75.571	12.298	10.117	1.00	0.00	LX0	N
ATOM	1840	H	CYS	215	75.033	12.166	10.951	1.00	0.00	LX0	H
ATOM	1841	CA	CYS	215	76.079	13.610	9.734	1.00	0.00	LX0	C
ATOM	1842	CB	CYS	215	75.253	14.725	10.373	1.00	0.00	LX0	C

ATOM	1843	SG	CYS	215	73.710	15.027	9.473	1.00	0.00	LX0	S
ATOM	1844	C	CYS	215	77.548	13.771	10.051	1.00	0.00	LX0	C
ATOM	1845	O	CYS	215	78.048	13.332	11.080	1.00	0.00	LX0	O
ATOM	1846	N	ALA	216	78.230	14.407	9.091	1.00	0.00	LX0	N
ATOM	1847	H	ALA	216	77.755	14.807	8.311	1.00	0.00	LX0	H
ATOM	1848	CA	ALA	216	79.641	14.655	9.354	1.00	0.00	LX0	C
ATOM	1849	CB	ALA	216	80.358	15.064	8.070	1.00	0.00	LX0	C
ATOM	1850	C	ALA	216	79.795	15.751	10.387	1.00	0.00	LX0	C
ATOM	1851	O	ALA	216	78.886	16.542	10.604	1.00	0.00	LX0	O
ATOM	1852	N	GLN	217	80.988	15.763	11.007	1.00	0.00	LX0	N
ATOM	1853	H	GLN	217	81.647	15.058	10.751	1.00	0.00	LX0	H
ATOM	1854	CA	GLN	217	81.240	16.700	12.111	1.00	0.00	LX0	C
ATOM	1855	CB	GLN	217	82.687	16.576	12.588	1.00	0.00	LX0	C
ATOM	1856	CG	GLN	217	83.022	15.182	13.125	1.00	0.00	LX0	C
ATOM	1857	CD	GLN	217	84.497	15.118	13.459	1.00	0.00	LX0	C
ATOM	1858	OE1	GLN	217	85.359	15.446	12.658	1.00	0.00	LX0	O
ATOM	1859	NE2	GLN	217	84.763	14.678	14.692	1.00	0.00	LX0	N
ATOM	1860	HE21	GLN	217	84.041	14.411	15.326	1.00	0.00	LX0	H
ATOM	1861	HE22	GLN	217	85.724	14.627	14.956	1.00	0.00	LX0	H
ATOM	1862	C	GLN	217	80.892	18.156	11.824	1.00	0.00	LX0	C
ATOM	1863	O	GLN	217	80.431	18.901	12.679	1.00	0.00	LX0	O
ATOM	1864	N	GLN	218	81.106	18.512	10.553	1.00	0.00	LX0	N
ATOM	1865	H	GLN	218	81.536	17.890	9.902	1.00	0.00	LX0	H
ATOM	1866	CA	GLN	218	80.377	19.675	10.074	1.00	0.00	LX0	C
ATOM	1867	CB	GLN	218	81.350	20.817	9.744	1.00	0.00	LX0	C
ATOM	1868	CG	GLN	218	80.685	22.149	9.371	1.00	0.00	LX0	C
ATOM	1869	CD	GLN	218	79.650	22.521	10.415	1.00	0.00	LX0	C
ATOM	1870	OE1	GLN	218	78.469	22.241	10.267	1.00	0.00	LX0	O
ATOM	1871	NE2	GLN	218	80.141	23.134	11.494	1.00	0.00	LX0	N
ATOM	1872	HE21	GLN	218	81.108	23.361	11.587	1.00	0.00	LX0	H
ATOM	1873	HE22	GLN	218	79.502	23.349	12.230	1.00	0.00	LX0	H
ATOM	1874	C	GLN	218	79.528	19.243	8.898	1.00	0.00	LX0	C
ATOM	1875	O	GLN	218	79.998	18.512	8.030	1.00	0.00	LX0	O
ATOM	1876	N	CYS	219	78.265	19.682	8.932	1.00	0.00	LX0	N
ATOM	1877	H	CYS	219	77.965	20.329	9.639	1.00	0.00	LX0	H
ATOM	1878	CA	CYS	219	77.308	19.146	7.969	1.00	0.00	LX0	C
ATOM	1879	CB	CYS	219	76.640	17.894	8.547	1.00	0.00	LX0	C
ATOM	1880	SG	CYS	219	76.006	16.727	7.313	1.00	0.00	LX0	S
ATOM	1881	C	CYS	219	76.273	20.190	7.616	1.00	0.00	LX0	C
ATOM	1882	O	CYS	219	75.494	20.630	8.450	1.00	0.00	LX0	O
ATOM	1883	N	SER	220	76.294	20.596	6.342	1.00	0.00	LX0	N
ATOM	1884	H	SER	220	76.894	20.186	5.654	1.00	0.00	LX0	H
ATOM	1885	CA	SER	220	75.479	21.746	5.945	1.00	0.00	LX0	C
ATOM	1886	CB	SER	220	76.113	22.338	4.689	1.00	0.00	LX0	C
ATOM	1887	OG	SER	220	76.395	21.273	3.770	1.00	0.00	LX0	O
ATOM	1888	HG	SER	220	75.589	21.147	3.270	1.00	0.00	LX0	H
ATOM	1889	C	SER	220	73.987	21.502	5.731	1.00	0.00	LX0	C
ATOM	1890	O	SER	220	73.330	22.233	4.998	1.00	0.00	LX0	O
ATOM	1891	N	GLY	221	73.486	20.435	6.360	1.00	0.00	LX0	N
ATOM	1892	H	GLY	221	74.012	19.944	7.056	1.00	0.00	LX0	H
ATOM	1893	CA	GLY	221	72.140	19.966	6.058	1.00	0.00	LX0	C
ATOM	1894	C	GLY	221	72.146	18.467	6.234	1.00	0.00	LX0	C
ATOM	1895	O	GLY	221	73.041	17.931	6.873	1.00	0.00	LX0	O
ATOM	1896	N	ARG	222	71.146	17.812	5.620	1.00	0.00	LX0	N
ATOM	1897	H	ARG	222	70.434	18.326	5.144	1.00	0.00	LX0	H
ATOM	1898	CA	ARG	222	71.118	16.343	5.663	1.00	0.00	LX0	C
ATOM	1899	CB	ARG	222	69.992	15.823	4.764	1.00	0.00	LX0	C
ATOM	1900	CG	ARG	222	68.561	16.219	5.146	1.00	0.00	LX0	C
ATOM	1901	CD	ARG	222	67.953	15.437	6.315	1.00	0.00	LX0	C
ATOM	1902	NE	ARG	222	67.889	13.998	6.046	1.00	0.00	LX0	N
ATOM	1903	HE	ARG	222	68.673	13.589	5.577	1.00	0.00	LX0	H

ATOM	1904	CZ	ARG	222	66.933	13.213	6.595	1.00	0.00	LX0	C
ATOM	1905	NH1	ARG	222	65.850	13.735	7.147	1.00	0.00	LX0	N
ATOM	1906	HH11	ARG	222	65.161	13.122	7.546	1.00	0.00	LX0	H
ATOM	1907	HH12	ARG	222	65.647	14.725	7.148	1.00	0.00	LX0	H
ATOM	1908	NH2	ARG	222	67.054	11.892	6.594	1.00	0.00	LX0	N
ATOM	1909	HH21	ARG	222	66.457	11.341	7.190	1.00	0.00	LX0	H
ATOM	1910	HH22	ARG	222	67.719	11.393	6.033	1.00	0.00	LX0	H
ATOM	1911	C	ARG	222	72.433	15.708	5.216	1.00	0.00	LX0	C
ATOM	1912	O	ARG	222	73.100	16.200	4.309	1.00	0.00	LX0	O
ATOM	1913	N	CYS	223	72.782	14.590	5.861	1.00	0.00	LX0	N
ATOM	1914	H	CYS	223	72.196	14.242	6.594	1.00	0.00	LX0	H
ATOM	1915	CA	CYS	223	73.941	13.844	5.373	1.00	0.00	LX0	C
ATOM	1916	CB	CYS	223	74.604	13.040	6.488	1.00	0.00	LX0	C
ATOM	1917	SG	CYS	223	73.489	11.921	7.373	1.00	0.00	LX0	S
ATOM	1918	C	CYS	223	73.662	12.986	4.152	1.00	0.00	LX0	C
ATOM	1919	O	CYS	223	73.541	11.763	4.176	1.00	0.00	LX0	O
ATOM	1920	N	ARG	224	73.590	13.730	3.042	1.00	0.00	LX0	N
ATOM	1921	H	ARG	224	73.620	14.723	3.161	1.00	0.00	LX0	H
ATOM	1922	CA	ARG	224	73.528	13.147	1.705	1.00	0.00	LX0	C
ATOM	1923	CB	ARG	224	73.456	14.335	0.749	1.00	0.00	LX0	C
ATOM	1924	CG	ARG	224	73.610	14.111	-0.748	1.00	0.00	LX0	C
ATOM	1925	CD	ARG	224	73.573	15.485	-1.415	1.00	0.00	LX0	C
ATOM	1926	NE	ARG	224	74.285	15.482	-2.686	1.00	0.00	LX0	N
ATOM	1927	HE	ARG	224	73.824	15.147	-3.513	1.00	0.00	LX0	H
ATOM	1928	CZ	ARG	224	75.598	15.786	-2.669	1.00	0.00	LX0	C
ATOM	1929	NH1	ARG	224	76.149	16.334	-1.599	1.00	0.00	LX0	N
ATOM	1930	HH11	ARG	224	77.151	16.446	-1.583	1.00	0.00	LX0	H
ATOM	1931	HH12	ARG	224	75.638	16.646	-0.796	1.00	0.00	LX0	H
ATOM	1932	NH2	ARG	224	76.362	15.536	-3.720	1.00	0.00	LX0	N
ATOM	1933	HH21	ARG	224	77.366	15.570	-3.586	1.00	0.00	LX0	H
ATOM	1934	HH22	ARG	224	75.971	15.301	-4.609	1.00	0.00	LX0	H
ATOM	1935	C	ARG	224	74.707	12.213	1.468	1.00	0.00	LX0	C
ATOM	1936	O	ARG	224	74.555	11.001	1.320	1.00	0.00	LX0	O
ATOM	1937	N	GLY	225	75.900	12.830	1.549	1.00	0.00	LX0	N
ATOM	1938	H	GLY	225	75.959	13.829	1.564	1.00	0.00	LX0	H
ATOM	1939	CA	GLY	225	77.140	12.060	1.616	1.00	0.00	LX0	C
ATOM	1940	C	GLY	225	77.126	11.078	2.769	1.00	0.00	LX0	C
ATOM	1941	O	GLY	225	76.264	11.109	3.642	1.00	0.00	LX0	O
ATOM	1942	N	LYS	226	78.070	10.139	2.709	1.00	0.00	LX0	N
ATOM	1943	H	LYS	226	78.779	10.141	2.004	1.00	0.00	LX0	H
ATOM	1944	CA	LYS	226	77.871	9.045	3.655	1.00	0.00	LX0	C
ATOM	1945	CB	LYS	226	77.681	7.735	2.882	1.00	0.00	LX0	C
ATOM	1946	CG	LYS	226	76.586	7.853	1.804	1.00	0.00	LX0	C
ATOM	1947	CD	LYS	226	75.167	7.403	2.187	1.00	0.00	LX0	C
ATOM	1948	CE	LYS	226	74.512	7.992	3.444	1.00	0.00	LX0	C
ATOM	1949	NZ	LYS	226	74.192	9.428	3.354	1.00	0.00	LX0	N
ATOM	1950	HZ1	LYS	226	73.347	9.640	3.933	1.00	0.00	LX0	H
ATOM	1951	HZ2	LYS	226	74.942	10.039	3.738	1.00	0.00	LX0	H
ATOM	1952	HZ3	LYS	226	74.017	9.750	2.380	1.00	0.00	LX0	H
ATOM	1953	C	LYS	226	78.878	8.945	4.786	1.00	0.00	LX0	C
ATOM	1954	O	LYS	226	78.833	8.030	5.597	1.00	0.00	LX0	O
ATOM	1955	N	SER	227	79.782	9.938	4.788	1.00	0.00	LX0	N
ATOM	1956	H	SER	227	79.724	10.648	4.091	1.00	0.00	LX0	H
ATOM	1957	CA	SER	227	80.771	10.124	5.857	1.00	0.00	LX0	C
ATOM	1958	CB	SER	227	81.775	8.961	6.026	1.00	0.00	LX0	C
ATOM	1959	OG	SER	227	81.460	7.884	5.136	1.00	0.00	LX0	O
ATOM	1960	HG	SER	227	80.661	7.494	5.485	1.00	0.00	LX0	H
ATOM	1961	C	SER	227	81.492	11.472	5.794	1.00	0.00	LX0	C
ATOM	1962	O	SER	227	81.462	12.227	6.757	1.00	0.00	LX0	O
ATOM	1963	N	PRO	228	82.130	11.810	4.632	1.00	0.00	LX0	N
ATOM	1964	CD	PRO	228	82.387	11.048	3.404	1.00	0.00	LX0	C

ATOM	1965	CA	PRO	228	82.656	13.177	4.549	1.00	0.00	LX0	C
ATOM	1966	CB	PRO	228	83.618	13.079	3.361	1.00	0.00	LX0	C
ATOM	1967	CG	PRO	228	82.967	12.062	2.427	1.00	0.00	LX0	C
ATOM	1968	C	PRO	228	81.525	14.167	4.318	1.00	0.00	LX0	C
ATOM	1969	O	PRO	228	80.396	13.791	4.016	1.00	0.00	LX0	O
ATOM	1970	N	SER	229	81.889	15.448	4.445	1.00	0.00	LX0	N
ATOM	1971	H	SER	229	82.810	15.694	4.744	1.00	0.00	LX0	H
ATOM	1972	CA	SER	229	80.890	16.510	4.343	1.00	0.00	LX0	C
ATOM	1973	CB	SER	229	81.431	17.749	5.056	1.00	0.00	LX0	C
ATOM	1974	OG	SER	229	81.956	17.362	6.333	1.00	0.00	LX0	O
ATOM	1975	HG	SER	229	81.300	17.659	6.964	1.00	0.00	LX0	H
ATOM	1976	C	SER	229	80.349	16.878	2.962	1.00	0.00	LX0	C
ATOM	1977	O	SER	229	80.139	18.047	2.661	1.00	0.00	LX0	O
ATOM	1978	N	ASP	230	80.089	15.852	2.131	1.00	0.00	LX0	N
ATOM	1979	H	ASP	230	80.197	14.910	2.440	1.00	0.00	LX0	H
ATOM	1980	CA	ASP	230	79.325	16.157	0.919	1.00	0.00	LX0	C
ATOM	1981	CB	ASP	230	79.667	15.183	-0.224	1.00	0.00	LX0	C
ATOM	1982	CG	ASP	230	79.028	15.626	-1.538	1.00	0.00	LX0	C
ATOM	1983	OD1	ASP	230	78.606	14.780	-2.318	1.00	0.00	LX0	O
ATOM	1984	OD2	ASP	230	78.863	16.822	-1.763	1.00	0.00	LX0	O
ATOM	1985	C	ASP	230	77.838	16.185	1.242	1.00	0.00	LX0	C
ATOM	1986	O	ASP	230	77.046	15.296	0.953	1.00	0.00	LX0	O
ATOM	1987	N	CYS	231	77.498	17.248	1.965	1.00	0.00	LX0	N
ATOM	1988	H	CYS	231	78.164	17.988	2.081	1.00	0.00	LX0	H
ATOM	1989	CA	CYS	231	76.185	17.235	2.595	1.00	0.00	LX0	C
ATOM	1990	CB	CYS	231	76.242	18.060	3.877	1.00	0.00	LX0	C
ATOM	1991	SG	CYS	231	77.685	17.655	4.890	1.00	0.00	LX0	S
ATOM	1992	C	CYS	231	75.071	17.682	1.669	1.00	0.00	LX0	C
ATOM	1993	O	CYS	231	75.250	17.848	0.467	1.00	0.00	LX0	O
ATOM	1994	N	CYS	232	73.901	17.881	2.276	1.00	0.00	LX0	N
ATOM	1995	H	CYS	232	73.760	17.648	3.238	1.00	0.00	LX0	H
ATOM	1996	CA	CYS	232	72.900	18.658	1.562	1.00	0.00	LX0	C
ATOM	1997	CB	CYS	232	71.501	18.242	2.010	1.00	0.00	LX0	C
ATOM	1998	SG	CYS	232	71.100	16.540	1.549	1.00	0.00	LX0	S
ATOM	1999	C	CYS	232	73.120	20.130	1.823	1.00	0.00	LX0	C
ATOM	2000	O	CYS	232	73.924	20.508	2.668	1.00	0.00	LX0	O
ATOM	2001	N	HIS	233	72.362	20.943	1.078	1.00	0.00	LX0	N
ATOM	2002	H	HIS	233	71.747	20.565	0.386	1.00	0.00	LX0	H
ATOM	2003	CA	HIS	233	72.192	22.317	1.540	1.00	0.00	LX0	C
ATOM	2004	CB	HIS	233	71.750	23.162	0.333	1.00	0.00	LX0	C
ATOM	2005	CG	HIS	233	71.308	24.571	0.667	1.00	0.00	LX0	C
ATOM	2006	ND1	HIS	233	70.161	25.100	0.203	1.00	0.00	LX0	N
ATOM	2007	HD1	HIS	233	69.528	24.678	-0.419	1.00	0.00	LX0	H
ATOM	2008	CD2	HIS	233	71.951	25.525	1.464	1.00	0.00	LX0	C
ATOM	2009	NE2	HIS	233	71.166	26.629	1.478	1.00	0.00	LX0	N
ATOM	2010	CE1	HIS	233	70.066	26.374	0.699	1.00	0.00	LX0	C
ATOM	2011	C	HIS	233	71.159	22.300	2.653	1.00	0.00	LX0	C
ATOM	2012	O	HIS	233	70.281	21.448	2.667	1.00	0.00	LX0	O
ATOM	2013	N	ASN	234	71.275	23.285	3.551	1.00	0.00	LX0	N
ATOM	2014	H	ASN	234	72.173	23.722	3.605	1.00	0.00	LX0	H
ATOM	2015	CA	ASN	234	70.299	23.482	4.628	1.00	0.00	LX0	C
ATOM	2016	CB	ASN	234	70.643	24.816	5.340	1.00	0.00	LX0	C
ATOM	2017	CG	ASN	234	69.461	25.763	5.484	1.00	0.00	LX0	C
ATOM	2018	OD1	ASN	234	68.644	25.642	6.389	1.00	0.00	LX0	O
ATOM	2019	ND2	ASN	234	69.346	26.646	4.490	1.00	0.00	LX0	N
ATOM	2020	HD21	ASN	234	70.068	26.830	3.823	1.00	0.00	LX0	H
ATOM	2021	HD22	ASN	234	68.466	27.122	4.405	1.00	0.00	LX0	H
ATOM	2022	C	ASN	234	68.825	23.321	4.245	1.00	0.00	LX0	C
ATOM	2023	O	ASN	234	68.009	22.807	4.998	1.00	0.00	LX0	O
ATOM	2024	N	GLN	235	68.524	23.813	3.034	1.00	0.00	LX0	N
ATOM	2025	H	GLN	235	69.239	24.180	2.443	1.00	0.00	LX0	H

ATOM	2026	CA	GLN	235	67.121	23.802	2.633	1.00	0.00	LX0	C
ATOM	2027	CB	GLN	235	66.857	24.965	1.680	1.00	0.00	LX0	C
ATOM	2028	CG	GLN	235	65.539	25.691	1.950	1.00	0.00	LX0	C
ATOM	2029	CD	GLN	235	65.566	26.364	3.308	1.00	0.00	LX0	C
ATOM	2030	OE1	GLN	235	66.544	26.366	4.045	1.00	0.00	LX0	O
ATOM	2031	NE2	GLN	235	64.403	26.918	3.626	1.00	0.00	LX0	N
ATOM	2032	HE21	GLN	235	63.693	27.074	2.941	1.00	0.00	LX0	H
ATOM	2033	HE22	GLN	235	64.281	27.278	4.552	1.00	0.00	LX0	H
ATOM	2034	C	GLN	235	66.594	22.496	2.072	1.00	0.00	LX0	C
ATOM	2035	O	GLN	235	65.396	22.302	1.890	1.00	0.00	LX0	O
ATOM	2036	N	CYS	236	67.558	21.613	1.797	1.00	0.00	LX0	N
ATOM	2037	H	CYS	236	68.502	21.771	2.075	1.00	0.00	LX0	H
ATOM	2038	CA	CYS	236	67.191	20.300	1.293	1.00	0.00	LX0	C
ATOM	2039	CB	CYS	236	68.363	19.658	0.568	1.00	0.00	LX0	C
ATOM	2040	SG	CYS	236	69.316	20.824	-0.439	1.00	0.00	LX0	S
ATOM	2041	C	CYS	236	66.773	19.391	2.415	1.00	0.00	LX0	C
ATOM	2042	O	CYS	236	67.593	18.865	3.159	1.00	0.00	LX0	O
ATOM	2043	N	ALA	237	65.453	19.229	2.500	1.00	0.00	LX0	N
ATOM	2044	H	ALA	237	64.869	19.594	1.773	1.00	0.00	LX0	H
ATOM	2045	CA	ALA	237	64.994	18.175	3.389	1.00	0.00	LX0	C
ATOM	2046	CB	ALA	237	63.512	18.379	3.682	1.00	0.00	LX0	C
ATOM	2047	C	ALA	237	65.230	16.849	2.693	1.00	0.00	LX0	C
ATOM	2048	O	ALA	237	65.305	16.813	1.469	1.00	0.00	LX0	O
ATOM	2049	N	ALA	238	65.382	15.790	3.504	1.00	0.00	LX0	N
ATOM	2050	H	ALA	238	65.283	15.954	4.490	1.00	0.00	LX0	H
ATOM	2051	CA	ALA	238	65.746	14.458	3.000	1.00	0.00	LX0	C
ATOM	2052	CB	ALA	238	64.647	13.851	2.113	1.00	0.00	LX0	C
ATOM	2053	C	ALA	238	67.101	14.343	2.306	1.00	0.00	LX0	C
ATOM	2054	O	ALA	238	68.039	13.770	2.849	1.00	0.00	LX0	O
ATOM	2055	N	GLY	239	67.171	14.897	1.087	1.00	0.00	LX0	N
ATOM	2056	H	GLY	239	66.405	15.430	0.721	1.00	0.00	LX0	H
ATOM	2057	CA	GLY	239	68.407	14.808	0.322	1.00	0.00	LX0	C
ATOM	2058	C	GLY	239	68.413	15.809	-0.816	1.00	0.00	LX0	C
ATOM	2059	O	GLY	239	67.478	16.584	-1.004	1.00	0.00	LX0	O
ATOM	2060	N	CYS	240	69.509	15.756	-1.583	1.00	0.00	LX0	N
ATOM	2061	H	CYS	240	70.255	15.113	-1.411	1.00	0.00	LX0	H
ATOM	2062	CA	CYS	240	69.621	16.672	-2.716	1.00	0.00	LX0	C
ATOM	2063	CB	CYS	240	70.072	18.058	-2.252	1.00	0.00	LX0	C
ATOM	2064	SG	CYS	240	71.647	18.092	-1.369	1.00	0.00	LX0	S
ATOM	2065	C	CYS	240	70.577	16.136	-3.754	1.00	0.00	LX0	C
ATOM	2066	O	CYS	240	71.296	15.175	-3.507	1.00	0.00	LX0	O
ATOM	2067	N	THR	241	70.585	16.808	-4.908	1.00	0.00	LX0	N
ATOM	2068	H	THR	241	69.912	17.521	-5.119	1.00	0.00	LX0	H
ATOM	2069	CA	THR	241	71.616	16.486	-5.888	1.00	0.00	LX0	C
ATOM	2070	CB	THR	241	71.210	17.110	-7.224	1.00	0.00	LX0	C
ATOM	2071	OG1	THR	241	69.800	16.938	-7.414	1.00	0.00	LX0	O
ATOM	2072	HG1	THR	241	69.550	17.478	-8.154	1.00	0.00	LX0	H
ATOM	2073	CG2	THR	241	71.998	16.542	-8.407	1.00	0.00	LX0	C
ATOM	2074	C	THR	241	72.997	16.950	-5.437	1.00	0.00	LX0	C
ATOM	2075	O	THR	241	73.972	16.202	-5.392	1.00	0.00	LX0	O
ATOM	2076	N	GLY	242	73.012	18.232	-5.050	1.00	0.00	LX0	N
ATOM	2077	H	GLY	242	72.190	18.805	-5.077	1.00	0.00	LX0	H
ATOM	2078	CA	GLY	242	74.241	18.809	-4.521	1.00	0.00	LX0	C
ATOM	2079	C	GLY	242	73.897	19.772	-3.407	1.00	0.00	LX0	C
ATOM	2080	O	GLY	242	72.743	20.149	-3.239	1.00	0.00	LX0	O
ATOM	2081	N	PRO	243	74.934	20.156	-2.632	1.00	0.00	LX0	N
ATOM	2082	CD	PRO	243	76.319	19.703	-2.740	1.00	0.00	LX0	C
ATOM	2083	CA	PRO	243	74.715	21.094	-1.525	1.00	0.00	LX0	C
ATOM	2084	CB	PRO	243	75.980	20.882	-0.686	1.00	0.00	LX0	C
ATOM	2085	CG	PRO	243	77.071	20.519	-1.694	1.00	0.00	LX0	C
ATOM	2086	C	PRO	243	74.519	22.536	-1.987	1.00	0.00	LX0	C

ATOM	2087	O	PRO	243	75.356	23.405	-1.782	1.00	0.00	LX0	O
ATOM	2088	N	ARG	244	73.364	22.766	-2.621	1.00	0.00	LX0	N
ATOM	2089	H	ARG	244	72.688	22.040	-2.771	1.00	0.00	LX0	H
ATOM	2090	CA	ARG	244	73.165	24.077	-3.221	1.00	0.00	LX0	C
ATOM	2091	CB	ARG	244	73.572	24.000	-4.686	1.00	0.00	LX0	C
ATOM	2092	CG	ARG	244	74.548	25.021	-5.288	1.00	0.00	LX0	C
ATOM	2093	CD	ARG	244	74.181	26.517	-5.262	1.00	0.00	LX0	C
ATOM	2094	NE	ARG	244	72.944	27.005	-5.912	1.00	0.00	LX0	N
ATOM	2095	HE	ARG	244	72.717	27.959	-5.690	1.00	0.00	LX0	H
ATOM	2096	CZ	ARG	244	72.053	26.454	-6.781	1.00	0.00	LX0	C
ATOM	2097	NH1	ARG	244	72.111	25.206	-7.243	1.00	0.00	LX0	N
ATOM	2098	HH11	ARG	244	71.377	24.790	-7.797	1.00	0.00	LX0	H
ATOM	2099	HH12	ARG	244	72.848	24.552	-7.045	1.00	0.00	LX0	H
ATOM	2100	NH2	ARG	244	71.030	27.211	-7.154	1.00	0.00	LX0	N
ATOM	2101	HH21	ARG	244	70.317	26.819	-7.748	1.00	0.00	LX0	H
ATOM	2102	HH22	ARG	244	70.903	28.163	-6.840	1.00	0.00	LX0	H
ATOM	2103	C	ARG	244	71.717	24.511	-3.106	1.00	0.00	LX0	C
ATOM	2104	O	ARG	244	70.796	23.710	-3.027	1.00	0.00	LX0	O
ATOM	2105	N	GLU	245	71.572	25.841	-3.119	1.00	0.00	LX0	N
ATOM	2106	H	GLU	245	72.412	26.380	-3.102	1.00	0.00	LX0	H
ATOM	2107	CA	GLU	245	70.284	26.535	-3.042	1.00	0.00	LX0	C
ATOM	2108	CB	GLU	245	70.533	27.957	-3.537	1.00	0.00	LX0	C
ATOM	2109	CG	GLU	245	69.312	28.863	-3.699	1.00	0.00	LX0	C
ATOM	2110	CD	GLU	245	69.527	29.789	-4.885	1.00	0.00	LX0	C
ATOM	2111	OE1	GLU	245	68.871	30.825	-4.940	1.00	0.00	LX0	O
ATOM	2112	OE2	GLU	245	70.320	29.462	-5.771	1.00	0.00	LX0	O
ATOM	2113	C	GLU	245	69.073	25.900	-3.732	1.00	0.00	LX0	C
ATOM	2114	O	GLU	245	67.996	25.814	-3.158	1.00	0.00	LX0	O
ATOM	2115	N	SER	246	69.267	25.491	-4.993	1.00	0.00	LX0	N
ATOM	2116	H	SER	246	70.153	25.479	-5.447	1.00	0.00	LX0	H
ATOM	2117	CA	SER	246	68.080	24.986	-5.680	1.00	0.00	LX0	C
ATOM	2118	CB	SER	246	68.020	25.524	-7.111	1.00	0.00	LX0	C
ATOM	2119	OG	SER	246	69.296	25.392	-7.755	1.00	0.00	LX0	O
ATOM	2120	HG	SER	246	69.339	24.466	-8.020	1.00	0.00	LX0	H
ATOM	2121	C	SER	246	67.931	23.483	-5.689	1.00	0.00	LX0	C
ATOM	2122	O	SER	246	66.897	22.945	-6.060	1.00	0.00	LX0	O
ATOM	2123	N	ASP	247	69.024	22.813	-5.313	1.00	0.00	LX0	N
ATOM	2124	H	ASP	247	69.767	23.222	-4.784	1.00	0.00	LX0	H
ATOM	2125	CA	ASP	247	69.160	21.464	-5.858	1.00	0.00	LX0	C
ATOM	2126	CB	ASP	247	70.629	21.191	-6.237	1.00	0.00	LX0	C
ATOM	2127	CG	ASP	247	71.230	22.285	-7.126	1.00	0.00	LX0	C
ATOM	2128	OD1	ASP	247	70.507	23.001	-7.816	1.00	0.00	LX0	O
ATOM	2129	OD2	ASP	247	72.444	22.459	-7.109	1.00	0.00	LX0	O
ATOM	2130	C	ASP	247	68.580	20.383	-4.961	1.00	0.00	LX0	C
ATOM	2131	O	ASP	247	69.103	19.283	-4.823	1.00	0.00	LX0	O
ATOM	2132	N	CYS	248	67.476	20.776	-4.313	1.00	0.00	LX0	N
ATOM	2133	H	CYS	248	66.998	21.581	-4.662	1.00	0.00	LX0	H
ATOM	2134	CA	CYS	248	66.846	19.939	-3.300	1.00	0.00	LX0	C
ATOM	2135	CB	CYS	248	65.998	20.816	-2.378	1.00	0.00	LX0	C
ATOM	2136	SG	CYS	248	66.887	22.253	-1.720	1.00	0.00	LX0	S
ATOM	2137	C	CYS	248	65.956	18.889	-3.922	1.00	0.00	LX0	C
ATOM	2138	O	CYS	248	65.285	19.144	-4.911	1.00	0.00	LX0	O
ATOM	2139	N	LEU	249	65.934	17.704	-3.291	1.00	0.00	LX0	N
ATOM	2140	H	LEU	249	66.497	17.515	-2.483	1.00	0.00	LX0	H
ATOM	2141	CA	LEU	249	64.892	16.780	-3.743	1.00	0.00	LX0	C
ATOM	2142	CB	LEU	249	65.205	15.344	-3.319	1.00	0.00	LX0	C
ATOM	2143	CG	LEU	249	66.518	14.793	-3.882	1.00	0.00	LX0	C
ATOM	2144	CD1	LEU	249	66.819	13.400	-3.326	1.00	0.00	LX0	C
ATOM	2145	CD2	LEU	249	66.560	14.820	-5.412	1.00	0.00	LX0	C
ATOM	2146	C	LEU	249	63.531	17.210	-3.222	1.00	0.00	LX0	C
ATOM	2147	O	LEU	249	62.550	17.406	-3.939	1.00	0.00	LX0	O

ATOM	2148	N	VAL	250	63.530	17.403	-1.899	1.00	0.00	LX0	N
ATOM	2149	H	VAL	250	64.327	17.210	-1.323	1.00	0.00	LX0	H
ATOM	2150	CA	VAL	250	62.346	18.019	-1.323	1.00	0.00	LX0	C
ATOM	2151	CB	VAL	250	61.522	17.005	-0.520	1.00	0.00	LX0	C
ATOM	2152	CG1	VAL	250	60.901	15.963	-1.451	1.00	0.00	LX0	C
ATOM	2153	CG2	VAL	250	62.332	16.333	0.583	1.00	0.00	LX0	C
ATOM	2154	C	VAL	250	62.719	19.241	-0.513	1.00	0.00	LX0	C
ATOM	2155	O	VAL	250	63.810	19.355	0.036	1.00	0.00	LX0	O
ATOM	2156	N	CYS	251	61.781	20.189	-0.513	1.00	0.00	LX0	N
ATOM	2157	H	CYS	251	60.889	20.012	-0.932	1.00	0.00	LX0	H
ATOM	2158	CA	CYS	251	62.169	21.472	0.068	1.00	0.00	LX0	C
ATOM	2159	CB	CYS	251	61.651	22.611	-0.811	1.00	0.00	LX0	C
ATOM	2160	SG	CYS	251	61.877	22.264	-2.574	1.00	0.00	LX0	S
ATOM	2161	C	CYS	251	61.673	21.639	1.486	1.00	0.00	LX0	C
ATOM	2162	O	CYS	251	60.551	21.247	1.794	1.00	0.00	LX0	O
ATOM	2163	N	ARG	252	62.524	22.257	2.325	1.00	0.00	LX0	N
ATOM	2164	H	ARG	252	63.469	22.432	2.040	1.00	0.00	LX0	H
ATOM	2165	CA	ARG	252	62.014	22.634	3.645	1.00	0.00	LX0	C
ATOM	2166	CB	ARG	252	63.151	23.044	4.602	1.00	0.00	LX0	C
ATOM	2167	CG	ARG	252	62.659	23.268	6.042	1.00	0.00	LX0	C
ATOM	2168	CD	ARG	252	63.737	23.601	7.082	1.00	0.00	LX0	C
ATOM	2169	NE	ARG	252	64.267	24.964	6.976	1.00	0.00	LX0	N
ATOM	2170	HE	ARG	252	63.670	25.758	7.116	1.00	0.00	LX0	H
ATOM	2171	CZ	ARG	252	65.598	25.165	6.864	1.00	0.00	LX0	C
ATOM	2172	NH1	ARG	252	66.425	24.142	6.697	1.00	0.00	LX0	N
ATOM	2173	HH11	ARG	252	67.408	24.324	6.584	1.00	0.00	LX0	H
ATOM	2174	HH12	ARG	252	66.121	23.193	6.651	1.00	0.00	LX0	H
ATOM	2175	NH2	ARG	252	66.103	26.388	6.913	1.00	0.00	LX0	N
ATOM	2176	HH21	ARG	252	67.095	26.518	6.827	1.00	0.00	LX0	H
ATOM	2177	HH22	ARG	252	65.520	27.205	7.006	1.00	0.00	LX0	H
ATOM	2178	C	ARG	252	60.953	23.722	3.560	1.00	0.00	LX0	C
ATOM	2179	O	ARG	252	59.788	23.521	3.871	1.00	0.00	LX0	O
ATOM	2180	N	LYS	253	61.433	24.894	3.114	1.00	0.00	LX0	N
ATOM	2181	H	LYS	253	62.381	24.966	2.817	1.00	0.00	LX0	H
ATOM	2182	CA	LYS	253	60.514	26.026	3.060	1.00	0.00	LX0	C
ATOM	2183	CB	LYS	253	60.932	27.090	4.082	1.00	0.00	LX0	C
ATOM	2184	CG	LYS	253	60.565	26.712	5.519	1.00	0.00	LX0	C
ATOM	2185	CD	LYS	253	61.282	27.573	6.556	1.00	0.00	LX0	C
ATOM	2186	CE	LYS	253	61.051	29.076	6.392	1.00	0.00	LX0	C
ATOM	2187	NZ	LYS	253	62.111	29.820	7.085	1.00	0.00	LX0	N
ATOM	2188	HZ1	LYS	253	62.351	30.681	6.546	1.00	0.00	LX0	H
ATOM	2189	HZ2	LYS	253	61.888	30.069	8.064	1.00	0.00	LX0	H
ATOM	2190	HZ3	LYS	253	63.013	29.287	7.081	1.00	0.00	LX0	H
ATOM	2191	C	LYS	253	60.307	26.568	1.654	1.00	0.00	LX0	C
ATOM	2192	O	LYS	253	59.670	25.927	0.830	1.00	0.00	LX0	O
ATOM	2193	N	PHE	254	60.834	27.779	1.410	1.00	0.00	LX0	N
ATOM	2194	H	PHE	254	61.590	28.191	1.919	1.00	0.00	LX0	H
ATOM	2195	CA	PHE	254	60.317	28.485	0.243	1.00	0.00	LX0	C
ATOM	2196	CB	PHE	254	60.551	29.992	0.372	1.00	0.00	LX0	C
ATOM	2197	CG	PHE	254	59.841	30.549	1.586	1.00	0.00	LX0	C
ATOM	2198	CD1	PHE	254	60.607	31.097	2.636	1.00	0.00	LX0	C
ATOM	2199	CD2	PHE	254	58.429	30.520	1.651	1.00	0.00	LX0	C
ATOM	2200	CE1	PHE	254	59.955	31.621	3.769	1.00	0.00	LX0	C
ATOM	2201	CE2	PHE	254	57.778	31.041	2.786	1.00	0.00	LX0	C
ATOM	2202	CZ	PHE	254	58.547	31.585	3.835	1.00	0.00	LX0	C
ATOM	2203	C	PHE	254	60.858	28.002	-1.081	1.00	0.00	LX0	C
ATOM	2204	O	PHE	254	62.057	28.023	-1.334	1.00	0.00	LX0	O
ATOM	2205	N	ARG	255	59.912	27.604	-1.937	1.00	0.00	LX0	N
ATOM	2206	H	ARG	255	58.956	27.652	-1.645	1.00	0.00	LX0	H
ATOM	2207	CA	ARG	255	60.299	27.465	-3.334	1.00	0.00	LX0	C
ATOM	2208	CB	ARG	255	59.369	26.524	-4.099	1.00	0.00	LX0	C

ATOM	2209	CG	ARG	255	60.143	25.466	-4.890	1.00	0.00	LX0	C
ATOM	2210	CD	ARG	255	59.343	24.877	-6.056	1.00	0.00	LX0	C
ATOM	2211	NE	ARG	255	59.214	25.872	-7.119	1.00	0.00	LX0	N
ATOM	2212	HE	ARG	255	59.844	26.651	-7.149	1.00	0.00	LX0	H
ATOM	2213	CZ	ARG	255	58.375	25.728	-8.164	1.00	0.00	LX0	C
ATOM	2214	NH1	ARG	255	57.569	24.690	-8.288	1.00	0.00	LX0	N
ATOM	2215	HH11	ARG	255	56.931	24.696	-9.070	1.00	0.00	LX0	H
ATOM	2216	HH12	ARG	255	57.593	23.888	-7.689	1.00	0.00	LX0	H
ATOM	2217	NH2	ARG	255	58.364	26.636	-9.121	1.00	0.00	LX0	N
ATOM	2218	HH21	ARG	255	57.769	26.475	-9.927	1.00	0.00	LX0	H
ATOM	2219	HH22	ARG	255	58.964	27.436	-9.089	1.00	0.00	LX0	H
ATOM	2220	C	ARG	255	60.327	28.824	-4.004	1.00	0.00	LX0	C
ATOM	2221	O	ARG	255	59.332	29.322	-4.520	1.00	0.00	LX0	O
ATOM	2222	N	ASP	256	61.518	29.420	-3.942	1.00	0.00	LX0	N
ATOM	2223	H	ASP	256	62.280	28.881	-3.578	1.00	0.00	LX0	H
ATOM	2224	CA	ASP	256	61.712	30.692	-4.629	1.00	0.00	LX0	C
ATOM	2225	CB	ASP	256	62.921	31.432	-4.053	1.00	0.00	LX0	C
ATOM	2226	CG	ASP	256	63.118	32.771	-4.743	1.00	0.00	LX0	C
ATOM	2227	OD1	ASP	256	62.123	33.406	-5.041	1.00	0.00	LX0	O
ATOM	2228	OD2	ASP	256	64.245	33.168	-5.032	1.00	0.00	LX0	O
ATOM	2229	C	ASP	256	61.880	30.490	-6.117	1.00	0.00	LX0	C
ATOM	2230	O	ASP	256	62.984	30.277	-6.611	1.00	0.00	LX0	O
ATOM	2231	N	GLU	257	60.730	30.511	-6.802	1.00	0.00	LX0	N
ATOM	2232	H	GLU	257	59.912	30.800	-6.299	1.00	0.00	LX0	H
ATOM	2233	CA	GLU	257	60.680	30.201	-8.230	1.00	0.00	LX0	C
ATOM	2234	CB	GLU	257	61.201	31.377	-9.061	1.00	0.00	LX0	C
ATOM	2235	CG	GLU	257	60.343	32.620	-8.809	1.00	0.00	LX0	C
ATOM	2236	CD	GLU	257	61.013	33.879	-9.319	1.00	0.00	LX0	C
ATOM	2237	OE1	GLU	257	62.238	33.950	-9.352	1.00	0.00	LX0	O
ATOM	2238	OE2	GLU	257	60.318	34.837	-9.636	1.00	0.00	LX0	O
ATOM	2239	C	GLU	257	61.312	28.860	-8.568	1.00	0.00	LX0	C
ATOM	2240	O	GLU	257	60.631	27.846	-8.522	1.00	0.00	LX0	O
ATOM	2241	N	ALA	258	62.617	28.866	-8.871	1.00	0.00	LX0	N
ATOM	2242	H	ALA	258	63.141	29.714	-8.791	1.00	0.00	LX0	H
ATOM	2243	CA	ALA	258	63.260	27.574	-9.105	1.00	0.00	LX0	C
ATOM	2244	CB	ALA	258	63.870	27.534	-10.508	1.00	0.00	LX0	C
ATOM	2245	C	ALA	258	64.336	27.229	-8.083	1.00	0.00	LX0	C
ATOM	2246	O	ALA	258	65.249	26.459	-8.341	1.00	0.00	LX0	O
ATOM	2247	N	THR	259	64.222	27.870	-6.914	1.00	0.00	LX0	N
ATOM	2248	H	THR	259	63.424	28.431	-6.708	1.00	0.00	LX0	H
ATOM	2249	CA	THR	259	65.259	27.708	-5.898	1.00	0.00	LX0	C
ATOM	2250	CB	THR	259	66.164	28.953	-5.844	1.00	0.00	LX0	C
ATOM	2251	OG1	THR	259	65.447	30.103	-5.368	1.00	0.00	LX0	O
ATOM	2252	HG1	THR	259	64.726	30.251	-5.986	1.00	0.00	LX0	H
ATOM	2253	CG2	THR	259	66.828	29.267	-7.188	1.00	0.00	LX0	C
ATOM	2254	C	THR	259	64.619	27.437	-4.549	1.00	0.00	LX0	C
ATOM	2255	O	THR	259	63.400	27.494	-4.430	1.00	0.00	LX0	O
ATOM	2256	N	CYS	260	65.459	27.179	-3.537	1.00	0.00	LX0	N
ATOM	2257	H	CYS	260	66.444	27.036	-3.646	1.00	0.00	LX0	H
ATOM	2258	CA	CYS	260	64.858	27.006	-2.219	1.00	0.00	LX0	C
ATOM	2259	CB	CYS	260	64.966	25.546	-1.784	1.00	0.00	LX0	C
ATOM	2260	SG	CYS	260	64.286	24.394	-3.004	1.00	0.00	LX0	S
ATOM	2261	C	CYS	260	65.436	27.937	-1.168	1.00	0.00	LX0	C
ATOM	2262	O	CYS	260	66.534	27.744	-0.659	1.00	0.00	LX0	O
ATOM	2263	N	LYS	261	64.653	28.982	-0.869	1.00	0.00	LX0	N
ATOM	2264	H	LYS	261	63.714	28.998	-1.215	1.00	0.00	LX0	H
ATOM	2265	CA	LYS	261	65.111	29.910	0.164	1.00	0.00	LX0	C
ATOM	2266	CB	LYS	261	64.759	31.361	-0.177	1.00	0.00	LX0	C
ATOM	2267	CG	LYS	261	65.220	31.902	-1.533	1.00	0.00	LX0	C
ATOM	2268	CD	LYS	261	66.724	31.903	-1.798	1.00	0.00	LX0	C
ATOM	2269	CE	LYS	261	67.087	32.699	-3.060	1.00	0.00	LX0	C

ATOM	2270	NZ	LYS	261	66.604	32.059	-4.293	1.00	0.00	LX0	N
ATOM	2271	HZ1	LYS	261	66.110	31.158	-4.137	1.00	0.00	LX0	H
ATOM	2272	HZ2	LYS	261	65.929	32.670	-4.802	1.00	0.00	LX0	H
ATOM	2273	HZ3	LYS	261	67.419	31.824	-4.902	1.00	0.00	LX0	H
ATOM	2274	C	LYS	261	64.542	29.584	1.535	1.00	0.00	LX0	C
ATOM	2275	O	LYS	261	63.632	28.770	1.689	1.00	0.00	LX0	O
ATOM	2276	N	ASP	262	65.120	30.277	2.530	1.00	0.00	LX0	N
ATOM	2277	H	ASP	262	65.873	30.904	2.344	1.00	0.00	LX0	H
ATOM	2278	CA	ASP	262	64.518	30.180	3.858	1.00	0.00	LX0	C
ATOM	2279	CB	ASP	262	65.559	29.845	4.935	1.00	0.00	LX0	C
ATOM	2280	CG	ASP	262	64.825	29.400	6.186	1.00	0.00	LX0	C
ATOM	2281	OD1	ASP	262	64.305	28.291	6.230	1.00	0.00	LX0	O
ATOM	2282	OD2	ASP	262	64.661	30.192	7.101	1.00	0.00	LX0	O
ATOM	2283	C	ASP	262	63.687	31.386	4.258	1.00	0.00	LX0	C
ATOM	2284	O	ASP	262	62.698	31.280	4.979	1.00	0.00	LX0	O
ATOM	2285	N	THR	263	64.121	32.537	3.748	1.00	0.00	LX0	N
ATOM	2286	H	THR	263	64.941	32.658	3.194	1.00	0.00	LX0	H
ATOM	2287	CA	THR	263	63.318	33.729	3.966	1.00	0.00	LX0	C
ATOM	2288	CB	THR	263	64.234	34.805	4.536	1.00	0.00	LX0	C
ATOM	2289	OG1	THR	263	65.447	34.870	3.776	1.00	0.00	LX0	O
ATOM	2290	HG1	THR	263	65.783	35.754	3.956	1.00	0.00	LX0	H
ATOM	2291	CG2	THR	263	64.561	34.523	6.001	1.00	0.00	LX0	C
ATOM	2292	C	THR	263	62.709	34.164	2.652	1.00	0.00	LX0	C
ATOM	2293	O	THR	263	63.026	33.607	1.604	1.00	0.00	LX0	O
ATOM	2294	N	CYS	264	61.852	35.183	2.739	1.00	0.00	LX0	N
ATOM	2295	H	CYS	264	61.544	35.543	3.623	1.00	0.00	LX0	H
ATOM	2296	CA	CYS	264	61.454	35.825	1.492	1.00	0.00	LX0	C
ATOM	2297	CB	CYS	264	59.935	35.885	1.392	1.00	0.00	LX0	C
ATOM	2298	SG	CYS	264	59.148	34.275	1.658	1.00	0.00	LX0	S
ATOM	2299	C	CYS	264	62.041	37.219	1.397	1.00	0.00	LX0	C
ATOM	2300	O	CYS	264	61.566	38.144	2.041	1.00	0.00	LX0	O
ATOM	2301	N	PRO	265	63.120	37.340	0.581	1.00	0.00	LX0	N
ATOM	2302	CD	PRO	265	63.740	36.285	-0.213	1.00	0.00	LX0	C
ATOM	2303	CA	PRO	265	63.817	38.629	0.454	1.00	0.00	LX0	C
ATOM	2304	CB	PRO	265	64.869	38.339	-0.623	1.00	0.00	LX0	C
ATOM	2305	CG	PRO	265	65.121	36.835	-0.540	1.00	0.00	LX0	C
ATOM	2306	C	PRO	265	62.909	39.797	0.091	1.00	0.00	LX0	C
ATOM	2307	O	PRO	265	62.325	39.837	-0.986	1.00	0.00	LX0	O
ATOM	2308	N	PRO	266	62.813	40.755	1.043	1.00	0.00	LX0	N
ATOM	2309	CD	PRO	266	63.514	40.782	2.320	1.00	0.00	LX0	C
ATOM	2310	CA	PRO	266	61.896	41.889	0.879	1.00	0.00	LX0	C
ATOM	2311	CB	PRO	266	62.162	42.730	2.131	1.00	0.00	LX0	C
ATOM	2312	CG	PRO	266	62.679	41.733	3.161	1.00	0.00	LX0	C
ATOM	2313	C	PRO	266	62.086	42.678	-0.403	1.00	0.00	LX0	C
ATOM	2314	O	PRO	266	63.093	42.580	-1.096	1.00	0.00	LX0	O
ATOM	2315	N	LEU	267	61.058	43.492	-0.682	1.00	0.00	LX0	N
ATOM	2316	H	LEU	267	60.239	43.464	-0.103	1.00	0.00	LX0	H
ATOM	2317	CA	LEU	267	61.114	44.317	-1.893	1.00	0.00	LX0	C
ATOM	2318	CB	LEU	267	59.802	45.085	-2.045	1.00	0.00	LX0	C
ATOM	2319	CG	LEU	267	58.815	44.454	-3.035	1.00	0.00	LX0	C
ATOM	2320	CD1	LEU	267	58.661	42.938	-2.877	1.00	0.00	LX0	C
ATOM	2321	CD2	LEU	267	57.467	45.173	-2.980	1.00	0.00	LX0	C
ATOM	2322	C	LEU	267	62.313	45.252	-1.988	1.00	0.00	LX0	C
ATOM	2323	O	LEU	267	62.876	45.501	-3.050	1.00	0.00	LX0	O
ATOM	2324	N	MET	268	62.698	45.750	-0.809	1.00	0.00	LX0	N
ATOM	2325	H	MET	268	62.242	45.501	0.043	1.00	0.00	LX0	H
ATOM	2326	CA	MET	268	63.980	46.439	-0.786	1.00	0.00	LX0	C
ATOM	2327	CB	MET	268	63.830	47.838	-0.182	1.00	0.00	LX0	C
ATOM	2328	CG	MET	268	62.668	48.648	-0.771	1.00	0.00	LX0	C
ATOM	2329	SD	MET	268	62.871	49.086	-2.505	1.00	0.00	LX0	S
ATOM	2330	CE	MET	268	63.764	50.628	-2.258	1.00	0.00	LX0	C

ATOM	2331	C	MET	268	64.982	45.588	-0.031	1.00	0.00	LX0	C
ATOM	2332	O	MET	268	64.618	44.662	0.686	1.00	0.00	LX0	O
ATOM	2333	N	LEU	269	66.259	45.909	-0.245	1.00	0.00	LX0	N
ATOM	2334	H	LEU	269	66.504	46.704	-0.802	1.00	0.00	LX0	H
ATOM	2335	CA	LEU	269	67.307	45.133	0.401	1.00	0.00	LX0	C
ATOM	2336	CB	LEU	269	67.922	44.142	-0.586	1.00	0.00	LX0	C
ATOM	2337	CG	LEU	269	68.234	42.771	0.019	1.00	0.00	LX0	C
ATOM	2338	CD1	LEU	269	66.977	42.076	0.549	1.00	0.00	LX0	C
ATOM	2339	CD2	LEU	269	68.990	41.885	-0.970	1.00	0.00	LX0	C
ATOM	2340	C	LEU	269	68.355	46.078	0.934	1.00	0.00	LX0	C
ATOM	2341	O	LEU	269	68.463	47.215	0.495	1.00	0.00	LX0	O
ATOM	2342	N	TYR	270	69.112	45.591	1.920	1.00	0.00	LX0	N
ATOM	2343	H	TYR	270	69.106	44.623	2.162	1.00	0.00	LX0	H
ATOM	2344	CA	TYR	270	70.050	46.544	2.497	1.00	0.00	LX0	C
ATOM	2345	CB	TYR	270	70.413	46.136	3.927	1.00	0.00	LX0	C
ATOM	2346	CG	TYR	270	71.012	47.319	4.648	1.00	0.00	LX0	C
ATOM	2347	CD1	TYR	270	70.154	48.194	5.341	1.00	0.00	LX0	C
ATOM	2348	CE1	TYR	270	70.705	49.303	5.997	1.00	0.00	LX0	C
ATOM	2349	CD2	TYR	270	72.406	47.521	4.597	1.00	0.00	LX0	C
ATOM	2350	CE2	TYR	270	72.956	48.629	5.256	1.00	0.00	LX0	C
ATOM	2351	CZ	TYR	270	72.097	49.503	5.947	1.00	0.00	LX0	C
ATOM	2352	OH	TYR	270	72.637	50.593	6.597	1.00	0.00	LX0	O
ATOM	2353	HH	TYR	270	73.575	50.615	6.457	1.00	0.00	LX0	H
ATOM	2354	C	TYR	270	71.292	46.765	1.654	1.00	0.00	LX0	C
ATOM	2355	O	TYR	270	72.029	45.840	1.338	1.00	0.00	LX0	O
ATOM	2356	N	ASN	271	71.500	48.044	1.334	1.00	0.00	LX0	N
ATOM	2357	H	ASN	271	70.833	48.742	1.601	1.00	0.00	LX0	H
ATOM	2358	CA	ASN	271	72.778	48.428	0.745	1.00	0.00	LX0	C
ATOM	2359	CB	ASN	271	72.545	49.254	-0.520	1.00	0.00	LX0	C
ATOM	2360	CG	ASN	271	73.839	49.645	-1.213	1.00	0.00	LX0	C
ATOM	2361	OD1	ASN	271	74.950	49.373	-0.764	1.00	0.00	LX0	O
ATOM	2362	ND2	ASN	271	73.649	50.294	-2.360	1.00	0.00	LX0	N
ATOM	2363	HD21	ASN	271	72.714	50.579	-2.602	1.00	0.00	LX0	H
ATOM	2364	HD22	ASN	271	74.385	50.563	-2.972	1.00	0.00	LX0	H
ATOM	2365	C	ASN	271	73.626	49.183	1.751	1.00	0.00	LX0	C
ATOM	2366	O	ASN	271	73.329	50.307	2.150	1.00	0.00	LX0	O
ATOM	2367	N	PRO	272	74.732	48.512	2.160	1.00	0.00	LX0	N
ATOM	2368	CD	PRO	272	75.117	47.153	1.791	1.00	0.00	LX0	C
ATOM	2369	CA	PRO	272	75.688	49.140	3.078	1.00	0.00	LX0	C
ATOM	2370	CB	PRO	272	76.831	48.121	3.127	1.00	0.00	LX0	C
ATOM	2371	CG	PRO	272	76.169	46.778	2.827	1.00	0.00	LX0	C
ATOM	2372	C	PRO	272	76.137	50.532	2.665	1.00	0.00	LX0	C
ATOM	2373	O	PRO	272	76.187	51.453	3.468	1.00	0.00	LX0	O
ATOM	2374	N	THR	273	76.437	50.640	1.363	1.00	0.00	LX0	N
ATOM	2375	H	THR	273	76.339	49.855	0.751	1.00	0.00	LX0	H
ATOM	2376	CA	THR	273	76.937	51.898	0.809	1.00	0.00	LX0	C
ATOM	2377	CB	THR	273	77.197	51.710	-0.688	1.00	0.00	LX0	C
ATOM	2378	OG1	THR	273	77.531	50.341	-0.964	1.00	0.00	LX0	O
ATOM	2379	HG1	THR	273	76.703	49.897	-1.140	1.00	0.00	LX0	H
ATOM	2380	CG2	THR	273	78.279	52.660	-1.205	1.00	0.00	LX0	C
ATOM	2381	C	THR	273	76.037	53.101	1.048	1.00	0.00	LX0	C
ATOM	2382	O	THR	273	76.457	54.172	1.463	1.00	0.00	LX0	O
ATOM	2383	N	THR	274	74.750	52.852	0.790	1.00	0.00	LX0	N
ATOM	2384	H	THR	274	74.415	51.994	0.398	1.00	0.00	LX0	H
ATOM	2385	CA	THR	274	73.772	53.916	0.988	1.00	0.00	LX0	C
ATOM	2386	CB	THR	274	72.576	53.631	0.077	1.00	0.00	LX0	C
ATOM	2387	OG1	THR	274	72.944	52.695	-0.946	1.00	0.00	LX0	O
ATOM	2388	HG1	THR	274	72.111	52.427	-1.361	1.00	0.00	LX0	H
ATOM	2389	CG2	THR	274	71.992	54.907	-0.533	1.00	0.00	LX0	C
ATOM	2390	C	THR	274	73.318	54.096	2.435	1.00	0.00	LX0	C
ATOM	2391	O	THR	274	72.535	54.981	2.767	1.00	0.00	LX0	O

ATOM	2392	N	TYR	275	73.805	53.159	3.278	1.00	0.00	LX0	N
ATOM	2393	H	TYR	275	74.538	52.557	2.966	1.00	0.00	LX0	H
ATOM	2394	CA	TYR	275	73.278	52.957	4.629	1.00	0.00	LX0	C
ATOM	2395	CB	TYR	275	73.809	54.009	5.612	1.00	0.00	LX0	C
ATOM	2396	CG	TYR	275	74.274	53.326	6.879	1.00	0.00	LX0	C
ATOM	2397	CD1	TYR	275	73.437	53.316	8.015	1.00	0.00	LX0	C
ATOM	2398	CE1	TYR	275	73.878	52.667	9.182	1.00	0.00	LX0	C
ATOM	2399	CD2	TYR	275	75.542	52.710	6.884	1.00	0.00	LX0	C
ATOM	2400	CE2	TYR	275	75.983	52.066	8.051	1.00	0.00	LX0	C
ATOM	2401	CZ	TYR	275	75.147	52.053	9.186	1.00	0.00	LX0	C
ATOM	2402	OH	TYR	275	75.599	51.420	10.331	1.00	0.00	LX0	O
ATOM	2403	HH	TYR	275	76.543	51.330	10.266	1.00	0.00	LX0	H
ATOM	2404	C	TYR	275	71.766	52.811	4.697	1.00	0.00	LX0	C
ATOM	2405	O	TYR	275	71.091	53.267	5.611	1.00	0.00	LX0	O
ATOM	2406	N	GLN	276	71.269	52.181	3.626	1.00	0.00	LX0	N
ATOM	2407	H	GLN	276	71.864	51.673	2.999	1.00	0.00	LX0	H
ATOM	2408	CA	GLN	276	69.849	52.311	3.338	1.00	0.00	LX0	C
ATOM	2409	CB	GLN	276	69.641	53.462	2.347	1.00	0.00	LX0	C
ATOM	2410	CG	GLN	276	68.551	54.477	2.713	1.00	0.00	LX0	C
ATOM	2411	CD	GLN	276	68.835	55.141	4.050	1.00	0.00	LX0	C
ATOM	2412	OE1	GLN	276	67.986	55.184	4.929	1.00	0.00	LX0	O
ATOM	2413	NE2	GLN	276	70.059	55.663	4.175	1.00	0.00	LX0	N
ATOM	2414	HE21	GLN	276	70.764	55.622	3.463	1.00	0.00	LX0	H
ATOM	2415	HE22	GLN	276	70.294	56.068	5.055	1.00	0.00	LX0	H
ATOM	2416	C	GLN	276	69.313	51.012	2.791	1.00	0.00	LX0	C
ATOM	2417	O	GLN	276	70.013	50.007	2.734	1.00	0.00	LX0	O
ATOM	2418	N	MET	277	68.040	51.071	2.398	1.00	0.00	LX0	N
ATOM	2419	H	MET	277	67.505	51.912	2.440	1.00	0.00	LX0	H
ATOM	2420	CA	MET	277	67.491	49.903	1.734	1.00	0.00	LX0	C
ATOM	2421	CB	MET	277	66.259	49.392	2.475	1.00	0.00	LX0	C
ATOM	2422	CG	MET	277	66.573	48.825	3.863	1.00	0.00	LX0	C
ATOM	2423	SD	MET	277	67.113	50.015	5.107	1.00	0.00	LX0	S
ATOM	2424	CE	MET	277	65.669	51.090	5.107	1.00	0.00	LX0	C
ATOM	2425	C	MET	277	67.181	50.241	0.298	1.00	0.00	LX0	C
ATOM	2426	O	MET	277	66.200	50.908	-0.010	1.00	0.00	LX0	O
ATOM	2427	N	ASP	278	68.099	49.780	-0.548	1.00	0.00	LX0	N
ATOM	2428	H	ASP	278	68.795	49.147	-0.213	1.00	0.00	LX0	H
ATOM	2429	CA	ASP	278	67.989	50.081	-1.970	1.00	0.00	LX0	C
ATOM	2430	CB	ASP	278	69.378	50.070	-2.627	1.00	0.00	LX0	C
ATOM	2431	CG	ASP	278	70.288	51.205	-2.156	1.00	0.00	LX0	C
ATOM	2432	OD1	ASP	278	70.257	51.592	-0.990	1.00	0.00	LX0	O
ATOM	2433	OD2	ASP	278	71.088	51.677	-2.956	1.00	0.00	LX0	O
ATOM	2434	C	ASP	278	67.068	49.064	-2.612	1.00	0.00	LX0	C
ATOM	2435	O	ASP	278	66.587	48.144	-1.961	1.00	0.00	LX0	O
ATOM	2436	N	VAL	279	66.807	49.247	-3.913	1.00	0.00	LX0	N
ATOM	2437	H	VAL	279	67.315	49.930	-4.433	1.00	0.00	LX0	H
ATOM	2438	CA	VAL	279	65.880	48.285	-4.513	1.00	0.00	LX0	C
ATOM	2439	CB	VAL	279	65.332	48.829	-5.849	1.00	0.00	LX0	C
ATOM	2440	CG1	VAL	279	66.432	49.068	-6.890	1.00	0.00	LX0	C
ATOM	2441	CG2	VAL	279	64.183	47.971	-6.388	1.00	0.00	LX0	C
ATOM	2442	C	VAL	279	66.470	46.884	-4.639	1.00	0.00	LX0	C
ATOM	2443	O	VAL	279	67.589	46.701	-5.102	1.00	0.00	LX0	O
ATOM	2444	N	ASN	280	65.677	45.901	-4.188	1.00	0.00	LX0	N
ATOM	2445	H	ASN	280	64.741	46.086	-3.878	1.00	0.00	LX0	H
ATOM	2446	CA	ASN	280	66.125	44.524	-4.389	1.00	0.00	LX0	C
ATOM	2447	CB	ASN	280	65.512	43.625	-3.303	1.00	0.00	LX0	C
ATOM	2448	CG	ASN	280	65.824	42.151	-3.509	1.00	0.00	LX0	C
ATOM	2449	OD1	ASN	280	66.676	41.753	-4.294	1.00	0.00	LX0	O
ATOM	2450	ND2	ASN	280	65.027	41.345	-2.812	1.00	0.00	LX0	N
ATOM	2451	HD21	ASN	280	64.400	41.713	-2.122	1.00	0.00	LX0	H
ATOM	2452	HD22	ASN	280	64.995	40.364	-3.011	1.00	0.00	LX0	H

ATOM	2453	C	ASN	280	65.753	44.043	-5.781	1.00	0.00	LX0	C
ATOM	2454	O	ASN	280	64.581	43.989	-6.131	1.00	0.00	LX0	O
ATOM	2455	N	PRO	281	66.791	43.680	-6.576	1.00	0.00	LX0	N
ATOM	2456	CD	PRO	281	68.216	43.791	-6.278	1.00	0.00	LX0	C
ATOM	2457	CA	PRO	281	66.529	43.096	-7.898	1.00	0.00	LX0	C
ATOM	2458	CB	PRO	281	67.929	42.678	-8.364	1.00	0.00	LX0	C
ATOM	2459	CG	PRO	281	68.887	43.616	-7.634	1.00	0.00	LX0	C
ATOM	2460	C	PRO	281	65.538	41.940	-7.872	1.00	0.00	LX0	C
ATOM	2461	O	PRO	281	64.581	41.884	-8.632	1.00	0.00	LX0	O
ATOM	2462	N	GLU	282	65.813	41.023	-6.936	1.00	0.00	LX0	N
ATOM	2463	H	GLU	282	66.534	41.176	-6.258	1.00	0.00	LX0	H
ATOM	2464	CA	GLU	282	64.859	39.937	-6.755	1.00	0.00	LX0	C
ATOM	2465	CB	GLU	282	65.588	38.605	-6.549	1.00	0.00	LX0	C
ATOM	2466	CG	GLU	282	65.864	37.842	-7.854	1.00	0.00	LX0	C
ATOM	2467	CD	GLU	282	64.597	37.193	-8.392	1.00	0.00	LX0	C
ATOM	2468	OE1	GLU	282	63.561	37.832	-8.485	1.00	0.00	LX0	O
ATOM	2469	OE2	GLU	282	64.607	36.015	-8.722	1.00	0.00	LX0	O
ATOM	2470	C	GLU	282	63.891	40.214	-5.627	1.00	0.00	LX0	C
ATOM	2471	O	GLU	282	63.874	39.537	-4.604	1.00	0.00	LX0	O
ATOM	2472	N	GLY	283	63.094	41.272	-5.850	1.00	0.00	LX0	N
ATOM	2473	H	GLY	283	63.173	41.758	-6.723	1.00	0.00	LX0	H
ATOM	2474	CA	GLY	283	62.081	41.632	-4.857	1.00	0.00	LX0	C
ATOM	2475	C	GLY	283	61.028	40.551	-4.711	1.00	0.00	LX0	C
ATOM	2476	O	GLY	283	60.190	40.333	-5.579	1.00	0.00	LX0	O
ATOM	2477	N	LYS	284	61.145	39.833	-3.593	1.00	0.00	LX0	N
ATOM	2478	H	LYS	284	61.760	40.080	-2.843	1.00	0.00	LX0	H
ATOM	2479	CA	LYS	284	60.461	38.553	-3.617	1.00	0.00	LX0	C
ATOM	2480	CB	LYS	284	61.403	37.445	-3.154	1.00	0.00	LX0	C
ATOM	2481	CG	LYS	284	61.493	36.371	-4.231	1.00	0.00	LX0	C
ATOM	2482	CD	LYS	284	61.878	36.945	-5.595	1.00	0.00	LX0	C
ATOM	2483	CE	LYS	284	61.706	35.963	-6.749	1.00	0.00	LX0	C
ATOM	2484	NZ	LYS	284	62.856	35.059	-6.906	1.00	0.00	LX0	N
ATOM	2485	HZ1	LYS	284	62.629	34.357	-7.641	1.00	0.00	LX0	H
ATOM	2486	HZ2	LYS	284	63.692	35.584	-7.240	1.00	0.00	LX0	H
ATOM	2487	HZ3	LYS	284	63.067	34.529	-6.029	1.00	0.00	LX0	H
ATOM	2488	C	LYS	284	59.106	38.491	-2.962	1.00	0.00	LX0	C
ATOM	2489	O	LYS	284	58.939	38.496	-1.749	1.00	0.00	LX0	O
ATOM	2490	N	TYR	285	58.118	38.420	-3.857	1.00	0.00	LX0	N
ATOM	2491	H	TYR	285	58.339	38.336	-4.831	1.00	0.00	LX0	H
ATOM	2492	CA	TYR	285	56.753	38.298	-3.365	1.00	0.00	LX0	C
ATOM	2493	CB	TYR	285	55.751	38.644	-4.467	1.00	0.00	LX0	C
ATOM	2494	CG	TYR	285	55.943	40.078	-4.907	1.00	0.00	LX0	C
ATOM	2495	CD1	TYR	285	56.779	40.356	-6.009	1.00	0.00	LX0	C
ATOM	2496	CE1	TYR	285	56.936	41.686	-6.432	1.00	0.00	LX0	C
ATOM	2497	CD2	TYR	285	55.276	41.102	-4.206	1.00	0.00	LX0	C
ATOM	2498	CE2	TYR	285	55.427	42.432	-4.631	1.00	0.00	LX0	C
ATOM	2499	CZ	TYR	285	56.252	42.706	-5.741	1.00	0.00	LX0	C
ATOM	2500	OH	TYR	285	56.393	44.012	-6.164	1.00	0.00	LX0	O
ATOM	2501	HH	TYR	285	56.017	44.602	-5.524	1.00	0.00	LX0	H
ATOM	2502	C	TYR	285	56.480	36.933	-2.769	1.00	0.00	LX0	C
ATOM	2503	O	TYR	285	56.765	35.885	-3.333	1.00	0.00	LX0	O
ATOM	2504	N	SER	286	55.931	37.006	-1.563	1.00	0.00	LX0	N
ATOM	2505	H	SER	286	55.653	37.899	-1.219	1.00	0.00	LX0	H
ATOM	2506	CA	SER	286	55.813	35.796	-0.766	1.00	0.00	LX0	C
ATOM	2507	CB	SER	286	56.055	36.141	0.704	1.00	0.00	LX0	C
ATOM	2508	OG	SER	286	57.106	37.110	0.847	1.00	0.00	LX0	O
ATOM	2509	HG	SER	286	57.006	37.796	0.190	1.00	0.00	LX0	H
ATOM	2510	C	SER	286	54.457	35.145	-0.935	1.00	0.00	LX0	C
ATOM	2511	O	SER	286	53.546	35.350	-0.137	1.00	0.00	LX0	O
ATOM	2512	N	PHE	287	54.331	34.371	-2.017	1.00	0.00	LX0	N
ATOM	2513	H	PHE	287	55.122	34.150	-2.595	1.00	0.00	LX0	H

ATOM	2514	CA	PHE	287	53.063	33.662	-2.156	1.00	0.00	LX0	C
ATOM	2515	CB	PHE	287	52.662	33.513	-3.632	1.00	0.00	LX0	C
ATOM	2516	CG	PHE	287	51.296	32.868	-3.762	1.00	0.00	LX0	C
ATOM	2517	CD1	PHE	287	50.186	33.401	-3.064	1.00	0.00	LX0	C
ATOM	2518	CD2	PHE	287	51.158	31.728	-4.583	1.00	0.00	LX0	C
ATOM	2519	CE1	PHE	287	48.928	32.782	-3.183	1.00	0.00	LX0	C
ATOM	2520	CE2	PHE	287	49.900	31.108	-4.704	1.00	0.00	LX0	C
ATOM	2521	CZ	PHE	287	48.798	31.639	-4.001	1.00	0.00	LX0	C
ATOM	2522	C	PHE	287	53.070	32.334	-1.426	1.00	0.00	LX0	C
ATOM	2523	O	PHE	287	53.260	31.261	-1.990	1.00	0.00	LX0	O
ATOM	2524	N	GLY	288	52.840	32.460	-0.113	1.00	0.00	LX0	N
ATOM	2525	H	GLY	288	52.796	33.379	0.281	1.00	0.00	LX0	H
ATOM	2526	CA	GLY	288	52.849	31.249	0.703	1.00	0.00	LX0	C
ATOM	2527	C	GLY	288	54.216	30.593	0.718	1.00	0.00	LX0	C
ATOM	2528	O	GLY	288	55.205	31.193	1.109	1.00	0.00	LX0	O
ATOM	2529	N	ALA	289	54.225	29.341	0.242	1.00	0.00	LX0	N
ATOM	2530	H	ALA	289	53.395	28.957	-0.158	1.00	0.00	LX0	H
ATOM	2531	CA	ALA	289	55.522	28.673	0.150	1.00	0.00	LX0	C
ATOM	2532	CB	ALA	289	55.350	27.153	0.173	1.00	0.00	LX0	C
ATOM	2533	C	ALA	289	56.329	29.055	-1.081	1.00	0.00	LX0	C
ATOM	2534	O	ALA	289	57.535	28.864	-1.155	1.00	0.00	LX0	O
ATOM	2535	N	THR	290	55.606	29.590	-2.065	1.00	0.00	LX0	N
ATOM	2536	H	THR	290	54.629	29.786	-1.984	1.00	0.00	LX0	H
ATOM	2537	CA	THR	290	56.302	29.994	-3.278	1.00	0.00	LX0	C
ATOM	2538	CB	THR	290	55.413	29.692	-4.481	1.00	0.00	LX0	C
ATOM	2539	OG1	THR	290	54.029	29.682	-4.090	1.00	0.00	LX0	O
ATOM	2540	HG1	THR	290	53.855	30.517	-3.659	1.00	0.00	LX0	H
ATOM	2541	CG2	THR	290	55.790	28.353	-5.117	1.00	0.00	LX0	C
ATOM	2542	C	THR	290	56.701	31.452	-3.253	1.00	0.00	LX0	C
ATOM	2543	O	THR	290	55.865	32.345	-3.187	1.00	0.00	LX0	O
ATOM	2544	N	CYS	291	58.016	31.665	-3.318	1.00	0.00	LX0	N
ATOM	2545	H	CYS	291	58.645	30.902	-3.466	1.00	0.00	LX0	H
ATOM	2546	CA	CYS	291	58.409	33.050	-3.551	1.00	0.00	LX0	C
ATOM	2547	CB	CYS	291	59.726	33.364	-2.853	1.00	0.00	LX0	C
ATOM	2548	SG	CYS	291	59.694	32.945	-1.096	1.00	0.00	LX0	S
ATOM	2549	C	CYS	291	58.496	33.278	-5.042	1.00	0.00	LX0	C
ATOM	2550	O	CYS	291	58.919	32.398	-5.781	1.00	0.00	LX0	O
ATOM	2551	N	VAL	292	58.004	34.449	-5.458	1.00	0.00	LX0	N
ATOM	2552	H	VAL	292	57.701	35.132	-4.792	1.00	0.00	LX0	H
ATOM	2553	CA	VAL	292	57.803	34.704	-6.883	1.00	0.00	LX0	C
ATOM	2554	CB	VAL	292	56.391	34.284	-7.337	1.00	0.00	LX0	C
ATOM	2555	CG1	VAL	292	56.282	32.781	-7.606	1.00	0.00	LX0	C
ATOM	2556	CG2	VAL	292	55.317	34.777	-6.361	1.00	0.00	LX0	C
ATOM	2557	C	VAL	292	58.015	36.171	-7.188	1.00	0.00	LX0	C
ATOM	2558	O	VAL	292	58.022	37.012	-6.299	1.00	0.00	LX0	O
ATOM	2559	N	LYS	293	58.157	36.452	-8.488	1.00	0.00	LX0	N
ATOM	2560	H	LYS	293	58.327	35.702	-9.131	1.00	0.00	LX0	H
ATOM	2561	CA	LYS	293	58.322	37.854	-8.871	1.00	0.00	LX0	C
ATOM	2562	CB	LYS	293	59.153	37.952	-10.152	1.00	0.00	LX0	C
ATOM	2563	CG	LYS	293	60.557	38.483	-9.855	1.00	0.00	LX0	C
ATOM	2564	CD	LYS	293	61.531	38.443	-11.041	1.00	0.00	LX0	C
ATOM	2565	CE	LYS	293	61.939	37.034	-11.495	1.00	0.00	LX0	C
ATOM	2566	NZ	LYS	293	62.456	36.264	-10.359	1.00	0.00	LX0	N
ATOM	2567	HZ1	LYS	293	62.830	35.324	-10.595	1.00	0.00	LX0	H
ATOM	2568	HZ2	LYS	293	63.188	36.763	-9.808	1.00	0.00	LX0	H
ATOM	2569	HZ3	LYS	293	61.676	36.020	-9.715	1.00	0.00	LX0	H
ATOM	2570	C	LYS	293	57.072	38.724	-8.960	1.00	0.00	LX0	C
ATOM	2571	O	LYS	293	57.155	39.889	-9.324	1.00	0.00	LX0	O
ATOM	2572	N	LYS	294	55.908	38.128	-8.628	1.00	0.00	LX0	N
ATOM	2573	H	LYS	294	55.876	37.169	-8.352	1.00	0.00	LX0	H
ATOM	2574	CA	LYS	294	54.693	38.944	-8.522	1.00	0.00	LX0	C

ATOM	2575	CB	LYS	294	54.188	39.456	-9.881	1.00	0.00	LX0	C
ATOM	2576	CG	LYS	294	53.855	38.388	-10.924	1.00	0.00	LX0	C
ATOM	2577	CD	LYS	294	53.409	39.043	-12.231	1.00	0.00	LX0	C
ATOM	2578	CE	LYS	294	53.074	38.038	-13.333	1.00	0.00	LX0	C
ATOM	2579	NZ	LYS	294	52.685	38.777	-14.542	1.00	0.00	LX0	N
ATOM	2580	HZ1	LYS	294	52.446	38.108	-15.301	1.00	0.00	LX0	H
ATOM	2581	HZ2	LYS	294	51.860	39.374	-14.331	1.00	0.00	LX0	H
ATOM	2582	HZ3	LYS	294	53.476	39.380	-14.847	1.00	0.00	LX0	H
ATOM	2583	C	LYS	294	53.572	38.241	-7.786	1.00	0.00	LX0	C
ATOM	2584	O	LYS	294	53.470	37.022	-7.795	1.00	0.00	LX0	O
ATOM	2585	N	CYS	295	52.735	39.070	-7.149	1.00	0.00	LX0	N
ATOM	2586	H	CYS	295	52.846	40.058	-7.234	1.00	0.00	LX0	H
ATOM	2587	CA	CYS	295	51.593	38.501	-6.432	1.00	0.00	LX0	C
ATOM	2588	CB	CYS	295	51.067	39.501	-5.402	1.00	0.00	LX0	C
ATOM	2589	SG	CYS	295	52.332	40.038	-4.227	1.00	0.00	LX0	S
ATOM	2590	C	CYS	295	50.451	38.097	-7.350	1.00	0.00	LX0	C
ATOM	2591	O	CYS	295	50.165	38.764	-8.339	1.00	0.00	LX0	O
ATOM	2592	N	PRO	296	49.787	36.972	-6.988	1.00	0.00	LX0	N
ATOM	2593	CD	PRO	296	50.186	35.992	-5.985	1.00	0.00	LX0	C
ATOM	2594	CA	PRO	296	48.522	36.624	-7.644	1.00	0.00	LX0	C
ATOM	2595	CB	PRO	296	48.087	35.354	-6.907	1.00	0.00	LX0	C
ATOM	2596	CG	PRO	296	49.383	34.757	-6.367	1.00	0.00	LX0	C
ATOM	2597	C	PRO	296	47.479	37.727	-7.550	1.00	0.00	LX0	C
ATOM	2598	O	PRO	296	47.325	38.388	-6.531	1.00	0.00	LX0	O
ATOM	2599	N	ARG	297	46.761	37.881	-8.674	1.00	0.00	LX0	N
ATOM	2600	H	ARG	297	46.953	37.251	-9.423	1.00	0.00	LX0	H
ATOM	2601	CA	ARG	297	45.862	39.023	-8.880	1.00	0.00	LX0	C
ATOM	2602	CB	ARG	297	45.327	38.928	-10.321	1.00	0.00	LX0	C
ATOM	2603	CG	ARG	297	44.881	40.215	-11.042	1.00	0.00	LX0	C
ATOM	2604	CD	ARG	297	43.447	40.694	-10.767	1.00	0.00	LX0	C
ATOM	2605	NE	ARG	297	42.463	39.689	-11.167	1.00	0.00	LX0	N
ATOM	2606	HE	ARG	297	42.375	39.474	-12.143	1.00	0.00	LX0	H
ATOM	2607	CZ	ARG	297	41.708	39.033	-10.259	1.00	0.00	LX0	C
ATOM	2608	NH1	ARG	297	41.780	39.311	-8.961	1.00	0.00	LX0	N
ATOM	2609	HH11	ARG	297	41.199	38.818	-8.312	1.00	0.00	LX0	H
ATOM	2610	HH12	ARG	297	42.421	40.007	-8.611	1.00	0.00	LX0	H
ATOM	2611	NH2	ARG	297	40.893	38.082	-10.677	1.00	0.00	LX0	N
ATOM	2612	HH21	ARG	297	40.357	37.500	-10.051	1.00	0.00	LX0	H
ATOM	2613	HH22	ARG	297	40.787	37.877	-11.654	1.00	0.00	LX0	H
ATOM	2614	C	ARG	297	44.756	39.256	-7.846	1.00	0.00	LX0	C
ATOM	2615	O	ARG	297	44.076	40.276	-7.839	1.00	0.00	LX0	O
ATOM	2616	N	ASN	298	44.543	38.264	-6.980	1.00	0.00	LX0	N
ATOM	2617	H	ASN	298	45.078	37.418	-6.958	1.00	0.00	LX0	H
ATOM	2618	CA	ASN	298	43.548	38.533	-5.946	1.00	0.00	LX0	C
ATOM	2619	CB	ASN	298	42.455	37.454	-5.903	1.00	0.00	LX0	C
ATOM	2620	CG	ASN	298	43.038	36.119	-5.487	1.00	0.00	LX0	C
ATOM	2621	OD1	ASN	298	44.206	35.835	-5.715	1.00	0.00	LX0	O
ATOM	2622	ND2	ASN	298	42.162	35.307	-4.905	1.00	0.00	LX0	N
ATOM	2623	HD21	ASN	298	41.226	35.548	-4.635	1.00	0.00	LX0	H
ATOM	2624	HD22	ASN	298	42.410	34.339	-4.844	1.00	0.00	LX0	H
ATOM	2625	C	ASN	298	44.098	38.772	-4.556	1.00	0.00	LX0	C
ATOM	2626	O	ASN	298	43.420	39.361	-3.721	1.00	0.00	LX0	O
ATOM	2627	N	TYR	299	45.311	38.256	-4.313	1.00	0.00	LX0	N
ATOM	2628	H	TYR	299	45.906	37.982	-5.069	1.00	0.00	LX0	H
ATOM	2629	CA	TYR	299	45.807	38.285	-2.937	1.00	0.00	LX0	C
ATOM	2630	CB	TYR	299	46.975	37.301	-2.768	1.00	0.00	LX0	C
ATOM	2631	CG	TYR	299	46.494	35.930	-2.328	1.00	0.00	LX0	C
ATOM	2632	CD1	TYR	299	45.571	35.211	-3.116	1.00	0.00	LX0	C
ATOM	2633	CE1	TYR	299	45.111	33.959	-2.674	1.00	0.00	LX0	C
ATOM	2634	CD2	TYR	299	46.993	35.401	-1.120	1.00	0.00	LX0	C
ATOM	2635	CE2	TYR	299	46.541	34.144	-0.680	1.00	0.00	LX0	C

ATOM	2636	CZ	TYR	299	45.587	33.447	-1.449	1.00	0.00	LX0	C
ATOM	2637	OH	TYR	299	45.096	32.238	-0.980	1.00	0.00	LX0	O
ATOM	2638	HH	TYR	299	44.388	31.941	-1.550	1.00	0.00	LX0	H
ATOM	2639	C	TYR	299	46.181	39.683	-2.483	1.00	0.00	LX0	C
ATOM	2640	O	TYR	299	46.372	40.591	-3.282	1.00	0.00	LX0	O
ATOM	2641	N	VAL	300	46.247	39.822	-1.154	1.00	0.00	LX0	N
ATOM	2642	H	VAL	300	46.194	39.031	-0.544	1.00	0.00	LX0	H
ATOM	2643	CA	VAL	300	46.688	41.117	-0.647	1.00	0.00	LX0	C
ATOM	2644	CB	VAL	300	46.241	41.352	0.799	1.00	0.00	LX0	C
ATOM	2645	CG1	VAL	300	46.476	42.796	1.254	1.00	0.00	LX0	C
ATOM	2646	CG2	VAL	300	44.801	40.927	1.012	1.00	0.00	LX0	C
ATOM	2647	C	VAL	300	48.192	41.188	-0.701	1.00	0.00	LX0	C
ATOM	2648	O	VAL	300	48.890	40.305	-0.215	1.00	0.00	LX0	O
ATOM	2649	N	VAL	301	48.660	42.281	-1.295	1.00	0.00	LX0	N
ATOM	2650	H	VAL	301	48.021	42.969	-1.642	1.00	0.00	LX0	H
ATOM	2651	CA	VAL	301	50.078	42.540	-1.122	1.00	0.00	LX0	C
ATOM	2652	CB	VAL	301	50.655	43.287	-2.331	1.00	0.00	LX0	C
ATOM	2653	CG1	VAL	301	52.185	43.236	-2.329	1.00	0.00	LX0	C
ATOM	2654	CG2	VAL	301	50.070	42.777	-3.651	1.00	0.00	LX0	C
ATOM	2655	C	VAL	301	50.276	43.337	0.151	1.00	0.00	LX0	C
ATOM	2656	O	VAL	301	49.745	44.429	0.324	1.00	0.00	LX0	O
ATOM	2657	N	THR	302	51.057	42.745	1.053	1.00	0.00	LX0	N
ATOM	2658	H	THR	302	51.464	41.856	0.849	1.00	0.00	LX0	H
ATOM	2659	CA	THR	302	51.549	43.628	2.103	1.00	0.00	LX0	C
ATOM	2660	CB	THR	302	51.889	42.832	3.361	1.00	0.00	LX0	C
ATOM	2661	OG1	THR	302	52.457	41.562	3.009	1.00	0.00	LX0	O
ATOM	2662	HG1	THR	302	51.746	41.067	2.602	1.00	0.00	LX0	H
ATOM	2663	CG2	THR	302	50.652	42.628	4.235	1.00	0.00	LX0	C
ATOM	2664	C	THR	302	52.753	44.381	1.586	1.00	0.00	LX0	C
ATOM	2665	O	THR	302	53.440	43.902	0.692	1.00	0.00	LX0	O
ATOM	2666	N	ASP	303	52.961	45.559	2.179	1.00	0.00	LX0	N
ATOM	2667	H	ASP	303	52.410	45.820	2.974	1.00	0.00	LX0	H
ATOM	2668	CA	ASP	303	54.099	46.431	1.872	1.00	0.00	LX0	C
ATOM	2669	CB	ASP	303	54.150	47.553	2.927	1.00	0.00	LX0	C
ATOM	2670	CG	ASP	303	54.368	47.018	4.342	1.00	0.00	LX0	C
ATOM	2671	OD1	ASP	303	55.197	47.572	5.051	1.00	0.00	LX0	O
ATOM	2672	OD2	ASP	303	53.744	46.030	4.731	1.00	0.00	LX0	O
ATOM	2673	C	ASP	303	55.444	45.724	1.725	1.00	0.00	LX0	C
ATOM	2674	O	ASP	303	56.188	45.921	0.775	1.00	0.00	LX0	O
ATOM	2675	N	HIS	304	55.681	44.831	2.700	1.00	0.00	LX0	N
ATOM	2676	H	HIS	304	55.067	44.911	3.488	1.00	0.00	LX0	H
ATOM	2677	CA	HIS	304	56.846	43.940	2.699	1.00	0.00	LX0	C
ATOM	2678	CB	HIS	304	56.630	42.935	3.837	1.00	0.00	LX0	C
ATOM	2679	CG	HIS	304	57.730	41.905	3.910	1.00	0.00	LX0	C
ATOM	2680	ND1	HIS	304	57.590	40.640	3.475	1.00	0.00	LX0	N
ATOM	2681	HD1	HIS	304	56.777	40.239	3.094	1.00	0.00	LX0	H
ATOM	2682	CD2	HIS	304	59.015	42.067	4.427	1.00	0.00	LX0	C
ATOM	2683	NE2	HIS	304	59.644	40.874	4.301	1.00	0.00	LX0	N
ATOM	2684	CE1	HIS	304	58.774	39.993	3.708	1.00	0.00	LX0	C
ATOM	2685	C	HIS	304	57.135	43.229	1.375	1.00	0.00	LX0	C
ATOM	2686	O	HIS	304	58.273	43.048	0.950	1.00	0.00	LX0	O
ATOM	2687	N	GLY	305	56.017	42.799	0.780	1.00	0.00	LX0	N
ATOM	2688	H	GLY	305	55.142	43.132	1.124	1.00	0.00	LX0	H
ATOM	2689	CA	GLY	305	56.089	41.888	-0.352	1.00	0.00	LX0	C
ATOM	2690	C	GLY	305	55.534	40.523	-0.006	1.00	0.00	LX0	C
ATOM	2691	O	GLY	305	56.074	39.488	-0.370	1.00	0.00	LX0	O
ATOM	2692	N	SER	306	54.409	40.541	0.719	1.00	0.00	LX0	N
ATOM	2693	H	SER	306	53.990	41.391	1.035	1.00	0.00	LX0	H
ATOM	2694	CA	SER	306	53.902	39.232	1.128	1.00	0.00	LX0	C
ATOM	2695	CB	SER	306	54.237	38.974	2.605	1.00	0.00	LX0	C
ATOM	2696	OG	SER	306	54.815	40.152	3.191	1.00	0.00	LX0	O

ATOM	2697	HG	SER	306	54.083	40.752	3.333	1.00	0.00	LX0	H
ATOM	2698	C	SER	306	52.429	39.046	0.829	1.00	0.00	LX0	C
ATOM	2699	O	SER	306	51.610	39.914	1.111	1.00	0.00	LX0	O
ATOM	2700	N	CYS	307	52.135	37.896	0.211	1.00	0.00	LX0	N
ATOM	2701	H	CYS	307	52.804	37.157	0.115	1.00	0.00	LX0	H
ATOM	2702	CA	CYS	307	50.769	37.686	-0.264	1.00	0.00	LX0	C
ATOM	2703	CB	CYS	307	50.769	36.798	-1.505	1.00	0.00	LX0	C
ATOM	2704	SG	CYS	307	52.033	37.269	-2.714	1.00	0.00	LX0	S
ATOM	2705	C	CYS	307	49.860	37.093	0.791	1.00	0.00	LX0	C
ATOM	2706	O	CYS	307	49.777	35.884	0.971	1.00	0.00	LX0	O
ATOM	2707	N	VAL	308	49.195	38.002	1.504	1.00	0.00	LX0	N
ATOM	2708	H	VAL	308	49.224	38.960	1.209	1.00	0.00	LX0	H
ATOM	2709	CA	VAL	308	48.290	37.500	2.534	1.00	0.00	LX0	C
ATOM	2710	CB	VAL	308	48.398	38.361	3.801	1.00	0.00	LX0	C
ATOM	2711	CG1	VAL	308	49.788	38.219	4.424	1.00	0.00	LX0	C
ATOM	2712	CG2	VAL	308	48.068	39.830	3.533	1.00	0.00	LX0	C
ATOM	2713	C	VAL	308	46.861	37.389	2.029	1.00	0.00	LX0	C
ATOM	2714	O	VAL	308	46.519	37.906	0.971	1.00	0.00	LX0	O
ATOM	2715	N	ARG	309	46.030	36.687	2.816	1.00	0.00	LX0	N
ATOM	2716	H	ARG	309	46.336	36.267	3.669	1.00	0.00	LX0	H
ATOM	2717	CA	ARG	309	44.637	36.634	2.372	1.00	0.00	LX0	C
ATOM	2718	CB	ARG	309	43.921	35.360	2.783	1.00	0.00	LX0	C
ATOM	2719	CG	ARG	309	44.439	34.122	2.077	1.00	0.00	LX0	C
ATOM	2720	CD	ARG	309	43.492	32.960	2.326	1.00	0.00	LX0	C
ATOM	2721	NE	ARG	309	43.953	31.764	1.635	1.00	0.00	LX0	N
ATOM	2722	HE	ARG	309	44.074	31.811	0.638	1.00	0.00	LX0	H
ATOM	2723	CZ	ARG	309	44.261	30.668	2.351	1.00	0.00	LX0	C
ATOM	2724	NH1	ARG	309	44.155	30.665	3.676	1.00	0.00	LX0	N
ATOM	2725	HH11	ARG	309	44.388	29.876	4.249	1.00	0.00	LX0	H
ATOM	2726	HH12	ARG	309	43.887	31.488	4.198	1.00	0.00	LX0	H
ATOM	2727	NH2	ARG	309	44.692	29.588	1.708	1.00	0.00	LX0	N
ATOM	2728	HH21	ARG	309	44.939	28.753	2.199	1.00	0.00	LX0	H
ATOM	2729	HH22	ARG	309	44.778	29.611	0.711	1.00	0.00	LX0	H
ATOM	2730	C	ARG	309	43.767	37.792	2.792	1.00	0.00	LX0	C
ATOM	2731	O	ARG	309	42.728	38.040	2.198	1.00	0.00	LX0	O
ATOM	2732	N	ALA	310	44.230	38.491	3.826	1.00	0.00	LX0	N
ATOM	2733	H	ALA	310	45.058	38.258	4.334	1.00	0.00	LX0	H
ATOM	2734	CA	ALA	310	43.491	39.676	4.230	1.00	0.00	LX0	C
ATOM	2735	CB	ALA	310	42.335	39.321	5.163	1.00	0.00	LX0	C
ATOM	2736	C	ALA	310	44.443	40.569	4.970	1.00	0.00	LX0	C
ATOM	2737	O	ALA	310	45.479	40.120	5.449	1.00	0.00	LX0	O
ATOM	2738	N	CYS	311	44.062	41.844	5.043	1.00	0.00	LX0	N
ATOM	2739	H	CYS	311	43.199	42.134	4.630	1.00	0.00	LX0	H
ATOM	2740	CA	CYS	311	44.845	42.685	5.939	1.00	0.00	LX0	C
ATOM	2741	CB	CYS	311	44.597	44.152	5.620	1.00	0.00	LX0	C
ATOM	2742	SG	CYS	311	45.239	44.595	3.991	1.00	0.00	LX0	S
ATOM	2743	C	CYS	311	44.545	42.388	7.394	1.00	0.00	LX0	C
ATOM	2744	O	CYS	311	43.505	41.836	7.734	1.00	0.00	LX0	O
ATOM	2745	N	GLY	312	45.512	42.777	8.243	1.00	0.00	LX0	N
ATOM	2746	H	GLY	312	46.328	43.236	7.901	1.00	0.00	LX0	H
ATOM	2747	CA	GLY	312	45.227	42.686	9.672	1.00	0.00	LX0	C
ATOM	2748	C	GLY	312	44.160	43.687	10.068	1.00	0.00	LX0	C
ATOM	2749	O	GLY	312	43.810	44.570	9.297	1.00	0.00	LX0	O
ATOM	2750	N	ALA	313	43.660	43.514	11.300	1.00	0.00	LX0	N
ATOM	2751	H	ALA	313	44.024	42.784	11.873	1.00	0.00	LX0	H
ATOM	2752	CA	ALA	313	42.513	44.319	11.729	1.00	0.00	LX0	C
ATOM	2753	CB	ALA	313	42.126	43.968	13.167	1.00	0.00	LX0	C
ATOM	2754	C	ALA	313	42.674	45.830	11.627	1.00	0.00	LX0	C
ATOM	2755	O	ALA	313	41.718	46.569	11.442	1.00	0.00	LX0	O
ATOM	2756	N	ASP	314	43.930	46.259	11.761	1.00	0.00	LX0	N
ATOM	2757	H	ASP	314	44.710	45.639	11.862	1.00	0.00	LX0	H

ATOM	2758	CA	ASP	314	44.248	47.684	11.694	1.00	0.00	LX0	C
ATOM	2759	CB	ASP	314	45.322	48.016	12.744	1.00	0.00	LX0	C
ATOM	2760	CG	ASP	314	46.632	47.246	12.557	1.00	0.00	LX0	C
ATOM	2761	OD1	ASP	314	46.688	46.268	11.812	1.00	0.00	LX0	O
ATOM	2762	OD2	ASP	314	47.613	47.630	13.183	1.00	0.00	LX0	O
ATOM	2763	C	ASP	314	44.643	48.211	10.319	1.00	0.00	LX0	C
ATOM	2764	O	ASP	314	45.050	49.356	10.155	1.00	0.00	LX0	O
ATOM	2765	N	SER	315	44.507	47.341	9.314	1.00	0.00	LX0	N
ATOM	2766	H	SER	315	44.105	46.435	9.440	1.00	0.00	LX0	H
ATOM	2767	CA	SER	315	44.967	47.770	8.001	1.00	0.00	LX0	C
ATOM	2768	CB	SER	315	46.196	46.946	7.609	1.00	0.00	LX0	C
ATOM	2769	OG	SER	315	46.977	47.622	6.612	1.00	0.00	LX0	O
ATOM	2770	HG	SER	315	47.820	47.777	7.027	1.00	0.00	LX0	H
ATOM	2771	C	SER	315	43.872	47.690	6.957	1.00	0.00	LX0	C
ATOM	2772	O	SER	315	43.096	46.745	6.888	1.00	0.00	LX0	O
ATOM	2773	N	TYR	316	43.829	48.740	6.132	1.00	0.00	LX0	N
ATOM	2774	H	TYR	316	44.509	49.466	6.220	1.00	0.00	LX0	H
ATOM	2775	CA	TYR	316	42.894	48.684	5.016	1.00	0.00	LX0	C
ATOM	2776	CB	TYR	316	42.588	50.082	4.473	1.00	0.00	LX0	C
ATOM	2777	CG	TYR	316	41.607	50.795	5.371	1.00	0.00	LX0	C
ATOM	2778	CD1	TYR	316	42.081	51.787	6.252	1.00	0.00	LX0	C
ATOM	2779	CE1	TYR	316	41.166	52.439	7.094	1.00	0.00	LX0	C
ATOM	2780	CD2	TYR	316	40.242	50.447	5.300	1.00	0.00	LX0	C
ATOM	2781	CE2	TYR	316	39.328	51.103	6.140	1.00	0.00	LX0	C
ATOM	2782	CZ	TYR	316	39.804	52.085	7.032	1.00	0.00	LX0	C
ATOM	2783	OH	TYR	316	38.911	52.718	7.879	1.00	0.00	LX0	O
ATOM	2784	HH	TYR	316	38.106	52.870	7.389	1.00	0.00	LX0	H
ATOM	2785	C	TYR	316	43.393	47.814	3.888	1.00	0.00	LX0	C
ATOM	2786	O	TYR	316	44.428	48.068	3.281	1.00	0.00	LX0	O
ATOM	2787	N	GLU	317	42.575	46.794	3.610	1.00	0.00	LX0	N
ATOM	2788	H	GLU	317	41.769	46.627	4.175	1.00	0.00	LX0	H
ATOM	2789	CA	GLU	317	42.752	46.132	2.324	1.00	0.00	LX0	C
ATOM	2790	CB	GLU	317	42.124	44.740	2.338	1.00	0.00	LX0	C
ATOM	2791	CG	GLU	317	42.505	43.932	1.096	1.00	0.00	LX0	C
ATOM	2792	CD	GLU	317	41.691	42.656	1.017	1.00	0.00	LX0	C
ATOM	2793	OE1	GLU	317	41.054	42.408	-0.006	1.00	0.00	LX0	O
ATOM	2794	OE2	GLU	317	41.716	41.874	1.957	1.00	0.00	LX0	O
ATOM	2795	C	GLU	317	42.123	46.979	1.242	1.00	0.00	LX0	C
ATOM	2796	O	GLU	317	40.908	47.030	1.103	1.00	0.00	LX0	O
ATOM	2797	N	MET	318	42.996	47.673	0.512	1.00	0.00	LX0	N
ATOM	2798	H	MET	318	43.986	47.610	0.658	1.00	0.00	LX0	H
ATOM	2799	CA	MET	318	42.424	48.566	-0.485	1.00	0.00	LX0	C
ATOM	2800	CB	MET	318	42.239	49.965	0.114	1.00	0.00	LX0	C
ATOM	2801	CG	MET	318	41.216	50.856	-0.595	1.00	0.00	LX0	C
ATOM	2802	SD	MET	318	39.529	50.247	-0.432	1.00	0.00	LX0	S
ATOM	2803	CE	MET	318	39.417	50.243	1.365	1.00	0.00	LX0	C
ATOM	2804	C	MET	318	43.288	48.575	-1.722	1.00	0.00	LX0	C
ATOM	2805	O	MET	318	44.511	48.546	-1.631	1.00	0.00	LX0	O
ATOM	2806	N	GLU	319	42.587	48.559	-2.853	1.00	0.00	LX0	N
ATOM	2807	H	GLU	319	41.603	48.720	-2.796	1.00	0.00	LX0	H
ATOM	2808	CA	GLU	319	43.196	48.450	-4.171	1.00	0.00	LX0	C
ATOM	2809	CB	GLU	319	42.235	47.750	-5.152	1.00	0.00	LX0	C
ATOM	2810	CG	GLU	319	40.830	48.359	-5.359	1.00	0.00	LX0	C
ATOM	2811	CD	GLU	319	39.850	47.926	-4.274	1.00	0.00	LX0	C
ATOM	2812	OE1	GLU	319	39.199	46.896	-4.422	1.00	0.00	LX0	O
ATOM	2813	OE2	GLU	319	39.738	48.594	-3.253	1.00	0.00	LX0	O
ATOM	2814	C	GLU	319	43.658	49.775	-4.736	1.00	0.00	LX0	C
ATOM	2815	O	GLU	319	43.086	50.828	-4.484	1.00	0.00	LX0	O
ATOM	2816	N	GLU	320	44.728	49.662	-5.531	1.00	0.00	LX0	N
ATOM	2817	H	GLU	320	45.246	48.807	-5.499	1.00	0.00	LX0	H
ATOM	2818	CA	GLU	320	45.202	50.852	-6.229	1.00	0.00	LX0	C

ATOM	2819	CB	GLU	320	46.521	51.380	-5.625	1.00	0.00	LX0	C
ATOM	2820	CG	GLU	320	46.618	51.428	-4.094	1.00	0.00	LX0	C
ATOM	2821	CD	GLU	320	47.200	50.146	-3.510	1.00	0.00	LX0	C
ATOM	2822	OE1	GLU	320	46.596	49.086	-3.553	1.00	0.00	LX0	O
ATOM	2823	OE2	GLU	320	48.269	50.179	-2.924	1.00	0.00	LX0	O
ATOM	2824	C	GLU	320	45.365	50.562	-7.709	1.00	0.00	LX0	C
ATOM	2825	O	GLU	320	44.561	50.906	-8.565	1.00	0.00	LX0	O
ATOM	2826	N	ASP	321	46.455	49.837	-7.955	1.00	0.00	LX0	N
ATOM	2827	H	ASP	321	47.001	49.507	-7.187	1.00	0.00	LX0	H
ATOM	2828	CA	ASP	321	46.882	49.338	-9.265	1.00	0.00	LX0	C
ATOM	2829	CB	ASP	321	48.399	49.080	-9.202	1.00	0.00	LX0	C
ATOM	2830	CG	ASP	321	48.778	48.394	-7.890	1.00	0.00	LX0	C
ATOM	2831	OD1	ASP	321	49.750	48.794	-7.261	1.00	0.00	LX0	O
ATOM	2832	OD2	ASP	321	48.039	47.533	-7.419	1.00	0.00	LX0	O
ATOM	2833	C	ASP	321	46.159	48.066	-9.690	1.00	0.00	LX0	C
ATOM	2834	O	ASP	321	46.706	47.166	-10.317	1.00	0.00	LX0	O
ATOM	2835	N	GLY	322	44.888	47.991	-9.281	1.00	0.00	LX0	N
ATOM	2836	H	GLY	322	44.437	48.780	-8.866	1.00	0.00	LX0	H
ATOM	2837	CA	GLY	322	44.255	46.684	-9.400	1.00	0.00	LX0	C
ATOM	2838	C	GLY	322	44.461	45.808	-8.177	1.00	0.00	LX0	C
ATOM	2839	O	GLY	322	43.511	45.441	-7.496	1.00	0.00	LX0	O
ATOM	2840	N	VAL	323	45.732	45.459	-7.916	1.00	0.00	LX0	N
ATOM	2841	H	VAL	323	46.503	45.937	-8.346	1.00	0.00	LX0	H
ATOM	2842	CA	VAL	323	45.882	44.582	-6.757	1.00	0.00	LX0	C
ATOM	2843	CB	VAL	323	47.100	43.645	-6.857	1.00	0.00	LX0	C
ATOM	2844	CG1	VAL	323	46.884	42.612	-7.963	1.00	0.00	LX0	C
ATOM	2845	CG2	VAL	323	48.430	44.380	-7.029	1.00	0.00	LX0	C
ATOM	2846	C	VAL	323	45.847	45.335	-5.444	1.00	0.00	LX0	C
ATOM	2847	O	VAL	323	46.294	46.473	-5.316	1.00	0.00	LX0	O
ATOM	2848	N	ARG	324	45.247	44.654	-4.463	1.00	0.00	LX0	N
ATOM	2849	H	ARG	324	45.032	43.684	-4.564	1.00	0.00	LX0	H
ATOM	2850	CA	ARG	324	45.039	45.381	-3.218	1.00	0.00	LX0	C
ATOM	2851	CB	ARG	324	43.802	44.897	-2.457	1.00	0.00	LX0	C
ATOM	2852	CG	ARG	324	42.648	44.450	-3.353	1.00	0.00	LX0	C
ATOM	2853	CD	ARG	324	41.350	44.235	-2.578	1.00	0.00	LX0	C
ATOM	2854	NE	ARG	324	40.613	45.483	-2.397	1.00	0.00	LX0	N
ATOM	2855	HE	ARG	324	40.567	46.143	-3.156	1.00	0.00	LX0	H
ATOM	2856	CZ	ARG	324	39.704	45.655	-1.416	1.00	0.00	LX0	C
ATOM	2857	NH1	ARG	324	39.637	44.831	-0.378	1.00	0.00	LX0	N
ATOM	2858	HH11	ARG	324	38.886	44.914	0.291	1.00	0.00	LX0	H
ATOM	2859	HH12	ARG	324	40.305	44.095	-0.230	1.00	0.00	LX0	H
ATOM	2860	NH2	ARG	324	38.839	46.650	-1.518	1.00	0.00	LX0	N
ATOM	2861	HH21	ARG	324	38.099	46.756	-0.854	1.00	0.00	LX0	H
ATOM	2862	HH22	ARG	324	38.916	47.298	-2.293	1.00	0.00	LX0	H
ATOM	2863	C	ARG	324	46.244	45.302	-2.320	1.00	0.00	LX0	C
ATOM	2864	O	ARG	324	46.626	44.234	-1.857	1.00	0.00	LX0	O
ATOM	2865	N	LYS	325	46.841	46.472	-2.090	1.00	0.00	LX0	N
ATOM	2866	H	LYS	325	46.444	47.311	-2.474	1.00	0.00	LX0	H
ATOM	2867	CA	LYS	325	47.821	46.451	-1.011	1.00	0.00	LX0	C
ATOM	2868	CB	LYS	325	48.957	47.460	-1.221	1.00	0.00	LX0	C
ATOM	2869	CG	LYS	325	50.212	46.966	-1.948	1.00	0.00	LX0	C
ATOM	2870	CD	LYS	325	50.373	47.440	-3.395	1.00	0.00	LX0	C
ATOM	2871	CE	LYS	325	49.343	46.847	-4.348	1.00	0.00	LX0	C
ATOM	2872	NZ	LYS	325	48.551	47.914	-4.956	1.00	0.00	LX0	N
ATOM	2873	HZ1	LYS	325	47.706	47.514	-5.422	1.00	0.00	LX0	H
ATOM	2874	HZ2	LYS	325	48.254	48.639	-4.268	1.00	0.00	LX0	H
ATOM	2875	HZ3	LYS	325	49.082	48.352	-5.739	1.00	0.00	LX0	H
ATOM	2876	C	LYS	325	47.128	46.775	0.289	1.00	0.00	LX0	C
ATOM	2877	O	LYS	325	46.068	47.396	0.304	1.00	0.00	LX0	O
ATOM	2878	N	CYS	326	47.803	46.403	1.377	1.00	0.00	LX0	N
ATOM	2879	H	CYS	326	48.650	45.877	1.269	1.00	0.00	LX0	H

ATOM	2880	CA	CYS	326	47.385	46.977	2.656	1.00	0.00	LX0	C
ATOM	2881	CB	CYS	326	48.178	46.320	3.787	1.00	0.00	LX0	C
ATOM	2882	SG	CYS	326	49.968	46.357	3.516	1.00	0.00	LX0	S
ATOM	2883	C	CYS	326	47.542	48.494	2.666	1.00	0.00	LX0	C
ATOM	2884	O	CYS	326	48.077	49.069	1.718	1.00	0.00	LX0	O
ATOM	2885	N	LYS	327	47.056	49.098	3.761	1.00	0.00	LX0	N
ATOM	2886	H	LYS	327	46.439	48.570	4.349	1.00	0.00	LX0	H
ATOM	2887	CA	LYS	327	47.361	50.479	4.145	1.00	0.00	LX0	C
ATOM	2888	CB	LYS	327	46.486	51.547	3.464	1.00	0.00	LX0	C
ATOM	2889	CG	LYS	327	46.668	51.757	1.962	1.00	0.00	LX0	C
ATOM	2890	CD	LYS	327	45.570	51.093	1.134	1.00	0.00	LX0	C
ATOM	2891	CE	LYS	327	45.929	51.028	-0.351	1.00	0.00	LX0	C
ATOM	2892	NZ	LYS	327	46.621	49.767	-0.641	1.00	0.00	LX0	N
ATOM	2893	HZ1	LYS	327	47.125	49.834	-1.555	1.00	0.00	LX0	H
ATOM	2894	HZ2	LYS	327	47.282	49.507	0.120	1.00	0.00	LX0	H
ATOM	2895	HZ3	LYS	327	45.918	49.000	-0.748	1.00	0.00	LX0	H
ATOM	2896	C	LYS	327	47.085	50.586	5.627	1.00	0.00	LX0	C
ATOM	2897	O	LYS	327	45.930	50.643	6.033	1.00	0.00	LX0	O
ATOM	2898	N	LYS	328	48.157	50.574	6.436	1.00	0.00	LX0	N
ATOM	2899	H	LYS	328	49.091	50.643	6.093	1.00	0.00	LX0	H
ATOM	2900	CA	LYS	328	47.831	50.589	7.861	1.00	0.00	LX0	C
ATOM	2901	CB	LYS	328	49.013	50.214	8.754	1.00	0.00	LX0	C
ATOM	2902	CG	LYS	328	48.549	49.288	9.884	1.00	0.00	LX0	C
ATOM	2903	CD	LYS	328	49.558	49.117	11.020	1.00	0.00	LX0	C
ATOM	2904	CE	LYS	328	49.537	50.252	12.053	1.00	0.00	LX0	C
ATOM	2905	NZ	LYS	328	48.271	50.276	12.800	1.00	0.00	LX0	N
ATOM	2906	HZ1	LYS	328	48.358	50.906	13.633	1.00	0.00	LX0	H
ATOM	2907	HZ2	LYS	328	47.494	50.671	12.236	1.00	0.00	LX0	H
ATOM	2908	HZ3	LYS	328	48.001	49.328	13.136	1.00	0.00	LX0	H
ATOM	2909	C	LYS	328	47.208	51.881	8.339	1.00	0.00	LX0	C
ATOM	2910	O	LYS	328	47.764	52.964	8.219	1.00	0.00	LX0	O
ATOM	2911	N	CYS	329	46.005	51.702	8.877	1.00	0.00	LX0	N
ATOM	2912	H	CYS	329	45.666	50.772	9.023	1.00	0.00	LX0	H
ATOM	2913	CA	CYS	329	45.406	52.819	9.583	1.00	0.00	LX0	C
ATOM	2914	CB	CYS	329	43.888	52.681	9.525	1.00	0.00	LX0	C
ATOM	2915	SG	CYS	329	42.988	54.145	10.100	1.00	0.00	LX0	S
ATOM	2916	C	CYS	329	45.895	52.770	11.010	1.00	0.00	LX0	C
ATOM	2917	O	CYS	329	46.509	51.790	11.429	1.00	0.00	LX0	O
ATOM	2918	N	GLU	330	45.580	53.839	11.747	1.00	0.00	LX0	N
ATOM	2919	H	GLU	330	45.122	54.650	11.389	1.00	0.00	LX0	H
ATOM	2920	CA	GLU	330	45.664	53.560	13.165	1.00	0.00	LX0	C
ATOM	2921	CB	GLU	330	46.569	54.549	13.911	1.00	0.00	LX0	C
ATOM	2922	CG	GLU	330	47.254	53.902	15.130	1.00	0.00	LX0	C
ATOM	2923	CD	GLU	330	47.854	52.557	14.739	1.00	0.00	LX0	C
ATOM	2924	OE1	GLU	330	47.201	51.530	14.915	1.00	0.00	LX0	O
ATOM	2925	OE2	GLU	330	48.953	52.519	14.202	1.00	0.00	LX0	O
ATOM	2926	C	GLU	330	44.303	53.332	13.782	1.00	0.00	LX0	C
ATOM	2927	O	GLU	330	43.281	53.860	13.338	1.00	0.00	LX0	O
ATOM	2928	N	GLY	331	44.329	52.446	14.783	1.00	0.00	LX0	N
ATOM	2929	H	GLY	331	45.220	52.048	15.023	1.00	0.00	LX0	H
ATOM	2930	CA	GLY	331	43.067	51.914	15.278	1.00	0.00	LX0	C
ATOM	2931	C	GLY	331	42.416	51.000	14.254	1.00	0.00	LX0	C
ATOM	2932	O	GLY	331	42.585	51.154	13.048	1.00	0.00	LX0	O
ATOM	2933	N	PRO	332	41.636	50.033	14.783	1.00	0.00	LX0	N
ATOM	2934	CD	PRO	332	41.325	49.837	16.195	1.00	0.00	LX0	C
ATOM	2935	CA	PRO	332	41.004	49.035	13.914	1.00	0.00	LX0	C
ATOM	2936	CB	PRO	332	40.053	48.310	14.870	1.00	0.00	LX0	C
ATOM	2937	CG	PRO	332	40.698	48.449	16.247	1.00	0.00	LX0	C
ATOM	2938	C	PRO	332	40.283	49.631	12.721	1.00	0.00	LX0	C
ATOM	2939	O	PRO	332	39.437	50.512	12.856	1.00	0.00	LX0	O
ATOM	2940	N	CYS	333	40.661	49.111	11.546	1.00	0.00	LX0	N

ATOM	2941	H	CYS	333	41.354	48.392	11.541	1.00	0.00	LX0	H
ATOM	2942	CA	CYS	333	39.859	49.362	10.356	1.00	0.00	LX0	C
ATOM	2943	CB	CYS	333	40.500	48.715	9.134	1.00	0.00	LX0	C
ATOM	2944	SG	CYS	333	42.033	49.578	8.721	1.00	0.00	LX0	S
ATOM	2945	C	CYS	333	38.442	48.888	10.569	1.00	0.00	LX0	C
ATOM	2946	O	CYS	333	38.167	47.965	11.327	1.00	0.00	LX0	O
ATOM	2947	N	ARG	334	37.537	49.656	9.964	1.00	0.00	LX0	N
ATOM	2948	H	ARG	334	37.749	50.250	9.191	1.00	0.00	LX0	H
ATOM	2949	CA	ARG	334	36.253	49.670	10.645	1.00	0.00	LX0	C
ATOM	2950	CB	ARG	334	35.513	50.994	10.403	1.00	0.00	LX0	C
ATOM	2951	CG	ARG	334	36.100	52.168	11.217	1.00	0.00	LX0	C
ATOM	2952	CD	ARG	334	37.425	52.761	10.707	1.00	0.00	LX0	C
ATOM	2953	NE	ARG	334	38.432	52.859	11.767	1.00	0.00	LX0	N
ATOM	2954	HE	ARG	334	38.151	52.669	12.710	1.00	0.00	LX0	H
ATOM	2955	CZ	ARG	334	39.737	53.104	11.495	1.00	0.00	LX0	C
ATOM	2956	NH1	ARG	334	40.143	53.279	10.241	1.00	0.00	LX0	N
ATOM	2957	HH11	ARG	334	41.100	53.484	10.041	1.00	0.00	LX0	H
ATOM	2958	HH12	ARG	334	39.509	53.205	9.462	1.00	0.00	LX0	H
ATOM	2959	NH2	ARG	334	40.620	53.159	12.493	1.00	0.00	LX0	N
ATOM	2960	HH21	ARG	334	41.605	53.343	12.369	1.00	0.00	LX0	H
ATOM	2961	HH22	ARG	334	40.320	52.985	13.428	1.00	0.00	LX0	H
ATOM	2962	C	ARG	334	35.411	48.426	10.438	1.00	0.00	LX0	C
ATOM	2963	O	ARG	334	34.914	48.125	9.360	1.00	0.00	LX0	O
ATOM	2964	N	LYS	335	35.293	47.722	11.580	1.00	0.00	LX0	N
ATOM	2965	H	LYS	335	35.871	48.030	12.335	1.00	0.00	LX0	H
ATOM	2966	CA	LYS	335	34.459	46.528	11.746	1.00	0.00	LX0	C
ATOM	2967	CB	LYS	335	33.030	46.914	12.177	1.00	0.00	LX0	C
ATOM	2968	CG	LYS	335	32.277	47.755	11.149	1.00	0.00	LX0	C
ATOM	2969	CD	LYS	335	30.883	48.216	11.542	1.00	0.00	LX0	C
ATOM	2970	CE	LYS	335	30.088	48.503	10.270	1.00	0.00	LX0	C
ATOM	2971	NZ	LYS	335	29.867	47.226	9.580	1.00	0.00	LX0	N
ATOM	2972	HZ1	LYS	335	28.857	47.083	9.376	1.00	0.00	LX0	H
ATOM	2973	HZ2	LYS	335	30.437	47.100	8.714	1.00	0.00	LX0	H
ATOM	2974	HZ3	LYS	335	30.133	46.438	10.203	1.00	0.00	LX0	H
ATOM	2975	C	LYS	335	34.529	45.490	10.630	1.00	0.00	LX0	C
ATOM	2976	O	LYS	335	33.580	45.180	9.913	1.00	0.00	LX0	O
ATOM	2977	N	VAL	336	35.751	44.955	10.531	1.00	0.00	LX0	N
ATOM	2978	H	VAL	336	36.465	45.242	11.167	1.00	0.00	LX0	H
ATOM	2979	CA	VAL	336	35.910	43.744	9.733	1.00	0.00	LX0	C
ATOM	2980	CB	VAL	336	37.246	43.770	8.974	1.00	0.00	LX0	C
ATOM	2981	CG1	VAL	336	37.261	44.938	7.985	1.00	0.00	LX0	C
ATOM	2982	CG2	VAL	336	38.465	43.807	9.905	1.00	0.00	LX0	C
ATOM	2983	C	VAL	336	35.794	42.527	10.634	1.00	0.00	LX0	C
ATOM	2984	O	VAL	336	35.952	42.637	11.844	1.00	0.00	LX0	O
ATOM	2985	N	CYS	337	35.503	41.379	10.017	1.00	0.00	LX0	N
ATOM	2986	H	CYS	337	35.395	41.324	9.022	1.00	0.00	LX0	H
ATOM	2987	CA	CYS	337	35.360	40.192	10.853	1.00	0.00	LX0	C
ATOM	2988	CB	CYS	337	33.883	39.838	11.030	1.00	0.00	LX0	C
ATOM	2989	SG	CYS	337	32.853	41.233	11.554	1.00	0.00	LX0	S
ATOM	2990	C	CYS	337	36.106	39.029	10.242	1.00	0.00	LX0	C
ATOM	2991	O	CYS	337	36.006	38.797	9.045	1.00	0.00	LX0	O
ATOM	2992	N	ASN	338	36.861	38.316	11.099	1.00	0.00	LX0	N
ATOM	2993	H	ASN	338	36.901	38.586	12.061	1.00	0.00	LX0	H
ATOM	2994	CA	ASN	338	37.777	37.293	10.569	1.00	0.00	LX0	C
ATOM	2995	CB	ASN	338	38.557	36.631	11.718	1.00	0.00	LX0	C
ATOM	2996	CG	ASN	338	39.384	35.438	11.255	1.00	0.00	LX0	C
ATOM	2997	OD1	ASN	338	39.020	34.290	11.466	1.00	0.00	LX0	O
ATOM	2998	ND2	ASN	338	40.477	35.744	10.559	1.00	0.00	LX0	N
ATOM	2999	HD21	ASN	338	40.917	36.642	10.654	1.00	0.00	LX0	H
ATOM	3000	HD22	ASN	338	40.920	35.073	9.970	1.00	0.00	LX0	H
ATOM	3001	C	ASN	338	37.179	36.291	9.584	1.00	0.00	LX0	C

ATOM	3002	O	ASN	338	37.674	36.133	8.479	1.00	0.00	LX0	O
ATOM	3003	N	GLY	339	36.081	35.653	9.994	1.00	0.00	LX0	N
ATOM	3004	H	GLY	339	35.743	35.722	10.930	1.00	0.00	LX0	H
ATOM	3005	CA	GLY	339	35.453	34.817	8.977	1.00	0.00	LX0	C
ATOM	3006	C	GLY	339	35.085	33.439	9.462	1.00	0.00	LX0	C
ATOM	3007	O	GLY	339	35.869	32.728	10.075	1.00	0.00	LX0	O
ATOM	3008	N	ILE	340	33.823	33.104	9.177	1.00	0.00	LX0	N
ATOM	3009	H	ILE	340	33.395	33.602	8.422	1.00	0.00	LX0	H
ATOM	3010	CA	ILE	340	33.275	31.835	9.645	1.00	0.00	LX0	C
ATOM	3011	CB	ILE	340	31.768	31.814	9.335	1.00	0.00	LX0	C
ATOM	3012	CG2	ILE	340	31.122	30.441	9.495	1.00	0.00	LX0	C
ATOM	3013	CG1	ILE	340	31.035	32.854	10.185	1.00	0.00	LX0	C
ATOM	3014	CD1	ILE	340	30.978	32.489	11.673	1.00	0.00	LX0	C
ATOM	3015	C	ILE	340	34.008	30.643	9.047	1.00	0.00	LX0	C
ATOM	3016	O	ILE	340	33.842	30.291	7.882	1.00	0.00	LX0	O
ATOM	3017	N	GLY	341	34.836	30.050	9.916	1.00	0.00	LX0	N
ATOM	3018	H	GLY	341	34.953	30.406	10.847	1.00	0.00	LX0	H
ATOM	3019	CA	GLY	341	35.605	28.901	9.464	1.00	0.00	LX0	C
ATOM	3020	C	GLY	341	37.103	29.093	9.564	1.00	0.00	LX0	C
ATOM	3021	O	GLY	341	37.854	28.151	9.817	1.00	0.00	LX0	O
ATOM	3022	N	ILE	342	37.514	30.341	9.320	1.00	0.00	LX0	N
ATOM	3023	H	ILE	342	36.872	31.108	9.384	1.00	0.00	LX0	H
ATOM	3024	CA	ILE	342	38.945	30.583	9.187	1.00	0.00	LX0	C
ATOM	3025	CB	ILE	342	39.162	31.516	7.977	1.00	0.00	LX0	C
ATOM	3026	CG2	ILE	342	38.830	32.973	8.308	1.00	0.00	LX0	C
ATOM	3027	CG1	ILE	342	40.528	31.319	7.314	1.00	0.00	LX0	C
ATOM	3028	CD1	ILE	342	40.726	32.152	6.050	1.00	0.00	LX0	C
ATOM	3029	C	ILE	342	39.535	31.097	10.499	1.00	0.00	LX0	C
ATOM	3030	O	ILE	342	38.807	31.304	11.459	1.00	0.00	LX0	O
ATOM	3031	N	GLY	343	40.873	31.271	10.506	1.00	0.00	LX0	N
ATOM	3032	H	GLY	343	41.389	31.026	9.690	1.00	0.00	LX0	H
ATOM	3033	CA	GLY	343	41.560	31.984	11.586	1.00	0.00	LX0	C
ATOM	3034	C	GLY	343	41.049	31.773	12.998	1.00	0.00	LX0	C
ATOM	3035	O	GLY	343	41.091	30.678	13.547	1.00	0.00	LX0	O
ATOM	3036	N	GLU	344	40.577	32.896	13.553	1.00	0.00	LX0	N
ATOM	3037	H	GLU	344	40.399	33.683	12.961	1.00	0.00	LX0	H
ATOM	3038	CA	GLU	344	40.095	32.817	14.928	1.00	0.00	LX0	C
ATOM	3039	CB	GLU	344	40.236	34.164	15.657	1.00	0.00	LX0	C
ATOM	3040	CG	GLU	344	39.640	35.374	14.931	1.00	0.00	LX0	C
ATOM	3041	CD	GLU	344	39.824	36.661	15.718	1.00	0.00	LX0	C
ATOM	3042	OE1	GLU	344	40.893	36.893	16.281	1.00	0.00	LX0	O
ATOM	3043	OE2	GLU	344	38.884	37.446	15.784	1.00	0.00	LX0	O
ATOM	3044	C	GLU	344	38.711	32.206	15.069	1.00	0.00	LX0	C
ATOM	3045	O	GLU	344	38.361	31.651	16.103	1.00	0.00	LX0	O
ATOM	3046	N	PHE	345	37.944	32.266	13.968	1.00	0.00	LX0	N
ATOM	3047	H	PHE	345	38.305	32.613	13.098	1.00	0.00	LX0	H
ATOM	3048	CA	PHE	345	36.692	31.507	13.994	1.00	0.00	LX0	C
ATOM	3049	CB	PHE	345	35.619	32.191	13.152	1.00	0.00	LX0	C
ATOM	3050	CG	PHE	345	35.303	33.546	13.733	1.00	0.00	LX0	C
ATOM	3051	CD1	PHE	345	34.395	33.639	14.807	1.00	0.00	LX0	C
ATOM	3052	CD2	PHE	345	35.930	34.692	13.203	1.00	0.00	LX0	C
ATOM	3053	CE1	PHE	345	34.128	34.896	15.376	1.00	0.00	LX0	C
ATOM	3054	CE2	PHE	345	35.664	35.952	13.769	1.00	0.00	LX0	C
ATOM	3055	CZ	PHE	345	34.773	36.038	14.858	1.00	0.00	LX0	C
ATOM	3056	C	PHE	345	36.902	30.070	13.553	1.00	0.00	LX0	C
ATOM	3057	O	PHE	345	36.326	29.543	12.603	1.00	0.00	LX0	O
ATOM	3058	N	LYS	346	37.821	29.436	14.297	1.00	0.00	LX0	N
ATOM	3059	H	LYS	346	38.167	29.885	15.123	1.00	0.00	LX0	H
ATOM	3060	CA	LYS	346	38.239	28.115	13.854	1.00	0.00	LX0	C
ATOM	3061	CB	LYS	346	39.570	27.710	14.495	1.00	0.00	LX0	C
ATOM	3062	CG	LYS	346	40.297	26.618	13.701	1.00	0.00	LX0	C

ATOM	3063	CD	LYS	346	40.642	27.052	12.271	1.00	0.00	LX0	C
ATOM	3064	CE	LYS	346	40.119	26.095	11.191	1.00	0.00	LX0	C
ATOM	3065	NZ	LYS	346	38.651	26.145	11.129	1.00	0.00	LX0	N
ATOM	3066	HZ1	LYS	346	38.294	25.661	10.285	1.00	0.00	LX0	H
ATOM	3067	HZ2	LYS	346	38.204	25.738	11.979	1.00	0.00	LX0	H
ATOM	3068	HZ3	LYS	346	38.345	27.138	11.049	1.00	0.00	LX0	H
ATOM	3069	C	LYS	346	37.182	27.038	14.005	1.00	0.00	LX0	C
ATOM	3070	O	LYS	346	37.176	26.054	13.266	1.00	0.00	LX0	O
ATOM	3071	N	ASP	347	36.291	27.300	14.967	1.00	0.00	LX0	N
ATOM	3072	H	ASP	347	36.318	28.176	15.453	1.00	0.00	LX0	H
ATOM	3073	CA	ASP	347	35.120	26.460	15.214	1.00	0.00	LX0	C
ATOM	3074	CB	ASP	347	34.316	27.031	16.402	1.00	0.00	LX0	C
ATOM	3075	CG	ASP	347	33.763	28.421	16.078	1.00	0.00	LX0	C
ATOM	3076	OD1	ASP	347	34.548	29.345	15.890	1.00	0.00	LX0	O
ATOM	3077	OD2	ASP	347	32.553	28.574	15.923	1.00	0.00	LX0	O
ATOM	3078	C	ASP	347	34.204	26.303	14.011	1.00	0.00	LX0	C
ATOM	3079	O	ASP	347	33.619	25.254	13.763	1.00	0.00	LX0	O
ATOM	3080	N	SER	348	34.085	27.424	13.289	1.00	0.00	LX0	N
ATOM	3081	H	SER	348	34.641	28.232	13.491	1.00	0.00	LX0	H
ATOM	3082	CA	SER	348	32.839	27.598	12.564	1.00	0.00	LX0	C
ATOM	3083	CB	SER	348	32.464	29.078	12.523	1.00	0.00	LX0	C
ATOM	3084	OG	SER	348	33.336	29.864	13.347	1.00	0.00	LX0	O
ATOM	3085	HG	SER	348	33.279	29.521	14.245	1.00	0.00	LX0	H
ATOM	3086	C	SER	348	32.716	26.940	11.206	1.00	0.00	LX0	C
ATOM	3087	O	SER	348	32.754	27.566	10.155	1.00	0.00	LX0	O
ATOM	3088	N	LEU	349	32.483	25.622	11.281	1.00	0.00	LX0	N
ATOM	3089	H	LEU	349	32.545	25.191	12.182	1.00	0.00	LX0	H
ATOM	3090	CA	LEU	349	32.022	24.920	10.082	1.00	0.00	LX0	C
ATOM	3091	CB	LEU	349	32.188	23.399	10.230	1.00	0.00	LX0	C
ATOM	3092	CG	LEU	349	31.762	22.753	11.559	1.00	0.00	LX0	C
ATOM	3093	CD1	LEU	349	30.258	22.815	11.835	1.00	0.00	LX0	C
ATOM	3094	CD2	LEU	349	32.278	21.316	11.646	1.00	0.00	LX0	C
ATOM	3095	C	LEU	349	30.619	25.319	9.636	1.00	0.00	LX0	C
ATOM	3096	O	LEU	349	30.161	25.013	8.543	1.00	0.00	LX0	O
ATOM	3097	N	SER	350	29.968	26.058	10.541	1.00	0.00	LX0	N
ATOM	3098	H	SER	350	30.306	26.195	11.470	1.00	0.00	LX0	H
ATOM	3099	CA	SER	350	28.784	26.783	10.130	1.00	0.00	LX0	C
ATOM	3100	CB	SER	350	27.532	25.957	10.408	1.00	0.00	LX0	C
ATOM	3101	OG	SER	350	26.410	26.536	9.736	1.00	0.00	LX0	O
ATOM	3102	HG	SER	350	26.042	27.181	10.342	1.00	0.00	LX0	H
ATOM	3103	C	SER	350	28.725	28.075	10.900	1.00	0.00	LX0	C
ATOM	3104	O	SER	350	29.154	28.150	12.048	1.00	0.00	LX0	O
ATOM	3105	N	ILE	351	28.129	29.072	10.239	1.00	0.00	LX0	N
ATOM	3106	H	ILE	351	27.929	28.949	9.266	1.00	0.00	LX0	H
ATOM	3107	CA	ILE	351	27.647	30.223	10.991	1.00	0.00	LX0	C
ATOM	3108	CB	ILE	351	27.244	31.341	10.009	1.00	0.00	LX0	C
ATOM	3109	CG2	ILE	351	26.037	30.953	9.150	1.00	0.00	LX0	C
ATOM	3110	CG1	ILE	351	27.095	32.704	10.694	1.00	0.00	LX0	C
ATOM	3111	CD1	ILE	351	26.988	33.856	9.693	1.00	0.00	LX0	C
ATOM	3112	C	ILE	351	26.520	29.756	11.899	1.00	0.00	LX0	C
ATOM	3113	O	ILE	351	25.895	28.736	11.628	1.00	0.00	LX0	O
ATOM	3114	N	ASN	352	26.358	30.484	13.007	1.00	0.00	LX0	N
ATOM	3115	H	ASN	352	26.881	31.325	13.147	1.00	0.00	LX0	H
ATOM	3116	CA	ASN	352	25.503	30.027	14.102	1.00	0.00	LX0	C
ATOM	3117	CB	ASN	352	26.101	28.772	14.770	1.00	0.00	LX0	C
ATOM	3118	CG	ASN	352	27.436	29.074	15.437	1.00	0.00	LX0	C
ATOM	3119	OD1	ASN	352	27.489	29.724	16.470	1.00	0.00	LX0	O
ATOM	3120	ND2	ASN	352	28.511	28.578	14.819	1.00	0.00	LX0	N
ATOM	3121	HD21	ASN	352	28.475	28.120	13.929	1.00	0.00	LX0	H
ATOM	3122	HD22	ASN	352	29.412	28.658	15.249	1.00	0.00	LX0	H
ATOM	3123	C	ASN	352	25.403	31.178	15.079	1.00	0.00	LX0	C

ATOM	3124	O	ASN	352	26.234	32.079	15.013	1.00	0.00	LX0	O
ATOM	3125	N	ALA	353	24.401	31.140	15.974	1.00	0.00	LX0	N
ATOM	3126	H	ALA	353	23.731	30.394	16.001	1.00	0.00	LX0	H
ATOM	3127	CA	ALA	353	24.291	32.296	16.869	1.00	0.00	LX0	C
ATOM	3128	CB	ALA	353	23.047	32.199	17.749	1.00	0.00	LX0	C
ATOM	3129	C	ALA	353	25.511	32.551	17.747	1.00	0.00	LX0	C
ATOM	3130	O	ALA	353	26.021	33.663	17.850	1.00	0.00	LX0	O
ATOM	3131	N	THR	354	25.995	31.446	18.336	1.00	0.00	LX0	N
ATOM	3132	H	THR	354	25.557	30.552	18.241	1.00	0.00	LX0	H
ATOM	3133	CA	THR	354	27.173	31.511	19.201	1.00	0.00	LX0	C
ATOM	3134	CB	THR	354	27.510	30.090	19.674	1.00	0.00	LX0	C
ATOM	3135	OG1	THR	354	26.290	29.371	19.900	1.00	0.00	LX0	O
ATOM	3136	HG1	THR	354	26.502	28.603	20.435	1.00	0.00	LX0	H
ATOM	3137	CG2	THR	354	28.388	30.069	20.928	1.00	0.00	LX0	C
ATOM	3138	C	THR	354	28.392	32.228	18.621	1.00	0.00	LX0	C
ATOM	3139	O	THR	354	29.191	32.836	19.333	1.00	0.00	LX0	O
ATOM	3140	N	ASN	355	28.502	32.188	17.285	1.00	0.00	LX0	N
ATOM	3141	H	ASN	355	27.889	31.620	16.731	1.00	0.00	LX0	H
ATOM	3142	CA	ASN	355	29.585	33.000	16.747	1.00	0.00	LX0	C
ATOM	3143	CB	ASN	355	30.674	32.149	16.093	1.00	0.00	LX0	C
ATOM	3144	CG	ASN	355	31.944	32.413	16.880	1.00	0.00	LX0	C
ATOM	3145	OD1	ASN	355	32.057	33.400	17.607	1.00	0.00	LX0	O
ATOM	3146	ND2	ASN	355	32.889	31.487	16.735	1.00	0.00	LX0	N
ATOM	3147	HD21	ASN	355	32.776	30.617	16.237	1.00	0.00	LX0	H
ATOM	3148	HD22	ASN	355	33.812	31.560	17.107	1.00	0.00	LX0	H
ATOM	3149	C	ASN	355	29.226	34.232	15.941	1.00	0.00	LX0	C
ATOM	3150	O	ASN	355	30.037	35.136	15.763	1.00	0.00	LX0	O
ATOM	3151	N	ILE	356	27.951	34.289	15.508	1.00	0.00	LX0	N
ATOM	3152	H	ILE	356	27.302	33.551	15.705	1.00	0.00	LX0	H
ATOM	3153	CA	ILE	356	27.543	35.469	14.737	1.00	0.00	LX0	C
ATOM	3154	CB	ILE	356	26.149	35.309	14.095	1.00	0.00	LX0	C
ATOM	3155	CG2	ILE	356	25.013	35.470	15.112	1.00	0.00	LX0	C
ATOM	3156	CG1	ILE	356	26.011	36.253	12.890	1.00	0.00	LX0	C
ATOM	3157	CD1	ILE	356	24.662	36.224	12.171	1.00	0.00	LX0	C
ATOM	3158	C	ILE	356	27.676	36.804	15.469	1.00	0.00	LX0	C
ATOM	3159	O	ILE	356	27.754	37.859	14.856	1.00	0.00	LX0	O
ATOM	3160	N	LYS	357	27.778	36.704	16.812	1.00	0.00	LX0	N
ATOM	3161	H	LYS	357	27.494	35.827	17.198	1.00	0.00	LX0	H
ATOM	3162	CA	LYS	357	28.146	37.845	17.670	1.00	0.00	LX0	C
ATOM	3163	CB	LYS	357	28.951	37.389	18.895	1.00	0.00	LX0	C
ATOM	3164	CG	LYS	357	28.542	36.075	19.563	1.00	0.00	LX0	C
ATOM	3165	CD	LYS	357	29.415	35.754	20.784	1.00	0.00	LX0	C
ATOM	3166	CE	LYS	357	30.923	35.647	20.503	1.00	0.00	LX0	C
ATOM	3167	NZ	LYS	357	31.244	34.477	19.674	1.00	0.00	LX0	N
ATOM	3168	HZ1	LYS	357	31.782	34.716	18.808	1.00	0.00	LX0	H
ATOM	3169	HZ2	LYS	357	31.821	33.792	20.195	1.00	0.00	LX0	H
ATOM	3170	HZ3	LYS	357	30.383	33.981	19.372	1.00	0.00	LX0	H
ATOM	3171	C	LYS	357	28.954	38.966	17.014	1.00	0.00	LX0	C
ATOM	3172	O	LYS	357	28.624	40.141	17.071	1.00	0.00	LX0	O
ATOM	3173	N	HIS	358	30.052	38.514	16.387	1.00	0.00	LX0	N
ATOM	3174	H	HIS	358	30.160	37.525	16.277	1.00	0.00	LX0	H
ATOM	3175	CA	HIS	358	30.985	39.461	15.770	1.00	0.00	LX0	C
ATOM	3176	CB	HIS	358	32.359	38.803	15.537	1.00	0.00	LX0	C
ATOM	3177	CG	HIS	358	32.911	38.101	16.766	1.00	0.00	LX0	C
ATOM	3178	ND1	HIS	358	34.029	38.480	17.413	1.00	0.00	LX0	N
ATOM	3179	HD1	HIS	358	34.618	39.228	17.180	1.00	0.00	LX0	H
ATOM	3180	CD2	HIS	358	32.412	36.959	17.404	1.00	0.00	LX0	C
ATOM	3181	NE2	HIS	358	33.232	36.655	18.437	1.00	0.00	LX0	N
ATOM	3182	CE1	HIS	358	34.235	37.595	18.441	1.00	0.00	LX0	C
ATOM	3183	C	HIS	358	30.454	39.986	14.443	1.00	0.00	LX0	C
ATOM	3184	O	HIS	358	30.483	41.163	14.108	1.00	0.00	LX0	O

ATOM	3185	N	PHE	359	29.944	39.004	13.689	1.00	0.00	LX0	N
ATOM	3186	H	PHE	359	29.782	38.116	14.115	1.00	0.00	LX0	H
ATOM	3187	CA	PHE	359	29.454	39.245	12.332	1.00	0.00	LX0	C
ATOM	3188	CB	PHE	359	29.195	37.906	11.629	1.00	0.00	LX0	C
ATOM	3189	CG	PHE	359	30.476	37.107	11.543	1.00	0.00	LX0	C
ATOM	3190	CD1	PHE	359	31.246	37.172	10.363	1.00	0.00	LX0	C
ATOM	3191	CD2	PHE	359	30.893	36.328	12.646	1.00	0.00	LX0	C
ATOM	3192	CE1	PHE	359	32.474	36.488	10.299	1.00	0.00	LX0	C
ATOM	3193	CE2	PHE	359	32.124	35.650	12.587	1.00	0.00	LX0	C
ATOM	3194	CZ	PHE	359	32.910	35.753	11.421	1.00	0.00	LX0	C
ATOM	3195	C	PHE	359	28.229	40.142	12.253	1.00	0.00	LX0	C
ATOM	3196	O	PHE	359	27.887	40.688	11.211	1.00	0.00	LX0	O
ATOM	3197	N	LYS	360	27.601	40.308	13.429	1.00	0.00	LX0	N
ATOM	3198	H	LYS	360	27.893	39.756	14.210	1.00	0.00	LX0	H
ATOM	3199	CA	LYS	360	26.426	41.167	13.546	1.00	0.00	LX0	C
ATOM	3200	CB	LYS	360	25.913	41.118	14.987	1.00	0.00	LX0	C
ATOM	3201	CG	LYS	360	24.419	41.423	15.099	1.00	0.00	LX0	C
ATOM	3202	CD	LYS	360	23.884	41.275	16.523	1.00	0.00	LX0	C
ATOM	3203	CE	LYS	360	22.387	41.582	16.618	1.00	0.00	LX0	C
ATOM	3204	NZ	LYS	360	21.640	40.661	15.759	1.00	0.00	LX0	N
ATOM	3205	HZ1	LYS	360	21.897	40.675	14.756	1.00	0.00	LX0	H
ATOM	3206	HZ2	LYS	360	21.738	39.654	16.016	1.00	0.00	LX0	H
ATOM	3207	HZ3	LYS	360	20.603	40.782	15.776	1.00	0.00	LX0	H
ATOM	3208	C	LYS	360	26.563	42.596	13.023	1.00	0.00	LX0	C
ATOM	3209	O	LYS	360	25.574	43.277	12.765	1.00	0.00	LX0	O
ATOM	3210	N	ASN	361	27.824	43.022	12.825	1.00	0.00	LX0	N
ATOM	3211	H	ASN	361	28.617	42.518	13.173	1.00	0.00	LX0	H
ATOM	3212	CA	ASN	361	27.942	44.087	11.832	1.00	0.00	LX0	C
ATOM	3213	CB	ASN	361	27.673	45.480	12.396	1.00	0.00	LX0	C
ATOM	3214	CG	ASN	361	26.912	46.211	11.314	1.00	0.00	LX0	C
ATOM	3215	OD1	ASN	361	27.468	46.913	10.475	1.00	0.00	LX0	O
ATOM	3216	ND2	ASN	361	25.598	45.986	11.356	1.00	0.00	LX0	N
ATOM	3217	HD21	ASN	361	25.239	45.312	12.009	1.00	0.00	LX0	H
ATOM	3218	HD22	ASN	361	24.976	46.451	10.731	1.00	0.00	LX0	H
ATOM	3219	C	ASN	361	29.196	44.098	10.991	1.00	0.00	LX0	C
ATOM	3220	O	ASN	361	30.010	45.014	11.062	1.00	0.00	LX0	O
ATOM	3221	N	CYS	362	29.297	43.055	10.161	1.00	0.00	LX0	N
ATOM	3222	H	CYS	362	28.589	42.344	10.181	1.00	0.00	LX0	H
ATOM	3223	CA	CYS	362	30.424	42.955	9.230	1.00	0.00	LX0	C
ATOM	3224	CB	CYS	362	30.230	41.747	8.320	1.00	0.00	LX0	C
ATOM	3225	SG	CYS	362	30.156	40.183	9.217	1.00	0.00	LX0	S
ATOM	3226	C	CYS	362	30.672	44.176	8.356	1.00	0.00	LX0	C
ATOM	3227	O	CYS	362	29.853	45.087	8.255	1.00	0.00	LX0	O
ATOM	3228	N	THR	363	31.846	44.131	7.710	1.00	0.00	LX0	N
ATOM	3229	H	THR	363	32.551	43.486	8.002	1.00	0.00	LX0	H
ATOM	3230	CA	THR	363	32.040	44.985	6.541	1.00	0.00	LX0	C
ATOM	3231	CB	THR	363	32.607	46.356	6.930	1.00	0.00	LX0	C
ATOM	3232	OG1	THR	363	32.064	46.778	8.191	1.00	0.00	LX0	O
ATOM	3233	HG1	THR	363	32.653	46.369	8.829	1.00	0.00	LX0	H
ATOM	3234	CG2	THR	363	32.344	47.404	5.847	1.00	0.00	LX0	C
ATOM	3235	C	THR	363	32.889	44.280	5.494	1.00	0.00	LX0	C
ATOM	3236	O	THR	363	32.454	43.970	4.393	1.00	0.00	LX0	O
ATOM	3237	N	SER	364	34.120	43.969	5.913	1.00	0.00	LX0	N
ATOM	3238	H	SER	364	34.475	44.223	6.811	1.00	0.00	LX0	H
ATOM	3239	CA	SER	364	34.828	42.978	5.112	1.00	0.00	LX0	C
ATOM	3240	CB	SER	364	36.129	43.559	4.538	1.00	0.00	LX0	C
ATOM	3241	OG	SER	364	36.696	42.659	3.582	1.00	0.00	LX0	O
ATOM	3242	HG	SER	364	36.986	43.187	2.833	1.00	0.00	LX0	H
ATOM	3243	C	SER	364	35.049	41.755	5.977	1.00	0.00	LX0	C
ATOM	3244	O	SER	364	34.976	41.837	7.201	1.00	0.00	LX0	O
ATOM	3245	N	ILE	365	35.268	40.632	5.293	1.00	0.00	LX0	N

ATOM	3246	H	ILE	365	35.327	40.691	4.293	1.00	0.00	LX0	H
ATOM	3247	CA	ILE	365	35.444	39.352	5.966	1.00	0.00	LX0	C
ATOM	3248	CB	ILE	365	34.344	38.365	5.529	1.00	0.00	LX0	C
ATOM	3249	CG2	ILE	365	34.541	36.954	6.089	1.00	0.00	LX0	C
ATOM	3250	CG1	ILE	365	32.957	38.895	5.890	1.00	0.00	LX0	C
ATOM	3251	CD1	ILE	365	32.726	38.950	7.398	1.00	0.00	LX0	C
ATOM	3252	C	ILE	365	36.811	38.813	5.622	1.00	0.00	LX0	C
ATOM	3253	O	ILE	365	37.106	38.530	4.466	1.00	0.00	LX0	O
ATOM	3254	N	SER	366	37.627	38.671	6.669	1.00	0.00	LX0	N
ATOM	3255	H	SER	366	37.312	38.896	7.592	1.00	0.00	LX0	H
ATOM	3256	CA	SER	366	39.016	38.243	6.497	1.00	0.00	LX0	C
ATOM	3257	CB	SER	366	39.769	38.547	7.792	1.00	0.00	LX0	C
ATOM	3258	OG	SER	366	39.297	39.794	8.329	1.00	0.00	LX0	O
ATOM	3259	HG	SER	366	39.930	40.447	8.052	1.00	0.00	LX0	H
ATOM	3260	C	SER	366	39.260	36.804	6.038	1.00	0.00	LX0	C
ATOM	3261	O	SER	366	40.392	36.352	5.915	1.00	0.00	LX0	O
ATOM	3262	N	GLY	367	38.142	36.116	5.770	1.00	0.00	LX0	N
ATOM	3263	H	GLY	367	37.262	36.476	6.067	1.00	0.00	LX0	H
ATOM	3264	CA	GLY	367	38.225	34.785	5.194	1.00	0.00	LX0	C
ATOM	3265	C	GLY	367	36.869	34.322	4.720	1.00	0.00	LX0	C
ATOM	3266	O	GLY	367	36.200	34.999	3.945	1.00	0.00	LX0	O
ATOM	3267	N	ASP	368	36.507	33.141	5.227	1.00	0.00	LX0	N
ATOM	3268	H	ASP	368	37.103	32.661	5.867	1.00	0.00	LX0	H
ATOM	3269	CA	ASP	368	35.363	32.429	4.661	1.00	0.00	LX0	C
ATOM	3270	CB	ASP	368	35.654	30.927	4.653	1.00	0.00	LX0	C
ATOM	3271	CG	ASP	368	36.939	30.644	3.906	1.00	0.00	LX0	C
ATOM	3272	OD1	ASP	368	37.018	30.961	2.725	1.00	0.00	LX0	O
ATOM	3273	OD2	ASP	368	37.870	30.114	4.502	1.00	0.00	LX0	O
ATOM	3274	C	ASP	368	34.041	32.668	5.359	1.00	0.00	LX0	C
ATOM	3275	O	ASP	368	33.971	33.221	6.453	1.00	0.00	LX0	O
ATOM	3276	N	LEU	369	32.990	32.180	4.681	1.00	0.00	LX0	N
ATOM	3277	H	LEU	369	33.122	31.906	3.726	1.00	0.00	LX0	H
ATOM	3278	CA	LEU	369	31.680	32.045	5.314	1.00	0.00	LX0	C
ATOM	3279	CB	LEU	369	30.698	33.118	4.823	1.00	0.00	LX0	C
ATOM	3280	CG	LEU	369	31.009	34.556	5.253	1.00	0.00	LX0	C
ATOM	3281	CD1	LEU	369	30.044	35.550	4.609	1.00	0.00	LX0	C
ATOM	3282	CD2	LEU	369	31.023	34.728	6.773	1.00	0.00	LX0	C
ATOM	3283	C	LEU	369	31.083	30.666	5.076	1.00	0.00	LX0	C
ATOM	3284	O	LEU	369	30.410	30.409	4.083	1.00	0.00	LX0	O
ATOM	3285	N	HIS	370	31.352	29.778	6.044	1.00	0.00	LX0	N
ATOM	3286	H	HIS	370	31.954	30.041	6.800	1.00	0.00	LX0	H
ATOM	3287	CA	HIS	370	30.676	28.476	6.016	1.00	0.00	LX0	C
ATOM	3288	CB	HIS	370	31.385	27.474	6.921	1.00	0.00	LX0	C
ATOM	3289	CG	HIS	370	32.733	27.047	6.402	1.00	0.00	LX0	C
ATOM	3290	ND1	HIS	370	33.868	27.741	6.590	1.00	0.00	LX0	N
ATOM	3291	HD1	HIS	370	33.957	28.604	7.050	1.00	0.00	LX0	H
ATOM	3292	CD2	HIS	370	33.033	25.879	5.700	1.00	0.00	LX0	C
ATOM	3293	NE2	HIS	370	34.368	25.877	5.469	1.00	0.00	LX0	N
ATOM	3294	CE1	HIS	370	34.884	27.024	6.020	1.00	0.00	LX0	C
ATOM	3295	C	HIS	370	29.232	28.547	6.494	1.00	0.00	LX0	C
ATOM	3296	O	HIS	370	28.957	28.956	7.614	1.00	0.00	LX0	O
ATOM	3297	N	ILE	371	28.312	28.116	5.625	1.00	0.00	LX0	N
ATOM	3298	H	ILE	371	28.545	27.877	4.681	1.00	0.00	LX0	H
ATOM	3299	CA	ILE	371	26.914	28.077	6.062	1.00	0.00	LX0	C
ATOM	3300	CB	ILE	371	26.073	29.151	5.343	1.00	0.00	LX0	C
ATOM	3301	CG2	ILE	371	24.661	29.248	5.930	1.00	0.00	LX0	C
ATOM	3302	CG1	ILE	371	26.775	30.513	5.341	1.00	0.00	LX0	C
ATOM	3303	CD1	ILE	371	26.036	31.572	4.529	1.00	0.00	LX0	C
ATOM	3304	C	ILE	371	26.309	26.691	5.877	1.00	0.00	LX0	C
ATOM	3305	O	ILE	371	25.601	26.397	4.921	1.00	0.00	LX0	O
ATOM	3306	N	LEU	372	26.641	25.823	6.838	1.00	0.00	LX0	N

ATOM	3307	H	LEU	372	27.050	26.165	7.685	1.00	0.00	LX0	H
ATOM	3308	CA	LEU	372	26.075	24.474	6.772	1.00	0.00	LX0	C
ATOM	3309	CB	LEU	372	26.910	23.542	7.662	1.00	0.00	LX0	C
ATOM	3310	CG	LEU	372	27.819	22.573	6.899	1.00	0.00	LX0	C
ATOM	3311	CD1	LEU	372	28.792	23.278	5.953	1.00	0.00	LX0	C
ATOM	3312	CD2	LEU	372	28.540	21.624	7.858	1.00	0.00	LX0	C
ATOM	3313	C	LEU	372	24.612	24.501	7.200	1.00	0.00	LX0	C
ATOM	3314	O	LEU	372	24.209	25.396	7.930	1.00	0.00	LX0	O
ATOM	3315	N	PRO	373	23.804	23.500	6.745	1.00	0.00	LX0	N
ATOM	3316	CD	PRO	373	24.158	22.388	5.864	1.00	0.00	LX0	C
ATOM	3317	CA	PRO	373	22.380	23.472	7.126	1.00	0.00	LX0	C
ATOM	3318	CB	PRO	373	21.944	22.069	6.693	1.00	0.00	LX0	C
ATOM	3319	CG	PRO	373	22.832	21.734	5.499	1.00	0.00	LX0	C
ATOM	3320	C	PRO	373	22.083	23.768	8.590	1.00	0.00	LX0	C
ATOM	3321	O	PRO	373	21.208	24.551	8.945	1.00	0.00	LX0	O
ATOM	3322	N	VAL	374	22.910	23.113	9.425	1.00	0.00	LX0	N
ATOM	3323	H	VAL	374	23.561	22.459	9.048	1.00	0.00	LX0	H
ATOM	3324	CA	VAL	374	22.803	23.267	10.874	1.00	0.00	LX0	C
ATOM	3325	CB	VAL	374	23.938	22.492	11.555	1.00	0.00	LX0	C
ATOM	3326	CG1	VAL	374	25.311	23.106	11.281	1.00	0.00	LX0	C
ATOM	3327	CG2	VAL	374	23.646	22.265	13.038	1.00	0.00	LX0	C
ATOM	3328	C	VAL	374	22.673	24.690	11.411	1.00	0.00	LX0	C
ATOM	3329	O	VAL	374	22.007	24.920	12.411	1.00	0.00	LX0	O
ATOM	3330	N	ALA	375	23.288	25.642	10.677	1.00	0.00	LX0	N
ATOM	3331	H	ALA	375	23.820	25.377	9.871	1.00	0.00	LX0	H
ATOM	3332	CA	ALA	375	23.219	27.062	11.034	1.00	0.00	LX0	C
ATOM	3333	CB	ALA	375	23.485	27.931	9.802	1.00	0.00	LX0	C
ATOM	3334	C	ALA	375	21.897	27.522	11.612	1.00	0.00	LX0	C
ATOM	3335	O	ALA	375	21.808	28.148	12.659	1.00	0.00	LX0	O
ATOM	3336	N	PHE	376	20.857	27.170	10.852	1.00	0.00	LX0	N
ATOM	3337	H	PHE	376	20.989	26.531	10.091	1.00	0.00	LX0	H
ATOM	3338	CA	PHE	376	19.551	27.718	11.205	1.00	0.00	LX0	C
ATOM	3339	CB	PHE	376	18.699	27.896	9.945	1.00	0.00	LX0	C
ATOM	3340	CG	PHE	376	19.569	28.348	8.792	1.00	0.00	LX0	C
ATOM	3341	CD1	PHE	376	19.838	27.433	7.750	1.00	0.00	LX0	C
ATOM	3342	CD2	PHE	376	20.110	29.653	8.783	1.00	0.00	LX0	C
ATOM	3343	CE1	PHE	376	20.671	27.825	6.687	1.00	0.00	LX0	C
ATOM	3344	CE2	PHE	376	20.941	30.046	7.717	1.00	0.00	LX0	C
ATOM	3345	CZ	PHE	376	21.212	29.127	6.681	1.00	0.00	LX0	C
ATOM	3346	C	PHE	376	18.805	26.905	12.242	1.00	0.00	LX0	C
ATOM	3347	O	PHE	376	17.881	27.376	12.886	1.00	0.00	LX0	O
ATOM	3348	N	ARG	377	19.247	25.645	12.345	1.00	0.00	LX0	N
ATOM	3349	H	ARG	377	20.126	25.387	11.949	1.00	0.00	LX0	H
ATOM	3350	CA	ARG	377	18.489	24.687	13.146	1.00	0.00	LX0	C
ATOM	3351	CB	ARG	377	18.581	23.265	12.567	1.00	0.00	LX0	C
ATOM	3352	CG	ARG	377	19.017	23.079	11.101	1.00	0.00	LX0	C
ATOM	3353	CD	ARG	377	18.207	23.776	9.998	1.00	0.00	LX0	C
ATOM	3354	NE	ARG	377	16.799	23.407	10.021	1.00	0.00	LX0	N
ATOM	3355	HE	ARG	377	16.443	22.766	10.719	1.00	0.00	LX0	H
ATOM	3356	CZ	ARG	377	15.914	23.922	9.148	1.00	0.00	LX0	C
ATOM	3357	NH1	ARG	377	16.302	24.717	8.159	1.00	0.00	LX0	N
ATOM	3358	HH11	ARG	377	15.599	25.044	7.504	1.00	0.00	LX0	H
ATOM	3359	HH12	ARG	377	17.252	24.979	8.031	1.00	0.00	LX0	H
ATOM	3360	NH2	ARG	377	14.632	23.632	9.270	1.00	0.00	LX0	N
ATOM	3361	HH21	ARG	377	13.949	24.074	8.678	1.00	0.00	LX0	H
ATOM	3362	HH22	ARG	377	14.366	22.949	9.967	1.00	0.00	LX0	H
ATOM	3363	C	ARG	377	18.938	24.682	14.596	1.00	0.00	LX0	C
ATOM	3364	O	ARG	377	18.168	24.573	15.539	1.00	0.00	LX0	O
ATOM	3365	N	GLY	378	20.261	24.849	14.722	1.00	0.00	LX0	N
ATOM	3366	H	GLY	378	20.849	24.859	13.916	1.00	0.00	LX0	H
ATOM	3367	CA	GLY	378	20.838	24.781	16.053	1.00	0.00	LX0	C

ATOM	3368	C	GLY	378	21.472	23.435	16.312	1.00	0.00	LX0	C
ATOM	3369	O	GLY	378	20.960	22.397	15.911	1.00	0.00	LX0	O
ATOM	3370	N	ASP	379	22.627	23.491	16.982	1.00	0.00	LX0	N
ATOM	3371	H	ASP	379	23.030	24.360	17.281	1.00	0.00	LX0	H
ATOM	3372	CA	ASP	379	23.259	22.208	17.260	1.00	0.00	LX0	C
ATOM	3373	CB	ASP	379	24.616	22.108	16.554	1.00	0.00	LX0	C
ATOM	3374	CG	ASP	379	25.335	20.781	16.775	1.00	0.00	LX0	C
ATOM	3375	OD1	ASP	379	24.694	19.758	16.978	1.00	0.00	LX0	O
ATOM	3376	OD2	ASP	379	26.559	20.774	16.772	1.00	0.00	LX0	O
ATOM	3377	C	ASP	379	23.373	21.898	18.734	1.00	0.00	LX0	C
ATOM	3378	O	ASP	379	23.743	22.716	19.574	1.00	0.00	LX0	O
ATOM	3379	N	SER	380	23.047	20.625	18.984	1.00	0.00	LX0	N
ATOM	3380	H	SER	380	22.932	20.069	18.160	1.00	0.00	LX0	H
ATOM	3381	CA	SER	380	23.257	20.038	20.299	1.00	0.00	LX0	C
ATOM	3382	CB	SER	380	22.747	18.594	20.305	1.00	0.00	LX0	C
ATOM	3383	OG	SER	380	21.434	18.530	19.732	1.00	0.00	LX0	O
ATOM	3384	HG	SER	380	21.542	18.497	18.790	1.00	0.00	LX0	H
ATOM	3385	C	SER	380	24.715	20.075	20.728	1.00	0.00	LX0	C
ATOM	3386	O	SER	380	25.059	20.553	21.800	1.00	0.00	LX0	O
ATOM	3387	N	PHE	381	25.564	19.547	19.832	1.00	0.00	LX0	N
ATOM	3388	H	PHE	381	25.266	19.375	18.888	1.00	0.00	LX0	H
ATOM	3389	CA	PHE	381	26.963	19.363	20.209	1.00	0.00	LX0	C
ATOM	3390	CB	PHE	381	27.630	18.390	19.222	1.00	0.00	LX0	C
ATOM	3391	CG	PHE	381	29.097	18.173	19.533	1.00	0.00	LX0	C
ATOM	3392	CD1	PHE	381	29.496	17.732	20.816	1.00	0.00	LX0	C
ATOM	3393	CD2	PHE	381	30.047	18.429	18.521	1.00	0.00	LX0	C
ATOM	3394	CE1	PHE	381	30.867	17.567	21.094	1.00	0.00	LX0	C
ATOM	3395	CE2	PHE	381	31.417	18.258	18.796	1.00	0.00	LX0	C
ATOM	3396	CZ	PHE	381	31.814	17.835	20.082	1.00	0.00	LX0	C
ATOM	3397	C	PHE	381	27.741	20.660	20.366	1.00	0.00	LX0	C
ATOM	3398	O	PHE	381	28.484	20.857	21.318	1.00	0.00	LX0	O
ATOM	3399	N	THR	382	27.538	21.549	19.391	1.00	0.00	LX0	N
ATOM	3400	H	THR	382	26.906	21.354	18.639	1.00	0.00	LX0	H
ATOM	3401	CA	THR	382	28.305	22.788	19.481	1.00	0.00	LX0	C
ATOM	3402	CB	THR	382	28.618	23.327	18.084	1.00	0.00	LX0	C
ATOM	3403	OG1	THR	382	27.419	23.436	17.306	1.00	0.00	LX0	O
ATOM	3404	HG1	THR	382	27.234	22.553	16.983	1.00	0.00	LX0	H
ATOM	3405	CG2	THR	382	29.641	22.450	17.360	1.00	0.00	LX0	C
ATOM	3406	C	THR	382	27.691	23.869	20.357	1.00	0.00	LX0	C
ATOM	3407	O	THR	382	28.244	24.950	20.513	1.00	0.00	LX0	O
ATOM	3408	N	HIS	383	26.508	23.533	20.916	1.00	0.00	LX0	N
ATOM	3409	H	HIS	383	26.107	22.640	20.719	1.00	0.00	LX0	H
ATOM	3410	CA	HIS	383	25.785	24.475	21.777	1.00	0.00	LX0	C
ATOM	3411	CB	HIS	383	26.497	24.699	23.123	1.00	0.00	LX0	C
ATOM	3412	CG	HIS	383	26.852	23.378	23.772	1.00	0.00	LX0	C
ATOM	3413	ND1	HIS	383	26.160	22.236	23.604	1.00	0.00	LX0	N
ATOM	3414	HD1	HIS	383	25.381	22.069	23.030	1.00	0.00	LX0	H
ATOM	3415	CD2	HIS	383	27.932	23.118	24.623	1.00	0.00	LX0	C
ATOM	3416	NE2	HIS	383	27.880	21.804	24.962	1.00	0.00	LX0	N
ATOM	3417	CE1	HIS	383	26.788	21.268	24.330	1.00	0.00	LX0	C
ATOM	3418	C	HIS	383	25.468	25.780	21.071	1.00	0.00	LX0	C
ATOM	3419	O	HIS	383	25.780	26.891	21.491	1.00	0.00	LX0	O
ATOM	3420	N	THR	384	24.840	25.569	19.916	1.00	0.00	LX0	N
ATOM	3421	H	THR	384	24.574	24.654	19.602	1.00	0.00	LX0	H
ATOM	3422	CA	THR	384	24.642	26.722	19.055	1.00	0.00	LX0	C
ATOM	3423	CB	THR	384	25.418	26.476	17.762	1.00	0.00	LX0	C
ATOM	3424	OG1	THR	384	25.199	25.133	17.311	1.00	0.00	LX0	O
ATOM	3425	HG1	THR	384	26.056	24.709	17.244	1.00	0.00	LX0	H
ATOM	3426	CG2	THR	384	26.916	26.716	17.956	1.00	0.00	LX0	C
ATOM	3427	C	THR	384	23.174	27.006	18.802	1.00	0.00	LX0	C
ATOM	3428	O	THR	384	22.494	26.289	18.081	1.00	0.00	LX0	O

ATOM	3429	N	PRO	385	22.673	28.092	19.440	1.00	0.00	LX0	N
ATOM	3430	CD	PRO	385	23.330	28.915	20.446	1.00	0.00	LX0	C
ATOM	3431	CA	PRO	385	21.305	28.534	19.153	1.00	0.00	LX0	C
ATOM	3432	CB	PRO	385	21.179	29.809	20.000	1.00	0.00	LX0	C
ATOM	3433	CG	PRO	385	22.184	29.639	21.135	1.00	0.00	LX0	C
ATOM	3434	C	PRO	385	21.046	28.770	17.668	1.00	0.00	LX0	C
ATOM	3435	O	PRO	385	21.920	29.228	16.933	1.00	0.00	LX0	O
ATOM	3436	N	PRO	386	19.798	28.424	17.264	1.00	0.00	LX0	N
ATOM	3437	CD	PRO	386	18.756	27.827	18.098	1.00	0.00	LX0	C
ATOM	3438	CA	PRO	386	19.360	28.624	15.878	1.00	0.00	LX0	C
ATOM	3439	CB	PRO	386	17.857	28.345	15.959	1.00	0.00	LX0	C
ATOM	3440	CG	PRO	386	17.692	27.365	17.115	1.00	0.00	LX0	C
ATOM	3441	C	PRO	386	19.647	30.009	15.331	1.00	0.00	LX0	C
ATOM	3442	O	PRO	386	19.292	31.026	15.917	1.00	0.00	LX0	O
ATOM	3443	N	LEU	387	20.297	30.012	14.164	1.00	0.00	LX0	N
ATOM	3444	H	LEU	387	20.582	29.158	13.728	1.00	0.00	LX0	H
ATOM	3445	CA	LEU	387	20.431	31.300	13.501	1.00	0.00	LX0	C
ATOM	3446	CB	LEU	387	21.642	31.316	12.568	1.00	0.00	LX0	C
ATOM	3447	CG	LEU	387	21.996	32.718	12.075	1.00	0.00	LX0	C
ATOM	3448	CD1	LEU	387	22.150	33.707	13.232	1.00	0.00	LX0	C
ATOM	3449	CD2	LEU	387	23.224	32.692	11.170	1.00	0.00	LX0	C
ATOM	3450	C	LEU	387	19.157	31.713	12.793	1.00	0.00	LX0	C
ATOM	3451	O	LEU	387	18.856	31.307	11.676	1.00	0.00	LX0	O
ATOM	3452	N	ASP	388	18.428	32.553	13.538	1.00	0.00	LX0	N
ATOM	3453	H	ASP	388	18.733	32.713	14.477	1.00	0.00	LX0	H
ATOM	3454	CA	ASP	388	17.165	33.113	13.062	1.00	0.00	LX0	C
ATOM	3455	CB	ASP	388	16.700	34.180	14.074	1.00	0.00	LX0	C
ATOM	3456	CG	ASP	388	15.526	35.016	13.583	1.00	0.00	LX0	C
ATOM	3457	OD1	ASP	388	14.578	34.482	13.017	1.00	0.00	LX0	O
ATOM	3458	OD2	ASP	388	15.587	36.230	13.703	1.00	0.00	LX0	O
ATOM	3459	C	ASP	388	17.171	33.614	11.617	1.00	0.00	LX0	C
ATOM	3460	O	ASP	388	17.963	34.457	11.199	1.00	0.00	LX0	O
ATOM	3461	N	PRO	389	16.202	33.055	10.854	1.00	0.00	LX0	N
ATOM	3462	CD	PRO	389	15.366	31.919	11.234	1.00	0.00	LX0	C
ATOM	3463	CA	PRO	389	15.912	33.543	9.502	1.00	0.00	LX0	C
ATOM	3464	CB	PRO	389	14.733	32.666	9.068	1.00	0.00	LX0	C
ATOM	3465	CG	PRO	389	14.826	31.399	9.913	1.00	0.00	LX0	C
ATOM	3466	C	PRO	389	15.601	35.031	9.335	1.00	0.00	LX0	C
ATOM	3467	O	PRO	389	15.413	35.497	8.213	1.00	0.00	LX0	O
ATOM	3468	N	GLN	390	15.496	35.763	10.451	1.00	0.00	LX0	N
ATOM	3469	H	GLN	390	15.536	35.355	11.368	1.00	0.00	LX0	H
ATOM	3470	CA	GLN	390	15.425	37.216	10.325	1.00	0.00	LX0	C
ATOM	3471	CB	GLN	390	14.348	37.784	11.246	1.00	0.00	LX0	C
ATOM	3472	CG	GLN	390	13.038	37.003	11.127	1.00	0.00	LX0	C
ATOM	3473	CD	GLN	390	12.165	37.286	12.329	1.00	0.00	LX0	C
ATOM	3474	OE1	GLN	390	11.102	37.882	12.226	1.00	0.00	LX0	O
ATOM	3475	NE2	GLN	390	12.653	36.812	13.476	1.00	0.00	LX0	N
ATOM	3476	HE21	GLN	390	13.530	36.308	13.494	1.00	0.00	LX0	H
ATOM	3477	HE22	GLN	390	12.174	36.942	14.338	1.00	0.00	LX0	H
ATOM	3478	C	GLN	390	16.776	37.866	10.570	1.00	0.00	LX0	C
ATOM	3479	O	GLN	390	17.238	38.669	9.771	1.00	0.00	LX0	O
ATOM	3480	N	GLU	391	17.425	37.422	11.667	1.00	0.00	LX0	N
ATOM	3481	H	GLU	391	16.921	36.864	12.332	1.00	0.00	LX0	H
ATOM	3482	CA	GLU	391	18.820	37.799	11.950	1.00	0.00	LX0	C
ATOM	3483	CB	GLU	391	19.415	36.884	13.023	1.00	0.00	LX0	C
ATOM	3484	CG	GLU	391	19.150	37.317	14.466	1.00	0.00	LX0	C
ATOM	3485	CD	GLU	391	20.029	38.499	14.825	1.00	0.00	LX0	C
ATOM	3486	OE1	GLU	391	21.246	38.337	14.931	1.00	0.00	LX0	O
ATOM	3487	OE2	GLU	391	19.512	39.597	15.027	1.00	0.00	LX0	O
ATOM	3488	C	GLU	391	19.734	37.777	10.735	1.00	0.00	LX0	C
ATOM	3489	O	GLU	391	20.532	38.675	10.487	1.00	0.00	LX0	O

ATOM	3490	N	LEU	392	19.525	36.707	9.944	1.00	0.00	LX0	N
ATOM	3491	H	LEU	392	18.936	35.983	10.310	1.00	0.00	LX0	H
ATOM	3492	CA	LEU	392	20.196	36.580	8.648	1.00	0.00	LX0	C
ATOM	3493	CB	LEU	392	19.472	35.570	7.761	1.00	0.00	LX0	C
ATOM	3494	CG	LEU	392	19.730	34.113	8.133	1.00	0.00	LX0	C
ATOM	3495	CD1	LEU	392	18.893	33.179	7.264	1.00	0.00	LX0	C
ATOM	3496	CD2	LEU	392	21.215	33.759	8.058	1.00	0.00	LX0	C
ATOM	3497	C	LEU	392	20.399	37.858	7.849	1.00	0.00	LX0	C
ATOM	3498	O	LEU	392	21.484	38.126	7.352	1.00	0.00	LX0	O
ATOM	3499	N	ASP	393	19.314	38.640	7.742	1.00	0.00	LX0	N
ATOM	3500	H	ASP	393	18.489	38.441	8.274	1.00	0.00	LX0	H
ATOM	3501	CA	ASP	393	19.351	39.793	6.840	1.00	0.00	LX0	C
ATOM	3502	CB	ASP	393	17.981	40.472	6.798	1.00	0.00	LX0	C
ATOM	3503	CG	ASP	393	17.817	41.174	5.468	1.00	0.00	LX0	C
ATOM	3504	OD1	ASP	393	17.819	42.400	5.425	1.00	0.00	LX0	O
ATOM	3505	OD2	ASP	393	17.715	40.501	4.448	1.00	0.00	LX0	O
ATOM	3506	C	ASP	393	20.470	40.801	7.085	1.00	0.00	LX0	C
ATOM	3507	O	ASP	393	21.000	41.445	6.185	1.00	0.00	LX0	O
ATOM	3508	N	ILE	394	20.872	40.862	8.367	1.00	0.00	LX0	N
ATOM	3509	H	ILE	394	20.453	40.237	9.031	1.00	0.00	LX0	H
ATOM	3510	CA	ILE	394	21.975	41.756	8.738	1.00	0.00	LX0	C
ATOM	3511	CB	ILE	394	22.183	41.721	10.263	1.00	0.00	LX0	C
ATOM	3512	CG2	ILE	394	23.213	42.750	10.738	1.00	0.00	LX0	C
ATOM	3513	CG1	ILE	394	20.851	41.927	10.994	1.00	0.00	LX0	C
ATOM	3514	CD1	ILE	394	20.955	41.695	12.501	1.00	0.00	LX0	C
ATOM	3515	C	ILE	394	23.282	41.513	7.972	1.00	0.00	LX0	C
ATOM	3516	O	ILE	394	24.129	42.390	7.814	1.00	0.00	LX0	O
ATOM	3517	N	LEU	395	23.397	40.283	7.446	1.00	0.00	LX0	N
ATOM	3518	H	LEU	395	22.668	39.612	7.587	1.00	0.00	LX0	H
ATOM	3519	CA	LEU	395	24.545	39.952	6.602	1.00	0.00	LX0	C
ATOM	3520	CB	LEU	395	24.615	38.444	6.358	1.00	0.00	LX0	C
ATOM	3521	CG	LEU	395	24.759	37.642	7.657	1.00	0.00	LX0	C
ATOM	3522	CD1	LEU	395	24.550	36.145	7.426	1.00	0.00	LX0	C
ATOM	3523	CD2	LEU	395	26.075	37.937	8.381	1.00	0.00	LX0	C
ATOM	3524	C	LEU	395	24.701	40.731	5.299	1.00	0.00	LX0	C
ATOM	3525	O	LEU	395	25.702	40.600	4.610	1.00	0.00	LX0	O
ATOM	3526	N	LYS	396	23.736	41.630	5.031	1.00	0.00	LX0	N
ATOM	3527	H	LYS	396	22.849	41.553	5.490	1.00	0.00	LX0	H
ATOM	3528	CA	LYS	396	23.976	42.674	4.024	1.00	0.00	LX0	C
ATOM	3529	CB	LYS	396	22.751	43.583	3.930	1.00	0.00	LX0	C
ATOM	3530	CG	LYS	396	21.524	42.952	3.262	1.00	0.00	LX0	C
ATOM	3531	CD	LYS	396	20.317	43.889	3.360	1.00	0.00	LX0	C
ATOM	3532	CE	LYS	396	19.137	43.530	2.451	1.00	0.00	LX0	C
ATOM	3533	NZ	LYS	396	18.547	42.233	2.786	1.00	0.00	LX0	N
ATOM	3534	HZ1	LYS	396	17.822	41.960	2.094	1.00	0.00	LX0	H
ATOM	3535	HZ2	LYS	396	19.236	41.458	2.812	1.00	0.00	LX0	H
ATOM	3536	HZ3	LYS	396	18.093	42.217	3.727	1.00	0.00	LX0	H
ATOM	3537	C	LYS	396	25.228	43.521	4.271	1.00	0.00	LX0	C
ATOM	3538	O	LYS	396	25.815	44.129	3.381	1.00	0.00	LX0	O
ATOM	3539	N	THR	397	25.629	43.525	5.553	1.00	0.00	LX0	N
ATOM	3540	H	THR	397	25.101	43.027	6.239	1.00	0.00	LX0	H
ATOM	3541	CA	THR	397	26.874	44.200	5.909	1.00	0.00	LX0	C
ATOM	3542	CB	THR	397	26.918	44.427	7.421	1.00	0.00	LX0	C
ATOM	3543	OG1	THR	397	26.768	43.197	8.144	1.00	0.00	LX0	O
ATOM	3544	HG1	THR	397	25.836	42.989	8.162	1.00	0.00	LX0	H
ATOM	3545	CG2	THR	397	25.843	45.428	7.846	1.00	0.00	LX0	C
ATOM	3546	C	THR	397	28.154	43.541	5.403	1.00	0.00	LX0	C
ATOM	3547	O	THR	397	29.234	44.121	5.423	1.00	0.00	LX0	O
ATOM	3548	N	VAL	398	27.997	42.300	4.921	1.00	0.00	LX0	N
ATOM	3549	H	VAL	398	27.099	41.867	4.860	1.00	0.00	LX0	H
ATOM	3550	CA	VAL	398	29.129	41.676	4.245	1.00	0.00	LX0	C

ATOM	3551	CB	VAL	398	28.950	40.152	4.184	1.00	0.00	LX0	C
ATOM	3552	CG1	VAL	398	30.155	39.480	3.530	1.00	0.00	LX0	C
ATOM	3553	CG2	VAL	398	28.683	39.559	5.569	1.00	0.00	LX0	C
ATOM	3554	C	VAL	398	29.324	42.265	2.858	1.00	0.00	LX0	C
ATOM	3555	O	VAL	398	28.694	41.883	1.883	1.00	0.00	LX0	O
ATOM	3556	N	LYS	399	30.239	43.236	2.830	1.00	0.00	LX0	N
ATOM	3557	H	LYS	399	30.692	43.513	3.678	1.00	0.00	LX0	H
ATOM	3558	CA	LYS	399	30.594	43.812	1.537	1.00	0.00	LX0	C
ATOM	3559	CB	LYS	399	31.139	45.237	1.702	1.00	0.00	LX0	C
ATOM	3560	CG	LYS	399	30.049	46.313	1.685	1.00	0.00	LX0	C
ATOM	3561	CD	LYS	399	29.011	46.181	2.800	1.00	0.00	LX0	C
ATOM	3562	CE	LYS	399	27.613	46.594	2.348	1.00	0.00	LX0	C
ATOM	3563	NZ	LYS	399	27.118	45.672	1.321	1.00	0.00	LX0	N
ATOM	3564	HZ1	LYS	399	27.784	45.543	0.528	1.00	0.00	LX0	H
ATOM	3565	HZ2	LYS	399	26.829	44.731	1.669	1.00	0.00	LX0	H
ATOM	3566	HZ3	LYS	399	26.285	46.085	0.866	1.00	0.00	LX0	H
ATOM	3567	C	LYS	399	31.577	42.934	0.799	1.00	0.00	LX0	C
ATOM	3568	O	LYS	399	31.373	42.515	-0.335	1.00	0.00	LX0	O
ATOM	3569	N	GLU	400	32.667	42.657	1.525	1.00	0.00	LX0	N
ATOM	3570	H	GLU	400	32.767	42.995	2.462	1.00	0.00	LX0	H
ATOM	3571	CA	GLU	400	33.638	41.754	0.922	1.00	0.00	LX0	C
ATOM	3572	CB	GLU	400	35.027	42.387	0.804	1.00	0.00	LX0	C
ATOM	3573	CG	GLU	400	35.074	43.904	0.583	1.00	0.00	LX0	C
ATOM	3574	CD	GLU	400	36.501	44.322	0.280	1.00	0.00	LX0	C
ATOM	3575	OE1	GLU	400	36.758	44.851	-0.795	1.00	0.00	LX0	O
ATOM	3576	OE2	GLU	400	37.398	44.052	1.070	1.00	0.00	LX0	O
ATOM	3577	C	GLU	400	33.765	40.477	1.715	1.00	0.00	LX0	C
ATOM	3578	O	GLU	400	33.907	40.498	2.930	1.00	0.00	LX0	O
ATOM	3579	N	ILE	401	33.741	39.368	0.979	1.00	0.00	LX0	N
ATOM	3580	H	ILE	401	33.537	39.429	0.003	1.00	0.00	LX0	H
ATOM	3581	CA	ILE	401	34.275	38.131	1.535	1.00	0.00	LX0	C
ATOM	3582	CB	ILE	401	33.398	36.929	1.164	1.00	0.00	LX0	C
ATOM	3583	CG2	ILE	401	33.824	35.659	1.907	1.00	0.00	LX0	C
ATOM	3584	CG1	ILE	401	31.921	37.221	1.378	1.00	0.00	LX0	C
ATOM	3585	CD1	ILE	401	31.045	36.067	0.908	1.00	0.00	LX0	C
ATOM	3586	C	ILE	401	35.630	37.952	0.900	1.00	0.00	LX0	C
ATOM	3587	O	ILE	401	35.772	38.138	-0.304	1.00	0.00	LX0	O
ATOM	3588	N	THR	402	36.624	37.595	1.711	1.00	0.00	LX0	N
ATOM	3589	H	THR	402	36.517	37.467	2.700	1.00	0.00	LX0	H
ATOM	3590	CA	THR	402	37.868	37.333	0.998	1.00	0.00	LX0	C
ATOM	3591	CB	THR	402	39.057	37.837	1.805	1.00	0.00	LX0	C
ATOM	3592	OG1	THR	402	39.083	37.212	3.084	1.00	0.00	LX0	O
ATOM	3593	HG1	THR	402	38.463	37.697	3.621	1.00	0.00	LX0	H
ATOM	3594	CG2	THR	402	38.991	39.358	1.971	1.00	0.00	LX0	C
ATOM	3595	C	THR	402	38.023	35.889	0.547	1.00	0.00	LX0	C
ATOM	3596	O	THR	402	38.637	35.586	-0.471	1.00	0.00	LX0	O
ATOM	3597	N	GLY	403	37.398	35.006	1.332	1.00	0.00	LX0	N
ATOM	3598	H	GLY	403	36.911	35.299	2.156	1.00	0.00	LX0	H
ATOM	3599	CA	GLY	403	37.353	33.615	0.905	1.00	0.00	LX0	C
ATOM	3600	C	GLY	403	36.097	33.289	0.129	1.00	0.00	LX0	C
ATOM	3601	O	GLY	403	35.750	33.957	-0.843	1.00	0.00	LX0	O
ATOM	3602	N	PHE	404	35.436	32.231	0.604	1.00	0.00	LX0	N
ATOM	3603	H	PHE	404	35.770	31.774	1.432	1.00	0.00	LX0	H
ATOM	3604	CA	PHE	404	34.259	31.752	-0.109	1.00	0.00	LX0	C
ATOM	3605	CB	PHE	404	34.434	30.275	-0.502	1.00	0.00	LX0	C
ATOM	3606	CG	PHE	404	34.695	29.381	0.691	1.00	0.00	LX0	C
ATOM	3607	CD1	PHE	404	33.666	29.098	1.617	1.00	0.00	LX0	C
ATOM	3608	CD2	PHE	404	35.983	28.830	0.847	1.00	0.00	LX0	C
ATOM	3609	CE1	PHE	404	33.932	28.259	2.714	1.00	0.00	LX0	C
ATOM	3610	CE2	PHE	404	36.253	27.981	1.935	1.00	0.00	LX0	C
ATOM	3611	CZ	PHE	404	35.223	27.708	2.856	1.00	0.00	LX0	C

ATOM	3612	C	PHE	404	32.944	31.972	0.615	1.00	0.00	LX0	C
ATOM	3613	O	PHE	404	32.881	32.244	1.808	1.00	0.00	LX0	O
ATOM	3614	N	LEU	405	31.888	31.803	-0.186	1.00	0.00	LX0	N
ATOM	3615	H	LEU	405	32.030	31.568	-1.147	1.00	0.00	LX0	H
ATOM	3616	CA	LEU	405	30.531	31.817	0.340	1.00	0.00	LX0	C
ATOM	3617	CB	LEU	405	29.724	32.869	-0.423	1.00	0.00	LX0	C
ATOM	3618	CG	LEU	405	28.299	33.100	0.082	1.00	0.00	LX0	C
ATOM	3619	CD1	LEU	405	28.255	33.559	1.541	1.00	0.00	LX0	C
ATOM	3620	CD2	LEU	405	27.529	34.039	-0.846	1.00	0.00	LX0	C
ATOM	3621	C	LEU	405	29.909	30.441	0.188	1.00	0.00	LX0	C
ATOM	3622	O	LEU	405	29.398	30.084	-0.867	1.00	0.00	LX0	O
ATOM	3623	N	LEU	406	29.992	29.672	1.279	1.00	0.00	LX0	N
ATOM	3624	H	LEU	406	30.333	30.048	2.144	1.00	0.00	LX0	H
ATOM	3625	CA	LEU	406	29.394	28.340	1.220	1.00	0.00	LX0	C
ATOM	3626	CB	LEU	406	30.282	27.333	1.969	1.00	0.00	LX0	C
ATOM	3627	CG	LEU	406	29.757	25.889	2.051	1.00	0.00	LX0	C
ATOM	3628	CD1	LEU	406	29.616	25.212	0.688	1.00	0.00	LX0	C
ATOM	3629	CD2	LEU	406	30.581	25.048	3.023	1.00	0.00	LX0	C
ATOM	3630	C	LEU	406	27.979	28.340	1.765	1.00	0.00	LX0	C
ATOM	3631	O	LEU	406	27.736	27.977	2.909	1.00	0.00	LX0	O
ATOM	3632	N	ILE	407	27.042	28.762	0.912	1.00	0.00	LX0	N
ATOM	3633	H	ILE	407	27.267	28.898	-0.056	1.00	0.00	LX0	H
ATOM	3634	CA	ILE	407	25.674	28.610	1.398	1.00	0.00	LX0	C
ATOM	3635	CB	ILE	407	24.718	29.673	0.852	1.00	0.00	LX0	C
ATOM	3636	CG2	ILE	407	23.475	29.729	1.741	1.00	0.00	LX0	C
ATOM	3637	CG1	ILE	407	25.365	31.049	0.748	1.00	0.00	LX0	C
ATOM	3638	CD1	ILE	407	24.408	32.097	0.181	1.00	0.00	LX0	C
ATOM	3639	C	ILE	407	25.141	27.228	1.091	1.00	0.00	LX0	C
ATOM	3640	O	ILE	407	24.744	26.925	-0.025	1.00	0.00	LX0	O
ATOM	3641	N	GLN	408	25.176	26.398	2.136	1.00	0.00	LX0	N
ATOM	3642	H	GLN	408	25.507	26.727	3.022	1.00	0.00	LX0	H
ATOM	3643	CA	GLN	408	24.776	25.013	1.917	1.00	0.00	LX0	C
ATOM	3644	CB	GLN	408	25.663	24.080	2.738	1.00	0.00	LX0	C
ATOM	3645	CG	GLN	408	26.198	22.922	1.903	1.00	0.00	LX0	C
ATOM	3646	CD	GLN	408	25.743	21.590	2.461	1.00	0.00	LX0	C
ATOM	3647	OE1	GLN	408	26.536	20.802	2.975	1.00	0.00	LX0	O
ATOM	3648	NE2	GLN	408	24.437	21.346	2.303	1.00	0.00	LX0	N
ATOM	3649	HE21	GLN	408	23.841	22.005	1.829	1.00	0.00	LX0	H
ATOM	3650	HE22	GLN	408	23.994	20.504	2.606	1.00	0.00	LX0	H
ATOM	3651	C	GLN	408	23.304	24.700	2.127	1.00	0.00	LX0	C
ATOM	3652	O	GLN	408	22.795	23.699	1.637	1.00	0.00	LX0	O
ATOM	3653	N	ALA	409	22.646	25.588	2.887	1.00	0.00	LX0	N
ATOM	3654	H	ALA	409	23.116	26.370	3.298	1.00	0.00	LX0	H
ATOM	3655	CA	ALA	409	21.192	25.495	3.007	1.00	0.00	LX0	C
ATOM	3656	CB	ALA	409	20.751	24.552	4.122	1.00	0.00	LX0	C
ATOM	3657	C	ALA	409	20.620	26.863	3.289	1.00	0.00	LX0	C
ATOM	3658	O	ALA	409	21.354	27.794	3.591	1.00	0.00	LX0	O
ATOM	3659	N	TRP	410	19.290	26.946	3.156	1.00	0.00	LX0	N
ATOM	3660	H	TRP	410	18.739	26.139	2.943	1.00	0.00	LX0	H
ATOM	3661	CA	TRP	410	18.631	28.240	3.305	1.00	0.00	LX0	C
ATOM	3662	CB	TRP	410	18.604	28.944	1.946	1.00	0.00	LX0	C
ATOM	3663	CG	TRP	410	18.691	30.446	2.083	1.00	0.00	LX0	C
ATOM	3664	CD2	TRP	410	19.654	31.247	2.806	1.00	0.00	LX0	C
ATOM	3665	CE2	TRP	410	19.319	32.623	2.572	1.00	0.00	LX0	C
ATOM	3666	CE3	TRP	410	20.760	30.925	3.622	1.00	0.00	LX0	C
ATOM	3667	CD1	TRP	410	17.839	31.369	1.467	1.00	0.00	LX0	C
ATOM	3668	NE1	TRP	410	18.198	32.647	1.744	1.00	0.00	LX0	N
ATOM	3669	HE1	TRP	410	17.737	33.435	1.380	1.00	0.00	LX0	H
ATOM	3670	CZ2	TRP	410	20.105	33.642	3.152	1.00	0.00	LX0	C
ATOM	3671	CZ3	TRP	410	21.535	31.953	4.198	1.00	0.00	LX0	C
ATOM	3672	CH2	TRP	410	21.209	33.305	3.963	1.00	0.00	LX0	C

ATOM	3673	C	TRP	410	17.229	28.001	3.824	1.00	0.00	LX0	C
ATOM	3674	O	TRP	410	16.525	27.159	3.282	1.00	0.00	LX0	O
ATOM	3675	N	PRO	411	16.865	28.720	4.912	1.00	0.00	LX0	N
ATOM	3676	CD	PRO	411	17.596	29.830	5.510	1.00	0.00	LX0	C
ATOM	3677	CA	PRO	411	15.655	28.372	5.668	1.00	0.00	LX0	C
ATOM	3678	CB	PRO	411	15.647	29.374	6.821	1.00	0.00	LX0	C
ATOM	3679	CG	PRO	411	17.071	29.896	6.934	1.00	0.00	LX0	C
ATOM	3680	C	PRO	411	14.354	28.436	4.896	1.00	0.00	LX0	C
ATOM	3681	O	PRO	411	13.712	29.476	4.829	1.00	0.00	LX0	O
ATOM	3682	N	GLU	412	13.990	27.267	4.359	1.00	0.00	LX0	N
ATOM	3683	H	GLU	412	14.640	26.520	4.517	1.00	0.00	LX0	H
ATOM	3684	CA	GLU	412	12.645	26.941	3.884	1.00	0.00	LX0	C
ATOM	3685	CB	GLU	412	11.879	26.185	4.985	1.00	0.00	LX0	C
ATOM	3686	CG	GLU	412	12.534	24.878	5.479	1.00	0.00	LX0	C
ATOM	3687	CD	GLU	412	13.796	25.132	6.294	1.00	0.00	LX0	C
ATOM	3688	OE1	GLU	412	13.698	25.512	7.454	1.00	0.00	LX0	O
ATOM	3689	OE2	GLU	412	14.897	24.965	5.776	1.00	0.00	LX0	O
ATOM	3690	C	GLU	412	11.844	28.084	3.271	1.00	0.00	LX0	C
ATOM	3691	O	GLU	412	11.836	28.277	2.062	1.00	0.00	LX0	O
ATOM	3692	N	ASN	413	11.192	28.868	4.142	1.00	0.00	LX0	N
ATOM	3693	H	ASN	413	11.331	28.723	5.121	1.00	0.00	LX0	H
ATOM	3694	CA	ASN	413	10.517	30.061	3.624	1.00	0.00	LX0	C
ATOM	3695	CB	ASN	413	9.324	30.461	4.498	1.00	0.00	LX0	C
ATOM	3696	CG	ASN	413	8.298	29.350	4.537	1.00	0.00	LX0	C
ATOM	3697	OD1	ASN	413	8.308	28.495	5.408	1.00	0.00	LX0	O
ATOM	3698	ND2	ASN	413	7.394	29.395	3.555	1.00	0.00	LX0	N
ATOM	3699	HD21	ASN	413	7.397	30.099	2.849	1.00	0.00	LX0	H
ATOM	3700	HD22	ASN	413	6.703	28.674	3.554	1.00	0.00	LX0	H
ATOM	3701	C	ASN	413	11.440	31.257	3.443	1.00	0.00	LX0	C
ATOM	3702	O	ASN	413	11.247	32.326	4.023	1.00	0.00	LX0	O
ATOM	3703	N	ARG	414	12.455	31.033	2.599	1.00	0.00	LX0	N
ATOM	3704	H	ARG	414	12.559	30.117	2.204	1.00	0.00	LX0	H
ATOM	3705	CA	ARG	414	13.395	32.089	2.237	1.00	0.00	LX0	C
ATOM	3706	CB	ARG	414	14.678	32.047	3.074	1.00	0.00	LX0	C
ATOM	3707	CG	ARG	414	14.531	32.632	4.480	1.00	0.00	LX0	C
ATOM	3708	CD	ARG	414	13.982	34.059	4.426	1.00	0.00	LX0	C
ATOM	3709	NE	ARG	414	13.960	34.687	5.744	1.00	0.00	LX0	N
ATOM	3710	HE	ARG	414	14.817	35.088	6.096	1.00	0.00	LX0	H
ATOM	3711	CZ	ARG	414	12.815	34.754	6.452	1.00	0.00	LX0	C
ATOM	3712	NH1	ARG	414	11.706	34.145	6.025	1.00	0.00	LX0	N
ATOM	3713	HH11	ARG	414	10.841	34.205	6.519	1.00	0.00	LX0	H
ATOM	3714	HH12	ARG	414	11.714	33.589	5.182	1.00	0.00	LX0	H
ATOM	3715	NH2	ARG	414	12.818	35.441	7.590	1.00	0.00	LX0	N
ATOM	3716	HH21	ARG	414	12.010	35.568	8.164	1.00	0.00	LX0	H
ATOM	3717	HH22	ARG	414	13.691	35.848	7.888	1.00	0.00	LX0	H
ATOM	3718	C	ARG	414	13.730	32.018	0.766	1.00	0.00	LX0	C
ATOM	3719	O	ARG	414	14.782	31.563	0.341	1.00	0.00	LX0	O
ATOM	3720	N	THR	415	12.758	32.509	-0.003	1.00	0.00	LX0	N
ATOM	3721	H	THR	415	11.884	32.812	0.377	1.00	0.00	LX0	H
ATOM	3722	CA	THR	415	12.855	32.391	-1.455	1.00	0.00	LX0	C
ATOM	3723	CB	THR	415	11.476	32.684	-2.047	1.00	0.00	LX0	C
ATOM	3724	OG1	THR	415	10.469	32.615	-1.020	1.00	0.00	LX0	O
ATOM	3725	HG1	THR	415	9.634	32.711	-1.464	1.00	0.00	LX0	H
ATOM	3726	CG2	THR	415	11.141	31.728	-3.193	1.00	0.00	LX0	C
ATOM	3727	C	THR	415	13.939	33.214	-2.149	1.00	0.00	LX0	C
ATOM	3728	O	THR	415	14.235	33.016	-3.324	1.00	0.00	LX0	O
ATOM	3729	N	ASP	416	14.494	34.155	-1.370	1.00	0.00	LX0	N
ATOM	3730	H	ASP	416	14.281	34.208	-0.398	1.00	0.00	LX0	H
ATOM	3731	CA	ASP	416	15.528	35.043	-1.892	1.00	0.00	LX0	C
ATOM	3732	CB	ASP	416	14.956	36.466	-2.017	1.00	0.00	LX0	C
ATOM	3733	CG	ASP	416	15.886	37.401	-2.775	1.00	0.00	LX0	C

ATOM	3734	OD1	ASP	416	15.639	37.675	-3.940	1.00	0.00	LX0	O
ATOM	3735	OD2	ASP	416	16.855	37.882	-2.201	1.00	0.00	LX0	O
ATOM	3736	C	ASP	416	16.724	34.993	-0.958	1.00	0.00	LX0	C
ATOM	3737	O	ASP	416	16.616	34.613	0.209	1.00	0.00	LX0	O
ATOM	3738	N	LEU	417	17.881	35.381	-1.507	1.00	0.00	LX0	N
ATOM	3739	H	LEU	417	17.840	35.868	-2.382	1.00	0.00	LX0	H
ATOM	3740	CA	LEU	417	19.061	35.359	-0.650	1.00	0.00	LX0	C
ATOM	3741	CB	LEU	417	20.365	35.376	-1.451	1.00	0.00	LX0	C
ATOM	3742	CG	LEU	417	20.813	33.963	-1.838	1.00	0.00	LX0	C
ATOM	3743	CD1	LEU	417	22.197	33.951	-2.483	1.00	0.00	LX0	C
ATOM	3744	CD2	LEU	417	20.784	33.014	-0.643	1.00	0.00	LX0	C
ATOM	3745	C	LEU	417	19.117	36.379	0.471	1.00	0.00	LX0	C
ATOM	3746	O	LEU	417	19.943	36.235	1.359	1.00	0.00	LX0	O
ATOM	3747	N	HIS	418	18.206	37.372	0.415	1.00	0.00	LX0	N
ATOM	3748	H	HIS	418	17.646	37.431	-0.415	1.00	0.00	LX0	H
ATOM	3749	CA	HIS	418	17.995	38.408	1.442	1.00	0.00	LX0	C
ATOM	3750	CB	HIS	418	16.718	38.138	2.268	1.00	0.00	LX0	C
ATOM	3751	CG	HIS	418	16.882	37.012	3.261	1.00	0.00	LX0	C
ATOM	3752	ND1	HIS	418	17.141	35.742	2.910	1.00	0.00	LX0	N
ATOM	3753	HD1	HIS	418	17.194	35.391	1.993	1.00	0.00	LX0	H
ATOM	3754	CD2	HIS	418	16.821	37.094	4.656	1.00	0.00	LX0	C
ATOM	3755	NE2	HIS	418	17.055	35.849	5.139	1.00	0.00	LX0	N
ATOM	3756	CE1	HIS	418	17.256	35.023	4.067	1.00	0.00	LX0	C
ATOM	3757	C	HIS	418	19.192	38.918	2.252	1.00	0.00	LX0	C
ATOM	3758	O	HIS	418	19.688	40.020	2.030	1.00	0.00	LX0	O
ATOM	3759	N	ALA	419	19.700	38.063	3.153	1.00	0.00	LX0	N
ATOM	3760	H	ALA	419	19.268	37.167	3.239	1.00	0.00	LX0	H
ATOM	3761	CA	ALA	419	20.979	38.322	3.812	1.00	0.00	LX0	C
ATOM	3762	CB	ALA	419	21.473	37.036	4.471	1.00	0.00	LX0	C
ATOM	3763	C	ALA	419	22.065	38.795	2.860	1.00	0.00	LX0	C
ATOM	3764	O	ALA	419	22.753	39.783	3.067	1.00	0.00	LX0	O
ATOM	3765	N	PHE	420	22.171	38.038	1.764	1.00	0.00	LX0	N
ATOM	3766	H	PHE	420	21.544	37.272	1.618	1.00	0.00	LX0	H
ATOM	3767	CA	PHE	420	23.255	38.363	0.847	1.00	0.00	LX0	C
ATOM	3768	CB	PHE	420	23.994	37.083	0.454	1.00	0.00	LX0	C
ATOM	3769	CG	PHE	420	24.551	36.421	1.694	1.00	0.00	LX0	C
ATOM	3770	CD1	PHE	420	25.603	37.040	2.406	1.00	0.00	LX0	C
ATOM	3771	CD2	PHE	420	24.001	35.195	2.125	1.00	0.00	LX0	C
ATOM	3772	CE1	PHE	420	26.118	36.424	3.562	1.00	0.00	LX0	C
ATOM	3773	CE2	PHE	420	24.512	34.579	3.283	1.00	0.00	LX0	C
ATOM	3774	CZ	PHE	420	25.566	35.198	3.988	1.00	0.00	LX0	C
ATOM	3775	C	PHE	420	22.875	39.204	-0.358	1.00	0.00	LX0	C
ATOM	3776	O	PHE	420	23.584	39.252	-1.354	1.00	0.00	LX0	O
ATOM	3777	N	GLU	421	21.732	39.907	-0.228	1.00	0.00	LX0	N
ATOM	3778	H	GLU	421	21.174	39.832	0.599	1.00	0.00	LX0	H
ATOM	3779	CA	GLU	421	21.357	40.806	-1.327	1.00	0.00	LX0	C
ATOM	3780	CB	GLU	421	20.086	41.582	-1.019	1.00	0.00	LX0	C
ATOM	3781	CG	GLU	421	18.826	40.743	-0.937	1.00	0.00	LX0	C
ATOM	3782	CD	GLU	421	17.670	41.674	-0.661	1.00	0.00	LX0	C
ATOM	3783	OE1	GLU	421	17.200	42.306	-1.592	1.00	0.00	LX0	O
ATOM	3784	OE2	GLU	421	17.243	41.794	0.477	1.00	0.00	LX0	O
ATOM	3785	C	GLU	421	22.408	41.851	-1.625	1.00	0.00	LX0	C
ATOM	3786	O	GLU	421	22.810	42.125	-2.753	1.00	0.00	LX0	O
ATOM	3787	N	ASN	422	22.821	42.449	-0.504	1.00	0.00	LX0	N
ATOM	3788	H	ASN	422	22.527	42.096	0.384	1.00	0.00	LX0	H
ATOM	3789	CA	ASN	422	23.796	43.519	-0.619	1.00	0.00	LX0	C
ATOM	3790	CB	ASN	422	23.312	44.738	0.180	1.00	0.00	LX0	C
ATOM	3791	CG	ASN	422	24.217	45.937	-0.029	1.00	0.00	LX0	C
ATOM	3792	OD1	ASN	422	24.675	46.563	0.917	1.00	0.00	LX0	O
ATOM	3793	ND2	ASN	422	24.537	46.188	-1.296	1.00	0.00	LX0	N
ATOM	3794	HD21	ASN	422	24.066	45.788	-2.085	1.00	0.00	LX0	H

ATOM	3795	HD22	ASN	422	25.348	46.750	-1.480	1.00	0.00	LX0	H
ATOM	3796	C	ASN	422	25.191	43.043	-0.266	1.00	0.00	LX0	C
ATOM	3797	O	ASN	422	25.886	43.608	0.567	1.00	0.00	LX0	O
ATOM	3798	N	LEU	423	25.528	41.943	-0.951	1.00	0.00	LX0	N
ATOM	3799	H	LEU	423	24.906	41.553	-1.630	1.00	0.00	LX0	H
ATOM	3800	CA	LEU	423	26.899	41.452	-0.929	1.00	0.00	LX0	C
ATOM	3801	CB	LEU	423	26.879	39.928	-0.802	1.00	0.00	LX0	C
ATOM	3802	CG	LEU	423	28.039	39.242	-0.069	1.00	0.00	LX0	C
ATOM	3803	CD1	LEU	423	27.864	37.730	-0.137	1.00	0.00	LX0	C
ATOM	3804	CD2	LEU	423	29.434	39.626	-0.555	1.00	0.00	LX0	C
ATOM	3805	C	LEU	423	27.502	41.846	-2.259	1.00	0.00	LX0	C
ATOM	3806	O	LEU	423	26.882	41.657	-3.301	1.00	0.00	LX0	O
ATOM	3807	N	GLU	424	28.710	42.401	-2.189	1.00	0.00	LX0	N
ATOM	3808	H	GLU	424	29.103	42.649	-1.300	1.00	0.00	LX0	H
ATOM	3809	CA	GLU	424	29.286	42.887	-3.433	1.00	0.00	LX0	C
ATOM	3810	CB	GLU	424	29.692	44.366	-3.306	1.00	0.00	LX0	C
ATOM	3811	CG	GLU	424	28.570	45.389	-3.023	1.00	0.00	LX0	C
ATOM	3812	CD	GLU	424	28.157	45.486	-1.555	1.00	0.00	LX0	C
ATOM	3813	OE1	GLU	424	28.611	44.706	-0.728	1.00	0.00	LX0	O
ATOM	3814	OE2	GLU	424	27.379	46.370	-1.207	1.00	0.00	LX0	O
ATOM	3815	C	GLU	424	30.445	42.049	-3.947	1.00	0.00	LX0	C
ATOM	3816	O	GLU	424	30.666	41.920	-5.149	1.00	0.00	LX0	O
ATOM	3817	N	ILE	425	31.215	41.504	-2.995	1.00	0.00	LX0	N
ATOM	3818	H	ILE	425	30.986	41.612	-2.024	1.00	0.00	LX0	H
ATOM	3819	CA	ILE	425	32.510	40.937	-3.376	1.00	0.00	LX0	C
ATOM	3820	CB	ILE	425	33.613	41.926	-2.959	1.00	0.00	LX0	C
ATOM	3821	CG2	ILE	425	35.005	41.383	-3.262	1.00	0.00	LX0	C
ATOM	3822	CG1	ILE	425	33.397	43.311	-3.576	1.00	0.00	LX0	C
ATOM	3823	CD1	ILE	425	34.194	44.439	-2.929	1.00	0.00	LX0	C
ATOM	3824	C	ILE	425	32.764	39.569	-2.755	1.00	0.00	LX0	C
ATOM	3825	O	ILE	425	32.554	39.373	-1.567	1.00	0.00	LX0	O
ATOM	3826	N	ILE	426	33.251	38.638	-3.594	1.00	0.00	LX0	N
ATOM	3827	H	ILE	426	33.341	38.851	-4.567	1.00	0.00	LX0	H
ATOM	3828	CA	ILE	426	33.746	37.349	-3.102	1.00	0.00	LX0	C
ATOM	3829	CB	ILE	426	32.741	36.218	-3.392	1.00	0.00	LX0	C
ATOM	3830	CG2	ILE	426	33.198	34.890	-2.779	1.00	0.00	LX0	C
ATOM	3831	CG1	ILE	426	31.329	36.583	-2.924	1.00	0.00	LX0	C
ATOM	3832	CD1	ILE	426	30.269	35.564	-3.324	1.00	0.00	LX0	C
ATOM	3833	C	ILE	426	35.097	37.035	-3.738	1.00	0.00	LX0	C
ATOM	3834	O	ILE	426	35.208	36.831	-4.942	1.00	0.00	LX0	O
ATOM	3835	N	ARG	427	36.131	37.027	-2.884	1.00	0.00	LX0	N
ATOM	3836	H	ARG	427	35.971	37.144	-1.904	1.00	0.00	LX0	H
ATOM	3837	CA	ARG	427	37.474	36.964	-3.460	1.00	0.00	LX0	C
ATOM	3838	CB	ARG	427	38.460	37.887	-2.742	1.00	0.00	LX0	C
ATOM	3839	CG	ARG	427	38.022	39.334	-2.877	1.00	0.00	LX0	C
ATOM	3840	CD	ARG	427	39.165	40.340	-2.827	1.00	0.00	LX0	C
ATOM	3841	NE	ARG	427	38.698	41.603	-3.387	1.00	0.00	LX0	N
ATOM	3842	HE	ARG	427	38.573	41.698	-4.380	1.00	0.00	LX0	H
ATOM	3843	CZ	ARG	427	38.168	42.560	-2.609	1.00	0.00	LX0	C
ATOM	3844	NH1	ARG	427	38.291	42.497	-1.286	1.00	0.00	LX0	N
ATOM	3845	HH11	ARG	427	37.817	43.174	-0.704	1.00	0.00	LX0	H
ATOM	3846	HH12	ARG	427	38.856	41.814	-0.823	1.00	0.00	LX0	H
ATOM	3847	NH2	ARG	427	37.511	43.561	-3.181	1.00	0.00	LX0	N
ATOM	3848	HH21	ARG	427	37.141	44.303	-2.610	1.00	0.00	LX0	H
ATOM	3849	HH22	ARG	427	37.356	43.575	-4.182	1.00	0.00	LX0	H
ATOM	3850	C	ARG	427	38.121	35.613	-3.665	1.00	0.00	LX0	C
ATOM	3851	O	ARG	427	39.168	35.523	-4.300	1.00	0.00	LX0	O
ATOM	3852	N	GLY	428	37.485	34.570	-3.108	1.00	0.00	LX0	N
ATOM	3853	H	GLY	428	36.698	34.722	-2.508	1.00	0.00	LX0	H
ATOM	3854	CA	GLY	428	37.945	33.207	-3.395	1.00	0.00	LX0	C
ATOM	3855	C	GLY	428	39.411	32.898	-3.119	1.00	0.00	LX0	C

ATOM	3856	O	GLY	428	40.027	32.047	-3.758	1.00	0.00	LX0	O
ATOM	3857	N	ARG	429	39.949	33.630	-2.131	1.00	0.00	LX0	N
ATOM	3858	H	ARG	429	39.362	34.291	-1.659	1.00	0.00	LX0	H
ATOM	3859	CA	ARG	429	41.343	33.419	-1.740	1.00	0.00	LX0	C
ATOM	3860	CB	ARG	429	41.818	34.569	-0.858	1.00	0.00	LX0	C
ATOM	3861	CG	ARG	429	41.837	35.907	-1.592	1.00	0.00	LX0	C
ATOM	3862	CD	ARG	429	42.240	37.054	-0.675	1.00	0.00	LX0	C
ATOM	3863	NE	ARG	429	42.382	38.295	-1.427	1.00	0.00	LX0	N
ATOM	3864	HE	ARG	429	42.692	38.250	-2.377	1.00	0.00	LX0	H
ATOM	3865	CZ	ARG	429	42.178	39.505	-0.872	1.00	0.00	LX0	C
ATOM	3866	NH1	ARG	429	41.799	39.632	0.386	1.00	0.00	LX0	N
ATOM	3867	HH11	ARG	429	41.580	40.541	0.774	1.00	0.00	LX0	H
ATOM	3868	HH12	ARG	429	41.754	38.852	1.017	1.00	0.00	LX0	H
ATOM	3869	NH2	ARG	429	42.368	40.597	-1.595	1.00	0.00	LX0	N
ATOM	3870	HH21	ARG	429	42.158	41.493	-1.184	1.00	0.00	LX0	H
ATOM	3871	HH22	ARG	429	42.720	40.530	-2.534	1.00	0.00	LX0	H
ATOM	3872	C	ARG	429	41.557	32.093	-1.036	1.00	0.00	LX0	C
ATOM	3873	O	ARG	429	42.565	31.410	-1.193	1.00	0.00	LX0	O
ATOM	3874	N	THR	430	40.524	31.759	-0.264	1.00	0.00	LX0	N
ATOM	3875	H	THR	430	39.744	32.376	-0.164	1.00	0.00	LX0	H
ATOM	3876	CA	THR	430	40.332	30.368	0.112	1.00	0.00	LX0	C
ATOM	3877	CB	THR	430	40.259	30.217	1.653	1.00	0.00	LX0	C
ATOM	3878	OG1	THR	430	39.321	29.219	2.063	1.00	0.00	LX0	O
ATOM	3879	HG1	THR	430	38.685	29.654	2.654	1.00	0.00	LX0	H
ATOM	3880	CG2	THR	430	40.073	31.542	2.394	1.00	0.00	LX0	C
ATOM	3881	C	THR	430	39.106	29.907	-0.657	1.00	0.00	LX0	C
ATOM	3882	O	THR	430	38.319	30.728	-1.122	1.00	0.00	LX0	O
ATOM	3883	N	LYS	431	39.053	28.585	-0.877	1.00	0.00	LX0	N
ATOM	3884	H	LYS	431	39.614	27.970	-0.324	1.00	0.00	LX0	H
ATOM	3885	CA	LYS	431	38.069	28.053	-1.817	1.00	0.00	LX0	C
ATOM	3886	CB	LYS	431	38.666	27.788	-3.206	1.00	0.00	LX0	C
ATOM	3887	CG	LYS	431	39.610	28.832	-3.807	1.00	0.00	LX0	C
ATOM	3888	CD	LYS	431	40.110	28.421	-5.196	1.00	0.00	LX0	C
ATOM	3889	CE	LYS	431	41.319	29.228	-5.681	1.00	0.00	LX0	C
ATOM	3890	NZ	LYS	431	40.986	30.647	-5.812	1.00	0.00	LX0	N
ATOM	3891	HZ1	LYS	431	41.834	31.246	-5.913	1.00	0.00	LX0	H
ATOM	3892	HZ2	LYS	431	40.475	30.882	-6.689	1.00	0.00	LX0	H
ATOM	3893	HZ3	LYS	431	40.465	31.036	-5.002	1.00	0.00	LX0	H
ATOM	3894	C	LYS	431	37.543	26.729	-1.305	1.00	0.00	LX0	C
ATOM	3895	O	LYS	431	38.320	25.856	-0.932	1.00	0.00	LX0	O
ATOM	3896	N	GLN	432	36.210	26.581	-1.310	1.00	0.00	LX0	N
ATOM	3897	H	GLN	432	35.641	27.304	-1.707	1.00	0.00	LX0	H
ATOM	3898	CA	GLN	432	35.690	25.295	-0.847	1.00	0.00	LX0	C
ATOM	3899	CB	GLN	432	34.169	25.304	-0.683	1.00	0.00	LX0	C
ATOM	3900	CG	GLN	432	33.713	25.295	0.781	1.00	0.00	LX0	C
ATOM	3901	CD	GLN	432	34.290	24.110	1.539	1.00	0.00	LX0	C
ATOM	3902	OE1	GLN	432	34.624	23.072	0.980	1.00	0.00	LX0	O
ATOM	3903	NE2	GLN	432	34.422	24.314	2.850	1.00	0.00	LX0	N
ATOM	3904	HE21	GLN	432	34.179	25.173	3.304	1.00	0.00	LX0	H
ATOM	3905	HE22	GLN	432	34.790	23.589	3.430	1.00	0.00	LX0	H
ATOM	3906	C	GLN	432	36.102	24.149	-1.743	1.00	0.00	LX0	C
ATOM	3907	O	GLN	432	36.094	24.271	-2.961	1.00	0.00	LX0	O
ATOM	3908	N	HIS	433	36.538	23.062	-1.086	1.00	0.00	LX0	N
ATOM	3909	H	HIS	433	36.399	23.045	-0.094	1.00	0.00	LX0	H
ATOM	3910	CA	HIS	433	37.147	21.930	-1.798	1.00	0.00	LX0	C
ATOM	3911	CB	HIS	433	36.094	21.105	-2.565	1.00	0.00	LX0	C
ATOM	3912	CG	HIS	433	35.216	20.259	-1.662	1.00	0.00	LX0	C
ATOM	3913	ND1	HIS	433	34.767	20.622	-0.445	1.00	0.00	LX0	N
ATOM	3914	HD1	HIS	433	34.916	21.471	0.024	1.00	0.00	LX0	H
ATOM	3915	CD2	HIS	433	34.718	18.982	-1.946	1.00	0.00	LX0	C
ATOM	3916	NE2	HIS	433	33.968	18.582	-0.888	1.00	0.00	LX0	N

ATOM	3917	CE1	HIS	433	34.002	19.594	0.037	1.00	0.00	LX0	C
ATOM	3918	C	HIS	433	38.305	22.301	-2.727	1.00	0.00	LX0	C
ATOM	3919	O	HIS	433	38.596	21.618	-3.700	1.00	0.00	LX0	O
ATOM	3920	N	GLY	434	38.957	23.433	-2.397	1.00	0.00	LX0	N
ATOM	3921	H	GLY	434	38.680	23.975	-1.602	1.00	0.00	LX0	H
ATOM	3922	CA	GLY	434	40.027	23.916	-3.275	1.00	0.00	LX0	C
ATOM	3923	C	GLY	434	39.570	24.444	-4.631	1.00	0.00	LX0	C
ATOM	3924	O	GLY	434	40.347	24.592	-5.564	1.00	0.00	LX0	O
ATOM	3925	N	GLN	435	38.263	24.721	-4.699	1.00	0.00	LX0	N
ATOM	3926	H	GLN	435	37.695	24.624	-3.885	1.00	0.00	LX0	H
ATOM	3927	CA	GLN	435	37.663	24.984	-6.001	1.00	0.00	LX0	C
ATOM	3928	CB	GLN	435	36.915	23.716	-6.434	1.00	0.00	LX0	C
ATOM	3929	CG	GLN	435	37.069	23.284	-7.896	1.00	0.00	LX0	C
ATOM	3930	CD	GLN	435	36.343	24.218	-8.842	1.00	0.00	LX0	C
ATOM	3931	OE1	GLN	435	35.180	24.573	-8.670	1.00	0.00	LX0	O
ATOM	3932	NE2	GLN	435	37.086	24.575	-9.889	1.00	0.00	LX0	N
ATOM	3933	HE21	GLN	435	38.033	24.279	-9.994	1.00	0.00	LX0	H
ATOM	3934	HE22	GLN	435	36.667	25.156	-10.591	1.00	0.00	LX0	H
ATOM	3935	C	GLN	435	36.755	26.204	-5.994	1.00	0.00	LX0	C
ATOM	3936	O	GLN	435	36.945	27.175	-6.717	1.00	0.00	LX0	O
ATOM	3937	N	PHE	436	35.736	26.097	-5.135	1.00	0.00	LX0	N
ATOM	3938	H	PHE	436	35.719	25.355	-4.466	1.00	0.00	LX0	H
ATOM	3939	CA	PHE	436	34.618	27.031	-5.236	1.00	0.00	LX0	C
ATOM	3940	CB	PHE	436	33.314	26.382	-4.755	1.00	0.00	LX0	C
ATOM	3941	CG	PHE	436	33.244	24.896	-5.039	1.00	0.00	LX0	C
ATOM	3942	CD1	PHE	436	33.142	24.007	-3.948	1.00	0.00	LX0	C
ATOM	3943	CD2	PHE	436	33.262	24.417	-6.368	1.00	0.00	LX0	C
ATOM	3944	CE1	PHE	436	33.059	22.622	-4.183	1.00	0.00	LX0	C
ATOM	3945	CE2	PHE	436	33.179	23.031	-6.605	1.00	0.00	LX0	C
ATOM	3946	CZ	PHE	436	33.080	22.147	-5.511	1.00	0.00	LX0	C
ATOM	3947	C	PHE	436	34.795	28.313	-4.451	1.00	0.00	LX0	C
ATOM	3948	O	PHE	436	35.321	28.305	-3.346	1.00	0.00	LX0	O
ATOM	3949	N	SER	437	34.280	29.401	-5.030	1.00	0.00	LX0	N
ATOM	3950	H	SER	437	33.962	29.388	-5.979	1.00	0.00	LX0	H
ATOM	3951	CA	SER	437	34.015	30.540	-4.155	1.00	0.00	LX0	C
ATOM	3952	CB	SER	437	34.641	31.813	-4.713	1.00	0.00	LX0	C
ATOM	3953	OG	SER	437	34.322	31.921	-6.105	1.00	0.00	LX0	O
ATOM	3954	HG	SER	437	34.907	32.595	-6.457	1.00	0.00	LX0	H
ATOM	3955	C	SER	437	32.528	30.718	-3.926	1.00	0.00	LX0	C
ATOM	3956	O	SER	437	32.024	30.668	-2.811	1.00	0.00	LX0	O
ATOM	3957	N	LEU	438	31.836	30.904	-5.056	1.00	0.00	LX0	N
ATOM	3958	H	LEU	438	32.345	30.962	-5.918	1.00	0.00	LX0	H
ATOM	3959	CA	LEU	438	30.384	31.044	-4.988	1.00	0.00	LX0	C
ATOM	3960	CB	LEU	438	29.876	31.774	-6.229	1.00	0.00	LX0	C
ATOM	3961	CG	LEU	438	29.167	33.091	-5.924	1.00	0.00	LX0	C
ATOM	3962	CD1	LEU	438	28.615	33.721	-7.198	1.00	0.00	LX0	C
ATOM	3963	CD2	LEU	438	28.079	32.943	-4.861	1.00	0.00	LX0	C
ATOM	3964	C	LEU	438	29.656	29.723	-4.829	1.00	0.00	LX0	C
ATOM	3965	O	LEU	438	29.086	29.189	-5.772	1.00	0.00	LX0	O
ATOM	3966	N	ALA	439	29.718	29.204	-3.602	1.00	0.00	LX0	N
ATOM	3967	H	ALA	439	30.098	29.727	-2.834	1.00	0.00	LX0	H
ATOM	3968	CA	ALA	439	29.101	27.903	-3.385	1.00	0.00	LX0	C
ATOM	3969	CB	ALA	439	29.965	27.067	-2.442	1.00	0.00	LX0	C
ATOM	3970	C	ALA	439	27.684	28.005	-2.856	1.00	0.00	LX0	C
ATOM	3971	O	ALA	439	27.397	27.772	-1.688	1.00	0.00	LX0	O
ATOM	3972	N	VAL	440	26.805	28.386	-3.788	1.00	0.00	LX0	N
ATOM	3973	H	VAL	440	27.109	28.411	-4.743	1.00	0.00	LX0	H
ATOM	3974	CA	VAL	440	25.390	28.430	-3.429	1.00	0.00	LX0	C
ATOM	3975	CB	VAL	440	24.734	29.671	-4.052	1.00	0.00	LX0	C
ATOM	3976	CG1	VAL	440	23.210	29.703	-3.932	1.00	0.00	LX0	C
ATOM	3977	CG2	VAL	440	25.351	30.931	-3.449	1.00	0.00	LX0	C

ATOM	3978	C	VAL	440	24.725	27.134	-3.849	1.00	0.00	LX0	C
ATOM	3979	O	VAL	440	24.401	26.899	-5.011	1.00	0.00	LX0	O
ATOM	3980	N	VAL	441	24.600	26.274	-2.841	1.00	0.00	LX0	N
ATOM	3981	H	VAL	441	24.790	26.548	-1.896	1.00	0.00	LX0	H
ATOM	3982	CA	VAL	441	24.206	24.908	-3.147	1.00	0.00	LX0	C
ATOM	3983	CB	VAL	441	25.411	23.963	-3.034	1.00	0.00	LX0	C
ATOM	3984	CG1	VAL	441	26.325	24.070	-4.256	1.00	0.00	LX0	C
ATOM	3985	CG2	VAL	441	26.180	24.168	-1.725	1.00	0.00	LX0	C
ATOM	3986	C	VAL	441	23.052	24.415	-2.302	1.00	0.00	LX0	C
ATOM	3987	O	VAL	441	22.865	24.793	-1.153	1.00	0.00	LX0	O
ATOM	3988	N	SER	442	22.272	23.530	-2.937	1.00	0.00	LX0	N
ATOM	3989	H	SER	442	22.511	23.333	-3.889	1.00	0.00	LX0	H
ATOM	3990	CA	SER	442	21.146	22.859	-2.274	1.00	0.00	LX0	C
ATOM	3991	CB	SER	442	21.609	21.840	-1.210	1.00	0.00	LX0	C
ATOM	3992	OG	SER	442	22.884	22.182	-0.640	1.00	0.00	LX0	O
ATOM	3993	HG	SER	442	22.766	23.036	-0.216	1.00	0.00	LX0	H
ATOM	3994	C	SER	442	19.979	23.702	-1.762	1.00	0.00	LX0	C
ATOM	3995	O	SER	442	19.061	23.193	-1.119	1.00	0.00	LX0	O
ATOM	3996	N	LEU	443	20.026	25.010	-2.068	1.00	0.00	LX0	N
ATOM	3997	H	LEU	443	20.757	25.353	-2.661	1.00	0.00	LX0	H
ATOM	3998	CA	LEU	443	19.107	25.940	-1.408	1.00	0.00	LX0	C
ATOM	3999	CB	LEU	443	19.478	27.406	-1.674	1.00	0.00	LX0	C
ATOM	4000	CG	LEU	443	20.887	27.867	-1.305	1.00	0.00	LX0	C
ATOM	4001	CD1	LEU	443	20.937	29.385	-1.206	1.00	0.00	LX0	C
ATOM	4002	CD2	LEU	443	21.403	27.260	-0.014	1.00	0.00	LX0	C
ATOM	4003	C	LEU	443	17.628	25.756	-1.710	1.00	0.00	LX0	C
ATOM	4004	O	LEU	443	17.204	24.871	-2.453	1.00	0.00	LX0	O
ATOM	4005	N	ASN	444	16.862	26.665	-1.080	1.00	0.00	LX0	N
ATOM	4006	H	ASN	444	17.271	27.342	-0.470	1.00	0.00	LX0	H
ATOM	4007	CA	ASN	444	15.412	26.706	-1.285	1.00	0.00	LX0	C
ATOM	4008	CB	ASN	444	14.675	26.535	0.052	1.00	0.00	LX0	C
ATOM	4009	CG	ASN	444	14.974	25.176	0.661	1.00	0.00	LX0	C
ATOM	4010	OD1	ASN	444	15.131	24.172	-0.023	1.00	0.00	LX0	O
ATOM	4011	ND2	ASN	444	15.079	25.177	1.989	1.00	0.00	LX0	N
ATOM	4012	HD21	ASN	444	15.064	26.035	2.507	1.00	0.00	LX0	H
ATOM	4013	HD22	ASN	444	15.203	24.342	2.526	1.00	0.00	LX0	H
ATOM	4014	C	ASN	444	14.978	28.001	-1.962	1.00	0.00	LX0	C
ATOM	4015	O	ASN	444	13.889	28.526	-1.757	1.00	0.00	LX0	O
ATOM	4016	N	ILE	445	15.918	28.531	-2.759	1.00	0.00	LX0	N
ATOM	4017	H	ILE	445	16.694	27.979	-3.049	1.00	0.00	LX0	H
ATOM	4018	CA	ILE	445	15.640	29.830	-3.367	1.00	0.00	LX0	C
ATOM	4019	CB	ILE	445	16.889	30.723	-3.376	1.00	0.00	LX0	C
ATOM	4020	CG2	ILE	445	17.225	31.184	-1.962	1.00	0.00	LX0	C
ATOM	4021	CG1	ILE	445	18.083	30.062	-4.073	1.00	0.00	LX0	C
ATOM	4022	CD1	ILE	445	19.299	30.982	-4.198	1.00	0.00	LX0	C
ATOM	4023	C	ILE	445	15.072	29.725	-4.766	1.00	0.00	LX0	C
ATOM	4024	O	ILE	445	15.345	28.787	-5.504	1.00	0.00	LX0	O
ATOM	4025	N	THR	446	14.284	30.752	-5.097	1.00	0.00	LX0	N
ATOM	4026	H	THR	446	14.080	31.438	-4.400	1.00	0.00	LX0	H
ATOM	4027	CA	THR	446	13.931	30.962	-6.497	1.00	0.00	LX0	C
ATOM	4028	CB	THR	446	12.411	31.102	-6.654	1.00	0.00	LX0	C
ATOM	4029	OG1	THR	446	11.802	29.865	-6.275	1.00	0.00	LX0	O
ATOM	4030	HG1	THR	446	12.250	29.215	-6.815	1.00	0.00	LX0	H
ATOM	4031	CG2	THR	446	11.957	31.477	-8.070	1.00	0.00	LX0	C
ATOM	4032	C	THR	446	14.680	32.135	-7.096	1.00	0.00	LX0	C
ATOM	4033	O	THR	446	15.080	32.118	-8.256	1.00	0.00	LX0	O
ATOM	4034	N	SER	447	14.874	33.141	-6.245	1.00	0.00	LX0	N
ATOM	4035	H	SER	447	14.577	33.149	-5.290	1.00	0.00	LX0	H
ATOM	4036	CA	SER	447	15.725	34.222	-6.708	1.00	0.00	LX0	C
ATOM	4037	CB	SER	447	15.211	35.540	-6.155	1.00	0.00	LX0	C
ATOM	4038	OG	SER	447	13.779	35.575	-6.218	1.00	0.00	LX0	O

ATOM	4039	HG	SER	447	13.540	36.263	-5.603	1.00	0.00	LX0	H
ATOM	4040	C	SER	447	17.145	33.982	-6.255	1.00	0.00	LX0	C
ATOM	4041	O	SER	447	17.394	33.545	-5.140	1.00	0.00	LX0	O
ATOM	4042	N	LEU	448	18.068	34.272	-7.174	1.00	0.00	LX0	N
ATOM	4043	H	LEU	448	17.791	34.681	-8.044	1.00	0.00	LX0	H
ATOM	4044	CA	LEU	448	19.469	34.200	-6.780	1.00	0.00	LX0	C
ATOM	4045	CB	LEU	448	20.364	34.173	-8.017	1.00	0.00	LX0	C
ATOM	4046	CG	LEU	448	21.226	32.912	-8.095	1.00	0.00	LX0	C
ATOM	4047	CD1	LEU	448	22.037	32.866	-9.388	1.00	0.00	LX0	C
ATOM	4048	CD2	LEU	448	22.106	32.727	-6.857	1.00	0.00	LX0	C
ATOM	4049	C	LEU	448	19.889	35.295	-5.816	1.00	0.00	LX0	C
ATOM	4050	O	LEU	448	20.793	35.133	-5.012	1.00	0.00	LX0	O
ATOM	4051	N	GLY	449	19.185	36.431	-5.914	1.00	0.00	LX0	N
ATOM	4052	H	GLY	449	18.561	36.563	-6.686	1.00	0.00	LX0	H
ATOM	4053	CA	GLY	449	19.234	37.384	-4.803	1.00	0.00	LX0	C
ATOM	4054	C	GLY	449	20.521	38.159	-4.537	1.00	0.00	LX0	C
ATOM	4055	O	GLY	449	20.525	39.101	-3.756	1.00	0.00	LX0	O
ATOM	4056	N	LEU	450	21.613	37.767	-5.215	1.00	0.00	LX0	N
ATOM	4057	H	LEU	450	21.556	36.948	-5.782	1.00	0.00	LX0	H
ATOM	4058	CA	LEU	450	22.884	38.481	-5.053	1.00	0.00	LX0	C
ATOM	4059	CB	LEU	450	24.047	37.572	-5.453	1.00	0.00	LX0	C
ATOM	4060	CG	LEU	450	24.274	36.382	-4.524	1.00	0.00	LX0	C
ATOM	4061	CD1	LEU	450	25.021	35.251	-5.228	1.00	0.00	LX0	C
ATOM	4062	CD2	LEU	450	24.974	36.803	-3.232	1.00	0.00	LX0	C
ATOM	4063	C	LEU	450	22.963	39.777	-5.844	1.00	0.00	LX0	C
ATOM	4064	O	LEU	450	23.866	40.010	-6.636	1.00	0.00	LX0	O
ATOM	4065	N	ARG	451	21.956	40.620	-5.606	1.00	0.00	LX0	N
ATOM	4066	H	ARG	451	21.363	40.419	-4.827	1.00	0.00	LX0	H
ATOM	4067	CA	ARG	451	21.748	41.773	-6.480	1.00	0.00	LX0	C
ATOM	4068	CB	ARG	451	20.313	42.302	-6.310	1.00	0.00	LX0	C
ATOM	4069	CG	ARG	451	19.812	42.363	-4.862	1.00	0.00	LX0	C
ATOM	4070	CD	ARG	451	18.310	42.635	-4.720	1.00	0.00	LX0	C
ATOM	4071	NE	ARG	451	17.496	41.655	-5.438	1.00	0.00	LX0	N
ATOM	4072	HE	ARG	451	17.424	41.692	-6.442	1.00	0.00	LX0	H
ATOM	4073	CZ	ARG	451	16.888	40.585	-4.877	1.00	0.00	LX0	C
ATOM	4074	NH1	ARG	451	16.914	40.347	-3.575	1.00	0.00	LX0	N
ATOM	4075	HH11	ARG	451	16.561	39.468	-3.217	1.00	0.00	LX0	H
ATOM	4076	HH12	ARG	451	17.277	41.010	-2.915	1.00	0.00	LX0	H
ATOM	4077	NH2	ARG	451	16.256	39.735	-5.662	1.00	0.00	LX0	N
ATOM	4078	HH21	ARG	451	15.790	38.937	-5.267	1.00	0.00	LX0	H
ATOM	4079	HH22	ARG	451	16.242	39.914	-6.657	1.00	0.00	LX0	H
ATOM	4080	C	ARG	451	22.810	42.865	-6.442	1.00	0.00	LX0	C
ATOM	4081	O	ARG	451	22.837	43.765	-7.270	1.00	0.00	LX0	O
ATOM	4082	N	SER	452	23.716	42.743	-5.461	1.00	0.00	LX0	N
ATOM	4083	H	SER	452	23.663	42.014	-4.779	1.00	0.00	LX0	H
ATOM	4084	CA	SER	452	24.816	43.705	-5.485	1.00	0.00	LX0	C
ATOM	4085	CB	SER	452	24.964	44.351	-4.114	1.00	0.00	LX0	C
ATOM	4086	OG	SER	452	23.665	44.634	-3.563	1.00	0.00	LX0	O
ATOM	4087	HG	SER	452	23.212	43.793	-3.558	1.00	0.00	LX0	H
ATOM	4088	C	SER	452	26.160	43.169	-5.959	1.00	0.00	LX0	C
ATOM	4089	O	SER	452	27.177	43.853	-5.917	1.00	0.00	LX0	O
ATOM	4090	N	LEU	453	26.139	41.899	-6.395	1.00	0.00	LX0	N
ATOM	4091	H	LEU	453	25.284	41.400	-6.540	1.00	0.00	LX0	H
ATOM	4092	CA	LEU	453	27.428	41.258	-6.637	1.00	0.00	LX0	C
ATOM	4093	CB	LEU	453	27.234	39.746	-6.756	1.00	0.00	LX0	C
ATOM	4094	CG	LEU	453	28.457	38.923	-6.349	1.00	0.00	LX0	C
ATOM	4095	CD1	LEU	453	28.770	39.061	-4.859	1.00	0.00	LX0	C
ATOM	4096	CD2	LEU	453	28.293	37.457	-6.739	1.00	0.00	LX0	C
ATOM	4097	C	LEU	453	28.202	41.822	-7.819	1.00	0.00	LX0	C
ATOM	4098	O	LEU	453	27.841	41.656	-8.976	1.00	0.00	LX0	O
ATOM	4099	N	LYS	454	29.298	42.503	-7.467	1.00	0.00	LX0	N

ATOM	4100	H	LYS	454	29.487	42.624	-6.493	1.00	0.00	LX0	H
ATOM	4101	CA	LYS	454	30.142	43.054	-8.522	1.00	0.00	LX0	C
ATOM	4102	CB	LYS	454	30.507	44.522	-8.240	1.00	0.00	LX0	C
ATOM	4103	CG	LYS	454	31.553	44.736	-7.134	1.00	0.00	LX0	C
ATOM	4104	CD	LYS	454	32.121	46.157	-7.107	1.00	0.00	LX0	C
ATOM	4105	CE	LYS	454	33.309	46.339	-6.151	1.00	0.00	LX0	C
ATOM	4106	NZ	LYS	454	34.469	45.549	-6.592	1.00	0.00	LX0	N
ATOM	4107	HZ1	LYS	454	35.316	45.736	-6.013	1.00	0.00	LX0	H
ATOM	4108	HZ2	LYS	454	34.339	44.524	-6.441	1.00	0.00	LX0	H
ATOM	4109	HZ3	LYS	454	34.713	45.705	-7.586	1.00	0.00	LX0	H
ATOM	4110	C	LYS	454	31.405	42.263	-8.831	1.00	0.00	LX0	C
ATOM	4111	O	LYS	454	32.010	42.420	-9.883	1.00	0.00	LX0	O
ATOM	4112	N	GLU	455	31.828	41.456	-7.847	1.00	0.00	LX0	N
ATOM	4113	H	GLU	455	31.297	41.325	-7.007	1.00	0.00	LX0	H
ATOM	4114	CA	GLU	455	33.209	40.991	-7.952	1.00	0.00	LX0	C
ATOM	4115	CB	GLU	455	34.095	41.887	-7.072	1.00	0.00	LX0	C
ATOM	4116	CG	GLU	455	35.596	41.935	-7.388	1.00	0.00	LX0	C
ATOM	4117	CD	GLU	455	36.364	42.679	-6.294	1.00	0.00	LX0	C
ATOM	4118	OE1	GLU	455	35.917	43.719	-5.823	1.00	0.00	LX0	O
ATOM	4119	OE2	GLU	455	37.423	42.216	-5.889	1.00	0.00	LX0	O
ATOM	4120	C	GLU	455	33.360	39.540	-7.550	1.00	0.00	LX0	C
ATOM	4121	O	GLU	455	33.357	39.221	-6.370	1.00	0.00	LX0	O
ATOM	4122	N	ILE	456	33.506	38.666	-8.556	1.00	0.00	LX0	N
ATOM	4123	H	ILE	456	33.496	38.965	-9.513	1.00	0.00	LX0	H
ATOM	4124	CA	ILE	456	33.959	37.331	-8.165	1.00	0.00	LX0	C
ATOM	4125	CB	ILE	456	33.154	36.200	-8.820	1.00	0.00	LX0	C
ATOM	4126	CG2	ILE	456	33.501	34.849	-8.177	1.00	0.00	LX0	C
ATOM	4127	CG1	ILE	456	31.649	36.460	-8.772	1.00	0.00	LX0	C
ATOM	4128	CD1	ILE	456	30.869	35.457	-9.625	1.00	0.00	LX0	C
ATOM	4129	C	ILE	456	35.437	37.194	-8.473	1.00	0.00	LX0	C
ATOM	4130	O	ILE	456	35.871	36.744	-9.532	1.00	0.00	LX0	O
ATOM	4131	N	SER	457	36.204	37.664	-7.492	1.00	0.00	LX0	N
ATOM	4132	H	SER	457	35.813	37.918	-6.600	1.00	0.00	LX0	H
ATOM	4133	CA	SER	457	37.607	37.951	-7.761	1.00	0.00	LX0	C
ATOM	4134	CB	SER	457	38.160	38.844	-6.655	1.00	0.00	LX0	C
ATOM	4135	OG	SER	457	37.070	39.388	-5.901	1.00	0.00	LX0	O
ATOM	4136	HG	SER	457	37.118	40.341	-5.981	1.00	0.00	LX0	H
ATOM	4137	C	SER	457	38.524	36.765	-8.009	1.00	0.00	LX0	C
ATOM	4138	O	SER	457	39.626	36.938	-8.531	1.00	0.00	LX0	O
ATOM	4139	N	ASP	458	38.013	35.581	-7.613	1.00	0.00	LX0	N
ATOM	4140	H	ASP	458	37.128	35.575	-7.142	1.00	0.00	LX0	H
ATOM	4141	CA	ASP	458	38.667	34.279	-7.778	1.00	0.00	LX0	C
ATOM	4142	CB	ASP	458	40.017	34.286	-7.036	1.00	0.00	LX0	C
ATOM	4143	CG	ASP	458	40.913	33.061	-7.177	1.00	0.00	LX0	C
ATOM	4144	OD1	ASP	458	40.666	32.175	-7.990	1.00	0.00	LX0	O
ATOM	4145	OD2	ASP	458	41.859	32.944	-6.406	1.00	0.00	LX0	O
ATOM	4146	C	ASP	458	37.699	33.223	-7.260	1.00	0.00	LX0	C
ATOM	4147	O	ASP	458	36.701	33.541	-6.620	1.00	0.00	LX0	O
ATOM	4148	N	GLY	459	38.011	31.963	-7.577	1.00	0.00	LX0	N
ATOM	4149	H	GLY	459	38.776	31.831	-8.206	1.00	0.00	LX0	H
ATOM	4150	CA	GLY	459	37.171	30.860	-7.135	1.00	0.00	LX0	C
ATOM	4151	C	GLY	459	36.068	30.594	-8.131	1.00	0.00	LX0	C
ATOM	4152	O	GLY	459	35.448	31.499	-8.674	1.00	0.00	LX0	O
ATOM	4153	N	ASP	460	35.901	29.303	-8.390	1.00	0.00	LX0	N
ATOM	4154	H	ASP	460	36.348	28.608	-7.826	1.00	0.00	LX0	H
ATOM	4155	CA	ASP	460	34.942	28.905	-9.409	1.00	0.00	LX0	C
ATOM	4156	CB	ASP	460	35.482	27.581	-9.965	1.00	0.00	LX0	C
ATOM	4157	CG	ASP	460	34.681	26.928	-11.078	1.00	0.00	LX0	C
ATOM	4158	OD1	ASP	460	35.130	25.927	-11.619	1.00	0.00	LX0	O
ATOM	4159	OD2	ASP	460	33.552	27.305	-11.346	1.00	0.00	LX0	O
ATOM	4160	C	ASP	460	33.547	28.854	-8.806	1.00	0.00	LX0	C

ATOM	4161	O	ASP	460	33.361	28.572	-7.626	1.00	0.00	LX0	O
ATOM	4162	N	VAL	461	32.568	29.187	-9.648	1.00	0.00	LX0	N
ATOM	4163	H	VAL	461	32.767	29.211	-10.631	1.00	0.00	LX0	H
ATOM	4164	CA	VAL	461	31.198	29.181	-9.152	1.00	0.00	LX0	C
ATOM	4165	CB	VAL	461	30.348	30.034	-10.113	1.00	0.00	LX0	C
ATOM	4166	CG1	VAL	461	28.862	30.095	-9.758	1.00	0.00	LX0	C
ATOM	4167	CG2	VAL	461	30.939	31.443	-10.227	1.00	0.00	LX0	C
ATOM	4168	C	VAL	461	30.677	27.754	-9.010	1.00	0.00	LX0	C
ATOM	4169	O	VAL	461	30.962	26.877	-9.824	1.00	0.00	LX0	O
ATOM	4170	N	ILE	462	29.889	27.560	-7.947	1.00	0.00	LX0	N
ATOM	4171	H	ILE	462	29.809	28.260	-7.237	1.00	0.00	LX0	H
ATOM	4172	CA	ILE	462	28.975	26.425	-7.930	1.00	0.00	LX0	C
ATOM	4173	CB	ILE	462	29.597	25.148	-7.326	1.00	0.00	LX0	C
ATOM	4174	CG2	ILE	462	29.931	25.264	-5.840	1.00	0.00	LX0	C
ATOM	4175	CG1	ILE	462	28.724	23.929	-7.641	1.00	0.00	LX0	C
ATOM	4176	CD1	ILE	462	29.353	22.603	-7.211	1.00	0.00	LX0	C
ATOM	4177	C	ILE	462	27.633	26.801	-7.319	1.00	0.00	LX0	C
ATOM	4178	O	ILE	462	27.345	26.669	-6.135	1.00	0.00	LX0	O
ATOM	4179	N	ILE	463	26.798	27.312	-8.220	1.00	0.00	LX0	N
ATOM	4180	H	ILE	463	27.036	27.318	-9.192	1.00	0.00	LX0	H
ATOM	4181	CA	ILE	463	25.438	27.552	-7.768	1.00	0.00	LX0	C
ATOM	4182	CB	ILE	463	24.989	28.968	-8.151	1.00	0.00	LX0	C
ATOM	4183	CG2	ILE	463	23.534	29.254	-7.774	1.00	0.00	LX0	C
ATOM	4184	CG1	ILE	463	25.942	29.956	-7.471	1.00	0.00	LX0	C
ATOM	4185	CD1	ILE	463	25.522	31.419	-7.562	1.00	0.00	LX0	C
ATOM	4186	C	ILE	463	24.537	26.443	-8.271	1.00	0.00	LX0	C
ATOM	4187	O	ILE	463	23.922	26.508	-9.328	1.00	0.00	LX0	O
ATOM	4188	N	SER	464	24.551	25.370	-7.478	1.00	0.00	LX0	N
ATOM	4189	H	SER	464	24.944	25.438	-6.557	1.00	0.00	LX0	H
ATOM	4190	CA	SER	464	23.918	24.153	-7.971	1.00	0.00	LX0	C
ATOM	4191	CB	SER	464	24.988	23.154	-8.412	1.00	0.00	LX0	C
ATOM	4192	OG	SER	464	25.680	23.636	-9.573	1.00	0.00	LX0	O
ATOM	4193	HG	SER	464	26.026	24.501	-9.362	1.00	0.00	LX0	H
ATOM	4194	C	SER	464	22.980	23.507	-6.974	1.00	0.00	LX0	C
ATOM	4195	O	SER	464	23.136	23.612	-5.764	1.00	0.00	LX0	O
ATOM	4196	N	GLY	465	21.991	22.807	-7.542	1.00	0.00	LX0	N
ATOM	4197	H	GLY	465	21.862	22.855	-8.534	1.00	0.00	LX0	H
ATOM	4198	CA	GLY	465	21.066	22.076	-6.678	1.00	0.00	LX0	C
ATOM	4199	C	GLY	465	20.105	22.961	-5.913	1.00	0.00	LX0	C
ATOM	4200	O	GLY	465	19.567	22.610	-4.869	1.00	0.00	LX0	O
ATOM	4201	N	ASN	466	19.923	24.160	-6.467	1.00	0.00	LX0	N
ATOM	4202	H	ASN	466	20.249	24.374	-7.390	1.00	0.00	LX0	H
ATOM	4203	CA	ASN	466	19.029	25.057	-5.753	1.00	0.00	LX0	C
ATOM	4204	CB	ASN	466	19.495	26.507	-5.817	1.00	0.00	LX0	C
ATOM	4205	CG	ASN	466	20.927	26.588	-5.343	1.00	0.00	LX0	C
ATOM	4206	OD1	ASN	466	21.229	26.500	-4.162	1.00	0.00	LX0	O
ATOM	4207	ND2	ASN	466	21.811	26.730	-6.325	1.00	0.00	LX0	N
ATOM	4208	HD21	ASN	466	21.521	26.740	-7.286	1.00	0.00	LX0	H
ATOM	4209	HD22	ASN	466	22.772	26.807	-6.055	1.00	0.00	LX0	H
ATOM	4210	C	ASN	466	17.644	24.859	-6.298	1.00	0.00	LX0	C
ATOM	4211	O	ASN	466	17.305	25.184	-7.431	1.00	0.00	LX0	O
ATOM	4212	N	LYS	467	16.879	24.199	-5.429	1.00	0.00	LX0	N
ATOM	4213	H	LYS	467	17.198	24.156	-4.482	1.00	0.00	LX0	H
ATOM	4214	CA	LYS	467	15.809	23.340	-5.938	1.00	0.00	LX0	C
ATOM	4215	CB	LYS	467	15.302	22.468	-4.781	1.00	0.00	LX0	C
ATOM	4216	CG	LYS	467	16.483	21.688	-4.178	1.00	0.00	LX0	C
ATOM	4217	CD	LYS	467	16.192	20.797	-2.967	1.00	0.00	LX0	C
ATOM	4218	CE	LYS	467	15.692	21.529	-1.717	1.00	0.00	LX0	C
ATOM	4219	NZ	LYS	467	16.654	22.529	-1.235	1.00	0.00	LX0	N
ATOM	4220	HZ1	LYS	467	16.255	22.986	-0.389	1.00	0.00	LX0	H
ATOM	4221	HZ2	LYS	467	17.592	22.150	-0.984	1.00	0.00	LX0	H

ATOM	4222	HZ3	LYS	467	16.803	23.288	-1.931	1.00	0.00	LX0	H
ATOM	4223	C	LYS	467	14.705	24.039	-6.719	1.00	0.00	LX0	C
ATOM	4224	O	LYS	467	14.046	23.479	-7.584	1.00	0.00	LX0	O
ATOM	4225	N	ASN	468	14.559	25.322	-6.387	1.00	0.00	LX0	N
ATOM	4226	H	ASN	468	15.082	25.705	-5.624	1.00	0.00	LX0	H
ATOM	4227	CA	ASN	468	13.525	26.131	-7.024	1.00	0.00	LX0	C
ATOM	4228	CB	ASN	468	12.416	26.498	-6.014	1.00	0.00	LX0	C
ATOM	4229	CG	ASN	468	12.962	26.991	-4.678	1.00	0.00	LX0	C
ATOM	4230	OD1	ASN	468	13.846	26.395	-4.074	1.00	0.00	LX0	O
ATOM	4231	ND2	ASN	468	12.360	28.081	-4.197	1.00	0.00	LX0	N
ATOM	4232	HD21	ASN	468	11.708	28.625	-4.727	1.00	0.00	LX0	H
ATOM	4233	HD22	ASN	468	12.597	28.362	-3.264	1.00	0.00	LX0	H
ATOM	4234	C	ASN	468	14.053	27.353	-7.758	1.00	0.00	LX0	C
ATOM	4235	O	ASN	468	13.316	28.293	-8.047	1.00	0.00	LX0	O
ATOM	4236	N	LEU	469	15.364	27.295	-8.051	1.00	0.00	LX0	N
ATOM	4237	H	LEU	469	15.876	26.460	-7.850	1.00	0.00	LX0	H
ATOM	4238	CA	LEU	469	16.070	28.473	-8.558	1.00	0.00	LX0	C
ATOM	4239	CB	LEU	469	17.559	28.332	-8.250	1.00	0.00	LX0	C
ATOM	4240	CG	LEU	469	18.422	29.548	-8.586	1.00	0.00	LX0	C
ATOM	4241	CD1	LEU	469	18.038	30.762	-7.751	1.00	0.00	LX0	C
ATOM	4242	CD2	LEU	469	19.911	29.238	-8.491	1.00	0.00	LX0	C
ATOM	4243	C	LEU	469	15.878	28.784	-10.029	1.00	0.00	LX0	C
ATOM	4244	O	LEU	469	16.133	27.971	-10.910	1.00	0.00	LX0	O
ATOM	4245	N	CYS	470	15.451	30.027	-10.251	1.00	0.00	LX0	N
ATOM	4246	H	CYS	470	15.328	30.645	-9.473	1.00	0.00	LX0	H
ATOM	4247	CA	CYS	470	15.484	30.562	-11.607	1.00	0.00	LX0	C
ATOM	4248	CB	CYS	470	14.192	31.324	-11.864	1.00	0.00	LX0	C
ATOM	4249	SG	CYS	470	12.768	30.221	-11.912	1.00	0.00	LX0	S
ATOM	4250	C	CYS	470	16.698	31.457	-11.778	1.00	0.00	LX0	C
ATOM	4251	O	CYS	470	17.555	31.506	-10.907	1.00	0.00	LX0	O
ATOM	4252	N	TYR	471	16.736	32.178	-12.916	1.00	0.00	LX0	N
ATOM	4253	H	TYR	471	16.077	31.993	-13.644	1.00	0.00	LX0	H
ATOM	4254	CA	TYR	471	17.676	33.294	-13.113	1.00	0.00	LX0	C
ATOM	4255	CB	TYR	471	17.518	34.388	-12.039	1.00	0.00	LX0	C
ATOM	4256	CG	TYR	471	16.088	34.880	-12.009	1.00	0.00	LX0	C
ATOM	4257	CD1	TYR	471	15.283	34.575	-10.892	1.00	0.00	LX0	C
ATOM	4258	CE1	TYR	471	13.940	34.986	-10.888	1.00	0.00	LX0	C
ATOM	4259	CD2	TYR	471	15.592	35.618	-13.104	1.00	0.00	LX0	C
ATOM	4260	CE2	TYR	471	14.252	36.035	-13.096	1.00	0.00	LX0	C
ATOM	4261	CZ	TYR	471	13.440	35.705	-11.993	1.00	0.00	LX0	C
ATOM	4262	OH	TYR	471	12.114	36.093	-11.992	1.00	0.00	LX0	O
ATOM	4263	HH	TYR	471	11.974	36.667	-12.740	1.00	0.00	LX0	H
ATOM	4264	C	TYR	471	19.144	32.981	-13.372	1.00	0.00	LX0	C
ATOM	4265	O	TYR	471	19.782	33.639	-14.181	1.00	0.00	LX0	O
ATOM	4266	N	ALA	472	19.651	31.926	-12.707	1.00	0.00	LX0	N
ATOM	4267	H	ALA	472	19.093	31.528	-11.981	1.00	0.00	LX0	H
ATOM	4268	CA	ALA	472	21.059	31.524	-12.826	1.00	0.00	LX0	C
ATOM	4269	CB	ALA	472	21.265	30.145	-12.194	1.00	0.00	LX0	C
ATOM	4270	C	ALA	472	21.678	31.503	-14.219	1.00	0.00	LX0	C
ATOM	4271	O	ALA	472	22.859	31.755	-14.413	1.00	0.00	LX0	O
ATOM	4272	N	ASN	473	20.823	31.185	-15.198	1.00	0.00	LX0	N
ATOM	4273	H	ASN	473	19.846	31.120	-14.996	1.00	0.00	LX0	H
ATOM	4274	CA	ASN	473	21.297	31.227	-16.582	1.00	0.00	LX0	C
ATOM	4275	CB	ASN	473	20.274	30.558	-17.523	1.00	0.00	LX0	C
ATOM	4276	CG	ASN	473	18.968	31.338	-17.565	1.00	0.00	LX0	C
ATOM	4277	OD1	ASN	473	18.474	31.827	-16.553	1.00	0.00	LX0	O
ATOM	4278	ND2	ASN	473	18.469	31.504	-18.789	1.00	0.00	LX0	N
ATOM	4279	HD21	ASN	473	18.834	31.047	-19.599	1.00	0.00	LX0	H
ATOM	4280	HD22	ASN	473	17.709	32.145	-18.906	1.00	0.00	LX0	H
ATOM	4281	C	ASN	473	21.677	32.624	-17.070	1.00	0.00	LX0	C
ATOM	4282	O	ASN	473	22.704	32.850	-17.697	1.00	0.00	LX0	O

ATOM	4283	N	THR	474	20.793	33.570	-16.749	1.00	0.00	LX0	N
ATOM	4284	H	THR	474	20.086	33.382	-16.067	1.00	0.00	LX0	H
ATOM	4285	CA	THR	474	20.895	34.875	-17.393	1.00	0.00	LX0	C
ATOM	4286	CB	THR	474	19.479	35.387	-17.705	1.00	0.00	LX0	C
ATOM	4287	OG1	THR	474	18.593	34.285	-17.968	1.00	0.00	LX0	O
ATOM	4288	HG1	THR	474	18.482	33.806	-17.152	1.00	0.00	LX0	H
ATOM	4289	CG2	THR	474	19.471	36.345	-18.901	1.00	0.00	LX0	C
ATOM	4290	C	THR	474	21.731	35.892	-16.619	1.00	0.00	LX0	C
ATOM	4291	O	THR	474	21.447	37.081	-16.557	1.00	0.00	LX0	O
ATOM	4292	N	ILE	475	22.793	35.360	-16.000	1.00	0.00	LX0	N
ATOM	4293	H	ILE	475	23.010	34.392	-16.127	1.00	0.00	LX0	H
ATOM	4294	CA	ILE	475	23.592	36.248	-15.154	1.00	0.00	LX0	C
ATOM	4295	CB	ILE	475	24.051	35.477	-13.900	1.00	0.00	LX0	C
ATOM	4296	CG2	ILE	475	24.936	36.294	-12.951	1.00	0.00	LX0	C
ATOM	4297	CG1	ILE	475	22.845	34.909	-13.148	1.00	0.00	LX0	C
ATOM	4298	CD1	ILE	475	21.959	35.975	-12.503	1.00	0.00	LX0	C
ATOM	4299	C	ILE	475	24.759	36.910	-15.880	1.00	0.00	LX0	C
ATOM	4300	O	ILE	475	25.255	37.952	-15.482	1.00	0.00	LX0	O
ATOM	4301	N	ASN	476	25.204	36.234	-16.959	1.00	0.00	LX0	N
ATOM	4302	H	ASN	476	24.670	35.461	-17.294	1.00	0.00	LX0	H
ATOM	4303	CA	ASN	476	26.495	36.593	-17.571	1.00	0.00	LX0	C
ATOM	4304	CB	ASN	476	26.468	37.908	-18.368	1.00	0.00	LX0	C
ATOM	4305	CG	ASN	476	27.812	38.127	-19.051	1.00	0.00	LX0	C
ATOM	4306	OD1	ASN	476	28.582	37.201	-19.291	1.00	0.00	LX0	O
ATOM	4307	ND2	ASN	476	28.068	39.399	-19.353	1.00	0.00	LX0	N
ATOM	4308	HD21	ASN	476	27.461	40.115	-18.994	1.00	0.00	LX0	H
ATOM	4309	HD22	ASN	476	28.857	39.663	-19.899	1.00	0.00	LX0	H
ATOM	4310	C	ASN	476	27.657	36.561	-16.591	1.00	0.00	LX0	C
ATOM	4311	O	ASN	476	28.341	37.529	-16.280	1.00	0.00	LX0	O
ATOM	4312	N	TRP	477	27.866	35.326	-16.131	1.00	0.00	LX0	N
ATOM	4313	H	TRP	477	27.246	34.605	-16.433	1.00	0.00	LX0	H
ATOM	4314	CA	TRP	477	28.880	35.088	-15.107	1.00	0.00	LX0	C
ATOM	4315	CB	TRP	477	28.985	33.589	-14.847	1.00	0.00	LX0	C
ATOM	4316	CG	TRP	477	27.677	33.091	-14.286	1.00	0.00	LX0	C
ATOM	4317	CD2	TRP	477	27.228	33.168	-12.918	1.00	0.00	LX0	C
ATOM	4318	CE2	TRP	477	25.927	32.568	-12.869	1.00	0.00	LX0	C
ATOM	4319	CE3	TRP	477	27.807	33.700	-11.747	1.00	0.00	LX0	C
ATOM	4320	CD1	TRP	477	26.636	32.459	-14.986	1.00	0.00	LX0	C
ATOM	4321	NE1	TRP	477	25.608	32.149	-14.156	1.00	0.00	LX0	N
ATOM	4322	HE1	TRP	477	24.753	31.730	-14.414	1.00	0.00	LX0	H
ATOM	4323	CZ2	TRP	477	25.226	32.510	-11.648	1.00	0.00	LX0	C
ATOM	4324	CZ3	TRP	477	27.096	33.639	-10.532	1.00	0.00	LX0	C
ATOM	4325	CH2	TRP	477	25.814	33.049	-10.485	1.00	0.00	LX0	C
ATOM	4326	C	TRP	477	30.252	35.696	-15.354	1.00	0.00	LX0	C
ATOM	4327	O	TRP	477	30.915	36.177	-14.443	1.00	0.00	LX0	O
ATOM	4328	N	LYS	478	30.668	35.684	-16.635	1.00	0.00	LX0	N
ATOM	4329	H	LYS	478	30.053	35.430	-17.381	1.00	0.00	LX0	H
ATOM	4330	CA	LYS	478	32.009	36.225	-16.842	1.00	0.00	LX0	C
ATOM	4331	CB	LYS	478	32.624	35.839	-18.189	1.00	0.00	LX0	C
ATOM	4332	CG	LYS	478	34.095	35.440	-17.995	1.00	0.00	LX0	C
ATOM	4333	CD	LYS	478	35.145	36.179	-18.838	1.00	0.00	LX0	C
ATOM	4334	CE	LYS	478	35.310	37.679	-18.548	1.00	0.00	LX0	C
ATOM	4335	NZ	LYS	478	35.643	37.926	-17.134	1.00	0.00	LX0	N
ATOM	4336	HZ1	LYS	478	34.865	37.635	-16.496	1.00	0.00	LX0	H
ATOM	4337	HZ2	LYS	478	36.476	37.402	-16.812	1.00	0.00	LX0	H
ATOM	4338	HZ3	LYS	478	35.765	38.940	-16.958	1.00	0.00	LX0	H
ATOM	4339	C	LYS	478	32.172	37.714	-16.599	1.00	0.00	LX0	C
ATOM	4340	O	LYS	478	33.270	38.183	-16.324	1.00	0.00	LX0	O
ATOM	4341	N	LYS	479	31.055	38.444	-16.684	1.00	0.00	LX0	N
ATOM	4342	H	LYS	479	30.164	38.031	-16.888	1.00	0.00	LX0	H
ATOM	4343	CA	LYS	479	31.141	39.858	-16.317	1.00	0.00	LX0	C

ATOM	4344	CB	LYS	479	29.797	40.513	-16.648	1.00	0.00	LX0	C
ATOM	4345	CG	LYS	479	29.609	42.013	-16.404	1.00	0.00	LX0	C
ATOM	4346	CD	LYS	479	28.966	42.316	-15.048	1.00	0.00	LX0	C
ATOM	4347	CE	LYS	479	28.069	43.556	-15.085	1.00	0.00	LX0	C
ATOM	4348	NZ	LYS	479	26.953	43.319	-16.008	1.00	0.00	LX0	N
ATOM	4349	HZ1	LYS	479	26.145	43.969	-15.871	1.00	0.00	LX0	H
ATOM	4350	HZ2	LYS	479	27.233	43.371	-17.010	1.00	0.00	LX0	H
ATOM	4351	HZ3	LYS	479	26.594	42.343	-15.943	1.00	0.00	LX0	H
ATOM	4352	C	LYS	479	31.586	40.067	-14.875	1.00	0.00	LX0	C
ATOM	4353	O	LYS	479	32.313	40.992	-14.544	1.00	0.00	LX0	O
ATOM	4354	N	LEU	480	31.133	39.119	-14.044	1.00	0.00	LX0	N
ATOM	4355	H	LEU	480	30.578	38.362	-14.390	1.00	0.00	LX0	H
ATOM	4356	CA	LEU	480	31.511	39.180	-12.638	1.00	0.00	LX0	C
ATOM	4357	CB	LEU	480	30.445	38.528	-11.761	1.00	0.00	LX0	C
ATOM	4358	CG	LEU	480	29.040	39.129	-11.798	1.00	0.00	LX0	C
ATOM	4359	CD1	LEU	480	28.168	38.614	-12.948	1.00	0.00	LX0	C
ATOM	4360	CD2	LEU	480	28.359	38.877	-10.461	1.00	0.00	LX0	C
ATOM	4361	C	LEU	480	32.851	38.537	-12.328	1.00	0.00	LX0	C
ATOM	4362	O	LEU	480	33.611	38.990	-11.479	1.00	0.00	LX0	O
ATOM	4363	N	PHE	481	33.106	37.423	-13.033	1.00	0.00	LX0	N
ATOM	4364	H	PHE	481	32.469	37.083	-13.723	1.00	0.00	LX0	H
ATOM	4365	CA	PHE	481	34.334	36.733	-12.666	1.00	0.00	LX0	C
ATOM	4366	CB	PHE	481	34.058	35.256	-12.322	1.00	0.00	LX0	C
ATOM	4367	CG	PHE	481	33.813	34.303	-13.475	1.00	0.00	LX0	C
ATOM	4368	CD1	PHE	481	34.822	34.047	-14.431	1.00	0.00	LX0	C
ATOM	4369	CD2	PHE	481	32.576	33.626	-13.532	1.00	0.00	LX0	C
ATOM	4370	CE1	PHE	481	34.607	33.080	-15.432	1.00	0.00	LX0	C
ATOM	4371	CE2	PHE	481	32.358	32.659	-14.533	1.00	0.00	LX0	C
ATOM	4372	CZ	PHE	481	33.379	32.388	-15.466	1.00	0.00	LX0	C
ATOM	4373	C	PHE	481	35.528	36.985	-13.570	1.00	0.00	LX0	C
ATOM	4374	O	PHE	481	35.431	37.095	-14.795	1.00	0.00	LX0	O
ATOM	4375	N	GLY	482	36.680	37.120	-12.903	1.00	0.00	LX0	N
ATOM	4376	H	GLY	482	36.700	36.939	-11.916	1.00	0.00	LX0	H
ATOM	4377	CA	GLY	482	37.824	37.633	-13.651	1.00	0.00	LX0	C
ATOM	4378	C	GLY	482	39.152	36.962	-13.361	1.00	0.00	LX0	C
ATOM	4379	O	GLY	482	40.184	37.612	-13.221	1.00	0.00	LX0	O
ATOM	4380	N	THR	483	39.085	35.635	-13.296	1.00	0.00	LX0	N
ATOM	4381	H	THR	483	38.205	35.159	-13.318	1.00	0.00	LX0	H
ATOM	4382	CA	THR	483	40.294	34.873	-13.018	1.00	0.00	LX0	C
ATOM	4383	CB	THR	483	40.319	34.477	-11.537	1.00	0.00	LX0	C
ATOM	4384	OG1	THR	483	40.001	35.620	-10.735	1.00	0.00	LX0	O
ATOM	4385	HG1	THR	483	39.979	35.313	-9.831	1.00	0.00	LX0	H
ATOM	4386	CG2	THR	483	41.658	33.876	-11.099	1.00	0.00	LX0	C
ATOM	4387	C	THR	483	40.369	33.657	-13.917	1.00	0.00	LX0	C
ATOM	4388	O	THR	483	39.445	32.853	-13.999	1.00	0.00	LX0	O
ATOM	4389	N	SER	484	41.513	33.575	-14.607	1.00	0.00	LX0	N
ATOM	4390	H	SER	484	42.202	34.300	-14.566	1.00	0.00	LX0	H
ATOM	4391	CA	SER	484	41.770	32.501	-15.564	1.00	0.00	LX0	C
ATOM	4392	CB	SER	484	43.099	32.803	-16.253	1.00	0.00	LX0	C
ATOM	4393	OG	SER	484	43.208	34.225	-16.422	1.00	0.00	LX0	O
ATOM	4394	HG	SER	484	43.958	34.368	-16.987	1.00	0.00	LX0	H
ATOM	4395	C	SER	484	41.743	31.091	-14.992	1.00	0.00	LX0	C
ATOM	4396	O	SER	484	42.758	30.492	-14.670	1.00	0.00	LX0	O
ATOM	4397	N	GLY	485	40.509	30.592	-14.880	1.00	0.00	LX0	N
ATOM	4398	H	GLY	485	39.744	31.201	-15.092	1.00	0.00	LX0	H
ATOM	4399	CA	GLY	485	40.319	29.304	-14.227	1.00	0.00	LX0	C
ATOM	4400	C	GLY	485	38.851	29.038	-13.987	1.00	0.00	LX0	C
ATOM	4401	O	GLY	485	38.320	27.987	-14.340	1.00	0.00	LX0	O
ATOM	4402	N	GLN	486	38.210	30.070	-13.411	1.00	0.00	LX0	N
ATOM	4403	H	GLN	486	38.724	30.916	-13.254	1.00	0.00	LX0	H
ATOM	4404	CA	GLN	486	36.799	29.982	-13.013	1.00	0.00	LX0	C

ATOM	4405	CB	GLN	486	36.313	31.351	-12.538	1.00	0.00	LX0	C
ATOM	4406	CG	GLN	486	37.119	31.955	-11.387	1.00	0.00	LX0	C
ATOM	4407	CD	GLN	486	36.786	33.429	-11.277	1.00	0.00	LX0	C
ATOM	4408	OE1	GLN	486	37.055	34.211	-12.175	1.00	0.00	LX0	O
ATOM	4409	NE2	GLN	486	36.205	33.800	-10.138	1.00	0.00	LX0	N
ATOM	4410	HE21	GLN	486	35.903	33.127	-9.461	1.00	0.00	LX0	H
ATOM	4411	HE22	GLN	486	36.036	34.772	-9.966	1.00	0.00	LX0	H
ATOM	4412	C	GLN	486	35.861	29.459	-14.094	1.00	0.00	LX0	C
ATOM	4413	O	GLN	486	36.140	29.540	-15.291	1.00	0.00	LX0	O
ATOM	4414	N	LYS	487	34.747	28.900	-13.614	1.00	0.00	LX0	N
ATOM	4415	H	LYS	487	34.601	28.769	-12.629	1.00	0.00	LX0	H
ATOM	4416	CA	LYS	487	33.640	28.485	-14.470	1.00	0.00	LX0	C
ATOM	4417	CB	LYS	487	33.606	26.956	-14.642	1.00	0.00	LX0	C
ATOM	4418	CG	LYS	487	34.838	26.361	-15.320	1.00	0.00	LX0	C
ATOM	4419	CD	LYS	487	34.950	26.780	-16.785	1.00	0.00	LX0	C
ATOM	4420	CE	LYS	487	36.379	26.643	-17.306	1.00	0.00	LX0	C
ATOM	4421	NZ	LYS	487	37.233	27.567	-16.552	1.00	0.00	LX0	N
ATOM	4422	HZ1	LYS	487	38.164	27.677	-16.991	1.00	0.00	LX0	H
ATOM	4423	HZ2	LYS	487	36.772	28.499	-16.464	1.00	0.00	LX0	H
ATOM	4424	HZ3	LYS	487	37.378	27.241	-15.570	1.00	0.00	LX0	H
ATOM	4425	C	LYS	487	32.364	28.943	-13.793	1.00	0.00	LX0	C
ATOM	4426	O	LYS	487	32.399	29.663	-12.801	1.00	0.00	LX0	O
ATOM	4427	N	THR	488	31.242	28.473	-14.348	1.00	0.00	LX0	N
ATOM	4428	H	THR	488	31.224	27.961	-15.205	1.00	0.00	LX0	H
ATOM	4429	CA	THR	488	30.030	28.566	-13.552	1.00	0.00	LX0	C
ATOM	4430	CB	THR	488	29.122	29.705	-14.044	1.00	0.00	LX0	C
ATOM	4431	OG1	THR	488	27.980	29.838	-13.189	1.00	0.00	LX0	O
ATOM	4432	HG1	THR	488	27.445	30.543	-13.536	1.00	0.00	LX0	H
ATOM	4433	CG2	THR	488	28.718	29.580	-15.518	1.00	0.00	LX0	C
ATOM	4434	C	THR	488	29.339	27.218	-13.513	1.00	0.00	LX0	C
ATOM	4435	O	THR	488	28.843	26.690	-14.502	1.00	0.00	LX0	O
ATOM	4436	N	LYS	489	29.373	26.640	-12.312	1.00	0.00	LX0	N
ATOM	4437	H	LYS	489	29.801	27.086	-11.525	1.00	0.00	LX0	H
ATOM	4438	CA	LYS	489	28.682	25.364	-12.211	1.00	0.00	LX0	C
ATOM	4439	CB	LYS	489	29.554	24.358	-11.463	1.00	0.00	LX0	C
ATOM	4440	CG	LYS	489	30.907	24.215	-12.168	1.00	0.00	LX0	C
ATOM	4441	CD	LYS	489	31.894	23.283	-11.468	1.00	0.00	LX0	C
ATOM	4442	CE	LYS	489	32.309	23.744	-10.069	1.00	0.00	LX0	C
ATOM	4443	NZ	LYS	489	33.002	25.035	-10.118	1.00	0.00	LX0	N
ATOM	4444	HZ1	LYS	489	32.356	25.856	-10.123	1.00	0.00	LX0	H
ATOM	4445	HZ2	LYS	489	33.674	25.125	-9.326	1.00	0.00	LX0	H
ATOM	4446	HZ3	LYS	489	33.592	25.158	-10.972	1.00	0.00	LX0	H
ATOM	4447	C	LYS	489	27.307	25.547	-11.616	1.00	0.00	LX0	C
ATOM	4448	O	LYS	489	27.125	25.674	-10.410	1.00	0.00	LX0	O
ATOM	4449	N	ILE	490	26.349	25.607	-12.546	1.00	0.00	LX0	N
ATOM	4450	H	ILE	490	26.606	25.497	-13.507	1.00	0.00	LX0	H
ATOM	4451	CA	ILE	490	24.980	25.932	-12.147	1.00	0.00	LX0	C
ATOM	4452	CB	ILE	490	24.559	27.296	-12.725	1.00	0.00	LX0	C
ATOM	4453	CG2	ILE	490	25.218	28.437	-11.945	1.00	0.00	LX0	C
ATOM	4454	CG1	ILE	490	24.831	27.393	-14.233	1.00	0.00	LX0	C
ATOM	4455	CD1	ILE	490	24.345	28.704	-14.854	1.00	0.00	LX0	C
ATOM	4456	C	ILE	490	23.965	24.846	-12.471	1.00	0.00	LX0	C
ATOM	4457	O	ILE	490	22.901	25.078	-13.039	1.00	0.00	LX0	O
ATOM	4458	N	ILE	491	24.359	23.619	-12.108	1.00	0.00	LX0	N
ATOM	4459	H	ILE	491	25.106	23.522	-11.448	1.00	0.00	LX0	H
ATOM	4460	CA	ILE	491	23.476	22.507	-12.456	1.00	0.00	LX0	C
ATOM	4461	CB	ILE	491	24.270	21.201	-12.619	1.00	0.00	LX0	C
ATOM	4462	CG2	ILE	491	25.295	21.346	-13.747	1.00	0.00	LX0	C
ATOM	4463	CG1	ILE	491	24.903	20.724	-11.306	1.00	0.00	LX0	C
ATOM	4464	CD1	ILE	491	25.520	19.328	-11.402	1.00	0.00	LX0	C
ATOM	4465	C	ILE	491	22.317	22.336	-11.489	1.00	0.00	LX0	C

ATOM	4466	O	ILE	491	22.285	22.913	-10.408	1.00	0.00	LX0 O
ATOM	4467	N	SER	492	21.352	21.516	-11.939	1.00	0.00	LX0 N
ATOM	4468	H	SER	492	21.513	21.006	-12.780	1.00	0.00	LX0 H
ATOM	4469	CA	SER	492	20.233	21.125	-11.076	1.00	0.00	LX0 C
ATOM	4470	CB	SER	492	20.692	20.022	-10.123	1.00	0.00	LX0 C
ATOM	4471	OG	SER	492	21.535	19.113	-10.847	1.00	0.00	LX0 O
ATOM	4472	HG	SER	492	21.644	18.354	-10.288	1.00	0.00	LX0 H
ATOM	4473	C	SER	492	19.506	22.244	-10.343	1.00	0.00	LX0 C
ATOM	4474	O	SER	492	19.189	22.177	-9.163	1.00	0.00	LX0 O
ATOM	4475	N	ASN	493	19.267	23.297	-11.125	1.00	0.00	LX0 N
ATOM	4476	H	ASN	493	19.471	23.251	-12.101	1.00	0.00	LX0 H
ATOM	4477	CA	ASN	493	18.417	24.361	-10.603	1.00	0.00	LX0 C
ATOM	4478	CB	ASN	493	19.091	25.728	-10.770	1.00	0.00	LX0 C
ATOM	4479	CG	ASN	493	20.388	25.807	-9.985	1.00	0.00	LX0 C
ATOM	4480	OD1	ASN	493	20.433	25.786	-8.762	1.00	0.00	LX0 O
ATOM	4481	ND2	ASN	493	21.470	25.936	-10.750	1.00	0.00	LX0 N
ATOM	4482	HD21	ASN	493	21.477	25.876	-11.749	1.00	0.00	LX0 H
ATOM	4483	HD22	ASN	493	22.349	26.078	-10.293	1.00	0.00	LX0 H
ATOM	4484	C	ASN	493	17.147	24.296	-11.417	1.00	0.00	LX0 C
ATOM	4485	O	ASN	493	17.133	23.635	-12.448	1.00	0.00	LX0 O
ATOM	4486	N	ARG	494	16.099	25.002	-10.959	1.00	0.00	LX0 N
ATOM	4487	H	ARG	494	16.196	25.528	-10.115	1.00	0.00	LX0 H
ATOM	4488	CA	ARG	494	14.858	25.019	-11.750	1.00	0.00	LX0 C
ATOM	4489	CB	ARG	494	13.794	25.831	-10.992	1.00	0.00	LX0 C
ATOM	4490	CG	ARG	494	12.524	26.191	-11.763	1.00	0.00	LX0 C
ATOM	4491	CD	ARG	494	11.483	26.943	-10.933	1.00	0.00	LX0 C
ATOM	4492	NE	ARG	494	10.475	27.544	-11.807	1.00	0.00	LX0 N
ATOM	4493	HE	ARG	494	10.159	27.067	-12.630	1.00	0.00	LX0 H
ATOM	4494	CZ	ARG	494	10.022	28.792	-11.574	1.00	0.00	LX0 C
ATOM	4495	NH1	ARG	494	10.367	29.460	-10.480	1.00	0.00	LX0 N
ATOM	4496	HH11	ARG	494	10.050	30.410	-10.381	1.00	0.00	LX0 H
ATOM	4497	HH12	ARG	494	10.944	29.054	-9.772	1.00	0.00	LX0 H
ATOM	4498	NH2	ARG	494	9.230	29.379	-12.454	1.00	0.00	LX0 N
ATOM	4499	HH21	ARG	494	8.951	30.334	-12.310	1.00	0.00	LX0 H
ATOM	4500	HH22	ARG	494	8.889	28.907	-13.279	1.00	0.00	LX0 H
ATOM	4501	C	ARG	494	15.056	25.471	-13.201	1.00	0.00	LX0 C
ATOM	4502	O	ARG	494	14.473	24.941	-14.142	1.00	0.00	LX0 O
ATOM	4503	N	GLY	495	15.969	26.443	-13.334	1.00	0.00	LX0 N
ATOM	4504	H	GLY	495	16.254	26.940	-12.514	1.00	0.00	LX0 H
ATOM	4505	CA	GLY	495	16.570	26.672	-14.644	1.00	0.00	LX0 C
ATOM	4506	C	GLY	495	15.699	27.361	-15.676	1.00	0.00	LX0 C
ATOM	4507	O	GLY	495	14.551	27.726	-15.450	1.00	0.00	LX0 O
ATOM	4508	N	GLU	496	16.353	27.531	-16.840	1.00	0.00	LX0 N
ATOM	4509	H	GLU	496	17.272	27.148	-16.906	1.00	0.00	LX0 H
ATOM	4510	CA	GLU	496	15.817	28.314	-17.958	1.00	0.00	LX0 C
ATOM	4511	CB	GLU	496	16.754	28.115	-19.165	1.00	0.00	LX0 C
ATOM	4512	CG	GLU	496	16.576	29.030	-20.387	1.00	0.00	LX0 C
ATOM	4513	CD	GLU	496	15.254	28.767	-21.080	1.00	0.00	LX0 C
ATOM	4514	OE1	GLU	496	14.347	29.576	-20.943	1.00	0.00	LX0 O
ATOM	4515	OE2	GLU	496	15.096	27.739	-21.731	1.00	0.00	LX0 O
ATOM	4516	C	GLU	496	14.354	28.034	-18.281	1.00	0.00	LX0 C
ATOM	4517	O	GLU	496	13.460	28.834	-18.024	1.00	0.00	LX0 O
ATOM	4518	N	ASN	497	14.137	26.823	-18.826	1.00	0.00	LX0 N
ATOM	4519	H	ASN	497	14.921	26.267	-19.093	1.00	0.00	LX0 H
ATOM	4520	CA	ASN	497	12.790	26.538	-19.321	1.00	0.00	LX0 C
ATOM	4521	CB	ASN	497	12.713	25.151	-19.957	1.00	0.00	LX0 C
ATOM	4522	CG	ASN	497	11.546	25.147	-20.921	1.00	0.00	LX0 C
ATOM	4523	OD1	ASN	497	11.441	26.004	-21.791	1.00	0.00	LX0 O
ATOM	4524	ND2	ASN	497	10.666	24.162	-20.728	1.00	0.00	LX0 N
ATOM	4525	HD21	ASN	497	10.787	23.475	-20.012	1.00	0.00	LX0 H
ATOM	4526	HD22	ASN	497	9.865	24.116	-21.323	1.00	0.00	LX0 H

ATOM	4527	C	ASN	497	11.685	26.716	-18.300	1.00	0.00	LX0	C
ATOM	4528	O	ASN	497	10.660	27.339	-18.537	1.00	0.00	LX0	O
ATOM	4529	N	SER	498	11.974	26.165	-17.122	1.00	0.00	LX0	N
ATOM	4530	H	SER	498	12.810	25.637	-16.966	1.00	0.00	LX0	H
ATOM	4531	CA	SER	498	11.007	26.244	-16.035	1.00	0.00	LX0	C
ATOM	4532	CB	SER	498	11.375	25.182	-15.004	1.00	0.00	LX0	C
ATOM	4533	OG	SER	498	12.246	24.213	-15.608	1.00	0.00	LX0	O
ATOM	4534	HG	SER	498	13.026	24.176	-15.051	1.00	0.00	LX0	H
ATOM	4535	C	SER	498	10.831	27.628	-15.412	1.00	0.00	LX0	C
ATOM	4536	O	SER	498	9.874	27.905	-14.695	1.00	0.00	LX0	O
ATOM	4537	N	CYS	499	11.797	28.500	-15.738	1.00	0.00	LX0	N
ATOM	4538	H	CYS	499	12.607	28.197	-16.245	1.00	0.00	LX0	H
ATOM	4539	CA	CYS	499	11.647	29.919	-15.428	1.00	0.00	LX0	C
ATOM	4540	CB	CYS	499	13.021	30.594	-15.467	1.00	0.00	LX0	C
ATOM	4541	SG	CYS	499	13.061	32.298	-14.866	1.00	0.00	LX0	S
ATOM	4542	C	CYS	499	10.670	30.600	-16.372	1.00	0.00	LX0	C
ATOM	4543	O	CYS	499	9.677	31.196	-15.963	1.00	0.00	LX0	O
ATOM	4544	N	LYS	500	10.964	30.452	-17.678	1.00	0.00	LX0	N
ATOM	4545	H	LYS	500	11.763	29.920	-17.972	1.00	0.00	LX0	H
ATOM	4546	CA	LYS	500	10.061	31.106	-18.627	1.00	0.00	LX0	C
ATOM	4547	CB	LYS	500	10.681	31.251	-20.022	1.00	0.00	LX0	C
ATOM	4548	CG	LYS	500	10.862	29.976	-20.847	1.00	0.00	LX0	C
ATOM	4549	CD	LYS	500	11.408	30.308	-22.240	1.00	0.00	LX0	C
ATOM	4550	CE	LYS	500	11.720	29.076	-23.094	1.00	0.00	LX0	C
ATOM	4551	NZ	LYS	500	12.712	28.245	-22.410	1.00	0.00	LX0	N
ATOM	4552	HZ1	LYS	500	13.666	28.261	-22.833	1.00	0.00	LX0	H
ATOM	4553	HZ2	LYS	500	12.938	28.628	-21.467	1.00	0.00	LX0	H
ATOM	4554	HZ3	LYS	500	12.404	27.260	-22.309	1.00	0.00	LX0	H
ATOM	4555	C	LYS	500	8.654	30.532	-18.686	1.00	0.00	LX0	C
ATOM	4556	O	LYS	500	7.692	31.218	-19.000	1.00	0.00	LX0	O
ATOM	4557	N	ALA	501	8.564	29.252	-18.290	1.00	0.00	LX0	N
ATOM	4558	H	ALA	501	9.402	28.720	-18.166	1.00	0.00	LX0	H
ATOM	4559	CA	ALA	501	7.249	28.631	-18.129	1.00	0.00	LX0	C
ATOM	4560	CB	ALA	501	7.397	27.166	-17.718	1.00	0.00	LX0	C
ATOM	4561	C	ALA	501	6.339	29.322	-17.123	1.00	0.00	LX0	C
ATOM	4562	O	ALA	501	5.122	29.222	-17.168	1.00	0.00	LX0	O
ATOM	4563	N	THR	502	6.983	30.049	-16.204	1.00	0.00	LX0	N
ATOM	4564	H	THR	502	7.980	30.090	-16.146	1.00	0.00	LX0	H
ATOM	4565	CA	THR	502	6.160	30.841	-15.299	1.00	0.00	LX0	C
ATOM	4566	CB	THR	502	6.484	30.447	-13.858	1.00	0.00	LX0	C
ATOM	4567	OG1	THR	502	6.951	29.086	-13.820	1.00	0.00	LX0	O
ATOM	4568	HG1	THR	502	6.245	28.571	-14.199	1.00	0.00	LX0	H
ATOM	4569	CG2	THR	502	5.292	30.630	-12.915	1.00	0.00	LX0	C
ATOM	4570	C	THR	502	6.312	32.338	-15.527	1.00	0.00	LX0	C
ATOM	4571	O	THR	502	6.214	33.157	-14.624	1.00	0.00	LX0	O
ATOM	4572	N	GLY	503	6.602	32.659	-16.798	1.00	0.00	LX0	N
ATOM	4573	H	GLY	503	6.653	31.949	-17.502	1.00	0.00	LX0	H
ATOM	4574	CA	GLY	503	6.736	34.062	-17.186	1.00	0.00	LX0	C
ATOM	4575	C	GLY	503	7.836	34.860	-16.503	1.00	0.00	LX0	C
ATOM	4576	O	GLY	503	7.826	36.083	-16.512	1.00	0.00	LX0	O
ATOM	4577	N	GLN	504	8.809	34.139	-15.919	1.00	0.00	LX0	N
ATOM	4578	H	GLN	504	8.849	33.144	-16.009	1.00	0.00	LX0	H
ATOM	4579	CA	GLN	504	9.808	34.885	-15.151	1.00	0.00	LX0	C
ATOM	4580	CB	GLN	504	10.307	34.050	-13.968	1.00	0.00	LX0	C
ATOM	4581	CG	GLN	504	9.223	33.723	-12.938	1.00	0.00	LX0	C
ATOM	4582	CD	GLN	504	9.835	33.013	-11.745	1.00	0.00	LX0	C
ATOM	4583	OE1	GLN	504	9.542	31.858	-11.442	1.00	0.00	LX0	O
ATOM	4584	NE2	GLN	504	10.696	33.760	-11.049	1.00	0.00	LX0	N
ATOM	4585	HE21	GLN	504	10.946	34.684	-11.357	1.00	0.00	LX0	H
ATOM	4586	HE22	GLN	504	11.119	33.432	-10.209	1.00	0.00	LX0	H
ATOM	4587	C	GLN	504	10.974	35.432	-15.964	1.00	0.00	LX0	C

ATOM	4588	O	GLN	504	12.137	35.289	-15.609	1.00	0.00	LX0	O
ATOM	4589	N	VAL	505	10.614	36.053	-17.094	1.00	0.00	LX0	N
ATOM	4590	H	VAL	505	9.655	36.314	-17.207	1.00	0.00	LX0	H
ATOM	4591	CA	VAL	505	11.670	36.632	-17.918	1.00	0.00	LX0	C
ATOM	4592	CB	VAL	505	11.349	36.485	-19.411	1.00	0.00	LX0	C
ATOM	4593	CG1	VAL	505	11.404	35.014	-19.821	1.00	0.00	LX0	C
ATOM	4594	CG2	VAL	505	10.018	37.142	-19.794	1.00	0.00	LX0	C
ATOM	4595	C	VAL	505	11.927	38.079	-17.551	1.00	0.00	LX0	C
ATOM	4596	O	VAL	505	11.120	38.725	-16.899	1.00	0.00	LX0	O
ATOM	4597	N	CYS	506	13.093	38.562	-17.996	1.00	0.00	LX0	N
ATOM	4598	H	CYS	506	13.752	37.957	-18.438	1.00	0.00	LX0	H
ATOM	4599	CA	CYS	506	13.453	39.894	-17.514	1.00	0.00	LX0	C
ATOM	4600	CB	CYS	506	14.471	39.739	-16.393	1.00	0.00	LX0	C
ATOM	4601	SG	CYS	506	15.931	38.823	-16.960	1.00	0.00	LX0	S
ATOM	4602	C	CYS	506	13.956	40.874	-18.562	1.00	0.00	LX0	C
ATOM	4603	O	CYS	506	14.120	42.064	-18.309	1.00	0.00	LX0	O
ATOM	4604	N	HIS	507	14.214	40.321	-19.761	1.00	0.00	LX0	N
ATOM	4605	H	HIS	507	13.947	39.379	-19.946	1.00	0.00	LX0	H
ATOM	4606	CA	HIS	507	14.801	41.139	-20.824	1.00	0.00	LX0	C
ATOM	4607	CB	HIS	507	15.319	40.225	-21.944	1.00	0.00	LX0	C
ATOM	4608	CG	HIS	507	15.978	41.033	-23.039	1.00	0.00	LX0	C
ATOM	4609	ND1	HIS	507	15.290	41.743	-23.950	1.00	0.00	LX0	N
ATOM	4610	HD1	HIS	507	14.310	41.823	-23.991	1.00	0.00	LX0	H
ATOM	4611	CD2	HIS	507	17.342	41.194	-23.283	1.00	0.00	LX0	C
ATOM	4612	NE2	HIS	507	17.466	42.015	-24.356	1.00	0.00	LX0	N
ATOM	4613	CE1	HIS	507	16.203	42.353	-24.768	1.00	0.00	LX0	C
ATOM	4614	C	HIS	507	13.850	42.203	-21.354	1.00	0.00	LX0	C
ATOM	4615	O	HIS	507	13.069	41.975	-22.271	1.00	0.00	LX0	O
ATOM	4616	N	ALA	508	13.970	43.365	-20.703	1.00	0.00	LX0	N
ATOM	4617	H	ALA	508	14.749	43.434	-20.075	1.00	0.00	LX0	H
ATOM	4618	CA	ALA	508	13.082	44.514	-20.884	1.00	0.00	LX0	C
ATOM	4619	CB	ALA	508	11.605	44.162	-20.652	1.00	0.00	LX0	C
ATOM	4620	C	ALA	508	13.461	45.570	-19.868	1.00	0.00	LX0	C
ATOM	4621	O	ALA	508	13.742	46.715	-20.187	1.00	0.00	LX0	O
ATOM	4622	N	LEU	509	13.492	45.095	-18.613	1.00	0.00	LX0	N
ATOM	4623	H	LEU	509	13.255	44.136	-18.457	1.00	0.00	LX0	H
ATOM	4624	CA	LEU	509	14.090	45.919	-17.562	1.00	0.00	LX0	C
ATOM	4625	CB	LEU	509	13.343	45.757	-16.226	1.00	0.00	LX0	C
ATOM	4626	CG	LEU	509	13.207	44.330	-15.670	1.00	0.00	LX0	C
ATOM	4627	CD1	LEU	509	13.385	44.295	-14.154	1.00	0.00	LX0	C
ATOM	4628	CD2	LEU	509	11.906	43.645	-16.096	1.00	0.00	LX0	C
ATOM	4629	C	LEU	509	15.572	45.609	-17.420	1.00	0.00	LX0	C
ATOM	4630	O	LEU	509	16.438	46.457	-17.222	1.00	0.00	LX0	O
ATOM	4631	N	CYS	510	15.826	44.309	-17.594	1.00	0.00	LX0	N
ATOM	4632	H	CYS	510	15.085	43.651	-17.718	1.00	0.00	LX0	H
ATOM	4633	CA	CYS	510	17.206	43.876	-17.706	1.00	0.00	LX0	C
ATOM	4634	CB	CYS	510	17.336	42.448	-17.207	1.00	0.00	LX0	C
ATOM	4635	SG	CYS	510	16.503	42.186	-15.627	1.00	0.00	LX0	S
ATOM	4636	C	CYS	510	17.678	43.945	-19.136	1.00	0.00	LX0	C
ATOM	4637	O	CYS	510	16.891	43.973	-20.079	1.00	0.00	LX0	O
ATOM	4638	N	SER	511	19.006	43.969	-19.238	1.00	0.00	LX0	N
ATOM	4639	H	SER	511	19.544	43.902	-18.396	1.00	0.00	LX0	H
ATOM	4640	CA	SER	511	19.639	43.843	-20.545	1.00	0.00	LX0	C
ATOM	4641	CB	SER	511	20.987	44.556	-20.393	1.00	0.00	LX0	C
ATOM	4642	OG	SER	511	21.842	43.814	-19.515	1.00	0.00	LX0	O
ATOM	4643	HG	SER	511	21.340	43.577	-18.732	1.00	0.00	LX0	H
ATOM	4644	C	SER	511	19.736	42.354	-20.904	1.00	0.00	LX0	C
ATOM	4645	O	SER	511	19.217	41.531	-20.158	1.00	0.00	LX0	O
ATOM	4646	N	PRO	512	20.443	41.987	-22.016	1.00	0.00	LX0	N
ATOM	4647	CD	PRO	512	20.816	42.780	-23.191	1.00	0.00	LX0	C
ATOM	4648	CA	PRO	512	20.908	40.594	-22.128	1.00	0.00	LX0	C

ATOM	4649	CB	PRO	512	21.846	40.641	-23.337	1.00	0.00	LX0	C
ATOM	4650	CG	PRO	512	21.280	41.752	-24.215	1.00	0.00	LX0	C
ATOM	4651	C	PRO	512	21.575	40.032	-20.873	1.00	0.00	LX0	C
ATOM	4652	O	PRO	512	21.420	38.869	-20.522	1.00	0.00	LX0	O
ATOM	4653	N	GLU	513	22.302	40.929	-20.182	1.00	0.00	LX0	N
ATOM	4654	H	GLU	513	22.396	41.882	-20.466	1.00	0.00	LX0	H
ATOM	4655	CA	GLU	513	22.616	40.558	-18.808	1.00	0.00	LX0	C
ATOM	4656	CB	GLU	513	23.796	41.347	-18.261	1.00	0.00	LX0	C
ATOM	4657	CG	GLU	513	25.019	41.374	-19.177	1.00	0.00	LX0	C
ATOM	4658	CD	GLU	513	26.203	41.849	-18.364	1.00	0.00	LX0	C
ATOM	4659	OE1	GLU	513	26.532	41.208	-17.376	1.00	0.00	LX0	O
ATOM	4660	OE2	GLU	513	26.804	42.867	-18.684	1.00	0.00	LX0	O
ATOM	4661	C	GLU	513	21.401	40.791	-17.938	1.00	0.00	LX0	C
ATOM	4662	O	GLU	513	20.980	41.925	-17.709	1.00	0.00	LX0	O
ATOM	4663	N	GLY	514	20.844	39.645	-17.534	1.00	0.00	LX0	N
ATOM	4664	H	GLY	514	21.330	38.782	-17.677	1.00	0.00	LX0	H
ATOM	4665	CA	GLY	514	19.531	39.634	-16.909	1.00	0.00	LX0	C
ATOM	4666	C	GLY	514	19.530	40.068	-15.465	1.00	0.00	LX0	C
ATOM	4667	O	GLY	514	19.827	41.208	-15.130	1.00	0.00	LX0	O
ATOM	4668	N	CYS	515	19.149	39.114	-14.613	1.00	0.00	LX0	N
ATOM	4669	H	CYS	515	18.984	38.168	-14.889	1.00	0.00	LX0	H
ATOM	4670	CA	CYS	515	18.828	39.537	-13.256	1.00	0.00	LX0	C
ATOM	4671	CB	CYS	515	17.376	40.007	-13.200	1.00	0.00	LX0	C
ATOM	4672	SG	CYS	515	16.236	38.734	-13.796	1.00	0.00	LX0	S
ATOM	4673	C	CYS	515	19.047	38.432	-12.255	1.00	0.00	LX0	C
ATOM	4674	O	CYS	515	18.976	37.256	-12.588	1.00	0.00	LX0	O
ATOM	4675	N	TRP	516	19.294	38.857	-11.011	1.00	0.00	LX0	N
ATOM	4676	H	TRP	516	19.373	39.836	-10.809	1.00	0.00	LX0	H
ATOM	4677	CA	TRP	516	19.357	37.870	-9.937	1.00	0.00	LX0	C
ATOM	4678	CB	TRP	516	20.204	38.377	-8.767	1.00	0.00	LX0	C
ATOM	4679	CG	TRP	516	21.618	38.703	-9.184	1.00	0.00	LX0	C
ATOM	4680	CD2	TRP	516	22.766	37.833	-9.157	1.00	0.00	LX0	C
ATOM	4681	CE2	TRP	516	23.891	38.601	-9.605	1.00	0.00	LX0	C
ATOM	4682	CE3	TRP	516	22.939	36.483	-8.788	1.00	0.00	LX0	C
ATOM	4683	CD1	TRP	516	22.101	39.938	-9.646	1.00	0.00	LX0	C
ATOM	4684	NE1	TRP	516	23.437	39.883	-9.894	1.00	0.00	LX0	N
ATOM	4685	HE1	TRP	516	23.991	40.632	-10.200	1.00	0.00	LX0	H
ATOM	4686	CZ2	TRP	516	25.165	38.002	-9.669	1.00	0.00	LX0	C
ATOM	4687	CZ3	TRP	516	24.220	35.895	-8.859	1.00	0.00	LX0	C
ATOM	4688	CH2	TRP	516	25.328	36.652	-9.295	1.00	0.00	LX0	C
ATOM	4689	C	TRP	516	17.993	37.435	-9.413	1.00	0.00	LX0	C
ATOM	4690	O	TRP	516	17.884	36.523	-8.601	1.00	0.00	LX0	O
ATOM	4691	N	GLY	517	16.954	38.110	-9.906	1.00	0.00	LX0	N
ATOM	4692	H	GLY	517	17.065	38.915	-10.486	1.00	0.00	LX0	H
ATOM	4693	CA	GLY	517	15.623	37.844	-9.384	1.00	0.00	LX0	C
ATOM	4694	C	GLY	517	14.613	38.683	-10.137	1.00	0.00	LX0	C
ATOM	4695	O	GLY	517	14.932	39.314	-11.136	1.00	0.00	LX0	O
ATOM	4696	N	PRO	518	13.360	38.641	-9.639	1.00	0.00	LX0	N
ATOM	4697	CD	PRO	518	12.910	37.887	-8.477	1.00	0.00	LX0	C
ATOM	4698	CA	PRO	518	12.266	39.331	-10.329	1.00	0.00	LX0	C
ATOM	4699	CB	PRO	518	11.018	38.790	-9.615	1.00	0.00	LX0	C
ATOM	4700	CG	PRO	518	11.464	37.563	-8.818	1.00	0.00	LX0	C
ATOM	4701	C	PRO	518	12.307	40.855	-10.287	1.00	0.00	LX0	C
ATOM	4702	O	PRO	518	11.611	41.534	-11.032	1.00	0.00	LX0	O
ATOM	4703	N	GLU	519	13.081	41.373	-9.327	1.00	0.00	LX0	N
ATOM	4704	H	GLU	519	13.840	40.877	-8.899	1.00	0.00	LX0	H
ATOM	4705	CA	GLU	519	12.888	42.780	-9.006	1.00	0.00	LX0	C
ATOM	4706	CB	GLU	519	13.263	43.003	-7.538	1.00	0.00	LX0	C
ATOM	4707	CG	GLU	519	12.409	42.163	-6.586	1.00	0.00	LX0	C
ATOM	4708	CD	GLU	519	12.915	42.335	-5.170	1.00	0.00	LX0	C
ATOM	4709	OE1	GLU	519	13.712	41.520	-4.722	1.00	0.00	LX0	O

ATOM	4710	OE2	GLU	519	12.532	43.292	-4.505	1.00	0.00	LX0	O
ATOM	4711	C	GLU	519	13.658	43.708	-9.929	1.00	0.00	LX0	C
ATOM	4712	O	GLU	519	14.725	43.387	-10.434	1.00	0.00	LX0	O
ATOM	4713	N	PRO	520	13.106	44.931	-10.129	1.00	0.00	LX0	N
ATOM	4714	CD	PRO	520	11.781	45.393	-9.724	1.00	0.00	LX0	C
ATOM	4715	CA	PRO	520	13.859	45.954	-10.870	1.00	0.00	LX0	C
ATOM	4716	CB	PRO	520	12.970	47.194	-10.734	1.00	0.00	LX0	C
ATOM	4717	CG	PRO	520	11.556	46.643	-10.565	1.00	0.00	LX0	C
ATOM	4718	C	PRO	520	15.294	46.197	-10.407	1.00	0.00	LX0	C
ATOM	4719	O	PRO	520	16.152	46.623	-11.163	1.00	0.00	LX0	O
ATOM	4720	N	ARG	521	15.525	45.918	-9.118	1.00	0.00	LX0	N
ATOM	4721	H	ARG	521	14.809	45.493	-8.570	1.00	0.00	LX0	H
ATOM	4722	CA	ARG	521	16.872	46.129	-8.586	1.00	0.00	LX0	C
ATOM	4723	CB	ARG	521	16.755	46.548	-7.117	1.00	0.00	LX0	C
ATOM	4724	CG	ARG	521	16.011	45.501	-6.288	1.00	0.00	LX0	C
ATOM	4725	CD	ARG	521	15.596	45.964	-4.893	1.00	0.00	LX0	C
ATOM	4726	NE	ARG	521	14.878	44.875	-4.239	1.00	0.00	LX0	N
ATOM	4727	HE	ARG	521	14.043	44.488	-4.650	1.00	0.00	LX0	H
ATOM	4728	CZ	ARG	521	15.396	44.202	-3.199	1.00	0.00	LX0	C
ATOM	4729	NH1	ARG	521	16.465	44.664	-2.562	1.00	0.00	LX0	N
ATOM	4730	HH11	ARG	521	16.876	44.072	-1.856	1.00	0.00	LX0	H
ATOM	4731	HH12	ARG	521	16.868	45.548	-2.778	1.00	0.00	LX0	H
ATOM	4732	NH2	ARG	521	14.854	43.056	-2.823	1.00	0.00	LX0	N
ATOM	4733	HH21	ARG	521	15.243	42.516	-2.070	1.00	0.00	LX0	H
ATOM	4734	HH22	ARG	521	14.057	42.688	-3.336	1.00	0.00	LX0	H
ATOM	4735	C	ARG	521	17.843	44.964	-8.768	1.00	0.00	LX0	C
ATOM	4736	O	ARG	521	18.921	44.939	-8.190	1.00	0.00	LX0	O
ATOM	4737	N	ASP	522	17.409	43.990	-9.579	1.00	0.00	LX0	N
ATOM	4738	H	ASP	522	16.531	44.044	-10.055	1.00	0.00	LX0	H
ATOM	4739	CA	ASP	522	18.194	42.764	-9.723	1.00	0.00	LX0	C
ATOM	4740	CB	ASP	522	17.256	41.571	-9.858	1.00	0.00	LX0	C
ATOM	4741	CG	ASP	522	16.943	40.924	-8.534	1.00	0.00	LX0	C
ATOM	4742	OD1	ASP	522	15.795	40.937	-8.116	1.00	0.00	LX0	O
ATOM	4743	OD2	ASP	522	17.831	40.340	-7.931	1.00	0.00	LX0	O
ATOM	4744	C	ASP	522	19.117	42.716	-10.926	1.00	0.00	LX0	C
ATOM	4745	O	ASP	522	19.766	41.709	-11.192	1.00	0.00	LX0	O
ATOM	4746	N	CYS	523	19.097	43.797	-11.711	1.00	0.00	LX0	N
ATOM	4747	H	CYS	523	18.686	44.661	-11.425	1.00	0.00	LX0	H
ATOM	4748	CA	CYS	523	19.644	43.584	-13.049	1.00	0.00	LX0	C
ATOM	4749	CB	CYS	523	18.931	44.460	-14.067	1.00	0.00	LX0	C
ATOM	4750	SG	CYS	523	17.137	44.441	-13.824	1.00	0.00	LX0	S
ATOM	4751	C	CYS	523	21.146	43.703	-13.194	1.00	0.00	LX0	C
ATOM	4752	O	CYS	523	21.749	44.738	-12.943	1.00	0.00	LX0	O
ATOM	4753	N	VAL	524	21.720	42.580	-13.652	1.00	0.00	LX0	N
ATOM	4754	H	VAL	524	21.101	41.823	-13.861	1.00	0.00	LX0	H
ATOM	4755	CA	VAL	524	23.169	42.488	-13.853	1.00	0.00	LX0	C
ATOM	4756	CB	VAL	524	23.575	41.066	-14.279	1.00	0.00	LX0	C
ATOM	4757	CG1	VAL	524	25.090	40.876	-14.178	1.00	0.00	LX0	C
ATOM	4758	CG2	VAL	524	22.850	39.987	-13.469	1.00	0.00	LX0	C
ATOM	4759	C	VAL	524	23.722	43.532	-14.822	1.00	0.00	LX0	C
ATOM	4760	O	VAL	524	24.827	44.044	-14.664	1.00	0.00	LX0	O
ATOM	4761	N	SER	525	22.876	43.852	-15.812	1.00	0.00	LX0	N
ATOM	4762	H	SER	525	22.068	43.293	-16.009	1.00	0.00	LX0	H
ATOM	4763	CA	SER	525	23.026	45.148	-16.465	1.00	0.00	LX0	C
ATOM	4764	CB	SER	525	23.941	45.094	-17.690	1.00	0.00	LX0	C
ATOM	4765	OG	SER	525	25.239	44.616	-17.328	1.00	0.00	LX0	O
ATOM	4766	HG	SER	525	25.551	44.169	-18.118	1.00	0.00	LX0	H
ATOM	4767	C	SER	525	21.660	45.679	-16.836	1.00	0.00	LX0	C
ATOM	4768	O	SER	525	20.718	44.933	-17.090	1.00	0.00	LX0	O
ATOM	4769	N	CYS	526	21.579	47.010	-16.803	1.00	0.00	LX0	N
ATOM	4770	H	CYS	526	22.394	47.582	-16.720	1.00	0.00	LX0	H

ATOM	4771	CA	CYS	526	20.255	47.616	-16.834	1.00	0.00	LX0	C
ATOM	4772	CB	CYS	526	20.244	48.767	-15.836	1.00	0.00	LX0	C
ATOM	4773	SG	CYS	526	21.611	49.935	-16.072	1.00	0.00	LX0	S
ATOM	4774	C	CYS	526	19.807	48.086	-18.202	1.00	0.00	LX0	C
ATOM	4775	O	CYS	526	20.600	48.432	-19.072	1.00	0.00	LX0	O
ATOM	4776	N	ARG	527	18.478	48.116	-18.362	1.00	0.00	LX0	N
ATOM	4777	H	ARG	527	17.842	47.766	-17.669	1.00	0.00	LX0	H
ATOM	4778	CA	ARG	527	17.983	48.800	-19.550	1.00	0.00	LX0	C
ATOM	4779	CB	ARG	527	16.605	48.273	-19.948	1.00	0.00	LX0	C
ATOM	4780	CG	ARG	527	16.050	48.729	-21.306	1.00	0.00	LX0	C
ATOM	4781	CD	ARG	527	16.874	48.311	-22.529	1.00	0.00	LX0	C
ATOM	4782	NE	ARG	527	17.900	49.303	-22.852	1.00	0.00	LX0	N
ATOM	4783	HE	ARG	527	17.858	50.203	-22.408	1.00	0.00	LX0	H
ATOM	4784	CZ	ARG	527	18.827	49.093	-23.807	1.00	0.00	LX0	C
ATOM	4785	NH1	ARG	527	18.892	47.923	-24.444	1.00	0.00	LX0	N
ATOM	4786	HH11	ARG	527	19.566	47.745	-25.161	1.00	0.00	LX0	H
ATOM	4787	HH12	ARG	527	18.249	47.193	-24.208	1.00	0.00	LX0	H
ATOM	4788	NH2	ARG	527	19.667	50.080	-24.104	1.00	0.00	LX0	N
ATOM	4789	HH21	ARG	527	20.391	50.001	-24.788	1.00	0.00	LX0	H
ATOM	4790	HH22	ARG	527	19.564	50.957	-23.615	1.00	0.00	LX0	H
ATOM	4791	C	ARG	527	17.992	50.309	-19.388	1.00	0.00	LX0	C
ATOM	4792	O	ARG	527	17.052	50.932	-18.909	1.00	0.00	LX0	O
ATOM	4793	N	ASN	528	19.125	50.852	-19.863	1.00	0.00	LX0	N
ATOM	4794	H	ASN	528	19.869	50.197	-20.009	1.00	0.00	LX0	H
ATOM	4795	CA	ASN	528	19.399	52.297	-19.858	1.00	0.00	LX0	C
ATOM	4796	CB	ASN	528	18.274	53.170	-20.455	1.00	0.00	LX0	C
ATOM	4797	CG	ASN	528	17.974	52.797	-21.894	1.00	0.00	LX0	C
ATOM	4798	OD1	ASN	528	18.663	52.010	-22.537	1.00	0.00	LX0	O
ATOM	4799	ND2	ASN	528	16.882	53.388	-22.385	1.00	0.00	LX0	N
ATOM	4800	HD21	ASN	528	16.330	54.003	-21.823	1.00	0.00	LX0	H
ATOM	4801	HD22	ASN	528	16.598	53.224	-23.328	1.00	0.00	LX0	H
ATOM	4802	C	ASN	528	19.775	52.787	-18.475	1.00	0.00	LX0	C
ATOM	4803	O	ASN	528	19.728	52.042	-17.502	1.00	0.00	LX0	O
ATOM	4804	N	VAL	529	20.188	54.068	-18.437	1.00	0.00	LX0	N
ATOM	4805	H	VAL	529	20.143	54.629	-19.261	1.00	0.00	LX0	H
ATOM	4806	CA	VAL	529	20.758	54.617	-17.203	1.00	0.00	LX0	C
ATOM	4807	CB	VAL	529	21.272	56.052	-17.419	1.00	0.00	LX0	C
ATOM	4808	CG1	VAL	529	21.992	56.595	-16.178	1.00	0.00	LX0	C
ATOM	4809	CG2	VAL	529	22.182	56.132	-18.648	1.00	0.00	LX0	C
ATOM	4810	C	VAL	529	19.841	54.531	-15.991	1.00	0.00	LX0	C
ATOM	4811	O	VAL	529	18.936	55.329	-15.758	1.00	0.00	LX0	O
ATOM	4812	N	SER	530	20.141	53.493	-15.213	1.00	0.00	LX0	N
ATOM	4813	H	SER	530	20.888	52.872	-15.449	1.00	0.00	LX0	H
ATOM	4814	CA	SER	530	19.316	53.284	-14.039	1.00	0.00	LX0	C
ATOM	4815	CB	SER	530	19.122	51.792	-13.832	1.00	0.00	LX0	C
ATOM	4816	OG	SER	530	18.510	51.259	-15.020	1.00	0.00	LX0	O
ATOM	4817	HG	SER	530	19.019	51.571	-15.769	1.00	0.00	LX0	H
ATOM	4818	C	SER	530	19.802	54.029	-12.818	1.00	0.00	LX0	C
ATOM	4819	O	SER	530	20.467	53.523	-11.924	1.00	0.00	LX0	O
ATOM	4820	N	ARG	531	19.397	55.306	-12.851	1.00	0.00	LX0	N
ATOM	4821	H	ARG	531	18.969	55.603	-13.703	1.00	0.00	LX0	H
ATOM	4822	CA	ARG	531	19.419	56.113	-11.632	1.00	0.00	LX0	C
ATOM	4823	CB	ARG	531	18.981	57.557	-11.941	1.00	0.00	LX0	C
ATOM	4824	CG	ARG	531	17.911	57.777	-13.029	1.00	0.00	LX0	C
ATOM	4825	CD	ARG	531	16.420	57.594	-12.683	1.00	0.00	LX0	C
ATOM	4826	NE	ARG	531	16.030	56.245	-12.261	1.00	0.00	LX0	N
ATOM	4827	HE	ARG	531	15.731	56.073	-11.313	1.00	0.00	LX0	H
ATOM	4828	CZ	ARG	531	15.979	55.159	-13.062	1.00	0.00	LX0	C
ATOM	4829	NH1	ARG	531	16.357	55.189	-14.339	1.00	0.00	LX0	N
ATOM	4830	HH11	ARG	531	16.287	54.369	-14.921	1.00	0.00	LX0	H
ATOM	4831	HH12	ARG	531	16.723	56.019	-14.757	1.00	0.00	LX0	H

ATOM	4832	NH2	ARG	531	15.541	54.039	-12.530	1.00	0.00	LX0	N
ATOM	4833	HH21	ARG	531	15.568	53.141	-12.974	1.00	0.00	LX0	H
ATOM	4834	HH22	ARG	531	15.149	54.060	-11.596	1.00	0.00	LX0	H
ATOM	4835	C	ARG	531	18.588	55.488	-10.526	1.00	0.00	LX0	C
ATOM	4836	O	ARG	531	17.853	54.537	-10.757	1.00	0.00	LX0	O
ATOM	4837	N	GLY	532	18.738	56.057	-9.322	1.00	0.00	LX0	N
ATOM	4838	H	GLY	532	19.409	56.780	-9.182	1.00	0.00	LX0	H
ATOM	4839	CA	GLY	532	17.984	55.478	-8.212	1.00	0.00	LX0	C
ATOM	4840	C	GLY	532	18.426	54.061	-7.901	1.00	0.00	LX0	C
ATOM	4841	O	GLY	532	19.606	53.787	-7.686	1.00	0.00	LX0	O
ATOM	4842	N	ARG	533	17.429	53.173	-7.890	1.00	0.00	LX0	N
ATOM	4843	H	ARG	533	16.503	53.482	-8.128	1.00	0.00	LX0	H
ATOM	4844	CA	ARG	533	17.746	51.763	-7.687	1.00	0.00	LX0	C
ATOM	4845	CB	ARG	533	17.463	51.333	-6.242	1.00	0.00	LX0	C
ATOM	4846	CG	ARG	533	18.719	50.840	-5.516	1.00	0.00	LX0	C
ATOM	4847	CD	ARG	533	19.671	51.980	-5.147	1.00	0.00	LX0	C
ATOM	4848	NE	ARG	533	21.018	51.488	-4.867	1.00	0.00	LX0	N
ATOM	4849	HE	ARG	533	21.148	50.976	-4.017	1.00	0.00	LX0	H
ATOM	4850	CZ	ARG	533	22.008	51.685	-5.768	1.00	0.00	LX0	C
ATOM	4851	NH1	ARG	533	21.789	52.382	-6.886	1.00	0.00	LX0	N
ATOM	4852	HH11	ARG	533	22.489	52.519	-7.585	1.00	0.00	LX0	H
ATOM	4853	HH12	ARG	533	20.890	52.803	-7.068	1.00	0.00	LX0	H
ATOM	4854	NH2	ARG	533	23.214	51.171	-5.526	1.00	0.00	LX0	N
ATOM	4855	HH21	ARG	533	23.973	51.289	-6.168	1.00	0.00	LX0	H
ATOM	4856	HH22	ARG	533	23.387	50.648	-4.690	1.00	0.00	LX0	H
ATOM	4857	C	ARG	533	17.038	50.827	-8.651	1.00	0.00	LX0	C
ATOM	4858	O	ARG	533	17.071	49.612	-8.502	1.00	0.00	LX0	O
ATOM	4859	N	GLU	534	16.361	51.439	-9.626	1.00	0.00	LX0	N
ATOM	4860	H	GLU	534	16.410	52.427	-9.788	1.00	0.00	LX0	H
ATOM	4861	CA	GLU	534	15.466	50.619	-10.431	1.00	0.00	LX0	C
ATOM	4862	CB	GLU	534	14.007	51.106	-10.344	1.00	0.00	LX0	C
ATOM	4863	CG	GLU	534	13.690	52.248	-9.358	1.00	0.00	LX0	C
ATOM	4864	CD	GLU	534	14.142	53.583	-9.926	1.00	0.00	LX0	C
ATOM	4865	OE1	GLU	534	13.461	54.125	-10.787	1.00	0.00	LX0	O
ATOM	4866	OE2	GLU	534	15.209	54.071	-9.569	1.00	0.00	LX0	O
ATOM	4867	C	GLU	534	15.914	50.512	-11.870	1.00	0.00	LX0	C
ATOM	4868	O	GLU	534	16.027	51.494	-12.594	1.00	0.00	LX0	O
ATOM	4869	N	CYS	535	16.181	49.268	-12.272	1.00	0.00	LX0	N
ATOM	4870	H	CYS	535	16.115	48.483	-11.656	1.00	0.00	LX0	H
ATOM	4871	CA	CYS	535	16.637	49.102	-13.647	1.00	0.00	LX0	C
ATOM	4872	CB	CYS	535	17.290	47.747	-13.842	1.00	0.00	LX0	C
ATOM	4873	SG	CYS	535	18.760	47.581	-12.794	1.00	0.00	LX0	S
ATOM	4874	C	CYS	535	15.601	49.365	-14.715	1.00	0.00	LX0	C
ATOM	4875	O	CYS	535	14.742	48.550	-15.025	1.00	0.00	LX0	O
ATOM	4876	N	VAL	536	15.750	50.578	-15.256	1.00	0.00	LX0	N
ATOM	4877	H	VAL	536	16.492	51.148	-14.894	1.00	0.00	LX0	H
ATOM	4878	CA	VAL	536	14.926	51.067	-16.355	1.00	0.00	LX0	C
ATOM	4879	CB	VAL	536	13.459	51.278	-15.921	1.00	0.00	LX0	C
ATOM	4880	CG1	VAL	536	13.314	52.246	-14.740	1.00	0.00	LX0	C
ATOM	4881	CG2	VAL	536	12.580	51.659	-17.114	1.00	0.00	LX0	C
ATOM	4882	C	VAL	536	15.534	52.344	-16.908	1.00	0.00	LX0	C
ATOM	4883	O	VAL	536	16.075	53.174	-16.175	1.00	0.00	LX0	O
ATOM	4884	N	SER	22	41.445	32.477	38.080	1.00	0.00	LX1	N
ATOM	4885	H	SER	22	42.425	32.293	37.976	1.00	0.00	LX1	H
ATOM	4886	CA	SER	22	41.025	33.847	38.377	1.00	0.00	LX1	C
ATOM	4887	CB	SER	22	41.181	34.121	39.881	1.00	0.00	LX1	C
ATOM	4888	OG	SER	22	40.586	35.373	40.242	1.00	0.00	LX1	O
ATOM	4889	HG	SER	22	41.007	36.026	39.685	1.00	0.00	LX1	H
ATOM	4890	C	SER	22	41.786	34.870	37.547	1.00	0.00	LX1	C
ATOM	4891	O	SER	22	41.991	36.010	37.948	1.00	0.00	LX1	O
ATOM	4892	N	THR	23	42.222	34.385	36.383	1.00	0.00	LX1	N

ATOM	4893	H	THR	23	41.997	33.459	36.080	1.00	0.00	LX1	H
ATOM	4894	CA	THR	23	43.158	35.145	35.565	1.00	0.00	LX1	C
ATOM	4895	CB	THR	23	44.587	34.743	35.963	1.00	0.00	LX1	C
ATOM	4896	OG1	THR	23	44.606	33.384	36.432	1.00	0.00	LX1	O
ATOM	4897	HG1	THR	23	45.517	33.156	36.563	1.00	0.00	LX1	H
ATOM	4898	CG2	THR	23	45.183	35.663	37.031	1.00	0.00	LX1	C
ATOM	4899	C	THR	23	42.882	34.816	34.111	1.00	0.00	LX1	C
ATOM	4900	O	THR	23	42.157	33.874	33.813	1.00	0.00	LX1	O
ATOM	4901	N	GLN	24	43.491	35.604	33.213	1.00	0.00	LX1	N
ATOM	4902	H	GLN	24	44.033	36.388	33.517	1.00	0.00	LX1	H
ATOM	4903	CA	GLN	24	43.433	35.185	31.813	1.00	0.00	LX1	C
ATOM	4904	CB	GLN	24	43.780	36.369	30.892	1.00	0.00	LX1	C
ATOM	4905	CG	GLN	24	44.089	36.080	29.410	1.00	0.00	LX1	C
ATOM	4906	CD	GLN	24	42.947	35.418	28.660	1.00	0.00	LX1	C
ATOM	4907	OE1	GLN	24	42.134	34.679	29.199	1.00	0.00	LX1	O
ATOM	4908	NE2	GLN	24	42.945	35.694	27.355	1.00	0.00	LX1	N
ATOM	4909	HE21	GLN	24	43.623	36.317	26.956	1.00	0.00	LX1	H
ATOM	4910	HE22	GLN	24	42.267	35.263	26.763	1.00	0.00	LX1	H
ATOM	4911	C	GLN	24	44.290	33.955	31.559	1.00	0.00	LX1	C
ATOM	4912	O	GLN	24	45.517	33.998	31.567	1.00	0.00	LX1	O
ATOM	4913	N	VAL	25	43.566	32.848	31.378	1.00	0.00	LX1	N
ATOM	4914	H	VAL	25	42.568	32.920	31.344	1.00	0.00	LX1	H
ATOM	4915	CA	VAL	25	44.215	31.554	31.200	1.00	0.00	LX1	C
ATOM	4916	CB	VAL	25	43.907	30.637	32.400	1.00	0.00	LX1	C
ATOM	4917	CG1	VAL	25	44.524	29.245	32.244	1.00	0.00	LX1	C
ATOM	4918	CG2	VAL	25	44.332	31.269	33.726	1.00	0.00	LX1	C
ATOM	4919	C	VAL	25	43.719	30.927	29.913	1.00	0.00	LX1	C
ATOM	4920	O	VAL	25	42.526	30.733	29.721	1.00	0.00	LX1	O
ATOM	4921	N	CYS	26	44.675	30.615	29.038	1.00	0.00	LX1	N
ATOM	4922	H	CYS	26	45.646	30.756	29.246	1.00	0.00	LX1	H
ATOM	4923	CA	CYS	26	44.251	29.957	27.807	1.00	0.00	LX1	C
ATOM	4924	CB	CYS	26	44.232	30.944	26.635	1.00	0.00	LX1	C
ATOM	4925	SG	CYS	26	45.872	31.577	26.215	1.00	0.00	LX1	S
ATOM	4926	C	CYS	26	45.150	28.779	27.525	1.00	0.00	LX1	C
ATOM	4927	O	CYS	26	46.168	28.599	28.181	1.00	0.00	LX1	O
ATOM	4928	N	THR	27	44.754	27.971	26.539	1.00	0.00	LX1	N
ATOM	4929	H	THR	27	43.939	28.122	25.980	1.00	0.00	LX1	H
ATOM	4930	CA	THR	27	45.695	26.935	26.128	1.00	0.00	LX1	C
ATOM	4931	CB	THR	27	44.918	25.870	25.366	1.00	0.00	LX1	C
ATOM	4932	OG1	THR	27	43.948	26.506	24.519	1.00	0.00	LX1	O
ATOM	4933	HG1	THR	27	44.449	26.992	23.870	1.00	0.00	LX1	H
ATOM	4934	CG2	THR	27	44.221	24.904	26.326	1.00	0.00	LX1	C
ATOM	4935	C	THR	27	46.789	27.525	25.263	1.00	0.00	LX1	C
ATOM	4936	O	THR	27	46.543	28.442	24.491	1.00	0.00	LX1	O
ATOM	4937	N	GLY	28	47.992	26.973	25.424	1.00	0.00	LX1	N
ATOM	4938	H	GLY	28	48.081	26.161	26.000	1.00	0.00	LX1	H
ATOM	4939	CA	GLY	28	49.018	27.362	24.464	1.00	0.00	LX1	C
ATOM	4940	C	GLY	28	48.938	26.461	23.259	1.00	0.00	LX1	C
ATOM	4941	O	GLY	28	48.052	25.615	23.169	1.00	0.00	LX1	O
ATOM	4942	N	THR	29	49.883	26.652	22.341	1.00	0.00	LX1	N
ATOM	4943	H	THR	29	50.633	27.310	22.466	1.00	0.00	LX1	H
ATOM	4944	CA	THR	29	49.864	25.658	21.280	1.00	0.00	LX1	C
ATOM	4945	CB	THR	29	50.131	26.314	19.917	1.00	0.00	LX1	C
ATOM	4946	OG1	THR	29	51.205	27.257	19.978	1.00	0.00	LX1	O
ATOM	4947	HG1	THR	29	51.974	26.780	20.296	1.00	0.00	LX1	H
ATOM	4948	CG2	THR	29	48.873	27.001	19.388	1.00	0.00	LX1	C
ATOM	4949	C	THR	29	50.782	24.480	21.557	1.00	0.00	LX1	C
ATOM	4950	O	THR	29	51.467	24.416	22.573	1.00	0.00	LX1	O
ATOM	4951	N	ASP	30	50.751	23.570	20.573	1.00	0.00	LX1	N
ATOM	4952	H	ASP	30	49.999	23.578	19.919	1.00	0.00	LX1	H
ATOM	4953	CA	ASP	30	51.886	22.688	20.316	1.00	0.00	LX1	C

ATOM	4954	CB	ASP	30	51.583	21.263	20.789	1.00	0.00	LX1	C
ATOM	4955	CG	ASP	30	52.819	20.383	20.705	1.00	0.00	LX1	C
ATOM	4956	OD1	ASP	30	52.851	19.504	19.844	1.00	0.00	LX1	O
ATOM	4957	OD2	ASP	30	53.736	20.568	21.506	1.00	0.00	LX1	O
ATOM	4958	C	ASP	30	52.172	22.793	18.825	1.00	0.00	LX1	C
ATOM	4959	O	ASP	30	52.020	21.889	18.009	1.00	0.00	LX1	O
ATOM	4960	N	MET	31	52.478	24.044	18.468	1.00	0.00	LX1	N
ATOM	4961	H	MET	31	52.774	24.659	19.201	1.00	0.00	LX1	H
ATOM	4962	CA	MET	31	52.653	24.341	17.052	1.00	0.00	LX1	C
ATOM	4963	CB	MET	31	52.347	25.813	16.756	1.00	0.00	LX1	C
ATOM	4964	CG	MET	31	52.046	26.069	15.276	1.00	0.00	LX1	C
ATOM	4965	SD	MET	31	51.572	27.765	14.904	1.00	0.00	LX1	S
ATOM	4966	CE	MET	31	53.172	28.536	15.186	1.00	0.00	LX1	C
ATOM	4967	C	MET	31	54.015	23.923	16.536	1.00	0.00	LX1	C
ATOM	4968	O	MET	31	54.164	23.482	15.403	1.00	0.00	LX1	O
ATOM	4969	N	LYS	32	54.994	24.031	17.444	1.00	0.00	LX1	N
ATOM	4970	H	LYS	32	54.774	24.373	18.357	1.00	0.00	LX1	H
ATOM	4971	CA	LYS	32	56.353	23.580	17.162	1.00	0.00	LX1	C
ATOM	4972	CB	LYS	32	56.364	22.049	17.051	1.00	0.00	LX1	C
ATOM	4973	CG	LYS	32	55.901	21.373	18.341	1.00	0.00	LX1	C
ATOM	4974	CD	LYS	32	56.018	19.850	18.296	1.00	0.00	LX1	C
ATOM	4975	CE	LYS	32	56.233	19.239	19.684	1.00	0.00	LX1	C
ATOM	4976	NZ	LYS	32	57.424	19.839	20.296	1.00	0.00	LX1	N
ATOM	4977	HZ1	LYS	32	57.154	20.588	20.964	1.00	0.00	LX1	H
ATOM	4978	HZ2	LYS	32	58.050	19.141	20.742	1.00	0.00	LX1	H
ATOM	4979	HZ3	LYS	32	58.038	20.293	19.584	1.00	0.00	LX1	H
ATOM	4980	C	LYS	32	56.962	24.309	15.972	1.00	0.00	LX1	C
ATOM	4981	O	LYS	32	56.553	25.417	15.638	1.00	0.00	LX1	O
ATOM	4982	N	LEU	33	57.929	23.652	15.313	1.00	0.00	LX1	N
ATOM	4983	H	LEU	33	58.279	22.773	15.637	1.00	0.00	LX1	H
ATOM	4984	CA	LEU	33	58.445	24.259	14.084	1.00	0.00	LX1	C
ATOM	4985	CB	LEU	33	59.795	23.644	13.710	1.00	0.00	LX1	C
ATOM	4986	CG	LEU	33	61.000	24.208	14.458	1.00	0.00	LX1	C
ATOM	4987	CD1	LEU	33	62.247	23.350	14.234	1.00	0.00	LX1	C
ATOM	4988	CD2	LEU	33	61.255	25.670	14.098	1.00	0.00	LX1	C
ATOM	4989	C	LEU	33	57.540	24.175	12.863	1.00	0.00	LX1	C
ATOM	4990	O	LEU	33	58.008	24.363	11.744	1.00	0.00	LX1	O
ATOM	4991	N	ARG	34	56.259	23.832	13.084	1.00	0.00	LX1	N
ATOM	4992	H	ARG	34	55.817	23.906	13.978	1.00	0.00	LX1	H
ATOM	4993	CA	ARG	34	55.501	23.460	11.896	1.00	0.00	LX1	C
ATOM	4994	CB	ARG	34	54.233	22.664	12.243	1.00	0.00	LX1	C
ATOM	4995	CG	ARG	34	52.989	23.515	12.511	1.00	0.00	LX1	C
ATOM	4996	CD	ARG	34	51.770	22.692	12.900	1.00	0.00	LX1	C
ATOM	4997	NE	ARG	34	51.938	22.057	14.204	1.00	0.00	LX1	N
ATOM	4998	HE	ARG	34	52.673	22.408	14.794	1.00	0.00	LX1	H
ATOM	4999	CZ	ARG	34	51.065	21.110	14.598	1.00	0.00	LX1	C
ATOM	5000	NH1	ARG	34	50.189	20.606	13.729	1.00	0.00	LX1	N
ATOM	5001	HH11	ARG	34	49.506	19.937	14.013	1.00	0.00	LX1	H
ATOM	5002	HH12	ARG	34	50.214	20.884	12.758	1.00	0.00	LX1	H
ATOM	5003	NH2	ARG	34	51.075	20.687	15.858	1.00	0.00	LX1	N
ATOM	5004	HH21	ARG	34	50.449	19.981	16.208	1.00	0.00	LX1	H
ATOM	5005	HH22	ARG	34	51.710	21.075	16.539	1.00	0.00	LX1	H
ATOM	5006	C	ARG	34	55.204	24.590	10.936	1.00	0.00	LX1	C
ATOM	5007	O	ARG	34	54.893	25.713	11.316	1.00	0.00	LX1	O
ATOM	5008	N	LEU	35	55.279	24.218	9.655	1.00	0.00	LX1	N
ATOM	5009	H	LEU	35	55.617	23.306	9.425	1.00	0.00	LX1	H
ATOM	5010	CA	LEU	35	54.724	25.125	8.655	1.00	0.00	LX1	C
ATOM	5011	CB	LEU	35	55.023	24.582	7.257	1.00	0.00	LX1	C
ATOM	5012	CG	LEU	35	56.514	24.610	6.920	1.00	0.00	LX1	C
ATOM	5013	CD1	LEU	35	56.837	23.768	5.686	1.00	0.00	LX1	C
ATOM	5014	CD2	LEU	35	57.026	26.043	6.779	1.00	0.00	LX1	C

ATOM	5015	C	LEU	35	53.230	25.337	8.867	1.00	0.00	LX1	C
ATOM	5016	O	LEU	35	52.462	24.391	9.012	1.00	0.00	LX1	O
ATOM	5017	N	PRO	36	52.844	26.632	8.925	1.00	0.00	LX1	N
ATOM	5018	CD	PRO	36	53.701	27.810	8.871	1.00	0.00	LX1	C
ATOM	5019	CA	PRO	36	51.428	26.965	9.103	1.00	0.00	LX1	C
ATOM	5020	CB	PRO	36	51.447	28.497	9.165	1.00	0.00	LX1	C
ATOM	5021	CG	PRO	36	52.870	28.882	9.561	1.00	0.00	LX1	C
ATOM	5022	C	PRO	36	50.560	26.453	7.966	1.00	0.00	LX1	C
ATOM	5023	O	PRO	36	51.041	25.917	6.974	1.00	0.00	LX1	O
ATOM	5024	N	ALA	37	49.248	26.674	8.135	1.00	0.00	LX1	N
ATOM	5025	H	ALA	37	48.901	27.118	8.959	1.00	0.00	LX1	H
ATOM	5026	CA	ALA	37	48.411	26.438	6.962	1.00	0.00	LX1	C
ATOM	5027	CB	ALA	37	46.956	26.198	7.367	1.00	0.00	LX1	C
ATOM	5028	C	ALA	37	48.497	27.598	5.983	1.00	0.00	LX1	C
ATOM	5029	O	ALA	37	48.768	27.434	4.801	1.00	0.00	LX1	O
ATOM	5030	N	SER	38	48.293	28.793	6.558	1.00	0.00	LX1	N
ATOM	5031	H	SER	38	47.924	28.874	7.486	1.00	0.00	LX1	H
ATOM	5032	CA	SER	38	48.395	30.016	5.767	1.00	0.00	LX1	C
ATOM	5033	CB	SER	38	47.201	30.051	4.801	1.00	0.00	LX1	C
ATOM	5034	OG	SER	38	45.960	29.911	5.509	1.00	0.00	LX1	O
ATOM	5035	HG	SER	38	45.655	30.819	5.630	1.00	0.00	LX1	H
ATOM	5036	C	SER	38	48.473	31.211	6.715	1.00	0.00	LX1	C
ATOM	5037	O	SER	38	48.121	31.074	7.882	1.00	0.00	LX1	O
ATOM	5038	N	PRO	39	48.973	32.379	6.220	1.00	0.00	LX1	N
ATOM	5039	CD	PRO	39	49.451	32.647	4.863	1.00	0.00	LX1	C
ATOM	5040	CA	PRO	39	49.131	33.553	7.094	1.00	0.00	LX1	C
ATOM	5041	CB	PRO	39	49.378	34.685	6.093	1.00	0.00	LX1	C
ATOM	5042	CG	PRO	39	50.129	34.009	4.950	1.00	0.00	LX1	C
ATOM	5043	C	PRO	39	48.022	33.847	8.098	1.00	0.00	LX1	C
ATOM	5044	O	PRO	39	48.270	33.991	9.285	1.00	0.00	LX1	O
ATOM	5045	N	GLU	40	46.792	33.927	7.575	1.00	0.00	LX1	N
ATOM	5046	H	GLU	40	46.676	33.711	6.605	1.00	0.00	LX1	H
ATOM	5047	CA	GLU	40	45.645	34.173	8.460	1.00	0.00	LX1	C
ATOM	5048	CB	GLU	40	44.329	34.463	7.716	1.00	0.00	LX1	C
ATOM	5049	CG	GLU	40	44.275	34.258	6.197	1.00	0.00	LX1	C
ATOM	5050	CD	GLU	40	44.731	32.867	5.787	1.00	0.00	LX1	C
ATOM	5051	OE1	GLU	40	44.128	31.876	6.173	1.00	0.00	LX1	O
ATOM	5052	OE2	GLU	40	45.728	32.759	5.086	1.00	0.00	LX1	O
ATOM	5053	C	GLU	40	45.376	33.077	9.475	1.00	0.00	LX1	C
ATOM	5054	O	GLU	40	45.320	33.314	10.675	1.00	0.00	LX1	O
ATOM	5055	N	THR	41	45.234	31.845	8.952	1.00	0.00	LX1	N
ATOM	5056	H	THR	41	45.210	31.752	7.955	1.00	0.00	LX1	H
ATOM	5057	CA	THR	41	44.973	30.721	9.860	1.00	0.00	LX1	C
ATOM	5058	CB	THR	41	44.910	29.393	9.097	1.00	0.00	LX1	C
ATOM	5059	OG1	THR	41	46.034	29.236	8.222	1.00	0.00	LX1	O
ATOM	5060	HG1	THR	41	45.789	29.629	7.385	1.00	0.00	LX1	H
ATOM	5061	CG2	THR	41	43.604	29.250	8.317	1.00	0.00	LX1	C
ATOM	5062	C	THR	41	45.934	30.614	11.037	1.00	0.00	LX1	C
ATOM	5063	O	THR	41	45.564	30.307	12.161	1.00	0.00	LX1	O
ATOM	5064	N	HIS	42	47.188	30.940	10.706	1.00	0.00	LX1	N
ATOM	5065	H	HIS	42	47.380	31.114	9.738	1.00	0.00	LX1	H
ATOM	5066	CA	HIS	42	48.214	31.144	11.722	1.00	0.00	LX1	C
ATOM	5067	CB	HIS	42	49.528	31.246	10.934	1.00	0.00	LX1	C
ATOM	5068	CG	HIS	42	50.717	31.739	11.719	1.00	0.00	LX1	C
ATOM	5069	ND1	HIS	42	51.325	31.060	12.708	1.00	0.00	LX1	N
ATOM	5070	HD1	HIS	42	51.087	30.183	13.074	1.00	0.00	LX1	H
ATOM	5071	CD2	HIS	42	51.392	32.944	11.512	1.00	0.00	LX1	C
ATOM	5072	NE2	HIS	42	52.418	32.985	12.389	1.00	0.00	LX1	N
ATOM	5073	CE1	HIS	42	52.381	31.828	13.124	1.00	0.00	LX1	C
ATOM	5074	C	HIS	42	47.913	32.324	12.654	1.00	0.00	LX1	C
ATOM	5075	O	HIS	42	47.596	32.164	13.827	1.00	0.00	LX1	O

ATOM	5076	N	LEU	43	48.040	33.527	12.078	1.00	0.00	LX1	N
ATOM	5077	H	LEU	43	48.155	33.584	11.088	1.00	0.00	LX1	H
ATOM	5078	CA	LEU	43	48.044	34.738	12.896	1.00	0.00	LX1	C
ATOM	5079	CB	LEU	43	48.386	35.952	12.026	1.00	0.00	LX1	C
ATOM	5080	CG	LEU	43	48.720	37.214	12.826	1.00	0.00	LX1	C
ATOM	5081	CD1	LEU	43	49.955	37.018	13.706	1.00	0.00	LX1	C
ATOM	5082	CD2	LEU	43	48.846	38.443	11.925	1.00	0.00	LX1	C
ATOM	5083	C	LEU	43	46.775	34.985	13.690	1.00	0.00	LX1	C
ATOM	5084	O	LEU	43	46.796	35.207	14.895	1.00	0.00	LX1	O
ATOM	5085	N	ASP	44	45.657	34.932	12.960	1.00	0.00	LX1	N
ATOM	5086	H	ASP	44	45.684	34.756	11.974	1.00	0.00	LX1	H
ATOM	5087	CA	ASP	44	44.381	35.237	13.603	1.00	0.00	LX1	C
ATOM	5088	CB	ASP	44	43.268	35.324	12.555	1.00	0.00	LX1	C
ATOM	5089	CG	ASP	44	43.309	36.679	11.868	1.00	0.00	LX1	C
ATOM	5090	OD1	ASP	44	44.344	37.068	11.338	1.00	0.00	LX1	O
ATOM	5091	OD2	ASP	44	42.329	37.413	11.935	1.00	0.00	LX1	O
ATOM	5092	C	ASP	44	44.030	34.292	14.733	1.00	0.00	LX1	C
ATOM	5093	O	ASP	44	43.609	34.695	15.810	1.00	0.00	LX1	O
ATOM	5094	N	MET	45	44.288	32.998	14.470	1.00	0.00	LX1	N
ATOM	5095	H	MET	45	44.691	32.724	13.596	1.00	0.00	LX1	H
ATOM	5096	CA	MET	45	44.069	32.052	15.566	1.00	0.00	LX1	C
ATOM	5097	CB	MET	45	44.229	30.615	15.063	1.00	0.00	LX1	C
ATOM	5098	CG	MET	45	43.532	29.548	15.916	1.00	0.00	LX1	C
ATOM	5099	SD	MET	45	44.285	29.260	17.526	1.00	0.00	LX1	S
ATOM	5100	CE	MET	45	45.840	28.557	16.955	1.00	0.00	LX1	C
ATOM	5101	C	MET	45	44.935	32.341	16.784	1.00	0.00	LX1	C
ATOM	5102	O	MET	45	44.476	32.389	17.921	1.00	0.00	LX1	O
ATOM	5103	N	LEU	46	46.222	32.602	16.484	1.00	0.00	LX1	N
ATOM	5104	H	LEU	46	46.552	32.541	15.539	1.00	0.00	LX1	H
ATOM	5105	CA	LEU	46	47.111	32.995	17.578	1.00	0.00	LX1	C
ATOM	5106	CB	LEU	46	48.561	33.181	17.120	1.00	0.00	LX1	C
ATOM	5107	CG	LEU	46	49.242	31.941	16.531	1.00	0.00	LX1	C
ATOM	5108	CD1	LEU	46	50.721	32.216	16.267	1.00	0.00	LX1	C
ATOM	5109	CD2	LEU	46	49.058	30.683	17.382	1.00	0.00	LX1	C
ATOM	5110	C	LEU	46	46.670	34.228	18.349	1.00	0.00	LX1	C
ATOM	5111	O	LEU	46	46.869	34.321	19.553	1.00	0.00	LX1	O
ATOM	5112	N	ARG	47	46.039	35.161	17.615	1.00	0.00	LX1	N
ATOM	5113	H	ARG	47	45.926	35.012	16.632	1.00	0.00	LX1	H
ATOM	5114	CA	ARG	47	45.504	36.358	18.262	1.00	0.00	LX1	C
ATOM	5115	CB	ARG	47	44.861	37.288	17.230	1.00	0.00	LX1	C
ATOM	5116	CG	ARG	47	44.410	38.641	17.793	1.00	0.00	LX1	C
ATOM	5117	CD	ARG	47	43.493	39.416	16.844	1.00	0.00	LX1	C
ATOM	5118	NE	ARG	47	44.130	39.595	15.540	1.00	0.00	LX1	N
ATOM	5119	HE	ARG	47	44.984	40.113	15.504	1.00	0.00	LX1	H
ATOM	5120	CZ	ARG	47	43.622	38.974	14.454	1.00	0.00	LX1	C
ATOM	5121	NH1	ARG	47	42.498	38.267	14.525	1.00	0.00	LX1	N
ATOM	5122	HH11	ARG	47	42.141	37.829	13.692	1.00	0.00	LX1	H
ATOM	5123	HH12	ARG	47	41.967	38.109	15.370	1.00	0.00	LX1	H
ATOM	5124	NH2	ARG	47	44.272	39.054	13.297	1.00	0.00	LX1	N
ATOM	5125	HH21	ARG	47	43.916	38.533	12.500	1.00	0.00	LX1	H
ATOM	5126	HH22	ARG	47	45.116	39.567	13.172	1.00	0.00	LX1	H
ATOM	5127	C	ARG	47	44.523	36.040	19.372	1.00	0.00	LX1	C
ATOM	5128	O	ARG	47	44.771	36.296	20.546	1.00	0.00	LX1	O
ATOM	5129	N	HIS	48	43.396	35.446	18.948	1.00	0.00	LX1	N
ATOM	5130	H	HIS	48	43.262	35.217	17.979	1.00	0.00	LX1	H
ATOM	5131	CA	HIS	48	42.369	35.212	19.959	1.00	0.00	LX1	C
ATOM	5132	CB	HIS	48	41.045	34.796	19.322	1.00	0.00	LX1	C
ATOM	5133	CG	HIS	48	39.989	35.813	19.692	1.00	0.00	LX1	C
ATOM	5134	ND1	HIS	48	39.465	36.701	18.830	1.00	0.00	LX1	N
ATOM	5135	HD1	HIS	48	39.687	36.799	17.872	1.00	0.00	LX1	H
ATOM	5136	CD2	HIS	48	39.402	36.016	20.945	1.00	0.00	LX1	C

ATOM	5137	NE2	HIS	48	38.521	37.039	20.819	1.00	0.00	LX1	N
ATOM	5138	CE1	HIS	48	38.559	37.461	19.513	1.00	0.00	LX1	C
ATOM	5139	C	HIS	48	42.768	34.261	21.072	1.00	0.00	LX1	C
ATOM	5140	O	HIS	48	42.359	34.406	22.217	1.00	0.00	LX1	O
ATOM	5141	N	LEU	49	43.619	33.296	20.684	1.00	0.00	LX1	N
ATOM	5142	H	LEU	49	43.891	33.222	19.722	1.00	0.00	LX1	H
ATOM	5143	CA	LEU	49	44.130	32.373	21.694	1.00	0.00	LX1	C
ATOM	5144	CB	LEU	49	44.832	31.203	20.999	1.00	0.00	LX1	C
ATOM	5145	CG	LEU	49	45.182	30.038	21.924	1.00	0.00	LX1	C
ATOM	5146	CD1	LEU	49	43.933	29.400	22.536	1.00	0.00	LX1	C
ATOM	5147	CD2	LEU	49	46.071	29.013	21.221	1.00	0.00	LX1	C
ATOM	5148	C	LEU	49	45.041	33.022	22.730	1.00	0.00	LX1	C
ATOM	5149	O	LEU	49	44.791	33.009	23.927	1.00	0.00	LX1	O
ATOM	5150	N	TYR	50	46.140	33.578	22.206	1.00	0.00	LX1	N
ATOM	5151	H	TYR	50	46.228	33.688	21.216	1.00	0.00	LX1	H
ATOM	5152	CA	TYR	50	47.194	34.033	23.110	1.00	0.00	LX1	C
ATOM	5153	CB	TYR	50	48.525	34.139	22.369	1.00	0.00	LX1	C
ATOM	5154	CG	TYR	50	49.162	32.785	22.154	1.00	0.00	LX1	C
ATOM	5155	CD1	TYR	50	49.376	31.932	23.258	1.00	0.00	LX1	C
ATOM	5156	CE1	TYR	50	50.059	30.721	23.061	1.00	0.00	LX1	C
ATOM	5157	CD2	TYR	50	49.562	32.430	20.851	1.00	0.00	LX1	C
ATOM	5158	CE2	TYR	50	50.248	31.220	20.656	1.00	0.00	LX1	C
ATOM	5159	CZ	TYR	50	50.513	30.398	21.768	1.00	0.00	LX1	C
ATOM	5160	OH	TYR	50	51.259	29.253	21.593	1.00	0.00	LX1	O
ATOM	5161	HH	TYR	50	51.085	28.887	20.728	1.00	0.00	LX1	H
ATOM	5162	C	TYR	50	46.932	35.331	23.848	1.00	0.00	LX1	C
ATOM	5163	O	TYR	50	47.574	35.628	24.848	1.00	0.00	LX1	O
ATOM	5164	N	GLN	51	45.974	36.094	23.296	1.00	0.00	LX1	N
ATOM	5165	H	GLN	51	45.550	35.774	22.447	1.00	0.00	LX1	H
ATOM	5166	CA	GLN	51	45.590	37.414	23.804	1.00	0.00	LX1	C
ATOM	5167	CB	GLN	51	44.142	37.659	23.383	1.00	0.00	LX1	C
ATOM	5168	CG	GLN	51	43.823	39.112	23.035	1.00	0.00	LX1	C
ATOM	5169	CD	GLN	51	43.076	39.168	21.712	1.00	0.00	LX1	C
ATOM	5170	OE1	GLN	51	43.465	39.867	20.787	1.00	0.00	LX1	O
ATOM	5171	NE2	GLN	51	41.973	38.412	21.654	1.00	0.00	LX1	N
ATOM	5172	HE21	GLN	51	41.675	37.831	22.409	1.00	0.00	LX1	H
ATOM	5173	HE22	GLN	51	41.431	38.421	20.814	1.00	0.00	LX1	H
ATOM	5174	C	GLN	51	45.806	37.715	25.287	1.00	0.00	LX1	C
ATOM	5175	O	GLN	51	44.961	37.447	26.135	1.00	0.00	LX1	O
ATOM	5176	N	GLY	52	46.989	38.299	25.552	1.00	0.00	LX1	N
ATOM	5177	H	GLY	52	47.629	38.432	24.792	1.00	0.00	LX1	H
ATOM	5178	CA	GLY	52	47.327	38.733	26.910	1.00	0.00	LX1	C
ATOM	5179	C	GLY	52	47.290	37.660	27.992	1.00	0.00	LX1	C
ATOM	5180	O	GLY	52	46.911	37.895	29.133	1.00	0.00	LX1	O
ATOM	5181	N	CYS	53	47.692	36.451	27.582	1.00	0.00	LX1	N
ATOM	5182	H	CYS	53	47.990	36.307	26.637	1.00	0.00	LX1	H
ATOM	5183	CA	CYS	53	47.514	35.342	28.514	1.00	0.00	LX1	C
ATOM	5184	CB	CYS	53	47.611	34.004	27.796	1.00	0.00	LX1	C
ATOM	5185	SG	CYS	53	46.974	32.673	28.840	1.00	0.00	LX1	S
ATOM	5186	C	CYS	53	48.403	35.345	29.742	1.00	0.00	LX1	C
ATOM	5187	O	CYS	53	49.614	35.177	29.688	1.00	0.00	LX1	O
ATOM	5188	N	GLN	54	47.737	35.518	30.886	1.00	0.00	LX1	N
ATOM	5189	H	GLN	54	46.737	35.545	30.867	1.00	0.00	LX1	H
ATOM	5190	CA	GLN	54	48.496	35.519	32.133	1.00	0.00	LX1	C
ATOM	5191	CB	GLN	54	47.636	36.015	33.289	1.00	0.00	LX1	C
ATOM	5192	CG	GLN	54	47.007	37.373	32.988	1.00	0.00	LX1	C
ATOM	5193	CD	GLN	54	46.312	37.872	34.228	1.00	0.00	LX1	C
ATOM	5194	OE1	GLN	54	45.118	37.689	34.416	1.00	0.00	LX1	O
ATOM	5195	NE2	GLN	54	47.118	38.507	35.083	1.00	0.00	LX1	N
ATOM	5196	HE21	GLN	54	48.102	38.601	34.903	1.00	0.00	LX1	H
ATOM	5197	HE22	GLN	54	46.727	38.887	35.919	1.00	0.00	LX1	H

ATOM	5198	C	GLN	54	49.117	34.180	32.483	1.00	0.00	LX1	C
ATOM	5199	O	GLN	54	50.234	34.100	32.991	1.00	0.00	LX1	O
ATOM	5200	N	VAL	55	48.323	33.139	32.185	1.00	0.00	LX1	N
ATOM	5201	H	VAL	55	47.404	33.304	31.817	1.00	0.00	LX1	H
ATOM	5202	CA	VAL	55	48.795	31.768	32.356	1.00	0.00	LX1	C
ATOM	5203	CB	VAL	55	48.125	31.095	33.572	1.00	0.00	LX1	C
ATOM	5204	CG1	VAL	55	48.626	29.662	33.782	1.00	0.00	LX1	C
ATOM	5205	CG2	VAL	55	48.281	31.918	34.855	1.00	0.00	LX1	C
ATOM	5206	C	VAL	55	48.523	30.972	31.091	1.00	0.00	LX1	C
ATOM	5207	O	VAL	55	47.415	30.497	30.862	1.00	0.00	LX1	O
ATOM	5208	N	VAL	56	49.571	30.852	30.264	1.00	0.00	LX1	N
ATOM	5209	H	VAL	56	50.483	31.210	30.467	1.00	0.00	LX1	H
ATOM	5210	CA	VAL	56	49.313	30.024	29.095	1.00	0.00	LX1	C
ATOM	5211	CB	VAL	56	49.853	30.658	27.794	1.00	0.00	LX1	C
ATOM	5212	CG1	VAL	56	51.322	30.390	27.469	1.00	0.00	LX1	C
ATOM	5213	CG2	VAL	56	48.945	30.276	26.634	1.00	0.00	LX1	C
ATOM	5214	C	VAL	56	49.667	28.558	29.295	1.00	0.00	LX1	C
ATOM	5215	O	VAL	56	50.809	28.132	29.449	1.00	0.00	LX1	O
ATOM	5216	N	GLN	57	48.576	27.795	29.343	1.00	0.00	LX1	N
ATOM	5217	H	GLN	57	47.690	28.226	29.173	1.00	0.00	LX1	H
ATOM	5218	CA	GLN	57	48.708	26.363	29.570	1.00	0.00	LX1	C
ATOM	5219	CB	GLN	57	47.487	25.817	30.309	1.00	0.00	LX1	C
ATOM	5220	CG	GLN	57	47.171	26.687	31.527	1.00	0.00	LX1	C
ATOM	5221	CD	GLN	57	46.440	25.889	32.583	1.00	0.00	LX1	C
ATOM	5222	OE1	GLN	57	46.953	25.629	33.662	1.00	0.00	LX1	O
ATOM	5223	NE2	GLN	57	45.203	25.520	32.239	1.00	0.00	LX1	N
ATOM	5224	HE21	GLN	57	44.818	25.759	31.350	1.00	0.00	LX1	H
ATOM	5225	HE22	GLN	57	44.678	24.995	32.906	1.00	0.00	LX1	H
ATOM	5226	C	GLN	57	48.996	25.601	28.299	1.00	0.00	LX1	C
ATOM	5227	O	GLN	57	48.116	25.095	27.612	1.00	0.00	LX1	O
ATOM	5228	N	GLY	58	50.294	25.588	28.003	1.00	0.00	LX1	N
ATOM	5229	H	GLY	58	50.932	26.102	28.581	1.00	0.00	LX1	H
ATOM	5230	CA	GLY	58	50.775	24.952	26.785	1.00	0.00	LX1	C
ATOM	5231	C	GLY	58	52.043	25.664	26.390	1.00	0.00	LX1	C
ATOM	5232	O	GLY	58	52.601	26.410	27.190	1.00	0.00	LX1	O
ATOM	5233	N	ASN	59	52.487	25.413	25.155	1.00	0.00	LX1	N
ATOM	5234	H	ASN	59	51.965	24.900	24.469	1.00	0.00	LX1	H
ATOM	5235	CA	ASN	59	53.677	26.165	24.766	1.00	0.00	LX1	C
ATOM	5236	CB	ASN	59	54.530	25.468	23.708	1.00	0.00	LX1	C
ATOM	5237	CG	ASN	59	54.687	23.984	23.919	1.00	0.00	LX1	C
ATOM	5238	OD1	ASN	59	55.260	23.508	24.895	1.00	0.00	LX1	O
ATOM	5239	ND2	ASN	59	54.159	23.273	22.921	1.00	0.00	LX1	N
ATOM	5240	HD21	ASN	59	53.788	23.806	22.150	1.00	0.00	LX1	H
ATOM	5241	HD22	ASN	59	54.094	22.281	22.793	1.00	0.00	LX1	H
ATOM	5242	C	ASN	59	53.282	27.511	24.206	1.00	0.00	LX1	C
ATOM	5243	O	ASN	59	52.112	27.770	23.951	1.00	0.00	LX1	O
ATOM	5244	N	LEU	60	54.309	28.343	24.021	1.00	0.00	LX1	N
ATOM	5245	H	LEU	60	55.231	28.060	24.288	1.00	0.00	LX1	H
ATOM	5246	CA	LEU	60	54.119	29.568	23.258	1.00	0.00	LX1	C
ATOM	5247	CB	LEU	60	54.519	30.774	24.114	1.00	0.00	LX1	C
ATOM	5248	CG	LEU	60	54.494	32.150	23.438	1.00	0.00	LX1	C
ATOM	5249	CD1	LEU	60	53.140	32.490	22.828	1.00	0.00	LX1	C
ATOM	5250	CD2	LEU	60	54.956	33.247	24.395	1.00	0.00	LX1	C
ATOM	5251	C	LEU	60	54.949	29.462	21.999	1.00	0.00	LX1	C
ATOM	5252	O	LEU	60	56.138	29.763	21.980	1.00	0.00	LX1	O
ATOM	5253	N	GLU	61	54.281	28.982	20.949	1.00	0.00	LX1	N
ATOM	5254	H	GLU	61	53.304	28.769	21.012	1.00	0.00	LX1	H
ATOM	5255	CA	GLU	61	55.066	28.844	19.729	1.00	0.00	LX1	C
ATOM	5256	CB	GLU	61	54.887	27.482	19.079	1.00	0.00	LX1	C
ATOM	5257	CG	GLU	61	55.560	26.429	19.955	1.00	0.00	LX1	C
ATOM	5258	CD	GLU	61	54.560	25.377	20.367	1.00	0.00	LX1	C

ATOM	5259	OE1	GLU	61	53.435	25.716	20.714	1.00	0.00	LX1	O
ATOM	5260	OE2	GLU	61	54.888	24.199	20.320	1.00	0.00	LX1	O
ATOM	5261	C	GLU	61	54.904	29.971	18.749	1.00	0.00	LX1	C
ATOM	5262	O	GLU	61	54.009	30.046	17.916	1.00	0.00	LX1	O
ATOM	5263	N	LEU	62	55.863	30.871	18.914	1.00	0.00	LX1	N
ATOM	5264	H	LEU	62	56.561	30.699	19.611	1.00	0.00	LX1	H
ATOM	5265	CA	LEU	62	56.029	31.947	17.954	1.00	0.00	LX1	C
ATOM	5266	CB	LEU	62	56.591	33.171	18.672	1.00	0.00	LX1	C
ATOM	5267	CG	LEU	62	55.711	33.556	19.860	1.00	0.00	LX1	C
ATOM	5268	CD1	LEU	62	56.327	34.683	20.681	1.00	0.00	LX1	C
ATOM	5269	CD2	LEU	62	54.270	33.843	19.433	1.00	0.00	LX1	C
ATOM	5270	C	LEU	62	56.913	31.503	16.812	1.00	0.00	LX1	C
ATOM	5271	O	LEU	62	58.116	31.744	16.773	1.00	0.00	LX1	O
ATOM	5272	N	THR	63	56.241	30.817	15.891	1.00	0.00	LX1	N
ATOM	5273	H	THR	63	55.274	30.595	16.016	1.00	0.00	LX1	H
ATOM	5274	CA	THR	63	56.985	30.313	14.749	1.00	0.00	LX1	C
ATOM	5275	CB	THR	63	56.941	28.781	14.751	1.00	0.00	LX1	C
ATOM	5276	OG1	THR	63	56.747	28.269	16.079	1.00	0.00	LX1	O
ATOM	5277	HG1	THR	63	56.613	27.326	15.978	1.00	0.00	LX1	H
ATOM	5278	CG2	THR	63	58.206	28.184	14.136	1.00	0.00	LX1	C
ATOM	5279	C	THR	63	56.426	30.874	13.454	1.00	0.00	LX1	C
ATOM	5280	O	THR	63	55.217	30.925	13.275	1.00	0.00	LX1	O
ATOM	5281	N	TYR	64	57.344	31.291	12.564	1.00	0.00	LX1	N
ATOM	5282	H	TYR	64	58.294	31.310	12.885	1.00	0.00	LX1	H
ATOM	5283	CA	TYR	64	56.972	31.782	11.226	1.00	0.00	LX1	C
ATOM	5284	CB	TYR	64	56.275	30.727	10.351	1.00	0.00	LX1	C
ATOM	5285	CG	TYR	64	57.092	29.469	10.202	1.00	0.00	LX1	C
ATOM	5286	CD1	TYR	64	58.183	29.451	9.312	1.00	0.00	LX1	C
ATOM	5287	CE1	TYR	64	58.923	28.265	9.177	1.00	0.00	LX1	C
ATOM	5288	CD2	TYR	64	56.721	28.339	10.955	1.00	0.00	LX1	C
ATOM	5289	CE2	TYR	64	57.468	27.160	10.821	1.00	0.00	LX1	C
ATOM	5290	CZ	TYR	64	58.560	27.136	9.936	1.00	0.00	LX1	C
ATOM	5291	OH	TYR	64	59.298	25.977	9.804	1.00	0.00	LX1	O
ATOM	5292	HH	TYR	64	58.913	25.300	10.362	1.00	0.00	LX1	H
ATOM	5293	C	TYR	64	56.154	33.065	11.133	1.00	0.00	LX1	C
ATOM	5294	O	TYR	64	55.635	33.403	10.075	1.00	0.00	LX1	O
ATOM	5295	N	LEU	65	56.072	33.788	12.260	1.00	0.00	LX1	N
ATOM	5296	H	LEU	65	56.493	33.437	13.095	1.00	0.00	LX1	H
ATOM	5297	CA	LEU	65	55.357	35.067	12.246	1.00	0.00	LX1	C
ATOM	5298	CB	LEU	65	55.477	35.769	13.598	1.00	0.00	LX1	C
ATOM	5299	CG	LEU	65	54.904	35.023	14.802	1.00	0.00	LX1	C
ATOM	5300	CD1	LEU	65	55.457	35.600	16.102	1.00	0.00	LX1	C
ATOM	5301	CD2	LEU	65	53.377	35.010	14.811	1.00	0.00	LX1	C
ATOM	5302	C	LEU	65	55.834	36.010	11.148	1.00	0.00	LX1	C
ATOM	5303	O	LEU	65	57.021	36.321	11.030	1.00	0.00	LX1	O
ATOM	5304	N	PRO	66	54.849	36.441	10.325	1.00	0.00	LX1	N
ATOM	5305	CD	PRO	66	53.424	36.134	10.427	1.00	0.00	LX1	C
ATOM	5306	CA	PRO	66	55.173	37.285	9.174	1.00	0.00	LX1	C
ATOM	5307	CB	PRO	66	53.844	37.326	8.409	1.00	0.00	LX1	C
ATOM	5308	CG	PRO	66	52.760	37.110	9.465	1.00	0.00	LX1	C
ATOM	5309	C	PRO	66	55.691	38.653	9.577	1.00	0.00	LX1	C
ATOM	5310	O	PRO	66	55.289	39.244	10.572	1.00	0.00	LX1	O
ATOM	5311	N	THR	67	56.613	39.127	8.732	1.00	0.00	LX1	N
ATOM	5312	H	THR	67	56.906	38.597	7.935	1.00	0.00	LX1	H
ATOM	5313	CA	THR	67	57.396	40.329	9.010	1.00	0.00	LX1	C
ATOM	5314	CB	THR	67	58.060	40.753	7.706	1.00	0.00	LX1	C
ATOM	5315	OG1	THR	67	58.347	39.591	6.914	1.00	0.00	LX1	O
ATOM	5316	HG1	THR	67	58.925	39.878	6.210	1.00	0.00	LX1	H
ATOM	5317	CG2	THR	67	59.318	41.575	7.954	1.00	0.00	LX1	C
ATOM	5318	C	THR	67	56.707	41.505	9.689	1.00	0.00	LX1	C
ATOM	5319	O	THR	67	57.144	42.028	10.710	1.00	0.00	LX1	O

ATOM	5320	N	ASN	68	55.593	41.893	9.057	1.00	0.00	LX1	N
ATOM	5321	H	ASN	68	55.230	41.355	8.299	1.00	0.00	LX1	H
ATOM	5322	CA	ASN	68	54.945	43.114	9.527	1.00	0.00	LX1	C
ATOM	5323	CB	ASN	68	54.729	44.112	8.379	1.00	0.00	LX1	C
ATOM	5324	CG	ASN	68	56.022	44.467	7.666	1.00	0.00	LX1	C
ATOM	5325	OD1	ASN	68	57.138	44.232	8.117	1.00	0.00	LX1	O
ATOM	5326	ND2	ASN	68	55.826	45.027	6.478	1.00	0.00	LX1	N
ATOM	5327	HD21	ASN	68	54.933	45.349	6.144	1.00	0.00	LX1	H
ATOM	5328	HD22	ASN	68	56.581	45.209	5.850	1.00	0.00	LX1	H
ATOM	5329	C	ASN	68	53.625	42.817	10.208	1.00	0.00	LX1	C
ATOM	5330	O	ASN	68	52.607	43.451	9.965	1.00	0.00	LX1	O
ATOM	5331	N	ALA	69	53.665	41.777	11.051	1.00	0.00	LX1	N
ATOM	5332	H	ALA	69	54.524	41.305	11.256	1.00	0.00	LX1	H
ATOM	5333	CA	ALA	69	52.412	41.417	11.708	1.00	0.00	LX1	C
ATOM	5334	CB	ALA	69	52.510	40.017	12.316	1.00	0.00	LX1	C
ATOM	5335	C	ALA	69	52.000	42.389	12.799	1.00	0.00	LX1	C
ATOM	5336	O	ALA	69	52.806	42.836	13.605	1.00	0.00	LX1	O
ATOM	5337	N	SER	70	50.687	42.668	12.813	1.00	0.00	LX1	N
ATOM	5338	H	SER	70	50.094	42.303	12.099	1.00	0.00	LX1	H
ATOM	5339	CA	SER	70	50.145	43.397	13.960	1.00	0.00	LX1	C
ATOM	5340	CB	SER	70	48.803	44.042	13.584	1.00	0.00	LX1	C
ATOM	5341	OG	SER	70	48.527	45.163	14.428	1.00	0.00	LX1	O
ATOM	5342	HG	SER	70	48.122	45.835	13.868	1.00	0.00	LX1	H
ATOM	5343	C	SER	70	50.066	42.480	15.176	1.00	0.00	LX1	C
ATOM	5344	O	SER	70	49.104	41.761	15.421	1.00	0.00	LX1	O
ATOM	5345	N	LEU	71	51.203	42.483	15.883	1.00	0.00	LX1	N
ATOM	5346	H	LEU	71	51.919	43.128	15.610	1.00	0.00	LX1	H
ATOM	5347	CA	LEU	71	51.479	41.384	16.805	1.00	0.00	LX1	C
ATOM	5348	CB	LEU	71	52.957	41.020	16.650	1.00	0.00	LX1	C
ATOM	5349	CG	LEU	71	53.306	39.571	16.979	1.00	0.00	LX1	C
ATOM	5350	CD1	LEU	71	52.516	38.580	16.127	1.00	0.00	LX1	C
ATOM	5351	CD2	LEU	71	54.810	39.328	16.884	1.00	0.00	LX1	C
ATOM	5352	C	LEU	71	51.095	41.612	18.262	1.00	0.00	LX1	C
ATOM	5353	O	LEU	71	51.272	40.764	19.131	1.00	0.00	LX1	O
ATOM	5354	N	SER	72	50.559	42.817	18.499	1.00	0.00	LX1	N
ATOM	5355	H	SER	72	50.403	43.469	17.755	1.00	0.00	LX1	H
ATOM	5356	CA	SER	72	50.372	43.339	19.856	1.00	0.00	LX1	C
ATOM	5357	CB	SER	72	49.552	44.624	19.752	1.00	0.00	LX1	C
ATOM	5358	OG	SER	72	49.886	45.263	18.510	1.00	0.00	LX1	O
ATOM	5359	HG	SER	72	49.393	46.076	18.472	1.00	0.00	LX1	H
ATOM	5360	C	SER	72	49.829	42.402	20.929	1.00	0.00	LX1	C
ATOM	5361	O	SER	72	50.251	42.424	22.076	1.00	0.00	LX1	O
ATOM	5362	N	PHE	73	48.893	41.541	20.497	1.00	0.00	LX1	N
ATOM	5363	H	PHE	73	48.651	41.563	19.529	1.00	0.00	LX1	H
ATOM	5364	CA	PHE	73	48.250	40.593	21.417	1.00	0.00	LX1	C
ATOM	5365	CB	PHE	73	47.263	39.698	20.647	1.00	0.00	LX1	C
ATOM	5366	CG	PHE	73	47.945	38.883	19.566	1.00	0.00	LX1	C
ATOM	5367	CD1	PHE	73	48.424	37.589	19.869	1.00	0.00	LX1	C
ATOM	5368	CD2	PHE	73	48.081	39.423	18.267	1.00	0.00	LX1	C
ATOM	5369	CE1	PHE	73	49.044	36.826	18.860	1.00	0.00	LX1	C
ATOM	5370	CE2	PHE	73	48.700	38.661	17.258	1.00	0.00	LX1	C
ATOM	5371	CZ	PHE	73	49.175	37.369	17.565	1.00	0.00	LX1	C
ATOM	5372	C	PHE	73	49.145	39.760	22.337	1.00	0.00	LX1	C
ATOM	5373	O	PHE	73	48.719	39.293	23.389	1.00	0.00	LX1	O
ATOM	5374	N	LEU	74	50.403	39.589	21.900	1.00	0.00	LX1	N
ATOM	5375	H	LEU	74	50.707	40.015	21.046	1.00	0.00	LX1	H
ATOM	5376	CA	LEU	74	51.324	38.818	22.734	1.00	0.00	LX1	C
ATOM	5377	CB	LEU	74	52.564	38.429	21.938	1.00	0.00	LX1	C
ATOM	5378	CG	LEU	74	52.307	37.551	20.720	1.00	0.00	LX1	C
ATOM	5379	CD1	LEU	74	53.582	37.413	19.898	1.00	0.00	LX1	C
ATOM	5380	CD2	LEU	74	51.732	36.186	21.095	1.00	0.00	LX1	C

ATOM	5381	C	LEU	74	51.759	39.468	24.040	1.00	0.00	LX1	C
ATOM	5382	O	LEU	74	52.087	38.784	25.002	1.00	0.00	LX1	O
ATOM	5383	N	GLN	75	51.753	40.815	24.041	1.00	0.00	LX1	N
ATOM	5384	H	GLN	75	51.406	41.289	23.231	1.00	0.00	LX1	H
ATOM	5385	CA	GLN	75	52.448	41.582	25.087	1.00	0.00	LX1	C
ATOM	5386	CB	GLN	75	52.022	43.054	25.047	1.00	0.00	LX1	C
ATOM	5387	CG	GLN	75	50.510	43.273	25.159	1.00	0.00	LX1	C
ATOM	5388	CD	GLN	75	50.220	44.742	25.351	1.00	0.00	LX1	C
ATOM	5389	OE1	GLN	75	50.226	45.550	24.432	1.00	0.00	LX1	O
ATOM	5390	NE2	GLN	75	49.951	45.063	26.617	1.00	0.00	LX1	N
ATOM	5391	HE21	GLN	75	49.948	44.342	27.321	1.00	0.00	LX1	H
ATOM	5392	HE22	GLN	75	49.748	46.008	26.846	1.00	0.00	LX1	H
ATOM	5393	C	GLN	75	52.430	41.060	26.521	1.00	0.00	LX1	C
ATOM	5394	O	GLN	75	53.436	41.057	27.227	1.00	0.00	LX1	O
ATOM	5395	N	ASP	76	51.232	40.618	26.920	1.00	0.00	LX1	N
ATOM	5396	H	ASP	76	50.482	40.553	26.267	1.00	0.00	LX1	H
ATOM	5397	CA	ASP	76	51.067	40.313	28.334	1.00	0.00	LX1	C
ATOM	5398	CB	ASP	76	49.826	41.015	28.899	1.00	0.00	LX1	C
ATOM	5399	CG	ASP	76	49.908	42.529	28.788	1.00	0.00	LX1	C
ATOM	5400	OD1	ASP	76	50.991	43.102	28.817	1.00	0.00	LX1	O
ATOM	5401	OD2	ASP	76	48.874	43.180	28.722	1.00	0.00	LX1	O
ATOM	5402	C	ASP	76	51.023	38.837	28.665	1.00	0.00	LX1	C
ATOM	5403	O	ASP	76	50.415	38.432	29.650	1.00	0.00	LX1	O
ATOM	5404	N	ILE	77	51.685	38.031	27.819	1.00	0.00	LX1	N
ATOM	5405	H	ILE	77	52.160	38.391	27.013	1.00	0.00	LX1	H
ATOM	5406	CA	ILE	77	51.753	36.619	28.200	1.00	0.00	LX1	C
ATOM	5407	CB	ILE	77	52.037	35.711	26.999	1.00	0.00	LX1	C
ATOM	5408	CG2	ILE	77	52.124	34.229	27.388	1.00	0.00	LX1	C
ATOM	5409	CG1	ILE	77	50.953	35.929	25.945	1.00	0.00	LX1	C
ATOM	5410	CD1	ILE	77	51.241	35.165	24.660	1.00	0.00	LX1	C
ATOM	5411	C	ILE	77	52.701	36.360	29.357	1.00	0.00	LX1	C
ATOM	5412	O	ILE	77	53.912	36.273	29.226	1.00	0.00	LX1	O
ATOM	5413	N	GLN	78	52.064	36.276	30.526	1.00	0.00	LX1	N
ATOM	5414	H	GLN	78	51.063	36.279	30.485	1.00	0.00	LX1	H
ATOM	5415	CA	GLN	78	52.837	36.298	31.760	1.00	0.00	LX1	C
ATOM	5416	CB	GLN	78	51.959	36.860	32.876	1.00	0.00	LX1	C
ATOM	5417	CG	GLN	78	51.698	38.347	32.638	1.00	0.00	LX1	C
ATOM	5418	CD	GLN	78	50.342	38.753	33.163	1.00	0.00	LX1	C
ATOM	5419	OE1	GLN	78	49.984	38.554	34.318	1.00	0.00	LX1	O
ATOM	5420	NE2	GLN	78	49.586	39.350	32.241	1.00	0.00	LX1	N
ATOM	5421	HE21	GLN	78	49.900	39.364	31.288	1.00	0.00	LX1	H
ATOM	5422	HE22	GLN	78	48.700	39.756	32.450	1.00	0.00	LX1	H
ATOM	5423	C	GLN	78	53.538	35.005	32.135	1.00	0.00	LX1	C
ATOM	5424	O	GLN	78	54.604	35.015	32.740	1.00	0.00	LX1	O
ATOM	5425	N	GLU	79	52.928	33.882	31.751	1.00	0.00	LX1	N
ATOM	5426	H	GLU	79	52.021	33.870	31.327	1.00	0.00	LX1	H
ATOM	5427	CA	GLU	79	53.721	32.665	31.888	1.00	0.00	LX1	C
ATOM	5428	CB	GLU	79	53.565	31.985	33.250	1.00	0.00	LX1	C
ATOM	5429	CG	GLU	79	52.185	31.422	33.574	1.00	0.00	LX1	C
ATOM	5430	CD	GLU	79	52.195	30.817	34.962	1.00	0.00	LX1	C
ATOM	5431	OE1	GLU	79	51.862	29.647	35.086	1.00	0.00	LX1	O
ATOM	5432	OE2	GLU	79	52.554	31.490	35.929	1.00	0.00	LX1	O
ATOM	5433	C	GLU	79	53.442	31.674	30.802	1.00	0.00	LX1	C
ATOM	5434	O	GLU	79	52.347	31.617	30.259	1.00	0.00	LX1	O
ATOM	5435	N	VAL	80	54.489	30.898	30.525	1.00	0.00	LX1	N
ATOM	5436	H	VAL	80	55.352	31.022	31.022	1.00	0.00	LX1	H
ATOM	5437	CA	VAL	80	54.320	29.815	29.568	1.00	0.00	LX1	C
ATOM	5438	CB	VAL	80	55.274	30.013	28.389	1.00	0.00	LX1	C
ATOM	5439	CG1	VAL	80	55.154	28.887	27.359	1.00	0.00	LX1	C
ATOM	5440	CG2	VAL	80	55.066	31.394	27.766	1.00	0.00	LX1	C
ATOM	5441	C	VAL	80	54.602	28.510	30.265	1.00	0.00	LX1	C

ATOM	5442	O	VAL	80	55.666	28.333	30.842	1.00	0.00	LX1	O
ATOM	5443	N	GLN	81	53.617	27.606	30.215	1.00	0.00	LX1	N
ATOM	5444	H	GLN	81	52.769	27.793	29.711	1.00	0.00	LX1	H
ATOM	5445	CA	GLN	81	53.888	26.343	30.896	1.00	0.00	LX1	C
ATOM	5446	CB	GLN	81	52.588	25.642	31.272	1.00	0.00	LX1	C
ATOM	5447	CG	GLN	81	51.782	26.456	32.288	1.00	0.00	LX1	C
ATOM	5448	CD	GLN	81	50.517	25.706	32.642	1.00	0.00	LX1	C
ATOM	5449	OE1	GLN	81	50.161	24.711	32.027	1.00	0.00	LX1	O
ATOM	5450	NE2	GLN	81	49.834	26.245	33.654	1.00	0.00	LX1	N
ATOM	5451	HE21	GLN	81	50.155	27.050	34.150	1.00	0.00	LX1	H
ATOM	5452	HE22	GLN	81	48.952	25.831	33.900	1.00	0.00	LX1	H
ATOM	5453	C	GLN	81	54.836	25.416	30.156	1.00	0.00	LX1	C
ATOM	5454	O	GLN	81	55.704	24.769	30.734	1.00	0.00	LX1	O
ATOM	5455	N	GLY	82	54.640	25.382	28.837	1.00	0.00	LX1	N
ATOM	5456	H	GLY	82	53.972	25.995	28.418	1.00	0.00	LX1	H
ATOM	5457	CA	GLY	82	55.579	24.611	28.031	1.00	0.00	LX1	C
ATOM	5458	C	GLY	82	56.821	25.419	27.726	1.00	0.00	LX1	C
ATOM	5459	O	GLY	82	57.294	26.205	28.542	1.00	0.00	LX1	O
ATOM	5460	N	TYR	83	57.314	25.193	26.509	1.00	0.00	LX1	N
ATOM	5461	H	TYR	83	56.820	24.592	25.877	1.00	0.00	LX1	H
ATOM	5462	CA	TYR	83	58.453	26.001	26.083	1.00	0.00	LX1	C
ATOM	5463	CB	TYR	83	59.443	25.152	25.263	1.00	0.00	LX1	C
ATOM	5464	CG	TYR	83	58.754	24.363	24.170	1.00	0.00	LX1	C
ATOM	5465	CD1	TYR	83	58.355	25.008	22.982	1.00	0.00	LX1	C
ATOM	5466	CE1	TYR	83	57.680	24.266	22.000	1.00	0.00	LX1	C
ATOM	5467	CD2	TYR	83	58.530	22.988	24.380	1.00	0.00	LX1	C
ATOM	5468	CE2	TYR	83	57.871	22.242	23.393	1.00	0.00	LX1	C
ATOM	5469	CZ	TYR	83	57.434	22.897	22.227	1.00	0.00	LX1	C
ATOM	5470	OH	TYR	83	56.730	22.169	21.291	1.00	0.00	LX1	O
ATOM	5471	HH	TYR	83	56.034	22.740	20.942	1.00	0.00	LX1	H
ATOM	5472	C	TYR	83	58.006	27.234	25.322	1.00	0.00	LX1	C
ATOM	5473	O	TYR	83	56.892	27.299	24.816	1.00	0.00	LX1	O
ATOM	5474	N	VAL	84	58.917	28.207	25.244	1.00	0.00	LX1	N
ATOM	5475	H	VAL	84	59.804	28.110	25.693	1.00	0.00	LX1	H
ATOM	5476	CA	VAL	84	58.681	29.273	24.278	1.00	0.00	LX1	C
ATOM	5477	CB	VAL	84	58.963	30.660	24.866	1.00	0.00	LX1	C
ATOM	5478	CG1	VAL	84	58.520	31.771	23.912	1.00	0.00	LX1	C
ATOM	5479	CG2	VAL	84	58.331	30.836	26.239	1.00	0.00	LX1	C
ATOM	5480	C	VAL	84	59.560	29.046	23.069	1.00	0.00	LX1	C
ATOM	5481	O	VAL	84	60.785	29.066	23.144	1.00	0.00	LX1	O
ATOM	5482	N	LEU	85	58.882	28.816	21.949	1.00	0.00	LX1	N
ATOM	5483	H	LEU	85	57.883	28.898	21.933	1.00	0.00	LX1	H
ATOM	5484	CA	LEU	85	59.655	28.765	20.720	1.00	0.00	LX1	C
ATOM	5485	CB	LEU	85	59.137	27.634	19.836	1.00	0.00	LX1	C
ATOM	5486	CG	LEU	85	59.901	27.487	18.522	1.00	0.00	LX1	C
ATOM	5487	CD1	LEU	85	61.326	26.993	18.743	1.00	0.00	LX1	C
ATOM	5488	CD2	LEU	85	59.144	26.621	17.526	1.00	0.00	LX1	C
ATOM	5489	C	LEU	85	59.529	30.093	20.013	1.00	0.00	LX1	C
ATOM	5490	O	LEU	85	58.433	30.537	19.717	1.00	0.00	LX1	O
ATOM	5491	N	ILE	86	60.679	30.716	19.758	1.00	0.00	LX1	N
ATOM	5492	H	ILE	86	61.550	30.317	20.042	1.00	0.00	LX1	H
ATOM	5493	CA	ILE	86	60.615	31.883	18.887	1.00	0.00	LX1	C
ATOM	5494	CB	ILE	86	61.034	33.172	19.614	1.00	0.00	LX1	C
ATOM	5495	CG2	ILE	86	60.702	34.394	18.754	1.00	0.00	LX1	C
ATOM	5496	CG1	ILE	86	60.393	33.290	21.000	1.00	0.00	LX1	C
ATOM	5497	CD1	ILE	86	60.855	34.517	21.789	1.00	0.00	LX1	C
ATOM	5498	C	ILE	86	61.485	31.632	17.673	1.00	0.00	LX1	C
ATOM	5499	O	ILE	86	62.665	31.964	17.653	1.00	0.00	LX1	O
ATOM	5500	N	ALA	87	60.868	30.981	16.681	1.00	0.00	LX1	N
ATOM	5501	H	ALA	87	59.877	30.827	16.691	1.00	0.00	LX1	H
ATOM	5502	CA	ALA	87	61.709	30.517	15.585	1.00	0.00	LX1	C

ATOM	5503	CB	ALA	87	61.911	29.010	15.665	1.00	0.00	LX1	C
ATOM	5504	C	ALA	87	61.194	30.834	14.201	1.00	0.00	LX1	C
ATOM	5505	O	ALA	87	59.996	30.923	13.951	1.00	0.00	LX1	O
ATOM	5506	N	HIS	88	62.176	30.966	13.292	1.00	0.00	LX1	N
ATOM	5507	H	HIS	88	63.118	30.999	13.631	1.00	0.00	LX1	H
ATOM	5508	CA	HIS	88	61.887	31.111	11.863	1.00	0.00	LX1	C
ATOM	5509	CB	HIS	88	61.387	29.789	11.271	1.00	0.00	LX1	C
ATOM	5510	CG	HIS	88	62.488	29.007	10.588	1.00	0.00	LX1	C
ATOM	5511	ND1	HIS	88	63.741	29.450	10.369	1.00	0.00	LX1	N
ATOM	5512	HD1	HIS	88	64.144	30.305	10.650	1.00	0.00	LX1	H
ATOM	5513	CD2	HIS	88	62.374	27.723	10.047	1.00	0.00	LX1	C
ATOM	5514	NE2	HIS	88	63.567	27.399	9.494	1.00	0.00	LX1	N
ATOM	5515	CE1	HIS	88	64.412	28.461	9.696	1.00	0.00	LX1	C
ATOM	5516	C	HIS	88	60.950	32.249	11.502	1.00	0.00	LX1	C
ATOM	5517	O	HIS	88	60.225	32.211	10.514	1.00	0.00	LX1	O
ATOM	5518	N	ASN	89	60.986	33.258	12.376	1.00	0.00	LX1	N
ATOM	5519	H	ASN	89	61.695	33.267	13.082	1.00	0.00	LX1	H
ATOM	5520	CA	ASN	89	60.076	34.383	12.213	1.00	0.00	LX1	C
ATOM	5521	CB	ASN	89	59.709	35.018	13.554	1.00	0.00	LX1	C
ATOM	5522	CG	ASN	89	59.055	34.018	14.476	1.00	0.00	LX1	C
ATOM	5523	OD1	ASN	89	57.906	33.635	14.324	1.00	0.00	LX1	O
ATOM	5524	ND2	ASN	89	59.838	33.619	15.470	1.00	0.00	LX1	N
ATOM	5525	HD21	ASN	89	60.802	33.885	15.529	1.00	0.00	LX1	H
ATOM	5526	HD22	ASN	89	59.415	32.999	16.130	1.00	0.00	LX1	H
ATOM	5527	C	ASN	89	60.741	35.446	11.390	1.00	0.00	LX1	C
ATOM	5528	O	ASN	89	61.940	35.670	11.501	1.00	0.00	LX1	O
ATOM	5529	N	GLN	90	59.912	36.109	10.581	1.00	0.00	LX1	N
ATOM	5530	H	GLN	90	58.942	35.868	10.504	1.00	0.00	LX1	H
ATOM	5531	CA	GLN	90	60.467	37.319	9.985	1.00	0.00	LX1	C
ATOM	5532	CB	GLN	90	60.295	37.317	8.460	1.00	0.00	LX1	C
ATOM	5533	CG	GLN	90	61.054	36.151	7.812	1.00	0.00	LX1	C
ATOM	5534	CD	GLN	90	61.302	36.395	6.333	1.00	0.00	LX1	C
ATOM	5535	OE1	GLN	90	60.840	35.673	5.459	1.00	0.00	LX1	O
ATOM	5536	NE2	GLN	90	62.126	37.414	6.091	1.00	0.00	LX1	N
ATOM	5537	HE21	GLN	90	62.504	37.974	6.828	1.00	0.00	LX1	H
ATOM	5538	HE22	GLN	90	62.478	37.680	5.190	1.00	0.00	LX1	H
ATOM	5539	C	GLN	90	59.960	38.598	10.633	1.00	0.00	LX1	C
ATOM	5540	O	GLN	90	60.384	39.698	10.303	1.00	0.00	LX1	O
ATOM	5541	N	VAL	91	59.021	38.405	11.585	1.00	0.00	LX1	N
ATOM	5542	H	VAL	91	58.644	37.496	11.749	1.00	0.00	LX1	H
ATOM	5543	CA	VAL	91	58.484	39.560	12.305	1.00	0.00	LX1	C
ATOM	5544	CB	VAL	91	57.347	39.132	13.253	1.00	0.00	LX1	C
ATOM	5545	CG1	VAL	91	57.858	38.364	14.476	1.00	0.00	LX1	C
ATOM	5546	CG2	VAL	91	56.453	40.315	13.633	1.00	0.00	LX1	C
ATOM	5547	C	VAL	91	59.528	40.404	13.018	1.00	0.00	LX1	C
ATOM	5548	O	VAL	91	60.422	39.906	13.689	1.00	0.00	LX1	O
ATOM	5549	N	ARG	92	59.360	41.717	12.828	1.00	0.00	LX1	N
ATOM	5550	H	ARG	92	58.585	42.017	12.267	1.00	0.00	LX1	H
ATOM	5551	CA	ARG	92	60.396	42.618	13.326	1.00	0.00	LX1	C
ATOM	5552	CB	ARG	92	60.232	43.992	12.675	1.00	0.00	LX1	C
ATOM	5553	CG	ARG	92	60.110	43.852	11.155	1.00	0.00	LX1	C
ATOM	5554	CD	ARG	92	59.987	45.183	10.415	1.00	0.00	LX1	C
ATOM	5555	NE	ARG	92	59.776	44.986	8.980	1.00	0.00	LX1	N
ATOM	5556	HE	ARG	92	58.825	44.979	8.654	1.00	0.00	LX1	H
ATOM	5557	CZ	ARG	92	60.788	44.741	8.119	1.00	0.00	LX1	C
ATOM	5558	NH1	ARG	92	62.035	44.575	8.554	1.00	0.00	LX1	N
ATOM	5559	HH11	ARG	92	62.788	44.342	7.924	1.00	0.00	LX1	H
ATOM	5560	HH12	ARG	92	62.249	44.648	9.526	1.00	0.00	LX1	H
ATOM	5561	NH2	ARG	92	60.530	44.650	6.823	1.00	0.00	LX1	N
ATOM	5562	HH21	ARG	92	61.270	44.473	6.166	1.00	0.00	LX1	H
ATOM	5563	HH22	ARG	92	59.600	44.780	6.460	1.00	0.00	LX1	H

ATOM	5564	C	ARG	92	60.527	42.687	14.842	1.00	0.00	LX1	C
ATOM	5565	O	ARG	92	61.611	42.580	15.402	1.00	0.00	LX1	O
ATOM	5566	N	GLN	93	59.373	42.835	15.504	1.00	0.00	LX1	N
ATOM	5567	H	GLN	93	58.499	42.932	15.032	1.00	0.00	LX1	H
ATOM	5568	CA	GLN	93	59.451	42.821	16.962	1.00	0.00	LX1	C
ATOM	5569	CB	GLN	93	59.289	44.224	17.563	1.00	0.00	LX1	C
ATOM	5570	CG	GLN	93	60.591	44.976	17.878	1.00	0.00	LX1	C
ATOM	5571	CD	GLN	93	61.182	45.657	16.657	1.00	0.00	LX1	C
ATOM	5572	OE1	GLN	93	60.661	45.616	15.549	1.00	0.00	LX1	O
ATOM	5573	NE2	GLN	93	62.297	46.339	16.920	1.00	0.00	LX1	N
ATOM	5574	HE21	GLN	93	62.732	46.335	17.830	1.00	0.00	LX1	H
ATOM	5575	HE22	GLN	93	62.743	46.875	16.213	1.00	0.00	LX1	H
ATOM	5576	C	GLN	93	58.414	41.904	17.569	1.00	0.00	LX1	C
ATOM	5577	O	GLN	93	57.267	41.868	17.146	1.00	0.00	LX1	O
ATOM	5578	N	VAL	94	58.862	41.177	18.603	1.00	0.00	LX1	N
ATOM	5579	H	VAL	94	59.822	41.224	18.879	1.00	0.00	LX1	H
ATOM	5580	CA	VAL	94	57.878	40.451	19.406	1.00	0.00	LX1	C
ATOM	5581	CB	VAL	94	58.337	39.002	19.657	1.00	0.00	LX1	C
ATOM	5582	CG1	VAL	94	57.440	38.264	20.654	1.00	0.00	LX1	C
ATOM	5583	CG2	VAL	94	58.414	38.220	18.346	1.00	0.00	LX1	C
ATOM	5584	C	VAL	94	57.638	41.184	20.716	1.00	0.00	LX1	C
ATOM	5585	O	VAL	94	58.556	41.393	21.502	1.00	0.00	LX1	O
ATOM	5586	N	PRO	95	56.372	41.607	20.916	1.00	0.00	LX1	N
ATOM	5587	CD	PRO	95	55.280	41.606	19.946	1.00	0.00	LX1	C
ATOM	5588	CA	PRO	95	55.993	42.169	22.213	1.00	0.00	LX1	C
ATOM	5589	CB	PRO	95	54.781	43.020	21.816	1.00	0.00	LX1	C
ATOM	5590	CG	PRO	95	54.107	42.257	20.673	1.00	0.00	LX1	C
ATOM	5591	C	PRO	95	55.671	41.076	23.228	1.00	0.00	LX1	C
ATOM	5592	O	PRO	95	54.655	40.401	23.140	1.00	0.00	LX1	O
ATOM	5593	N	LEU	96	56.573	40.924	24.199	1.00	0.00	LX1	N
ATOM	5594	H	LEU	96	57.426	41.450	24.208	1.00	0.00	LX1	H
ATOM	5595	CA	LEU	96	56.261	40.036	25.319	1.00	0.00	LX1	C
ATOM	5596	CB	LEU	96	56.959	38.682	25.192	1.00	0.00	LX1	C
ATOM	5597	CG	LEU	96	56.246	37.638	24.337	1.00	0.00	LX1	C
ATOM	5598	CD1	LEU	96	57.134	36.418	24.096	1.00	0.00	LX1	C
ATOM	5599	CD2	LEU	96	54.908	37.230	24.949	1.00	0.00	LX1	C
ATOM	5600	C	LEU	96	56.662	40.679	26.627	1.00	0.00	LX1	C
ATOM	5601	O	LEU	96	57.306	40.092	27.489	1.00	0.00	LX1	O
ATOM	5602	N	GLN	97	56.261	41.950	26.731	1.00	0.00	LX1	N
ATOM	5603	H	GLN	97	55.697	42.322	25.993	1.00	0.00	LX1	H
ATOM	5604	CA	GLN	97	56.689	42.787	27.852	1.00	0.00	LX1	C
ATOM	5605	CB	GLN	97	55.945	44.125	27.801	1.00	0.00	LX1	C
ATOM	5606	CG	GLN	97	56.474	45.149	26.788	1.00	0.00	LX1	C
ATOM	5607	CD	GLN	97	56.403	44.659	25.353	1.00	0.00	LX1	C
ATOM	5608	OE1	GLN	97	55.489	43.960	24.930	1.00	0.00	LX1	O
ATOM	5609	NE2	GLN	97	57.411	45.089	24.599	1.00	0.00	LX1	N
ATOM	5610	HE21	GLN	97	58.188	45.548	25.043	1.00	0.00	LX1	H
ATOM	5611	HE22	GLN	97	57.435	44.968	23.612	1.00	0.00	LX1	H
ATOM	5612	C	GLN	97	56.511	42.141	29.216	1.00	0.00	LX1	C
ATOM	5613	O	GLN	97	57.409	42.086	30.053	1.00	0.00	LX1	O
ATOM	5614	N	ARG	98	55.290	41.635	29.407	1.00	0.00	LX1	N
ATOM	5615	H	ARG	98	54.619	41.605	28.662	1.00	0.00	LX1	H
ATOM	5616	CA	ARG	98	55.055	41.023	30.706	1.00	0.00	LX1	C
ATOM	5617	CB	ARG	98	53.724	41.491	31.292	1.00	0.00	LX1	C
ATOM	5618	CG	ARG	98	53.876	42.888	31.898	1.00	0.00	LX1	C
ATOM	5619	CD	ARG	98	52.595	43.430	32.534	1.00	0.00	LX1	C
ATOM	5620	NE	ARG	98	51.584	43.707	31.518	1.00	0.00	LX1	N
ATOM	5621	HE	ARG	98	51.576	43.166	30.667	1.00	0.00	LX1	H
ATOM	5622	CZ	ARG	98	50.645	44.660	31.670	1.00	0.00	LX1	C
ATOM	5623	NH1	ARG	98	50.613	45.404	32.775	1.00	0.00	LX1	N
ATOM	5624	HH11	ARG	98	49.922	46.112	32.908	1.00	0.00	LX1	H

ATOM	5625	HH12	ARG	98	51.294	45.254	33.491	1.00	0.00	LX1	H
ATOM	5626	NH2	ARG	98	49.752	44.847	30.708	1.00	0.00	LX1	N
ATOM	5627	HH21	ARG	98	49.021	45.523	30.727	1.00	0.00	LX1	H
ATOM	5628	HH22	ARG	98	49.798	44.250	29.886	1.00	0.00	LX1	H
ATOM	5629	C	ARG	98	55.221	39.519	30.774	1.00	0.00	LX1	C
ATOM	5630	O	ARG	98	54.664	38.863	31.646	1.00	0.00	LX1	O
ATOM	5631	N	LEU	99	56.051	38.995	29.856	1.00	0.00	LX1	N
ATOM	5632	H	LEU	99	56.496	39.562	29.165	1.00	0.00	LX1	H
ATOM	5633	CA	LEU	99	56.499	37.622	30.081	1.00	0.00	LX1	C
ATOM	5634	CB	LEU	99	57.287	37.094	28.874	1.00	0.00	LX1	C
ATOM	5635	CG	LEU	99	57.817	35.657	29.000	1.00	0.00	LX1	C
ATOM	5636	CD1	LEU	99	56.726	34.630	29.314	1.00	0.00	LX1	C
ATOM	5637	CD2	LEU	99	58.626	35.257	27.767	1.00	0.00	LX1	C
ATOM	5638	C	LEU	99	57.311	37.554	31.355	1.00	0.00	LX1	C
ATOM	5639	O	LEU	99	58.317	38.226	31.509	1.00	0.00	LX1	O
ATOM	5640	N	ARG	100	56.774	36.762	32.279	1.00	0.00	LX1	N
ATOM	5641	H	ARG	100	55.953	36.245	32.041	1.00	0.00	LX1	H
ATOM	5642	CA	ARG	100	57.342	36.722	33.618	1.00	0.00	LX1	C
ATOM	5643	CB	ARG	100	56.170	36.943	34.594	1.00	0.00	LX1	C
ATOM	5644	CG	ARG	100	56.159	36.229	35.949	1.00	0.00	LX1	C
ATOM	5645	CD	ARG	100	54.818	36.388	36.684	1.00	0.00	LX1	C
ATOM	5646	NE	ARG	100	53.674	35.919	35.888	1.00	0.00	LX1	N
ATOM	5647	HE	ARG	100	53.288	36.564	35.226	1.00	0.00	LX1	H
ATOM	5648	CZ	ARG	100	53.194	34.657	35.994	1.00	0.00	LX1	C
ATOM	5649	NH1	ARG	100	53.751	33.792	36.833	1.00	0.00	LX1	N
ATOM	5650	HH11	ARG	100	53.412	32.842	36.836	1.00	0.00	LX1	H
ATOM	5651	HH12	ARG	100	54.498	34.055	37.440	1.00	0.00	LX1	H
ATOM	5652	NH2	ARG	100	52.161	34.243	35.264	1.00	0.00	LX1	N
ATOM	5653	HH21	ARG	100	51.881	33.276	35.335	1.00	0.00	LX1	H
ATOM	5654	HH22	ARG	100	51.649	34.818	34.623	1.00	0.00	LX1	H
ATOM	5655	C	ARG	100	58.152	35.454	33.830	1.00	0.00	LX1	C
ATOM	5656	O	ARG	100	59.302	35.480	34.256	1.00	0.00	LX1	O
ATOM	5657	N	ILE	101	57.508	34.328	33.486	1.00	0.00	LX1	N
ATOM	5658	H	ILE	101	56.572	34.350	33.128	1.00	0.00	LX1	H
ATOM	5659	CA	ILE	101	58.207	33.069	33.730	1.00	0.00	LX1	C
ATOM	5660	CB	ILE	101	57.870	32.558	35.150	1.00	0.00	LX1	C
ATOM	5661	CG2	ILE	101	56.374	32.336	35.348	1.00	0.00	LX1	C
ATOM	5662	CG1	ILE	101	58.672	31.330	35.577	1.00	0.00	LX1	C
ATOM	5663	CD1	ILE	101	58.430	30.997	37.050	1.00	0.00	LX1	C
ATOM	5664	C	ILE	101	57.964	32.039	32.639	1.00	0.00	LX1	C
ATOM	5665	O	ILE	101	56.841	31.790	32.210	1.00	0.00	LX1	O
ATOM	5666	N	VAL	102	59.084	31.460	32.192	1.00	0.00	LX1	N
ATOM	5667	H	VAL	102	59.954	31.670	32.645	1.00	0.00	LX1	H
ATOM	5668	CA	VAL	102	58.942	30.293	31.324	1.00	0.00	LX1	C
ATOM	5669	CB	VAL	102	60.041	30.277	30.248	1.00	0.00	LX1	C
ATOM	5670	CG1	VAL	102	59.926	29.076	29.301	1.00	0.00	LX1	C
ATOM	5671	CG2	VAL	102	60.060	31.591	29.468	1.00	0.00	LX1	C
ATOM	5672	C	VAL	102	59.047	29.073	32.211	1.00	0.00	LX1	C
ATOM	5673	O	VAL	102	59.864	29.047	33.122	1.00	0.00	LX1	O
ATOM	5674	N	ARG	103	58.196	28.079	31.949	1.00	0.00	LX1	N
ATOM	5675	H	ARG	103	57.528	28.139	31.206	1.00	0.00	LX1	H
ATOM	5676	CA	ARG	103	58.338	26.903	32.796	1.00	0.00	LX1	C
ATOM	5677	CB	ARG	103	56.977	26.389	33.268	1.00	0.00	LX1	C
ATOM	5678	CG	ARG	103	56.173	27.463	34.007	1.00	0.00	LX1	C
ATOM	5679	CD	ARG	103	54.886	26.907	34.615	1.00	0.00	LX1	C
ATOM	5680	NE	ARG	103	54.127	27.948	35.309	1.00	0.00	LX1	N
ATOM	5681	HE	ARG	103	53.534	28.570	34.782	1.00	0.00	LX1	H
ATOM	5682	CZ	ARG	103	54.169	28.101	36.648	1.00	0.00	LX1	C
ATOM	5683	NH1	ARG	103	54.922	27.295	37.395	1.00	0.00	LX1	N
ATOM	5684	HH11	ARG	103	54.968	27.384	38.389	1.00	0.00	LX1	H
ATOM	5685	HH12	ARG	103	55.457	26.574	36.953	1.00	0.00	LX1	H

ATOM	5686	NH2	ARG	103	53.446	29.063	37.212	1.00	0.00	LX1	N
ATOM	5687	HH21	ARG	103	53.411	29.241	38.191	1.00	0.00	LX1	H
ATOM	5688	HH22	ARG	103	52.879	29.651	36.606	1.00	0.00	LX1	H
ATOM	5689	C	ARG	103	59.186	25.805	32.190	1.00	0.00	LX1	C
ATOM	5690	O	ARG	103	60.079	25.265	32.832	1.00	0.00	LX1	O
ATOM	5691	N	GLY	104	58.885	25.509	30.917	1.00	0.00	LX1	N
ATOM	5692	H	GLY	104	58.137	25.962	30.426	1.00	0.00	LX1	H
ATOM	5693	CA	GLY	104	59.650	24.433	30.287	1.00	0.00	LX1	C
ATOM	5694	C	GLY	104	59.245	23.048	30.762	1.00	0.00	LX1	C
ATOM	5695	O	GLY	104	60.050	22.147	30.951	1.00	0.00	LX1	O
ATOM	5696	N	THR	105	57.922	22.912	30.944	1.00	0.00	LX1	N
ATOM	5697	H	THR	105	57.313	23.683	30.768	1.00	0.00	LX1	H
ATOM	5698	CA	THR	105	57.439	21.603	31.388	1.00	0.00	LX1	C
ATOM	5699	CB	THR	105	55.994	21.719	31.893	1.00	0.00	LX1	C
ATOM	5700	OG1	THR	105	55.856	22.879	32.728	1.00	0.00	LX1	O
ATOM	5701	HG1	THR	105	55.872	23.629	32.145	1.00	0.00	LX1	H
ATOM	5702	CG2	THR	105	55.531	20.469	32.647	1.00	0.00	LX1	C
ATOM	5703	C	THR	105	57.601	20.507	30.336	1.00	0.00	LX1	C
ATOM	5704	O	THR	105	57.879	19.350	30.624	1.00	0.00	LX1	O
ATOM	5705	N	GLN	106	57.453	20.946	29.077	1.00	0.00	LX1	N
ATOM	5706	H	GLN	106	57.243	21.900	28.878	1.00	0.00	LX1	H
ATOM	5707	CA	GLN	106	58.014	20.107	28.025	1.00	0.00	LX1	C
ATOM	5708	CB	GLN	106	56.942	19.548	27.084	1.00	0.00	LX1	C
ATOM	5709	CG	GLN	106	56.073	20.577	26.357	1.00	0.00	LX1	C
ATOM	5710	CD	GLN	106	55.230	19.855	25.323	1.00	0.00	LX1	C
ATOM	5711	OE1	GLN	106	54.909	18.682	25.451	1.00	0.00	LX1	O
ATOM	5712	NE2	GLN	106	54.922	20.607	24.267	1.00	0.00	LX1	N
ATOM	5713	HE21	GLN	106	55.180	21.574	24.261	1.00	0.00	LX1	H
ATOM	5714	HE22	GLN	106	54.432	20.232	23.472	1.00	0.00	LX1	H
ATOM	5715	C	GLN	106	59.057	20.912	27.288	1.00	0.00	LX1	C
ATOM	5716	O	GLN	106	59.056	22.136	27.362	1.00	0.00	LX1	O
ATOM	5717	N	LEU	107	59.954	20.179	26.617	1.00	0.00	LX1	N
ATOM	5718	H	LEU	107	59.850	19.192	26.505	1.00	0.00	LX1	H
ATOM	5719	CA	LEU	107	61.128	20.855	26.075	1.00	0.00	LX1	C
ATOM	5720	CB	LEU	107	62.390	20.326	26.755	1.00	0.00	LX1	C
ATOM	5721	CG	LEU	107	62.437	20.555	28.268	1.00	0.00	LX1	C
ATOM	5722	CD1	LEU	107	63.628	19.851	28.915	1.00	0.00	LX1	C
ATOM	5723	CD2	LEU	107	62.399	22.041	28.619	1.00	0.00	LX1	C
ATOM	5724	C	LEU	107	61.250	20.701	24.575	1.00	0.00	LX1	C
ATOM	5725	O	LEU	107	61.066	19.623	24.025	1.00	0.00	LX1	O
ATOM	5726	N	PHE	108	61.578	21.835	23.945	1.00	0.00	LX1	N
ATOM	5727	H	PHE	108	61.744	22.651	24.496	1.00	0.00	LX1	H
ATOM	5728	CA	PHE	108	61.833	21.838	22.506	1.00	0.00	LX1	C
ATOM	5729	CB	PHE	108	61.854	23.294	22.022	1.00	0.00	LX1	C
ATOM	5730	CG	PHE	108	61.613	23.372	20.534	1.00	0.00	LX1	C
ATOM	5731	CD1	PHE	108	60.286	23.445	20.062	1.00	0.00	LX1	C
ATOM	5732	CD2	PHE	108	62.708	23.362	19.643	1.00	0.00	LX1	C
ATOM	5733	CE1	PHE	108	60.044	23.471	18.677	1.00	0.00	LX1	C
ATOM	5734	CE2	PHE	108	62.468	23.386	18.256	1.00	0.00	LX1	C
ATOM	5735	CZ	PHE	108	61.138	23.421	17.790	1.00	0.00	LX1	C
ATOM	5736	C	PHE	108	63.143	21.134	22.194	1.00	0.00	LX1	C
ATOM	5737	O	PHE	108	64.087	21.239	22.972	1.00	0.00	LX1	O
ATOM	5738	N	GLU	109	63.163	20.399	21.063	1.00	0.00	LX1	N
ATOM	5739	H	GLU	109	62.323	20.409	20.514	1.00	0.00	LX1	H
ATOM	5740	CA	GLU	109	64.349	19.614	20.662	1.00	0.00	LX1	C
ATOM	5741	CB	GLU	109	65.327	20.446	19.819	1.00	0.00	LX1	C
ATOM	5742	CG	GLU	109	64.945	20.614	18.337	1.00	0.00	LX1	C
ATOM	5743	CD	GLU	109	65.184	19.340	17.532	1.00	0.00	LX1	C
ATOM	5744	OE1	GLU	109	66.301	18.829	17.539	1.00	0.00	LX1	O
ATOM	5745	OE2	GLU	109	64.260	18.855	16.882	1.00	0.00	LX1	O
ATOM	5746	C	GLU	109	65.053	18.928	21.828	1.00	0.00	LX1	C

ATOM	5747	O	GLU	109	66.226	19.126	22.138	1.00	0.00	LX1	O
ATOM	5748	N	ASP	110	64.190	18.170	22.523	1.00	0.00	LX1	N
ATOM	5749	H	ASP	110	63.275	18.058	22.141	1.00	0.00	LX1	H
ATOM	5750	CA	ASP	110	64.536	17.405	23.725	1.00	0.00	LX1	C
ATOM	5751	CB	ASP	110	65.540	16.260	23.445	1.00	0.00	LX1	C
ATOM	5752	CG	ASP	110	65.305	15.467	22.159	1.00	0.00	LX1	C
ATOM	5753	OD1	ASP	110	64.170	15.212	21.764	1.00	0.00	LX1	O
ATOM	5754	OD2	ASP	110	66.284	15.063	21.539	1.00	0.00	LX1	O
ATOM	5755	C	ASP	110	64.974	18.179	24.976	1.00	0.00	LX1	C
ATOM	5756	O	ASP	110	64.907	17.661	26.083	1.00	0.00	LX1	O
ATOM	5757	N	ASN	111	65.448	19.429	24.795	1.00	0.00	LX1	N
ATOM	5758	H	ASN	111	65.393	19.861	23.893	1.00	0.00	LX1	H
ATOM	5759	CA	ASN	111	66.252	20.003	25.886	1.00	0.00	LX1	C
ATOM	5760	CB	ASN	111	67.745	20.022	25.539	1.00	0.00	LX1	C
ATOM	5761	CG	ASN	111	68.335	18.639	25.373	1.00	0.00	LX1	C
ATOM	5762	OD1	ASN	111	68.910	18.066	26.287	1.00	0.00	LX1	O
ATOM	5763	ND2	ASN	111	68.213	18.137	24.140	1.00	0.00	LX1	N
ATOM	5764	HD21	ASN	111	67.711	18.601	23.404	1.00	0.00	LX1	H
ATOM	5765	HD22	ASN	111	68.608	17.242	23.948	1.00	0.00	LX1	H
ATOM	5766	C	ASN	111	65.906	21.420	26.318	1.00	0.00	LX1	C
ATOM	5767	O	ASN	111	66.381	21.925	27.332	1.00	0.00	LX1	O
ATOM	5768	N	TYR	112	65.118	22.095	25.477	1.00	0.00	LX1	N
ATOM	5769	H	TYR	112	64.629	21.649	24.728	1.00	0.00	LX1	H
ATOM	5770	CA	TYR	112	65.136	23.548	25.620	1.00	0.00	LX1	C
ATOM	5771	CB	TYR	112	65.643	24.201	24.331	1.00	0.00	LX1	C
ATOM	5772	CG	TYR	112	66.957	23.575	23.924	1.00	0.00	LX1	C
ATOM	5773	CD1	TYR	112	68.130	23.907	24.634	1.00	0.00	LX1	C
ATOM	5774	CE1	TYR	112	69.346	23.320	24.248	1.00	0.00	LX1	C
ATOM	5775	CD2	TYR	112	66.969	22.668	22.844	1.00	0.00	LX1	C
ATOM	5776	CE2	TYR	112	68.183	22.075	22.465	1.00	0.00	LX1	C
ATOM	5777	CZ	TYR	112	69.356	22.412	23.170	1.00	0.00	LX1	C
ATOM	5778	OH	TYR	112	70.554	21.839	22.797	1.00	0.00	LX1	O
ATOM	5779	HH	TYR	112	70.403	21.212	22.100	1.00	0.00	LX1	H
ATOM	5780	C	TYR	112	63.826	24.165	26.047	1.00	0.00	LX1	C
ATOM	5781	O	TYR	112	62.759	23.824	25.557	1.00	0.00	LX1	O
ATOM	5782	N	ALA	113	63.963	25.104	26.988	1.00	0.00	LX1	N
ATOM	5783	H	ALA	113	64.878	25.373	27.286	1.00	0.00	LX1	H
ATOM	5784	CA	ALA	113	62.777	25.844	27.402	1.00	0.00	LX1	C
ATOM	5785	CB	ALA	113	62.863	26.239	28.870	1.00	0.00	LX1	C
ATOM	5786	C	ALA	113	62.555	27.104	26.595	1.00	0.00	LX1	C
ATOM	5787	O	ALA	113	61.433	27.519	26.341	1.00	0.00	LX1	O
ATOM	5788	N	LEU	114	63.678	27.706	26.191	1.00	0.00	LX1	N
ATOM	5789	H	LEU	114	64.586	27.353	26.423	1.00	0.00	LX1	H
ATOM	5790	CA	LEU	114	63.529	28.887	25.351	1.00	0.00	LX1	C
ATOM	5791	CB	LEU	114	63.905	30.141	26.149	1.00	0.00	LX1	C
ATOM	5792	CG	LEU	114	63.811	31.469	25.388	1.00	0.00	LX1	C
ATOM	5793	CD1	LEU	114	62.430	31.717	24.784	1.00	0.00	LX1	C
ATOM	5794	CD2	LEU	114	64.246	32.641	26.263	1.00	0.00	LX1	C
ATOM	5795	C	LEU	114	64.363	28.721	24.104	1.00	0.00	LX1	C
ATOM	5796	O	LEU	114	65.586	28.705	24.152	1.00	0.00	LX1	O
ATOM	5797	N	ALA	115	63.655	28.559	22.986	1.00	0.00	LX1	N
ATOM	5798	H	ALA	115	62.654	28.627	22.994	1.00	0.00	LX1	H
ATOM	5799	CA	ALA	115	64.410	28.318	21.765	1.00	0.00	LX1	C
ATOM	5800	CB	ALA	115	64.082	26.936	21.197	1.00	0.00	LX1	C
ATOM	5801	C	ALA	115	64.181	29.396	20.727	1.00	0.00	LX1	C
ATOM	5802	O	ALA	115	63.197	29.409	19.998	1.00	0.00	LX1	O
ATOM	5803	N	VAL	116	65.130	30.335	20.717	1.00	0.00	LX1	N
ATOM	5804	H	VAL	116	65.972	30.163	21.231	1.00	0.00	LX1	H
ATOM	5805	CA	VAL	116	65.020	31.435	19.763	1.00	0.00	LX1	C
ATOM	5806	CB	VAL	116	65.469	32.753	20.414	1.00	0.00	LX1	C
ATOM	5807	CG1	VAL	116	65.204	33.955	19.506	1.00	0.00	LX1	C

ATOM	5808	CG2	VAL	116	64.814	32.949	21.783	1.00	0.00	LX1	C
ATOM	5809	C	VAL	116	65.830	31.128	18.515	1.00	0.00	LX1	C
ATOM	5810	O	VAL	116	67.036	31.335	18.465	1.00	0.00	LX1	O
ATOM	5811	N	LEU	117	65.130	30.575	17.521	1.00	0.00	LX1	N
ATOM	5812	H	LEU	117	64.129	30.582	17.545	1.00	0.00	LX1	H
ATOM	5813	CA	LEU	117	65.894	30.026	16.401	1.00	0.00	LX1	C
ATOM	5814	CB	LEU	117	65.537	28.558	16.146	1.00	0.00	LX1	C
ATOM	5815	CG	LEU	117	65.406	27.672	17.387	1.00	0.00	LX1	C
ATOM	5816	CD1	LEU	117	64.899	26.275	17.027	1.00	0.00	LX1	C
ATOM	5817	CD2	LEU	117	66.695	27.611	18.199	1.00	0.00	LX1	C
ATOM	5818	C	LEU	117	65.696	30.793	15.113	1.00	0.00	LX1	C
ATOM	5819	O	LEU	117	64.607	31.266	14.817	1.00	0.00	LX1	O
ATOM	5820	N	ASP	118	66.795	30.868	14.358	1.00	0.00	LX1	N
ATOM	5821	H	ASP	118	67.646	30.487	14.725	1.00	0.00	LX1	H
ATOM	5822	CA	ASP	118	66.791	31.113	12.912	1.00	0.00	LX1	C
ATOM	5823	CB	ASP	118	66.656	29.773	12.164	1.00	0.00	LX1	C
ATOM	5824	CG	ASP	118	67.760	28.762	12.493	1.00	0.00	LX1	C
ATOM	5825	OD1	ASP	118	68.085	27.937	11.646	1.00	0.00	LX1	O
ATOM	5826	OD2	ASP	118	68.310	28.764	13.591	1.00	0.00	LX1	O
ATOM	5827	C	ASP	118	65.793	32.154	12.422	1.00	0.00	LX1	C
ATOM	5828	O	ASP	118	64.913	31.906	11.600	1.00	0.00	LX1	O
ATOM	5829	N	ASN	119	65.943	33.345	13.020	1.00	0.00	LX1	N
ATOM	5830	H	ASN	119	66.794	33.511	13.521	1.00	0.00	LX1	H
ATOM	5831	CA	ASN	119	64.868	34.328	12.860	1.00	0.00	LX1	C
ATOM	5832	CB	ASN	119	64.533	35.055	14.161	1.00	0.00	LX1	C
ATOM	5833	CG	ASN	119	63.655	34.222	15.065	1.00	0.00	LX1	C
ATOM	5834	OD1	ASN	119	62.611	33.701	14.691	1.00	0.00	LX1	O
ATOM	5835	ND2	ASN	119	64.127	34.121	16.306	1.00	0.00	LX1	N
ATOM	5836	HD21	ASN	119	64.920	34.672	16.580	1.00	0.00	LX1	H
ATOM	5837	HD22	ASN	119	63.670	33.496	16.941	1.00	0.00	LX1	H
ATOM	5838	C	ASN	119	65.082	35.369	11.787	1.00	0.00	LX1	C
ATOM	5839	O	ASN	119	65.306	36.545	12.061	1.00	0.00	LX1	O
ATOM	5840	N	GLY	120	64.949	34.883	10.549	1.00	0.00	LX1	N
ATOM	5841	H	GLY	120	64.924	33.894	10.411	1.00	0.00	LX1	H
ATOM	5842	CA	GLY	120	64.785	35.843	9.462	1.00	0.00	LX1	C
ATOM	5843	C	GLY	120	66.056	36.474	8.924	1.00	0.00	LX1	C
ATOM	5844	O	GLY	120	67.175	36.088	9.244	1.00	0.00	LX1	O
ATOM	5845	N	ASP	121	65.805	37.460	8.052	1.00	0.00	LX1	N
ATOM	5846	H	ASP	121	64.860	37.738	7.879	1.00	0.00	LX1	H
ATOM	5847	CA	ASP	121	66.883	38.083	7.284	1.00	0.00	LX1	C
ATOM	5848	CB	ASP	121	66.335	39.092	6.274	1.00	0.00	LX1	C
ATOM	5849	CG	ASP	121	65.449	38.459	5.227	1.00	0.00	LX1	C
ATOM	5850	OD1	ASP	121	65.911	37.577	4.511	1.00	0.00	LX1	O
ATOM	5851	OD2	ASP	121	64.301	38.874	5.114	1.00	0.00	LX1	O
ATOM	5852	C	ASP	121	67.931	38.808	8.107	1.00	0.00	LX1	C
ATOM	5853	O	ASP	121	67.639	39.507	9.073	1.00	0.00	LX1	O
ATOM	5854	N	PRO	122	69.197	38.639	7.657	1.00	0.00	LX1	N
ATOM	5855	CD	PRO	122	69.638	37.680	6.650	1.00	0.00	LX1	C
ATOM	5856	CA	PRO	122	70.308	39.413	8.220	1.00	0.00	LX1	C
ATOM	5857	CB	PRO	122	71.541	38.742	7.595	1.00	0.00	LX1	C
ATOM	5858	CG	PRO	122	71.077	37.394	7.048	1.00	0.00	LX1	C
ATOM	5859	C	PRO	122	70.282	40.914	7.930	1.00	0.00	LX1	C
ATOM	5860	O	PRO	122	71.096	41.428	7.172	1.00	0.00	LX1	O
ATOM	5861	N	LEU	123	69.346	41.626	8.575	1.00	0.00	LX1	N
ATOM	5862	H	LEU	123	68.697	41.166	9.182	1.00	0.00	LX1	H
ATOM	5863	CA	LEU	123	69.467	43.082	8.483	1.00	0.00	LX1	C
ATOM	5864	CB	LEU	123	68.115	43.754	8.754	1.00	0.00	LX1	C
ATOM	5865	CG	LEU	123	68.123	45.285	8.884	1.00	0.00	LX1	C
ATOM	5866	CD1	LEU	123	68.425	45.988	7.563	1.00	0.00	LX1	C
ATOM	5867	CD2	LEU	123	66.843	45.811	9.532	1.00	0.00	LX1	C
ATOM	5868	C	LEU	123	70.536	43.597	9.427	1.00	0.00	LX1	C

ATOM	5869	O	LEU	123	70.594	43.246	10.602	1.00	0.00	LX1	O
ATOM	5870	N	ASN	124	71.389	44.448	8.858	1.00	0.00	LX1	N
ATOM	5871	H	ASN	124	71.321	44.701	7.890	1.00	0.00	LX1	H
ATOM	5872	CA	ASN	124	72.326	45.155	9.722	1.00	0.00	LX1	C
ATOM	5873	CB	ASN	124	73.725	45.237	9.093	1.00	0.00	LX1	C
ATOM	5874	CG	ASN	124	73.716	46.009	7.790	1.00	0.00	LX1	C
ATOM	5875	OD1	ASN	124	72.853	45.841	6.943	1.00	0.00	LX1	O
ATOM	5876	ND2	ASN	124	74.731	46.866	7.659	1.00	0.00	LX1	N
ATOM	5877	HD21	ASN	124	75.423	46.969	8.371	1.00	0.00	LX1	H
ATOM	5878	HD22	ASN	124	74.777	47.393	6.813	1.00	0.00	LX1	H
ATOM	5879	C	ASN	124	71.814	46.526	10.111	1.00	0.00	LX1	C
ATOM	5880	O	ASN	124	71.173	47.223	9.340	1.00	0.00	LX1	O
ATOM	5881	N	ASN	125	72.138	46.861	11.367	1.00	0.00	LX1	N
ATOM	5882	H	ASN	125	72.719	46.217	11.862	1.00	0.00	LX1	H
ATOM	5883	CA	ASN	125	71.864	48.158	11.997	1.00	0.00	LX1	C
ATOM	5884	CB	ASN	125	73.016	49.140	11.753	1.00	0.00	LX1	C
ATOM	5885	CG	ASN	125	74.222	48.711	12.558	1.00	0.00	LX1	C
ATOM	5886	OD1	ASN	125	74.246	47.661	13.189	1.00	0.00	LX1	O
ATOM	5887	ND2	ASN	125	75.233	49.579	12.515	1.00	0.00	LX1	N
ATOM	5888	HD21	ASN	125	75.167	50.400	11.942	1.00	0.00	LX1	H
ATOM	5889	HD22	ASN	125	76.052	49.402	13.055	1.00	0.00	LX1	H
ATOM	5890	C	ASN	125	70.526	48.847	11.784	1.00	0.00	LX1	C
ATOM	5891	O	ASN	125	69.661	48.851	12.655	1.00	0.00	LX1	O
ATOM	5892	N	THR	126	70.421	49.481	10.617	1.00	0.00	LX1	N
ATOM	5893	H	THR	126	71.089	49.303	9.891	1.00	0.00	LX1	H
ATOM	5894	CA	THR	126	69.404	50.485	10.320	1.00	0.00	LX1	C
ATOM	5895	CB	THR	126	69.683	50.897	8.865	1.00	0.00	LX1	C
ATOM	5896	OG1	THR	126	71.109	50.996	8.733	1.00	0.00	LX1	O
ATOM	5897	HG1	THR	126	71.312	51.158	7.811	1.00	0.00	LX1	H
ATOM	5898	CG2	THR	126	69.028	52.199	8.391	1.00	0.00	LX1	C
ATOM	5899	C	THR	126	67.956	50.095	10.634	1.00	0.00	LX1	C
ATOM	5900	O	THR	126	67.605	48.934	10.832	1.00	0.00	LX1	O
ATOM	5901	N	THR	127	67.132	51.150	10.742	1.00	0.00	LX1	N
ATOM	5902	H	THR	127	67.498	52.062	10.571	1.00	0.00	LX1	H
ATOM	5903	CA	THR	127	65.722	50.973	11.090	1.00	0.00	LX1	C
ATOM	5904	CB	THR	127	65.042	52.356	11.130	1.00	0.00	LX1	C
ATOM	5905	OG1	THR	127	63.706	52.257	11.629	1.00	0.00	LX1	O
ATOM	5906	HG1	THR	127	63.372	53.143	11.705	1.00	0.00	LX1	H
ATOM	5907	CG2	THR	127	65.079	53.093	9.787	1.00	0.00	LX1	C
ATOM	5908	C	THR	127	64.977	49.970	10.207	1.00	0.00	LX1	C
ATOM	5909	O	THR	127	65.134	49.928	8.989	1.00	0.00	LX1	O
ATOM	5910	N	PRO	128	64.178	49.113	10.884	1.00	0.00	LX1	N
ATOM	5911	CD	PRO	128	63.989	49.031	12.331	1.00	0.00	LX1	C
ATOM	5912	CA	PRO	128	63.421	48.097	10.153	1.00	0.00	LX1	C
ATOM	5913	CB	PRO	128	63.191	47.059	11.255	1.00	0.00	LX1	C
ATOM	5914	CG	PRO	128	62.999	47.888	12.525	1.00	0.00	LX1	C
ATOM	5915	C	PRO	128	62.135	48.642	9.538	1.00	0.00	LX1	C
ATOM	5916	O	PRO	128	61.033	48.331	9.971	1.00	0.00	LX1	O
ATOM	5917	N	VAL	129	62.311	49.456	8.488	1.00	0.00	LX1	N
ATOM	5918	H	VAL	129	63.233	49.585	8.117	1.00	0.00	LX1	H
ATOM	5919	CA	VAL	129	61.122	49.937	7.782	1.00	0.00	LX1	C
ATOM	5920	CB	VAL	129	61.510	50.967	6.710	1.00	0.00	LX1	C
ATOM	5921	CG1	VAL	129	60.287	51.575	6.012	1.00	0.00	LX1	C
ATOM	5922	CG2	VAL	129	62.396	52.062	7.303	1.00	0.00	LX1	C
ATOM	5923	C	VAL	129	60.349	48.775	7.179	1.00	0.00	LX1	C
ATOM	5924	O	VAL	129	60.914	47.876	6.564	1.00	0.00	LX1	O
ATOM	5925	N	THR	130	59.036	48.815	7.419	1.00	0.00	LX1	N
ATOM	5926	H	THR	130	58.597	49.617	7.824	1.00	0.00	LX1	H
ATOM	5927	CA	THR	130	58.215	47.631	7.193	1.00	0.00	LX1	C
ATOM	5928	CB	THR	130	56.796	47.975	7.627	1.00	0.00	LX1	C
ATOM	5929	OG1	THR	130	56.397	49.203	7.009	1.00	0.00	LX1	O

ATOM	5930	HG1	THR	130	55.762	48.928	6.340	1.00	0.00	LX1	H
ATOM	5931	CG2	THR	130	56.681	48.078	9.149	1.00	0.00	LX1	C
ATOM	5932	C	THR	130	58.281	47.031	5.793	1.00	0.00	LX1	C
ATOM	5933	O	THR	130	58.481	45.831	5.620	1.00	0.00	LX1	O
ATOM	5934	N	GLY	131	58.168	47.923	4.802	1.00	0.00	LX1	N
ATOM	5935	H	GLY	131	57.903	48.863	5.016	1.00	0.00	LX1	H
ATOM	5936	CA	GLY	131	58.272	47.426	3.430	1.00	0.00	LX1	C
ATOM	5937	C	GLY	131	59.625	47.631	2.775	1.00	0.00	LX1	C
ATOM	5938	O	GLY	131	59.781	47.553	1.564	1.00	0.00	LX1	O
ATOM	5939	N	ALA	132	60.615	47.925	3.628	1.00	0.00	LX1	N
ATOM	5940	H	ALA	132	60.484	47.886	4.617	1.00	0.00	LX1	H
ATOM	5941	CA	ALA	132	61.902	48.227	3.015	1.00	0.00	LX1	C
ATOM	5942	CB	ALA	132	62.269	49.699	3.209	1.00	0.00	LX1	C
ATOM	5943	C	ALA	132	63.023	47.356	3.528	1.00	0.00	LX1	C
ATOM	5944	O	ALA	132	63.730	46.686	2.790	1.00	0.00	LX1	O
ATOM	5945	N	SER	133	63.169	47.391	4.850	1.00	0.00	LX1	N
ATOM	5946	H	SER	133	62.576	47.911	5.463	1.00	0.00	LX1	H
ATOM	5947	CA	SER	133	64.280	46.627	5.395	1.00	0.00	LX1	C
ATOM	5948	CB	SER	133	64.585	47.147	6.796	1.00	0.00	LX1	C
ATOM	5949	OG	SER	133	64.379	48.566	6.826	1.00	0.00	LX1	O
ATOM	5950	HG	SER	133	65.079	48.943	7.359	1.00	0.00	LX1	H
ATOM	5951	C	SER	133	64.025	45.134	5.406	1.00	0.00	LX1	C
ATOM	5952	O	SER	133	62.908	44.697	5.676	1.00	0.00	LX1	O
ATOM	5953	N	PRO	134	65.108	44.359	5.142	1.00	0.00	LX1	N
ATOM	5954	CD	PRO	134	66.384	44.808	4.594	1.00	0.00	LX1	C
ATOM	5955	CA	PRO	134	65.092	42.916	5.422	1.00	0.00	LX1	C
ATOM	5956	CB	PRO	134	66.584	42.580	5.398	1.00	0.00	LX1	C
ATOM	5957	CG	PRO	134	67.164	43.523	4.349	1.00	0.00	LX1	C
ATOM	5958	C	PRO	134	64.367	42.545	6.711	1.00	0.00	LX1	C
ATOM	5959	O	PRO	134	64.247	43.355	7.628	1.00	0.00	LX1	O
ATOM	5960	N	GLY	135	63.811	41.333	6.710	1.00	0.00	LX1	N
ATOM	5961	H	GLY	135	63.995	40.649	5.996	1.00	0.00	LX1	H
ATOM	5962	CA	GLY	135	62.921	41.023	7.819	1.00	0.00	LX1	C
ATOM	5963	C	GLY	135	63.410	39.905	8.703	1.00	0.00	LX1	C
ATOM	5964	O	GLY	135	63.226	38.726	8.425	1.00	0.00	LX1	O
ATOM	5965	N	GLY	136	64.029	40.326	9.801	1.00	0.00	LX1	N
ATOM	5966	H	GLY	136	64.243	41.292	9.956	1.00	0.00	LX1	H
ATOM	5967	CA	GLY	136	64.244	39.326	10.835	1.00	0.00	LX1	C
ATOM	5968	C	GLY	136	63.578	39.757	12.117	1.00	0.00	LX1	C
ATOM	5969	O	GLY	136	62.943	40.806	12.180	1.00	0.00	LX1	O
ATOM	5970	N	LEU	137	63.803	38.939	13.151	1.00	0.00	LX1	N
ATOM	5971	H	LEU	137	64.354	38.114	13.005	1.00	0.00	LX1	H
ATOM	5972	CA	LEU	137	63.467	39.449	14.478	1.00	0.00	LX1	C
ATOM	5973	CB	LEU	137	63.216	38.275	15.426	1.00	0.00	LX1	C
ATOM	5974	CG	LEU	137	62.747	38.648	16.834	1.00	0.00	LX1	C
ATOM	5975	CD1	LEU	137	61.495	39.520	16.827	1.00	0.00	LX1	C
ATOM	5976	CD2	LEU	137	62.568	37.407	17.702	1.00	0.00	LX1	C
ATOM	5977	C	LEU	137	64.567	40.374	14.968	1.00	0.00	LX1	C
ATOM	5978	O	LEU	137	65.743	40.124	14.744	1.00	0.00	LX1	O
ATOM	5979	N	ARG	138	64.149	41.484	15.587	1.00	0.00	LX1	N
ATOM	5980	H	ARG	138	63.170	41.622	15.740	1.00	0.00	LX1	H
ATOM	5981	CA	ARG	138	65.140	42.500	15.932	1.00	0.00	LX1	C
ATOM	5982	CB	ARG	138	64.641	43.891	15.519	1.00	0.00	LX1	C
ATOM	5983	CG	ARG	138	65.789	44.884	15.334	1.00	0.00	LX1	C
ATOM	5984	CD	ARG	138	65.361	46.311	14.999	1.00	0.00	LX1	C
ATOM	5985	NE	ARG	138	66.416	47.270	15.334	1.00	0.00	LX1	N
ATOM	5986	HE	ARG	138	66.533	47.511	16.308	1.00	0.00	LX1	H
ATOM	5987	CZ	ARG	138	67.243	47.824	14.420	1.00	0.00	LX1	C
ATOM	5988	NH1	ARG	138	67.153	47.512	13.129	1.00	0.00	LX1	N
ATOM	5989	HH11	ARG	138	67.745	47.964	12.446	1.00	0.00	LX1	H
ATOM	5990	HH12	ARG	138	66.497	46.832	12.811	1.00	0.00	LX1	H

ATOM	5991	NH2	ARG	138	68.158	48.695	14.820	1.00	0.00	LX1	N
ATOM	5992	HH21	ARG	138	68.814	49.096	14.174	1.00	0.00	LX1	H
ATOM	5993	HH22	ARG	138	68.216	48.961	15.792	1.00	0.00	LX1	H
ATOM	5994	C	ARG	138	65.634	42.467	17.366	1.00	0.00	LX1	C
ATOM	5995	O	ARG	138	66.801	42.726	17.660	1.00	0.00	LX1	O
ATOM	5996	N	GLU	139	64.690	42.118	18.250	1.00	0.00	LX1	N
ATOM	5997	H	GLU	139	63.746	41.955	17.965	1.00	0.00	LX1	H
ATOM	5998	CA	GLU	139	65.019	42.074	19.672	1.00	0.00	LX1	C
ATOM	5999	CB	GLU	139	65.093	43.475	20.259	1.00	0.00	LX1	C
ATOM	6000	CG	GLU	139	63.810	44.256	19.998	1.00	0.00	LX1	C
ATOM	6001	CD	GLU	139	64.168	45.567	19.351	1.00	0.00	LX1	C
ATOM	6002	OE1	GLU	139	64.979	45.574	18.430	1.00	0.00	LX1	O
ATOM	6003	OE2	GLU	139	63.612	46.582	19.751	1.00	0.00	LX1	O
ATOM	6004	C	GLU	139	64.012	41.253	20.440	1.00	0.00	LX1	C
ATOM	6005	O	GLU	139	62.946	40.903	19.942	1.00	0.00	LX1	O
ATOM	6006	N	LEU	140	64.408	40.956	21.679	1.00	0.00	LX1	N
ATOM	6007	H	LEU	140	65.227	41.378	22.077	1.00	0.00	LX1	H
ATOM	6008	CA	LEU	140	63.502	40.183	22.512	1.00	0.00	LX1	C
ATOM	6009	CB	LEU	140	64.263	39.053	23.210	1.00	0.00	LX1	C
ATOM	6010	CG	LEU	140	64.808	37.974	22.276	1.00	0.00	LX1	C
ATOM	6011	CD1	LEU	140	65.719	36.997	23.021	1.00	0.00	LX1	C
ATOM	6012	CD2	LEU	140	63.689	37.249	21.525	1.00	0.00	LX1	C
ATOM	6013	C	LEU	140	62.858	41.079	23.541	1.00	0.00	LX1	C
ATOM	6014	O	LEU	140	63.354	41.210	24.646	1.00	0.00	LX1	O
ATOM	6015	N	GLN	141	61.734	41.699	23.158	1.00	0.00	LX1	N
ATOM	6016	H	GLN	141	61.323	41.495	22.271	1.00	0.00	LX1	H
ATOM	6017	CA	GLN	141	61.088	42.573	24.144	1.00	0.00	LX1	C
ATOM	6018	CB	GLN	141	60.145	43.552	23.460	1.00	0.00	LX1	C
ATOM	6019	CG	GLN	141	60.756	44.445	22.384	1.00	0.00	LX1	C
ATOM	6020	CD	GLN	141	59.622	45.171	21.689	1.00	0.00	LX1	C
ATOM	6021	OE1	GLN	141	59.513	46.386	21.674	1.00	0.00	LX1	O
ATOM	6022	NE2	GLN	141	58.741	44.353	21.104	1.00	0.00	LX1	N
ATOM	6023	HE21	GLN	141	58.844	43.360	21.179	1.00	0.00	LX1	H
ATOM	6024	HE22	GLN	141	57.990	44.776	20.602	1.00	0.00	LX1	H
ATOM	6025	C	GLN	141	60.303	41.813	25.204	1.00	0.00	LX1	C
ATOM	6026	O	GLN	141	59.079	41.759	25.177	1.00	0.00	LX1	O
ATOM	6027	N	LEU	142	61.057	41.187	26.115	1.00	0.00	LX1	N
ATOM	6028	H	LEU	142	62.053	41.284	26.089	1.00	0.00	LX1	H
ATOM	6029	CA	LEU	142	60.381	40.333	27.088	1.00	0.00	LX1	C
ATOM	6030	CB	LEU	142	60.778	38.847	26.959	1.00	0.00	LX1	C
ATOM	6031	CG	LEU	142	61.354	38.304	25.638	1.00	0.00	LX1	C
ATOM	6032	CD1	LEU	142	61.840	36.866	25.815	1.00	0.00	LX1	C
ATOM	6033	CD2	LEU	142	60.419	38.395	24.432	1.00	0.00	LX1	C
ATOM	6034	C	LEU	142	60.647	40.794	28.508	1.00	0.00	LX1	C
ATOM	6035	O	LEU	142	61.083	40.027	29.358	1.00	0.00	LX1	O
ATOM	6036	N	ARG	143	60.429	42.105	28.728	1.00	0.00	LX1	N
ATOM	6037	H	ARG	143	60.030	42.671	28.002	1.00	0.00	LX1	H
ATOM	6038	CA	ARG	143	61.070	42.730	29.893	1.00	0.00	LX1	C
ATOM	6039	CB	ARG	143	60.715	44.215	30.019	1.00	0.00	LX1	C
ATOM	6040	CG	ARG	143	59.339	44.509	30.613	1.00	0.00	LX1	C
ATOM	6041	CD	ARG	143	59.001	45.991	30.626	1.00	0.00	LX1	C
ATOM	6042	NE	ARG	143	58.805	46.494	29.270	1.00	0.00	LX1	N
ATOM	6043	HE	ARG	143	59.062	45.938	28.467	1.00	0.00	LX1	H
ATOM	6044	CZ	ARG	143	58.340	47.744	29.086	1.00	0.00	LX1	C
ATOM	6045	NH1	ARG	143	58.094	48.542	30.118	1.00	0.00	LX1	N
ATOM	6046	HH11	ARG	143	57.803	49.501	29.945	1.00	0.00	LX1	H
ATOM	6047	HH12	ARG	143	58.220	48.260	31.063	1.00	0.00	LX1	H
ATOM	6048	NH2	ARG	143	58.138	48.190	27.859	1.00	0.00	LX1	N
ATOM	6049	HH21	ARG	143	57.747	49.111	27.729	1.00	0.00	LX1	H
ATOM	6050	HH22	ARG	143	58.391	47.635	27.058	1.00	0.00	LX1	H
ATOM	6051	C	ARG	143	61.021	42.060	31.260	1.00	0.00	LX1	C

ATOM	6052	O	ARG	143	61.957	42.174	32.047	1.00	0.00	LX1	O
ATOM	6053	N	SER	144	59.895	41.390	31.525	1.00	0.00	LX1	N
ATOM	6054	H	SER	144	59.222	41.225	30.803	1.00	0.00	LX1	H
ATOM	6055	CA	SER	144	59.723	40.884	32.883	1.00	0.00	LX1	C
ATOM	6056	CB	SER	144	58.242	40.951	33.253	1.00	0.00	LX1	C
ATOM	6057	OG	SER	144	57.717	42.231	32.865	1.00	0.00	LX1	O
ATOM	6058	HG	SER	144	57.679	42.221	31.910	1.00	0.00	LX1	H
ATOM	6059	C	SER	144	60.321	39.514	33.175	1.00	0.00	LX1	C
ATOM	6060	O	SER	144	60.251	39.009	34.294	1.00	0.00	LX1	O
ATOM	6061	N	LEU	145	60.919	38.930	32.122	1.00	0.00	LX1	N
ATOM	6062	H	LEU	145	60.961	39.381	31.230	1.00	0.00	LX1	H
ATOM	6063	CA	LEU	145	61.369	37.550	32.265	1.00	0.00	LX1	C
ATOM	6064	CB	LEU	145	61.780	37.004	30.895	1.00	0.00	LX1	C
ATOM	6065	CG	LEU	145	62.031	35.495	30.843	1.00	0.00	LX1	C
ATOM	6066	CD1	LEU	145	60.846	34.690	31.371	1.00	0.00	LX1	C
ATOM	6067	CD2	LEU	145	62.448	35.047	29.443	1.00	0.00	LX1	C
ATOM	6068	C	LEU	145	62.441	37.385	33.322	1.00	0.00	LX1	C
ATOM	6069	O	LEU	145	63.514	37.974	33.270	1.00	0.00	LX1	O
ATOM	6070	N	THR	146	62.060	36.578	34.313	1.00	0.00	LX1	N
ATOM	6071	H	THR	146	61.147	36.166	34.317	1.00	0.00	LX1	H
ATOM	6072	CA	THR	146	62.931	36.480	35.475	1.00	0.00	LX1	C
ATOM	6073	CB	THR	146	62.227	37.114	36.676	1.00	0.00	LX1	C
ATOM	6074	OG1	THR	146	60.806	37.160	36.459	1.00	0.00	LX1	O
ATOM	6075	HG1	THR	146	60.655	37.778	35.744	1.00	0.00	LX1	H
ATOM	6076	CG2	THR	146	62.761	38.525	36.938	1.00	0.00	LX1	C
ATOM	6077	C	THR	146	63.417	35.072	35.759	1.00	0.00	LX1	C
ATOM	6078	O	THR	146	64.602	34.833	35.958	1.00	0.00	LX1	O
ATOM	6079	N	GLU	147	62.450	34.142	35.722	1.00	0.00	LX1	N
ATOM	6080	H	GLU	147	61.495	34.392	35.561	1.00	0.00	LX1	H
ATOM	6081	CA	GLU	147	62.878	32.752	35.835	1.00	0.00	LX1	C
ATOM	6082	CB	GLU	147	62.330	32.070	37.099	1.00	0.00	LX1	C
ATOM	6083	CG	GLU	147	62.537	32.769	38.454	1.00	0.00	LX1	C
ATOM	6084	CD	GLU	147	64.000	32.877	38.858	1.00	0.00	LX1	C
ATOM	6085	OE1	GLU	147	64.468	33.985	39.089	1.00	0.00	LX1	O
ATOM	6086	OE2	GLU	147	64.697	31.870	38.979	1.00	0.00	LX1	O
ATOM	6087	C	GLU	147	62.480	31.937	34.617	1.00	0.00	LX1	C
ATOM	6088	O	GLU	147	61.384	32.050	34.077	1.00	0.00	LX1	O
ATOM	6089	N	ILE	148	63.424	31.079	34.225	1.00	0.00	LX1	N
ATOM	6090	H	ILE	148	64.342	31.225	34.594	1.00	0.00	LX1	H
ATOM	6091	CA	ILE	148	63.133	30.037	33.243	1.00	0.00	LX1	C
ATOM	6092	CB	ILE	148	64.035	30.209	32.010	1.00	0.00	LX1	C
ATOM	6093	CG2	ILE	148	63.825	29.102	30.974	1.00	0.00	LX1	C
ATOM	6094	CG1	ILE	148	63.845	31.594	31.389	1.00	0.00	LX1	C
ATOM	6095	CD1	ILE	148	64.860	31.898	30.289	1.00	0.00	LX1	C
ATOM	6096	C	ILE	148	63.349	28.688	33.904	1.00	0.00	LX1	C
ATOM	6097	O	ILE	148	64.481	28.243	34.073	1.00	0.00	LX1	O
ATOM	6098	N	LEU	149	62.215	28.099	34.330	1.00	0.00	LX1	N
ATOM	6099	H	LEU	149	61.349	28.527	34.078	1.00	0.00	LX1	H
ATOM	6100	CA	LEU	149	62.252	26.990	35.286	1.00	0.00	LX1	C
ATOM	6101	CB	LEU	149	60.862	26.456	35.649	1.00	0.00	LX1	C
ATOM	6102	CG	LEU	149	59.951	27.435	36.392	1.00	0.00	LX1	C
ATOM	6103	CD1	LEU	149	58.631	26.770	36.779	1.00	0.00	LX1	C
ATOM	6104	CD2	LEU	149	60.613	28.039	37.627	1.00	0.00	LX1	C
ATOM	6105	C	LEU	149	63.188	25.840	34.985	1.00	0.00	LX1	C
ATOM	6106	O	LEU	149	64.158	25.629	35.706	1.00	0.00	LX1	O
ATOM	6107	N	LYS	150	62.861	25.093	33.921	1.00	0.00	LX1	N
ATOM	6108	H	LYS	150	62.036	25.258	33.371	1.00	0.00	LX1	H
ATOM	6109	CA	LYS	150	63.776	24.024	33.532	1.00	0.00	LX1	C
ATOM	6110	CB	LYS	150	63.354	22.663	34.110	1.00	0.00	LX1	C
ATOM	6111	CG	LYS	150	63.590	22.623	35.623	1.00	0.00	LX1	C
ATOM	6112	CD	LYS	150	63.232	21.334	36.356	1.00	0.00	LX1	C

ATOM	6113	CE	LYS	150	63.465	21.482	37.867	1.00	0.00	LX1	C
ATOM	6114	NZ	LYS	150	64.868	21.824	38.145	1.00	0.00	LX1	N
ATOM	6115	HZ1	LYS	150	65.090	21.752	39.154	1.00	0.00	LX1	H
ATOM	6116	HZ2	LYS	150	65.501	21.193	37.614	1.00	0.00	LX1	H
ATOM	6117	HZ3	LYS	150	65.100	22.802	37.862	1.00	0.00	LX1	H
ATOM	6118	C	LYS	150	63.946	23.968	32.034	1.00	0.00	LX1	C
ATOM	6119	O	LYS	150	63.022	24.233	31.280	1.00	0.00	LX1	O
ATOM	6120	N	GLY	151	65.176	23.618	31.642	1.00	0.00	LX1	N
ATOM	6121	H	GLY	151	65.915	23.573	32.315	1.00	0.00	LX1	H
ATOM	6122	CA	GLY	151	65.436	23.570	30.207	1.00	0.00	LX1	C
ATOM	6123	C	GLY	151	66.237	24.765	29.739	1.00	0.00	LX1	C
ATOM	6124	O	GLY	151	66.178	25.845	30.315	1.00	0.00	LX1	O
ATOM	6125	N	GLY	152	67.018	24.508	28.683	1.00	0.00	LX1	N
ATOM	6126	H	GLY	152	66.947	23.624	28.215	1.00	0.00	LX1	H
ATOM	6127	CA	GLY	152	67.982	25.532	28.291	1.00	0.00	LX1	C
ATOM	6128	C	GLY	152	67.457	26.600	27.356	1.00	0.00	LX1	C
ATOM	6129	O	GLY	152	66.366	26.504	26.801	1.00	0.00	LX1	O
ATOM	6130	N	VAL	153	68.306	27.623	27.196	1.00	0.00	LX1	N
ATOM	6131	H	VAL	153	69.207	27.620	27.631	1.00	0.00	LX1	H
ATOM	6132	CA	VAL	153	68.018	28.626	26.178	1.00	0.00	LX1	C
ATOM	6133	CB	VAL	153	68.257	30.048	26.715	1.00	0.00	LX1	C
ATOM	6134	CG1	VAL	153	67.950	31.123	25.669	1.00	0.00	LX1	C
ATOM	6135	CG2	VAL	153	67.428	30.297	27.974	1.00	0.00	LX1	C
ATOM	6136	C	VAL	153	68.846	28.353	24.936	1.00	0.00	LX1	C
ATOM	6137	O	VAL	153	70.024	28.676	24.841	1.00	0.00	LX1	O
ATOM	6138	N	LEU	154	68.161	27.709	23.988	1.00	0.00	LX1	N
ATOM	6139	H	LEU	154	67.175	27.595	24.117	1.00	0.00	LX1	H
ATOM	6140	CA	LEU	154	68.784	27.523	22.686	1.00	0.00	LX1	C
ATOM	6141	CB	LEU	154	68.263	26.247	22.023	1.00	0.00	LX1	C
ATOM	6142	CG	LEU	154	68.940	25.865	20.702	1.00	0.00	LX1	C
ATOM	6143	CD1	LEU	154	70.424	25.562	20.881	1.00	0.00	LX1	C
ATOM	6144	CD2	LEU	154	68.215	24.716	20.001	1.00	0.00	LX1	C
ATOM	6145	C	LEU	154	68.497	28.708	21.795	1.00	0.00	LX1	C
ATOM	6146	O	LEU	154	67.424	28.828	21.217	1.00	0.00	LX1	O
ATOM	6147	N	ILE	155	69.497	29.576	21.685	1.00	0.00	LX1	N
ATOM	6148	H	ILE	155	70.365	29.480	22.179	1.00	0.00	LX1	H
ATOM	6149	CA	ILE	155	69.371	30.497	20.565	1.00	0.00	LX1	C
ATOM	6150	CB	ILE	155	69.859	31.897	20.973	1.00	0.00	LX1	C
ATOM	6151	CG2	ILE	155	69.590	32.962	19.904	1.00	0.00	LX1	C
ATOM	6152	CG1	ILE	155	69.235	32.294	22.314	1.00	0.00	LX1	C
ATOM	6153	CD1	ILE	155	69.941	33.462	23.001	1.00	0.00	LX1	C
ATOM	6154	C	ILE	155	70.153	29.902	19.407	1.00	0.00	LX1	C
ATOM	6155	O	ILE	155	71.169	29.244	19.607	1.00	0.00	LX1	O
ATOM	6156	N	GLN	156	69.630	30.118	18.198	1.00	0.00	LX1	N
ATOM	6157	H	GLN	156	68.768	30.621	18.102	1.00	0.00	LX1	H
ATOM	6158	CA	GLN	156	70.365	29.723	17.002	1.00	0.00	LX1	C
ATOM	6159	CB	GLN	156	69.916	28.350	16.497	1.00	0.00	LX1	C
ATOM	6160	CG	GLN	156	70.555	27.154	17.209	1.00	0.00	LX1	C
ATOM	6161	CD	GLN	156	72.021	27.074	16.838	1.00	0.00	LX1	C
ATOM	6162	OE1	GLN	156	72.398	26.423	15.867	1.00	0.00	LX1	O
ATOM	6163	NE2	GLN	156	72.825	27.799	17.618	1.00	0.00	LX1	N
ATOM	6164	HE21	GLN	156	72.454	28.291	18.413	1.00	0.00	LX1	H
ATOM	6165	HE22	GLN	156	73.794	27.925	17.416	1.00	0.00	LX1	H
ATOM	6166	C	GLN	156	70.136	30.762	15.940	1.00	0.00	LX1	C
ATOM	6167	O	GLN	156	69.047	31.314	15.870	1.00	0.00	LX1	O
ATOM	6168	N	ARG	157	71.192	31.007	15.146	1.00	0.00	LX1	N
ATOM	6169	H	ARG	157	72.076	30.643	15.449	1.00	0.00	LX1	H
ATOM	6170	CA	ARG	157	71.151	31.914	13.994	1.00	0.00	LX1	C
ATOM	6171	CB	ARG	157	71.006	31.120	12.701	1.00	0.00	LX1	C
ATOM	6172	CG	ARG	157	72.262	30.286	12.448	1.00	0.00	LX1	C
ATOM	6173	CD	ARG	157	72.025	29.157	11.443	1.00	0.00	LX1	C

ATOM	6174	NE	ARG	157	70.942	28.293	11.908	1.00	0.00	LX1	N
ATOM	6175	HE	ARG	157	69.989	28.500	11.646	1.00	0.00	LX1	H
ATOM	6176	CZ	ARG	157	71.110	27.348	12.852	1.00	0.00	LX1	C
ATOM	6177	NH1	ARG	157	72.322	27.051	13.312	1.00	0.00	LX1	N
ATOM	6178	HH11	ARG	157	72.441	26.449	14.111	1.00	0.00	LX1	H
ATOM	6179	HH12	ARG	157	73.133	27.452	12.892	1.00	0.00	LX1	H
ATOM	6180	NH2	ARG	157	70.038	26.737	13.331	1.00	0.00	LX1	N
ATOM	6181	HH21	ARG	157	70.062	25.980	13.979	1.00	0.00	LX1	H
ATOM	6182	HH22	ARG	157	69.137	27.105	13.031	1.00	0.00	LX1	H
ATOM	6183	C	ARG	157	70.161	33.057	14.068	1.00	0.00	LX1	C
ATOM	6184	O	ARG	157	69.114	33.090	13.429	1.00	0.00	LX1	O
ATOM	6185	N	ASN	158	70.545	34.016	14.911	1.00	0.00	LX1	N
ATOM	6186	H	ASN	158	71.407	33.926	15.419	1.00	0.00	LX1	H
ATOM	6187	CA	ASN	158	69.618	35.135	15.037	1.00	0.00	LX1	C
ATOM	6188	CB	ASN	158	69.024	35.132	16.444	1.00	0.00	LX1	C
ATOM	6189	CG	ASN	158	67.547	34.792	16.375	1.00	0.00	LX1	C
ATOM	6190	OD1	ASN	158	66.668	35.639	16.424	1.00	0.00	LX1	O
ATOM	6191	ND2	ASN	158	67.286	33.491	16.274	1.00	0.00	LX1	N
ATOM	6192	HD21	ASN	158	68.027	32.818	16.240	1.00	0.00	LX1	H
ATOM	6193	HD22	ASN	158	66.351	33.148	16.237	1.00	0.00	LX1	H
ATOM	6194	C	ASN	158	70.226	36.473	14.650	1.00	0.00	LX1	C
ATOM	6195	O	ASN	158	70.650	37.259	15.489	1.00	0.00	LX1	O
ATOM	6196	N	PRO	159	70.271	36.710	13.313	1.00	0.00	LX1	N
ATOM	6197	CD	PRO	159	69.606	35.952	12.253	1.00	0.00	LX1	C
ATOM	6198	CA	PRO	159	71.131	37.780	12.795	1.00	0.00	LX1	C
ATOM	6199	CB	PRO	159	71.231	37.425	11.309	1.00	0.00	LX1	C
ATOM	6200	CG	PRO	159	69.930	36.702	10.970	1.00	0.00	LX1	C
ATOM	6201	C	PRO	159	70.664	39.197	13.087	1.00	0.00	LX1	C
ATOM	6202	O	PRO	159	71.417	40.058	13.532	1.00	0.00	LX1	O
ATOM	6203	N	GLN	160	69.368	39.413	12.834	1.00	0.00	LX1	N
ATOM	6204	H	GLN	160	68.772	38.691	12.486	1.00	0.00	LX1	H
ATOM	6205	CA	GLN	160	68.850	40.735	13.161	1.00	0.00	LX1	C
ATOM	6206	CB	GLN	160	67.633	41.016	12.281	1.00	0.00	LX1	C
ATOM	6207	CG	GLN	160	67.322	42.499	12.078	1.00	0.00	LX1	C
ATOM	6208	CD	GLN	160	65.896	42.629	11.592	1.00	0.00	LX1	C
ATOM	6209	OE1	GLN	160	65.574	42.856	10.436	1.00	0.00	LX1	O
ATOM	6210	NE2	GLN	160	65.023	42.452	12.568	1.00	0.00	LX1	N
ATOM	6211	HE21	GLN	160	65.359	42.255	13.486	1.00	0.00	LX1	H
ATOM	6212	HE22	GLN	160	64.034	42.439	12.401	1.00	0.00	LX1	H
ATOM	6213	C	GLN	160	68.549	40.917	14.648	1.00	0.00	LX1	C
ATOM	6214	O	GLN	160	68.149	41.982	15.106	1.00	0.00	LX1	O
ATOM	6215	N	LEU	161	68.756	39.818	15.392	1.00	0.00	LX1	N
ATOM	6216	H	LEU	161	69.238	39.018	15.044	1.00	0.00	LX1	H
ATOM	6217	CA	LEU	161	68.447	39.877	16.811	1.00	0.00	LX1	C
ATOM	6218	CB	LEU	161	68.062	38.490	17.312	1.00	0.00	LX1	C
ATOM	6219	CG	LEU	161	67.398	38.456	18.686	1.00	0.00	LX1	C
ATOM	6220	CD1	LEU	161	65.948	38.889	18.582	1.00	0.00	LX1	C
ATOM	6221	CD2	LEU	161	67.494	37.092	19.362	1.00	0.00	LX1	C
ATOM	6222	C	LEU	161	69.624	40.389	17.603	1.00	0.00	LX1	C
ATOM	6223	O	LEU	161	70.718	39.837	17.572	1.00	0.00	LX1	O
ATOM	6224	N	CYS	162	69.348	41.464	18.335	1.00	0.00	LX1	N
ATOM	6225	H	CYS	162	68.427	41.856	18.306	1.00	0.00	LX1	H
ATOM	6226	CA	CYS	162	70.312	41.775	19.378	1.00	0.00	LX1	C
ATOM	6227	CB	CYS	162	70.620	43.266	19.407	1.00	0.00	LX1	C
ATOM	6228	SG	CYS	162	71.736	43.742	18.072	1.00	0.00	LX1	S
ATOM	6229	C	CYS	162	69.823	41.269	20.715	1.00	0.00	LX1	C
ATOM	6230	O	CYS	162	68.786	40.624	20.807	1.00	0.00	LX1	O
ATOM	6231	N	TYR	163	70.618	41.599	21.749	1.00	0.00	LX1	N
ATOM	6232	H	TYR	163	71.500	42.043	21.599	1.00	0.00	LX1	H
ATOM	6233	CA	TYR	163	70.198	41.410	23.141	1.00	0.00	LX1	C
ATOM	6234	CB	TYR	163	68.879	42.146	23.450	1.00	0.00	LX1	C

ATOM	6235	CG	TYR	163	69.017	43.597	23.029	1.00	0.00	LX1	C
ATOM	6236	CD1	TYR	163	69.968	44.415	23.671	1.00	0.00	LX1	C
ATOM	6237	CE1	TYR	163	70.152	45.734	23.233	1.00	0.00	LX1	C
ATOM	6238	CD2	TYR	163	68.210	44.088	21.983	1.00	0.00	LX1	C
ATOM	6239	CE2	TYR	163	68.394	45.408	21.542	1.00	0.00	LX1	C
ATOM	6240	CZ	TYR	163	69.374	46.210	22.164	1.00	0.00	LX1	C
ATOM	6241	OH	TYR	163	69.595	47.493	21.709	1.00	0.00	LX1	O
ATOM	6242	HH	TYR	163	68.861	47.740	21.149	1.00	0.00	LX1	H
ATOM	6243	C	TYR	163	70.268	40.009	23.730	1.00	0.00	LX1	C
ATOM	6244	O	TYR	163	70.116	39.804	24.926	1.00	0.00	LX1	O
ATOM	6245	N	GLN	164	70.614	39.051	22.847	1.00	0.00	LX1	N
ATOM	6246	H	GLN	164	70.459	39.249	21.881	1.00	0.00	LX1	H
ATOM	6247	CA	GLN	164	70.992	37.706	23.311	1.00	0.00	LX1	C
ATOM	6248	CB	GLN	164	71.599	36.844	22.192	1.00	0.00	LX1	C
ATOM	6249	CG	GLN	164	70.784	36.593	20.923	1.00	0.00	LX1	C
ATOM	6250	CD	GLN	164	71.127	37.615	19.862	1.00	0.00	LX1	C
ATOM	6251	OE1	GLN	164	71.192	38.810	20.122	1.00	0.00	LX1	O
ATOM	6252	NE2	GLN	164	71.320	37.108	18.644	1.00	0.00	LX1	N
ATOM	6253	HE21	GLN	164	71.471	36.121	18.541	1.00	0.00	LX1	H
ATOM	6254	HE22	GLN	164	71.317	37.694	17.828	1.00	0.00	LX1	H
ATOM	6255	C	GLN	164	71.994	37.651	24.462	1.00	0.00	LX1	C
ATOM	6256	O	GLN	164	72.002	36.730	25.266	1.00	0.00	LX1	O
ATOM	6257	N	ASP	165	72.870	38.660	24.439	1.00	0.00	LX1	N
ATOM	6258	H	ASP	165	72.742	39.421	23.808	1.00	0.00	LX1	H
ATOM	6259	CA	ASP	165	73.999	38.761	25.361	1.00	0.00	LX1	C
ATOM	6260	CB	ASP	165	75.283	39.188	24.612	1.00	0.00	LX1	C
ATOM	6261	CG	ASP	165	75.047	40.157	23.449	1.00	0.00	LX1	C
ATOM	6262	OD1	ASP	165	74.086	40.926	23.445	1.00	0.00	LX1	O
ATOM	6263	OD2	ASP	165	75.777	40.090	22.467	1.00	0.00	LX1	O
ATOM	6264	C	ASP	165	73.735	39.678	26.541	1.00	0.00	LX1	C
ATOM	6265	O	ASP	165	73.937	39.327	27.698	1.00	0.00	LX1	O
ATOM	6266	N	THR	166	73.240	40.877	26.197	1.00	0.00	LX1	N
ATOM	6267	H	THR	166	73.143	41.107	25.228	1.00	0.00	LX1	H
ATOM	6268	CA	THR	166	72.896	41.879	27.208	1.00	0.00	LX1	C
ATOM	6269	CB	THR	166	72.290	43.081	26.493	1.00	0.00	LX1	C
ATOM	6270	OG1	THR	166	71.244	42.638	25.625	1.00	0.00	LX1	O
ATOM	6271	HG1	THR	166	70.422	43.009	25.948	1.00	0.00	LX1	H
ATOM	6272	CG2	THR	166	73.338	43.860	25.694	1.00	0.00	LX1	C
ATOM	6273	C	THR	166	71.940	41.350	28.262	1.00	0.00	LX1	C
ATOM	6274	O	THR	166	72.042	41.586	29.464	1.00	0.00	LX1	O
ATOM	6275	N	ILE	167	70.998	40.560	27.733	1.00	0.00	LX1	N
ATOM	6276	H	ILE	167	70.942	40.436	26.738	1.00	0.00	LX1	H
ATOM	6277	CA	ILE	167	70.189	39.764	28.638	1.00	0.00	LX1	C
ATOM	6278	CB	ILE	167	68.871	39.390	27.957	1.00	0.00	LX1	C
ATOM	6279	CG2	ILE	167	68.018	38.419	28.770	1.00	0.00	LX1	C
ATOM	6280	CG1	ILE	167	68.106	40.684	27.678	1.00	0.00	LX1	C
ATOM	6281	CD1	ILE	167	67.863	41.503	28.952	1.00	0.00	LX1	C
ATOM	6282	C	ILE	167	70.958	38.579	29.184	1.00	0.00	LX1	C
ATOM	6283	O	ILE	167	70.968	37.466	28.675	1.00	0.00	LX1	O
ATOM	6284	N	LEU	168	71.615	38.893	30.303	1.00	0.00	LX1	N
ATOM	6285	H	LEU	168	71.664	39.862	30.550	1.00	0.00	LX1	H
ATOM	6286	CA	LEU	168	72.393	37.853	30.959	1.00	0.00	LX1	C
ATOM	6287	CB	LEU	168	73.292	38.487	32.023	1.00	0.00	LX1	C
ATOM	6288	CG	LEU	168	74.460	37.598	32.456	1.00	0.00	LX1	C
ATOM	6289	CD1	LEU	168	75.322	37.156	31.271	1.00	0.00	LX1	C
ATOM	6290	CD2	LEU	168	75.297	38.263	33.549	1.00	0.00	LX1	C
ATOM	6291	C	LEU	168	71.554	36.708	31.508	1.00	0.00	LX1	C
ATOM	6292	O	LEU	168	71.046	36.732	32.625	1.00	0.00	LX1	O
ATOM	6293	N	TRP	169	71.468	35.664	30.667	1.00	0.00	LX1	N
ATOM	6294	H	TRP	169	71.728	35.856	29.716	1.00	0.00	LX1	H
ATOM	6295	CA	TRP	169	70.703	34.468	31.037	1.00	0.00	LX1	C

ATOM	6296	CB	TRP	169	70.692	33.429	29.912	1.00	0.00	LX1	C
ATOM	6297	CG	TRP	169	70.211	34.057	28.626	1.00	0.00	LX1	C
ATOM	6298	CD2	TRP	169	68.870	34.443	28.252	1.00	0.00	LX1	C
ATOM	6299	CE2	TRP	169	68.948	35.025	26.942	1.00	0.00	LX1	C
ATOM	6300	CE3	TRP	169	67.625	34.361	28.913	1.00	0.00	LX1	C
ATOM	6301	CD1	TRP	169	71.014	34.415	27.535	1.00	0.00	LX1	C
ATOM	6302	NE1	TRP	169	70.281	34.984	26.547	1.00	0.00	LX1	N
ATOM	6303	HE1	TRP	169	70.665	35.374	25.730	1.00	0.00	LX1	H
ATOM	6304	CZ2	TRP	169	67.779	35.507	26.315	1.00	0.00	LX1	C
ATOM	6305	CZ3	TRP	169	66.464	34.849	28.277	1.00	0.00	LX1	C
ATOM	6306	CH2	TRP	169	66.541	35.419	26.986	1.00	0.00	LX1	C
ATOM	6307	C	TRP	169	71.119	33.814	32.343	1.00	0.00	LX1	C
ATOM	6308	O	TRP	169	70.328	33.180	33.024	1.00	0.00	LX1	O
ATOM	6309	N	LYS	170	72.387	34.069	32.713	1.00	0.00	LX1	N
ATOM	6310	H	LYS	170	72.969	34.539	32.053	1.00	0.00	LX1	H
ATOM	6311	CA	LYS	170	72.900	33.698	34.039	1.00	0.00	LX1	C
ATOM	6312	CB	LYS	170	74.309	34.283	34.193	1.00	0.00	LX1	C
ATOM	6313	CG	LYS	170	75.153	33.772	35.369	1.00	0.00	LX1	C
ATOM	6314	CD	LYS	170	75.862	34.909	36.117	1.00	0.00	LX1	C
ATOM	6315	CE	LYS	170	75.219	35.319	37.454	1.00	0.00	LX1	C
ATOM	6316	NZ	LYS	170	73.798	35.645	37.294	1.00	0.00	LX1	N
ATOM	6317	HZ1	LYS	170	73.418	36.254	38.052	1.00	0.00	LX1	H
ATOM	6318	HZ2	LYS	170	73.616	36.225	36.446	1.00	0.00	LX1	H
ATOM	6319	HZ3	LYS	170	73.203	34.793	37.236	1.00	0.00	LX1	H
ATOM	6320	C	LYS	170	72.043	34.112	35.242	1.00	0.00	LX1	C
ATOM	6321	O	LYS	170	72.252	33.675	36.370	1.00	0.00	LX1	O
ATOM	6322	N	ASP	171	71.108	35.026	34.988	1.00	0.00	LX1	N
ATOM	6323	H	ASP	171	71.070	35.490	34.104	1.00	0.00	LX1	H
ATOM	6324	CA	ASP	171	70.235	35.444	36.080	1.00	0.00	LX1	C
ATOM	6325	CB	ASP	171	70.346	36.959	36.301	1.00	0.00	LX1	C
ATOM	6326	CG	ASP	171	71.799	37.368	36.496	1.00	0.00	LX1	C
ATOM	6327	OD1	ASP	171	72.217	37.631	37.612	1.00	0.00	LX1	O
ATOM	6328	OD2	ASP	171	72.568	37.376	35.541	1.00	0.00	LX1	O
ATOM	6329	C	ASP	171	68.794	35.036	35.847	1.00	0.00	LX1	C
ATOM	6330	O	ASP	171	68.016	34.829	36.770	1.00	0.00	LX1	O
ATOM	6331	N	ILE	172	68.482	34.927	34.548	1.00	0.00	LX1	N
ATOM	6332	H	ILE	172	69.189	35.001	33.849	1.00	0.00	LX1	H
ATOM	6333	CA	ILE	172	67.101	34.658	34.157	1.00	0.00	LX1	C
ATOM	6334	CB	ILE	172	66.799	35.476	32.893	1.00	0.00	LX1	C
ATOM	6335	CG2	ILE	172	65.391	35.270	32.329	1.00	0.00	LX1	C
ATOM	6336	CG1	ILE	172	67.045	36.951	33.235	1.00	0.00	LX1	C
ATOM	6337	CD1	ILE	172	66.785	37.914	32.083	1.00	0.00	LX1	C
ATOM	6338	C	ILE	172	66.763	33.173	34.049	1.00	0.00	LX1	C
ATOM	6339	O	ILE	172	65.618	32.738	34.054	1.00	0.00	LX1	O
ATOM	6340	N	PHE	173	67.832	32.362	34.021	1.00	0.00	LX1	N
ATOM	6341	H	PHE	173	68.758	32.728	33.975	1.00	0.00	LX1	H
ATOM	6342	CA	PHE	173	67.585	30.953	34.319	1.00	0.00	LX1	C
ATOM	6343	CB	PHE	173	68.864	30.124	34.204	1.00	0.00	LX1	C
ATOM	6344	CG	PHE	173	69.079	29.641	32.790	1.00	0.00	LX1	C
ATOM	6345	CD1	PHE	173	68.185	28.698	32.238	1.00	0.00	LX1	C
ATOM	6346	CD2	PHE	173	70.183	30.120	32.054	1.00	0.00	LX1	C
ATOM	6347	CE1	PHE	173	68.410	28.207	30.939	1.00	0.00	LX1	C
ATOM	6348	CE2	PHE	173	70.409	29.631	30.754	1.00	0.00	LX1	C
ATOM	6349	CZ	PHE	173	69.528	28.671	30.216	1.00	0.00	LX1	C
ATOM	6350	C	PHE	173	67.038	30.804	35.721	1.00	0.00	LX1	C
ATOM	6351	O	PHE	173	67.420	31.530	36.638	1.00	0.00	LX1	O
ATOM	6352	N	HIS	174	66.119	29.840	35.854	1.00	0.00	LX1	N
ATOM	6353	H	HIS	174	65.864	29.243	35.093	1.00	0.00	LX1	H
ATOM	6354	CA	HIS	174	65.663	29.592	37.215	1.00	0.00	LX1	C
ATOM	6355	CB	HIS	174	64.474	28.636	37.240	1.00	0.00	LX1	C
ATOM	6356	CG	HIS	174	63.965	28.359	38.636	1.00	0.00	LX1	C

ATOM	6357	ND1	HIS	174	63.634	29.309	39.528	1.00	0.00	LX1	N
ATOM	6358	HD1	HIS	174	63.645	30.285	39.384	1.00	0.00	LX1	H
ATOM	6359	CD2	HIS	174	63.780	27.109	39.228	1.00	0.00	LX1	C
ATOM	6360	NE2	HIS	174	63.343	27.321	40.494	1.00	0.00	LX1	N
ATOM	6361	CE1	HIS	174	63.249	28.679	40.679	1.00	0.00	LX1	C
ATOM	6362	C	HIS	174	66.792	29.085	38.073	1.00	0.00	LX1	C
ATOM	6363	O	HIS	174	67.677	28.366	37.632	1.00	0.00	LX1	O
ATOM	6364	N	LYS	175	66.724	29.508	39.332	1.00	0.00	LX1	N
ATOM	6365	H	LYS	175	65.956	30.104	39.582	1.00	0.00	LX1	H
ATOM	6366	CA	LYS	175	67.848	29.187	40.207	1.00	0.00	LX1	C
ATOM	6367	CB	LYS	175	67.759	30.052	41.471	1.00	0.00	LX1	C
ATOM	6368	CG	LYS	175	67.422	31.520	41.141	1.00	0.00	LX1	C
ATOM	6369	CD	LYS	175	68.430	32.211	40.210	1.00	0.00	LX1	C
ATOM	6370	CE	LYS	175	67.947	33.543	39.616	1.00	0.00	LX1	C
ATOM	6371	NZ	LYS	175	66.940	33.338	38.564	1.00	0.00	LX1	N
ATOM	6372	HZ1	LYS	175	66.831	34.190	37.973	1.00	0.00	LX1	H
ATOM	6373	HZ2	LYS	175	67.178	32.563	37.920	1.00	0.00	LX1	H
ATOM	6374	HZ3	LYS	175	65.995	33.140	38.964	1.00	0.00	LX1	H
ATOM	6375	C	LYS	175	68.038	27.691	40.470	1.00	0.00	LX1	C
ATOM	6376	O	LYS	175	69.107	27.219	40.821	1.00	0.00	LX1	O
ATOM	6377	N	ASN	176	66.941	26.954	40.220	1.00	0.00	LX1	N
ATOM	6378	H	ASN	176	66.101	27.431	39.976	1.00	0.00	LX1	H
ATOM	6379	CA	ASN	176	67.026	25.490	40.228	1.00	0.00	LX1	C
ATOM	6380	CB	ASN	176	65.925	24.863	41.093	1.00	0.00	LX1	C
ATOM	6381	CG	ASN	176	66.038	25.288	42.539	1.00	0.00	LX1	C
ATOM	6382	OD1	ASN	176	66.865	24.809	43.296	1.00	0.00	LX1	O
ATOM	6383	ND2	ASN	176	65.138	26.206	42.903	1.00	0.00	LX1	N
ATOM	6384	HD21	ASN	176	64.453	26.565	42.267	1.00	0.00	LX1	H
ATOM	6385	HD22	ASN	176	65.167	26.519	43.851	1.00	0.00	LX1	H
ATOM	6386	C	ASN	176	66.938	24.871	38.837	1.00	0.00	LX1	C
ATOM	6387	O	ASN	176	66.335	23.813	38.635	1.00	0.00	LX1	O
ATOM	6388	N	ASN	177	67.528	25.573	37.860	1.00	0.00	LX1	N
ATOM	6389	H	ASN	177	68.037	26.419	38.037	1.00	0.00	LX1	H
ATOM	6390	CA	ASN	177	67.496	24.995	36.516	1.00	0.00	LX1	C
ATOM	6391	CB	ASN	177	67.235	26.048	35.432	1.00	0.00	LX1	C
ATOM	6392	CG	ASN	177	66.873	25.377	34.115	1.00	0.00	LX1	C
ATOM	6393	OD1	ASN	177	67.035	24.175	33.911	1.00	0.00	LX1	O
ATOM	6394	ND2	ASN	177	66.391	26.223	33.205	1.00	0.00	LX1	N
ATOM	6395	HD21	ASN	177	66.070	27.132	33.476	1.00	0.00	LX1	H
ATOM	6396	HD22	ASN	177	66.323	25.989	32.233	1.00	0.00	LX1	H
ATOM	6397	C	ASN	177	68.753	24.223	36.188	1.00	0.00	LX1	C
ATOM	6398	O	ASN	177	69.782	24.772	35.824	1.00	0.00	LX1	O
ATOM	6399	N	GLN	178	68.606	22.897	36.312	1.00	0.00	LX1	N
ATOM	6400	H	GLN	178	67.725	22.535	36.610	1.00	0.00	LX1	H
ATOM	6401	CA	GLN	178	69.734	22.029	35.971	1.00	0.00	LX1	C
ATOM	6402	CB	GLN	178	69.435	20.569	36.364	1.00	0.00	LX1	C
ATOM	6403	CG	GLN	178	68.478	19.748	35.478	1.00	0.00	LX1	C
ATOM	6404	CD	GLN	178	67.093	20.364	35.417	1.00	0.00	LX1	C
ATOM	6405	OE1	GLN	178	66.560	20.893	36.389	1.00	0.00	LX1	O
ATOM	6406	NE2	GLN	178	66.528	20.297	34.210	1.00	0.00	LX1	N
ATOM	6407	HE21	GLN	178	66.999	19.840	33.454	1.00	0.00	LX1	H
ATOM	6408	HE22	GLN	178	65.628	20.690	34.032	1.00	0.00	LX1	H
ATOM	6409	C	GLN	178	70.220	22.152	34.531	1.00	0.00	LX1	C
ATOM	6410	O	GLN	178	71.397	22.033	34.221	1.00	0.00	LX1	O
ATOM	6411	N	LEU	179	69.247	22.412	33.649	1.00	0.00	LX1	N
ATOM	6412	H	LEU	179	68.327	22.667	33.948	1.00	0.00	LX1	H
ATOM	6413	CA	LEU	179	69.665	22.661	32.277	1.00	0.00	LX1	C
ATOM	6414	CB	LEU	179	68.681	22.053	31.277	1.00	0.00	LX1	C
ATOM	6415	CG	LEU	179	68.628	20.525	31.291	1.00	0.00	LX1	C
ATOM	6416	CD1	LEU	179	67.535	20.000	30.360	1.00	0.00	LX1	C
ATOM	6417	CD2	LEU	179	69.989	19.896	30.984	1.00	0.00	LX1	C

ATOM	6418	C	LEU	179	69.833	24.141	32.028	1.00	0.00	LX1	C
ATOM	6419	O	LEU	179	69.101	24.761	31.267	1.00	0.00	LX1	O
ATOM	6420	N	ALA	180	70.852	24.686	32.701	1.00	0.00	LX1	N
ATOM	6421	H	ALA	180	71.412	24.110	33.300	1.00	0.00	LX1	H
ATOM	6422	CA	ALA	180	71.170	26.091	32.460	1.00	0.00	LX1	C
ATOM	6423	CB	ALA	180	71.886	26.694	33.671	1.00	0.00	LX1	C
ATOM	6424	C	ALA	180	72.007	26.293	31.205	1.00	0.00	LX1	C
ATOM	6425	O	ALA	180	73.123	26.793	31.217	1.00	0.00	LX1	O
ATOM	6426	N	LEU	181	71.411	25.844	30.094	1.00	0.00	LX1	N
ATOM	6427	H	LEU	181	70.449	25.575	30.137	1.00	0.00	LX1	H
ATOM	6428	CA	LEU	181	72.156	25.876	28.841	1.00	0.00	LX1	C
ATOM	6429	CB	LEU	181	71.624	24.813	27.876	1.00	0.00	LX1	C
ATOM	6430	CG	LEU	181	71.542	23.409	28.488	1.00	0.00	LX1	C
ATOM	6431	CD1	LEU	181	70.756	22.446	27.598	1.00	0.00	LX1	C
ATOM	6432	CD2	LEU	181	72.917	22.854	28.865	1.00	0.00	LX1	C
ATOM	6433	C	LEU	181	72.114	27.249	28.208	1.00	0.00	LX1	C
ATOM	6434	O	LEU	181	71.162	27.621	27.533	1.00	0.00	LX1	O
ATOM	6435	N	THR	182	73.190	27.987	28.470	1.00	0.00	LX1	N
ATOM	6436	H	THR	182	73.908	27.662	29.091	1.00	0.00	LX1	H
ATOM	6437	CA	THR	182	73.337	29.323	27.903	1.00	0.00	LX1	C
ATOM	6438	CB	THR	182	74.331	30.090	28.778	1.00	0.00	LX1	C
ATOM	6439	OG1	THR	182	75.227	29.167	29.417	1.00	0.00	LX1	O
ATOM	6440	HG1	THR	182	75.919	29.683	29.810	1.00	0.00	LX1	H
ATOM	6441	CG2	THR	182	73.629	30.945	29.832	1.00	0.00	LX1	C
ATOM	6442	C	THR	182	73.786	29.339	26.449	1.00	0.00	LX1	C
ATOM	6443	O	THR	182	74.875	29.798	26.123	1.00	0.00	LX1	O
ATOM	6444	N	LEU	183	72.919	28.823	25.564	1.00	0.00	LX1	N
ATOM	6445	H	LEU	183	71.981	28.558	25.810	1.00	0.00	LX1	H
ATOM	6446	CA	LEU	183	73.376	28.850	24.177	1.00	0.00	LX1	C
ATOM	6447	CB	LEU	183	72.893	27.618	23.409	1.00	0.00	LX1	C
ATOM	6448	CG	LEU	183	73.945	27.119	22.410	1.00	0.00	LX1	C
ATOM	6449	CD1	LEU	183	73.885	25.602	22.233	1.00	0.00	LX1	C
ATOM	6450	CD2	LEU	183	73.905	27.861	21.072	1.00	0.00	LX1	C
ATOM	6451	C	LEU	183	73.092	30.167	23.476	1.00	0.00	LX1	C
ATOM	6452	O	LEU	183	72.147	30.346	22.717	1.00	0.00	LX1	O
ATOM	6453	N	ILE	184	73.988	31.103	23.803	1.00	0.00	LX1	N
ATOM	6454	H	ILE	184	74.751	30.834	24.394	1.00	0.00	LX1	H
ATOM	6455	CA	ILE	184	73.863	32.461	23.288	1.00	0.00	LX1	C
ATOM	6456	CB	ILE	184	74.547	33.440	24.259	1.00	0.00	LX1	C
ATOM	6457	CG2	ILE	184	74.382	34.900	23.831	1.00	0.00	LX1	C
ATOM	6458	CG1	ILE	184	74.036	33.223	25.688	1.00	0.00	LX1	C
ATOM	6459	CD1	ILE	184	74.777	34.062	26.732	1.00	0.00	LX1	C
ATOM	6460	C	ILE	184	74.396	32.592	21.869	1.00	0.00	LX1	C
ATOM	6461	O	ILE	184	75.538	32.957	21.623	1.00	0.00	LX1	O
ATOM	6462	N	ASP	185	73.494	32.281	20.931	1.00	0.00	LX1	N
ATOM	6463	H	ASP	185	72.608	31.905	21.204	1.00	0.00	LX1	H
ATOM	6464	CA	ASP	185	73.850	32.538	19.537	1.00	0.00	LX1	C
ATOM	6465	CB	ASP	185	72.915	31.769	18.608	1.00	0.00	LX1	C
ATOM	6466	CG	ASP	185	73.341	31.815	17.154	1.00	0.00	LX1	C
ATOM	6467	OD1	ASP	185	73.732	30.780	16.622	1.00	0.00	LX1	O
ATOM	6468	OD2	ASP	185	73.238	32.867	16.527	1.00	0.00	LX1	O
ATOM	6469	C	ASP	185	73.853	34.019	19.209	1.00	0.00	LX1	C
ATOM	6470	O	ASP	185	72.839	34.712	19.230	1.00	0.00	LX1	O
ATOM	6471	N	THR	186	75.068	34.474	18.916	1.00	0.00	LX1	N
ATOM	6472	H	THR	186	75.880	33.904	19.044	1.00	0.00	LX1	H
ATOM	6473	CA	THR	186	75.224	35.871	18.550	1.00	0.00	LX1	C
ATOM	6474	CB	THR	186	76.209	36.505	19.528	1.00	0.00	LX1	C
ATOM	6475	OG1	THR	186	77.232	35.556	19.865	1.00	0.00	LX1	O
ATOM	6476	HG1	THR	186	77.679	35.899	20.629	1.00	0.00	LX1	H
ATOM	6477	CG2	THR	186	75.495	36.976	20.796	1.00	0.00	LX1	C
ATOM	6478	C	THR	186	75.652	36.071	17.108	1.00	0.00	LX1	C

ATOM	6479	O	THR	186	76.379	37.000	16.775	1.00	0.00	LX1	O
ATOM	6480	N	ASN	187	75.164	35.155	16.252	1.00	0.00	LX1	N
ATOM	6481	H	ASN	187	74.529	34.447	16.574	1.00	0.00	LX1	H
ATOM	6482	CA	ASN	187	75.461	35.293	14.823	1.00	0.00	LX1	C
ATOM	6483	CB	ASN	187	75.178	34.001	14.052	1.00	0.00	LX1	C
ATOM	6484	CG	ASN	187	76.264	32.987	14.321	1.00	0.00	LX1	C
ATOM	6485	OD1	ASN	187	77.361	33.046	13.782	1.00	0.00	LX1	O
ATOM	6486	ND2	ASN	187	75.913	32.033	15.181	1.00	0.00	LX1	N
ATOM	6487	HD21	ASN	187	74.996	32.009	15.598	1.00	0.00	LX1	H
ATOM	6488	HD22	ASN	187	76.552	31.315	15.438	1.00	0.00	LX1	H
ATOM	6489	C	ASN	187	74.680	36.424	14.193	1.00	0.00	LX1	C
ATOM	6490	O	ASN	187	73.621	36.239	13.607	1.00	0.00	LX1	O
ATOM	6491	N	ARG	188	75.241	37.620	14.384	1.00	0.00	LX1	N
ATOM	6492	H	ARG	188	76.133	37.678	14.835	1.00	0.00	LX1	H
ATOM	6493	CA	ARG	188	74.486	38.807	14.019	1.00	0.00	LX1	C
ATOM	6494	CB	ARG	188	74.543	39.852	15.123	1.00	0.00	LX1	C
ATOM	6495	CG	ARG	188	73.867	39.440	16.417	1.00	0.00	LX1	C
ATOM	6496	CD	ARG	188	73.989	40.574	17.423	1.00	0.00	LX1	C
ATOM	6497	NE	ARG	188	73.347	40.205	18.674	1.00	0.00	LX1	N
ATOM	6498	HE	ARG	188	72.408	39.838	18.618	1.00	0.00	LX1	H
ATOM	6499	CZ	ARG	188	73.993	40.358	19.840	1.00	0.00	LX1	C
ATOM	6500	NH1	ARG	188	75.222	40.856	19.908	1.00	0.00	LX1	N
ATOM	6501	HH11	ARG	188	75.669	40.885	20.816	1.00	0.00	LX1	H
ATOM	6502	HH12	ARG	188	75.709	41.185	19.102	1.00	0.00	LX1	H
ATOM	6503	NH2	ARG	188	73.392	39.996	20.952	1.00	0.00	LX1	N
ATOM	6504	HH21	ARG	188	73.866	40.146	21.833	1.00	0.00	LX1	H
ATOM	6505	HH22	ARG	188	72.487	39.571	20.930	1.00	0.00	LX1	H
ATOM	6506	C	ARG	188	74.931	39.483	12.753	1.00	0.00	LX1	C
ATOM	6507	O	ARG	188	76.085	39.456	12.348	1.00	0.00	LX1	O
ATOM	6508	N	SER	189	73.936	40.167	12.195	1.00	0.00	LX1	N
ATOM	6509	H	SER	189	72.991	40.029	12.485	1.00	0.00	LX1	H
ATOM	6510	CA	SER	189	74.223	41.243	11.265	1.00	0.00	LX1	C
ATOM	6511	CB	SER	189	73.208	41.159	10.131	1.00	0.00	LX1	C
ATOM	6512	OG	SER	189	71.916	40.808	10.662	1.00	0.00	LX1	O
ATOM	6513	HG	SER	189	71.483	41.628	10.895	1.00	0.00	LX1	H
ATOM	6514	C	SER	189	74.191	42.602	11.949	1.00	0.00	LX1	C
ATOM	6515	O	SER	189	74.848	43.550	11.536	1.00	0.00	LX1	O
ATOM	6516	N	ARG	190	73.372	42.681	13.012	1.00	0.00	LX1	N
ATOM	6517	H	ARG	190	72.897	41.887	13.396	1.00	0.00	LX1	H
ATOM	6518	CA	ARG	190	73.250	44.015	13.588	1.00	0.00	LX1	C
ATOM	6519	CB	ARG	190	71.786	44.451	13.682	1.00	0.00	LX1	C
ATOM	6520	CG	ARG	190	70.961	43.858	14.822	1.00	0.00	LX1	C
ATOM	6521	CD	ARG	190	69.782	44.790	15.078	1.00	0.00	LX1	C
ATOM	6522	NE	ARG	190	68.982	44.417	16.243	1.00	0.00	LX1	N
ATOM	6523	HE	ARG	190	68.446	43.564	16.212	1.00	0.00	LX1	H
ATOM	6524	CZ	ARG	190	68.786	45.350	17.199	1.00	0.00	LX1	C
ATOM	6525	NH1	ARG	190	69.596	46.396	17.294	1.00	0.00	LX1	N
ATOM	6526	HH11	ARG	190	69.295	47.170	17.875	1.00	0.00	LX1	H
ATOM	6527	HH12	ARG	190	70.482	46.454	16.822	1.00	0.00	LX1	H
ATOM	6528	NH2	ARG	190	67.766	45.249	18.039	1.00	0.00	LX1	N
ATOM	6529	HH21	ARG	190	67.580	45.991	18.699	1.00	0.00	LX1	H
ATOM	6530	HH22	ARG	190	67.124	44.474	18.030	1.00	0.00	LX1	H
ATOM	6531	C	ARG	190	73.965	44.251	14.904	1.00	0.00	LX1	C
ATOM	6532	O	ARG	190	74.283	43.334	15.651	1.00	0.00	LX1	O
ATOM	6533	N	ALA	191	74.171	45.549	15.158	1.00	0.00	LX1	N
ATOM	6534	H	ALA	191	73.940	46.245	14.477	1.00	0.00	LX1	H
ATOM	6535	CA	ALA	191	74.562	45.951	16.501	1.00	0.00	LX1	C
ATOM	6536	CB	ALA	191	75.571	47.101	16.437	1.00	0.00	LX1	C
ATOM	6537	C	ALA	191	73.352	46.390	17.306	1.00	0.00	LX1	C
ATOM	6538	O	ALA	191	72.256	46.584	16.783	1.00	0.00	LX1	O
ATOM	6539	N	CYS	192	73.603	46.526	18.614	1.00	0.00	LX1	N

ATOM	6540	H	CYS	192	74.534	46.416	18.954	1.00	0.00	LX1	H
ATOM	6541	CA	CYS	192	72.550	47.034	19.492	1.00	0.00	LX1	C
ATOM	6542	CB	CYS	192	72.831	46.606	20.932	1.00	0.00	LX1	C
ATOM	6543	SG	CYS	192	73.126	44.824	21.084	1.00	0.00	LX1	S
ATOM	6544	C	CYS	192	72.456	48.543	19.414	1.00	0.00	LX1	C
ATOM	6545	O	CYS	192	73.394	49.209	18.987	1.00	0.00	LX1	O
ATOM	6546	N	HIS	193	71.301	49.060	19.846	1.00	0.00	LX1	N
ATOM	6547	H	HIS	193	70.594	48.479	20.256	1.00	0.00	LX1	H
ATOM	6548	CA	HIS	193	71.262	50.509	20.033	1.00	0.00	LX1	C
ATOM	6549	CB	HIS	193	69.810	51.002	19.954	1.00	0.00	LX1	C
ATOM	6550	CG	HIS	193	69.370	51.205	18.517	1.00	0.00	LX1	C
ATOM	6551	ND1	HIS	193	68.147	51.662	18.191	1.00	0.00	LX1	N
ATOM	6552	HD1	HIS	193	67.420	51.862	18.817	1.00	0.00	LX1	H
ATOM	6553	CD2	HIS	193	70.083	50.978	17.332	1.00	0.00	LX1	C
ATOM	6554	NE2	HIS	193	69.270	51.302	16.293	1.00	0.00	LX1	N
ATOM	6555	CE1	HIS	193	68.079	51.724	16.826	1.00	0.00	LX1	C
ATOM	6556	C	HIS	193	71.921	50.866	21.359	1.00	0.00	LX1	C
ATOM	6557	O	HIS	193	71.983	50.040	22.262	1.00	0.00	LX1	O
ATOM	6558	N	PRO	194	72.457	52.110	21.457	1.00	0.00	LX1	N
ATOM	6559	CD	PRO	194	72.562	53.127	20.412	1.00	0.00	LX1	C
ATOM	6560	CA	PRO	194	73.059	52.544	22.725	1.00	0.00	LX1	C
ATOM	6561	CB	PRO	194	73.351	54.026	22.470	1.00	0.00	LX1	C
ATOM	6562	CG	PRO	194	73.574	54.123	20.963	1.00	0.00	LX1	C
ATOM	6563	C	PRO	194	72.204	52.305	23.962	1.00	0.00	LX1	C
ATOM	6564	O	PRO	194	71.103	52.824	24.106	1.00	0.00	LX1	O
ATOM	6565	N	CYS	195	72.782	51.487	24.853	1.00	0.00	LX1	N
ATOM	6566	H	CYS	195	73.640	51.038	24.617	1.00	0.00	LX1	H
ATOM	6567	CA	CYS	195	72.078	51.145	26.089	1.00	0.00	LX1	C
ATOM	6568	CB	CYS	195	72.938	50.202	26.928	1.00	0.00	LX1	C
ATOM	6569	SG	CYS	195	73.627	48.834	25.961	1.00	0.00	LX1	S
ATOM	6570	C	CYS	195	71.670	52.341	26.930	1.00	0.00	LX1	C
ATOM	6571	O	CYS	195	72.468	53.215	27.247	1.00	0.00	LX1	O
ATOM	6572	N	SER	196	70.382	52.339	27.286	1.00	0.00	LX1	N
ATOM	6573	H	SER	196	69.786	51.580	27.027	1.00	0.00	LX1	H
ATOM	6574	CA	SER	196	69.925	53.397	28.181	1.00	0.00	LX1	C
ATOM	6575	CB	SER	196	68.398	53.484	28.054	1.00	0.00	LX1	C
ATOM	6576	OG	SER	196	67.781	52.331	28.644	1.00	0.00	LX1	O
ATOM	6577	HG	SER	196	67.582	51.729	27.922	1.00	0.00	LX1	H
ATOM	6578	C	SER	196	70.375	53.121	29.615	1.00	0.00	LX1	C
ATOM	6579	O	SER	196	70.750	51.999	29.939	1.00	0.00	LX1	O
ATOM	6580	N	PRO	197	70.285	54.137	30.514	1.00	0.00	LX1	N
ATOM	6581	CD	PRO	197	70.026	55.556	30.277	1.00	0.00	LX1	C
ATOM	6582	CA	PRO	197	70.455	53.819	31.941	1.00	0.00	LX1	C
ATOM	6583	CB	PRO	197	70.318	55.191	32.614	1.00	0.00	LX1	C
ATOM	6584	CG	PRO	197	69.544	56.067	31.628	1.00	0.00	LX1	C
ATOM	6585	C	PRO	197	69.481	52.761	32.468	1.00	0.00	LX1	C
ATOM	6586	O	PRO	197	69.776	51.988	33.372	1.00	0.00	LX1	O
ATOM	6587	N	MET	198	68.294	52.717	31.832	1.00	0.00	LX1	N
ATOM	6588	H	MET	198	68.105	53.299	31.043	1.00	0.00	LX1	H
ATOM	6589	CA	MET	198	67.352	51.670	32.229	1.00	0.00	LX1	C
ATOM	6590	CB	MET	198	65.955	51.958	31.688	1.00	0.00	LX1	C
ATOM	6591	CG	MET	198	65.379	53.277	32.204	1.00	0.00	LX1	C
ATOM	6592	SD	MET	198	65.246	53.314	33.998	1.00	0.00	LX1	S
ATOM	6593	CE	MET	198	64.589	54.981	34.162	1.00	0.00	LX1	C
ATOM	6594	C	MET	198	67.789	50.253	31.887	1.00	0.00	LX1	C
ATOM	6595	O	MET	198	67.273	49.269	32.413	1.00	0.00	LX1	O
ATOM	6596	N	CYS	199	68.818	50.194	31.031	1.00	0.00	LX1	N
ATOM	6597	H	CYS	199	69.145	51.014	30.562	1.00	0.00	LX1	H
ATOM	6598	CA	CYS	199	69.542	48.944	30.825	1.00	0.00	LX1	C
ATOM	6599	CB	CYS	199	70.166	48.918	29.432	1.00	0.00	LX1	C
ATOM	6600	SG	CYS	199	69.089	49.626	28.163	1.00	0.00	LX1	S

ATOM	6601	C	CYS	199	70.630	48.659	31.850	1.00	0.00	LX1	C
ATOM	6602	O	CYS	199	71.635	48.030	31.543	1.00	0.00	LX1	O
ATOM	6603	N	LYS	200	70.393	49.122	33.094	1.00	0.00	LX1	N
ATOM	6604	H	LYS	200	69.699	49.827	33.228	1.00	0.00	LX1	H
ATOM	6605	CA	LYS	200	71.270	48.759	34.214	1.00	0.00	LX1	C
ATOM	6606	CB	LYS	200	70.611	49.055	35.579	1.00	0.00	LX1	C
ATOM	6607	CG	LYS	200	69.416	48.171	35.979	1.00	0.00	LX1	C
ATOM	6608	CD	LYS	200	68.120	48.520	35.248	1.00	0.00	LX1	C
ATOM	6609	CE	LYS	200	67.118	47.370	35.164	1.00	0.00	LX1	C
ATOM	6610	NZ	LYS	200	66.033	47.739	34.242	1.00	0.00	LX1	N
ATOM	6611	HZ1	LYS	200	65.464	46.898	33.984	1.00	0.00	LX1	H
ATOM	6612	HZ2	LYS	200	65.397	48.443	34.654	1.00	0.00	LX1	H
ATOM	6613	HZ3	LYS	200	66.415	48.119	33.350	1.00	0.00	LX1	H
ATOM	6614	C	LYS	200	71.808	47.334	34.180	1.00	0.00	LX1	C
ATOM	6615	O	LYS	200	71.104	46.374	33.884	1.00	0.00	LX1	O
ATOM	6616	N	GLY	201	73.118	47.257	34.447	1.00	0.00	LX1	N
ATOM	6617	H	GLY	201	73.630	48.088	34.657	1.00	0.00	LX1	H
ATOM	6618	CA	GLY	201	73.770	45.949	34.384	1.00	0.00	LX1	C
ATOM	6619	C	GLY	201	73.773	45.280	33.015	1.00	0.00	LX1	C
ATOM	6620	O	GLY	201	73.858	44.060	32.920	1.00	0.00	LX1	O
ATOM	6621	N	SER	202	73.692	46.146	31.984	1.00	0.00	LX1	N
ATOM	6622	H	SER	202	73.526	47.110	32.184	1.00	0.00	LX1	H
ATOM	6623	CA	SER	202	73.691	45.782	30.559	1.00	0.00	LX1	C
ATOM	6624	CB	SER	202	74.837	44.833	30.174	1.00	0.00	LX1	C
ATOM	6625	OG	SER	202	75.908	44.957	31.120	1.00	0.00	LX1	O
ATOM	6626	HG	SER	202	75.707	44.311	31.789	1.00	0.00	LX1	H
ATOM	6627	C	SER	202	72.373	45.268	29.999	1.00	0.00	LX1	C
ATOM	6628	O	SER	202	72.268	44.877	28.846	1.00	0.00	LX1	O
ATOM	6629	N	ARG	203	71.353	45.253	30.864	1.00	0.00	LX1	N
ATOM	6630	H	ARG	203	71.402	45.766	31.722	1.00	0.00	LX1	H
ATOM	6631	CA	ARG	203	70.221	44.395	30.524	1.00	0.00	LX1	C
ATOM	6632	CB	ARG	203	69.697	43.762	31.805	1.00	0.00	LX1	C
ATOM	6633	CG	ARG	203	70.811	42.969	32.478	1.00	0.00	LX1	C
ATOM	6634	CD	ARG	203	70.506	42.609	33.924	1.00	0.00	LX1	C
ATOM	6635	NE	ARG	203	71.670	41.976	34.535	1.00	0.00	LX1	N
ATOM	6636	HE	ARG	203	72.470	42.564	34.673	1.00	0.00	LX1	H
ATOM	6637	CZ	ARG	203	71.678	40.657	34.815	1.00	0.00	LX1	C
ATOM	6638	NH1	ARG	203	70.645	39.874	34.507	1.00	0.00	LX1	N
ATOM	6639	HH11	ARG	203	70.702	38.885	34.658	1.00	0.00	LX1	H
ATOM	6640	HH12	ARG	203	69.791	40.246	34.118	1.00	0.00	LX1	H
ATOM	6641	NH2	ARG	203	72.742	40.136	35.414	1.00	0.00	LX1	N
ATOM	6642	HH21	ARG	203	72.750	39.158	35.664	1.00	0.00	LX1	H
ATOM	6643	HH22	ARG	203	73.543	40.687	35.636	1.00	0.00	LX1	H
ATOM	6644	C	ARG	203	69.111	45.018	29.697	1.00	0.00	LX1	C
ATOM	6645	O	ARG	203	67.983	45.200	30.144	1.00	0.00	LX1	O
ATOM	6646	N	CYS	204	69.472	45.327	28.449	1.00	0.00	LX1	N
ATOM	6647	H	CYS	204	70.409	45.142	28.142	1.00	0.00	LX1	H
ATOM	6648	CA	CYS	204	68.421	45.708	27.507	1.00	0.00	LX1	C
ATOM	6649	CB	CYS	204	68.934	46.744	26.507	1.00	0.00	LX1	C
ATOM	6650	SG	CYS	204	70.737	46.718	26.321	1.00	0.00	LX1	S
ATOM	6651	C	CYS	204	67.861	44.505	26.783	1.00	0.00	LX1	C
ATOM	6652	O	CYS	204	68.605	43.633	26.350	1.00	0.00	LX1	O
ATOM	6653	N	TRP	205	66.522	44.502	26.708	1.00	0.00	LX1	N
ATOM	6654	H	TRP	205	66.020	45.285	27.077	1.00	0.00	LX1	H
ATOM	6655	CA	TRP	205	65.792	43.485	25.946	1.00	0.00	LX1	C
ATOM	6656	CB	TRP	205	64.420	43.229	26.568	1.00	0.00	LX1	C
ATOM	6657	CG	TRP	205	64.432	42.353	27.795	1.00	0.00	LX1	C
ATOM	6658	CD2	TRP	205	64.483	40.911	27.879	1.00	0.00	LX1	C
ATOM	6659	CE2	TRP	205	64.364	40.564	29.266	1.00	0.00	LX1	C
ATOM	6660	CE3	TRP	205	64.613	39.889	26.914	1.00	0.00	LX1	C
ATOM	6661	CD1	TRP	205	64.300	42.790	29.118	1.00	0.00	LX1	C

ATOM	6662	NE1	TRP	205	64.254	41.745	29.988	1.00	0.00	LX1	N
ATOM	6663	HE1	TRP	205	64.106	41.785	30.957	1.00	0.00	LX1	H
ATOM	6664	CZ2	TRP	205	64.368	39.210	29.658	1.00	0.00	LX1	C
ATOM	6665	CZ3	TRP	205	64.620	38.538	27.317	1.00	0.00	LX1	C
ATOM	6666	CH2	TRP	205	64.494	38.200	28.682	1.00	0.00	LX1	C
ATOM	6667	C	TRP	205	65.546	43.930	24.511	1.00	0.00	LX1	C
ATOM	6668	O	TRP	205	65.682	43.181	23.549	1.00	0.00	LX1	O
ATOM	6669	N	GLY	206	65.155	45.211	24.428	1.00	0.00	LX1	N
ATOM	6670	H	GLY	206	65.055	45.763	25.257	1.00	0.00	LX1	H
ATOM	6671	CA	GLY	206	64.929	45.805	23.114	1.00	0.00	LX1	C
ATOM	6672	C	GLY	206	65.825	47.005	22.910	1.00	0.00	LX1	C
ATOM	6673	O	GLY	206	66.674	47.283	23.753	1.00	0.00	LX1	O
ATOM	6674	N	GLU	207	65.583	47.728	21.802	1.00	0.00	LX1	N
ATOM	6675	H	GLU	207	64.911	47.394	21.131	1.00	0.00	LX1	H
ATOM	6676	CA	GLU	207	66.245	49.028	21.611	1.00	0.00	LX1	C
ATOM	6677	CB	GLU	207	66.031	49.573	20.192	1.00	0.00	LX1	C
ATOM	6678	CG	GLU	207	66.117	48.605	19.011	1.00	0.00	LX1	C
ATOM	6679	CD	GLU	207	67.535	48.258	18.619	1.00	0.00	LX1	C
ATOM	6680	OE1	GLU	207	68.266	47.647	19.384	1.00	0.00	LX1	O
ATOM	6681	OE2	GLU	207	67.912	48.525	17.490	1.00	0.00	LX1	O
ATOM	6682	C	GLU	207	65.692	50.078	22.567	1.00	0.00	LX1	C
ATOM	6683	O	GLU	207	64.936	50.963	22.183	1.00	0.00	LX1	O
ATOM	6684	N	SER	208	66.027	49.919	23.851	1.00	0.00	LX1	N
ATOM	6685	H	SER	208	66.703	49.240	24.141	1.00	0.00	LX1	H
ATOM	6686	CA	SER	208	65.041	50.489	24.755	1.00	0.00	LX1	C
ATOM	6687	CB	SER	208	63.980	49.420	25.052	1.00	0.00	LX1	C
ATOM	6688	OG	SER	208	62.706	50.024	25.311	1.00	0.00	LX1	O
ATOM	6689	HG	SER	208	62.268	50.074	24.465	1.00	0.00	LX1	H
ATOM	6690	C	SER	208	65.555	51.122	26.030	1.00	0.00	LX1	C
ATOM	6691	O	SER	208	66.700	50.995	26.453	1.00	0.00	LX1	O
ATOM	6692	N	SER	209	64.588	51.809	26.638	1.00	0.00	LX1	N
ATOM	6693	H	SER	209	63.734	51.983	26.146	1.00	0.00	LX1	H
ATOM	6694	CA	SER	209	64.631	52.083	28.065	1.00	0.00	LX1	C
ATOM	6695	CB	SER	209	64.296	53.561	28.265	1.00	0.00	LX1	C
ATOM	6696	OG	SER	209	63.252	53.922	27.346	1.00	0.00	LX1	O
ATOM	6697	HG	SER	209	63.044	54.835	27.512	1.00	0.00	LX1	H
ATOM	6698	C	SER	209	63.626	51.170	28.750	1.00	0.00	LX1	C
ATOM	6699	O	SER	209	63.926	50.401	29.654	1.00	0.00	LX1	O
ATOM	6700	N	GLU	210	62.406	51.286	28.202	1.00	0.00	LX1	N
ATOM	6701	H	GLU	210	62.281	52.017	27.531	1.00	0.00	LX1	H
ATOM	6702	CA	GLU	210	61.249	50.477	28.577	1.00	0.00	LX1	C
ATOM	6703	CB	GLU	210	60.161	50.761	27.537	1.00	0.00	LX1	C
ATOM	6704	CG	GLU	210	59.167	51.853	27.943	1.00	0.00	LX1	C
ATOM	6705	CD	GLU	210	58.162	51.252	28.903	1.00	0.00	LX1	C
ATOM	6706	OE1	GLU	210	57.181	50.670	28.448	1.00	0.00	LX1	O
ATOM	6707	OE2	GLU	210	58.384	51.277	30.107	1.00	0.00	LX1	O
ATOM	6708	C	GLU	210	61.513	48.983	28.739	1.00	0.00	LX1	C
ATOM	6709	O	GLU	210	61.459	48.415	29.826	1.00	0.00	LX1	O
ATOM	6710	N	ASP	211	61.817	48.350	27.599	1.00	0.00	LX1	N
ATOM	6711	H	ASP	211	61.894	48.853	26.737	1.00	0.00	LX1	H
ATOM	6712	CA	ASP	211	62.133	46.925	27.694	1.00	0.00	LX1	C
ATOM	6713	CB	ASP	211	61.738	46.157	26.428	1.00	0.00	LX1	C
ATOM	6714	CG	ASP	211	60.260	45.825	26.476	1.00	0.00	LX1	C
ATOM	6715	OD1	ASP	211	59.897	44.754	26.953	1.00	0.00	LX1	O
ATOM	6716	OD2	ASP	211	59.457	46.647	26.055	1.00	0.00	LX1	O
ATOM	6717	C	ASP	211	63.578	46.667	28.053	1.00	0.00	LX1	C
ATOM	6718	O	ASP	211	64.419	46.330	27.224	1.00	0.00	LX1	O
ATOM	6719	N	CYS	212	63.829	46.839	29.357	1.00	0.00	LX1	N
ATOM	6720	H	CYS	212	63.106	47.241	29.926	1.00	0.00	LX1	H
ATOM	6721	CA	CYS	212	65.177	46.615	29.869	1.00	0.00	LX1	C
ATOM	6722	CB	CYS	212	65.995	47.900	29.786	1.00	0.00	LX1	C

```
ATOM   6723  SG  CYS   212       66.166  48.529  28.100  1.00  0.00       LX1  S
ATOM   6724  C   CYS   212       65.156  46.085  31.289  1.00  0.00       LX1  C
ATOM   6725  O   CYS   212       64.853  46.802  32.237  1.00  0.00       LX1  O
ATOM   6726  N   GLN   213       65.458  44.777  31.379  1.00  0.00       LX1  N
ATOM   6727  H   GLN   213       65.934  44.401  30.582  1.00  0.00       LX1  H
ATOM   6728  CA  GLN   213       65.118  43.889  32.501  1.00  0.00       LX1  C
ATOM   6729  CB  GLN   213       66.322  43.056  32.951  1.00  0.00       LX1  C
ATOM   6730  CG  GLN   213       65.927  41.633  33.365  1.00  0.00       LX1  C
ATOM   6731  CD  GLN   213       66.977  41.029  34.280  1.00  0.00       LX1  C
ATOM   6732  OE1 GLN   213       68.079  40.651  33.894  1.00  0.00       LX1  O
ATOM   6733  NE2 GLN   213       66.573  40.952  35.549  1.00  0.00       LX1  N
ATOM   6734 HE21 GLN   213       65.673  41.314  35.810  1.00  0.00       LX1  H
ATOM   6735 HE22 GLN   213       67.144  40.531  36.248  1.00  0.00       LX1  H
ATOM   6736  C   GLN   213       64.405  44.447  33.724  1.00  0.00       LX1  C
ATOM   6737  O   GLN   213       64.884  45.339  34.420  1.00  0.00       LX1  O
ATOM   6738  N   SER   214       63.236  43.847  33.957  1.00  0.00       LX1  N
ATOM   6739  H   SER   214       62.930  43.110  33.355  1.00  0.00       LX1  H
ATOM   6740  CA  SER   214       62.561  44.086  35.227  1.00  0.00       LX1  C
ATOM   6741  CB  SER   214       61.068  43.791  35.070  1.00  0.00       LX1  C
ATOM   6742  OG  SER   214       60.346  44.270  36.207  1.00  0.00       LX1  O
ATOM   6743  HG  SER   214       59.426  44.117  36.023  1.00  0.00       LX1  H
ATOM   6744  C   SER   214       63.180  43.252  36.336  1.00  0.00       LX1  C
ATOM   6745  O   SER   214       64.183  42.568  36.134  1.00  0.00       LX1  O
TER
```
