# Supplementary material for: Structural analysis of hubs in human NR-RTK network
Source: Biol Direct. 2011 Oct 5;6:49. doi: 10.1186/1745-6150-6-49 (PMC3220635; doi:10.1186/1745-6150-6-49)
Supplement: Additional file 17 — ESR1-EGFR-Erbb2-PGR. ESR1-EGFR-Erbb2-PGR complex structure. [file 1745-6150-6-49-S17.PDF]

```
HEADER ESR1-EGFR-ERBB2-PGR
REMARK original generated coordinate pdb file
ATOM      1  N   ALA   156      10.627  12.174   8.322  1.00  0.00      RX0  N
ATOM      2  H   ALA   156      11.195  11.352   8.272  1.00  0.00      RX0  H
ATOM      3  CA  ALA   156       9.864  12.527   9.538  1.00  0.00      RX0  C
ATOM      4  CB  ALA   156      10.757  12.402  10.765  1.00  0.00      RX0  C
ATOM      5  C   ALA   156       9.377  13.991   9.496  1.00  0.00      RX0  C
ATOM      6  O   ALA   156       9.121  14.644  10.500  1.00  0.00      RX0  O
ATOM      7  N   LEU   157       9.039  14.416   8.289  1.00  0.00      RX0  N
ATOM      8  H   LEU   157       9.131  13.767   7.531  1.00  0.00      RX0  H
ATOM      9  CA  LEU   157       8.850  15.849   7.979  1.00  0.00      RX0  C
ATOM     10  CB  LEU   157       9.701  16.239   6.777  1.00  0.00      RX0  C
ATOM     11  CG  LEU   157      11.056  15.541   6.734  1.00  0.00      RX0  C
ATOM     12  CD1 LEU   157      11.687  15.681   5.354  1.00  0.00      RX0  C
ATOM     13  CD2 LEU   157      11.986  15.985   7.862  1.00  0.00      RX0  C
ATOM     14  C   LEU   157       7.387  16.184   7.642  1.00  0.00      RX0  C
ATOM     15  O   LEU   157       7.075  17.216   7.039  1.00  0.00      RX0  O
ATOM     16  N   SER   158       6.522  15.244   7.955  1.00  0.00      RX0  N
ATOM     17  H   SER   158       6.875  14.326   8.100  1.00  0.00      RX0  H
ATOM     18  CA  SER   158       5.051  15.362   7.847  1.00  0.00      RX0  C
ATOM     19  CB  SER   158       4.729  14.894   6.436  1.00  0.00      RX0  C
ATOM     20  OG  SER   158       5.939  15.034   5.680  1.00  0.00      RX0  O
ATOM     21  HG  SER   158       6.097  15.974   5.622  1.00  0.00      RX0  H
ATOM     22  C   SER   158       4.335  14.560   8.949  1.00  0.00      RX0  C
ATOM     23  O   SER   158       3.148  14.670   9.188  1.00  0.00      RX0  O
ATOM     24  N   LEU   159       5.132  13.681   9.591  1.00  0.00      RX0  N
ATOM     25  H   LEU   159       6.083  13.586   9.324  1.00  0.00      RX0  H
ATOM     26  CA  LEU   159       4.759  12.951  10.797  1.00  0.00      RX0  C
ATOM     27  CB  LEU   159       5.860  11.985  11.247  1.00  0.00      RX0  C
ATOM     28  CG  LEU   159       5.905  10.626  10.537  1.00  0.00      RX0  C
ATOM     29  CD1 LEU   159       6.370  10.707   9.081  1.00  0.00      RX0  C
ATOM     30  CD2 LEU   159       6.746   9.630  11.338  1.00  0.00      RX0  C
ATOM     31  C   LEU   159       4.518  13.965  11.920  1.00  0.00      RX0  C
ATOM     32  O   LEU   159       5.291  14.932  12.058  1.00  0.00      RX0  O
ATOM     33  N   THR   160       3.434  13.807  12.646  1.00  0.00      RX0  N
ATOM     34  H   THR   160       2.846  13.020  12.457  1.00  0.00      RX0  H
ATOM     35  CA  THR   160       3.156  14.665  13.825  1.00  0.00      RX0  C
ATOM     36  CB  THR   160       1.666  14.645  14.226  1.00  0.00      RX0  C
ATOM     37  OG1 THR   160       1.374  15.732  15.110  1.00  0.00      RX0  O
ATOM     38  HG1 THR   160       0.426  15.817  15.130  1.00  0.00      RX0  H
ATOM     39  CG2 THR   160       1.176  13.330  14.815  1.00  0.00      RX0  C
ATOM     40  C   THR   160       4.203  14.411  14.921  1.00  0.00      RX0  C
ATOM     41  O   THR   160       4.902  13.383  14.913  1.00  0.00      RX0  O
ATOM     42  N   ALA   161       4.153  15.229  15.953  1.00  0.00      RX0  N
ATOM     43  H   ALA   161       3.463  15.955  15.915  1.00  0.00      RX0  H
ATOM     44  CA  ALA   161       4.942  15.044  17.184  1.00  0.00      RX0  C
ATOM     45  CB  ALA   161       4.756  16.235  18.116  1.00  0.00      RX0  C
ATOM     46  C   ALA   161       4.543  13.746  17.920  1.00  0.00      RX0  C
ATOM     47  O   ALA   161       5.387  12.950  18.288  1.00  0.00      RX0  O
ATOM     48  N   ASP   162       3.226  13.461  17.917  1.00  0.00      RX0  N
ATOM     49  H   ASP   162       2.574  14.184  17.698  1.00  0.00      RX0  H
ATOM     50  CA  ASP   162       2.690  12.196  18.469  1.00  0.00      RX0  C
ATOM     51  CB  ASP   162       1.209  12.322  18.853  1.00  0.00      RX0  C
ATOM     52  CG  ASP   162       1.112  13.150  20.136  1.00  0.00      RX0  C
ATOM     53  OD1 ASP   162       2.044  13.885  20.453  1.00  0.00      RX0  O
ATOM     54  OD2 ASP   162       0.120  13.060  20.857  1.00  0.00      RX0  O
ATOM     55  C   ASP   162       3.088  10.948  17.668  1.00  0.00      RX0  C
ATOM     56  O   ASP   162       3.397   9.903  18.257  1.00  0.00      RX0  O
ATOM     57  N   GLN   163       3.164  11.087  16.353  1.00  0.00      RX0  N
ATOM     58  H   GLN   163       3.083  12.015  15.998  1.00  0.00      RX0  H
ATOM     59  CA  GLN   163       3.593   9.998  15.449  1.00  0.00      RX0  C
```

ATOM	60	CB	GLN	163	3.252	10.287	13.995	1.00	0.00	RX0	C
ATOM	61	CG	GLN	163	1.834	9.863	13.620	1.00	0.00	RX0	C
ATOM	62	CD	GLN	163	1.568	10.371	12.225	1.00	0.00	RX0	C
ATOM	63	OE1	GLN	163	2.087	11.409	11.830	1.00	0.00	RX0	O
ATOM	64	NE2	GLN	163	0.744	9.592	11.503	1.00	0.00	RX0	N
ATOM	65	HE21	GLN	163	0.360	8.753	11.890	1.00	0.00	RX0	H
ATOM	66	HE22	GLN	163	0.492	9.826	10.563	1.00	0.00	RX0	H
ATOM	67	C	GLN	163	5.089	9.698	15.572	1.00	0.00	RX0	C
ATOM	68	O	GLN	163	5.477	8.537	15.545	1.00	0.00	RX0	O
ATOM	69	N	MET	164	5.882	10.740	15.840	1.00	0.00	RX0	N
ATOM	70	H	MET	164	5.496	11.664	15.885	1.00	0.00	RX0	H
ATOM	71	CA	MET	164	7.331	10.587	16.060	1.00	0.00	RX0	C
ATOM	72	CB	MET	164	8.015	11.955	16.082	1.00	0.00	RX0	C
ATOM	73	CG	MET	164	9.451	11.879	16.605	1.00	0.00	RX0	C
ATOM	74	SD	MET	164	10.535	10.863	15.593	1.00	0.00	RX0	S
ATOM	75	CE	MET	164	11.026	12.133	14.425	1.00	0.00	RX0	C
ATOM	76	C	MET	164	7.610	9.825	17.366	1.00	0.00	RX0	C
ATOM	77	O	MET	164	8.404	8.887	17.381	1.00	0.00	RX0	O
ATOM	78	N	VAL	165	6.828	10.145	18.396	1.00	0.00	RX0	N
ATOM	79	H	VAL	165	6.180	10.901	18.283	1.00	0.00	RX0	H
ATOM	80	CA	VAL	165	6.992	9.563	19.744	1.00	0.00	RX0	C
ATOM	81	CB	VAL	165	6.101	10.259	20.779	1.00	0.00	RX0	C
ATOM	82	CG1	VAL	165	6.185	9.568	22.139	1.00	0.00	RX0	C
ATOM	83	CG2	VAL	165	6.449	11.733	20.917	1.00	0.00	RX0	C
ATOM	84	C	VAL	165	6.649	8.067	19.731	1.00	0.00	RX0	C
ATOM	85	O	VAL	165	7.442	7.255	20.191	1.00	0.00	RX0	O
ATOM	86	N	SER	166	5.467	7.742	19.205	1.00	0.00	RX0	N
ATOM	87	H	SER	166	4.827	8.453	18.916	1.00	0.00	RX0	H
ATOM	88	CA	SER	166	5.029	6.335	19.106	1.00	0.00	RX0	C
ATOM	89	CB	SER	166	3.571	6.419	18.696	1.00	0.00	RX0	C
ATOM	90	OG	SER	166	3.004	7.490	19.465	1.00	0.00	RX0	O
ATOM	91	HG	SER	166	2.932	8.253	18.899	1.00	0.00	RX0	H
ATOM	92	C	SER	166	5.941	5.501	18.195	1.00	0.00	RX0	C
ATOM	93	O	SER	166	6.295	4.379	18.542	1.00	0.00	RX0	O
ATOM	94	N	ALA	167	6.456	6.129	17.133	1.00	0.00	RX0	N
ATOM	95	H	ALA	167	6.183	7.068	16.918	1.00	0.00	RX0	H
ATOM	96	CA	ALA	167	7.397	5.466	16.208	1.00	0.00	RX0	C
ATOM	97	CB	ALA	167	7.731	6.357	15.012	1.00	0.00	RX0	C
ATOM	98	C	ALA	167	8.706	5.103	16.927	1.00	0.00	RX0	C
ATOM	99	O	ALA	167	9.113	3.946	16.932	1.00	0.00	RX0	O
ATOM	100	N	LEU	168	9.179	6.054	17.734	1.00	0.00	RX0	N
ATOM	101	H	LEU	168	8.732	6.950	17.742	1.00	0.00	RX0	H
ATOM	102	CA	LEU	168	10.385	5.875	18.562	1.00	0.00	RX0	C
ATOM	103	CB	LEU	168	10.907	7.213	19.074	1.00	0.00	RX0	C
ATOM	104	CG	LEU	168	11.570	8.041	17.977	1.00	0.00	RX0	C
ATOM	105	CD1	LEU	168	12.102	9.369	18.518	1.00	0.00	RX0	C
ATOM	106	CD2	LEU	168	12.651	7.239	17.253	1.00	0.00	RX0	C
ATOM	107	C	LEU	168	10.197	4.896	19.724	1.00	0.00	RX0	C
ATOM	108	O	LEU	168	11.077	4.078	19.994	1.00	0.00	RX0	O
ATOM	109	N	LEU	169	9.007	4.918	20.317	1.00	0.00	RX0	N
ATOM	110	H	LEU	169	8.331	5.588	20.014	1.00	0.00	RX0	H
ATOM	111	CA	LEU	169	8.640	3.970	21.384	1.00	0.00	RX0	C
ATOM	112	CB	LEU	169	7.359	4.393	22.101	1.00	0.00	RX0	C
ATOM	113	CG	LEU	169	7.540	5.638	22.971	1.00	0.00	RX0	C
ATOM	114	CD1	LEU	169	6.229	6.046	23.643	1.00	0.00	RX0	C
ATOM	115	CD2	LEU	169	8.669	5.468	23.988	1.00	0.00	RX0	C
ATOM	116	C	LEU	169	8.505	2.536	20.864	1.00	0.00	RX0	C
ATOM	117	O	LEU	169	9.003	1.602	21.486	1.00	0.00	RX0	O
ATOM	118	N	ASP	170	7.977	2.423	19.645	1.00	0.00	RX0	N
ATOM	119	H	ASP	170	7.668	3.241	19.162	1.00	0.00	RX0	H
ATOM	120	CA	ASP	170	7.822	1.133	18.952	1.00	0.00	RX0	C

ATOM	121	CB	ASP	170	6.881	1.394	17.759	1.00	0.00	RX0	C
ATOM	122	CG	ASP	170	6.641	0.242	16.793	1.00	0.00	RX0	C
ATOM	123	OD1	ASP	170	7.093	-0.878	16.998	1.00	0.00	RX0	O
ATOM	124	OD2	ASP	170	6.024	0.476	15.756	1.00	0.00	RX0	O
ATOM	125	C	ASP	170	9.164	0.506	18.541	1.00	0.00	RX0	C
ATOM	126	O	ASP	170	9.313	-0.704	18.571	1.00	0.00	RX0	O
ATOM	127	N	ALA	171	10.119	1.387	18.228	1.00	0.00	RX0	N
ATOM	128	H	ALA	171	9.894	2.361	18.291	1.00	0.00	RX0	H
ATOM	129	CA	ALA	171	11.447	1.008	17.717	1.00	0.00	RX0	C
ATOM	130	CB	ALA	171	12.102	2.218	17.053	1.00	0.00	RX0	C
ATOM	131	C	ALA	171	12.418	0.479	18.779	1.00	0.00	RX0	C
ATOM	132	O	ALA	171	13.427	-0.136	18.431	1.00	0.00	RX0	O
ATOM	133	N	GLU	172	12.125	0.739	20.058	1.00	0.00	RX0	N
ATOM	134	H	GLU	172	11.281	1.230	20.277	1.00	0.00	RX0	H
ATOM	135	CA	GLU	172	13.017	0.374	21.170	1.00	0.00	RX0	C
ATOM	136	CB	GLU	172	12.365	0.709	22.510	1.00	0.00	RX0	C
ATOM	137	CG	GLU	172	12.324	2.228	22.680	1.00	0.00	RX0	C
ATOM	138	CD	GLU	172	13.730	2.778	22.516	1.00	0.00	RX0	C
ATOM	139	OE1	GLU	172	14.554	2.617	23.416	1.00	0.00	RX0	O
ATOM	140	OE2	GLU	172	14.037	3.383	21.488	1.00	0.00	RX0	O
ATOM	141	C	GLU	172	13.554	-1.065	21.099	1.00	0.00	RX0	C
ATOM	142	O	GLU	172	12.785	-2.004	20.837	1.00	0.00	RX0	O
ATOM	143	N	PRO	173	14.865	-1.209	21.269	1.00	0.00	RX0	N
ATOM	144	CD	PRO	173	15.789	-0.096	21.453	1.00	0.00	RX0	C
ATOM	145	CA	PRO	173	15.538	-2.517	21.328	1.00	0.00	RX0	C
ATOM	146	CB	PRO	173	17.014	-2.108	21.199	1.00	0.00	RX0	C
ATOM	147	CG	PRO	173	17.103	-0.741	21.869	1.00	0.00	RX0	C
ATOM	148	C	PRO	173	15.206	-3.249	22.640	1.00	0.00	RX0	C
ATOM	149	O	PRO	173	14.829	-2.595	23.631	1.00	0.00	RX0	O
ATOM	150	N	PRO	174	15.294	-4.574	22.646	1.00	0.00	RX0	N
ATOM	151	CD	PRO	174	15.605	-5.388	21.475	1.00	0.00	RX0	C
ATOM	152	CA	PRO	174	15.084	-5.400	23.852	1.00	0.00	RX0	C
ATOM	153	CB	PRO	174	14.968	-6.812	23.273	1.00	0.00	RX0	C
ATOM	154	CG	PRO	174	15.853	-6.784	22.032	1.00	0.00	RX0	C
ATOM	155	C	PRO	174	16.250	-5.248	24.838	1.00	0.00	RX0	C
ATOM	156	O	PRO	174	17.379	-4.922	24.444	1.00	0.00	RX0	O
ATOM	157	N	ILE	175	15.956	-5.464	26.106	1.00	0.00	RX0	N
ATOM	158	H	ILE	175	15.039	-5.788	26.327	1.00	0.00	RX0	H
ATOM	159	CA	ILE	175	16.988	-5.556	27.159	1.00	0.00	RX0	C
ATOM	160	CB	ILE	175	16.446	-5.212	28.551	1.00	0.00	RX0	C
ATOM	161	CG2	ILE	175	17.623	-5.099	29.520	1.00	0.00	RX0	C
ATOM	162	CG1	ILE	175	15.624	-3.918	28.562	1.00	0.00	RX0	C
ATOM	163	CD1	ILE	175	14.111	-4.135	28.454	1.00	0.00	RX0	C
ATOM	164	C	ILE	175	17.586	-6.969	27.112	1.00	0.00	RX0	C
ATOM	165	O	ILE	175	16.886	-7.963	27.343	1.00	0.00	RX0	O
ATOM	166	N	LEU	176	18.884	-7.017	26.884	1.00	0.00	RX0	N
ATOM	167	H	LEU	176	19.406	-6.165	26.836	1.00	0.00	RX0	H
ATOM	168	CA	LEU	176	19.617	-8.291	26.770	1.00	0.00	RX0	C
ATOM	169	CB	LEU	176	20.650	-8.227	25.647	1.00	0.00	RX0	C
ATOM	170	CG	LEU	176	20.014	-7.998	24.276	1.00	0.00	RX0	C
ATOM	171	CD1	LEU	176	21.072	-7.947	23.177	1.00	0.00	RX0	C
ATOM	172	CD2	LEU	176	18.929	-9.030	23.964	1.00	0.00	RX0	C
ATOM	173	C	LEU	176	20.277	-8.690	28.089	1.00	0.00	RX0	C
ATOM	174	O	LEU	176	20.563	-7.852	28.952	1.00	0.00	RX0	O
ATOM	175	N	TYR	177	20.459	-9.989	28.237	1.00	0.00	RX0	N
ATOM	176	H	TYR	177	20.223	-10.604	27.489	1.00	0.00	RX0	H
ATOM	177	CA	TYR	177	21.114	-10.573	29.420	1.00	0.00	RX0	C
ATOM	178	CB	TYR	177	20.420	-11.864	29.855	1.00	0.00	RX0	C
ATOM	179	CG	TYR	177	19.153	-11.561	30.618	1.00	0.00	RX0	C
ATOM	180	CD1	TYR	177	18.030	-11.069	29.964	1.00	0.00	RX0	C
ATOM	181	CE1	TYR	177	16.866	-10.811	30.678	1.00	0.00	RX0	C

ATOM	182	CD2	TYR	177	19.114	-11.790	31.988	1.00	0.00	RX0	C
ATOM	183	CE2	TYR	177	17.949	-11.540	32.702	1.00	0.00	RX0	C
ATOM	184	CZ	TYR	177	16.825	-11.049	32.047	1.00	0.00	RX0	C
ATOM	185	OH	TYR	177	15.671	-10.793	32.758	1.00	0.00	RX0	O
ATOM	186	HH	TYR	177	15.780	-11.075	33.657	1.00	0.00	RX0	H
ATOM	187	C	TYR	177	22.589	-10.858	29.163	1.00	0.00	RX0	C
ATOM	188	O	TYR	177	22.985	-11.163	28.046	1.00	0.00	RX0	O
ATOM	189	N	SER	178	23.381	-10.750	30.220	1.00	0.00	RX0	N
ATOM	190	H	SER	178	23.027	-10.493	31.123	1.00	0.00	RX0	H
ATOM	191	CA	SER	178	24.788	-11.188	30.183	1.00	0.00	RX0	C
ATOM	192	CB	SER	178	25.490	-10.621	31.401	1.00	0.00	RX0	C
ATOM	193	OG	SER	178	25.211	-9.221	31.419	1.00	0.00	RX0	O
ATOM	194	HG	SER	178	25.672	-8.842	30.681	1.00	0.00	RX0	H
ATOM	195	C	SER	178	24.834	-12.718	30.070	1.00	0.00	RX0	C
ATOM	196	O	SER	178	23.999	-13.413	30.674	1.00	0.00	RX0	O
ATOM	197	N	GLU	179	25.827	-13.218	29.362	1.00	0.00	RX0	N
ATOM	198	H	GLU	179	26.520	-12.641	28.923	1.00	0.00	RX0	H
ATOM	199	CA	GLU	179	26.033	-14.670	29.175	1.00	0.00	RX0	C
ATOM	200	CB	GLU	179	26.319	-15.085	27.732	1.00	0.00	RX0	C
ATOM	201	CG	GLU	179	25.809	-14.134	26.663	1.00	0.00	RX0	C
ATOM	202	CD	GLU	179	26.879	-13.119	26.306	1.00	0.00	RX0	C
ATOM	203	OE1	GLU	179	27.337	-13.142	25.174	1.00	0.00	RX0	O
ATOM	204	OE2	GLU	179	27.200	-12.247	27.112	1.00	0.00	RX0	O
ATOM	205	C	GLU	179	27.192	-15.208	30.012	1.00	0.00	RX0	C
ATOM	206	O	GLU	179	28.361	-15.227	29.589	1.00	0.00	RX0	O
ATOM	207	N	TYR	180	26.873	-15.475	31.254	1.00	0.00	RX0	N
ATOM	208	H	TYR	180	25.922	-15.393	31.560	1.00	0.00	RX0	H
ATOM	209	CA	TYR	180	27.735	-16.233	32.177	1.00	0.00	RX0	C
ATOM	210	CB	TYR	180	28.490	-15.317	33.152	1.00	0.00	RX0	C
ATOM	211	CG	TYR	180	27.547	-14.668	34.139	1.00	0.00	RX0	C
ATOM	212	CD1	TYR	180	26.920	-13.467	33.829	1.00	0.00	RX0	C
ATOM	213	CE1	TYR	180	26.025	-12.902	34.730	1.00	0.00	RX0	C
ATOM	214	CD2	TYR	180	27.305	-15.281	35.362	1.00	0.00	RX0	C
ATOM	215	CE2	TYR	180	26.403	-14.724	36.256	1.00	0.00	RX0	C
ATOM	216	CZ	TYR	180	25.750	-13.543	35.932	1.00	0.00	RX0	C
ATOM	217	OH	TYR	180	24.824	-13.020	36.807	1.00	0.00	RX0	O
ATOM	218	HH	TYR	180	24.692	-12.096	36.606	1.00	0.00	RX0	H
ATOM	219	C	TYR	180	26.838	-17.226	32.909	1.00	0.00	RX0	C
ATOM	220	O	TYR	180	25.642	-16.953	33.094	1.00	0.00	RX0	O
ATOM	221	N	ASP	181	27.404	-18.345	33.318	1.00	0.00	RX0	N
ATOM	222	H	ASP	181	28.379	-18.498	33.174	1.00	0.00	RX0	H
ATOM	223	CA	ASP	181	26.630	-19.347	34.059	1.00	0.00	RX0	C
ATOM	224	CB	ASP	181	27.266	-20.726	34.055	1.00	0.00	RX0	C
ATOM	225	CG	ASP	181	26.455	-21.527	35.041	1.00	0.00	RX0	C
ATOM	226	OD1	ASP	181	25.244	-21.599	34.885	1.00	0.00	RX0	O
ATOM	227	OD2	ASP	181	27.015	-22.018	36.008	1.00	0.00	RX0	O
ATOM	228	C	ASP	181	26.420	-18.851	35.504	1.00	0.00	RX0	C
ATOM	229	O	ASP	181	27.391	-18.832	36.273	1.00	0.00	RX0	O
ATOM	230	N	PRO	182	25.185	-18.489	35.856	1.00	0.00	RX0	N
ATOM	231	CD	PRO	182	24.008	-18.600	34.996	1.00	0.00	RX0	C
ATOM	232	CA	PRO	182	24.825	-17.989	37.201	1.00	0.00	RX0	C
ATOM	233	CB	PRO	182	23.394	-17.487	36.999	1.00	0.00	RX0	C
ATOM	234	CG	PRO	182	22.821	-18.409	35.929	1.00	0.00	RX0	C
ATOM	235	C	PRO	182	24.941	-19.052	38.308	1.00	0.00	RX0	C
ATOM	236	O	PRO	182	24.654	-18.763	39.474	1.00	0.00	RX0	O
ATOM	237	N	THR	183	25.345	-20.259	37.948	1.00	0.00	RX0	N
ATOM	238	H	THR	183	25.529	-20.541	37.004	1.00	0.00	RX0	H
ATOM	239	CA	THR	183	25.568	-21.363	38.913	1.00	0.00	RX0	C
ATOM	240	CB	THR	183	24.905	-22.561	38.269	1.00	0.00	RX0	C
ATOM	241	OG1	THR	183	23.924	-22.061	37.349	1.00	0.00	RX0	O
ATOM	242	HG1	THR	183	24.374	-22.047	36.502	1.00	0.00	RX0	H

ATOM	243	CG2	THR	183	24.283	-23.515	39.290	1.00	0.00	RX0	C
ATOM	244	C	THR	183	27.063	-21.532	39.218	1.00	0.00	RX0	C
ATOM	245	O	THR	183	27.455	-22.345	40.058	1.00	0.00	RX0	O
ATOM	246	N	ARG	184	27.887	-20.699	38.573	1.00	0.00	RX0	N
ATOM	247	H	ARG	184	27.503	-20.038	37.930	1.00	0.00	RX0	H
ATOM	248	CA	ARG	184	29.343	-20.701	38.681	1.00	0.00	RX0	C
ATOM	249	CB	ARG	184	29.796	-20.961	37.238	1.00	0.00	RX0	C
ATOM	250	CG	ARG	184	31.218	-20.711	36.751	1.00	0.00	RX0	C
ATOM	251	CD	ARG	184	31.079	-20.139	35.337	1.00	0.00	RX0	C
ATOM	252	NE	ARG	184	30.059	-19.092	35.400	1.00	0.00	RX0	N
ATOM	253	HE	ARG	184	29.103	-19.401	35.358	1.00	0.00	RX0	H
ATOM	254	CZ	ARG	184	30.439	-17.947	36.026	1.00	0.00	RX0	C
ATOM	255	NH1	ARG	184	31.729	-17.597	36.017	1.00	0.00	RX0	N
ATOM	256	HH11	ARG	184	32.126	-16.922	36.645	1.00	0.00	RX0	H
ATOM	257	HH12	ARG	184	32.445	-18.080	35.486	1.00	0.00	RX0	H
ATOM	258	NH2	ARG	184	29.522	-17.229	36.702	1.00	0.00	RX0	N
ATOM	259	HH21	ARG	184	29.762	-16.407	37.220	1.00	0.00	RX0	H
ATOM	260	HH22	ARG	184	28.567	-17.552	36.749	1.00	0.00	RX0	H
ATOM	261	C	ARG	184	29.836	-19.410	39.407	1.00	0.00	RX0	C
ATOM	262	O	ARG	184	29.116	-18.390	39.334	1.00	0.00	RX0	O
ATOM	263	N	PRO	185	30.940	-19.460	40.113	1.00	0.00	RX0	N
ATOM	264	CD	PRO	185	31.732	-20.668	40.314	1.00	0.00	RX0	C
ATOM	265	CA	PRO	185	31.574	-18.285	40.781	1.00	0.00	RX0	C
ATOM	266	CB	PRO	185	32.857	-18.869	41.385	1.00	0.00	RX0	C
ATOM	267	CG	PRO	185	33.134	-20.147	40.600	1.00	0.00	RX0	C
ATOM	268	C	PRO	185	31.820	-17.125	39.813	1.00	0.00	RX0	C
ATOM	269	O	PRO	185	31.836	-17.275	38.592	1.00	0.00	RX0	O
ATOM	270	N	PHE	186	32.164	-15.998	40.422	1.00	0.00	RX0	N
ATOM	271	H	PHE	186	32.267	-15.999	41.415	1.00	0.00	RX0	H
ATOM	272	CA	PHE	186	32.333	-14.726	39.697	1.00	0.00	RX0	C
ATOM	273	CB	PHE	186	31.513	-13.613	40.351	1.00	0.00	RX0	C
ATOM	274	CG	PHE	186	31.391	-12.458	39.384	1.00	0.00	RX0	C
ATOM	275	CD1	PHE	186	30.829	-12.665	38.130	1.00	0.00	RX0	C
ATOM	276	CD2	PHE	186	31.842	-11.193	39.743	1.00	0.00	RX0	C
ATOM	277	CE1	PHE	186	30.720	-11.609	37.233	1.00	0.00	RX0	C
ATOM	278	CE2	PHE	186	31.733	-10.137	38.846	1.00	0.00	RX0	C
ATOM	279	CZ	PHE	186	31.174	-10.345	37.590	1.00	0.00	RX0	C
ATOM	280	C	PHE	186	33.791	-14.305	39.507	1.00	0.00	RX0	C
ATOM	281	O	PHE	186	34.127	-13.678	38.496	1.00	0.00	RX0	O
ATOM	282	N	SER	187	34.655	-14.802	40.380	1.00	0.00	RX0	N
ATOM	283	H	SER	187	34.320	-15.367	41.129	1.00	0.00	RX0	H
ATOM	284	CA	SER	187	36.113	-14.542	40.380	1.00	0.00	RX0	C
ATOM	285	CB	SER	187	36.607	-15.272	41.615	1.00	0.00	RX0	C
ATOM	286	OG	SER	187	35.490	-15.345	42.520	1.00	0.00	RX0	O
ATOM	287	HG	SER	187	35.869	-15.398	43.390	1.00	0.00	RX0	H
ATOM	288	C	SER	187	36.764	-14.980	39.057	1.00	0.00	RX0	C
ATOM	289	O	SER	187	37.834	-14.531	38.683	1.00	0.00	RX0	O
ATOM	290	N	GLU	188	36.054	-15.878	38.369	1.00	0.00	RX0	N
ATOM	291	H	GLU	188	35.131	-16.153	38.631	1.00	0.00	RX0	H
ATOM	292	CA	GLU	188	36.561	-16.586	37.191	1.00	0.00	RX0	C
ATOM	293	CB	GLU	188	36.534	-18.109	37.433	1.00	0.00	RX0	C
ATOM	294	CG	GLU	188	36.017	-19.012	36.318	1.00	0.00	RX0	C
ATOM	295	CD	GLU	188	34.554	-18.705	36.193	1.00	0.00	RX0	C
ATOM	296	OE1	GLU	188	33.965	-18.355	37.210	1.00	0.00	RX0	O
ATOM	297	OE2	GLU	188	34.017	-18.752	35.089	1.00	0.00	RX0	O
ATOM	298	C	GLU	188	36.028	-16.018	35.856	1.00	0.00	RX0	C
ATOM	299	O	GLU	188	36.494	-16.416	34.788	1.00	0.00	RX0	O
ATOM	300	N	ALA	189	35.058	-15.107	35.914	1.00	0.00	RX0	N
ATOM	301	H	ALA	189	34.824	-14.710	36.803	1.00	0.00	RX0	H
ATOM	302	CA	ALA	189	34.543	-14.432	34.708	1.00	0.00	RX0	C
ATOM	303	CB	ALA	189	33.065	-14.087	34.891	1.00	0.00	RX0	C

ATOM	304	C	ALA	189	35.336	-13.151	34.407	1.00	0.00	RX0	C
ATOM	305	O	ALA	189	35.533	-12.292	35.270	1.00	0.00	RX0	O
ATOM	306	N	SER	190	35.819	-13.065	33.173	1.00	0.00	RX0	N
ATOM	307	H	SER	190	35.614	-13.822	32.554	1.00	0.00	RX0	H
ATOM	308	CA	SER	190	36.430	-11.825	32.646	1.00	0.00	RX0	C
ATOM	309	CB	SER	190	37.088	-12.202	31.294	1.00	0.00	RX0	C
ATOM	310	OG	SER	190	38.226	-11.380	30.934	1.00	0.00	RX0	O
ATOM	311	HG	SER	190	38.774	-11.413	31.719	1.00	0.00	RX0	H
ATOM	312	C	SER	190	35.341	-10.761	32.513	1.00	0.00	RX0	C
ATOM	313	O	SER	190	34.465	-10.869	31.639	1.00	0.00	RX0	O
ATOM	314	N	MET	191	35.401	-9.751	33.361	1.00	0.00	RX0	N
ATOM	315	H	MET	191	36.035	-9.825	34.132	1.00	0.00	RX0	H
ATOM	316	CA	MET	191	34.414	-8.652	33.337	1.00	0.00	RX0	C
ATOM	317	CB	MET	191	34.641	-7.665	34.478	1.00	0.00	RX0	C
ATOM	318	CG	MET	191	33.544	-6.600	34.498	1.00	0.00	RX0	C
ATOM	319	SD	MET	191	33.632	-5.545	35.947	1.00	0.00	RX0	S
ATOM	320	CE	MET	191	33.279	-6.807	37.182	1.00	0.00	RX0	C
ATOM	321	C	MET	191	34.384	-7.939	31.976	1.00	0.00	RX0	C
ATOM	322	O	MET	191	33.329	-7.841	31.363	1.00	0.00	RX0	O
ATOM	323	N	MET	192	35.577	-7.654	31.438	1.00	0.00	RX0	N
ATOM	324	H	MET	192	36.384	-7.746	32.018	1.00	0.00	RX0	H
ATOM	325	CA	MET	192	35.697	-7.082	30.089	1.00	0.00	RX0	C
ATOM	326	CB	MET	192	37.136	-6.648	29.804	1.00	0.00	RX0	C
ATOM	327	CG	MET	192	37.279	-5.924	28.461	1.00	0.00	RX0	C
ATOM	328	SD	MET	192	36.171	-4.513	28.294	1.00	0.00	RX0	S
ATOM	329	CE	MET	192	36.770	-3.522	29.673	1.00	0.00	RX0	C
ATOM	330	C	MET	192	35.151	-8.021	28.999	1.00	0.00	RX0	C
ATOM	331	O	MET	192	34.484	-7.587	28.093	1.00	0.00	RX0	O
ATOM	332	N	GLY	193	35.358	-9.342	29.220	1.00	0.00	RX0	N
ATOM	333	H	GLY	193	35.684	-9.614	30.121	1.00	0.00	RX0	H
ATOM	334	CA	GLY	193	34.804	-10.378	28.330	1.00	0.00	RX0	C
ATOM	335	C	GLY	193	33.267	-10.338	28.334	1.00	0.00	RX0	C
ATOM	336	O	GLY	193	32.637	-10.184	27.296	1.00	0.00	RX0	O
ATOM	337	N	LEU	194	32.696	-10.293	29.537	1.00	0.00	RX0	N
ATOM	338	H	LEU	194	33.263	-10.322	30.355	1.00	0.00	RX0	H
ATOM	339	CA	LEU	194	31.235	-10.169	29.722	1.00	0.00	RX0	C
ATOM	340	CB	LEU	194	30.847	-10.228	31.198	1.00	0.00	RX0	C
ATOM	341	CG	LEU	194	31.121	-11.580	31.850	1.00	0.00	RX0	C
ATOM	342	CD1	LEU	194	30.676	-11.582	33.312	1.00	0.00	RX0	C
ATOM	343	CD2	LEU	194	30.498	-12.728	31.055	1.00	0.00	RX0	C
ATOM	344	C	LEU	194	30.647	-8.891	29.116	1.00	0.00	RX0	C
ATOM	345	O	LEU	194	29.706	-8.959	28.317	1.00	0.00	RX0	O
ATOM	346	N	LEU	195	31.327	-7.782	29.364	1.00	0.00	RX0	N
ATOM	347	H	LEU	195	32.143	-7.848	29.934	1.00	0.00	RX0	H
ATOM	348	CA	LEU	195	30.920	-6.462	28.846	1.00	0.00	RX0	C
ATOM	349	CB	LEU	195	31.731	-5.338	29.492	1.00	0.00	RX0	C
ATOM	350	CG	LEU	195	31.536	-5.241	31.006	1.00	0.00	RX0	C
ATOM	351	CD1	LEU	195	32.371	-4.111	31.611	1.00	0.00	RX0	C
ATOM	352	CD2	LEU	195	30.061	-5.133	31.389	1.00	0.00	RX0	C
ATOM	353	C	LEU	195	31.020	-6.357	27.321	1.00	0.00	RX0	C
ATOM	354	O	LEU	195	30.051	-5.942	26.671	1.00	0.00	RX0	O
ATOM	355	N	THR	196	32.075	-6.931	26.767	1.00	0.00	RX0	N
ATOM	356	H	THR	196	32.792	-7.348	27.324	1.00	0.00	RX0	H
ATOM	357	CA	THR	196	32.335	-6.901	25.309	1.00	0.00	RX0	C
ATOM	358	CB	THR	196	33.783	-7.303	25.037	1.00	0.00	RX0	C
ATOM	359	OG1	THR	196	34.661	-6.379	25.696	1.00	0.00	RX0	O
ATOM	360	HG1	THR	196	34.333	-5.515	25.484	1.00	0.00	RX0	H
ATOM	361	CG2	THR	196	34.092	-7.362	23.540	1.00	0.00	RX0	C
ATOM	362	C	THR	196	31.317	-7.765	24.552	1.00	0.00	RX0	C
ATOM	363	O	THR	196	30.772	-7.327	23.532	1.00	0.00	RX0	O
ATOM	364	N	ASN	197	31.003	-8.928	25.107	1.00	0.00	RX0	N

ATOM	365	H	ASN	197	31.473	-9.232	25.940	1.00	0.00	RX0	H
ATOM	366	CA	ASN	197	30.010	-9.840	24.504	1.00	0.00	RX0	C
ATOM	367	CB	ASN	197	29.844	-11.072	25.390	1.00	0.00	RX0	C
ATOM	368	CG	ASN	197	30.830	-12.193	25.153	1.00	0.00	RX0	C
ATOM	369	OD1	ASN	197	32.024	-12.015	24.914	1.00	0.00	RX0	O
ATOM	370	ND2	ASN	197	30.239	-13.393	25.299	1.00	0.00	RX0	N
ATOM	371	HD21	ASN	197	29.241	-13.397	25.446	1.00	0.00	RX0	H
ATOM	372	HD22	ASN	197	30.680	-14.287	25.304	1.00	0.00	RX0	H
ATOM	373	C	ASN	197	28.594	-9.255	24.528	1.00	0.00	RX0	C
ATOM	374	O	ASN	197	27.900	-9.272	23.514	1.00	0.00	RX0	O
ATOM	375	N	LEU	198	28.277	-8.575	25.633	1.00	0.00	RX0	N
ATOM	376	H	LEU	198	28.919	-8.582	26.404	1.00	0.00	RX0	H
ATOM	377	CA	LEU	198	27.002	-7.850	25.760	1.00	0.00	RX0	C
ATOM	378	CB	LEU	198	26.867	-7.330	27.188	1.00	0.00	RX0	C
ATOM	379	CG	LEU	198	25.539	-6.636	27.483	1.00	0.00	RX0	C
ATOM	380	CD1	LEU	198	24.330	-7.549	27.269	1.00	0.00	RX0	C
ATOM	381	CD2	LEU	198	25.559	-6.043	28.887	1.00	0.00	RX0	C
ATOM	382	C	LEU	198	26.885	-6.719	24.724	1.00	0.00	RX0	C
ATOM	383	O	LEU	198	25.930	-6.676	23.947	1.00	0.00	RX0	O
ATOM	384	N	ALA	199	27.942	-5.914	24.641	1.00	0.00	RX0	N
ATOM	385	H	ALA	199	28.702	-6.063	25.276	1.00	0.00	RX0	H
ATOM	386	CA	ALA	199	28.029	-4.784	23.694	1.00	0.00	RX0	C
ATOM	387	CB	ALA	199	29.343	-4.024	23.882	1.00	0.00	RX0	C
ATOM	388	C	ALA	199	27.921	-5.230	22.227	1.00	0.00	RX0	C
ATOM	389	O	ALA	199	27.138	-4.660	21.467	1.00	0.00	RX0	O
ATOM	390	N	ASP	200	28.555	-6.360	21.908	1.00	0.00	RX0	N
ATOM	391	H	ASP	200	29.144	-6.806	22.584	1.00	0.00	RX0	H
ATOM	392	CA	ASP	200	28.494	-6.940	20.550	1.00	0.00	RX0	C
ATOM	393	CB	ASP	200	29.529	-8.059	20.384	1.00	0.00	RX0	C
ATOM	394	CG	ASP	200	30.188	-7.983	19.014	1.00	0.00	RX0	C
ATOM	395	OD1	ASP	200	30.499	-6.887	18.549	1.00	0.00	RX0	O
ATOM	396	OD2	ASP	200	30.455	-9.020	18.409	1.00	0.00	RX0	O
ATOM	397	C	ASP	200	27.084	-7.410	20.171	1.00	0.00	RX0	C
ATOM	398	O	ASP	200	26.604	-7.102	19.080	1.00	0.00	RX0	O
ATOM	399	N	ARG	201	26.390	-7.998	21.143	1.00	0.00	RX0	N
ATOM	400	H	ARG	201	26.847	-8.145	22.024	1.00	0.00	RX0	H
ATOM	401	CA	ARG	201	24.992	-8.434	20.957	1.00	0.00	RX0	C
ATOM	402	CB	ARG	201	24.583	-9.425	22.034	1.00	0.00	RX0	C
ATOM	403	CG	ARG	201	25.244	-10.780	21.787	1.00	0.00	RX0	C
ATOM	404	CD	ARG	201	24.802	-11.840	22.791	1.00	0.00	RX0	C
ATOM	405	NE	ARG	201	25.312	-11.542	24.124	1.00	0.00	RX0	N
ATOM	406	HE	ARG	201	26.283	-11.773	24.293	1.00	0.00	RX0	H
ATOM	407	CZ	ARG	201	24.495	-11.099	25.117	1.00	0.00	RX0	C
ATOM	408	NH1	ARG	201	23.186	-10.901	24.869	1.00	0.00	RX0	N
ATOM	409	HH11	ARG	201	22.563	-10.625	25.603	1.00	0.00	RX0	H
ATOM	410	HH12	ARG	201	22.809	-11.034	23.949	1.00	0.00	RX0	H
ATOM	411	NH2	ARG	201	24.996	-10.883	26.343	1.00	0.00	RX0	N
ATOM	412	HH21	ARG	201	24.430	-10.594	27.121	1.00	0.00	RX0	H
ATOM	413	HH22	ARG	201	25.975	-11.072	26.529	1.00	0.00	RX0	H
ATOM	414	C	ARG	201	23.991	-7.279	20.827	1.00	0.00	RX0	C
ATOM	415	O	ARG	201	23.123	-7.308	19.955	1.00	0.00	RX0	O
ATOM	416	N	GLU	202	24.240	-6.201	21.568	1.00	0.00	RX0	N
ATOM	417	H	GLU	202	25.009	-6.214	22.213	1.00	0.00	RX0	H
ATOM	418	CA	GLU	202	23.401	-4.988	21.493	1.00	0.00	RX0	C
ATOM	419	CB	GLU	202	23.558	-4.077	22.732	1.00	0.00	RX0	C
ATOM	420	CG	GLU	202	22.908	-4.685	23.995	1.00	0.00	RX0	C
ATOM	421	CD	GLU	202	22.863	-3.731	25.192	1.00	0.00	RX0	C
ATOM	422	OE1	GLU	202	21.841	-3.084	25.423	1.00	0.00	RX0	O
ATOM	423	OE2	GLU	202	23.820	-3.677	25.958	1.00	0.00	RX0	O
ATOM	424	C	GLU	202	23.526	-4.262	20.149	1.00	0.00	RX0	C
ATOM	425	O	GLU	202	22.539	-3.754	19.625	1.00	0.00	RX0	O

ATOM	426	N	LEU	203	24.712	-4.358	19.546	1.00	0.00	RX0	N
ATOM	427	H	LEU	203	25.464	-4.809	20.035	1.00	0.00	RX0	H
ATOM	428	CA	LEU	203	25.004	-3.680	18.270	1.00	0.00	RX0	C
ATOM	429	CB	LEU	203	26.480	-3.871	17.916	1.00	0.00	RX0	C
ATOM	430	CG	LEU	203	26.920	-3.128	16.652	1.00	0.00	RX0	C
ATOM	431	CD1	LEU	203	26.718	-1.617	16.769	1.00	0.00	RX0	C
ATOM	432	CD2	LEU	203	28.356	-3.483	16.265	1.00	0.00	RX0	C
ATOM	433	C	LEU	203	24.099	-4.160	17.127	1.00	0.00	RX0	C
ATOM	434	O	LEU	203	23.593	-3.349	16.346	1.00	0.00	RX0	O
ATOM	435	N	VAL	204	23.782	-5.447	17.151	1.00	0.00	RX0	N
ATOM	436	H	VAL	204	24.175	-6.003	17.887	1.00	0.00	RX0	H
ATOM	437	CA	VAL	204	22.925	-6.083	16.127	1.00	0.00	RX0	C
ATOM	438	CB	VAL	204	22.899	-7.597	16.333	1.00	0.00	RX0	C
ATOM	439	CG1	VAL	204	22.010	-8.279	15.292	1.00	0.00	RX0	C
ATOM	440	CG2	VAL	204	24.321	-8.162	16.349	1.00	0.00	RX0	C
ATOM	441	C	VAL	204	21.502	-5.497	16.213	1.00	0.00	RX0	C
ATOM	442	O	VAL	204	20.938	-5.041	15.221	1.00	0.00	RX0	O
ATOM	443	N	HIS	205	21.015	-5.393	17.448	1.00	0.00	RX0	N
ATOM	444	H	HIS	205	21.603	-5.676	18.208	1.00	0.00	RX0	H
ATOM	445	CA	HIS	205	19.703	-4.786	17.746	1.00	0.00	RX0	C
ATOM	446	CB	HIS	205	19.275	-5.097	19.182	1.00	0.00	RX0	C
ATOM	447	CG	HIS	205	19.002	-6.576	19.315	1.00	0.00	RX0	C
ATOM	448	ND1	HIS	205	17.806	-7.139	19.059	1.00	0.00	RX0	N
ATOM	449	HD1	HIS	205	16.990	-6.674	18.780	1.00	0.00	RX0	H
ATOM	450	CD2	HIS	205	19.896	-7.579	19.700	1.00	0.00	RX0	C
ATOM	451	NE2	HIS	205	19.223	-8.756	19.674	1.00	0.00	RX0	N
ATOM	452	CE1	HIS	205	17.938	-8.486	19.279	1.00	0.00	RX0	C
ATOM	453	C	HIS	205	19.668	-3.277	17.476	1.00	0.00	RX0	C
ATOM	454	O	HIS	205	18.642	-2.756	17.030	1.00	0.00	RX0	O
ATOM	455	N	MET	206	20.820	-2.627	17.609	1.00	0.00	RX0	N
ATOM	456	H	MET	206	21.610	-3.125	17.973	1.00	0.00	RX0	H
ATOM	457	CA	MET	206	20.969	-1.185	17.340	1.00	0.00	RX0	C
ATOM	458	CB	MET	206	22.357	-0.695	17.746	1.00	0.00	RX0	C
ATOM	459	CG	MET	206	22.542	0.804	17.511	1.00	0.00	RX0	C
ATOM	460	SD	MET	206	24.244	1.324	17.769	1.00	0.00	RX0	S
ATOM	461	CE	MET	206	24.502	0.512	19.351	1.00	0.00	RX0	C
ATOM	462	C	MET	206	20.721	-0.870	15.856	1.00	0.00	RX0	C
ATOM	463	O	MET	206	20.035	0.103	15.544	1.00	0.00	RX0	O
ATOM	464	N	ILE	207	21.183	-1.758	14.977	1.00	0.00	RX0	N
ATOM	465	H	ILE	207	21.731	-2.517	15.339	1.00	0.00	RX0	H
ATOM	466	CA	ILE	207	20.975	-1.628	13.516	1.00	0.00	RX0	C
ATOM	467	CB	ILE	207	21.672	-2.790	12.802	1.00	0.00	RX0	C
ATOM	468	CG2	ILE	207	21.421	-2.771	11.296	1.00	0.00	RX0	C
ATOM	469	CG1	ILE	207	23.165	-2.809	13.125	1.00	0.00	RX0	C
ATOM	470	CD1	ILE	207	23.907	-1.600	12.558	1.00	0.00	RX0	C
ATOM	471	C	ILE	207	19.470	-1.621	13.197	1.00	0.00	RX0	C
ATOM	472	O	ILE	207	18.988	-0.755	12.467	1.00	0.00	RX0	O
ATOM	473	N	ASN	208	18.761	-2.558	13.816	1.00	0.00	RX0	N
ATOM	474	H	ASN	208	19.238	-3.129	14.488	1.00	0.00	RX0	H
ATOM	475	CA	ASN	208	17.313	-2.734	13.586	1.00	0.00	RX0	C
ATOM	476	CB	ASN	208	16.798	-4.071	14.116	1.00	0.00	RX0	C
ATOM	477	CG	ASN	208	17.007	-5.132	13.049	1.00	0.00	RX0	C
ATOM	478	OD1	ASN	208	17.465	-4.868	11.933	1.00	0.00	RX0	O
ATOM	479	ND2	ASN	208	16.652	-6.364	13.455	1.00	0.00	RX0	N
ATOM	480	HD21	ASN	208	16.285	-6.508	14.376	1.00	0.00	RX0	H
ATOM	481	HD22	ASN	208	16.738	-7.179	12.879	1.00	0.00	RX0	H
ATOM	482	C	ASN	208	16.516	-1.532	14.103	1.00	0.00	RX0	C
ATOM	483	O	ASN	208	15.637	-1.017	13.417	1.00	0.00	RX0	O
ATOM	484	N	TRP	209	16.982	-1.008	15.238	1.00	0.00	RX0	N
ATOM	485	H	TRP	209	17.725	-1.474	15.722	1.00	0.00	RX0	H
ATOM	486	CA	TRP	209	16.425	0.201	15.864	1.00	0.00	RX0	C

ATOM	487	CB	TRP	209	17.093	0.419	17.231	1.00	0.00	RX0	C
ATOM	488	CG	TRP	209	16.696	1.748	17.837	1.00	0.00	RX0	C
ATOM	489	CD2	TRP	209	17.415	3.000	17.806	1.00	0.00	RX0	C
ATOM	490	CE2	TRP	209	16.636	3.960	18.496	1.00	0.00	RX0	C
ATOM	491	CE3	TRP	209	18.635	3.370	17.253	1.00	0.00	RX0	C
ATOM	492	CD1	TRP	209	15.528	2.029	18.554	1.00	0.00	RX0	C
ATOM	493	NE1	TRP	209	15.481	3.327	18.950	1.00	0.00	RX0	N
ATOM	494	HE1	TRP	209	14.725	3.719	19.454	1.00	0.00	RX0	H
ATOM	495	CZ2	TRP	209	17.099	5.266	18.598	1.00	0.00	RX0	C
ATOM	496	CZ3	TRP	209	19.088	4.678	17.370	1.00	0.00	RX0	C
ATOM	497	CH2	TRP	209	18.319	5.625	18.036	1.00	0.00	RX0	C
ATOM	498	C	TRP	209	16.619	1.438	14.972	1.00	0.00	RX0	C
ATOM	499	O	TRP	209	15.652	2.120	14.634	1.00	0.00	RX0	O
ATOM	500	N	ALA	210	17.853	1.624	14.503	1.00	0.00	RX0	N
ATOM	501	H	ALA	210	18.567	0.988	14.791	1.00	0.00	RX0	H
ATOM	502	CA	ALA	210	18.233	2.756	13.635	1.00	0.00	RX0	C
ATOM	503	CB	ALA	210	19.715	2.665	13.273	1.00	0.00	RX0	C
ATOM	504	C	ALA	210	17.400	2.800	12.347	1.00	0.00	RX0	C
ATOM	505	O	ALA	210	16.892	3.855	11.979	1.00	0.00	RX0	O
ATOM	506	N	LYS	211	17.095	1.613	11.820	1.00	0.00	RX0	N
ATOM	507	H	LYS	211	17.503	0.799	12.236	1.00	0.00	RX0	H
ATOM	508	CA	LYS	211	16.258	1.472	10.614	1.00	0.00	RX0	C
ATOM	509	CB	LYS	211	16.406	0.069	10.022	1.00	0.00	RX0	C
ATOM	510	CG	LYS	211	17.793	-0.087	9.388	1.00	0.00	RX0	C
ATOM	511	CD	LYS	211	18.068	-1.494	8.854	1.00	0.00	RX0	C
ATOM	512	CE	LYS	211	17.866	-2.509	9.972	1.00	0.00	RX0	C
ATOM	513	NZ	LYS	211	18.343	-3.850	9.607	1.00	0.00	RX0	N
ATOM	514	HZ1	LYS	211	18.159	-4.476	10.425	1.00	0.00	RX0	H
ATOM	515	HZ2	LYS	211	17.828	-4.204	8.780	1.00	0.00	RX0	H
ATOM	516	HZ3	LYS	211	19.364	-3.835	9.404	1.00	0.00	RX0	H
ATOM	517	C	LYS	211	14.795	1.898	10.823	1.00	0.00	RX0	C
ATOM	518	O	LYS	211	14.129	2.337	9.881	1.00	0.00	RX0	O
ATOM	519	N	ARG	212	14.357	1.870	12.071	1.00	0.00	RX0	N
ATOM	520	H	ARG	212	14.980	1.608	12.809	1.00	0.00	RX0	H
ATOM	521	CA	ARG	212	13.005	2.320	12.466	1.00	0.00	RX0	C
ATOM	522	CB	ARG	212	12.393	1.398	13.520	1.00	0.00	RX0	C
ATOM	523	CG	ARG	212	12.555	-0.088	13.194	1.00	0.00	RX0	C
ATOM	524	CD	ARG	212	11.678	-0.985	14.070	1.00	0.00	RX0	C
ATOM	525	NE	ARG	212	10.272	-0.783	13.726	1.00	0.00	RX0	N
ATOM	526	HE	ARG	212	10.085	-0.545	12.769	1.00	0.00	RX0	H
ATOM	527	CZ	ARG	212	9.295	-0.890	14.676	1.00	0.00	RX0	C
ATOM	528	NH1	ARG	212	9.611	-1.221	15.943	1.00	0.00	RX0	N
ATOM	529	HH11	ARG	212	8.888	-1.242	16.664	1.00	0.00	RX0	H
ATOM	530	HH12	ARG	212	10.534	-1.437	16.263	1.00	0.00	RX0	H
ATOM	531	NH2	ARG	212	8.018	-0.648	14.335	1.00	0.00	RX0	N
ATOM	532	HH21	ARG	212	7.297	-0.640	15.059	1.00	0.00	RX0	H
ATOM	533	HH22	ARG	212	7.695	-0.430	13.416	1.00	0.00	RX0	H
ATOM	534	C	ARG	212	12.933	3.790	12.894	1.00	0.00	RX0	C
ATOM	535	O	ARG	212	11.827	4.344	12.989	1.00	0.00	RX0	O
ATOM	536	N	VAL	213	14.074	4.417	13.148	1.00	0.00	RX0	N
ATOM	537	H	VAL	213	14.933	3.928	12.994	1.00	0.00	RX0	H
ATOM	538	CA	VAL	213	14.155	5.874	13.374	1.00	0.00	RX0	C
ATOM	539	CB	VAL	213	15.581	6.281	13.748	1.00	0.00	RX0	C
ATOM	540	CG1	VAL	213	15.751	7.800	13.838	1.00	0.00	RX0	C
ATOM	541	CG2	VAL	213	15.979	5.583	15.047	1.00	0.00	RX0	C
ATOM	542	C	VAL	213	13.672	6.590	12.095	1.00	0.00	RX0	C
ATOM	543	O	VAL	213	14.288	6.431	11.023	1.00	0.00	RX0	O
ATOM	544	N	PRO	214	12.622	7.395	12.213	1.00	0.00	RX0	N
ATOM	545	CD	PRO	214	11.895	7.609	13.458	1.00	0.00	RX0	C
ATOM	546	CA	PRO	214	12.035	8.145	11.084	1.00	0.00	RX0	C
ATOM	547	CB	PRO	214	10.927	8.954	11.761	1.00	0.00	RX0	C

ATOM	548	CG	PRO	214	10.552	8.163	13.010	1.00	0.00	RX0	C
ATOM	549	C	PRO	214	13.102	9.001	10.387	1.00	0.00	RX0	C
ATOM	550	O	PRO	214	13.853	9.727	11.025	1.00	0.00	RX0	O
ATOM	551	N	GLY	215	13.244	8.730	9.080	1.00	0.00	RX0	N
ATOM	552	H	GLY	215	12.750	7.982	8.636	1.00	0.00	RX0	H
ATOM	553	CA	GLY	215	14.194	9.473	8.227	1.00	0.00	RX0	C
ATOM	554	C	GLY	215	15.511	8.732	7.950	1.00	0.00	RX0	C
ATOM	555	O	GLY	215	16.085	8.889	6.862	1.00	0.00	RX0	O
ATOM	556	N	PHE	216	15.917	7.845	8.848	1.00	0.00	RX0	N
ATOM	557	H	PHE	216	15.329	7.653	9.637	1.00	0.00	RX0	H
ATOM	558	CA	PHE	216	17.224	7.160	8.764	1.00	0.00	RX0	C
ATOM	559	CB	PHE	216	17.453	6.293	9.997	1.00	0.00	RX0	C
ATOM	560	CG	PHE	216	18.893	5.846	10.056	1.00	0.00	RX0	C
ATOM	561	CD1	PHE	216	19.896	6.777	10.297	1.00	0.00	RX0	C
ATOM	562	CD2	PHE	216	19.216	4.505	9.883	1.00	0.00	RX0	C
ATOM	563	CE1	PHE	216	21.218	6.362	10.404	1.00	0.00	RX0	C
ATOM	564	CE2	PHE	216	20.537	4.090	9.992	1.00	0.00	RX0	C
ATOM	565	CZ	PHE	216	21.535	5.016	10.273	1.00	0.00	RX0	C
ATOM	566	C	PHE	216	17.435	6.347	7.474	1.00	0.00	RX0	C
ATOM	567	O	PHE	216	18.358	6.641	6.711	1.00	0.00	RX0	O
ATOM	568	N	VAL	217	16.482	5.483	7.151	1.00	0.00	RX0	N
ATOM	569	H	VAL	217	15.672	5.448	7.735	1.00	0.00	RX0	H
ATOM	570	CA	VAL	217	16.570	4.608	5.958	1.00	0.00	RX0	C
ATOM	571	CB	VAL	217	15.527	3.496	6.007	1.00	0.00	RX0	C
ATOM	572	CG1	VAL	217	15.894	2.482	7.081	1.00	0.00	RX0	C
ATOM	573	CG2	VAL	217	14.110	4.054	6.166	1.00	0.00	RX0	C
ATOM	574	C	VAL	217	16.469	5.342	4.608	1.00	0.00	RX0	C
ATOM	575	O	VAL	217	16.660	4.747	3.556	1.00	0.00	RX0	O
ATOM	576	N	ASP	218	16.058	6.613	4.671	1.00	0.00	RX0	N
ATOM	577	H	ASP	218	15.766	7.061	5.519	1.00	0.00	RX0	H
ATOM	578	CA	ASP	218	16.006	7.470	3.472	1.00	0.00	RX0	C
ATOM	579	CB	ASP	218	15.091	8.670	3.760	1.00	0.00	RX0	C
ATOM	580	CG	ASP	218	13.742	8.250	4.347	1.00	0.00	RX0	C
ATOM	581	OD1	ASP	218	12.734	8.402	3.660	1.00	0.00	RX0	O
ATOM	582	OD2	ASP	218	13.683	7.808	5.500	1.00	0.00	RX0	O
ATOM	583	C	ASP	218	17.401	7.924	3.023	1.00	0.00	RX0	C
ATOM	584	O	ASP	218	17.595	8.369	1.896	1.00	0.00	RX0	O
ATOM	585	N	LEU	219	18.344	7.857	3.967	1.00	0.00	RX0	N
ATOM	586	H	LEU	219	18.095	7.488	4.863	1.00	0.00	RX0	H
ATOM	587	CA	LEU	219	19.767	8.083	3.697	1.00	0.00	RX0	C
ATOM	588	CB	LEU	219	20.543	8.275	4.997	1.00	0.00	RX0	C
ATOM	589	CG	LEU	219	19.964	9.430	5.816	1.00	0.00	RX0	C
ATOM	590	CD1	LEU	219	20.542	9.468	7.228	1.00	0.00	RX0	C
ATOM	591	CD2	LEU	219	20.095	10.772	5.092	1.00	0.00	RX0	C
ATOM	592	C	LEU	219	20.350	6.970	2.832	1.00	0.00	RX0	C
ATOM	593	O	LEU	219	19.843	5.836	2.789	1.00	0.00	RX0	O
ATOM	594	N	THR	220	21.442	7.293	2.191	1.00	0.00	RX0	N
ATOM	595	H	THR	220	21.736	8.253	2.249	1.00	0.00	RX0	H
ATOM	596	CA	THR	220	22.263	6.312	1.453	1.00	0.00	RX0	C
ATOM	597	CB	THR	220	23.357	6.946	0.577	1.00	0.00	RX0	C
ATOM	598	OG1	THR	220	24.593	7.091	1.276	1.00	0.00	RX0	O
ATOM	599	HG1	THR	220	24.634	7.988	1.625	1.00	0.00	RX0	H
ATOM	600	CG2	THR	220	22.916	8.244	-0.089	1.00	0.00	RX0	C
ATOM	601	C	THR	220	22.829	5.303	2.454	1.00	0.00	RX0	C
ATOM	602	O	THR	220	23.112	5.646	3.611	1.00	0.00	RX0	O
ATOM	603	N	LEU	221	23.130	4.116	1.957	1.00	0.00	RX0	N
ATOM	604	H	LEU	221	22.878	3.918	1.012	1.00	0.00	RX0	H
ATOM	605	CA	LEU	221	23.750	3.065	2.781	1.00	0.00	RX0	C
ATOM	606	CB	LEU	221	23.995	1.842	1.901	1.00	0.00	RX0	C
ATOM	607	CG	LEU	221	24.904	0.806	2.562	1.00	0.00	RX0	C
ATOM	608	CD1	LEU	221	24.198	0.076	3.701	1.00	0.00	RX0	C

ATOM	609	CD2	LEU	221	25.521	-0.143	1.536	1.00	0.00	RX0	C
ATOM	610	C	LEU	221	25.092	3.509	3.394	1.00	0.00	RX0	C
ATOM	611	O	LEU	221	25.324	3.347	4.578	1.00	0.00	RX0	O
ATOM	612	N	HIS	222	25.854	4.270	2.593	1.00	0.00	RX0	N
ATOM	613	H	HIS	222	25.505	4.542	1.698	1.00	0.00	RX0	H
ATOM	614	CA	HIS	222	27.131	4.847	3.045	1.00	0.00	RX0	C
ATOM	615	CB	HIS	222	27.792	5.634	1.912	1.00	0.00	RX0	C
ATOM	616	CG	HIS	222	28.870	6.547	2.458	1.00	0.00	RX0	C
ATOM	617	ND1	HIS	222	29.815	6.170	3.340	1.00	0.00	RX0	N
ATOM	618	HD1	HIS	222	29.939	5.278	3.743	1.00	0.00	RX0	H
ATOM	619	CD2	HIS	222	29.073	7.894	2.141	1.00	0.00	RX0	C
ATOM	620	NE2	HIS	222	30.154	8.320	2.842	1.00	0.00	RX0	N
ATOM	621	CE1	HIS	222	30.606	7.260	3.585	1.00	0.00	RX0	C
ATOM	622	C	HIS	222	26.942	5.765	4.264	1.00	0.00	RX0	C
ATOM	623	O	HIS	222	27.673	5.642	5.246	1.00	0.00	RX0	O
ATOM	624	N	ASP	223	25.958	6.658	4.167	1.00	0.00	RX0	N
ATOM	625	H	ASP	223	25.447	6.779	3.313	1.00	0.00	RX0	H
ATOM	626	CA	ASP	223	25.694	7.636	5.242	1.00	0.00	RX0	C
ATOM	627	CB	ASP	223	24.751	8.742	4.742	1.00	0.00	RX0	C
ATOM	628	CG	ASP	223	25.418	9.603	3.676	1.00	0.00	RX0	C
ATOM	629	OD1	ASP	223	25.562	10.805	3.889	1.00	0.00	RX0	O
ATOM	630	OD2	ASP	223	25.780	9.081	2.623	1.00	0.00	RX0	O
ATOM	631	C	ASP	223	25.148	6.996	6.516	1.00	0.00	RX0	C
ATOM	632	O	ASP	223	25.558	7.375	7.616	1.00	0.00	RX0	O
ATOM	633	N	GLN	224	24.393	5.921	6.332	1.00	0.00	RX0	N
ATOM	634	H	GLN	224	24.153	5.689	5.386	1.00	0.00	RX0	H
ATOM	635	CA	GLN	224	23.868	5.118	7.452	1.00	0.00	RX0	C
ATOM	636	CB	GLN	224	22.846	4.093	6.958	1.00	0.00	RX0	C
ATOM	637	CG	GLN	224	21.595	4.761	6.381	1.00	0.00	RX0	C
ATOM	638	CD	GLN	224	20.552	3.707	6.078	1.00	0.00	RX0	C
ATOM	639	OE1	GLN	224	20.401	2.729	6.806	1.00	0.00	RX0	O
ATOM	640	NE2	GLN	224	19.843	3.952	4.963	1.00	0.00	RX0	N
ATOM	641	HE21	GLN	224	19.998	4.773	4.405	1.00	0.00	RX0	H
ATOM	642	HE22	GLN	224	19.116	3.359	4.618	1.00	0.00	RX0	H
ATOM	643	C	GLN	224	25.003	4.453	8.243	1.00	0.00	RX0	C
ATOM	644	O	GLN	224	25.073	4.591	9.468	1.00	0.00	RX0	O
ATOM	645	N	VAL	225	25.993	3.956	7.505	1.00	0.00	RX0	N
ATOM	646	H	VAL	225	25.895	3.978	6.507	1.00	0.00	RX0	H
ATOM	647	CA	VAL	225	27.191	3.319	8.093	1.00	0.00	RX0	C
ATOM	648	CB	VAL	225	28.054	2.613	7.044	1.00	0.00	RX0	C
ATOM	649	CG1	VAL	225	29.267	1.975	7.716	1.00	0.00	RX0	C
ATOM	650	CG2	VAL	225	27.264	1.565	6.263	1.00	0.00	RX0	C
ATOM	651	C	VAL	225	28.021	4.368	8.852	1.00	0.00	RX0	C
ATOM	652	O	VAL	225	28.415	4.141	9.995	1.00	0.00	RX0	O
ATOM	653	N	HIS	226	28.182	5.534	8.231	1.00	0.00	RX0	N
ATOM	654	H	HIS	226	27.807	5.643	7.307	1.00	0.00	RX0	H
ATOM	655	CA	HIS	226	28.959	6.641	8.815	1.00	0.00	RX0	C
ATOM	656	CB	HIS	226	29.142	7.831	7.874	1.00	0.00	RX0	C
ATOM	657	CG	HIS	226	30.027	8.850	8.565	1.00	0.00	RX0	C
ATOM	658	ND1	HIS	226	31.225	8.560	9.108	1.00	0.00	RX0	N
ATOM	659	HD1	HIS	226	31.658	7.680	9.152	1.00	0.00	RX0	H
ATOM	660	CD2	HIS	226	29.776	10.212	8.759	1.00	0.00	RX0	C
ATOM	661	NE2	HIS	226	30.833	10.745	9.421	1.00	0.00	RX0	N
ATOM	662	CE1	HIS	226	31.725	9.724	9.635	1.00	0.00	RX0	C
ATOM	663	C	HIS	226	28.363	7.118	10.150	1.00	0.00	RX0	C
ATOM	664	O	HIS	226	29.071	7.189	11.155	1.00	0.00	RX0	O
ATOM	665	N	LEU	227	27.047	7.317	10.166	1.00	0.00	RX0	N
ATOM	666	H	LEU	227	26.530	7.186	9.317	1.00	0.00	RX0	H
ATOM	667	CA	LEU	227	26.344	7.787	11.375	1.00	0.00	RX0	C
ATOM	668	CB	LEU	227	24.877	8.090	11.075	1.00	0.00	RX0	C
ATOM	669	CG	LEU	227	24.685	9.357	10.244	1.00	0.00	RX0	C

ATOM	670	CD1	LEU	227	23.217	9.576	9.890	1.00	0.00	RX0	C
ATOM	671	CD2	LEU	227	25.276	10.585	10.937	1.00	0.00	RX0	C
ATOM	672	C	LEU	227	26.435	6.799	12.540	1.00	0.00	RX0	C
ATOM	673	O	LEU	227	26.853	7.165	13.635	1.00	0.00	RX0	O
ATOM	674	N	LEU	228	26.270	5.522	12.200	1.00	0.00	RX0	N
ATOM	675	H	LEU	228	26.039	5.298	11.249	1.00	0.00	RX0	H
ATOM	676	CA	LEU	228	26.384	4.431	13.181	1.00	0.00	RX0	C
ATOM	677	CB	LEU	228	25.764	3.155	12.619	1.00	0.00	RX0	C
ATOM	678	CG	LEU	228	24.258	3.145	12.873	1.00	0.00	RX0	C
ATOM	679	CD1	LEU	228	23.517	2.138	12.000	1.00	0.00	RX0	C
ATOM	680	CD2	LEU	228	23.959	2.937	14.358	1.00	0.00	RX0	C
ATOM	681	C	LEU	228	27.805	4.188	13.685	1.00	0.00	RX0	C
ATOM	682	O	LEU	228	28.004	4.019	14.891	1.00	0.00	RX0	O
ATOM	683	N	GLU	229	28.784	4.376	12.809	1.00	0.00	RX0	N
ATOM	684	H	GLU	229	28.578	4.587	11.852	1.00	0.00	RX0	H
ATOM	685	CA	GLU	229	30.199	4.229	13.197	1.00	0.00	RX0	C
ATOM	686	CB	GLU	229	31.154	4.095	11.991	1.00	0.00	RX0	C
ATOM	687	CG	GLU	229	32.502	3.470	12.403	1.00	0.00	RX0	C
ATOM	688	CD	GLU	229	33.382	3.106	11.211	1.00	0.00	RX0	C
ATOM	689	OE1	GLU	229	33.341	3.794	10.196	1.00	0.00	RX0	O
ATOM	690	OE2	GLU	229	34.133	2.132	11.301	1.00	0.00	RX0	O
ATOM	691	C	GLU	229	30.618	5.338	14.175	1.00	0.00	RX0	C
ATOM	692	O	GLU	229	31.393	5.088	15.099	1.00	0.00	RX0	O
ATOM	693	N	CYS	230	30.060	6.523	13.970	1.00	0.00	RX0	N
ATOM	694	H	CYS	230	29.475	6.652	13.165	1.00	0.00	RX0	H
ATOM	695	CA	CYS	230	30.321	7.692	14.829	1.00	0.00	RX0	C
ATOM	696	CB	CYS	230	30.013	8.970	14.063	1.00	0.00	RX0	C
ATOM	697	SG	CYS	230	31.129	9.174	12.661	1.00	0.00	RX0	S
ATOM	698	C	CYS	230	29.592	7.653	16.182	1.00	0.00	RX0	C
ATOM	699	O	CYS	230	30.123	8.119	17.188	1.00	0.00	RX0	O
ATOM	700	N	ALA	231	28.434	7.000	16.215	1.00	0.00	RX0	N
ATOM	701	H	ALA	231	28.113	6.533	15.389	1.00	0.00	RX0	H
ATOM	702	CA	ALA	231	27.494	7.141	17.345	1.00	0.00	RX0	C
ATOM	703	CB	ALA	231	26.145	7.669	16.853	1.00	0.00	RX0	C
ATOM	704	C	ALA	231	27.249	5.885	18.186	1.00	0.00	RX0	C
ATOM	705	O	ALA	231	26.768	6.021	19.321	1.00	0.00	RX0	O
ATOM	706	N	TRP	232	27.687	4.719	17.731	1.00	0.00	RX0	N
ATOM	707	H	TRP	232	28.107	4.686	16.821	1.00	0.00	RX0	H
ATOM	708	CA	TRP	232	27.348	3.433	18.379	1.00	0.00	RX0	C
ATOM	709	CB	TRP	232	27.971	2.236	17.648	1.00	0.00	RX0	C
ATOM	710	CG	TRP	232	29.475	2.279	17.763	1.00	0.00	RX0	C
ATOM	711	CD2	TRP	232	30.331	1.539	18.658	1.00	0.00	RX0	C
ATOM	712	CE2	TRP	232	31.663	1.948	18.411	1.00	0.00	RX0	C
ATOM	713	CE3	TRP	232	30.074	0.583	19.633	1.00	0.00	RX0	C
ATOM	714	CD1	TRP	232	30.342	3.088	17.024	1.00	0.00	RX0	C
ATOM	715	NE1	TRP	232	31.631	2.902	17.399	1.00	0.00	RX0	N
ATOM	716	HE1	TRP	232	32.391	3.374	16.992	1.00	0.00	RX0	H
ATOM	717	CZ2	TRP	232	32.700	1.390	19.148	1.00	0.00	RX0	C
ATOM	718	CZ3	TRP	232	31.121	0.033	20.363	1.00	0.00	RX0	C
ATOM	719	CH2	TRP	232	32.429	0.435	20.122	1.00	0.00	RX0	C
ATOM	720	C	TRP	232	27.676	3.373	19.884	1.00	0.00	RX0	C
ATOM	721	O	TRP	232	26.862	2.914	20.672	1.00	0.00	RX0	O
ATOM	722	N	LEU	233	28.801	3.989	20.277	1.00	0.00	RX0	N
ATOM	723	H	LEU	233	29.361	4.452	19.591	1.00	0.00	RX0	H
ATOM	724	CA	LEU	233	29.211	3.960	21.691	1.00	0.00	RX0	C
ATOM	725	CB	LEU	233	30.717	4.176	21.833	1.00	0.00	RX0	C
ATOM	726	CG	LEU	233	31.201	3.788	23.232	1.00	0.00	RX0	C
ATOM	727	CD1	LEU	233	30.812	2.352	23.586	1.00	0.00	RX0	C
ATOM	728	CD2	LEU	233	32.699	4.026	23.411	1.00	0.00	RX0	C
ATOM	729	C	LEU	233	28.415	4.936	22.566	1.00	0.00	RX0	C
ATOM	730	O	LEU	233	27.943	4.566	23.634	1.00	0.00	RX0	O

ATOM	731	N	GLU	234	28.150	6.122	22.016	1.00	0.00	RX0	N
ATOM	732	H	GLU	234	28.490	6.337	21.103	1.00	0.00	RX0	H
ATOM	733	CA	GLU	234	27.227	7.090	22.644	1.00	0.00	RX0	C
ATOM	734	CB	GLU	234	27.211	8.360	21.786	1.00	0.00	RX0	C
ATOM	735	CG	GLU	234	26.616	9.555	22.522	1.00	0.00	RX0	C
ATOM	736	CD	GLU	234	26.649	10.811	21.676	1.00	0.00	RX0	C
ATOM	737	OE1	GLU	234	26.433	11.892	22.215	1.00	0.00	RX0	O
ATOM	738	OE2	GLU	234	26.893	10.733	20.477	1.00	0.00	RX0	O
ATOM	739	C	GLU	234	25.830	6.482	22.841	1.00	0.00	RX0	C
ATOM	740	O	GLU	234	25.253	6.598	23.926	1.00	0.00	RX0	O
ATOM	741	N	ILE	235	25.389	5.711	21.848	1.00	0.00	RX0	N
ATOM	742	H	ILE	235	25.962	5.643	21.030	1.00	0.00	RX0	H
ATOM	743	CA	ILE	235	24.069	5.045	21.863	1.00	0.00	RX0	C
ATOM	744	CB	ILE	235	23.701	4.470	20.490	1.00	0.00	RX0	C
ATOM	745	CG2	ILE	235	22.414	3.645	20.565	1.00	0.00	RX0	C
ATOM	746	CG1	ILE	235	23.567	5.580	19.450	1.00	0.00	RX0	C
ATOM	747	CD1	ILE	235	23.230	5.043	18.059	1.00	0.00	RX0	C
ATOM	748	C	ILE	235	24.018	3.945	22.939	1.00	0.00	RX0	C
ATOM	749	O	ILE	235	23.068	3.902	23.724	1.00	0.00	RX0	O
ATOM	750	N	LEU	236	25.072	3.140	23.020	1.00	0.00	RX0	N
ATOM	751	H	LEU	236	25.813	3.237	22.352	1.00	0.00	RX0	H
ATOM	752	CA	LEU	236	25.176	2.114	24.078	1.00	0.00	RX0	C
ATOM	753	CB	LEU	236	26.441	1.278	23.892	1.00	0.00	RX0	C
ATOM	754	CG	LEU	236	26.337	0.309	22.717	1.00	0.00	RX0	C
ATOM	755	CD1	LEU	236	27.665	-0.388	22.424	1.00	0.00	RX0	C
ATOM	756	CD2	LEU	236	25.201	-0.691	22.932	1.00	0.00	RX0	C
ATOM	757	C	LEU	236	25.182	2.733	25.479	1.00	0.00	RX0	C
ATOM	758	O	LEU	236	24.381	2.362	26.336	1.00	0.00	RX0	O
ATOM	759	N	MET	237	25.933	3.823	25.600	1.00	0.00	RX0	N
ATOM	760	H	MET	237	26.454	4.138	24.804	1.00	0.00	RX0	H
ATOM	761	CA	MET	237	26.132	4.523	26.881	1.00	0.00	RX0	C
ATOM	762	CB	MET	237	27.281	5.528	26.806	1.00	0.00	RX0	C
ATOM	763	CG	MET	237	28.653	4.854	26.821	1.00	0.00	RX0	C
ATOM	764	SD	MET	237	30.000	6.045	26.842	1.00	0.00	RX0	S
ATOM	765	CE	MET	237	31.349	4.891	27.132	1.00	0.00	RX0	C
ATOM	766	C	MET	237	24.875	5.215	27.409	1.00	0.00	RX0	C
ATOM	767	O	MET	237	24.517	5.003	28.572	1.00	0.00	RX0	O
ATOM	768	N	ILE	238	24.128	5.878	26.531	1.00	0.00	RX0	N
ATOM	769	H	ILE	238	24.460	5.966	25.588	1.00	0.00	RX0	H
ATOM	770	CA	ILE	238	22.871	6.546	26.925	1.00	0.00	RX0	C
ATOM	771	CB	ILE	238	22.340	7.525	25.865	1.00	0.00	RX0	C
ATOM	772	CG2	ILE	238	21.911	6.840	24.567	1.00	0.00	RX0	C
ATOM	773	CG1	ILE	238	21.211	8.366	26.468	1.00	0.00	RX0	C
ATOM	774	CD1	ILE	238	20.566	9.333	25.475	1.00	0.00	RX0	C
ATOM	775	C	ILE	238	21.800	5.514	27.357	1.00	0.00	RX0	C
ATOM	776	O	ILE	238	21.031	5.731	28.268	1.00	0.00	RX0	O
ATOM	777	N	GLY	239	21.845	4.355	26.660	1.00	0.00	RX0	N
ATOM	778	H	GLY	239	22.520	4.245	25.926	1.00	0.00	RX0	H
ATOM	779	CA	GLY	239	20.969	3.213	26.975	1.00	0.00	RX0	C
ATOM	780	C	GLY	239	21.301	2.634	28.356	1.00	0.00	RX0	C
ATOM	781	O	GLY	239	20.417	2.460	29.193	1.00	0.00	RX0	O
ATOM	782	N	LEU	240	22.605	2.583	28.639	1.00	0.00	RX0	N
ATOM	783	H	LEU	240	23.260	2.805	27.913	1.00	0.00	RX0	H
ATOM	784	CA	LEU	240	23.124	2.082	29.919	1.00	0.00	RX0	C
ATOM	785	CB	LEU	240	24.644	1.943	29.849	1.00	0.00	RX0	C
ATOM	786	CG	LEU	240	25.285	1.612	31.198	1.00	0.00	RX0	C
ATOM	787	CD1	LEU	240	24.822	0.265	31.750	1.00	0.00	RX0	C
ATOM	788	CD2	LEU	240	26.806	1.714	31.129	1.00	0.00	RX0	C
ATOM	789	C	LEU	240	22.728	2.995	31.086	1.00	0.00	RX0	C
ATOM	790	O	LEU	240	22.214	2.535	32.097	1.00	0.00	RX0	O
ATOM	791	N	VAL	241	22.901	4.295	30.880	1.00	0.00	RX0	N

ATOM	792	H	VAL	241	23.279	4.586	29.999	1.00	0.00	RX0	H
ATOM	793	CA	VAL	241	22.596	5.307	31.912	1.00	0.00	RX0	C
ATOM	794	CB	VAL	241	23.251	6.672	31.660	1.00	0.00	RX0	C
ATOM	795	CG1	VAL	241	24.769	6.508	31.590	1.00	0.00	RX0	C
ATOM	796	CG2	VAL	241	22.692	7.409	30.450	1.00	0.00	RX0	C
ATOM	797	C	VAL	241	21.084	5.392	32.193	1.00	0.00	RX0	C
ATOM	798	O	VAL	241	20.670	5.516	33.338	1.00	0.00	RX0	O
ATOM	799	N	TRP	242	20.290	5.181	31.134	1.00	0.00	RX0	N
ATOM	800	H	TRP	242	20.699	5.072	30.225	1.00	0.00	RX0	H
ATOM	801	CA	TRP	242	18.822	5.192	31.222	1.00	0.00	RX0	C
ATOM	802	CB	TRP	242	18.253	5.149	29.801	1.00	0.00	RX0	C
ATOM	803	CG	TRP	242	16.824	4.661	29.780	1.00	0.00	RX0	C
ATOM	804	CD2	TRP	242	15.637	5.341	30.238	1.00	0.00	RX0	C
ATOM	805	CE2	TRP	242	14.541	4.476	30.021	1.00	0.00	RX0	C
ATOM	806	CE3	TRP	242	15.425	6.592	30.801	1.00	0.00	RX0	C
ATOM	807	CD1	TRP	242	16.374	3.420	29.305	1.00	0.00	RX0	C
ATOM	808	NE1	TRP	242	15.027	3.306	29.447	1.00	0.00	RX0	N
ATOM	809	HE1	TRP	242	14.483	2.532	29.190	1.00	0.00	RX0	H
ATOM	810	CZ2	TRP	242	13.264	4.882	30.388	1.00	0.00	RX0	C
ATOM	811	CZ3	TRP	242	14.144	6.989	31.160	1.00	0.00	RX0	C
ATOM	812	CH2	TRP	242	13.069	6.134	30.958	1.00	0.00	RX0	C
ATOM	813	C	TRP	242	18.281	4.038	32.076	1.00	0.00	RX0	C
ATOM	814	O	TRP	242	17.477	4.269	32.979	1.00	0.00	RX0	O
ATOM	815	N	ARG	243	18.818	2.841	31.865	1.00	0.00	RX0	N
ATOM	816	H	ARG	243	19.515	2.737	31.151	1.00	0.00	RX0	H
ATOM	817	CA	ARG	243	18.359	1.661	32.627	1.00	0.00	RX0	C
ATOM	818	CB	ARG	243	18.486	0.403	31.737	1.00	0.00	RX0	C
ATOM	819	CG	ARG	243	19.891	-0.064	31.298	1.00	0.00	RX0	C
ATOM	820	CD	ARG	243	19.830	-1.101	30.153	1.00	0.00	RX0	C
ATOM	821	NE	ARG	243	21.114	-1.760	29.863	1.00	0.00	RX0	N
ATOM	822	HE	ARG	243	21.664	-2.016	30.671	1.00	0.00	RX0	H
ATOM	823	CZ	ARG	243	21.431	-2.131	28.569	1.00	0.00	RX0	C
ATOM	824	NH1	ARG	243	20.665	-1.725	27.537	1.00	0.00	RX0	N
ATOM	825	HH11	ARG	243	20.910	-2.039	26.601	1.00	0.00	RX0	H
ATOM	826	HH12	ARG	243	19.865	-1.134	27.639	1.00	0.00	RX0	H
ATOM	827	NH2	ARG	243	22.496	-2.913	28.304	1.00	0.00	RX0	N
ATOM	828	HH21	ARG	243	22.777	-3.131	27.347	1.00	0.00	RX0	H
ATOM	829	HH22	ARG	243	23.054	-3.343	29.023	1.00	0.00	RX0	H
ATOM	830	C	ARG	243	19.043	1.510	33.998	1.00	0.00	RX0	C
ATOM	831	O	ARG	243	18.610	0.722	34.836	1.00	0.00	RX0	O
ATOM	832	N	SER	244	20.027	2.366	34.245	1.00	0.00	RX0	N
ATOM	833	H	SER	244	20.316	3.010	33.538	1.00	0.00	RX0	H
ATOM	834	CA	SER	244	20.722	2.464	35.548	1.00	0.00	RX0	C
ATOM	835	CB	SER	244	22.206	2.697	35.311	1.00	0.00	RX0	C
ATOM	836	OG	SER	244	22.700	1.648	34.476	1.00	0.00	RX0	O
ATOM	837	HG	SER	244	22.226	1.717	33.654	1.00	0.00	RX0	H
ATOM	838	C	SER	244	20.112	3.547	36.442	1.00	0.00	RX0	C
ATOM	839	O	SER	244	20.448	3.642	37.630	1.00	0.00	RX0	O
ATOM	840	N	MET	245	19.184	4.322	35.895	1.00	0.00	RX0	N
ATOM	841	H	MET	245	18.884	4.151	34.954	1.00	0.00	RX0	H
ATOM	842	CA	MET	245	18.600	5.504	36.550	1.00	0.00	RX0	C
ATOM	843	CB	MET	245	17.596	6.196	35.632	1.00	0.00	RX0	C
ATOM	844	CG	MET	245	17.037	7.467	36.271	1.00	0.00	RX0	C
ATOM	845	SD	MET	245	15.733	8.229	35.302	1.00	0.00	RX0	S
ATOM	846	CE	MET	245	16.551	8.109	33.710	1.00	0.00	RX0	C
ATOM	847	C	MET	245	17.925	5.204	37.895	1.00	0.00	RX0	C
ATOM	848	O	MET	245	18.105	5.946	38.853	1.00	0.00	RX0	O
ATOM	849	N	GLU	246	17.212	4.082	37.945	1.00	0.00	RX0	N
ATOM	850	H	GLU	246	17.124	3.484	37.147	1.00	0.00	RX0	H
ATOM	851	CA	GLU	246	16.494	3.692	39.178	1.00	0.00	RX0	C
ATOM	852	CB	GLU	246	15.244	2.900	38.818	1.00	0.00	RX0	C

ATOM	853	CG	GLU	246	14.342	3.654	37.845	1.00	0.00	RX0	C
ATOM	854	CD	GLU	246	13.166	2.769	37.506	1.00	0.00	RX0	C
ATOM	855	OE1	GLU	246	12.977	1.766	38.193	1.00	0.00	RX0	O
ATOM	856	OE2	GLU	246	12.452	3.077	36.556	1.00	0.00	RX0	O
ATOM	857	C	GLU	246	17.379	2.867	40.123	1.00	0.00	RX0	C
ATOM	858	O	GLU	246	16.897	2.295	41.108	1.00	0.00	RX0	O
ATOM	859	N	HIS	247	18.674	2.844	39.836	1.00	0.00	RX0	N
ATOM	860	H	HIS	247	19.044	3.323	39.041	1.00	0.00	RX0	H
ATOM	861	CA	HIS	247	19.658	2.055	40.601	1.00	0.00	RX0	C
ATOM	862	CB	HIS	247	20.174	0.865	39.788	1.00	0.00	RX0	C
ATOM	863	CG	HIS	247	19.081	-0.138	39.489	1.00	0.00	RX0	C
ATOM	864	ND1	HIS	247	17.866	-0.170	40.076	1.00	0.00	RX0	N
ATOM	865	HD1	HIS	247	17.501	0.452	40.746	1.00	0.00	RX0	H
ATOM	866	CD2	HIS	247	19.155	-1.190	38.574	1.00	0.00	RX0	C
ATOM	867	NE2	HIS	247	17.978	-1.858	38.613	1.00	0.00	RX0	N
ATOM	868	CE1	HIS	247	17.182	-1.229	39.536	1.00	0.00	RX0	C
ATOM	869	C	HIS	247	20.841	2.947	41.015	1.00	0.00	RX0	C
ATOM	870	O	HIS	247	21.962	2.788	40.490	1.00	0.00	RX0	O
ATOM	871	N	PRO	248	20.632	3.827	41.991	1.00	0.00	RX0	N
ATOM	872	CD	PRO	248	19.384	3.973	42.733	1.00	0.00	RX0	C
ATOM	873	CA	PRO	248	21.659	4.767	42.484	1.00	0.00	RX0	C
ATOM	874	CB	PRO	248	20.980	5.449	43.676	1.00	0.00	RX0	C
ATOM	875	CG	PRO	248	19.487	5.344	43.385	1.00	0.00	RX0	C
ATOM	876	C	PRO	248	22.939	4.013	42.877	1.00	0.00	RX0	C
ATOM	877	O	PRO	248	22.892	2.963	43.503	1.00	0.00	RX0	O
ATOM	878	N	GLY	249	24.055	4.541	42.350	1.00	0.00	RX0	N
ATOM	879	H	GLY	249	23.974	5.301	41.711	1.00	0.00	RX0	H
ATOM	880	CA	GLY	249	25.407	3.996	42.610	1.00	0.00	RX0	C
ATOM	881	C	GLY	249	25.783	2.749	41.794	1.00	0.00	RX0	C
ATOM	882	O	GLY	249	26.914	2.250	41.927	1.00	0.00	RX0	O
ATOM	883	N	LYS	250	24.877	2.267	40.961	1.00	0.00	RX0	N
ATOM	884	H	LYS	250	23.982	2.694	40.809	1.00	0.00	RX0	H
ATOM	885	CA	LYS	250	25.097	1.050	40.158	1.00	0.00	RX0	C
ATOM	886	CB	LYS	250	24.373	-0.148	40.775	1.00	0.00	RX0	C
ATOM	887	CG	LYS	250	25.231	-0.851	41.836	1.00	0.00	RX0	C
ATOM	888	CD	LYS	250	24.616	-2.142	42.383	1.00	0.00	RX0	C
ATOM	889	CE	LYS	250	25.555	-2.970	43.269	1.00	0.00	RX0	C
ATOM	890	NZ	LYS	250	26.707	-3.460	42.498	1.00	0.00	RX0	N
ATOM	891	HZ1	LYS	250	27.420	-3.869	43.144	1.00	0.00	RX0	H
ATOM	892	HZ2	LYS	250	26.446	-4.158	41.767	1.00	0.00	RX0	H
ATOM	893	HZ3	LYS	250	27.213	-2.655	42.082	1.00	0.00	RX0	H
ATOM	894	C	LYS	250	24.802	1.266	38.671	1.00	0.00	RX0	C
ATOM	895	O	LYS	250	24.040	2.166	38.282	1.00	0.00	RX0	O
ATOM	896	N	LEU	251	25.472	0.476	37.863	1.00	0.00	RX0	N
ATOM	897	H	LEU	251	26.052	-0.237	38.243	1.00	0.00	RX0	H
ATOM	898	CA	LEU	251	25.292	0.430	36.401	1.00	0.00	RX0	C
ATOM	899	CB	LEU	251	26.626	0.592	35.683	1.00	0.00	RX0	C
ATOM	900	CG	LEU	251	27.162	2.016	35.766	1.00	0.00	RX0	C
ATOM	901	CD1	LEU	251	28.583	2.116	35.215	1.00	0.00	RX0	C
ATOM	902	CD2	LEU	251	26.208	3.013	35.105	1.00	0.00	RX0	C
ATOM	903	C	LEU	251	24.646	-0.892	36.017	1.00	0.00	RX0	C
ATOM	904	O	LEU	251	25.224	-1.976	36.286	1.00	0.00	RX0	O
ATOM	905	N	LEU	252	23.437	-0.808	35.533	1.00	0.00	RX0	N
ATOM	906	H	LEU	252	23.084	0.097	35.312	1.00	0.00	RX0	H
ATOM	907	CA	LEU	252	22.659	-1.981	35.114	1.00	0.00	RX0	C
ATOM	908	CB	LEU	252	21.157	-1.712	35.230	1.00	0.00	RX0	C
ATOM	909	CG	LEU	252	20.298	-2.957	34.975	1.00	0.00	RX0	C
ATOM	910	CD1	LEU	252	20.449	-3.993	36.086	1.00	0.00	RX0	C
ATOM	911	CD2	LEU	252	18.831	-2.608	34.727	1.00	0.00	RX0	C
ATOM	912	C	LEU	252	23.007	-2.330	33.663	1.00	0.00	RX0	C
ATOM	913	O	LEU	252	22.274	-2.025	32.731	1.00	0.00	RX0	O

ATOM	914	N	PHE	253	24.144	-3.005	33.506	1.00	0.00	RX0	N
ATOM	915	H	PHE	253	24.631	-3.308	34.330	1.00	0.00	RX0	H
ATOM	916	CA	PHE	253	24.599	-3.457	32.174	1.00	0.00	RX0	C
ATOM	917	CB	PHE	253	25.969	-4.123	32.270	1.00	0.00	RX0	C
ATOM	918	CG	PHE	253	27.033	-3.091	32.538	1.00	0.00	RX0	C
ATOM	919	CD1	PHE	253	27.562	-2.362	31.480	1.00	0.00	RX0	C
ATOM	920	CD2	PHE	253	27.490	-2.877	33.831	1.00	0.00	RX0	C
ATOM	921	CE1	PHE	253	28.560	-1.425	31.713	1.00	0.00	RX0	C
ATOM	922	CE2	PHE	253	28.487	-1.939	34.063	1.00	0.00	RX0	C
ATOM	923	CZ	PHE	253	29.024	-1.216	33.005	1.00	0.00	RX0	C
ATOM	924	C	PHE	253	23.603	-4.446	31.564	1.00	0.00	RX0	C
ATOM	925	O	PHE	253	23.259	-4.379	30.390	1.00	0.00	RX0	O
ATOM	926	N	ALA	254	23.094	-5.300	32.445	1.00	0.00	RX0	N
ATOM	927	H	ALA	254	23.408	-5.311	33.400	1.00	0.00	RX0	H
ATOM	928	CA	ALA	254	22.050	-6.280	32.141	1.00	0.00	RX0	C
ATOM	929	CB	ALA	254	22.697	-7.549	31.592	1.00	0.00	RX0	C
ATOM	930	C	ALA	254	21.288	-6.584	33.440	1.00	0.00	RX0	C
ATOM	931	O	ALA	254	21.887	-6.418	34.526	1.00	0.00	RX0	O
ATOM	932	N	PRO	255	20.056	-7.064	33.372	1.00	0.00	RX0	N
ATOM	933	CD	PRO	255	19.307	-7.243	32.131	1.00	0.00	RX0	C
ATOM	934	CA	PRO	255	19.236	-7.444	34.545	1.00	0.00	RX0	C
ATOM	935	CB	PRO	255	17.987	-8.068	33.924	1.00	0.00	RX0	C
ATOM	936	CG	PRO	255	17.856	-7.385	32.569	1.00	0.00	RX0	C
ATOM	937	C	PRO	255	19.972	-8.395	35.506	1.00	0.00	RX0	C
ATOM	938	O	PRO	255	19.756	-8.342	36.714	1.00	0.00	RX0	O
ATOM	939	N	ASN	256	20.900	-9.179	34.970	1.00	0.00	RX0	N
ATOM	940	H	ASN	256	21.115	-9.136	33.993	1.00	0.00	RX0	H
ATOM	941	CA	ASN	256	21.722	-10.125	35.761	1.00	0.00	RX0	C
ATOM	942	CB	ASN	256	21.709	-11.529	35.150	1.00	0.00	RX0	C
ATOM	943	CG	ASN	256	22.399	-11.535	33.795	1.00	0.00	RX0	C
ATOM	944	OD1	ASN	256	22.294	-10.594	33.008	1.00	0.00	RX0	O
ATOM	945	ND2	ASN	256	23.022	-12.695	33.515	1.00	0.00	RX0	N
ATOM	946	HD21	ASN	256	23.136	-13.382	34.236	1.00	0.00	RX0	H
ATOM	947	HD22	ASN	256	23.376	-12.939	32.606	1.00	0.00	RX0	H
ATOM	948	C	ASN	256	23.191	-9.669	35.876	1.00	0.00	RX0	C
ATOM	949	O	ASN	256	24.101	-10.505	36.011	1.00	0.00	RX0	O
ATOM	950	N	LEU	257	23.439	-8.384	35.742	1.00	0.00	RX0	N
ATOM	951	H	LEU	257	22.692	-7.726	35.628	1.00	0.00	RX0	H
ATOM	952	CA	LEU	257	24.796	-7.805	35.783	1.00	0.00	RX0	C
ATOM	953	CB	LEU	257	25.513	-7.966	34.443	1.00	0.00	RX0	C
ATOM	954	CG	LEU	257	27.024	-7.763	34.565	1.00	0.00	RX0	C
ATOM	955	CD1	LEU	257	27.654	-8.808	35.488	1.00	0.00	RX0	C
ATOM	956	CD2	LEU	257	27.713	-7.723	33.201	1.00	0.00	RX0	C
ATOM	957	C	LEU	257	24.720	-6.327	36.168	1.00	0.00	RX0	C
ATOM	958	O	LEU	257	24.738	-5.412	35.328	1.00	0.00	RX0	O
ATOM	959	N	LEU	258	24.604	-6.148	37.469	1.00	0.00	RX0	N
ATOM	960	H	LEU	258	24.689	-6.942	38.070	1.00	0.00	RX0	H
ATOM	961	CA	LEU	258	24.482	-4.834	38.118	1.00	0.00	RX0	C
ATOM	962	CB	LEU	258	23.222	-4.903	38.981	1.00	0.00	RX0	C
ATOM	963	CG	LEU	258	22.814	-3.612	39.678	1.00	0.00	RX0	C
ATOM	964	CD1	LEU	258	22.656	-2.462	38.693	1.00	0.00	RX0	C
ATOM	965	CD2	LEU	258	21.556	-3.804	40.525	1.00	0.00	RX0	C
ATOM	966	C	LEU	258	25.743	-4.564	38.938	1.00	0.00	RX0	C
ATOM	967	O	LEU	258	26.013	-5.237	39.948	1.00	0.00	RX0	O
ATOM	968	N	LEU	259	26.528	-3.622	38.460	1.00	0.00	RX0	N
ATOM	969	H	LEU	259	26.218	-3.062	37.684	1.00	0.00	RX0	H
ATOM	970	CA	LEU	259	27.862	-3.349	39.027	1.00	0.00	RX0	C
ATOM	971	CB	LEU	259	28.937	-3.489	37.946	1.00	0.00	RX0	C
ATOM	972	CG	LEU	259	28.872	-4.796	37.149	1.00	0.00	RX0	C
ATOM	973	CD1	LEU	259	29.885	-4.807	36.004	1.00	0.00	RX0	C
ATOM	974	CD2	LEU	259	29.018	-6.031	38.037	1.00	0.00	RX0	C

ATOM	975	C	LEU	259	27.958	-1.956	39.652	1.00	0.00	RX0	C
ATOM	976	O	LEU	259	27.419	-0.984	39.137	1.00	0.00	RX0	O
ATOM	977	N	ASP	260	28.645	-1.912	40.785	1.00	0.00	RX0	N
ATOM	978	H	ASP	260	29.225	-2.688	41.045	1.00	0.00	RX0	H
ATOM	979	CA	ASP	260	29.043	-0.657	41.454	1.00	0.00	RX0	C
ATOM	980	CB	ASP	260	29.070	-0.858	42.963	1.00	0.00	RX0	C
ATOM	981	CG	ASP	260	29.780	-2.163	43.228	1.00	0.00	RX0	C
ATOM	982	OD1	ASP	260	31.008	-2.177	43.263	1.00	0.00	RX0	O
ATOM	983	OD2	ASP	260	29.093	-3.177	43.353	1.00	0.00	RX0	O
ATOM	984	C	ASP	260	30.443	-0.222	40.970	1.00	0.00	RX0	C
ATOM	985	O	ASP	260	31.127	-1.008	40.295	1.00	0.00	RX0	O
ATOM	986	N	ARG	261	30.963	0.873	41.504	1.00	0.00	RX0	N
ATOM	987	H	ARG	261	30.383	1.401	42.122	1.00	0.00	RX0	H
ATOM	988	CA	ARG	261	32.263	1.408	41.044	1.00	0.00	RX0	C
ATOM	989	CB	ARG	261	32.393	2.884	41.425	1.00	0.00	RX0	C
ATOM	990	CG	ARG	261	32.377	3.160	42.926	1.00	0.00	RX0	C
ATOM	991	CD	ARG	261	31.998	4.608	43.240	1.00	0.00	RX0	C
ATOM	992	NE	ARG	261	32.846	5.570	42.540	1.00	0.00	RX0	N
ATOM	993	HE	ARG	261	32.836	5.626	41.533	1.00	0.00	RX0	H
ATOM	994	CZ	ARG	261	33.576	6.480	43.209	1.00	0.00	RX0	C
ATOM	995	NH1	ARG	261	33.635	6.428	44.534	1.00	0.00	RX0	N
ATOM	996	HH11	ARG	261	34.104	7.148	45.061	1.00	0.00	RX0	H
ATOM	997	HH12	ARG	261	33.193	5.693	45.041	1.00	0.00	RX0	H
ATOM	998	NH2	ARG	261	34.222	7.428	42.543	1.00	0.00	RX0	N
ATOM	999	HH21	ARG	261	34.775	8.137	42.976	1.00	0.00	RX0	H
ATOM	1000	HH22	ARG	261	34.159	7.453	41.527	1.00	0.00	RX0	H
ATOM	1001	C	ARG	261	33.476	0.540	41.436	1.00	0.00	RX0	C
ATOM	1002	O	ARG	261	34.378	0.347	40.637	1.00	0.00	RX0	O
ATOM	1003	N	ASN	262	33.410	-0.067	42.632	1.00	0.00	RX0	N
ATOM	1004	H	ASN	262	32.529	-0.059	43.108	1.00	0.00	RX0	H
ATOM	1005	CA	ASN	262	34.456	-1.000	43.094	1.00	0.00	RX0	C
ATOM	1006	CB	ASN	262	34.154	-1.490	44.503	1.00	0.00	RX0	C
ATOM	1007	CG	ASN	262	35.244	-2.458	44.906	1.00	0.00	RX0	C
ATOM	1008	OD1	ASN	262	36.349	-2.039	45.257	1.00	0.00	RX0	O
ATOM	1009	ND2	ASN	262	34.872	-3.750	44.880	1.00	0.00	RX0	N
ATOM	1010	HD21	ASN	262	33.940	-4.006	44.612	1.00	0.00	RX0	H
ATOM	1011	HD22	ASN	262	35.489	-4.504	45.110	1.00	0.00	RX0	H
ATOM	1012	C	ASN	262	34.635	-2.233	42.204	1.00	0.00	RX0	C
ATOM	1013	O	ASN	262	35.755	-2.665	41.964	1.00	0.00	RX0	O
ATOM	1014	N	GLN	263	33.530	-2.679	41.603	1.00	0.00	RX0	N
ATOM	1015	H	GLN	263	32.638	-2.271	41.825	1.00	0.00	RX0	H
ATOM	1016	CA	GLN	263	33.559	-3.756	40.599	1.00	0.00	RX0	C
ATOM	1017	CB	GLN	263	32.195	-4.418	40.448	1.00	0.00	RX0	C
ATOM	1018	CG	GLN	263	31.901	-5.142	41.760	1.00	0.00	RX0	C
ATOM	1019	CD	GLN	263	30.681	-6.020	41.631	1.00	0.00	RX0	C
ATOM	1020	OE1	GLN	263	30.571	-6.865	40.751	1.00	0.00	RX0	O
ATOM	1021	NE2	GLN	263	29.784	-5.814	42.606	1.00	0.00	RX0	N
ATOM	1022	HE21	GLN	263	29.917	-5.015	43.207	1.00	0.00	RX0	H
ATOM	1023	HE22	GLN	263	29.014	-6.432	42.751	1.00	0.00	RX0	H
ATOM	1024	C	GLN	263	34.189	-3.308	39.265	1.00	0.00	RX0	C
ATOM	1025	O	GLN	263	34.644	-4.116	38.479	1.00	0.00	RX0	O
ATOM	1026	N	GLY	264	34.180	-1.978	39.039	1.00	0.00	RX0	N
ATOM	1027	H	GLY	264	33.765	-1.377	39.721	1.00	0.00	RX0	H
ATOM	1028	CA	GLY	264	34.831	-1.337	37.881	1.00	0.00	RX0	C
ATOM	1029	C	GLY	264	36.364	-1.310	37.978	1.00	0.00	RX0	C
ATOM	1030	O	GLY	264	37.050	-1.510	36.977	1.00	0.00	RX0	O
ATOM	1031	N	LYS	265	36.881	-1.155	39.202	1.00	0.00	RX0	N
ATOM	1032	H	LYS	265	36.232	-1.017	39.951	1.00	0.00	RX0	H
ATOM	1033	CA	LYS	265	38.336	-1.184	39.469	1.00	0.00	RX0	C
ATOM	1034	CB	LYS	265	38.649	-1.087	40.954	1.00	0.00	RX0	C
ATOM	1035	CG	LYS	265	38.076	0.065	41.769	1.00	0.00	RX0	C

ATOM	1036	CD	LYS	265	38.417	-0.266	43.220	1.00	0.00	RX0	C
ATOM	1037	CE	LYS	265	37.843	0.663	44.282	1.00	0.00	RX0	C
ATOM	1038	NZ	LYS	265	38.015	-0.017	45.573	1.00	0.00	RX0	N
ATOM	1039	HZ1	LYS	265	37.565	0.519	46.339	1.00	0.00	RX0	H
ATOM	1040	HZ2	LYS	265	37.555	-0.953	45.510	1.00	0.00	RX0	H
ATOM	1041	HZ3	LYS	265	39.023	-0.161	45.773	1.00	0.00	RX0	H
ATOM	1042	C	LYS	265	38.994	-2.500	39.030	1.00	0.00	RX0	C
ATOM	1043	O	LYS	265	40.184	-2.535	38.765	1.00	0.00	RX0	O
ATOM	1044	N	CYS	266	38.153	-3.548	38.917	1.00	0.00	RX0	N
ATOM	1045	H	CYS	266	37.198	-3.456	39.196	1.00	0.00	RX0	H
ATOM	1046	CA	CYS	266	38.538	-4.854	38.355	1.00	0.00	RX0	C
ATOM	1047	CB	CYS	266	37.315	-5.759	38.262	1.00	0.00	RX0	C
ATOM	1048	SG	CYS	266	36.553	-5.950	39.895	1.00	0.00	RX0	S
ATOM	1049	C	CYS	266	39.318	-4.732	37.033	1.00	0.00	RX0	C
ATOM	1050	O	CYS	266	40.108	-5.603	36.695	1.00	0.00	RX0	O
ATOM	1051	N	VAL	267	39.075	-3.636	36.304	1.00	0.00	RX0	N
ATOM	1052	H	VAL	267	38.469	-2.903	36.621	1.00	0.00	RX0	H
ATOM	1053	CA	VAL	267	39.804	-3.345	35.058	1.00	0.00	RX0	C
ATOM	1054	CB	VAL	267	38.857	-3.352	33.851	1.00	0.00	RX0	C
ATOM	1055	CG1	VAL	267	39.587	-3.008	32.550	1.00	0.00	RX0	C
ATOM	1056	CG2	VAL	267	38.128	-4.694	33.738	1.00	0.00	RX0	C
ATOM	1057	C	VAL	267	40.557	-2.013	35.191	1.00	0.00	RX0	C
ATOM	1058	O	VAL	267	39.969	-0.956	35.468	1.00	0.00	RX0	O
ATOM	1059	N	GLU	268	41.829	-2.078	34.821	1.00	0.00	RX0	N
ATOM	1060	H	GLU	268	42.209	-2.964	34.565	1.00	0.00	RX0	H
ATOM	1061	CA	GLU	268	42.726	-0.908	34.727	1.00	0.00	RX0	C
ATOM	1062	CB	GLU	268	44.098	-1.330	34.210	1.00	0.00	RX0	C
ATOM	1063	CG	GLU	268	45.133	-0.212	34.332	1.00	0.00	RX0	C
ATOM	1064	CD	GLU	268	46.502	-0.783	34.037	1.00	0.00	RX0	C
ATOM	1065	OE1	GLU	268	46.600	-1.996	33.861	1.00	0.00	RX0	O
ATOM	1066	OE2	GLU	268	47.463	-0.017	33.998	1.00	0.00	RX0	O
ATOM	1067	C	GLU	268	42.079	0.194	33.866	1.00	0.00	RX0	C
ATOM	1068	O	GLU	268	41.697	-0.027	32.727	1.00	0.00	RX0	O
ATOM	1069	N	GLY	269	41.924	1.355	34.524	1.00	0.00	RX0	N
ATOM	1070	H	GLY	269	42.198	1.393	35.483	1.00	0.00	RX0	H
ATOM	1071	CA	GLY	269	41.377	2.576	33.902	1.00	0.00	RX0	C
ATOM	1072	C	GLY	269	39.898	2.490	33.494	1.00	0.00	RX0	C
ATOM	1073	O	GLY	269	39.424	3.343	32.745	1.00	0.00	RX0	O
ATOM	1074	N	MET	270	39.146	1.598	34.134	1.00	0.00	RX0	N
ATOM	1075	H	MET	270	39.580	0.948	34.761	1.00	0.00	RX0	H
ATOM	1076	CA	MET	270	37.704	1.465	33.841	1.00	0.00	RX0	C
ATOM	1077	CB	MET	270	37.280	0.008	33.653	1.00	0.00	RX0	C
ATOM	1078	CG	MET	270	35.924	-0.098	32.950	1.00	0.00	RX0	C
ATOM	1079	SD	MET	270	35.417	-1.787	32.584	1.00	0.00	RX0	S
ATOM	1080	CE	MET	270	35.156	-2.355	34.269	1.00	0.00	RX0	C
ATOM	1081	C	MET	270	36.825	2.181	34.877	1.00	0.00	RX0	C
ATOM	1082	O	MET	270	35.781	2.734	34.514	1.00	0.00	RX0	O
ATOM	1083	N	VAL	271	37.310	2.290	36.107	1.00	0.00	RX0	N
ATOM	1084	H	VAL	271	38.225	1.928	36.281	1.00	0.00	RX0	H
ATOM	1085	CA	VAL	271	36.591	3.043	37.171	1.00	0.00	RX0	C
ATOM	1086	CB	VAL	271	37.252	2.870	38.550	1.00	0.00	RX0	C
ATOM	1087	CG1	VAL	271	38.731	3.244	38.560	1.00	0.00	RX0	C
ATOM	1088	CG2	VAL	271	36.444	3.570	39.646	1.00	0.00	RX0	C
ATOM	1089	C	VAL	271	36.343	4.502	36.749	1.00	0.00	RX0	C
ATOM	1090	O	VAL	271	35.261	5.071	37.095	1.00	0.00	RX0	O
ATOM	1091	N	GLU	272	37.228	5.074	36.011	1.00	0.00	RX0	N
ATOM	1092	H	GLU	272	38.114	4.616	35.905	1.00	0.00	RX0	H
ATOM	1093	CA	GLU	272	37.141	6.453	35.460	1.00	0.00	RX0	C
ATOM	1094	CB	GLU	272	38.390	6.761	34.625	1.00	0.00	RX0	C
ATOM	1095	CG	GLU	272	39.682	7.054	35.394	1.00	0.00	RX0	C
ATOM	1096	CD	GLU	272	39.967	5.954	36.390	1.00	0.00	RX0	C

ATOM	1097	OE1	GLU	272	40.124	4.804	35.978	1.00	0.00	RX0	O
ATOM	1098	OE2	GLU	272	39.975	6.243	37.583	1.00	0.00	RX0	O
ATOM	1099	C	GLU	272	35.927	6.588	34.526	1.00	0.00	RX0	C
ATOM	1100	O	GLU	272	35.142	7.521	34.681	1.00	0.00	RX0	O
ATOM	1101	N	ILE	273	35.702	5.552	33.729	1.00	0.00	RX0	N
ATOM	1102	H	ILE	273	36.301	4.755	33.821	1.00	0.00	RX0	H
ATOM	1103	CA	ILE	273	34.533	5.484	32.820	1.00	0.00	RX0	C
ATOM	1104	CB	ILE	273	34.734	4.378	31.779	1.00	0.00	RX0	C
ATOM	1105	CG2	ILE	273	33.623	4.410	30.729	1.00	0.00	RX0	C
ATOM	1106	CG1	ILE	273	36.127	4.436	31.145	1.00	0.00	RX0	C
ATOM	1107	CD1	ILE	273	36.340	5.668	30.265	1.00	0.00	RX0	C
ATOM	1108	C	ILE	273	33.249	5.235	33.628	1.00	0.00	RX0	C
ATOM	1109	O	ILE	273	32.257	5.951	33.452	1.00	0.00	RX0	O
ATOM	1110	N	PHE	274	33.320	4.302	34.575	1.00	0.00	RX0	N
ATOM	1111	H	PHE	274	34.185	3.809	34.680	1.00	0.00	RX0	H
ATOM	1112	CA	PHE	274	32.191	3.976	35.472	1.00	0.00	RX0	C
ATOM	1113	CB	PHE	274	32.607	2.931	36.510	1.00	0.00	RX0	C
ATOM	1114	CG	PHE	274	32.270	1.533	36.056	1.00	0.00	RX0	C
ATOM	1115	CD1	PHE	274	32.531	1.127	34.754	1.00	0.00	RX0	C
ATOM	1116	CD2	PHE	274	31.693	0.648	36.960	1.00	0.00	RX0	C
ATOM	1117	CE1	PHE	274	32.222	-0.169	34.360	1.00	0.00	RX0	C
ATOM	1118	CE2	PHE	274	31.385	-0.648	36.567	1.00	0.00	RX0	C
ATOM	1119	CZ	PHE	274	31.658	-1.058	35.268	1.00	0.00	RX0	C
ATOM	1120	C	PHE	274	31.669	5.203	36.222	1.00	0.00	RX0	C
ATOM	1121	O	PHE	274	30.484	5.521	36.143	1.00	0.00	RX0	O
ATOM	1122	N	ASP	275	32.607	5.983	36.757	1.00	0.00	RX0	N
ATOM	1123	H	ASP	275	33.557	5.670	36.777	1.00	0.00	RX0	H
ATOM	1124	CA	ASP	275	32.273	7.222	37.483	1.00	0.00	RX0	C
ATOM	1125	CB	ASP	275	33.563	7.901	37.973	1.00	0.00	RX0	C
ATOM	1126	CG	ASP	275	33.918	7.612	39.421	1.00	0.00	RX0	C
ATOM	1127	OD1	ASP	275	33.420	6.654	40.001	1.00	0.00	RX0	O
ATOM	1128	OD2	ASP	275	34.705	8.364	39.996	1.00	0.00	RX0	O
ATOM	1129	C	ASP	275	31.555	8.270	36.629	1.00	0.00	RX0	C
ATOM	1130	O	ASP	275	30.604	8.890	37.102	1.00	0.00	RX0	O
ATOM	1131	N	MET	276	31.921	8.336	35.353	1.00	0.00	RX0	N
ATOM	1132	H	MET	276	32.641	7.718	35.035	1.00	0.00	RX0	H
ATOM	1133	CA	MET	276	31.257	9.254	34.408	1.00	0.00	RX0	C
ATOM	1134	CB	MET	276	32.108	9.473	33.159	1.00	0.00	RX0	C
ATOM	1135	CG	MET	276	33.409	10.219	33.465	1.00	0.00	RX0	C
ATOM	1136	SD	MET	276	34.327	10.636	31.974	1.00	0.00	RX0	S
ATOM	1137	CE	MET	276	34.437	8.975	31.294	1.00	0.00	RX0	C
ATOM	1138	C	MET	276	29.833	8.798	34.061	1.00	0.00	RX0	C
ATOM	1139	O	MET	276	28.893	9.589	34.158	1.00	0.00	RX0	O
ATOM	1140	N	LEU	277	29.673	7.488	33.891	1.00	0.00	RX0	N
ATOM	1141	H	LEU	277	30.488	6.907	33.946	1.00	0.00	RX0	H
ATOM	1142	CA	LEU	277	28.362	6.866	33.606	1.00	0.00	RX0	C
ATOM	1143	CB	LEU	277	28.562	5.403	33.223	1.00	0.00	RX0	C
ATOM	1144	CG	LEU	277	29.434	5.227	31.980	1.00	0.00	RX0	C
ATOM	1145	CD1	LEU	277	29.922	3.787	31.825	1.00	0.00	RX0	C
ATOM	1146	CD2	LEU	277	28.736	5.738	30.722	1.00	0.00	RX0	C
ATOM	1147	C	LEU	277	27.393	6.992	34.787	1.00	0.00	RX0	C
ATOM	1148	O	LEU	277	26.257	7.447	34.627	1.00	0.00	RX0	O
ATOM	1149	N	LEU	278	27.939	6.795	35.983	1.00	0.00	RX0	N
ATOM	1150	H	LEU	278	28.893	6.487	36.003	1.00	0.00	RX0	H
ATOM	1151	CA	LEU	278	27.196	6.926	37.250	1.00	0.00	RX0	C
ATOM	1152	CB	LEU	278	28.053	6.458	38.425	1.00	0.00	RX0	C
ATOM	1153	CG	LEU	278	28.232	4.944	38.482	1.00	0.00	RX0	C
ATOM	1154	CD1	LEU	278	29.297	4.527	39.497	1.00	0.00	RX0	C
ATOM	1155	CD2	LEU	278	26.899	4.250	38.742	1.00	0.00	RX0	C
ATOM	1156	C	LEU	278	26.716	8.359	37.508	1.00	0.00	RX0	C
ATOM	1157	O	LEU	278	25.554	8.575	37.840	1.00	0.00	RX0	O

ATOM	1158	N	ALA	279	27.582	9.317	37.175	1.00	0.00	RX0	N
ATOM	1159	H	ALA	279	28.514	9.042	36.921	1.00	0.00	RX0	H
ATOM	1160	CA	ALA	279	27.275	10.754	37.296	1.00	0.00	RX0	C
ATOM	1161	CB	ALA	279	28.524	11.570	36.983	1.00	0.00	RX0	C
ATOM	1162	C	ALA	279	26.182	11.196	36.310	1.00	0.00	RX0	C
ATOM	1163	O	ALA	279	25.263	11.922	36.684	1.00	0.00	RX0	O
ATOM	1164	N	THR	280	26.210	10.618	35.109	1.00	0.00	RX0	N
ATOM	1165	H	THR	280	26.995	10.031	34.902	1.00	0.00	RX0	H
ATOM	1166	CA	THR	280	25.203	10.899	34.059	1.00	0.00	RX0	C
ATOM	1167	CB	THR	280	25.701	10.368	32.712	1.00	0.00	RX0	C
ATOM	1168	OG1	THR	280	27.025	10.859	32.461	1.00	0.00	RX0	O
ATOM	1169	HG1	THR	280	27.631	10.381	33.020	1.00	0.00	RX0	H
ATOM	1170	CG2	THR	280	24.771	10.757	31.558	1.00	0.00	RX0	C
ATOM	1171	C	THR	280	23.835	10.327	34.462	1.00	0.00	RX0	C
ATOM	1172	O	THR	280	22.822	11.023	34.397	1.00	0.00	RX0	O
ATOM	1173	N	SER	281	23.868	9.112	35.003	1.00	0.00	RX0	N
ATOM	1174	H	SER	281	24.738	8.615	35.051	1.00	0.00	RX0	H
ATOM	1175	CA	SER	281	22.669	8.413	35.507	1.00	0.00	RX0	C
ATOM	1176	CB	SER	281	23.140	6.978	35.861	1.00	0.00	RX0	C
ATOM	1177	OG	SER	281	22.402	6.342	36.923	1.00	0.00	RX0	O
ATOM	1178	HG	SER	281	22.620	5.415	36.848	1.00	0.00	RX0	H
ATOM	1179	C	SER	281	22.019	9.180	36.675	1.00	0.00	RX0	C
ATOM	1180	O	SER	281	20.814	9.399	36.693	1.00	0.00	RX0	O
ATOM	1181	N	SER	282	22.889	9.788	37.491	1.00	0.00	RX0	N
ATOM	1182	H	SER	282	23.866	9.617	37.351	1.00	0.00	RX0	H
ATOM	1183	CA	SER	282	22.489	10.660	38.613	1.00	0.00	RX0	C
ATOM	1184	CB	SER	282	23.661	10.790	39.574	1.00	0.00	RX0	C
ATOM	1185	OG	SER	282	23.980	9.444	39.974	1.00	0.00	RX0	O
ATOM	1186	HG	SER	282	24.726	9.188	39.427	1.00	0.00	RX0	H
ATOM	1187	C	SER	282	21.828	11.963	38.130	1.00	0.00	RX0	C
ATOM	1188	O	SER	282	20.788	12.371	38.639	1.00	0.00	RX0	O
ATOM	1189	N	ARG	283	22.365	12.516	37.039	1.00	0.00	RX0	N
ATOM	1190	H	ARG	283	23.205	12.117	36.665	1.00	0.00	RX0	H
ATOM	1191	CA	ARG	283	21.822	13.735	36.412	1.00	0.00	RX0	C
ATOM	1192	CB	ARG	283	22.820	14.245	35.368	1.00	0.00	RX0	C
ATOM	1193	CG	ARG	283	22.444	15.560	34.686	1.00	0.00	RX0	C
ATOM	1194	CD	ARG	283	22.258	16.723	35.661	1.00	0.00	RX0	C
ATOM	1195	NE	ARG	283	22.067	17.963	34.916	1.00	0.00	RX0	N
ATOM	1196	HE	ARG	283	22.597	18.108	34.074	1.00	0.00	RX0	H
ATOM	1197	CZ	ARG	283	21.297	18.974	35.360	1.00	0.00	RX0	C
ATOM	1198	NH1	ARG	283	20.635	18.884	36.509	1.00	0.00	RX0	N
ATOM	1199	HH11	ARG	283	20.163	19.683	36.887	1.00	0.00	RX0	H
ATOM	1200	HH12	ARG	283	20.624	18.032	37.030	1.00	0.00	RX0	H
ATOM	1201	NH2	ARG	283	21.232	20.072	34.627	1.00	0.00	RX0	N
ATOM	1202	HH21	ARG	283	20.580	20.821	34.801	1.00	0.00	RX0	H
ATOM	1203	HH22	ARG	283	21.866	20.172	33.855	1.00	0.00	RX0	H
ATOM	1204	C	ARG	283	20.432	13.477	35.812	1.00	0.00	RX0	C
ATOM	1205	O	ARG	283	19.498	14.246	36.035	1.00	0.00	RX0	O
ATOM	1206	N	PHE	284	20.293	12.325	35.169	1.00	0.00	RX0	N
ATOM	1207	H	PHE	284	21.097	11.741	35.032	1.00	0.00	RX0	H
ATOM	1208	CA	PHE	284	19.003	11.899	34.606	1.00	0.00	RX0	C
ATOM	1209	CB	PHE	284	19.202	10.645	33.762	1.00	0.00	RX0	C
ATOM	1210	CG	PHE	284	19.642	11.031	32.372	1.00	0.00	RX0	C
ATOM	1211	CD1	PHE	284	19.201	12.223	31.812	1.00	0.00	RX0	C
ATOM	1212	CD2	PHE	284	20.465	10.182	31.642	1.00	0.00	RX0	C
ATOM	1213	CE1	PHE	284	19.550	12.549	30.507	1.00	0.00	RX0	C
ATOM	1214	CE2	PHE	284	20.817	10.511	30.338	1.00	0.00	RX0	C
ATOM	1215	CZ	PHE	284	20.345	11.685	29.764	1.00	0.00	RX0	C
ATOM	1216	C	PHE	284	17.921	11.680	35.654	1.00	0.00	RX0	C
ATOM	1217	O	PHE	284	16.817	12.216	35.524	1.00	0.00	RX0	O
ATOM	1218	N	ARG	285	18.348	11.087	36.760	1.00	0.00	RX0	N

ATOM	1219	H	ARG	285	19.281	10.722	36.764	1.00	0.00	RX0	H
ATOM	1220	CA	ARG	285	17.485	10.841	37.923	1.00	0.00	RX0	C
ATOM	1221	CB	ARG	285	18.335	10.060	38.926	1.00	0.00	RX0	C
ATOM	1222	CG	ARG	285	17.771	9.791	40.322	1.00	0.00	RX0	C
ATOM	1223	CD	ARG	285	18.802	9.035	41.172	1.00	0.00	RX0	C
ATOM	1224	NE	ARG	285	19.225	7.830	40.461	1.00	0.00	RX0	N
ATOM	1225	HE	ARG	285	18.472	7.232	40.157	1.00	0.00	RX0	H
ATOM	1226	CZ	ARG	285	20.519	7.658	40.057	1.00	0.00	RX0	C
ATOM	1227	NH1	ARG	285	21.483	8.436	40.596	1.00	0.00	RX0	N
ATOM	1228	HH11	ARG	285	22.451	8.435	40.297	1.00	0.00	RX0	H
ATOM	1229	HH12	ARG	285	21.269	9.085	41.331	1.00	0.00	RX0	H
ATOM	1230	NH2	ARG	285	20.786	6.726	39.117	1.00	0.00	RX0	N
ATOM	1231	HH21	ARG	285	21.675	6.595	38.656	1.00	0.00	RX0	H
ATOM	1232	HH22	ARG	285	20.045	6.118	38.806	1.00	0.00	RX0	H
ATOM	1233	C	ARG	285	17.003	12.164	38.534	1.00	0.00	RX0	C
ATOM	1234	O	ARG	285	15.822	12.321	38.816	1.00	0.00	RX0	O
ATOM	1235	N	MET	286	17.922	13.130	38.591	1.00	0.00	RX0	N
ATOM	1236	H	MET	286	18.855	12.926	38.289	1.00	0.00	RX0	H
ATOM	1237	CA	MET	286	17.643	14.467	39.144	1.00	0.00	RX0	C
ATOM	1238	CB	MET	286	18.971	15.217	39.288	1.00	0.00	RX0	C
ATOM	1239	CG	MET	286	18.863	16.640	39.841	1.00	0.00	RX0	C
ATOM	1240	SD	MET	286	20.422	17.531	39.727	1.00	0.00	RX0	S
ATOM	1241	CE	MET	286	19.849	19.095	40.410	1.00	0.00	RX0	C
ATOM	1242	C	MET	286	16.681	15.257	38.243	1.00	0.00	RX0	C
ATOM	1243	O	MET	286	15.799	15.962	38.735	1.00	0.00	RX0	O
ATOM	1244	N	MET	287	16.911	15.161	36.942	1.00	0.00	RX0	N
ATOM	1245	H	MET	287	17.637	14.546	36.628	1.00	0.00	RX0	H
ATOM	1246	CA	MET	287	16.049	15.814	35.941	1.00	0.00	RX0	C
ATOM	1247	CB	MET	287	16.714	15.895	34.570	1.00	0.00	RX0	C
ATOM	1248	CG	MET	287	17.899	16.859	34.585	1.00	0.00	RX0	C
ATOM	1249	SD	MET	287	18.366	17.355	32.924	1.00	0.00	RX0	S
ATOM	1250	CE	MET	287	16.750	17.975	32.428	1.00	0.00	RX0	C
ATOM	1251	C	MET	287	14.674	15.156	35.849	1.00	0.00	RX0	C
ATOM	1252	O	MET	287	13.755	15.729	35.264	1.00	0.00	RX0	O
ATOM	1253	N	ASN	288	14.583	13.920	36.346	1.00	0.00	RX0	N
ATOM	1254	H	ASN	288	15.394	13.491	36.745	1.00	0.00	RX0	H
ATOM	1255	CA	ASN	288	13.391	13.066	36.242	1.00	0.00	RX0	C
ATOM	1256	CB	ASN	288	12.160	13.611	36.968	1.00	0.00	RX0	C
ATOM	1257	CG	ASN	288	11.010	12.650	36.723	1.00	0.00	RX0	C
ATOM	1258	OD1	ASN	288	11.180	11.438	36.654	1.00	0.00	RX0	O
ATOM	1259	ND2	ASN	288	9.820	13.262	36.572	1.00	0.00	RX0	N
ATOM	1260	HD21	ASN	288	9.739	14.255	36.656	1.00	0.00	RX0	H
ATOM	1261	HD22	ASN	288	8.996	12.739	36.350	1.00	0.00	RX0	H
ATOM	1262	C	ASN	288	13.076	12.836	34.753	1.00	0.00	RX0	C
ATOM	1263	O	ASN	288	11.986	13.121	34.256	1.00	0.00	RX0	O
ATOM	1264	N	LEU	289	14.125	12.439	34.028	1.00	0.00	RX0	N
ATOM	1265	H	LEU	289	14.957	12.176	34.521	1.00	0.00	RX0	H
ATOM	1266	CA	LEU	289	14.030	12.183	32.583	1.00	0.00	RX0	C
ATOM	1267	CB	LEU	289	15.373	11.629	32.098	1.00	0.00	RX0	C
ATOM	1268	CG	LEU	289	15.415	11.224	30.622	1.00	0.00	RX0	C
ATOM	1269	CD1	LEU	289	15.553	12.435	29.706	1.00	0.00	RX0	C
ATOM	1270	CD2	LEU	289	16.499	10.185	30.336	1.00	0.00	RX0	C
ATOM	1271	C	LEU	289	12.926	11.152	32.321	1.00	0.00	RX0	C
ATOM	1272	O	LEU	289	12.814	10.137	33.014	1.00	0.00	RX0	O
ATOM	1273	N	GLN	290	12.165	11.420	31.281	1.00	0.00	RX0	N
ATOM	1274	H	GLN	290	12.393	12.178	30.664	1.00	0.00	RX0	H
ATOM	1275	CA	GLN	290	11.011	10.584	30.916	1.00	0.00	RX0	C
ATOM	1276	CB	GLN	290	9.778	11.454	30.677	1.00	0.00	RX0	C
ATOM	1277	CG	GLN	290	9.417	12.307	31.901	1.00	0.00	RX0	C
ATOM	1278	CD	GLN	290	8.988	11.441	33.078	1.00	0.00	RX0	C
ATOM	1279	OE1	GLN	290	7.825	11.089	33.232	1.00	0.00	RX0	O

ATOM	1280	NE2	GLN	290	9.979	11.153	33.939	1.00	0.00	RX0	N
ATOM	1281	HE21	GLN	290	10.909	11.496	33.790	1.00	0.00	RX0	H
ATOM	1282	HE22	GLN	290	9.883	10.606	34.773	1.00	0.00	RX0	H
ATOM	1283	C	GLN	290	11.379	9.701	29.727	1.00	0.00	RX0	C
ATOM	1284	O	GLN	290	12.115	10.141	28.832	1.00	0.00	RX0	O
ATOM	1285	N	GLY	291	10.739	8.533	29.672	1.00	0.00	RX0	N
ATOM	1286	H	GLY	291	10.024	8.355	30.349	1.00	0.00	RX0	H
ATOM	1287	CA	GLY	291	10.952	7.542	28.589	1.00	0.00	RX0	C
ATOM	1288	C	GLY	291	10.769	8.134	27.181	1.00	0.00	RX0	C
ATOM	1289	O	GLY	291	11.559	7.865	26.269	1.00	0.00	RX0	O
ATOM	1290	N	GLU	292	9.854	9.086	27.078	1.00	0.00	RX0	N
ATOM	1291	H	GLU	292	9.262	9.255	27.870	1.00	0.00	RX0	H
ATOM	1292	CA	GLU	292	9.541	9.789	25.813	1.00	0.00	RX0	C
ATOM	1293	CB	GLU	292	8.214	10.550	25.942	1.00	0.00	RX0	C
ATOM	1294	CG	GLU	292	6.980	9.709	26.311	1.00	0.00	RX0	C
ATOM	1295	CD	GLU	292	7.112	9.165	27.722	1.00	0.00	RX0	C
ATOM	1296	OE1	GLU	292	7.503	9.921	28.609	1.00	0.00	RX0	O
ATOM	1297	OE2	GLU	292	6.932	7.967	27.915	1.00	0.00	RX0	O
ATOM	1298	C	GLU	292	10.671	10.744	25.400	1.00	0.00	RX0	C
ATOM	1299	O	GLU	292	11.065	10.802	24.241	1.00	0.00	RX0	O
ATOM	1300	N	GLU	293	11.241	11.417	26.395	1.00	0.00	RX0	N
ATOM	1301	H	GLU	293	10.949	11.212	27.330	1.00	0.00	RX0	H
ATOM	1302	CA	GLU	293	12.389	12.320	26.184	1.00	0.00	RX0	C
ATOM	1303	CB	GLU	293	12.635	13.156	27.435	1.00	0.00	RX0	C
ATOM	1304	CG	GLU	293	11.404	13.910	27.931	1.00	0.00	RX0	C
ATOM	1305	CD	GLU	293	11.720	14.503	29.287	1.00	0.00	RX0	C
ATOM	1306	OE1	GLU	293	12.636	14.031	29.953	1.00	0.00	RX0	O
ATOM	1307	OE2	GLU	293	11.050	15.440	29.703	1.00	0.00	RX0	O
ATOM	1308	C	GLU	293	13.669	11.544	25.843	1.00	0.00	RX0	C
ATOM	1309	O	GLU	293	14.364	11.888	24.886	1.00	0.00	RX0	O
ATOM	1310	N	PHE	294	13.855	10.409	26.517	1.00	0.00	RX0	N
ATOM	1311	H	PHE	294	13.194	10.200	27.241	1.00	0.00	RX0	H
ATOM	1312	CA	PHE	294	14.999	9.503	26.300	1.00	0.00	RX0	C
ATOM	1313	CB	PHE	294	14.904	8.308	27.253	1.00	0.00	RX0	C
ATOM	1314	CG	PHE	294	15.862	7.224	26.816	1.00	0.00	RX0	C
ATOM	1315	CD1	PHE	294	17.234	7.444	26.839	1.00	0.00	RX0	C
ATOM	1316	CD2	PHE	294	15.362	6.004	26.374	1.00	0.00	RX0	C
ATOM	1317	CE1	PHE	294	18.101	6.453	26.394	1.00	0.00	RX0	C
ATOM	1318	CE2	PHE	294	16.230	5.013	25.931	1.00	0.00	RX0	C
ATOM	1319	CZ	PHE	294	17.601	5.242	25.933	1.00	0.00	RX0	C
ATOM	1320	C	PHE	294	15.115	9.012	24.847	1.00	0.00	RX0	C
ATOM	1321	O	PHE	294	16.186	9.132	24.238	1.00	0.00	RX0	O
ATOM	1322	N	VAL	295	14.000	8.581	24.280	1.00	0.00	RX0	N
ATOM	1323	H	VAL	295	13.158	8.569	24.828	1.00	0.00	RX0	H
ATOM	1324	CA	VAL	295	13.976	8.026	22.907	1.00	0.00	RX0	C
ATOM	1325	CB	VAL	295	12.686	7.251	22.616	1.00	0.00	RX0	C
ATOM	1326	CG1	VAL	295	12.586	6.064	23.571	1.00	0.00	RX0	C
ATOM	1327	CG2	VAL	295	11.430	8.119	22.660	1.00	0.00	RX0	C
ATOM	1328	C	VAL	295	14.286	9.097	21.847	1.00	0.00	RX0	C
ATOM	1329	O	VAL	295	14.999	8.845	20.884	1.00	0.00	RX0	O
ATOM	1330	N	CYS	296	13.866	10.330	22.156	1.00	0.00	RX0	N
ATOM	1331	H	CYS	296	13.305	10.480	22.973	1.00	0.00	RX0	H
ATOM	1332	CA	CYS	296	14.167	11.498	21.314	1.00	0.00	RX0	C
ATOM	1333	CB	CYS	296	13.264	12.653	21.730	1.00	0.00	RX0	C
ATOM	1334	SG	CYS	296	11.519	12.261	21.461	1.00	0.00	RX0	S
ATOM	1335	C	CYS	296	15.661	11.843	21.352	1.00	0.00	RX0	C
ATOM	1336	O	CYS	296	16.290	11.969	20.305	1.00	0.00	RX0	O
ATOM	1337	N	LEU	297	16.247	11.750	22.547	1.00	0.00	RX0	N
ATOM	1338	H	LEU	297	15.681	11.549	23.351	1.00	0.00	RX0	H
ATOM	1339	CA	LEU	297	17.681	12.031	22.752	1.00	0.00	RX0	C
ATOM	1340	CB	LEU	297	18.018	12.110	24.239	1.00	0.00	RX0	C

ATOM	1341	CG	LEU	297	17.351	13.287	24.947	1.00	0.00	RX0	C
ATOM	1342	CD1	LEU	297	17.640	13.265	26.447	1.00	0.00	RX0	C
ATOM	1343	CD2	LEU	297	17.726	14.626	24.309	1.00	0.00	RX0	C
ATOM	1344	C	LEU	297	18.589	11.009	22.068	1.00	0.00	RX0	C
ATOM	1345	O	LEU	297	19.526	11.385	21.359	1.00	0.00	RX0	O
ATOM	1346	N	LYS	298	18.173	9.750	22.123	1.00	0.00	RX0	N
ATOM	1347	H	LYS	298	17.360	9.546	22.672	1.00	0.00	RX0	H
ATOM	1348	CA	LYS	298	18.945	8.654	21.515	1.00	0.00	RX0	C
ATOM	1349	CB	LYS	298	18.432	7.320	22.046	1.00	0.00	RX0	C
ATOM	1350	CG	LYS	298	19.255	6.122	21.580	1.00	0.00	RX0	C
ATOM	1351	CD	LYS	298	18.615	4.835	22.086	1.00	0.00	RX0	C
ATOM	1352	CE	LYS	298	17.099	5.002	22.049	1.00	0.00	RX0	C
ATOM	1353	NZ	LYS	298	16.431	3.709	22.163	1.00	0.00	RX0	N
ATOM	1354	HZ1	LYS	298	15.438	3.866	22.460	1.00	0.00	RX0	H
ATOM	1355	HZ2	LYS	298	16.295	3.224	21.255	1.00	0.00	RX0	H
ATOM	1356	HZ3	LYS	298	16.791	3.061	22.885	1.00	0.00	RX0	H
ATOM	1357	C	LYS	298	18.925	8.733	19.978	1.00	0.00	RX0	C
ATOM	1358	O	LYS	298	19.964	8.557	19.332	1.00	0.00	RX0	O
ATOM	1359	N	SER	299	17.791	9.152	19.431	1.00	0.00	RX0	N
ATOM	1360	H	SER	299	16.965	9.268	19.982	1.00	0.00	RX0	H
ATOM	1361	CA	SER	299	17.645	9.383	17.977	1.00	0.00	RX0	C
ATOM	1362	CB	SER	299	16.155	9.361	17.703	1.00	0.00	RX0	C
ATOM	1363	OG	SER	299	15.660	8.203	18.383	1.00	0.00	RX0	O
ATOM	1364	HG	SER	299	15.127	8.511	19.109	1.00	0.00	RX0	H
ATOM	1365	C	SER	299	18.416	10.621	17.504	1.00	0.00	RX0	C
ATOM	1366	O	SER	299	19.051	10.583	16.444	1.00	0.00	RX0	O
ATOM	1367	N	ILE	300	18.478	11.644	18.354	1.00	0.00	RX0	N
ATOM	1368	H	ILE	300	17.957	11.595	19.209	1.00	0.00	RX0	H
ATOM	1369	CA	ILE	300	19.283	12.860	18.096	1.00	0.00	RX0	C
ATOM	1370	CB	ILE	300	19.081	13.940	19.163	1.00	0.00	RX0	C
ATOM	1371	CG2	ILE	300	20.113	15.062	19.022	1.00	0.00	RX0	C
ATOM	1372	CG1	ILE	300	17.672	14.515	19.097	1.00	0.00	RX0	C
ATOM	1373	CD1	ILE	300	17.419	15.545	20.196	1.00	0.00	RX0	C
ATOM	1374	C	ILE	300	20.773	12.490	18.013	1.00	0.00	RX0	C
ATOM	1375	O	ILE	300	21.456	12.924	17.087	1.00	0.00	RX0	O
ATOM	1376	N	ILE	301	21.227	11.630	18.921	1.00	0.00	RX0	N
ATOM	1377	H	ILE	301	20.606	11.327	19.647	1.00	0.00	RX0	H
ATOM	1378	CA	ILE	301	22.629	11.156	18.925	1.00	0.00	RX0	C
ATOM	1379	CB	ILE	301	22.796	10.176	20.077	1.00	0.00	RX0	C
ATOM	1380	CG2	ILE	301	23.998	9.260	19.878	1.00	0.00	RX0	C
ATOM	1381	CG1	ILE	301	22.827	10.933	21.399	1.00	0.00	RX0	C
ATOM	1382	CD1	ILE	301	22.872	9.983	22.591	1.00	0.00	RX0	C
ATOM	1383	C	ILE	301	22.948	10.474	17.584	1.00	0.00	RX0	C
ATOM	1384	O	ILE	301	23.927	10.823	16.926	1.00	0.00	RX0	O
ATOM	1385	N	LEU	302	22.047	9.582	17.175	1.00	0.00	RX0	N
ATOM	1386	H	LEU	302	21.273	9.375	17.776	1.00	0.00	RX0	H
ATOM	1387	CA	LEU	302	22.205	8.836	15.917	1.00	0.00	RX0	C
ATOM	1388	CB	LEU	302	20.992	7.929	15.709	1.00	0.00	RX0	C
ATOM	1389	CG	LEU	302	21.000	7.209	14.360	1.00	0.00	RX0	C
ATOM	1390	CD1	LEU	302	22.181	6.250	14.225	1.00	0.00	RX0	C
ATOM	1391	CD2	LEU	302	19.665	6.525	14.080	1.00	0.00	RX0	C
ATOM	1392	C	LEU	302	22.391	9.757	14.699	1.00	0.00	RX0	C
ATOM	1393	O	LEU	302	23.285	9.544	13.882	1.00	0.00	RX0	O
ATOM	1394	N	LEU	303	21.581	10.806	14.658	1.00	0.00	RX0	N
ATOM	1395	H	LEU	303	20.946	10.957	15.419	1.00	0.00	RX0	H
ATOM	1396	CA	LEU	303	21.546	11.719	13.502	1.00	0.00	RX0	C
ATOM	1397	CB	LEU	303	20.125	12.232	13.312	1.00	0.00	RX0	C
ATOM	1398	CG	LEU	303	19.162	11.067	13.085	1.00	0.00	RX0	C
ATOM	1399	CD1	LEU	303	17.707	11.489	13.253	1.00	0.00	RX0	C
ATOM	1400	CD2	LEU	303	19.418	10.367	11.748	1.00	0.00	RX0	C
ATOM	1401	C	LEU	303	22.576	12.849	13.543	1.00	0.00	RX0	C

ATOM	1402	O	LEU	303	23.073	13.268	12.494	1.00	0.00	RX0	O
ATOM	1403	N	ASN	304	22.971	13.240	14.745	1.00	0.00	RX0	N
ATOM	1404	H	ASN	304	22.646	12.743	15.552	1.00	0.00	RX0	H
ATOM	1405	CA	ASN	304	23.832	14.419	14.940	1.00	0.00	RX0	C
ATOM	1406	CB	ASN	304	23.536	15.135	16.254	1.00	0.00	RX0	C
ATOM	1407	CG	ASN	304	24.772	15.901	16.713	1.00	0.00	RX0	C
ATOM	1408	OD1	ASN	304	25.562	15.448	17.549	1.00	0.00	RX0	O
ATOM	1409	ND2	ASN	304	24.836	17.134	16.188	1.00	0.00	RX0	N
ATOM	1410	HD21	ASN	304	24.194	17.457	15.492	1.00	0.00	RX0	H
ATOM	1411	HD22	ASN	304	25.446	17.883	16.471	1.00	0.00	RX0	H
ATOM	1412	C	ASN	304	25.330	14.097	14.987	1.00	0.00	RX0	C
ATOM	1413	O	ASN	304	26.132	14.783	14.344	1.00	0.00	RX0	O
ATOM	1414	N	SER	305	25.693	13.015	15.656	1.00	0.00	RX0	N
ATOM	1415	H	SER	305	25.043	12.424	16.144	1.00	0.00	RX0	H
ATOM	1416	CA	SER	305	27.104	12.765	16.012	1.00	0.00	RX0	C
ATOM	1417	CB	SER	305	27.112	11.577	16.959	1.00	0.00	RX0	C
ATOM	1418	OG	SER	305	26.124	11.870	17.950	1.00	0.00	RX0	O
ATOM	1419	HG	SER	305	26.290	11.268	18.680	1.00	0.00	RX0	H
ATOM	1420	C	SER	305	28.103	12.695	14.846	1.00	0.00	RX0	C
ATOM	1421	O	SER	305	29.198	13.228	14.946	1.00	0.00	RX0	O
ATOM	1422	N	GLY	306	27.634	12.177	13.693	1.00	0.00	RX0	N
ATOM	1423	H	GLY	306	26.685	11.868	13.637	1.00	0.00	RX0	H
ATOM	1424	CA	GLY	306	28.500	12.079	12.501	1.00	0.00	RX0	C
ATOM	1425	C	GLY	306	28.091	12.961	11.315	1.00	0.00	RX0	C
ATOM	1426	O	GLY	306	28.756	12.897	10.274	1.00	0.00	RX0	O
ATOM	1427	N	VAL	307	27.242	13.954	11.536	1.00	0.00	RX0	N
ATOM	1428	H	VAL	307	26.877	14.103	12.458	1.00	0.00	RX0	H
ATOM	1429	CA	VAL	307	26.760	14.815	10.435	1.00	0.00	RX0	C
ATOM	1430	CB	VAL	307	25.417	15.480	10.776	1.00	0.00	RX0	C
ATOM	1431	CG1	VAL	307	25.552	16.616	11.793	1.00	0.00	RX0	C
ATOM	1432	CG2	VAL	307	24.714	15.936	9.498	1.00	0.00	RX0	C
ATOM	1433	C	VAL	307	27.809	15.841	9.948	1.00	0.00	RX0	C
ATOM	1434	O	VAL	307	27.790	16.280	8.811	1.00	0.00	RX0	O
ATOM	1435	N	TYR	308	28.719	16.199	10.859	1.00	0.00	RX0	N
ATOM	1436	H	TYR	308	28.744	15.712	11.731	1.00	0.00	RX0	H
ATOM	1437	CA	TYR	308	29.761	17.211	10.584	1.00	0.00	RX0	C
ATOM	1438	CB	TYR	308	30.231	17.855	11.880	1.00	0.00	RX0	C
ATOM	1439	CG	TYR	308	29.186	18.774	12.439	1.00	0.00	RX0	C
ATOM	1440	CD1	TYR	308	28.363	19.492	11.583	1.00	0.00	RX0	C
ATOM	1441	CE1	TYR	308	27.463	20.404	12.112	1.00	0.00	RX0	C
ATOM	1442	CD2	TYR	308	29.079	18.916	13.815	1.00	0.00	RX0	C
ATOM	1443	CE2	TYR	308	28.181	19.829	14.340	1.00	0.00	RX0	C
ATOM	1444	CZ	TYR	308	27.395	20.589	13.486	1.00	0.00	RX0	C
ATOM	1445	OH	TYR	308	26.552	21.540	14.007	1.00	0.00	RX0	O
ATOM	1446	HH	TYR	308	26.409	21.322	14.927	1.00	0.00	RX0	H
ATOM	1447	C	TYR	308	31.032	16.660	9.940	1.00	0.00	RX0	C
ATOM	1448	O	TYR	308	31.912	17.411	9.537	1.00	0.00	RX0	O
ATOM	1449	N	THR	309	31.091	15.334	9.861	1.00	0.00	RX0	N
ATOM	1450	H	THR	309	30.340	14.755	10.174	1.00	0.00	RX0	H
ATOM	1451	CA	THR	309	32.303	14.643	9.386	1.00	0.00	RX0	C
ATOM	1452	CB	THR	309	32.580	13.743	10.568	1.00	0.00	RX0	C
ATOM	1453	OG1	THR	309	31.328	13.585	11.262	1.00	0.00	RX0	O
ATOM	1454	HG1	THR	309	30.861	12.892	10.792	1.00	0.00	RX0	H
ATOM	1455	CG2	THR	309	33.628	14.336	11.511	1.00	0.00	RX0	C
ATOM	1456	C	THR	309	32.183	13.866	8.071	1.00	0.00	RX0	C
ATOM	1457	O	THR	309	33.137	13.195	7.681	1.00	0.00	RX0	O
ATOM	1458	N	PHE	310	31.041	13.953	7.382	1.00	0.00	RX0	N
ATOM	1459	H	PHE	310	30.308	14.532	7.733	1.00	0.00	RX0	H
ATOM	1460	CA	PHE	310	30.972	13.467	5.988	1.00	0.00	RX0	C
ATOM	1461	CB	PHE	310	29.594	13.727	5.375	1.00	0.00	RX0	C
ATOM	1462	CG	PHE	310	28.526	12.909	6.059	1.00	0.00	RX0	C

ATOM	1463	CD1	PHE	310	28.423	11.548	5.800	1.00	0.00	RX0	C
ATOM	1464	CD2	PHE	310	27.635	13.519	6.935	1.00	0.00	RX0	C
ATOM	1465	CE1	PHE	310	27.425	10.801	6.413	1.00	0.00	RX0	C
ATOM	1466	CE2	PHE	310	26.637	12.769	7.546	1.00	0.00	RX0	C
ATOM	1467	CZ	PHE	310	26.529	11.409	7.283	1.00	0.00	RX0	C
ATOM	1468	C	PHE	310	32.019	14.245	5.180	1.00	0.00	RX0	C
ATOM	1469	O	PHE	310	32.102	15.468	5.301	1.00	0.00	RX0	O
ATOM	1470	N	LEU	311	32.854	13.508	4.462	1.00	0.00	RX0	N
ATOM	1471	H	LEU	311	32.726	12.519	4.500	1.00	0.00	RX0	H
ATOM	1472	CA	LEU	311	33.988	14.087	3.701	1.00	0.00	RX0	C
ATOM	1473	CB	LEU	311	35.004	13.092	3.120	1.00	0.00	RX0	C
ATOM	1474	CG	LEU	311	34.645	11.987	2.130	1.00	0.00	RX0	C
ATOM	1475	CD1	LEU	311	35.944	11.379	1.600	1.00	0.00	RX0	C
ATOM	1476	CD2	LEU	311	33.710	10.917	2.695	1.00	0.00	RX0	C
ATOM	1477	C	LEU	311	33.468	15.166	2.739	1.00	0.00	RX0	C
ATOM	1478	O	LEU	311	33.554	16.351	2.969	1.00	0.00	RX0	O
ATOM	1479	N	SER	312	32.784	14.619	1.713	1.00	0.00	RX0	N
ATOM	1480	H	SER	312	32.567	13.644	1.743	1.00	0.00	RX0	H
ATOM	1481	CA	SER	312	32.345	15.333	0.526	1.00	0.00	RX0	C
ATOM	1482	CB	SER	312	31.775	14.217	-0.322	1.00	0.00	RX0	C
ATOM	1483	OG	SER	312	32.590	13.070	-0.057	1.00	0.00	RX0	O
ATOM	1484	HG	SER	312	33.476	13.305	-0.305	1.00	0.00	RX0	H
ATOM	1485	C	SER	312	31.423	16.517	0.816	1.00	0.00	RX0	C
ATOM	1486	O	SER	312	31.042	16.847	1.948	1.00	0.00	RX0	O
ATOM	1487	N	SER	313	30.837	16.923	-0.276	1.00	0.00	RX0	N
ATOM	1488	H	SER	313	31.223	16.627	-1.150	1.00	0.00	RX0	H
ATOM	1489	CA	SER	313	29.838	17.983	-0.455	1.00	0.00	RX0	C
ATOM	1490	CB	SER	313	30.367	19.320	0.071	1.00	0.00	RX0	C
ATOM	1491	OG	SER	313	30.336	19.257	1.515	1.00	0.00	RX0	O
ATOM	1492	HG	SER	313	31.083	18.702	1.755	1.00	0.00	RX0	H
ATOM	1493	C	SER	313	29.353	17.880	-1.909	1.00	0.00	RX0	C
ATOM	1494	O	SER	313	29.168	18.836	-2.628	1.00	0.00	RX0	O
ATOM	1495	N	THR	314	29.278	16.602	-2.358	1.00	0.00	RX0	N
ATOM	1496	H	THR	314	29.452	15.840	-1.739	1.00	0.00	RX0	H
ATOM	1497	CA	THR	314	28.609	16.250	-3.614	1.00	0.00	RX0	C
ATOM	1498	CB	THR	314	28.747	14.743	-3.669	1.00	0.00	RX0	C
ATOM	1499	OG1	THR	314	29.852	14.389	-2.824	1.00	0.00	RX0	O
ATOM	1500	HG1	THR	314	30.024	13.464	-2.973	1.00	0.00	RX0	H
ATOM	1501	CG2	THR	314	28.927	14.209	-5.091	1.00	0.00	RX0	C
ATOM	1502	C	THR	314	27.167	16.747	-3.490	1.00	0.00	RX0	C
ATOM	1503	O	THR	314	26.675	16.978	-2.368	1.00	0.00	RX0	O
ATOM	1504	N	LEU	315	26.451	16.801	-4.589	1.00	0.00	RX0	N
ATOM	1505	H	LEU	315	26.881	16.640	-5.476	1.00	0.00	RX0	H
ATOM	1506	CA	LEU	315	25.050	17.256	-4.537	1.00	0.00	RX0	C
ATOM	1507	CB	LEU	315	24.460	17.346	-5.942	1.00	0.00	RX0	C
ATOM	1508	CG	LEU	315	23.042	17.919	-5.927	1.00	0.00	RX0	C
ATOM	1509	CD1	LEU	315	22.991	19.281	-5.234	1.00	0.00	RX0	C
ATOM	1510	CD2	LEU	315	22.431	17.959	-7.327	1.00	0.00	RX0	C
ATOM	1511	C	LEU	315	24.190	16.343	-3.639	1.00	0.00	RX0	C
ATOM	1512	O	LEU	315	23.484	16.800	-2.757	1.00	0.00	RX0	O
ATOM	1513	N	LYS	316	24.524	15.049	-3.730	1.00	0.00	RX0	N
ATOM	1514	H	LYS	316	25.101	14.784	-4.496	1.00	0.00	RX0	H
ATOM	1515	CA	LYS	316	23.912	13.990	-2.918	1.00	0.00	RX0	C
ATOM	1516	CB	LYS	316	24.324	12.613	-3.438	1.00	0.00	RX0	C
ATOM	1517	CG	LYS	316	23.097	11.737	-3.706	1.00	0.00	RX0	C
ATOM	1518	CD	LYS	316	22.464	11.152	-2.443	1.00	0.00	RX0	C
ATOM	1519	CE	LYS	316	20.939	11.278	-2.400	1.00	0.00	RX0	C
ATOM	1520	NZ	LYS	316	20.600	12.632	-1.962	1.00	0.00	RX0	N
ATOM	1521	HZ1	LYS	316	19.592	12.862	-2.081	1.00	0.00	RX0	H
ATOM	1522	HZ2	LYS	316	20.845	12.753	-0.955	1.00	0.00	RX0	H
ATOM	1523	HZ3	LYS	316	21.070	13.362	-2.539	1.00	0.00	RX0	H

ATOM	1524	C	LYS	316	24.213	14.187	-1.422	1.00	0.00	RX0	C
ATOM	1525	O	LYS	316	23.297	14.194	-0.611	1.00	0.00	RX0	O
ATOM	1526	N	SER	317	25.467	14.540	-1.122	1.00	0.00	RX0	N
ATOM	1527	H	SER	317	26.106	14.717	-1.864	1.00	0.00	RX0	H
ATOM	1528	CA	SER	317	25.934	14.787	0.260	1.00	0.00	RX0	C
ATOM	1529	CB	SER	317	27.465	14.858	0.205	1.00	0.00	RX0	C
ATOM	1530	OG	SER	317	28.073	14.598	1.471	1.00	0.00	RX0	O
ATOM	1531	HG	SER	317	27.679	13.796	1.811	1.00	0.00	RX0	H
ATOM	1532	C	SER	317	25.242	16.001	0.898	1.00	0.00	RX0	C
ATOM	1533	O	SER	317	24.716	15.915	2.007	1.00	0.00	RX0	O
ATOM	1534	N	LEU	318	25.067	17.043	0.088	1.00	0.00	RX0	N
ATOM	1535	H	LEU	318	25.367	16.979	-0.866	1.00	0.00	RX0	H
ATOM	1536	CA	LEU	318	24.405	18.288	0.525	1.00	0.00	RX0	C
ATOM	1537	CB	LEU	318	24.449	19.337	-0.584	1.00	0.00	RX0	C
ATOM	1538	CG	LEU	318	25.790	19.566	-1.272	1.00	0.00	RX0	C
ATOM	1539	CD1	LEU	318	25.632	20.381	-2.552	1.00	0.00	RX0	C
ATOM	1540	CD2	LEU	318	26.841	20.157	-0.350	1.00	0.00	RX0	C
ATOM	1541	C	LEU	318	22.918	18.061	0.823	1.00	0.00	RX0	C
ATOM	1542	O	LEU	318	22.412	18.453	1.877	1.00	0.00	RX0	O
ATOM	1543	N	GLU	319	22.299	17.244	-0.024	1.00	0.00	RX0	N
ATOM	1544	H	GLU	319	22.776	16.930	-0.848	1.00	0.00	RX0	H
ATOM	1545	CA	GLU	319	20.900	16.808	0.157	1.00	0.00	RX0	C
ATOM	1546	CB	GLU	319	20.419	15.895	-0.960	1.00	0.00	RX0	C
ATOM	1547	CG	GLU	319	20.363	16.362	-2.407	1.00	0.00	RX0	C
ATOM	1548	CD	GLU	319	20.118	15.086	-3.180	1.00	0.00	RX0	C
ATOM	1549	OE1	GLU	319	21.026	14.600	-3.847	1.00	0.00	RX0	O
ATOM	1550	OE2	GLU	319	19.079	14.462	-2.983	1.00	0.00	RX0	O
ATOM	1551	C	GLU	319	20.725	15.924	1.402	1.00	0.00	RX0	C
ATOM	1552	O	GLU	319	19.808	16.153	2.196	1.00	0.00	RX0	O
ATOM	1553	N	GLU	320	21.706	15.058	1.645	1.00	0.00	RX0	N
ATOM	1554	H	GLU	320	22.411	14.920	0.950	1.00	0.00	RX0	H
ATOM	1555	CA	GLU	320	21.731	14.149	2.812	1.00	0.00	RX0	C
ATOM	1556	CB	GLU	320	22.834	13.085	2.770	1.00	0.00	RX0	C
ATOM	1557	CG	GLU	320	22.806	12.144	1.558	1.00	0.00	RX0	C
ATOM	1558	CD	GLU	320	21.423	11.570	1.293	1.00	0.00	RX0	C
ATOM	1559	OE1	GLU	320	20.607	12.250	0.669	1.00	0.00	RX0	O
ATOM	1560	OE2	GLU	320	21.181	10.418	1.646	1.00	0.00	RX0	O
ATOM	1561	C	GLU	320	21.765	14.931	4.131	1.00	0.00	RX0	C
ATOM	1562	O	GLU	320	20.881	14.754	4.973	1.00	0.00	RX0	O
ATOM	1563	N	LYS	321	22.647	15.926	4.188	1.00	0.00	RX0	N
ATOM	1564	H	LYS	321	23.258	16.020	3.399	1.00	0.00	RX0	H
ATOM	1565	CA	LYS	321	22.801	16.771	5.391	1.00	0.00	RX0	C
ATOM	1566	CB	LYS	321	24.009	17.707	5.250	1.00	0.00	RX0	C
ATOM	1567	CG	LYS	321	25.332	17.050	4.834	1.00	0.00	RX0	C
ATOM	1568	CD	LYS	321	26.398	18.108	4.517	1.00	0.00	RX0	C
ATOM	1569	CE	LYS	321	27.593	17.632	3.673	1.00	0.00	RX0	C
ATOM	1570	NZ	LYS	321	28.427	18.795	3.321	1.00	0.00	RX0	N
ATOM	1571	HZ1	LYS	321	29.099	18.577	2.550	1.00	0.00	RX0	H
ATOM	1572	HZ2	LYS	321	27.819	19.591	3.023	1.00	0.00	RX0	H
ATOM	1573	HZ3	LYS	321	28.984	19.093	4.143	1.00	0.00	RX0	H
ATOM	1574	C	LYS	321	21.549	17.612	5.656	1.00	0.00	RX0	C
ATOM	1575	O	LYS	321	21.102	17.724	6.798	1.00	0.00	RX0	O
ATOM	1576	N	ASP	322	20.935	18.099	4.575	1.00	0.00	RX0	N
ATOM	1577	H	ASP	322	21.303	17.912	3.660	1.00	0.00	RX0	H
ATOM	1578	CA	ASP	322	19.715	18.915	4.684	1.00	0.00	RX0	C
ATOM	1579	CB	ASP	322	19.417	19.447	3.274	1.00	0.00	RX0	C
ATOM	1580	CG	ASP	322	18.142	20.261	3.208	1.00	0.00	RX0	C
ATOM	1581	OD1	ASP	322	17.773	20.872	4.200	1.00	0.00	RX0	O
ATOM	1582	OD2	ASP	322	17.486	20.256	2.166	1.00	0.00	RX0	O
ATOM	1583	C	ASP	322	18.561	18.097	5.282	1.00	0.00	RX0	C
ATOM	1584	O	ASP	322	17.955	18.512	6.263	1.00	0.00	RX0	O

ATOM	1585	N	HIS	323	18.423	16.872	4.772	1.00	0.00	RX0	N
ATOM	1586	H	HIS	323	19.046	16.590	4.036	1.00	0.00	RX0	H
ATOM	1587	CA	HIS	323	17.423	15.916	5.274	1.00	0.00	RX0	C
ATOM	1588	CB	HIS	323	17.457	14.619	4.464	1.00	0.00	RX0	C
ATOM	1589	CG	HIS	323	16.282	13.762	4.870	1.00	0.00	RX0	C
ATOM	1590	ND1	HIS	323	16.298	12.418	4.939	1.00	0.00	RX0	N
ATOM	1591	HD1	HIS	323	17.049	11.815	4.736	1.00	0.00	RX0	H
ATOM	1592	CD2	HIS	323	15.007	14.211	5.219	1.00	0.00	RX0	C
ATOM	1593	NE2	HIS	323	14.250	13.129	5.501	1.00	0.00	RX0	N
ATOM	1594	CE1	HIS	323	15.043	12.019	5.329	1.00	0.00	RX0	C
ATOM	1595	C	HIS	323	17.630	15.607	6.766	1.00	0.00	RX0	C
ATOM	1596	O	HIS	323	16.677	15.663	7.540	1.00	0.00	RX0	O
ATOM	1597	N	ILE	324	18.888	15.420	7.164	1.00	0.00	RX0	N
ATOM	1598	H	ILE	324	19.611	15.414	6.468	1.00	0.00	RX0	H
ATOM	1599	CA	ILE	324	19.235	15.107	8.570	1.00	0.00	RX0	C
ATOM	1600	CB	ILE	324	20.719	14.765	8.706	1.00	0.00	RX0	C
ATOM	1601	CG2	ILE	324	21.119	14.623	10.174	1.00	0.00	RX0	C
ATOM	1602	CG1	ILE	324	21.042	13.496	7.917	1.00	0.00	RX0	C
ATOM	1603	CD1	ILE	324	22.532	13.151	7.917	1.00	0.00	RX0	C
ATOM	1604	C	ILE	324	18.844	16.271	9.496	1.00	0.00	RX0	C
ATOM	1605	O	ILE	324	18.200	16.053	10.527	1.00	0.00	RX0	O
ATOM	1606	N	HIS	325	19.159	17.485	9.071	1.00	0.00	RX0	N
ATOM	1607	H	HIS	325	19.606	17.582	8.179	1.00	0.00	RX0	H
ATOM	1608	CA	HIS	325	18.840	18.694	9.856	1.00	0.00	RX0	C
ATOM	1609	CB	HIS	325	19.608	19.892	9.319	1.00	0.00	RX0	C
ATOM	1610	CG	HIS	325	21.065	19.674	9.639	1.00	0.00	RX0	C
ATOM	1611	ND1	HIS	325	21.994	19.384	8.715	1.00	0.00	RX0	N
ATOM	1612	HD1	HIS	325	21.813	19.227	7.763	1.00	0.00	RX0	H
ATOM	1613	CD2	HIS	325	21.675	19.708	10.895	1.00	0.00	RX0	C
ATOM	1614	NE2	HIS	325	22.991	19.434	10.707	1.00	0.00	RX0	N
ATOM	1615	CE1	HIS	325	23.187	19.233	9.367	1.00	0.00	RX0	C
ATOM	1616	C	HIS	325	17.335	18.955	9.948	1.00	0.00	RX0	C
ATOM	1617	O	HIS	325	16.820	19.290	11.021	1.00	0.00	RX0	O
ATOM	1618	N	ARG	326	16.631	18.584	8.887	1.00	0.00	RX0	N
ATOM	1619	H	ARG	326	17.122	18.332	8.052	1.00	0.00	RX0	H
ATOM	1620	CA	ARG	326	15.160	18.594	8.873	1.00	0.00	RX0	C
ATOM	1621	CB	ARG	326	14.649	18.482	7.439	1.00	0.00	RX0	C
ATOM	1622	CG	ARG	326	14.692	19.852	6.755	1.00	0.00	RX0	C
ATOM	1623	CD	ARG	326	14.190	19.850	5.308	1.00	0.00	RX0	C
ATOM	1624	NE	ARG	326	15.229	19.451	4.360	1.00	0.00	RX0	N
ATOM	1625	HE	ARG	326	15.976	20.122	4.211	1.00	0.00	RX0	H
ATOM	1626	CZ	ARG	326	15.193	18.251	3.715	1.00	0.00	RX0	C
ATOM	1627	NH1	ARG	326	14.227	17.363	4.030	1.00	0.00	RX0	N
ATOM	1628	HH11	ARG	326	14.142	16.468	3.585	1.00	0.00	RX0	H
ATOM	1629	HH12	ARG	326	13.555	17.578	4.744	1.00	0.00	RX0	H
ATOM	1630	NH2	ARG	326	16.117	17.971	2.775	1.00	0.00	RX0	N
ATOM	1631	HH21	ARG	326	16.205	17.103	2.283	1.00	0.00	RX0	H
ATOM	1632	HH22	ARG	326	16.790	18.690	2.535	1.00	0.00	RX0	H
ATOM	1633	C	ARG	326	14.537	17.576	9.843	1.00	0.00	RX0	C
ATOM	1634	O	ARG	326	13.617	17.928	10.589	1.00	0.00	RX0	O
ATOM	1635	N	VAL	327	15.171	16.419	9.987	1.00	0.00	RX0	N
ATOM	1636	H	VAL	327	15.979	16.246	9.419	1.00	0.00	RX0	H
ATOM	1637	CA	VAL	327	14.709	15.376	10.935	1.00	0.00	RX0	C
ATOM	1638	CB	VAL	327	15.276	13.994	10.605	1.00	0.00	RX0	C
ATOM	1639	CG1	VAL	327	14.753	12.957	11.599	1.00	0.00	RX0	C
ATOM	1640	CG2	VAL	327	14.933	13.580	9.174	1.00	0.00	RX0	C
ATOM	1641	C	VAL	327	15.041	15.781	12.381	1.00	0.00	RX0	C
ATOM	1642	O	VAL	327	14.187	15.661	13.270	1.00	0.00	RX0	O
ATOM	1643	N	LEU	328	16.212	16.371	12.574	1.00	0.00	RX0	N
ATOM	1644	H	LEU	328	16.820	16.498	11.789	1.00	0.00	RX0	H
ATOM	1645	CA	LEU	328	16.631	16.899	13.887	1.00	0.00	RX0	C

ATOM	1646	CB	LEU	328	18.069	17.412	13.829	1.00	0.00	RX0	C
ATOM	1647	CG	LEU	328	19.087	16.274	13.772	1.00	0.00	RX0	C
ATOM	1648	CD1	LEU	328	20.508	16.784	13.529	1.00	0.00	RX0	C
ATOM	1649	CD2	LEU	328	18.998	15.396	15.020	1.00	0.00	RX0	C
ATOM	1650	C	LEU	328	15.692	18.005	14.386	1.00	0.00	RX0	C
ATOM	1651	O	LEU	328	15.231	17.955	15.519	1.00	0.00	RX0	O
ATOM	1652	N	ASP	329	15.222	18.821	13.436	1.00	0.00	RX0	N
ATOM	1653	H	ASP	329	15.637	18.860	12.523	1.00	0.00	RX0	H
ATOM	1654	CA	ASP	329	14.223	19.870	13.722	1.00	0.00	RX0	C
ATOM	1655	CB	ASP	329	13.760	20.672	12.500	1.00	0.00	RX0	C
ATOM	1656	CG	ASP	329	14.807	21.471	11.771	1.00	0.00	RX0	C
ATOM	1657	OD1	ASP	329	15.475	22.297	12.378	1.00	0.00	RX0	O
ATOM	1658	OD2	ASP	329	14.898	21.326	10.557	1.00	0.00	RX0	O
ATOM	1659	C	ASP	329	12.864	19.312	14.154	1.00	0.00	RX0	C
ATOM	1660	O	ASP	329	12.272	19.788	15.128	1.00	0.00	RX0	O
ATOM	1661	N	LYS	330	12.463	18.218	13.512	1.00	0.00	RX0	N
ATOM	1662	H	LYS	330	13.013	17.923	12.728	1.00	0.00	RX0	H
ATOM	1663	CA	LYS	330	11.217	17.525	13.871	1.00	0.00	RX0	C
ATOM	1664	CB	LYS	330	10.800	16.459	12.860	1.00	0.00	RX0	C
ATOM	1665	CG	LYS	330	9.624	15.604	13.368	1.00	0.00	RX0	C
ATOM	1666	CD	LYS	330	8.369	16.381	13.797	1.00	0.00	RX0	C
ATOM	1667	CE	LYS	330	7.826	17.306	12.716	1.00	0.00	RX0	C
ATOM	1668	NZ	LYS	330	7.439	16.476	11.577	1.00	0.00	RX0	N
ATOM	1669	HZ1	LYS	330	7.128	17.094	10.807	1.00	0.00	RX0	H
ATOM	1670	HZ2	LYS	330	8.249	15.889	11.280	1.00	0.00	RX0	H
ATOM	1671	HZ3	LYS	330	6.658	15.857	11.878	1.00	0.00	RX0	H
ATOM	1672	C	LYS	330	11.283	16.915	15.278	1.00	0.00	RX0	C
ATOM	1673	O	LYS	330	10.354	17.095	16.067	1.00	0.00	RX0	O
ATOM	1674	N	ILE	331	12.441	16.375	15.625	1.00	0.00	RX0	N
ATOM	1675	H	ILE	331	13.182	16.369	14.948	1.00	0.00	RX0	H
ATOM	1676	CA	ILE	331	12.656	15.802	16.972	1.00	0.00	RX0	C
ATOM	1677	CB	ILE	331	13.953	14.996	17.053	1.00	0.00	RX0	C
ATOM	1678	CG2	ILE	331	14.062	14.329	18.421	1.00	0.00	RX0	C
ATOM	1679	CG1	ILE	331	14.043	13.952	15.943	1.00	0.00	RX0	C
ATOM	1680	CD1	ILE	331	15.358	13.172	15.985	1.00	0.00	RX0	C
ATOM	1681	C	ILE	331	12.642	16.922	18.027	1.00	0.00	RX0	C
ATOM	1682	O	ILE	331	12.078	16.732	19.120	1.00	0.00	RX0	O
ATOM	1683	N	THR	332	13.158	18.084	17.675	1.00	0.00	RX0	N
ATOM	1684	H	THR	332	13.546	18.205	16.761	1.00	0.00	RX0	H
ATOM	1685	CA	THR	332	13.155	19.265	18.570	1.00	0.00	RX0	C
ATOM	1686	CB	THR	332	14.035	20.319	17.919	1.00	0.00	RX0	C
ATOM	1687	OG1	THR	332	15.311	19.724	17.648	1.00	0.00	RX0	O
ATOM	1688	HG1	THR	332	15.230	19.250	16.827	1.00	0.00	RX0	H
ATOM	1689	CG2	THR	332	14.188	21.563	18.796	1.00	0.00	RX0	C
ATOM	1690	C	THR	332	11.706	19.696	18.836	1.00	0.00	RX0	C
ATOM	1691	O	THR	332	11.302	19.832	19.995	1.00	0.00	RX0	O
ATOM	1692	N	ASP	333	10.912	19.714	17.772	1.00	0.00	RX0	N
ATOM	1693	H	ASP	333	11.278	19.676	16.836	1.00	0.00	RX0	H
ATOM	1694	CA	ASP	333	9.466	20.017	17.863	1.00	0.00	RX0	C
ATOM	1695	CB	ASP	333	8.673	19.801	16.565	1.00	0.00	RX0	C
ATOM	1696	CG	ASP	333	9.129	20.568	15.350	1.00	0.00	RX0	C
ATOM	1697	OD1	ASP	333	9.566	21.705	15.497	1.00	0.00	RX0	O
ATOM	1698	OD2	ASP	333	8.998	20.024	14.249	1.00	0.00	RX0	O
ATOM	1699	C	ASP	333	8.732	19.000	18.747	1.00	0.00	RX0	C
ATOM	1700	O	ASP	333	7.880	19.374	19.559	1.00	0.00	RX0	O
ATOM	1701	N	THR	334	9.187	17.759	18.682	1.00	0.00	RX0	N
ATOM	1702	H	THR	334	9.940	17.582	18.047	1.00	0.00	RX0	H
ATOM	1703	CA	THR	334	8.631	16.638	19.462	1.00	0.00	RX0	C
ATOM	1704	CB	THR	334	9.176	15.373	18.821	1.00	0.00	RX0	C
ATOM	1705	OG1	THR	334	8.792	15.338	17.438	1.00	0.00	RX0	O
ATOM	1706	HG1	THR	334	9.147	16.124	17.032	1.00	0.00	RX0	H

ATOM	1707	CG2	THR	334	8.726	14.119	19.558	1.00	0.00	RX0	C
ATOM	1708	C	THR	334	8.961	16.782	20.953	1.00	0.00	RX0	C
ATOM	1709	O	THR	334	8.059	16.689	21.789	1.00	0.00	RX0	O
ATOM	1710	N	LEU	335	10.217	17.081	21.263	1.00	0.00	RX0	N
ATOM	1711	H	LEU	335	10.880	17.204	20.523	1.00	0.00	RX0	H
ATOM	1712	CA	LEU	335	10.648	17.349	22.650	1.00	0.00	RX0	C
ATOM	1713	CB	LEU	335	12.150	17.611	22.696	1.00	0.00	RX0	C
ATOM	1714	CG	LEU	335	12.959	16.321	22.780	1.00	0.00	RX0	C
ATOM	1715	CD1	LEU	335	14.457	16.567	22.608	1.00	0.00	RX0	C
ATOM	1716	CD2	LEU	335	12.650	15.572	24.077	1.00	0.00	RX0	C
ATOM	1717	C	LEU	335	9.903	18.522	23.297	1.00	0.00	RX0	C
ATOM	1718	O	LEU	335	9.384	18.379	24.399	1.00	0.00	RX0	O
ATOM	1719	N	ILE	336	9.668	19.567	22.501	1.00	0.00	RX0	N
ATOM	1720	H	ILE	336	10.071	19.579	21.582	1.00	0.00	RX0	H
ATOM	1721	CA	ILE	336	8.897	20.743	22.962	1.00	0.00	RX0	C
ATOM	1722	CB	ILE	336	9.048	21.912	21.989	1.00	0.00	RX0	C
ATOM	1723	CG2	ILE	336	8.092	23.058	22.328	1.00	0.00	RX0	C
ATOM	1724	CG1	ILE	336	10.502	22.387	21.993	1.00	0.00	RX0	C
ATOM	1725	CD1	ILE	336	10.935	22.867	23.381	1.00	0.00	RX0	C
ATOM	1726	C	ILE	336	7.427	20.365	23.180	1.00	0.00	RX0	C
ATOM	1727	O	ILE	336	6.836	20.730	24.203	1.00	0.00	RX0	O
ATOM	1728	N	HIS	337	6.891	19.571	22.265	1.00	0.00	RX0	N
ATOM	1729	H	HIS	337	7.438	19.318	21.465	1.00	0.00	RX0	H
ATOM	1730	CA	HIS	337	5.501	19.096	22.359	1.00	0.00	RX0	C
ATOM	1731	CB	HIS	337	5.081	18.322	21.119	1.00	0.00	RX0	C
ATOM	1732	CG	HIS	337	3.606	18.034	21.230	1.00	0.00	RX0	C
ATOM	1733	ND1	HIS	337	2.645	18.946	20.995	1.00	0.00	RX0	N
ATOM	1734	HD1	HIS	337	2.784	19.876	20.713	1.00	0.00	RX0	H
ATOM	1735	CD2	HIS	337	3.003	16.833	21.605	1.00	0.00	RX0	C
ATOM	1736	NE2	HIS	337	1.660	17.030	21.600	1.00	0.00	RX0	N
ATOM	1737	CE1	HIS	337	1.441	18.332	21.221	1.00	0.00	RX0	C
ATOM	1738	C	HIS	337	5.301	18.263	23.631	1.00	0.00	RX0	C
ATOM	1739	O	HIS	337	4.339	18.490	24.365	1.00	0.00	RX0	O
ATOM	1740	N	LEU	338	6.274	17.405	23.914	1.00	0.00	RX0	N
ATOM	1741	H	LEU	338	7.051	17.335	23.286	1.00	0.00	RX0	H
ATOM	1742	CA	LEU	338	6.246	16.526	25.097	1.00	0.00	RX0	C
ATOM	1743	CB	LEU	338	7.441	15.575	25.089	1.00	0.00	RX0	C
ATOM	1744	CG	LEU	338	7.340	14.530	23.981	1.00	0.00	RX0	C
ATOM	1745	CD1	LEU	338	8.642	13.747	23.813	1.00	0.00	RX0	C
ATOM	1746	CD2	LEU	338	6.121	13.629	24.181	1.00	0.00	RX0	C
ATOM	1747	C	LEU	338	6.240	17.331	26.400	1.00	0.00	RX0	C
ATOM	1748	O	LEU	338	5.410	17.098	27.277	1.00	0.00	RX0	O
ATOM	1749	N	MET	339	7.027	18.402	26.394	1.00	0.00	RX0	N
ATOM	1750	H	MET	339	7.615	18.548	25.595	1.00	0.00	RX0	H
ATOM	1751	CA	MET	339	7.168	19.308	27.550	1.00	0.00	RX0	C
ATOM	1752	CB	MET	339	8.406	20.190	27.400	1.00	0.00	RX0	C
ATOM	1753	CG	MET	339	9.705	19.388	27.372	1.00	0.00	RX0	C
ATOM	1754	SD	MET	339	11.138	20.425	27.053	1.00	0.00	RX0	S
ATOM	1755	CE	MET	339	12.272	19.111	26.583	1.00	0.00	RX0	C
ATOM	1756	C	MET	339	5.924	20.183	27.753	1.00	0.00	RX0	C
ATOM	1757	O	MET	339	5.433	20.322	28.878	1.00	0.00	RX0	O
ATOM	1758	N	ALA	340	5.370	20.672	26.646	1.00	0.00	RX0	N
ATOM	1759	H	ALA	340	5.814	20.472	25.772	1.00	0.00	RX0	H
ATOM	1760	CA	ALA	340	4.120	21.456	26.636	1.00	0.00	RX0	C
ATOM	1761	CB	ALA	340	3.835	21.992	25.232	1.00	0.00	RX0	C
ATOM	1762	C	ALA	340	2.921	20.619	27.100	1.00	0.00	RX0	C
ATOM	1763	O	ALA	340	2.176	21.064	27.967	1.00	0.00	RX0	O
ATOM	1764	N	LYS	341	2.865	19.360	26.655	1.00	0.00	RX0	N
ATOM	1765	H	LYS	341	3.552	19.058	25.994	1.00	0.00	RX0	H
ATOM	1766	CA	LYS	341	1.846	18.397	27.117	1.00	0.00	RX0	C
ATOM	1767	CB	LYS	341	1.970	17.109	26.278	1.00	0.00	RX0	C

ATOM	1768	CG	LYS	341	0.690	16.269	26.166	1.00	0.00	RX0	C
ATOM	1769	CD	LYS	341	0.393	15.766	24.738	1.00	0.00	RX0	C
ATOM	1770	CE	LYS	341	1.378	14.743	24.147	1.00	0.00	RX0	C
ATOM	1771	NZ	LYS	341	1.076	14.512	22.723	1.00	0.00	RX0	N
ATOM	1772	HZ1	LYS	341	1.802	13.951	22.224	1.00	0.00	RX0	H
ATOM	1773	HZ2	LYS	341	0.210	13.973	22.505	1.00	0.00	RX0	H
ATOM	1774	HZ3	LYS	341	1.037	15.387	22.165	1.00	0.00	RX0	H
ATOM	1775	C	LYS	341	1.927	18.179	28.637	1.00	0.00	RX0	C
ATOM	1776	O	LYS	341	0.908	18.032	29.304	1.00	0.00	RX0	O
ATOM	1777	N	ALA	342	3.166	18.103	29.120	1.00	0.00	RX0	N
ATOM	1778	H	ALA	342	3.940	18.170	28.488	1.00	0.00	RX0	H
ATOM	1779	CA	ALA	342	3.459	17.942	30.556	1.00	0.00	RX0	C
ATOM	1780	CB	ALA	342	4.953	17.706	30.786	1.00	0.00	RX0	C
ATOM	1781	C	ALA	342	3.007	19.166	31.371	1.00	0.00	RX0	C
ATOM	1782	O	ALA	342	2.879	19.099	32.588	1.00	0.00	RX0	O
ATOM	1783	N	GLY	343	2.900	20.305	30.669	1.00	0.00	RX0	N
ATOM	1784	H	GLY	343	3.153	20.317	29.702	1.00	0.00	RX0	H
ATOM	1785	CA	GLY	343	2.393	21.560	31.244	1.00	0.00	RX0	C
ATOM	1786	C	GLY	343	3.505	22.433	31.829	1.00	0.00	RX0	C
ATOM	1787	O	GLY	343	3.244	23.282	32.678	1.00	0.00	RX0	O
ATOM	1788	N	LEU	344	4.736	22.226	31.356	1.00	0.00	RX0	N
ATOM	1789	H	LEU	344	4.854	21.555	30.623	1.00	0.00	RX0	H
ATOM	1790	CA	LEU	344	5.823	23.174	31.634	1.00	0.00	RX0	C
ATOM	1791	CB	LEU	344	7.151	22.611	31.138	1.00	0.00	RX0	C
ATOM	1792	CG	LEU	344	7.554	21.338	31.877	1.00	0.00	RX0	C
ATOM	1793	CD1	LEU	344	8.836	20.742	31.301	1.00	0.00	RX0	C
ATOM	1794	CD2	LEU	344	7.656	21.564	33.387	1.00	0.00	RX0	C
ATOM	1795	C	LEU	344	5.508	24.488	30.929	1.00	0.00	RX0	C
ATOM	1796	O	LEU	344	4.977	24.500	29.792	1.00	0.00	RX0	O
ATOM	1797	N	THR	345	5.822	25.577	31.575	1.00	0.00	RX0	N
ATOM	1798	H	THR	345	6.292	25.484	32.452	1.00	0.00	RX0	H
ATOM	1799	CA	THR	345	5.705	26.912	30.948	1.00	0.00	RX0	C
ATOM	1800	CB	THR	345	5.998	27.965	32.010	1.00	0.00	RX0	C
ATOM	1801	OG1	THR	345	7.176	27.610	32.733	1.00	0.00	RX0	O
ATOM	1802	HG1	THR	345	6.909	26.968	33.388	1.00	0.00	RX0	H
ATOM	1803	CG2	THR	345	4.823	28.120	32.976	1.00	0.00	RX0	C
ATOM	1804	C	THR	345	6.639	26.971	29.731	1.00	0.00	RX0	C
ATOM	1805	O	THR	345	7.615	26.233	29.623	1.00	0.00	RX0	O
ATOM	1806	N	LEU	346	6.390	27.971	28.898	1.00	0.00	RX0	N
ATOM	1807	H	LEU	346	5.563	28.512	29.044	1.00	0.00	RX0	H
ATOM	1808	CA	LEU	346	7.214	28.242	27.708	1.00	0.00	RX0	C
ATOM	1809	CB	LEU	346	6.672	29.451	26.948	1.00	0.00	RX0	C
ATOM	1810	CG	LEU	346	7.379	29.658	25.607	1.00	0.00	RX0	C
ATOM	1811	CD1	LEU	346	7.248	28.433	24.700	1.00	0.00	RX0	C
ATOM	1812	CD2	LEU	346	6.916	30.940	24.913	1.00	0.00	RX0	C
ATOM	1813	C	LEU	346	8.705	28.433	28.051	1.00	0.00	RX0	C
ATOM	1814	O	LEU	346	9.594	27.857	27.432	1.00	0.00	RX0	O
ATOM	1815	N	GLN	347	8.927	29.083	29.198	1.00	0.00	RX0	N
ATOM	1816	H	GLN	347	8.145	29.437	29.706	1.00	0.00	RX0	H
ATOM	1817	CA	GLN	347	10.275	29.275	29.754	1.00	0.00	RX0	C
ATOM	1818	CB	GLN	347	10.216	30.285	30.896	1.00	0.00	RX0	C
ATOM	1819	CG	GLN	347	11.597	30.636	31.446	1.00	0.00	RX0	C
ATOM	1820	CD	GLN	347	11.429	31.603	32.594	1.00	0.00	RX0	C
ATOM	1821	OE1	GLN	347	10.500	31.502	33.384	1.00	0.00	RX0	O
ATOM	1822	NE2	GLN	347	12.376	32.557	32.636	1.00	0.00	RX0	N
ATOM	1823	HE21	GLN	347	13.105	32.593	31.952	1.00	0.00	RX0	H
ATOM	1824	HE22	GLN	347	12.362	33.257	33.351	1.00	0.00	RX0	H
ATOM	1825	C	GLN	347	10.911	27.956	30.229	1.00	0.00	RX0	C
ATOM	1826	O	GLN	347	12.052	27.652	29.875	1.00	0.00	RX0	O
ATOM	1827	N	GLN	348	10.120	27.140	30.905	1.00	0.00	RX0	N
ATOM	1828	H	GLN	348	9.171	27.397	31.088	1.00	0.00	RX0	H

ATOM	1829	CA	GLN	348	10.582	25.823	31.394	1.00	0.00	RX0	C
ATOM	1830	CB	GLN	348	9.593	25.217	32.377	1.00	0.00	RX0	C
ATOM	1831	CG	GLN	348	9.647	25.884	33.748	1.00	0.00	RX0	C
ATOM	1832	CD	GLN	348	8.479	25.371	34.557	1.00	0.00	RX0	C
ATOM	1833	OE1	GLN	348	7.353	25.294	34.070	1.00	0.00	RX0	O
ATOM	1834	NE2	GLN	348	8.802	25.021	35.812	1.00	0.00	RX0	N
ATOM	1835	HE21	GLN	348	9.743	25.128	36.137	1.00	0.00	RX0	H
ATOM	1836	HE22	GLN	348	8.123	24.655	36.449	1.00	0.00	RX0	H
ATOM	1837	C	GLN	348	10.871	24.847	30.248	1.00	0.00	RX0	C
ATOM	1838	O	GLN	348	11.861	24.115	30.296	1.00	0.00	RX0	O
ATOM	1839	N	GLN	349	10.121	24.983	29.160	1.00	0.00	RX0	N
ATOM	1840	H	GLN	349	9.375	25.648	29.187	1.00	0.00	RX0	H
ATOM	1841	CA	GLN	349	10.299	24.172	27.940	1.00	0.00	RX0	C
ATOM	1842	CB	GLN	349	9.205	24.481	26.921	1.00	0.00	RX0	C
ATOM	1843	CG	GLN	349	7.821	24.032	27.384	1.00	0.00	RX0	C
ATOM	1844	CD	GLN	349	6.788	24.591	26.435	1.00	0.00	RX0	C
ATOM	1845	OE1	GLN	349	7.076	24.922	25.291	1.00	0.00	RX0	O
ATOM	1846	NE2	GLN	349	5.563	24.697	26.977	1.00	0.00	RX0	N
ATOM	1847	HE21	GLN	349	5.398	24.431	27.933	1.00	0.00	RX0	H
ATOM	1848	HE22	GLN	349	4.780	25.035	26.458	1.00	0.00	RX0	H
ATOM	1849	C	GLN	349	11.682	24.372	27.306	1.00	0.00	RX0	C
ATOM	1850	O	GLN	349	12.447	23.422	27.181	1.00	0.00	RX0	O
ATOM	1851	N	HIS	350	12.056	25.641	27.113	1.00	0.00	RX0	N
ATOM	1852	H	HIS	350	11.422	26.375	27.365	1.00	0.00	RX0	H
ATOM	1853	CA	HIS	350	13.344	25.953	26.463	1.00	0.00	RX0	C
ATOM	1854	CB	HIS	350	13.380	27.326	25.780	1.00	0.00	RX0	C
ATOM	1855	CG	HIS	350	13.574	28.469	26.744	1.00	0.00	RX0	C
ATOM	1856	ND1	HIS	350	12.557	29.184	27.252	1.00	0.00	RX0	N
ATOM	1857	HD1	HIS	350	11.600	29.024	27.093	1.00	0.00	RX0	H
ATOM	1858	CD2	HIS	350	14.781	28.994	27.218	1.00	0.00	RX0	C
ATOM	1859	NE2	HIS	350	14.481	30.042	28.022	1.00	0.00	RX0	N
ATOM	1860	CE1	HIS	350	13.116	30.159	28.039	1.00	0.00	RX0	C
ATOM	1861	C	HIS	350	14.540	25.670	27.386	1.00	0.00	RX0	C
ATOM	1862	O	HIS	350	15.573	25.182	26.934	1.00	0.00	RX0	O
ATOM	1863	N	GLN	351	14.320	25.854	28.691	1.00	0.00	RX0	N
ATOM	1864	H	GLN	351	13.440	26.231	28.991	1.00	0.00	RX0	H
ATOM	1865	CA	GLN	351	15.341	25.538	29.706	1.00	0.00	RX0	C
ATOM	1866	CB	GLN	351	14.916	26.061	31.072	1.00	0.00	RX0	C
ATOM	1867	CG	GLN	351	14.893	27.584	31.149	1.00	0.00	RX0	C
ATOM	1868	CD	GLN	351	14.201	27.981	32.432	1.00	0.00	RX0	C
ATOM	1869	OE1	GLN	351	13.388	27.245	32.984	1.00	0.00	RX0	O
ATOM	1870	NE2	GLN	351	14.581	29.186	32.891	1.00	0.00	RX0	N
ATOM	1871	HE21	GLN	351	15.249	29.726	32.379	1.00	0.00	RX0	H
ATOM	1872	HE22	GLN	351	14.208	29.555	33.743	1.00	0.00	RX0	H
ATOM	1873	C	GLN	351	15.597	24.030	29.805	1.00	0.00	RX0	C
ATOM	1874	O	GLN	351	16.752	23.608	29.740	1.00	0.00	RX0	O
ATOM	1875	N	ARG	352	14.527	23.242	29.744	1.00	0.00	RX0	N
ATOM	1876	H	ARG	352	13.617	23.654	29.660	1.00	0.00	RX0	H
ATOM	1877	CA	ARG	352	14.627	21.772	29.816	1.00	0.00	RX0	C
ATOM	1878	CB	ARG	352	13.312	21.109	30.248	1.00	0.00	RX0	C
ATOM	1879	CG	ARG	352	13.429	19.588	30.444	1.00	0.00	RX0	C
ATOM	1880	CD	ARG	352	12.235	18.992	31.199	1.00	0.00	RX0	C
ATOM	1881	NE	ARG	352	12.217	17.527	31.177	1.00	0.00	RX0	N
ATOM	1882	HE	ARG	352	12.007	17.060	30.303	1.00	0.00	RX0	H
ATOM	1883	CZ	ARG	352	12.353	16.775	32.312	1.00	0.00	RX0	C
ATOM	1884	NH1	ARG	352	12.655	17.382	33.477	1.00	0.00	RX0	N
ATOM	1885	HH11	ARG	352	12.834	16.840	34.311	1.00	0.00	RX0	H
ATOM	1886	HH12	ARG	352	12.726	18.377	33.552	1.00	0.00	RX0	H
ATOM	1887	NH2	ARG	352	12.179	15.439	32.254	1.00	0.00	RX0	N
ATOM	1888	HH21	ARG	352	12.218	14.806	33.035	1.00	0.00	RX0	H
ATOM	1889	HH22	ARG	352	11.983	15.018	31.346	1.00	0.00	RX0	H

ATOM	1890	C	ARG	352	15.192	21.188	28.514	1.00	0.00	RX0	C
ATOM	1891	O	ARG	352	16.048	20.305	28.551	1.00	0.00	RX0	O
ATOM	1892	N	LEU	353	14.843	21.816	27.391	1.00	0.00	RX0	N
ATOM	1893	H	LEU	353	14.133	22.521	27.430	1.00	0.00	RX0	H
ATOM	1894	CA	LEU	353	15.400	21.446	26.079	1.00	0.00	RX0	C
ATOM	1895	CB	LEU	353	14.767	22.300	24.982	1.00	0.00	RX0	C
ATOM	1896	CG	LEU	353	15.252	21.927	23.581	1.00	0.00	RX0	C
ATOM	1897	CD1	LEU	353	14.832	20.509	23.193	1.00	0.00	RX0	C
ATOM	1898	CD2	LEU	353	14.827	22.961	22.540	1.00	0.00	RX0	C
ATOM	1899	C	LEU	353	16.930	21.610	26.063	1.00	0.00	RX0	C
ATOM	1900	O	LEU	353	17.658	20.692	25.694	1.00	0.00	RX0	O
ATOM	1901	N	ALA	354	17.375	22.739	26.614	1.00	0.00	RX0	N
ATOM	1902	H	ALA	354	16.705	23.419	26.923	1.00	0.00	RX0	H
ATOM	1903	CA	ALA	354	18.808	23.073	26.716	1.00	0.00	RX0	C
ATOM	1904	CB	ALA	354	18.994	24.518	27.181	1.00	0.00	RX0	C
ATOM	1905	C	ALA	354	19.540	22.141	27.690	1.00	0.00	RX0	C
ATOM	1906	O	ALA	354	20.574	21.567	27.334	1.00	0.00	RX0	O
ATOM	1907	N	GLN	355	18.893	21.844	28.811	1.00	0.00	RX0	N
ATOM	1908	H	GLN	355	18.018	22.304	28.974	1.00	0.00	RX0	H
ATOM	1909	CA	GLN	355	19.427	20.920	29.833	1.00	0.00	RX0	C
ATOM	1910	CB	GLN	355	18.617	20.926	31.124	1.00	0.00	RX0	C
ATOM	1911	CG	GLN	355	18.966	22.145	31.978	1.00	0.00	RX0	C
ATOM	1912	CD	GLN	355	18.358	21.990	33.354	1.00	0.00	RX0	C
ATOM	1913	OE1	GLN	355	18.955	21.448	34.279	1.00	0.00	RX0	O
ATOM	1914	NE2	GLN	355	17.133	22.509	33.457	1.00	0.00	RX0	N
ATOM	1915	HE21	GLN	355	16.701	22.934	32.661	1.00	0.00	RX0	H
ATOM	1916	HE22	GLN	355	16.659	22.476	34.334	1.00	0.00	RX0	H
ATOM	1917	C	GLN	355	19.631	19.504	29.273	1.00	0.00	RX0	C
ATOM	1918	O	GLN	355	20.705	18.919	29.430	1.00	0.00	RX0	O
ATOM	1919	N	LEU	356	18.681	19.086	28.443	1.00	0.00	RX0	N
ATOM	1920	H	LEU	356	17.885	19.670	28.288	1.00	0.00	RX0	H
ATOM	1921	CA	LEU	356	18.716	17.770	27.778	1.00	0.00	RX0	C
ATOM	1922	CB	LEU	356	17.348	17.400	27.207	1.00	0.00	RX0	C
ATOM	1923	CG	LEU	356	16.353	17.037	28.307	1.00	0.00	RX0	C
ATOM	1924	CD1	LEU	356	14.994	16.627	27.740	1.00	0.00	RX0	C
ATOM	1925	CD2	LEU	356	16.923	15.970	29.241	1.00	0.00	RX0	C
ATOM	1926	C	LEU	356	19.795	17.661	26.699	1.00	0.00	RX0	C
ATOM	1927	O	LEU	356	20.593	16.720	26.700	1.00	0.00	RX0	O
ATOM	1928	N	LEU	357	19.916	18.722	25.913	1.00	0.00	RX0	N
ATOM	1929	H	LEU	357	19.258	19.473	26.028	1.00	0.00	RX0	H
ATOM	1930	CA	LEU	357	20.885	18.765	24.803	1.00	0.00	RX0	C
ATOM	1931	CB	LEU	357	20.543	19.872	23.806	1.00	0.00	RX0	C
ATOM	1932	CG	LEU	357	19.190	19.686	23.118	1.00	0.00	RX0	C
ATOM	1933	CD1	LEU	357	18.890	20.836	22.156	1.00	0.00	RX0	C
ATOM	1934	CD2	LEU	357	19.069	18.325	22.435	1.00	0.00	RX0	C
ATOM	1935	C	LEU	357	22.335	18.932	25.264	1.00	0.00	RX0	C
ATOM	1936	O	LEU	357	23.247	18.345	24.683	1.00	0.00	RX0	O
ATOM	1937	N	LEU	358	22.501	19.595	26.404	1.00	0.00	RX0	N
ATOM	1938	H	LEU	358	21.702	20.041	26.814	1.00	0.00	RX0	H
ATOM	1939	CA	LEU	358	23.826	19.754	27.029	1.00	0.00	RX0	C
ATOM	1940	CB	LEU	358	23.776	20.840	28.110	1.00	0.00	RX0	C
ATOM	1941	CG	LEU	358	23.506	22.246	27.560	1.00	0.00	RX0	C
ATOM	1942	CD1	LEU	358	23.044	23.227	28.637	1.00	0.00	RX0	C
ATOM	1943	CD2	LEU	358	24.691	22.805	26.785	1.00	0.00	RX0	C
ATOM	1944	C	LEU	358	24.390	18.455	27.609	1.00	0.00	RX0	C
ATOM	1945	O	LEU	358	25.603	18.227	27.557	1.00	0.00	RX0	O
ATOM	1946	N	ILE	359	23.510	17.559	28.043	1.00	0.00	RX0	N
ATOM	1947	H	ILE	359	22.543	17.819	28.067	1.00	0.00	RX0	H
ATOM	1948	CA	ILE	359	23.928	16.212	28.495	1.00	0.00	RX0	C
ATOM	1949	CB	ILE	359	22.762	15.471	29.164	1.00	0.00	RX0	C
ATOM	1950	CG2	ILE	359	23.144	14.058	29.619	1.00	0.00	RX0	C

ATOM	1951	CG1	ILE	359	22.257	16.300	30.349	1.00	0.00	RX0	C
ATOM	1952	CD1	ILE	359	20.950	15.784	30.948	1.00	0.00	RX0	C
ATOM	1953	C	ILE	359	24.559	15.428	27.334	1.00	0.00	RX0	C
ATOM	1954	O	ILE	359	25.552	14.723	27.543	1.00	0.00	RX0	O
ATOM	1955	N	LEU	360	24.038	15.620	26.133	1.00	0.00	RX0	N
ATOM	1956	H	LEU	360	23.233	16.211	26.060	1.00	0.00	RX0	H
ATOM	1957	CA	LEU	360	24.601	14.974	24.928	1.00	0.00	RX0	C
ATOM	1958	CB	LEU	360	23.767	15.284	23.683	1.00	0.00	RX0	C
ATOM	1959	CG	LEU	360	22.277	14.962	23.843	1.00	0.00	RX0	C
ATOM	1960	CD1	LEU	360	21.494	15.312	22.580	1.00	0.00	RX0	C
ATOM	1961	CD2	LEU	360	22.025	13.518	24.277	1.00	0.00	RX0	C
ATOM	1962	C	LEU	360	26.079	15.308	24.694	1.00	0.00	RX0	C
ATOM	1963	O	LEU	360	26.843	14.455	24.246	1.00	0.00	RX0	O
ATOM	1964	N	SER	361	26.491	16.472	25.202	1.00	0.00	RX0	N
ATOM	1965	H	SER	361	25.805	17.074	25.613	1.00	0.00	RX0	H
ATOM	1966	CA	SER	361	27.906	16.894	25.202	1.00	0.00	RX0	C
ATOM	1967	CB	SER	361	28.000	18.370	25.489	1.00	0.00	RX0	C
ATOM	1968	OG	SER	361	28.819	18.982	24.484	1.00	0.00	RX0	O
ATOM	1969	HG	SER	361	28.252	19.240	23.762	1.00	0.00	RX0	H
ATOM	1970	C	SER	361	28.769	15.985	26.099	1.00	0.00	RX0	C
ATOM	1971	O	SER	361	29.797	15.456	25.681	1.00	0.00	RX0	O
ATOM	1972	N	HIS	362	28.237	15.704	27.286	1.00	0.00	RX0	N
ATOM	1973	H	HIS	362	27.337	16.093	27.492	1.00	0.00	RX0	H
ATOM	1974	CA	HIS	362	28.854	14.793	28.272	1.00	0.00	RX0	C
ATOM	1975	CB	HIS	362	28.130	14.836	29.615	1.00	0.00	RX0	C
ATOM	1976	CG	HIS	362	28.890	15.694	30.593	1.00	0.00	RX0	C
ATOM	1977	ND1	HIS	362	28.487	16.915	30.966	1.00	0.00	RX0	N
ATOM	1978	HD1	HIS	362	27.674	17.366	30.653	1.00	0.00	RX0	H
ATOM	1979	CD2	HIS	362	30.085	15.395	31.252	1.00	0.00	RX0	C
ATOM	1980	NE2	HIS	362	30.394	16.463	32.030	1.00	0.00	RX0	N
ATOM	1981	CE1	HIS	362	29.409	17.401	31.852	1.00	0.00	RX0	C
ATOM	1982	C	HIS	362	28.890	13.339	27.777	1.00	0.00	RX0	C
ATOM	1983	O	HIS	362	29.902	12.656	27.936	1.00	0.00	RX0	O
ATOM	1984	N	ILE	363	27.856	12.940	27.039	1.00	0.00	RX0	N
ATOM	1985	H	ILE	363	27.105	13.589	26.897	1.00	0.00	RX0	H
ATOM	1986	CA	ILE	363	27.773	11.584	26.446	1.00	0.00	RX0	C
ATOM	1987	CB	ILE	363	26.355	11.169	26.048	1.00	0.00	RX0	C
ATOM	1988	CG2	ILE	363	26.270	9.643	25.970	1.00	0.00	RX0	C
ATOM	1989	CG1	ILE	363	25.347	11.645	27.091	1.00	0.00	RX0	C
ATOM	1990	CD1	ILE	363	23.916	11.224	26.767	1.00	0.00	RX0	C
ATOM	1991	C	ILE	363	28.830	11.416	25.343	1.00	0.00	RX0	C
ATOM	1992	O	ILE	363	29.487	10.370	25.263	1.00	0.00	RX0	O
ATOM	1993	N	ARG	364	29.035	12.469	24.564	1.00	0.00	RX0	N
ATOM	1994	H	ARG	364	28.385	13.231	24.611	1.00	0.00	RX0	H
ATOM	1995	CA	ARG	364	30.102	12.498	23.547	1.00	0.00	RX0	C
ATOM	1996	CB	ARG	364	30.001	13.839	22.813	1.00	0.00	RX0	C
ATOM	1997	CG	ARG	364	30.621	13.977	21.419	1.00	0.00	RX0	C
ATOM	1998	CD	ARG	364	29.902	13.186	20.322	1.00	0.00	RX0	C
ATOM	1999	NE	ARG	364	28.458	13.152	20.541	1.00	0.00	RX0	N
ATOM	2000	HE	ARG	364	28.110	12.490	21.224	1.00	0.00	RX0	H
ATOM	2001	CZ	ARG	364	27.524	13.821	19.801	1.00	0.00	RX0	C
ATOM	2002	NH1	ARG	364	27.889	14.669	18.816	1.00	0.00	RX0	N
ATOM	2003	HH11	ARG	364	27.181	15.121	18.250	1.00	0.00	RX0	H
ATOM	2004	HH12	ARG	364	28.848	14.859	18.606	1.00	0.00	RX0	H
ATOM	2005	NH2	ARG	364	26.224	13.598	20.063	1.00	0.00	RX0	N
ATOM	2006	HH21	ARG	364	25.477	14.021	19.545	1.00	0.00	RX0	H
ATOM	2007	HH22	ARG	364	25.991	12.928	20.790	1.00	0.00	RX0	H
ATOM	2008	C	ARG	364	31.469	12.319	24.223	1.00	0.00	RX0	C
ATOM	2009	O	ARG	364	32.264	11.465	23.831	1.00	0.00	RX0	O
ATOM	2010	N	HIS	365	31.645	13.058	25.316	1.00	0.00	RX0	N
ATOM	2011	H	HIS	365	30.914	13.697	25.566	1.00	0.00	RX0	H

ATOM	2012	CA	HIS	365	32.879	13.027	26.115	1.00	0.00	RX0	C
ATOM	2013	CB	HIS	365	32.749	14.056	27.244	1.00	0.00	RX0	C
ATOM	2014	CG	HIS	365	34.058	14.359	27.940	1.00	0.00	RX0	C
ATOM	2015	ND1	HIS	365	34.954	13.436	28.341	1.00	0.00	RX0	N
ATOM	2016	HD1	HIS	365	34.904	12.465	28.204	1.00	0.00	RX0	H
ATOM	2017	CD2	HIS	365	34.534	15.623	28.304	1.00	0.00	RX0	C
ATOM	2018	NE2	HIS	365	35.724	15.450	28.932	1.00	0.00	RX0	N
ATOM	2019	CE1	HIS	365	35.981	14.103	28.953	1.00	0.00	RX0	C
ATOM	2020	C	HIS	365	33.175	11.617	26.649	1.00	0.00	RX0	C
ATOM	2021	O	HIS	365	34.274	11.105	26.425	1.00	0.00	RX0	O
ATOM	2022	N	MET	366	32.138	10.946	27.137	1.00	0.00	RX0	N
ATOM	2023	H	MET	366	31.249	11.408	27.187	1.00	0.00	RX0	H
ATOM	2024	CA	MET	366	32.267	9.572	27.662	1.00	0.00	RX0	C
ATOM	2025	CB	MET	366	31.020	9.149	28.439	1.00	0.00	RX0	C
ATOM	2026	CG	MET	366	30.752	10.030	29.658	1.00	0.00	RX0	C
ATOM	2027	SD	MET	366	29.423	9.403	30.696	1.00	0.00	RX0	S
ATOM	2028	CE	MET	366	28.116	9.393	29.463	1.00	0.00	RX0	C
ATOM	2029	C	MET	366	32.567	8.557	26.555	1.00	0.00	RX0	C
ATOM	2030	O	MET	366	33.398	7.674	26.741	1.00	0.00	RX0	O
ATOM	2031	N	SER	367	31.985	8.782	25.378	1.00	0.00	RX0	N
ATOM	2032	H	SER	367	31.321	9.523	25.278	1.00	0.00	RX0	H
ATOM	2033	CA	SER	367	32.231	7.934	24.196	1.00	0.00	RX0	C
ATOM	2034	CB	SER	367	31.158	8.257	23.175	1.00	0.00	RX0	C
ATOM	2035	OG	SER	367	29.912	8.077	23.850	1.00	0.00	RX0	O
ATOM	2036	HG	SER	367	29.647	8.926	24.190	1.00	0.00	RX0	H
ATOM	2037	C	SER	367	33.681	8.046	23.711	1.00	0.00	RX0	C
ATOM	2038	O	SER	367	34.361	7.038	23.567	1.00	0.00	RX0	O
ATOM	2039	N	ASN	368	34.193	9.278	23.688	1.00	0.00	RX0	N
ATOM	2040	H	ASN	368	33.594	10.049	23.913	1.00	0.00	RX0	H
ATOM	2041	CA	ASN	368	35.583	9.549	23.266	1.00	0.00	RX0	C
ATOM	2042	CB	ASN	368	35.906	11.009	22.986	1.00	0.00	RX0	C
ATOM	2043	CG	ASN	368	37.046	10.970	21.985	1.00	0.00	RX0	C
ATOM	2044	OD1	ASN	368	37.010	10.236	21.008	1.00	0.00	RX0	O
ATOM	2045	ND2	ASN	368	38.052	11.816	22.225	1.00	0.00	RX0	N
ATOM	2046	HD21	ASN	368	38.103	12.394	23.037	1.00	0.00	RX0	H
ATOM	2047	HD22	ASN	368	38.782	11.834	21.543	1.00	0.00	RX0	H
ATOM	2048	C	ASN	368	36.615	8.948	24.226	1.00	0.00	RX0	C
ATOM	2049	O	ASN	368	37.514	8.215	23.806	1.00	0.00	RX0	O
ATOM	2050	N	LYS	369	36.353	9.119	25.515	1.00	0.00	RX0	N
ATOM	2051	H	LYS	369	35.555	9.672	25.763	1.00	0.00	RX0	H
ATOM	2052	CA	LYS	369	37.205	8.554	26.580	1.00	0.00	RX0	C
ATOM	2053	CB	LYS	369	36.741	8.992	27.970	1.00	0.00	RX0	C
ATOM	2054	CG	LYS	369	36.928	10.460	28.330	1.00	0.00	RX0	C
ATOM	2055	CD	LYS	369	38.354	10.922	28.073	1.00	0.00	RX0	C
ATOM	2056	CE	LYS	369	39.436	10.026	28.678	1.00	0.00	RX0	C
ATOM	2057	NZ	LYS	369	40.710	10.480	28.124	1.00	0.00	RX0	N
ATOM	2058	HZ1	LYS	369	41.371	9.689	27.975	1.00	0.00	RX0	H
ATOM	2059	HZ2	LYS	369	40.565	10.789	27.136	1.00	0.00	RX0	H
ATOM	2060	HZ3	LYS	369	41.121	11.241	28.700	1.00	0.00	RX0	H
ATOM	2061	C	LYS	369	37.117	7.021	26.620	1.00	0.00	RX0	C
ATOM	2062	O	LYS	369	38.123	6.337	26.771	1.00	0.00	RX0	O
ATOM	2063	N	GLY	370	35.908	6.527	26.299	1.00	0.00	RX0	N
ATOM	2064	H	GLY	370	35.157	7.163	26.119	1.00	0.00	RX0	H
ATOM	2065	CA	GLY	370	35.597	5.090	26.225	1.00	0.00	RX0	C
ATOM	2066	C	GLY	370	36.324	4.429	25.048	1.00	0.00	RX0	C
ATOM	2067	O	GLY	370	36.946	3.386	25.214	1.00	0.00	RX0	O
ATOM	2068	N	MET	371	36.380	5.143	23.928	1.00	0.00	RX0	N
ATOM	2069	H	MET	371	35.921	6.032	23.908	1.00	0.00	RX0	H
ATOM	2070	CA	MET	371	37.103	4.709	22.719	1.00	0.00	RX0	C
ATOM	2071	CB	MET	371	36.785	5.595	21.512	1.00	0.00	RX0	C
ATOM	2072	CG	MET	371	35.354	5.439	20.997	1.00	0.00	RX0	C

ATOM	2073	SD	MET	371	34.986	3.770	20.433	1.00	0.00	RX0	S
ATOM	2074	CE	MET	371	36.010	3.774	18.954	1.00	0.00	RX0	C
ATOM	2075	C	MET	371	38.619	4.671	22.937	1.00	0.00	RX0	C
ATOM	2076	O	MET	371	39.258	3.669	22.631	1.00	0.00	RX0	O
ATOM	2077	N	GLU	372	39.117	5.692	23.643	1.00	0.00	RX0	N
ATOM	2078	H	GLU	372	38.534	6.484	23.838	1.00	0.00	RX0	H
ATOM	2079	CA	GLU	372	40.525	5.740	24.082	1.00	0.00	RX0	C
ATOM	2080	CB	GLU	372	40.809	6.969	24.957	1.00	0.00	RX0	C
ATOM	2081	CG	GLU	372	40.832	8.365	24.326	1.00	0.00	RX0	C
ATOM	2082	CD	GLU	372	41.009	9.412	25.423	1.00	0.00	RX0	C
ATOM	2083	OE1	GLU	372	40.222	10.353	25.485	1.00	0.00	RX0	O
ATOM	2084	OE2	GLU	372	41.907	9.293	26.259	1.00	0.00	RX0	O
ATOM	2085	C	GLU	372	40.884	4.541	24.968	1.00	0.00	RX0	C
ATOM	2086	O	GLU	372	41.865	3.853	24.723	1.00	0.00	RX0	O
ATOM	2087	N	HIS	373	40.006	4.291	25.945	1.00	0.00	RX0	N
ATOM	2088	H	HIS	373	39.220	4.903	26.040	1.00	0.00	RX0	H
ATOM	2089	CA	HIS	373	40.194	3.212	26.919	1.00	0.00	RX0	C
ATOM	2090	CB	HIS	373	39.263	3.436	28.120	1.00	0.00	RX0	C
ATOM	2091	CG	HIS	373	38.386	2.246	28.433	1.00	0.00	RX0	C
ATOM	2092	ND1	HIS	373	37.278	1.932	27.735	1.00	0.00	RX0	N
ATOM	2093	HD1	HIS	373	36.946	2.393	26.932	1.00	0.00	RX0	H
ATOM	2094	CD2	HIS	373	38.533	1.332	29.481	1.00	0.00	RX0	C
ATOM	2095	NE2	HIS	373	37.494	0.462	29.408	1.00	0.00	RX0	N
ATOM	2096	CE1	HIS	373	36.724	0.832	28.336	1.00	0.00	RX0	C
ATOM	2097	C	HIS	373	40.078	1.832	26.262	1.00	0.00	RX0	C
ATOM	2098	O	HIS	373	40.934	1.001	26.470	1.00	0.00	RX0	O
ATOM	2099	N	LEU	374	39.111	1.679	25.351	1.00	0.00	RX0	N
ATOM	2100	H	LEU	374	38.558	2.475	25.114	1.00	0.00	RX0	H
ATOM	2101	CA	LEU	374	38.919	0.408	24.629	1.00	0.00	RX0	C
ATOM	2102	CB	LEU	374	37.661	0.451	23.762	1.00	0.00	RX0	C
ATOM	2103	CG	LEU	374	36.365	0.293	24.555	1.00	0.00	RX0	C
ATOM	2104	CD1	LEU	374	35.135	0.605	23.700	1.00	0.00	RX0	C
ATOM	2105	CD2	LEU	374	36.275	-1.085	25.213	1.00	0.00	RX0	C
ATOM	2106	C	LEU	374	40.113	0.051	23.744	1.00	0.00	RX0	C
ATOM	2107	O	LEU	374	40.555	-1.109	23.754	1.00	0.00	RX0	O
ATOM	2108	N	TYR	375	40.664	1.068	23.103	1.00	0.00	RX0	N
ATOM	2109	H	TYR	375	40.304	1.989	23.272	1.00	0.00	RX0	H
ATOM	2110	CA	TYR	375	41.854	0.983	22.222	1.00	0.00	RX0	C
ATOM	2111	CB	TYR	375	41.895	2.119	21.202	1.00	0.00	RX0	C
ATOM	2112	CG	TYR	375	41.097	1.644	20.009	1.00	0.00	RX0	C
ATOM	2113	CD1	TYR	375	41.670	0.738	19.123	1.00	0.00	RX0	C
ATOM	2114	CE1	TYR	375	40.933	0.237	18.056	1.00	0.00	RX0	C
ATOM	2115	CD2	TYR	375	39.791	2.074	19.807	1.00	0.00	RX0	C
ATOM	2116	CE2	TYR	375	39.052	1.572	18.740	1.00	0.00	RX0	C
ATOM	2117	CZ	TYR	375	39.617	0.646	17.869	1.00	0.00	RX0	C
ATOM	2118	OH	TYR	375	38.875	0.130	16.823	1.00	0.00	RX0	O
ATOM	2119	HH	TYR	375	38.015	0.537	16.836	1.00	0.00	RX0	H
ATOM	2120	C	TYR	375	43.165	0.750	22.977	1.00	0.00	RX0	C
ATOM	2121	O	TYR	375	44.176	1.452	22.753	1.00	0.00	RX0	O
ATOM	2122	N	SER	376	43.162	-0.226	23.845	1.00	0.00	RX0	N
ATOM	2123	H	SER	376	42.296	-0.613	24.146	1.00	0.00	RX0	H
ATOM	2124	CA	SER	376	44.345	-0.646	24.634	1.00	0.00	RX0	C
ATOM	2125	CB	SER	376	44.672	0.409	25.703	1.00	0.00	RX0	C
ATOM	2126	OG	SER	376	44.167	1.701	25.352	1.00	0.00	RX0	O
ATOM	2127	HG	SER	376	44.505	1.916	24.488	1.00	0.00	RX0	H
ATOM	2128	C	SER	376	44.183	-1.992	25.350	1.00	0.00	RX0	C
ATOM	2129	O	SER	376	44.985	-2.369	26.199	1.00	0.00	RX0	O
ATOM	2130	N	MET	377	43.156	-2.746	24.938	1.00	0.00	RX0	N
ATOM	2131	H	MET	377	42.554	-2.427	24.205	1.00	0.00	RX0	H
ATOM	2132	CA	MET	377	42.915	-4.112	25.422	1.00	0.00	RX0	C
ATOM	2133	CB	MET	377	41.735	-4.071	26.399	1.00	0.00	RX0	C

ATOM	2134	CG	MET	377	42.037	-3.406	27.743	1.00	0.00	RX0	C
ATOM	2135	SD	MET	377	40.548	-3.241	28.735	1.00	0.00	RX0	S
ATOM	2136	CE	MET	377	39.728	-2.014	27.704	1.00	0.00	RX0	C
ATOM	2137	C	MET	377	42.647	-5.080	24.257	1.00	0.00	RX0	C
ATOM	2138	O	MET	377	43.092	-4.854	23.130	1.00	0.00	RX0	O
ATOM	2139	N	LYS	378	41.856	-6.107	24.527	1.00	0.00	RX0	N
ATOM	2140	H	LYS	378	41.482	-6.208	25.443	1.00	0.00	RX0	H
ATOM	2141	CA	LYS	378	41.570	-7.191	23.576	1.00	0.00	RX0	C
ATOM	2142	CB	LYS	378	41.428	-8.519	24.362	1.00	0.00	RX0	C
ATOM	2143	CG	LYS	378	40.424	-8.511	25.541	1.00	0.00	RX0	C
ATOM	2144	CD	LYS	378	40.320	-9.816	26.358	1.00	0.00	RX0	C
ATOM	2145	CE	LYS	378	39.355	-9.724	27.561	1.00	0.00	RX0	C
ATOM	2146	NZ	LYS	378	39.310	-10.990	28.323	1.00	0.00	RX0	N
ATOM	2147	HZ1	LYS	378	38.759	-10.897	29.209	1.00	0.00	RX0	H
ATOM	2148	HZ2	LYS	378	38.873	-11.730	27.738	1.00	0.00	RX0	H
ATOM	2149	HZ3	LYS	378	40.276	-11.282	28.571	1.00	0.00	RX0	H
ATOM	2150	C	LYS	378	40.316	-6.920	22.720	1.00	0.00	RX0	C
ATOM	2151	O	LYS	378	40.245	-7.352	21.611	1.00	0.00	RX0	O
ATOM	2152	N	CYS	379	39.320	-6.266	23.410	1.00	0.00	RX0	N
ATOM	2153	H	CYS	379	39.561	-5.894	24.301	1.00	0.00	RX0	H
ATOM	2154	CA	CYS	379	37.939	-6.053	22.985	1.00	0.00	RX0	C
ATOM	2155	CB	CYS	379	37.614	-4.559	23.098	1.00	0.00	RX0	C
ATOM	2156	SG	CYS	379	35.844	-4.181	23.071	1.00	0.00	RX0	S
ATOM	2157	C	CYS	379	37.599	-6.654	21.614	1.00	0.00	RX0	C
ATOM	2158	O	CYS	379	37.115	-7.773	21.559	1.00	0.00	RX0	O
ATOM	2159	N	LYS	380	38.012	-5.930	20.563	1.00	0.00	RX0	N
ATOM	2160	H	LYS	380	38.375	-5.002	20.645	1.00	0.00	RX0	H
ATOM	2161	CA	LYS	380	37.949	-6.383	19.165	1.00	0.00	RX0	C
ATOM	2162	CB	LYS	380	36.477	-6.758	18.912	1.00	0.00	RX0	C
ATOM	2163	CG	LYS	380	35.908	-7.174	17.554	1.00	0.00	RX0	C
ATOM	2164	CD	LYS	380	34.397	-7.375	17.756	1.00	0.00	RX0	C
ATOM	2165	CE	LYS	380	33.577	-7.761	16.522	1.00	0.00	RX0	C
ATOM	2166	NZ	LYS	380	32.164	-7.877	16.908	1.00	0.00	RX0	N
ATOM	2167	HZ1	LYS	380	31.520	-8.148	16.146	1.00	0.00	RX0	H
ATOM	2168	HZ2	LYS	380	31.993	-8.567	17.674	1.00	0.00	RX0	H
ATOM	2169	HZ3	LYS	380	31.774	-7.011	17.342	1.00	0.00	RX0	H
ATOM	2170	C	LYS	380	38.311	-5.215	18.248	1.00	0.00	RX0	C
ATOM	2171	O	LYS	380	37.971	-4.057	18.518	1.00	0.00	RX0	O
ATOM	2172	N	ASN	381	38.881	-5.592	17.123	1.00	0.00	RX0	N
ATOM	2173	H	ASN	381	39.166	-6.547	17.045	1.00	0.00	RX0	H
ATOM	2174	CA	ASN	381	39.208	-4.700	15.996	1.00	0.00	RX0	C
ATOM	2175	CB	ASN	381	40.308	-3.727	16.461	1.00	0.00	RX0	C
ATOM	2176	CG	ASN	381	40.838	-2.831	15.357	1.00	0.00	RX0	C
ATOM	2177	OD1	ASN	381	41.935	-3.049	14.847	1.00	0.00	RX0	O
ATOM	2178	ND2	ASN	381	40.041	-1.795	15.048	1.00	0.00	RX0	N
ATOM	2179	HD21	ASN	381	39.255	-1.542	15.623	1.00	0.00	RX0	H
ATOM	2180	HD22	ASN	381	40.208	-1.200	14.263	1.00	0.00	RX0	H
ATOM	2181	C	ASN	381	39.620	-5.523	14.759	1.00	0.00	RX0	C
ATOM	2182	O	ASN	381	40.286	-5.062	13.834	1.00	0.00	RX0	O
ATOM	2183	N	VAL	382	39.176	-6.776	14.737	1.00	0.00	RX0	N
ATOM	2184	H	VAL	382	38.426	-7.026	15.344	1.00	0.00	RX0	H
ATOM	2185	CA	VAL	382	39.338	-7.659	13.572	1.00	0.00	RX0	C
ATOM	2186	CB	VAL	382	39.215	-9.093	14.087	1.00	0.00	RX0	C
ATOM	2187	CG1	VAL	382	38.861	-10.101	12.999	1.00	0.00	RX0	C
ATOM	2188	CG2	VAL	382	40.481	-9.468	14.859	1.00	0.00	RX0	C
ATOM	2189	C	VAL	382	38.195	-7.277	12.635	1.00	0.00	RX0	C
ATOM	2190	O	VAL	382	37.049	-7.276	13.059	1.00	0.00	RX0	O
ATOM	2191	N	VAL	383	38.551	-7.063	11.363	1.00	0.00	RX0	N
ATOM	2192	H	VAL	383	39.523	-7.101	11.128	1.00	0.00	RX0	H
ATOM	2193	CA	VAL	383	37.600	-6.539	10.366	1.00	0.00	RX0	C
ATOM	2194	CB	VAL	383	36.417	-7.483	10.110	1.00	0.00	RX0	C

ATOM	2195	CG1	VAL	383	35.366	-6.828	9.211	1.00	0.00	RX0	C
ATOM	2196	CG2	VAL	383	36.894	-8.814	9.537	1.00	0.00	RX0	C
ATOM	2197	C	VAL	383	37.102	-5.173	10.877	1.00	0.00	RX0	C
ATOM	2198	O	VAL	383	36.355	-5.097	11.860	1.00	0.00	RX0	O
ATOM	2199	N	PRO	384	37.530	-4.088	10.236	1.00	0.00	RX0	N
ATOM	2200	CD	PRO	384	38.440	-4.092	9.097	1.00	0.00	RX0	C
ATOM	2201	CA	PRO	384	37.142	-2.731	10.663	1.00	0.00	RX0	C
ATOM	2202	CB	PRO	384	37.676	-1.869	9.516	1.00	0.00	RX0	C
ATOM	2203	CG	PRO	384	38.879	-2.639	8.969	1.00	0.00	RX0	C
ATOM	2204	C	PRO	384	35.624	-2.628	10.828	1.00	0.00	RX0	C
ATOM	2205	O	PRO	384	34.847	-3.282	10.112	1.00	0.00	RX0	O
ATOM	2206	N	LEU	385	35.228	-1.769	11.750	1.00	0.00	RX0	N
ATOM	2207	H	LEU	385	35.906	-1.178	12.181	1.00	0.00	RX0	H
ATOM	2208	CA	LEU	385	33.812	-1.621	12.127	1.00	0.00	RX0	C
ATOM	2209	CB	LEU	385	33.676	-0.667	13.313	1.00	0.00	RX0	C
ATOM	2210	CG	LEU	385	32.278	-0.703	13.932	1.00	0.00	RX0	C
ATOM	2211	CD1	LEU	385	31.896	-2.107	14.405	1.00	0.00	RX0	C
ATOM	2212	CD2	LEU	385	32.125	0.335	15.041	1.00	0.00	RX0	C
ATOM	2213	C	LEU	385	32.896	-1.213	10.964	1.00	0.00	RX0	C
ATOM	2214	O	LEU	385	31.809	-1.761	10.815	1.00	0.00	RX0	O
ATOM	2215	N	TYR	386	33.449	-0.406	10.055	1.00	0.00	RX0	N
ATOM	2216	H	TYR	386	34.245	0.131	10.339	1.00	0.00	RX0	H
ATOM	2217	CA	TYR	386	32.718	0.069	8.867	1.00	0.00	RX0	C
ATOM	2218	CB	TYR	386	33.647	0.912	7.988	1.00	0.00	RX0	C
ATOM	2219	CG	TYR	386	32.907	1.653	6.895	1.00	0.00	RX0	C
ATOM	2220	CD1	TYR	386	32.723	3.027	7.000	1.00	0.00	RX0	C
ATOM	2221	CE1	TYR	386	32.089	3.727	5.979	1.00	0.00	RX0	C
ATOM	2222	CD2	TYR	386	32.429	0.981	5.775	1.00	0.00	RX0	C
ATOM	2223	CE2	TYR	386	31.795	1.678	4.755	1.00	0.00	RX0	C
ATOM	2224	CZ	TYR	386	31.639	3.054	4.849	1.00	0.00	RX0	C
ATOM	2225	OH	TYR	386	31.046	3.753	3.819	1.00	0.00	RX0	O
ATOM	2226	HH	TYR	386	31.330	3.375	2.982	1.00	0.00	RX0	H
ATOM	2227	C	TYR	386	32.144	-1.103	8.049	1.00	0.00	RX0	C
ATOM	2228	O	TYR	386	30.957	-1.141	7.757	1.00	0.00	RX0	O
ATOM	2229	N	ASP	387	32.969	-2.141	7.903	1.00	0.00	RX0	N
ATOM	2230	H	ASP	387	33.922	-2.094	8.209	1.00	0.00	RX0	H
ATOM	2231	CA	ASP	387	32.619	-3.323	7.093	1.00	0.00	RX0	C
ATOM	2232	CB	ASP	387	33.851	-4.187	6.792	1.00	0.00	RX0	C
ATOM	2233	CG	ASP	387	35.105	-3.387	6.472	1.00	0.00	RX0	C
ATOM	2234	OD1	ASP	387	35.025	-2.306	5.892	1.00	0.00	RX0	O
ATOM	2235	OD2	ASP	387	36.187	-3.865	6.797	1.00	0.00	RX0	O
ATOM	2236	C	ASP	387	31.563	-4.207	7.765	1.00	0.00	RX0	C
ATOM	2237	O	ASP	387	30.619	-4.649	7.114	1.00	0.00	RX0	O
ATOM	2238	N	LEU	388	31.677	-4.332	9.088	1.00	0.00	RX0	N
ATOM	2239	H	LEU	388	32.453	-3.867	9.520	1.00	0.00	RX0	H
ATOM	2240	CA	LEU	388	30.691	-5.072	9.891	1.00	0.00	RX0	C
ATOM	2241	CB	LEU	388	31.207	-5.218	11.323	1.00	0.00	RX0	C
ATOM	2242	CG	LEU	388	30.284	-6.048	12.217	1.00	0.00	RX0	C
ATOM	2243	CD1	LEU	388	30.090	-7.466	11.676	1.00	0.00	RX0	C
ATOM	2244	CD2	LEU	388	30.752	-6.046	13.673	1.00	0.00	RX0	C
ATOM	2245	C	LEU	388	29.315	-4.386	9.871	1.00	0.00	RX0	C
ATOM	2246	O	LEU	388	28.299	-5.023	9.588	1.00	0.00	RX0	O
ATOM	2247	N	LEU	389	29.339	-3.070	10.049	1.00	0.00	RX0	N
ATOM	2248	H	LEU	389	30.229	-2.626	10.176	1.00	0.00	RX0	H
ATOM	2249	CA	LEU	389	28.129	-2.229	9.970	1.00	0.00	RX0	C
ATOM	2250	CB	LEU	389	28.429	-0.784	10.350	1.00	0.00	RX0	C
ATOM	2251	CG	LEU	389	28.869	-0.621	11.802	1.00	0.00	RX0	C
ATOM	2252	CD1	LEU	389	29.265	0.821	12.087	1.00	0.00	RX0	C
ATOM	2253	CD2	LEU	389	27.815	-1.116	12.790	1.00	0.00	RX0	C
ATOM	2254	C	LEU	389	27.489	-2.268	8.583	1.00	0.00	RX0	C
ATOM	2255	O	LEU	389	26.284	-2.473	8.458	1.00	0.00	RX0	O

ATOM	2256	N	LEU	390	28.350	-2.246	7.565	1.00	0.00	RX0	N
ATOM	2257	H	LEU	390	29.327	-2.139	7.756	1.00	0.00	RX0	H
ATOM	2258	CA	LEU	390	27.929	-2.335	6.162	1.00	0.00	RX0	C
ATOM	2259	CB	LEU	390	29.109	-2.096	5.216	1.00	0.00	RX0	C
ATOM	2260	CG	LEU	390	28.679	-1.880	3.761	1.00	0.00	RX0	C
ATOM	2261	CD1	LEU	390	29.337	-0.640	3.156	1.00	0.00	RX0	C
ATOM	2262	CD2	LEU	390	28.890	-3.126	2.897	1.00	0.00	RX0	C
ATOM	2263	C	LEU	390	27.202	-3.653	5.862	1.00	0.00	RX0	C
ATOM	2264	O	LEU	390	26.123	-3.627	5.302	1.00	0.00	RX0	O
ATOM	2265	N	GLU	391	27.733	-4.754	6.404	1.00	0.00	RX0	N
ATOM	2266	H	GLU	391	28.641	-4.703	6.824	1.00	0.00	RX0	H
ATOM	2267	CA	GLU	391	27.124	-6.090	6.264	1.00	0.00	RX0	C
ATOM	2268	CB	GLU	391	27.999	-7.128	6.971	1.00	0.00	RX0	C
ATOM	2269	CG	GLU	391	29.277	-7.520	6.229	1.00	0.00	RX0	C
ATOM	2270	CD	GLU	391	28.950	-8.553	5.170	1.00	0.00	RX0	C
ATOM	2271	OE1	GLU	391	28.294	-9.540	5.502	1.00	0.00	RX0	O
ATOM	2272	OE2	GLU	391	29.363	-8.378	4.026	1.00	0.00	RX0	O
ATOM	2273	C	GLU	391	25.713	-6.159	6.867	1.00	0.00	RX0	C
ATOM	2274	O	GLU	391	24.778	-6.600	6.214	1.00	0.00	RX0	O
ATOM	2275	N	MET	392	25.589	-5.588	8.070	1.00	0.00	RX0	N
ATOM	2276	H	MET	392	26.420	-5.190	8.466	1.00	0.00	RX0	H
ATOM	2277	CA	MET	392	24.313	-5.555	8.807	1.00	0.00	RX0	C
ATOM	2278	CB	MET	392	24.545	-5.227	10.281	1.00	0.00	RX0	C
ATOM	2279	CG	MET	392	25.244	-6.380	11.004	1.00	0.00	RX0	C
ATOM	2280	SD	MET	392	25.410	-6.113	12.777	1.00	0.00	RX0	S
ATOM	2281	CE	MET	392	26.596	-4.765	12.713	1.00	0.00	RX0	C
ATOM	2282	C	MET	392	23.266	-4.630	8.173	1.00	0.00	RX0	C
ATOM	2283	O	MET	392	22.107	-5.006	8.028	1.00	0.00	RX0	O
ATOM	2284	N	LEU	393	23.721	-3.452	7.754	1.00	0.00	RX0	N
ATOM	2285	H	LEU	393	24.708	-3.285	7.787	1.00	0.00	RX0	H
ATOM	2286	CA	LEU	393	22.852	-2.457	7.099	1.00	0.00	RX0	C
ATOM	2287	CB	LEU	393	23.511	-1.082	7.150	1.00	0.00	RX0	C
ATOM	2288	CG	LEU	393	23.453	-0.451	8.538	1.00	0.00	RX0	C
ATOM	2289	CD1	LEU	393	24.516	0.631	8.711	1.00	0.00	RX0	C
ATOM	2290	CD2	LEU	393	22.052	0.071	8.858	1.00	0.00	RX0	C
ATOM	2291	C	LEU	393	22.462	-2.815	5.663	1.00	0.00	RX0	C
ATOM	2292	O	LEU	393	21.313	-2.601	5.257	1.00	0.00	RX0	O
ATOM	2293	N	ASP	394	23.400	-3.384	4.923	1.00	0.00	RX0	N
ATOM	2294	H	ASP	394	24.269	-3.667	5.326	1.00	0.00	RX0	H
ATOM	2295	CA	ASP	394	23.187	-3.768	3.516	1.00	0.00	RX0	C
ATOM	2296	CB	ASP	394	24.208	-3.557	2.400	1.00	0.00	RX0	C
ATOM	2297	CG	ASP	394	23.379	-3.395	1.104	1.00	0.00	RX0	C
ATOM	2298	OD1	ASP	394	22.137	-3.399	1.149	1.00	0.00	RX0	O
ATOM	2299	OD2	ASP	394	23.966	-3.235	0.036	1.00	0.00	RX0	O
ATOM	2300	C	ASP	394	22.492	-5.132	3.432	1.00	0.00	RX0	C
ATOM	2301	O	ASP	394	23.034	-6.108	2.888	1.00	0.00	RX0	O
ATOM	2302	N	ALA	395	21.256	-5.134	3.874	1.00	0.00	RX0	N
ATOM	2303	H	ALA	395	20.894	-4.232	4.112	1.00	0.00	RX0	H
ATOM	2304	CA	ALA	395	20.412	-6.338	3.880	1.00	0.00	RX0	C
ATOM	2305	CB	ALA	395	19.422	-6.295	5.045	1.00	0.00	RX0	C
ATOM	2306	C	ALA	395	19.639	-6.454	2.565	1.00	0.00	RX0	C
ATOM	2307	O	ALA	395	19.303	-5.463	1.911	1.00	0.00	RX0	O
ATOM	2308	N	HIS	396	19.441	-7.696	2.156	1.00	0.00	RX0	N
ATOM	2309	H	HIS	396	19.739	-8.408	2.789	1.00	0.00	RX0	H
ATOM	2310	CA	HIS	396	18.536	-7.999	1.035	1.00	0.00	RX0	C
ATOM	2311	CB	HIS	396	18.856	-9.401	0.513	1.00	0.00	RX0	C
ATOM	2312	CG	HIS	396	19.016	-10.346	1.685	1.00	0.00	RX0	C
ATOM	2313	ND1	HIS	396	20.211	-10.765	2.145	1.00	0.00	RX0	N
ATOM	2314	HD1	HIS	396	21.098	-10.534	1.787	1.00	0.00	RX0	H
ATOM	2315	CD2	HIS	396	18.015	-10.907	2.483	1.00	0.00	RX0	C
ATOM	2316	NE2	HIS	396	18.615	-11.665	3.428	1.00	0.00	RX0	N

ATOM	2317	CE1	HIS	396	19.970	-11.580	3.221	1.00	0.00	RX0	C
ATOM	2318	C	HIS	396	17.077	-7.909	1.523	1.00	0.00	RX0	C
ATOM	2319	O	HIS	396	16.821	-7.755	2.721	1.00	0.00	RX0	O
ATOM	2320	N	ARG	397	16.142	-8.174	0.629	1.00	0.00	RX0	N
ATOM	2321	H	ARG	397	16.383	-8.386	-0.317	1.00	0.00	RX0	H
ATOM	2322	CA	ARG	397	14.704	-8.151	0.982	1.00	0.00	RX0	C
ATOM	2323	CB	ARG	397	14.159	-6.973	0.134	1.00	0.00	RX0	C
ATOM	2324	CG	ARG	397	15.016	-5.658	0.194	1.00	0.00	RX0	C
ATOM	2325	CD	ARG	397	16.203	-5.414	-0.801	1.00	0.00	RX0	C
ATOM	2326	NE	ARG	397	17.402	-4.832	-0.150	1.00	0.00	RX0	N
ATOM	2327	HE	ARG	397	17.475	-4.922	0.853	1.00	0.00	RX0	H
ATOM	2328	CZ	ARG	397	18.480	-4.268	-0.821	1.00	0.00	RX0	C
ATOM	2329	NH1	ARG	397	18.420	-4.050	-2.155	1.00	0.00	RX0	N
ATOM	2330	HH11	ARG	397	19.201	-3.647	-2.644	1.00	0.00	RX0	H
ATOM	2331	HH12	ARG	397	17.610	-4.278	-2.700	1.00	0.00	RX0	H
ATOM	2332	NH2	ARG	397	19.612	-3.947	-0.143	1.00	0.00	RX0	N
ATOM	2333	HH21	ARG	397	20.452	-3.516	-0.505	1.00	0.00	RX0	H
ATOM	2334	HH22	ARG	397	19.711	-4.169	0.840	1.00	0.00	RX0	H
ATOM	2335	C	ARG	397	14.082	-9.540	0.774	1.00	0.00	RX0	C
ATOM	2336	O	ARG	397	12.875	-9.706	0.613	1.00	0.00	RX0	O
ATOM	2337	N	LEU	398	14.938	-10.556	0.835	1.00	0.00	RX0	N
ATOM	2338	H	LEU	398	15.871	-10.365	1.128	1.00	0.00	RX0	H
ATOM	2339	CA	LEU	398	14.565	-11.951	0.542	1.00	0.00	RX0	C
ATOM	2340	CB	LEU	398	15.799	-12.794	0.228	1.00	0.00	RX0	C
ATOM	2341	CG	LEU	398	16.576	-12.268	-0.980	1.00	0.00	RX0	C
ATOM	2342	CD1	LEU	398	17.867	-13.057	-1.208	1.00	0.00	RX0	C
ATOM	2343	CD2	LEU	398	15.707	-12.208	-2.237	1.00	0.00	RX0	C
ATOM	2344	C	LEU	398	13.757	-12.584	1.680	1.00	0.00	RX0	C
ATOM	2345	O	LEU	398	12.772	-13.259	1.439	1.00	0.00	RX0	O
ATOM	2346	N	HIS	399	14.100	-12.169	2.904	1.00	0.00	RX0	N
ATOM	2347	H	HIS	399	14.843	-11.514	3.012	1.00	0.00	RX0	H
ATOM	2348	CA	HIS	399	13.392	-12.616	4.118	1.00	0.00	RX0	C
ATOM	2349	CB	HIS	399	14.353	-12.614	5.307	1.00	0.00	RX0	C
ATOM	2350	CG	HIS	399	15.405	-13.671	5.069	1.00	0.00	RX0	C
ATOM	2351	ND1	HIS	399	16.708	-13.412	4.840	1.00	0.00	RX0	N
ATOM	2352	HD1	HIS	399	17.152	-12.537	4.799	1.00	0.00	RX0	H
ATOM	2353	CD2	HIS	399	15.209	-15.054	5.031	1.00	0.00	RX0	C
ATOM	2354	NE2	HIS	399	16.409	-15.627	4.775	1.00	0.00	RX0	N
ATOM	2355	CE1	HIS	399	17.331	-14.620	4.658	1.00	0.00	RX0	C
ATOM	2356	C	HIS	399	12.131	-11.786	4.401	1.00	0.00	RX0	C
ATOM	2357	O	HIS	399	11.630	-11.743	5.524	1.00	0.00	RX0	O
ATOM	2358	N	ALA	400	11.619	-11.143	3.351	1.00	0.00	RX0	N
ATOM	2359	H	ALA	400	11.998	-11.313	2.443	1.00	0.00	RX0	H
ATOM	2360	CA	ALA	400	10.356	-10.385	3.402	1.00	0.00	RX0	C
ATOM	2361	CB	ALA	400	10.144	-9.553	2.137	1.00	0.00	RX0	C
ATOM	2362	C	ALA	400	9.147	-11.323	3.579	1.00	0.00	RX0	C
ATOM	2363	O	ALA	400	8.508	-11.220	4.647	1.00	0.00	RX0	O
ATOM	2364	N	GLU	26	60.738	22.011	-9.048	1.00	0.00	RX1	N
ATOM	2365	H	GLU	26	61.696	21.749	-9.139	1.00	0.00	RX1	H
ATOM	2366	CA	GLU	26	59.774	21.589	-10.066	1.00	0.00	RX1	C
ATOM	2367	CB	GLU	26	59.877	20.061	-10.191	1.00	0.00	RX1	C
ATOM	2368	CG	GLU	26	60.545	19.481	-8.932	1.00	0.00	RX1	C
ATOM	2369	CD	GLU	26	60.148	18.042	-8.639	1.00	0.00	RX1	C
ATOM	2370	OE1	GLU	26	59.225	17.532	-9.265	1.00	0.00	RX1	O
ATOM	2371	OE2	GLU	26	60.724	17.445	-7.728	1.00	0.00	RX1	O
ATOM	2372	C	GLU	26	58.345	21.943	-9.709	1.00	0.00	RX1	C
ATOM	2373	O	GLU	26	57.989	22.117	-8.543	1.00	0.00	RX1	O
ATOM	2374	N	GLU	27	57.517	22.009	-10.763	1.00	0.00	RX1	N
ATOM	2375	H	GLU	27	57.843	21.827	-11.688	1.00	0.00	RX1	H
ATOM	2376	CA	GLU	27	56.095	22.223	-10.502	1.00	0.00	RX1	C
ATOM	2377	CB	GLU	27	55.385	22.910	-11.672	1.00	0.00	RX1	C

ATOM	2378	CG	GLU	27	56.192	24.017	-12.355	1.00	0.00	RX1	C
ATOM	2379	CD	GLU	27	56.797	24.957	-11.333	1.00	0.00	RX1	C
ATOM	2380	OE1	GLU	27	56.092	25.473	-10.471	1.00	0.00	RX1	O
ATOM	2381	OE2	GLU	27	58.005	25.153	-11.369	1.00	0.00	RX1	O
ATOM	2382	C	GLU	27	55.373	20.944	-10.136	1.00	0.00	RX1	C
ATOM	2383	O	GLU	27	54.536	20.421	-10.861	1.00	0.00	RX1	O
ATOM	2384	N	LYS	28	55.770	20.455	-8.954	1.00	0.00	RX1	N
ATOM	2385	H	LYS	28	56.401	21.002	-8.407	1.00	0.00	RX1	H
ATOM	2386	CA	LYS	28	55.200	19.217	-8.438	1.00	0.00	RX1	C
ATOM	2387	CB	LYS	28	55.846	18.822	-7.106	1.00	0.00	RX1	C
ATOM	2388	CG	LYS	28	57.306	19.217	-6.884	1.00	0.00	RX1	C
ATOM	2389	CD	LYS	28	57.915	18.588	-5.625	1.00	0.00	RX1	C
ATOM	2390	CE	LYS	28	59.363	19.026	-5.371	1.00	0.00	RX1	C
ATOM	2391	NZ	LYS	28	60.031	18.052	-4.500	1.00	0.00	RX1	N
ATOM	2392	HZ1	LYS	28	61.064	18.193	-4.483	1.00	0.00	RX1	H
ATOM	2393	HZ2	LYS	28	59.640	18.108	-3.534	1.00	0.00	RX1	H
ATOM	2394	HZ3	LYS	28	59.887	17.102	-4.898	1.00	0.00	RX1	H
ATOM	2395	C	LYS	28	53.709	19.383	-8.215	1.00	0.00	RX1	C
ATOM	2396	O	LYS	28	53.274	20.265	-7.481	1.00	0.00	RX1	O
ATOM	2397	N	LYS	29	52.941	18.547	-8.919	1.00	0.00	RX1	N
ATOM	2398	H	LYS	29	53.327	17.725	-9.346	1.00	0.00	RX1	H
ATOM	2399	CA	LYS	29	51.509	18.829	-8.938	1.00	0.00	RX1	C
ATOM	2400	CB	LYS	29	50.823	18.112	-10.098	1.00	0.00	RX1	C
ATOM	2401	CG	LYS	29	51.666	18.218	-11.371	1.00	0.00	RX1	C
ATOM	2402	CD	LYS	29	51.076	17.456	-12.557	1.00	0.00	RX1	C
ATOM	2403	CE	LYS	29	50.683	16.018	-12.211	1.00	0.00	RX1	C
ATOM	2404	NZ	LYS	29	51.818	15.245	-11.695	1.00	0.00	RX1	N
ATOM	2405	HZ1	LYS	29	51.562	14.852	-10.760	1.00	0.00	RX1	H
ATOM	2406	HZ2	LYS	29	52.055	14.430	-12.291	1.00	0.00	RX1	H
ATOM	2407	HZ3	LYS	29	52.669	15.804	-11.468	1.00	0.00	RX1	H
ATOM	2408	C	LYS	29	50.845	18.540	-7.608	1.00	0.00	RX1	C
ATOM	2409	O	LYS	29	51.117	17.546	-6.943	1.00	0.00	RX1	O
ATOM	2410	N	VAL	30	49.997	19.497	-7.230	1.00	0.00	RX1	N
ATOM	2411	H	VAL	30	49.689	20.203	-7.863	1.00	0.00	RX1	H
ATOM	2412	CA	VAL	30	49.432	19.415	-5.890	1.00	0.00	RX1	C
ATOM	2413	CB	VAL	30	49.502	20.781	-5.206	1.00	0.00	RX1	C
ATOM	2414	CG1	VAL	30	50.953	21.248	-5.081	1.00	0.00	RX1	C
ATOM	2415	CG2	VAL	30	48.633	21.814	-5.928	1.00	0.00	RX1	C
ATOM	2416	C	VAL	30	48.012	18.887	-5.892	1.00	0.00	RX1	C
ATOM	2417	O	VAL	30	47.307	18.969	-6.891	1.00	0.00	RX1	O
ATOM	2418	N	CYS	31	47.615	18.373	-4.725	1.00	0.00	RX1	N
ATOM	2419	H	CYS	31	48.235	18.256	-3.945	1.00	0.00	RX1	H
ATOM	2420	CA	CYS	31	46.214	17.993	-4.583	1.00	0.00	RX1	C
ATOM	2421	CB	CYS	31	46.056	16.492	-4.799	1.00	0.00	RX1	C
ATOM	2422	SG	CYS	31	46.910	15.538	-3.519	1.00	0.00	RX1	S
ATOM	2423	C	CYS	31	45.679	18.415	-3.235	1.00	0.00	RX1	C
ATOM	2424	O	CYS	31	46.433	18.694	-2.308	1.00	0.00	RX1	O
ATOM	2425	N	GLN	32	44.341	18.443	-3.154	1.00	0.00	RX1	N
ATOM	2426	H	GLN	32	43.797	18.151	-3.941	1.00	0.00	RX1	H
ATOM	2427	CA	GLN	32	43.704	18.848	-1.901	1.00	0.00	RX1	C
ATOM	2428	CB	GLN	32	42.181	18.994	-2.049	1.00	0.00	RX1	C
ATOM	2429	CG	GLN	32	41.690	20.019	-3.082	1.00	0.00	RX1	C
ATOM	2430	CD	GLN	32	41.490	19.386	-4.449	1.00	0.00	RX1	C
ATOM	2431	OE1	GLN	32	42.269	18.560	-4.910	1.00	0.00	RX1	O
ATOM	2432	NE2	GLN	32	40.386	19.817	-5.079	1.00	0.00	RX1	N
ATOM	2433	HE21	GLN	32	39.774	20.488	-4.649	1.00	0.00	RX1	H
ATOM	2434	HE22	GLN	32	40.134	19.490	-5.989	1.00	0.00	RX1	H
ATOM	2435	C	GLN	32	44.023	17.913	-0.746	1.00	0.00	RX1	C
ATOM	2436	O	GLN	32	44.412	18.329	0.336	1.00	0.00	RX1	O
ATOM	2437	N	GLY	33	43.875	16.619	-1.050	1.00	0.00	RX1	N
ATOM	2438	H	GLY	33	43.449	16.345	-1.909	1.00	0.00	RX1	H

ATOM	2439	CA	GLY	33	44.194	15.634	-0.023	1.00	0.00	RX1	C
ATOM	2440	C	GLY	33	42.978	15.117	0.714	1.00	0.00	RX1	C
ATOM	2441	O	GLY	33	42.059	15.858	1.053	1.00	0.00	RX1	O
ATOM	2442	N	THR	34	42.989	13.795	0.930	1.00	0.00	RX1	N
ATOM	2443	H	THR	34	43.817	13.236	0.869	1.00	0.00	RX1	H
ATOM	2444	CA	THR	34	41.899	13.220	1.709	1.00	0.00	RX1	C
ATOM	2445	CB	THR	34	41.971	11.706	1.538	1.00	0.00	RX1	C
ATOM	2446	OG1	THR	34	43.332	11.302	1.327	1.00	0.00	RX1	O
ATOM	2447	HG1	THR	34	43.334	10.362	1.513	1.00	0.00	RX1	H
ATOM	2448	CG2	THR	34	41.105	11.232	0.369	1.00	0.00	RX1	C
ATOM	2449	C	THR	34	42.004	13.654	3.155	1.00	0.00	RX1	C
ATOM	2450	O	THR	34	42.912	13.278	3.879	1.00	0.00	RX1	O
ATOM	2451	N	SER	35	41.048	14.511	3.517	1.00	0.00	RX1	N
ATOM	2452	H	SER	35	40.324	14.821	2.905	1.00	0.00	RX1	H
ATOM	2453	CA	SER	35	41.174	15.121	4.833	1.00	0.00	RX1	C
ATOM	2454	CB	SER	35	41.648	16.554	4.614	1.00	0.00	RX1	C
ATOM	2455	OG	SER	35	42.699	16.545	3.636	1.00	0.00	RX1	O
ATOM	2456	HG	SER	35	42.269	16.463	2.788	1.00	0.00	RX1	H
ATOM	2457	C	SER	35	39.903	14.988	5.649	1.00	0.00	RX1	C
ATOM	2458	O	SER	35	39.109	15.913	5.755	1.00	0.00	RX1	O
ATOM	2459	N	ASN	36	39.723	13.775	6.186	1.00	0.00	RX1	N
ATOM	2460	H	ASN	36	40.467	13.098	6.191	1.00	0.00	RX1	H
ATOM	2461	CA	ASN	36	38.469	13.489	6.883	1.00	0.00	RX1	C
ATOM	2462	CB	ASN	36	37.396	12.965	5.928	1.00	0.00	RX1	C
ATOM	2463	CG	ASN	36	37.539	11.527	5.441	1.00	0.00	RX1	C
ATOM	2464	OD1	ASN	36	36.583	11.006	4.881	1.00	0.00	RX1	O
ATOM	2465	ND2	ASN	36	38.712	10.895	5.622	1.00	0.00	RX1	N
ATOM	2466	HD21	ASN	36	39.555	11.267	6.017	1.00	0.00	RX1	H
ATOM	2467	HD22	ASN	36	38.763	9.934	5.334	1.00	0.00	RX1	H
ATOM	2468	C	ASN	36	38.586	12.660	8.152	1.00	0.00	RX1	C
ATOM	2469	O	ASN	36	37.704	11.892	8.526	1.00	0.00	RX1	O
ATOM	2470	N	LYS	37	39.748	12.878	8.787	1.00	0.00	RX1	N
ATOM	2471	H	LYS	37	40.426	13.422	8.290	1.00	0.00	RX1	H
ATOM	2472	CA	LYS	37	40.245	12.229	10.002	1.00	0.00	RX1	C
ATOM	2473	CB	LYS	37	40.192	13.175	11.211	1.00	0.00	RX1	C
ATOM	2474	CG	LYS	37	38.821	13.416	11.857	1.00	0.00	RX1	C
ATOM	2475	CD	LYS	37	38.907	13.632	13.378	1.00	0.00	RX1	C
ATOM	2476	CE	LYS	37	39.141	12.367	14.234	1.00	0.00	RX1	C
ATOM	2477	NZ	LYS	37	40.441	11.742	13.954	1.00	0.00	RX1	N
ATOM	2478	HZ1	LYS	37	40.656	10.937	14.576	1.00	0.00	RX1	H
ATOM	2479	HZ2	LYS	37	40.452	11.351	12.988	1.00	0.00	RX1	H
ATOM	2480	HZ3	LYS	37	41.213	12.432	14.084	1.00	0.00	RX1	H
ATOM	2481	C	LYS	37	39.811	10.820	10.392	1.00	0.00	RX1	C
ATOM	2482	O	LYS	37	39.699	10.541	11.585	1.00	0.00	RX1	O
ATOM	2483	N	LEU	38	39.618	9.985	9.343	1.00	0.00	RX1	N
ATOM	2484	H	LEU	38	39.810	10.372	8.436	1.00	0.00	RX1	H
ATOM	2485	CA	LEU	38	39.290	8.547	9.361	1.00	0.00	RX1	C
ATOM	2486	CB	LEU	38	38.608	8.062	10.646	1.00	0.00	RX1	C
ATOM	2487	CG	LEU	38	38.615	6.542	10.806	1.00	0.00	RX1	C
ATOM	2488	CD1	LEU	38	40.039	5.984	10.825	1.00	0.00	RX1	C
ATOM	2489	CD2	LEU	38	37.814	6.101	12.032	1.00	0.00	RX1	C
ATOM	2490	C	LEU	38	38.398	8.118	8.206	1.00	0.00	RX1	C
ATOM	2491	O	LEU	38	38.615	7.102	7.558	1.00	0.00	RX1	O
ATOM	2492	N	THR	39	37.363	8.938	8.003	1.00	0.00	RX1	N
ATOM	2493	H	THR	39	37.277	9.789	8.520	1.00	0.00	RX1	H
ATOM	2494	CA	THR	39	36.221	8.546	7.172	1.00	0.00	RX1	C
ATOM	2495	CB	THR	39	35.319	9.769	7.210	1.00	0.00	RX1	C
ATOM	2496	OG1	THR	39	35.430	10.327	8.528	1.00	0.00	RX1	O
ATOM	2497	HG1	THR	39	35.874	11.166	8.423	1.00	0.00	RX1	H
ATOM	2498	CG2	THR	39	33.867	9.511	6.805	1.00	0.00	RX1	C
ATOM	2499	C	THR	39	36.530	7.947	5.790	1.00	0.00	RX1	C

ATOM	2500	O	THR	39	37.549	8.223	5.165	1.00	0.00	RX1	O
ATOM	2501	N	GLN	40	35.602	7.066	5.373	1.00	0.00	RX1	N
ATOM	2502	H	GLN	40	34.747	6.972	5.881	1.00	0.00	RX1	H
ATOM	2503	CA	GLN	40	35.669	6.439	4.053	1.00	0.00	RX1	C
ATOM	2504	CB	GLN	40	35.245	4.971	4.191	1.00	0.00	RX1	C
ATOM	2505	CG	GLN	40	35.293	4.148	2.900	1.00	0.00	RX1	C
ATOM	2506	CD	GLN	40	34.672	2.778	3.098	1.00	0.00	RX1	C
ATOM	2507	OE1	GLN	40	33.899	2.302	2.277	1.00	0.00	RX1	O
ATOM	2508	NE2	GLN	40	35.051	2.152	4.225	1.00	0.00	RX1	N
ATOM	2509	HE21	GLN	40	34.749	1.214	4.416	1.00	0.00	RX1	H
ATOM	2510	HE22	GLN	40	35.652	2.561	4.910	1.00	0.00	RX1	H
ATOM	2511	C	GLN	40	34.730	7.158	3.100	1.00	0.00	RX1	C
ATOM	2512	O	GLN	40	33.649	7.567	3.495	1.00	0.00	RX1	O
ATOM	2513	N	LEU	41	35.155	7.270	1.833	1.00	0.00	RX1	N
ATOM	2514	H	LEU	41	36.034	6.893	1.551	1.00	0.00	RX1	H
ATOM	2515	CA	LEU	41	34.161	7.676	0.840	1.00	0.00	RX1	C
ATOM	2516	CB	LEU	41	34.797	8.493	-0.295	1.00	0.00	RX1	C
ATOM	2517	CG	LEU	41	33.818	9.275	-1.194	1.00	0.00	RX1	C
ATOM	2518	CD1	LEU	41	34.463	10.542	-1.754	1.00	0.00	RX1	C
ATOM	2519	CD2	LEU	41	33.220	8.449	-2.337	1.00	0.00	RX1	C
ATOM	2520	C	LEU	41	33.427	6.487	0.268	1.00	0.00	RX1	C
ATOM	2521	O	LEU	41	33.995	5.693	-0.469	1.00	0.00	RX1	O
ATOM	2522	N	GLY	42	32.131	6.451	0.594	1.00	0.00	RX1	N
ATOM	2523	H	GLY	42	31.780	7.041	1.322	1.00	0.00	RX1	H
ATOM	2524	CA	GLY	42	31.235	5.545	-0.119	1.00	0.00	RX1	C
ATOM	2525	C	GLY	42	31.517	4.067	0.050	1.00	0.00	RX1	C
ATOM	2526	O	GLY	42	31.522	3.515	1.145	1.00	0.00	RX1	O
ATOM	2527	N	THR	43	31.711	3.468	-1.123	1.00	0.00	RX1	N
ATOM	2528	H	THR	43	31.751	4.003	-1.964	1.00	0.00	RX1	H
ATOM	2529	CA	THR	43	32.022	2.050	-1.228	1.00	0.00	RX1	C
ATOM	2530	CB	THR	43	31.638	1.767	-2.668	1.00	0.00	RX1	C
ATOM	2531	OG1	THR	43	30.812	2.861	-3.105	1.00	0.00	RX1	O
ATOM	2532	HG1	THR	43	31.155	3.150	-3.956	1.00	0.00	RX1	H
ATOM	2533	CG2	THR	43	30.940	0.420	-2.856	1.00	0.00	RX1	C
ATOM	2534	C	THR	43	33.491	1.800	-0.912	1.00	0.00	RX1	C
ATOM	2535	O	THR	43	34.312	2.705	-1.002	1.00	0.00	RX1	O
ATOM	2536	N	PHE	44	33.823	0.539	-0.598	1.00	0.00	RX1	N
ATOM	2537	H	PHE	44	33.152	-0.201	-0.604	1.00	0.00	RX1	H
ATOM	2538	CA	PHE	44	35.247	0.252	-0.376	1.00	0.00	RX1	C
ATOM	2539	CB	PHE	44	35.416	-1.207	0.028	1.00	0.00	RX1	C
ATOM	2540	CG	PHE	44	34.500	-1.533	1.180	1.00	0.00	RX1	C
ATOM	2541	CD1	PHE	44	34.569	-0.794	2.354	1.00	0.00	RX1	C
ATOM	2542	CD2	PHE	44	33.587	-2.574	1.064	1.00	0.00	RX1	C
ATOM	2543	CE1	PHE	44	33.715	-1.092	3.407	1.00	0.00	RX1	C
ATOM	2544	CE2	PHE	44	32.737	-2.874	2.122	1.00	0.00	RX1	C
ATOM	2545	CZ	PHE	44	32.800	-2.131	3.293	1.00	0.00	RX1	C
ATOM	2546	C	PHE	44	36.101	0.558	-1.602	1.00	0.00	RX1	C
ATOM	2547	O	PHE	44	37.105	1.269	-1.577	1.00	0.00	RX1	O
ATOM	2548	N	GLU	45	35.550	0.061	-2.718	1.00	0.00	RX1	N
ATOM	2549	H	GLU	45	34.817	-0.618	-2.634	1.00	0.00	RX1	H
ATOM	2550	CA	GLU	45	36.005	0.429	-4.057	1.00	0.00	RX1	C
ATOM	2551	CB	GLU	45	35.060	-0.120	-5.133	1.00	0.00	RX1	C
ATOM	2552	CG	GLU	45	35.046	-1.642	-5.314	1.00	0.00	RX1	C
ATOM	2553	CD	GLU	45	34.507	-2.330	-4.078	1.00	0.00	RX1	C
ATOM	2554	OE1	GLU	45	33.542	-1.836	-3.494	1.00	0.00	RX1	O
ATOM	2555	OE2	GLU	45	35.085	-3.333	-3.674	1.00	0.00	RX1	O
ATOM	2556	C	GLU	45	36.120	1.927	-4.242	1.00	0.00	RX1	C
ATOM	2557	O	GLU	45	37.110	2.441	-4.740	1.00	0.00	RX1	O
ATOM	2558	N	ASP	46	35.069	2.619	-3.782	1.00	0.00	RX1	N
ATOM	2559	H	ASP	46	34.325	2.163	-3.301	1.00	0.00	RX1	H
ATOM	2560	CA	ASP	46	35.097	4.075	-3.880	1.00	0.00	RX1	C

ATOM	2561	CB	ASP	46	33.731	4.727	-3.697	1.00	0.00	RX1	C
ATOM	2562	CG	ASP	46	33.071	4.939	-5.041	1.00	0.00	RX1	C
ATOM	2563	OD1	ASP	46	32.097	4.247	-5.317	1.00	0.00	RX1	O
ATOM	2564	OD2	ASP	46	33.517	5.803	-5.799	1.00	0.00	RX1	O
ATOM	2565	C	ASP	46	36.098	4.791	-3.009	1.00	0.00	RX1	C
ATOM	2566	O	ASP	46	36.506	5.900	-3.333	1.00	0.00	RX1	O
ATOM	2567	N	HIS	47	36.548	4.145	-1.922	1.00	0.00	RX1	N
ATOM	2568	H	HIS	47	36.177	3.265	-1.613	1.00	0.00	RX1	H
ATOM	2569	CA	HIS	47	37.658	4.837	-1.276	1.00	0.00	RX1	C
ATOM	2570	CB	HIS	47	37.851	4.474	0.191	1.00	0.00	RX1	C
ATOM	2571	CG	HIS	47	38.330	5.736	0.869	1.00	0.00	RX1	C
ATOM	2572	ND1	HIS	47	38.899	5.791	2.084	1.00	0.00	RX1	N
ATOM	2573	HD1	HIS	47	39.118	5.029	2.665	1.00	0.00	RX1	H
ATOM	2574	CD2	HIS	47	38.242	7.036	0.365	1.00	0.00	RX1	C
ATOM	2575	NE2	HIS	47	38.759	7.875	1.290	1.00	0.00	RX1	N
ATOM	2576	CE1	HIS	47	39.169	7.109	2.354	1.00	0.00	RX1	C
ATOM	2577	C	HIS	47	38.961	4.749	-2.040	1.00	0.00	RX1	C
ATOM	2578	O	HIS	47	39.641	5.747	-2.242	1.00	0.00	RX1	O
ATOM	2579	N	PHE	48	39.224	3.537	-2.562	1.00	0.00	RX1	N
ATOM	2580	H	PHE	48	38.616	2.764	-2.357	1.00	0.00	RX1	H
ATOM	2581	CA	PHE	48	40.294	3.424	-3.565	1.00	0.00	RX1	C
ATOM	2582	CB	PHE	48	40.325	1.991	-4.108	1.00	0.00	RX1	C
ATOM	2583	CG	PHE	48	40.771	1.946	-5.554	1.00	0.00	RX1	C
ATOM	2584	CD1	PHE	48	42.084	2.240	-5.908	1.00	0.00	RX1	C
ATOM	2585	CD2	PHE	48	39.849	1.612	-6.540	1.00	0.00	RX1	C
ATOM	2586	CE1	PHE	48	42.464	2.227	-7.244	1.00	0.00	RX1	C
ATOM	2587	CE2	PHE	48	40.229	1.597	-7.876	1.00	0.00	RX1	C
ATOM	2588	CZ	PHE	48	41.535	1.913	-8.228	1.00	0.00	RX1	C
ATOM	2589	C	PHE	48	40.148	4.451	-4.688	1.00	0.00	RX1	C
ATOM	2590	O	PHE	48	41.060	5.175	-5.070	1.00	0.00	RX1	O
ATOM	2591	N	LEU	49	38.907	4.506	-5.174	1.00	0.00	RX1	N
ATOM	2592	H	LEU	49	38.201	3.902	-4.811	1.00	0.00	RX1	H
ATOM	2593	CA	LEU	49	38.608	5.419	-6.262	1.00	0.00	RX1	C
ATOM	2594	CB	LEU	49	37.251	5.071	-6.871	1.00	0.00	RX1	C
ATOM	2595	CG	LEU	49	37.140	5.478	-8.338	1.00	0.00	RX1	C
ATOM	2596	CD1	LEU	49	38.320	4.951	-9.154	1.00	0.00	RX1	C
ATOM	2597	CD2	LEU	49	35.802	5.050	-8.942	1.00	0.00	RX1	C
ATOM	2598	C	LEU	49	38.720	6.881	-5.880	1.00	0.00	RX1	C
ATOM	2599	O	LEU	49	39.001	7.726	-6.711	1.00	0.00	RX1	O
ATOM	2600	N	SER	50	38.538	7.167	-4.587	1.00	0.00	RX1	N
ATOM	2601	H	SER	50	38.316	6.470	-3.906	1.00	0.00	RX1	H
ATOM	2602	CA	SER	50	38.816	8.532	-4.148	1.00	0.00	RX1	C
ATOM	2603	CB	SER	50	38.092	8.805	-2.842	1.00	0.00	RX1	C
ATOM	2604	OG	SER	50	36.730	8.419	-3.029	1.00	0.00	RX1	O
ATOM	2605	HG	SER	50	36.650	7.568	-2.597	1.00	0.00	RX1	H
ATOM	2606	C	SER	50	40.288	8.853	-4.074	1.00	0.00	RX1	C
ATOM	2607	O	SER	50	40.728	9.930	-4.451	1.00	0.00	RX1	O
ATOM	2608	N	LEU	51	41.048	7.840	-3.640	1.00	0.00	RX1	N
ATOM	2609	H	LEU	51	40.656	6.969	-3.340	1.00	0.00	RX1	H
ATOM	2610	CA	LEU	51	42.498	7.988	-3.723	1.00	0.00	RX1	C
ATOM	2611	CB	LEU	51	43.204	6.798	-3.071	1.00	0.00	RX1	C
ATOM	2612	CG	LEU	51	43.570	6.989	-1.595	1.00	0.00	RX1	C
ATOM	2613	CD1	LEU	51	42.359	7.142	-0.670	1.00	0.00	RX1	C
ATOM	2614	CD2	LEU	51	44.500	5.873	-1.126	1.00	0.00	RX1	C
ATOM	2615	C	LEU	51	42.985	8.210	-5.148	1.00	0.00	RX1	C
ATOM	2616	O	LEU	51	43.910	8.964	-5.406	1.00	0.00	RX1	O
ATOM	2617	N	GLN	52	42.270	7.577	-6.089	1.00	0.00	RX1	N
ATOM	2618	H	GLN	52	41.574	6.907	-5.828	1.00	0.00	RX1	H
ATOM	2619	CA	GLN	52	42.525	7.929	-7.484	1.00	0.00	RX1	C
ATOM	2620	CB	GLN	52	41.850	6.917	-8.404	1.00	0.00	RX1	C
ATOM	2621	CG	GLN	52	42.315	7.039	-9.852	1.00	0.00	RX1	C

ATOM	2622	CD	GLN	52	41.621	5.976	-10.671	1.00	0.00	RX1	C
ATOM	2623	OE1	GLN	52	40.685	6.244	-11.413	1.00	0.00	RX1	O
ATOM	2624	NE2	GLN	52	42.127	4.743	-10.485	1.00	0.00	RX1	N
ATOM	2625	HE21	GLN	52	42.894	4.581	-9.863	1.00	0.00	RX1	H
ATOM	2626	HE22	GLN	52	41.747	3.955	-10.969	1.00	0.00	RX1	H
ATOM	2627	C	GLN	52	42.135	9.362	-7.835	1.00	0.00	RX1	C
ATOM	2628	O	GLN	52	42.951	10.173	-8.246	1.00	0.00	RX1	O
ATOM	2629	N	ARG	53	40.842	9.647	-7.613	1.00	0.00	RX1	N
ATOM	2630	H	ARG	53	40.255	8.928	-7.254	1.00	0.00	RX1	H
ATOM	2631	CA	ARG	53	40.266	10.962	-7.915	1.00	0.00	RX1	C
ATOM	2632	CB	ARG	53	38.780	10.993	-7.508	1.00	0.00	RX1	C
ATOM	2633	CG	ARG	53	37.896	10.071	-8.367	1.00	0.00	RX1	C
ATOM	2634	CD	ARG	53	36.425	9.941	-7.927	1.00	0.00	RX1	C
ATOM	2635	NE	ARG	53	36.286	9.314	-6.609	1.00	0.00	RX1	N
ATOM	2636	HE	ARG	53	36.887	9.660	-5.883	1.00	0.00	RX1	H
ATOM	2637	CZ	ARG	53	35.371	8.314	-6.386	1.00	0.00	RX1	C
ATOM	2638	NH1	ARG	53	34.577	7.856	-7.374	1.00	0.00	RX1	N
ATOM	2639	HH11	ARG	53	33.938	7.091	-7.171	1.00	0.00	RX1	H
ATOM	2640	HH12	ARG	53	34.586	8.220	-8.307	1.00	0.00	RX1	H
ATOM	2641	NH2	ARG	53	35.261	7.766	-5.165	1.00	0.00	RX1	N
ATOM	2642	HH21	ARG	53	34.612	7.004	-5.005	1.00	0.00	RX1	H
ATOM	2643	HH22	ARG	53	35.803	8.059	-4.369	1.00	0.00	RX1	H
ATOM	2644	C	ARG	53	41.027	12.156	-7.345	1.00	0.00	RX1	C
ATOM	2645	O	ARG	53	41.035	13.241	-7.908	1.00	0.00	RX1	O
ATOM	2646	N	MET	54	41.668	11.898	-6.199	1.00	0.00	RX1	N
ATOM	2647	H	MET	54	41.637	10.988	-5.788	1.00	0.00	RX1	H
ATOM	2648	CA	MET	54	42.454	12.952	-5.571	1.00	0.00	RX1	C
ATOM	2649	CB	MET	54	42.236	12.899	-4.061	1.00	0.00	RX1	C
ATOM	2650	CG	MET	54	42.870	14.075	-3.327	1.00	0.00	RX1	C
ATOM	2651	SD	MET	54	42.110	15.650	-3.741	1.00	0.00	RX1	S
ATOM	2652	CE	MET	54	40.487	15.339	-3.027	1.00	0.00	RX1	C
ATOM	2653	C	MET	54	43.940	12.906	-5.904	1.00	0.00	RX1	C
ATOM	2654	O	MET	54	44.586	13.916	-6.142	1.00	0.00	RX1	O
ATOM	2655	N	PHE	55	44.477	11.679	-5.873	1.00	0.00	RX1	N
ATOM	2656	H	PHE	55	43.936	10.839	-5.828	1.00	0.00	RX1	H
ATOM	2657	CA	PHE	55	45.936	11.592	-5.935	1.00	0.00	RX1	C
ATOM	2658	CB	PHE	55	46.504	10.712	-4.821	1.00	0.00	RX1	C
ATOM	2659	CG	PHE	55	45.982	11.175	-3.487	1.00	0.00	RX1	C
ATOM	2660	CD1	PHE	55	46.432	12.372	-2.949	1.00	0.00	RX1	C
ATOM	2661	CD2	PHE	55	45.049	10.406	-2.804	1.00	0.00	RX1	C
ATOM	2662	CE1	PHE	55	45.934	12.810	-1.730	1.00	0.00	RX1	C
ATOM	2663	CE2	PHE	55	44.549	10.843	-1.586	1.00	0.00	RX1	C
ATOM	2664	CZ	PHE	55	44.990	12.048	-1.056	1.00	0.00	RX1	C
ATOM	2665	C	PHE	55	46.503	11.099	-7.249	1.00	0.00	RX1	C
ATOM	2666	O	PHE	55	47.674	10.741	-7.338	1.00	0.00	RX1	O
ATOM	2667	N	ASN	56	45.641	11.077	-8.278	1.00	0.00	RX1	N
ATOM	2668	H	ASN	56	44.683	11.346	-8.168	1.00	0.00	RX1	H
ATOM	2669	CA	ASN	56	46.129	10.570	-9.563	1.00	0.00	RX1	C
ATOM	2670	CB	ASN	56	45.078	10.554	-10.657	1.00	0.00	RX1	C
ATOM	2671	CG	ASN	56	45.830	10.204	-11.922	1.00	0.00	RX1	C
ATOM	2672	OD1	ASN	56	46.579	9.232	-11.973	1.00	0.00	RX1	O
ATOM	2673	ND2	ASN	56	45.581	11.036	-12.948	1.00	0.00	RX1	N
ATOM	2674	HD21	ASN	56	44.991	11.833	-12.805	1.00	0.00	RX1	H
ATOM	2675	HD22	ASN	56	45.975	10.897	-13.855	1.00	0.00	RX1	H
ATOM	2676	C	ASN	56	47.307	11.341	-10.130	1.00	0.00	RX1	C
ATOM	2677	O	ASN	56	47.186	12.472	-10.581	1.00	0.00	RX1	O
ATOM	2678	N	ASN	57	48.464	10.665	-10.047	1.00	0.00	RX1	N
ATOM	2679	H	ASN	57	48.438	9.772	-9.603	1.00	0.00	RX1	H
ATOM	2680	CA	ASN	57	49.742	11.285	-10.404	1.00	0.00	RX1	C
ATOM	2681	CB	ASN	57	49.862	11.461	-11.922	1.00	0.00	RX1	C
ATOM	2682	CG	ASN	57	51.286	11.812	-12.312	1.00	0.00	RX1	C

ATOM	2683	OD1	ASN	57	51.584	12.896	-12.813	1.00	0.00	RX1	O
ATOM	2684	ND2	ASN	57	52.152	10.802	-12.122	1.00	0.00	RX1	N
ATOM	2685	HD21	ASN	57	51.899	9.971	-11.618	1.00	0.00	RX1	H
ATOM	2686	HD22	ASN	57	53.102	10.847	-12.433	1.00	0.00	RX1	H
ATOM	2687	C	ASN	57	50.027	12.593	-9.677	1.00	0.00	RX1	C
ATOM	2688	O	ASN	57	50.534	13.550	-10.247	1.00	0.00	RX1	O
ATOM	2689	N	CYS	58	49.667	12.595	-8.387	1.00	0.00	RX1	N
ATOM	2690	H	CYS	58	49.320	11.781	-7.918	1.00	0.00	RX1	H
ATOM	2691	CA	CYS	58	49.987	13.788	-7.606	1.00	0.00	RX1	C
ATOM	2692	CB	CYS	58	48.862	14.078	-6.617	1.00	0.00	RX1	C
ATOM	2693	SG	CYS	58	49.177	15.550	-5.614	1.00	0.00	RX1	S
ATOM	2694	C	CYS	58	51.320	13.650	-6.906	1.00	0.00	RX1	C
ATOM	2695	O	CYS	58	51.731	12.557	-6.537	1.00	0.00	RX1	O
ATOM	2696	N	GLU	59	51.969	14.803	-6.739	1.00	0.00	RX1	N
ATOM	2697	H	GLU	59	51.617	15.655	-7.133	1.00	0.00	RX1	H
ATOM	2698	CA	GLU	59	53.285	14.801	-6.121	1.00	0.00	RX1	C
ATOM	2699	CB	GLU	59	54.319	15.537	-6.982	1.00	0.00	RX1	C
ATOM	2700	CG	GLU	59	54.633	14.982	-8.381	1.00	0.00	RX1	C
ATOM	2701	CD	GLU	59	53.677	15.496	-9.448	1.00	0.00	RX1	C
ATOM	2702	OE1	GLU	59	54.121	15.954	-10.494	1.00	0.00	RX1	O
ATOM	2703	OE2	GLU	59	52.471	15.457	-9.268	1.00	0.00	RX1	O
ATOM	2704	C	GLU	59	53.262	15.402	-4.735	1.00	0.00	RX1	C
ATOM	2705	O	GLU	59	53.931	14.922	-3.827	1.00	0.00	RX1	O
ATOM	2706	N	VAL	60	52.475	16.480	-4.608	1.00	0.00	RX1	N
ATOM	2707	H	VAL	60	51.901	16.816	-5.358	1.00	0.00	RX1	H
ATOM	2708	CA	VAL	60	52.366	17.111	-3.297	1.00	0.00	RX1	C
ATOM	2709	CB	VAL	60	52.908	18.541	-3.291	1.00	0.00	RX1	C
ATOM	2710	CG1	VAL	60	52.823	19.147	-1.888	1.00	0.00	RX1	C
ATOM	2711	CG2	VAL	60	54.330	18.603	-3.834	1.00	0.00	RX1	C
ATOM	2712	C	VAL	60	50.942	17.114	-2.787	1.00	0.00	RX1	C
ATOM	2713	O	VAL	60	50.065	17.818	-3.275	1.00	0.00	RX1	O
ATOM	2714	N	VAL	61	50.752	16.302	-1.748	1.00	0.00	RX1	N
ATOM	2715	H	VAL	61	51.543	15.857	-1.325	1.00	0.00	RX1	H
ATOM	2716	CA	VAL	61	49.477	16.413	-1.050	1.00	0.00	RX1	C
ATOM	2717	CB	VAL	61	49.219	15.138	-0.260	1.00	0.00	RX1	C
ATOM	2718	CG1	VAL	61	47.853	15.178	0.414	1.00	0.00	RX1	C
ATOM	2719	CG2	VAL	61	49.385	13.915	-1.154	1.00	0.00	RX1	C
ATOM	2720	C	VAL	61	49.518	17.612	-0.121	1.00	0.00	RX1	C
ATOM	2721	O	VAL	61	50.380	17.702	0.743	1.00	0.00	RX1	O
ATOM	2722	N	LEU	62	48.577	18.537	-0.345	1.00	0.00	RX1	N
ATOM	2723	H	LEU	62	47.869	18.406	-1.041	1.00	0.00	RX1	H
ATOM	2724	CA	LEU	62	48.563	19.702	0.538	1.00	0.00	RX1	C
ATOM	2725	CB	LEU	62	47.805	20.858	-0.108	1.00	0.00	RX1	C
ATOM	2726	CG	LEU	62	48.437	21.318	-1.420	1.00	0.00	RX1	C
ATOM	2727	CD1	LEU	62	47.568	22.363	-2.120	1.00	0.00	RX1	C
ATOM	2728	CD2	LEU	62	49.874	21.800	-1.218	1.00	0.00	RX1	C
ATOM	2729	C	LEU	62	47.970	19.400	1.899	1.00	0.00	RX1	C
ATOM	2730	O	LEU	62	48.598	19.554	2.940	1.00	0.00	RX1	O
ATOM	2731	N	GLY	63	46.711	18.949	1.836	1.00	0.00	RX1	N
ATOM	2732	H	GLY	63	46.245	18.766	0.970	1.00	0.00	RX1	H
ATOM	2733	CA	GLY	63	46.085	18.504	3.072	1.00	0.00	RX1	C
ATOM	2734	C	GLY	63	46.553	17.118	3.441	1.00	0.00	RX1	C
ATOM	2735	O	GLY	63	47.715	16.758	3.279	1.00	0.00	RX1	O
ATOM	2736	N	ASN	64	45.598	16.348	3.958	1.00	0.00	RX1	N
ATOM	2737	H	ASN	64	44.622	16.558	3.876	1.00	0.00	RX1	H
ATOM	2738	CA	ASN	64	46.053	15.060	4.462	1.00	0.00	RX1	C
ATOM	2739	CB	ASN	64	45.192	14.516	5.595	1.00	0.00	RX1	C
ATOM	2740	CG	ASN	64	45.115	15.474	6.751	1.00	0.00	RX1	C
ATOM	2741	OD1	ASN	64	46.072	16.149	7.125	1.00	0.00	RX1	O
ATOM	2742	ND2	ASN	64	43.891	15.501	7.299	1.00	0.00	RX1	N
ATOM	2743	HD21	ASN	64	43.224	14.816	6.980	1.00	0.00	RX1	H

ATOM	2744	HD22	ASN	64	43.601	16.112	8.033	1.00	0.00	RX1	H
ATOM	2745	C	ASN	64	46.118	14.005	3.392	1.00	0.00	RX1	C
ATOM	2746	O	ASN	64	45.396	14.010	2.402	1.00	0.00	RX1	O
ATOM	2747	N	LEU	65	47.032	13.077	3.652	1.00	0.00	RX1	N
ATOM	2748	H	LEU	65	47.582	13.126	4.485	1.00	0.00	RX1	H
ATOM	2749	CA	LEU	65	46.992	11.839	2.899	1.00	0.00	RX1	C
ATOM	2750	CB	LEU	65	48.412	11.498	2.454	1.00	0.00	RX1	C
ATOM	2751	CG	LEU	65	48.563	10.243	1.598	1.00	0.00	RX1	C
ATOM	2752	CD1	LEU	65	47.561	10.169	0.450	1.00	0.00	RX1	C
ATOM	2753	CD2	LEU	65	49.994	10.132	1.088	1.00	0.00	RX1	C
ATOM	2754	C	LEU	65	46.356	10.774	3.768	1.00	0.00	RX1	C
ATOM	2755	O	LEU	65	47.007	9.944	4.395	1.00	0.00	RX1	O
ATOM	2756	N	GLU	66	45.023	10.862	3.808	1.00	0.00	RX1	N
ATOM	2757	H	GLU	66	44.506	11.531	3.266	1.00	0.00	RX1	H
ATOM	2758	CA	GLU	66	44.332	9.799	4.521	1.00	0.00	RX1	C
ATOM	2759	CB	GLU	66	43.075	10.313	5.218	1.00	0.00	RX1	C
ATOM	2760	CG	GLU	66	43.455	11.305	6.317	1.00	0.00	RX1	C
ATOM	2761	CD	GLU	66	42.218	11.893	6.953	1.00	0.00	RX1	C
ATOM	2762	OE1	GLU	66	41.208	11.204	7.040	1.00	0.00	RX1	O
ATOM	2763	OE2	GLU	66	42.244	13.055	7.352	1.00	0.00	RX1	O
ATOM	2764	C	GLU	66	44.040	8.630	3.617	1.00	0.00	RX1	C
ATOM	2765	O	GLU	66	43.251	8.687	2.679	1.00	0.00	RX1	O
ATOM	2766	N	ILE	67	44.764	7.564	3.944	1.00	0.00	RX1	N
ATOM	2767	H	ILE	67	45.331	7.587	4.767	1.00	0.00	RX1	H
ATOM	2768	CA	ILE	67	44.494	6.272	3.338	1.00	0.00	RX1	C
ATOM	2769	CB	ILE	67	45.812	5.599	2.968	1.00	0.00	RX1	C
ATOM	2770	CG2	ILE	67	45.583	4.274	2.239	1.00	0.00	RX1	C
ATOM	2771	CG1	ILE	67	46.693	6.562	2.174	1.00	0.00	RX1	C
ATOM	2772	CD1	ILE	67	48.079	5.985	1.903	1.00	0.00	RX1	C
ATOM	2773	C	ILE	67	43.759	5.453	4.373	1.00	0.00	RX1	C
ATOM	2774	O	ILE	67	44.358	4.905	5.293	1.00	0.00	RX1	O
ATOM	2775	N	THR	68	42.437	5.428	4.216	1.00	0.00	RX1	N
ATOM	2776	H	THR	68	41.915	5.974	3.563	1.00	0.00	RX1	H
ATOM	2777	CA	THR	68	41.689	4.676	5.211	1.00	0.00	RX1	C
ATOM	2778	CB	THR	68	40.977	5.779	5.952	1.00	0.00	RX1	C
ATOM	2779	OG1	THR	68	40.965	6.927	5.088	1.00	0.00	RX1	O
ATOM	2780	HG1	THR	68	40.288	7.492	5.450	1.00	0.00	RX1	H
ATOM	2781	CG2	THR	68	41.694	6.136	7.254	1.00	0.00	RX1	C
ATOM	2782	C	THR	68	40.782	3.643	4.582	1.00	0.00	RX1	C
ATOM	2783	O	THR	68	40.254	3.832	3.491	1.00	0.00	RX1	O
ATOM	2784	N	TYR	69	40.617	2.532	5.320	1.00	0.00	RX1	N
ATOM	2785	H	TYR	69	41.183	2.421	6.140	1.00	0.00	RX1	H
ATOM	2786	CA	TYR	69	39.654	1.492	4.929	1.00	0.00	RX1	C
ATOM	2787	CB	TYR	69	38.211	1.992	5.040	1.00	0.00	RX1	C
ATOM	2788	CG	TYR	69	37.809	2.413	6.436	1.00	0.00	RX1	C
ATOM	2789	CD1	TYR	69	37.399	1.463	7.363	1.00	0.00	RX1	C
ATOM	2790	CE1	TYR	69	36.933	1.862	8.612	1.00	0.00	RX1	C
ATOM	2791	CD2	TYR	69	37.800	3.759	6.782	1.00	0.00	RX1	C
ATOM	2792	CE2	TYR	69	37.335	4.156	8.030	1.00	0.00	RX1	C
ATOM	2793	CZ	TYR	69	36.887	3.210	8.944	1.00	0.00	RX1	C
ATOM	2794	OH	TYR	69	36.419	3.615	10.177	1.00	0.00	RX1	O
ATOM	2795	HH	TYR	69	35.511	3.312	10.319	1.00	0.00	RX1	H
ATOM	2796	C	TYR	69	39.826	0.861	3.547	1.00	0.00	RX1	C
ATOM	2797	O	TYR	69	38.927	0.224	3.011	1.00	0.00	RX1	O
ATOM	2798	N	VAL	70	41.016	1.064	2.966	1.00	0.00	RX1	N
ATOM	2799	H	VAL	70	41.754	1.531	3.449	1.00	0.00	RX1	H
ATOM	2800	CA	VAL	70	41.237	0.492	1.641	1.00	0.00	RX1	C
ATOM	2801	CB	VAL	70	42.423	1.175	0.954	1.00	0.00	RX1	C
ATOM	2802	CG1	VAL	70	42.731	0.551	-0.406	1.00	0.00	RX1	C
ATOM	2803	CG2	VAL	70	42.165	2.679	0.832	1.00	0.00	RX1	C
ATOM	2804	C	VAL	70	41.407	-1.016	1.723	1.00	0.00	RX1	C

ATOM	2805	O	VAL	70	42.326	-1.543	2.344	1.00	0.00	RX1	O
ATOM	2806	N	GLN	71	40.428	-1.678	1.097	1.00	0.00	RX1	N
ATOM	2807	H	GLN	71	39.727	-1.172	0.598	1.00	0.00	RX1	H
ATOM	2808	CA	GLN	71	40.361	-3.130	1.210	1.00	0.00	RX1	C
ATOM	2809	CB	GLN	71	38.957	-3.623	0.855	1.00	0.00	RX1	C
ATOM	2810	CG	GLN	71	37.889	-3.198	1.867	1.00	0.00	RX1	C
ATOM	2811	CD	GLN	71	38.040	-4.008	3.135	1.00	0.00	RX1	C
ATOM	2812	OE1	GLN	71	38.810	-4.968	3.184	1.00	0.00	RX1	O
ATOM	2813	NE2	GLN	71	37.273	-3.576	4.148	1.00	0.00	RX1	N
ATOM	2814	HE21	GLN	71	36.638	-2.798	4.118	1.00	0.00	RX1	H
ATOM	2815	HE22	GLN	71	37.194	-3.974	5.070	1.00	0.00	RX1	H
ATOM	2816	C	GLN	71	41.425	-3.910	0.452	1.00	0.00	RX1	C
ATOM	2817	O	GLN	71	42.089	-3.424	-0.458	1.00	0.00	RX1	O
ATOM	2818	N	ARG	72	41.532	-5.170	0.906	1.00	0.00	RX1	N
ATOM	2819	H	ARG	72	40.883	-5.387	1.638	1.00	0.00	RX1	H
ATOM	2820	CA	ARG	72	42.622	-6.114	0.644	1.00	0.00	RX1	C
ATOM	2821	CB	ARG	72	42.108	-7.557	0.709	1.00	0.00	RX1	C
ATOM	2822	CG	ARG	72	43.226	-8.557	1.027	1.00	0.00	RX1	C
ATOM	2823	CD	ARG	72	43.724	-8.463	2.474	1.00	0.00	RX1	C
ATOM	2824	NE	ARG	72	45.047	-9.066	2.616	1.00	0.00	RX1	N
ATOM	2825	HE	ARG	72	45.747	-8.869	1.914	1.00	0.00	RX1	H
ATOM	2826	CZ	ARG	72	45.413	-9.752	3.738	1.00	0.00	RX1	C
ATOM	2827	NH1	ARG	72	44.509	-10.016	4.699	1.00	0.00	RX1	N
ATOM	2828	HH11	ARG	72	44.771	-10.441	5.579	1.00	0.00	RX1	H
ATOM	2829	HH12	ARG	72	43.536	-9.789	4.630	1.00	0.00	RX1	H
ATOM	2830	NH2	ARG	72	46.686	-10.162	3.877	1.00	0.00	RX1	N
ATOM	2831	HH21	ARG	72	46.998	-10.700	4.662	1.00	0.00	RX1	H
ATOM	2832	HH22	ARG	72	47.389	-9.923	3.175	1.00	0.00	RX1	H
ATOM	2833	C	ARG	72	43.551	-5.932	-0.547	1.00	0.00	RX1	C
ATOM	2834	O	ARG	72	44.768	-6.028	-0.425	1.00	0.00	RX1	O
ATOM	2835	N	ASN	73	42.947	-5.723	-1.721	1.00	0.00	RX1	N
ATOM	2836	H	ASN	73	41.977	-5.504	-1.819	1.00	0.00	RX1	H
ATOM	2837	CA	ASN	73	43.850	-5.779	-2.865	1.00	0.00	RX1	C
ATOM	2838	CB	ASN	73	43.941	-7.181	-3.459	1.00	0.00	RX1	C
ATOM	2839	CG	ASN	73	45.289	-7.303	-4.139	1.00	0.00	RX1	C
ATOM	2840	OD1	ASN	73	45.408	-7.676	-5.301	1.00	0.00	RX1	O
ATOM	2841	ND2	ASN	73	46.318	-7.001	-3.322	1.00	0.00	RX1	N
ATOM	2842	HD21	ASN	73	46.174	-6.759	-2.358	1.00	0.00	RX1	H
ATOM	2843	HD22	ASN	73	47.259	-7.030	-3.657	1.00	0.00	RX1	H
ATOM	2844	C	ASN	73	43.616	-4.761	-3.953	1.00	0.00	RX1	C
ATOM	2845	O	ASN	73	43.744	-5.038	-5.137	1.00	0.00	RX1	O
ATOM	2846	N	TYR	74	43.259	-3.547	-3.512	1.00	0.00	RX1	N
ATOM	2847	H	TYR	74	43.245	-3.301	-2.541	1.00	0.00	RX1	H
ATOM	2848	CA	TYR	74	43.283	-2.524	-4.556	1.00	0.00	RX1	C
ATOM	2849	CB	TYR	74	42.369	-1.338	-4.252	1.00	0.00	RX1	C
ATOM	2850	CG	TYR	74	41.015	-1.739	-3.717	1.00	0.00	RX1	C
ATOM	2851	CD1	TYR	74	40.173	-2.610	-4.403	1.00	0.00	RX1	C
ATOM	2852	CE1	TYR	74	38.923	-2.925	-3.874	1.00	0.00	RX1	C
ATOM	2853	CD2	TYR	74	40.604	-1.189	-2.513	1.00	0.00	RX1	C
ATOM	2854	CE2	TYR	74	39.359	-1.493	-1.992	1.00	0.00	RX1	C
ATOM	2855	CZ	TYR	74	38.523	-2.374	-2.660	1.00	0.00	RX1	C
ATOM	2856	OH	TYR	74	37.316	-2.691	-2.078	1.00	0.00	RX1	O
ATOM	2857	HH	TYR	74	36.672	-2.972	-2.733	1.00	0.00	RX1	H
ATOM	2858	C	TYR	74	44.685	-1.979	-4.724	1.00	0.00	RX1	C
ATOM	2859	O	TYR	74	45.292	-1.524	-3.762	1.00	0.00	RX1	O
ATOM	2860	N	ASP	75	45.174	-2.027	-5.971	1.00	0.00	RX1	N
ATOM	2861	H	ASP	75	44.628	-2.360	-6.736	1.00	0.00	RX1	H
ATOM	2862	CA	ASP	75	46.403	-1.264	-6.196	1.00	0.00	RX1	C
ATOM	2863	CB	ASP	75	47.038	-1.656	-7.529	1.00	0.00	RX1	C
ATOM	2864	CG	ASP	75	48.202	-0.734	-7.808	1.00	0.00	RX1	C
ATOM	2865	OD1	ASP	75	49.146	-0.687	-7.023	1.00	0.00	RX1	O

ATOM	2866	OD2	ASP	75	48.147	-0.002	-8.786	1.00	0.00	RX1	O
ATOM	2867	C	ASP	75	46.126	0.228	-6.110	1.00	0.00	RX1	C
ATOM	2868	O	ASP	75	45.037	0.714	-6.393	1.00	0.00	RX1	O
ATOM	2869	N	LEU	76	47.172	0.919	-5.660	1.00	0.00	RX1	N
ATOM	2870	H	LEU	76	48.050	0.440	-5.598	1.00	0.00	RX1	H
ATOM	2871	CA	LEU	76	47.057	2.356	-5.478	1.00	0.00	RX1	C
ATOM	2872	CB	LEU	76	47.049	2.708	-3.988	1.00	0.00	RX1	C
ATOM	2873	CG	LEU	76	45.995	1.995	-3.136	1.00	0.00	RX1	C
ATOM	2874	CD1	LEU	76	46.327	2.072	-1.645	1.00	0.00	RX1	C
ATOM	2875	CD2	LEU	76	44.582	2.500	-3.423	1.00	0.00	RX1	C
ATOM	2876	C	LEU	76	48.203	3.076	-6.162	1.00	0.00	RX1	C
ATOM	2877	O	LEU	76	48.724	4.067	-5.667	1.00	0.00	RX1	O
ATOM	2878	N	SER	77	48.618	2.536	-7.321	1.00	0.00	RX1	N
ATOM	2879	H	SER	77	48.206	1.698	-7.694	1.00	0.00	RX1	H
ATOM	2880	CA	SER	77	49.868	3.028	-7.906	1.00	0.00	RX1	C
ATOM	2881	CB	SER	77	50.315	2.139	-9.058	1.00	0.00	RX1	C
ATOM	2882	OG	SER	77	50.837	0.929	-8.480	1.00	0.00	RX1	O
ATOM	2883	HG	SER	77	50.054	0.368	-8.362	1.00	0.00	RX1	H
ATOM	2884	C	SER	77	50.062	4.512	-8.164	1.00	0.00	RX1	C
ATOM	2885	O	SER	77	51.191	4.980	-8.226	1.00	0.00	RX1	O
ATOM	2886	N	PHE	78	48.942	5.244	-8.254	1.00	0.00	RX1	N
ATOM	2887	H	PHE	78	48.065	4.772	-8.191	1.00	0.00	RX1	H
ATOM	2888	CA	PHE	78	49.040	6.710	-8.278	1.00	0.00	RX1	C
ATOM	2889	CB	PHE	78	47.648	7.349	-8.369	1.00	0.00	RX1	C
ATOM	2890	CG	PHE	78	46.635	6.574	-7.559	1.00	0.00	RX1	C
ATOM	2891	CD1	PHE	78	46.564	6.736	-6.182	1.00	0.00	RX1	C
ATOM	2892	CD2	PHE	78	45.774	5.688	-8.199	1.00	0.00	RX1	C
ATOM	2893	CE1	PHE	78	45.648	5.998	-5.445	1.00	0.00	RX1	C
ATOM	2894	CE2	PHE	78	44.857	4.950	-7.462	1.00	0.00	RX1	C
ATOM	2895	CZ	PHE	78	44.797	5.104	-6.084	1.00	0.00	RX1	C
ATOM	2896	C	PHE	78	49.898	7.333	-7.174	1.00	0.00	RX1	C
ATOM	2897	O	PHE	78	50.643	8.284	-7.393	1.00	0.00	RX1	O
ATOM	2898	N	LEU	79	49.812	6.696	-5.989	1.00	0.00	RX1	N
ATOM	2899	H	LEU	79	49.216	5.899	-5.890	1.00	0.00	RX1	H
ATOM	2900	CA	LEU	79	50.618	7.123	-4.840	1.00	0.00	RX1	C
ATOM	2901	CB	LEU	79	50.304	6.291	-3.595	1.00	0.00	RX1	C
ATOM	2902	CG	LEU	79	48.838	6.239	-3.161	1.00	0.00	RX1	C
ATOM	2903	CD1	LEU	79	48.679	5.366	-1.919	1.00	0.00	RX1	C
ATOM	2904	CD2	LEU	79	48.221	7.618	-2.943	1.00	0.00	RX1	C
ATOM	2905	C	LEU	79	52.128	7.129	-5.053	1.00	0.00	RX1	C
ATOM	2906	O	LEU	79	52.873	7.807	-4.358	1.00	0.00	RX1	O
ATOM	2907	N	LYS	80	52.565	6.378	-6.082	1.00	0.00	RX1	N
ATOM	2908	H	LYS	80	51.925	5.864	-6.652	1.00	0.00	RX1	H
ATOM	2909	CA	LYS	80	53.986	6.424	-6.435	1.00	0.00	RX1	C
ATOM	2910	CB	LYS	80	54.397	5.412	-7.506	1.00	0.00	RX1	C
ATOM	2911	CG	LYS	80	53.981	3.971	-7.228	1.00	0.00	RX1	C
ATOM	2912	CD	LYS	80	54.750	2.979	-8.104	1.00	0.00	RX1	C
ATOM	2913	CE	LYS	80	53.915	1.778	-8.548	1.00	0.00	RX1	C
ATOM	2914	NZ	LYS	80	53.202	1.175	-7.416	1.00	0.00	RX1	N
ATOM	2915	HZ1	LYS	80	52.364	0.649	-7.757	1.00	0.00	RX1	H
ATOM	2916	HZ2	LYS	80	53.844	0.566	-6.865	1.00	0.00	RX1	H
ATOM	2917	HZ3	LYS	80	52.872	1.907	-6.762	1.00	0.00	RX1	H
ATOM	2918	C	LYS	80	54.527	7.784	-6.825	1.00	0.00	RX1	C
ATOM	2919	O	LYS	80	55.731	8.013	-6.814	1.00	0.00	RX1	O
ATOM	2920	N	THR	81	53.593	8.664	-7.190	1.00	0.00	RX1	N
ATOM	2921	H	THR	81	52.614	8.483	-7.134	1.00	0.00	RX1	H
ATOM	2922	CA	THR	81	54.051	9.994	-7.563	1.00	0.00	RX1	C
ATOM	2923	CB	THR	81	53.019	10.563	-8.515	1.00	0.00	RX1	C
ATOM	2924	OG1	THR	81	52.396	9.497	-9.259	1.00	0.00	RX1	O
ATOM	2925	HG1	THR	81	51.784	9.090	-8.651	1.00	0.00	RX1	H
ATOM	2926	CG2	THR	81	53.663	11.604	-9.428	1.00	0.00	RX1	C

ATOM	2927	C	THR	81	54.348	10.907	-6.378	1.00	0.00	RX1	C
ATOM	2928	O	THR	81	55.082	11.886	-6.469	1.00	0.00	RX1	O
ATOM	2929	N	ILE	82	53.737	10.536	-5.239	1.00	0.00	RX1	N
ATOM	2930	H	ILE	82	53.338	9.630	-5.104	1.00	0.00	RX1	H
ATOM	2931	CA	ILE	82	53.790	11.470	-4.121	1.00	0.00	RX1	C
ATOM	2932	CB	ILE	82	52.733	11.125	-3.077	1.00	0.00	RX1	C
ATOM	2933	CG2	ILE	82	52.719	12.162	-1.954	1.00	0.00	RX1	C
ATOM	2934	CG1	ILE	82	51.366	10.991	-3.745	1.00	0.00	RX1	C
ATOM	2935	CD1	ILE	82	50.261	10.655	-2.752	1.00	0.00	RX1	C
ATOM	2936	C	ILE	82	55.164	11.582	-3.495	1.00	0.00	RX1	C
ATOM	2937	O	ILE	82	55.693	10.675	-2.866	1.00	0.00	RX1	O
ATOM	2938	N	GLN	83	55.719	12.773	-3.715	1.00	0.00	RX1	N
ATOM	2939	H	GLN	83	55.191	13.464	-4.209	1.00	0.00	RX1	H
ATOM	2940	CA	GLN	83	56.992	13.087	-3.085	1.00	0.00	RX1	C
ATOM	2941	CB	GLN	83	57.785	14.095	-3.911	1.00	0.00	RX1	C
ATOM	2942	CG	GLN	83	57.935	13.863	-5.413	1.00	0.00	RX1	C
ATOM	2943	CD	GLN	83	58.859	14.962	-5.890	1.00	0.00	RX1	C
ATOM	2944	OE1	GLN	83	59.651	15.468	-5.094	1.00	0.00	RX1	O
ATOM	2945	NE2	GLN	83	58.702	15.324	-7.174	1.00	0.00	RX1	N
ATOM	2946	HE21	GLN	83	58.035	14.905	-7.792	1.00	0.00	RX1	H
ATOM	2947	HE22	GLN	83	59.235	16.058	-7.630	1.00	0.00	RX1	H
ATOM	2948	C	GLN	83	56.812	13.691	-1.706	1.00	0.00	RX1	C
ATOM	2949	O	GLN	83	57.517	13.373	-0.753	1.00	0.00	RX1	O
ATOM	2950	N	GLU	84	55.848	14.622	-1.670	1.00	0.00	RX1	N
ATOM	2951	H	GLU	84	55.192	14.764	-2.414	1.00	0.00	RX1	H
ATOM	2952	CA	GLU	84	55.726	15.491	-0.508	1.00	0.00	RX1	C
ATOM	2953	CB	GLU	84	56.139	16.922	-0.869	1.00	0.00	RX1	C
ATOM	2954	CG	GLU	84	57.447	17.040	-1.660	1.00	0.00	RX1	C
ATOM	2955	CD	GLU	84	57.863	18.490	-1.773	1.00	0.00	RX1	C
ATOM	2956	OE1	GLU	84	57.010	19.357	-1.638	1.00	0.00	RX1	O
ATOM	2957	OE2	GLU	84	59.048	18.775	-1.944	1.00	0.00	RX1	O
ATOM	2958	C	GLU	84	54.309	15.510	0.023	1.00	0.00	RX1	C
ATOM	2959	O	GLU	84	53.363	15.748	-0.715	1.00	0.00	RX1	O
ATOM	2960	N	VAL	85	54.185	15.278	1.331	1.00	0.00	RX1	N
ATOM	2961	H	VAL	85	54.965	15.072	1.927	1.00	0.00	RX1	H
ATOM	2962	CA	VAL	85	52.886	15.583	1.930	1.00	0.00	RX1	C
ATOM	2963	CB	VAL	85	52.338	14.364	2.681	1.00	0.00	RX1	C
ATOM	2964	CG1	VAL	85	50.903	14.579	3.165	1.00	0.00	RX1	C
ATOM	2965	CG2	VAL	85	52.431	13.113	1.812	1.00	0.00	RX1	C
ATOM	2966	C	VAL	85	53.073	16.760	2.867	1.00	0.00	RX1	C
ATOM	2967	O	VAL	85	54.131	16.903	3.465	1.00	0.00	RX1	O
ATOM	2968	N	ALA	86	52.043	17.605	2.964	1.00	0.00	RX1	N
ATOM	2969	H	ALA	86	51.198	17.464	2.445	1.00	0.00	RX1	H
ATOM	2970	CA	ALA	86	52.114	18.640	3.986	1.00	0.00	RX1	C
ATOM	2971	CB	ALA	86	51.687	19.995	3.420	1.00	0.00	RX1	C
ATOM	2972	C	ALA	86	51.286	18.287	5.207	1.00	0.00	RX1	C
ATOM	2973	O	ALA	86	51.746	18.362	6.341	1.00	0.00	RX1	O
ATOM	2974	N	GLY	87	50.046	17.855	4.932	1.00	0.00	RX1	N
ATOM	2975	H	GLY	87	49.666	17.837	4.004	1.00	0.00	RX1	H
ATOM	2976	CA	GLY	87	49.246	17.332	6.037	1.00	0.00	RX1	C
ATOM	2977	C	GLY	87	49.743	16.003	6.580	1.00	0.00	RX1	C
ATOM	2978	O	GLY	87	50.834	15.536	6.270	1.00	0.00	RX1	O
ATOM	2979	N	TYR	88	48.890	15.417	7.429	1.00	0.00	RX1	N
ATOM	2980	H	TYR	88	47.939	15.723	7.493	1.00	0.00	RX1	H
ATOM	2981	CA	TYR	88	49.323	14.149	8.006	1.00	0.00	RX1	C
ATOM	2982	CB	TYR	88	48.791	13.976	9.436	1.00	0.00	RX1	C
ATOM	2983	CG	TYR	88	47.282	13.929	9.521	1.00	0.00	RX1	C
ATOM	2984	CD1	TYR	88	46.586	12.812	9.059	1.00	0.00	RX1	C
ATOM	2985	CE1	TYR	88	45.201	12.825	9.207	1.00	0.00	RX1	C
ATOM	2986	CD2	TYR	88	46.606	15.007	10.104	1.00	0.00	RX1	C
ATOM	2987	CE2	TYR	88	45.222	15.013	10.274	1.00	0.00	RX1	C

ATOM	2988	CZ	TYR	88	44.530	13.898	9.802	1.00	0.00	RX1	C
ATOM	2989	OH	TYR	88	43.156	13.859	9.909	1.00	0.00	RX1	O
ATOM	2990	HH	TYR	88	42.826	13.362	9.158	1.00	0.00	RX1	H
ATOM	2991	C	TYR	88	49.004	12.966	7.113	1.00	0.00	RX1	C
ATOM	2992	O	TYR	88	48.122	13.029	6.264	1.00	0.00	RX1	O
ATOM	2993	N	VAL	89	49.751	11.880	7.333	1.00	0.00	RX1	N
ATOM	2994	H	VAL	89	50.380	11.820	8.109	1.00	0.00	RX1	H
ATOM	2995	CA	VAL	89	49.435	10.682	6.563	1.00	0.00	RX1	C
ATOM	2996	CB	VAL	89	50.681	10.122	5.880	1.00	0.00	RX1	C
ATOM	2997	CG1	VAL	89	50.325	8.941	4.977	1.00	0.00	RX1	C
ATOM	2998	CG2	VAL	89	51.410	11.219	5.109	1.00	0.00	RX1	C
ATOM	2999	C	VAL	89	48.780	9.636	7.445	1.00	0.00	RX1	C
ATOM	3000	O	VAL	89	49.422	8.906	8.195	1.00	0.00	RX1	O
ATOM	3001	N	LEU	90	47.450	9.621	7.341	1.00	0.00	RX1	N
ATOM	3002	H	LEU	90	47.005	10.082	6.571	1.00	0.00	RX1	H
ATOM	3003	CA	LEU	90	46.726	8.658	8.162	1.00	0.00	RX1	C
ATOM	3004	CB	LEU	90	45.401	9.251	8.632	1.00	0.00	RX1	C
ATOM	3005	CG	LEU	90	44.555	8.312	9.491	1.00	0.00	RX1	C
ATOM	3006	CD1	LEU	90	45.244	7.955	10.807	1.00	0.00	RX1	C
ATOM	3007	CD2	LEU	90	43.153	8.874	9.715	1.00	0.00	RX1	C
ATOM	3008	C	LEU	90	46.493	7.370	7.406	1.00	0.00	RX1	C
ATOM	3009	O	LEU	90	45.585	7.249	6.597	1.00	0.00	RX1	O
ATOM	3010	N	ILE	91	47.366	6.410	7.707	1.00	0.00	RX1	N
ATOM	3011	H	ILE	91	48.000	6.524	8.474	1.00	0.00	RX1	H
ATOM	3012	CA	ILE	91	47.146	5.089	7.136	1.00	0.00	RX1	C
ATOM	3013	CB	ILE	91	48.488	4.470	6.744	1.00	0.00	RX1	C
ATOM	3014	CG2	ILE	91	48.325	3.054	6.198	1.00	0.00	RX1	C
ATOM	3015	CG1	ILE	91	49.216	5.374	5.752	1.00	0.00	RX1	C
ATOM	3016	CD1	ILE	91	50.557	4.792	5.304	1.00	0.00	RX1	C
ATOM	3017	C	ILE	91	46.419	4.222	8.147	1.00	0.00	RX1	C
ATOM	3018	O	ILE	91	47.028	3.595	9.007	1.00	0.00	RX1	O
ATOM	3019	N	ALA	92	45.089	4.232	8.032	1.00	0.00	RX1	N
ATOM	3020	H	ALA	92	44.603	4.680	7.275	1.00	0.00	RX1	H
ATOM	3021	CA	ALA	92	44.362	3.482	9.048	1.00	0.00	RX1	C
ATOM	3022	CB	ALA	92	43.725	4.419	10.074	1.00	0.00	RX1	C
ATOM	3023	C	ALA	92	43.300	2.562	8.496	1.00	0.00	RX1	C
ATOM	3024	O	ALA	92	42.631	2.853	7.511	1.00	0.00	RX1	O
ATOM	3025	N	LEU	93	43.163	1.427	9.204	1.00	0.00	RX1	N
ATOM	3026	H	LEU	93	43.828	1.229	9.926	1.00	0.00	RX1	H
ATOM	3027	CA	LEU	93	42.093	0.467	8.912	1.00	0.00	RX1	C
ATOM	3028	CB	LEU	93	40.739	1.041	9.334	1.00	0.00	RX1	C
ATOM	3029	CG	LEU	93	40.643	1.200	10.853	1.00	0.00	RX1	C
ATOM	3030	CD1	LEU	93	39.470	2.080	11.276	1.00	0.00	RX1	C
ATOM	3031	CD2	LEU	93	40.602	-0.157	11.554	1.00	0.00	RX1	C
ATOM	3032	C	LEU	93	42.062	-0.059	7.486	1.00	0.00	RX1	C
ATOM	3033	O	LEU	93	41.032	-0.414	6.931	1.00	0.00	RX1	O
ATOM	3034	N	ASN	94	43.262	-0.079	6.900	1.00	0.00	RX1	N
ATOM	3035	H	ASN	94	44.093	0.098	7.425	1.00	0.00	RX1	H
ATOM	3036	CA	ASN	94	43.355	-0.603	5.542	1.00	0.00	RX1	C
ATOM	3037	CB	ASN	94	44.475	0.049	4.730	1.00	0.00	RX1	C
ATOM	3038	CG	ASN	94	44.298	1.544	4.639	1.00	0.00	RX1	C
ATOM	3039	OD1	ASN	94	43.485	2.068	3.886	1.00	0.00	RX1	O
ATOM	3040	ND2	ASN	94	45.128	2.213	5.445	1.00	0.00	RX1	N
ATOM	3041	HD21	ASN	94	45.734	1.716	6.070	1.00	0.00	RX1	H
ATOM	3042	HD22	ASN	94	45.133	3.214	5.465	1.00	0.00	RX1	H
ATOM	3043	C	ASN	94	43.665	-2.069	5.606	1.00	0.00	RX1	C
ATOM	3044	O	ASN	94	44.506	-2.507	6.379	1.00	0.00	RX1	O
ATOM	3045	N	THR	95	42.964	-2.817	4.762	1.00	0.00	RX1	N
ATOM	3046	H	THR	95	42.285	-2.470	4.113	1.00	0.00	RX1	H
ATOM	3047	CA	THR	95	43.336	-4.222	4.718	1.00	0.00	RX1	C
ATOM	3048	CB	THR	95	41.974	-4.862	4.609	1.00	0.00	RX1	C

ATOM	3049	OG1	THR	95	41.178	-3.961	3.849	1.00	0.00	RX1	O
ATOM	3050	HG1	THR	95	40.279	-4.255	3.982	1.00	0.00	RX1	H
ATOM	3051	CG2	THR	95	41.277	-4.986	5.955	1.00	0.00	RX1	C
ATOM	3052	C	THR	95	44.280	-4.547	3.565	1.00	0.00	RX1	C
ATOM	3053	O	THR	95	44.816	-5.641	3.457	1.00	0.00	RX1	O
ATOM	3054	N	VAL	96	44.470	-3.534	2.699	1.00	0.00	RX1	N
ATOM	3055	H	VAL	96	43.959	-2.680	2.798	1.00	0.00	RX1	H
ATOM	3056	CA	VAL	96	45.344	-3.729	1.543	1.00	0.00	RX1	C
ATOM	3057	CB	VAL	96	45.255	-2.514	0.608	1.00	0.00	RX1	C
ATOM	3058	CG1	VAL	96	45.672	-1.226	1.313	1.00	0.00	RX1	C
ATOM	3059	CG2	VAL	96	46.016	-2.740	-0.700	1.00	0.00	RX1	C
ATOM	3060	C	VAL	96	46.790	-4.123	1.839	1.00	0.00	RX1	C
ATOM	3061	O	VAL	96	47.526	-3.455	2.556	1.00	0.00	RX1	O
ATOM	3062	N	GLU	97	47.145	-5.251	1.199	1.00	0.00	RX1	N
ATOM	3063	H	GLU	97	46.424	-5.739	0.705	1.00	0.00	RX1	H
ATOM	3064	CA	GLU	97	48.462	-5.877	1.346	1.00	0.00	RX1	C
ATOM	3065	CB	GLU	97	48.603	-7.011	0.331	1.00	0.00	RX1	C
ATOM	3066	CG	GLU	97	49.490	-8.160	0.811	1.00	0.00	RX1	C
ATOM	3067	CD	GLU	97	48.668	-9.057	1.711	1.00	0.00	RX1	C
ATOM	3068	OE1	GLU	97	49.122	-9.422	2.792	1.00	0.00	RX1	O
ATOM	3069	OE2	GLU	97	47.553	-9.400	1.332	1.00	0.00	RX1	O
ATOM	3070	C	GLU	97	49.661	-4.945	1.202	1.00	0.00	RX1	C
ATOM	3071	O	GLU	97	50.631	-4.955	1.953	1.00	0.00	RX1	O
ATOM	3072	N	ARG	98	49.568	-4.136	0.143	1.00	0.00	RX1	N
ATOM	3073	H	ARG	98	48.736	-4.038	-0.402	1.00	0.00	RX1	H
ATOM	3074	CA	ARG	98	50.716	-3.298	-0.175	1.00	0.00	RX1	C
ATOM	3075	CB	ARG	98	51.434	-3.914	-1.374	1.00	0.00	RX1	C
ATOM	3076	CG	ARG	98	52.801	-3.310	-1.675	1.00	0.00	RX1	C
ATOM	3077	CD	ARG	98	53.488	-4.034	-2.835	1.00	0.00	RX1	C
ATOM	3078	NE	ARG	98	54.924	-3.755	-2.870	1.00	0.00	RX1	N
ATOM	3079	HE	ARG	98	55.499	-4.407	-2.360	1.00	0.00	RX1	H
ATOM	3080	CZ	ARG	98	55.390	-2.638	-3.510	1.00	0.00	RX1	C
ATOM	3081	NH1	ARG	98	54.525	-1.795	-4.110	1.00	0.00	RX1	N
ATOM	3082	HH11	ARG	98	54.864	-0.927	-4.522	1.00	0.00	RX1	H
ATOM	3083	HH12	ARG	98	53.540	-1.967	-4.166	1.00	0.00	RX1	H
ATOM	3084	NH2	ARG	98	56.713	-2.373	-3.537	1.00	0.00	RX1	N
ATOM	3085	HH21	ARG	98	57.034	-1.518	-3.979	1.00	0.00	RX1	H
ATOM	3086	HH22	ARG	98	57.414	-2.964	-3.133	1.00	0.00	RX1	H
ATOM	3087	C	ARG	98	50.248	-1.902	-0.493	1.00	0.00	RX1	C
ATOM	3088	O	ARG	98	49.416	-1.713	-1.368	1.00	0.00	RX1	O
ATOM	3089	N	ILE	99	50.814	-0.930	0.229	1.00	0.00	RX1	N
ATOM	3090	H	ILE	99	51.564	-1.114	0.871	1.00	0.00	RX1	H
ATOM	3091	CA	ILE	99	50.470	0.432	-0.168	1.00	0.00	RX1	C
ATOM	3092	CB	ILE	99	50.097	1.283	1.046	1.00	0.00	RX1	C
ATOM	3093	CG2	ILE	99	49.966	2.764	0.691	1.00	0.00	RX1	C
ATOM	3094	CG1	ILE	99	48.803	0.748	1.652	1.00	0.00	RX1	C
ATOM	3095	CD1	ILE	99	48.328	1.566	2.848	1.00	0.00	RX1	C
ATOM	3096	C	ILE	99	51.597	1.061	-0.961	1.00	0.00	RX1	C
ATOM	3097	O	ILE	99	52.687	1.303	-0.459	1.00	0.00	RX1	O
ATOM	3098	N	PRO	100	51.308	1.310	-2.259	1.00	0.00	RX1	N
ATOM	3099	CD	PRO	100	50.082	0.931	-2.936	1.00	0.00	RX1	C
ATOM	3100	CA	PRO	100	52.302	1.902	-3.160	1.00	0.00	RX1	C
ATOM	3101	CB	PRO	100	51.632	1.722	-4.527	1.00	0.00	RX1	C
ATOM	3102	CG	PRO	100	50.566	0.648	-4.345	1.00	0.00	RX1	C
ATOM	3103	C	PRO	100	52.664	3.369	-2.944	1.00	0.00	RX1	C
ATOM	3104	O	PRO	100	52.866	4.100	-3.906	1.00	0.00	RX1	O
ATOM	3105	N	LEU	101	52.818	3.773	-1.673	1.00	0.00	RX1	N
ATOM	3106	H	LEU	101	52.771	3.118	-0.921	1.00	0.00	RX1	H
ATOM	3107	CA	LEU	101	53.398	5.087	-1.380	1.00	0.00	RX1	C
ATOM	3108	CB	LEU	101	53.035	5.485	0.043	1.00	0.00	RX1	C
ATOM	3109	CG	LEU	101	52.060	6.648	0.148	1.00	0.00	RX1	C

ATOM	3110	CD1	LEU	101	51.799	6.983	1.616	1.00	0.00	RX1	C
ATOM	3111	CD2	LEU	101	52.533	7.862	-0.654	1.00	0.00	RX1	C
ATOM	3112	C	LEU	101	54.916	5.093	-1.494	1.00	0.00	RX1	C
ATOM	3113	O	LEU	101	55.647	5.782	-0.799	1.00	0.00	RX1	O
ATOM	3114	N	GLU	102	55.379	4.221	-2.390	1.00	0.00	RX1	N
ATOM	3115	H	GLU	102	54.784	3.962	-3.147	1.00	0.00	RX1	H
ATOM	3116	CA	GLU	102	56.745	3.722	-2.282	1.00	0.00	RX1	C
ATOM	3117	CB	GLU	102	56.885	2.447	-3.134	1.00	0.00	RX1	C
ATOM	3118	CG	GLU	102	56.152	2.431	-4.481	1.00	0.00	RX1	C
ATOM	3119	CD	GLU	102	55.745	1.008	-4.841	1.00	0.00	RX1	C
ATOM	3120	OE1	GLU	102	56.530	0.090	-4.663	1.00	0.00	RX1	O
ATOM	3121	OE2	GLU	102	54.620	0.782	-5.279	1.00	0.00	RX1	O
ATOM	3122	C	GLU	102	57.832	4.761	-2.531	1.00	0.00	RX1	C
ATOM	3123	O	GLU	102	58.976	4.631	-2.110	1.00	0.00	RX1	O
ATOM	3124	N	ASN	103	57.411	5.830	-3.221	1.00	0.00	RX1	N
ATOM	3125	H	ASN	103	56.448	5.979	-3.438	1.00	0.00	RX1	H
ATOM	3126	CA	ASN	103	58.410	6.855	-3.511	1.00	0.00	RX1	C
ATOM	3127	CB	ASN	103	58.324	7.381	-4.940	1.00	0.00	RX1	C
ATOM	3128	CG	ASN	103	58.933	6.405	-5.915	1.00	0.00	RX1	C
ATOM	3129	OD1	ASN	103	59.970	5.787	-5.677	1.00	0.00	RX1	O
ATOM	3130	ND2	ASN	103	58.207	6.296	-7.040	1.00	0.00	RX1	N
ATOM	3131	HD21	ASN	103	57.397	6.886	-7.140	1.00	0.00	RX1	H
ATOM	3132	HD22	ASN	103	58.432	5.670	-7.785	1.00	0.00	RX1	H
ATOM	3133	C	ASN	103	58.405	8.059	-2.590	1.00	0.00	RX1	C
ATOM	3134	O	ASN	103	59.214	8.963	-2.775	1.00	0.00	RX1	O
ATOM	3135	N	LEU	104	57.477	8.036	-1.609	1.00	0.00	RX1	N
ATOM	3136	H	LEU	104	56.907	7.231	-1.445	1.00	0.00	RX1	H
ATOM	3137	CA	LEU	104	57.319	9.180	-0.703	1.00	0.00	RX1	C
ATOM	3138	CB	LEU	104	56.280	8.834	0.367	1.00	0.00	RX1	C
ATOM	3139	CG	LEU	104	55.995	9.950	1.374	1.00	0.00	RX1	C
ATOM	3140	CD1	LEU	104	55.440	11.202	0.698	1.00	0.00	RX1	C
ATOM	3141	CD2	LEU	104	55.088	9.473	2.508	1.00	0.00	RX1	C
ATOM	3142	C	LEU	104	58.621	9.597	-0.050	1.00	0.00	RX1	C
ATOM	3143	O	LEU	104	59.367	8.754	0.429	1.00	0.00	RX1	O
ATOM	3144	N	GLN	105	58.870	10.914	-0.083	1.00	0.00	RX1	N
ATOM	3145	H	GLN	105	58.191	11.559	-0.435	1.00	0.00	RX1	H
ATOM	3146	CA	GLN	105	60.135	11.394	0.470	1.00	0.00	RX1	C
ATOM	3147	CB	GLN	105	60.867	12.321	-0.498	1.00	0.00	RX1	C
ATOM	3148	CG	GLN	105	60.971	11.804	-1.932	1.00	0.00	RX1	C
ATOM	3149	CD	GLN	105	62.013	12.621	-2.665	1.00	0.00	RX1	C
ATOM	3150	OE1	GLN	105	63.195	12.588	-2.333	1.00	0.00	RX1	O
ATOM	3151	NE2	GLN	105	61.516	13.359	-3.675	1.00	0.00	RX1	N
ATOM	3152	HE21	GLN	105	60.540	13.370	-3.895	1.00	0.00	RX1	H
ATOM	3153	HE22	GLN	105	62.082	13.947	-4.254	1.00	0.00	RX1	H
ATOM	3154	C	GLN	105	60.014	12.087	1.813	1.00	0.00	RX1	C
ATOM	3155	O	GLN	105	60.810	11.886	2.729	1.00	0.00	RX1	O
ATOM	3156	N	ILE	106	58.977	12.938	1.869	1.00	0.00	RX1	N
ATOM	3157	H	ILE	106	58.313	13.046	1.125	1.00	0.00	RX1	H
ATOM	3158	CA	ILE	106	58.830	13.790	3.045	1.00	0.00	RX1	C
ATOM	3159	CB	ILE	106	59.407	15.183	2.760	1.00	0.00	RX1	C
ATOM	3160	CG2	ILE	106	58.699	15.822	1.579	1.00	0.00	RX1	C
ATOM	3161	CG1	ILE	106	59.354	16.127	3.957	1.00	0.00	RX1	C
ATOM	3162	CD1	ILE	106	59.804	17.530	3.556	1.00	0.00	RX1	C
ATOM	3163	C	ILE	106	57.392	13.873	3.519	1.00	0.00	RX1	C
ATOM	3164	O	ILE	106	56.455	14.046	2.745	1.00	0.00	RX1	O
ATOM	3165	N	ILE	107	57.266	13.778	4.844	1.00	0.00	RX1	N
ATOM	3166	H	ILE	107	58.067	13.629	5.430	1.00	0.00	RX1	H
ATOM	3167	CA	ILE	107	56.049	14.316	5.435	1.00	0.00	RX1	C
ATOM	3168	CB	ILE	107	55.449	13.311	6.414	1.00	0.00	RX1	C
ATOM	3169	CG2	ILE	107	54.128	13.815	7.000	1.00	0.00	RX1	C
ATOM	3170	CG1	ILE	107	55.304	11.952	5.731	1.00	0.00	RX1	C

ATOM	3171	CD1	ILE	107	54.887	10.848	6.698	1.00	0.00	RX1	C
ATOM	3172	C	ILE	107	56.423	15.606	6.136	1.00	0.00	RX1	C
ATOM	3173	O	ILE	107	57.348	15.648	6.939	1.00	0.00	RX1	O
ATOM	3174	N	ARG	108	55.701	16.669	5.767	1.00	0.00	RX1	N
ATOM	3175	H	ARG	108	54.925	16.580	5.143	1.00	0.00	RX1	H
ATOM	3176	CA	ARG	108	55.996	17.930	6.435	1.00	0.00	RX1	C
ATOM	3177	CB	ARG	108	55.583	19.132	5.588	1.00	0.00	RX1	C
ATOM	3178	CG	ARG	108	56.550	19.319	4.421	1.00	0.00	RX1	C
ATOM	3179	CD	ARG	108	56.271	20.555	3.567	1.00	0.00	RX1	C
ATOM	3180	NE	ARG	108	57.376	20.781	2.636	1.00	0.00	RX1	N
ATOM	3181	HE	ARG	108	58.213	21.198	3.015	1.00	0.00	RX1	H
ATOM	3182	CZ	ARG	108	57.266	20.356	1.343	1.00	0.00	RX1	C
ATOM	3183	NH1	ARG	108	56.096	19.862	0.890	1.00	0.00	RX1	N
ATOM	3184	HH11	ARG	108	56.038	19.564	-0.077	1.00	0.00	RX1	H
ATOM	3185	HH12	ARG	108	55.277	19.775	1.460	1.00	0.00	RX1	H
ATOM	3186	NH2	ARG	108	58.329	20.426	0.524	1.00	0.00	RX1	N
ATOM	3187	HH21	ARG	108	58.269	20.097	-0.438	1.00	0.00	RX1	H
ATOM	3188	HH22	ARG	108	59.210	20.790	0.844	1.00	0.00	RX1	H
ATOM	3189	C	ARG	108	55.413	18.006	7.824	1.00	0.00	RX1	C
ATOM	3190	O	ARG	108	56.127	18.244	8.782	1.00	0.00	RX1	O
ATOM	3191	N	GLY	109	54.102	17.749	7.905	1.00	0.00	RX1	N
ATOM	3192	H	GLY	109	53.502	17.685	7.106	1.00	0.00	RX1	H
ATOM	3193	CA	GLY	109	53.531	17.745	9.248	1.00	0.00	RX1	C
ATOM	3194	C	GLY	109	52.968	19.087	9.676	1.00	0.00	RX1	C
ATOM	3195	O	GLY	109	53.021	19.498	10.827	1.00	0.00	RX1	O
ATOM	3196	N	ASN	110	52.369	19.763	8.686	1.00	0.00	RX1	N
ATOM	3197	H	ASN	110	52.425	19.444	7.739	1.00	0.00	RX1	H
ATOM	3198	CA	ASN	110	51.547	20.928	9.024	1.00	0.00	RX1	C
ATOM	3199	CB	ASN	110	50.983	21.625	7.775	1.00	0.00	RX1	C
ATOM	3200	CG	ASN	110	52.054	22.011	6.771	1.00	0.00	RX1	C
ATOM	3201	OD1	ASN	110	52.862	21.194	6.335	1.00	0.00	RX1	O
ATOM	3202	ND2	ASN	110	51.975	23.287	6.359	1.00	0.00	RX1	N
ATOM	3203	HD21	ASN	110	51.383	23.957	6.817	1.00	0.00	RX1	H
ATOM	3204	HD22	ASN	110	52.516	23.649	5.602	1.00	0.00	RX1	H
ATOM	3205	C	ASN	110	50.364	20.477	9.868	1.00	0.00	RX1	C
ATOM	3206	O	ASN	110	50.084	20.934	10.975	1.00	0.00	RX1	O
ATOM	3207	N	MET	111	49.682	19.485	9.284	1.00	0.00	RX1	N
ATOM	3208	H	MET	111	50.046	19.039	8.466	1.00	0.00	RX1	H
ATOM	3209	CA	MET	111	48.661	18.833	10.092	1.00	0.00	RX1	C
ATOM	3210	CB	MET	111	47.501	18.356	9.223	1.00	0.00	RX1	C
ATOM	3211	CG	MET	111	46.602	19.495	8.747	1.00	0.00	RX1	C
ATOM	3212	SD	MET	111	45.752	20.284	10.123	1.00	0.00	RX1	S
ATOM	3213	CE	MET	111	44.818	18.851	10.687	1.00	0.00	RX1	C
ATOM	3214	C	MET	111	49.254	17.682	10.870	1.00	0.00	RX1	C
ATOM	3215	O	MET	111	50.192	17.028	10.435	1.00	0.00	RX1	O
ATOM	3216	N	TYR	112	48.669	17.492	12.056	1.00	0.00	RX1	N
ATOM	3217	H	TYR	112	47.850	18.007	12.305	1.00	0.00	RX1	H
ATOM	3218	CA	TYR	112	49.080	16.376	12.901	1.00	0.00	RX1	C
ATOM	3219	CB	TYR	112	49.585	16.881	14.254	1.00	0.00	RX1	C
ATOM	3220	CG	TYR	112	51.025	17.345	14.347	1.00	0.00	RX1	C
ATOM	3221	CD1	TYR	112	51.770	17.774	13.254	1.00	0.00	RX1	C
ATOM	3222	CE1	TYR	112	53.061	18.253	13.450	1.00	0.00	RX1	C
ATOM	3223	CD2	TYR	112	51.600	17.366	15.610	1.00	0.00	RX1	C
ATOM	3224	CE2	TYR	112	52.887	17.840	15.809	1.00	0.00	RX1	C
ATOM	3225	CZ	TYR	112	53.618	18.294	14.723	1.00	0.00	RX1	C
ATOM	3226	OH	TYR	112	54.899	18.772	14.918	1.00	0.00	RX1	O
ATOM	3227	HH	TYR	112	55.026	18.943	15.844	1.00	0.00	RX1	H
ATOM	3228	C	TYR	112	47.825	15.582	13.188	1.00	0.00	RX1	C
ATOM	3229	O	TYR	112	46.805	16.177	13.517	1.00	0.00	RX1	O
ATOM	3230	N	TYR	113	47.913	14.252	13.061	1.00	0.00	RX1	N
ATOM	3231	H	TYR	113	48.812	13.837	12.918	1.00	0.00	RX1	H

ATOM	3232	CA	TYR	113	46.747	13.464	13.456	1.00	0.00	RX1	C
ATOM	3233	CB	TYR	113	46.848	12.035	12.894	1.00	0.00	RX1	C
ATOM	3234	CG	TYR	113	45.540	11.299	13.097	1.00	0.00	RX1	C
ATOM	3235	CD1	TYR	113	44.417	11.689	12.341	1.00	0.00	RX1	C
ATOM	3236	CE1	TYR	113	43.177	11.089	12.600	1.00	0.00	RX1	C
ATOM	3237	CD2	TYR	113	45.470	10.263	14.051	1.00	0.00	RX1	C
ATOM	3238	CE2	TYR	113	44.227	9.660	14.311	1.00	0.00	RX1	C
ATOM	3239	CZ	TYR	113	43.089	10.116	13.614	1.00	0.00	RX1	C
ATOM	3240	OH	TYR	113	41.840	9.621	13.950	1.00	0.00	RX1	O
ATOM	3241	HH	TYR	113	41.955	8.725	14.258	1.00	0.00	RX1	H
ATOM	3242	C	TYR	113	46.585	13.459	14.962	1.00	0.00	RX1	C
ATOM	3243	O	TYR	113	47.461	13.004	15.680	1.00	0.00	RX1	O
ATOM	3244	N	GLU	114	45.455	14.052	15.387	1.00	0.00	RX1	N
ATOM	3245	H	GLU	114	44.738	14.194	14.705	1.00	0.00	RX1	H
ATOM	3246	CA	GLU	114	45.156	14.311	16.803	1.00	0.00	RX1	C
ATOM	3247	CB	GLU	114	44.455	13.114	17.467	1.00	0.00	RX1	C
ATOM	3248	CG	GLU	114	43.463	12.323	16.581	1.00	0.00	RX1	C
ATOM	3249	CD	GLU	114	42.480	13.193	15.798	1.00	0.00	RX1	C
ATOM	3250	OE1	GLU	114	42.640	13.356	14.593	1.00	0.00	RX1	O
ATOM	3251	OE2	GLU	114	41.522	13.711	16.354	1.00	0.00	RX1	O
ATOM	3252	C	GLU	114	46.268	14.960	17.632	1.00	0.00	RX1	C
ATOM	3253	O	GLU	114	46.390	14.783	18.838	1.00	0.00	RX1	O
ATOM	3254	N	ASN	115	47.078	15.751	16.900	1.00	0.00	RX1	N
ATOM	3255	H	ASN	115	46.862	15.827	15.929	1.00	0.00	RX1	H
ATOM	3256	CA	ASN	115	48.358	16.259	17.411	1.00	0.00	RX1	C
ATOM	3257	CB	ASN	115	48.197	17.366	18.465	1.00	0.00	RX1	C
ATOM	3258	CG	ASN	115	49.312	18.374	18.254	1.00	0.00	RX1	C
ATOM	3259	OD1	ASN	115	49.479	18.939	17.176	1.00	0.00	RX1	O
ATOM	3260	ND2	ASN	115	50.126	18.513	19.301	1.00	0.00	RX1	N
ATOM	3261	HD21	ASN	115	49.957	18.089	20.190	1.00	0.00	RX1	H
ATOM	3262	HD22	ASN	115	50.970	19.064	19.264	1.00	0.00	RX1	H
ATOM	3263	C	ASN	115	49.366	15.174	17.789	1.00	0.00	RX1	C
ATOM	3264	O	ASN	115	49.062	13.993	17.739	1.00	0.00	RX1	O
ATOM	3265	N	SER	116	50.605	15.607	18.101	1.00	0.00	RX1	N
ATOM	3266	H	SER	116	50.830	16.575	18.141	1.00	0.00	RX1	H
ATOM	3267	CA	SER	116	51.743	14.683	18.165	1.00	0.00	RX1	C
ATOM	3268	CB	SER	116	51.553	13.635	19.290	1.00	0.00	RX1	C
ATOM	3269	OG	SER	116	52.735	12.856	19.497	1.00	0.00	RX1	O
ATOM	3270	HG	SER	116	52.733	12.631	20.426	1.00	0.00	RX1	H
ATOM	3271	C	SER	116	52.133	14.106	16.808	1.00	0.00	RX1	C
ATOM	3272	O	SER	116	53.132	14.501	16.217	1.00	0.00	RX1	O
ATOM	3273	N	TYR	117	51.324	13.157	16.327	1.00	0.00	RX1	N
ATOM	3274	H	TYR	117	50.402	13.014	16.696	1.00	0.00	RX1	H
ATOM	3275	CA	TYR	117	51.794	12.384	15.183	1.00	0.00	RX1	C
ATOM	3276	CB	TYR	117	51.206	10.978	15.252	1.00	0.00	RX1	C
ATOM	3277	CG	TYR	117	51.621	10.363	16.563	1.00	0.00	RX1	C
ATOM	3278	CD1	TYR	117	52.944	9.994	16.750	1.00	0.00	RX1	C
ATOM	3279	CE1	TYR	117	53.349	9.433	17.951	1.00	0.00	RX1	C
ATOM	3280	CD2	TYR	117	50.695	10.173	17.583	1.00	0.00	RX1	C
ATOM	3281	CE2	TYR	117	51.100	9.612	18.788	1.00	0.00	RX1	C
ATOM	3282	CZ	TYR	117	52.426	9.233	18.967	1.00	0.00	RX1	C
ATOM	3283	OH	TYR	117	52.826	8.632	20.144	1.00	0.00	RX1	O
ATOM	3284	HH	TYR	117	53.332	9.284	20.639	1.00	0.00	RX1	H
ATOM	3285	C	TYR	117	51.506	13.011	13.836	1.00	0.00	RX1	C
ATOM	3286	O	TYR	117	50.552	13.755	13.662	1.00	0.00	RX1	O
ATOM	3287	N	ALA	118	52.369	12.663	12.877	1.00	0.00	RX1	N
ATOM	3288	H	ALA	118	53.138	12.063	13.091	1.00	0.00	RX1	H
ATOM	3289	CA	ALA	118	52.067	13.009	11.492	1.00	0.00	RX1	C
ATOM	3290	CB	ALA	118	53.209	13.795	10.851	1.00	0.00	RX1	C
ATOM	3291	C	ALA	118	51.819	11.766	10.665	1.00	0.00	RX1	C
ATOM	3292	O	ALA	118	50.823	11.631	9.966	1.00	0.00	RX1	O

ATOM	3293	N	LEU	119	52.767	10.829	10.788	1.00	0.00	RX1	N
ATOM	3294	H	LEU	119	53.511	10.915	11.451	1.00	0.00	RX1	H
ATOM	3295	CA	LEU	119	52.471	9.535	10.181	1.00	0.00	RX1	C
ATOM	3296	CB	LEU	119	53.779	8.870	9.746	1.00	0.00	RX1	C
ATOM	3297	CG	LEU	119	53.615	7.530	9.022	1.00	0.00	RX1	C
ATOM	3298	CD1	LEU	119	52.692	7.635	7.810	1.00	0.00	RX1	C
ATOM	3299	CD2	LEU	119	54.967	6.933	8.631	1.00	0.00	RX1	C
ATOM	3300	C	LEU	119	51.727	8.683	11.187	1.00	0.00	RX1	C
ATOM	3301	O	LEU	119	52.151	8.570	12.329	1.00	0.00	RX1	O
ATOM	3302	N	ALA	120	50.608	8.110	10.735	1.00	0.00	RX1	N
ATOM	3303	H	ALA	120	50.265	8.253	9.803	1.00	0.00	RX1	H
ATOM	3304	CA	ALA	120	49.845	7.289	11.668	1.00	0.00	RX1	C
ATOM	3305	CB	ALA	120	48.697	8.095	12.274	1.00	0.00	RX1	C
ATOM	3306	C	ALA	120	49.306	6.037	11.011	1.00	0.00	RX1	C
ATOM	3307	O	ALA	120	48.259	6.026	10.376	1.00	0.00	RX1	O
ATOM	3308	N	VAL	121	50.098	4.974	11.178	1.00	0.00	RX1	N
ATOM	3309	H	VAL	121	50.895	5.032	11.780	1.00	0.00	RX1	H
ATOM	3310	CA	VAL	121	49.751	3.695	10.564	1.00	0.00	RX1	C
ATOM	3311	CB	VAL	121	51.015	3.066	9.979	1.00	0.00	RX1	C
ATOM	3312	CG1	VAL	121	50.736	1.775	9.209	1.00	0.00	RX1	C
ATOM	3313	CG2	VAL	121	51.734	4.090	9.105	1.00	0.00	RX1	C
ATOM	3314	C	VAL	121	49.059	2.764	11.551	1.00	0.00	RX1	C
ATOM	3315	O	VAL	121	49.679	1.940	12.219	1.00	0.00	RX1	O
ATOM	3316	N	LEU	122	47.738	2.966	11.627	1.00	0.00	RX1	N
ATOM	3317	H	LEU	122	47.282	3.536	10.939	1.00	0.00	RX1	H
ATOM	3318	CA	LEU	122	46.944	2.316	12.671	1.00	0.00	RX1	C
ATOM	3319	CB	LEU	122	46.069	3.355	13.371	1.00	0.00	RX1	C
ATOM	3320	CG	LEU	122	46.795	4.656	13.714	1.00	0.00	RX1	C
ATOM	3321	CD1	LEU	122	45.823	5.740	14.180	1.00	0.00	RX1	C
ATOM	3322	CD2	LEU	122	47.924	4.437	14.718	1.00	0.00	RX1	C
ATOM	3323	C	LEU	122	46.038	1.212	12.151	1.00	0.00	RX1	C
ATOM	3324	O	LEU	122	45.374	1.369	11.134	1.00	0.00	RX1	O
ATOM	3325	N	SER	123	46.009	0.100	12.905	1.00	0.00	RX1	N
ATOM	3326	H	SER	123	46.579	0.036	13.723	1.00	0.00	RX1	H
ATOM	3327	CA	SER	123	44.998	-0.941	12.671	1.00	0.00	RX1	C
ATOM	3328	CB	SER	123	43.784	-0.423	13.408	1.00	0.00	RX1	C
ATOM	3329	OG	SER	123	44.298	0.125	14.634	1.00	0.00	RX1	O
ATOM	3330	HG	SER	123	43.608	0.682	14.973	1.00	0.00	RX1	H
ATOM	3331	C	SER	123	44.799	-1.410	11.237	1.00	0.00	RX1	C
ATOM	3332	O	SER	123	43.701	-1.442	10.699	1.00	0.00	RX1	O
ATOM	3333	N	ASN	124	45.929	-1.761	10.621	1.00	0.00	RX1	N
ATOM	3334	H	ASN	124	46.757	-1.941	11.151	1.00	0.00	RX1	H
ATOM	3335	CA	ASN	124	45.809	-2.037	9.189	1.00	0.00	RX1	C
ATOM	3336	CB	ASN	124	46.942	-1.391	8.396	1.00	0.00	RX1	C
ATOM	3337	CG	ASN	124	46.755	0.104	8.348	1.00	0.00	RX1	C
ATOM	3338	OD1	ASN	124	45.919	0.639	7.628	1.00	0.00	RX1	O
ATOM	3339	ND2	ASN	124	47.578	0.765	9.171	1.00	0.00	RX1	N
ATOM	3340	HD21	ASN	124	48.274	0.290	9.708	1.00	0.00	RX1	H
ATOM	3341	HD22	ASN	124	47.485	1.760	9.267	1.00	0.00	RX1	H
ATOM	3342	C	ASN	124	45.790	-3.511	8.871	1.00	0.00	RX1	C
ATOM	3343	O	ASN	124	46.829	-4.080	8.553	1.00	0.00	RX1	O
ATOM	3344	N	TYR	125	44.588	-4.104	9.001	1.00	0.00	RX1	N
ATOM	3345	H	TYR	125	43.744	-3.603	9.206	1.00	0.00	RX1	H
ATOM	3346	CA	TYR	125	44.476	-5.552	8.804	1.00	0.00	RX1	C
ATOM	3347	CB	TYR	125	45.192	-6.312	9.915	1.00	0.00	RX1	C
ATOM	3348	CG	TYR	125	44.496	-6.071	11.220	1.00	0.00	RX1	C
ATOM	3349	CD1	TYR	125	43.490	-6.933	11.631	1.00	0.00	RX1	C
ATOM	3350	CE1	TYR	125	42.869	-6.742	12.852	1.00	0.00	RX1	C
ATOM	3351	CD2	TYR	125	44.868	-4.991	12.004	1.00	0.00	RX1	C
ATOM	3352	CE2	TYR	125	44.255	-4.805	13.228	1.00	0.00	RX1	C
ATOM	3353	CZ	TYR	125	43.291	-5.701	13.661	1.00	0.00	RX1	C

ATOM	3354	OH	TYR	125	42.772	-5.570	14.930	1.00	0.00	RX1	O
ATOM	3355	HH	TYR	125	42.099	-4.893	14.814	1.00	0.00	RX1	H
ATOM	3356	C	TYR	125	43.054	-6.061	8.638	1.00	0.00	RX1	C
ATOM	3357	O	TYR	125	42.092	-5.384	8.977	1.00	0.00	RX1	O
ATOM	3358	N	ASP	126	42.989	-7.294	8.114	1.00	0.00	RX1	N
ATOM	3359	H	ASP	126	43.808	-7.819	7.877	1.00	0.00	RX1	H
ATOM	3360	CA	ASP	126	41.717	-7.967	7.862	1.00	0.00	RX1	C
ATOM	3361	CB	ASP	126	41.857	-8.809	6.586	1.00	0.00	RX1	C
ATOM	3362	CG	ASP	126	42.538	-10.141	6.859	1.00	0.00	RX1	C
ATOM	3363	OD1	ASP	126	42.035	-11.156	6.399	1.00	0.00	RX1	O
ATOM	3364	OD2	ASP	126	43.542	-10.194	7.558	1.00	0.00	RX1	O
ATOM	3365	C	ASP	126	41.170	-8.752	9.059	1.00	0.00	RX1	C
ATOM	3366	O	ASP	126	41.419	-8.422	10.214	1.00	0.00	RX1	O
ATOM	3367	N	ALA	127	40.428	-9.834	8.748	1.00	0.00	RX1	N
ATOM	3368	H	ALA	127	40.303	-10.132	7.802	1.00	0.00	RX1	H
ATOM	3369	CA	ALA	127	40.104	-10.797	9.796	1.00	0.00	RX1	C
ATOM	3370	CB	ALA	127	39.159	-11.872	9.257	1.00	0.00	RX1	C
ATOM	3371	C	ALA	127	41.328	-11.490	10.367	1.00	0.00	RX1	C
ATOM	3372	O	ALA	127	41.509	-11.635	11.569	1.00	0.00	RX1	O
ATOM	3373	N	ASN	128	42.186	-11.910	9.434	1.00	0.00	RX1	N
ATOM	3374	H	ASN	128	42.128	-11.578	8.489	1.00	0.00	RX1	H
ATOM	3375	CA	ASN	128	43.316	-12.757	9.817	1.00	0.00	RX1	C
ATOM	3376	CB	ASN	128	43.809	-13.615	8.648	1.00	0.00	RX1	C
ATOM	3377	CG	ASN	128	42.729	-14.540	8.139	1.00	0.00	RX1	C
ATOM	3378	OD1	ASN	128	42.509	-15.633	8.651	1.00	0.00	RX1	O
ATOM	3379	ND2	ASN	128	42.069	-14.048	7.074	1.00	0.00	RX1	N
ATOM	3380	HD21	ASN	128	42.277	-13.124	6.729	1.00	0.00	RX1	H
ATOM	3381	HD22	ASN	128	41.357	-14.560	6.597	1.00	0.00	RX1	H
ATOM	3382	C	ASN	128	44.524	-11.984	10.307	1.00	0.00	RX1	C
ATOM	3383	O	ASN	128	45.659	-12.383	10.085	1.00	0.00	RX1	O
ATOM	3384	N	LYS	129	44.252	-10.824	10.943	1.00	0.00	RX1	N
ATOM	3385	H	LYS	129	43.300	-10.610	11.161	1.00	0.00	RX1	H
ATOM	3386	CA	LYS	129	45.333	-9.917	11.348	1.00	0.00	RX1	C
ATOM	3387	CB	LYS	129	45.996	-10.381	12.653	1.00	0.00	RX1	C
ATOM	3388	CG	LYS	129	45.282	-10.066	13.976	1.00	0.00	RX1	C
ATOM	3389	CD	LYS	129	45.335	-8.584	14.371	1.00	0.00	RX1	C
ATOM	3390	CE	LYS	129	44.888	-8.301	15.813	1.00	0.00	RX1	C
ATOM	3391	NZ	LYS	129	44.823	-6.853	16.079	1.00	0.00	RX1	N
ATOM	3392	HZ1	LYS	129	43.967	-6.440	15.653	1.00	0.00	RX1	H
ATOM	3393	HZ2	LYS	129	44.787	-6.661	17.097	1.00	0.00	RX1	H
ATOM	3394	HZ3	LYS	129	45.638	-6.321	15.700	1.00	0.00	RX1	H
ATOM	3395	C	LYS	129	46.411	-9.658	10.295	1.00	0.00	RX1	C
ATOM	3396	O	LYS	129	47.585	-9.514	10.603	1.00	0.00	RX1	O
ATOM	3397	N	THR	130	45.966	-9.591	9.034	1.00	0.00	RX1	N
ATOM	3398	H	THR	130	45.009	-9.680	8.756	1.00	0.00	RX1	H
ATOM	3399	CA	THR	130	46.942	-9.233	8.012	1.00	0.00	RX1	C
ATOM	3400	CB	THR	130	47.284	-10.487	7.196	1.00	0.00	RX1	C
ATOM	3401	OG1	THR	130	46.088	-11.130	6.724	1.00	0.00	RX1	O
ATOM	3402	HG1	THR	130	45.483	-11.160	7.466	1.00	0.00	RX1	H
ATOM	3403	CG2	THR	130	48.161	-11.477	7.967	1.00	0.00	RX1	C
ATOM	3404	C	THR	130	46.413	-8.103	7.157	1.00	0.00	RX1	C
ATOM	3405	O	THR	130	45.295	-8.153	6.663	1.00	0.00	RX1	O
ATOM	3406	N	GLY	131	47.227	-7.068	7.008	1.00	0.00	RX1	N
ATOM	3407	H	GLY	131	48.077	-6.935	7.522	1.00	0.00	RX1	H
ATOM	3408	CA	GLY	131	46.764	-6.058	6.068	1.00	0.00	RX1	C
ATOM	3409	C	GLY	131	47.922	-5.412	5.375	1.00	0.00	RX1	C
ATOM	3410	O	GLY	131	48.439	-5.896	4.380	1.00	0.00	RX1	O
ATOM	3411	N	LEU	132	48.345	-4.302	5.982	1.00	0.00	RX1	N
ATOM	3412	H	LEU	132	47.965	-4.046	6.872	1.00	0.00	RX1	H
ATOM	3413	CA	LEU	132	49.520	-3.666	5.404	1.00	0.00	RX1	C
ATOM	3414	CB	LEU	132	49.641	-2.229	5.903	1.00	0.00	RX1	C

ATOM	3415	CG	LEU	132	50.811	-1.475	5.275	1.00	0.00	RX1	C
ATOM	3416	CD1	LEU	132	50.806	-1.583	3.752	1.00	0.00	RX1	C
ATOM	3417	CD2	LEU	132	50.861	-0.026	5.753	1.00	0.00	RX1	C
ATOM	3418	C	LEU	132	50.792	-4.450	5.663	1.00	0.00	RX1	C
ATOM	3419	O	LEU	132	51.445	-4.304	6.687	1.00	0.00	RX1	O
ATOM	3420	N	LYS	133	51.115	-5.280	4.669	1.00	0.00	RX1	N
ATOM	3421	H	LYS	133	50.491	-5.369	3.887	1.00	0.00	RX1	H
ATOM	3422	CA	LYS	133	52.412	-5.939	4.673	1.00	0.00	RX1	C
ATOM	3423	CB	LYS	133	52.373	-7.183	3.784	1.00	0.00	RX1	C
ATOM	3424	CG	LYS	133	53.721	-7.898	3.756	1.00	0.00	RX1	C
ATOM	3425	CD	LYS	133	53.760	-9.091	2.807	1.00	0.00	RX1	C
ATOM	3426	CE	LYS	133	55.129	-9.772	2.821	1.00	0.00	RX1	C
ATOM	3427	NZ	LYS	133	56.163	-8.826	2.390	1.00	0.00	RX1	N
ATOM	3428	HZ1	LYS	133	57.107	-9.254	2.359	1.00	0.00	RX1	H
ATOM	3429	HZ2	LYS	133	55.964	-8.439	1.444	1.00	0.00	RX1	H
ATOM	3430	HZ3	LYS	133	56.228	-7.967	2.979	1.00	0.00	RX1	H
ATOM	3431	C	LYS	133	53.510	-5.001	4.213	1.00	0.00	RX1	C
ATOM	3432	O	LYS	133	54.417	-4.622	4.947	1.00	0.00	RX1	O
ATOM	3433	N	GLU	134	53.390	-4.642	2.933	1.00	0.00	RX1	N
ATOM	3434	H	GLU	134	52.548	-4.811	2.415	1.00	0.00	RX1	H
ATOM	3435	CA	GLU	134	54.481	-3.841	2.409	1.00	0.00	RX1	C
ATOM	3436	CB	GLU	134	54.907	-4.288	1.017	1.00	0.00	RX1	C
ATOM	3437	CG	GLU	134	56.384	-4.666	0.924	1.00	0.00	RX1	C
ATOM	3438	CD	GLU	134	56.548	-6.110	1.339	1.00	0.00	RX1	C
ATOM	3439	OE1	GLU	134	56.257	-6.990	0.537	1.00	0.00	RX1	O
ATOM	3440	OE2	GLU	134	56.993	-6.386	2.447	1.00	0.00	RX1	O
ATOM	3441	C	GLU	134	54.163	-2.369	2.398	1.00	0.00	RX1	C
ATOM	3442	O	GLU	134	53.351	-1.879	1.618	1.00	0.00	RX1	O
ATOM	3443	N	LEU	135	54.885	-1.678	3.286	1.00	0.00	RX1	N
ATOM	3444	H	LEU	135	55.494	-2.154	3.922	1.00	0.00	RX1	H
ATOM	3445	CA	LEU	135	54.944	-0.225	3.157	1.00	0.00	RX1	C
ATOM	3446	CB	LEU	135	54.604	0.420	4.499	1.00	0.00	RX1	C
ATOM	3447	CG	LEU	135	54.347	1.923	4.405	1.00	0.00	RX1	C
ATOM	3448	CD1	LEU	135	53.242	2.247	3.397	1.00	0.00	RX1	C
ATOM	3449	CD2	LEU	135	54.072	2.529	5.782	1.00	0.00	RX1	C
ATOM	3450	C	LEU	135	56.324	0.196	2.670	1.00	0.00	RX1	C
ATOM	3451	O	LEU	135	57.226	0.510	3.437	1.00	0.00	RX1	O
ATOM	3452	N	PRO	136	56.491	0.150	1.331	1.00	0.00	RX1	N
ATOM	3453	CD	PRO	136	55.471	-0.025	0.311	1.00	0.00	RX1	C
ATOM	3454	CA	PRO	136	57.845	0.166	0.791	1.00	0.00	RX1	C
ATOM	3455	CB	PRO	136	57.651	-0.670	-0.472	1.00	0.00	RX1	C
ATOM	3456	CG	PRO	136	56.255	-0.285	-0.962	1.00	0.00	RX1	C
ATOM	3457	C	PRO	136	58.374	1.558	0.513	1.00	0.00	RX1	C
ATOM	3458	O	PRO	136	58.881	1.826	-0.571	1.00	0.00	RX1	O
ATOM	3459	N	MET	137	58.221	2.448	1.503	1.00	0.00	RX1	N
ATOM	3460	H	MET	137	57.994	2.140	2.429	1.00	0.00	RX1	H
ATOM	3461	CA	MET	137	58.536	3.848	1.213	1.00	0.00	RX1	C
ATOM	3462	CB	MET	137	57.749	4.792	2.122	1.00	0.00	RX1	C
ATOM	3463	CG	MET	137	56.256	4.475	2.101	1.00	0.00	RX1	C
ATOM	3464	SD	MET	137	55.238	5.730	2.895	1.00	0.00	RX1	S
ATOM	3465	CE	MET	137	55.991	5.689	4.524	1.00	0.00	RX1	C
ATOM	3466	C	MET	137	60.015	4.189	1.248	1.00	0.00	RX1	C
ATOM	3467	O	MET	137	60.508	4.864	2.140	1.00	0.00	RX1	O
ATOM	3468	N	ARG	138	60.722	3.701	0.218	1.00	0.00	RX1	N
ATOM	3469	H	ARG	138	60.217	3.307	-0.555	1.00	0.00	RX1	H
ATOM	3470	CA	ARG	138	62.185	3.816	0.222	1.00	0.00	RX1	C
ATOM	3471	CB	ARG	138	62.857	2.983	-0.886	1.00	0.00	RX1	C
ATOM	3472	CG	ARG	138	62.802	3.444	-2.357	1.00	0.00	RX1	C
ATOM	3473	CD	ARG	138	61.723	2.761	-3.214	1.00	0.00	RX1	C
ATOM	3474	NE	ARG	138	62.009	2.821	-4.657	1.00	0.00	RX1	N
ATOM	3475	HE	ARG	138	61.734	3.671	-5.123	1.00	0.00	RX1	H

ATOM	3476	CZ	ARG	138	62.581	1.732	-5.285	1.00	0.00	RX1	C
ATOM	3477	NH1	ARG	138	62.907	0.629	-4.589	1.00	0.00	RX1	N
ATOM	3478	HH11	ARG	138	63.429	-0.120	-5.034	1.00	0.00	RX1	H
ATOM	3479	HH12	ARG	138	62.629	0.477	-3.634	1.00	0.00	RX1	H
ATOM	3480	NH2	ARG	138	62.823	1.732	-6.612	1.00	0.00	RX1	N
ATOM	3481	HH21	ARG	138	63.240	0.899	-7.025	1.00	0.00	RX1	H
ATOM	3482	HH22	ARG	138	62.612	2.487	-7.231	1.00	0.00	RX1	H
ATOM	3483	C	ARG	138	62.755	5.219	0.219	1.00	0.00	RX1	C
ATOM	3484	O	ARG	138	63.956	5.422	0.372	1.00	0.00	RX1	O
ATOM	3485	N	ASN	139	61.871	6.191	-0.011	1.00	0.00	RX1	N
ATOM	3486	H	ASN	139	60.881	6.055	-0.061	1.00	0.00	RX1	H
ATOM	3487	CA	ASN	139	62.434	7.531	-0.005	1.00	0.00	RX1	C
ATOM	3488	CB	ASN	139	62.089	8.322	-1.263	1.00	0.00	RX1	C
ATOM	3489	CG	ASN	139	62.669	7.669	-2.492	1.00	0.00	RX1	C
ATOM	3490	OD1	ASN	139	63.727	7.034	-2.460	1.00	0.00	RX1	O
ATOM	3491	ND2	ASN	139	61.889	7.831	-3.574	1.00	0.00	RX1	N
ATOM	3492	HD21	ASN	139	61.058	8.392	-3.476	1.00	0.00	RX1	H
ATOM	3493	HD22	ASN	139	62.051	7.428	-4.475	1.00	0.00	RX1	H
ATOM	3494	C	ASN	139	62.066	8.345	1.208	1.00	0.00	RX1	C
ATOM	3495	O	ASN	139	62.447	9.506	1.305	1.00	0.00	RX1	O
ATOM	3496	N	LEU	140	61.325	7.706	2.135	1.00	0.00	RX1	N
ATOM	3497	H	LEU	140	61.105	6.731	2.100	1.00	0.00	RX1	H
ATOM	3498	CA	LEU	140	60.948	8.475	3.315	1.00	0.00	RX1	C
ATOM	3499	CB	LEU	140	59.799	7.837	4.095	1.00	0.00	RX1	C
ATOM	3500	CG	LEU	140	59.320	8.775	5.210	1.00	0.00	RX1	C
ATOM	3501	CD1	LEU	140	58.731	10.068	4.648	1.00	0.00	RX1	C
ATOM	3502	CD2	LEU	140	58.361	8.096	6.185	1.00	0.00	RX1	C
ATOM	3503	C	LEU	140	62.141	8.692	4.211	1.00	0.00	RX1	C
ATOM	3504	O	LEU	140	62.556	7.847	4.992	1.00	0.00	RX1	O
ATOM	3505	N	GLN	141	62.696	9.881	3.995	1.00	0.00	RX1	N
ATOM	3506	H	GLN	141	62.268	10.493	3.324	1.00	0.00	RX1	H
ATOM	3507	CA	GLN	141	63.893	10.254	4.728	1.00	0.00	RX1	C
ATOM	3508	CB	GLN	141	65.016	10.555	3.737	1.00	0.00	RX1	C
ATOM	3509	CG	GLN	141	65.354	9.344	2.866	1.00	0.00	RX1	C
ATOM	3510	CD	GLN	141	65.891	9.808	1.527	1.00	0.00	RX1	C
ATOM	3511	OE1	GLN	141	67.018	9.515	1.141	1.00	0.00	RX1	O
ATOM	3512	NE2	GLN	141	65.009	10.535	0.820	1.00	0.00	RX1	N
ATOM	3513	HE21	GLN	141	64.062	10.629	1.144	1.00	0.00	RX1	H
ATOM	3514	HE22	GLN	141	65.227	10.991	-0.044	1.00	0.00	RX1	H
ATOM	3515	C	GLN	141	63.620	11.428	5.640	1.00	0.00	RX1	C
ATOM	3516	O	GLN	141	64.281	11.628	6.651	1.00	0.00	RX1	O
ATOM	3517	N	GLU	142	62.599	12.201	5.248	1.00	0.00	RX1	N
ATOM	3518	H	GLU	142	61.984	12.019	4.477	1.00	0.00	RX1	H
ATOM	3519	CA	GLU	142	62.315	13.351	6.089	1.00	0.00	RX1	C
ATOM	3520	CB	GLU	142	62.524	14.631	5.293	1.00	0.00	RX1	C
ATOM	3521	CG	GLU	142	62.520	15.873	6.177	1.00	0.00	RX1	C
ATOM	3522	CD	GLU	142	63.780	15.868	7.004	1.00	0.00	RX1	C
ATOM	3523	OE1	GLU	142	64.824	16.252	6.490	1.00	0.00	RX1	O
ATOM	3524	OE2	GLU	142	63.744	15.432	8.148	1.00	0.00	RX1	O
ATOM	3525	C	GLU	142	60.915	13.335	6.649	1.00	0.00	RX1	C
ATOM	3526	O	GLU	142	59.930	13.255	5.923	1.00	0.00	RX1	O
ATOM	3527	N	ILE	143	60.867	13.452	7.976	1.00	0.00	RX1	N
ATOM	3528	H	ILE	143	61.703	13.542	8.523	1.00	0.00	RX1	H
ATOM	3529	CA	ILE	143	59.628	13.985	8.522	1.00	0.00	RX1	C
ATOM	3530	CB	ILE	143	58.874	12.980	9.399	1.00	0.00	RX1	C
ATOM	3531	CG2	ILE	143	57.621	13.631	9.976	1.00	0.00	RX1	C
ATOM	3532	CG1	ILE	143	58.492	11.717	8.628	1.00	0.00	RX1	C
ATOM	3533	CD1	ILE	143	57.708	10.725	9.490	1.00	0.00	RX1	C
ATOM	3534	C	ILE	143	59.987	15.234	9.292	1.00	0.00	RX1	C
ATOM	3535	O	ILE	143	60.666	15.202	10.316	1.00	0.00	RX1	O
ATOM	3536	N	LEU	144	59.538	16.352	8.712	1.00	0.00	RX1	N

ATOM	3537	H	LEU	144	58.876	16.292	7.962	1.00	0.00	RX1	H
ATOM	3538	CA	LEU	144	59.893	17.625	9.330	1.00	0.00	RX1	C
ATOM	3539	CB	LEU	144	59.499	18.788	8.421	1.00	0.00	RX1	C
ATOM	3540	CG	LEU	144	60.458	19.036	7.260	1.00	0.00	RX1	C
ATOM	3541	CD1	LEU	144	59.954	20.162	6.357	1.00	0.00	RX1	C
ATOM	3542	CD2	LEU	144	61.880	19.306	7.754	1.00	0.00	RX1	C
ATOM	3543	C	LEU	144	59.273	17.804	10.700	1.00	0.00	RX1	C
ATOM	3544	O	LEU	144	59.938	18.140	11.674	1.00	0.00	RX1	O
ATOM	3545	N	HIS	145	57.958	17.566	10.720	1.00	0.00	RX1	N
ATOM	3546	H	HIS	145	57.425	17.262	9.926	1.00	0.00	RX1	H
ATOM	3547	CA	HIS	145	57.195	17.789	11.939	1.00	0.00	RX1	C
ATOM	3548	CB	HIS	145	56.399	19.099	11.879	1.00	0.00	RX1	C
ATOM	3549	CG	HIS	145	57.146	20.185	11.137	1.00	0.00	RX1	C
ATOM	3550	ND1	HIS	145	58.381	20.619	11.452	1.00	0.00	RX1	N
ATOM	3551	HD1	HIS	145	58.967	20.244	12.147	1.00	0.00	RX1	H
ATOM	3552	CD2	HIS	145	56.691	20.907	10.029	1.00	0.00	RX1	C
ATOM	3553	NE2	HIS	145	57.661	21.784	9.679	1.00	0.00	RX1	N
ATOM	3554	CE1	HIS	145	58.703	21.606	10.555	1.00	0.00	RX1	C
ATOM	3555	C	HIS	145	56.242	16.632	12.140	1.00	0.00	RX1	C
ATOM	3556	O	HIS	145	55.860	15.957	11.194	1.00	0.00	RX1	O
ATOM	3557	N	GLY	146	55.881	16.424	13.411	1.00	0.00	RX1	N
ATOM	3558	H	GLY	146	56.182	17.043	14.135	1.00	0.00	RX1	H
ATOM	3559	CA	GLY	146	55.036	15.269	13.702	1.00	0.00	RX1	C
ATOM	3560	C	GLY	146	55.797	13.986	13.957	1.00	0.00	RX1	C
ATOM	3561	O	GLY	146	56.588	13.516	13.154	1.00	0.00	RX1	O
ATOM	3562	N	ALA	147	55.522	13.434	15.144	1.00	0.00	RX1	N
ATOM	3563	H	ALA	147	54.809	13.831	15.722	1.00	0.00	RX1	H
ATOM	3564	CA	ALA	147	56.107	12.137	15.469	1.00	0.00	RX1	C
ATOM	3565	CB	ALA	147	55.897	11.815	16.947	1.00	0.00	RX1	C
ATOM	3566	C	ALA	147	55.527	11.014	14.625	1.00	0.00	RX1	C
ATOM	3567	O	ALA	147	54.577	11.193	13.870	1.00	0.00	RX1	O
ATOM	3568	N	VAL	148	56.139	9.838	14.775	1.00	0.00	RX1	N
ATOM	3569	H	VAL	148	56.812	9.650	15.489	1.00	0.00	RX1	H
ATOM	3570	CA	VAL	148	55.640	8.747	13.950	1.00	0.00	RX1	C
ATOM	3571	CB	VAL	148	56.788	8.190	13.104	1.00	0.00	RX1	C
ATOM	3572	CG1	VAL	148	58.005	7.852	13.961	1.00	0.00	RX1	C
ATOM	3573	CG2	VAL	148	56.332	7.024	12.234	1.00	0.00	RX1	C
ATOM	3574	C	VAL	148	54.930	7.683	14.771	1.00	0.00	RX1	C
ATOM	3575	O	VAL	148	55.403	7.237	15.808	1.00	0.00	RX1	O
ATOM	3576	N	ARG	149	53.748	7.323	14.267	1.00	0.00	RX1	N
ATOM	3577	H	ARG	149	53.409	7.675	13.394	1.00	0.00	RX1	H
ATOM	3578	CA	ARG	149	52.950	6.320	14.954	1.00	0.00	RX1	C
ATOM	3579	CB	ARG	149	51.580	6.896	15.312	1.00	0.00	RX1	C
ATOM	3580	CG	ARG	149	50.837	6.043	16.335	1.00	0.00	RX1	C
ATOM	3581	CD	ARG	149	51.544	6.053	17.686	1.00	0.00	RX1	C
ATOM	3582	NE	ARG	149	51.023	5.017	18.570	1.00	0.00	RX1	N
ATOM	3583	HE	ARG	149	50.579	4.221	18.140	1.00	0.00	RX1	H
ATOM	3584	CZ	ARG	149	51.364	5.049	19.890	1.00	0.00	RX1	C
ATOM	3585	NH1	ARG	149	51.966	6.133	20.418	1.00	0.00	RX1	N
ATOM	3586	HH11	ARG	149	52.377	6.075	21.336	1.00	0.00	RX1	H
ATOM	3587	HH12	ARG	149	52.079	7.017	19.945	1.00	0.00	RX1	H
ATOM	3588	NH2	ARG	149	51.122	3.981	20.668	1.00	0.00	RX1	N
ATOM	3589	HH21	ARG	149	51.246	3.975	21.661	1.00	0.00	RX1	H
ATOM	3590	HH22	ARG	149	50.874	3.088	20.258	1.00	0.00	RX1	H
ATOM	3591	C	ARG	149	52.782	5.080	14.107	1.00	0.00	RX1	C
ATOM	3592	O	ARG	149	52.326	5.135	12.971	1.00	0.00	RX1	O
ATOM	3593	N	PHE	150	53.145	3.953	14.717	1.00	0.00	RX1	N
ATOM	3594	H	PHE	150	53.478	3.927	15.661	1.00	0.00	RX1	H
ATOM	3595	CA	PHE	150	52.736	2.681	14.132	1.00	0.00	RX1	C
ATOM	3596	CB	PHE	150	53.946	1.873	13.660	1.00	0.00	RX1	C
ATOM	3597	CG	PHE	150	54.390	2.303	12.280	1.00	0.00	RX1	C

ATOM	3598	CD1	PHE	150	54.129	1.481	11.191	1.00	0.00	RX1	C
ATOM	3599	CD2	PHE	150	55.066	3.501	12.091	1.00	0.00	RX1	C
ATOM	3600	CE1	PHE	150	54.547	1.850	9.918	1.00	0.00	RX1	C
ATOM	3601	CE2	PHE	150	55.482	3.873	10.819	1.00	0.00	RX1	C
ATOM	3602	CZ	PHE	150	55.223	3.049	9.732	1.00	0.00	RX1	C
ATOM	3603	C	PHE	150	52.036	1.918	15.226	1.00	0.00	RX1	C
ATOM	3604	O	PHE	150	52.535	1.889	16.343	1.00	0.00	RX1	O
ATOM	3605	N	SER	151	50.884	1.333	14.879	1.00	0.00	RX1	N
ATOM	3606	H	SER	151	50.434	1.435	13.990	1.00	0.00	RX1	H
ATOM	3607	CA	SER	151	50.154	0.590	15.904	1.00	0.00	RX1	C
ATOM	3608	CB	SER	151	49.346	1.592	16.730	1.00	0.00	RX1	C
ATOM	3609	OG	SER	151	50.165	2.724	17.043	1.00	0.00	RX1	O
ATOM	3610	HG	SER	151	50.932	2.325	17.471	1.00	0.00	RX1	H
ATOM	3611	C	SER	151	49.241	-0.449	15.290	1.00	0.00	RX1	C
ATOM	3612	O	SER	151	48.473	-0.149	14.381	1.00	0.00	RX1	O
ATOM	3613	N	ASN	152	49.333	-1.680	15.831	1.00	0.00	RX1	N
ATOM	3614	H	ASN	152	50.095	-1.883	16.451	1.00	0.00	RX1	H
ATOM	3615	CA	ASN	152	48.349	-2.724	15.492	1.00	0.00	RX1	C
ATOM	3616	CB	ASN	152	47.024	-2.445	16.212	1.00	0.00	RX1	C
ATOM	3617	CG	ASN	152	45.940	-3.452	15.861	1.00	0.00	RX1	C
ATOM	3618	OD1	ASN	152	46.108	-4.677	15.878	1.00	0.00	RX1	O
ATOM	3619	ND2	ASN	152	44.769	-2.843	15.596	1.00	0.00	RX1	N
ATOM	3620	HD21	ASN	152	44.739	-1.838	15.566	1.00	0.00	RX1	H
ATOM	3621	HD22	ASN	152	43.889	-3.274	15.376	1.00	0.00	RX1	H
ATOM	3622	C	ASN	152	48.152	-2.936	13.994	1.00	0.00	RX1	C
ATOM	3623	O	ASN	152	47.059	-2.935	13.442	1.00	0.00	RX1	O
ATOM	3624	N	ASN	153	49.301	-3.107	13.343	1.00	0.00	RX1	N
ATOM	3625	H	ASN	153	50.161	-3.201	13.852	1.00	0.00	RX1	H
ATOM	3626	CA	ASN	153	49.304	-3.297	11.893	1.00	0.00	RX1	C
ATOM	3627	CB	ASN	153	49.965	-2.097	11.213	1.00	0.00	RX1	C
ATOM	3628	CG	ASN	153	51.305	-1.796	11.861	1.00	0.00	RX1	C
ATOM	3629	OD1	ASN	153	52.086	-2.665	12.237	1.00	0.00	RX1	O
ATOM	3630	ND2	ASN	153	51.516	-0.476	12.022	1.00	0.00	RX1	N
ATOM	3631	HD21	ASN	153	50.821	0.212	11.792	1.00	0.00	RX1	H
ATOM	3632	HD22	ASN	153	52.383	-0.146	12.393	1.00	0.00	RX1	H
ATOM	3633	C	ASN	153	49.964	-4.591	11.417	1.00	0.00	RX1	C
ATOM	3634	O	ASN	153	50.934	-4.579	10.669	1.00	0.00	RX1	O
ATOM	3635	N	PRO	154	49.431	-5.756	11.869	1.00	0.00	RX1	N
ATOM	3636	CD	PRO	154	48.266	-6.009	12.700	1.00	0.00	RX1	C
ATOM	3637	CA	PRO	154	50.112	-6.991	11.492	1.00	0.00	RX1	C
ATOM	3638	CB	PRO	154	49.478	-8.044	12.407	1.00	0.00	RX1	C
ATOM	3639	CG	PRO	154	48.646	-7.282	13.437	1.00	0.00	RX1	C
ATOM	3640	C	PRO	154	49.988	-7.290	10.007	1.00	0.00	RX1	C
ATOM	3641	O	PRO	154	49.039	-6.897	9.338	1.00	0.00	RX1	O
ATOM	3642	N	ALA	155	51.061	-7.972	9.568	1.00	0.00	RX1	N
ATOM	3643	H	ALA	155	51.740	-8.180	10.269	1.00	0.00	RX1	H
ATOM	3644	CA	ALA	155	51.509	-8.223	8.190	1.00	0.00	RX1	C
ATOM	3645	CB	ALA	155	50.462	-8.033	7.091	1.00	0.00	RX1	C
ATOM	3646	C	ALA	155	52.732	-7.399	7.850	1.00	0.00	RX1	C
ATOM	3647	O	ALA	155	53.592	-7.829	7.094	1.00	0.00	RX1	O
ATOM	3648	N	LEU	156	52.797	-6.219	8.493	1.00	0.00	RX1	N
ATOM	3649	H	LEU	156	52.021	-5.861	9.014	1.00	0.00	RX1	H
ATOM	3650	CA	LEU	156	53.915	-5.311	8.242	1.00	0.00	RX1	C
ATOM	3651	CB	LEU	156	53.850	-4.144	9.225	1.00	0.00	RX1	C
ATOM	3652	CG	LEU	156	54.693	-2.942	8.803	1.00	0.00	RX1	C
ATOM	3653	CD1	LEU	156	54.224	-2.352	7.471	1.00	0.00	RX1	C
ATOM	3654	CD2	LEU	156	54.737	-1.887	9.903	1.00	0.00	RX1	C
ATOM	3655	C	LEU	156	55.303	-5.928	8.242	1.00	0.00	RX1	C
ATOM	3656	O	LEU	156	55.717	-6.629	9.160	1.00	0.00	RX1	O
ATOM	3657	N	CYS	157	56.002	-5.616	7.151	1.00	0.00	RX1	N
ATOM	3658	H	CYS	157	55.615	-5.047	6.422	1.00	0.00	RX1	H

ATOM	3659	CA	CYS	157	57.382	-6.061	7.057	1.00	0.00	RX1	C
ATOM	3660	CB	CYS	157	57.583	-6.812	5.745	1.00	0.00	RX1	C
ATOM	3661	SG	CYS	157	56.522	-8.268	5.584	1.00	0.00	RX1	S
ATOM	3662	C	CYS	157	58.346	-4.905	7.144	1.00	0.00	RX1	C
ATOM	3663	O	CYS	157	58.213	-3.904	6.450	1.00	0.00	RX1	O
ATOM	3664	N	ASN	158	59.362	-5.109	7.996	1.00	0.00	RX1	N
ATOM	3665	H	ASN	158	59.271	-5.747	8.764	1.00	0.00	RX1	H
ATOM	3666	CA	ASN	158	60.661	-4.438	7.836	1.00	0.00	RX1	C
ATOM	3667	CB	ASN	158	61.248	-4.618	6.438	1.00	0.00	RX1	C
ATOM	3668	CG	ASN	158	61.629	-6.056	6.198	1.00	0.00	RX1	C
ATOM	3669	OD1	ASN	158	61.409	-6.927	7.039	1.00	0.00	RX1	O
ATOM	3670	ND2	ASN	158	62.167	-6.264	4.992	1.00	0.00	RX1	N
ATOM	3671	HD21	ASN	158	62.384	-5.483	4.398	1.00	0.00	RX1	H
ATOM	3672	HD22	ASN	158	62.397	-7.168	4.625	1.00	0.00	RX1	H
ATOM	3673	C	ASN	158	60.786	-2.996	8.286	1.00	0.00	RX1	C
ATOM	3674	O	ASN	158	61.858	-2.529	8.657	1.00	0.00	RX1	O
ATOM	3675	N	VAL	159	59.629	-2.314	8.311	1.00	0.00	RX1	N
ATOM	3676	H	VAL	159	58.808	-2.733	7.929	1.00	0.00	RX1	H
ATOM	3677	CA	VAL	159	59.615	-0.941	8.821	1.00	0.00	RX1	C
ATOM	3678	CB	VAL	159	58.222	-0.334	8.623	1.00	0.00	RX1	C
ATOM	3679	CG1	VAL	159	58.167	1.134	9.048	1.00	0.00	RX1	C
ATOM	3680	CG2	VAL	159	57.781	-0.499	7.166	1.00	0.00	RX1	C
ATOM	3681	C	VAL	159	60.109	-0.802	10.265	1.00	0.00	RX1	C
ATOM	3682	O	VAL	159	60.579	0.243	10.705	1.00	0.00	RX1	O
ATOM	3683	N	GLU	160	60.027	-1.939	10.980	1.00	0.00	RX1	N
ATOM	3684	H	GLU	160	59.759	-2.807	10.564	1.00	0.00	RX1	H
ATOM	3685	CA	GLU	160	60.561	-1.971	12.340	1.00	0.00	RX1	C
ATOM	3686	CB	GLU	160	60.214	-3.311	13.008	1.00	0.00	RX1	C
ATOM	3687	CG	GLU	160	61.152	-4.505	12.767	1.00	0.00	RX1	C
ATOM	3688	CD	GLU	160	61.249	-4.901	11.304	1.00	0.00	RX1	C
ATOM	3689	OE1	GLU	160	60.424	-5.667	10.813	1.00	0.00	RX1	O
ATOM	3690	OE2	GLU	160	62.206	-4.515	10.647	1.00	0.00	RX1	O
ATOM	3691	C	GLU	160	62.036	-1.603	12.485	1.00	0.00	RX1	C
ATOM	3692	O	GLU	160	62.497	-1.184	13.538	1.00	0.00	RX1	O
ATOM	3693	N	SER	161	62.757	-1.756	11.368	1.00	0.00	RX1	N
ATOM	3694	H	SER	161	62.358	-2.086	10.513	1.00	0.00	RX1	H
ATOM	3695	CA	SER	161	64.184	-1.474	11.432	1.00	0.00	RX1	C
ATOM	3696	CB	SER	161	64.923	-2.634	10.765	1.00	0.00	RX1	C
ATOM	3697	OG	SER	161	64.334	-2.943	9.496	1.00	0.00	RX1	O
ATOM	3698	HG	SER	161	63.433	-3.223	9.668	1.00	0.00	RX1	H
ATOM	3699	C	SER	161	64.620	-0.087	10.977	1.00	0.00	RX1	C
ATOM	3700	O	SER	161	65.806	0.205	10.879	1.00	0.00	RX1	O
ATOM	3701	N	ILE	162	63.628	0.768	10.674	1.00	0.00	RX1	N
ATOM	3702	H	ILE	162	62.669	0.569	10.882	1.00	0.00	RX1	H
ATOM	3703	CA	ILE	162	64.044	2.113	10.268	1.00	0.00	RX1	C
ATOM	3704	CB	ILE	162	62.934	2.826	9.472	1.00	0.00	RX1	C
ATOM	3705	CG2	ILE	162	63.054	4.348	9.545	1.00	0.00	RX1	C
ATOM	3706	CG1	ILE	162	62.950	2.405	8.001	1.00	0.00	RX1	C
ATOM	3707	CD1	ILE	162	62.387	1.020	7.705	1.00	0.00	RX1	C
ATOM	3708	C	ILE	162	64.489	2.930	11.472	1.00	0.00	RX1	C
ATOM	3709	O	ILE	162	63.831	2.987	12.504	1.00	0.00	RX1	O
ATOM	3710	N	GLN	163	65.655	3.566	11.312	1.00	0.00	RX1	N
ATOM	3711	H	GLN	163	66.089	3.597	10.409	1.00	0.00	RX1	H
ATOM	3712	CA	GLN	163	66.047	4.406	12.438	1.00	0.00	RX1	C
ATOM	3713	CB	GLN	163	67.560	4.425	12.638	1.00	0.00	RX1	C
ATOM	3714	CG	GLN	163	68.399	4.803	11.423	1.00	0.00	RX1	C
ATOM	3715	CD	GLN	163	69.842	4.671	11.840	1.00	0.00	RX1	C
ATOM	3716	OE1	GLN	163	70.361	5.455	12.625	1.00	0.00	RX1	O
ATOM	3717	NE2	GLN	163	70.441	3.596	11.309	1.00	0.00	RX1	N
ATOM	3718	HE21	GLN	163	69.965	3.011	10.654	1.00	0.00	RX1	H
ATOM	3719	HE22	GLN	163	71.375	3.324	11.563	1.00	0.00	RX1	H

ATOM	3720	C	GLN	163	65.414	5.784	12.396	1.00	0.00	RX1	C
ATOM	3721	O	GLN	163	65.879	6.726	11.768	1.00	0.00	RX1	O
ATOM	3722	N	TRP	164	64.285	5.855	13.109	1.00	0.00	RX1	N
ATOM	3723	H	TRP	164	63.946	5.006	13.523	1.00	0.00	RX1	H
ATOM	3724	CA	TRP	164	63.422	7.026	12.951	1.00	0.00	RX1	C
ATOM	3725	CB	TRP	164	62.088	6.798	13.662	1.00	0.00	RX1	C
ATOM	3726	CG	TRP	164	61.287	5.791	12.872	1.00	0.00	RX1	C
ATOM	3727	CD2	TRP	164	60.450	6.042	11.725	1.00	0.00	RX1	C
ATOM	3728	CE2	TRP	164	59.910	4.803	11.309	1.00	0.00	RX1	C
ATOM	3729	CE3	TRP	164	60.130	7.203	11.032	1.00	0.00	RX1	C
ATOM	3730	CD1	TRP	164	61.205	4.407	13.085	1.00	0.00	RX1	C
ATOM	3731	NE1	TRP	164	60.395	3.819	12.163	1.00	0.00	RX1	N
ATOM	3732	HE1	TRP	164	60.212	2.855	12.101	1.00	0.00	RX1	H
ATOM	3733	CZ2	TRP	164	59.059	4.762	10.212	1.00	0.00	RX1	C
ATOM	3734	CZ3	TRP	164	59.280	7.148	9.935	1.00	0.00	RX1	C
ATOM	3735	CH2	TRP	164	58.745	5.932	9.530	1.00	0.00	RX1	C
ATOM	3736	C	TRP	164	63.967	8.416	13.256	1.00	0.00	RX1	C
ATOM	3737	O	TRP	164	63.458	9.400	12.735	1.00	0.00	RX1	O
ATOM	3738	N	ARG	165	65.029	8.495	14.079	1.00	0.00	RX1	N
ATOM	3739	H	ARG	165	65.491	7.673	14.407	1.00	0.00	RX1	H
ATOM	3740	CA	ARG	165	65.584	9.843	14.278	1.00	0.00	RX1	C
ATOM	3741	CB	ARG	165	66.345	9.930	15.620	1.00	0.00	RX1	C
ATOM	3742	CG	ARG	165	66.757	11.356	16.033	1.00	0.00	RX1	C
ATOM	3743	CD	ARG	165	67.139	11.558	17.511	1.00	0.00	RX1	C
ATOM	3744	NE	ARG	165	65.973	11.766	18.380	1.00	0.00	RX1	N
ATOM	3745	HE	ARG	165	65.194	11.144	18.270	1.00	0.00	RX1	H
ATOM	3746	CZ	ARG	165	66.002	12.783	19.305	1.00	0.00	RX1	C
ATOM	3747	NH1	ARG	165	67.100	13.559	19.404	1.00	0.00	RX1	N
ATOM	3748	HH11	ARG	165	67.109	14.302	20.097	1.00	0.00	RX1	H
ATOM	3749	HH12	ARG	165	67.910	13.467	18.824	1.00	0.00	RX1	H
ATOM	3750	NH2	ARG	165	64.946	13.037	20.113	1.00	0.00	RX1	N
ATOM	3751	HH21	ARG	165	65.001	13.798	20.792	1.00	0.00	RX1	H
ATOM	3752	HH22	ARG	165	64.058	12.559	20.116	1.00	0.00	RX1	H
ATOM	3753	C	ARG	165	66.342	10.413	13.071	1.00	0.00	RX1	C
ATOM	3754	O	ARG	165	66.558	11.608	12.930	1.00	0.00	RX1	O
ATOM	3755	N	ASP	166	66.682	9.489	12.159	1.00	0.00	RX1	N
ATOM	3756	H	ASP	166	66.502	8.514	12.289	1.00	0.00	RX1	H
ATOM	3757	CA	ASP	166	67.158	9.907	10.837	1.00	0.00	RX1	C
ATOM	3758	CB	ASP	166	67.762	8.676	10.172	1.00	0.00	RX1	C
ATOM	3759	CG	ASP	166	68.500	8.955	8.883	1.00	0.00	RX1	C
ATOM	3760	OD1	ASP	166	67.872	9.190	7.856	1.00	0.00	RX1	O
ATOM	3761	OD2	ASP	166	69.722	8.867	8.889	1.00	0.00	RX1	O
ATOM	3762	C	ASP	166	66.058	10.533	9.984	1.00	0.00	RX1	C
ATOM	3763	O	ASP	166	66.257	11.370	9.109	1.00	0.00	RX1	O
ATOM	3764	N	ILE	167	64.839	10.090	10.301	1.00	0.00	RX1	N
ATOM	3765	H	ILE	167	64.672	9.521	11.102	1.00	0.00	RX1	H
ATOM	3766	CA	ILE	167	63.730	10.614	9.518	1.00	0.00	RX1	C
ATOM	3767	CB	ILE	167	62.647	9.542	9.382	1.00	0.00	RX1	C
ATOM	3768	CG2	ILE	167	61.652	9.897	8.281	1.00	0.00	RX1	C
ATOM	3769	CG1	ILE	167	63.268	8.166	9.146	1.00	0.00	RX1	C
ATOM	3770	CD1	ILE	167	63.981	8.059	7.800	1.00	0.00	RX1	C
ATOM	3771	C	ILE	167	63.179	11.895	10.125	1.00	0.00	RX1	C
ATOM	3772	O	ILE	167	63.347	13.000	9.617	1.00	0.00	RX1	O
ATOM	3773	N	VAL	168	62.509	11.688	11.266	1.00	0.00	RX1	N
ATOM	3774	H	VAL	168	62.654	10.819	11.739	1.00	0.00	RX1	H
ATOM	3775	CA	VAL	168	61.809	12.787	11.932	1.00	0.00	RX1	C
ATOM	3776	CB	VAL	168	60.809	12.200	12.939	1.00	0.00	RX1	C
ATOM	3777	CG1	VAL	168	59.666	13.161	13.266	1.00	0.00	RX1	C
ATOM	3778	CG2	VAL	168	60.251	10.865	12.443	1.00	0.00	RX1	C
ATOM	3779	C	VAL	168	62.812	13.741	12.585	1.00	0.00	RX1	C
ATOM	3780	O	VAL	168	64.014	13.505	12.517	1.00	0.00	RX1	O

ATOM	3781	N	SER	169	62.325	14.820	13.203	1.00	0.00	RX1	N
ATOM	3782	H	SER	169	61.358	15.062	13.243	1.00	0.00	RX1	H
ATOM	3783	CA	SER	169	63.265	15.636	13.967	1.00	0.00	RX1	C
ATOM	3784	CB	SER	169	63.272	16.953	13.209	1.00	0.00	RX1	C
ATOM	3785	OG	SER	169	63.162	16.591	11.815	1.00	0.00	RX1	O
ATOM	3786	HG	SER	169	62.230	16.676	11.602	1.00	0.00	RX1	H
ATOM	3787	C	SER	169	62.908	15.626	15.450	1.00	0.00	RX1	C
ATOM	3788	O	SER	169	61.784	15.282	15.808	1.00	0.00	RX1	O
ATOM	3789	N	SER	170	63.909	15.917	16.297	1.00	0.00	RX1	N
ATOM	3790	H	SER	170	64.727	16.421	16.019	1.00	0.00	RX1	H
ATOM	3791	CA	SER	170	63.841	15.515	17.708	1.00	0.00	RX1	C
ATOM	3792	CB	SER	170	65.127	16.022	18.324	1.00	0.00	RX1	C
ATOM	3793	OG	SER	170	66.082	16.103	17.258	1.00	0.00	RX1	O
ATOM	3794	HG	SER	170	66.274	17.048	17.218	1.00	0.00	RX1	H
ATOM	3795	C	SER	170	62.589	15.909	18.470	1.00	0.00	RX1	C
ATOM	3796	O	SER	170	61.843	15.076	18.976	1.00	0.00	RX1	O
ATOM	3797	N	ASP	171	62.373	17.232	18.453	1.00	0.00	RX1	N
ATOM	3798	H	ASP	171	63.056	17.814	18.002	1.00	0.00	RX1	H
ATOM	3799	CA	ASP	171	61.212	17.902	19.046	1.00	0.00	RX1	C
ATOM	3800	CB	ASP	171	61.159	19.301	18.450	1.00	0.00	RX1	C
ATOM	3801	CG	ASP	171	59.971	20.028	19.007	1.00	0.00	RX1	C
ATOM	3802	OD1	ASP	171	59.881	20.190	20.220	1.00	0.00	RX1	O
ATOM	3803	OD2	ASP	171	59.109	20.423	18.234	1.00	0.00	RX1	O
ATOM	3804	C	ASP	171	59.872	17.204	18.843	1.00	0.00	RX1	C
ATOM	3805	O	ASP	171	58.958	17.227	19.665	1.00	0.00	RX1	O
ATOM	3806	N	PHE	172	59.809	16.595	17.658	1.00	0.00	RX1	N
ATOM	3807	H	PHE	172	60.632	16.520	17.096	1.00	0.00	RX1	H
ATOM	3808	CA	PHE	172	58.586	15.934	17.246	1.00	0.00	RX1	C
ATOM	3809	CB	PHE	172	58.447	16.079	15.732	1.00	0.00	RX1	C
ATOM	3810	CG	PHE	172	58.819	17.489	15.333	1.00	0.00	RX1	C
ATOM	3811	CD1	PHE	172	57.901	18.519	15.477	1.00	0.00	RX1	C
ATOM	3812	CD2	PHE	172	60.087	17.763	14.832	1.00	0.00	RX1	C
ATOM	3813	CE1	PHE	172	58.255	19.819	15.138	1.00	0.00	RX1	C
ATOM	3814	CE2	PHE	172	60.446	19.064	14.505	1.00	0.00	RX1	C
ATOM	3815	CZ	PHE	172	59.531	20.095	14.665	1.00	0.00	RX1	C
ATOM	3816	C	PHE	172	58.596	14.476	17.659	1.00	0.00	RX1	C
ATOM	3817	O	PHE	172	57.722	13.977	18.358	1.00	0.00	RX1	O
ATOM	3818	N	LEU	173	59.655	13.792	17.192	1.00	0.00	RX1	N
ATOM	3819	H	LEU	173	60.428	14.275	16.777	1.00	0.00	RX1	H
ATOM	3820	CA	LEU	173	59.660	12.342	17.374	1.00	0.00	RX1	C
ATOM	3821	CB	LEU	173	60.757	11.660	16.556	1.00	0.00	RX1	C
ATOM	3822	CG	LEU	173	62.182	11.823	17.080	1.00	0.00	RX1	C
ATOM	3823	CD1	LEU	173	62.649	10.608	17.884	1.00	0.00	RX1	C
ATOM	3824	CD2	LEU	173	63.139	12.128	15.935	1.00	0.00	RX1	C
ATOM	3825	C	LEU	173	59.655	11.846	18.804	1.00	0.00	RX1	C
ATOM	3826	O	LEU	173	59.170	10.764	19.096	1.00	0.00	RX1	O
ATOM	3827	N	SER	174	60.187	12.679	19.702	1.00	0.00	RX1	N
ATOM	3828	H	SER	174	60.648	13.543	19.487	1.00	0.00	RX1	H
ATOM	3829	CA	SER	174	60.233	12.200	21.079	1.00	0.00	RX1	C
ATOM	3830	CB	SER	174	61.294	13.055	21.747	1.00	0.00	RX1	C
ATOM	3831	OG	SER	174	62.356	13.199	20.788	1.00	0.00	RX1	O
ATOM	3832	HG	SER	174	62.680	14.093	20.940	1.00	0.00	RX1	H
ATOM	3833	C	SER	174	58.897	12.057	21.814	1.00	0.00	RX1	C
ATOM	3834	O	SER	174	58.844	11.699	22.982	1.00	0.00	RX1	O
ATOM	3835	N	ASN	175	57.798	12.311	21.078	1.00	0.00	RX1	N
ATOM	3836	H	ASN	175	57.838	12.643	20.133	1.00	0.00	RX1	H
ATOM	3837	CA	ASN	175	56.510	11.922	21.652	1.00	0.00	RX1	C
ATOM	3838	CB	ASN	175	55.481	13.047	21.579	1.00	0.00	RX1	C
ATOM	3839	CG	ASN	175	54.351	12.727	22.541	1.00	0.00	RX1	C
ATOM	3840	OD1	ASN	175	53.184	12.630	22.174	1.00	0.00	RX1	O
ATOM	3841	ND2	ASN	175	54.760	12.591	23.817	1.00	0.00	RX1	N

ATOM	3842	HD21	ASN	175	55.723	12.697	24.072	1.00	0.00	RX1	H
ATOM	3843	HD22	ASN	175	54.110	12.387	24.548	1.00	0.00	RX1	H
ATOM	3844	C	ASN	175	55.932	10.618	21.098	1.00	0.00	RX1	C
ATOM	3845	O	ASN	175	54.756	10.302	21.246	1.00	0.00	RX1	O
ATOM	3846	N	MET	176	56.802	9.858	20.420	1.00	0.00	RX1	N
ATOM	3847	H	MET	176	57.782	10.054	20.367	1.00	0.00	RX1	H
ATOM	3848	CA	MET	176	56.270	8.626	19.844	1.00	0.00	RX1	C
ATOM	3849	CB	MET	176	56.958	8.293	18.515	1.00	0.00	RX1	C
ATOM	3850	CG	MET	176	58.372	7.730	18.677	1.00	0.00	RX1	C
ATOM	3851	SD	MET	176	59.214	7.463	17.112	1.00	0.00	RX1	S
ATOM	3852	CE	MET	176	60.772	6.852	17.770	1.00	0.00	RX1	C
ATOM	3853	C	MET	176	56.301	7.424	20.769	1.00	0.00	RX1	C
ATOM	3854	O	MET	176	57.222	7.223	21.549	1.00	0.00	RX1	O
ATOM	3855	N	SER	177	55.277	6.587	20.586	1.00	0.00	RX1	N
ATOM	3856	H	SER	177	54.486	6.784	20.008	1.00	0.00	RX1	H
ATOM	3857	CA	SER	177	55.603	5.177	20.738	1.00	0.00	RX1	C
ATOM	3858	CB	SER	177	54.960	4.601	22.013	1.00	0.00	RX1	C
ATOM	3859	OG	SER	177	53.528	4.579	21.934	1.00	0.00	RX1	O
ATOM	3860	HG	SER	177	53.346	3.715	21.543	1.00	0.00	RX1	H
ATOM	3861	C	SER	177	55.224	4.515	19.436	1.00	0.00	RX1	C
ATOM	3862	O	SER	177	54.285	4.947	18.776	1.00	0.00	RX1	O
ATOM	3863	N	MET	178	56.002	3.501	19.062	1.00	0.00	RX1	N
ATOM	3864	H	MET	178	56.731	3.097	19.613	1.00	0.00	RX1	H
ATOM	3865	CA	MET	178	55.628	2.872	17.805	1.00	0.00	RX1	C
ATOM	3866	CB	MET	178	56.555	3.343	16.683	1.00	0.00	RX1	C
ATOM	3867	CG	MET	178	58.046	3.194	16.985	1.00	0.00	RX1	C
ATOM	3868	SD	MET	178	59.021	4.032	15.734	1.00	0.00	RX1	S
ATOM	3869	CE	MET	178	57.756	4.037	14.456	1.00	0.00	RX1	C
ATOM	3870	C	MET	178	55.530	1.376	17.950	1.00	0.00	RX1	C
ATOM	3871	O	MET	178	56.488	0.614	17.901	1.00	0.00	RX1	O
ATOM	3872	N	ASP	179	54.281	1.018	18.211	1.00	0.00	RX1	N
ATOM	3873	H	ASP	179	53.523	1.658	18.068	1.00	0.00	RX1	H
ATOM	3874	CA	ASP	179	53.939	-0.319	18.669	1.00	0.00	RX1	C
ATOM	3875	CB	ASP	179	52.682	-0.247	19.550	1.00	0.00	RX1	C
ATOM	3876	CG	ASP	179	52.312	1.201	19.868	1.00	0.00	RX1	C
ATOM	3877	OD1	ASP	179	51.368	1.700	19.264	1.00	0.00	RX1	O
ATOM	3878	OD2	ASP	179	52.947	1.850	20.706	1.00	0.00	RX1	O
ATOM	3879	C	ASP	179	53.823	-1.295	17.515	1.00	0.00	RX1	C
ATOM	3880	O	ASP	179	52.753	-1.668	17.041	1.00	0.00	RX1	O
ATOM	3881	N	PHE	180	55.028	-1.682	17.068	1.00	0.00	RX1	N
ATOM	3882	H	PHE	180	55.838	-1.320	17.531	1.00	0.00	RX1	H
ATOM	3883	CA	PHE	180	55.123	-2.764	16.094	1.00	0.00	RX1	C
ATOM	3884	CB	PHE	180	56.536	-2.868	15.514	1.00	0.00	RX1	C
ATOM	3885	CG	PHE	180	56.894	-1.666	14.673	1.00	0.00	RX1	C
ATOM	3886	CD1	PHE	180	57.760	-0.700	15.173	1.00	0.00	RX1	C
ATOM	3887	CD2	PHE	180	56.382	-1.540	13.386	1.00	0.00	RX1	C
ATOM	3888	CE1	PHE	180	58.137	0.374	14.377	1.00	0.00	RX1	C
ATOM	3889	CE2	PHE	180	56.761	-0.466	12.591	1.00	0.00	RX1	C
ATOM	3890	CZ	PHE	180	57.647	0.485	13.082	1.00	0.00	RX1	C
ATOM	3891	C	PHE	180	54.774	-4.101	16.718	1.00	0.00	RX1	C
ATOM	3892	O	PHE	180	54.767	-4.274	17.930	1.00	0.00	RX1	O
ATOM	3893	N	GLN	181	54.515	-5.050	15.816	1.00	0.00	RX1	N
ATOM	3894	H	GLN	181	54.555	-4.896	14.828	1.00	0.00	RX1	H
ATOM	3895	CA	GLN	181	54.228	-6.411	16.248	1.00	0.00	RX1	C
ATOM	3896	CB	GLN	181	52.744	-6.603	16.570	1.00	0.00	RX1	C
ATOM	3897	CG	GLN	181	51.840	-6.698	15.337	1.00	0.00	RX1	C
ATOM	3898	CD	GLN	181	51.718	-5.366	14.625	1.00	0.00	RX1	C
ATOM	3899	OE1	GLN	181	50.928	-4.517	15.019	1.00	0.00	RX1	O
ATOM	3900	NE2	GLN	181	52.493	-5.248	13.530	1.00	0.00	RX1	N
ATOM	3901	HE21	GLN	181	53.155	-5.961	13.280	1.00	0.00	RX1	H
ATOM	3902	HE22	GLN	181	52.459	-4.445	12.922	1.00	0.00	RX1	H

ATOM	3903	C	GLN	181	54.644	-7.360	15.149	1.00	0.00	RX1	C
ATOM	3904	O	GLN	181	54.778	-6.951	14.001	1.00	0.00	RX1	O
ATOM	3905	N	ASN	182	54.819	-8.633	15.532	1.00	0.00	RX1	N
ATOM	3906	H	ASN	182	54.583	-8.929	16.455	1.00	0.00	RX1	H
ATOM	3907	CA	ASN	182	55.123	-9.595	14.472	1.00	0.00	RX1	C
ATOM	3908	CB	ASN	182	55.558	-10.935	15.066	1.00	0.00	RX1	C
ATOM	3909	CG	ASN	182	56.033	-11.818	13.934	1.00	0.00	RX1	C
ATOM	3910	OD1	ASN	182	56.684	-11.350	13.007	1.00	0.00	RX1	O
ATOM	3911	ND2	ASN	182	55.652	-13.101	14.039	1.00	0.00	RX1	N
ATOM	3912	HD21	ASN	182	55.158	-13.454	14.833	1.00	0.00	RX1	H
ATOM	3913	HD22	ASN	182	55.823	-13.734	13.281	1.00	0.00	RX1	H
ATOM	3914	C	ASN	182	53.954	-9.801	13.521	1.00	0.00	RX1	C
ATOM	3915	O	ASN	182	52.791	-9.684	13.891	1.00	0.00	RX1	O
ATOM	3916	N	HIS	183	54.320	-10.101	12.275	1.00	0.00	RX1	N
ATOM	3917	H	HIS	183	55.288	-10.287	12.092	1.00	0.00	RX1	H
ATOM	3918	CA	HIS	183	53.294	-10.512	11.324	1.00	0.00	RX1	C
ATOM	3919	CB	HIS	183	53.748	-10.234	9.891	1.00	0.00	RX1	C
ATOM	3920	CG	HIS	183	55.232	-10.444	9.743	1.00	0.00	RX1	C
ATOM	3921	ND1	HIS	183	56.123	-9.437	9.779	1.00	0.00	RX1	N
ATOM	3922	HD1	HIS	183	55.930	-8.471	9.849	1.00	0.00	RX1	H
ATOM	3923	CD2	HIS	183	55.915	-11.650	9.566	1.00	0.00	RX1	C
ATOM	3924	NE2	HIS	183	57.236	-11.359	9.496	1.00	0.00	RX1	N
ATOM	3925	CE1	HIS	183	57.362	-9.997	9.633	1.00	0.00	RX1	C
ATOM	3926	C	HIS	183	52.921	-11.969	11.488	1.00	0.00	RX1	C
ATOM	3927	O	HIS	183	53.690	-12.782	11.986	1.00	0.00	RX1	O
ATOM	3928	N	LEU	184	51.701	-12.276	11.028	1.00	0.00	RX1	N
ATOM	3929	H	LEU	184	51.113	-11.615	10.566	1.00	0.00	RX1	H
ATOM	3930	CA	LEU	184	51.359	-13.695	10.999	1.00	0.00	RX1	C
ATOM	3931	CB	LEU	184	49.847	-13.903	11.070	1.00	0.00	RX1	C
ATOM	3932	CG	LEU	184	49.238	-13.392	12.376	1.00	0.00	RX1	C
ATOM	3933	CD1	LEU	184	47.746	-13.713	12.452	1.00	0.00	RX1	C
ATOM	3934	CD2	LEU	184	49.988	-13.913	13.604	1.00	0.00	RX1	C
ATOM	3935	C	LEU	184	51.933	-14.385	9.779	1.00	0.00	RX1	C
ATOM	3936	O	LEU	184	51.297	-14.519	8.744	1.00	0.00	RX1	O
ATOM	3937	N	GLY	185	53.191	-14.795	9.950	1.00	0.00	RX1	N
ATOM	3938	H	GLY	185	53.727	-14.605	10.777	1.00	0.00	RX1	H
ATOM	3939	CA	GLY	185	53.846	-15.479	8.844	1.00	0.00	RX1	C
ATOM	3940	C	GLY	185	55.334	-15.239	8.896	1.00	0.00	RX1	C
ATOM	3941	O	GLY	185	55.968	-15.366	9.937	1.00	0.00	RX1	O
ATOM	3942	N	SER	186	55.868	-14.869	7.731	1.00	0.00	RX1	N
ATOM	3943	H	SER	186	55.372	-14.748	6.871	1.00	0.00	RX1	H
ATOM	3944	CA	SER	186	57.289	-14.548	7.687	1.00	0.00	RX1	C
ATOM	3945	CB	SER	186	58.052	-15.852	7.454	1.00	0.00	RX1	C
ATOM	3946	OG	SER	186	57.461	-16.884	8.253	1.00	0.00	RX1	O
ATOM	3947	HG	SER	186	57.298	-16.496	9.111	1.00	0.00	RX1	H
ATOM	3948	C	SER	186	57.537	-13.508	6.618	1.00	0.00	RX1	C
ATOM	3949	O	SER	186	56.720	-13.327	5.722	1.00	0.00	RX1	O
ATOM	3950	N	CYS	187	58.673	-12.820	6.743	1.00	0.00	RX1	N
ATOM	3951	H	CYS	187	59.367	-12.968	7.446	1.00	0.00	RX1	H
ATOM	3952	CA	CYS	187	58.902	-11.779	5.751	1.00	0.00	RX1	C
ATOM	3953	CB	CYS	187	58.910	-10.428	6.453	1.00	0.00	RX1	C
ATOM	3954	SG	CYS	187	60.107	-10.379	7.809	1.00	0.00	RX1	S
ATOM	3955	C	CYS	187	60.176	-11.985	4.976	1.00	0.00	RX1	C
ATOM	3956	O	CYS	187	61.100	-12.666	5.407	1.00	0.00	RX1	O
ATOM	3957	N	GLN	188	60.207	-11.315	3.819	1.00	0.00	RX1	N
ATOM	3958	H	GLN	188	59.425	-10.770	3.523	1.00	0.00	RX1	H
ATOM	3959	CA	GLN	188	61.501	-11.132	3.177	1.00	0.00	RX1	C
ATOM	3960	CB	GLN	188	61.331	-10.874	1.663	1.00	0.00	RX1	C
ATOM	3961	CG	GLN	188	60.895	-9.503	1.111	1.00	0.00	RX1	C
ATOM	3962	CD	GLN	188	59.465	-9.086	1.426	1.00	0.00	RX1	C
ATOM	3963	OE1	GLN	188	58.699	-9.721	2.154	1.00	0.00	RX1	O

ATOM	3964	NE2	GLN	188	59.136	-7.942	0.813	1.00	0.00	RX1	N
ATOM	3965	HE21	GLN	188	59.775	-7.496	0.179	1.00	0.00	RX1	H
ATOM	3966	HE22	GLN	188	58.269	-7.443	0.921	1.00	0.00	RX1	H
ATOM	3967	C	GLN	188	62.330	-10.106	3.944	1.00	0.00	RX1	C
ATOM	3968	O	GLN	188	62.241	-8.901	3.762	1.00	0.00	RX1	O
ATOM	3969	N	LYS	189	63.086	-10.662	4.903	1.00	0.00	RX1	N
ATOM	3970	H	LYS	189	63.081	-11.658	5.004	1.00	0.00	RX1	H
ATOM	3971	CA	LYS	189	63.565	-9.799	5.984	1.00	0.00	RX1	C
ATOM	3972	CB	LYS	189	64.130	-10.654	7.130	1.00	0.00	RX1	C
ATOM	3973	CG	LYS	189	64.381	-9.847	8.410	1.00	0.00	RX1	C
ATOM	3974	CD	LYS	189	63.135	-9.064	8.833	1.00	0.00	RX1	C
ATOM	3975	CE	LYS	189	63.454	-7.849	9.706	1.00	0.00	RX1	C
ATOM	3976	NZ	LYS	189	62.381	-6.866	9.541	1.00	0.00	RX1	N
ATOM	3977	HZ1	LYS	189	62.729	-5.897	9.708	1.00	0.00	RX1	H
ATOM	3978	HZ2	LYS	189	61.992	-6.907	8.580	1.00	0.00	RX1	H
ATOM	3979	HZ3	LYS	189	61.597	-6.950	10.223	1.00	0.00	RX1	H
ATOM	3980	C	LYS	189	64.484	-8.637	5.615	1.00	0.00	RX1	C
ATOM	3981	O	LYS	189	64.471	-7.598	6.264	1.00	0.00	RX1	O
ATOM	3982	N	CYS	190	65.279	-8.842	4.560	1.00	0.00	RX1	N
ATOM	3983	H	CYS	190	65.317	-9.654	3.979	1.00	0.00	RX1	H
ATOM	3984	CA	CYS	190	66.233	-7.777	4.268	1.00	0.00	RX1	C
ATOM	3985	CB	CYS	190	67.468	-7.999	5.139	1.00	0.00	RX1	C
ATOM	3986	SG	CYS	190	67.580	-9.728	5.678	1.00	0.00	RX1	S
ATOM	3987	C	CYS	190	66.571	-7.685	2.802	1.00	0.00	RX1	C
ATOM	3988	O	CYS	190	66.624	-8.684	2.095	1.00	0.00	RX1	O
ATOM	3989	N	ASP	191	66.799	-6.430	2.406	1.00	0.00	RX1	N
ATOM	3990	H	ASP	191	66.739	-5.666	3.044	1.00	0.00	RX1	H
ATOM	3991	CA	ASP	191	67.459	-6.152	1.135	1.00	0.00	RX1	C
ATOM	3992	CB	ASP	191	67.109	-4.720	0.728	1.00	0.00	RX1	C
ATOM	3993	CG	ASP	191	65.847	-4.747	-0.105	1.00	0.00	RX1	C
ATOM	3994	OD1	ASP	191	64.872	-4.096	0.252	1.00	0.00	RX1	O
ATOM	3995	OD2	ASP	191	65.832	-5.406	-1.137	1.00	0.00	RX1	O
ATOM	3996	C	ASP	191	68.958	-6.364	1.292	1.00	0.00	RX1	C
ATOM	3997	O	ASP	191	69.461	-6.327	2.411	1.00	0.00	RX1	O
ATOM	3998	N	PRO	192	69.660	-6.642	0.161	1.00	0.00	RX1	N
ATOM	3999	CD	PRO	192	69.137	-6.722	-1.201	1.00	0.00	RX1	C
ATOM	4000	CA	PRO	192	71.089	-6.981	0.229	1.00	0.00	RX1	C
ATOM	4001	CB	PRO	192	71.509	-6.960	-1.244	1.00	0.00	RX1	C
ATOM	4002	CG	PRO	192	70.252	-7.385	-2.000	1.00	0.00	RX1	C
ATOM	4003	C	PRO	192	71.988	-6.143	1.131	1.00	0.00	RX1	C
ATOM	4004	O	PRO	192	72.781	-6.676	1.899	1.00	0.00	RX1	O
ATOM	4005	N	SER	193	71.860	-4.821	0.994	1.00	0.00	RX1	N
ATOM	4006	H	SER	193	71.152	-4.378	0.437	1.00	0.00	RX1	H
ATOM	4007	CA	SER	193	72.792	-3.994	1.752	1.00	0.00	RX1	C
ATOM	4008	CB	SER	193	73.246	-2.903	0.797	1.00	0.00	RX1	C
ATOM	4009	OG	SER	193	73.252	-3.480	-0.516	1.00	0.00	RX1	O
ATOM	4010	HG	SER	193	72.338	-3.458	-0.807	1.00	0.00	RX1	H
ATOM	4011	C	SER	193	72.272	-3.529	3.098	1.00	0.00	RX1	C
ATOM	4012	O	SER	193	73.025	-3.408	4.056	1.00	0.00	RX1	O
ATOM	4013	N	CYS	194	70.948	-3.275	3.135	1.00	0.00	RX1	N
ATOM	4014	H	CYS	194	70.428	-3.339	2.278	1.00	0.00	RX1	H
ATOM	4015	CA	CYS	194	70.242	-2.982	4.395	1.00	0.00	RX1	C
ATOM	4016	CB	CYS	194	69.603	-4.264	4.916	1.00	0.00	RX1	C
ATOM	4017	SG	CYS	194	67.871	-4.428	4.427	1.00	0.00	RX1	S
ATOM	4018	C	CYS	194	71.018	-2.306	5.527	1.00	0.00	RX1	C
ATOM	4019	O	CYS	194	71.322	-2.922	6.544	1.00	0.00	RX1	O
ATOM	4020	N	PRO	195	71.318	-0.993	5.327	1.00	0.00	RX1	N
ATOM	4021	CD	PRO	195	70.747	-0.143	4.288	1.00	0.00	RX1	C
ATOM	4022	CA	PRO	195	72.306	-0.284	6.158	1.00	0.00	RX1	C
ATOM	4023	CB	PRO	195	71.974	1.181	5.861	1.00	0.00	RX1	C
ATOM	4024	CG	PRO	195	71.516	1.169	4.403	1.00	0.00	RX1	C

ATOM	4025	C	PRO	195	72.366	-0.630	7.642	1.00	0.00	RX1	C
ATOM	4026	O	PRO	195	73.375	-1.082	8.169	1.00	0.00	RX1	O
ATOM	4027	N	ASN	196	71.228	-0.407	8.305	1.00	0.00	RX1	N
ATOM	4028	H	ASN	196	70.418	-0.013	7.874	1.00	0.00	RX1	H
ATOM	4029	CA	ASN	196	71.141	-0.915	9.673	1.00	0.00	RX1	C
ATOM	4030	CB	ASN	196	71.123	0.200	10.720	1.00	0.00	RX1	C
ATOM	4031	CG	ASN	196	72.492	0.749	11.093	1.00	0.00	RX1	C
ATOM	4032	OD1	ASN	196	72.576	1.798	11.722	1.00	0.00	RX1	O
ATOM	4033	ND2	ASN	196	73.560	0.023	10.715	1.00	0.00	RX1	N
ATOM	4034	HD21	ASN	196	73.530	-0.770	10.099	1.00	0.00	RX1	H
ATOM	4035	HD22	ASN	196	74.477	0.280	11.018	1.00	0.00	RX1	H
ATOM	4036	C	ASN	196	69.854	-1.685	9.824	1.00	0.00	RX1	C
ATOM	4037	O	ASN	196	68.998	-1.394	10.648	1.00	0.00	RX1	O
ATOM	4038	N	GLY	197	69.713	-2.653	8.910	1.00	0.00	RX1	N
ATOM	4039	H	GLY	197	70.440	-2.840	8.247	1.00	0.00	RX1	H
ATOM	4040	CA	GLY	197	68.347	-3.077	8.626	1.00	0.00	RX1	C
ATOM	4041	C	GLY	197	67.706	-2.022	7.748	1.00	0.00	RX1	C
ATOM	4042	O	GLY	197	67.900	-1.982	6.538	1.00	0.00	RX1	O
ATOM	4043	N	SER	198	66.996	-1.109	8.426	1.00	0.00	RX1	N
ATOM	4044	H	SER	198	66.977	-1.132	9.427	1.00	0.00	RX1	H
ATOM	4045	CA	SER	198	66.438	0.064	7.750	1.00	0.00	RX1	C
ATOM	4046	CB	SER	198	67.562	1.094	7.598	1.00	0.00	RX1	C
ATOM	4047	OG	SER	198	68.779	0.434	7.232	1.00	0.00	RX1	O
ATOM	4048	HG	SER	198	68.522	-0.156	6.524	1.00	0.00	RX1	H
ATOM	4049	C	SER	198	65.645	-0.212	6.476	1.00	0.00	RX1	C
ATOM	4050	O	SER	198	65.707	0.524	5.498	1.00	0.00	RX1	O
ATOM	4051	N	CYS	199	64.918	-1.334	6.510	1.00	0.00	RX1	N
ATOM	4052	H	CYS	199	64.735	-1.871	7.337	1.00	0.00	RX1	H
ATOM	4053	CA	CYS	199	64.329	-1.745	5.239	1.00	0.00	RX1	C
ATOM	4054	CB	CYS	199	64.628	-3.228	5.033	1.00	0.00	RX1	C
ATOM	4055	SG	CYS	199	64.775	-4.114	6.607	1.00	0.00	RX1	S
ATOM	4056	C	CYS	199	62.867	-1.373	5.090	1.00	0.00	RX1	C
ATOM	4057	O	CYS	199	62.005	-1.717	5.887	1.00	0.00	RX1	O
ATOM	4058	N	TRP	200	62.610	-0.599	4.031	1.00	0.00	RX1	N
ATOM	4059	H	TRP	200	63.274	-0.488	3.290	1.00	0.00	RX1	H
ATOM	4060	CA	TRP	200	61.222	-0.201	3.814	1.00	0.00	RX1	C
ATOM	4061	CB	TRP	200	61.161	1.103	3.027	1.00	0.00	RX1	C
ATOM	4062	CG	TRP	200	61.342	2.297	3.927	1.00	0.00	RX1	C
ATOM	4063	CD2	TRP	200	60.357	2.867	4.808	1.00	0.00	RX1	C
ATOM	4064	CE2	TRP	200	60.937	3.999	5.421	1.00	0.00	RX1	C
ATOM	4065	CE3	TRP	200	59.049	2.508	5.108	1.00	0.00	RX1	C
ATOM	4066	CD1	TRP	200	62.473	3.117	4.058	1.00	0.00	RX1	C
ATOM	4067	NE1	TRP	200	62.236	4.127	4.940	1.00	0.00	RX1	N
ATOM	4068	HE1	TRP	200	62.853	4.849	5.186	1.00	0.00	RX1	H
ATOM	4069	CZ2	TRP	200	60.189	4.744	6.324	1.00	0.00	RX1	C
ATOM	4070	CZ3	TRP	200	58.312	3.260	6.010	1.00	0.00	RX1	C
ATOM	4071	CH2	TRP	200	58.882	4.373	6.615	1.00	0.00	RX1	C
ATOM	4072	C	TRP	200	60.461	-1.281	3.073	1.00	0.00	RX1	C
ATOM	4073	O	TRP	200	60.305	-1.238	1.860	1.00	0.00	RX1	O
ATOM	4074	N	GLY	201	60.022	-2.284	3.844	1.00	0.00	RX1	N
ATOM	4075	H	GLY	201	60.293	-2.296	4.807	1.00	0.00	RX1	H
ATOM	4076	CA	GLY	201	59.679	-3.518	3.136	1.00	0.00	RX1	C
ATOM	4077	C	GLY	201	60.980	-4.161	2.693	1.00	0.00	RX1	C
ATOM	4078	O	GLY	201	62.009	-3.928	3.318	1.00	0.00	RX1	O
ATOM	4079	N	ALA	202	60.911	-4.929	1.601	1.00	0.00	RX1	N
ATOM	4080	H	ALA	202	60.070	-5.152	1.106	1.00	0.00	RX1	H
ATOM	4081	CA	ALA	202	62.162	-5.326	0.956	1.00	0.00	RX1	C
ATOM	4082	CB	ALA	202	62.879	-6.451	1.705	1.00	0.00	RX1	C
ATOM	4083	C	ALA	202	61.886	-5.800	-0.446	1.00	0.00	RX1	C
ATOM	4084	O	ALA	202	60.752	-6.153	-0.760	1.00	0.00	RX1	O
ATOM	4085	N	GLY	203	62.943	-5.803	-1.258	1.00	0.00	RX1	N

ATOM	4086	H	GLY	203	63.853	-5.503	-0.945	1.00	0.00	RX1	H
ATOM	4087	CA	GLY	203	62.804	-6.360	-2.599	1.00	0.00	RX1	C
ATOM	4088	C	GLY	203	63.927	-5.984	-3.548	1.00	0.00	RX1	C
ATOM	4089	O	GLY	203	64.461	-6.818	-4.268	1.00	0.00	RX1	O
ATOM	4090	N	GLU	204	64.222	-4.676	-3.539	1.00	0.00	RX1	N
ATOM	4091	H	GLU	204	63.900	-4.077	-2.808	1.00	0.00	RX1	H
ATOM	4092	CA	GLU	204	65.129	-4.128	-4.546	1.00	0.00	RX1	C
ATOM	4093	CB	GLU	204	64.229	-3.602	-5.681	1.00	0.00	RX1	C
ATOM	4094	CG	GLU	204	64.835	-2.923	-6.920	1.00	0.00	RX1	C
ATOM	4095	CD	GLU	204	63.918	-1.777	-7.335	1.00	0.00	RX1	C
ATOM	4096	OE1	GLU	204	63.986	-0.713	-6.725	1.00	0.00	RX1	O
ATOM	4097	OE2	GLU	204	63.124	-1.937	-8.256	1.00	0.00	RX1	O
ATOM	4098	C	GLU	204	65.934	-2.974	-3.968	1.00	0.00	RX1	C
ATOM	4099	O	GLU	204	65.810	-1.817	-4.350	1.00	0.00	RX1	O
ATOM	4100	N	GLU	205	66.734	-3.345	-2.951	1.00	0.00	RX1	N
ATOM	4101	H	GLU	205	66.714	-4.295	-2.630	1.00	0.00	RX1	H
ATOM	4102	CA	GLU	205	67.441	-2.360	-2.121	1.00	0.00	RX1	C
ATOM	4103	CB	GLU	205	68.748	-1.882	-2.755	1.00	0.00	RX1	C
ATOM	4104	CG	GLU	205	69.739	-1.417	-1.681	1.00	0.00	RX1	C
ATOM	4105	CD	GLU	205	70.088	-2.583	-0.777	1.00	0.00	RX1	C
ATOM	4106	OE1	GLU	205	69.993	-2.457	0.442	1.00	0.00	RX1	O
ATOM	4107	OE2	GLU	205	70.492	-3.622	-1.288	1.00	0.00	RX1	O
ATOM	4108	C	GLU	205	66.559	-1.209	-1.655	1.00	0.00	RX1	C
ATOM	4109	O	GLU	205	66.847	-0.016	-1.731	1.00	0.00	RX1	O
ATOM	4110	N	ASN	206	65.390	-1.660	-1.189	1.00	0.00	RX1	N
ATOM	4111	H	ASN	206	65.339	-2.632	-0.946	1.00	0.00	RX1	H
ATOM	4112	CA	ASN	206	64.277	-0.762	-0.906	1.00	0.00	RX1	C
ATOM	4113	CB	ASN	206	62.953	-1.515	-0.972	1.00	0.00	RX1	C
ATOM	4114	CG	ASN	206	61.871	-0.529	-1.326	1.00	0.00	RX1	C
ATOM	4115	OD1	ASN	206	61.617	-0.287	-2.499	1.00	0.00	RX1	O
ATOM	4116	ND2	ASN	206	61.257	0.059	-0.301	1.00	0.00	RX1	N
ATOM	4117	HD21	ASN	206	61.481	-0.251	0.629	1.00	0.00	RX1	H
ATOM	4118	HD22	ASN	206	60.568	0.775	-0.416	1.00	0.00	RX1	H
ATOM	4119	C	ASN	206	64.384	-0.134	0.476	1.00	0.00	RX1	C
ATOM	4120	O	ASN	206	63.506	-0.251	1.324	1.00	0.00	RX1	O
ATOM	4121	N	CYS	207	65.522	0.537	0.685	1.00	0.00	RX1	N
ATOM	4122	H	CYS	207	66.195	0.678	-0.043	1.00	0.00	RX1	H
ATOM	4123	CA	CYS	207	65.849	0.905	2.057	1.00	0.00	RX1	C
ATOM	4124	CB	CYS	207	67.055	0.078	2.498	1.00	0.00	RX1	C
ATOM	4125	SG	CYS	207	66.695	-1.690	2.428	1.00	0.00	RX1	S
ATOM	4126	C	CYS	207	66.036	2.388	2.315	1.00	0.00	RX1	C
ATOM	4127	O	CYS	207	66.173	3.220	1.420	1.00	0.00	RX1	O
ATOM	4128	N	GLN	208	66.047	2.667	3.626	1.00	0.00	RX1	N
ATOM	4129	H	GLN	208	65.944	1.919	4.282	1.00	0.00	RX1	H
ATOM	4130	CA	GLN	208	66.420	3.977	4.144	1.00	0.00	RX1	C
ATOM	4131	CB	GLN	208	66.112	4.018	5.642	1.00	0.00	RX1	C
ATOM	4132	CG	GLN	208	66.616	5.272	6.354	1.00	0.00	RX1	C
ATOM	4133	CD	GLN	208	66.484	5.089	7.853	1.00	0.00	RX1	C
ATOM	4134	OE1	GLN	208	66.183	4.016	8.370	1.00	0.00	RX1	O
ATOM	4135	NE2	GLN	208	66.724	6.216	8.532	1.00	0.00	RX1	N
ATOM	4136	HE21	GLN	208	67.030	7.060	8.072	1.00	0.00	RX1	H
ATOM	4137	HE22	GLN	208	66.627	6.308	9.525	1.00	0.00	RX1	H
ATOM	4138	C	GLN	208	67.891	4.264	3.900	1.00	0.00	RX1	C
ATOM	4139	O	GLN	208	68.785	3.639	4.455	1.00	0.00	RX1	O
ATOM	4140	N	LYS	209	68.106	5.267	3.047	1.00	0.00	RX1	N
ATOM	4141	H	LYS	209	67.347	5.812	2.697	1.00	0.00	RX1	H
ATOM	4142	CA	LYS	209	69.479	5.747	2.979	1.00	0.00	RX1	C
ATOM	4143	CB	LYS	209	69.931	6.151	1.564	1.00	0.00	RX1	C
ATOM	4144	CG	LYS	209	68.958	6.947	0.686	1.00	0.00	RX1	C
ATOM	4145	CD	LYS	209	67.899	6.071	0.011	1.00	0.00	RX1	C
ATOM	4146	CE	LYS	209	66.880	6.856	-0.807	1.00	0.00	RX1	C

ATOM	4147	NZ	LYS	209	65.843	5.921	-1.252	1.00	0.00	RX1	N
ATOM	4148	HZ1	LYS	209	65.086	6.435	-1.747	1.00	0.00	RX1	H
ATOM	4149	HZ2	LYS	209	66.231	5.184	-1.868	1.00	0.00	RX1	H
ATOM	4150	HZ3	LYS	209	65.415	5.473	-0.413	1.00	0.00	RX1	H
ATOM	4151	C	LYS	209	69.706	6.843	3.994	1.00	0.00	RX1	C
ATOM	4152	O	LYS	209	69.201	7.955	3.891	1.00	0.00	RX1	O
ATOM	4153	N	LEU	210	70.485	6.438	5.007	1.00	0.00	RX1	N
ATOM	4154	H	LEU	210	70.778	5.483	5.024	1.00	0.00	RX1	H
ATOM	4155	CA	LEU	210	70.776	7.320	6.140	1.00	0.00	RX1	C
ATOM	4156	CB	LEU	210	71.842	6.701	7.045	1.00	0.00	RX1	C
ATOM	4157	CG	LEU	210	71.306	5.845	8.199	1.00	0.00	RX1	C
ATOM	4158	CD1	LEU	210	70.512	4.618	7.744	1.00	0.00	RX1	C
ATOM	4159	CD2	LEU	210	72.437	5.468	9.155	1.00	0.00	RX1	C
ATOM	4160	C	LEU	210	71.184	8.724	5.737	1.00	0.00	RX1	C
ATOM	4161	O	LEU	210	72.149	8.943	5.006	1.00	0.00	RX1	O
ATOM	4162	N	THR	211	70.380	9.664	6.217	1.00	0.00	RX1	N
ATOM	4163	H	THR	211	69.598	9.440	6.809	1.00	0.00	RX1	H
ATOM	4164	CA	THR	211	70.602	11.036	5.791	1.00	0.00	RX1	C
ATOM	4165	CB	THR	211	69.660	11.291	4.622	1.00	0.00	RX1	C
ATOM	4166	OG1	THR	211	68.507	10.461	4.802	1.00	0.00	RX1	O
ATOM	4167	HG1	THR	211	68.737	9.603	4.448	1.00	0.00	RX1	H
ATOM	4168	CG2	THR	211	70.291	11.065	3.245	1.00	0.00	RX1	C
ATOM	4169	C	THR	211	70.479	12.073	6.900	1.00	0.00	RX1	C
ATOM	4170	O	THR	211	70.543	13.267	6.644	1.00	0.00	RX1	O
ATOM	4171	N	LYS	212	70.326	11.589	8.139	1.00	0.00	RX1	N
ATOM	4172	H	LYS	212	70.166	10.614	8.288	1.00	0.00	RX1	H
ATOM	4173	CA	LYS	212	70.497	12.466	9.299	1.00	0.00	RX1	C
ATOM	4174	CB	LYS	212	69.177	12.693	10.043	1.00	0.00	RX1	C
ATOM	4175	CG	LYS	212	68.479	14.042	9.829	1.00	0.00	RX1	C
ATOM	4176	CD	LYS	212	67.078	14.048	10.462	1.00	0.00	RX1	C
ATOM	4177	CE	LYS	212	66.247	15.314	10.225	1.00	0.00	RX1	C
ATOM	4178	NZ	LYS	212	64.832	15.022	10.492	1.00	0.00	RX1	N
ATOM	4179	HZ1	LYS	212	64.303	15.874	10.775	1.00	0.00	RX1	H
ATOM	4180	HZ2	LYS	212	64.350	14.656	9.638	1.00	0.00	RX1	H
ATOM	4181	HZ3	LYS	212	64.738	14.307	11.239	1.00	0.00	RX1	H
ATOM	4182	C	LYS	212	71.532	11.941	10.282	1.00	0.00	RX1	C
ATOM	4183	O	LYS	212	72.413	12.660	10.729	1.00	0.00	RX1	O
ATOM	4184	N	ILE	213	71.393	10.644	10.616	1.00	0.00	RX1	N
ATOM	4185	H	ILE	213	70.704	10.065	10.175	1.00	0.00	RX1	H
ATOM	4186	CA	ILE	213	72.231	10.109	11.697	1.00	0.00	RX1	C
ATOM	4187	CB	ILE	213	71.825	8.674	12.038	1.00	0.00	RX1	C
ATOM	4188	CG2	ILE	213	72.816	7.997	12.988	1.00	0.00	RX1	C
ATOM	4189	CG1	ILE	213	70.417	8.683	12.631	1.00	0.00	RX1	C
ATOM	4190	CD1	ILE	213	70.314	9.513	13.912	1.00	0.00	RX1	C
ATOM	4191	C	ILE	213	73.729	10.226	11.456	1.00	0.00	RX1	C
ATOM	4192	O	ILE	213	74.489	10.684	12.299	1.00	0.00	RX1	O
ATOM	4193	N	ILE	214	74.131	9.809	10.250	1.00	0.00	RX1	N
ATOM	4194	H	ILE	214	73.464	9.584	9.543	1.00	0.00	RX1	H
ATOM	4195	CA	ILE	214	75.568	9.905	9.995	1.00	0.00	RX1	C
ATOM	4196	CB	ILE	214	76.068	8.700	9.182	1.00	0.00	RX1	C
ATOM	4197	CG2	ILE	214	76.207	7.509	10.130	1.00	0.00	RX1	C
ATOM	4198	CG1	ILE	214	75.173	8.325	7.994	1.00	0.00	RX1	C
ATOM	4199	CD1	ILE	214	75.298	9.213	6.758	1.00	0.00	RX1	C
ATOM	4200	C	ILE	214	76.041	11.242	9.441	1.00	0.00	RX1	C
ATOM	4201	O	ILE	214	76.780	11.340	8.467	1.00	0.00	RX1	O
ATOM	4202	N	CYS	215	75.571	12.298	10.117	1.00	0.00	RX1	N
ATOM	4203	H	CYS	215	75.053	12.186	10.967	1.00	0.00	RX1	H
ATOM	4204	CA	CYS	215	76.079	13.610	9.734	1.00	0.00	RX1	C
ATOM	4205	CB	CYS	215	75.226	14.707	10.370	1.00	0.00	RX1	C
ATOM	4206	SG	CYS	215	73.675	14.978	9.473	1.00	0.00	RX1	S
ATOM	4207	C	CYS	215	77.548	13.771	10.051	1.00	0.00	RX1	C

ATOM	4208	O	CYS	215	78.048	13.332	11.080	1.00	0.00	RX1	O
ATOM	4209	N	ALA	216	78.230	14.407	9.091	1.00	0.00	RX1	N
ATOM	4210	H	ALA	216	77.763	14.841	8.324	1.00	0.00	RX1	H
ATOM	4211	CA	ALA	216	79.641	14.655	9.354	1.00	0.00	RX1	C
ATOM	4212	CB	ALA	216	80.359	15.067	8.072	1.00	0.00	RX1	C
ATOM	4213	C	ALA	216	79.795	15.751	10.387	1.00	0.00	RX1	C
ATOM	4214	O	ALA	216	78.886	16.542	10.604	1.00	0.00	RX1	O
ATOM	4215	N	GLN	217	80.988	15.763	11.007	1.00	0.00	RX1	N
ATOM	4216	H	GLN	217	81.664	15.076	10.746	1.00	0.00	RX1	H
ATOM	4217	CA	GLN	217	81.240	16.700	12.111	1.00	0.00	RX1	C
ATOM	4218	CB	GLN	217	82.689	16.583	12.572	1.00	0.00	RX1	C
ATOM	4219	CG	GLN	217	83.013	15.181	13.086	1.00	0.00	RX1	C
ATOM	4220	CD	GLN	217	84.488	15.113	13.409	1.00	0.00	RX1	C
ATOM	4221	OE1	GLN	217	85.335	15.492	12.610	1.00	0.00	RX1	O
ATOM	4222	NE2	GLN	217	84.748	14.611	14.629	1.00	0.00	RX1	N
ATOM	4223	HE21	GLN	217	84.005	14.318	15.231	1.00	0.00	RX1	H
ATOM	4224	HE22	GLN	217	85.689	14.521	14.956	1.00	0.00	RX1	H
ATOM	4225	C	GLN	217	80.892	18.156	11.824	1.00	0.00	RX1	C
ATOM	4226	O	GLN	217	80.431	18.901	12.679	1.00	0.00	RX1	O
ATOM	4227	N	GLN	218	81.106	18.512	10.553	1.00	0.00	RX1	N
ATOM	4228	H	GLN	218	81.472	17.890	9.864	1.00	0.00	RX1	H
ATOM	4229	CA	GLN	218	80.377	19.675	10.074	1.00	0.00	RX1	C
ATOM	4230	CB	GLN	218	81.364	20.785	9.706	1.00	0.00	RX1	C
ATOM	4231	CG	GLN	218	80.676	22.086	9.295	1.00	0.00	RX1	C
ATOM	4232	CD	GLN	218	79.654	22.447	10.352	1.00	0.00	RX1	C
ATOM	4233	OE1	GLN	218	78.460	22.231	10.180	1.00	0.00	RX1	O
ATOM	4234	NE2	GLN	218	80.188	22.989	11.460	1.00	0.00	RX1	N
ATOM	4235	HE21	GLN	218	81.173	23.144	11.537	1.00	0.00	RX1	H
ATOM	4236	HE22	GLN	218	79.613	23.238	12.240	1.00	0.00	RX1	H
ATOM	4237	C	GLN	218	79.528	19.243	8.898	1.00	0.00	RX1	C
ATOM	4238	O	GLN	218	79.998	18.512	8.030	1.00	0.00	RX1	O
ATOM	4239	N	CYS	219	78.265	19.682	8.932	1.00	0.00	RX1	N
ATOM	4240	H	CYS	219	77.926	20.346	9.605	1.00	0.00	RX1	H
ATOM	4241	CA	CYS	219	77.308	19.146	7.969	1.00	0.00	RX1	C
ATOM	4242	CB	CYS	219	76.685	17.880	8.561	1.00	0.00	RX1	C
ATOM	4243	SG	CYS	219	76.227	16.616	7.344	1.00	0.00	RX1	S
ATOM	4244	C	CYS	219	76.273	20.190	7.616	1.00	0.00	RX1	C
ATOM	4245	O	CYS	219	75.494	20.630	8.450	1.00	0.00	RX1	O
ATOM	4246	N	SER	220	76.294	20.596	6.342	1.00	0.00	RX1	N
ATOM	4247	H	SER	220	76.897	20.209	5.644	1.00	0.00	RX1	H
ATOM	4248	CA	SER	220	75.479	21.746	5.945	1.00	0.00	RX1	C
ATOM	4249	CB	SER	220	76.184	22.279	4.712	1.00	0.00	RX1	C
ATOM	4250	OG	SER	220	76.645	21.141	3.977	1.00	0.00	RX1	O
ATOM	4251	HG	SER	220	75.935	20.955	3.363	1.00	0.00	RX1	H
ATOM	4252	C	SER	220	73.987	21.502	5.731	1.00	0.00	RX1	C
ATOM	4253	O	SER	220	73.330	22.233	4.998	1.00	0.00	RX1	O
ATOM	4254	N	GLY	221	73.486	20.435	6.360	1.00	0.00	RX1	N
ATOM	4255	H	GLY	221	73.996	19.882	7.022	1.00	0.00	RX1	H
ATOM	4256	CA	GLY	221	72.140	19.966	6.058	1.00	0.00	RX1	C
ATOM	4257	C	GLY	221	72.146	18.467	6.234	1.00	0.00	RX1	C
ATOM	4258	O	GLY	221	73.041	17.931	6.873	1.00	0.00	RX1	O
ATOM	4259	N	ARG	222	71.146	17.812	5.620	1.00	0.00	RX1	N
ATOM	4260	H	ARG	222	70.445	18.315	5.116	1.00	0.00	RX1	H
ATOM	4261	CA	ARG	222	71.118	16.343	5.663	1.00	0.00	RX1	C
ATOM	4262	CB	ARG	222	69.988	15.819	4.770	1.00	0.00	RX1	C
ATOM	4263	CG	ARG	222	68.557	16.217	5.148	1.00	0.00	RX1	C
ATOM	4264	CD	ARG	222	67.937	15.423	6.304	1.00	0.00	RX1	C
ATOM	4265	NE	ARG	222	67.912	13.982	6.035	1.00	0.00	RX1	N
ATOM	4266	HE	ARG	222	68.726	13.614	5.578	1.00	0.00	RX1	H
ATOM	4267	CZ	ARG	222	66.941	13.190	6.588	1.00	0.00	RX1	C
ATOM	4268	NH1	ARG	222	65.843	13.748	7.123	1.00	0.00	RX1	N

ATOM	4269	HH11	ARG	222	65.126	13.164	7.525	1.00	0.00	RX1	H
ATOM	4270	HH12	ARG	222	65.638	14.740	7.114	1.00	0.00	RX1	H
ATOM	4271	NH2	ARG	222	67.067	11.848	6.609	1.00	0.00	RX1	N
ATOM	4272	HH21	ARG	222	66.473	11.290	7.209	1.00	0.00	RX1	H
ATOM	4273	HH22	ARG	222	67.733	11.320	6.068	1.00	0.00	RX1	H
ATOM	4274	C	ARG	222	72.433	15.708	5.216	1.00	0.00	RX1	C
ATOM	4275	O	ARG	222	73.100	16.200	4.309	1.00	0.00	RX1	O
ATOM	4276	N	CYS	223	72.782	14.590	5.861	1.00	0.00	RX1	N
ATOM	4277	H	CYS	223	72.203	14.209	6.583	1.00	0.00	RX1	H
ATOM	4278	CA	CYS	223	73.941	13.844	5.373	1.00	0.00	RX1	C
ATOM	4279	CB	CYS	223	74.600	13.068	6.507	1.00	0.00	RX1	C
ATOM	4280	SG	CYS	223	73.455	12.045	7.460	1.00	0.00	RX1	S
ATOM	4281	C	CYS	223	73.662	12.986	4.152	1.00	0.00	RX1	C
ATOM	4282	O	CYS	223	73.541	11.763	4.176	1.00	0.00	RX1	O
ATOM	4283	N	ARG	224	73.590	13.730	3.042	1.00	0.00	RX1	N
ATOM	4284	H	ARG	224	73.643	14.724	3.149	1.00	0.00	RX1	H
ATOM	4285	CA	ARG	224	73.528	13.147	1.705	1.00	0.00	RX1	C
ATOM	4286	CB	ARG	224	73.481	14.338	0.763	1.00	0.00	RX1	C
ATOM	4287	CG	ARG	224	73.602	14.105	-0.731	1.00	0.00	RX1	C
ATOM	4288	CD	ARG	224	73.547	15.482	-1.385	1.00	0.00	RX1	C
ATOM	4289	NE	ARG	224	74.174	15.467	-2.696	1.00	0.00	RX1	N
ATOM	4290	HE	ARG	224	73.639	15.132	-3.483	1.00	0.00	RX1	H
ATOM	4291	CZ	ARG	224	75.501	15.769	-2.702	1.00	0.00	RX1	C
ATOM	4292	NH1	ARG	224	76.055	16.303	-1.604	1.00	0.00	RX1	N
ATOM	4293	HH11	ARG	224	77.059	16.420	-1.566	1.00	0.00	RX1	H
ATOM	4294	HH12	ARG	224	75.562	16.613	-0.784	1.00	0.00	RX1	H
ATOM	4295	NH2	ARG	224	76.258	15.534	-3.787	1.00	0.00	RX1	N
ATOM	4296	HH21	ARG	224	77.268	15.562	-3.689	1.00	0.00	RX1	H
ATOM	4297	HH22	ARG	224	75.853	15.321	-4.682	1.00	0.00	RX1	H
ATOM	4298	C	ARG	224	74.707	12.213	1.468	1.00	0.00	RX1	C
ATOM	4299	O	ARG	224	74.555	11.001	1.320	1.00	0.00	RX1	O
ATOM	4300	N	GLY	225	75.900	12.830	1.549	1.00	0.00	RX1	N
ATOM	4301	H	GLY	225	75.972	13.827	1.587	1.00	0.00	RX1	H
ATOM	4302	CA	GLY	225	77.140	12.060	1.616	1.00	0.00	RX1	C
ATOM	4303	C	GLY	225	77.126	11.078	2.769	1.00	0.00	RX1	C
ATOM	4304	O	GLY	225	76.264	11.109	3.642	1.00	0.00	RX1	O
ATOM	4305	N	LYS	226	78.070	10.139	2.709	1.00	0.00	RX1	N
ATOM	4306	H	LYS	226	78.818	10.142	2.045	1.00	0.00	RX1	H
ATOM	4307	CA	LYS	226	77.871	9.045	3.655	1.00	0.00	RX1	C
ATOM	4308	CB	LYS	226	77.682	7.739	2.879	1.00	0.00	RX1	C
ATOM	4309	CG	LYS	226	76.597	7.884	1.798	1.00	0.00	RX1	C
ATOM	4310	CD	LYS	226	75.178	7.423	2.169	1.00	0.00	RX1	C
ATOM	4311	CE	LYS	226	74.524	7.994	3.435	1.00	0.00	RX1	C
ATOM	4312	NZ	LYS	226	74.197	9.429	3.357	1.00	0.00	RX1	N
ATOM	4313	HZ1	LYS	226	73.356	9.637	3.942	1.00	0.00	RX1	H
ATOM	4314	HZ2	LYS	226	74.949	10.041	3.737	1.00	0.00	RX1	H
ATOM	4315	HZ3	LYS	226	74.016	9.756	2.386	1.00	0.00	RX1	H
ATOM	4316	C	LYS	226	78.878	8.945	4.786	1.00	0.00	RX1	C
ATOM	4317	O	LYS	226	78.833	8.030	5.597	1.00	0.00	RX1	O
ATOM	4318	N	SER	227	79.782	9.938	4.788	1.00	0.00	RX1	N
ATOM	4319	H	SER	227	79.766	10.666	4.107	1.00	0.00	RX1	H
ATOM	4320	CA	SER	227	80.771	10.124	5.857	1.00	0.00	RX1	C
ATOM	4321	CB	SER	227	81.728	8.920	5.951	1.00	0.00	RX1	C
ATOM	4322	OG	SER	227	81.388	7.958	4.945	1.00	0.00	RX1	O
ATOM	4323	HG	SER	227	80.635	7.492	5.302	1.00	0.00	RX1	H
ATOM	4324	C	SER	227	81.492	11.472	5.794	1.00	0.00	RX1	C
ATOM	4325	O	SER	227	81.462	12.227	6.757	1.00	0.00	RX1	O
ATOM	4326	N	PRO	228	82.130	11.810	4.632	1.00	0.00	RX1	N
ATOM	4327	CD	PRO	228	82.375	11.062	3.394	1.00	0.00	RX1	C
ATOM	4328	CA	PRO	228	82.656	13.177	4.549	1.00	0.00	RX1	C
ATOM	4329	CB	PRO	228	83.609	13.091	3.356	1.00	0.00	RX1	C

ATOM	4330	CG	PRO	228	82.938	12.090	2.421	1.00	0.00	RX1	C
ATOM	4331	C	PRO	228	81.525	14.167	4.318	1.00	0.00	RX1	C
ATOM	4332	O	PRO	228	80.396	13.791	4.016	1.00	0.00	RX1	O
ATOM	4333	N	SER	229	81.889	15.448	4.445	1.00	0.00	RX1	N
ATOM	4334	H	SER	229	82.785	15.744	4.776	1.00	0.00	RX1	H
ATOM	4335	CA	SER	229	80.890	16.510	4.343	1.00	0.00	RX1	C
ATOM	4336	CB	SER	229	81.512	17.674	5.086	1.00	0.00	RX1	C
ATOM	4337	OG	SER	229	82.175	17.107	6.221	1.00	0.00	RX1	O
ATOM	4338	HG	SER	229	81.609	17.349	6.950	1.00	0.00	RX1	H
ATOM	4339	C	SER	229	80.349	16.878	2.962	1.00	0.00	RX1	C
ATOM	4340	O	SER	229	80.139	18.047	2.661	1.00	0.00	RX1	O
ATOM	4341	N	ASP	230	80.089	15.852	2.131	1.00	0.00	RX1	N
ATOM	4342	H	ASP	230	80.154	14.907	2.446	1.00	0.00	RX1	H
ATOM	4343	CA	ASP	230	79.325	16.157	0.919	1.00	0.00	RX1	C
ATOM	4344	CB	ASP	230	79.629	15.192	-0.231	1.00	0.00	RX1	C
ATOM	4345	CG	ASP	230	78.948	15.722	-1.480	1.00	0.00	RX1	C
ATOM	4346	OD1	ASP	230	78.532	14.940	-2.330	1.00	0.00	RX1	O
ATOM	4347	OD2	ASP	230	78.749	16.929	-1.580	1.00	0.00	RX1	O
ATOM	4348	C	ASP	230	77.838	16.185	1.242	1.00	0.00	RX1	C
ATOM	4349	O	ASP	230	77.046	15.296	0.953	1.00	0.00	RX1	O
ATOM	4350	N	CYS	231	77.498	17.248	1.965	1.00	0.00	RX1	N
ATOM	4351	H	CYS	231	78.140	18.010	2.073	1.00	0.00	RX1	H
ATOM	4352	CA	CYS	231	76.185	17.235	2.595	1.00	0.00	RX1	C
ATOM	4353	CB	CYS	231	76.259	18.034	3.887	1.00	0.00	RX1	C
ATOM	4354	SG	CYS	231	77.730	17.595	4.841	1.00	0.00	RX1	S
ATOM	4355	C	CYS	231	75.071	17.682	1.669	1.00	0.00	RX1	C
ATOM	4356	O	CYS	231	75.250	17.848	0.467	1.00	0.00	RX1	O
ATOM	4357	N	CYS	232	73.901	17.881	2.276	1.00	0.00	RX1	N
ATOM	4358	H	CYS	232	73.743	17.686	3.244	1.00	0.00	RX1	H
ATOM	4359	CA	CYS	232	72.900	18.658	1.562	1.00	0.00	RX1	C
ATOM	4360	CB	CYS	232	71.512	18.223	2.020	1.00	0.00	RX1	C
ATOM	4361	SG	CYS	232	71.163	16.496	1.620	1.00	0.00	RX1	S
ATOM	4362	C	CYS	232	73.120	20.130	1.823	1.00	0.00	RX1	C
ATOM	4363	O	CYS	232	73.924	20.508	2.668	1.00	0.00	RX1	O
ATOM	4364	N	HIS	233	72.362	20.943	1.078	1.00	0.00	RX1	N
ATOM	4365	H	HIS	233	71.701	20.572	0.426	1.00	0.00	RX1	H
ATOM	4366	CA	HIS	233	72.192	22.317	1.540	1.00	0.00	RX1	C
ATOM	4367	CB	HIS	233	71.744	23.150	0.333	1.00	0.00	RX1	C
ATOM	4368	CG	HIS	233	71.264	24.542	0.675	1.00	0.00	RX1	C
ATOM	4369	ND1	HIS	233	70.115	25.052	0.195	1.00	0.00	RX1	N
ATOM	4370	HD1	HIS	233	69.500	24.620	-0.443	1.00	0.00	RX1	H
ATOM	4371	CD2	HIS	233	71.878	25.502	1.485	1.00	0.00	RX1	C
ATOM	4372	NE2	HIS	233	71.077	26.595	1.494	1.00	0.00	RX1	N
ATOM	4373	CE1	HIS	233	69.994	26.324	0.696	1.00	0.00	RX1	C
ATOM	4374	C	HIS	233	71.159	22.300	2.653	1.00	0.00	RX1	C
ATOM	4375	O	HIS	233	70.281	21.448	2.667	1.00	0.00	RX1	O
ATOM	4376	N	ASN	234	71.275	23.285	3.551	1.00	0.00	RX1	N
ATOM	4377	H	ASN	234	72.159	23.749	3.601	1.00	0.00	RX1	H
ATOM	4378	CA	ASN	234	70.299	23.482	4.628	1.00	0.00	RX1	C
ATOM	4379	CB	ASN	234	70.549	24.827	5.308	1.00	0.00	RX1	C
ATOM	4380	CG	ASN	234	69.250	25.595	5.400	1.00	0.00	RX1	C
ATOM	4381	OD1	ASN	234	68.412	25.358	6.266	1.00	0.00	RX1	O
ATOM	4382	ND2	ASN	234	69.128	26.536	4.446	1.00	0.00	RX1	N
ATOM	4383	HD21	ASN	234	69.871	26.778	3.817	1.00	0.00	RX1	H
ATOM	4384	HD22	ASN	234	68.252	27.012	4.322	1.00	0.00	RX1	H
ATOM	4385	C	ASN	234	68.825	23.321	4.245	1.00	0.00	RX1	C
ATOM	4386	O	ASN	234	68.009	22.807	4.998	1.00	0.00	RX1	O
ATOM	4387	N	GLN	235	68.524	23.813	3.034	1.00	0.00	RX1	N
ATOM	4388	H	GLN	235	69.230	24.148	2.413	1.00	0.00	RX1	H
ATOM	4389	CA	GLN	235	67.121	23.802	2.633	1.00	0.00	RX1	C
ATOM	4390	CB	GLN	235	66.825	24.975	1.700	1.00	0.00	RX1	C

ATOM	4391	CG	GLN	235	65.469	25.631	1.962	1.00	0.00	RX1	C
ATOM	4392	CD	GLN	235	65.466	26.284	3.328	1.00	0.00	RX1	C
ATOM	4393	OE1	GLN	235	66.419	26.219	4.095	1.00	0.00	RX1	O
ATOM	4394	NE2	GLN	235	64.319	26.909	3.606	1.00	0.00	RX1	N
ATOM	4395	HE21	GLN	235	63.648	27.104	2.887	1.00	0.00	RX1	H
ATOM	4396	HE22	GLN	235	64.141	27.300	4.514	1.00	0.00	RX1	H
ATOM	4397	C	GLN	235	66.594	22.496	2.072	1.00	0.00	RX1	C
ATOM	4398	O	GLN	235	65.396	22.302	1.890	1.00	0.00	RX1	O
ATOM	4399	N	CYS	236	67.558	21.613	1.797	1.00	0.00	RX1	N
ATOM	4400	H	CYS	236	68.502	21.735	2.093	1.00	0.00	RX1	H
ATOM	4401	CA	CYS	236	67.191	20.300	1.293	1.00	0.00	RX1	C
ATOM	4402	CB	CYS	236	68.375	19.702	0.558	1.00	0.00	RX1	C
ATOM	4403	SG	CYS	236	69.285	20.948	-0.386	1.00	0.00	RX1	S
ATOM	4404	C	CYS	236	66.773	19.391	2.415	1.00	0.00	RX1	C
ATOM	4405	O	CYS	236	67.593	18.865	3.159	1.00	0.00	RX1	O
ATOM	4406	N	ALA	237	65.453	19.229	2.500	1.00	0.00	RX1	N
ATOM	4407	H	ALA	237	64.867	19.551	1.755	1.00	0.00	RX1	H
ATOM	4408	CA	ALA	237	64.994	18.175	3.389	1.00	0.00	RX1	C
ATOM	4409	CB	ALA	237	63.510	18.374	3.686	1.00	0.00	RX1	C
ATOM	4410	C	ALA	237	65.230	16.849	2.693	1.00	0.00	RX1	C
ATOM	4411	O	ALA	237	65.305	16.813	1.469	1.00	0.00	RX1	O
ATOM	4412	N	ALA	238	65.382	15.790	3.504	1.00	0.00	RX1	N
ATOM	4413	H	ALA	238	65.306	15.945	4.494	1.00	0.00	RX1	H
ATOM	4414	CA	ALA	238	65.746	14.458	3.000	1.00	0.00	RX1	C
ATOM	4415	CB	ALA	238	64.657	13.874	2.092	1.00	0.00	RX1	C
ATOM	4416	C	ALA	238	67.101	14.343	2.306	1.00	0.00	RX1	C
ATOM	4417	O	ALA	238	68.039	13.770	2.849	1.00	0.00	RX1	O
ATOM	4418	N	GLY	239	67.171	14.897	1.087	1.00	0.00	RX1	N
ATOM	4419	H	GLY	239	66.435	15.453	0.691	1.00	0.00	RX1	H
ATOM	4420	CA	GLY	239	68.407	14.808	0.322	1.00	0.00	RX1	C
ATOM	4421	C	GLY	239	68.413	15.809	-0.816	1.00	0.00	RX1	C
ATOM	4422	O	GLY	239	67.478	16.584	-1.004	1.00	0.00	RX1	O
ATOM	4423	N	CYS	240	69.509	15.756	-1.583	1.00	0.00	RX1	N
ATOM	4424	H	CYS	240	70.271	15.117	-1.470	1.00	0.00	RX1	H
ATOM	4425	CA	CYS	240	69.621	16.672	-2.716	1.00	0.00	RX1	C
ATOM	4426	CB	CYS	240	70.038	18.056	-2.226	1.00	0.00	RX1	C
ATOM	4427	SG	CYS	240	71.586	18.081	-1.303	1.00	0.00	RX1	S
ATOM	4428	C	CYS	240	70.577	16.136	-3.754	1.00	0.00	RX1	C
ATOM	4429	O	CYS	240	71.296	15.175	-3.507	1.00	0.00	RX1	O
ATOM	4430	N	THR	241	70.585	16.808	-4.908	1.00	0.00	RX1	N
ATOM	4431	H	THR	241	69.938	17.539	-5.138	1.00	0.00	RX1	H
ATOM	4432	CA	THR	241	71.616	16.486	-5.888	1.00	0.00	RX1	C
ATOM	4433	CB	THR	241	71.098	17.164	-7.134	1.00	0.00	RX1	C
ATOM	4434	OG1	THR	241	69.668	17.171	-7.023	1.00	0.00	RX1	O
ATOM	4435	HG1	THR	241	69.341	17.757	-7.695	1.00	0.00	RX1	H
ATOM	4436	CG2	THR	241	71.570	16.502	-8.429	1.00	0.00	RX1	C
ATOM	4437	C	THR	241	72.997	16.950	-5.437	1.00	0.00	RX1	C
ATOM	4438	O	THR	241	73.972	16.202	-5.392	1.00	0.00	RX1	O
ATOM	4439	N	GLY	242	73.012	18.232	-5.050	1.00	0.00	RX1	N
ATOM	4440	H	GLY	242	72.203	18.825	-5.022	1.00	0.00	RX1	H
ATOM	4441	CA	GLY	242	74.241	18.809	-4.521	1.00	0.00	RX1	C
ATOM	4442	C	GLY	242	73.897	19.772	-3.407	1.00	0.00	RX1	C
ATOM	4443	O	GLY	242	72.743	20.149	-3.239	1.00	0.00	RX1	O
ATOM	4444	N	PRO	243	74.934	20.156	-2.632	1.00	0.00	RX1	N
ATOM	4445	CD	PRO	243	76.315	19.705	-2.752	1.00	0.00	RX1	C
ATOM	4446	CA	PRO	243	74.715	21.094	-1.525	1.00	0.00	RX1	C
ATOM	4447	CB	PRO	243	75.988	20.892	-0.697	1.00	0.00	RX1	C
ATOM	4448	CG	PRO	243	77.069	20.533	-1.717	1.00	0.00	RX1	C
ATOM	4449	C	PRO	243	74.519	22.536	-1.987	1.00	0.00	RX1	C
ATOM	4450	O	PRO	243	75.356	23.405	-1.782	1.00	0.00	RX1	O
ATOM	4451	N	ARG	244	73.364	22.766	-2.621	1.00	0.00	RX1	N

ATOM	4452	H	ARG	244	72.655	22.071	-2.766	1.00	0.00	RX1	H
ATOM	4453	CA	ARG	244	73.165	24.077	-3.221	1.00	0.00	RX1	C
ATOM	4454	CB	ARG	244	73.580	23.983	-4.677	1.00	0.00	RX1	C
ATOM	4455	CG	ARG	244	74.637	24.934	-5.249	1.00	0.00	RX1	C
ATOM	4456	CD	ARG	244	74.371	26.447	-5.184	1.00	0.00	RX1	C
ATOM	4457	NE	ARG	244	73.142	27.019	-5.776	1.00	0.00	RX1	N
ATOM	4458	HE	ARG	244	73.001	27.970	-5.477	1.00	0.00	RX1	H
ATOM	4459	CZ	ARG	244	72.200	26.534	-6.662	1.00	0.00	RX1	C
ATOM	4460	NH1	ARG	244	72.226	25.285	-7.187	1.00	0.00	RX1	N
ATOM	4461	HH11	ARG	244	71.482	24.892	-7.748	1.00	0.00	RX1	H
ATOM	4462	HH12	ARG	244	72.949	24.600	-7.032	1.00	0.00	RX1	H
ATOM	4463	NH2	ARG	244	71.179	27.356	-6.975	1.00	0.00	RX1	N
ATOM	4464	HH21	ARG	244	70.425	27.035	-7.565	1.00	0.00	RX1	H
ATOM	4465	HH22	ARG	244	71.076	28.299	-6.615	1.00	0.00	RX1	H
ATOM	4466	C	ARG	244	71.717	24.511	-3.106	1.00	0.00	RX1	C
ATOM	4467	O	ARG	244	70.796	23.710	-3.027	1.00	0.00	RX1	O
ATOM	4468	N	GLU	245	71.572	25.841	-3.119	1.00	0.00	RX1	N
ATOM	4469	H	GLU	245	72.404	26.392	-3.126	1.00	0.00	RX1	H
ATOM	4470	CA	GLU	245	70.284	26.535	-3.042	1.00	0.00	RX1	C
ATOM	4471	CB	GLU	245	70.505	27.948	-3.558	1.00	0.00	RX1	C
ATOM	4472	CG	GLU	245	69.259	28.820	-3.630	1.00	0.00	RX1	C
ATOM	4473	CD	GLU	245	69.502	29.795	-4.754	1.00	0.00	RX1	C
ATOM	4474	OE1	GLU	245	68.759	30.766	-4.873	1.00	0.00	RX1	O
ATOM	4475	OE2	GLU	245	70.429	29.559	-5.526	1.00	0.00	RX1	O
ATOM	4476	C	GLU	245	69.073	25.900	-3.732	1.00	0.00	RX1	C
ATOM	4477	O	GLU	245	67.996	25.814	-3.158	1.00	0.00	RX1	O
ATOM	4478	N	SER	246	69.267	25.491	-4.993	1.00	0.00	RX1	N
ATOM	4479	H	SER	246	70.129	25.466	-5.492	1.00	0.00	RX1	H
ATOM	4480	CA	SER	246	68.080	24.986	-5.680	1.00	0.00	RX1	C
ATOM	4481	CB	SER	246	68.079	25.588	-7.078	1.00	0.00	RX1	C
ATOM	4482	OG	SER	246	69.421	25.592	-7.575	1.00	0.00	RX1	O
ATOM	4483	HG	SER	246	69.529	24.688	-7.888	1.00	0.00	RX1	H
ATOM	4484	C	SER	246	67.931	23.483	-5.689	1.00	0.00	RX1	C
ATOM	4485	O	SER	246	66.897	22.945	-6.060	1.00	0.00	RX1	O
ATOM	4486	N	ASP	247	69.024	22.813	-5.313	1.00	0.00	RX1	N
ATOM	4487	H	ASP	247	69.768	23.204	-4.773	1.00	0.00	RX1	H
ATOM	4488	CA	ASP	247	69.160	21.464	-5.858	1.00	0.00	RX1	C
ATOM	4489	CB	ASP	247	70.632	21.210	-6.231	1.00	0.00	RX1	C
ATOM	4490	CG	ASP	247	71.226	22.341	-7.080	1.00	0.00	RX1	C
ATOM	4491	OD1	ASP	247	70.497	23.108	-7.703	1.00	0.00	RX1	O
ATOM	4492	OD2	ASP	247	72.444	22.488	-7.107	1.00	0.00	RX1	O
ATOM	4493	C	ASP	247	68.580	20.383	-4.961	1.00	0.00	RX1	C
ATOM	4494	O	ASP	247	69.103	19.283	-4.823	1.00	0.00	RX1	O
ATOM	4495	N	CYS	248	67.476	20.776	-4.313	1.00	0.00	RX1	N
ATOM	4496	H	CYS	248	66.972	21.570	-4.652	1.00	0.00	RX1	H
ATOM	4497	CA	CYS	248	66.846	19.939	-3.300	1.00	0.00	RX1	C
ATOM	4498	CB	CYS	248	66.036	20.854	-2.385	1.00	0.00	RX1	C
ATOM	4499	SG	CYS	248	66.969	22.325	-1.886	1.00	0.00	RX1	S
ATOM	4500	C	CYS	248	65.956	18.889	-3.922	1.00	0.00	RX1	C
ATOM	4501	O	CYS	248	65.285	19.144	-4.911	1.00	0.00	RX1	O
ATOM	4502	N	LEU	249	65.934	17.704	-3.291	1.00	0.00	RX1	N
ATOM	4503	H	LEU	249	66.466	17.515	-2.463	1.00	0.00	RX1	H
ATOM	4504	CA	LEU	249	64.892	16.780	-3.743	1.00	0.00	RX1	C
ATOM	4505	CB	LEU	249	65.195	15.348	-3.303	1.00	0.00	RX1	C
ATOM	4506	CG	LEU	249	66.513	14.816	-3.865	1.00	0.00	RX1	C
ATOM	4507	CD1	LEU	249	66.815	13.409	-3.350	1.00	0.00	RX1	C
ATOM	4508	CD2	LEU	249	66.566	14.898	-5.392	1.00	0.00	RX1	C
ATOM	4509	C	LEU	249	63.531	17.210	-3.222	1.00	0.00	RX1	C
ATOM	4510	O	LEU	249	62.550	17.406	-3.939	1.00	0.00	RX1	O
ATOM	4511	N	VAL	250	63.530	17.403	-1.899	1.00	0.00	RX1	N
ATOM	4512	H	VAL	250	64.327	17.274	-1.306	1.00	0.00	RX1	H

ATOM	4513	CA	VAL	250	62.346	18.019	-1.323	1.00	0.00	RX1	C
ATOM	4514	CB	VAL	250	61.527	17.011	-0.518	1.00	0.00	RX1	C
ATOM	4515	CG1	VAL	250	60.941	15.941	-1.436	1.00	0.00	RX1	C
ATOM	4516	CG2	VAL	250	62.341	16.383	0.602	1.00	0.00	RX1	C
ATOM	4517	C	VAL	250	62.719	19.241	-0.513	1.00	0.00	RX1	C
ATOM	4518	O	VAL	250	63.810	19.355	0.036	1.00	0.00	RX1	O
ATOM	4519	N	CYS	251	61.781	20.189	-0.513	1.00	0.00	RX1	N
ATOM	4520	H	CYS	251	60.876	20.033	-0.912	1.00	0.00	RX1	H
ATOM	4521	CA	CYS	251	62.169	21.472	0.068	1.00	0.00	RX1	C
ATOM	4522	CB	CYS	251	61.674	22.571	-0.865	1.00	0.00	RX1	C
ATOM	4523	SG	CYS	251	61.852	22.040	-2.586	1.00	0.00	RX1	S
ATOM	4524	C	CYS	251	61.673	21.639	1.486	1.00	0.00	RX1	C
ATOM	4525	O	CYS	251	60.551	21.247	1.794	1.00	0.00	RX1	O
ATOM	4526	N	ARG	252	62.524	22.257	2.325	1.00	0.00	RX1	N
ATOM	4527	H	ARG	252	63.463	22.465	2.042	1.00	0.00	RX1	H
ATOM	4528	CA	ARG	252	62.014	22.634	3.645	1.00	0.00	RX1	C
ATOM	4529	CB	ARG	252	63.119	23.046	4.627	1.00	0.00	RX1	C
ATOM	4530	CG	ARG	252	62.522	23.232	6.030	1.00	0.00	RX1	C
ATOM	4531	CD	ARG	252	63.511	23.536	7.162	1.00	0.00	RX1	C
ATOM	4532	NE	ARG	252	64.059	24.892	7.109	1.00	0.00	RX1	N
ATOM	4533	HE	ARG	252	63.461	25.667	7.348	1.00	0.00	RX1	H
ATOM	4534	CZ	ARG	252	65.390	25.047	6.853	1.00	0.00	RX1	C
ATOM	4535	NH1	ARG	252	66.140	23.960	6.580	1.00	0.00	RX1	N
ATOM	4536	HH11	ARG	252	67.117	24.062	6.336	1.00	0.00	RX1	H
ATOM	4537	HH12	ARG	252	65.792	23.022	6.585	1.00	0.00	RX1	H
ATOM	4538	NH2	ARG	252	65.946	26.272	6.864	1.00	0.00	RX1	N
ATOM	4539	HH21	ARG	252	66.934	26.378	6.692	1.00	0.00	RX1	H
ATOM	4540	HH22	ARG	252	65.415	27.121	7.008	1.00	0.00	RX1	H
ATOM	4541	C	ARG	252	60.953	23.722	3.560	1.00	0.00	RX1	C
ATOM	4542	O	ARG	252	59.788	23.521	3.871	1.00	0.00	RX1	O
ATOM	4543	N	LYS	253	61.433	24.894	3.114	1.00	0.00	RX1	N
ATOM	4544	H	LYS	253	62.367	24.983	2.778	1.00	0.00	RX1	H
ATOM	4545	CA	LYS	253	60.514	26.026	3.060	1.00	0.00	RX1	C
ATOM	4546	CB	LYS	253	60.926	27.105	4.065	1.00	0.00	RX1	C
ATOM	4547	CG	LYS	253	60.550	26.720	5.497	1.00	0.00	RX1	C
ATOM	4548	CD	LYS	253	61.253	27.582	6.540	1.00	0.00	RX1	C
ATOM	4549	CE	LYS	253	61.023	29.082	6.359	1.00	0.00	RX1	C
ATOM	4550	NZ	LYS	253	62.068	29.832	7.069	1.00	0.00	RX1	N
ATOM	4551	HZ1	LYS	253	62.313	30.695	6.536	1.00	0.00	RX1	H
ATOM	4552	HZ2	LYS	253	61.834	30.077	8.046	1.00	0.00	RX1	H
ATOM	4553	HZ3	LYS	253	62.976	29.308	7.072	1.00	0.00	RX1	H
ATOM	4554	C	LYS	253	60.307	26.568	1.654	1.00	0.00	RX1	C
ATOM	4555	O	LYS	253	59.670	25.927	0.830	1.00	0.00	RX1	O
ATOM	4556	N	PHE	254	60.834	27.779	1.410	1.00	0.00	RX1	N
ATOM	4557	H	PHE	254	61.601	28.198	1.897	1.00	0.00	RX1	H
ATOM	4558	CA	PHE	254	60.317	28.485	0.243	1.00	0.00	RX1	C
ATOM	4559	CB	PHE	254	60.550	29.984	0.398	1.00	0.00	RX1	C
ATOM	4560	CG	PHE	254	59.845	30.456	1.645	1.00	0.00	RX1	C
ATOM	4561	CD1	PHE	254	60.542	31.170	2.612	1.00	0.00	RX1	C
ATOM	4562	CD2	PHE	254	58.496	30.174	1.828	1.00	0.00	RX1	C
ATOM	4563	CE1	PHE	254	59.890	31.597	3.763	1.00	0.00	RX1	C
ATOM	4564	CE2	PHE	254	57.844	30.599	2.979	1.00	0.00	RX1	C
ATOM	4565	CZ	PHE	254	58.544	31.308	3.948	1.00	0.00	RX1	C
ATOM	4566	C	PHE	254	60.858	28.002	-1.081	1.00	0.00	RX1	C
ATOM	4567	O	PHE	254	62.057	28.023	-1.334	1.00	0.00	RX1	O
ATOM	4568	N	ARG	255	59.912	27.604	-1.937	1.00	0.00	RX1	N
ATOM	4569	H	ARG	255	58.951	27.685	-1.671	1.00	0.00	RX1	H
ATOM	4570	CA	ARG	255	60.299	27.465	-3.334	1.00	0.00	RX1	C
ATOM	4571	CB	ARG	255	59.353	26.545	-4.105	1.00	0.00	RX1	C
ATOM	4572	CG	ARG	255	60.103	25.461	-4.883	1.00	0.00	RX1	C
ATOM	4573	CD	ARG	255	59.294	24.903	-6.058	1.00	0.00	RX1	C

ATOM	4574	NE	ARG	255	59.203	25.906	-7.116	1.00	0.00	RX1	N
ATOM	4575	HE	ARG	255	59.848	26.678	-7.098	1.00	0.00	RX1	H
ATOM	4576	CZ	ARG	255	58.373	25.741	-8.188	1.00	0.00	RX1	C
ATOM	4577	NH1	ARG	255	57.570	24.674	-8.283	1.00	0.00	RX1	N
ATOM	4578	HH11	ARG	255	56.925	24.633	-9.064	1.00	0.00	RX1	H
ATOM	4579	HH12	ARG	255	57.593	23.878	-7.670	1.00	0.00	RX1	H
ATOM	4580	NH2	ARG	255	58.373	26.644	-9.178	1.00	0.00	RX1	N
ATOM	4581	HH21	ARG	255	57.796	26.473	-10.001	1.00	0.00	RX1	H
ATOM	4582	HH22	ARG	255	58.965	27.455	-9.164	1.00	0.00	RX1	H
ATOM	4583	C	ARG	255	60.327	28.824	-4.004	1.00	0.00	RX1	C
ATOM	4584	O	ARG	255	59.332	29.322	-4.520	1.00	0.00	RX1	O
ATOM	4585	N	ASP	256	61.518	29.420	-3.942	1.00	0.00	RX1	N
ATOM	4586	H	ASP	256	62.292	28.883	-3.605	1.00	0.00	RX1	H
ATOM	4587	CA	ASP	256	61.712	30.692	-4.629	1.00	0.00	RX1	C
ATOM	4588	CB	ASP	256	62.937	31.414	-4.103	1.00	0.00	RX1	C
ATOM	4589	CG	ASP	256	63.052	32.726	-4.839	1.00	0.00	RX1	C
ATOM	4590	OD1	ASP	256	62.017	33.300	-5.126	1.00	0.00	RX1	O
ATOM	4591	OD2	ASP	256	64.154	33.181	-5.145	1.00	0.00	RX1	O
ATOM	4592	C	ASP	256	61.880	30.490	-6.117	1.00	0.00	RX1	C
ATOM	4593	O	ASP	256	62.984	30.277	-6.611	1.00	0.00	RX1	O
ATOM	4594	N	GLU	257	60.730	30.511	-6.802	1.00	0.00	RX1	N
ATOM	4595	H	GLU	257	59.903	30.783	-6.303	1.00	0.00	RX1	H
ATOM	4596	CA	GLU	257	60.680	30.201	-8.230	1.00	0.00	RX1	C
ATOM	4597	CB	GLU	257	61.221	31.380	-9.039	1.00	0.00	RX1	C
ATOM	4598	CG	GLU	257	60.297	32.581	-8.841	1.00	0.00	RX1	C
ATOM	4599	CD	GLU	257	60.973	33.849	-9.306	1.00	0.00	RX1	C
ATOM	4600	OE1	GLU	257	62.196	33.896	-9.346	1.00	0.00	RX1	O
ATOM	4601	OE2	GLU	257	60.290	34.827	-9.586	1.00	0.00	RX1	O
ATOM	4602	C	GLU	257	61.312	28.860	-8.568	1.00	0.00	RX1	C
ATOM	4603	O	GLU	257	60.631	27.846	-8.522	1.00	0.00	RX1	O
ATOM	4604	N	ALA	258	62.617	28.866	-8.871	1.00	0.00	RX1	N
ATOM	4605	H	ALA	258	63.167	29.696	-8.761	1.00	0.00	RX1	H
ATOM	4606	CA	ALA	258	63.260	27.574	-9.105	1.00	0.00	RX1	C
ATOM	4607	CB	ALA	258	63.881	27.533	-10.502	1.00	0.00	RX1	C
ATOM	4608	C	ALA	258	64.336	27.229	-8.083	1.00	0.00	RX1	C
ATOM	4609	O	ALA	258	65.249	26.459	-8.341	1.00	0.00	RX1	O
ATOM	4610	N	THR	259	64.222	27.870	-6.914	1.00	0.00	RX1	N
ATOM	4611	H	THR	259	63.429	28.417	-6.653	1.00	0.00	RX1	H
ATOM	4612	CA	THR	259	65.259	27.708	-5.898	1.00	0.00	RX1	C
ATOM	4613	CB	THR	259	66.173	28.949	-5.896	1.00	0.00	RX1	C
ATOM	4614	OG1	THR	259	65.446	30.135	-5.549	1.00	0.00	RX1	O
ATOM	4615	HG1	THR	259	64.746	30.225	-6.197	1.00	0.00	RX1	H
ATOM	4616	CG2	THR	259	66.898	29.163	-7.228	1.00	0.00	RX1	C
ATOM	4617	C	THR	259	64.619	27.437	-4.549	1.00	0.00	RX1	C
ATOM	4618	O	THR	259	63.400	27.494	-4.430	1.00	0.00	RX1	O
ATOM	4619	N	CYS	260	65.459	27.179	-3.537	1.00	0.00	RX1	N
ATOM	4620	H	CYS	260	66.451	27.057	-3.604	1.00	0.00	RX1	H
ATOM	4621	CA	CYS	260	64.858	27.006	-2.219	1.00	0.00	RX1	C
ATOM	4622	CB	CYS	260	64.950	25.533	-1.830	1.00	0.00	RX1	C
ATOM	4623	SG	CYS	260	64.298	24.444	-3.120	1.00	0.00	RX1	S
ATOM	4624	C	CYS	260	65.436	27.937	-1.168	1.00	0.00	RX1	C
ATOM	4625	O	CYS	260	66.534	27.744	-0.659	1.00	0.00	RX1	O
ATOM	4626	N	LYS	261	64.653	28.982	-0.869	1.00	0.00	RX1	N
ATOM	4627	H	LYS	261	63.700	28.997	-1.175	1.00	0.00	RX1	H
ATOM	4628	CA	LYS	261	65.111	29.910	0.164	1.00	0.00	RX1	C
ATOM	4629	CB	LYS	261	64.751	31.358	-0.167	1.00	0.00	RX1	C
ATOM	4630	CG	LYS	261	65.162	31.889	-1.541	1.00	0.00	RX1	C
ATOM	4631	CD	LYS	261	66.657	31.917	-1.853	1.00	0.00	RX1	C
ATOM	4632	CE	LYS	261	66.958	32.731	-3.121	1.00	0.00	RX1	C
ATOM	4633	NZ	LYS	261	66.490	32.072	-4.350	1.00	0.00	RX1	N
ATOM	4634	HZ1	LYS	261	65.955	31.194	-4.196	1.00	0.00	RX1	H

ATOM	4635	HZ2	LYS	261	65.856	32.691	-4.900	1.00	0.00	RX1	H
ATOM	4636	HZ3	LYS	261	67.315	31.788	-4.924	1.00	0.00	RX1	H
ATOM	4637	C	LYS	261	64.542	29.584	1.535	1.00	0.00	RX1	C
ATOM	4638	O	LYS	261	63.632	28.770	1.689	1.00	0.00	RX1	O
ATOM	4639	N	ASP	262	65.120	30.277	2.530	1.00	0.00	RX1	N
ATOM	4640	H	ASP	262	65.838	30.951	2.363	1.00	0.00	RX1	H
ATOM	4641	CA	ASP	262	64.518	30.180	3.858	1.00	0.00	RX1	C
ATOM	4642	CB	ASP	262	65.553	29.868	4.936	1.00	0.00	RX1	C
ATOM	4643	CG	ASP	262	64.786	29.450	6.171	1.00	0.00	RX1	C
ATOM	4644	OD1	ASP	262	64.282	28.334	6.221	1.00	0.00	RX1	O
ATOM	4645	OD2	ASP	262	64.602	30.254	7.073	1.00	0.00	RX1	O
ATOM	4646	C	ASP	262	63.687	31.386	4.258	1.00	0.00	RX1	C
ATOM	4647	O	ASP	262	62.698	31.280	4.979	1.00	0.00	RX1	O
ATOM	4648	N	THR	263	64.121	32.537	3.748	1.00	0.00	RX1	N
ATOM	4649	H	THR	263	64.948	32.710	3.217	1.00	0.00	RX1	H
ATOM	4650	CA	THR	263	63.318	33.729	3.966	1.00	0.00	RX1	C
ATOM	4651	CB	THR	263	64.277	34.719	4.584	1.00	0.00	RX1	C
ATOM	4652	OG1	THR	263	65.576	34.541	4.006	1.00	0.00	RX1	O
ATOM	4653	HG1	THR	263	65.980	35.403	4.148	1.00	0.00	RX1	H
ATOM	4654	CG2	THR	263	64.372	34.493	6.091	1.00	0.00	RX1	C
ATOM	4655	C	THR	263	62.709	34.164	2.652	1.00	0.00	RX1	C
ATOM	4656	O	THR	263	63.026	33.607	1.604	1.00	0.00	RX1	O
ATOM	4657	N	CYS	264	61.852	35.183	2.739	1.00	0.00	RX1	N
ATOM	4658	H	CYS	264	61.552	35.587	3.607	1.00	0.00	RX1	H
ATOM	4659	CA	CYS	264	61.454	35.825	1.492	1.00	0.00	RX1	C
ATOM	4660	CB	CYS	264	59.935	35.848	1.425	1.00	0.00	RX1	C
ATOM	4661	SG	CYS	264	59.214	34.246	1.863	1.00	0.00	RX1	S
ATOM	4662	C	CYS	264	62.041	37.219	1.397	1.00	0.00	RX1	C
ATOM	4663	O	CYS	264	61.566	38.144	2.041	1.00	0.00	RX1	O
ATOM	4664	N	PRO	265	63.120	37.340	0.581	1.00	0.00	RX1	N
ATOM	4665	CD	PRO	265	63.726	36.280	-0.212	1.00	0.00	RX1	C
ATOM	4666	CA	PRO	265	63.817	38.629	0.454	1.00	0.00	RX1	C
ATOM	4667	CB	PRO	265	64.861	38.335	-0.630	1.00	0.00	RX1	C
ATOM	4668	CG	PRO	265	65.105	36.828	-0.555	1.00	0.00	RX1	C
ATOM	4669	C	PRO	265	62.909	39.797	0.091	1.00	0.00	RX1	C
ATOM	4670	O	PRO	265	62.325	39.837	-0.986	1.00	0.00	RX1	O
ATOM	4671	N	PRO	266	62.813	40.755	1.043	1.00	0.00	RX1	N
ATOM	4672	CD	PRO	266	63.519	40.771	2.315	1.00	0.00	RX1	C
ATOM	4673	CA	PRO	266	61.896	41.889	0.879	1.00	0.00	RX1	C
ATOM	4674	CB	PRO	266	62.169	42.729	2.130	1.00	0.00	RX1	C
ATOM	4675	CG	PRO	266	62.695	41.731	3.157	1.00	0.00	RX1	C
ATOM	4676	C	PRO	266	62.086	42.678	-0.403	1.00	0.00	RX1	C
ATOM	4677	O	PRO	266	63.093	42.580	-1.096	1.00	0.00	RX1	O
ATOM	4678	N	LEU	267	61.058	43.492	-0.682	1.00	0.00	RX1	N
ATOM	4679	H	LEU	267	60.244	43.495	-0.095	1.00	0.00	RX1	H
ATOM	4680	CA	LEU	267	61.114	44.317	-1.893	1.00	0.00	RX1	C
ATOM	4681	CB	LEU	267	59.814	45.108	-2.022	1.00	0.00	RX1	C
ATOM	4682	CG	LEU	267	58.795	44.478	-2.976	1.00	0.00	RX1	C
ATOM	4683	CD1	LEU	267	58.711	42.955	-2.854	1.00	0.00	RX1	C
ATOM	4684	CD2	LEU	267	57.429	45.148	-2.827	1.00	0.00	RX1	C
ATOM	4685	C	LEU	267	62.313	45.252	-1.988	1.00	0.00	RX1	C
ATOM	4686	O	LEU	267	62.876	45.501	-3.050	1.00	0.00	RX1	O
ATOM	4687	N	MET	268	62.698	45.750	-0.809	1.00	0.00	RX1	N
ATOM	4688	H	MET	268	62.295	45.472	0.062	1.00	0.00	RX1	H
ATOM	4689	CA	MET	268	63.980	46.439	-0.786	1.00	0.00	RX1	C
ATOM	4690	CB	MET	268	63.827	47.833	-0.178	1.00	0.00	RX1	C
ATOM	4691	CG	MET	268	62.639	48.609	-0.751	1.00	0.00	RX1	C
ATOM	4692	SD	MET	268	62.817	49.053	-2.486	1.00	0.00	RX1	S
ATOM	4693	CE	MET	268	63.736	50.581	-2.246	1.00	0.00	RX1	C
ATOM	4694	C	MET	268	64.982	45.588	-0.031	1.00	0.00	RX1	C
ATOM	4695	O	MET	268	64.618	44.662	0.686	1.00	0.00	RX1	O

ATOM	4696	N	LEU	269	66.259	45.909	-0.245	1.00	0.00	RX1	N
ATOM	4697	H	LEU	269	66.540	46.721	-0.760	1.00	0.00	RX1	H
ATOM	4698	CA	LEU	269	67.307	45.133	0.401	1.00	0.00	RX1	C
ATOM	4699	CB	LEU	269	67.920	44.145	-0.590	1.00	0.00	RX1	C
ATOM	4700	CG	LEU	269	68.212	42.774	0.020	1.00	0.00	RX1	C
ATOM	4701	CD1	LEU	269	66.949	42.128	0.593	1.00	0.00	RX1	C
ATOM	4702	CD2	LEU	269	68.911	41.853	-0.981	1.00	0.00	RX1	C
ATOM	4703	C	LEU	269	68.355	46.078	0.934	1.00	0.00	RX1	C
ATOM	4704	O	LEU	269	68.463	47.215	0.495	1.00	0.00	RX1	O
ATOM	4705	N	TYR	270	69.112	45.591	1.920	1.00	0.00	RX1	N
ATOM	4706	H	TYR	270	69.147	44.619	2.142	1.00	0.00	RX1	H
ATOM	4707	CA	TYR	270	70.050	46.544	2.497	1.00	0.00	RX1	C
ATOM	4708	CB	TYR	270	70.422	46.119	3.915	1.00	0.00	RX1	C
ATOM	4709	CG	TYR	270	71.025	47.303	4.624	1.00	0.00	RX1	C
ATOM	4710	CD1	TYR	270	70.204	48.164	5.337	1.00	0.00	RX1	C
ATOM	4711	CE1	TYR	270	70.750	49.262	5.982	1.00	0.00	RX1	C
ATOM	4712	CD2	TYR	270	72.390	47.542	4.552	1.00	0.00	RX1	C
ATOM	4713	CE2	TYR	270	72.933	48.652	5.181	1.00	0.00	RX1	C
ATOM	4714	CZ	TYR	270	72.114	49.500	5.910	1.00	0.00	RX1	C
ATOM	4715	OH	TYR	270	72.666	50.578	6.568	1.00	0.00	RX1	O
ATOM	4716	HH	TYR	270	73.604	50.557	6.422	1.00	0.00	RX1	H
ATOM	4717	C	TYR	270	71.292	46.765	1.654	1.00	0.00	RX1	C
ATOM	4718	O	TYR	270	72.029	45.840	1.338	1.00	0.00	RX1	O
ATOM	4719	N	ASN	271	71.500	48.044	1.334	1.00	0.00	RX1	N
ATOM	4720	H	ASN	271	70.869	48.764	1.627	1.00	0.00	RX1	H
ATOM	4721	CA	ASN	271	72.778	48.428	0.745	1.00	0.00	RX1	C
ATOM	4722	CB	ASN	271	72.566	49.261	-0.505	1.00	0.00	RX1	C
ATOM	4723	CG	ASN	271	73.887	49.598	-1.163	1.00	0.00	RX1	C
ATOM	4724	OD1	ASN	271	74.977	49.289	-0.684	1.00	0.00	RX1	O
ATOM	4725	ND2	ASN	271	73.730	50.222	-2.339	1.00	0.00	RX1	N
ATOM	4726	HD21	ASN	271	72.796	50.504	-2.598	1.00	0.00	RX1	H
ATOM	4727	HD22	ASN	271	74.447	50.481	-2.982	1.00	0.00	RX1	H
ATOM	4728	C	ASN	271	73.626	49.183	1.751	1.00	0.00	RX1	C
ATOM	4729	O	ASN	271	73.329	50.307	2.150	1.00	0.00	RX1	O
ATOM	4730	N	PRO	272	74.732	48.512	2.160	1.00	0.00	RX1	N
ATOM	4731	CD	PRO	272	75.105	47.154	1.784	1.00	0.00	RX1	C
ATOM	4732	CA	PRO	272	75.688	49.140	3.078	1.00	0.00	RX1	C
ATOM	4733	CB	PRO	272	76.831	48.121	3.112	1.00	0.00	RX1	C
ATOM	4734	CG	PRO	272	76.170	46.777	2.805	1.00	0.00	RX1	C
ATOM	4735	C	PRO	272	76.137	50.532	2.665	1.00	0.00	RX1	C
ATOM	4736	O	PRO	272	76.187	51.453	3.468	1.00	0.00	RX1	O
ATOM	4737	N	THR	273	76.437	50.640	1.363	1.00	0.00	RX1	N
ATOM	4738	H	THR	273	76.332	49.868	0.738	1.00	0.00	RX1	H
ATOM	4739	CA	THR	273	76.937	51.898	0.809	1.00	0.00	RX1	C
ATOM	4740	CB	THR	273	77.192	51.600	-0.655	1.00	0.00	RX1	C
ATOM	4741	OG1	THR	273	77.524	50.207	-0.766	1.00	0.00	RX1	O
ATOM	4742	HG1	THR	273	76.722	49.768	-1.039	1.00	0.00	RX1	H
ATOM	4743	CG2	THR	273	78.278	52.492	-1.258	1.00	0.00	RX1	C
ATOM	4744	C	THR	273	76.037	53.101	1.048	1.00	0.00	RX1	C
ATOM	4745	O	THR	273	76.457	54.172	1.463	1.00	0.00	RX1	O
ATOM	4746	N	THR	274	74.750	52.852	0.790	1.00	0.00	RX1	N
ATOM	4747	H	THR	274	74.389	51.984	0.448	1.00	0.00	RX1	H
ATOM	4748	CA	THR	274	73.772	53.916	0.988	1.00	0.00	RX1	C
ATOM	4749	CB	THR	274	72.634	53.552	0.057	1.00	0.00	RX1	C
ATOM	4750	OG1	THR	274	73.110	52.560	-0.859	1.00	0.00	RX1	O
ATOM	4751	HG1	THR	274	72.313	52.280	-1.334	1.00	0.00	RX1	H
ATOM	4752	CG2	THR	274	72.057	54.763	-0.678	1.00	0.00	RX1	C
ATOM	4753	C	THR	274	73.318	54.096	2.435	1.00	0.00	RX1	C
ATOM	4754	O	THR	274	72.535	54.981	2.767	1.00	0.00	RX1	O
ATOM	4755	N	TYR	275	73.805	53.159	3.278	1.00	0.00	RX1	N
ATOM	4756	H	TYR	275	74.506	52.520	2.963	1.00	0.00	RX1	H

ATOM	4757	CA	TYR	275	73.278	52.957	4.629	1.00	0.00	RX1	C
ATOM	4758	CB	TYR	275	73.797	54.004	5.617	1.00	0.00	RX1	C
ATOM	4759	CG	TYR	275	74.199	53.293	6.887	1.00	0.00	RX1	C
ATOM	4760	CD1	TYR	275	73.404	53.359	8.027	1.00	0.00	RX1	C
ATOM	4761	CE1	TYR	275	73.782	52.678	9.180	1.00	0.00	RX1	C
ATOM	4762	CD2	TYR	275	75.371	52.550	6.905	1.00	0.00	RX1	C
ATOM	4763	CE2	TYR	275	75.750	51.873	8.057	1.00	0.00	RX1	C
ATOM	4764	CZ	TYR	275	74.958	51.936	9.196	1.00	0.00	RX1	C
ATOM	4765	OH	TYR	275	75.357	51.262	10.336	1.00	0.00	RX1	O
ATOM	4766	HH	TYR	275	76.279	51.056	10.242	1.00	0.00	RX1	H
ATOM	4767	C	TYR	275	71.766	52.811	4.697	1.00	0.00	RX1	C
ATOM	4768	O	TYR	275	71.091	53.267	5.611	1.00	0.00	RX1	O
ATOM	4769	N	GLN	276	71.269	52.181	3.626	1.00	0.00	RX1	N
ATOM	4770	H	GLN	276	71.830	51.638	2.995	1.00	0.00	RX1	H
ATOM	4771	CA	GLN	276	69.849	52.311	3.338	1.00	0.00	RX1	C
ATOM	4772	CB	GLN	276	69.645	53.445	2.333	1.00	0.00	RX1	C
ATOM	4773	CG	GLN	276	68.542	54.438	2.697	1.00	0.00	RX1	C
ATOM	4774	CD	GLN	276	68.813	55.029	4.064	1.00	0.00	RX1	C
ATOM	4775	OE1	GLN	276	67.958	54.995	4.945	1.00	0.00	RX1	O
ATOM	4776	NE2	GLN	276	70.038	55.568	4.198	1.00	0.00	RX1	N
ATOM	4777	HE21	GLN	276	70.724	55.580	3.463	1.00	0.00	RX1	H
ATOM	4778	HE22	GLN	276	70.345	55.924	5.079	1.00	0.00	RX1	H
ATOM	4779	C	GLN	276	69.313	51.012	2.791	1.00	0.00	RX1	C
ATOM	4780	O	GLN	276	70.013	50.007	2.734	1.00	0.00	RX1	O
ATOM	4781	N	MET	277	68.040	51.071	2.398	1.00	0.00	RX1	N
ATOM	4782	H	MET	277	67.499	51.910	2.380	1.00	0.00	RX1	H
ATOM	4783	CA	MET	277	67.491	49.903	1.734	1.00	0.00	RX1	C
ATOM	4784	CB	MET	277	66.257	49.399	2.474	1.00	0.00	RX1	C
ATOM	4785	CG	MET	277	66.575	48.822	3.855	1.00	0.00	RX1	C
ATOM	4786	SD	MET	277	67.123	50.003	5.104	1.00	0.00	RX1	S
ATOM	4787	CE	MET	277	65.708	51.116	5.065	1.00	0.00	RX1	C
ATOM	4788	C	MET	277	67.181	50.241	0.298	1.00	0.00	RX1	C
ATOM	4789	O	MET	277	66.200	50.908	-0.010	1.00	0.00	RX1	O
ATOM	4790	N	ASP	278	68.099	49.780	-0.548	1.00	0.00	RX1	N
ATOM	4791	H	ASP	278	68.778	49.118	-0.235	1.00	0.00	RX1	H
ATOM	4792	CA	ASP	278	67.989	50.081	-1.970	1.00	0.00	RX1	C
ATOM	4793	CB	ASP	278	69.375	50.073	-2.639	1.00	0.00	RX1	C
ATOM	4794	CG	ASP	278	70.313	51.172	-2.135	1.00	0.00	RX1	C
ATOM	4795	OD1	ASP	278	70.240	51.577	-0.977	1.00	0.00	RX1	O
ATOM	4796	OD2	ASP	278	71.179	51.596	-2.895	1.00	0.00	RX1	O
ATOM	4797	C	ASP	278	67.068	49.064	-2.612	1.00	0.00	RX1	C
ATOM	4798	O	ASP	278	66.587	48.144	-1.961	1.00	0.00	RX1	O
ATOM	4799	N	VAL	279	66.807	49.247	-3.913	1.00	0.00	RX1	N
ATOM	4800	H	VAL	279	67.331	49.905	-4.449	1.00	0.00	RX1	H
ATOM	4801	CA	VAL	279	65.880	48.285	-4.513	1.00	0.00	RX1	C
ATOM	4802	CB	VAL	279	65.349	48.817	-5.854	1.00	0.00	RX1	C
ATOM	4803	CG1	VAL	279	66.474	49.081	-6.856	1.00	0.00	RX1	C
ATOM	4804	CG2	VAL	279	64.252	47.912	-6.421	1.00	0.00	RX1	C
ATOM	4805	C	VAL	279	66.470	46.884	-4.639	1.00	0.00	RX1	C
ATOM	4806	O	VAL	279	67.589	46.701	-5.102	1.00	0.00	RX1	O
ATOM	4807	N	ASN	280	65.677	45.901	-4.188	1.00	0.00	RX1	N
ATOM	4808	H	ASN	280	64.727	46.064	-3.908	1.00	0.00	RX1	H
ATOM	4809	CA	ASN	280	66.125	44.524	-4.389	1.00	0.00	RX1	C
ATOM	4810	CB	ASN	280	65.496	43.630	-3.326	1.00	0.00	RX1	C
ATOM	4811	CG	ASN	280	65.793	42.171	-3.585	1.00	0.00	RX1	C
ATOM	4812	OD1	ASN	280	66.662	41.788	-4.365	1.00	0.00	RX1	O
ATOM	4813	ND2	ASN	280	64.987	41.363	-2.885	1.00	0.00	RX1	N
ATOM	4814	HD21	ASN	280	64.358	41.745	-2.201	1.00	0.00	RX1	H
ATOM	4815	HD22	ASN	280	64.917	40.374	-3.042	1.00	0.00	RX1	H
ATOM	4816	C	ASN	280	65.753	44.043	-5.781	1.00	0.00	RX1	C
ATOM	4817	O	ASN	280	64.581	43.989	-6.131	1.00	0.00	RX1	O

ATOM	4818	N	PRO	281	66.791	43.680	-6.576	1.00	0.00	RX1	N
ATOM	4819	CD	PRO	281	68.212	43.801	-6.273	1.00	0.00	RX1	C
ATOM	4820	CA	PRO	281	66.529	43.096	-7.898	1.00	0.00	RX1	C
ATOM	4821	CB	PRO	281	67.930	42.674	-8.355	1.00	0.00	RX1	C
ATOM	4822	CG	PRO	281	68.887	43.617	-7.626	1.00	0.00	RX1	C
ATOM	4823	C	PRO	281	65.538	41.940	-7.872	1.00	0.00	RX1	C
ATOM	4824	O	PRO	281	64.581	41.884	-8.632	1.00	0.00	RX1	O
ATOM	4825	N	GLU	282	65.813	41.023	-6.936	1.00	0.00	RX1	N
ATOM	4826	H	GLU	282	66.508	41.176	-6.232	1.00	0.00	RX1	H
ATOM	4827	CA	GLU	282	64.859	39.937	-6.755	1.00	0.00	RX1	C
ATOM	4828	CB	GLU	282	65.577	38.606	-6.518	1.00	0.00	RX1	C
ATOM	4829	CG	GLU	282	65.857	37.803	-7.795	1.00	0.00	RX1	C
ATOM	4830	CD	GLU	282	64.582	37.153	-8.306	1.00	0.00	RX1	C
ATOM	4831	OE1	GLU	282	63.539	37.784	-8.355	1.00	0.00	RX1	O
ATOM	4832	OE2	GLU	282	64.588	35.980	-8.655	1.00	0.00	RX1	O
ATOM	4833	C	GLU	282	63.891	40.214	-5.627	1.00	0.00	RX1	C
ATOM	4834	O	GLU	282	63.874	39.537	-4.604	1.00	0.00	RX1	O
ATOM	4835	N	GLY	283	63.094	41.272	-5.850	1.00	0.00	RX1	N
ATOM	4836	H	GLY	283	63.142	41.744	-6.732	1.00	0.00	RX1	H
ATOM	4837	CA	GLY	283	62.081	41.632	-4.857	1.00	0.00	RX1	C
ATOM	4838	C	GLY	283	61.028	40.551	-4.711	1.00	0.00	RX1	C
ATOM	4839	O	GLY	283	60.190	40.333	-5.579	1.00	0.00	RX1	O
ATOM	4840	N	LYS	284	61.145	39.833	-3.593	1.00	0.00	RX1	N
ATOM	4841	H	LYS	284	61.728	40.080	-2.817	1.00	0.00	RX1	H
ATOM	4842	CA	LYS	284	60.461	38.553	-3.617	1.00	0.00	RX1	C
ATOM	4843	CB	LYS	284	61.397	37.448	-3.135	1.00	0.00	RX1	C
ATOM	4844	CG	LYS	284	61.461	36.345	-4.185	1.00	0.00	RX1	C
ATOM	4845	CD	LYS	284	61.770	36.927	-5.562	1.00	0.00	RX1	C
ATOM	4846	CE	LYS	284	61.603	35.936	-6.712	1.00	0.00	RX1	C
ATOM	4847	NZ	LYS	284	62.769	35.056	-6.890	1.00	0.00	RX1	N
ATOM	4848	HZ1	LYS	284	62.562	34.372	-7.648	1.00	0.00	RX1	H
ATOM	4849	HZ2	LYS	284	63.605	35.599	-7.193	1.00	0.00	RX1	H
ATOM	4850	HZ3	LYS	284	62.976	34.494	-6.032	1.00	0.00	RX1	H
ATOM	4851	C	LYS	284	59.106	38.491	-2.962	1.00	0.00	RX1	C
ATOM	4852	O	LYS	284	58.939	38.496	-1.749	1.00	0.00	RX1	O
ATOM	4853	N	TYR	285	58.118	38.420	-3.857	1.00	0.00	RX1	N
ATOM	4854	H	TYR	285	58.328	38.308	-4.830	1.00	0.00	RX1	H
ATOM	4855	CA	TYR	285	56.753	38.298	-3.365	1.00	0.00	RX1	C
ATOM	4856	CB	TYR	285	55.757	38.640	-4.469	1.00	0.00	RX1	C
ATOM	4857	CG	TYR	285	55.962	40.076	-4.885	1.00	0.00	RX1	C
ATOM	4858	CD1	TYR	285	56.725	40.384	-6.005	1.00	0.00	RX1	C
ATOM	4859	CE1	TYR	285	56.881	41.705	-6.402	1.00	0.00	RX1	C
ATOM	4860	CD2	TYR	285	55.379	41.097	-4.146	1.00	0.00	RX1	C
ATOM	4861	CE2	TYR	285	55.527	42.418	-4.547	1.00	0.00	RX1	C
ATOM	4862	CZ	TYR	285	56.274	42.723	-5.677	1.00	0.00	RX1	C
ATOM	4863	OH	TYR	285	56.412	44.037	-6.074	1.00	0.00	RX1	O
ATOM	4864	HH	TYR	285	56.088	44.608	-5.391	1.00	0.00	RX1	H
ATOM	4865	C	TYR	285	56.480	36.933	-2.769	1.00	0.00	RX1	C
ATOM	4866	O	TYR	285	56.765	35.885	-3.333	1.00	0.00	RX1	O
ATOM	4867	N	SER	286	55.931	37.006	-1.563	1.00	0.00	RX1	N
ATOM	4868	H	SER	286	55.622	37.878	-1.194	1.00	0.00	RX1	H
ATOM	4869	CA	SER	286	55.813	35.796	-0.766	1.00	0.00	RX1	C
ATOM	4870	CB	SER	286	56.128	36.210	0.663	1.00	0.00	RX1	C
ATOM	4871	OG	SER	286	57.214	37.150	0.655	1.00	0.00	RX1	O
ATOM	4872	HG	SER	286	57.037	37.858	0.040	1.00	0.00	RX1	H
ATOM	4873	C	SER	286	54.457	35.145	-0.935	1.00	0.00	RX1	C
ATOM	4874	O	SER	286	53.546	35.350	-0.137	1.00	0.00	RX1	O
ATOM	4875	N	PHE	287	54.331	34.371	-2.017	1.00	0.00	RX1	N
ATOM	4876	H	PHE	287	55.120	34.115	-2.583	1.00	0.00	RX1	H
ATOM	4877	CA	PHE	287	53.063	33.662	-2.156	1.00	0.00	RX1	C
ATOM	4878	CB	PHE	287	52.685	33.478	-3.625	1.00	0.00	RX1	C

ATOM	4879	CG	PHE	287	51.310	32.857	-3.698	1.00	0.00	RX1	C
ATOM	4880	CD1	PHE	287	50.267	33.387	-2.946	1.00	0.00	RX1	C
ATOM	4881	CD2	PHE	287	51.090	31.751	-4.511	1.00	0.00	RX1	C
ATOM	4882	CE1	PHE	287	49.004	32.809	-3.003	1.00	0.00	RX1	C
ATOM	4883	CE2	PHE	287	49.827	31.174	-4.569	1.00	0.00	RX1	C
ATOM	4884	CZ	PHE	287	48.785	31.701	-3.813	1.00	0.00	RX1	C
ATOM	4885	C	PHE	287	53.070	32.334	-1.426	1.00	0.00	RX1	C
ATOM	4886	O	PHE	287	53.260	31.261	-1.990	1.00	0.00	RX1	O
ATOM	4887	N	GLY	288	52.840	32.460	-0.113	1.00	0.00	RX1	N
ATOM	4888	H	GLY	288	52.828	33.374	0.296	1.00	0.00	RX1	H
ATOM	4889	CA	GLY	288	52.849	31.249	0.703	1.00	0.00	RX1	C
ATOM	4890	C	GLY	288	54.216	30.593	0.718	1.00	0.00	RX1	C
ATOM	4891	O	GLY	288	55.205	31.193	1.109	1.00	0.00	RX1	O
ATOM	4892	N	ALA	289	54.225	29.341	0.242	1.00	0.00	RX1	N
ATOM	4893	H	ALA	289	53.416	28.957	-0.198	1.00	0.00	RX1	H
ATOM	4894	CA	ALA	289	55.522	28.673	0.150	1.00	0.00	RX1	C
ATOM	4895	CB	ALA	289	55.351	27.154	0.172	1.00	0.00	RX1	C
ATOM	4896	C	ALA	289	56.329	29.055	-1.081	1.00	0.00	RX1	C
ATOM	4897	O	ALA	289	57.535	28.864	-1.155	1.00	0.00	RX1	O
ATOM	4898	N	THR	290	55.606	29.590	-2.065	1.00	0.00	RX1	N
ATOM	4899	H	THR	290	54.645	29.832	-1.965	1.00	0.00	RX1	H
ATOM	4900	CA	THR	290	56.302	29.994	-3.278	1.00	0.00	RX1	C
ATOM	4901	CB	THR	290	55.307	29.648	-4.362	1.00	0.00	RX1	C
ATOM	4902	OG1	THR	290	54.097	29.230	-3.705	1.00	0.00	RX1	O
ATOM	4903	HG1	THR	290	53.679	30.035	-3.404	1.00	0.00	RX1	H
ATOM	4904	CG2	THR	290	55.830	28.548	-5.287	1.00	0.00	RX1	C
ATOM	4905	C	THR	290	56.701	31.452	-3.253	1.00	0.00	RX1	C
ATOM	4906	O	THR	290	55.865	32.345	-3.187	1.00	0.00	RX1	O
ATOM	4907	N	CYS	291	58.016	31.665	-3.318	1.00	0.00	RX1	N
ATOM	4908	H	CYS	291	58.660	30.924	-3.508	1.00	0.00	RX1	H
ATOM	4909	CA	CYS	291	58.409	33.050	-3.551	1.00	0.00	RX1	C
ATOM	4910	CB	CYS	291	59.699	33.342	-2.799	1.00	0.00	RX1	C
ATOM	4911	SG	CYS	291	59.547	32.896	-1.055	1.00	0.00	RX1	S
ATOM	4912	C	CYS	291	58.496	33.278	-5.042	1.00	0.00	RX1	C
ATOM	4913	O	CYS	291	58.919	32.398	-5.781	1.00	0.00	RX1	O
ATOM	4914	N	VAL	292	58.004	34.449	-5.458	1.00	0.00	RX1	N
ATOM	4915	H	VAL	292	57.718	35.169	-4.822	1.00	0.00	RX1	H
ATOM	4916	CA	VAL	292	57.803	34.704	-6.883	1.00	0.00	RX1	C
ATOM	4917	CB	VAL	292	56.387	34.300	-7.316	1.00	0.00	RX1	C
ATOM	4918	CG1	VAL	292	56.235	32.794	-7.548	1.00	0.00	RX1	C
ATOM	4919	CG2	VAL	292	55.360	34.840	-6.320	1.00	0.00	RX1	C
ATOM	4920	C	VAL	292	58.015	36.171	-7.188	1.00	0.00	RX1	C
ATOM	4921	O	VAL	292	58.022	37.012	-6.299	1.00	0.00	RX1	O
ATOM	4922	N	LYS	293	58.157	36.452	-8.488	1.00	0.00	RX1	N
ATOM	4923	H	LYS	293	58.291	35.710	-9.149	1.00	0.00	RX1	H
ATOM	4924	CA	LYS	293	58.322	37.854	-8.871	1.00	0.00	RX1	C
ATOM	4925	CB	LYS	293	59.169	37.960	-10.142	1.00	0.00	RX1	C
ATOM	4926	CG	LYS	293	60.575	38.466	-9.814	1.00	0.00	RX1	C
ATOM	4927	CD	LYS	293	61.568	38.435	-10.982	1.00	0.00	RX1	C
ATOM	4928	CE	LYS	293	61.968	37.022	-11.426	1.00	0.00	RX1	C
ATOM	4929	NZ	LYS	293	62.436	36.251	-10.272	1.00	0.00	RX1	N
ATOM	4930	HZ1	LYS	293	62.807	35.306	-10.499	1.00	0.00	RX1	H
ATOM	4931	HZ2	LYS	293	63.162	36.740	-9.704	1.00	0.00	RX1	H
ATOM	4932	HZ3	LYS	293	61.634	36.015	-9.653	1.00	0.00	RX1	H
ATOM	4933	C	LYS	293	57.072	38.724	-8.960	1.00	0.00	RX1	C
ATOM	4934	O	LYS	293	57.155	39.889	-9.324	1.00	0.00	RX1	O
ATOM	4935	N	LYS	294	55.908	38.128	-8.628	1.00	0.00	RX1	N
ATOM	4936	H	LYS	294	55.831	37.179	-8.322	1.00	0.00	RX1	H
ATOM	4937	CA	LYS	294	54.693	38.944	-8.522	1.00	0.00	RX1	C
ATOM	4938	CB	LYS	294	54.194	39.402	-9.896	1.00	0.00	RX1	C
ATOM	4939	CG	LYS	294	53.885	38.252	-10.855	1.00	0.00	RX1	C

ATOM	4940	CD	LYS	294	53.463	38.769	-12.230	1.00	0.00	RX1	C
ATOM	4941	CE	LYS	294	53.176	37.643	-13.223	1.00	0.00	RX1	C
ATOM	4942	NZ	LYS	294	52.821	38.229	-14.523	1.00	0.00	RX1	N
ATOM	4943	HZ1	LYS	294	52.620	37.469	-15.204	1.00	0.00	RX1	H
ATOM	4944	HZ2	LYS	294	51.981	38.831	-14.413	1.00	0.00	RX1	H
ATOM	4945	HZ3	LYS	294	53.615	38.804	-14.870	1.00	0.00	RX1	H
ATOM	4946	C	LYS	294	53.572	38.241	-7.786	1.00	0.00	RX1	C
ATOM	4947	O	LYS	294	53.470	37.022	-7.795	1.00	0.00	RX1	O
ATOM	4948	N	CYS	295	52.735	39.070	-7.149	1.00	0.00	RX1	N
ATOM	4949	H	CYS	295	52.807	40.061	-7.237	1.00	0.00	RX1	H
ATOM	4950	CA	CYS	295	51.593	38.501	-6.432	1.00	0.00	RX1	C
ATOM	4951	CB	CYS	295	51.102	39.513	-5.401	1.00	0.00	RX1	C
ATOM	4952	SG	CYS	295	52.442	40.133	-4.361	1.00	0.00	RX1	S
ATOM	4953	C	CYS	295	50.451	38.097	-7.350	1.00	0.00	RX1	C
ATOM	4954	O	CYS	295	50.165	38.764	-8.339	1.00	0.00	RX1	O
ATOM	4955	N	PRO	296	49.787	36.972	-6.988	1.00	0.00	RX1	N
ATOM	4956	CD	PRO	296	50.184	36.012	-5.967	1.00	0.00	RX1	C
ATOM	4957	CA	PRO	296	48.522	36.624	-7.644	1.00	0.00	RX1	C
ATOM	4958	CB	PRO	296	48.088	35.359	-6.895	1.00	0.00	RX1	C
ATOM	4959	CG	PRO	296	49.376	34.772	-6.324	1.00	0.00	RX1	C
ATOM	4960	C	PRO	296	47.479	37.727	-7.550	1.00	0.00	RX1	C
ATOM	4961	O	PRO	296	47.325	38.388	-6.531	1.00	0.00	RX1	O
ATOM	4962	N	ARG	297	46.761	37.881	-8.674	1.00	0.00	RX1	N
ATOM	4963	H	ARG	297	46.927	37.246	-9.426	1.00	0.00	RX1	H
ATOM	4964	CA	ARG	297	45.862	39.023	-8.880	1.00	0.00	RX1	C
ATOM	4965	CB	ARG	297	45.328	38.931	-10.321	1.00	0.00	RX1	C
ATOM	4966	CG	ARG	297	44.902	40.226	-11.035	1.00	0.00	RX1	C
ATOM	4967	CD	ARG	297	43.463	40.695	-10.779	1.00	0.00	RX1	C
ATOM	4968	NE	ARG	297	42.496	39.683	-11.200	1.00	0.00	RX1	N
ATOM	4969	HE	ARG	297	42.457	39.473	-12.183	1.00	0.00	RX1	H
ATOM	4970	CZ	ARG	297	41.707	39.062	-10.272	1.00	0.00	RX1	C
ATOM	4971	NH1	ARG	297	41.780	39.411	-8.971	1.00	0.00	RX1	N
ATOM	4972	HH11	ARG	297	41.187	38.982	-8.285	1.00	0.00	RX1	H
ATOM	4973	HH12	ARG	297	42.438	40.103	-8.635	1.00	0.00	RX1	H
ATOM	4974	NH2	ARG	297	40.871	38.092	-10.671	1.00	0.00	RX1	N
ATOM	4975	HH21	ARG	297	40.308	37.538	-10.039	1.00	0.00	RX1	H
ATOM	4976	HH22	ARG	297	40.763	37.838	-11.640	1.00	0.00	RX1	H
ATOM	4977	C	ARG	297	44.756	39.256	-7.846	1.00	0.00	RX1	C
ATOM	4978	O	ARG	297	44.076	40.276	-7.839	1.00	0.00	RX1	O
ATOM	4979	N	ASN	298	44.543	38.264	-6.980	1.00	0.00	RX1	N
ATOM	4980	H	ASN	298	45.095	37.432	-6.909	1.00	0.00	RX1	H
ATOM	4981	CA	ASN	298	43.548	38.533	-5.946	1.00	0.00	RX1	C
ATOM	4982	CB	ASN	298	42.453	37.469	-5.881	1.00	0.00	RX1	C
ATOM	4983	CG	ASN	298	43.047	36.180	-5.372	1.00	0.00	RX1	C
ATOM	4984	OD1	ASN	298	44.238	35.925	-5.534	1.00	0.00	RX1	O
ATOM	4985	ND2	ASN	298	42.140	35.351	-4.841	1.00	0.00	RX1	N
ATOM	4986	HD21	ASN	298	41.187	35.595	-4.632	1.00	0.00	RX1	H
ATOM	4987	HD22	ASN	298	42.329	34.369	-4.753	1.00	0.00	RX1	H
ATOM	4988	C	ASN	298	44.098	38.772	-4.556	1.00	0.00	RX1	C
ATOM	4989	O	ASN	298	43.420	39.361	-3.721	1.00	0.00	RX1	O
ATOM	4990	N	TYR	299	45.311	38.256	-4.313	1.00	0.00	RX1	N
ATOM	4991	H	TYR	299	45.921	37.988	-5.059	1.00	0.00	RX1	H
ATOM	4992	CA	TYR	299	45.807	38.285	-2.937	1.00	0.00	RX1	C
ATOM	4993	CB	TYR	299	46.987	37.323	-2.750	1.00	0.00	RX1	C
ATOM	4994	CG	TYR	299	46.507	35.965	-2.282	1.00	0.00	RX1	C
ATOM	4995	CD1	TYR	299	45.539	35.271	-2.995	1.00	0.00	RX1	C
ATOM	4996	CE1	TYR	299	45.085	34.033	-2.554	1.00	0.00	RX1	C
ATOM	4997	CD2	TYR	299	47.039	35.399	-1.129	1.00	0.00	RX1	C
ATOM	4998	CE2	TYR	299	46.589	34.159	-0.687	1.00	0.00	RX1	C
ATOM	4999	CZ	TYR	299	45.598	33.480	-1.388	1.00	0.00	RX1	C
ATOM	5000	OH	TYR	299	45.115	32.268	-0.924	1.00	0.00	RX1	O

ATOM	5001	HH	TYR	299	44.391	31.984	-1.482	1.00	0.00	RX1	H
ATOM	5002	C	TYR	299	46.181	39.683	-2.483	1.00	0.00	RX1	C
ATOM	5003	O	TYR	299	46.372	40.591	-3.282	1.00	0.00	RX1	O
ATOM	5004	N	VAL	300	46.247	39.822	-1.154	1.00	0.00	RX1	N
ATOM	5005	H	VAL	300	46.233	39.034	-0.538	1.00	0.00	RX1	H
ATOM	5006	CA	VAL	300	46.688	41.117	-0.647	1.00	0.00	RX1	C
ATOM	5007	CB	VAL	300	46.241	41.344	0.792	1.00	0.00	RX1	C
ATOM	5008	CG1	VAL	300	46.509	42.780	1.248	1.00	0.00	RX1	C
ATOM	5009	CG2	VAL	300	44.792	40.939	0.976	1.00	0.00	RX1	C
ATOM	5010	C	VAL	300	48.192	41.188	-0.701	1.00	0.00	RX1	C
ATOM	5011	O	VAL	300	48.890	40.305	-0.215	1.00	0.00	RX1	O
ATOM	5012	N	VAL	301	48.660	42.281	-1.295	1.00	0.00	RX1	N
ATOM	5013	H	VAL	301	48.033	42.996	-1.605	1.00	0.00	RX1	H
ATOM	5014	CA	VAL	301	50.078	42.540	-1.122	1.00	0.00	RX1	C
ATOM	5015	CB	VAL	301	50.620	43.301	-2.329	1.00	0.00	RX1	C
ATOM	5016	CG1	VAL	301	52.150	43.314	-2.336	1.00	0.00	RX1	C
ATOM	5017	CG2	VAL	301	50.026	42.749	-3.624	1.00	0.00	RX1	C
ATOM	5018	C	VAL	301	50.276	43.337	0.151	1.00	0.00	RX1	C
ATOM	5019	O	VAL	301	49.745	44.429	0.324	1.00	0.00	RX1	O
ATOM	5020	N	THR	302	51.057	42.745	1.053	1.00	0.00	RX1	N
ATOM	5021	H	THR	302	51.513	41.876	0.865	1.00	0.00	RX1	H
ATOM	5022	CA	THR	302	51.549	43.628	2.103	1.00	0.00	RX1	C
ATOM	5023	CB	THR	302	51.835	42.810	3.354	1.00	0.00	RX1	C
ATOM	5024	OG1	THR	302	52.270	41.500	2.978	1.00	0.00	RX1	O
ATOM	5025	HG1	THR	302	51.515	41.077	2.573	1.00	0.00	RX1	H
ATOM	5026	CG2	THR	302	50.597	42.707	4.245	1.00	0.00	RX1	C
ATOM	5027	C	THR	302	52.753	44.381	1.586	1.00	0.00	RX1	C
ATOM	5028	O	THR	302	53.440	43.902	0.692	1.00	0.00	RX1	O
ATOM	5029	N	ASP	303	52.961	45.559	2.179	1.00	0.00	RX1	N
ATOM	5030	H	ASP	303	52.433	45.822	2.989	1.00	0.00	RX1	H
ATOM	5031	CA	ASP	303	54.099	46.431	1.872	1.00	0.00	RX1	C
ATOM	5032	CB	ASP	303	54.169	47.557	2.916	1.00	0.00	RX1	C
ATOM	5033	CG	ASP	303	54.400	47.015	4.321	1.00	0.00	RX1	C
ATOM	5034	OD1	ASP	303	55.215	47.578	5.043	1.00	0.00	RX1	O
ATOM	5035	OD2	ASP	303	53.783	46.019	4.699	1.00	0.00	RX1	O
ATOM	5036	C	ASP	303	55.444	45.724	1.725	1.00	0.00	RX1	C
ATOM	5037	O	ASP	303	56.188	45.921	0.775	1.00	0.00	RX1	O
ATOM	5038	N	HIS	304	55.681	44.831	2.700	1.00	0.00	RX1	N
ATOM	5039	H	HIS	304	55.060	44.885	3.485	1.00	0.00	RX1	H
ATOM	5040	CA	HIS	304	56.846	43.940	2.699	1.00	0.00	RX1	C
ATOM	5041	CB	HIS	304	56.646	42.936	3.829	1.00	0.00	RX1	C
ATOM	5042	CG	HIS	304	57.741	41.902	3.833	1.00	0.00	RX1	C
ATOM	5043	ND1	HIS	304	57.601	40.664	3.330	1.00	0.00	RX1	N
ATOM	5044	HD1	HIS	304	56.790	40.299	2.907	1.00	0.00	RX1	H
ATOM	5045	CD2	HIS	304	59.022	42.032	4.367	1.00	0.00	RX1	C
ATOM	5046	NE2	HIS	304	59.652	40.847	4.187	1.00	0.00	RX1	N
ATOM	5047	CE1	HIS	304	58.782	40.001	3.541	1.00	0.00	RX1	C
ATOM	5048	C	HIS	304	57.135	43.229	1.375	1.00	0.00	RX1	C
ATOM	5049	O	HIS	304	58.273	43.048	0.950	1.00	0.00	RX1	O
ATOM	5050	N	GLY	305	56.017	42.799	0.780	1.00	0.00	RX1	N
ATOM	5051	H	GLY	305	55.127	43.070	1.140	1.00	0.00	RX1	H
ATOM	5052	CA	GLY	305	56.089	41.888	-0.352	1.00	0.00	RX1	C
ATOM	5053	C	GLY	305	55.534	40.523	-0.006	1.00	0.00	RX1	C
ATOM	5054	O	GLY	305	56.074	39.488	-0.370	1.00	0.00	RX1	O
ATOM	5055	N	SER	306	54.409	40.541	0.719	1.00	0.00	RX1	N
ATOM	5056	H	SER	306	53.965	41.366	1.062	1.00	0.00	RX1	H
ATOM	5057	CA	SER	306	53.902	39.232	1.128	1.00	0.00	RX1	C
ATOM	5058	CB	SER	306	54.309	39.143	2.583	1.00	0.00	RX1	C
ATOM	5059	OG	SER	306	54.822	40.445	2.899	1.00	0.00	RX1	O
ATOM	5060	HG	SER	306	54.045	40.955	3.130	1.00	0.00	RX1	H
ATOM	5061	C	SER	306	52.429	39.046	0.829	1.00	0.00	RX1	C

ATOM	5062	O	SER	306	51.610	39.914	1.111	1.00	0.00	RX1	O
ATOM	5063	N	CYS	307	52.135	37.896	0.211	1.00	0.00	RX1	N
ATOM	5064	H	CYS	307	52.777	37.134	0.118	1.00	0.00	RX1	H
ATOM	5065	CA	CYS	307	50.769	37.686	-0.264	1.00	0.00	RX1	C
ATOM	5066	CB	CYS	307	50.822	36.838	-1.528	1.00	0.00	RX1	C
ATOM	5067	SG	CYS	307	52.180	37.362	-2.606	1.00	0.00	RX1	S
ATOM	5068	C	CYS	307	49.860	37.093	0.791	1.00	0.00	RX1	C
ATOM	5069	O	CYS	307	49.777	35.884	0.971	1.00	0.00	RX1	O
ATOM	5070	N	VAL	308	49.195	38.002	1.504	1.00	0.00	RX1	N
ATOM	5071	H	VAL	308	49.186	38.957	1.199	1.00	0.00	RX1	H
ATOM	5072	CA	VAL	308	48.290	37.500	2.534	1.00	0.00	RX1	C
ATOM	5073	CB	VAL	308	48.397	38.365	3.790	1.00	0.00	RX1	C
ATOM	5074	CG1	VAL	308	49.802	38.260	4.387	1.00	0.00	RX1	C
ATOM	5075	CG2	VAL	308	48.037	39.818	3.496	1.00	0.00	RX1	C
ATOM	5076	C	VAL	308	46.861	37.389	2.029	1.00	0.00	RX1	C
ATOM	5077	O	VAL	308	46.519	37.906	0.971	1.00	0.00	RX1	O
ATOM	5078	N	ARG	309	46.030	36.687	2.816	1.00	0.00	RX1	N
ATOM	5079	H	ARG	309	46.307	36.295	3.692	1.00	0.00	RX1	H
ATOM	5080	CA	ARG	309	44.637	36.634	2.372	1.00	0.00	RX1	C
ATOM	5081	CB	ARG	309	43.932	35.349	2.782	1.00	0.00	RX1	C
ATOM	5082	CG	ARG	309	44.488	34.101	2.113	1.00	0.00	RX1	C
ATOM	5083	CD	ARG	309	43.618	32.887	2.423	1.00	0.00	RX1	C
ATOM	5084	NE	ARG	309	44.100	31.706	1.718	1.00	0.00	RX1	N
ATOM	5085	HE	ARG	309	44.203	31.790	0.718	1.00	0.00	RX1	H
ATOM	5086	CZ	ARG	309	44.427	30.605	2.452	1.00	0.00	RX1	C
ATOM	5087	NH1	ARG	309	44.319	30.631	3.794	1.00	0.00	RX1	N
ATOM	5088	HH11	ARG	309	44.570	29.852	4.380	1.00	0.00	RX1	H
ATOM	5089	HH12	ARG	309	44.054	31.452	4.329	1.00	0.00	RX1	H
ATOM	5090	NH2	ARG	309	44.878	29.499	1.824	1.00	0.00	RX1	N
ATOM	5091	HH21	ARG	309	45.154	28.680	2.337	1.00	0.00	RX1	H
ATOM	5092	HH22	ARG	309	44.960	29.470	0.824	1.00	0.00	RX1	H
ATOM	5093	C	ARG	309	43.767	37.792	2.792	1.00	0.00	RX1	C
ATOM	5094	O	ARG	309	42.728	38.040	2.198	1.00	0.00	RX1	O
ATOM	5095	N	ALA	310	44.230	38.491	3.826	1.00	0.00	RX1	N
ATOM	5096	H	ALA	310	45.070	38.322	4.343	1.00	0.00	RX1	H
ATOM	5097	CA	ALA	310	43.491	39.676	4.230	1.00	0.00	RX1	C
ATOM	5098	CB	ALA	310	42.334	39.316	5.158	1.00	0.00	RX1	C
ATOM	5099	C	ALA	310	44.443	40.569	4.970	1.00	0.00	RX1	C
ATOM	5100	O	ALA	310	45.479	40.120	5.449	1.00	0.00	RX1	O
ATOM	5101	N	CYS	311	44.062	41.844	5.043	1.00	0.00	RX1	N
ATOM	5102	H	CYS	311	43.182	42.147	4.678	1.00	0.00	RX1	H
ATOM	5103	CA	CYS	311	44.845	42.685	5.939	1.00	0.00	RX1	C
ATOM	5104	CB	CYS	311	44.604	44.144	5.584	1.00	0.00	RX1	C
ATOM	5105	SG	CYS	311	45.229	44.522	3.932	1.00	0.00	RX1	S
ATOM	5106	C	CYS	311	44.545	42.388	7.394	1.00	0.00	RX1	C
ATOM	5107	O	CYS	311	43.505	41.836	7.734	1.00	0.00	RX1	O
ATOM	5108	N	GLY	312	45.512	42.777	8.243	1.00	0.00	RX1	N
ATOM	5109	H	GLY	312	46.314	43.274	7.922	1.00	0.00	RX1	H
ATOM	5110	CA	GLY	312	45.227	42.686	9.672	1.00	0.00	RX1	C
ATOM	5111	C	GLY	312	44.160	43.687	10.068	1.00	0.00	RX1	C
ATOM	5112	O	GLY	312	43.810	44.570	9.297	1.00	0.00	RX1	O
ATOM	5113	N	ALA	313	43.660	43.514	11.300	1.00	0.00	RX1	N
ATOM	5114	H	ALA	313	44.026	42.796	11.887	1.00	0.00	RX1	H
ATOM	5115	CA	ALA	313	42.513	44.319	11.729	1.00	0.00	RX1	C
ATOM	5116	CB	ALA	313	42.128	43.970	13.168	1.00	0.00	RX1	C
ATOM	5117	C	ALA	313	42.674	45.830	11.627	1.00	0.00	RX1	C
ATOM	5118	O	ALA	313	41.718	46.569	11.442	1.00	0.00	RX1	O
ATOM	5119	N	ASP	314	43.930	46.259	11.761	1.00	0.00	RX1	N
ATOM	5120	H	ASP	314	44.727	45.654	11.805	1.00	0.00	RX1	H
ATOM	5121	CA	ASP	314	44.248	47.684	11.694	1.00	0.00	RX1	C
ATOM	5122	CB	ASP	314	45.343	48.005	12.716	1.00	0.00	RX1	C

ATOM	5123	CG	ASP	314	46.646	47.250	12.452	1.00	0.00	RX1	C
ATOM	5124	OD1	ASP	314	46.687	46.322	11.642	1.00	0.00	RX1	O
ATOM	5125	OD2	ASP	314	47.639	47.593	13.085	1.00	0.00	RX1	O
ATOM	5126	C	ASP	314	44.643	48.211	10.319	1.00	0.00	RX1	C
ATOM	5127	O	ASP	314	45.050	49.356	10.155	1.00	0.00	RX1	O
ATOM	5128	N	SER	315	44.507	47.341	9.314	1.00	0.00	RX1	N
ATOM	5129	H	SER	315	44.101	46.432	9.399	1.00	0.00	RX1	H
ATOM	5130	CA	SER	315	44.967	47.770	8.001	1.00	0.00	RX1	C
ATOM	5131	CB	SER	315	46.197	46.913	7.675	1.00	0.00	RX1	C
ATOM	5132	OG	SER	315	47.135	47.612	6.845	1.00	0.00	RX1	O
ATOM	5133	HG	SER	315	47.898	47.741	7.398	1.00	0.00	RX1	H
ATOM	5134	C	SER	315	43.872	47.690	6.957	1.00	0.00	RX1	C
ATOM	5135	O	SER	315	43.096	46.745	6.888	1.00	0.00	RX1	O
ATOM	5136	N	TYR	316	43.829	48.740	6.132	1.00	0.00	RX1	N
ATOM	5137	H	TYR	316	44.518	49.460	6.191	1.00	0.00	RX1	H
ATOM	5138	CA	TYR	316	42.894	48.684	5.016	1.00	0.00	RX1	C
ATOM	5139	CB	TYR	316	42.621	50.081	4.461	1.00	0.00	RX1	C
ATOM	5140	CG	TYR	316	41.658	50.841	5.339	1.00	0.00	RX1	C
ATOM	5141	CD1	TYR	316	42.116	51.852	6.174	1.00	0.00	RX1	C
ATOM	5142	CE1	TYR	316	41.213	52.585	6.937	1.00	0.00	RX1	C
ATOM	5143	CD2	TYR	316	40.303	50.542	5.294	1.00	0.00	RX1	C
ATOM	5144	CE2	TYR	316	39.400	51.278	6.052	1.00	0.00	RX1	C
ATOM	5145	CZ	TYR	316	39.854	52.309	6.865	1.00	0.00	RX1	C
ATOM	5146	OH	TYR	316	38.951	53.060	7.596	1.00	0.00	RX1	O
ATOM	5147	HH	TYR	316	38.138	53.093	7.098	1.00	0.00	RX1	H
ATOM	5148	C	TYR	316	43.393	47.814	3.888	1.00	0.00	RX1	C
ATOM	5149	O	TYR	316	44.428	48.068	3.281	1.00	0.00	RX1	O
ATOM	5150	N	GLU	317	42.575	46.794	3.610	1.00	0.00	RX1	N
ATOM	5151	H	GLU	317	41.742	46.641	4.139	1.00	0.00	RX1	H
ATOM	5152	CA	GLU	317	42.752	46.132	2.324	1.00	0.00	RX1	C
ATOM	5153	CB	GLU	317	42.120	44.741	2.334	1.00	0.00	RX1	C
ATOM	5154	CG	GLU	317	42.462	43.966	1.063	1.00	0.00	RX1	C
ATOM	5155	CD	GLU	317	41.660	42.682	0.999	1.00	0.00	RX1	C
ATOM	5156	OE1	GLU	317	41.022	42.417	-0.020	1.00	0.00	RX1	O
ATOM	5157	OE2	GLU	317	41.690	41.917	1.952	1.00	0.00	RX1	O
ATOM	5158	C	GLU	317	42.123	46.979	1.242	1.00	0.00	RX1	C
ATOM	5159	O	GLU	317	40.908	47.030	1.103	1.00	0.00	RX1	O
ATOM	5160	N	MET	318	42.996	47.673	0.512	1.00	0.00	RX1	N
ATOM	5161	H	MET	318	43.993	47.596	0.599	1.00	0.00	RX1	H
ATOM	5162	CA	MET	318	42.424	48.566	-0.485	1.00	0.00	RX1	C
ATOM	5163	CB	MET	318	42.237	49.967	0.098	1.00	0.00	RX1	C
ATOM	5164	CG	MET	318	41.193	50.808	-0.637	1.00	0.00	RX1	C
ATOM	5165	SD	MET	318	39.533	50.138	-0.444	1.00	0.00	RX1	S
ATOM	5166	CE	MET	318	39.437	50.200	1.353	1.00	0.00	RX1	C
ATOM	5167	C	MET	318	43.288	48.575	-1.722	1.00	0.00	RX1	C
ATOM	5168	O	MET	318	44.511	48.546	-1.631	1.00	0.00	RX1	O
ATOM	5169	N	GLU	319	42.587	48.559	-2.853	1.00	0.00	RX1	N
ATOM	5170	H	GLU	319	41.609	48.773	-2.824	1.00	0.00	RX1	H
ATOM	5171	CA	GLU	319	43.196	48.450	-4.171	1.00	0.00	RX1	C
ATOM	5172	CB	GLU	319	42.260	47.750	-5.171	1.00	0.00	RX1	C
ATOM	5173	CG	GLU	319	40.878	48.383	-5.408	1.00	0.00	RX1	C
ATOM	5174	CD	GLU	319	39.881	47.955	-4.341	1.00	0.00	RX1	C
ATOM	5175	OE1	GLU	319	39.249	46.915	-4.490	1.00	0.00	RX1	O
ATOM	5176	OE2	GLU	319	39.739	48.637	-3.334	1.00	0.00	RX1	O
ATOM	5177	C	GLU	319	43.658	49.775	-4.736	1.00	0.00	RX1	C
ATOM	5178	O	GLU	319	43.086	50.828	-4.484	1.00	0.00	RX1	O
ATOM	5179	N	GLU	320	44.728	49.662	-5.531	1.00	0.00	RX1	N
ATOM	5180	H	GLU	320	45.217	48.792	-5.601	1.00	0.00	RX1	H
ATOM	5181	CA	GLU	320	45.202	50.852	-6.229	1.00	0.00	RX1	C
ATOM	5182	CB	GLU	320	46.538	51.339	-5.643	1.00	0.00	RX1	C
ATOM	5183	CG	GLU	320	46.627	51.430	-4.114	1.00	0.00	RX1	C

ATOM	5184	CD	GLU	320	47.199	50.162	-3.491	1.00	0.00	RX1	C
ATOM	5185	OE1	GLU	320	46.587	49.106	-3.513	1.00	0.00	RX1	O
ATOM	5186	OE2	GLU	320	48.263	50.205	-2.898	1.00	0.00	RX1	O
ATOM	5187	C	GLU	320	45.365	50.562	-7.709	1.00	0.00	RX1	C
ATOM	5188	O	GLU	320	44.561	50.906	-8.565	1.00	0.00	RX1	O
ATOM	5189	N	ASP	321	46.455	49.837	-7.955	1.00	0.00	RX1	N
ATOM	5190	H	ASP	321	46.968	49.455	-7.188	1.00	0.00	RX1	H
ATOM	5191	CA	ASP	321	46.882	49.338	-9.265	1.00	0.00	RX1	C
ATOM	5192	CB	ASP	321	48.385	49.060	-9.193	1.00	0.00	RX1	C
ATOM	5193	CG	ASP	321	48.706	48.385	-7.866	1.00	0.00	RX1	C
ATOM	5194	OD1	ASP	321	49.678	48.764	-7.222	1.00	0.00	RX1	O
ATOM	5195	OD2	ASP	321	47.938	47.544	-7.404	1.00	0.00	RX1	O
ATOM	5196	C	ASP	321	46.159	48.066	-9.690	1.00	0.00	RX1	C
ATOM	5197	O	ASP	321	46.706	47.166	-10.317	1.00	0.00	RX1	O
ATOM	5198	N	GLY	322	44.888	47.991	-9.281	1.00	0.00	RX1	N
ATOM	5199	H	GLY	322	44.442	48.750	-8.810	1.00	0.00	RX1	H
ATOM	5200	CA	GLY	322	44.255	46.684	-9.400	1.00	0.00	RX1	C
ATOM	5201	C	GLY	322	44.461	45.808	-8.177	1.00	0.00	RX1	C
ATOM	5202	O	GLY	322	43.511	45.441	-7.496	1.00	0.00	RX1	O
ATOM	5203	N	VAL	323	45.732	45.459	-7.916	1.00	0.00	RX1	N
ATOM	5204	H	VAL	323	46.511	45.938	-8.332	1.00	0.00	RX1	H
ATOM	5205	CA	VAL	323	45.882	44.582	-6.757	1.00	0.00	RX1	C
ATOM	5206	CB	VAL	323	47.089	43.642	-6.875	1.00	0.00	RX1	C
ATOM	5207	CG1	VAL	323	46.882	42.686	-8.049	1.00	0.00	RX1	C
ATOM	5208	CG2	VAL	323	48.422	44.378	-6.974	1.00	0.00	RX1	C
ATOM	5209	C	VAL	323	45.847	45.335	-5.444	1.00	0.00	RX1	C
ATOM	5210	O	VAL	323	46.294	46.473	-5.316	1.00	0.00	RX1	O
ATOM	5211	N	ARG	324	45.247	44.654	-4.463	1.00	0.00	RX1	N
ATOM	5212	H	ARG	324	45.058	43.677	-4.541	1.00	0.00	RX1	H
ATOM	5213	CA	ARG	324	45.039	45.381	-3.218	1.00	0.00	RX1	C
ATOM	5214	CB	ARG	324	43.811	44.886	-2.457	1.00	0.00	RX1	C
ATOM	5215	CG	ARG	324	42.675	44.422	-3.363	1.00	0.00	RX1	C
ATOM	5216	CD	ARG	324	41.367	44.240	-2.597	1.00	0.00	RX1	C
ATOM	5217	NE	ARG	324	40.639	45.498	-2.465	1.00	0.00	RX1	N
ATOM	5218	HE	ARG	324	40.652	46.134	-3.250	1.00	0.00	RX1	H
ATOM	5219	CZ	ARG	324	39.687	45.677	-1.502	1.00	0.00	RX1	C
ATOM	5220	NH1	ARG	324	39.614	44.844	-0.445	1.00	0.00	RX1	N
ATOM	5221	HH11	ARG	324	38.850	44.903	0.218	1.00	0.00	RX1	H
ATOM	5222	HH12	ARG	324	40.289	44.117	-0.264	1.00	0.00	RX1	H
ATOM	5223	NH2	ARG	324	38.799	46.675	-1.645	1.00	0.00	RX1	N
ATOM	5224	HH21	ARG	324	38.024	46.794	-1.017	1.00	0.00	RX1	H
ATOM	5225	HH22	ARG	324	38.888	47.323	-2.424	1.00	0.00	RX1	H
ATOM	5226	C	ARG	324	46.244	45.302	-2.320	1.00	0.00	RX1	C
ATOM	5227	O	ARG	324	46.626	44.234	-1.857	1.00	0.00	RX1	O
ATOM	5228	N	LYS	325	46.841	46.472	-2.090	1.00	0.00	RX1	N
ATOM	5229	H	LYS	325	46.437	47.323	-2.439	1.00	0.00	RX1	H
ATOM	5230	CA	LYS	325	47.821	46.451	-1.011	1.00	0.00	RX1	C
ATOM	5231	CB	LYS	325	48.954	47.464	-1.213	1.00	0.00	RX1	C
ATOM	5232	CG	LYS	325	50.220	46.978	-1.925	1.00	0.00	RX1	C
ATOM	5233	CD	LYS	325	50.386	47.452	-3.372	1.00	0.00	RX1	C
ATOM	5234	CE	LYS	325	49.367	46.846	-4.332	1.00	0.00	RX1	C
ATOM	5235	NZ	LYS	325	48.543	47.896	-4.929	1.00	0.00	RX1	N
ATOM	5236	HZ1	LYS	325	47.760	47.473	-5.477	1.00	0.00	RX1	H
ATOM	5237	HZ2	LYS	325	48.153	48.559	-4.227	1.00	0.00	RX1	H
ATOM	5238	HZ3	LYS	325	49.090	48.418	-5.645	1.00	0.00	RX1	H
ATOM	5239	C	LYS	325	47.128	46.775	0.289	1.00	0.00	RX1	C
ATOM	5240	O	LYS	325	46.068	47.396	0.304	1.00	0.00	RX1	O
ATOM	5241	N	CYS	326	47.803	46.403	1.377	1.00	0.00	RX1	N
ATOM	5242	H	CYS	326	48.665	45.898	1.291	1.00	0.00	RX1	H
ATOM	5243	CA	CYS	326	47.385	46.977	2.656	1.00	0.00	RX1	C
ATOM	5244	CB	CYS	326	48.188	46.303	3.764	1.00	0.00	RX1	C

ATOM	5245	SG	CYS	326	49.953	46.266	3.374	1.00	0.00	RX1	S
ATOM	5246	C	CYS	326	47.542	48.494	2.666	1.00	0.00	RX1	C
ATOM	5247	O	CYS	326	48.077	49.069	1.718	1.00	0.00	RX1	O
ATOM	5248	N	LYS	327	47.056	49.098	3.761	1.00	0.00	RX1	N
ATOM	5249	H	LYS	327	46.480	48.591	4.407	1.00	0.00	RX1	H
ATOM	5250	CA	LYS	327	47.361	50.479	4.145	1.00	0.00	RX1	C
ATOM	5251	CB	LYS	327	46.473	51.531	3.460	1.00	0.00	RX1	C
ATOM	5252	CG	LYS	327	46.713	51.755	1.966	1.00	0.00	RX1	C
ATOM	5253	CD	LYS	327	45.570	51.216	1.107	1.00	0.00	RX1	C
ATOM	5254	CE	LYS	327	45.951	51.089	-0.368	1.00	0.00	RX1	C
ATOM	5255	NZ	LYS	327	46.645	49.817	-0.608	1.00	0.00	RX1	N
ATOM	5256	HZ1	LYS	327	47.153	49.863	-1.520	1.00	0.00	RX1	H
ATOM	5257	HZ2	LYS	327	47.314	49.592	0.159	1.00	0.00	RX1	H
ATOM	5258	HZ3	LYS	327	45.947	49.043	-0.698	1.00	0.00	RX1	H
ATOM	5259	C	LYS	327	47.085	50.586	5.627	1.00	0.00	RX1	C
ATOM	5260	O	LYS	327	45.930	50.643	6.033	1.00	0.00	RX1	O
ATOM	5261	N	LYS	328	48.157	50.574	6.436	1.00	0.00	RX1	N
ATOM	5262	H	LYS	328	49.092	50.681	6.104	1.00	0.00	RX1	H
ATOM	5263	CA	LYS	328	47.831	50.589	7.861	1.00	0.00	RX1	C
ATOM	5264	CB	LYS	328	49.019	50.224	8.749	1.00	0.00	RX1	C
ATOM	5265	CG	LYS	328	48.556	49.307	9.883	1.00	0.00	RX1	C
ATOM	5266	CD	LYS	328	49.579	49.129	11.003	1.00	0.00	RX1	C
ATOM	5267	CE	LYS	328	49.537	50.251	12.046	1.00	0.00	RX1	C
ATOM	5268	NZ	LYS	328	48.253	50.258	12.763	1.00	0.00	RX1	N
ATOM	5269	HZ1	LYS	328	48.304	50.905	13.586	1.00	0.00	RX1	H
ATOM	5270	HZ2	LYS	328	47.485	50.632	12.173	1.00	0.00	RX1	H
ATOM	5271	HZ3	LYS	328	47.993	49.306	13.099	1.00	0.00	RX1	H
ATOM	5272	C	LYS	328	47.208	51.881	8.339	1.00	0.00	RX1	C
ATOM	5273	O	LYS	328	47.764	52.964	8.219	1.00	0.00	RX1	O
ATOM	5274	N	CYS	329	46.005	51.702	8.877	1.00	0.00	RX1	N
ATOM	5275	H	CYS	329	45.661	50.782	9.064	1.00	0.00	RX1	H
ATOM	5276	CA	CYS	329	45.406	52.819	9.583	1.00	0.00	RX1	C
ATOM	5277	CB	CYS	329	43.888	52.677	9.485	1.00	0.00	RX1	C
ATOM	5278	SG	CYS	329	42.953	54.149	9.986	1.00	0.00	RX1	S
ATOM	5279	C	CYS	329	45.895	52.770	11.010	1.00	0.00	RX1	C
ATOM	5280	O	CYS	329	46.509	51.790	11.429	1.00	0.00	RX1	O
ATOM	5281	N	GLU	330	45.580	53.839	11.747	1.00	0.00	RX1	N
ATOM	5282	H	GLU	330	45.062	54.628	11.423	1.00	0.00	RX1	H
ATOM	5283	CA	GLU	330	45.664	53.560	13.165	1.00	0.00	RX1	C
ATOM	5284	CB	GLU	330	46.533	54.576	13.907	1.00	0.00	RX1	C
ATOM	5285	CG	GLU	330	47.198	53.909	15.117	1.00	0.00	RX1	C
ATOM	5286	CD	GLU	330	47.783	52.579	14.670	1.00	0.00	RX1	C
ATOM	5287	OE1	GLU	330	47.179	51.533	14.901	1.00	0.00	RX1	O
ATOM	5288	OE2	GLU	330	48.838	52.571	14.050	1.00	0.00	RX1	O
ATOM	5289	C	GLU	330	44.303	53.332	13.782	1.00	0.00	RX1	C
ATOM	5290	O	GLU	330	43.281	53.860	13.338	1.00	0.00	RX1	O
ATOM	5291	N	GLY	331	44.329	52.446	14.783	1.00	0.00	RX1	N
ATOM	5292	H	GLY	331	45.209	52.017	15.006	1.00	0.00	RX1	H
ATOM	5293	CA	GLY	331	43.067	51.914	15.278	1.00	0.00	RX1	C
ATOM	5294	C	GLY	331	42.416	51.000	14.254	1.00	0.00	RX1	C
ATOM	5295	O	GLY	331	42.585	51.154	13.048	1.00	0.00	RX1	O
ATOM	5296	N	PRO	332	41.636	50.033	14.783	1.00	0.00	RX1	N
ATOM	5297	CD	PRO	332	41.359	49.825	16.197	1.00	0.00	RX1	C
ATOM	5298	CA	PRO	332	41.004	49.035	13.914	1.00	0.00	RX1	C
ATOM	5299	CB	PRO	332	40.077	48.295	14.882	1.00	0.00	RX1	C
ATOM	5300	CG	PRO	332	40.760	48.423	16.244	1.00	0.00	RX1	C
ATOM	5301	C	PRO	332	40.283	49.631	12.721	1.00	0.00	RX1	C
ATOM	5302	O	PRO	332	39.437	50.512	12.856	1.00	0.00	RX1	O
ATOM	5303	N	CYS	333	40.661	49.111	11.546	1.00	0.00	RX1	N
ATOM	5304	H	CYS	333	41.329	48.370	11.523	1.00	0.00	RX1	H
ATOM	5305	CA	CYS	333	39.859	49.362	10.356	1.00	0.00	RX1	C

ATOM	5306	CB	CYS	333	40.539	48.734	9.145	1.00	0.00	RX1 C
ATOM	5307	SG	CYS	333	42.069	49.626	8.786	1.00	0.00	RX1 S
ATOM	5308	C	CYS	333	38.442	48.888	10.569	1.00	0.00	RX1 C
ATOM	5309	O	CYS	333	38.167	47.965	11.327	1.00	0.00	RX1 O
ATOM	5310	N	ARG	334	37.537	49.656	9.964	1.00	0.00	RX1 N
ATOM	5311	H	ARG	334	37.728	50.241	9.179	1.00	0.00	RX1 H
ATOM	5312	CA	ARG	334	36.253	49.670	10.645	1.00	0.00	RX1 C
ATOM	5313	CB	ARG	334	35.488	50.951	10.288	1.00	0.00	RX1 C
ATOM	5314	CG	ARG	334	36.010	52.285	10.868	1.00	0.00	RX1 C
ATOM	5315	CD	ARG	334	37.354	52.843	10.359	1.00	0.00	RX1 C
ATOM	5316	NE	ARG	334	38.476	52.494	11.235	1.00	0.00	RX1 N
ATOM	5317	HE	ARG	334	38.293	51.865	12.003	1.00	0.00	RX1 H
ATOM	5318	CZ	ARG	334	39.715	53.057	11.062	1.00	0.00	RX1 C
ATOM	5319	NH1	ARG	334	39.965	53.805	9.967	1.00	0.00	RX1 N
ATOM	5320	HH11	ARG	334	40.818	54.323	9.857	1.00	0.00	RX1 H
ATOM	5321	HH12	ARG	334	39.317	53.847	9.193	1.00	0.00	RX1 H
ATOM	5322	NH2	ARG	334	40.674	52.843	11.990	1.00	0.00	RX1 N
ATOM	5323	HH21	ARG	334	41.593	53.265	11.998	1.00	0.00	RX1 H
ATOM	5324	HH22	ARG	334	40.504	52.196	12.741	1.00	0.00	RX1 H
ATOM	5325	C	ARG	334	35.411	48.426	10.438	1.00	0.00	RX1 C
ATOM	5326	O	ARG	334	34.914	48.125	9.360	1.00	0.00	RX1 O
ATOM	5327	N	LYS	335	35.293	47.722	11.580	1.00	0.00	RX1 N
ATOM	5328	H	LYS	335	35.877	48.011	12.337	1.00	0.00	RX1 H
ATOM	5329	CA	LYS	335	34.459	46.528	11.746	1.00	0.00	RX1 C
ATOM	5330	CB	LYS	335	33.033	46.917	12.175	1.00	0.00	RX1 C
ATOM	5331	CG	LYS	335	32.275	47.737	11.137	1.00	0.00	RX1 C
ATOM	5332	CD	LYS	335	30.900	48.235	11.546	1.00	0.00	RX1 C
ATOM	5333	CE	LYS	335	30.104	48.522	10.278	1.00	0.00	RX1 C
ATOM	5334	NZ	LYS	335	29.895	47.242	9.592	1.00	0.00	RX1 N
ATOM	5335	HZ1	LYS	335	28.887	47.097	9.373	1.00	0.00	RX1 H
ATOM	5336	HZ2	LYS	335	30.467	47.117	8.728	1.00	0.00	RX1 H
ATOM	5337	HZ3	LYS	335	30.151	46.452	10.216	1.00	0.00	RX1 H
ATOM	5338	C	LYS	335	34.529	45.490	10.630	1.00	0.00	RX1 C
ATOM	5339	O	LYS	335	33.580	45.180	9.913	1.00	0.00	RX1 O
ATOM	5340	N	VAL	336	35.751	44.955	10.531	1.00	0.00	RX1 N
ATOM	5341	H	VAL	336	36.458	45.191	11.196	1.00	0.00	RX1 H
ATOM	5342	CA	VAL	336	35.910	43.744	9.733	1.00	0.00	RX1 C
ATOM	5343	CB	VAL	336	37.249	43.767	8.991	1.00	0.00	RX1 C
ATOM	5344	CG1	VAL	336	37.308	44.981	8.063	1.00	0.00	RX1 C
ATOM	5345	CG2	VAL	336	38.438	43.727	9.954	1.00	0.00	RX1 C
ATOM	5346	C	VAL	336	35.794	42.527	10.634	1.00	0.00	RX1 C
ATOM	5347	O	VAL	336	35.952	42.637	11.844	1.00	0.00	RX1 O
ATOM	5348	N	CYS	337	35.503	41.379	10.017	1.00	0.00	RX1 N
ATOM	5349	H	CYS	337	35.412	41.276	9.024	1.00	0.00	RX1 H
ATOM	5350	CA	CYS	337	35.360	40.192	10.853	1.00	0.00	RX1 C
ATOM	5351	CB	CYS	337	33.873	39.896	11.037	1.00	0.00	RX1 C
ATOM	5352	SG	CYS	337	32.904	41.378	11.417	1.00	0.00	RX1 S
ATOM	5353	C	CYS	337	36.106	39.029	10.242	1.00	0.00	RX1 C
ATOM	5354	O	CYS	337	36.006	38.797	9.045	1.00	0.00	RX1 O
ATOM	5355	N	ASN	338	36.861	38.316	11.099	1.00	0.00	RX1 N
ATOM	5356	H	ASN	338	36.917	38.573	12.063	1.00	0.00	RX1 H
ATOM	5357	CA	ASN	338	37.777	37.293	10.569	1.00	0.00	RX1 C
ATOM	5358	CB	ASN	338	38.521	36.587	11.694	1.00	0.00	RX1 C
ATOM	5359	CG	ASN	338	39.222	35.360	11.151	1.00	0.00	RX1 C
ATOM	5360	OD1	ASN	338	38.785	34.235	11.369	1.00	0.00	RX1 O
ATOM	5361	ND2	ASN	338	40.336	35.625	10.449	1.00	0.00	RX1 N
ATOM	5362	HD21	ASN	338	40.791	36.521	10.520	1.00	0.00	RX1 H
ATOM	5363	HD22	ASN	338	40.814	34.966	9.870	1.00	0.00	RX1 H
ATOM	5364	C	ASN	338	37.179	36.291	9.584	1.00	0.00	RX1 C
ATOM	5365	O	ASN	338	37.674	36.133	8.479	1.00	0.00	RX1 O
ATOM	5366	N	GLY	339	36.081	35.653	9.994	1.00	0.00	RX1 N

ATOM	5367	H	GLY	339	35.741	35.663	10.931	1.00	0.00	RX1	H
ATOM	5368	CA	GLY	339	35.453	34.817	8.977	1.00	0.00	RX1	C
ATOM	5369	C	GLY	339	35.085	33.439	9.462	1.00	0.00	RX1	C
ATOM	5370	O	GLY	339	35.869	32.728	10.075	1.00	0.00	RX1	O
ATOM	5371	N	ILE	340	33.823	33.104	9.177	1.00	0.00	RX1	N
ATOM	5372	H	ILE	340	33.392	33.583	8.411	1.00	0.00	RX1	H
ATOM	5373	CA	ILE	340	33.275	31.835	9.645	1.00	0.00	RX1	C
ATOM	5374	CB	ILE	340	31.781	31.800	9.323	1.00	0.00	RX1	C
ATOM	5375	CG2	ILE	340	31.178	30.418	9.527	1.00	0.00	RX1	C
ATOM	5376	CG1	ILE	340	31.035	32.838	10.156	1.00	0.00	RX1	C
ATOM	5377	CD1	ILE	340	30.987	32.447	11.634	1.00	0.00	RX1	C
ATOM	5378	C	ILE	340	34.008	30.643	9.047	1.00	0.00	RX1	C
ATOM	5379	O	ILE	340	33.842	30.291	7.882	1.00	0.00	RX1	O
ATOM	5380	N	GLY	341	34.836	30.050	9.916	1.00	0.00	RX1	N
ATOM	5381	H	GLY	341	35.004	30.404	10.841	1.00	0.00	RX1	H
ATOM	5382	CA	GLY	341	35.605	28.901	9.464	1.00	0.00	RX1	C
ATOM	5383	C	GLY	341	37.103	29.093	9.564	1.00	0.00	RX1	C
ATOM	5384	O	GLY	341	37.854	28.151	9.817	1.00	0.00	RX1	O
ATOM	5385	N	ILE	342	37.514	30.341	9.320	1.00	0.00	RX1	N
ATOM	5386	H	ILE	342	36.895	31.124	9.412	1.00	0.00	RX1	H
ATOM	5387	CA	ILE	342	38.945	30.583	9.187	1.00	0.00	RX1	C
ATOM	5388	CB	ILE	342	39.174	31.509	7.983	1.00	0.00	RX1	C
ATOM	5389	CG2	ILE	342	38.906	32.966	8.346	1.00	0.00	RX1	C
ATOM	5390	CG1	ILE	342	40.524	31.279	7.299	1.00	0.00	RX1	C
ATOM	5391	CD1	ILE	342	40.699	32.153	6.060	1.00	0.00	RX1	C
ATOM	5392	C	ILE	342	39.535	31.097	10.499	1.00	0.00	RX1	C
ATOM	5393	O	ILE	342	38.807	31.304	11.459	1.00	0.00	RX1	O
ATOM	5394	N	GLY	343	40.873	31.271	10.506	1.00	0.00	RX1	N
ATOM	5395	H	GLY	343	41.393	31.033	9.691	1.00	0.00	RX1	H
ATOM	5396	CA	GLY	343	41.560	31.984	11.586	1.00	0.00	RX1	C
ATOM	5397	C	GLY	343	41.049	31.773	12.998	1.00	0.00	RX1	C
ATOM	5398	O	GLY	343	41.091	30.678	13.547	1.00	0.00	RX1	O
ATOM	5399	N	GLU	344	40.577	32.896	13.553	1.00	0.00	RX1	N
ATOM	5400	H	GLU	344	40.386	33.696	12.984	1.00	0.00	RX1	H
ATOM	5401	CA	GLU	344	40.095	32.817	14.928	1.00	0.00	RX1	C
ATOM	5402	CB	GLU	344	40.260	34.169	15.629	1.00	0.00	RX1	C
ATOM	5403	CG	GLU	344	39.513	35.331	14.982	1.00	0.00	RX1	C
ATOM	5404	CD	GLU	344	40.124	36.638	15.447	1.00	0.00	RX1	C
ATOM	5405	OE1	GLU	344	41.110	36.617	16.176	1.00	0.00	RX1	O
ATOM	5406	OE2	GLU	344	39.645	37.698	15.061	1.00	0.00	RX1	O
ATOM	5407	C	GLU	344	38.711	32.206	15.069	1.00	0.00	RX1	C
ATOM	5408	O	GLU	344	38.361	31.651	16.103	1.00	0.00	RX1	O
ATOM	5409	N	PHE	345	37.944	32.266	13.968	1.00	0.00	RX1	N
ATOM	5410	H	PHE	345	38.289	32.588	13.082	1.00	0.00	RX1	H
ATOM	5411	CA	PHE	345	36.692	31.507	13.994	1.00	0.00	RX1	C
ATOM	5412	CB	PHE	345	35.614	32.258	13.226	1.00	0.00	RX1	C
ATOM	5413	CG	PHE	345	35.492	33.607	13.888	1.00	0.00	RX1	C
ATOM	5414	CD1	PHE	345	35.200	33.684	15.244	1.00	0.00	RX1	C
ATOM	5415	CD2	PHE	345	35.696	34.768	13.158	1.00	0.00	RX1	C
ATOM	5416	CE1	PHE	345	35.138	34.920	15.874	1.00	0.00	RX1	C
ATOM	5417	CE2	PHE	345	35.632	36.006	13.785	1.00	0.00	RX1	C
ATOM	5418	CZ	PHE	345	35.365	36.081	15.146	1.00	0.00	RX1	C
ATOM	5419	C	PHE	345	36.902	30.070	13.553	1.00	0.00	RX1	C
ATOM	5420	O	PHE	345	36.326	29.543	12.603	1.00	0.00	RX1	O
ATOM	5421	N	LYS	346	37.821	29.436	14.297	1.00	0.00	RX1	N
ATOM	5422	H	LYS	346	38.149	29.864	15.142	1.00	0.00	RX1	H
ATOM	5423	CA	LYS	346	38.239	28.115	13.854	1.00	0.00	RX1	C
ATOM	5424	CB	LYS	346	39.562	27.714	14.507	1.00	0.00	RX1	C
ATOM	5425	CG	LYS	346	40.265	26.621	13.701	1.00	0.00	RX1	C
ATOM	5426	CD	LYS	346	40.603	27.081	12.279	1.00	0.00	RX1	C
ATOM	5427	CE	LYS	346	40.113	26.115	11.194	1.00	0.00	RX1	C

ATOM	5428	NZ	LYS	346	38.645	26.130	11.135	1.00	0.00	RX1	N
ATOM	5429	HZ1	LYS	346	38.296	25.620	10.304	1.00	0.00	RX1	H
ATOM	5430	HZ2	LYS	346	38.210	25.734	11.996	1.00	0.00	RX1	H
ATOM	5431	HZ3	LYS	346	38.318	27.116	11.037	1.00	0.00	RX1	H
ATOM	5432	C	LYS	346	37.182	27.038	14.005	1.00	0.00	RX1	C
ATOM	5433	O	LYS	346	37.176	26.054	13.266	1.00	0.00	RX1	O
ATOM	5434	N	ASP	347	36.291	27.300	14.967	1.00	0.00	RX1	N
ATOM	5435	H	ASP	347	36.288	28.180	15.448	1.00	0.00	RX1	H
ATOM	5436	CA	ASP	347	35.120	26.460	15.214	1.00	0.00	RX1	C
ATOM	5437	CB	ASP	347	34.325	27.026	16.403	1.00	0.00	RX1	C
ATOM	5438	CG	ASP	347	33.817	28.428	16.089	1.00	0.00	RX1	C
ATOM	5439	OD1	ASP	347	34.623	29.346	15.965	1.00	0.00	RX1	O
ATOM	5440	OD2	ASP	347	32.617	28.616	15.901	1.00	0.00	RX1	O
ATOM	5441	C	ASP	347	34.204	26.303	14.011	1.00	0.00	RX1	C
ATOM	5442	O	ASP	347	33.619	25.254	13.763	1.00	0.00	RX1	O
ATOM	5443	N	SER	348	34.085	27.424	13.289	1.00	0.00	RX1	N
ATOM	5444	H	SER	348	34.671	28.229	13.404	1.00	0.00	RX1	H
ATOM	5445	CA	SER	348	32.839	27.598	12.564	1.00	0.00	RX1	C
ATOM	5446	CB	SER	348	32.543	29.085	12.574	1.00	0.00	RX1	C
ATOM	5447	OG	SER	348	33.535	29.737	13.371	1.00	0.00	RX1	O
ATOM	5448	HG	SER	348	33.411	29.436	14.275	1.00	0.00	RX1	H
ATOM	5449	C	SER	348	32.716	26.940	11.206	1.00	0.00	RX1	C
ATOM	5450	O	SER	348	32.754	27.566	10.155	1.00	0.00	RX1	O
ATOM	5451	N	LEU	349	32.483	25.622	11.281	1.00	0.00	RX1	N
ATOM	5452	H	LEU	349	32.531	25.180	12.179	1.00	0.00	RX1	H
ATOM	5453	CA	LEU	349	32.022	24.920	10.082	1.00	0.00	RX1	C
ATOM	5454	CB	LEU	349	32.171	23.401	10.256	1.00	0.00	RX1	C
ATOM	5455	CG	LEU	349	31.730	22.812	11.606	1.00	0.00	RX1	C
ATOM	5456	CD1	LEU	349	30.218	22.848	11.845	1.00	0.00	RX1	C
ATOM	5457	CD2	LEU	349	32.278	21.396	11.786	1.00	0.00	RX1	C
ATOM	5458	C	LEU	349	30.619	25.319	9.636	1.00	0.00	RX1	C
ATOM	5459	O	LEU	349	30.161	25.013	8.543	1.00	0.00	RX1	O
ATOM	5460	N	SER	350	29.968	26.058	10.541	1.00	0.00	RX1	N
ATOM	5461	H	SER	350	30.283	26.256	11.468	1.00	0.00	RX1	H
ATOM	5462	CA	SER	350	28.784	26.783	10.130	1.00	0.00	RX1	C
ATOM	5463	CB	SER	350	27.573	25.893	10.398	1.00	0.00	RX1	C
ATOM	5464	OG	SER	350	26.423	26.381	9.699	1.00	0.00	RX1	O
ATOM	5465	HG	SER	350	26.046	27.057	10.263	1.00	0.00	RX1	H
ATOM	5466	C	SER	350	28.725	28.075	10.900	1.00	0.00	RX1	C
ATOM	5467	O	SER	350	29.154	28.150	12.048	1.00	0.00	RX1	O
ATOM	5468	N	ILE	351	28.129	29.072	10.239	1.00	0.00	RX1	N
ATOM	5469	H	ILE	351	27.899	28.951	9.272	1.00	0.00	RX1	H
ATOM	5470	CA	ILE	351	27.647	30.223	10.991	1.00	0.00	RX1	C
ATOM	5471	CB	ILE	351	27.231	31.337	10.018	1.00	0.00	RX1	C
ATOM	5472	CG2	ILE	351	26.000	30.938	9.206	1.00	0.00	RX1	C
ATOM	5473	CG1	ILE	351	27.088	32.696	10.705	1.00	0.00	RX1	C
ATOM	5474	CD1	ILE	351	26.904	33.834	9.700	1.00	0.00	RX1	C
ATOM	5475	C	ILE	351	26.520	29.756	11.899	1.00	0.00	RX1	C
ATOM	5476	O	ILE	351	25.895	28.736	11.628	1.00	0.00	RX1	O
ATOM	5477	N	ASN	352	26.358	30.484	13.007	1.00	0.00	RX1	N
ATOM	5478	H	ASN	352	26.838	31.345	13.188	1.00	0.00	RX1	H
ATOM	5479	CA	ASN	352	25.503	30.027	14.102	1.00	0.00	RX1	C
ATOM	5480	CB	ASN	352	26.113	28.801	14.775	1.00	0.00	RX1	C
ATOM	5481	CG	ASN	352	27.401	29.197	15.465	1.00	0.00	RX1	C
ATOM	5482	OD1	ASN	352	27.386	29.803	16.528	1.00	0.00	RX1	O
ATOM	5483	ND2	ASN	352	28.520	28.797	14.833	1.00	0.00	RX1	N
ATOM	5484	HD21	ASN	352	28.507	28.356	13.930	1.00	0.00	RX1	H
ATOM	5485	HD22	ASN	352	29.438	28.893	15.229	1.00	0.00	RX1	H
ATOM	5486	C	ASN	352	25.403	31.178	15.079	1.00	0.00	RX1	C
ATOM	5487	O	ASN	352	26.234	32.079	15.013	1.00	0.00	RX1	O
ATOM	5488	N	ALA	353	24.401	31.140	15.974	1.00	0.00	RX1	N

ATOM	5489	H	ALA	353	23.742	30.385	16.035	1.00	0.00	RX1	H
ATOM	5490	CA	ALA	353	24.291	32.296	16.869	1.00	0.00	RX1	C
ATOM	5491	CB	ALA	353	23.050	32.196	17.753	1.00	0.00	RX1	C
ATOM	5492	C	ALA	353	25.511	32.551	17.747	1.00	0.00	RX1	C
ATOM	5493	O	ALA	353	26.021	33.663	17.850	1.00	0.00	RX1	O
ATOM	5494	N	THR	354	25.995	31.446	18.336	1.00	0.00	RX1	N
ATOM	5495	H	THR	354	25.612	30.530	18.218	1.00	0.00	RX1	H
ATOM	5496	CA	THR	354	27.173	31.511	19.201	1.00	0.00	RX1	C
ATOM	5497	CB	THR	354	27.446	30.082	19.634	1.00	0.00	RX1	C
ATOM	5498	OG1	THR	354	26.184	29.412	19.724	1.00	0.00	RX1	O
ATOM	5499	HG1	THR	354	26.329	28.646	20.283	1.00	0.00	RX1	H
ATOM	5500	CG2	THR	354	28.219	29.993	20.951	1.00	0.00	RX1	C
ATOM	5501	C	THR	354	28.392	32.228	18.621	1.00	0.00	RX1	C
ATOM	5502	O	THR	354	29.191	32.836	19.333	1.00	0.00	RX1	O
ATOM	5503	N	ASN	355	28.502	32.188	17.285	1.00	0.00	RX1	N
ATOM	5504	H	ASN	355	27.859	31.675	16.711	1.00	0.00	RX1	H
ATOM	5505	CA	ASN	355	29.585	33.000	16.747	1.00	0.00	RX1	C
ATOM	5506	CB	ASN	355	30.636	32.147	16.043	1.00	0.00	RX1	C
ATOM	5507	CG	ASN	355	31.955	32.528	16.675	1.00	0.00	RX1	C
ATOM	5508	OD1	ASN	355	32.050	33.550	17.358	1.00	0.00	RX1	O
ATOM	5509	ND2	ASN	355	32.936	31.631	16.475	1.00	0.00	RX1	N
ATOM	5510	HD21	ASN	355	32.807	30.736	16.024	1.00	0.00	RX1	H
ATOM	5511	HD22	ASN	355	33.892	31.694	16.765	1.00	0.00	RX1	H
ATOM	5512	C	ASN	355	29.226	34.232	15.941	1.00	0.00	RX1	C
ATOM	5513	O	ASN	355	30.037	35.136	15.763	1.00	0.00	RX1	O
ATOM	5514	N	ILE	356	27.951	34.289	15.508	1.00	0.00	RX1	N
ATOM	5515	H	ILE	356	27.284	33.573	15.724	1.00	0.00	RX1	H
ATOM	5516	CA	ILE	356	27.543	35.469	14.737	1.00	0.00	RX1	C
ATOM	5517	CB	ILE	356	26.139	35.320	14.129	1.00	0.00	RX1	C
ATOM	5518	CG2	ILE	356	25.047	35.553	15.172	1.00	0.00	RX1	C
ATOM	5519	CG1	ILE	356	25.984	36.248	12.918	1.00	0.00	RX1	C
ATOM	5520	CD1	ILE	356	24.571	36.309	12.341	1.00	0.00	RX1	C
ATOM	5521	C	ILE	356	27.676	36.804	15.469	1.00	0.00	RX1	C
ATOM	5522	O	ILE	356	27.754	37.859	14.856	1.00	0.00	RX1	O
ATOM	5523	N	LYS	357	27.778	36.704	16.812	1.00	0.00	RX1	N
ATOM	5524	H	LYS	357	27.498	35.827	17.202	1.00	0.00	RX1	H
ATOM	5525	CA	LYS	357	28.146	37.845	17.670	1.00	0.00	RX1	C
ATOM	5526	CB	LYS	357	28.974	37.382	18.873	1.00	0.00	RX1	C
ATOM	5527	CG	LYS	357	28.568	36.048	19.494	1.00	0.00	RX1	C
ATOM	5528	CD	LYS	357	29.429	35.687	20.711	1.00	0.00	RX1	C
ATOM	5529	CE	LYS	357	30.940	35.577	20.449	1.00	0.00	RX1	C
ATOM	5530	NZ	LYS	357	31.276	34.480	19.527	1.00	0.00	RX1	N
ATOM	5531	HZ1	LYS	357	31.330	34.775	18.527	1.00	0.00	RX1	H
ATOM	5532	HZ2	LYS	357	32.221	34.101	19.727	1.00	0.00	RX1	H
ATOM	5533	HZ3	LYS	357	30.584	33.706	19.596	1.00	0.00	RX1	H
ATOM	5534	C	LYS	357	28.954	38.966	17.014	1.00	0.00	RX1	C
ATOM	5535	O	LYS	357	28.624	40.141	17.071	1.00	0.00	RX1	O
ATOM	5536	N	HIS	358	30.052	38.514	16.387	1.00	0.00	RX1	N
ATOM	5537	H	HIS	358	30.159	37.529	16.248	1.00	0.00	RX1	H
ATOM	5538	CA	HIS	358	30.985	39.461	15.770	1.00	0.00	RX1	C
ATOM	5539	CB	HIS	358	32.348	38.797	15.533	1.00	0.00	RX1	C
ATOM	5540	CG	HIS	358	32.838	38.088	16.776	1.00	0.00	RX1	C
ATOM	5541	ND1	HIS	358	33.700	38.622	17.661	1.00	0.00	RX1	N
ATOM	5542	HD1	HIS	358	34.123	39.506	17.605	1.00	0.00	RX1	H
ATOM	5543	CD2	HIS	358	32.497	36.803	17.210	1.00	0.00	RX1	C
ATOM	5544	NE2	HIS	358	33.157	36.566	18.369	1.00	0.00	RX1	N
ATOM	5545	CE1	HIS	358	33.903	37.688	18.645	1.00	0.00	RX1	C
ATOM	5546	C	HIS	358	30.454	39.986	14.443	1.00	0.00	RX1	C
ATOM	5547	O	HIS	358	30.483	41.163	14.108	1.00	0.00	RX1	O
ATOM	5548	N	PHE	359	29.944	39.004	13.689	1.00	0.00	RX1	N
ATOM	5549	H	PHE	359	29.747	38.122	14.112	1.00	0.00	RX1	H

ATOM	5550	CA	PHE	359	29.454	39.245	12.332	1.00	0.00	RX1 C
ATOM	5551	CB	PHE	359	29.195	37.902	11.643	1.00	0.00	RX1 C
ATOM	5552	CG	PHE	359	30.478	37.104	11.600	1.00	0.00	RX1 C
ATOM	5553	CD1	PHE	359	31.286	37.168	10.472	1.00	0.00	RX1 C
ATOM	5554	CD2	PHE	359	30.861	36.319	12.684	1.00	0.00	RX1 C
ATOM	5555	CE1	PHE	359	32.494	36.483	10.442	1.00	0.00	RX1 C
ATOM	5556	CE2	PHE	359	32.072	35.642	12.661	1.00	0.00	RX1 C
ATOM	5557	CZ	PHE	359	32.894	35.740	11.545	1.00	0.00	RX1 C
ATOM	5558	C	PHE	359	28.229	40.142	12.253	1.00	0.00	RX1 C
ATOM	5559	O	PHE	359	27.887	40.688	11.211	1.00	0.00	RX1 O
ATOM	5560	N	LYS	360	27.601	40.308	13.429	1.00	0.00	RX1 N
ATOM	5561	H	LYS	360	27.895	39.777	14.224	1.00	0.00	RX1 H
ATOM	5562	CA	LYS	360	26.426	41.167	13.546	1.00	0.00	RX1 C
ATOM	5563	CB	LYS	360	25.906	41.115	14.984	1.00	0.00	RX1 C
ATOM	5564	CG	LYS	360	24.415	41.438	15.079	1.00	0.00	RX1 C
ATOM	5565	CD	LYS	360	23.857	41.274	16.491	1.00	0.00	RX1 C
ATOM	5566	CE	LYS	360	22.364	41.597	16.545	1.00	0.00	RX1 C
ATOM	5567	NZ	LYS	360	21.647	40.729	15.610	1.00	0.00	RX1 N
ATOM	5568	HZ1	LYS	360	21.964	40.769	14.625	1.00	0.00	RX1 H
ATOM	5569	HZ2	LYS	360	21.697	39.713	15.843	1.00	0.00	RX1 H
ATOM	5570	HZ3	LYS	360	20.614	40.875	15.587	1.00	0.00	RX1 H
ATOM	5571	C	LYS	360	26.563	42.596	13.023	1.00	0.00	RX1 C
ATOM	5572	O	LYS	360	25.574	43.277	12.765	1.00	0.00	RX1 O
ATOM	5573	N	ASN	361	27.824	43.022	12.825	1.00	0.00	RX1 N
ATOM	5574	H	ASN	361	28.638	42.518	13.124	1.00	0.00	RX1 H
ATOM	5575	CA	ASN	361	27.942	44.087	11.832	1.00	0.00	RX1 C
ATOM	5576	CB	ASN	361	27.688	45.484	12.367	1.00	0.00	RX1 C
ATOM	5577	CG	ASN	361	26.955	46.168	11.241	1.00	0.00	RX1 C
ATOM	5578	OD1	ASN	361	27.517	46.897	10.426	1.00	0.00	RX1 O
ATOM	5579	ND2	ASN	361	25.644	45.872	11.238	1.00	0.00	RX1 N
ATOM	5580	HD21	ASN	361	25.304	45.186	11.894	1.00	0.00	RX1 H
ATOM	5581	HD22	ASN	361	24.978	46.268	10.607	1.00	0.00	RX1 H
ATOM	5582	C	ASN	361	29.196	44.098	10.991	1.00	0.00	RX1 C
ATOM	5583	O	ASN	361	30.010	45.014	11.062	1.00	0.00	RX1 O
ATOM	5584	N	CYS	362	29.297	43.055	10.161	1.00	0.00	RX1 N
ATOM	5585	H	CYS	362	28.590	42.342	10.149	1.00	0.00	RX1 H
ATOM	5586	CA	CYS	362	30.424	42.955	9.230	1.00	0.00	RX1 C
ATOM	5587	CB	CYS	362	30.227	41.732	8.342	1.00	0.00	RX1 C
ATOM	5588	SG	CYS	362	30.174	40.187	9.270	1.00	0.00	RX1 S
ATOM	5589	C	CYS	362	30.672	44.176	8.356	1.00	0.00	RX1 C
ATOM	5590	O	CYS	362	29.853	45.087	8.255	1.00	0.00	RX1 O
ATOM	5591	N	THR	363	31.846	44.131	7.710	1.00	0.00	RX1 N
ATOM	5592	H	THR	363	32.559	43.468	7.935	1.00	0.00	RX1 H
ATOM	5593	CA	THR	363	32.040	44.985	6.541	1.00	0.00	RX1 C
ATOM	5594	CB	THR	363	32.600	46.338	6.965	1.00	0.00	RX1 C
ATOM	5595	OG1	THR	363	32.036	46.709	8.228	1.00	0.00	RX1 O
ATOM	5596	HG1	THR	363	32.588	46.239	8.853	1.00	0.00	RX1 H
ATOM	5597	CG2	THR	363	32.344	47.411	5.907	1.00	0.00	RX1 C
ATOM	5598	C	THR	363	32.889	44.280	5.494	1.00	0.00	RX1 C
ATOM	5599	O	THR	363	32.454	43.970	4.393	1.00	0.00	RX1 O
ATOM	5600	N	SER	364	34.120	43.969	5.913	1.00	0.00	RX1 N
ATOM	5601	H	SER	364	34.495	44.164	6.818	1.00	0.00	RX1 H
ATOM	5602	CA	SER	364	34.828	42.978	5.112	1.00	0.00	RX1 C
ATOM	5603	CB	SER	364	36.101	43.622	4.537	1.00	0.00	RX1 C
ATOM	5604	OG	SER	364	36.698	42.778	3.548	1.00	0.00	RX1 O
ATOM	5605	HG	SER	364	36.930	43.330	2.796	1.00	0.00	RX1 H
ATOM	5606	C	SER	364	35.049	41.755	5.977	1.00	0.00	RX1 C
ATOM	5607	O	SER	364	34.976	41.837	7.201	1.00	0.00	RX1 O
ATOM	5608	N	ILE	365	35.268	40.632	5.293	1.00	0.00	RX1 N
ATOM	5609	H	ILE	365	35.354	40.664	4.294	1.00	0.00	RX1 H
ATOM	5610	CA	ILE	365	35.444	39.352	5.966	1.00	0.00	RX1 C

ATOM	5611	CB	ILE	365	34.356	38.370	5.519	1.00	0.00	RX1	C
ATOM	5612	CG2	ILE	365	34.581	36.971	6.085	1.00	0.00	RX1	C
ATOM	5613	CG1	ILE	365	32.973	38.883	5.903	1.00	0.00	RX1	C
ATOM	5614	CD1	ILE	365	32.784	38.882	7.417	1.00	0.00	RX1	C
ATOM	5615	C	ILE	365	36.811	38.813	5.622	1.00	0.00	RX1	C
ATOM	5616	O	ILE	365	37.106	38.530	4.466	1.00	0.00	RX1	O
ATOM	5617	N	SER	366	37.627	38.671	6.669	1.00	0.00	RX1	N
ATOM	5618	H	SER	366	37.315	38.819	7.610	1.00	0.00	RX1	H
ATOM	5619	CA	SER	366	39.016	38.243	6.497	1.00	0.00	RX1	C
ATOM	5620	CB	SER	366	39.650	38.653	7.809	1.00	0.00	RX1	C
ATOM	5621	OG	SER	366	38.862	39.767	8.264	1.00	0.00	RX1	O
ATOM	5622	HG	SER	366	39.094	40.483	7.686	1.00	0.00	RX1	H
ATOM	5623	C	SER	366	39.260	36.804	6.038	1.00	0.00	RX1	C
ATOM	5624	O	SER	366	40.392	36.352	5.915	1.00	0.00	RX1	O
ATOM	5625	N	GLY	367	38.142	36.116	5.770	1.00	0.00	RX1	N
ATOM	5626	H	GLY	367	37.233	36.458	5.994	1.00	0.00	RX1	H
ATOM	5627	CA	GLY	367	38.225	34.785	5.194	1.00	0.00	RX1	C
ATOM	5628	C	GLY	367	36.869	34.322	4.720	1.00	0.00	RX1	C
ATOM	5629	O	GLY	367	36.200	34.999	3.945	1.00	0.00	RX1	O
ATOM	5630	N	ASP	368	36.507	33.141	5.227	1.00	0.00	RX1	N
ATOM	5631	H	ASP	368	37.079	32.654	5.885	1.00	0.00	RX1	H
ATOM	5632	CA	ASP	368	35.363	32.429	4.661	1.00	0.00	RX1	C
ATOM	5633	CB	ASP	368	35.663	30.936	4.656	1.00	0.00	RX1	C
ATOM	5634	CG	ASP	368	36.965	30.702	3.928	1.00	0.00	RX1	C
ATOM	5635	OD1	ASP	368	37.040	31.011	2.745	1.00	0.00	RX1	O
ATOM	5636	OD2	ASP	368	37.912	30.216	4.537	1.00	0.00	RX1	O
ATOM	5637	C	ASP	368	34.041	32.668	5.359	1.00	0.00	RX1	C
ATOM	5638	O	ASP	368	33.971	33.221	6.453	1.00	0.00	RX1	O
ATOM	5639	N	LEU	369	32.990	32.180	4.681	1.00	0.00	RX1	N
ATOM	5640	H	LEU	369	33.098	31.858	3.737	1.00	0.00	RX1	H
ATOM	5641	CA	LEU	369	31.680	32.045	5.314	1.00	0.00	RX1	C
ATOM	5642	CB	LEU	369	30.700	33.111	4.812	1.00	0.00	RX1	C
ATOM	5643	CG	LEU	369	31.019	34.549	5.237	1.00	0.00	RX1	C
ATOM	5644	CD1	LEU	369	30.051	35.548	4.605	1.00	0.00	RX1	C
ATOM	5645	CD2	LEU	369	31.050	34.713	6.756	1.00	0.00	RX1	C
ATOM	5646	C	LEU	369	31.083	30.666	5.076	1.00	0.00	RX1	C
ATOM	5647	O	LEU	369	30.410	30.409	4.083	1.00	0.00	RX1	O
ATOM	5648	N	HIS	370	31.352	29.778	6.044	1.00	0.00	RX1	N
ATOM	5649	H	HIS	370	31.916	30.042	6.828	1.00	0.00	RX1	H
ATOM	5650	CA	HIS	370	30.676	28.476	6.016	1.00	0.00	RX1	C
ATOM	5651	CB	HIS	370	31.389	27.475	6.919	1.00	0.00	RX1	C
ATOM	5652	CG	HIS	370	32.739	27.056	6.395	1.00	0.00	RX1	C
ATOM	5653	ND1	HIS	370	33.874	27.746	6.596	1.00	0.00	RX1	N
ATOM	5654	HD1	HIS	370	33.960	28.603	7.071	1.00	0.00	RX1	H
ATOM	5655	CD2	HIS	370	33.039	25.900	5.673	1.00	0.00	RX1	C
ATOM	5656	NE2	HIS	370	34.375	25.898	5.441	1.00	0.00	RX1	N
ATOM	5657	CE1	HIS	370	34.889	27.037	6.013	1.00	0.00	RX1	C
ATOM	5658	C	HIS	370	29.232	28.547	6.494	1.00	0.00	RX1	C
ATOM	5659	O	HIS	370	28.957	28.956	7.614	1.00	0.00	RX1	O
ATOM	5660	N	ILE	371	28.312	28.116	5.625	1.00	0.00	RX1	N
ATOM	5661	H	ILE	371	28.521	27.830	4.688	1.00	0.00	RX1	H
ATOM	5662	CA	ILE	371	26.914	28.077	6.062	1.00	0.00	RX1	C
ATOM	5663	CB	ILE	371	26.076	29.133	5.330	1.00	0.00	RX1	C
ATOM	5664	CG2	ILE	371	24.669	29.227	5.920	1.00	0.00	RX1	C
ATOM	5665	CG1	ILE	371	26.772	30.494	5.319	1.00	0.00	RX1	C
ATOM	5666	CD1	ILE	371	25.964	31.548	4.570	1.00	0.00	RX1	C
ATOM	5667	C	ILE	371	26.309	26.691	5.877	1.00	0.00	RX1	C
ATOM	5668	O	ILE	371	25.601	26.397	4.921	1.00	0.00	RX1	O
ATOM	5669	N	LEU	372	26.641	25.823	6.838	1.00	0.00	RX1	N
ATOM	5670	H	LEU	372	27.015	26.153	7.705	1.00	0.00	RX1	H
ATOM	5671	CA	LEU	372	26.075	24.474	6.772	1.00	0.00	RX1	C

ATOM	5672	CB	LEU	372	26.906	23.541	7.664	1.00	0.00	RX1	C
ATOM	5673	CG	LEU	372	27.830	22.587	6.900	1.00	0.00	RX1	C
ATOM	5674	CD1	LEU	372	28.756	23.312	5.925	1.00	0.00	RX1	C
ATOM	5675	CD2	LEU	372	28.600	21.677	7.859	1.00	0.00	RX1	C
ATOM	5676	C	LEU	372	24.612	24.501	7.200	1.00	0.00	RX1	C
ATOM	5677	O	LEU	372	24.209	25.396	7.930	1.00	0.00	RX1	O
ATOM	5678	N	PRO	373	23.804	23.500	6.745	1.00	0.00	RX1	N
ATOM	5679	CD	PRO	373	24.165	22.389	5.868	1.00	0.00	RX1	C
ATOM	5680	CA	PRO	373	22.380	23.472	7.126	1.00	0.00	RX1	C
ATOM	5681	CB	PRO	373	21.951	22.066	6.699	1.00	0.00	RX1	C
ATOM	5682	CG	PRO	373	22.841	21.729	5.506	1.00	0.00	RX1	C
ATOM	5683	C	PRO	373	22.083	23.768	8.590	1.00	0.00	RX1	C
ATOM	5684	O	PRO	373	21.208	24.551	8.945	1.00	0.00	RX1	O
ATOM	5685	N	VAL	374	22.910	23.113	9.425	1.00	0.00	RX1	N
ATOM	5686	H	VAL	374	23.583	22.474	9.060	1.00	0.00	RX1	H
ATOM	5687	CA	VAL	374	22.803	23.267	10.874	1.00	0.00	RX1	C
ATOM	5688	CB	VAL	374	23.924	22.497	11.571	1.00	0.00	RX1	C
ATOM	5689	CG1	VAL	374	25.295	23.117	11.311	1.00	0.00	RX1	C
ATOM	5690	CG2	VAL	374	23.602	22.308	13.052	1.00	0.00	RX1	C
ATOM	5691	C	VAL	374	22.673	24.690	11.411	1.00	0.00	RX1	C
ATOM	5692	O	VAL	374	22.007	24.920	12.411	1.00	0.00	RX1	O
ATOM	5693	N	ALA	375	23.288	25.642	10.677	1.00	0.00	RX1	N
ATOM	5694	H	ALA	375	23.811	25.388	9.862	1.00	0.00	RX1	H
ATOM	5695	CA	ALA	375	23.219	27.062	11.034	1.00	0.00	RX1	C
ATOM	5696	CB	ALA	375	23.484	27.927	9.802	1.00	0.00	RX1	C
ATOM	5697	C	ALA	375	21.897	27.522	11.612	1.00	0.00	RX1	C
ATOM	5698	O	ALA	375	21.808	28.148	12.659	1.00	0.00	RX1	O
ATOM	5699	N	PHE	376	20.857	27.170	10.852	1.00	0.00	RX1	N
ATOM	5700	H	PHE	376	20.968	26.509	10.107	1.00	0.00	RX1	H
ATOM	5701	CA	PHE	376	19.551	27.718	11.205	1.00	0.00	RX1	C
ATOM	5702	CB	PHE	376	18.705	27.867	9.946	1.00	0.00	RX1	C
ATOM	5703	CG	PHE	376	19.598	28.307	8.812	1.00	0.00	RX1	C
ATOM	5704	CD1	PHE	376	19.871	27.425	7.773	1.00	0.00	RX1	C
ATOM	5705	CD2	PHE	376	20.150	29.584	8.810	1.00	0.00	RX1	C
ATOM	5706	CE1	PHE	376	20.695	27.819	6.728	1.00	0.00	RX1	C
ATOM	5707	CE2	PHE	376	20.972	29.978	7.762	1.00	0.00	RX1	C
ATOM	5708	CZ	PHE	376	21.238	29.098	6.721	1.00	0.00	RX1	C
ATOM	5709	C	PHE	376	18.805	26.905	12.242	1.00	0.00	RX1	C
ATOM	5710	O	PHE	376	17.881	27.376	12.886	1.00	0.00	RX1	O
ATOM	5711	N	ARG	377	19.247	25.645	12.345	1.00	0.00	RX1	N
ATOM	5712	H	ARG	377	20.139	25.381	11.983	1.00	0.00	RX1	H
ATOM	5713	CA	ARG	377	18.489	24.687	13.146	1.00	0.00	RX1	C
ATOM	5714	CB	ARG	377	18.587	23.274	12.554	1.00	0.00	RX1	C
ATOM	5715	CG	ARG	377	19.010	23.132	11.081	1.00	0.00	RX1	C
ATOM	5716	CD	ARG	377	18.167	23.825	9.999	1.00	0.00	RX1	C
ATOM	5717	NE	ARG	377	16.773	23.409	10.021	1.00	0.00	RX1	N
ATOM	5718	HE	ARG	377	16.452	22.762	10.736	1.00	0.00	RX1	H
ATOM	5719	CZ	ARG	377	15.888	23.918	9.117	1.00	0.00	RX1	C
ATOM	5720	NH1	ARG	377	16.318	24.722	8.127	1.00	0.00	RX1	N
ATOM	5721	HH11	ARG	377	15.641	25.059	7.443	1.00	0.00	RX1	H
ATOM	5722	HH12	ARG	377	17.271	24.992	8.012	1.00	0.00	RX1	H
ATOM	5723	NH2	ARG	377	14.586	23.613	9.213	1.00	0.00	RX1	N
ATOM	5724	HH21	ARG	377	13.900	24.049	8.614	1.00	0.00	RX1	H
ATOM	5725	HH22	ARG	377	14.294	22.926	9.899	1.00	0.00	RX1	H
ATOM	5726	C	ARG	377	18.938	24.682	14.596	1.00	0.00	RX1	C
ATOM	5727	O	ARG	377	18.168	24.573	15.539	1.00	0.00	RX1	O
ATOM	5728	N	GLY	378	20.261	24.849	14.722	1.00	0.00	RX1	N
ATOM	5729	H	GLY	378	20.867	24.814	13.930	1.00	0.00	RX1	H
ATOM	5730	CA	GLY	378	20.838	24.781	16.053	1.00	0.00	RX1	C
ATOM	5731	C	GLY	378	21.472	23.435	16.312	1.00	0.00	RX1	C
ATOM	5732	O	GLY	378	20.960	22.397	15.911	1.00	0.00	RX1	O

ATOM	5733	N	ASP	379	22.627	23.491	16.982	1.00	0.00	RX1	N
ATOM	5734	H	ASP	379	22.997	24.334	17.377	1.00	0.00	RX1	H
ATOM	5735	CA	ASP	379	23.259	22.208	17.260	1.00	0.00	RX1	C
ATOM	5736	CB	ASP	379	24.627	22.132	16.586	1.00	0.00	RX1	C
ATOM	5737	CG	ASP	379	25.320	20.797	16.813	1.00	0.00	RX1	C
ATOM	5738	OD1	ASP	379	24.663	19.780	16.972	1.00	0.00	RX1	O
ATOM	5739	OD2	ASP	379	26.543	20.764	16.821	1.00	0.00	RX1	O
ATOM	5740	C	ASP	379	23.373	21.898	18.734	1.00	0.00	RX1	C
ATOM	5741	O	ASP	379	23.743	22.716	19.574	1.00	0.00	RX1	O
ATOM	5742	N	SER	380	23.047	20.625	18.984	1.00	0.00	RX1	N
ATOM	5743	H	SER	380	22.898	20.021	18.195	1.00	0.00	RX1	H
ATOM	5744	CA	SER	380	23.257	20.038	20.299	1.00	0.00	RX1	C
ATOM	5745	CB	SER	380	22.672	18.657	20.187	1.00	0.00	RX1	C
ATOM	5746	OG	SER	380	21.780	18.732	19.072	1.00	0.00	RX1	O
ATOM	5747	HG	SER	380	21.175	19.443	19.249	1.00	0.00	RX1	H
ATOM	5748	C	SER	380	24.715	20.075	20.728	1.00	0.00	RX1	C
ATOM	5749	O	SER	380	25.059	20.553	21.800	1.00	0.00	RX1	O
ATOM	5750	N	PHE	381	25.564	19.547	19.832	1.00	0.00	RX1	N
ATOM	5751	H	PHE	381	25.247	19.392	18.891	1.00	0.00	RX1	H
ATOM	5752	CA	PHE	381	26.963	19.363	20.209	1.00	0.00	RX1	C
ATOM	5753	CB	PHE	381	27.666	18.375	19.262	1.00	0.00	RX1	C
ATOM	5754	CG	PHE	381	29.103	18.154	19.691	1.00	0.00	RX1	C
ATOM	5755	CD1	PHE	381	29.390	17.677	20.991	1.00	0.00	RX1	C
ATOM	5756	CD2	PHE	381	30.140	18.457	18.781	1.00	0.00	RX1	C
ATOM	5757	CE1	PHE	381	30.731	17.538	21.398	1.00	0.00	RX1	C
ATOM	5758	CE2	PHE	381	31.481	18.311	19.185	1.00	0.00	RX1	C
ATOM	5759	CZ	PHE	381	31.763	17.864	20.493	1.00	0.00	RX1	C
ATOM	5760	C	PHE	381	27.741	20.660	20.366	1.00	0.00	RX1	C
ATOM	5761	O	PHE	381	28.484	20.857	21.318	1.00	0.00	RX1	O
ATOM	5762	N	THR	382	27.538	21.549	19.391	1.00	0.00	RX1	N
ATOM	5763	H	THR	382	26.887	21.371	18.652	1.00	0.00	RX1	H
ATOM	5764	CA	THR	382	28.305	22.788	19.481	1.00	0.00	RX1	C
ATOM	5765	CB	THR	382	28.704	23.303	18.092	1.00	0.00	RX1	C
ATOM	5766	OG1	THR	382	27.579	23.360	17.209	1.00	0.00	RX1	O
ATOM	5767	HG1	THR	382	27.392	22.457	16.949	1.00	0.00	RX1	H
ATOM	5768	CG2	THR	382	29.799	22.426	17.481	1.00	0.00	RX1	C
ATOM	5769	C	THR	382	27.691	23.869	20.357	1.00	0.00	RX1	C
ATOM	5770	O	THR	382	28.244	24.950	20.513	1.00	0.00	RX1	O
ATOM	5771	N	HIS	383	26.508	23.533	20.916	1.00	0.00	RX1	N
ATOM	5772	H	HIS	383	26.073	22.674	20.656	1.00	0.00	RX1	H
ATOM	5773	CA	HIS	383	25.785	24.475	21.777	1.00	0.00	RX1	C
ATOM	5774	CB	HIS	383	26.512	24.772	23.098	1.00	0.00	RX1	C
ATOM	5775	CG	HIS	383	26.904	23.509	23.825	1.00	0.00	RX1	C
ATOM	5776	ND1	HIS	383	26.342	22.302	23.628	1.00	0.00	RX1	N
ATOM	5777	HD1	HIS	383	25.642	22.049	22.986	1.00	0.00	RX1	H
ATOM	5778	CD2	HIS	383	27.893	23.390	24.806	1.00	0.00	RX1	C
ATOM	5779	NE2	HIS	383	27.919	22.095	25.200	1.00	0.00	RX1	N
ATOM	5780	CE1	HIS	383	26.965	21.434	24.472	1.00	0.00	RX1	C
ATOM	5781	C	HIS	383	25.468	25.780	21.071	1.00	0.00	RX1	C
ATOM	5782	O	HIS	383	25.780	26.891	21.491	1.00	0.00	RX1	O
ATOM	5783	N	THR	384	24.840	25.569	19.916	1.00	0.00	RX1	N
ATOM	5784	H	THR	384	24.559	24.665	19.585	1.00	0.00	RX1	H
ATOM	5785	CA	THR	384	24.642	26.722	19.055	1.00	0.00	RX1	C
ATOM	5786	CB	THR	384	25.435	26.258	17.862	1.00	0.00	RX1	C
ATOM	5787	OG1	THR	384	25.486	24.832	17.985	1.00	0.00	RX1	O
ATOM	5788	HG1	THR	384	26.213	24.529	17.443	1.00	0.00	RX1	H
ATOM	5789	CG2	THR	384	26.871	26.778	17.880	1.00	0.00	RX1	C
ATOM	5790	C	THR	384	23.174	27.006	18.802	1.00	0.00	RX1	C
ATOM	5791	O	THR	384	22.494	26.289	18.081	1.00	0.00	RX1	O
ATOM	5792	N	PRO	385	22.673	28.092	19.440	1.00	0.00	RX1	N
ATOM	5793	CD	PRO	385	23.328	28.901	20.455	1.00	0.00	RX1	C

ATOM	5794	CA	PRO	385	21.305	28.534	19.153	1.00	0.00	RX1	C
ATOM	5795	CB	PRO	385	21.183	29.808	20.002	1.00	0.00	RX1	C
ATOM	5796	CG	PRO	385	22.183	29.630	21.141	1.00	0.00	RX1	C
ATOM	5797	C	PRO	385	21.046	28.770	17.668	1.00	0.00	RX1	C
ATOM	5798	O	PRO	385	21.920	29.228	16.933	1.00	0.00	RX1	O
ATOM	5799	N	PRO	386	19.798	28.424	17.264	1.00	0.00	RX1	N
ATOM	5800	CD	PRO	386	18.767	27.832	18.109	1.00	0.00	RX1	C
ATOM	5801	CA	PRO	386	19.360	28.624	15.878	1.00	0.00	RX1	C
ATOM	5802	CB	PRO	386	17.857	28.343	15.967	1.00	0.00	RX1	C
ATOM	5803	CG	PRO	386	17.696	27.371	17.132	1.00	0.00	RX1	C
ATOM	5804	C	PRO	386	19.647	30.009	15.331	1.00	0.00	RX1	C
ATOM	5805	O	PRO	386	19.292	31.026	15.917	1.00	0.00	RX1	O
ATOM	5806	N	LEU	387	20.297	30.012	14.164	1.00	0.00	RX1	N
ATOM	5807	H	LEU	387	20.550	29.165	13.695	1.00	0.00	RX1	H
ATOM	5808	CA	LEU	387	20.431	31.300	13.501	1.00	0.00	RX1	C
ATOM	5809	CB	LEU	387	21.632	31.320	12.562	1.00	0.00	RX1	C
ATOM	5810	CG	LEU	387	21.981	32.744	12.149	1.00	0.00	RX1	C
ATOM	5811	CD1	LEU	387	22.103	33.659	13.368	1.00	0.00	RX1	C
ATOM	5812	CD2	LEU	387	23.229	32.781	11.272	1.00	0.00	RX1	C
ATOM	5813	C	LEU	387	19.157	31.713	12.793	1.00	0.00	RX1	C
ATOM	5814	O	LEU	387	18.856	31.307	11.676	1.00	0.00	RX1	O
ATOM	5815	N	ASP	388	18.428	32.553	13.538	1.00	0.00	RX1	N
ATOM	5816	H	ASP	388	18.734	32.739	14.472	1.00	0.00	RX1	H
ATOM	5817	CA	ASP	388	17.165	33.113	13.062	1.00	0.00	RX1	C
ATOM	5818	CB	ASP	388	16.720	34.183	14.063	1.00	0.00	RX1	C
ATOM	5819	CG	ASP	388	15.560	34.997	13.535	1.00	0.00	RX1	C
ATOM	5820	OD1	ASP	388	14.616	34.448	12.979	1.00	0.00	RX1	O
ATOM	5821	OD2	ASP	388	15.605	36.212	13.647	1.00	0.00	RX1	O
ATOM	5822	C	ASP	388	17.171	33.614	11.617	1.00	0.00	RX1	C
ATOM	5823	O	ASP	388	17.963	34.457	11.199	1.00	0.00	RX1	O
ATOM	5824	N	PRO	389	16.202	33.055	10.854	1.00	0.00	RX1	N
ATOM	5825	CD	PRO	389	15.379	31.916	11.244	1.00	0.00	RX1	C
ATOM	5826	CA	PRO	389	15.912	33.543	9.502	1.00	0.00	RX1	C
ATOM	5827	CB	PRO	389	14.731	32.664	9.075	1.00	0.00	RX1	C
ATOM	5828	CG	PRO	389	14.828	31.399	9.925	1.00	0.00	RX1	C
ATOM	5829	C	PRO	389	15.601	35.031	9.335	1.00	0.00	RX1	C
ATOM	5830	O	PRO	389	15.413	35.497	8.213	1.00	0.00	RX1	O
ATOM	5831	N	GLN	390	15.496	35.763	10.451	1.00	0.00	RX1	N
ATOM	5832	H	GLN	390	15.577	35.378	11.374	1.00	0.00	RX1	H
ATOM	5833	CA	GLN	390	15.425	37.216	10.325	1.00	0.00	RX1	C
ATOM	5834	CB	GLN	390	14.336	37.767	11.235	1.00	0.00	RX1	C
ATOM	5835	CG	GLN	390	13.072	36.923	11.087	1.00	0.00	RX1	C
ATOM	5836	CD	GLN	390	12.158	37.175	12.261	1.00	0.00	RX1	C
ATOM	5837	OE1	GLN	390	11.067	37.720	12.115	1.00	0.00	RX1	O
ATOM	5838	NE2	GLN	390	12.659	36.735	13.426	1.00	0.00	RX1	N
ATOM	5839	HE21	GLN	390	13.557	36.265	13.441	1.00	0.00	RX1	H
ATOM	5840	HE22	GLN	390	12.215	36.828	14.313	1.00	0.00	RX1	H
ATOM	5841	C	GLN	390	16.776	37.866	10.570	1.00	0.00	RX1	C
ATOM	5842	O	GLN	390	17.238	38.669	9.771	1.00	0.00	RX1	O
ATOM	5843	N	GLU	391	17.425	37.422	11.667	1.00	0.00	RX1	N
ATOM	5844	H	GLU	391	16.942	36.839	12.324	1.00	0.00	RX1	H
ATOM	5845	CA	GLU	391	18.820	37.799	11.950	1.00	0.00	RX1	C
ATOM	5846	CB	GLU	391	19.410	36.884	13.023	1.00	0.00	RX1	C
ATOM	5847	CG	GLU	391	19.140	37.347	14.453	1.00	0.00	RX1	C
ATOM	5848	CD	GLU	391	20.009	38.549	14.757	1.00	0.00	RX1	C
ATOM	5849	OE1	GLU	391	21.234	38.433	14.703	1.00	0.00	RX1	O
ATOM	5850	OE2	GLU	391	19.482	39.614	15.074	1.00	0.00	RX1	O
ATOM	5851	C	GLU	391	19.734	37.777	10.735	1.00	0.00	RX1	C
ATOM	5852	O	GLU	391	20.532	38.675	10.487	1.00	0.00	RX1	O
ATOM	5853	N	LEU	392	19.525	36.707	9.944	1.00	0.00	RX1	N
ATOM	5854	H	LEU	392	18.924	35.985	10.294	1.00	0.00	RX1	H

ATOM	5855	CA	LEU	392	20.196	36.580	8.648	1.00	0.00	RX1	C
ATOM	5856	CB	LEU	392	19.469	35.572	7.763	1.00	0.00	RX1	C
ATOM	5857	CG	LEU	392	19.716	34.123	8.167	1.00	0.00	RX1	C
ATOM	5858	CD1	LEU	392	18.926	33.166	7.279	1.00	0.00	RX1	C
ATOM	5859	CD2	LEU	392	21.206	33.787	8.163	1.00	0.00	RX1	C
ATOM	5860	C	LEU	392	20.399	37.858	7.849	1.00	0.00	RX1	C
ATOM	5861	O	LEU	392	21.484	38.126	7.352	1.00	0.00	RX1	O
ATOM	5862	N	ASP	393	19.314	38.640	7.742	1.00	0.00	RX1	N
ATOM	5863	H	ASP	393	18.490	38.462	8.282	1.00	0.00	RX1	H
ATOM	5864	CA	ASP	393	19.351	39.793	6.840	1.00	0.00	RX1	C
ATOM	5865	CB	ASP	393	17.996	40.492	6.804	1.00	0.00	RX1	C
ATOM	5866	CG	ASP	393	17.875	41.197	5.474	1.00	0.00	RX1	C
ATOM	5867	OD1	ASP	393	17.872	42.423	5.428	1.00	0.00	RX1	O
ATOM	5868	OD2	ASP	393	17.795	40.519	4.455	1.00	0.00	RX1	O
ATOM	5869	C	ASP	393	20.470	40.801	7.085	1.00	0.00	RX1	C
ATOM	5870	O	ASP	393	21.000	41.445	6.185	1.00	0.00	RX1	O
ATOM	5871	N	ILE	394	20.872	40.862	8.367	1.00	0.00	RX1	N
ATOM	5872	H	ILE	394	20.480	40.221	9.031	1.00	0.00	RX1	H
ATOM	5873	CA	ILE	394	21.975	41.756	8.738	1.00	0.00	RX1	C
ATOM	5874	CB	ILE	394	22.173	41.705	10.255	1.00	0.00	RX1	C
ATOM	5875	CG2	ILE	394	23.223	42.704	10.738	1.00	0.00	RX1	C
ATOM	5876	CG1	ILE	394	20.833	41.944	10.953	1.00	0.00	RX1	C
ATOM	5877	CD1	ILE	394	20.932	41.805	12.470	1.00	0.00	RX1	C
ATOM	5878	C	ILE	394	23.282	41.513	7.972	1.00	0.00	RX1	C
ATOM	5879	O	ILE	394	24.129	42.390	7.814	1.00	0.00	RX1	O
ATOM	5880	N	LEU	395	23.397	40.283	7.446	1.00	0.00	RX1	N
ATOM	5881	H	LEU	395	22.660	39.614	7.553	1.00	0.00	RX1	H
ATOM	5882	CA	LEU	395	24.545	39.952	6.602	1.00	0.00	RX1	C
ATOM	5883	CB	LEU	395	24.611	38.446	6.363	1.00	0.00	RX1	C
ATOM	5884	CG	LEU	395	24.723	37.669	7.677	1.00	0.00	RX1	C
ATOM	5885	CD1	LEU	395	24.557	36.164	7.466	1.00	0.00	RX1	C
ATOM	5886	CD2	LEU	395	26.005	38.013	8.438	1.00	0.00	RX1	C
ATOM	5887	C	LEU	395	24.701	40.731	5.299	1.00	0.00	RX1	C
ATOM	5888	O	LEU	395	25.702	40.600	4.610	1.00	0.00	RX1	O
ATOM	5889	N	LYS	396	23.736	41.630	5.031	1.00	0.00	RX1	N
ATOM	5890	H	LYS	396	22.856	41.589	5.508	1.00	0.00	RX1	H
ATOM	5891	CA	LYS	396	23.976	42.674	4.024	1.00	0.00	RX1	C
ATOM	5892	CB	LYS	396	22.751	43.584	3.929	1.00	0.00	RX1	C
ATOM	5893	CG	LYS	396	21.524	42.953	3.265	1.00	0.00	RX1	C
ATOM	5894	CD	LYS	396	20.328	43.905	3.339	1.00	0.00	RX1	C
ATOM	5895	CE	LYS	396	19.153	43.541	2.427	1.00	0.00	RX1	C
ATOM	5896	NZ	LYS	396	18.561	42.248	2.773	1.00	0.00	RX1	N
ATOM	5897	HZ1	LYS	396	17.830	41.974	2.087	1.00	0.00	RX1	H
ATOM	5898	HZ2	LYS	396	19.246	41.469	2.801	1.00	0.00	RX1	H
ATOM	5899	HZ3	LYS	396	18.116	42.239	3.719	1.00	0.00	RX1	H
ATOM	5900	C	LYS	396	25.228	43.521	4.271	1.00	0.00	RX1	C
ATOM	5901	O	LYS	396	25.815	44.129	3.381	1.00	0.00	RX1	O
ATOM	5902	N	THR	397	25.629	43.525	5.553	1.00	0.00	RX1	N
ATOM	5903	H	THR	397	25.148	43.003	6.256	1.00	0.00	RX1	H
ATOM	5904	CA	THR	397	26.874	44.200	5.909	1.00	0.00	RX1	C
ATOM	5905	CB	THR	397	26.870	44.467	7.414	1.00	0.00	RX1	C
ATOM	5906	OG1	THR	397	26.654	43.265	8.163	1.00	0.00	RX1	O
ATOM	5907	HG1	THR	397	25.715	43.111	8.184	1.00	0.00	RX1	H
ATOM	5908	CG2	THR	397	25.798	45.500	7.756	1.00	0.00	RX1	C
ATOM	5909	C	THR	397	28.154	43.541	5.403	1.00	0.00	RX1	C
ATOM	5910	O	THR	397	29.234	44.121	5.423	1.00	0.00	RX1	O
ATOM	5911	N	VAL	398	27.997	42.300	4.921	1.00	0.00	RX1	N
ATOM	5912	H	VAL	398	27.100	41.874	4.815	1.00	0.00	RX1	H
ATOM	5913	CA	VAL	398	29.129	41.676	4.245	1.00	0.00	RX1	C
ATOM	5914	CB	VAL	398	28.954	40.161	4.170	1.00	0.00	RX1	C
ATOM	5915	CG1	VAL	398	30.166	39.527	3.497	1.00	0.00	RX1	C

ATOM	5916	CG2	VAL	398	28.695	39.559	5.551	1.00	0.00	RX1	C
ATOM	5917	C	VAL	398	29.324	42.265	2.858	1.00	0.00	RX1	C
ATOM	5918	O	VAL	398	28.694	41.883	1.883	1.00	0.00	RX1	O
ATOM	5919	N	LYS	399	30.239	43.236	2.830	1.00	0.00	RX1	N
ATOM	5920	H	LYS	399	30.727	43.496	3.664	1.00	0.00	RX1	H
ATOM	5921	CA	LYS	399	30.594	43.812	1.537	1.00	0.00	RX1	C
ATOM	5922	CB	LYS	399	31.168	45.225	1.707	1.00	0.00	RX1	C
ATOM	5923	CG	LYS	399	30.102	46.323	1.705	1.00	0.00	RX1	C
ATOM	5924	CD	LYS	399	29.063	46.194	2.817	1.00	0.00	RX1	C
ATOM	5925	CE	LYS	399	27.666	46.562	2.330	1.00	0.00	RX1	C
ATOM	5926	NZ	LYS	399	27.221	45.609	1.308	1.00	0.00	RX1	N
ATOM	5927	HZ1	LYS	399	27.944	45.391	0.590	1.00	0.00	RX1	H
ATOM	5928	HZ2	LYS	399	26.821	44.717	1.677	1.00	0.00	RX1	H
ATOM	5929	HZ3	LYS	399	26.471	46.047	0.738	1.00	0.00	RX1	H
ATOM	5930	C	LYS	399	31.577	42.934	0.799	1.00	0.00	RX1	C
ATOM	5931	O	LYS	399	31.373	42.515	-0.335	1.00	0.00	RX1	O
ATOM	5932	N	GLU	400	32.667	42.657	1.525	1.00	0.00	RX1	N
ATOM	5933	H	GLU	400	32.771	42.948	2.478	1.00	0.00	RX1	H
ATOM	5934	CA	GLU	400	33.638	41.754	0.922	1.00	0.00	RX1	C
ATOM	5935	CB	GLU	400	35.022	42.398	0.810	1.00	0.00	RX1	C
ATOM	5936	CG	GLU	400	35.033	43.899	0.513	1.00	0.00	RX1	C
ATOM	5937	CD	GLU	400	36.457	44.327	0.216	1.00	0.00	RX1	C
ATOM	5938	OE1	GLU	400	36.714	44.899	-0.837	1.00	0.00	RX1	O
ATOM	5939	OE2	GLU	400	37.355	44.015	0.988	1.00	0.00	RX1	O
ATOM	5940	C	GLU	400	33.765	40.477	1.715	1.00	0.00	RX1	C
ATOM	5941	O	GLU	400	33.907	40.498	2.930	1.00	0.00	RX1	O
ATOM	5942	N	ILE	401	33.741	39.368	0.979	1.00	0.00	RX1	N
ATOM	5943	H	ILE	401	33.596	39.411	-0.010	1.00	0.00	RX1	H
ATOM	5944	CA	ILE	401	34.275	38.131	1.535	1.00	0.00	RX1	C
ATOM	5945	CB	ILE	401	33.405	36.939	1.142	1.00	0.00	RX1	C
ATOM	5946	CG2	ILE	401	33.819	35.665	1.877	1.00	0.00	RX1	C
ATOM	5947	CG1	ILE	401	31.932	37.238	1.347	1.00	0.00	RX1	C
ATOM	5948	CD1	ILE	401	31.086	36.040	0.937	1.00	0.00	RX1	C
ATOM	5949	C	ILE	401	35.630	37.952	0.900	1.00	0.00	RX1	C
ATOM	5950	O	ILE	401	35.772	38.138	-0.304	1.00	0.00	RX1	O
ATOM	5951	N	THR	402	36.624	37.595	1.711	1.00	0.00	RX1	N
ATOM	5952	H	THR	402	36.544	37.431	2.696	1.00	0.00	RX1	H
ATOM	5953	CA	THR	402	37.868	37.333	0.998	1.00	0.00	RX1	C
ATOM	5954	CB	THR	402	39.037	37.885	1.800	1.00	0.00	RX1	C
ATOM	5955	OG1	THR	402	39.064	37.305	3.096	1.00	0.00	RX1	O
ATOM	5956	HG1	THR	402	38.447	37.807	3.612	1.00	0.00	RX1	H
ATOM	5957	CG2	THR	402	38.921	39.407	1.920	1.00	0.00	RX1	C
ATOM	5958	C	THR	402	38.023	35.889	0.547	1.00	0.00	RX1	C
ATOM	5959	O	THR	402	38.637	35.586	-0.471	1.00	0.00	RX1	O
ATOM	5960	N	GLY	403	37.398	35.006	1.332	1.00	0.00	RX1	N
ATOM	5961	H	GLY	403	36.872	35.282	2.138	1.00	0.00	RX1	H
ATOM	5962	CA	GLY	403	37.353	33.615	0.905	1.00	0.00	RX1	C
ATOM	5963	C	GLY	403	36.097	33.289	0.129	1.00	0.00	RX1	C
ATOM	5964	O	GLY	403	35.750	33.957	-0.843	1.00	0.00	RX1	O
ATOM	5965	N	PHE	404	35.436	32.231	0.604	1.00	0.00	RX1	N
ATOM	5966	H	PHE	404	35.739	31.780	1.449	1.00	0.00	RX1	H
ATOM	5967	CA	PHE	404	34.259	31.752	-0.109	1.00	0.00	RX1	C
ATOM	5968	CB	PHE	404	34.429	30.283	-0.511	1.00	0.00	RX1	C
ATOM	5969	CG	PHE	404	34.710	29.407	0.686	1.00	0.00	RX1	C
ATOM	5970	CD1	PHE	404	33.716	29.121	1.615	1.00	0.00	RX1	C
ATOM	5971	CD2	PHE	404	35.980	28.872	0.845	1.00	0.00	RX1	C
ATOM	5972	CE1	PHE	404	33.996	28.300	2.698	1.00	0.00	RX1	C
ATOM	5973	CE2	PHE	404	36.264	28.042	1.919	1.00	0.00	RX1	C
ATOM	5974	CZ	PHE	404	35.269	27.760	2.843	1.00	0.00	RX1	C
ATOM	5975	C	PHE	404	32.944	31.972	0.615	1.00	0.00	RX1	C
ATOM	5976	O	PHE	404	32.881	32.244	1.808	1.00	0.00	RX1	O

ATOM	5977	N	LEU	405	31.888	31.803	-0.186	1.00	0.00	RX1	N
ATOM	5978	H	LEU	405	32.003	31.523	-1.139	1.00	0.00	RX1	H
ATOM	5979	CA	LEU	405	30.531	31.817	0.340	1.00	0.00	RX1	C
ATOM	5980	CB	LEU	405	29.720	32.859	-0.425	1.00	0.00	RX1	C
ATOM	5981	CG	LEU	405	28.304	33.070	0.103	1.00	0.00	RX1	C
ATOM	5982	CD1	LEU	405	28.286	33.476	1.578	1.00	0.00	RX1	C
ATOM	5983	CD2	LEU	405	27.523	34.047	-0.777	1.00	0.00	RX1	C
ATOM	5984	C	LEU	405	29.909	30.441	0.188	1.00	0.00	RX1	C
ATOM	5985	O	LEU	405	29.398	30.084	-0.867	1.00	0.00	RX1	O
ATOM	5986	N	LEU	406	29.992	29.672	1.279	1.00	0.00	RX1	N
ATOM	5987	H	LEU	406	30.294	30.043	2.160	1.00	0.00	RX1	H
ATOM	5988	CA	LEU	406	29.394	28.340	1.220	1.00	0.00	RX1	C
ATOM	5989	CB	LEU	406	30.273	27.340	1.982	1.00	0.00	RX1	C
ATOM	5990	CG	LEU	406	29.727	25.906	2.065	1.00	0.00	RX1	C
ATOM	5991	CD1	LEU	406	29.544	25.247	0.700	1.00	0.00	RX1	C
ATOM	5992	CD2	LEU	406	30.565	25.043	3.004	1.00	0.00	RX1	C
ATOM	5993	C	LEU	406	27.979	28.340	1.765	1.00	0.00	RX1	C
ATOM	5994	O	LEU	406	27.736	27.977	2.909	1.00	0.00	RX1	O
ATOM	5995	N	ILE	407	27.042	28.762	0.912	1.00	0.00	RX1	N
ATOM	5996	H	ILE	407	27.246	28.868	-0.064	1.00	0.00	RX1	H
ATOM	5997	CA	ILE	407	25.674	28.610	1.398	1.00	0.00	RX1	C
ATOM	5998	CB	ILE	407	24.725	29.651	0.822	1.00	0.00	RX1	C
ATOM	5999	CG2	ILE	407	23.468	29.712	1.683	1.00	0.00	RX1	C
ATOM	6000	CG1	ILE	407	25.369	31.023	0.716	1.00	0.00	RX1	C
ATOM	6001	CD1	ILE	407	24.370	32.049	0.185	1.00	0.00	RX1	C
ATOM	6002	C	ILE	407	25.141	27.228	1.091	1.00	0.00	RX1	C
ATOM	6003	O	ILE	407	24.744	26.925	-0.025	1.00	0.00	RX1	O
ATOM	6004	N	GLN	408	25.176	26.398	2.136	1.00	0.00	RX1	N
ATOM	6005	H	GLN	408	25.472	26.719	3.038	1.00	0.00	RX1	H
ATOM	6006	CA	GLN	408	24.776	25.013	1.917	1.00	0.00	RX1	C
ATOM	6007	CB	GLN	408	25.654	24.078	2.746	1.00	0.00	RX1	C
ATOM	6008	CG	GLN	408	26.217	22.930	1.918	1.00	0.00	RX1	C
ATOM	6009	CD	GLN	408	25.796	21.594	2.494	1.00	0.00	RX1	C
ATOM	6010	OE1	GLN	408	26.620	20.816	2.985	1.00	0.00	RX1	O
ATOM	6011	NE2	GLN	408	24.478	21.347	2.365	1.00	0.00	RX1	N
ATOM	6012	HE21	GLN	408	23.873	22.020	1.920	1.00	0.00	RX1	H
ATOM	6013	HE22	GLN	408	24.004	20.512	2.650	1.00	0.00	RX1	H
ATOM	6014	C	GLN	408	23.304	24.700	2.127	1.00	0.00	RX1	C
ATOM	6015	O	GLN	408	22.795	23.699	1.637	1.00	0.00	RX1	O
ATOM	6016	N	ALA	409	22.646	25.588	2.887	1.00	0.00	RX1	N
ATOM	6017	H	ALA	409	23.065	26.409	3.281	1.00	0.00	RX1	H
ATOM	6018	CA	ALA	409	21.192	25.495	3.007	1.00	0.00	RX1	C
ATOM	6019	CB	ALA	409	20.751	24.554	4.122	1.00	0.00	RX1	C
ATOM	6020	C	ALA	409	20.620	26.863	3.289	1.00	0.00	RX1	C
ATOM	6021	O	ALA	409	21.354	27.794	3.591	1.00	0.00	RX1	O
ATOM	6022	N	TRP	410	19.290	26.946	3.156	1.00	0.00	RX1	N
ATOM	6023	H	TRP	410	18.695	26.161	2.974	1.00	0.00	RX1	H
ATOM	6024	CA	TRP	410	18.631	28.240	3.305	1.00	0.00	RX1	C
ATOM	6025	CB	TRP	410	18.610	28.933	1.945	1.00	0.00	RX1	C
ATOM	6026	CG	TRP	410	18.683	30.433	2.079	1.00	0.00	RX1	C
ATOM	6027	CD2	TRP	410	19.643	31.240	2.798	1.00	0.00	RX1	C
ATOM	6028	CE2	TRP	410	19.309	32.596	2.574	1.00	0.00	RX1	C
ATOM	6029	CE3	TRP	410	20.741	30.929	3.591	1.00	0.00	RX1	C
ATOM	6030	CD1	TRP	410	17.822	31.352	1.472	1.00	0.00	RX1	C
ATOM	6031	NE1	TRP	410	18.182	32.629	1.755	1.00	0.00	RX1	N
ATOM	6032	HE1	TRP	410	17.720	33.419	1.397	1.00	0.00	RX1	H
ATOM	6033	CZ2	TRP	410	20.088	33.594	3.148	1.00	0.00	RX1	C
ATOM	6034	CZ3	TRP	410	21.508	31.936	4.162	1.00	0.00	RX1	C
ATOM	6035	CH2	TRP	410	21.183	33.268	3.938	1.00	0.00	RX1	C
ATOM	6036	C	TRP	410	17.229	28.001	3.824	1.00	0.00	RX1	C
ATOM	6037	O	TRP	410	16.525	27.159	3.282	1.00	0.00	RX1	O

ATOM	6038	N	PRO	411	16.865	28.720	4.912	1.00	0.00	RX1	N
ATOM	6039	CD	PRO	411	17.609	29.817	5.511	1.00	0.00	RX1	C
ATOM	6040	CA	PRO	411	15.655	28.372	5.668	1.00	0.00	RX1	C
ATOM	6041	CB	PRO	411	15.648	29.383	6.813	1.00	0.00	RX1	C
ATOM	6042	CG	PRO	411	17.081	29.884	6.935	1.00	0.00	RX1	C
ATOM	6043	C	PRO	411	14.354	28.436	4.896	1.00	0.00	RX1	C
ATOM	6044	O	PRO	411	13.712	29.476	4.829	1.00	0.00	RX1	O
ATOM	6045	N	GLU	412	13.990	27.267	4.359	1.00	0.00	RX1	N
ATOM	6046	H	GLU	412	14.636	26.515	4.507	1.00	0.00	RX1	H
ATOM	6047	CA	GLU	412	12.645	26.941	3.884	1.00	0.00	RX1	C
ATOM	6048	CB	GLU	412	11.874	26.207	4.991	1.00	0.00	RX1	C
ATOM	6049	CG	GLU	412	12.520	24.892	5.472	1.00	0.00	RX1	C
ATOM	6050	CD	GLU	412	13.795	25.137	6.266	1.00	0.00	RX1	C
ATOM	6051	OE1	GLU	412	13.715	25.550	7.416	1.00	0.00	RX1	O
ATOM	6052	OE2	GLU	412	14.886	24.935	5.741	1.00	0.00	RX1	O
ATOM	6053	C	GLU	412	11.844	28.084	3.271	1.00	0.00	RX1	C
ATOM	6054	O	GLU	412	11.836	28.277	2.062	1.00	0.00	RX1	O
ATOM	6055	N	ASN	413	11.192	28.868	4.142	1.00	0.00	RX1	N
ATOM	6056	H	ASN	413	11.387	28.775	5.118	1.00	0.00	RX1	H
ATOM	6057	CA	ASN	413	10.517	30.061	3.624	1.00	0.00	RX1	C
ATOM	6058	CB	ASN	413	9.351	30.507	4.506	1.00	0.00	RX1	C
ATOM	6059	CG	ASN	413	8.132	29.647	4.259	1.00	0.00	RX1	C
ATOM	6060	OD1	ASN	413	7.761	28.817	5.080	1.00	0.00	RX1	O
ATOM	6061	ND2	ASN	413	7.514	29.894	3.089	1.00	0.00	RX1	N
ATOM	6062	HD21	ASN	413	7.848	30.583	2.446	1.00	0.00	RX1	H
ATOM	6063	HD22	ASN	413	6.696	29.376	2.836	1.00	0.00	RX1	H
ATOM	6064	C	ASN	413	11.440	31.257	3.443	1.00	0.00	RX1	C
ATOM	6065	O	ASN	413	11.247	32.326	4.023	1.00	0.00	RX1	O
ATOM	6066	N	ARG	414	12.455	31.033	2.599	1.00	0.00	RX1	N
ATOM	6067	H	ARG	414	12.561	30.140	2.154	1.00	0.00	RX1	H
ATOM	6068	CA	ARG	414	13.395	32.089	2.237	1.00	0.00	RX1	C
ATOM	6069	CB	ARG	414	14.683	32.030	3.061	1.00	0.00	RX1	C
ATOM	6070	CG	ARG	414	14.545	32.621	4.464	1.00	0.00	RX1	C
ATOM	6071	CD	ARG	414	13.980	34.039	4.398	1.00	0.00	RX1	C
ATOM	6072	NE	ARG	414	14.000	34.696	5.702	1.00	0.00	RX1	N
ATOM	6073	HE	ARG	414	14.885	35.075	6.014	1.00	0.00	RX1	H
ATOM	6074	CZ	ARG	414	12.838	34.798	6.410	1.00	0.00	RX1	C
ATOM	6075	NH1	ARG	414	11.717	34.189	5.956	1.00	0.00	RX1	N
ATOM	6076	HH11	ARG	414	10.832	34.258	6.419	1.00	0.00	RX1	H
ATOM	6077	HH12	ARG	414	11.721	33.613	5.123	1.00	0.00	RX1	H
ATOM	6078	NH2	ARG	414	12.839	35.511	7.555	1.00	0.00	RX1	N
ATOM	6079	HH21	ARG	414	12.031	35.671	8.128	1.00	0.00	RX1	H
ATOM	6080	HH22	ARG	414	13.712	35.911	7.876	1.00	0.00	RX1	H
ATOM	6081	C	ARG	414	13.730	32.018	0.766	1.00	0.00	RX1	C
ATOM	6082	O	ARG	414	14.782	31.563	0.341	1.00	0.00	RX1	O
ATOM	6083	N	THR	415	12.758	32.509	-0.003	1.00	0.00	RX1	N
ATOM	6084	H	THR	415	11.915	32.877	0.383	1.00	0.00	RX1	H
ATOM	6085	CA	THR	415	12.855	32.391	-1.455	1.00	0.00	RX1	C
ATOM	6086	CB	THR	415	11.447	32.686	-1.926	1.00	0.00	RX1	C
ATOM	6087	OG1	THR	415	10.566	32.486	-0.806	1.00	0.00	RX1	O
ATOM	6088	HG1	THR	415	9.687	32.501	-1.165	1.00	0.00	RX1	H
ATOM	6089	CG2	THR	415	11.036	31.819	-3.117	1.00	0.00	RX1	C
ATOM	6090	C	THR	415	13.939	33.214	-2.149	1.00	0.00	RX1	C
ATOM	6091	O	THR	415	14.235	33.016	-3.324	1.00	0.00	RX1	O
ATOM	6092	N	ASP	416	14.494	34.155	-1.370	1.00	0.00	RX1	N
ATOM	6093	H	ASP	416	14.353	34.196	-0.383	1.00	0.00	RX1	H
ATOM	6094	CA	ASP	416	15.528	35.043	-1.892	1.00	0.00	RX1	C
ATOM	6095	CB	ASP	416	14.976	36.469	-2.035	1.00	0.00	RX1	C
ATOM	6096	CG	ASP	416	15.954	37.373	-2.767	1.00	0.00	RX1	C
ATOM	6097	OD1	ASP	416	15.824	37.550	-3.971	1.00	0.00	RX1	O
ATOM	6098	OD2	ASP	416	16.845	37.928	-2.139	1.00	0.00	RX1	O

ATOM	6099	C	ASP	416	16.724	34.993	-0.958	1.00	0.00	RX1	C
ATOM	6100	O	ASP	416	16.616	34.613	0.209	1.00	0.00	RX1	O
ATOM	6101	N	LEU	417	17.881	35.381	-1.507	1.00	0.00	RX1	N
ATOM	6102	H	LEU	417	17.851	35.887	-2.373	1.00	0.00	RX1	H
ATOM	6103	CA	LEU	417	19.061	35.359	-0.650	1.00	0.00	RX1	C
ATOM	6104	CB	LEU	417	20.359	35.402	-1.456	1.00	0.00	RX1	C
ATOM	6105	CG	LEU	417	20.789	33.994	-1.863	1.00	0.00	RX1	C
ATOM	6106	CD1	LEU	417	22.196	33.958	-2.456	1.00	0.00	RX1	C
ATOM	6107	CD2	LEU	417	20.679	33.036	-0.683	1.00	0.00	RX1	C
ATOM	6108	C	LEU	417	19.117	36.379	0.471	1.00	0.00	RX1	C
ATOM	6109	O	LEU	417	19.943	36.235	1.359	1.00	0.00	RX1	O
ATOM	6110	N	HIS	418	18.206	37.372	0.415	1.00	0.00	RX1	N
ATOM	6111	H	HIS	418	17.641	37.443	-0.410	1.00	0.00	RX1	H
ATOM	6112	CA	HIS	418	17.995	38.408	1.442	1.00	0.00	RX1	C
ATOM	6113	CB	HIS	418	16.725	38.141	2.275	1.00	0.00	RX1	C
ATOM	6114	CG	HIS	418	16.898	37.020	3.270	1.00	0.00	RX1	C
ATOM	6115	ND1	HIS	418	17.161	35.748	2.922	1.00	0.00	RX1	N
ATOM	6116	HD1	HIS	418	17.217	35.398	2.004	1.00	0.00	RX1	H
ATOM	6117	CD2	HIS	418	16.832	37.104	4.665	1.00	0.00	RX1	C
ATOM	6118	NE2	HIS	418	17.064	35.861	5.155	1.00	0.00	RX1	N
ATOM	6119	CE1	HIS	418	17.270	35.033	4.083	1.00	0.00	RX1	C
ATOM	6120	C	HIS	418	19.192	38.918	2.252	1.00	0.00	RX1	C
ATOM	6121	O	HIS	418	19.688	40.020	2.030	1.00	0.00	RX1	O
ATOM	6122	N	ALA	419	19.700	38.063	3.153	1.00	0.00	RX1	N
ATOM	6123	H	ALA	419	19.294	37.154	3.225	1.00	0.00	RX1	H
ATOM	6124	CA	ALA	419	20.979	38.322	3.812	1.00	0.00	RX1	C
ATOM	6125	CB	ALA	419	21.477	37.033	4.460	1.00	0.00	RX1	C
ATOM	6126	C	ALA	419	22.065	38.795	2.860	1.00	0.00	RX1	C
ATOM	6127	O	ALA	419	22.753	39.783	3.067	1.00	0.00	RX1	O
ATOM	6128	N	PHE	420	22.171	38.038	1.764	1.00	0.00	RX1	N
ATOM	6129	H	PHE	420	21.534	37.287	1.586	1.00	0.00	RX1	H
ATOM	6130	CA	PHE	420	23.255	38.363	0.847	1.00	0.00	RX1	C
ATOM	6131	CB	PHE	420	23.956	37.076	0.428	1.00	0.00	RX1	C
ATOM	6132	CG	PHE	420	24.456	36.386	1.673	1.00	0.00	RX1	C
ATOM	6133	CD1	PHE	420	25.418	37.002	2.465	1.00	0.00	RX1	C
ATOM	6134	CD2	PHE	420	23.952	35.142	2.030	1.00	0.00	RX1	C
ATOM	6135	CE1	PHE	420	25.888	36.371	3.609	1.00	0.00	RX1	C
ATOM	6136	CE2	PHE	420	24.418	34.512	3.176	1.00	0.00	RX1	C
ATOM	6137	CZ	PHE	420	25.387	35.124	3.962	1.00	0.00	RX1	C
ATOM	6138	C	PHE	420	22.875	39.204	-0.358	1.00	0.00	RX1	C
ATOM	6139	O	PHE	420	23.584	39.252	-1.354	1.00	0.00	RX1	O
ATOM	6140	N	GLU	421	21.732	39.907	-0.228	1.00	0.00	RX1	N
ATOM	6141	H	GLU	421	21.182	39.865	0.607	1.00	0.00	RX1	H
ATOM	6142	CA	GLU	421	21.357	40.806	-1.327	1.00	0.00	RX1	C
ATOM	6143	CB	GLU	421	20.097	41.593	-1.015	1.00	0.00	RX1	C
ATOM	6144	CG	GLU	421	18.842	40.754	-0.901	1.00	0.00	RX1	C
ATOM	6145	CD	GLU	421	17.691	41.694	-0.644	1.00	0.00	RX1	C
ATOM	6146	OE1	GLU	421	17.274	42.359	-1.577	1.00	0.00	RX1	O
ATOM	6147	OE2	GLU	421	17.221	41.791	0.480	1.00	0.00	RX1	O
ATOM	6148	C	GLU	421	22.408	41.851	-1.625	1.00	0.00	RX1	C
ATOM	6149	O	GLU	421	22.810	42.125	-2.753	1.00	0.00	RX1	O
ATOM	6150	N	ASN	422	22.821	42.449	-0.504	1.00	0.00	RX1	N
ATOM	6151	H	ASN	422	22.571	42.087	0.393	1.00	0.00	RX1	H
ATOM	6152	CA	ASN	422	23.796	43.519	-0.619	1.00	0.00	RX1	C
ATOM	6153	CB	ASN	422	23.348	44.726	0.198	1.00	0.00	RX1	C
ATOM	6154	CG	ASN	422	24.305	45.871	-0.034	1.00	0.00	RX1	C
ATOM	6155	OD1	ASN	422	24.822	46.467	0.907	1.00	0.00	RX1	O
ATOM	6156	ND2	ASN	422	24.556	46.121	-1.326	1.00	0.00	RX1	N
ATOM	6157	HD21	ASN	422	24.023	45.741	-2.088	1.00	0.00	RX1	H
ATOM	6158	HD22	ASN	422	25.359	46.666	-1.594	1.00	0.00	RX1	H
ATOM	6159	C	ASN	422	25.191	43.043	-0.266	1.00	0.00	RX1	C

ATOM	6160	O	ASN	422	25.886	43.608	0.567	1.00	0.00	RX1	O
ATOM	6161	N	LEU	423	25.528	41.943	-0.951	1.00	0.00	RX1	N
ATOM	6162	H	LEU	423	24.944	41.573	-1.674	1.00	0.00	RX1	H
ATOM	6163	CA	LEU	423	26.899	41.452	-0.929	1.00	0.00	RX1	C
ATOM	6164	CB	LEU	423	26.880	39.929	-0.783	1.00	0.00	RX1	C
ATOM	6165	CG	LEU	423	28.044	39.277	-0.027	1.00	0.00	RX1	C
ATOM	6166	CD1	LEU	423	27.841	37.769	0.058	1.00	0.00	RX1	C
ATOM	6167	CD2	LEU	423	29.425	39.590	-0.596	1.00	0.00	RX1	C
ATOM	6168	C	LEU	423	27.502	41.846	-2.259	1.00	0.00	RX1	C
ATOM	6169	O	LEU	423	26.882	41.657	-3.301	1.00	0.00	RX1	O
ATOM	6170	N	GLU	424	28.710	42.401	-2.189	1.00	0.00	RX1	N
ATOM	6171	H	GLU	424	29.148	42.625	-1.314	1.00	0.00	RX1	H
ATOM	6172	CA	GLU	424	29.286	42.887	-3.433	1.00	0.00	RX1	C
ATOM	6173	CB	GLU	424	29.698	44.363	-3.310	1.00	0.00	RX1	C
ATOM	6174	CG	GLU	424	28.574	45.381	-3.045	1.00	0.00	RX1	C
ATOM	6175	CD	GLU	424	28.208	45.466	-1.572	1.00	0.00	RX1	C
ATOM	6176	OE1	GLU	424	28.768	44.736	-0.768	1.00	0.00	RX1	O
ATOM	6177	OE2	GLU	424	27.371	46.282	-1.200	1.00	0.00	RX1	O
ATOM	6178	C	GLU	424	30.445	42.049	-3.947	1.00	0.00	RX1	C
ATOM	6179	O	GLU	424	30.666	41.920	-5.149	1.00	0.00	RX1	O
ATOM	6180	N	ILE	425	31.215	41.504	-2.995	1.00	0.00	RX1	N
ATOM	6181	H	ILE	425	31.002	41.571	-2.017	1.00	0.00	RX1	H
ATOM	6182	CA	ILE	425	32.510	40.937	-3.376	1.00	0.00	RX1	C
ATOM	6183	CB	ILE	425	33.594	41.924	-2.943	1.00	0.00	RX1	C
ATOM	6184	CG2	ILE	425	34.979	41.341	-3.171	1.00	0.00	RX1	C
ATOM	6185	CG1	ILE	425	33.403	43.287	-3.612	1.00	0.00	RX1	C
ATOM	6186	CD1	ILE	425	34.161	44.438	-2.964	1.00	0.00	RX1	C
ATOM	6187	C	ILE	425	32.764	39.569	-2.755	1.00	0.00	RX1	C
ATOM	6188	O	ILE	425	32.554	39.373	-1.567	1.00	0.00	RX1	O
ATOM	6189	N	ILE	426	33.251	38.638	-3.594	1.00	0.00	RX1	N
ATOM	6190	H	ILE	426	33.403	38.831	-4.564	1.00	0.00	RX1	H
ATOM	6191	CA	ILE	426	33.746	37.349	-3.102	1.00	0.00	RX1	C
ATOM	6192	CB	ILE	426	32.750	36.227	-3.416	1.00	0.00	RX1	C
ATOM	6193	CG2	ILE	426	33.234	34.891	-2.851	1.00	0.00	RX1	C
ATOM	6194	CG1	ILE	426	31.346	36.571	-2.920	1.00	0.00	RX1	C
ATOM	6195	CD1	ILE	426	30.313	35.516	-3.292	1.00	0.00	RX1	C
ATOM	6196	C	ILE	426	35.097	37.035	-3.738	1.00	0.00	RX1	C
ATOM	6197	O	ILE	426	35.208	36.831	-4.942	1.00	0.00	RX1	O
ATOM	6198	N	ARG	427	36.131	37.027	-2.884	1.00	0.00	RX1	N
ATOM	6199	H	ARG	427	35.987	37.113	-1.898	1.00	0.00	RX1	H
ATOM	6200	CA	ARG	427	37.474	36.964	-3.460	1.00	0.00	RX1	C
ATOM	6201	CB	ARG	427	38.456	37.889	-2.745	1.00	0.00	RX1	C
ATOM	6202	CG	ARG	427	38.025	39.332	-2.933	1.00	0.00	RX1	C
ATOM	6203	CD	ARG	427	39.152	40.348	-2.798	1.00	0.00	RX1	C
ATOM	6204	NE	ARG	427	38.707	41.601	-3.392	1.00	0.00	RX1	N
ATOM	6205	HE	ARG	427	38.618	41.657	-4.395	1.00	0.00	RX1	H
ATOM	6206	CZ	ARG	427	38.150	42.554	-2.603	1.00	0.00	RX1	C
ATOM	6207	NH1	ARG	427	38.296	42.471	-1.263	1.00	0.00	RX1	N
ATOM	6208	HH11	ARG	427	37.810	43.116	-0.649	1.00	0.00	RX1	H
ATOM	6209	HH12	ARG	427	38.895	41.812	-0.801	1.00	0.00	RX1	H
ATOM	6210	NH2	ARG	427	37.455	43.555	-3.179	1.00	0.00	RX1	N
ATOM	6211	HH21	ARG	427	37.069	44.302	-2.618	1.00	0.00	RX1	H
ATOM	6212	HH22	ARG	427	37.279	43.571	-4.179	1.00	0.00	RX1	H
ATOM	6213	C	ARG	427	38.121	35.613	-3.665	1.00	0.00	RX1	C
ATOM	6214	O	ARG	427	39.168	35.523	-4.300	1.00	0.00	RX1	O
ATOM	6215	N	GLY	428	37.485	34.570	-3.108	1.00	0.00	RX1	N
ATOM	6216	H	GLY	428	36.709	34.710	-2.490	1.00	0.00	RX1	H
ATOM	6217	CA	GLY	428	37.945	33.207	-3.395	1.00	0.00	RX1	C
ATOM	6218	C	GLY	428	39.411	32.898	-3.119	1.00	0.00	RX1	C
ATOM	6219	O	GLY	428	40.027	32.047	-3.758	1.00	0.00	RX1	O
ATOM	6220	N	ARG	429	39.949	33.630	-2.131	1.00	0.00	RX1	N

ATOM	6221	H	ARG	429	39.366	34.272	-1.631	1.00	0.00	RX1	H
ATOM	6222	CA	ARG	429	41.343	33.419	-1.740	1.00	0.00	RX1	C
ATOM	6223	CB	ARG	429	41.807	34.567	-0.847	1.00	0.00	RX1	C
ATOM	6224	CG	ARG	429	41.802	35.908	-1.574	1.00	0.00	RX1	C
ATOM	6225	CD	ARG	429	42.196	37.071	-0.670	1.00	0.00	RX1	C
ATOM	6226	NE	ARG	429	42.357	38.297	-1.443	1.00	0.00	RX1	N
ATOM	6227	HE	ARG	429	42.673	38.202	-2.392	1.00	0.00	RX1	H
ATOM	6228	CZ	ARG	429	42.162	39.525	-0.880	1.00	0.00	RX1	C
ATOM	6229	NH1	ARG	429	41.783	39.634	0.400	1.00	0.00	RX1	N
ATOM	6230	HH11	ARG	429	41.564	40.536	0.815	1.00	0.00	RX1	H
ATOM	6231	HH12	ARG	429	41.736	38.852	1.035	1.00	0.00	RX1	H
ATOM	6232	NH2	ARG	429	42.362	40.633	-1.616	1.00	0.00	RX1	N
ATOM	6233	HH21	ARG	429	42.156	41.541	-1.219	1.00	0.00	RX1	H
ATOM	6234	HH22	ARG	429	42.718	40.577	-2.558	1.00	0.00	RX1	H
ATOM	6235	C	ARG	429	41.557	32.093	-1.036	1.00	0.00	RX1	C
ATOM	6236	O	ARG	429	42.565	31.410	-1.193	1.00	0.00	RX1	O
ATOM	6237	N	THR	430	40.524	31.759	-0.264	1.00	0.00	RX1	N
ATOM	6238	H	THR	430	39.703	32.323	-0.193	1.00	0.00	RX1	H
ATOM	6239	CA	THR	430	40.332	30.368	0.112	1.00	0.00	RX1	C
ATOM	6240	CB	THR	430	40.300	30.289	1.654	1.00	0.00	RX1	C
ATOM	6241	OG1	THR	430	39.399	29.298	2.152	1.00	0.00	RX1	O
ATOM	6242	HG1	THR	430	38.758	29.757	2.717	1.00	0.00	RX1	H
ATOM	6243	CG2	THR	430	40.093	31.657	2.298	1.00	0.00	RX1	C
ATOM	6244	C	THR	430	39.106	29.907	-0.657	1.00	0.00	RX1	C
ATOM	6245	O	THR	430	38.319	30.728	-1.122	1.00	0.00	RX1	O
ATOM	6246	N	LYS	431	39.053	28.585	-0.877	1.00	0.00	RX1	N
ATOM	6247	H	LYS	431	39.587	27.933	-0.338	1.00	0.00	RX1	H
ATOM	6248	CA	LYS	431	38.069	28.053	-1.817	1.00	0.00	RX1	C
ATOM	6249	CB	LYS	431	38.669	27.786	-3.203	1.00	0.00	RX1	C
ATOM	6250	CG	LYS	431	39.625	28.822	-3.797	1.00	0.00	RX1	C
ATOM	6251	CD	LYS	431	40.127	28.400	-5.181	1.00	0.00	RX1	C
ATOM	6252	CE	LYS	431	41.326	29.217	-5.671	1.00	0.00	RX1	C
ATOM	6253	NZ	LYS	431	40.975	30.633	-5.789	1.00	0.00	RX1	N
ATOM	6254	HZ1	LYS	431	41.816	31.244	-5.880	1.00	0.00	RX1	H
ATOM	6255	HZ2	LYS	431	40.463	30.874	-6.663	1.00	0.00	RX1	H
ATOM	6256	HZ3	LYS	431	40.450	31.008	-4.976	1.00	0.00	RX1	H
ATOM	6257	C	LYS	431	37.543	26.729	-1.305	1.00	0.00	RX1	C
ATOM	6258	O	LYS	431	38.320	25.856	-0.932	1.00	0.00	RX1	O
ATOM	6259	N	GLN	432	36.210	26.581	-1.310	1.00	0.00	RX1	N
ATOM	6260	H	GLN	432	35.633	27.283	-1.732	1.00	0.00	RX1	H
ATOM	6261	CA	GLN	432	35.690	25.295	-0.847	1.00	0.00	RX1	C
ATOM	6262	CB	GLN	432	34.170	25.310	-0.697	1.00	0.00	RX1	C
ATOM	6263	CG	GLN	432	33.725	25.328	0.765	1.00	0.00	RX1	C
ATOM	6264	CD	GLN	432	34.343	24.166	1.520	1.00	0.00	RX1	C
ATOM	6265	OE1	GLN	432	34.651	23.117	0.959	1.00	0.00	RX1	O
ATOM	6266	NE2	GLN	432	34.523	24.411	2.828	1.00	0.00	RX1	N
ATOM	6267	HE21	GLN	432	34.280	25.288	3.253	1.00	0.00	RX1	H
ATOM	6268	HE22	GLN	432	34.909	23.744	3.466	1.00	0.00	RX1	H
ATOM	6269	C	GLN	432	36.102	24.149	-1.743	1.00	0.00	RX1	C
ATOM	6270	O	GLN	432	36.094	24.271	-2.961	1.00	0.00	RX1	O
ATOM	6271	N	HIS	433	36.538	23.062	-1.086	1.00	0.00	RX1	N
ATOM	6272	H	HIS	433	36.431	23.046	-0.091	1.00	0.00	RX1	H
ATOM	6273	CA	HIS	433	37.147	21.930	-1.798	1.00	0.00	RX1	C
ATOM	6274	CB	HIS	433	36.097	21.116	-2.571	1.00	0.00	RX1	C
ATOM	6275	CG	HIS	433	35.225	20.273	-1.662	1.00	0.00	RX1	C
ATOM	6276	ND1	HIS	433	34.779	20.646	-0.447	1.00	0.00	RX1	N
ATOM	6277	HD1	HIS	433	34.928	21.503	0.011	1.00	0.00	RX1	H
ATOM	6278	CD2	HIS	433	34.732	18.990	-1.932	1.00	0.00	RX1	C
ATOM	6279	NE2	HIS	433	33.987	18.595	-0.869	1.00	0.00	RX1	N
ATOM	6280	CE1	HIS	433	34.021	19.617	0.046	1.00	0.00	RX1	C
ATOM	6281	C	HIS	433	38.305	22.301	-2.727	1.00	0.00	RX1	C

ATOM	6282	O	HIS	433	38.596	21.618	-3.700	1.00	0.00	RX1	O
ATOM	6283	N	GLY	434	38.957	23.433	-2.397	1.00	0.00	RX1	N
ATOM	6284	H	GLY	434	38.673	23.999	-1.621	1.00	0.00	RX1	H
ATOM	6285	CA	GLY	434	40.027	23.916	-3.275	1.00	0.00	RX1	C
ATOM	6286	C	GLY	434	39.570	24.444	-4.631	1.00	0.00	RX1	C
ATOM	6287	O	GLY	434	40.347	24.592	-5.564	1.00	0.00	RX1	O
ATOM	6288	N	GLN	435	38.263	24.721	-4.699	1.00	0.00	RX1	N
ATOM	6289	H	GLN	435	37.675	24.660	-3.896	1.00	0.00	RX1	H
ATOM	6290	CA	GLN	435	37.663	24.984	-6.001	1.00	0.00	RX1	C
ATOM	6291	CB	GLN	435	36.913	23.717	-6.420	1.00	0.00	RX1	C
ATOM	6292	CG	GLN	435	37.061	23.292	-7.882	1.00	0.00	RX1	C
ATOM	6293	CD	GLN	435	36.326	24.232	-8.812	1.00	0.00	RX1	C
ATOM	6294	OE1	GLN	435	35.155	24.565	-8.625	1.00	0.00	RX1	O
ATOM	6295	NE2	GLN	435	37.073	24.606	-9.860	1.00	0.00	RX1	N
ATOM	6296	HE21	GLN	435	38.030	24.328	-9.947	1.00	0.00	RX1	H
ATOM	6297	HE22	GLN	435	36.686	25.174	-10.595	1.00	0.00	RX1	H
ATOM	6298	C	GLN	435	36.755	26.204	-5.994	1.00	0.00	RX1	C
ATOM	6299	O	GLN	435	36.945	27.175	-6.717	1.00	0.00	RX1	O
ATOM	6300	N	PHE	436	35.736	26.097	-5.135	1.00	0.00	RX1	N
ATOM	6301	H	PHE	436	35.713	25.377	-4.443	1.00	0.00	RX1	H
ATOM	6302	CA	PHE	436	34.618	27.031	-5.236	1.00	0.00	RX1	C
ATOM	6303	CB	PHE	436	33.325	26.391	-4.734	1.00	0.00	RX1	C
ATOM	6304	CG	PHE	436	33.268	24.910	-5.025	1.00	0.00	RX1	C
ATOM	6305	CD1	PHE	436	33.171	24.012	-3.969	1.00	0.00	RX1	C
ATOM	6306	CD2	PHE	436	33.287	24.437	-6.331	1.00	0.00	RX1	C
ATOM	6307	CE1	PHE	436	33.094	22.646	-4.212	1.00	0.00	RX1	C
ATOM	6308	CE2	PHE	436	33.210	23.071	-6.577	1.00	0.00	RX1	C
ATOM	6309	CZ	PHE	436	33.115	22.176	-5.519	1.00	0.00	RX1	C
ATOM	6310	C	PHE	436	34.795	28.313	-4.451	1.00	0.00	RX1	C
ATOM	6311	O	PHE	436	35.321	28.305	-3.346	1.00	0.00	RX1	O
ATOM	6312	N	SER	437	34.280	29.401	-5.030	1.00	0.00	RX1	N
ATOM	6313	H	SER	437	33.941	29.430	-5.972	1.00	0.00	RX1	H
ATOM	6314	CA	SER	437	34.015	30.540	-4.155	1.00	0.00	RX1	C
ATOM	6315	CB	SER	437	34.626	31.767	-4.793	1.00	0.00	RX1	C
ATOM	6316	OG	SER	437	34.360	31.669	-6.193	1.00	0.00	RX1	O
ATOM	6317	HG	SER	437	34.858	32.378	-6.604	1.00	0.00	RX1	H
ATOM	6318	C	SER	437	32.528	30.718	-3.926	1.00	0.00	RX1	C
ATOM	6319	O	SER	437	32.024	30.668	-2.811	1.00	0.00	RX1	O
ATOM	6320	N	LEU	438	31.836	30.904	-5.056	1.00	0.00	RX1	N
ATOM	6321	H	LEU	438	32.324	30.900	-5.932	1.00	0.00	RX1	H
ATOM	6322	CA	LEU	438	30.384	31.044	-4.988	1.00	0.00	RX1	C
ATOM	6323	CB	LEU	438	29.874	31.756	-6.236	1.00	0.00	RX1	C
ATOM	6324	CG	LEU	438	29.156	33.064	-5.920	1.00	0.00	RX1	C
ATOM	6325	CD1	LEU	438	28.549	33.683	-7.175	1.00	0.00	RX1	C
ATOM	6326	CD2	LEU	438	28.115	32.896	-4.816	1.00	0.00	RX1	C
ATOM	6327	C	LEU	438	29.656	29.723	-4.829	1.00	0.00	RX1	C
ATOM	6328	O	LEU	438	29.086	29.189	-5.772	1.00	0.00	RX1	O
ATOM	6329	N	ALA	439	29.718	29.204	-3.602	1.00	0.00	RX1	N
ATOM	6330	H	ALA	439	30.059	29.726	-2.814	1.00	0.00	RX1	H
ATOM	6331	CA	ALA	439	29.101	27.903	-3.385	1.00	0.00	RX1	C
ATOM	6332	CB	ALA	439	29.955	27.070	-2.432	1.00	0.00	RX1	C
ATOM	6333	C	ALA	439	27.684	28.005	-2.856	1.00	0.00	RX1	C
ATOM	6334	O	ALA	439	27.397	27.772	-1.688	1.00	0.00	RX1	O
ATOM	6335	N	VAL	440	26.805	28.386	-3.788	1.00	0.00	RX1	N
ATOM	6336	H	VAL	440	27.082	28.371	-4.751	1.00	0.00	RX1	H
ATOM	6337	CA	VAL	440	25.390	28.430	-3.429	1.00	0.00	RX1	C
ATOM	6338	CB	VAL	440	24.727	29.651	-4.066	1.00	0.00	RX1	C
ATOM	6339	CG1	VAL	440	23.206	29.633	-3.944	1.00	0.00	RX1	C
ATOM	6340	CG2	VAL	440	25.321	30.926	-3.473	1.00	0.00	RX1	C
ATOM	6341	C	VAL	440	24.725	27.134	-3.849	1.00	0.00	RX1	C
ATOM	6342	O	VAL	440	24.401	26.899	-5.011	1.00	0.00	RX1	O

ATOM	6343	N	VAL	441	24.600	26.274	-2.841	1.00	0.00	RX1	N
ATOM	6344	H	VAL	441	24.744	26.530	-1.882	1.00	0.00	RX1	H
ATOM	6345	CA	VAL	441	24.206	24.908	-3.147	1.00	0.00	RX1	C
ATOM	6346	CB	VAL	441	25.411	23.974	-3.023	1.00	0.00	RX1	C
ATOM	6347	CG1	VAL	441	26.346	24.090	-4.227	1.00	0.00	RX1	C
ATOM	6348	CG2	VAL	441	26.147	24.209	-1.704	1.00	0.00	RX1	C
ATOM	6349	C	VAL	441	23.052	24.415	-2.302	1.00	0.00	RX1	C
ATOM	6350	O	VAL	441	22.865	24.793	-1.153	1.00	0.00	RX1	O
ATOM	6351	N	SER	442	22.272	23.530	-2.937	1.00	0.00	RX1	N
ATOM	6352	H	SER	442	22.492	23.325	-3.891	1.00	0.00	RX1	H
ATOM	6353	CA	SER	442	21.146	22.859	-2.274	1.00	0.00	RX1	C
ATOM	6354	CB	SER	442	21.671	21.866	-1.221	1.00	0.00	RX1	C
ATOM	6355	OG	SER	442	23.018	22.174	-0.831	1.00	0.00	RX1	O
ATOM	6356	HG	SER	442	22.952	23.005	-0.353	1.00	0.00	RX1	H
ATOM	6357	C	SER	442	19.979	23.702	-1.762	1.00	0.00	RX1	C
ATOM	6358	O	SER	442	19.061	23.193	-1.119	1.00	0.00	RX1	O
ATOM	6359	N	LEU	443	20.026	25.010	-2.068	1.00	0.00	RX1	N
ATOM	6360	H	LEU	443	20.744	25.363	-2.672	1.00	0.00	RX1	H
ATOM	6361	CA	LEU	443	19.107	25.940	-1.408	1.00	0.00	RX1	C
ATOM	6362	CB	LEU	443	19.449	27.407	-1.681	1.00	0.00	RX1	C
ATOM	6363	CG	LEU	443	20.856	27.867	-1.315	1.00	0.00	RX1	C
ATOM	6364	CD1	LEU	443	20.917	29.385	-1.211	1.00	0.00	RX1	C
ATOM	6365	CD2	LEU	443	21.362	27.242	-0.030	1.00	0.00	RX1	C
ATOM	6366	C	LEU	443	17.628	25.756	-1.710	1.00	0.00	RX1	C
ATOM	6367	O	LEU	443	17.204	24.871	-2.453	1.00	0.00	RX1	O
ATOM	6368	N	ASN	444	16.862	26.665	-1.080	1.00	0.00	RX1	N
ATOM	6369	H	ASN	444	17.247	27.386	-0.503	1.00	0.00	RX1	H
ATOM	6370	CA	ASN	444	15.412	26.706	-1.285	1.00	0.00	RX1	C
ATOM	6371	CB	ASN	444	14.666	26.558	0.047	1.00	0.00	RX1	C
ATOM	6372	CG	ASN	444	14.926	25.194	0.656	1.00	0.00	RX1	C
ATOM	6373	OD1	ASN	444	15.063	24.189	-0.040	1.00	0.00	RX1	O
ATOM	6374	ND2	ASN	444	15.022	25.203	1.995	1.00	0.00	RX1	N
ATOM	6375	HD21	ASN	444	15.028	26.075	2.493	1.00	0.00	RX1	H
ATOM	6376	HD22	ASN	444	15.120	24.400	2.587	1.00	0.00	RX1	H
ATOM	6377	C	ASN	444	14.978	28.001	-1.962	1.00	0.00	RX1	C
ATOM	6378	O	ASN	444	13.889	28.526	-1.757	1.00	0.00	RX1	O
ATOM	6379	N	ILE	445	15.918	28.531	-2.759	1.00	0.00	RX1	N
ATOM	6380	H	ILE	445	16.671	27.975	-3.100	1.00	0.00	RX1	H
ATOM	6381	CA	ILE	445	15.640	29.830	-3.367	1.00	0.00	RX1	C
ATOM	6382	CB	ILE	445	16.886	30.717	-3.383	1.00	0.00	RX1	C
ATOM	6383	CG2	ILE	445	17.271	31.137	-1.971	1.00	0.00	RX1	C
ATOM	6384	CG1	ILE	445	18.047	30.049	-4.121	1.00	0.00	RX1	C
ATOM	6385	CD1	ILE	445	19.279	30.950	-4.208	1.00	0.00	RX1	C
ATOM	6386	C	ILE	445	15.072	29.725	-4.766	1.00	0.00	RX1	C
ATOM	6387	O	ILE	445	15.345	28.787	-5.504	1.00	0.00	RX1	O
ATOM	6388	N	THR	446	14.284	30.752	-5.097	1.00	0.00	RX1	N
ATOM	6389	H	THR	446	14.091	31.479	-4.440	1.00	0.00	RX1	H
ATOM	6390	CA	THR	446	13.931	30.962	-6.497	1.00	0.00	RX1	C
ATOM	6391	CB	THR	446	12.419	31.108	-6.598	1.00	0.00	RX1	C
ATOM	6392	OG1	THR	446	11.830	29.865	-6.211	1.00	0.00	RX1	O
ATOM	6393	HG1	THR	446	12.310	29.226	-6.736	1.00	0.00	RX1	H
ATOM	6394	CG2	THR	446	11.951	31.500	-8.002	1.00	0.00	RX1	C
ATOM	6395	C	THR	446	14.680	32.135	-7.096	1.00	0.00	RX1	C
ATOM	6396	O	THR	446	15.080	32.118	-8.256	1.00	0.00	RX1	O
ATOM	6397	N	SER	447	14.874	33.141	-6.245	1.00	0.00	RX1	N
ATOM	6398	H	SER	447	14.564	33.211	-5.296	1.00	0.00	RX1	H
ATOM	6399	CA	SER	447	15.725	34.222	-6.708	1.00	0.00	RX1	C
ATOM	6400	CB	SER	447	15.034	35.427	-6.133	1.00	0.00	RX1	C
ATOM	6401	OG	SER	447	13.708	34.981	-5.802	1.00	0.00	RX1	O
ATOM	6402	HG	SER	447	13.447	35.559	-5.092	1.00	0.00	RX1	H
ATOM	6403	C	SER	447	17.145	33.982	-6.255	1.00	0.00	RX1	C

ATOM	6404	O	SER	447	17.394	33.545	-5.140	1.00	0.00	RX1	O
ATOM	6405	N	LEU	448	18.068	34.272	-7.174	1.00	0.00	RX1	N
ATOM	6406	H	LEU	448	17.805	34.706	-8.036	1.00	0.00	RX1	H
ATOM	6407	CA	LEU	448	19.469	34.200	-6.780	1.00	0.00	RX1	C
ATOM	6408	CB	LEU	448	20.362	34.185	-8.017	1.00	0.00	RX1	C
ATOM	6409	CG	LEU	448	21.181	32.900	-8.117	1.00	0.00	RX1	C
ATOM	6410	CD1	LEU	448	22.055	32.878	-9.370	1.00	0.00	RX1	C
ATOM	6411	CD2	LEU	448	21.980	32.634	-6.842	1.00	0.00	RX1	C
ATOM	6412	C	LEU	448	19.889	35.295	-5.816	1.00	0.00	RX1	C
ATOM	6413	O	LEU	448	20.793	35.133	-5.012	1.00	0.00	RX1	O
ATOM	6414	N	GLY	449	19.185	36.431	-5.914	1.00	0.00	RX1	N
ATOM	6415	H	GLY	449	18.563	36.583	-6.684	1.00	0.00	RX1	H
ATOM	6416	CA	GLY	449	19.234	37.384	-4.803	1.00	0.00	RX1	C
ATOM	6417	C	GLY	449	20.521	38.159	-4.537	1.00	0.00	RX1	C
ATOM	6418	O	GLY	449	20.525	39.101	-3.756	1.00	0.00	RX1	O
ATOM	6419	N	LEU	450	21.613	37.767	-5.215	1.00	0.00	RX1	N
ATOM	6420	H	LEU	450	21.561	36.975	-5.820	1.00	0.00	RX1	H
ATOM	6421	CA	LEU	450	22.884	38.481	-5.053	1.00	0.00	RX1	C
ATOM	6422	CB	LEU	450	24.046	37.583	-5.472	1.00	0.00	RX1	C
ATOM	6423	CG	LEU	450	24.264	36.380	-4.560	1.00	0.00	RX1	C
ATOM	6424	CD1	LEU	450	25.031	35.263	-5.266	1.00	0.00	RX1	C
ATOM	6425	CD2	LEU	450	24.939	36.790	-3.252	1.00	0.00	RX1	C
ATOM	6426	C	LEU	450	22.963	39.777	-5.844	1.00	0.00	RX1	C
ATOM	6427	O	LEU	450	23.866	40.010	-6.636	1.00	0.00	RX1	O
ATOM	6428	N	ARG	451	21.956	40.620	-5.606	1.00	0.00	RX1	N
ATOM	6429	H	ARG	451	21.371	40.433	-4.818	1.00	0.00	RX1	H
ATOM	6430	CA	ARG	451	21.748	41.773	-6.480	1.00	0.00	RX1	C
ATOM	6431	CB	ARG	451	20.318	42.303	-6.307	1.00	0.00	RX1	C
ATOM	6432	CG	ARG	451	19.839	42.354	-4.855	1.00	0.00	RX1	C
ATOM	6433	CD	ARG	451	18.339	42.626	-4.707	1.00	0.00	RX1	C
ATOM	6434	NE	ARG	451	17.531	41.658	-5.445	1.00	0.00	RX1	N
ATOM	6435	HE	ARG	451	17.480	41.732	-6.452	1.00	0.00	RX1	H
ATOM	6436	CZ	ARG	451	16.931	40.568	-4.872	1.00	0.00	RX1	C
ATOM	6437	NH1	ARG	451	16.977	40.359	-3.544	1.00	0.00	RX1	N
ATOM	6438	HH11	ARG	451	16.647	39.485	-3.146	1.00	0.00	RX1	H
ATOM	6439	HH12	ARG	451	17.334	41.036	-2.890	1.00	0.00	RX1	H
ATOM	6440	NH2	ARG	451	16.297	39.690	-5.660	1.00	0.00	RX1	N
ATOM	6441	HH21	ARG	451	15.852	38.871	-5.275	1.00	0.00	RX1	H
ATOM	6442	HH22	ARG	451	16.257	39.860	-6.659	1.00	0.00	RX1	H
ATOM	6443	C	ARG	451	22.810	42.865	-6.442	1.00	0.00	RX1	C
ATOM	6444	O	ARG	451	22.837	43.765	-7.270	1.00	0.00	RX1	O
ATOM	6445	N	SER	452	23.716	42.743	-5.461	1.00	0.00	RX1	N
ATOM	6446	H	SER	452	23.709	42.011	-4.780	1.00	0.00	RX1	H
ATOM	6447	CA	SER	452	24.816	43.705	-5.485	1.00	0.00	RX1	C
ATOM	6448	CB	SER	452	24.890	44.352	-4.115	1.00	0.00	RX1	C
ATOM	6449	OG	SER	452	23.552	44.575	-3.635	1.00	0.00	RX1	O
ATOM	6450	HG	SER	452	23.143	43.714	-3.629	1.00	0.00	RX1	H
ATOM	6451	C	SER	452	26.160	43.169	-5.959	1.00	0.00	RX1	C
ATOM	6452	O	SER	452	27.177	43.853	-5.917	1.00	0.00	RX1	O
ATOM	6453	N	LEU	453	26.139	41.899	-6.395	1.00	0.00	RX1	N
ATOM	6454	H	LEU	453	25.291	41.396	-6.568	1.00	0.00	RX1	H
ATOM	6455	CA	LEU	453	27.428	41.258	-6.637	1.00	0.00	RX1	C
ATOM	6456	CB	LEU	453	27.228	39.749	-6.771	1.00	0.00	RX1	C
ATOM	6457	CG	LEU	453	28.471	38.927	-6.432	1.00	0.00	RX1	C
ATOM	6458	CD1	LEU	453	28.858	39.069	-4.961	1.00	0.00	RX1	C
ATOM	6459	CD2	LEU	453	28.288	37.460	-6.810	1.00	0.00	RX1	C
ATOM	6460	C	LEU	453	28.202	41.822	-7.819	1.00	0.00	RX1	C
ATOM	6461	O	LEU	453	27.841	41.656	-8.976	1.00	0.00	RX1	O
ATOM	6462	N	LYS	454	29.298	42.503	-7.467	1.00	0.00	RX1	N
ATOM	6463	H	LYS	454	29.522	42.609	-6.499	1.00	0.00	RX1	H
ATOM	6464	CA	LYS	454	30.142	43.054	-8.522	1.00	0.00	RX1	C

ATOM	6465	CB	LYS	454	30.507	44.519	-8.246	1.00	0.00	RX1	C
ATOM	6466	CG	LYS	454	31.601	44.706	-7.187	1.00	0.00	RX1	C
ATOM	6467	CD	LYS	454	32.153	46.132	-7.138	1.00	0.00	RX1	C
ATOM	6468	CE	LYS	454	33.356	46.299	-6.201	1.00	0.00	RX1	C
ATOM	6469	NZ	LYS	454	34.490	45.479	-6.653	1.00	0.00	RX1	N
ATOM	6470	HZ1	LYS	454	35.352	45.660	-6.095	1.00	0.00	RX1	H
ATOM	6471	HZ2	LYS	454	34.344	44.460	-6.475	1.00	0.00	RX1	H
ATOM	6472	HZ3	LYS	454	34.715	45.611	-7.656	1.00	0.00	RX1	H
ATOM	6473	C	LYS	454	31.405	42.263	-8.831	1.00	0.00	RX1	C
ATOM	6474	O	LYS	454	32.010	42.420	-9.883	1.00	0.00	RX1	O
ATOM	6475	N	GLU	455	31.828	41.456	-7.847	1.00	0.00	RX1	N
ATOM	6476	H	GLU	455	31.311	41.282	-7.005	1.00	0.00	RX1	H
ATOM	6477	CA	GLU	455	33.209	40.991	-7.952	1.00	0.00	RX1	C
ATOM	6478	CB	GLU	455	34.093	41.880	-7.069	1.00	0.00	RX1	C
ATOM	6479	CG	GLU	455	35.586	41.923	-7.406	1.00	0.00	RX1	C
ATOM	6480	CD	GLU	455	36.349	42.620	-6.285	1.00	0.00	RX1	C
ATOM	6481	OE1	GLU	455	35.916	43.663	-5.809	1.00	0.00	RX1	O
ATOM	6482	OE2	GLU	455	37.381	42.115	-5.861	1.00	0.00	RX1	O
ATOM	6483	C	GLU	455	33.360	39.540	-7.550	1.00	0.00	RX1	C
ATOM	6484	O	GLU	455	33.357	39.221	-6.370	1.00	0.00	RX1	O
ATOM	6485	N	ILE	456	33.506	38.666	-8.556	1.00	0.00	RX1	N
ATOM	6486	H	ILE	456	33.546	38.952	-9.516	1.00	0.00	RX1	H
ATOM	6487	CA	ILE	456	33.959	37.331	-8.165	1.00	0.00	RX1	C
ATOM	6488	CB	ILE	456	33.162	36.213	-8.835	1.00	0.00	RX1	C
ATOM	6489	CG2	ILE	456	33.517	34.858	-8.213	1.00	0.00	RX1	C
ATOM	6490	CG1	ILE	456	31.660	36.476	-8.770	1.00	0.00	RX1	C
ATOM	6491	CD1	ILE	456	30.877	35.435	-9.570	1.00	0.00	RX1	C
ATOM	6492	C	ILE	456	35.437	37.194	-8.473	1.00	0.00	RX1	C
ATOM	6493	O	ILE	456	35.871	36.744	-9.532	1.00	0.00	RX1	O
ATOM	6494	N	SER	457	36.204	37.664	-7.492	1.00	0.00	RX1	N
ATOM	6495	H	SER	457	35.817	37.869	-6.588	1.00	0.00	RX1	H
ATOM	6496	CA	SER	457	37.607	37.951	-7.761	1.00	0.00	RX1	C
ATOM	6497	CB	SER	457	38.033	38.852	-6.626	1.00	0.00	RX1	C
ATOM	6498	OG	SER	457	36.823	39.305	-6.010	1.00	0.00	RX1	O
ATOM	6499	HG	SER	457	36.898	40.256	-5.933	1.00	0.00	RX1	H
ATOM	6500	C	SER	457	38.524	36.765	-8.009	1.00	0.00	RX1	C
ATOM	6501	O	SER	457	39.626	36.938	-8.531	1.00	0.00	RX1	O
ATOM	6502	N	ASP	458	38.013	35.581	-7.613	1.00	0.00	RX1	N
ATOM	6503	H	ASP	458	37.127	35.518	-7.145	1.00	0.00	RX1	H
ATOM	6504	CA	ASP	458	38.667	34.279	-7.778	1.00	0.00	RX1	C
ATOM	6505	CB	ASP	458	40.008	34.308	-7.030	1.00	0.00	RX1	C
ATOM	6506	CG	ASP	458	40.886	33.070	-7.128	1.00	0.00	RX1	C
ATOM	6507	OD1	ASP	458	40.654	32.184	-7.945	1.00	0.00	RX1	O
ATOM	6508	OD2	ASP	458	41.811	32.952	-6.330	1.00	0.00	RX1	O
ATOM	6509	C	ASP	458	37.699	33.223	-7.260	1.00	0.00	RX1	C
ATOM	6510	O	ASP	458	36.701	33.541	-6.620	1.00	0.00	RX1	O
ATOM	6511	N	GLY	459	38.011	31.963	-7.577	1.00	0.00	RX1	N
ATOM	6512	H	GLY	459	38.761	31.815	-8.221	1.00	0.00	RX1	H
ATOM	6513	CA	GLY	459	37.171	30.860	-7.135	1.00	0.00	RX1	C
ATOM	6514	C	GLY	459	36.068	30.594	-8.131	1.00	0.00	RX1	C
ATOM	6515	O	GLY	459	35.448	31.499	-8.674	1.00	0.00	RX1	O
ATOM	6516	N	ASP	460	35.901	29.303	-8.390	1.00	0.00	RX1	N
ATOM	6517	H	ASP	460	36.313	28.599	-7.811	1.00	0.00	RX1	H
ATOM	6518	CA	ASP	460	34.942	28.905	-9.409	1.00	0.00	RX1	C
ATOM	6519	CB	ASP	460	35.486	27.586	-9.966	1.00	0.00	RX1	C
ATOM	6520	CG	ASP	460	34.670	26.930	-11.062	1.00	0.00	RX1	C
ATOM	6521	OD1	ASP	460	35.105	25.919	-11.598	1.00	0.00	RX1	O
ATOM	6522	OD2	ASP	460	33.545	27.316	-11.327	1.00	0.00	RX1	O
ATOM	6523	C	ASP	460	33.547	28.854	-8.806	1.00	0.00	RX1	C
ATOM	6524	O	ASP	460	33.361	28.572	-7.626	1.00	0.00	RX1	O
ATOM	6525	N	VAL	461	32.568	29.187	-9.648	1.00	0.00	RX1	N

ATOM	6526	H	VAL	461	32.750	29.185	-10.635	1.00	0.00	RX1	H
ATOM	6527	CA	VAL	461	31.198	29.181	-9.152	1.00	0.00	RX1	C
ATOM	6528	CB	VAL	461	30.352	30.014	-10.122	1.00	0.00	RX1	C
ATOM	6529	CG1	VAL	461	28.870	30.068	-9.759	1.00	0.00	RX1	C
ATOM	6530	CG2	VAL	461	30.956	31.413	-10.259	1.00	0.00	RX1	C
ATOM	6531	C	VAL	461	30.677	27.754	-9.010	1.00	0.00	RX1	C
ATOM	6532	O	VAL	461	30.962	26.877	-9.824	1.00	0.00	RX1	O
ATOM	6533	N	ILE	462	29.889	27.560	-7.947	1.00	0.00	RX1	N
ATOM	6534	H	ILE	462	29.744	28.262	-7.248	1.00	0.00	RX1	H
ATOM	6535	CA	ILE	462	28.975	26.425	-7.930	1.00	0.00	RX1	C
ATOM	6536	CB	ILE	462	29.595	25.159	-7.319	1.00	0.00	RX1	C
ATOM	6537	CG2	ILE	462	29.945	25.302	-5.843	1.00	0.00	RX1	C
ATOM	6538	CG1	ILE	462	28.694	23.954	-7.581	1.00	0.00	RX1	C
ATOM	6539	CD1	ILE	462	29.295	22.650	-7.055	1.00	0.00	RX1	C
ATOM	6540	C	ILE	462	27.633	26.801	-7.319	1.00	0.00	RX1	C
ATOM	6541	O	ILE	462	27.345	26.669	-6.135	1.00	0.00	RX1	O
ATOM	6542	N	ILE	463	26.798	27.312	-8.220	1.00	0.00	RX1	N
ATOM	6543	H	ILE	463	27.003	27.286	-9.199	1.00	0.00	RX1	H
ATOM	6544	CA	ILE	463	25.438	27.552	-7.768	1.00	0.00	RX1	C
ATOM	6545	CB	ILE	463	24.991	28.951	-8.184	1.00	0.00	RX1	C
ATOM	6546	CG2	ILE	463	23.542	29.240	-7.805	1.00	0.00	RX1	C
ATOM	6547	CG1	ILE	463	25.937	29.954	-7.527	1.00	0.00	RX1	C
ATOM	6548	CD1	ILE	463	25.427	31.388	-7.592	1.00	0.00	RX1	C
ATOM	6549	C	ILE	463	24.537	26.443	-8.271	1.00	0.00	RX1	C
ATOM	6550	O	ILE	463	23.922	26.508	-9.328	1.00	0.00	RX1	O
ATOM	6551	N	SER	464	24.551	25.370	-7.478	1.00	0.00	RX1	N
ATOM	6552	H	SER	464	24.924	25.402	-6.547	1.00	0.00	RX1	H
ATOM	6553	CA	SER	464	23.918	24.153	-7.971	1.00	0.00	RX1	C
ATOM	6554	CB	SER	464	25.010	23.190	-8.423	1.00	0.00	RX1	C
ATOM	6555	OG	SER	464	25.696	23.723	-9.562	1.00	0.00	RX1	O
ATOM	6556	HG	SER	464	26.009	24.592	-9.324	1.00	0.00	RX1	H
ATOM	6557	C	SER	464	22.980	23.507	-6.974	1.00	0.00	RX1	C
ATOM	6558	O	SER	464	23.136	23.612	-5.764	1.00	0.00	RX1	O
ATOM	6559	N	GLY	465	21.991	22.807	-7.542	1.00	0.00	RX1	N
ATOM	6560	H	GLY	465	21.834	22.869	-8.530	1.00	0.00	RX1	H
ATOM	6561	CA	GLY	465	21.066	22.076	-6.678	1.00	0.00	RX1	C
ATOM	6562	C	GLY	465	20.105	22.961	-5.913	1.00	0.00	RX1	C
ATOM	6563	O	GLY	465	19.567	22.610	-4.869	1.00	0.00	RX1	O
ATOM	6564	N	ASN	466	19.923	24.160	-6.467	1.00	0.00	RX1	N
ATOM	6565	H	ASN	466	20.203	24.375	-7.404	1.00	0.00	RX1	H
ATOM	6566	CA	ASN	466	19.029	25.057	-5.753	1.00	0.00	RX1	C
ATOM	6567	CB	ASN	466	19.487	26.509	-5.824	1.00	0.00	RX1	C
ATOM	6568	CG	ASN	466	20.915	26.595	-5.344	1.00	0.00	RX1	C
ATOM	6569	OD1	ASN	466	21.201	26.510	-4.156	1.00	0.00	RX1	O
ATOM	6570	ND2	ASN	466	21.802	26.747	-6.334	1.00	0.00	RX1	N
ATOM	6571	HD21	ASN	466	21.497	26.752	-7.293	1.00	0.00	RX1	H
ATOM	6572	HD22	ASN	466	22.778	26.839	-6.119	1.00	0.00	RX1	H
ATOM	6573	C	ASN	466	17.644	24.859	-6.298	1.00	0.00	RX1	C
ATOM	6574	O	ASN	466	17.305	25.184	-7.431	1.00	0.00	RX1	O
ATOM	6575	N	LYS	467	16.879	24.199	-5.429	1.00	0.00	RX1	N
ATOM	6576	H	LYS	467	17.176	24.174	-4.474	1.00	0.00	RX1	H
ATOM	6577	CA	LYS	467	15.809	23.340	-5.938	1.00	0.00	RX1	C
ATOM	6578	CB	LYS	467	15.306	22.474	-4.777	1.00	0.00	RX1	C
ATOM	6579	CG	LYS	467	16.510	21.745	-4.160	1.00	0.00	RX1	C
ATOM	6580	CD	LYS	467	16.235	20.827	-2.967	1.00	0.00	RX1	C
ATOM	6581	CE	LYS	467	15.699	21.532	-1.719	1.00	0.00	RX1	C
ATOM	6582	NZ	LYS	467	16.628	22.557	-1.228	1.00	0.00	RX1	N
ATOM	6583	HZ1	LYS	467	16.205	23.010	-0.392	1.00	0.00	RX1	H
ATOM	6584	HZ2	LYS	467	17.572	22.200	-0.967	1.00	0.00	RX1	H
ATOM	6585	HZ3	LYS	467	16.768	23.311	-1.930	1.00	0.00	RX1	H
ATOM	6586	C	LYS	467	14.705	24.039	-6.719	1.00	0.00	RX1	C

ATOM	6587	O	LYS	467	14.046	23.479	-7.584	1.00	0.00	RX1	O
ATOM	6588	N	ASN	468	14.559	25.322	-6.387	1.00	0.00	RX1	N
ATOM	6589	H	ASN	468	15.102	25.744	-5.659	1.00	0.00	RX1	H
ATOM	6590	CA	ASN	468	13.525	26.131	-7.024	1.00	0.00	RX1	C
ATOM	6591	CB	ASN	468	12.433	26.520	-6.010	1.00	0.00	RX1	C
ATOM	6592	CG	ASN	468	13.008	27.036	-4.696	1.00	0.00	RX1	C
ATOM	6593	OD1	ASN	468	13.936	26.464	-4.126	1.00	0.00	RX1	O
ATOM	6594	ND2	ASN	468	12.363	28.097	-4.185	1.00	0.00	RX1	N
ATOM	6595	HD21	ASN	468	11.678	28.614	-4.706	1.00	0.00	RX1	H
ATOM	6596	HD22	ASN	468	12.568	28.419	-3.255	1.00	0.00	RX1	H
ATOM	6597	C	ASN	468	14.053	27.353	-7.758	1.00	0.00	RX1	C
ATOM	6598	O	ASN	468	13.316	28.293	-8.047	1.00	0.00	RX1	O
ATOM	6599	N	LEU	469	15.364	27.295	-8.051	1.00	0.00	RX1	N
ATOM	6600	H	LEU	469	15.885	26.458	-7.879	1.00	0.00	RX1	H
ATOM	6601	CA	LEU	469	16.070	28.473	-8.558	1.00	0.00	RX1	C
ATOM	6602	CB	LEU	469	17.558	28.321	-8.264	1.00	0.00	RX1	C
ATOM	6603	CG	LEU	469	18.402	29.538	-8.624	1.00	0.00	RX1	C
ATOM	6604	CD1	LEU	469	18.017	30.750	-7.786	1.00	0.00	RX1	C
ATOM	6605	CD2	LEU	469	19.895	29.244	-8.545	1.00	0.00	RX1	C
ATOM	6606	C	LEU	469	15.878	28.784	-10.029	1.00	0.00	RX1	C
ATOM	6607	O	LEU	469	16.133	27.971	-10.910	1.00	0.00	RX1	O
ATOM	6608	N	CYS	470	15.451	30.027	-10.251	1.00	0.00	RX1	N
ATOM	6609	H	CYS	470	15.356	30.676	-9.495	1.00	0.00	RX1	H
ATOM	6610	CA	CYS	470	15.484	30.562	-11.607	1.00	0.00	RX1	C
ATOM	6611	CB	CYS	470	14.187	31.313	-11.845	1.00	0.00	RX1	C
ATOM	6612	SG	CYS	470	12.785	30.186	-11.824	1.00	0.00	RX1	S
ATOM	6613	C	CYS	470	16.698	31.457	-11.778	1.00	0.00	RX1	C
ATOM	6614	O	CYS	470	17.555	31.506	-10.907	1.00	0.00	RX1	O
ATOM	6615	N	TYR	471	16.736	32.178	-12.916	1.00	0.00	RX1	N
ATOM	6616	H	TYR	471	16.096	31.991	-13.660	1.00	0.00	RX1	H
ATOM	6617	CA	TYR	471	17.676	33.294	-13.113	1.00	0.00	RX1	C
ATOM	6618	CB	TYR	471	17.527	34.356	-12.016	1.00	0.00	RX1	C
ATOM	6619	CG	TYR	471	16.104	34.855	-12.018	1.00	0.00	RX1	C
ATOM	6620	CD1	TYR	471	15.274	34.609	-10.931	1.00	0.00	RX1	C
ATOM	6621	CE1	TYR	471	13.951	35.033	-10.962	1.00	0.00	RX1	C
ATOM	6622	CD2	TYR	471	15.620	35.549	-13.119	1.00	0.00	RX1	C
ATOM	6623	CE2	TYR	471	14.300	35.979	-13.146	1.00	0.00	RX1	C
ATOM	6624	CZ	TYR	471	13.464	35.712	-12.071	1.00	0.00	RX1	C
ATOM	6625	OH	TYR	471	12.145	36.119	-12.105	1.00	0.00	RX1	O
ATOM	6626	HH	TYR	471	12.028	36.642	-12.893	1.00	0.00	RX1	H
ATOM	6627	C	TYR	471	19.144	32.981	-13.372	1.00	0.00	RX1	C
ATOM	6628	O	TYR	471	19.782	33.639	-14.181	1.00	0.00	RX1	O
ATOM	6629	N	ALA	472	19.651	31.926	-12.707	1.00	0.00	RX1	N
ATOM	6630	H	ALA	472	19.097	31.507	-11.989	1.00	0.00	RX1	H
ATOM	6631	CA	ALA	472	21.059	31.524	-12.826	1.00	0.00	RX1	C
ATOM	6632	CB	ALA	472	21.267	30.145	-12.196	1.00	0.00	RX1	C
ATOM	6633	C	ALA	472	21.678	31.503	-14.219	1.00	0.00	RX1	C
ATOM	6634	O	ALA	472	22.859	31.755	-14.413	1.00	0.00	RX1	O
ATOM	6635	N	ASN	473	20.823	31.185	-15.198	1.00	0.00	RX1	N
ATOM	6636	H	ASN	473	19.841	31.150	-15.016	1.00	0.00	RX1	H
ATOM	6637	CA	ASN	473	21.297	31.227	-16.582	1.00	0.00	RX1	C
ATOM	6638	CB	ASN	473	20.314	30.542	-17.540	1.00	0.00	RX1	C
ATOM	6639	CG	ASN	473	19.043	31.354	-17.637	1.00	0.00	RX1	C
ATOM	6640	OD1	ASN	473	18.511	31.819	-16.627	1.00	0.00	RX1	O
ATOM	6641	ND2	ASN	473	18.609	31.536	-18.894	1.00	0.00	RX1	N
ATOM	6642	HD21	ASN	473	19.021	31.078	-19.683	1.00	0.00	RX1	H
ATOM	6643	HD22	ASN	473	17.867	32.182	-19.092	1.00	0.00	RX1	H
ATOM	6644	C	ASN	473	21.677	32.624	-17.070	1.00	0.00	RX1	C
ATOM	6645	O	ASN	473	22.704	32.850	-17.697	1.00	0.00	RX1	O
ATOM	6646	N	THR	474	20.793	33.570	-16.749	1.00	0.00	RX1	N
ATOM	6647	H	THR	474	20.081	33.418	-16.064	1.00	0.00	RX1	H

ATOM	6648	CA	THR	474	20.895	34.875	-17.393	1.00	0.00	RX1	C
ATOM	6649	CB	THR	474	19.468	35.334	-17.679	1.00	0.00	RX1	C
ATOM	6650	OG1	THR	474	18.654	34.184	-17.947	1.00	0.00	RX1	O
ATOM	6651	HG1	THR	474	18.528	33.723	-17.123	1.00	0.00	RX1	H
ATOM	6652	CG2	THR	474	19.401	36.309	-18.858	1.00	0.00	RX1	C
ATOM	6653	C	THR	474	21.731	35.892	-16.619	1.00	0.00	RX1	C
ATOM	6654	O	THR	474	21.447	37.081	-16.557	1.00	0.00	RX1	O
ATOM	6655	N	ILE	475	22.793	35.360	-16.000	1.00	0.00	RX1	N
ATOM	6656	H	ILE	475	23.042	34.402	-16.144	1.00	0.00	RX1	H
ATOM	6657	CA	ILE	475	23.592	36.248	-15.154	1.00	0.00	RX1	C
ATOM	6658	CB	ILE	475	24.058	35.473	-13.916	1.00	0.00	RX1	C
ATOM	6659	CG2	ILE	475	24.929	36.303	-12.974	1.00	0.00	RX1	C
ATOM	6660	CG1	ILE	475	22.850	34.914	-13.170	1.00	0.00	RX1	C
ATOM	6661	CD1	ILE	475	22.005	36.013	-12.531	1.00	0.00	RX1	C
ATOM	6662	C	ILE	475	24.759	36.910	-15.880	1.00	0.00	RX1	C
ATOM	6663	O	ILE	475	25.255	37.952	-15.482	1.00	0.00	RX1	O
ATOM	6664	N	ASN	476	25.204	36.234	-16.959	1.00	0.00	RX1	N
ATOM	6665	H	ASN	476	24.702	35.432	-17.276	1.00	0.00	RX1	H
ATOM	6666	CA	ASN	476	26.495	36.593	-17.571	1.00	0.00	RX1	C
ATOM	6667	CB	ASN	476	26.510	37.903	-18.344	1.00	0.00	RX1	C
ATOM	6668	CG	ASN	476	27.902	38.074	-18.928	1.00	0.00	RX1	C
ATOM	6669	OD1	ASN	476	28.665	37.123	-19.111	1.00	0.00	RX1	O
ATOM	6670	ND2	ASN	476	28.177	39.346	-19.250	1.00	0.00	RX1	N
ATOM	6671	HD21	ASN	476	27.544	40.061	-18.926	1.00	0.00	RX1	H
ATOM	6672	HD22	ASN	476	28.973	39.655	-19.766	1.00	0.00	RX1	H
ATOM	6673	C	ASN	476	27.657	36.561	-16.591	1.00	0.00	RX1	C
ATOM	6674	O	ASN	476	28.341	37.529	-16.280	1.00	0.00	RX1	O
ATOM	6675	N	TRP	477	27.866	35.326	-16.131	1.00	0.00	RX1	N
ATOM	6676	H	TRP	477	27.272	34.591	-16.451	1.00	0.00	RX1	H
ATOM	6677	CA	TRP	477	28.880	35.088	-15.107	1.00	0.00	RX1	C
ATOM	6678	CB	TRP	477	28.983	33.592	-14.858	1.00	0.00	RX1	C
ATOM	6679	CG	TRP	477	27.675	33.114	-14.287	1.00	0.00	RX1	C
ATOM	6680	CD2	TRP	477	27.250	33.198	-12.914	1.00	0.00	RX1	C
ATOM	6681	CE2	TRP	477	25.963	32.622	-12.836	1.00	0.00	RX1	C
ATOM	6682	CE3	TRP	477	27.852	33.715	-11.775	1.00	0.00	RX1	C
ATOM	6683	CD1	TRP	477	26.617	32.494	-14.969	1.00	0.00	RX1	C
ATOM	6684	NE1	TRP	477	25.606	32.201	-14.112	1.00	0.00	RX1	N
ATOM	6685	HE1	TRP	477	24.743	31.787	-14.347	1.00	0.00	RX1	H
ATOM	6686	CZ2	TRP	477	25.306	32.577	-11.614	1.00	0.00	RX1	C
ATOM	6687	CZ3	TRP	477	27.183	33.667	-10.560	1.00	0.00	RX1	C
ATOM	6688	CH2	TRP	477	25.917	33.102	-10.483	1.00	0.00	RX1	C
ATOM	6689	C	TRP	477	30.252	35.696	-15.354	1.00	0.00	RX1	C
ATOM	6690	O	TRP	477	30.915	36.177	-14.443	1.00	0.00	RX1	O
ATOM	6691	N	LYS	478	30.668	35.684	-16.635	1.00	0.00	RX1	N
ATOM	6692	H	LYS	478	30.046	35.485	-17.393	1.00	0.00	RX1	H
ATOM	6693	CA	LYS	478	32.009	36.225	-16.842	1.00	0.00	RX1	C
ATOM	6694	CB	LYS	478	32.629	35.846	-18.189	1.00	0.00	RX1	C
ATOM	6695	CG	LYS	478	34.102	35.462	-17.988	1.00	0.00	RX1	C
ATOM	6696	CD	LYS	478	35.144	36.188	-18.848	1.00	0.00	RX1	C
ATOM	6697	CE	LYS	478	35.311	37.685	-18.554	1.00	0.00	RX1	C
ATOM	6698	NZ	LYS	478	35.644	37.919	-17.138	1.00	0.00	RX1	N
ATOM	6699	HZ1	LYS	478	34.862	37.633	-16.502	1.00	0.00	RX1	H
ATOM	6700	HZ2	LYS	478	36.471	37.384	-16.816	1.00	0.00	RX1	H
ATOM	6701	HZ3	LYS	478	35.778	38.930	-16.955	1.00	0.00	RX1	H
ATOM	6702	C	LYS	478	32.172	37.714	-16.599	1.00	0.00	RX1	C
ATOM	6703	O	LYS	478	33.270	38.183	-16.324	1.00	0.00	RX1	O
ATOM	6704	N	LYS	479	31.055	38.444	-16.684	1.00	0.00	RX1	N
ATOM	6705	H	LYS	479	30.156	38.038	-16.864	1.00	0.00	RX1	H
ATOM	6706	CA	LYS	479	31.141	39.858	-16.317	1.00	0.00	RX1	C
ATOM	6707	CB	LYS	479	29.796	40.511	-16.631	1.00	0.00	RX1	C
ATOM	6708	CG	LYS	479	29.609	42.005	-16.360	1.00	0.00	RX1	C

ATOM	6709	CD	LYS	479	28.984	42.286	-14.994	1.00	0.00	RX1	C
ATOM	6710	CE	LYS	479	28.053	43.497	-15.039	1.00	0.00	RX1	C
ATOM	6711	NZ	LYS	479	26.965	43.221	-15.984	1.00	0.00	RX1	N
ATOM	6712	HZ1	LYS	479	26.138	43.850	-15.860	1.00	0.00	RX1	H
ATOM	6713	HZ2	LYS	479	27.253	43.283	-16.984	1.00	0.00	RX1	H
ATOM	6714	HZ3	LYS	479	26.626	42.238	-15.923	1.00	0.00	RX1	H
ATOM	6715	C	LYS	479	31.586	40.067	-14.875	1.00	0.00	RX1	C
ATOM	6716	O	LYS	479	32.313	40.992	-14.544	1.00	0.00	RX1	O
ATOM	6717	N	LEU	480	31.133	39.119	-14.044	1.00	0.00	RX1	N
ATOM	6718	H	LEU	480	30.610	38.335	-14.378	1.00	0.00	RX1	H
ATOM	6719	CA	LEU	480	31.511	39.180	-12.638	1.00	0.00	RX1	C
ATOM	6720	CB	LEU	480	30.440	38.529	-11.765	1.00	0.00	RX1	C
ATOM	6721	CG	LEU	480	29.030	39.117	-11.841	1.00	0.00	RX1	C
ATOM	6722	CD1	LEU	480	28.179	38.563	-12.988	1.00	0.00	RX1	C
ATOM	6723	CD2	LEU	480	28.325	38.900	-10.510	1.00	0.00	RX1	C
ATOM	6724	C	LEU	480	32.851	38.537	-12.328	1.00	0.00	RX1	C
ATOM	6725	O	LEU	480	33.611	38.990	-11.479	1.00	0.00	RX1	O
ATOM	6726	N	PHE	481	33.106	37.423	-13.033	1.00	0.00	RX1	N
ATOM	6727	H	PHE	481	32.498	37.083	-13.749	1.00	0.00	RX1	H
ATOM	6728	CA	PHE	481	34.334	36.733	-12.666	1.00	0.00	RX1	C
ATOM	6729	CB	PHE	481	34.054	35.262	-12.318	1.00	0.00	RX1	C
ATOM	6730	CG	PHE	481	33.804	34.319	-13.475	1.00	0.00	RX1	C
ATOM	6731	CD1	PHE	481	34.783	34.070	-14.431	1.00	0.00	RX1	C
ATOM	6732	CD2	PHE	481	32.595	33.638	-13.542	1.00	0.00	RX1	C
ATOM	6733	CE1	PHE	481	34.572	33.118	-15.421	1.00	0.00	RX1	C
ATOM	6734	CE2	PHE	481	32.382	32.687	-14.533	1.00	0.00	RX1	C
ATOM	6735	CZ	PHE	481	33.376	32.414	-15.464	1.00	0.00	RX1	C
ATOM	6736	C	PHE	481	35.528	36.985	-13.570	1.00	0.00	RX1	C
ATOM	6737	O	PHE	481	35.431	37.095	-14.795	1.00	0.00	RX1	O
ATOM	6738	N	GLY	482	36.680	37.120	-12.903	1.00	0.00	RX1	N
ATOM	6739	H	GLY	482	36.743	36.915	-11.923	1.00	0.00	RX1	H
ATOM	6740	CA	GLY	482	37.824	37.633	-13.651	1.00	0.00	RX1	C
ATOM	6741	C	GLY	482	39.152	36.962	-13.361	1.00	0.00	RX1	C
ATOM	6742	O	GLY	482	40.184	37.612	-13.221	1.00	0.00	RX1	O
ATOM	6743	N	THR	483	39.085	35.635	-13.296	1.00	0.00	RX1	N
ATOM	6744	H	THR	483	38.230	35.117	-13.335	1.00	0.00	RX1	H
ATOM	6745	CA	THR	483	40.294	34.873	-13.018	1.00	0.00	RX1	C
ATOM	6746	CB	THR	483	40.251	34.547	-11.533	1.00	0.00	RX1	C
ATOM	6747	OG1	THR	483	39.759	35.698	-10.832	1.00	0.00	RX1	O
ATOM	6748	HG1	THR	483	39.717	35.443	-9.914	1.00	0.00	RX1	H
ATOM	6749	CG2	THR	483	41.607	34.100	-10.986	1.00	0.00	RX1	C
ATOM	6750	C	THR	483	40.369	33.657	-13.917	1.00	0.00	RX1	C
ATOM	6751	O	THR	483	39.445	32.853	-13.999	1.00	0.00	RX1	O
ATOM	6752	N	SER	484	41.513	33.575	-14.607	1.00	0.00	RX1	N
ATOM	6753	H	SER	484	42.236	34.255	-14.497	1.00	0.00	RX1	H
ATOM	6754	CA	SER	484	41.770	32.501	-15.564	1.00	0.00	RX1	C
ATOM	6755	CB	SER	484	43.073	32.925	-16.212	1.00	0.00	RX1	C
ATOM	6756	OG	SER	484	43.100	34.360	-16.120	1.00	0.00	RX1	O
ATOM	6757	HG	SER	484	43.800	34.644	-16.697	1.00	0.00	RX1	H
ATOM	6758	C	SER	484	41.743	31.091	-14.992	1.00	0.00	RX1	C
ATOM	6759	O	SER	484	42.758	30.492	-14.670	1.00	0.00	RX1	O
ATOM	6760	N	GLY	485	40.509	30.592	-14.880	1.00	0.00	RX1	N
ATOM	6761	H	GLY	485	39.716	31.171	-15.080	1.00	0.00	RX1	H
ATOM	6762	CA	GLY	485	40.319	29.304	-14.227	1.00	0.00	RX1	C
ATOM	6763	C	GLY	485	38.851	29.038	-13.987	1.00	0.00	RX1	C
ATOM	6764	O	GLY	485	38.320	27.987	-14.340	1.00	0.00	RX1	O
ATOM	6765	N	GLN	486	38.210	30.070	-13.411	1.00	0.00	RX1	N
ATOM	6766	H	GLN	486	38.708	30.928	-13.267	1.00	0.00	RX1	H
ATOM	6767	CA	GLN	486	36.799	29.982	-13.013	1.00	0.00	RX1	C
ATOM	6768	CB	GLN	486	36.307	31.348	-12.545	1.00	0.00	RX1	C
ATOM	6769	CG	GLN	486	37.119	31.949	-11.401	1.00	0.00	RX1	C

ATOM	6770	CD	GLN	486	36.784	33.421	-11.309	1.00	0.00	RX1	C
ATOM	6771	OE1	GLN	486	37.034	34.177	-12.240	1.00	0.00	RX1	O
ATOM	6772	NE2	GLN	486	36.226	33.802	-10.152	1.00	0.00	RX1	N
ATOM	6773	HE21	GLN	486	35.945	33.122	-9.469	1.00	0.00	RX1	H
ATOM	6774	HE22	GLN	486	36.032	34.764	-9.938	1.00	0.00	RX1	H
ATOM	6775	C	GLN	486	35.861	29.459	-14.094	1.00	0.00	RX1	C
ATOM	6776	O	GLN	486	36.140	29.540	-15.291	1.00	0.00	RX1	O
ATOM	6777	N	LYS	487	34.747	28.900	-13.614	1.00	0.00	RX1	N
ATOM	6778	H	LYS	487	34.539	28.802	-12.636	1.00	0.00	RX1	H
ATOM	6779	CA	LYS	487	33.640	28.485	-14.470	1.00	0.00	RX1	C
ATOM	6780	CB	LYS	487	33.590	26.960	-14.628	1.00	0.00	RX1	C
ATOM	6781	CG	LYS	487	34.823	26.366	-15.301	1.00	0.00	RX1	C
ATOM	6782	CD	LYS	487	34.920	26.771	-16.769	1.00	0.00	RX1	C
ATOM	6783	CE	LYS	487	36.346	26.621	-17.288	1.00	0.00	RX1	C
ATOM	6784	NZ	LYS	487	37.203	27.534	-16.524	1.00	0.00	RX1	N
ATOM	6785	HZ1	LYS	487	38.139	27.633	-16.954	1.00	0.00	RX1	H
ATOM	6786	HZ2	LYS	487	36.754	28.472	-16.446	1.00	0.00	RX1	H
ATOM	6787	HZ3	LYS	487	37.338	27.213	-15.540	1.00	0.00	RX1	H
ATOM	6788	C	LYS	487	32.364	28.943	-13.793	1.00	0.00	RX1	C
ATOM	6789	O	LYS	487	32.399	29.663	-12.801	1.00	0.00	RX1	O
ATOM	6790	N	THR	488	31.242	28.473	-14.348	1.00	0.00	RX1	N
ATOM	6791	H	THR	488	31.182	27.922	-15.178	1.00	0.00	RX1	H
ATOM	6792	CA	THR	488	30.030	28.566	-13.552	1.00	0.00	RX1	C
ATOM	6793	CB	THR	488	29.177	29.743	-14.050	1.00	0.00	RX1	C
ATOM	6794	OG1	THR	488	28.112	30.027	-13.135	1.00	0.00	RX1	O
ATOM	6795	HG1	THR	488	27.566	30.694	-13.533	1.00	0.00	RX1	H
ATOM	6796	CG2	THR	488	28.668	29.559	-15.482	1.00	0.00	RX1	C
ATOM	6797	C	THR	488	29.339	27.218	-13.513	1.00	0.00	RX1	C
ATOM	6798	O	THR	488	28.843	26.690	-14.502	1.00	0.00	RX1	O
ATOM	6799	N	LYS	489	29.373	26.640	-12.312	1.00	0.00	RX1	N
ATOM	6800	H	LYS	489	29.761	27.085	-11.503	1.00	0.00	RX1	H
ATOM	6801	CA	LYS	489	28.682	25.364	-12.211	1.00	0.00	RX1	C
ATOM	6802	CB	LYS	489	29.555	24.368	-11.453	1.00	0.00	RX1	C
ATOM	6803	CG	LYS	489	30.921	24.270	-12.139	1.00	0.00	RX1	C
ATOM	6804	CD	LYS	489	31.896	23.318	-11.453	1.00	0.00	RX1	C
ATOM	6805	CE	LYS	489	32.289	23.761	-10.045	1.00	0.00	RX1	C
ATOM	6806	NZ	LYS	489	32.987	25.049	-10.085	1.00	0.00	RX1	N
ATOM	6807	HZ1	LYS	489	32.348	25.873	-10.091	1.00	0.00	RX1	H
ATOM	6808	HZ2	LYS	489	33.660	25.132	-9.293	1.00	0.00	RX1	H
ATOM	6809	HZ3	LYS	489	33.577	25.169	-10.939	1.00	0.00	RX1	H
ATOM	6810	C	LYS	489	27.307	25.547	-11.616	1.00	0.00	RX1	C
ATOM	6811	O	LYS	489	27.125	25.674	-10.410	1.00	0.00	RX1	O
ATOM	6812	N	ILE	490	26.349	25.607	-12.546	1.00	0.00	RX1	N
ATOM	6813	H	ILE	490	26.572	25.457	-13.510	1.00	0.00	RX1	H
ATOM	6814	CA	ILE	490	24.980	25.932	-12.147	1.00	0.00	RX1	C
ATOM	6815	CB	ILE	490	24.567	27.288	-12.734	1.00	0.00	RX1	C
ATOM	6816	CG2	ILE	490	25.268	28.435	-12.003	1.00	0.00	RX1	C
ATOM	6817	CG1	ILE	490	24.828	27.350	-14.241	1.00	0.00	RX1	C
ATOM	6818	CD1	ILE	490	24.441	28.702	-14.841	1.00	0.00	RX1	C
ATOM	6819	C	ILE	490	23.965	24.846	-12.471	1.00	0.00	RX1	C
ATOM	6820	O	ILE	490	22.901	25.078	-13.039	1.00	0.00	RX1	O
ATOM	6821	N	ILE	491	24.359	23.619	-12.108	1.00	0.00	RX1	N
ATOM	6822	H	ILE	491	25.081	23.517	-11.421	1.00	0.00	RX1	H
ATOM	6823	CA	ILE	491	23.476	22.507	-12.456	1.00	0.00	RX1	C
ATOM	6824	CB	ILE	491	24.284	21.216	-12.603	1.00	0.00	RX1	C
ATOM	6825	CG2	ILE	491	25.345	21.385	-13.692	1.00	0.00	RX1	C
ATOM	6826	CG1	ILE	491	24.896	20.772	-11.273	1.00	0.00	RX1	C
ATOM	6827	CD1	ILE	491	25.634	19.438	-11.377	1.00	0.00	RX1	C
ATOM	6828	C	ILE	491	22.317	22.336	-11.489	1.00	0.00	RX1	C
ATOM	6829	O	ILE	491	22.285	22.913	-10.408	1.00	0.00	RX1	O
ATOM	6830	N	SER	492	21.352	21.516	-11.939	1.00	0.00	RX1	N

ATOM	6831	H	SER	492	21.531	20.947	-12.737	1.00	0.00	RX1	H
ATOM	6832	CA	SER	492	20.233	21.125	-11.076	1.00	0.00	RX1	C
ATOM	6833	CB	SER	492	20.831	20.021	-10.234	1.00	0.00	RX1	C
ATOM	6834	OG	SER	492	21.821	19.419	-11.084	1.00	0.00	RX1	O
ATOM	6835	HG	SER	492	22.103	18.632	-10.634	1.00	0.00	RX1	H
ATOM	6836	C	SER	492	19.506	22.244	-10.343	1.00	0.00	RX1	C
ATOM	6837	O	SER	492	19.189	22.177	-9.163	1.00	0.00	RX1	O
ATOM	6838	N	ASN	493	19.267	23.297	-11.125	1.00	0.00	RX1	N
ATOM	6839	H	ASN	493	19.407	23.251	-12.113	1.00	0.00	RX1	H
ATOM	6840	CA	ASN	493	18.417	24.361	-10.603	1.00	0.00	RX1	C
ATOM	6841	CB	ASN	493	19.081	25.730	-10.785	1.00	0.00	RX1	C
ATOM	6842	CG	ASN	493	20.366	25.836	-9.984	1.00	0.00	RX1	C
ATOM	6843	OD1	ASN	493	20.382	25.823	-8.757	1.00	0.00	RX1	O
ATOM	6844	ND2	ASN	493	21.460	25.978	-10.748	1.00	0.00	RX1	N
ATOM	6845	HD21	ASN	493	21.461	25.902	-11.749	1.00	0.00	RX1	H
ATOM	6846	HD22	ASN	493	22.360	26.143	-10.337	1.00	0.00	RX1	H
ATOM	6847	C	ASN	493	17.147	24.296	-11.417	1.00	0.00	RX1	C
ATOM	6848	O	ASN	493	17.133	23.635	-12.448	1.00	0.00	RX1	O
ATOM	6849	N	ARG	494	16.099	25.002	-10.959	1.00	0.00	RX1	N
ATOM	6850	H	ARG	494	16.197	25.566	-10.140	1.00	0.00	RX1	H
ATOM	6851	CA	ARG	494	14.858	25.019	-11.750	1.00	0.00	RX1	C
ATOM	6852	CB	ARG	494	13.793	25.826	-10.998	1.00	0.00	RX1	C
ATOM	6853	CG	ARG	494	12.560	26.210	-11.811	1.00	0.00	RX1	C
ATOM	6854	CD	ARG	494	11.479	26.901	-10.978	1.00	0.00	RX1	C
ATOM	6855	NE	ARG	494	10.472	27.502	-11.851	1.00	0.00	RX1	N
ATOM	6856	HE	ARG	494	10.173	27.005	-12.674	1.00	0.00	RX1	H
ATOM	6857	CZ	ARG	494	10.039	28.767	-11.590	1.00	0.00	RX1	C
ATOM	6858	NH1	ARG	494	10.406	29.392	-10.453	1.00	0.00	RX1	N
ATOM	6859	HH11	ARG	494	10.124	30.350	-10.304	1.00	0.00	RX1	H
ATOM	6860	HH12	ARG	494	10.970	28.956	-9.746	1.00	0.00	RX1	H
ATOM	6861	NH2	ARG	494	9.259	29.402	-12.479	1.00	0.00	RX1	N
ATOM	6862	HH21	ARG	494	8.994	30.363	-12.332	1.00	0.00	RX1	H
ATOM	6863	HH22	ARG	494	8.910	28.963	-13.325	1.00	0.00	RX1	H
ATOM	6864	C	ARG	494	15.056	25.471	-13.201	1.00	0.00	RX1	C
ATOM	6865	O	ARG	494	14.473	24.941	-14.142	1.00	0.00	RX1	O
ATOM	6866	N	GLY	495	15.969	26.443	-13.334	1.00	0.00	RX1	N
ATOM	6867	H	GLY	495	16.247	26.965	-12.528	1.00	0.00	RX1	H
ATOM	6868	CA	GLY	495	16.570	26.672	-14.644	1.00	0.00	RX1	C
ATOM	6869	C	GLY	495	15.699	27.361	-15.676	1.00	0.00	RX1	C
ATOM	6870	O	GLY	495	14.551	27.726	-15.450	1.00	0.00	RX1	O
ATOM	6871	N	GLU	496	16.353	27.531	-16.840	1.00	0.00	RX1	N
ATOM	6872	H	GLU	496	17.268	27.141	-16.923	1.00	0.00	RX1	H
ATOM	6873	CA	GLU	496	15.817	28.314	-17.958	1.00	0.00	RX1	C
ATOM	6874	CB	GLU	496	16.731	28.099	-19.178	1.00	0.00	RX1	C
ATOM	6875	CG	GLU	496	16.530	29.011	-20.398	1.00	0.00	RX1	C
ATOM	6876	CD	GLU	496	15.193	28.753	-21.063	1.00	0.00	RX1	C
ATOM	6877	OE1	GLU	496	14.310	29.592	-20.954	1.00	0.00	RX1	O
ATOM	6878	OE2	GLU	496	14.996	27.695	-21.655	1.00	0.00	RX1	O
ATOM	6879	C	GLU	496	14.354	28.034	-18.281	1.00	0.00	RX1	C
ATOM	6880	O	GLU	496	13.460	28.834	-18.024	1.00	0.00	RX1	O
ATOM	6881	N	ASN	497	14.137	26.823	-18.826	1.00	0.00	RX1	N
ATOM	6882	H	ASN	497	14.911	26.247	-19.082	1.00	0.00	RX1	H
ATOM	6883	CA	ASN	497	12.790	26.538	-19.321	1.00	0.00	RX1	C
ATOM	6884	CB	ASN	497	12.637	25.147	-19.899	1.00	0.00	RX1	C
ATOM	6885	CG	ASN	497	11.286	25.191	-20.566	1.00	0.00	RX1	C
ATOM	6886	OD1	ASN	497	11.064	26.036	-21.431	1.00	0.00	RX1	O
ATOM	6887	ND2	ASN	497	10.414	24.264	-20.131	1.00	0.00	RX1	N
ATOM	6888	HD21	ASN	497	10.672	23.604	-19.423	1.00	0.00	RX1	H
ATOM	6889	HD22	ASN	497	9.484	24.201	-20.495	1.00	0.00	RX1	H
ATOM	6890	C	ASN	497	11.685	26.716	-18.300	1.00	0.00	RX1	C
ATOM	6891	O	ASN	497	10.660	27.339	-18.537	1.00	0.00	RX1	O

ATOM	6892	N	SER	498	11.974	26.165	-17.122	1.00	0.00	RX1	N
ATOM	6893	H	SER	498	12.863	25.748	-16.944	1.00	0.00	RX1	H
ATOM	6894	CA	SER	498	11.007	26.244	-16.035	1.00	0.00	RX1	C
ATOM	6895	CB	SER	498	11.457	25.135	-15.111	1.00	0.00	RX1	C
ATOM	6896	OG	SER	498	12.401	24.359	-15.864	1.00	0.00	RX1	O
ATOM	6897	HG	SER	498	13.146	24.230	-15.274	1.00	0.00	RX1	H
ATOM	6898	C	SER	498	10.831	27.628	-15.412	1.00	0.00	RX1	C
ATOM	6899	O	SER	498	9.874	27.905	-14.695	1.00	0.00	RX1	O
ATOM	6900	N	CYS	499	11.797	28.500	-15.738	1.00	0.00	RX1	N
ATOM	6901	H	CYS	499	12.602	28.229	-16.271	1.00	0.00	RX1	H
ATOM	6902	CA	CYS	499	11.647	29.919	-15.428	1.00	0.00	RX1	C
ATOM	6903	CB	CYS	499	13.033	30.569	-15.457	1.00	0.00	RX1	C
ATOM	6904	SG	CYS	499	13.111	32.266	-14.838	1.00	0.00	RX1	S
ATOM	6905	C	CYS	499	10.670	30.600	-16.372	1.00	0.00	RX1	C
ATOM	6906	O	CYS	499	9.677	31.196	-15.963	1.00	0.00	RX1	O
ATOM	6907	N	LYS	500	10.964	30.452	-17.678	1.00	0.00	RX1	N
ATOM	6908	H	LYS	500	11.747	29.904	-17.986	1.00	0.00	RX1	H
ATOM	6909	CA	LYS	500	10.061	31.106	-18.627	1.00	0.00	RX1	C
ATOM	6910	CB	LYS	500	10.673	31.236	-20.025	1.00	0.00	RX1	C
ATOM	6911	CG	LYS	500	10.800	29.948	-20.835	1.00	0.00	RX1	C
ATOM	6912	CD	LYS	500	11.348	30.245	-22.234	1.00	0.00	RX1	C
ATOM	6913	CE	LYS	500	11.668	28.983	-23.036	1.00	0.00	RX1	C
ATOM	6914	NZ	LYS	500	12.560	28.140	-22.239	1.00	0.00	RX1	N
ATOM	6915	HZ1	LYS	500	13.467	27.915	-22.702	1.00	0.00	RX1	H
ATOM	6916	HZ2	LYS	500	12.906	28.662	-21.404	1.00	0.00	RX1	H
ATOM	6917	HZ3	LYS	500	12.091	27.267	-21.935	1.00	0.00	RX1	H
ATOM	6918	C	LYS	500	8.654	30.532	-18.686	1.00	0.00	RX1	C
ATOM	6919	O	LYS	500	7.692	31.218	-19.000	1.00	0.00	RX1	O
ATOM	6920	N	ALA	501	8.564	29.252	-18.290	1.00	0.00	RX1	N
ATOM	6921	H	ALA	501	9.393	28.716	-18.130	1.00	0.00	RX1	H
ATOM	6922	CA	ALA	501	7.249	28.631	-18.129	1.00	0.00	RX1	C
ATOM	6923	CB	ALA	501	7.399	27.166	-17.717	1.00	0.00	RX1	C
ATOM	6924	C	ALA	501	6.339	29.322	-17.123	1.00	0.00	RX1	C
ATOM	6925	O	ALA	501	5.122	29.222	-17.168	1.00	0.00	RX1	O
ATOM	6926	N	THR	502	6.983	30.049	-16.204	1.00	0.00	RX1	N
ATOM	6927	H	THR	502	7.976	30.113	-16.108	1.00	0.00	RX1	H
ATOM	6928	CA	THR	502	6.160	30.841	-15.299	1.00	0.00	RX1	C
ATOM	6929	CB	THR	502	6.584	30.371	-13.934	1.00	0.00	RX1	C
ATOM	6930	OG1	THR	502	7.174	29.072	-14.113	1.00	0.00	RX1	O
ATOM	6931	HG1	THR	502	6.496	28.554	-14.537	1.00	0.00	RX1	H
ATOM	6932	CG2	THR	502	5.436	30.331	-12.923	1.00	0.00	RX1	C
ATOM	6933	C	THR	502	6.312	32.338	-15.527	1.00	0.00	RX1	C
ATOM	6934	O	THR	502	6.214	33.157	-14.624	1.00	0.00	RX1	O
ATOM	6935	N	GLY	503	6.602	32.659	-16.798	1.00	0.00	RX1	N
ATOM	6936	H	GLY	503	6.682	31.957	-17.506	1.00	0.00	RX1	H
ATOM	6937	CA	GLY	503	6.736	34.062	-17.186	1.00	0.00	RX1	C
ATOM	6938	C	GLY	503	7.836	34.860	-16.503	1.00	0.00	RX1	C
ATOM	6939	O	GLY	503	7.826	36.083	-16.512	1.00	0.00	RX1	O
ATOM	6940	N	GLN	504	8.809	34.139	-15.919	1.00	0.00	RX1	N
ATOM	6941	H	GLN	504	8.880	33.147	-16.025	1.00	0.00	RX1	H
ATOM	6942	CA	GLN	504	9.808	34.885	-15.151	1.00	0.00	RX1	C
ATOM	6943	CB	GLN	504	10.328	34.049	-13.980	1.00	0.00	RX1	C
ATOM	6944	CG	GLN	504	9.247	33.663	-12.970	1.00	0.00	RX1	C
ATOM	6945	CD	GLN	504	9.887	32.991	-11.769	1.00	0.00	RX1	C
ATOM	6946	OE1	GLN	504	9.600	31.840	-11.431	1.00	0.00	RX1	O
ATOM	6947	NE2	GLN	504	10.766	33.777	-11.123	1.00	0.00	RX1	N
ATOM	6948	HE21	GLN	504	10.987	34.697	-11.469	1.00	0.00	RX1	H
ATOM	6949	HE22	GLN	504	11.249	33.524	-10.286	1.00	0.00	RX1	H
ATOM	6950	C	GLN	504	10.974	35.432	-15.964	1.00	0.00	RX1	C
ATOM	6951	O	GLN	504	12.137	35.289	-15.609	1.00	0.00	RX1	O
ATOM	6952	N	VAL	505	10.614	36.053	-17.094	1.00	0.00	RX1	N

ATOM	6953	H	VAL	505	9.664	36.350	-17.203	1.00	0.00	RX1	H
ATOM	6954	CA	VAL	505	11.670	36.632	-17.918	1.00	0.00	RX1	C
ATOM	6955	CB	VAL	505	11.322	36.507	-19.401	1.00	0.00	RX1	C
ATOM	6956	CG1	VAL	505	11.222	35.036	-19.800	1.00	0.00	RX1	C
ATOM	6957	CG2	VAL	505	10.051	37.287	-19.745	1.00	0.00	RX1	C
ATOM	6958	C	VAL	505	11.927	38.079	-17.551	1.00	0.00	RX1	C
ATOM	6959	O	VAL	505	11.120	38.725	-16.899	1.00	0.00	RX1	O
ATOM	6960	N	CYS	506	13.093	38.562	-17.996	1.00	0.00	RX1	N
ATOM	6961	H	CYS	506	13.775	37.991	-18.449	1.00	0.00	RX1	H
ATOM	6962	CA	CYS	506	13.453	39.894	-17.514	1.00	0.00	RX1	C
ATOM	6963	CB	CYS	506	14.459	39.672	-16.401	1.00	0.00	RX1	C
ATOM	6964	SG	CYS	506	15.737	38.520	-16.972	1.00	0.00	RX1	S
ATOM	6965	C	CYS	506	13.956	40.874	-18.562	1.00	0.00	RX1	C
ATOM	6966	O	CYS	506	14.120	42.064	-18.309	1.00	0.00	RX1	O
ATOM	6967	N	HIS	507	14.214	40.321	-19.761	1.00	0.00	RX1	N
ATOM	6968	H	HIS	507	13.915	39.395	-19.973	1.00	0.00	RX1	H
ATOM	6969	CA	HIS	507	14.801	41.139	-20.824	1.00	0.00	RX1	C
ATOM	6970	CB	HIS	507	15.312	40.275	-21.976	1.00	0.00	RX1	C
ATOM	6971	CG	HIS	507	15.922	41.177	-23.024	1.00	0.00	RX1	C
ATOM	6972	ND1	HIS	507	15.211	41.820	-23.967	1.00	0.00	RX1	N
ATOM	6973	HD1	HIS	507	14.229	41.795	-24.055	1.00	0.00	RX1	H
ATOM	6974	CD2	HIS	507	17.268	41.508	-23.180	1.00	0.00	RX1	C
ATOM	6975	NE2	HIS	507	17.364	42.363	-24.229	1.00	0.00	RX1	N
ATOM	6976	CE1	HIS	507	16.094	42.553	-24.715	1.00	0.00	RX1	C
ATOM	6977	C	HIS	507	13.850	42.203	-21.354	1.00	0.00	RX1	C
ATOM	6978	O	HIS	507	13.069	41.975	-22.271	1.00	0.00	RX1	O
ATOM	6979	N	ALA	508	13.970	43.365	-20.703	1.00	0.00	RX1	N
ATOM	6980	H	ALA	508	14.727	43.473	-20.054	1.00	0.00	RX1	H
ATOM	6981	CA	ALA	508	13.082	44.514	-20.884	1.00	0.00	RX1	C
ATOM	6982	CB	ALA	508	11.617	44.145	-20.632	1.00	0.00	RX1	C
ATOM	6983	C	ALA	508	13.461	45.570	-19.868	1.00	0.00	RX1	C
ATOM	6984	O	ALA	508	13.742	46.715	-20.187	1.00	0.00	RX1	O
ATOM	6985	N	LEU	509	13.492	45.095	-18.613	1.00	0.00	RX1	N
ATOM	6986	H	LEU	509	13.291	44.129	-18.443	1.00	0.00	RX1	H
ATOM	6987	CA	LEU	509	14.090	45.919	-17.562	1.00	0.00	RX1	C
ATOM	6988	CB	LEU	509	13.346	45.755	-16.225	1.00	0.00	RX1	C
ATOM	6989	CG	LEU	509	13.227	44.326	-15.675	1.00	0.00	RX1	C
ATOM	6990	CD1	LEU	509	13.419	44.286	-14.160	1.00	0.00	RX1	C
ATOM	6991	CD2	LEU	509	11.925	43.634	-16.089	1.00	0.00	RX1	C
ATOM	6992	C	LEU	509	15.572	45.609	-17.420	1.00	0.00	RX1	C
ATOM	6993	O	LEU	509	16.438	46.457	-17.222	1.00	0.00	RX1	O
ATOM	6994	N	CYS	510	15.826	44.309	-17.594	1.00	0.00	RX1	N
ATOM	6995	H	CYS	510	15.107	43.632	-17.742	1.00	0.00	RX1	H
ATOM	6996	CA	CYS	510	17.206	43.876	-17.706	1.00	0.00	RX1	C
ATOM	6997	CB	CYS	510	17.300	42.468	-17.153	1.00	0.00	RX1	C
ATOM	6998	SG	CYS	510	16.328	42.310	-15.639	1.00	0.00	RX1	S
ATOM	6999	C	CYS	510	17.678	43.945	-19.136	1.00	0.00	RX1	C
ATOM	7000	O	CYS	510	16.891	43.973	-20.079	1.00	0.00	RX1	O
ATOM	7001	N	SER	511	19.006	43.969	-19.238	1.00	0.00	RX1	N
ATOM	7002	H	SER	511	19.560	43.868	-18.410	1.00	0.00	RX1	H
ATOM	7003	CA	SER	511	19.639	43.843	-20.545	1.00	0.00	RX1	C
ATOM	7004	CB	SER	511	20.958	44.599	-20.379	1.00	0.00	RX1	C
ATOM	7005	OG	SER	511	21.776	43.954	-19.400	1.00	0.00	RX1	O
ATOM	7006	HG	SER	511	21.228	43.686	-18.660	1.00	0.00	RX1	H
ATOM	7007	C	SER	511	19.736	42.354	-20.904	1.00	0.00	RX1	C
ATOM	7008	O	SER	511	19.217	41.531	-20.158	1.00	0.00	RX1	O
ATOM	7009	N	PRO	512	20.443	41.987	-22.016	1.00	0.00	RX1	N
ATOM	7010	CD	PRO	512	20.830	42.788	-23.178	1.00	0.00	RX1	C
ATOM	7011	CA	PRO	512	20.908	40.594	-22.128	1.00	0.00	RX1	C
ATOM	7012	CB	PRO	512	21.847	40.644	-23.336	1.00	0.00	RX1	C
ATOM	7013	CG	PRO	512	21.289	41.765	-24.208	1.00	0.00	RX1	C

ATOM	7014	C	PRO	512	21.575	40.032	-20.873	1.00	0.00	RX1	C
ATOM	7015	O	PRO	512	21.420	38.869	-20.522	1.00	0.00	RX1	O
ATOM	7016	N	GLU	513	22.302	40.929	-20.182	1.00	0.00	RX1	N
ATOM	7017	H	GLU	513	22.370	41.893	-20.435	1.00	0.00	RX1	H
ATOM	7018	CA	GLU	513	22.616	40.558	-18.808	1.00	0.00	RX1	C
ATOM	7019	CB	GLU	513	23.809	41.330	-18.266	1.00	0.00	RX1	C
ATOM	7020	CG	GLU	513	25.020	41.337	-19.193	1.00	0.00	RX1	C
ATOM	7021	CD	GLU	513	26.207	41.776	-18.369	1.00	0.00	RX1	C
ATOM	7022	OE1	GLU	513	26.492	41.133	-17.370	1.00	0.00	RX1	O
ATOM	7023	OE2	GLU	513	26.842	42.777	-18.677	1.00	0.00	RX1	O
ATOM	7024	C	GLU	513	21.401	40.791	-17.938	1.00	0.00	RX1	C
ATOM	7025	O	GLU	513	20.980	41.925	-17.709	1.00	0.00	RX1	O
ATOM	7026	N	GLY	514	20.844	39.645	-17.534	1.00	0.00	RX1	N
ATOM	7027	H	GLY	514	21.331	38.777	-17.642	1.00	0.00	RX1	H
ATOM	7028	CA	GLY	514	19.531	39.634	-16.909	1.00	0.00	RX1	C
ATOM	7029	C	GLY	514	19.530	40.068	-15.465	1.00	0.00	RX1	C
ATOM	7030	O	GLY	514	19.827	41.208	-15.130	1.00	0.00	RX1	O
ATOM	7031	N	CYS	515	19.149	39.114	-14.613	1.00	0.00	RX1	N
ATOM	7032	H	CYS	515	18.985	38.153	-14.835	1.00	0.00	RX1	H
ATOM	7033	CA	CYS	515	18.828	39.537	-13.256	1.00	0.00	RX1	C
ATOM	7034	CB	CYS	515	17.383	40.019	-13.249	1.00	0.00	RX1	C
ATOM	7035	SG	CYS	515	16.291	38.730	-13.887	1.00	0.00	RX1	S
ATOM	7036	C	CYS	515	19.047	38.432	-12.255	1.00	0.00	RX1	C
ATOM	7037	O	CYS	515	18.976	37.256	-12.588	1.00	0.00	RX1	O
ATOM	7038	N	TRP	516	19.294	38.857	-11.011	1.00	0.00	RX1	N
ATOM	7039	H	TRP	516	19.338	39.833	-10.786	1.00	0.00	RX1	H
ATOM	7040	CA	TRP	516	19.357	37.870	-9.937	1.00	0.00	RX1	C
ATOM	7041	CB	TRP	516	20.206	38.376	-8.770	1.00	0.00	RX1	C
ATOM	7042	CG	TRP	516	21.613	38.710	-9.196	1.00	0.00	RX1	C
ATOM	7043	CD2	TRP	516	22.762	37.842	-9.177	1.00	0.00	RX1	C
ATOM	7044	CE2	TRP	516	23.872	38.594	-9.619	1.00	0.00	RX1	C
ATOM	7045	CE3	TRP	516	22.932	36.512	-8.817	1.00	0.00	RX1	C
ATOM	7046	CD1	TRP	516	22.093	39.945	-9.656	1.00	0.00	RX1	C
ATOM	7047	NE1	TRP	516	23.428	39.881	-9.906	1.00	0.00	RX1	N
ATOM	7048	HE1	TRP	516	23.989	40.624	-10.212	1.00	0.00	RX1	H
ATOM	7049	CZ2	TRP	516	25.121	37.993	-9.683	1.00	0.00	RX1	C
ATOM	7050	CZ3	TRP	516	24.187	35.922	-8.889	1.00	0.00	RX1	C
ATOM	7051	CH2	TRP	516	25.281	36.662	-9.319	1.00	0.00	RX1	C
ATOM	7052	C	TRP	516	17.993	37.435	-9.413	1.00	0.00	RX1	C
ATOM	7053	O	TRP	516	17.884	36.523	-8.601	1.00	0.00	RX1	O
ATOM	7054	N	GLY	517	16.954	38.110	-9.906	1.00	0.00	RX1	N
ATOM	7055	H	GLY	517	17.009	38.909	-10.505	1.00	0.00	RX1	H
ATOM	7056	CA	GLY	517	15.623	37.844	-9.384	1.00	0.00	RX1	C
ATOM	7057	C	GLY	517	14.613	38.683	-10.137	1.00	0.00	RX1	C
ATOM	7058	O	GLY	517	14.932	39.314	-11.136	1.00	0.00	RX1	O
ATOM	7059	N	PRO	518	13.360	38.641	-9.639	1.00	0.00	RX1	N
ATOM	7060	CD	PRO	518	12.926	37.881	-8.479	1.00	0.00	RX1	C
ATOM	7061	CA	PRO	518	12.266	39.331	-10.329	1.00	0.00	RX1	C
ATOM	7062	CB	PRO	518	11.024	38.784	-9.610	1.00	0.00	RX1	C
ATOM	7063	CG	PRO	518	11.478	37.556	-8.816	1.00	0.00	RX1	C
ATOM	7064	C	PRO	518	12.307	40.855	-10.287	1.00	0.00	RX1	C
ATOM	7065	O	PRO	518	11.611	41.534	-11.032	1.00	0.00	RX1	O
ATOM	7066	N	GLU	519	13.081	41.373	-9.327	1.00	0.00	RX1	N
ATOM	7067	H	GLU	519	13.860	40.898	-8.910	1.00	0.00	RX1	H
ATOM	7068	CA	GLU	519	12.888	42.780	-9.006	1.00	0.00	RX1	C
ATOM	7069	CB	GLU	519	13.264	42.988	-7.537	1.00	0.00	RX1	C
ATOM	7070	CG	GLU	519	12.488	42.038	-6.624	1.00	0.00	RX1	C
ATOM	7071	CD	GLU	519	12.954	42.222	-5.197	1.00	0.00	RX1	C
ATOM	7072	OE1	GLU	519	13.723	41.402	-4.711	1.00	0.00	RX1	O
ATOM	7073	OE2	GLU	519	12.567	43.197	-4.561	1.00	0.00	RX1	O
ATOM	7074	C	GLU	519	13.658	43.708	-9.929	1.00	0.00	RX1	C

ATOM	7075	O	GLU	519	14.725	43.387	-10.434	1.00	0.00	RX1	O
ATOM	7076	N	PRO	520	13.106	44.931	-10.129	1.00	0.00	RX1	N
ATOM	7077	CD	PRO	520	11.785	45.386	-9.711	1.00	0.00	RX1	C
ATOM	7078	CA	PRO	520	13.859	45.954	-10.870	1.00	0.00	RX1	C
ATOM	7079	CB	PRO	520	12.967	47.191	-10.729	1.00	0.00	RX1	C
ATOM	7080	CG	PRO	520	11.554	46.640	-10.546	1.00	0.00	RX1	C
ATOM	7081	C	PRO	520	15.294	46.197	-10.407	1.00	0.00	RX1	C
ATOM	7082	O	PRO	520	16.152	46.623	-11.163	1.00	0.00	RX1	O
ATOM	7083	N	ARG	521	15.525	45.918	-9.118	1.00	0.00	RX1	N
ATOM	7084	H	ARG	521	14.831	45.451	-8.574	1.00	0.00	RX1	H
ATOM	7085	CA	ARG	521	16.872	46.129	-8.586	1.00	0.00	RX1	C
ATOM	7086	CB	ARG	521	16.761	46.538	-7.118	1.00	0.00	RX1	C
ATOM	7087	CG	ARG	521	16.020	45.471	-6.319	1.00	0.00	RX1	C
ATOM	7088	CD	ARG	521	15.598	45.905	-4.917	1.00	0.00	RX1	C
ATOM	7089	NE	ARG	521	14.870	44.809	-4.290	1.00	0.00	RX1	N
ATOM	7090	HE	ARG	521	14.040	44.442	-4.736	1.00	0.00	RX1	H
ATOM	7091	CZ	ARG	521	15.397	44.150	-3.224	1.00	0.00	RX1	C
ATOM	7092	NH1	ARG	521	16.462	44.673	-2.586	1.00	0.00	RX1	N
ATOM	7093	HH11	ARG	521	16.907	44.117	-1.865	1.00	0.00	RX1	H
ATOM	7094	HH12	ARG	521	16.846	45.570	-2.801	1.00	0.00	RX1	H
ATOM	7095	NH2	ARG	521	14.869	42.978	-2.832	1.00	0.00	RX1	N
ATOM	7096	HH21	ARG	521	15.248	42.445	-2.066	1.00	0.00	RX1	H
ATOM	7097	HH22	ARG	521	14.083	42.575	-3.344	1.00	0.00	RX1	H
ATOM	7098	C	ARG	521	17.843	44.964	-8.768	1.00	0.00	RX1	C
ATOM	7099	O	ARG	521	18.921	44.939	-8.190	1.00	0.00	RX1	O
ATOM	7100	N	ASP	522	17.409	43.990	-9.579	1.00	0.00	RX1	N
ATOM	7101	H	ASP	522	16.547	44.032	-10.086	1.00	0.00	RX1	H
ATOM	7102	CA	ASP	522	18.194	42.764	-9.723	1.00	0.00	RX1	C
ATOM	7103	CB	ASP	522	17.257	41.572	-9.862	1.00	0.00	RX1	C
ATOM	7104	CG	ASP	522	16.959	40.917	-8.539	1.00	0.00	RX1	C
ATOM	7105	OD1	ASP	522	15.809	40.898	-8.121	1.00	0.00	RX1	O
ATOM	7106	OD2	ASP	522	17.863	40.360	-7.935	1.00	0.00	RX1	O
ATOM	7107	C	ASP	522	19.117	42.716	-10.926	1.00	0.00	RX1	C
ATOM	7108	O	ASP	522	19.766	41.709	-11.192	1.00	0.00	RX1	O
ATOM	7109	N	CYS	523	19.097	43.797	-11.711	1.00	0.00	RX1	N
ATOM	7110	H	CYS	523	18.660	44.659	-11.463	1.00	0.00	RX1	H
ATOM	7111	CA	CYS	523	19.644	43.584	-13.049	1.00	0.00	RX1	C
ATOM	7112	CB	CYS	523	18.874	44.447	-14.031	1.00	0.00	RX1	C
ATOM	7113	SG	CYS	523	17.120	44.437	-13.586	1.00	0.00	RX1	S
ATOM	7114	C	CYS	523	21.146	43.703	-13.194	1.00	0.00	RX1	C
ATOM	7115	O	CYS	523	21.749	44.738	-12.943	1.00	0.00	RX1	O
ATOM	7116	N	VAL	524	21.720	42.580	-13.652	1.00	0.00	RX1	N
ATOM	7117	H	VAL	524	21.110	41.826	-13.893	1.00	0.00	RX1	H
ATOM	7118	CA	VAL	524	23.169	42.488	-13.853	1.00	0.00	RX1	C
ATOM	7119	CB	VAL	524	23.562	41.071	-14.285	1.00	0.00	RX1	C
ATOM	7120	CG1	VAL	524	25.076	40.883	-14.195	1.00	0.00	RX1	C
ATOM	7121	CG2	VAL	524	22.829	40.008	-13.466	1.00	0.00	RX1	C
ATOM	7122	C	VAL	524	23.722	43.532	-14.822	1.00	0.00	RX1	C
ATOM	7123	O	VAL	524	24.827	44.044	-14.664	1.00	0.00	RX1	O
ATOM	7124	N	SER	525	22.876	43.852	-15.812	1.00	0.00	RX1	N
ATOM	7125	H	SER	525	22.043	43.340	-16.032	1.00	0.00	RX1	H
ATOM	7126	CA	SER	525	23.026	45.148	-16.465	1.00	0.00	RX1	C
ATOM	7127	CB	SER	525	23.977	45.059	-17.657	1.00	0.00	RX1	C
ATOM	7128	OG	SER	525	25.189	44.409	-17.270	1.00	0.00	RX1	O
ATOM	7129	HG	SER	525	25.473	43.950	-18.064	1.00	0.00	RX1	H
ATOM	7130	C	SER	525	21.660	45.679	-16.836	1.00	0.00	RX1	C
ATOM	7131	O	SER	525	20.718	44.933	-17.090	1.00	0.00	RX1	O
ATOM	7132	N	CYS	526	21.579	47.010	-16.803	1.00	0.00	RX1	N
ATOM	7133	H	CYS	526	22.380	47.605	-16.754	1.00	0.00	RX1	H
ATOM	7134	CA	CYS	526	20.255	47.616	-16.834	1.00	0.00	RX1	C
ATOM	7135	CB	CYS	526	20.273	48.737	-15.808	1.00	0.00	RX1	C

ATOM	7136	SG	CYS	526	21.825	49.671	-15.839	1.00	0.00	RX1	S
ATOM	7137	C	CYS	526	19.807	48.086	-18.202	1.00	0.00	RX1	C
ATOM	7138	O	CYS	526	20.600	48.432	-19.072	1.00	0.00	RX1	O
ATOM	7139	N	ARG	527	18.478	48.116	-18.362	1.00	0.00	RX1	N
ATOM	7140	H	ARG	527	17.828	47.805	-17.663	1.00	0.00	RX1	H
ATOM	7141	CA	ARG	527	17.983	48.800	-19.550	1.00	0.00	RX1	C
ATOM	7142	CB	ARG	527	16.583	48.298	-19.900	1.00	0.00	RX1	C
ATOM	7143	CG	ARG	527	15.900	48.900	-21.137	1.00	0.00	RX1	C
ATOM	7144	CD	ARG	527	16.569	48.591	-22.478	1.00	0.00	RX1	C
ATOM	7145	NE	ARG	527	17.705	49.476	-22.731	1.00	0.00	RX1	N
ATOM	7146	HE	ARG	527	17.589	50.452	-22.506	1.00	0.00	RX1	H
ATOM	7147	CZ	ARG	527	18.828	48.997	-23.334	1.00	0.00	RX1	C
ATOM	7148	NH1	ARG	527	18.903	47.683	-23.650	1.00	0.00	RX1	N
ATOM	7149	HH11	ARG	527	19.702	47.277	-24.101	1.00	0.00	RX1	H
ATOM	7150	HH12	ARG	527	18.144	47.057	-23.443	1.00	0.00	RX1	H
ATOM	7151	NH2	ARG	527	19.834	49.857	-23.599	1.00	0.00	RX1	N
ATOM	7152	HH21	ARG	527	20.700	49.600	-24.036	1.00	0.00	RX1	H
ATOM	7153	HH22	ARG	527	19.721	50.831	-23.347	1.00	0.00	RX1	H
ATOM	7154	C	ARG	527	17.992	50.309	-19.388	1.00	0.00	RX1	C
ATOM	7155	O	ARG	527	17.052	50.932	-18.909	1.00	0.00	RX1	O
ATOM	7156	N	ASN	528	19.125	50.852	-19.863	1.00	0.00	RX1	N
ATOM	7157	H	ASN	528	19.884	50.209	-19.984	1.00	0.00	RX1	H
ATOM	7158	CA	ASN	528	19.399	52.297	-19.858	1.00	0.00	RX1	C
ATOM	7159	CB	ASN	528	18.284	53.165	-20.466	1.00	0.00	RX1	C
ATOM	7160	CG	ASN	528	18.332	53.095	-21.978	1.00	0.00	RX1	C
ATOM	7161	OD1	ASN	528	18.769	52.106	-22.569	1.00	0.00	RX1	O
ATOM	7162	ND2	ASN	528	17.860	54.201	-22.581	1.00	0.00	RX1	N
ATOM	7163	HD21	ASN	528	17.497	54.967	-22.048	1.00	0.00	RX1	H
ATOM	7164	HD22	ASN	528	17.841	54.312	-23.577	1.00	0.00	RX1	H
ATOM	7165	C	ASN	528	19.775	52.787	-18.475	1.00	0.00	RX1	C
ATOM	7166	O	ASN	528	19.728	52.042	-17.502	1.00	0.00	RX1	O
ATOM	7167	N	VAL	529	20.188	54.068	-18.437	1.00	0.00	RX1	N
ATOM	7168	H	VAL	529	20.119	54.652	-19.243	1.00	0.00	RX1	H
ATOM	7169	CA	VAL	529	20.758	54.617	-17.203	1.00	0.00	RX1	C
ATOM	7170	CB	VAL	529	21.233	56.057	-17.416	1.00	0.00	RX1	C
ATOM	7171	CG1	VAL	529	21.900	56.610	-16.153	1.00	0.00	RX1	C
ATOM	7172	CG2	VAL	529	22.157	56.149	-18.632	1.00	0.00	RX1	C
ATOM	7173	C	VAL	529	19.841	54.531	-15.991	1.00	0.00	RX1	C
ATOM	7174	O	VAL	529	18.936	55.329	-15.758	1.00	0.00	RX1	O
ATOM	7175	N	SER	530	20.141	53.493	-15.213	1.00	0.00	RX1	N
ATOM	7176	H	SER	530	20.900	52.875	-15.413	1.00	0.00	RX1	H
ATOM	7177	CA	SER	530	19.316	53.284	-14.039	1.00	0.00	RX1	C
ATOM	7178	CB	SER	530	19.135	51.788	-13.873	1.00	0.00	RX1	C
ATOM	7179	OG	SER	530	18.563	51.278	-15.087	1.00	0.00	RX1	O
ATOM	7180	HG	SER	530	19.033	51.674	-15.819	1.00	0.00	RX1	H
ATOM	7181	C	SER	530	19.802	54.029	-12.818	1.00	0.00	RX1	C
ATOM	7182	O	SER	530	20.467	53.523	-11.924	1.00	0.00	RX1	O
ATOM	7183	N	ARG	531	19.397	55.306	-12.851	1.00	0.00	RX1	N
ATOM	7184	H	ARG	531	18.925	55.588	-13.685	1.00	0.00	RX1	H
ATOM	7185	CA	ARG	531	19.419	56.113	-11.632	1.00	0.00	RX1	C
ATOM	7186	CB	ARG	531	18.995	57.560	-11.931	1.00	0.00	RX1	C
ATOM	7187	CG	ARG	531	17.983	57.777	-13.070	1.00	0.00	RX1	C
ATOM	7188	CD	ARG	531	16.478	57.624	-12.776	1.00	0.00	RX1	C
ATOM	7189	NE	ARG	531	16.068	56.299	-12.308	1.00	0.00	RX1	N
ATOM	7190	HE	ARG	531	15.779	56.194	-11.345	1.00	0.00	RX1	H
ATOM	7191	CZ	ARG	531	16.003	55.194	-13.111	1.00	0.00	RX1	C
ATOM	7192	NH1	ARG	531	16.388	55.238	-14.407	1.00	0.00	RX1	N
ATOM	7193	HH11	ARG	531	16.314	54.431	-15.012	1.00	0.00	RX1	H
ATOM	7194	HH12	ARG	531	16.768	56.063	-14.831	1.00	0.00	RX1	H
ATOM	7195	NH2	ARG	531	15.551	54.059	-12.569	1.00	0.00	RX1	N
ATOM	7196	HH21	ARG	531	15.557	53.152	-13.003	1.00	0.00	RX1	H

ATOM	7197	HH22	ARG	531	15.157	54.068	-11.632	1.00	0.00	RX1	H
ATOM	7198	C	ARG	531	18.588	55.488	-10.526	1.00	0.00	RX1	C
ATOM	7199	O	ARG	531	17.853	54.537	-10.757	1.00	0.00	RX1	O
ATOM	7200	N	GLY	532	18.738	56.057	-9.322	1.00	0.00	RX1	N
ATOM	7201	H	GLY	532	19.431	56.755	-9.154	1.00	0.00	RX1	H
ATOM	7202	CA	GLY	532	17.984	55.478	-8.212	1.00	0.00	RX1	C
ATOM	7203	C	GLY	532	18.426	54.061	-7.901	1.00	0.00	RX1	C
ATOM	7204	O	GLY	532	19.606	53.787	-7.686	1.00	0.00	RX1	O
ATOM	7205	N	ARG	533	17.429	53.173	-7.890	1.00	0.00	RX1	N
ATOM	7206	H	ARG	533	16.496	53.455	-8.140	1.00	0.00	RX1	H
ATOM	7207	CA	ARG	533	17.746	51.763	-7.687	1.00	0.00	RX1	C
ATOM	7208	CB	ARG	533	17.616	51.363	-6.218	1.00	0.00	RX1	C
ATOM	7209	CG	ARG	533	18.657	50.304	-5.835	1.00	0.00	RX1	C
ATOM	7210	CD	ARG	533	20.055	50.620	-6.387	1.00	0.00	RX1	C
ATOM	7211	NE	ARG	533	20.383	49.749	-7.519	1.00	0.00	RX1	N
ATOM	7212	HE	ARG	533	20.349	48.764	-7.311	1.00	0.00	RX1	H
ATOM	7213	CZ	ARG	533	20.693	50.260	-8.753	1.00	0.00	RX1	C
ATOM	7214	NH1	ARG	533	20.696	51.604	-8.958	1.00	0.00	RX1	N
ATOM	7215	HH11	ARG	533	20.905	52.030	-9.846	1.00	0.00	RX1	H
ATOM	7216	HH12	ARG	533	20.470	52.267	-8.224	1.00	0.00	RX1	H
ATOM	7217	NH2	ARG	533	20.985	49.396	-9.755	1.00	0.00	RX1	N
ATOM	7218	HH21	ARG	533	21.217	49.698	-10.684	1.00	0.00	RX1	H
ATOM	7219	HH22	ARG	533	20.976	48.400	-9.607	1.00	0.00	RX1	H
ATOM	7220	C	ARG	533	17.038	50.827	-8.651	1.00	0.00	RX1	C
ATOM	7221	O	ARG	533	17.071	49.612	-8.502	1.00	0.00	RX1	O
ATOM	7222	N	GLU	534	16.361	51.439	-9.626	1.00	0.00	RX1	N
ATOM	7223	H	GLU	534	16.403	52.424	-9.820	1.00	0.00	RX1	H
ATOM	7224	CA	GLU	534	15.466	50.619	-10.431	1.00	0.00	RX1	C
ATOM	7225	CB	GLU	534	14.012	51.104	-10.330	1.00	0.00	RX1	C
ATOM	7226	CG	GLU	534	13.752	52.245	-9.335	1.00	0.00	RX1	C
ATOM	7227	CD	GLU	534	14.182	53.566	-9.945	1.00	0.00	RX1	C
ATOM	7228	OE1	GLU	534	13.488	54.068	-10.818	1.00	0.00	RX1	O
ATOM	7229	OE2	GLU	534	15.244	54.078	-9.610	1.00	0.00	RX1	O
ATOM	7230	C	GLU	534	15.914	50.512	-11.870	1.00	0.00	RX1	C
ATOM	7231	O	GLU	534	16.027	51.494	-12.594	1.00	0.00	RX1	O
ATOM	7232	N	CYS	535	16.181	49.268	-12.272	1.00	0.00	RX1	N
ATOM	7233	H	CYS	535	16.056	48.458	-11.699	1.00	0.00	RX1	H
ATOM	7234	CA	CYS	535	16.637	49.102	-13.647	1.00	0.00	RX1	C
ATOM	7235	CB	CYS	535	17.330	47.762	-13.792	1.00	0.00	RX1	C
ATOM	7236	SG	CYS	535	18.730	47.660	-12.639	1.00	0.00	RX1	S
ATOM	7237	C	CYS	535	15.601	49.365	-14.715	1.00	0.00	RX1	C
ATOM	7238	O	CYS	535	14.742	48.550	-15.025	1.00	0.00	RX1	O
ATOM	7239	N	VAL	536	15.750	50.578	-15.256	1.00	0.00	RX1	N
ATOM	7240	H	VAL	536	16.490	51.179	-14.943	1.00	0.00	RX1	H
ATOM	7241	CA	VAL	536	14.926	51.067	-16.355	1.00	0.00	RX1	C
ATOM	7242	CB	VAL	536	13.473	51.294	-15.921	1.00	0.00	RX1	C
ATOM	7243	CG1	VAL	536	13.361	52.228	-14.713	1.00	0.00	RX1	C
ATOM	7244	CG2	VAL	536	12.639	51.770	-17.111	1.00	0.00	RX1	C
ATOM	7245	C	VAL	536	15.534	52.344	-16.908	1.00	0.00	RX1	C
ATOM	7246	O	VAL	536	16.075	53.174	-16.175	1.00	0.00	RX1	O
ATOM	7247	N	SER	22	41.445	32.477	38.080	1.00	0.00	RX2	N
ATOM	7248	H	SER	22	42.392	32.392	37.760	1.00	0.00	RX2	H
ATOM	7249	CA	SER	22	41.025	33.847	38.377	1.00	0.00	RX2	C
ATOM	7250	CB	SER	22	41.216	34.138	39.873	1.00	0.00	RX2	C
ATOM	7251	OG	SER	22	40.649	35.414	40.231	1.00	0.00	RX2	O
ATOM	7252	HG	SER	22	41.068	36.021	39.610	1.00	0.00	RX2	H
ATOM	7253	C	SER	22	41.786	34.870	37.547	1.00	0.00	RX2	C
ATOM	7254	O	SER	22	41.991	36.010	37.948	1.00	0.00	RX2	O
ATOM	7255	N	THR	23	42.222	34.385	36.383	1.00	0.00	RX2	N
ATOM	7256	H	THR	23	41.984	33.470	36.057	1.00	0.00	RX2	H
ATOM	7257	CA	THR	23	43.158	35.145	35.565	1.00	0.00	RX2	C

ATOM	7258	CB	THR	23	44.573	34.732	35.986	1.00	0.00	RX2	C
ATOM	7259	OG1	THR	23	44.562	33.392	36.512	1.00	0.00	RX2	O
ATOM	7260	HG1	THR	23	45.471	33.151	36.646	1.00	0.00	RX2	H
ATOM	7261	CG2	THR	23	45.181	35.685	37.018	1.00	0.00	RX2	C
ATOM	7262	C	THR	23	42.882	34.816	34.111	1.00	0.00	RX2	C
ATOM	7263	O	THR	23	42.157	33.874	33.813	1.00	0.00	RX2	O
ATOM	7264	N	GLN	24	43.491	35.604	33.213	1.00	0.00	RX2	N
ATOM	7265	H	GLN	24	44.014	36.402	33.516	1.00	0.00	RX2	H
ATOM	7266	CA	GLN	24	43.433	35.185	31.813	1.00	0.00	RX2	C
ATOM	7267	CB	GLN	24	43.778	36.378	30.901	1.00	0.00	RX2	C
ATOM	7268	CG	GLN	24	44.120	36.121	29.421	1.00	0.00	RX2	C
ATOM	7269	CD	GLN	24	43.034	35.392	28.653	1.00	0.00	RX2	C
ATOM	7270	OE1	GLN	24	42.060	34.887	29.195	1.00	0.00	RX2	O
ATOM	7271	NE2	GLN	24	43.275	35.311	27.345	1.00	0.00	RX2	N
ATOM	7272	HE21	GLN	24	44.001	35.851	26.908	1.00	0.00	RX2	H
ATOM	7273	HE22	GLN	24	42.720	34.684	26.793	1.00	0.00	RX2	H
ATOM	7274	C	GLN	24	44.290	33.955	31.559	1.00	0.00	RX2	C
ATOM	7275	O	GLN	24	45.517	33.998	31.567	1.00	0.00	RX2	O
ATOM	7276	N	VAL	25	43.566	32.848	31.378	1.00	0.00	RX2	N
ATOM	7277	H	VAL	25	42.567	32.916	31.374	1.00	0.00	RX2	H
ATOM	7278	CA	VAL	25	44.215	31.554	31.200	1.00	0.00	RX2	C
ATOM	7279	CB	VAL	25	43.918	30.630	32.396	1.00	0.00	RX2	C
ATOM	7280	CG1	VAL	25	44.614	29.274	32.258	1.00	0.00	RX2	C
ATOM	7281	CG2	VAL	25	44.279	31.281	33.733	1.00	0.00	RX2	C
ATOM	7282	C	VAL	25	43.719	30.927	29.913	1.00	0.00	RX2	C
ATOM	7283	O	VAL	25	42.526	30.733	29.721	1.00	0.00	RX2	O
ATOM	7284	N	CYS	26	44.675	30.615	29.038	1.00	0.00	RX2	N
ATOM	7285	H	CYS	26	45.646	30.761	29.239	1.00	0.00	RX2	H
ATOM	7286	CA	CYS	26	44.251	29.957	27.807	1.00	0.00	RX2	C
ATOM	7287	CB	CYS	26	44.224	30.942	26.634	1.00	0.00	RX2	C
ATOM	7288	SG	CYS	26	45.861	31.582	26.209	1.00	0.00	RX2	S
ATOM	7289	C	CYS	26	45.150	28.779	27.525	1.00	0.00	RX2	C
ATOM	7290	O	CYS	26	46.168	28.599	28.181	1.00	0.00	RX2	O
ATOM	7291	N	THR	27	44.754	27.971	26.539	1.00	0.00	RX2	N
ATOM	7292	H	THR	27	43.936	28.160	25.993	1.00	0.00	RX2	H
ATOM	7293	CA	THR	27	45.695	26.935	26.128	1.00	0.00	RX2	C
ATOM	7294	CB	THR	27	44.998	25.816	25.361	1.00	0.00	RX2	C
ATOM	7295	OG1	THR	27	44.288	26.350	24.237	1.00	0.00	RX2	O
ATOM	7296	HG1	THR	27	44.840	27.048	23.883	1.00	0.00	RX2	H
ATOM	7297	CG2	THR	27	44.059	25.011	26.258	1.00	0.00	RX2	C
ATOM	7298	C	THR	27	46.789	27.525	25.263	1.00	0.00	RX2	C
ATOM	7299	O	THR	27	46.543	28.442	24.491	1.00	0.00	RX2	O
ATOM	7300	N	GLY	28	47.992	26.973	25.424	1.00	0.00	RX2	N
ATOM	7301	H	GLY	28	48.082	26.162	25.999	1.00	0.00	RX2	H
ATOM	7302	CA	GLY	28	49.018	27.362	24.464	1.00	0.00	RX2	C
ATOM	7303	C	GLY	28	48.938	26.461	23.259	1.00	0.00	RX2	C
ATOM	7304	O	GLY	28	48.052	25.615	23.169	1.00	0.00	RX2	O
ATOM	7305	N	THR	29	49.883	26.652	22.341	1.00	0.00	RX2	N
ATOM	7306	H	THR	29	50.627	27.319	22.458	1.00	0.00	RX2	H
ATOM	7307	CA	THR	29	49.864	25.658	21.280	1.00	0.00	RX2	C
ATOM	7308	CB	THR	29	50.127	26.308	19.913	1.00	0.00	RX2	C
ATOM	7309	OG1	THR	29	51.182	27.273	19.975	1.00	0.00	RX2	O
ATOM	7310	HG1	THR	29	51.962	26.808	20.282	1.00	0.00	RX2	H
ATOM	7311	CG2	THR	29	48.859	26.962	19.367	1.00	0.00	RX2	C
ATOM	7312	C	THR	29	50.782	24.480	21.557	1.00	0.00	RX2	C
ATOM	7313	O	THR	29	51.467	24.416	22.573	1.00	0.00	RX2	O
ATOM	7314	N	ASP	30	50.751	23.570	20.573	1.00	0.00	RX2	N
ATOM	7315	H	ASP	30	50.000	23.579	19.918	1.00	0.00	RX2	H
ATOM	7316	CA	ASP	30	51.886	22.688	20.316	1.00	0.00	RX2	C
ATOM	7317	CB	ASP	30	51.582	21.261	20.761	1.00	0.00	RX2	C
ATOM	7318	CG	ASP	30	52.828	20.408	20.641	1.00	0.00	RX2	C

ATOM	7319	OD1	ASP	30	52.847	19.517	19.791	1.00	0.00	RX2	O
ATOM	7320	OD2	ASP	30	53.767	20.621	21.409	1.00	0.00	RX2	O
ATOM	7321	C	ASP	30	52.172	22.793	18.825	1.00	0.00	RX2	C
ATOM	7322	O	ASP	30	52.020	21.889	18.009	1.00	0.00	RX2	O
ATOM	7323	N	MET	31	52.478	24.044	18.468	1.00	0.00	RX2	N
ATOM	7324	H	MET	31	52.801	24.660	19.189	1.00	0.00	RX2	H
ATOM	7325	CA	MET	31	52.653	24.341	17.052	1.00	0.00	RX2	C
ATOM	7326	CB	MET	31	52.345	25.808	16.742	1.00	0.00	RX2	C
ATOM	7327	CG	MET	31	52.112	26.032	15.245	1.00	0.00	RX2	C
ATOM	7328	SD	MET	31	51.591	27.702	14.821	1.00	0.00	RX2	S
ATOM	7329	CE	MET	31	53.139	28.540	15.187	1.00	0.00	RX2	C
ATOM	7330	C	MET	31	54.015	23.923	16.536	1.00	0.00	RX2	C
ATOM	7331	O	MET	31	54.164	23.482	15.403	1.00	0.00	RX2	O
ATOM	7332	N	LYS	32	54.994	24.031	17.444	1.00	0.00	RX2	N
ATOM	7333	H	LYS	32	54.793	24.397	18.352	1.00	0.00	RX2	H
ATOM	7334	CA	LYS	32	56.353	23.580	17.162	1.00	0.00	RX2	C
ATOM	7335	CB	LYS	32	56.348	22.056	17.024	1.00	0.00	RX2	C
ATOM	7336	CG	LYS	32	55.887	21.372	18.311	1.00	0.00	RX2	C
ATOM	7337	CD	LYS	32	55.957	19.848	18.236	1.00	0.00	RX2	C
ATOM	7338	CE	LYS	32	56.165	19.217	19.613	1.00	0.00	RX2	C
ATOM	7339	NZ	LYS	32	57.331	19.843	20.244	1.00	0.00	RX2	N
ATOM	7340	HZ1	LYS	32	57.042	20.597	20.899	1.00	0.00	RX2	H
ATOM	7341	HZ2	LYS	32	57.957	19.159	20.712	1.00	0.00	RX2	H
ATOM	7342	HZ3	LYS	32	57.962	20.284	19.540	1.00	0.00	RX2	H
ATOM	7343	C	LYS	32	56.962	24.309	15.972	1.00	0.00	RX2	C
ATOM	7344	O	LYS	32	56.553	25.417	15.638	1.00	0.00	RX2	O
ATOM	7345	N	LEU	33	57.929	23.652	15.313	1.00	0.00	RX2	N
ATOM	7346	H	LEU	33	58.258	22.752	15.598	1.00	0.00	RX2	H
ATOM	7347	CA	LEU	33	58.445	24.259	14.084	1.00	0.00	RX2	C
ATOM	7348	CB	LEU	33	59.786	23.638	13.695	1.00	0.00	RX2	C
ATOM	7349	CG	LEU	33	60.982	24.146	14.492	1.00	0.00	RX2	C
ATOM	7350	CD1	LEU	33	62.230	23.299	14.237	1.00	0.00	RX2	C
ATOM	7351	CD2	LEU	33	61.241	25.626	14.223	1.00	0.00	RX2	C
ATOM	7352	C	LEU	33	57.540	24.175	12.863	1.00	0.00	RX2	C
ATOM	7353	O	LEU	33	58.008	24.363	11.744	1.00	0.00	RX2	O
ATOM	7354	N	ARG	34	56.259	23.832	13.084	1.00	0.00	RX2	N
ATOM	7355	H	ARG	34	55.800	23.931	13.967	1.00	0.00	RX2	H
ATOM	7356	CA	ARG	34	55.501	23.460	11.896	1.00	0.00	RX2	C
ATOM	7357	CB	ARG	34	54.242	22.657	12.239	1.00	0.00	RX2	C
ATOM	7358	CG	ARG	34	52.999	23.514	12.455	1.00	0.00	RX2	C
ATOM	7359	CD	ARG	34	51.787	22.689	12.871	1.00	0.00	RX2	C
ATOM	7360	NE	ARG	34	51.976	22.075	14.183	1.00	0.00	RX2	N
ATOM	7361	HE	ARG	34	52.731	22.443	14.739	1.00	0.00	RX2	H
ATOM	7362	CZ	ARG	34	51.084	21.127	14.586	1.00	0.00	RX2	C
ATOM	7363	NH1	ARG	34	50.193	20.648	13.691	1.00	0.00	RX2	N
ATOM	7364	HH11	ARG	34	49.487	19.987	13.948	1.00	0.00	RX2	H
ATOM	7365	HH12	ARG	34	50.215	20.928	12.718	1.00	0.00	RX2	H
ATOM	7366	NH2	ARG	34	51.094	20.687	15.862	1.00	0.00	RX2	N
ATOM	7367	HH21	ARG	34	50.464	19.985	16.223	1.00	0.00	RX2	H
ATOM	7368	HH22	ARG	34	51.733	21.055	16.555	1.00	0.00	RX2	H
ATOM	7369	C	ARG	34	55.204	24.590	10.936	1.00	0.00	RX2	C
ATOM	7370	O	ARG	34	54.893	25.713	11.316	1.00	0.00	RX2	O
ATOM	7371	N	LEU	35	55.279	24.218	9.655	1.00	0.00	RX2	N
ATOM	7372	H	LEU	35	55.569	23.291	9.419	1.00	0.00	RX2	H
ATOM	7373	CA	LEU	35	54.724	25.125	8.655	1.00	0.00	RX2	C
ATOM	7374	CB	LEU	35	55.017	24.571	7.262	1.00	0.00	RX2	C
ATOM	7375	CG	LEU	35	56.513	24.561	6.958	1.00	0.00	RX2	C
ATOM	7376	CD1	LEU	35	56.843	23.752	5.705	1.00	0.00	RX2	C
ATOM	7377	CD2	LEU	35	57.064	25.983	6.879	1.00	0.00	RX2	C
ATOM	7378	C	LEU	35	53.230	25.337	8.867	1.00	0.00	RX2	C
ATOM	7379	O	LEU	35	52.462	24.391	9.012	1.00	0.00	RX2	O

ATOM	7380	N	PRO	36	52.844	26.632	8.925	1.00	0.00	RX2	N
ATOM	7381	CD	PRO	36	53.708	27.802	8.868	1.00	0.00	RX2	C
ATOM	7382	CA	PRO	36	51.428	26.965	9.103	1.00	0.00	RX2	C
ATOM	7383	CB	PRO	36	51.449	28.497	9.162	1.00	0.00	RX2	C
ATOM	7384	CG	PRO	36	52.877	28.880	9.550	1.00	0.00	RX2	C
ATOM	7385	C	PRO	36	50.560	26.453	7.966	1.00	0.00	RX2	C
ATOM	7386	O	PRO	36	51.041	25.917	6.974	1.00	0.00	RX2	O
ATOM	7387	N	ALA	37	49.248	26.674	8.135	1.00	0.00	RX2	N
ATOM	7388	H	ALA	37	48.900	27.138	8.946	1.00	0.00	RX2	H
ATOM	7389	CA	ALA	37	48.411	26.438	6.962	1.00	0.00	RX2	C
ATOM	7390	CB	ALA	37	46.951	26.240	7.374	1.00	0.00	RX2	C
ATOM	7391	C	ALA	37	48.497	27.598	5.983	1.00	0.00	RX2	C
ATOM	7392	O	ALA	37	48.768	27.434	4.801	1.00	0.00	RX2	O
ATOM	7393	N	SER	38	48.293	28.793	6.558	1.00	0.00	RX2	N
ATOM	7394	H	SER	38	48.012	28.942	7.505	1.00	0.00	RX2	H
ATOM	7395	CA	SER	38	48.395	30.016	5.767	1.00	0.00	RX2	C
ATOM	7396	CB	SER	38	47.227	29.994	4.775	1.00	0.00	RX2	C
ATOM	7397	OG	SER	38	46.180	29.113	5.222	1.00	0.00	RX2	O
ATOM	7398	HG	SER	38	45.973	29.409	6.108	1.00	0.00	RX2	H
ATOM	7399	C	SER	38	48.473	31.211	6.715	1.00	0.00	RX2	C
ATOM	7400	O	SER	38	48.121	31.074	7.882	1.00	0.00	RX2	O
ATOM	7401	N	PRO	39	48.973	32.379	6.220	1.00	0.00	RX2	N
ATOM	7402	CD	PRO	39	49.435	32.643	4.859	1.00	0.00	RX2	C
ATOM	7403	CA	PRO	39	49.131	33.553	7.094	1.00	0.00	RX2	C
ATOM	7404	CB	PRO	39	49.361	34.684	6.089	1.00	0.00	RX2	C
ATOM	7405	CG	PRO	39	50.100	34.012	4.935	1.00	0.00	RX2	C
ATOM	7406	C	PRO	39	48.022	33.847	8.098	1.00	0.00	RX2	C
ATOM	7407	O	PRO	39	48.270	33.991	9.285	1.00	0.00	RX2	O
ATOM	7408	N	GLU	40	46.792	33.927	7.575	1.00	0.00	RX2	N
ATOM	7409	H	GLU	40	46.664	33.664	6.619	1.00	0.00	RX2	H
ATOM	7410	CA	GLU	40	45.645	34.173	8.460	1.00	0.00	RX2	C
ATOM	7411	CB	GLU	40	44.330	34.484	7.730	1.00	0.00	RX2	C
ATOM	7412	CG	GLU	40	44.294	34.273	6.218	1.00	0.00	RX2	C
ATOM	7413	CD	GLU	40	44.690	32.857	5.853	1.00	0.00	RX2	C
ATOM	7414	OE1	GLU	40	44.010	31.912	6.232	1.00	0.00	RX2	O
ATOM	7415	OE2	GLU	40	45.703	32.688	5.186	1.00	0.00	RX2	O
ATOM	7416	C	GLU	40	45.376	33.077	9.475	1.00	0.00	RX2	C
ATOM	7417	O	GLU	40	45.320	33.314	10.675	1.00	0.00	RX2	O
ATOM	7418	N	THR	41	45.234	31.845	8.952	1.00	0.00	RX2	N
ATOM	7419	H	THR	41	45.352	31.733	7.964	1.00	0.00	RX2	H
ATOM	7420	CA	THR	41	44.973	30.721	9.860	1.00	0.00	RX2	C
ATOM	7421	CB	THR	41	44.950	29.479	8.990	1.00	0.00	RX2	C
ATOM	7422	OG1	THR	41	45.127	29.870	7.622	1.00	0.00	RX2	O
ATOM	7423	HG1	THR	41	44.394	30.448	7.392	1.00	0.00	RX2	H
ATOM	7424	CG2	THR	41	43.655	28.684	9.167	1.00	0.00	RX2	C
ATOM	7425	C	THR	41	45.934	30.614	11.037	1.00	0.00	RX2	C
ATOM	7426	O	THR	41	45.564	30.307	12.161	1.00	0.00	RX2	O
ATOM	7427	N	HIS	42	47.188	30.940	10.706	1.00	0.00	RX2	N
ATOM	7428	H	HIS	42	47.380	31.216	9.762	1.00	0.00	RX2	H
ATOM	7429	CA	HIS	42	48.214	31.144	11.722	1.00	0.00	RX2	C
ATOM	7430	CB	HIS	42	49.527	31.275	10.946	1.00	0.00	RX2	C
ATOM	7431	CG	HIS	42	50.690	31.786	11.757	1.00	0.00	RX2	C
ATOM	7432	ND1	HIS	42	51.307	31.104	12.738	1.00	0.00	RX2	N
ATOM	7433	HD1	HIS	42	51.087	30.212	13.083	1.00	0.00	RX2	H
ATOM	7434	CD2	HIS	42	51.337	33.012	11.579	1.00	0.00	RX2	C
ATOM	7435	NE2	HIS	42	52.358	33.062	12.462	1.00	0.00	RX2	N
ATOM	7436	CE1	HIS	42	52.343	31.890	13.174	1.00	0.00	RX2	C
ATOM	7437	C	HIS	42	47.913	32.324	12.654	1.00	0.00	RX2	C
ATOM	7438	O	HIS	42	47.596	32.164	13.827	1.00	0.00	RX2	O
ATOM	7439	N	LEU	43	48.040	33.527	12.078	1.00	0.00	RX2	N
ATOM	7440	H	LEU	43	48.119	33.608	11.087	1.00	0.00	RX2	H

ATOM	7441	CA	LEU	43	48.044	34.738	12.896	1.00	0.00	RX2	C
ATOM	7442	CB	LEU	43	48.363	35.955	12.028	1.00	0.00	RX2	C
ATOM	7443	CG	LEU	43	48.618	37.215	12.855	1.00	0.00	RX2	C
ATOM	7444	CD1	LEU	43	49.828	37.044	13.774	1.00	0.00	RX2	C
ATOM	7445	CD2	LEU	43	48.730	38.464	11.980	1.00	0.00	RX2	C
ATOM	7446	C	LEU	43	46.775	34.985	13.690	1.00	0.00	RX2	C
ATOM	7447	O	LEU	43	46.796	35.207	14.895	1.00	0.00	RX2	O
ATOM	7448	N	ASP	44	45.657	34.932	12.960	1.00	0.00	RX2	N
ATOM	7449	H	ASP	44	45.666	34.749	11.975	1.00	0.00	RX2	H
ATOM	7450	CA	ASP	44	44.381	35.237	13.603	1.00	0.00	RX2	C
ATOM	7451	CB	ASP	44	43.259	35.268	12.558	1.00	0.00	RX2	C
ATOM	7452	CG	ASP	44	43.317	36.558	11.759	1.00	0.00	RX2	C
ATOM	7453	OD1	ASP	44	44.376	36.915	11.255	1.00	0.00	RX2	O
ATOM	7454	OD2	ASP	44	42.323	37.277	11.712	1.00	0.00	RX2	O
ATOM	7455	C	ASP	44	44.030	34.292	14.733	1.00	0.00	RX2	C
ATOM	7456	O	ASP	44	43.609	34.695	15.810	1.00	0.00	RX2	O
ATOM	7457	N	MET	45	44.288	32.998	14.470	1.00	0.00	RX2	N
ATOM	7458	H	MET	45	44.693	32.726	13.594	1.00	0.00	RX2	H
ATOM	7459	CA	MET	45	44.069	32.052	15.566	1.00	0.00	RX2	C
ATOM	7460	CB	MET	45	44.230	30.611	15.075	1.00	0.00	RX2	C
ATOM	7461	CG	MET	45	43.476	29.563	15.903	1.00	0.00	RX2	C
ATOM	7462	SD	MET	45	44.140	29.284	17.553	1.00	0.00	RX2	S
ATOM	7463	CE	MET	45	45.705	28.542	17.070	1.00	0.00	RX2	C
ATOM	7464	C	MET	45	44.935	32.341	16.784	1.00	0.00	RX2	C
ATOM	7465	O	MET	45	44.476	32.389	17.921	1.00	0.00	RX2	O
ATOM	7466	N	LEU	46	46.222	32.602	16.484	1.00	0.00	RX2	N
ATOM	7467	H	LEU	46	46.544	32.579	15.537	1.00	0.00	RX2	H
ATOM	7468	CA	LEU	46	47.111	32.995	17.578	1.00	0.00	RX2	C
ATOM	7469	CB	LEU	46	48.558	33.174	17.117	1.00	0.00	RX2	C
ATOM	7470	CG	LEU	46	49.211	31.898	16.584	1.00	0.00	RX2	C
ATOM	7471	CD1	LEU	46	50.709	32.105	16.366	1.00	0.00	RX2	C
ATOM	7472	CD2	LEU	46	48.938	30.680	17.466	1.00	0.00	RX2	C
ATOM	7473	C	LEU	46	46.670	34.228	18.349	1.00	0.00	RX2	C
ATOM	7474	O	LEU	46	46.869	34.321	19.553	1.00	0.00	RX2	O
ATOM	7475	N	ARG	47	46.039	35.161	17.615	1.00	0.00	RX2	N
ATOM	7476	H	ARG	47	45.902	35.015	16.634	1.00	0.00	RX2	H
ATOM	7477	CA	ARG	47	45.504	36.358	18.262	1.00	0.00	RX2	C
ATOM	7478	CB	ARG	47	44.859	37.262	17.209	1.00	0.00	RX2	C
ATOM	7479	CG	ARG	47	44.236	38.551	17.748	1.00	0.00	RX2	C
ATOM	7480	CD	ARG	47	43.339	39.215	16.700	1.00	0.00	RX2	C
ATOM	7481	NE	ARG	47	44.066	39.411	15.448	1.00	0.00	RX2	N
ATOM	7482	HE	ARG	47	44.930	39.916	15.511	1.00	0.00	RX2	H
ATOM	7483	CZ	ARG	47	43.571	38.847	14.302	1.00	0.00	RX2	C
ATOM	7484	NH1	ARG	47	42.396	38.190	14.313	1.00	0.00	RX2	N
ATOM	7485	HH11	ARG	47	42.026	37.785	13.464	1.00	0.00	RX2	H
ATOM	7486	HH12	ARG	47	41.806	38.015	15.123	1.00	0.00	RX2	H
ATOM	7487	NH2	ARG	47	44.280	38.935	13.158	1.00	0.00	RX2	N
ATOM	7488	HH21	ARG	47	43.959	38.436	12.329	1.00	0.00	RX2	H
ATOM	7489	HH22	ARG	47	45.141	39.432	13.065	1.00	0.00	RX2	H
ATOM	7490	C	ARG	47	44.523	36.040	19.372	1.00	0.00	RX2	C
ATOM	7491	O	ARG	47	44.771	36.296	20.546	1.00	0.00	RX2	O
ATOM	7492	N	HIS	48	43.396	35.446	18.948	1.00	0.00	RX2	N
ATOM	7493	H	HIS	48	43.259	35.220	17.978	1.00	0.00	RX2	H
ATOM	7494	CA	HIS	48	42.369	35.212	19.959	1.00	0.00	RX2	C
ATOM	7495	CB	HIS	48	41.040	34.807	19.327	1.00	0.00	RX2	C
ATOM	7496	CG	HIS	48	40.035	35.908	19.575	1.00	0.00	RX2	C
ATOM	7497	ND1	HIS	48	39.645	36.789	18.640	1.00	0.00	RX2	N
ATOM	7498	HD1	HIS	48	39.943	36.819	17.700	1.00	0.00	RX2	H
ATOM	7499	CD2	HIS	48	39.372	36.210	20.767	1.00	0.00	RX2	C
ATOM	7500	NE2	HIS	48	38.581	37.285	20.532	1.00	0.00	RX2	N
ATOM	7501	CE1	HIS	48	38.748	37.642	19.218	1.00	0.00	RX2	C

ATOM	7502	C	HIS	48	42.768	34.261	21.072	1.00	0.00	RX2	C
ATOM	7503	O	HIS	48	42.359	34.406	22.217	1.00	0.00	RX2	O
ATOM	7504	N	LEU	49	43.619	33.296	20.684	1.00	0.00	RX2	N
ATOM	7505	H	LEU	49	43.892	33.215	19.722	1.00	0.00	RX2	H
ATOM	7506	CA	LEU	49	44.130	32.373	21.694	1.00	0.00	RX2	C
ATOM	7507	CB	LEU	49	44.858	31.227	20.986	1.00	0.00	RX2	C
ATOM	7508	CG	LEU	49	45.304	30.093	21.907	1.00	0.00	RX2	C
ATOM	7509	CD1	LEU	49	44.114	29.297	22.438	1.00	0.00	RX2	C
ATOM	7510	CD2	LEU	49	46.357	29.211	21.240	1.00	0.00	RX2	C
ATOM	7511	C	LEU	49	45.041	33.022	22.730	1.00	0.00	RX2	C
ATOM	7512	O	LEU	49	44.791	33.009	23.927	1.00	0.00	RX2	O
ATOM	7513	N	TYR	50	46.140	33.578	22.206	1.00	0.00	RX2	N
ATOM	7514	H	TYR	50	46.229	33.684	21.215	1.00	0.00	RX2	H
ATOM	7515	CA	TYR	50	47.194	34.033	23.110	1.00	0.00	RX2	C
ATOM	7516	CB	TYR	50	48.507	34.168	22.348	1.00	0.00	RX2	C
ATOM	7517	CG	TYR	50	49.133	32.816	22.123	1.00	0.00	RX2	C
ATOM	7518	CD1	TYR	50	49.234	31.918	23.178	1.00	0.00	RX2	C
ATOM	7519	CE1	TYR	50	49.899	30.713	22.998	1.00	0.00	RX2	C
ATOM	7520	CD2	TYR	50	49.635	32.485	20.872	1.00	0.00	RX2	C
ATOM	7521	CE2	TYR	50	50.298	31.278	20.692	1.00	0.00	RX2	C
ATOM	7522	CZ	TYR	50	50.457	30.409	21.763	1.00	0.00	RX2	C
ATOM	7523	OH	TYR	50	51.188	29.253	21.605	1.00	0.00	RX2	O
ATOM	7524	HH	TYR	50	51.044	28.904	20.727	1.00	0.00	RX2	H
ATOM	7525	C	TYR	50	46.932	35.331	23.848	1.00	0.00	RX2	C
ATOM	7526	O	TYR	50	47.574	35.628	24.848	1.00	0.00	RX2	O
ATOM	7527	N	GLN	51	45.974	36.094	23.296	1.00	0.00	RX2	N
ATOM	7528	H	GLN	51	45.550	35.783	22.443	1.00	0.00	RX2	H
ATOM	7529	CA	GLN	51	45.590	37.414	23.804	1.00	0.00	RX2	C
ATOM	7530	CB	GLN	51	44.108	37.613	23.486	1.00	0.00	RX2	C
ATOM	7531	CG	GLN	51	43.671	39.058	23.252	1.00	0.00	RX2	C
ATOM	7532	CD	GLN	51	43.499	39.295	21.764	1.00	0.00	RX2	C
ATOM	7533	OE1	GLN	51	44.158	40.132	21.157	1.00	0.00	RX2	O
ATOM	7534	NE2	GLN	51	42.543	38.527	21.210	1.00	0.00	RX2	N
ATOM	7535	HE21	GLN	51	42.040	37.855	21.755	1.00	0.00	RX2	H
ATOM	7536	HE22	GLN	51	42.303	38.596	20.241	1.00	0.00	RX2	H
ATOM	7537	C	GLN	51	45.806	37.715	25.287	1.00	0.00	RX2	C
ATOM	7538	O	GLN	51	44.961	37.447	26.135	1.00	0.00	RX2	O
ATOM	7539	N	GLY	52	46.989	38.299	25.552	1.00	0.00	RX2	N
ATOM	7540	H	GLY	52	47.645	38.425	24.804	1.00	0.00	RX2	H
ATOM	7541	CA	GLY	52	47.327	38.733	26.910	1.00	0.00	RX2	C
ATOM	7542	C	GLY	52	47.290	37.660	27.992	1.00	0.00	RX2	C
ATOM	7543	O	GLY	52	46.911	37.895	29.133	1.00	0.00	RX2	O
ATOM	7544	N	CYS	53	47.692	36.451	27.582	1.00	0.00	RX2	N
ATOM	7545	H	CYS	53	48.014	36.296	26.649	1.00	0.00	RX2	H
ATOM	7546	CA	CYS	53	47.514	35.342	28.514	1.00	0.00	RX2	C
ATOM	7547	CB	CYS	53	47.591	34.015	27.774	1.00	0.00	RX2	C
ATOM	7548	SG	CYS	53	46.964	32.679	28.817	1.00	0.00	RX2	S
ATOM	7549	C	CYS	53	48.403	35.345	29.742	1.00	0.00	RX2	C
ATOM	7550	O	CYS	53	49.614	35.177	29.688	1.00	0.00	RX2	O
ATOM	7551	N	GLN	54	47.737	35.518	30.886	1.00	0.00	RX2	N
ATOM	7552	H	GLN	54	46.737	35.548	30.867	1.00	0.00	RX2	H
ATOM	7553	CA	GLN	54	48.496	35.519	32.133	1.00	0.00	RX2	C
ATOM	7554	CB	GLN	54	47.633	36.010	33.285	1.00	0.00	RX2	C
ATOM	7555	CG	GLN	54	46.989	37.348	32.951	1.00	0.00	RX2	C
ATOM	7556	CD	GLN	54	46.310	37.867	34.190	1.00	0.00	RX2	C
ATOM	7557	OE1	GLN	54	45.120	37.680	34.399	1.00	0.00	RX2	O
ATOM	7558	NE2	GLN	54	47.125	38.517	35.023	1.00	0.00	RX2	N
ATOM	7559	HE21	GLN	54	48.106	38.607	34.833	1.00	0.00	RX2	H
ATOM	7560	HE22	GLN	54	46.742	38.914	35.855	1.00	0.00	RX2	H
ATOM	7561	C	GLN	54	49.117	34.180	32.483	1.00	0.00	RX2	C
ATOM	7562	O	GLN	54	50.234	34.100	32.991	1.00	0.00	RX2	O

ATOM	7563	N	VAL	55	48.323	33.139	32.185	1.00	0.00	RX2	N
ATOM	7564	H	VAL	55	47.415	33.272	31.778	1.00	0.00	RX2	H
ATOM	7565	CA	VAL	55	48.795	31.768	32.356	1.00	0.00	RX2	C
ATOM	7566	CB	VAL	55	48.100	31.104	33.552	1.00	0.00	RX2	C
ATOM	7567	CG1	VAL	55	48.535	29.646	33.704	1.00	0.00	RX2	C
ATOM	7568	CG2	VAL	55	48.304	31.890	34.849	1.00	0.00	RX2	C
ATOM	7569	C	VAL	55	48.523	30.972	31.091	1.00	0.00	RX2	C
ATOM	7570	O	VAL	55	47.415	30.497	30.862	1.00	0.00	RX2	O
ATOM	7571	N	VAL	56	49.571	30.852	30.264	1.00	0.00	RX2	N
ATOM	7572	H	VAL	56	50.496	31.179	30.462	1.00	0.00	RX2	H
ATOM	7573	CA	VAL	56	49.313	30.024	29.095	1.00	0.00	RX2	C
ATOM	7574	CB	VAL	56	49.856	30.651	27.796	1.00	0.00	RX2	C
ATOM	7575	CG1	VAL	56	51.319	30.353	27.476	1.00	0.00	RX2	C
ATOM	7576	CG2	VAL	56	48.943	30.273	26.638	1.00	0.00	RX2	C
ATOM	7577	C	VAL	56	49.667	28.558	29.295	1.00	0.00	RX2	C
ATOM	7578	O	VAL	56	50.809	28.132	29.449	1.00	0.00	RX2	O
ATOM	7579	N	GLN	57	48.576	27.795	29.343	1.00	0.00	RX2	N
ATOM	7580	H	GLN	57	47.690	28.230	29.169	1.00	0.00	RX2	H
ATOM	7581	CA	GLN	57	48.708	26.363	29.570	1.00	0.00	RX2	C
ATOM	7582	CB	GLN	57	47.475	25.786	30.262	1.00	0.00	RX2	C
ATOM	7583	CG	GLN	57	47.195	26.384	31.638	1.00	0.00	RX2	C
ATOM	7584	CD	GLN	57	46.259	25.458	32.382	1.00	0.00	RX2	C
ATOM	7585	OE1	GLN	57	46.678	24.510	33.038	1.00	0.00	RX2	O
ATOM	7586	NE2	GLN	57	44.968	25.758	32.256	1.00	0.00	RX2	N
ATOM	7587	HE21	GLN	57	44.666	26.538	31.711	1.00	0.00	RX2	H
ATOM	7588	HE22	GLN	57	44.296	25.182	32.726	1.00	0.00	RX2	H
ATOM	7589	C	GLN	57	48.996	25.601	28.299	1.00	0.00	RX2	C
ATOM	7590	O	GLN	57	48.116	25.095	27.612	1.00	0.00	RX2	O
ATOM	7591	N	GLY	58	50.294	25.588	28.003	1.00	0.00	RX2	N
ATOM	7592	H	GLY	58	50.960	26.147	28.502	1.00	0.00	RX2	H
ATOM	7593	CA	GLY	58	50.775	24.952	26.785	1.00	0.00	RX2	C
ATOM	7594	C	GLY	58	52.043	25.664	26.390	1.00	0.00	RX2	C
ATOM	7595	O	GLY	58	52.601	26.410	27.190	1.00	0.00	RX2	O
ATOM	7596	N	ASN	59	52.487	25.413	25.155	1.00	0.00	RX2	N
ATOM	7597	H	ASN	59	51.960	24.939	24.445	1.00	0.00	RX2	H
ATOM	7598	CA	ASN	59	53.677	26.165	24.766	1.00	0.00	RX2	C
ATOM	7599	CB	ASN	59	54.520	25.487	23.693	1.00	0.00	RX2	C
ATOM	7600	CG	ASN	59	54.694	24.006	23.901	1.00	0.00	RX2	C
ATOM	7601	OD1	ASN	59	55.279	23.541	24.881	1.00	0.00	RX2	O
ATOM	7602	ND2	ASN	59	54.165	23.292	22.895	1.00	0.00	RX2	N
ATOM	7603	HD21	ASN	59	53.787	23.831	22.129	1.00	0.00	RX2	H
ATOM	7604	HD22	ASN	59	54.092	22.305	22.724	1.00	0.00	RX2	H
ATOM	7605	C	ASN	59	53.282	27.511	24.206	1.00	0.00	RX2	C
ATOM	7606	O	ASN	59	52.112	27.770	23.951	1.00	0.00	RX2	O
ATOM	7607	N	LEU	60	54.309	28.343	24.021	1.00	0.00	RX2	N
ATOM	7608	H	LEU	60	55.248	28.065	24.231	1.00	0.00	RX2	H
ATOM	7609	CA	LEU	60	54.119	29.568	23.258	1.00	0.00	RX2	C
ATOM	7610	CB	LEU	60	54.542	30.766	24.107	1.00	0.00	RX2	C
ATOM	7611	CG	LEU	60	54.495	32.124	23.404	1.00	0.00	RX2	C
ATOM	7612	CD1	LEU	60	53.122	32.428	22.818	1.00	0.00	RX2	C
ATOM	7613	CD2	LEU	60	54.964	33.246	24.328	1.00	0.00	RX2	C
ATOM	7614	C	LEU	60	54.949	29.462	21.999	1.00	0.00	RX2	C
ATOM	7615	O	LEU	60	56.138	29.763	21.980	1.00	0.00	RX2	O
ATOM	7616	N	GLU	61	54.281	28.982	20.949	1.00	0.00	RX2	N
ATOM	7617	H	GLU	61	53.298	28.793	20.981	1.00	0.00	RX2	H
ATOM	7618	CA	GLU	61	55.066	28.844	19.729	1.00	0.00	RX2	C
ATOM	7619	CB	GLU	61	54.867	27.485	19.084	1.00	0.00	RX2	C
ATOM	7620	CG	GLU	61	55.574	26.440	19.935	1.00	0.00	RX2	C
ATOM	7621	CD	GLU	61	54.569	25.403	20.351	1.00	0.00	RX2	C
ATOM	7622	OE1	GLU	61	53.446	25.764	20.669	1.00	0.00	RX2	O
ATOM	7623	OE2	GLU	61	54.885	24.222	20.318	1.00	0.00	RX2	O

ATOM	7624	C	GLU	61	54.904	29.971	18.749	1.00	0.00	RX2	C
ATOM	7625	O	GLU	61	54.009	30.046	17.916	1.00	0.00	RX2	O
ATOM	7626	N	LEU	62	55.863	30.871	18.914	1.00	0.00	RX2	N
ATOM	7627	H	LEU	62	56.584	30.692	19.583	1.00	0.00	RX2	H
ATOM	7628	CA	LEU	62	56.029	31.947	17.954	1.00	0.00	RX2	C
ATOM	7629	CB	LEU	62	56.618	33.158	18.665	1.00	0.00	RX2	C
ATOM	7630	CG	LEU	62	55.745	33.533	19.857	1.00	0.00	RX2	C
ATOM	7631	CD1	LEU	62	56.347	34.679	20.666	1.00	0.00	RX2	C
ATOM	7632	CD2	LEU	62	54.301	33.797	19.428	1.00	0.00	RX2	C
ATOM	7633	C	LEU	62	56.913	31.503	16.812	1.00	0.00	RX2	C
ATOM	7634	O	LEU	62	58.116	31.744	16.773	1.00	0.00	RX2	O
ATOM	7635	N	THR	63	56.241	30.817	15.891	1.00	0.00	RX2	N
ATOM	7636	H	THR	63	55.273	30.583	15.978	1.00	0.00	RX2	H
ATOM	7637	CA	THR	63	56.985	30.313	14.749	1.00	0.00	RX2	C
ATOM	7638	CB	THR	63	56.906	28.800	14.840	1.00	0.00	RX2	C
ATOM	7639	OG1	THR	63	56.701	28.415	16.208	1.00	0.00	RX2	O
ATOM	7640	HG1	THR	63	56.568	27.469	16.181	1.00	0.00	RX2	H
ATOM	7641	CG2	THR	63	58.153	28.135	14.258	1.00	0.00	RX2	C
ATOM	7642	C	THR	63	56.426	30.874	13.454	1.00	0.00	RX2	C
ATOM	7643	O	THR	63	55.217	30.925	13.275	1.00	0.00	RX2	O
ATOM	7644	N	TYR	64	57.344	31.291	12.564	1.00	0.00	RX2	N
ATOM	7645	H	TYR	64	58.294	31.340	12.882	1.00	0.00	RX2	H
ATOM	7646	CA	TYR	64	56.972	31.782	11.226	1.00	0.00	RX2	C
ATOM	7647	CB	TYR	64	56.256	30.726	10.378	1.00	0.00	RX2	C
ATOM	7648	CG	TYR	64	57.081	29.474	10.240	1.00	0.00	RX2	C
ATOM	7649	CD1	TYR	64	58.152	29.438	9.358	1.00	0.00	RX2	C
ATOM	7650	CE1	TYR	64	58.893	28.272	9.224	1.00	0.00	RX2	C
ATOM	7651	CD2	TYR	64	56.756	28.352	10.987	1.00	0.00	RX2	C
ATOM	7652	CE2	TYR	64	57.505	27.191	10.856	1.00	0.00	RX2	C
ATOM	7653	CZ	TYR	64	58.574	27.148	9.974	1.00	0.00	RX2	C
ATOM	7654	OH	TYR	64	59.317	25.994	9.841	1.00	0.00	RX2	O
ATOM	7655	HH	TYR	64	58.923	25.317	10.393	1.00	0.00	RX2	H
ATOM	7656	C	TYR	64	56.154	33.065	11.133	1.00	0.00	RX2	C
ATOM	7657	O	TYR	64	55.635	33.403	10.075	1.00	0.00	RX2	O
ATOM	7658	N	LEU	65	56.072	33.788	12.260	1.00	0.00	RX2	N
ATOM	7659	H	LEU	65	56.500	33.455	13.098	1.00	0.00	RX2	H
ATOM	7660	CA	LEU	65	55.357	35.067	12.246	1.00	0.00	RX2	C
ATOM	7661	CB	LEU	65	55.491	35.777	13.593	1.00	0.00	RX2	C
ATOM	7662	CG	LEU	65	54.930	35.037	14.806	1.00	0.00	RX2	C
ATOM	7663	CD1	LEU	65	55.514	35.604	16.097	1.00	0.00	RX2	C
ATOM	7664	CD2	LEU	65	53.403	35.050	14.843	1.00	0.00	RX2	C
ATOM	7665	C	LEU	65	55.834	36.010	11.148	1.00	0.00	RX2	C
ATOM	7666	O	LEU	65	57.021	36.321	11.030	1.00	0.00	RX2	O
ATOM	7667	N	PRO	66	54.849	36.441	10.325	1.00	0.00	RX2	N
ATOM	7668	CD	PRO	66	53.429	36.135	10.443	1.00	0.00	RX2	C
ATOM	7669	CA	PRO	66	55.173	37.285	9.174	1.00	0.00	RX2	C
ATOM	7670	CB	PRO	66	53.840	37.325	8.417	1.00	0.00	RX2	C
ATOM	7671	CG	PRO	66	52.763	37.112	9.482	1.00	0.00	RX2	C
ATOM	7672	C	PRO	66	55.691	38.653	9.577	1.00	0.00	RX2	C
ATOM	7673	O	PRO	66	55.289	39.244	10.572	1.00	0.00	RX2	O
ATOM	7674	N	THR	67	56.613	39.127	8.732	1.00	0.00	RX2	N
ATOM	7675	H	THR	67	56.890	38.601	7.928	1.00	0.00	RX2	H
ATOM	7676	CA	THR	67	57.396	40.329	9.010	1.00	0.00	RX2	C
ATOM	7677	CB	THR	67	58.002	40.691	7.675	1.00	0.00	RX2	C
ATOM	7678	OG1	THR	67	58.168	39.481	6.919	1.00	0.00	RX2	O
ATOM	7679	HG1	THR	67	58.733	39.705	6.183	1.00	0.00	RX2	H
ATOM	7680	CG2	THR	67	59.310	41.453	7.838	1.00	0.00	RX2	C
ATOM	7681	C	THR	67	56.707	41.505	9.689	1.00	0.00	RX2	C
ATOM	7682	O	THR	67	57.144	42.028	10.710	1.00	0.00	RX2	O
ATOM	7683	N	ASN	68	55.593	41.893	9.057	1.00	0.00	RX2	N
ATOM	7684	H	ASN	68	55.185	41.349	8.327	1.00	0.00	RX2	H

ATOM	7685	CA	ASN	68	54.945	43.114	9.527	1.00	0.00	RX2	C
ATOM	7686	CB	ASN	68	54.704	44.105	8.382	1.00	0.00	RX2	C
ATOM	7687	CG	ASN	68	55.995	44.485	7.683	1.00	0.00	RX2	C
ATOM	7688	OD1	ASN	68	57.108	44.220	8.135	1.00	0.00	RX2	O
ATOM	7689	ND2	ASN	68	55.790	45.094	6.511	1.00	0.00	RX2	N
ATOM	7690	HD21	ASN	68	54.887	45.418	6.201	1.00	0.00	RX2	H
ATOM	7691	HD22	ASN	68	56.497	45.331	5.842	1.00	0.00	RX2	H
ATOM	7692	C	ASN	68	53.625	42.817	10.208	1.00	0.00	RX2	C
ATOM	7693	O	ASN	68	52.607	43.451	9.965	1.00	0.00	RX2	O
ATOM	7694	N	ALA	69	53.665	41.777	11.051	1.00	0.00	RX2	N
ATOM	7695	H	ALA	69	54.523	41.321	11.293	1.00	0.00	RX2	H
ATOM	7696	CA	ALA	69	52.412	41.417	11.708	1.00	0.00	RX2	C
ATOM	7697	CB	ALA	69	52.505	40.016	12.315	1.00	0.00	RX2	C
ATOM	7698	C	ALA	69	52.000	42.389	12.799	1.00	0.00	RX2	C
ATOM	7699	O	ALA	69	52.806	42.836	13.605	1.00	0.00	RX2	O
ATOM	7700	N	SER	70	50.687	42.668	12.813	1.00	0.00	RX2	N
ATOM	7701	H	SER	70	50.049	42.467	12.071	1.00	0.00	RX2	H
ATOM	7702	CA	SER	70	50.145	43.397	13.960	1.00	0.00	RX2	C
ATOM	7703	CB	SER	70	48.799	43.949	13.511	1.00	0.00	RX2	C
ATOM	7704	OG	SER	70	48.864	44.167	12.095	1.00	0.00	RX2	O
ATOM	7705	HG	SER	70	48.382	44.989	11.953	1.00	0.00	RX2	H
ATOM	7706	C	SER	70	50.066	42.480	15.176	1.00	0.00	RX2	C
ATOM	7707	O	SER	70	49.104	41.761	15.421	1.00	0.00	RX2	O
ATOM	7708	N	LEU	71	51.203	42.483	15.883	1.00	0.00	RX2	N
ATOM	7709	H	LEU	71	51.934	43.119	15.625	1.00	0.00	RX2	H
ATOM	7710	CA	LEU	71	51.479	41.384	16.805	1.00	0.00	RX2	C
ATOM	7711	CB	LEU	71	52.958	41.027	16.658	1.00	0.00	RX2	C
ATOM	7712	CG	LEU	71	53.288	39.575	16.982	1.00	0.00	RX2	C
ATOM	7713	CD1	LEU	71	52.471	38.609	16.127	1.00	0.00	RX2	C
ATOM	7714	CD2	LEU	71	54.787	39.304	16.875	1.00	0.00	RX2	C
ATOM	7715	C	LEU	71	51.095	41.612	18.262	1.00	0.00	RX2	C
ATOM	7716	O	LEU	71	51.272	40.764	19.131	1.00	0.00	RX2	O
ATOM	7717	N	SER	72	50.559	42.817	18.499	1.00	0.00	RX2	N
ATOM	7718	H	SER	72	50.380	43.433	17.735	1.00	0.00	RX2	H
ATOM	7719	CA	SER	72	50.372	43.339	19.856	1.00	0.00	RX2	C
ATOM	7720	CB	SER	72	49.572	44.616	19.658	1.00	0.00	RX2	C
ATOM	7721	OG	SER	72	49.837	45.058	18.315	1.00	0.00	RX2	O
ATOM	7722	HG	SER	72	49.541	45.961	18.275	1.00	0.00	RX2	H
ATOM	7723	C	SER	72	49.829	42.402	20.929	1.00	0.00	RX2	C
ATOM	7724	O	SER	72	50.251	42.424	22.076	1.00	0.00	RX2	O
ATOM	7725	N	PHE	73	48.893	41.541	20.497	1.00	0.00	RX2	N
ATOM	7726	H	PHE	73	48.653	41.540	19.529	1.00	0.00	RX2	H
ATOM	7727	CA	PHE	73	48.250	40.593	21.417	1.00	0.00	RX2	C
ATOM	7728	CB	PHE	73	47.280	39.688	20.648	1.00	0.00	RX2	C
ATOM	7729	CG	PHE	73	47.985	38.859	19.598	1.00	0.00	RX2	C
ATOM	7730	CD1	PHE	73	48.451	37.587	19.909	1.00	0.00	RX2	C
ATOM	7731	CD2	PHE	73	48.151	39.358	18.310	1.00	0.00	RX2	C
ATOM	7732	CE1	PHE	73	49.075	36.816	18.935	1.00	0.00	RX2	C
ATOM	7733	CE2	PHE	73	48.776	38.588	17.336	1.00	0.00	RX2	C
ATOM	7734	CZ	PHE	73	49.236	37.315	17.649	1.00	0.00	RX2	C
ATOM	7735	C	PHE	73	49.145	39.760	22.337	1.00	0.00	RX2	C
ATOM	7736	O	PHE	73	48.719	39.293	23.389	1.00	0.00	RX2	O
ATOM	7737	N	LEU	74	50.403	39.589	21.900	1.00	0.00	RX2	N
ATOM	7738	H	LEU	74	50.728	40.034	21.063	1.00	0.00	RX2	H
ATOM	7739	CA	LEU	74	51.324	38.818	22.734	1.00	0.00	RX2	C
ATOM	7740	CB	LEU	74	52.563	38.438	21.934	1.00	0.00	RX2	C
ATOM	7741	CG	LEU	74	52.294	37.516	20.754	1.00	0.00	RX2	C
ATOM	7742	CD1	LEU	74	53.544	37.371	19.896	1.00	0.00	RX2	C
ATOM	7743	CD2	LEU	74	51.758	36.158	21.204	1.00	0.00	RX2	C
ATOM	7744	C	LEU	74	51.759	39.468	24.040	1.00	0.00	RX2	C
ATOM	7745	O	LEU	74	52.087	38.784	25.002	1.00	0.00	RX2	O

ATOM	7746	N	GLN	75	51.753	40.815	24.041	1.00	0.00	RX2	N
ATOM	7747	H	GLN	75	51.401	41.300	23.240	1.00	0.00	RX2	H
ATOM	7748	CA	GLN	75	52.448	41.582	25.087	1.00	0.00	RX2	C
ATOM	7749	CB	GLN	75	52.014	43.048	25.047	1.00	0.00	RX2	C
ATOM	7750	CG	GLN	75	50.499	43.232	25.136	1.00	0.00	RX2	C
ATOM	7751	CD	GLN	75	50.178	44.697	25.302	1.00	0.00	RX2	C
ATOM	7752	OE1	GLN	75	50.189	45.486	24.361	1.00	0.00	RX2	O
ATOM	7753	NE2	GLN	75	49.878	45.017	26.570	1.00	0.00	RX2	N
ATOM	7754	HE21	GLN	75	49.887	44.286	27.267	1.00	0.00	RX2	H
ATOM	7755	HE22	GLN	75	49.637	45.941	26.853	1.00	0.00	RX2	H
ATOM	7756	C	GLN	75	52.430	41.060	26.521	1.00	0.00	RX2	C
ATOM	7757	O	GLN	75	53.436	41.057	27.227	1.00	0.00	RX2	O
ATOM	7758	N	ASP	76	51.232	40.618	26.920	1.00	0.00	RX2	N
ATOM	7759	H	ASP	76	50.478	40.511	26.275	1.00	0.00	RX2	H
ATOM	7760	CA	ASP	76	51.067	40.313	28.334	1.00	0.00	RX2	C
ATOM	7761	CB	ASP	76	49.810	40.985	28.891	1.00	0.00	RX2	C
ATOM	7762	CG	ASP	76	49.879	42.493	28.759	1.00	0.00	RX2	C
ATOM	7763	OD1	ASP	76	50.962	43.064	28.771	1.00	0.00	RX2	O
ATOM	7764	OD2	ASP	76	48.842	43.139	28.690	1.00	0.00	RX2	O
ATOM	7765	C	ASP	76	51.023	38.837	28.665	1.00	0.00	RX2	C
ATOM	7766	O	ASP	76	50.415	38.432	29.650	1.00	0.00	RX2	O
ATOM	7767	N	ILE	77	51.685	38.031	27.819	1.00	0.00	RX2	N
ATOM	7768	H	ILE	77	52.191	38.383	27.028	1.00	0.00	RX2	H
ATOM	7769	CA	ILE	77	51.753	36.619	28.200	1.00	0.00	RX2	C
ATOM	7770	CB	ILE	77	52.055	35.715	27.011	1.00	0.00	RX2	C
ATOM	7771	CG2	ILE	77	52.160	34.248	27.435	1.00	0.00	RX2	C
ATOM	7772	CG1	ILE	77	50.971	35.902	25.956	1.00	0.00	RX2	C
ATOM	7773	CD1	ILE	77	51.200	34.985	24.764	1.00	0.00	RX2	C
ATOM	7774	C	ILE	77	52.701	36.360	29.357	1.00	0.00	RX2	C
ATOM	7775	O	ILE	77	53.912	36.273	29.226	1.00	0.00	RX2	O
ATOM	7776	N	GLN	78	52.064	36.276	30.526	1.00	0.00	RX2	N
ATOM	7777	H	GLN	78	51.063	36.251	30.498	1.00	0.00	RX2	H
ATOM	7778	CA	GLN	78	52.837	36.298	31.760	1.00	0.00	RX2	C
ATOM	7779	CB	GLN	78	51.957	36.863	32.870	1.00	0.00	RX2	C
ATOM	7780	CG	GLN	78	51.668	38.333	32.582	1.00	0.00	RX2	C
ATOM	7781	CD	GLN	78	50.326	38.738	33.138	1.00	0.00	RX2	C
ATOM	7782	OE1	GLN	78	49.998	38.516	34.301	1.00	0.00	RX2	O
ATOM	7783	NE2	GLN	78	49.558	39.354	32.225	1.00	0.00	RX2	N
ATOM	7784	HE21	GLN	78	49.876	39.371	31.271	1.00	0.00	RX2	H
ATOM	7785	HE22	GLN	78	48.668	39.776	32.393	1.00	0.00	RX2	H
ATOM	7786	C	GLN	78	53.538	35.005	32.135	1.00	0.00	RX2	C
ATOM	7787	O	GLN	78	54.604	35.015	32.740	1.00	0.00	RX2	O
ATOM	7788	N	GLU	79	52.928	33.882	31.751	1.00	0.00	RX2	N
ATOM	7789	H	GLU	79	52.048	33.835	31.275	1.00	0.00	RX2	H
ATOM	7790	CA	GLU	79	53.721	32.665	31.888	1.00	0.00	RX2	C
ATOM	7791	CB	GLU	79	53.538	31.969	33.234	1.00	0.00	RX2	C
ATOM	7792	CG	GLU	79	52.200	31.263	33.420	1.00	0.00	RX2	C
ATOM	7793	CD	GLU	79	52.138	30.673	34.811	1.00	0.00	RX2	C
ATOM	7794	OE1	GLU	79	51.792	29.505	34.940	1.00	0.00	RX2	O
ATOM	7795	OE2	GLU	79	52.463	31.362	35.777	1.00	0.00	RX2	O
ATOM	7796	C	GLU	79	53.442	31.674	30.802	1.00	0.00	RX2	C
ATOM	7797	O	GLU	79	52.347	31.617	30.259	1.00	0.00	RX2	O
ATOM	7798	N	VAL	80	54.489	30.898	30.525	1.00	0.00	RX2	N
ATOM	7799	H	VAL	80	55.351	30.976	31.033	1.00	0.00	RX2	H
ATOM	7800	CA	VAL	80	54.320	29.815	29.568	1.00	0.00	RX2	C
ATOM	7801	CB	VAL	80	55.282	30.008	28.404	1.00	0.00	RX2	C
ATOM	7802	CG1	VAL	80	55.168	28.863	27.400	1.00	0.00	RX2	C
ATOM	7803	CG2	VAL	80	55.074	31.385	27.775	1.00	0.00	RX2	C
ATOM	7804	C	VAL	80	54.602	28.510	30.265	1.00	0.00	RX2	C
ATOM	7805	O	VAL	80	55.666	28.333	30.842	1.00	0.00	RX2	O
ATOM	7806	N	GLN	81	53.617	27.606	30.215	1.00	0.00	RX2	N

ATOM	7807	H	GLN	81	52.786	27.769	29.675	1.00	0.00	RX2	H
ATOM	7808	CA	GLN	81	53.888	26.343	30.896	1.00	0.00	RX2	C
ATOM	7809	CB	GLN	81	52.594	25.677	31.379	1.00	0.00	RX2	C
ATOM	7810	CG	GLN	81	51.824	26.692	32.233	1.00	0.00	RX2	C
ATOM	7811	CD	GLN	81	50.795	26.046	33.145	1.00	0.00	RX2	C
ATOM	7812	OE1	GLN	81	50.329	24.920	32.977	1.00	0.00	RX2	O
ATOM	7813	NE2	GLN	81	50.461	26.838	34.167	1.00	0.00	RX2	N
ATOM	7814	HE21	GLN	81	50.867	27.755	34.258	1.00	0.00	RX2	H
ATOM	7815	HE22	GLN	81	49.798	26.567	34.860	1.00	0.00	RX2	H
ATOM	7816	C	GLN	81	54.836	25.416	30.156	1.00	0.00	RX2	C
ATOM	7817	O	GLN	81	55.704	24.769	30.734	1.00	0.00	RX2	O
ATOM	7818	N	GLY	82	54.640	25.382	28.837	1.00	0.00	RX2	N
ATOM	7819	H	GLY	82	54.014	26.024	28.394	1.00	0.00	RX2	H
ATOM	7820	CA	GLY	82	55.579	24.611	28.031	1.00	0.00	RX2	C
ATOM	7821	C	GLY	82	56.821	25.419	27.726	1.00	0.00	RX2	C
ATOM	7822	O	GLY	82	57.294	26.205	28.542	1.00	0.00	RX2	O
ATOM	7823	N	TYR	83	57.314	25.193	26.509	1.00	0.00	RX2	N
ATOM	7824	H	TYR	83	56.811	24.626	25.852	1.00	0.00	RX2	H
ATOM	7825	CA	TYR	83	58.453	26.001	26.083	1.00	0.00	RX2	C
ATOM	7826	CB	TYR	83	59.443	25.157	25.269	1.00	0.00	RX2	C
ATOM	7827	CG	TYR	83	58.745	24.377	24.179	1.00	0.00	RX2	C
ATOM	7828	CD1	TYR	83	58.316	25.004	23.015	1.00	0.00	RX2	C
ATOM	7829	CE1	TYR	83	57.653	24.273	22.038	1.00	0.00	RX2	C
ATOM	7830	CD2	TYR	83	58.538	23.013	24.342	1.00	0.00	RX2	C
ATOM	7831	CE2	TYR	83	57.890	22.278	23.359	1.00	0.00	RX2	C
ATOM	7832	CZ	TYR	83	57.426	22.914	22.217	1.00	0.00	RX2	C
ATOM	7833	OH	TYR	83	56.726	22.192	21.272	1.00	0.00	RX2	O
ATOM	7834	HH	TYR	83	56.039	22.771	20.921	1.00	0.00	RX2	H
ATOM	7835	C	TYR	83	58.006	27.234	25.322	1.00	0.00	RX2	C
ATOM	7836	O	TYR	83	56.892	27.299	24.816	1.00	0.00	RX2	O
ATOM	7837	N	VAL	84	58.917	28.207	25.244	1.00	0.00	RX2	N
ATOM	7838	H	VAL	84	59.829	28.107	25.641	1.00	0.00	RX2	H
ATOM	7839	CA	VAL	84	58.681	29.273	24.278	1.00	0.00	RX2	C
ATOM	7840	CB	VAL	84	58.983	30.647	24.868	1.00	0.00	RX2	C
ATOM	7841	CG1	VAL	84	58.565	31.754	23.901	1.00	0.00	RX2	C
ATOM	7842	CG2	VAL	84	58.344	30.816	26.237	1.00	0.00	RX2	C
ATOM	7843	C	VAL	84	59.560	29.046	23.069	1.00	0.00	RX2	C
ATOM	7844	O	VAL	84	60.785	29.066	23.144	1.00	0.00	RX2	O
ATOM	7845	N	LEU	85	58.882	28.816	21.949	1.00	0.00	RX2	N
ATOM	7846	H	LEU	85	57.888	28.937	21.903	1.00	0.00	RX2	H
ATOM	7847	CA	LEU	85	59.655	28.765	20.720	1.00	0.00	RX2	C
ATOM	7848	CB	LEU	85	59.120	27.642	19.843	1.00	0.00	RX2	C
ATOM	7849	CG	LEU	85	59.878	27.500	18.531	1.00	0.00	RX2	C
ATOM	7850	CD1	LEU	85	61.306	27.017	18.757	1.00	0.00	RX2	C
ATOM	7851	CD2	LEU	85	59.131	26.605	17.553	1.00	0.00	RX2	C
ATOM	7852	C	LEU	85	59.529	30.093	20.013	1.00	0.00	RX2	C
ATOM	7853	O	LEU	85	58.433	30.537	19.717	1.00	0.00	RX2	O
ATOM	7854	N	ILE	86	60.679	30.716	19.758	1.00	0.00	RX2	N
ATOM	7855	H	ILE	86	61.567	30.318	19.987	1.00	0.00	RX2	H
ATOM	7856	CA	ILE	86	60.615	31.883	18.887	1.00	0.00	RX2	C
ATOM	7857	CB	ILE	86	61.066	33.153	19.613	1.00	0.00	RX2	C
ATOM	7858	CG2	ILE	86	60.775	34.382	18.753	1.00	0.00	RX2	C
ATOM	7859	CG1	ILE	86	60.418	33.282	20.991	1.00	0.00	RX2	C
ATOM	7860	CD1	ILE	86	60.856	34.542	21.737	1.00	0.00	RX2	C
ATOM	7861	C	ILE	86	61.485	31.632	17.673	1.00	0.00	RX2	C
ATOM	7862	O	ILE	86	62.665	31.964	17.653	1.00	0.00	RX2	O
ATOM	7863	N	ALA	87	60.868	30.981	16.681	1.00	0.00	RX2	N
ATOM	7864	H	ALA	87	59.875	30.838	16.649	1.00	0.00	RX2	H
ATOM	7865	CA	ALA	87	61.709	30.517	15.585	1.00	0.00	RX2	C
ATOM	7866	CB	ALA	87	61.910	29.011	15.665	1.00	0.00	RX2	C
ATOM	7867	C	ALA	87	61.194	30.834	14.201	1.00	0.00	RX2	C

ATOM	7868	O	ALA	87	59.996	30.923	13.951	1.00	0.00	RX2	O
ATOM	7869	N	HIS	88	62.176	30.966	13.292	1.00	0.00	RX2	N
ATOM	7870	H	HIS	88	63.121	31.019	13.620	1.00	0.00	RX2	H
ATOM	7871	CA	HIS	88	61.887	31.111	11.863	1.00	0.00	RX2	C
ATOM	7872	CB	HIS	88	61.368	29.795	11.285	1.00	0.00	RX2	C
ATOM	7873	CG	HIS	88	62.459	29.026	10.575	1.00	0.00	RX2	C
ATOM	7874	ND1	HIS	88	63.708	29.479	10.352	1.00	0.00	RX2	N
ATOM	7875	HD1	HIS	88	64.110	30.333	10.642	1.00	0.00	RX2	H
ATOM	7876	CD2	HIS	88	62.343	27.750	10.016	1.00	0.00	RX2	C
ATOM	7877	NE2	HIS	88	63.531	27.436	9.446	1.00	0.00	RX2	N
ATOM	7878	CE1	HIS	88	64.374	28.500	9.657	1.00	0.00	RX2	C
ATOM	7879	C	HIS	88	60.950	32.249	11.502	1.00	0.00	RX2	C
ATOM	7880	O	HIS	88	60.225	32.211	10.514	1.00	0.00	RX2	O
ATOM	7881	N	ASN	89	60.986	33.258	12.376	1.00	0.00	RX2	N
ATOM	7882	H	ASN	89	61.714	33.306	13.060	1.00	0.00	RX2	H
ATOM	7883	CA	ASN	89	60.076	34.383	12.213	1.00	0.00	RX2	C
ATOM	7884	CB	ASN	89	59.714	35.026	13.552	1.00	0.00	RX2	C
ATOM	7885	CG	ASN	89	59.052	34.037	14.480	1.00	0.00	RX2	C
ATOM	7886	OD1	ASN	89	57.899	33.660	14.313	1.00	0.00	RX2	O
ATOM	7887	ND2	ASN	89	59.836	33.649	15.491	1.00	0.00	RX2	N
ATOM	7888	HD21	ASN	89	60.800	33.925	15.534	1.00	0.00	RX2	H
ATOM	7889	HD22	ASN	89	59.458	33.036	16.188	1.00	0.00	RX2	H
ATOM	7890	C	ASN	89	60.741	35.446	11.390	1.00	0.00	RX2	C
ATOM	7891	O	ASN	89	61.940	35.670	11.501	1.00	0.00	RX2	O
ATOM	7892	N	GLN	90	59.912	36.109	10.581	1.00	0.00	RX2	N
ATOM	7893	H	GLN	90	58.932	35.906	10.518	1.00	0.00	RX2	H
ATOM	7894	CA	GLN	90	60.467	37.319	9.985	1.00	0.00	RX2	C
ATOM	7895	CB	GLN	90	60.290	37.325	8.462	1.00	0.00	RX2	C
ATOM	7896	CG	GLN	90	61.004	36.136	7.808	1.00	0.00	RX2	C
ATOM	7897	CD	GLN	90	61.263	36.391	6.332	1.00	0.00	RX2	C
ATOM	7898	OE1	GLN	90	60.783	35.681	5.452	1.00	0.00	RX2	O
ATOM	7899	NE2	GLN	90	62.111	37.406	6.115	1.00	0.00	RX2	N
ATOM	7900	HE21	GLN	90	62.484	37.945	6.872	1.00	0.00	RX2	H
ATOM	7901	HE22	GLN	90	62.507	37.708	5.240	1.00	0.00	RX2	H
ATOM	7902	C	GLN	90	59.960	38.598	10.633	1.00	0.00	RX2	C
ATOM	7903	O	GLN	90	60.384	39.698	10.303	1.00	0.00	RX2	O
ATOM	7904	N	VAL	91	59.021	38.405	11.585	1.00	0.00	RX2	N
ATOM	7905	H	VAL	91	58.659	37.496	11.784	1.00	0.00	RX2	H
ATOM	7906	CA	VAL	91	58.484	39.560	12.305	1.00	0.00	RX2	C
ATOM	7907	CB	VAL	91	57.360	39.137	13.261	1.00	0.00	RX2	C
ATOM	7908	CG1	VAL	91	57.901	38.385	14.477	1.00	0.00	RX2	C
ATOM	7909	CG2	VAL	91	56.482	40.327	13.655	1.00	0.00	RX2	C
ATOM	7910	C	VAL	91	59.528	40.404	13.018	1.00	0.00	RX2	C
ATOM	7911	O	VAL	91	60.422	39.906	13.689	1.00	0.00	RX2	O
ATOM	7912	N	ARG	92	59.360	41.717	12.828	1.00	0.00	RX2	N
ATOM	7913	H	ARG	92	58.570	42.030	12.296	1.00	0.00	RX2	H
ATOM	7914	CA	ARG	92	60.396	42.618	13.326	1.00	0.00	RX2	C
ATOM	7915	CB	ARG	92	60.227	43.987	12.676	1.00	0.00	RX2	C
ATOM	7916	CG	ARG	92	60.065	43.819	11.165	1.00	0.00	RX2	C
ATOM	7917	CD	ARG	92	59.978	45.146	10.414	1.00	0.00	RX2	C
ATOM	7918	NE	ARG	92	59.740	44.944	8.985	1.00	0.00	RX2	N
ATOM	7919	HE	ARG	92	58.773	44.957	8.697	1.00	0.00	RX2	H
ATOM	7920	CZ	ARG	92	60.771	44.695	8.124	1.00	0.00	RX2	C
ATOM	7921	NH1	ARG	92	62.024	44.528	8.600	1.00	0.00	RX2	N
ATOM	7922	HH11	ARG	92	62.805	44.300	7.998	1.00	0.00	RX2	H
ATOM	7923	HH12	ARG	92	62.231	44.595	9.578	1.00	0.00	RX2	H
ATOM	7924	NH2	ARG	92	60.522	44.611	6.804	1.00	0.00	RX2	N
ATOM	7925	HH21	ARG	92	61.261	44.437	6.139	1.00	0.00	RX2	H
ATOM	7926	HH22	ARG	92	59.599	44.746	6.419	1.00	0.00	RX2	H
ATOM	7927	C	ARG	92	60.527	42.687	14.842	1.00	0.00	RX2	C
ATOM	7928	O	ARG	92	61.611	42.580	15.402	1.00	0.00	RX2	O

ATOM	7929	N	GLN	93	59.373	42.835	15.504	1.00	0.00	RX2	N
ATOM	7930	H	GLN	93	58.477	42.891	15.066	1.00	0.00	RX2	H
ATOM	7931	CA	GLN	93	59.451	42.821	16.962	1.00	0.00	RX2	C
ATOM	7932	CB	GLN	93	59.278	44.220	17.564	1.00	0.00	RX2	C
ATOM	7933	CG	GLN	93	60.574	44.968	17.902	1.00	0.00	RX2	C
ATOM	7934	CD	GLN	93	61.178	45.634	16.682	1.00	0.00	RX2	C
ATOM	7935	OE1	GLN	93	60.650	45.586	15.574	1.00	0.00	RX2	O
ATOM	7936	NE2	GLN	93	62.309	46.303	16.961	1.00	0.00	RX2	N
ATOM	7937	HE21	GLN	93	62.712	46.289	17.887	1.00	0.00	RX2	H
ATOM	7938	HE22	GLN	93	62.823	46.844	16.302	1.00	0.00	RX2	H
ATOM	7939	C	GLN	93	58.414	41.904	17.569	1.00	0.00	RX2	C
ATOM	7940	O	GLN	93	57.267	41.868	17.146	1.00	0.00	RX2	O
ATOM	7941	N	VAL	94	58.862	41.177	18.603	1.00	0.00	RX2	N
ATOM	7942	H	VAL	94	59.805	41.248	18.927	1.00	0.00	RX2	H
ATOM	7943	CA	VAL	94	57.878	40.451	19.406	1.00	0.00	RX2	C
ATOM	7944	CB	VAL	94	58.347	39.017	19.676	1.00	0.00	RX2	C
ATOM	7945	CG1	VAL	94	57.464	38.334	20.719	1.00	0.00	RX2	C
ATOM	7946	CG2	VAL	94	58.403	38.203	18.384	1.00	0.00	RX2	C
ATOM	7947	C	VAL	94	57.638	41.184	20.716	1.00	0.00	RX2	C
ATOM	7948	O	VAL	94	58.556	41.393	21.502	1.00	0.00	RX2	O
ATOM	7949	N	PRO	95	56.372	41.607	20.916	1.00	0.00	RX2	N
ATOM	7950	CD	PRO	95	55.282	41.589	19.948	1.00	0.00	RX2	C
ATOM	7951	CA	PRO	95	55.993	42.169	22.213	1.00	0.00	RX2	C
ATOM	7952	CB	PRO	95	54.776	43.009	21.813	1.00	0.00	RX2	C
ATOM	7953	CG	PRO	95	54.107	42.231	20.678	1.00	0.00	RX2	C
ATOM	7954	C	PRO	95	55.671	41.076	23.228	1.00	0.00	RX2	C
ATOM	7955	O	PRO	95	54.655	40.401	23.140	1.00	0.00	RX2	O
ATOM	7956	N	LEU	96	56.573	40.924	24.199	1.00	0.00	RX2	N
ATOM	7957	H	LEU	96	57.414	41.466	24.262	1.00	0.00	RX2	H
ATOM	7958	CA	LEU	96	56.261	40.036	25.319	1.00	0.00	RX2	C
ATOM	7959	CB	LEU	96	56.971	38.690	25.207	1.00	0.00	RX2	C
ATOM	7960	CG	LEU	96	56.268	37.642	24.350	1.00	0.00	RX2	C
ATOM	7961	CD1	LEU	96	57.149	36.410	24.139	1.00	0.00	RX2	C
ATOM	7962	CD2	LEU	96	54.915	37.257	24.941	1.00	0.00	RX2	C
ATOM	7963	C	LEU	96	56.662	40.679	26.627	1.00	0.00	RX2	C
ATOM	7964	O	LEU	96	57.306	40.092	27.489	1.00	0.00	RX2	O
ATOM	7965	N	GLN	97	56.261	41.950	26.731	1.00	0.00	RX2	N
ATOM	7966	H	GLN	97	55.667	42.321	26.016	1.00	0.00	RX2	H
ATOM	7967	CA	GLN	97	56.689	42.787	27.852	1.00	0.00	RX2	C
ATOM	7968	CB	GLN	97	55.934	44.116	27.818	1.00	0.00	RX2	C
ATOM	7969	CG	GLN	97	56.450	45.139	26.801	1.00	0.00	RX2	C
ATOM	7970	CD	GLN	97	56.382	44.624	25.378	1.00	0.00	RX2	C
ATOM	7971	OE1	GLN	97	55.439	43.953	24.963	1.00	0.00	RX2	O
ATOM	7972	NE2	GLN	97	57.428	45.007	24.637	1.00	0.00	RX2	N
ATOM	7973	HE21	GLN	97	58.210	45.439	25.106	1.00	0.00	RX2	H
ATOM	7974	HE22	GLN	97	57.520	44.899	23.650	1.00	0.00	RX2	H
ATOM	7975	C	GLN	97	56.511	42.141	29.216	1.00	0.00	RX2	C
ATOM	7976	O	GLN	97	57.409	42.086	30.053	1.00	0.00	RX2	O
ATOM	7977	N	ARG	98	55.290	41.635	29.407	1.00	0.00	RX2	N
ATOM	7978	H	ARG	98	54.619	41.570	28.664	1.00	0.00	RX2	H
ATOM	7979	CA	ARG	98	55.055	41.023	30.706	1.00	0.00	RX2	C
ATOM	7980	CB	ARG	98	53.725	41.489	31.292	1.00	0.00	RX2	C
ATOM	7981	CG	ARG	98	53.878	42.901	31.861	1.00	0.00	RX2	C
ATOM	7982	CD	ARG	98	52.595	43.456	32.477	1.00	0.00	RX2	C
ATOM	7983	NE	ARG	98	51.587	43.693	31.449	1.00	0.00	RX2	N
ATOM	7984	HE	ARG	98	51.603	43.118	30.617	1.00	0.00	RX2	H
ATOM	7985	CZ	ARG	98	50.643	44.664	31.605	1.00	0.00	RX2	C
ATOM	7986	NH1	ARG	98	50.652	45.422	32.725	1.00	0.00	RX2	N
ATOM	7987	HH11	ARG	98	49.977	46.145	32.887	1.00	0.00	RX2	H
ATOM	7988	HH12	ARG	98	51.345	45.282	33.438	1.00	0.00	RX2	H
ATOM	7989	NH2	ARG	98	49.721	44.847	30.641	1.00	0.00	RX2	N

ATOM	7990	HH21	ARG	98	48.986	45.525	30.651	1.00	0.00	RX2	H
ATOM	7991	HH22	ARG	98	49.741	44.244	29.818	1.00	0.00	RX2	H
ATOM	7992	C	ARG	98	55.221	39.519	30.774	1.00	0.00	RX2	C
ATOM	7993	O	ARG	98	54.664	38.863	31.646	1.00	0.00	RX2	O
ATOM	7994	N	LEU	99	56.051	38.995	29.856	1.00	0.00	RX2	N
ATOM	7995	H	LEU	99	56.534	39.560	29.188	1.00	0.00	RX2	H
ATOM	7996	CA	LEU	99	56.499	37.622	30.081	1.00	0.00	RX2	C
ATOM	7997	CB	LEU	99	57.298	37.100	28.885	1.00	0.00	RX2	C
ATOM	7998	CG	LEU	99	57.840	35.675	29.057	1.00	0.00	RX2	C
ATOM	7999	CD1	LEU	99	56.747	34.650	29.368	1.00	0.00	RX2	C
ATOM	8000	CD2	LEU	99	58.678	35.253	27.850	1.00	0.00	RX2	C
ATOM	8001	C	LEU	99	57.311	37.554	31.355	1.00	0.00	RX2	C
ATOM	8002	O	LEU	99	58.317	38.226	31.509	1.00	0.00	RX2	O
ATOM	8003	N	ARG	100	56.774	36.762	32.279	1.00	0.00	RX2	N
ATOM	8004	H	ARG	100	55.975	36.206	32.051	1.00	0.00	RX2	H
ATOM	8005	CA	ARG	100	57.342	36.722	33.618	1.00	0.00	RX2	C
ATOM	8006	CB	ARG	100	56.180	36.938	34.606	1.00	0.00	RX2	C
ATOM	8007	CG	ARG	100	56.133	36.083	35.874	1.00	0.00	RX2	C
ATOM	8008	CD	ARG	100	54.795	36.197	36.624	1.00	0.00	RX2	C
ATOM	8009	NE	ARG	100	53.645	35.809	35.797	1.00	0.00	RX2	N
ATOM	8010	HE	ARG	100	53.311	36.513	35.162	1.00	0.00	RX2	H
ATOM	8011	CZ	ARG	100	53.124	34.544	35.884	1.00	0.00	RX2	C
ATOM	8012	NH1	ARG	100	53.682	33.655	36.726	1.00	0.00	RX2	N
ATOM	8013	HH11	ARG	100	53.343	32.701	36.730	1.00	0.00	RX2	H
ATOM	8014	HH12	ARG	100	54.437	33.889	37.342	1.00	0.00	RX2	H
ATOM	8015	NH2	ARG	100	52.063	34.159	35.139	1.00	0.00	RX2	N
ATOM	8016	HH21	ARG	100	51.745	33.199	35.183	1.00	0.00	RX2	H
ATOM	8017	HH22	ARG	100	51.551	34.744	34.499	1.00	0.00	RX2	H
ATOM	8018	C	ARG	100	58.152	35.454	33.830	1.00	0.00	RX2	C
ATOM	8019	O	ARG	100	59.302	35.480	34.256	1.00	0.00	RX2	O
ATOM	8020	N	ILE	101	57.508	34.328	33.486	1.00	0.00	RX2	N
ATOM	8021	H	ILE	101	56.584	34.316	33.097	1.00	0.00	RX2	H
ATOM	8022	CA	ILE	101	58.207	33.069	33.730	1.00	0.00	RX2	C
ATOM	8023	CB	ILE	101	57.864	32.548	35.137	1.00	0.00	RX2	C
ATOM	8024	CG2	ILE	101	56.372	32.306	35.308	1.00	0.00	RX2	C
ATOM	8025	CG1	ILE	101	58.644	31.303	35.538	1.00	0.00	RX2	C
ATOM	8026	CD1	ILE	101	58.255	30.881	36.953	1.00	0.00	RX2	C
ATOM	8027	C	ILE	101	57.964	32.039	32.639	1.00	0.00	RX2	C
ATOM	8028	O	ILE	101	56.841	31.790	32.210	1.00	0.00	RX2	O
ATOM	8029	N	VAL	102	59.084	31.460	32.192	1.00	0.00	RX2	N
ATOM	8030	H	VAL	102	59.955	31.626	32.663	1.00	0.00	RX2	H
ATOM	8031	CA	VAL	102	58.942	30.293	31.324	1.00	0.00	RX2	C
ATOM	8032	CB	VAL	102	60.049	30.279	30.266	1.00	0.00	RX2	C
ATOM	8033	CG1	VAL	102	59.947	29.065	29.338	1.00	0.00	RX2	C
ATOM	8034	CG2	VAL	102	60.063	31.593	29.489	1.00	0.00	RX2	C
ATOM	8035	C	VAL	102	59.047	29.073	32.211	1.00	0.00	RX2	C
ATOM	8036	O	VAL	102	59.864	29.047	33.122	1.00	0.00	RX2	O
ATOM	8037	N	ARG	103	58.196	28.079	31.949	1.00	0.00	RX2	N
ATOM	8038	H	ARG	103	57.546	28.106	31.187	1.00	0.00	RX2	H
ATOM	8039	CA	ARG	103	58.338	26.903	32.796	1.00	0.00	RX2	C
ATOM	8040	CB	ARG	103	56.985	26.380	33.266	1.00	0.00	RX2	C
ATOM	8041	CG	ARG	103	56.179	27.461	33.985	1.00	0.00	RX2	C
ATOM	8042	CD	ARG	103	54.918	26.888	34.627	1.00	0.00	RX2	C
ATOM	8043	NE	ARG	103	54.123	27.936	35.261	1.00	0.00	RX2	N
ATOM	8044	HE	ARG	103	53.547	28.522	34.673	1.00	0.00	RX2	H
ATOM	8045	CZ	ARG	103	54.145	28.111	36.613	1.00	0.00	RX2	C
ATOM	8046	NH1	ARG	103	54.944	27.328	37.373	1.00	0.00	RX2	N
ATOM	8047	HH11	ARG	103	54.994	27.410	38.371	1.00	0.00	RX2	H
ATOM	8048	HH12	ARG	103	55.525	26.625	36.951	1.00	0.00	RX2	H
ATOM	8049	NH2	ARG	103	53.360	29.062	37.161	1.00	0.00	RX2	N
ATOM	8050	HH21	ARG	103	53.294	29.262	38.139	1.00	0.00	RX2	H

ATOM	8051	HH22	ARG	103	52.775	29.629	36.546	1.00	0.00	RX2	H
ATOM	8052	C	ARG	103	59.186	25.805	32.190	1.00	0.00	RX2	C
ATOM	8053	O	ARG	103	60.079	25.265	32.832	1.00	0.00	RX2	O
ATOM	8054	N	GLY	104	58.885	25.509	30.917	1.00	0.00	RX2	N
ATOM	8055	H	GLY	104	58.123	25.933	30.423	1.00	0.00	RX2	H
ATOM	8056	CA	GLY	104	59.650	24.433	30.287	1.00	0.00	RX2	C
ATOM	8057	C	GLY	104	59.245	23.048	30.762	1.00	0.00	RX2	C
ATOM	8058	O	GLY	104	60.050	22.147	30.951	1.00	0.00	RX2	O
ATOM	8059	N	THR	105	57.922	22.912	30.944	1.00	0.00	RX2	N
ATOM	8060	H	THR	105	57.289	23.648	30.716	1.00	0.00	RX2	H
ATOM	8061	CA	THR	105	57.439	21.603	31.388	1.00	0.00	RX2	C
ATOM	8062	CB	THR	105	56.010	21.779	31.892	1.00	0.00	RX2	C
ATOM	8063	OG1	THR	105	55.943	22.950	32.721	1.00	0.00	RX2	O
ATOM	8064	HG1	THR	105	56.075	23.692	32.143	1.00	0.00	RX2	H
ATOM	8065	CG2	THR	105	55.503	20.549	32.645	1.00	0.00	RX2	C
ATOM	8066	C	THR	105	57.601	20.507	30.336	1.00	0.00	RX2	C
ATOM	8067	O	THR	105	57.879	19.350	30.624	1.00	0.00	RX2	O
ATOM	8068	N	GLN	106	57.453	20.946	29.077	1.00	0.00	RX2	N
ATOM	8069	H	GLN	106	57.315	21.906	28.842	1.00	0.00	RX2	H
ATOM	8070	CA	GLN	106	58.014	20.107	28.025	1.00	0.00	RX2	C
ATOM	8071	CB	GLN	106	56.950	19.554	27.076	1.00	0.00	RX2	C
ATOM	8072	CG	GLN	106	56.182	20.598	26.267	1.00	0.00	RX2	C
ATOM	8073	CD	GLN	106	55.353	19.861	25.236	1.00	0.00	RX2	C
ATOM	8074	OE1	GLN	106	55.097	18.669	25.367	1.00	0.00	RX2	O
ATOM	8075	NE2	GLN	106	54.989	20.625	24.194	1.00	0.00	RX2	N
ATOM	8076	HE21	GLN	106	55.212	21.604	24.211	1.00	0.00	RX2	H
ATOM	8077	HE22	GLN	106	54.500	20.290	23.379	1.00	0.00	RX2	H
ATOM	8078	C	GLN	106	59.057	20.912	27.288	1.00	0.00	RX2	C
ATOM	8079	O	GLN	106	59.056	22.136	27.362	1.00	0.00	RX2	O
ATOM	8080	N	LEU	107	59.954	20.179	26.617	1.00	0.00	RX2	N
ATOM	8081	H	LEU	107	59.855	19.198	26.451	1.00	0.00	RX2	H
ATOM	8082	CA	LEU	107	61.128	20.855	26.075	1.00	0.00	RX2	C
ATOM	8083	CB	LEU	107	62.387	20.318	26.751	1.00	0.00	RX2	C
ATOM	8084	CG	LEU	107	62.424	20.555	28.261	1.00	0.00	RX2	C
ATOM	8085	CD1	LEU	107	63.619	19.867	28.921	1.00	0.00	RX2	C
ATOM	8086	CD2	LEU	107	62.372	22.043	28.594	1.00	0.00	RX2	C
ATOM	8087	C	LEU	107	61.250	20.701	24.575	1.00	0.00	RX2	C
ATOM	8088	O	LEU	107	61.066	19.623	24.025	1.00	0.00	RX2	O
ATOM	8089	N	PHE	108	61.578	21.835	23.945	1.00	0.00	RX2	N
ATOM	8090	H	PHE	108	61.777	22.651	24.487	1.00	0.00	RX2	H
ATOM	8091	CA	PHE	108	61.833	21.838	22.506	1.00	0.00	RX2	C
ATOM	8092	CB	PHE	108	61.858	23.288	22.019	1.00	0.00	RX2	C
ATOM	8093	CG	PHE	108	61.573	23.344	20.539	1.00	0.00	RX2	C
ATOM	8094	CD1	PHE	108	60.256	23.380	20.101	1.00	0.00	RX2	C
ATOM	8095	CD2	PHE	108	62.614	23.353	19.620	1.00	0.00	RX2	C
ATOM	8096	CE1	PHE	108	59.974	23.393	18.743	1.00	0.00	RX2	C
ATOM	8097	CE2	PHE	108	62.335	23.364	18.259	1.00	0.00	RX2	C
ATOM	8098	CZ	PHE	108	61.015	23.364	17.825	1.00	0.00	RX2	C
ATOM	8099	C	PHE	108	63.143	21.134	22.194	1.00	0.00	RX2	C
ATOM	8100	O	PHE	108	64.087	21.239	22.972	1.00	0.00	RX2	O
ATOM	8101	N	GLU	109	63.163	20.399	21.063	1.00	0.00	RX2	N
ATOM	8102	H	GLU	109	62.324	20.381	20.513	1.00	0.00	RX2	H
ATOM	8103	CA	GLU	109	64.349	19.614	20.662	1.00	0.00	RX2	C
ATOM	8104	CB	GLU	109	65.322	20.467	19.841	1.00	0.00	RX2	C
ATOM	8105	CG	GLU	109	64.907	20.658	18.375	1.00	0.00	RX2	C
ATOM	8106	CD	GLU	109	65.176	19.395	17.573	1.00	0.00	RX2	C
ATOM	8107	OE1	GLU	109	66.302	18.910	17.602	1.00	0.00	RX2	O
ATOM	8108	OE2	GLU	109	64.271	18.889	16.911	1.00	0.00	RX2	O
ATOM	8109	C	GLU	109	65.053	18.928	21.828	1.00	0.00	RX2	C
ATOM	8110	O	GLU	109	66.226	19.126	22.138	1.00	0.00	RX2	O
ATOM	8111	N	ASP	110	64.190	18.170	22.523	1.00	0.00	RX2	N

ATOM	8112	H	ASP	110	63.262	18.071	22.168	1.00	0.00	RX2	H
ATOM	8113	CA	ASP	110	64.536	17.405	23.725	1.00	0.00	RX2	C
ATOM	8114	CB	ASP	110	65.525	16.259	23.415	1.00	0.00	RX2	C
ATOM	8115	CG	ASP	110	65.251	15.489	22.120	1.00	0.00	RX2	C
ATOM	8116	OD1	ASP	110	64.104	15.220	21.762	1.00	0.00	RX2	O
ATOM	8117	OD2	ASP	110	66.212	15.107	21.458	1.00	0.00	RX2	O
ATOM	8118	C	ASP	110	64.974	18.179	24.976	1.00	0.00	RX2	C
ATOM	8119	O	ASP	110	64.907	17.661	26.083	1.00	0.00	RX2	O
ATOM	8120	N	ASN	111	65.448	19.429	24.795	1.00	0.00	RX2	N
ATOM	8121	H	ASN	111	65.388	19.891	23.908	1.00	0.00	RX2	H
ATOM	8122	CA	ASN	111	66.252	20.003	25.886	1.00	0.00	RX2	C
ATOM	8123	CB	ASN	111	67.745	20.040	25.542	1.00	0.00	RX2	C
ATOM	8124	CG	ASN	111	68.366	18.668	25.415	1.00	0.00	RX2	C
ATOM	8125	OD1	ASN	111	68.971	18.147	26.346	1.00	0.00	RX2	O
ATOM	8126	ND2	ASN	111	68.226	18.129	24.188	1.00	0.00	RX2	N
ATOM	8127	HD21	ASN	111	67.698	18.582	23.459	1.00	0.00	RX2	H
ATOM	8128	HD22	ASN	111	68.612	17.238	23.951	1.00	0.00	RX2	H
ATOM	8129	C	ASN	111	65.906	21.420	26.318	1.00	0.00	RX2	C
ATOM	8130	O	ASN	111	66.381	21.925	27.332	1.00	0.00	RX2	O
ATOM	8131	N	TYR	112	65.118	22.095	25.477	1.00	0.00	RX2	N
ATOM	8132	H	TYR	112	64.596	21.669	24.738	1.00	0.00	RX2	H
ATOM	8133	CA	TYR	112	65.136	23.548	25.620	1.00	0.00	RX2	C
ATOM	8134	CB	TYR	112	65.611	24.194	24.321	1.00	0.00	RX2	C
ATOM	8135	CG	TYR	112	66.899	23.541	23.889	1.00	0.00	RX2	C
ATOM	8136	CD1	TYR	112	68.078	23.805	24.575	1.00	0.00	RX2	C
ATOM	8137	CE1	TYR	112	69.264	23.209	24.166	1.00	0.00	RX2	C
ATOM	8138	CD2	TYR	112	66.904	22.672	22.805	1.00	0.00	RX2	C
ATOM	8139	CE2	TYR	112	68.088	22.066	22.404	1.00	0.00	RX2	C
ATOM	8140	CZ	TYR	112	69.268	22.339	23.082	1.00	0.00	RX2	C
ATOM	8141	OH	TYR	112	70.446	21.747	22.679	1.00	0.00	RX2	O
ATOM	8142	HH	TYR	112	70.261	21.145	21.968	1.00	0.00	RX2	H
ATOM	8143	C	TYR	112	63.826	24.165	26.047	1.00	0.00	RX2	C
ATOM	8144	O	TYR	112	62.759	23.824	25.557	1.00	0.00	RX2	O
ATOM	8145	N	ALA	113	63.963	25.104	26.988	1.00	0.00	RX2	N
ATOM	8146	H	ALA	113	64.871	25.409	27.274	1.00	0.00	RX2	H
ATOM	8147	CA	ALA	113	62.777	25.844	27.402	1.00	0.00	RX2	C
ATOM	8148	CB	ALA	113	62.866	26.242	28.869	1.00	0.00	RX2	C
ATOM	8149	C	ALA	113	62.555	27.104	26.595	1.00	0.00	RX2	C
ATOM	8150	O	ALA	113	61.433	27.519	26.341	1.00	0.00	RX2	O
ATOM	8151	N	LEU	114	63.678	27.706	26.191	1.00	0.00	RX2	N
ATOM	8152	H	LEU	114	64.600	27.355	26.368	1.00	0.00	RX2	H
ATOM	8153	CA	LEU	114	63.529	28.887	25.351	1.00	0.00	RX2	C
ATOM	8154	CB	LEU	114	63.923	30.136	26.141	1.00	0.00	RX2	C
ATOM	8155	CG	LEU	114	63.852	31.438	25.339	1.00	0.00	RX2	C
ATOM	8156	CD1	LEU	114	62.471	31.681	24.734	1.00	0.00	RX2	C
ATOM	8157	CD2	LEU	114	64.307	32.634	26.172	1.00	0.00	RX2	C
ATOM	8158	C	LEU	114	64.363	28.721	24.104	1.00	0.00	RX2	C
ATOM	8159	O	LEU	114	65.586	28.705	24.152	1.00	0.00	RX2	O
ATOM	8160	N	ALA	115	63.655	28.559	22.986	1.00	0.00	RX2	N
ATOM	8161	H	ALA	115	62.657	28.659	22.960	1.00	0.00	RX2	H
ATOM	8162	CA	ALA	115	64.410	28.318	21.765	1.00	0.00	RX2	C
ATOM	8163	CB	ALA	115	64.076	26.940	21.193	1.00	0.00	RX2	C
ATOM	8164	C	ALA	115	64.181	29.396	20.727	1.00	0.00	RX2	C
ATOM	8165	O	ALA	115	63.197	29.409	19.998	1.00	0.00	RX2	O
ATOM	8166	N	VAL	116	65.130	30.335	20.717	1.00	0.00	RX2	N
ATOM	8167	H	VAL	116	65.993	30.163	21.195	1.00	0.00	RX2	H
ATOM	8168	CA	VAL	116	65.020	31.435	19.763	1.00	0.00	RX2	C
ATOM	8169	CB	VAL	116	65.493	32.737	20.409	1.00	0.00	RX2	C
ATOM	8170	CG1	VAL	116	65.250	33.934	19.491	1.00	0.00	RX2	C
ATOM	8171	CG2	VAL	116	64.840	32.929	21.778	1.00	0.00	RX2	C
ATOM	8172	C	VAL	116	65.830	31.128	18.515	1.00	0.00	RX2	C

ATOM	8173	O	VAL	116	67.036	31.335	18.465	1.00	0.00	RX2	O
ATOM	8174	N	LEU	117	65.130	30.575	17.521	1.00	0.00	RX2	N
ATOM	8175	H	LEU	117	64.129	30.602	17.506	1.00	0.00	RX2	H
ATOM	8176	CA	LEU	117	65.894	30.026	16.401	1.00	0.00	RX2	C
ATOM	8177	CB	LEU	117	65.530	28.562	16.147	1.00	0.00	RX2	C
ATOM	8178	CG	LEU	117	65.405	27.700	17.402	1.00	0.00	RX2	C
ATOM	8179	CD1	LEU	117	64.884	26.300	17.075	1.00	0.00	RX2	C
ATOM	8180	CD2	LEU	117	66.708	27.645	18.191	1.00	0.00	RX2	C
ATOM	8181	C	LEU	117	65.696	30.793	15.113	1.00	0.00	RX2	C
ATOM	8182	O	LEU	117	64.607	31.266	14.817	1.00	0.00	RX2	O
ATOM	8183	N	ASP	118	66.795	30.868	14.358	1.00	0.00	RX2	N
ATOM	8184	H	ASP	118	67.650	30.489	14.716	1.00	0.00	RX2	H
ATOM	8185	CA	ASP	118	66.791	31.113	12.912	1.00	0.00	RX2	C
ATOM	8186	CB	ASP	118	66.635	29.778	12.167	1.00	0.00	RX2	C
ATOM	8187	CG	ASP	118	67.748	28.779	12.485	1.00	0.00	RX2	C
ATOM	8188	OD1	ASP	118	68.101	27.981	11.622	1.00	0.00	RX2	O
ATOM	8189	OD2	ASP	118	68.279	28.763	13.592	1.00	0.00	RX2	O
ATOM	8190	C	ASP	118	65.793	32.154	12.422	1.00	0.00	RX2	C
ATOM	8191	O	ASP	118	64.913	31.906	11.600	1.00	0.00	RX2	O
ATOM	8192	N	ASN	119	65.943	33.345	13.020	1.00	0.00	RX2	N
ATOM	8193	H	ASN	119	66.802	33.541	13.497	1.00	0.00	RX2	H
ATOM	8194	CA	ASN	119	64.868	34.328	12.860	1.00	0.00	RX2	C
ATOM	8195	CB	ASN	119	64.547	35.078	14.150	1.00	0.00	RX2	C
ATOM	8196	CG	ASN	119	63.637	34.281	15.058	1.00	0.00	RX2	C
ATOM	8197	OD1	ASN	119	62.585	33.781	14.669	1.00	0.00	RX2	O
ATOM	8198	ND2	ASN	119	64.108	34.180	16.311	1.00	0.00	RX2	N
ATOM	8199	HD21	ASN	119	64.912	34.724	16.577	1.00	0.00	RX2	H
ATOM	8200	HD22	ASN	119	63.679	33.575	16.987	1.00	0.00	RX2	H
ATOM	8201	C	ASN	119	65.082	35.369	11.787	1.00	0.00	RX2	C
ATOM	8202	O	ASN	119	65.306	36.545	12.061	1.00	0.00	RX2	O
ATOM	8203	N	GLY	120	64.949	34.883	10.549	1.00	0.00	RX2	N
ATOM	8204	H	GLY	120	64.949	33.896	10.388	1.00	0.00	RX2	H
ATOM	8205	CA	GLY	120	64.785	35.843	9.462	1.00	0.00	RX2	C
ATOM	8206	C	GLY	120	66.056	36.474	8.924	1.00	0.00	RX2	C
ATOM	8207	O	GLY	120	67.175	36.088	9.244	1.00	0.00	RX2	O
ATOM	8208	N	ASP	121	65.805	37.460	8.052	1.00	0.00	RX2	N
ATOM	8209	H	ASP	121	64.865	37.755	7.879	1.00	0.00	RX2	H
ATOM	8210	CA	ASP	121	66.883	38.083	7.284	1.00	0.00	RX2	C
ATOM	8211	CB	ASP	121	66.328	39.067	6.256	1.00	0.00	RX2	C
ATOM	8212	CG	ASP	121	65.431	38.382	5.254	1.00	0.00	RX2	C
ATOM	8213	OD1	ASP	121	65.858	37.404	4.644	1.00	0.00	RX2	O
ATOM	8214	OD2	ASP	121	64.308	38.846	5.077	1.00	0.00	RX2	O
ATOM	8215	C	ASP	121	67.931	38.808	8.107	1.00	0.00	RX2	C
ATOM	8216	O	ASP	121	67.639	39.507	9.073	1.00	0.00	RX2	O
ATOM	8217	N	PRO	122	69.197	38.639	7.657	1.00	0.00	RX2	N
ATOM	8218	CD	PRO	122	69.624	37.683	6.645	1.00	0.00	RX2	C
ATOM	8219	CA	PRO	122	70.308	39.413	8.220	1.00	0.00	RX2	C
ATOM	8220	CB	PRO	122	71.536	38.752	7.577	1.00	0.00	RX2	C
ATOM	8221	CG	PRO	122	71.070	37.405	7.025	1.00	0.00	RX2	C
ATOM	8222	C	PRO	122	70.282	40.914	7.930	1.00	0.00	RX2	C
ATOM	8223	O	PRO	122	71.096	41.428	7.172	1.00	0.00	RX2	O
ATOM	8224	N	LEU	123	69.346	41.626	8.575	1.00	0.00	RX2	N
ATOM	8225	H	LEU	123	68.709	41.181	9.208	1.00	0.00	RX2	H
ATOM	8226	CA	LEU	123	69.467	43.082	8.483	1.00	0.00	RX2	C
ATOM	8227	CB	LEU	123	68.122	43.757	8.759	1.00	0.00	RX2	C
ATOM	8228	CG	LEU	123	68.164	45.284	8.892	1.00	0.00	RX2	C
ATOM	8229	CD1	LEU	123	68.474	45.976	7.567	1.00	0.00	RX2	C
ATOM	8230	CD2	LEU	123	66.892	45.839	9.532	1.00	0.00	RX2	C
ATOM	8231	C	LEU	123	70.536	43.597	9.427	1.00	0.00	RX2	C
ATOM	8232	O	LEU	123	70.594	43.246	10.602	1.00	0.00	RX2	O
ATOM	8233	N	ASN	124	71.389	44.448	8.858	1.00	0.00	RX2	N

ATOM	8234	H	ASN	124	71.273	44.761	7.915	1.00	0.00	RX2	H
ATOM	8235	CA	ASN	124	72.326	45.155	9.722	1.00	0.00	RX2	C
ATOM	8236	CB	ASN	124	73.720	45.288	9.115	1.00	0.00	RX2	C
ATOM	8237	CG	ASN	124	73.865	46.662	8.503	1.00	0.00	RX2	C
ATOM	8238	OD1	ASN	124	73.319	46.938	7.445	1.00	0.00	RX2	O
ATOM	8239	ND2	ASN	124	74.647	47.502	9.207	1.00	0.00	RX2	N
ATOM	8240	HD21	ASN	124	75.123	47.194	10.033	1.00	0.00	RX2	H
ATOM	8241	HD22	ASN	124	74.767	48.452	8.915	1.00	0.00	RX2	H
ATOM	8242	C	ASN	124	71.814	46.526	10.111	1.00	0.00	RX2	C
ATOM	8243	O	ASN	124	71.173	47.223	9.340	1.00	0.00	RX2	O
ATOM	8244	N	ASN	125	72.138	46.861	11.367	1.00	0.00	RX2	N
ATOM	8245	H	ASN	125	72.685	46.207	11.886	1.00	0.00	RX2	H
ATOM	8246	CA	ASN	125	71.864	48.158	11.997	1.00	0.00	RX2	C
ATOM	8247	CB	ASN	125	72.981	49.165	11.738	1.00	0.00	RX2	C
ATOM	8248	CG	ASN	125	74.063	48.939	12.761	1.00	0.00	RX2	C
ATOM	8249	OD1	ASN	125	74.100	47.907	13.428	1.00	0.00	RX2	O
ATOM	8250	ND2	ASN	125	74.937	49.955	12.850	1.00	0.00	RX2	N
ATOM	8251	HD21	ASN	125	74.862	50.718	12.197	1.00	0.00	RX2	H
ATOM	8252	HD22	ASN	125	75.665	49.990	13.534	1.00	0.00	RX2	H
ATOM	8253	C	ASN	125	70.526	48.847	11.784	1.00	0.00	RX2	C
ATOM	8254	O	ASN	125	69.661	48.851	12.655	1.00	0.00	RX2	O
ATOM	8255	N	THR	126	70.421	49.481	10.617	1.00	0.00	RX2	N
ATOM	8256	H	THR	126	71.048	49.253	9.869	1.00	0.00	RX2	H
ATOM	8257	CA	THR	126	69.404	50.485	10.320	1.00	0.00	RX2	C
ATOM	8258	CB	THR	126	69.745	50.833	8.882	1.00	0.00	RX2	C
ATOM	8259	OG1	THR	126	71.177	50.767	8.815	1.00	0.00	RX2	O
ATOM	8260	HG1	THR	126	71.430	50.944	7.907	1.00	0.00	RX2	H
ATOM	8261	CG2	THR	126	69.236	52.195	8.399	1.00	0.00	RX2	C
ATOM	8262	C	THR	126	67.956	50.095	10.634	1.00	0.00	RX2	C
ATOM	8263	O	THR	126	67.605	48.934	10.832	1.00	0.00	RX2	O
ATOM	8264	N	THR	127	67.132	51.150	10.742	1.00	0.00	RX2	N
ATOM	8265	H	THR	127	67.459	52.069	10.530	1.00	0.00	RX2	H
ATOM	8266	CA	THR	127	65.722	50.973	11.090	1.00	0.00	RX2	C
ATOM	8267	CB	THR	127	65.114	52.387	11.158	1.00	0.00	RX2	C
ATOM	8268	OG1	THR	127	63.898	52.408	11.908	1.00	0.00	RX2	O
ATOM	8269	HG1	THR	127	63.632	53.317	11.977	1.00	0.00	RX2	H
ATOM	8270	CG2	THR	127	64.952	53.034	9.783	1.00	0.00	RX2	C
ATOM	8271	C	THR	127	64.977	49.970	10.207	1.00	0.00	RX2	C
ATOM	8272	O	THR	127	65.134	49.928	8.989	1.00	0.00	RX2	O
ATOM	8273	N	PRO	128	64.178	49.113	10.884	1.00	0.00	RX2	N
ATOM	8274	CD	PRO	128	63.983	49.051	12.328	1.00	0.00	RX2	C
ATOM	8275	CA	PRO	128	63.421	48.097	10.153	1.00	0.00	RX2	C
ATOM	8276	CB	PRO	128	63.176	47.071	11.261	1.00	0.00	RX2	C
ATOM	8277	CG	PRO	128	62.983	47.916	12.521	1.00	0.00	RX2	C
ATOM	8278	C	PRO	128	62.135	48.642	9.538	1.00	0.00	RX2	C
ATOM	8279	O	PRO	128	61.033	48.331	9.971	1.00	0.00	RX2	O
ATOM	8280	N	VAL	129	62.311	49.456	8.488	1.00	0.00	RX2	N
ATOM	8281	H	VAL	129	63.222	49.573	8.092	1.00	0.00	RX2	H
ATOM	8282	CA	VAL	129	61.122	49.937	7.782	1.00	0.00	RX2	C
ATOM	8283	CB	VAL	129	61.509	50.946	6.699	1.00	0.00	RX2	C
ATOM	8284	CG1	VAL	129	60.280	51.519	5.989	1.00	0.00	RX2	C
ATOM	8285	CG2	VAL	129	62.386	52.050	7.284	1.00	0.00	RX2	C
ATOM	8286	C	VAL	129	60.349	48.775	7.179	1.00	0.00	RX2	C
ATOM	8287	O	VAL	129	60.914	47.876	6.564	1.00	0.00	RX2	O
ATOM	8288	N	THR	130	59.036	48.815	7.419	1.00	0.00	RX2	N
ATOM	8289	H	THR	130	58.578	49.597	7.843	1.00	0.00	RX2	H
ATOM	8290	CA	THR	130	58.215	47.631	7.193	1.00	0.00	RX2	C
ATOM	8291	CB	THR	130	56.845	48.098	7.628	1.00	0.00	RX2	C
ATOM	8292	OG1	THR	130	56.761	49.500	7.340	1.00	0.00	RX2	O
ATOM	8293	HG1	THR	130	56.010	49.558	6.753	1.00	0.00	RX2	H
ATOM	8294	CG2	THR	130	56.616	47.863	9.123	1.00	0.00	RX2	C

ATOM	8295	C	THR	130	58.281	47.031	5.793	1.00	0.00	RX2	C
ATOM	8296	O	THR	130	58.481	45.831	5.620	1.00	0.00	RX2	O
ATOM	8297	N	GLY	131	58.168	47.923	4.802	1.00	0.00	RX2	N
ATOM	8298	H	GLY	131	57.975	48.884	4.991	1.00	0.00	RX2	H
ATOM	8299	CA	GLY	131	58.272	47.426	3.430	1.00	0.00	RX2	C
ATOM	8300	C	GLY	131	59.625	47.631	2.775	1.00	0.00	RX2	C
ATOM	8301	O	GLY	131	59.781	47.553	1.564	1.00	0.00	RX2	O
ATOM	8302	N	ALA	132	60.615	47.925	3.628	1.00	0.00	RX2	N
ATOM	8303	H	ALA	132	60.522	47.861	4.621	1.00	0.00	RX2	H
ATOM	8304	CA	ALA	132	61.902	48.227	3.015	1.00	0.00	RX2	C
ATOM	8305	CB	ALA	132	62.276	49.694	3.221	1.00	0.00	RX2	C
ATOM	8306	C	ALA	132	63.023	47.356	3.528	1.00	0.00	RX2	C
ATOM	8307	O	ALA	132	63.730	46.686	2.790	1.00	0.00	RX2	O
ATOM	8308	N	SER	133	63.169	47.391	4.850	1.00	0.00	RX2	N
ATOM	8309	H	SER	133	62.601	47.904	5.493	1.00	0.00	RX2	H
ATOM	8310	CA	SER	133	64.280	46.627	5.395	1.00	0.00	RX2	C
ATOM	8311	CB	SER	133	64.547	47.232	6.758	1.00	0.00	RX2	C
ATOM	8312	OG	SER	133	64.227	48.627	6.665	1.00	0.00	RX2	O
ATOM	8313	HG	SER	133	64.913	49.088	7.148	1.00	0.00	RX2	H
ATOM	8314	C	SER	133	64.025	45.134	5.406	1.00	0.00	RX2	C
ATOM	8315	O	SER	133	62.908	44.697	5.676	1.00	0.00	RX2	O
ATOM	8316	N	PRO	134	65.108	44.359	5.142	1.00	0.00	RX2	N
ATOM	8317	CD	PRO	134	66.384	44.822	4.613	1.00	0.00	RX2	C
ATOM	8318	CA	PRO	134	65.092	42.916	5.422	1.00	0.00	RX2	C
ATOM	8319	CB	PRO	134	66.585	42.583	5.401	1.00	0.00	RX2	C
ATOM	8320	CG	PRO	134	67.172	43.541	4.368	1.00	0.00	RX2	C
ATOM	8321	C	PRO	134	64.367	42.545	6.711	1.00	0.00	RX2	C
ATOM	8322	O	PRO	134	64.247	43.355	7.628	1.00	0.00	RX2	O
ATOM	8323	N	GLY	135	63.811	41.333	6.710	1.00	0.00	RX2	N
ATOM	8324	H	GLY	135	64.002	40.621	6.025	1.00	0.00	RX2	H
ATOM	8325	CA	GLY	135	62.921	41.023	7.819	1.00	0.00	RX2	C
ATOM	8326	C	GLY	135	63.410	39.905	8.703	1.00	0.00	RX2	C
ATOM	8327	O	GLY	135	63.226	38.726	8.425	1.00	0.00	RX2	O
ATOM	8328	N	GLY	136	64.029	40.326	9.801	1.00	0.00	RX2	N
ATOM	8329	H	GLY	136	64.202	41.290	10.009	1.00	0.00	RX2	H
ATOM	8330	CA	GLY	136	64.244	39.326	10.835	1.00	0.00	RX2	C
ATOM	8331	C	GLY	136	63.578	39.757	12.117	1.00	0.00	RX2	C
ATOM	8332	O	GLY	136	62.943	40.806	12.180	1.00	0.00	RX2	O
ATOM	8333	N	LEU	137	63.803	38.939	13.151	1.00	0.00	RX2	N
ATOM	8334	H	LEU	137	64.385	38.133	13.025	1.00	0.00	RX2	H
ATOM	8335	CA	LEU	137	63.467	39.449	14.478	1.00	0.00	RX2	C
ATOM	8336	CB	LEU	137	63.229	38.277	15.429	1.00	0.00	RX2	C
ATOM	8337	CG	LEU	137	62.763	38.673	16.829	1.00	0.00	RX2	C
ATOM	8338	CD1	LEU	137	61.499	39.524	16.801	1.00	0.00	RX2	C
ATOM	8339	CD2	LEU	137	62.597	37.448	17.724	1.00	0.00	RX2	C
ATOM	8340	C	LEU	137	64.567	40.374	14.968	1.00	0.00	RX2	C
ATOM	8341	O	LEU	137	65.743	40.124	14.744	1.00	0.00	RX2	O
ATOM	8342	N	ARG	138	64.149	41.484	15.587	1.00	0.00	RX2	N
ATOM	8343	H	ARG	138	63.177	41.630	15.779	1.00	0.00	RX2	H
ATOM	8344	CA	ARG	138	65.140	42.500	15.932	1.00	0.00	RX2	C
ATOM	8345	CB	ARG	138	64.641	43.891	15.520	1.00	0.00	RX2	C
ATOM	8346	CG	ARG	138	65.791	44.889	15.393	1.00	0.00	RX2	C
ATOM	8347	CD	ARG	138	65.380	46.311	15.017	1.00	0.00	RX2	C
ATOM	8348	NE	ARG	138	66.426	47.270	15.376	1.00	0.00	RX2	N
ATOM	8349	HE	ARG	138	66.515	47.482	16.364	1.00	0.00	RX2	H
ATOM	8350	CZ	ARG	138	67.256	47.835	14.447	1.00	0.00	RX2	C
ATOM	8351	NH1	ARG	138	67.133	47.514	13.140	1.00	0.00	RX2	N
ATOM	8352	HH11	ARG	138	67.708	47.956	12.430	1.00	0.00	RX2	H
ATOM	8353	HH12	ARG	138	66.470	46.837	12.819	1.00	0.00	RX2	H
ATOM	8354	NH2	ARG	138	68.194	48.713	14.849	1.00	0.00	RX2	N
ATOM	8355	HH21	ARG	138	68.857	49.122	14.209	1.00	0.00	RX2	H

ATOM	8356	HH22	ARG	138	68.270	48.983	15.824	1.00	0.00	RX2	H
ATOM	8357	C	ARG	138	65.634	42.467	17.366	1.00	0.00	RX2	C
ATOM	8358	O	ARG	138	66.801	42.726	17.660	1.00	0.00	RX2	O
ATOM	8359	N	GLU	139	64.690	42.118	18.250	1.00	0.00	RX2	N
ATOM	8360	H	GLU	139	63.740	41.914	18.012	1.00	0.00	RX2	H
ATOM	8361	CA	GLU	139	65.019	42.074	19.672	1.00	0.00	RX2	C
ATOM	8362	CB	GLU	139	65.102	43.469	20.249	1.00	0.00	RX2	C
ATOM	8363	CG	GLU	139	63.795	44.208	20.029	1.00	0.00	RX2	C
ATOM	8364	CD	GLU	139	64.160	45.520	19.401	1.00	0.00	RX2	C
ATOM	8365	OE1	GLU	139	65.023	45.520	18.531	1.00	0.00	RX2	O
ATOM	8366	OE2	GLU	139	63.584	46.534	19.773	1.00	0.00	RX2	O
ATOM	8367	C	GLU	139	64.012	41.253	20.440	1.00	0.00	RX2	C
ATOM	8368	O	GLU	139	62.946	40.903	19.942	1.00	0.00	RX2	O
ATOM	8369	N	LEU	140	64.408	40.956	21.679	1.00	0.00	RX2	N
ATOM	8370	H	LEU	140	65.197	41.402	22.110	1.00	0.00	RX2	H
ATOM	8371	CA	LEU	140	63.502	40.183	22.512	1.00	0.00	RX2	C
ATOM	8372	CB	LEU	140	64.268	39.072	23.227	1.00	0.00	RX2	C
ATOM	8373	CG	LEU	140	64.821	37.998	22.293	1.00	0.00	RX2	C
ATOM	8374	CD1	LEU	140	65.712	37.004	23.040	1.00	0.00	RX2	C
ATOM	8375	CD2	LEU	140	63.703	37.296	21.520	1.00	0.00	RX2	C
ATOM	8376	C	LEU	140	62.858	41.079	23.541	1.00	0.00	RX2	C
ATOM	8377	O	LEU	140	63.354	41.210	24.646	1.00	0.00	RX2	O
ATOM	8378	N	GLN	141	61.734	41.699	23.158	1.00	0.00	RX2	N
ATOM	8379	H	GLN	141	61.290	41.477	22.290	1.00	0.00	RX2	H
ATOM	8380	CA	GLN	141	61.088	42.573	24.144	1.00	0.00	RX2	C
ATOM	8381	CB	GLN	141	60.141	43.541	23.458	1.00	0.00	RX2	C
ATOM	8382	CG	GLN	141	60.772	44.439	22.401	1.00	0.00	RX2	C
ATOM	8383	CD	GLN	141	59.646	45.176	21.708	1.00	0.00	RX2	C
ATOM	8384	OE1	GLN	141	59.587	46.397	21.652	1.00	0.00	RX2	O
ATOM	8385	NE2	GLN	141	58.722	44.347	21.189	1.00	0.00	RX2	N
ATOM	8386	HE21	GLN	141	58.822	43.354	21.288	1.00	0.00	RX2	H
ATOM	8387	HE22	GLN	141	57.931	44.712	20.698	1.00	0.00	RX2	H
ATOM	8388	C	GLN	141	60.303	41.813	25.204	1.00	0.00	RX2	C
ATOM	8389	O	GLN	141	59.079	41.759	25.177	1.00	0.00	RX2	O
ATOM	8390	N	LEU	142	61.057	41.187	26.115	1.00	0.00	RX2	N
ATOM	8391	H	LEU	142	62.053	41.295	26.128	1.00	0.00	RX2	H
ATOM	8392	CA	LEU	142	60.381	40.333	27.088	1.00	0.00	RX2	C
ATOM	8393	CB	LEU	142	60.799	38.856	26.982	1.00	0.00	RX2	C
ATOM	8394	CG	LEU	142	61.376	38.320	25.661	1.00	0.00	RX2	C
ATOM	8395	CD1	LEU	142	61.884	36.888	25.840	1.00	0.00	RX2	C
ATOM	8396	CD2	LEU	142	60.432	38.390	24.462	1.00	0.00	RX2	C
ATOM	8397	C	LEU	142	60.647	40.794	28.508	1.00	0.00	RX2	C
ATOM	8398	O	LEU	142	61.083	40.027	29.358	1.00	0.00	RX2	O
ATOM	8399	N	ARG	143	60.429	42.105	28.728	1.00	0.00	RX2	N
ATOM	8400	H	ARG	143	60.032	42.682	28.009	1.00	0.00	RX2	H
ATOM	8401	CA	ARG	143	61.070	42.730	29.893	1.00	0.00	RX2	C
ATOM	8402	CB	ARG	143	60.728	44.215	30.011	1.00	0.00	RX2	C
ATOM	8403	CG	ARG	143	59.357	44.498	30.610	1.00	0.00	RX2	C
ATOM	8404	CD	ARG	143	59.013	45.978	30.603	1.00	0.00	RX2	C
ATOM	8405	NE	ARG	143	58.832	46.455	29.238	1.00	0.00	RX2	N
ATOM	8406	HE	ARG	143	59.085	45.859	28.458	1.00	0.00	RX2	H
ATOM	8407	CZ	ARG	143	58.377	47.725	29.063	1.00	0.00	RX2	C
ATOM	8408	NH1	ARG	143	58.171	48.517	30.134	1.00	0.00	RX2	N
ATOM	8409	HH11	ARG	143	57.882	49.486	29.998	1.00	0.00	RX2	H
ATOM	8410	HH12	ARG	143	58.325	48.235	31.079	1.00	0.00	RX2	H
ATOM	8411	NH2	ARG	143	58.148	48.184	27.823	1.00	0.00	RX2	N
ATOM	8412	HH21	ARG	143	57.757	49.108	27.688	1.00	0.00	RX2	H
ATOM	8413	HH22	ARG	143	58.382	47.640	27.005	1.00	0.00	RX2	H
ATOM	8414	C	ARG	143	61.021	42.060	31.260	1.00	0.00	RX2	C
ATOM	8415	O	ARG	143	61.957	42.174	32.047	1.00	0.00	RX2	O
ATOM	8416	N	SER	144	59.895	41.390	31.525	1.00	0.00	RX2	N

ATOM	8417	H	SER	144	59.214	41.193	30.819	1.00	0.00	RX2	H
ATOM	8418	CA	SER	144	59.723	40.884	32.883	1.00	0.00	RX2	C
ATOM	8419	CB	SER	144	58.256	41.080	33.216	1.00	0.00	RX2	C
ATOM	8420	OG	SER	144	57.925	42.405	32.770	1.00	0.00	RX2	O
ATOM	8421	HG	SER	144	57.803	42.335	31.826	1.00	0.00	RX2	H
ATOM	8422	C	SER	144	60.321	39.514	33.175	1.00	0.00	RX2	C
ATOM	8423	O	SER	144	60.251	39.009	34.294	1.00	0.00	RX2	O
ATOM	8424	N	LEU	145	60.919	38.930	32.122	1.00	0.00	RX2	N
ATOM	8425	H	LEU	145	61.005	39.385	31.235	1.00	0.00	RX2	H
ATOM	8426	CA	LEU	145	61.369	37.550	32.265	1.00	0.00	RX2	C
ATOM	8427	CB	LEU	145	61.794	37.013	30.899	1.00	0.00	RX2	C
ATOM	8428	CG	LEU	145	62.070	35.511	30.872	1.00	0.00	RX2	C
ATOM	8429	CD1	LEU	145	60.895	34.702	31.415	1.00	0.00	RX2	C
ATOM	8430	CD2	LEU	145	62.493	35.043	29.479	1.00	0.00	RX2	C
ATOM	8431	C	LEU	145	62.441	37.385	33.322	1.00	0.00	RX2	C
ATOM	8432	O	LEU	145	63.514	37.974	33.270	1.00	0.00	RX2	O
ATOM	8433	N	THR	146	62.060	36.578	34.313	1.00	0.00	RX2	N
ATOM	8434	H	THR	146	61.155	36.149	34.360	1.00	0.00	RX2	H
ATOM	8435	CA	THR	146	62.931	36.480	35.475	1.00	0.00	RX2	C
ATOM	8436	CB	THR	146	62.129	37.086	36.608	1.00	0.00	RX2	C
ATOM	8437	OG1	THR	146	60.750	37.096	36.215	1.00	0.00	RX2	O
ATOM	8438	HG1	THR	146	60.654	37.780	35.557	1.00	0.00	RX2	H
ATOM	8439	CG2	THR	146	62.594	38.506	36.941	1.00	0.00	RX2	C
ATOM	8440	C	THR	146	63.417	35.072	35.759	1.00	0.00	RX2	C
ATOM	8441	O	THR	146	64.602	34.833	35.958	1.00	0.00	RX2	O
ATOM	8442	N	GLU	147	62.450	34.142	35.722	1.00	0.00	RX2	N
ATOM	8443	H	GLU	147	61.496	34.360	35.512	1.00	0.00	RX2	H
ATOM	8444	CA	GLU	147	62.878	32.752	35.835	1.00	0.00	RX2	C
ATOM	8445	CB	GLU	147	62.312	32.075	37.090	1.00	0.00	RX2	C
ATOM	8446	CG	GLU	147	62.498	32.801	38.430	1.00	0.00	RX2	C
ATOM	8447	CD	GLU	147	63.958	32.900	38.838	1.00	0.00	RX2	C
ATOM	8448	OE1	GLU	147	64.425	33.997	39.114	1.00	0.00	RX2	O
ATOM	8449	OE2	GLU	147	64.655	31.890	38.911	1.00	0.00	RX2	O
ATOM	8450	C	GLU	147	62.480	31.937	34.617	1.00	0.00	RX2	C
ATOM	8451	O	GLU	147	61.384	32.050	34.077	1.00	0.00	RX2	O
ATOM	8452	N	ILE	148	63.424	31.079	34.225	1.00	0.00	RX2	N
ATOM	8453	H	ILE	148	64.339	31.167	34.619	1.00	0.00	RX2	H
ATOM	8454	CA	ILE	148	63.133	30.037	33.243	1.00	0.00	RX2	C
ATOM	8455	CB	ILE	148	64.048	30.200	32.028	1.00	0.00	RX2	C
ATOM	8456	CG2	ILE	148	63.849	29.074	31.016	1.00	0.00	RX2	C
ATOM	8457	CG1	ILE	148	63.852	31.575	31.391	1.00	0.00	RX2	C
ATOM	8458	CD1	ILE	148	64.830	31.830	30.247	1.00	0.00	RX2	C
ATOM	8459	C	ILE	148	63.349	28.688	33.904	1.00	0.00	RX2	C
ATOM	8460	O	ILE	148	64.481	28.243	34.073	1.00	0.00	RX2	O
ATOM	8461	N	LEU	149	62.215	28.099	34.330	1.00	0.00	RX2	N
ATOM	8462	H	LEU	149	61.342	28.509	34.073	1.00	0.00	RX2	H
ATOM	8463	CA	LEU	149	62.252	26.990	35.286	1.00	0.00	RX2	C
ATOM	8464	CB	LEU	149	60.866	26.445	35.632	1.00	0.00	RX2	C
ATOM	8465	CG	LEU	149	59.956	27.430	36.365	1.00	0.00	RX2	C
ATOM	8466	CD1	LEU	149	58.649	26.764	36.793	1.00	0.00	RX2	C
ATOM	8467	CD2	LEU	149	60.647	28.072	37.564	1.00	0.00	RX2	C
ATOM	8468	C	LEU	149	63.188	25.840	34.985	1.00	0.00	RX2	C
ATOM	8469	O	LEU	149	64.158	25.629	35.706	1.00	0.00	RX2	O
ATOM	8470	N	LYS	150	62.861	25.093	33.921	1.00	0.00	RX2	N
ATOM	8471	H	LYS	150	62.063	25.251	33.329	1.00	0.00	RX2	H
ATOM	8472	CA	LYS	150	63.776	24.024	33.532	1.00	0.00	RX2	C
ATOM	8473	CB	LYS	150	63.353	22.665	34.110	1.00	0.00	RX2	C
ATOM	8474	CG	LYS	150	63.606	22.615	35.621	1.00	0.00	RX2	C
ATOM	8475	CD	LYS	150	63.229	21.328	36.344	1.00	0.00	RX2	C
ATOM	8476	CE	LYS	150	63.483	21.473	37.849	1.00	0.00	RX2	C
ATOM	8477	NZ	LYS	150	64.886	21.838	38.089	1.00	0.00	RX2	N

ATOM	8478	HZ1	LYS	150	65.136	21.770	39.093	1.00	0.00	RX2	H
ATOM	8479	HZ2	LYS	150	65.516	21.214	37.546	1.00	0.00	RX2	H
ATOM	8480	HZ3	LYS	150	65.099	22.819	37.805	1.00	0.00	RX2	H
ATOM	8481	C	LYS	150	63.946	23.968	32.034	1.00	0.00	RX2	C
ATOM	8482	O	LYS	150	63.022	24.233	31.280	1.00	0.00	RX2	O
ATOM	8483	N	GLY	151	65.176	23.618	31.642	1.00	0.00	RX2	N
ATOM	8484	H	GLY	151	65.935	23.605	32.294	1.00	0.00	RX2	H
ATOM	8485	CA	GLY	151	65.436	23.570	30.207	1.00	0.00	RX2	C
ATOM	8486	C	GLY	151	66.237	24.765	29.739	1.00	0.00	RX2	C
ATOM	8487	O	GLY	151	66.178	25.845	30.315	1.00	0.00	RX2	O
ATOM	8488	N	GLY	152	67.018	24.508	28.683	1.00	0.00	RX2	N
ATOM	8489	H	GLY	152	66.940	23.640	28.187	1.00	0.00	RX2	H
ATOM	8490	CA	GLY	152	67.982	25.532	28.291	1.00	0.00	RX2	C
ATOM	8491	C	GLY	152	67.457	26.600	27.356	1.00	0.00	RX2	C
ATOM	8492	O	GLY	152	66.366	26.504	26.801	1.00	0.00	RX2	O
ATOM	8493	N	VAL	153	68.306	27.623	27.196	1.00	0.00	RX2	N
ATOM	8494	H	VAL	153	69.226	27.621	27.592	1.00	0.00	RX2	H
ATOM	8495	CA	VAL	153	68.018	28.626	26.178	1.00	0.00	RX2	C
ATOM	8496	CB	VAL	153	68.276	30.039	26.706	1.00	0.00	RX2	C
ATOM	8497	CG1	VAL	153	67.992	31.092	25.637	1.00	0.00	RX2	C
ATOM	8498	CG2	VAL	153	67.445	30.305	27.958	1.00	0.00	RX2	C
ATOM	8499	C	VAL	153	68.846	28.353	24.936	1.00	0.00	RX2	C
ATOM	8500	O	VAL	153	70.024	28.676	24.841	1.00	0.00	RX2	O
ATOM	8501	N	LEU	154	68.161	27.709	23.988	1.00	0.00	RX2	N
ATOM	8502	H	LEU	154	67.169	27.622	24.086	1.00	0.00	RX2	H
ATOM	8503	CA	LEU	154	68.784	27.523	22.686	1.00	0.00	RX2	C
ATOM	8504	CB	LEU	154	68.253	26.256	22.021	1.00	0.00	RX2	C
ATOM	8505	CG	LEU	154	68.924	25.921	20.689	1.00	0.00	RX2	C
ATOM	8506	CD1	LEU	154	70.420	25.679	20.852	1.00	0.00	RX2	C
ATOM	8507	CD2	LEU	154	68.241	24.750	19.982	1.00	0.00	RX2	C
ATOM	8508	C	LEU	154	68.497	28.708	21.795	1.00	0.00	RX2	C
ATOM	8509	O	LEU	154	67.424	28.828	21.217	1.00	0.00	RX2	O
ATOM	8510	N	ILE	155	69.497	29.576	21.685	1.00	0.00	RX2	N
ATOM	8511	H	ILE	155	70.389	29.465	22.131	1.00	0.00	RX2	H
ATOM	8512	CA	ILE	155	69.371	30.497	20.565	1.00	0.00	RX2	C
ATOM	8513	CB	ILE	155	69.871	31.885	20.976	1.00	0.00	RX2	C
ATOM	8514	CG2	ILE	155	69.583	32.960	19.924	1.00	0.00	RX2	C
ATOM	8515	CG1	ILE	155	69.270	32.250	22.332	1.00	0.00	RX2	C
ATOM	8516	CD1	ILE	155	69.928	33.466	22.981	1.00	0.00	RX2	C
ATOM	8517	C	ILE	155	70.153	29.902	19.407	1.00	0.00	RX2	C
ATOM	8518	O	ILE	155	71.169	29.244	19.607	1.00	0.00	RX2	O
ATOM	8519	N	GLN	156	69.630	30.118	18.198	1.00	0.00	RX2	N
ATOM	8520	H	GLN	156	68.791	30.651	18.057	1.00	0.00	RX2	H
ATOM	8521	CA	GLN	156	70.365	29.723	17.002	1.00	0.00	RX2	C
ATOM	8522	CB	GLN	156	69.934	28.343	16.507	1.00	0.00	RX2	C
ATOM	8523	CG	GLN	156	70.664	27.182	17.186	1.00	0.00	RX2	C
ATOM	8524	CD	GLN	156	72.121	27.216	16.778	1.00	0.00	RX2	C
ATOM	8525	OE1	GLN	156	72.508	26.605	15.781	1.00	0.00	RX2	O
ATOM	8526	NE2	GLN	156	72.890	27.992	17.560	1.00	0.00	RX2	N
ATOM	8527	HE21	GLN	156	72.498	28.411	18.388	1.00	0.00	RX2	H
ATOM	8528	HE22	GLN	156	73.833	28.265	17.363	1.00	0.00	RX2	H
ATOM	8529	C	GLN	156	70.136	30.762	15.940	1.00	0.00	RX2	C
ATOM	8530	O	GLN	156	69.047	31.314	15.870	1.00	0.00	RX2	O
ATOM	8531	N	ARG	157	71.192	31.007	15.146	1.00	0.00	RX2	N
ATOM	8532	H	ARG	157	72.084	30.660	15.447	1.00	0.00	RX2	H
ATOM	8533	CA	ARG	157	71.151	31.914	13.994	1.00	0.00	RX2	C
ATOM	8534	CB	ARG	157	70.995	31.125	12.702	1.00	0.00	RX2	C
ATOM	8535	CG	ARG	157	72.267	30.328	12.425	1.00	0.00	RX2	C
ATOM	8536	CD	ARG	157	72.038	29.234	11.384	1.00	0.00	RX2	C
ATOM	8537	NE	ARG	157	70.946	28.370	11.823	1.00	0.00	RX2	N
ATOM	8538	HE	ARG	157	70.003	28.592	11.527	1.00	0.00	RX2	H

ATOM	8539	CZ	ARG	157	71.136	27.419	12.780	1.00	0.00	RX2	C
ATOM	8540	NH1	ARG	157	72.384	27.143	13.215	1.00	0.00	RX2	N
ATOM	8541	HH11	ARG	157	72.547	26.562	14.025	1.00	0.00	RX2	H
ATOM	8542	HH12	ARG	157	73.193	27.538	12.775	1.00	0.00	RX2	H
ATOM	8543	NH2	ARG	157	70.062	26.790	13.287	1.00	0.00	RX2	N
ATOM	8544	HH21	ARG	157	70.089	26.033	13.939	1.00	0.00	RX2	H
ATOM	8545	HH22	ARG	157	69.143	27.139	13.009	1.00	0.00	RX2	H
ATOM	8546	C	ARG	157	70.161	33.057	14.068	1.00	0.00	RX2	C
ATOM	8547	O	ARG	157	69.114	33.090	13.429	1.00	0.00	RX2	O
ATOM	8548	N	ASN	158	70.545	34.016	14.911	1.00	0.00	RX2	N
ATOM	8549	H	ASN	158	71.419	33.965	15.405	1.00	0.00	RX2	H
ATOM	8550	CA	ASN	158	69.618	35.135	15.037	1.00	0.00	RX2	C
ATOM	8551	CB	ASN	158	69.011	35.141	16.436	1.00	0.00	RX2	C
ATOM	8552	CG	ASN	158	67.529	34.831	16.344	1.00	0.00	RX2	C
ATOM	8553	OD1	ASN	158	66.663	35.693	16.452	1.00	0.00	RX2	O
ATOM	8554	ND2	ASN	158	67.264	33.529	16.156	1.00	0.00	RX2	N
ATOM	8555	HD21	ASN	158	68.014	32.864	16.075	1.00	0.00	RX2	H
ATOM	8556	HD22	ASN	158	66.346	33.140	16.095	1.00	0.00	RX2	H
ATOM	8557	C	ASN	158	70.226	36.473	14.650	1.00	0.00	RX2	C
ATOM	8558	O	ASN	158	70.650	37.259	15.489	1.00	0.00	RX2	O
ATOM	8559	N	PRO	159	70.271	36.710	13.313	1.00	0.00	RX2	N
ATOM	8560	CD	PRO	159	69.602	35.950	12.262	1.00	0.00	RX2	C
ATOM	8561	CA	PRO	159	71.131	37.780	12.795	1.00	0.00	RX2	C
ATOM	8562	CB	PRO	159	71.222	37.421	11.309	1.00	0.00	RX2	C
ATOM	8563	CG	PRO	159	69.917	36.702	10.978	1.00	0.00	RX2	C
ATOM	8564	C	PRO	159	70.664	39.197	13.087	1.00	0.00	RX2	C
ATOM	8565	O	PRO	159	71.417	40.058	13.532	1.00	0.00	RX2	O
ATOM	8566	N	GLN	160	69.368	39.413	12.834	1.00	0.00	RX2	N
ATOM	8567	H	GLN	160	68.751	38.688	12.529	1.00	0.00	RX2	H
ATOM	8568	CA	GLN	160	68.850	40.735	13.161	1.00	0.00	RX2	C
ATOM	8569	CB	GLN	160	67.632	41.021	12.285	1.00	0.00	RX2	C
ATOM	8570	CG	GLN	160	67.342	42.506	12.082	1.00	0.00	RX2	C
ATOM	8571	CD	GLN	160	65.913	42.647	11.611	1.00	0.00	RX2	C
ATOM	8572	OE1	GLN	160	65.589	42.931	10.464	1.00	0.00	RX2	O
ATOM	8573	NE2	GLN	160	65.057	42.410	12.599	1.00	0.00	RX2	N
ATOM	8574	HE21	GLN	160	65.426	42.166	13.496	1.00	0.00	RX2	H
ATOM	8575	HE22	GLN	160	64.056	42.394	12.504	1.00	0.00	RX2	H
ATOM	8576	C	GLN	160	68.549	40.917	14.648	1.00	0.00	RX2	C
ATOM	8577	O	GLN	160	68.149	41.982	15.106	1.00	0.00	RX2	O
ATOM	8578	N	LEU	161	68.756	39.818	15.392	1.00	0.00	RX2	N
ATOM	8579	H	LEU	161	69.281	39.032	15.075	1.00	0.00	RX2	H
ATOM	8580	CA	LEU	161	68.447	39.877	16.811	1.00	0.00	RX2	C
ATOM	8581	CB	LEU	161	68.078	38.490	17.315	1.00	0.00	RX2	C
ATOM	8582	CG	LEU	161	67.429	38.469	18.694	1.00	0.00	RX2	C
ATOM	8583	CD1	LEU	161	65.983	38.914	18.592	1.00	0.00	RX2	C
ATOM	8584	CD2	LEU	161	67.512	37.108	19.377	1.00	0.00	RX2	C
ATOM	8585	C	LEU	161	69.624	40.389	17.603	1.00	0.00	RX2	C
ATOM	8586	O	LEU	161	70.718	39.837	17.572	1.00	0.00	RX2	O
ATOM	8587	N	CYS	162	69.348	41.464	18.335	1.00	0.00	RX2	N
ATOM	8588	H	CYS	162	68.425	41.851	18.353	1.00	0.00	RX2	H
ATOM	8589	CA	CYS	162	70.312	41.775	19.378	1.00	0.00	RX2	C
ATOM	8590	CB	CYS	162	70.626	43.262	19.397	1.00	0.00	RX2	C
ATOM	8591	SG	CYS	162	71.736	43.720	18.052	1.00	0.00	RX2	S
ATOM	8592	C	CYS	162	69.823	41.269	20.715	1.00	0.00	RX2	C
ATOM	8593	O	CYS	162	68.786	40.624	20.807	1.00	0.00	RX2	O
ATOM	8594	N	TYR	163	70.618	41.599	21.749	1.00	0.00	RX2	N
ATOM	8595	H	TYR	163	71.517	42.017	21.616	1.00	0.00	RX2	H
ATOM	8596	CA	TYR	163	70.198	41.410	23.141	1.00	0.00	RX2	C
ATOM	8597	CB	TYR	163	68.867	42.117	23.426	1.00	0.00	RX2	C
ATOM	8598	CG	TYR	163	69.019	43.563	23.016	1.00	0.00	RX2	C
ATOM	8599	CD1	TYR	163	69.977	44.358	23.630	1.00	0.00	RX2	C

ATOM	8600	CE1	TYR	163	70.174	45.668	23.217	1.00	0.00	RX2	C
ATOM	8601	CD2	TYR	163	68.226	44.097	22.009	1.00	0.00	RX2	C
ATOM	8602	CE2	TYR	163	68.424	45.406	21.592	1.00	0.00	RX2	C
ATOM	8603	CZ	TYR	163	69.409	46.187	22.184	1.00	0.00	RX2	C
ATOM	8604	OH	TYR	163	69.638	47.470	21.737	1.00	0.00	RX2	O
ATOM	8605	HH	TYR	163	68.904	47.716	21.174	1.00	0.00	RX2	H
ATOM	8606	C	TYR	163	70.268	40.009	23.730	1.00	0.00	RX2	C
ATOM	8607	O	TYR	163	70.116	39.804	24.926	1.00	0.00	RX2	O
ATOM	8608	N	GLN	164	70.614	39.051	22.847	1.00	0.00	RX2	N
ATOM	8609	H	GLN	164	70.486	39.246	21.876	1.00	0.00	RX2	H
ATOM	8610	CA	GLN	164	70.992	37.706	23.311	1.00	0.00	RX2	C
ATOM	8611	CB	GLN	164	71.606	36.851	22.192	1.00	0.00	RX2	C
ATOM	8612	CG	GLN	164	70.794	36.594	20.923	1.00	0.00	RX2	C
ATOM	8613	CD	GLN	164	71.133	37.618	19.863	1.00	0.00	RX2	C
ATOM	8614	OE1	GLN	164	71.200	38.813	20.138	1.00	0.00	RX2	O
ATOM	8615	NE2	GLN	164	71.317	37.100	18.639	1.00	0.00	RX2	N
ATOM	8616	HE21	GLN	164	71.461	36.108	18.547	1.00	0.00	RX2	H
ATOM	8617	HE22	GLN	164	71.319	37.642	17.791	1.00	0.00	RX2	H
ATOM	8618	C	GLN	164	71.994	37.651	24.462	1.00	0.00	RX2	C
ATOM	8619	O	GLN	164	72.002	36.730	25.266	1.00	0.00	RX2	O
ATOM	8620	N	ASP	165	72.870	38.660	24.439	1.00	0.00	RX2	N
ATOM	8621	H	ASP	165	72.735	39.452	23.848	1.00	0.00	RX2	H
ATOM	8622	CA	ASP	165	73.999	38.761	25.361	1.00	0.00	RX2	C
ATOM	8623	CB	ASP	165	75.274	39.195	24.612	1.00	0.00	RX2	C
ATOM	8624	CG	ASP	165	75.016	40.180	23.471	1.00	0.00	RX2	C
ATOM	8625	OD1	ASP	165	74.044	40.933	23.484	1.00	0.00	RX2	O
ATOM	8626	OD2	ASP	165	75.748	40.153	22.488	1.00	0.00	RX2	O
ATOM	8627	C	ASP	165	73.735	39.678	26.541	1.00	0.00	RX2	C
ATOM	8628	O	ASP	165	73.937	39.327	27.698	1.00	0.00	RX2	O
ATOM	8629	N	THR	166	73.240	40.877	26.197	1.00	0.00	RX2	N
ATOM	8630	H	THR	166	73.095	41.145	25.243	1.00	0.00	RX2	H
ATOM	8631	CA	THR	166	72.896	41.879	27.208	1.00	0.00	RX2	C
ATOM	8632	CB	THR	166	72.372	43.065	26.429	1.00	0.00	RX2	C
ATOM	8633	OG1	THR	166	71.560	42.563	25.373	1.00	0.00	RX2	O
ATOM	8634	HG1	THR	166	70.658	42.717	25.650	1.00	0.00	RX2	H
ATOM	8635	CG2	THR	166	73.509	43.902	25.840	1.00	0.00	RX2	C
ATOM	8636	C	THR	166	71.940	41.350	28.262	1.00	0.00	RX2	C
ATOM	8637	O	THR	166	72.042	41.586	29.464	1.00	0.00	RX2	O
ATOM	8638	N	ILE	167	70.998	40.560	27.733	1.00	0.00	RX2	N
ATOM	8639	H	ILE	167	70.979	40.361	26.750	1.00	0.00	RX2	H
ATOM	8640	CA	ILE	167	70.189	39.764	28.638	1.00	0.00	RX2	C
ATOM	8641	CB	ILE	167	68.890	39.365	27.955	1.00	0.00	RX2	C
ATOM	8642	CG2	ILE	167	68.054	38.424	28.813	1.00	0.00	RX2	C
ATOM	8643	CG1	ILE	167	68.128	40.649	27.655	1.00	0.00	RX2	C
ATOM	8644	CD1	ILE	167	67.911	41.456	28.938	1.00	0.00	RX2	C
ATOM	8645	C	ILE	167	70.958	38.579	29.184	1.00	0.00	RX2	C
ATOM	8646	O	ILE	167	70.968	37.466	28.675	1.00	0.00	RX2	O
ATOM	8647	N	LEU	168	71.615	38.893	30.303	1.00	0.00	RX2	N
ATOM	8648	H	LEU	168	71.642	39.854	30.582	1.00	0.00	RX2	H
ATOM	8649	CA	LEU	168	72.393	37.853	30.959	1.00	0.00	RX2	C
ATOM	8650	CB	LEU	168	73.286	38.478	32.028	1.00	0.00	RX2	C
ATOM	8651	CG	LEU	168	74.428	37.553	32.443	1.00	0.00	RX2	C
ATOM	8652	CD1	LEU	168	75.225	37.062	31.232	1.00	0.00	RX2	C
ATOM	8653	CD2	LEU	168	75.326	38.198	33.499	1.00	0.00	RX2	C
ATOM	8654	C	LEU	168	71.554	36.708	31.508	1.00	0.00	RX2	C
ATOM	8655	O	LEU	168	71.046	36.732	32.625	1.00	0.00	RX2	O
ATOM	8656	N	TRP	169	71.468	35.664	30.667	1.00	0.00	RX2	N
ATOM	8657	H	TRP	169	71.749	35.834	29.718	1.00	0.00	RX2	H
ATOM	8658	CA	TRP	169	70.703	34.468	31.037	1.00	0.00	RX2	C
ATOM	8659	CB	TRP	169	70.704	33.429	29.916	1.00	0.00	RX2	C
ATOM	8660	CG	TRP	169	70.226	34.065	28.636	1.00	0.00	RX2	C

ATOM	8661	CD2	TRP	169	68.884	34.452	28.270	1.00	0.00	RX2	C
ATOM	8662	CE2	TRP	169	68.946	35.029	26.980	1.00	0.00	RX2	C
ATOM	8663	CE3	TRP	169	67.663	34.361	28.927	1.00	0.00	RX2	C
ATOM	8664	CD1	TRP	169	71.022	34.429	27.544	1.00	0.00	RX2	C
ATOM	8665	NE1	TRP	169	70.274	35.000	26.568	1.00	0.00	RX2	N
ATOM	8666	HE1	TRP	169	70.646	35.395	25.748	1.00	0.00	RX2	H
ATOM	8667	CZ2	TRP	169	67.785	35.501	26.381	1.00	0.00	RX2	C
ATOM	8668	CZ3	TRP	169	66.509	34.838	28.317	1.00	0.00	RX2	C
ATOM	8669	CH2	TRP	169	66.570	35.406	27.049	1.00	0.00	RX2	C
ATOM	8670	C	TRP	169	71.119	33.814	32.343	1.00	0.00	RX2	C
ATOM	8671	O	TRP	169	70.328	33.180	33.024	1.00	0.00	RX2	O
ATOM	8672	N	LYS	170	72.387	34.069	32.713	1.00	0.00	RX2	N
ATOM	8673	H	LYS	170	72.970	34.566	32.074	1.00	0.00	RX2	H
ATOM	8674	CA	LYS	170	72.900	33.698	34.039	1.00	0.00	RX2	C
ATOM	8675	CB	LYS	170	74.310	34.277	34.201	1.00	0.00	RX2	C
ATOM	8676	CG	LYS	170	75.125	33.758	35.392	1.00	0.00	RX2	C
ATOM	8677	CD	LYS	170	75.876	34.879	36.120	1.00	0.00	RX2	C
ATOM	8678	CE	LYS	170	75.248	35.299	37.460	1.00	0.00	RX2	C
ATOM	8679	NZ	LYS	170	73.824	35.610	37.302	1.00	0.00	RX2	N
ATOM	8680	HZ1	LYS	170	73.441	36.221	38.056	1.00	0.00	RX2	H
ATOM	8681	HZ2	LYS	170	73.637	36.184	36.451	1.00	0.00	RX2	H
ATOM	8682	HZ3	LYS	170	73.234	34.753	37.247	1.00	0.00	RX2	H
ATOM	8683	C	LYS	170	72.043	34.112	35.242	1.00	0.00	RX2	C
ATOM	8684	O	LYS	170	72.252	33.675	36.370	1.00	0.00	RX2	O
ATOM	8685	N	ASP	171	71.108	35.026	34.988	1.00	0.00	RX2	N
ATOM	8686	H	ASP	171	71.023	35.476	34.099	1.00	0.00	RX2	H
ATOM	8687	CA	ASP	171	70.235	35.444	36.080	1.00	0.00	RX2	C
ATOM	8688	CB	ASP	171	70.364	36.956	36.292	1.00	0.00	RX2	C
ATOM	8689	CG	ASP	171	71.824	37.332	36.486	1.00	0.00	RX2	C
ATOM	8690	OD1	ASP	171	72.245	37.597	37.603	1.00	0.00	RX2	O
ATOM	8691	OD2	ASP	171	72.598	37.324	35.534	1.00	0.00	RX2	O
ATOM	8692	C	ASP	171	68.794	35.036	35.847	1.00	0.00	RX2	C
ATOM	8693	O	ASP	171	68.016	34.829	36.770	1.00	0.00	RX2	O
ATOM	8694	N	ILE	172	68.482	34.927	34.548	1.00	0.00	RX2	N
ATOM	8695	H	ILE	172	69.182	34.949	33.838	1.00	0.00	RX2	H
ATOM	8696	CA	ILE	172	67.101	34.658	34.157	1.00	0.00	RX2	C
ATOM	8697	CB	ILE	172	66.823	35.451	32.882	1.00	0.00	RX2	C
ATOM	8698	CG2	ILE	172	65.405	35.278	32.346	1.00	0.00	RX2	C
ATOM	8699	CG1	ILE	172	67.127	36.917	33.193	1.00	0.00	RX2	C
ATOM	8700	CD1	ILE	172	66.702	37.862	32.078	1.00	0.00	RX2	C
ATOM	8701	C	ILE	172	66.763	33.173	34.049	1.00	0.00	RX2	C
ATOM	8702	O	ILE	172	65.618	32.738	34.054	1.00	0.00	RX2	O
ATOM	8703	N	PHE	173	67.832	32.362	34.021	1.00	0.00	RX2	N
ATOM	8704	H	PHE	173	68.764	32.717	34.009	1.00	0.00	RX2	H
ATOM	8705	CA	PHE	173	67.585	30.953	34.319	1.00	0.00	RX2	C
ATOM	8706	CB	PHE	173	68.872	30.135	34.224	1.00	0.00	RX2	C
ATOM	8707	CG	PHE	173	69.137	29.702	32.804	1.00	0.00	RX2	C
ATOM	8708	CD1	PHE	173	68.235	28.865	32.160	1.00	0.00	RX2	C
ATOM	8709	CD2	PHE	173	70.293	30.118	32.153	1.00	0.00	RX2	C
ATOM	8710	CE1	PHE	173	68.503	28.417	30.873	1.00	0.00	RX2	C
ATOM	8711	CE2	PHE	173	70.561	29.673	30.865	1.00	0.00	RX2	C
ATOM	8712	CZ	PHE	173	69.671	28.813	30.233	1.00	0.00	RX2	C
ATOM	8713	C	PHE	173	67.038	30.804	35.721	1.00	0.00	RX2	C
ATOM	8714	O	PHE	173	67.420	31.530	36.638	1.00	0.00	RX2	O
ATOM	8715	N	HIS	174	66.119	29.840	35.854	1.00	0.00	RX2	N
ATOM	8716	H	HIS	174	65.882	29.215	35.110	1.00	0.00	RX2	H
ATOM	8717	CA	HIS	174	65.663	29.592	37.215	1.00	0.00	RX2	C
ATOM	8718	CB	HIS	174	64.483	28.631	37.239	1.00	0.00	RX2	C
ATOM	8719	CG	HIS	174	63.995	28.356	38.640	1.00	0.00	RX2	C
ATOM	8720	ND1	HIS	174	63.679	29.306	39.537	1.00	0.00	RX2	N
ATOM	8721	HD1	HIS	174	63.684	30.283	39.389	1.00	0.00	RX2	H

ATOM	8722	CD2	HIS	174	63.804	27.104	39.229	1.00	0.00	RX2	C
ATOM	8723	NE2	HIS	174	63.381	27.312	40.500	1.00	0.00	RX2	N
ATOM	8724	CE1	HIS	174	63.299	28.671	40.689	1.00	0.00	RX2	C
ATOM	8725	C	HIS	174	66.792	29.085	38.073	1.00	0.00	RX2	C
ATOM	8726	O	HIS	174	67.677	28.366	37.632	1.00	0.00	RX2	O
ATOM	8727	N	LYS	175	66.724	29.508	39.332	1.00	0.00	RX2	N
ATOM	8728	H	LYS	175	65.937	30.068	39.602	1.00	0.00	RX2	H
ATOM	8729	CA	LYS	175	67.848	29.187	40.207	1.00	0.00	RX2	C
ATOM	8730	CB	LYS	175	67.739	30.050	41.466	1.00	0.00	RX2	C
ATOM	8731	CG	LYS	175	67.368	31.499	41.099	1.00	0.00	RX2	C
ATOM	8732	CD	LYS	175	68.385	32.199	40.186	1.00	0.00	RX2	C
ATOM	8733	CE	LYS	175	67.905	33.537	39.601	1.00	0.00	RX2	C
ATOM	8734	NZ	LYS	175	66.899	33.345	38.547	1.00	0.00	RX2	N
ATOM	8735	HZ1	LYS	175	66.809	34.196	37.951	1.00	0.00	RX2	H
ATOM	8736	HZ2	LYS	175	67.128	32.563	37.908	1.00	0.00	RX2	H
ATOM	8737	HZ3	LYS	175	65.950	33.163	38.942	1.00	0.00	RX2	H
ATOM	8738	C	LYS	175	68.038	27.691	40.470	1.00	0.00	RX2	C
ATOM	8739	O	LYS	175	69.107	27.219	40.821	1.00	0.00	RX2	O
ATOM	8740	N	ASN	176	66.941	26.954	40.220	1.00	0.00	RX2	N
ATOM	8741	H	ASN	176	66.105	27.407	39.922	1.00	0.00	RX2	H
ATOM	8742	CA	ASN	176	67.026	25.490	40.228	1.00	0.00	RX2	C
ATOM	8743	CB	ASN	176	65.902	24.831	41.034	1.00	0.00	RX2	C
ATOM	8744	CG	ASN	176	66.022	25.100	42.514	1.00	0.00	RX2	C
ATOM	8745	OD1	ASN	176	66.813	24.487	43.218	1.00	0.00	RX2	O
ATOM	8746	ND2	ASN	176	65.160	26.037	42.953	1.00	0.00	RX2	N
ATOM	8747	HD21	ASN	176	64.516	26.483	42.326	1.00	0.00	RX2	H
ATOM	8748	HD22	ASN	176	65.137	26.301	43.917	1.00	0.00	RX2	H
ATOM	8749	C	ASN	176	66.938	24.871	38.837	1.00	0.00	RX2	C
ATOM	8750	O	ASN	176	66.335	23.813	38.635	1.00	0.00	RX2	O
ATOM	8751	N	ASN	177	67.528	25.573	37.860	1.00	0.00	RX2	N
ATOM	8752	H	ASN	177	68.070	26.402	38.023	1.00	0.00	RX2	H
ATOM	8753	CA	ASN	177	67.496	24.995	36.516	1.00	0.00	RX2	C
ATOM	8754	CB	ASN	177	67.266	26.042	35.433	1.00	0.00	RX2	C
ATOM	8755	CG	ASN	177	66.951	25.346	34.123	1.00	0.00	RX2	C
ATOM	8756	OD1	ASN	177	67.057	24.128	33.964	1.00	0.00	RX2	O
ATOM	8757	ND2	ASN	177	66.546	26.205	33.174	1.00	0.00	RX2	N
ATOM	8758	HD21	ASN	177	66.269	27.136	33.426	1.00	0.00	RX2	H
ATOM	8759	HD22	ASN	177	66.477	26.001	32.193	1.00	0.00	RX2	H
ATOM	8760	C	ASN	177	68.753	24.223	36.188	1.00	0.00	RX2	C
ATOM	8761	O	ASN	177	69.782	24.772	35.824	1.00	0.00	RX2	O
ATOM	8762	N	GLN	178	68.606	22.897	36.312	1.00	0.00	RX2	N
ATOM	8763	H	GLN	178	67.718	22.524	36.569	1.00	0.00	RX2	H
ATOM	8764	CA	GLN	178	69.734	22.029	35.971	1.00	0.00	RX2	C
ATOM	8765	CB	GLN	178	69.443	20.569	36.360	1.00	0.00	RX2	C
ATOM	8766	CG	GLN	178	68.516	19.759	35.439	1.00	0.00	RX2	C
ATOM	8767	CD	GLN	178	67.131	20.372	35.369	1.00	0.00	RX2	C
ATOM	8768	OE1	GLN	178	66.594	20.889	36.350	1.00	0.00	RX2	O
ATOM	8769	NE2	GLN	178	66.583	20.304	34.145	1.00	0.00	RX2	N
ATOM	8770	HE21	GLN	178	67.081	19.848	33.402	1.00	0.00	RX2	H
ATOM	8771	HE22	GLN	178	65.687	20.676	33.900	1.00	0.00	RX2	H
ATOM	8772	C	GLN	178	70.220	22.152	34.531	1.00	0.00	RX2	C
ATOM	8773	O	GLN	178	71.397	22.033	34.221	1.00	0.00	RX2	O
ATOM	8774	N	LEU	179	69.247	22.412	33.649	1.00	0.00	RX2	N
ATOM	8775	H	LEU	179	68.332	22.692	33.936	1.00	0.00	RX2	H
ATOM	8776	CA	LEU	179	69.665	22.661	32.277	1.00	0.00	RX2	C
ATOM	8777	CB	LEU	179	68.676	22.075	31.273	1.00	0.00	RX2	C
ATOM	8778	CG	LEU	179	68.647	20.549	31.266	1.00	0.00	RX2	C
ATOM	8779	CD1	LEU	179	67.602	20.014	30.286	1.00	0.00	RX2	C
ATOM	8780	CD2	LEU	179	70.033	19.957	31.008	1.00	0.00	RX2	C
ATOM	8781	C	LEU	179	69.833	24.141	32.028	1.00	0.00	RX2	C
ATOM	8782	O	LEU	179	69.101	24.761	31.267	1.00	0.00	RX2	O

ATOM	8783	N	ALA	180	70.852	24.686	32.701	1.00	0.00	RX2	N
ATOM	8784	H	ALA	180	71.443	24.114	33.273	1.00	0.00	RX2	H
ATOM	8785	CA	ALA	180	71.170	26.091	32.460	1.00	0.00	RX2	C
ATOM	8786	CB	ALA	180	71.905	26.691	33.659	1.00	0.00	RX2	C
ATOM	8787	C	ALA	180	72.007	26.293	31.205	1.00	0.00	RX2	C
ATOM	8788	O	ALA	180	73.123	26.793	31.217	1.00	0.00	RX2	O
ATOM	8789	N	LEU	181	71.411	25.844	30.094	1.00	0.00	RX2	N
ATOM	8790	H	LEU	181	70.440	25.604	30.119	1.00	0.00	RX2	H
ATOM	8791	CA	LEU	181	72.156	25.876	28.841	1.00	0.00	RX2	C
ATOM	8792	CB	LEU	181	71.613	24.825	27.873	1.00	0.00	RX2	C
ATOM	8793	CG	LEU	181	71.497	23.436	28.505	1.00	0.00	RX2	C
ATOM	8794	CD1	LEU	181	70.732	22.465	27.605	1.00	0.00	RX2	C
ATOM	8795	CD2	LEU	181	72.856	22.880	28.933	1.00	0.00	RX2	C
ATOM	8796	C	LEU	181	72.114	27.249	28.208	1.00	0.00	RX2	C
ATOM	8797	O	LEU	181	71.162	27.621	27.533	1.00	0.00	RX2	O
ATOM	8798	N	THR	182	73.190	27.987	28.470	1.00	0.00	RX2	N
ATOM	8799	H	THR	182	73.854	27.695	29.160	1.00	0.00	RX2	H
ATOM	8800	CA	THR	182	73.337	29.323	27.903	1.00	0.00	RX2	C
ATOM	8801	CB	THR	182	74.281	29.993	28.877	1.00	0.00	RX2	C
ATOM	8802	OG1	THR	182	74.106	29.319	30.129	1.00	0.00	RX2	O
ATOM	8803	HG1	THR	182	74.780	29.632	30.717	1.00	0.00	RX2	H
ATOM	8804	CG2	THR	182	74.035	31.493	29.032	1.00	0.00	RX2	C
ATOM	8805	C	THR	182	73.786	29.339	26.449	1.00	0.00	RX2	C
ATOM	8806	O	THR	182	74.875	29.798	26.123	1.00	0.00	RX2	O
ATOM	8807	N	LEU	183	72.919	28.823	25.564	1.00	0.00	RX2	N
ATOM	8808	H	LEU	183	71.968	28.586	25.790	1.00	0.00	RX2	H
ATOM	8809	CA	LEU	183	73.376	28.850	24.177	1.00	0.00	RX2	C
ATOM	8810	CB	LEU	183	72.892	27.622	23.403	1.00	0.00	RX2	C
ATOM	8811	CG	LEU	183	73.946	27.133	22.400	1.00	0.00	RX2	C
ATOM	8812	CD1	LEU	183	73.883	25.618	22.205	1.00	0.00	RX2	C
ATOM	8813	CD2	LEU	183	73.910	27.891	21.071	1.00	0.00	RX2	C
ATOM	8814	C	LEU	183	73.092	30.167	23.476	1.00	0.00	RX2	C
ATOM	8815	O	LEU	183	72.147	30.346	22.717	1.00	0.00	RX2	O
ATOM	8816	N	ILE	184	73.988	31.103	23.803	1.00	0.00	RX2	N
ATOM	8817	H	ILE	184	74.769	30.842	24.374	1.00	0.00	RX2	H
ATOM	8818	CA	ILE	184	73.863	32.461	23.288	1.00	0.00	RX2	C
ATOM	8819	CB	ILE	184	74.561	33.462	24.214	1.00	0.00	RX2	C
ATOM	8820	CG2	ILE	184	74.396	34.899	23.718	1.00	0.00	RX2	C
ATOM	8821	CG1	ILE	184	74.052	33.329	25.645	1.00	0.00	RX2	C
ATOM	8822	CD1	ILE	184	74.687	34.376	26.562	1.00	0.00	RX2	C
ATOM	8823	C	ILE	184	74.396	32.592	21.869	1.00	0.00	RX2	C
ATOM	8824	O	ILE	184	75.538	32.957	21.623	1.00	0.00	RX2	O
ATOM	8825	N	ASP	185	73.494	32.281	20.931	1.00	0.00	RX2	N
ATOM	8826	H	ASP	185	72.589	31.944	21.191	1.00	0.00	RX2	H
ATOM	8827	CA	ASP	185	73.850	32.538	19.537	1.00	0.00	RX2	C
ATOM	8828	CB	ASP	185	72.907	31.795	18.602	1.00	0.00	RX2	C
ATOM	8829	CG	ASP	185	73.341	31.882	17.154	1.00	0.00	RX2	C
ATOM	8830	OD1	ASP	185	73.763	30.865	16.610	1.00	0.00	RX2	O
ATOM	8831	OD2	ASP	185	73.202	32.935	16.537	1.00	0.00	RX2	O
ATOM	8832	C	ASP	185	73.853	34.019	19.209	1.00	0.00	RX2	C
ATOM	8833	O	ASP	185	72.839	34.712	19.230	1.00	0.00	RX2	O
ATOM	8834	N	THR	186	75.068	34.474	18.916	1.00	0.00	RX2	N
ATOM	8835	H	THR	186	75.882	33.892	18.914	1.00	0.00	RX2	H
ATOM	8836	CA	THR	186	75.224	35.871	18.550	1.00	0.00	RX2	C
ATOM	8837	CB	THR	186	76.244	36.295	19.577	1.00	0.00	RX2	C
ATOM	8838	OG1	THR	186	76.775	35.081	20.140	1.00	0.00	RX2	O
ATOM	8839	HG1	THR	186	76.153	34.745	20.781	1.00	0.00	RX2	H
ATOM	8840	CG2	THR	186	75.635	37.158	20.683	1.00	0.00	RX2	C
ATOM	8841	C	THR	186	75.652	36.071	17.108	1.00	0.00	RX2	C
ATOM	8842	O	THR	186	76.379	37.000	16.775	1.00	0.00	RX2	O
ATOM	8843	N	ASN	187	75.164	35.155	16.252	1.00	0.00	RX2	N

ATOM	8844	H	ASN	187	74.494	34.472	16.552	1.00	0.00	RX2	H
ATOM	8845	CA	ASN	187	75.461	35.293	14.823	1.00	0.00	RX2	C
ATOM	8846	CB	ASN	187	75.118	34.033	14.027	1.00	0.00	RX2	C
ATOM	8847	CG	ASN	187	76.198	32.991	14.174	1.00	0.00	RX2	C
ATOM	8848	OD1	ASN	187	77.246	33.045	13.537	1.00	0.00	RX2	O
ATOM	8849	ND2	ASN	187	75.880	32.024	15.047	1.00	0.00	RX2	N
ATOM	8850	HD21	ASN	187	74.989	32.038	15.525	1.00	0.00	RX2	H
ATOM	8851	HD22	ASN	187	76.469	31.254	15.282	1.00	0.00	RX2	H
ATOM	8852	C	ASN	187	74.680	36.424	14.193	1.00	0.00	RX2	C
ATOM	8853	O	ASN	187	73.621	36.239	13.607	1.00	0.00	RX2	O
ATOM	8854	N	ARG	188	75.241	37.620	14.384	1.00	0.00	RX2	N
ATOM	8855	H	ARG	188	76.147	37.700	14.803	1.00	0.00	RX2	H
ATOM	8856	CA	ARG	188	74.486	38.807	14.019	1.00	0.00	RX2	C
ATOM	8857	CB	ARG	188	74.550	39.855	15.116	1.00	0.00	RX2	C
ATOM	8858	CG	ARG	188	73.883	39.440	16.414	1.00	0.00	RX2	C
ATOM	8859	CD	ARG	188	74.031	40.563	17.429	1.00	0.00	RX2	C
ATOM	8860	NE	ARG	188	73.349	40.208	18.662	1.00	0.00	RX2	N
ATOM	8861	HE	ARG	188	72.405	39.857	18.561	1.00	0.00	RX2	H
ATOM	8862	CZ	ARG	188	73.997	40.371	19.844	1.00	0.00	RX2	C
ATOM	8863	NH1	ARG	188	75.253	40.858	19.889	1.00	0.00	RX2	N
ATOM	8864	HH11	ARG	188	75.722	40.901	20.789	1.00	0.00	RX2	H
ATOM	8865	HH12	ARG	188	75.753	41.169	19.079	1.00	0.00	RX2	H
ATOM	8866	NH2	ARG	188	73.373	40.032	20.974	1.00	0.00	RX2	N
ATOM	8867	HH21	ARG	188	73.831	40.188	21.866	1.00	0.00	RX2	H
ATOM	8868	HH22	ARG	188	72.461	39.613	20.968	1.00	0.00	RX2	H
ATOM	8869	C	ARG	188	74.931	39.483	12.753	1.00	0.00	RX2	C
ATOM	8870	O	ARG	188	76.085	39.456	12.348	1.00	0.00	RX2	O
ATOM	8871	N	SER	189	73.936	40.167	12.195	1.00	0.00	RX2	N
ATOM	8872	H	SER	189	72.982	40.064	12.469	1.00	0.00	RX2	H
ATOM	8873	CA	SER	189	74.223	41.243	11.265	1.00	0.00	RX2	C
ATOM	8874	CB	SER	189	73.188	41.090	10.167	1.00	0.00	RX2	C
ATOM	8875	OG	SER	189	71.964	40.641	10.772	1.00	0.00	RX2	O
ATOM	8876	HG	SER	189	71.473	41.436	10.982	1.00	0.00	RX2	H
ATOM	8877	C	SER	189	74.191	42.602	11.949	1.00	0.00	RX2	C
ATOM	8878	O	SER	189	74.848	43.550	11.536	1.00	0.00	RX2	O
ATOM	8879	N	ARG	190	73.372	42.681	13.012	1.00	0.00	RX2	N
ATOM	8880	H	ARG	190	72.920	41.894	13.435	1.00	0.00	RX2	H
ATOM	8881	CA	ARG	190	73.250	44.015	13.588	1.00	0.00	RX2	C
ATOM	8882	CB	ARG	190	71.791	44.457	13.691	1.00	0.00	RX2	C
ATOM	8883	CG	ARG	190	70.992	43.873	14.852	1.00	0.00	RX2	C
ATOM	8884	CD	ARG	190	69.819	44.809	15.135	1.00	0.00	RX2	C
ATOM	8885	NE	ARG	190	69.002	44.404	16.277	1.00	0.00	RX2	N
ATOM	8886	HE	ARG	190	68.493	43.533	16.209	1.00	0.00	RX2	H
ATOM	8887	CZ	ARG	190	68.788	45.352	17.234	1.00	0.00	RX2	C
ATOM	8888	NH1	ARG	190	69.606	46.418	17.299	1.00	0.00	RX2	N
ATOM	8889	HH11	ARG	190	69.318	47.211	17.867	1.00	0.00	RX2	H
ATOM	8890	HH12	ARG	190	70.492	46.486	16.823	1.00	0.00	RX2	H
ATOM	8891	NH2	ARG	190	67.755	45.244	18.091	1.00	0.00	RX2	N
ATOM	8892	HH21	ARG	190	67.549	45.980	18.756	1.00	0.00	RX2	H
ATOM	8893	HH22	ARG	190	67.109	44.468	18.096	1.00	0.00	RX2	H
ATOM	8894	C	ARG	190	73.965	44.251	14.904	1.00	0.00	RX2	C
ATOM	8895	O	ARG	190	74.283	43.334	15.651	1.00	0.00	RX2	O
ATOM	8896	N	ALA	191	74.171	45.549	15.158	1.00	0.00	RX2	N
ATOM	8897	H	ALA	191	73.890	46.272	14.524	1.00	0.00	RX2	H
ATOM	8898	CA	ALA	191	74.562	45.951	16.501	1.00	0.00	RX2	C
ATOM	8899	CB	ALA	191	75.570	47.101	16.449	1.00	0.00	RX2	C
ATOM	8900	C	ALA	191	73.352	46.390	17.306	1.00	0.00	RX2	C
ATOM	8901	O	ALA	191	72.256	46.584	16.783	1.00	0.00	RX2	O
ATOM	8902	N	CYS	192	73.603	46.526	18.614	1.00	0.00	RX2	N
ATOM	8903	H	CYS	192	74.528	46.451	18.978	1.00	0.00	RX2	H
ATOM	8904	CA	CYS	192	72.550	47.034	19.492	1.00	0.00	RX2	C

ATOM	8905	CB	CYS	192	72.855	46.560	20.909	1.00	0.00	RX2	C
ATOM	8906	SG	CYS	192	73.309	44.805	20.921	1.00	0.00	RX2	S
ATOM	8907	C	CYS	192	72.456	48.543	19.414	1.00	0.00	RX2	C
ATOM	8908	O	CYS	192	73.394	49.209	18.987	1.00	0.00	RX2	O
ATOM	8909	N	HIS	193	71.301	49.060	19.846	1.00	0.00	RX2	N
ATOM	8910	H	HIS	193	70.609	48.494	20.300	1.00	0.00	RX2	H
ATOM	8911	CA	HIS	193	71.262	50.509	20.033	1.00	0.00	RX2	C
ATOM	8912	CB	HIS	193	69.810	50.999	19.975	1.00	0.00	RX2	C
ATOM	8913	CG	HIS	193	69.386	51.207	18.538	1.00	0.00	RX2	C
ATOM	8914	ND1	HIS	193	68.169	51.661	18.187	1.00	0.00	RX2	N
ATOM	8915	HD1	HIS	193	67.428	51.858	18.798	1.00	0.00	RX2	H
ATOM	8916	CD2	HIS	193	70.131	50.981	17.375	1.00	0.00	RX2	C
ATOM	8917	NE2	HIS	193	69.345	51.302	16.315	1.00	0.00	RX2	N
ATOM	8918	CE1	HIS	193	68.139	51.721	16.819	1.00	0.00	RX2	C
ATOM	8919	C	HIS	193	71.921	50.866	21.359	1.00	0.00	RX2	C
ATOM	8920	O	HIS	193	71.983	50.040	22.262	1.00	0.00	RX2	O
ATOM	8921	N	PRO	194	72.457	52.110	21.457	1.00	0.00	RX2	N
ATOM	8922	CD	PRO	194	72.556	53.122	20.410	1.00	0.00	RX2	C
ATOM	8923	CA	PRO	194	73.059	52.544	22.725	1.00	0.00	RX2	C
ATOM	8924	CB	PRO	194	73.347	54.027	22.468	1.00	0.00	RX2	C
ATOM	8925	CG	PRO	194	73.564	54.124	20.959	1.00	0.00	RX2	C
ATOM	8926	C	PRO	194	72.204	52.305	23.962	1.00	0.00	RX2	C
ATOM	8927	O	PRO	194	71.103	52.824	24.106	1.00	0.00	RX2	O
ATOM	8928	N	CYS	195	72.782	51.487	24.853	1.00	0.00	RX2	N
ATOM	8929	H	CYS	195	73.662	51.058	24.661	1.00	0.00	RX2	H
ATOM	8930	CA	CYS	195	72.078	51.145	26.089	1.00	0.00	RX2	C
ATOM	8931	CB	CYS	195	72.956	50.176	26.873	1.00	0.00	RX2	C
ATOM	8932	SG	CYS	195	73.769	48.996	25.767	1.00	0.00	RX2	S
ATOM	8933	C	CYS	195	71.670	52.341	26.930	1.00	0.00	RX2	C
ATOM	8934	O	CYS	195	72.468	53.215	27.247	1.00	0.00	RX2	O
ATOM	8935	N	SER	196	70.382	52.339	27.286	1.00	0.00	RX2	N
ATOM	8936	H	SER	196	69.768	51.581	27.073	1.00	0.00	RX2	H
ATOM	8937	CA	SER	196	69.925	53.397	28.181	1.00	0.00	RX2	C
ATOM	8938	CB	SER	196	68.406	53.486	28.009	1.00	0.00	RX2	C
ATOM	8939	OG	SER	196	67.789	52.319	28.560	1.00	0.00	RX2	O
ATOM	8940	HG	SER	196	67.615	51.728	27.826	1.00	0.00	RX2	H
ATOM	8941	C	SER	196	70.375	53.121	29.615	1.00	0.00	RX2	C
ATOM	8942	O	SER	196	70.750	51.999	29.939	1.00	0.00	RX2	O
ATOM	8943	N	PRO	197	70.285	54.137	30.514	1.00	0.00	RX2	N
ATOM	8944	CD	PRO	197	70.010	55.550	30.273	1.00	0.00	RX2	C
ATOM	8945	CA	PRO	197	70.455	53.819	31.941	1.00	0.00	RX2	C
ATOM	8946	CB	PRO	197	70.312	55.193	32.611	1.00	0.00	RX2	C
ATOM	8947	CG	PRO	197	69.527	56.058	31.625	1.00	0.00	RX2	C
ATOM	8948	C	PRO	197	69.481	52.761	32.468	1.00	0.00	RX2	C
ATOM	8949	O	PRO	197	69.776	51.988	33.372	1.00	0.00	RX2	O
ATOM	8950	N	MET	198	68.294	52.717	31.832	1.00	0.00	RX2	N
ATOM	8951	H	MET	198	68.106	53.270	31.022	1.00	0.00	RX2	H
ATOM	8952	CA	MET	198	67.352	51.670	32.229	1.00	0.00	RX2	C
ATOM	8953	CB	MET	198	65.962	51.958	31.675	1.00	0.00	RX2	C
ATOM	8954	CG	MET	198	65.428	53.311	32.141	1.00	0.00	RX2	C
ATOM	8955	SD	MET	198	65.364	53.428	33.935	1.00	0.00	RX2	S
ATOM	8956	CE	MET	198	64.753	55.117	34.050	1.00	0.00	RX2	C
ATOM	8957	C	MET	198	67.789	50.253	31.887	1.00	0.00	RX2	C
ATOM	8958	O	MET	198	67.273	49.269	32.413	1.00	0.00	RX2	O
ATOM	8959	N	CYS	199	68.818	50.194	31.031	1.00	0.00	RX2	N
ATOM	8960	H	CYS	199	69.188	51.003	30.574	1.00	0.00	RX2	H
ATOM	8961	CA	CYS	199	69.542	48.944	30.825	1.00	0.00	RX2	C
ATOM	8962	CB	CYS	199	70.135	48.945	29.425	1.00	0.00	RX2	C
ATOM	8963	SG	CYS	199	68.996	49.669	28.225	1.00	0.00	RX2	S
ATOM	8964	C	CYS	199	70.630	48.659	31.850	1.00	0.00	RX2	C
ATOM	8965	O	CYS	199	71.635	48.030	31.543	1.00	0.00	RX2	O

ATOM	8966	N	LYS	200	70.393	49.122	33.094	1.00	0.00	RX2	N
ATOM	8967	H	LYS	200	69.683	49.808	33.246	1.00	0.00	RX2	H
ATOM	8968	CA	LYS	200	71.270	48.759	34.214	1.00	0.00	RX2	C
ATOM	8969	CB	LYS	200	70.613	49.045	35.579	1.00	0.00	RX2	C
ATOM	8970	CG	LYS	200	69.432	48.137	35.960	1.00	0.00	RX2	C
ATOM	8971	CD	LYS	200	68.131	48.495	35.242	1.00	0.00	RX2	C
ATOM	8972	CE	LYS	200	67.133	47.343	35.139	1.00	0.00	RX2	C
ATOM	8973	NZ	LYS	200	66.041	47.725	34.231	1.00	0.00	RX2	N
ATOM	8974	HZ1	LYS	200	65.466	46.889	33.969	1.00	0.00	RX2	H
ATOM	8975	HZ2	LYS	200	65.409	48.427	34.652	1.00	0.00	RX2	H
ATOM	8976	HZ3	LYS	200	66.415	48.110	33.337	1.00	0.00	RX2	H
ATOM	8977	C	LYS	200	71.808	47.334	34.180	1.00	0.00	RX2	C
ATOM	8978	O	LYS	200	71.104	46.374	33.884	1.00	0.00	RX2	O
ATOM	8979	N	GLY	201	73.118	47.257	34.447	1.00	0.00	RX2	N
ATOM	8980	H	GLY	201	73.649	48.085	34.619	1.00	0.00	RX2	H
ATOM	8981	CA	GLY	201	73.770	45.949	34.384	1.00	0.00	RX2	C
ATOM	8982	C	GLY	201	73.773	45.280	33.015	1.00	0.00	RX2	C
ATOM	8983	O	GLY	201	73.858	44.060	32.920	1.00	0.00	RX2	O
ATOM	8984	N	SER	202	73.692	46.146	31.984	1.00	0.00	RX2	N
ATOM	8985	H	SER	202	73.509	47.111	32.159	1.00	0.00	RX2	H
ATOM	8986	CA	SER	202	73.691	45.782	30.559	1.00	0.00	RX2	C
ATOM	8987	CB	SER	202	74.898	44.891	30.265	1.00	0.00	RX2	C
ATOM	8988	OG	SER	202	75.898	45.174	31.254	1.00	0.00	RX2	O
ATOM	8989	HG	SER	202	75.784	44.485	31.900	1.00	0.00	RX2	H
ATOM	8990	C	SER	202	72.373	45.268	29.999	1.00	0.00	RX2	C
ATOM	8991	O	SER	202	72.268	44.877	28.846	1.00	0.00	RX2	O
ATOM	8992	N	ARG	203	71.353	45.253	30.864	1.00	0.00	RX2	N
ATOM	8993	H	ARG	203	71.377	45.778	31.716	1.00	0.00	RX2	H
ATOM	8994	CA	ARG	203	70.221	44.395	30.524	1.00	0.00	RX2	C
ATOM	8995	CB	ARG	203	69.691	43.766	31.800	1.00	0.00	RX2	C
ATOM	8996	CG	ARG	203	70.831	43.008	32.463	1.00	0.00	RX2	C
ATOM	8997	CD	ARG	203	70.529	42.579	33.889	1.00	0.00	RX2	C
ATOM	8998	NE	ARG	203	71.711	41.965	34.479	1.00	0.00	RX2	N
ATOM	8999	HE	ARG	203	72.500	42.581	34.585	1.00	0.00	RX2	H
ATOM	9000	CZ	ARG	203	71.694	40.633	34.774	1.00	0.00	RX2	C
ATOM	9001	NH1	ARG	203	70.612	39.883	34.468	1.00	0.00	RX2	N
ATOM	9002	HH11	ARG	203	70.613	38.888	34.612	1.00	0.00	RX2	H
ATOM	9003	HH12	ARG	203	69.759	40.273	34.084	1.00	0.00	RX2	H
ATOM	9004	NH2	ARG	203	72.768	40.081	35.373	1.00	0.00	RX2	N
ATOM	9005	HH21	ARG	203	72.772	39.103	35.635	1.00	0.00	RX2	H
ATOM	9006	HH22	ARG	203	73.594	40.604	35.589	1.00	0.00	RX2	H
ATOM	9007	C	ARG	203	69.111	45.018	29.697	1.00	0.00	RX2	C
ATOM	9008	O	ARG	203	67.983	45.200	30.144	1.00	0.00	RX2	O
ATOM	9009	N	CYS	204	69.472	45.327	28.449	1.00	0.00	RX2	N
ATOM	9010	H	CYS	204	70.393	45.118	28.113	1.00	0.00	RX2	H
ATOM	9011	CA	CYS	204	68.421	45.708	27.507	1.00	0.00	RX2	C
ATOM	9012	CB	CYS	204	68.983	46.728	26.523	1.00	0.00	RX2	C
ATOM	9013	SG	CYS	204	70.777	46.885	26.713	1.00	0.00	RX2	S
ATOM	9014	C	CYS	204	67.861	44.505	26.783	1.00	0.00	RX2	C
ATOM	9015	O	CYS	204	68.605	43.633	26.350	1.00	0.00	RX2	O
ATOM	9016	N	TRP	205	66.522	44.502	26.708	1.00	0.00	RX2	N
ATOM	9017	H	TRP	205	66.007	45.292	27.041	1.00	0.00	RX2	H
ATOM	9018	CA	TRP	205	65.792	43.485	25.946	1.00	0.00	RX2	C
ATOM	9019	CB	TRP	205	64.419	43.236	26.564	1.00	0.00	RX2	C
ATOM	9020	CG	TRP	205	64.435	42.372	27.799	1.00	0.00	RX2	C
ATOM	9021	CD2	TRP	205	64.492	40.932	27.891	1.00	0.00	RX2	C
ATOM	9022	CE2	TRP	205	64.373	40.590	29.257	1.00	0.00	RX2	C
ATOM	9023	CE3	TRP	205	64.628	39.925	26.946	1.00	0.00	RX2	C
ATOM	9024	CD1	TRP	205	64.297	42.814	29.118	1.00	0.00	RX2	C
ATOM	9025	NE1	TRP	205	64.253	41.767	29.984	1.00	0.00	RX2	N
ATOM	9026	HE1	TRP	205	64.098	41.806	30.952	1.00	0.00	RX2	H

ATOM	9027	CZ2	TRP	205	64.382	39.255	29.637	1.00	0.00	RX2	C
ATOM	9028	CZ3	TRP	205	64.641	38.593	27.338	1.00	0.00	RX2	C
ATOM	9029	CH2	TRP	205	64.514	38.258	28.679	1.00	0.00	RX2	C
ATOM	9030	C	TRP	205	65.546	43.930	24.511	1.00	0.00	RX2	C
ATOM	9031	O	TRP	205	65.682	43.181	23.549	1.00	0.00	RX2	O
ATOM	9032	N	GLY	206	65.155	45.211	24.428	1.00	0.00	RX2	N
ATOM	9033	H	GLY	206	65.085	45.798	25.236	1.00	0.00	RX2	H
ATOM	9034	CA	GLY	206	64.929	45.805	23.114	1.00	0.00	RX2	C
ATOM	9035	C	GLY	206	65.825	47.005	22.910	1.00	0.00	RX2	C
ATOM	9036	O	GLY	206	66.674	47.283	23.753	1.00	0.00	RX2	O
ATOM	9037	N	GLU	207	65.583	47.728	21.802	1.00	0.00	RX2	N
ATOM	9038	H	GLU	207	64.890	47.418	21.141	1.00	0.00	RX2	H
ATOM	9039	CA	GLU	207	66.245	49.028	21.611	1.00	0.00	RX2	C
ATOM	9040	CB	GLU	207	66.016	49.575	20.200	1.00	0.00	RX2	C
ATOM	9041	CG	GLU	207	66.111	48.590	19.034	1.00	0.00	RX2	C
ATOM	9042	CD	GLU	207	67.536	48.262	18.648	1.00	0.00	RX2	C
ATOM	9043	OE1	GLU	207	68.268	47.656	19.416	1.00	0.00	RX2	O
ATOM	9044	OE2	GLU	207	67.916	48.539	17.522	1.00	0.00	RX2	O
ATOM	9045	C	GLU	207	65.692	50.078	22.567	1.00	0.00	RX2	C
ATOM	9046	O	GLU	207	64.936	50.963	22.183	1.00	0.00	RX2	O
ATOM	9047	N	SER	208	66.027	49.919	23.851	1.00	0.00	RX2	N
ATOM	9048	H	SER	208	66.691	49.245	24.178	1.00	0.00	RX2	H
ATOM	9049	CA	SER	208	65.041	50.489	24.755	1.00	0.00	RX2	C
ATOM	9050	CB	SER	208	64.000	49.391	25.006	1.00	0.00	RX2	C
ATOM	9051	OG	SER	208	62.678	49.935	25.107	1.00	0.00	RX2	O
ATOM	9052	HG	SER	208	62.376	50.036	24.208	1.00	0.00	RX2	H
ATOM	9053	C	SER	208	65.555	51.122	26.030	1.00	0.00	RX2	C
ATOM	9054	O	SER	208	66.700	50.995	26.453	1.00	0.00	RX2	O
ATOM	9055	N	SER	209	64.588	51.809	26.638	1.00	0.00	RX2	N
ATOM	9056	H	SER	209	63.736	52.009	26.157	1.00	0.00	RX2	H
ATOM	9057	CA	SER	209	64.631	52.083	28.065	1.00	0.00	RX2	C
ATOM	9058	CB	SER	209	64.295	53.554	28.081	1.00	0.00	RX2	C
ATOM	9059	OG	SER	209	63.953	53.848	26.717	1.00	0.00	RX2	O
ATOM	9060	HG	SER	209	63.939	54.795	26.641	1.00	0.00	RX2	H
ATOM	9061	C	SER	209	63.626	51.170	28.750	1.00	0.00	RX2	C
ATOM	9062	O	SER	209	63.926	50.401	29.654	1.00	0.00	RX2	O
ATOM	9063	N	GLU	210	62.406	51.286	28.202	1.00	0.00	RX2	N
ATOM	9064	H	GLU	210	62.273	51.943	27.462	1.00	0.00	RX2	H
ATOM	9065	CA	GLU	210	61.249	50.477	28.577	1.00	0.00	RX2	C
ATOM	9066	CB	GLU	210	60.173	50.748	27.526	1.00	0.00	RX2	C
ATOM	9067	CG	GLU	210	59.180	51.838	27.929	1.00	0.00	RX2	C
ATOM	9068	CD	GLU	210	58.205	51.234	28.914	1.00	0.00	RX2	C
ATOM	9069	OE1	GLU	210	57.216	50.650	28.484	1.00	0.00	RX2	O
ATOM	9070	OE2	GLU	210	58.459	51.261	30.113	1.00	0.00	RX2	O
ATOM	9071	C	GLU	210	61.513	48.983	28.739	1.00	0.00	RX2	C
ATOM	9072	O	GLU	210	61.459	48.415	29.826	1.00	0.00	RX2	O
ATOM	9073	N	ASP	211	61.817	48.350	27.599	1.00	0.00	RX2	N
ATOM	9074	H	ASP	211	61.921	48.836	26.729	1.00	0.00	RX2	H
ATOM	9075	CA	ASP	211	62.133	46.925	27.694	1.00	0.00	RX2	C
ATOM	9076	CB	ASP	211	61.749	46.153	26.427	1.00	0.00	RX2	C
ATOM	9077	CG	ASP	211	60.276	45.796	26.463	1.00	0.00	RX2	C
ATOM	9078	OD1	ASP	211	59.925	44.711	26.921	1.00	0.00	RX2	O
ATOM	9079	OD2	ASP	211	59.460	46.608	26.047	1.00	0.00	RX2	O
ATOM	9080	C	ASP	211	63.578	46.667	28.053	1.00	0.00	RX2	C
ATOM	9081	O	ASP	211	64.419	46.330	27.224	1.00	0.00	RX2	O
ATOM	9082	N	CYS	212	63.829	46.839	29.357	1.00	0.00	RX2	N
ATOM	9083	H	CYS	212	63.123	47.211	29.966	1.00	0.00	RX2	H
ATOM	9084	CA	CYS	212	65.177	46.615	29.869	1.00	0.00	RX2	C
ATOM	9085	CB	CYS	212	65.978	47.907	29.748	1.00	0.00	RX2	C
ATOM	9086	SG	CYS	212	66.085	48.524	28.051	1.00	0.00	RX2	S
ATOM	9087	C	CYS	212	65.156	46.085	31.289	1.00	0.00	RX2	C

```
ATOM   9088  O   CYS   212       64.853  46.802  32.237  1.00  0.00       RX2  O
ATOM   9089  N   GLN   213       65.458  44.777  31.379  1.00  0.00       RX2  N
ATOM   9090  H   GLN   213       65.916  44.386  30.579  1.00  0.00       RX2  H
ATOM   9091  CA  GLN   213       65.118  43.889  32.501  1.00  0.00       RX2  C
ATOM   9092  CB  GLN   213       66.318  43.054  32.949  1.00  0.00       RX2  C
ATOM   9093  CG  GLN   213       65.899  41.643  33.373  1.00  0.00       RX2  C
ATOM   9094  CD  GLN   213       66.953  41.021  34.268  1.00  0.00       RX2  C
ATOM   9095  OE1 GLN   213       68.055  40.658  33.856  1.00  0.00       RX2  O
ATOM   9096  NE2 GLN   213       66.540  40.919  35.541  1.00  0.00       RX2  N
ATOM   9097 HE21 GLN   213       65.634  41.282  35.787  1.00  0.00       RX2  H
ATOM   9098 HE22 GLN   213       67.065  40.493  36.277  1.00  0.00       RX2  H
ATOM   9099  C   GLN   213       64.405  44.447  33.724  1.00  0.00       RX2  C
ATOM   9100  O   GLN   213       64.884  45.339  34.420  1.00  0.00       RX2  O
ATOM   9101  N   SER   214       63.236  43.847  33.957  1.00  0.00       RX2  N
ATOM   9102  H   SER   214       62.817  43.180  33.344  1.00  0.00       RX2  H
ATOM   9103  CA  SER   214       62.561  44.086  35.227  1.00  0.00       RX2  C
ATOM   9104  CB  SER   214       61.100  43.770  34.982  1.00  0.00       RX2  C
ATOM   9105  OG  SER   214       60.803  44.193  33.648  1.00  0.00       RX2  O
ATOM   9106  HG  SER   214       59.973  43.785  33.414  1.00  0.00       RX2  H
ATOM   9107  C   SER   214       63.180  43.252  36.336  1.00  0.00       RX2  C
ATOM   9108  O   SER   214       64.183  42.568  36.134  1.00  0.00       RX2  O
TER
HEADER lig.000.00.pdb
ATOM      1  N   PRO   563       28.098   5.338  44.696  1.00  0.00       LX0  N
ATOM      2  CD  PRO   563       28.614   4.023  45.052  1.00  0.00       LX0  C
ATOM      3  CA  PRO   563       27.573   6.093  45.842  1.00  0.00       LX0  C
ATOM      4  CB  PRO   563       27.652   5.056  46.969  1.00  0.00       LX0  C
ATOM      5  CG  PRO   563       28.773   4.098  46.564  1.00  0.00       LX0  C
ATOM      6  C   PRO   563       28.341   7.378  46.132  1.00  0.00       LX0  C
ATOM      7  O   PRO   563       28.182   8.016  47.164  1.00  0.00       LX0  O
ATOM      8  N   GLN   564       29.198   7.736  45.166  1.00  0.00       LX0  N
ATOM      9  H   GLN   564       29.180   7.243  44.299  0.00  0.00       LX0  H
ATOM     10  CA  GLN   564       29.840   9.039  45.296  1.00  0.00       LX0  C
ATOM     11  CB  GLN   564       31.190   8.997  44.578  1.00  0.00       LX0  C
ATOM     12  CG  GLN   564       32.220  10.052  44.987  1.00  0.00       LX0  C
ATOM     13  CD  GLN   564       33.077   9.524  46.120  1.00  0.00       LX0  C
ATOM     14  OE1 GLN   564       33.849   8.587  45.950  1.00  0.00       LX0  O
ATOM     15  NE2 GLN   564       32.913  10.143  47.285  1.00  0.00       LX0  N
ATOM     16 HE21 GLN   564       32.317  10.946  47.377  0.00  0.00       LX0  H
ATOM     17 HE22 GLN   564       33.411   9.852  48.097  0.00  0.00       LX0  H
ATOM     18  C   GLN   564       28.922  10.080  44.676  1.00  0.00       LX0  C
ATOM     19  O   GLN   564       28.140   9.767  43.785  1.00  0.00       LX0  O
ATOM     20  N   LYS   565       29.036  11.322  45.158  1.00  0.00       LX0  N
ATOM     21  H   LYS   565       29.782  11.563  45.784  0.00  0.00       LX0  H
ATOM     22  CA  LYS   565       28.252  12.365  44.497  1.00  0.00       LX0  C
ATOM     23  CB  LYS   565       28.020  13.537  45.454  1.00  0.00       LX0  C
ATOM     24  CG  LYS   565       27.266  13.166  46.730  1.00  0.00       LX0  C
ATOM     25  CD  LYS   565       27.356  14.233  47.829  1.00  0.00       LX0  C
ATOM     26  CE  LYS   565       28.582  14.108  48.748  1.00  0.00       LX0  C
ATOM     27  NZ  LYS   565       29.841  14.387  48.050  1.00  0.00       LX0  N
ATOM     28  HZ1 LYS   565       30.666  14.041  48.586  0.00  0.00       LX0  H
ATOM     29  HZ2 LYS   565       29.951  13.864  47.156  0.00  0.00       LX0  H
ATOM     30  HZ3 LYS   565       29.987  15.393  47.836  0.00  0.00       LX0  H
ATOM     31  C   LYS   565       28.979  12.854  43.260  1.00  0.00       LX0  C
ATOM     32  O   LYS   565       29.587  13.912  43.258  1.00  0.00       LX0  O
ATOM     33  N   ILE   566       28.945  12.028  42.212  1.00  0.00       LX0  N
ATOM     34  H   ILE   566       28.382  11.201  42.246  0.00  0.00       LX0  H
ATOM     35  CA  ILE   566       29.888  12.343  41.138  1.00  0.00       LX0  C
ATOM     36  CB  ILE   566       30.185  11.111  40.270  1.00  0.00       LX0  C
ATOM     37  CG2 ILE   566       31.399  11.343  39.360  1.00  0.00       LX0  C
ATOM     38  CG1 ILE   566       30.403   9.887  41.160  1.00  0.00       LX0  C
```

ATOM	39	CD1	ILE	566	30.417	8.550	40.428	1.00	0.00	LX0	C
ATOM	40	C	ILE	566	29.559	13.576	40.303	1.00	0.00	LX0	C
ATOM	41	O	ILE	566	28.663	13.621	39.468	1.00	0.00	LX0	O
ATOM	42	N	CYS	567	30.378	14.592	40.571	1.00	0.00	LX0	N
ATOM	43	H	CYS	567	31.051	14.453	41.300	0.00	0.00	LX0	H
ATOM	44	CA	CYS	567	30.431	15.778	39.727	1.00	0.00	LX0	C
ATOM	45	CB	CYS	567	31.328	16.826	40.385	1.00	0.00	LX0	C
ATOM	46	SG	CYS	567	31.605	18.339	39.420	1.00	0.00	LX0	S
ATOM	47	C	CYS	567	30.962	15.472	38.345	1.00	0.00	LX0	C
ATOM	48	O	CYS	567	32.166	15.449	38.117	1.00	0.00	LX0	O
ATOM	49	N	LEU	568	30.010	15.304	37.412	1.00	0.00	LX0	N
ATOM	50	H	LEU	568	29.093	15.132	37.778	0.00	0.00	LX0	H
ATOM	51	CA	LEU	568	30.316	15.172	35.976	1.00	0.00	LX0	C
ATOM	52	CB	LEU	568	29.088	15.503	35.112	1.00	0.00	LX0	C
ATOM	53	CG	LEU	568	27.776	14.753	35.377	1.00	0.00	LX0	C
ATOM	54	CD1	LEU	568	26.874	15.458	36.397	1.00	0.00	LX0	C
ATOM	55	CD2	LEU	568	27.021	14.489	34.073	1.00	0.00	LX0	C
ATOM	56	C	LEU	568	31.475	16.042	35.482	1.00	0.00	LX0	C
ATOM	57	O	LEU	568	32.406	15.631	34.786	1.00	0.00	LX0	O
ATOM	58	N	ILE	569	31.372	17.307	35.932	1.00	0.00	LX0	N
ATOM	59	H	ILE	569	30.590	17.516	36.517	0.00	0.00	LX0	H
ATOM	60	CA	ILE	569	32.336	18.324	35.510	1.00	0.00	LX0	C
ATOM	61	CB	ILE	569	32.041	19.708	36.119	1.00	0.00	LX0	C
ATOM	62	CG2	ILE	569	32.762	20.790	35.306	1.00	0.00	LX0	C
ATOM	63	CG1	ILE	569	30.548	20.016	36.298	1.00	0.00	LX0	C
ATOM	64	CD1	ILE	569	29.745	20.047	34.999	1.00	0.00	LX0	C
ATOM	65	C	ILE	569	33.776	17.931	35.799	1.00	0.00	LX0	C
ATOM	66	O	ILE	569	34.619	17.835	34.914	1.00	0.00	LX0	O
ATOM	67	N	CYS	570	34.028	17.657	37.083	1.00	0.00	LX0	N
ATOM	68	H	CYS	570	33.296	17.621	37.766	0.00	0.00	LX0	H
ATOM	69	CA	CYS	570	35.394	17.230	37.374	1.00	0.00	LX0	C
ATOM	70	CB	CYS	570	35.774	17.560	38.815	1.00	0.00	LX0	C
ATOM	71	SG	CYS	570	34.723	16.726	40.025	1.00	0.00	LX0	S
ATOM	72	C	CYS	570	35.676	15.769	37.071	1.00	0.00	LX0	C
ATOM	73	O	CYS	570	36.821	15.346	36.990	1.00	0.00	LX0	O
ATOM	74	N	GLY	571	34.583	15.012	36.918	1.00	0.00	LX0	N
ATOM	75	H	GLY	571	33.677	15.419	37.019	0.00	0.00	LX0	H
ATOM	76	CA	GLY	571	34.704	13.562	36.813	1.00	0.00	LX0	C
ATOM	77	C	GLY	571	35.090	12.894	38.122	1.00	0.00	LX0	C
ATOM	78	O	GLY	571	35.832	11.921	38.125	1.00	0.00	LX0	O
ATOM	79	N	ASP	572	34.583	13.482	39.221	1.00	0.00	LX0	N
ATOM	80	H	ASP	572	33.907	14.218	39.151	0.00	0.00	LX0	H
ATOM	81	CA	ASP	572	34.935	12.999	40.562	1.00	0.00	LX0	C
ATOM	82	CB	ASP	572	36.364	13.395	40.959	1.00	0.00	LX0	C
ATOM	83	CG	ASP	572	37.248	12.166	40.892	1.00	0.00	LX0	C
ATOM	84	OD1	ASP	572	36.931	11.165	41.521	1.00	0.00	LX0	O
ATOM	85	OD2	ASP	572	38.236	12.171	40.166	1.00	0.00	LX0	O
ATOM	86	C	ASP	572	33.947	13.495	41.597	1.00	0.00	LX0	C
ATOM	87	O	ASP	572	32.935	14.082	41.240	1.00	0.00	LX0	O
ATOM	88	N	GLU	573	34.271	13.249	42.879	1.00	0.00	LX0	N
ATOM	89	H	GLU	573	35.106	12.732	43.069	0.00	0.00	LX0	H
ATOM	90	CA	GLU	573	33.405	13.706	43.974	1.00	0.00	LX0	C
ATOM	91	CB	GLU	573	34.068	13.350	45.316	1.00	0.00	LX0	C
ATOM	92	CG	GLU	573	33.398	13.836	46.617	1.00	0.00	LX0	C
ATOM	93	CD	GLU	573	32.055	13.177	46.906	1.00	0.00	LX0	C
ATOM	94	OE1	GLU	573	31.176	13.131	46.055	1.00	0.00	LX0	O
ATOM	95	OE2	GLU	573	31.828	12.764	48.037	1.00	0.00	LX0	O
ATOM	96	C	GLU	573	32.996	15.178	43.929	1.00	0.00	LX0	C
ATOM	97	O	GLU	573	33.787	16.078	43.665	1.00	0.00	LX0	O
ATOM	98	N	ALA	574	31.703	15.377	44.204	1.00	0.00	LX0	N
ATOM	99	H	ALA	574	31.140	14.580	44.439	0.00	0.00	LX0	H

ATOM	100	CA	ALA	574	31.193	16.732	44.343	1.00	0.00	LX0	C
ATOM	101	CB	ALA	574	29.812	16.880	43.714	1.00	0.00	LX0	C
ATOM	102	C	ALA	574	31.024	17.119	45.787	1.00	0.00	LX0	C
ATOM	103	O	ALA	574	30.751	16.304	46.661	1.00	0.00	LX0	O
ATOM	104	N	SER	575	31.134	18.431	45.998	1.00	0.00	LX0	N
ATOM	105	H	SER	575	31.339	19.064	45.253	0.00	0.00	LX0	H
ATOM	106	CA	SER	575	30.759	18.949	47.309	1.00	0.00	LX0	C
ATOM	107	CB	SER	575	31.158	20.428	47.398	1.00	0.00	LX0	C
ATOM	108	OG	SER	575	31.679	20.882	46.133	1.00	0.00	LX0	O
ATOM	109	HG	SER	575	30.915	21.035	45.576	0.00	0.00	LX0	H
ATOM	110	C	SER	575	29.280	18.723	47.606	1.00	0.00	LX0	C
ATOM	111	O	SER	575	28.890	18.148	48.614	1.00	0.00	LX0	O
ATOM	112	N	GLY	576	28.478	19.158	46.628	1.00	0.00	LX0	N
ATOM	113	H	GLY	576	28.840	19.585	45.795	0.00	0.00	LX0	H
ATOM	114	CA	GLY	576	27.039	18.955	46.724	1.00	0.00	LX0	C
ATOM	115	C	GLY	576	26.439	19.368	45.404	1.00	0.00	LX0	C
ATOM	116	O	GLY	576	27.146	19.447	44.409	1.00	0.00	LX0	O
ATOM	117	N	CYS	577	25.133	19.652	45.420	1.00	0.00	LX0	N
ATOM	118	H	CYS	577	24.602	19.643	46.265	0.00	0.00	LX0	H
ATOM	119	CA	CYS	577	24.615	20.233	44.184	1.00	0.00	LX0	C
ATOM	120	CB	CYS	577	23.163	19.820	43.941	1.00	0.00	LX0	C
ATOM	121	SG	CYS	577	22.545	20.334	42.318	1.00	0.00	LX0	S
ATOM	122	C	CYS	577	24.750	21.740	44.201	1.00	0.00	LX0	C
ATOM	123	O	CYS	577	24.264	22.418	45.101	1.00	0.00	LX0	O
ATOM	124	N	HIS	578	25.463	22.241	43.192	1.00	0.00	LX0	N
ATOM	125	H	HIS	578	25.878	21.664	42.487	0.00	0.00	LX0	H
ATOM	126	CA	HIS	578	25.591	23.687	43.095	1.00	0.00	LX0	C
ATOM	127	CB	HIS	578	27.006	24.155	43.459	1.00	0.00	LX0	C
ATOM	128	CG	HIS	578	27.482	23.541	44.760	1.00	0.00	LX0	C
ATOM	129	ND1	HIS	578	26.721	23.405	45.864	1.00	0.00	LX0	N
ATOM	130	HD1	HIS	578	25.756	23.589	45.930	0.00	0.00	LX0	H
ATOM	131	CD2	HIS	578	28.754	23.034	45.038	1.00	0.00	LX0	C
ATOM	132	NE2	HIS	578	28.749	22.601	46.323	1.00	0.00	LX0	N
ATOM	133	CE1	HIS	578	27.496	22.826	46.831	1.00	0.00	LX0	C
ATOM	134	C	HIS	578	25.204	24.143	41.707	1.00	0.00	LX0	C
ATOM	135	O	HIS	578	25.409	23.450	40.715	1.00	0.00	LX0	O
ATOM	136	N	TYR	579	24.589	25.339	41.699	1.00	0.00	LX0	N
ATOM	137	H	TYR	579	24.485	25.796	42.580	0.00	0.00	LX0	H
ATOM	138	CA	TYR	579	24.000	25.948	40.498	1.00	0.00	LX0	C
ATOM	139	CB	TYR	579	25.048	26.398	39.467	1.00	0.00	LX0	C
ATOM	140	CG	TYR	579	26.142	27.209	40.127	1.00	0.00	LX0	C
ATOM	141	CD1	TYR	579	25.888	28.534	40.540	1.00	0.00	LX0	C
ATOM	142	CE1	TYR	579	26.917	29.261	41.165	1.00	0.00	LX0	C
ATOM	143	CD2	TYR	579	27.399	26.602	40.310	1.00	0.00	LX0	C
ATOM	144	CE2	TYR	579	28.427	27.329	40.925	1.00	0.00	LX0	C
ATOM	145	CZ	TYR	579	28.172	28.646	41.353	1.00	0.00	LX0	C
ATOM	146	OH	TYR	579	29.184	29.344	41.980	1.00	0.00	LX0	O
ATOM	147	HH	TYR	579	30.020	29.015	41.655	0.00	0.00	LX0	H
ATOM	148	C	TYR	579	22.877	25.155	39.846	1.00	0.00	LX0	C
ATOM	149	O	TYR	579	21.724	25.570	39.886	1.00	0.00	LX0	O
ATOM	150	N	GLY	580	23.246	23.999	39.278	1.00	0.00	LX0	N
ATOM	151	H	GLY	580	24.219	23.770	39.241	0.00	0.00	LX0	H
ATOM	152	CA	GLY	580	22.210	23.083	38.811	1.00	0.00	LX0	C
ATOM	153	C	GLY	580	22.620	21.620	38.719	1.00	0.00	LX0	C
ATOM	154	O	GLY	580	21.847	20.789	38.250	1.00	0.00	LX0	O
ATOM	155	N	VAL	581	23.870	21.334	39.142	1.00	0.00	LX0	N
ATOM	156	H	VAL	581	24.445	22.001	39.621	0.00	0.00	LX0	H
ATOM	157	CA	VAL	581	24.364	19.955	39.046	1.00	0.00	LX0	C
ATOM	158	CB	VAL	581	25.198	19.698	37.769	1.00	0.00	LX0	C
ATOM	159	CG1	VAL	581	24.435	20.035	36.494	1.00	0.00	LX0	C
ATOM	160	CG2	VAL	581	26.565	20.388	37.798	1.00	0.00	LX0	C

ATOM	161	C	VAL	581	25.212	19.604	40.253	1.00	0.00	LX0	C
ATOM	162	O	VAL	581	25.620	20.473	41.013	1.00	0.00	LX0	O
ATOM	163	N	LEU	582	25.527	18.304	40.368	1.00	0.00	LX0	N
ATOM	164	H	LEU	582	25.175	17.640	39.712	0.00	0.00	LX0	H
ATOM	165	CA	LEU	582	26.587	17.947	41.314	1.00	0.00	LX0	C
ATOM	166	CB	LEU	582	26.756	16.428	41.375	1.00	0.00	LX0	C
ATOM	167	CG	LEU	582	25.554	15.692	41.966	1.00	0.00	LX0	C
ATOM	168	CD1	LEU	582	25.674	14.180	41.767	1.00	0.00	LX0	C
ATOM	169	CD2	LEU	582	25.322	16.062	43.433	1.00	0.00	LX0	C
ATOM	170	C	LEU	582	27.914	18.593	40.948	1.00	0.00	LX0	C
ATOM	171	O	LEU	582	28.459	18.365	39.875	1.00	0.00	LX0	O
ATOM	172	N	THR	583	28.393	19.427	41.872	1.00	0.00	LX0	N
ATOM	173	H	THR	583	27.886	19.627	42.709	0.00	0.00	LX0	H
ATOM	174	CA	THR	583	29.623	20.151	41.585	1.00	0.00	LX0	C
ATOM	175	CB	THR	583	29.309	21.597	41.217	1.00	0.00	LX0	C
ATOM	176	OG1	THR	583	27.944	21.924	41.514	1.00	0.00	LX0	O
ATOM	177	HG1	THR	583	27.382	21.408	40.941	0.00	0.00	LX0	H
ATOM	178	CG2	THR	583	29.637	21.861	39.752	1.00	0.00	LX0	C
ATOM	179	C	THR	583	30.649	20.096	42.698	1.00	0.00	LX0	C
ATOM	180	O	THR	583	30.361	20.245	43.883	1.00	0.00	LX0	O
ATOM	181	N	CYS	584	31.894	19.859	42.263	1.00	0.00	LX0	N
ATOM	182	H	CYS	584	32.078	19.751	41.287	0.00	0.00	LX0	H
ATOM	183	CA	CYS	584	32.979	19.969	43.234	1.00	0.00	LX0	C
ATOM	184	CB	CYS	584	34.197	19.161	42.779	1.00	0.00	LX0	C
ATOM	185	SG	CYS	584	34.985	19.851	41.297	1.00	0.00	LX0	S
ATOM	186	C	CYS	584	33.364	21.420	43.412	1.00	0.00	LX0	C
ATOM	187	O	CYS	584	33.201	22.212	42.490	1.00	0.00	LX0	O
ATOM	188	N	GLY	585	33.910	21.729	44.599	1.00	0.00	LX0	N
ATOM	189	H	GLY	585	33.784	21.065	45.338	0.00	0.00	LX0	H
ATOM	190	CA	GLY	585	34.368	23.090	44.906	1.00	0.00	LX0	C
ATOM	191	C	GLY	585	34.991	23.886	43.765	1.00	0.00	LX0	C
ATOM	192	O	GLY	585	34.560	24.983	43.429	1.00	0.00	LX0	O
ATOM	193	N	SER	586	36.018	23.273	43.151	1.00	0.00	LX0	N
ATOM	194	H	SER	586	36.304	22.356	43.419	0.00	0.00	LX0	H
ATOM	195	CA	SER	586	36.672	24.029	42.082	1.00	0.00	LX0	C
ATOM	196	CB	SER	586	38.014	23.391	41.698	1.00	0.00	LX0	C
ATOM	197	OG	SER	586	37.843	22.019	41.297	1.00	0.00	LX0	O
ATOM	198	HG	SER	586	38.201	21.497	42.012	0.00	0.00	LX0	H
ATOM	199	C	SER	586	35.802	24.322	40.864	1.00	0.00	LX0	C
ATOM	200	O	SER	586	35.907	25.360	40.227	1.00	0.00	LX0	O
ATOM	201	N	CYS	587	34.906	23.368	40.566	1.00	0.00	LX0	N
ATOM	202	H	CYS	587	34.763	22.591	41.181	0.00	0.00	LX0	H
ATOM	203	CA	CYS	587	33.974	23.670	39.480	1.00	0.00	LX0	C
ATOM	204	CB	CYS	587	33.335	22.394	38.942	1.00	0.00	LX0	C
ATOM	205	SG	CYS	587	34.585	21.218	38.360	1.00	0.00	LX0	S
ATOM	206	C	CYS	587	32.921	24.701	39.848	1.00	0.00	LX0	C
ATOM	207	O	CYS	587	32.535	25.537	39.046	1.00	0.00	LX0	O
ATOM	208	N	LYS	588	32.513	24.639	41.126	1.00	0.00	LX0	N
ATOM	209	H	LYS	588	32.874	23.926	41.726	0.00	0.00	LX0	H
ATOM	210	CA	LYS	588	31.604	25.654	41.663	1.00	0.00	LX0	C
ATOM	211	CB	LYS	588	31.353	25.354	43.145	1.00	0.00	LX0	C
ATOM	212	CG	LYS	588	30.480	26.364	43.894	1.00	0.00	LX0	C
ATOM	213	CD	LYS	588	30.504	26.102	45.399	1.00	0.00	LX0	C
ATOM	214	CE	LYS	588	29.583	27.030	46.189	1.00	0.00	LX0	C
ATOM	215	NZ	LYS	588	29.639	26.651	47.607	1.00	0.00	LX0	N
ATOM	216	HZ1	LYS	588	28.991	27.250	48.156	0.00	0.00	LX0	H
ATOM	217	HZ2	LYS	588	29.363	25.653	47.706	0.00	0.00	LX0	H
ATOM	218	HZ3	LYS	588	30.609	26.776	47.962	0.00	0.00	LX0	H
ATOM	219	C	LYS	588	32.131	27.071	41.483	1.00	0.00	LX0	C
ATOM	220	O	LYS	588	31.448	27.977	41.016	1.00	0.00	LX0	O
ATOM	221	N	VAL	589	33.405	27.217	41.876	1.00	0.00	LX0	N

ATOM	222	H	VAL	589	33.924	26.452	42.259	0.00	0.00	LX0	H
ATOM	223	CA	VAL	589	33.970	28.548	41.691	1.00	0.00	LX0	C
ATOM	224	CB	VAL	589	35.187	28.795	42.599	1.00	0.00	LX0	C
ATOM	225	CG1	VAL	589	34.761	28.695	44.065	1.00	0.00	LX0	C
ATOM	226	CG2	VAL	589	36.386	27.892	42.294	1.00	0.00	LX0	C
ATOM	227	C	VAL	589	34.234	28.913	40.241	1.00	0.00	LX0	C
ATOM	228	O	VAL	589	34.028	30.046	39.834	1.00	0.00	LX0	O
ATOM	229	N	PHE	590	34.647	27.901	39.459	1.00	0.00	LX0	N
ATOM	230	H	PHE	590	34.831	26.999	39.849	0.00	0.00	LX0	H
ATOM	231	CA	PHE	590	34.911	28.178	38.047	1.00	0.00	LX0	C
ATOM	232	CB	PHE	590	35.534	26.945	37.379	1.00	0.00	LX0	C
ATOM	233	CG	PHE	590	36.373	27.321	36.176	1.00	0.00	LX0	C
ATOM	234	CD1	PHE	590	37.472	28.196	36.331	1.00	0.00	LX0	C
ATOM	235	CD2	PHE	590	36.053	26.772	34.916	1.00	0.00	LX0	C
ATOM	236	CE1	PHE	590	38.265	28.522	35.213	1.00	0.00	LX0	C
ATOM	237	CE2	PHE	590	36.847	27.096	33.798	1.00	0.00	LX0	C
ATOM	238	CZ	PHE	590	37.944	27.967	33.956	1.00	0.00	LX0	C
ATOM	239	C	PHE	590	33.701	28.689	37.279	1.00	0.00	LX0	C
ATOM	240	O	PHE	590	33.738	29.725	36.631	1.00	0.00	LX0	O
ATOM	241	N	PHE	591	32.600	27.934	37.424	1.00	0.00	LX0	N
ATOM	242	H	PHE	591	32.640	27.104	37.979	0.00	0.00	LX0	H
ATOM	243	CA	PHE	591	31.369	28.304	36.721	1.00	0.00	LX0	C
ATOM	244	CB	PHE	591	30.269	27.280	37.032	1.00	0.00	LX0	C
ATOM	245	CG	PHE	591	29.012	27.544	36.228	1.00	0.00	LX0	C
ATOM	246	CD1	PHE	591	29.048	27.486	34.817	1.00	0.00	LX0	C
ATOM	247	CD2	PHE	591	27.815	27.845	36.913	1.00	0.00	LX0	C
ATOM	248	CE1	PHE	591	27.872	27.722	34.080	1.00	0.00	LX0	C
ATOM	249	CE2	PHE	591	26.636	28.080	36.180	1.00	0.00	LX0	C
ATOM	250	CZ	PHE	591	26.677	28.013	34.771	1.00	0.00	LX0	C
ATOM	251	C	PHE	591	30.889	29.726	36.977	1.00	0.00	LX0	C
ATOM	252	O	PHE	591	30.374	30.405	36.102	1.00	0.00	LX0	O
ATOM	253	N	LYS	592	31.087	30.148	38.232	1.00	0.00	LX0	N
ATOM	254	H	LYS	592	31.556	29.565	38.894	0.00	0.00	LX0	H
ATOM	255	CA	LYS	592	30.742	31.538	38.501	1.00	0.00	LX0	C
ATOM	256	CB	LYS	592	30.400	31.687	39.985	1.00	0.00	LX0	C
ATOM	257	CG	LYS	592	29.762	33.023	40.369	1.00	0.00	LX0	C
ATOM	258	CD	LYS	592	29.642	33.163	41.887	1.00	0.00	LX0	C
ATOM	259	CE	LYS	592	29.096	34.518	42.349	1.00	0.00	LX0	C
ATOM	260	NZ	LYS	592	29.960	35.602	41.873	1.00	0.00	LX0	N
ATOM	261	HZ1	LYS	592	29.949	36.468	42.439	0.00	0.00	LX0	H
ATOM	262	HZ2	LYS	592	30.953	35.324	41.713	0.00	0.00	LX0	H
ATOM	263	HZ3	LYS	592	29.711	35.915	40.907	0.00	0.00	LX0	H
ATOM	264	C	LYS	592	31.847	32.492	38.067	1.00	0.00	LX0	C
ATOM	265	O	LYS	592	31.733	33.268	37.128	1.00	0.00	LX0	O
ATOM	266	N	ARG	593	32.952	32.383	38.812	1.00	0.00	LX0	N
ATOM	267	H	ARG	593	33.041	31.617	39.446	0.00	0.00	LX0	H
ATOM	268	CA	ARG	593	34.008	33.386	38.721	1.00	0.00	LX0	C
ATOM	269	CB	ARG	593	35.028	33.117	39.828	1.00	0.00	LX0	C
ATOM	270	CG	ARG	593	36.097	34.189	40.025	1.00	0.00	LX0	C
ATOM	271	CD	ARG	593	36.969	33.858	41.232	1.00	0.00	LX0	C
ATOM	272	NE	ARG	593	38.101	34.775	41.332	1.00	0.00	LX0	N
ATOM	273	HE	ARG	593	38.769	34.790	40.582	0.00	0.00	LX0	H
ATOM	274	CZ	ARG	593	38.300	35.565	42.403	1.00	0.00	LX0	C
ATOM	275	NH1	ARG	593	37.405	35.613	43.389	1.00	0.00	LX0	N
ATOM	276	HH11	ARG	593	37.536	36.182	44.201	0.00	0.00	LX0	H
ATOM	277	HH12	ARG	593	36.568	35.068	43.321	0.00	0.00	LX0	H
ATOM	278	NH2	ARG	593	39.413	36.288	42.452	1.00	0.00	LX0	N
ATOM	279	HH21	ARG	593	39.632	36.921	43.193	0.00	0.00	LX0	H
ATOM	280	HH22	ARG	593	40.073	36.181	41.695	0.00	0.00	LX0	H
ATOM	281	C	ARG	593	34.651	33.540	37.353	1.00	0.00	LX0	C
ATOM	282	O	ARG	593	35.033	34.630	36.951	1.00	0.00	LX0	O

ATOM	283	N	ALA	594	34.739	32.412	36.633	1.00	0.00	LX0	N
ATOM	284	H	ALA	594	34.361	31.546	36.964	0.00	0.00	LX0	H
ATOM	285	CA	ALA	594	35.276	32.549	35.280	1.00	0.00	LX0	C
ATOM	286	CB	ALA	594	35.665	31.196	34.685	1.00	0.00	LX0	C
ATOM	287	C	ALA	594	34.340	33.260	34.319	1.00	0.00	LX0	C
ATOM	288	O	ALA	594	34.766	33.955	33.407	1.00	0.00	LX0	O
ATOM	289	N	MET	595	33.035	33.082	34.583	1.00	0.00	LX0	N
ATOM	290	H	MET	595	32.731	32.546	35.371	0.00	0.00	LX0	H
ATOM	291	CA	MET	595	32.073	33.857	33.801	1.00	0.00	LX0	C
ATOM	292	CB	MET	595	30.675	33.242	33.893	1.00	0.00	LX0	C
ATOM	293	CG	MET	595	30.549	31.898	33.172	1.00	0.00	LX0	C
ATOM	294	SD	MET	595	30.778	32.028	31.390	1.00	0.00	LX0	S
ATOM	295	CE	MET	595	29.366	33.082	31.021	1.00	0.00	LX0	C
ATOM	296	C	MET	595	32.020	35.327	34.181	1.00	0.00	LX0	C
ATOM	297	O	MET	595	31.706	36.190	33.372	1.00	0.00	LX0	O
ATOM	298	N	GLU	596	32.353	35.581	35.457	1.00	0.00	LX0	N
ATOM	299	H	GLU	596	32.518	34.819	36.083	0.00	0.00	LX0	H
ATOM	300	CA	GLU	596	32.421	36.974	35.899	1.00	0.00	LX0	C
ATOM	301	CB	GLU	596	32.431	37.050	37.428	1.00	0.00	LX0	C
ATOM	302	CG	GLU	596	31.215	36.359	38.043	1.00	0.00	LX0	C
ATOM	303	CD	GLU	596	31.253	36.447	39.554	1.00	0.00	LX0	C
ATOM	304	OE1	GLU	596	31.897	35.625	40.205	1.00	0.00	LX0	O
ATOM	305	OE2	GLU	596	30.551	37.283	40.117	1.00	0.00	LX0	O
ATOM	306	C	GLU	596	33.609	37.727	35.324	1.00	0.00	LX0	C
ATOM	307	O	GLU	596	33.499	38.784	34.712	1.00	0.00	LX0	O
ATOM	308	N	GLY	597	34.779	37.116	35.545	1.00	0.00	LX0	N
ATOM	309	H	GLY	597	34.793	36.209	35.963	0.00	0.00	LX0	H
ATOM	310	CA	GLY	597	35.985	37.715	34.991	1.00	0.00	LX0	C
ATOM	311	C	GLY	597	36.082	37.476	33.501	1.00	0.00	LX0	C
ATOM	312	O	GLY	597	36.268	36.358	33.041	1.00	0.00	LX0	O
ATOM	313	N	GLN	598	35.933	38.590	32.771	1.00	0.00	LX0	N
ATOM	314	H	GLN	598	35.796	39.454	33.250	0.00	0.00	LX0	H
ATOM	315	CA	GLN	598	35.951	38.509	31.310	1.00	0.00	LX0	C
ATOM	316	CB	GLN	598	35.344	39.777	30.695	1.00	0.00	LX0	C
ATOM	317	CG	GLN	598	33.946	40.140	31.211	1.00	0.00	LX0	C
ATOM	318	CD	GLN	598	32.969	39.025	30.905	1.00	0.00	LX0	C
ATOM	319	OE1	GLN	598	32.659	38.720	29.762	1.00	0.00	LX0	O
ATOM	320	NE2	GLN	598	32.514	38.413	31.996	1.00	0.00	LX0	N
ATOM	321	HE21	GLN	598	32.761	38.715	32.917	0.00	0.00	LX0	H
ATOM	322	HE22	GLN	598	31.928	37.601	31.947	0.00	0.00	LX0	H
ATOM	323	C	GLN	598	37.323	38.240	30.716	1.00	0.00	LX0	C
ATOM	324	O	GLN	598	37.987	39.117	30.178	1.00	0.00	LX0	O
ATOM	325	N	HIS	599	37.726	36.977	30.849	1.00	0.00	LX0	N
ATOM	326	H	HIS	599	37.125	36.299	31.275	0.00	0.00	LX0	H
ATOM	327	CA	HIS	599	39.007	36.597	30.278	1.00	0.00	LX0	C
ATOM	328	CB	HIS	599	39.879	35.891	31.313	1.00	0.00	LX0	C
ATOM	329	CG	HIS	599	39.965	36.653	32.613	1.00	0.00	LX0	C
ATOM	330	ND1	HIS	599	39.431	36.201	33.761	1.00	0.00	LX0	N
ATOM	331	HD1	HIS	599	38.914	35.369	33.870	0.00	0.00	LX0	H
ATOM	332	CD2	HIS	599	40.589	37.879	32.860	1.00	0.00	LX0	C
ATOM	333	NE2	HIS	599	40.422	38.152	34.179	1.00	0.00	LX0	N
ATOM	334	CE1	HIS	599	39.711	37.120	34.733	1.00	0.00	LX0	C
ATOM	335	C	HIS	599	38.763	35.673	29.111	1.00	0.00	LX0	C
ATOM	336	O	HIS	599	37.819	34.891	29.103	1.00	0.00	LX0	O
ATOM	337	N	ASN	600	39.628	35.795	28.103	1.00	0.00	LX0	N
ATOM	338	H	ASN	600	40.489	36.282	28.237	0.00	0.00	LX0	H
ATOM	339	CA	ASN	600	39.387	34.893	26.983	1.00	0.00	LX0	C
ATOM	340	CB	ASN	600	39.733	35.543	25.646	1.00	0.00	LX0	C
ATOM	341	CG	ASN	600	39.190	34.657	24.544	1.00	0.00	LX0	C
ATOM	342	OD1	ASN	600	38.094	34.121	24.617	1.00	0.00	LX0	O
ATOM	343	ND2	ASN	600	40.015	34.502	23.518	1.00	0.00	LX0	N

ATOM	344	HD21	ASN	600	40.954	34.850	23.491	0.00	0.00	LX0	H
ATOM	345	HD22	ASN	600	39.700	33.988	22.725	0.00	0.00	LX0	H
ATOM	346	C	ASN	600	40.095	33.564	27.143	1.00	0.00	LX0	C
ATOM	347	O	ASN	600	41.212	33.368	26.678	1.00	0.00	LX0	O
ATOM	348	N	TYR	601	39.371	32.658	27.809	1.00	0.00	LX0	N
ATOM	349	H	TYR	601	38.469	32.958	28.125	0.00	0.00	LX0	H
ATOM	350	CA	TYR	601	39.943	31.365	28.202	1.00	0.00	LX0	C
ATOM	351	CB	TYR	601	39.059	30.682	29.258	1.00	0.00	LX0	C
ATOM	352	CG	TYR	601	38.680	31.584	30.411	1.00	0.00	LX0	C
ATOM	353	CD1	TYR	601	39.610	31.830	31.442	1.00	0.00	LX0	C
ATOM	354	CE1	TYR	601	39.230	32.653	32.516	1.00	0.00	LX0	C
ATOM	355	CD2	TYR	601	37.385	32.139	30.426	1.00	0.00	LX0	C
ATOM	356	CE2	TYR	601	37.011	32.972	31.492	1.00	0.00	LX0	C
ATOM	357	CZ	TYR	601	37.940	33.224	32.521	1.00	0.00	LX0	C
ATOM	358	OH	TYR	601	37.583	34.059	33.562	1.00	0.00	LX0	O
ATOM	359	HH	TYR	601	36.727	34.457	33.364	0.00	0.00	LX0	H
ATOM	360	C	TYR	601	40.213	30.334	27.102	1.00	0.00	LX0	C
ATOM	361	O	TYR	601	40.178	29.136	27.363	1.00	0.00	LX0	O
ATOM	362	N	LEU	602	40.412	30.812	25.861	1.00	0.00	LX0	N
ATOM	363	H	LEU	602	40.646	31.775	25.743	0.00	0.00	LX0	H
ATOM	364	CA	LEU	602	40.302	29.914	24.708	1.00	0.00	LX0	C
ATOM	365	CB	LEU	602	40.503	30.665	23.389	1.00	0.00	LX0	C
ATOM	366	CG	LEU	602	39.207	31.190	22.765	1.00	0.00	LX0	C
ATOM	367	CD1	LEU	602	39.467	31.758	21.369	1.00	0.00	LX0	C
ATOM	368	CD2	LEU	602	38.095	30.138	22.731	1.00	0.00	LX0	C
ATOM	369	C	LEU	602	41.154	28.657	24.696	1.00	0.00	LX0	C
ATOM	370	O	LEU	602	42.188	28.542	25.343	1.00	0.00	LX0	O
ATOM	371	N	CYS	603	40.638	27.713	23.898	1.00	0.00	LX0	N
ATOM	372	H	CYS	603	39.842	27.907	23.326	0.00	0.00	LX0	H
ATOM	373	CA	CYS	603	41.381	26.483	23.662	1.00	0.00	LX0	C
ATOM	374	CB	CYS	603	40.551	25.270	24.080	1.00	0.00	LX0	C
ATOM	375	SG	CYS	603	41.444	23.699	23.935	1.00	0.00	LX0	S
ATOM	376	C	CYS	603	41.755	26.398	22.199	1.00	0.00	LX0	C
ATOM	377	O	CYS	603	41.066	26.958	21.357	1.00	0.00	LX0	O
ATOM	378	N	ALA	604	42.855	25.681	21.934	1.00	0.00	LX0	N
ATOM	379	H	ALA	604	43.400	25.337	22.701	0.00	0.00	LX0	H
ATOM	380	CA	ALA	604	43.250	25.480	20.540	1.00	0.00	LX0	C
ATOM	381	CB	ALA	604	44.739	25.783	20.362	1.00	0.00	LX0	C
ATOM	382	C	ALA	604	42.964	24.087	19.993	1.00	0.00	LX0	C
ATOM	383	O	ALA	604	43.450	23.695	18.938	1.00	0.00	LX0	O
ATOM	384	N	GLY	605	42.171	23.335	20.772	1.00	0.00	LX0	N
ATOM	385	H	GLY	605	41.719	23.738	21.563	0.00	0.00	LX0	H
ATOM	386	CA	GLY	605	41.733	22.047	20.239	1.00	0.00	LX0	C
ATOM	387	C	GLY	605	40.519	22.241	19.355	1.00	0.00	LX0	C
ATOM	388	O	GLY	605	40.038	23.354	19.188	1.00	0.00	LX0	O
ATOM	389	N	ARG	606	40.040	21.125	18.788	1.00	0.00	LX0	N
ATOM	390	H	ARG	606	40.363	20.225	19.096	0.00	0.00	LX0	H
ATOM	391	CA	ARG	606	38.860	21.292	17.939	1.00	0.00	LX0	C
ATOM	392	CB	ARG	606	38.710	20.199	16.868	1.00	0.00	LX0	C
ATOM	393	CG	ARG	606	39.870	19.902	15.905	1.00	0.00	LX0	C
ATOM	394	CD	ARG	606	41.052	19.162	16.540	1.00	0.00	LX0	C
ATOM	395	NE	ARG	606	41.678	18.245	15.593	1.00	0.00	LX0	N
ATOM	396	HE	ARG	606	42.110	18.636	14.781	0.00	0.00	LX0	H
ATOM	397	CZ	ARG	606	41.635	16.913	15.822	1.00	0.00	LX0	C
ATOM	398	NH1	ARG	606	41.057	16.403	16.907	1.00	0.00	LX0	N
ATOM	399	HH11	ARG	606	41.068	15.405	17.056	0.00	0.00	LX0	H
ATOM	400	HH12	ARG	606	40.595	16.975	17.600	0.00	0.00	LX0	H
ATOM	401	NH2	ARG	606	42.183	16.091	14.935	1.00	0.00	LX0	N
ATOM	402	HH21	ARG	606	42.149	15.093	15.106	0.00	0.00	LX0	H
ATOM	403	HH22	ARG	606	42.629	16.406	14.101	0.00	0.00	LX0	H
ATOM	404	C	ARG	606	37.598	21.324	18.779	1.00	0.00	LX0	C

ATOM	405	O	ARG	606	36.751	22.205	18.675	1.00	0.00	LX0	O
ATOM	406	N	ASN	607	37.536	20.296	19.644	1.00	0.00	LX0	N
ATOM	407	H	ASN	607	38.309	19.652	19.639	0.00	0.00	LX0	H
ATOM	408	CA	ASN	607	36.529	20.251	20.711	1.00	0.00	LX0	C
ATOM	409	CB	ASN	607	35.077	20.286	20.240	1.00	0.00	LX0	C
ATOM	410	CG	ASN	607	34.399	21.371	21.048	1.00	0.00	LX0	C
ATOM	411	OD1	ASN	607	33.935	21.173	22.166	1.00	0.00	LX0	O
ATOM	412	ND2	ASN	607	34.384	22.551	20.428	1.00	0.00	LX0	N
ATOM	413	HD21	ASN	607	34.897	22.659	19.570	0.00	0.00	LX0	H
ATOM	414	HD22	ASN	607	33.893	23.332	20.809	0.00	0.00	LX0	H
ATOM	415	C	ASN	607	36.701	19.073	21.634	1.00	0.00	LX0	C
ATOM	416	O	ASN	607	36.420	19.134	22.825	1.00	0.00	LX0	O
ATOM	417	N	ASP	608	37.246	18.012	21.019	1.00	0.00	LX0	N
ATOM	418	H	ASP	608	37.326	18.025	20.022	0.00	0.00	LX0	H
ATOM	419	CA	ASP	608	37.993	16.948	21.707	1.00	0.00	LX0	C
ATOM	420	CB	ASP	608	39.207	16.529	20.851	1.00	0.00	LX0	C
ATOM	421	CG	ASP	608	39.660	17.672	19.950	1.00	0.00	LX0	C
ATOM	422	OD1	ASP	608	39.371	17.621	18.763	1.00	0.00	LX0	O
ATOM	423	OD2	ASP	608	40.206	18.656	20.436	1.00	0.00	LX0	O
ATOM	424	C	ASP	608	38.422	17.242	23.139	1.00	0.00	LX0	C
ATOM	425	O	ASP	608	37.993	16.580	24.076	1.00	0.00	LX0	O
ATOM	426	N	CYS	609	39.242	18.307	23.253	1.00	0.00	LX0	N
ATOM	427	H	CYS	609	39.606	18.669	22.390	0.00	0.00	LX0	H
ATOM	428	CA	CYS	609	39.514	19.021	24.508	1.00	0.00	LX0	C
ATOM	429	CB	CYS	609	39.056	20.466	24.365	1.00	0.00	LX0	C
ATOM	430	SG	CYS	609	39.438	21.182	22.747	1.00	0.00	LX0	S
ATOM	431	C	CYS	609	38.981	18.415	25.804	1.00	0.00	LX0	C
ATOM	432	O	CYS	609	37.903	18.733	26.312	1.00	0.00	LX0	O
ATOM	433	N	ILE	610	39.815	17.483	26.278	1.00	0.00	LX0	N
ATOM	434	H	ILE	610	40.715	17.393	25.855	0.00	0.00	LX0	H
ATOM	435	CA	ILE	610	39.384	16.541	27.306	1.00	0.00	LX0	C
ATOM	436	CB	ILE	610	40.236	15.268	27.190	1.00	0.00	LX0	C
ATOM	437	CG2	ILE	610	39.796	14.206	28.190	1.00	0.00	LX0	C
ATOM	438	CG1	ILE	610	40.250	14.714	25.762	1.00	0.00	LX0	C
ATOM	439	CD1	ILE	610	41.265	13.584	25.580	1.00	0.00	LX0	C
ATOM	440	C	ILE	610	39.451	17.098	28.718	1.00	0.00	LX0	C
ATOM	441	O	ILE	610	40.475	17.601	29.162	1.00	0.00	LX0	O
ATOM	442	N	VAL	611	38.321	16.947	29.423	1.00	0.00	LX0	N
ATOM	443	H	VAL	611	37.499	16.585	28.981	0.00	0.00	LX0	H
ATOM	444	CA	VAL	611	38.365	17.214	30.859	1.00	0.00	LX0	C
ATOM	445	CB	VAL	611	37.462	18.405	31.231	1.00	0.00	LX0	C
ATOM	446	CG1	VAL	611	37.662	18.835	32.688	1.00	0.00	LX0	C
ATOM	447	CG2	VAL	611	37.664	19.594	30.286	1.00	0.00	LX0	C
ATOM	448	C	VAL	611	38.029	15.960	31.665	1.00	0.00	LX0	C
ATOM	449	O	VAL	611	36.917	15.759	32.162	1.00	0.00	LX0	O
ATOM	450	N	ASP	612	39.060	15.104	31.740	1.00	0.00	LX0	N
ATOM	451	H	ASP	612	39.942	15.339	31.333	0.00	0.00	LX0	H
ATOM	452	CA	ASP	612	38.941	13.898	32.560	1.00	0.00	LX0	C
ATOM	453	CB	ASP	612	39.364	12.634	31.776	1.00	0.00	LX0	C
ATOM	454	CG	ASP	612	40.834	12.594	31.358	1.00	0.00	LX0	C
ATOM	455	OD1	ASP	612	41.710	13.018	32.100	1.00	0.00	LX0	O
ATOM	456	OD2	ASP	612	41.134	12.102	30.277	1.00	0.00	LX0	O
ATOM	457	C	ASP	612	39.667	14.036	33.891	1.00	0.00	LX0	C
ATOM	458	O	ASP	612	40.186	15.101	34.213	1.00	0.00	LX0	O
ATOM	459	N	LYS	613	39.695	12.913	34.645	1.00	0.00	LX0	N
ATOM	460	H	LYS	613	39.273	12.078	34.298	0.00	0.00	LX0	H
ATOM	461	CA	LYS	613	40.322	12.924	35.973	1.00	0.00	LX0	C
ATOM	462	CB	LYS	613	40.293	11.541	36.642	1.00	0.00	LX0	C
ATOM	463	CG	LYS	613	38.924	10.974	37.049	1.00	0.00	LX0	C
ATOM	464	CD	LYS	613	39.110	9.753	37.966	1.00	0.00	LX0	C
ATOM	465	CE	LYS	613	37.849	8.982	38.396	1.00	0.00	LX0	C

ATOM	466	NZ	LYS	613	36.966	9.786	39.251	1.00	0.00	LX0	N
ATOM	467	HZ1	LYS	613	36.193	9.204	39.636	0.00	0.00	LX0	H
ATOM	468	HZ2	LYS	613	37.464	10.237	40.052	0.00	0.00	LX0	H
ATOM	469	HZ3	LYS	613	36.537	10.561	38.716	0.00	0.00	LX0	H
ATOM	470	C	LYS	613	41.743	13.475	36.061	1.00	0.00	LX0	C
ATOM	471	O	LYS	613	42.173	13.969	37.098	1.00	0.00	LX0	O
ATOM	472	N	ILE	614	42.457	13.367	34.934	1.00	0.00	LX0	N
ATOM	473	H	ILE	614	42.036	12.989	34.109	0.00	0.00	LX0	H
ATOM	474	CA	ILE	614	43.789	13.964	34.914	1.00	0.00	LX0	C
ATOM	475	CB	ILE	614	44.761	13.046	34.154	1.00	0.00	LX0	C
ATOM	476	CG2	ILE	614	46.197	13.585	34.175	1.00	0.00	LX0	C
ATOM	477	CG1	ILE	614	44.681	11.611	34.687	1.00	0.00	LX0	C
ATOM	478	CD1	ILE	614	45.502	10.616	33.864	1.00	0.00	LX0	C
ATOM	479	C	ILE	614	43.778	15.365	34.319	1.00	0.00	LX0	C
ATOM	480	O	ILE	614	44.295	16.331	34.865	1.00	0.00	LX0	O
ATOM	481	N	ARG	615	43.157	15.437	33.134	1.00	0.00	LX0	N
ATOM	482	H	ARG	615	42.620	14.657	32.810	0.00	0.00	LX0	H
ATOM	483	CA	ARG	615	43.296	16.665	32.352	1.00	0.00	LX0	C
ATOM	484	CB	ARG	615	43.038	16.358	30.880	1.00	0.00	LX0	C
ATOM	485	CG	ARG	615	44.319	15.854	30.210	1.00	0.00	LX0	C
ATOM	486	CD	ARG	615	44.066	14.765	29.169	1.00	0.00	LX0	C
ATOM	487	NE	ARG	615	43.546	13.572	29.831	1.00	0.00	LX0	N
ATOM	488	HE	ARG	615	42.603	13.573	30.191	0.00	0.00	LX0	H
ATOM	489	CZ	ARG	615	44.299	12.488	30.099	1.00	0.00	LX0	C
ATOM	490	NH1	ARG	615	45.543	12.388	29.635	1.00	0.00	LX0	N
ATOM	491	HH11	ARG	615	46.116	11.591	29.825	0.00	0.00	LX0	H
ATOM	492	HH12	ARG	615	45.921	13.124	29.072	0.00	0.00	LX0	H
ATOM	493	NH2	ARG	615	43.764	11.526	30.840	1.00	0.00	LX0	N
ATOM	494	HH21	ARG	615	44.227	10.680	31.096	0.00	0.00	LX0	H
ATOM	495	HH22	ARG	615	42.814	11.683	31.161	0.00	0.00	LX0	H
ATOM	496	C	ARG	615	42.534	17.890	32.828	1.00	0.00	LX0	C
ATOM	497	O	ARG	615	42.660	18.976	32.274	1.00	0.00	LX0	O
ATOM	498	N	ARG	616	41.773	17.695	33.916	1.00	0.00	LX0	N
ATOM	499	H	ARG	616	41.653	16.756	34.242	0.00	0.00	LX0	H
ATOM	500	CA	ARG	616	41.006	18.790	34.518	1.00	0.00	LX0	C
ATOM	501	CB	ARG	616	40.140	18.239	35.664	1.00	0.00	LX0	C
ATOM	502	CG	ARG	616	40.869	17.359	36.687	1.00	0.00	LX0	C
ATOM	503	CD	ARG	616	39.895	16.697	37.669	1.00	0.00	LX0	C
ATOM	504	NE	ARG	616	40.566	15.694	38.498	1.00	0.00	LX0	N
ATOM	505	HE	ARG	616	41.556	15.769	38.638	0.00	0.00	LX0	H
ATOM	506	CZ	ARG	616	39.898	14.623	38.984	1.00	0.00	LX0	C
ATOM	507	NH1	ARG	616	38.628	14.415	38.677	1.00	0.00	LX0	N
ATOM	508	HH11	ARG	616	38.148	13.630	39.090	0.00	0.00	LX0	H
ATOM	509	HH12	ARG	616	38.126	14.995	38.032	0.00	0.00	LX0	H
ATOM	510	NH2	ARG	616	40.504	13.734	39.763	1.00	0.00	LX0	N
ATOM	511	HH21	ARG	616	39.958	12.973	40.143	0.00	0.00	LX0	H
ATOM	512	HH22	ARG	616	41.479	13.790	39.973	0.00	0.00	LX0	H
ATOM	513	C	ARG	616	41.684	20.113	34.902	1.00	0.00	LX0	C
ATOM	514	O	ARG	616	41.006	21.033	35.358	1.00	0.00	LX0	O
ATOM	515	N	LYS	617	43.013	20.212	34.697	1.00	0.00	LX0	N
ATOM	516	H	LYS	617	43.531	19.422	34.373	0.00	0.00	LX0	H
ATOM	517	CA	LYS	617	43.567	21.568	34.741	1.00	0.00	LX0	C
ATOM	518	CB	LYS	617	44.917	21.659	35.494	1.00	0.00	LX0	C
ATOM	519	CG	LYS	617	46.214	21.440	34.686	1.00	0.00	LX0	C
ATOM	520	CD	LYS	617	47.476	21.939	35.402	1.00	0.00	LX0	C
ATOM	521	CE	LYS	617	48.757	21.937	34.548	1.00	0.00	LX0	C
ATOM	522	NZ	LYS	617	48.672	22.913	33.453	1.00	0.00	LX0	N
ATOM	523	HZ1	LYS	617	49.536	23.488	33.361	0.00	0.00	LX0	H
ATOM	524	HZ2	LYS	617	48.511	22.443	32.540	0.00	0.00	LX0	H
ATOM	525	HZ3	LYS	617	47.878	23.566	33.600	0.00	0.00	LX0	H
ATOM	526	C	LYS	617	43.659	22.282	33.394	1.00	0.00	LX0	C

ATOM	527	O	LYS	617	43.493	23.490	33.285	1.00	0.00	LX0	O
ATOM	528	N	ASN	618	43.995	21.492	32.362	1.00	0.00	LX0	N
ATOM	529	H	ASN	618	43.838	20.504	32.386	0.00	0.00	LX0	H
ATOM	530	CA	ASN	618	44.696	22.155	31.261	1.00	0.00	LX0	C
ATOM	531	CB	ASN	618	45.748	21.242	30.627	1.00	0.00	LX0	C
ATOM	532	CG	ASN	618	46.977	22.058	30.256	1.00	0.00	LX0	C
ATOM	533	OD1	ASN	618	47.914	22.181	31.039	1.00	0.00	LX0	O
ATOM	534	ND2	ASN	618	46.953	22.591	29.032	1.00	0.00	LX0	N
ATOM	535	HD21	ASN	618	46.113	22.570	28.487	0.00	0.00	LX0	H
ATOM	536	HD22	ASN	618	47.728	23.078	28.630	0.00	0.00	LX0	H
ATOM	537	C	ASN	618	43.849	22.796	30.186	1.00	0.00	LX0	C
ATOM	538	O	ASN	618	44.359	23.410	29.260	1.00	0.00	LX0	O
ATOM	539	N	CYS	619	42.532	22.629	30.320	1.00	0.00	LX0	N
ATOM	540	H	CYS	619	42.128	22.122	31.080	0.00	0.00	LX0	H
ATOM	541	CA	CYS	619	41.740	23.250	29.264	1.00	0.00	LX0	C
ATOM	542	CB	CYS	619	41.156	22.179	28.344	1.00	0.00	LX0	C
ATOM	543	SG	CYS	619	40.500	22.885	26.816	1.00	0.00	LX0	S
ATOM	544	C	CYS	619	40.669	24.182	29.789	1.00	0.00	LX0	C
ATOM	545	O	CYS	619	39.527	23.788	29.990	1.00	0.00	LX0	O
ATOM	546	N	PRO	620	41.071	25.459	30.007	1.00	0.00	LX0	N
ATOM	547	CD	PRO	620	42.365	26.036	29.664	1.00	0.00	LX0	C
ATOM	548	CA	PRO	620	40.170	26.418	30.661	1.00	0.00	LX0	C
ATOM	549	CB	PRO	620	41.024	27.692	30.737	1.00	0.00	LX0	C
ATOM	550	CG	PRO	620	42.113	27.530	29.677	1.00	0.00	LX0	C
ATOM	551	C	PRO	620	38.814	26.566	29.981	1.00	0.00	LX0	C
ATOM	552	O	PRO	620	37.785	26.218	30.552	1.00	0.00	LX0	O
ATOM	553	N	ALA	621	38.850	27.022	28.710	1.00	0.00	LX0	N
ATOM	554	H	ALA	621	39.682	27.447	28.343	0.00	0.00	LX0	H
ATOM	555	CA	ALA	621	37.604	27.116	27.941	1.00	0.00	LX0	C
ATOM	556	CB	ALA	621	37.865	27.387	26.459	1.00	0.00	LX0	C
ATOM	557	C	ALA	621	36.741	25.878	27.994	1.00	0.00	LX0	C
ATOM	558	O	ALA	621	35.531	25.936	28.137	1.00	0.00	LX0	O
ATOM	559	N	CYS	622	37.423	24.734	27.881	1.00	0.00	LX0	N
ATOM	560	H	CYS	622	38.421	24.717	27.860	0.00	0.00	LX0	H
ATOM	561	CA	CYS	622	36.613	23.528	27.829	1.00	0.00	LX0	C
ATOM	562	CB	CYS	622	37.260	22.500	26.922	1.00	0.00	LX0	C
ATOM	563	SG	CYS	622	37.386	23.160	25.242	1.00	0.00	LX0	S
ATOM	564	C	CYS	622	36.178	22.938	29.152	1.00	0.00	LX0	C
ATOM	565	O	CYS	622	35.303	22.082	29.196	1.00	0.00	LX0	O
ATOM	566	N	ARG	623	36.781	23.456	30.232	1.00	0.00	LX0	N
ATOM	567	H	ARG	623	37.530	24.116	30.147	0.00	0.00	LX0	H
ATOM	568	CA	ARG	623	36.229	23.127	31.542	1.00	0.00	LX0	C
ATOM	569	CB	ARG	623	37.311	23.210	32.624	1.00	0.00	LX0	C
ATOM	570	CG	ARG	623	36.829	22.683	33.978	1.00	0.00	LX0	C
ATOM	571	CD	ARG	623	37.915	22.658	35.053	1.00	0.00	LX0	C
ATOM	572	NE	ARG	623	37.323	22.333	36.350	1.00	0.00	LX0	N
ATOM	573	HE	ARG	623	36.326	22.411	36.414	0.00	0.00	LX0	H
ATOM	574	CZ	ARG	623	38.088	22.067	37.430	1.00	0.00	LX0	C
ATOM	575	NH1	ARG	623	39.410	21.941	37.318	1.00	0.00	LX0	N
ATOM	576	HH11	ARG	623	40.004	21.824	38.112	0.00	0.00	LX0	H
ATOM	577	HH12	ARG	623	39.858	21.945	36.413	0.00	0.00	LX0	H
ATOM	578	NH2	ARG	623	37.493	21.932	38.613	1.00	0.00	LX0	N
ATOM	579	HH21	ARG	623	37.991	21.794	39.477	0.00	0.00	LX0	H
ATOM	580	HH22	ARG	623	36.496	21.971	38.678	0.00	0.00	LX0	H
ATOM	581	C	ARG	623	35.012	23.978	31.866	1.00	0.00	LX0	C
ATOM	582	O	ARG	623	34.004	23.498	32.368	1.00	0.00	LX0	O
ATOM	583	N	LEU	624	35.124	25.263	31.488	1.00	0.00	LX0	N
ATOM	584	H	LEU	624	35.992	25.610	31.125	0.00	0.00	LX0	H
ATOM	585	CA	LEU	624	33.945	26.128	31.589	1.00	0.00	LX0	C
ATOM	586	CB	LEU	624	34.338	27.564	31.232	1.00	0.00	LX0	C
ATOM	587	CG	LEU	624	33.320	28.627	31.653	1.00	0.00	LX0	C

ATOM	588	CD1	LEU	624	32.987	28.559	33.146	1.00	0.00	LX0	C
ATOM	589	CD2	LEU	624	33.782	30.025	31.238	1.00	0.00	LX0	C
ATOM	590	C	LEU	624	32.787	25.621	30.738	1.00	0.00	LX0	C
ATOM	591	O	LEU	624	31.653	25.461	31.174	1.00	0.00	LX0	O
ATOM	592	N	ARG	625	33.182	25.268	29.503	1.00	0.00	LX0	N
ATOM	593	H	ARG	625	34.081	25.575	29.201	0.00	0.00	LX0	H
ATOM	594	CA	ARG	625	32.309	24.502	28.614	1.00	0.00	LX0	C
ATOM	595	CB	ARG	625	33.071	24.030	27.371	1.00	0.00	LX0	C
ATOM	596	CG	ARG	625	32.235	23.266	26.338	1.00	0.00	LX0	C
ATOM	597	CD	ARG	625	33.087	22.317	25.490	1.00	0.00	LX0	C
ATOM	598	NE	ARG	625	33.714	21.294	26.333	1.00	0.00	LX0	N
ATOM	599	HE	ARG	625	33.417	21.204	27.290	0.00	0.00	LX0	H
ATOM	600	CZ	ARG	625	34.635	20.444	25.837	1.00	0.00	LX0	C
ATOM	601	NH1	ARG	625	34.905	20.427	24.539	1.00	0.00	LX0	N
ATOM	602	HH11	ARG	625	35.610	19.828	24.147	0.00	0.00	LX0	H
ATOM	603	HH12	ARG	625	34.403	20.996	23.875	0.00	0.00	LX0	H
ATOM	604	NH2	ARG	625	35.280	19.625	26.665	1.00	0.00	LX0	N
ATOM	605	HH21	ARG	625	36.046	19.046	26.353	0.00	0.00	LX0	H
ATOM	606	HH22	ARG	625	35.016	19.587	27.630	0.00	0.00	LX0	H
ATOM	607	C	ARG	625	31.694	23.307	29.311	1.00	0.00	LX0	C
ATOM	608	O	ARG	625	30.490	23.127	29.294	1.00	0.00	LX0	O
ATOM	609	N	LYS	626	32.565	22.513	29.954	1.00	0.00	LX0	N
ATOM	610	H	LYS	626	33.545	22.698	29.927	0.00	0.00	LX0	H
ATOM	611	CA	LYS	626	32.055	21.326	30.637	1.00	0.00	LX0	C
ATOM	612	CB	LYS	626	33.196	20.475	31.186	1.00	0.00	LX0	C
ATOM	613	CG	LYS	626	32.844	18.989	31.173	1.00	0.00	LX0	C
ATOM	614	CD	LYS	626	33.914	18.161	31.865	1.00	0.00	LX0	C
ATOM	615	CE	LYS	626	33.607	16.667	31.903	1.00	0.00	LX0	C
ATOM	616	NZ	LYS	626	34.403	16.060	32.973	1.00	0.00	LX0	N
ATOM	617	HZ1	LYS	626	33.785	15.443	33.544	0.00	0.00	LX0	H
ATOM	618	HZ2	LYS	626	35.235	15.562	32.589	0.00	0.00	LX0	H
ATOM	619	HZ3	LYS	626	34.747	16.820	33.593	0.00	0.00	LX0	H
ATOM	620	C	LYS	626	30.993	21.584	31.690	1.00	0.00	LX0	C
ATOM	621	O	LYS	626	30.031	20.841	31.834	1.00	0.00	LX0	O
ATOM	622	N	CYS	627	31.177	22.718	32.381	1.00	0.00	LX0	N
ATOM	623	H	CYS	627	32.017	23.251	32.261	0.00	0.00	LX0	H
ATOM	624	CA	CYS	627	30.093	23.187	33.243	1.00	0.00	LX0	C
ATOM	625	CB	CYS	627	30.527	24.454	33.979	1.00	0.00	LX0	C
ATOM	626	SG	CYS	627	32.021	24.233	34.982	1.00	0.00	LX0	S
ATOM	627	C	CYS	627	28.776	23.381	32.498	1.00	0.00	LX0	C
ATOM	628	O	CYS	627	27.731	22.837	32.844	1.00	0.00	LX0	O
ATOM	629	N	CYS	628	28.879	24.126	31.388	1.00	0.00	LX0	N
ATOM	630	H	CYS	628	29.752	24.543	31.124	0.00	0.00	LX0	H
ATOM	631	CA	CYS	628	27.702	24.220	30.517	1.00	0.00	LX0	C
ATOM	632	CB	CYS	628	27.976	25.143	29.331	1.00	0.00	LX0	C
ATOM	633	SG	CYS	628	28.608	26.757	29.851	1.00	0.00	LX0	S
ATOM	634	C	CYS	628	27.151	22.881	30.039	1.00	0.00	LX0	C
ATOM	635	O	CYS	628	25.949	22.674	29.961	1.00	0.00	LX0	O
ATOM	636	N	GLN	629	28.083	21.948	29.794	1.00	0.00	LX0	N
ATOM	637	H	GLN	629	29.038	22.186	29.955	0.00	0.00	LX0	H
ATOM	638	CA	GLN	629	27.738	20.609	29.314	1.00	0.00	LX0	C
ATOM	639	CB	GLN	629	29.000	19.867	28.840	1.00	0.00	LX0	C
ATOM	640	CG	GLN	629	29.739	20.588	27.705	1.00	0.00	LX0	C
ATOM	641	CD	GLN	629	31.034	19.886	27.313	1.00	0.00	LX0	C
ATOM	642	OE1	GLN	629	32.005	19.786	28.054	1.00	0.00	LX0	O
ATOM	643	NE2	GLN	629	31.053	19.438	26.061	1.00	0.00	LX0	N
ATOM	644	HE21	GLN	629	30.277	19.608	25.448	0.00	0.00	LX0	H
ATOM	645	HE22	GLN	629	31.821	18.924	25.691	0.00	0.00	LX0	H
ATOM	646	C	GLN	629	26.945	19.744	30.289	1.00	0.00	LX0	C
ATOM	647	O	GLN	629	26.514	18.647	29.967	1.00	0.00	LX0	O
ATOM	648	N	ALA	630	26.741	20.252	31.512	1.00	0.00	LX0	N

ATOM	649	H	ALA	630	27.188	21.092	31.829	0.00	0.00	LX0	H
ATOM	650	CA	ALA	630	25.795	19.483	32.318	1.00	0.00	LX0	C
ATOM	651	CB	ALA	630	26.425	19.101	33.653	1.00	0.00	LX0	C
ATOM	652	C	ALA	630	24.454	20.166	32.542	1.00	0.00	LX0	C
ATOM	653	O	ALA	630	23.572	19.681	33.247	1.00	0.00	LX0	O
ATOM	654	N	GLY	631	24.322	21.343	31.918	1.00	0.00	LX0	N
ATOM	655	H	GLY	631	25.041	21.682	31.313	0.00	0.00	LX0	H
ATOM	656	CA	GLY	631	23.117	22.129	32.160	1.00	0.00	LX0	C
ATOM	657	C	GLY	631	23.119	22.790	33.522	1.00	0.00	LX0	C
ATOM	658	O	GLY	631	22.192	22.658	34.314	1.00	0.00	LX0	O
ATOM	659	N	MET	632	24.225	23.501	33.775	1.00	0.00	LX0	N
ATOM	660	H	MET	632	24.937	23.623	33.083	0.00	0.00	LX0	H
ATOM	661	CA	MET	632	24.267	24.193	35.057	1.00	0.00	LX0	C
ATOM	662	CB	MET	632	25.698	24.514	35.467	1.00	0.00	LX0	C
ATOM	663	CG	MET	632	26.512	23.239	35.646	1.00	0.00	LX0	C
ATOM	664	SD	MET	632	28.174	23.531	36.257	1.00	0.00	LX0	S
ATOM	665	CE	MET	632	27.704	24.238	37.835	1.00	0.00	LX0	C
ATOM	666	C	MET	632	23.401	25.432	35.100	1.00	0.00	LX0	C
ATOM	667	O	MET	632	23.742	26.501	34.615	1.00	0.00	LX0	O
ATOM	668	N	VAL	633	22.238	25.225	35.725	1.00	0.00	LX0	N
ATOM	669	H	VAL	633	22.005	24.285	35.974	0.00	0.00	LX0	H
ATOM	670	CA	VAL	633	21.308	26.344	35.866	1.00	0.00	LX0	C
ATOM	671	CB	VAL	633	19.986	25.877	36.500	1.00	0.00	LX0	C
ATOM	672	CG1	VAL	633	18.942	26.998	36.547	1.00	0.00	LX0	C
ATOM	673	CG2	VAL	633	19.432	24.646	35.779	1.00	0.00	LX0	C
ATOM	674	C	VAL	633	21.894	27.520	36.637	1.00	0.00	LX0	C
ATOM	675	O	VAL	633	22.078	27.474	37.851	1.00	0.00	LX0	O
ATOM	676	N	LEU	634	22.144	28.584	35.859	1.00	0.00	LX0	N
ATOM	677	H	LEU	634	22.125	28.431	34.871	0.00	0.00	LX0	H
ATOM	678	CA	LEU	634	22.590	29.863	36.413	1.00	0.00	LX0	C
ATOM	679	CB	LEU	634	22.680	30.901	35.291	1.00	0.00	LX0	C
ATOM	680	CG	LEU	634	23.988	30.826	34.502	1.00	0.00	LX0	C
ATOM	681	CD1	LEU	634	23.894	31.575	33.173	1.00	0.00	LX0	C
ATOM	682	CD2	LEU	634	25.173	31.313	35.339	1.00	0.00	LX0	C
ATOM	683	C	LEU	634	21.756	30.402	37.566	1.00	0.00	LX0	C
ATOM	684	O	LEU	634	20.723	29.858	37.950	1.00	0.00	LX0	O
ATOM	685	N	GLY	635	22.273	31.507	38.121	1.00	0.00	LX0	N
ATOM	686	H	GLY	635	23.143	31.867	37.789	0.00	0.00	LX0	H
ATOM	687	CA	GLY	635	21.624	32.041	39.313	1.00	0.00	LX0	C
ATOM	688	C	GLY	635	22.034	31.268	40.549	1.00	0.00	LX0	C
ATOM	689	O	GLY	635	22.118	30.043	40.546	1.00	0.00	LX0	O
ATOM	690	N	GLY	636	22.311	32.047	41.601	1.00	0.00	LX0	N
ATOM	691	H	GLY	636	22.163	33.033	41.545	0.00	0.00	LX0	H
ATOM	692	CA	GLY	636	22.757	31.398	42.830	1.00	0.00	LX0	C
ATOM	693	C	GLY	636	21.624	30.754	43.605	1.00	0.00	LX0	C
ATOM	694	O	GLY	636	21.106	29.697	43.261	1.00	0.00	LX0	O
ATOM	695	N	ARG	637	21.256	31.456	44.683	1.00	0.00	LX0	N
ATOM	696	H	ARG	637	21.633	32.363	44.865	0.00	0.00	LX0	H
ATOM	697	CA	ARG	637	20.243	30.877	45.558	1.00	0.00	LX0	C
ATOM	698	CB	ARG	637	20.461	31.345	47.001	1.00	0.00	LX0	C
ATOM	699	CG	ARG	637	20.263	30.247	48.055	1.00	0.00	LX0	C
ATOM	700	CD	ARG	637	18.834	29.710	48.179	1.00	0.00	LX0	C
ATOM	701	NE	ARG	637	18.788	28.596	49.123	1.00	0.00	LX0	N
ATOM	702	HE	ARG	637	19.099	28.793	50.055	0.00	0.00	LX0	H
ATOM	703	CZ	ARG	637	18.403	27.365	48.720	1.00	0.00	LX0	C
ATOM	704	NH1	ARG	637	17.942	27.162	47.486	1.00	0.00	LX0	N
ATOM	705	HH11	ARG	637	17.711	26.255	47.140	0.00	0.00	LX0	H
ATOM	706	HH12	ARG	637	17.800	27.933	46.849	0.00	0.00	LX0	H
ATOM	707	NH2	ARG	637	18.492	26.348	49.576	1.00	0.00	LX0	N
ATOM	708	HH21	ARG	637	18.222	25.419	49.322	0.00	0.00	LX0	H
ATOM	709	HH22	ARG	637	18.837	26.497	50.504	0.00	0.00	LX0	H

```
ATOM    710  C   ARG   637       18.813  31.101  45.100  1.00  0.00      LX0  C
ATOM    711  O   ARG   637       18.051  31.857  45.686  1.00  0.00      LX0  O
ATOM    712  N   LYS   638       18.465  30.367  44.036  1.00  0.00      LX0  N
ATOM    713  H   LYS   638       19.147  29.758  43.634  0.00  0.00      LX0  H
ATOM    714  CA  LYS   638       17.033  30.297  43.757  1.00  0.00      LX0  C
ATOM    715  CB  LYS   638       16.721  29.842  42.321  1.00  0.00      LX0  C
ATOM    716  CG  LYS   638       17.428  28.586  41.795  1.00  0.00      LX0  C
ATOM    717  CD  LYS   638       18.741  28.896  41.068  1.00  0.00      LX0  C
ATOM    718  CE  LYS   638       19.443  27.638  40.560  1.00  0.00      LX0  C
ATOM    719  NZ  LYS   638       20.688  28.011  39.881  1.00  0.00      LX0  N
ATOM    720  HZ1 LYS   638       21.196  27.171  39.525  0.00  0.00      LX0  H
ATOM    721  HZ2 LYS   638       20.512  28.614  39.053  0.00  0.00      LX0  H
ATOM    722  HZ3 LYS   638       21.324  28.543  40.509  0.00  0.00      LX0  H
ATOM    723  C   LYS   638       16.319  29.447  44.794  1.00  0.00      LX0  C
ATOM    724  O   LYS   638       16.928  28.652  45.509  1.00  0.00      LX0  O
ATOM    725  N   PHE   639       15.009  29.691  44.880  1.00  0.00      LX0  N
ATOM    726  H   PHE   639       14.549  30.264  44.204  0.00  0.00      LX0  H
ATOM    727  CA  PHE   639       14.268  29.040  45.953  1.00  0.00      LX0  C
ATOM    728  CB  PHE   639       13.412  30.068  46.702  1.00  0.00      LX0  C
ATOM    729  CG  PHE   639       14.297  31.158  47.267  1.00  0.00      LX0  C
ATOM    730  CD1 PHE   639       15.036  30.914  48.446  1.00  0.00      LX0  C
ATOM    731  CD2 PHE   639       14.377  32.400  46.599  1.00  0.00      LX0  C
ATOM    732  CE1 PHE   639       15.877  31.922  48.957  1.00  0.00      LX0  C
ATOM    733  CE2 PHE   639       15.219  33.408  47.108  1.00  0.00      LX0  C
ATOM    734  CZ  PHE   639       15.964  33.156  48.279  1.00  0.00      LX0  C
ATOM    735  C   PHE   639       13.441  27.884  45.436  1.00  0.00      LX0  C
ATOM    736  O   PHE   639       13.302  27.679  44.239  1.00  0.00      LX0  O
ATOM    737  N   LYS   640       12.895  27.116  46.393  1.00  0.00      LX0  N
ATOM    738  H   LYS   640       13.037  27.361  47.351  0.00  0.00      LX0  H
ATOM    739  CA  LYS   640       12.165  25.910  45.987  1.00  0.00      LX0  C
ATOM    740  CB  LYS   640       11.605  25.173  47.213  1.00  0.00      LX0  C
ATOM    741  CG  LYS   640       10.756  26.040  48.156  1.00  0.00      LX0  C
ATOM    742  CD  LYS   640        9.717  25.237  48.948  1.00  0.00      LX0  C
ATOM    743  CE  LYS   640        8.323  25.155  48.300  1.00  0.00      LX0  C
ATOM    744  NZ  LYS   640        8.352  24.532  46.967  1.00  0.00      LX0  N
ATOM    745  HZ1 LYS   640        7.383  24.369  46.631  0.00  0.00      LX0  H
ATOM    746  HZ2 LYS   640        8.826  25.164  46.287  0.00  0.00      LX0  H
ATOM    747  HZ3 LYS   640        8.865  23.630  46.996  0.00  0.00      LX0  H
ATOM    748  C   LYS   640       11.062  26.099  44.945  1.00  0.00      LX0  C
ATOM    749  O   LYS   640        9.917  25.693  45.166  1.00  0.00      LX0  O
TER
```
